# Unsupervised discovery of clinical disease signatures using probabilistic independence

**DOI:** 10.1016/j.jbi.2025.104837

**Published:** 2025-04-23

**Authors:** Thomas A. Lasko, William W. Stead, John M. Still, Thomas Z. Li, Michael Kammer, Marco Barbero-Mota, Eric V. Strobl, Bennett A. Landman, Fabien Maldonado

**Affiliations:** a Vanderbilt University Medical Center, 1211 Medical Center Dr, Nashville, TN 37232, USA; b Vanderbilt University, 2301 Vanderbilt Pl, Nashville, TN 37235, USA; c University of Pittsburgh, 4200 Fifth Ave, Pittsburgh, PA 15260, USA

**Keywords:** Phenotype discovery, Machine learning, Electronic health records, Causal inference, Indeterminate pulmonary nodule, Lung cancer

## Abstract

**Objective:**

This study uses probabilistic independence to disentangle patient-specific sources of disease and their signatures in Electronic Health Record (EHR) data.

**Materials and Methods:**

We model a disease source as an unobserved root node in the causal graph of observed EHR variables (laboratory test results, medication exposures, billing codes, and demographics), and a signature as the set of downstream effects that a given source has on those observed variables. We used probabilistic independence to infer 2000 sources and their signatures from 9195 variables in 630,000 cross-sectional training instances sampled at random times from 269,099 longitudinal patient records. We evaluated the learned sources by using them to infer and explain the causes of benign vs. malignant pulmonary nodules in 13,252 records, comparing the inferred causes to an external reference list and other medical literature. We compared models trained by three different algorithms and used corresponding models trained directly from the observed variables as baselines.

**Results:**

The model recovered 92% of malignant and 30% of benign causes in the reference standard. Of the top 20 inferred causes of malignancy, 14 were not listed in the reference standard, but had supporting evidence in the literature, as did 11 of the top 20 inferred causes of benign nodules. The model decomposed listed malignant causes by an average factor of 5.5 and benign causes by 4.1, with most stratifying by disease course or treatment regimen. Predictive accuracy of causal predictive models trained on source expressions (Random Forest AUC 0.788) was similar to (p = 0.058) their associational baselines (0.738).

**Discussion:**

Most of the unrecovered causes were due to the rarity of the condition or lack of sufficient detail in the input data. Surprisingly, the causal model found many patients with apparently undiagnosed cancer as the source of the malignant nodules. Causal model AUC also suggests that some sources remained undiscovered in this cohort.

**Conclusion:**

These promising results demonstrate the potential of using probabilistic independence to disentangle complex clinical signatures from noisy, asynchronous, and incomplete EHR data that represent the confluence of multiple simultaneous conditions, and to identify patient-specific causes that support precise treatment decisions.

## 1. Background and significance

A clinical disease label is an abstraction over a collection of unobserved pathophysiologic mechanisms that produce an overlapping set of clinical manifestations. The label can be an effective way to reduce the complexity of clinical practice, but the lack of precision can lead to treatment failure when the patient’s specific disease mechanism does not respond to the typical treatment [1–9].

Identifying the mechanistic pathways underlying a given disease label is an important but labor-intensive undertaking involving biochemical or clinical experiments. However, we may be able to get partway there by identifying the set of changes (or *signatures*) that each unobserved mechanism has on the observable variables in the Electronic Health Record (EHR). Our premise is that similar but distinct mechanisms leave similar but distinct signatures in the record.

Our goal is to computationally identify these signatures, and from them draw some inference about their unobserved mechanisms. We want to understand causes of disease at the individual patient level, and to perform the inference task at institutional scale in the number of variables, training instances, and learned causes. (We will use the term *cause* in its technical sense of an ancestor node in a causal graph. From a clinical perspective, a cause can be any factor that if manually changed would affect the downstream probability of a disease. It may include a direct cause, a risk factor, a protective factor, or a preventative measure. A cause is distinguished from an *association* or a *correlate*, which may have a common upstream cause with the disease in question, or may be a downstream *effect* of the disease, but if it were manually changed would not actually affect the disease.).

We require an unsupervised approach because our motivating observation is that the existing clinical labels are insufficiently precise. Instead, we want to allow the data to speak directly, modeling the unobserved disease mechanisms as unobserved root nodes (or *latent disease sources*) in the causal graph of observed EHR variables, and identifying a signature for each latent source. We have previously named this task *phenotype discovery*[10], and success requires overcoming both inherent and practical difficulties.

The major inherent difficulty arises from the fact that patients simultaneously experience multiple conditions that together employ a shared vocabulary of manifestations, creating a record that is a confluence of patterns that must be disentangled.

The major practical difficulty arises from the fact that medical records are neither clean nor complete[11]: data are observed sparsely, irregularly, and asynchronously, but not at random; billing codes and problem lists don’t always reflect the true or current patient state [12,13]; medication lists incompletely capture whether medications are prescribed, appropriately dosed, filled, and taken as prescribed[14,15]; and clinical measurements are subject to error in analytic, pre-analytic, and post-analytic processes[16].

### 1.1. Previous approaches

We are unaware of any prior work that achieves our goal of inferring patient-specific causes of disease from institutional-scale EHR data (much of it infers predictive instead of causal features), but we review major steps that have been taken in this direction.

A common approach to phenotype discovery has been to partition a set of patients into disjoint clusters and then identify the salient clinical pattern in each cluster[17–25], but it turns out that this *hard clustering* approach does not address the inherent difficulty of the problem. Hard clustering is appropriate when each record legitimately belongs to exactly one cluster, such as partitioning dwellings into geographic regions. But it fails for phenotype discovery because it identifies only one pattern per patient, allowing strong patterns to dominate subtle patterns. In medical record data, strong patterns tend to reflect demographics, common conditions unrelated to the disease of interest, or even cohort inclusion criteria.

A better approach is to adapt methods of *representation learning*[26], which structures raw elements of a record into multiple meaningful or explanatory features. Representation learning was originally designed to replace the practice of engaging experts to create predictive features manually. Phenotype discovery falls into the subproblem of *causal representation learning*[27], which imposes the additional constraint that the explanatory patterns should represent elements of the data’s cause-effect network. Disentangling a patient record into a causal representation would not only identify the precise causes of the patient’s diseases, but also allow us to answer interventional questions, such as ‘What would be the effect on the patient’s future health outcomes if we intervened to remove this cause?’ If treatment response data can be included in the model, this is the type of question that can lead to precision treatment decisions.

The work that began the popular wave of deep learning used a multi-layer autoencoder for disentangling[28]. The disentangling was guided by minimizing reconstruction error under a constrained number of explanatory factors. The main contribution of the work was to demonstrate learning nonlinear components one layer at a time that were intended to be a useful decomposition of the data space (with the meaning of *useful* determined by the constraints imposed on each hidden layer), but with no attention to causal properties.

The earliest phenotype discovery from EHR data that we know of used a similar autoencoder guided by sparsity, L_2_ regularization, and uncertainty-normalized reconstruction error to find explanatory features in irregular longitudinal observations of a single laboratory test result[10]. Other autoencoder variants use robustness to noise[29], factor decorrelation, hierarchical structure, and many other guiding principles[26,30]. Variational autoencoders are considered by some to be the state of the art for disentanglement, although using them to recover true latent sources remains an open problem, in part because of the difficulty of designing a loss function that achieves probabilistic independence of the learned explanatory factors[31], or one that can otherwise guide the learning toward causal rather than predictive features.

The question of how to judge a learned representation (and therefore how to guide the learning) is also an open one, and is more complex than judging the accuracy of a classification or a regression[26]. Early work, including applications in medicine, nevertheless used predictive accuracy on one or more supervised problems as a measure of representation quality[10,29,32,33–38]. Predictive accuracy is a reasonable baseline measure, particularly if the supervised application used for evaluation did not directly guide the feature learning. But the most predictive features may not necessarily be those with the greatest explanatory power, and may not actually represent the pathophysiologic pathways.

### 1.2. Using independence to guide disentangling

The degree of probabilistic independence between inferred sources can be a useful signal to guide disentangling[39,40], and has the additional benefit that it can produce sources with our desired causal properties[27,41]. In phenotype discovery, the intuition is that the true sources of different diseases act independently of each other, so their observable patterns in a patient record should also occur independently. Of course, some diseases are caused by others, such as myocardial infarction caused in part by atherosclerosis, and these relationships form a potentially dense causal network. However, we consider every disease to have an additional unobserved (or latent) cause of its own that is independent of all other causes, and our task is to find these mutually independent latent causes. These latent causes form the set of root nodes of that network because none of them have observed parents.

**Fig. 1.**
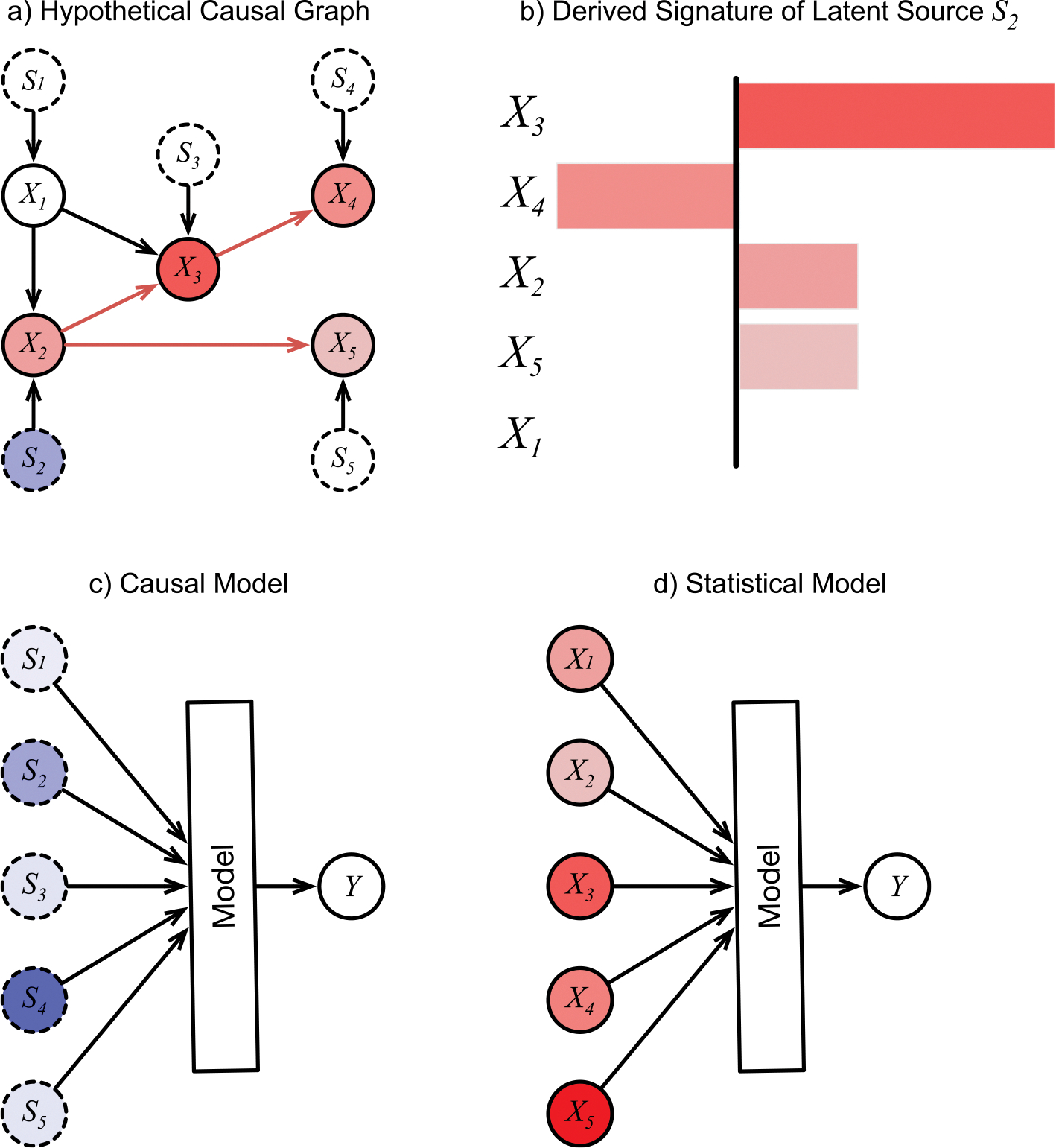
A hypothetical causal graph and structured derived from it. a) The causal graph inferred from observing the $X_{i}$ (solid circles) over many records. The $S_{i}$ are inferred latent sources (dotted circles). Colors of the nodes $X_{i}$ indicate the degree to which a unit change in source $S_{2}$ affects them. They are arbitrary here for illustration, except for $X_{1}$, which cannot be affected by $S_{2}$. b) Causal effects of source $S_{2}$ collected into a bar-graph signature c) Causal model of Y using latent sources $S_{i}$ as inputs. d) Statistical model of Y using observations $X_{i}$ as inputs. Color intensity of inputs represent their hypothetical importance values for the prediction in a single instance. For the causal model, the inputs are mutually independent root nodes, and therefore can be interpreted as the causal sources of Y, which may suggest treatment approaches that address the specific causes for this patient, and which may be manipulated to investigate different counterfactual scenarios. For the statistical model, the importance values remain entangled and cannot be interpreted this way.

More formally, a causal graph corresponding to a set of observed variables $X={\left\{X_{i}\right\}}_{i=0}^{n}$ can be estimated from a large set of observations made under different conditions or at different times in different patients (Fig. 1a). In such a graph, we consider the value of each observed variable $X_{i}$ to be a function $X_{i}=f_{i}\left({PA}_{i},S_{i}\right)$ of its observed parents ${PA}_{i}$, and an unobserved parent $S_{i}\in S$ representing a latent source.

The $S_{i}$ are often called error terms, but their function is much more important than simply adding noise to the observations. In effect, they are the locations in the graph where all outside information is injected before being distributed among the observed variables. We can think of the graph as existing in a steady state until a latent source changes its value, which then causes downstream changes in some observed variables[41]. For source $S_{i}$, the set of downstream changes follows a specific pattern or *signature $A_{i}$* that depends on the strength of causal relationships in the network (Fig. 1 a, b). The $S_{i}$ are considered *root causes* of the changes in the graph because they are the roots, or parent-less nodes, of the causal graph. In phenotype discovery from EHR data, each $S_{i}$ is in fact the output of an unobserved and potentially large upstream network of biochemical and environmental processes. The true pathophysiologic cause of disease is contained somewhere in that upstream network, but the latent $S_{i}$ is as close as we can get to that cause given the observed variables in the data.

The mutual independence of the $S_{i}$ values (or *source expressions*) is used to guide the disentangling. In general, if the functions $f_{i}$ and the distributions of the latent sources $S_{i}$ can be arbitrarily chosen, it is impossible to recover them from observations alone, but a solution can be found by imposing additional assumptions[27,31]. If we assume linear functions and non-Gaussian distributions for the source expressions, with a sufficiently comprehensive set of variables X and minimal confounding (which we achieve by using large-scale EHR data), methods such as ICA and Linear Non-Gaussian Acyclic Models (LiNGAM)[40,42] can recover both the sources S and the signatures A by the linear decomposition X=AS. The causal graph itself can then be recovered from A[43], or Direct LiNGAM can recover it directly from X.

Relaxing the requirement of linear $f_{i}$ can be done if additional assumptions are made, which is an active research direction [27,31,44–52,53].

Empirically, source independence has been demonstrated to be an objectively more accurate principle than other disentangling approaches. On a task in the genomic domain, objectively comparing 42 different methods of inferring groups of genes that are functionally related and coregulated, investigators found that independence-based methods outperformed all others tested[54]. A different group found that transcriptome structure inferred using ICA was conserved across five datasets, despite those datasets coming from different research groups using different technologies[55].

#### 1.2.1. Identifying causal sources

The fact that the latent sources $S_{i}$ are mutually independent root nodes implies that any learned model $Y=H_{c}(S)$ will be a causal model of Y given S, regardless of the architecture of $H_{c}$ (Fig. 1c). Specifically, $H_{c}$ may be a standard probabilistic statistical model such as a Random Forest or a deep architecture and still produce causal predictions of Y given S. The model $H_{c}$ does not explicitly receive the values of any $X_{i}$, and we do not assume that it internally reconstructs the causal relationships among the $X_{i}$, although a sufficiently powerful architecture may infer some version of them in constructing the effect of S on Y. This analysis depends on certain assumptions, some of which are common to causal inference, and others enable the ICA decomposition. Supplemental Section A1.6.1 details the assumptions and the formal causal definitions. Also see previous work[41] for the full rigorous development of the causal nature of $Y=H_{c}(S)$.

If Y is a disease label for a given patient, the elements of S that meaningfully affect the prediction are the estimated latent causes for that patient’s disease. If those causes have identifiable treatments, then those treatments would be the optimal and precise treatments for the patient.

In contrast, the elements of X are mutually dependent and represent entangled effects, so the common approach of building a predictive statistical model $Y=H_{c}(S)$ (Fig. 1d) would not identify causal sources without attention to recovering the causal graph among the $X_{i}$ as part of $H_{s}$. Specifically, we would not expect a Random Forest, a deep architecture, or any other standard machine learning approach to produce a causal model under the design $Y=H_{s}(X)$.

Identifying patient-specific latent sources is the first step in the direction of identifying patient-specific treatments. Understanding what those latent sources represent pathophysiologically and what treatments may affect them are important future steps.

### 1.3. Contributions

In this work, we present 1) the use of probabilistic independence to disentangle data signatures of latent sources of disease from routinely collected, episodic, and noisy EHR data at institutional scale, 2) an evaluation of this approach by estimating patient-specific causes of indeterminate pulmonary nodules found either incidentally or by screening, and 3) discovery of potential patient-specific signals of existing but undiagnosed cancer as causes of the nodules.

## 2. Materials and methods

**Fig. 2.**
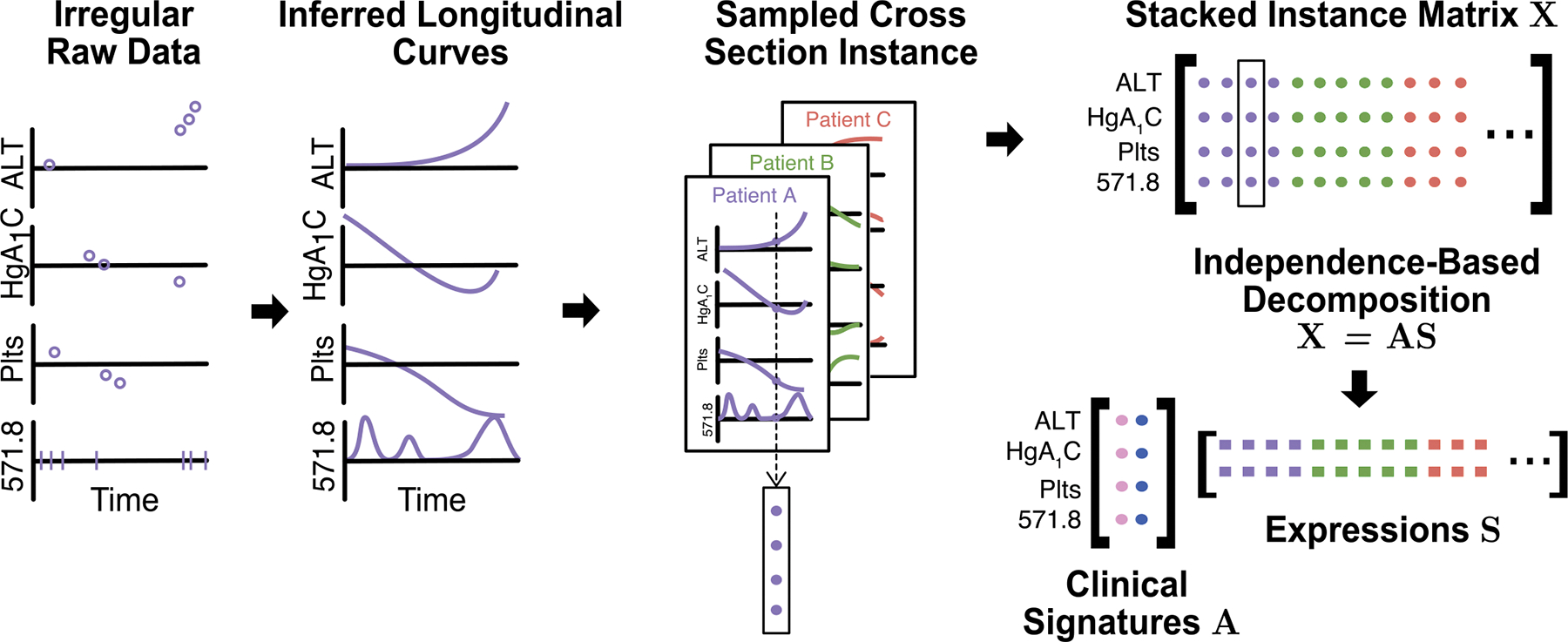
The pipeline for learning clinical signatures and their patient-level source expressions from noisy, asynchronous, and irregular EHR data. The first three steps transform the data into a dense, regular matrix X for machine learning. The final step infers the clinical signatures A and the expression levels S of latent disease sources using probabilistic independence.

The inference pipeline included data collection, transformation, and signature discovery components (Fig. 2). We summarize these methods here, with more detailed descriptions in the supplemental material Section A1.

This research was approved as non-human-subjects research by the Vanderbilt University Institutional Review Board (#210761).

### 2.1. Data collection

Vanderbilt University Medical Center is a tertiary care center with an extensive EHR covering about 3 million patients, with nearly all inpatient and outpatient records complete after 2005. We used two datasets drawn from our EHR: a *Discovery Set*, comprising 269,099 records of patients with a broad range of lung disease, used to learn the latent clinical signatures; and an *Evaluation Set* comprising 13,252 records of patients with an indeterminate pulmonary nodule and no prior history of any cancer, used to train and test the predictive model. The Evaluation Set is a subset of the Discovery Set.

We extracted *n* = 9,195 variables from all records, including clinical measurements, billing codes, medication mentions, and demographics. Records in the evaluation set were labeled positive for patients who received a billing code for any lung malignancy over the 3 years after the nodule appearance, and negative for patients who did not (Table 1). A set of 2651 records (20%) from the Evaluation Set were randomly partitioned into the final test set.

For the Evaluation Set, data from each record were collected from the time of the first pulmonary nodule code and earlier. No information except the label was included from after the nodule date. Labels were positive if the record included a code for malignant lung neoplasm on day 4 – 1095 following the pulmonary nodule date. This interval begins on day 4 to exclude patients diagnosed with cancer on the same day as the nodule discovery, allowing for variability in coding timing of the two types of codes. The intent is to identify the causes of benign vs. malignant nodules in patients for whom that was unknown at the time of nodule discovery.

Labels were validated in a separate study[56] by comparison with cancer registry records (all positive labeled records) and manual chart review (all mismatches with the cancer registry and a random sample of negative labeled records), and estimated to have 0.98 PPV, 0.99 NPV, 0.93 sensitivity, and 0.996 specificity (supplemental Section A1.1.2).

### 2.2. Data transformation

To overcome some of the practical difficulties arising from the messiness of EHR data (Section 1), our data were converted from point observations to probabilistic continuous longitudinal curves with a specific transformation for each data mode that preserves its essential characteristics but abstracts away the problems of sparse, irregular, and asynchronous observations (supplemental Section A1.2). Clinical measurements were fit with a smooth interpolation that maintains non-stationarity, or a constant population median if a patient had no observations of a given test. Billing codes were represented as a longitudinal intensity curve of code occurrence events per unit time, minimizing the effects of errors by treating the events as a probabilistic signal instead of ground truth. Medication mentions were transformed to piecewise-constant 0/1 curves representing the presence or absence of the medication in a record at a given date. Race and sex were represented as constant 0/1 curves, binarized from categorical variables when appropriate.

Next, curves for each patient were time-aligned and stacked, with cross sections sampled uniformly at random at a mean density of 1 sample per 3 record-years. An individual record may be randomly sampled once, more than once, or not at all, with longer records tending to be sampled more times. A total of t=630,000 sampled cross sections were stacked into a final data matrix $X\in R^{n\times t}$. Each column was standardized to place them all onto roughly the same scale.

### 2.3. Clinical signature discovery

The matrix X of stacked cross sections was decomposed by FastICA [57] into X=AS, where $A\in R^{n\times m}$, $S\in R^{m\times t}$, $X\in R^{n\times t}$, and m=2,000 (Fig. 2). The rows $S_{i*}$ are (approximately) mutually independent by construction, the column $A_{*i}$ represents the learned clinical signature of latent source i, and matrix element $S_{ij}$ represents the level at which cross section $X_{*j}$ expresses source i. The choice of the number of latent sources m was limited by computational complexity and available resources; we suspect the optimal m is much higher (Fig. A1 in supplemental methods).

**Fig. 3.**
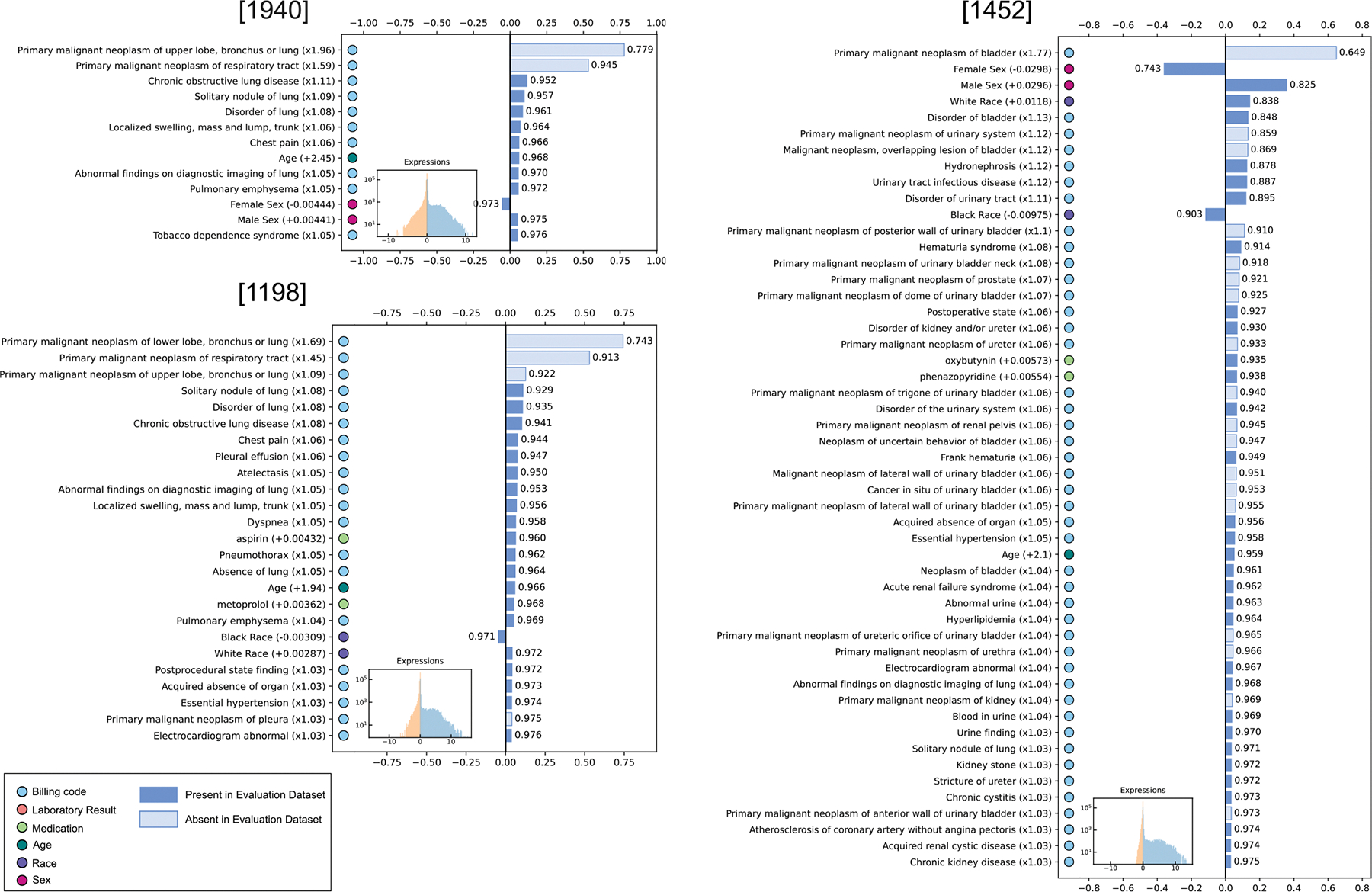
Three learned clinical signatures identified as common sources for malignant nodules. The signatures present the changes to input variables resulting from a one-unit increase in the expression level of the source. Numbers in parenthesis give the magnitude of the change in original data space: multiplicative for billing code intensities (indicated by operators × and /) and additive for other modes (operators + and −). Bar length indicates the size of the change in standardized space. Light-colored bars indicate billing codes for various malignancies, which were not present in any record of Evaluation Set by design. Numbers to the right of the bar give the fraction of the vector’s information, as judged by the L_2_ norm, contained in that and larger elements. Inset is a log-scaled histogram of inferred expression levels in the Discovery Set sample matrix. Expression units are individually scaled for each signature such that the mean is zero, and standard deviation 0.5, placing 95% of all expressions within the interval [−1,1].

The scale of each $S_{i,*}$ is non-identifiable under ICA, so expressions of each source are arbitrarily scaled individually to zero mean and 0.5 standard deviation in the Discovery Set (Fig. 3, insets), so a record may express a source either positively or negatively. Expressing a source negatively means the record manifests the opposite effect from what appears in the visualized signature.

Signatures in A were produced with only a sequential identifier, and no clinical interpretation or descriptive name. Where a name was needed for reporting purposes, we provided one using clinical expertise to interpret the pattern.

### 2.4. Evaluation

Causal models learned from observational data are notoriously difficult to evaluate for correctness[58], because they model interventions that have not been observed – in our case, how a patient record would change if we removed a given source. Likewise, evaluating an unsupervised disentangling is currently an unsolved problem[31,59] – in our case, the clinical sources are either unlabeled or an existing label may group them with (perhaps many) related sources.

Typically, causal inference approaches are evaluated using synthetic data in which all causal effects are known by design. ICA has been amply validated this way[39,41,57,60,61], but synthetic validation only goes so far, because synthetic data is typically simpler and cleaner than real data. Synthetic validation doesn’t, for example, assess whether the processes observed in a particular observational dataset violate the assumptions of the analysis, nor how far from truth the results of that analysis land under those violations.

In the current work, objective evaluation of the causal model was inspired by the technique of quantitative probing[62], in which verifiable elements are compared with externally known facts, with the difference that the original work evaluated edges internal to the causal graph, and we assess edges between root nodes and the predicted target.

#### 2.4.1. Clinical Background and Rationale

To implement the evaluation, we trained supervised models to predict whether an indeterminate pulmonary nodule appearing in a patient record would in the following 3 years be labeled malignant or benign, and to identify the latent sources for that labelled outcome. Indeterminate pulmonary nodules are abundant in clinical care, with over 1.5 million identified each year in the US alone[63]. There are many different potential causes of lung nodules, with the vast majority representing benign disease of little consequence for patients, but a small minority being extremely consequential as they represent early stage lung cancer (Table 2). Lung cancer remains one of the most common cancers, as well as the deadliest, accounting for over 120,000 deaths every year in the US – more than colon, breast, and prostate cancers combined. The best chance for cure is early detection and surgical resection, which makes accurate identification and characterization of malignant nodules a critically important healthcare project. Current management of lung nodules is imperfect, as human detection is error-prone due to lack of expertise, CT interpretation fatigue, and communication breakdowns. In addition, radiologist estimation of the probability of malignancy is often based on morphological features in the absence of clinical context, ignoring important patient-specific clinical variables. In addition, current methods of diagnosis are costly and carry morbidity[64], and even identifying the approximately 5% of nodules that are malignant is a clinical challenge[65].

#### 2.4.2. Source completeness and decomposition

We collected a reference list of the causes of malignant and benign lung nodules presented by a respected literature synthesis source[66] and identified the match(es) for each listed cause among our inferred latent sources. The first step in characterizing the inferred sources was to assess them for *completeness* and *decomposition* compared to this reference list. Ideally, this would be done for all possible sources for all conditions, but that would be intractable. Instead, we chose what from the discovery model’s perspective is an arbitrary subset of sources relating to indeterminate pulmonary nodules, unknown to the model during the discovery step, and assessed that subset.

To assess completeness, we identified the fraction of conditions on the reference list for which we recovered at least one match among the inferred signatures.

To assess decomposition, we computed the average number of recovered sources that matched a listed cause, when at least one such match was found.

#### 2.4.3. Causal source accuracy

We next assessed the degree to which a supervised model predicting malignant/benign labels Y from source expressions S (Section 1.2.1) identified (on average) accurate causes for the nodules by comparing the top 20 inferred causes to the reference list.

To extract inferred causes from the model, we used sample-specific Shapley additive explanation (SHAP) values[67,68]. We then aggregate these causes over all instances and compare the top causes to the reference list.

We used Random Forest, XGBoost, and Elastic Net architectures for the supervised models. One causal model $Y=H_{c}(S)$ of each type was trained to predict the binary malignancy label Y using as input the source expressions S, and a corresponding associative statistical model $Y\;=\;H_{s}(X)$ was trained using the original variable values X. Hyperparameters were optimized by cross validation independently for each of the six configurations. With the optimized hyperparameters fixed, training for each configuration was repeated with 100 different random seeds and evaluated on the common held-out test set to determine the extent of variation due to random choices made during training.

For each matched latent source, its patient-specific causal effect on the malignancy label was computed using the global mean positive sample-specific SHAP value (omitting negative values), which was visualized for comparison with the reference list. For causes of benign nodules, we computed the global mean negative SHAP value (omitting positive values). Polarity-specific means were used because for causes of malignant nodules, a positive SHAP value for a given patient indicates the source was generating disease, and a negative value indicates it was protecting against disease. Causes of benign nodules have the opposite polarity.

A SHAP value is a mathematical representation of the patient-specific causal effect of a source, taking into account (by marginalizing over) combinations with all other sources[41]. Because we allow H to include nonlinear and high-order effects, identical values of $S_{i}$ in two different records may have different causal effects, depending on other sources expressed in the record.

The relative performance between causal and statistical models could legitimately lean either way by a small amount. A causal model includes only causal predictors, and the omitted non-causal predictive associations may advantage the statistical model. Additionally, information is lost when reducing from 9195 to 2000 input variables, which is done by PCA as the first step of ICA. On the other hand, the causal model learns its signatures from the much larger Discovery Set, which allows it to borrow statistical strength. Our comparison is to ensure that one is not dramatically different from the other, which would suggest a pipeline problem; whichever way the balance leans in terms of predictive accuracy, the main advantage of the causal model is in illuminating the potentially treatable sources of disease for each patient.

#### 2.4.4. New candidate identification

We next considered any of the top 20 causes that were not on the reference list to be potential new candidate causes. We assessed them by evaluating literature evidence from PubMed searches that they may be causes of malignant or benign nodules, or lung cancer in general. The sources are cited and reasoning for each case are noted in Tables 2 and 3.

If literature indicating that a source has a mechanistic relationship in the correct direction to lung nodules or lung cancer, it is listed as strong evidence. For example, source 1936/COPD (Table 3) named: COPD is a known independent risk factor for lung cancer due to its effect on the cellular environment, in addition to the fact that both COPD and lung cancer have a common cause of smoking[69,70]. It is the size of independent causal effects like this that the analysis is designed to estimate, even in the presence of common upstream causes.

If the signature of an inferred cause contains elements that affect cancer risk, but the signature itself doesn’t appear to be the main cause, that was assessed as moderate evidence. For example, source 361/Treated Unstable Angina (Table 4): while treating unstable angina isn’t known to directly affect lung cancer risk, newly diagnosed cancer is associated with anatomical severity of coronary artery disease in patients with high levels of inflammation,[71] and the signature contains medications such as aspirin and simvastatin that possess known anti-inflammatory and anticancer properties.[72–74].

If there is some possible but unclear connection between some signature elements and the label, then that was labeled weak evidence. For example, source 227/Pregnancy Elevated Glucose Complications (Table 3): the signature is of women with pregnancy complications and abnormal glucose tolerance, with a small probability of diabetes mellitus. Diabetes and cancer are associated[75], but this signature is more specific than that, and no other diabetes signatures ranked highly as causes. Moreover, lung cancer is not high on the list of cancers associated with diabetes,[75] and the specific relationship of Gestational Diabetes to any cancer has been only sparsely studied, with inconsistent results.

If no literature was found linking the inferred source to pulmonary nodules or cancer, that was labeled absent evidence. Sources with no evidence may be model errors or they may be true but as-yet unrecognized causes. For example, source 7/Postpartum Pregnancy No Complications (Tables 3 and 4): the signature is a general postpartum pattern with a small race differential, and positive expression increases predicted malignancy risk. Lung cancer in pregnancy is rare[76], but we can find no estimates of lung cancer probability given a pulmonary nodule in or soon after pregnancy.

## 3. Results

### 3.1. Learned clinical signatures

The 2000 learned signatures in A (three relevant examples in Fig. 3, all 2000 in supplemental material) cover the full range of disease found in the Discovery Set, and most of them are unrelated to lung nodules. The signatures are sparse, most indicating near-zero changes for the vast majority of the 9195 variables. Our signature plots sort the variables by the size of the change and present the top k variables, where k is chosen such that 97.5% of the information in the original vector (as judged by the $L_{2}$ norm) is present in those k variables.

As with the signatures, source expressions are also sparse (Fig. 3, insets).

Looking in detail at some relevant examples (Fig. 3), we interpret signatures 1940 and 1198 to represent primary upper lobe and lower lobe lung cancer, respectively. They include well-established clinical manifestations[77], such as dyspnea, pneumothorax, localized swelling consistent with superior vena cava syndrome, and risk factors such as tobacco use.

We interpret signature 1452 to represent sources of primary malignancy in various locations of the genitourinary system, but predominantly bladder cancer. It includes appropriate differentials for sex and age, and known manifestations such as hydronephrosis, hematuria, and recurrent urinary tract infections.[78,79] It may represent multiple causes combined into a single source because the Discovery Set focused on lung disease and contained too few examples of genitourinary disease to resolve them.

### 3.2. Completeness

For malignant etiologies, matching signatures were found for 12 of 13 (92%) listed elements, with the caveat that the learned signatures for primary lung cancer were decomposed by anatomic location rather than cell type (Table 2). For benign etiologies, matching signatures were found for 7 of 23 (30%) listed elements.

### 3.3. Decomposition

Malignant etiologies with matching signatures had an average of 5.5 signatures per element (Table 2), with the maximum decomposition for Breast Cancer, which was decomposed by treatment approach and clinical course. Benign etiologies had an average of 4.1 signatures per element, with the maximum decomposition for Rheumatoid Arthritis, which was mainly decomposed by treatment approach.

### 3.4. Causal source accuracy and new candidate cause identification

Of the top 20 inferred sources of malignant nodules, 6 (30%) were directly listed as causes (Table 3). Of those not listed, 7 (35%) had at least moderate evidence in other literature, 3 (15%) had weak or mixed evidence, and 4 (20%) had no prior evidence found, although one of these (1622) turned out to be a proxy for the length of the patient record (details in supplemental information).

Of the top 20 inferred sources of benign nodules, 6 (30%) were directly listed as causes (Table 4). Of those not listed, 7 (35%) had at least moderate evidence, 4 (20%) had weak evidence, and 3 (15%) had no evidence found.

### 3.5. Model comparison

Predictive power of the causal models was not dramatically different from that of the associational models, although the performance of each causal model exceeded that of its corresponding associational model by 0.04 to 0.05 AUC (p = 0.058 for the Random Forest models) (supplemental Fig. A6). The modest but consistent improvement suggests that the causal predictors may have been cleaner than associational predictors, and there may have been some borrowing of statistical strength from the Discovery Set.

Performance of the XGBoost causal model (0.789) was similar to the Random Forest causal model (0.788), and both outperformed the linear causal model (0.757), suggesting that there were nonlinear effects in the causal network.

The associational XGBoost model (0.755) outperformed the associational Random Forest model (0.738), but the difference disappeared in the causal models, suggesting that the learned signatures effectively captured essential predictive information.

Of the three causal models, the top predictive signatures of the Random Forest model make the most clinical sense to our subjective clinical judgement, which we attribute to model mismatch for the linear model, and to the greediness of the XGBoost model, which also had the greatest variability over random seeds. See supplemental material Section A3.1 and Figs. A2 – A4 for more on this point.

## 4. Discussion

We have demonstrated the use of probabilistic independence to disentangle clinical signatures of latent disease sources from institutional scale, structured EHR data using an unsupervised learning algorithm. We have also inferred the estimated expression of each source in a given patient record, evaluating the process by using the estimated expressions to classify malignant vs. benign pulmonary nodules and identify the sources driving the classification. Achieving this source identification is a step toward making patient-specific treatment decisions.

In our evaluation, the discovery model recovered a total of 102 signatures matching causes directly listed in the external reference, including 92% of the malignant causes and 30% of the benign causes (Table 2). Many of the unrecovered benign conditions are rare, and simply may not have been present in our Discovery Set. Furthermore, if a nodule is proven benign by biopsy or judged benign based on radiologic follow-up, the specific benign cause is typically not elucidated, and the elements of what would have become their signatures are left unobserved. Finally, some unrecovered signatures were for very detailed conditions, such that the granularity of the input data was not sufficient to represent them.

Because sources are arbitrarily scaled such that the distribution of their expressions over the Discovery Set has zero mean and unit standard deviation, some with long tails in both directions, they can appear as either a benign (negative SHAP value) or a malignant (positive SHAP value) cause, depending on the expression polarity of a given patient. SHAP value polarity usually matches expression polarity, but not always (all panels of Fig. 6 show mismatched polarity).

The polarity mismatch is usually due to the sign ambiguity of ICA inference[57,68] (our heuristic is to assign the sign such that the dominant element is a positive change), but the polarity of signatures containing malignancy codes (signatures 886, 1940, 1198, and 1452) are a special case that is at first glance counterintuitive (Fig. 6). All signatures were learned from the broad Discovery Set comprising patients with many kinds of lung disease, including cancer, and the patients with lung cancer in the Discovery Set have positive expressions for the lung cancer sources, as expected. But the Evaluation Set from which the supervised model was trained *contained only patients with no history of any cancer*. Therefore, no record in the sets from which the predictive models were trained or tested contained any cancer billing codes (the light-colored bars in Fig. 3). We might expect these records to have near zero expression for these sources, but that is not what happened. Instead, we determined experimentally that if a record perfectly matches one of these cancer signatures, except it has no cancer codes, it produces a *negative* expression (data not shown). This behavior depends upon the other signatures in A, because the expressions $S=g(X)=A^{-1}X$, are a complex and dense mapping g from X to S (while the mapping in the reverse direction is simple and sparse). In the case of these specific signatures in our matrix A, records with a partial match to the signature, absent the major cancer billing code elements, express the source with a negative polarity.

**Fig. 4.**
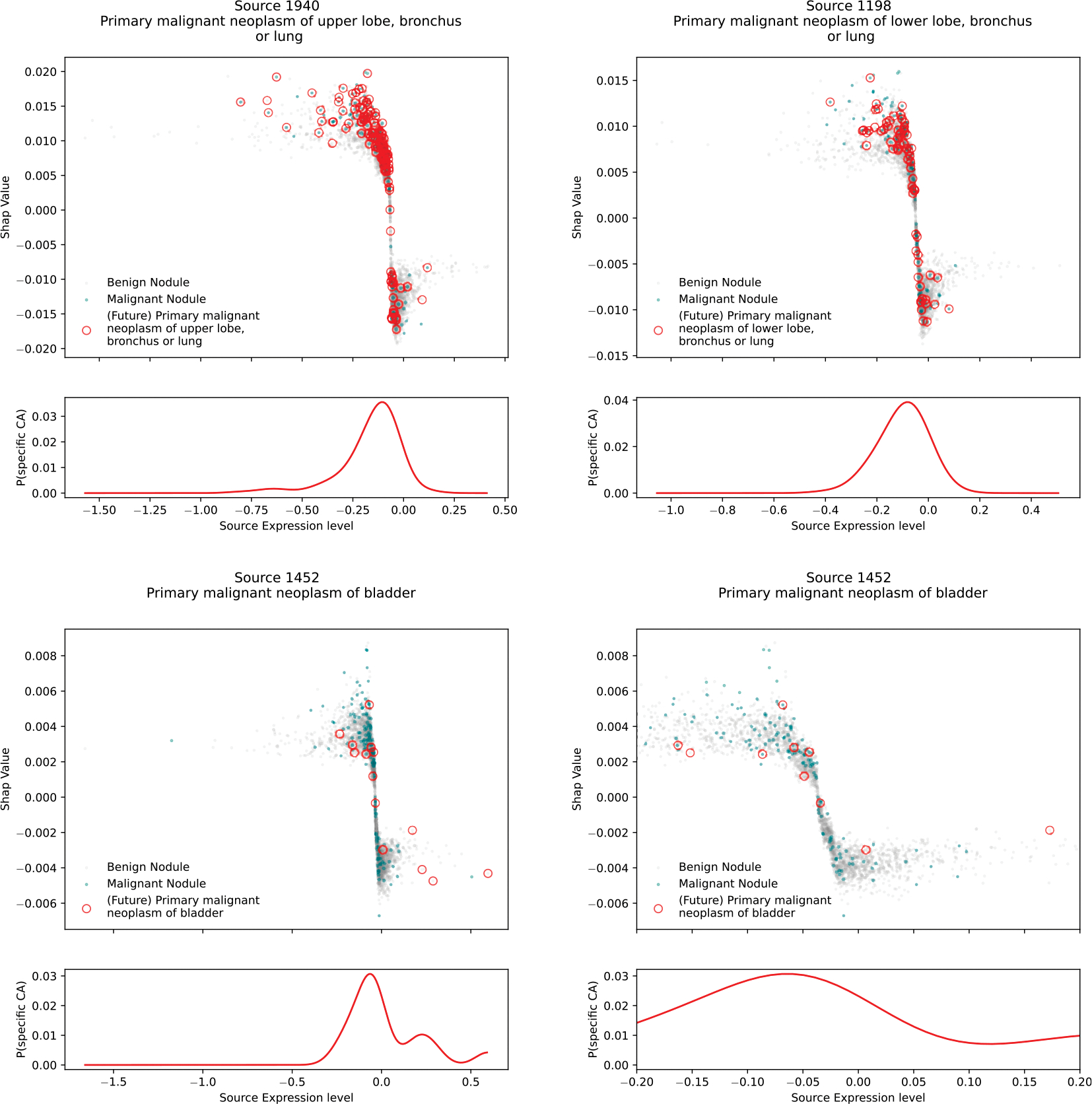
Patients who in the future develop specific cancers (red circles) do indeed express partial signatures of those cancers (without the billing codes for malignancy) at the time of lung nodule detection (positive SHAP Values, negative expression values). Many others who don’t develop cancer also express positive SHAP values, which is consistent with values in the 0.02 or lower probability ranges. In the lung malignancies (1940, 1198), a large fraction of future cancers appear in patients with negative expression away from zero (curve below the scatter plot), which the model picked up as predicting lung cancer (positive SHAP values). In the bladder cancer signature (1452, full plot on left, detail on right), patients with future bladder cancer fell on both sides of zero on both axes, but only those with negative expressions had malignant nodules. The small numbers make it hard to draw firm conclusions, but this suggests that the signature may differentiate between undiagnosed bladder cancer that has metastasized to the lung and undiagnosed bladder cancer that hasn’t. (For interpretation of the references to color in this figure legend, the reader is referred to the web version of this article.)

The negative expressions of these unusual sources were identified by the supervised model as top predictors of a malignant nodule (Fig. 4) suggesting that these records may be indicating the presence of undiagnosed cancer in a patient. This can’t be an artifact of future information leaking into the past by treatment being started without cancer codes entered, because cancer treatment elements are not included as important members of the signatures. (There are many signatures that contain cancer treatment elements, but these have near zero expression in all Evaluation Set patients.).

To investigate whether these partial matches to cancer signatures actually represent patients with undiagnosed cancer, we overlay markers in Fig. 4 to indicate the records that contain future primary malignancies of upper lobe, lower lobe, and bladder, and find that they fall overwhelmingly into the appropriate regions (quantified by the probability curve below the scatter plot), providing evidence that the model had indeed detected specific undiagnosed cancer. Many other records without cancer also fall into the same region, so a positive SHAP value for a single signature has low PPV for cancer. But this is consistent with the magnitude of the values themselves, up to about a 0.02 probability increase.

Additionally, the behavior of the bladder cancer source is interesting in an unexpected way. The plot shows records containing future bladder cancer falling on both sides of zero for both axes, although they are more concentrated in the appropriate region. However, *only* records in the appropriate region contained a malignant pulmonary nodule. The small numbers prevent firm conclusions, but these results suggest that the signature may distinguish undiagnosed bladder cancer that has metastasized to the lung from undiagnosed bladder cancer that has not, depending on whether the partial match produces a negative or positive expression.

It is curious that non-respiratory primary cancers in the reference list other than bladder were not higher on the list of inferred causes, although melanoma and kidney cancer are also ranked fairly high. Perhaps this indicates something different about the behavior of bladder cancer compared to the others, such as a greater tendency to metastasize before diagnosis, whereas malignancies such as breast cancer are the focus of intense screening efforts and may be more often diagnosed before metastasis.

### 4.1. Limitations and future directions

The discovery model produced an average decomposition of 5.5 signatures per listed cause when at least one match was found for the malignant nodules, and 4.1 per listed cause of benign nodules. A large fraction of the decompositions for common conditions like breast cancer and rheumatoid arthritis were by treatment approach or disease course, which is superficially exactly what we desired, but probably operationally insufficient. These decompositions were made from data recorded *after* treatment was decided and *after* the disease had run part of its course, but what we want is to distinguish mechanisms *before* that happens, to suggest optimal treatment. It may be that these decompositions can function the same way that our cancer signatures did for undiagnosed disease, where patients express a source in a salient way before the major, definitive elements are recorded. But it may also be that the signatures instead represent the effects of clinical processes, such as the different treatment preferences of different clinicians[97].

A striking result of the causal model is the multiplicity of sources that make causal contributions for a given record. This is most evident in the fact that the largest maximum causal effect over all test-set records, averaged over the 100 random seeds, was less than 0.03. Which means that in the best case, the expression of that source by a record increased the probability of malignancy by less than 0.03. Typically, tens of sources combine to produce the final prediction for a single record (data not shown). This seems reasonable for our selected clinical problem, given that human clinicians also have difficulty predicting the nature of an indeterminate pulmonary nodule using the patient’s clinical history. If there were just a few salient attributes that could predict malignancy with any reliability, we might expect clinicians to already be using them for diagnosis. The causal model’s average performance (AUC 0.788) is promising, but also demonstrates that there are nodule causes unknown to the model. These may be the etiologies in Table 2 that lack a matching signature, or they may be causes yet to be discovered.

Additionally, the absence of signatures corresponding to histological properties of lung nodules, such as non-small cell cancer or adenocarcinoma, implies that the signatures are built on insufficiently detailed observations. Extending the observed variables included in the Discovery Set to other data sources that capture these details may provide higher-resolution signatures.

Errors in source and signature inference may also have arisen from limitations inherent to ICA: linearity assumptions in source discovery, the possibility of converging to local minima, and allowing small residual dependency between sources. In addition, the dimensionality reduction from 9,195 original variables to 2,000 sources almost certainly affected some of the relevant signatures by combining important sources. Improving the implementation of ICA or Direct LiNGAM to scale up even further is an important direction for future work.

Our model is also subject to the standard assumptions of causal inference. For example, we assume no directed cycles in the causal graph, no unobserved single-cause confounders, and linear functions $f_{i}$ described in Section 1.2 for inferring the value of the latent sources from observations. While relaxation of these assumptions can be accommodated[45,46,95,] (and our experimental design tries to minimize the violations, such as by using nearly 10,000 variables for each record), the accommodations add complexity that we wanted to avoid here. They may be fruitful directions for future work.

Genetic variants, environmental factors, or social and behavioral determinants of health may be confounders in this analysis, and it may be useful to include them in future models. However, the risk of unobserved confounders is minimized by our design, which (approximately) eliminates *multiple-source* confounding among the inferred latent sources. The design retains the assumption of no *unobserved single-cause confounding*, which is a weaker assumption that there is no unobserved confounder that affects a *single* source $S_{i}$ and the label Y (supplemental Section A1.6.1).

Finally, while our models have produced clinically plausible sources for patients’ malignant or benign pulmonary nodules that align with the published literature on lung cancer and suggest the detection of undiagnosed disease, we have not established these sources as definitive. Caution is indicated by the fact that the three different model architectures produced different estimated causal effects for each, depending on the inductive bias of the model (Supplemental section A3.1 and Figs. A2–A4).

## 5. Conclusion

We have demonstrated the unsupervised learning of clinical signatures in routinely collected EHR data, using probabilistic independence as the guiding principle. This principle produces signatures corresponding to unobserved root nodes in the inferred causal graph of the observed data. Clinically, the signatures represent the imprint on the medical record of unobserved sources of disease, and a prediction model using the expression of those sources as input becomes a patient-specific causal model for the predicted target, which is a step in the direction of identifying patient-specific optimal treatment. In the absence of established definitive methods to evaluate disentangling or causal models, we evaluated the signatures by using them to predict the malignant or benign nature of pulmonary nodules in 13,252 patients, and then comparing the inferred patient-specific causes with known etiologies in the medical literature. The model left unrecovered some infrequently observed or narrowly detailed causes of benign nodules, but, surprisingly, it also identified apparently undiagnosed cancer in many patients.

## Supplementary Material

Supplement 2

Supplement 3

Supplement Appendix A

## Figures and Tables

**Table 1:** Data summary statistics.

	Discovery Set	Labeled Evaluation Set	
		Malignant	Benign

Number of records	269,065	767	12,485
Male sex	144,258 (53.6%)	371 (48.4%)	5,728 (45.9%)
Female sex	124,793 (46.4%)	396 (51.6%)	6,757 (54.1%)
White race	202,553 (75.3%)	674 (87.9%)	10,126 (81.1%)
Black race	36,814 (13.7%)	63 (8.2%)	1,512 (12.1%)
Unknown race	17,338 (6.4%)	13 (1.7%)	449 (3.6%)
Other race	12,360 (4.6%)	17 (2.2%)	398 (3.2%)
Age (years)	52 [16,68]	68 [61,74]	59 [47,68]
Per Record			
Length (days before SPN event)	1,911 [407,4348]	1,707 [90,4057]	2,252 [406,4429]
Number of billing codes	81 [27,224]	37 [11,125]	57 [22,149]
Number of laboratory results	210 [41,764]	87 [14,430]	139 [39,464]
Number of medication mentions	2 [0,28]	6 [0,50]	4 [0,35]
Unique billing codes	36 [16,71]	24 [10,51]	32 [16,61]
Unique laboratory tests	62 [14,94]	52 [7,77]	62 [23,86]
Unique medications	1 [0,7]	3 [0,9]	2 [0,8]

Per record values are Median [IQI]. SPN = Solitary Pulmonary Nodule billing code.

**Table 2:** Reference List of Malignant and Benign Etiologies of Indeterminate Pulmonary Nodules with the Corresponding Learned Signatures. Signature numbers in the middle column refer to the 2000 signatures given in the large supplementary file, where each signature is (searchably) labeled by its number. Signature number in bold has the largest causal effect and given rank.

List Element	Matching Learned Signatures	Largest Causal Effect	Highest Rank

**Malignant Etiologies**			
**Primary Lung Cancer**			
Adenocarcinoma	**886**, 1198, 1940 (Stratified by respiratory region, not by cell type)	0.0136	1
Squamous Cell Carcinoma			
Large Cell Carcinoma			
Small Cell Carcinoma			
**Metastatic Cancer**			
Breast	203, 236, 403, 546, **566**, 654, 880, 919, 971, 982, 1075, 1076, 1086, 1110, 1133, 1180, 1742, 1762, 1781	0.00035	183
Head and Neck	292, 360, **597**, 831, 927, 934, 974, 1607, 1802, 1938	0.00046	128
Melanoma	986, 1120, **1344**, 1470	0.00097	46
Colon	**1341**, 1342, 1945	0.00015	558
Kidney	25, 174, **991**, 1349, 1631, 1722	0.00058	93
Sarcoma	1457, **1886**, 1953	0.00013	677
Germ Cell Tumor	**1132** (Testicle)	0.000089	1062
Others	**1452** (Bladder/urogenital cancer)	0.0034	7
**Pulmonary Carcinoid tumor**	None		
**Benign Etiologies Infectious**			
Histoplasmosis	**760**, 995	−0.0011	44
Coccidiomycosis	None		
Mycobacteria	1096, **1432**	−0.000096	1059
Staph aureus Abscess	None		
Dirofilariasis	None		
**Benign tumors**			
Pulmonary Hamartoma	None		
Pulmonary Fibroma	None		
Pulmonary Leiomyoma	None		
Pulmonary Hemangioma	None		
Amyloidoma	None		
Pneumocytoma	None		
**Vascular**			
Pulmonary AVM	None		
Pulmonary Infarct	**1568**	−0.00011	908
Pulmonary Hematoma	None		
Pulmonary Varix	None		
**Other**			
Inflammatory Lesions			
Granulomatosis with Polyangiitis	**45**	−0.00030	312
Rheumatoid Arthritis	135, 140, 168, 320, **369**, 482, 494, 716, 948, 1049, 1072, 1296, 1317, 1357, 1383, 1581, 1738, 1845, 1846, 1866, 1992	−0.00064	130
Sarcoidosis	**1961**	−0.000059	1935
Rounded Atelectasis	None		
Intrapulmonary Lymph Nodes	None		
Developmental Lesions	1273	−0.0031	6
Loculated Fluid	None		
Mucoid Impaction	None		

**Table 3:** Top Inferred Causes of Malignant Nodules. IDs in parenthesis are latent sources that are documented as causes of *benign* nodules, but when expressed in their opposite polarity here, are predictive of malignancy. Supporting evidence levels: *Direct:* source is listed in the external literature synthesis. *Strong, Moderate, Weak:* source not listed in the synthesis but has the indicated amount of support in other literature. *Absent:* source has no identified literature support.

ID	Name	Mean Causal Effect	Evidence	Detail

1936	COPD	0.0152	Strong	Pathogenesis of COPD and Lung cancer is related, with strong cause-effect relationships between them.[69]
886	Respiratory tract primary malignancy	0.0136	Direct	Listed cause (as primary lung cancer)
1940	Upper lobe primary malignancy	0.0095	Direct	Listed cause (as primary lung cancer). Upper lobe cancers are more common than lower lobe.[80]
1198	Lower lobe primary malignancy	0.0067	Direct	Listed cause (as primary lung cancer)
1622	Indeterminate Pulmonary Nodule	0.0067	Absent	In our cohort, this signature becomes a proxy for (inverse) record length, and probably represents a workflow process. Records shorter than a threshold see a step increase in predicted risk. See text for details.
148	Hypertensive disorder	0.0044	Strong	Direct relationship between hypertension and cancer incidence [81]
1872	Nicotine dependence	0.0034	Strong	Smoking is a strong risk factor[77,82]
1452	Bladder primary malignancy	0.0034	Direct/Moderate	Metastasis from other organs is a listed cause. Bladder cancer is not listed, but included in other sources.[83] Other more common primary malignancies are ranked further down the list of learned causes, for unclear reasons. Alternatively, this source may reflect the fact that cigarette smoking is also the primary risk factor for bladder cancer.
638	Respiratory malignancy, multiple locations	0.0025	Direct	Listed cause (as primary lung cancer)
227	Pregnancy Elevated Glucose Complications	0.0022	Weak	Signature is women with pregnancy complications and abnormal glucose tolerance (and small probability of diabetes mellitus). Diabetes and cancer are associated [75], but this signature is more specific, and no other diabetes-related signatures rank highly. Lung cancer is not high on the list of cancers associated with diabetes[75], and the relationship between Gestational diabetes and any cancer has only been sparsely studied, with inconsistent results.
(343)	COPD ICS LABA	0.0021	Strong	Opposite polarity of benign source (Table 4).
5	Tobacco dependence syndrome	0.0021	Strong	Smoking is a strong risk factor.[77,82]
(760)	Histoplasmosis	0.0021	Direct	Opposite polarity of benign source. This cause doesn’t make the cutoff to be included in Table 4, but is a listed cause of benign nodules.
1066	Abnormal findings on lung imaging NOS	0.0020	Weak	Signature maps almost entirely onto time-intensity of this vague billing code. Expression is nearly linear with increased predicted malignancy risk. Clinically consistent, but weak validation.
(1616)	Unrecorded Race URI	0.0020	Absent	Opposite polarity of benign source (Table 4).
(1158)	Lovastatin	0.0020	Strong	Opposite polarity of benign source (Table 4).
979	Lung Disorder NOS	0.0019	Absent	Most of the signature maps onto the intensity of the vague billing code. Negative expression slightly increases predicted malignancy risk, and less so vice versa. May represent coding practices.
1305	Age with elevated Hematocrit and other CV parameters	0.0018	Mixed	Age is a strong risk factor,[77] agreeing with the causal direction learned for this source, but elevated Hematocrit reduces lung cancer risk, opposite to the learned causal direction.[84] Signature includes a complex mix of other CV parameters, which may be indicating a more specific process. Shap values indicate a threshold effect, with small constant average decrease for negative expressions, and linear positive increase for positive expressions.
467	Post-inflammatory vs. idiopathic pulmonary fibrosis	0.0017	Strong	Post-inflammatory pulmonary fibrosis is sometimes coded as Idiopathic pulmonary fibrosis [85], and this signature appears to be differentiating between them. Idiopathic pulmonary fibrosis (which would be a negative expression of this source) has common pathogenic mechanisms with lung cancer,[86] which agrees with the causal direction found here.
7	Postpartum Pregnancy No Complications	0.0017	Absent	Signature is a typical postpartum pattern with a race differential. Positive expression slightly increases predicted risk. Lung cancer incidence in pregnancy is rare.[76] We can find no estimates of the probability of lung cancer given a pulmonary nodule in pregnancy.

**Table 4:** Top Inferred Causes of Benign Nodules. IDs in parenthesis are latent sources that are documented as causes of *malignant* nodules, but when expressed in their opposite polarity here, are predictive of benign nodules. Validation evidence as in Table 3.

ID	Name	Mean Causal Effect	Evidence	Discussion

(1940)	Upper lobe primary malignancy	−0.0129	Direct	Opposite polarity of malignant source (Table 3)
(1198)	Lower lobeprimary malignancy	−0.0071	Direct	Opposite polarity of malignant source (Table 3)
(886)	Respiratory tract primary malignancy	−0.0061	Direct	Opposite polarity of malignant source (Table 3)
1616	Unrecorded Race URI	−0.0047	Absent	Lung cancer risk depends on race[87], but this signature is for unrecorded race, with a tiny component of an upper respiratory tract infection. Possibly represents a clinical or social process.
(227)	Pregnancy Elevated Glucose Complications	−0.0040	Weak	Signature is women with pregnancy complications and abnormal glucose tolerance. Diabetes and cancer are associated [75], but this signature is more specific, and no other diabetes-related signatures rank highly. This is clinically plausible but weak evidence – lung cancer is not high on the list of cancers associated with diabetes [75], and the relationship between Gestational diabetes and any cancer has only been sparsely studied, with inconsistent results.
(1452)	Bladder primary malignancy	−0.0033	Direct	Opposite polarity of malignant source (Table 3)
1273	Congenital Anomalies	−0.0031	Direct/Weak	Signature is broad list of congenital anomalies, and so may simply represent a source that increases the likelihood of any congenital anomaly, or may be a mixture of many sources. Certain anomalies are listed causes, with more described elsewhere, [88] but only pulmonary arteriovenous malformation is included here, and that with negligible weight.
1158	Lovastatin	−0.0030	Strong	Statins are known to reduce cancer mortality, [89] and Lovastatin causes human lung cancer cell apoptosis in vitro.[89]
361	Treated Unstable Angina	−0.0028	Moderate	Signature is largely medications used to treat atherosclerosis and angina. Newly diagnosed cancer is associated with anatomical severity of coronary artery disease in patients with high levels of inflammation[71]. The slight effect here may be driven by anti-inflammatory and anticancer properties of aspirin[98], including for lung cancer.[72] Signature also includes simvastatin, which has anti-inflammatory and anticancer properties. [73,74]
7	Postpartum Pregnancy No Complications	−0.0027	Absent	Signature is a typical postpartum pattern with a race differential. Lung cancer incidence in pregnancy is rare.[76] We can find no estimates of the probability of lung cancer given a pulmonary nodule after a normal pregnancy.
782	Croup vs. Infective Pneumonia	−0.0027	Weak	Negative expression corresponds to infective pneumonia, which increases probability of benign nodule, weakly matching known causes. See ID 1109 below for more discussion.
465	Adjustment Disorder with Anxiety	−0.0025	Weak	Signature is largely psychiatric disorders, but many smaller elements are known symptoms of lung cancer,[77] such as atelectasis, chest pain, dyspnea, pleural effusion, anemia, and hypoxemia, so negative expression would appropriately decrease predicted risk. There is some literature investigating these comorbidities, particularly with anxiety or depression following the respiratory disease [90,91].
362	Bacteremia	−0.0024	Weak	Signature is nonspecific bacteremia with small elements of cardiovascular, renal, and respiratory symptoms. Also includes multiple symptoms of lung cancer[77] (atelectasis, pleural effusion, dyspnea, abnormal breathing). Specific microbiome disturbances are linked to lung cancer, possibly via inflammation and signaling pathways.[92]
343	COPD ICS LABA	−0.0023	Strong	Signature is COPD treatment[93], which improves lung cancer prognosis[69], and may reduce incidence[94].
(638)	Respiratory malignancy, multiple locations	−0.0023	Direct	Opposite polarity of malignant source (Table 3)
1962	ADHD Black Male	−0.0021	Moderate	There are both genetic correlations and causal associations between ADHD and lung cancer risk, which persist after accounting for smoking. [95] Signature includes a gender and race differential, which was not studied.
1109	Infective Pneumonia	−0.0018	Moderate	Fungi and abscess-forming bacteria are listed causes, which may be coded as Infective Pneumonia.
(864)	COVID-19	−0.0018	Moderate	Opposite polarity of malignant source. There is a complex and incompletely understood causal relationship between COVID-19 and Lung Cancer.[96]
(1936)	COPD	−0.0018	Strong	Opposite polarity of malignant source (Table 3)
1552	Elevated venous blood oxygen	−0.0017	Absent	Signature is increased venous pO2 and decreased venous pCO2, which increases predicted malignancy risk (and a stronger effect vice versa)

## Statement of Significance.

**Table:**

Summary	Description

Problem	Clinical disease labels are insufficiently precise for precision diagnosis and treatment, with a single label covering potentially hundreds of distinct pathophysiologic mechanisms.
What is already known	Work to date on inferring clinical sources or signatures from EHR data are focused either on small-scale problems or on non-causal, predictive contexts.
What this paper adds	We present an unsupervised method to infer clinical signatures and their latent disease sources at institutional scale from routinely collected EHR data, and to use those latent sources to identify patient-specific causes of disease. We demonstrate its potential by estimating causes of indeterminate pulmonary nodules in patients with no history of cancer.

## Appendix A. Supplementary data

Supplementary data to this article can be found online at https://doi.org/10.1016/j.jbi.2025.104837.
