# Supplementary material for: Unsupervised discovery of clinical disease signatures using probabilistic independence
Source: J Biomed Inform. Author manuscript; Available in PMC 2026 Jan 5. (PMC12767692; doi:10.1016/j.jbi.2025.104837)
Supplement: Supplement Appendix A [file NIHMS2115438-supplement-Supplement_Appendix_A.pdf]

Unsupervised Discovery of Clinical Disease Signatures Using Probabilistic Independence

Appendix A - Supplemental Information

A1 DETAILED METHODS

A1.1 Data

All data were extracted from the research mirror of VUMC's EHR, which covers about 3 million patients, with nearly all inpatient and outpatient records complete after 2005. HIPAA-defined Personal Health Information (names, record numbers, absolute dates, etc.) was removed before processing. Original ICD-9 and ICD-10 billing codes were mapped onto the SNOMED condition code taxonomy, original medications were resolved into ingredients, and extreme values for clinical measurements that were deemed impossible or incompatible with life were removed. Any resulting variables with fewer than 1000 total observed instances in the database were excluded, and measurements present in fewer than 10 records of the Discovery Set (see below) were excluded. A final total of $n = 9195$ variables were recorded, including 7453 SNOMED condition codes, 771 medication ingredients, 949 clinical measurements, and 12 demographics (1 age continuous variable, 3 sex categories, and 8 race categories). Categorical variables were binarized into binary variables.

A1.1.1 Discovery Set

A set of 95 ICD10 codes was specified by a pulmonologist to include a broad scope of infectious, malignant, and other lung conditions that could produce an Indeterminate Pulmonary Nodule (Table 1). These ICD10 codes were mapped onto SNOMED condition codes. Patient records were included in the Discovery Set if they contained at least one ICD9 or ICD10 billing code mapping to any descendent of any specified SNOMED code prior to the cutoff date of 4/29/2022. Records were excluded if they contained fewer than two distinct dates containing an observation of a condition, measurement, or medication. A total of 269,099 records met these criteria.

Table 1 – ICD10 Billing Codes Defining the Discovery Set

Code	Description
A15.0	Tuberculosis of lung
B39.0	Acute pulmonary histoplasmosis capsulati
B39.1	Chronic pulmonary histoplasmosis capsulati
B39.2	Pulmonary histoplasmosis capsulati, unspecified
B39.4	Histoplasmosis capsulati, unspecified
B39.9	Histoplasmosis, unspecified
C34	Malignant neoplasm of bronchus and lung
C34.0	Malignant neoplasm of main bronchus
C34.00	Malignant neoplasm of unspecified main bronchus
C34.000	Malignant neoplasm: Main bronchus
C34.001	Hilar malignancies (machine translation)
C34.01	Malignant neoplasm of right main bronchus
C34.02	Malignant neoplasm of left main bronchus
C34.1	Malignant neoplasm of upper lobe, bronchus or lung
C34.10	Malignant neoplasm of upper lobe, unspecified bronchus or lung
C34.100	Malignant neoplasm: Upper lobe, bronchus or lung
C34.101	Upper lobe cancer (machine translation)
C34.102	Pancoast tumor (machine translation)
C34.11	Malignant neoplasm of upper lobe, right bronchus or lung
C34.12	Malignant neoplasm of upper lobe, left bronchus or lung
C34.2	Malignant neoplasm of middle lobe, bronchus or lung
C34.200	Malignant neoplasm: Middle lobe, bronchus or lung

C34.201	Mid lung cancer (machine translation)
C34.3	Malignant neoplasm of lower lobe, bronchus or lung
C34.30	Malignant neoplasm of lower lobe, unspecified bronchus or lung
C34.300	Malignant neoplasm: Lower lobe, bronchus or lung
C34.301	Lower lung lobe tumor (machine translation)
C34.31	Malignant neoplasm of lower lobe, right bronchus or lung
C34.32	Malignant neoplasm of lower lobe, left bronchus or lung
C34.8	Malignant neoplasm of overlapping sites of bronchus and lung
C34.80	Malignant neoplasm of overlapping sites of unspecified bronchus and lung
C34.800	Malignant neoplasm: Overlapping lesion of bronchus and lung
C34.801	Upper lobe lung cancer (machine translation)
C34.802	Lower lobe lung cancer (machine translation)
C34.803	The upper and lower lobe lung cancer (machine translation)
C34.81	Malignant neoplasm of overlapping sites of right bronchus and lung
C34.82	Malignant neoplasm of overlapping sites of left bronchus and lung
C34.9	Malignant neoplasm of unspecified part of bronchus or lung
C34.90	Malignant neoplasm of unspecified part of unspecified bronchus or lung
C34.900	Malignant neoplasm: Bronchus or lung, unspecified
C34.901	Bronchus cancer (machine translation)
C34.902	Bronchial cancer (machine translation)
C34.91	Malignant neoplasm of unspecified part of right bronchus or lung
C34.92	Malignant neoplasm of unspecified part of left bronchus or lung
C78.00	Secondary malignant neoplasm of unspecified lung
C78.01	Secondary malignant neoplasm of right lung
C78.02	Secondary malignant neoplasm of left lung
C78.1	Secondary malignant neoplasm of mediastinum
C78.100	Secondary malignant neoplasm of mediastinum
C78.2	Secondary malignant neoplasm of pleura
C78.200	Secondary malignant neoplasm of pleura
C78.201	Malignant pleural effusion (machine translation)
C78.3	Secondary malignant neoplasm of other and unspecified respiratory organs
C78.30	Secondary malignant neoplasm of unspecified respiratory organ
C78.300	Secondary malignant neoplasm of other and unspecified respiratory organs
C78.301	Secondary sinus carcinoma (machine translation)
C78.302	Secondary malignant nasal cavity (machine translation)
C78.303	Secondary malignant tumor of the middle ear (machine translation)
C78.304	Trachea secondary malignancies (machine translation)
C78.305	Secondary throat cancer (machine translation)
C78.306	Respiratory secondary malignancies (machine translation)
C78.39	Secondary malignant neoplasm of other respiratory organs
C7A.090	Malignant carcinoid tumor of the bronchus and lung
D02.20	Carcinoma in situ of unspecified bronchus and lung
D02.21	Carcinoma in situ of right bronchus and lung
D02.22	Carcinoma in situ of left bronchus and lung
D14.30	Benign neoplasm of unspecified bronchus and lung
D14.31	Benign neoplasm of right bronchus and lung
D14.32	Benign neoplasm of left bronchus and lung
D38.1	Neoplasm of uncertain behavior of trachea, bronchus and lung
D3A.090	Benign carcinoid tumor of the bronchus and lung
D86.0	Sarcoidosis of lung
J84.115	Respiratory bronchiolitis interstitial lung disease
J85.1	Abscess of lung with pneumonia
J85.2	Abscess of lung without pneumonia
M05.10	Rheumatoid lung disease with rheumatoid arthritis of unspecified site
M05.19	Rheumatoid lung disease with rheumatoid arthritis of multiple sites
R91	Abnormal findings on diagnostic imaging of lung
R91.1	Solitary pulmonary nodule
R91.8	Other nonspecific abnormal finding of lung field
R91.x00	Abnormal findings on diagnostic imaging of lung
R91.x01	Coin-shaped lung damage (machine translation)
R91.x02	Lung tumor (machine translation)
R91.x03	Lung lesions (machine translation)
Z85.118	Personal history of other malignant neoplasm of bronchus and lung

A1.1.2 Evaluation Set

A cohort of 13,252 records was collected comprising those with at least one billing code for a Solitary Pulmonary Nodule (SPN, which is how indeterminate pulmonary nodules are coded in the ICD taxonomies), and no codes for any type of malignancy preceding the SPN date. The Evaluation Set X^E was a subset of the Discovery Set X.

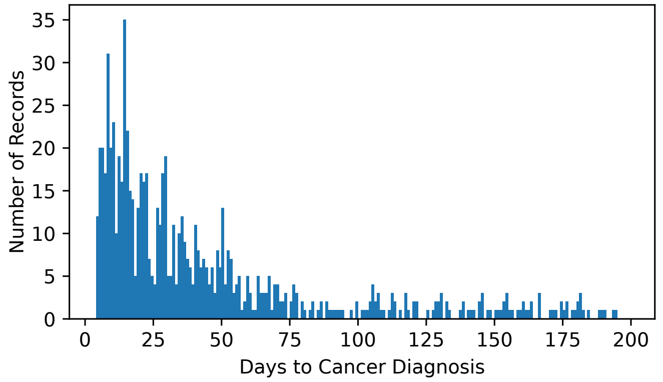

Figure A1 – Distribution of time between solitary pulmonary nodule code and lung cancer diagnosis code, truncated at 200 days. This separation enforces the requirement that the target node (cancer diagnosis) is a terminal node, because all information given to the predictive model comes from before the *nodule* code date, which itself precedes the *cancer* code date in time.

Label Y_i for Evaluation Set record X_i^E was positive if the record contained at least one code for a malignant lung neoplasm (including primary lung cancer or metastasis from other locations) on day 4 - 1095 following the SPN date, and negative if it had no lung malignancy code before day 1095, even if it had a malignancy in some other organ after the SPN date. Records with lung malignancy codes within 4 days of the SPN code were excluded because they represented cases where the malignancy status was presumed to be known at the time of the nodule detection. Distribution of time between the SPN date and malignancy date is presented in Figure A1. Labels were validated by comparison with cancer registry records (all positive labeled records) and manual chart review (all mismatches with the cancer registry and a random sample of negative labeled records) as detailed elsewhere[1], and estimated to have 0.98 PPV, 0.99 NPV, 0.93 sensitivity, and 0.996 specificity.

A random partition of 2651 records (20%) of the Evaluation Set were set aside as the final test set.

A1.2 Continuous Curve Generation

Continuous longitudinal curves were built with methods specific to each data mode. A curve was generated for each clinical variable, such as a specific laboratory test or billing code, with one-day resolution. The time constant of the actual information captured by the curve was usually not as small as one day, because observations were made in general at far larger intervals. Nevertheless, creating the longitudinal curves at this resolution allows us to estimate the value of a given variable on arbitrary dates, given the set of observations.

A1.2.1 Clinical Measurements

Curves for clinical measurements (mostly laboratory test results) were originally built using non-stationary Gaussian process regression [2], which provides a distribution over all possible paths that the measurand could have taken over time, given the observed values and assumptions on the smoothness of the path. However, that method is too inefficient for large-scale use. For this work, we used the univariate PCHIP method [3] that interpolates a curve with a continuous first derivative through all observations. This method adapts to non-stationarity, maintains monotonicity given monotonic data, and does not overshoot the maxima defined by the observations. Extrapolation beyond the first and last observation used the value of the nearest observation.

If a record listed no observations for a given clinical measurement, a constant curve at the population median was imputed.

A1.2.2 Billing Codes

Curves for billing code events were originally computed as the intensity curves of non-homogeneous gamma processes [4], which provide a distribution over all possible intensity curves, given smoothness assumptions, as well as a distribution over a shape parameter that models dependence between adjacent events. But as with clinical measurements curves, the time complexity of this method precluded use at this scale.

To trade approximation for complexity with this data mode, we replaced the gamma process with a simple density function that assumes memorylessness between events, but is still able to model nonstationarity over time. Specifically, we used a variation we developed for Random Average Shifted Histograms (RASH) [5]. RASH estimates an intensity curve by averaging many equally-spaced histograms over the data, with each histogram shifted by a random amount. It produces a smooth curve similar to kernel density estimation (KDE), but the constant bin width causes the same problems with nonstationarity that a constant KDE bandwidth causes. To accommodate nonstationarity, we modified RASH to use random-sized bins, with the random choice of bin size made in event space rather than data space. For example, a bin size of 5.7 would mean that the bin includes the next 5 events plus 70% of the interval between the 5th and 6th events. Bins were specified in increasing order by event time, with each bin size chosen uniformly at random between 3.0 and the number of remaining events. This naturally handles the non-stationarity in event density, with events spaced further apart in time allocated into wider time bins than events closer in time.

A1.2.3 Medication Mentions

Curves for medication mentions were binary curves intended to reflect whether the patient had been prescribed to take the medication at the indicated time. Creating the curve was challenging, however, because for much of the history of our EHR, the start and stop dates of medications are either missing or unreliable, and dispensing or refill data was not available. Instead, at some patient visits, a clinician would review the current medication list with the patient, confirming or removing entries. The modified list was then inserted into the database and marked with the date of the reconciliation.

Curves were computed by setting them at 1 (taking) for all medications mentioned on a given reconciliation, and 0 (not taking) otherwise. Times between reconciliations used the value of the nearest observation.

If a record included no mentions of a given medication, a constant curve of 0 was imputed.

A1.2.4 Demographics

Curves for demographics were either constant (race, sex) or linearly increasing (age). Categorical values were binarized into sets of binary curves.

A1.3 Smoothing

We found that capturing a small amount of history in the curves was useful for capturing short-term trajectories of disease and filling short documentation gaps. To do this, a one-year retrospective rolling mean was applied to each curve. For medications, the *taking* region of each curve was extended for 365 days in each direction.

A1.4 Cross-section Sampling

All curves from the ith record were aligned into a curveset, anchoring on the first date of the record. A number c_i of cross-sections were sampled uniformly at random from each record in the Discovery Set, where $c_i \sim \text{Bin}(l_i, d)$, l_i is the record length in days and the sampling density $d = 1/(3 * 365)$ was fixed at one sample per three record-years. Longer records therefore had a higher probability of being sampled multiple times, and some records were not sampled at all. A total of $t =$

$630{,}037$ cross sections from $175{,}711$ records were sampled and stacked into the matrix $X \in \mathbb{R}^{n\times t}$. The size of X (controlled by sampling density d) was limited by computational resources.

In the Evaluation Set, records were sampled exactly once, at the time of the first SPN billing code. No data observed after the SPN date were used to construct the curves for the Evaluation Set.

A1.5 Standardization

The discovery matrix X was transformed to bring variables from all modes onto roughly the same scale. Different transformations were used for different row subsets that correspond to data modes.

The row subset $X^C = X_{i,*}, i \in V^C$ of clinical measurement variables V^C and the row subset $X^M = X_{i,*}, i \in V^M$ of medication variables V^M were each transformed by subtracting the subset mean and dividing by two standard deviations.

The row subset $X^B = X_{i,*}, i \in V^B$ of billing code intensity variables V^B was first transformed to $\tilde{X}^B = \log(X^B + \epsilon)$, where the smoothing value $\epsilon = 1/(20 \times 365)$ represents an arbitrarily chosen prior of one code per twenty record-years, and then each row $\tilde{X}^B_{i,*}$ was scaled by dividing by $s = 2 \cdot \mathrm{std}(\tilde{X}^B_{i,*})$.

Demographics were not transformed, remaining as 0/1 variables.

The logarithmic transform affects the composition of the signatures and their expression values, but the scaling operations only affect the relative ranking of the different data modes in the signature visualizations.

Evaluation set cross sections were standardized using the transformation determined by the Discovery Set.

A1.6 Clinical Signature Discovery

The final discovery matrix $X \in \mathbb{R}^{n\times t}$ was decomposed by FastICA into a mixing matrix $A \in \mathbb{R}^{n\times m}$ and a source matrix $S \in \mathbb{R}^{m\times t}$, such that $X = AS$ the rows of S are (close to) mutually probabilistically independent, and m is the number of latent sources inferred. The Direct LiNGAM method [6] is generally a more accurate method for performing this decomposition [7], in part because of the risk of ICA finding a local minimum, but it is far too inefficient for the matrix size used here.

Mathematically, we can infer $m = n$ components, although optimally, we want to match m to the intrinsic dimension of X to avoid over- or under-learning [8]. In our case, we used $m = 2000$, which was limited by the computational complexity of the FastICA implementation we used and our computational resources. We suspect that the true number of components is much larger (Figure A2). To infer $m < n$, standard implementations use the modified ICA equation $VX = AS$, or $X = V^{-1}AS = \tilde{A}S$, where $V \in \mathbb{R}^{m\times n}$ is obtained from singular value decomposition and provides the additional benefit of whitening the data [8]. In this paper, when we refer to our computed results as A we are technically referring to $\tilde{A}$.

The matrix X^E of the Evaluation Set was created analagously to the discovery matrix X, with the exception of each record being sampled exactly once. The same standardizing transformations were applied (with the same parameters) as with the Discovery Set. Evaluation Set source expressions $S^E = \tilde{A}^{-1}X^E$ were computed, and the matrices S^E and X^E were partitioned into training and test sets, using the patient-level partitioning described above.

A1.6.1 Formal Causality Definitions and Assumptions

We consider a *structural equation model* over $p + 1$ random variables $Z = X \cup Y$ that is linear over X but potentially non-linear over Y:

$$X_i = \beta_i \mathrm{PA}_{X_i} + S_{X_i}, \quad \forall X_i \in X,$$
$$Y = f_Y(\mathrm{PA}_Y, S_Y),$$

where $\mathrm{PA}_i \subseteq Z \backslash Z_i$ refers to the *parents*, or *direct causes*, of Z_i. The matrix β contains linear coefficients, and β_i denotes the row vector associated with variable X_i. The sources, or noise terms, S are mutually independent and have no parents.

We can associate a *directed graph* with a structural equation model by drawing a directed edge from each member of PA_i to Z_i and from S_i to Z_i for each $Z_i \in Z$. We assume that the directed graph is *acyclic*, or contains no feedback loops. *Root vertices* correspond to vertices without any incoming directed edges. As a result, sources correspond to the root vertices in this formulation. A *root cause* of Y corresponds to a root vertex, or source, that causes Y. We define the *causal contribution* of S_{X_i} on Y as its Shapley additive explanation (SHAP) value [9] because the sources are mutually independent:

$$\mathrm{SHAP}_{S_{X_i}} = \frac{1}{p} \sum_{W \subseteq S_X \backslash S_{X_i}} \binom{p-1}{|W|}^{-1} \left(E\left(Y | S_{X_i}, W\right) - E(Y|W) \right).$$

The SHAP value accounts for a non-linear relation from X to Y and considers all possible ways in which clinicians can collect new source information [10].We also have $\mathrm{SHAP}_{S_{X_i}} = 0$ when S_{X_i} is not a root cause of Y. We are particularly interested in identifying sources with large causal contributions that induce disease.

We can isolate the sources and match them to their corresponding variables in X in the linear case by rewriting the structural equations over X in matrix form $X = \beta X + S$. As a result, we have $(I - \beta)X = S$.

ICA similarly recovers $A^{-1}X = S$ with the de-mixing matrix A^{-1} but only identified up to permutation and scaling. Fortunately, the matrix $I - \beta$ has a diagonal of ones, which we can exploit to identify the appropriate permutation and scaling of A^{-1}; we permute the rows of A^{-1} so that all diagonal elements are non-zero, and then scale the rows so that all diagonal elements are one [11]. The permutation thus allows us to match each member of S to its direct effect in X via the diagonal of ones. Ultimately, the methodology proposed in this paper can discover the root causes of Y by running ICA to identify the sources S and then identify the sources with non-zero SHAP values. Further, it can identify sources with large causal contributions and match each source S_{X_i} to its direct effect X_i for downstream interpretation.

This analysis depends on certain assumptions that are discussed in more detail in prior work [7]. The assumptions include that of unconfoundedness, which is standard in causal inference; the assumption that all but at most one of the S_i have a non-Gaussian distribution, necessary for the ICA decomposition; and the assumption that the target Y is a terminal node (has no children), necessary for the causal prediction.

The unconfoundedness assumed by this analysis is a weaker assumption than the usual causal-inference assumption of no unobserved confounders at all. Instead, this analysis is able to assume only that that there are no unobserved *single-cause* confounders, because any concern of confounding between sources is eliminated by the model's design.[12] The proof is that if an unobserved confounder affected at least two sources S_i and S_j (and possibly also Y), then S_i and S_j would exhibit mutual dependence. But S_i and S_j are independent by construction, and therefore there is no confounding between them.

However, there remains the possibility of a confounder between a *single* source S_i and Y. For example, a genetic variant may affect S_i and increase the risk of Y, even when the sources achieve perfect mutual independence. While the absence of unobserved confounders is in general impossible to prove from data, we minimize the risk by including as many variables as possible from the EHR, and noting that any remaining confounding must be limited to a variable affecting *exactly one* latent source S_i and the predictive target Y.

Confounding variables that are observed and included as inputs to the discovery model are appropriately incorporated and pose no problems to the causal interpretation.

Our target Y was designed to meet the terminal node assumption by selecting as input to the predictive model only information that preceded the appearance of the pulmonary nodule in time. Cases

where a cancer diagnosis code appeared within 3 days after the pulmonary nodule code were excluded, to allow for variability in code timing and remove the cases where a cancer diagnosis was made simultaneously with the nodule discovery. The distribution of time (up to 200 days) between nodule code appearance and lung cancer code appearance for positive records is in Figure A1.

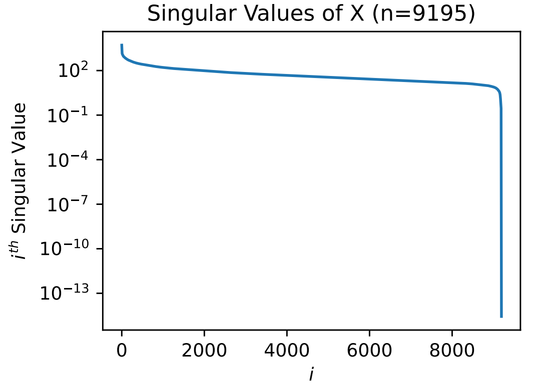

Figure A2 – Singular values of the input data X suggest that the optimal value for m is much larger than the value $m = 2000$ that we were able to use.

A1.7 Predictive Model Training

Predictive models were trained as $Y = H_i(S)$ and $Y = H_i(X)$, where the H_i were a Random Forest (Python 3.10, scikit-learn 1.1.3), a Gradient-Boosted Machine (Python 3.10, XGBoost 1.6), and an Elastic Net (Python 3.10, scikit-learn 1.0.2). Hyperparameters for all six models were optimized independently using $10\times$ cross validation under a combination of random search, grid search, and human guided search. After optimal hyperparameters were determined for each model, training on the full training set and testing on the test set were repeated with 100 different random seeds each to determine the extent of variation due to randomness in training.

Models were interrogated for causal effects using SHAP values as described in Section A1.6.1.

A2 ADDITIONAL RESULTS

The singular values of X suggest that the optimal value for m is likely to be much larger than the $m = 2000$ that we were able to use (Figure A2).

Ridgeline plots of results from the three model architectures, where the distribution is computed over all 100 causal models with different random seeds of a given architecture, are given in Figures A3 – A5. SHAP values of top predictors, filtered and ranked by mean of positive values are presented in Figure A3 (analogous to Table 3 in the main text), filtered and ranked by mean of negative values in Figure A4 (analogous to Table 4 in the main text), and mean of unfiltered absolute values in Figure A5.

A3 ADDITIONAL DISCUSSION

A3.1 Model Architecture Comparison.

While in theory we expect the predictors in S to reflect root nodes in the causal graph, in practice we found important differences in how the S_i were used between Elastic Net, Random Forest, and XGBoost models (Figures A3-A5), which urges caution in drawing causal conclusions. Random Forest and XGBoost produced sets of top sources with moderate overlap, but attributed different causal effect values to them. Elastic Net attributed very different causal effects compared to the tree ensemble models. We speculatively attribute these differences to interactions and nonlinearities in the true causal effects of S on Y, some of which are visible in the SHAP Scatter plots supplemental information. These can obviously be better modeled by tree ensembles than by a linear model.

From our perspective, the Random Forest results look clinically the most plausible. We hypothesize that the broader and more random search performed by Random Forest training may have produced a more representative model, while the greedy XGBoost approach may be more likely to find local minima. Some evidence for this interpretation lies in the disproportionately dominant use by XGBoost of the signature for *Upper lobe primary malignancy*, the most common location for lung malignancies.

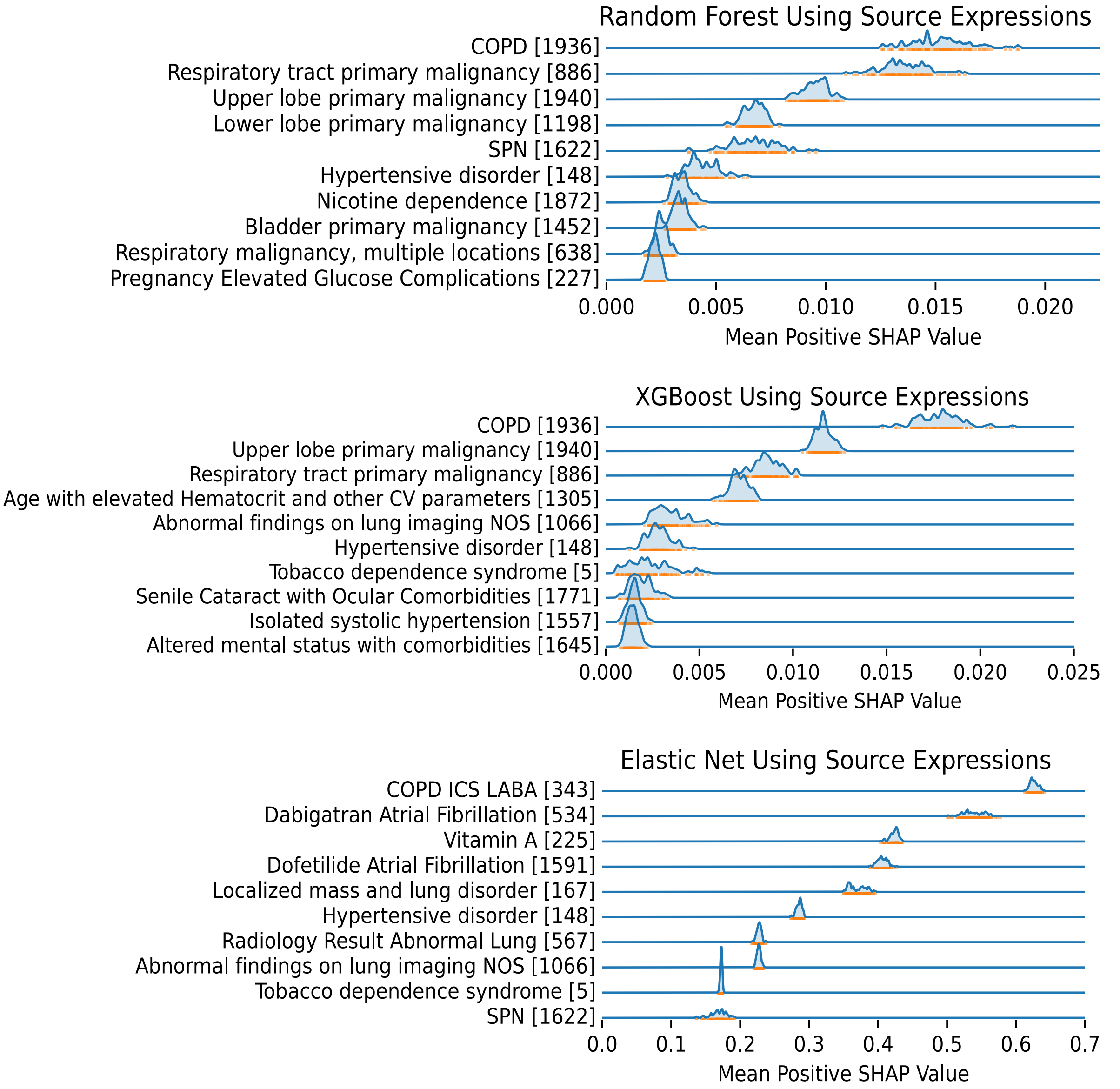

Figure A3 – Top predictors by ***positive*** SHAP value of the three causal models, analogous to Table 3 in main text. Estimated causal factors differ between architectures, with the largest differences between Elastic Net and the tree ensembles. We attribute the poor performance of Elastic Net to the mismatch between the linear model and the nonlinear causal effects found by the nonlinear models. The effect of the mismatch is magnified here by filtering to only positive SHAP values, which in the Elastic Net model tends to identify small groups of outliers with reduced clinical plausibility.

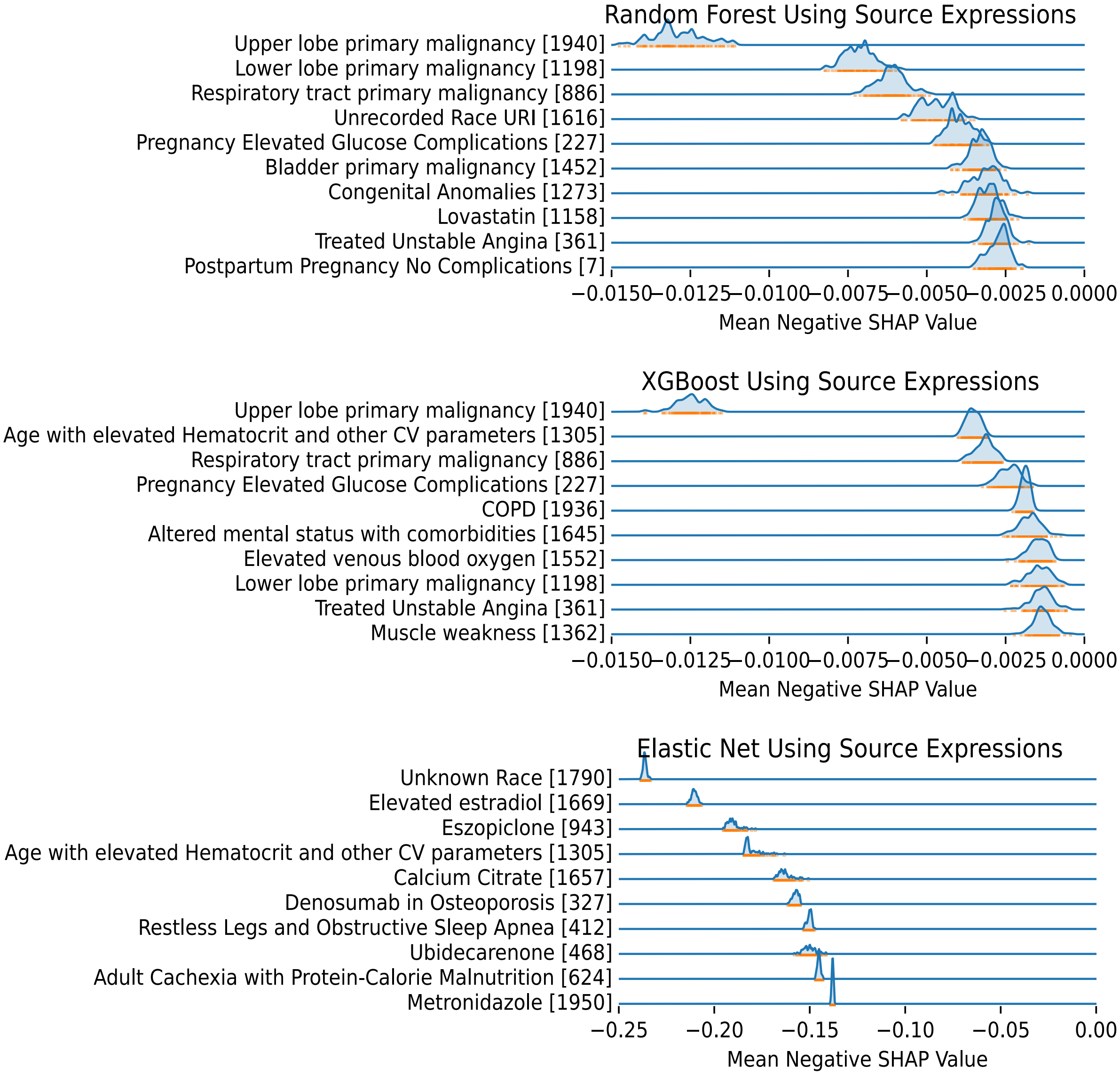

Figure A4 – Top predictors by ***negative*** SHAP value of the three causal models, each analogous to Table 4 in main text. The same effects as in Figure A3 are apparent here.

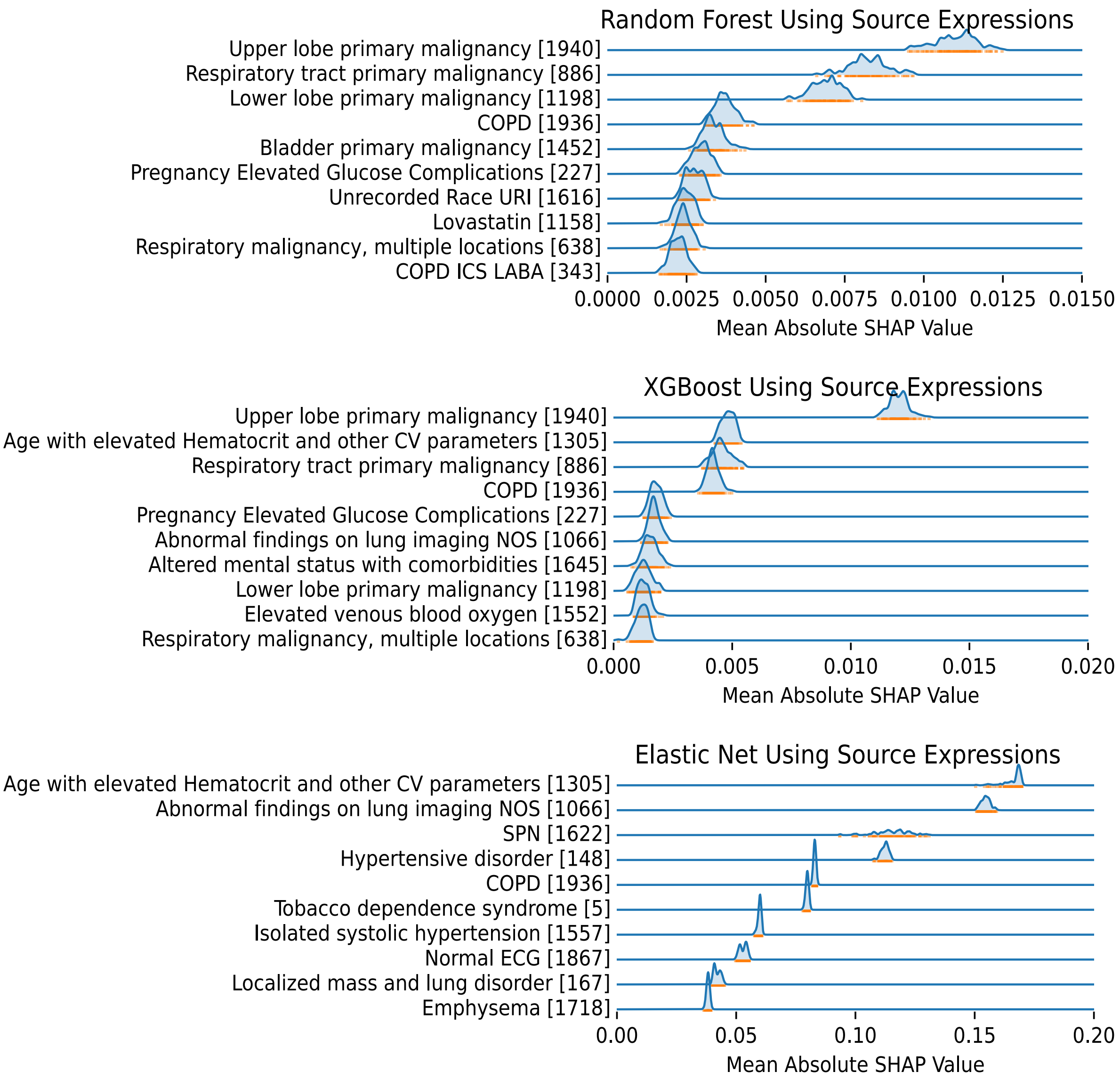

Figure A5 – Top predictors by ***absolute value*** of SHAP values of the three causal models. Similar problems with Elastic Net unable to model nonlinear causal effects are apparent here as in Figures A3 and A4. But because the data represented here are not filtered to positive or negative effects, and thus avoid the artifacts in the figures above, the Elastic Net estimated causes are more clinically plausible than in the filtered versions, although they still disagree with the tree ensemble estimates. In these plots, the ranking selects the sources with strongest overall effect, regardless of direction.

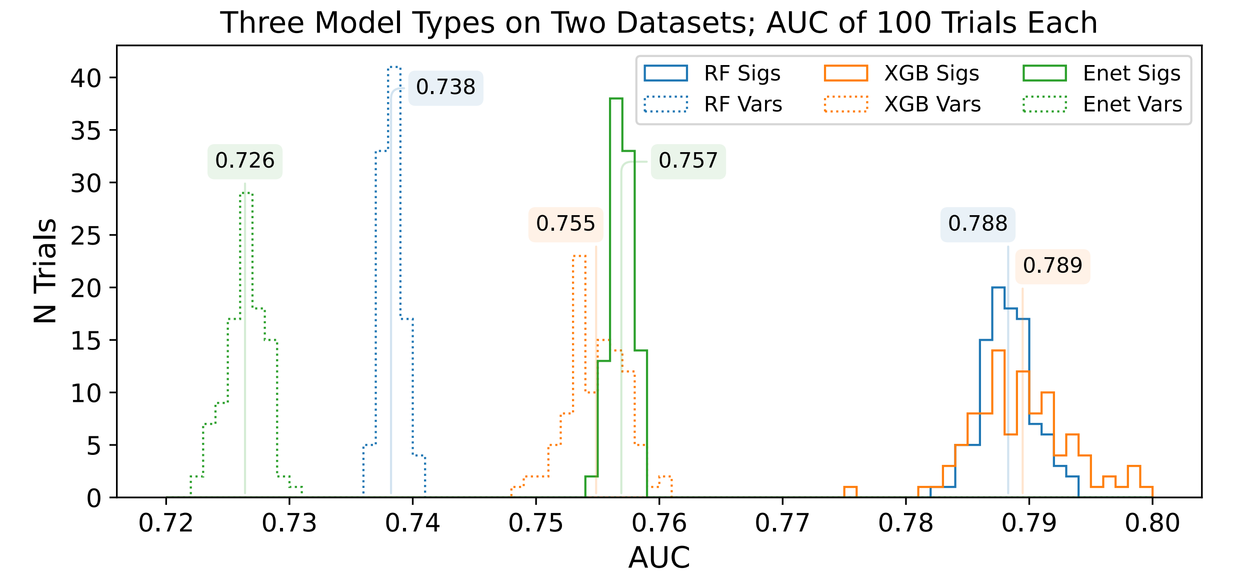

Figure A6 - Causal models outperformed statistical models for each architecture. Random Forest and Gradient Boosted Tree models performed approximately equivalently, and better than the linear models. RF: Random Forest. XGB: XG Boost. Enet: Elastic Net. Sigs: Models trained on learned signatures. Vars: Models trained on original variables. Annotations give the mean AUC for each model.

The greater clinical plausibility of the Random Forest model is maintained when we look at only the positive or negative SHAP values, filtering for causal effects in each given direction (Figures A3 and A4). The filtered Elastic Net results look particularly implausible from a clinical perspective. In many cases, the estimated effect is distorted due to the filter isolating small pockets of records on one or the other side of zero, allowing noise to have a greater effect on the mean.

Additional evidence of increased model instability produced by XGBoost is the wider range of discriminative performance of XGBoost models under different random seeds, compared to Random Forest models, although all models had higher variation than we anticipated. The range of AUC under different seeds was about 0.02 for XGBoost, 0.01 for Random Forest, and 0.005 for Elastic Net (Figure A6). This variation was typically at least as large as the change due to hyperparameter adjustment during tuning, which could easily mislead an algorithmic search for the best hyperparameters.

A3.2 Signature 1622 (SPN) is a proxy for the length of the clinical record.

The main element for signature 1622 is the code intensity for a Solitary Pulmonary Nodule (SPN). All data for the Evaluation Set was truncated at the date for the first SPN code, and curves were computed for all data prior to that time, which means that all records in the set had exactly one SPN code. For records with three or fewer events of a given ICD code, the curve generation algorithm produced a constant intensity curve of events per time, which resulted in the SPN intensity being inversely proportional for the length of the record preceding the first SPN code. This element was the dominant element for signature 1622, so the signature is a proxy for the length of the preceding record, which turned out to be predictive for malignancy.

REFERENCES

[1] Li TZ, Xu K, Chada NC, Chen H, Knight M, Antic S, et al. Curating retrospective multimodal and longitudinal data for community cohorts at risk for lung cancer. Cancer Biomark 2024;Preprint:1–9. https://doi.org/10.3233/CBM-230340.

[2] Lasko TA. Nonstationary gaussian process regression for evaluating clinical laboratory test sampling strategies. Proc. Twenty-Ninth AAAI Conf. Artif. Intell., 2015, p. 1777–83.
[3] Fritsch FN, Butland J. A Method for Constructing Local Monotone Piecewise Cubic Interpolants. SIAM J Sci Stat Comput 1984;5:300–4. https://doi.org/10.1137/0905021.
[4] Lasko TA. Efficient inference of Gaussian process modulated renewal processes with application to medical event data. Proc. Thirtieth Conf. Uncertain. Artif. Intell. UAI, 2014.
[5] Bourel M, Fraiman R, Ghattas B. Random average shifted histograms. Comput Stat Data Anal 2014;79:149–64. https://doi.org/10.1016/j.csda.2014.05.004.
[6] Shimizu S, Inazumi T, Sogawa Y, Hyvärinen A, Kawahara Y, Washio T, et al. DirectLiNGAM: A direct method for learning a linear non-gaussian structural equation model. J Mach Learn Res 2011;12:1225–48.
[7] Strobl EV, Lasko TA. Identifying patient-specific root causes of disease. Proc. 13th ACM Int. Conf. Bioinforma. Comput. Biol. Health Inform., New York, NY, USA: Association for Computing Machinery; 2022, p. 1–10. https://doi.org/10.1145/3535508.3545553.
[8] Hyvarinen A, Oja E. Independent component analysis: Algorithms and applications. Neural Netw 2000;13:411–30.
[9] Lundberg SM, Lee S-I. A unified approach to interpreting model predictions. In: Guyon I, Luxburg UV, Bengio S, Wallach H, Fergus R, Vishwanathan S, et al., editors. Adv. Neural Inf. Process. Syst. 30, Curran Associates, Inc.; 2017, p. 4765–74.
[10] Strobl EV. Counterfactual formulation of patient-specific root causes of disease. J Biomed Inform 2024;150:104585. https://doi.org/10.1016/j.jbi.2024.104585.
[11] Shimizu S, Hoyer PO, Hyvärinen A, Kerminen AJ. A linear non-gaussian acyclic model for causal discovery. J Mach Learn Res 2006;7:2003–30.
[12] Wang Y, Blei DM. The Blessings of Multiple Causes. J Am Stat Assoc 2019;114:1574–96. https://doi.org/10.1080/01621459.2019.1686987.
