# Supplementary material for: Unsupervised discovery of clinical disease signatures using probabilistic independence
Source: J Biomed Inform. Author manuscript; Available in PMC 2026 Jan 5. (PMC12767692; doi:10.1016/j.jbi.2025.104837)
Supplement: Supplement 2 [file NIHMS2115438-supplement-Supplement_2.pdf]

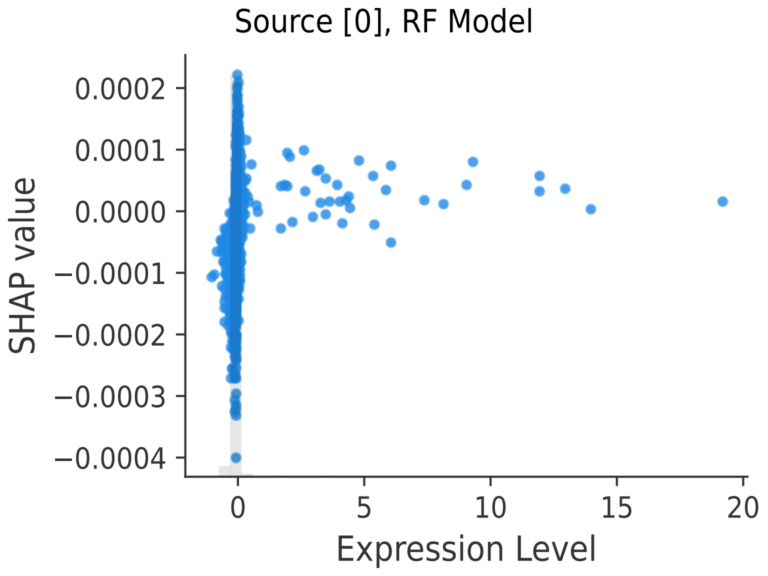
Source [0], RF Model
SHAP value
0.0002
0.0001
0.0000
−0.0001
−0.0002
−0.0003
−0.0004
0
5
10
15
20
Expression Level

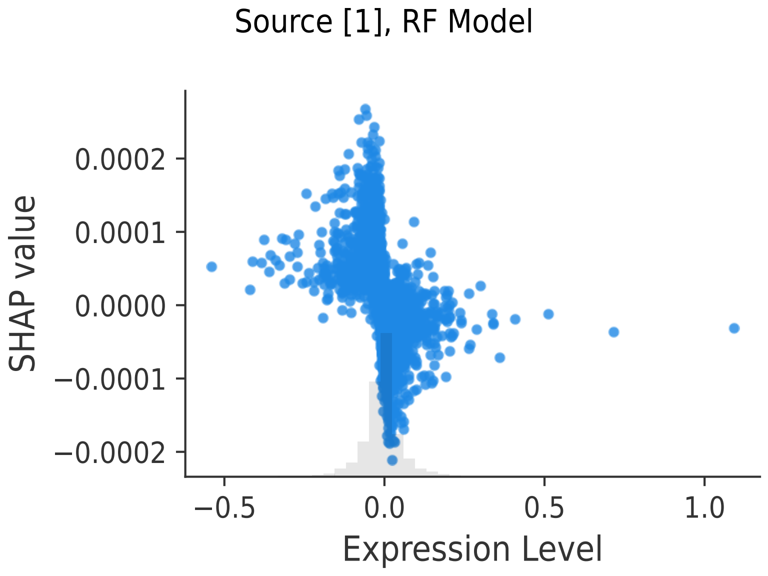
Source [1], RF Model
0.0002
0.0001
0.0000
−0.0001
−0.0002
SHAP value
−0.5
0.0
0.5
1.0
Expression Level

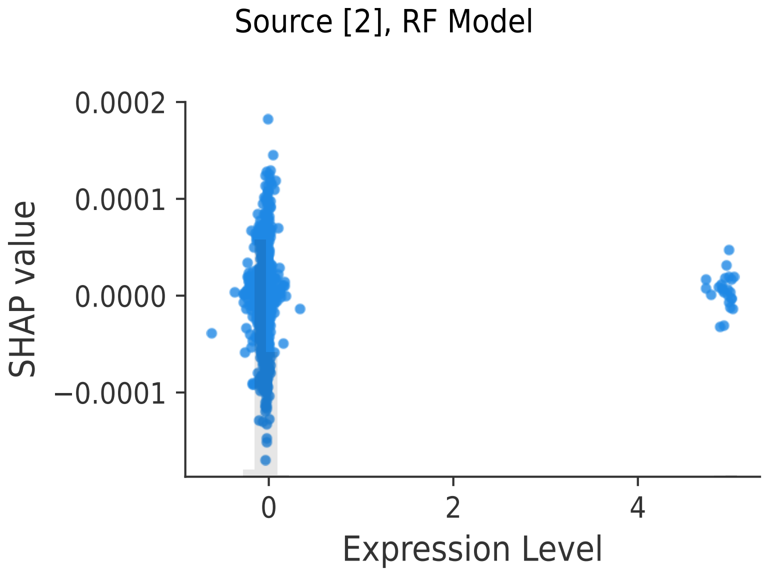
Source [2], RF Model
0.0002
0.0001
0.0000
−0.0001
SHAP value
0
2
4
Expression Level

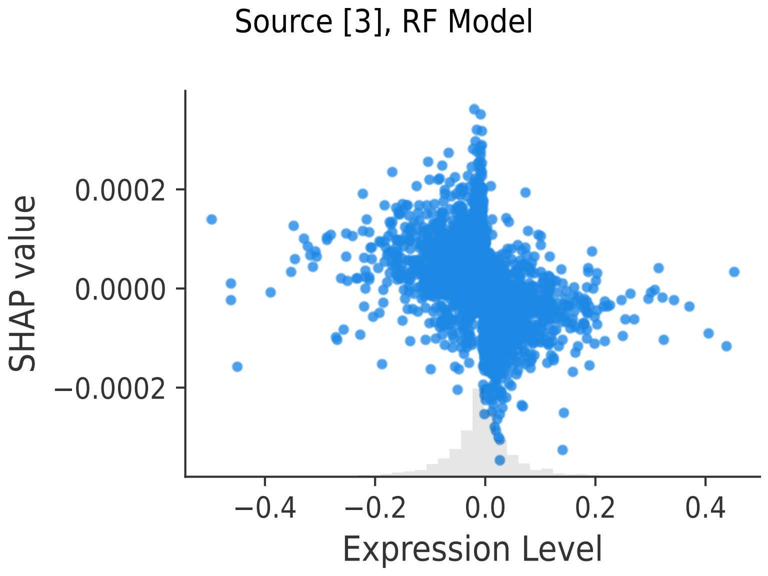
Source [3], RF Model
SHAP value
0.0002
0.0000
−0.0002
−0.4
−0.2
0.0
0.2
0.4
Expression Level

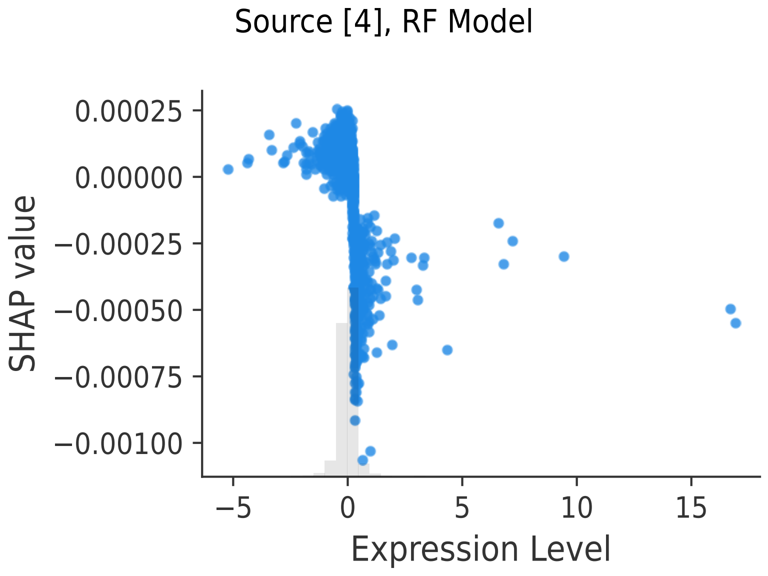
Source [4], RF Model
0.00025
0.00000
−0.00025
−0.00050
−0.00075
−0.00100
SHAP value
−5
0
5
10
15
Expression Level

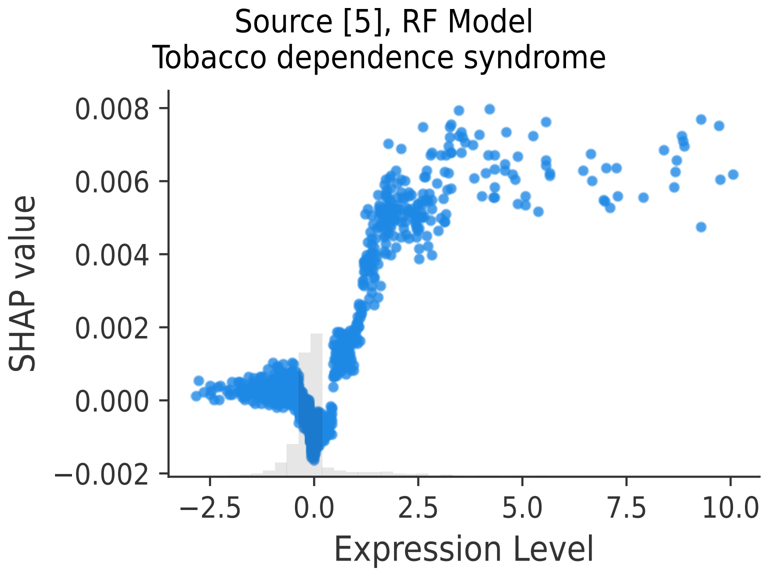
Source [5], RF Model
Tobacco dependence syndrome
SHAP value
0.008
0.006
0.004
0.002
0.000
−0.002
−2.5
0.0
2.5
5.0
7.5
10.0
Expression Level

Source [6], RF Model
Somatropin in pituitary dwarfism

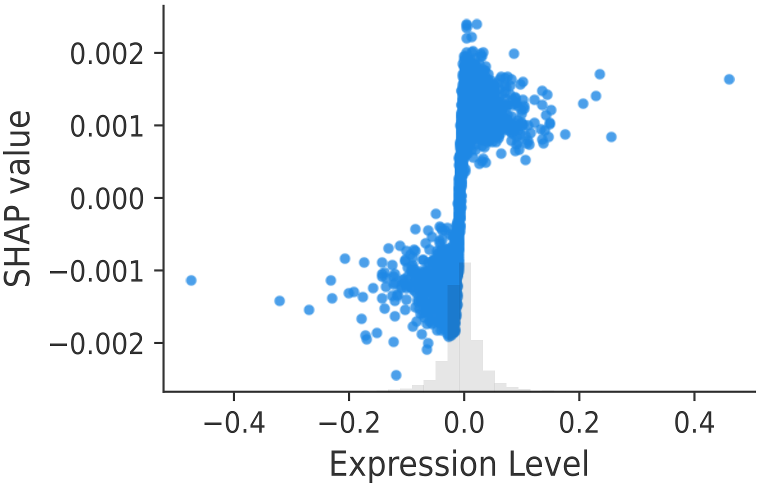

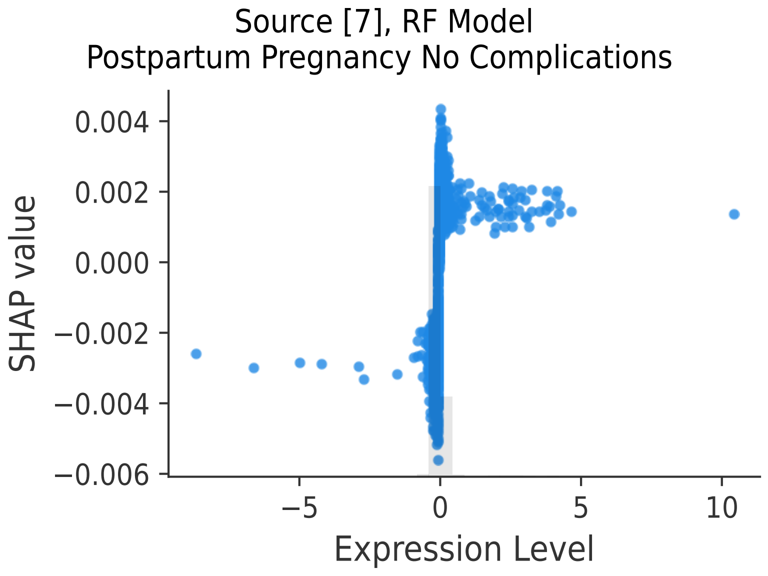

Source [7], RF Model
Postpartum Pregnancy No Complications
SHAP value
0.004
0.002
0.000
−0.002
−0.004
−0.006
−5
0
5
10
Expression Level

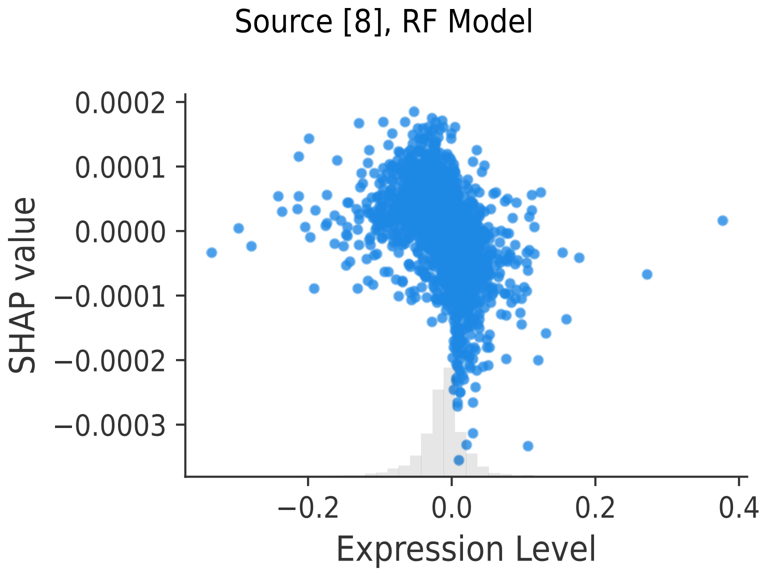

Source [8], RF Model
0.0002
0.0001
0.0000
−0.0001
−0.0002
−0.0003
SHAP value
−0.2
0.0
0.2
0.4
Expression Level

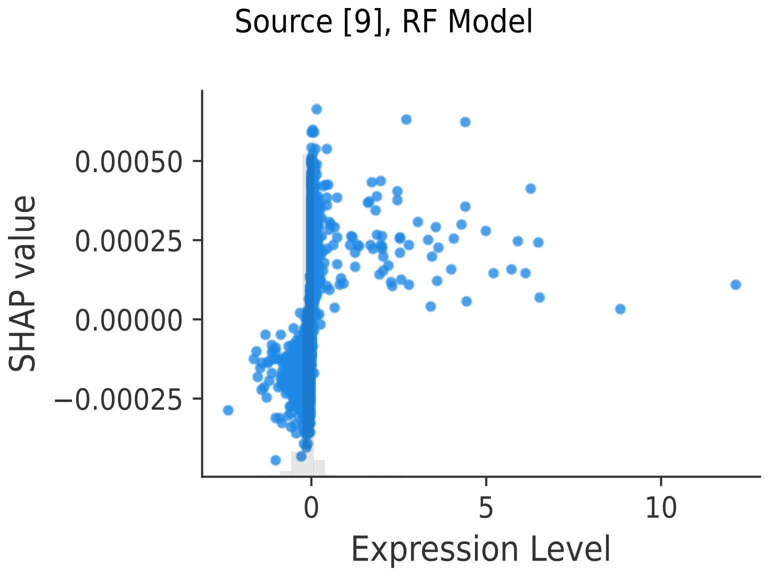
Source [9], RF Model
0.00050
0.00025
0.00000
−0.00025
SHAP value
0
5
10
Expression Level

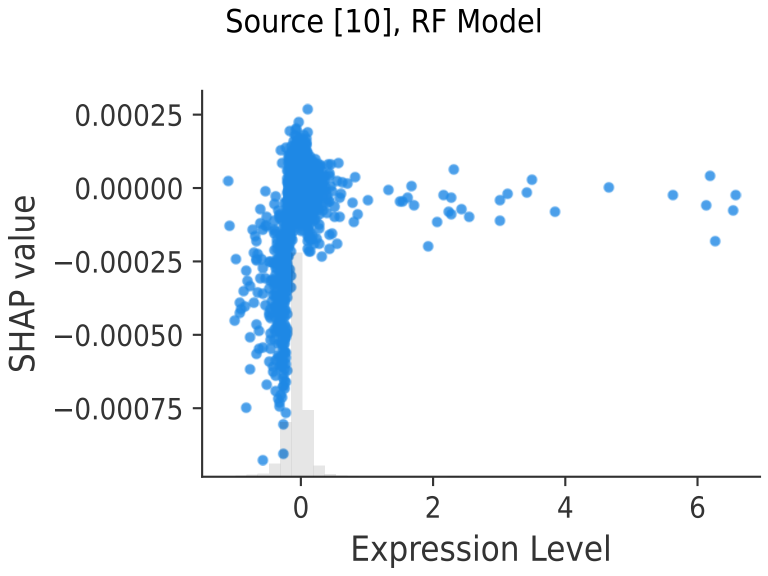
Source [10], RF Model
SHAP value
0.00025
0.00000
−0.00025
−0.00050
−0.00075
0
2
4
6
Expression Level

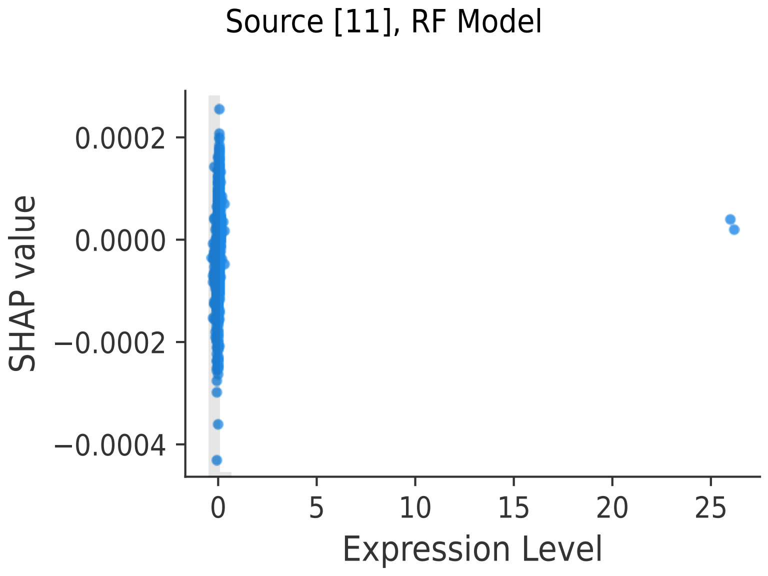
Source [11], RF Model
SHAP value
0.0002
0.0000
−0.0002
−0.0004
0
5
10
15
20
25
Expression Level

Source [12], RF Model

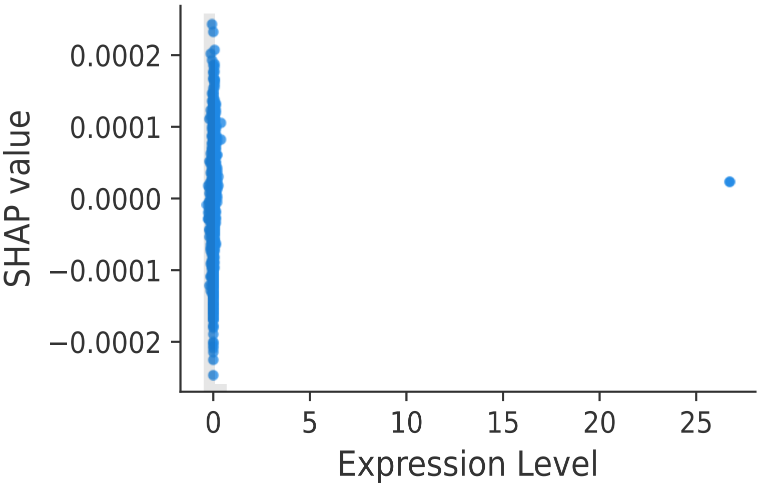

Source [13], RF Model

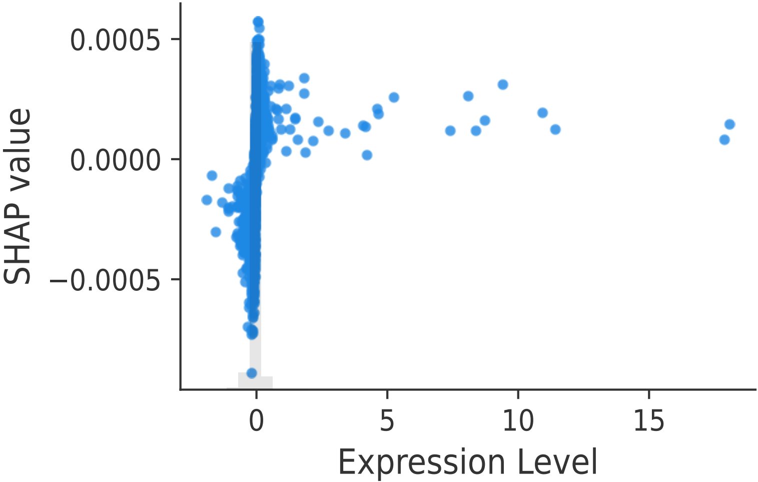

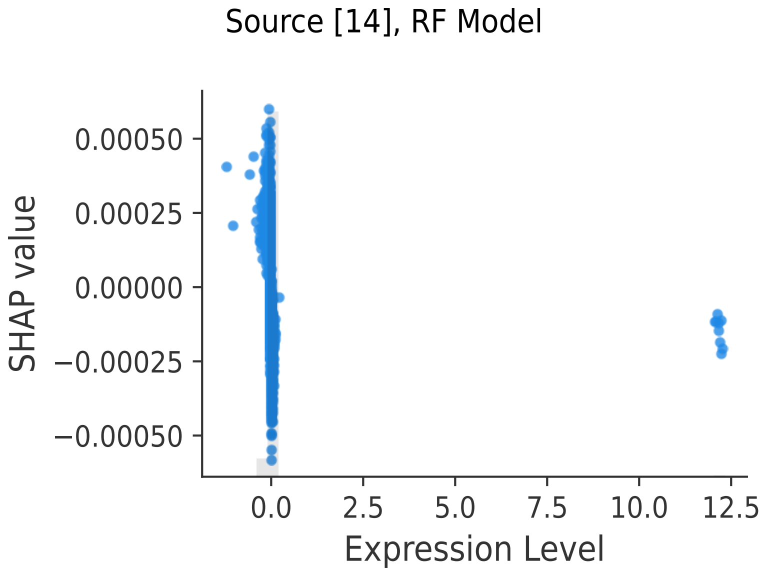

Source [14], RF Model
SHAP value
0.00050
0.00025
0.00000
−0.00025
−0.00050
0.0
2.5
5.0
7.5
10.0
12.5
Expression Level

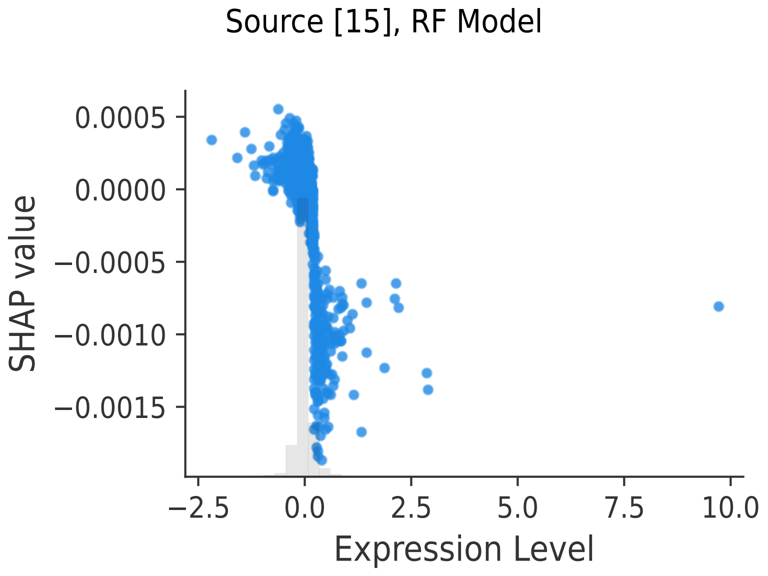

Source [15], RF Model
0.0005
0.0000
−0.0005
−0.0010
−0.0015
SHAP value
−2.5
0.0
2.5
5.0
7.5
10.0
Expression Level

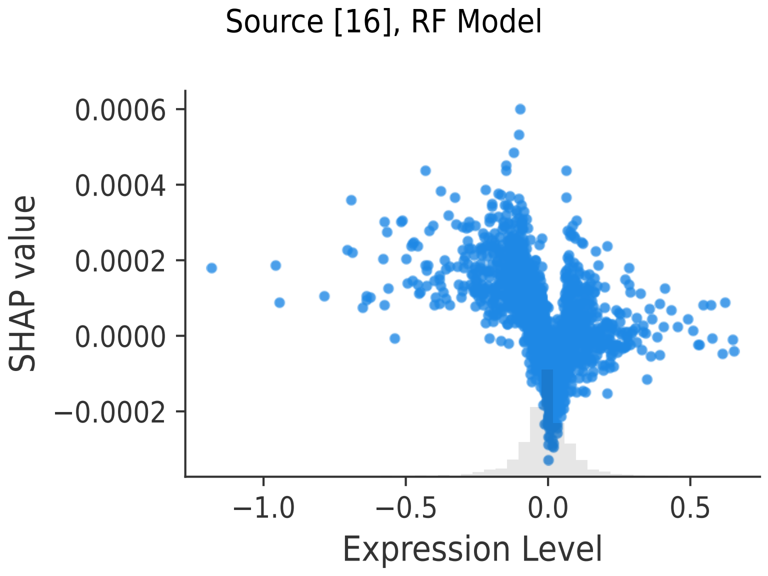
Source [16], RF Model
0.0006
0.0004
0.0002
0.0000
−0.0002
SHAP value
−1.0
−0.5
0.0
0.5
Expression Level

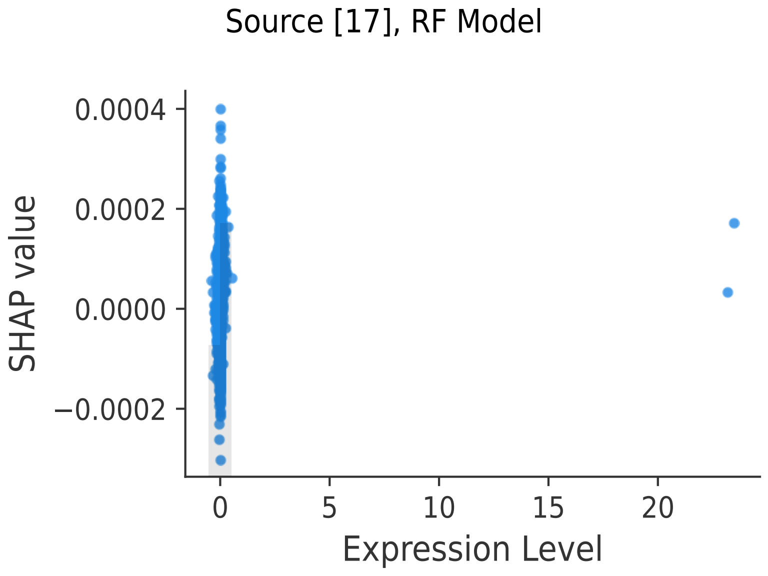

Source [17], RF Model
0.0004
0.0002
0.0000
−0.0002
SHAP value
0
5
10
15
20
Expression Level

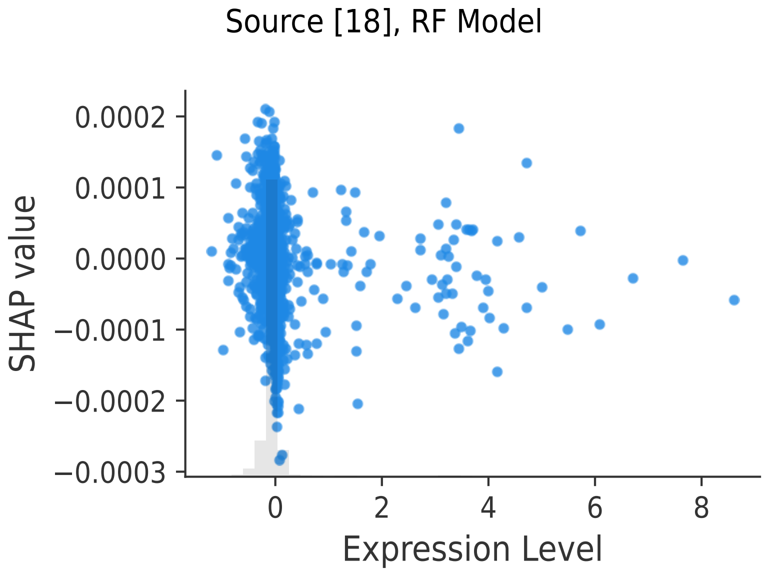
Source [18], RF Model
SHAP value
0.0002
0.0001
0.0000
−0.0001
−0.0002
−0.0003
0
2
4
6
8
Expression Level

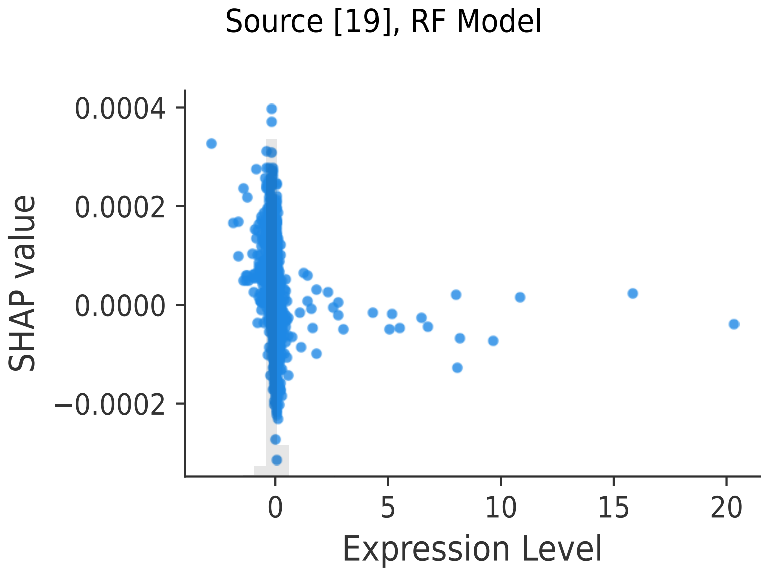
Source [19], RF Model
SHAP value
0.0004
0.0002
0.0000
−0.0002
0
5
10
15
20
Expression Level

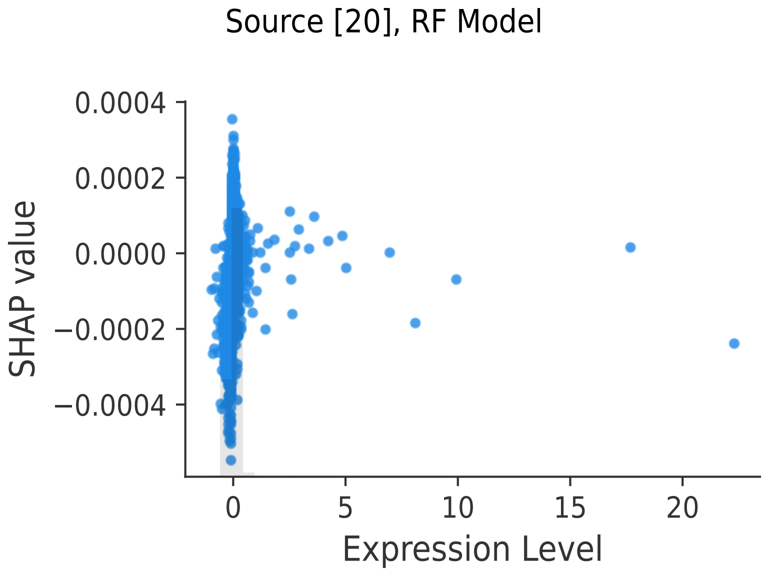
Source [20], RF Model
0.0004
0.0002
0.0000
−0.0002
−0.0004
SHAP value
0
5
10
15
20
Expression Level

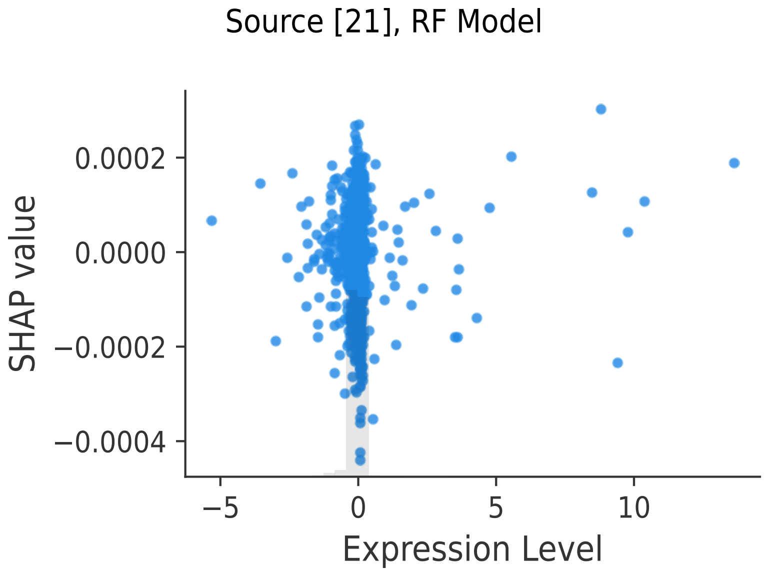
Source [21], RF Model
SHAP value
0.0002
0.0000
−0.0002
−0.0004
−5
0
5
10
Expression Level

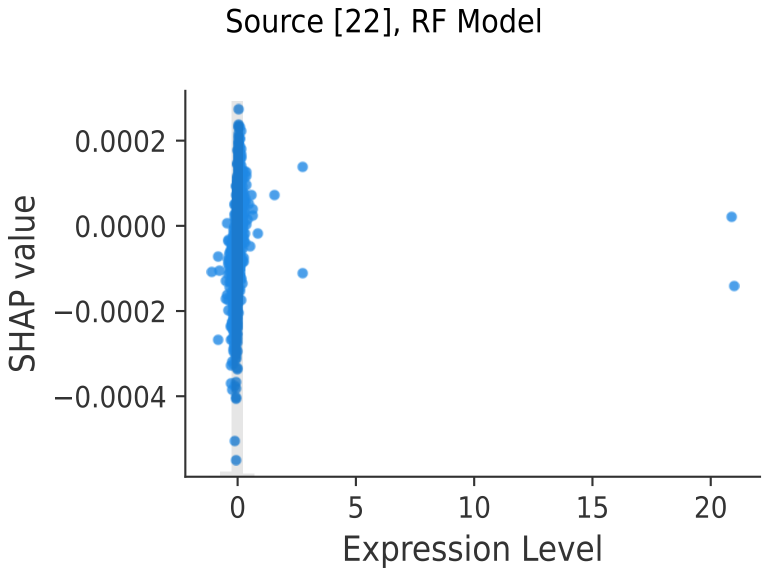
Source [22], RF Model
SHAP value
0.0002
0.0000
−0.0002
−0.0004
0
5
10
15
20
Expression Level

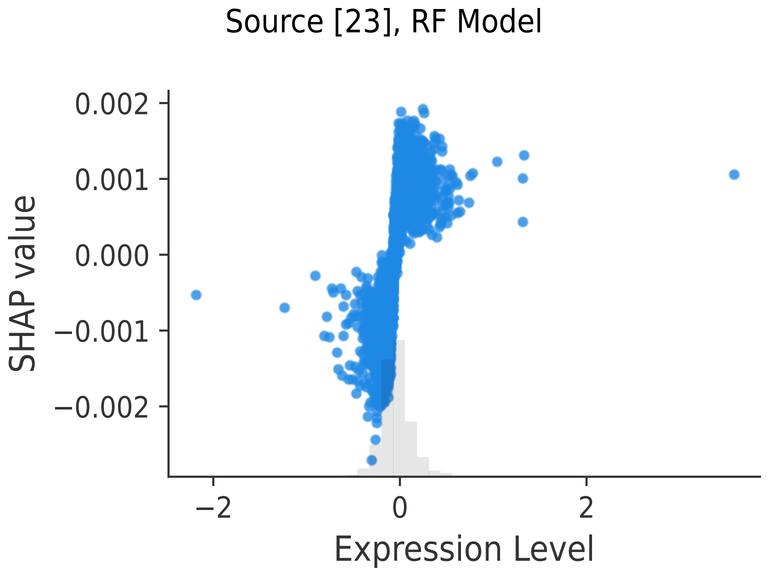
Source [23], RF Model
SHAP value
0.002
0.001
0.000
−0.001
−0.002
−2
0
2
Expression Level

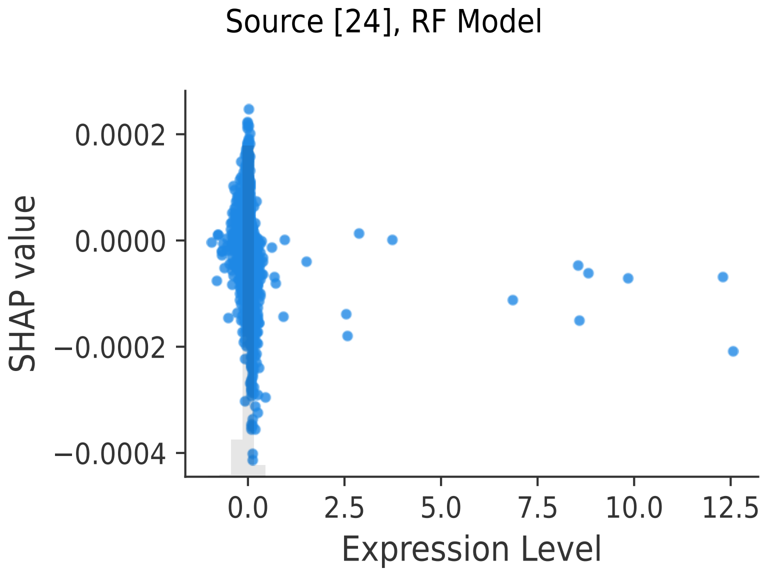

Source [24], RF Model
0.0002
0.0000
−0.0002
−0.0004
SHAP value
0.0
2.5
5.0
7.5
10.0
12.5
Expression Level

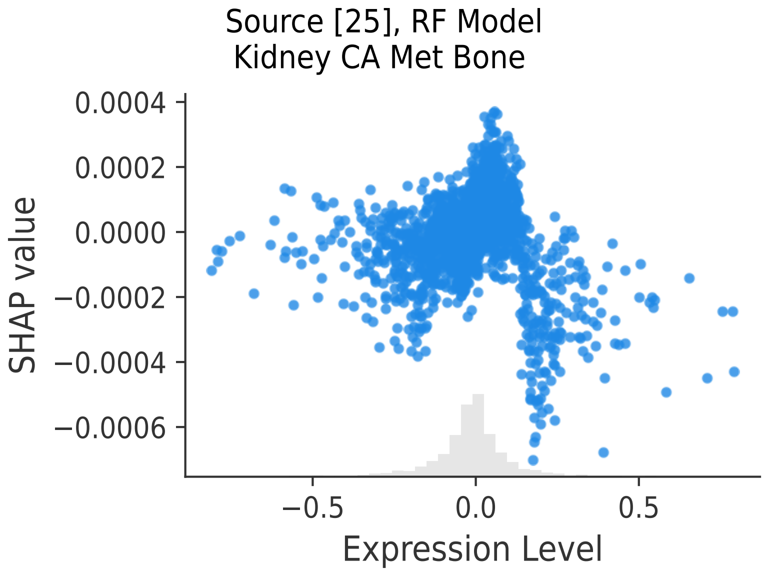
Source [25], RF Model
Kidney CA Met Bone
0.0004
0.0002
0.0000
−0.0002
−0.0004
−0.0006
SHAP value
−0.5
0.0
0.5
Expression Level

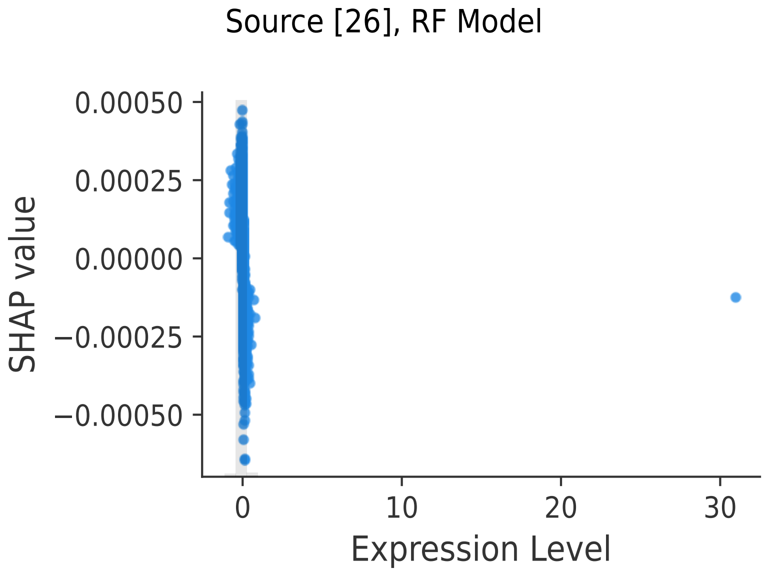
Source [26], RF Model
SHAP value
0.00050
0.00025
0.00000
−0.00025
−0.00050
0
10
20
30
Expression Level

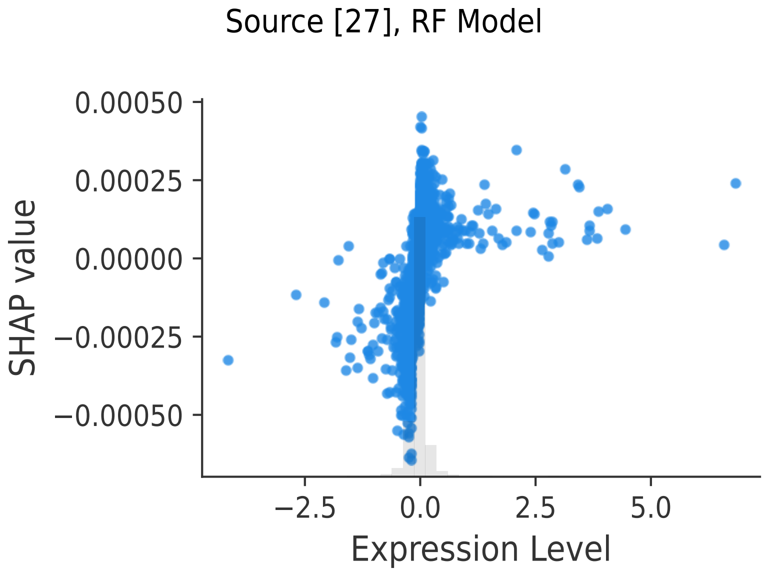
Source [27], RF Model
0.00050
0.00025
0.00000
−0.00025
−0.00050
SHAP value
−2.5
0.0
2.5
5.0
Expression Level

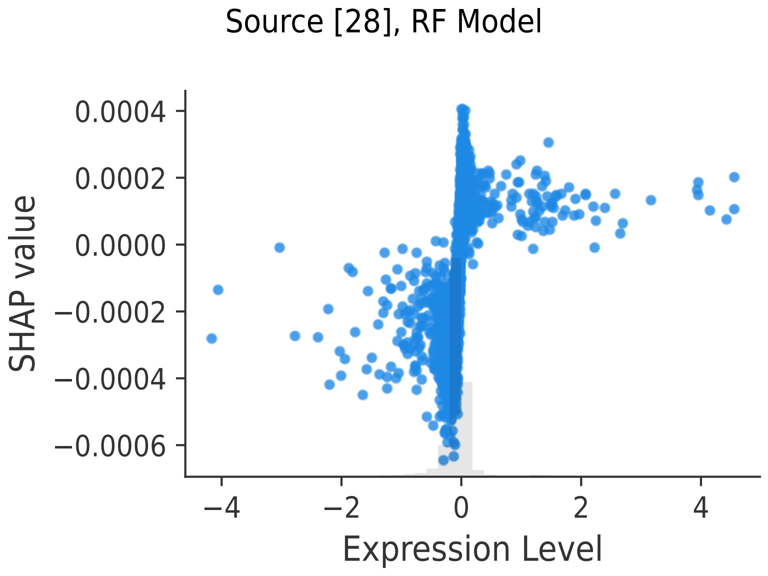

Source [28], RF Model
0.0004
0.0002
0.0000
−0.0002
−0.0004
−0.0006
SHAP value
−4
−2
0
2
4
Expression Level

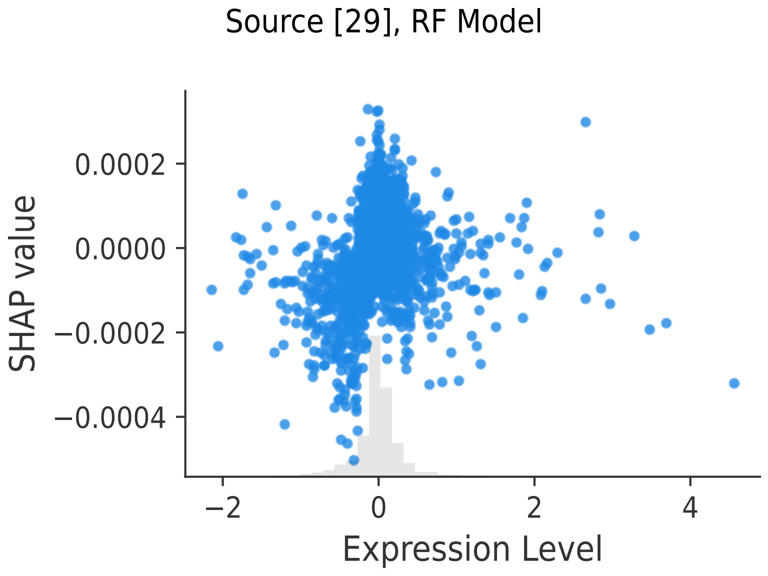
Source [29], RF Model
0.0002
0.0000
−0.0002
−0.0004
SHAP value
−2
0
2
4
Expression Level

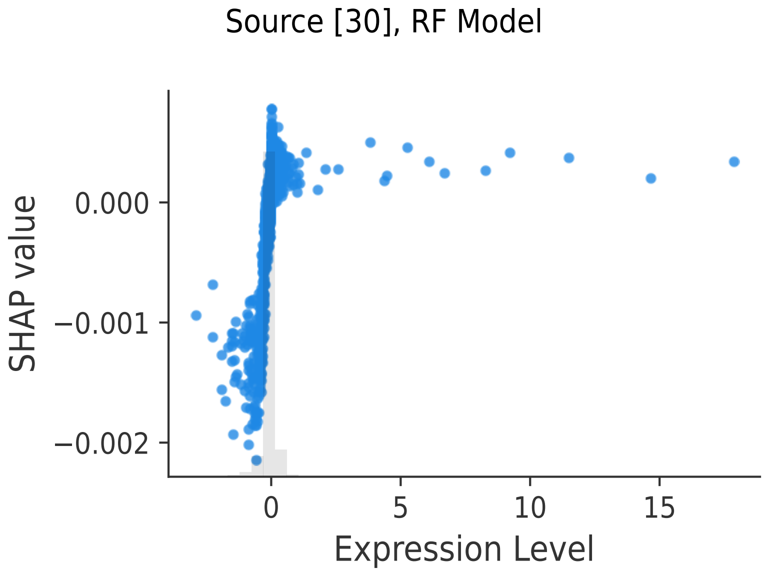
Source [30], RF Model
SHAP value
0.000
−0.001
−0.002
0
5
10
15
Expression Level

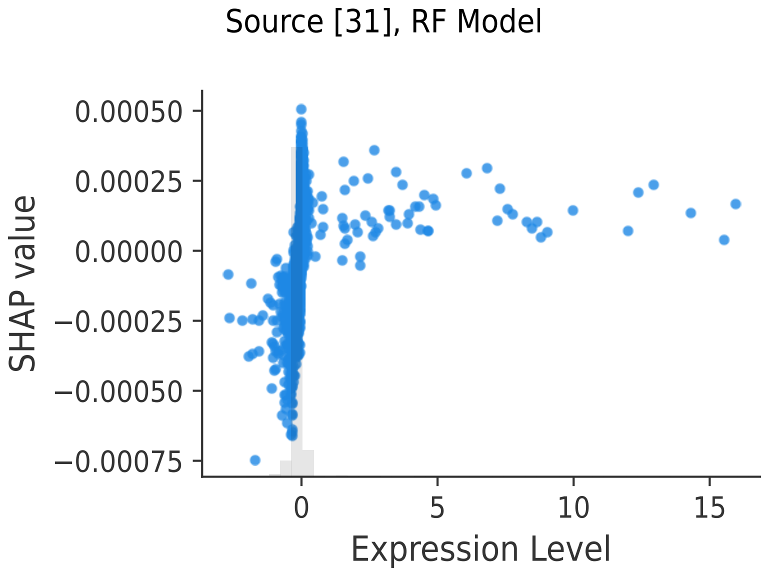

Source [31], RF Model
0.00050
0.00025
0.00000
−0.00025
−0.00050
−0.00075
SHAP value
0
5
10
15
Expression Level

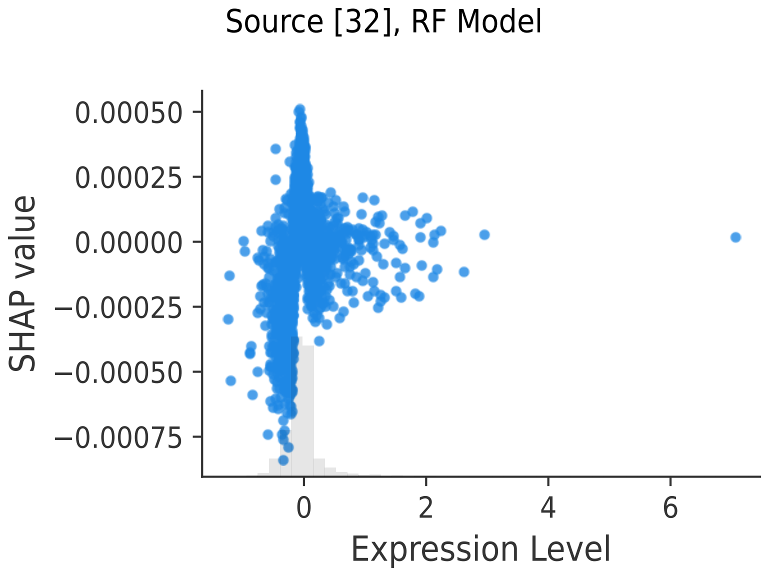

Source [32], RF Model
0.00050
0.00025
0.00000
−0.00025
−0.00050
−0.00075
SHAP value
0
2
4
6
Expression Level

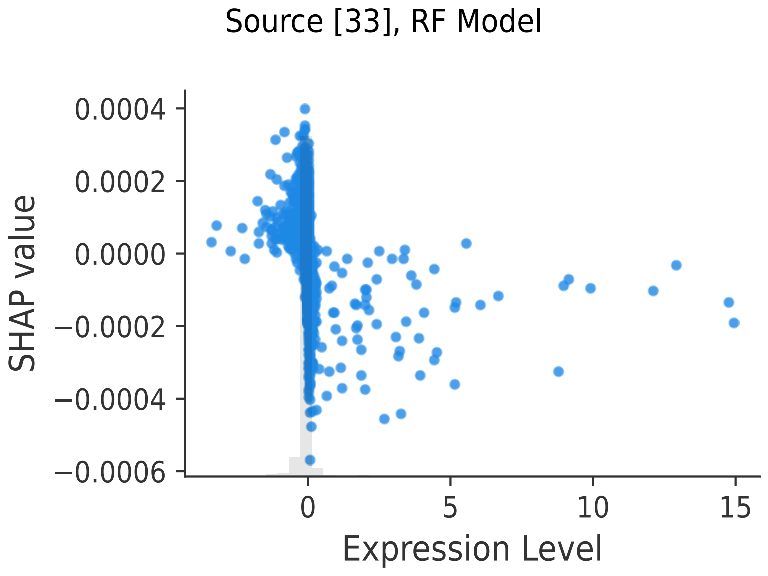
Source [33], RF Model
SHAP value
0.0004
0.0002
0.0000
−0.0002
−0.0004
−0.0006
0
5
10
15
Expression Level

Source [34], RF Model

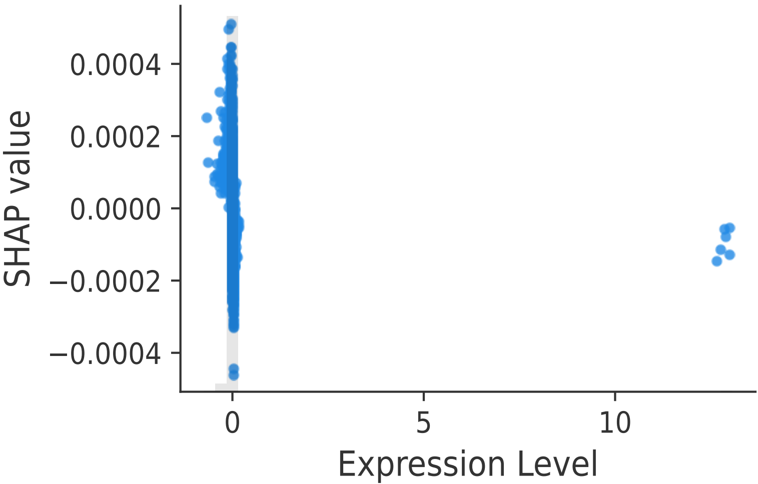

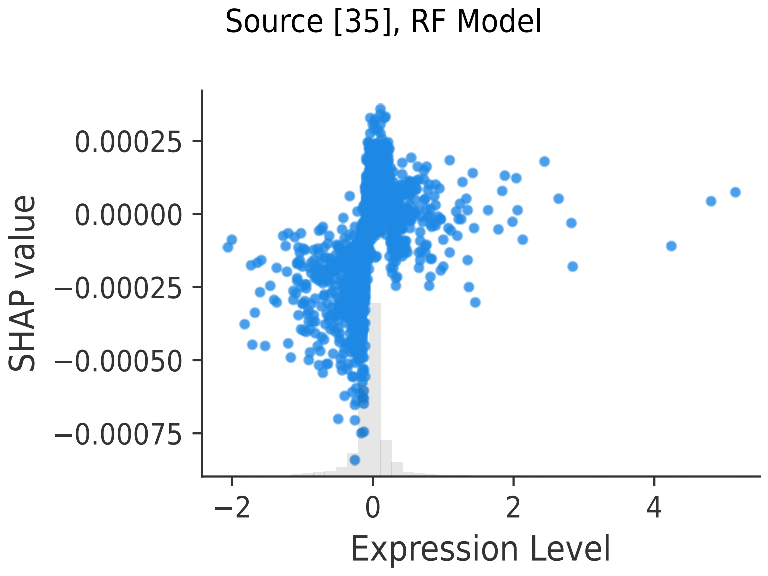
Source [35], RF Model
SHAP value
0.00025
0.00000
−0.00025
−0.00050
−0.00075
−2
0
2
4
Expression Level

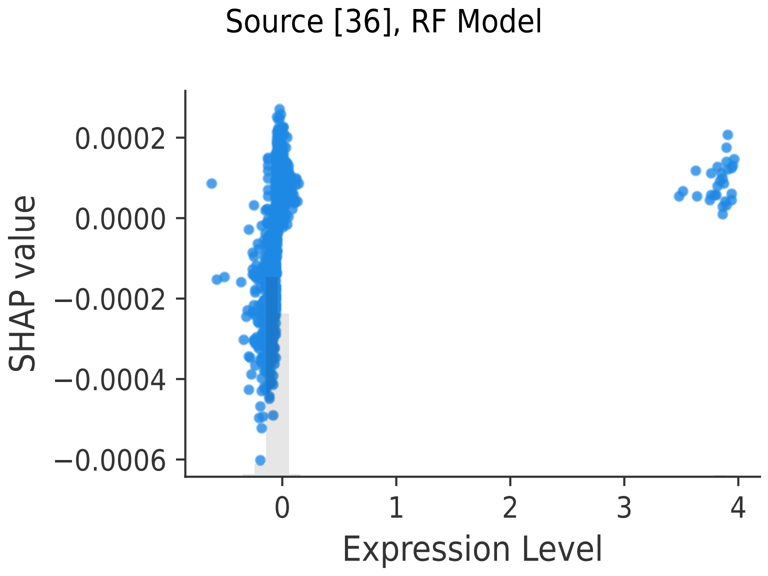
Source [36], RF Model
0.0002
0.0000
−0.0002
−0.0004
−0.0006
SHAP value
0
1
2
3
4
Expression Level

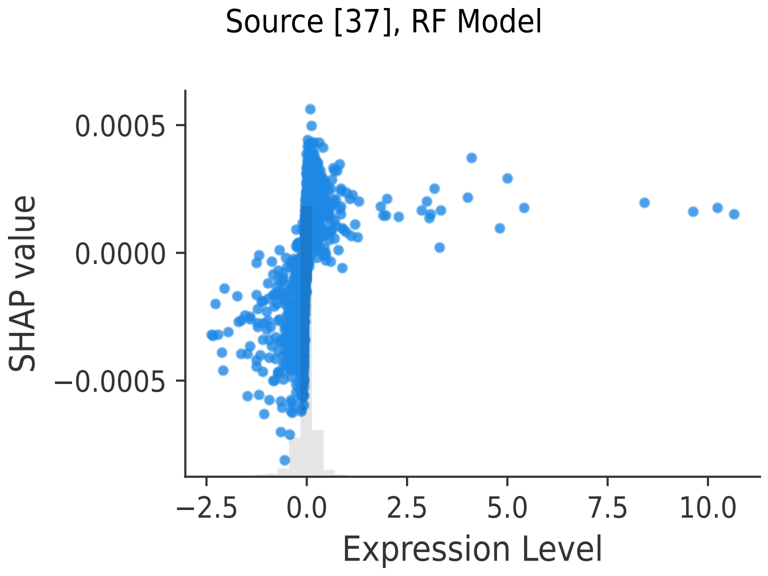
Source [37], RF Model
SHAP value
0.0005
0.0000
−0.0005
−2.5
0.0
2.5
5.0
7.5
10.0
Expression Level

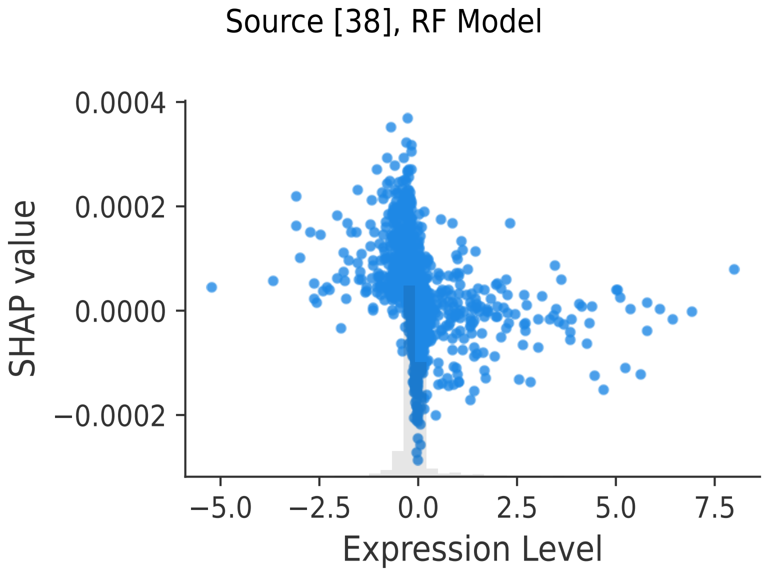
Source [38], RF Model
0.0004
0.0002
0.0000
−0.0002
SHAP value
−5.0
−2.5
0.0
2.5
5.0
7.5
Expression Level

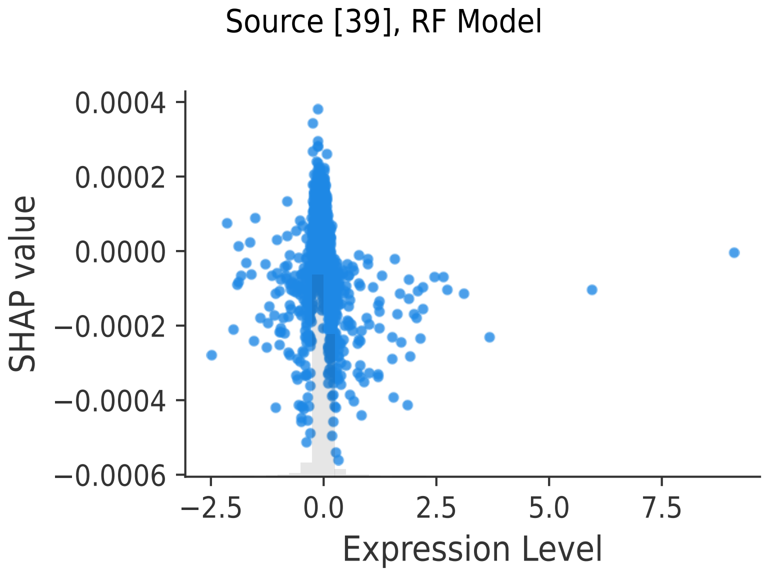
Source [39], RF Model
SHAP value
0.0004
0.0002
0.0000
−0.0002
−0.0004
−0.0006
−2.5
0.0
2.5
5.0
7.5
Expression Level

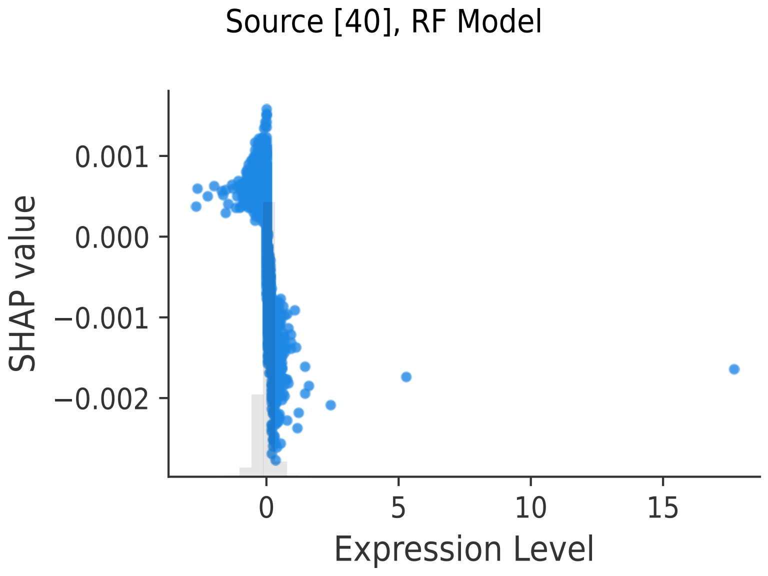
Source [40], RF Model
0.001
0.000
−0.001
−0.002
SHAP value
0
5
10
15
Expression Level

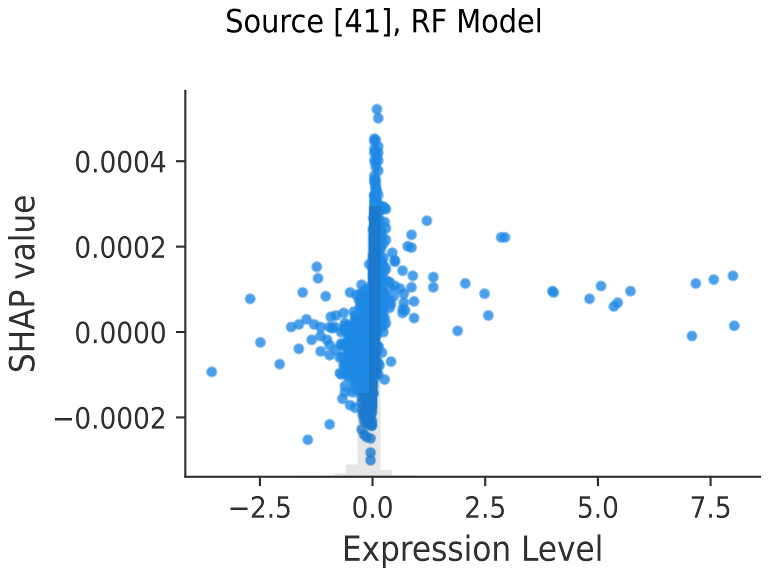
Source [41], RF Model
0.0004
0.0002
0.0000
−0.0002
SHAP value
−2.5
0.0
2.5
5.0
7.5
Expression Level

Source [42], RF Model

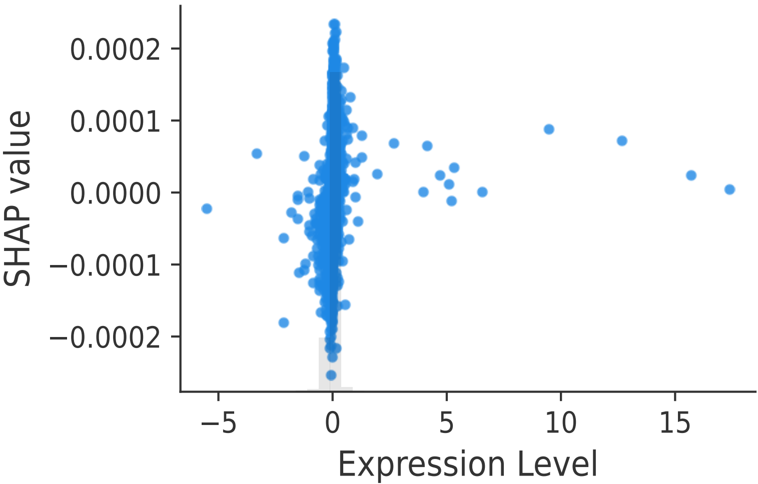

Source [43], RF Model

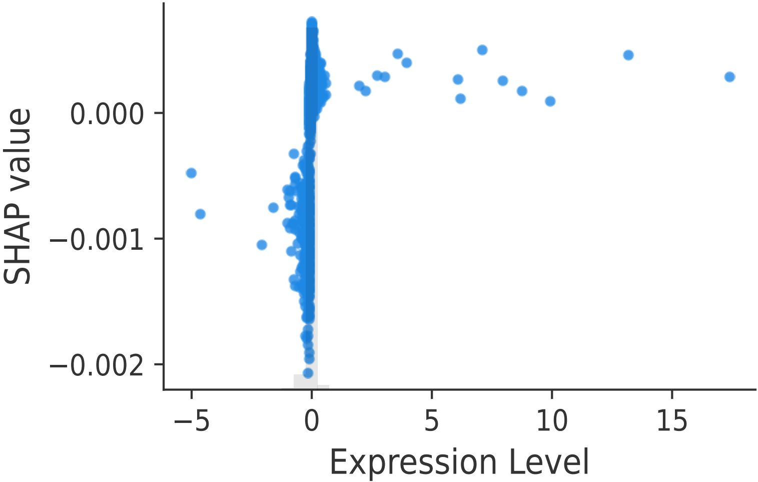

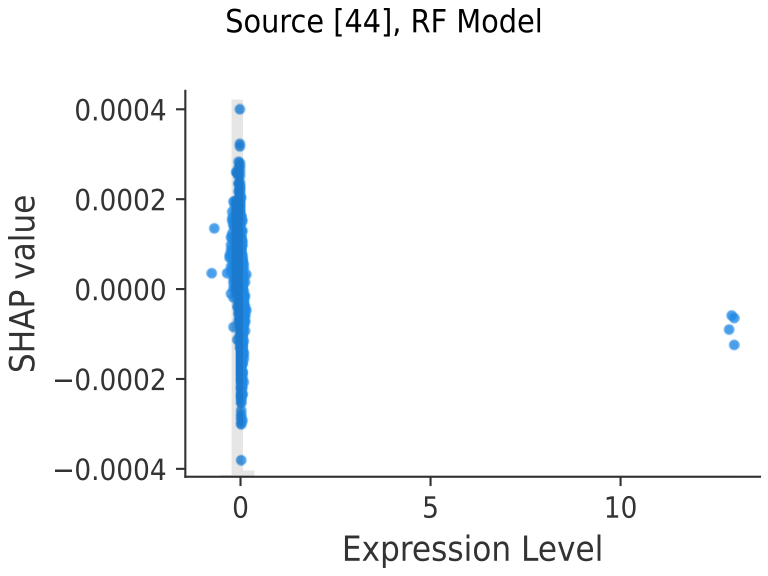
Source [44], RF Model
SHAP value
0.0004
0.0002
0.0000
−0.0002
−0.0004
0
5
10
Expression Level

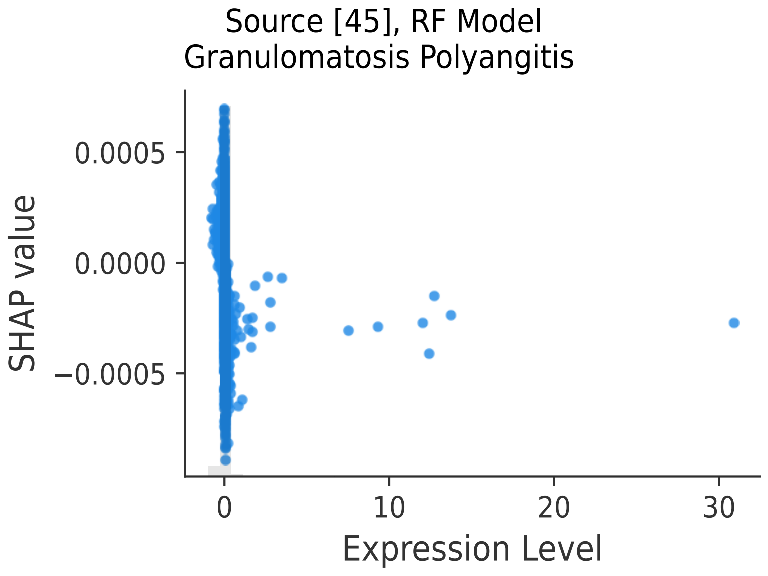

Source [45], RF Model
Granulomatosis Polyangitis
SHAP value
0.0005
0.0000
−0.0005
0
10
20
30
Expression Level

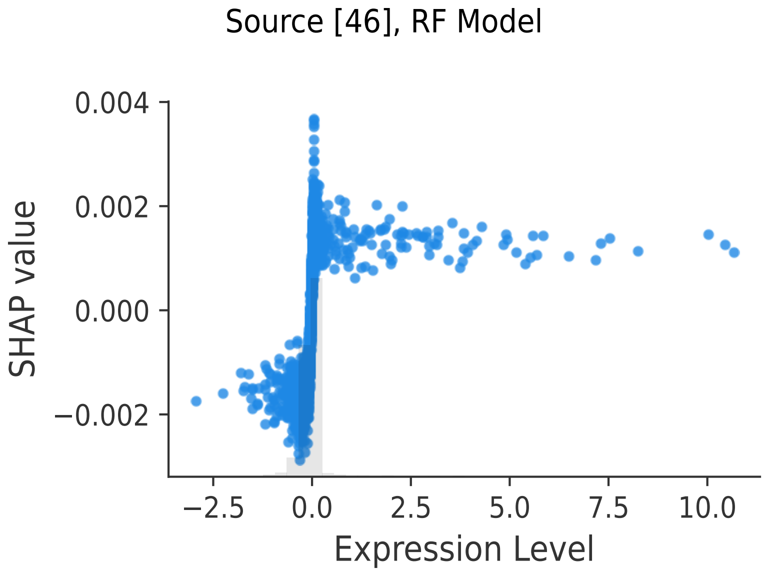
Source [46], RF Model
0.004
0.002
0.000
−0.002
SHAP value
−2.5
0.0
2.5
5.0
7.5
10.0
Expression Level

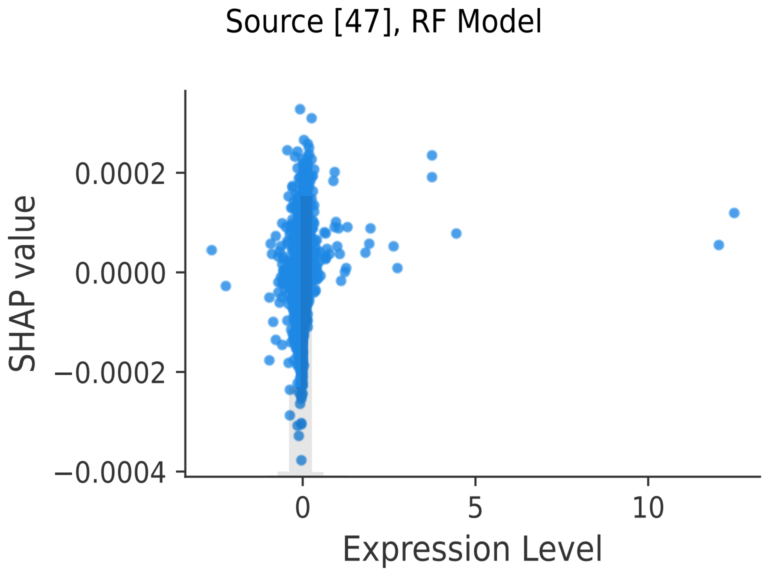

Source [47], RF Model
0.0002
0.0000
−0.0002
−0.0004
SHAP value
0
5
10
Expression Level

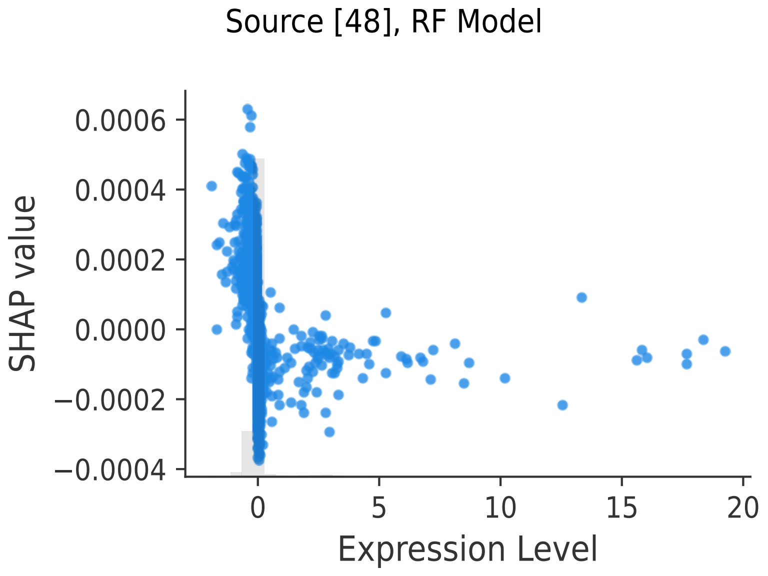
Source [48], RF Model
SHAP value
0.0006
0.0004
0.0002
0.0000
−0.0002
−0.0004
0
5
10
15
20
Expression Level

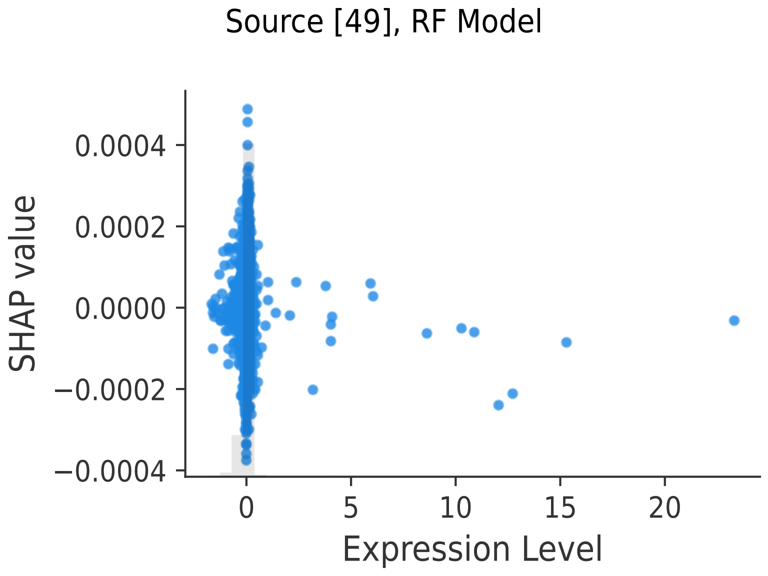
Source [49], RF Model
SHAP value
0.0004
0.0002
0.0000
−0.0002
−0.0004
0
5
10
15
20
Expression Level

Source [50], RF Model

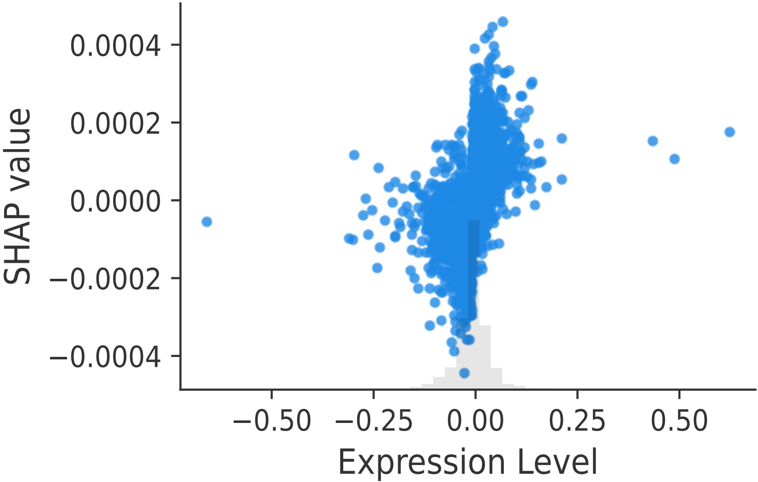

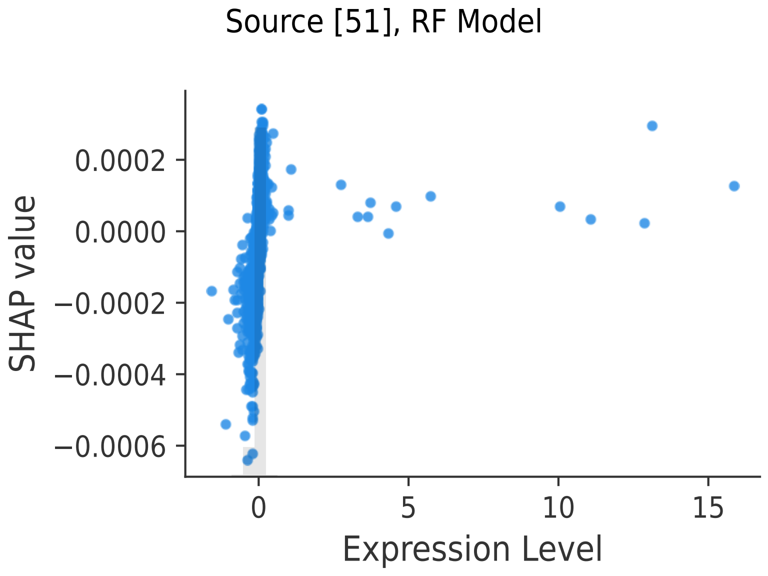
Source [51], RF Model
0.0002
0.0000
−0.0002
−0.0004
−0.0006
SHAP value
0
5
10
15
Expression Level

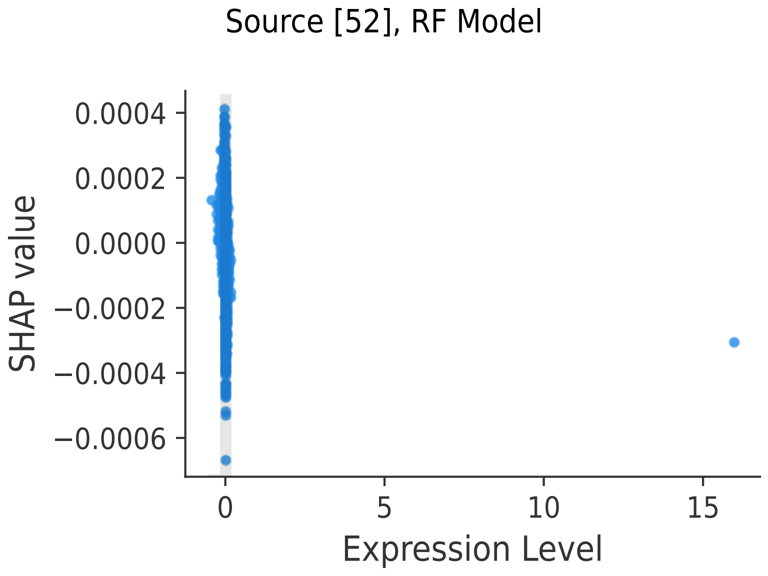

Source [52], RF Model
0.0004
0.0002
0.0000
−0.0002
−0.0004
−0.0006
SHAP value
0
5
10
15
Expression Level

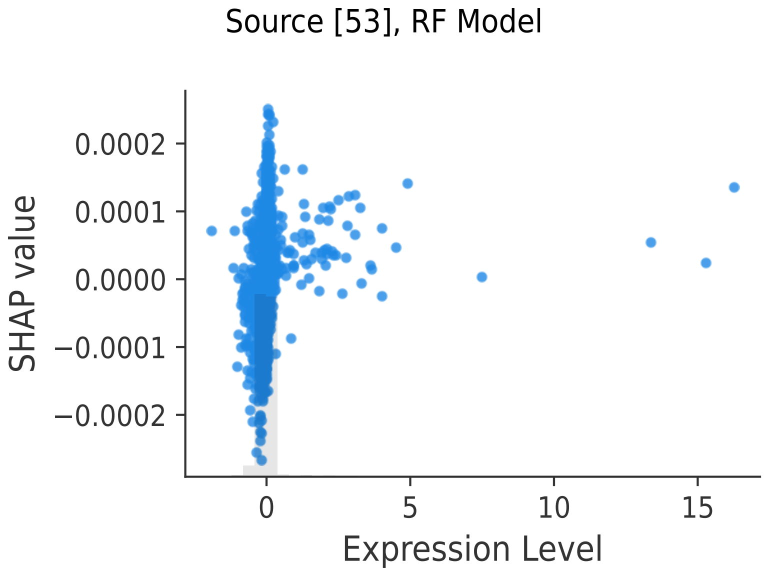

Source [53], RF Model
0.0002
0.0001
0.0000
−0.0001
−0.0002
SHAP value
0
5
10
15
Expression Level

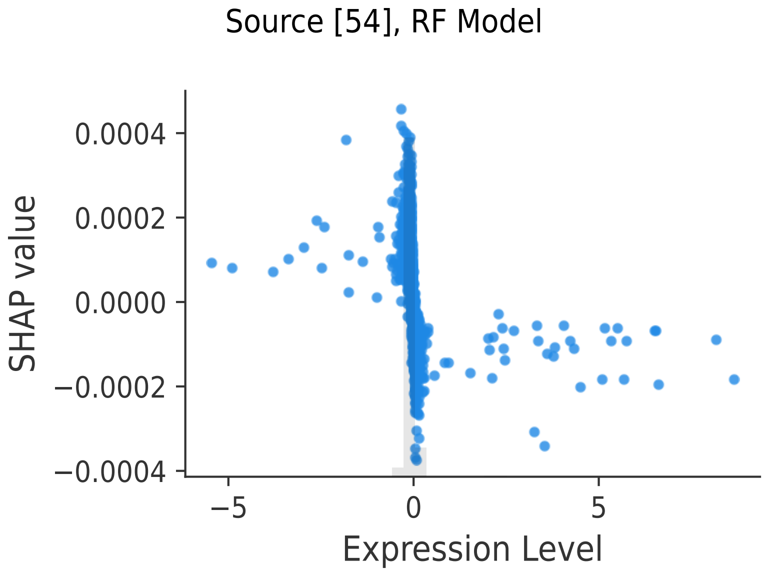
Source [54], RF Model
0.0004
0.0002
0.0000
−0.0002
−0.0004
SHAP value
−5
0
5
Expression Level

Source [55], RF Model

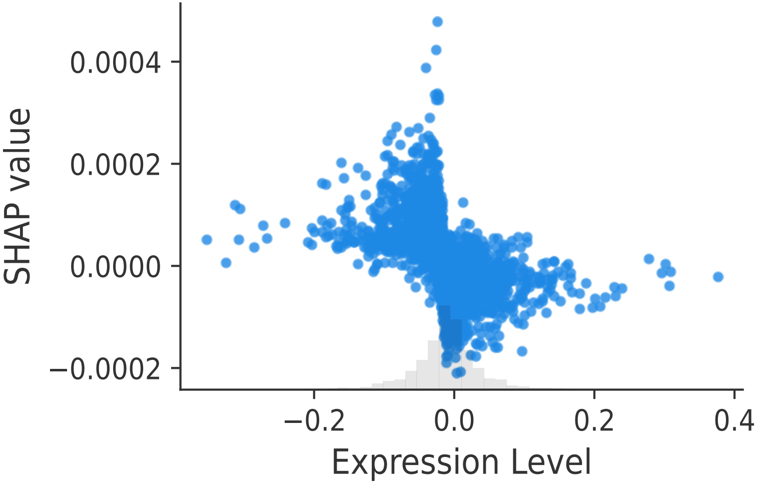

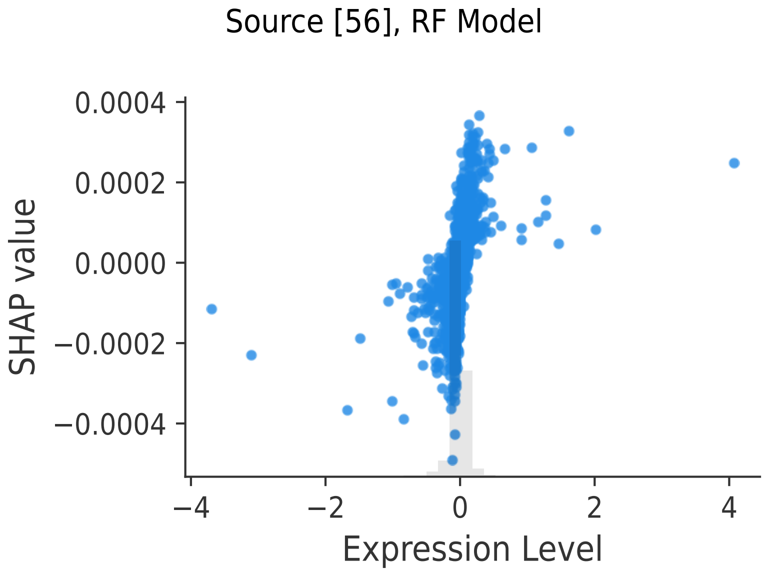

Source [56], RF Model
0.0004
0.0002
0.0000
−0.0002
−0.0004
SHAP value
−4
−2
0
2
4
Expression Level

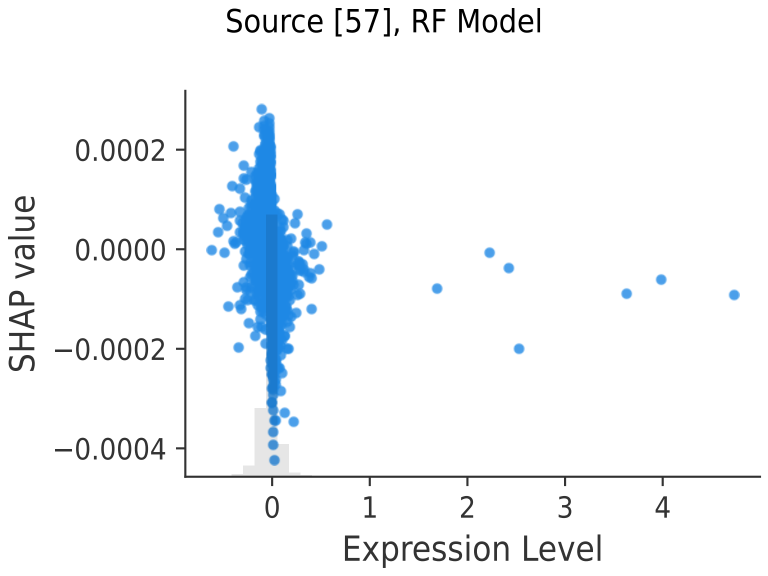

Source [57], RF Model
0.0002
0.0000
−0.0002
−0.0004
SHAP value
0
1
2
3
4
Expression Level

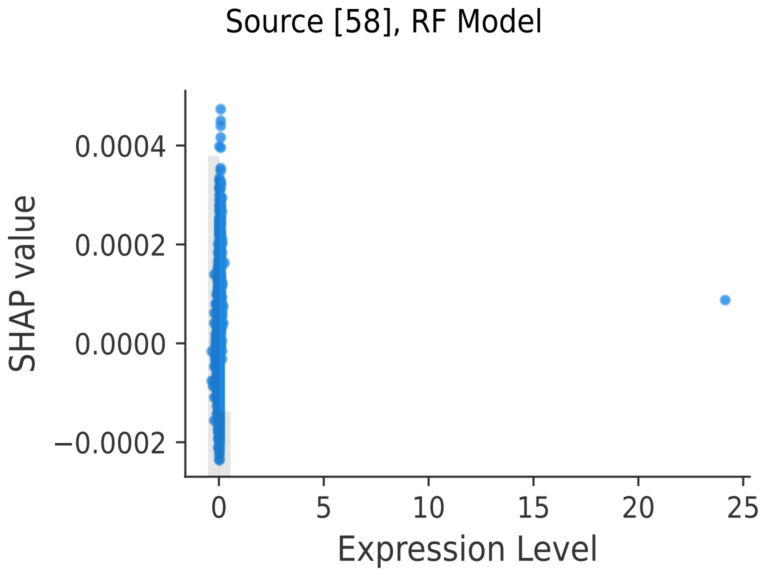
Source [58], RF Model
SHAP value
0.0004
0.0002
0.0000
−0.0002
0
5
10
15
20
25
Expression Level

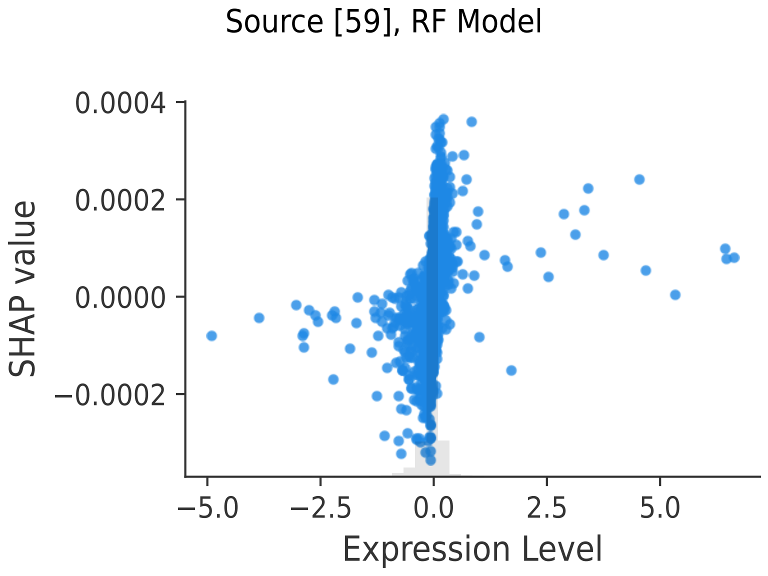

Source [59], RF Model
0.0004
0.0002
0.0000
−0.0002
SHAP value
−5.0
−2.5
0.0
2.5
5.0
Expression Level

Source [60], RF Model

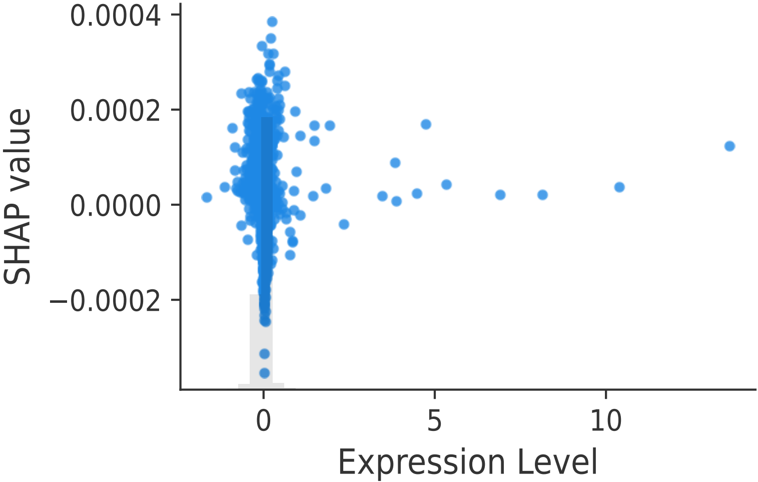

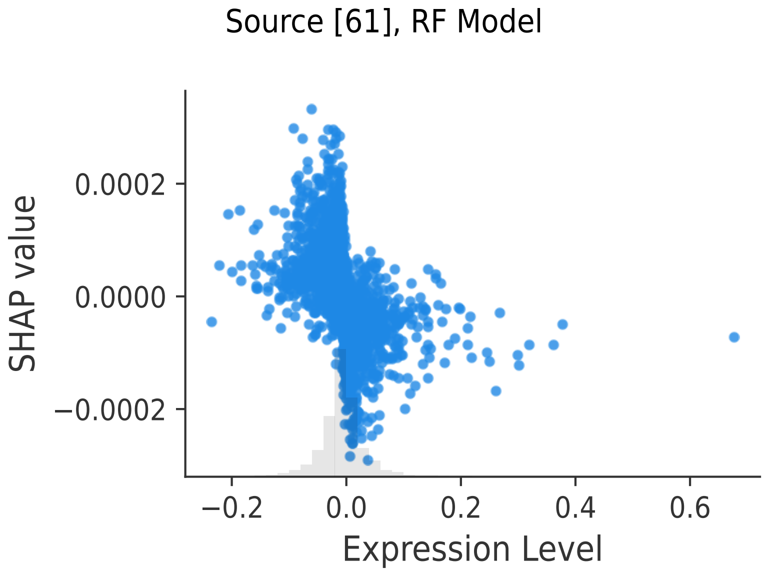
Source [61], RF Model
0.0002
0.0000
−0.0002
SHAP value
−0.2
0.0
0.2
0.4
0.6
Expression Level

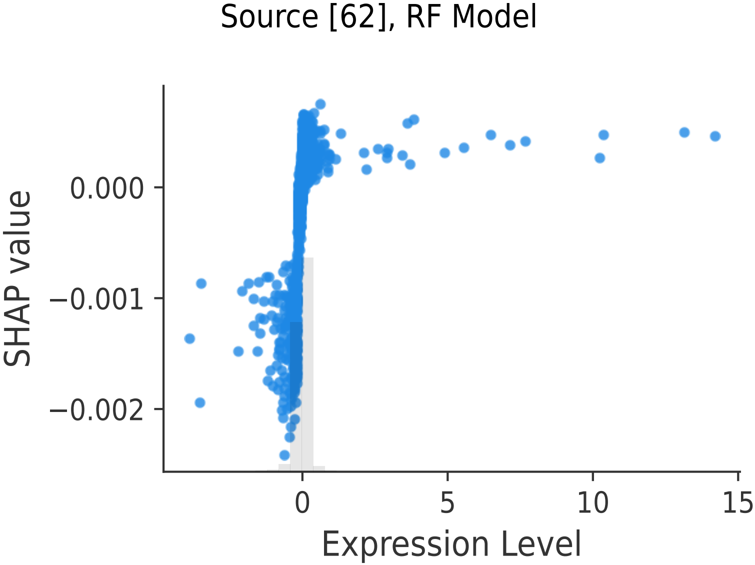
Source [62], RF Model
SHAP value
0.000
−0.001
−0.002
0
5
10
15
Expression Level

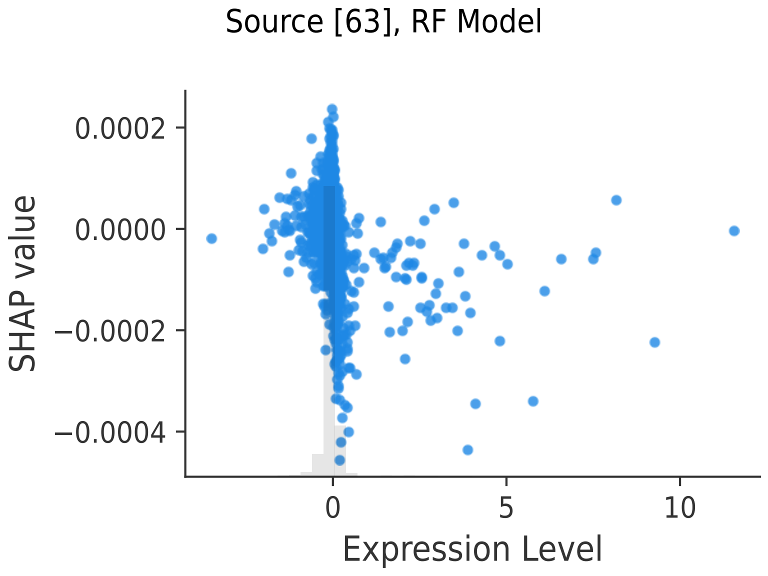

Source [63], RF Model
0.0002
0.0000
−0.0002
−0.0004
SHAP value
0
5
10
Expression Level

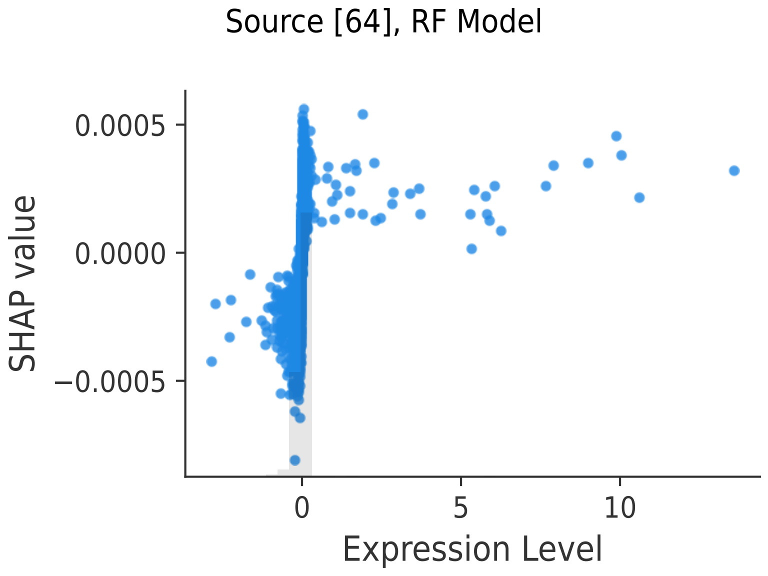
Source [64], RF Model
SHAP value
0.0005
0.0000
−0.0005
0
5
10
Expression Level

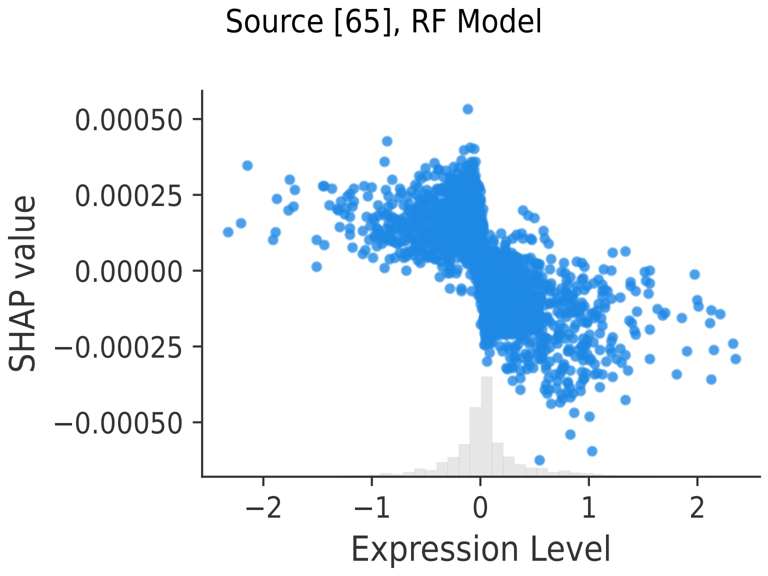
Source [65], RF Model
0.00050
0.00025
0.00000
−0.00025
−0.00050
SHAP value
−2
−1
0
1
2
Expression Level

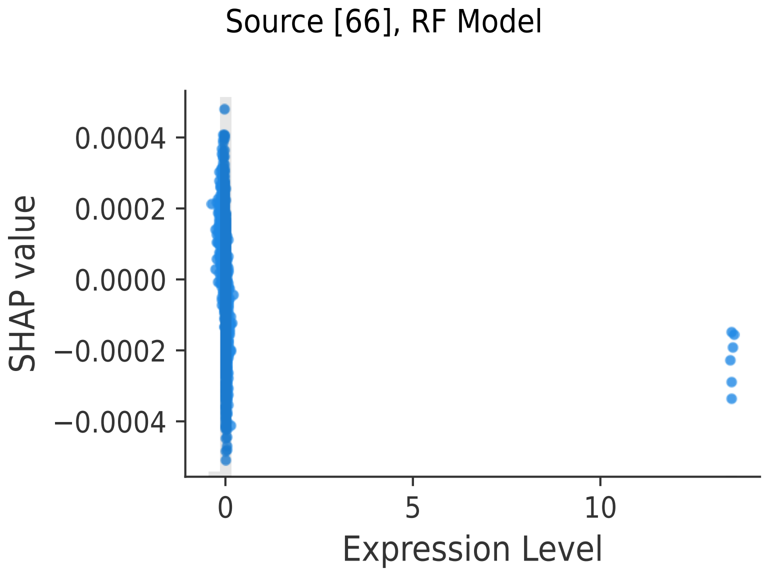
Source [66], RF Model
0.0004
0.0002
0.0000
−0.0002
−0.0004
SHAP value
0
5
10
Expression Level

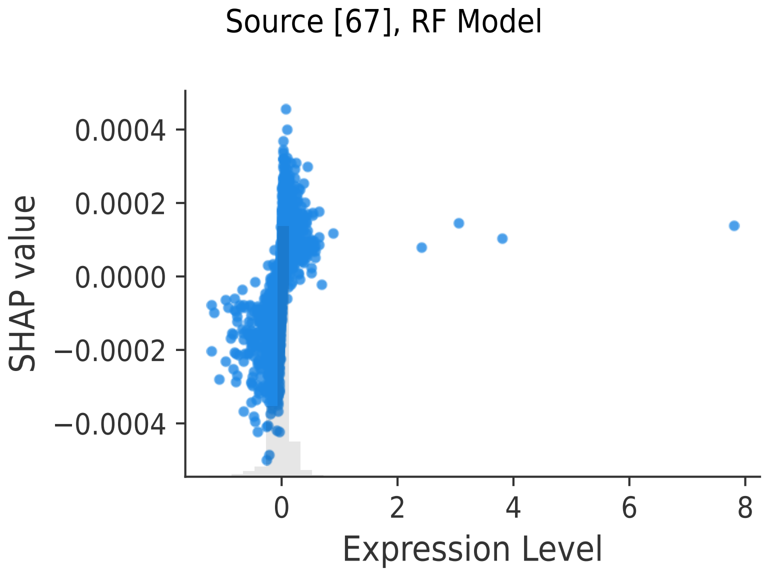

Source [67], RF Model
0.0004
0.0002
0.0000
−0.0002
−0.0004
SHAP value
0
2
4
6
8
Expression Level

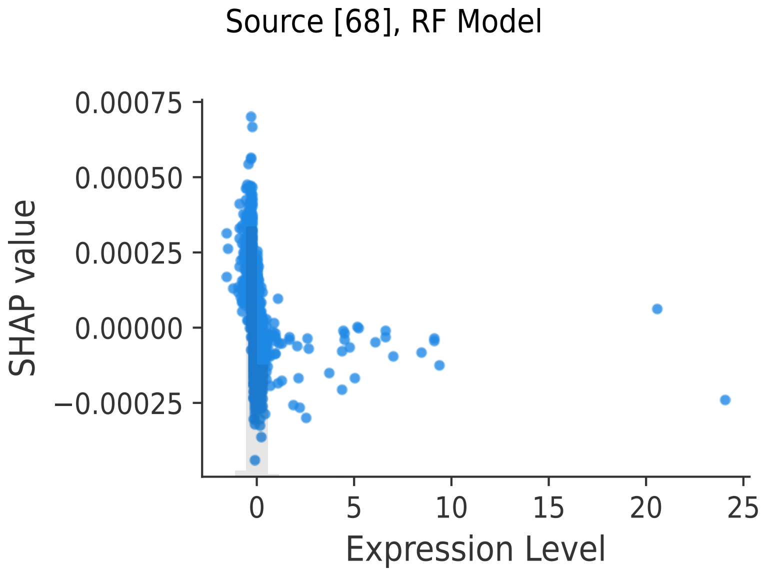
Source [68], RF Model
SHAP value
0.00075
0.00050
0.00025
0.00000
−0.00025
0
5
10
15
20
25
Expression Level

Source [69], RF Model

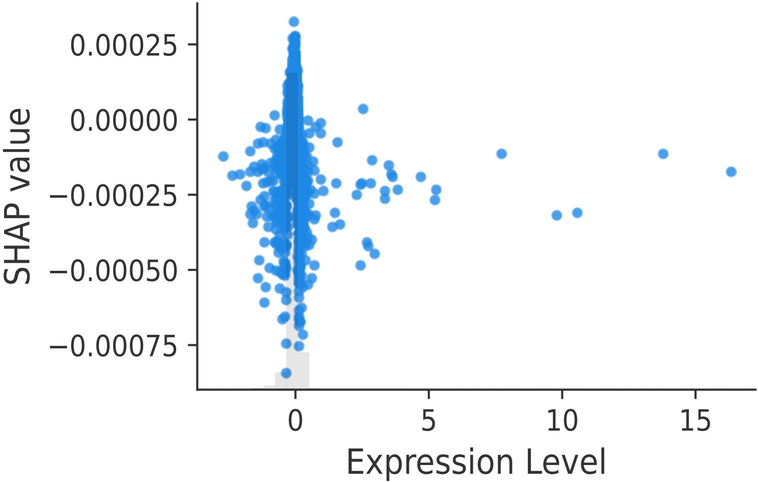

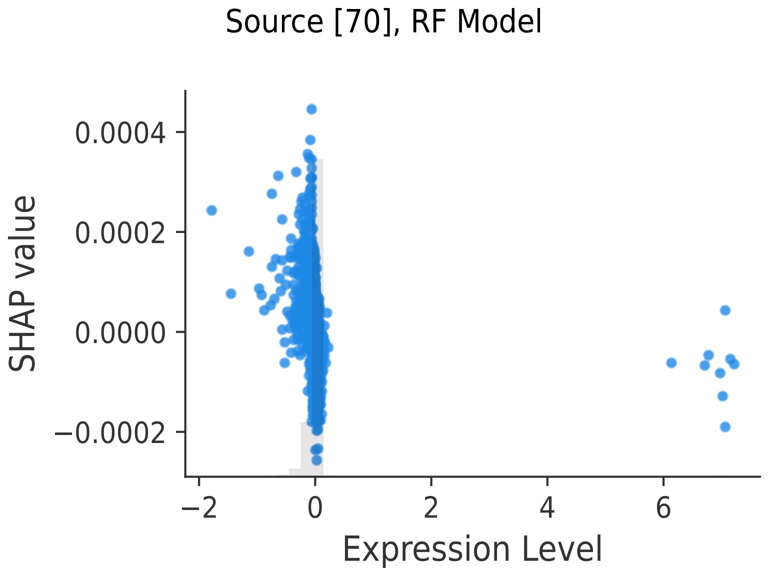
Source [70], RF Model
SHAP value
0.0004
0.0002
0.0000
−0.0002
−2
0
2
4
6
Expression Level

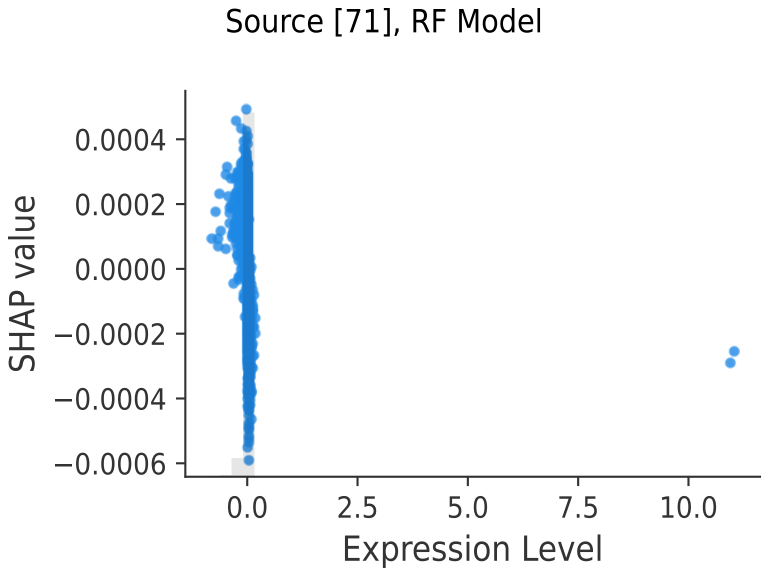
Source [71], RF Model
SHAP value
0.0004
0.0002
0.0000
−0.0002
−0.0004
−0.0006
0.0
2.5
5.0
7.5
10.0
Expression Level

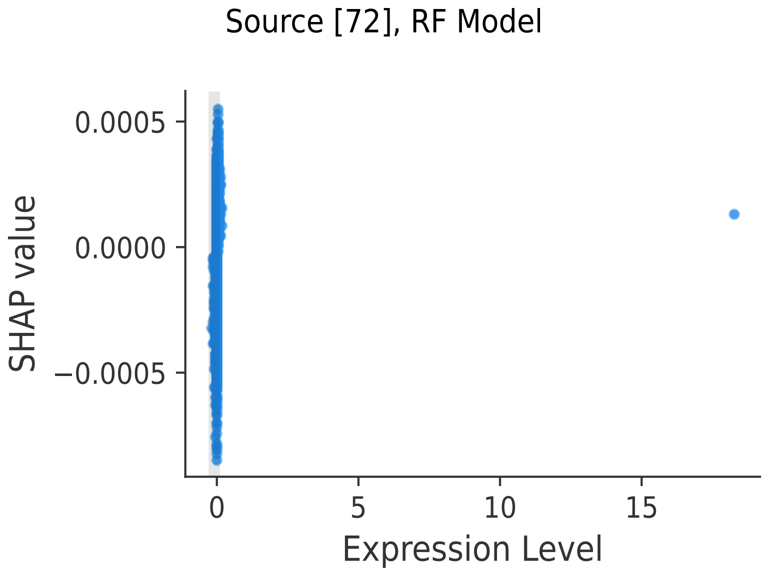
Source [72], RF Model
0.0005
0.0000
−0.0005
SHAP value
0
5
10
15
Expression Level

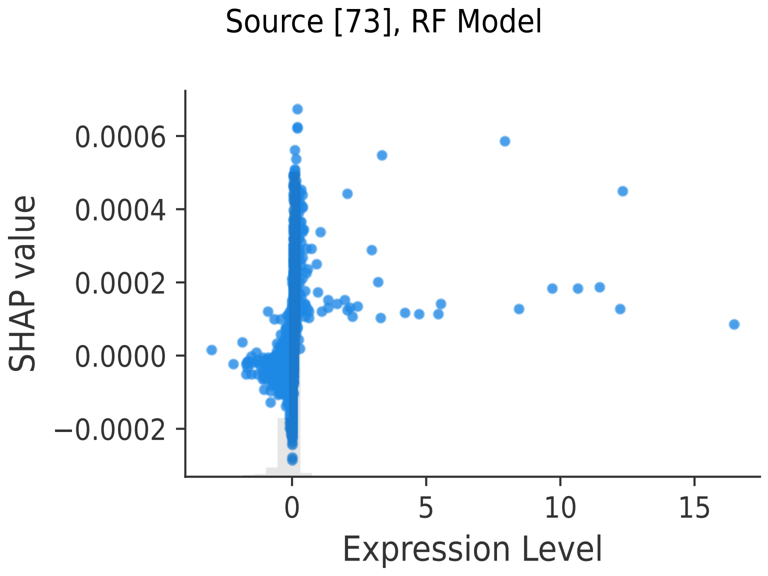
Source [73], RF Model
SHAP value
0.0006
0.0004
0.0002
0.0000
−0.0002
0
5
10
15
Expression Level

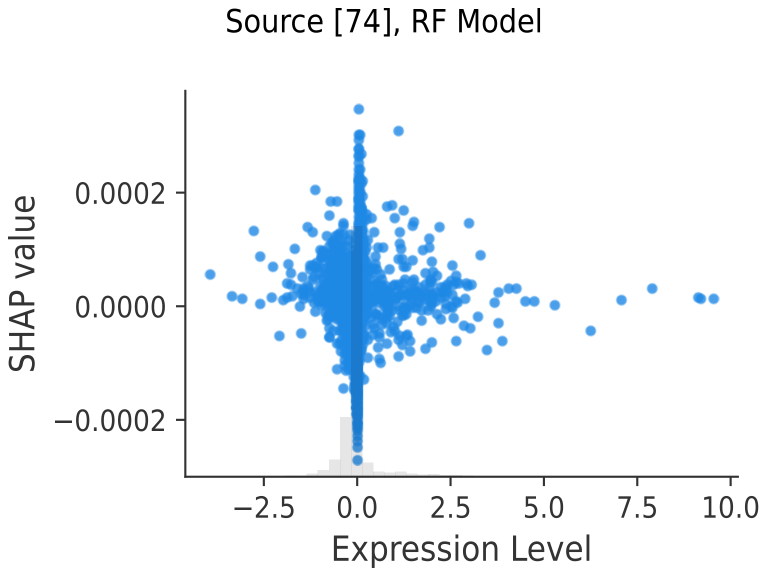
Source [74], RF Model
SHAP value
0.0002
0.0000
−0.0002
−2.5
0.0
2.5
5.0
7.5
10.0
Expression Level

Source [75], RF Model

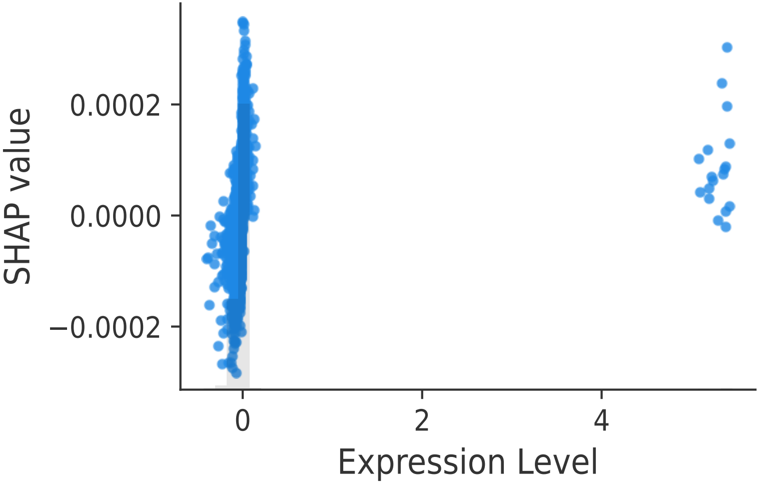

Source [76], RF Model

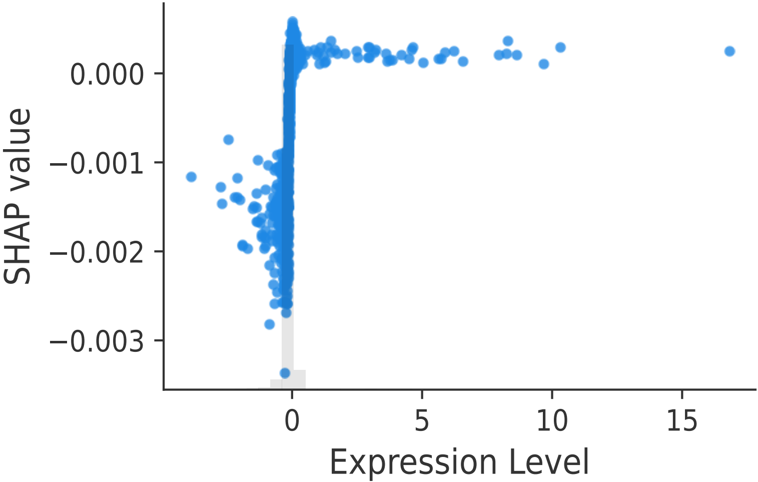

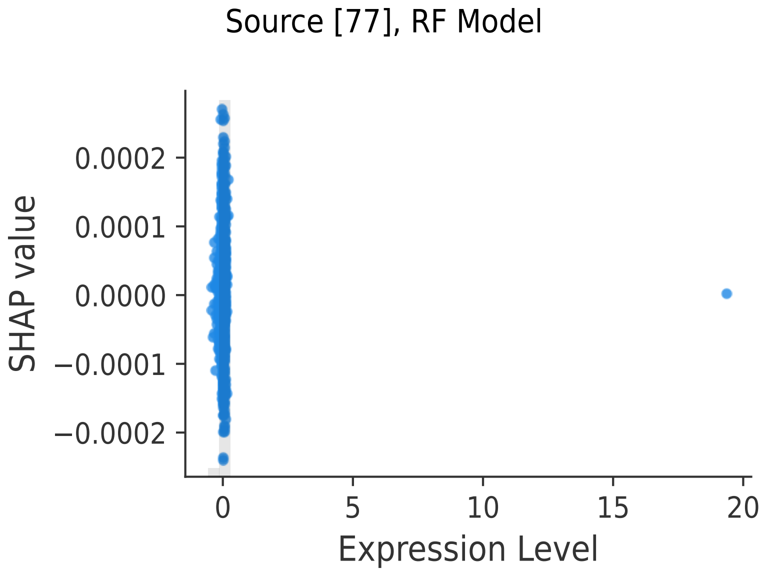

Source [77], RF Model
SHAP value
0.0002
0.0001
0.0000
−0.0001
−0.0002
0
5
10
15
20
Expression Level

Source [78], RF Model

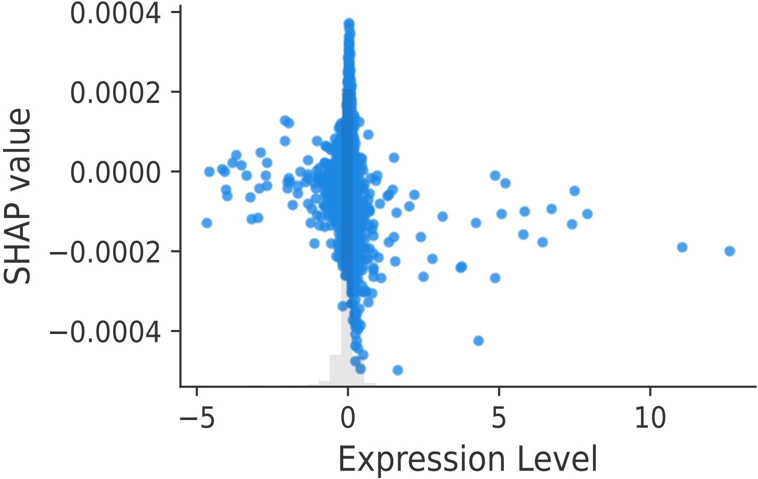

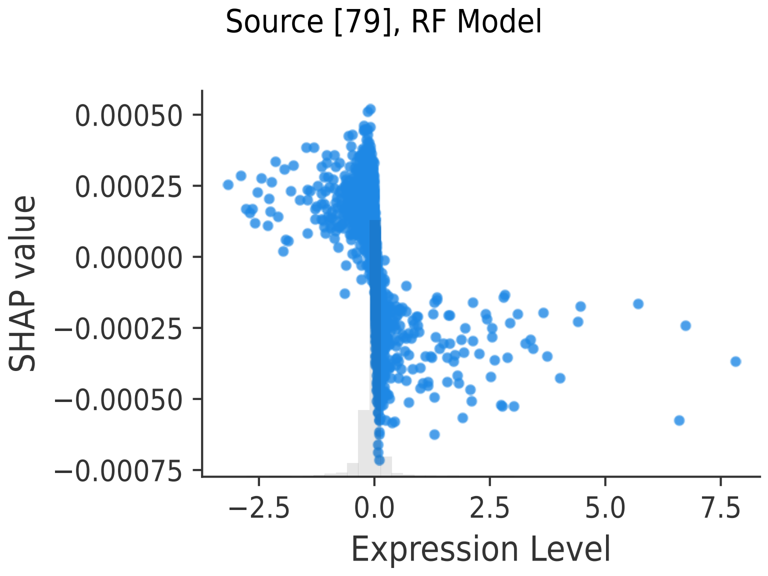

Source [79], RF Model
SHAP value
0.00050
0.00025
0.00000
−0.00025
−0.00050
−0.00075
−2.5
0.0
2.5
5.0
7.5
Expression Level

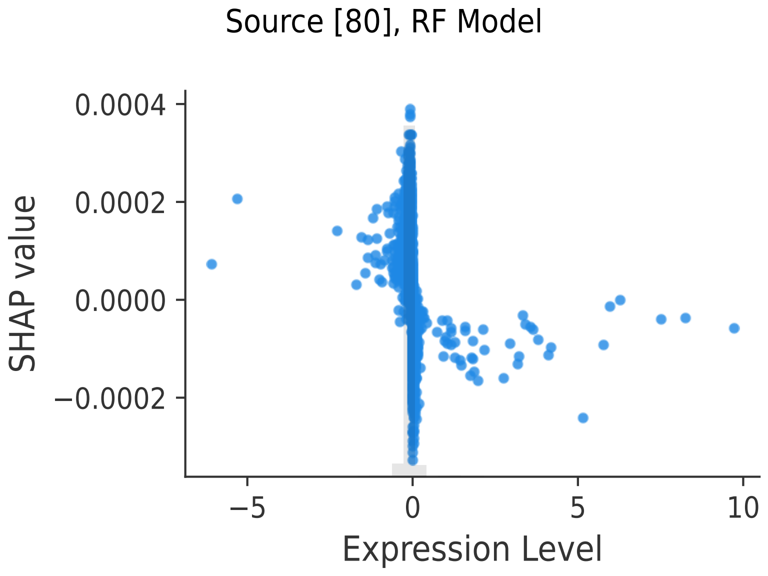
Source [80], RF Model
0.0004
0.0002
0.0000
−0.0002
SHAP value
−5
0
5
10
Expression Level

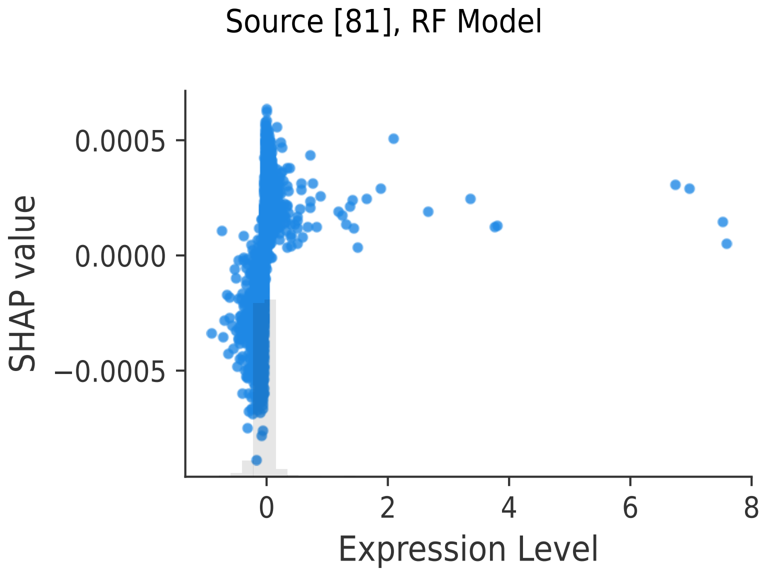
Source [81], RF Model
SHAP value
0.0005
0.0000
−0.0005
0
2
4
6
8
Expression Level

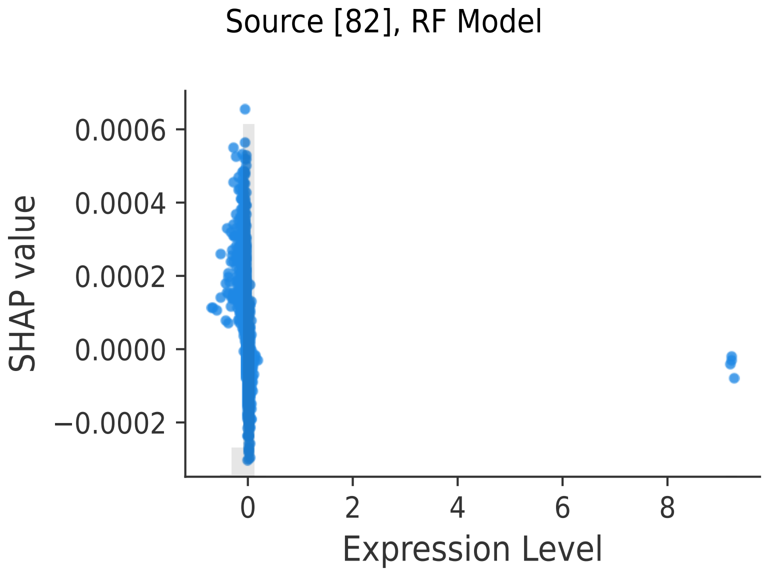
Source [82], RF Model
SHAP value
0.0006
0.0004
0.0002
0.0000
−0.0002
0
2
4
6
8
Expression Level

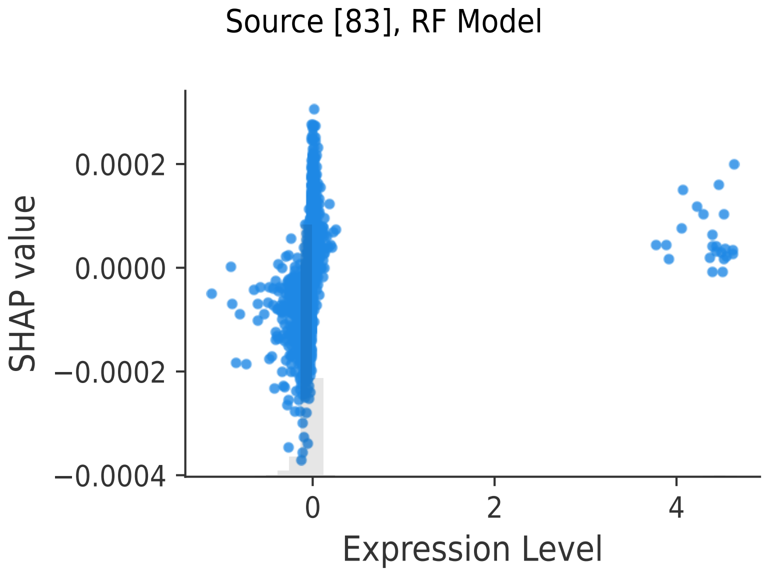
Source [83], RF Model
0.0002
0.0000
−0.0002
−0.0004
SHAP value
0
2
4
Expression Level

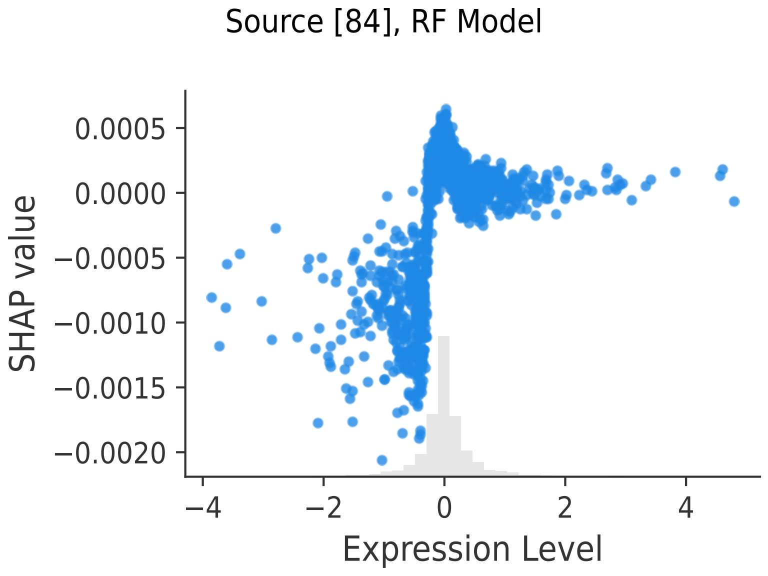
Source [84], RF Model
SHAP value
0.0005
0.0000
−0.0005
−0.0010
−0.0015
−0.0020
−4
−2
0
2
4
Expression Level

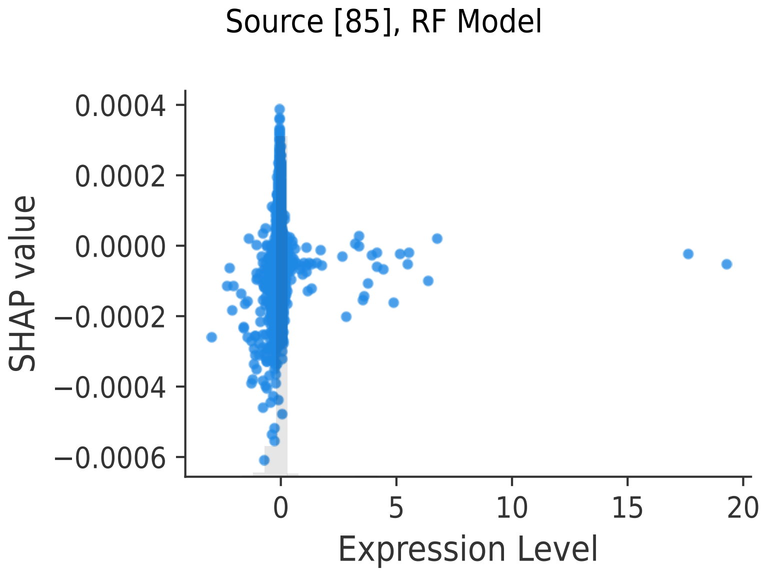

Source [85], RF Model
SHAP value
0.0004
0.0002
0.0000
−0.0002
−0.0004
−0.0006
0
5
10
15
20
Expression Level

Source [86], RF Model

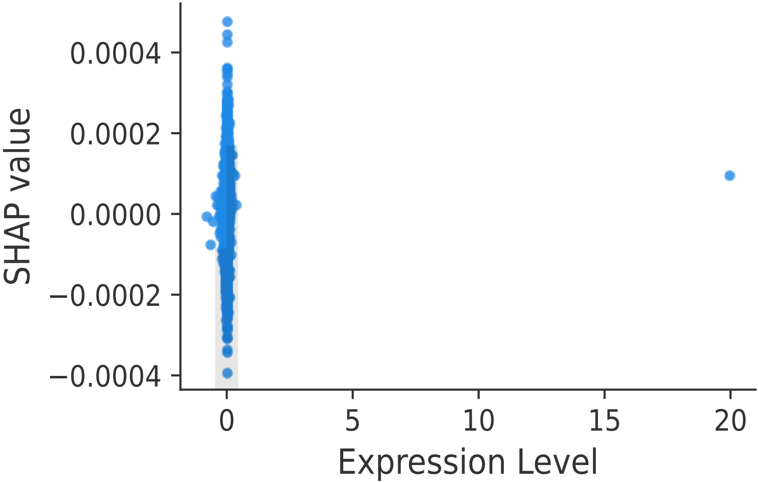

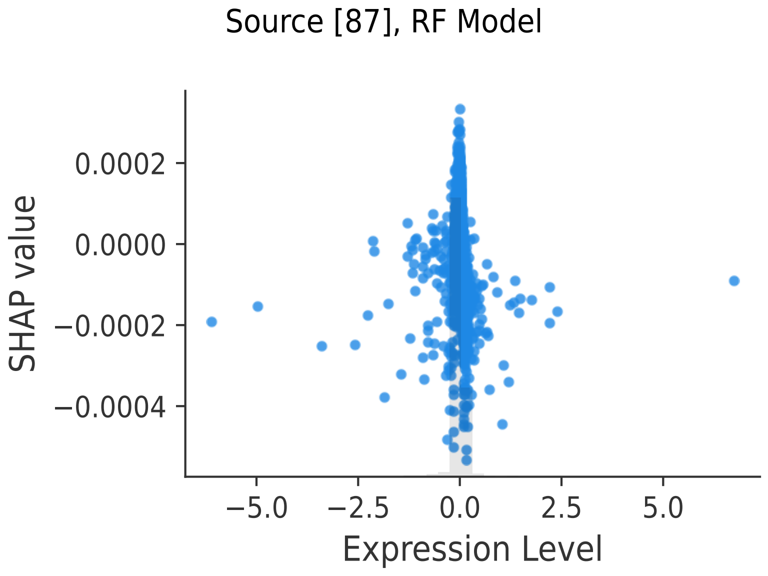
Source [87], RF Model
SHAP value
0.0002
0.0000
−0.0002
−0.0004
−5.0
−2.5
0.0
2.5
5.0
Expression Level

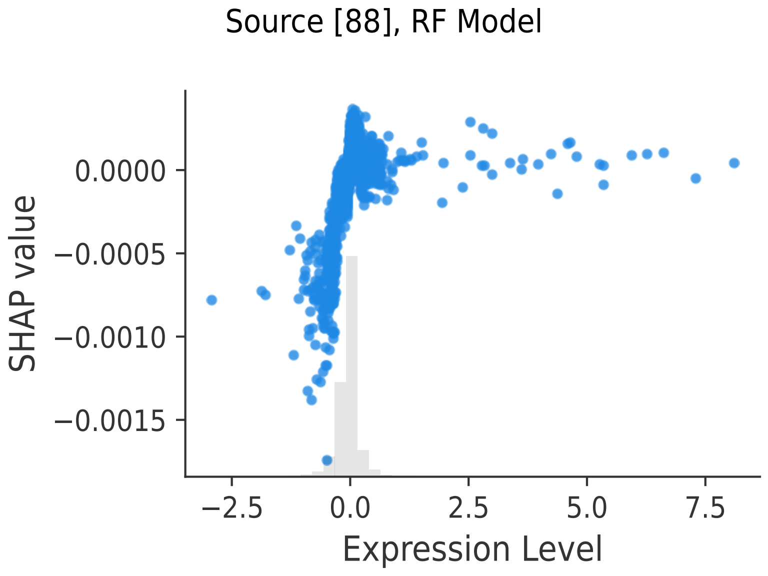

Source [88], RF Model
SHAP value
0.0000
−0.0005
−0.0010
−0.0015
−2.5
0.0
2.5
5.0
7.5
Expression Level

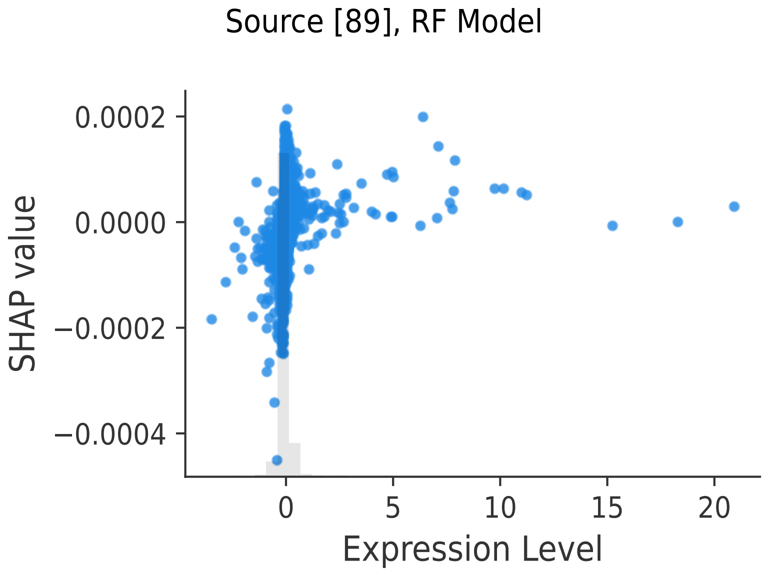
Source [89], RF Model
SHAP value
0.0002
0.0000
−0.0002
−0.0004
0
5
10
15
20
Expression Level

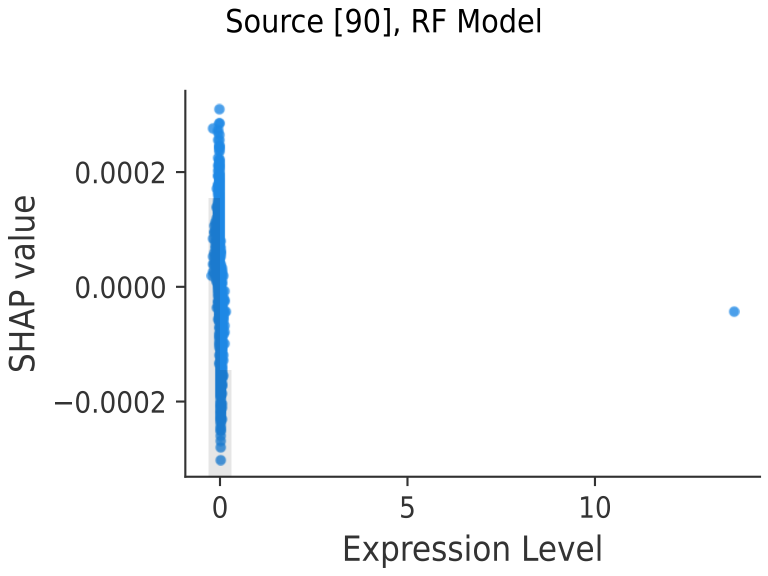
Source [90], RF Model
0.0002
0.0000
−0.0002
SHAP value
0
5
10
Expression Level

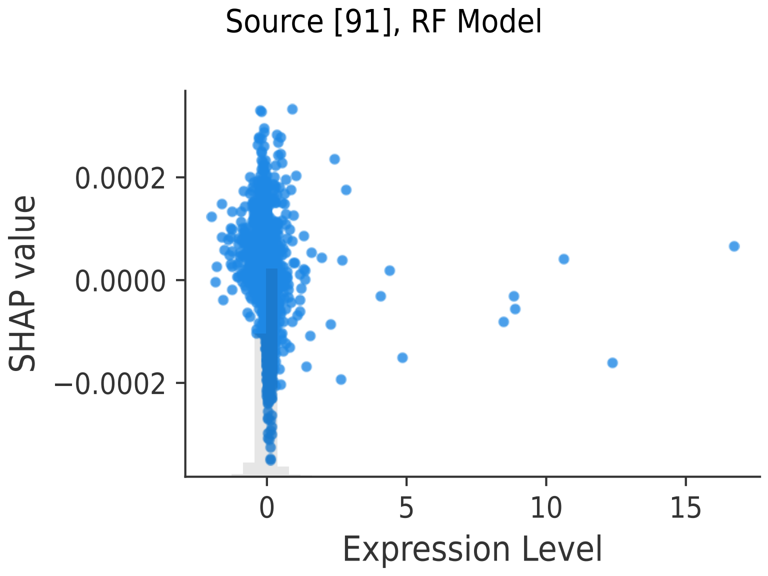
Source [91], RF Model
0.0002
0.0000
−0.0002
SHAP value
0
5
10
15
Expression Level

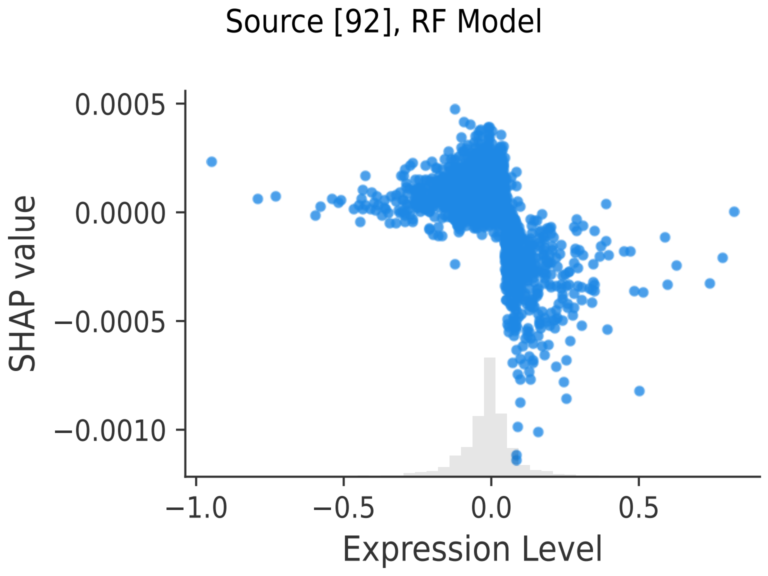

Source [92], RF Model
0.0005
0.0000
−0.0005
−0.0010
SHAP value
−1.0
−0.5
0.0
0.5
Expression Level

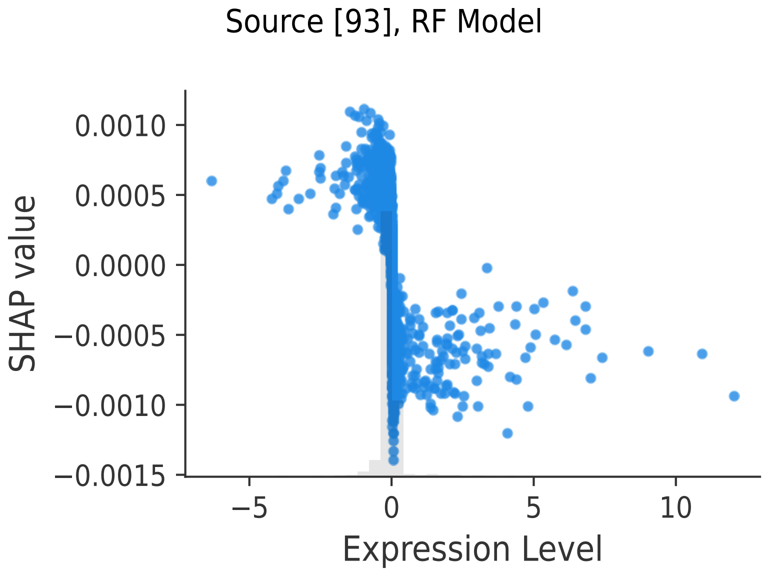
Source [93], RF Model
0.0010
0.0005
0.0000
−0.0005
−0.0010
−0.0015
SHAP value
−5
0
5
10
Expression Level

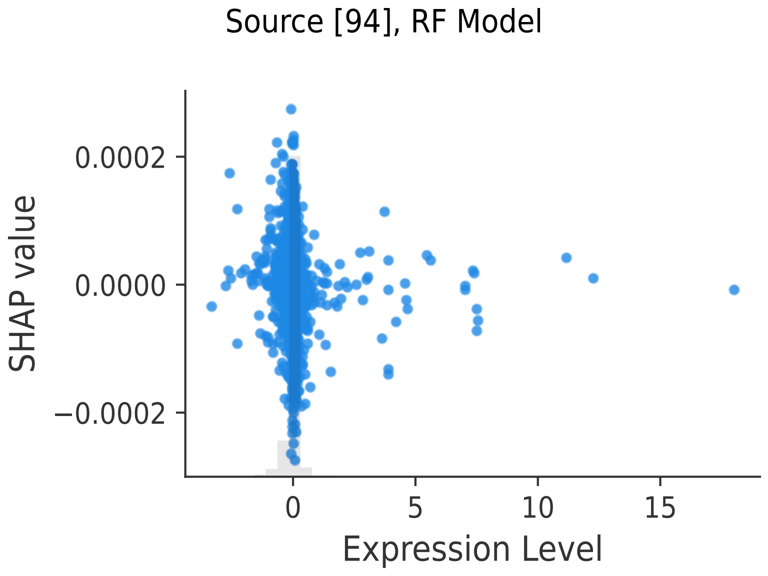

Source [94], RF Model
SHAP value
0.0002
0.0000
−0.0002
0
5
10
15
Expression Level

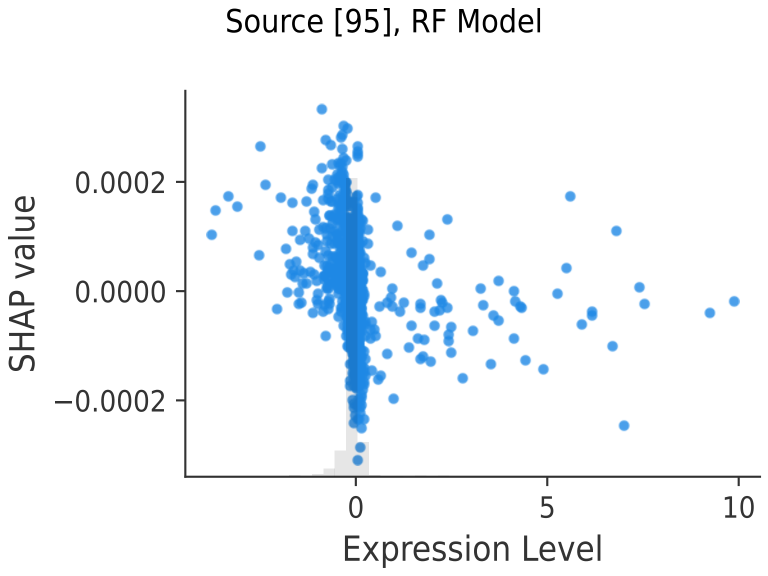
Source [95], RF Model
SHAP value
0.0002
0.0000
−0.0002
0
5
10
Expression Level

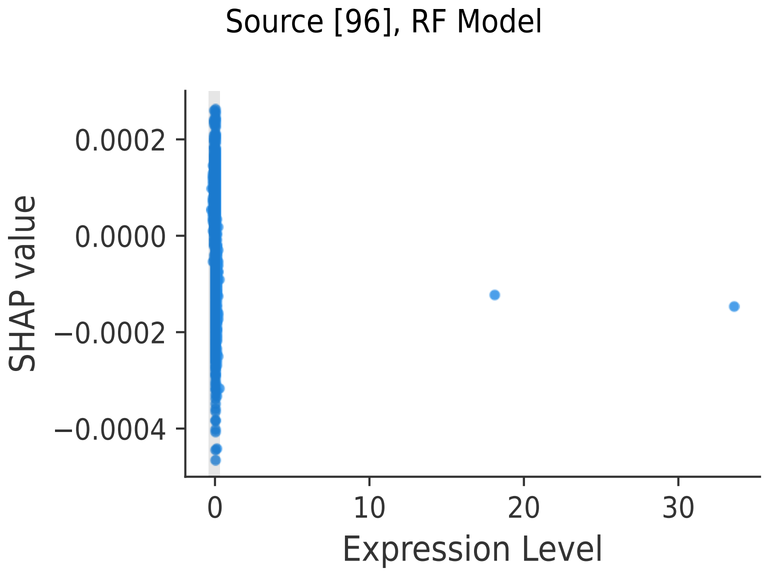

Source [96], RF Model
0.0002
0.0000
−0.0002
−0.0004
SHAP value
0
10
20
30
Expression Level

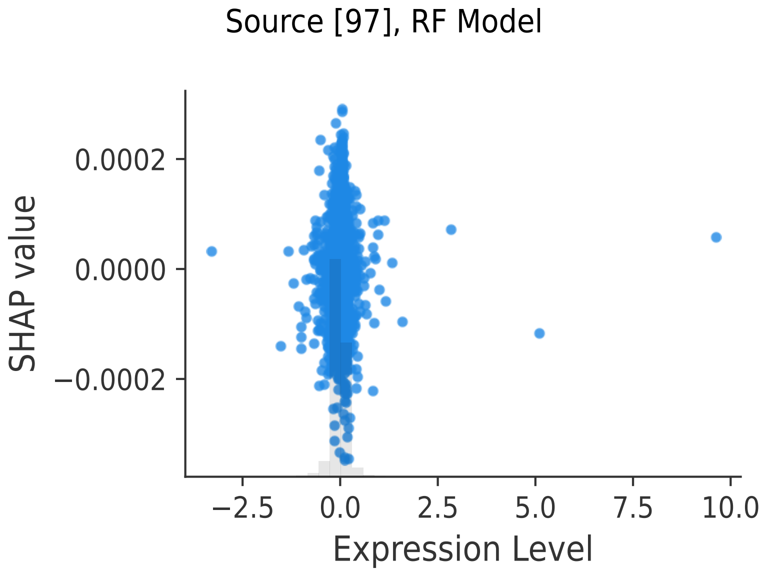
Source [97], RF Model
0.0002
0.0000
−0.0002
SHAP value
−2.5
0.0
2.5
5.0
7.5
10.0
Expression Level

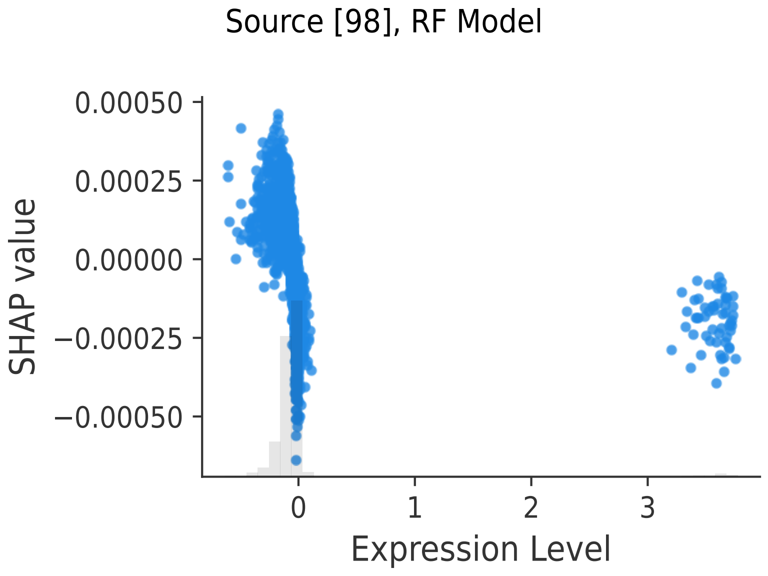

Source [98], RF Model
SHAP value
0.00050
0.00025
0.00000
−0.00025
−0.00050
0
1
2
3
Expression Level

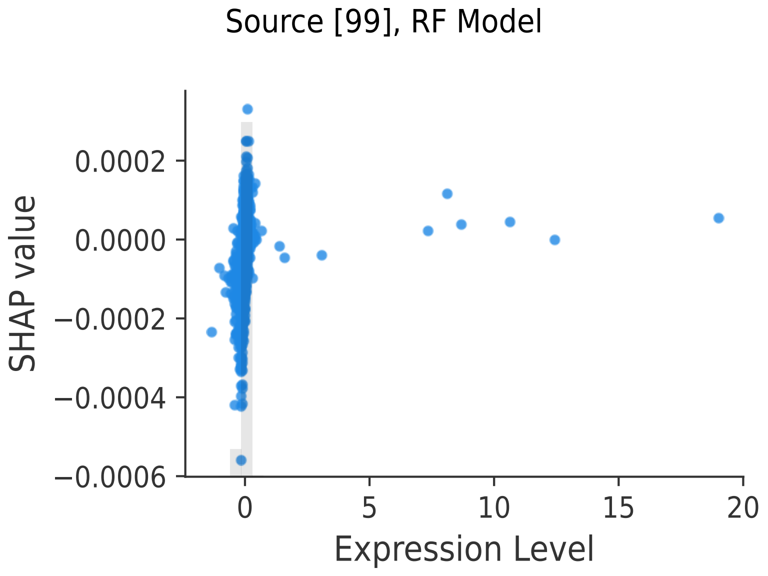
Source [99], RF Model
0.0002
0.0000
−0.0002
−0.0004
−0.0006
SHAP value
0
5
10
15
20
Expression Level

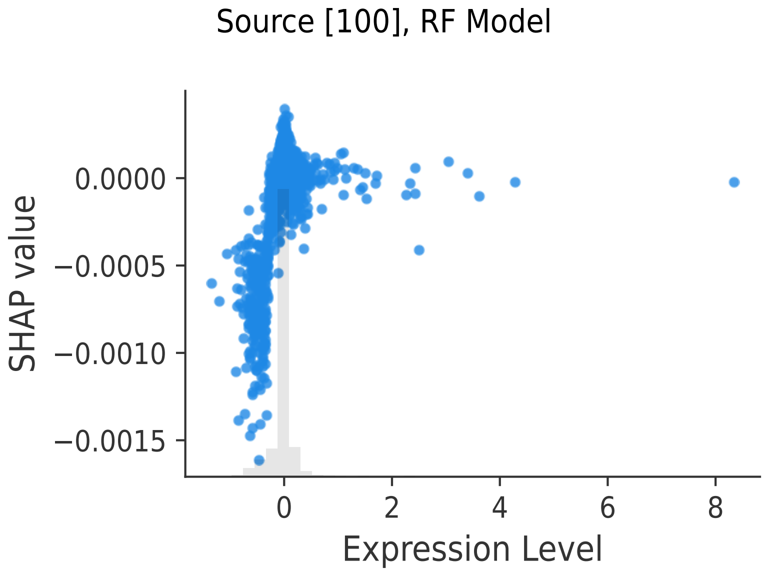
Source [100], RF Model
SHAP value
0.0000
−0.0005
−0.0010
−0.0015
0
2
4
6
8
Expression Level

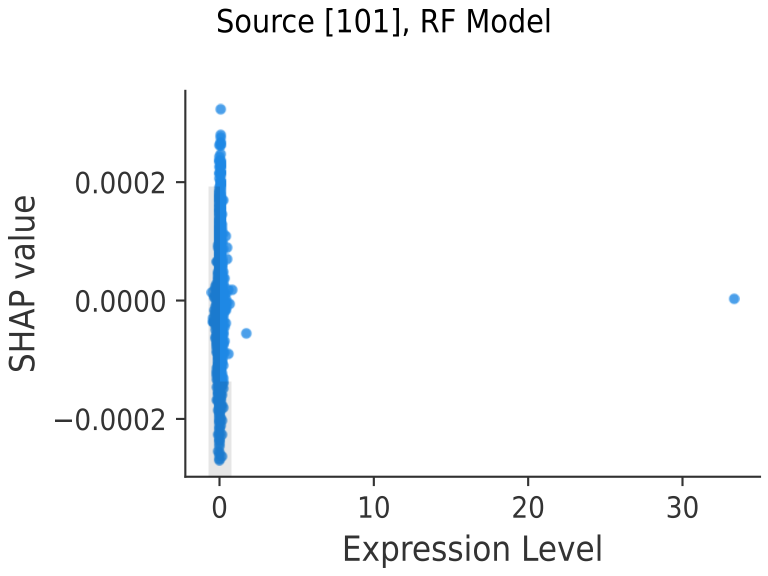
Source [101], RF Model
SHAP value
0.0002
0.0000
−0.0002
0
10
20
30
Expression Level

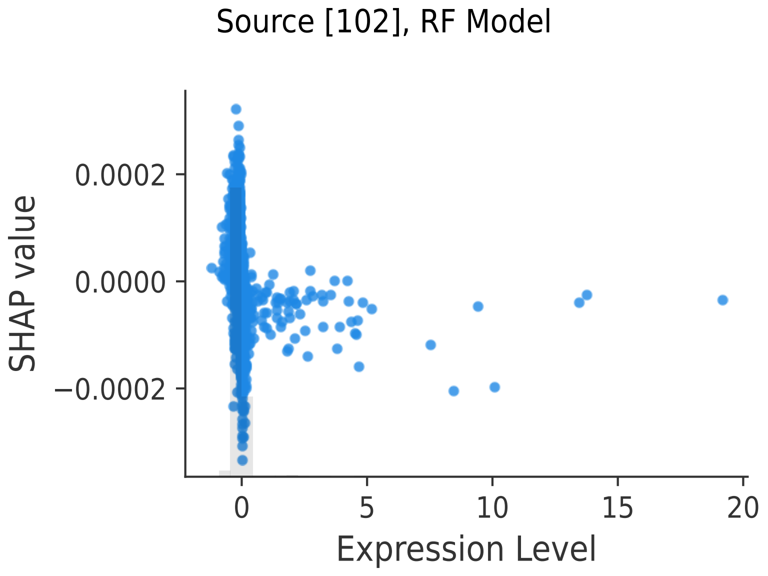
Source [102], RF Model
SHAP value
0.0002
0.0000
−0.0002
0
5
10
15
20
Expression Level

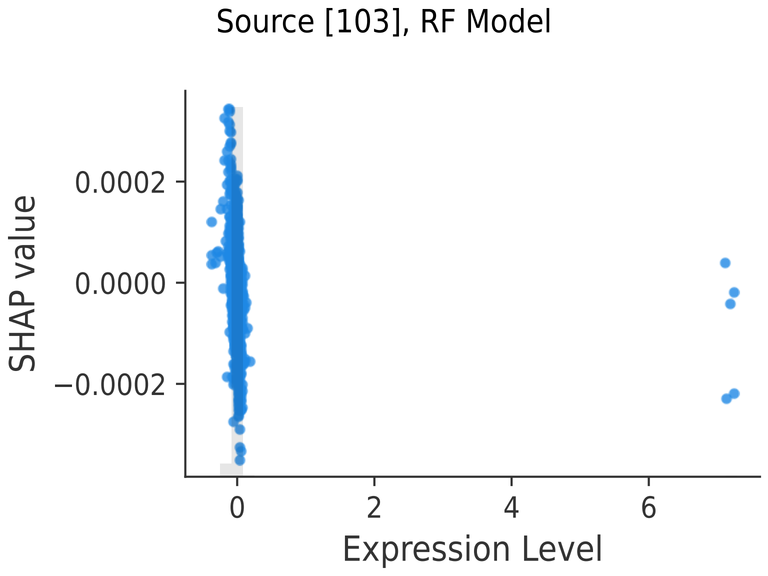

Source [103], RF Model
0.0002
0.0000
−0.0002
SHAP value
0
2
4
6
Expression Level

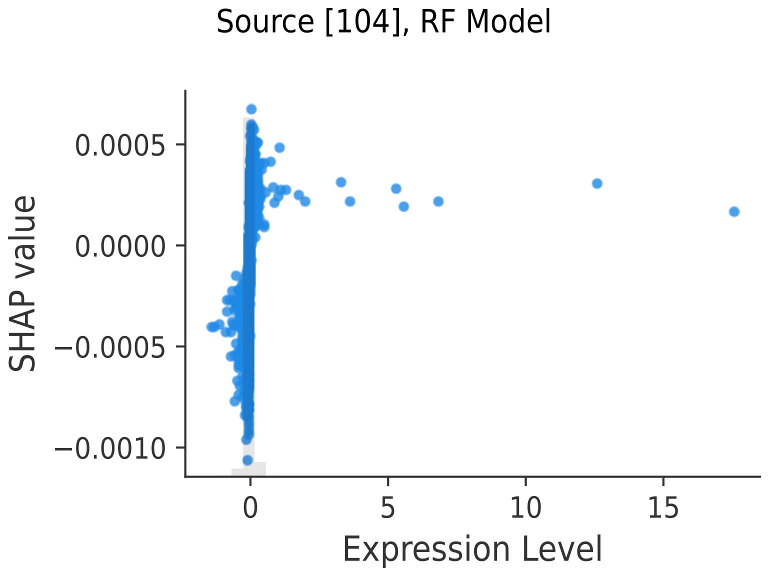
Source [104], RF Model
SHAP value
0.0005
0.0000
−0.0005
−0.0010
0
5
10
15
Expression Level

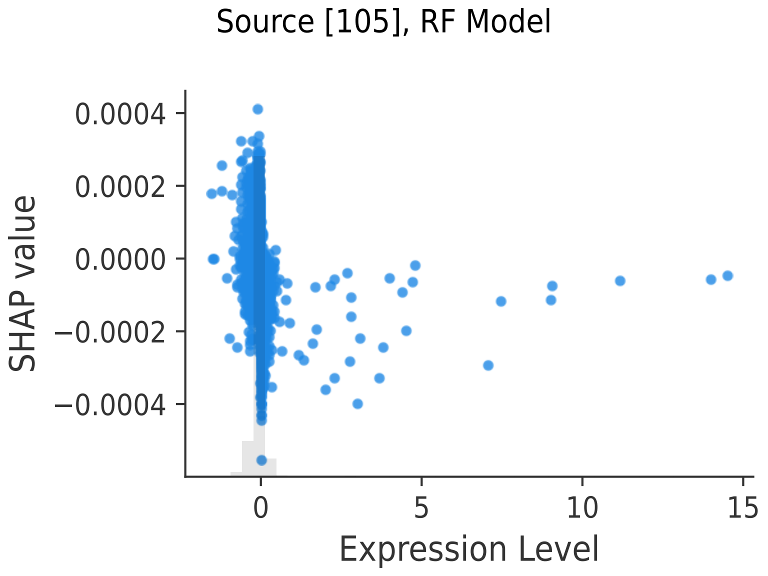
Source [105], RF Model
0.0004
0.0002
0.0000
−0.0002
−0.0004
SHAP value
0
5
10
15
Expression Level

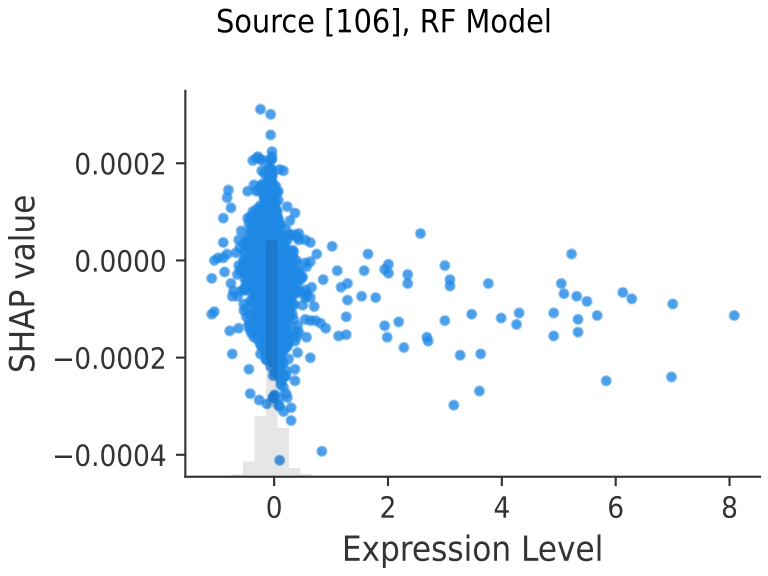

Source [106], RF Model
0.0002
0.0000
−0.0002
−0.0004
SHAP value
0
2
4
6
8
Expression Level

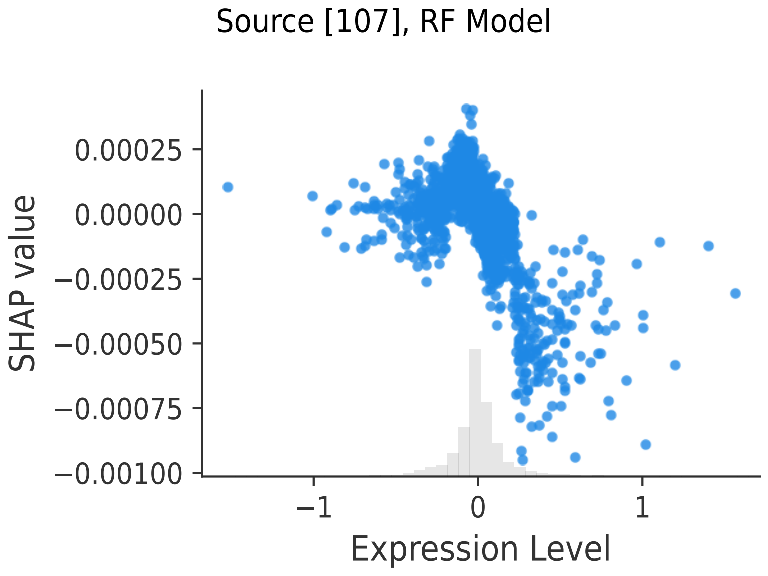

Source [107], RF Model
SHAP value
0.00025
0.00000
−0.00025
−0.00050
−0.00075
−0.00100
−1
0
1
Expression Level

Source [108], RF Model

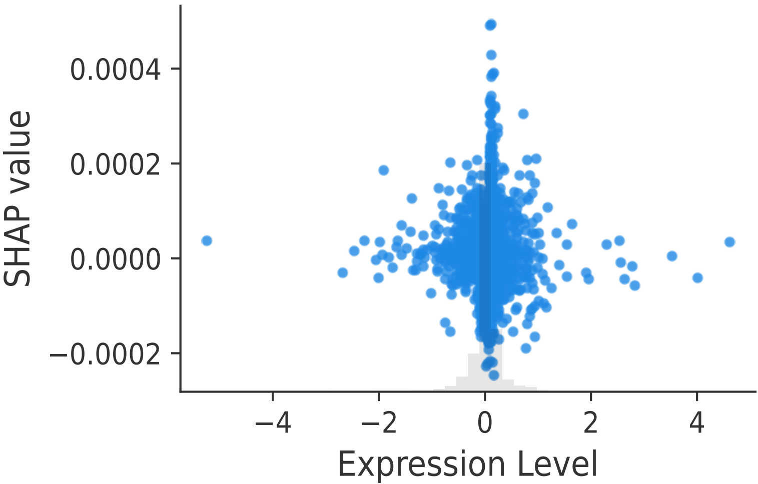

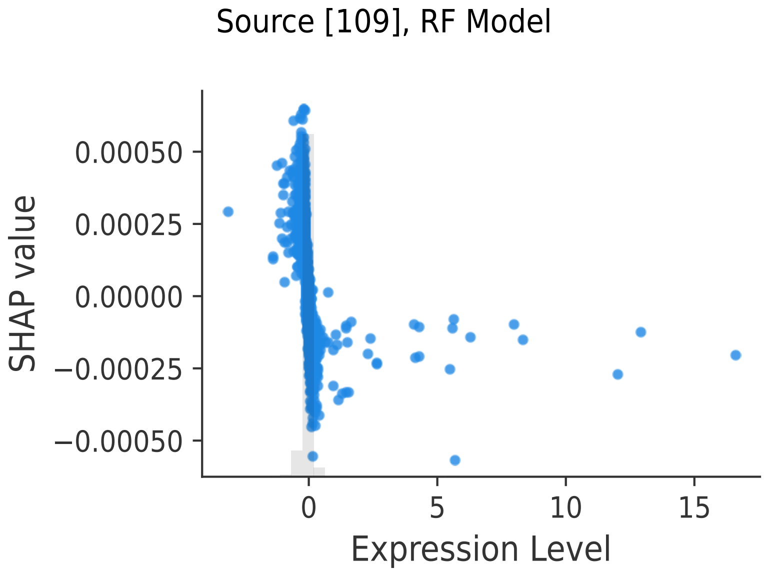

Source [109], RF Model
0.00050
0.00025
0.00000
−0.00025
−0.00050
SHAP value
0
5
10
15
Expression Level

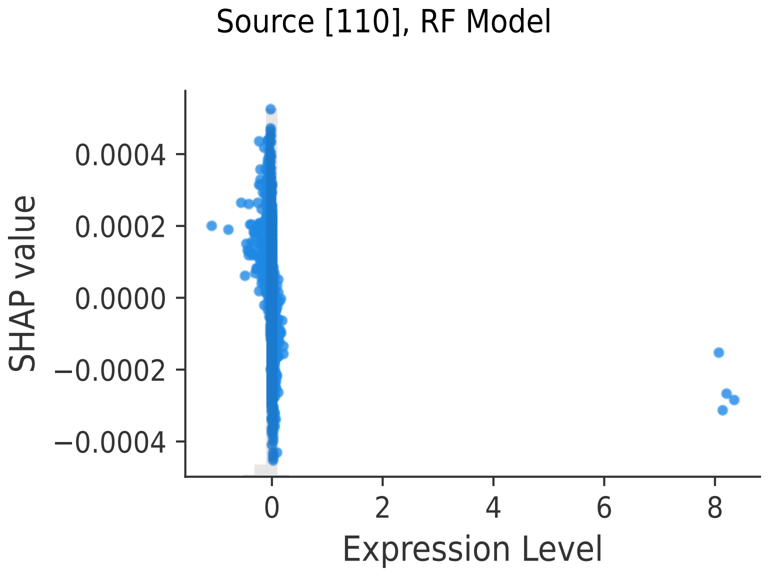
Source [110], RF Model
SHAP value
0.0004
0.0002
0.0000
−0.0002
−0.0004
0
2
4
6
8
Expression Level

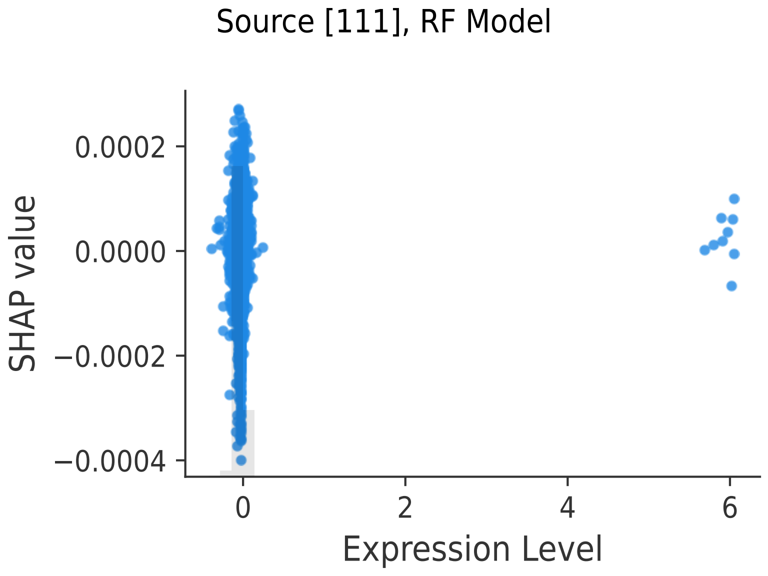
Source [111], RF Model
0.0002
0.0000
−0.0002
−0.0004
SHAP value
0
2
4
6
Expression Level

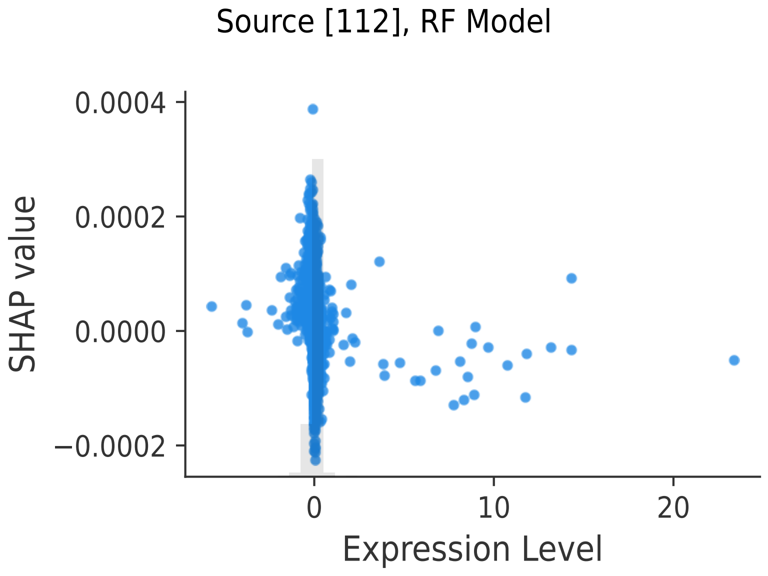
Source [112], RF Model
0.0004
0.0002
0.0000
−0.0002
SHAP value
0
10
20
Expression Level

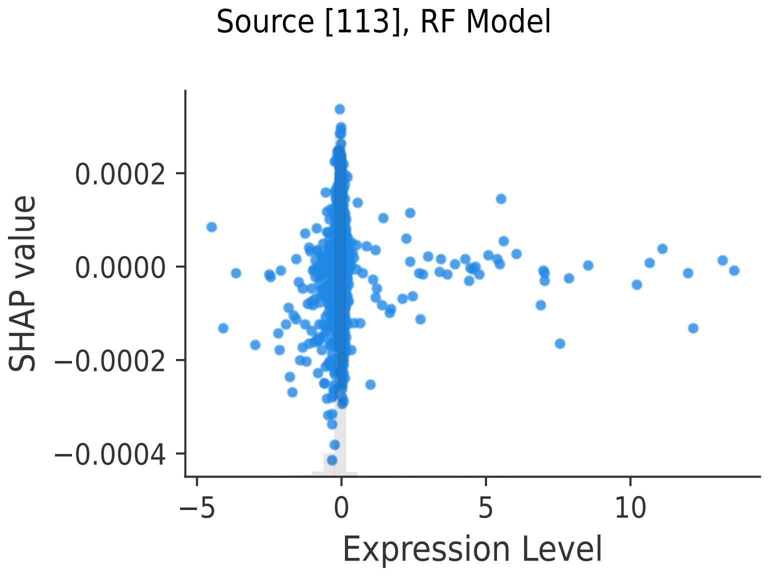

Source [113], RF Model
SHAP value
0.0002
0.0000
−0.0002
−0.0004
−5
0
5
10
Expression Level

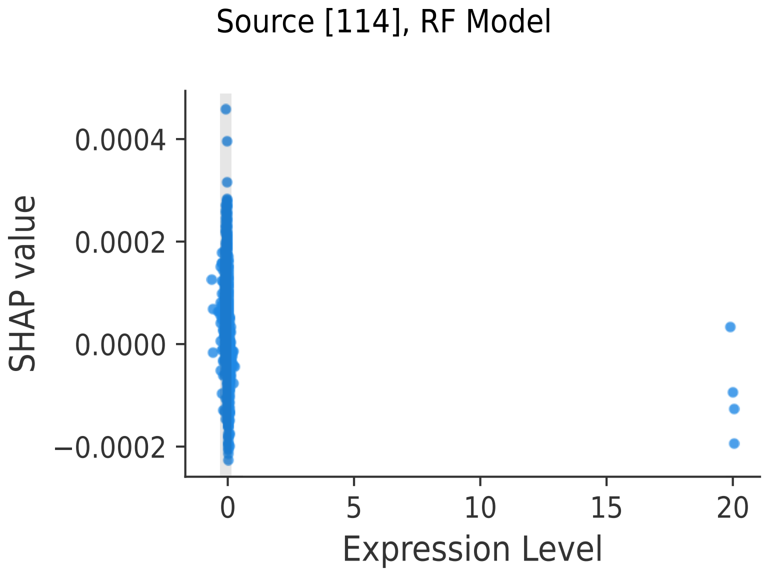
Source [114], RF Model
0.0004
0.0002
0.0000
−0.0002
SHAP value
0
5
10
15
20
Expression Level

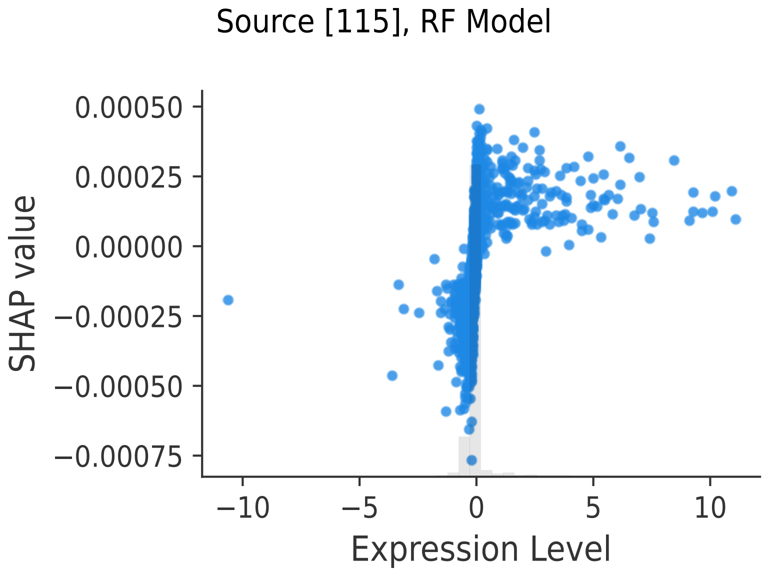
Source [115], RF Model
SHAP value
0.00050
0.00025
0.00000
−0.00025
−0.00050
−0.00075
−10
−5
0
5
10
Expression Level

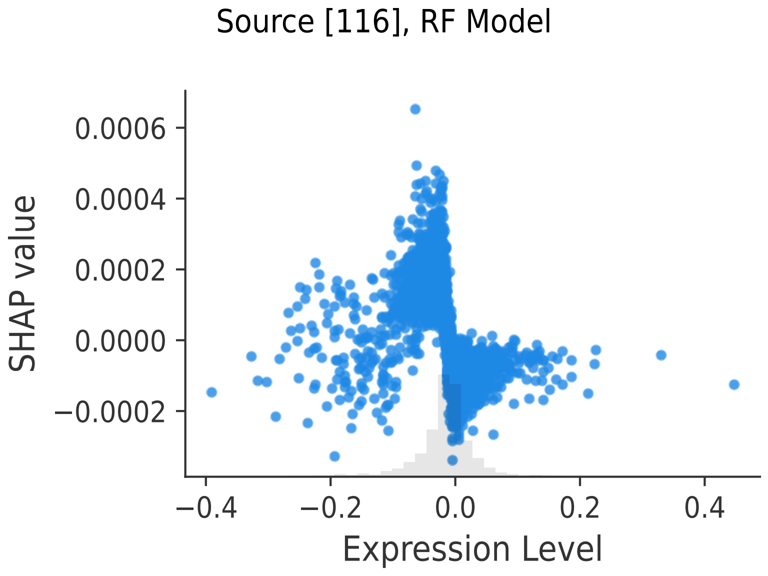
Source [116], RF Model
SHAP value
0.0006
0.0004
0.0002
0.0000
−0.0002
−0.4
−0.2
0.0
0.2
0.4
Expression Level

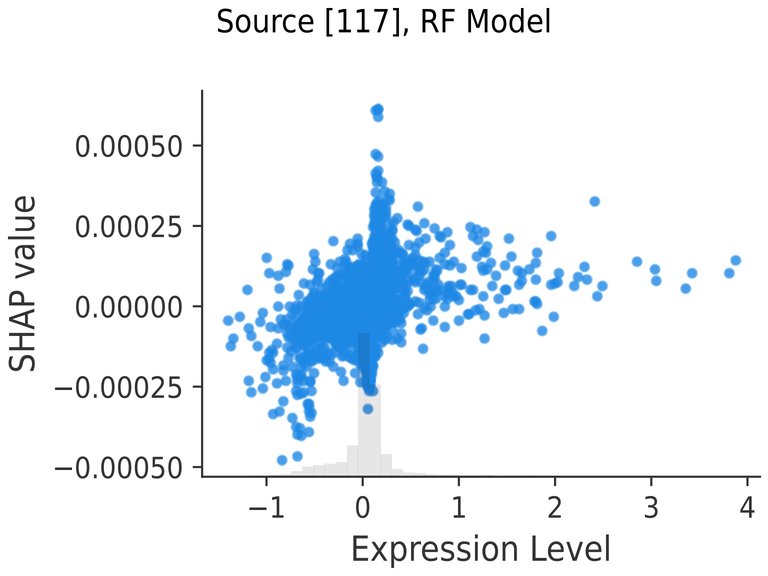

Source [117], RF Model
SHAP value
0.00050
0.00025
0.00000
−0.00025
−0.00050
−1
0
1
2
3
4
Expression Level

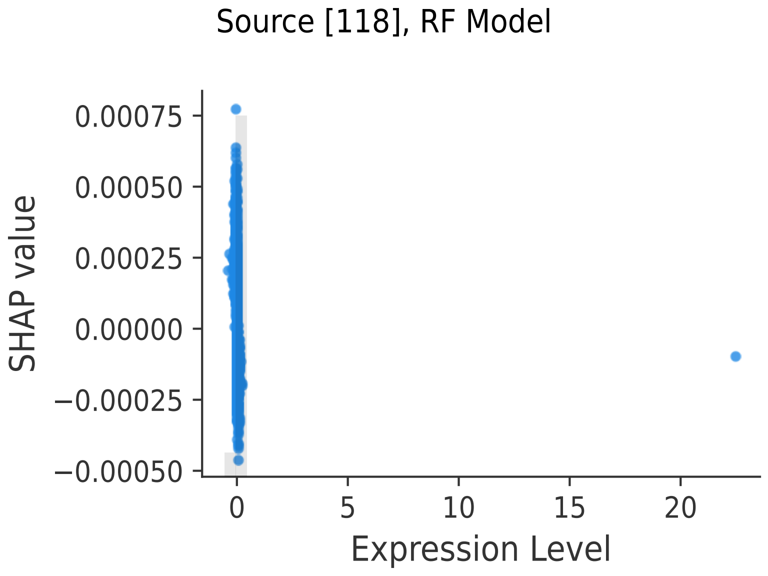

Source [118], RF Model
0.00075
0.00050
0.00025
0.00000
−0.00025
−0.00050
SHAP value
0
5
10
15
20
Expression Level

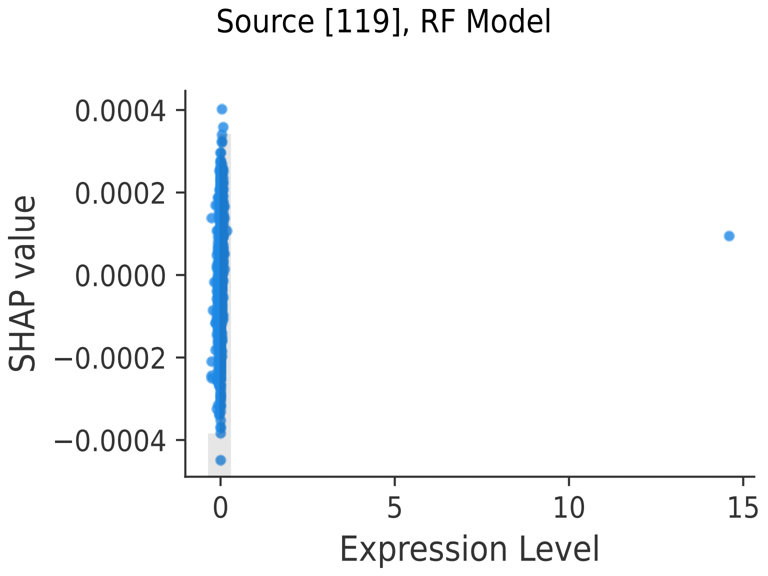

Source [119], RF Model
0.0004
0.0002
0.0000
−0.0002
−0.0004
SHAP value
0
5
10
15
Expression Level

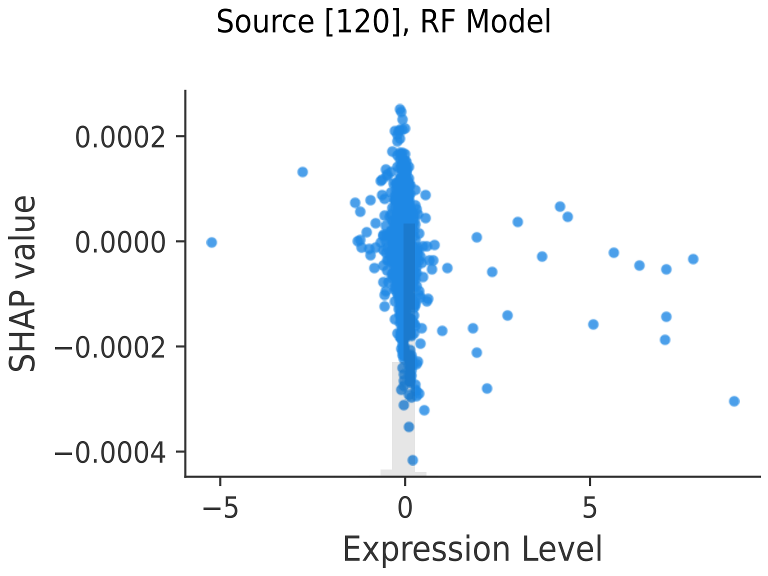
Source [120], RF Model
0.0002
0.0000
−0.0002
−0.0004
SHAP value
−5
0
5
Expression Level

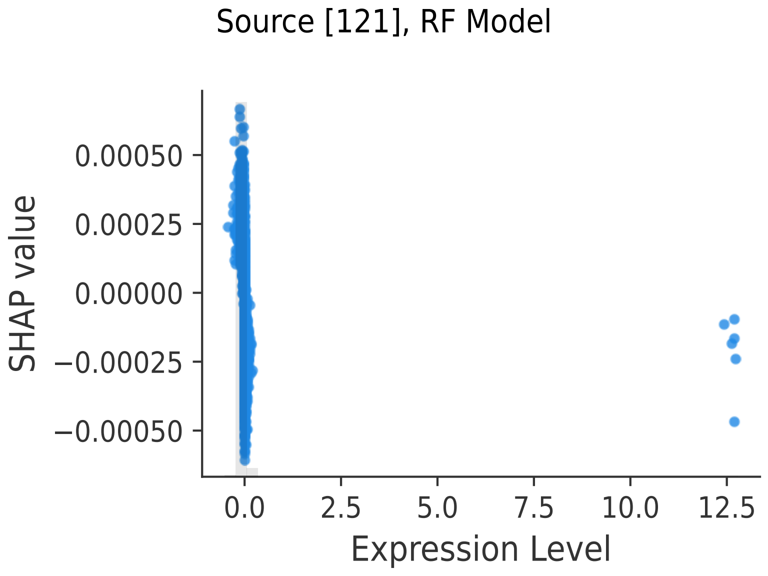

Source [121], RF Model
SHAP value
0.00050
0.00025
0.00000
−0.00025
−0.00050
0.0
2.5
5.0
7.5
10.0
12.5
Expression Level

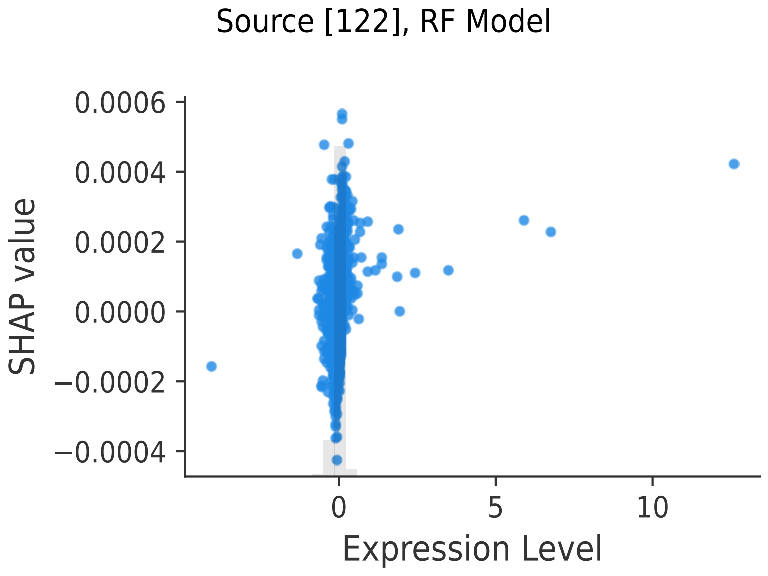
Source [122], RF Model
SHAP value
0.0006
0.0004
0.0002
0.0000
−0.0002
−0.0004
0
5
10
Expression Level

Source [123], RF Model

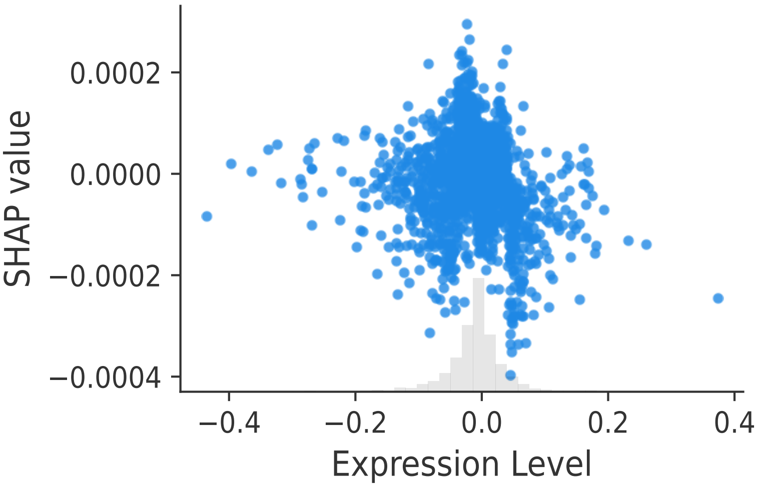

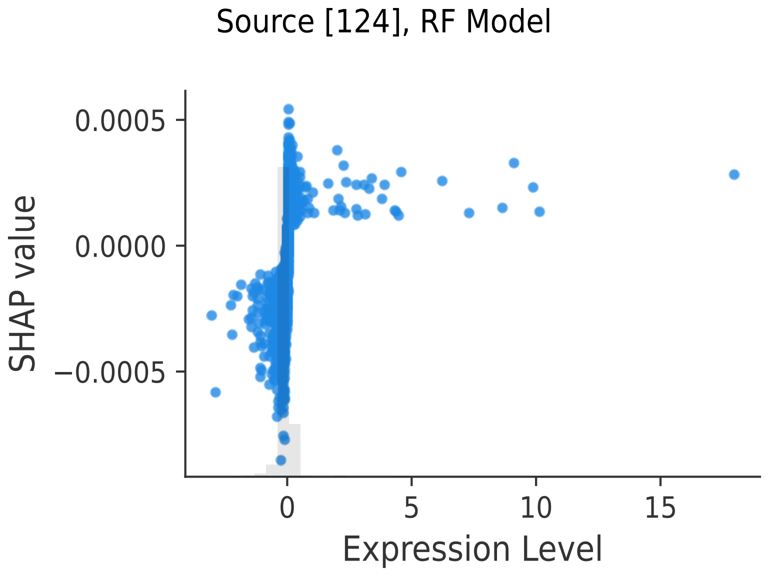

Source [124], RF Model
0.0005
0.0000
−0.0005
SHAP value
0
5
10
15
Expression Level

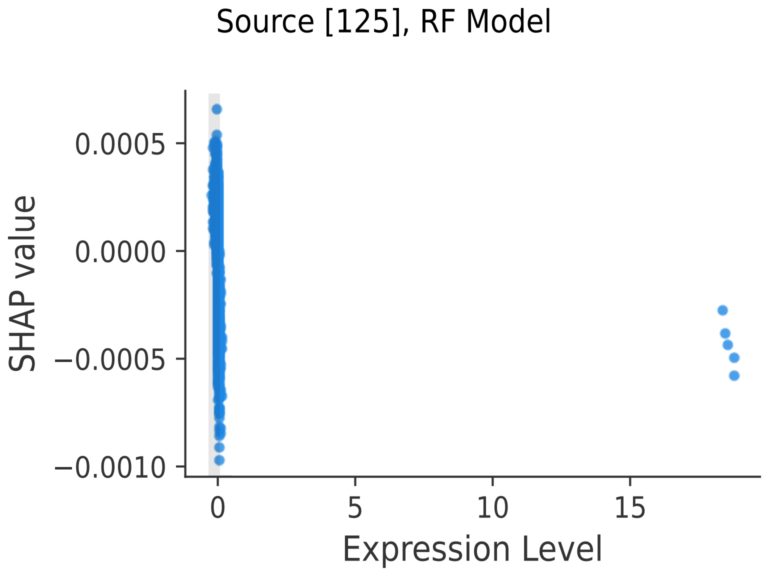

Source [125], RF Model
0.0005
0.0000
−0.0005
−0.0010
SHAP value
0
5
10
15
Expression Level

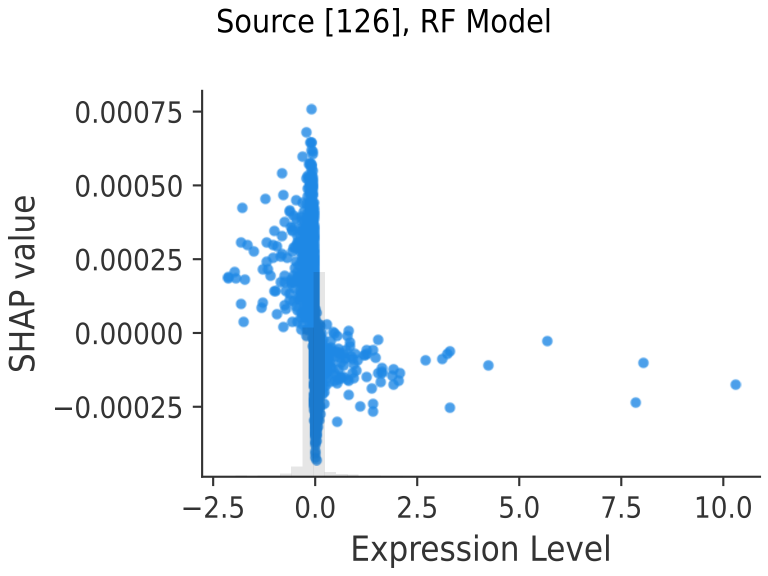
Source [126], RF Model
SHAP value
0.00075
0.00050
0.00025
0.00000
−0.00025
−2.5
0.0
2.5
5.0
7.5
10.0
Expression Level

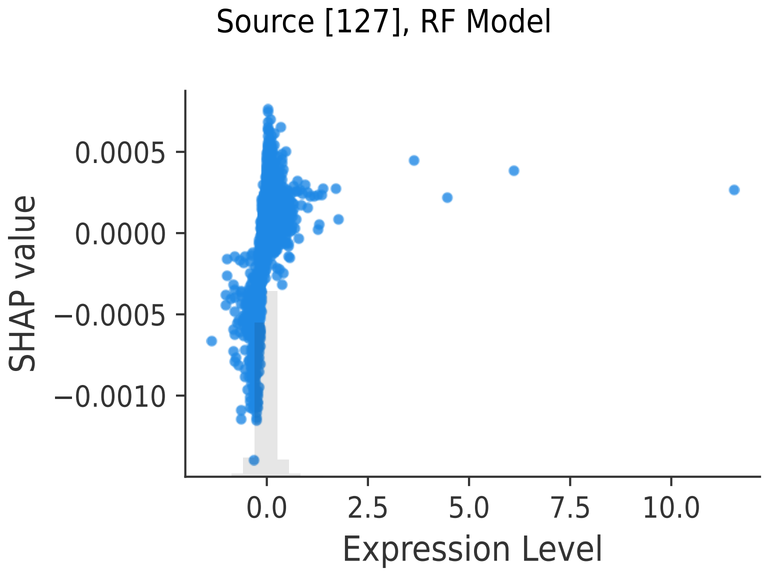
Source [127], RF Model
SHAP value
0.0005
0.0000
−0.0005
−0.0010
0.0
2.5
5.0
7.5
10.0
Expression Level

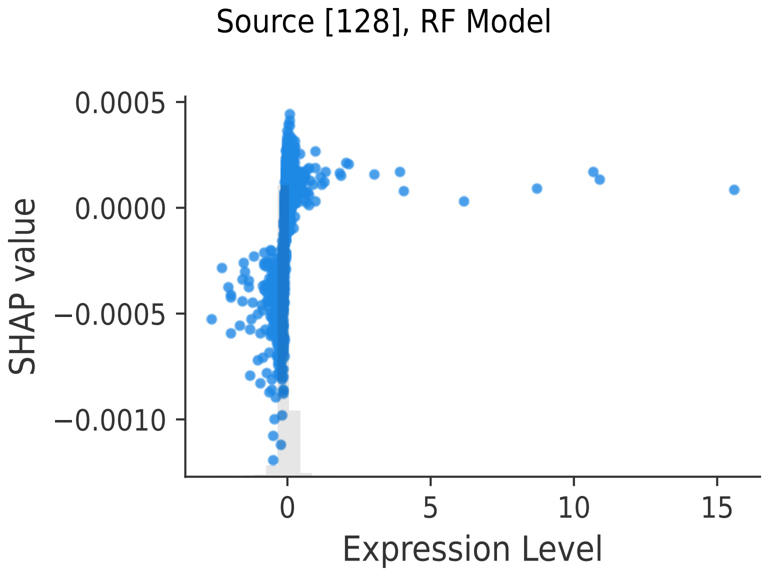
Source [128], RF Model
SHAP value
0.0005
0.0000
−0.0005
−0.0010
0
5
10
15
Expression Level

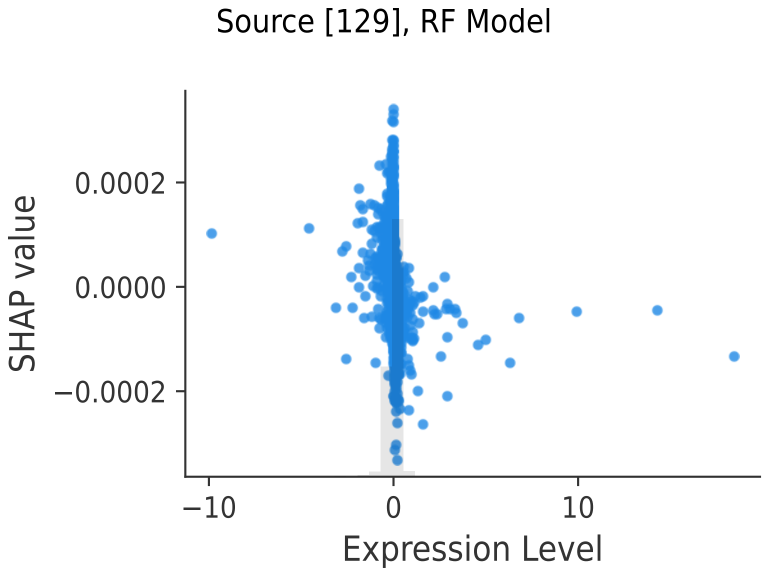
Source [129], RF Model
SHAP value
0.0002
0.0000
−0.0002
−10
0
10
Expression Level

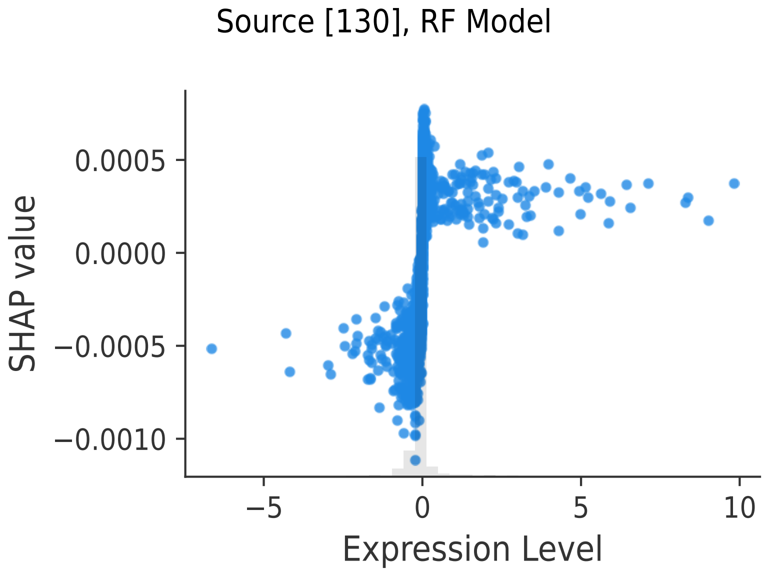
Source [130], RF Model
SHAP value
0.0005
0.0000
−0.0005
−0.0010
−5
0
5
10
Expression Level

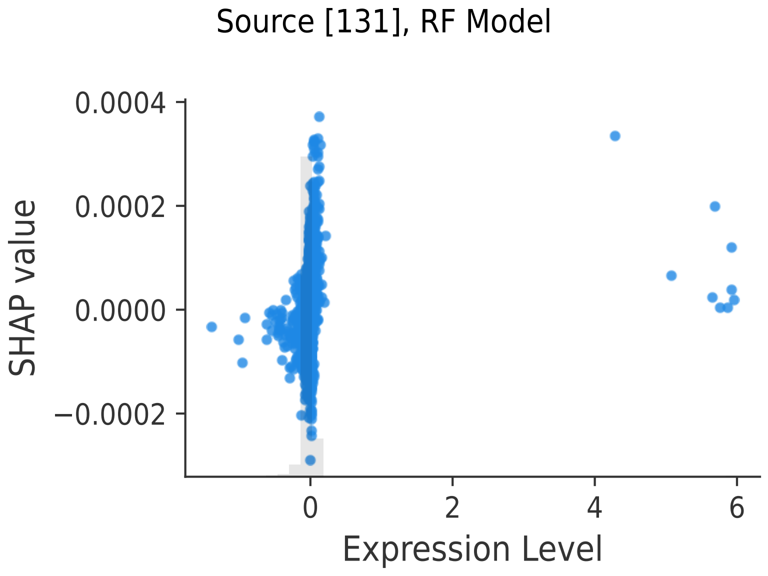
Source [131], RF Model
SHAP value
0.0004
0.0002
0.0000
−0.0002
0
2
4
6
Expression Level

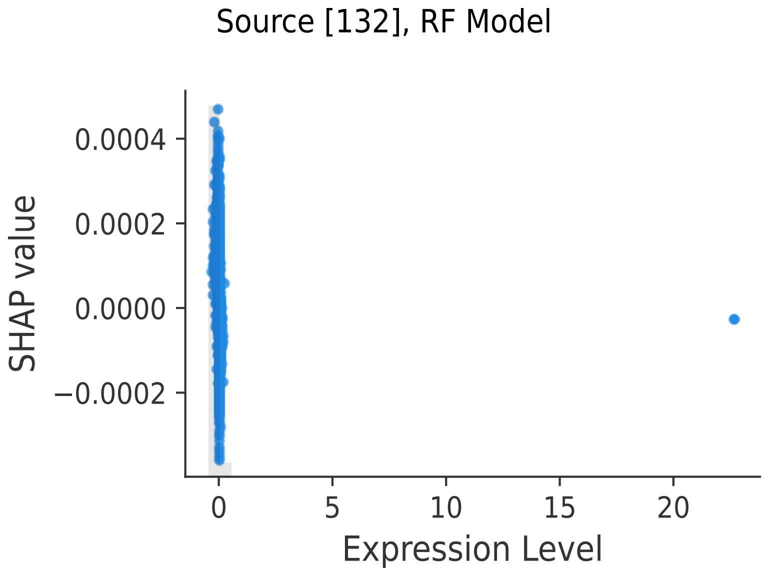

Source [132], RF Model
0.0004
0.0002
0.0000
−0.0002
SHAP value
0
5
10
15
20
Expression Level

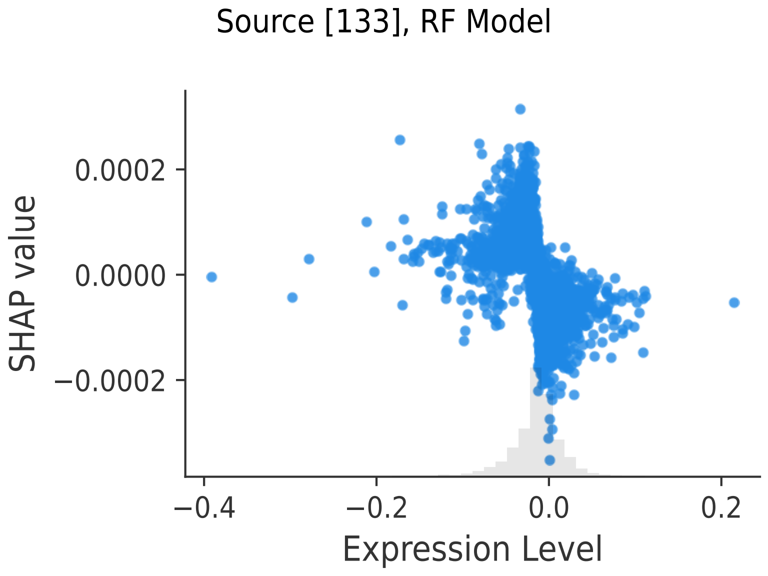
Source [133], RF Model
SHAP value
0.0002
0.0000
−0.0002
−0.4
−0.2
0.0
0.2
Expression Level

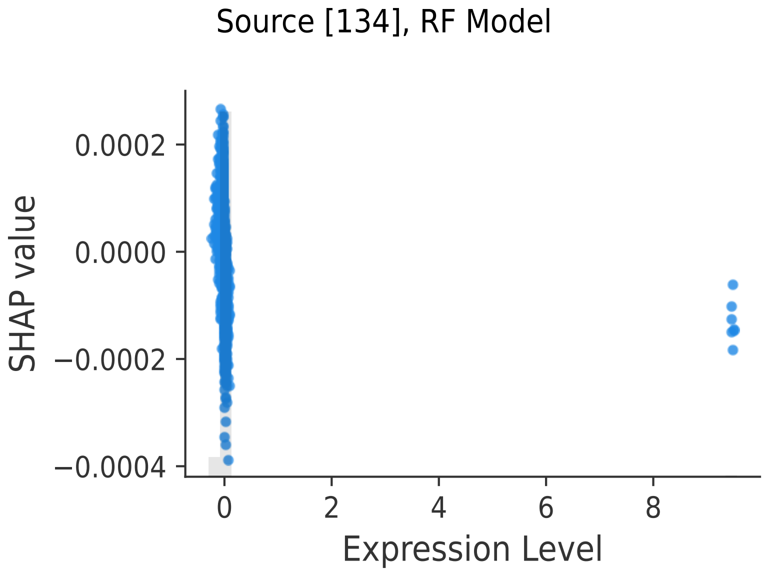
Source [134], RF Model
SHAP value
0.0002
0.0000
−0.0002
−0.0004
0
2
4
6
8
Expression Level

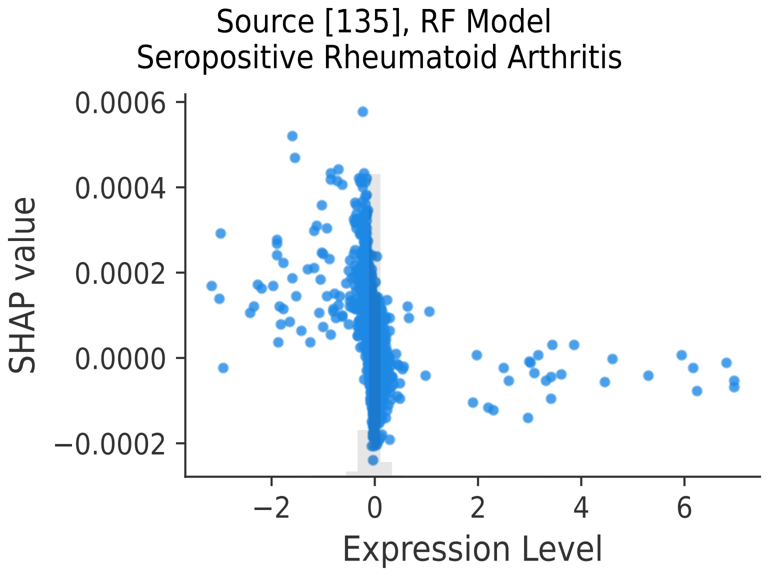
Source [135], RF Model
Seropositive Rheumatoid Arthritis
0.0006
0.0004
0.0002
0.0000
−0.0002
SHAP value
−2
0
2
4
6
Expression Level

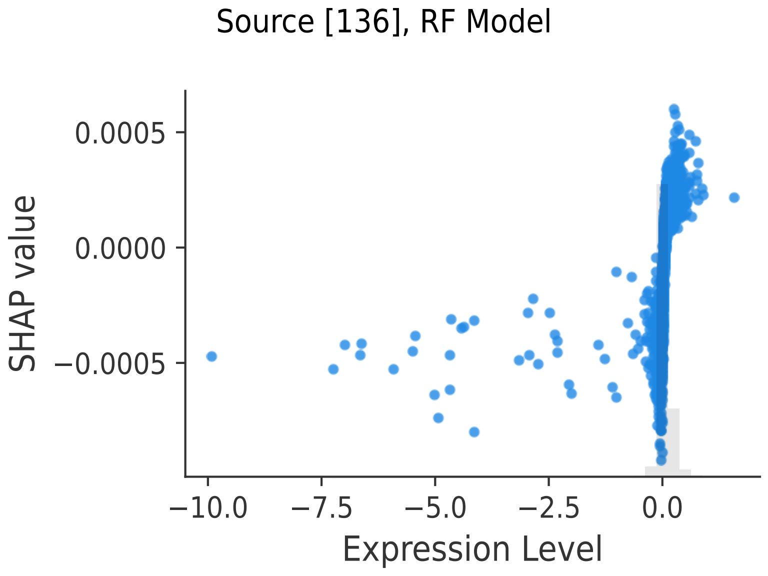
Source [136], RF Model
0.0005
0.0000
−0.0005
SHAP value
−10.0
−7.5
−5.0
−2.5
0.0
Expression Level

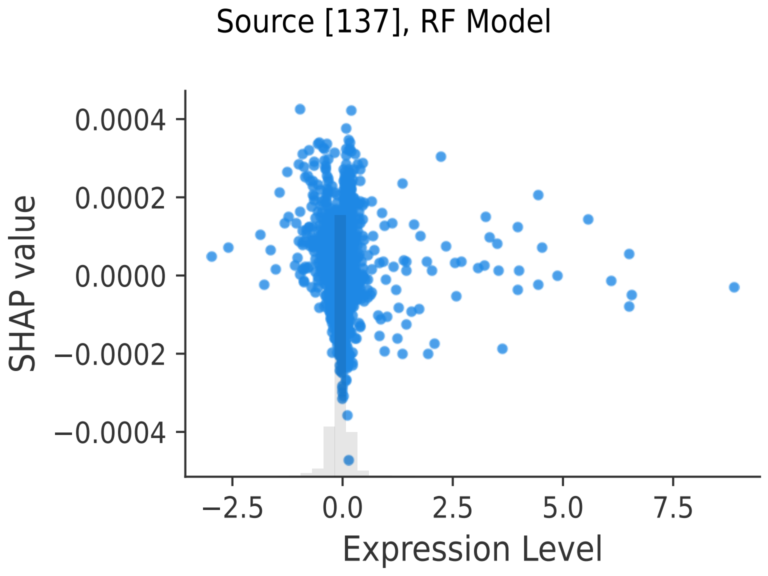
Source [137], RF Model
0.0004
0.0002
0.0000
−0.0002
−0.0004
SHAP value
−2.5
0.0
2.5
5.0
7.5
Expression Level

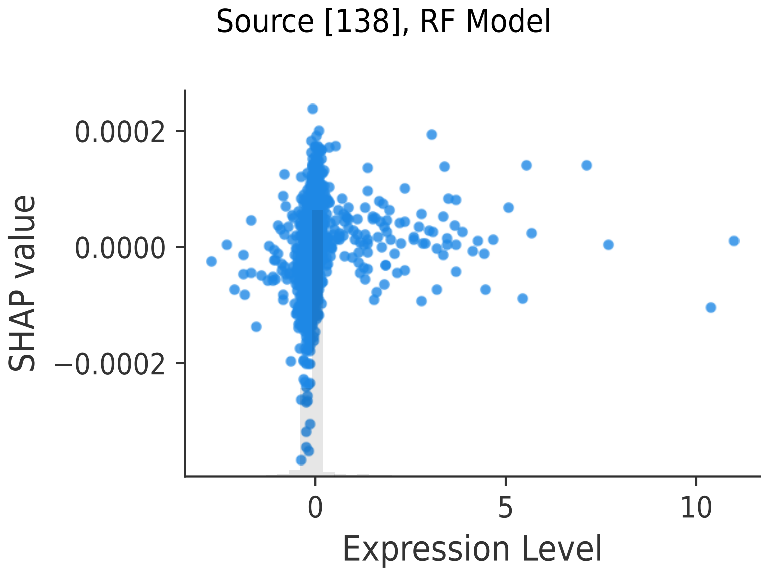
Source [138], RF Model
0.0002
0.0000
−0.0002
SHAP value
0
5
10
Expression Level

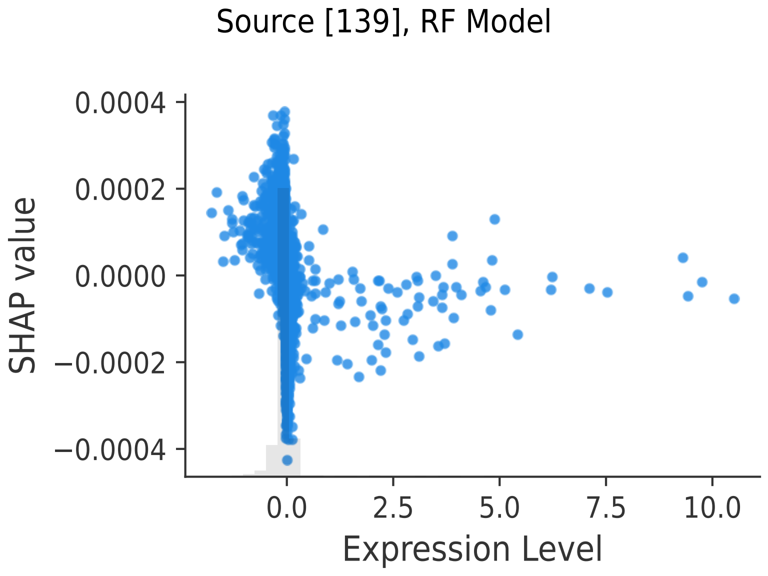
Source [139], RF Model
0.0004
0.0002
0.0000
−0.0002
−0.0004
SHAP value
0.0
2.5
5.0
7.5
10.0
Expression Level

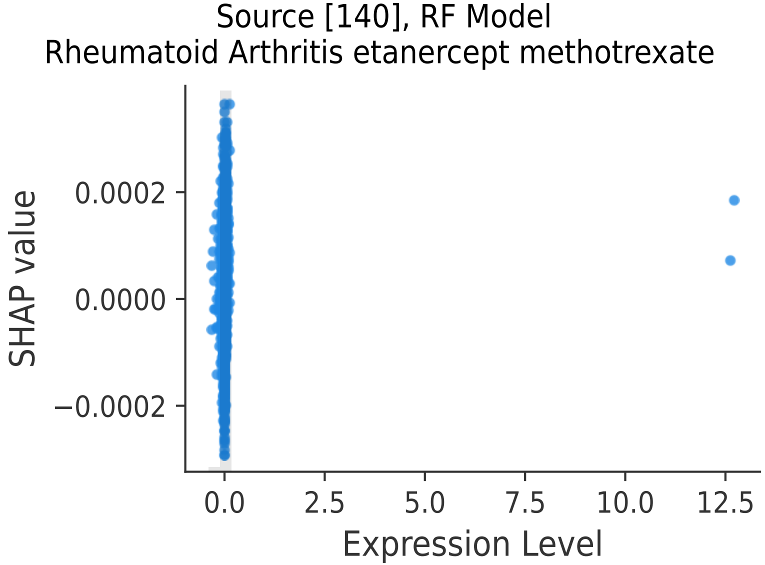
Source [140], RF Model
Rheumatoid Arthritis etanercept methotrexate
SHAP value
0.0002
0.0000
−0.0002
0.0
2.5
5.0
7.5
10.0
12.5
Expression Level

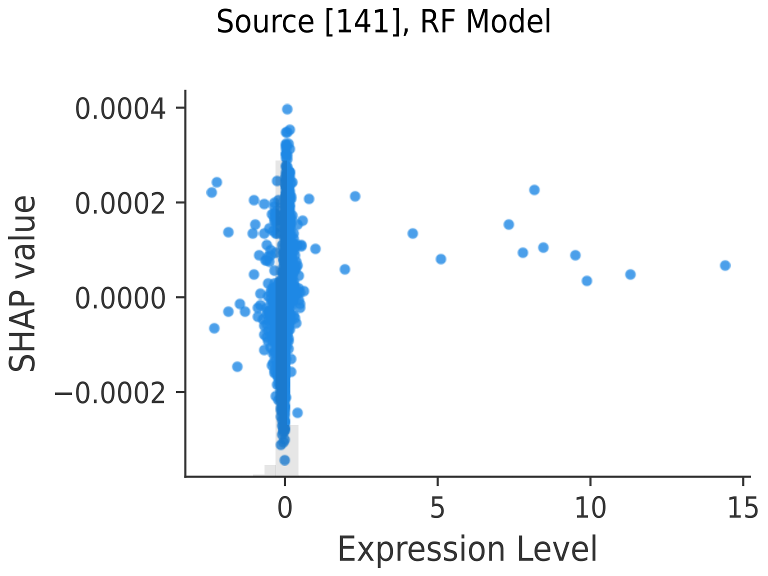

Source [141], RF Model
0.0004
0.0002
0.0000
−0.0002
SHAP value
0
5
10
15
Expression Level

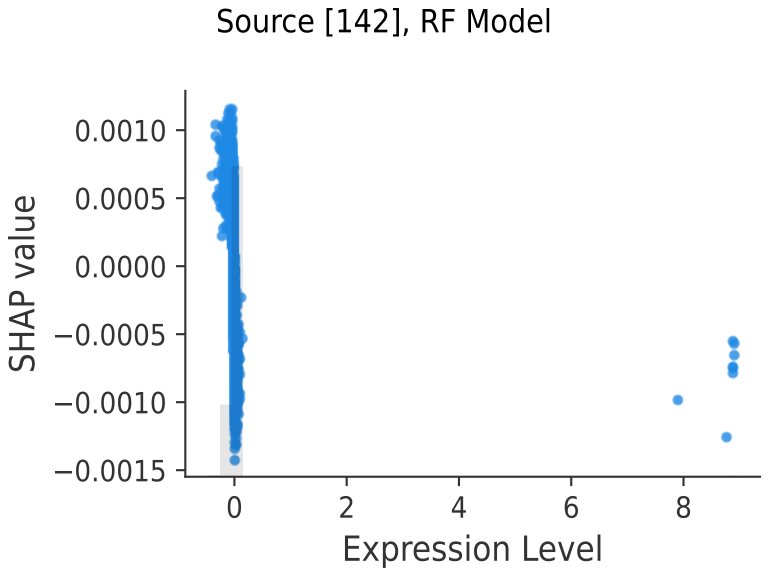

Source [142], RF Model
SHAP value
0.0010
0.0005
0.0000
−0.0005
−0.0010
−0.0015
0
2
4
6
8
Expression Level

Source [143], RF Model

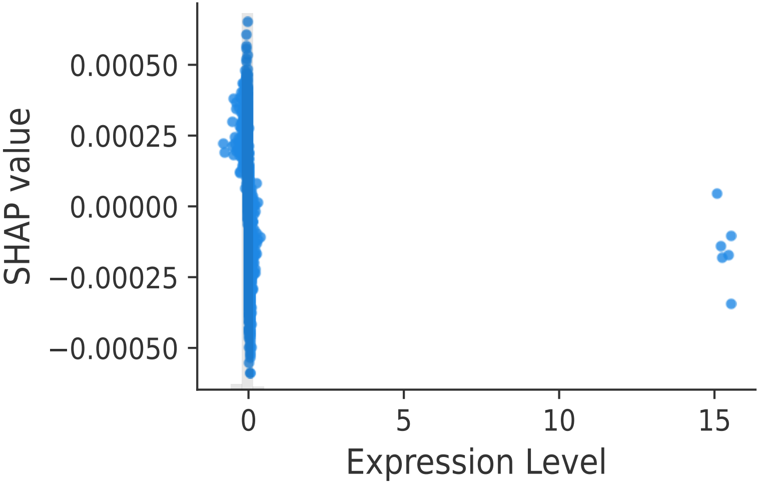

Source [144], RF Model

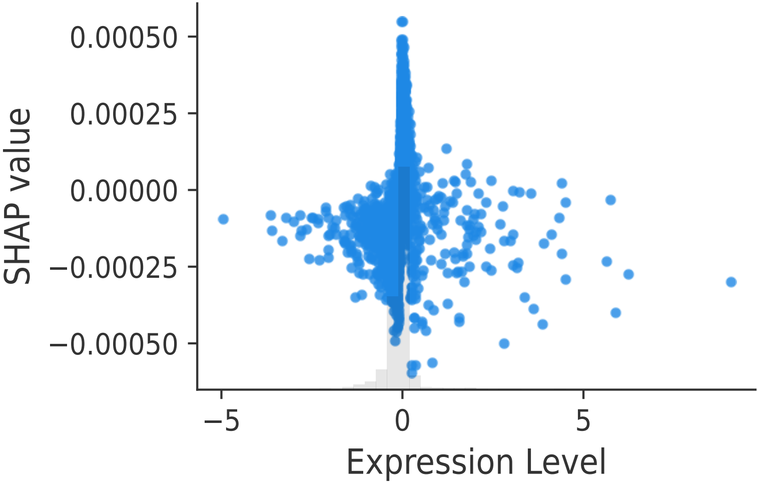

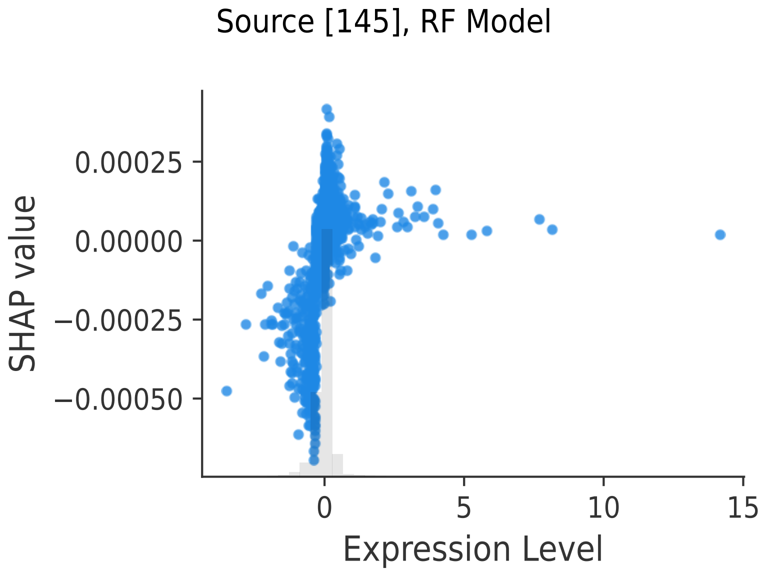
Source [145], RF Model
0.00025
0.00000
−0.00025
−0.00050
SHAP value
0
5
10
15
Expression Level

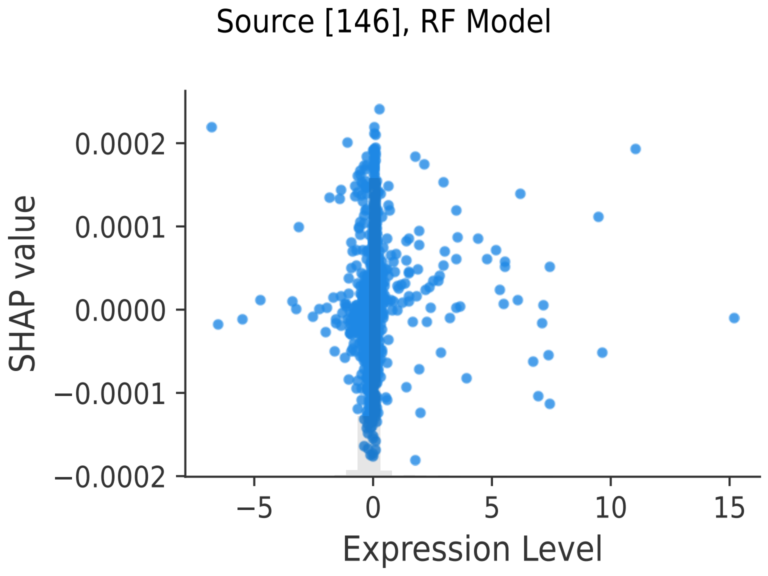
Source [146], RF Model
SHAP value
0.0002
0.0001
0.0000
−0.0001
−0.0002
−5
0
5
10
15
Expression Level

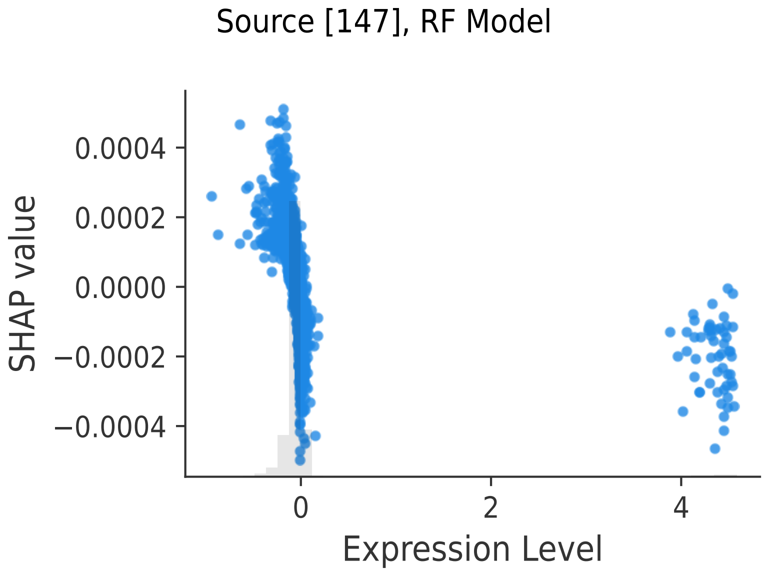
Source [147], RF Model
0.0004
0.0002
0.0000
−0.0002
−0.0004
SHAP value
0
2
4
Expression Level

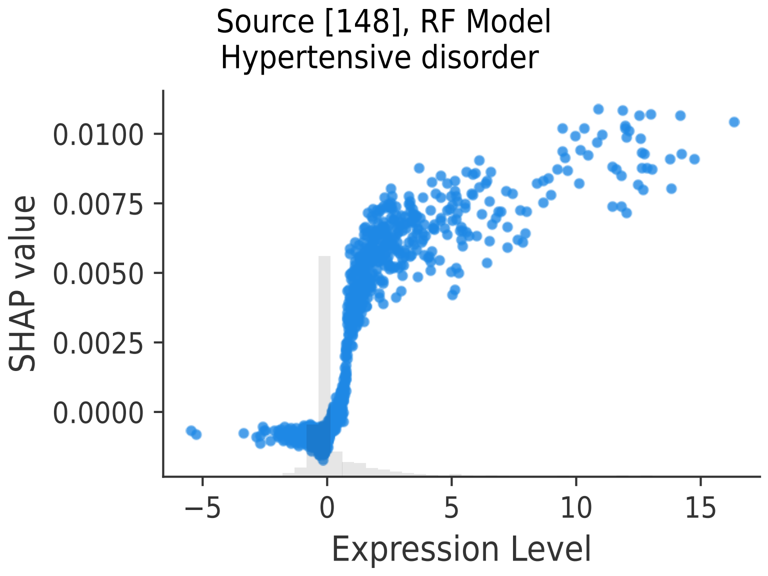
Source [148], RF Model
Hypertensive disorder
SHAP value
0.0100
0.0075
0.0050
0.0025
0.0000
−5
0
5
10
15
Expression Level

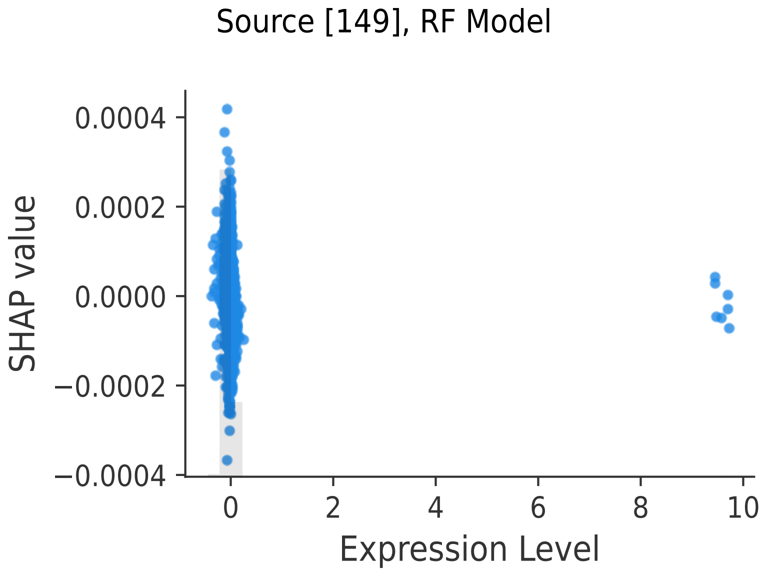

Source [149], RF Model
SHAP value
0.0004
0.0002
0.0000
−0.0002
−0.0004
0
2
4
6
8
10
Expression Level

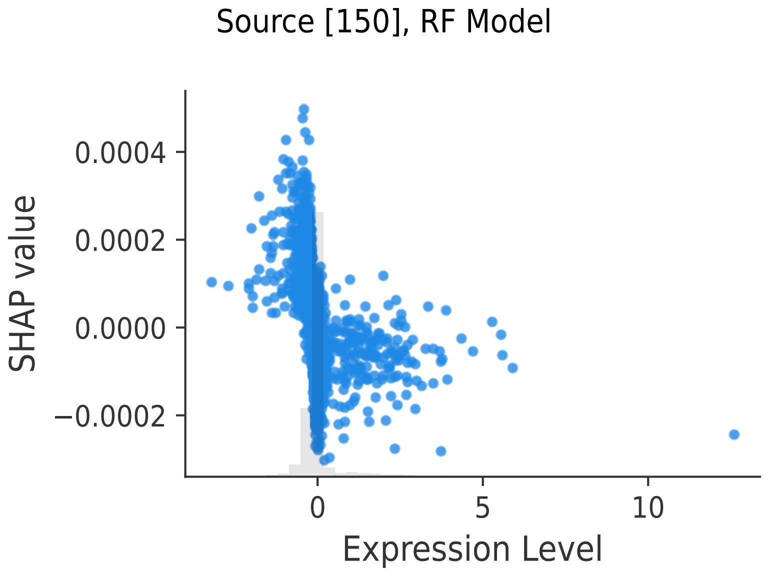
Source [150], RF Model
SHAP value
0.0004
0.0002
0.0000
−0.0002
0
5
10
Expression Level

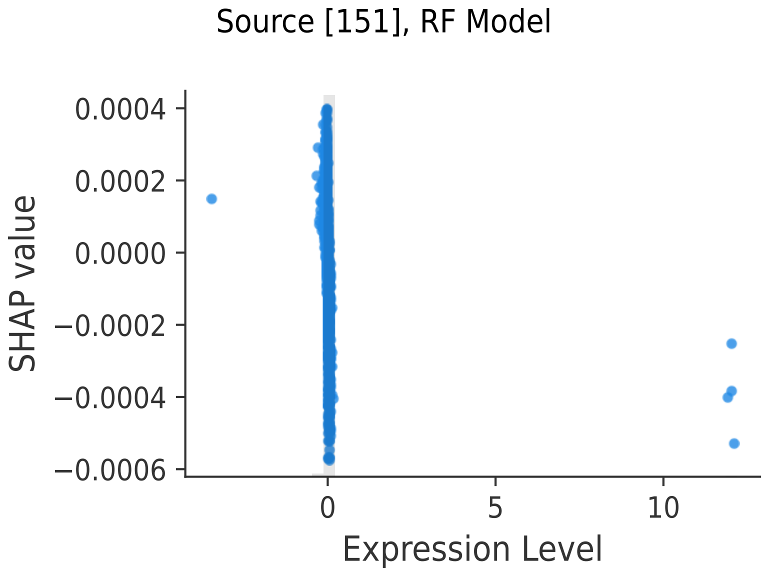

Source [151], RF Model
0.0004
0.0002
0.0000
−0.0002
−0.0004
−0.0006
SHAP value
0
5
10
Expression Level

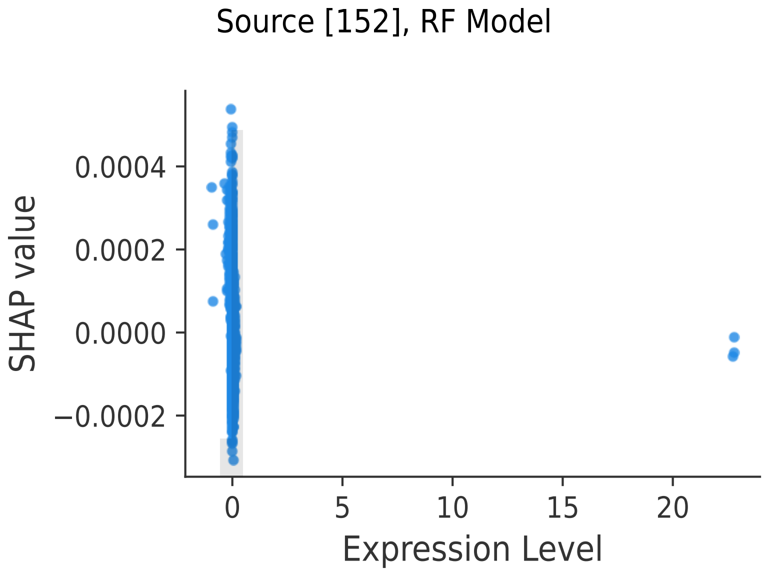

Source [152], RF Model
SHAP value
0.0004
0.0002
0.0000
−0.0002
0
5
10
15
20
Expression Level

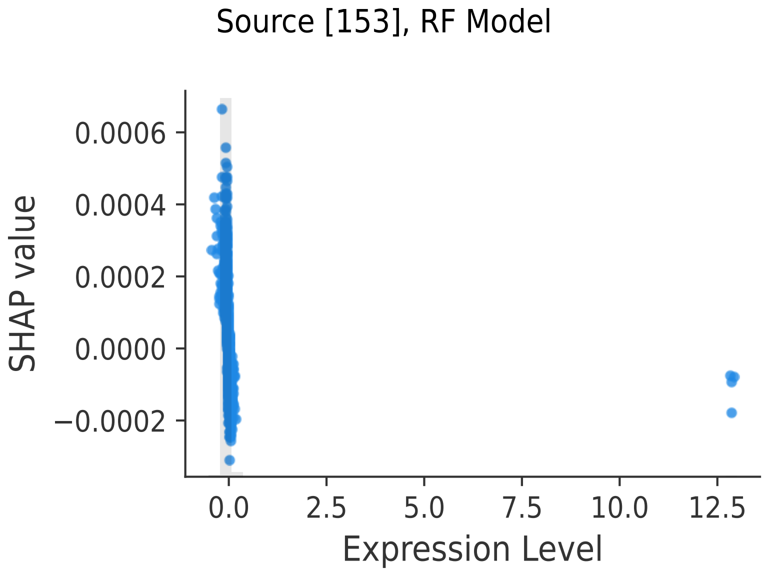
Source [153], RF Model
SHAP value
0.0006
0.0004
0.0002
0.0000
−0.0002
0.0
2.5
5.0
7.5
10.0
12.5
Expression Level

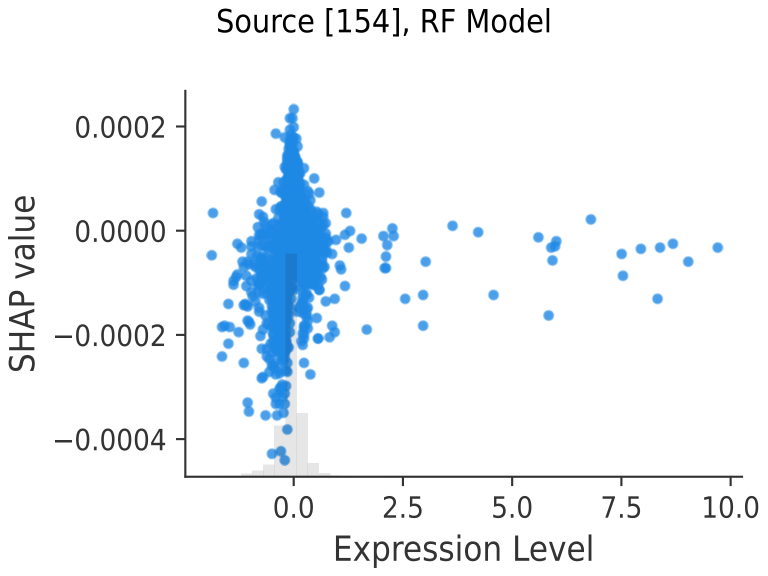
Source [154], RF Model
SHAP value
0.0002
0.0000
−0.0002
−0.0004
0.0
2.5
5.0
7.5
10.0
Expression Level

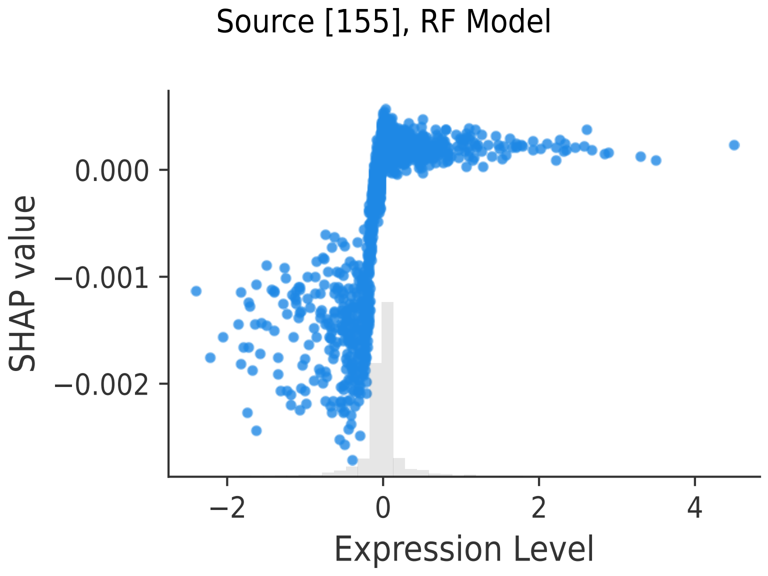
Source [155], RF Model
0.000
−0.001
−0.002
SHAP value
−2
0
2
4
Expression Level

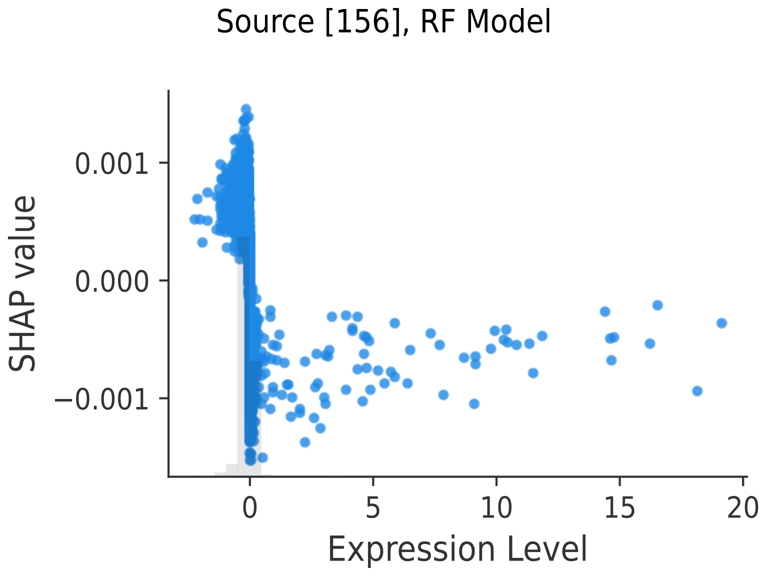

Source [156], RF Model
SHAP value
0.001
0.000
−0.001
0
5
10
15
20
Expression Level

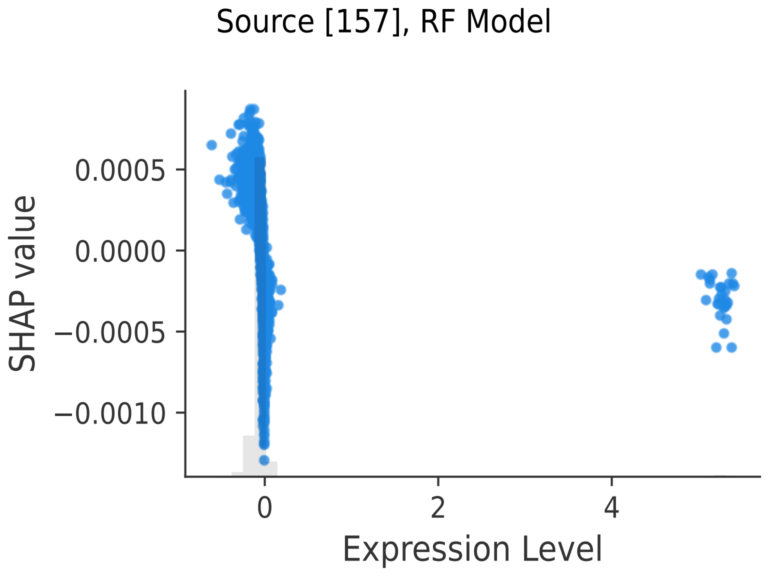
Source [157], RF Model
SHAP value
0.0005
0.0000
−0.0005
−0.0010
0
2
4
Expression Level

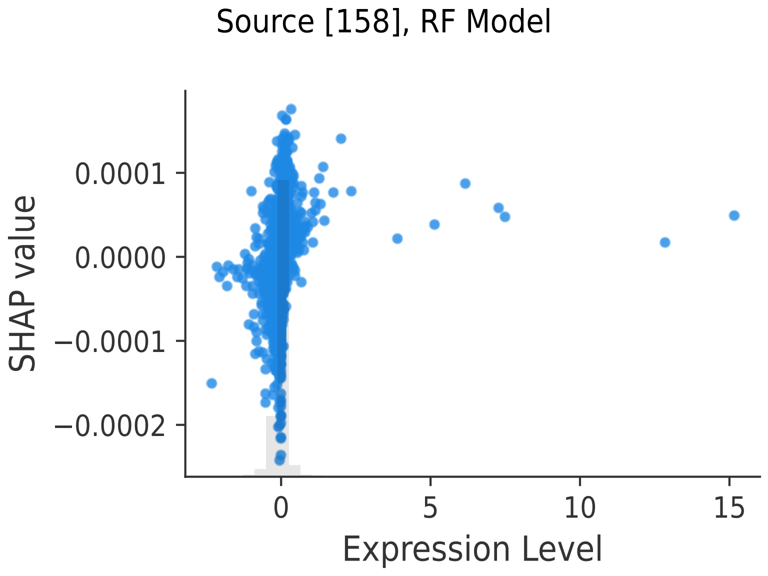
Source [158], RF Model
SHAP value
0.0001
0.0000
−0.0001
−0.0002
0
5
10
15
Expression Level

Source [159], RF Model

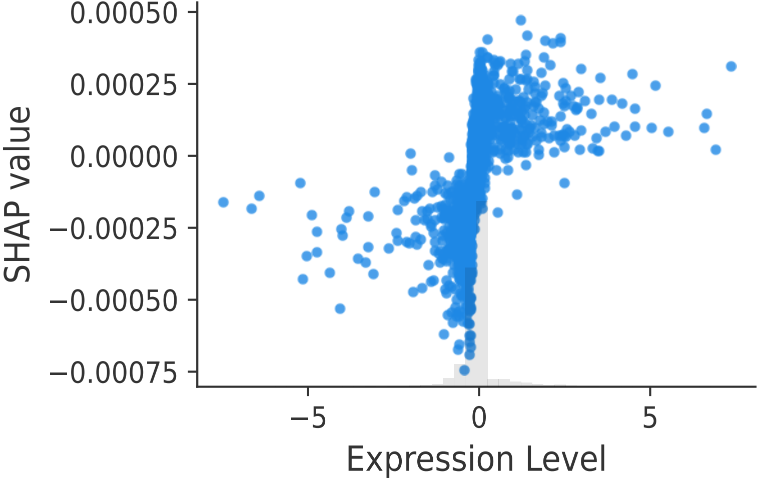

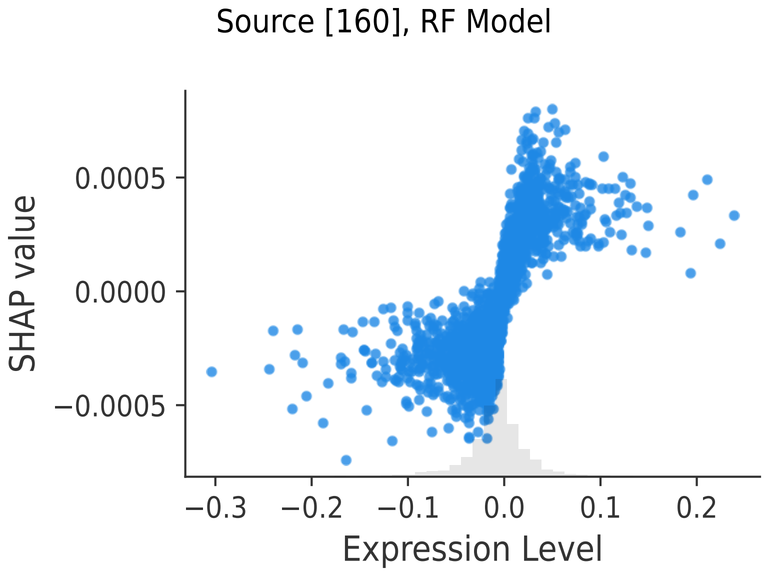
Source [160], RF Model
SHAP value
0.0005
0.0000
−0.0005
−0.3
−0.2
−0.1
0.0
0.1
0.2
Expression Level

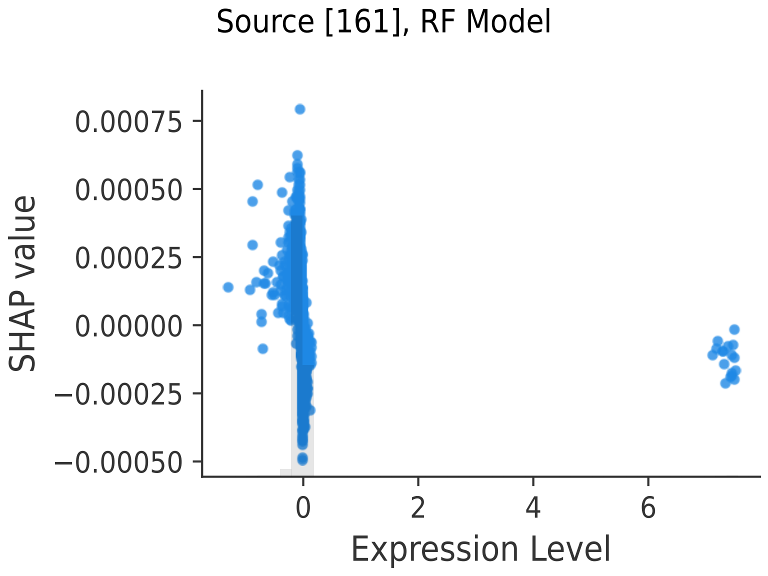

Source [161], RF Model
SHAP value
0.00075
0.00050
0.00025
0.00000
−0.00025
−0.00050
0
2
4
6
Expression Level

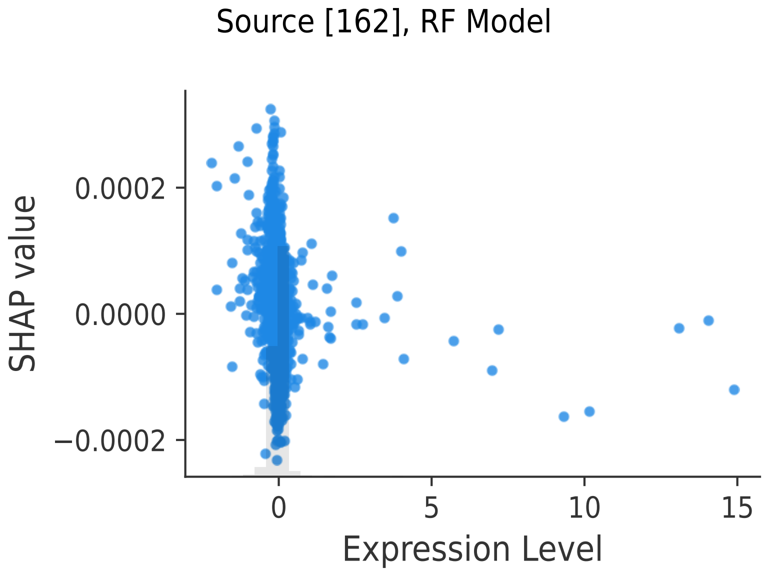
Source [162], RF Model
SHAP value
0.0002
0.0000
−0.0002
0
5
10
15
Expression Level

Source [163], RF Model

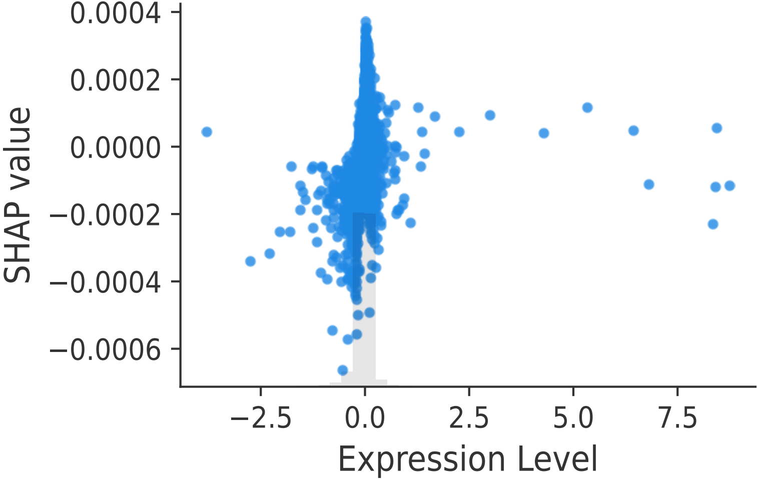

Source [164], RF Model

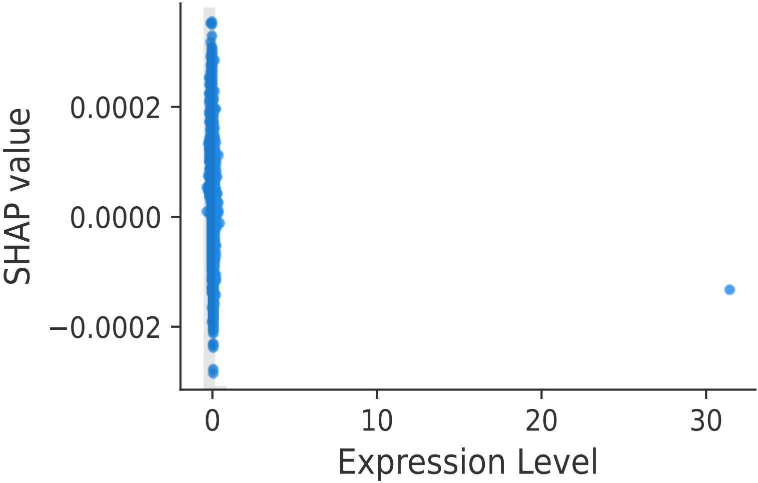

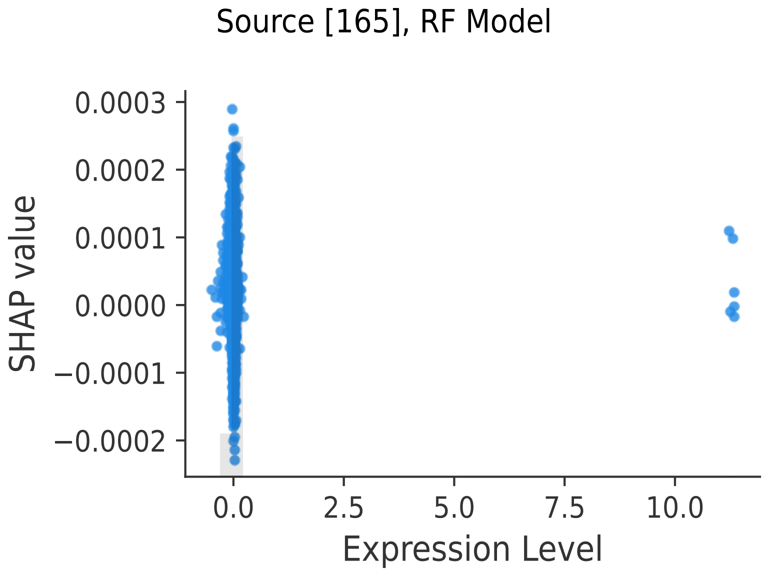

Source [165], RF Model
SHAP value
0.0003
0.0002
0.0001
0.0000
−0.0001
−0.0002
0.0
2.5
5.0
7.5
10.0
Expression Level

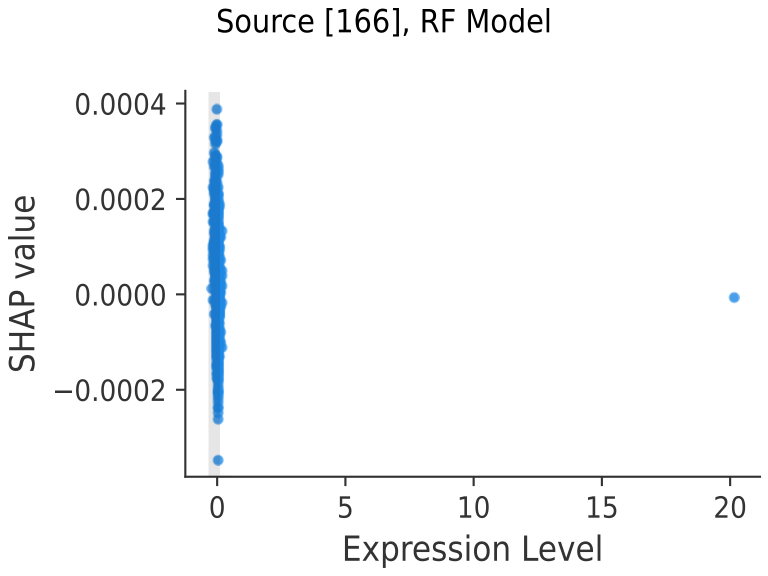

Source [166], RF Model
SHAP value
0.0004
0.0002
0.0000
−0.0002
0
5
10
15
20
Expression Level

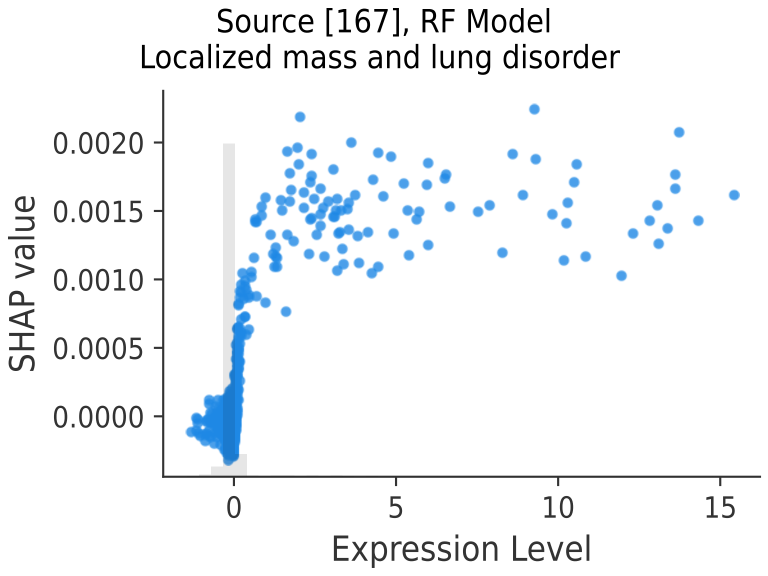
Source [167], RF Model
Localized mass and lung disorder
0.0020
0.0015
0.0010
0.0005
0.0000
SHAP value
0
5
10
15
Expression Level

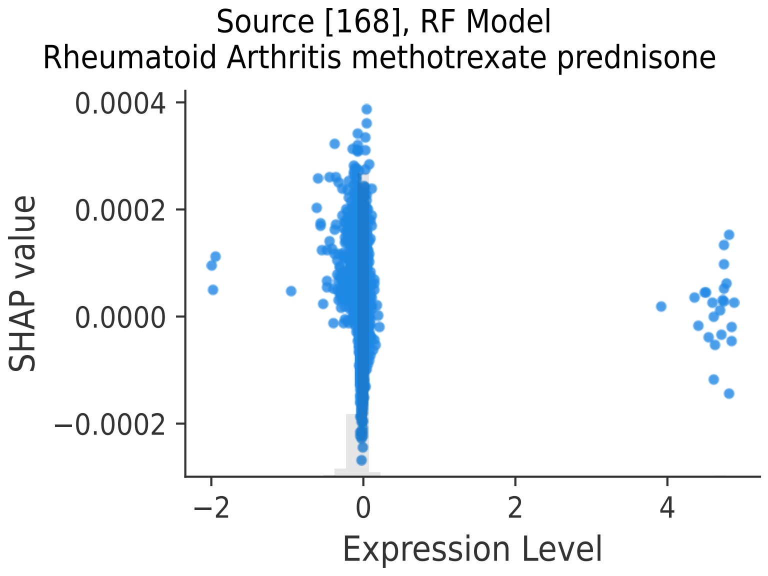
Source [168], RF Model
Rheumatoid Arthritis methotrexate prednisone
0.0004
0.0002
0.0000
−0.0002
SHAP value
−2
0
2
4
Expression Level

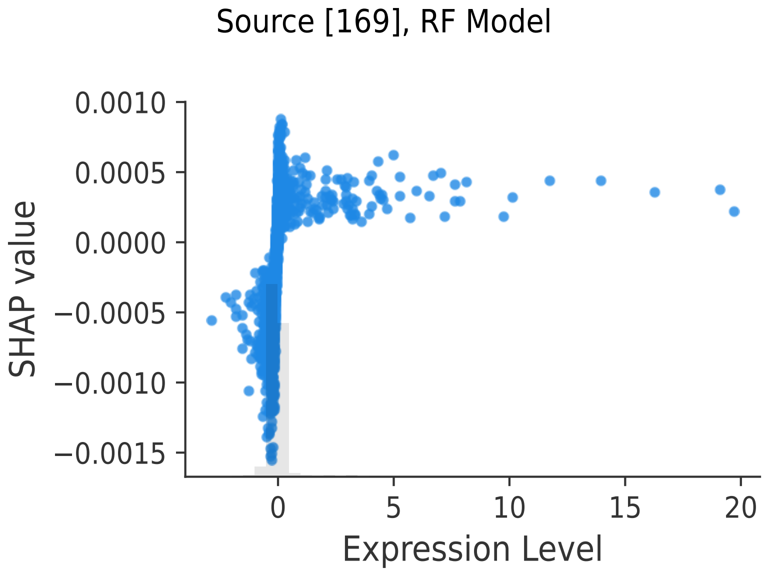
Source [169], RF Model
0.0010
0.0005
0.0000
−0.0005
−0.0010
−0.0015
SHAP value
0
5
10
15
20
Expression Level

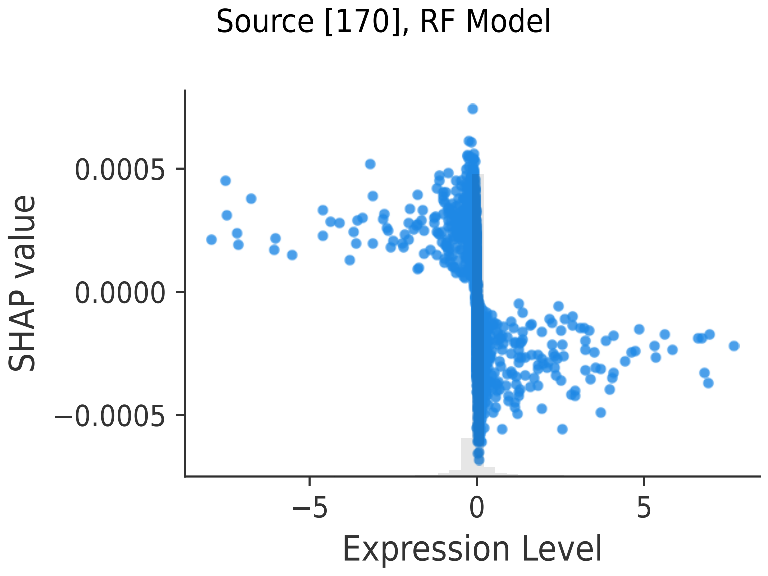

Source [170], RF Model
SHAP value
0.0005
0.0000
−0.0005
−5
0
5
Expression Level

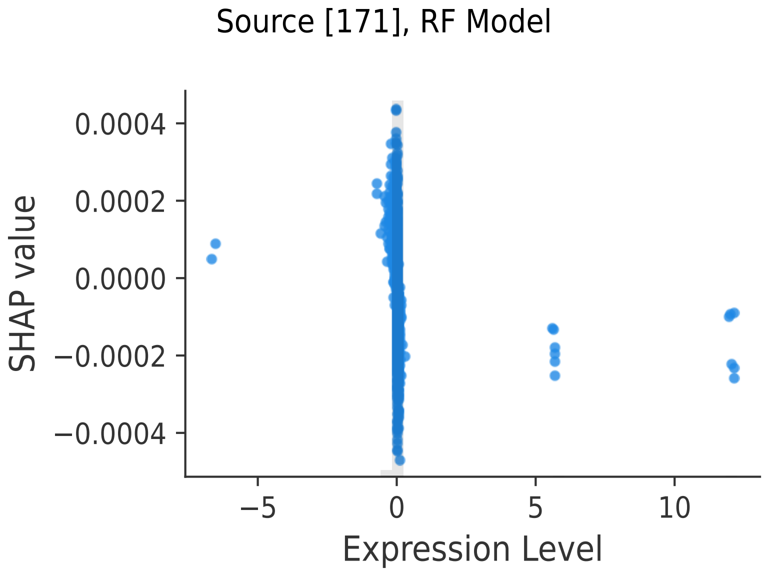
Source [171], RF Model
SHAP value
0.0004
0.0002
0.0000
−0.0002
−0.0004
−5
0
5
10
Expression Level

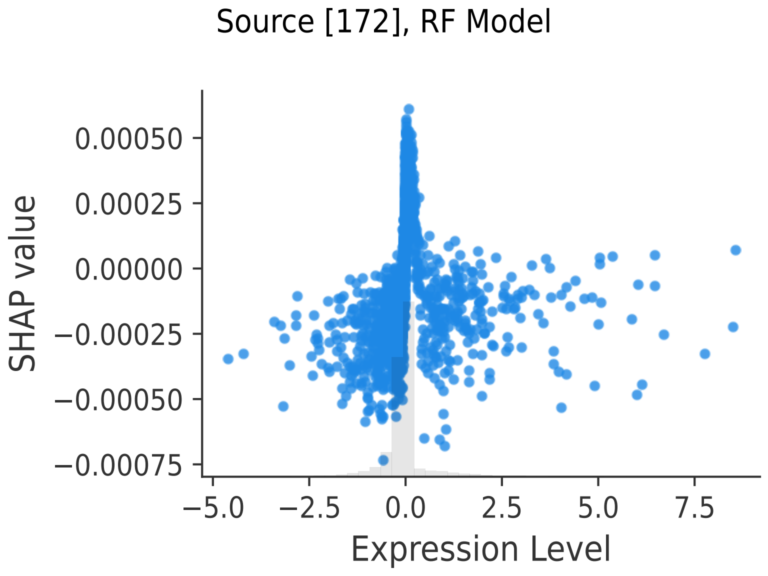
Source [172], RF Model
SHAP value
0.00050
0.00025
0.00000
−0.00025
−0.00050
−0.00075
−5.0
−2.5
0.0
2.5
5.0
7.5
Expression Level

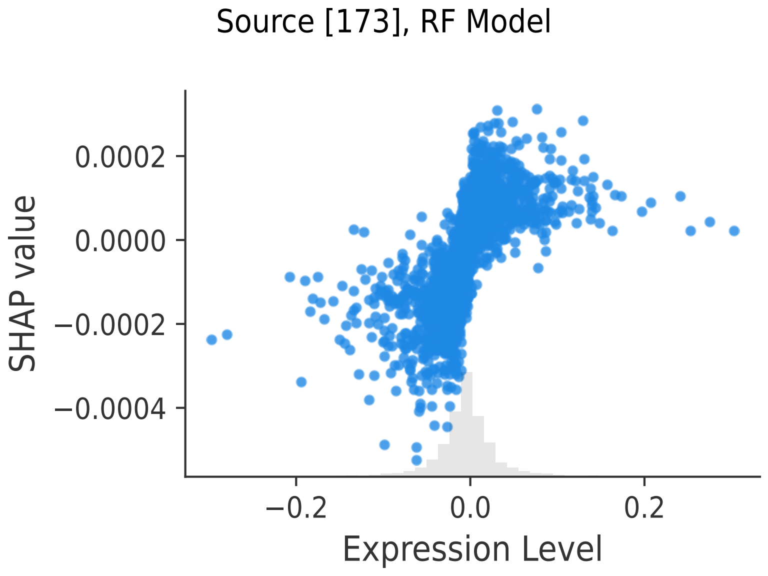
Source [173], RF Model
0.0002
0.0000
−0.0002
−0.0004
SHAP value
−0.2
0.0
0.2
Expression Level

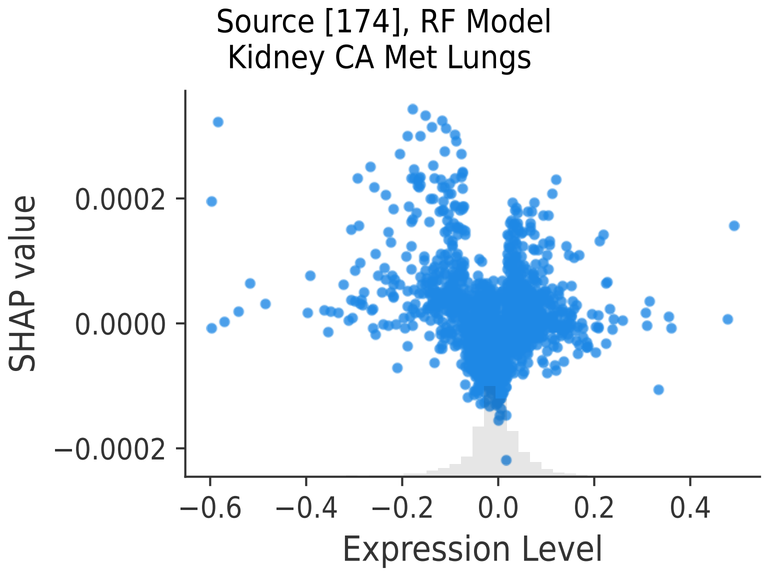
Source [174], RF Model
Kidney CA Met Lungs
SHAP value
0.0002
0.0000
−0.0002
−0.6
−0.4
−0.2
0.0
0.2
0.4
Expression Level

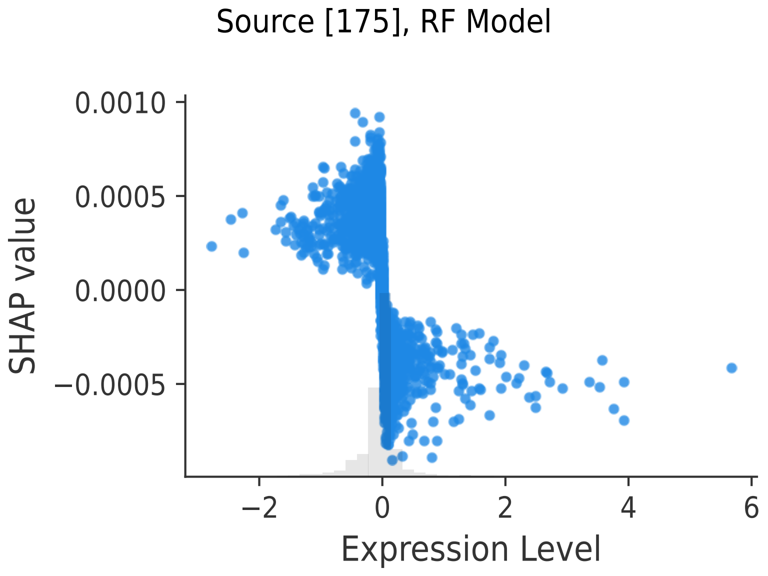
Source [175], RF Model
0.0010
0.0005
0.0000
−0.0005
SHAP value
−2
0
2
4
6
Expression Level

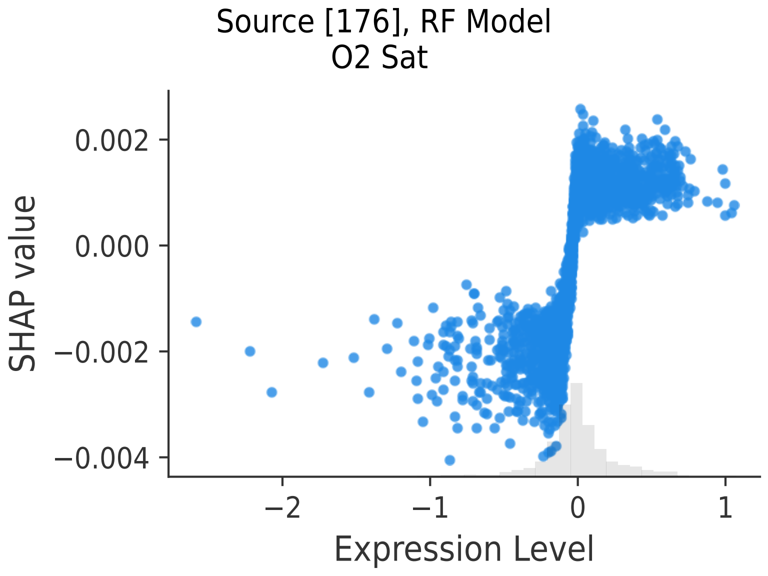

Source [176], RF Model
O2 Sat
0.002
0.000
−0.002
−0.004
SHAP value
−2
−1
0
1
Expression Level

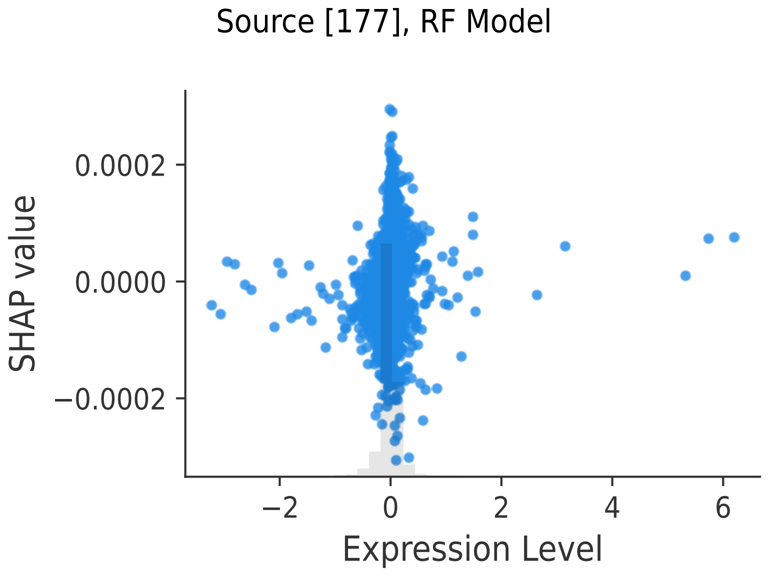
Source [177], RF Model
SHAP value
0.0002
0.0000
−0.0002
−2
0
2
4
6
Expression Level

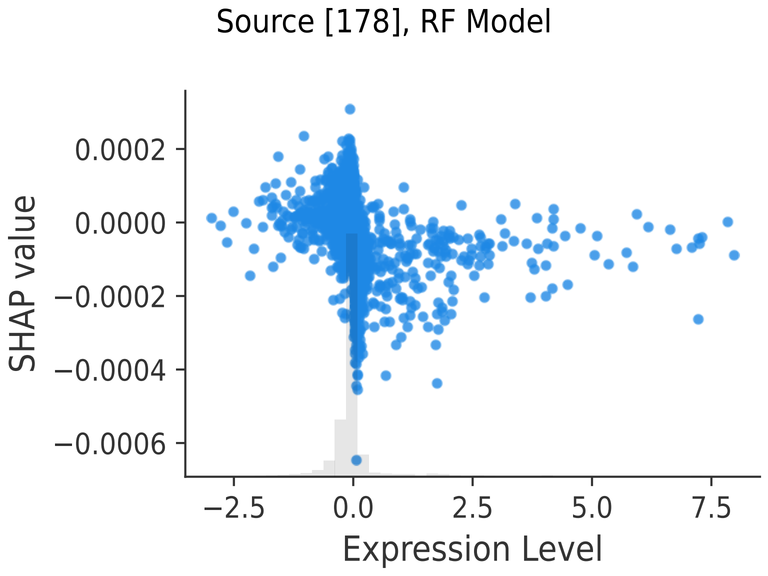
Source [178], RF Model
0.0002
0.0000
−0.0002
−0.0004
−0.0006
SHAP value
−2.5
0.0
2.5
5.0
7.5
Expression Level

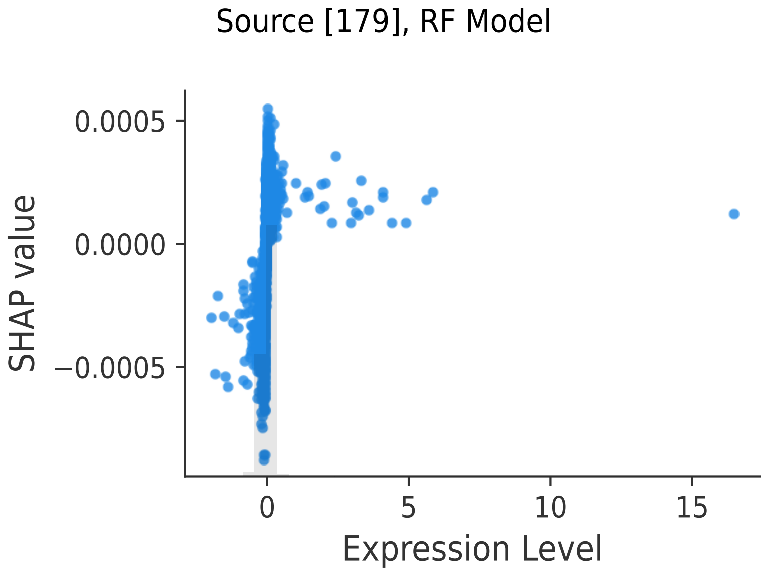
Source [179], RF Model
SHAP value
0.0005
0.0000
−0.0005
0
5
10
15
Expression Level

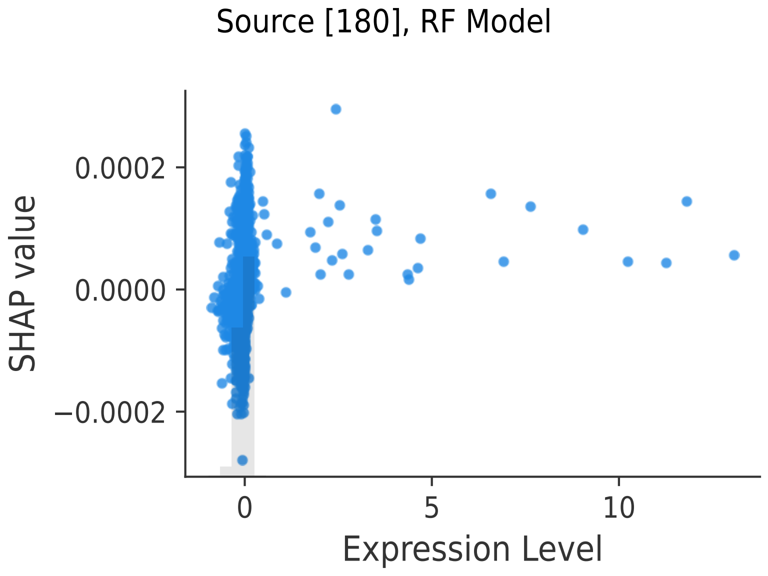

Source [180], RF Model
0.0002
0.0000
−0.0002
SHAP value
0
5
10
Expression Level

Source [181], RF Model

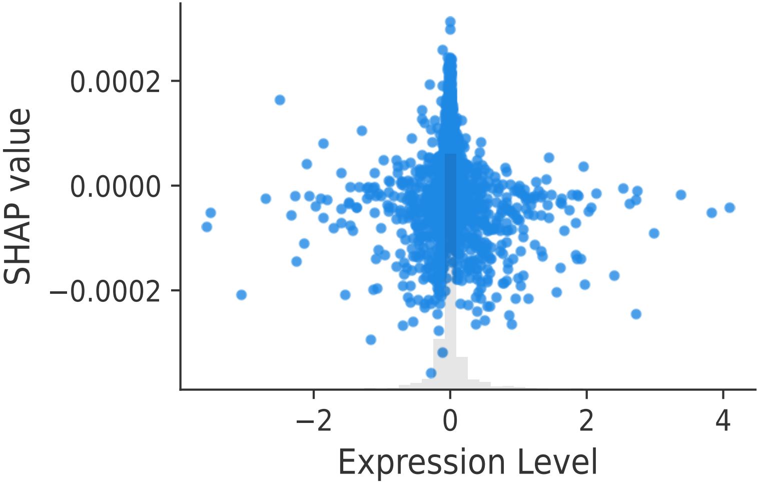

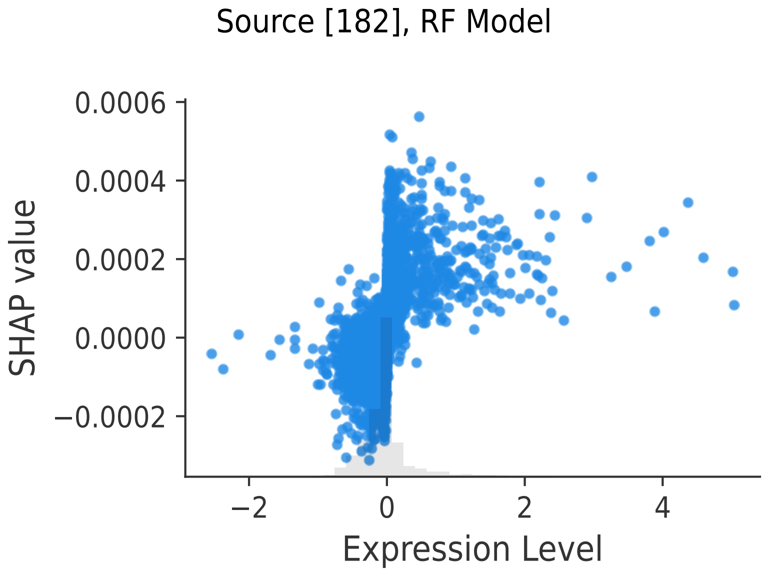

Source [182], RF Model
0.0006
0.0004
0.0002
0.0000
−0.0002
SHAP value
−2
0
2
4
Expression Level

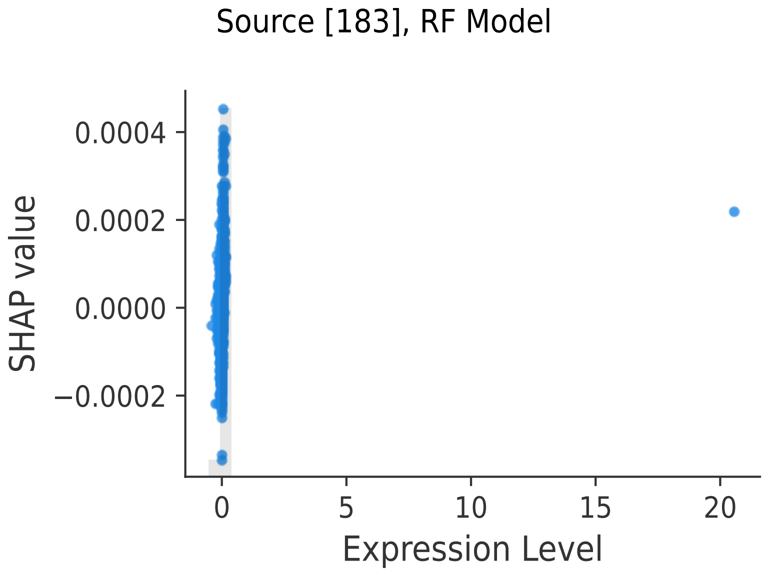
Source [183], RF Model
SHAP value
0.0004
0.0002
0.0000
−0.0002
0
5
10
15
20
Expression Level

Source [184], RF Model

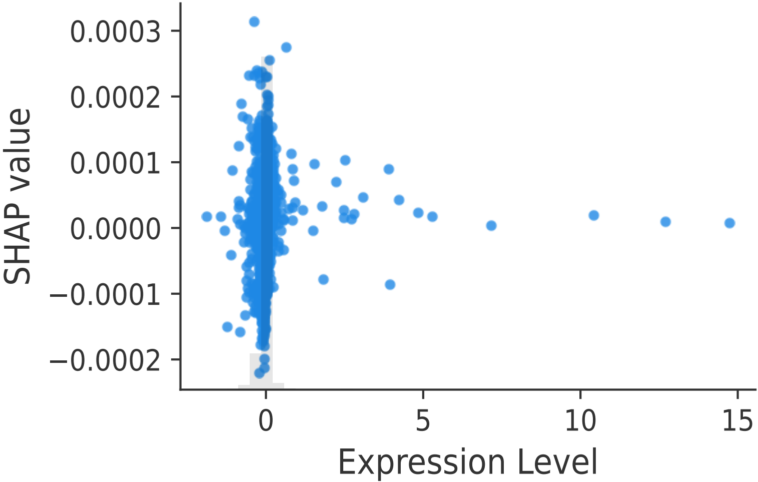

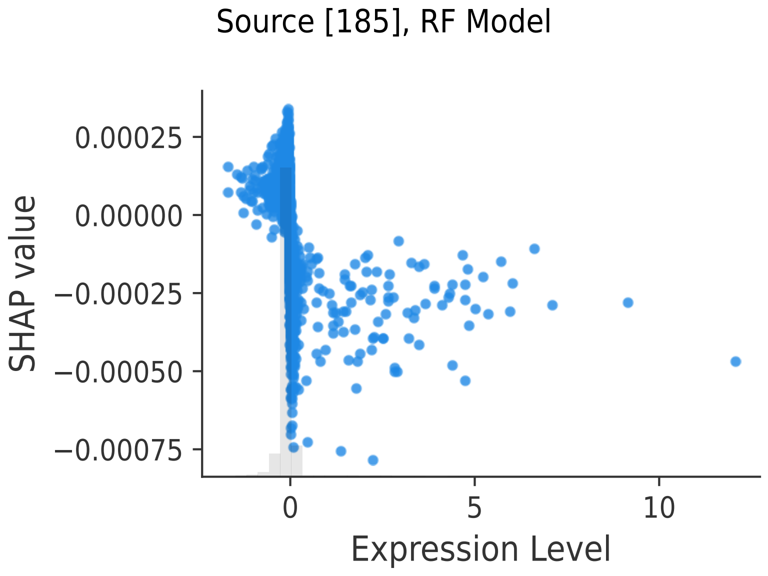
Source [185], RF Model
SHAP value
0.00025
0.00000
−0.00025
−0.00050
−0.00075
0
5
10
Expression Level

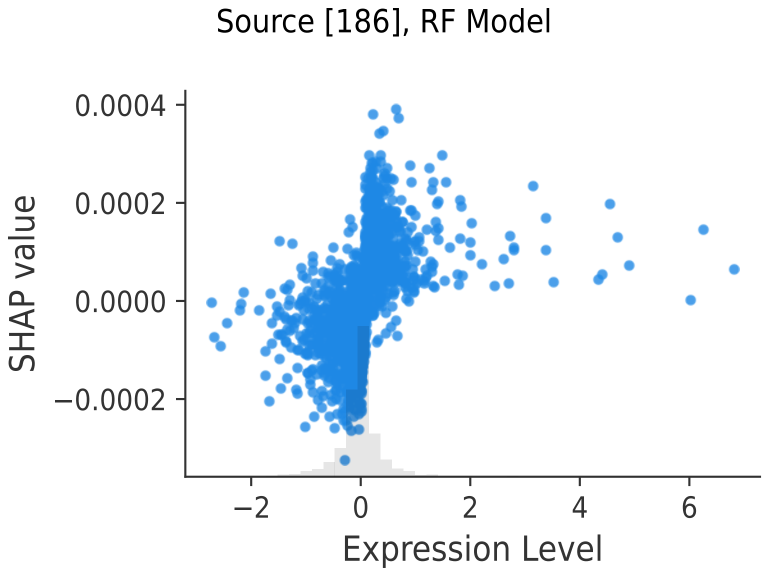
Source [186], RF Model
SHAP value
0.0004
0.0002
0.0000
−0.0002
−2
0
2
4
6
Expression Level

Source [187], RF Model

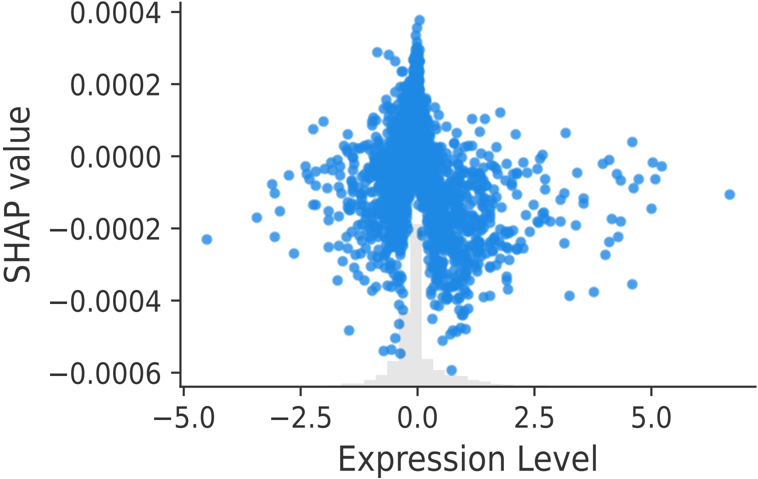

Source [188], RF Model

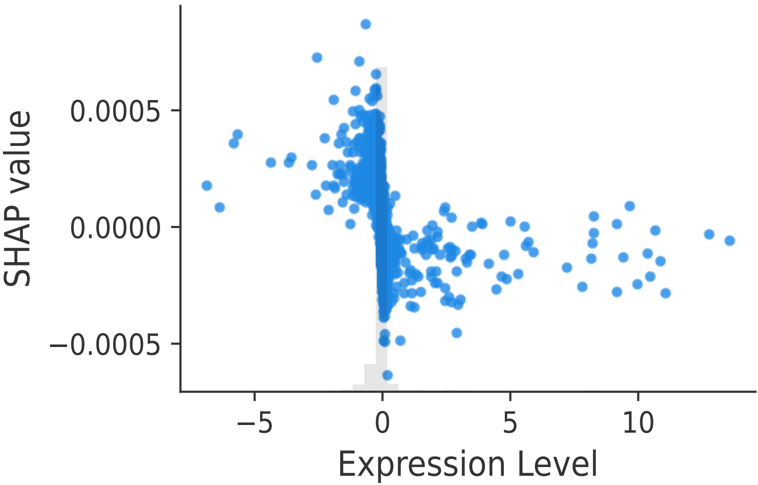

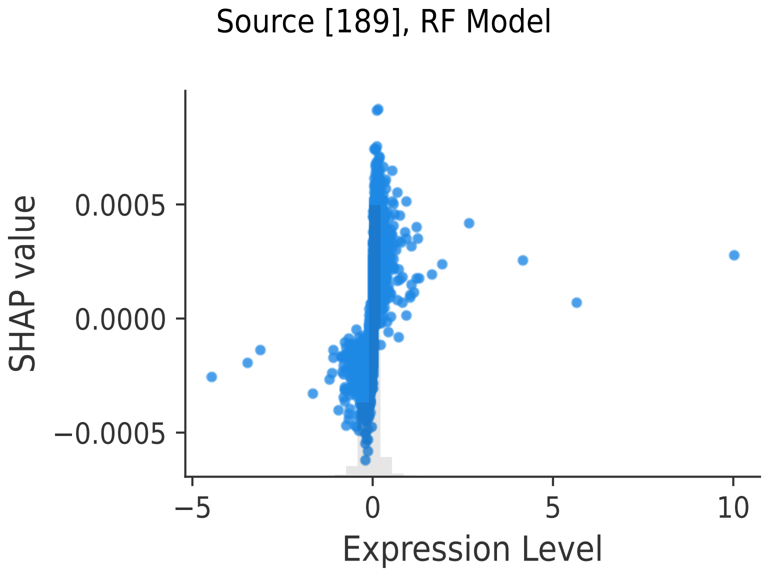
Source [189], RF Model
SHAP value
0.0005
0.0000
−0.0005
−5
0
5
10
Expression Level

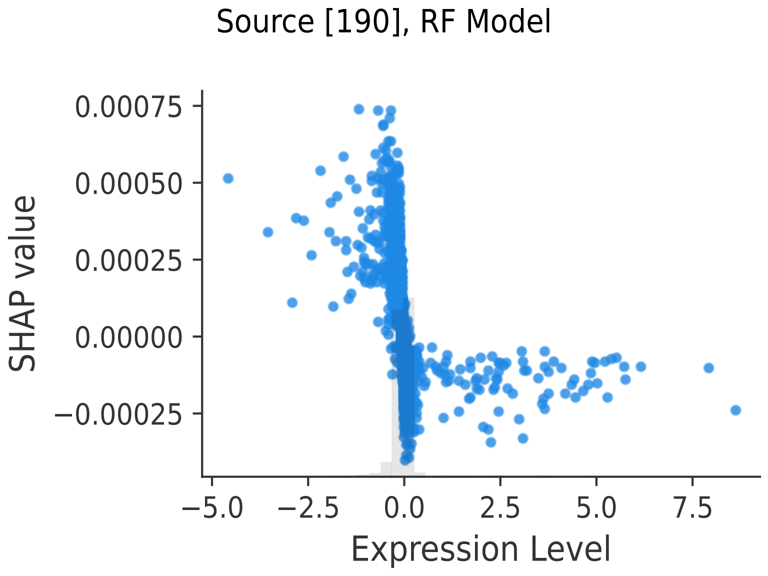
Source [190], RF Model
SHAP value
0.00075
0.00050
0.00025
0.00000
−0.00025
−5.0
−2.5
0.0
2.5
5.0
7.5
Expression Level

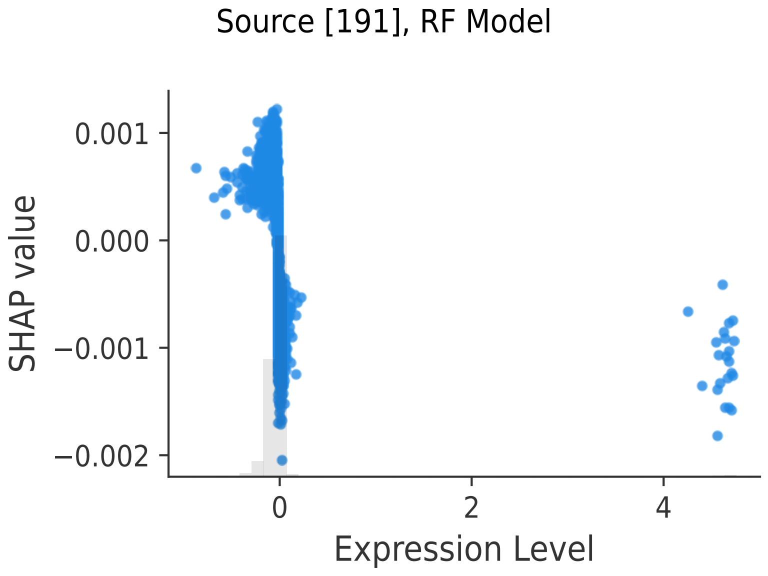
Source [191], RF Model
0.001
0.000
−0.001
−0.002
SHAP value
0
2
4
Expression Level

Source [192], RF Model

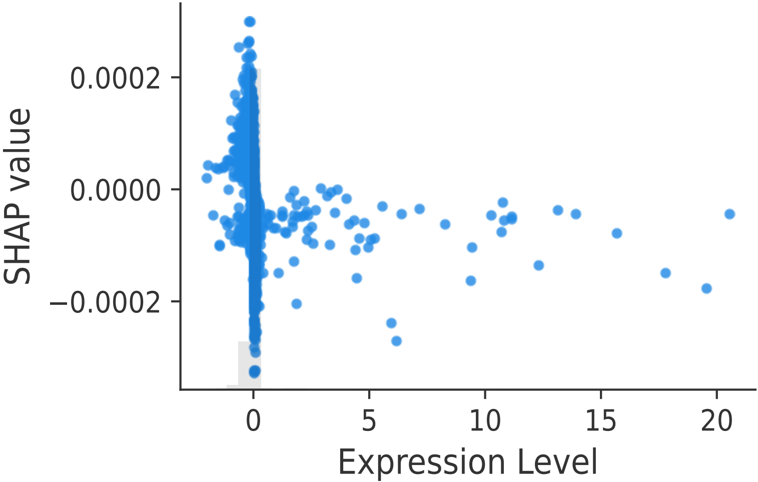

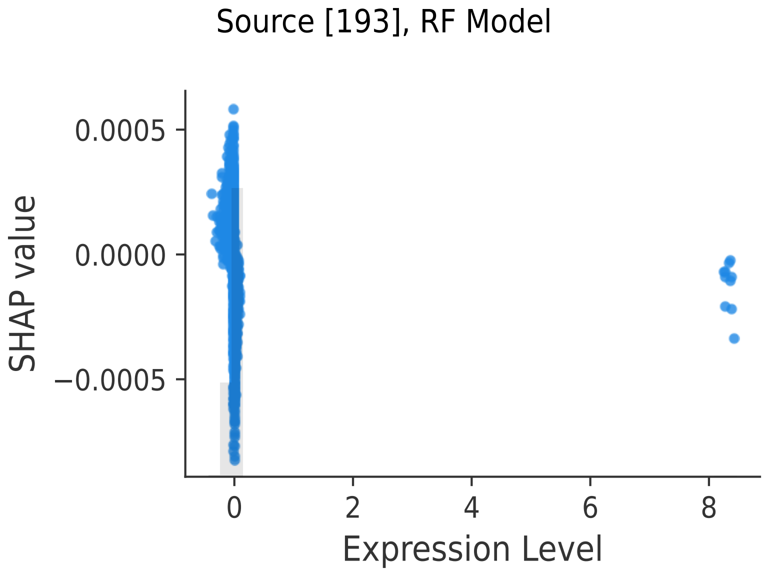

Source [193], RF Model
0.0005
0.0000
−0.0005
SHAP value
0
2
4
6
8
Expression Level

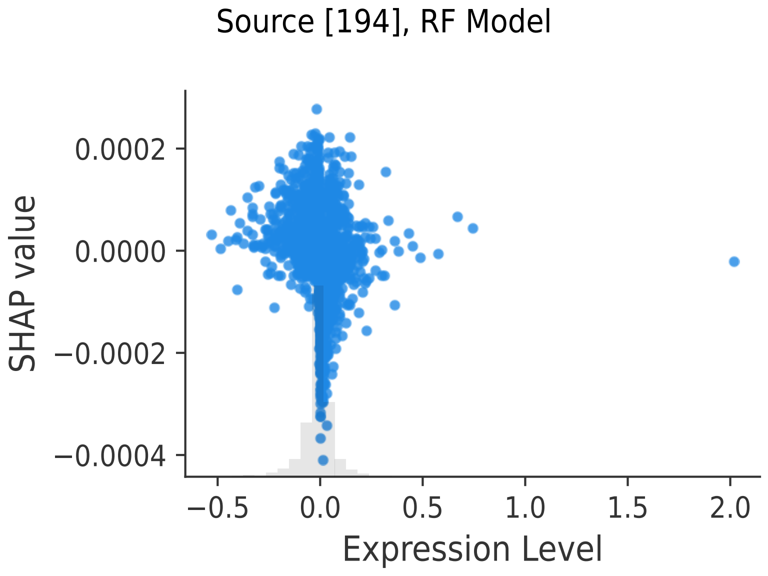
Source [194], RF Model
0.0002
0.0000
−0.0002
−0.0004
SHAP value
−0.5
0.0
0.5
1.0
1.5
2.0
Expression Level

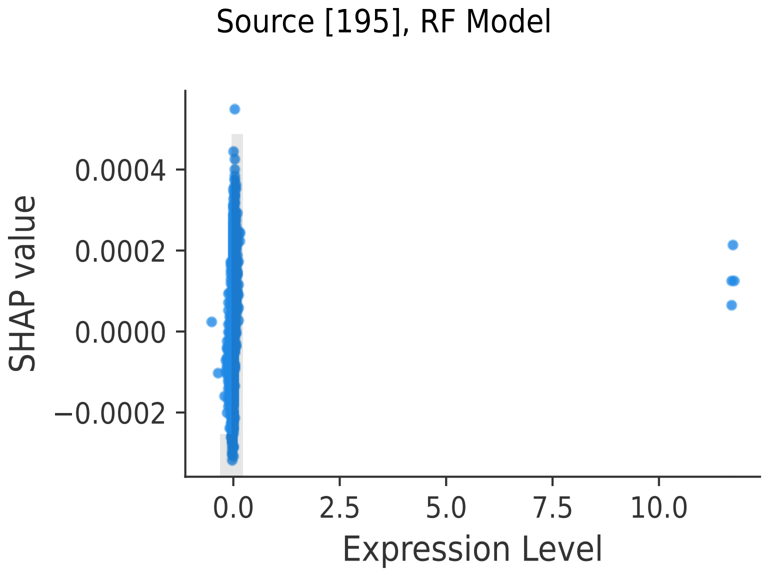
Source [195], RF Model
0.0004
0.0002
0.0000
−0.0002
SHAP value
0.0
2.5
5.0
7.5
10.0
Expression Level

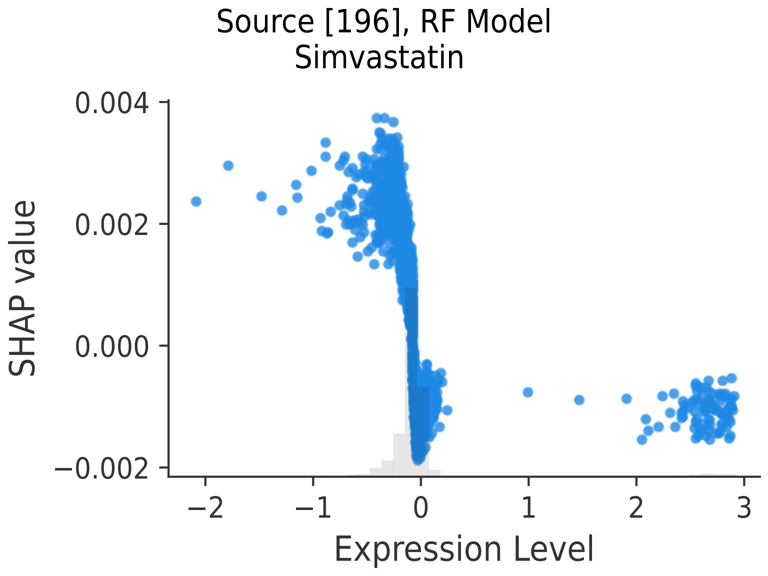

Source [196], RF Model
Simvastatin
SHAP value
0.004
0.002
0.000
−0.002
−2
−1
0
1
2
3
Expression Level

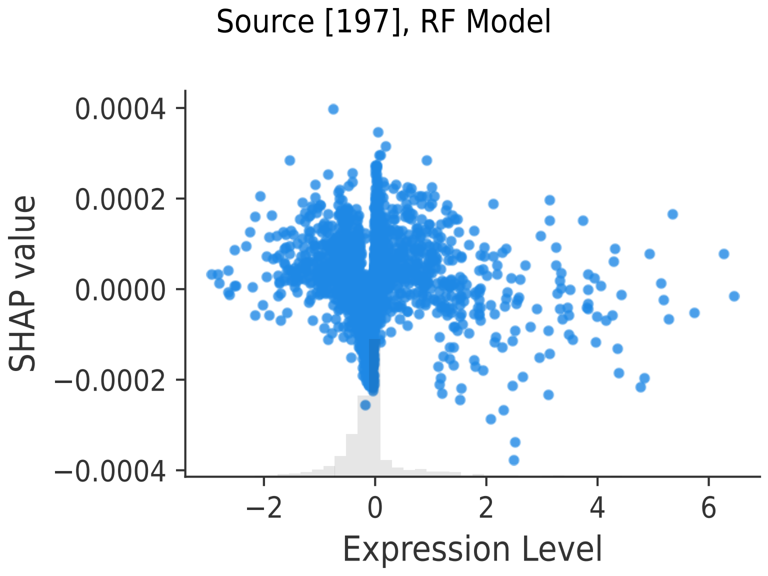
Source [197], RF Model
0.0004
0.0002
0.0000
−0.0002
−0.0004
SHAP value
−2
0
2
4
6
Expression Level

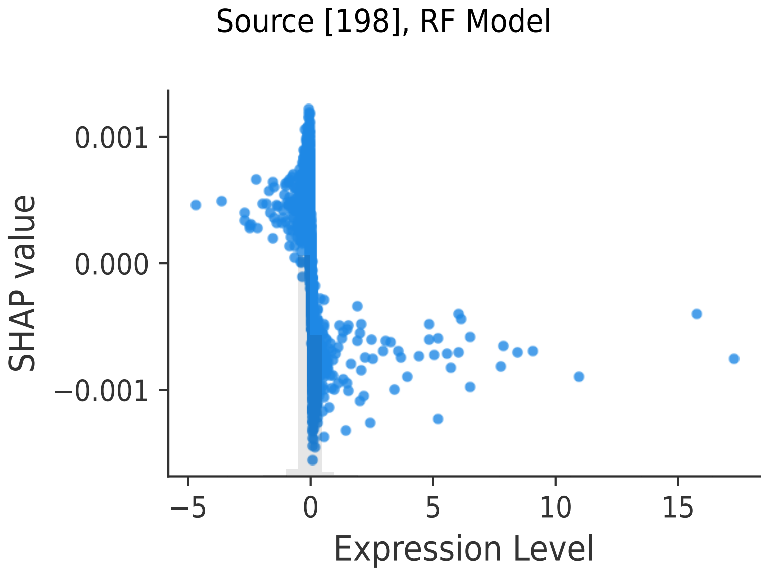

Source [198], RF Model
SHAP value
0.001
0.000
−0.001
−5
0
5
10
15
Expression Level

Source [199], RF Model

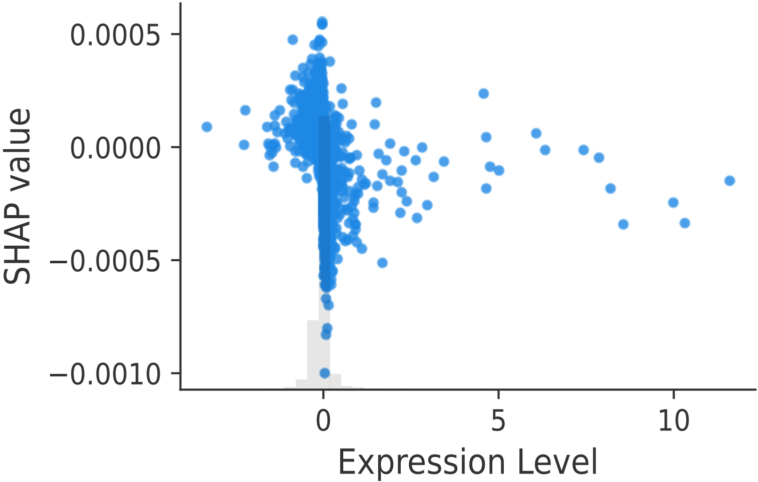

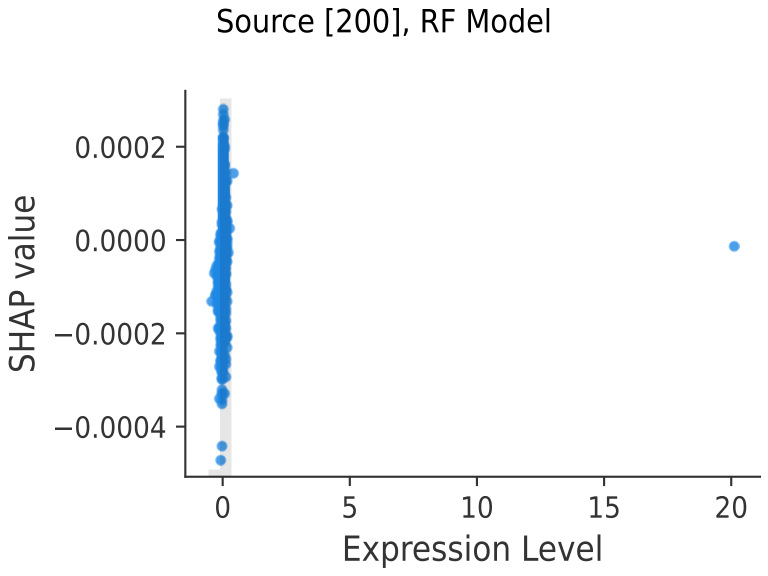
Source [200], RF Model
SHAP value
0.0002
0.0000
−0.0002
−0.0004
0
5
10
15
20
Expression Level

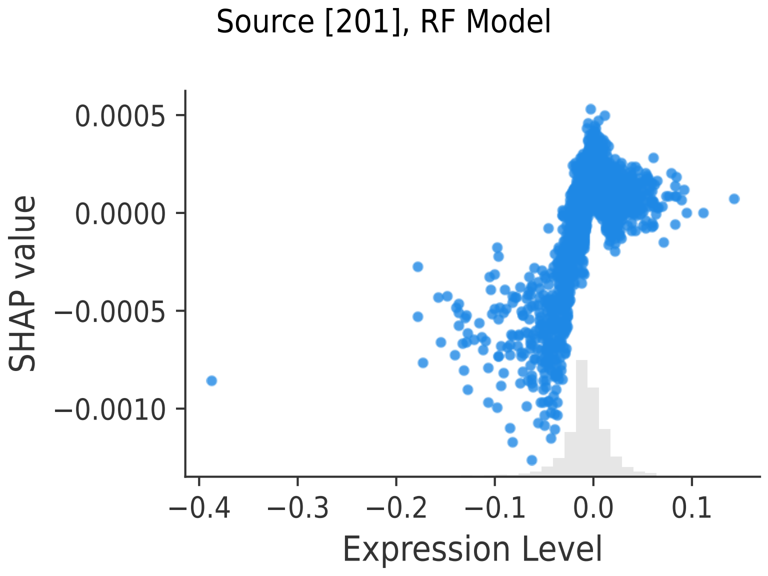
Source [201], RF Model
0.0005
0.0000
−0.0005
−0.0010
SHAP value
−0.4
−0.3
−0.2
−0.1
0.0
0.1
Expression Level

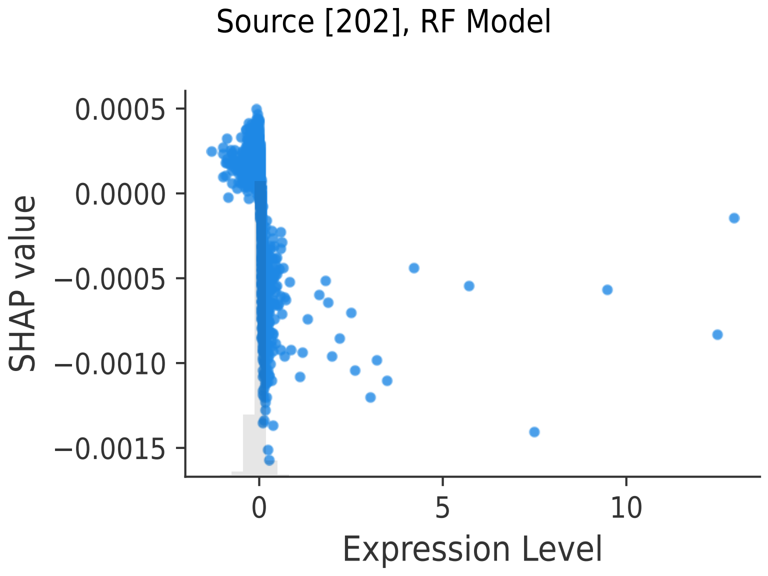
Source [202], RF Model
SHAP value
0.0005
0.0000
−0.0005
−0.0010
−0.0015
0
5
10
Expression Level

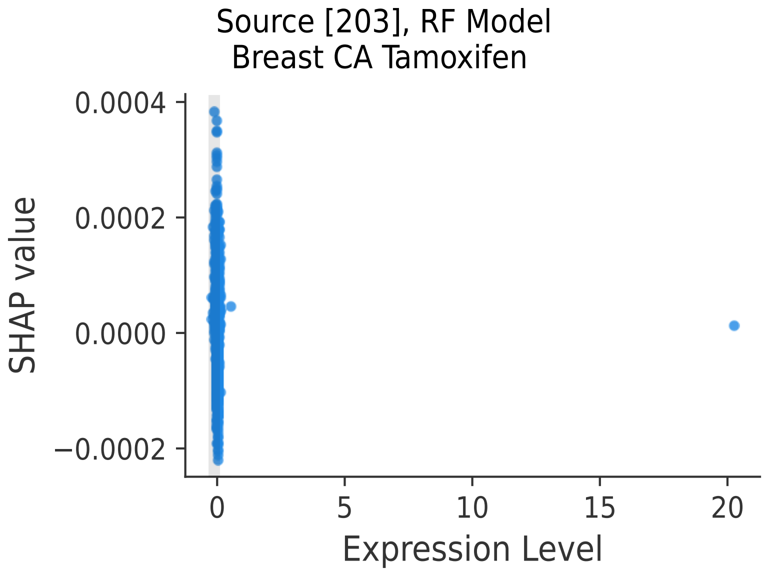

Source [203], RF Model
Breast CA Tamoxifen
0.0004
0.0002
0.0000
−0.0002
SHAP value
0
5
10
15
20
Expression Level

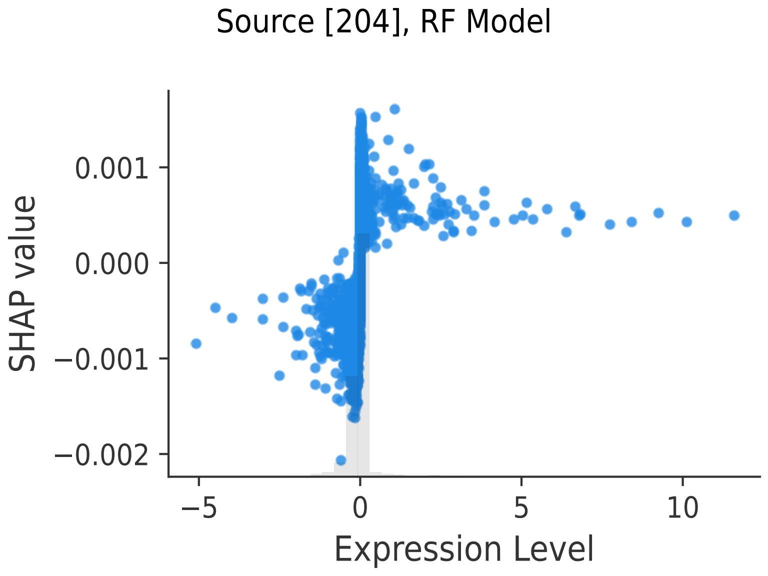
Source [204], RF Model
SHAP value
0.001
0.000
−0.001
−0.002
−5
0
5
10
Expression Level

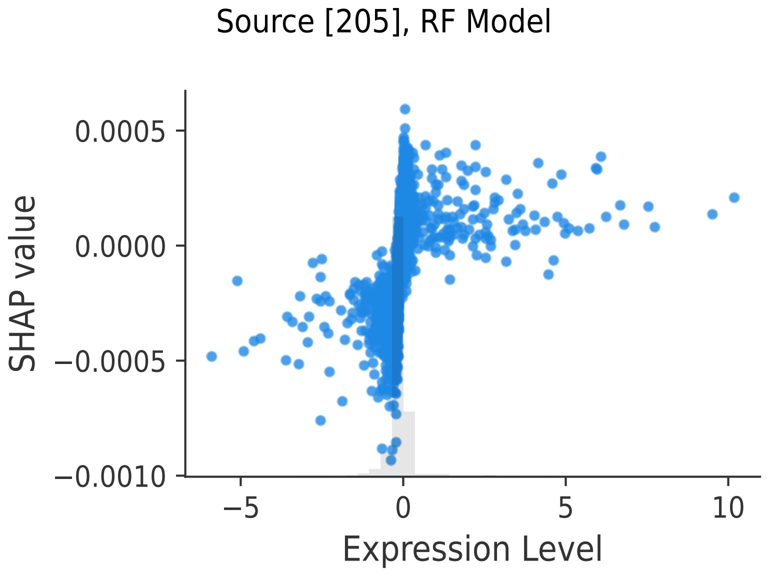
Source [205], RF Model
SHAP value
0.0005
0.0000
−0.0005
−0.0010
−5
0
5
10
Expression Level

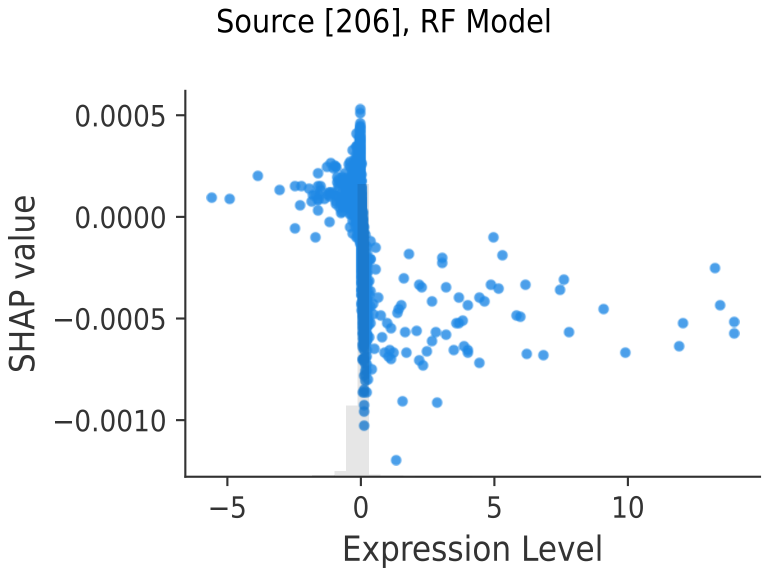
Source [206], RF Model
SHAP value
0.0005
0.0000
−0.0005
−0.0010
−5
0
5
10
Expression Level

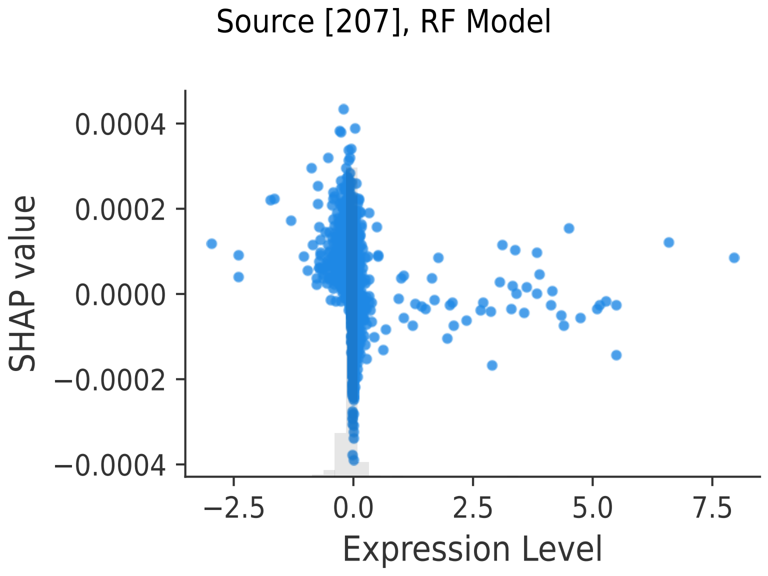
Source [207], RF Model
0.0004
0.0002
0.0000
−0.0002
−0.0004
SHAP value
−2.5
0.0
2.5
5.0
7.5
Expression Level

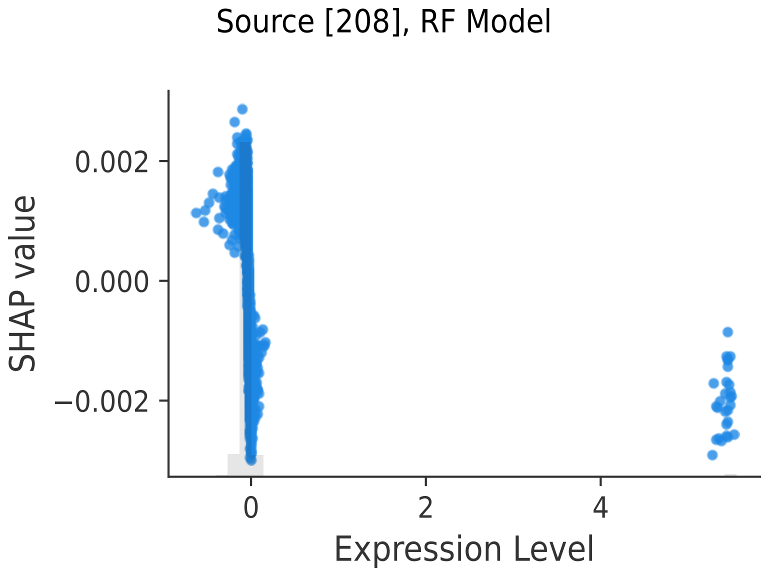

Source [208], RF Model
SHAP value
0.002
0.000
−0.002
0
2
4
Expression Level

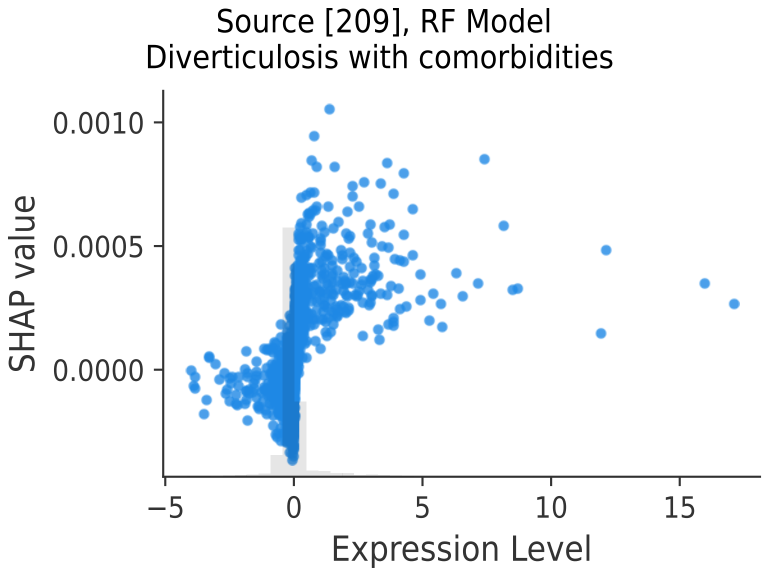

Source [209], RF Model
Diverticulosis with comorbidities
SHAP value
0.0010
0.0005
0.0000
−5
0
5
10
15
Expression Level

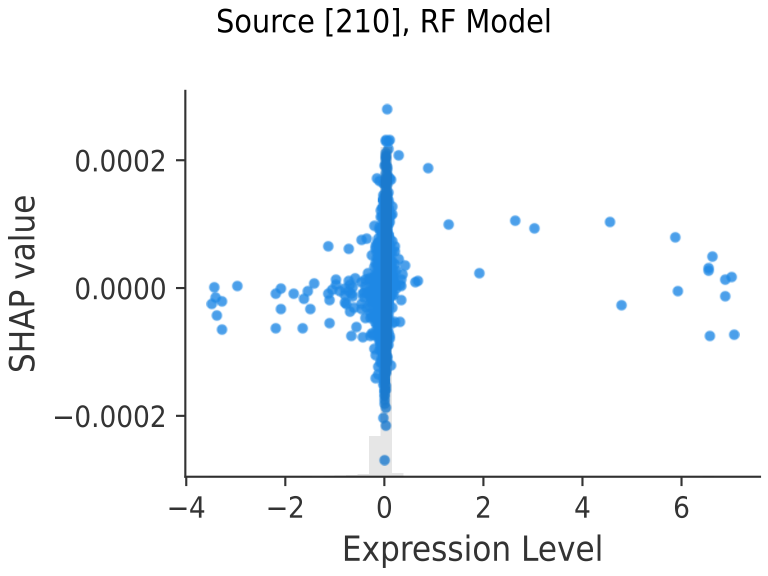

Source [210], RF Model
SHAP value
0.0002
0.0000
−0.0002
−4
−2
0
2
4
6
Expression Level

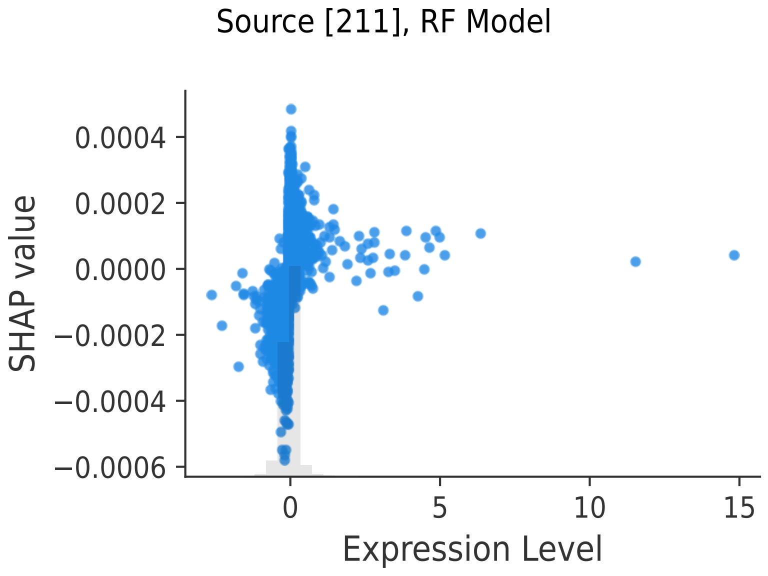

Source [211], RF Model
SHAP value
0.0004
0.0002
0.0000
−0.0002
−0.0004
−0.0006
0
5
10
15
Expression Level

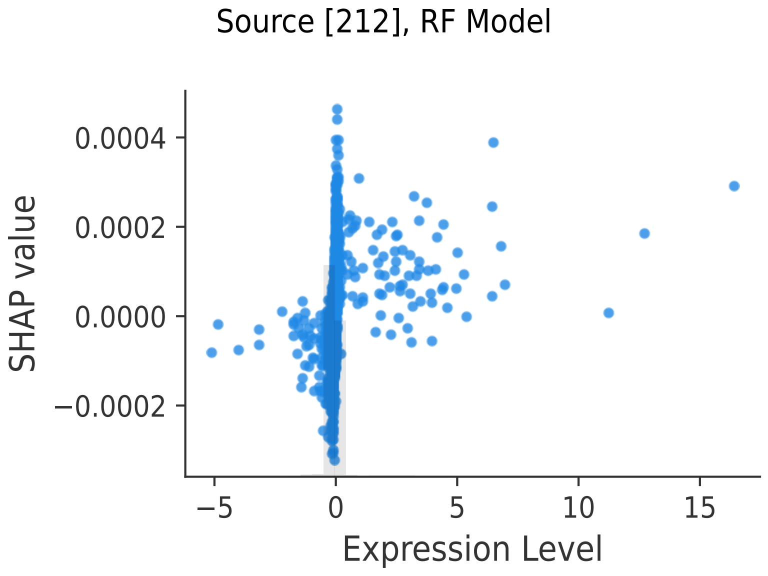
Source [212], RF Model
SHAP value
0.0004
0.0002
0.0000
−0.0002
−5
0
5
10
15
Expression Level

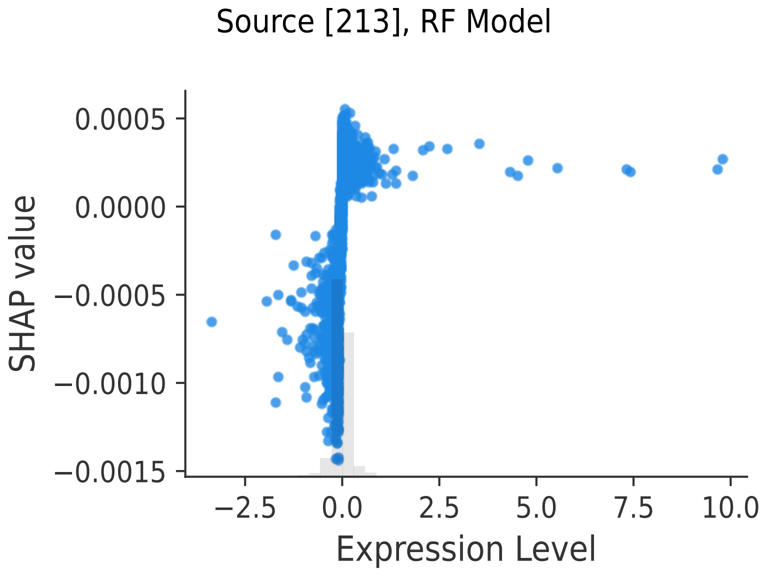

Source [213], RF Model
SHAP value
0.0005
0.0000
−0.0005
−0.0010
−0.0015
−2.5
0.0
2.5
5.0
7.5
10.0
Expression Level

Source [214], RF Model

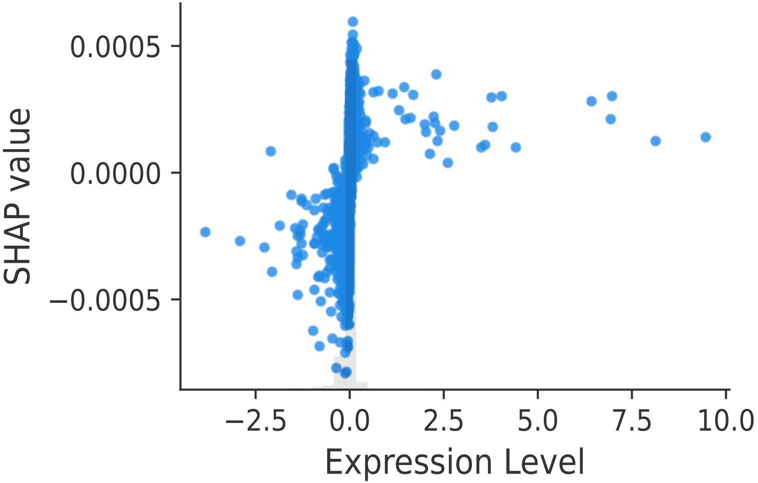

Source [215], RF Model

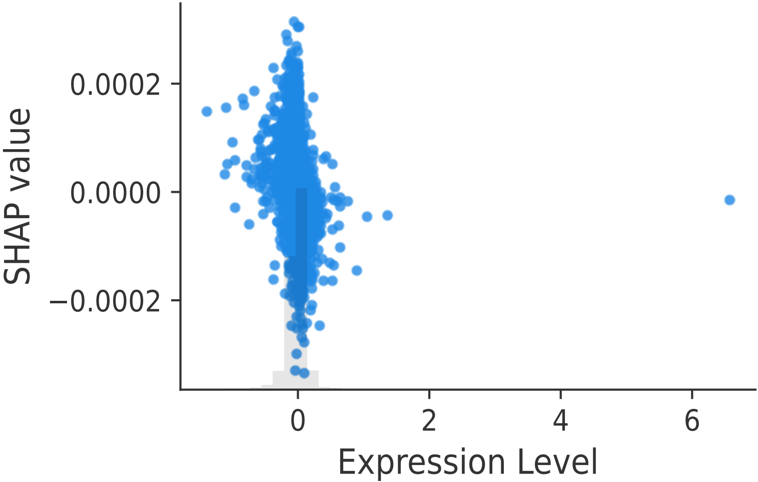

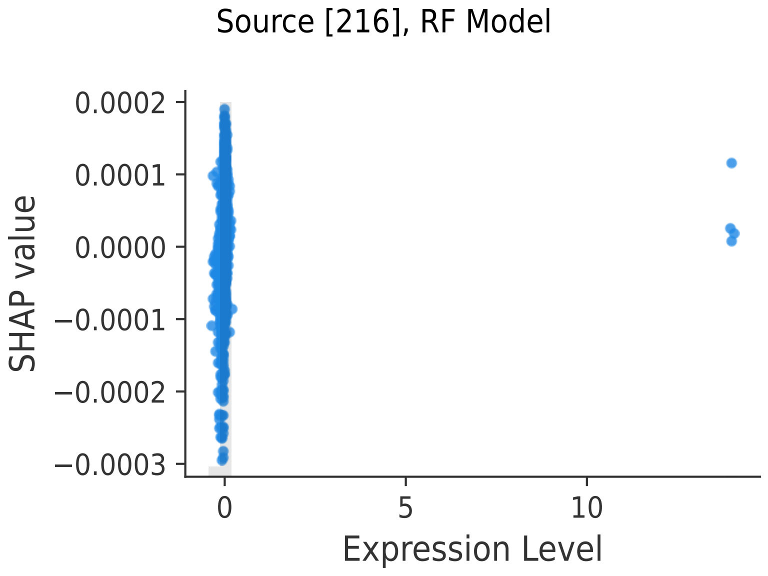
Source [216], RF Model
0.0002
0.0001
0.0000
−0.0001
−0.0002
−0.0003
SHAP value
0
5
10
Expression Level

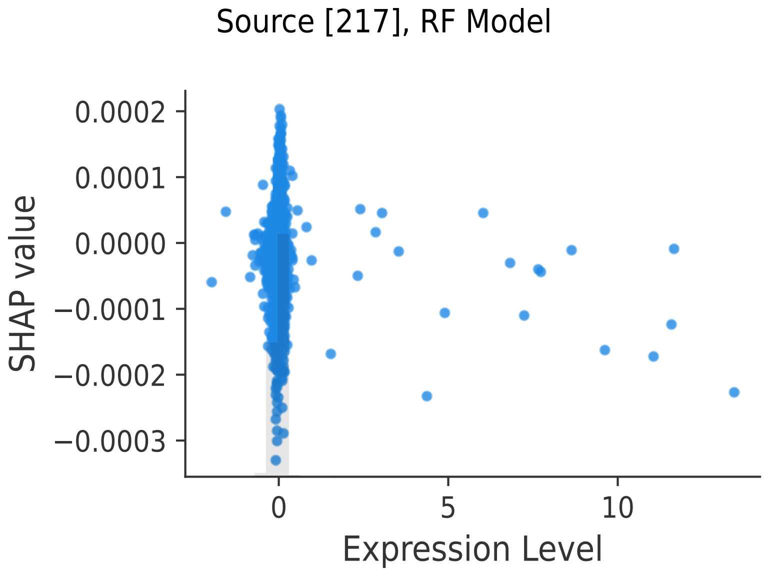
Source [217], RF Model
0.0002
0.0001
0.0000
−0.0001
−0.0002
−0.0003
SHAP value
0
5
10
Expression Level

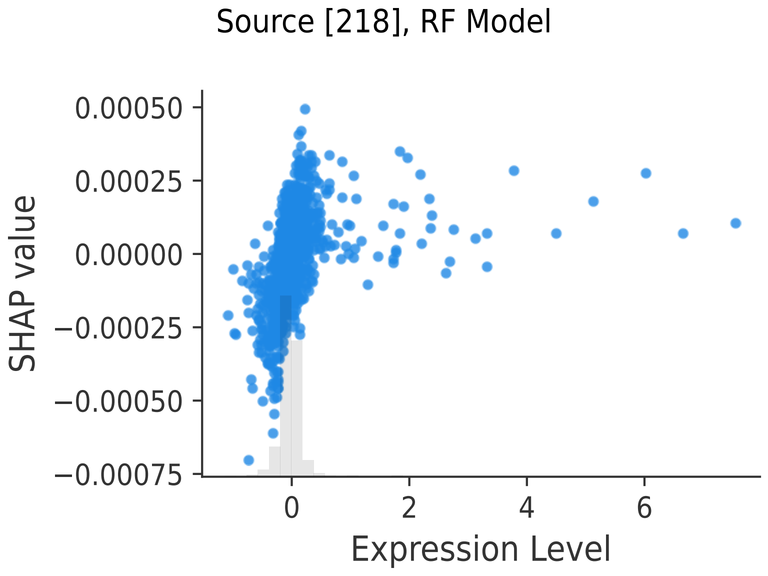
Source [218], RF Model
0.00050
0.00025
0.00000
−0.00025
−0.00050
−0.00075
SHAP value
0
2
4
6
Expression Level

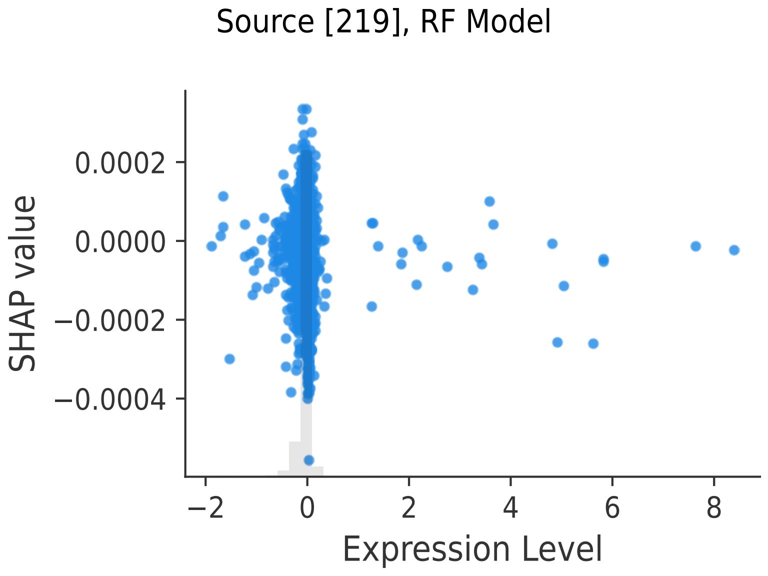
Source [219], RF Model
0.0002
0.0000
−0.0002
−0.0004
SHAP value
−2
0
2
4
6
8
Expression Level

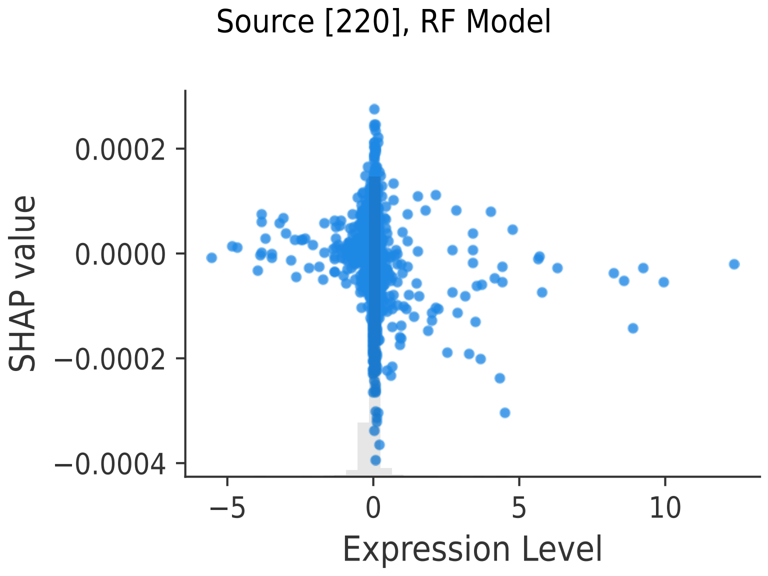
Source [220], RF Model
0.0002
0.0000
−0.0002
−0.0004
SHAP value
−5
0
5
10
Expression Level

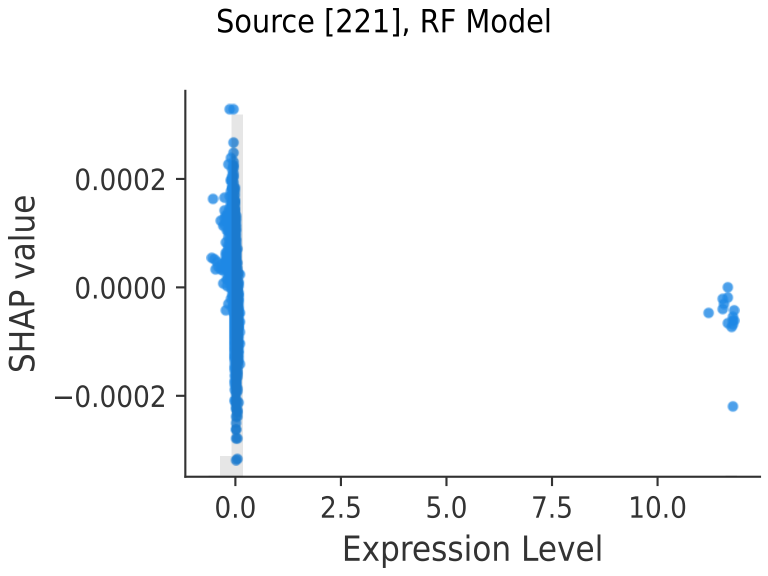

Source [221], RF Model
0.0002
0.0000
−0.0002
SHAP value
0.0
2.5
5.0
7.5
10.0
Expression Level

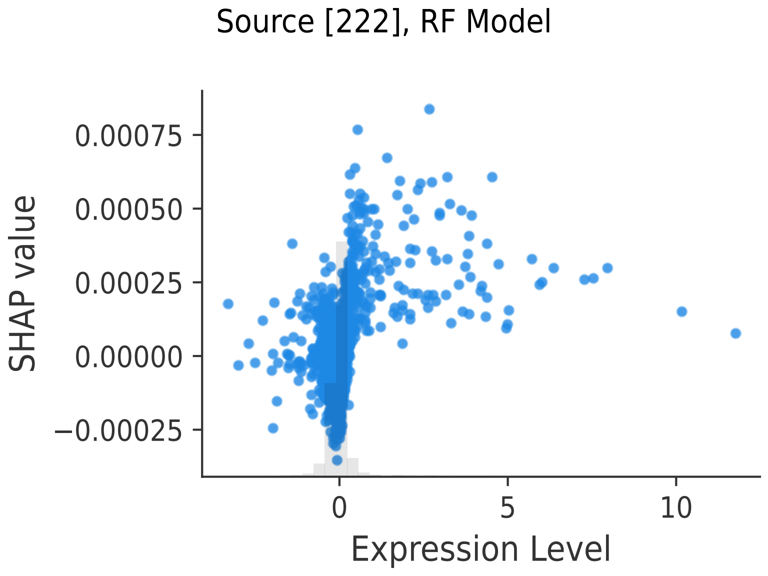
Source [222], RF Model
SHAP value
0.00075
0.00050
0.00025
0.00000
−0.00025
0
5
10
Expression Level

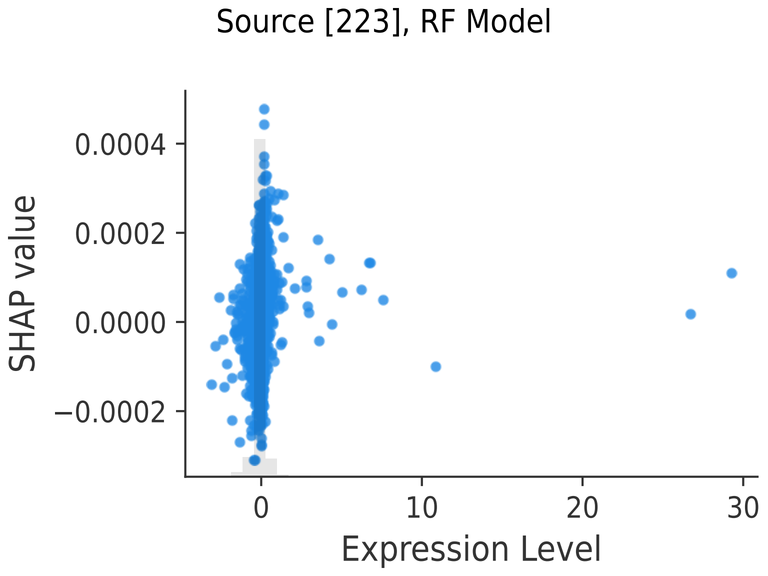
Source [223], RF Model
SHAP value
0.0004
0.0002
0.0000
−0.0002
0
10
20
30
Expression Level

Source [224], RF Model

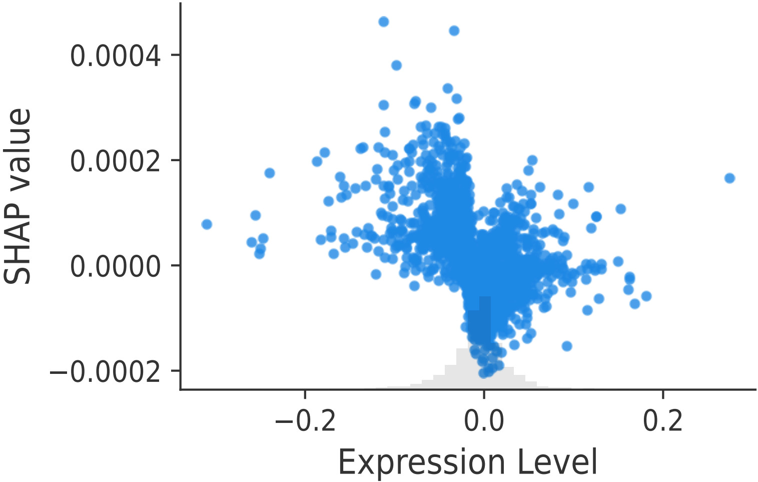

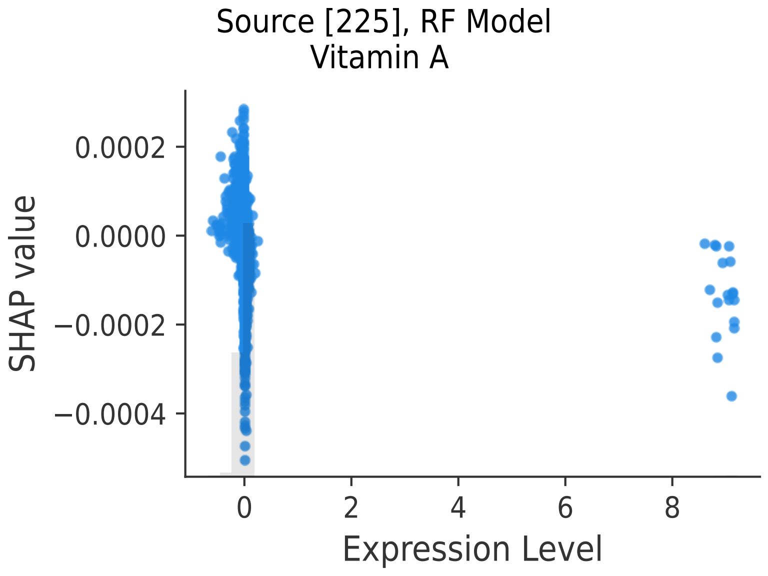

Source [225], RF Model
Vitamin A
SHAP value
0.0002
0.0000
−0.0002
−0.0004
0
2
4
6
8
Expression Level

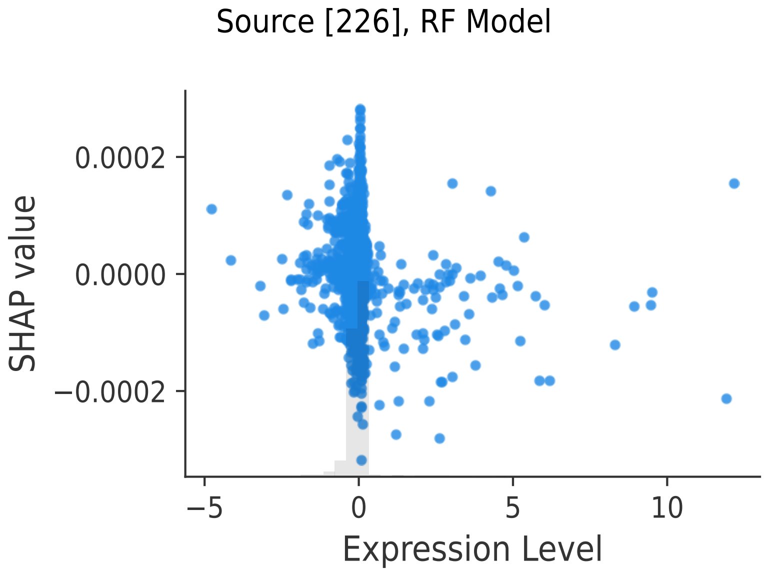

Source [226], RF Model
0.0002
0.0000
−0.0002
SHAP value
−5
0
5
10
Expression Level

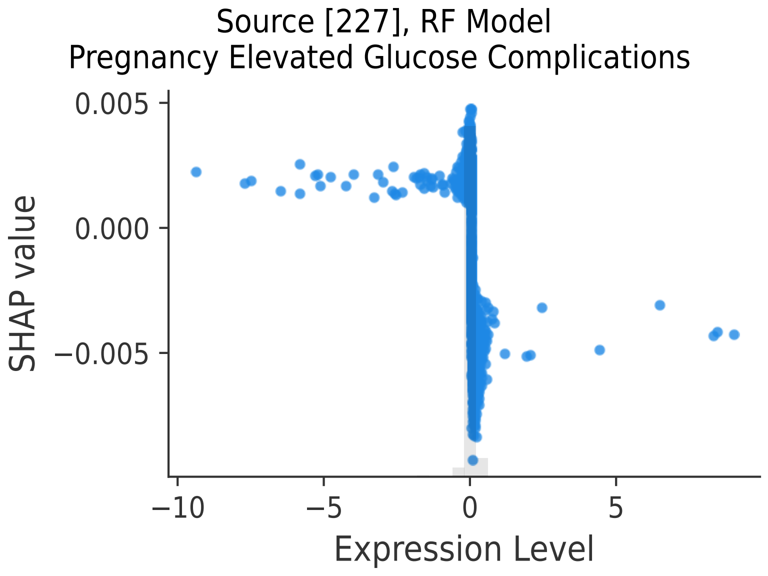
Source [227], RF Model
Pregnancy Elevated Glucose Complications
0.005
0.000
−0.005
SHAP value
−10
−5
0
5
Expression Level

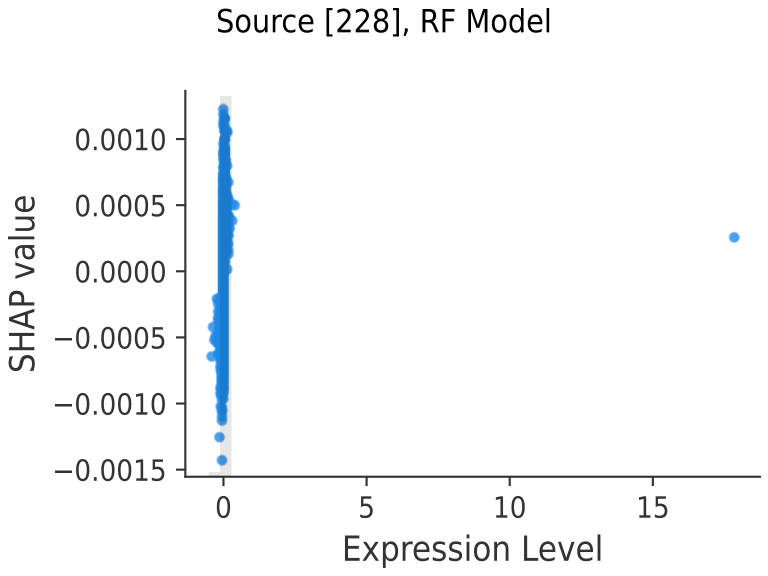
Source [228], RF Model
SHAP value
0.0010
0.0005
0.0000
−0.0005
−0.0010
−0.0015
0
5
10
15
Expression Level

Source [229], RF Model

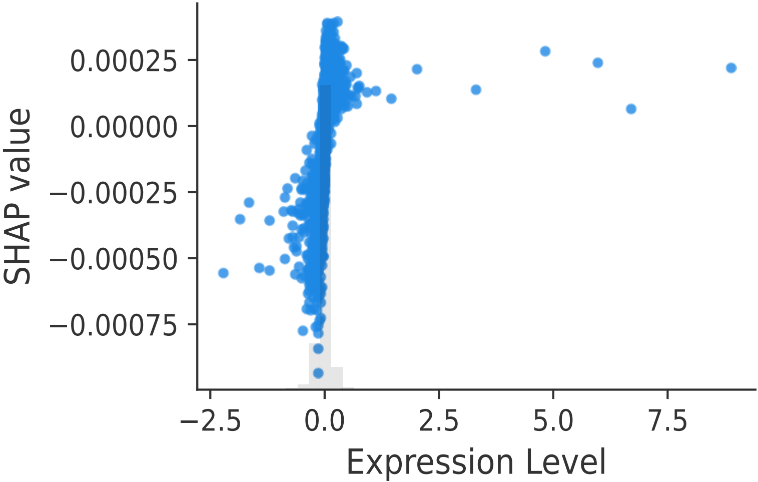

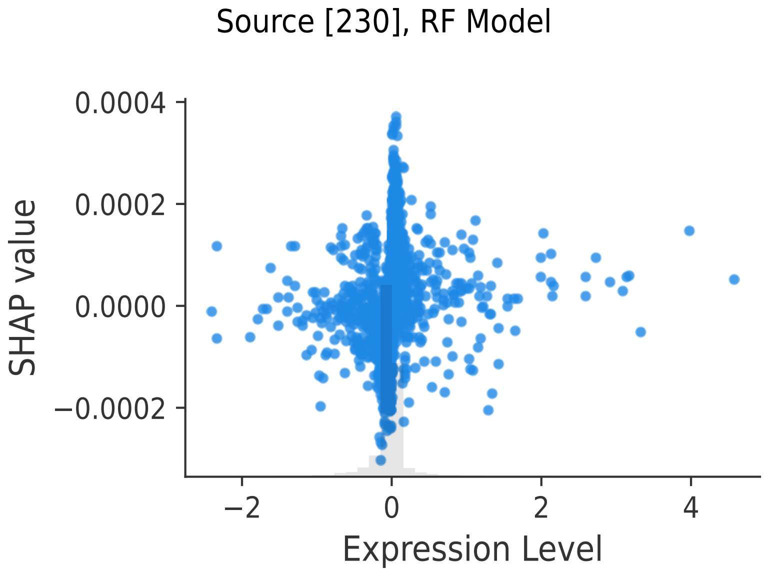

Source [230], RF Model
0.0004
0.0002
0.0000
−0.0002
SHAP value
−2
0
2
4
Expression Level

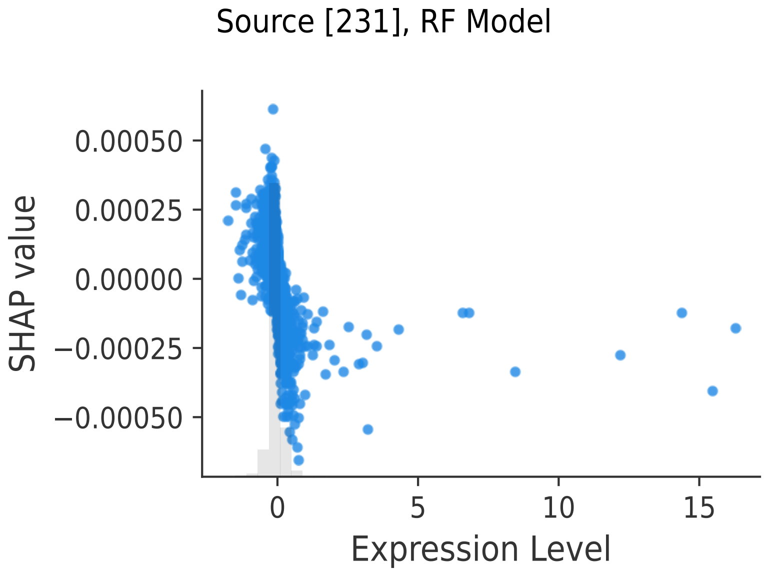

Source [231], RF Model
0.00050
0.00025
0.00000
−0.00025
−0.00050
SHAP value
0
5
10
15
Expression Level

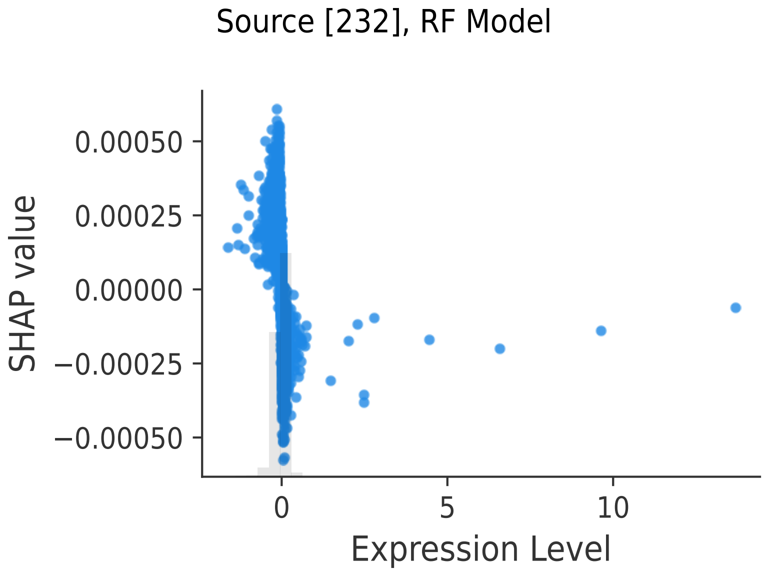
Source [232], RF Model
SHAP value
0.00050
0.00025
0.00000
−0.00025
−0.00050
0
5
10
Expression Level

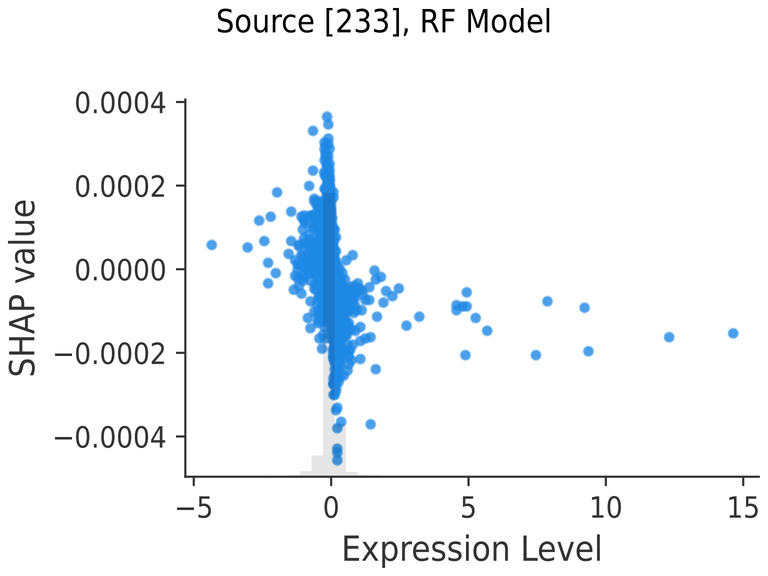
Source [233], RF Model
0.0004
0.0002
0.0000
−0.0002
−0.0004
SHAP value
−5
0
5
10
15
Expression Level

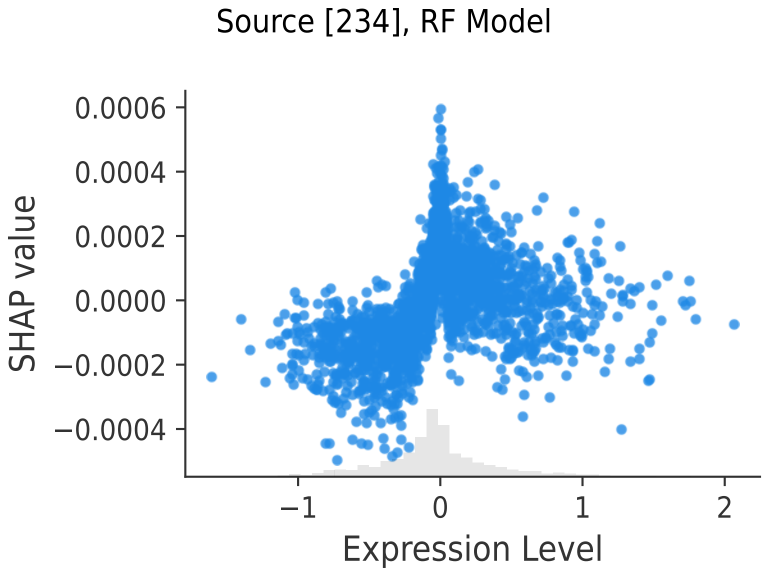

Source [234], RF Model
SHAP value
0.0006
0.0004
0.0002
0.0000
−0.0002
−0.0004
−1
0
1
2
Expression Level

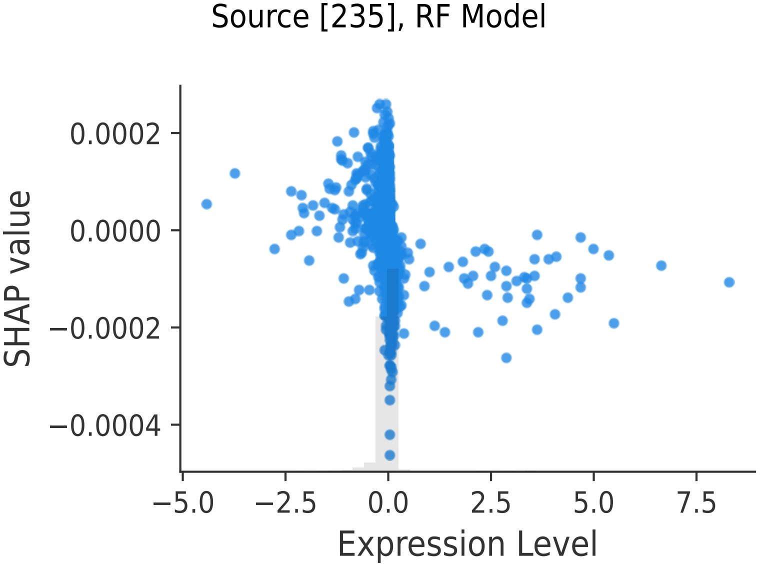
Source [235], RF Model
0.0002
0.0000
−0.0002
−0.0004
SHAP value
−5.0
−2.5
0.0
2.5
5.0
7.5
Expression Level

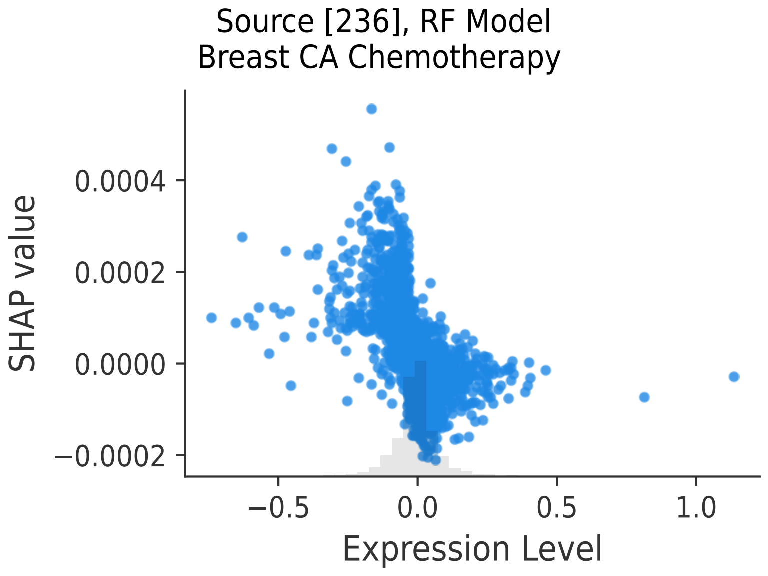
Source [236], RF Model
Breast CA Chemotherapy
SHAP value
0.0004
0.0002
0.0000
−0.0002
−0.5
0.0
0.5
1.0
Expression Level

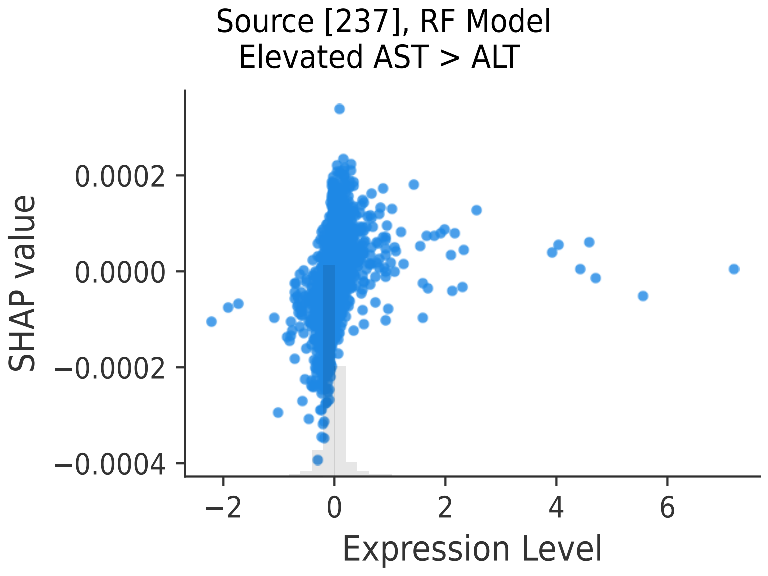
Source [237], RF Model
Elevated AST > ALT
SHAP value
0.0002
0.0000
−0.0002
−0.0004
−2
0
2
4
6
Expression Level

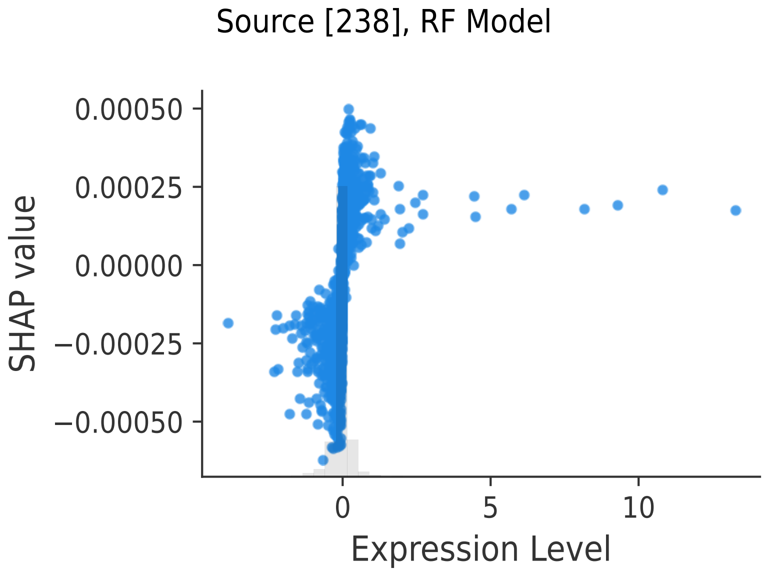
Source [238], RF Model
SHAP value
0.00050
0.00025
0.00000
−0.00025
−0.00050
0
5
10
Expression Level

Source [239], RF Model

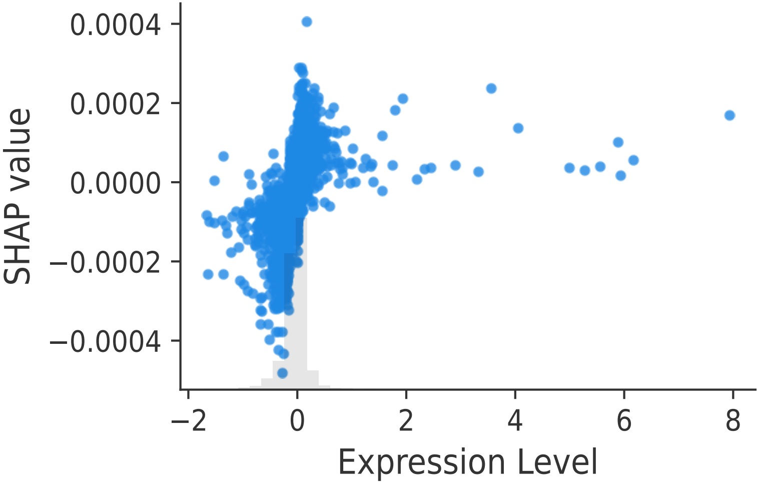

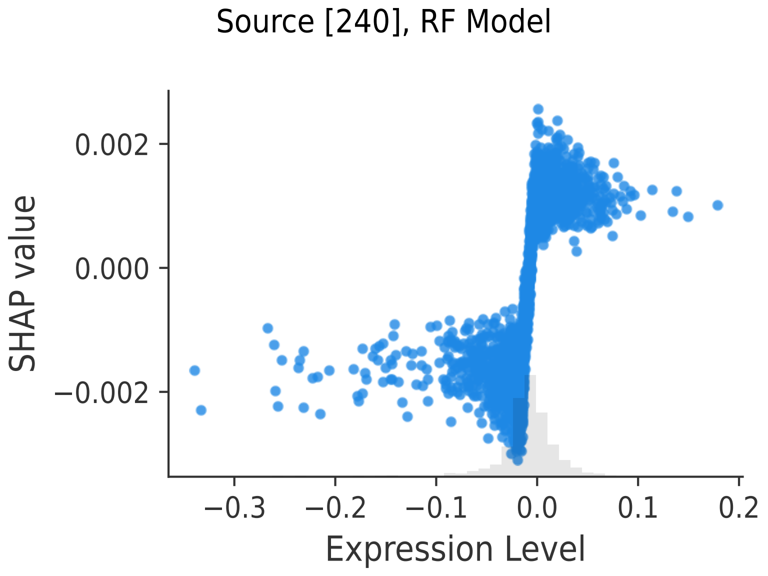
Source [240], RF Model
0.002
0.000
−0.002
SHAP value
−0.3
−0.2
−0.1
0.0
0.1
0.2
Expression Level

Source [241], RF Model

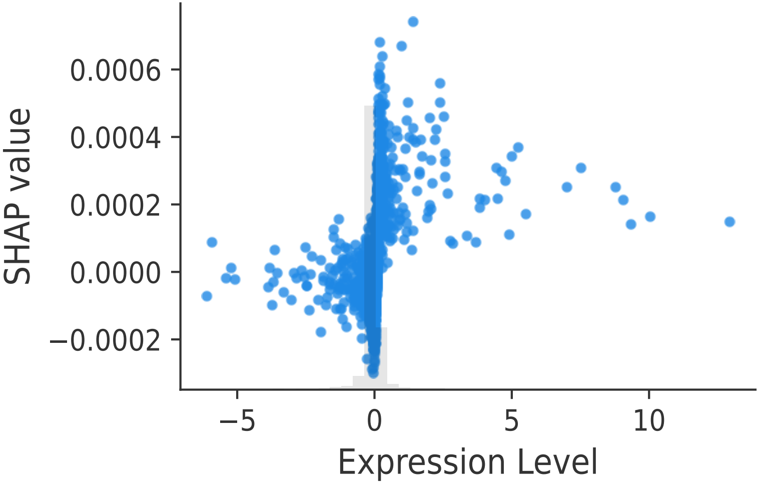

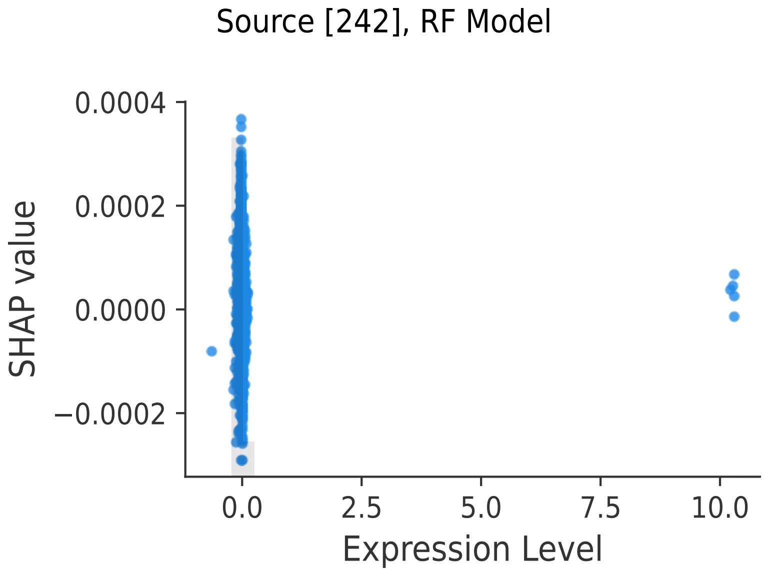
Source [242], RF Model
0.0004
0.0002
0.0000
−0.0002
SHAP value
0.0
2.5
5.0
7.5
10.0
Expression Level

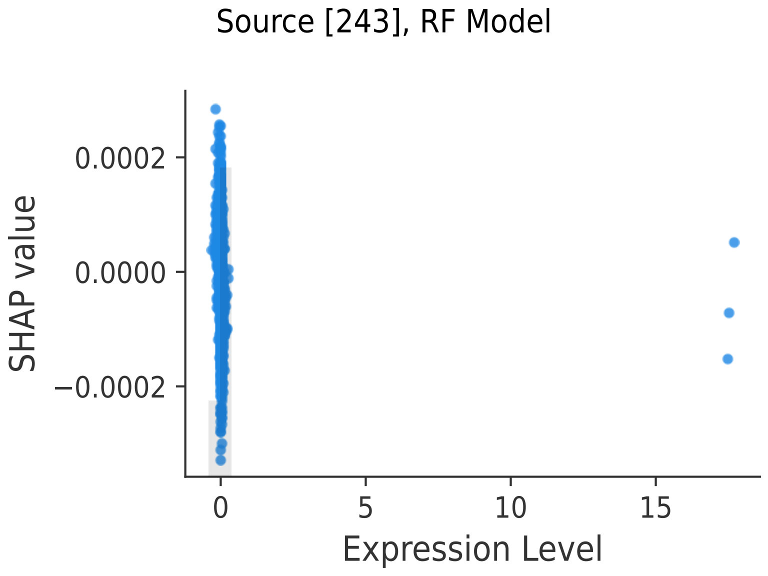

Source [243], RF Model
SHAP value
0.0002
0.0000
−0.0002
0
5
10
15
Expression Level

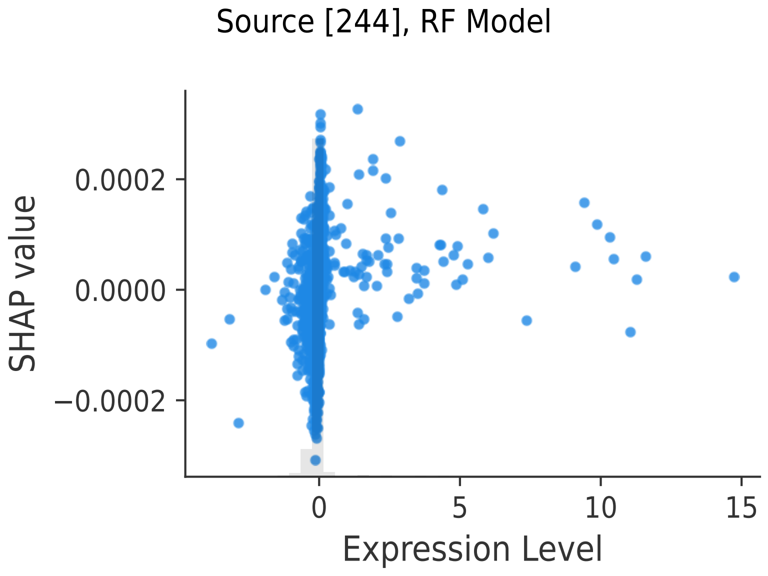
Source [244], RF Model
0.0002
0.0000
−0.0002
SHAP value
0
5
10
15
Expression Level

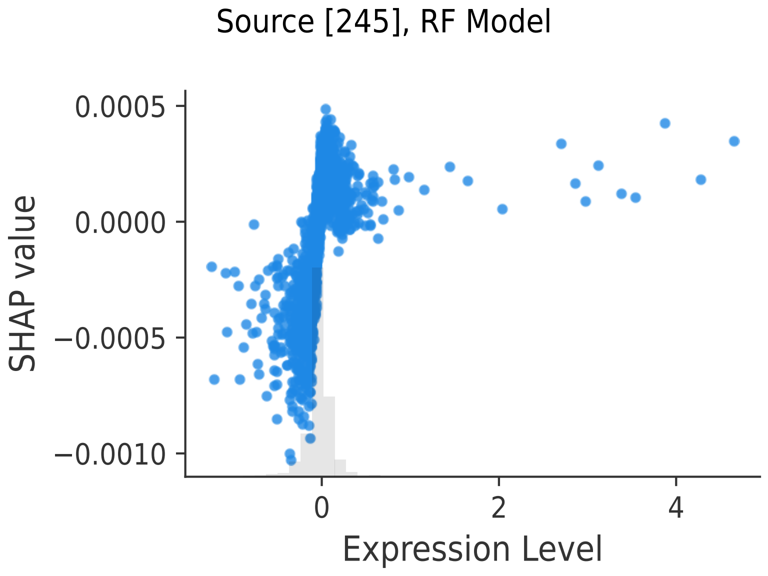
Source [245], RF Model
0.0005
0.0000
−0.0005
−0.0010
SHAP value
0
2
4
Expression Level

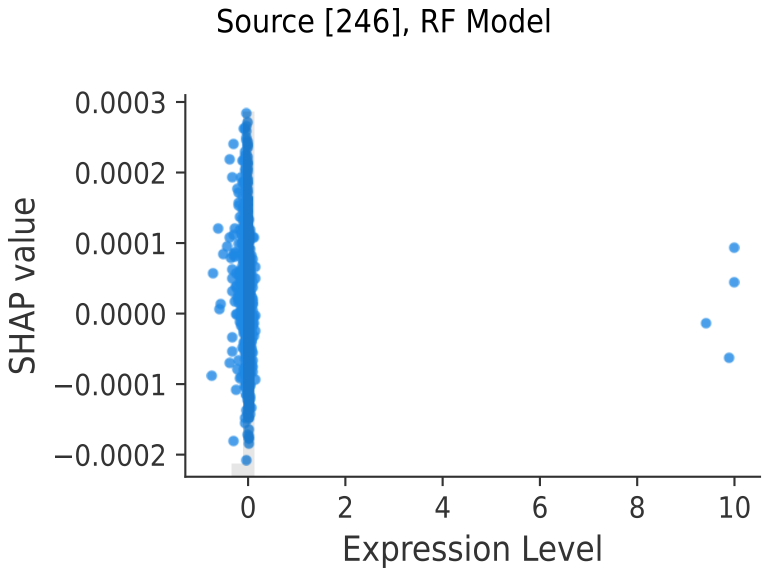
Source [246], RF Model
SHAP value
0.0003
0.0002
0.0001
0.0000
−0.0001
−0.0002
0
2
4
6
8
10
Expression Level

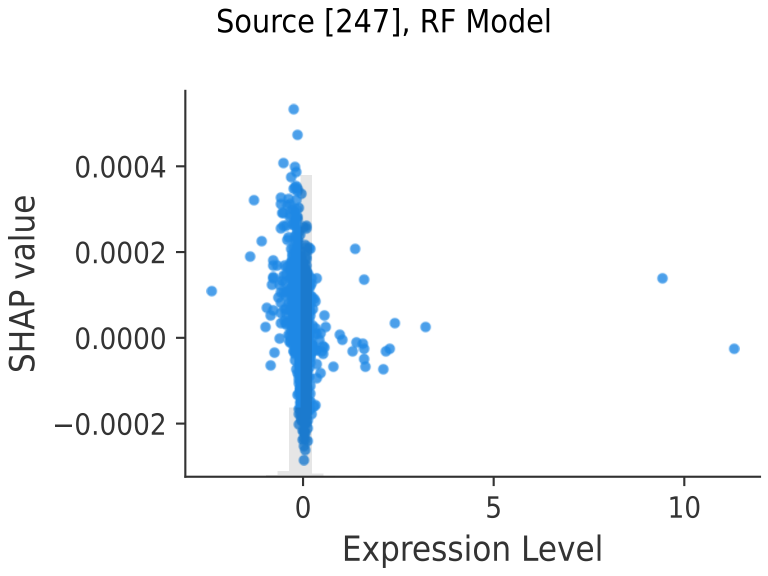
Source [247], RF Model
0.0004
0.0002
0.0000
−0.0002
SHAP value
0
5
10
Expression Level

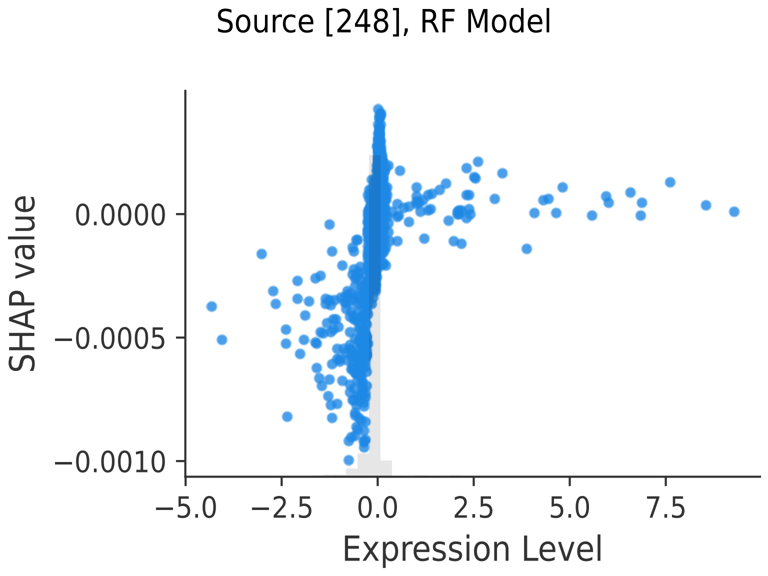
Source [248], RF Model
SHAP value
0.0000
−0.0005
−0.0010
−5.0
−2.5
0.0
2.5
5.0
7.5
Expression Level

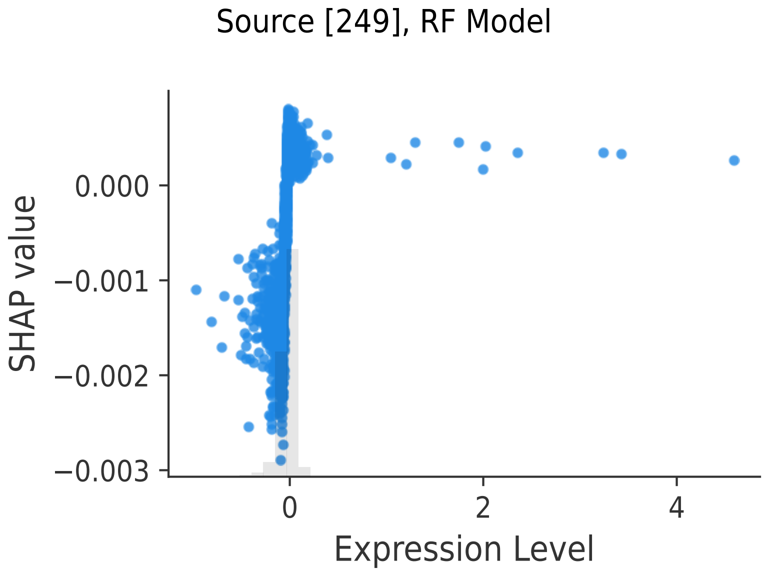
Source [249], RF Model
SHAP value
0.000
−0.001
−0.002
−0.003
0
2
4
Expression Level

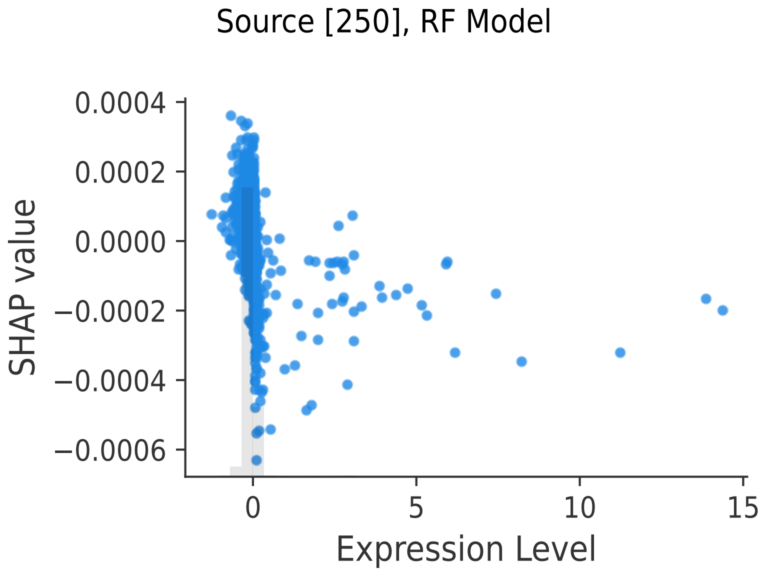

Source [250], RF Model
0.0004
0.0002
0.0000
−0.0002
−0.0004
−0.0006
SHAP value
0
5
10
15
Expression Level

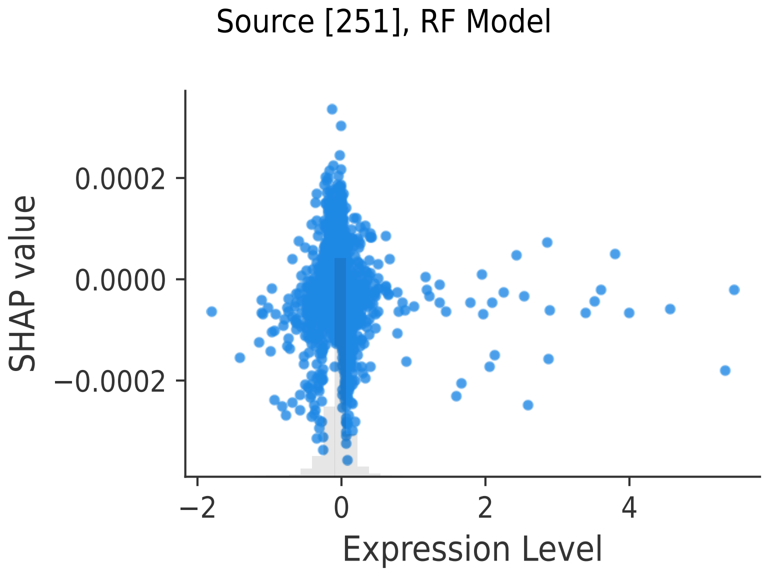

Source [251], RF Model
SHAP value
0.0002
0.0000
−0.0002
−2
0
2
4
Expression Level

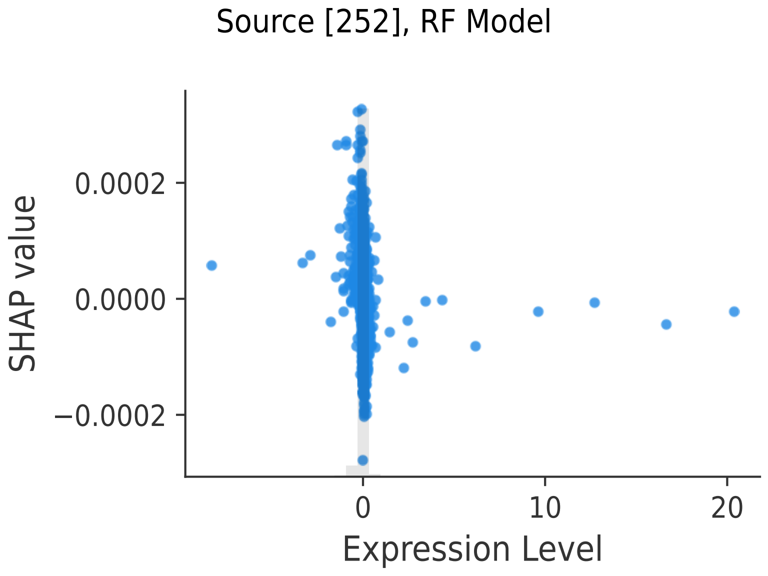

Source [252], RF Model
SHAP value
0.0002
0.0000
−0.0002
0
10
20
Expression Level

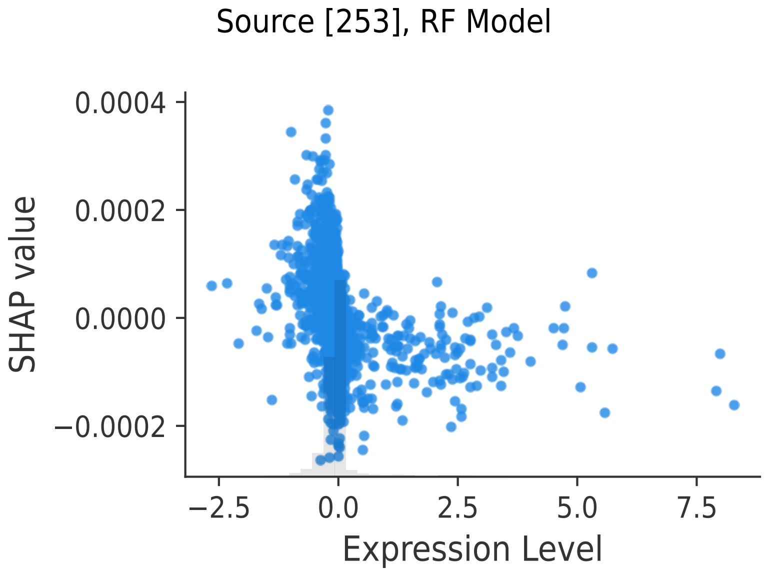
Source [253], RF Model
0.0004
0.0002
0.0000
−0.0002
SHAP value
−2.5
0.0
2.5
5.0
7.5
Expression Level

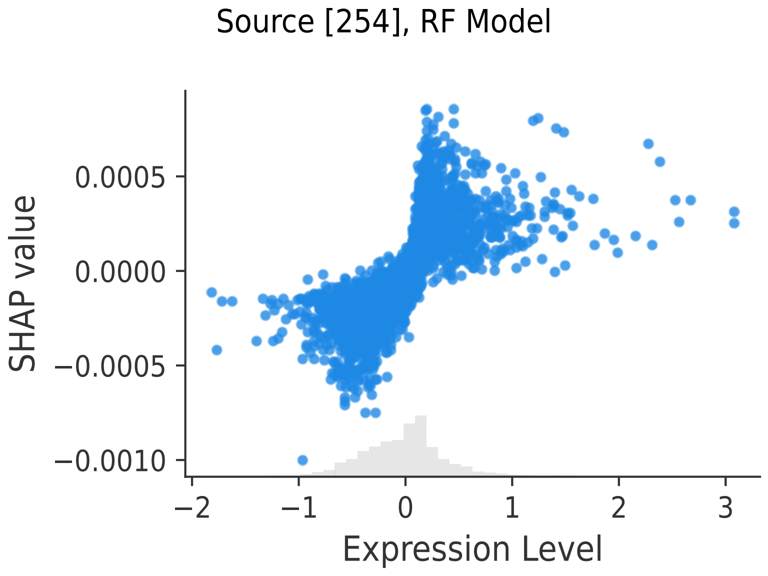
Source [254], RF Model
SHAP value
0.0005
0.0000
−0.0005
−0.0010
−2
−1
0
1
2
3
Expression Level

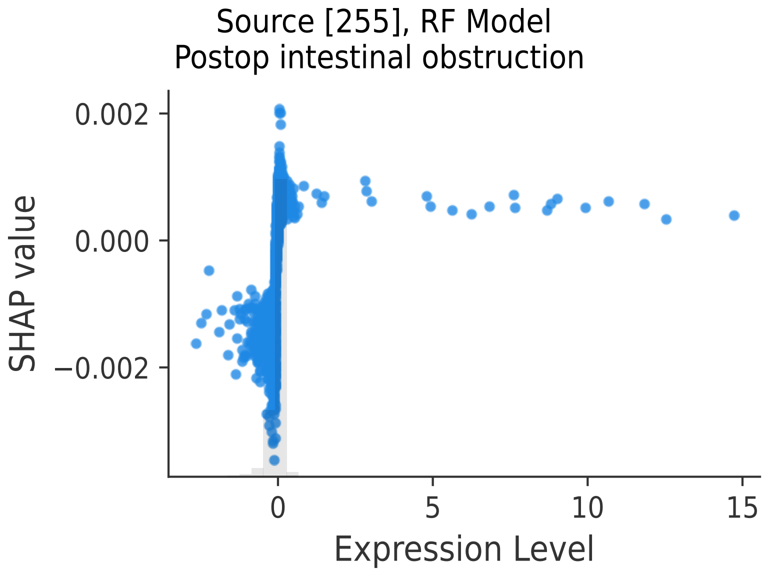

Source [255], RF Model
Postop intestinal obstruction
SHAP value
0.002
0.000
−0.002
0
5
10
15
Expression Level

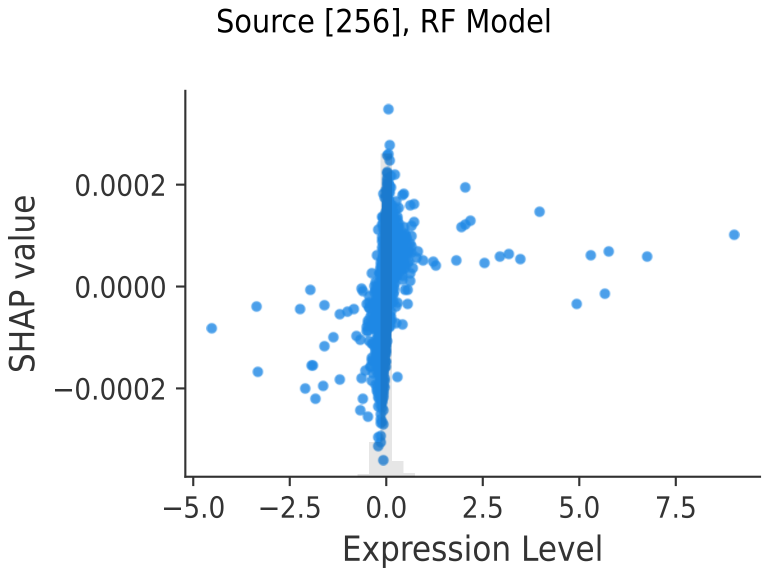

Source [256], RF Model
0.0002
0.0000
−0.0002
SHAP value
−5.0
−2.5
0.0
2.5
5.0
7.5
Expression Level

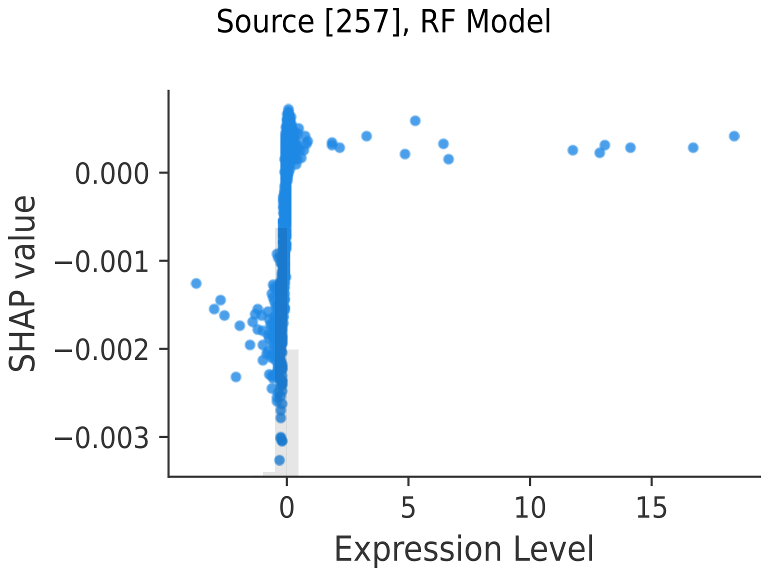
Source [257], RF Model
SHAP value
0.000
−0.001
−0.002
−0.003
0
5
10
15
Expression Level

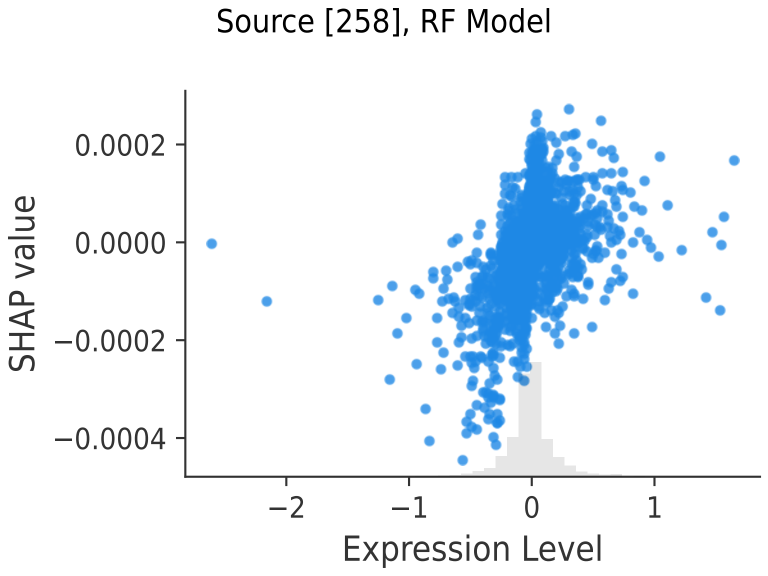
Source [258], RF Model
0.0002
0.0000
−0.0002
−0.0004
SHAP value
−2
−1
0
1
Expression Level

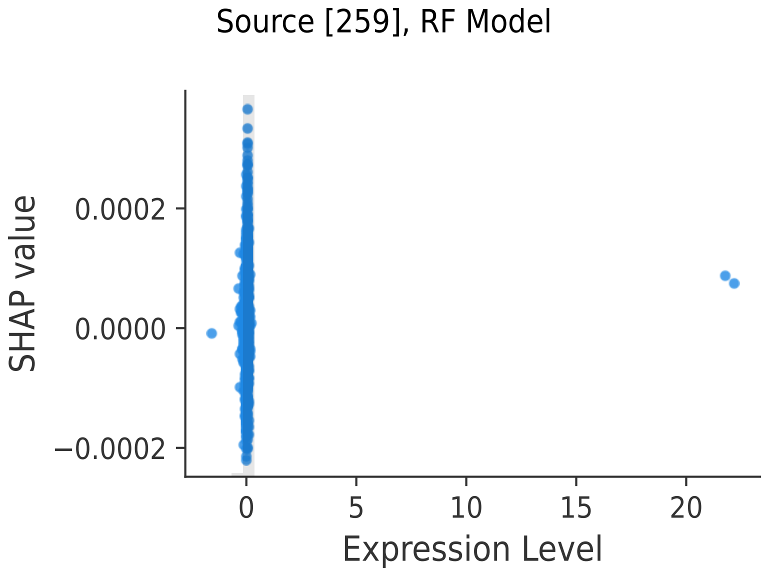
Source [259], RF Model
SHAP value
0.0002
0.0000
−0.0002
0
5
10
15
20
Expression Level

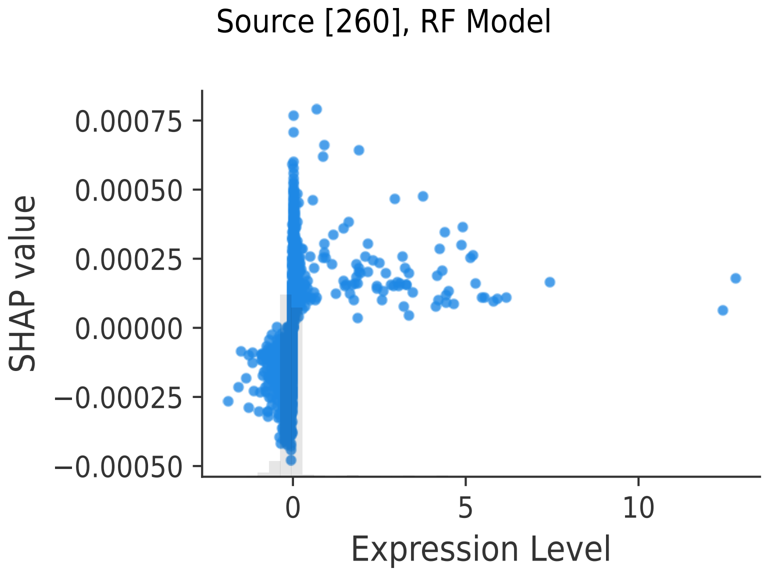
Source [260], RF Model
SHAP value
0.00075
0.00050
0.00025
0.00000
−0.00025
−0.00050
0
5
10
Expression Level

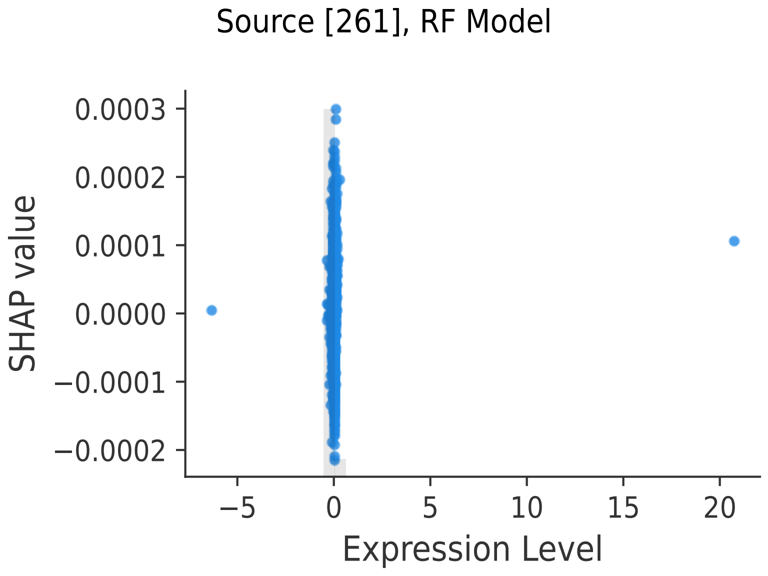
Source [261], RF Model
SHAP value
0.0003
0.0002
0.0001
0.0000
−0.0001
−0.0002
−5
0
5
10
15
20
Expression Level

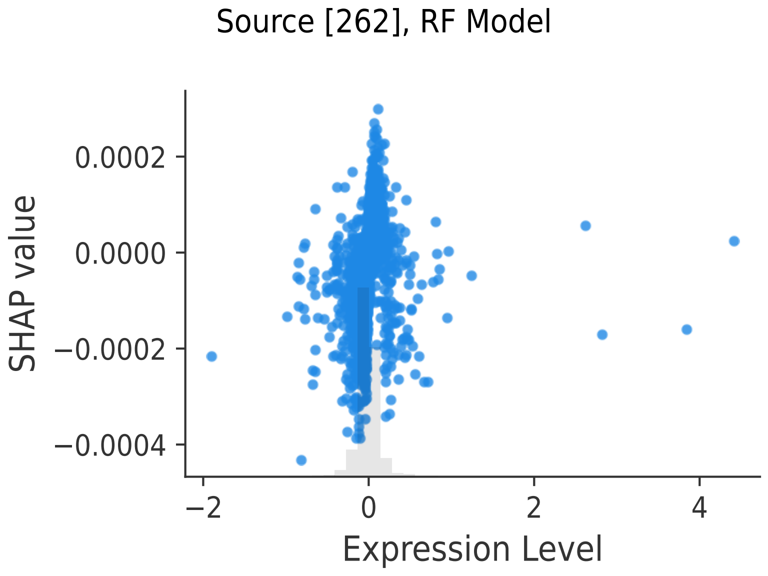
Source [262], RF Model
0.0002
0.0000
−0.0002
−0.0004
SHAP value
−2
0
2
4
Expression Level

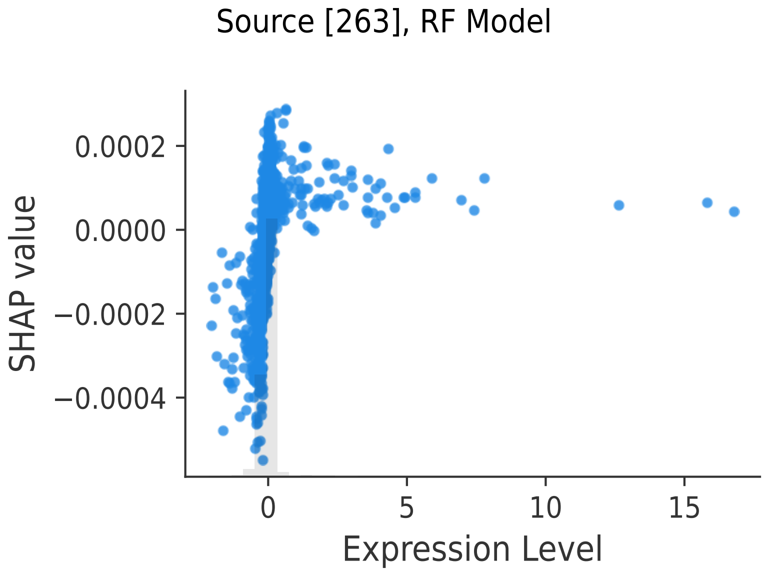
Source [263], RF Model
SHAP value
0.0002
0.0000
−0.0002
−0.0004
Expression Level
0
5
10
15

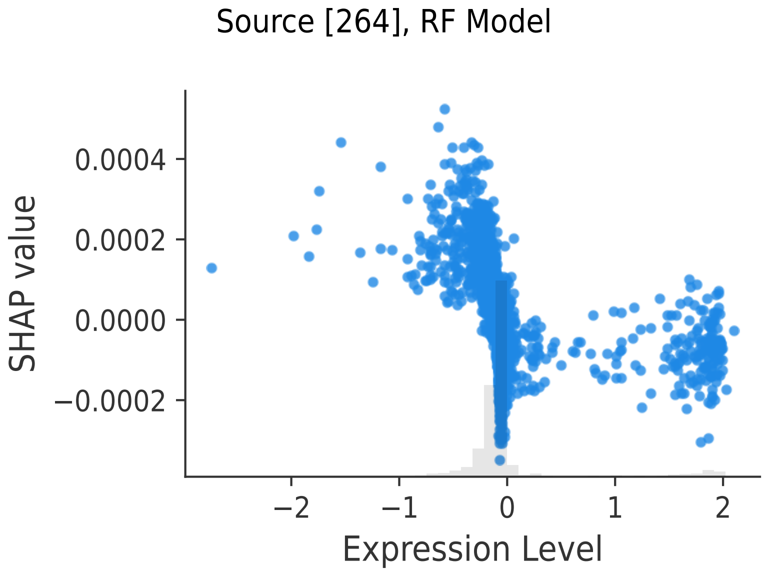

Source [264], RF Model
SHAP value
0.0004
0.0002
0.0000
−0.0002
−2
−1
0
1
2
Expression Level

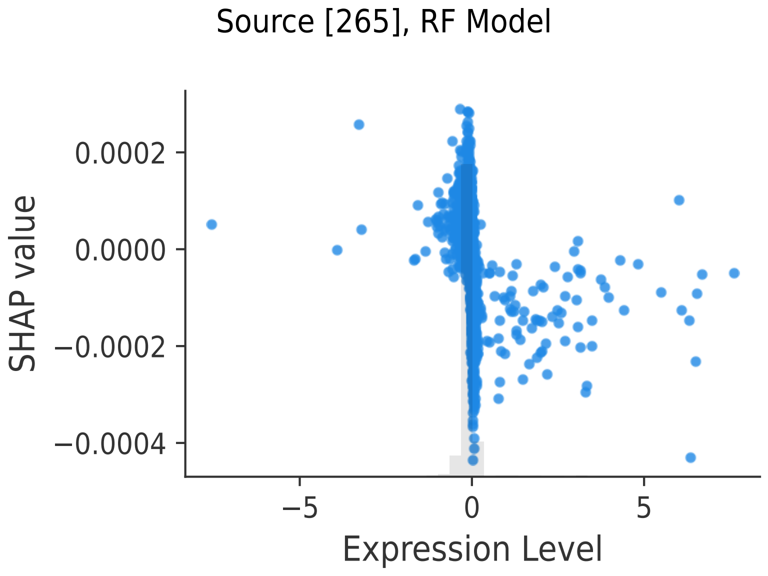

Source [265], RF Model
0.0002
0.0000
−0.0002
−0.0004
SHAP value
−5
0
5
Expression Level

Source [266], RF Model

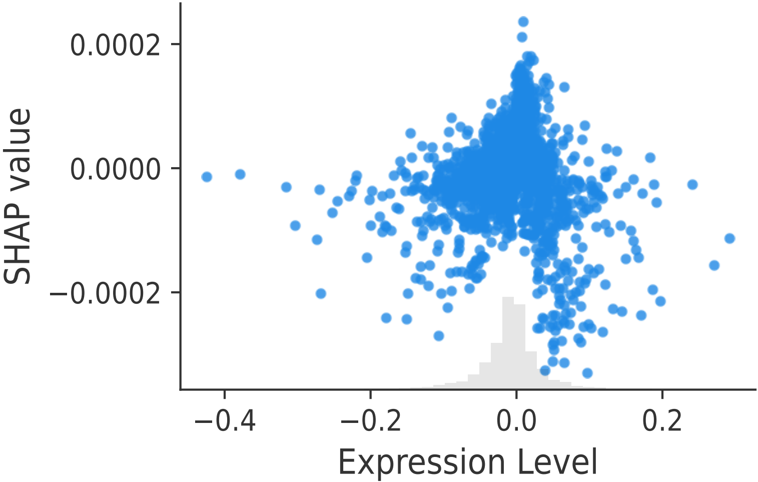

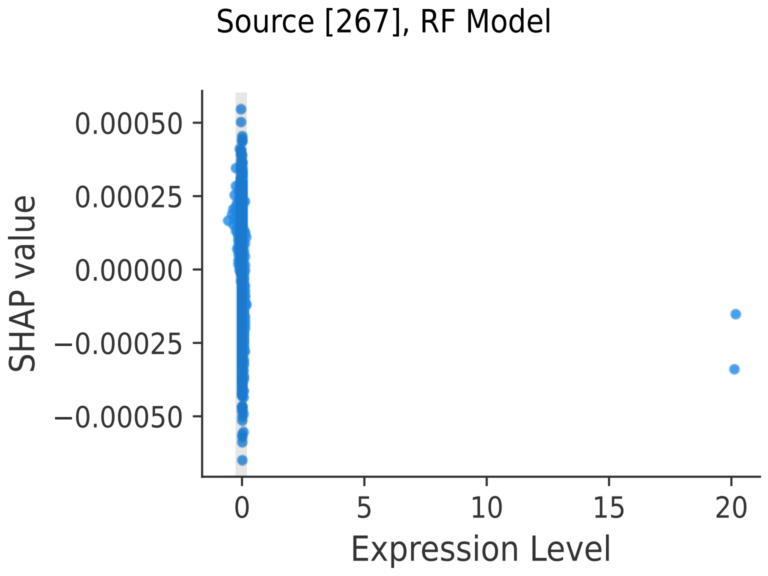

Source [267], RF Model
SHAP value
0.00050
0.00025
0.00000
−0.00025
−0.00050
0
5
10
15
20
Expression Level

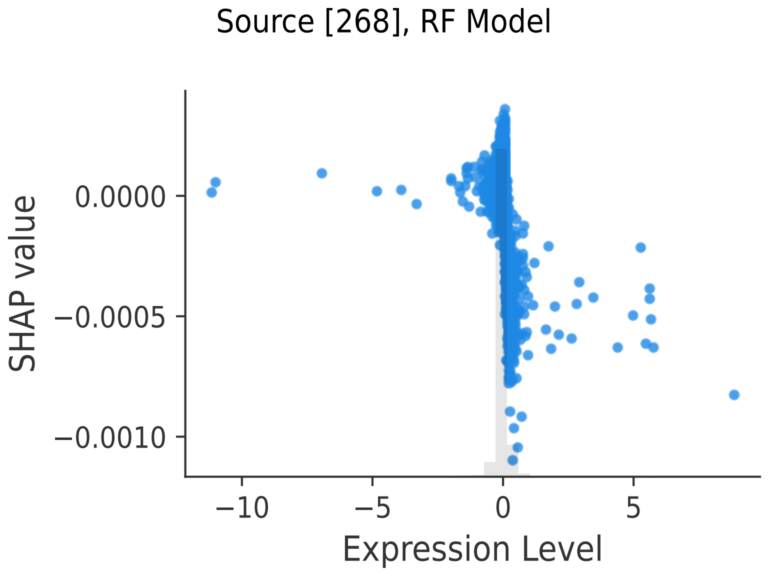

Source [268], RF Model
SHAP value
0.0000
−0.0005
−0.0010
−10
−5
0
5
Expression Level

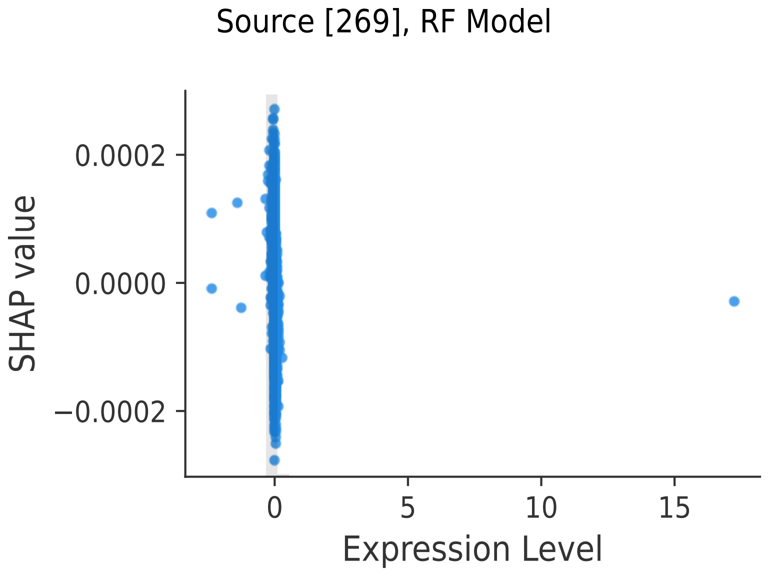
Source [269], RF Model
SHAP value
0.0002
0.0000
−0.0002
0
5
10
15
Expression Level

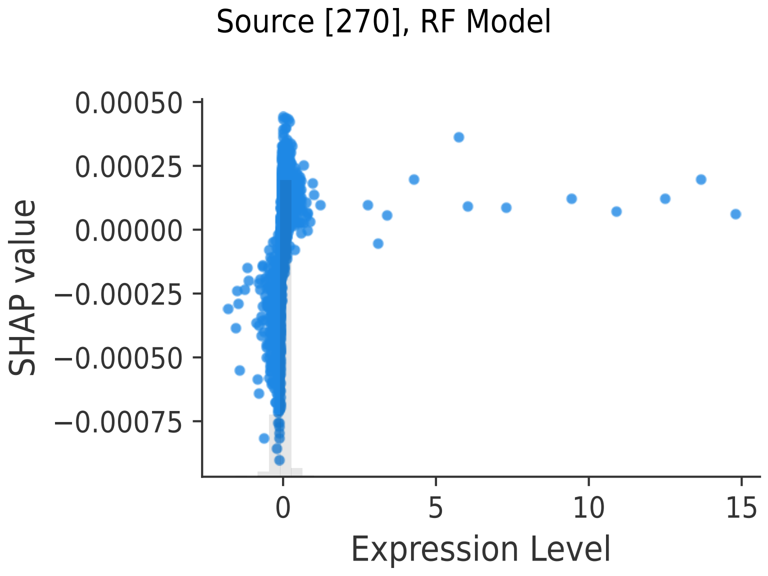
Source [270], RF Model
0.00050
0.00025
0.00000
−0.00025
−0.00050
−0.00075
SHAP value
0
5
10
15
Expression Level

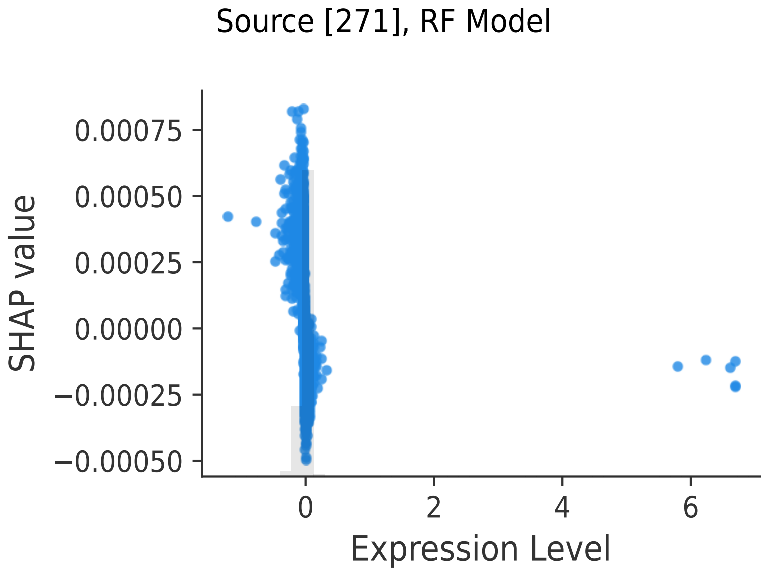

Source [271], RF Model
SHAP value
Expression Level
0.00075
0.00050
0.00025
0.00000
−0.00025
−0.00050
0
2
4
6

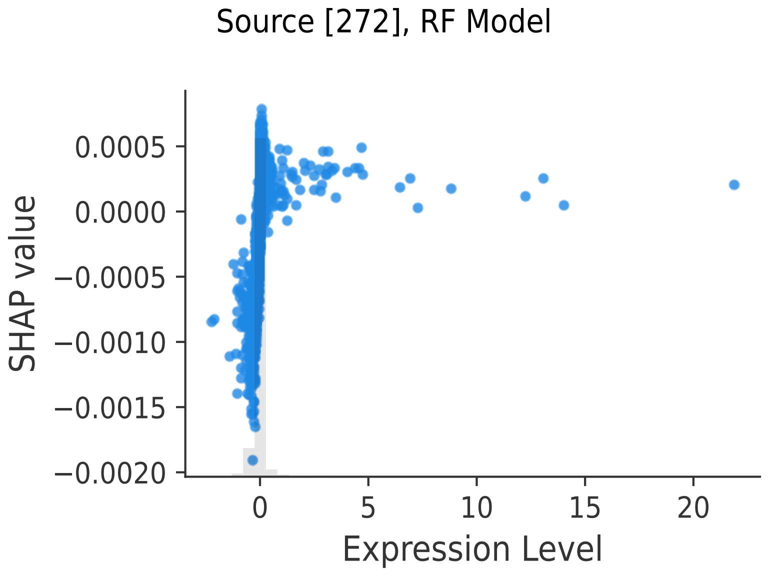

Source [272], RF Model
SHAP value
0.0005
0.0000
−0.0005
−0.0010
−0.0015
−0.0020
0
5
10
15
20
Expression Level

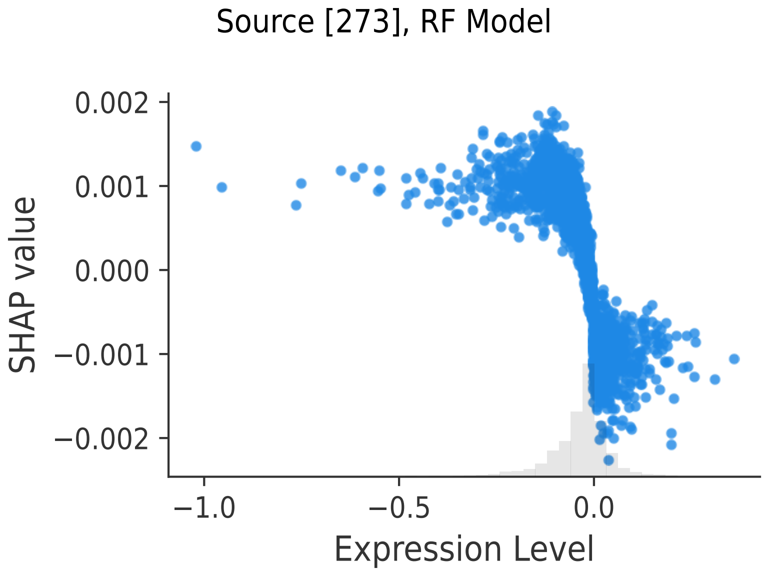

Source [273], RF Model
0.002
0.001
0.000
−0.001
−0.002
SHAP value
−1.0
−0.5
0.0
Expression Level

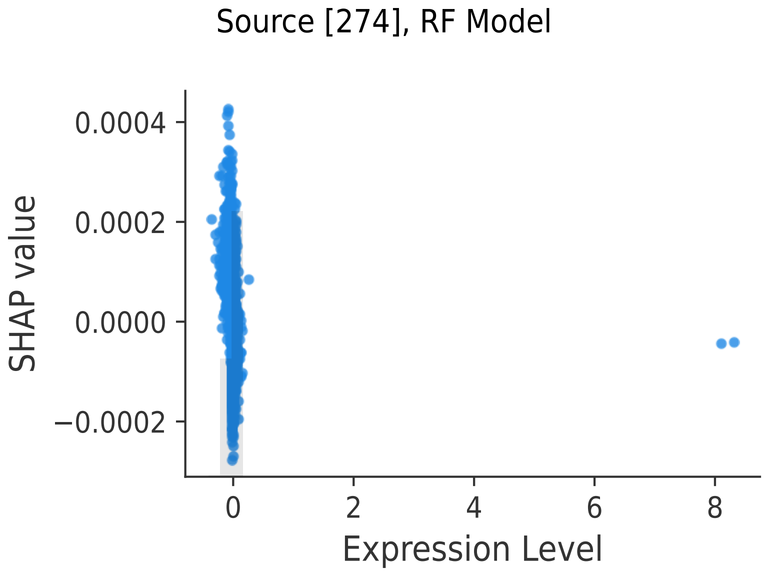
Source [274], RF Model
SHAP value
0.0004
0.0002
0.0000
−0.0002
0
2
4
6
8
Expression Level

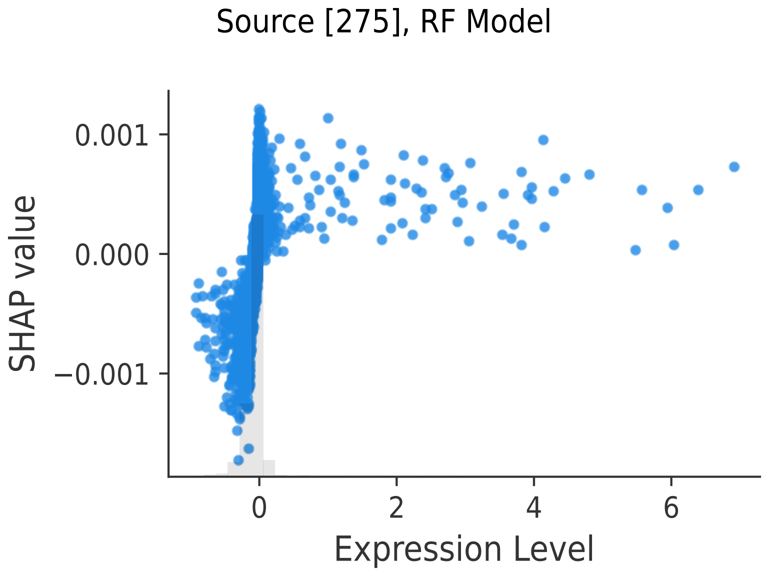

Source [275], RF Model
0.001
0.000
−0.001
SHAP value
0
2
4
6
Expression Level

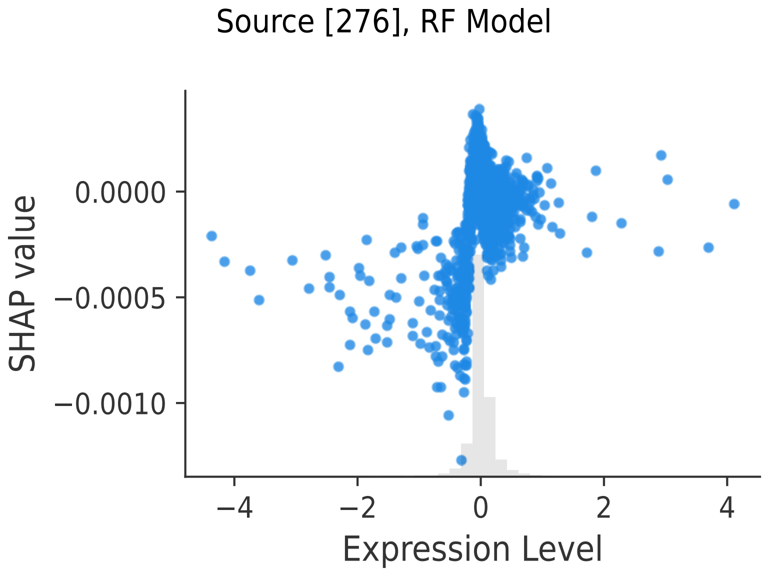

Source [276], RF Model
0.0000
−0.0005
−0.0010
SHAP value
−4
−2
0
2
4
Expression Level

Source [277], RF Model

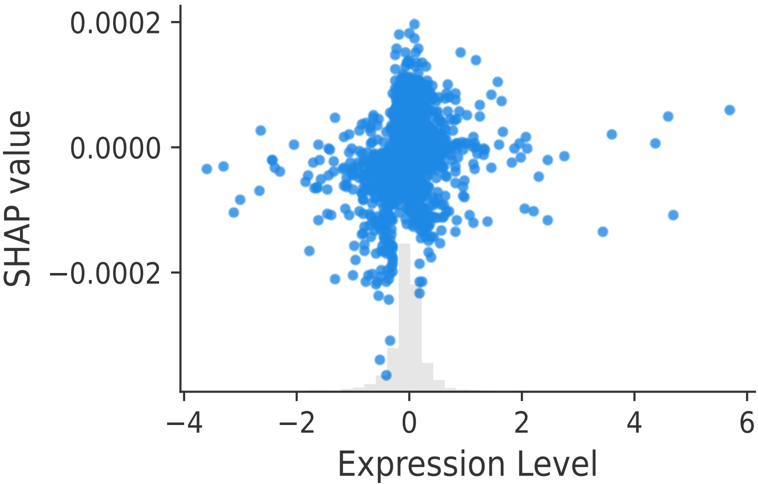

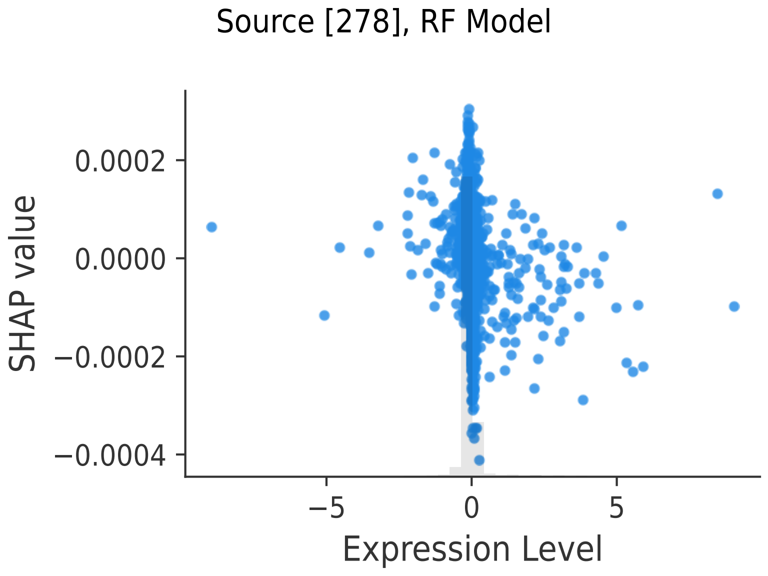
Source [278], RF Model
SHAP value
0.0002
0.0000
−0.0002
−0.0004
−5
0
5
Expression Level

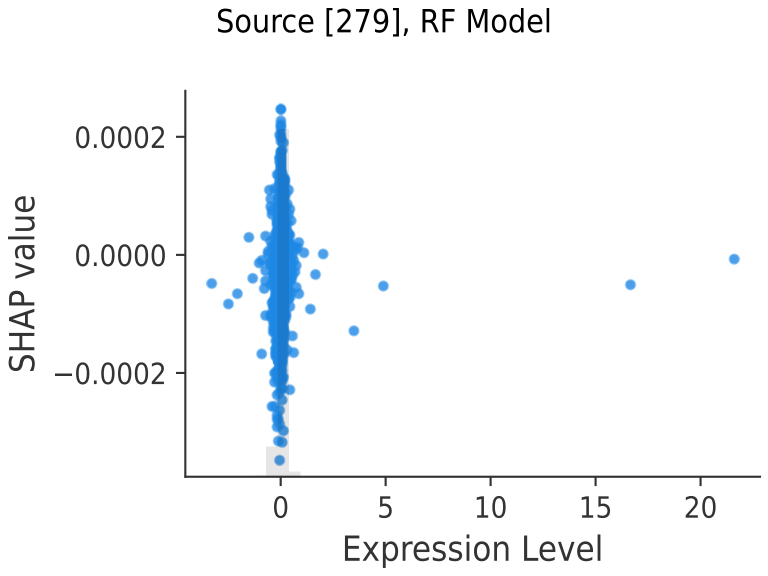
Source [279], RF Model
SHAP value
0.0002
0.0000
−0.0002
0
5
10
15
20
Expression Level

Source [280], RF Model

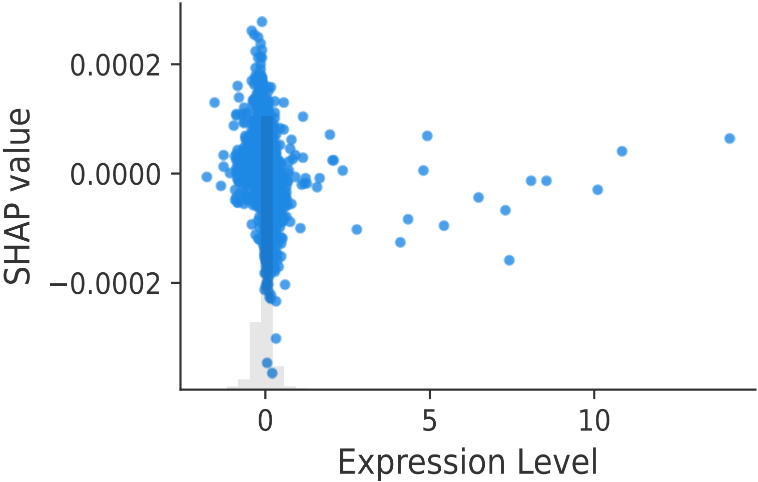

Source [281], RF Model

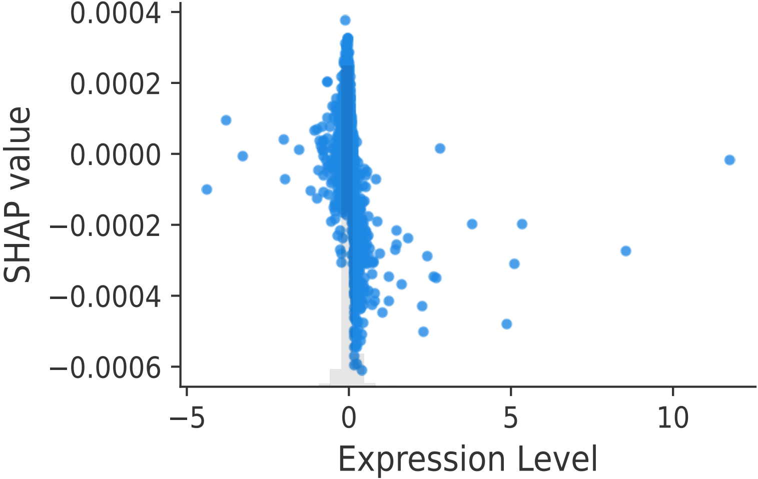

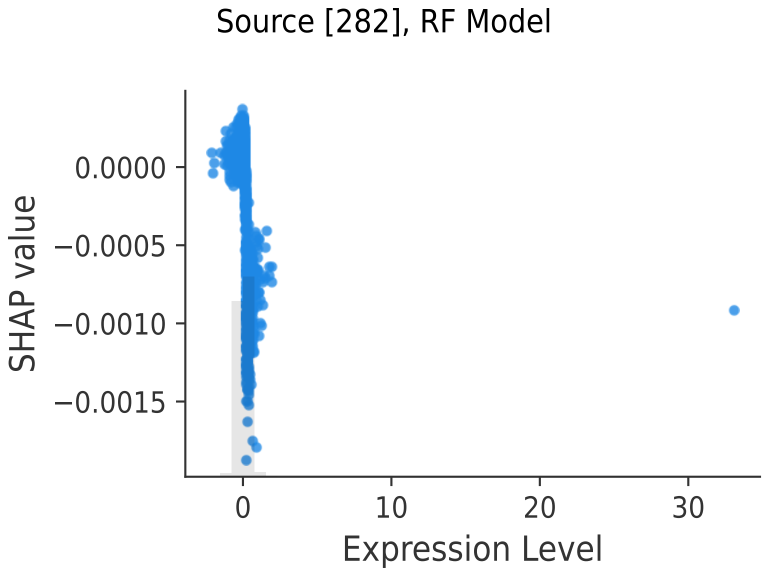
Source [282], RF Model
0.0000
−0.0005
−0.0010
−0.0015
SHAP value
0
10
20
30
Expression Level

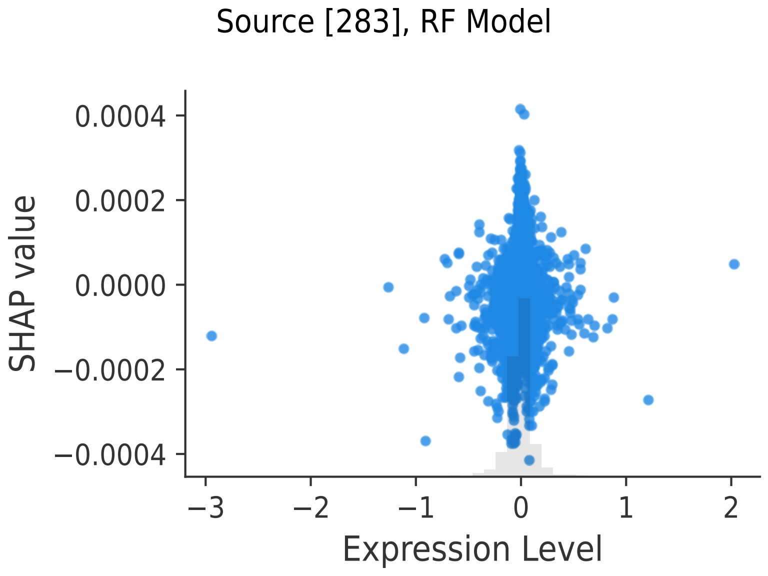
Source [283], RF Model
SHAP value
0.0004
0.0002
0.0000
−0.0002
−0.0004
−3
−2
−1
0
1
2
Expression Level

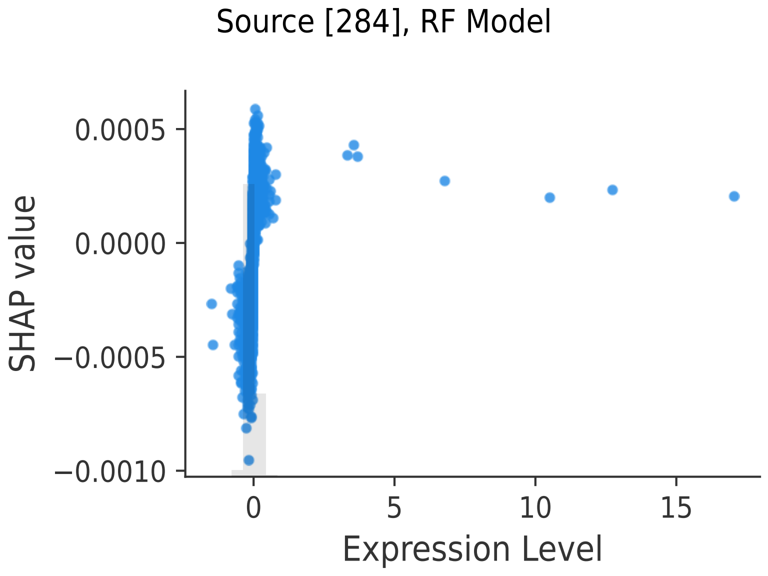

Source [284], RF Model
0.0005
0.0000
−0.0005
−0.0010
SHAP value
0
5
10
15
Expression Level

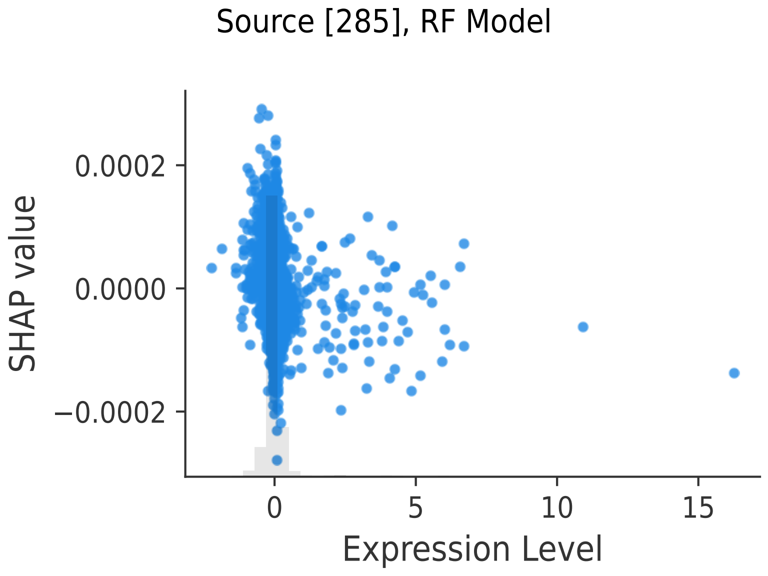
Source [285], RF Model
0.0002
0.0000
−0.0002
SHAP value
0
5
10
15
Expression Level

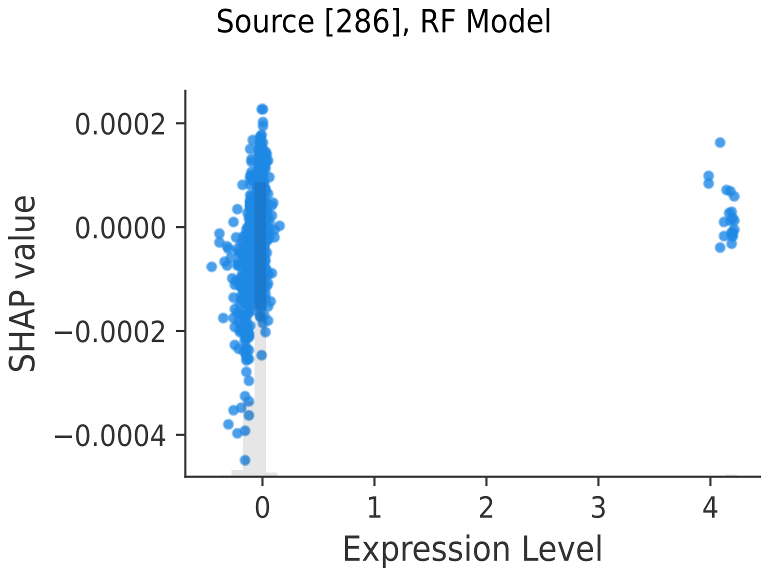
Source [286], RF Model
0.0002
0.0000
−0.0002
−0.0004
SHAP value
0
1
2
3
4
Expression Level

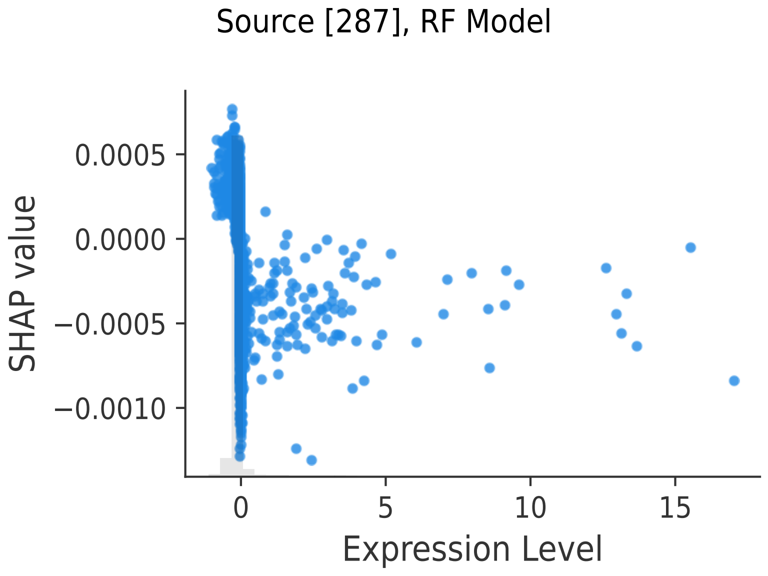
Source [287], RF Model
SHAP value
0.0005
0.0000
−0.0005
−0.0010
0
5
10
15
Expression Level

Source [288], RF Model

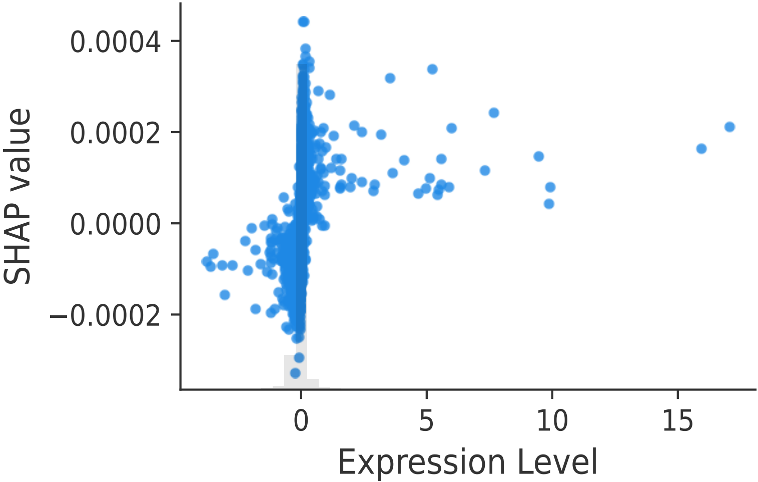

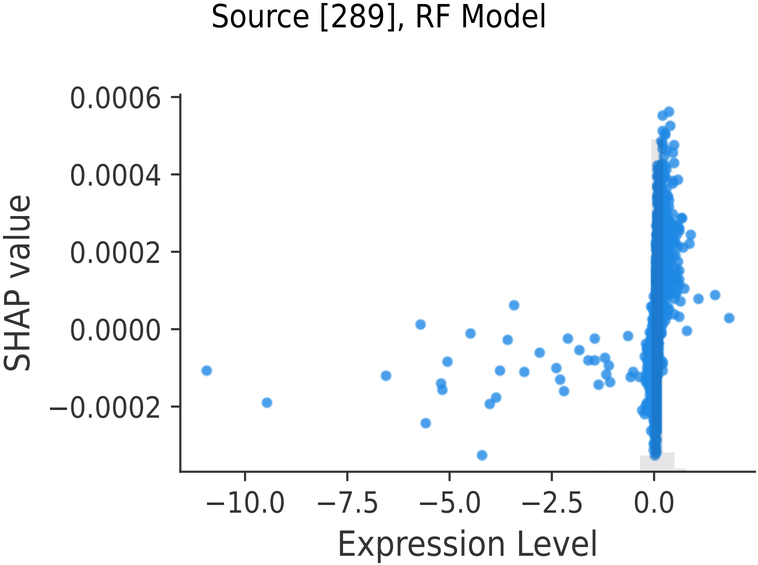
Source [289], RF Model
0.0006
0.0004
0.0002
0.0000
−0.0002
SHAP value
−10.0
−7.5
−5.0
−2.5
0.0
Expression Level

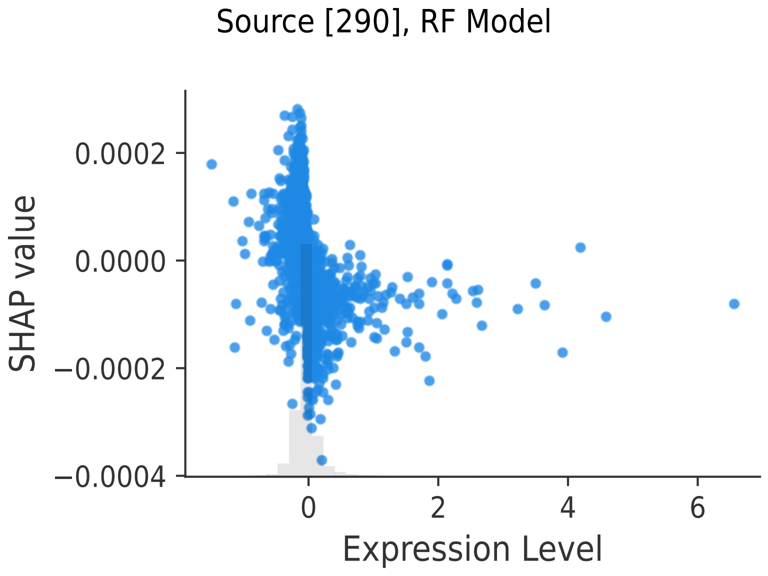

Source [290], RF Model
SHAP value
0.0002
0.0000
−0.0002
−0.0004
0
2
4
6
Expression Level

Source [291], RF Model

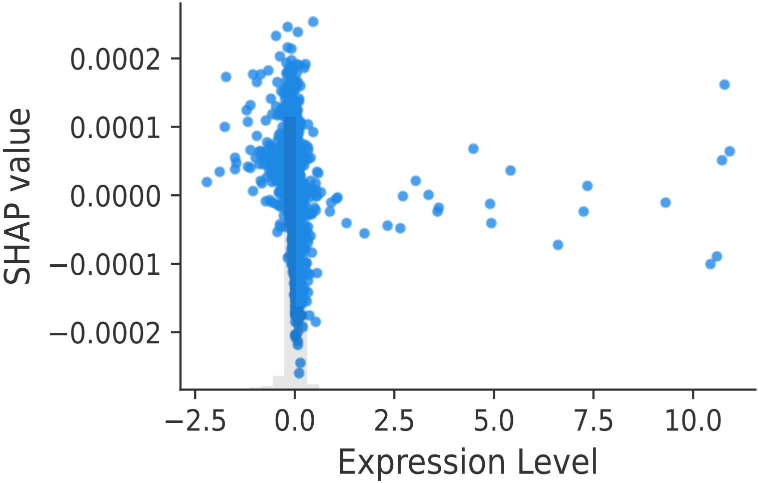

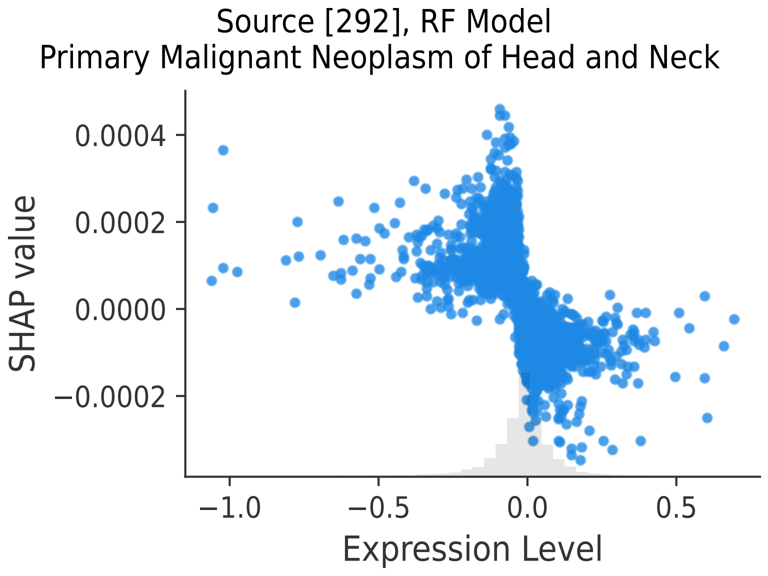
Source [292], RF Model
Primary Malignant Neoplasm of Head and Neck
SHAP value
0.0004
0.0002
0.0000
−0.0002
−1.0
−0.5
0.0
0.5
Expression Level

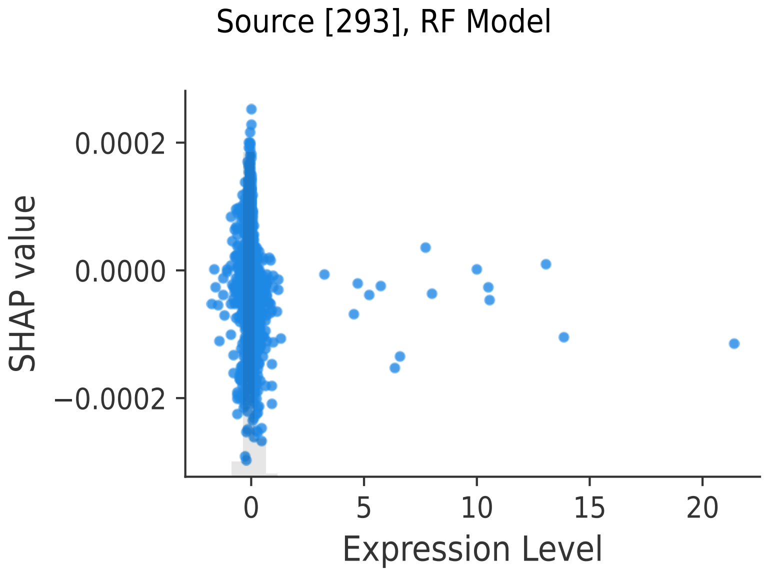
Source [293], RF Model
0.0002
0.0000
−0.0002
SHAP value
0
5
10
15
20
Expression Level

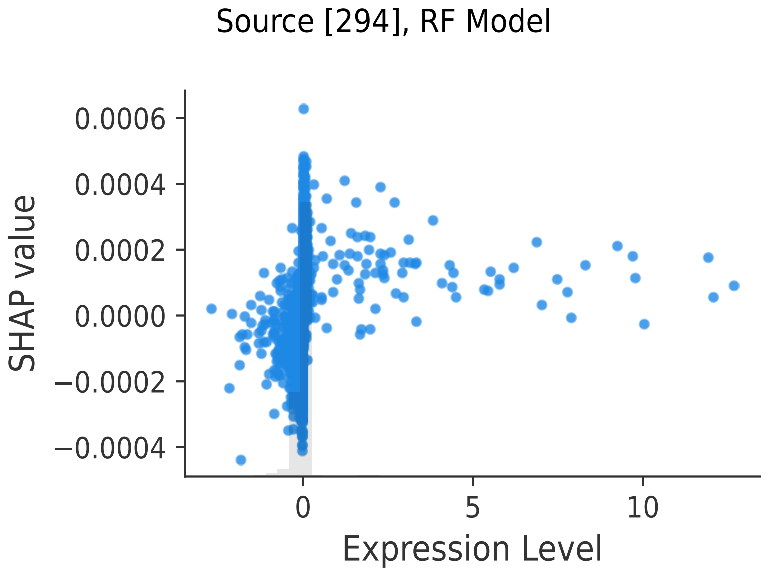

Source [294], RF Model
SHAP value
0.0006
0.0004
0.0002
0.0000
−0.0002
−0.0004
0
5
10
Expression Level

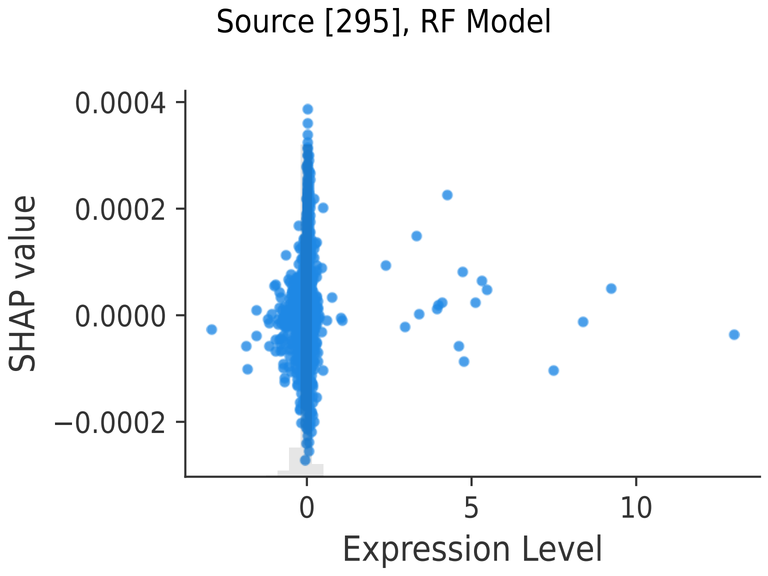

Source [295], RF Model
SHAP value
0.0004
0.0002
0.0000
−0.0002
0
5
10
Expression Level

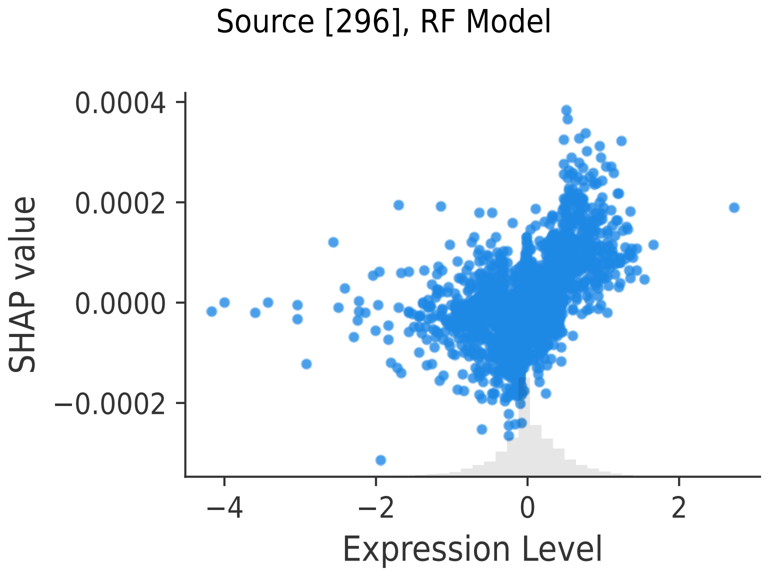

Source [296], RF Model
0.0004
0.0002
0.0000
−0.0002
SHAP value
−4
−2
0
2
Expression Level

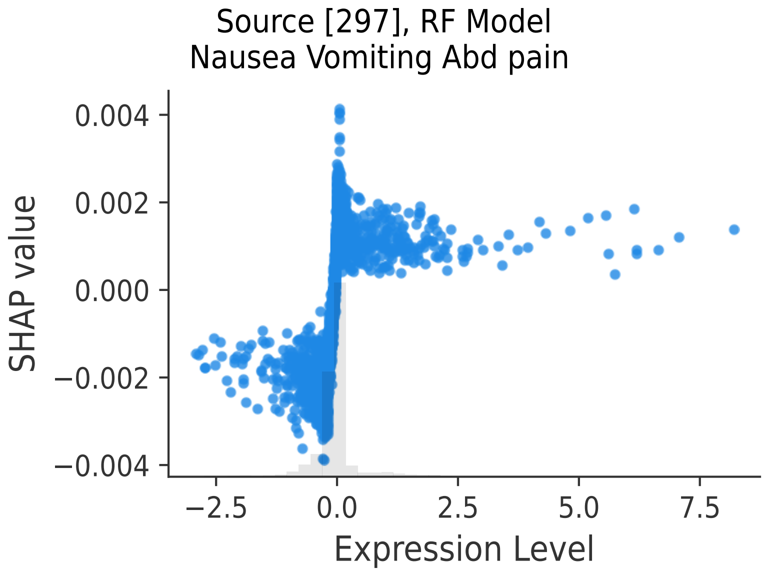

Source [297], RF Model
Nausea Vomiting Abd pain
SHAP value
0.004
0.002
0.000
−0.002
−0.004
−2.5
0.0
2.5
5.0
7.5
Expression Level

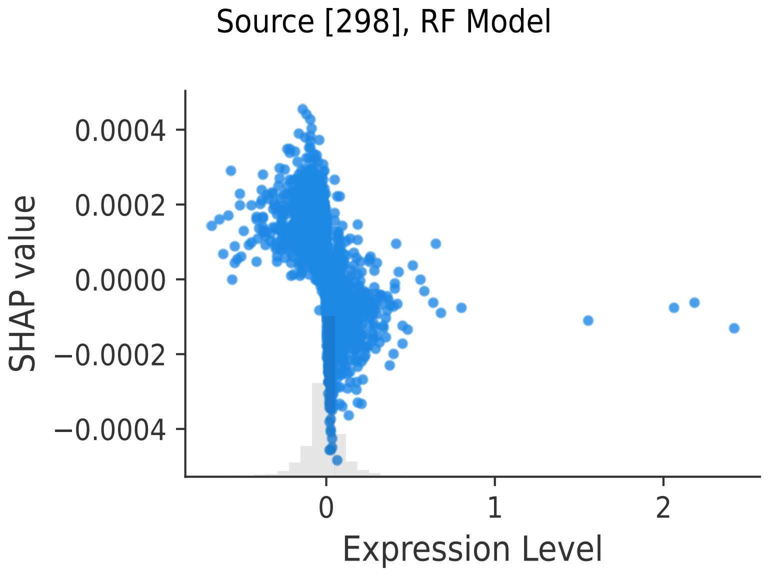
Source [298], RF Model
0.0004
0.0002
0.0000
−0.0002
−0.0004
SHAP value
0
1
2
Expression Level

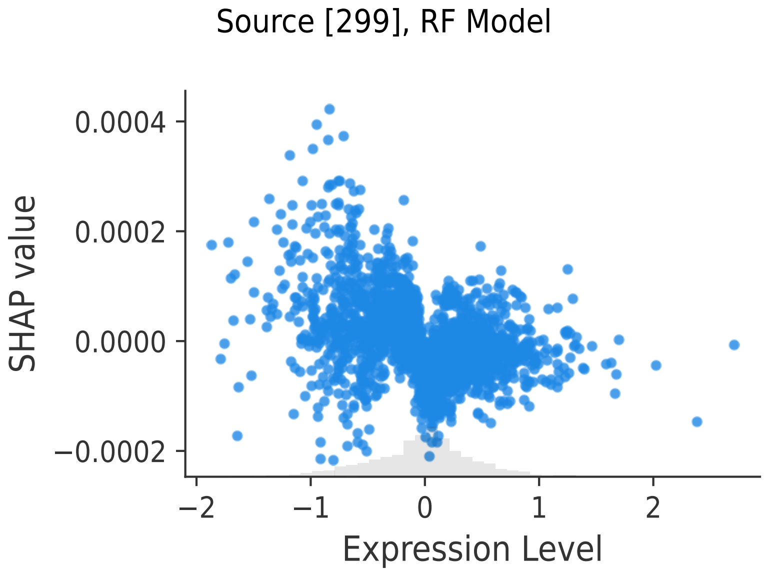
Source [299], RF Model
0.0004
0.0002
0.0000
−0.0002
SHAP value
−2
−1
0
1
2
Expression Level

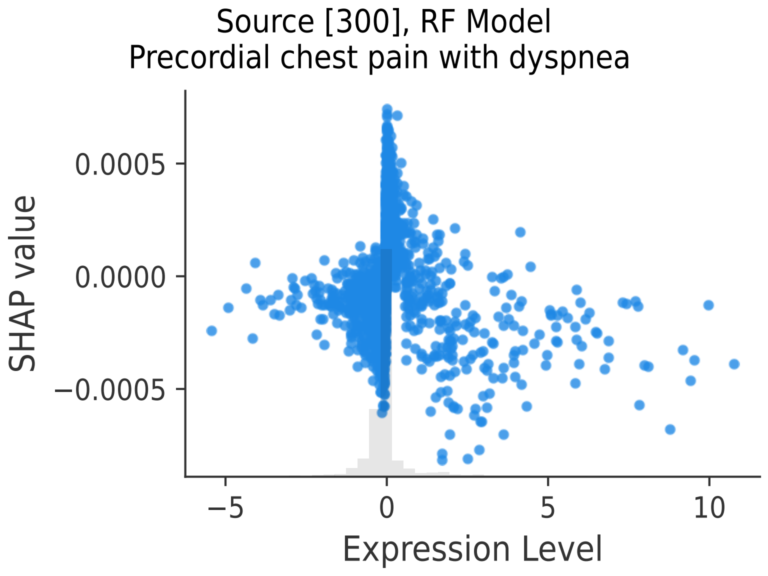
Source [300], RF Model
Precordial chest pain with dyspnea
SHAP value
0.0005
0.0000
−0.0005
−5
0
5
10
Expression Level

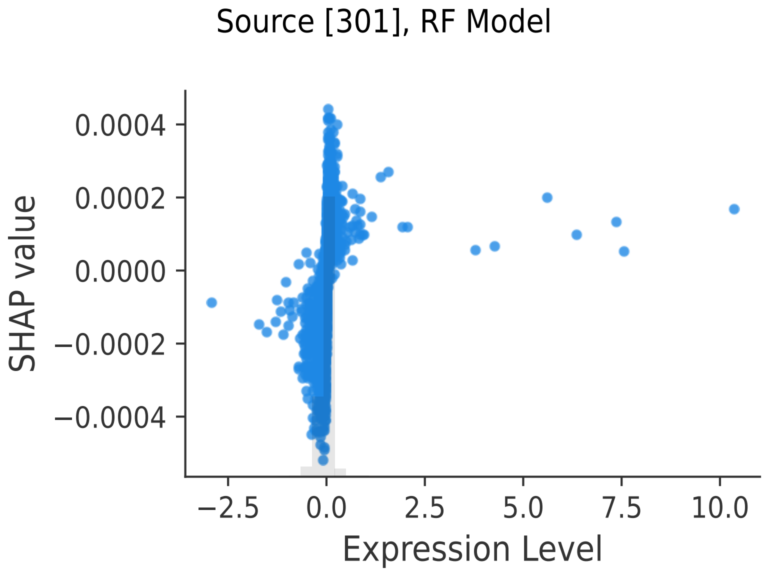
Source [301], RF Model
SHAP value
Expression Level
0.0004
0.0002
0.0000
−0.0002
−0.0004
−2.5
0.0
2.5
5.0
7.5
10.0

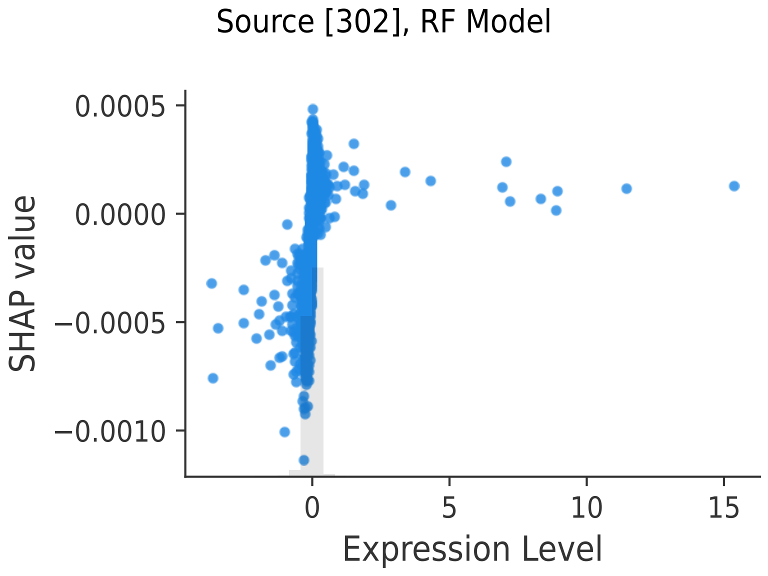
Source [302], RF Model
SHAP value
0.0005
0.0000
−0.0005
−0.0010
0
5
10
15
Expression Level

Source [303], RF Model

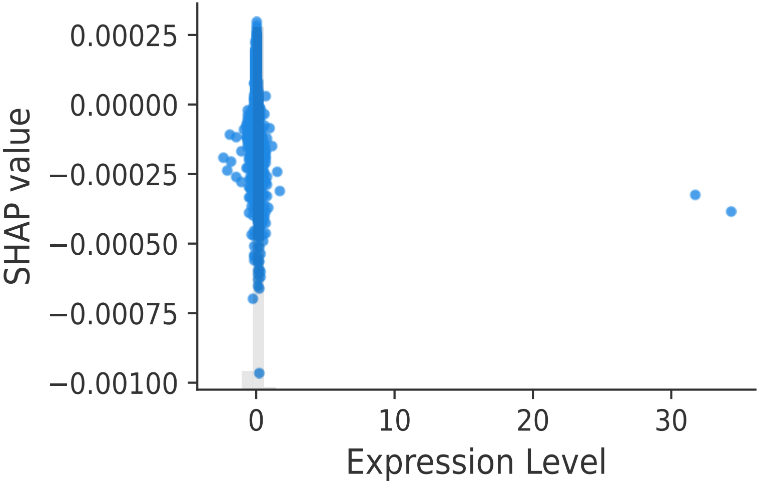

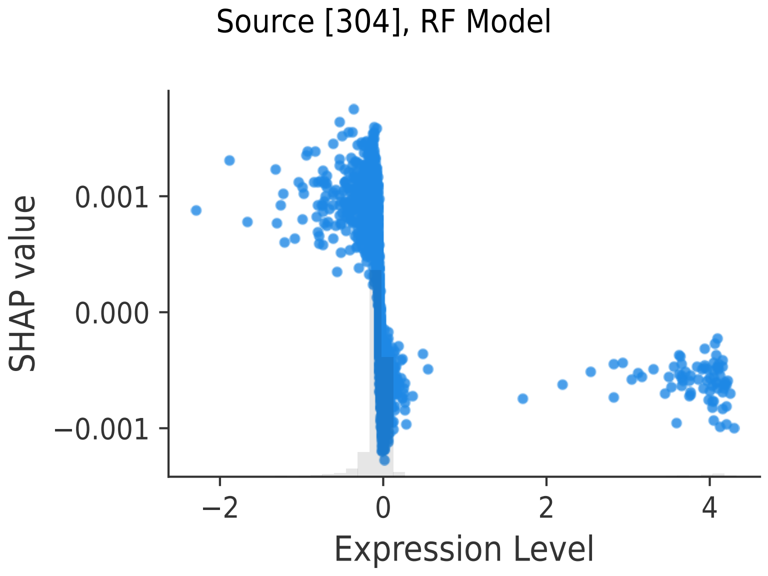
Source [304], RF Model
SHAP value
0.001
0.000
−0.001
−2
0
2
4
Expression Level

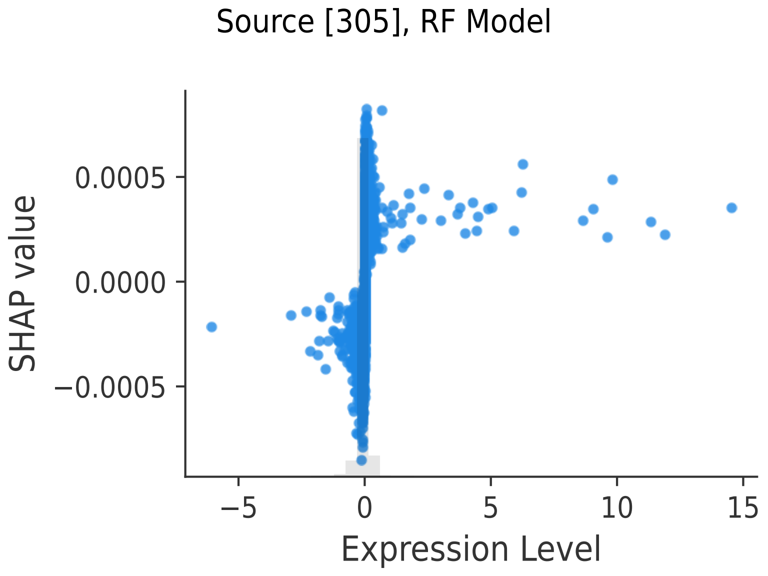
Source [305], RF Model
SHAP value
0.0005
0.0000
−0.0005
−5
0
5
10
15
Expression Level

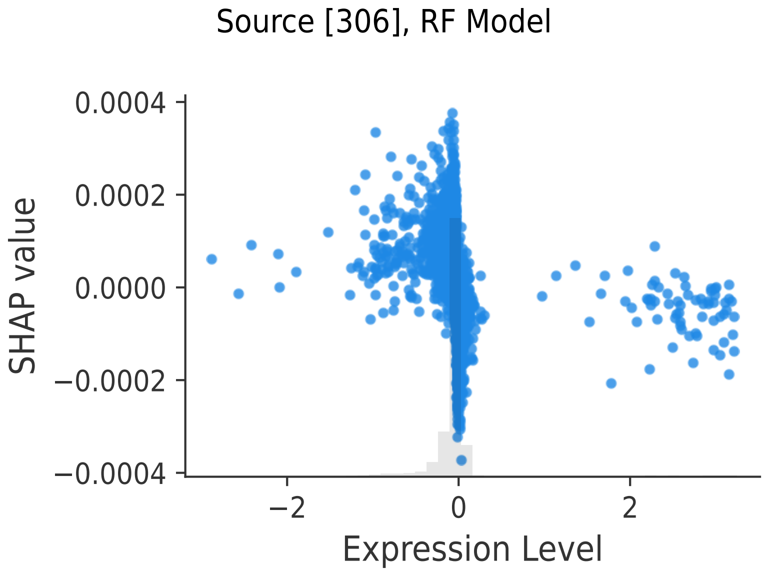
Source [306], RF Model
0.0004
0.0002
0.0000
−0.0002
−0.0004
SHAP value
−2
0
2
Expression Level

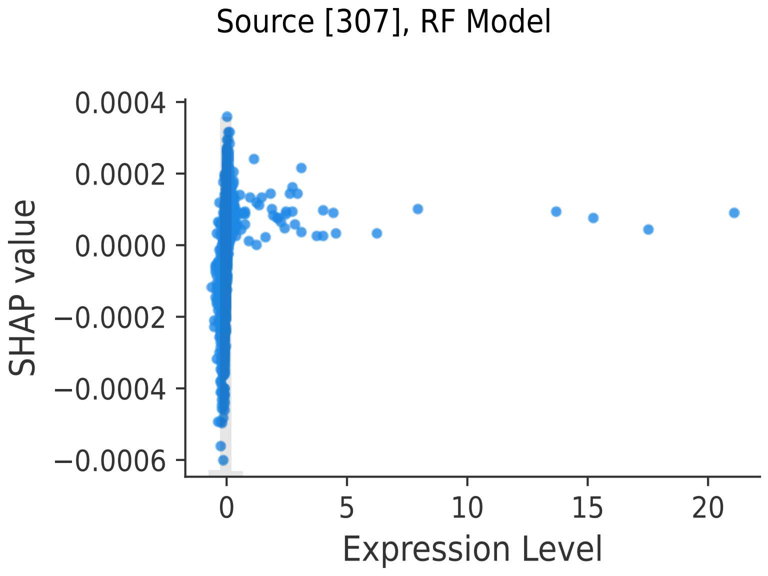

Source [307], RF Model
0.0004
0.0002
0.0000
−0.0002
−0.0004
−0.0006
SHAP value
0
5
10
15
20
Expression Level

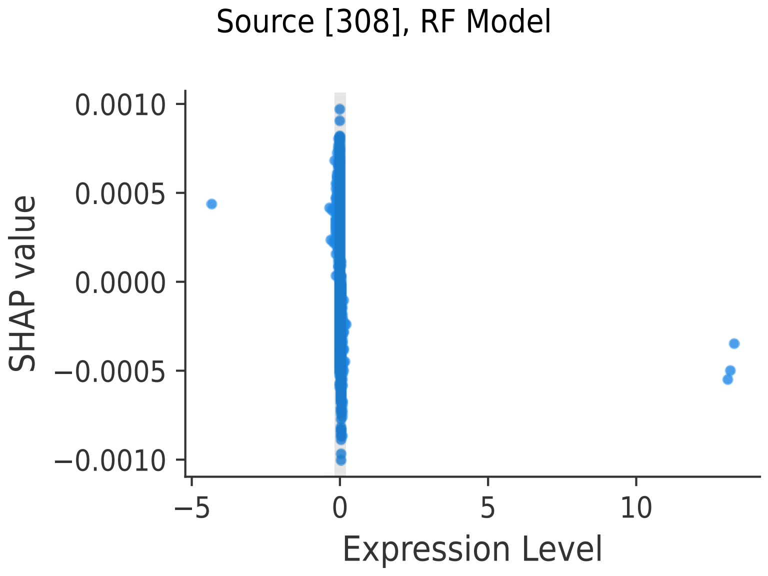
Source [308], RF Model
SHAP value
0.0010
0.0005
0.0000
−0.0005
−0.0010
−5
0
5
10
Expression Level

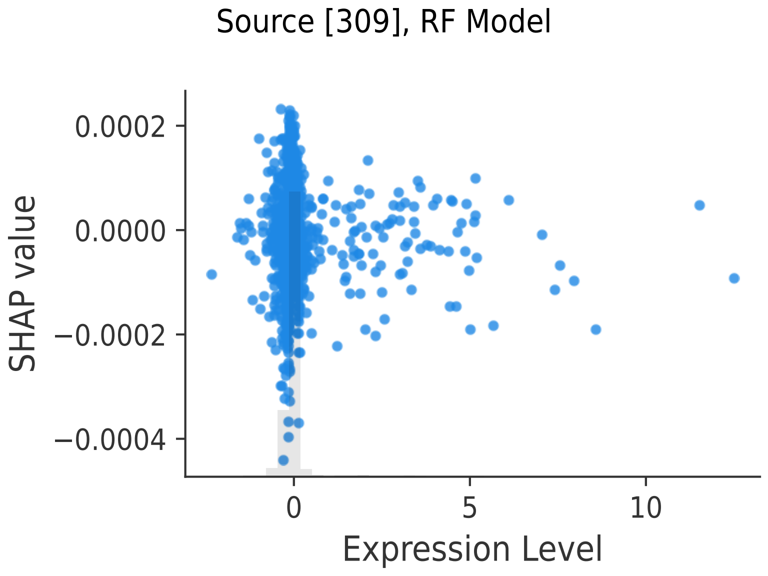
Source [309], RF Model
0.0002
0.0000
−0.0002
−0.0004
SHAP value
0
5
10
Expression Level

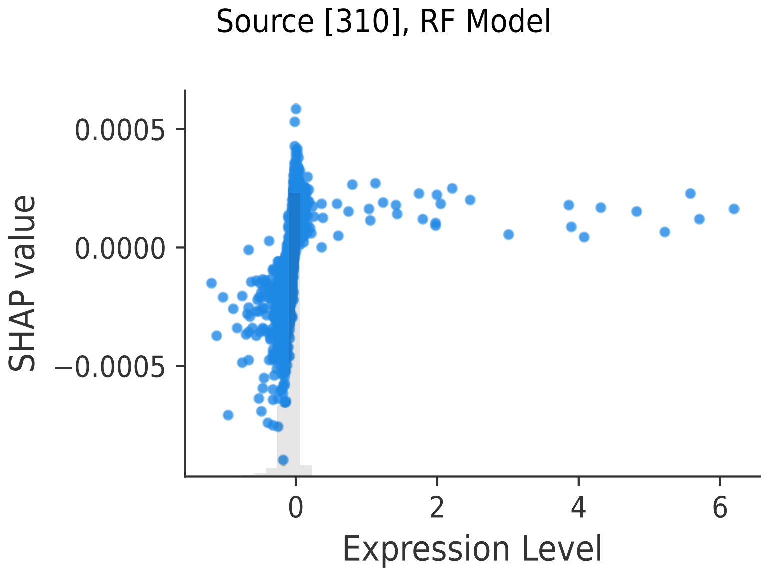
Source [310], RF Model
0.0005
0.0000
−0.0005
SHAP value
0
2
4
6
Expression Level

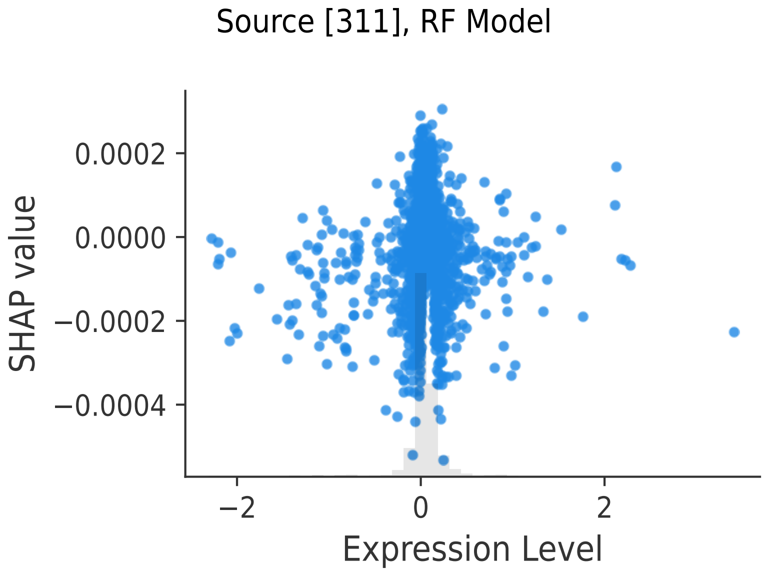
Source [311], RF Model
0.0002
0.0000
−0.0002
−0.0004
SHAP value
−2
0
2
Expression Level

Source [312], RF Model

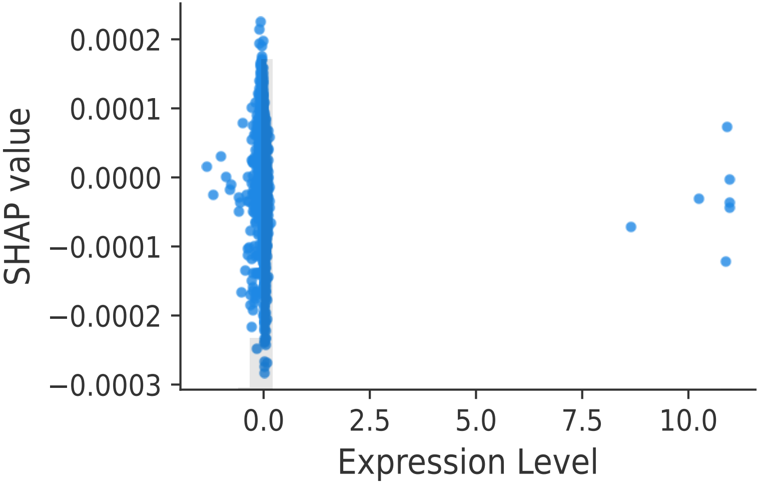

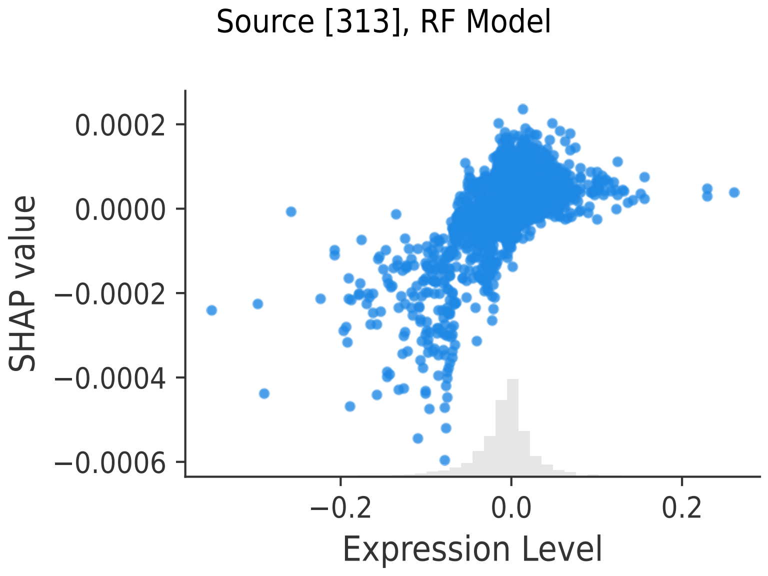
Source [313], RF Model
0.0002
0.0000
−0.0002
−0.0004
−0.0006
SHAP value
−0.2
0.0
0.2
Expression Level

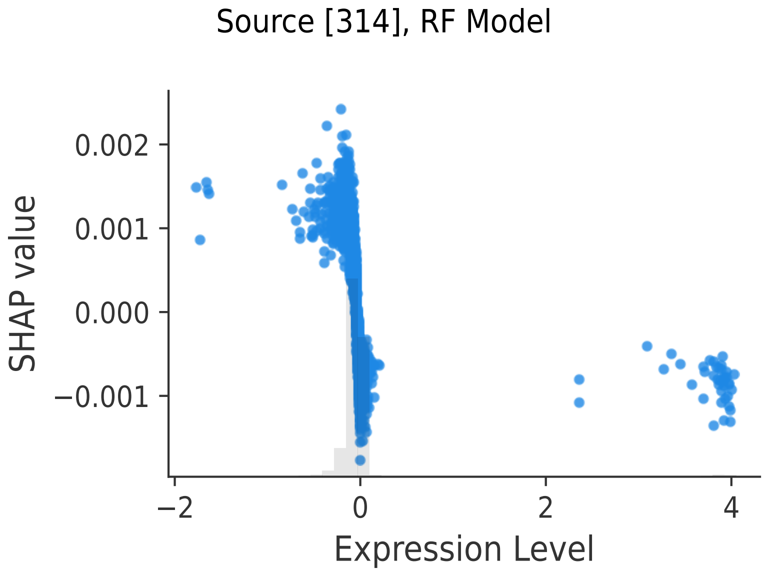

Source [314], RF Model
SHAP value
0.002
0.001
0.000
−0.001
−2
0
2
4
Expression Level

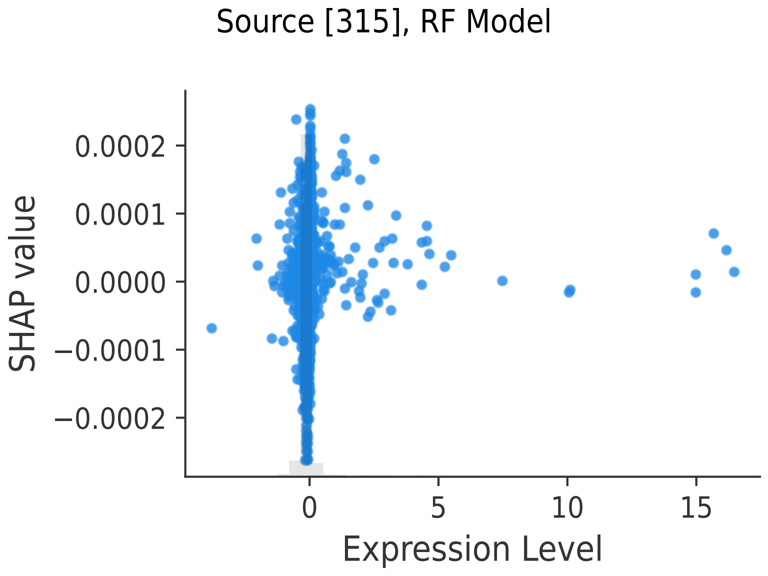
Source [315], RF Model
SHAP value
0.0002
0.0001
0.0000
−0.0001
−0.0002
0
5
10
15
Expression Level

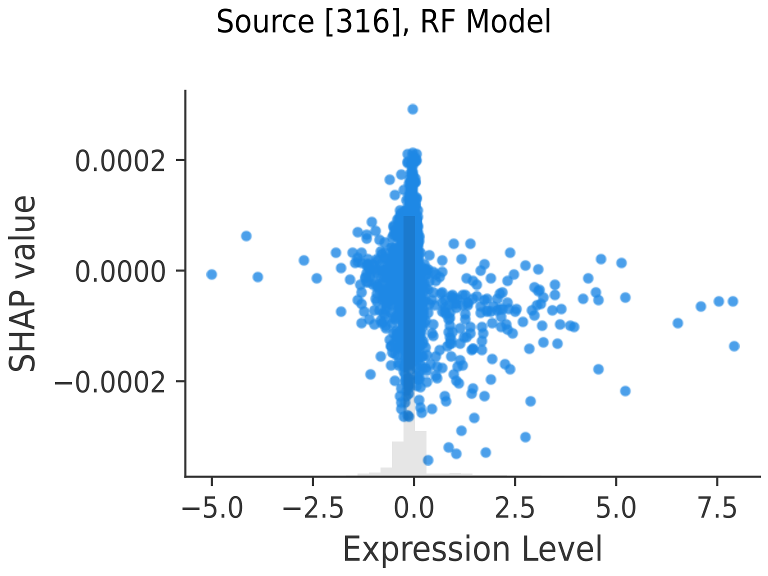
Source [316], RF Model
0.0002
0.0000
−0.0002
SHAP value
−5.0
−2.5
0.0
2.5
5.0
7.5
Expression Level

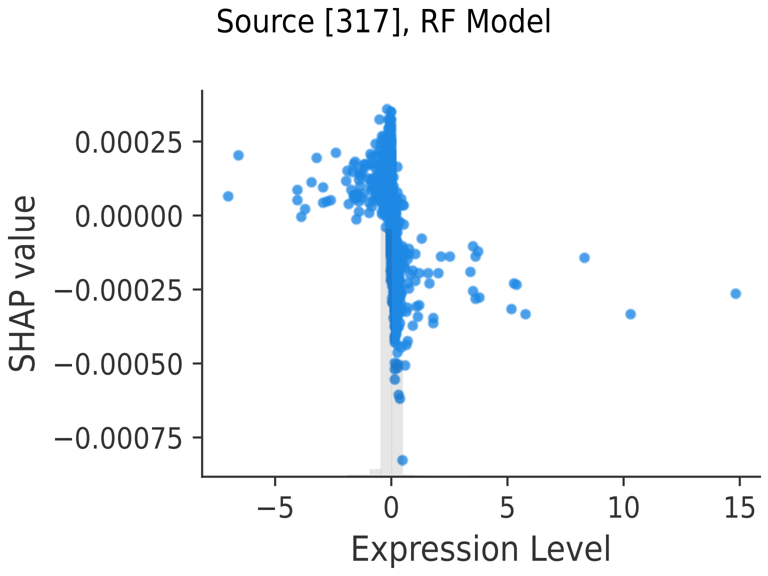
Source [317], RF Model
SHAP value
0.00025
0.00000
−0.00025
−0.00050
−0.00075
Expression Level
−5
0
5
10
15

Source [318], RF Model

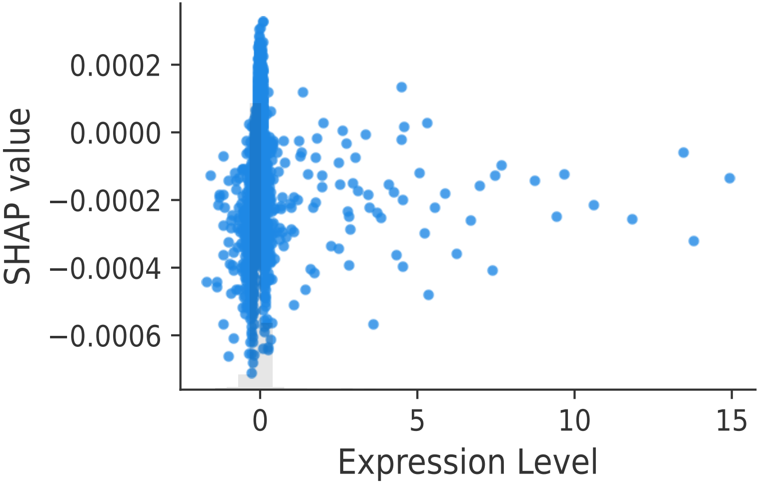

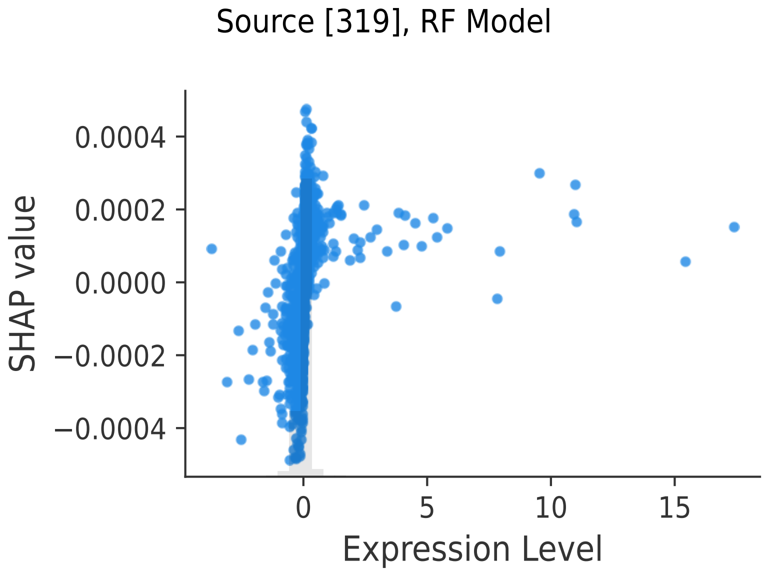

Source [319], RF Model
0.0004
0.0002
0.0000
−0.0002
−0.0004
SHAP value
0
5
10
15
Expression Level

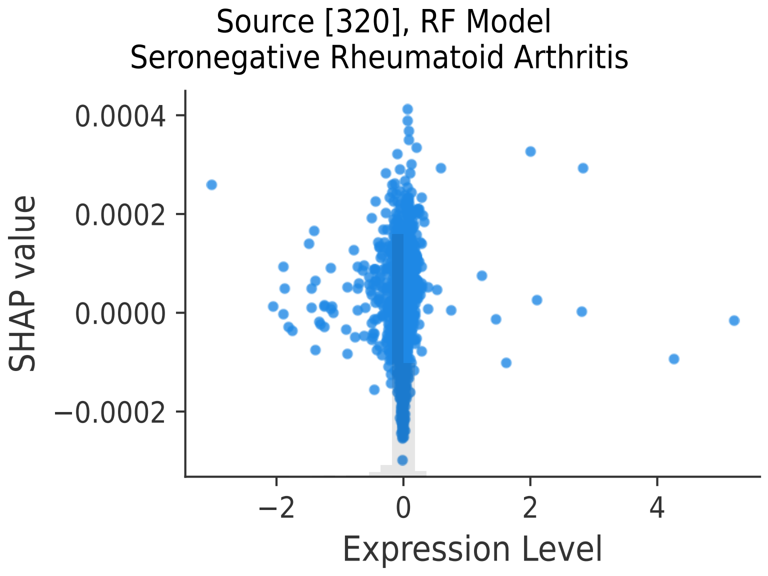
Source [320], RF Model
Seronegative Rheumatoid Arthritis
0.0004
0.0002
0.0000
−0.0002
SHAP value
−2
0
2
4
Expression Level

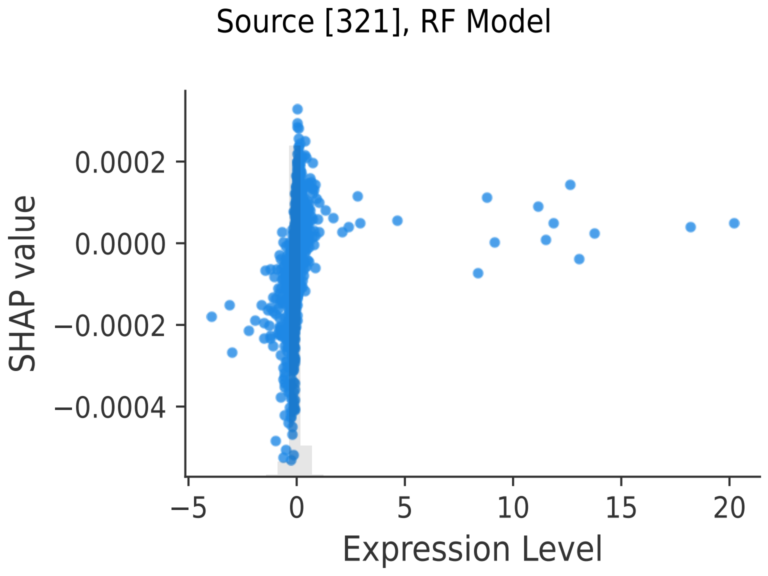

Source [321], RF Model
SHAP value
0.0002
0.0000
−0.0002
−0.0004
−5
0
5
10
15
20
Expression Level

Source [322], RF Model

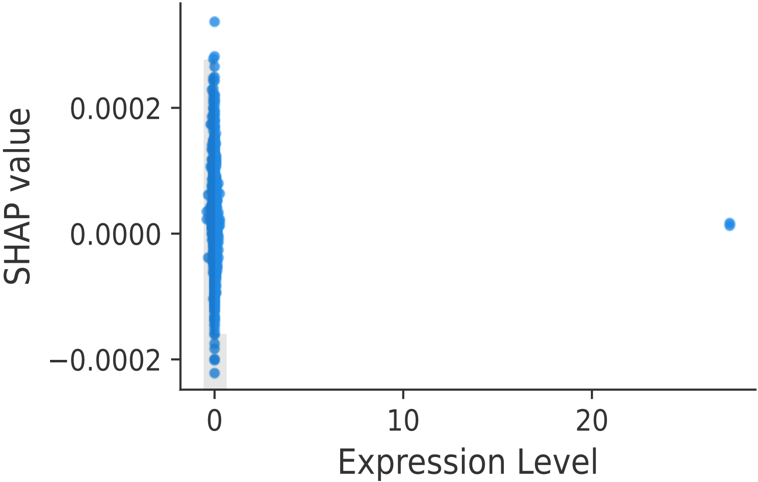

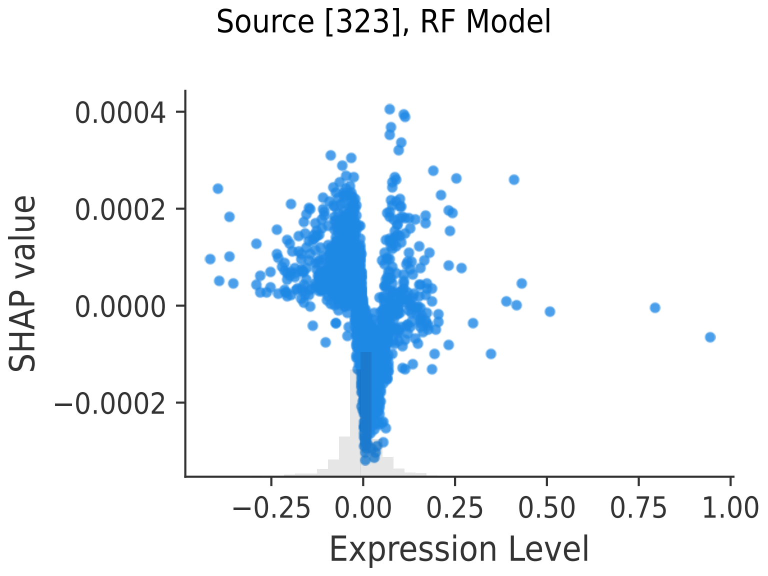

Source [323], RF Model
0.0004
0.0002
0.0000
−0.0002
SHAP value
−0.25
0.00
0.25
0.50
0.75
1.00
Expression Level

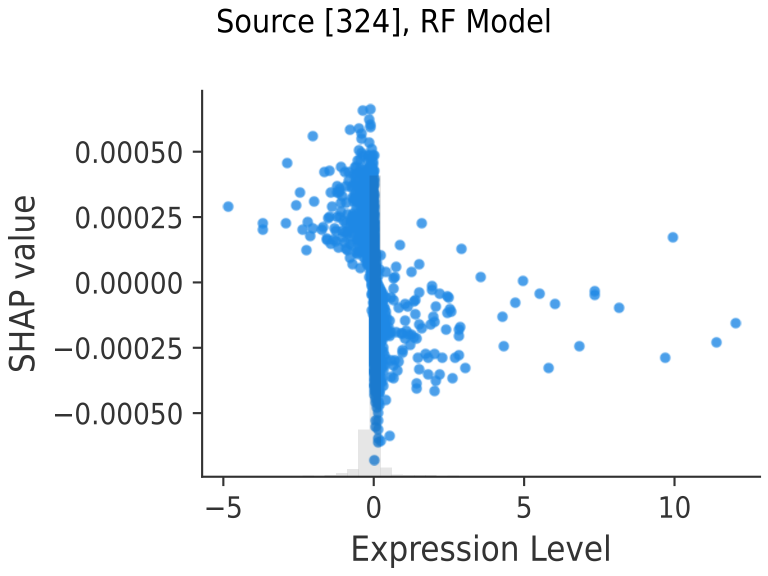
Source [324], RF Model
SHAP value
0.00050
0.00025
0.00000
−0.00025
−0.00050
−5
0
5
10
Expression Level

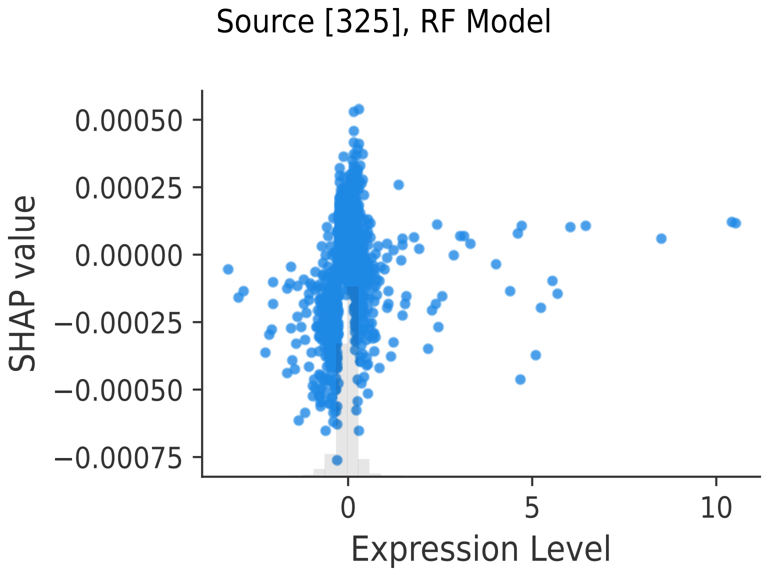
Source [325], RF Model
SHAP value
0.00050
0.00025
0.00000
−0.00025
−0.00050
−0.00075
0
5
10
Expression Level

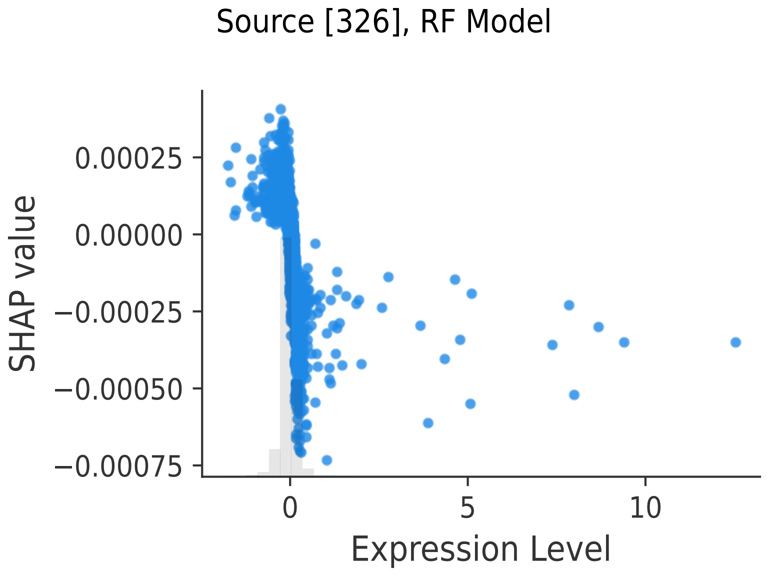

Source [326], RF Model
0.00025
0.00000
−0.00025
−0.00050
−0.00075
SHAP value
0
5
10
Expression Level

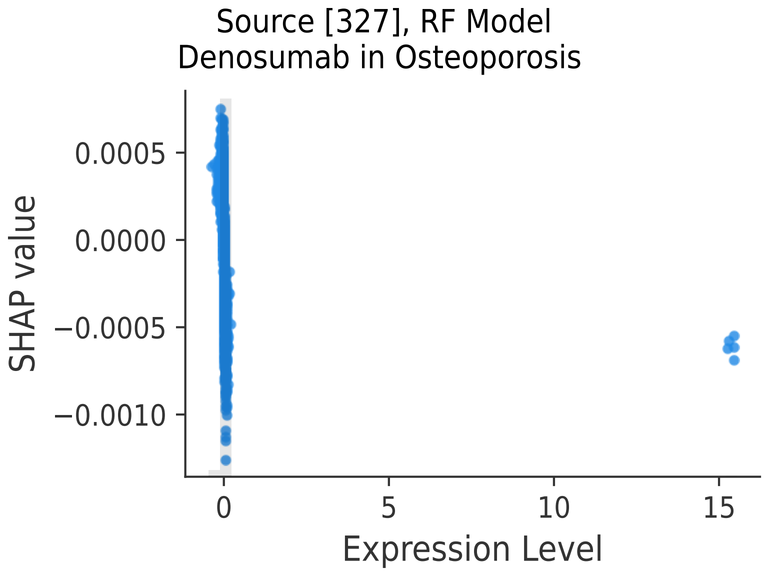

Source [327], RF Model
Denosumab in Osteoporosis
0.0005
0.0000
−0.0005
−0.0010
SHAP value
0
5
10
15
Expression Level

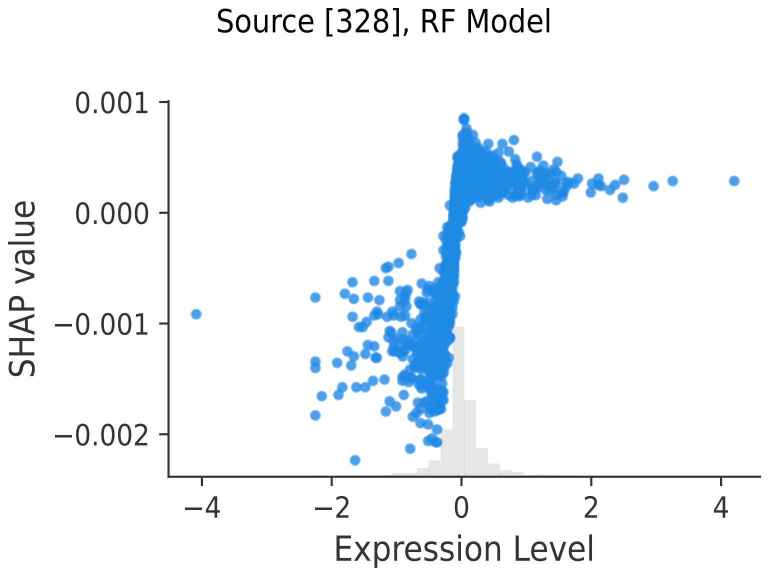

Source [328], RF Model
0.001
0.000
−0.001
−0.002
SHAP value
−4
−2
0
2
4
Expression Level

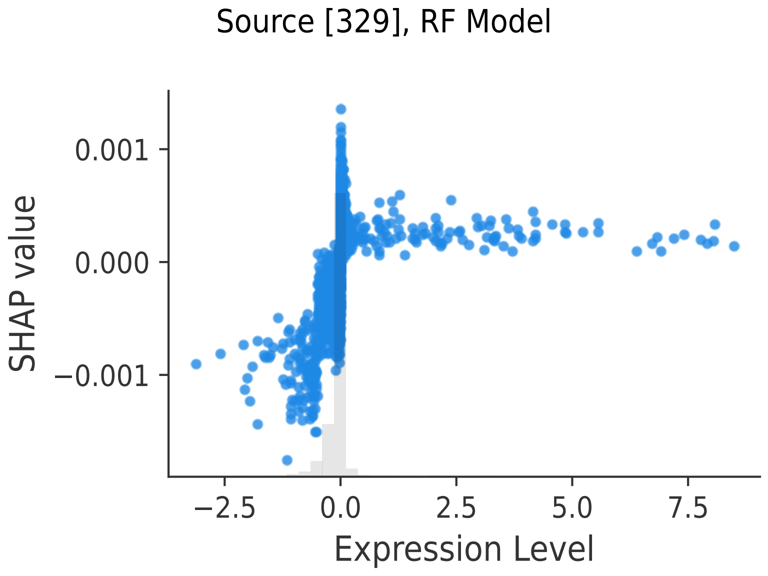

Source [329], RF Model
0.001
0.000
−0.001
SHAP value
−2.5
0.0
2.5
5.0
7.5
Expression Level

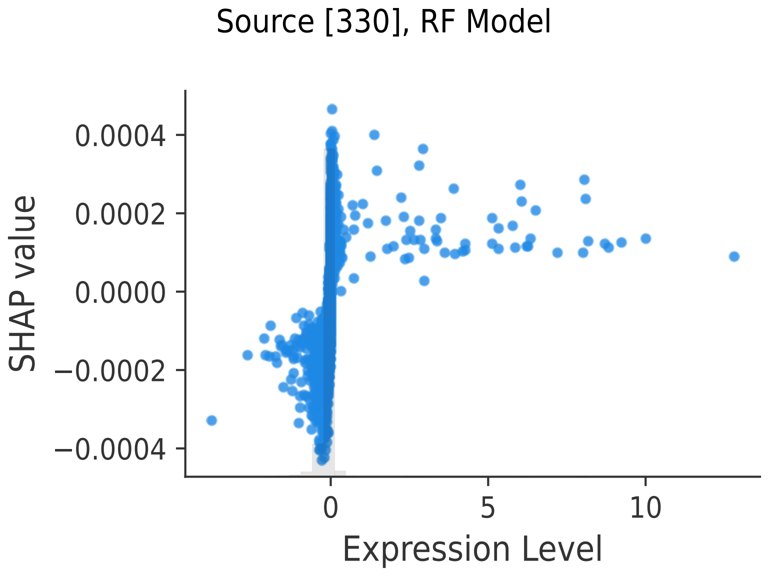

Source [330], RF Model
0.0004
0.0002
0.0000
−0.0002
−0.0004
SHAP value
0
5
10
Expression Level

Source [331], RF Model

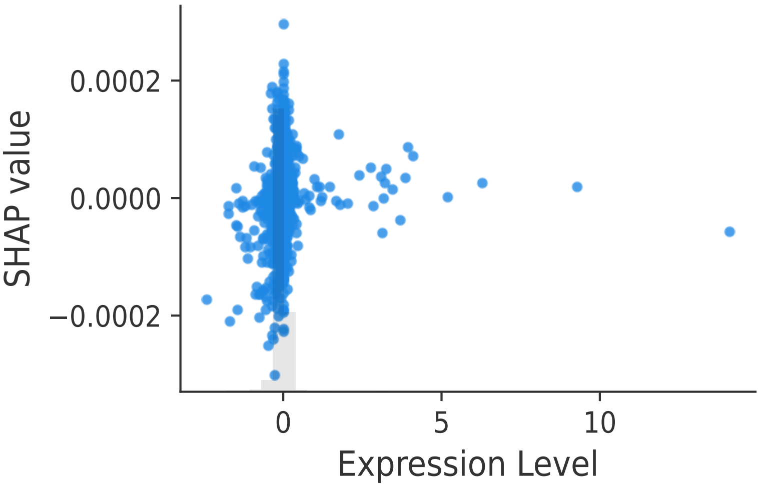

Source [332], RF Model

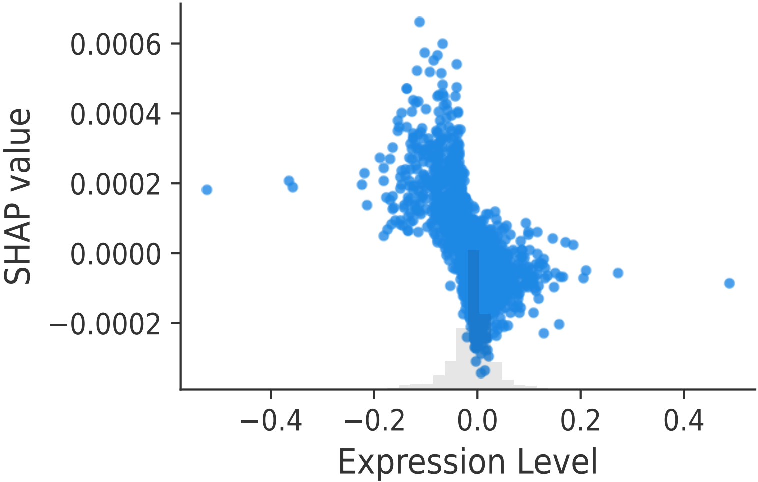

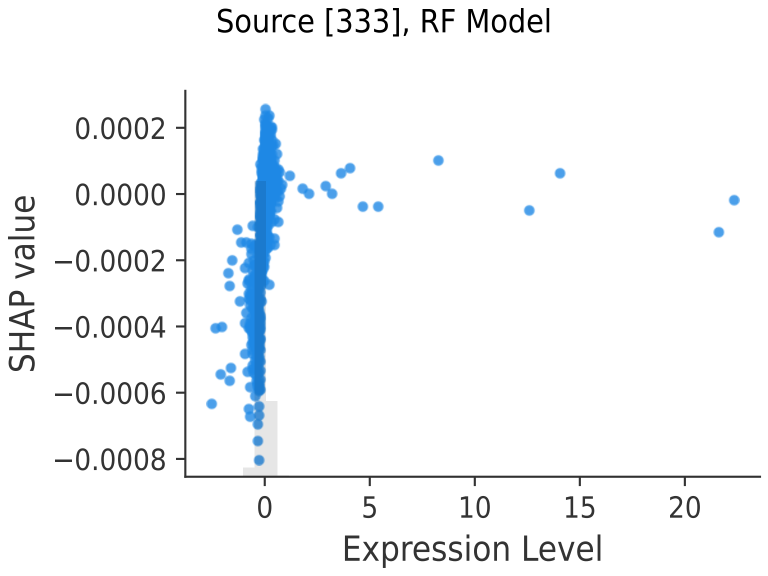

Source [333], RF Model
SHAP value
0.0002
0.0000
−0.0002
−0.0004
−0.0006
−0.0008
0
5
10
15
20
Expression Level

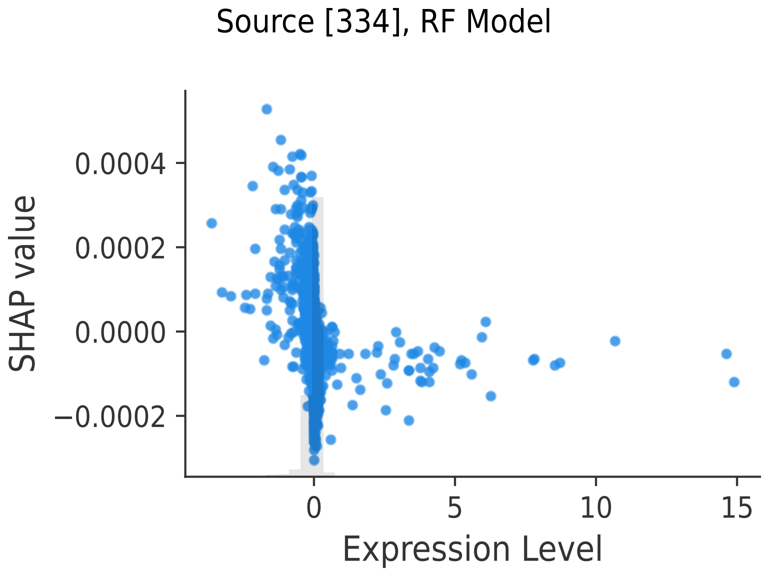

Source [334], RF Model
0.0004
0.0002
0.0000
−0.0002
SHAP value
0
5
10
15
Expression Level

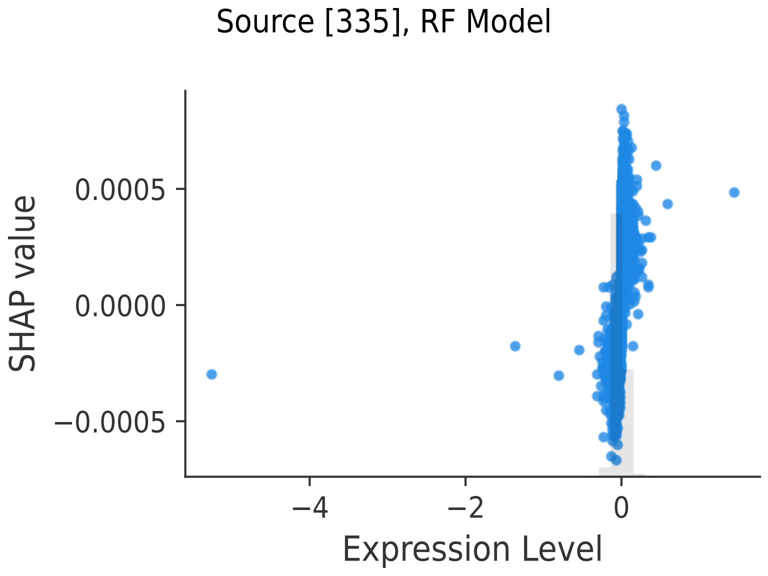
Source [335], RF Model
SHAP value
0.0005
0.0000
−0.0005
−4
−2
0
Expression Level

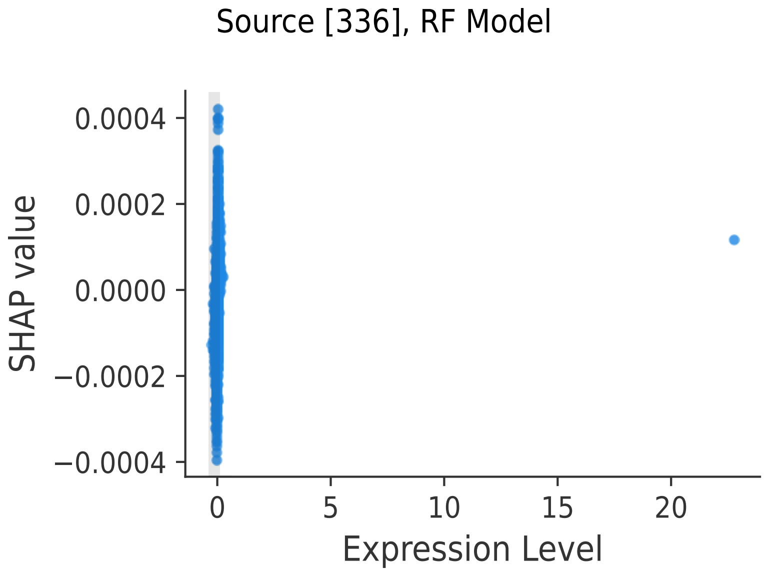
Source [336], RF Model
SHAP value
0.0004
0.0002
0.0000
−0.0002
−0.0004
0
5
10
15
20
Expression Level

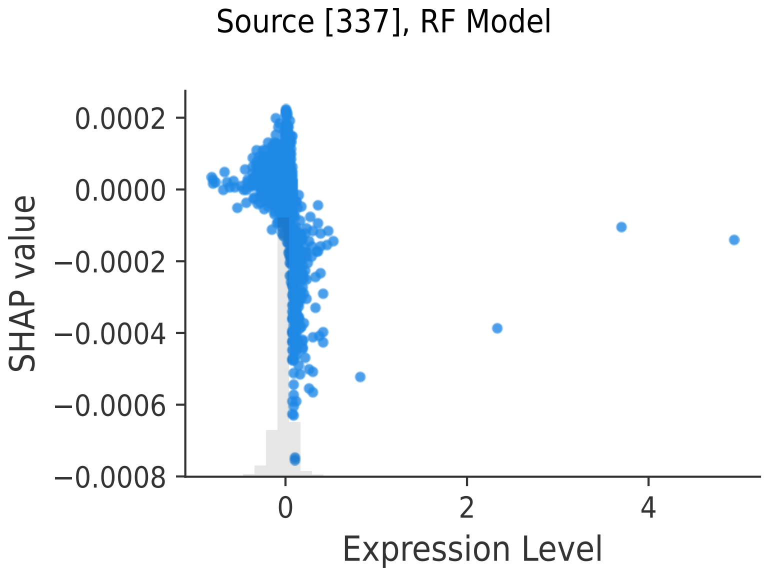
Source [337], RF Model
SHAP value
0.0002
0.0000
−0.0002
−0.0004
−0.0006
−0.0008
0
2
4
Expression Level

Source [338], RF Model

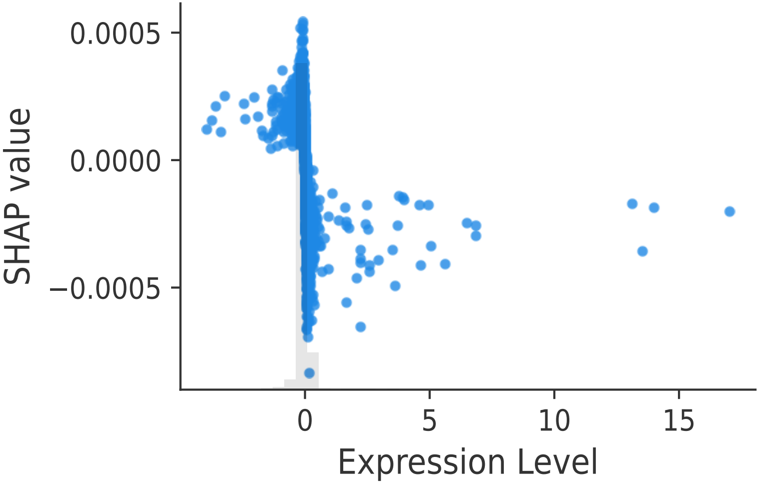

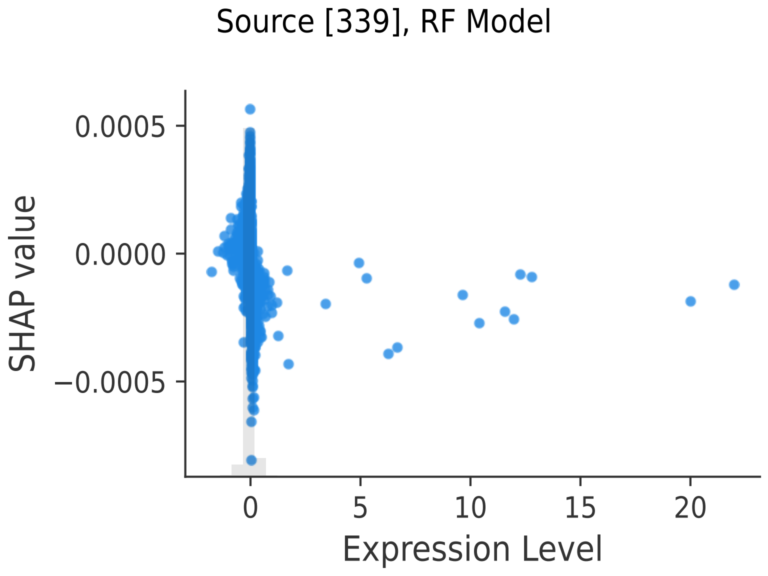
Source [339], RF Model
0.0005
0.0000
−0.0005
SHAP value
0
5
10
15
20
Expression Level

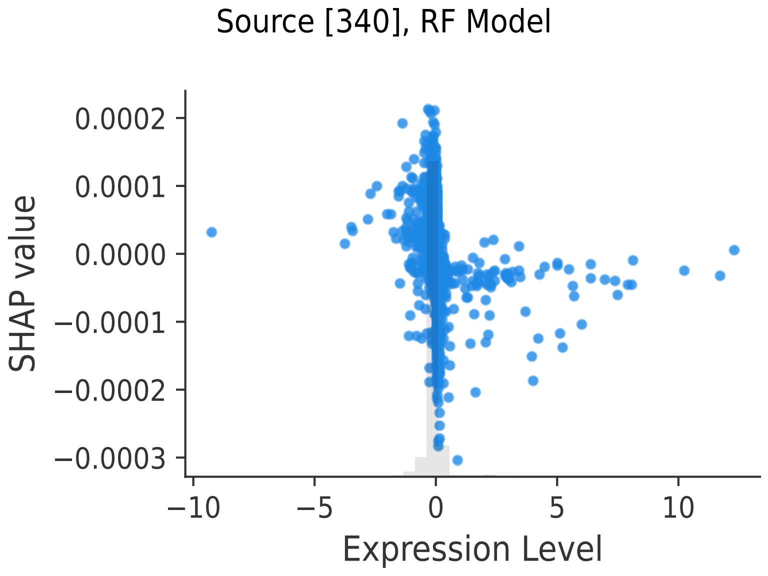
Source [340], RF Model
SHAP value
0.0002
0.0001
0.0000
−0.0001
−0.0002
−0.0003
−10
−5
0
5
10
Expression Level

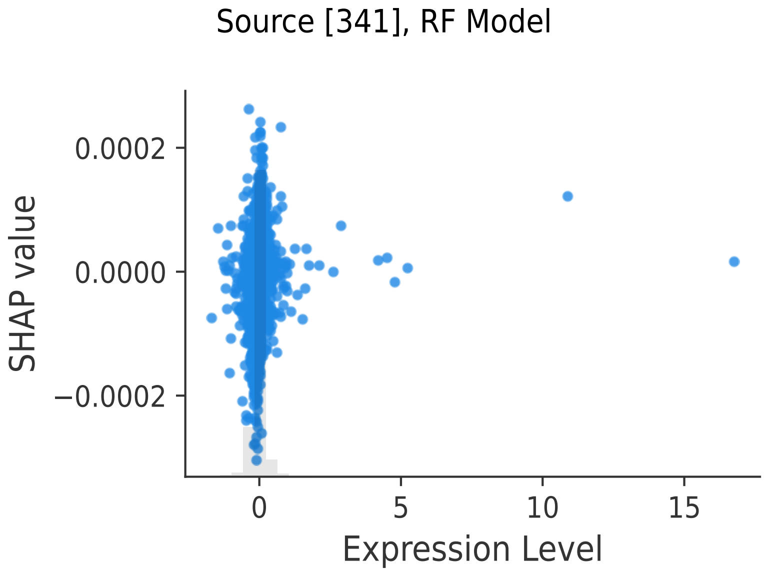

Source [341], RF Model
0.0002
0.0000
−0.0002
SHAP value
0
5
10
15
Expression Level

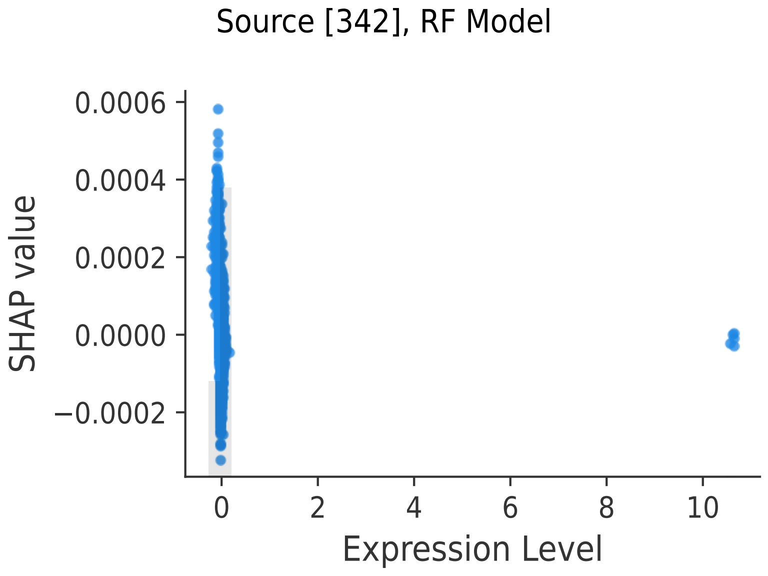
Source [342], RF Model
SHAP value
0.0006
0.0004
0.0002
0.0000
−0.0002
0
2
4
6
8
10
Expression Level

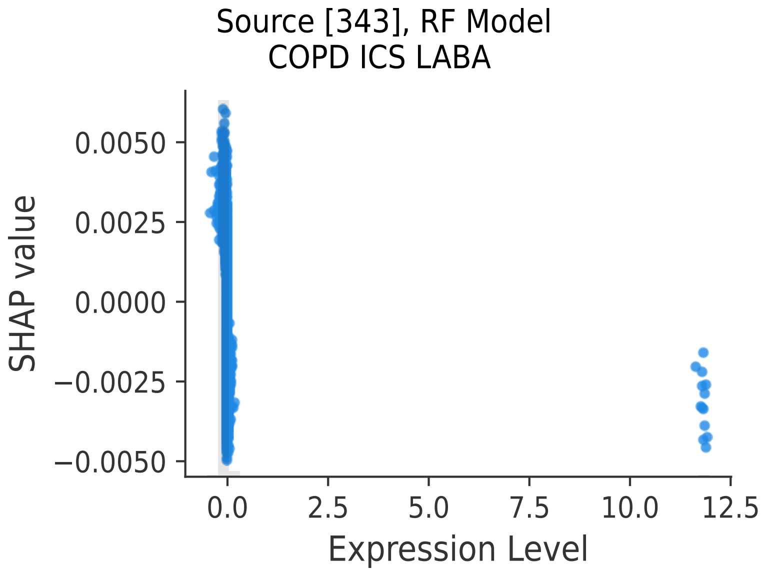

Source [343], RF Model
COPD ICS LABA
0.0050
0.0025
0.0000
−0.0025
−0.0050
SHAP value
0.0
2.5
5.0
7.5
10.0
12.5
Expression Level

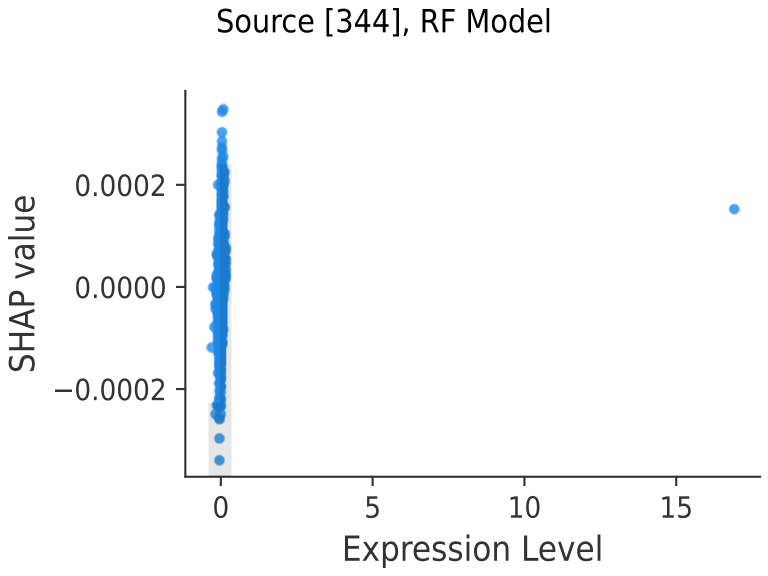
Source [344], RF Model
SHAP value
0.0002
0.0000
−0.0002
0
5
10
15
Expression Level

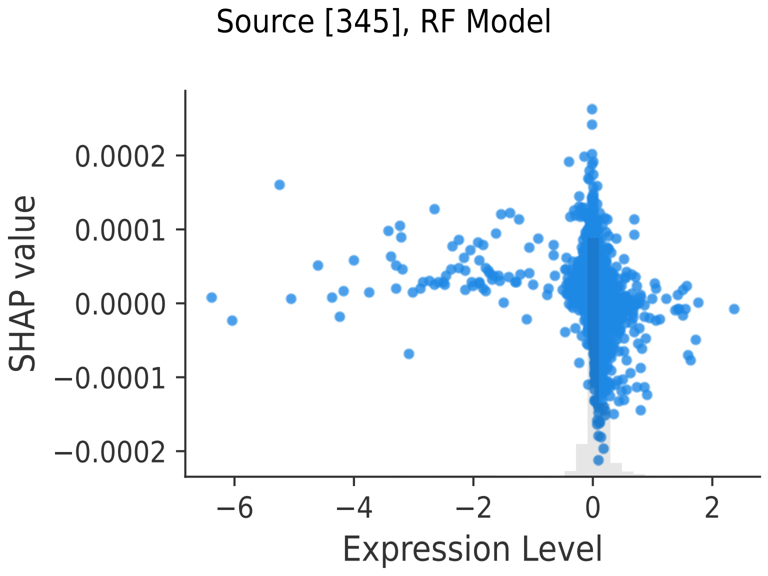
Source [345], RF Model
SHAP value
0.0002
0.0001
0.0000
−0.0001
−0.0002
−6
−4
−2
0
2
Expression Level

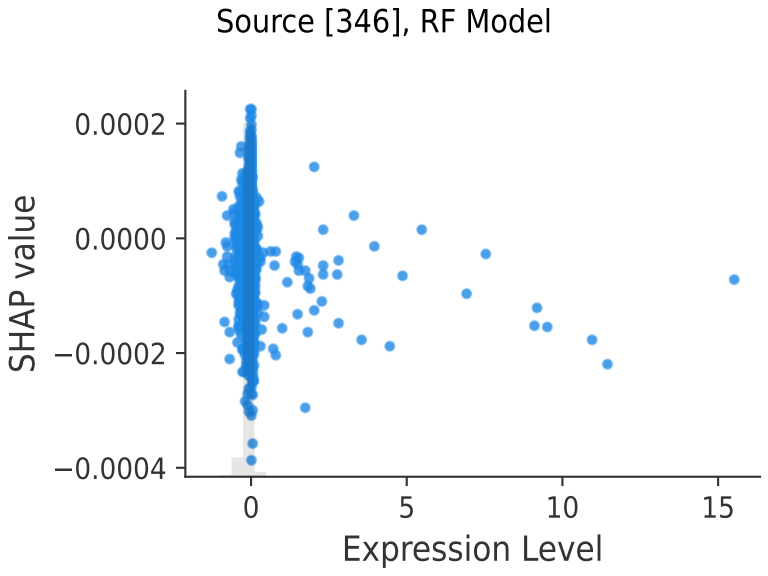

Source [346], RF Model
0.0002
0.0000
−0.0002
−0.0004
SHAP value
0
5
10
15
Expression Level

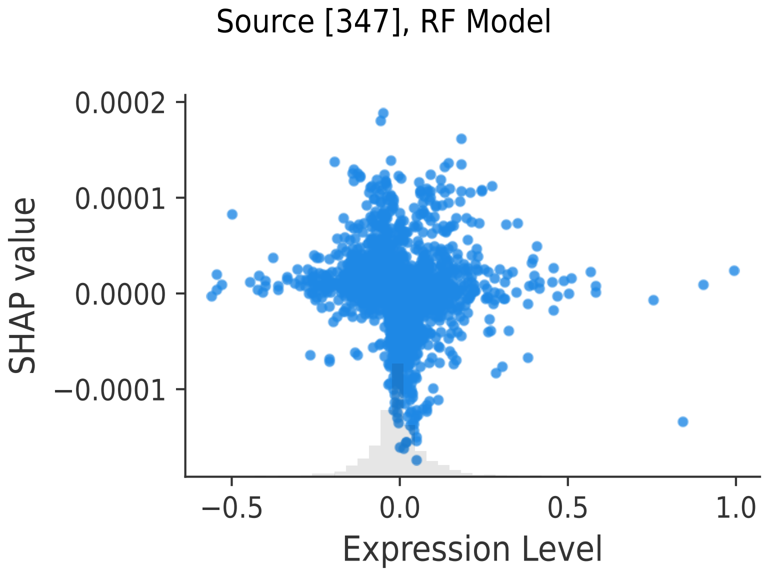
Source [347], RF Model
0.0002
0.0001
0.0000
−0.0001
SHAP value
−0.5
0.0
0.5
1.0
Expression Level

Source [348], RF Model

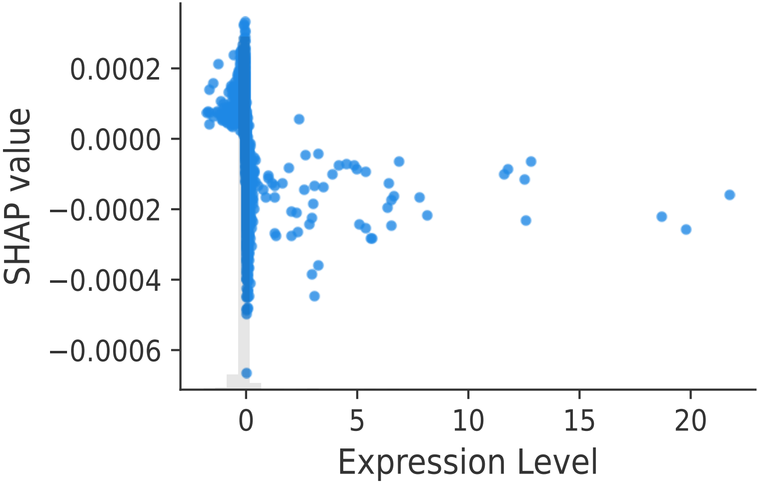

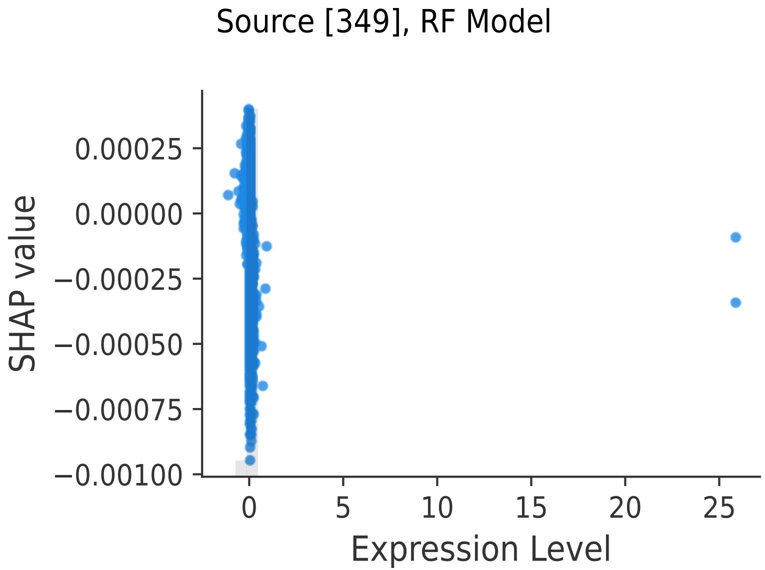
Source [349], RF Model
SHAP value
0.00025
0.00000
−0.00025
−0.00050
−0.00075
−0.00100
0
5
10
15
20
25
Expression Level

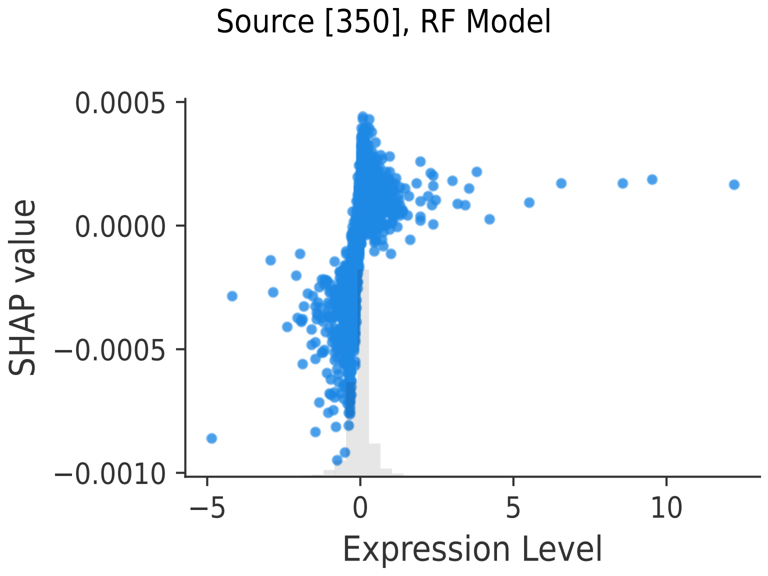
Source [350], RF Model
0.0005
0.0000
−0.0005
−0.0010
SHAP value
−5
0
5
10
Expression Level

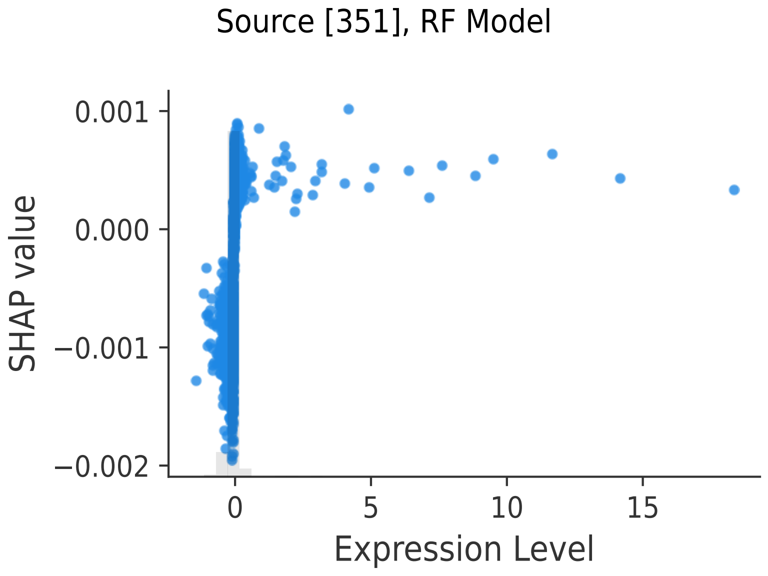

Source [351], RF Model
SHAP value
0.001
0.000
−0.001
−0.002
0
5
10
15
Expression Level

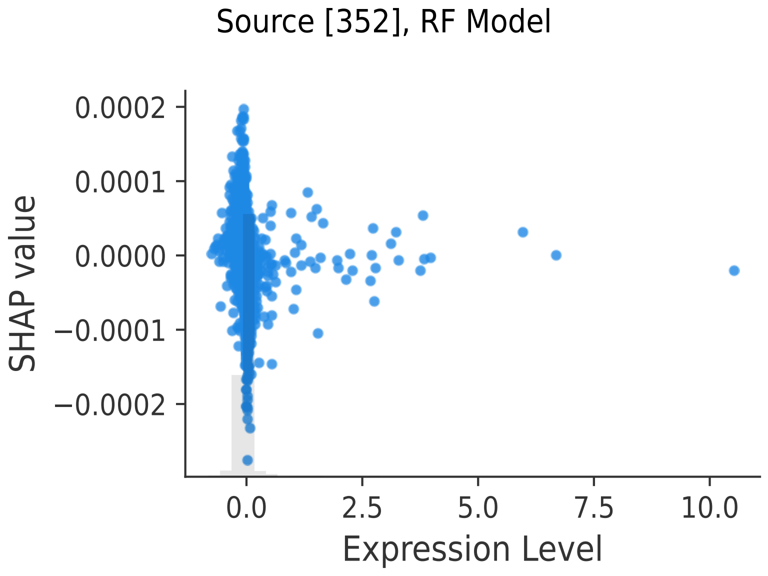

Source [352], RF Model
0.0002
0.0001
0.0000
−0.0001
−0.0002
SHAP value
0.0
2.5
5.0
7.5
10.0
Expression Level

Source [353], RF Model

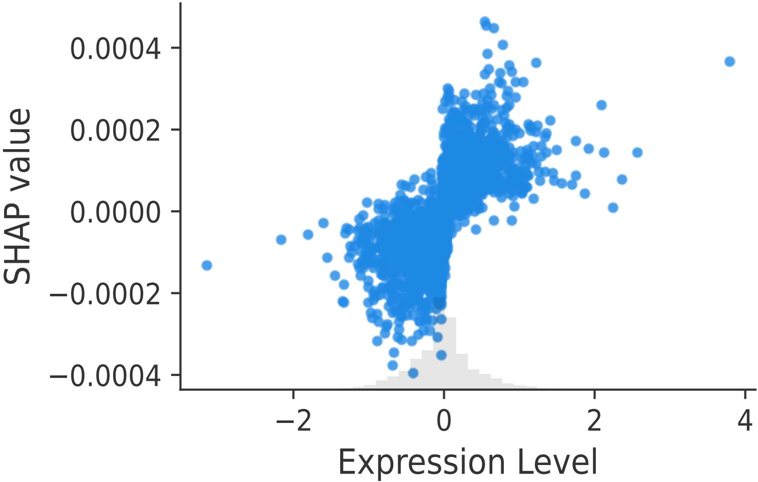

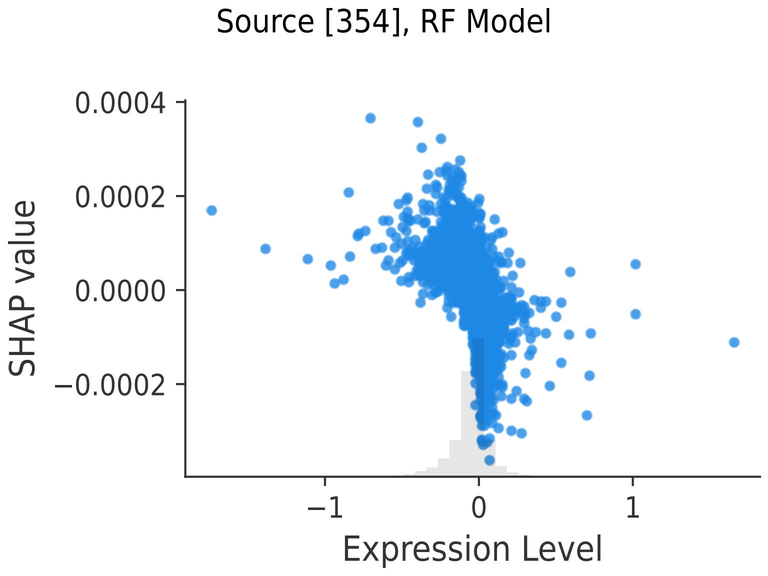

Source [354], RF Model
0.0004
0.0002
0.0000
−0.0002
SHAP value
−1
0
1
Expression Level

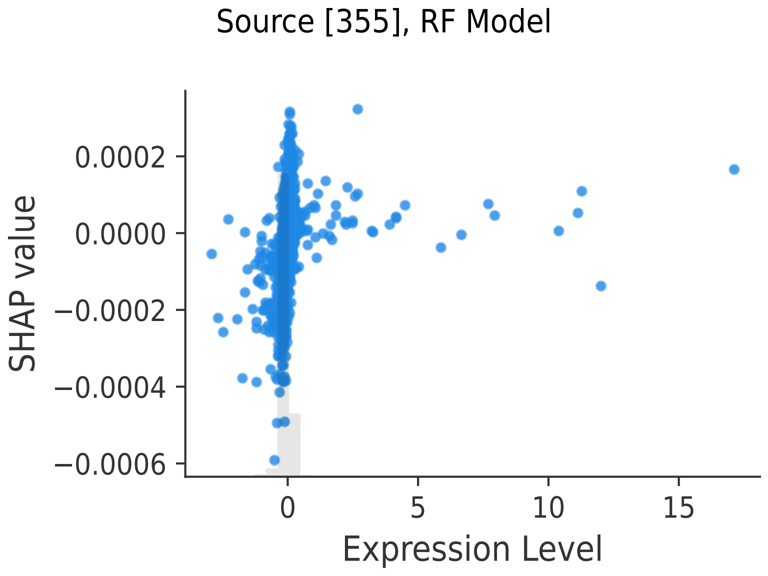

Source [355], RF Model
SHAP value
0.0002
0.0000
−0.0002
−0.0004
−0.0006
0
5
10
15
Expression Level

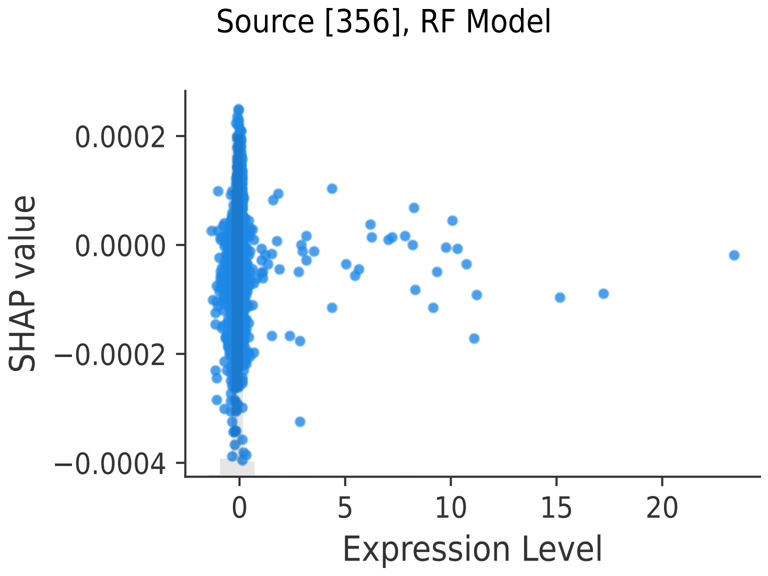
Source [356], RF Model
0.0002
0.0000
−0.0002
−0.0004
SHAP value
0
5
10
15
20
Expression Level

Source [357], RF Model

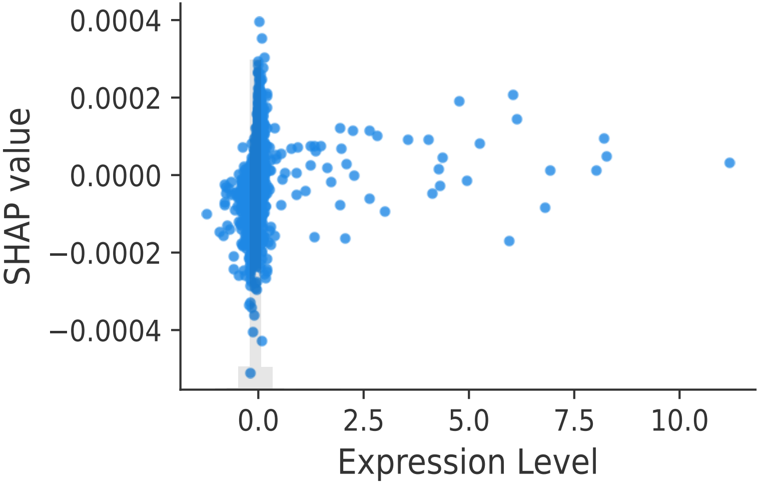

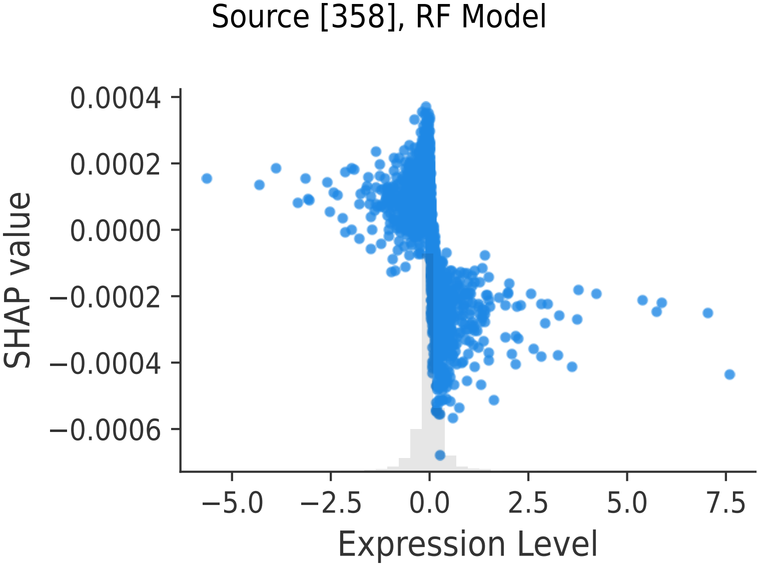
Source [358], RF Model
0.0004
0.0002
0.0000
−0.0002
−0.0004
−0.0006
SHAP value
−5.0
−2.5
0.0
2.5
5.0
7.5
Expression Level

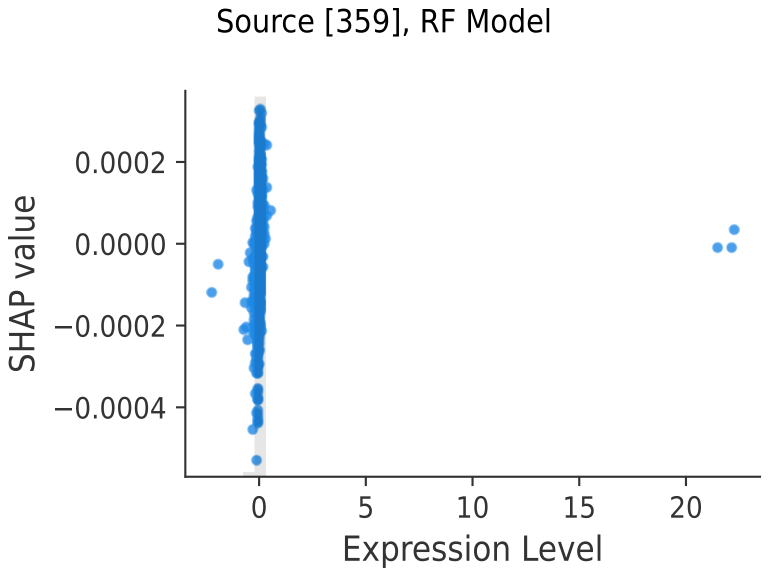

Source [359], RF Model
0.0002
0.0000
−0.0002
−0.0004
SHAP value
0
5
10
15
20
Expression Level

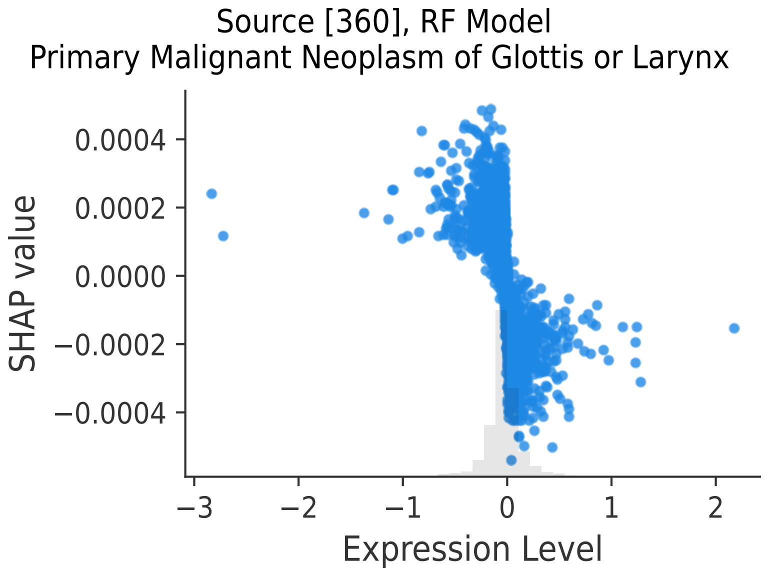
Source [360], RF Model
Primary Malignant Neoplasm of Glottis or Larynx
0.0004
0.0002
0.0000
−0.0002
−0.0004
SHAP value
−3
−2
−1
0
1
2
Expression Level

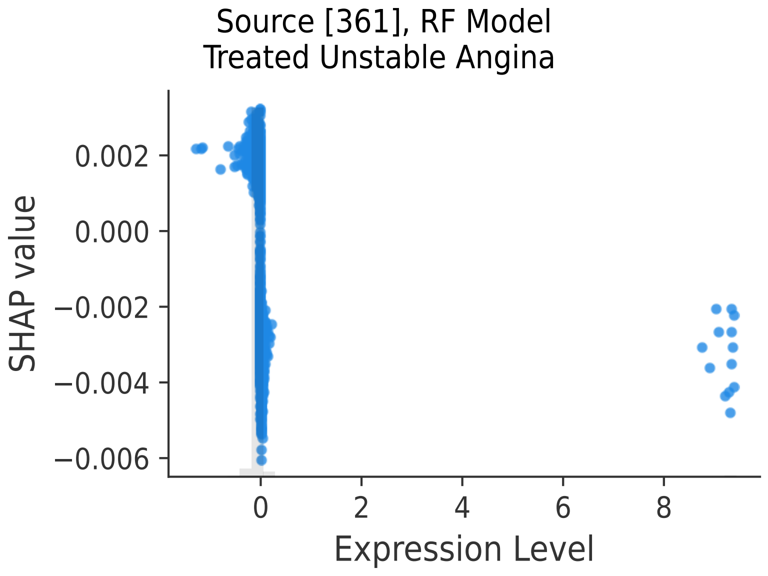

Source [361], RF Model
Treated Unstable Angina
0.002
0.000
−0.002
−0.004
−0.006
SHAP value
0
2
4
6
8
Expression Level

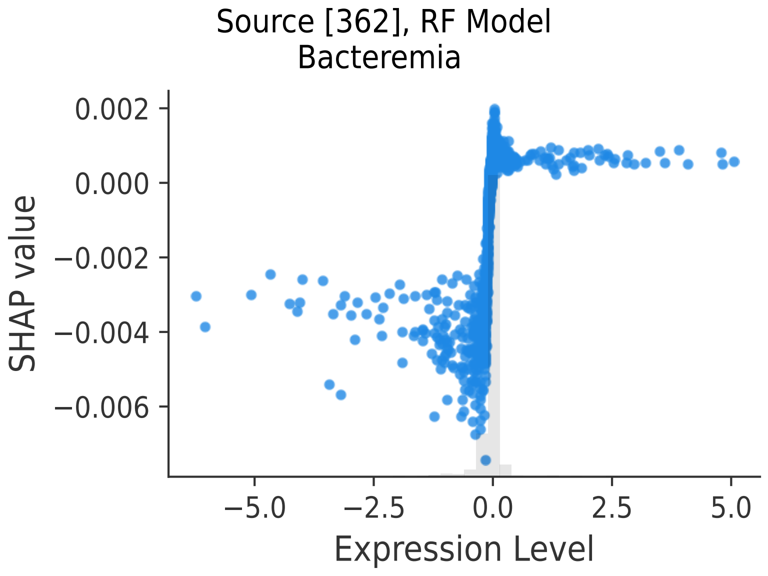

Source [362], RF Model
Bacteremia
0.002
0.000
−0.002
−0.004
−0.006
SHAP value
−5.0
−2.5
0.0
2.5
5.0
Expression Level

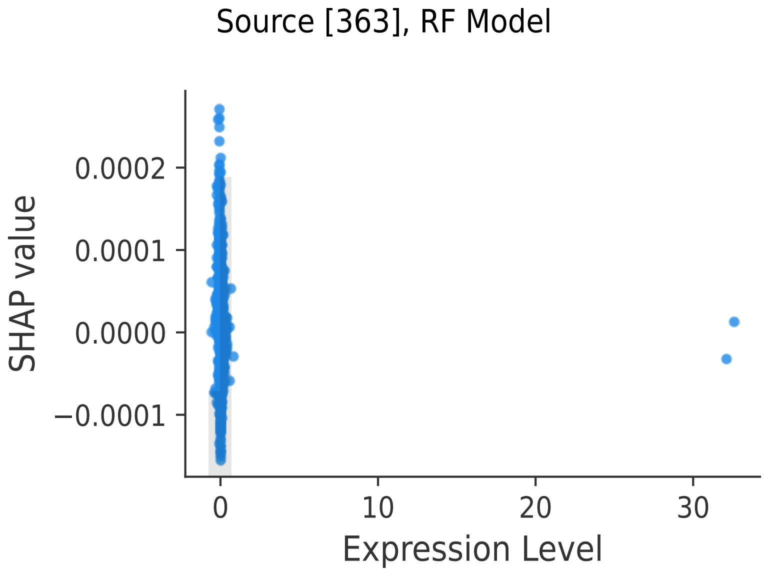
Source [363], RF Model
0.0002
0.0001
0.0000
−0.0001
SHAP value
0
10
20
30
Expression Level

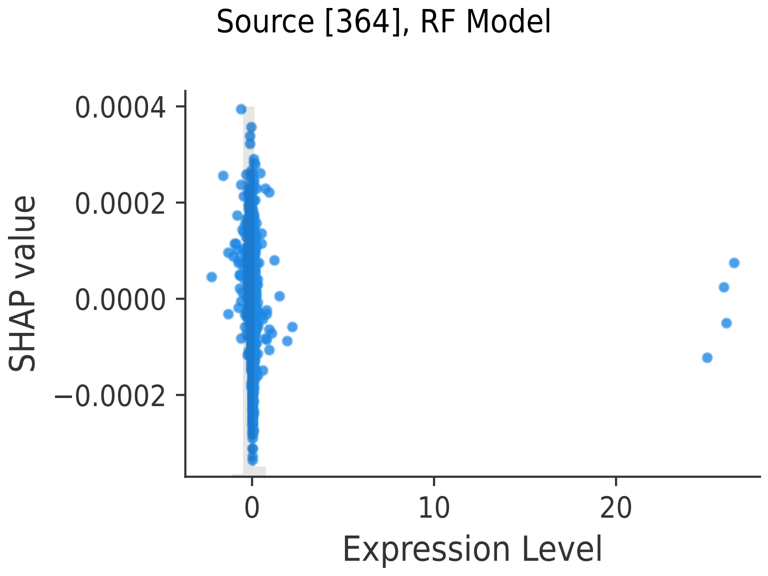
Source [364], RF Model
SHAP value
0.0004
0.0002
0.0000
−0.0002
0
10
20
Expression Level

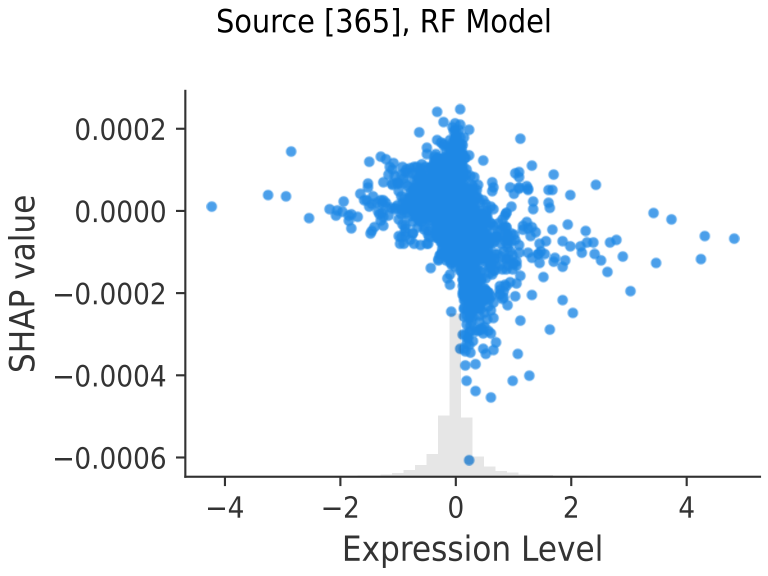
Source [365], RF Model
0.0002
0.0000
−0.0002
−0.0004
−0.0006
SHAP value
−4
−2
0
2
4
Expression Level

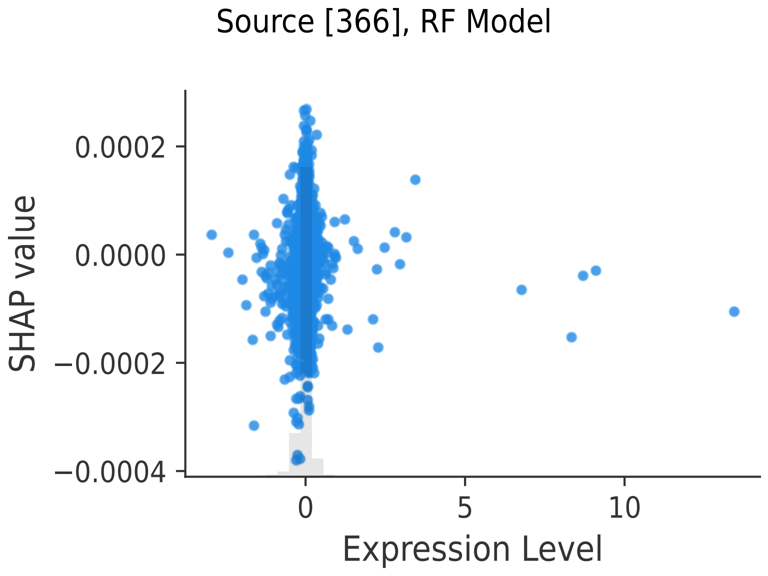

Source [366], RF Model
0.0002
0.0000
−0.0002
−0.0004
SHAP value
0
5
10
Expression Level

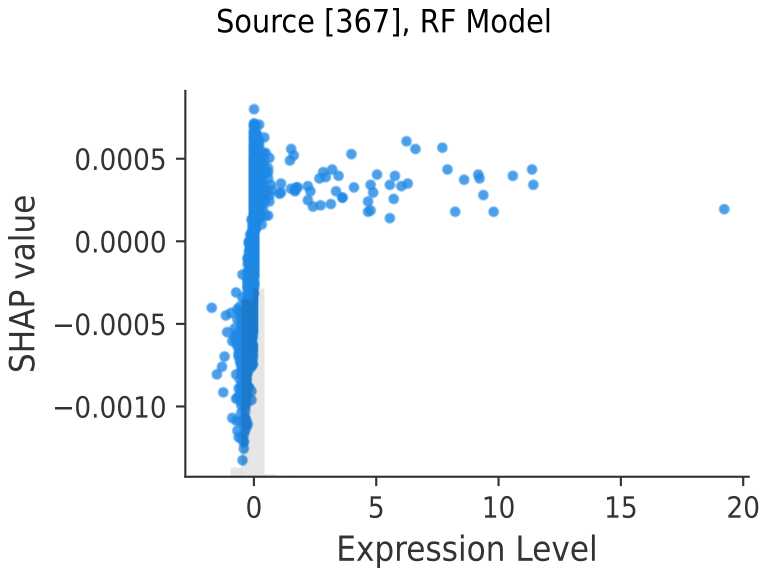
Source [367], RF Model
SHAP value
0.0005
0.0000
−0.0005
−0.0010
0
5
10
15
20
Expression Level

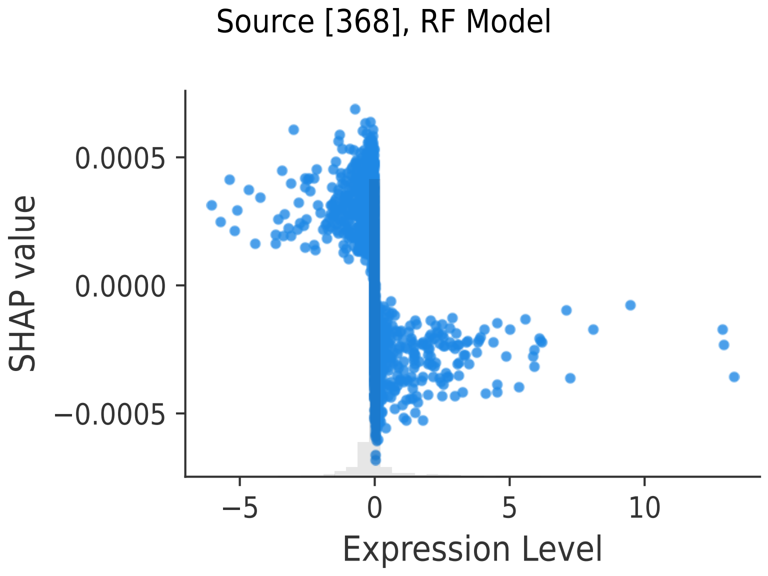
Source [368], RF Model
SHAP value
0.0005
0.0000
−0.0005
−5
0
5
10
Expression Level

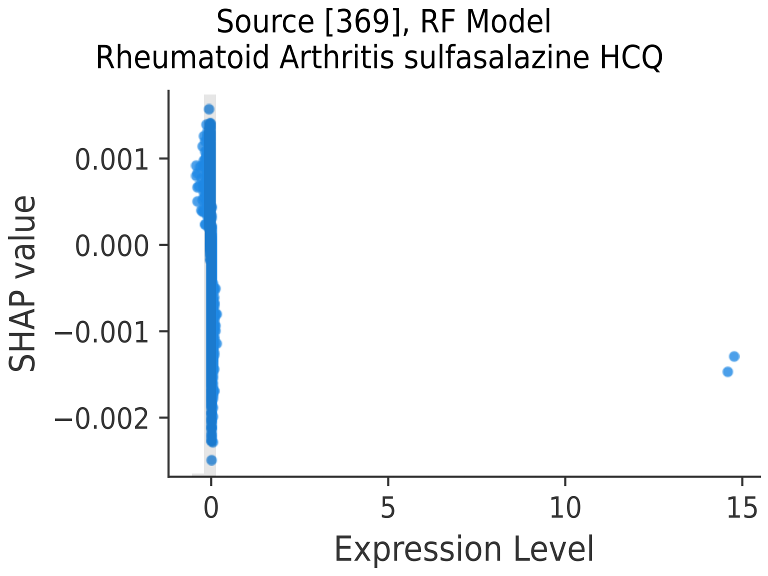
Source [369], RF Model
Rheumatoid Arthritis sulfasalazine HCQ
SHAP value
0.001
0.000
−0.001
−0.002
0
5
10
15
Expression Level

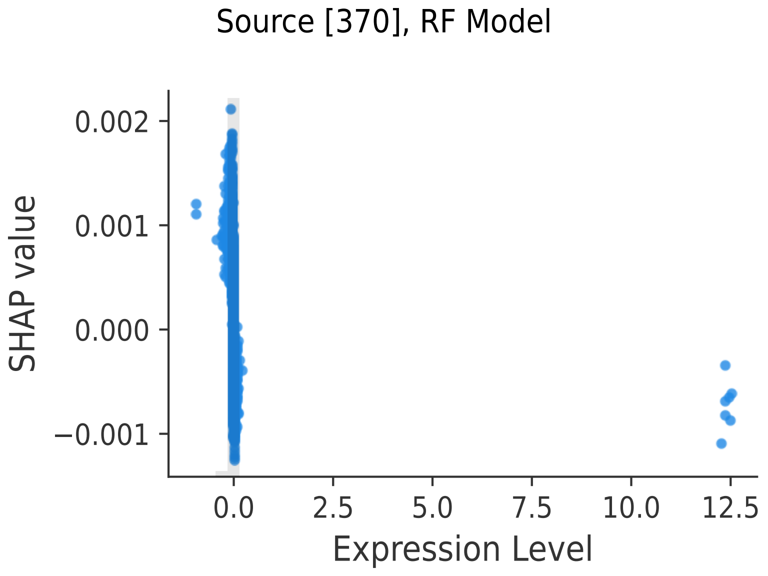
Source [370], RF Model
SHAP value
0.002
0.001
0.000
−0.001
0.0
2.5
5.0
7.5
10.0
12.5
Expression Level

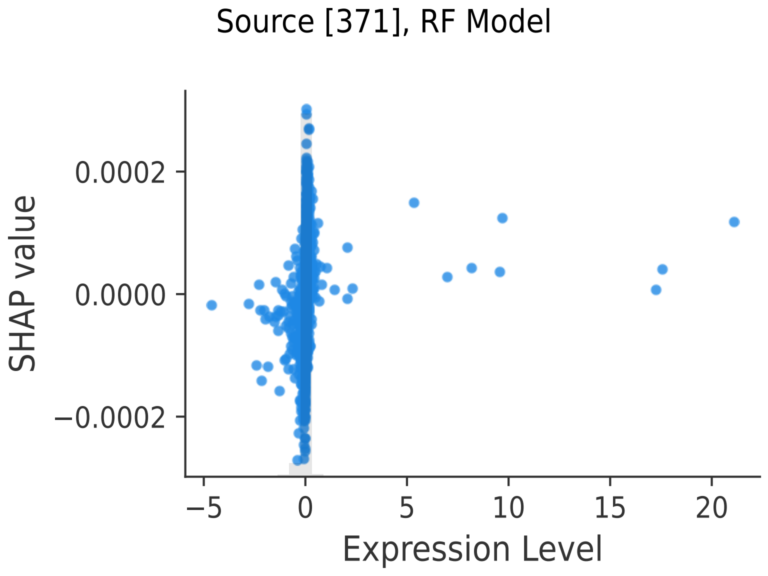

Source [371], RF Model
SHAP value
0.0002
0.0000
−0.0002
−5
0
5
10
15
20
Expression Level

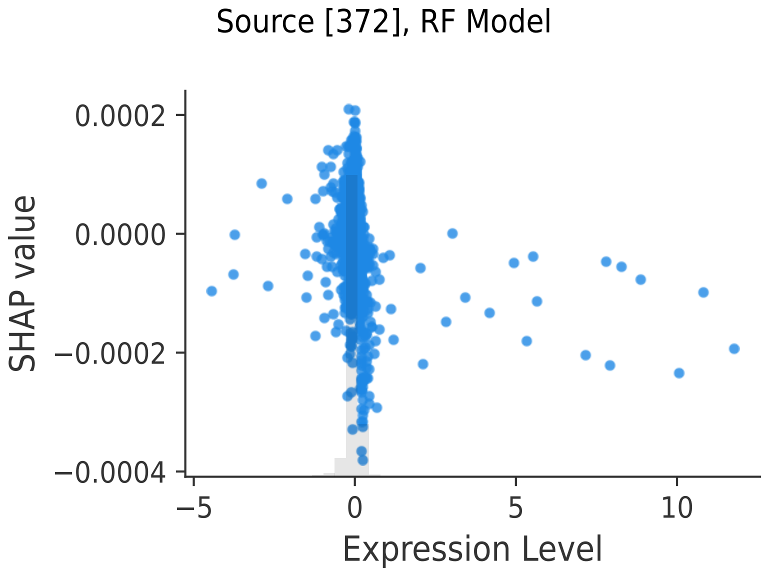
Source [372], RF Model
SHAP value
0.0002
0.0000
−0.0002
−0.0004
−5
0
5
10
Expression Level

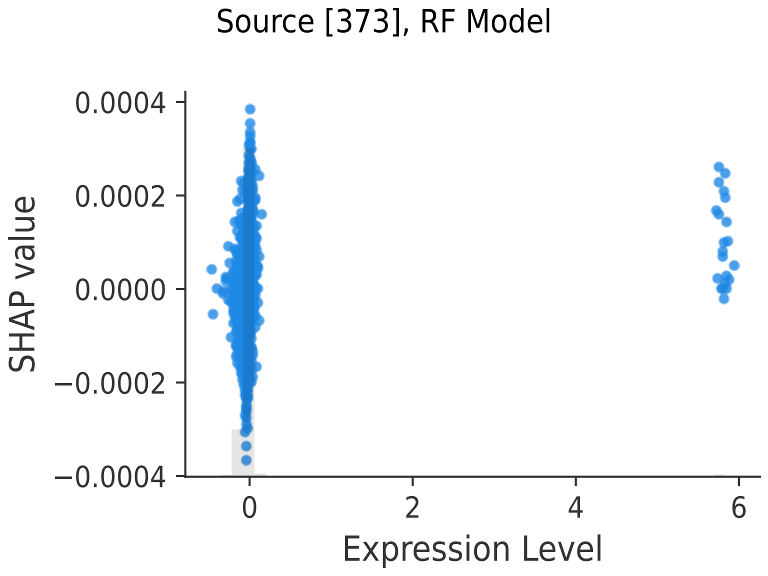

Source [373], RF Model
SHAP value
0.0004
0.0002
0.0000
−0.0002
−0.0004
0
2
4
6
Expression Level

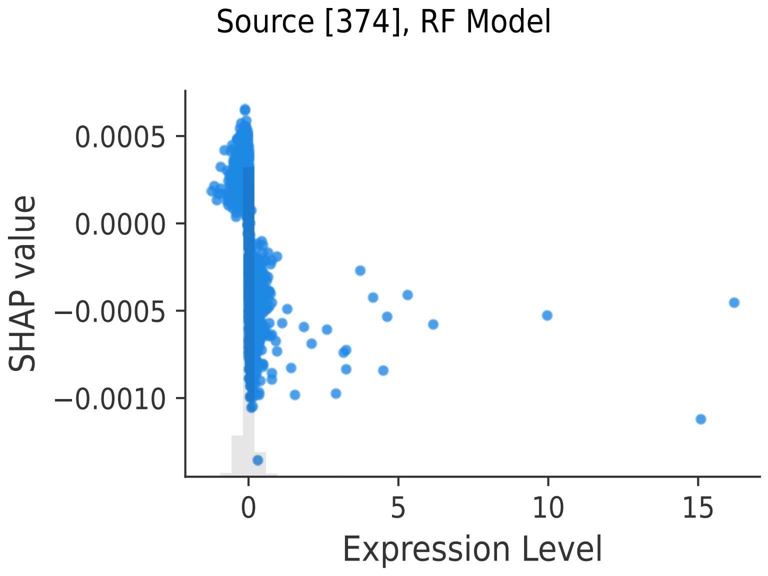
Source [374], RF Model
0.0005
0.0000
−0.0005
−0.0010
SHAP value
0
5
10
15
Expression Level

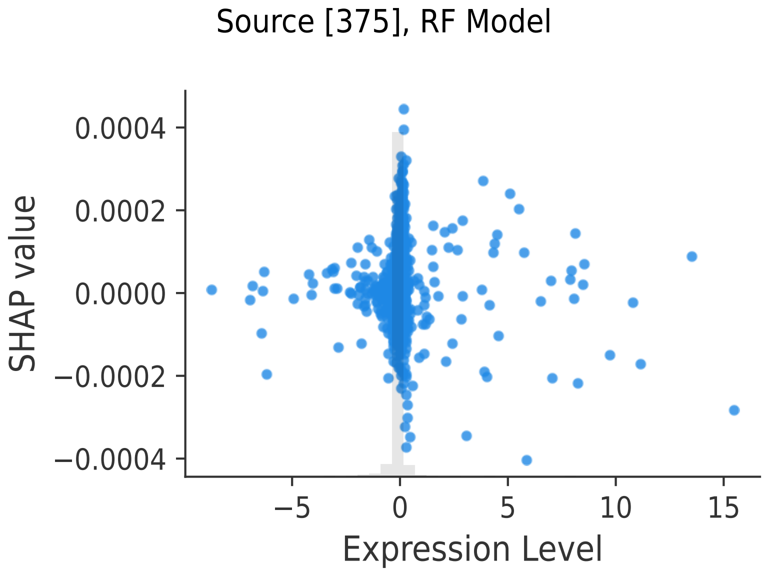

Source [375], RF Model
0.0004
0.0002
0.0000
−0.0002
−0.0004
SHAP value
−5
0
5
10
15
Expression Level

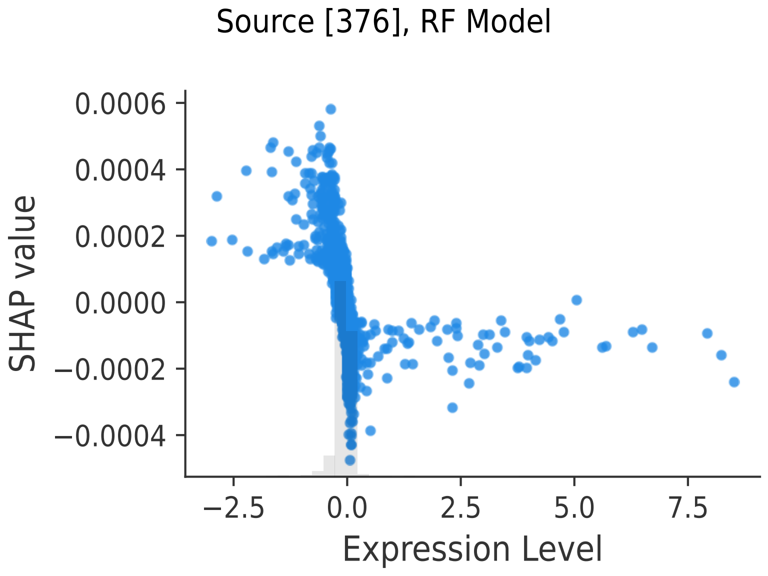

Source [376], RF Model
0.0006
0.0004
0.0002
0.0000
−0.0002
−0.0004
SHAP value
−2.5
0.0
2.5
5.0
7.5
Expression Level

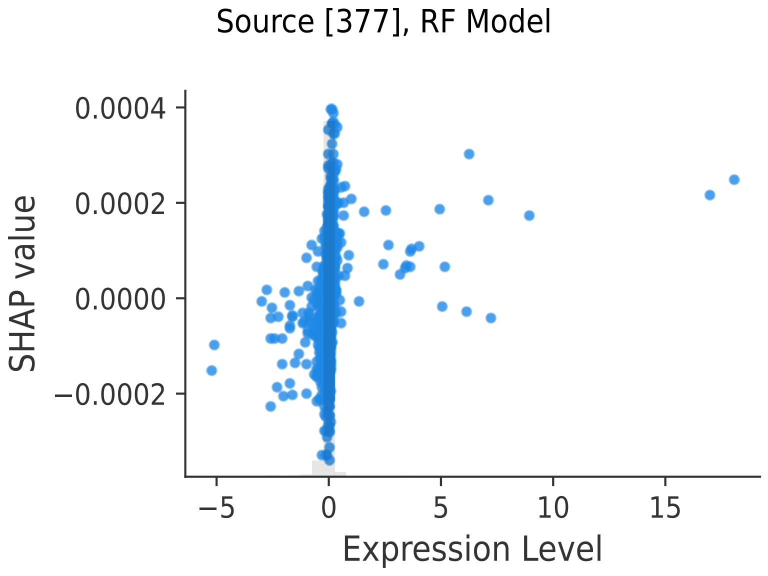
Source [377], RF Model
0.0004
0.0002
0.0000
−0.0002
SHAP value
−5
0
5
10
15
Expression Level

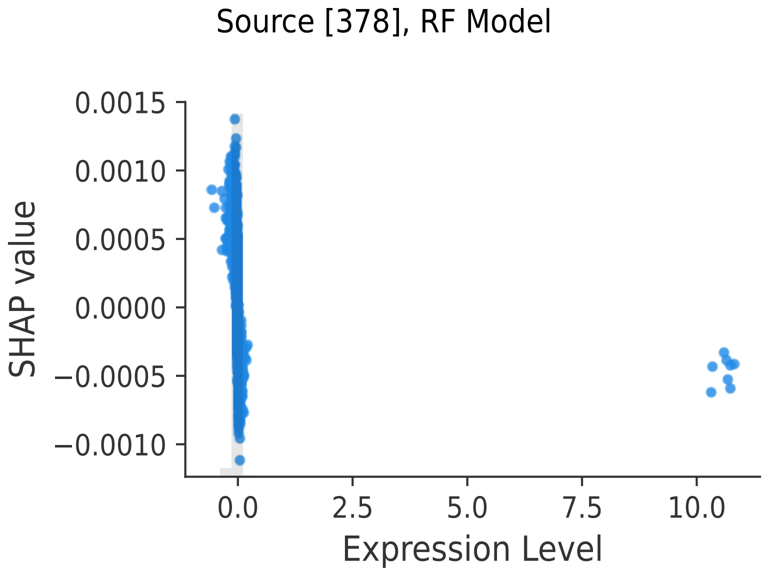
Source [378], RF Model
SHAP value
0.0015
0.0010
0.0005
0.0000
−0.0005
−0.0010
0.0
2.5
5.0
7.5
10.0
Expression Level

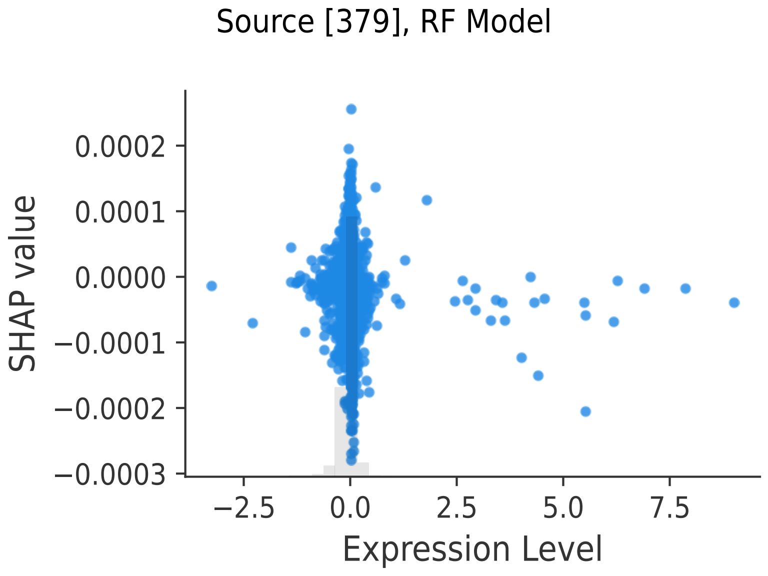
Source [379], RF Model
SHAP value
0.0002
0.0001
0.0000
−0.0001
−0.0002
−0.0003
−2.5
0.0
2.5
5.0
7.5
Expression Level

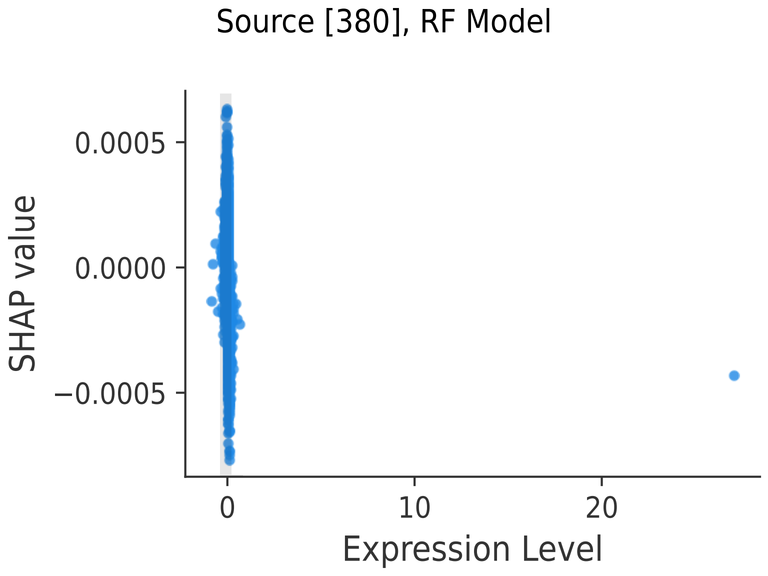
Source [380], RF Model
0.0005
0.0000
−0.0005
SHAP value
0
10
20
Expression Level

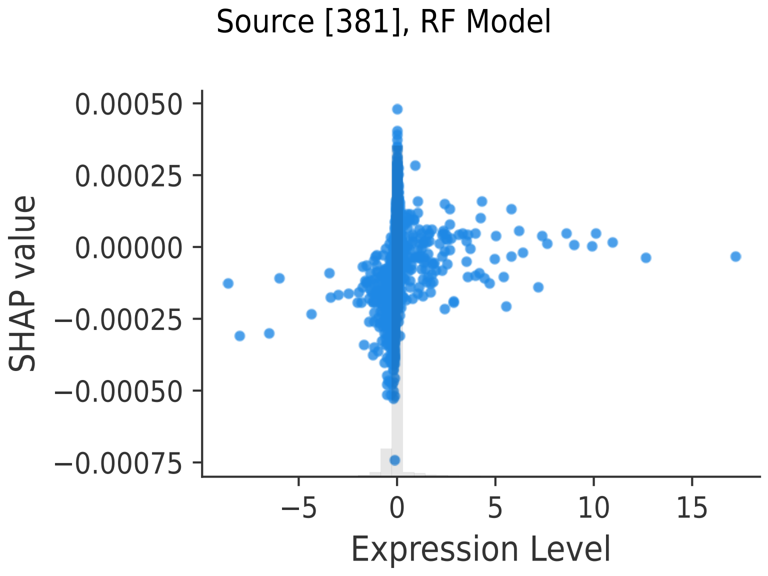

Source [381], RF Model
0.00050
0.00025
0.00000
−0.00025
−0.00050
−0.00075
SHAP value
−5
0
5
10
15
Expression Level

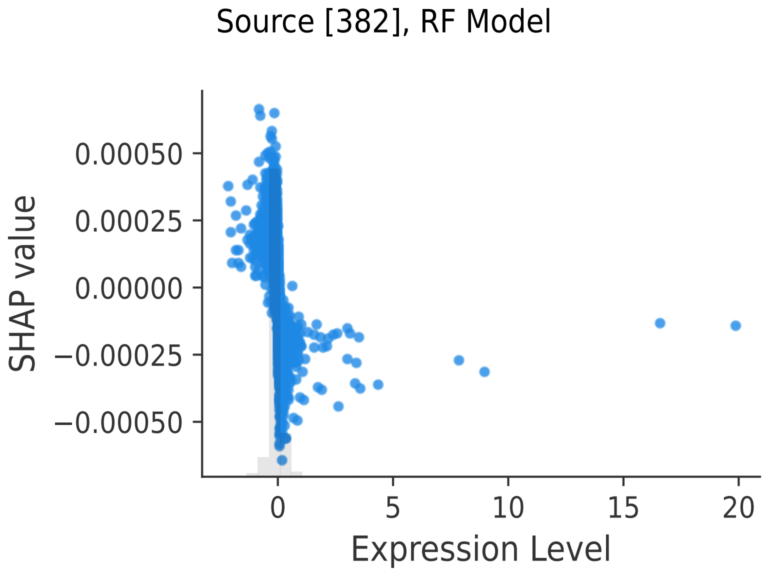
Source [382], RF Model
SHAP value
0.00050
0.00025
0.00000
−0.00025
−0.00050
0
5
10
15
20
Expression Level

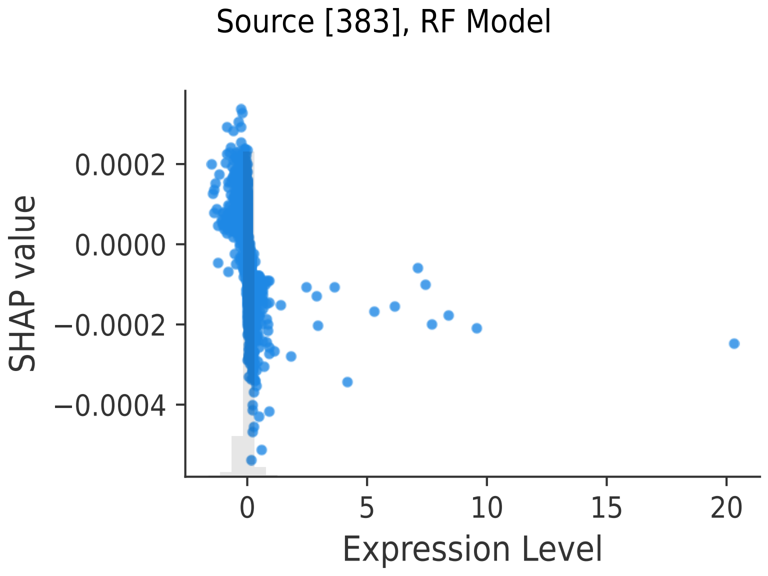

Source [383], RF Model
0.0002
0.0000
−0.0002
−0.0004
SHAP value
0
5
10
15
20
Expression Level

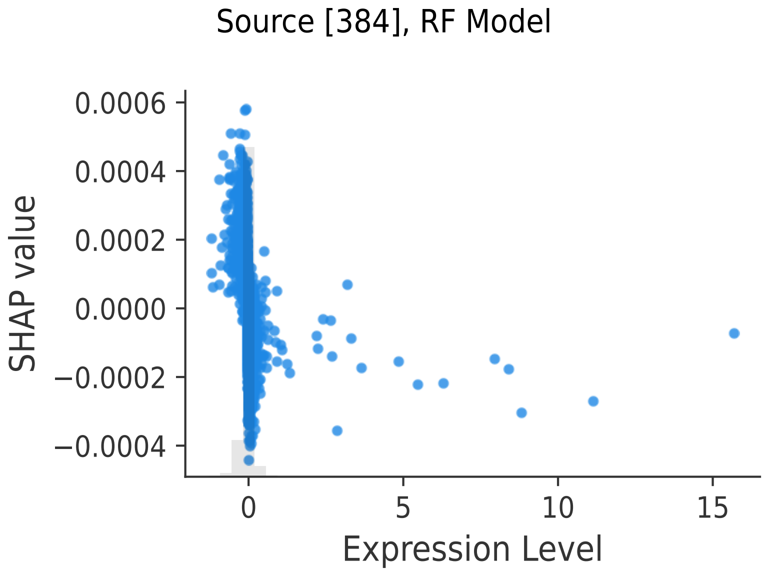
Source [384], RF Model
0.0006
0.0004
0.0002
0.0000
−0.0002
−0.0004
SHAP value
0
5
10
15
Expression Level

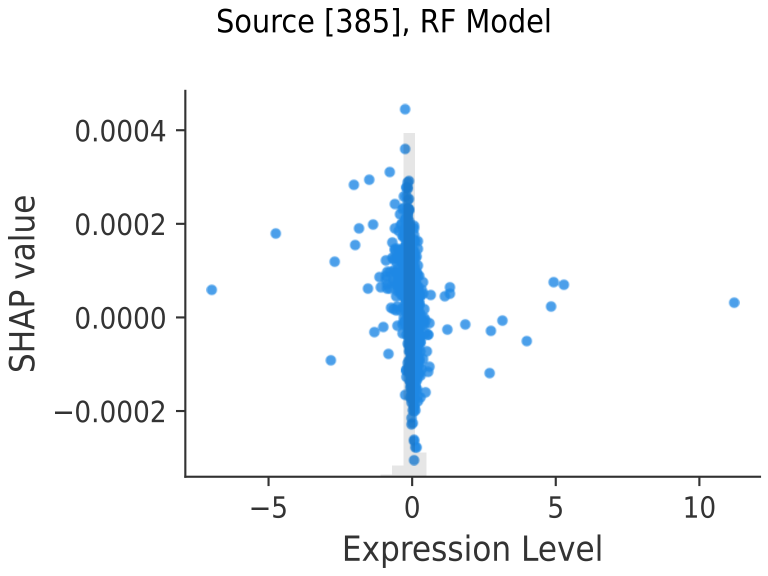
Source [385], RF Model
SHAP value
0.0004
0.0002
0.0000
−0.0002
−5
0
5
10
Expression Level

Source [386], RF Model

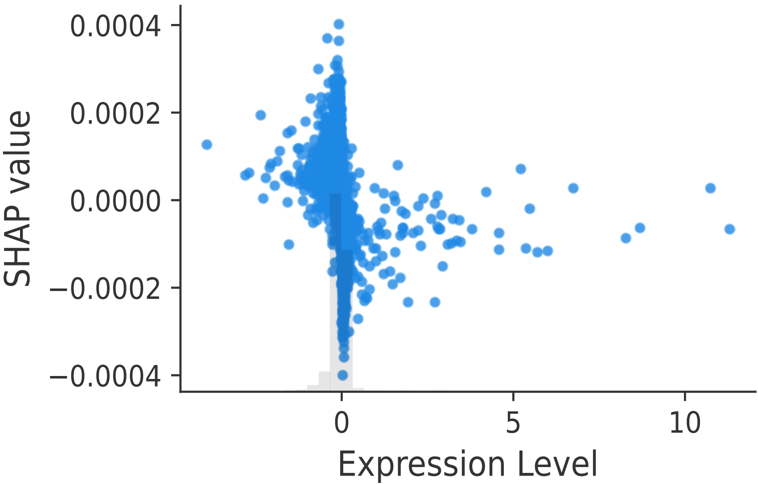

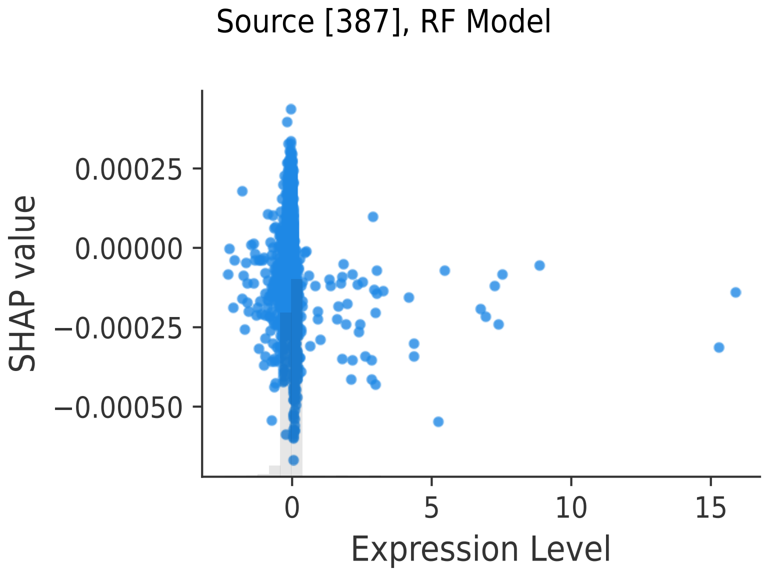
Source [387], RF Model
SHAP value
0.00025
0.00000
−0.00025
−0.00050
0
5
10
15
Expression Level

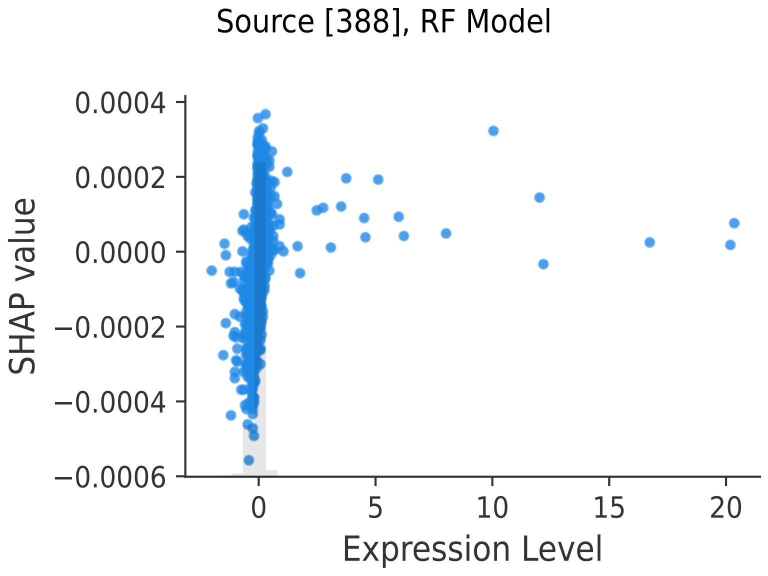

Source [388], RF Model
0.0004
0.0002
0.0000
−0.0002
−0.0004
−0.0006
SHAP value
0
5
10
15
20
Expression Level

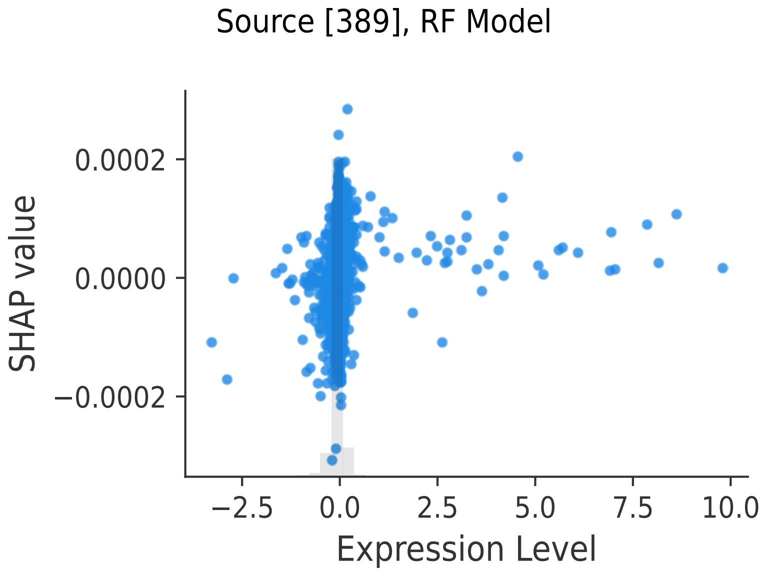

Source [389], RF Model
0.0002
0.0000
−0.0002
SHAP value
−2.5
0.0
2.5
5.0
7.5
10.0
Expression Level

Source [390], RF Model

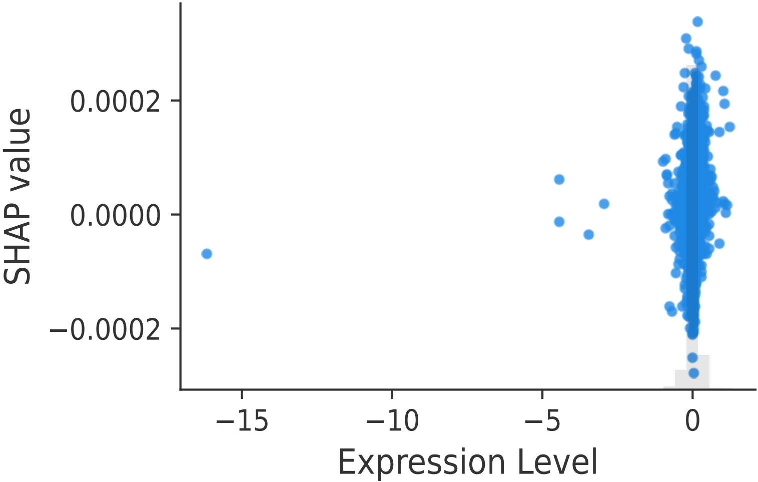

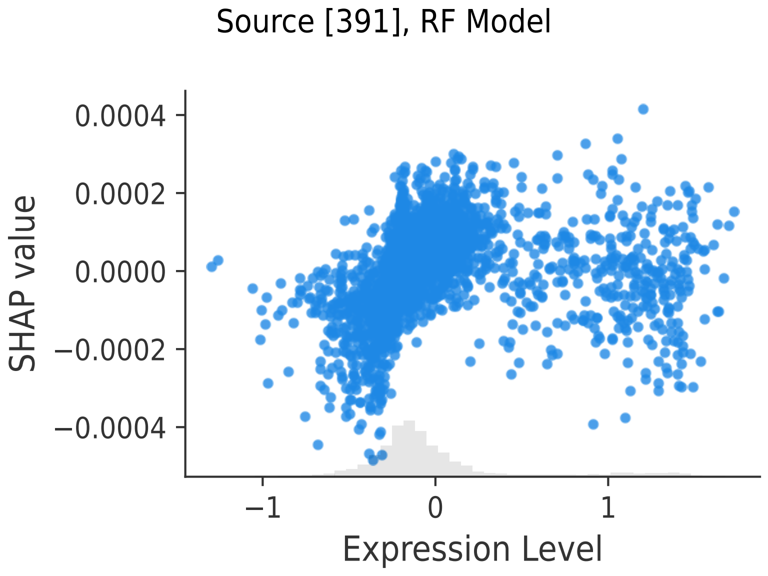
Source [391], RF Model
0.0004
0.0002
0.0000
−0.0002
−0.0004
SHAP value
−1
0
1
Expression Level

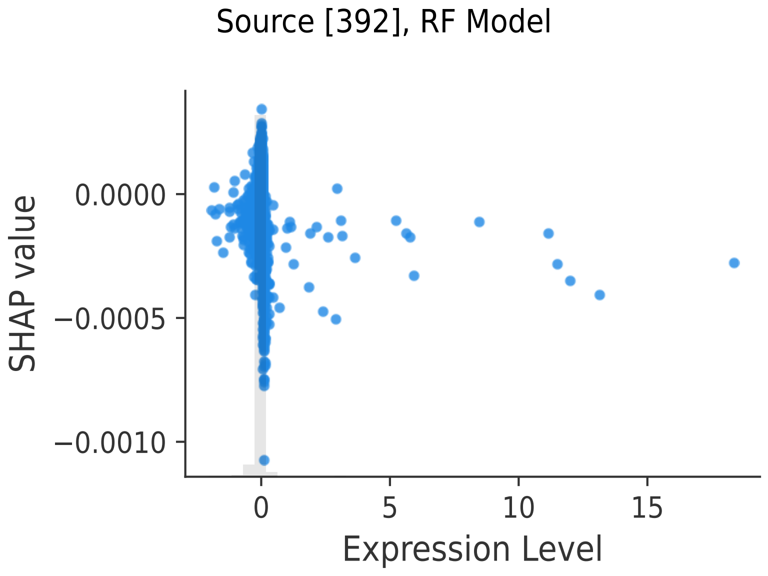
Source [392], RF Model
SHAP value
0.0000
−0.0005
−0.0010
0
5
10
15
Expression Level

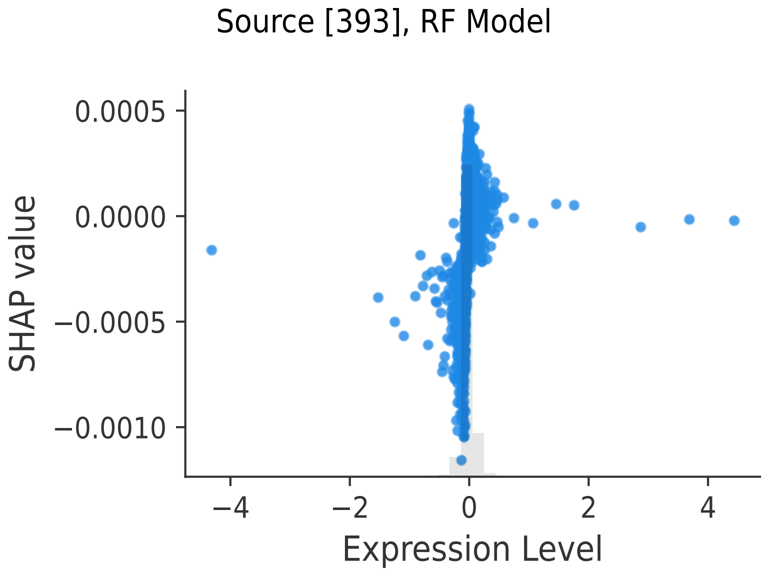
Source [393], RF Model
0.0005
0.0000
−0.0005
−0.0010
SHAP value
−4
−2
0
2
4
Expression Level

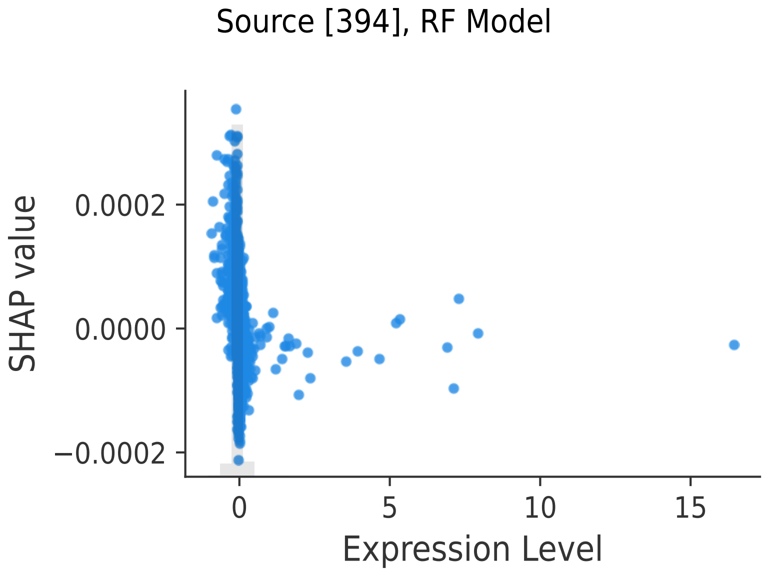

Source [394], RF Model
SHAP value
0.0002
0.0000
−0.0002
0
5
10
15
Expression Level

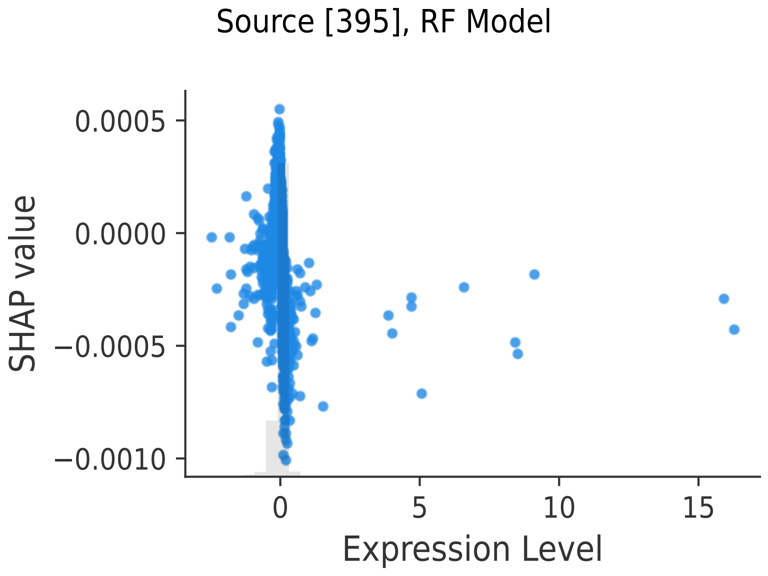
Source [395], RF Model
0.0005
0.0000
−0.0005
−0.0010
SHAP value
0
5
10
15
Expression Level

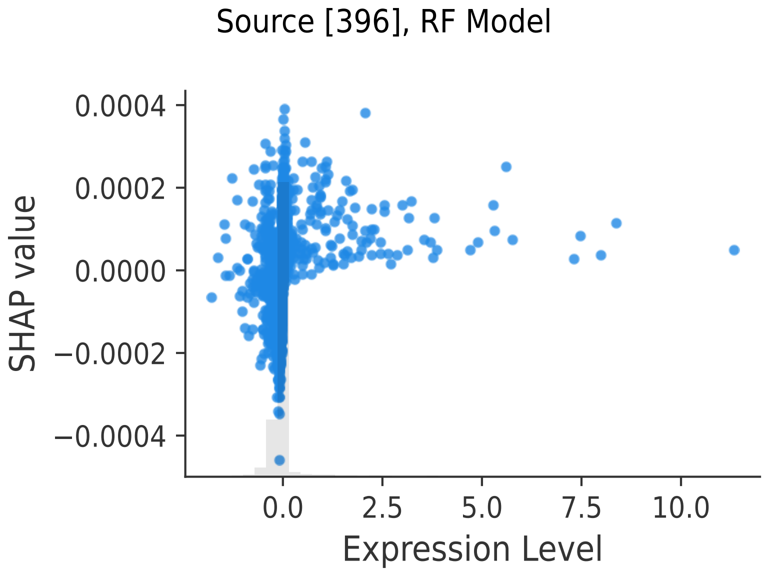

Source [396], RF Model
0.0004
0.0002
0.0000
−0.0002
−0.0004
SHAP value
0.0
2.5
5.0
7.5
10.0
Expression Level

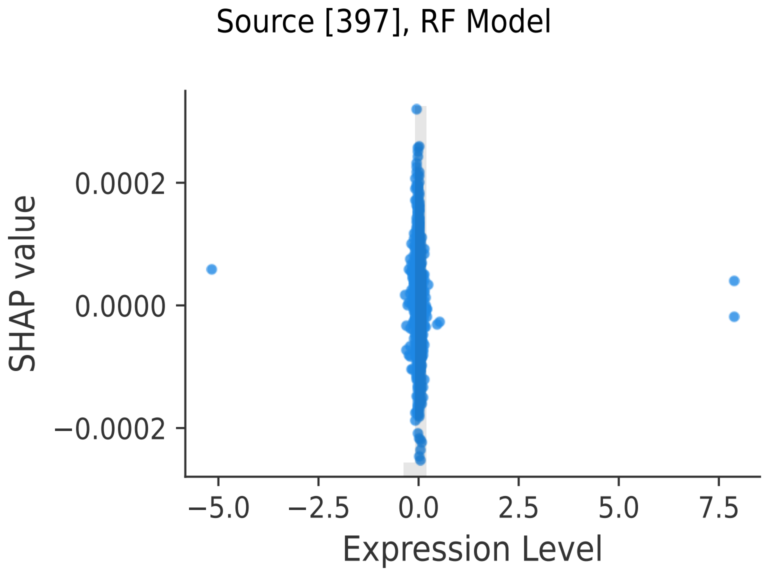
Source [397], RF Model
SHAP value
0.0002
0.0000
−0.0002
−5.0
−2.5
0.0
2.5
5.0
7.5
Expression Level

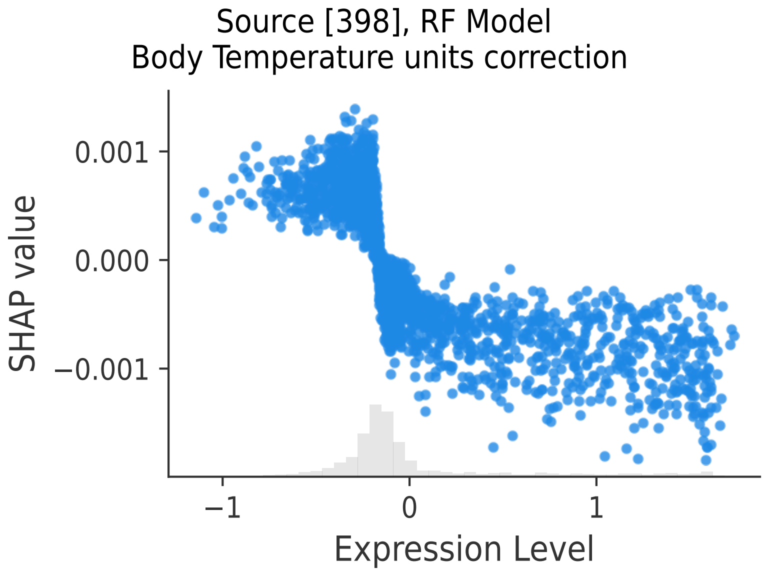
Source [398], RF Model
Body Temperature units correction
0.001
0.000
−0.001
SHAP value
−1
0
1
Expression Level

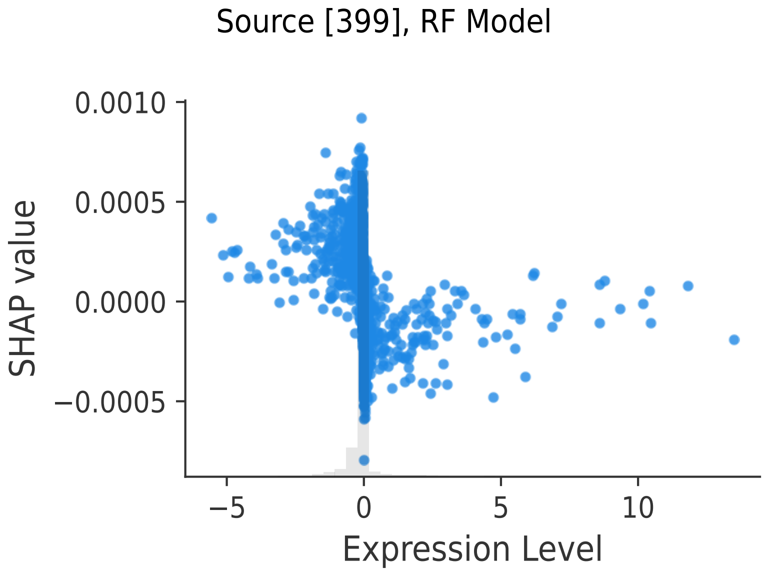
Source [399], RF Model
0.0010
0.0005
0.0000
−0.0005
SHAP value
−5
0
5
10
Expression Level

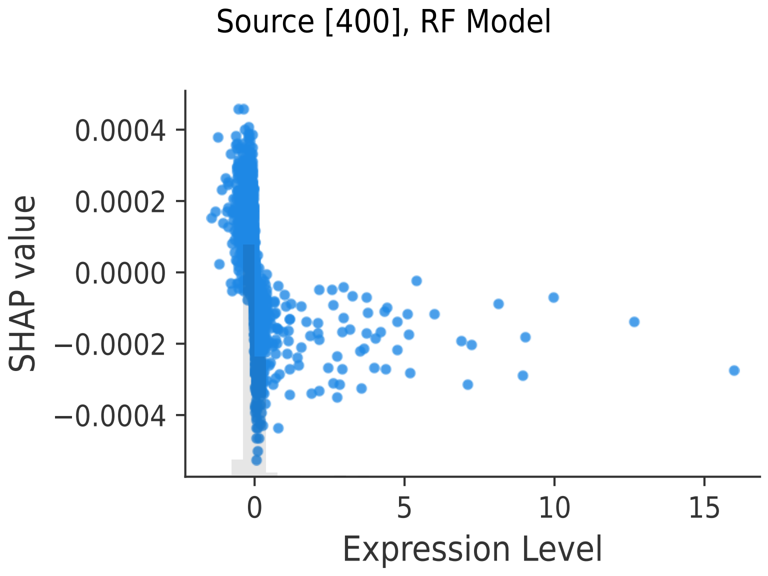
Source [400], RF Model
SHAP value
0.0004
0.0002
0.0000
−0.0002
−0.0004
0
5
10
15
Expression Level

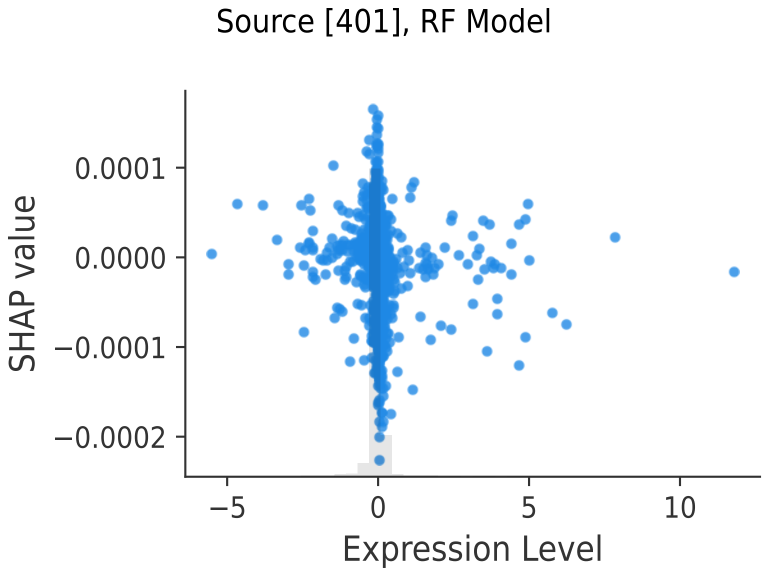

Source [401], RF Model
SHAP value
0.0001
0.0000
−0.0001
−0.0002
−5
0
5
10
Expression Level

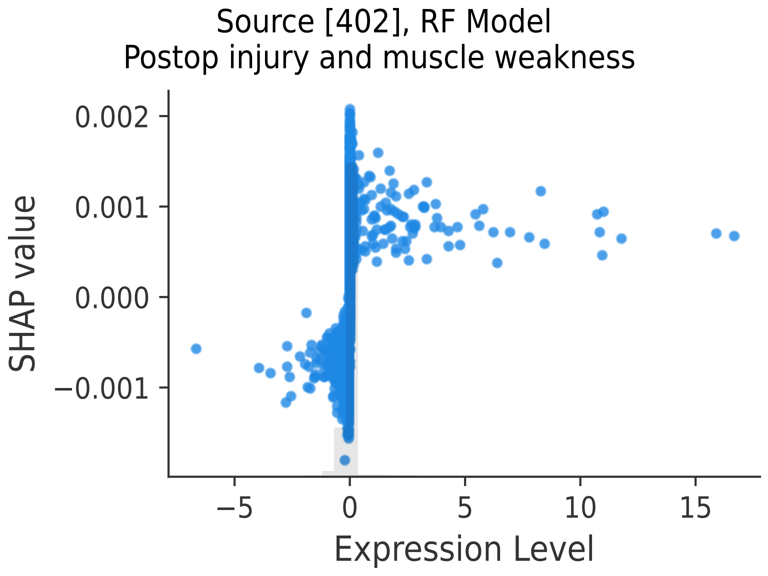
Source [402], RF Model
Postop injury and muscle weakness
SHAP value
0.002
0.001
0.000
−0.001
−5
0
5
10
15
Expression Level

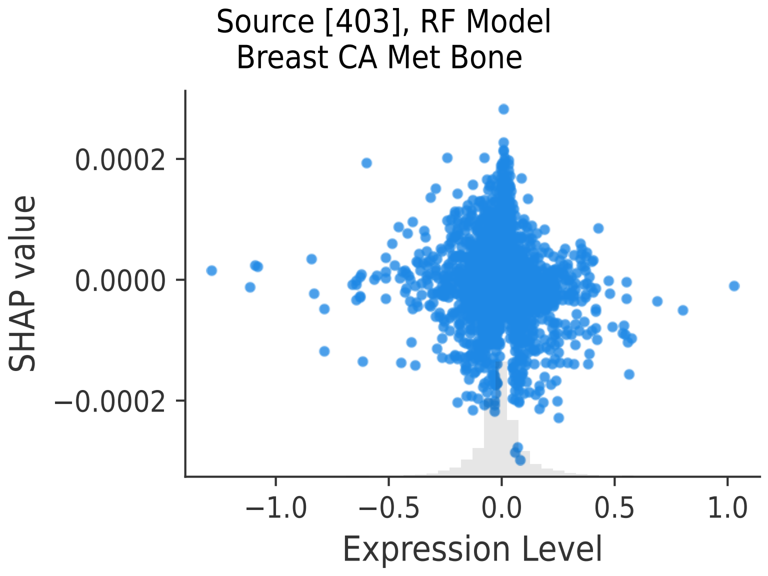

Source [403], RF Model
Breast CA Met Bone
0.0002
0.0000
−0.0002
SHAP value
−1.0
−0.5
0.0
0.5
1.0
Expression Level

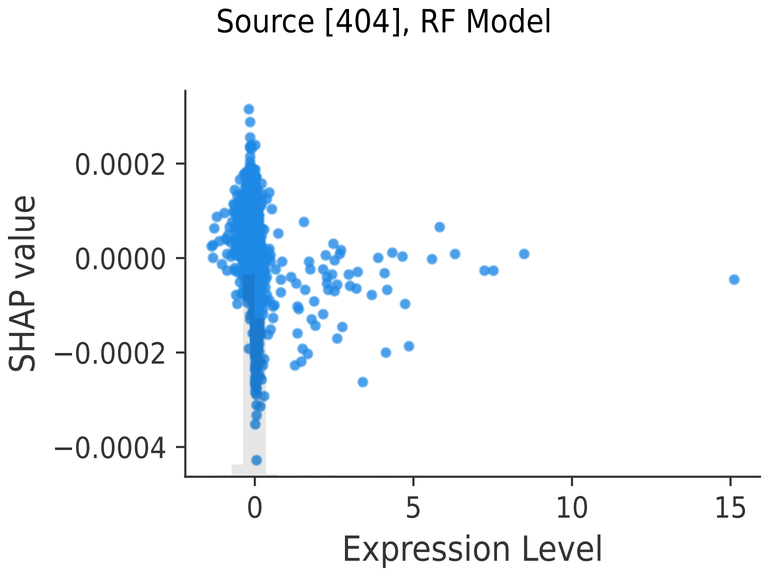

Source [404], RF Model
0.0002
0.0000
−0.0002
−0.0004
SHAP value
0
5
10
15
Expression Level

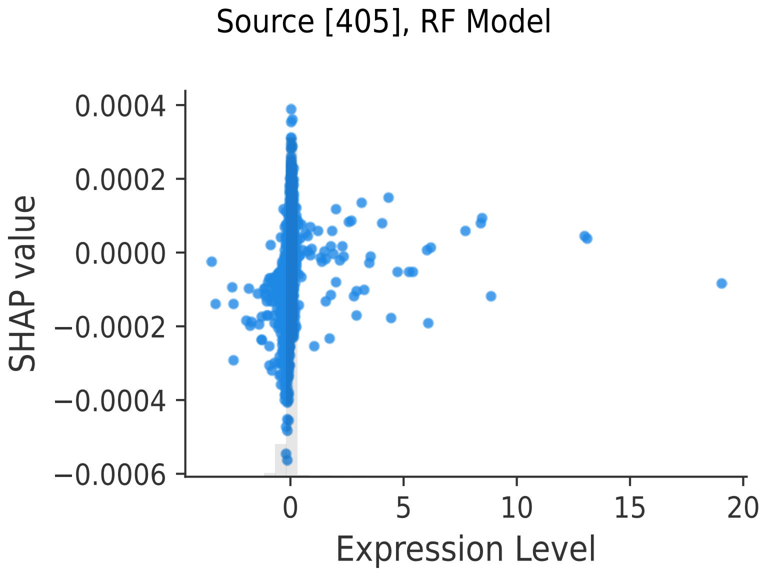

Source [405], RF Model
0.0004
0.0002
0.0000
−0.0002
−0.0004
−0.0006
SHAP value
0
5
10
15
20
Expression Level

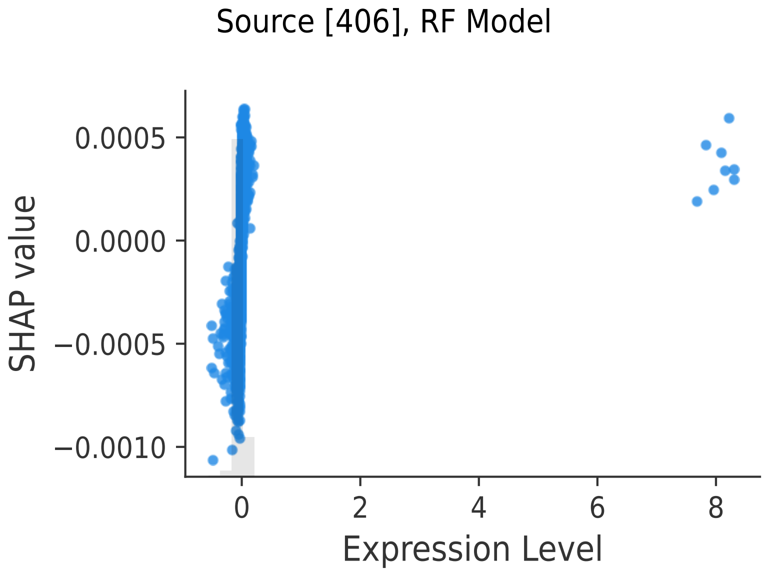
Source [406], RF Model
SHAP value
0.0005
0.0000
−0.0005
−0.0010
0
2
4
6
8
Expression Level

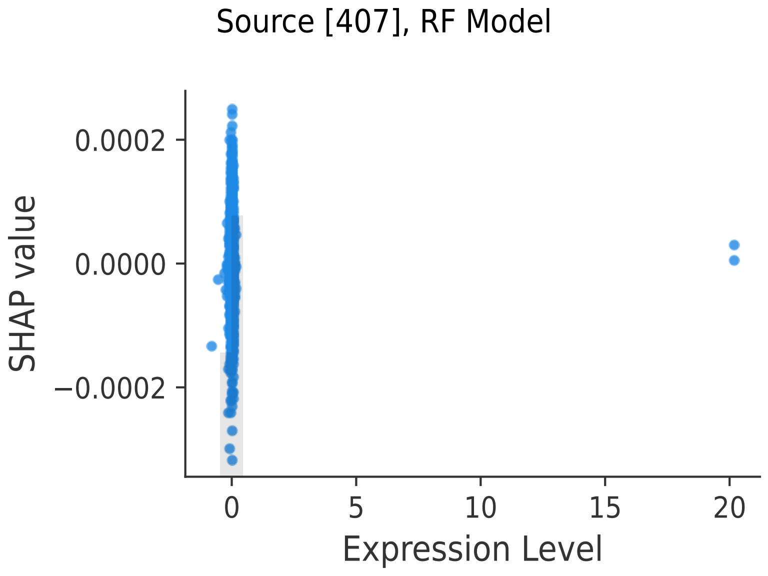

Source [407], RF Model
SHAP value
0.0002
0.0000
−0.0002
0
5
10
15
20
Expression Level

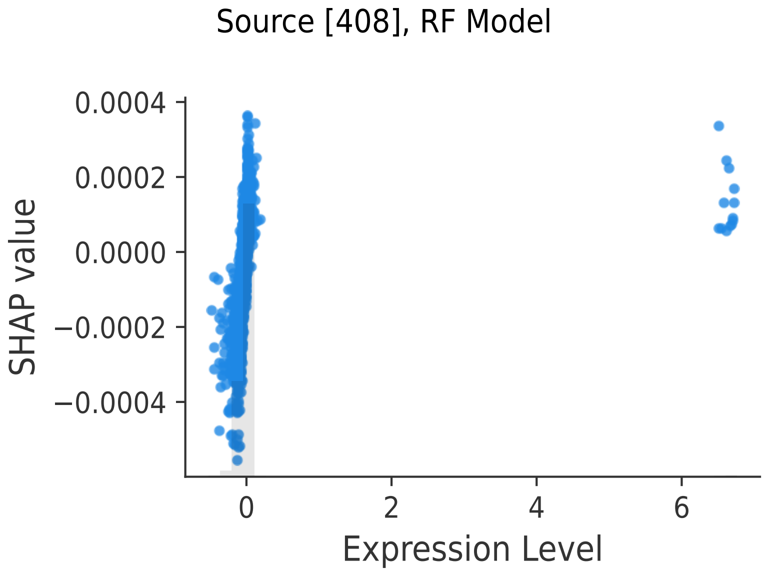
Source [408], RF Model
SHAP value
0.0004
0.0002
0.0000
−0.0002
−0.0004
0
2
4
6
Expression Level

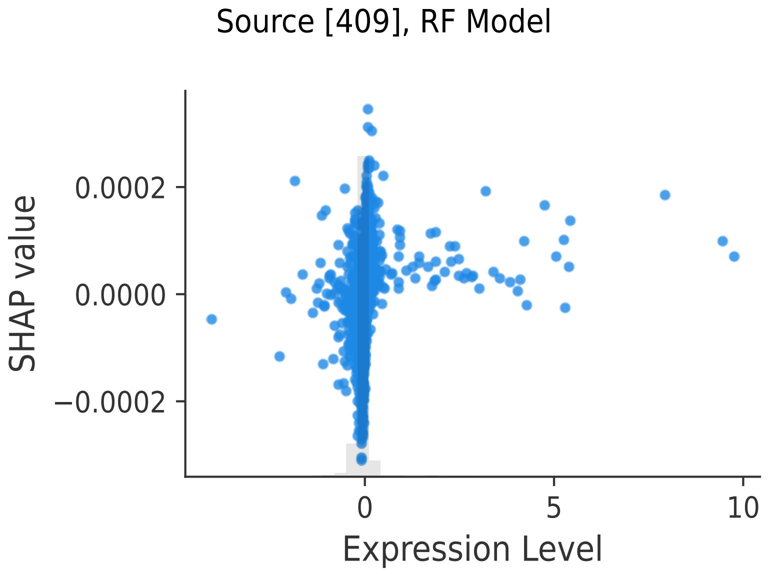
Source [409], RF Model
SHAP value
0.0002
0.0000
−0.0002
0
5
10
Expression Level

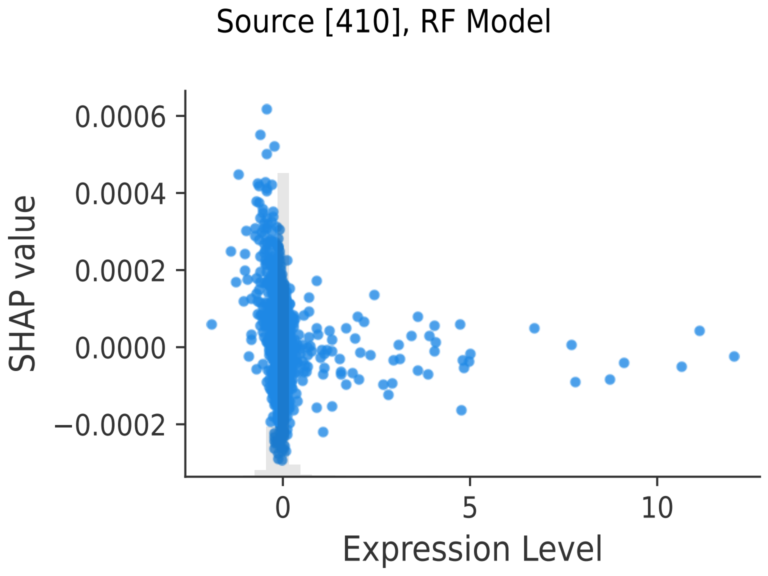

Source [410], RF Model
0.0006
0.0004
0.0002
0.0000
−0.0002
SHAP value
0
5
10
Expression Level

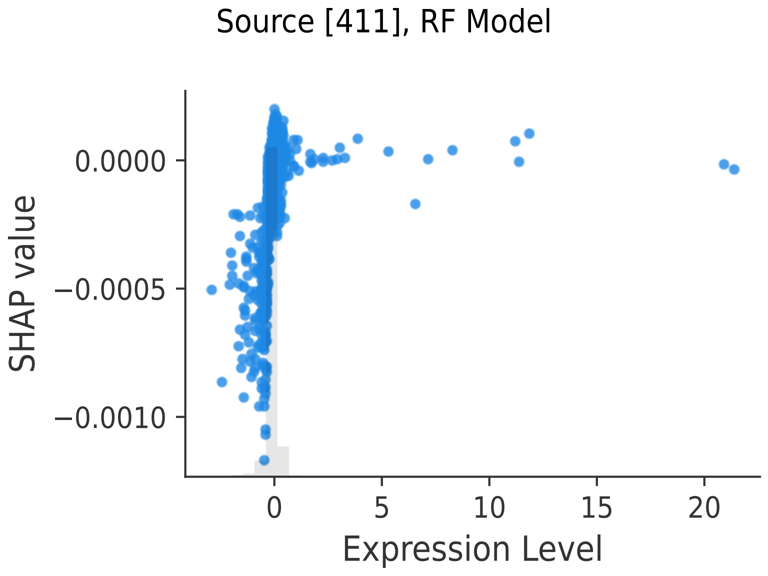
Source [411], RF Model
SHAP value
0.0000
−0.0005
−0.0010
Expression Level
0
5
10
15
20

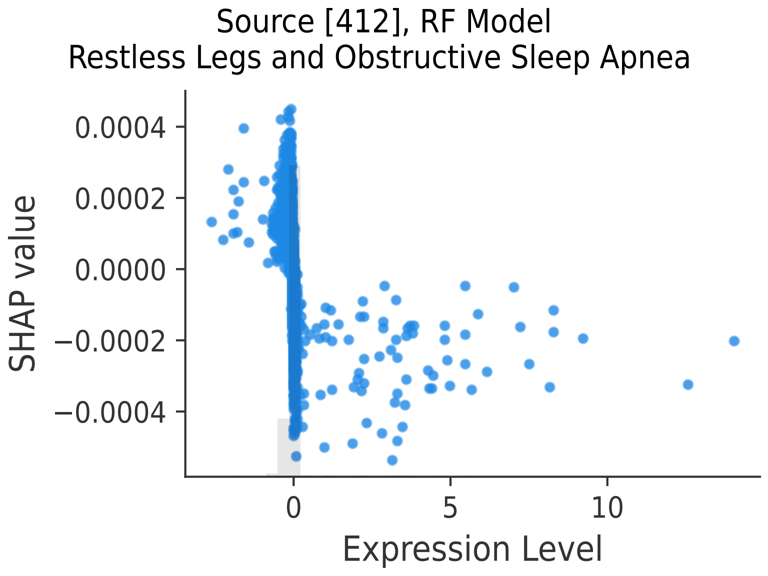
Source [412], RF Model
Restless Legs and Obstructive Sleep Apnea
0.0004
0.0002
0.0000
−0.0002
−0.0004
SHAP value
0
5
10
Expression Level

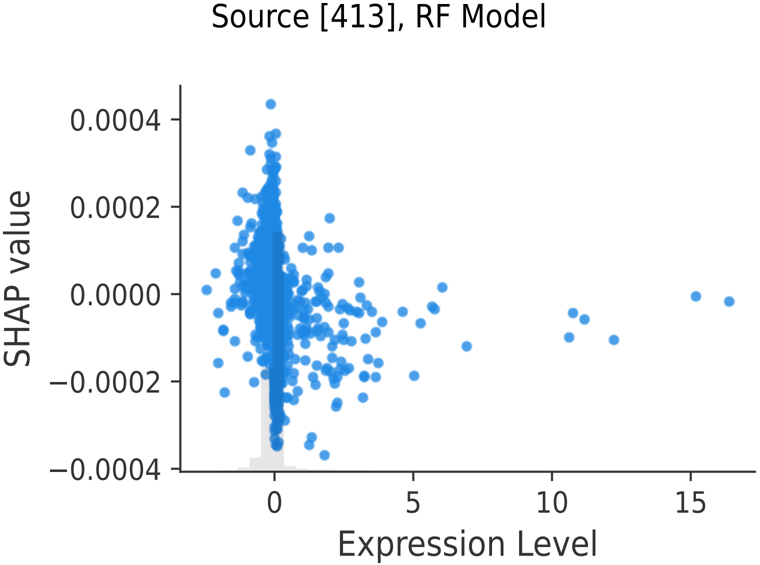

Source [413], RF Model
SHAP value
0.0004
0.0002
0.0000
−0.0002
−0.0004
0
5
10
15
Expression Level

Source [414], RF Model

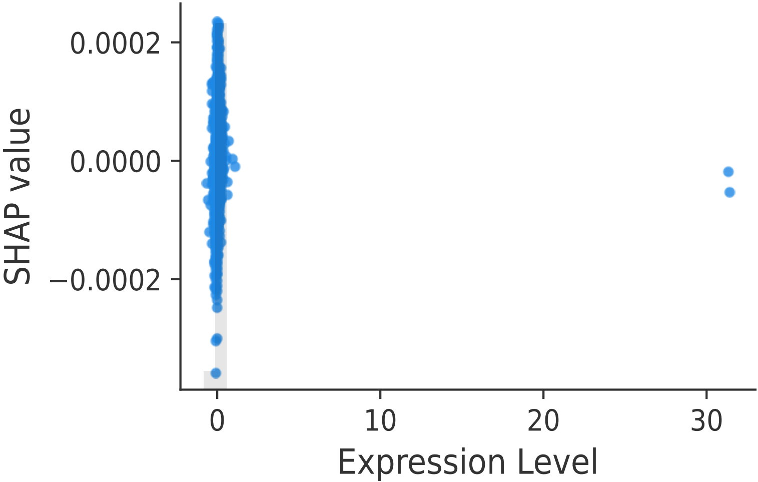

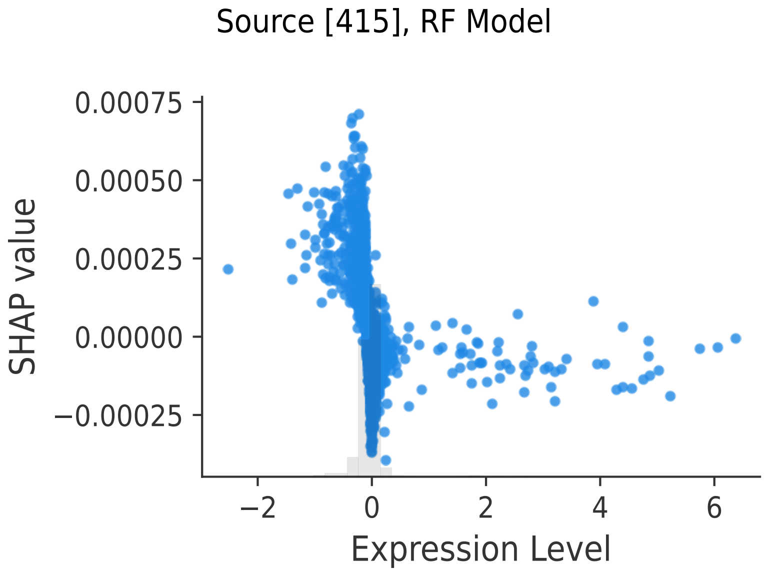
Source [415], RF Model
SHAP value
0.00075
0.00050
0.00025
0.00000
−0.00025
−2
0
2
4
6
Expression Level

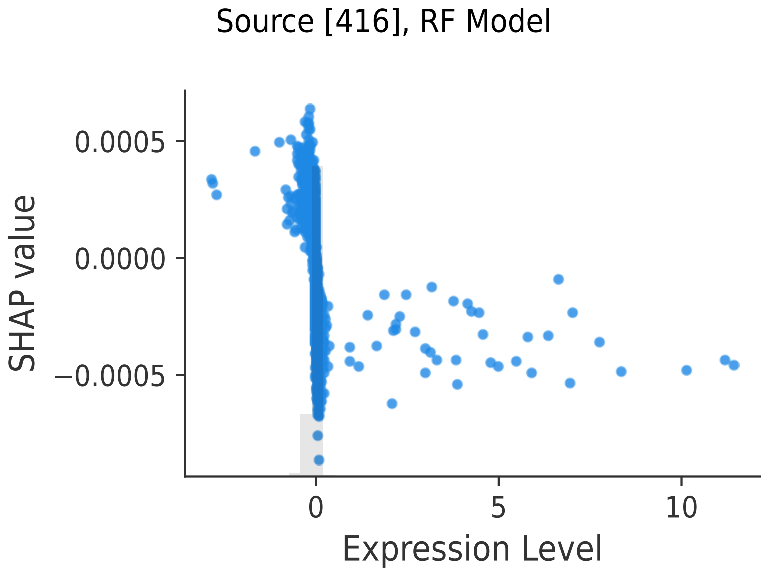

Source [416], RF Model
0.0005
0.0000
−0.0005
SHAP value
0
5
10
Expression Level

Source [417], RF Model

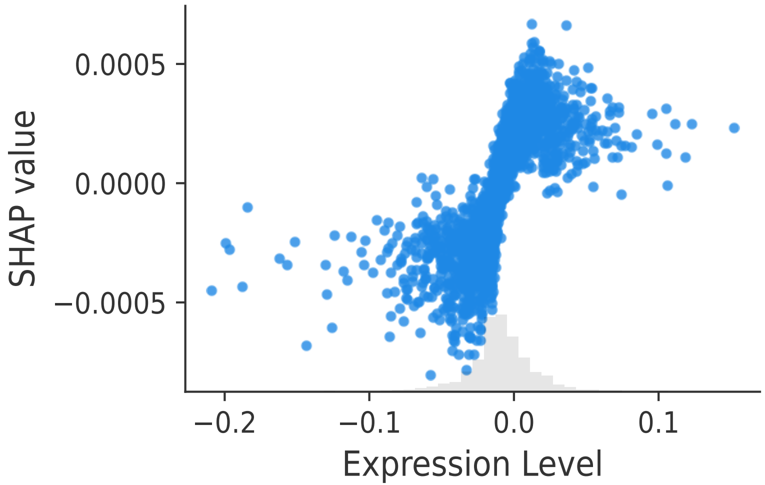

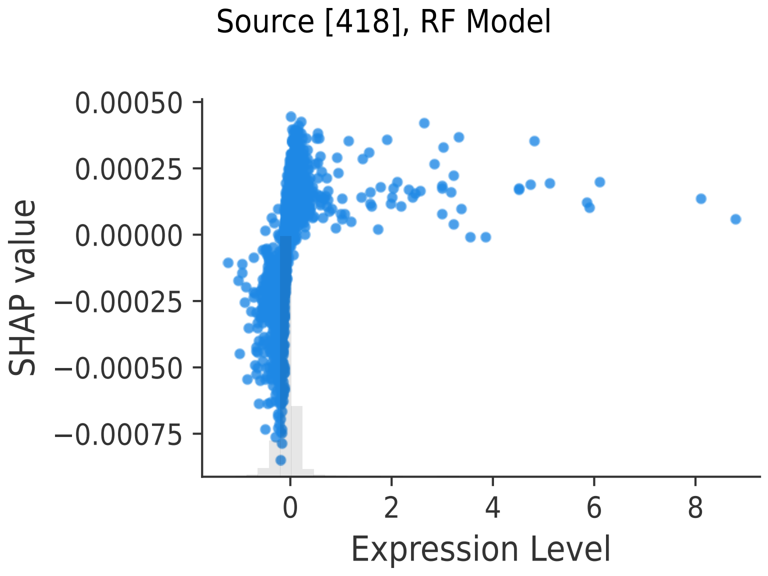

Source [418], RF Model
0.00050
0.00025
0.00000
−0.00025
−0.00050
−0.00075
SHAP value
0
2
4
6
8
Expression Level

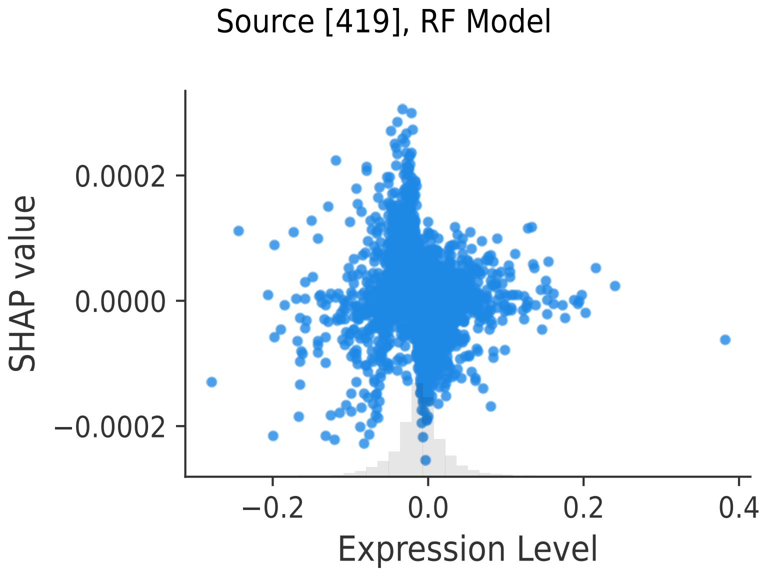
Source [419], RF Model
0.0002
0.0000
−0.0002
SHAP value
−0.2
0.0
0.2
0.4
Expression Level

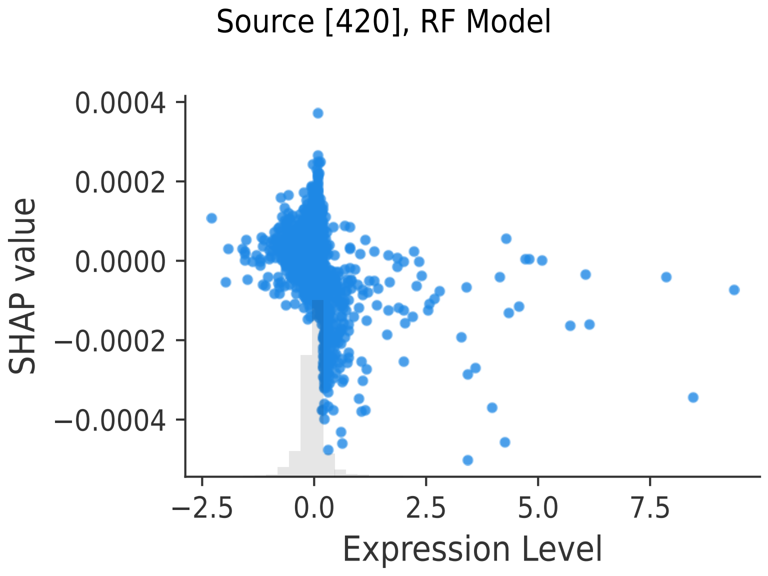

Source [420], RF Model
0.0004
0.0002
0.0000
−0.0002
−0.0004
SHAP value
−2.5
0.0
2.5
5.0
7.5
Expression Level

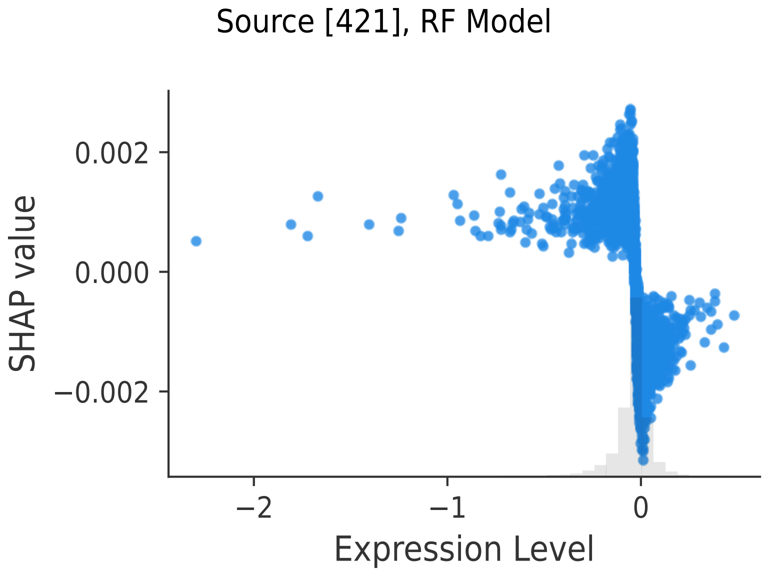
Source [421], RF Model
0.002
0.000
−0.002
SHAP value
−2
−1
0
Expression Level

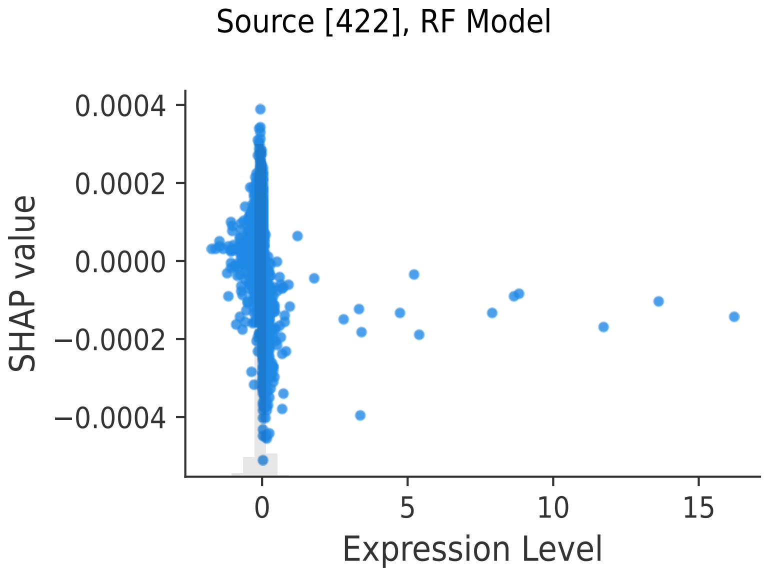

Source [422], RF Model
0.0004
0.0002
0.0000
−0.0002
−0.0004
SHAP value
0
5
10
15
Expression Level

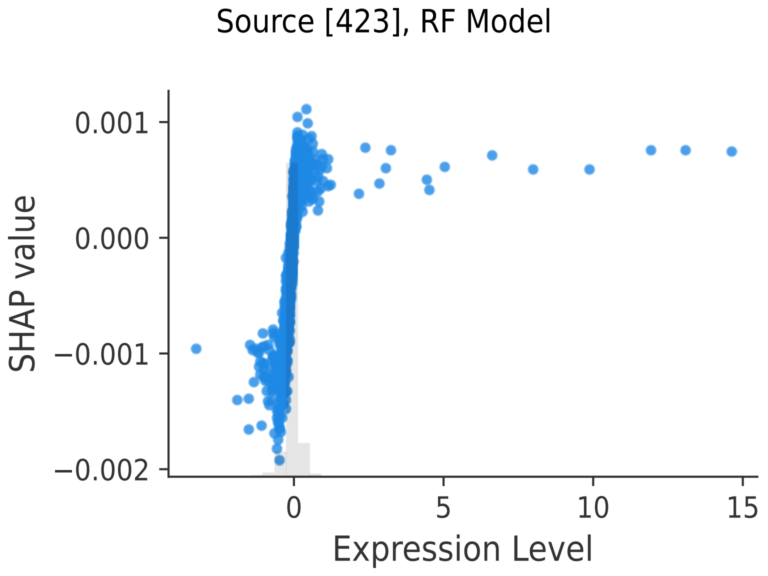

Source [423], RF Model
0.001
0.000
−0.001
−0.002
SHAP value
0
5
10
15
Expression Level

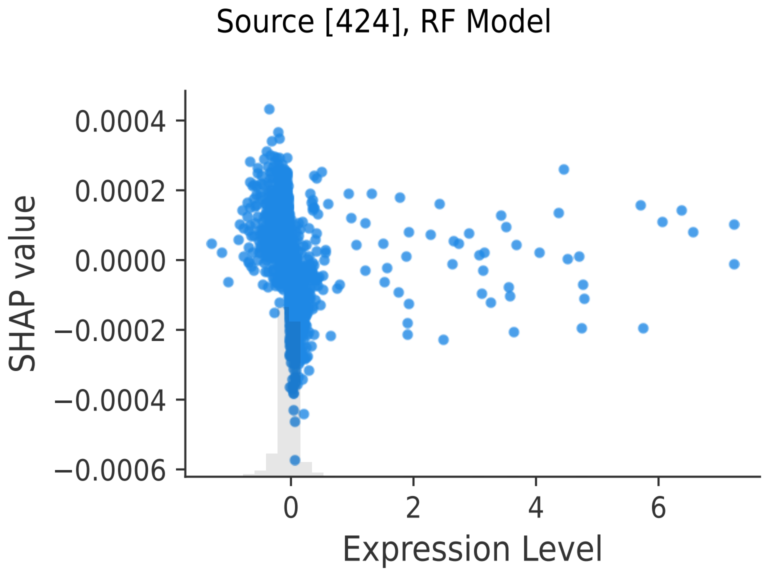

Source [424], RF Model
SHAP value
0.0004
0.0002
0.0000
−0.0002
−0.0004
−0.0006
0
2
4
6
Expression Level

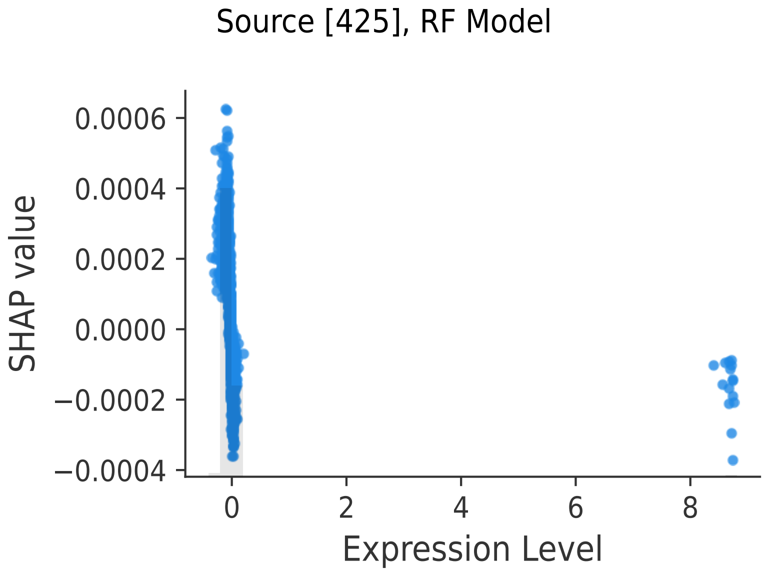

Source [425], RF Model
SHAP value
0.0006
0.0004
0.0002
0.0000
−0.0002
−0.0004
0
2
4
6
8
Expression Level

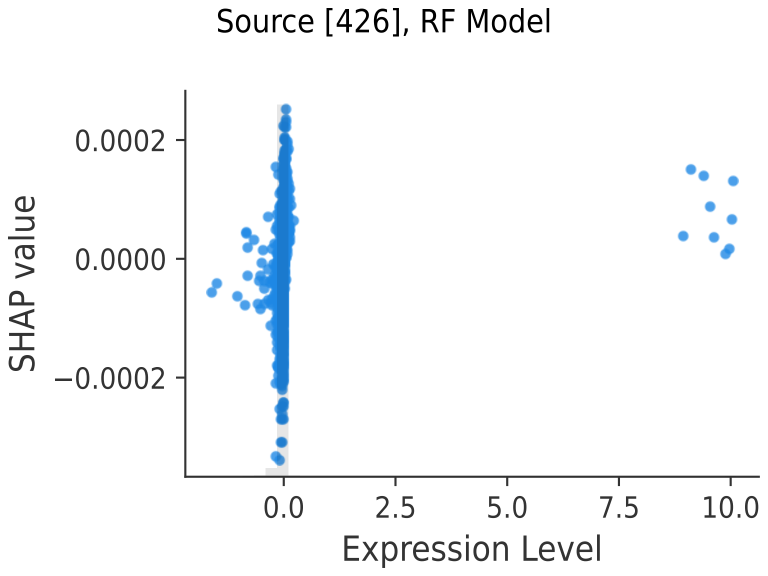
Source [426], RF Model
0.0002
0.0000
−0.0002
SHAP value
0.0
2.5
5.0
7.5
10.0
Expression Level

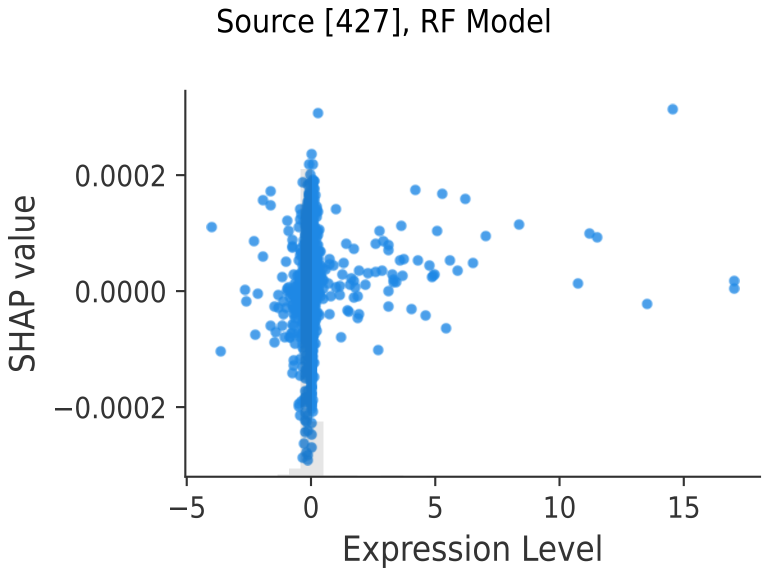

Source [427], RF Model
SHAP value
0.0002
0.0000
−0.0002
−5
0
5
10
15
Expression Level

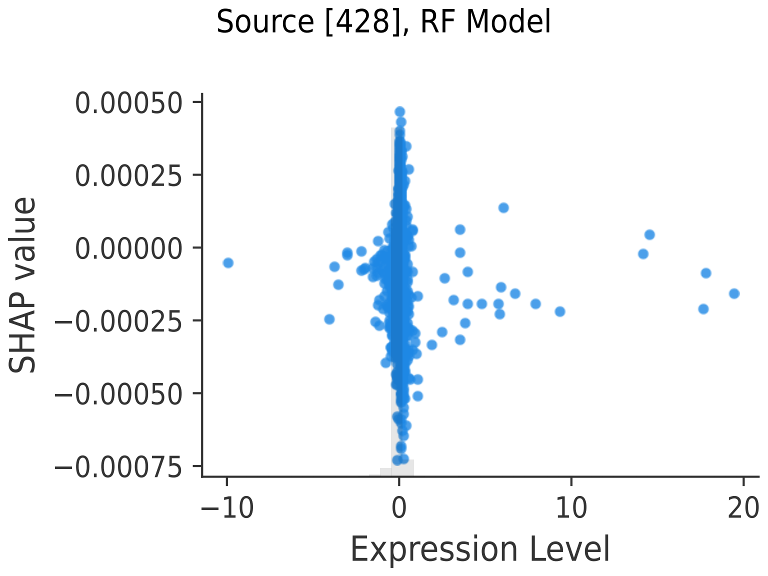
Source [428], RF Model
0.00050
0.00025
0.00000
−0.00025
−0.00050
−0.00075
SHAP value
−10
0
10
20
Expression Level

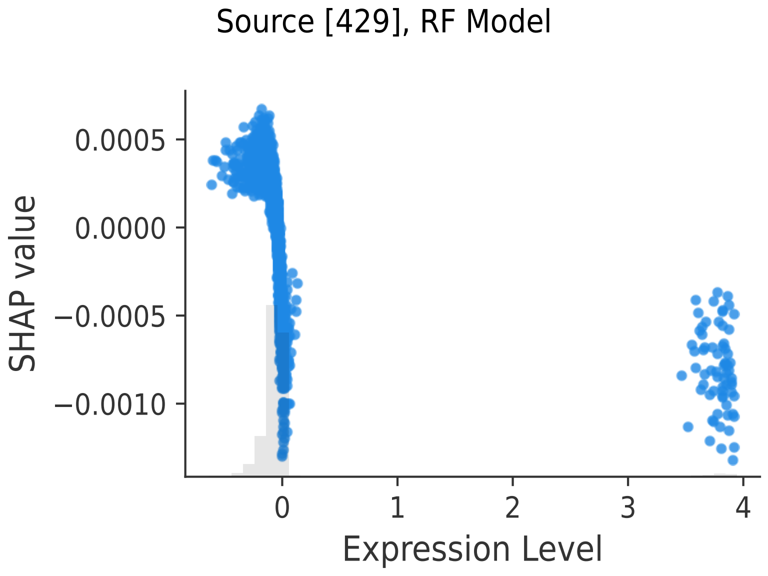

Source [429], RF Model
0.0005
0.0000
−0.0005
−0.0010
SHAP value
0
1
2
3
4
Expression Level

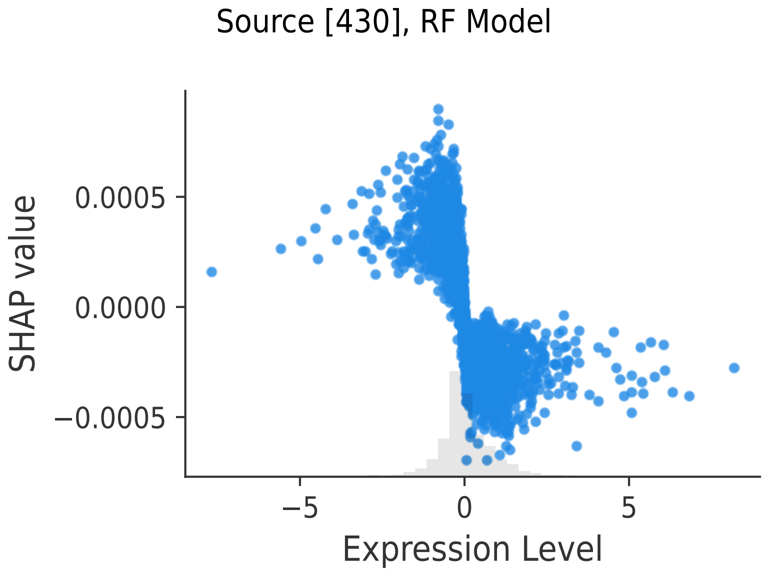
Source [430], RF Model
SHAP value
0.0005
0.0000
−0.0005
−5
0
5
Expression Level

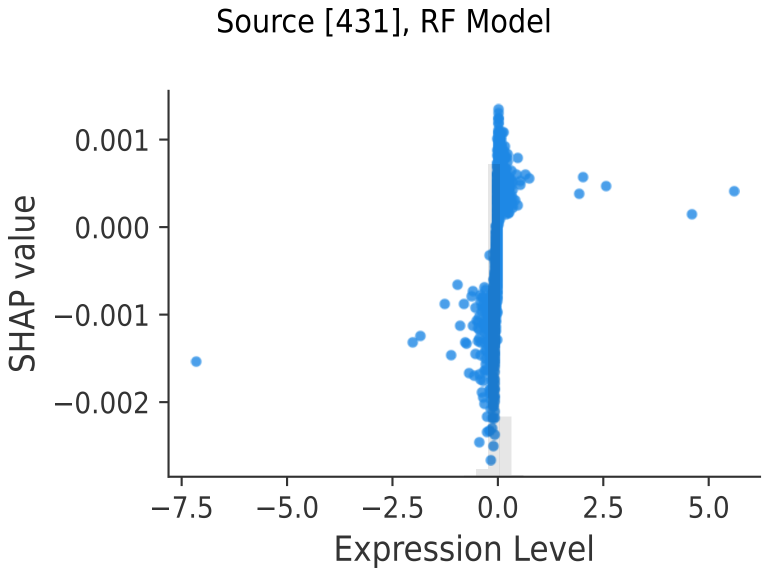
Source [431], RF Model
SHAP value
0.001
0.000
−0.001
−0.002
−7.5
−5.0
−2.5
0.0
2.5
5.0
Expression Level

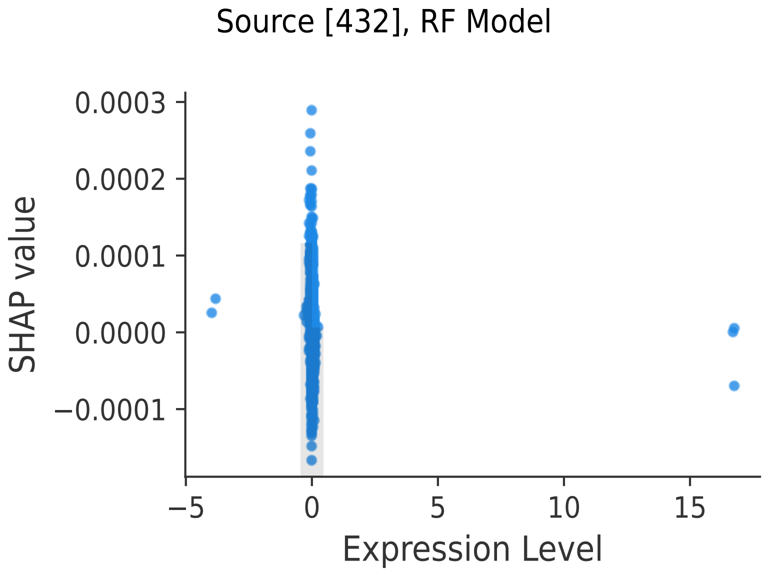
Source [432], RF Model
SHAP value
0.0003
0.0002
0.0001
0.0000
−0.0001
Expression Level
−5
0
5
10
15

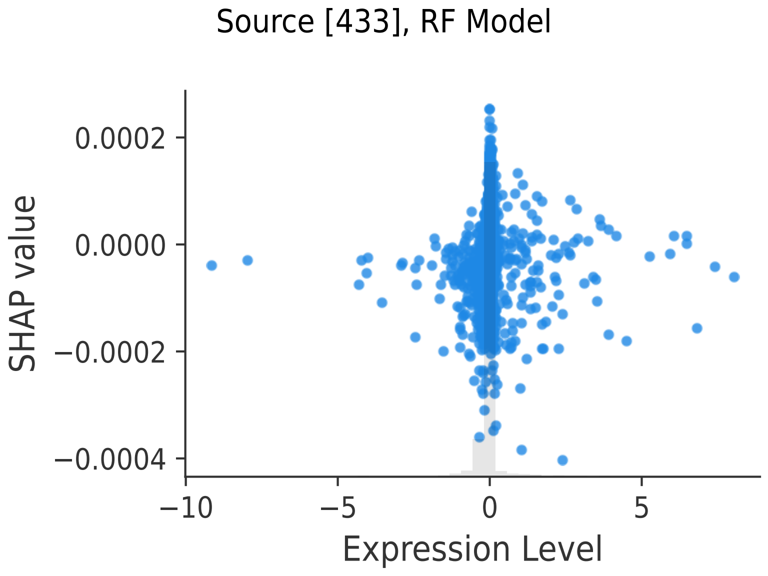
Source [433], RF Model
SHAP value
0.0002
0.0000
−0.0002
−0.0004
−10
−5
0
5
Expression Level

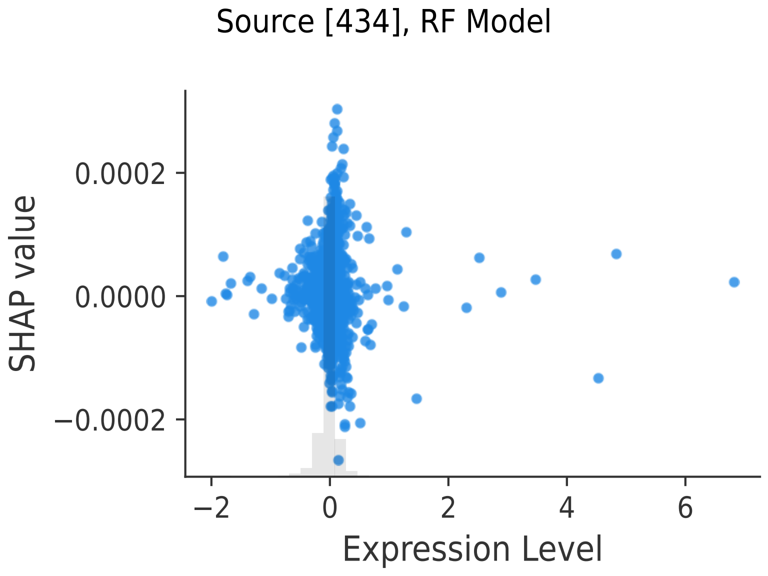
Source [434], RF Model
SHAP value
0.0002
0.0000
−0.0002
−2
0
2
4
6
Expression Level

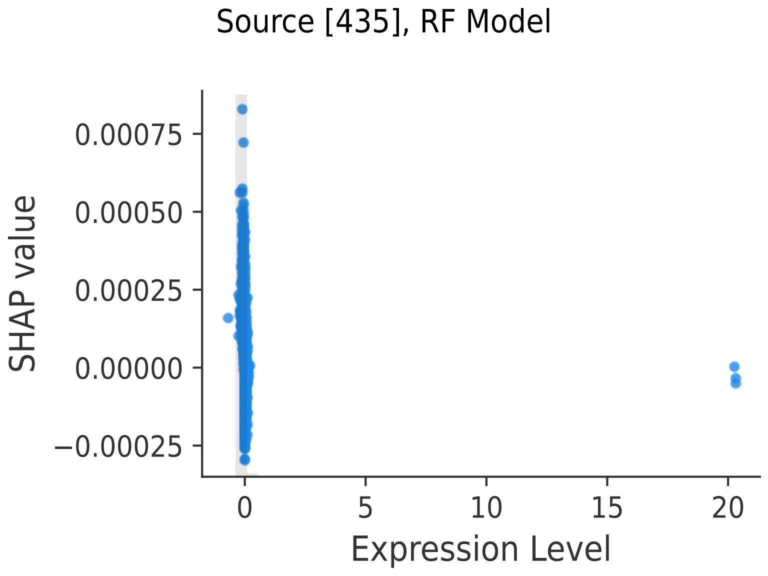

Source [435], RF Model
SHAP value
0.00075
0.00050
0.00025
0.00000
−0.00025
0
5
10
15
20
Expression Level

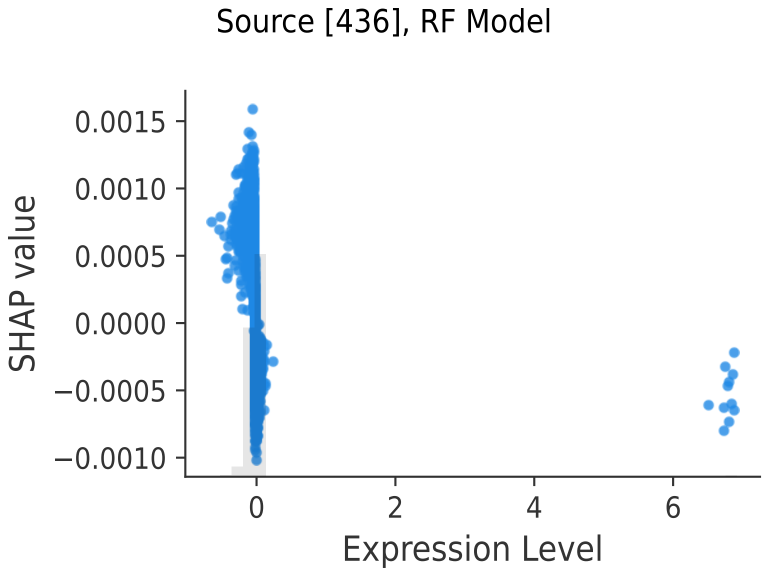
Source [436], RF Model
SHAP value
0.0015
0.0010
0.0005
0.0000
−0.0005
−0.0010
0
2
4
6
Expression Level

Source [437], RF Model

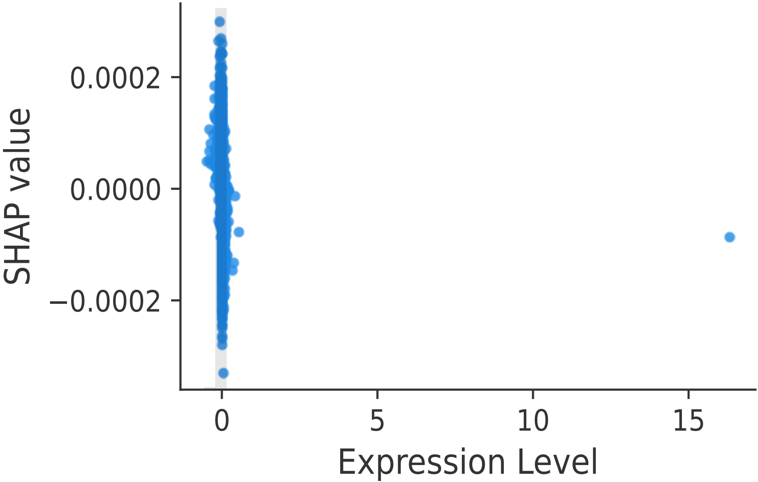

Source [438], RF Model

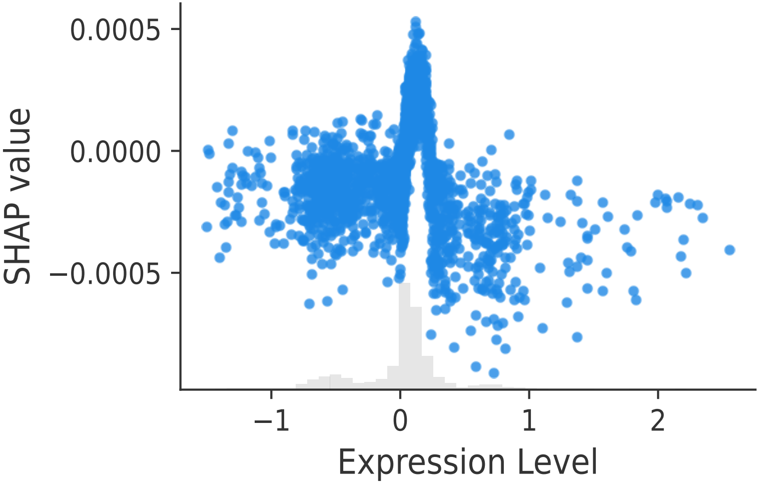

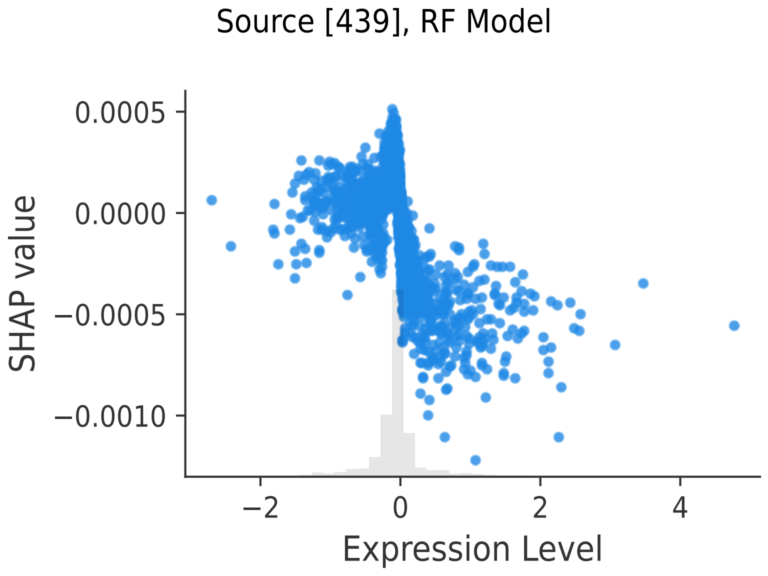
Source [439], RF Model
0.0005
0.0000
−0.0005
−0.0010
SHAP value
−2
0
2
4
Expression Level

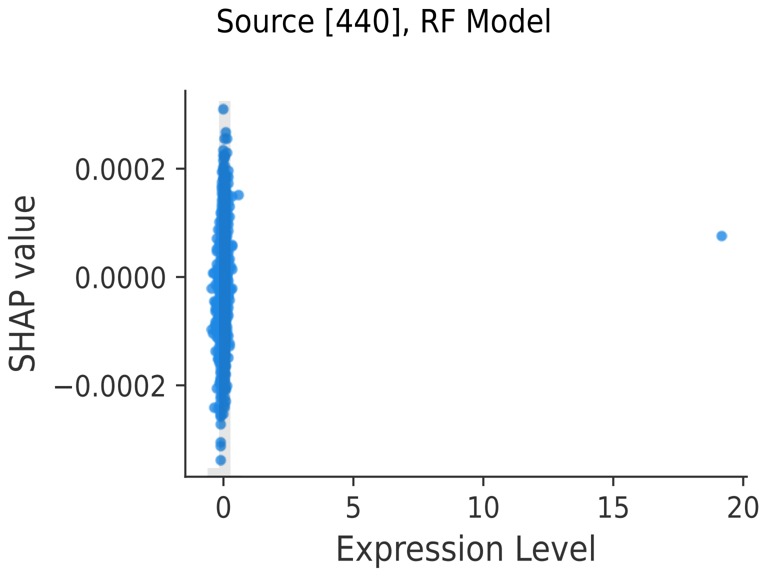
Source [440], RF Model
SHAP value
0.0002
0.0000
−0.0002
0
5
10
15
20
Expression Level

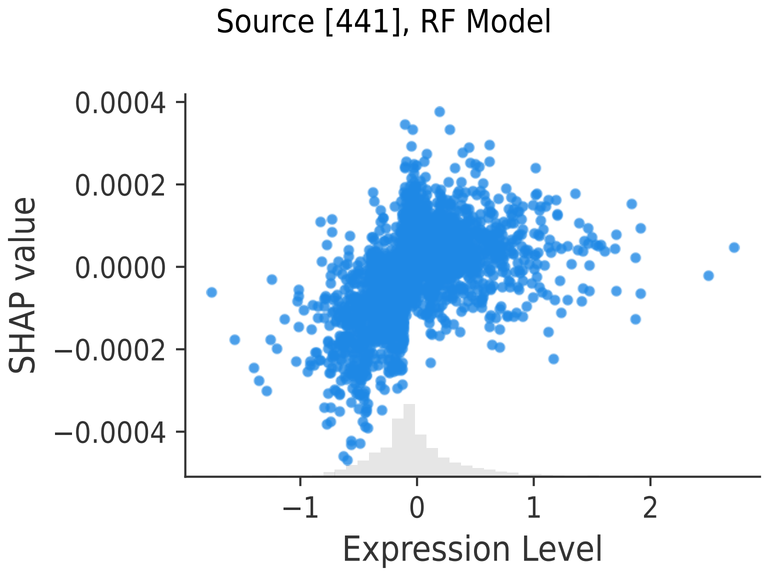
Source [441], RF Model
0.0004
0.0002
0.0000
−0.0002
−0.0004
SHAP value
−1
0
1
2
Expression Level

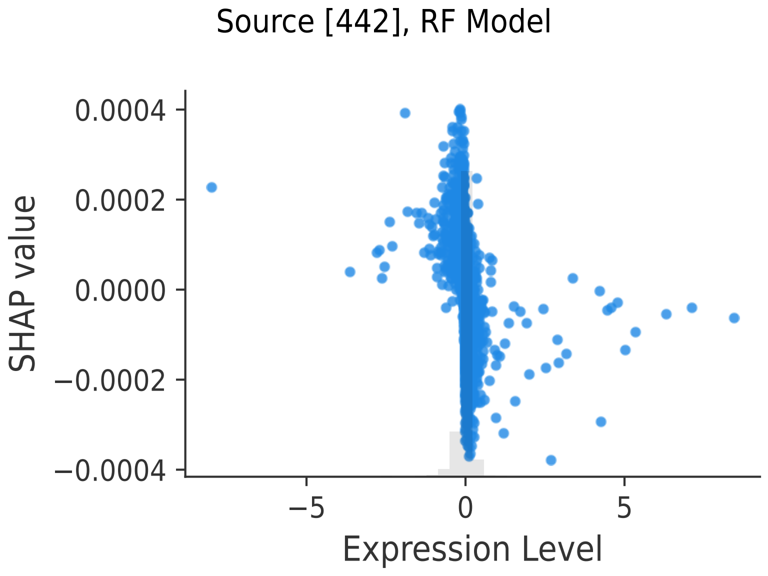
Source [442], RF Model
0.0004
0.0002
0.0000
−0.0002
−0.0004
SHAP value
−5
0
5
Expression Level

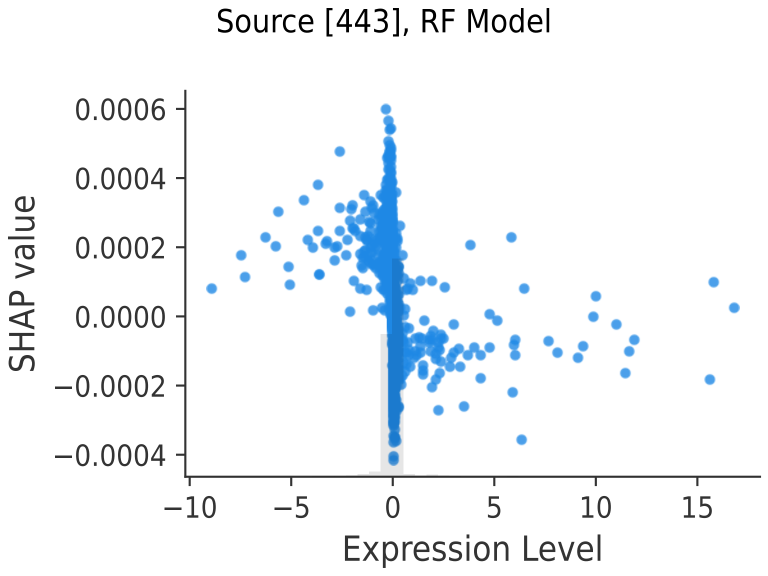
Source [443], RF Model
SHAP value
0.0006
0.0004
0.0002
0.0000
−0.0002
−0.0004
−10
−5
0
5
10
15
Expression Level

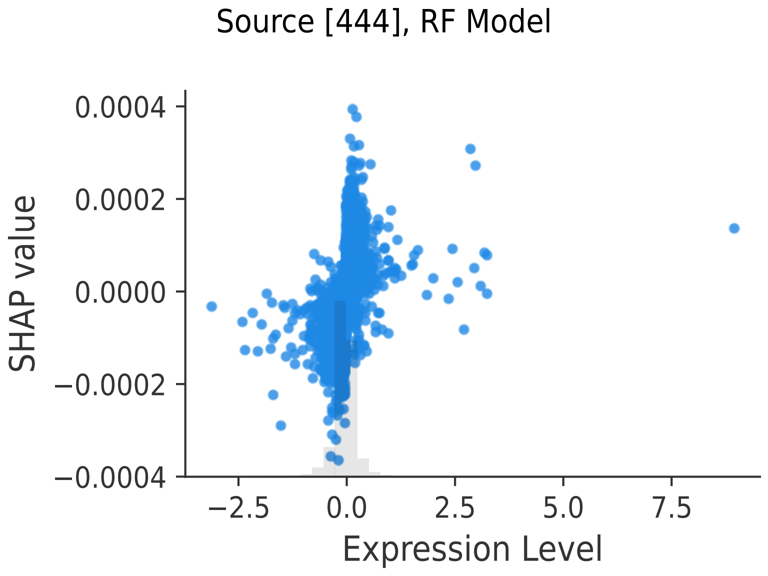
Source [444], RF Model
SHAP value
0.0004
0.0002
0.0000
−0.0002
−0.0004
−2.5
0.0
2.5
5.0
7.5
Expression Level

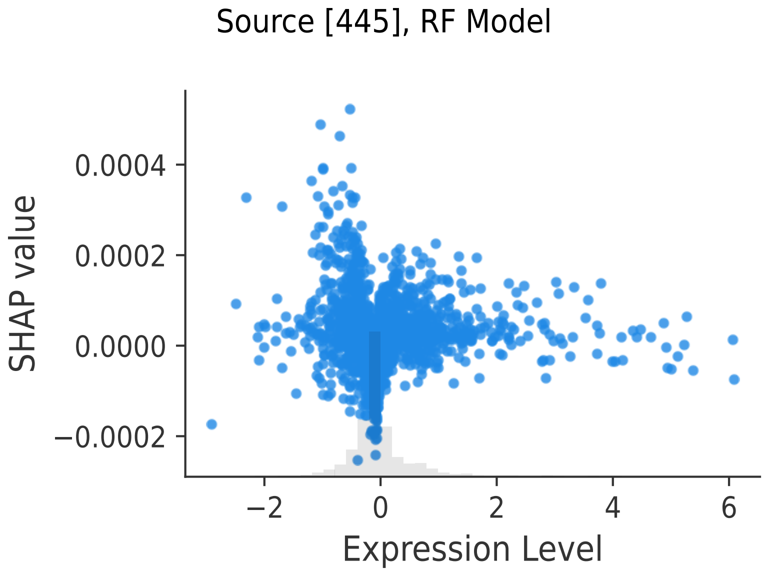

Source [445], RF Model
SHAP value
0.0004
0.0002
0.0000
−0.0002
−2
0
2
4
6
Expression Level

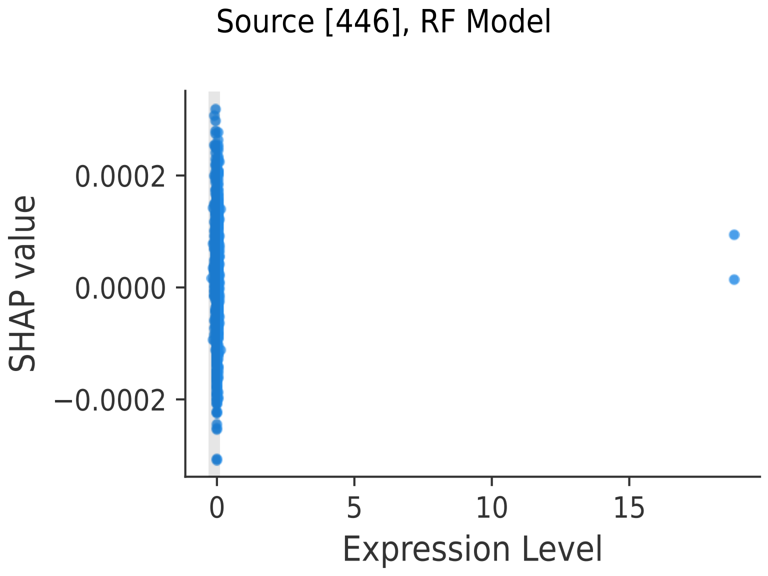
Source [446], RF Model
0.0002
0.0000
−0.0002
SHAP value
0
5
10
15
Expression Level

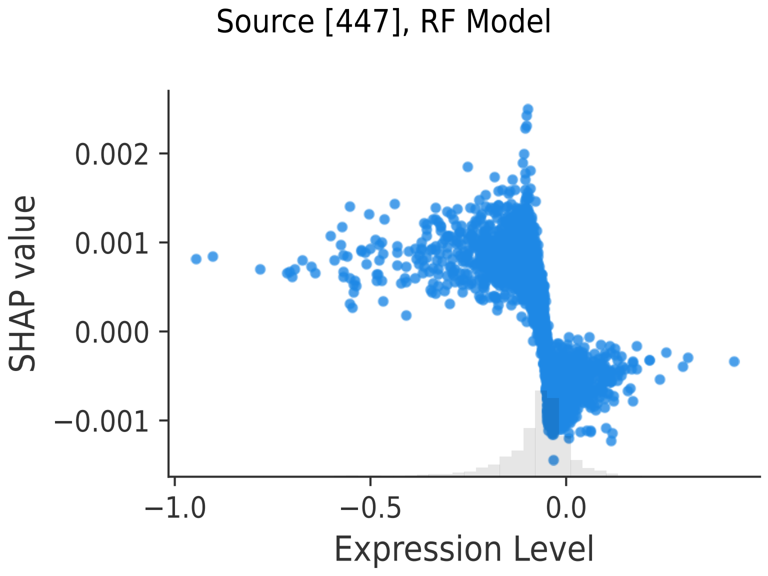

Source [447], RF Model
SHAP value
0.002
0.001
0.000
−0.001
−1.0
−0.5
0.0
Expression Level

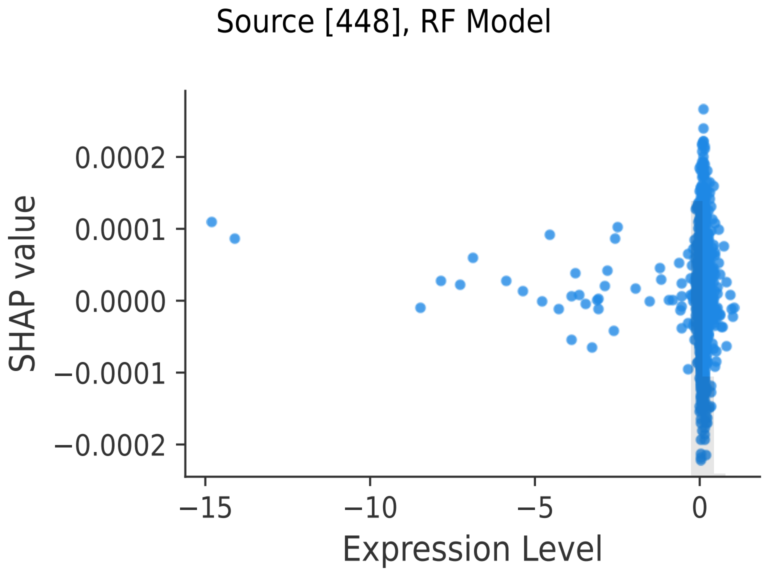
Source [448], RF Model
0.0002
0.0001
0.0000
−0.0001
−0.0002
SHAP value
−15
−10
−5
0
Expression Level

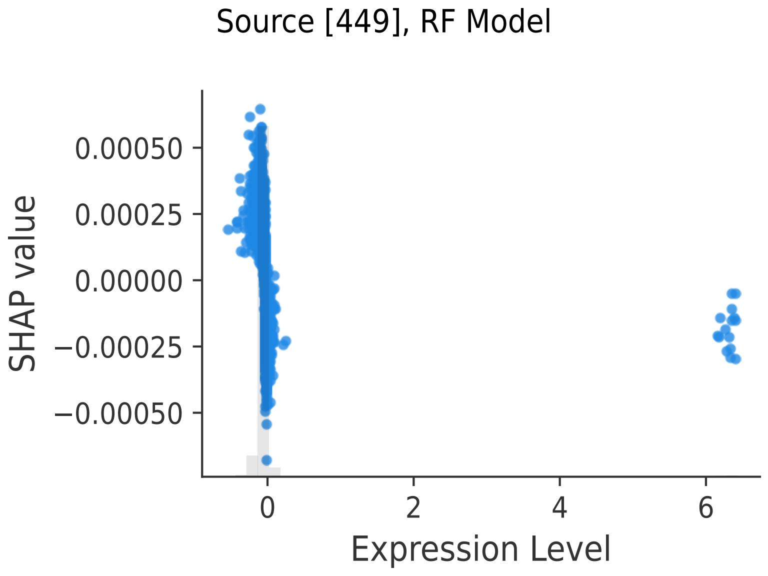

Source [449], RF Model
SHAP value
0.00050
0.00025
0.00000
−0.00025
−0.00050
0
2
4
6
Expression Level

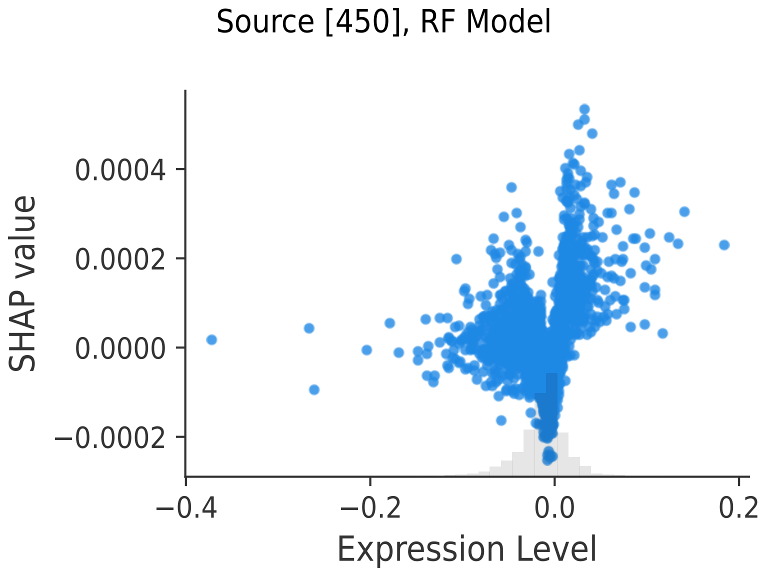
Source [450], RF Model
0.0004
0.0002
0.0000
−0.0002
SHAP value
−0.4
−0.2
0.0
0.2
Expression Level

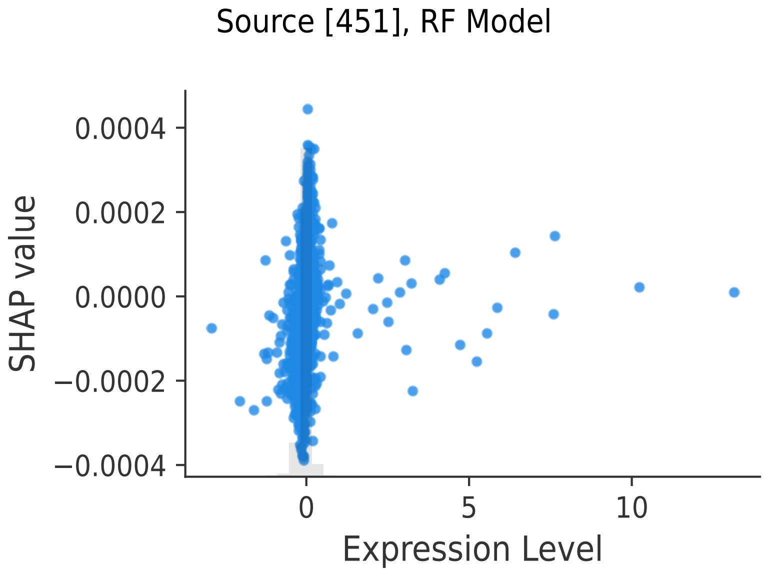

Source [451], RF Model
0.0004
0.0002
0.0000
−0.0002
−0.0004
SHAP value
0
5
10
Expression Level

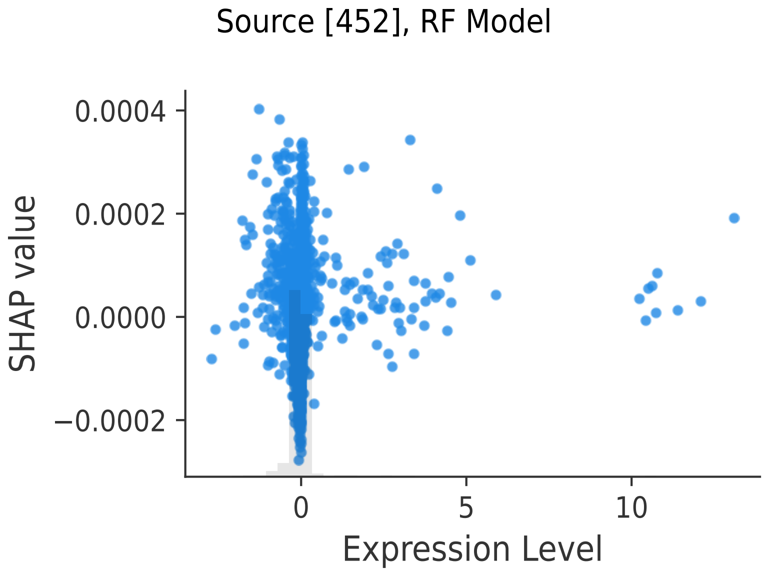

Source [452], RF Model
0.0004
0.0002
0.0000
−0.0002
SHAP value
0
5
10
Expression Level

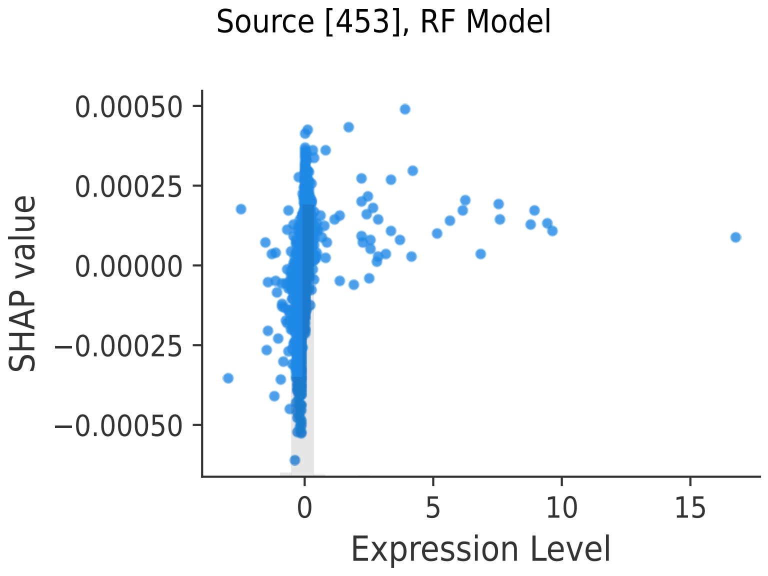
Source [453], RF Model
SHAP value
0.00050
0.00025
0.00000
−0.00025
−0.00050
Expression Level
0
5
10
15

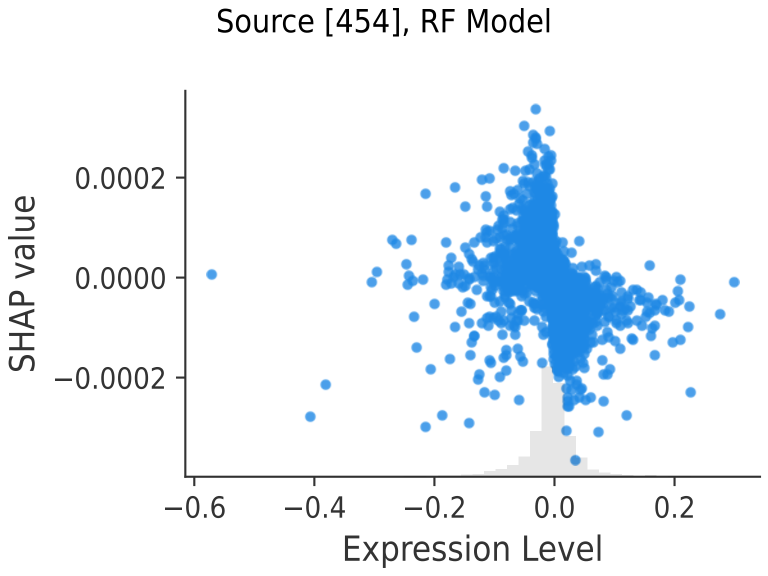

Source [454], RF Model
0.0002
0.0000
−0.0002
SHAP value
−0.6
−0.4
−0.2
0.0
0.2
Expression Level

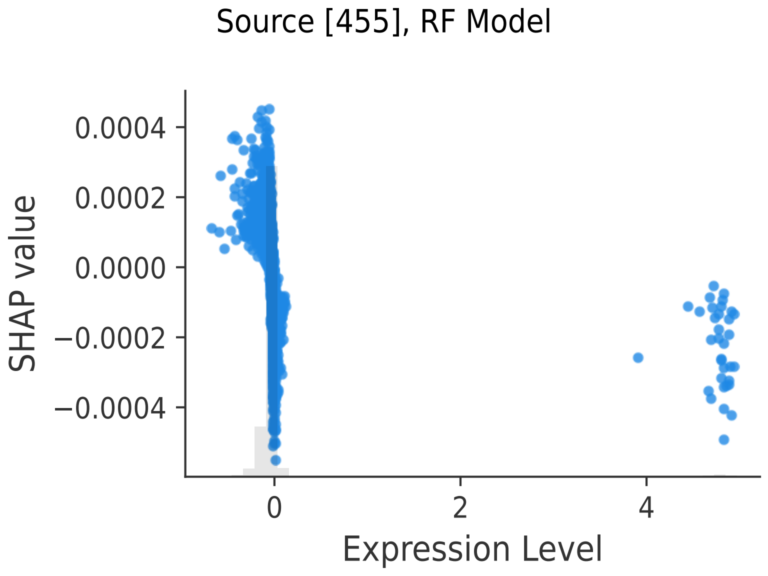
Source [455], RF Model
SHAP value
0.0004
0.0002
0.0000
−0.0002
−0.0004
0
2
4
Expression Level

Source [456], RF Model

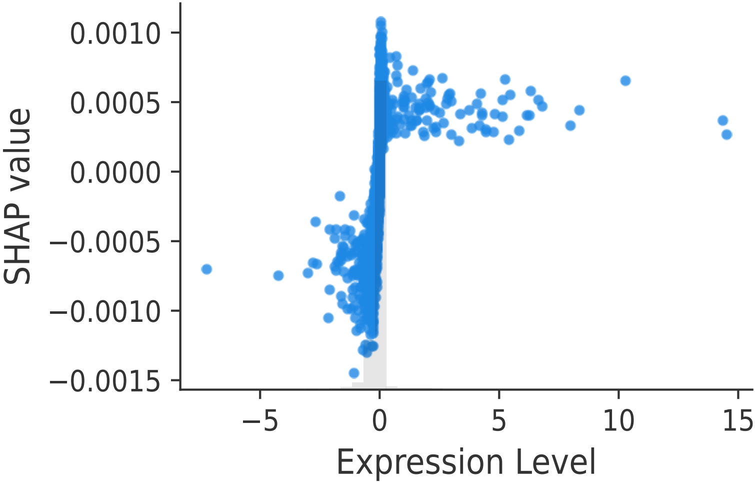

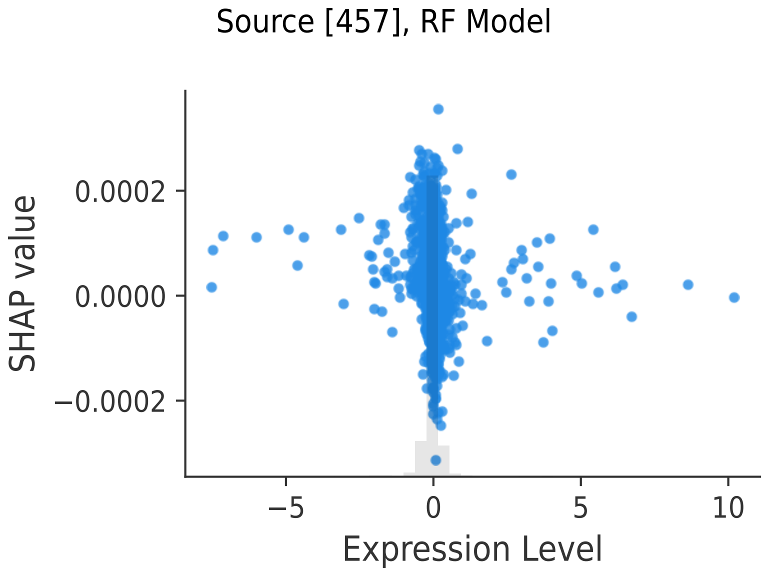

Source [457], RF Model
0.0002
0.0000
−0.0002
SHAP value
−5
0
5
10
Expression Level

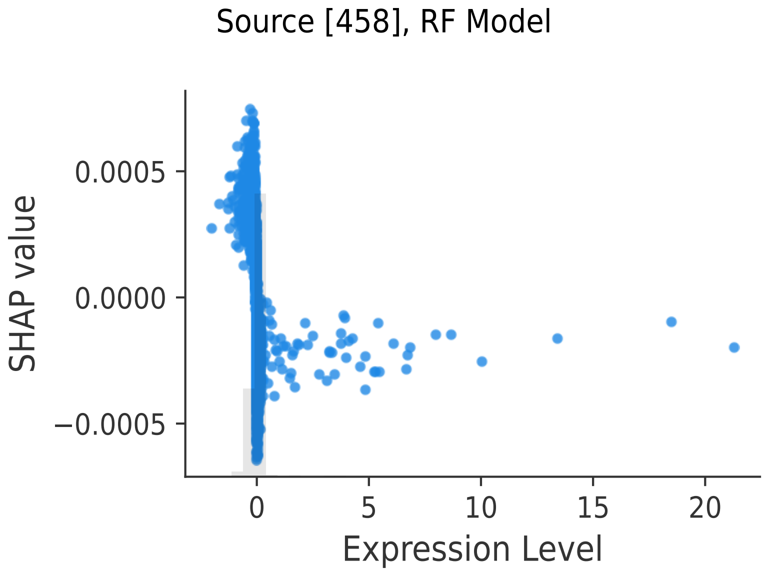

Source [458], RF Model
SHAP value
0.0005
0.0000
−0.0005
0
5
10
15
20
Expression Level

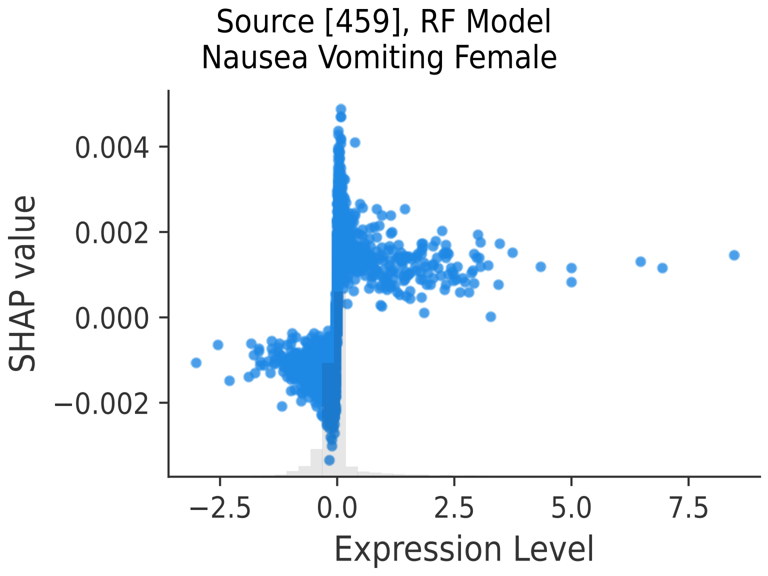

Source [459], RF Model
Nausea Vomiting Female
0.004
0.002
0.000
−0.002
SHAP value
−2.5
0.0
2.5
5.0
7.5
Expression Level

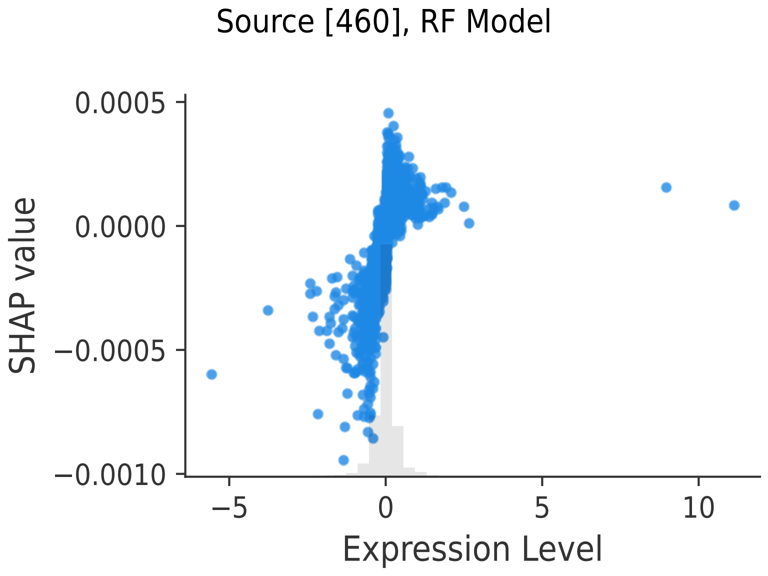

Source [460], RF Model
0.0005
0.0000
−0.0005
−0.0010
SHAP value
−5
0
5
10
Expression Level

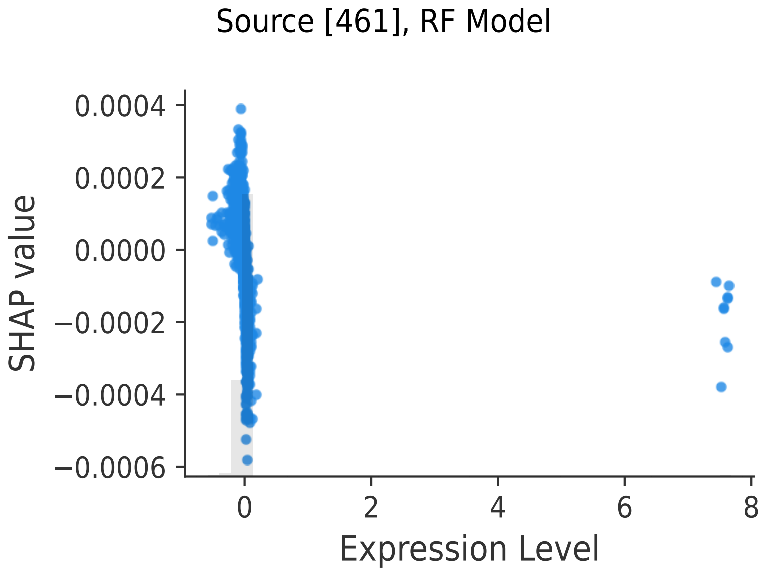

Source [461], RF Model
0.0004
0.0002
0.0000
−0.0002
−0.0004
−0.0006
SHAP value
0
2
4
6
8
Expression Level

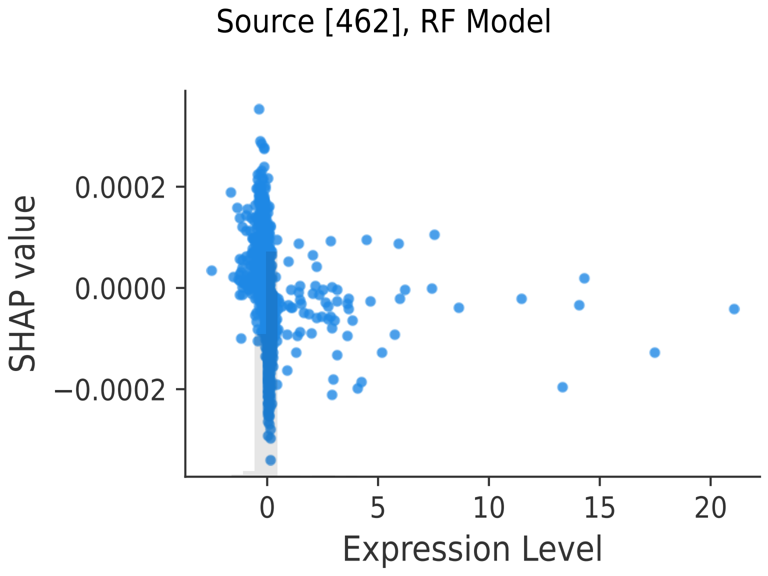
Source [462], RF Model
SHAP value
0.0002
0.0000
−0.0002
0
5
10
15
20
Expression Level

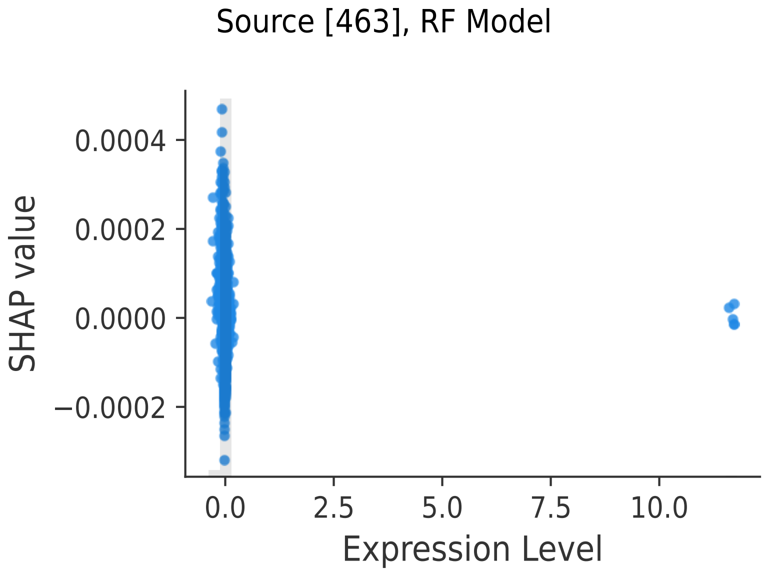
Source [463], RF Model
SHAP value
0.0004
0.0002
0.0000
−0.0002
0.0
2.5
5.0
7.5
10.0
Expression Level

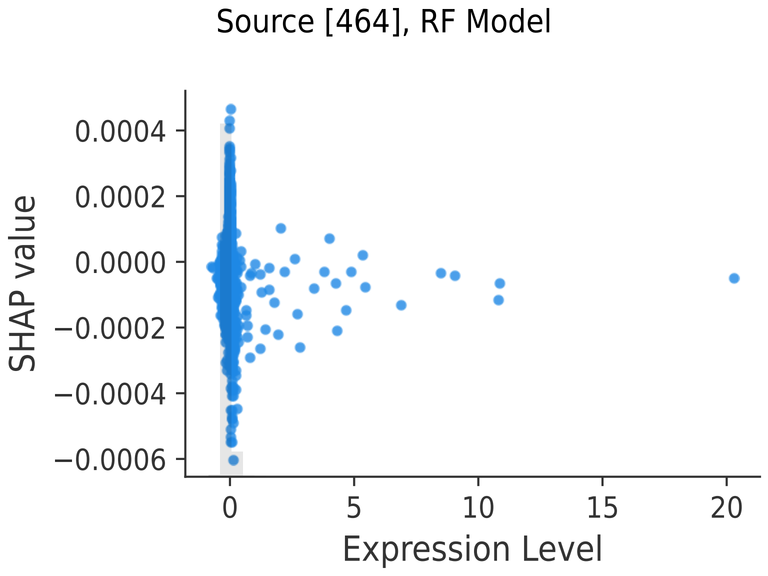

Source [464], RF Model
SHAP value
0.0004
0.0002
0.0000
−0.0002
−0.0004
−0.0006
0
5
10
15
20
Expression Level

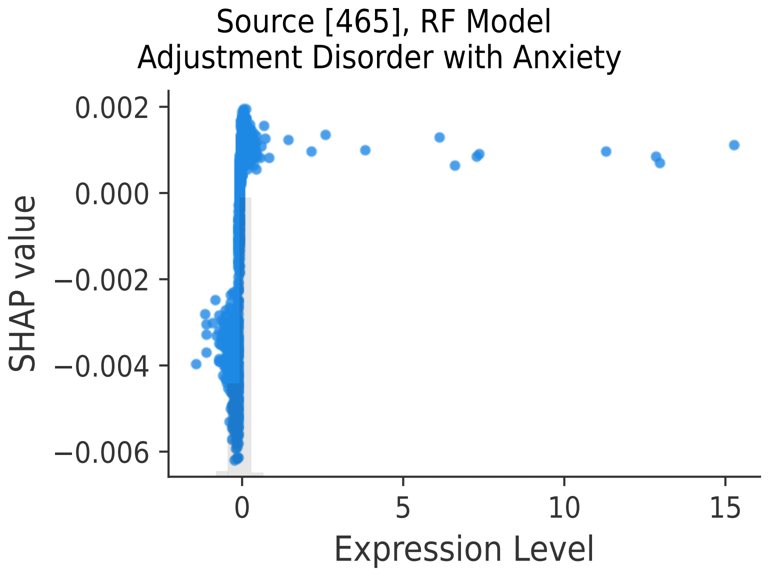

Source [465], RF Model
Adjustment Disorder with Anxiety
SHAP value
0.002
0.000
−0.002
−0.004
−0.006
0
5
10
15
Expression Level

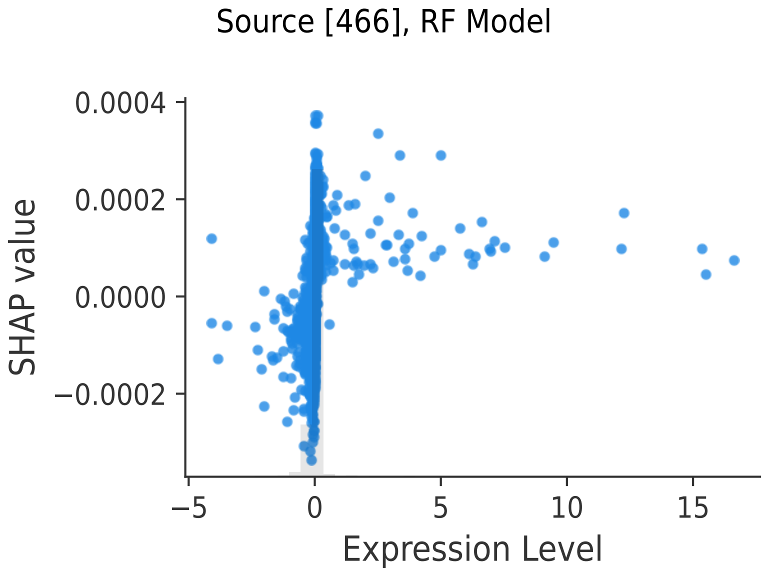

Source [466], RF Model
SHAP value
0.0004
0.0002
0.0000
−0.0002
−5
0
5
10
15
Expression Level

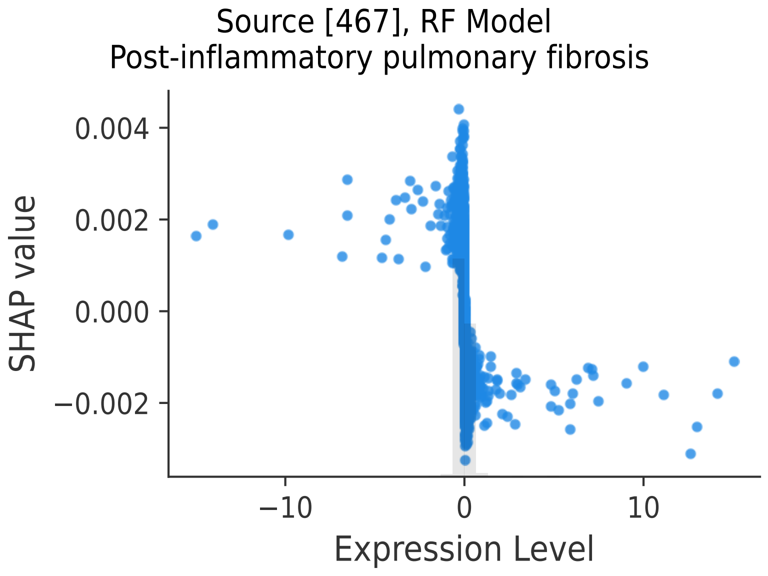

Source [467], RF Model
Post-inflammatory pulmonary fibrosis
SHAP value
0.004
0.002
0.000
−0.002
−10
0
10
Expression Level

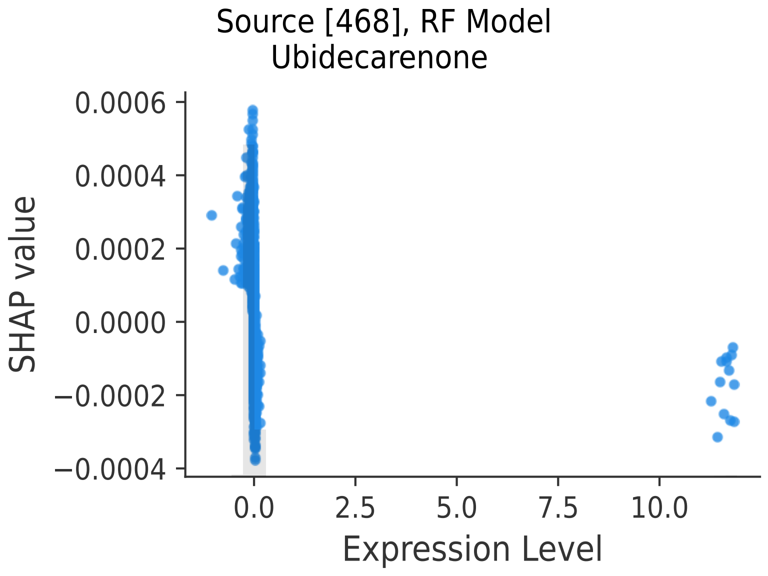

Source [468], RF Model
Ubidecarenone
SHAP value
0.0006
0.0004
0.0002
0.0000
−0.0002
−0.0004
0.0
2.5
5.0
7.5
10.0
Expression Level

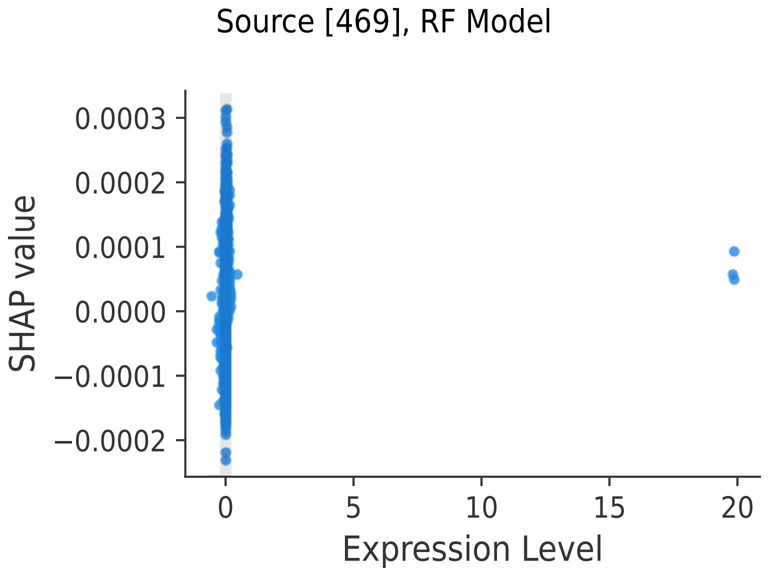
Source [469], RF Model
SHAP value
0.0003
0.0002
0.0001
0.0000
−0.0001
−0.0002
0
5
10
15
20
Expression Level

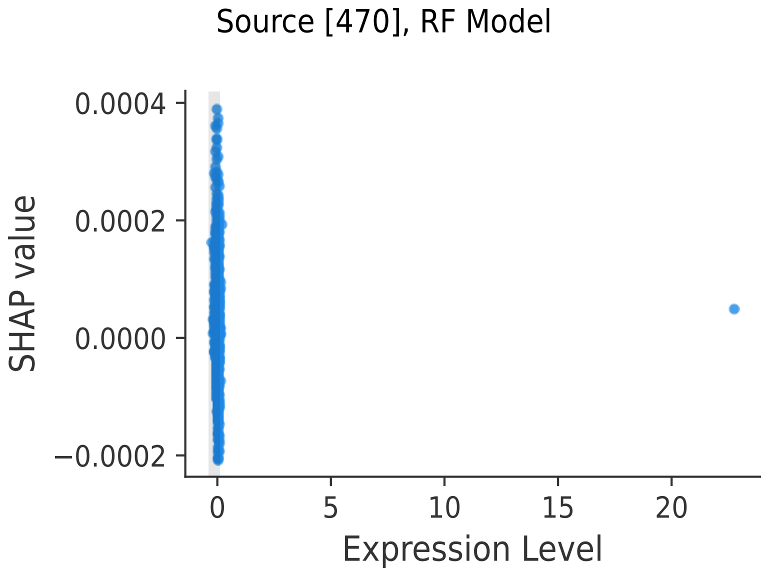
Source [470], RF Model
0.0004
0.0002
0.0000
−0.0002
SHAP value
0
5
10
15
20
Expression Level

Source [471], RF Model

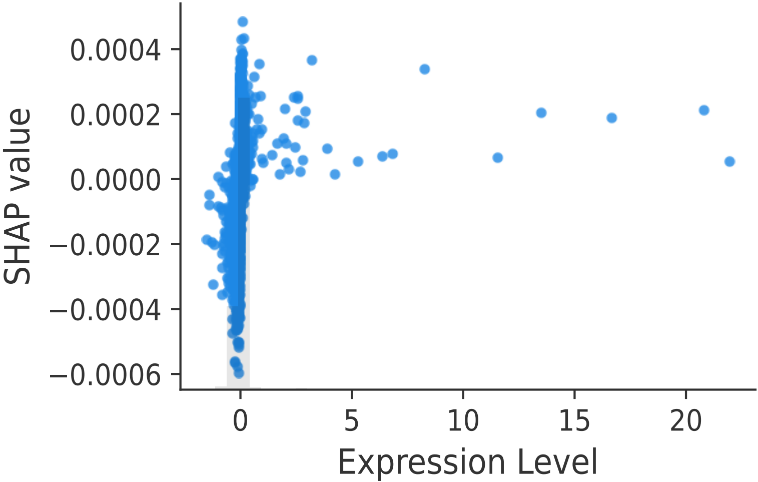

Source [472], RF Model

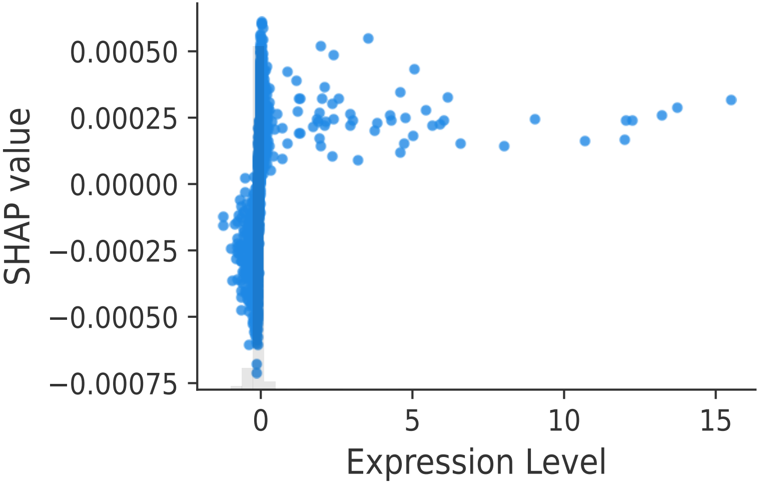

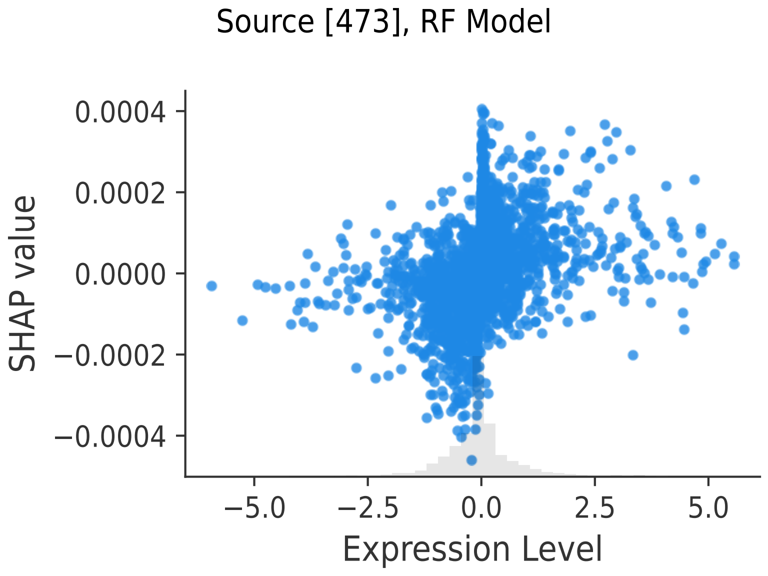
Source [473], RF Model
0.0004
0.0002
0.0000
−0.0002
−0.0004
SHAP value
−5.0
−2.5
0.0
2.5
5.0
Expression Level

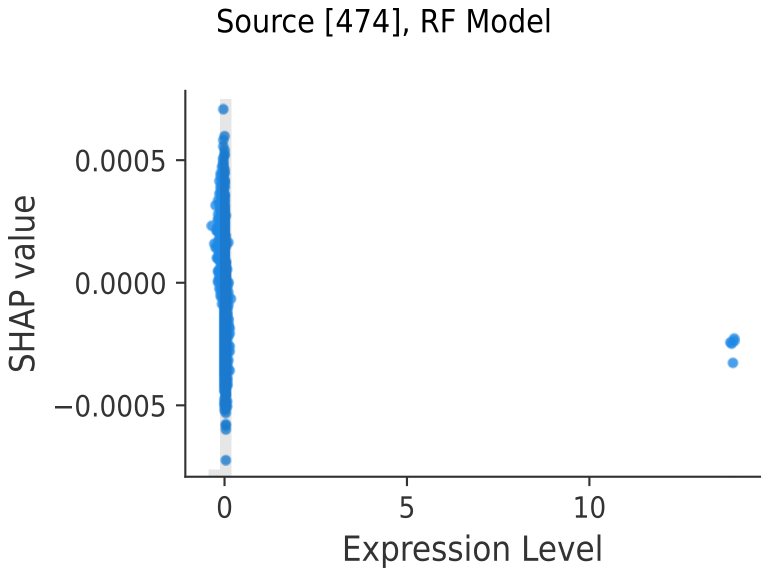
Source [474], RF Model
0.0005
0.0000
−0.0005
SHAP value
0
5
10
Expression Level

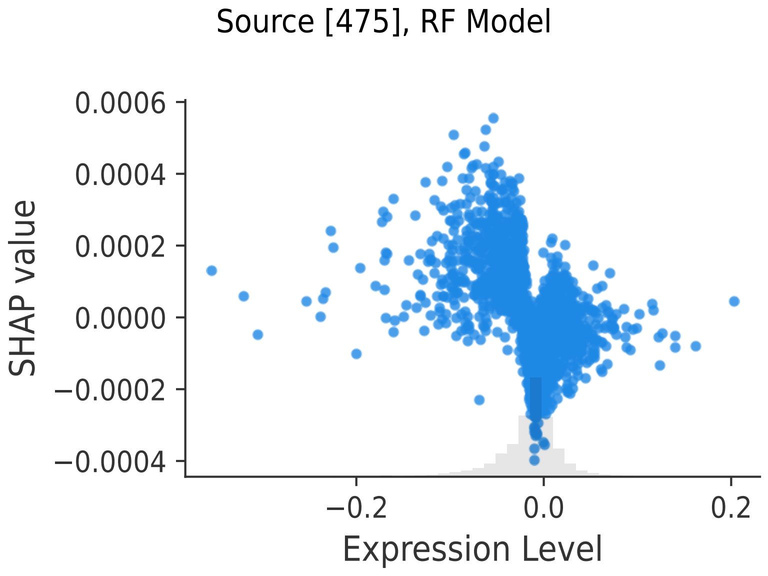
Source [475], RF Model
0.0006
0.0004
0.0002
0.0000
−0.0002
−0.0004
SHAP value
−0.2
0.0
0.2
Expression Level

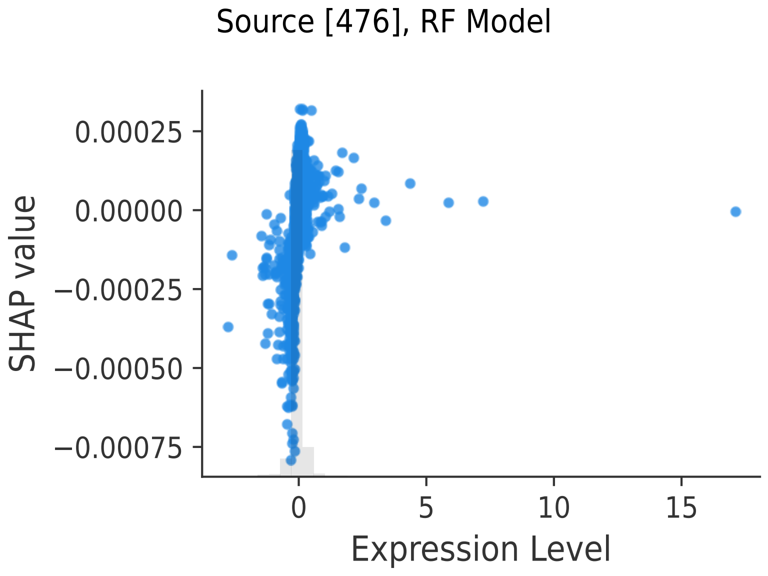

Source [476], RF Model
0.00025
0.00000
−0.00025
−0.00050
−0.00075
SHAP value
0
5
10
15
Expression Level

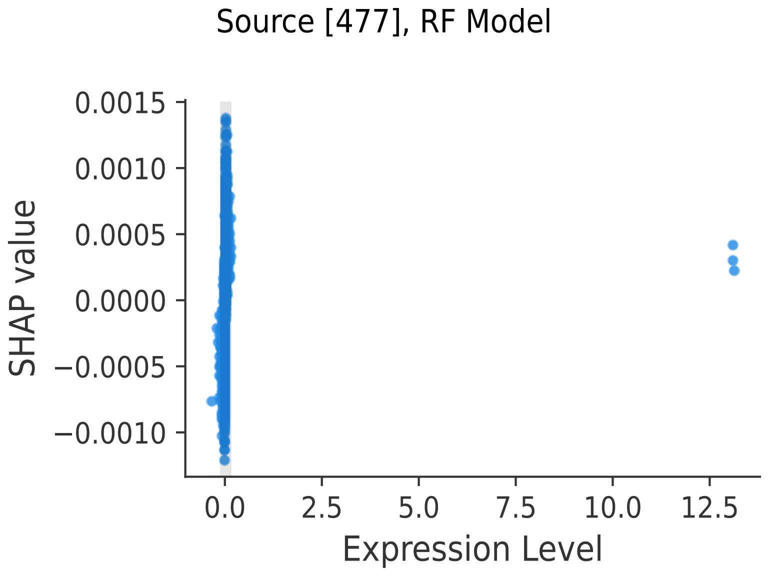
Source [477], RF Model
SHAP value
0.0015
0.0010
0.0005
0.0000
−0.0005
−0.0010
0.0
2.5
5.0
7.5
10.0
12.5
Expression Level

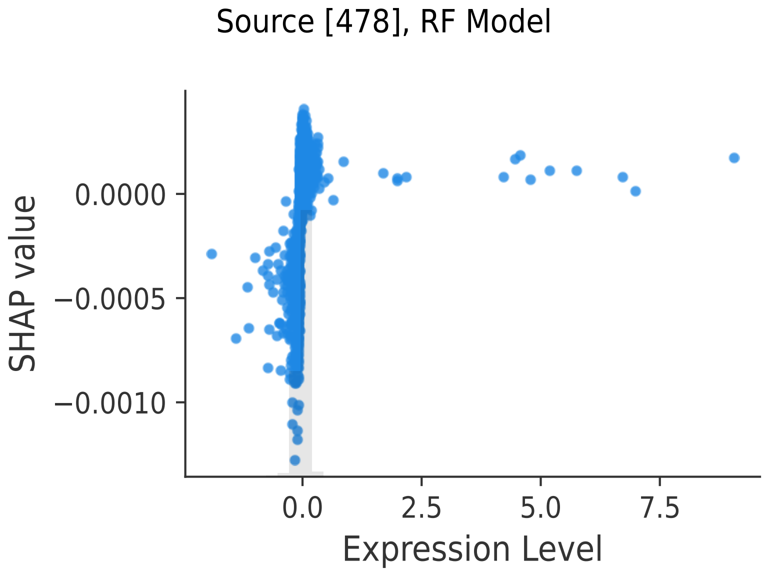

Source [478], RF Model
SHAP value
0.0000
−0.0005
−0.0010
0.0
2.5
5.0
7.5
Expression Level

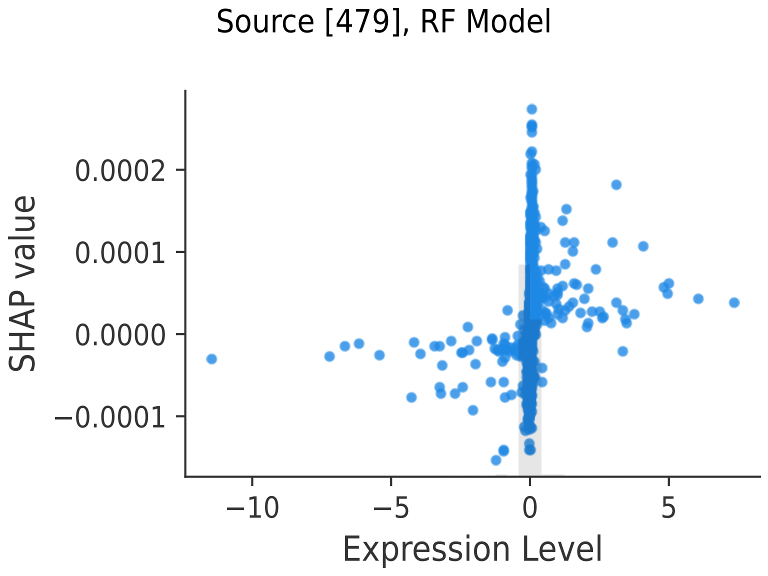
Source [479], RF Model
SHAP value
0.0002
0.0001
0.0000
−0.0001
−10
−5
0
5
Expression Level

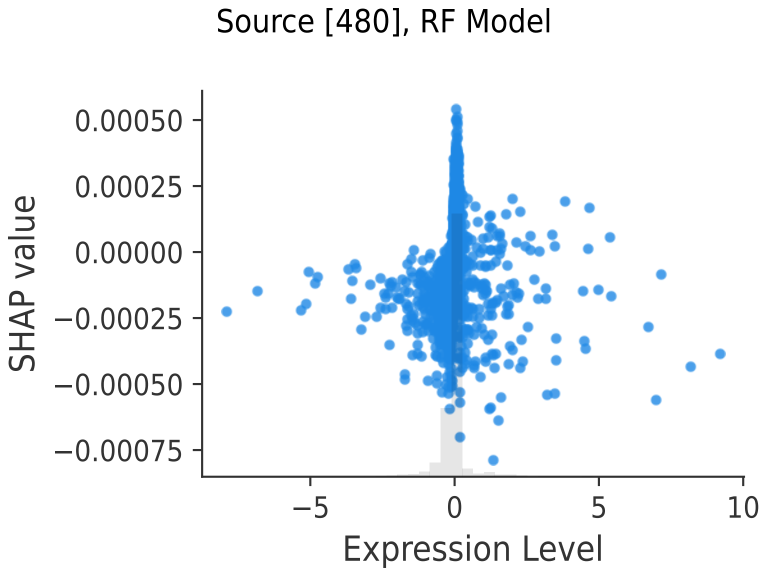
Source [480], RF Model
SHAP value
0.00050
0.00025
0.00000
−0.00025
−0.00050
−0.00075
−5
0
5
10
Expression Level

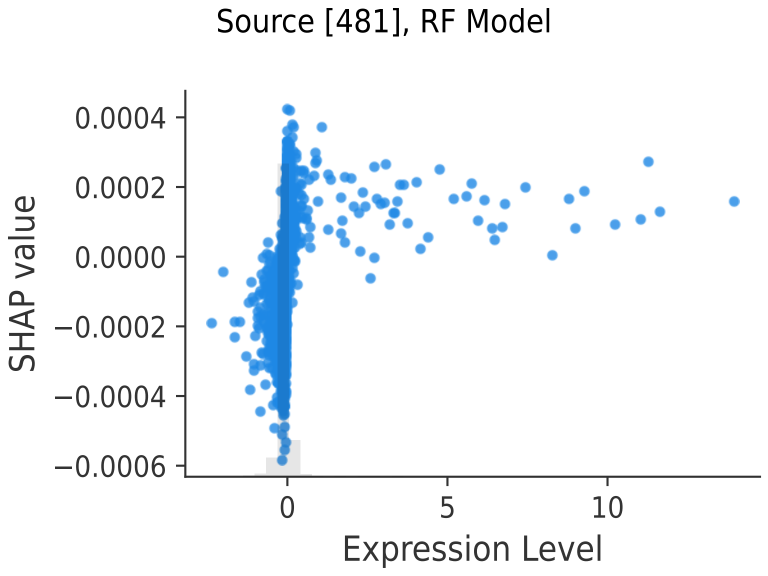
Source [481], RF Model
0.0004
0.0002
0.0000
−0.0002
−0.0004
−0.0006
SHAP value
0
5
10
Expression Level

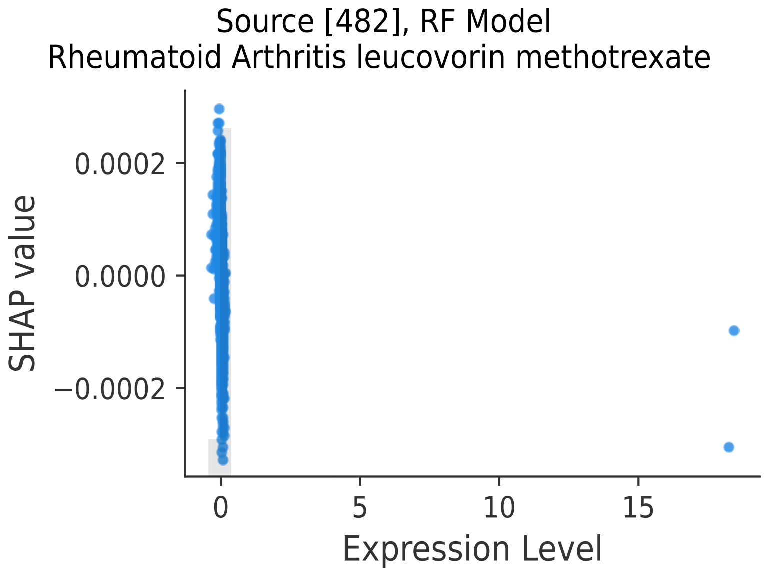

Source [482], RF Model
Rheumatoid Arthritis leucovorin methotrexate
SHAP value
0.0002
0.0000
−0.0002
0
5
10
15
Expression Level

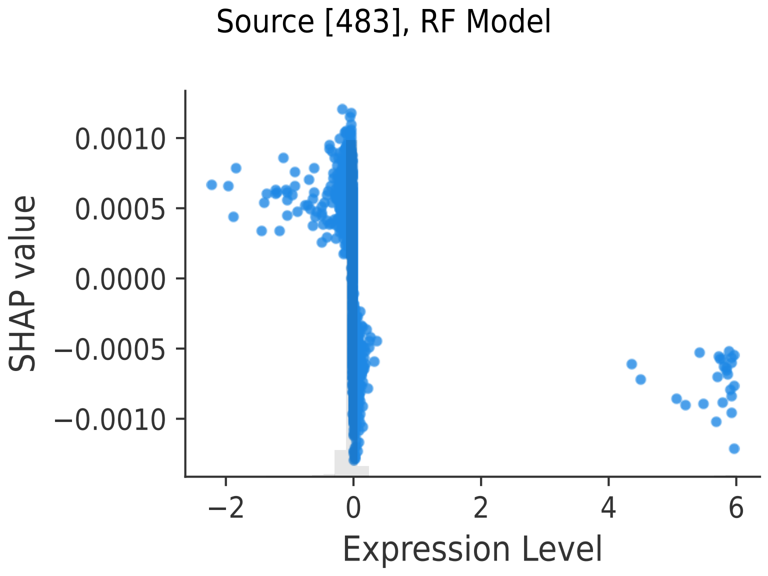
Source [483], RF Model
SHAP value
0.0010
0.0005
0.0000
−0.0005
−0.0010
Expression Level
−2
0
2
4
6

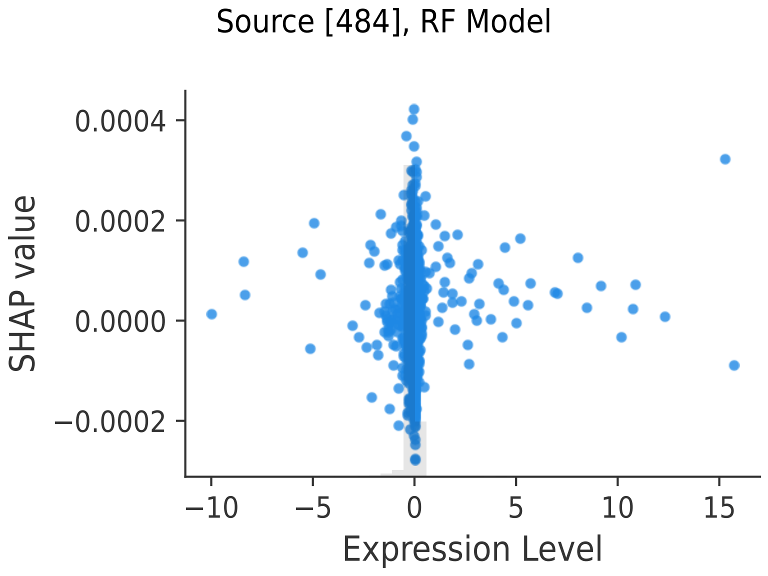

Source [484], RF Model
0.0004
0.0002
0.0000
−0.0002
SHAP value
−10
−5
0
5
10
15
Expression Level

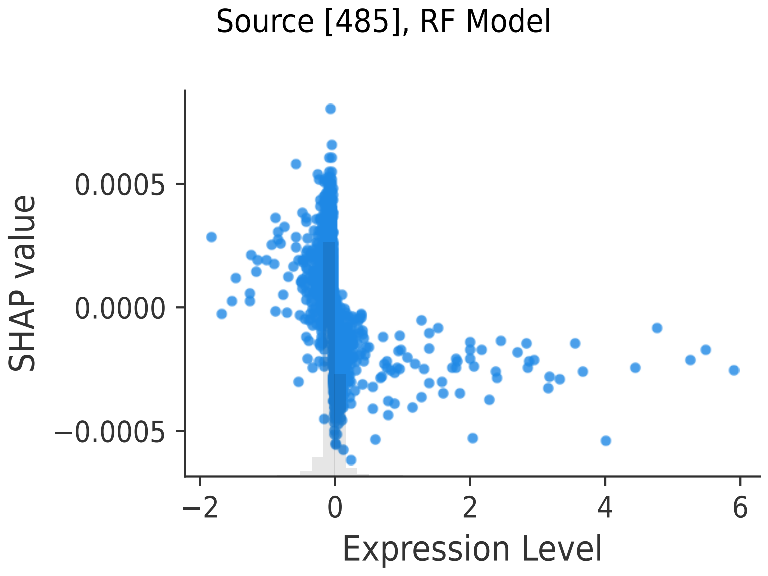

Source [485], RF Model
0.0005
0.0000
−0.0005
SHAP value
−2
0
2
4
6
Expression Level

Source [486], RF Model

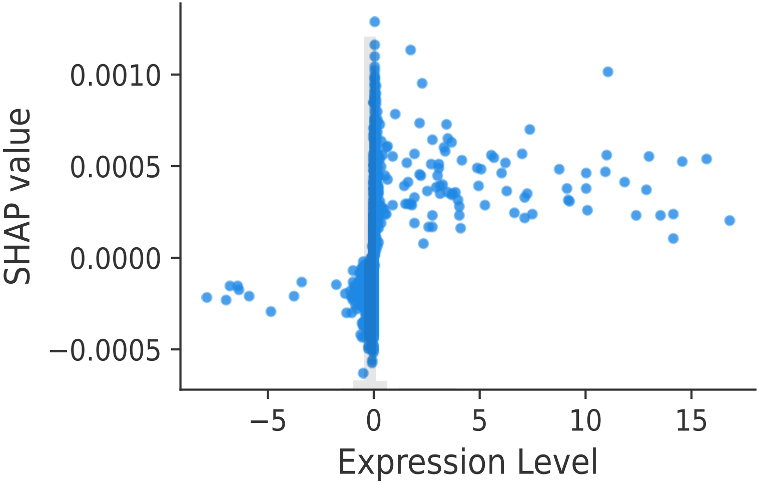

Source [487], RF Model

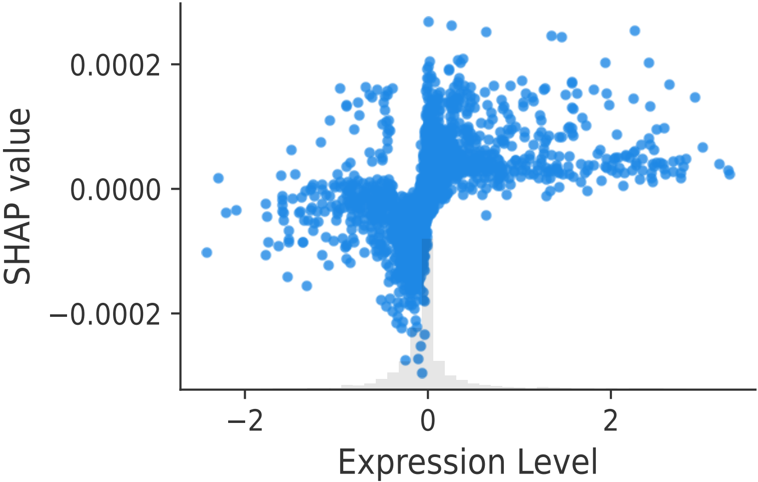

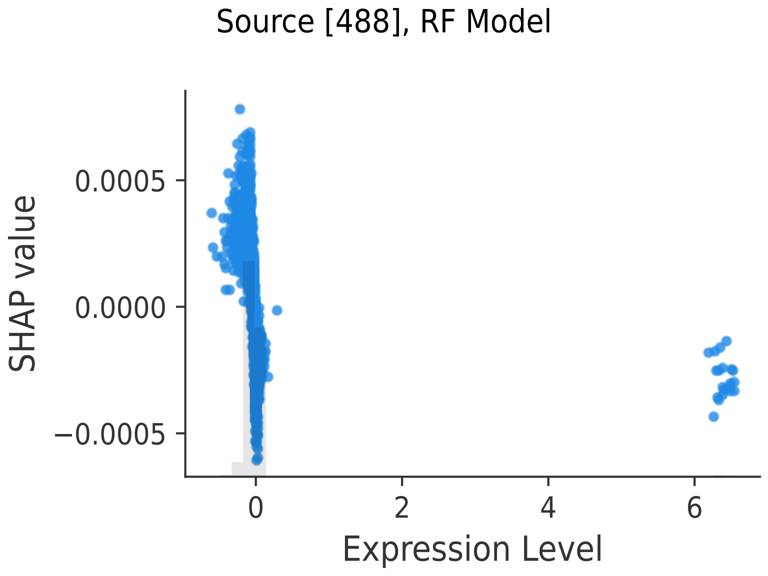

Source [488], RF Model
SHAP value
0.0005
0.0000
−0.0005
0
2
4
6
Expression Level

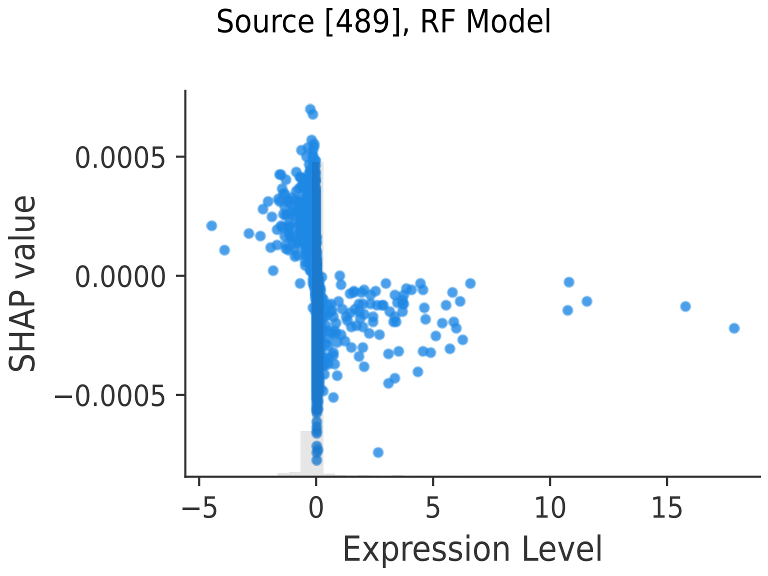

Source [489], RF Model
SHAP value
0.0005
0.0000
−0.0005
−5
0
5
10
15
Expression Level

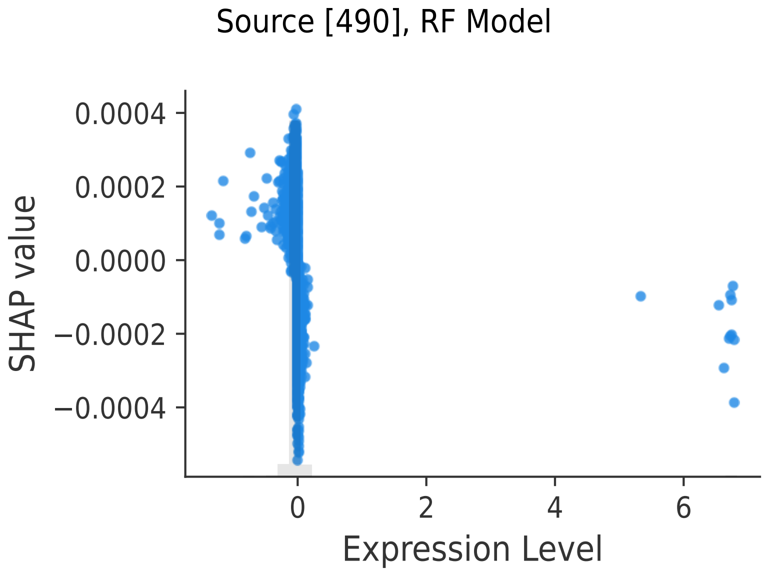
Source [490], RF Model
SHAP value
0.0004
0.0002
0.0000
−0.0002
−0.0004
0
2
4
6
Expression Level

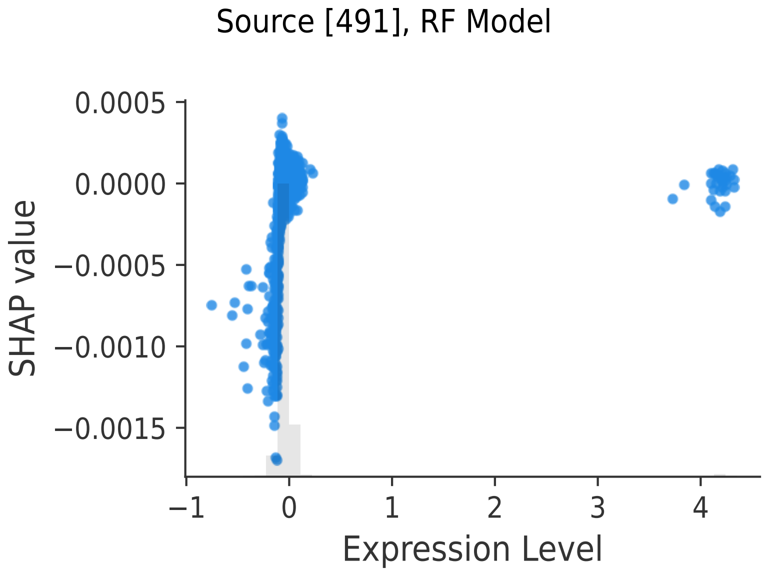
Source [491], RF Model
0.0005
0.0000
−0.0005
−0.0010
−0.0015
SHAP value
−1
0
1
2
3
4
Expression Level

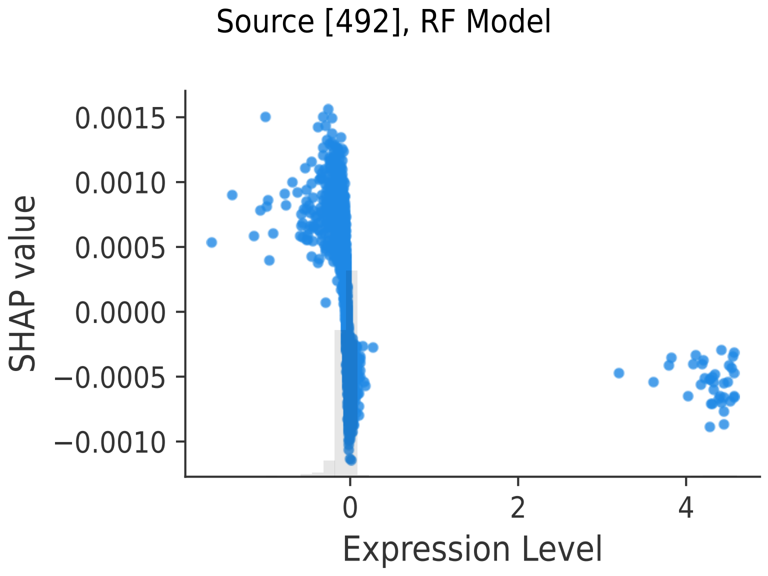

Source [492], RF Model
0.0015
0.0010
0.0005
0.0000
−0.0005
−0.0010
SHAP value
0
2
4
Expression Level

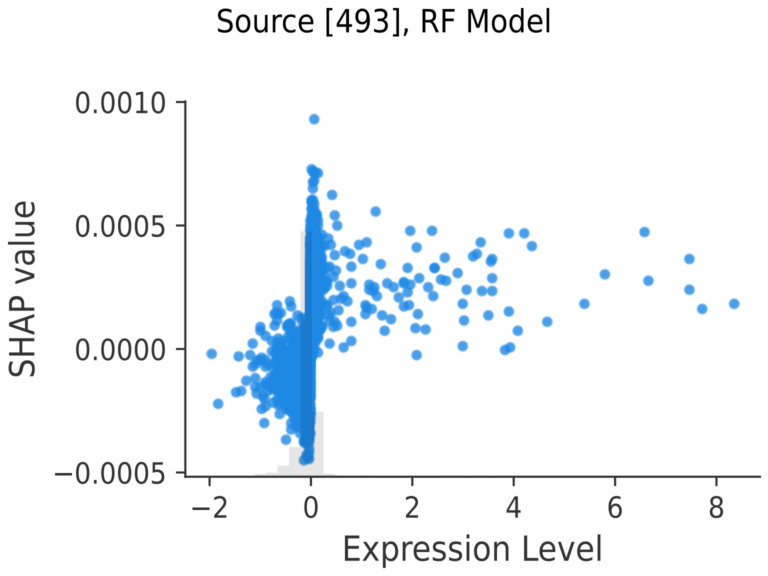
Source [493], RF Model
SHAP value
0.0010
0.0005
0.0000
−0.0005
−2
0
2
4
6
8
Expression Level

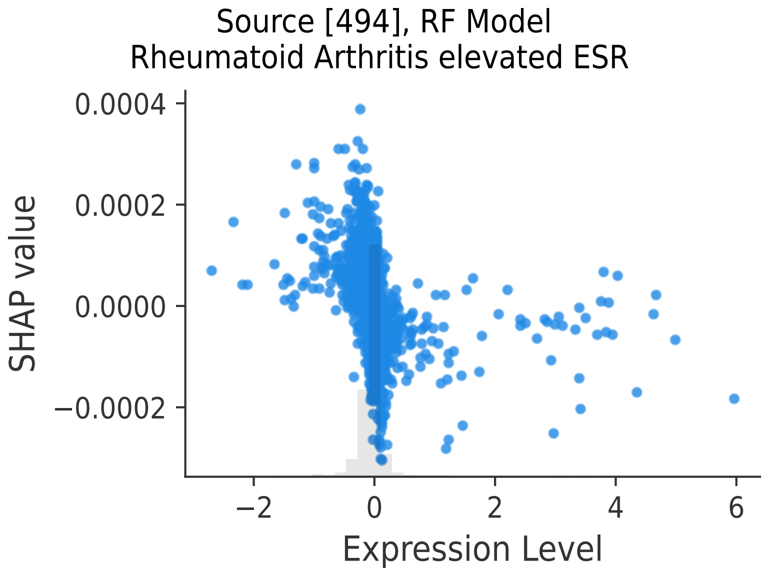

Source [494], RF Model
Rheumatoid Arthritis elevated ESR
0.0004
0.0002
0.0000
−0.0002
SHAP value
−2
0
2
4
6
Expression Level

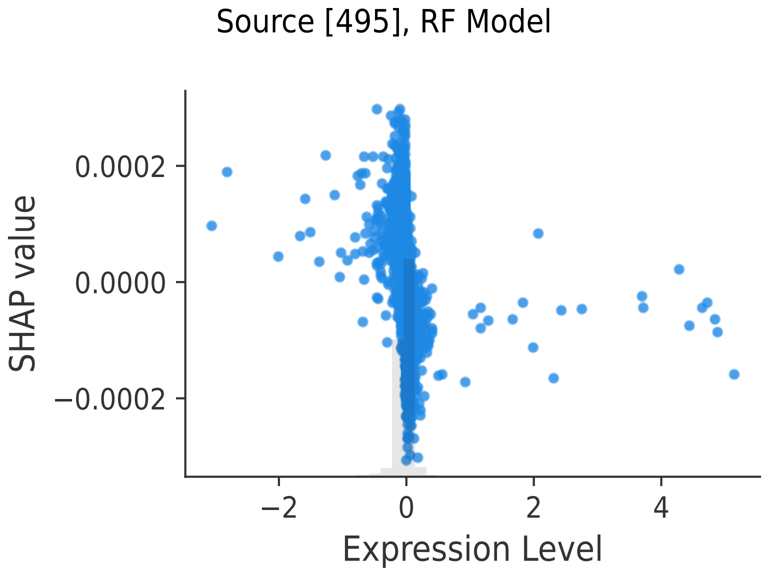
Source [495], RF Model
SHAP value
0.0002
0.0000
−0.0002
−2
0
2
4
Expression Level

Source [496], RF Model

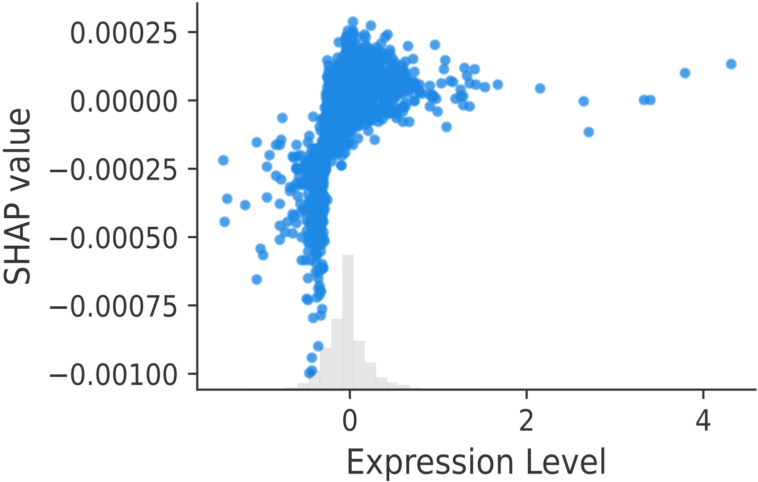

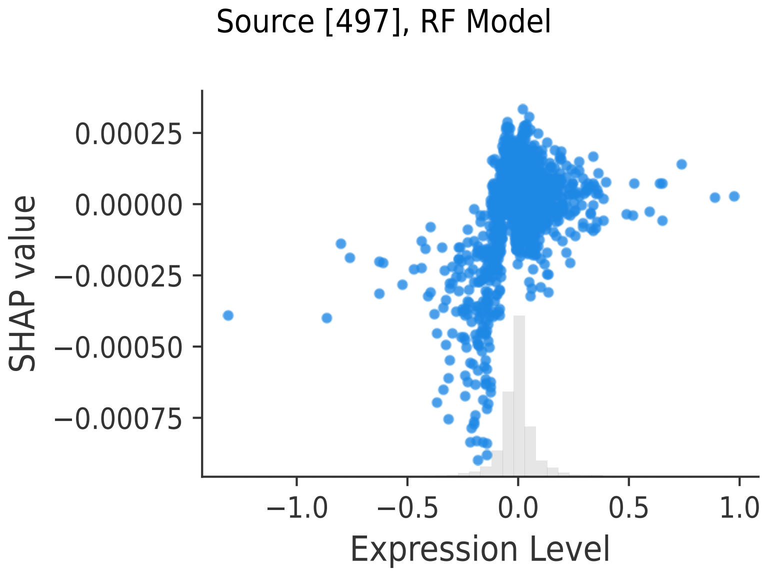
Source [497], RF Model
SHAP value
0.00025
0.00000
−0.00025
−0.00050
−0.00075
Expression Level
−1.0
−0.5
0.0
0.5
1.0

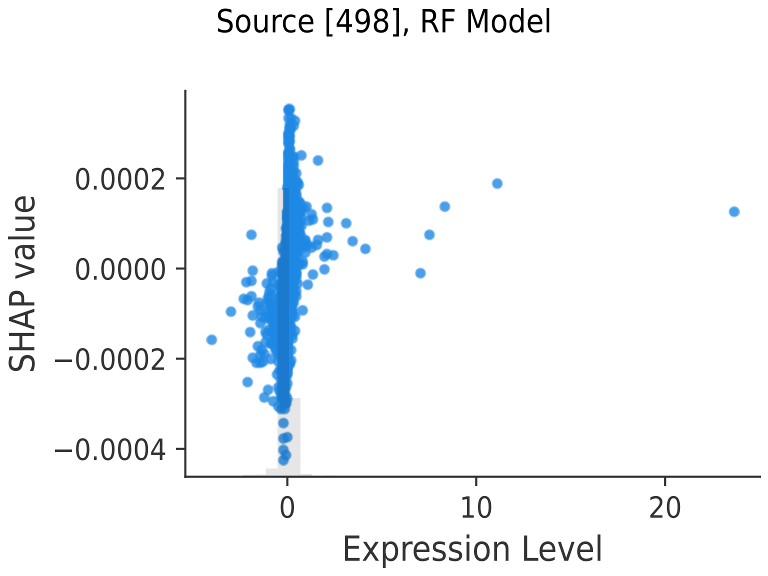
Source [498], RF Model
SHAP value
0.0002
0.0000
−0.0002
−0.0004
0
10
20
Expression Level

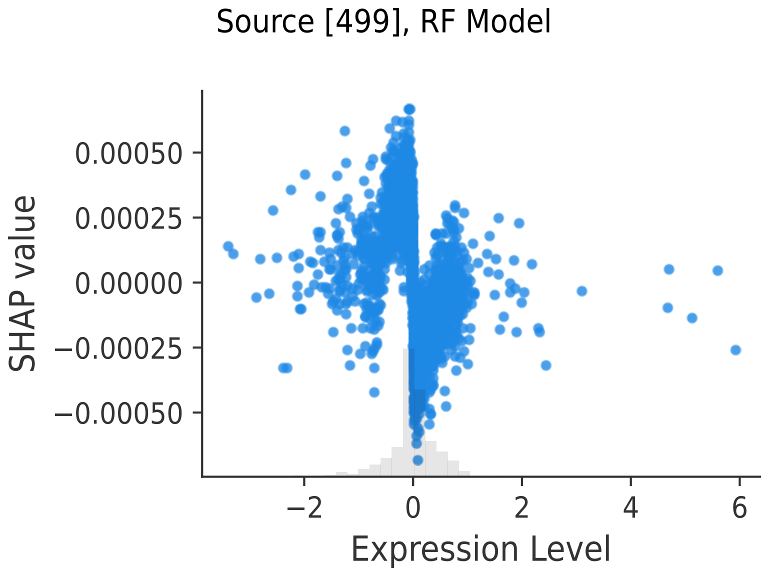

Source [499], RF Model
0.00050
0.00025
0.00000
−0.00025
−0.00050
SHAP value
−2
0
2
4
6
Expression Level

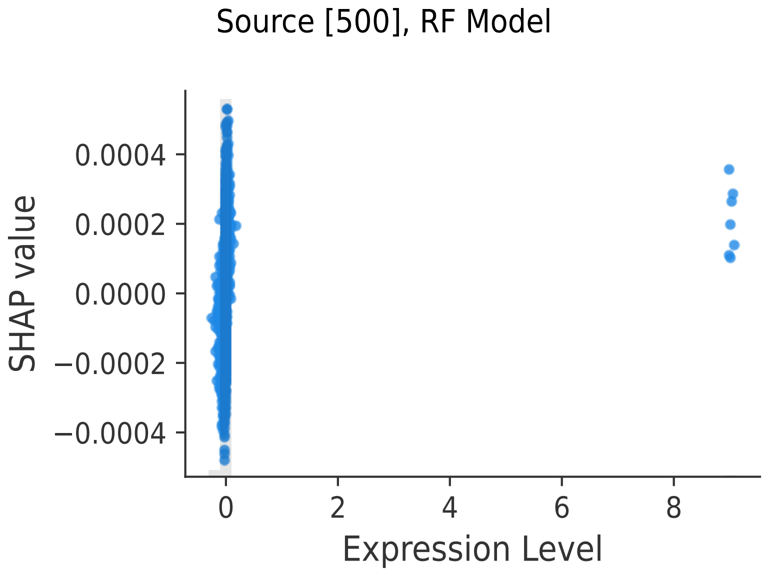

Source [500], RF Model
0.0004
0.0002
0.0000
−0.0002
−0.0004
SHAP value
0
2
4
6
8
Expression Level

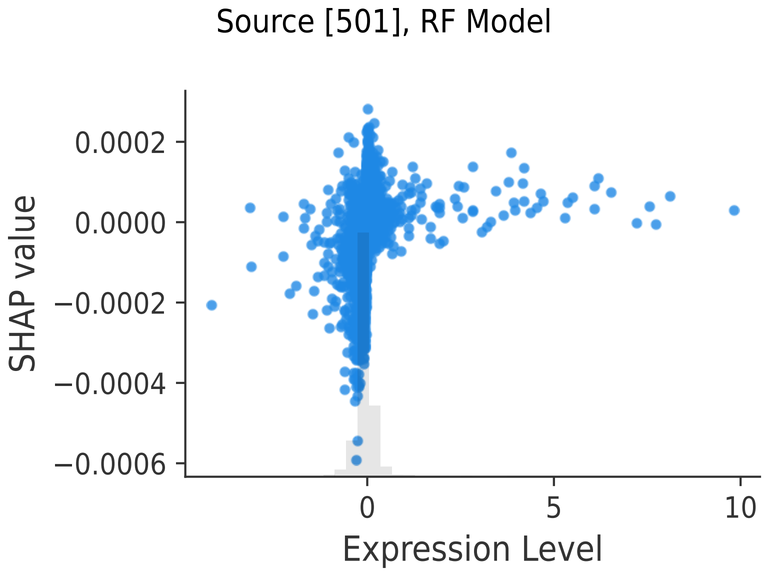
Source [501], RF Model
0.0002
0.0000
−0.0002
−0.0004
−0.0006
SHAP value
0
5
10
Expression Level

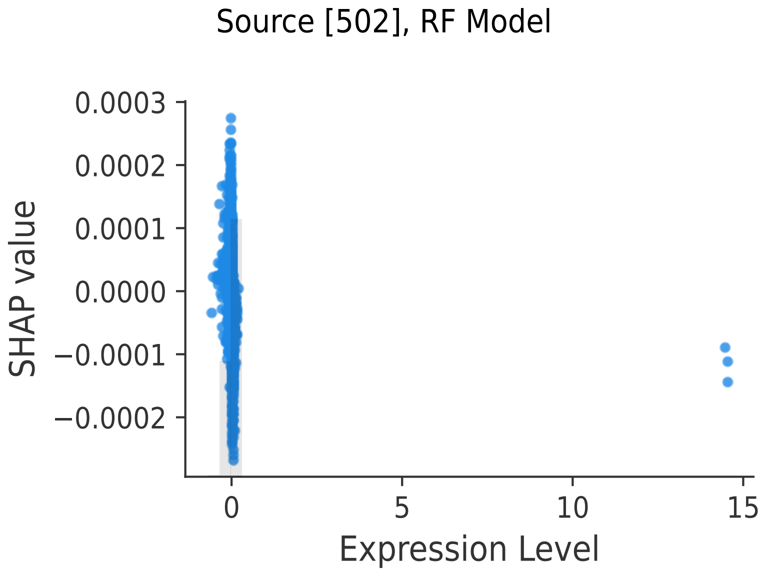

Source [502], RF Model
SHAP value
0.0003
0.0002
0.0001
0.0000
−0.0001
−0.0002
0
5
10
15
Expression Level

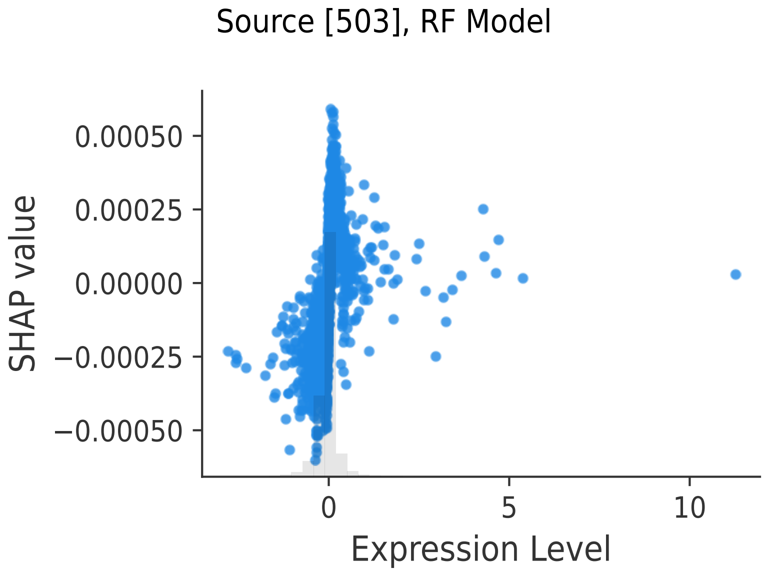

Source [503], RF Model
SHAP value
0.00050
0.00025
0.00000
−0.00025
−0.00050
0
5
10
Expression Level

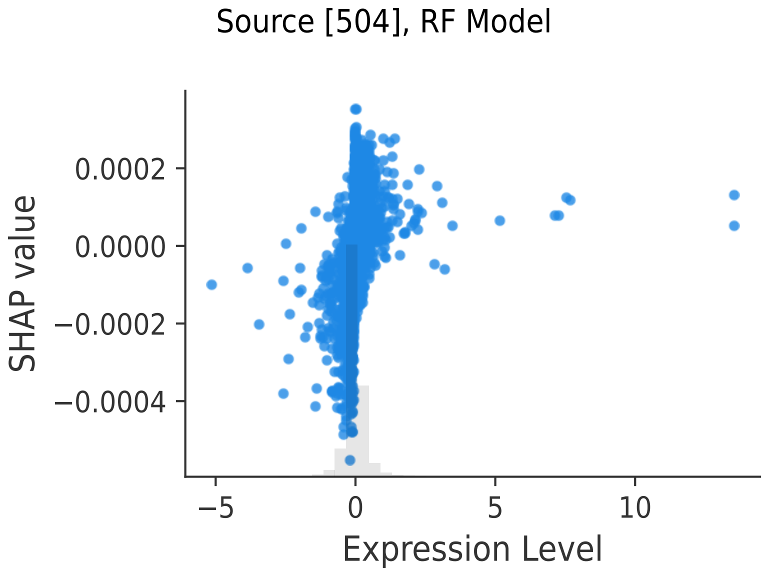

Source [504], RF Model
SHAP value
0.0002
0.0000
−0.0002
−0.0004
−5
0
5
10
Expression Level

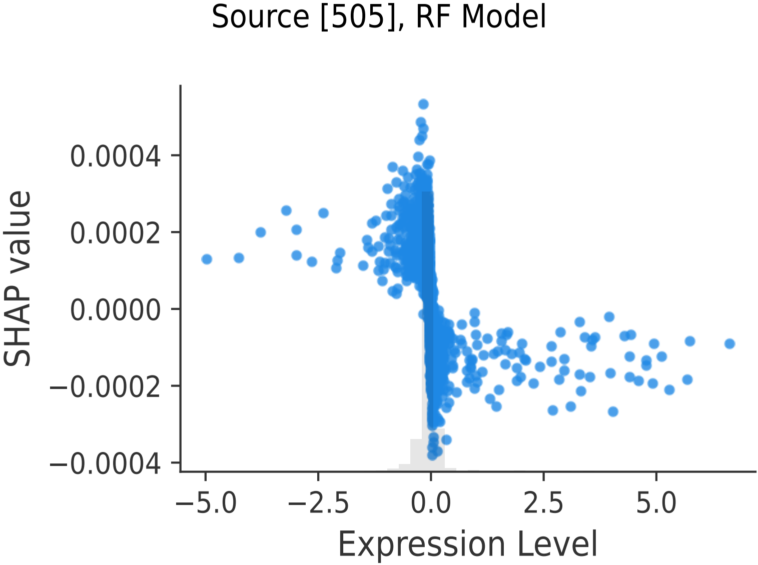
Source [505], RF Model
0.0004
0.0002
0.0000
−0.0002
−0.0004
SHAP value
−5.0
−2.5
0.0
2.5
5.0
Expression Level

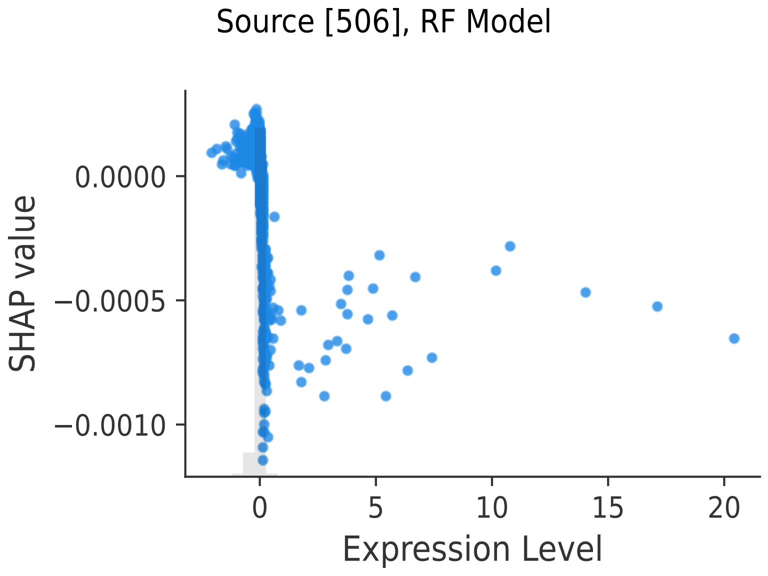

Source [506], RF Model
0.0000
−0.0005
−0.0010
SHAP value
0
5
10
15
20
Expression Level

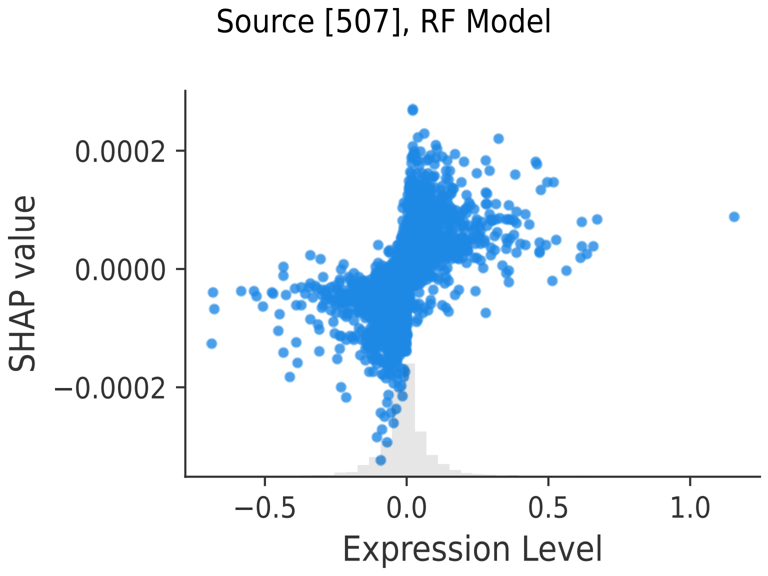

Source [507], RF Model
0.0002
0.0000
−0.0002
SHAP value
−0.5
0.0
0.5
1.0
Expression Level

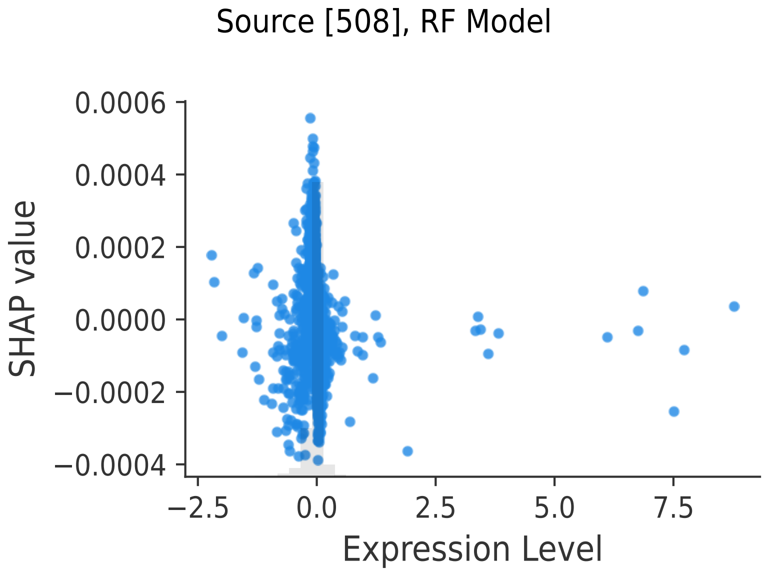
Source [508], RF Model
SHAP value
0.0006
0.0004
0.0002
0.0000
−0.0002
−0.0004
Expression Level
−2.5
0.0
2.5
5.0
7.5

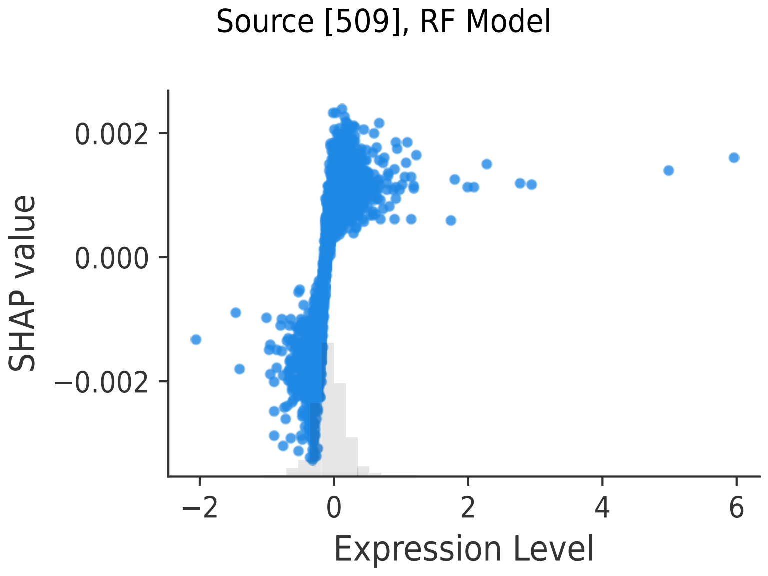

Source [509], RF Model
0.002
0.000
−0.002
SHAP value
−2
0
2
4
6
Expression Level

Source [510], RF Model

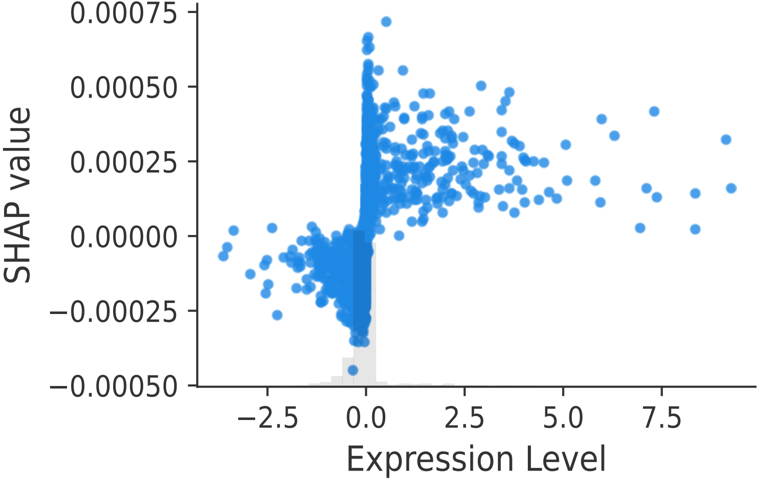

Source [511], RF Model

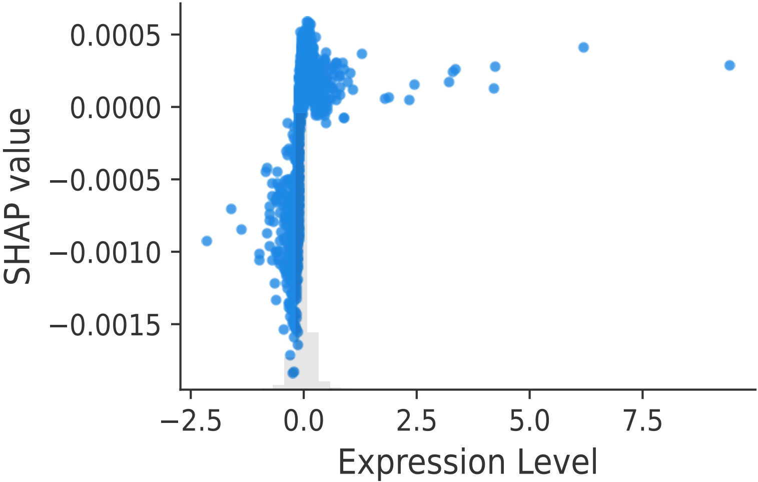

Source [512], RF Model

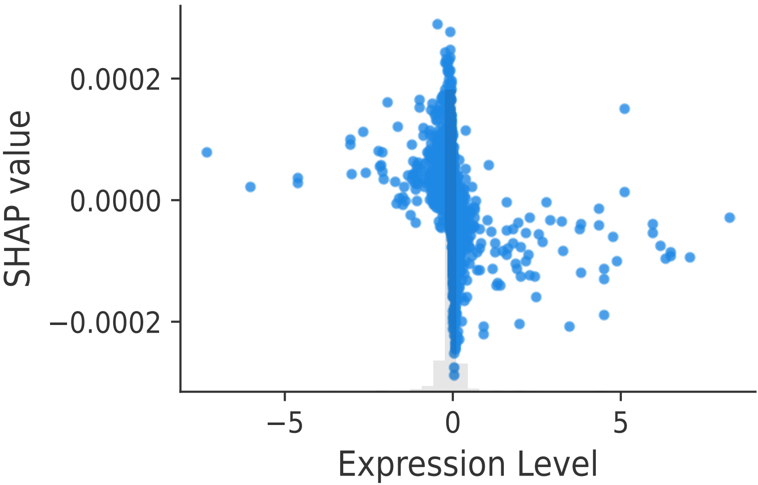

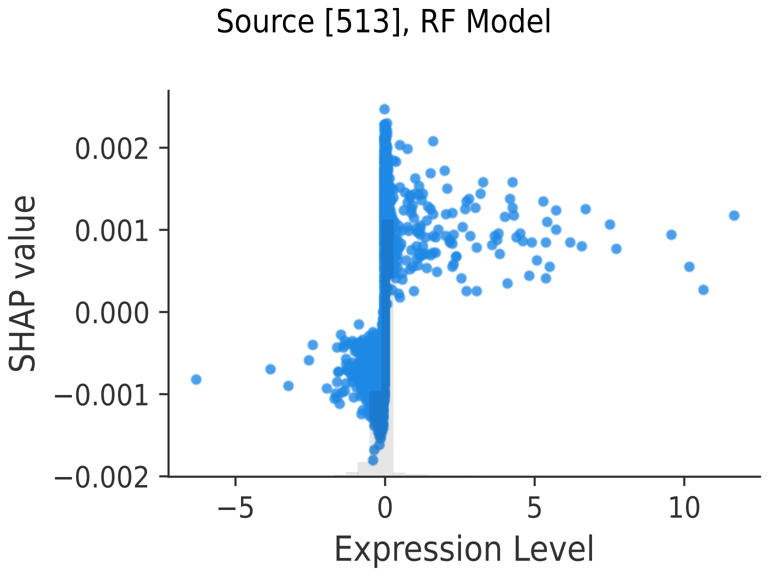

Source [513], RF Model
SHAP value
0.002
0.001
0.000
−0.001
−0.002
−5
0
5
10
Expression Level

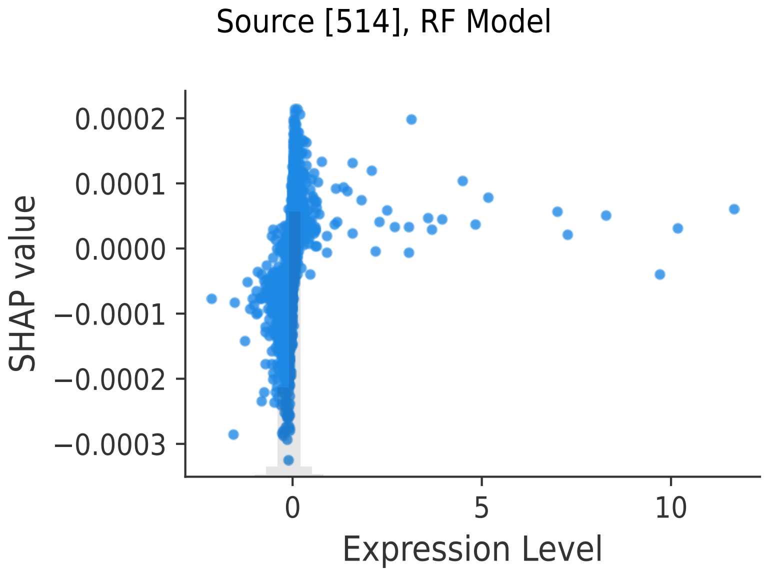

Source [514], RF Model
0.0002
0.0001
0.0000
−0.0001
−0.0002
−0.0003
SHAP value
0
5
10
Expression Level

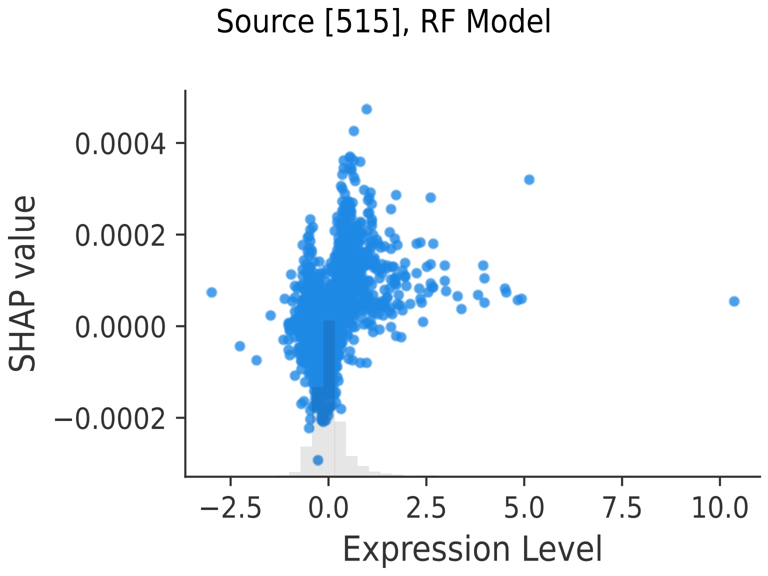
Source [515], RF Model
SHAP value
0.0004
0.0002
0.0000
−0.0002
−2.5
0.0
2.5
5.0
7.5
10.0
Expression Level

Source [516], RF Model

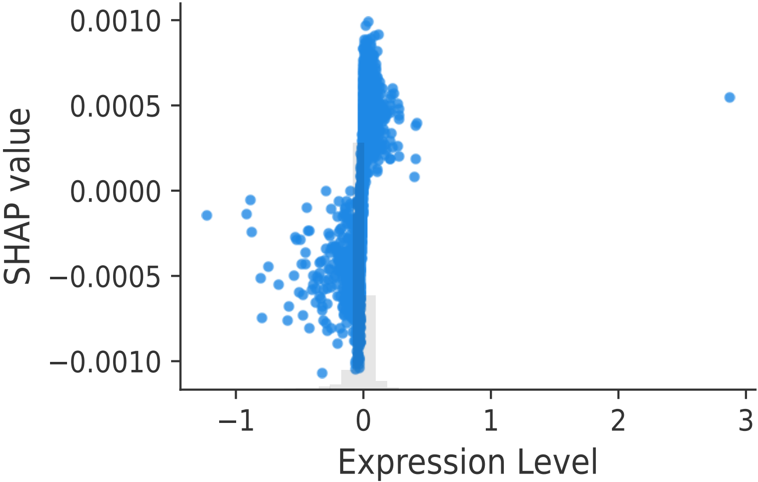

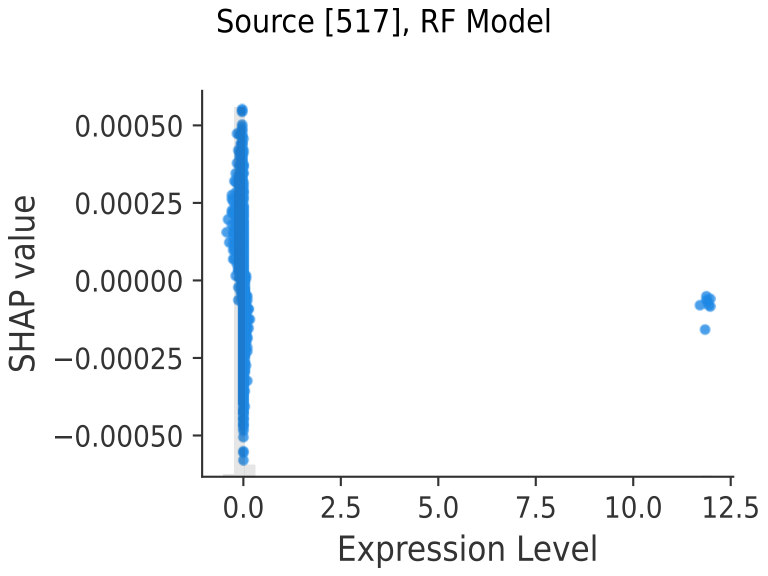
Source [517], RF Model
SHAP value
0.00050
0.00025
0.00000
−0.00025
−0.00050
0.0
2.5
5.0
7.5
10.0
12.5
Expression Level

Source [518], RF Model

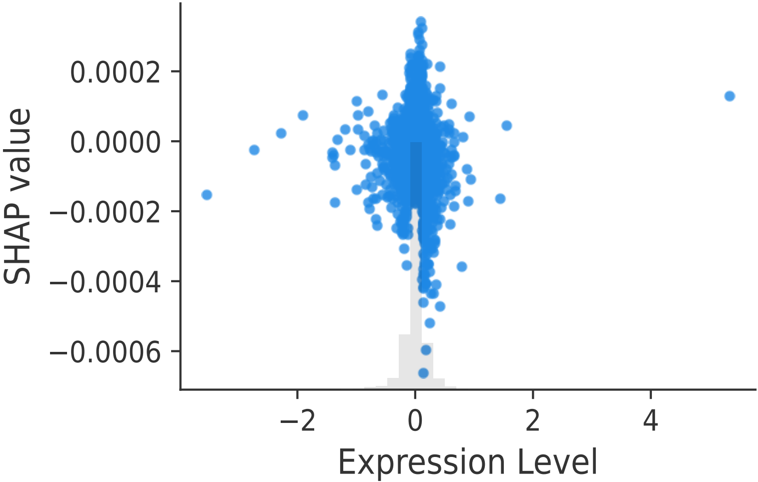

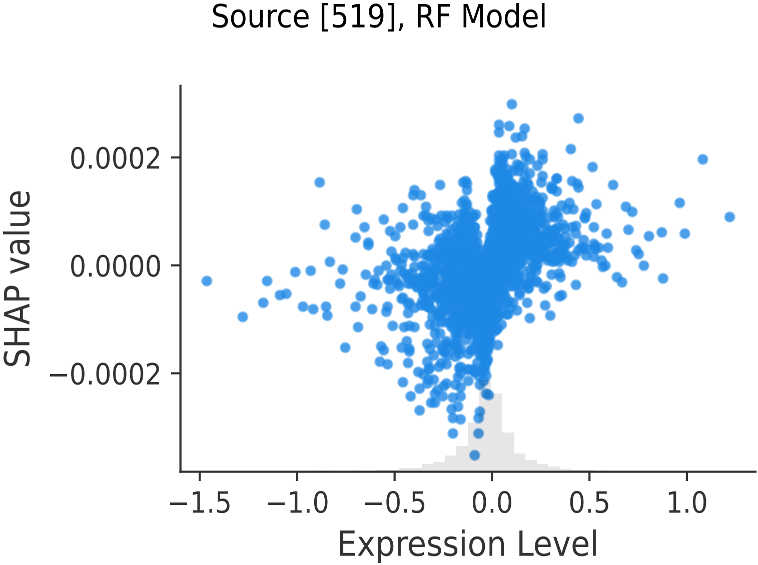

Source [519], RF Model
0.0002
0.0000
−0.0002
SHAP value
−1.5
−1.0
−0.5
0.0
0.5
1.0
Expression Level

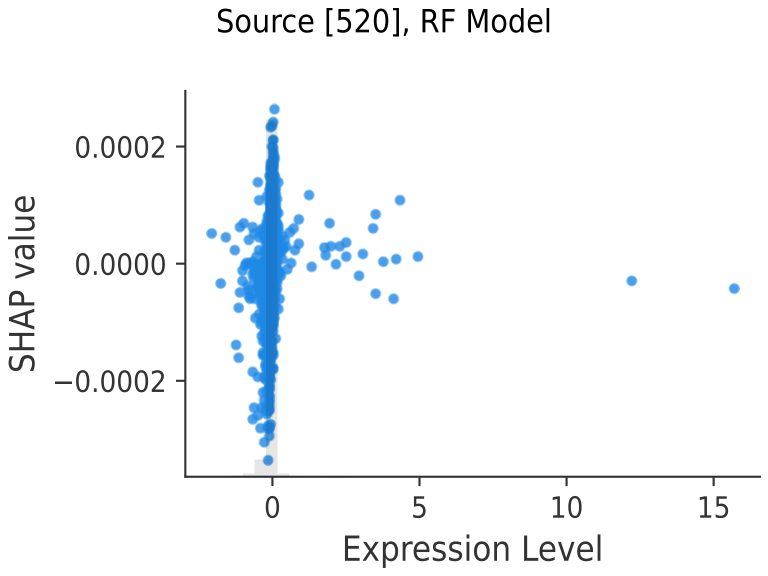

Source [520], RF Model
SHAP value
0.0002
0.0000
−0.0002
0
5
10
15
Expression Level

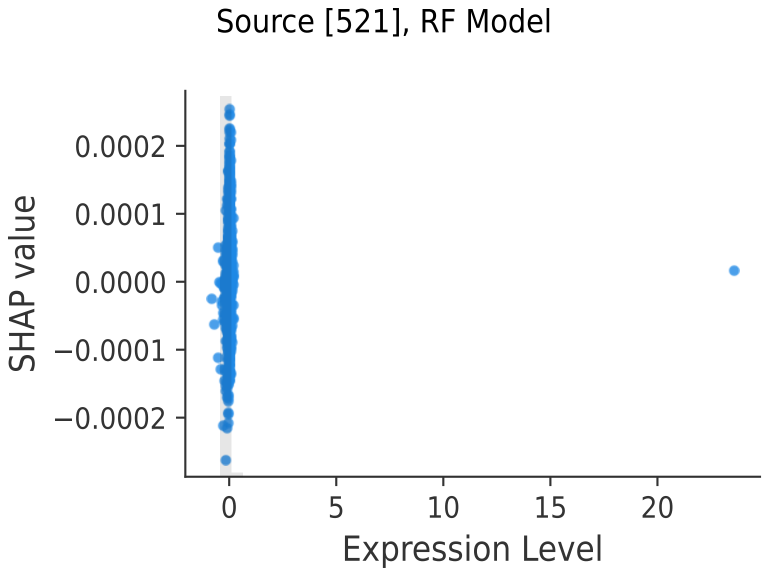
Source [521], RF Model
SHAP value
0.0002
0.0001
0.0000
−0.0001
−0.0002
0
5
10
15
20
Expression Level

Source [522], RF Model

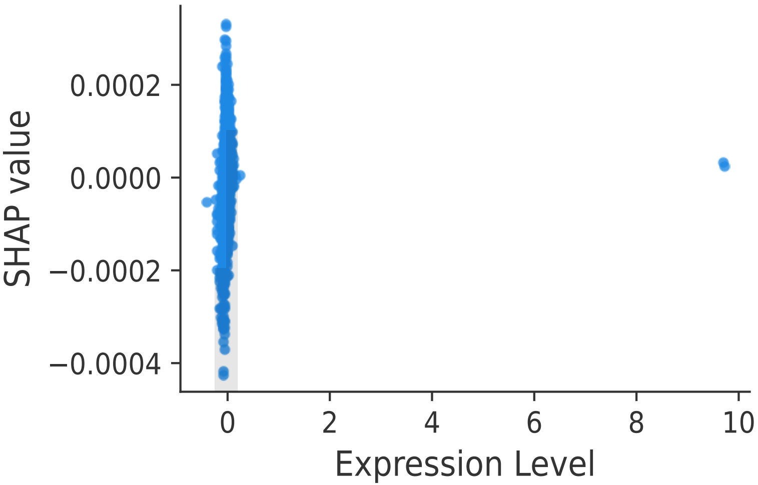

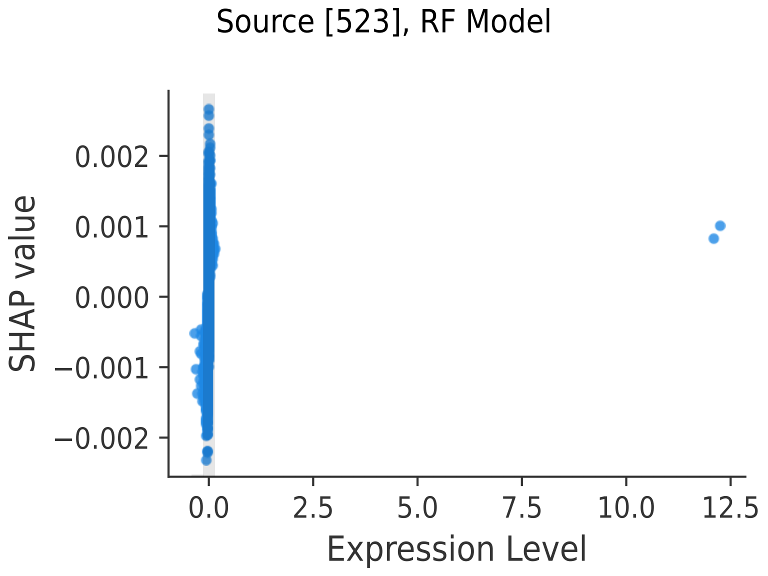
Source [523], RF Model
SHAP value
0.002
0.001
0.000
−0.001
−0.002
0.0
2.5
5.0
7.5
10.0
12.5
Expression Level

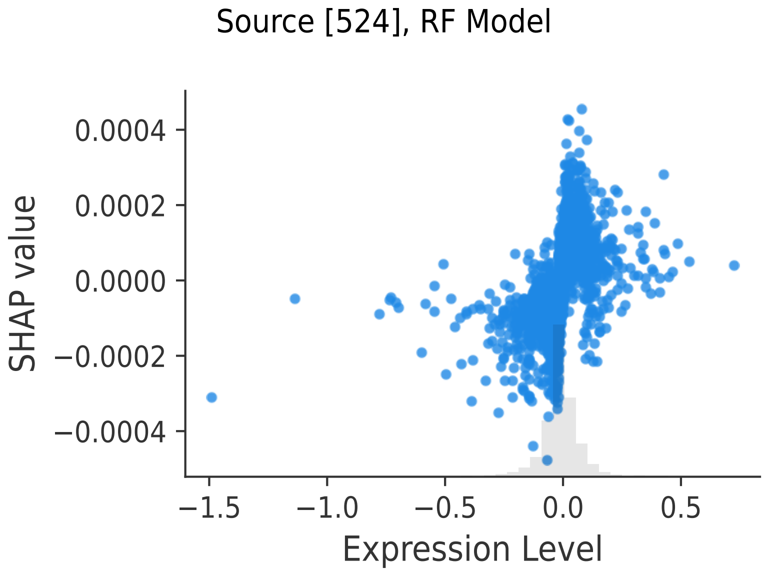

Source [524], RF Model
0.0004
0.0002
0.0000
−0.0002
−0.0004
SHAP value
−1.5
−1.0
−0.5
0.0
0.5
Expression Level

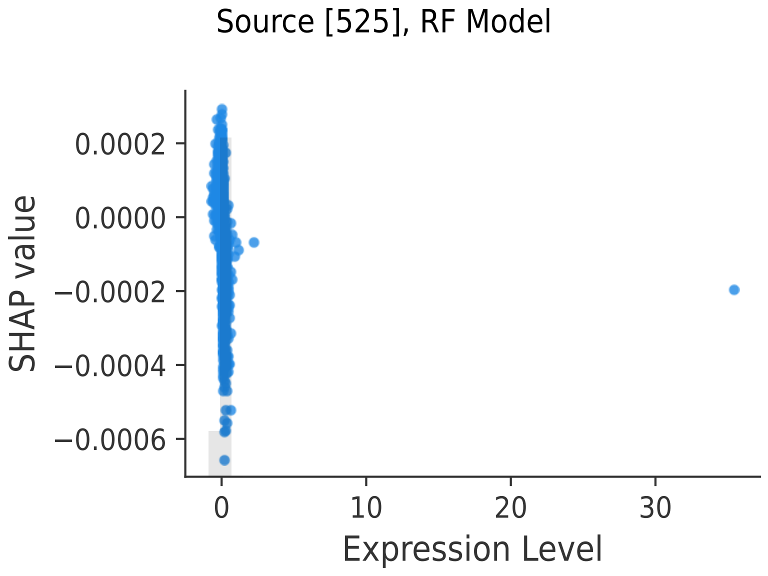
Source [525], RF Model
SHAP value
0.0002
0.0000
−0.0002
−0.0004
−0.0006
0
10
20
30
Expression Level

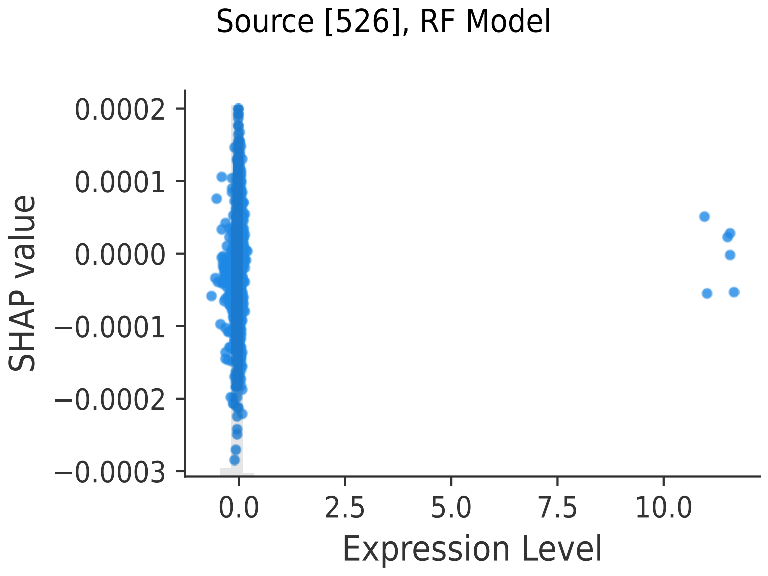
Source [526], RF Model
0.0002
0.0001
0.0000
−0.0001
−0.0002
−0.0003
SHAP value
0.0
2.5
5.0
7.5
10.0
Expression Level

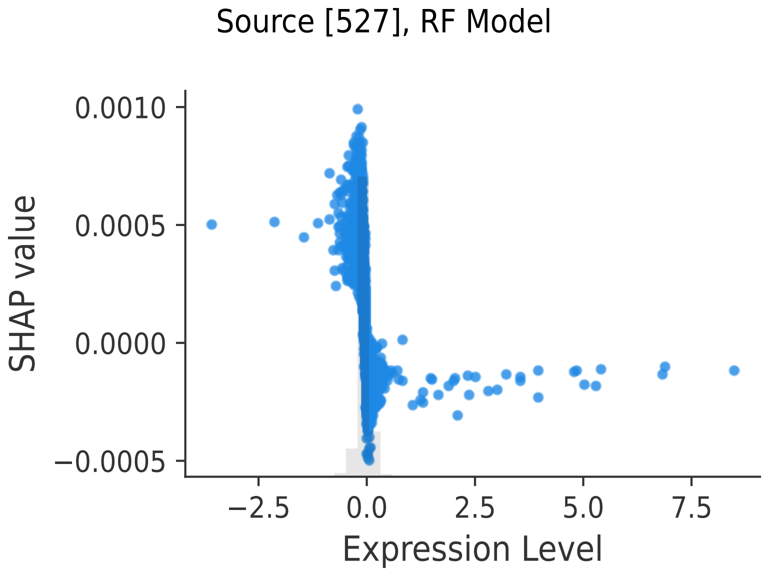

Source [527], RF Model
0.0010
0.0005
0.0000
−0.0005
SHAP value
−2.5
0.0
2.5
5.0
7.5
Expression Level

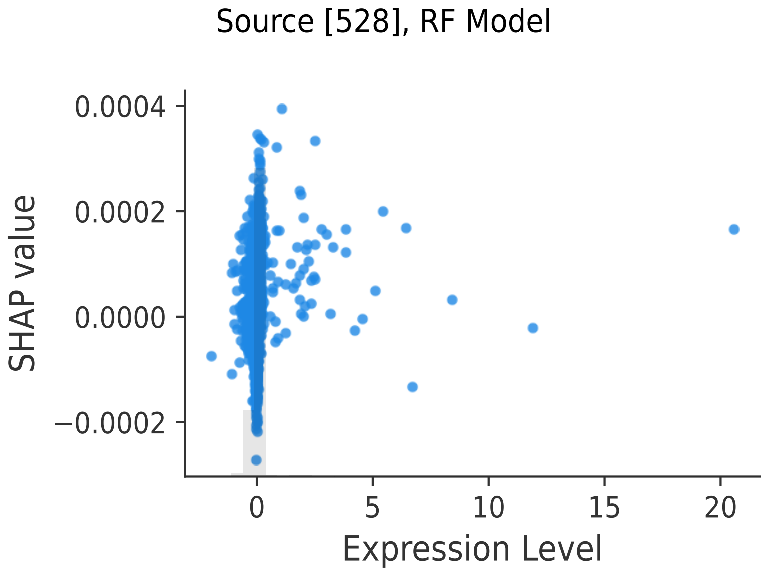

Source [528], RF Model
SHAP value
0.0004
0.0002
0.0000
−0.0002
0
5
10
15
20
Expression Level

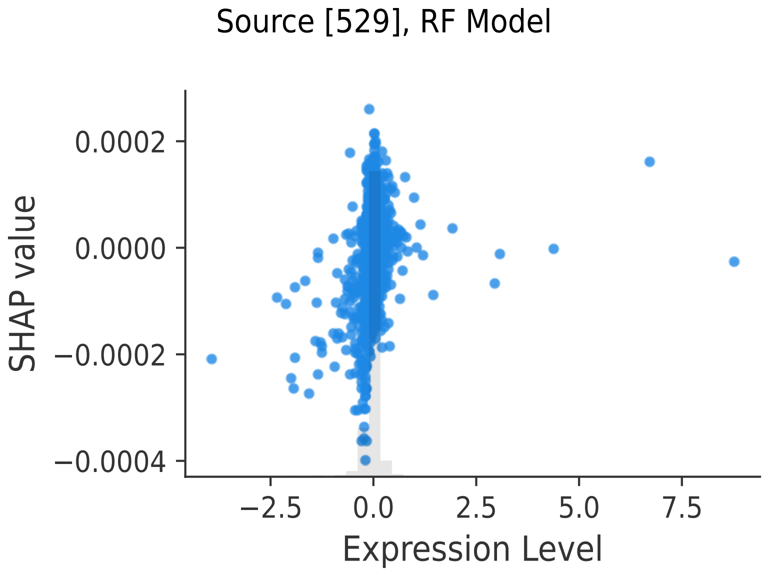
Source [529], RF Model
0.0002
0.0000
−0.0002
−0.0004
SHAP value
−2.5
0.0
2.5
5.0
7.5
Expression Level

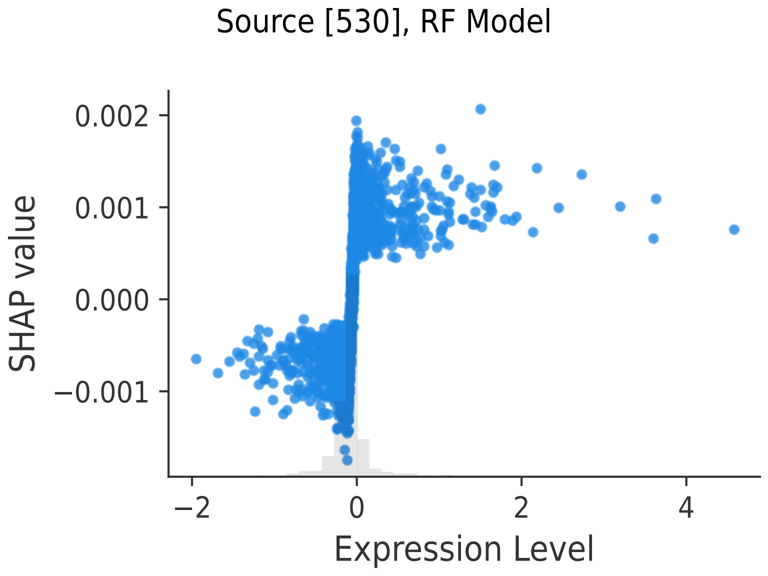
Source [530], RF Model
SHAP value
0.002
0.001
0.000
−0.001
−2
0
2
4
Expression Level

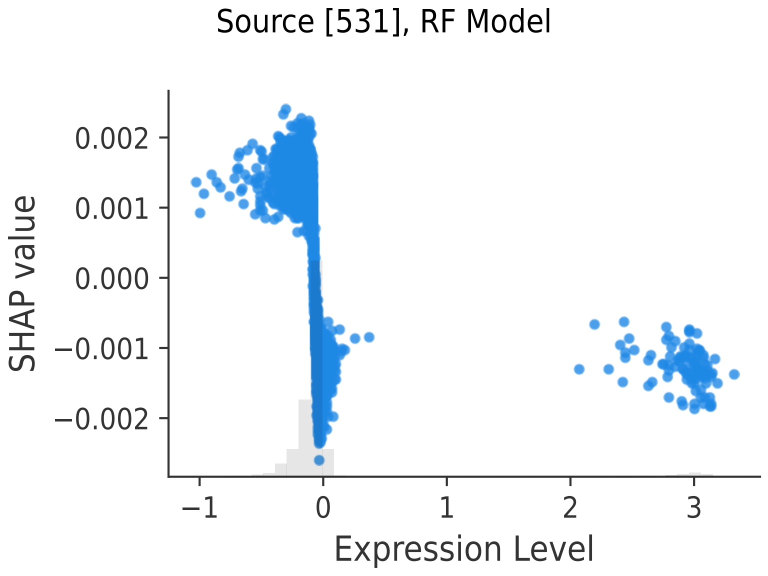
Source [531], RF Model
0.002
0.001
0.000
−0.001
−0.002
SHAP value
−1
0
1
2
3
Expression Level

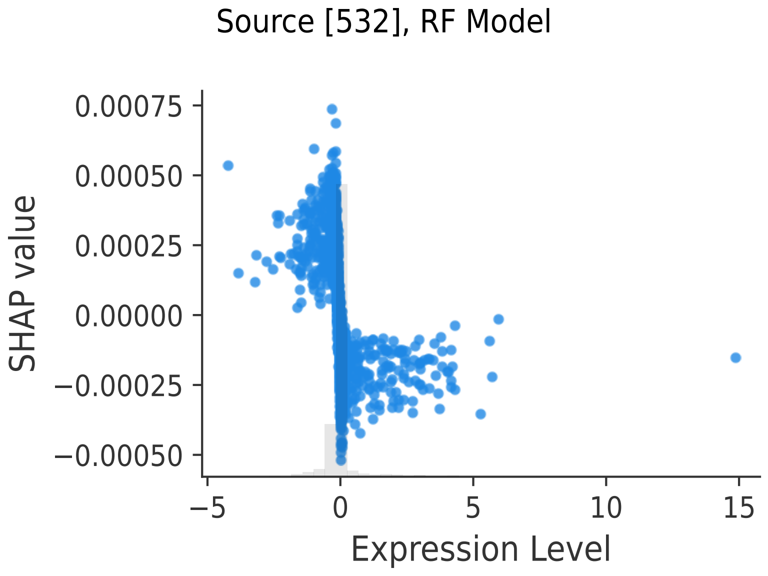
Source [532], RF Model
0.00075
0.00050
0.00025
0.00000
−0.00025
−0.00050
SHAP value
−5
0
5
10
15
Expression Level

Source [533], RF Model

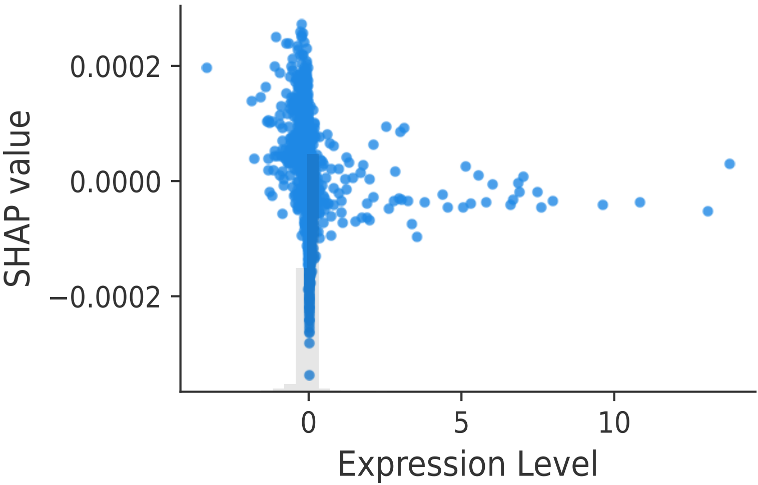

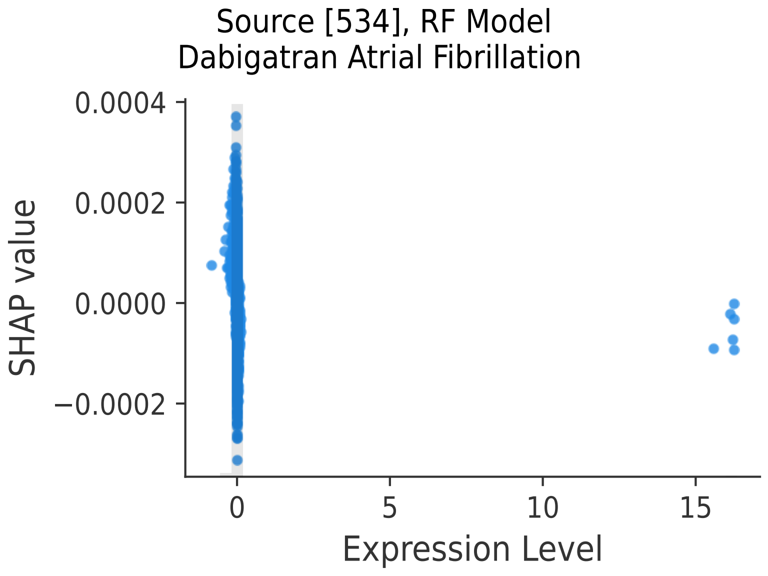

Source [534], RF Model
Dabigatran Atrial Fibrillation
0.0004
0.0002
0.0000
−0.0002
SHAP value
0
5
10
15
Expression Level

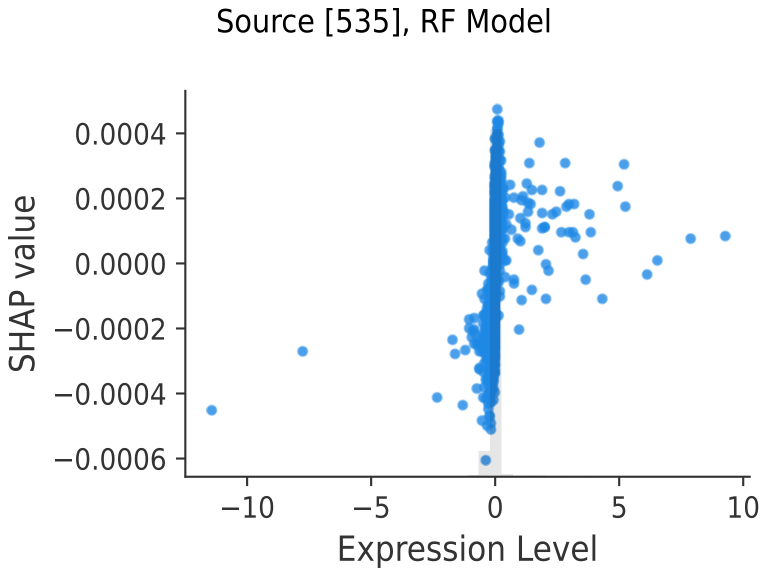
Source [535], RF Model
SHAP value
0.0004
0.0002
0.0000
−0.0002
−0.0004
−0.0006
−10
−5
0
5
10
Expression Level

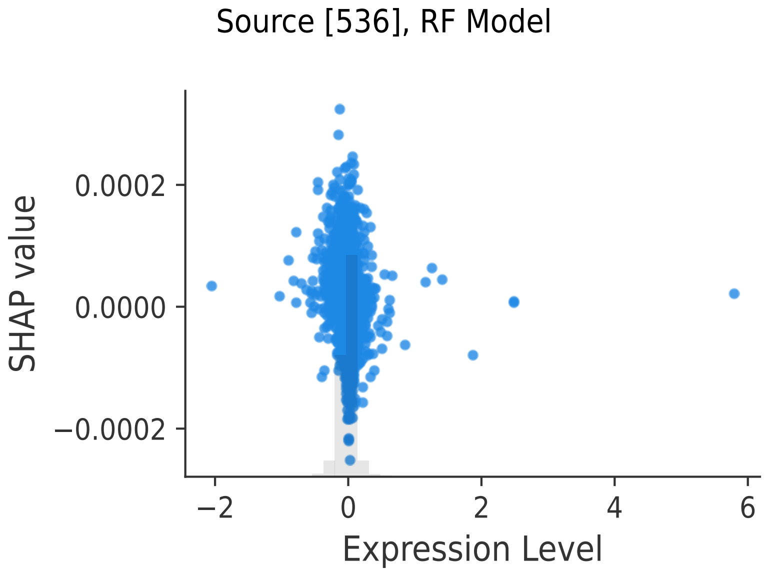
Source [536], RF Model
SHAP value
0.0002
0.0000
−0.0002
−2
0
2
4
6
Expression Level

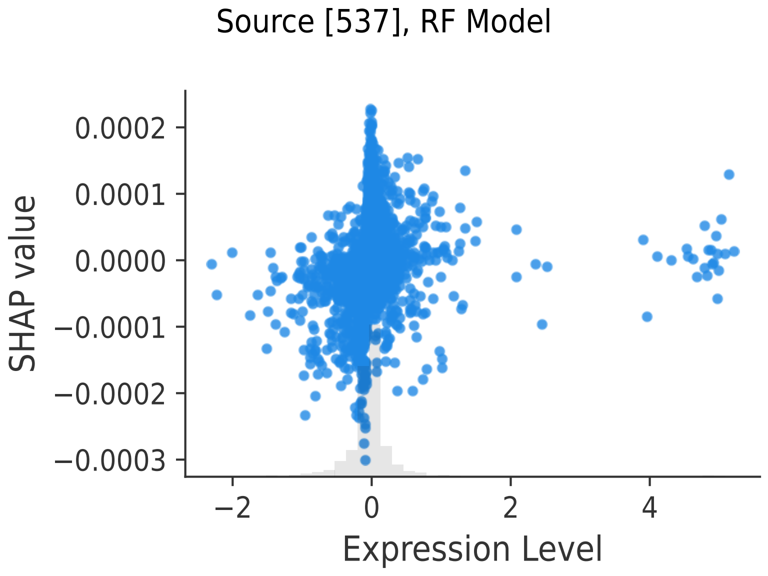

Source [537], RF Model
0.0002
0.0001
0.0000
−0.0001
−0.0002
−0.0003
SHAP value
−2
0
2
4
Expression Level

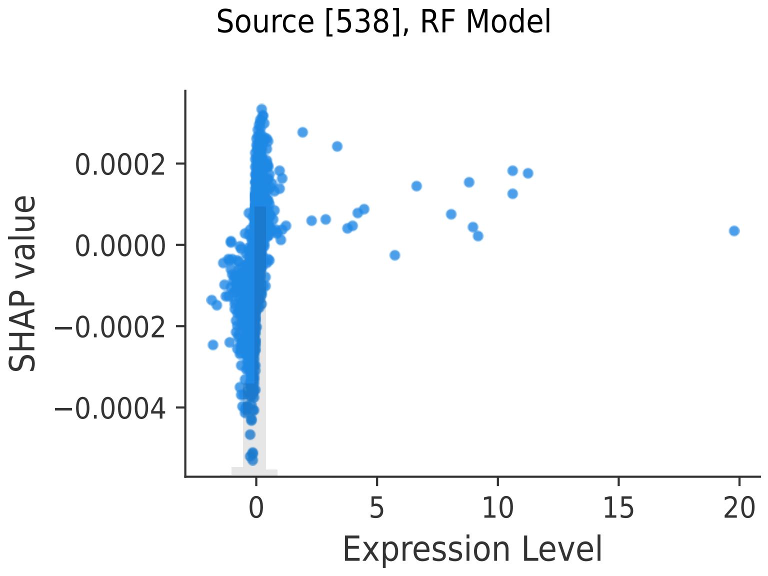
Source [538], RF Model
SHAP value
0.0002
0.0000
−0.0002
−0.0004
Expression Level
0
5
10
15
20

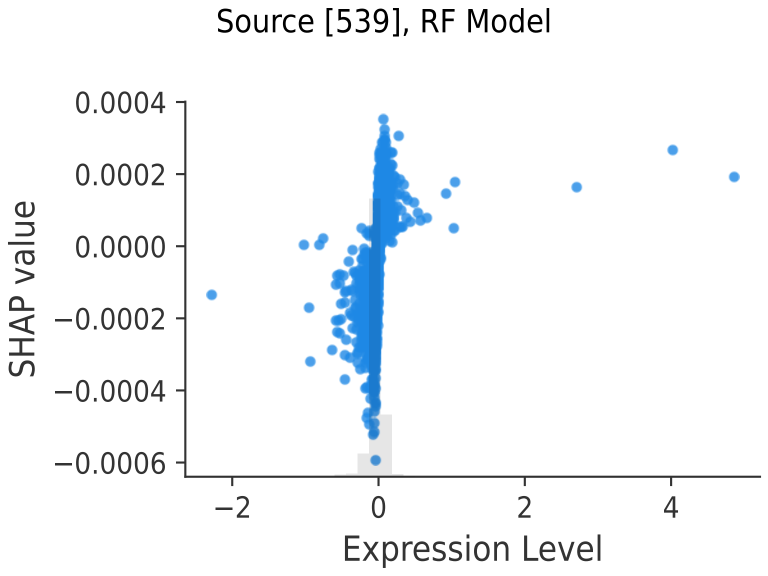

Source [539], RF Model
0.0004
0.0002
0.0000
−0.0002
−0.0004
−0.0006
SHAP value
−2
0
2
4
Expression Level

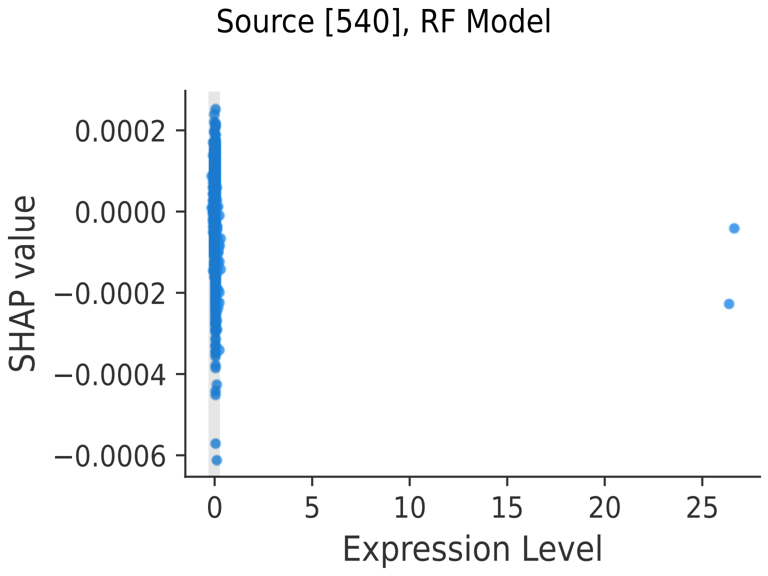
Source [540], RF Model
SHAP value
0.0002
0.0000
−0.0002
−0.0004
−0.0006
0
5
10
15
20
25
Expression Level

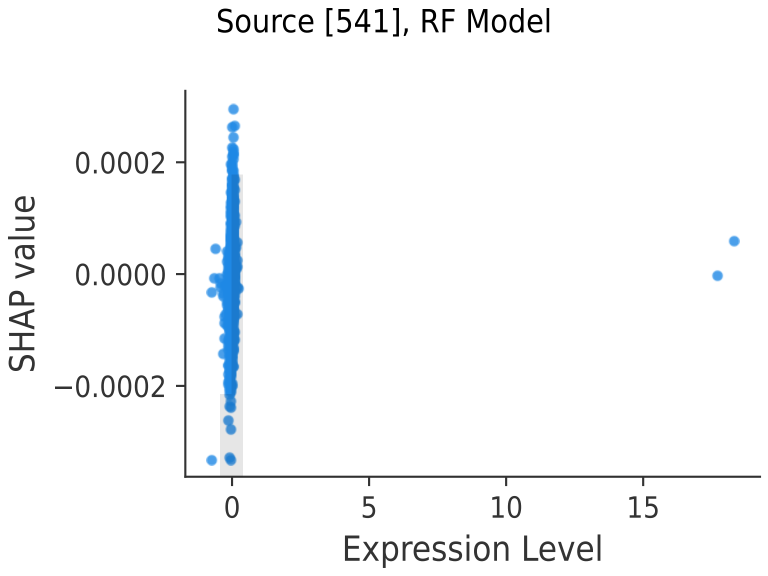
Source [541], RF Model
SHAP value
0.0002
0.0000
−0.0002
0
5
10
15
Expression Level

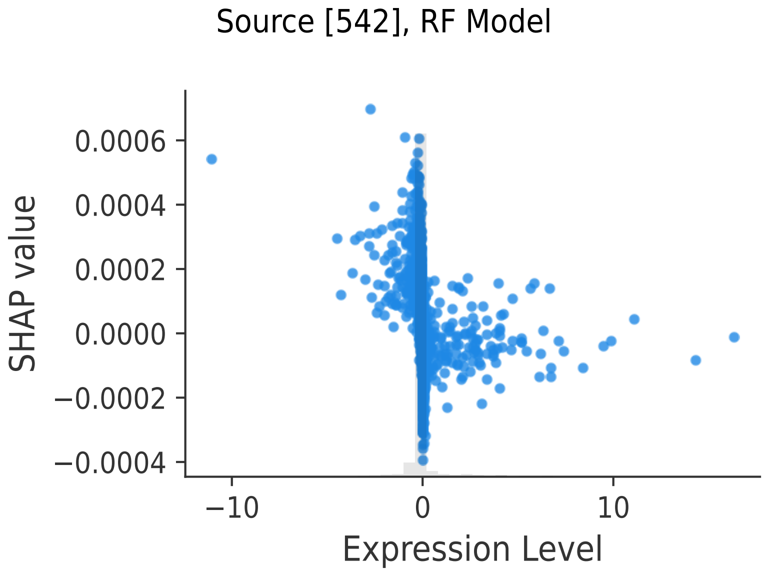

Source [542], RF Model
SHAP value
0.0006
0.0004
0.0002
0.0000
−0.0002
−0.0004
−10
0
10
Expression Level

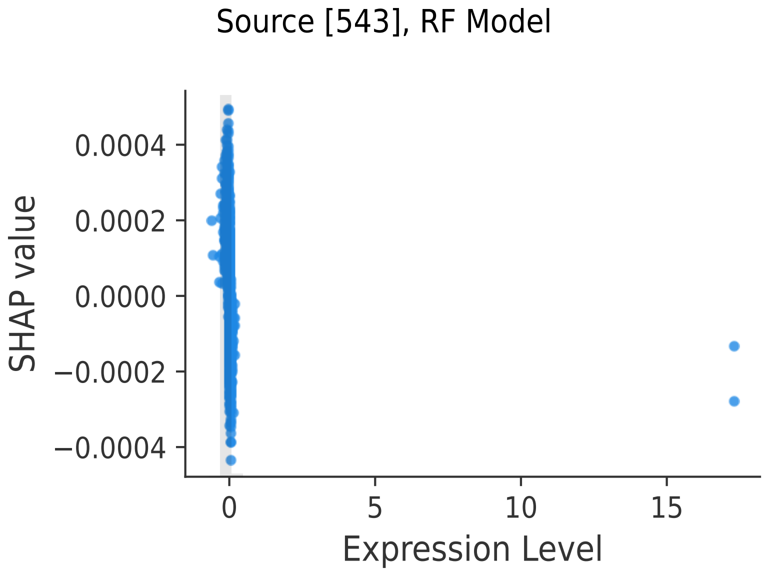
Source [543], RF Model
0.0004
0.0002
0.0000
−0.0002
−0.0004
SHAP value
0
5
10
15
Expression Level

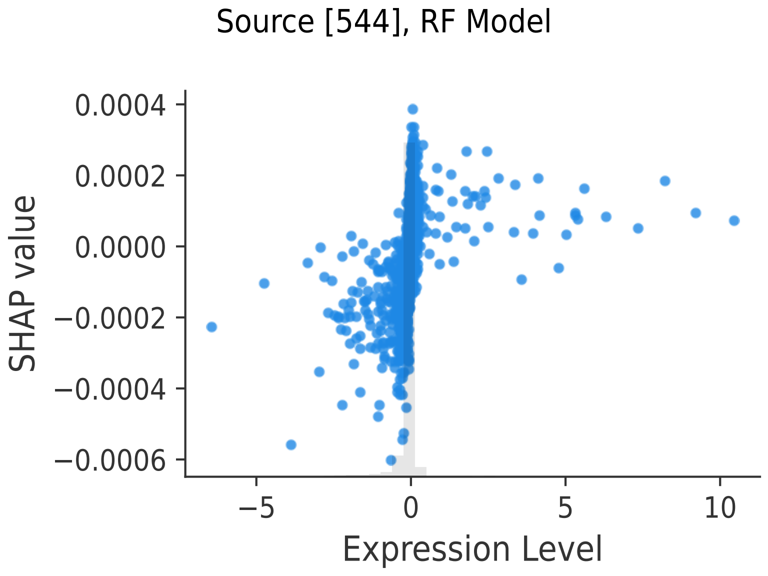

Source [544], RF Model
0.0004
0.0002
0.0000
−0.0002
−0.0004
−0.0006
SHAP value
−5
0
5
10
Expression Level

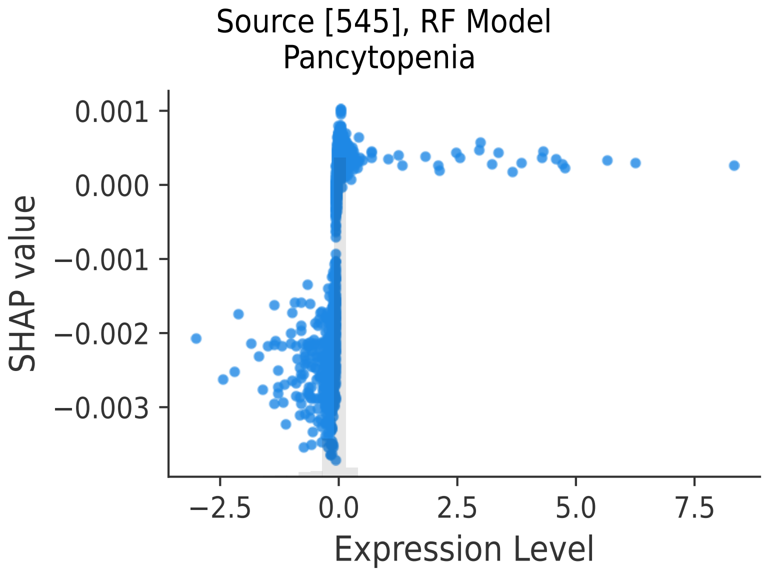
Source [545], RF Model
Pancytopenia
SHAP value
0.001
0.000
−0.001
−0.002
−0.003
−2.5
0.0
2.5
5.0
7.5
Expression Level

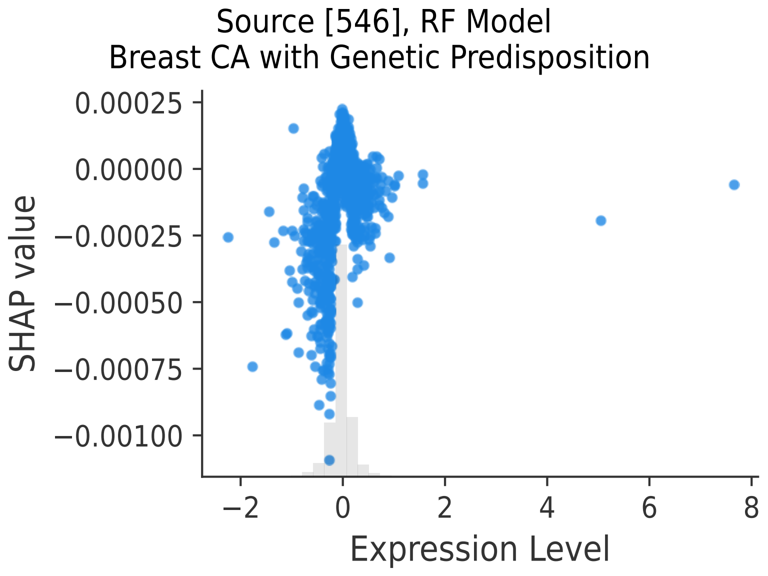

Source [546], RF Model
Breast CA with Genetic Predisposition
0.00025
0.00000
−0.00025
−0.00050
−0.00075
−0.00100
SHAP value
−2
0
2
4
6
8
Expression Level

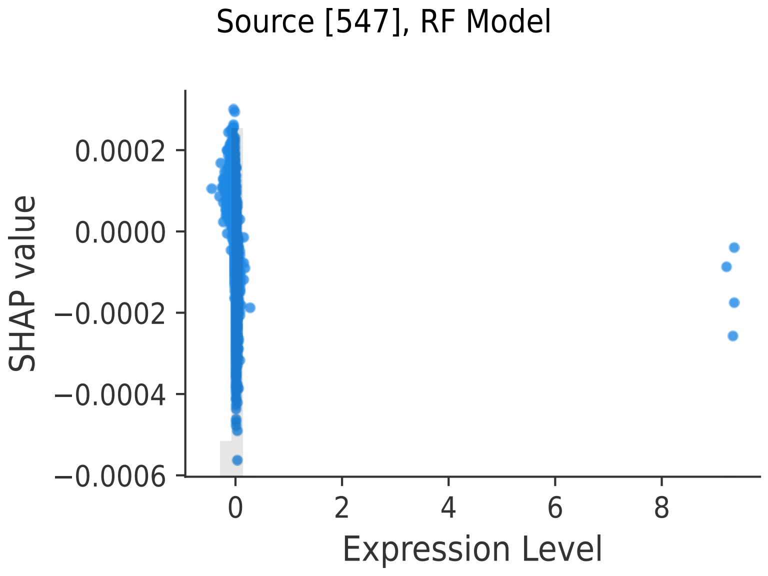
Source [547], RF Model
SHAP value
0.0002
0.0000
−0.0002
−0.0004
−0.0006
0
2
4
6
8
Expression Level

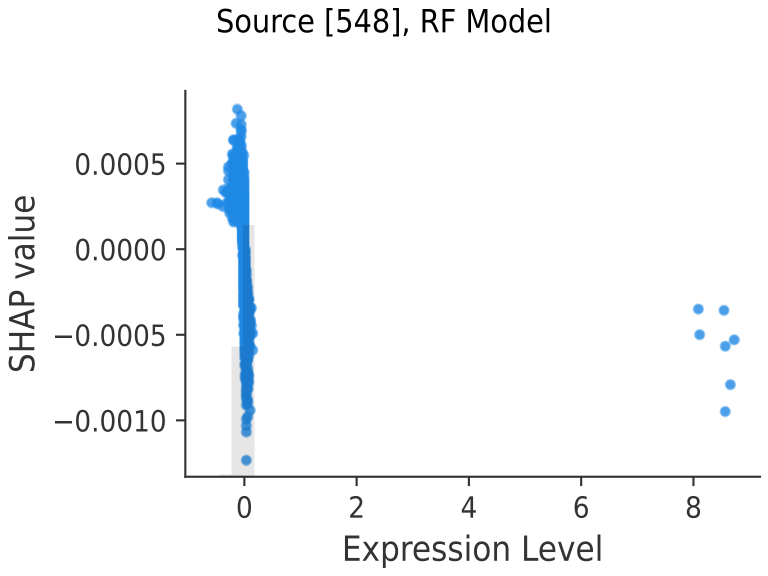
Source [548], RF Model
SHAP value
0.0005
0.0000
−0.0005
−0.0010
0
2
4
6
8
Expression Level

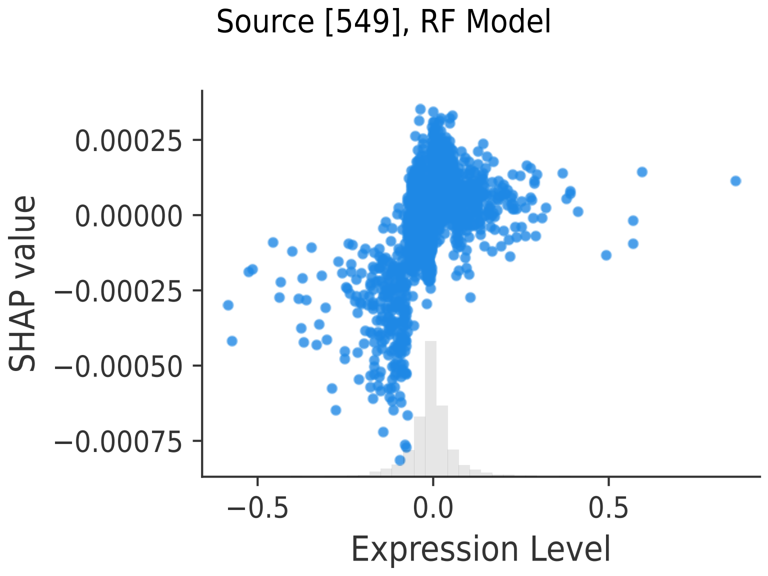
Source [549], RF Model
0.00025
0.00000
−0.00025
−0.00050
−0.00075
SHAP value
−0.5
0.0
0.5
Expression Level

Source [550], RF Model

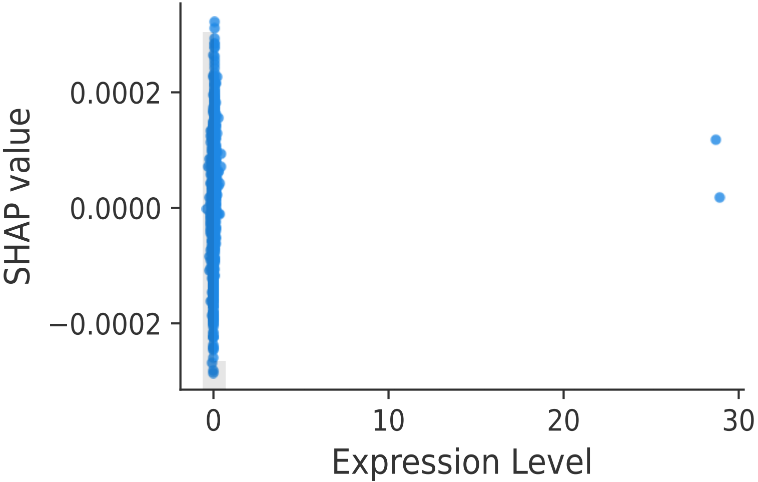

Source [551], RF Model

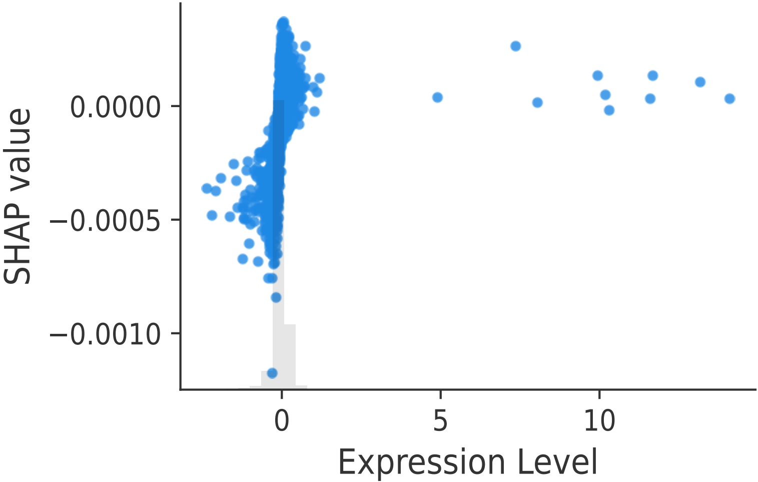

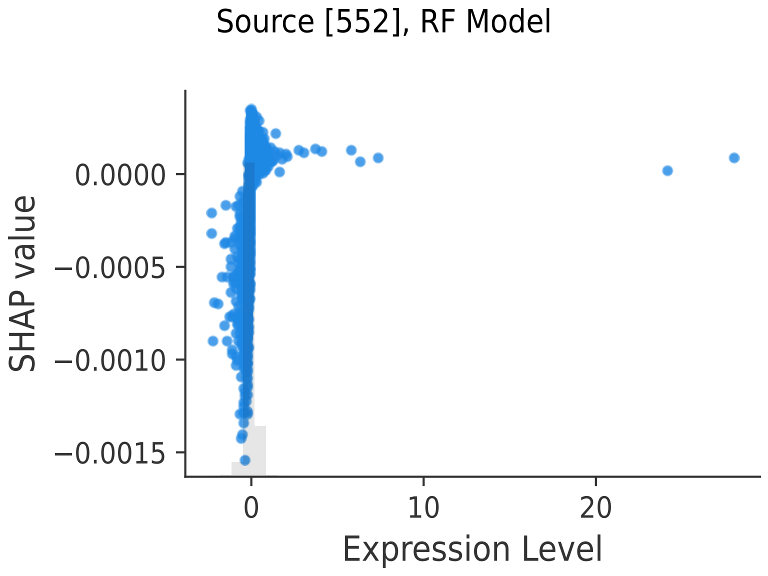

Source [552], RF Model
SHAP value
0.0000
−0.0005
−0.0010
−0.0015
0
10
20
Expression Level

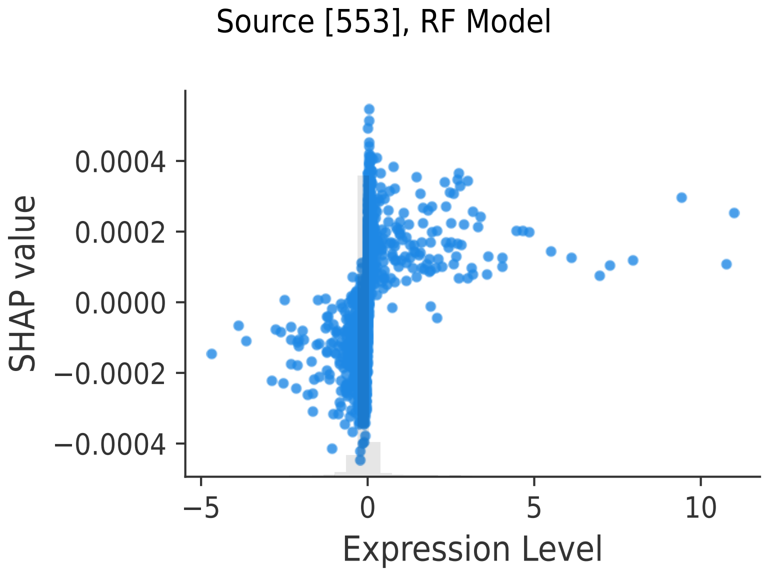
Source [553], RF Model
SHAP value
0.0004
0.0002
0.0000
−0.0002
−0.0004
−5
0
5
10
Expression Level

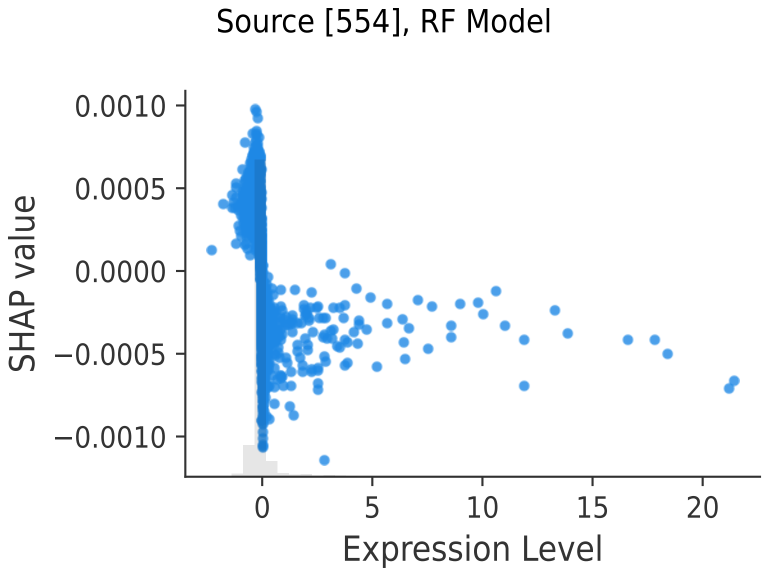
Source [554], RF Model
SHAP value
0.0010
0.0005
0.0000
−0.0005
−0.0010
0
5
10
15
20
Expression Level

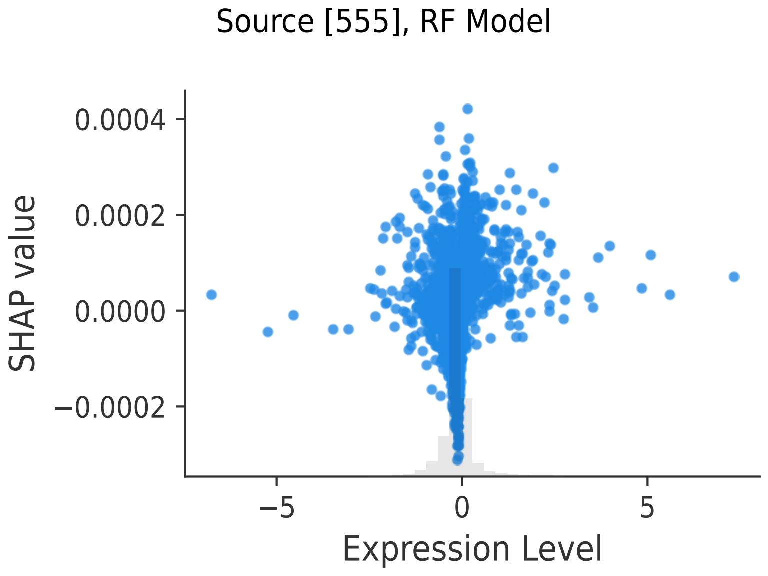

Source [555], RF Model
0.0004
0.0002
0.0000
−0.0002
SHAP value
−5
0
5
Expression Level

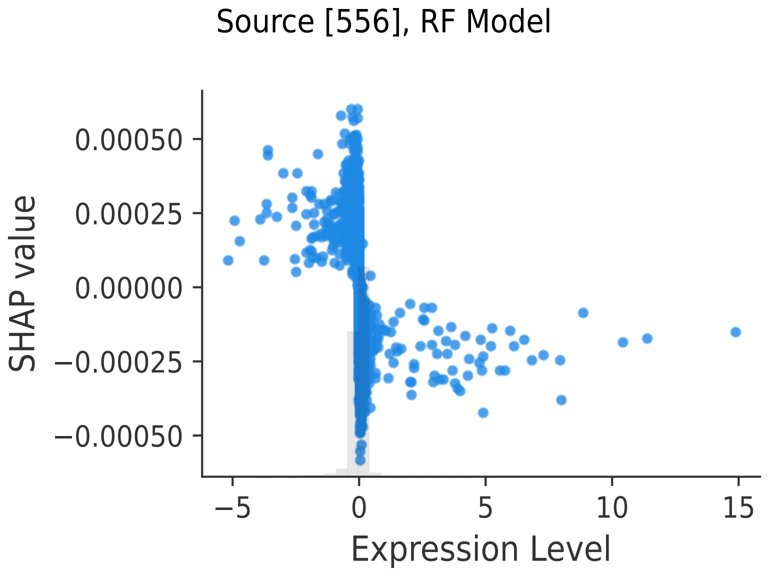

Source [556], RF Model
0.00050
0.00025
0.00000
−0.00025
−0.00050
SHAP value
−5
0
5
10
15
Expression Level

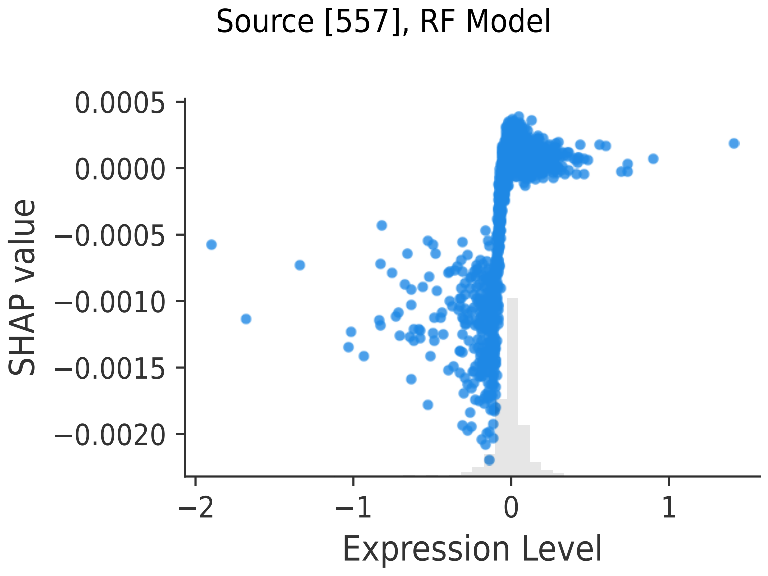

Source [557], RF Model
0.0005
0.0000
−0.0005
−0.0010
−0.0015
−0.0020
SHAP value
−2
−1
0
1
Expression Level

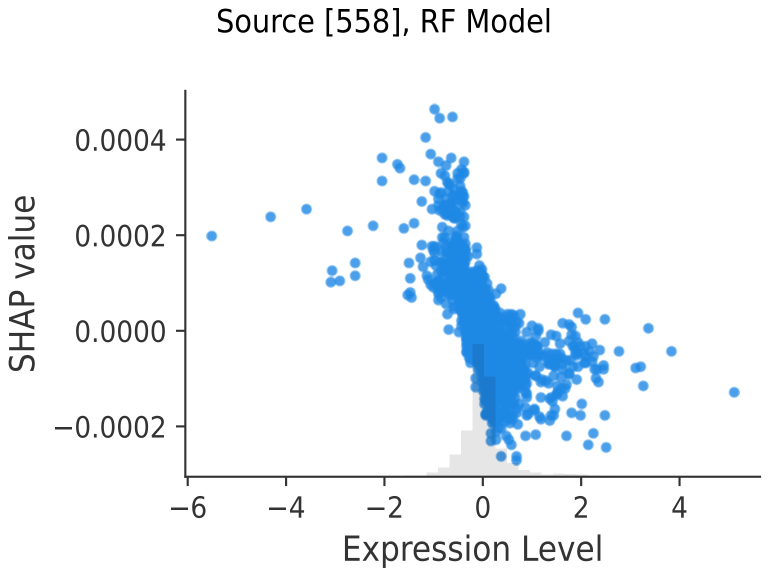

Source [558], RF Model
0.0004
0.0002
0.0000
−0.0002
SHAP value
−6
−4
−2
0
2
4
Expression Level

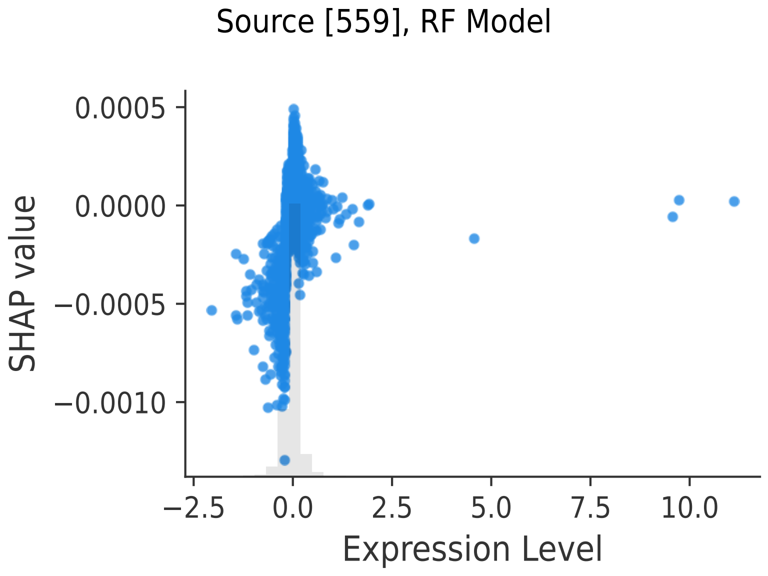
Source [559], RF Model
SHAP value
0.0005
0.0000
−0.0005
−0.0010
−2.5
0.0
2.5
5.0
7.5
10.0
Expression Level

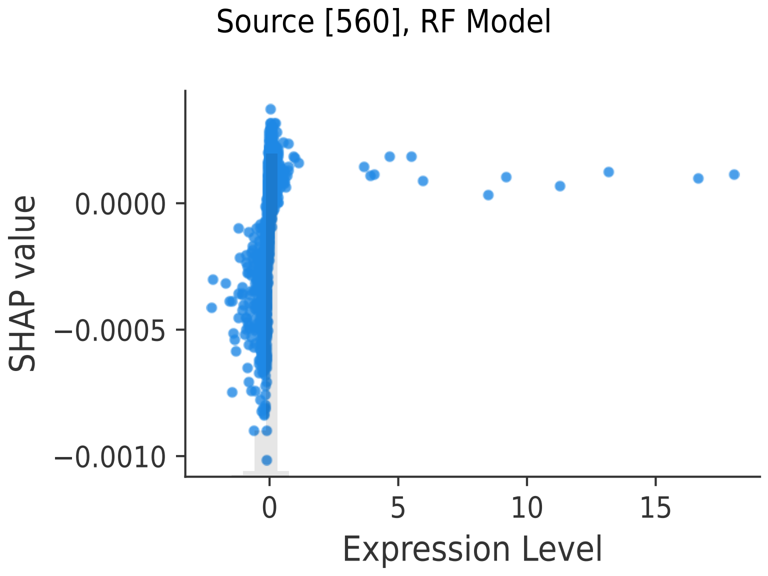
Source [560], RF Model
SHAP value
0.0000
−0.0005
−0.0010
0
5
10
15
Expression Level

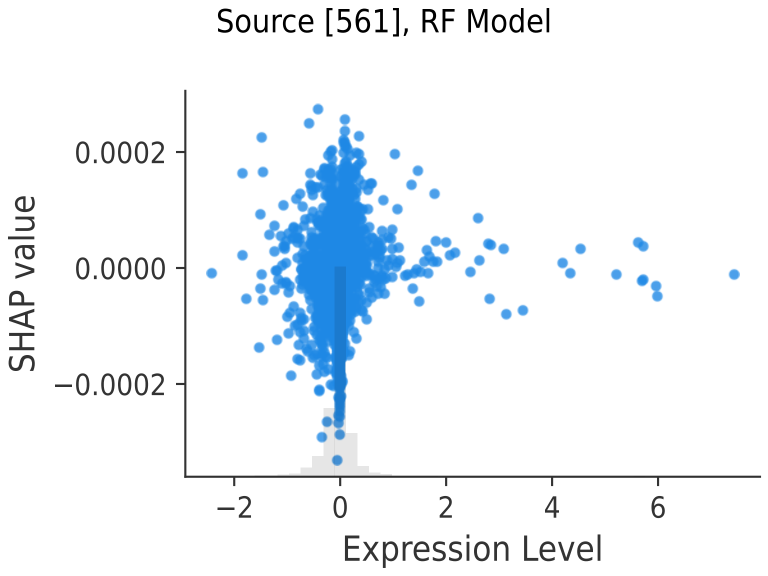

Source [561], RF Model
SHAP value
0.0002
0.0000
−0.0002
−2
0
2
4
6
Expression Level

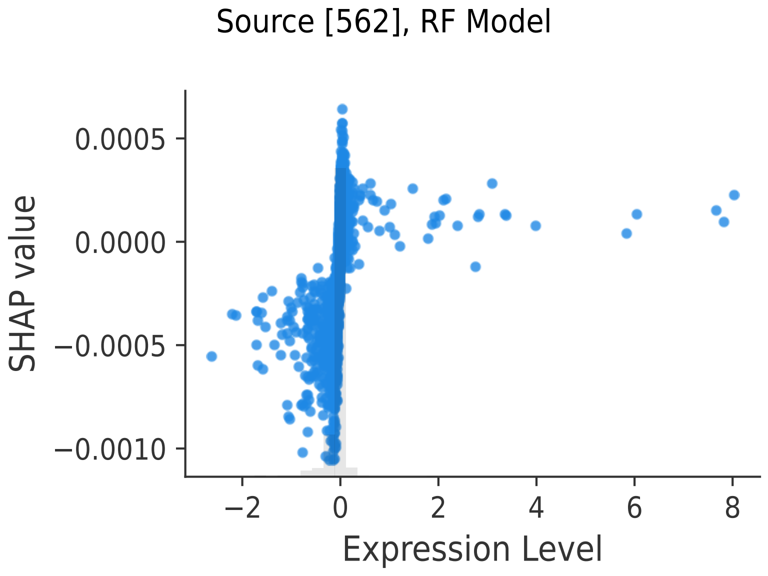

Source [562], RF Model
0.0005
0.0000
−0.0005
−0.0010
SHAP value
−2
0
2
4
6
8
Expression Level

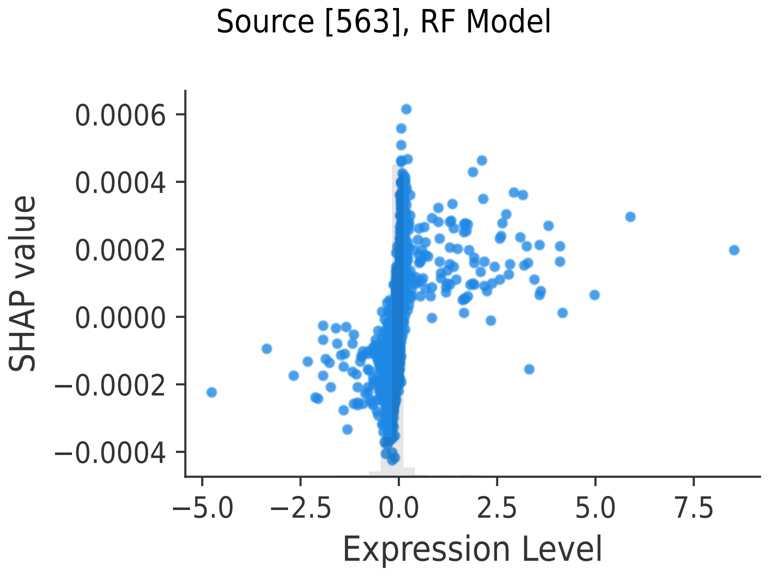

Source [563], RF Model
SHAP value
0.0006
0.0004
0.0002
0.0000
−0.0002
−0.0004
−5.0
−2.5
0.0
2.5
5.0
7.5
Expression Level

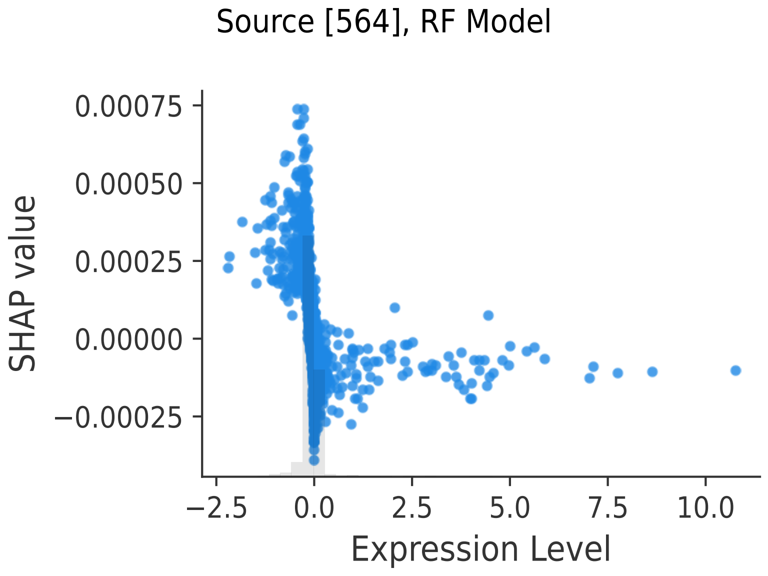
Source [564], RF Model
SHAP value
0.00075
0.00050
0.00025
0.00000
−0.00025
−2.5
0.0
2.5
5.0
7.5
10.0
Expression Level

Source [565], RF Model

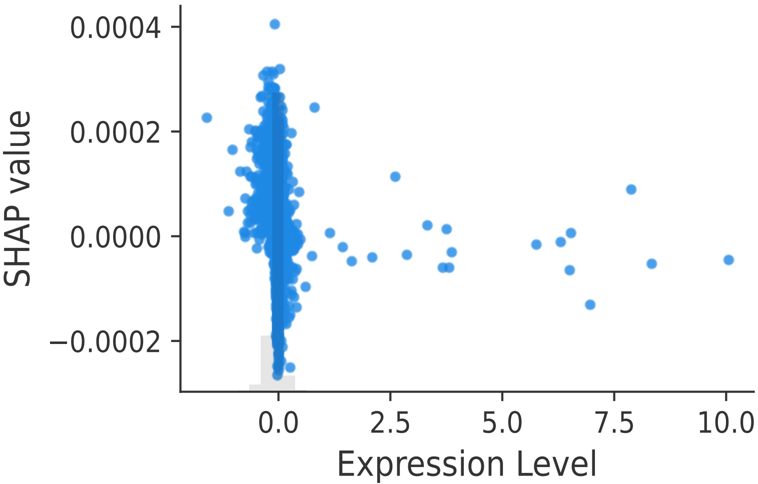

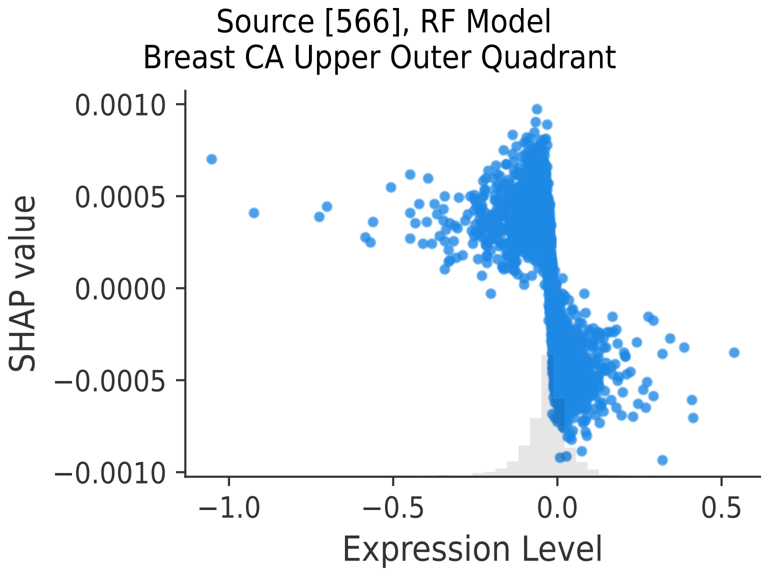
Source [566], RF Model
Breast CA Upper Outer Quadrant
0.0010
0.0005
0.0000
−0.0005
−0.0010
SHAP value
−1.0
−0.5
0.0
0.5
Expression Level

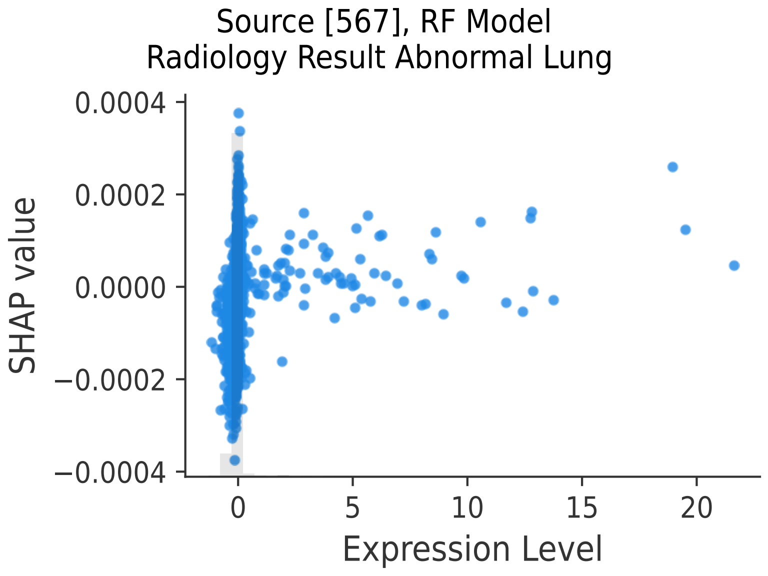
Source [567], RF Model
Radiology Result Abnormal Lung
0.0004
0.0002
0.0000
−0.0002
−0.0004
SHAP value
0
5
10
15
20
Expression Level

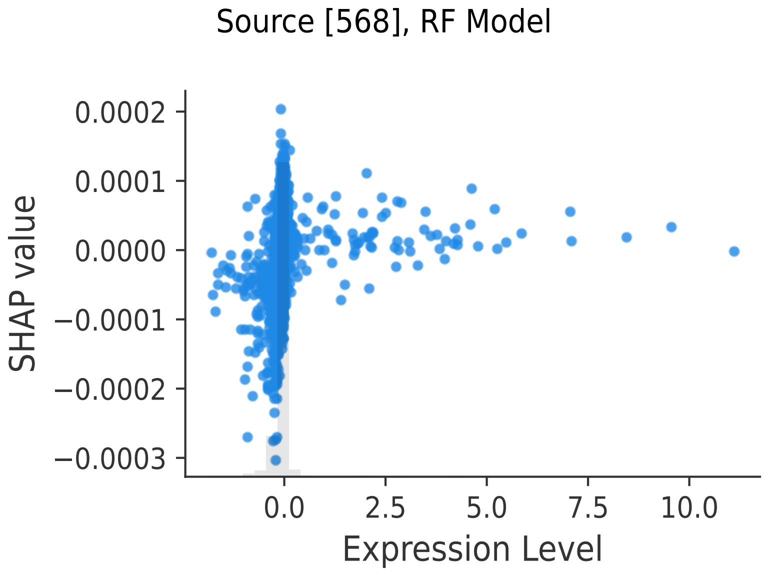

Source [568], RF Model
0.0002
0.0001
0.0000
−0.0001
−0.0002
−0.0003
SHAP value
0.0
2.5
5.0
7.5
10.0
Expression Level

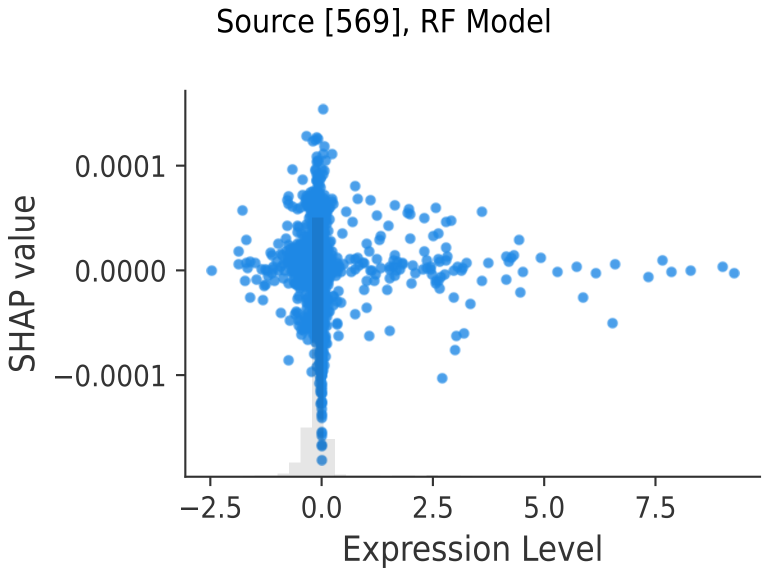

Source [569], RF Model
0.0001
0.0000
−0.0001
SHAP value
−2.5
0.0
2.5
5.0
7.5
Expression Level

Source [570], RF Model

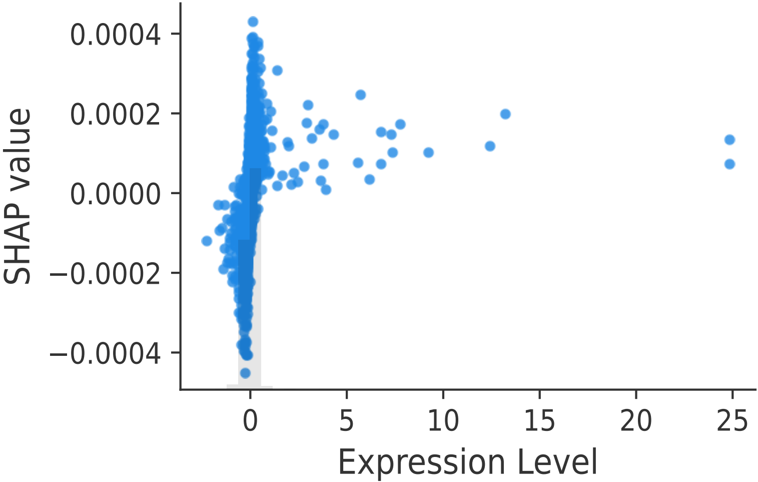

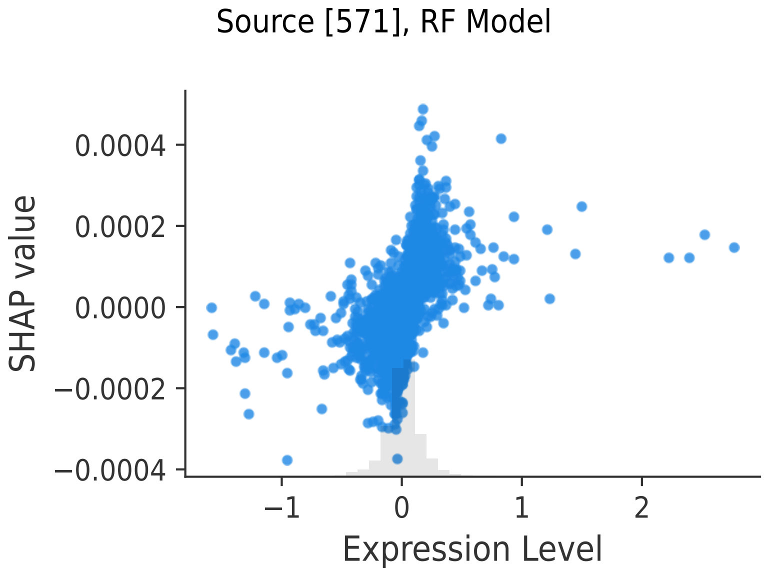
Source [571], RF Model
SHAP value
0.0004
0.0002
0.0000
−0.0002
−0.0004
−1
0
1
2
Expression Level

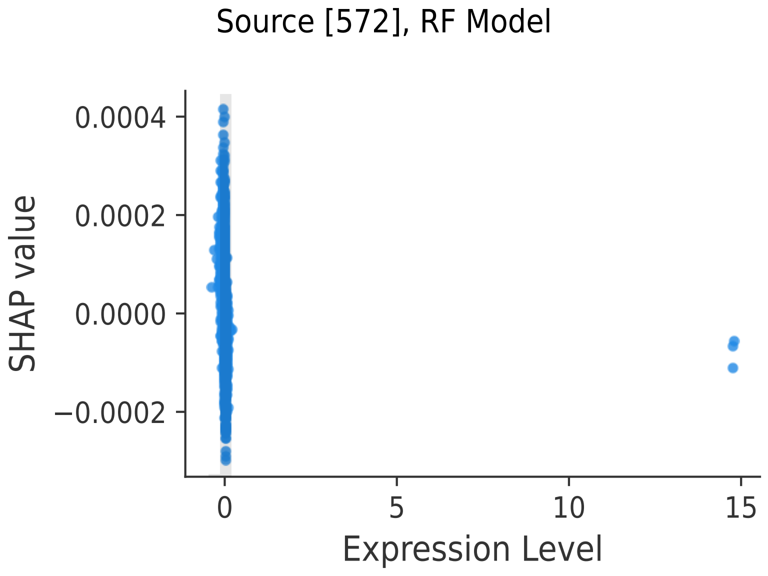

Source [572], RF Model
0.0004
0.0002
0.0000
−0.0002
SHAP value
0
5
10
15
Expression Level

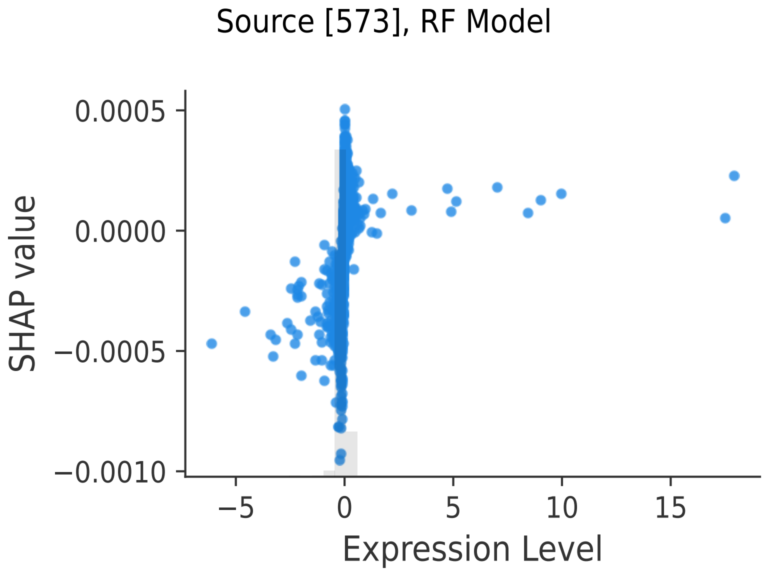

Source [573], RF Model
SHAP value
0.0005
0.0000
−0.0005
−0.0010
−5
0
5
10
15
Expression Level

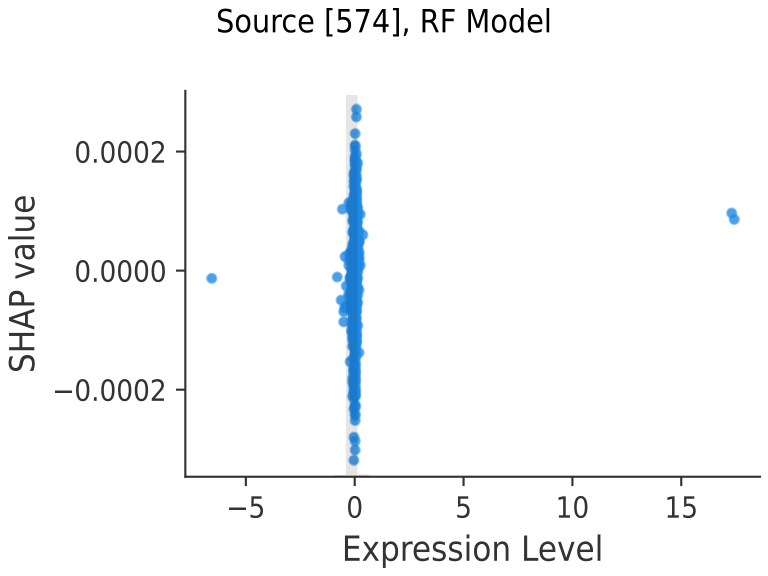
Source [574], RF Model
SHAP value
0.0002
0.0000
−0.0002
−5
0
5
10
15
Expression Level

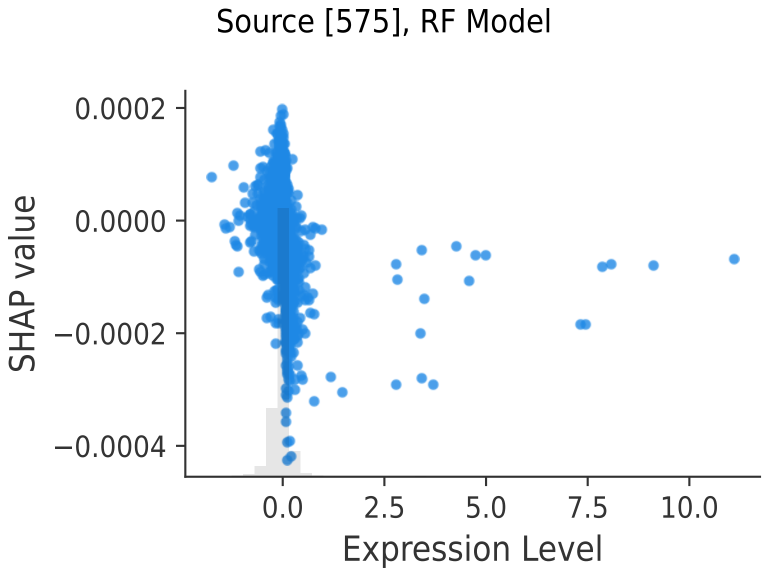
Source [575], RF Model
0.0002
0.0000
−0.0002
−0.0004
SHAP value
0.0
2.5
5.0
7.5
10.0
Expression Level

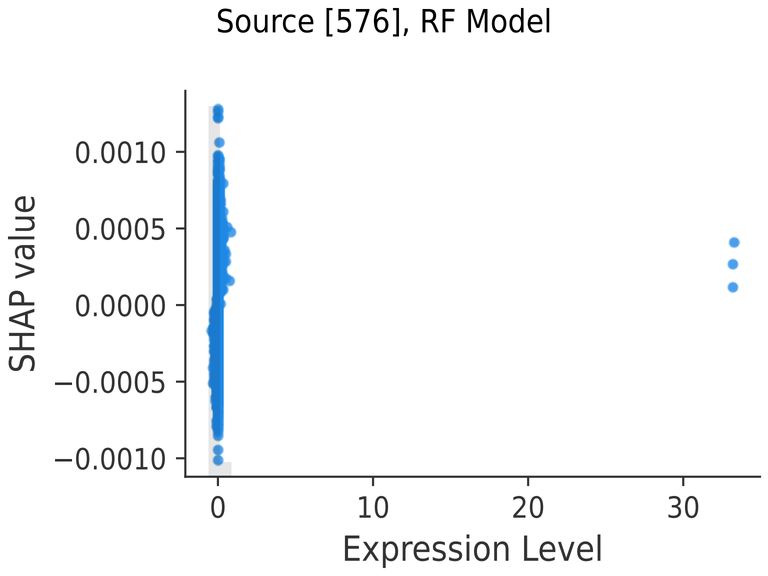
Source [576], RF Model
SHAP value
0.0010
0.0005
0.0000
−0.0005
−0.0010
0
10
20
30
Expression Level

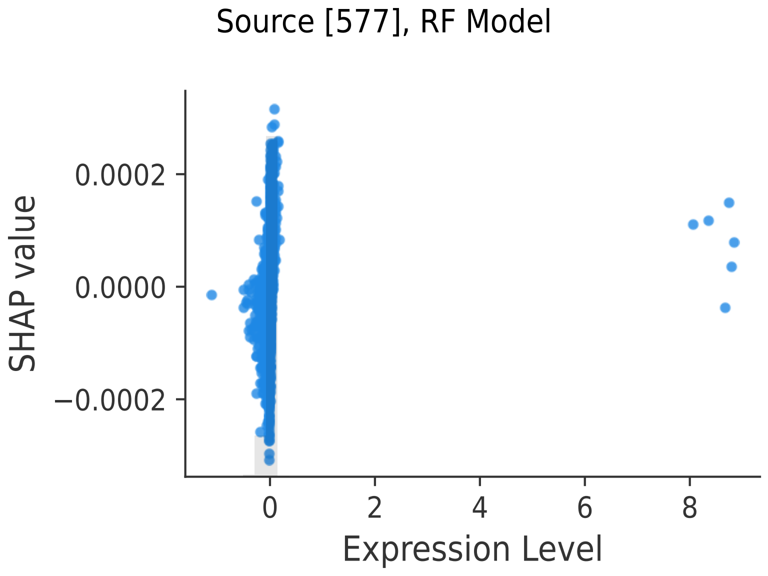
Source [577], RF Model
0.0002
0.0000
−0.0002
SHAP value
0
2
4
6
8
Expression Level

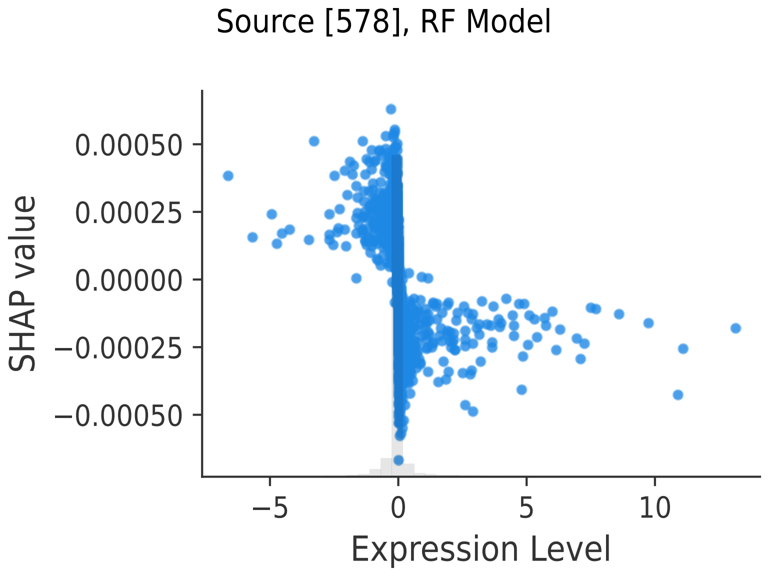
Source [578], RF Model
SHAP value
0.00050
0.00025
0.00000
−0.00025
−0.00050
−5
0
5
10
Expression Level

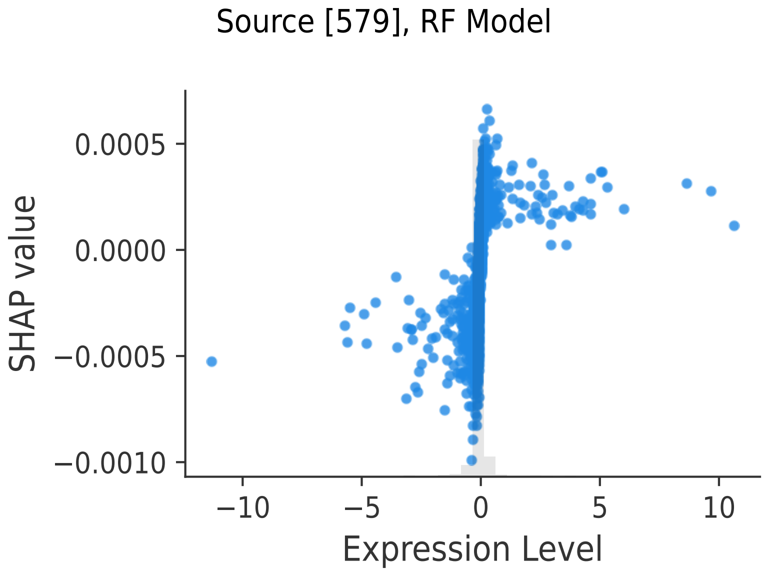
Source [579], RF Model
0.0005
0.0000
−0.0005
−0.0010
SHAP value
−10
−5
0
5
10
Expression Level

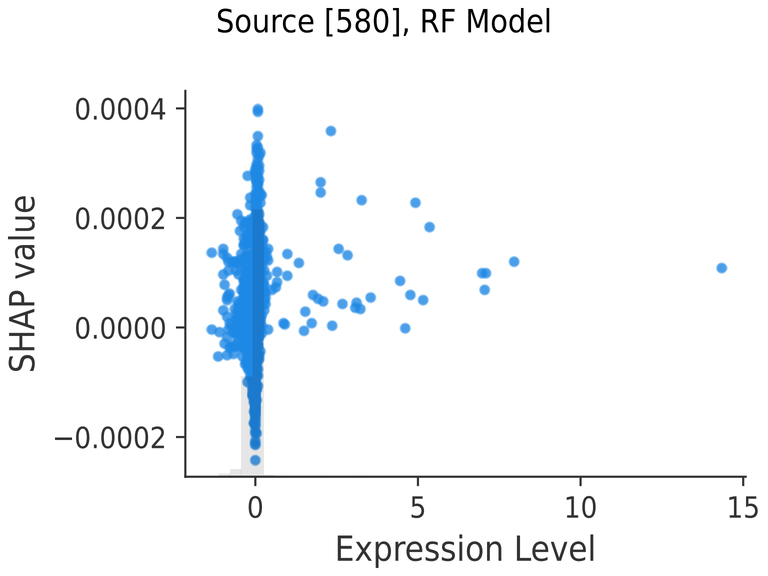

Source [580], RF Model
SHAP value
0.0004
0.0002
0.0000
−0.0002
0
5
10
15
Expression Level

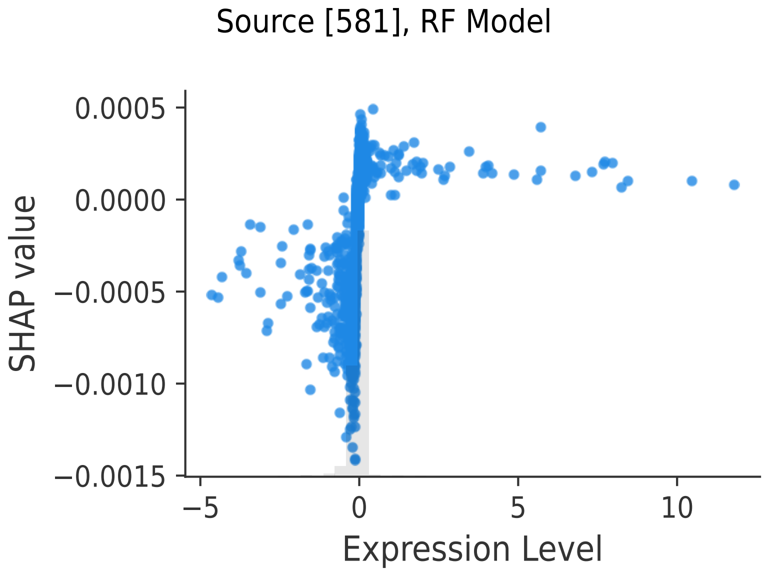

Source [581], RF Model
0.0005
0.0000
−0.0005
−0.0010
−0.0015
SHAP value
−5
0
5
10
Expression Level

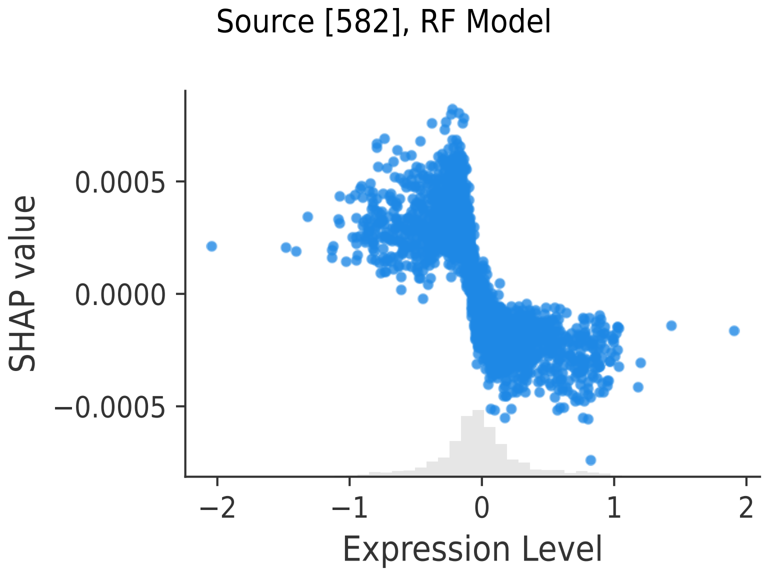
Source [582], RF Model
SHAP value
0.0005
0.0000
−0.0005
−2
−1
0
1
2
Expression Level

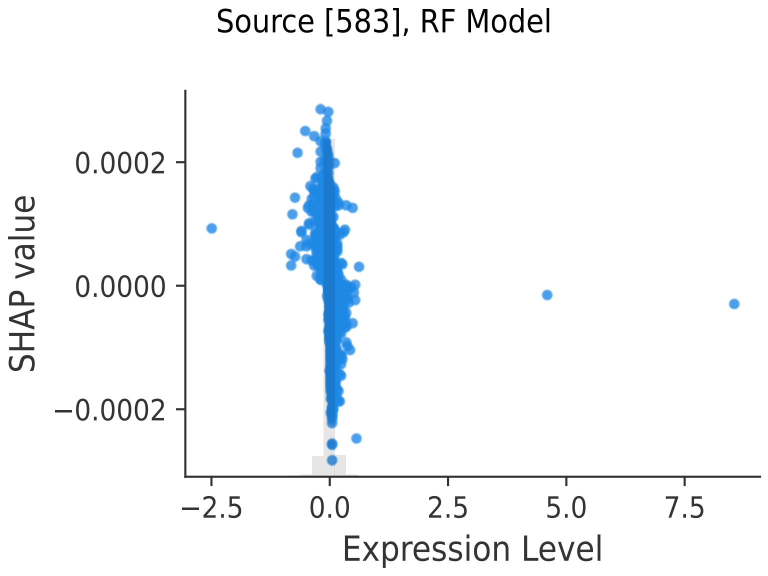

Source [583], RF Model
SHAP value
0.0002
0.0000
−0.0002
−2.5
0.0
2.5
5.0
7.5
Expression Level

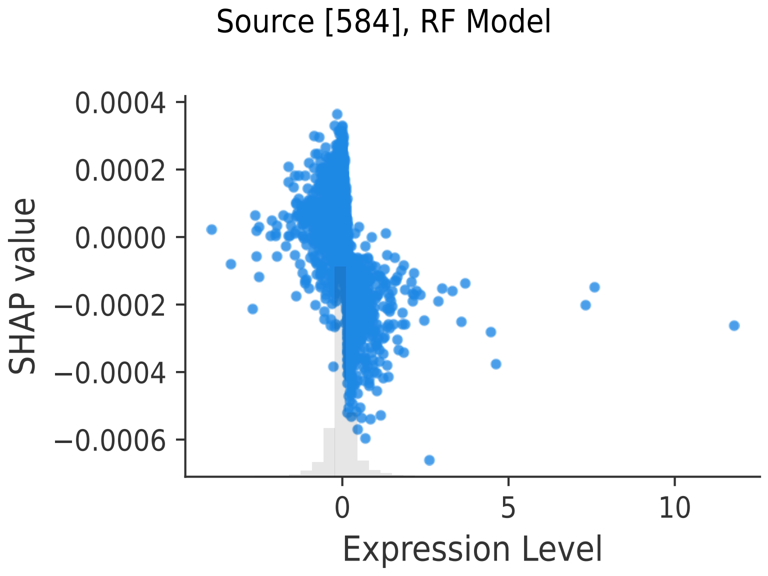

Source [584], RF Model
0.0004
0.0002
0.0000
−0.0002
−0.0004
−0.0006
SHAP value
0
5
10
Expression Level

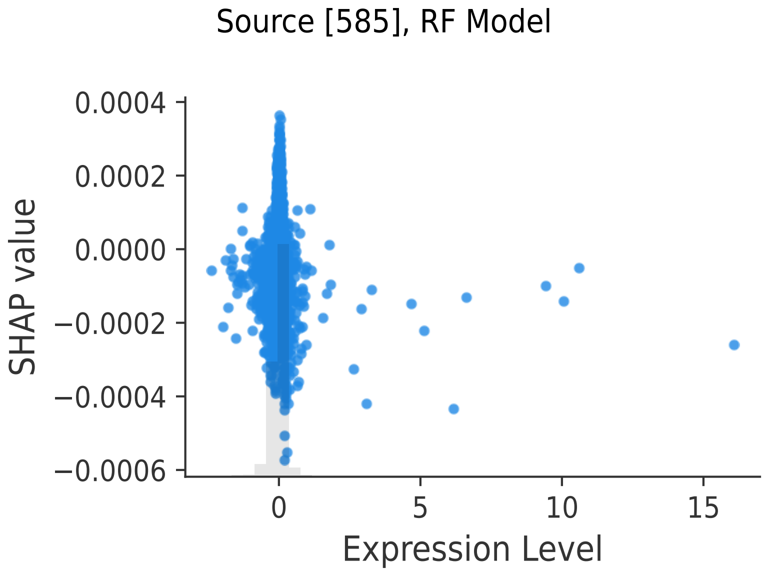
Source [585], RF Model
0.0004
0.0002
0.0000
−0.0002
−0.0004
−0.0006
SHAP value
0
5
10
15
Expression Level

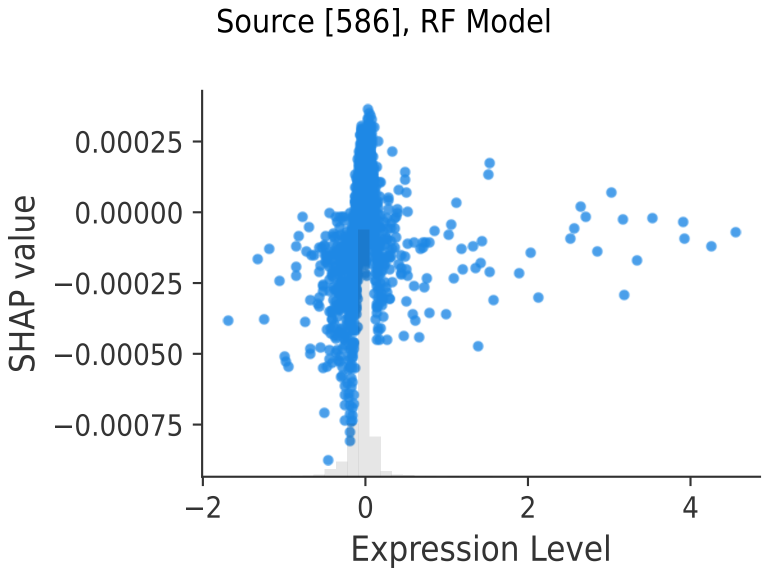
Source [586], RF Model
0.00025
0.00000
−0.00025
−0.00050
−0.00075
SHAP value
−2
0
2
4
Expression Level

Source [587], RF Model

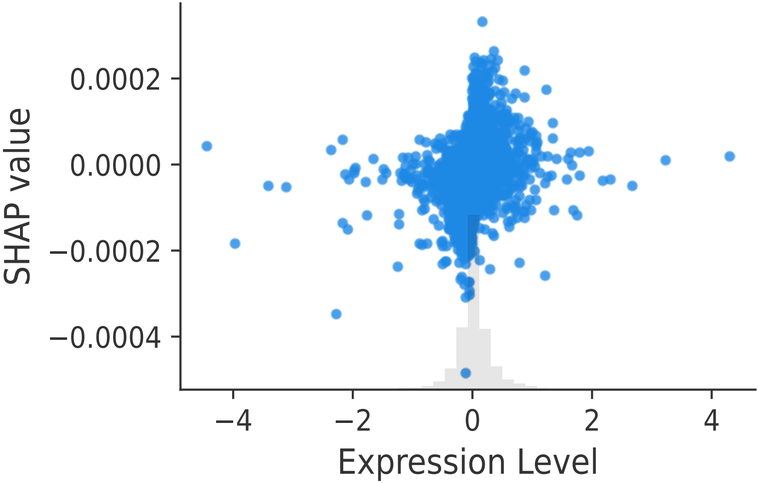

Source [588], RF Model

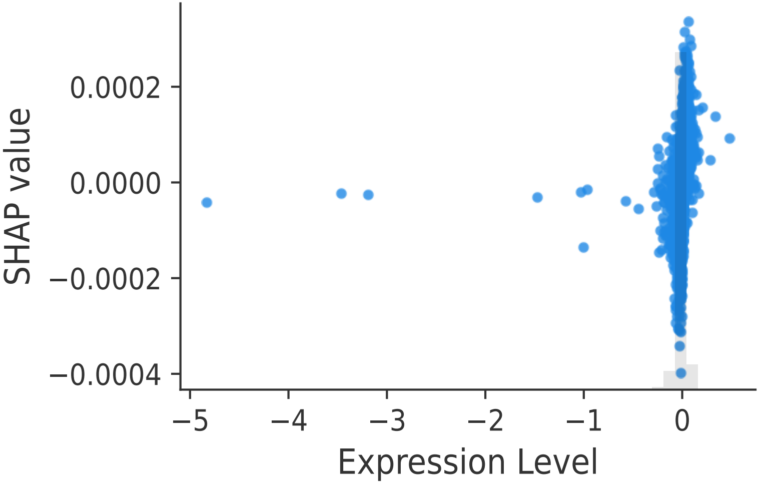

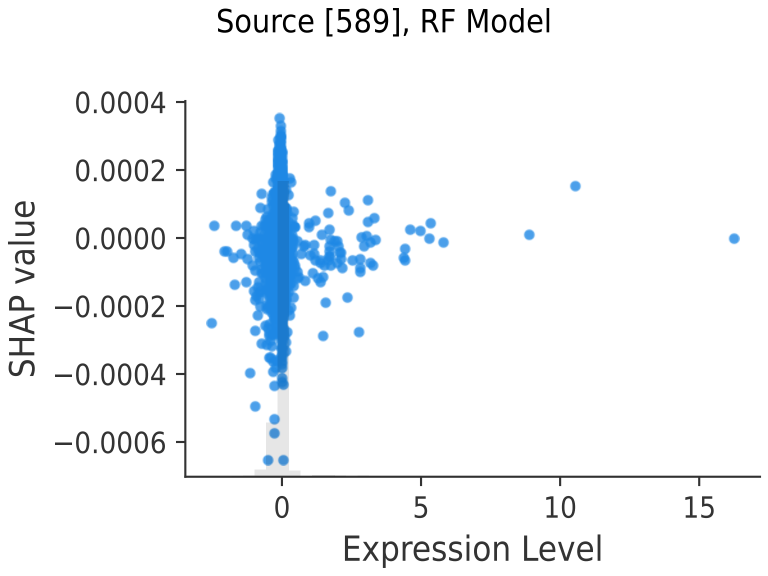
Source [589], RF Model
0.0004
0.0002
0.0000
−0.0002
−0.0004
−0.0006
SHAP value
0
5
10
15
Expression Level

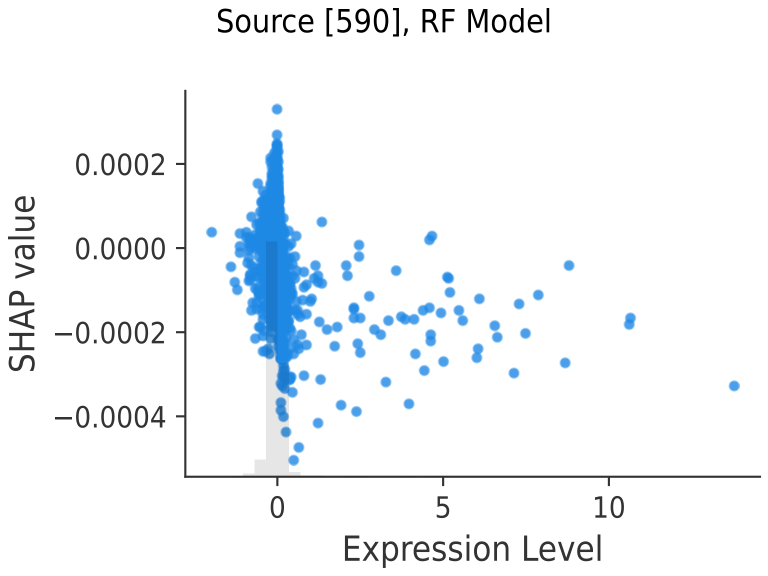
Source [590], RF Model
0.0002
0.0000
−0.0002
−0.0004
SHAP value
0
5
10
Expression Level

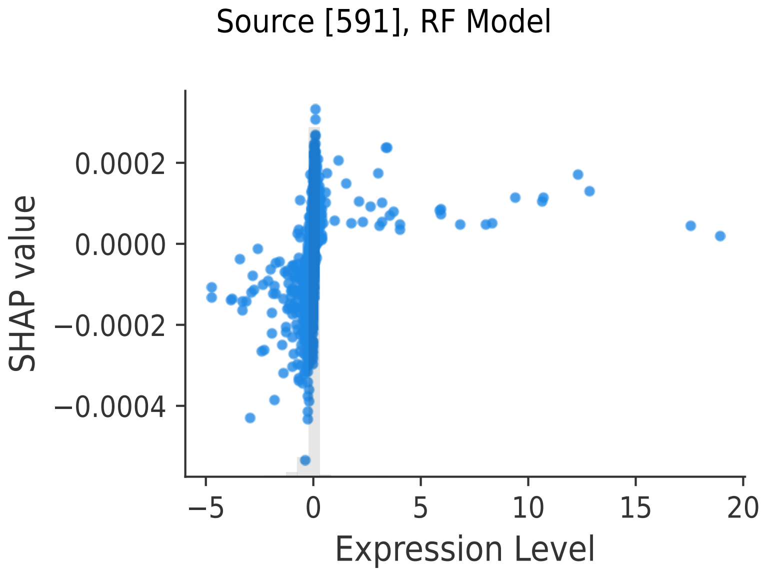
Source [591], RF Model
0.0002
0.0000
−0.0002
−0.0004
SHAP value
−5
0
5
10
15
20
Expression Level

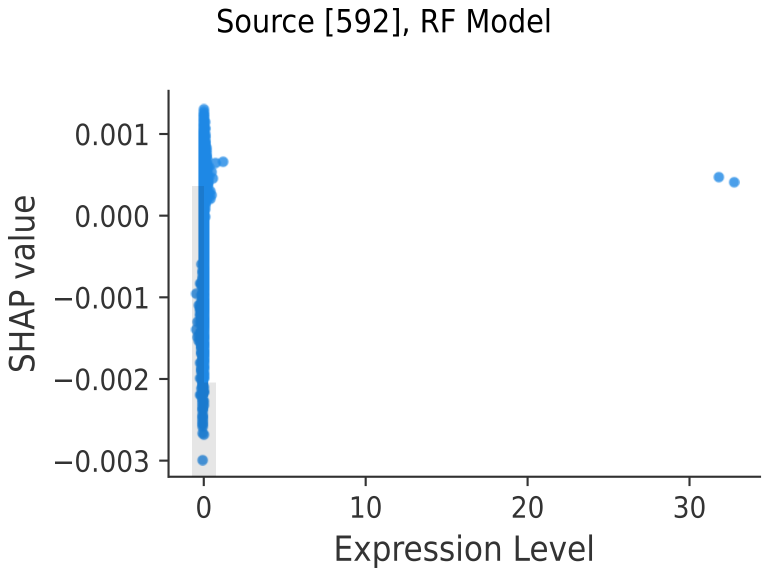
Source [592], RF Model
0.001
0.000
−0.001
−0.002
−0.003
SHAP value
0
10
20
30
Expression Level

Source [593], RF Model

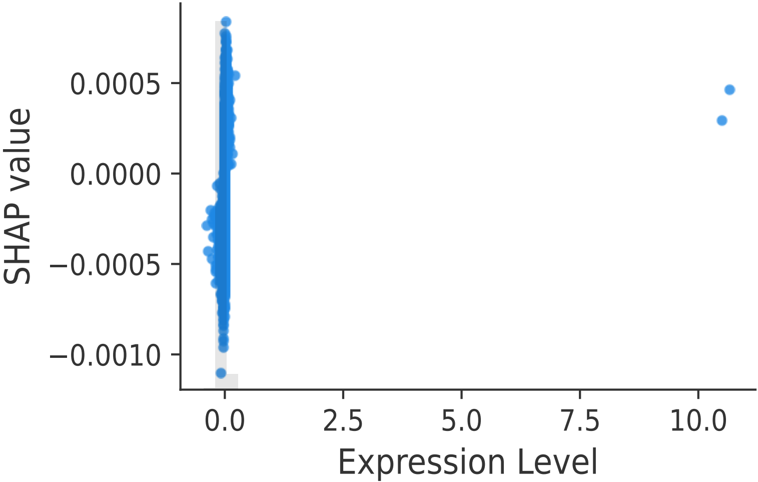

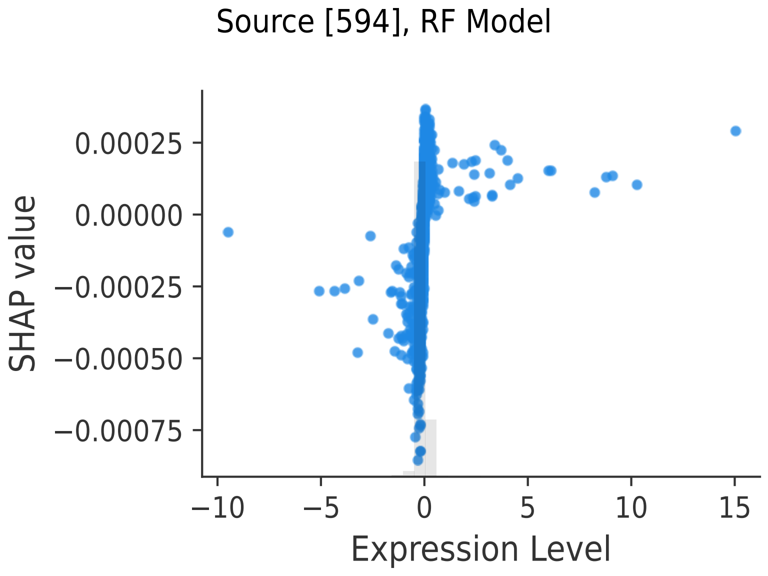
Source [594], RF Model
SHAP value
0.00025
0.00000
−0.00025
−0.00050
−0.00075
−10
−5
0
5
10
15
Expression Level

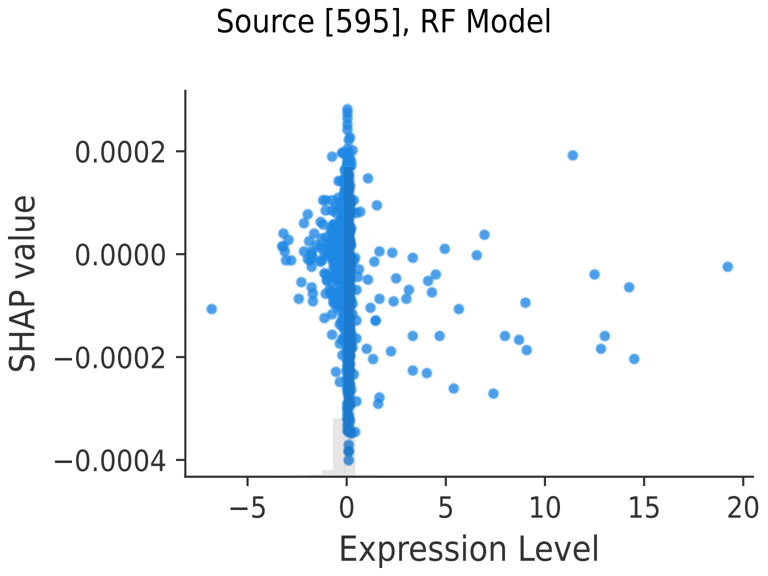

Source [595], RF Model
SHAP value
0.0002
0.0000
−0.0002
−0.0004
−5
0
5
10
15
20
Expression Level

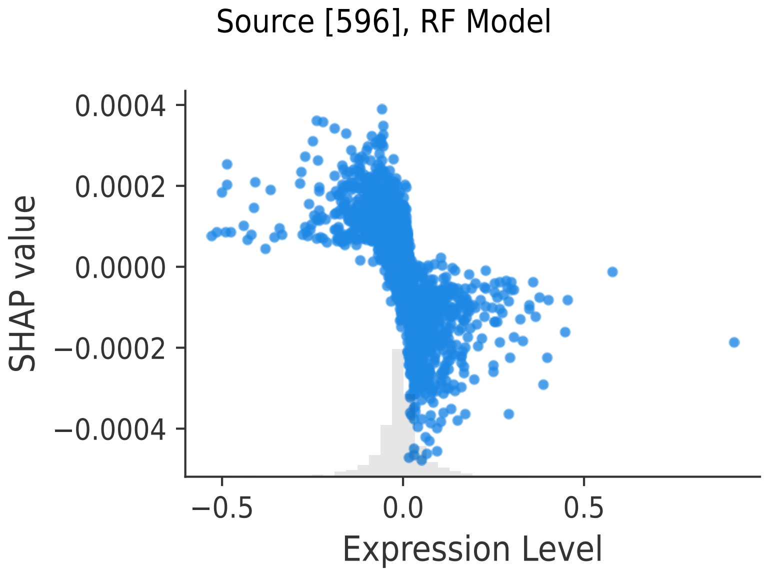

Source [596], RF Model
0.0004
0.0002
0.0000
−0.0002
−0.0004
SHAP value
−0.5
0.0
0.5
Expression Level

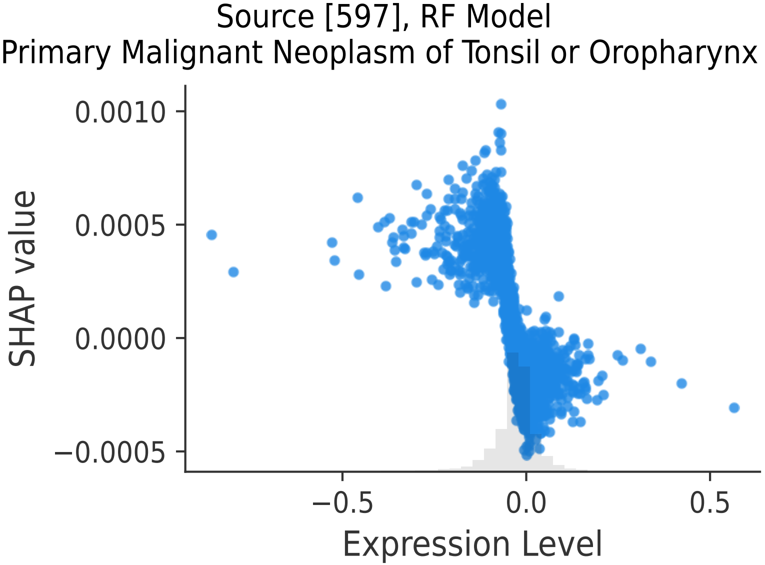

Source [597], RF Model
Primary Malignant Neoplasm of Tonsil or Oropharynx
0.0010
0.0005
0.0000
−0.0005
SHAP value
−0.5
0.0
0.5
Expression Level

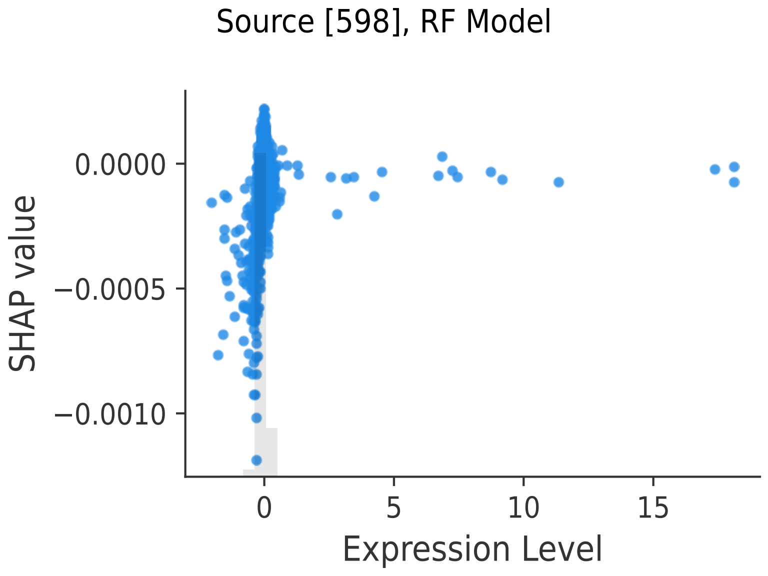

Source [598], RF Model
SHAP value
0.0000
−0.0005
−0.0010
0
5
10
15
Expression Level

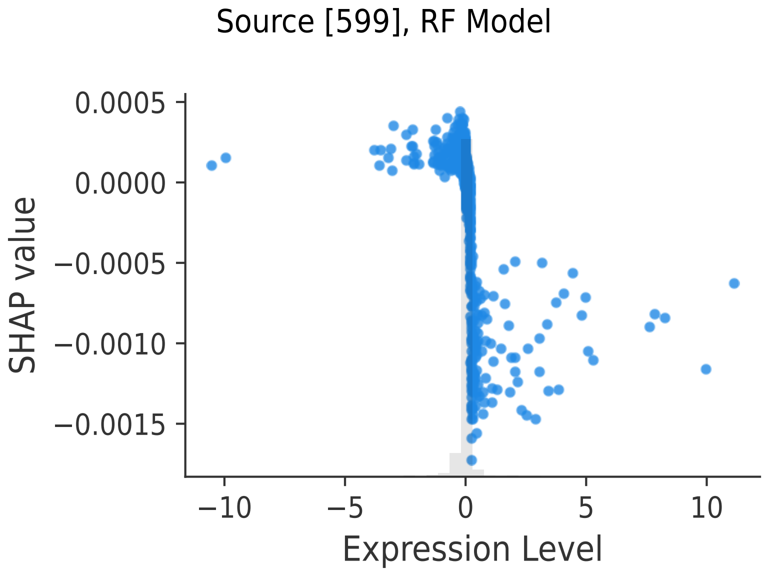

Source [599], RF Model
0.0005
0.0000
−0.0005
−0.0010
−0.0015
SHAP value
−10
−5
0
5
10
Expression Level

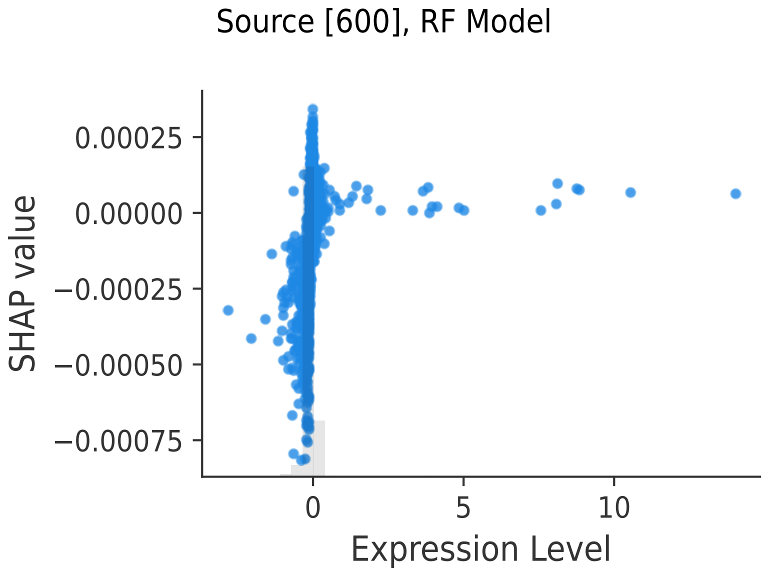
Source [600], RF Model
SHAP value
0.00025
0.00000
−0.00025
−0.00050
−0.00075
0
5
10
Expression Level

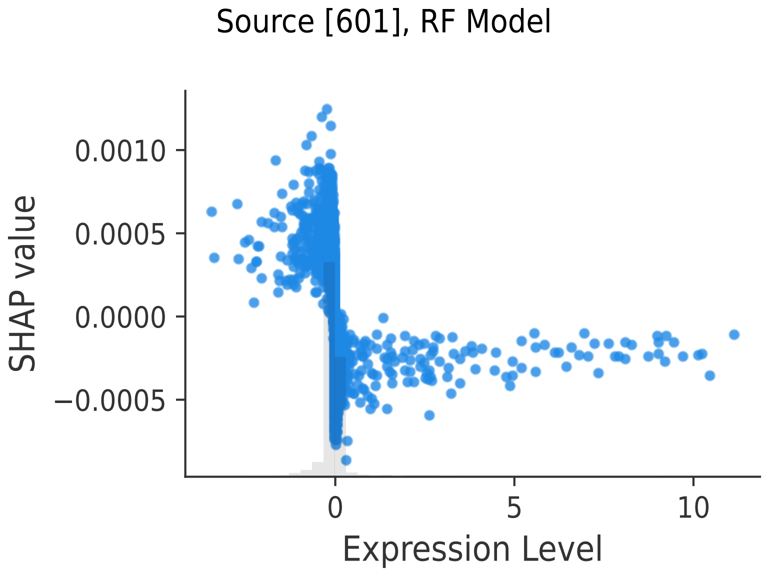
Source [601], RF Model
0.0010
0.0005
0.0000
−0.0005
SHAP value
0
5
10
Expression Level

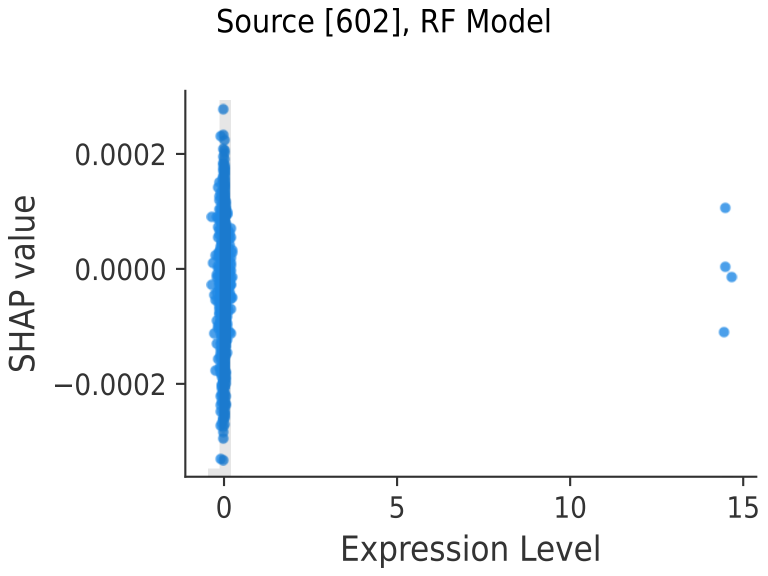

Source [602], RF Model
0.0002
0.0000
−0.0002
SHAP value
0
5
10
15
Expression Level

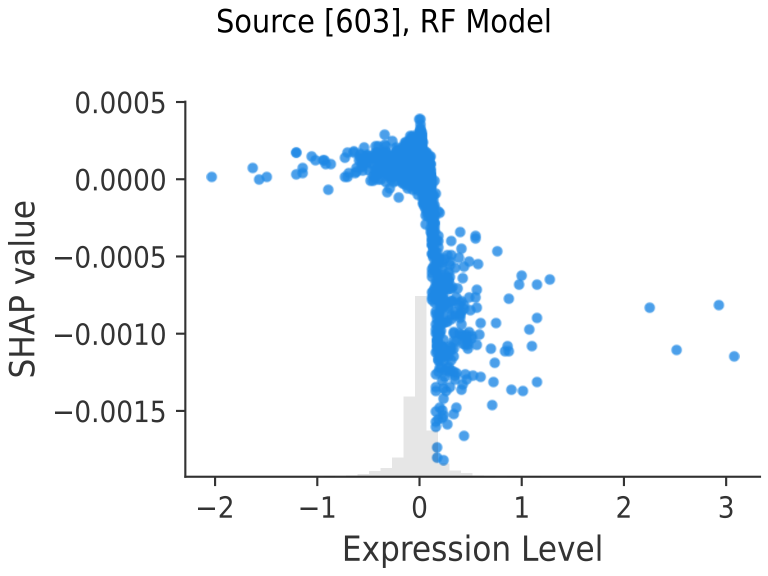
Source [603], RF Model
0.0005
0.0000
−0.0005
−0.0010
−0.0015
SHAP value
−2
−1
0
1
2
3
Expression Level

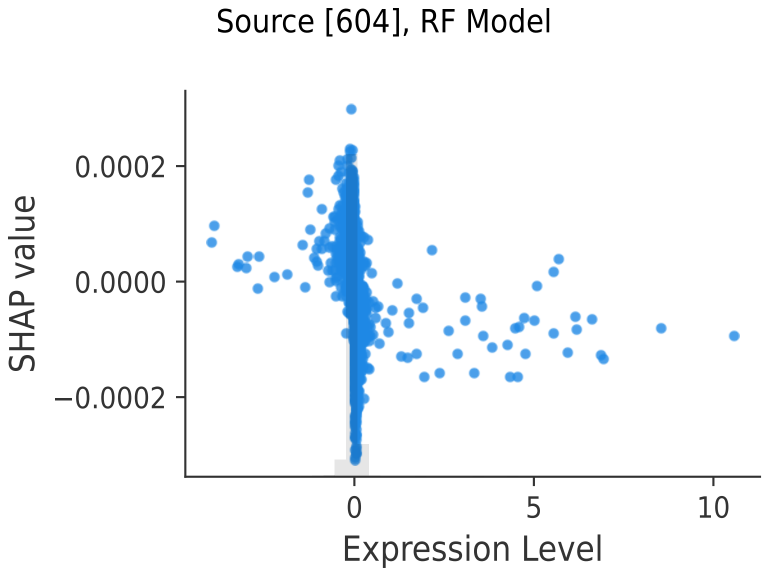
Source [604], RF Model
0.0002
0.0000
−0.0002
SHAP value
0
5
10
Expression Level

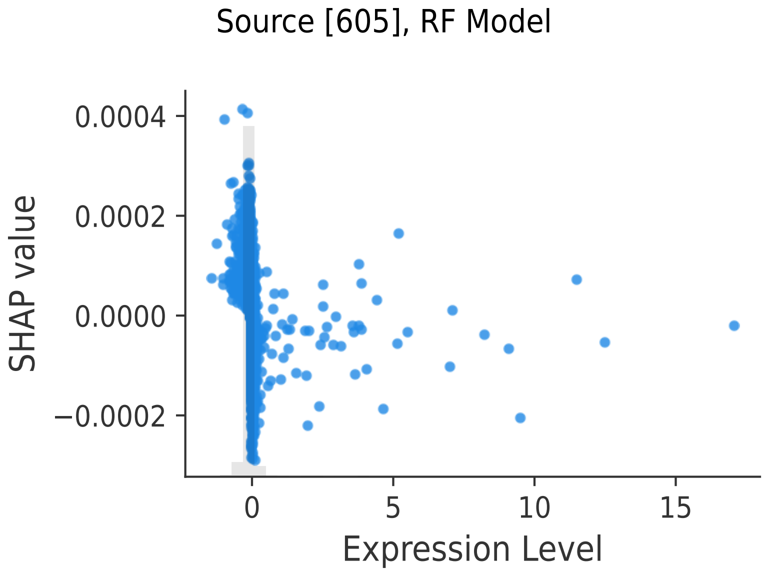
Source [605], RF Model
SHAP value
0.0004
0.0002
0.0000
−0.0002
0
5
10
15
Expression Level

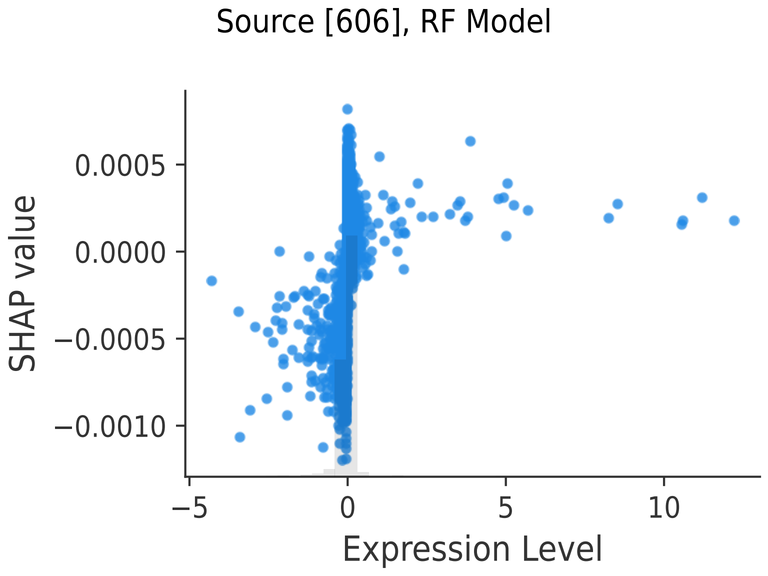
Source [606], RF Model
SHAP value
0.0005
0.0000
−0.0005
−0.0010
−5
0
5
10
Expression Level

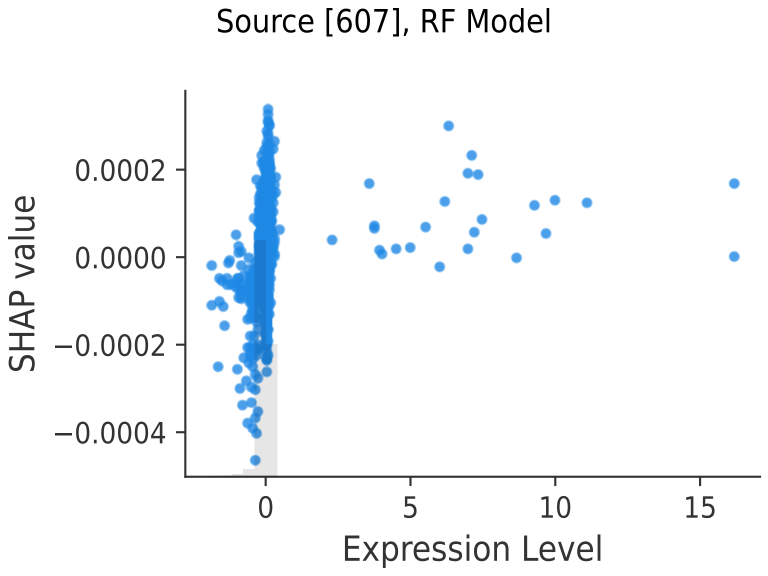

Source [607], RF Model
0.0002
0.0000
−0.0002
−0.0004
SHAP value
0
5
10
15
Expression Level

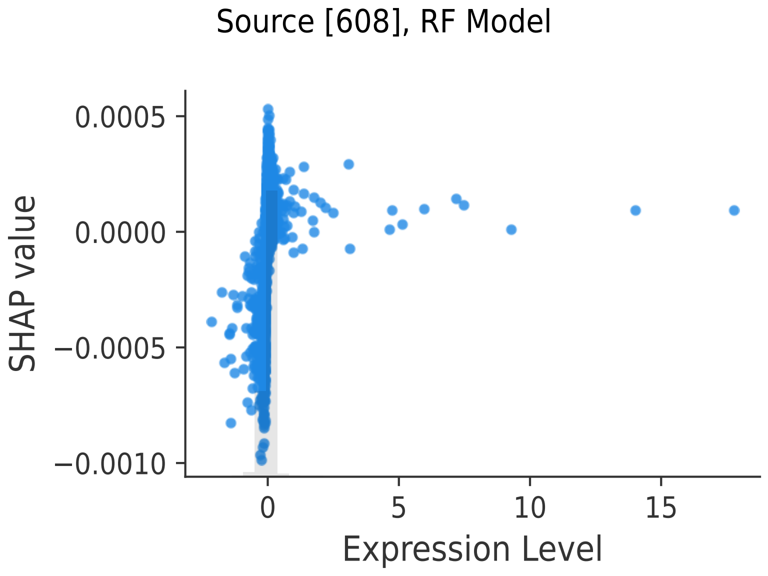

Source [608], RF Model
SHAP value
0.0005
0.0000
−0.0005
−0.0010
0
5
10
15
Expression Level

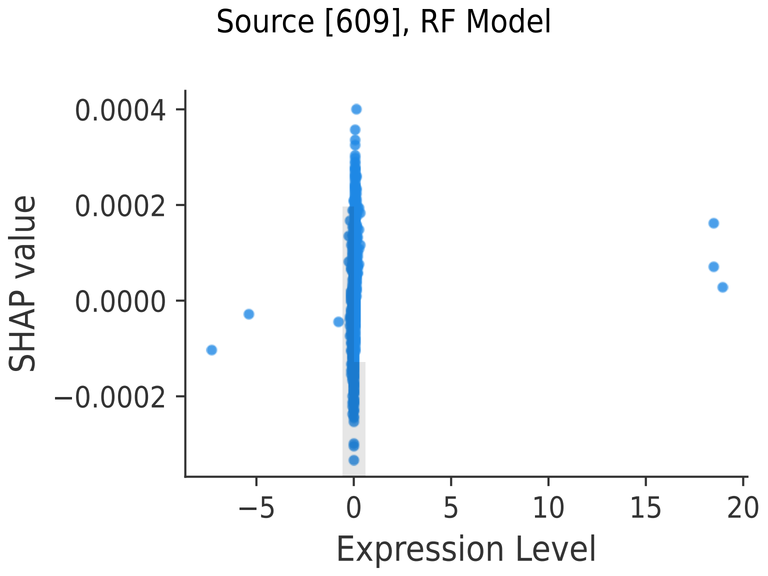

Source [609], RF Model
SHAP value
0.0004
0.0002
0.0000
−0.0002
−5
0
5
10
15
20
Expression Level

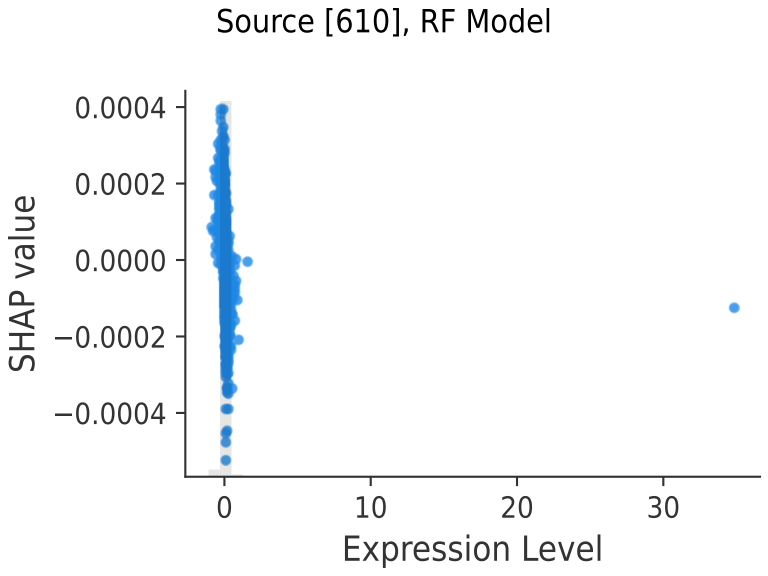
Source [610], RF Model
SHAP value
0.0004
0.0002
0.0000
−0.0002
−0.0004
0
10
20
30
Expression Level

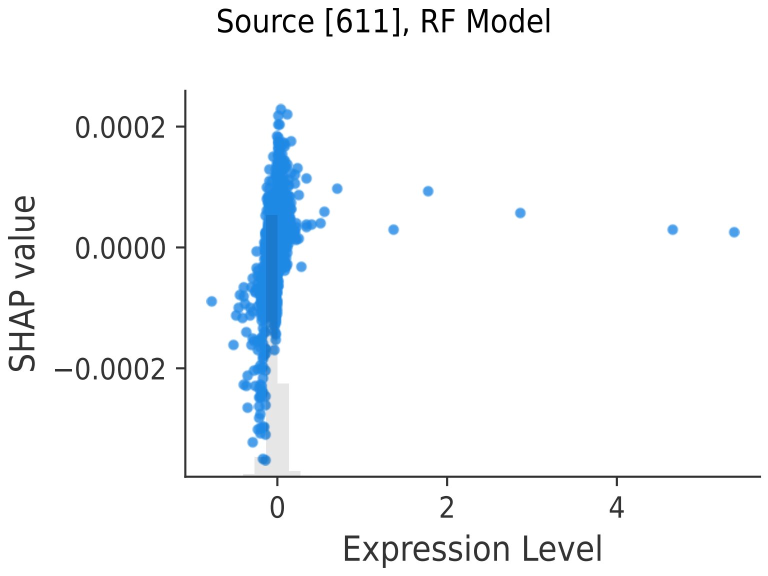
Source [611], RF Model
SHAP value
0.0002
0.0000
−0.0002
0
2
4
Expression Level

Source [612], RF Model

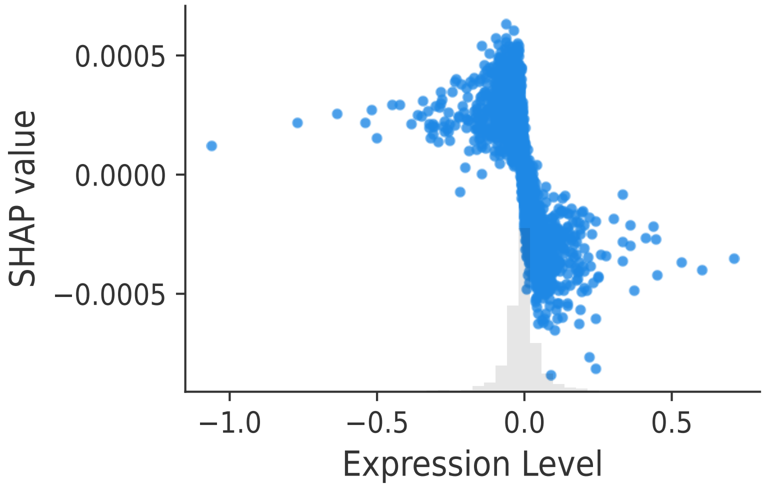

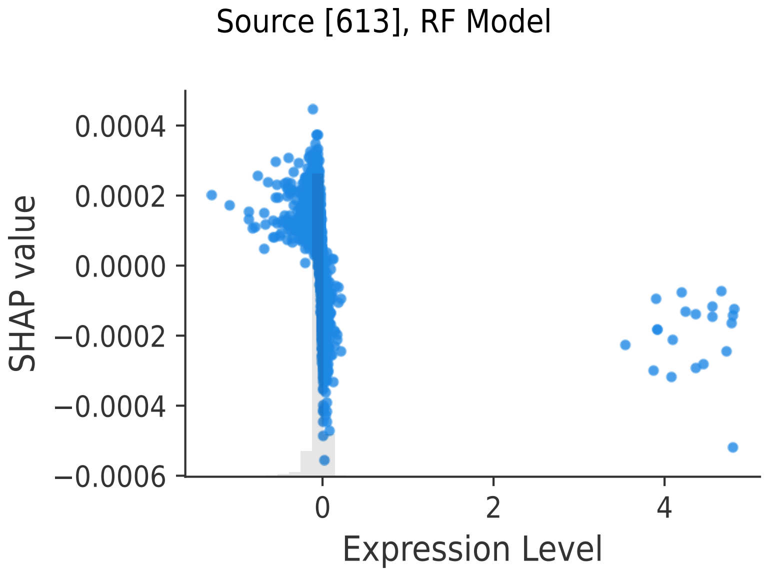
Source [613], RF Model
SHAP value
0.0004
0.0002
0.0000
−0.0002
−0.0004
−0.0006
0
2
4
Expression Level

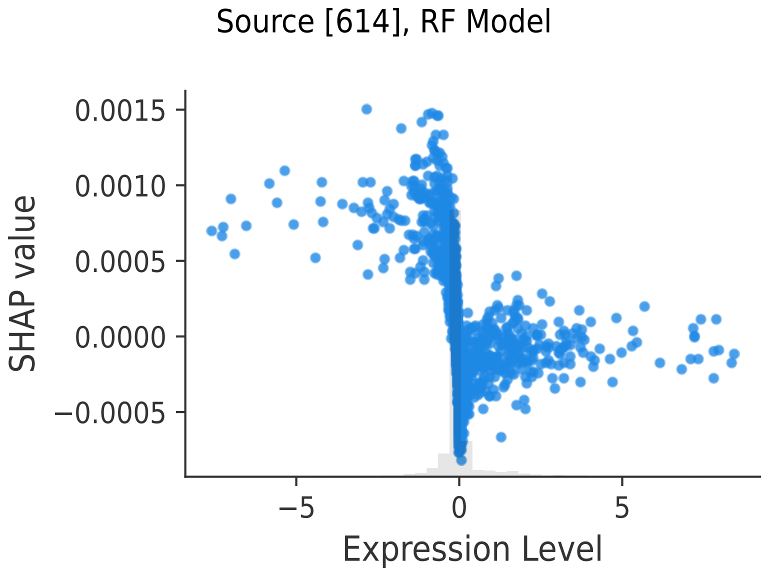
Source [614], RF Model
0.0015
0.0010
0.0005
0.0000
−0.0005
SHAP value
−5
0
5
Expression Level

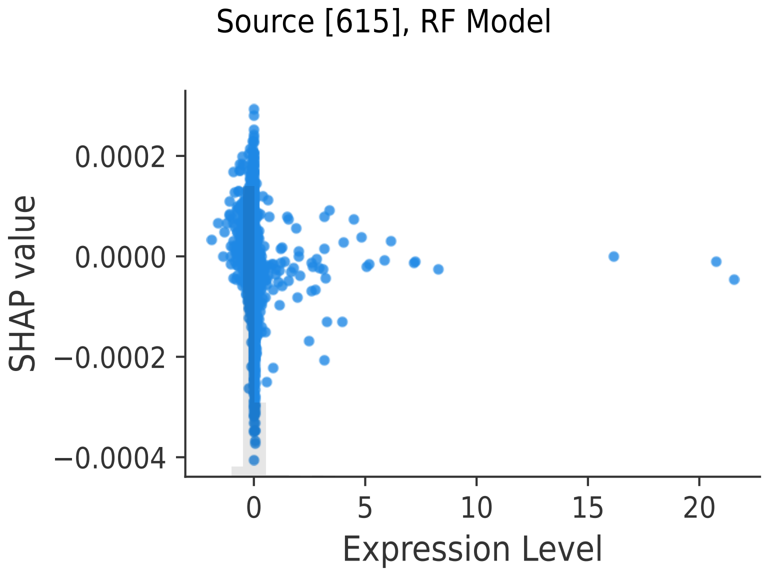

Source [615], RF Model
0.0002
0.0000
−0.0002
−0.0004
SHAP value
0
5
10
15
20
Expression Level

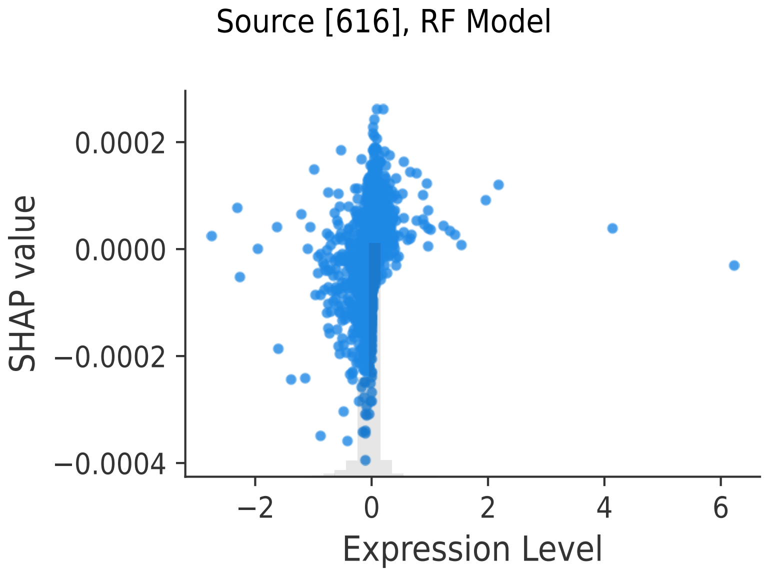

Source [616], RF Model
0.0002
0.0000
−0.0002
−0.0004
SHAP value
−2
0
2
4
6
Expression Level

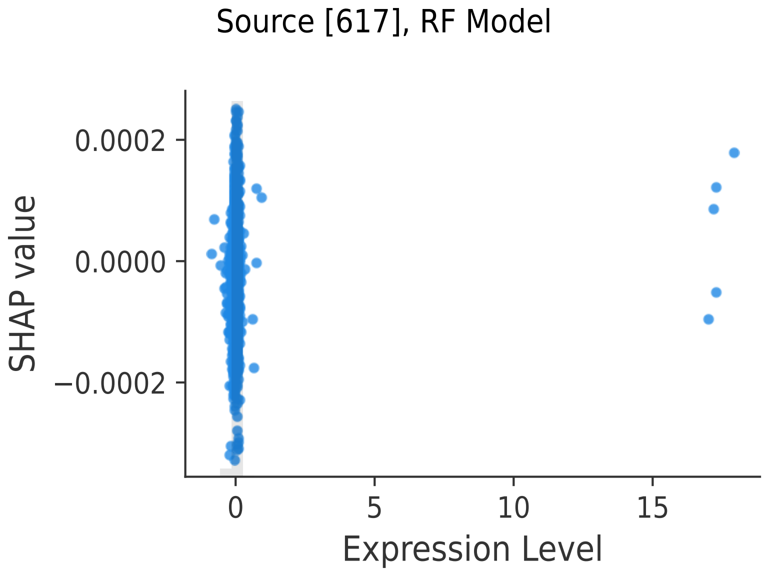

Source [617], RF Model
SHAP value
0.0002
0.0000
−0.0002
0
5
10
15
Expression Level

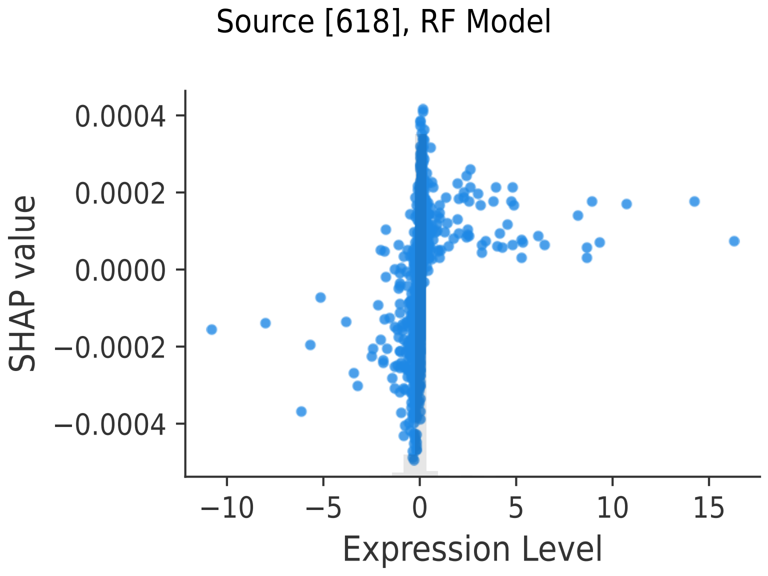

Source [618], RF Model
0.0004
0.0002
0.0000
−0.0002
−0.0004
SHAP value
−10
−5
0
5
10
15
Expression Level

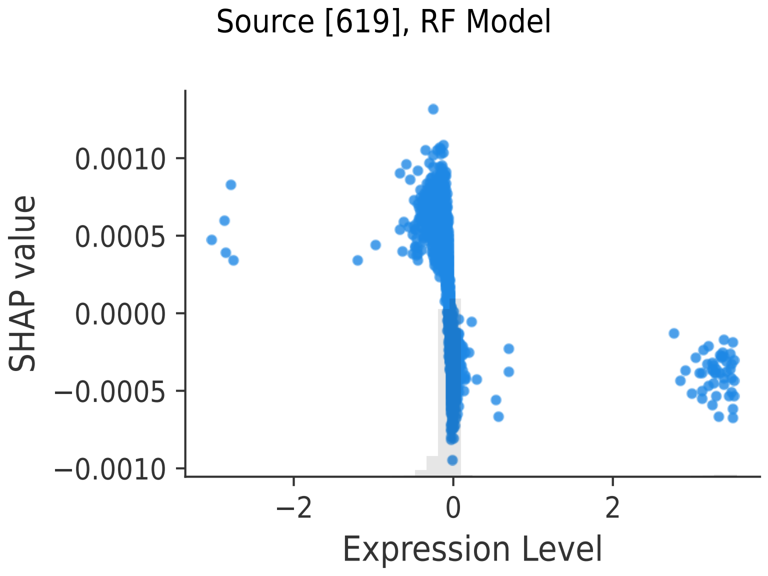

Source [619], RF Model
SHAP value
0.0010
0.0005
0.0000
−0.0005
−0.0010
−2
0
2
Expression Level

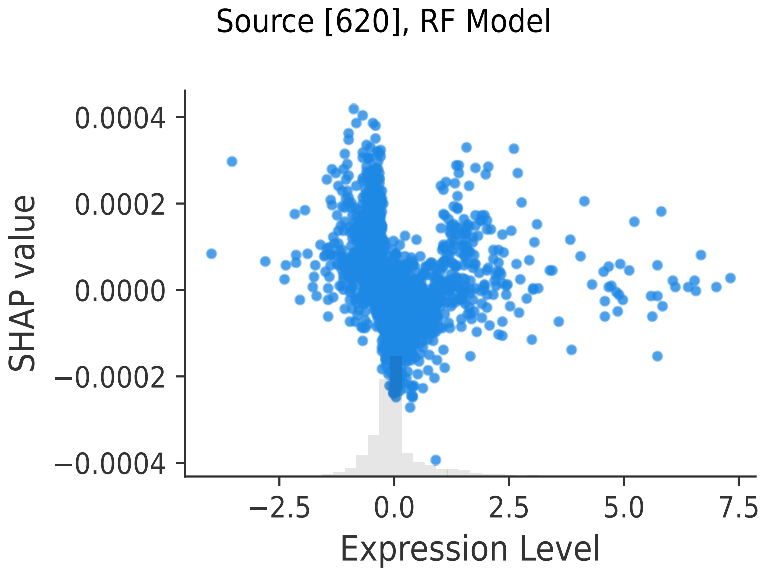
Source [620], RF Model
SHAP value
0.0004
0.0002
0.0000
−0.0002
−0.0004
−2.5
0.0
2.5
5.0
7.5
Expression Level

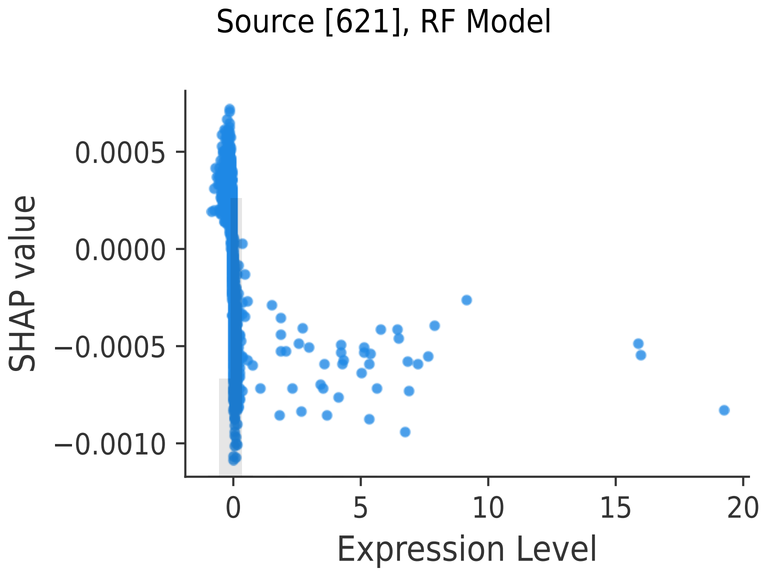
Source [621], RF Model
SHAP value
0.0005
0.0000
−0.0005
−0.0010
Expression Level
0
5
10
15
20

Source [622], RF Model

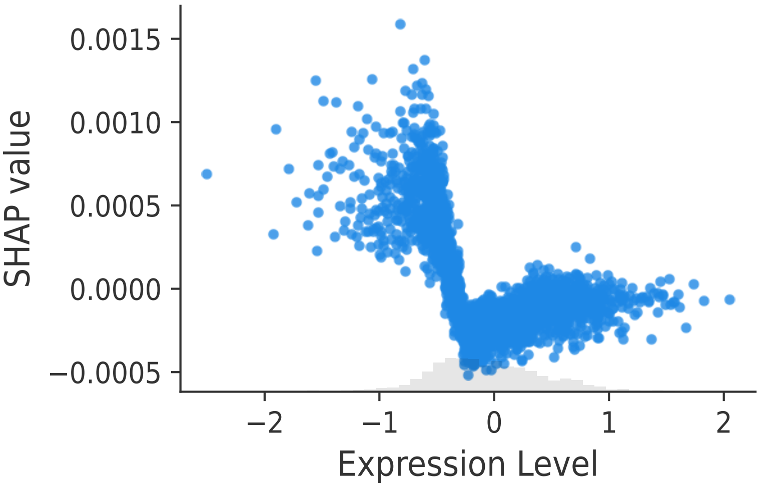

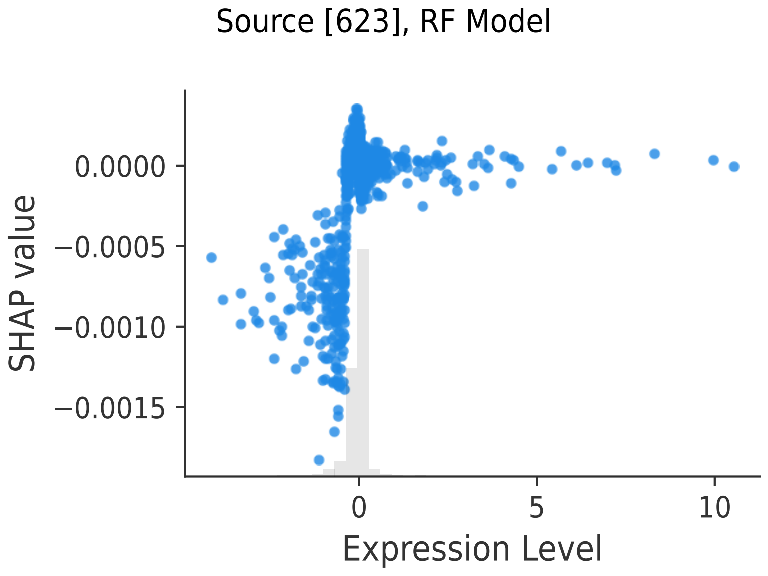

Source [623], RF Model
SHAP value
0.0000
−0.0005
−0.0010
−0.0015
0
5
10
Expression Level

Source [624], RF Model
Adult Cachexia with Protein-Calorie Malnutrition

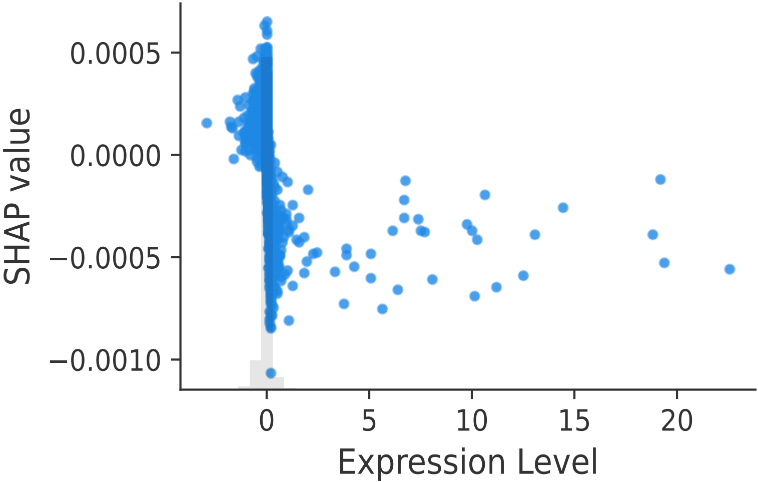

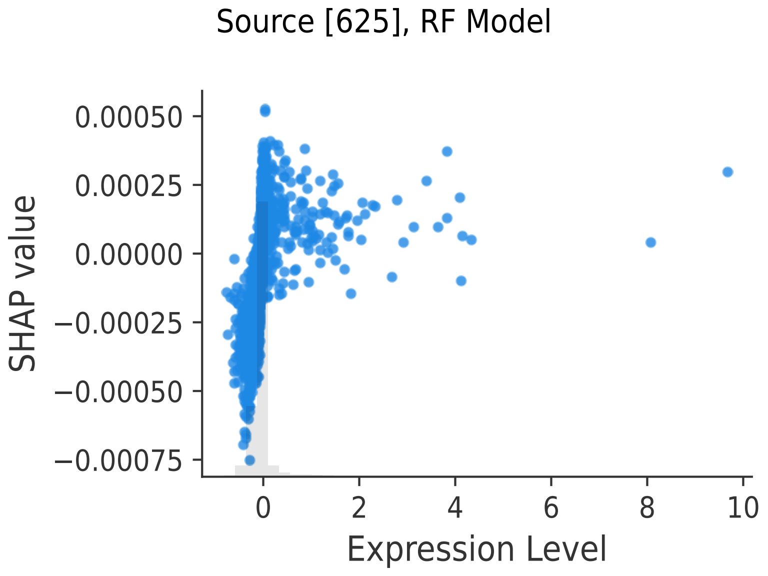

Source [625], RF Model
SHAP value
0.00050
0.00025
0.00000
−0.00025
−0.00050
−0.00075
0
2
4
6
8
10
Expression Level

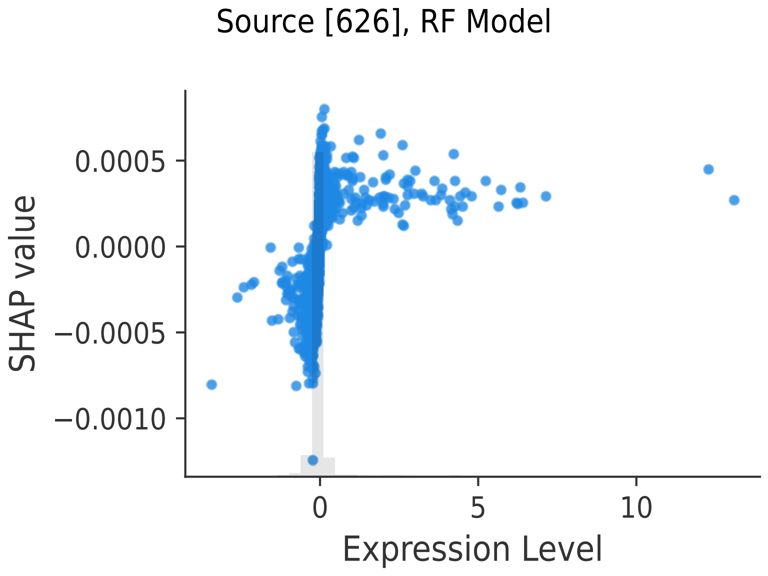

Source [626], RF Model
0.0005
0.0000
−0.0005
−0.0010
SHAP value
0
5
10
Expression Level

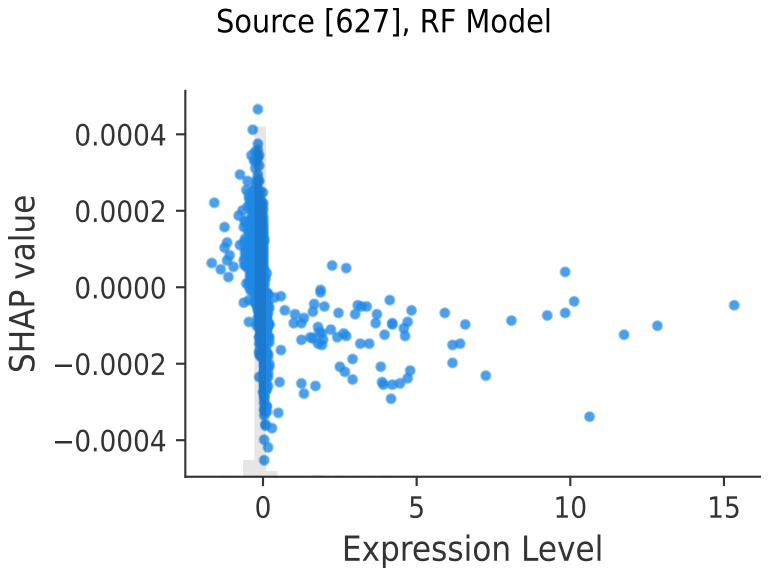

Source [627], RF Model
0.0004
0.0002
0.0000
−0.0002
−0.0004
SHAP value
0
5
10
15
Expression Level

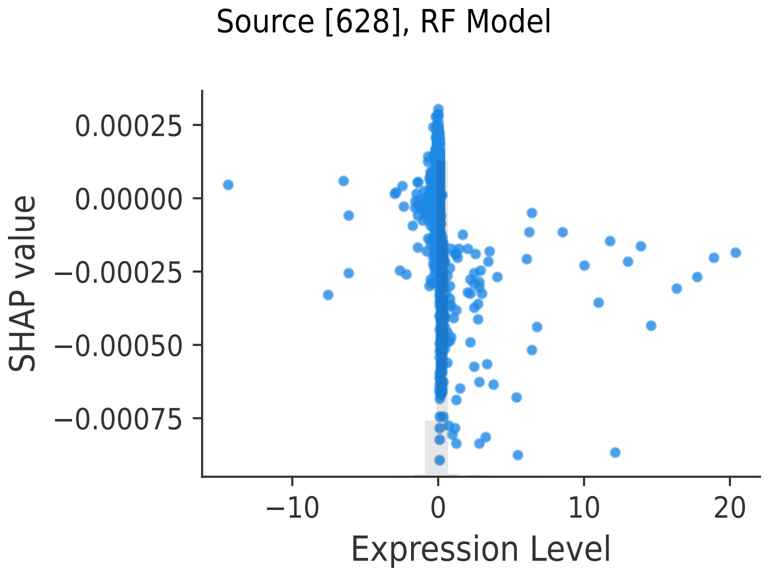

Source [628], RF Model
0.00025
0.00000
−0.00025
−0.00050
−0.00075
SHAP value
−10
0
10
20
Expression Level

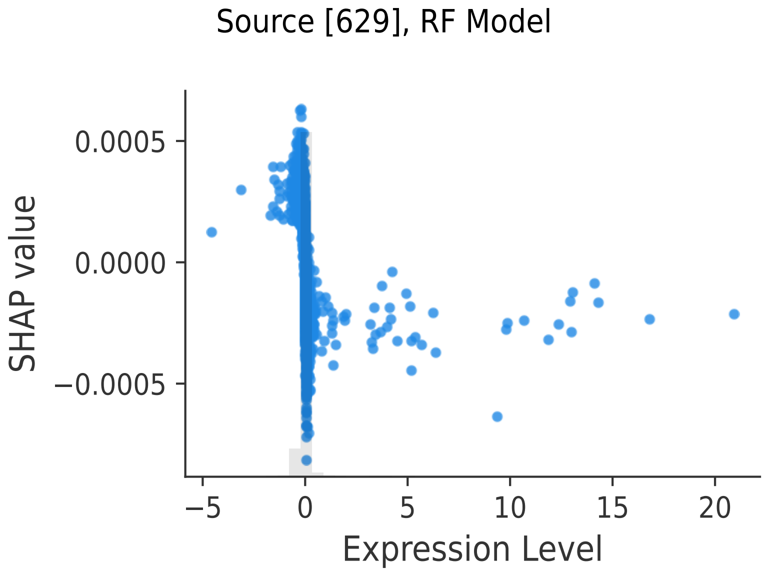

Source [629], RF Model
0.0005
0.0000
−0.0005
SHAP value
−5
0
5
10
15
20
Expression Level

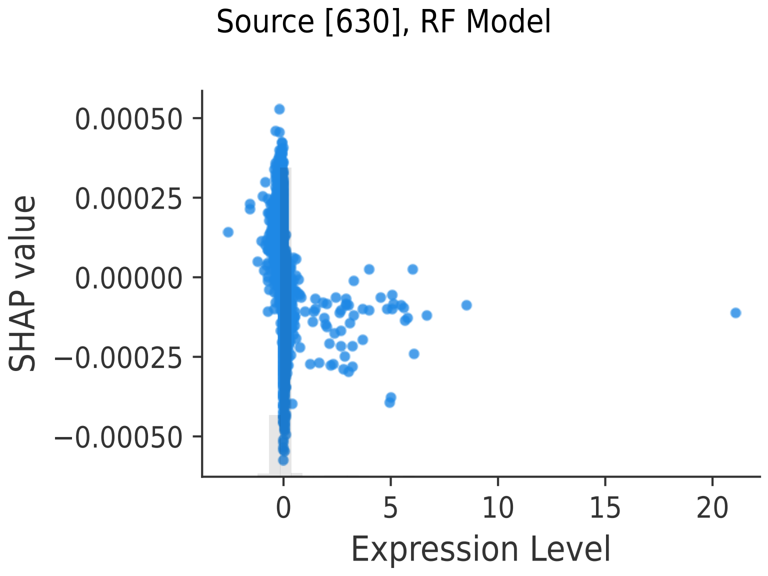

Source [630], RF Model
SHAP value
0.00050
0.00025
0.00000
−0.00025
−0.00050
0
5
10
15
20
Expression Level

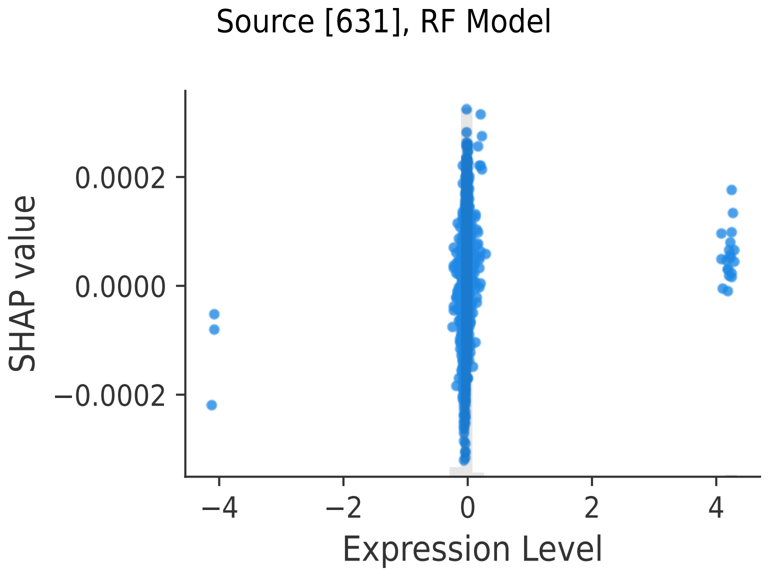

Source [631], RF Model
SHAP value
0.0002
0.0000
−0.0002
−4
−2
0
2
4
Expression Level

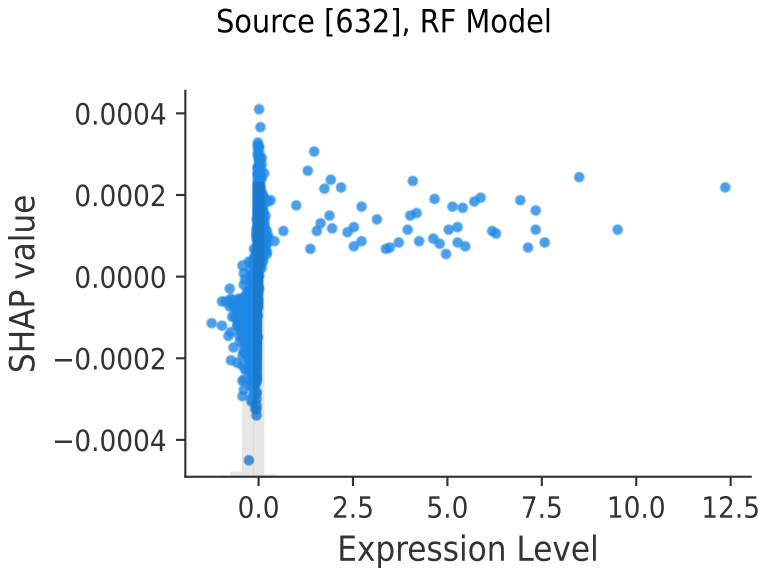

Source [632], RF Model
0.0004
0.0002
0.0000
−0.0002
−0.0004
SHAP value
0.0
2.5
5.0
7.5
10.0
12.5
Expression Level

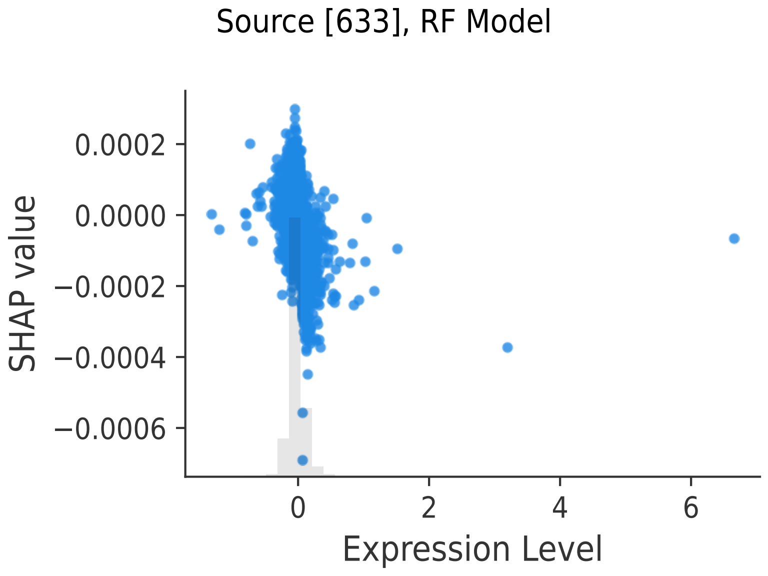

Source [633], RF Model
0.0002
0.0000
−0.0002
−0.0004
−0.0006
SHAP value
0
2
4
6
Expression Level

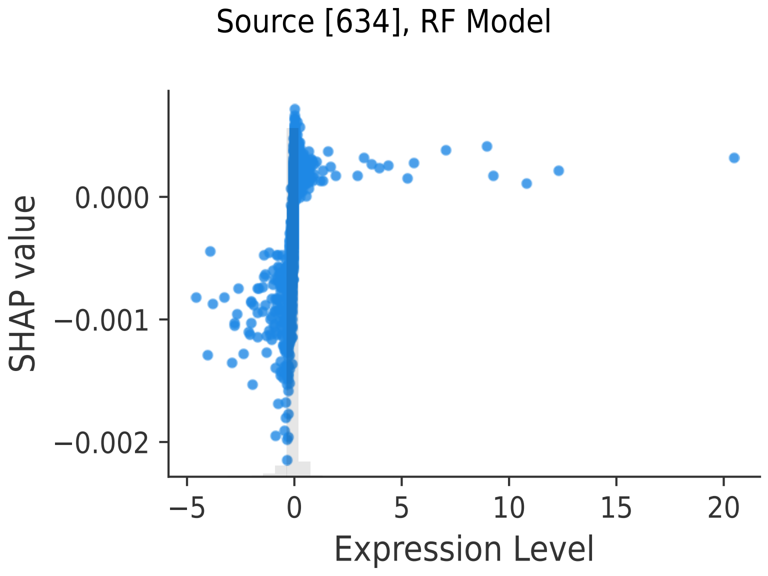
Source [634], RF Model
SHAP value
0.000
−0.001
−0.002
−5
0
5
10
15
20
Expression Level

Source [635], RF Model

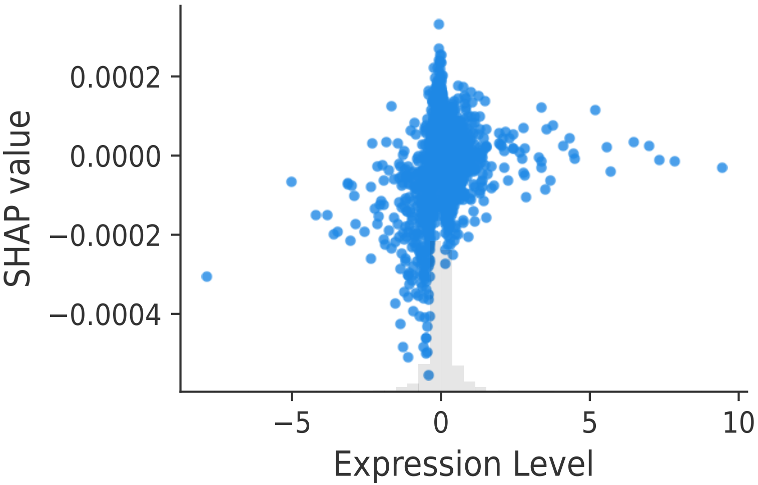

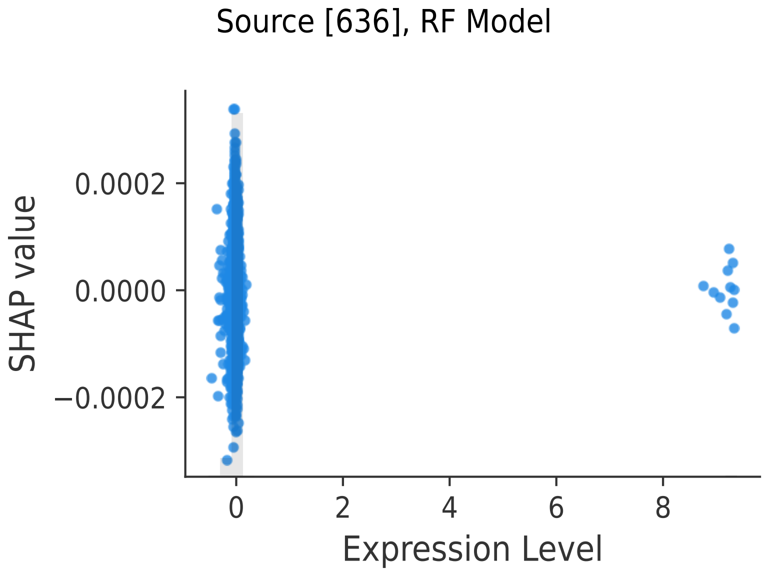
Source [636], RF Model
SHAP value
0.0002
0.0000
−0.0002
0
2
4
6
8
Expression Level

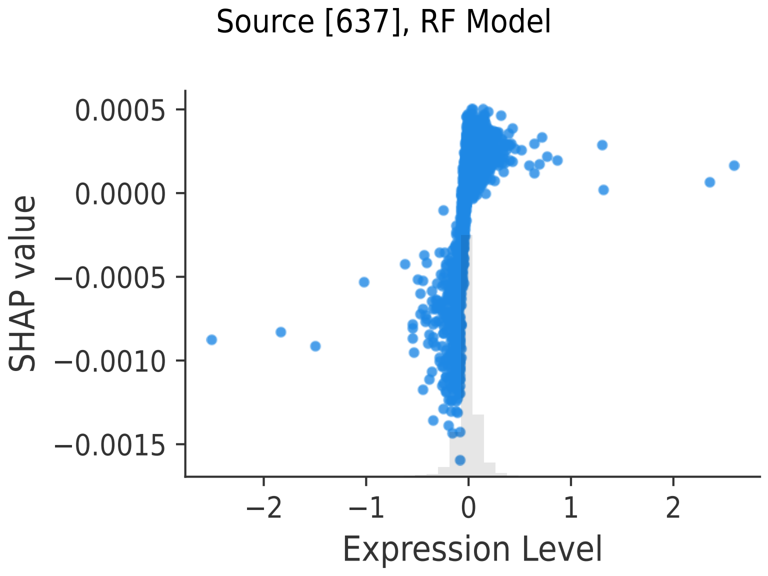

Source [637], RF Model
0.0005
0.0000
−0.0005
−0.0010
−0.0015
SHAP value
−2
−1
0
1
2
Expression Level

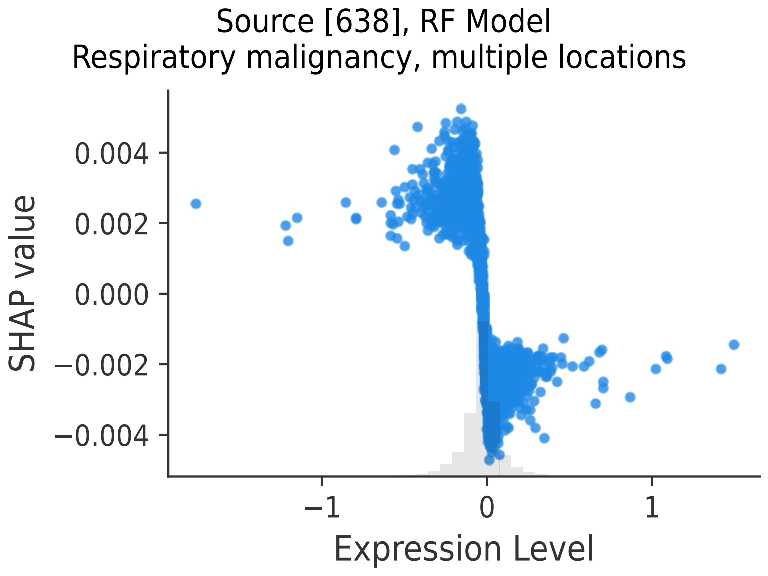

Source [638], RF Model
Respiratory malignancy, multiple locations
0.004
0.002
0.000
−0.002
−0.004
SHAP value
−1
0
1
Expression Level

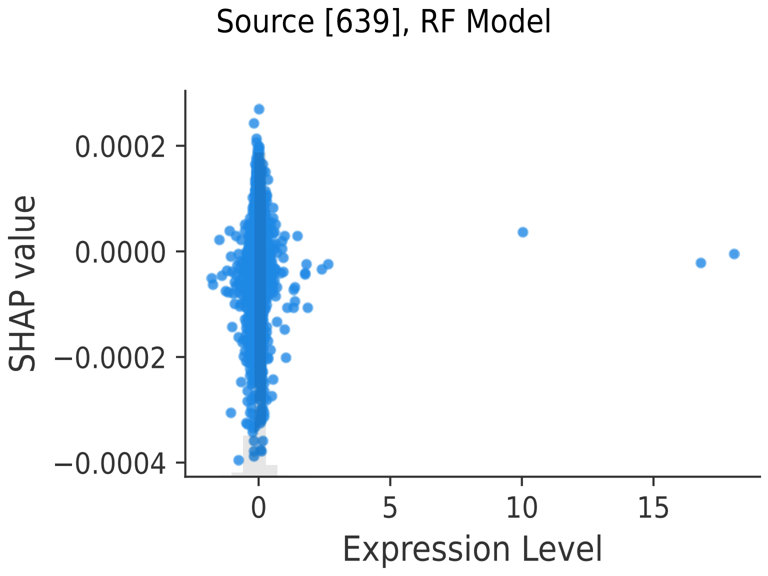
Source [639], RF Model
0.0002
0.0000
−0.0002
−0.0004
SHAP value
0
5
10
15
Expression Level

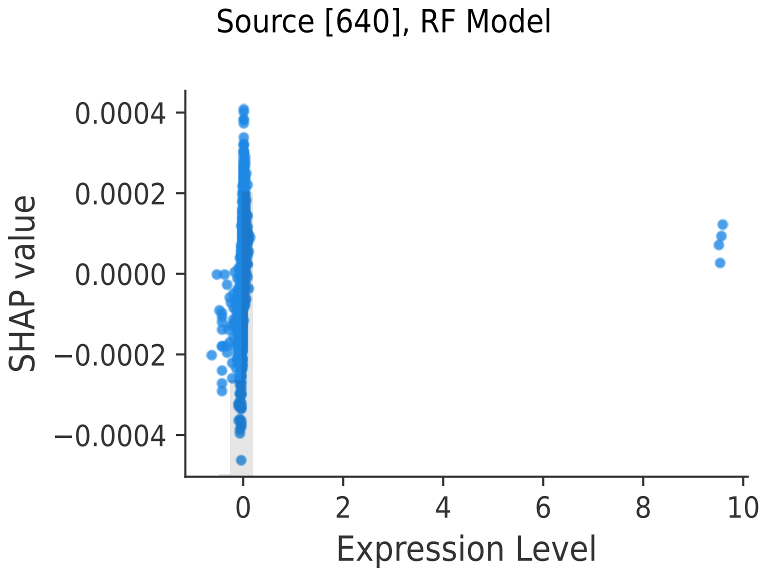

Source [640], RF Model
SHAP value
0.0004
0.0002
0.0000
−0.0002
−0.0004
0
2
4
6
8
10
Expression Level

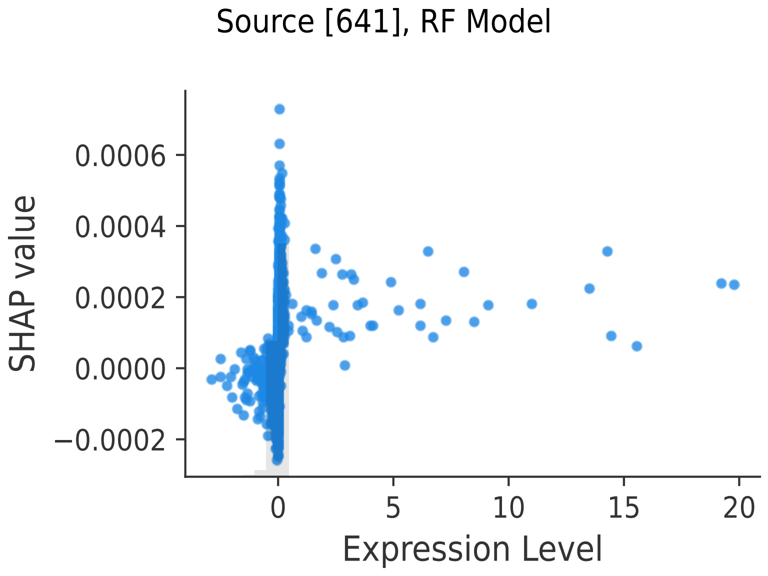
Source [641], RF Model
SHAP value
0.0006
0.0004
0.0002
0.0000
−0.0002
0
5
10
15
20
Expression Level

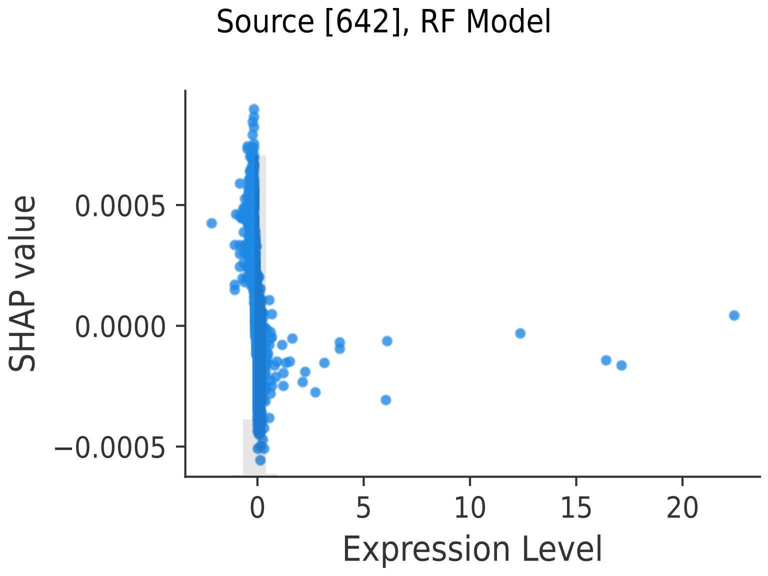
Source [642], RF Model
SHAP value
0.0005
0.0000
−0.0005
0
5
10
15
20
Expression Level

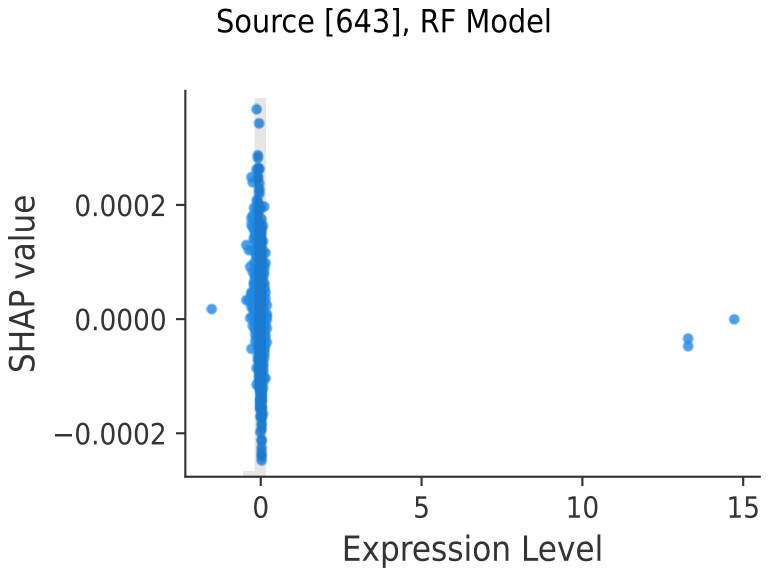

Source [643], RF Model
SHAP value
0.0002
0.0000
−0.0002
0
5
10
15
Expression Level

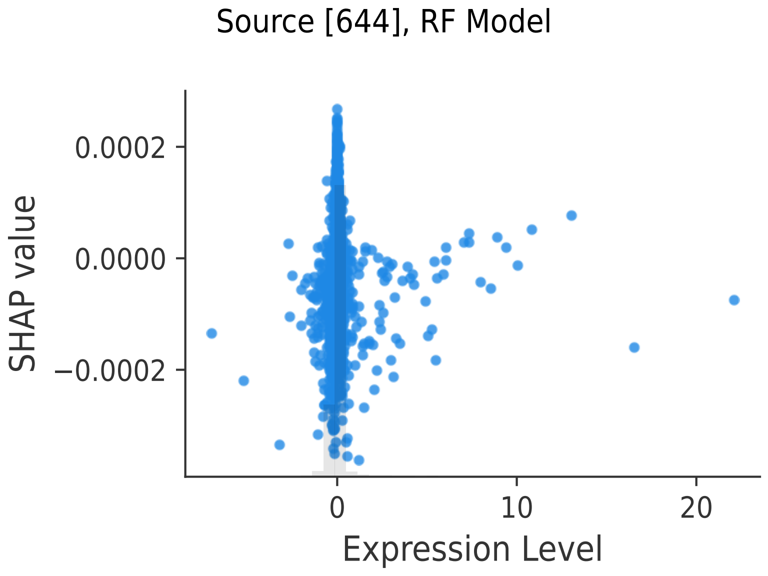

Source [644], RF Model
0.0002
0.0000
−0.0002
SHAP value
0
10
20
Expression Level

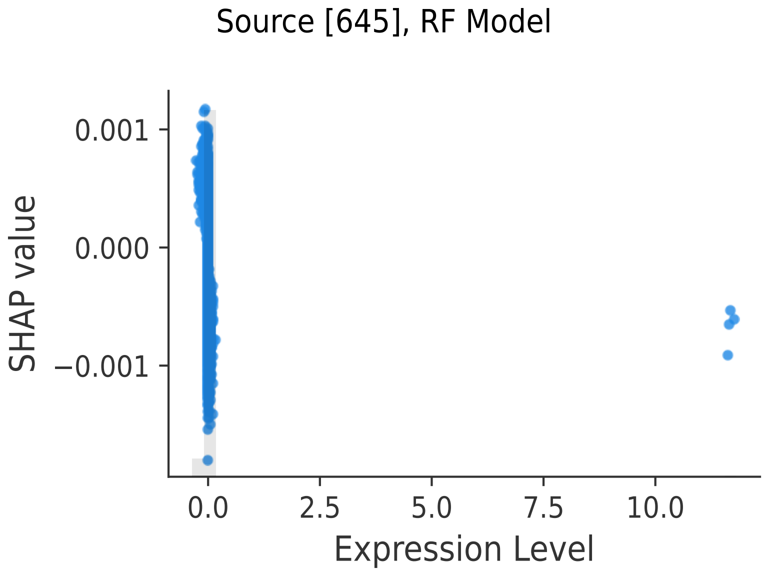

Source [645], RF Model
0.001
0.000
−0.001
SHAP value
0.0
2.5
5.0
7.5
10.0
Expression Level

Source [646], RF Model

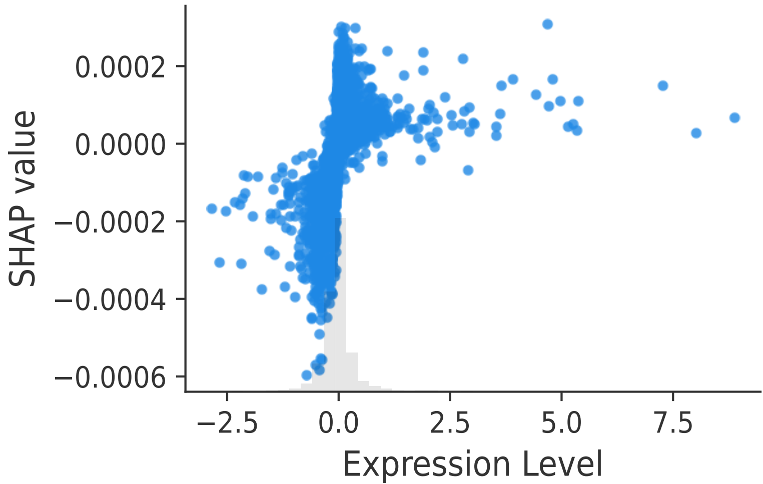

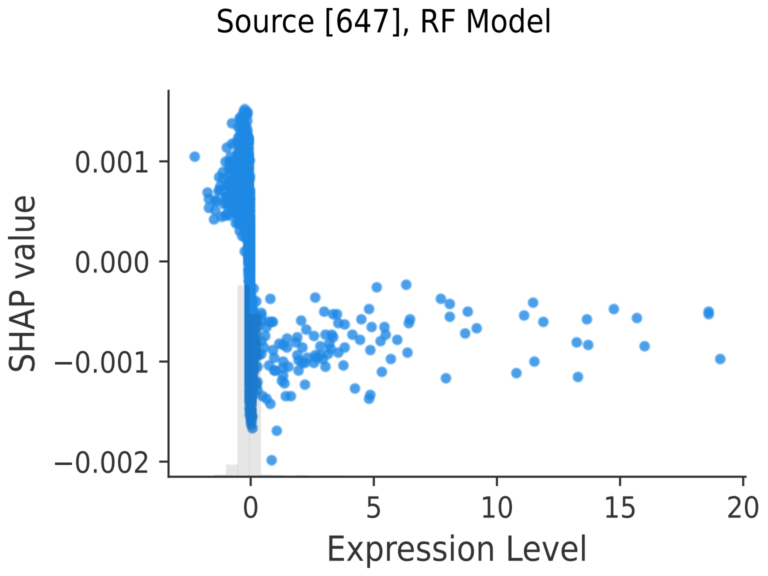
Source [647], RF Model
SHAP value
0.001
0.000
−0.001
−0.002
0
5
10
15
20
Expression Level

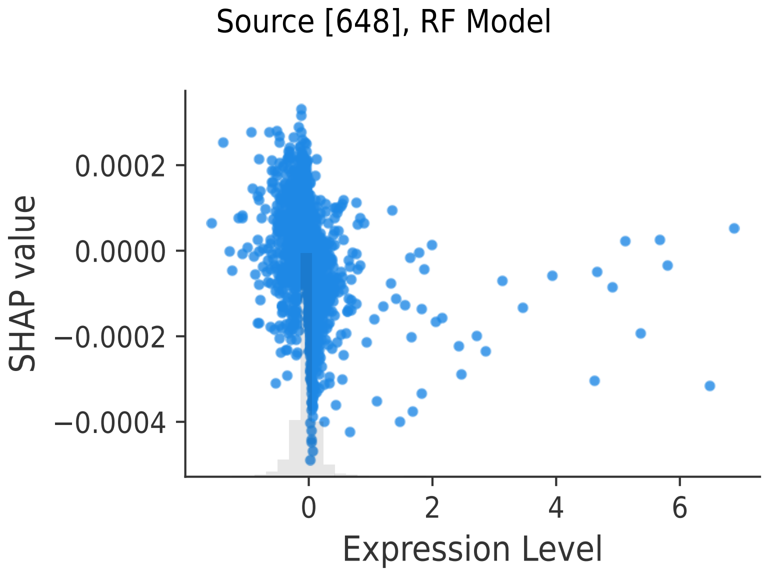
Source [648], RF Model
0.0002
0.0000
−0.0002
−0.0004
SHAP value
0
2
4
6
Expression Level

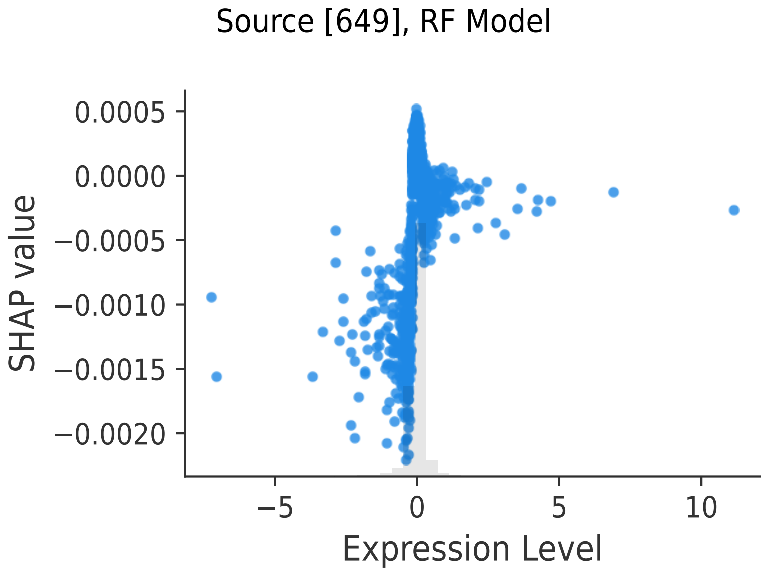
Source [649], RF Model
SHAP value
0.0005
0.0000
−0.0005
−0.0010
−0.0015
−0.0020
−5
0
5
10
Expression Level

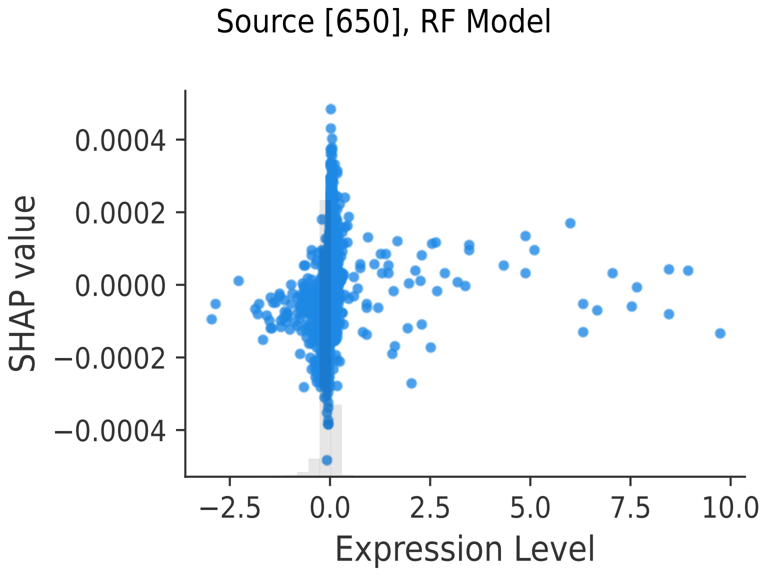
Source [650], RF Model
0.0004
0.0002
0.0000
−0.0002
−0.0004
SHAP value
−2.5
0.0
2.5
5.0
7.5
10.0
Expression Level

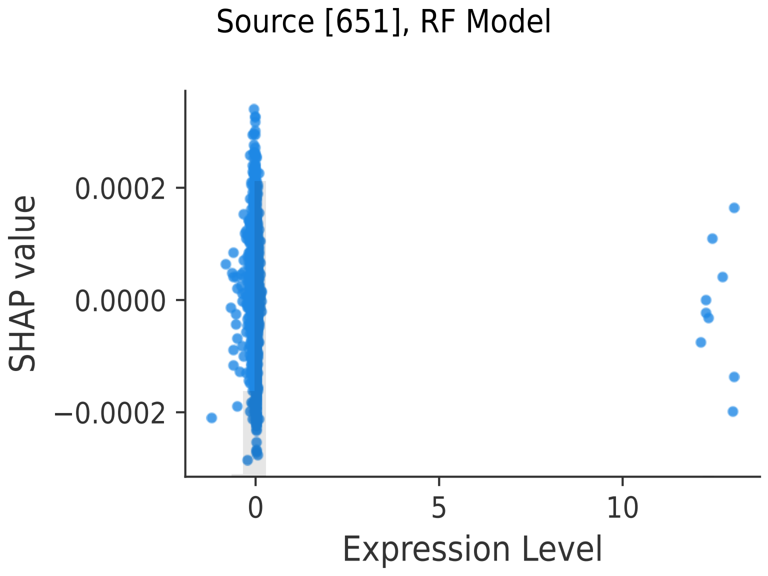

Source [651], RF Model
SHAP value
0.0002
0.0000
−0.0002
0
5
10
Expression Level

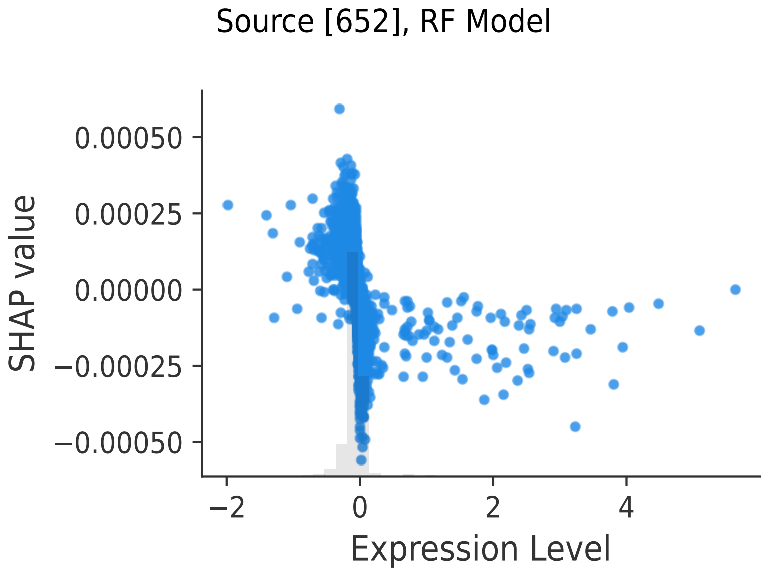
Source [652], RF Model
SHAP value
0.00050
0.00025
0.00000
−0.00025
−0.00050
−2
0
2
4
Expression Level

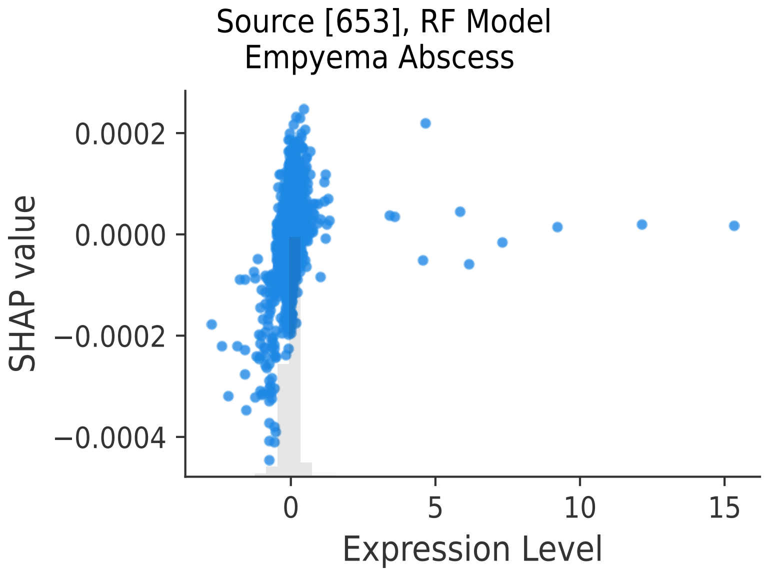

Source [653], RF Model
Empyema Abscess
SHAP value
0.0002
0.0000
−0.0002
−0.0004
0
5
10
15
Expression Level

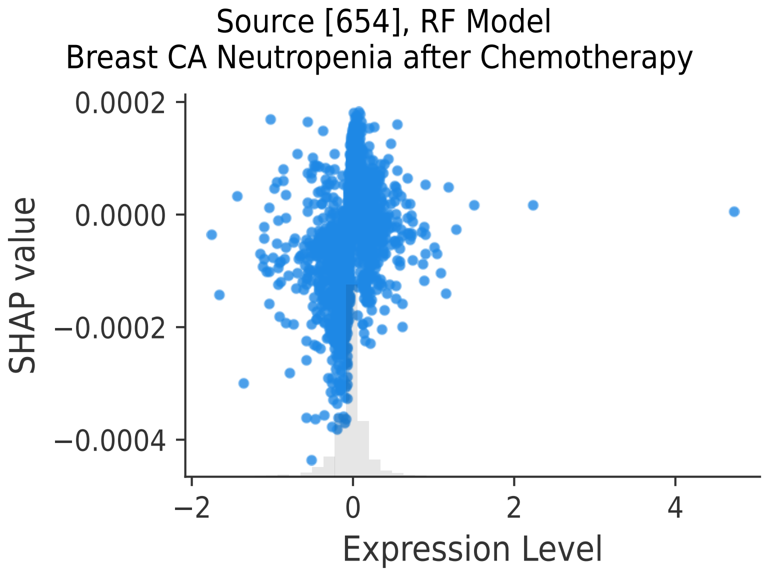
Source [654], RF Model
Breast CA Neutropenia after Chemotherapy
0.0002
0.0000
−0.0002
−0.0004
SHAP value
−2
0
2
4
Expression Level

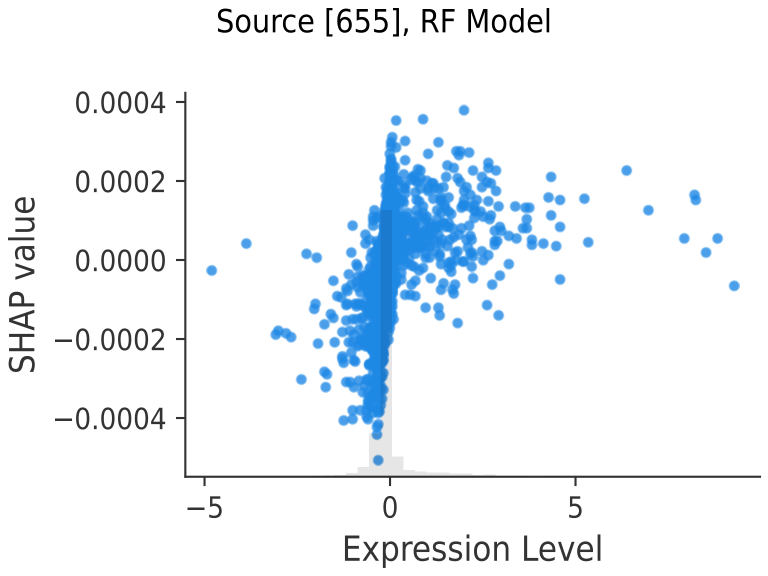
Source [655], RF Model
0.0004
0.0002
0.0000
−0.0002
−0.0004
SHAP value
−5
0
5
Expression Level

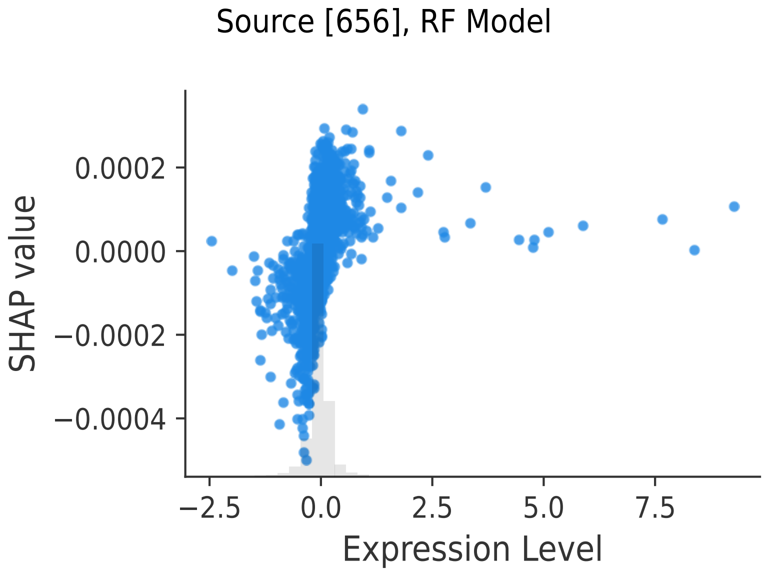
Source [656], RF Model
0.0002
0.0000
−0.0002
−0.0004
SHAP value
−2.5
0.0
2.5
5.0
7.5
Expression Level

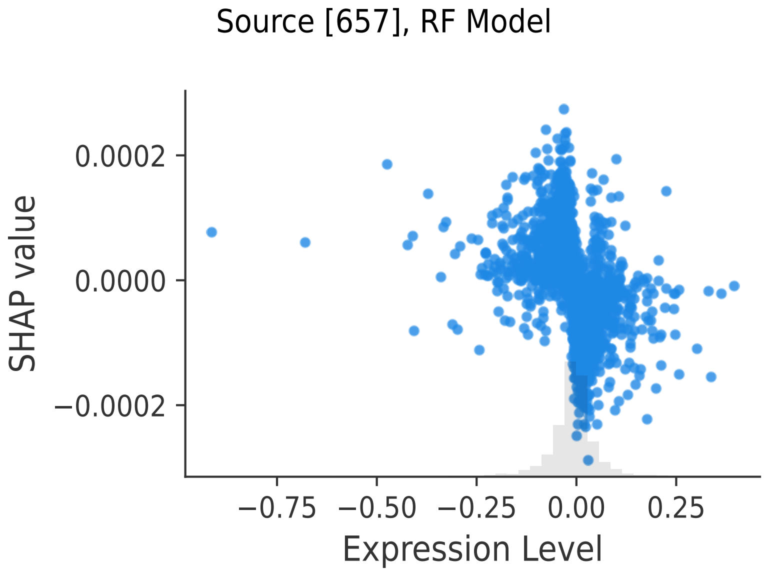
Source [657], RF Model
0.0002
0.0000
−0.0002
SHAP value
−0.75
−0.50
−0.25
0.00
0.25
Expression Level

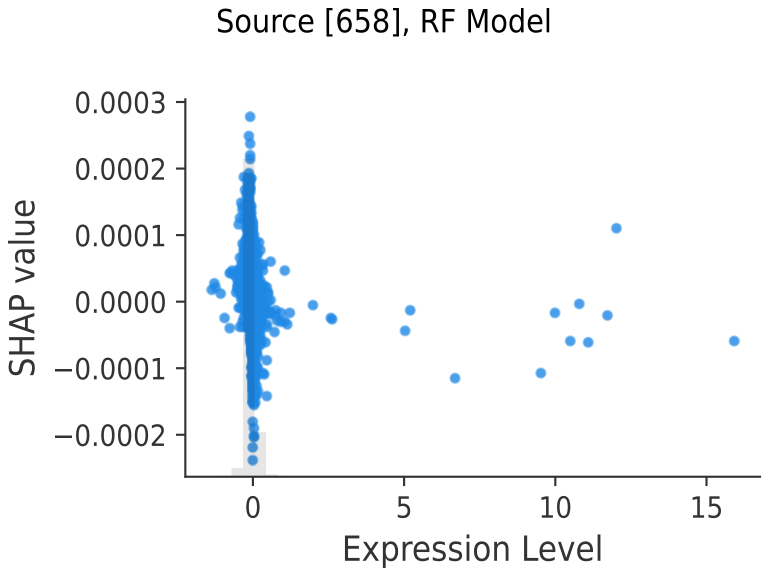
Source [658], RF Model
0.0003
0.0002
0.0001
0.0000
−0.0001
−0.0002
SHAP value
0
5
10
15
Expression Level

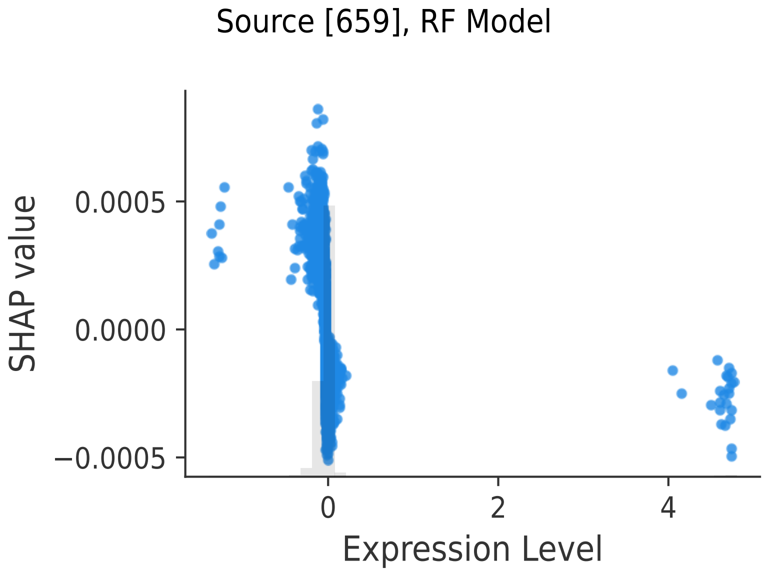

Source [659], RF Model
SHAP value
0.0005
0.0000
−0.0005
0
2
4
Expression Level

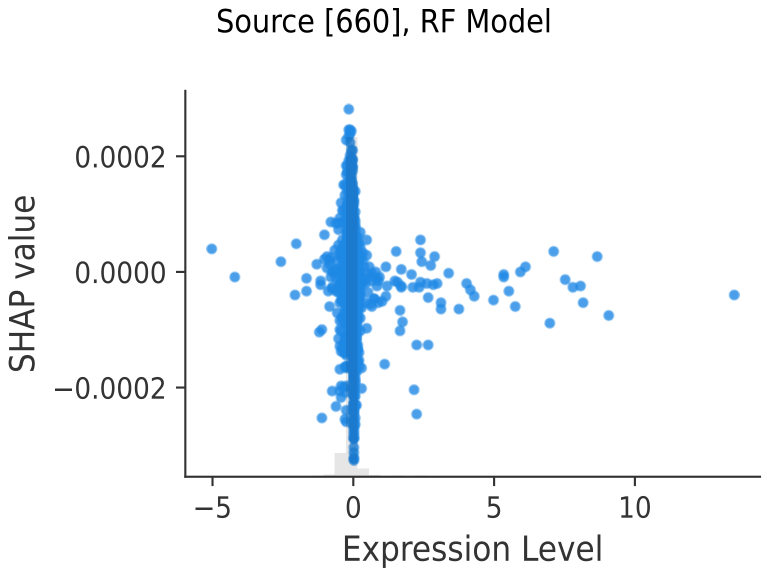
Source [660], RF Model
SHAP value
0.0002
0.0000
−0.0002
−5
0
5
10
Expression Level

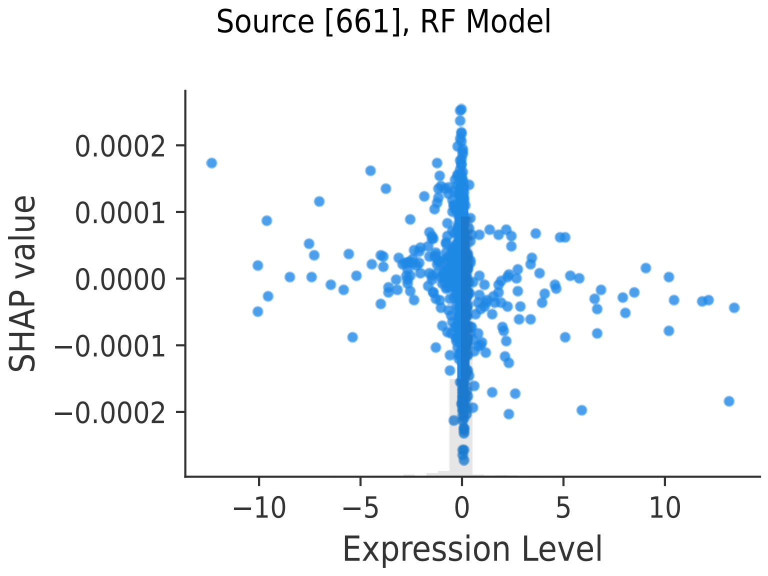
Source [661], RF Model
SHAP value
0.0002
0.0001
0.0000
−0.0001
−0.0002
−10
−5
0
5
10
Expression Level

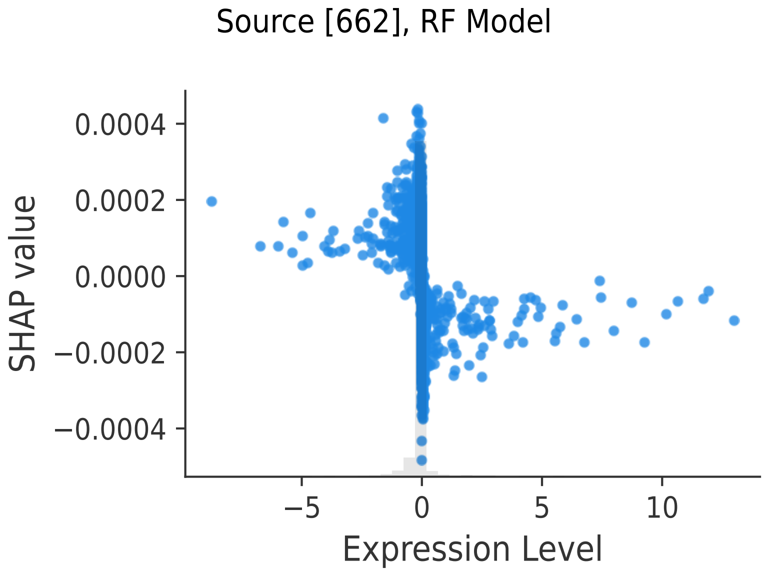
Source [662], RF Model
SHAP value
0.0004
0.0002
0.0000
−0.0002
−0.0004
−5
0
5
10
Expression Level

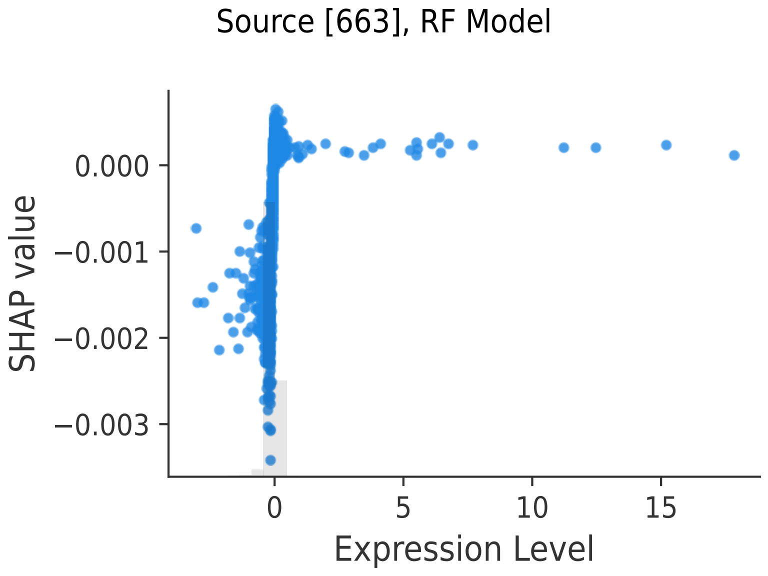

Source [663], RF Model
SHAP value
0.000
−0.001
−0.002
−0.003
0
5
10
15
Expression Level

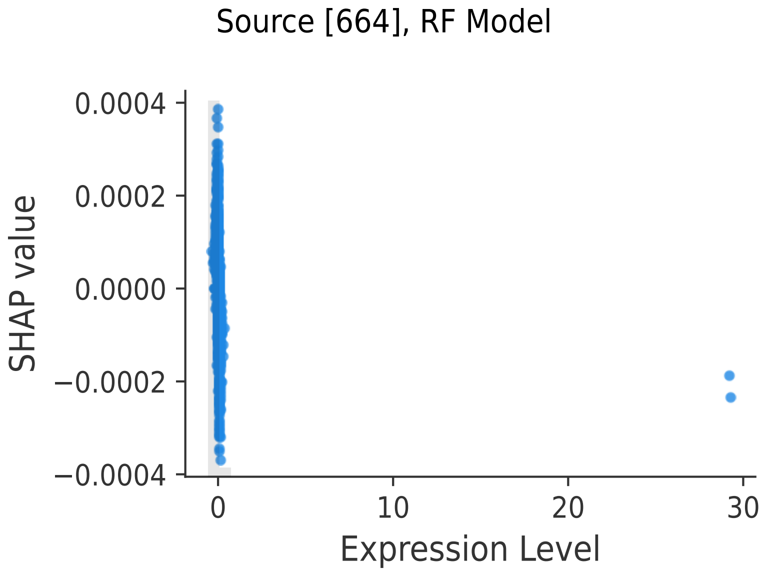
Source [664], RF Model
0.0004
0.0002
0.0000
−0.0002
−0.0004
SHAP value
0
10
20
30
Expression Level

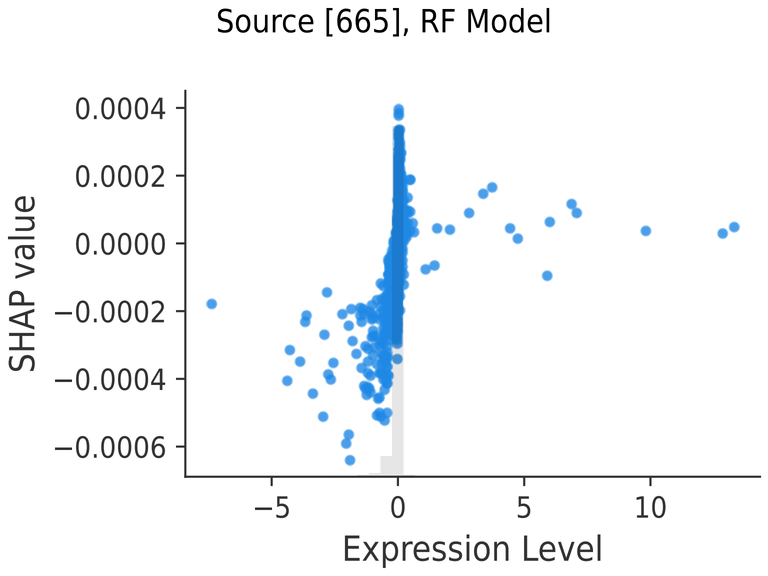
Source [665], RF Model
SHAP value
0.0004
0.0002
0.0000
−0.0002
−0.0004
−0.0006
−5
0
5
10
Expression Level

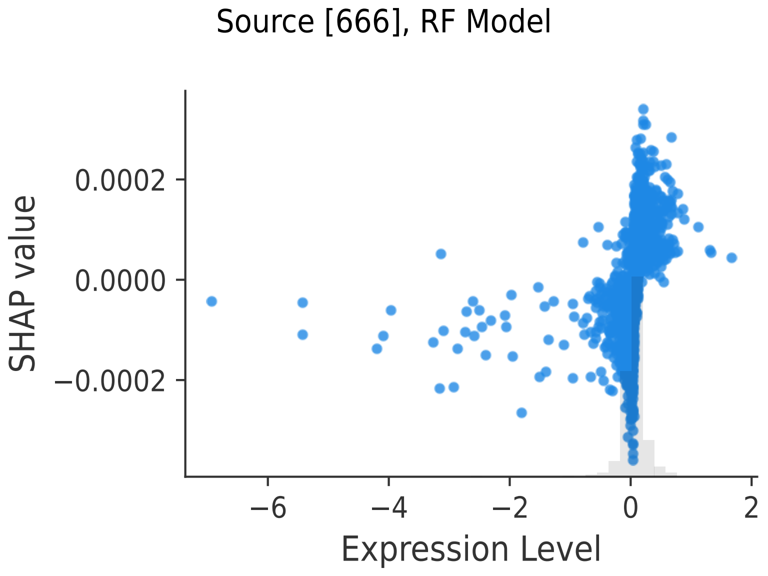
Source [666], RF Model
SHAP value
0.0002
0.0000
−0.0002
−6
−4
−2
0
2
Expression Level

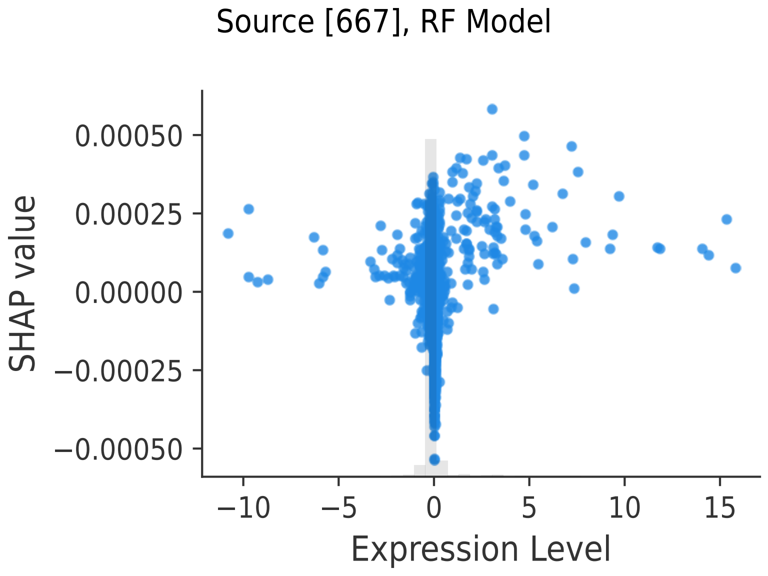

Source [667], RF Model
SHAP value
0.00050
0.00025
0.00000
−0.00025
−0.00050
−10
−5
0
5
10
15
Expression Level

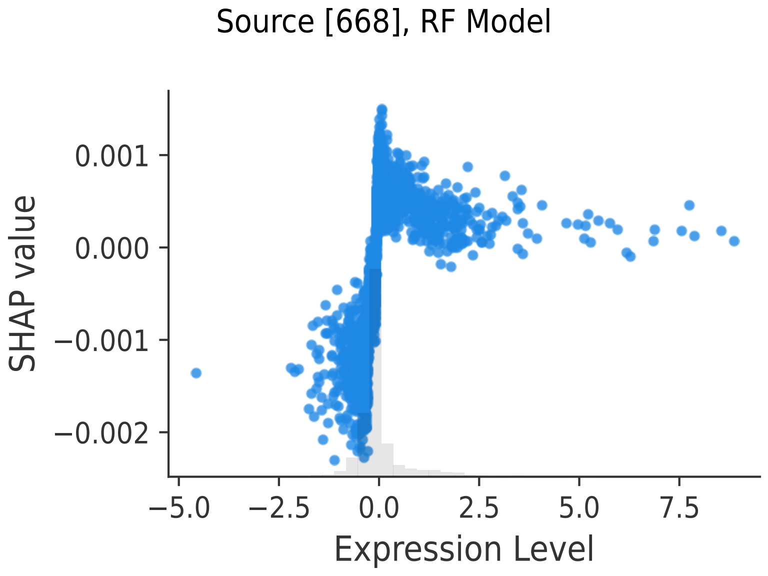
Source [668], RF Model
SHAP value
0.001
0.000
−0.001
−0.002
Expression Level
−5.0
−2.5
0.0
2.5
5.0
7.5

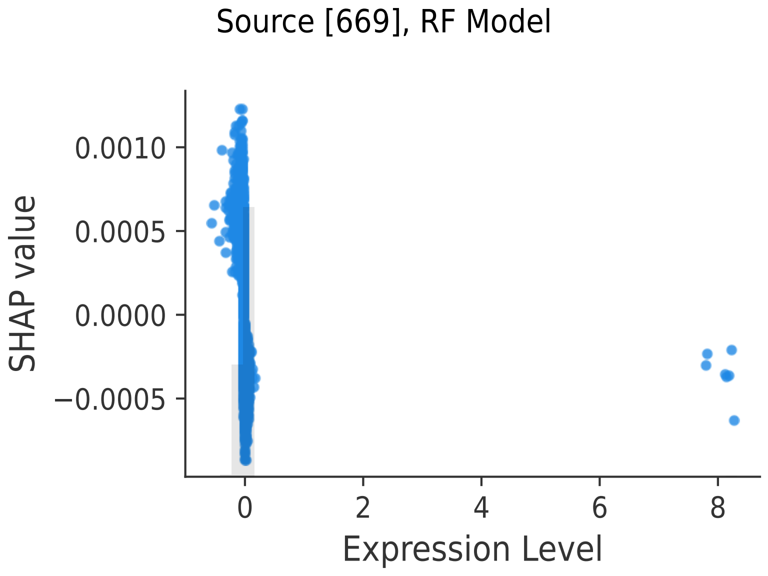
Source [669], RF Model
0.0010
0.0005
0.0000
−0.0005
SHAP value
0
2
4
6
8
Expression Level

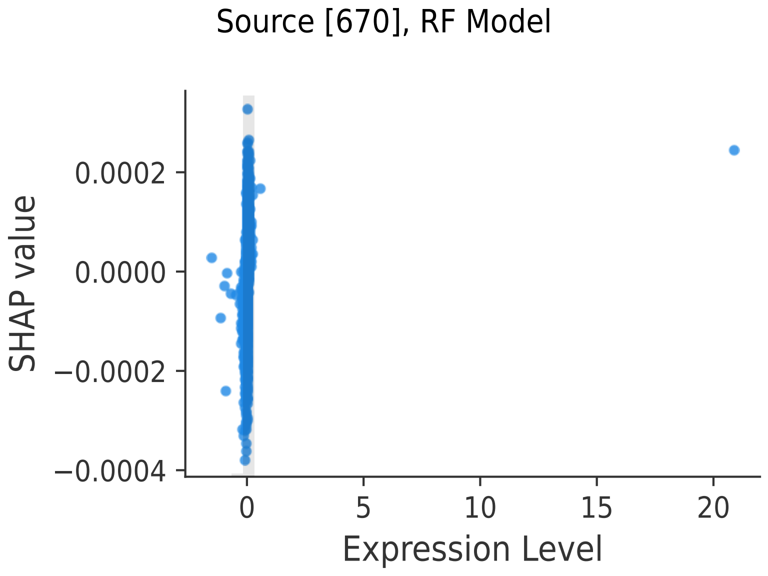
Source [670], RF Model
SHAP value
0.0002
0.0000
−0.0002
−0.0004
0
5
10
15
20
Expression Level

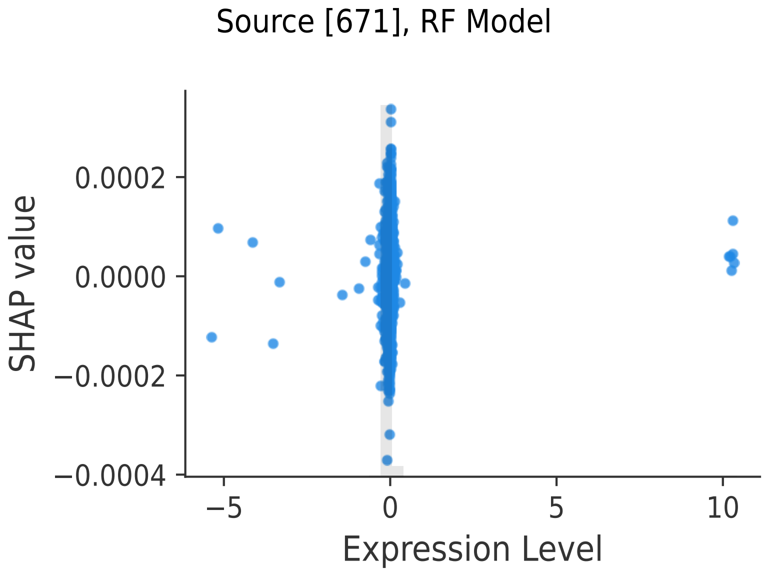
Source [671], RF Model
SHAP value
0.0002
0.0000
−0.0002
−0.0004
−5
0
5
10
Expression Level

Source [672], RF Model

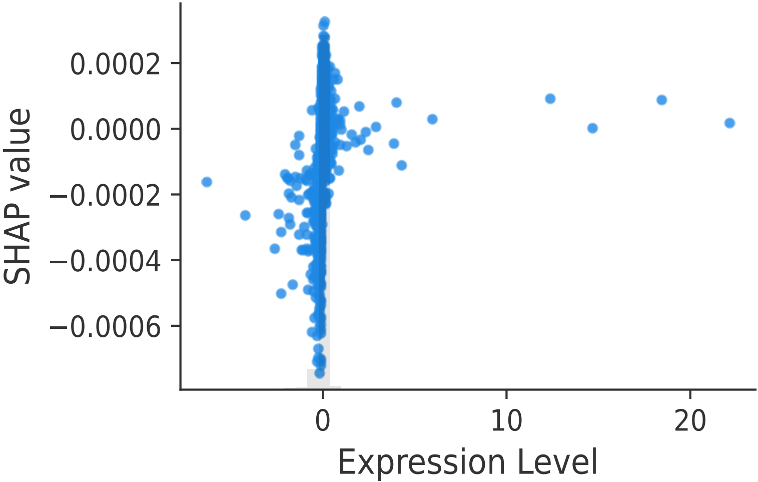

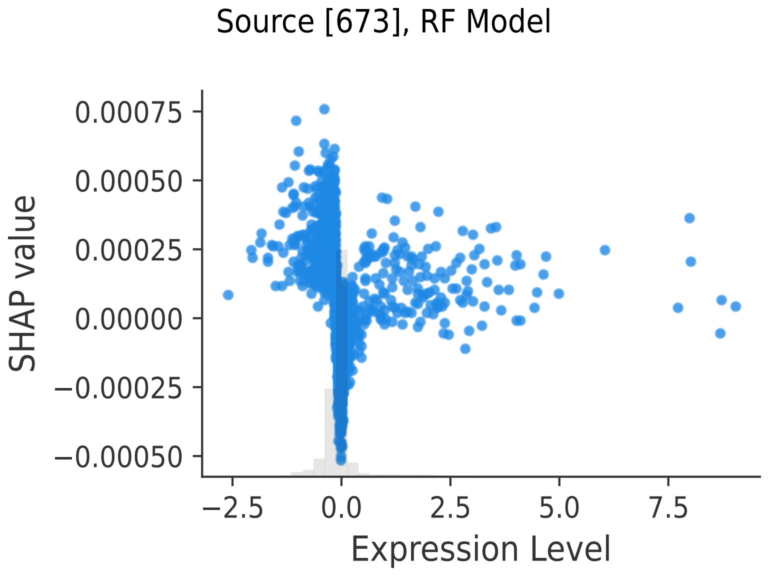
Source [673], RF Model
0.00075
0.00050
0.00025
0.00000
−0.00025
−0.00050
SHAP value
−2.5
0.0
2.5
5.0
7.5
Expression Level

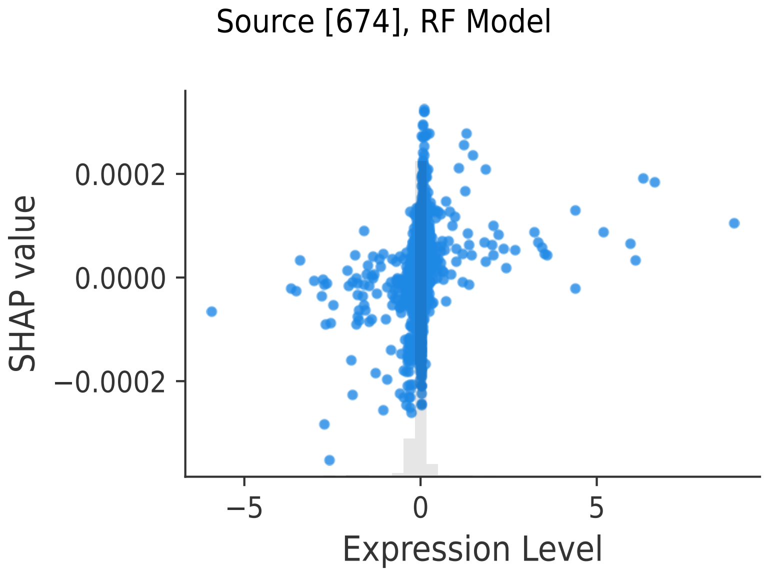
Source [674], RF Model
SHAP value
0.0002
0.0000
−0.0002
−5
0
5
Expression Level

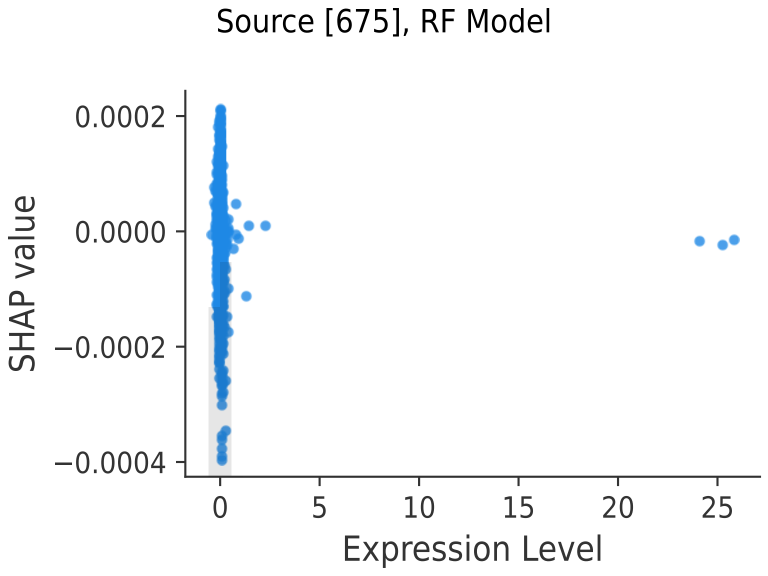

Source [675], RF Model
0.0002
0.0000
−0.0002
−0.0004
SHAP value
0
5
10
15
20
25
Expression Level

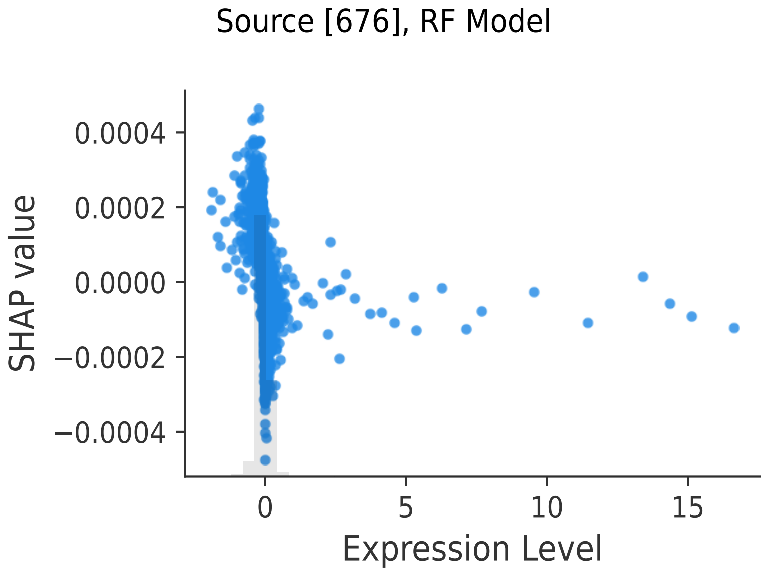

Source [676], RF Model
0.0004
0.0002
0.0000
−0.0002
−0.0004
SHAP value
0
5
10
15
Expression Level

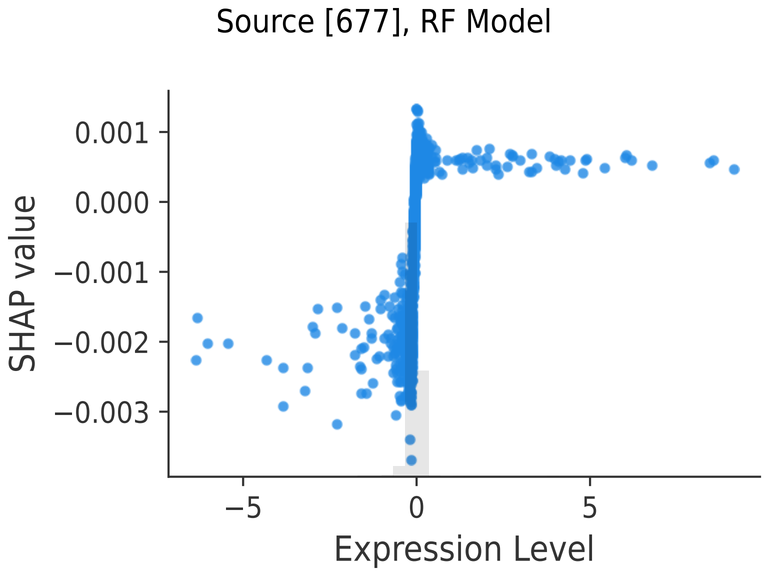

Source [677], RF Model
SHAP value
0.001
0.000
−0.001
−0.002
−0.003
−5
0
5
Expression Level

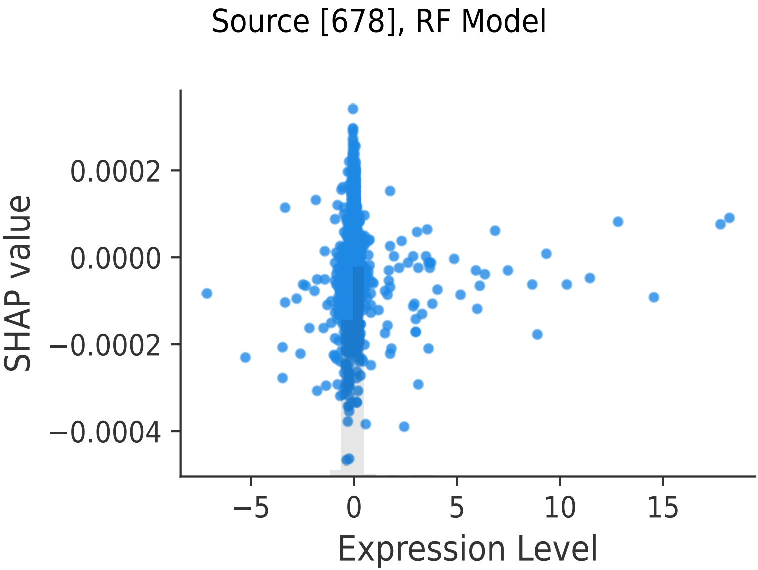
Source [678], RF Model
0.0002
0.0000
−0.0002
−0.0004
SHAP value
−5
0
5
10
15
Expression Level

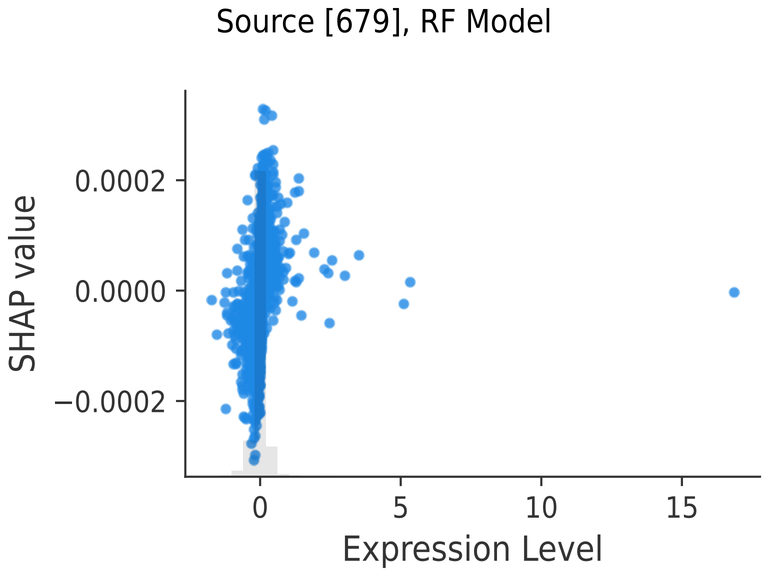

Source [679], RF Model
SHAP value
0.0002
0.0000
−0.0002
0
5
10
15
Expression Level

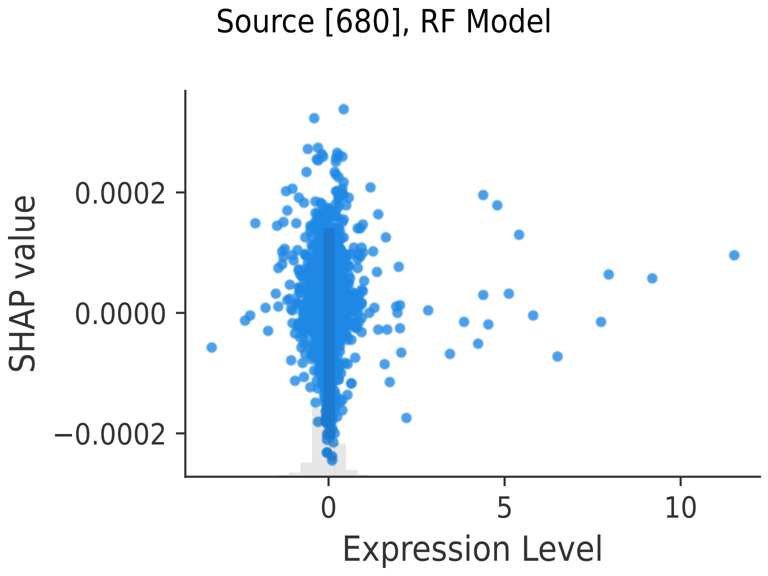

Source [680], RF Model
SHAP value
0.0002
0.0000
−0.0002
0
5
10
Expression Level

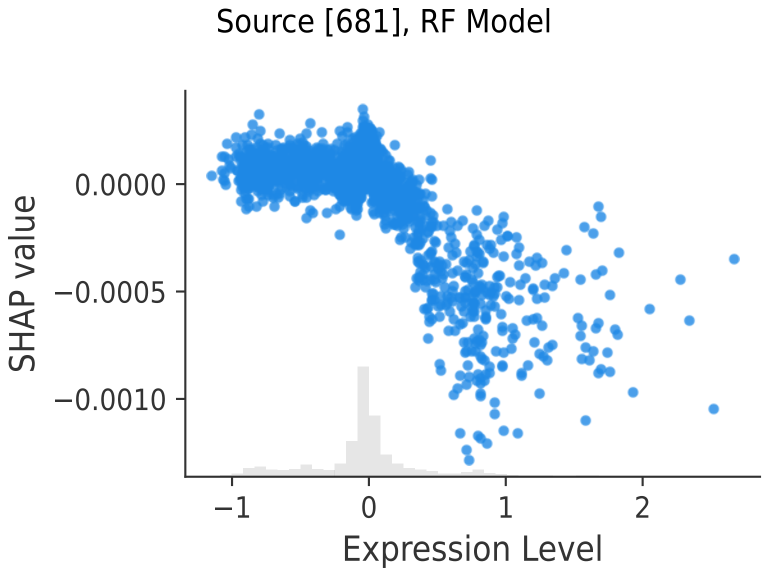
Source [681], RF Model
SHAP value
0.0000
−0.0005
−0.0010
−1
0
1
2
Expression Level

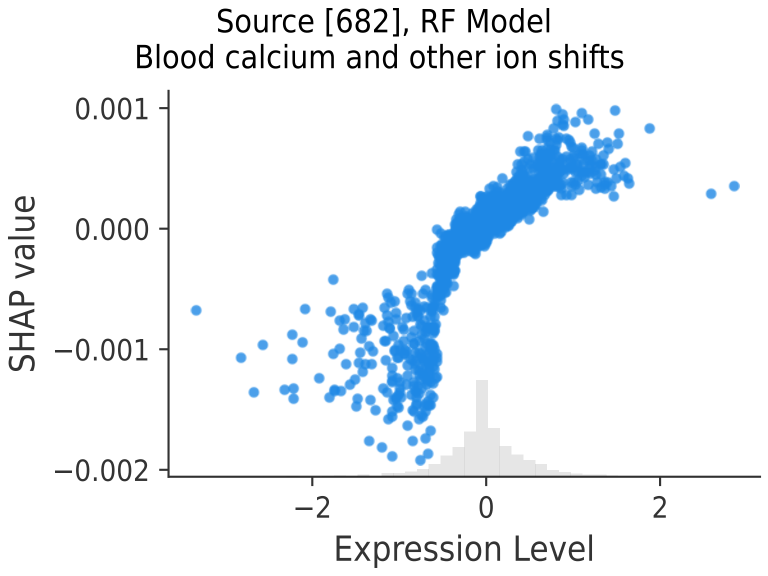
Source [682], RF Model
Blood calcium and other ion shifts
0.001
0.000
−0.001
−0.002
SHAP value
−2
0
2
Expression Level

Source [683], RF Model

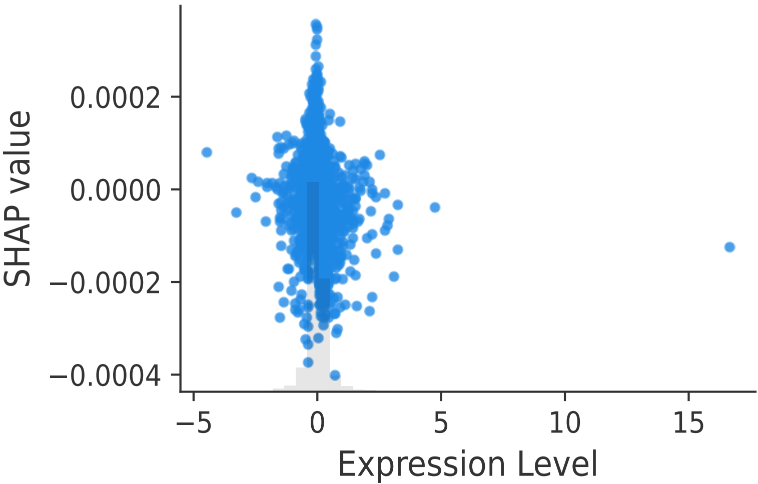

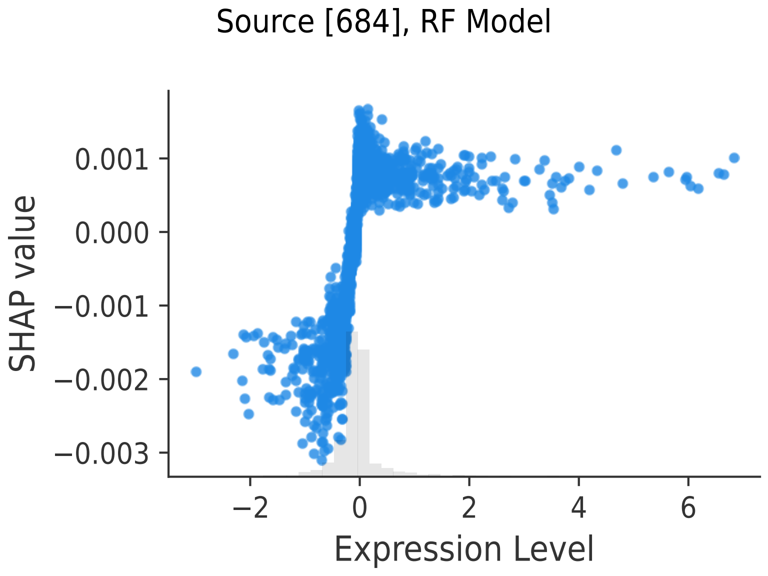
Source [684], RF Model
SHAP value
0.001
0.000
−0.001
−0.002
−0.003
−2
0
2
4
6
Expression Level

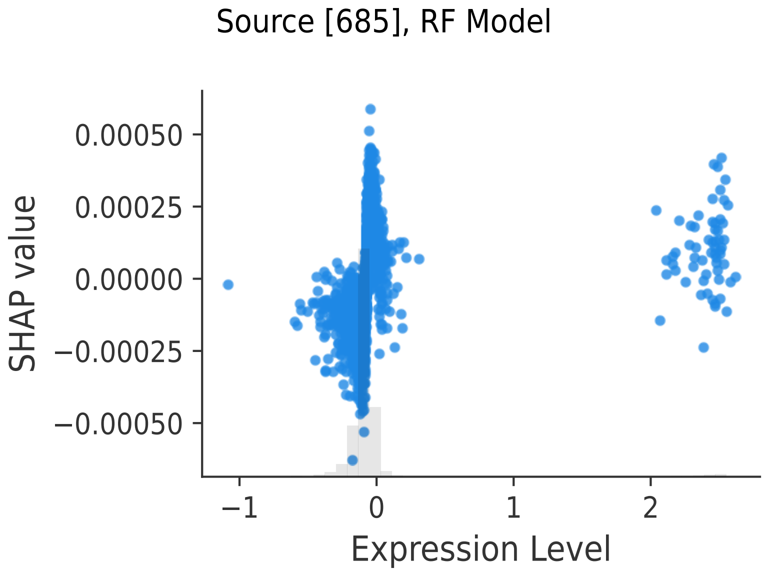
Source [685], RF Model
0.00050
0.00025
0.00000
−0.00025
−0.00050
SHAP value
−1
0
1
2
Expression Level

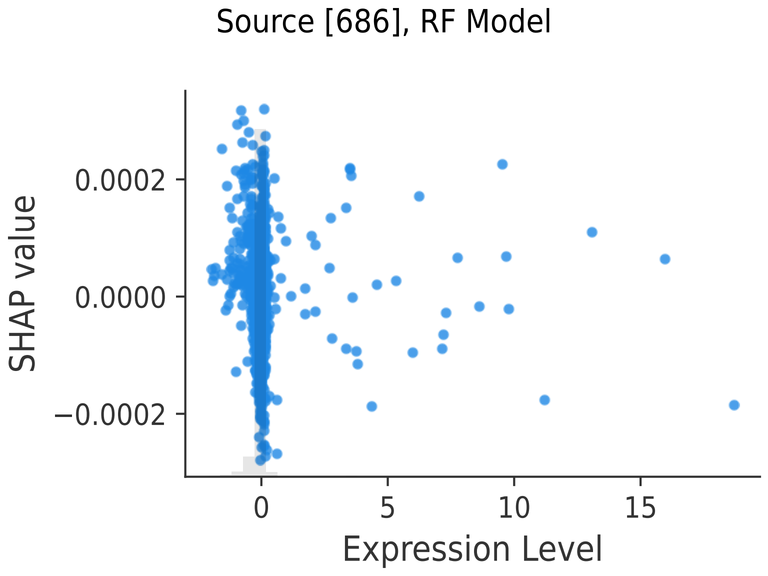
Source [686], RF Model
SHAP value
0.0002
0.0000
−0.0002
0
5
10
15
Expression Level

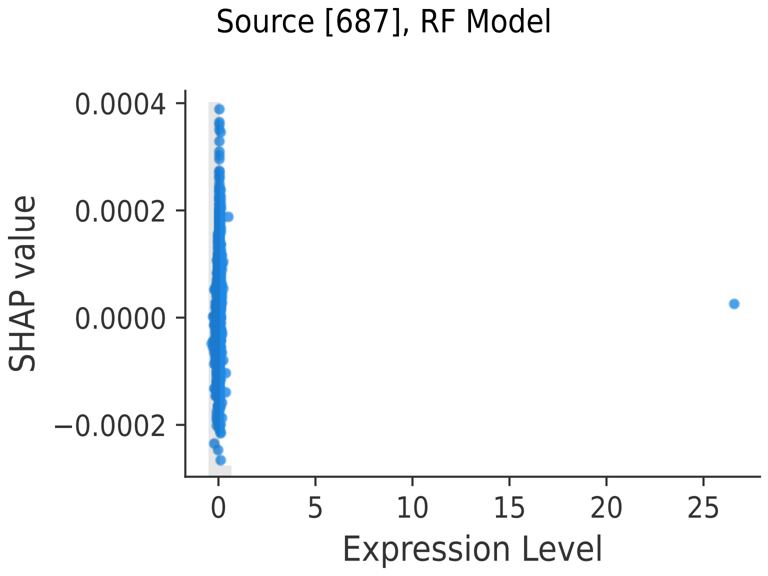

Source [687], RF Model
SHAP value
0.0004
0.0002
0.0000
−0.0002
0
5
10
15
20
25
Expression Level

Source [688], RF Model

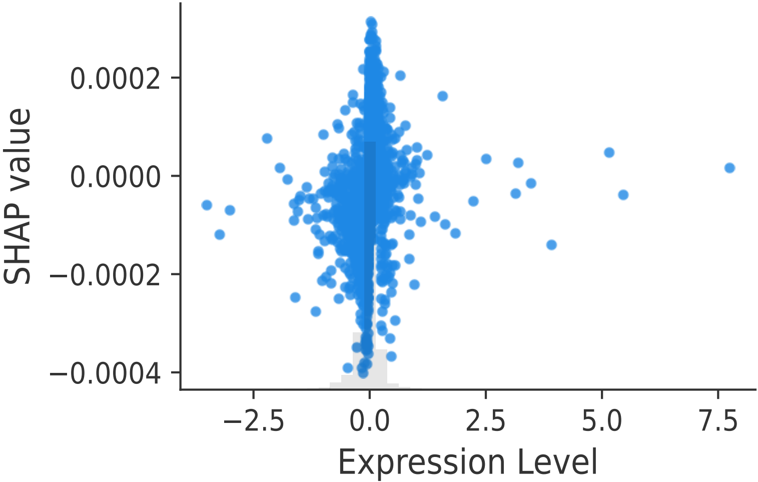

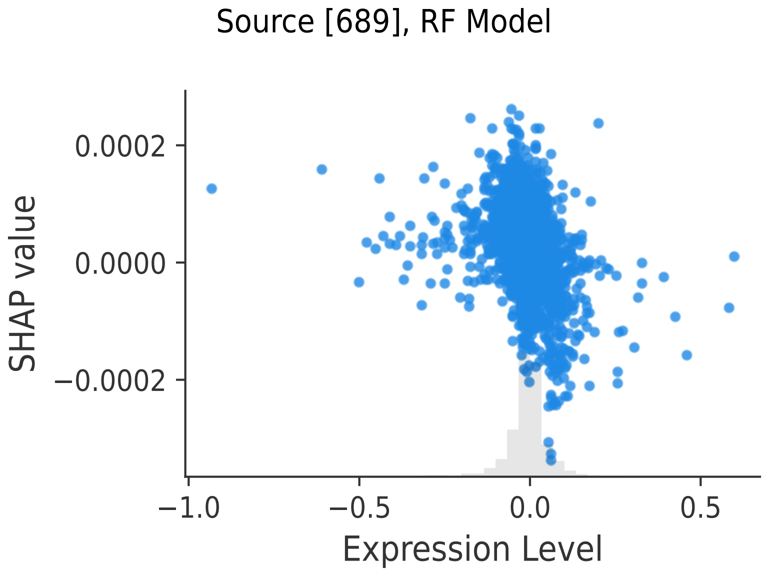

Source [689], RF Model
0.0002
0.0000
−0.0002
SHAP value
−1.0
−0.5
0.0
0.5
Expression Level

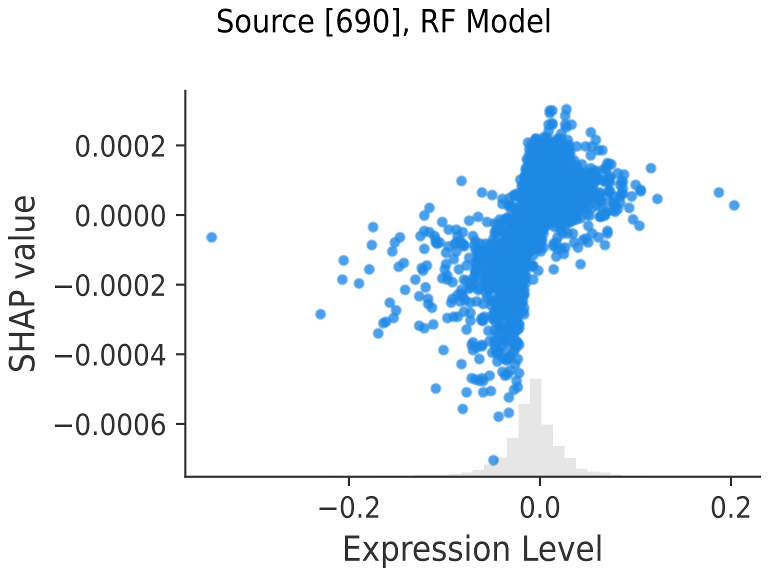
Source [690], RF Model
0.0002
0.0000
−0.0002
−0.0004
−0.0006
SHAP value
−0.2
0.0
0.2
Expression Level

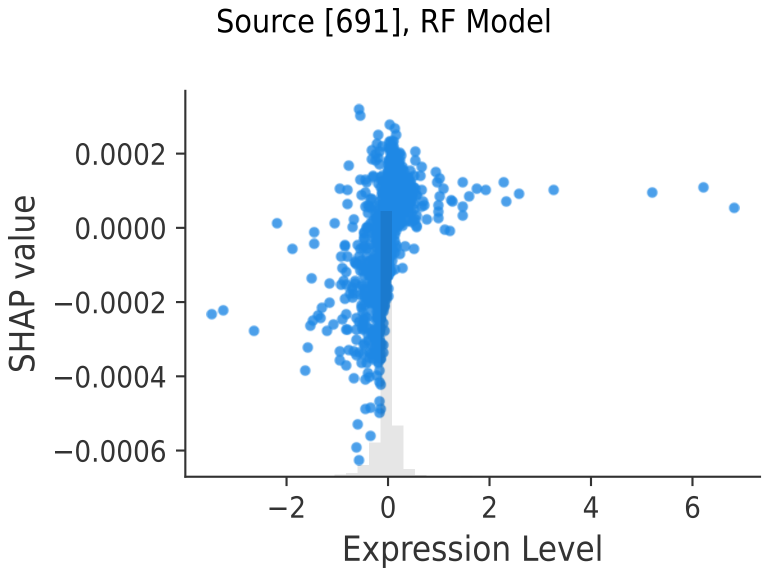
Source [691], RF Model
SHAP value
0.0002
0.0000
−0.0002
−0.0004
−0.0006
−2
0
2
4
6
Expression Level

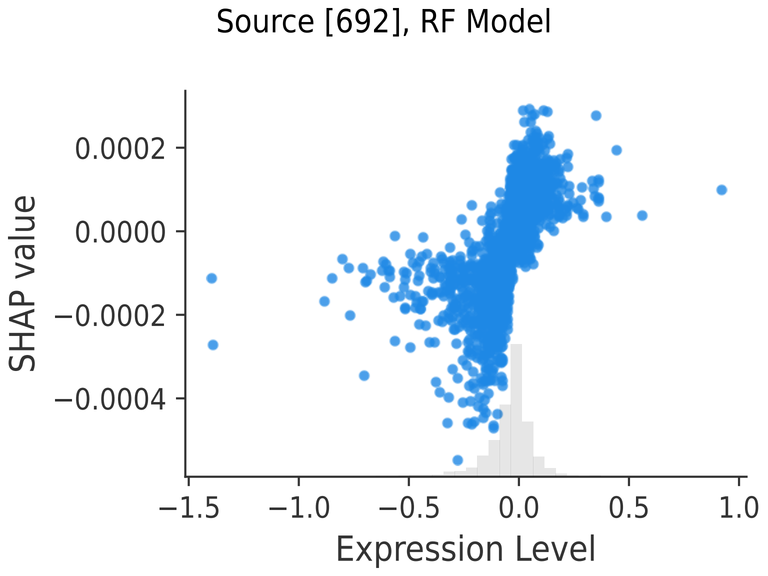

Source [692], RF Model
SHAP value
0.0002
0.0000
−0.0002
−0.0004
−1.5
−1.0
−0.5
0.0
0.5
1.0
Expression Level

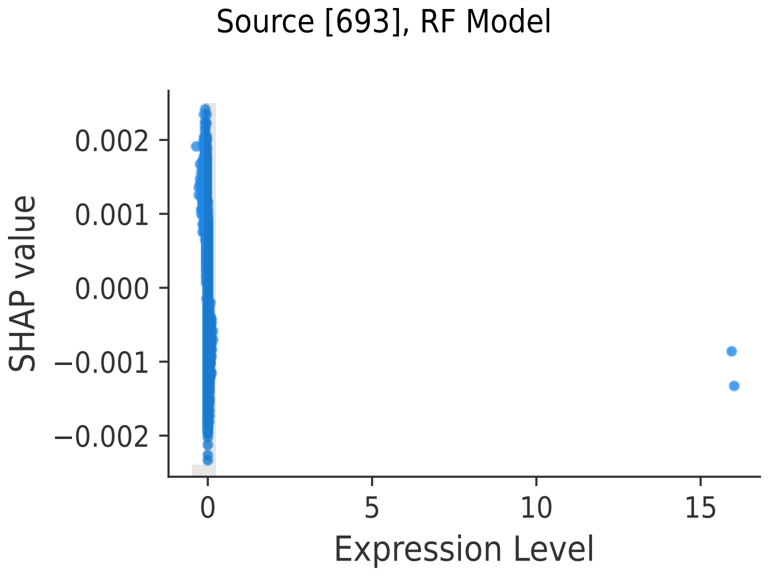
Source [693], RF Model
SHAP value
0.002
0.001
0.000
−0.001
−0.002
0
5
10
15
Expression Level

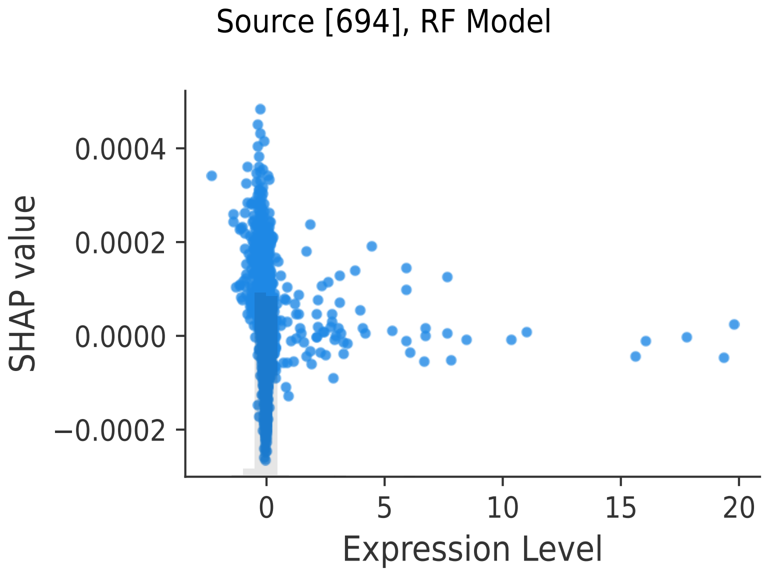
Source [694], RF Model
SHAP value
0.0004
0.0002
0.0000
−0.0002
0
5
10
15
20
Expression Level

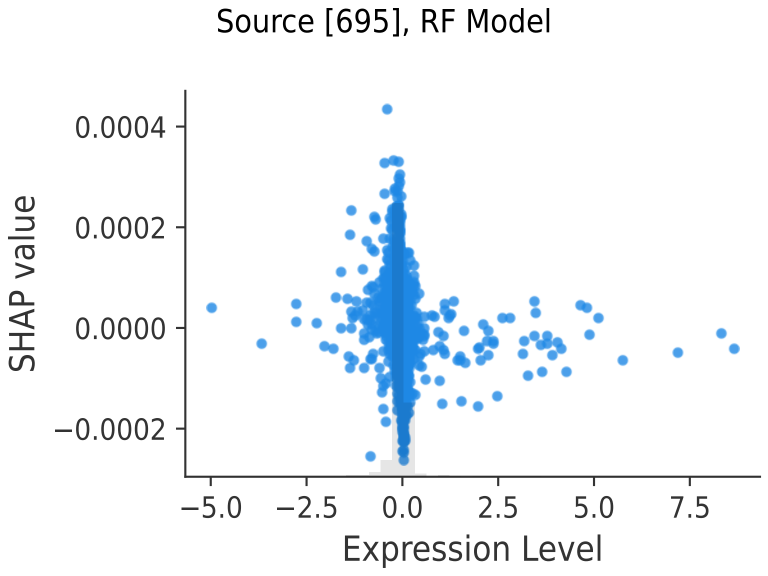
Source [695], RF Model
SHAP value
0.0004
0.0002
0.0000
−0.0002
−5.0
−2.5
0.0
2.5
5.0
7.5
Expression Level

Source [696], RF Model

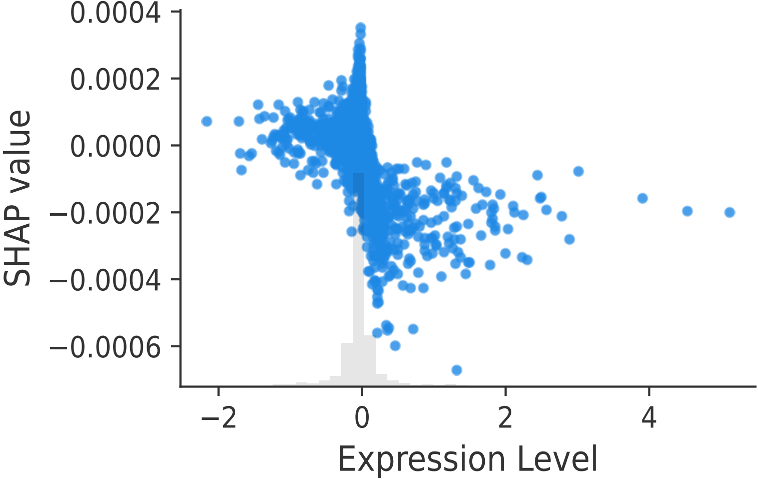

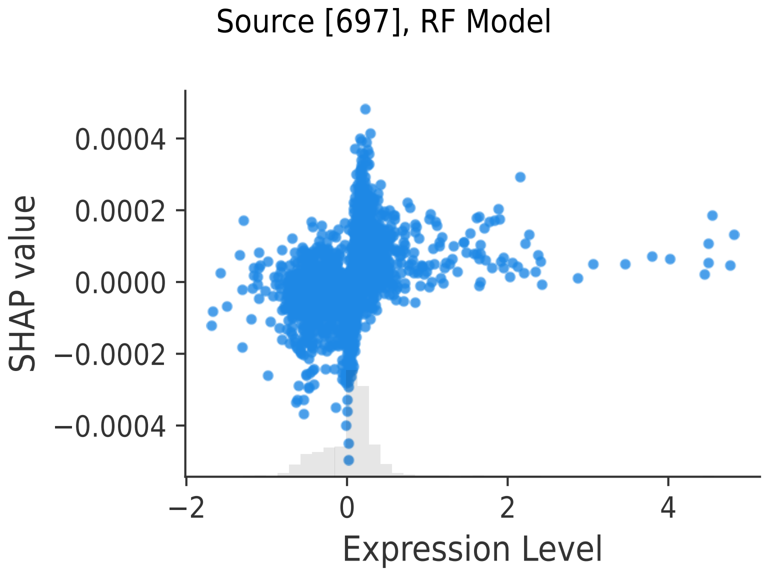

Source [697], RF Model
0.0004
0.0002
0.0000
−0.0002
−0.0004
SHAP value
−2
0
2
4
Expression Level

Source [698], RF Model

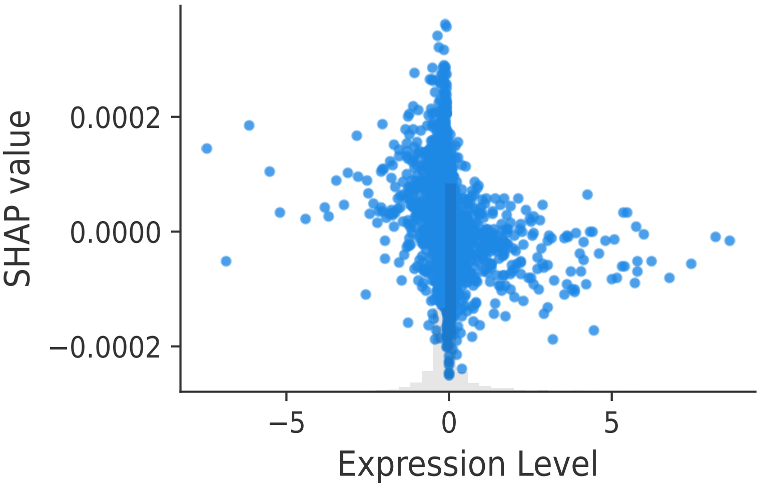

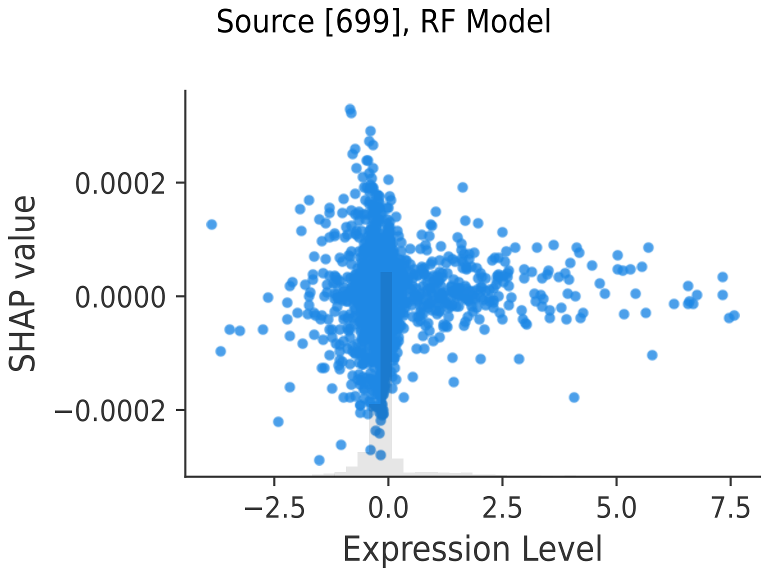
Source [699], RF Model
0.0002
0.0000
−0.0002
SHAP value
−2.5
0.0
2.5
5.0
7.5
Expression Level

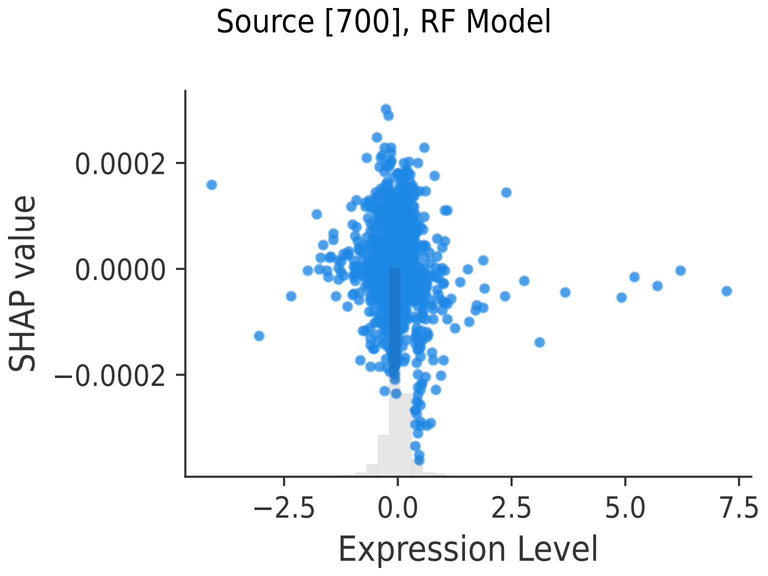
Source [700], RF Model
0.0002
0.0000
−0.0002
SHAP value
−2.5
0.0
2.5
5.0
7.5
Expression Level

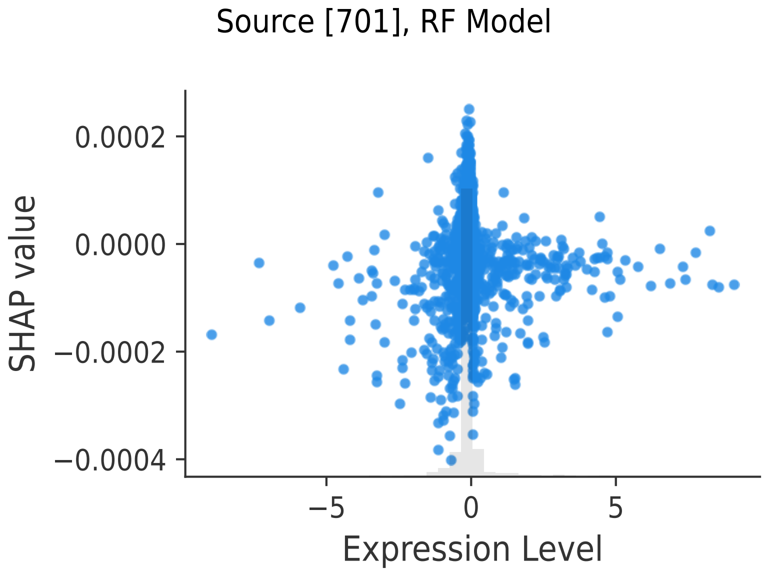
Source [701], RF Model
SHAP value
0.0002
0.0000
−0.0002
−0.0004
−5
0
5
Expression Level

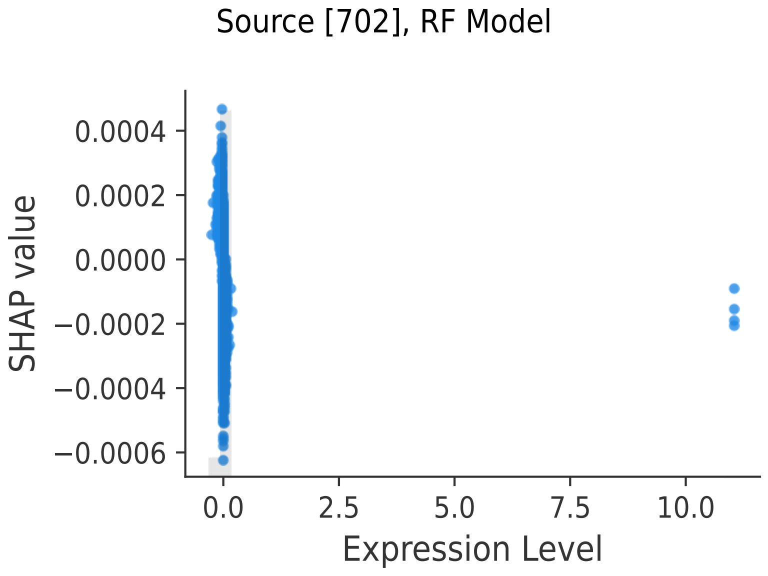
Source [702], RF Model
0.0004
0.0002
0.0000
−0.0002
−0.0004
−0.0006
SHAP value
0.0
2.5
5.0
7.5
10.0
Expression Level

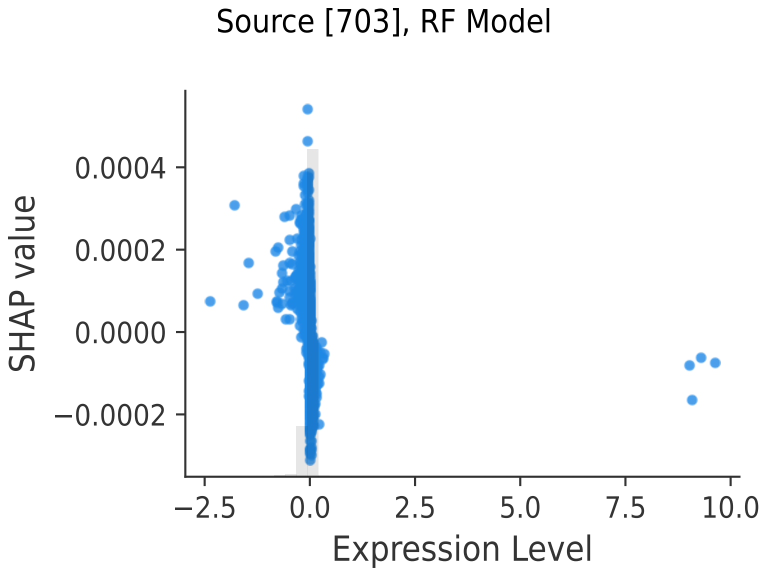

Source [703], RF Model
0.0004
0.0002
0.0000
−0.0002
SHAP value
−2.5
0.0
2.5
5.0
7.5
10.0
Expression Level

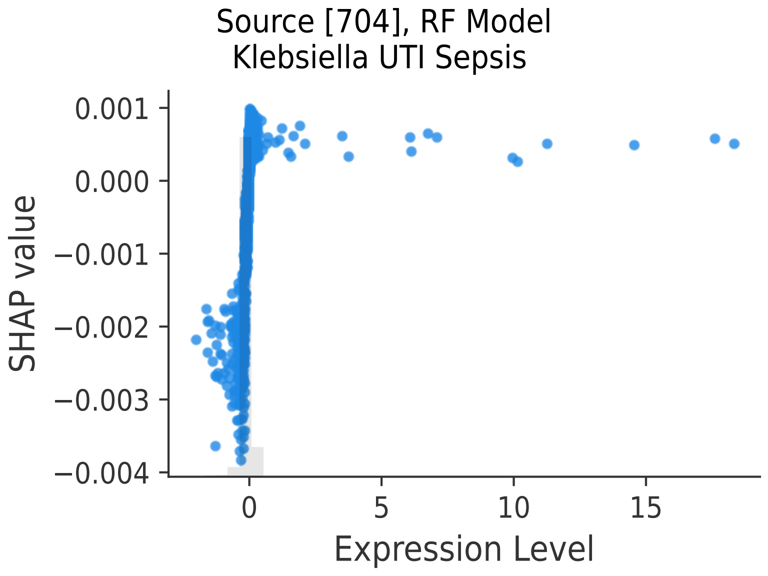

Source [704], RF Model
Klebsiella UTI Sepsis
SHAP value
0.001
0.000
−0.001
−0.002
−0.003
−0.004
0
5
10
15
Expression Level

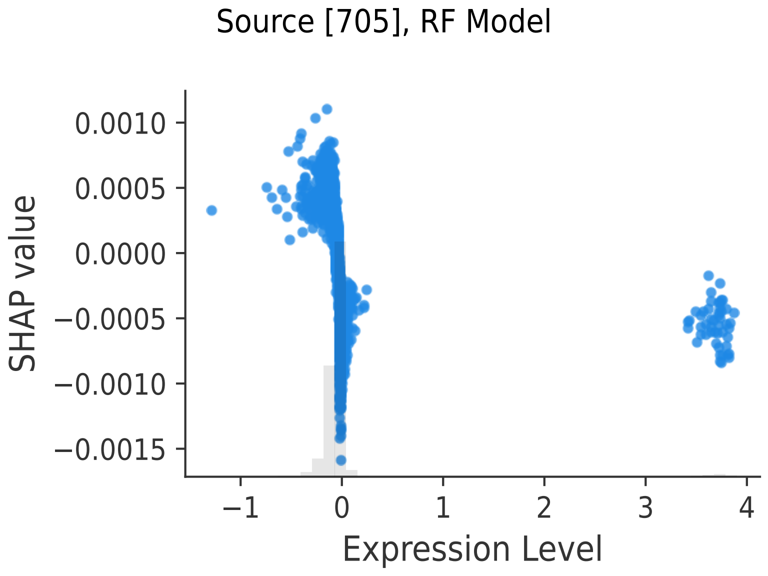
Source [705], RF Model
SHAP value
0.0010
0.0005
0.0000
−0.0005
−0.0010
−0.0015
Expression Level
−1
0
1
2
3
4

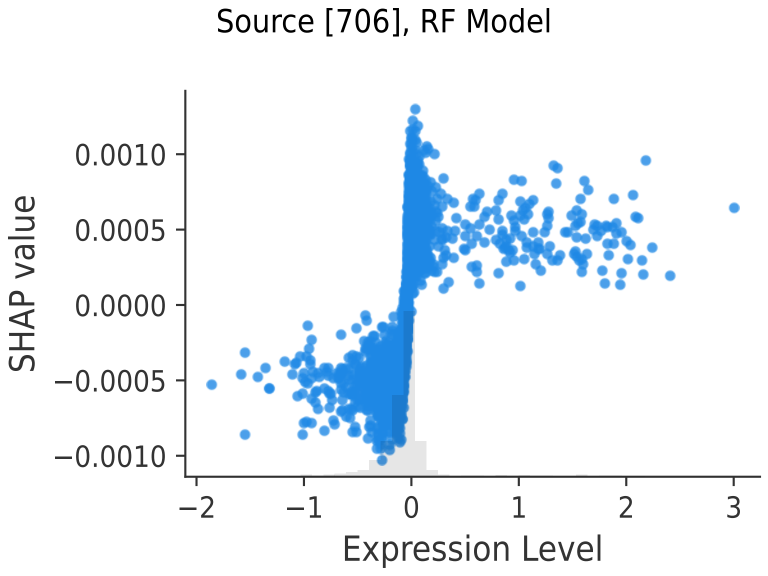
Source [706], RF Model
SHAP value
0.0010
0.0005
0.0000
−0.0005
−0.0010
−2
−1
0
1
2
3
Expression Level

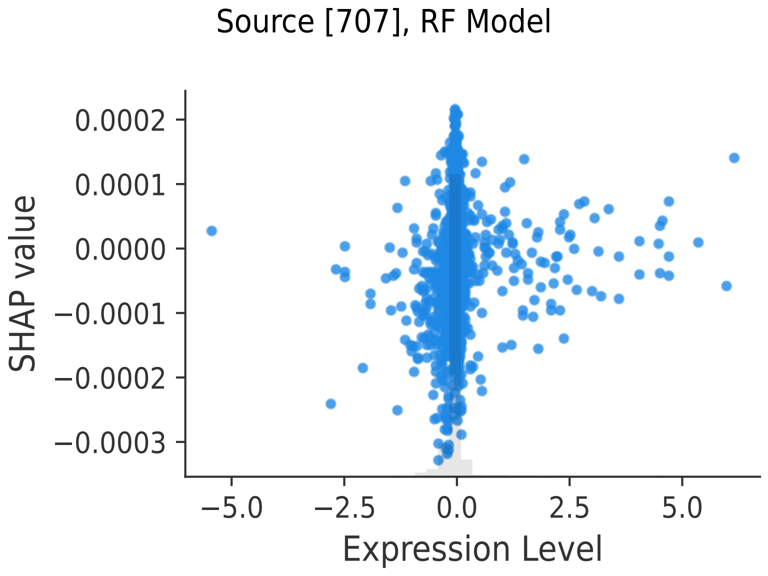

Source [707], RF Model
0.0002
0.0001
0.0000
−0.0001
−0.0002
−0.0003
SHAP value
−5.0
−2.5
0.0
2.5
5.0
Expression Level

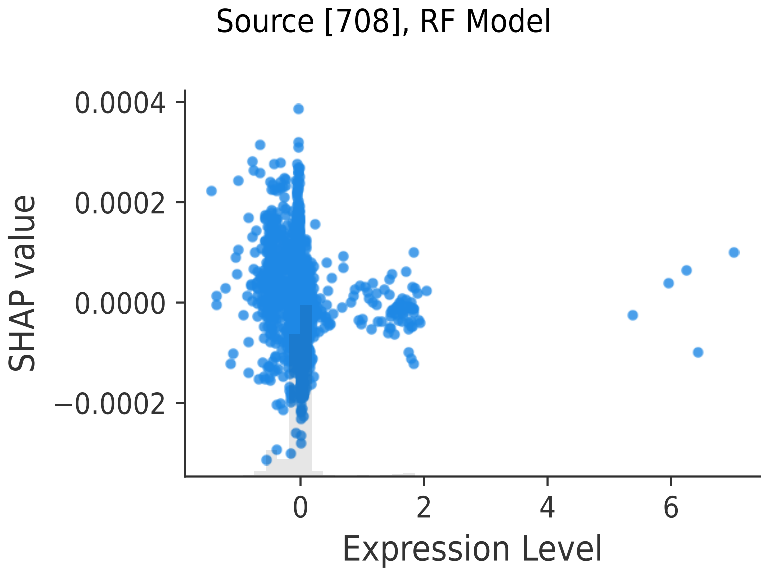
Source [708], RF Model
0.0004
0.0002
0.0000
−0.0002
SHAP value
0
2
4
6
Expression Level

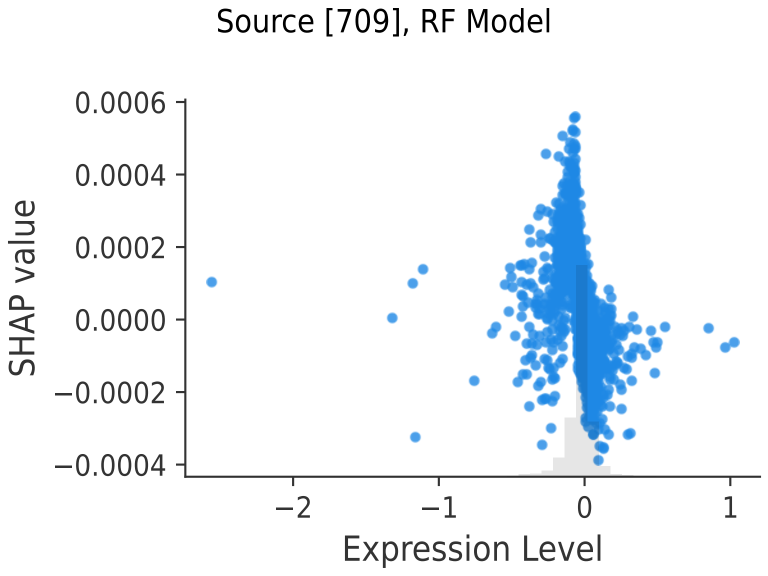
Source [709], RF Model
SHAP value
0.0006
0.0004
0.0002
0.0000
−0.0002
−0.0004
−2
−1
0
1
Expression Level

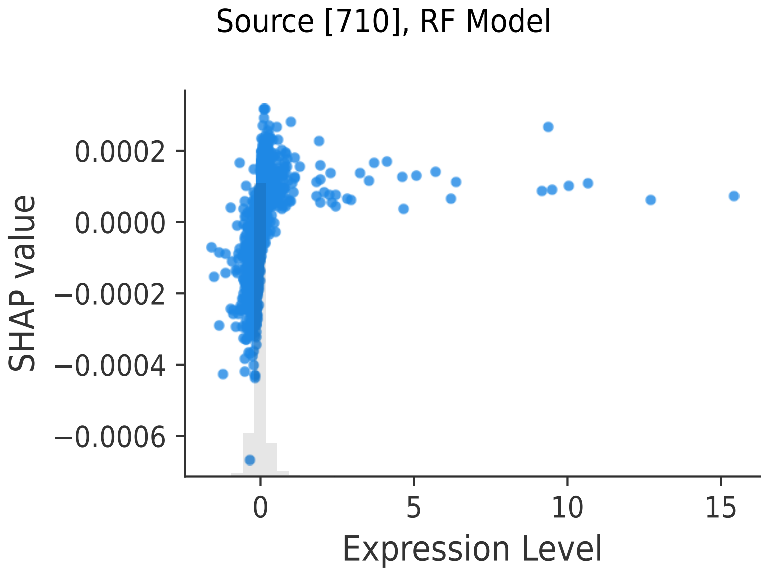
Source [710], RF Model
SHAP value
0.0002
0.0000
−0.0002
−0.0004
−0.0006
0
5
10
15
Expression Level

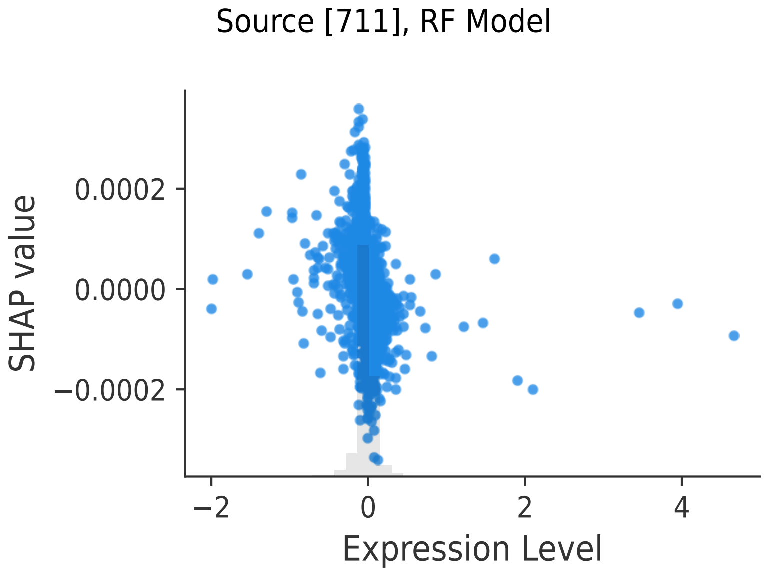

Source [711], RF Model
0.0002
0.0000
−0.0002
SHAP value
−2
0
2
4
Expression Level

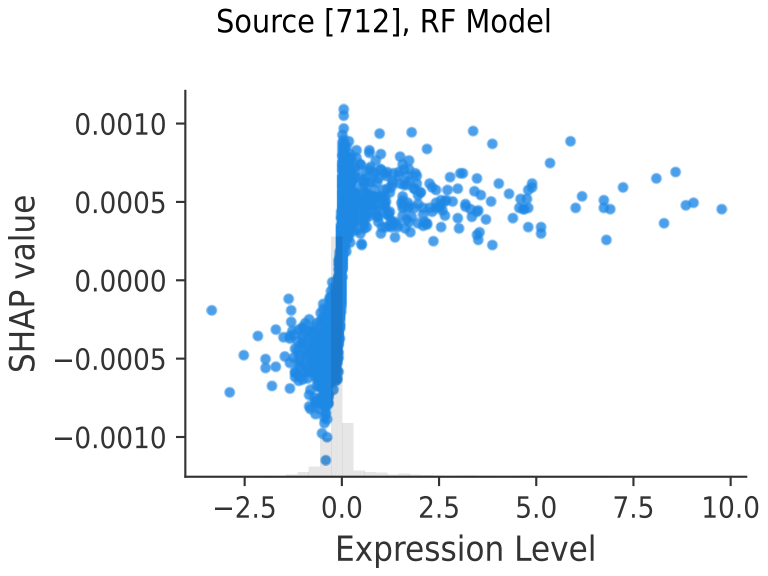
Source [712], RF Model
0.0010
0.0005
0.0000
−0.0005
−0.0010
SHAP value
−2.5
0.0
2.5
5.0
7.5
10.0
Expression Level

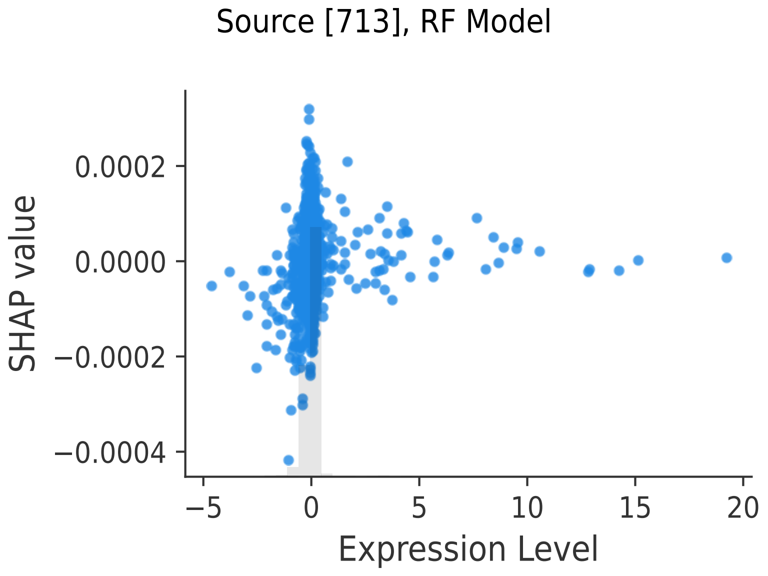
Source [713], RF Model
SHAP value
0.0002
0.0000
−0.0002
−0.0004
Expression Level
−5
0
5
10
15
20

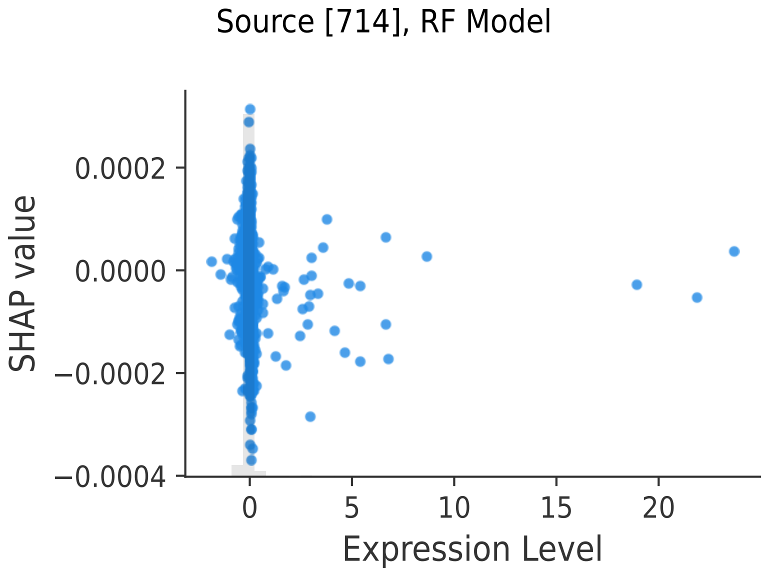
Source [714], RF Model
SHAP value
0.0002
0.0000
−0.0002
−0.0004
0
5
10
15
20
Expression Level

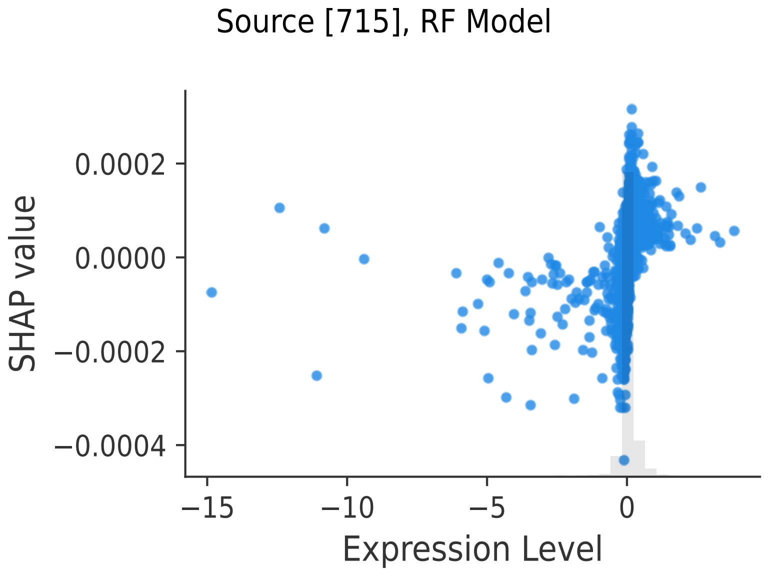
Source [715], RF Model
SHAP value
0.0002
0.0000
−0.0002
−0.0004
−15
−10
−5
0
Expression Level

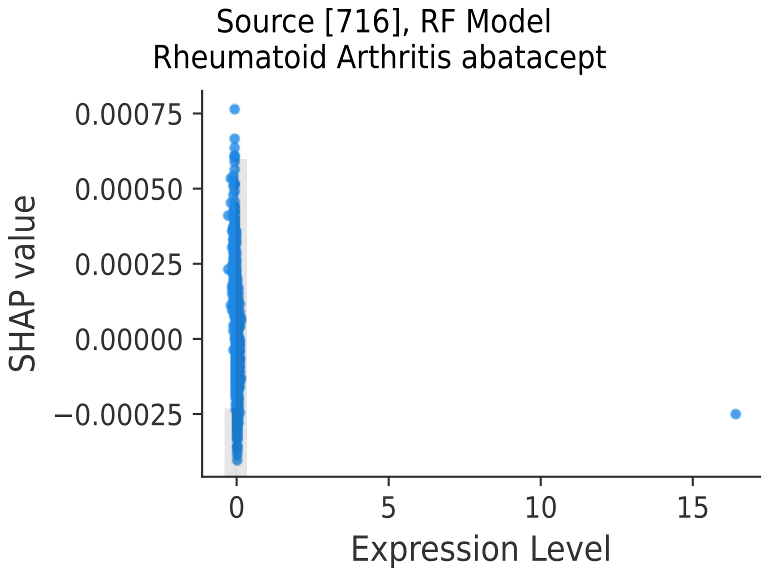

Source [716], RF Model
Rheumatoid Arthritis abatacept
0.00075
0.00050
0.00025
0.00000
−0.00025
SHAP value
0
5
10
15
Expression Level

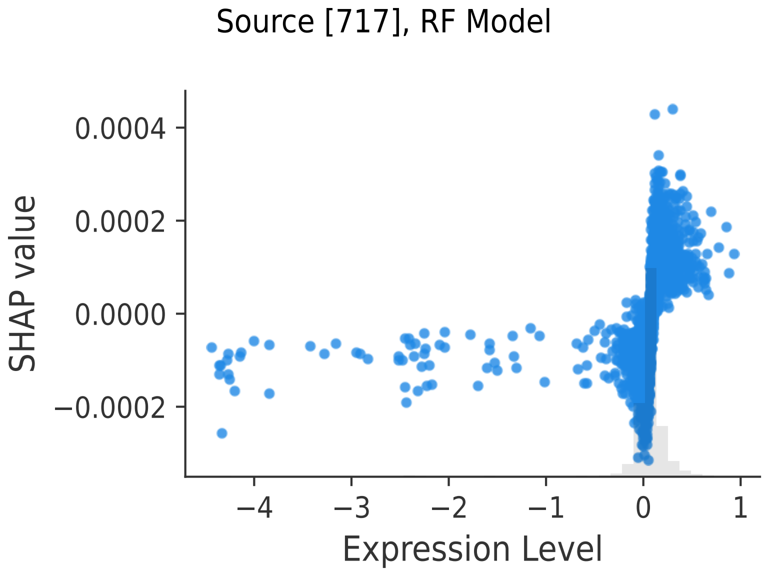
Source [717], RF Model
SHAP value
0.0004
0.0002
0.0000
−0.0002
−4
−3
−2
−1
0
1
Expression Level

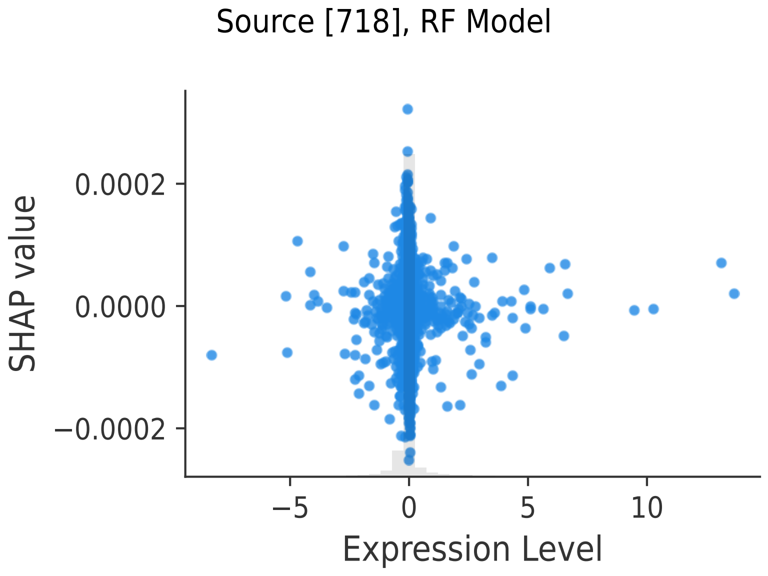

Source [718], RF Model
0.0002
0.0000
−0.0002
SHAP value
−5
0
5
10
Expression Level

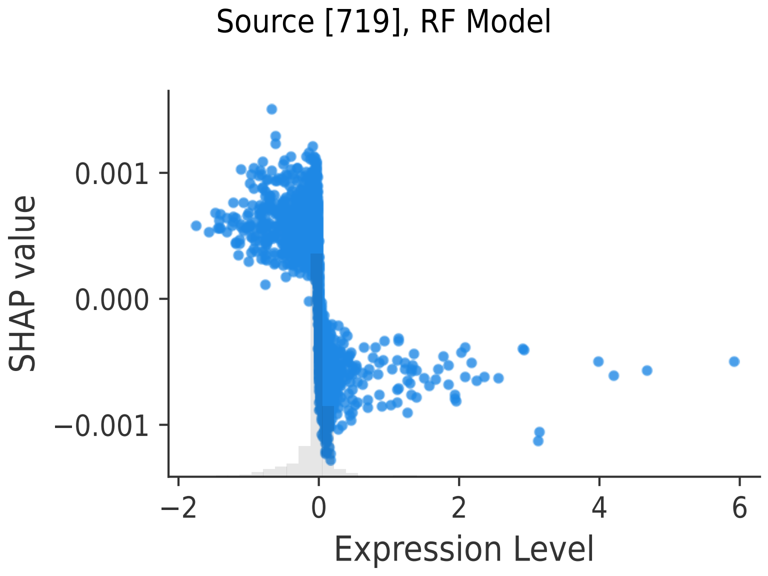
Source [719], RF Model
SHAP value
0.001
0.000
−0.001
−2
0
2
4
6
Expression Level

Source [720], RF Model

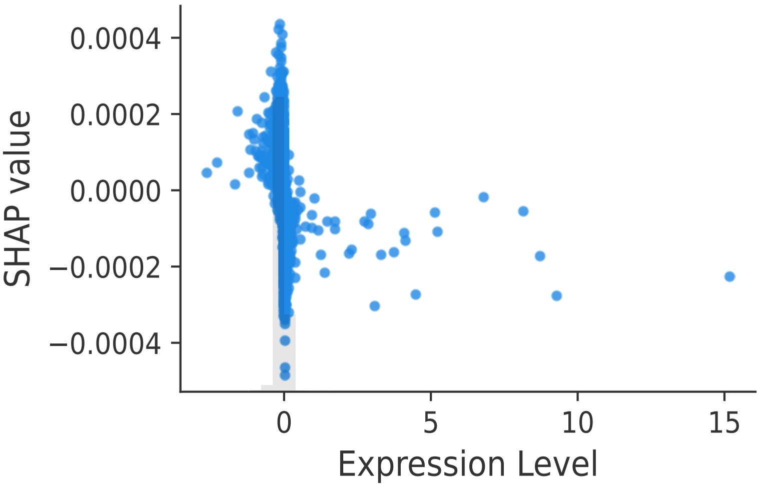

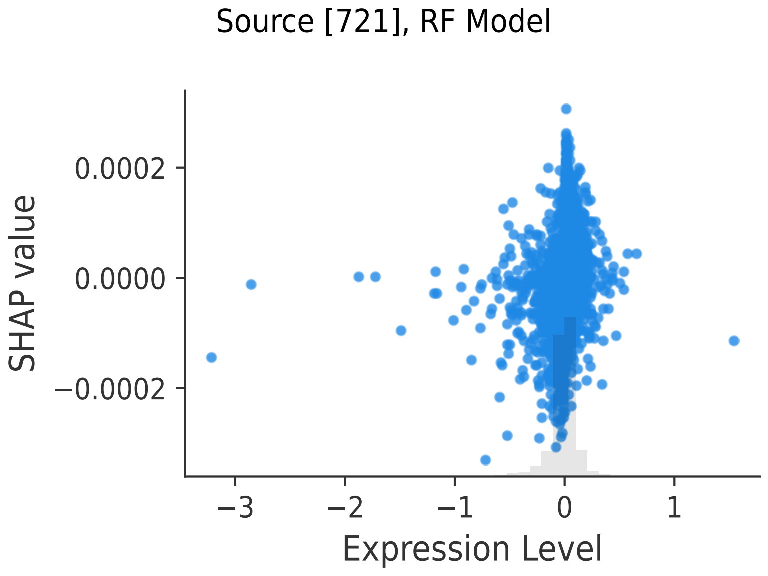
Source [721], RF Model
0.0002
0.0000
−0.0002
SHAP value
−3
−2
−1
0
1
Expression Level

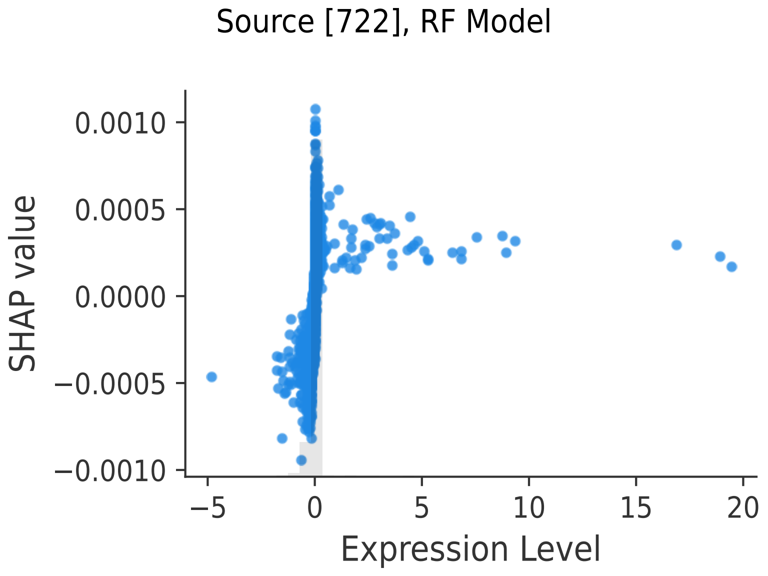
Source [722], RF Model
0.0010
0.0005
0.0000
−0.0005
−0.0010
SHAP value
−5
0
5
10
15
20
Expression Level

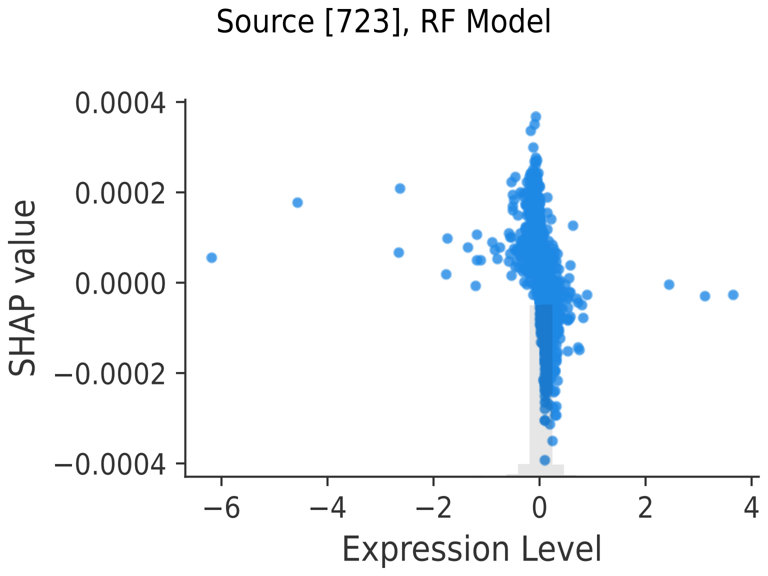

Source [723], RF Model
0.0004
0.0002
0.0000
−0.0002
−0.0004
SHAP value
−6
−4
−2
0
2
4
Expression Level

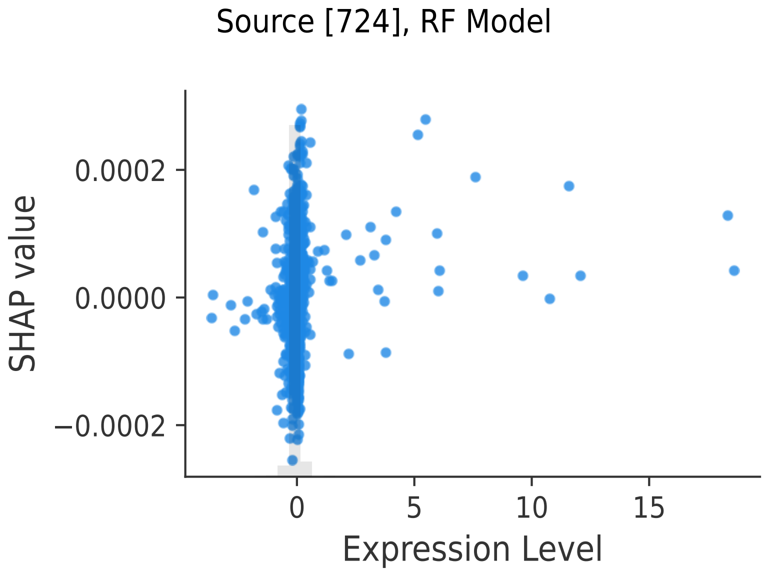

Source [724], RF Model
0.0002
0.0000
−0.0002
SHAP value
0
5
10
15
Expression Level

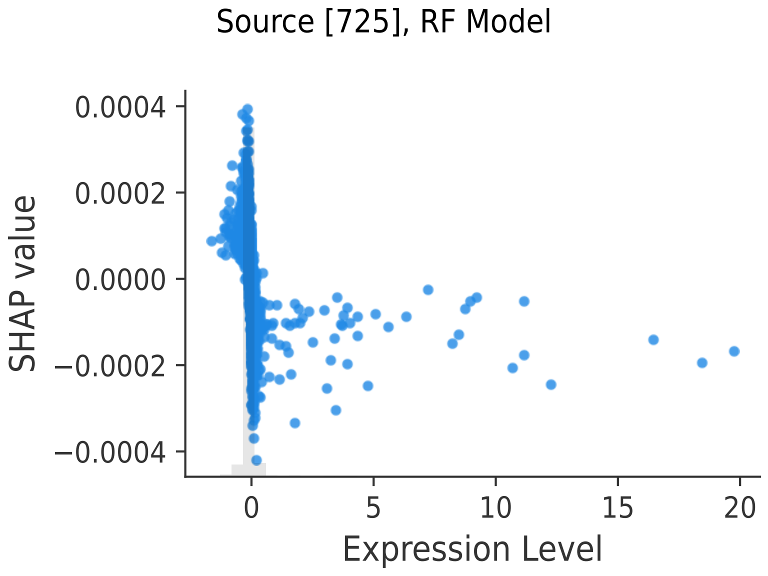
Source [725], RF Model
SHAP value
0.0004
0.0002
0.0000
−0.0002
−0.0004
0
5
10
15
20
Expression Level

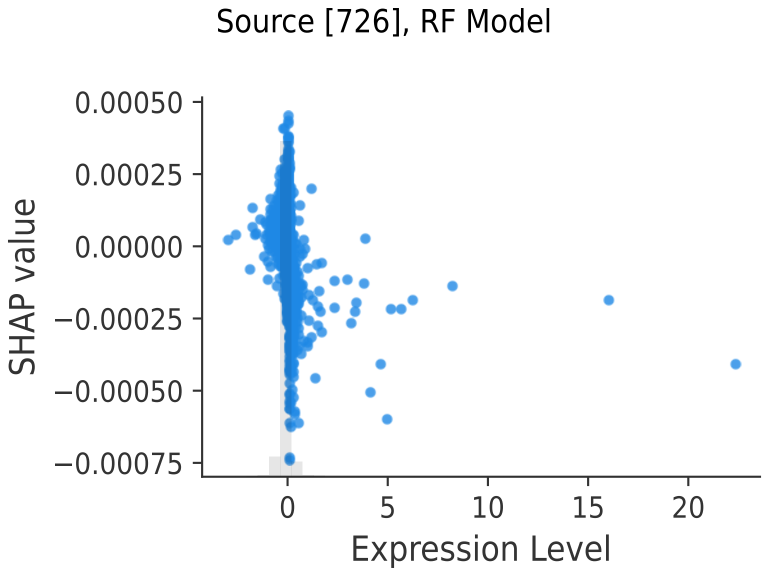

Source [726], RF Model
0.00050
0.00025
0.00000
−0.00025
−0.00050
−0.00075
SHAP value
0
5
10
15
20
Expression Level

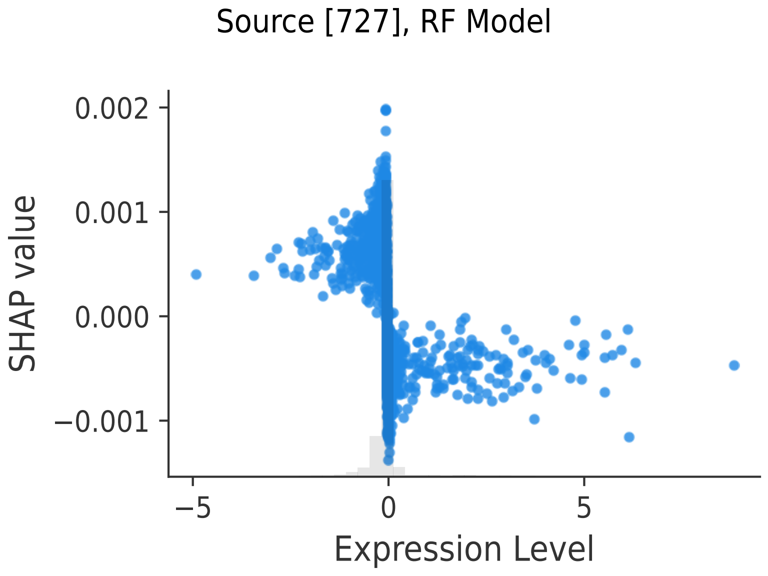
Source [727], RF Model
0.002
0.001
0.000
−0.001
SHAP value
−5
0
5
Expression Level

Source [728], RF Model

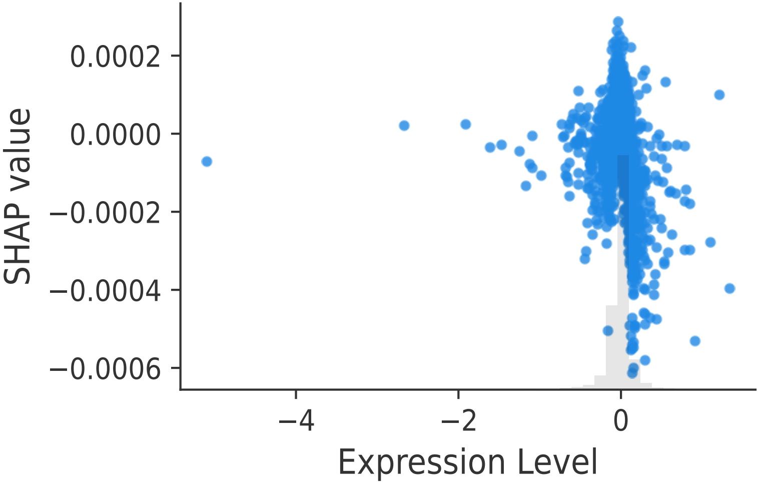

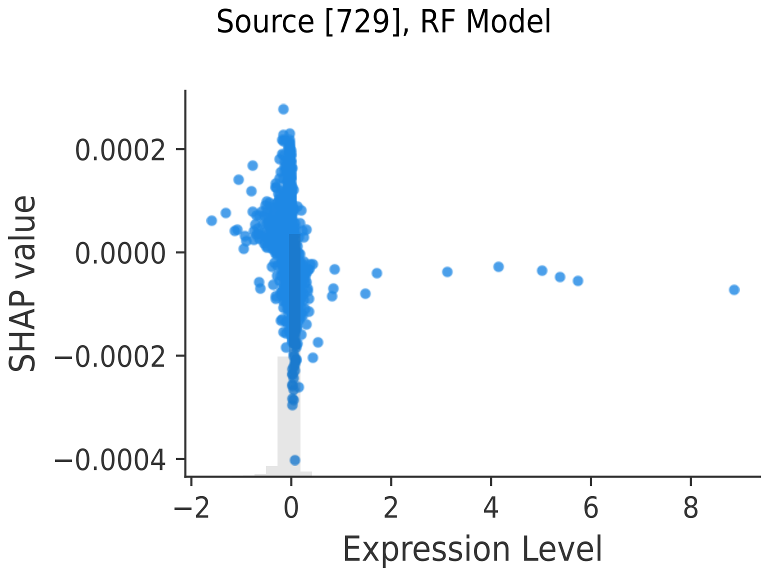
Source [729], RF Model
SHAP value
0.0002
0.0000
−0.0002
−0.0004
−2
0
2
4
6
8
Expression Level

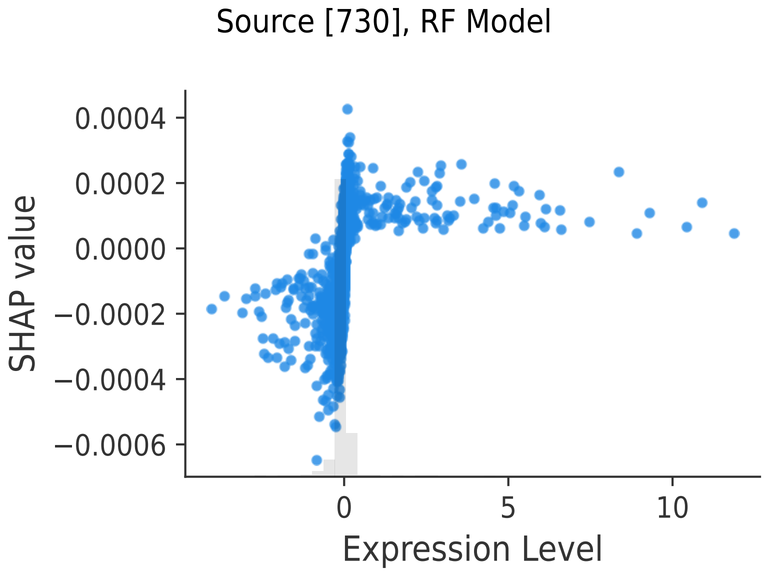
Source [730], RF Model
0.0004
0.0002
0.0000
−0.0002
−0.0004
−0.0006
SHAP value
0
5
10
Expression Level

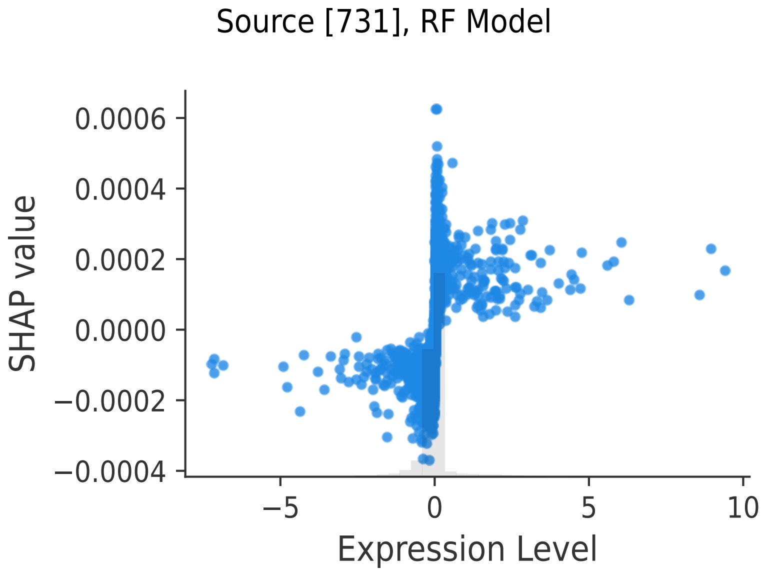
Source [731], RF Model
SHAP value
0.0006
0.0004
0.0002
0.0000
−0.0002
−0.0004
−5
0
5
10
Expression Level

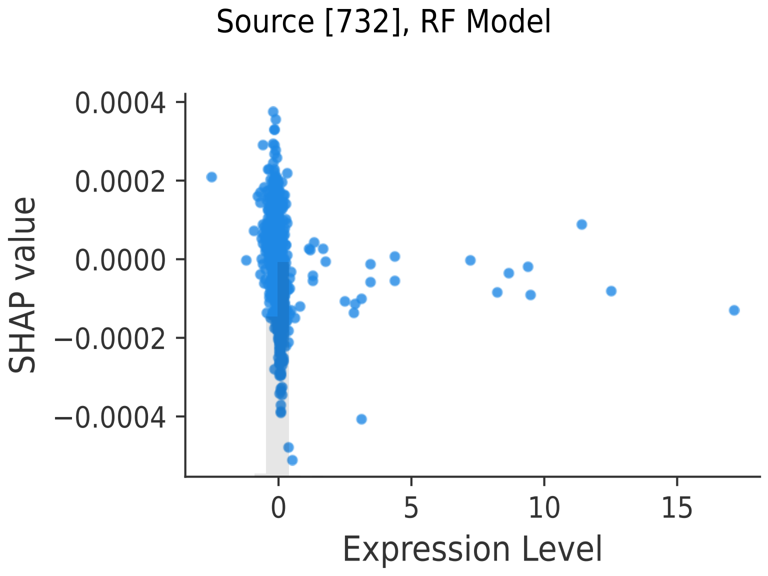

Source [732], RF Model
SHAP value
0.0004
0.0002
0.0000
−0.0002
−0.0004
0
5
10
15
Expression Level

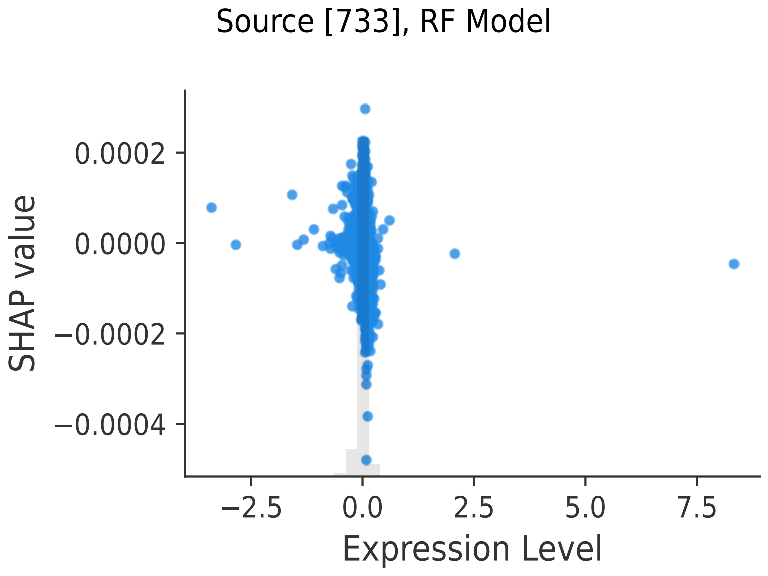

Source [733], RF Model
0.0002
0.0000
−0.0002
−0.0004
SHAP value
−2.5
0.0
2.5
5.0
7.5
Expression Level

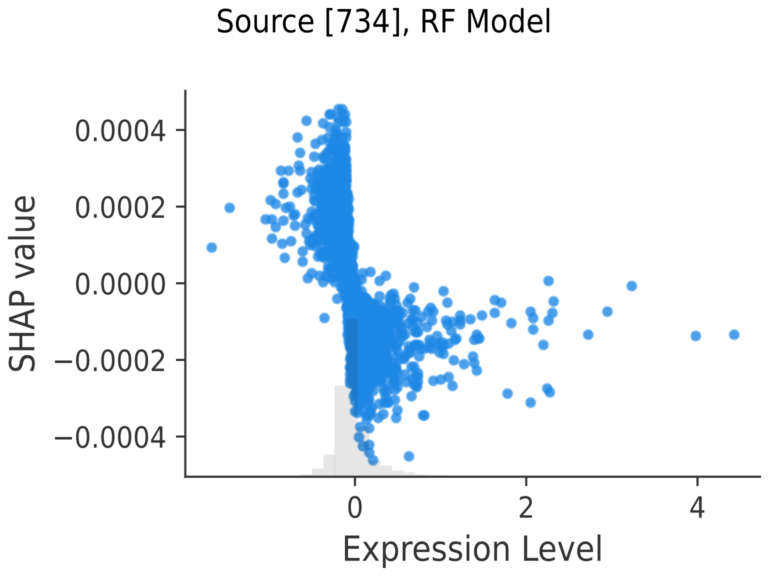
Source [734], RF Model
SHAP value
0.0004
0.0002
0.0000
−0.0002
−0.0004
0
2
4
Expression Level

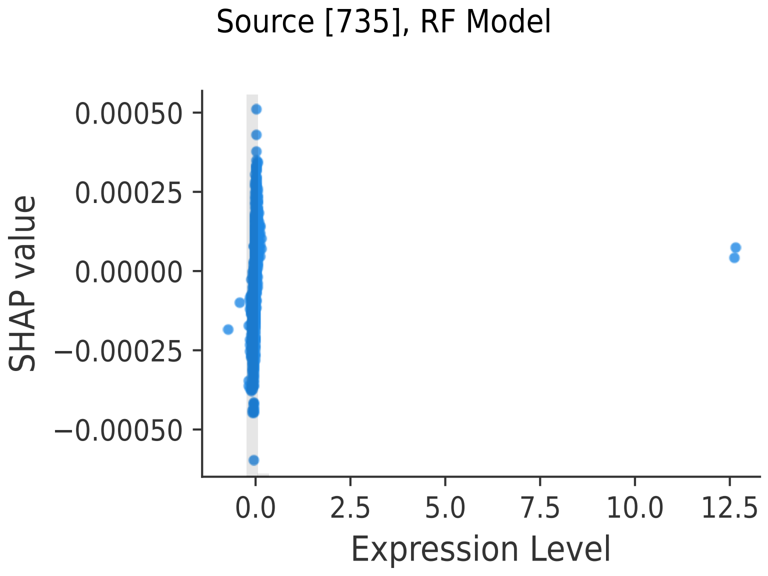
Source [735], RF Model
SHAP value
0.00050
0.00025
0.00000
−0.00025
−0.00050
0.0
2.5
5.0
7.5
10.0
12.5
Expression Level

Source [736], RF Model

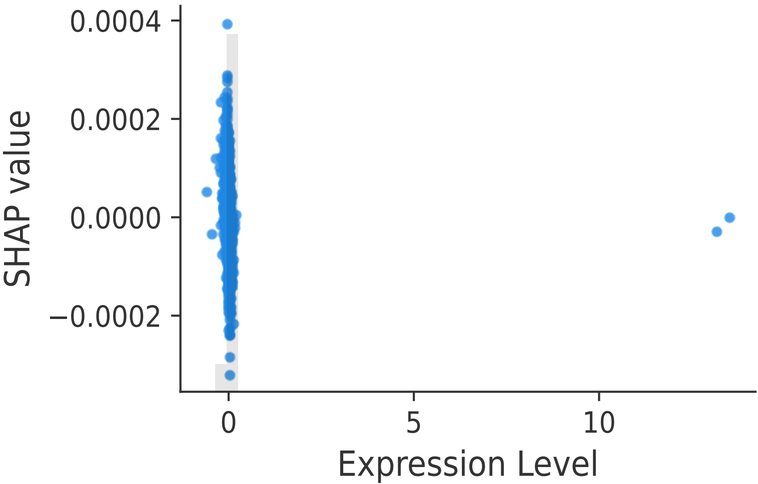

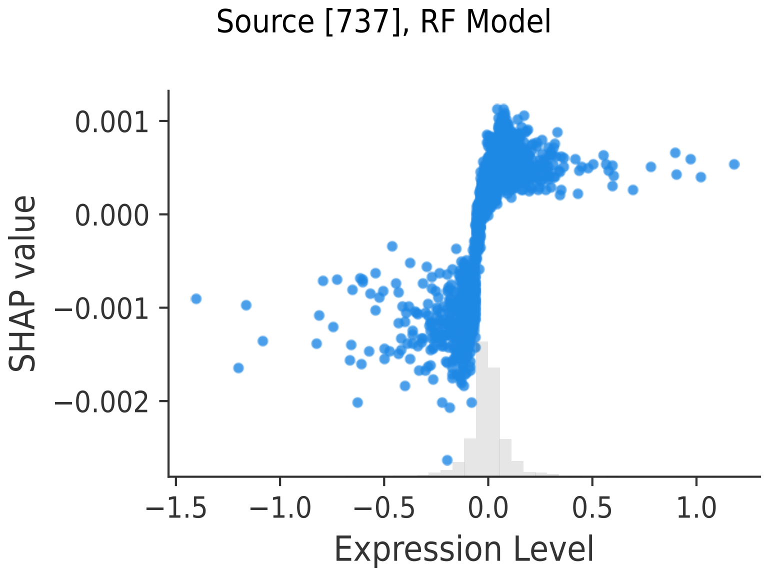

Source [737], RF Model
0.001
0.000
−0.001
−0.002
SHAP value
−1.5
−1.0
−0.5
0.0
0.5
1.0
Expression Level

Source [738], RF Model
Streptococcus URI

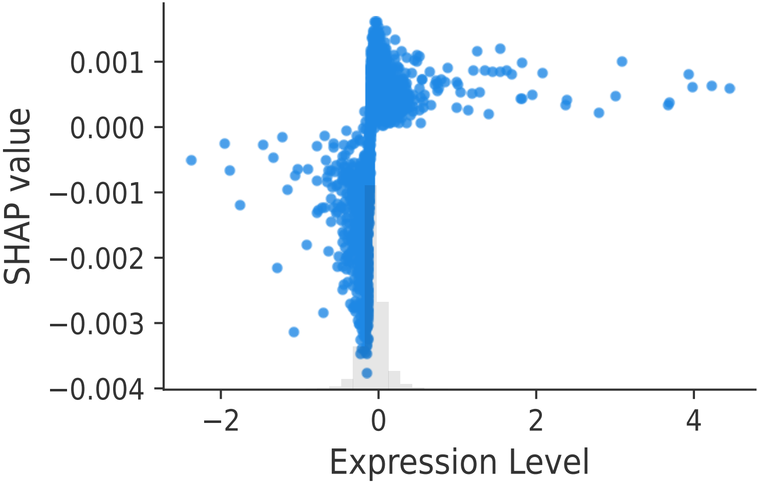

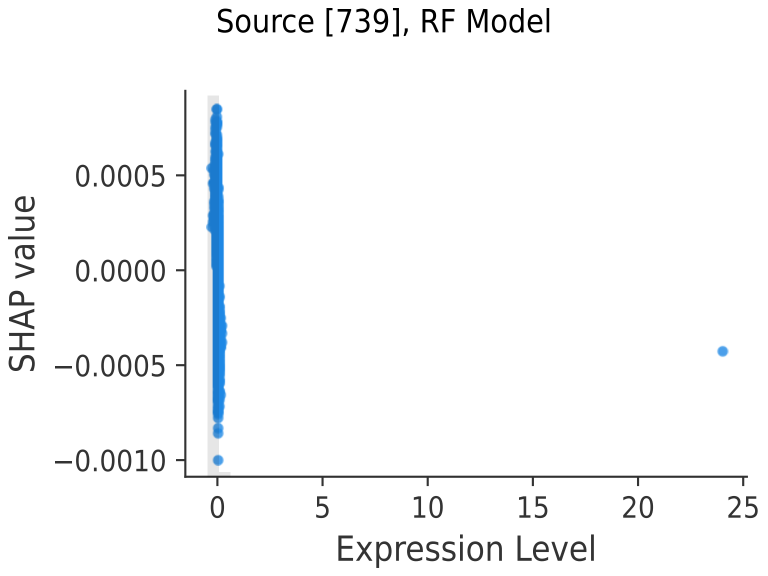

Source [739], RF Model
SHAP value
0.0005
0.0000
−0.0005
−0.0010
0
5
10
15
20
25
Expression Level

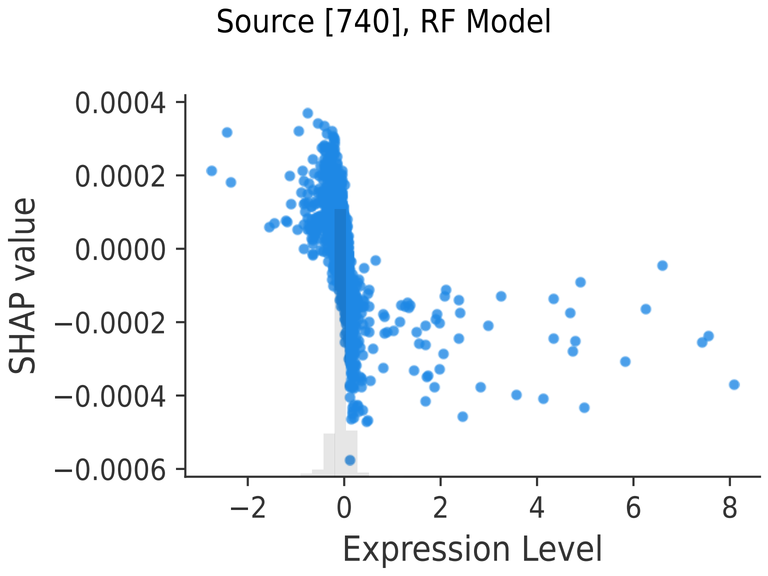
Source [740], RF Model
0.0004
0.0002
0.0000
−0.0002
−0.0004
−0.0006
SHAP value
−2
0
2
4
6
8
Expression Level

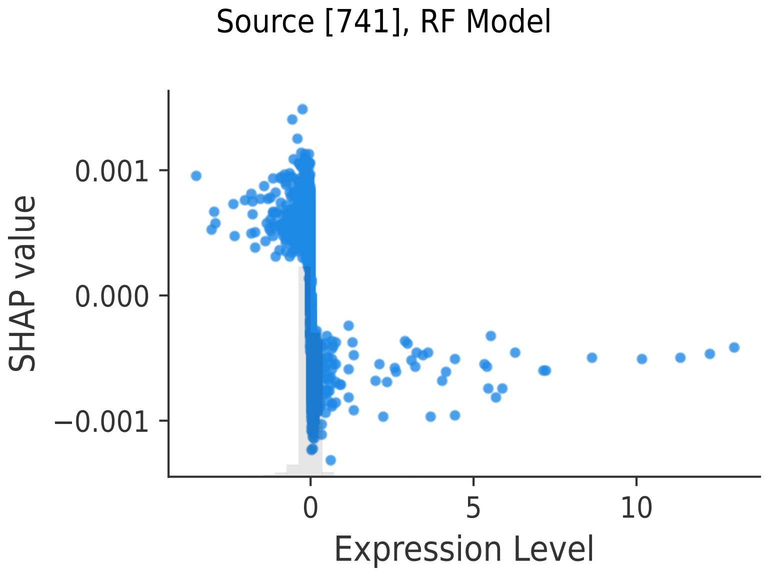
Source [741], RF Model
SHAP value
0.001
0.000
−0.001
0
5
10
Expression Level

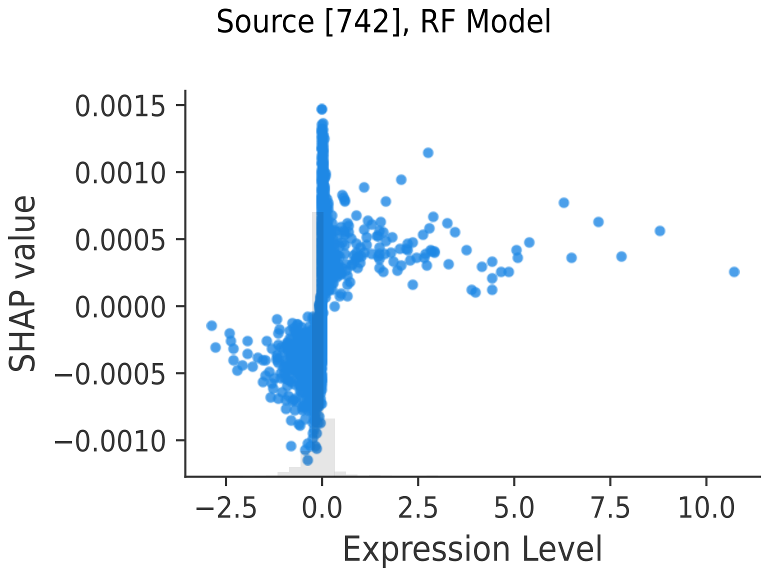
Source [742], RF Model
SHAP value
0.0015
0.0010
0.0005
0.0000
−0.0005
−0.0010
−2.5
0.0
2.5
5.0
7.5
10.0
Expression Level

Source [743], RF Model

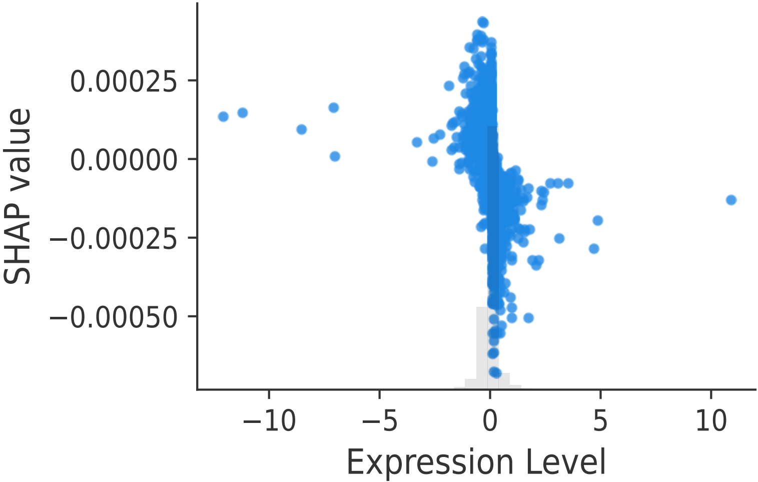

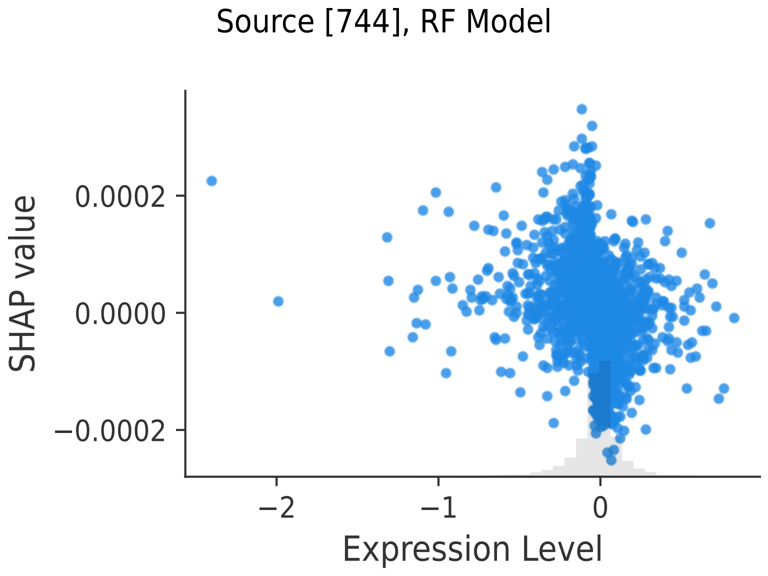
Source [744], RF Model
SHAP value
0.0002
0.0000
−0.0002
−2
−1
0
Expression Level

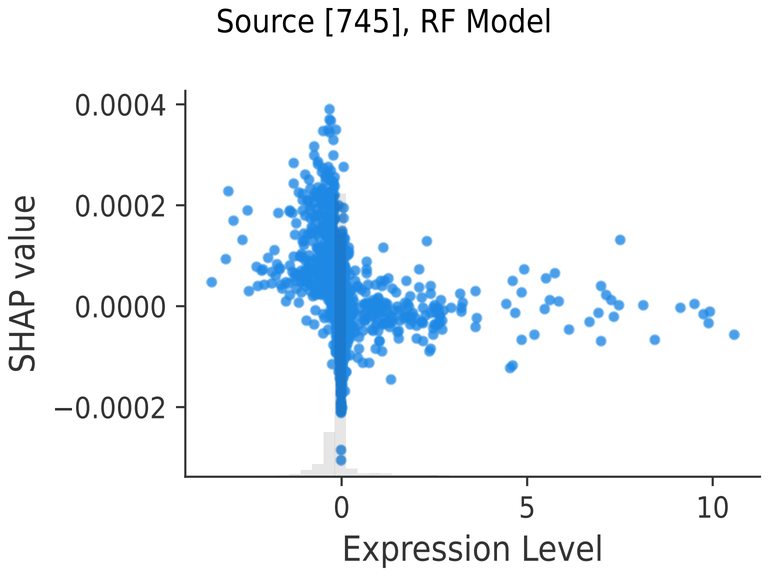
Source [745], RF Model
SHAP value
0.0004
0.0002
0.0000
−0.0002
0
5
10
Expression Level

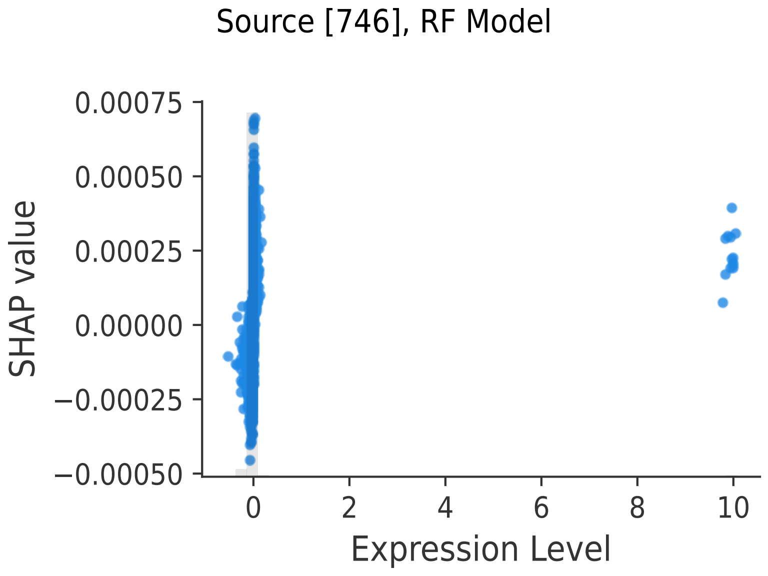

Source [746], RF Model
0.00075
0.00050
0.00025
0.00000
−0.00025
−0.00050
SHAP value
0
2
4
6
8
10
Expression Level

Source [747], RF Model

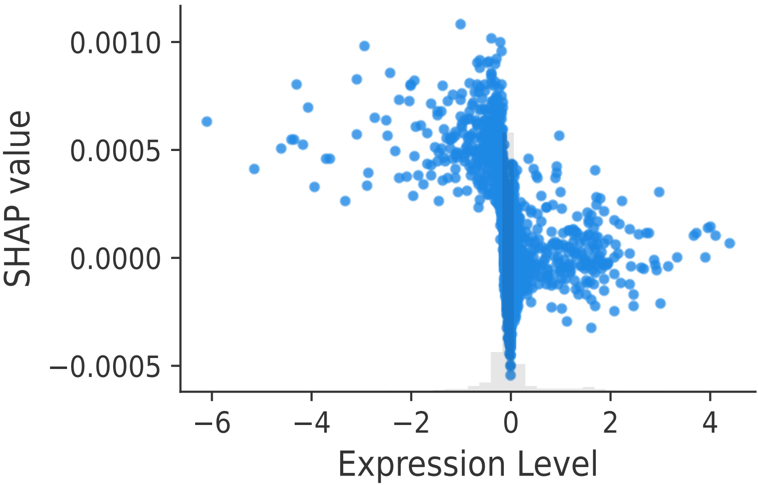

Source [748], RF Model

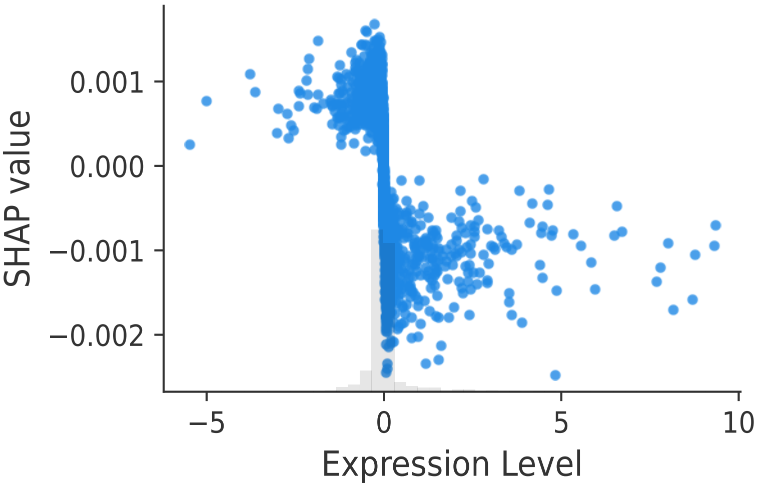

Source [749], RF Model

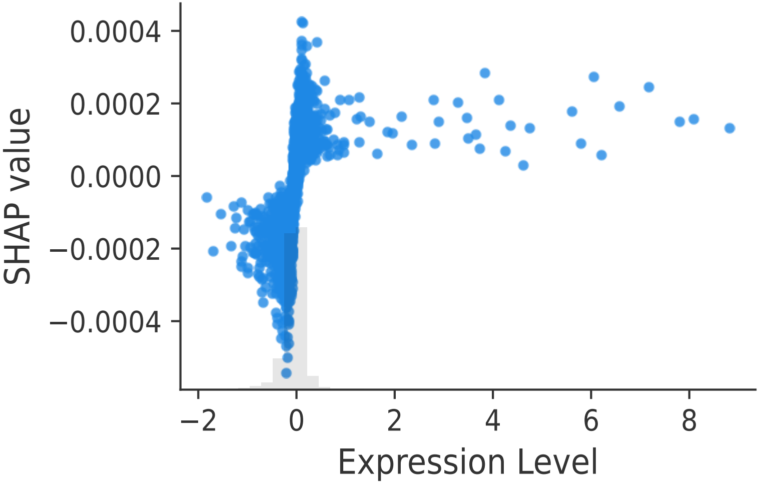

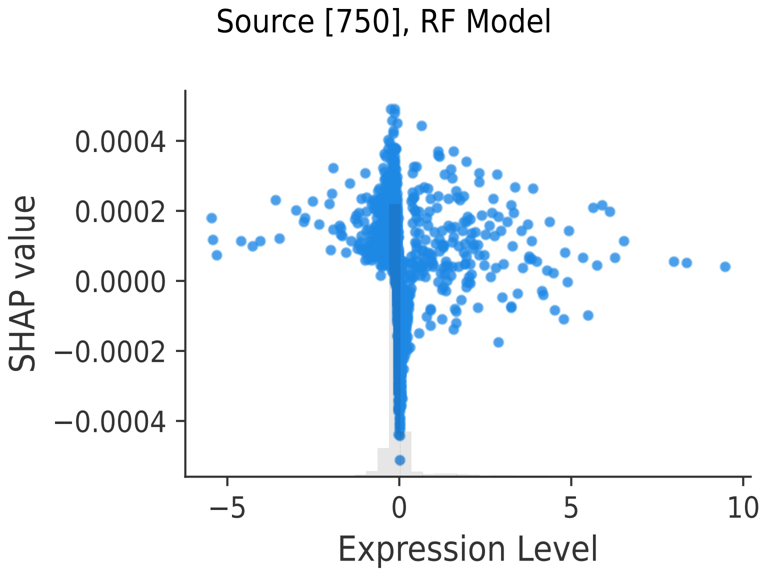

Source [750], RF Model
0.0004
0.0002
0.0000
−0.0002
−0.0004
SHAP value
−5
0
5
10
Expression Level

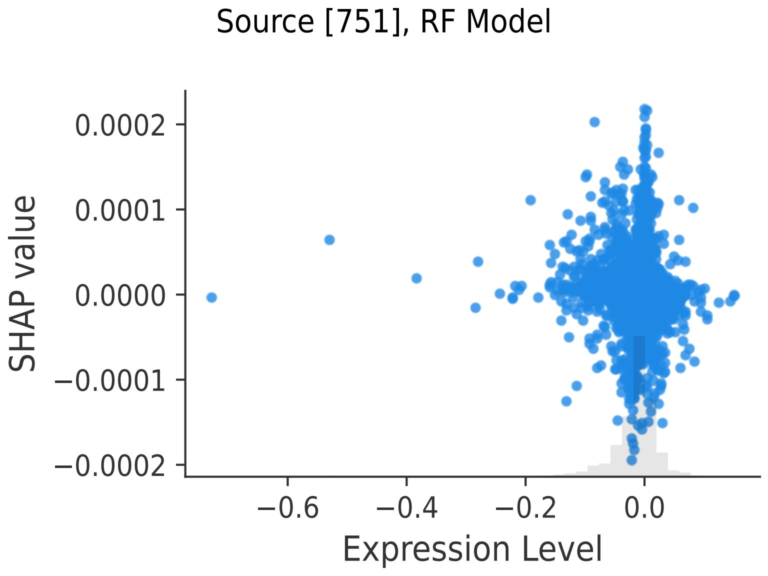
Source [751], RF Model
SHAP value
0.0002
0.0001
0.0000
−0.0001
−0.0002
−0.6
−0.4
−0.2
0.0
Expression Level

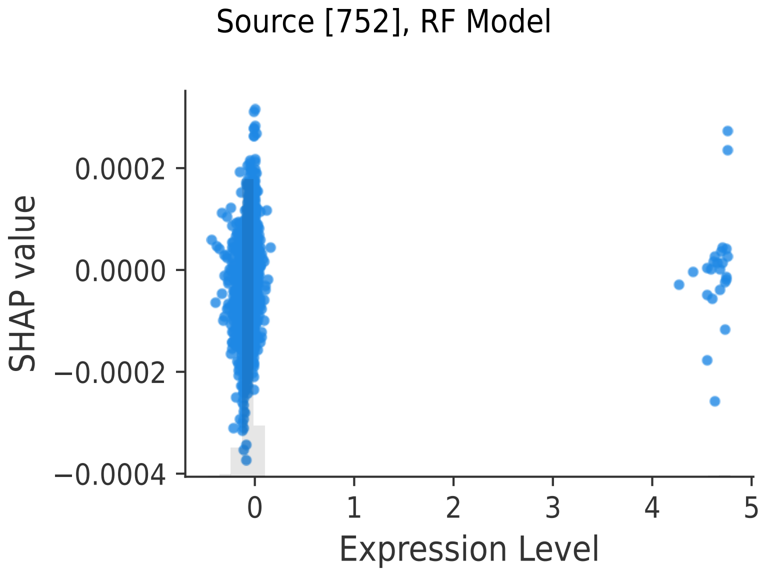
Source [752], RF Model
SHAP value
0.0002
0.0000
−0.0002
−0.0004
0
1
2
3
4
5
Expression Level

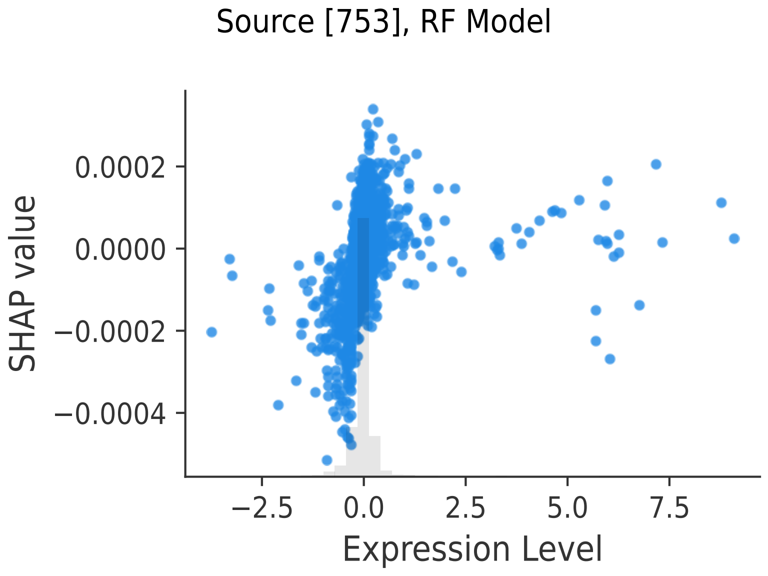
Source [753], RF Model
0.0002
0.0000
−0.0002
−0.0004
SHAP value
−2.5
0.0
2.5
5.0
7.5
Expression Level

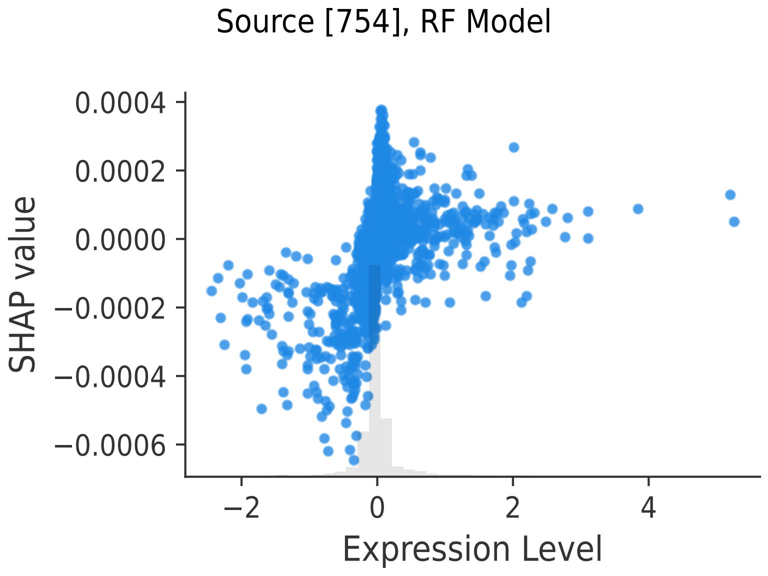
Source [754], RF Model
0.0004
0.0002
0.0000
−0.0002
−0.0004
−0.0006
SHAP value
−2
0
2
4
Expression Level

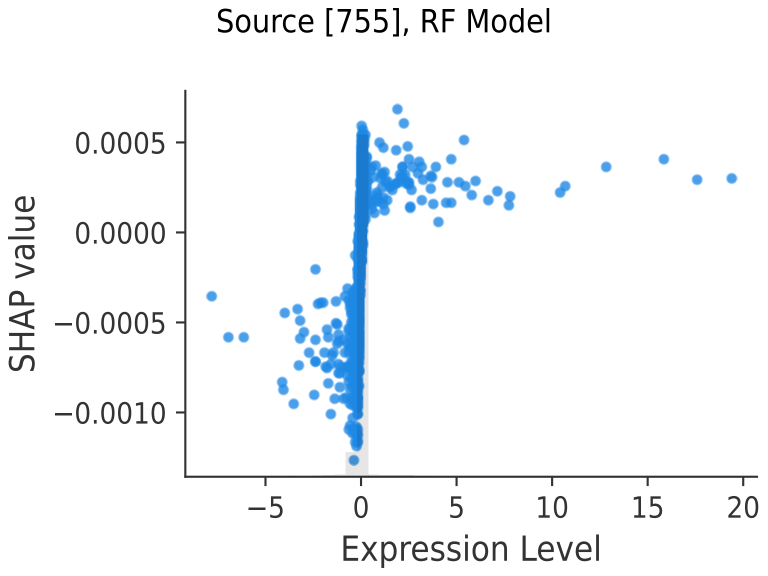
Source [755], RF Model
0.0005
0.0000
−0.0005
−0.0010
SHAP value
−5
0
5
10
15
20
Expression Level

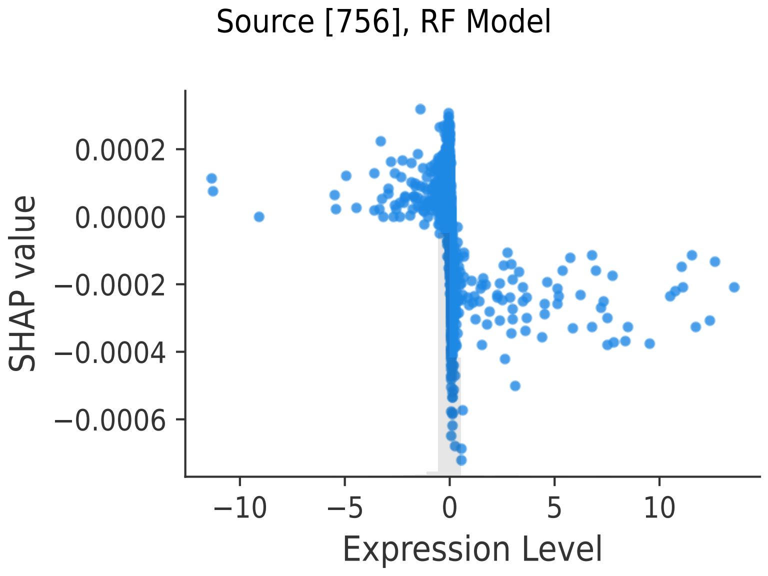
Source [756], RF Model
SHAP value
0.0002
0.0000
−0.0002
−0.0004
−0.0006
−10
−5
0
5
10
Expression Level

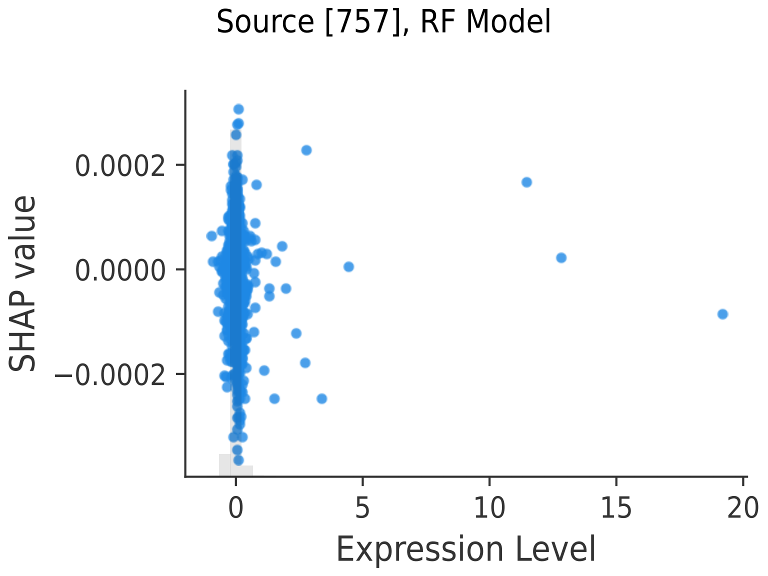

Source [757], RF Model
SHAP value
0.0002
0.0000
−0.0002
0
5
10
15
20
Expression Level

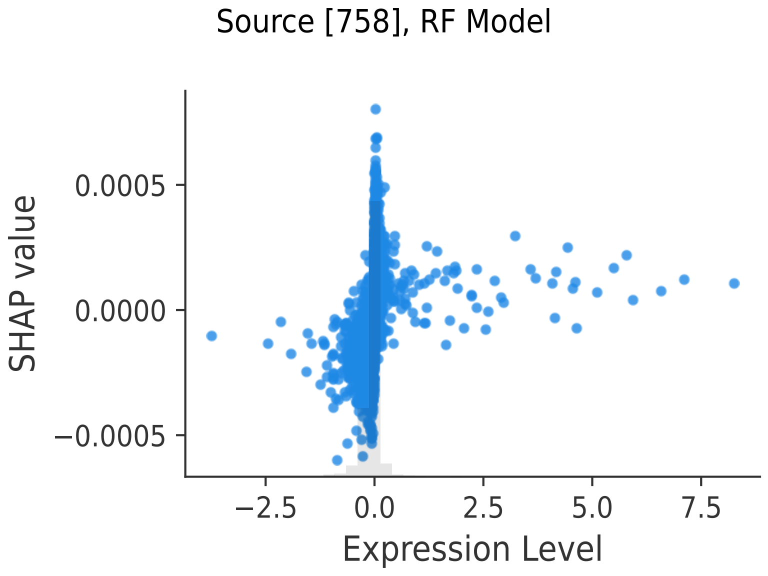
Source [758], RF Model
SHAP value
0.0005
0.0000
−0.0005
−2.5
0.0
2.5
5.0
7.5
Expression Level

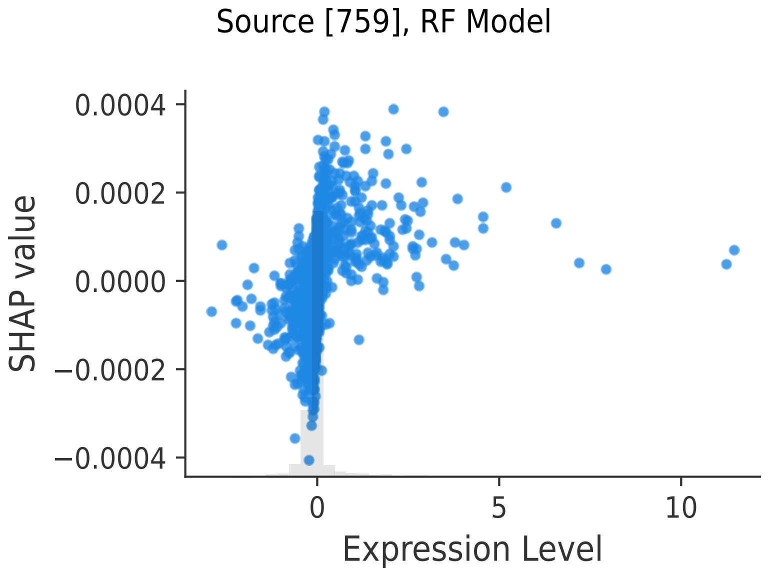
Source [759], RF Model
SHAP value
0.0004
0.0002
0.0000
−0.0002
−0.0004
0
5
10
Expression Level

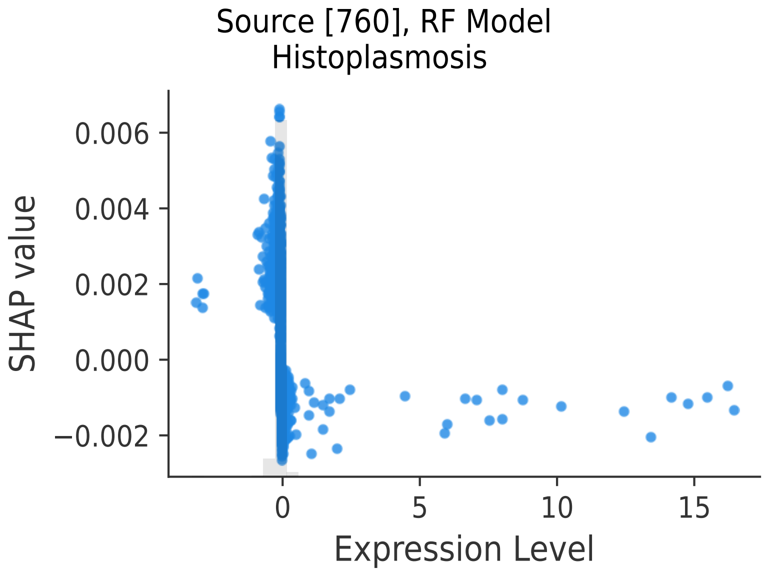
Source [760], RF Model
Histoplasmosis
SHAP value
0.006
0.004
0.002
0.000
−0.002
0
5
10
15
Expression Level

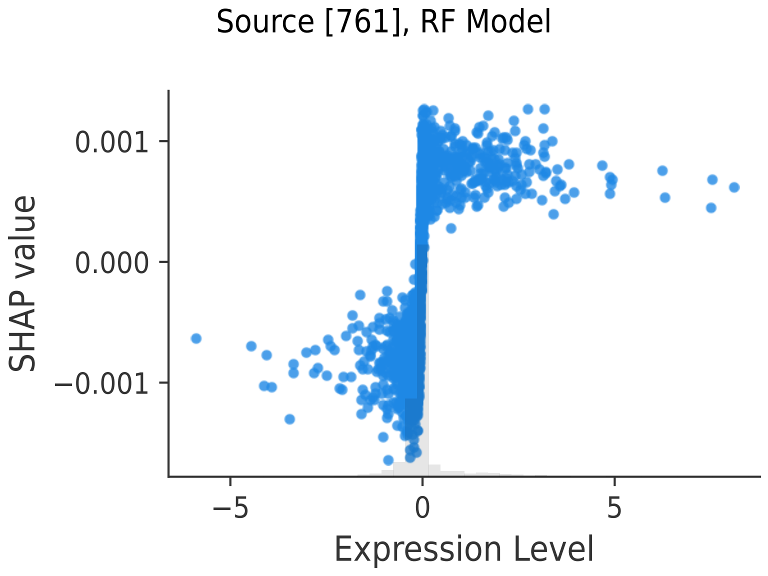
Source [761], RF Model
SHAP value
0.001
0.000
−0.001
−5
0
5
Expression Level

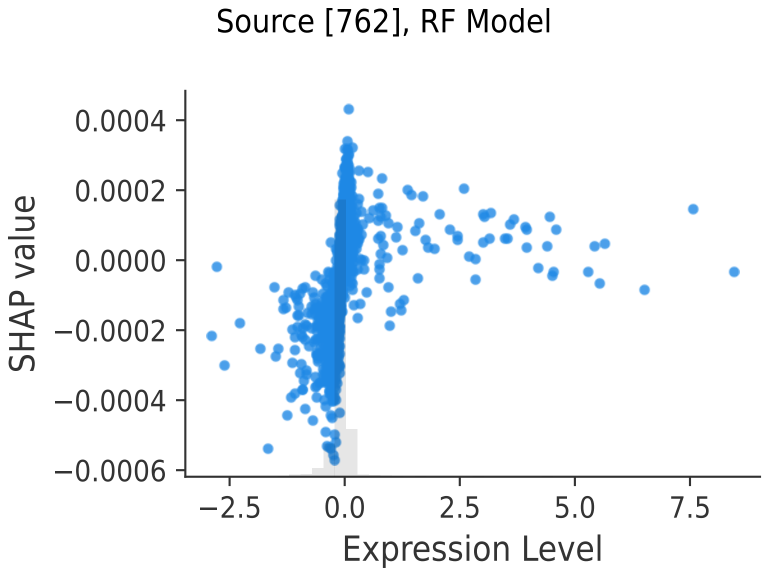

Source [762], RF Model
SHAP value
0.0004
0.0002
0.0000
−0.0002
−0.0004
−0.0006
−2.5
0.0
2.5
5.0
7.5
Expression Level

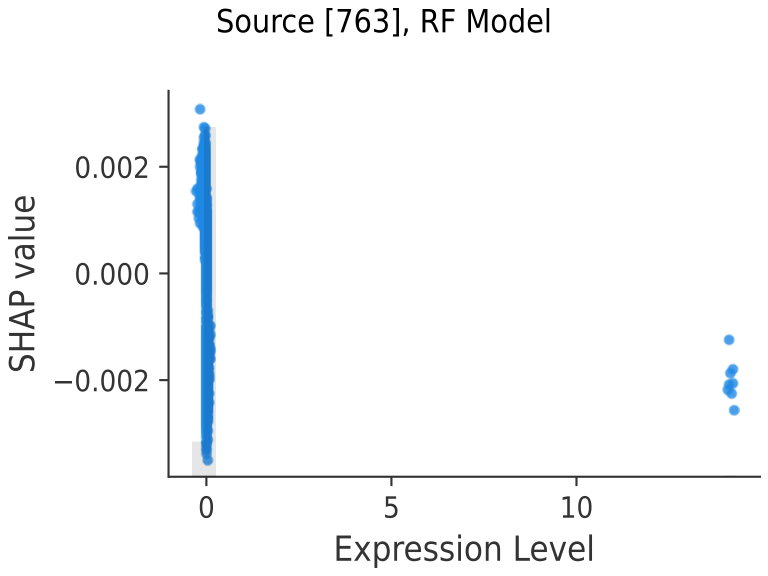

Source [763], RF Model
0.002
0.000
−0.002
SHAP value
0
5
10
Expression Level

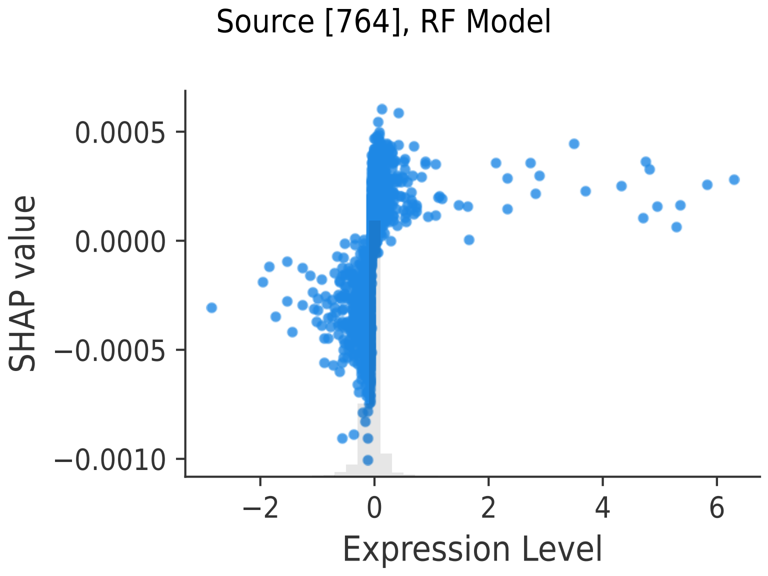

Source [764], RF Model
0.0005
0.0000
−0.0005
−0.0010
SHAP value
−2
0
2
4
6
Expression Level

Source [765], RF Model

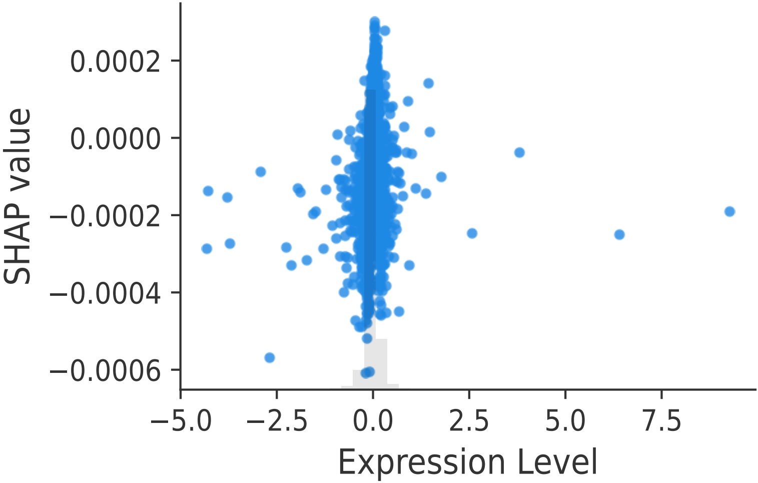

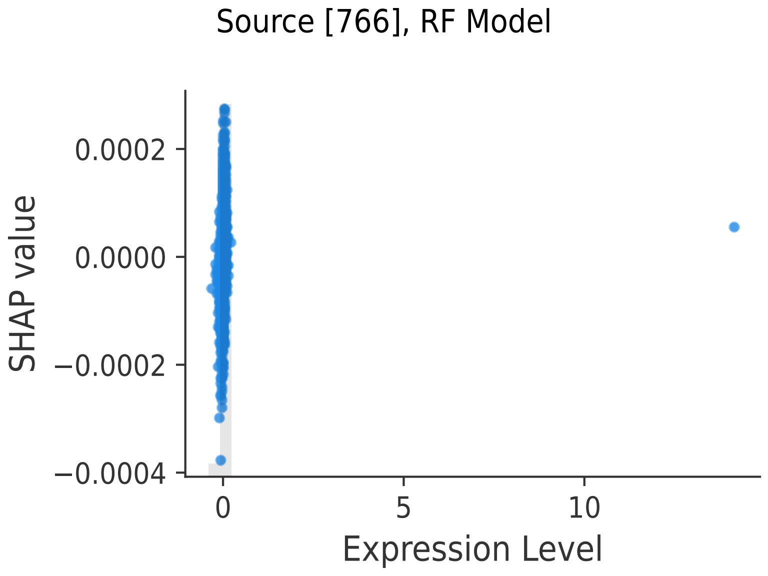

Source [766], RF Model
0.0002
0.0000
−0.0002
−0.0004
SHAP value
0
5
10
Expression Level

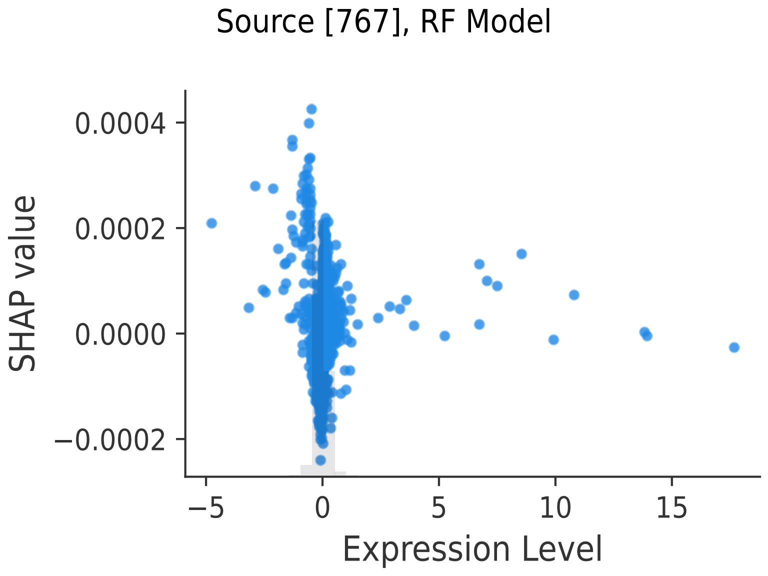
Source [767], RF Model
SHAP value
0.0004
0.0002
0.0000
−0.0002
−5
0
5
10
15
Expression Level

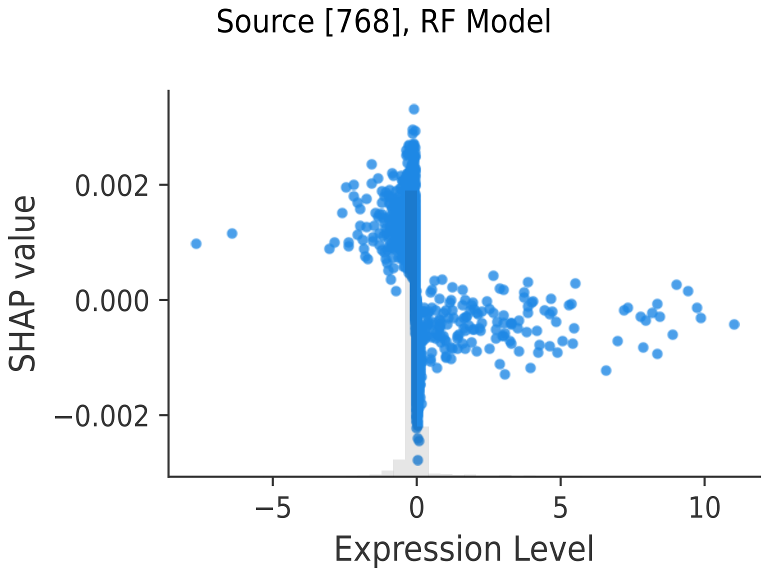
Source [768], RF Model
SHAP value
0.002
0.000
−0.002
−5
0
5
10
Expression Level

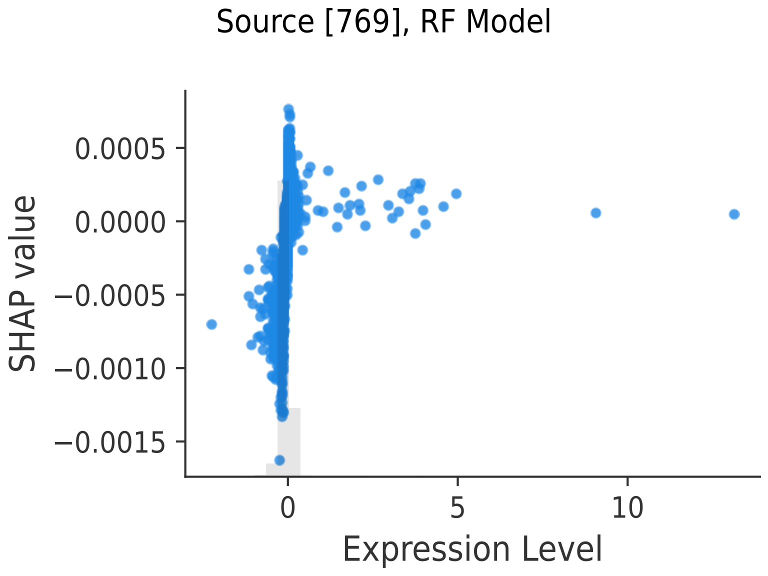

Source [769], RF Model
SHAP value
0.0005
0.0000
−0.0005
−0.0010
−0.0015
0
5
10
Expression Level

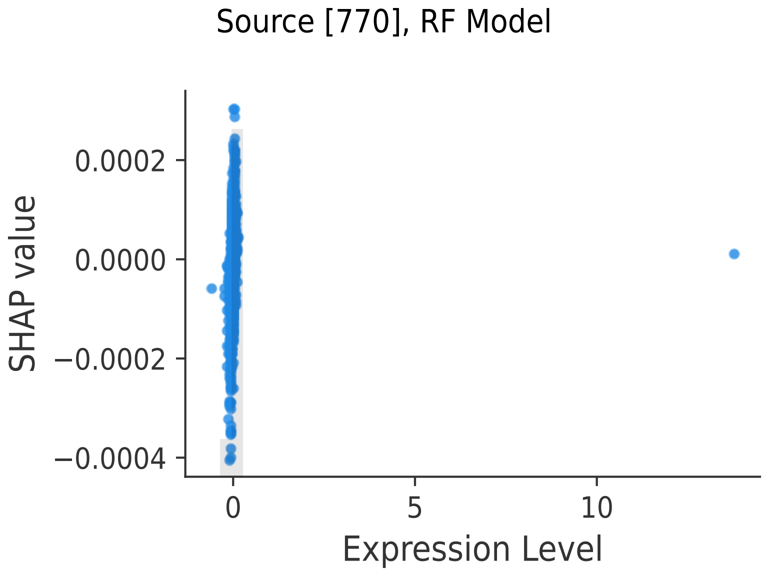

Source [770], RF Model
0.0002
0.0000
−0.0002
−0.0004
SHAP value
0
5
10
Expression Level

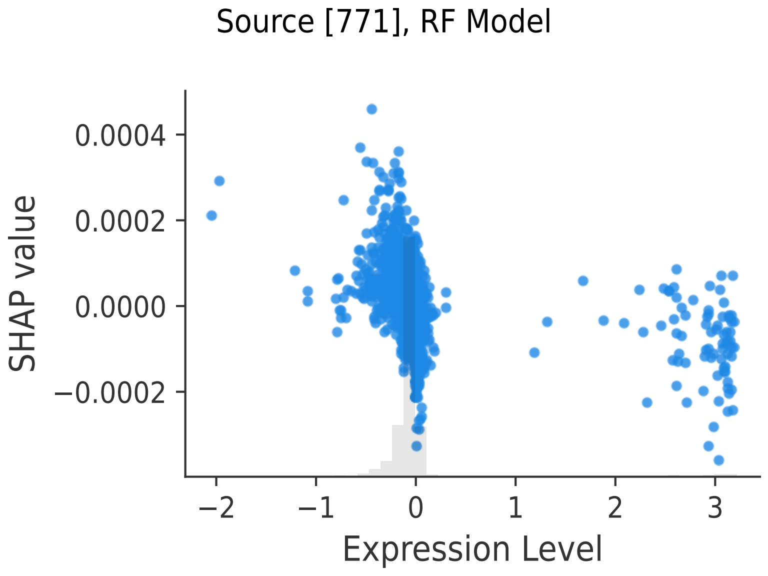
Source [771], RF Model
SHAP value
0.0004
0.0002
0.0000
−0.0002
−2
−1
0
1
2
3
Expression Level

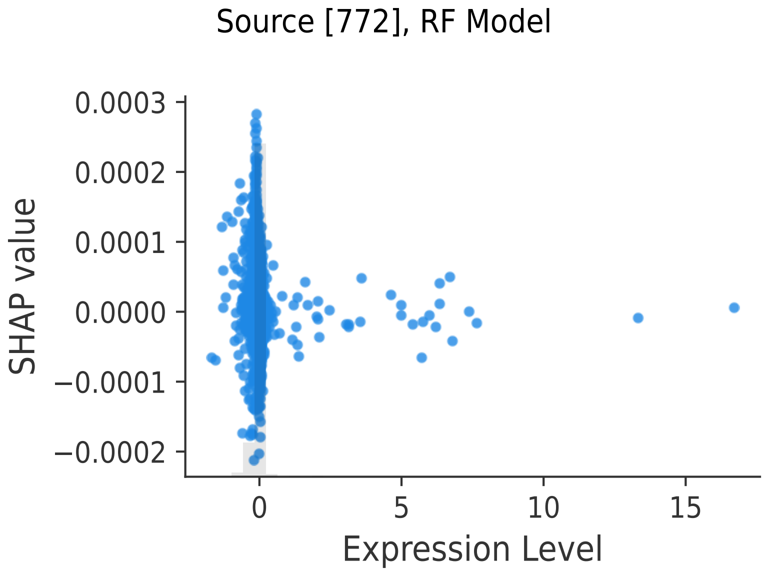
Source [772], RF Model
SHAP value
0.0003
0.0002
0.0001
0.0000
−0.0001
−0.0002
0
5
10
15
Expression Level

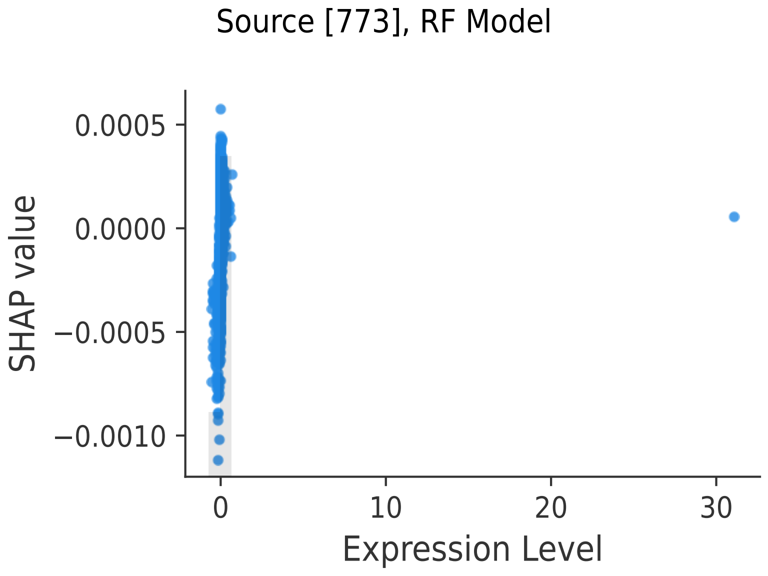
Source [773], RF Model
0.0005
0.0000
−0.0005
−0.0010
SHAP value
0
10
20
30
Expression Level

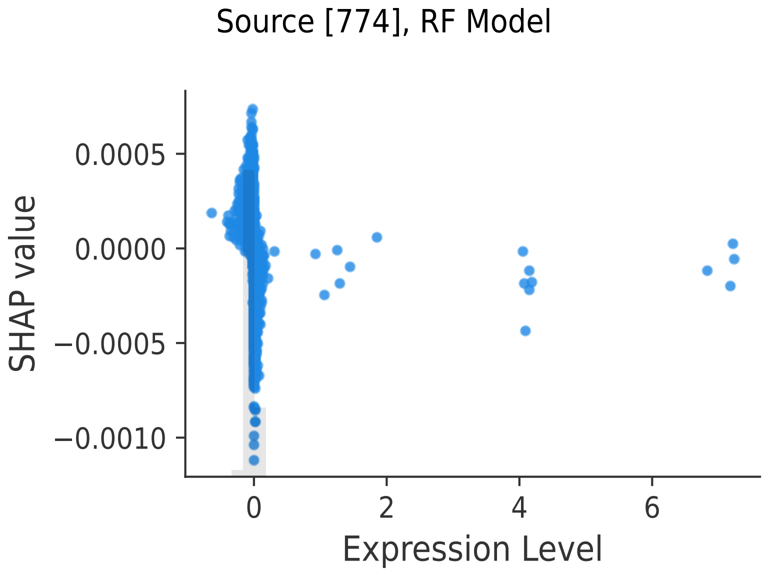
Source [774], RF Model
0.0005
0.0000
−0.0005
−0.0010
SHAP value
0
2
4
6
Expression Level

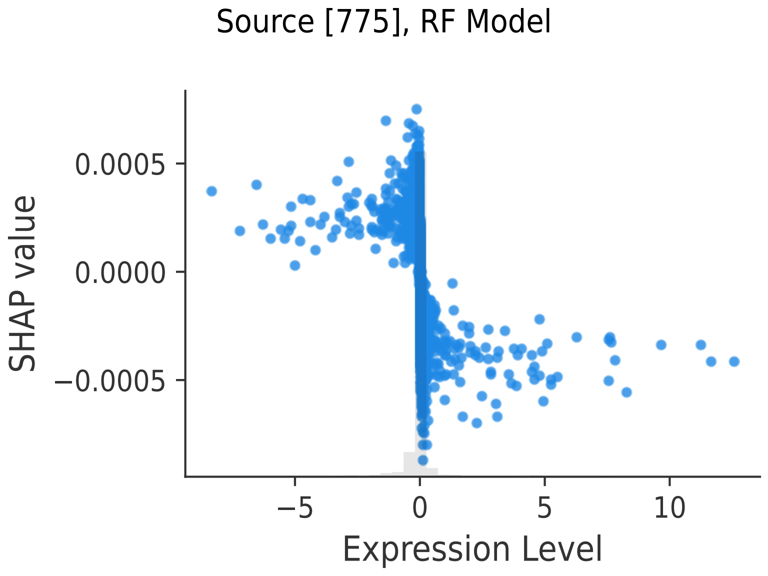
Source [775], RF Model
SHAP value
0.0005
0.0000
−0.0005
−5
0
5
10
Expression Level

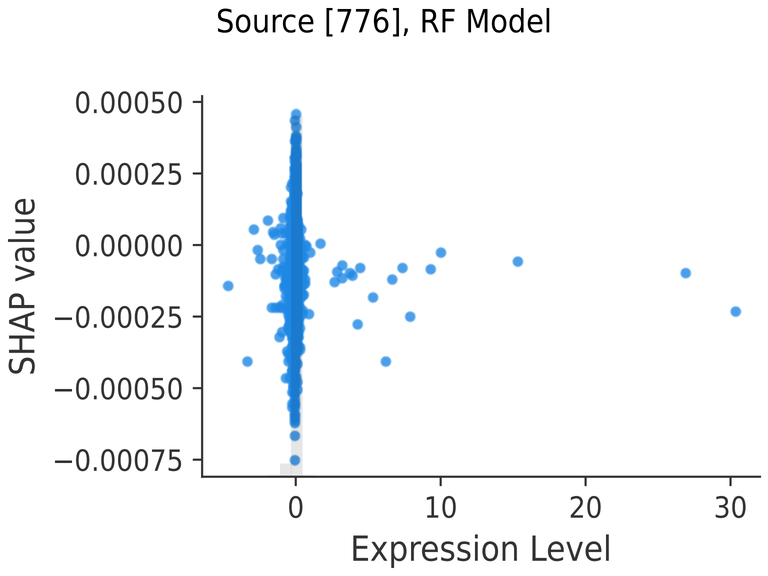

Source [776], RF Model
0.00050
0.00025
0.00000
−0.00025
−0.00050
−0.00075
SHAP value
0
10
20
30
Expression Level

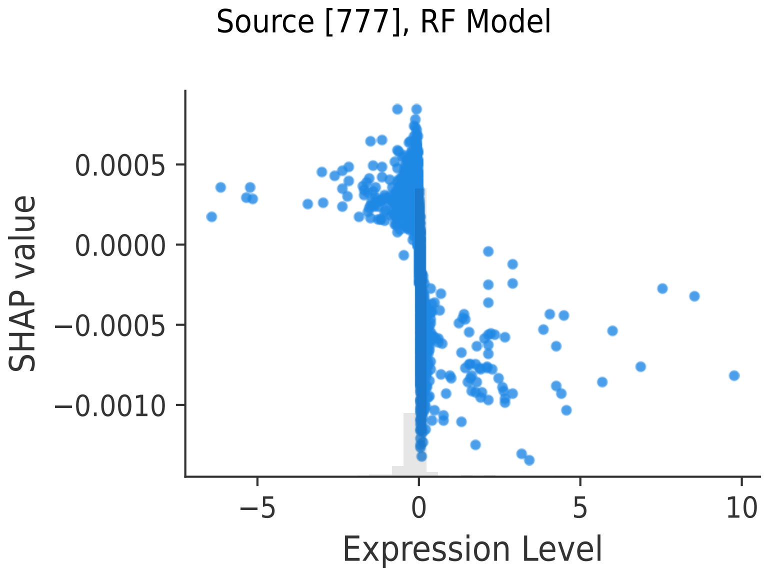
Source [777], RF Model
SHAP value
0.0005
0.0000
−0.0005
−0.0010
−5
0
5
10
Expression Level

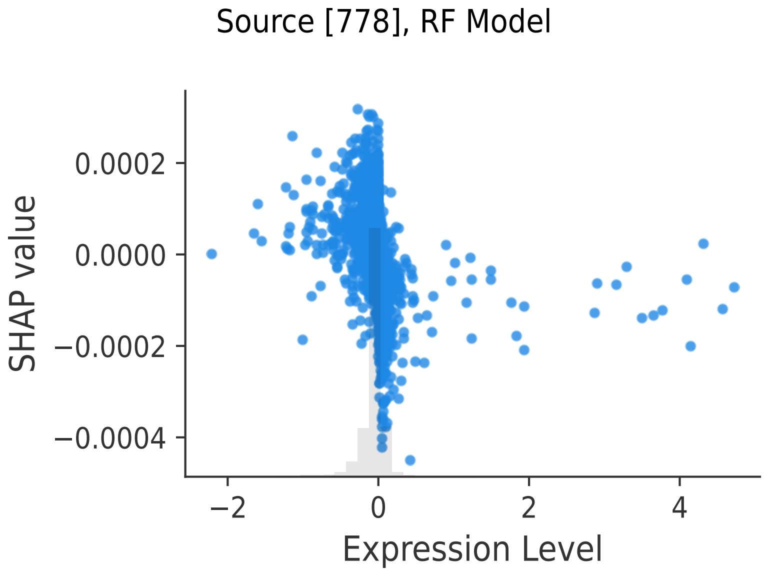
Source [778], RF Model
0.0002
0.0000
−0.0002
−0.0004
SHAP value
−2
0
2
4
Expression Level

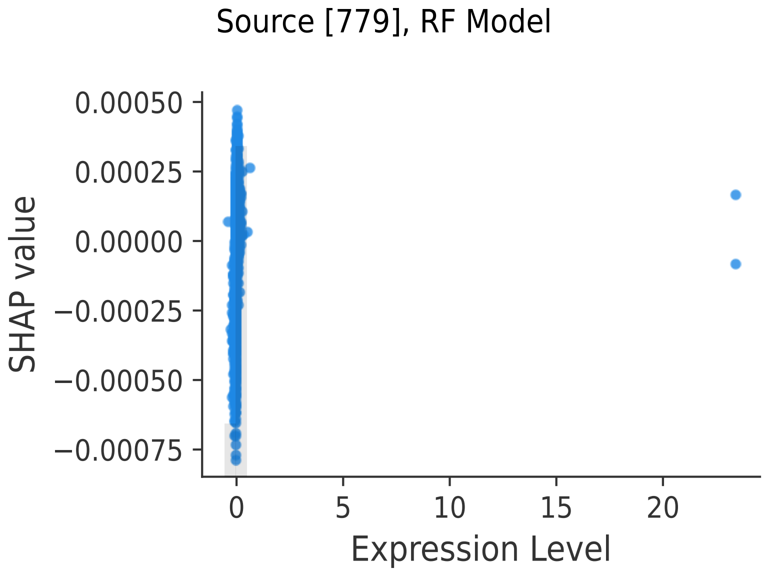

Source [779], RF Model
0.00050
0.00025
0.00000
−0.00025
−0.00050
−0.00075
SHAP value
0
5
10
15
20
Expression Level

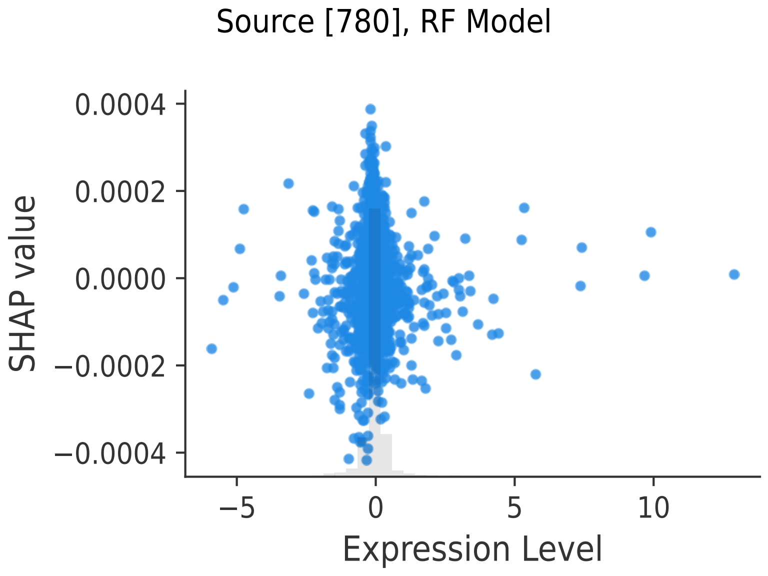
Source [780], RF Model
0.0004
0.0002
0.0000
−0.0002
−0.0004
SHAP value
−5
0
5
10
Expression Level

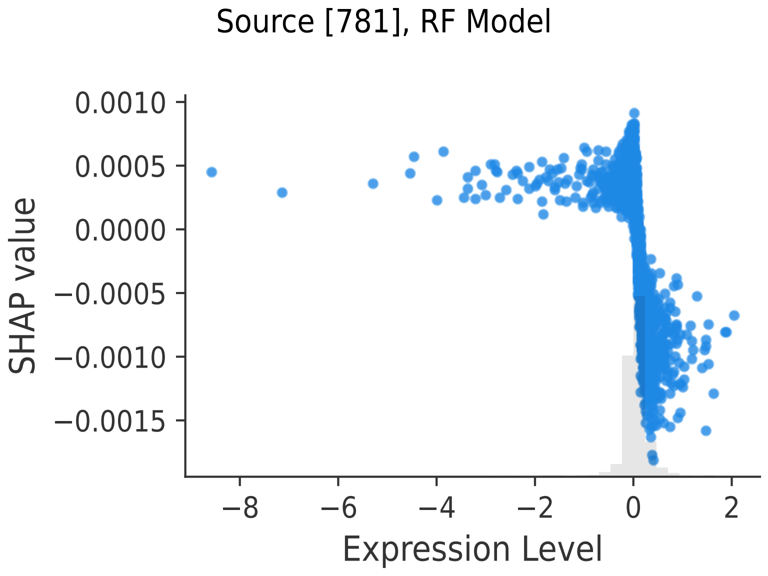
Source [781], RF Model
0.0010
0.0005
0.0000
−0.0005
−0.0010
−0.0015
SHAP value
−8
−6
−4
−2
0
2
Expression Level

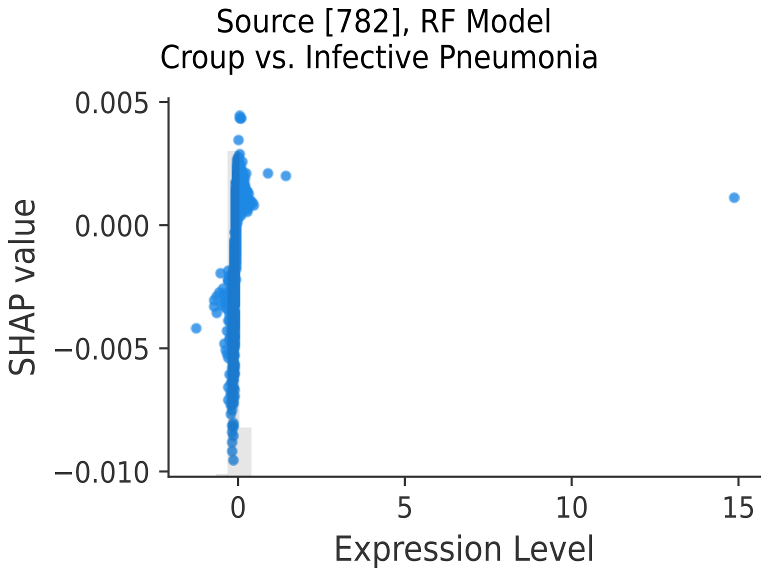

Source [782], RF Model
Croup vs. Infective Pneumonia
SHAP value
0.005
0.000
−0.005
−0.010
0
5
10
15
Expression Level

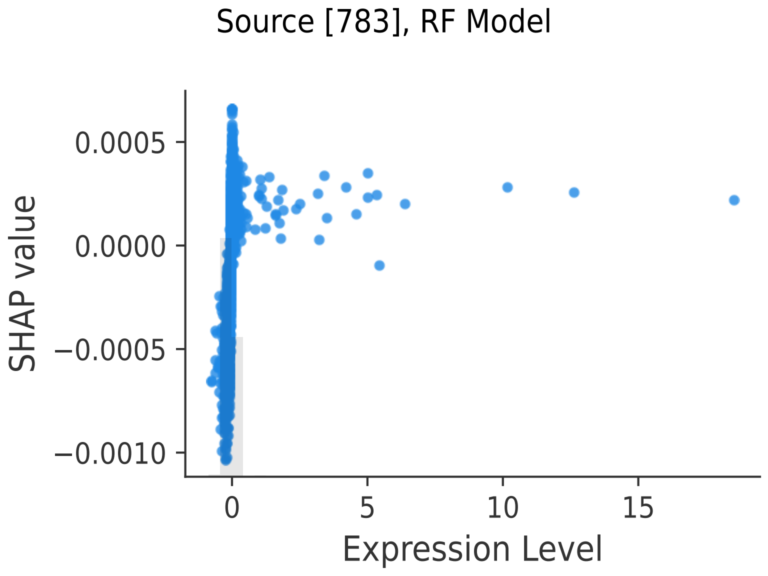

Source [783], RF Model
SHAP value
0.0005
0.0000
−0.0005
−0.0010
0
5
10
15
Expression Level

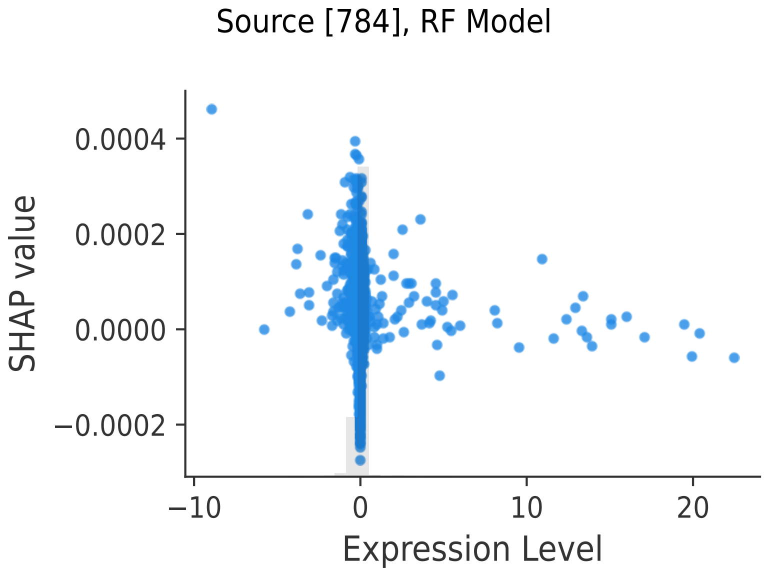
Source [784], RF Model
SHAP value
0.0004
0.0002
0.0000
−0.0002
−10
0
10
20
Expression Level

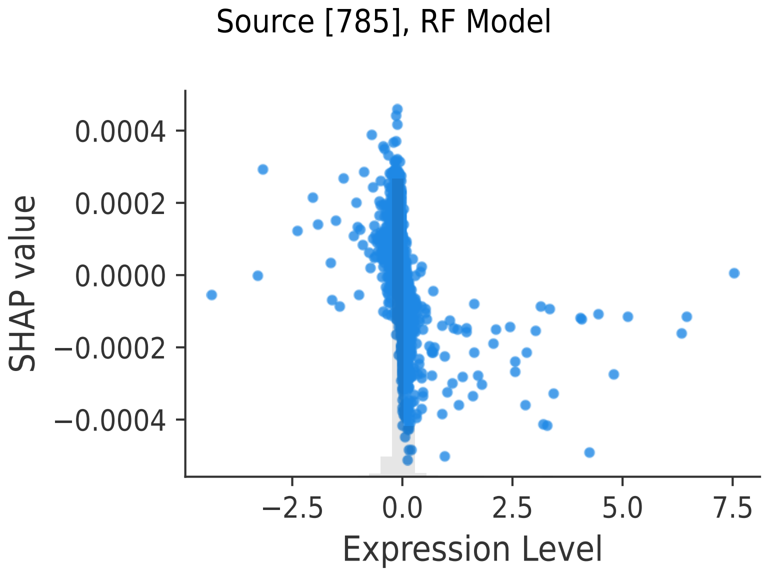
Source [785], RF Model
0.0004
0.0002
0.0000
−0.0002
−0.0004
SHAP value
−2.5
0.0
2.5
5.0
7.5
Expression Level

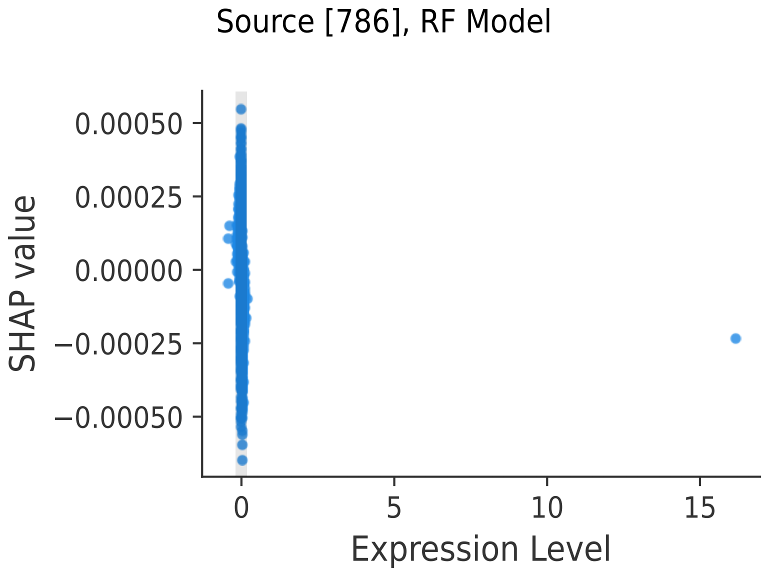
Source [786], RF Model
SHAP value
0.00050
0.00025
0.00000
−0.00025
−0.00050
0
5
10
15
Expression Level

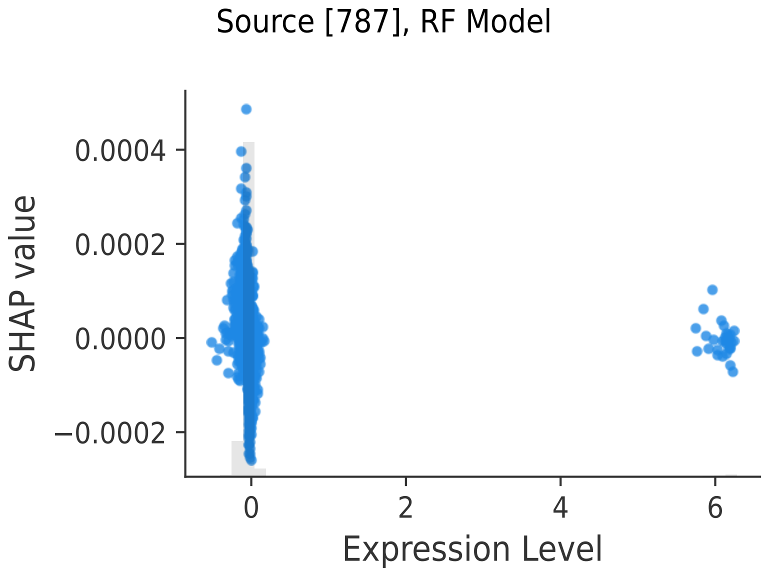

Source [787], RF Model
0.0004
0.0002
0.0000
−0.0002
SHAP value
0
2
4
6
Expression Level

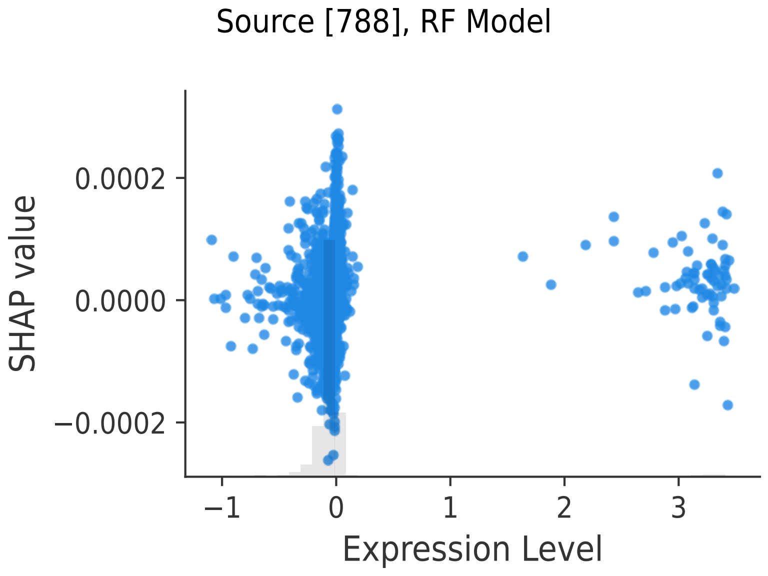
Source [788], RF Model
0.0002
0.0000
−0.0002
SHAP value
−1
0
1
2
3
Expression Level

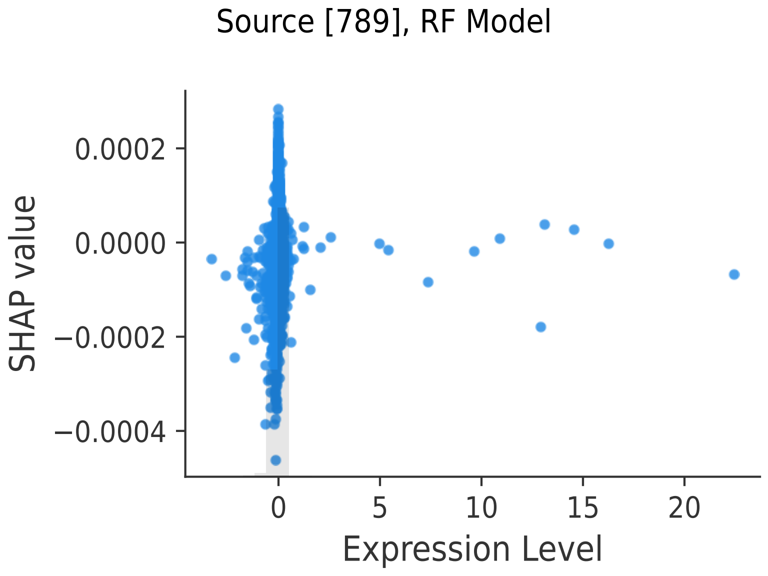
Source [789], RF Model
0.0002
0.0000
−0.0002
−0.0004
SHAP value
0
5
10
15
20
Expression Level

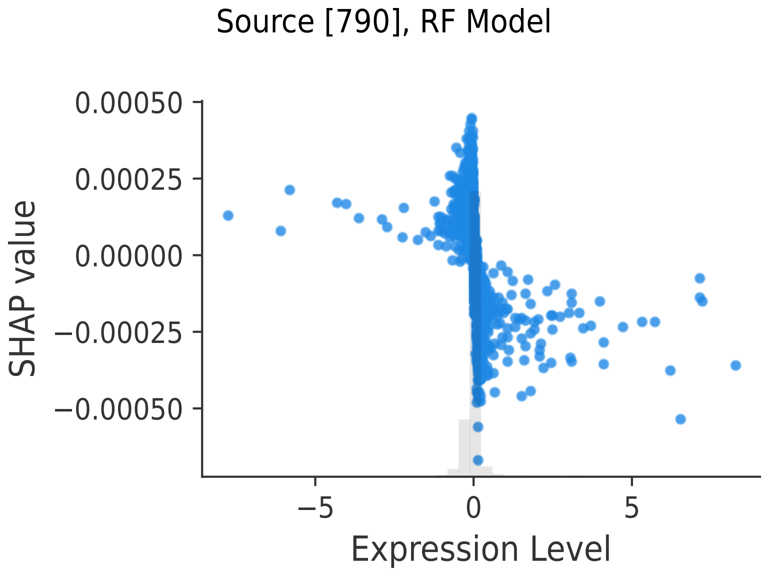

Source [790], RF Model
0.00050
0.00025
0.00000
−0.00025
−0.00050
SHAP value
−5
0
5
Expression Level

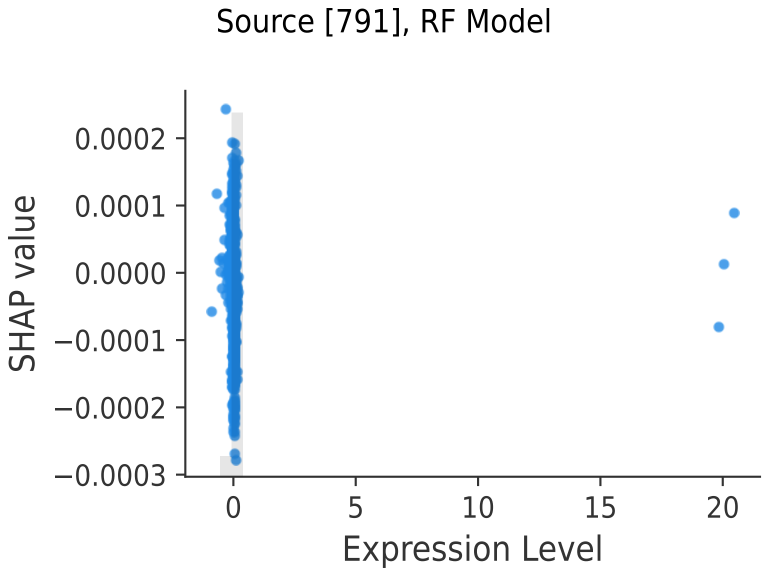

Source [791], RF Model
SHAP value
0.0002
0.0001
0.0000
−0.0001
−0.0002
−0.0003
0
5
10
15
20
Expression Level

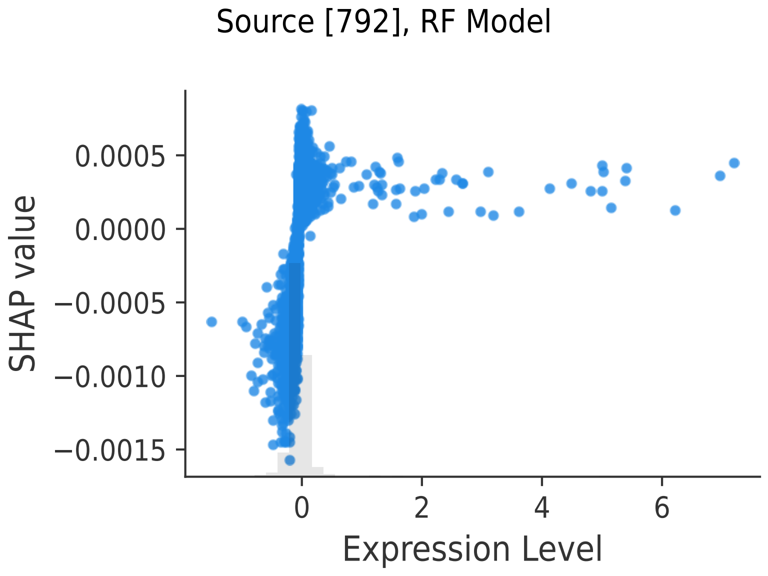
Source [792], RF Model
0.0005
0.0000
−0.0005
−0.0010
−0.0015
SHAP value
0
2
4
6
Expression Level

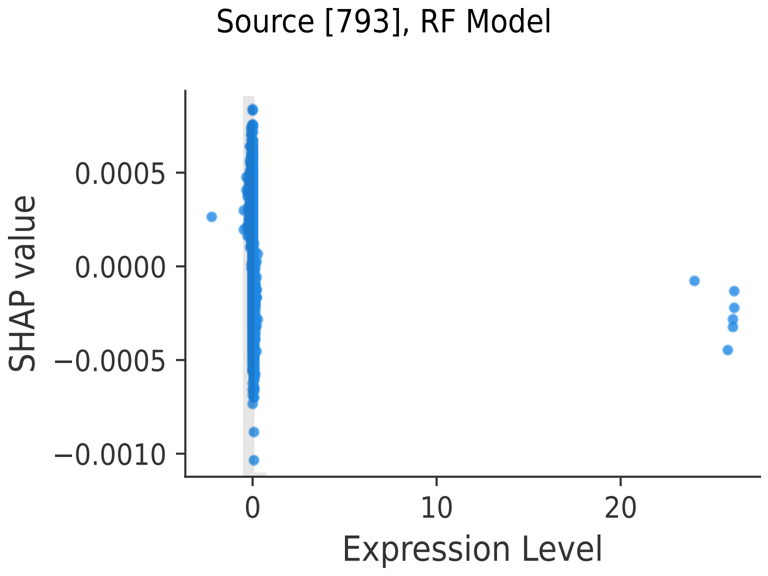
Source [793], RF Model
SHAP value
0.0005
0.0000
−0.0005
−0.0010
0
10
20
Expression Level

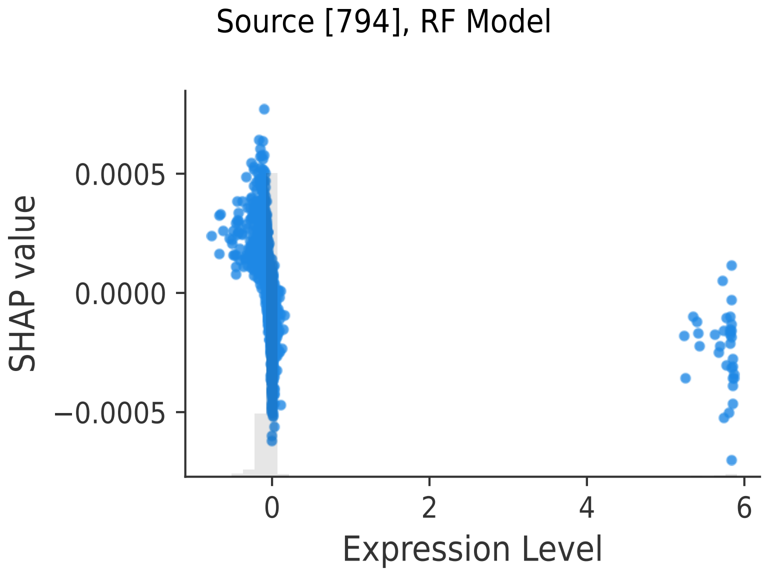
Source [794], RF Model
SHAP value
0.0005
0.0000
−0.0005
0
2
4
6
Expression Level

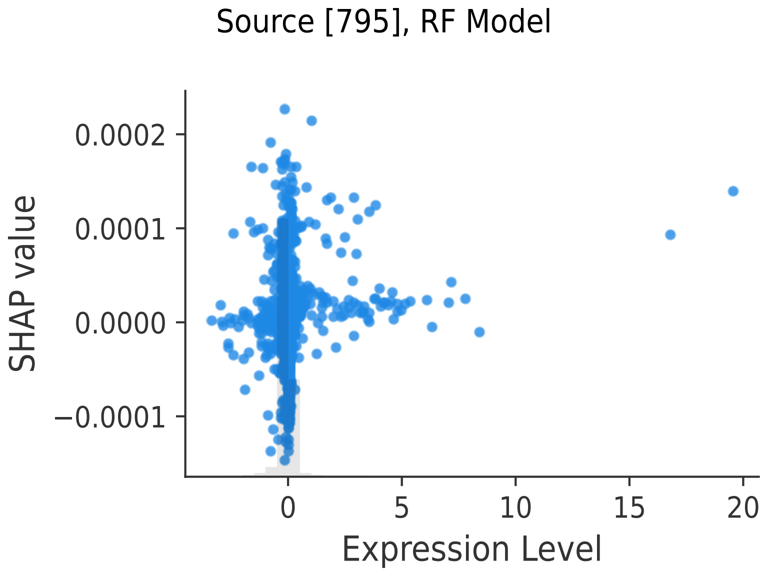

Source [795], RF Model
0.0002
0.0001
0.0000
−0.0001
SHAP value
0
5
10
15
20
Expression Level

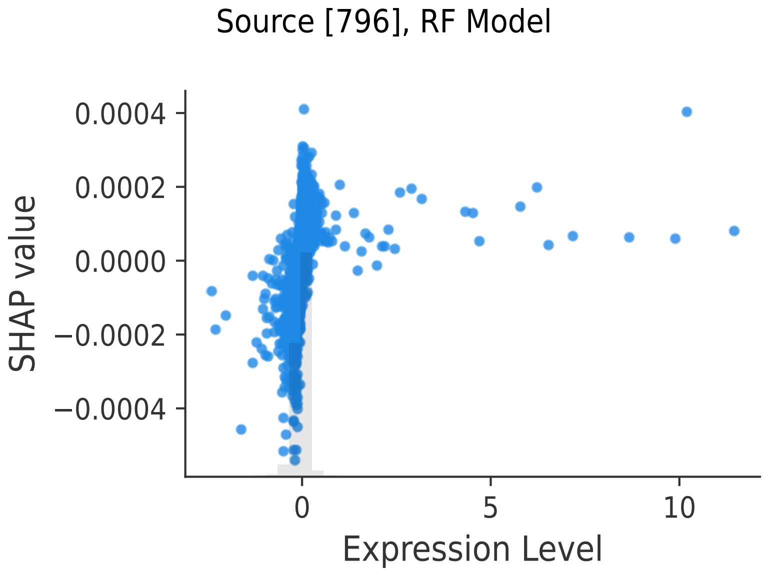
Source [796], RF Model
SHAP value
0.0004
0.0002
0.0000
−0.0002
−0.0004
0
5
10
Expression Level

Source [797], RF Model

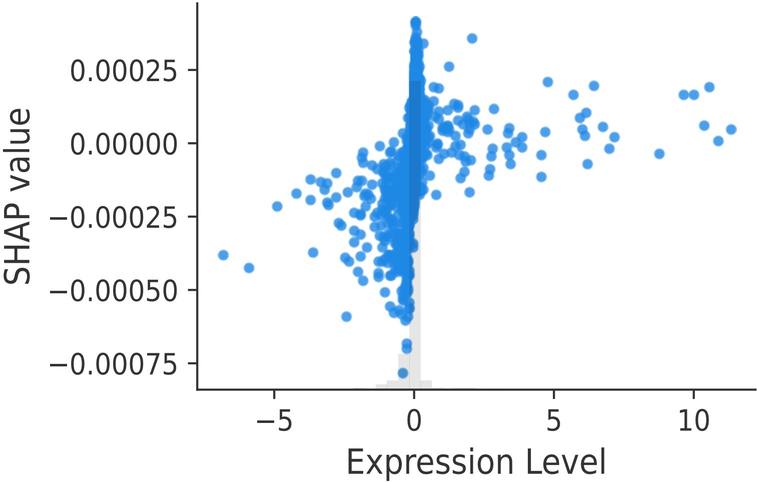

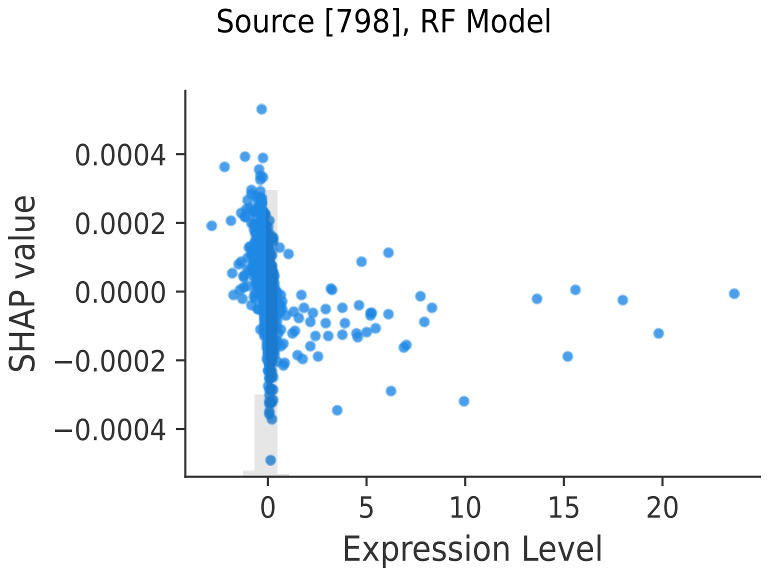

Source [798], RF Model
0.0004
0.0002
0.0000
−0.0002
−0.0004
SHAP value
0
5
10
15
20
Expression Level

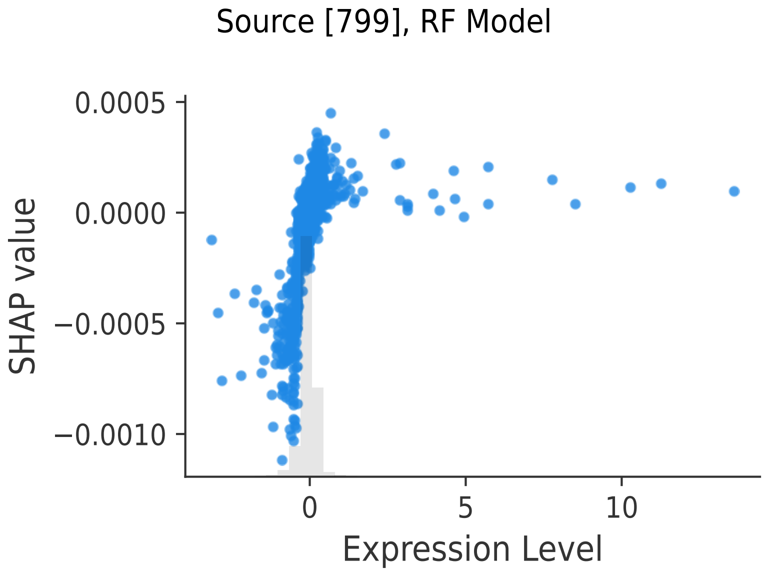
Source [799], RF Model
0.0005
0.0000
−0.0005
−0.0010
SHAP value
0
5
10
Expression Level

Source [800], RF Model

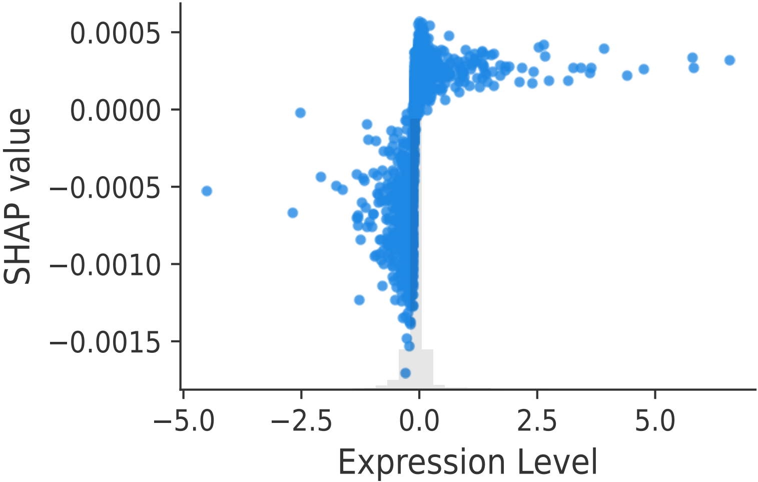

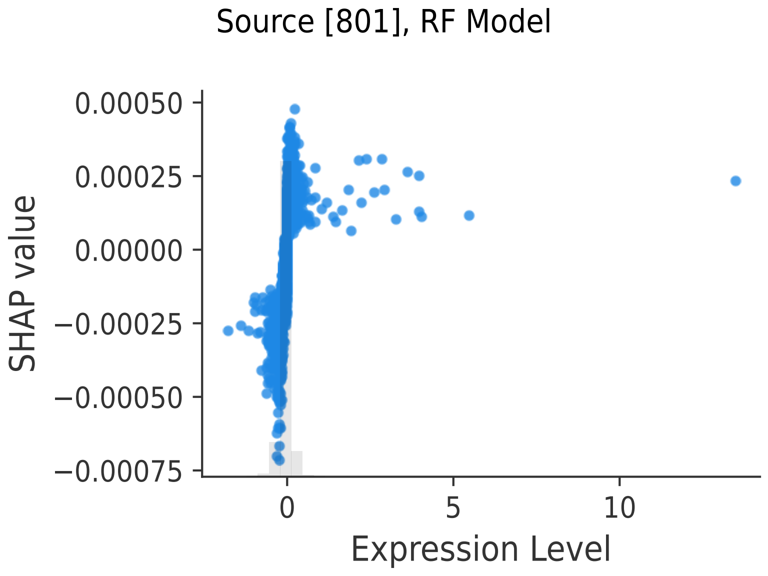
Source [801], RF Model
SHAP value
0.00050
0.00025
0.00000
−0.00025
−0.00050
−0.00075
Expression Level
0
5
10

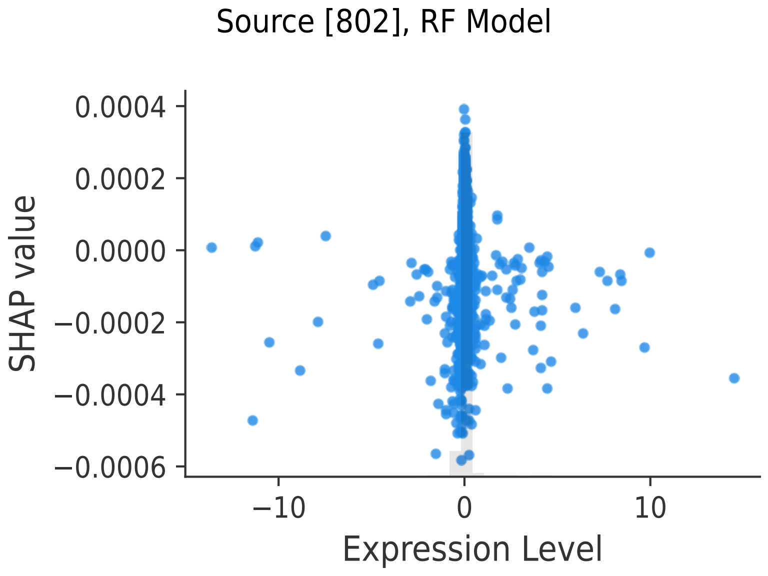
Source [802], RF Model
0.0004
0.0002
0.0000
−0.0002
−0.0004
−0.0006
SHAP value
−10
0
10
Expression Level

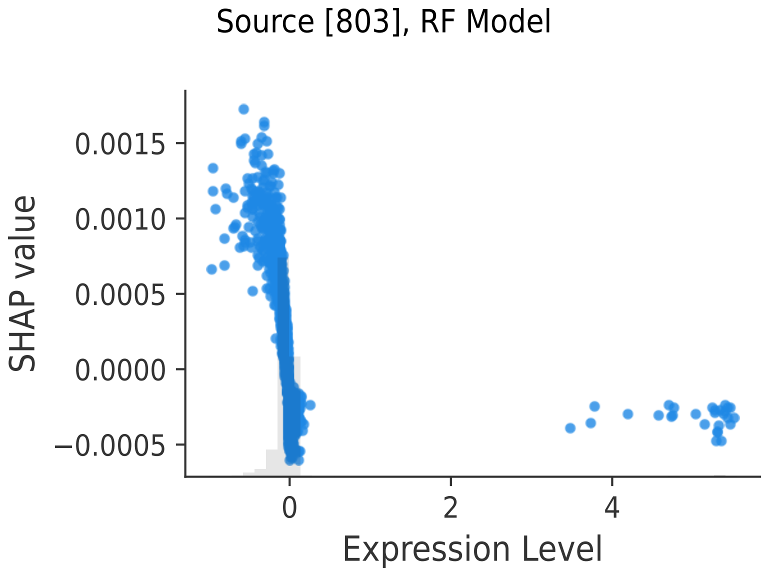

Source [803], RF Model
0.0015
0.0010
0.0005
0.0000
−0.0005
SHAP value
0
2
4
Expression Level

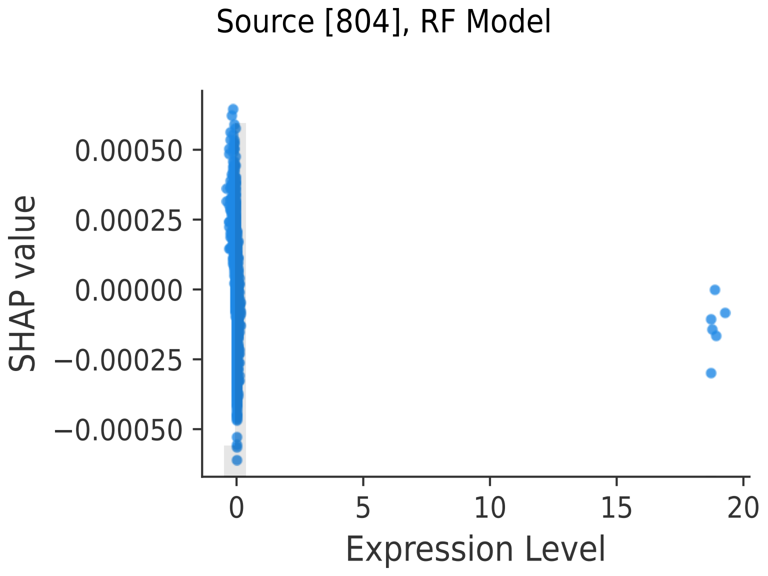
Source [804], RF Model
SHAP value
0.00050
0.00025
0.00000
−0.00025
−0.00050
0
5
10
15
20
Expression Level

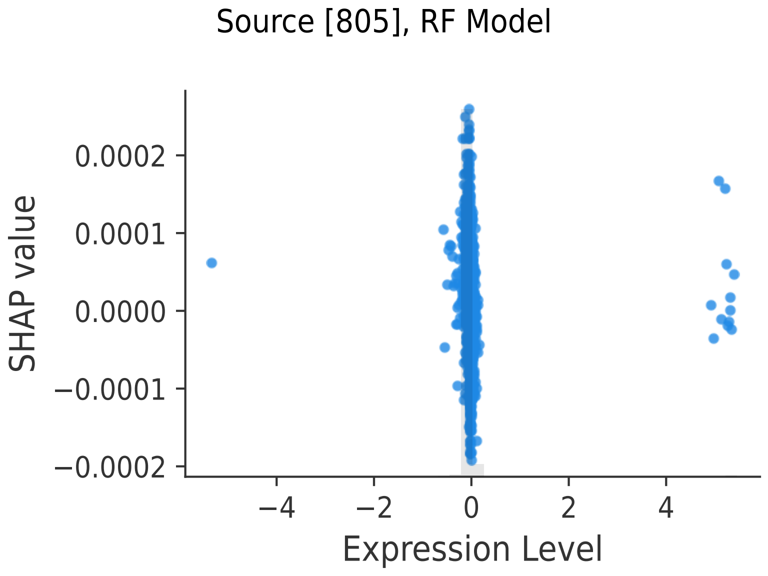
Source [805], RF Model
SHAP value
0.0002
0.0001
0.0000
−0.0001
−0.0002
−4
−2
0
2
4
Expression Level

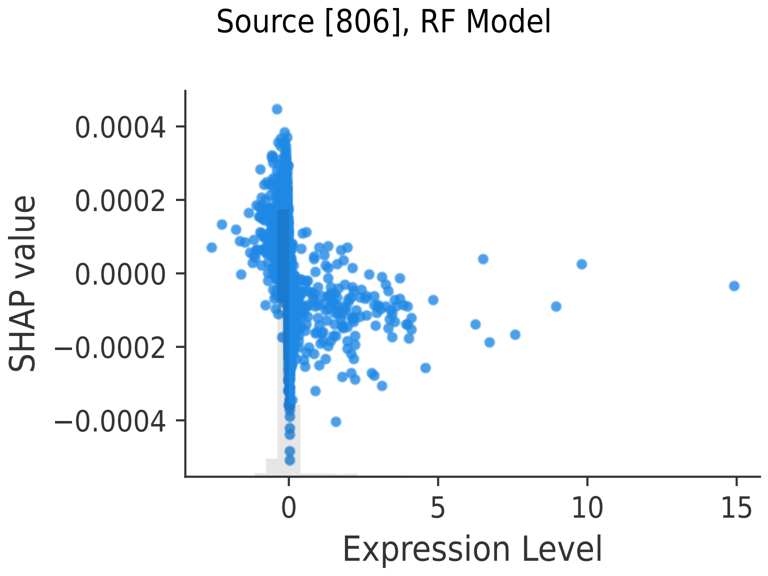

Source [806], RF Model
0.0004
0.0002
0.0000
−0.0002
−0.0004
SHAP value
0
5
10
15
Expression Level

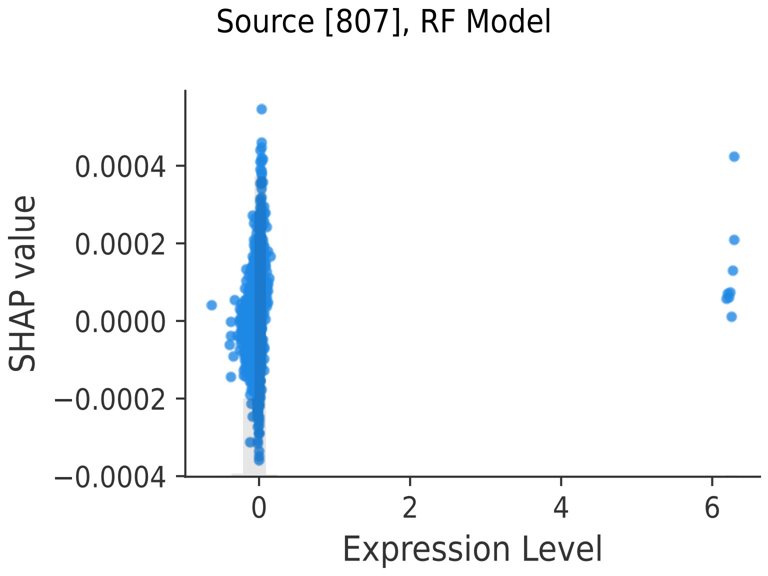
Source [807], RF Model
SHAP value
0.0004
0.0002
0.0000
−0.0002
−0.0004
0
2
4
6
Expression Level

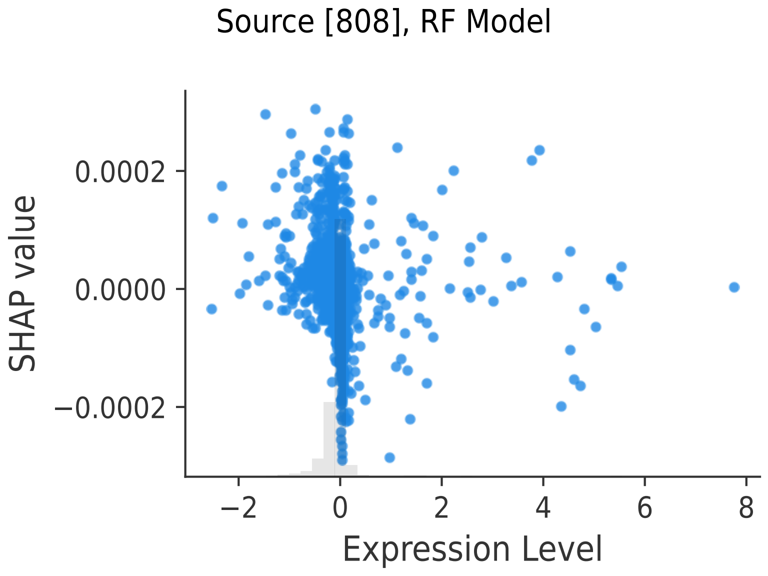
Source [808], RF Model
0.0002
0.0000
−0.0002
SHAP value
−2
0
2
4
6
8
Expression Level

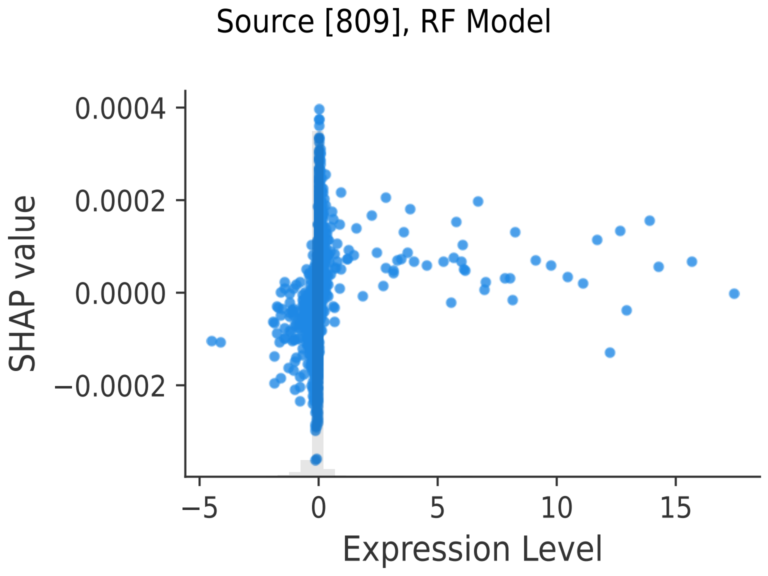
Source [809], RF Model
0.0004
0.0002
0.0000
−0.0002
SHAP value
−5
0
5
10
15
Expression Level

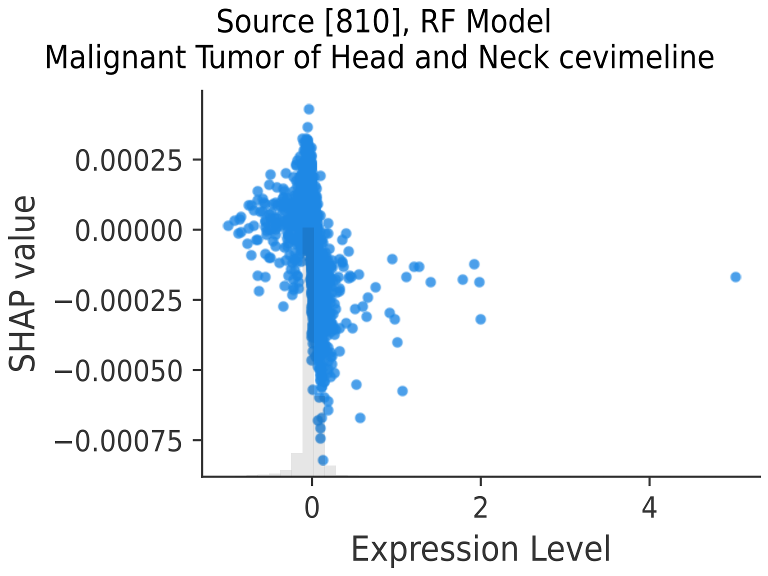

Source [810], RF Model
Malignant Tumor of Head and Neck cevimeline
0.00025
0.00000
−0.00025
−0.00050
−0.00075
SHAP value
0
2
4
Expression Level

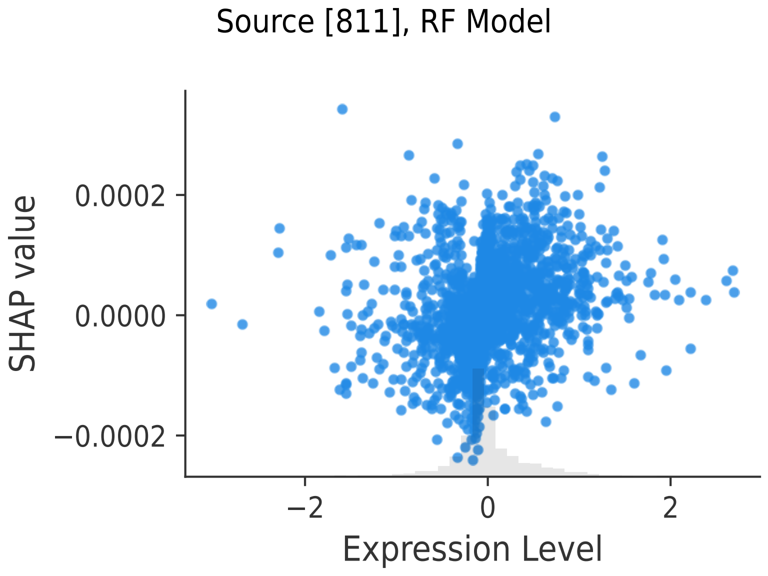
Source [811], RF Model
SHAP value
0.0002
0.0000
−0.0002
−2
0
2
Expression Level

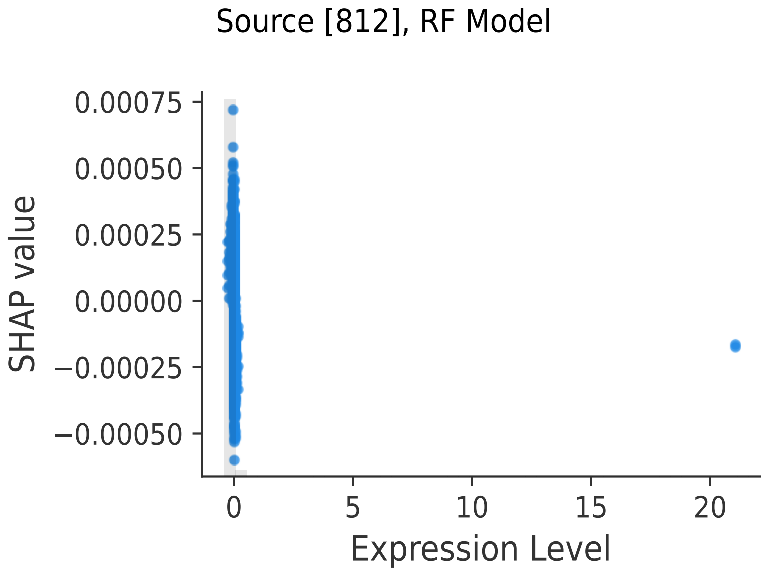
Source [812], RF Model
SHAP value
0.00075
0.00050
0.00025
0.00000
−0.00025
−0.00050
0
5
10
15
20
Expression Level

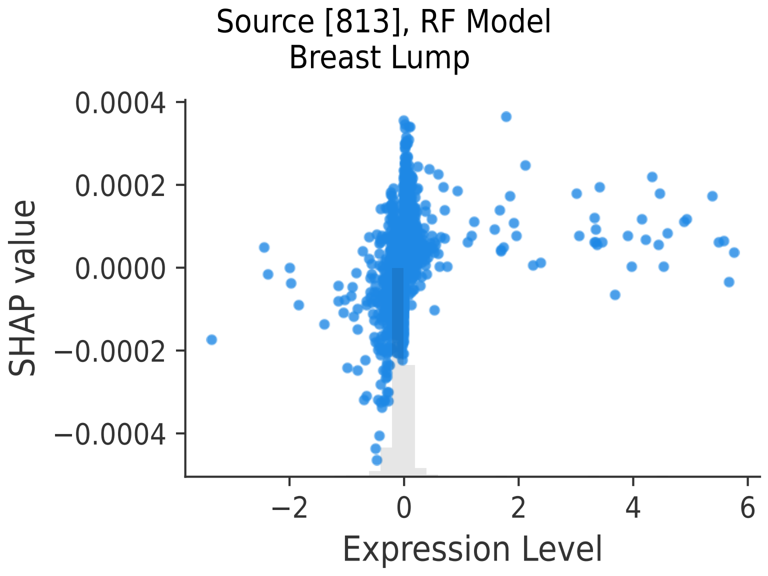
Source [813], RF Model
Breast Lump
0.0004
0.0002
0.0000
−0.0002
−0.0004
SHAP value
−2
0
2
4
6
Expression Level

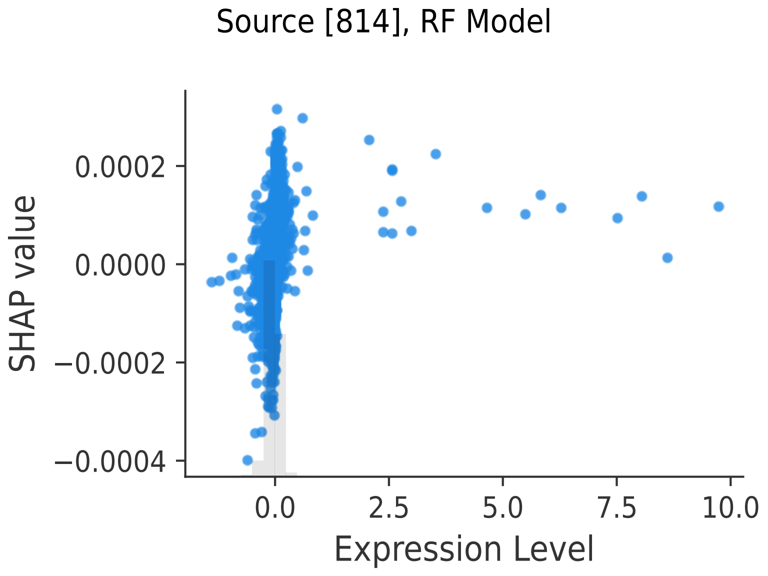

Source [814], RF Model
SHAP value
0.0002
0.0000
−0.0002
−0.0004
0.0
2.5
5.0
7.5
10.0
Expression Level

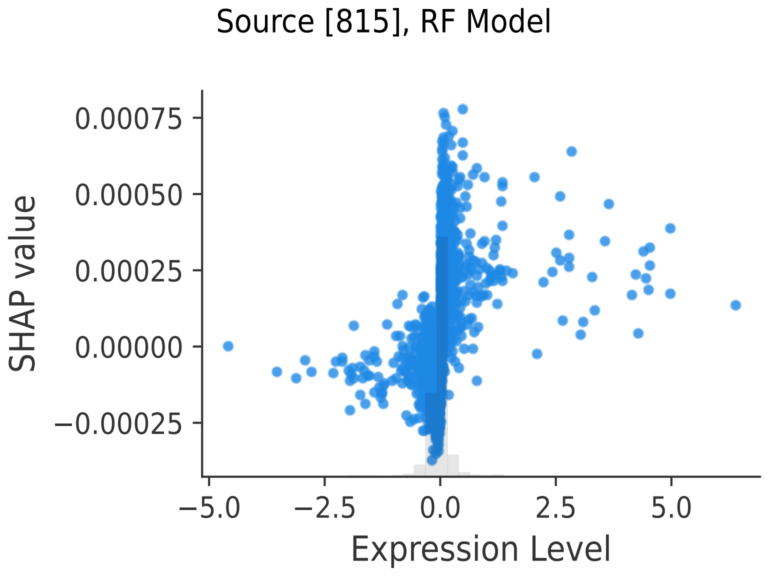
Source [815], RF Model
SHAP value
0.00075
0.00050
0.00025
0.00000
−0.00025
−5.0
−2.5
0.0
2.5
5.0
Expression Level

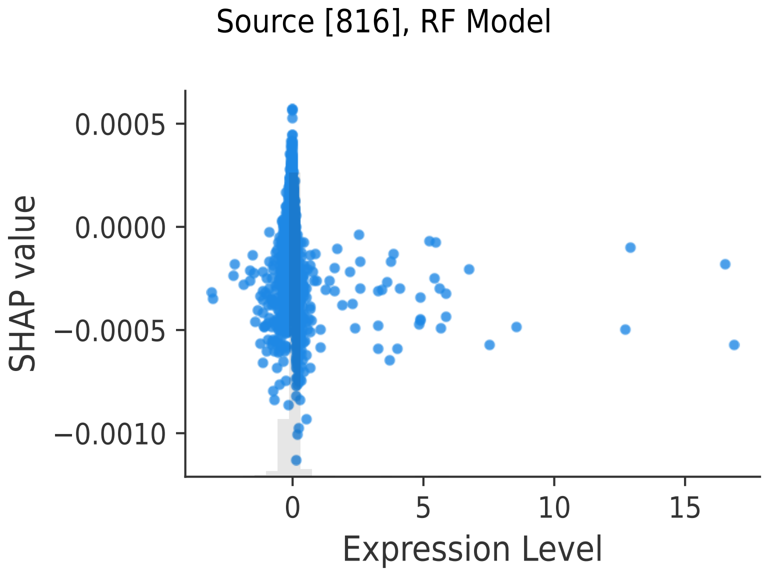

Source [816], RF Model
0.0005
0.0000
−0.0005
−0.0010
SHAP value
0
5
10
15
Expression Level

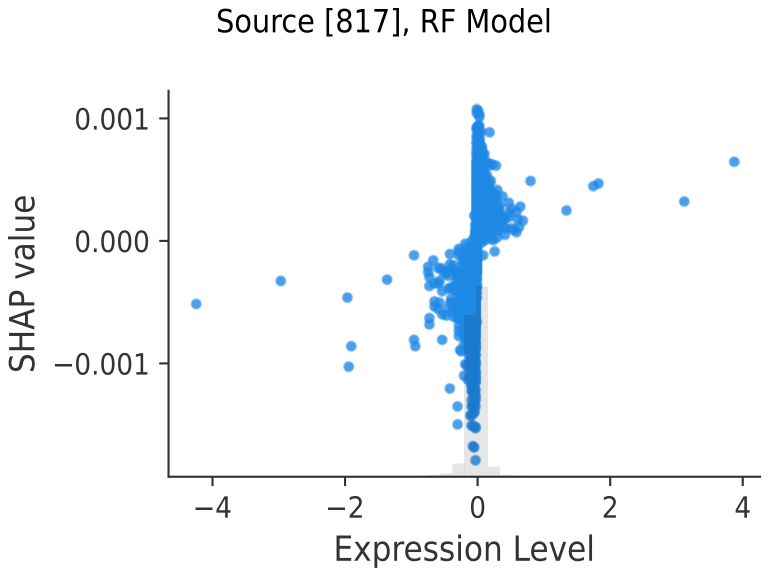

Source [817], RF Model
SHAP value
0.001
0.000
−0.001
−4
−2
0
2
4
Expression Level

Source [818], RF Model

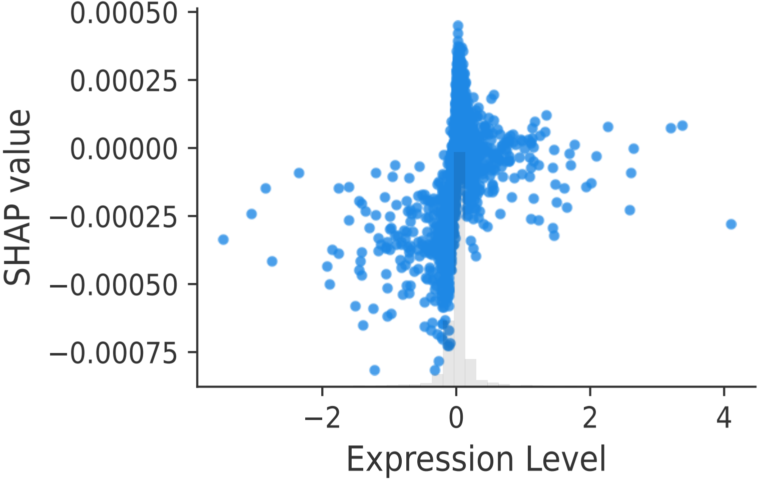

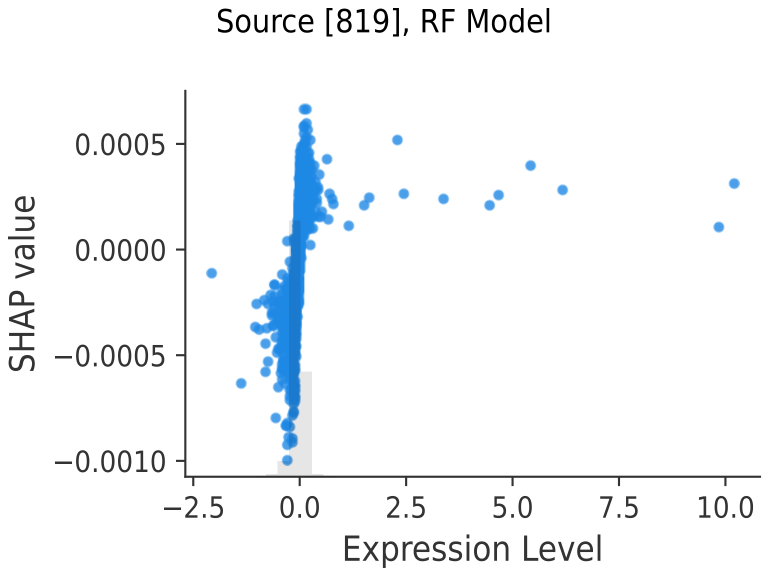

Source [819], RF Model
SHAP value
0.0005
0.0000
−0.0005
−0.0010
−2.5
0.0
2.5
5.0
7.5
10.0
Expression Level

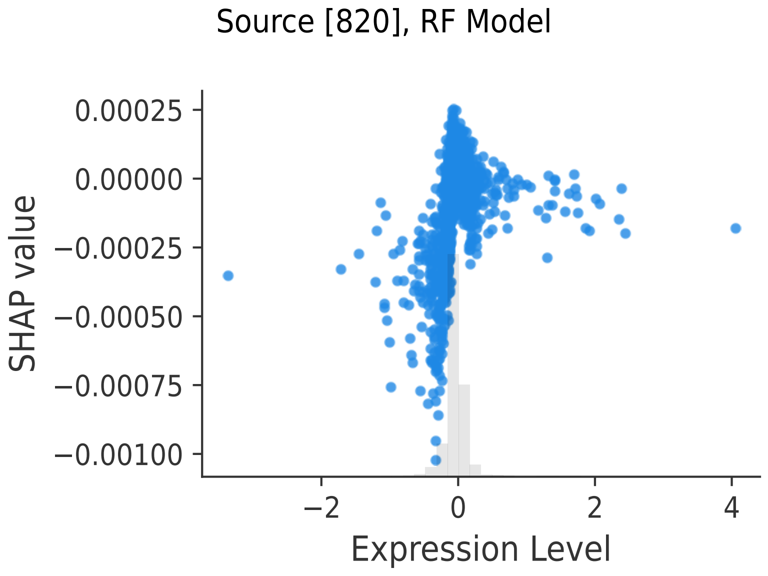

Source [820], RF Model
0.00025
0.00000
−0.00025
−0.00050
−0.00075
−0.00100
SHAP value
−2
0
2
4
Expression Level

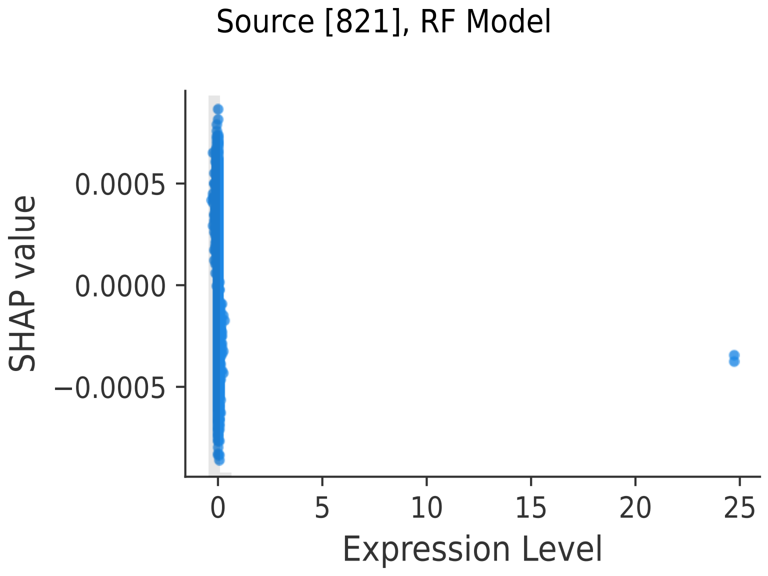

Source [821], RF Model
SHAP value
0.0005
0.0000
−0.0005
0
5
10
15
20
25
Expression Level

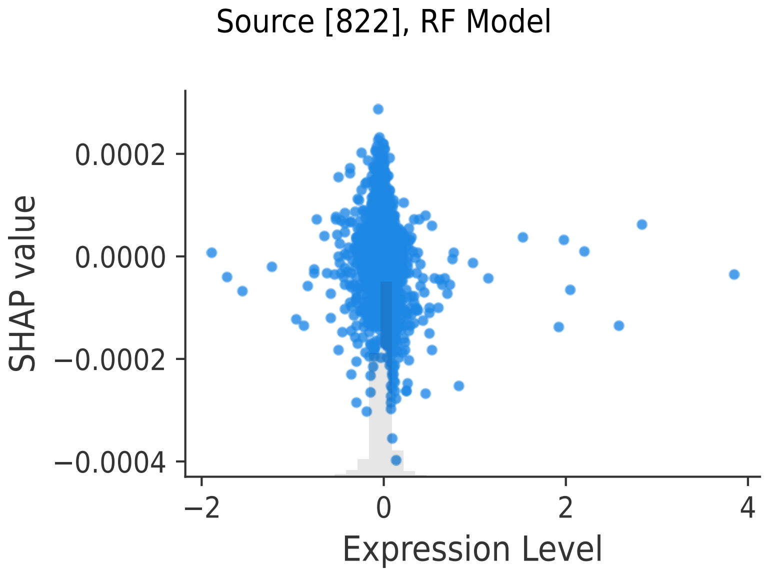
Source [822], RF Model
0.0002
0.0000
−0.0002
−0.0004
SHAP value
−2
0
2
4
Expression Level

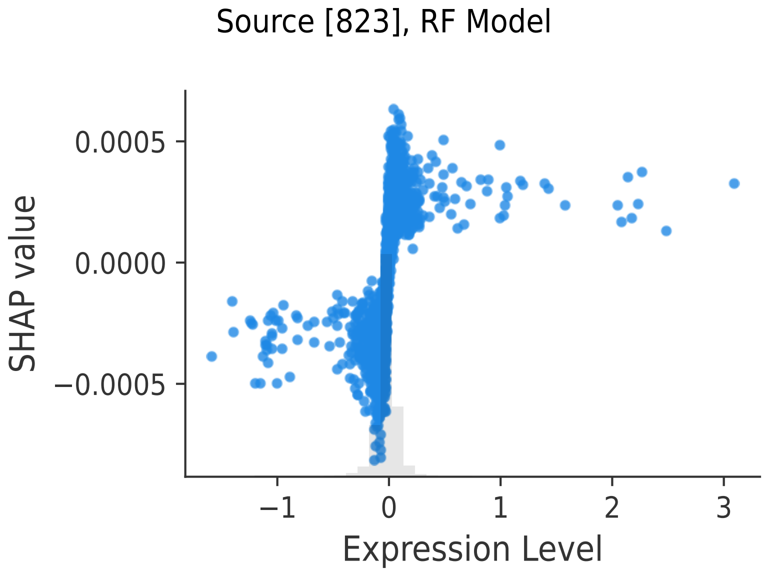
Source [823], RF Model
SHAP value
0.0005
0.0000
−0.0005
−1
0
1
2
3
Expression Level

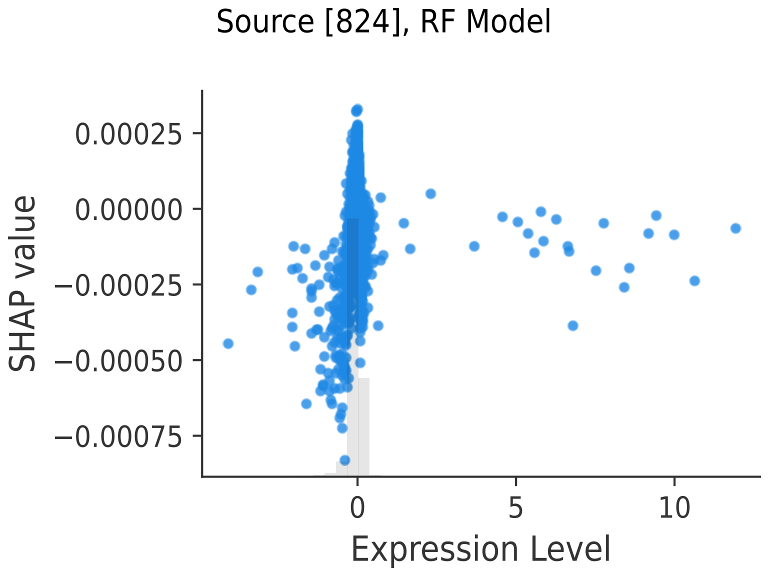
Source [824], RF Model
SHAP value
0.00025
0.00000
−0.00025
−0.00050
−0.00075
0
5
10
Expression Level

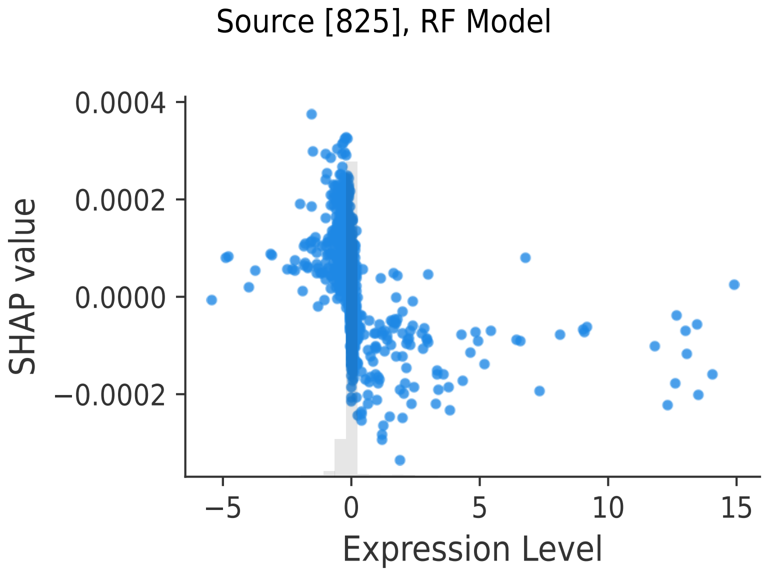
Source [825], RF Model
0.0004
0.0002
0.0000
−0.0002
SHAP value
−5
0
5
10
15
Expression Level

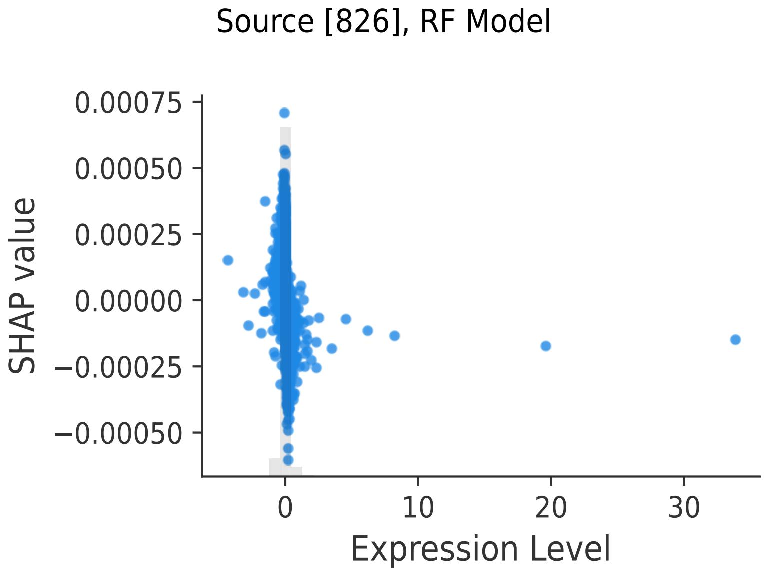
Source [826], RF Model
0.00075
0.00050
0.00025
0.00000
−0.00025
−0.00050
SHAP value
0
10
20
30
Expression Level

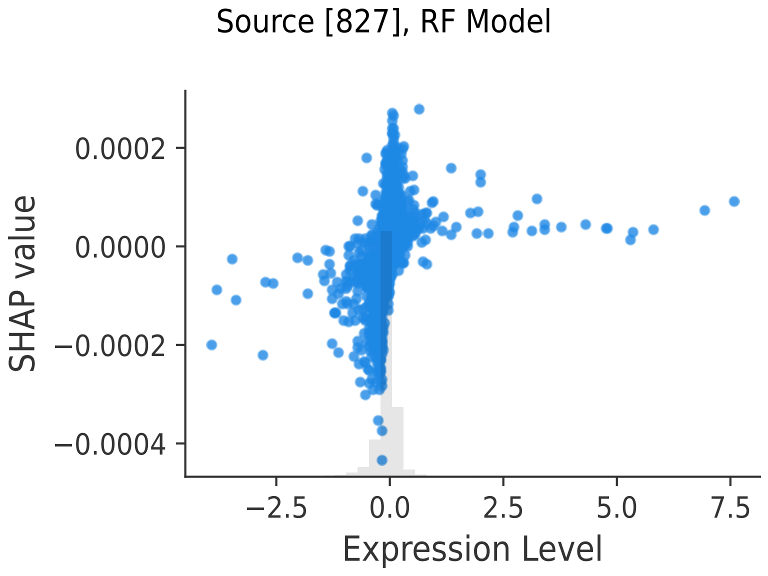
Source [827], RF Model
0.0002
0.0000
−0.0002
−0.0004
SHAP value
−2.5
0.0
2.5
5.0
7.5
Expression Level

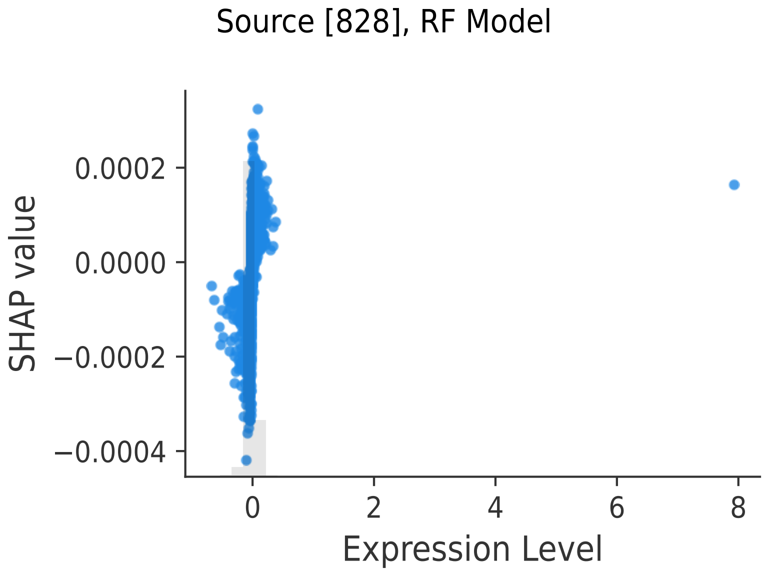

Source [828], RF Model
0.0002
0.0000
−0.0002
−0.0004
SHAP value
0
2
4
6
8
Expression Level

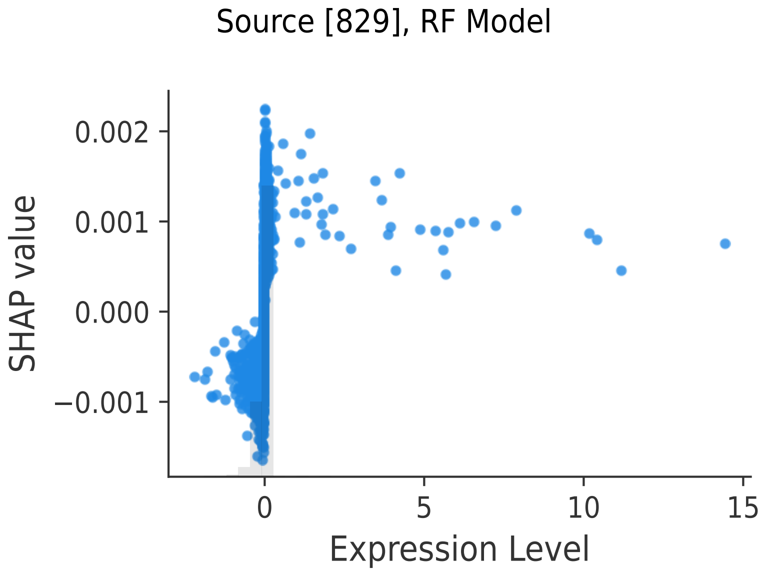

Source [829], RF Model
SHAP value
0.002
0.001
0.000
−0.001
Expression Level
0
5
10
15

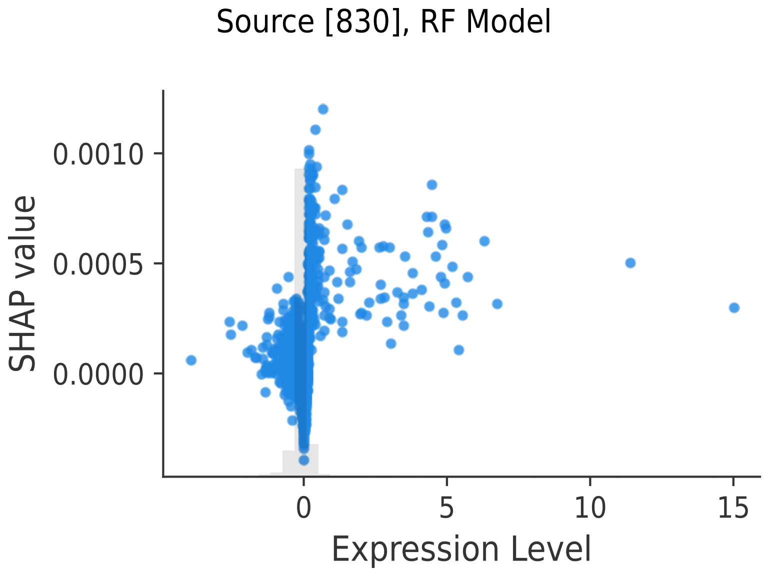
Source [830], RF Model
0.0010
0.0005
0.0000
SHAP value
0
5
10
15
Expression Level

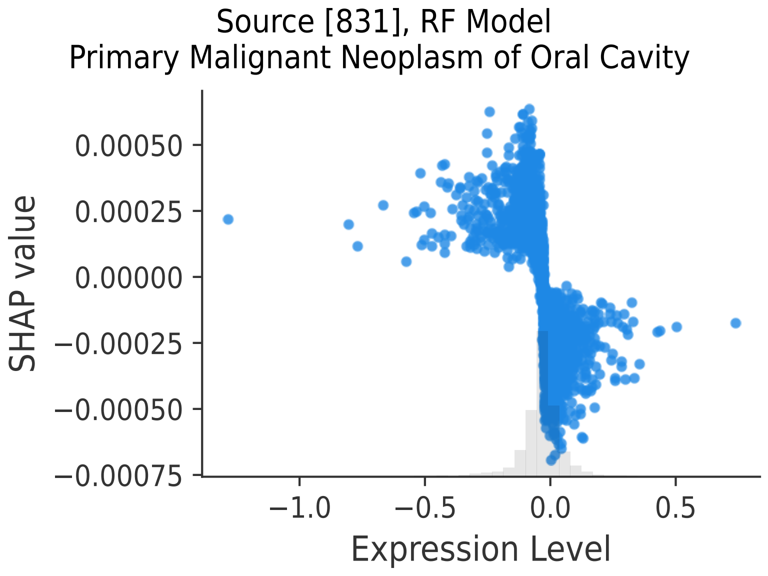

Source [831], RF Model
Primary Malignant Neoplasm of Oral Cavity
SHAP value
0.00050
0.00025
0.00000
−0.00025
−0.00050
−0.00075
−1.0
−0.5
0.0
0.5
Expression Level

Source [832], RF Model

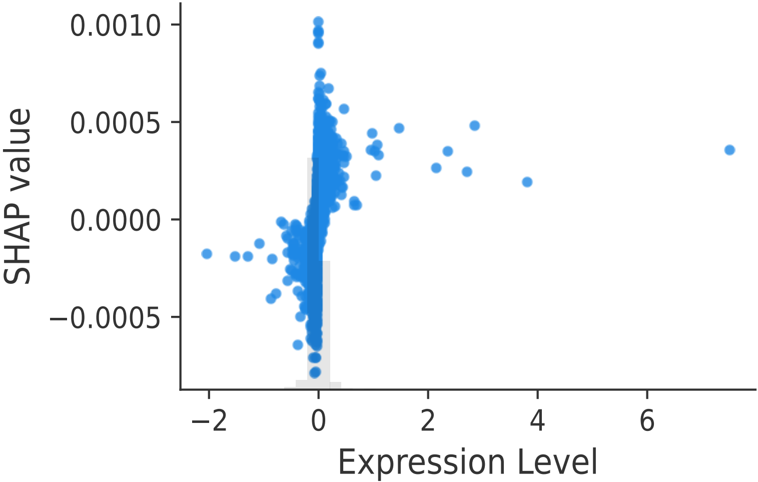

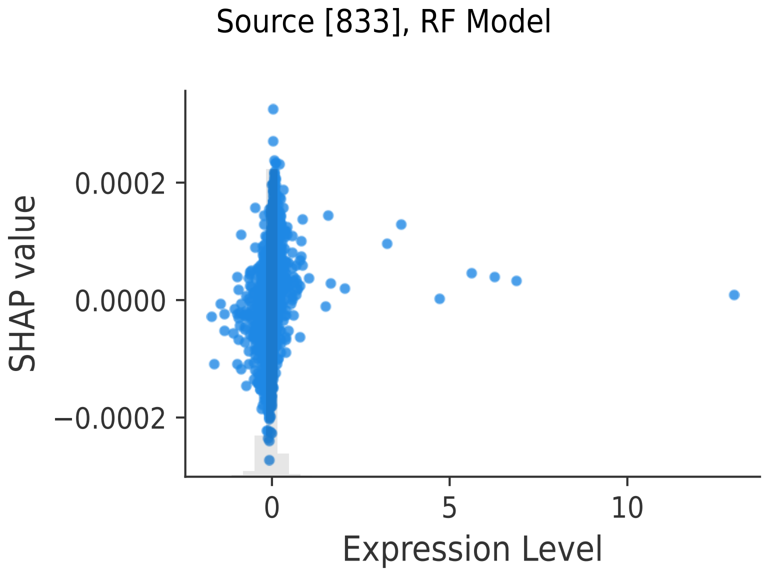

Source [833], RF Model
SHAP value
0.0002
0.0000
−0.0002
0
5
10
Expression Level

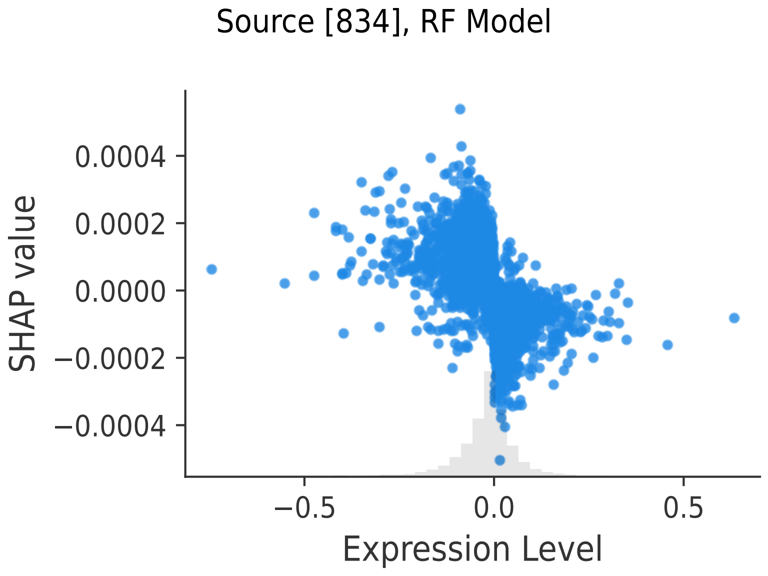
Source [834], RF Model
0.0004
0.0002
0.0000
−0.0002
−0.0004
SHAP value
−0.5
0.0
0.5
Expression Level

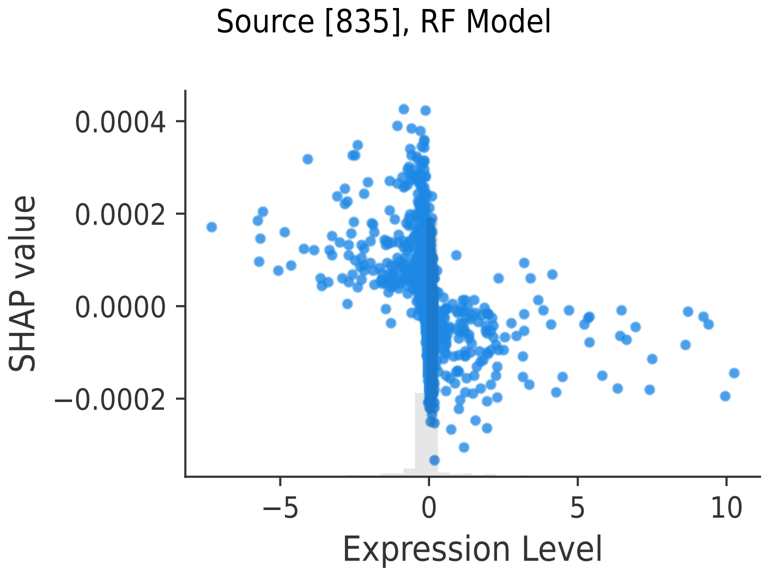
Source [835], RF Model
SHAP value
0.0004
0.0002
0.0000
−0.0002
−5
0
5
10
Expression Level

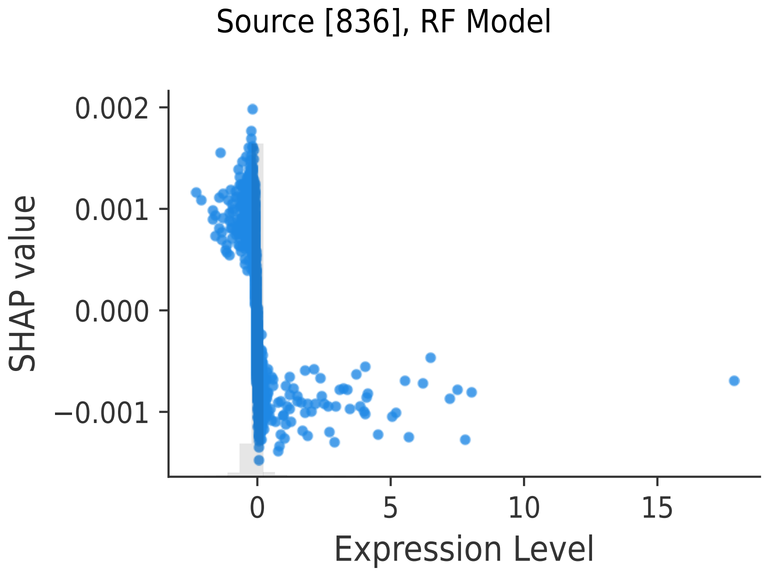

Source [836], RF Model
0.002
0.001
0.000
−0.001
SHAP value
0
5
10
15
Expression Level

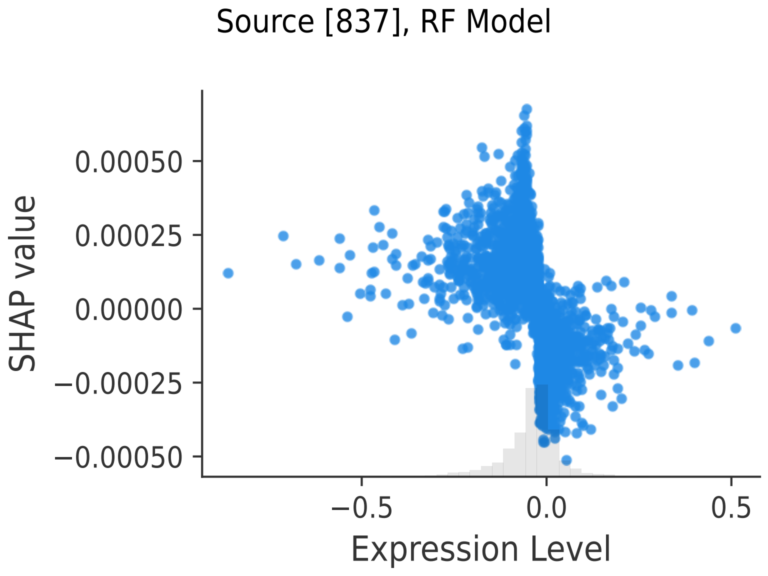
Source [837], RF Model
SHAP value
0.00050
0.00025
0.00000
−0.00025
−0.00050
−0.5
0.0
0.5
Expression Level

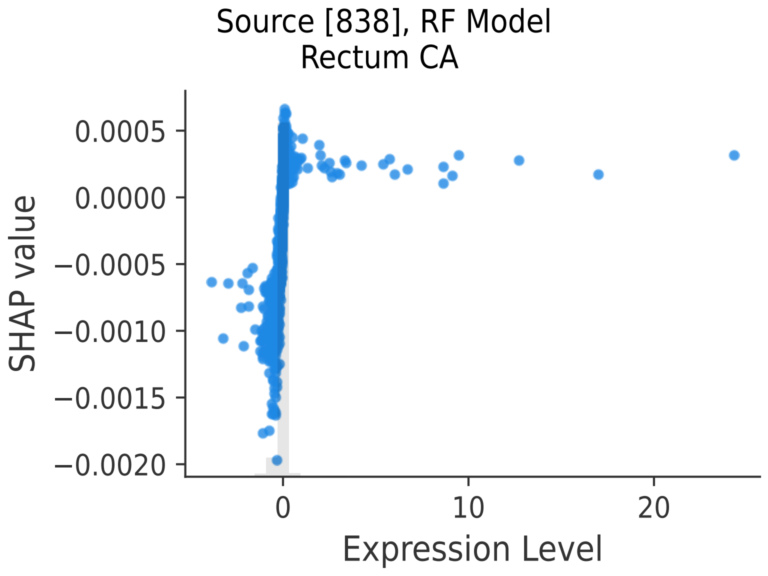
Source [838], RF Model
Rectum CA
SHAP value
0.0005
0.0000
−0.0005
−0.0010
−0.0015
−0.0020
0
10
20
Expression Level

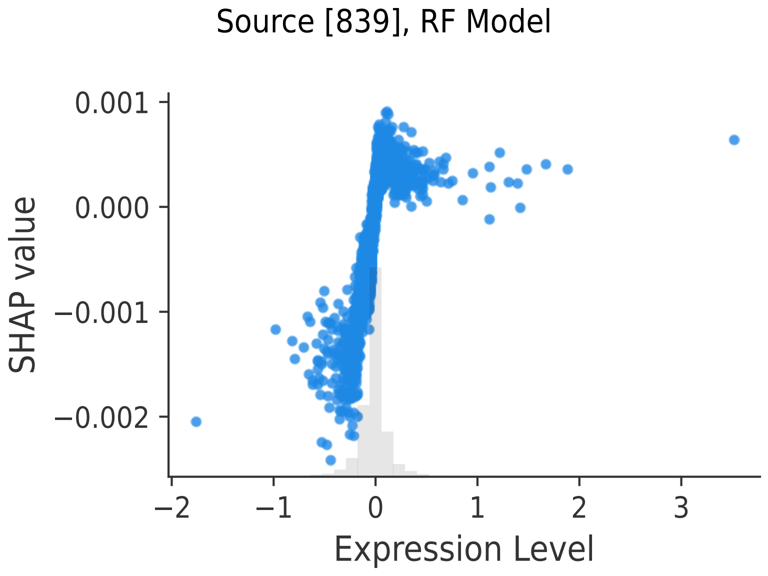

Source [839], RF Model
0.001
0.000
−0.001
−0.002
SHAP value
−2
−1
0
1
2
3
Expression Level

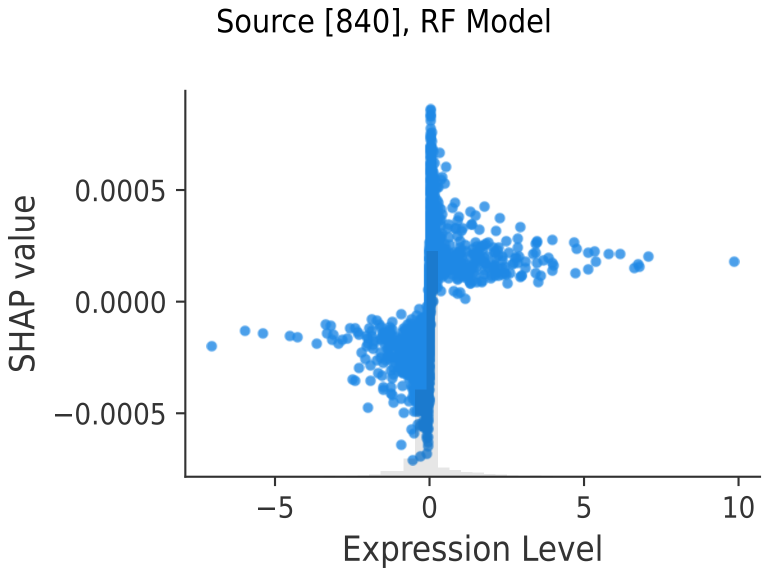

Source [840], RF Model
0.0005
0.0000
−0.0005
SHAP value
−5
0
5
10
Expression Level

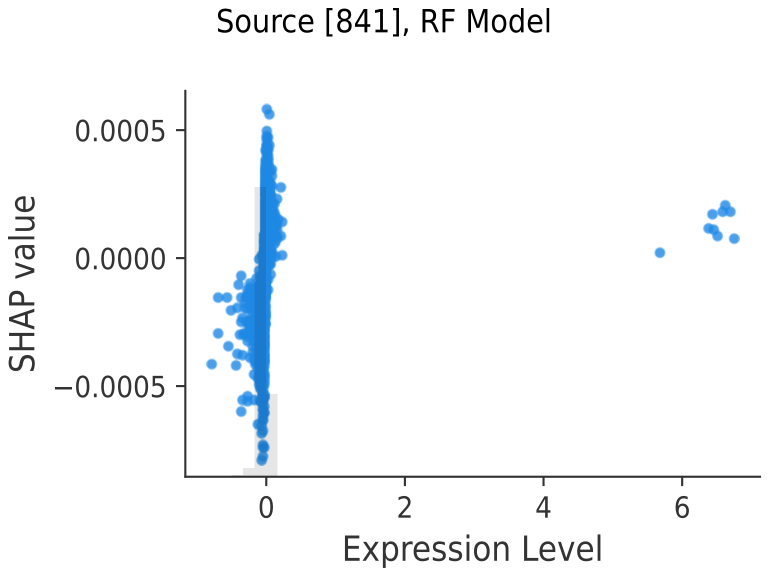

Source [841], RF Model
0.0005
0.0000
−0.0005
SHAP value
0
2
4
6
Expression Level

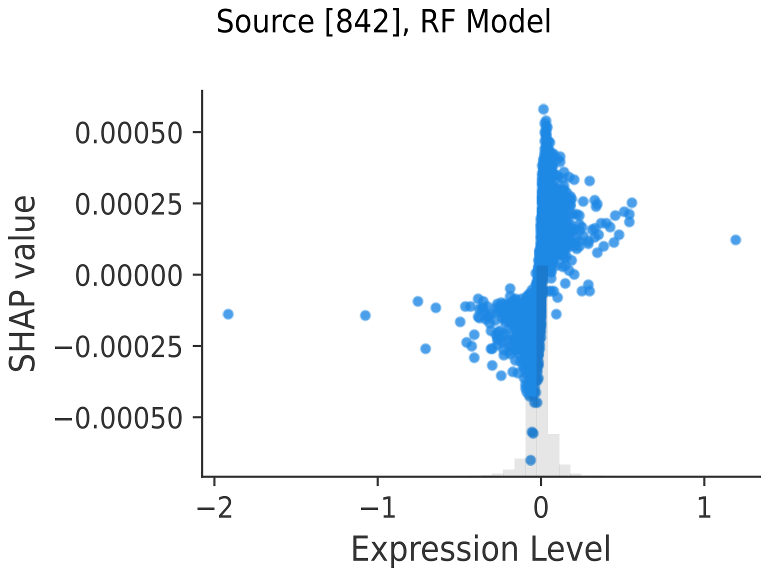
Source [842], RF Model
0.00050
0.00025
0.00000
−0.00025
−0.00050
SHAP value
−2
−1
0
1
Expression Level

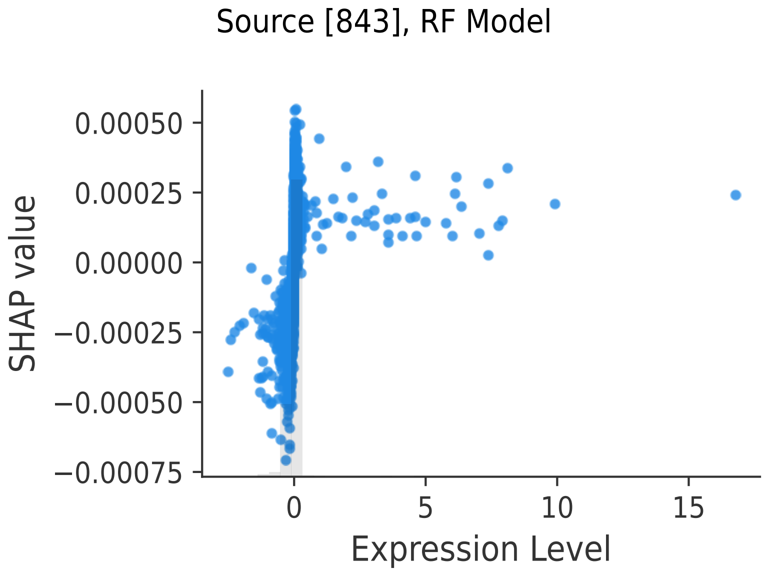
Source [843], RF Model
SHAP value
0.00050
0.00025
0.00000
−0.00025
−0.00050
−0.00075
0
5
10
15
Expression Level

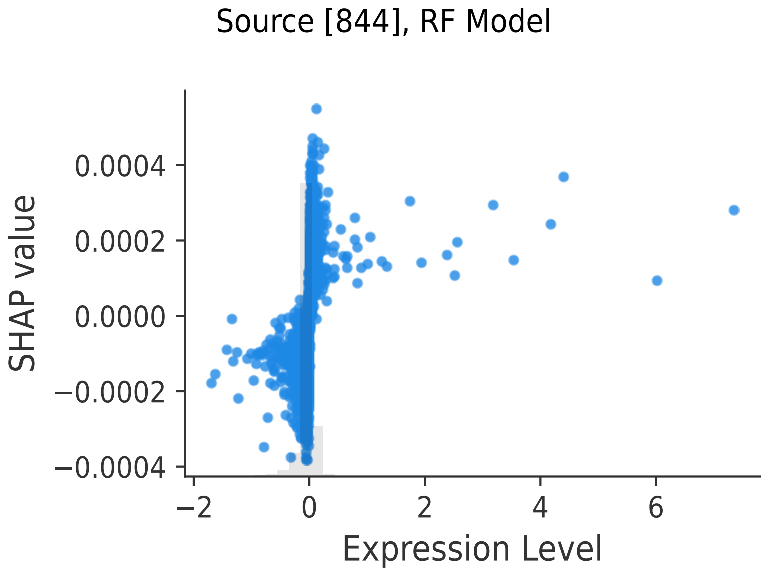

Source [844], RF Model
SHAP value
0.0004
0.0002
0.0000
−0.0002
−0.0004
−2
0
2
4
6
Expression Level

Source [845], RF Model

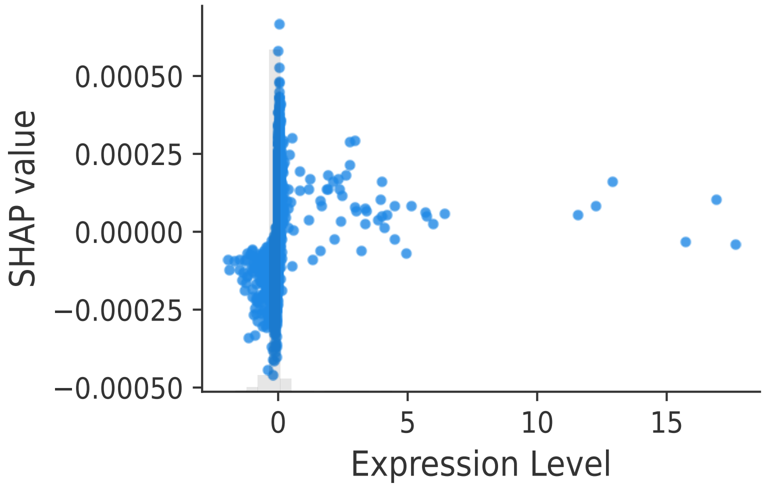

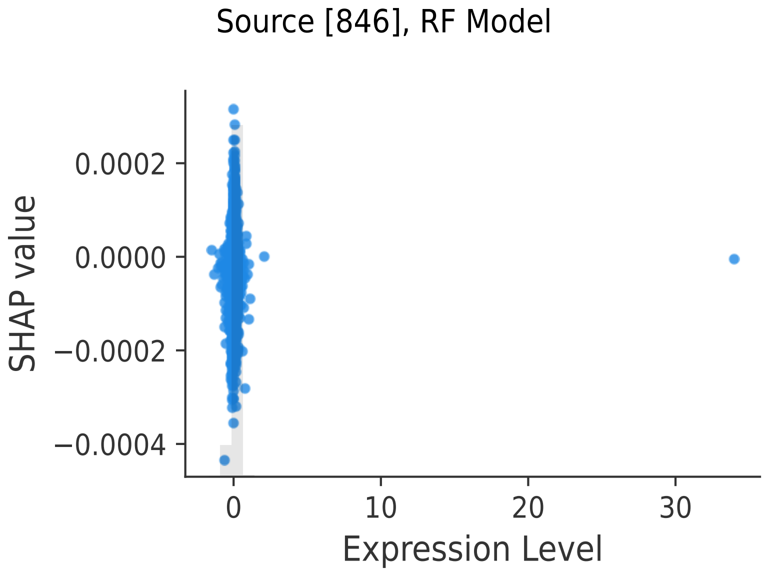

Source [846], RF Model
SHAP value
0.0002
0.0000
−0.0002
−0.0004
0
10
20
30
Expression Level

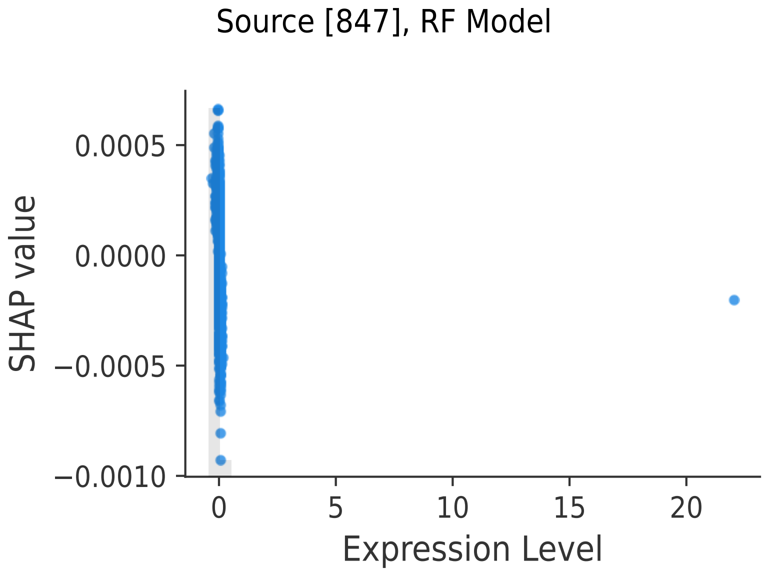
Source [847], RF Model
SHAP value
0.0005
0.0000
−0.0005
−0.0010
0
5
10
15
20
Expression Level

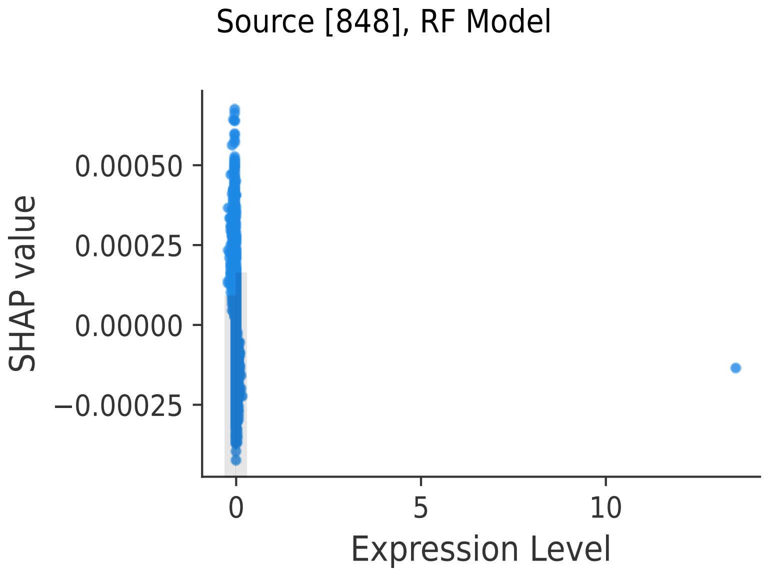
Source [848], RF Model
SHAP value
0.00050
0.00025
0.00000
−0.00025
0
5
10
Expression Level

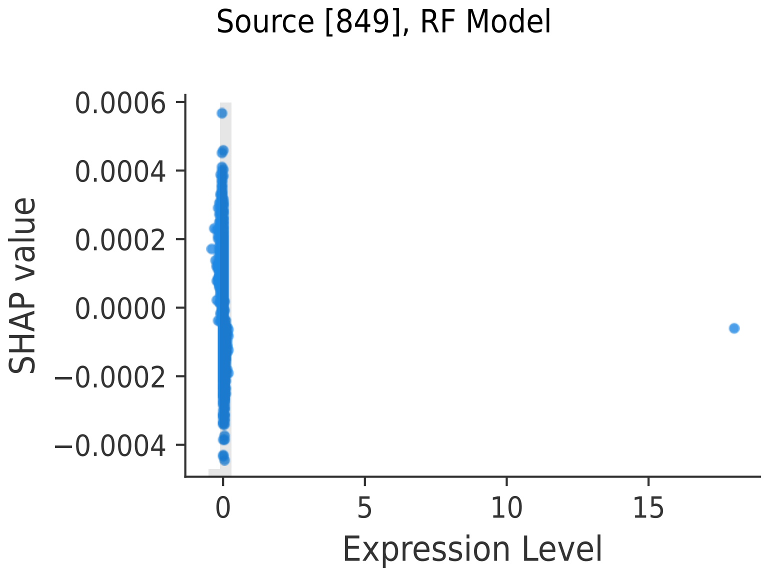
Source [849], RF Model
0.0006
0.0004
0.0002
0.0000
−0.0002
−0.0004
SHAP value
0
5
10
15
Expression Level

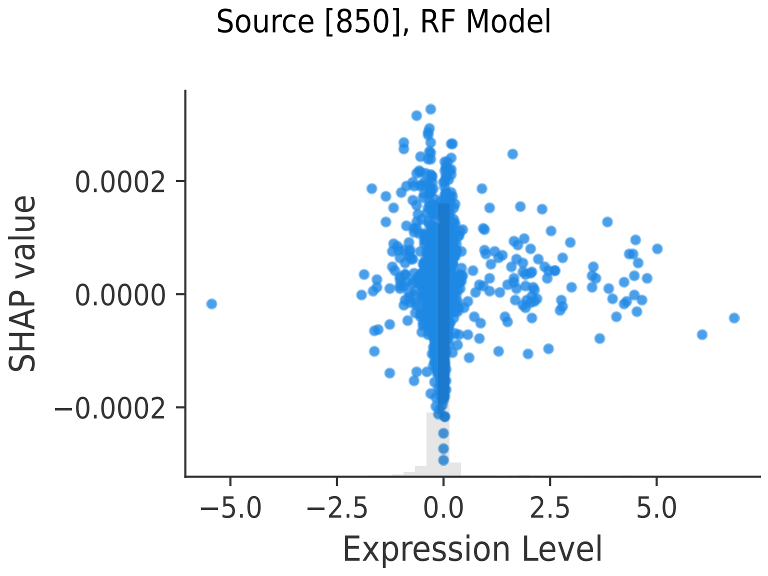
Source [850], RF Model
SHAP value
0.0002
0.0000
−0.0002
−5.0
−2.5
0.0
2.5
5.0
Expression Level

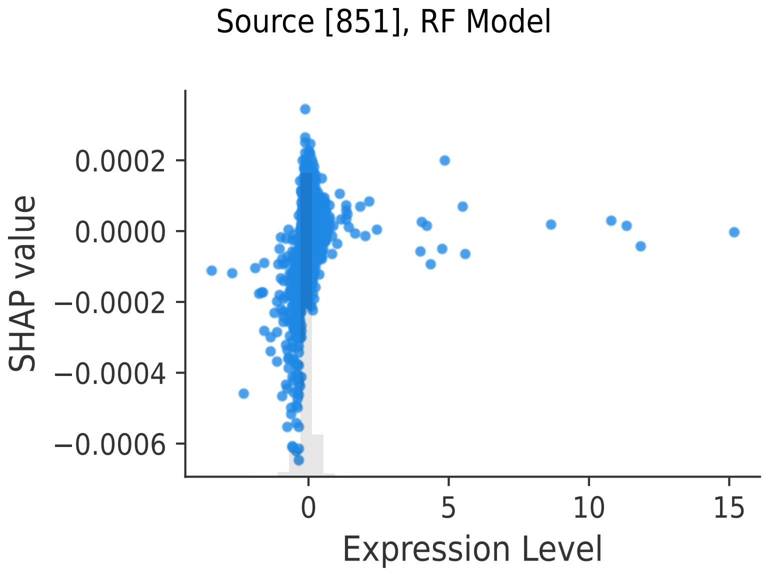

Source [851], RF Model
0.0002
0.0000
−0.0002
−0.0004
−0.0006
SHAP value
0
5
10
15
Expression Level

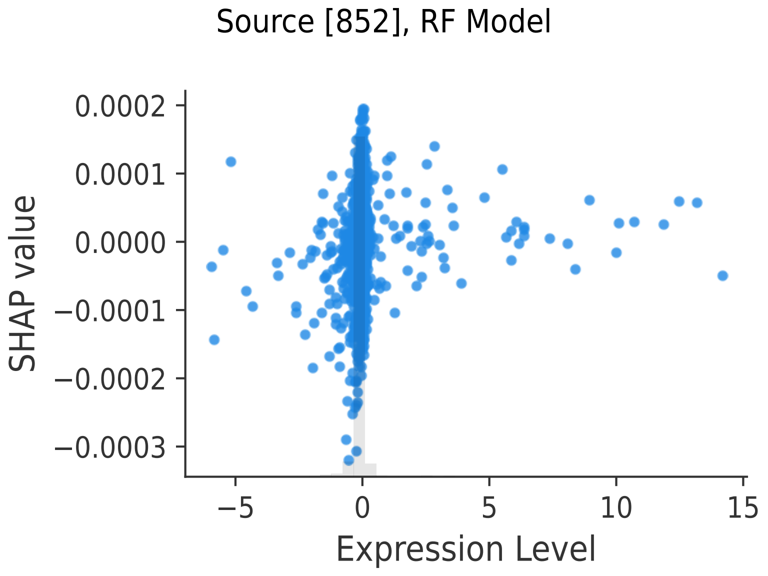

Source [852], RF Model
SHAP value
0.0002
0.0001
0.0000
−0.0001
−0.0002
−0.0003
−5
0
5
10
15
Expression Level

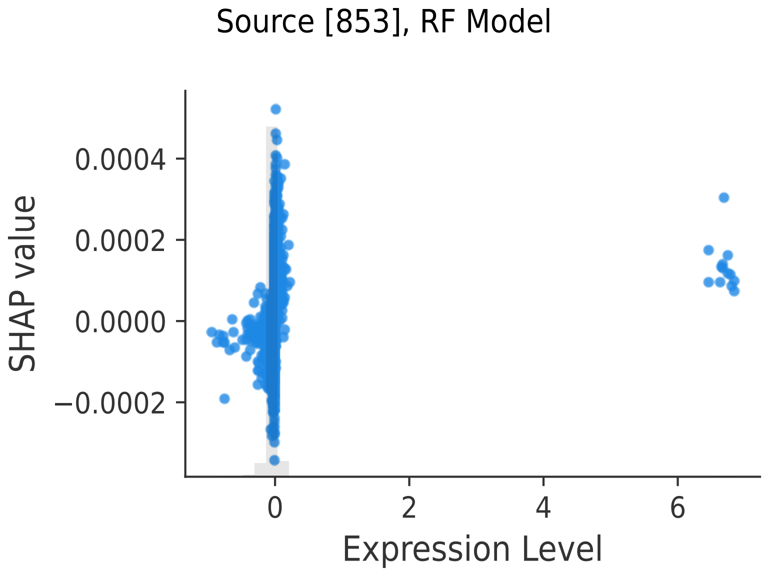
Source [853], RF Model
SHAP value
0.0004
0.0002
0.0000
−0.0002
Expression Level
0
2
4
6

Source [854], RF Model

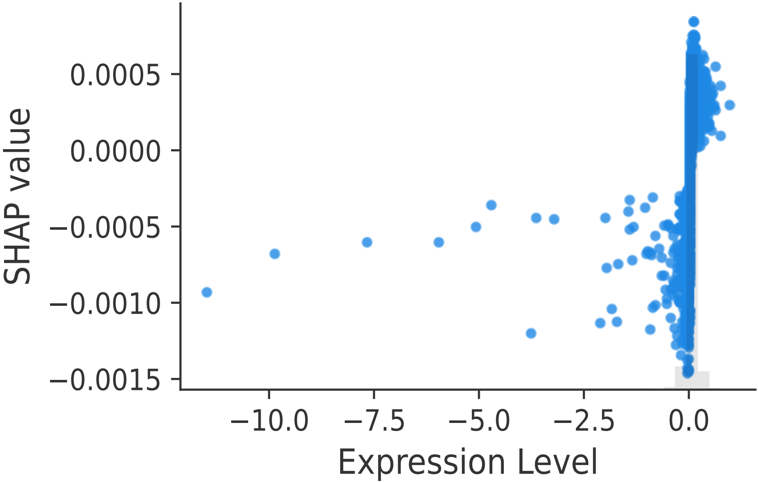

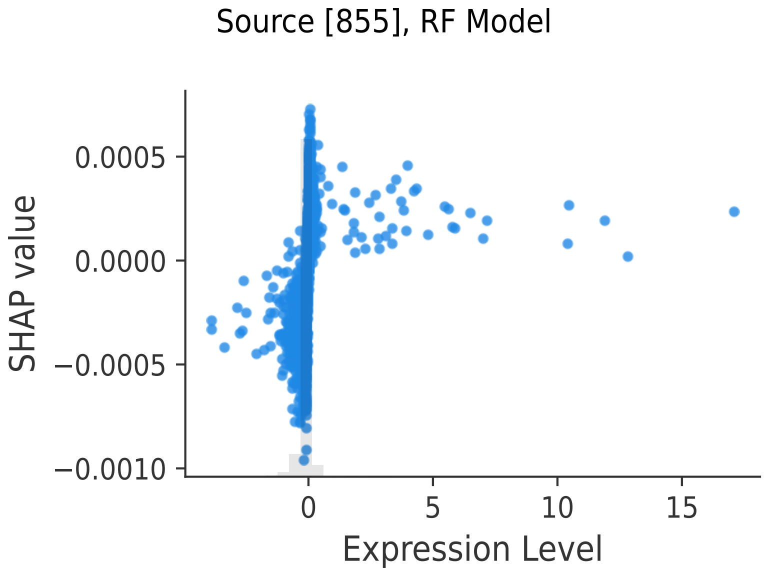

Source [855], RF Model
0.0005
0.0000
−0.0005
−0.0010
SHAP value
0
5
10
15
Expression Level

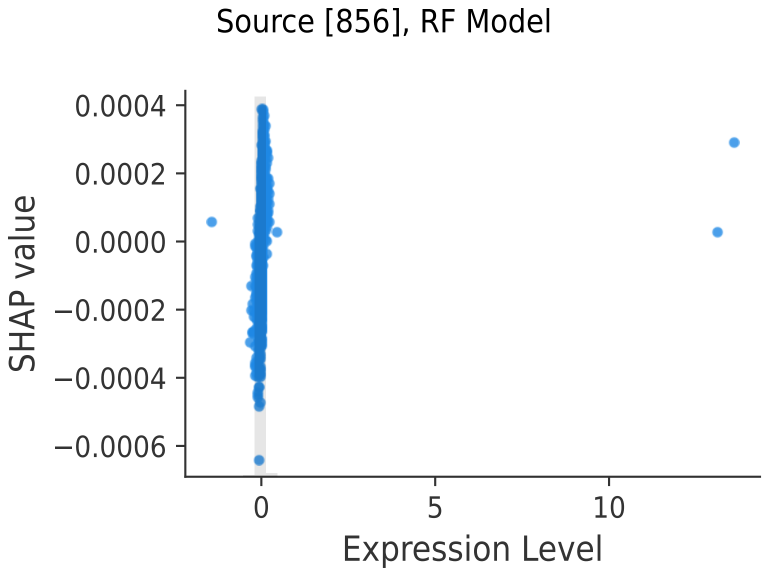
Source [856], RF Model
SHAP value
0.0004
0.0002
0.0000
−0.0002
−0.0004
−0.0006
0
5
10
Expression Level

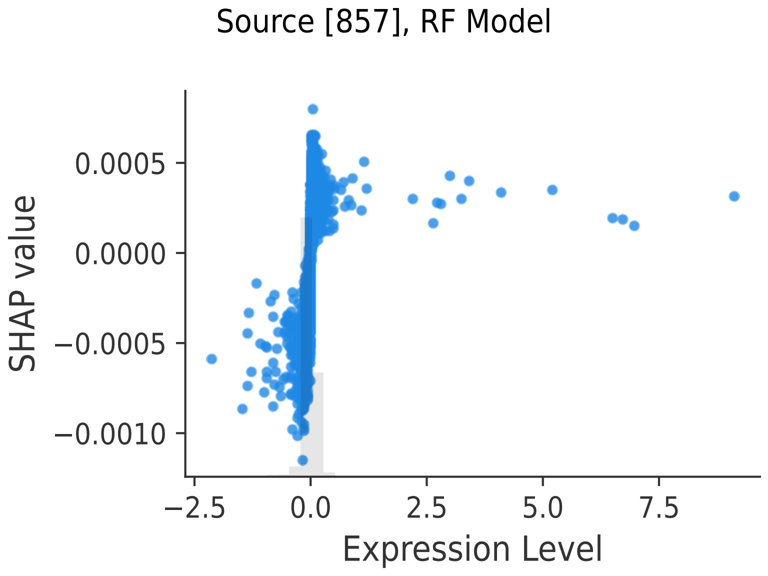

Source [857], RF Model
SHAP value
0.0005
0.0000
−0.0005
−0.0010
−2.5
0.0
2.5
5.0
7.5
Expression Level

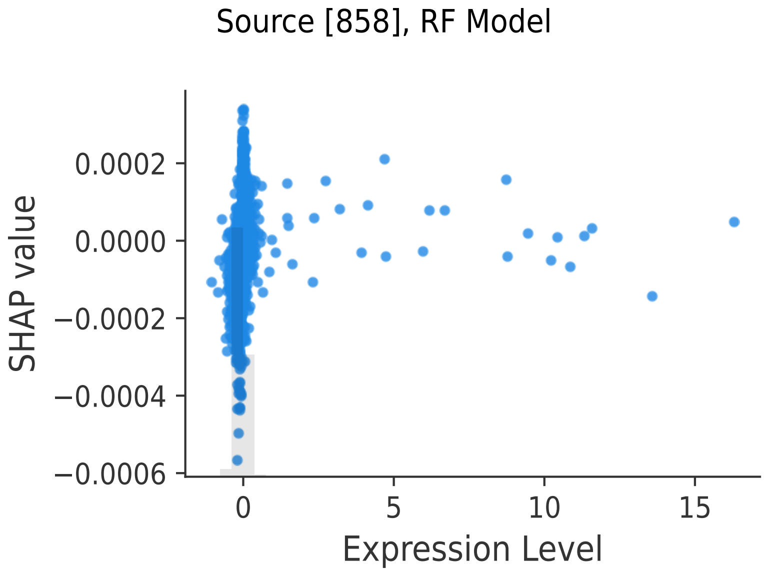
Source [858], RF Model
0.0002
0.0000
−0.0002
−0.0004
−0.0006
SHAP value
0
5
10
15
Expression Level

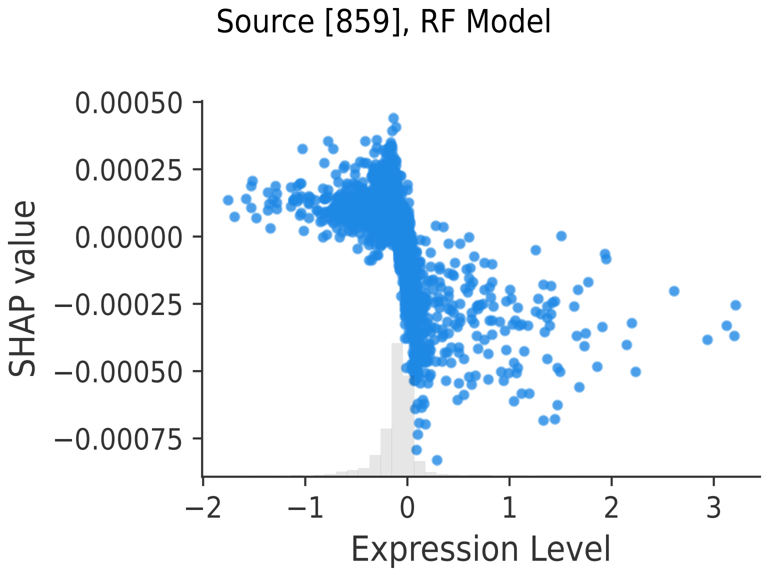
Source [859], RF Model
SHAP value
0.00050
0.00025
0.00000
−0.00025
−0.00050
−0.00075
Expression Level
−2
−1
0
1
2
3

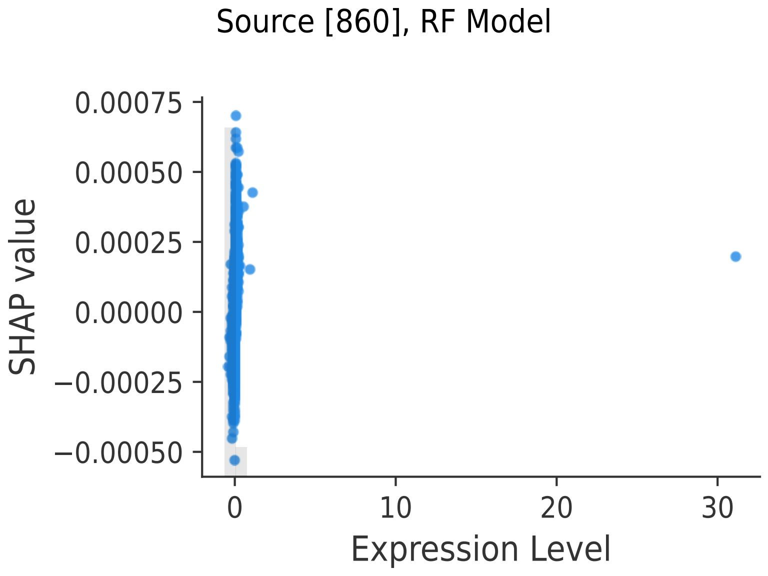

Source [860], RF Model
0.00075
0.00050
0.00025
0.00000
−0.00025
−0.00050
SHAP value
0
10
20
30
Expression Level

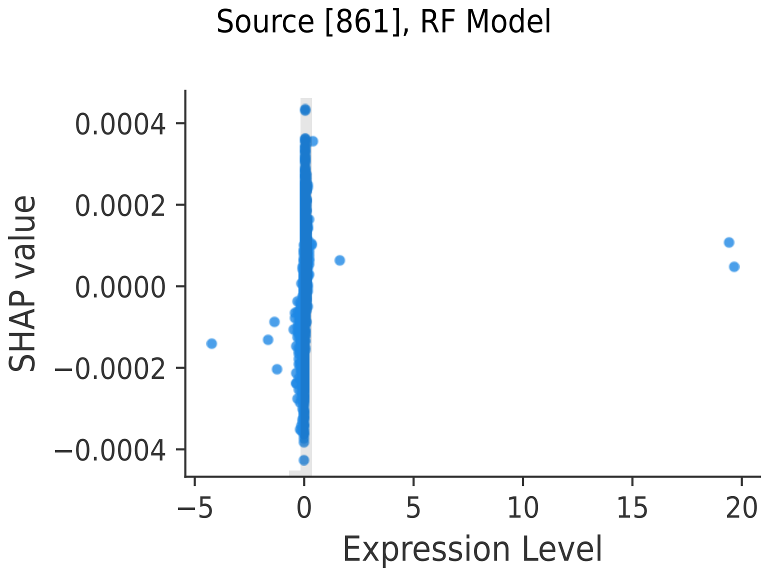
Source [861], RF Model
0.0004
0.0002
0.0000
−0.0002
−0.0004
SHAP value
−5
0
5
10
15
20
Expression Level

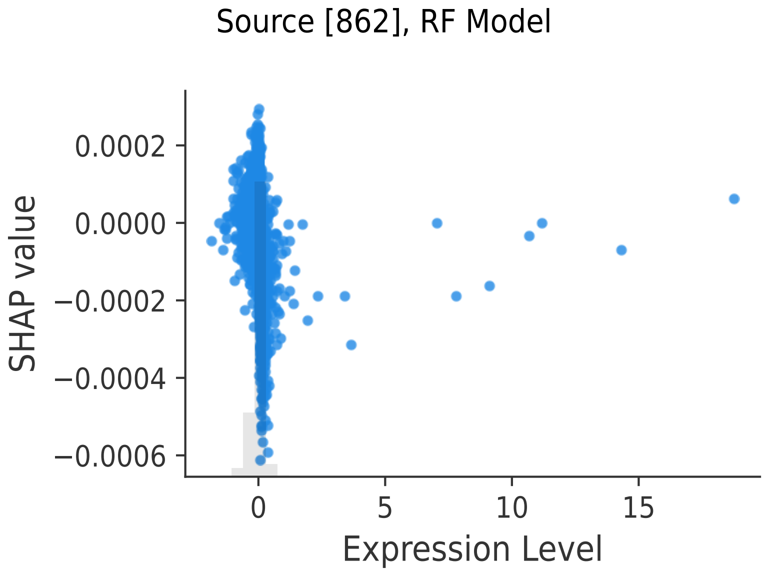
Source [862], RF Model
SHAP value
0.0002
0.0000
−0.0002
−0.0004
−0.0006
0
5
10
15
Expression Level

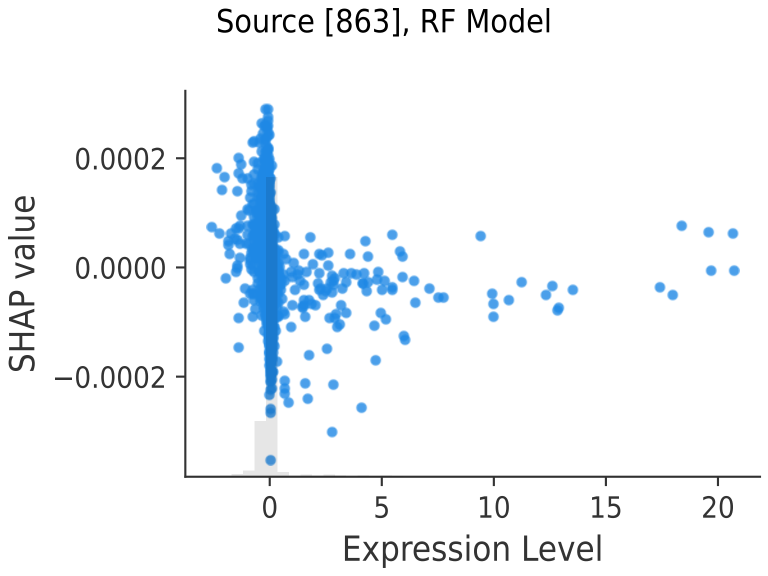
Source [863], RF Model
SHAP value
0.0002
0.0000
−0.0002
0
5
10
15
20
Expression Level

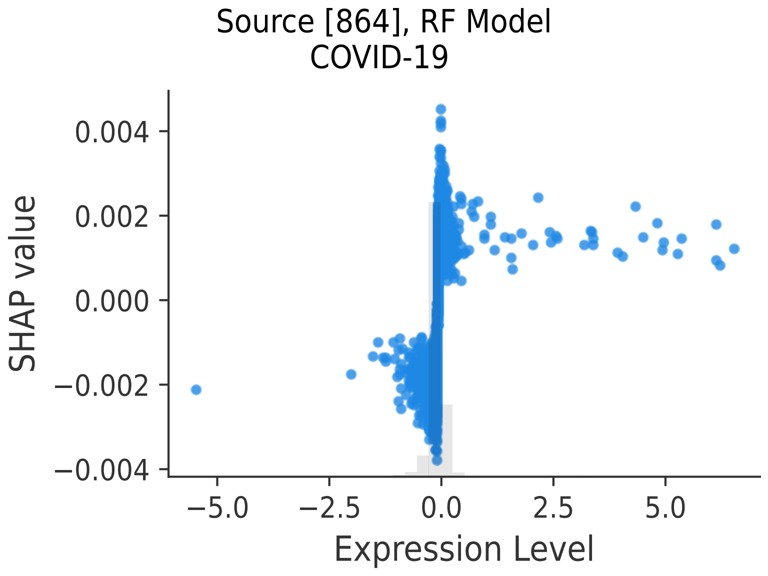
Source [864], RF Model
COVID-19
SHAP value
0.004
0.002
0.000
−0.002
−0.004
−5.0
−2.5
0.0
2.5
5.0
Expression Level

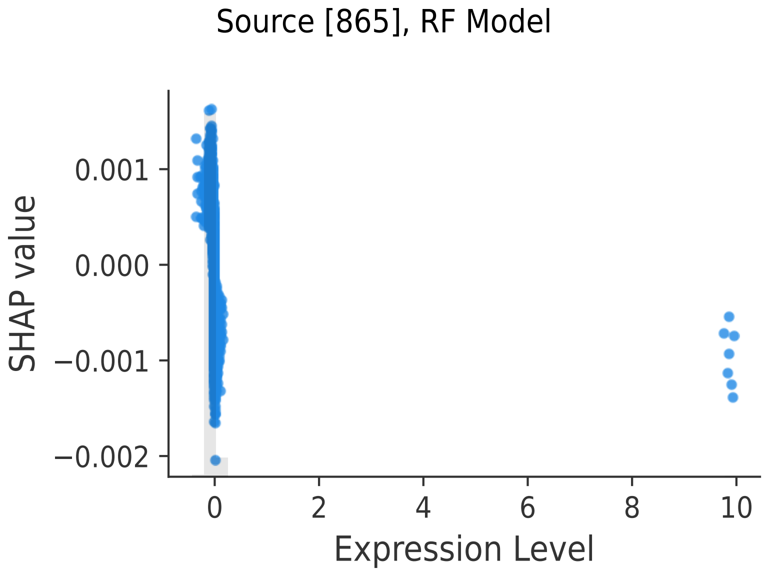

Source [865], RF Model
0.001
0.000
−0.001
−0.002
SHAP value
0
2
4
6
8
10
Expression Level

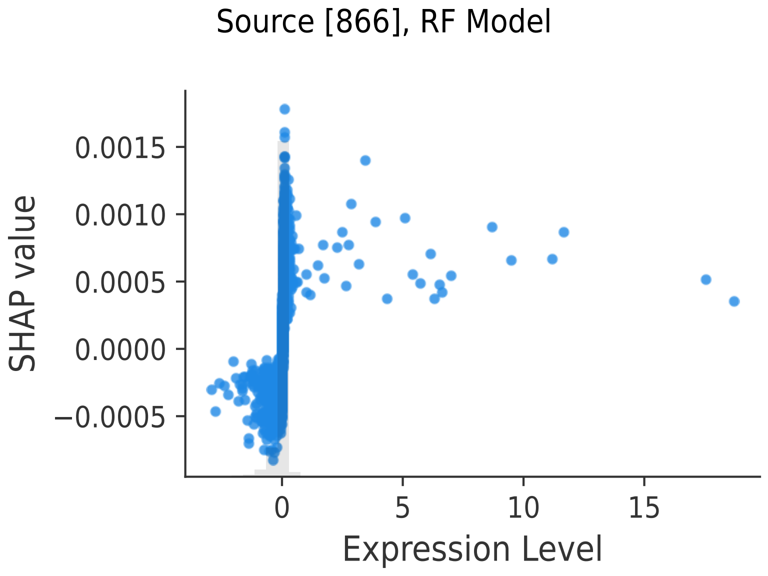
Source [866], RF Model
SHAP value
0.0015
0.0010
0.0005
0.0000
−0.0005
0
5
10
15
Expression Level

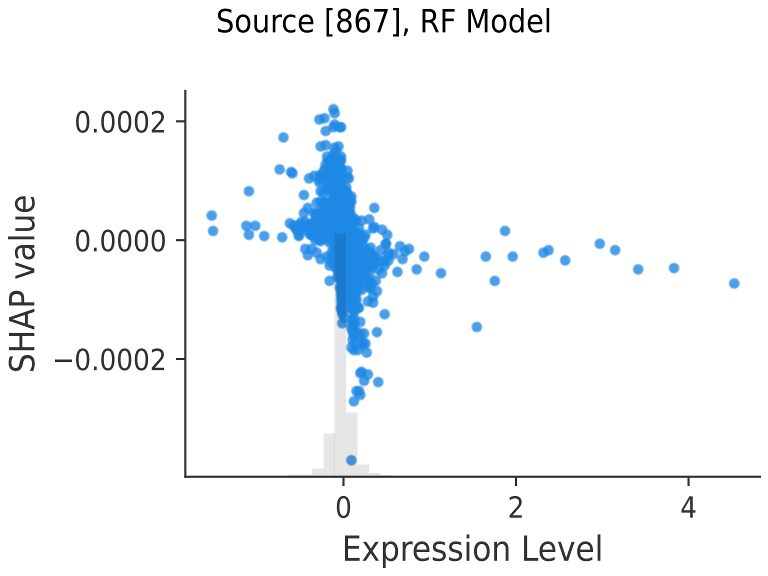
Source [867], RF Model
0.0002
0.0000
−0.0002
SHAP value
0
2
4
Expression Level

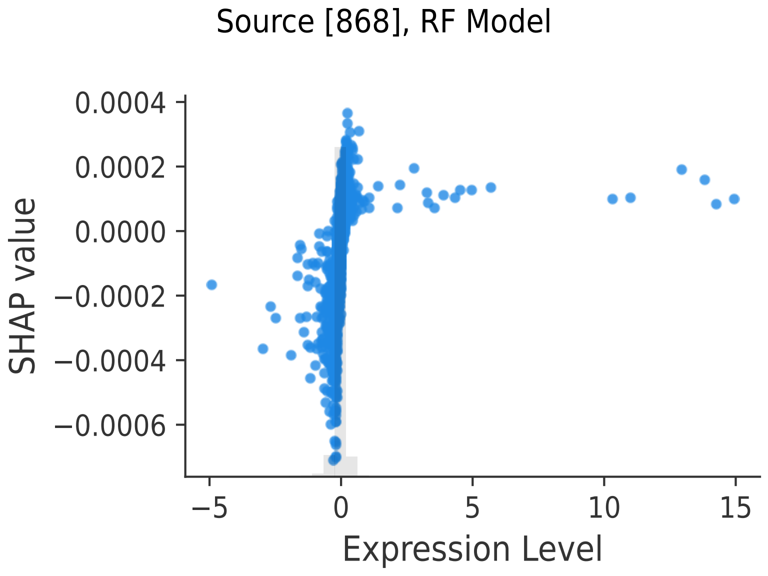

Source [868], RF Model
0.0004
0.0002
0.0000
−0.0002
−0.0004
−0.0006
SHAP value
−5
0
5
10
15
Expression Level

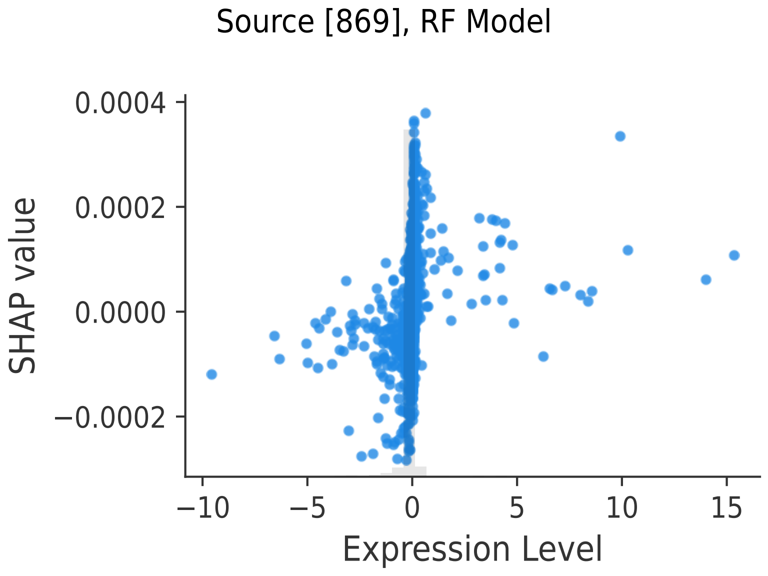

Source [869], RF Model
0.0004
0.0002
0.0000
−0.0002
SHAP value
−10
−5
0
5
10
15
Expression Level

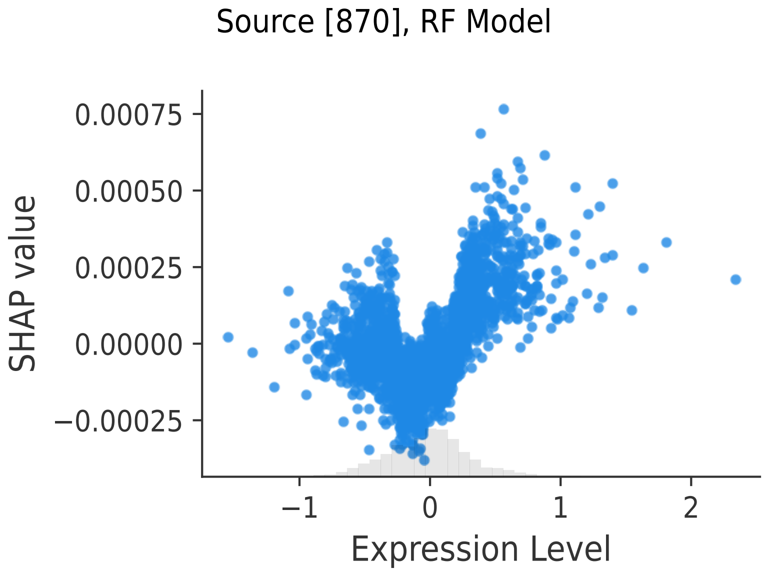

Source [870], RF Model
0.00075
0.00050
0.00025
0.00000
−0.00025
SHAP value
−1
0
1
2
Expression Level

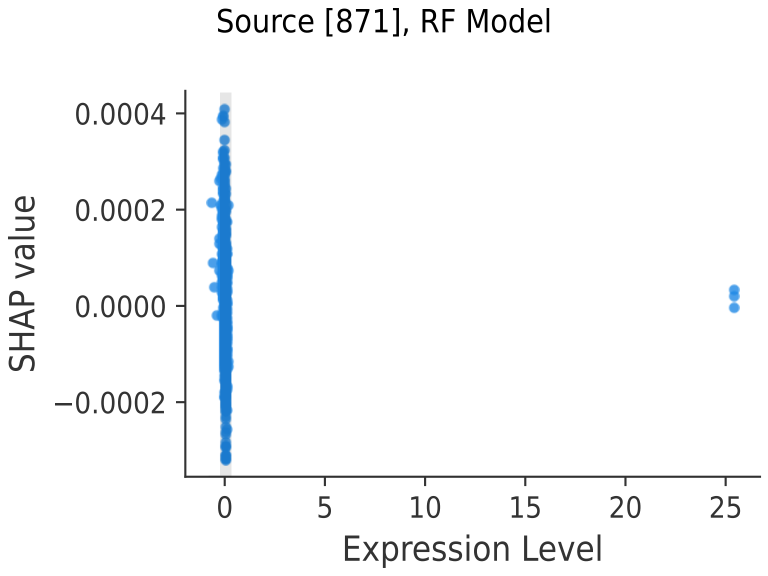
Source [871], RF Model
SHAP value
0.0004
0.0002
0.0000
−0.0002
0
5
10
15
20
25
Expression Level

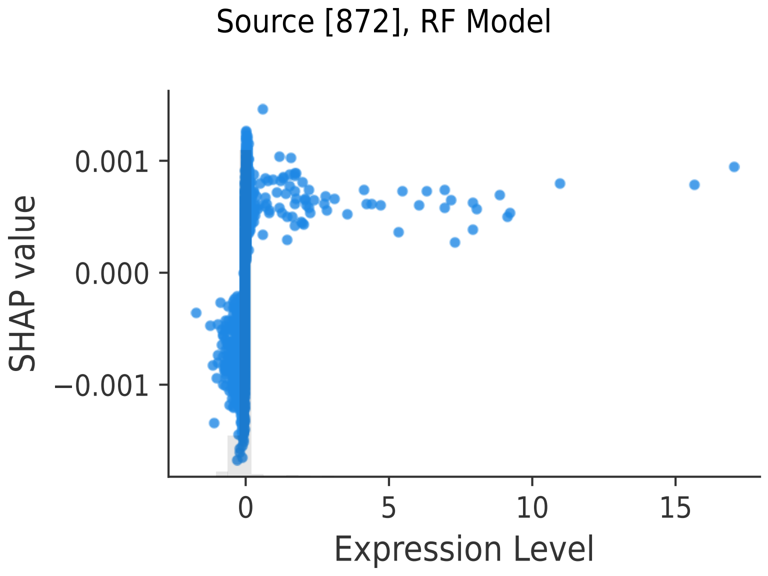
Source [872], RF Model
0.001
0.000
−0.001
SHAP value
0
5
10
15
Expression Level

Source [873], RF Model

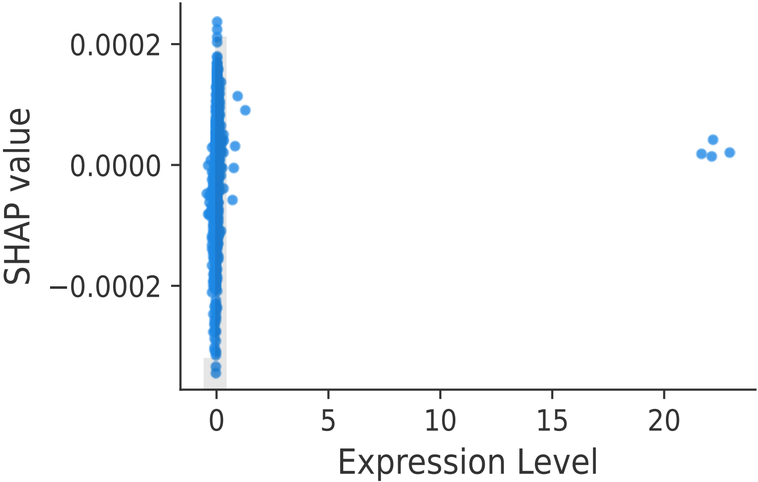

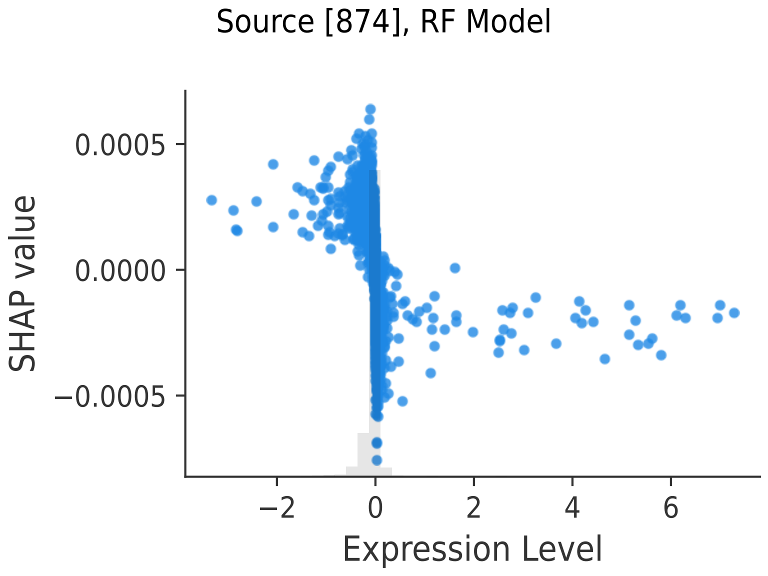

Source [874], RF Model
0.0005
0.0000
−0.0005
SHAP value
−2
0
2
4
6
Expression Level

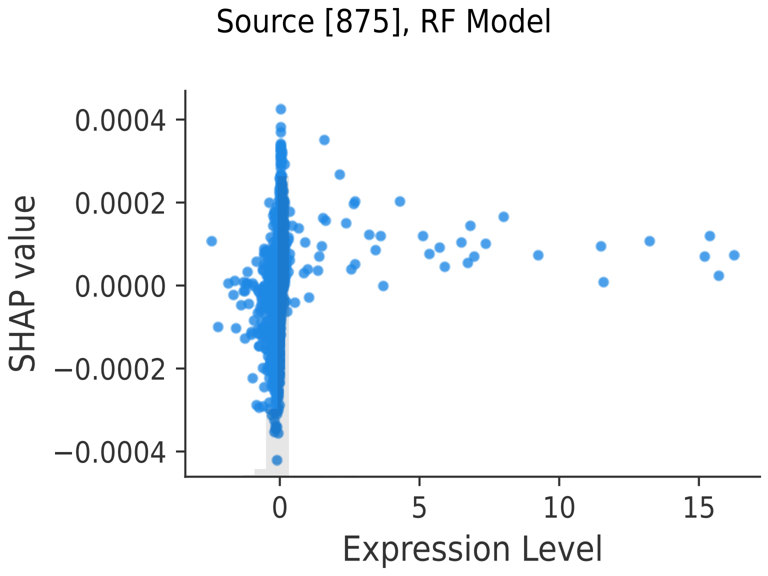
Source [875], RF Model
0.0004
0.0002
0.0000
−0.0002
−0.0004
SHAP value
0
5
10
15
Expression Level

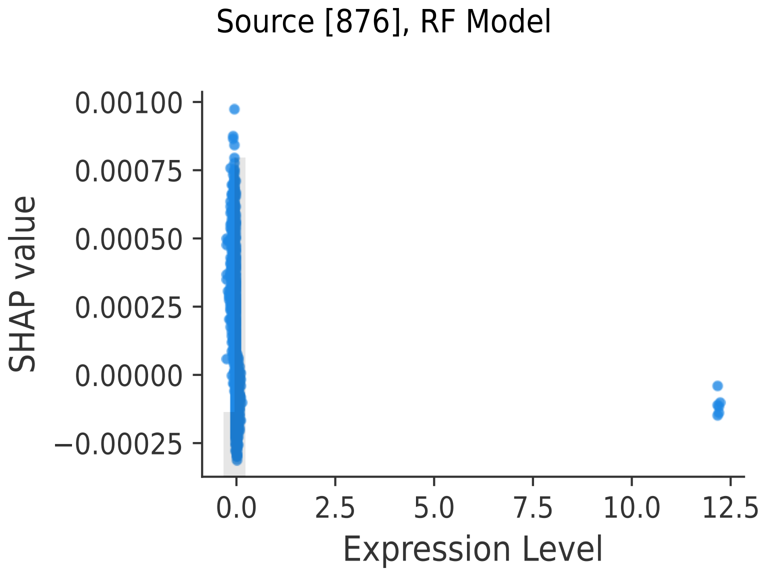
Source [876], RF Model
0.00100
0.00075
0.00050
0.00025
0.00000
−0.00025
SHAP value
0.0
2.5
5.0
7.5
10.0
12.5
Expression Level

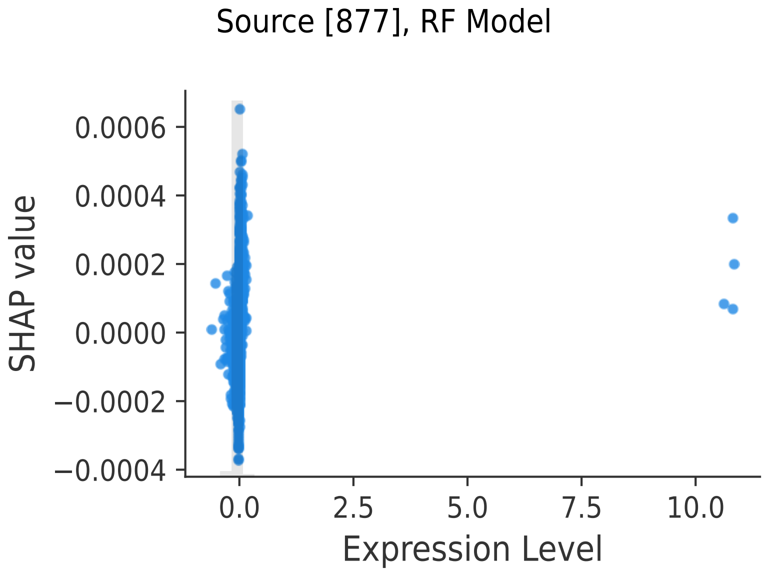
Source [877], RF Model
SHAP value
0.0006
0.0004
0.0002
0.0000
−0.0002
−0.0004
0.0
2.5
5.0
7.5
10.0
Expression Level

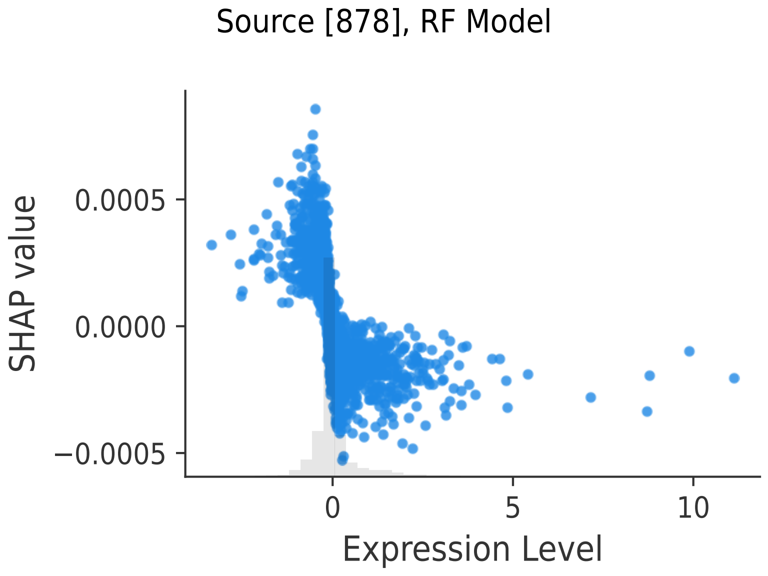
Source [878], RF Model
SHAP value
0.0005
0.0000
−0.0005
0
5
10
Expression Level

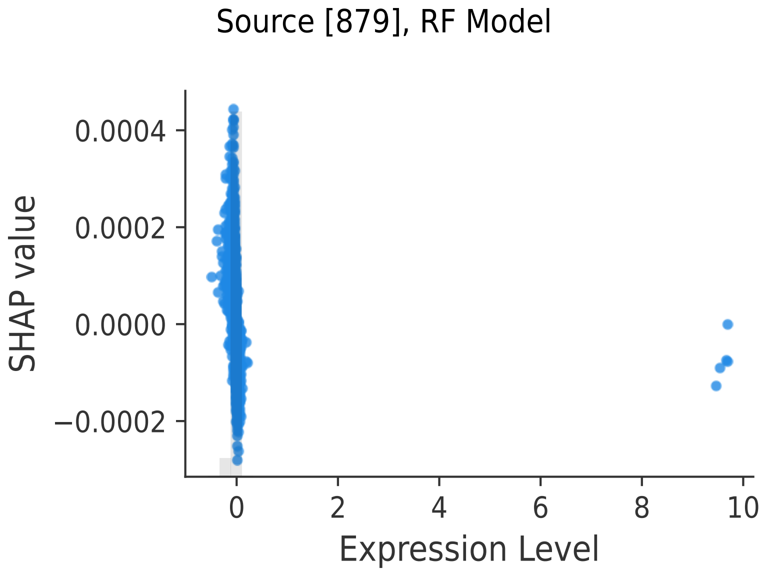
Source [879], RF Model
SHAP value
0.0004
0.0002
0.0000
−0.0002
0
2
4
6
8
10
Expression Level

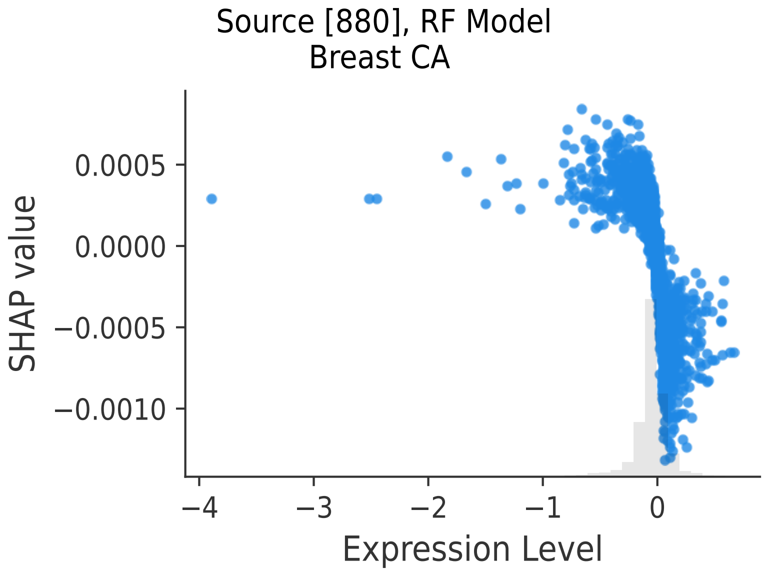
Source [880], RF Model
Breast CA
0.0005
0.0000
−0.0005
−0.0010
SHAP value
−4
−3
−2
−1
0
Expression Level

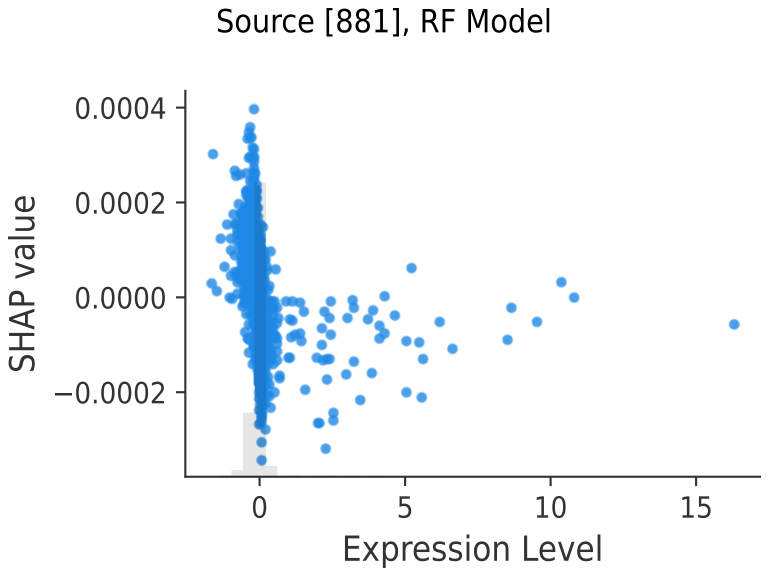
Source [881], RF Model
SHAP value
0.0004
0.0002
0.0000
−0.0002
0
5
10
15
Expression Level

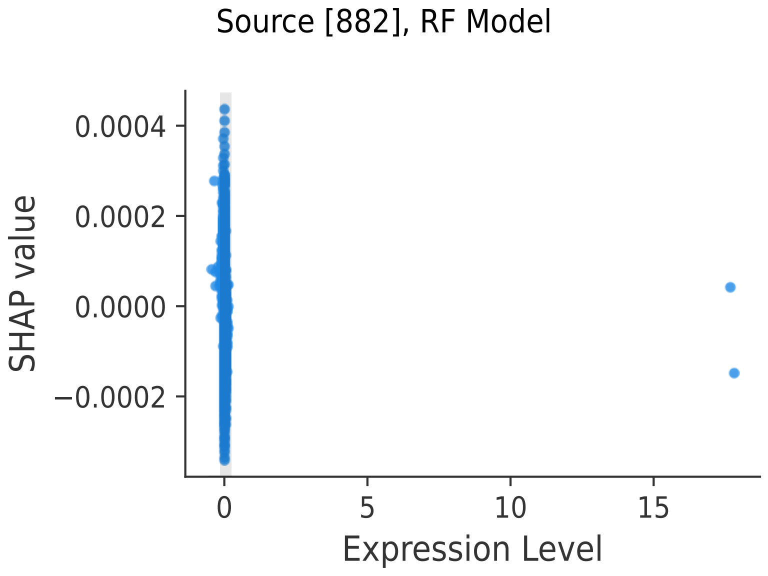

Source [882], RF Model
SHAP value
0.0004
0.0002
0.0000
−0.0002
0
5
10
15
Expression Level

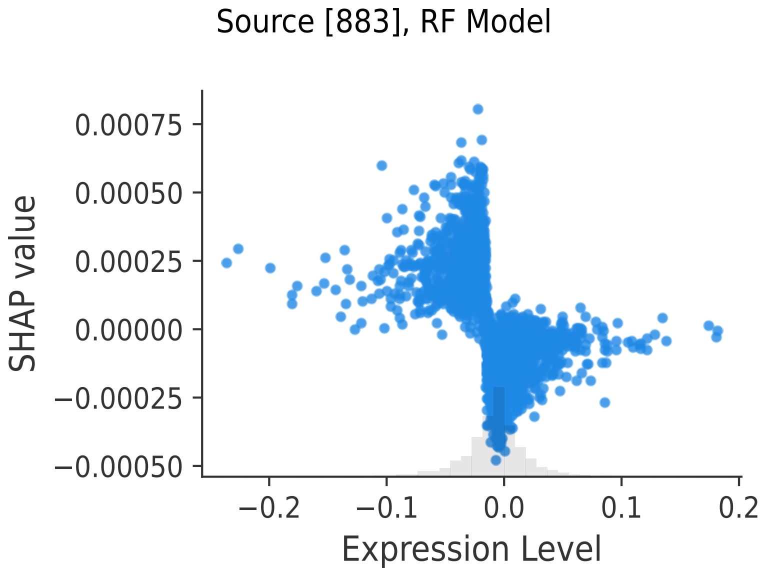
Source [883], RF Model
SHAP value
0.00075
0.00050
0.00025
0.00000
−0.00025
−0.00050
−0.2
−0.1
0.0
0.1
0.2
Expression Level

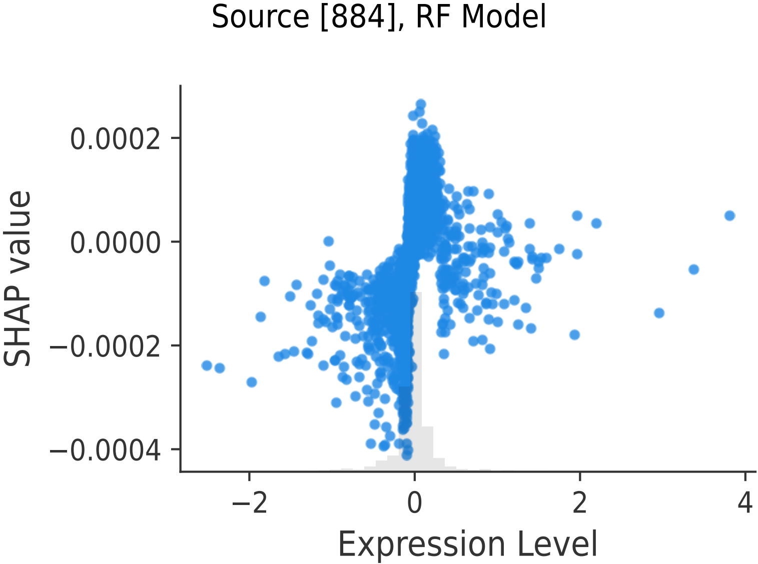

Source [884], RF Model
0.0002
0.0000
−0.0002
−0.0004
SHAP value
−2
0
2
4
Expression Level

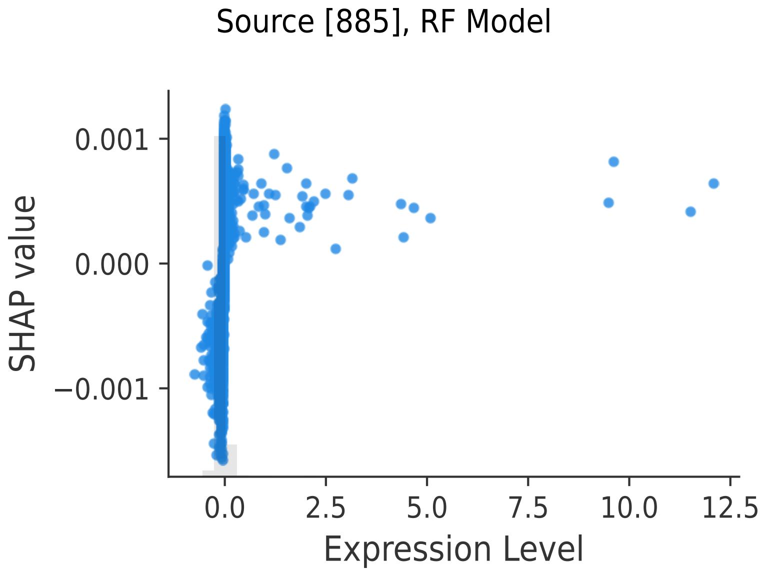
Source [885], RF Model
0.001
0.000
−0.001
SHAP value
0.0
2.5
5.0
7.5
10.0
12.5
Expression Level

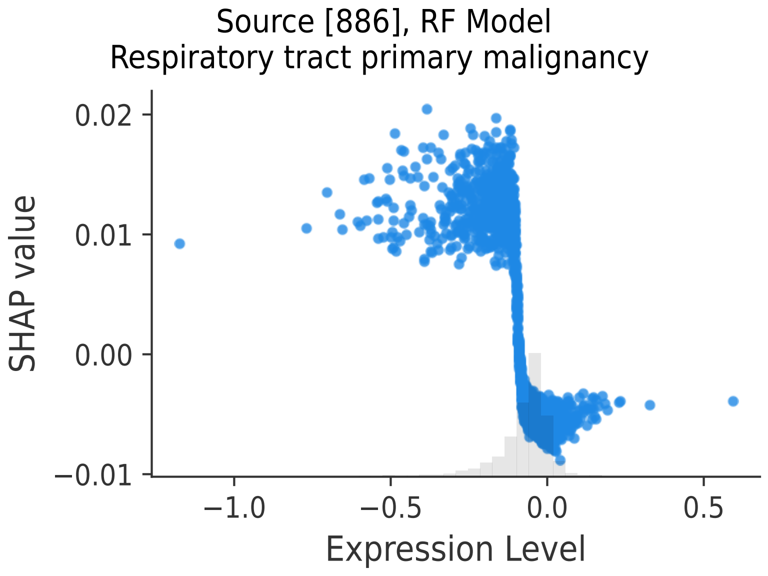
Source [886], RF Model
Respiratory tract primary malignancy
SHAP value
0.02
0.01
0.00
−0.01
−1.0
−0.5
0.0
0.5
Expression Level

Source [887], RF Model

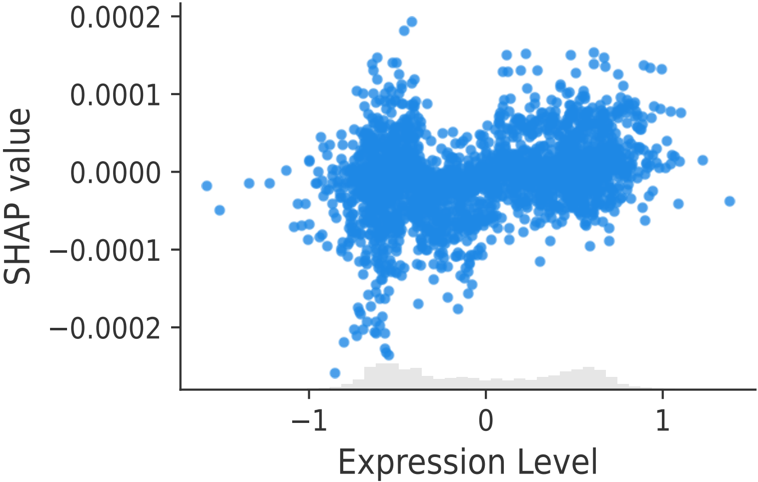

Source [888], RF Model

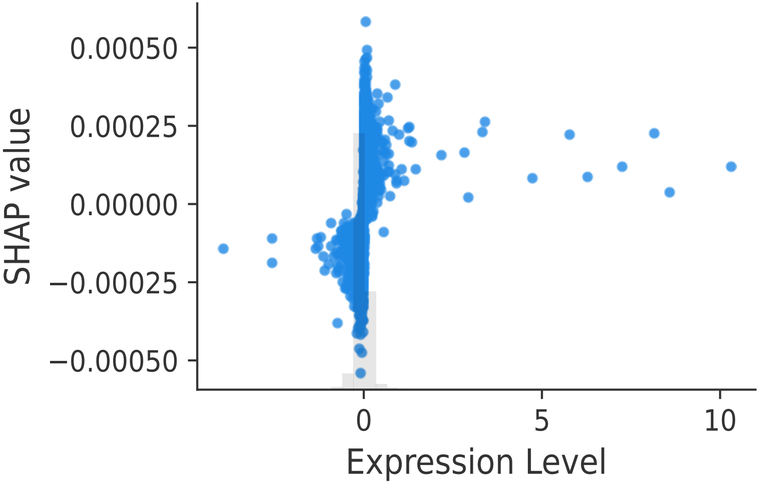

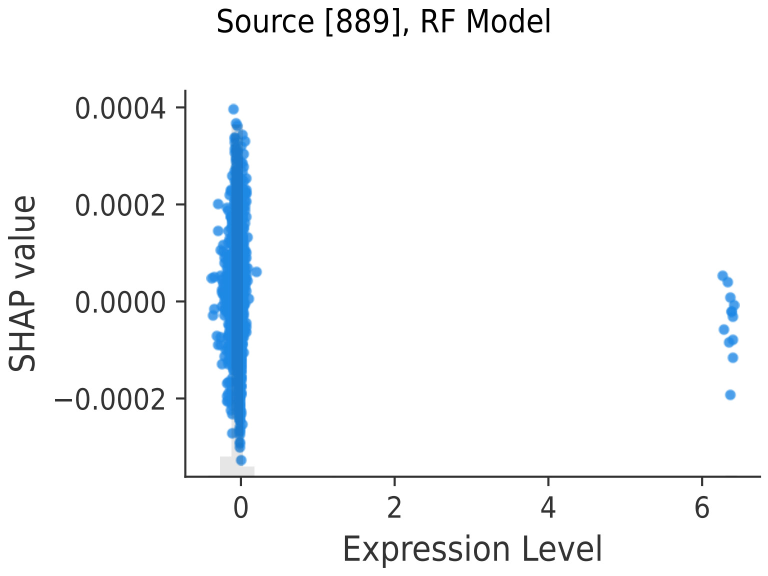

Source [889], RF Model
0.0004
0.0002
0.0000
−0.0002
SHAP value
0
2
4
6
Expression Level

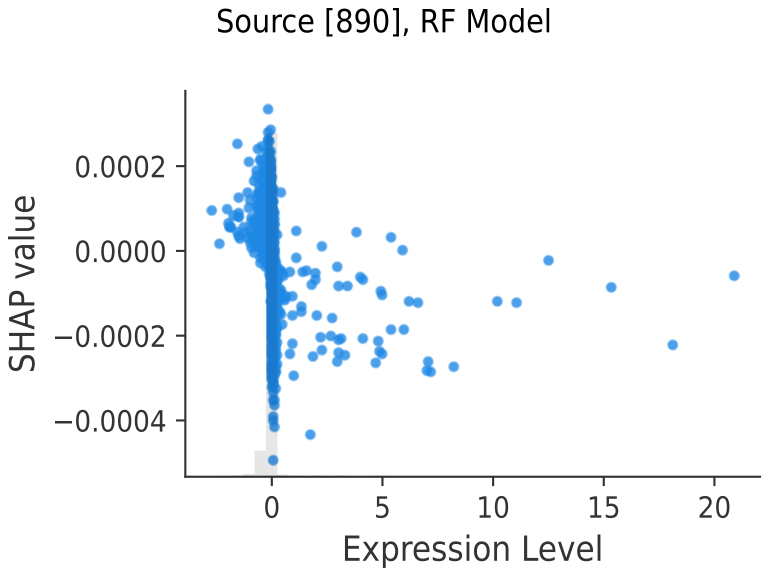
Source [890], RF Model
SHAP value
0.0002
0.0000
−0.0002
−0.0004
0
5
10
15
20
Expression Level

Source [891], RF Model

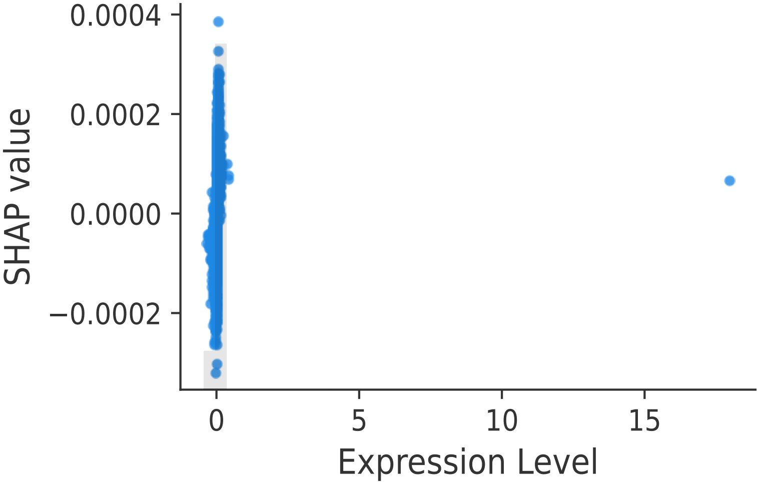

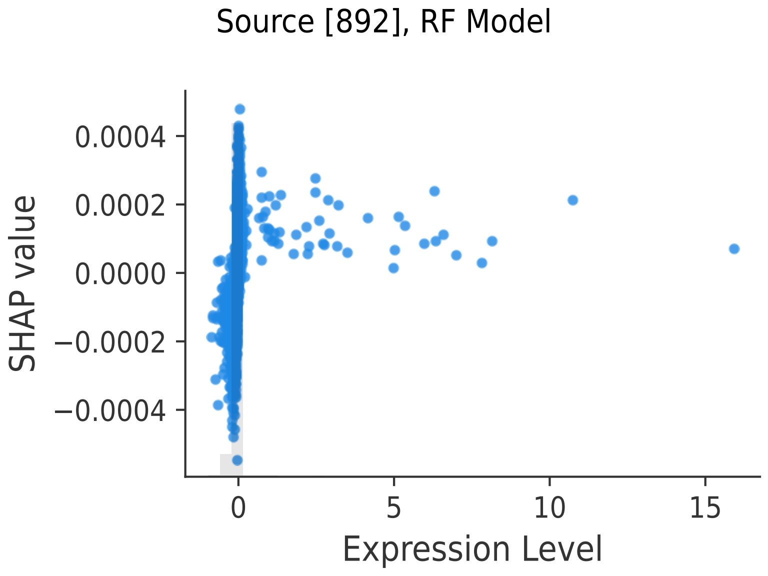

Source [892], RF Model
0.0004
0.0002
0.0000
−0.0002
−0.0004
SHAP value
0
5
10
15
Expression Level

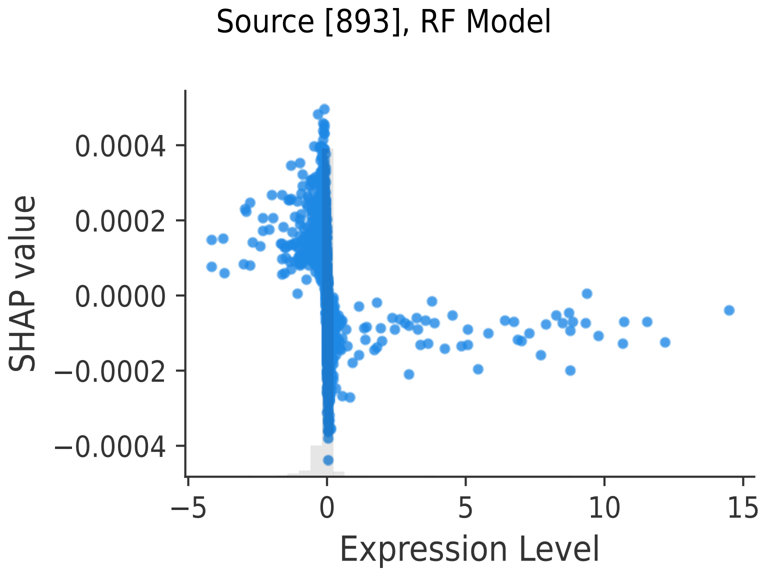
Source [893], RF Model
0.0004
0.0002
0.0000
−0.0002
−0.0004
SHAP value
−5
0
5
10
15
Expression Level

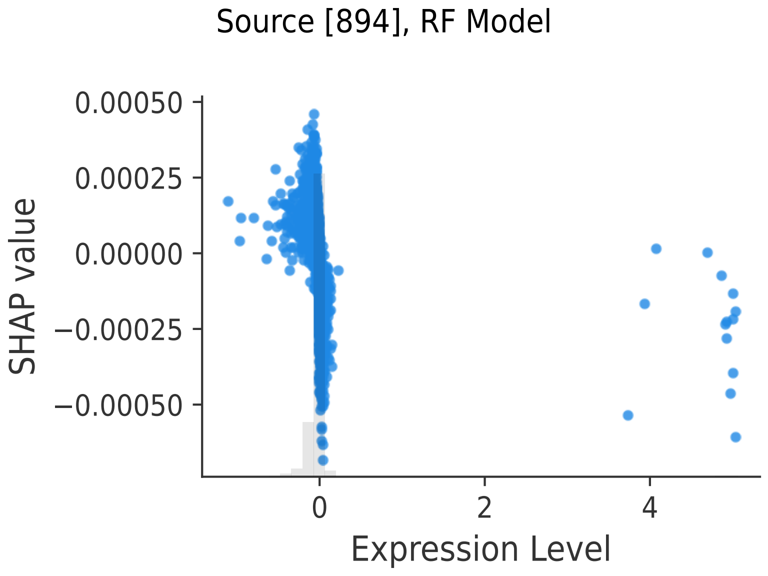

Source [894], RF Model
0.00050
0.00025
0.00000
−0.00025
−0.00050
SHAP value
0
2
4
Expression Level

Source [895], RF Model

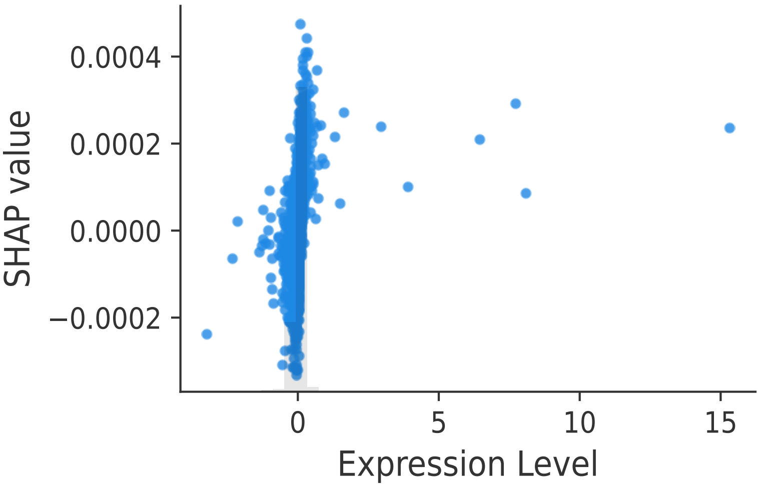

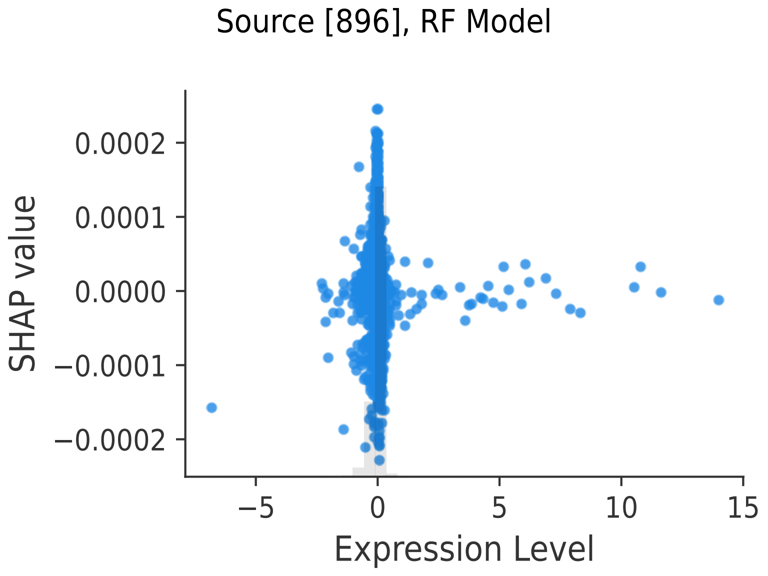

Source [896], RF Model
0.0002
0.0001
0.0000
−0.0001
−0.0002
SHAP value
−5
0
5
10
15
Expression Level

Source [897], RF Model

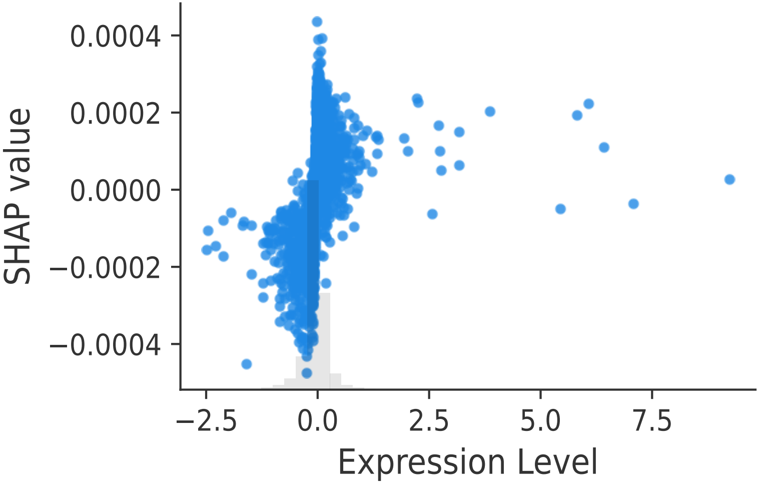

Source [898], RF Model

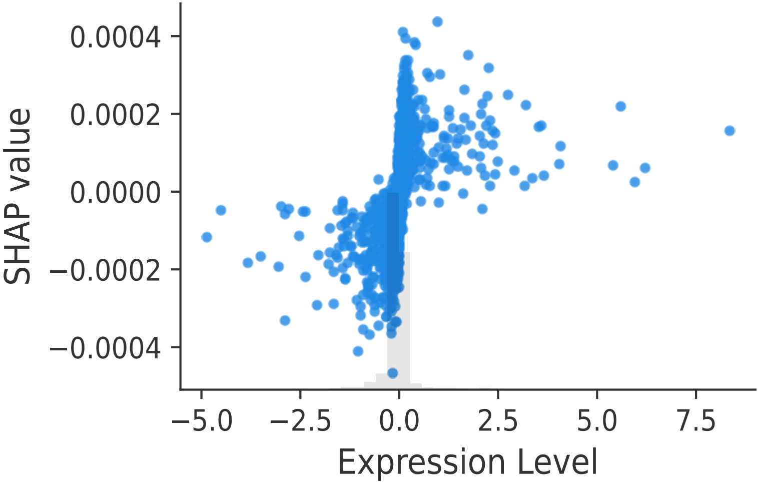

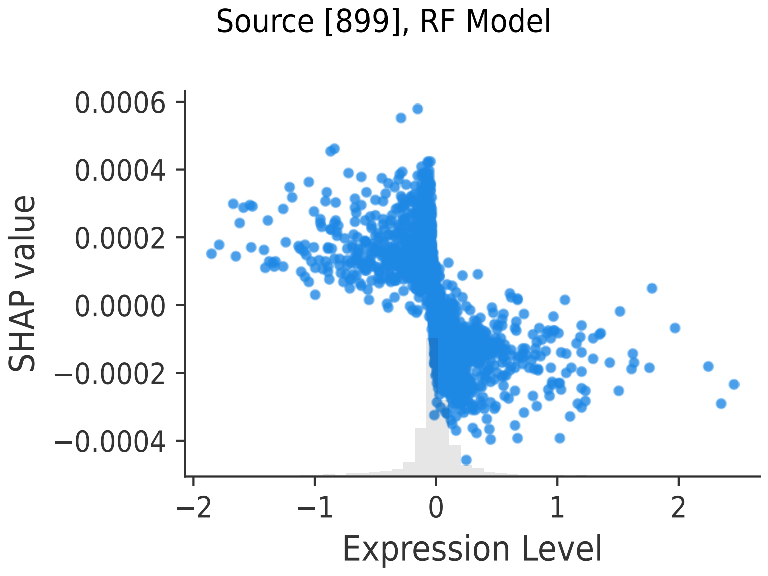
Source [899], RF Model
0.0006
0.0004
0.0002
0.0000
−0.0002
−0.0004
SHAP value
−2
−1
0
1
2
Expression Level

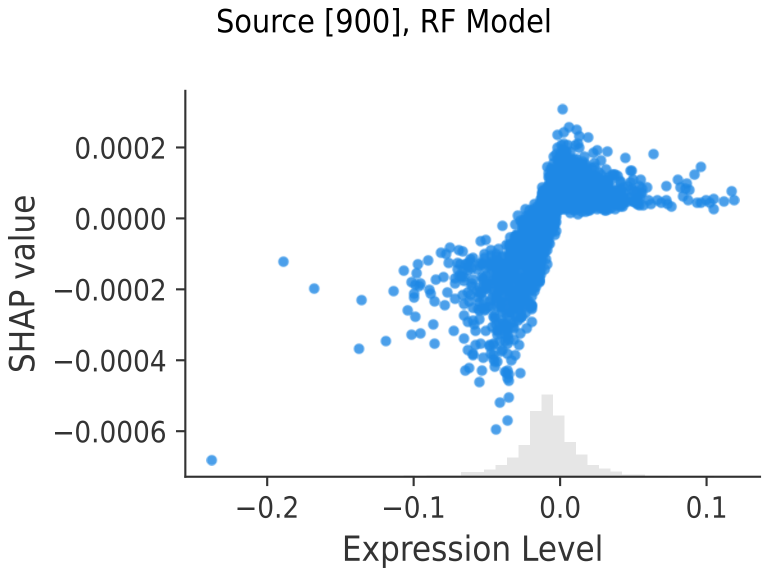

Source [900], RF Model
SHAP value
0.0002
0.0000
−0.0002
−0.0004
−0.0006
−0.2
−0.1
0.0
0.1
Expression Level

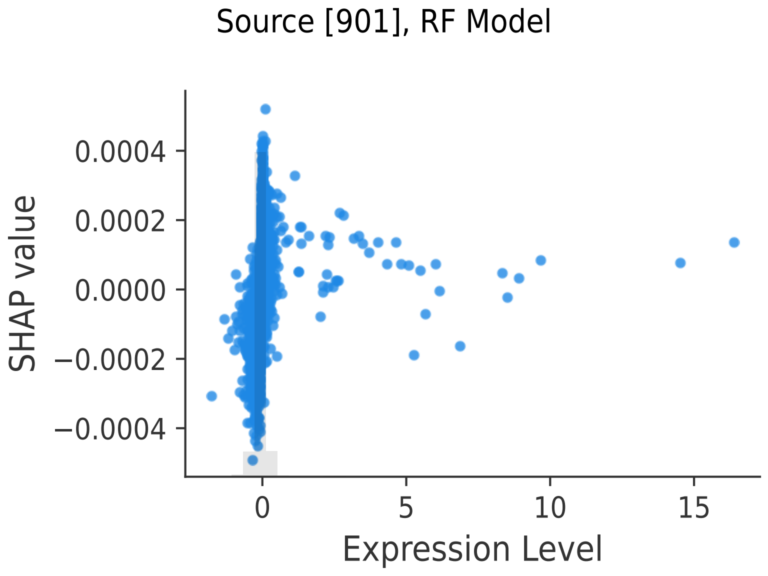

Source [901], RF Model
SHAP value
0.0004
0.0002
0.0000
−0.0002
−0.0004
0
5
10
15
Expression Level

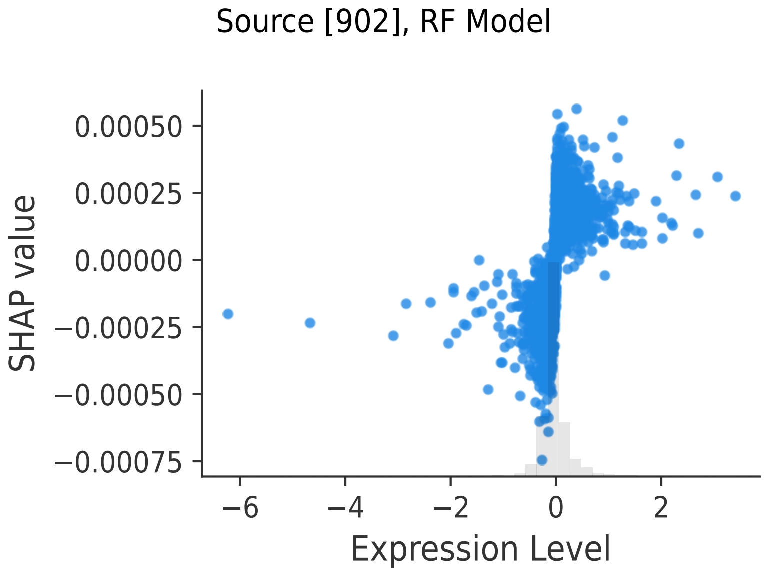
Source [902], RF Model
SHAP value
0.00050
0.00025
0.00000
−0.00025
−0.00050
−0.00075
−6
−4
−2
0
2
Expression Level

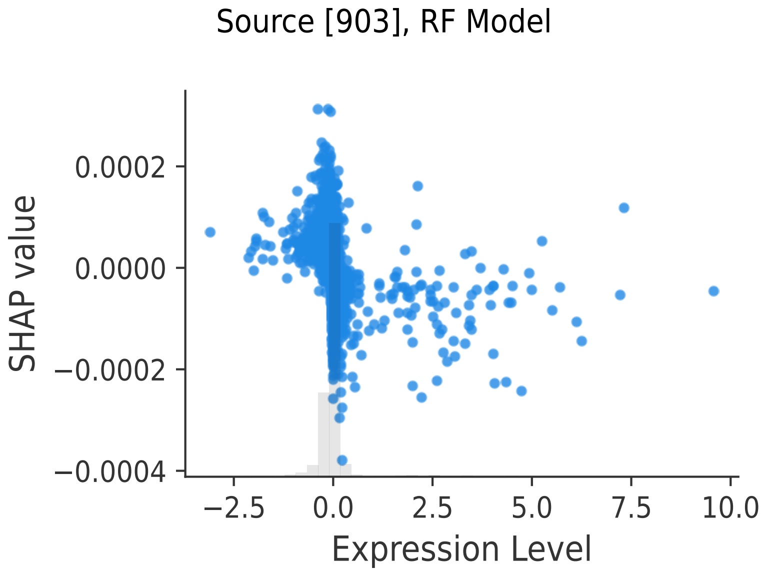

Source [903], RF Model
SHAP value
0.0002
0.0000
−0.0002
−0.0004
−2.5
0.0
2.5
5.0
7.5
10.0
Expression Level

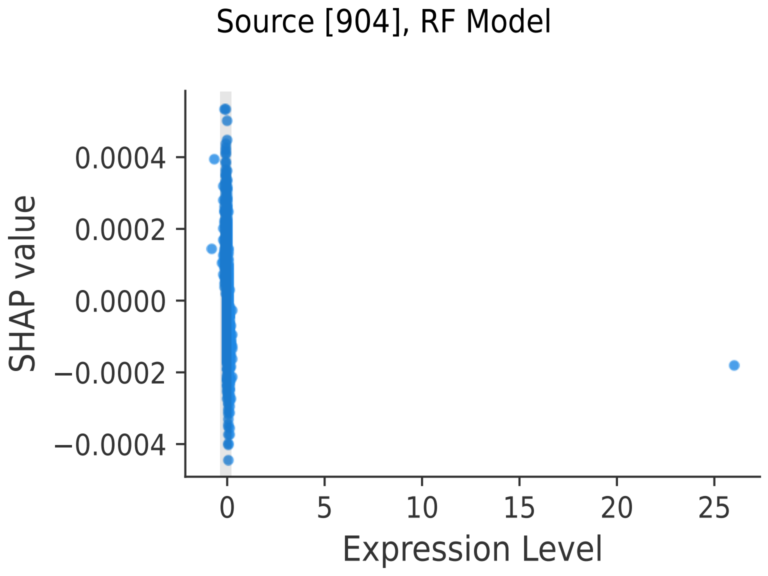
Source [904], RF Model
SHAP value
0.0004
0.0002
0.0000
−0.0002
−0.0004
0
5
10
15
20
25
Expression Level

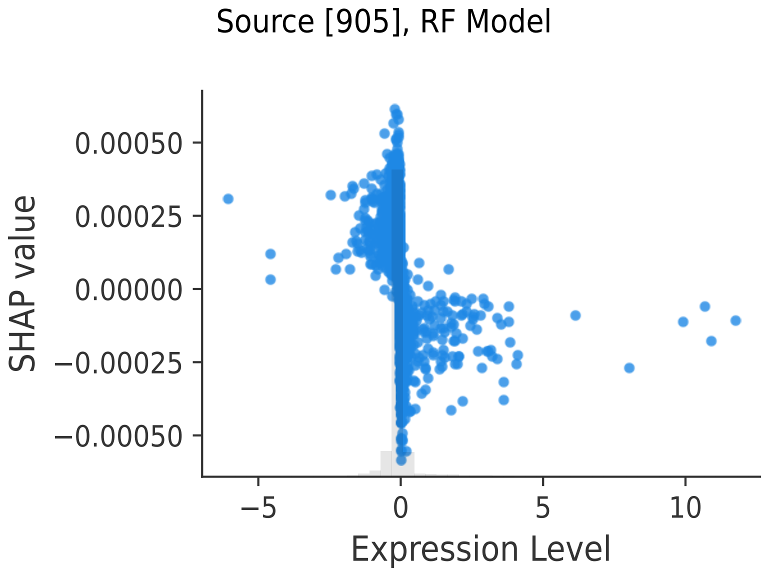
Source [905], RF Model
0.00050
0.00025
0.00000
−0.00025
−0.00050
SHAP value
−5
0
5
10
Expression Level

Source [906], RF Model

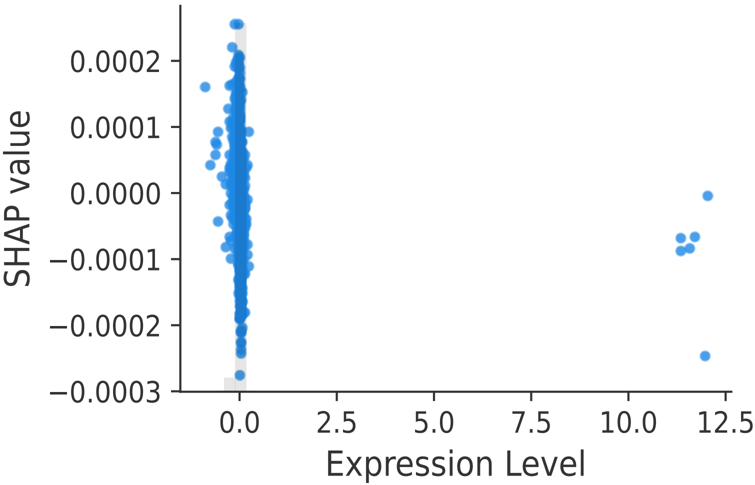

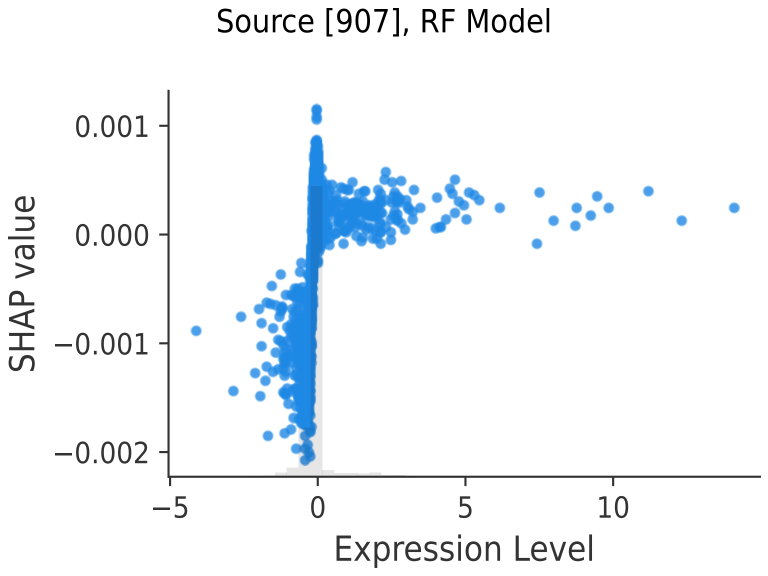
Source [907], RF Model
SHAP value
0.001
0.000
−0.001
−0.002
−5
0
5
10
Expression Level

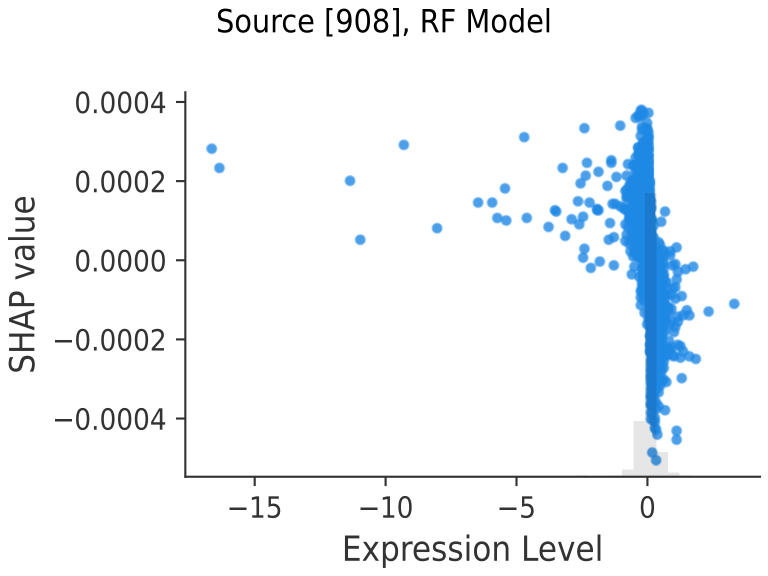
Source [908], RF Model
0.0004
0.0002
0.0000
−0.0002
−0.0004
SHAP value
−15
−10
−5
0
Expression Level

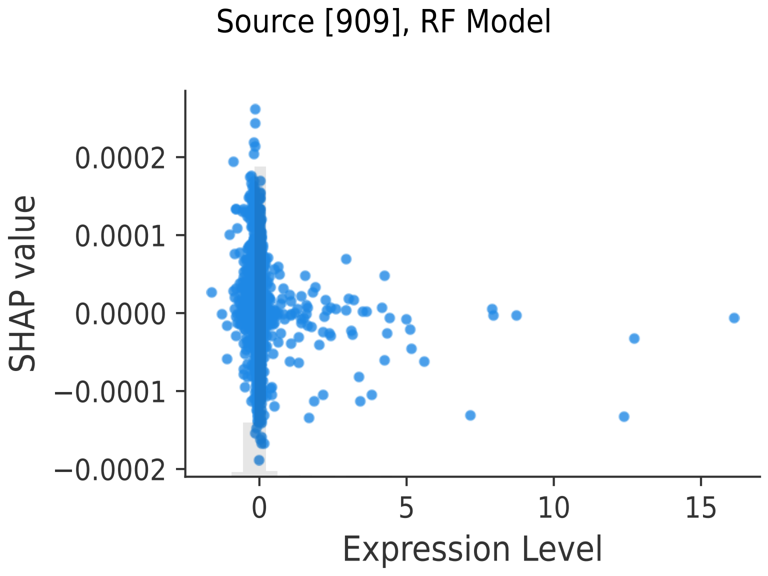

Source [909], RF Model
SHAP value
0.0002
0.0001
0.0000
−0.0001
−0.0002
0
5
10
15
Expression Level

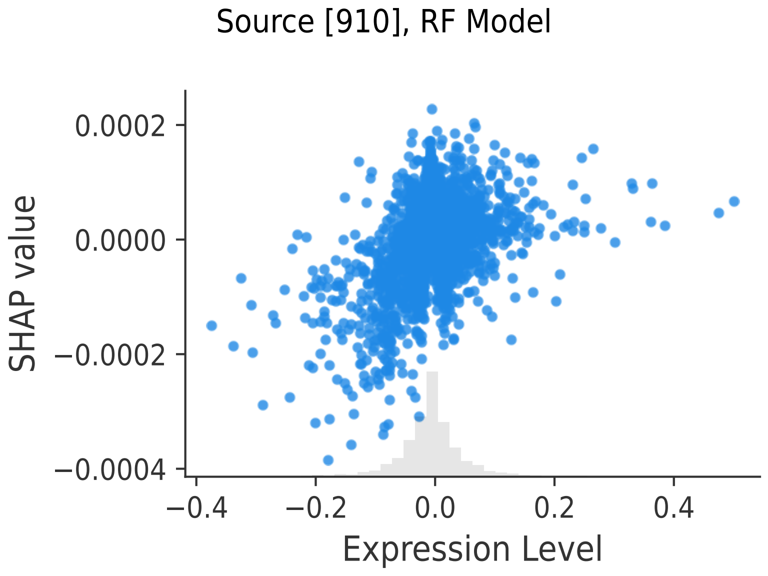
Source [910], RF Model
0.0002
0.0000
−0.0002
−0.0004
SHAP value
−0.4
−0.2
0.0
0.2
0.4
Expression Level

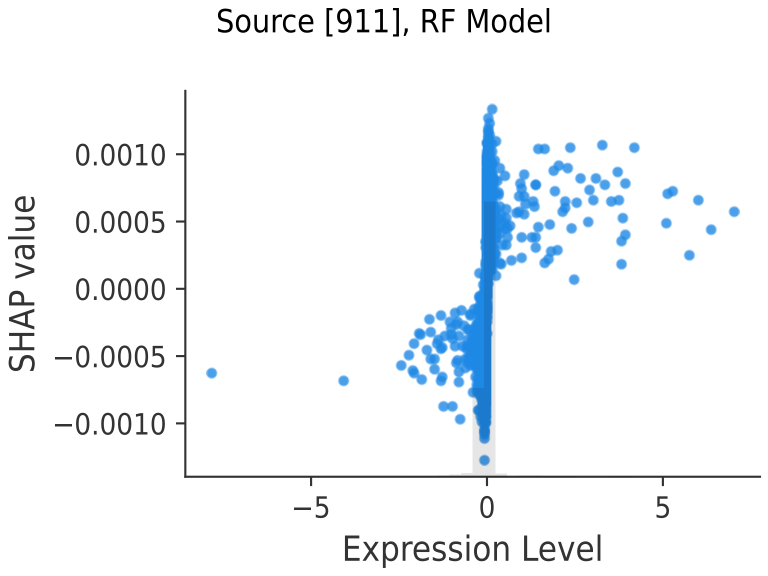

Source [911], RF Model
0.0010
0.0005
0.0000
−0.0005
−0.0010
SHAP value
−5
0
5
Expression Level

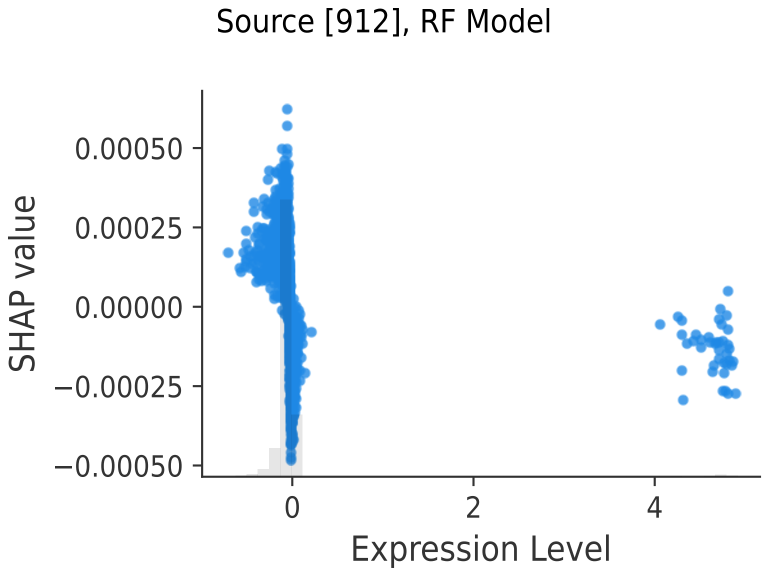
Source [912], RF Model
0.00050
0.00025
0.00000
−0.00025
−0.00050
SHAP value
0
2
4
Expression Level

Source [913], RF Model

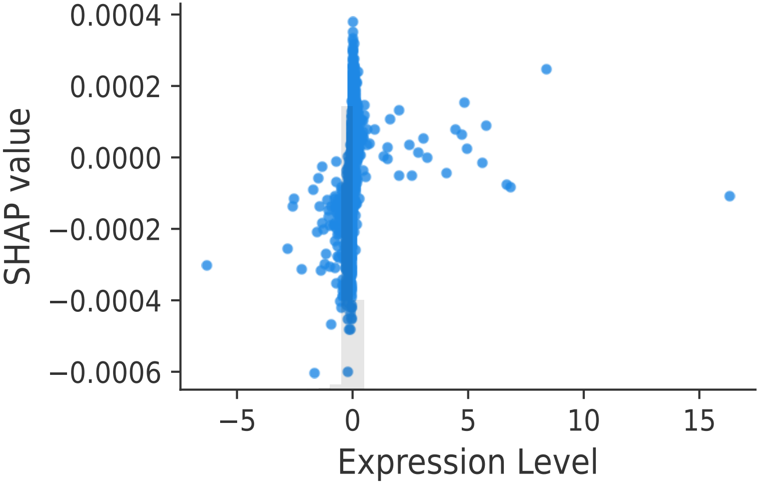

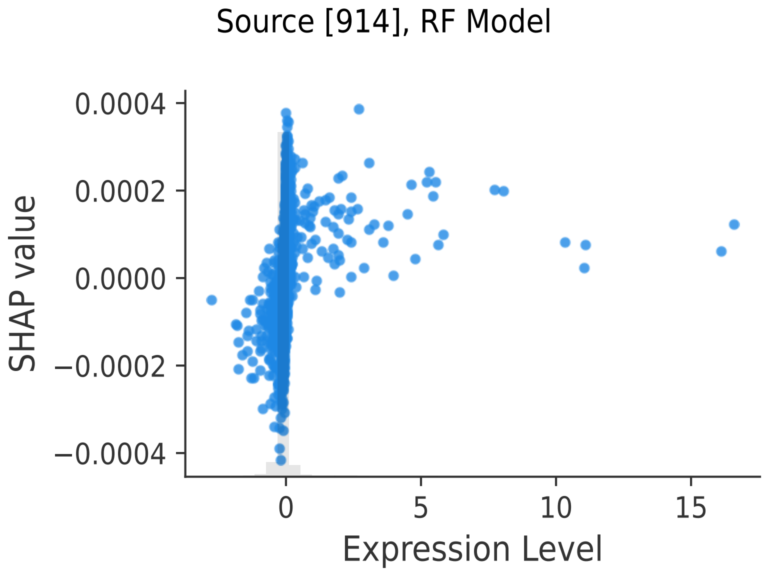

Source [914], RF Model
SHAP value
0.0004
0.0002
0.0000
−0.0002
−0.0004
0
5
10
15
Expression Level

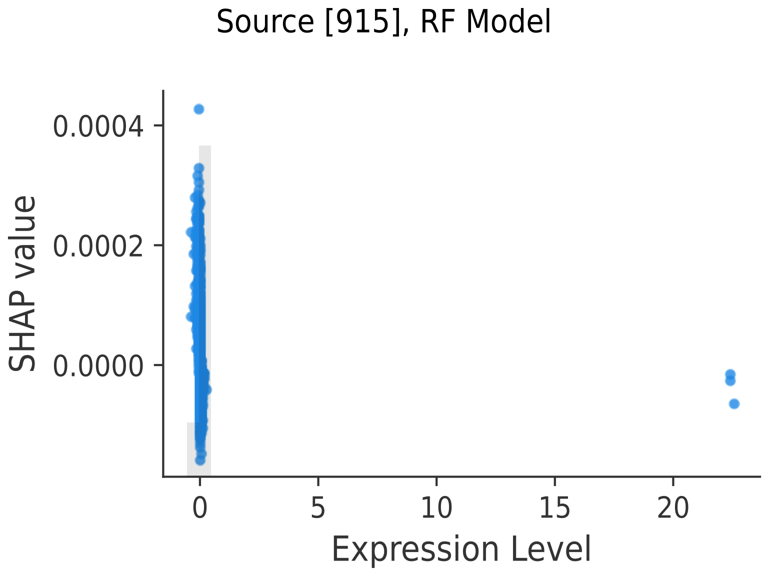
Source [915], RF Model
SHAP value
0.0004
0.0002
0.0000
0
5
10
15
20
Expression Level

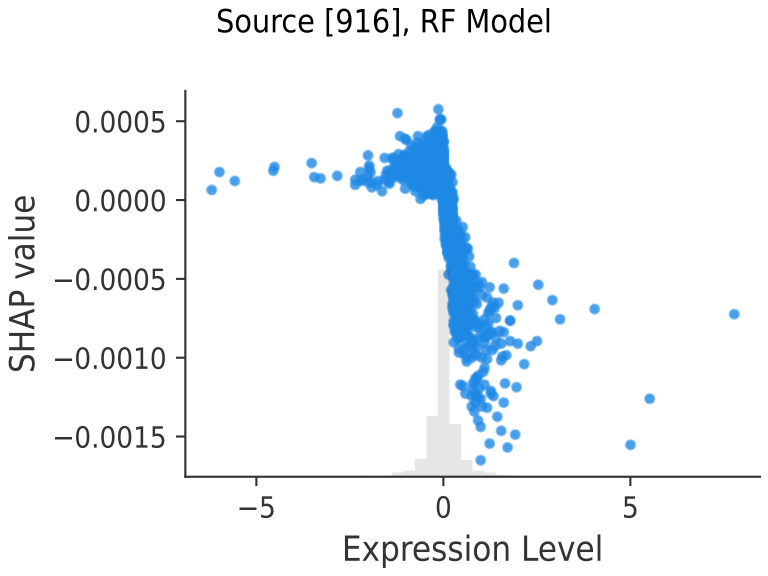
Source [916], RF Model
SHAP value
0.0005
0.0000
−0.0005
−0.0010
−0.0015
Expression Level
−5
0
5

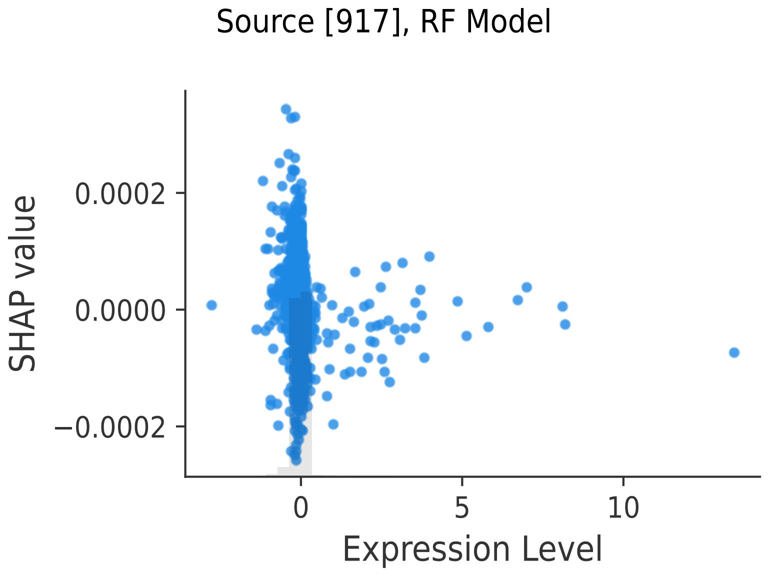
Source [917], RF Model
SHAP value
0.0002
0.0000
−0.0002
0
5
10
Expression Level

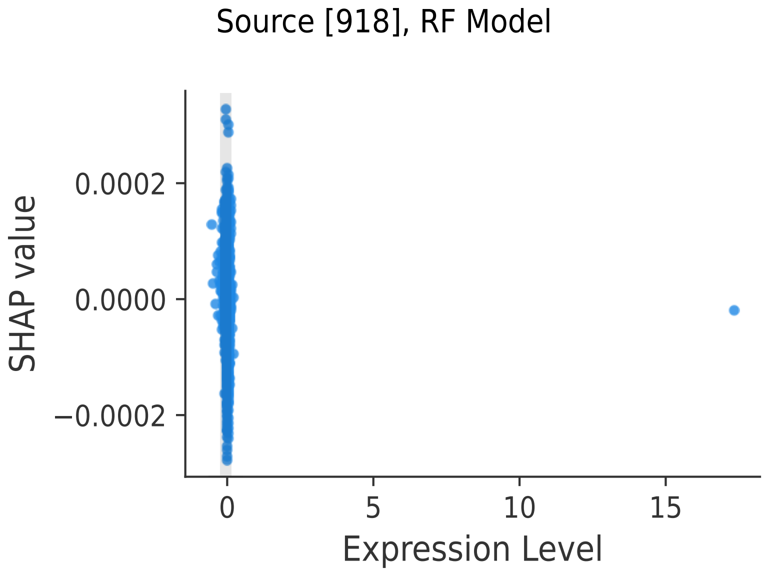

Source [918], RF Model
SHAP value
0.0002
0.0000
−0.0002
0
5
10
15
Expression Level

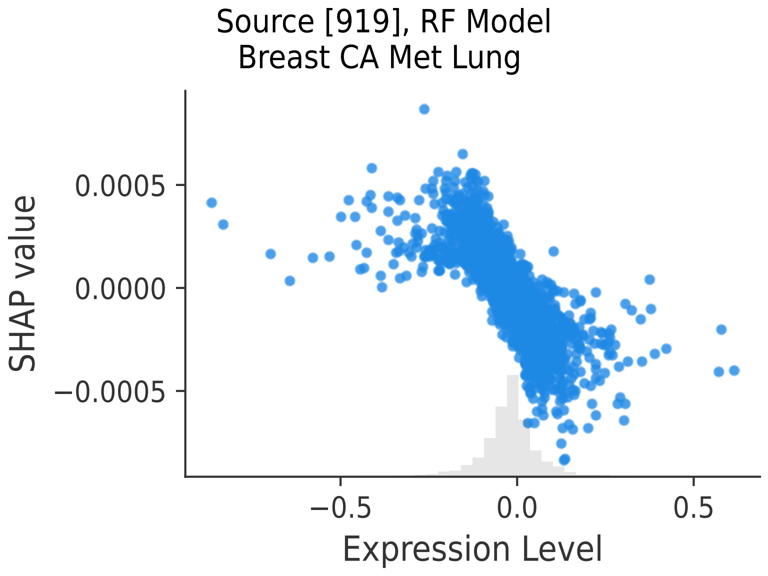

Source [919], RF Model
Breast CA Met Lung
0.0005
0.0000
−0.0005
SHAP value
−0.5
0.0
0.5
Expression Level

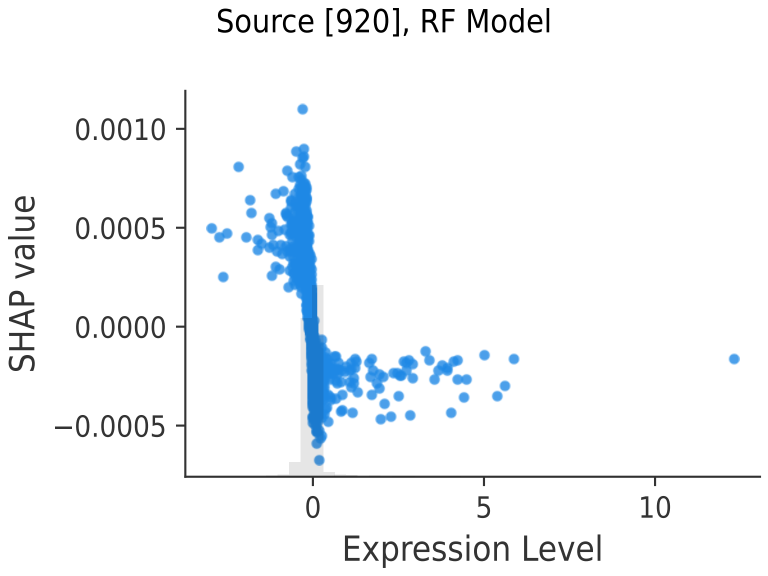

Source [920], RF Model
SHAP value
0.0010
0.0005
0.0000
−0.0005
0
5
10
Expression Level

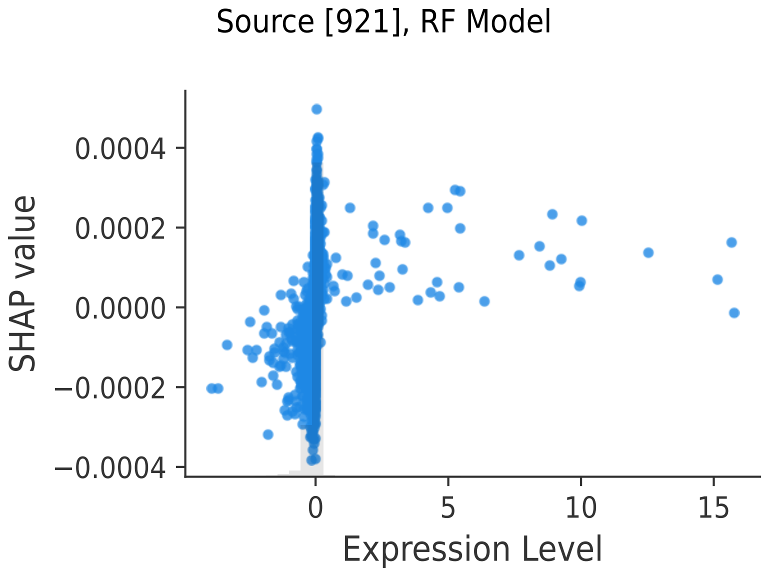
Source [921], RF Model
SHAP value
0.0004
0.0002
0.0000
−0.0002
−0.0004
0
5
10
15
Expression Level

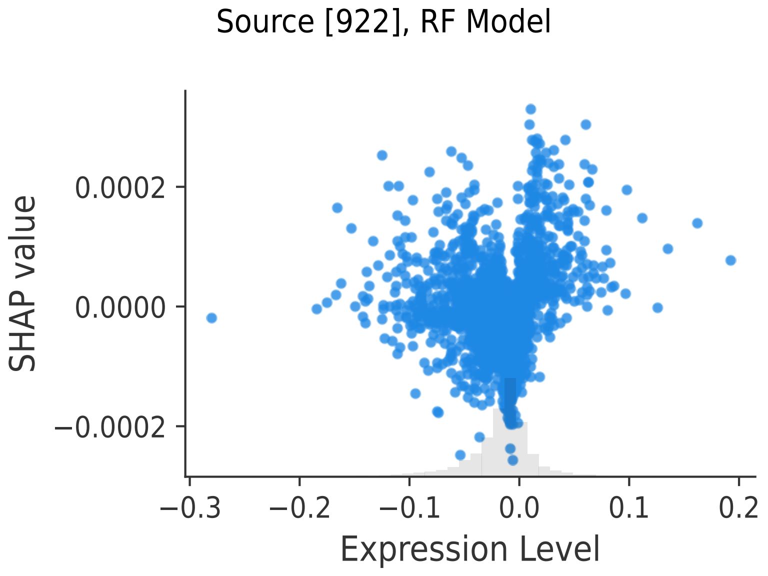
Source [922], RF Model
SHAP value
0.0002
0.0000
−0.0002
−0.3
−0.2
−0.1
0.0
0.1
0.2
Expression Level

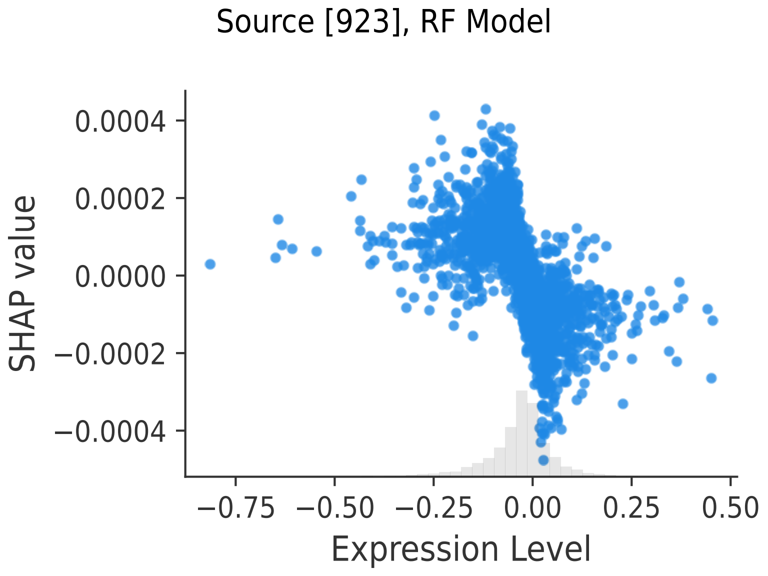
Source [923], RF Model
0.0004
0.0002
0.0000
−0.0002
−0.0004
SHAP value
−0.75
−0.50
−0.25
0.00
0.25
0.50
Expression Level

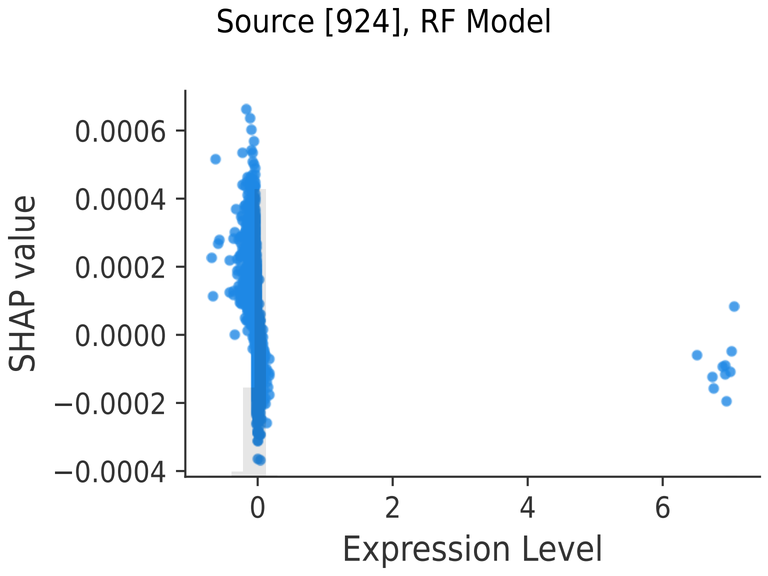

Source [924], RF Model
SHAP value
0.0006
0.0004
0.0002
0.0000
−0.0002
−0.0004
0
2
4
6
Expression Level

Source [925], RF Model

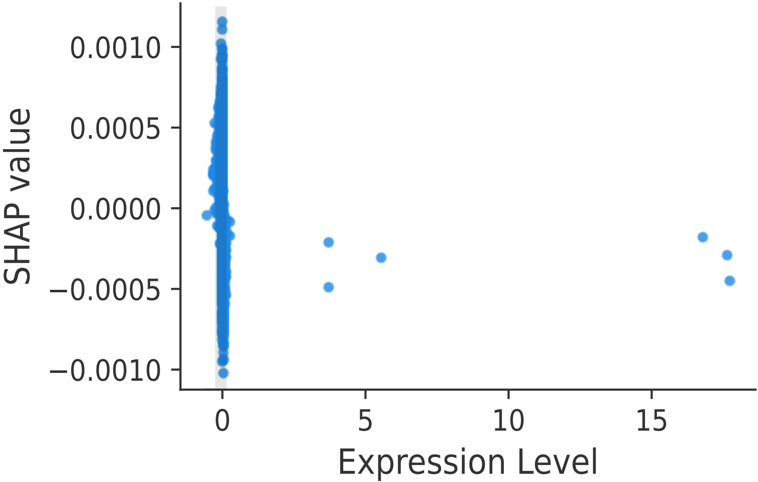

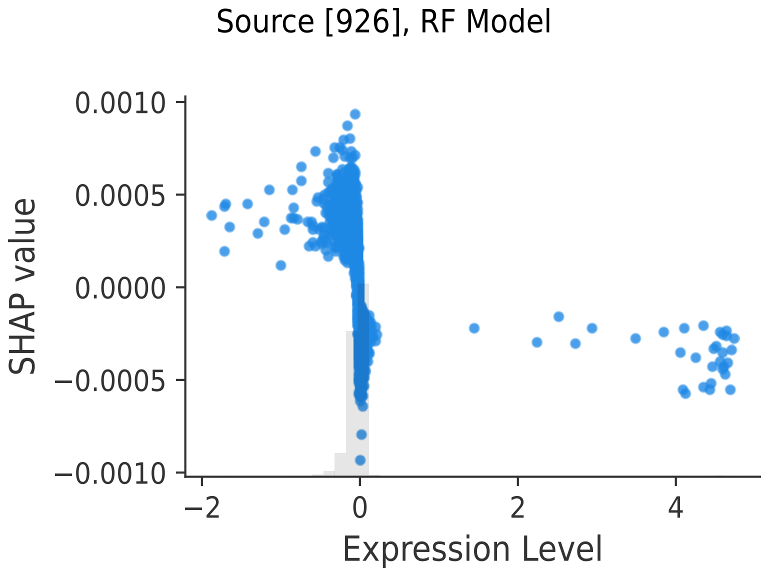

Source [926], RF Model
0.0010
0.0005
0.0000
−0.0005
−0.0010
SHAP value
−2
0
2
4
Expression Level

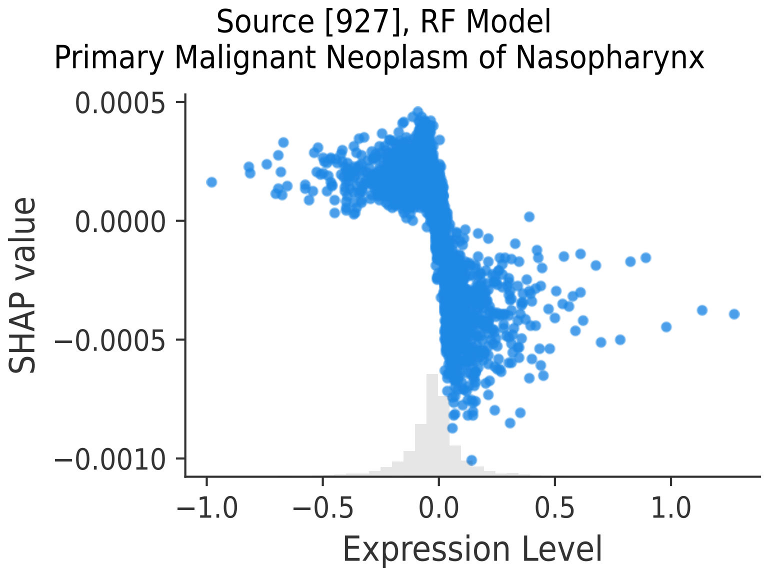

Source [927], RF Model
Primary Malignant Neoplasm of Nasopharynx
SHAP value
0.0005
0.0000
−0.0005
−0.0010
−1.0
−0.5
0.0
0.5
1.0
Expression Level

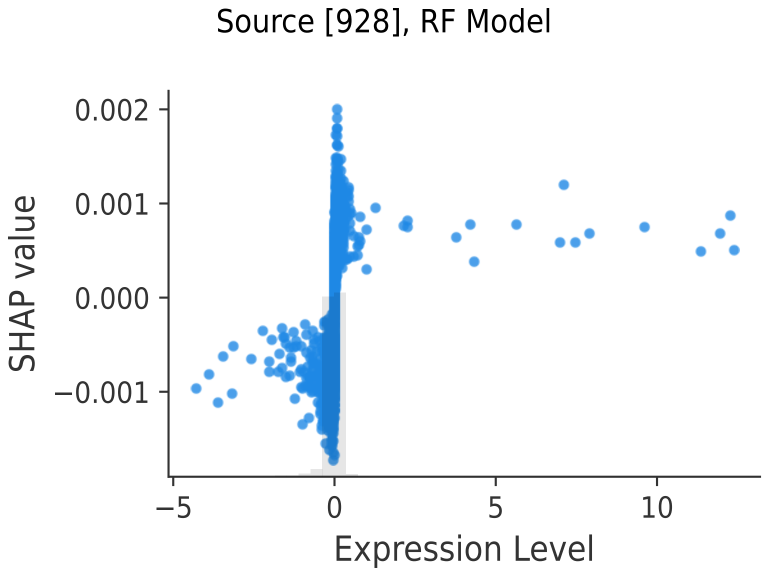
Source [928], RF Model
SHAP value
0.002
0.001
0.000
−0.001
−5
0
5
10
Expression Level

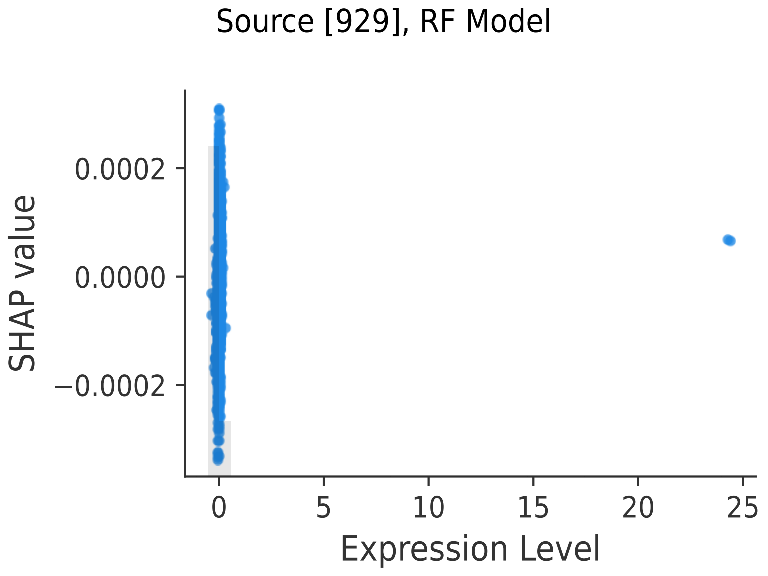
Source [929], RF Model
SHAP value
0.0002
0.0000
−0.0002
0
5
10
15
20
25
Expression Level

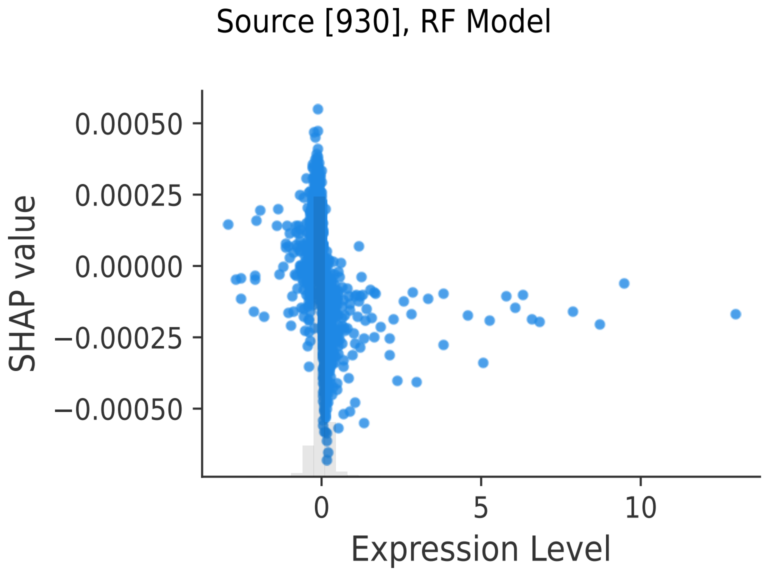
Source [930], RF Model
0.00050
0.00025
0.00000
−0.00025
−0.00050
SHAP value
0
5
10
Expression Level

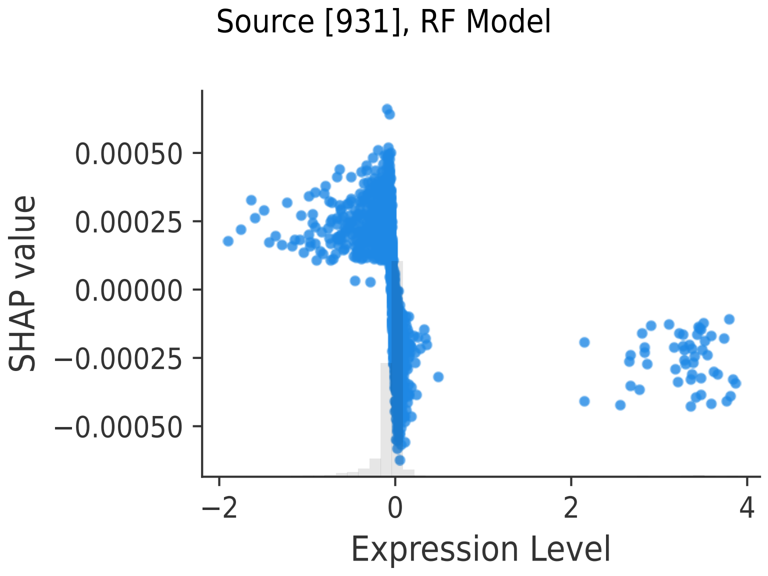
Source [931], RF Model
0.00050
0.00025
0.00000
−0.00025
−0.00050
SHAP value
−2
0
2
4
Expression Level

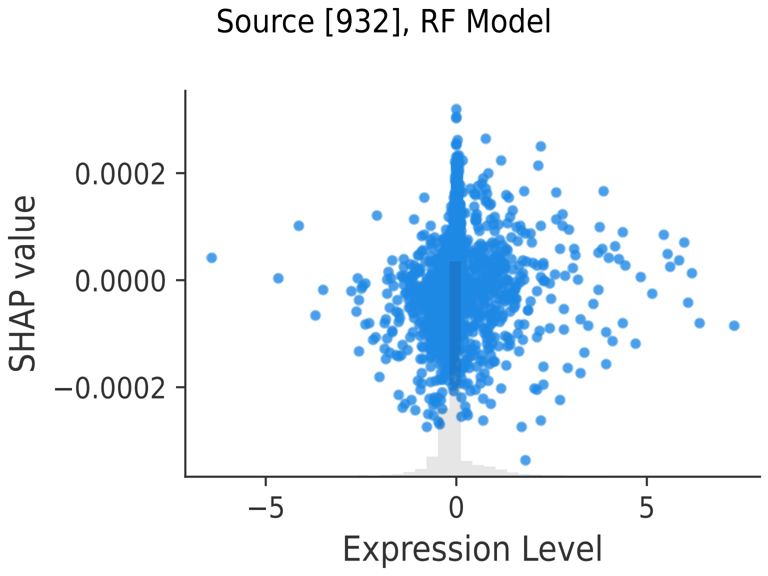
Source [932], RF Model
0.0002
0.0000
−0.0002
SHAP value
−5
0
5
Expression Level

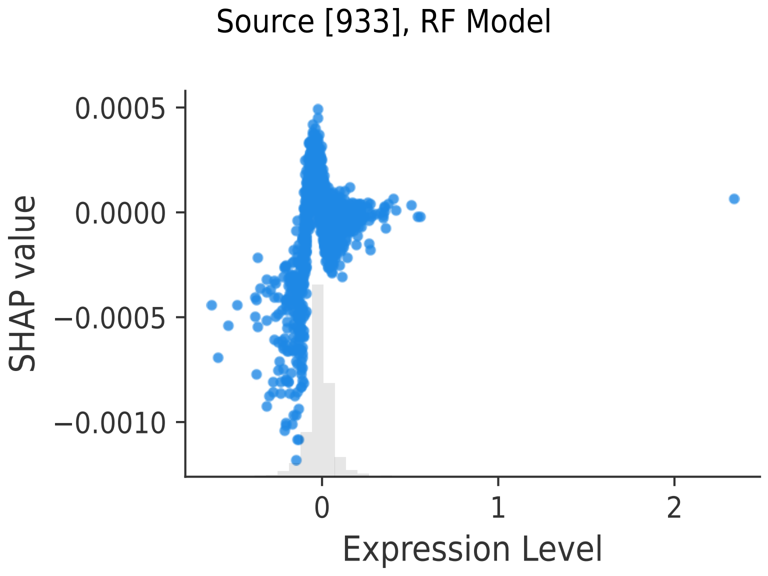

Source [933], RF Model
0.0005
0.0000
−0.0005
−0.0010
SHAP value
0
1
2
Expression Level

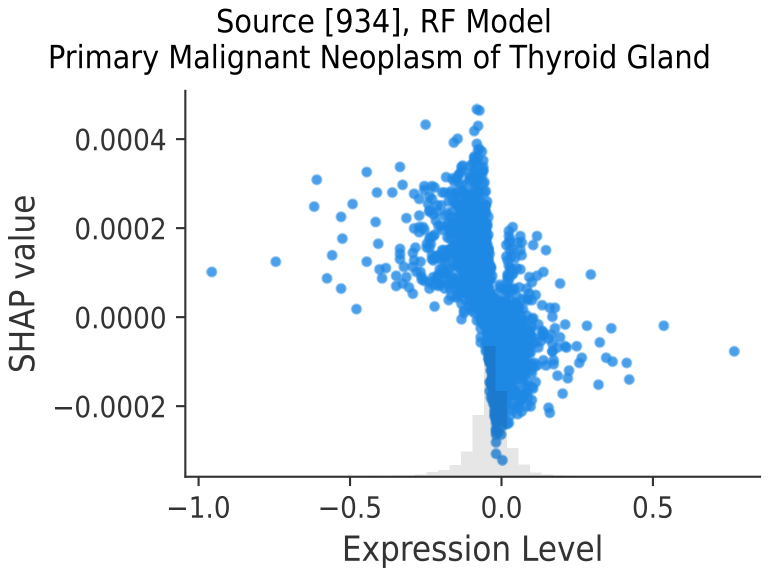
Source [934], RF Model
Primary Malignant Neoplasm of Thyroid Gland
SHAP value
0.0004
0.0002
0.0000
−0.0002
−1.0
−0.5
0.0
0.5
Expression Level

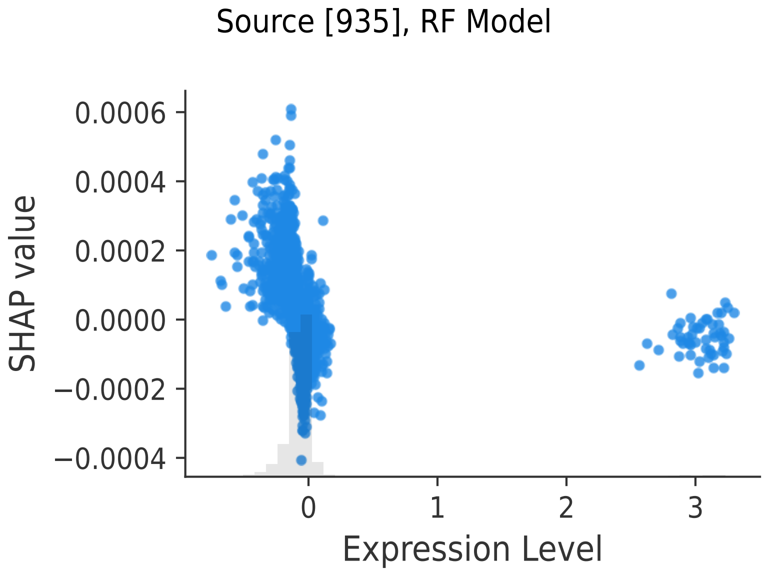

Source [935], RF Model
0.0006
0.0004
0.0002
0.0000
−0.0002
−0.0004
SHAP value
0
1
2
3
Expression Level

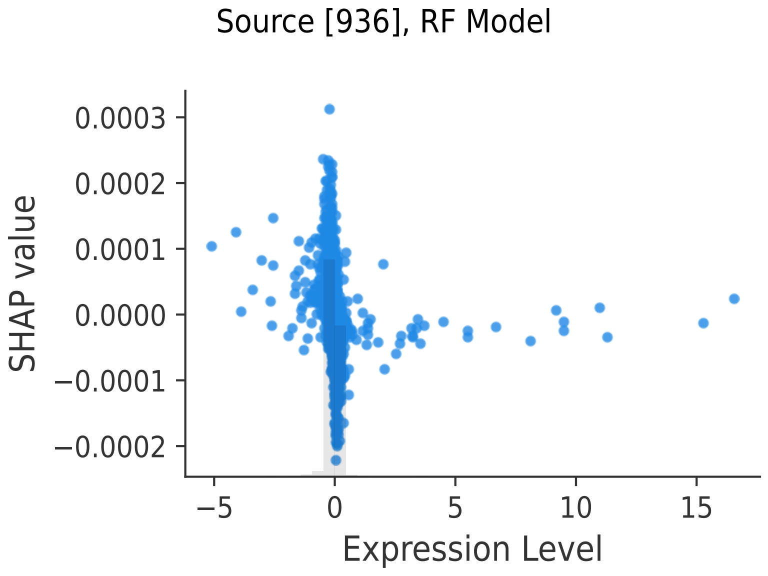

Source [936], RF Model
SHAP value
0.0003
0.0002
0.0001
0.0000
−0.0001
−0.0002
−5
0
5
10
15
Expression Level

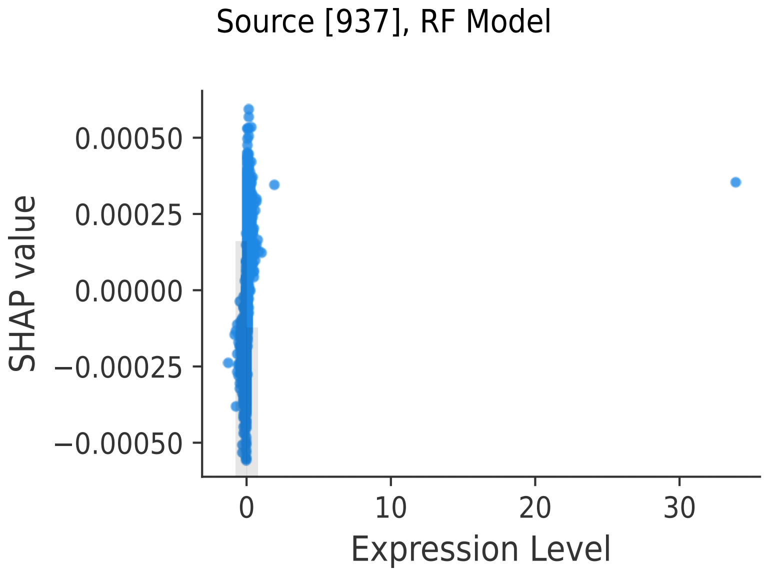

Source [937], RF Model
SHAP value
0.00050
0.00025
0.00000
−0.00025
−0.00050
0
10
20
30
Expression Level

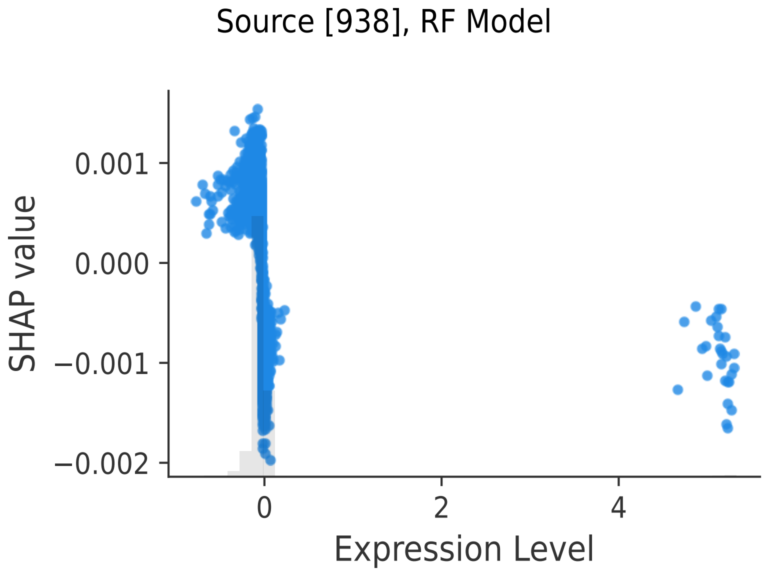

Source [938], RF Model
0.001
0.000
−0.001
−0.002
SHAP value
0
2
4
Expression Level

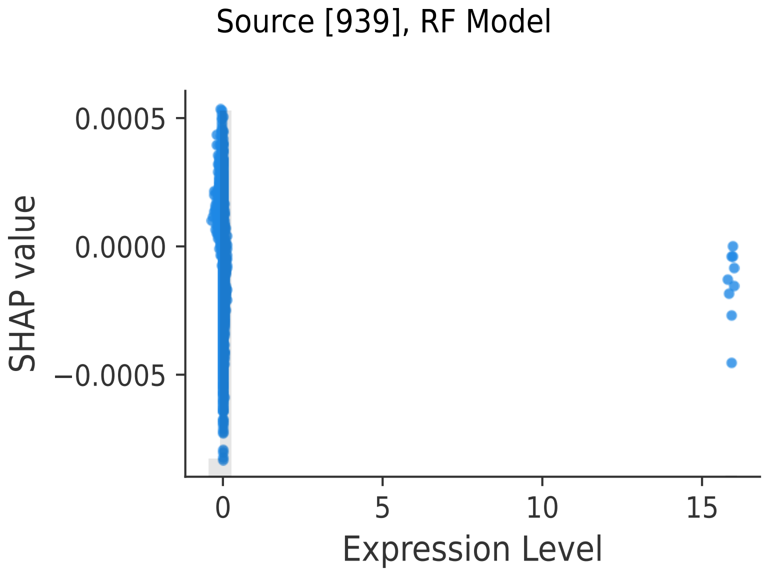
Source [939], RF Model
0.0005
0.0000
−0.0005
SHAP value
0
5
10
15
Expression Level

Source [940], RF Model

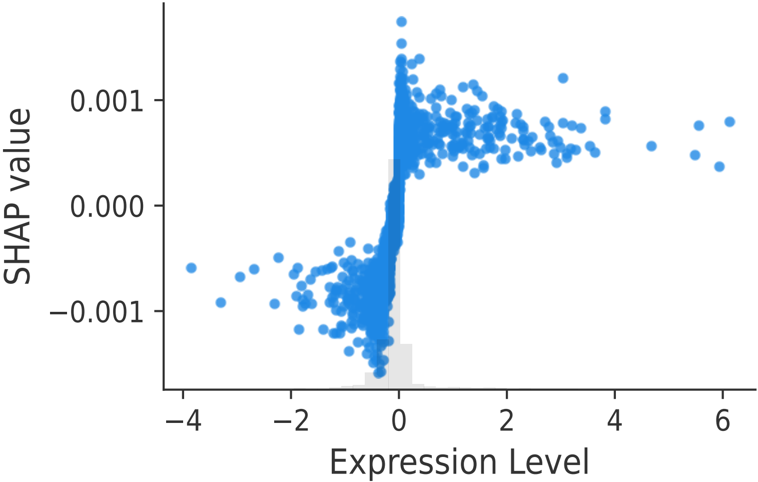

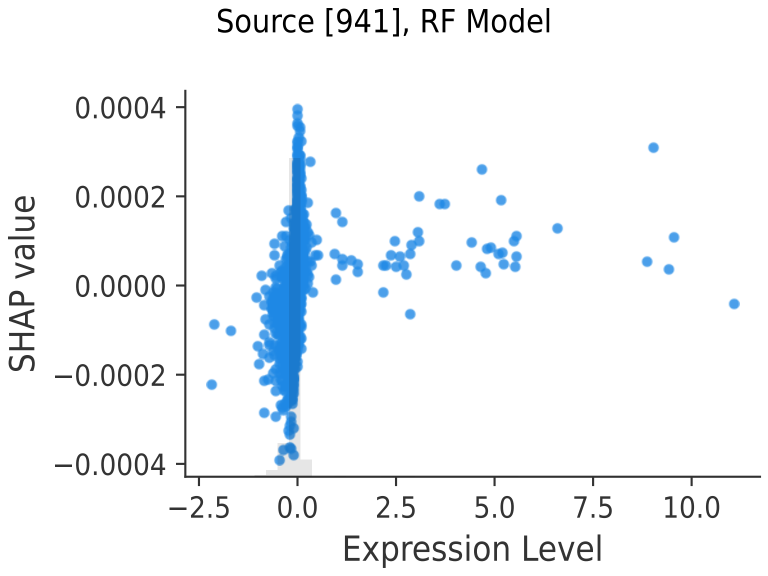
Source [941], RF Model
0.0004
0.0002
0.0000
−0.0002
−0.0004
SHAP value
−2.5
0.0
2.5
5.0
7.5
10.0
Expression Level

Source [942], RF Model

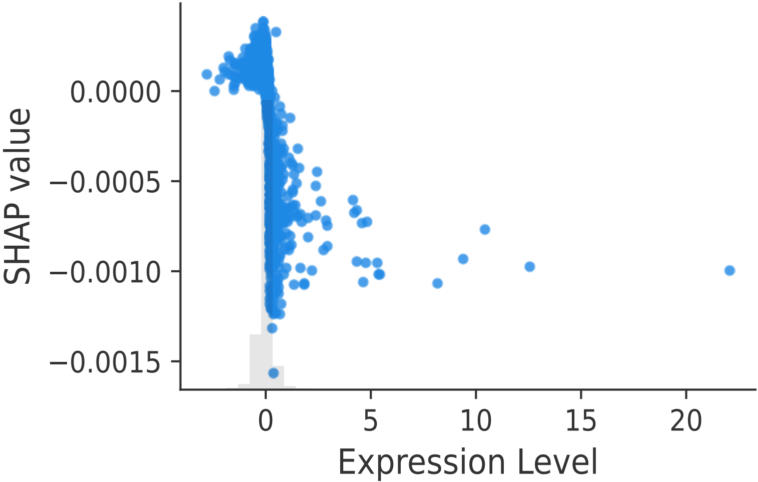

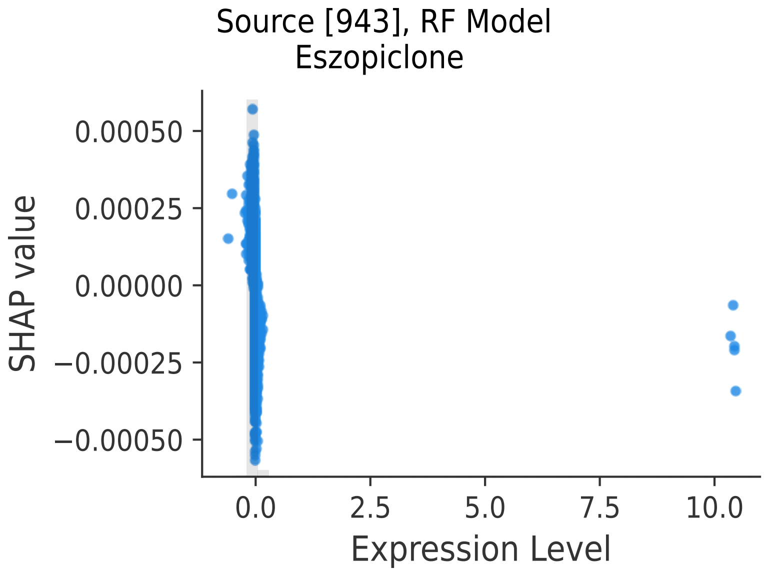

Source [943], RF Model
Eszopiclone
SHAP value
0.00050
0.00025
0.00000
−0.00025
−0.00050
0.0
2.5
5.0
7.5
10.0
Expression Level

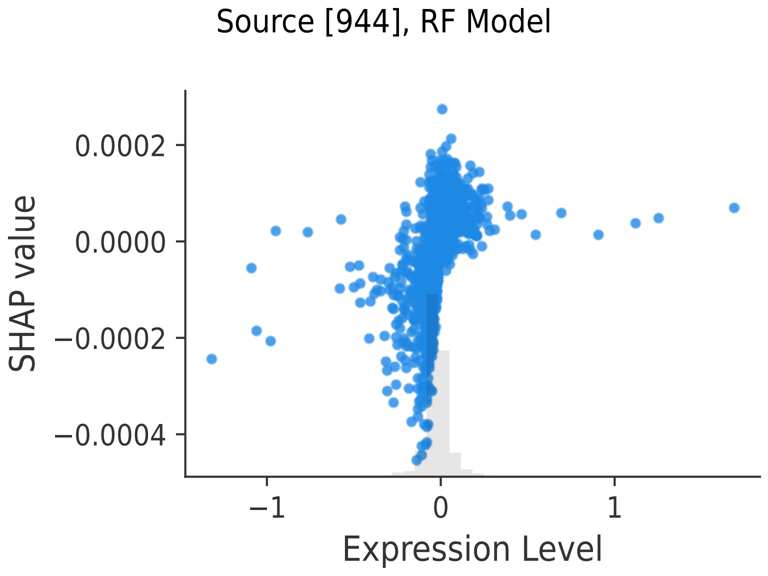

Source [944], RF Model
0.0002
0.0000
−0.0002
−0.0004
SHAP value
−1
0
1
Expression Level

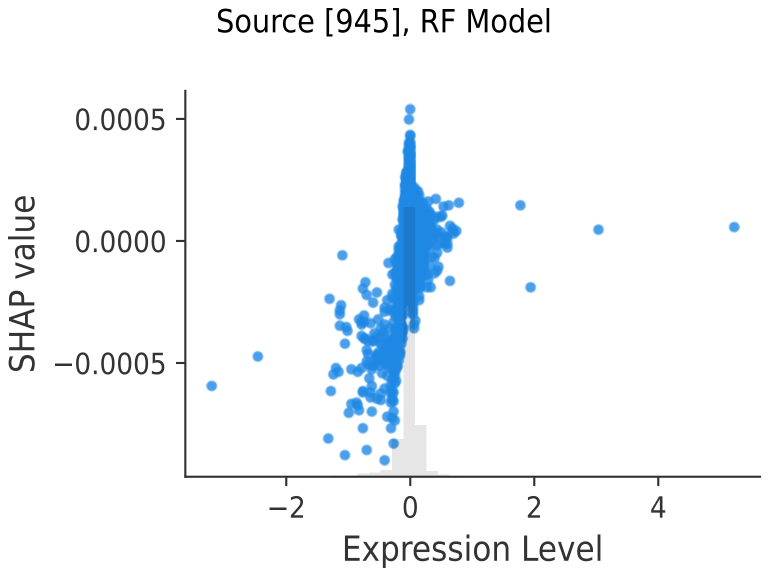
Source [945], RF Model
SHAP value
0.0005
0.0000
−0.0005
Expression Level
−2
0
2
4

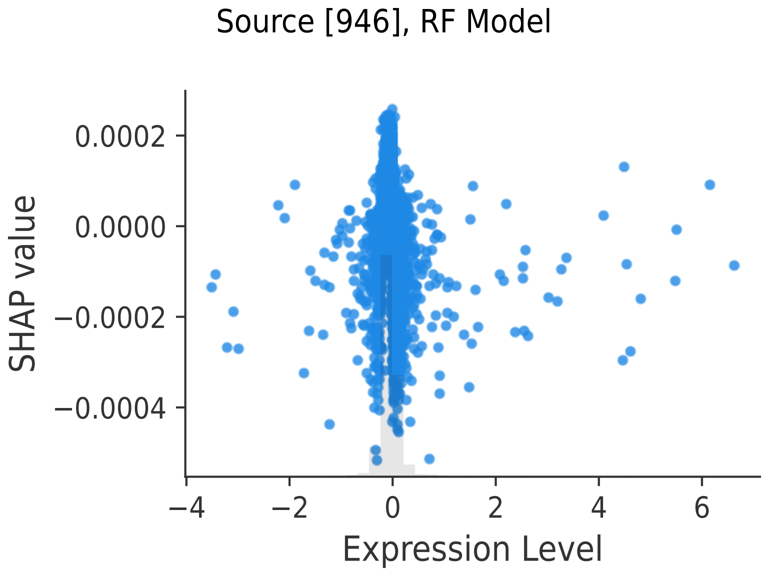

Source [946], RF Model
SHAP value
0.0002
0.0000
−0.0002
−0.0004
−4
−2
0
2
4
6
Expression Level

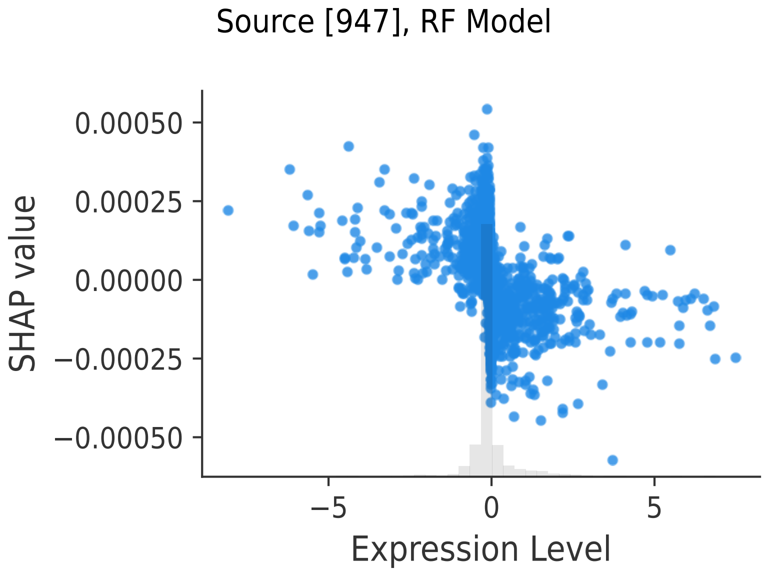

Source [947], RF Model
0.00050
0.00025
0.00000
−0.00025
−0.00050
SHAP value
−5
0
5
Expression Level

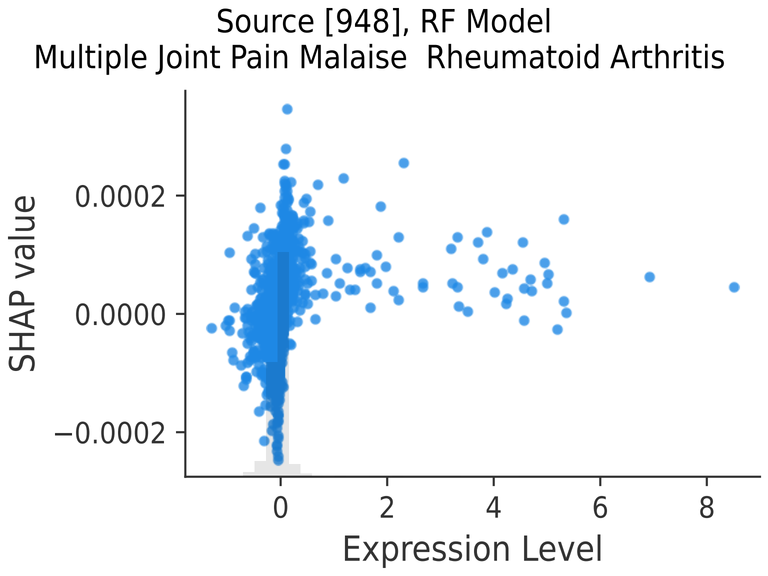

Source [948], RF Model
Multiple Joint Pain Malaise Rheumatoid Arthritis
SHAP value
0.0002
0.0000
−0.0002
0
2
4
6
8
Expression Level

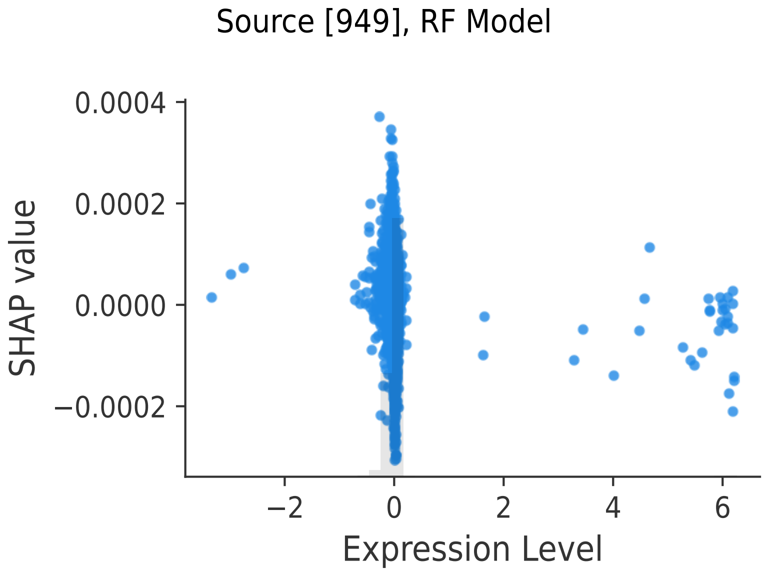
Source [949], RF Model
0.0004
0.0002
0.0000
−0.0002
SHAP value
−2
0
2
4
6
Expression Level

Source [950], RF Model

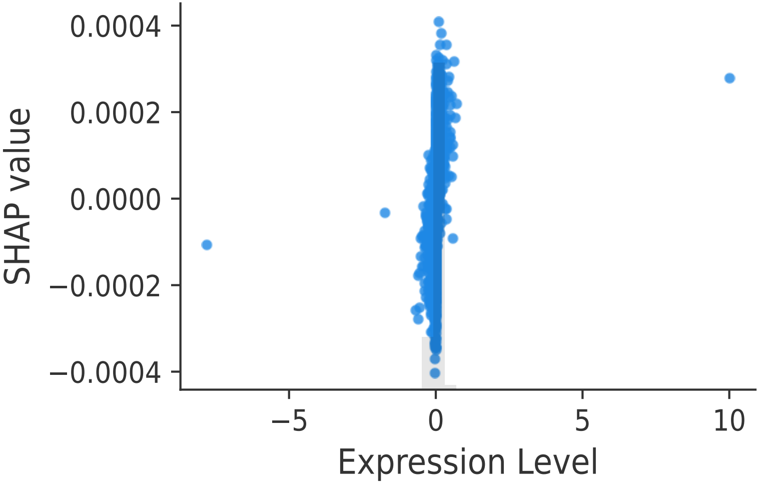

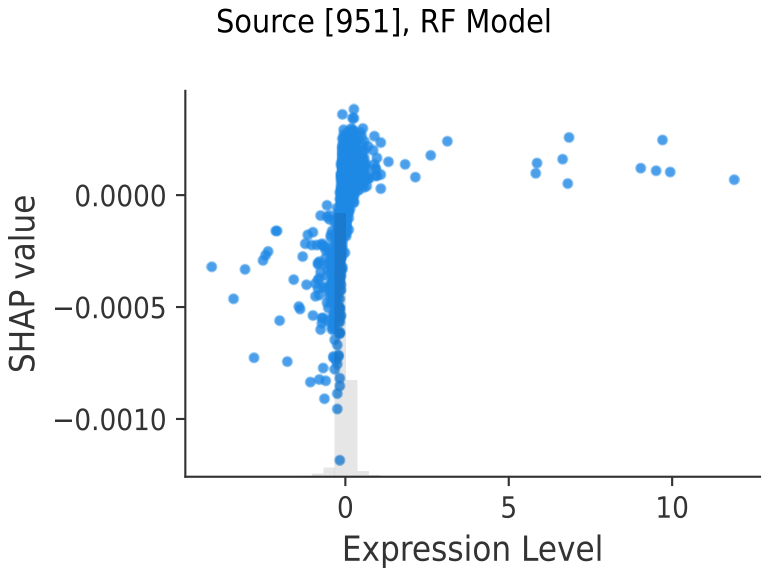

Source [951], RF Model
SHAP value
0.0000
−0.0005
−0.0010
0
5
10
Expression Level

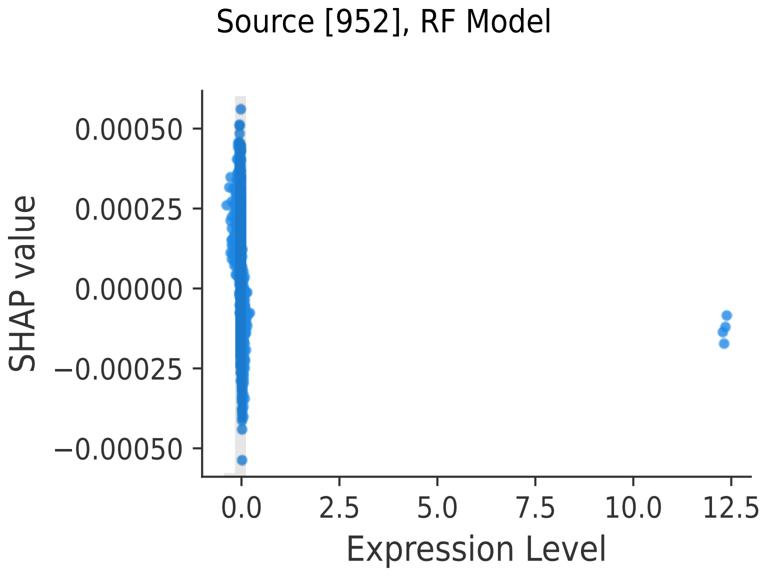
Source [952], RF Model
SHAP value
0.00050
0.00025
0.00000
−0.00025
−0.00050
0.0
2.5
5.0
7.5
10.0
12.5
Expression Level

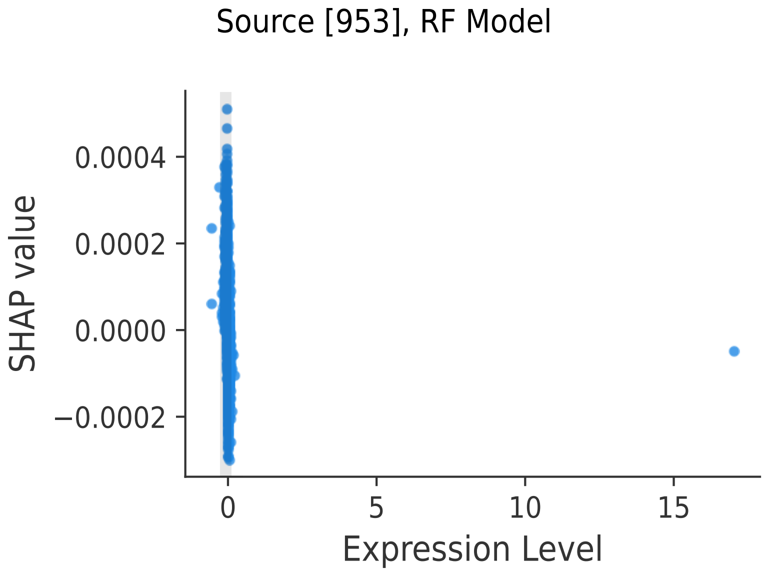

Source [953], RF Model
SHAP value
0.0004
0.0002
0.0000
−0.0002
0
5
10
15
Expression Level

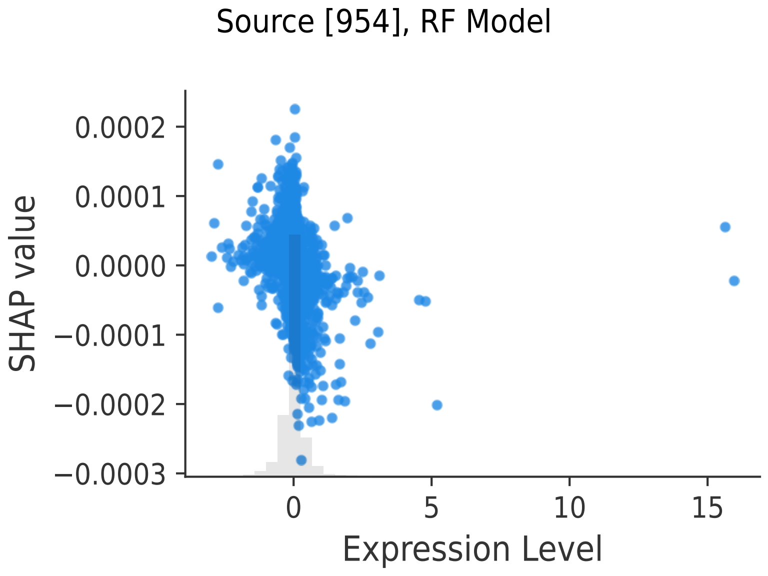

Source [954], RF Model
0.0002
0.0001
0.0000
−0.0001
−0.0002
−0.0003
SHAP value
0
5
10
15
Expression Level

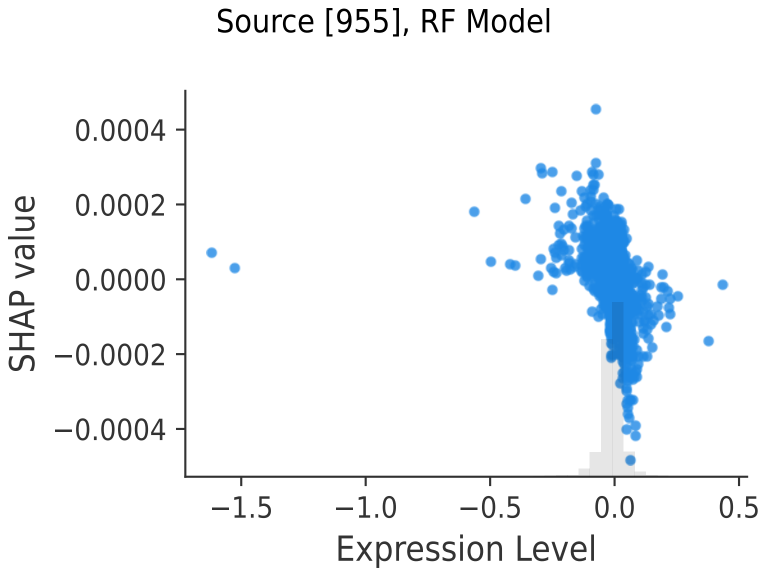

Source [955], RF Model
0.0004
0.0002
0.0000
−0.0002
−0.0004
SHAP value
−1.5
−1.0
−0.5
0.0
0.5
Expression Level

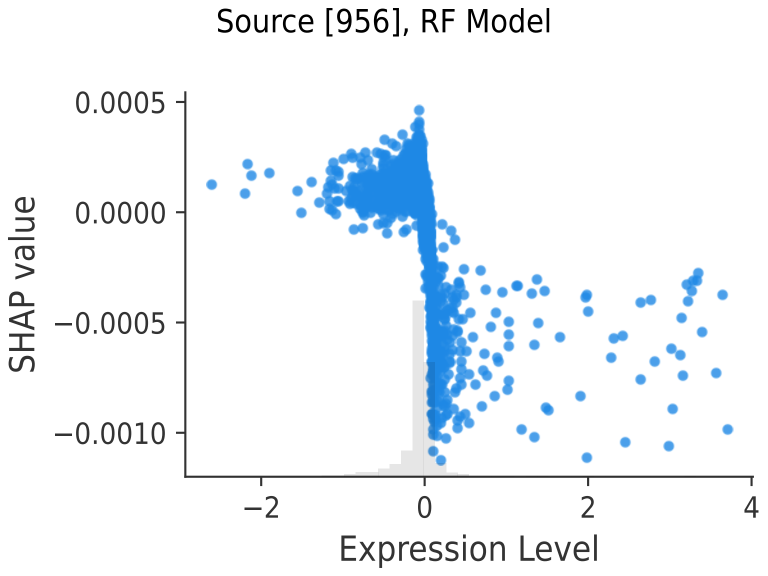
Source [956], RF Model
0.0005
0.0000
−0.0005
−0.0010
SHAP value
−2
0
2
4
Expression Level

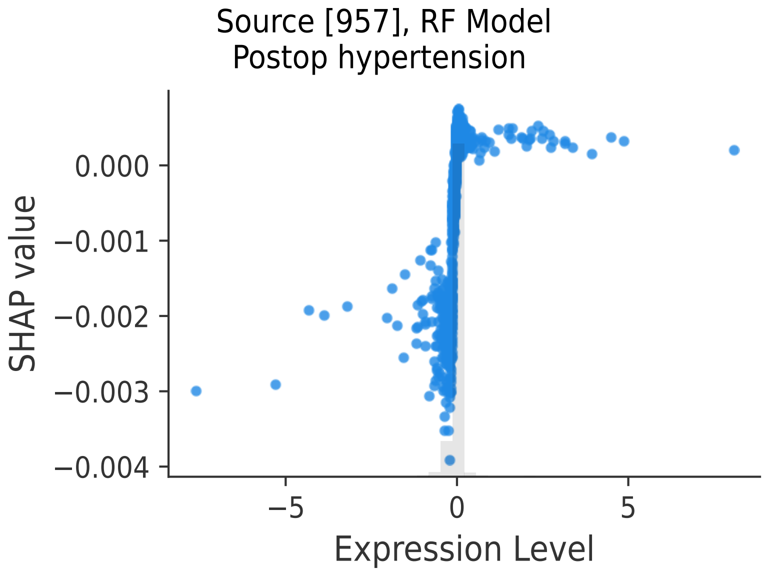
Source [957], RF Model
Postop hypertension
0.000
−0.001
−0.002
−0.003
−0.004
SHAP value
−5
0
5
Expression Level

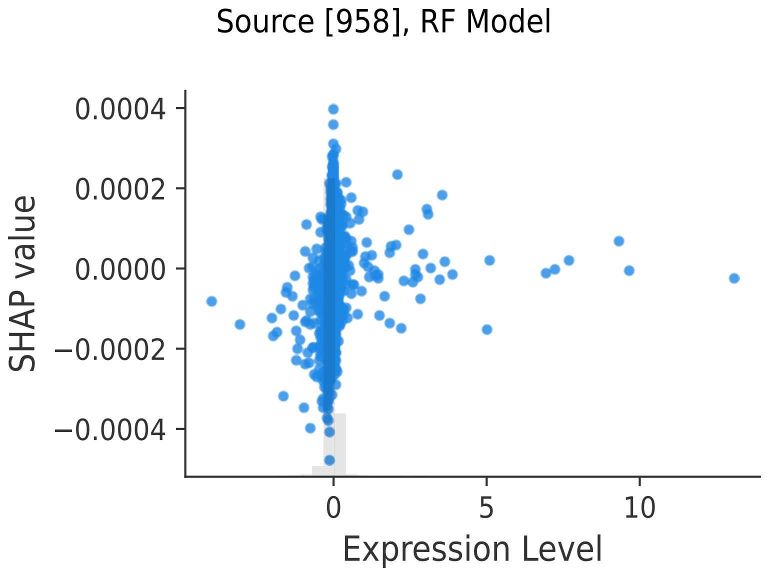

Source [958], RF Model
0.0004
0.0002
0.0000
−0.0002
−0.0004
SHAP value
0
5
10
Expression Level

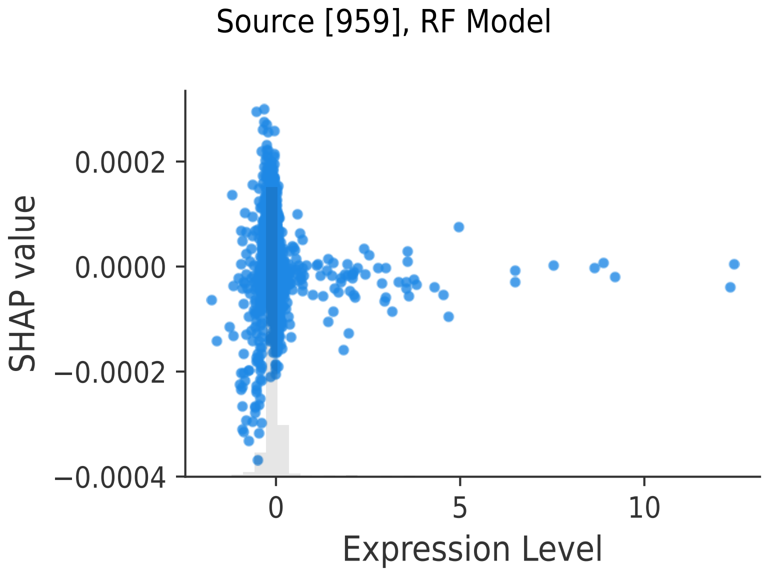
Source [959], RF Model
0.0002
0.0000
−0.0002
−0.0004
SHAP value
0
5
10
Expression Level

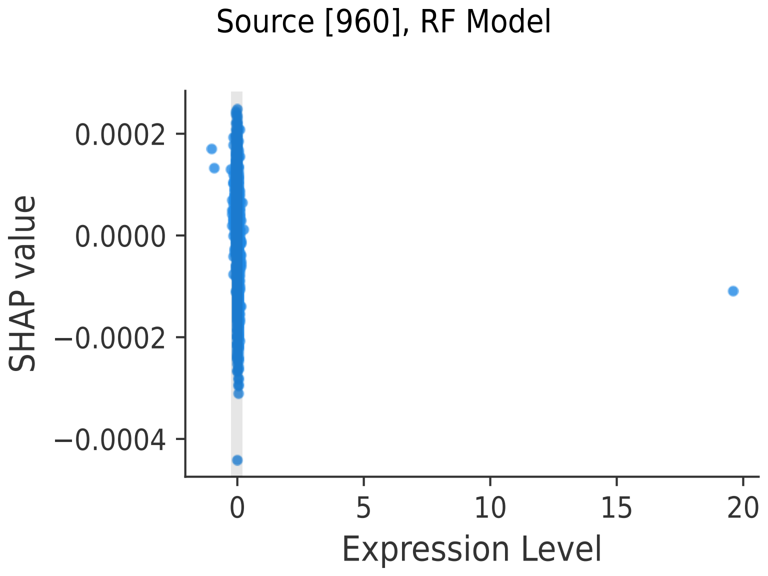

Source [960], RF Model
SHAP value
0.0002
0.0000
−0.0002
−0.0004
0
5
10
15
20
Expression Level

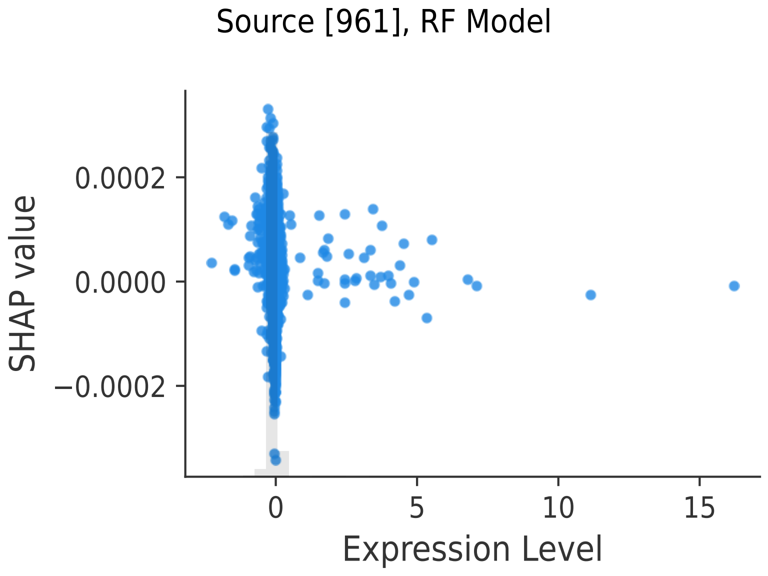
Source [961], RF Model
SHAP value
0.0002
0.0000
−0.0002
0
5
10
15
Expression Level

Source [962], RF Model

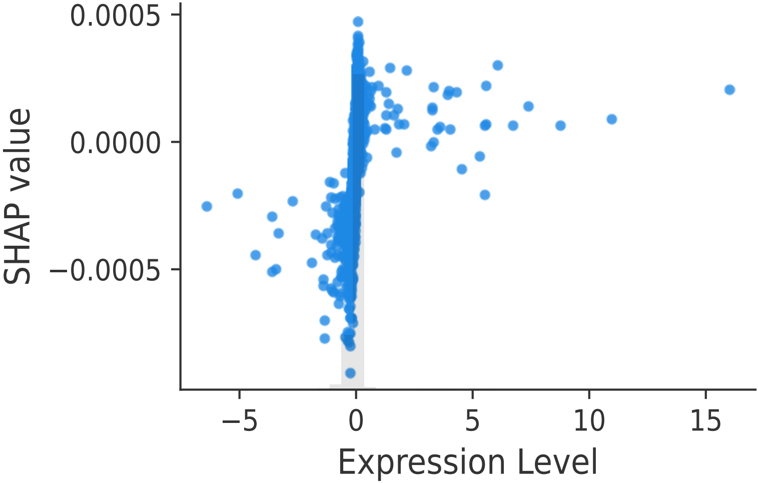

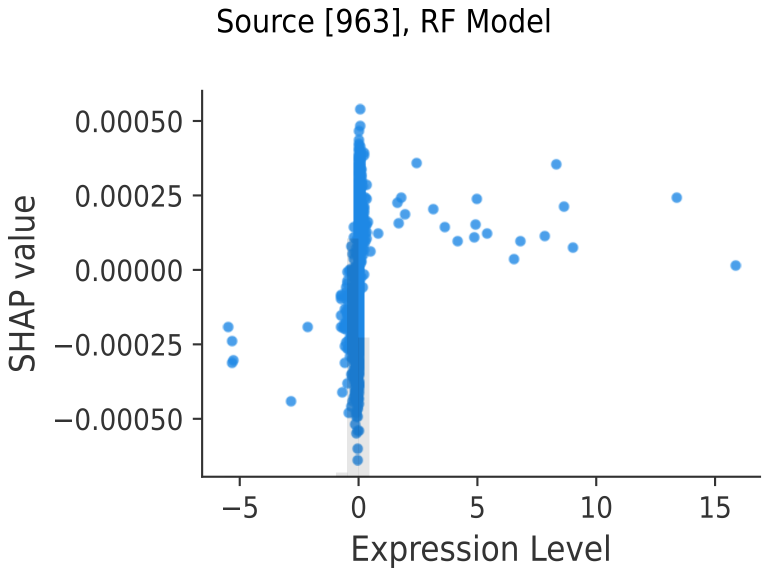
Source [963], RF Model
0.00050
0.00025
0.00000
−0.00025
−0.00050
SHAP value
−5
0
5
10
15
Expression Level

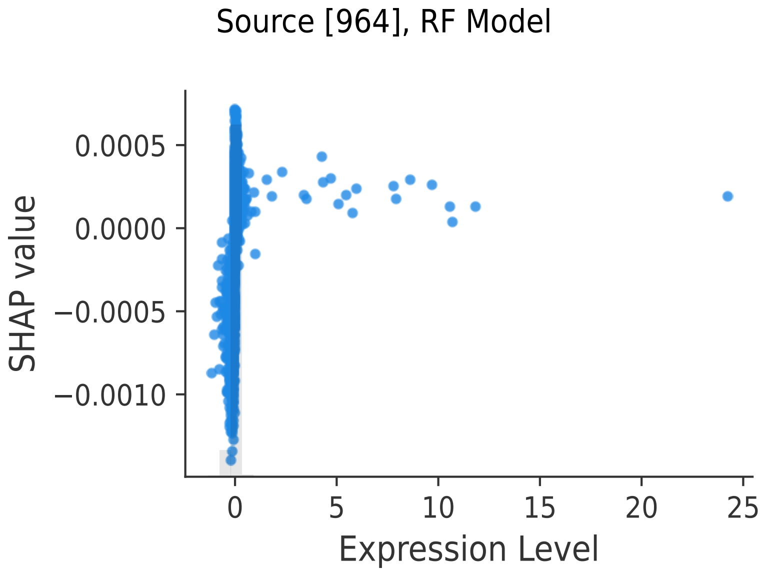

Source [964], RF Model
0.0005
0.0000
−0.0005
−0.0010
SHAP value
0
5
10
15
20
25
Expression Level

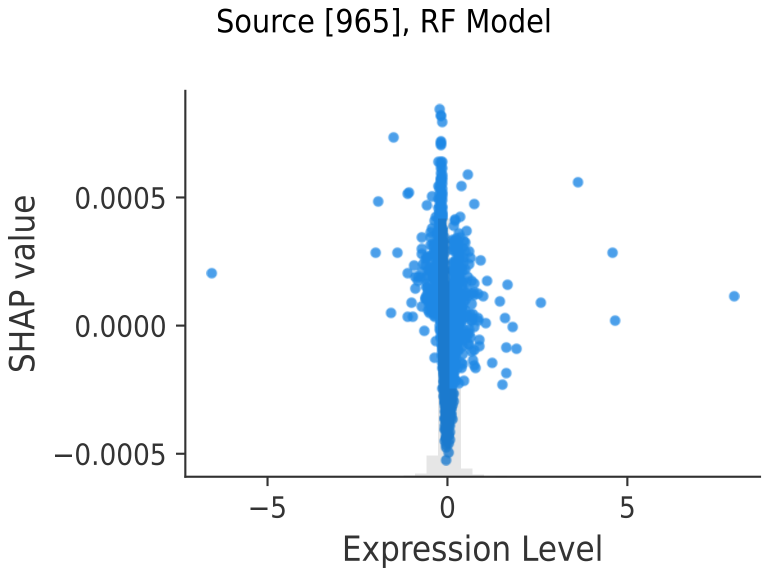

Source [965], RF Model
SHAP value
0.0005
0.0000
−0.0005
−5
0
5
Expression Level

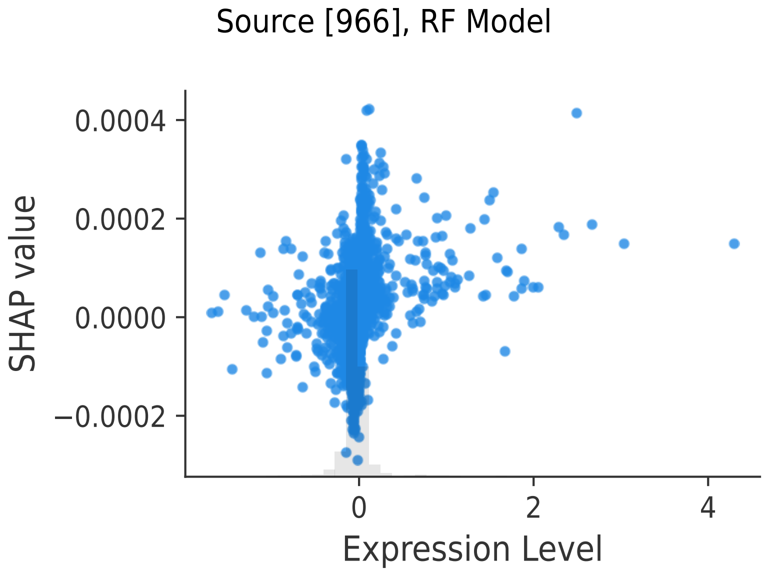

Source [966], RF Model
0.0004
0.0002
0.0000
−0.0002
SHAP value
0
2
4
Expression Level

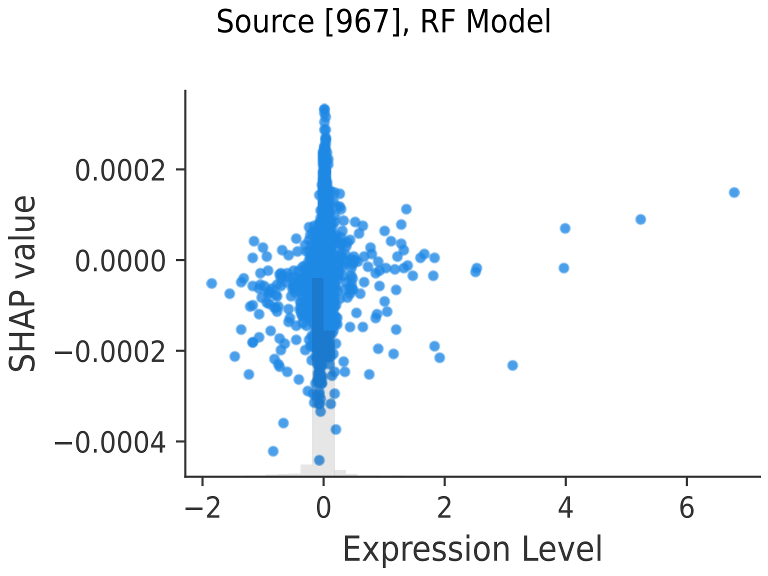

Source [967], RF Model
0.0002
0.0000
−0.0002
−0.0004
SHAP value
−2
0
2
4
6
Expression Level

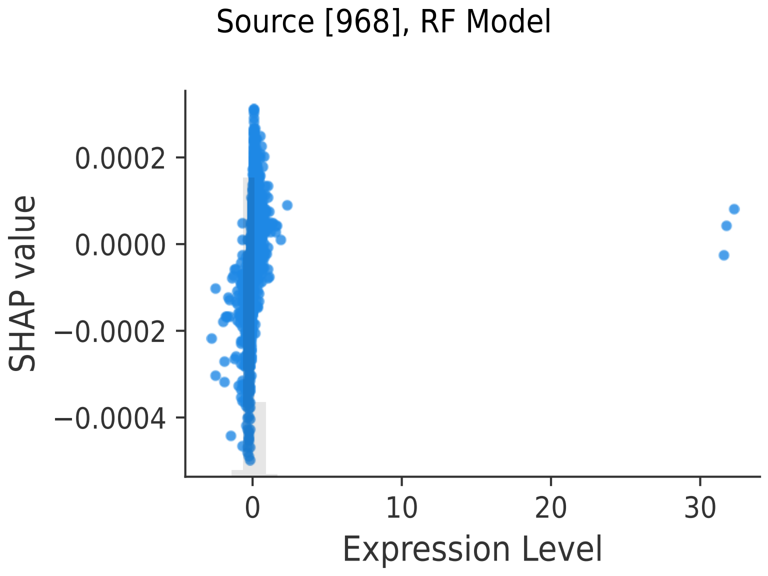

Source [968], RF Model
0.0002
0.0000
−0.0002
−0.0004
SHAP value
0
10
20
30
Expression Level

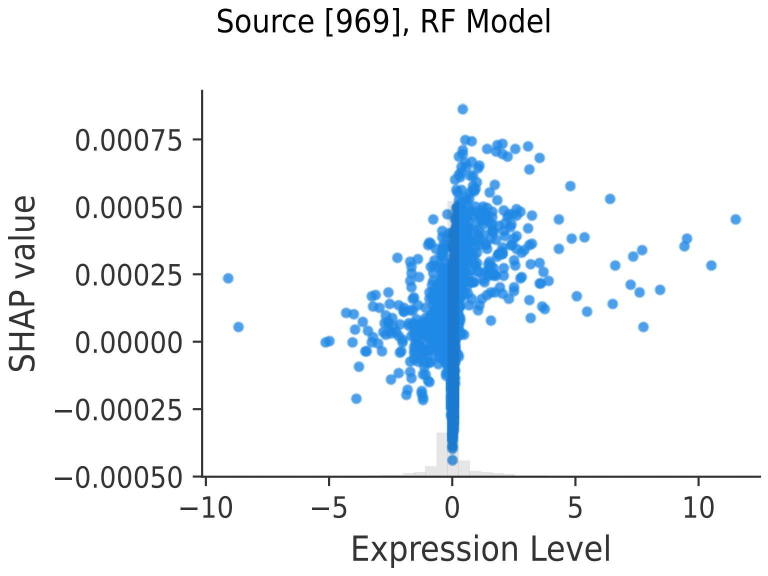
Source [969], RF Model
SHAP value
0.00075
0.00050
0.00025
0.00000
−0.00025
−0.00050
−10
−5
0
5
10
Expression Level

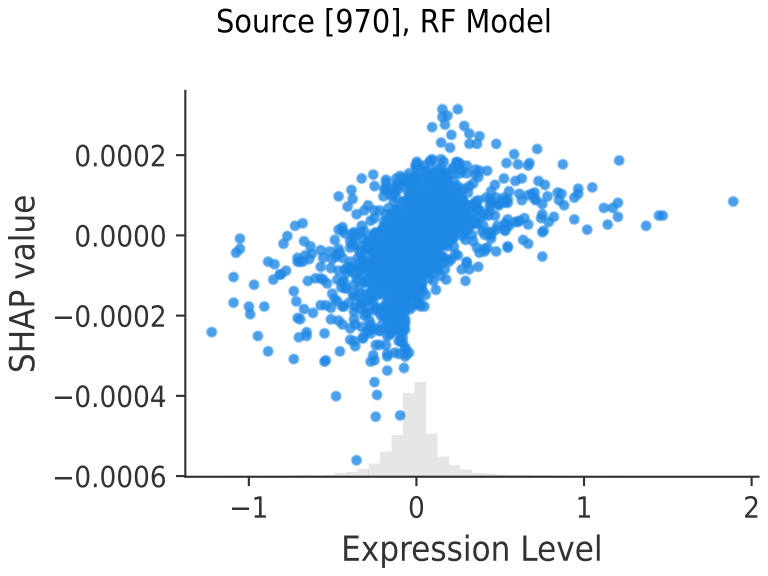
Source [970], RF Model
0.0002
0.0000
−0.0002
−0.0004
−0.0006
SHAP value
−1
0
1
2
Expression Level

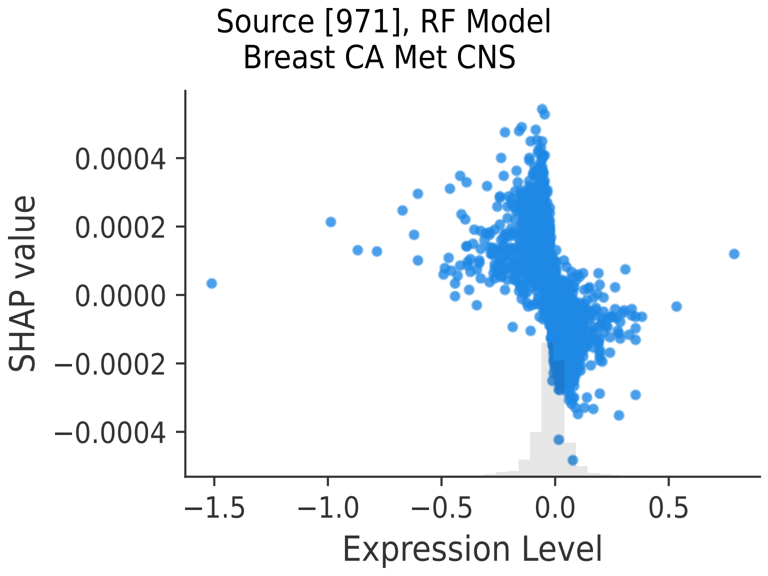
Source [971], RF Model
Breast CA Met CNS
SHAP value
0.0004
0.0002
0.0000
−0.0002
−0.0004
−1.5
−1.0
−0.5
0.0
0.5
Expression Level

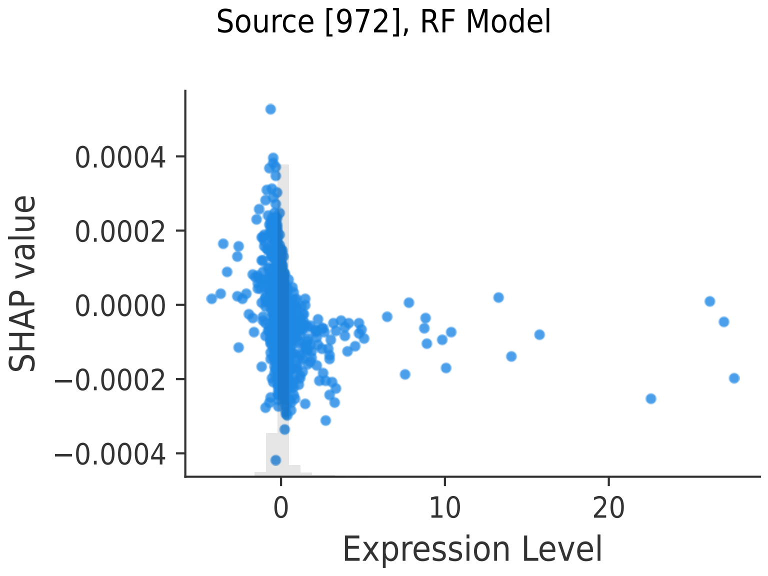

Source [972], RF Model
0.0004
0.0002
0.0000
−0.0002
−0.0004
SHAP value
0
10
20
Expression Level

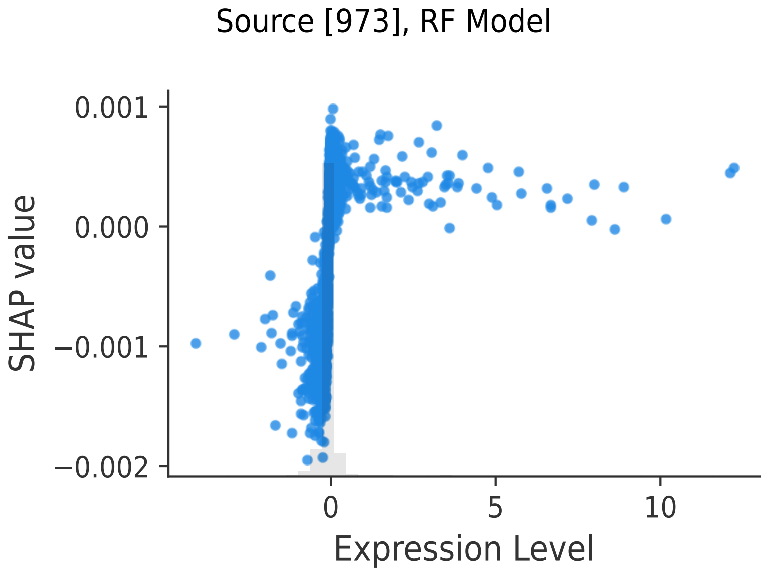
Source [973], RF Model
0.001
0.000
−0.001
−0.002
SHAP value
0
5
10
Expression Level

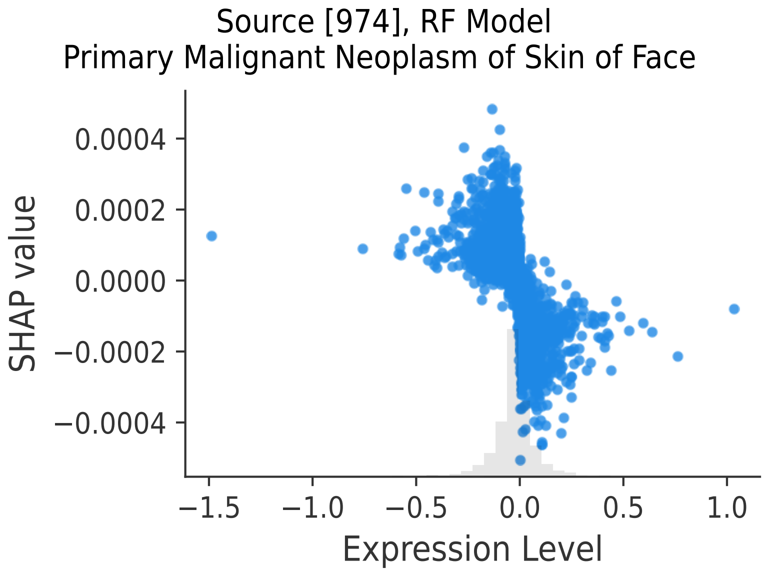

Source [974], RF Model
Primary Malignant Neoplasm of Skin of Face
SHAP value
0.0004
0.0002
0.0000
−0.0002
−0.0004
−1.5
−1.0
−0.5
0.0
0.5
1.0
Expression Level

Source [975], RF Model

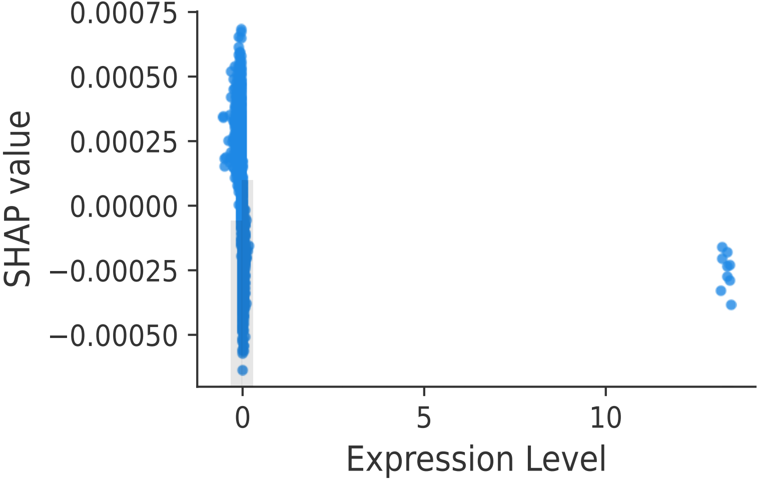

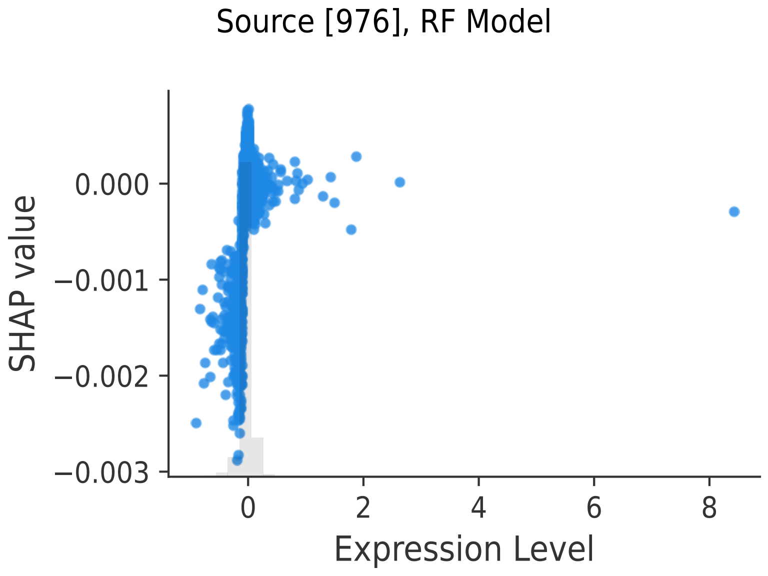
Source [976], RF Model
SHAP value
0.000
−0.001
−0.002
−0.003
0
2
4
6
8
Expression Level

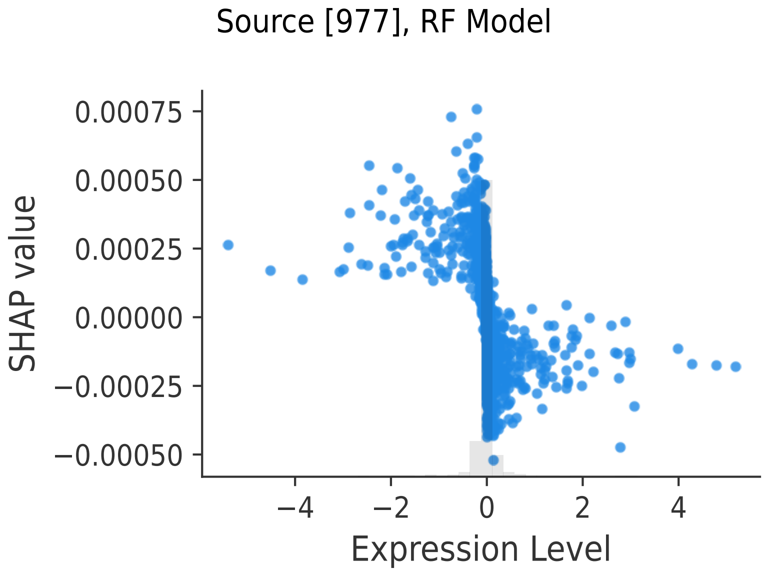

Source [977], RF Model
SHAP value
0.00075
0.00050
0.00025
0.00000
−0.00025
−0.00050
−4
−2
0
2
4
Expression Level

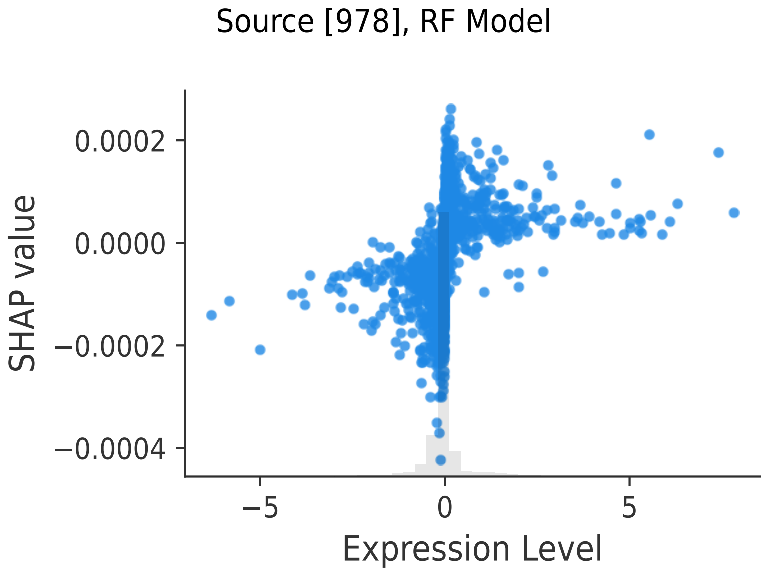

Source [978], RF Model
0.0002
0.0000
−0.0002
−0.0004
SHAP value
−5
0
5
Expression Level

Source [979], RF Model
Lung Disorder NOS

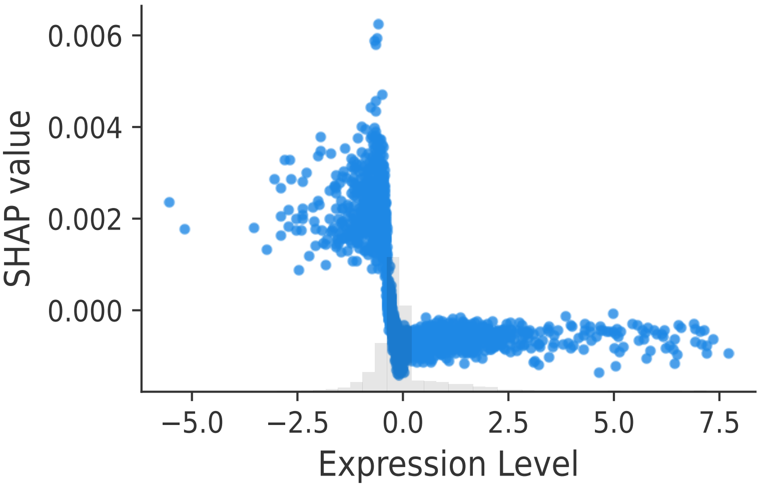

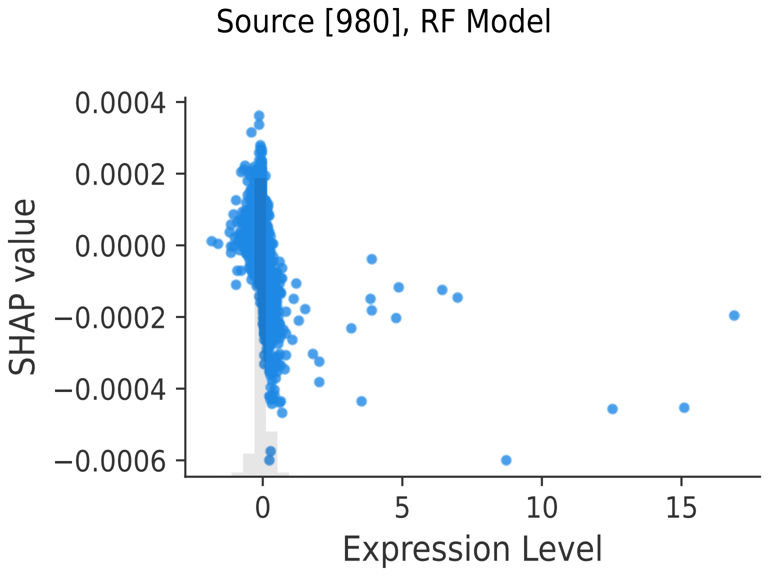

Source [980], RF Model
0.0004
0.0002
0.0000
−0.0002
−0.0004
−0.0006
SHAP value
0
5
10
15
Expression Level

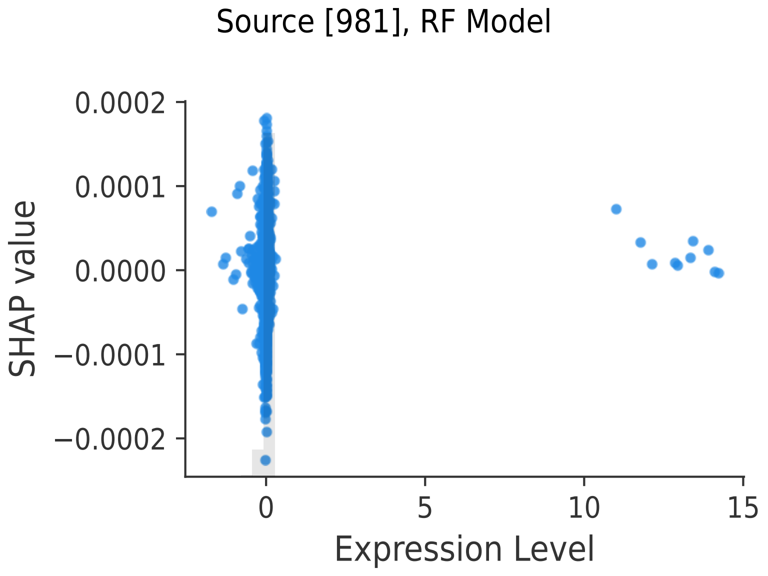
Source [981], RF Model
0.0002
0.0001
0.0000
−0.0001
−0.0002
SHAP value
0
5
10
15
Expression Level

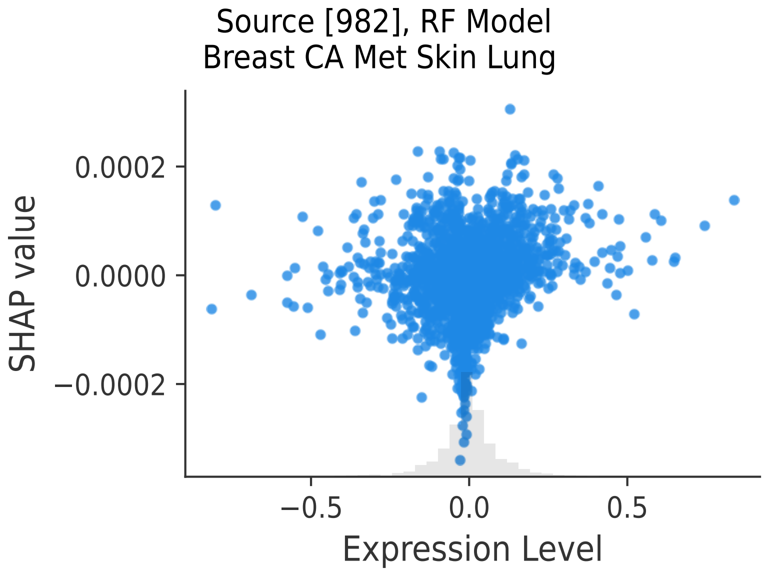

Source [982], RF Model
Breast CA Met Skin Lung
0.0002
0.0000
−0.0002
SHAP value
−0.5
0.0
0.5
Expression Level

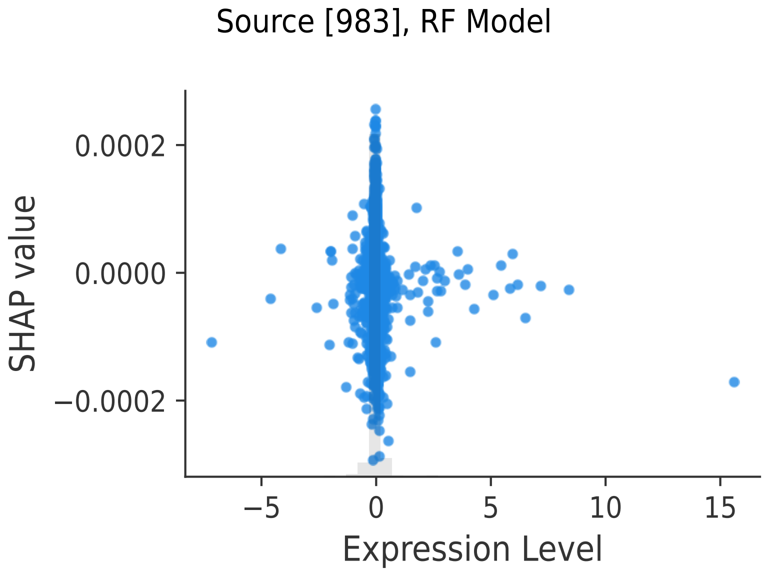

Source [983], RF Model
0.0002
0.0000
−0.0002
SHAP value
−5
0
5
10
15
Expression Level

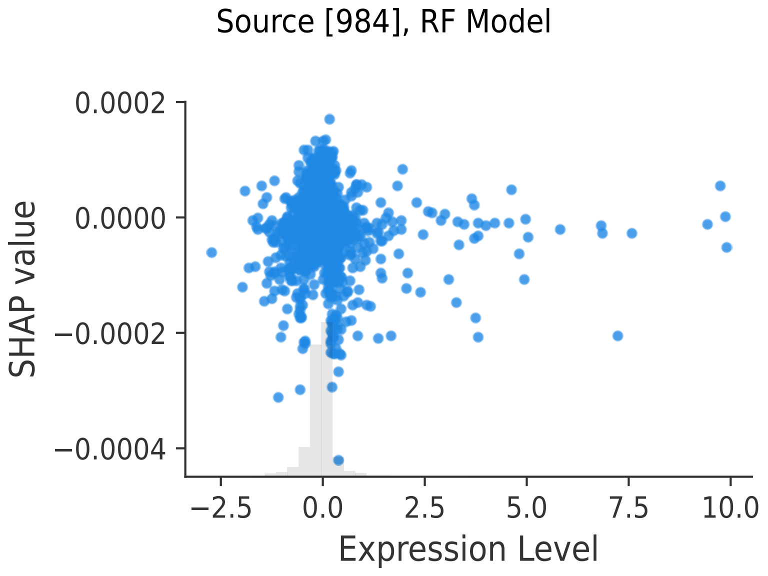

Source [984], RF Model
0.0002
0.0000
−0.0002
−0.0004
SHAP value
−2.5
0.0
2.5
5.0
7.5
10.0
Expression Level

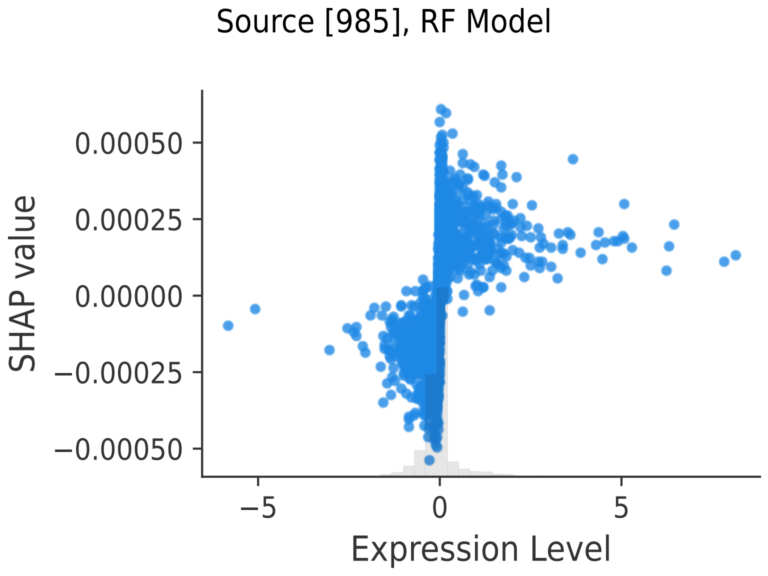

Source [985], RF Model
SHAP value
0.00050
0.00025
0.00000
−0.00025
−0.00050
−5
0
5
Expression Level

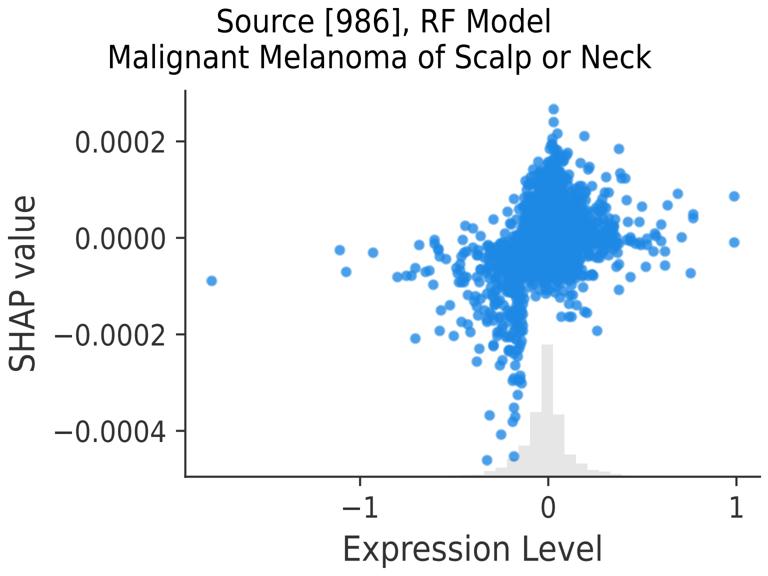

Source [986], RF Model
Malignant Melanoma of Scalp or Neck
SHAP value
0.0002
0.0000
−0.0002
−0.0004
−1
0
1
Expression Level

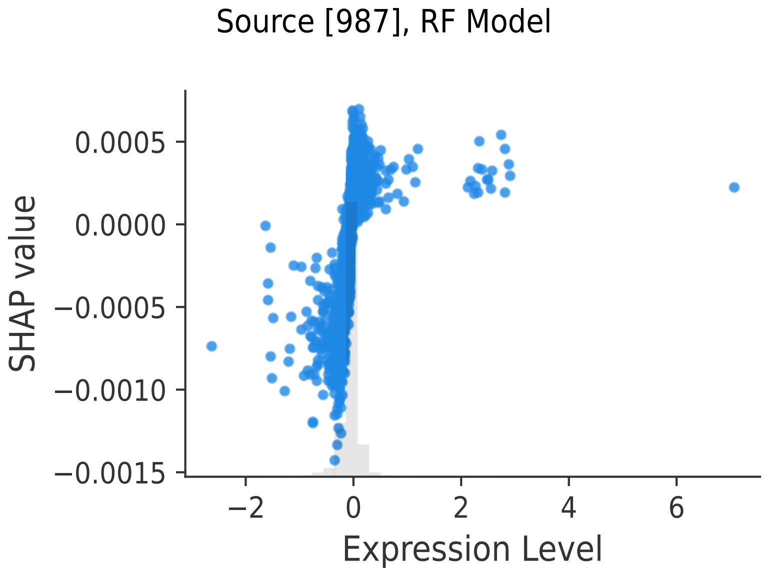

Source [987], RF Model
0.0005
0.0000
−0.0005
−0.0010
−0.0015
SHAP value
−2
0
2
4
6
Expression Level

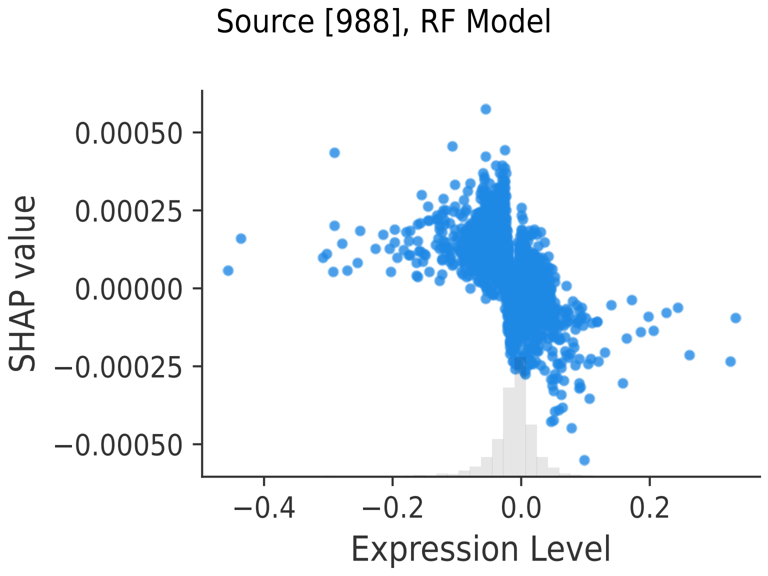

Source [988], RF Model
0.00050
0.00025
0.00000
−0.00025
−0.00050
SHAP value
−0.4
−0.2
0.0
0.2
Expression Level

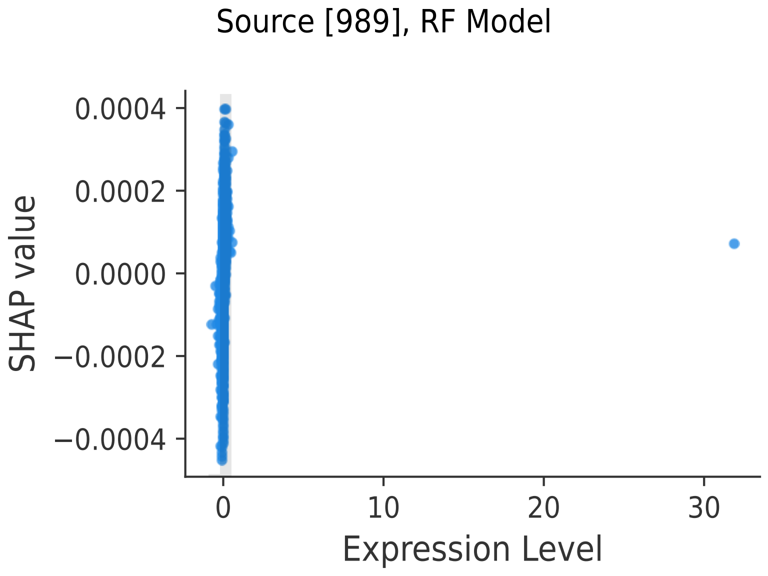
Source [989], RF Model
SHAP value
0.0004
0.0002
0.0000
−0.0002
−0.0004
0
10
20
30
Expression Level

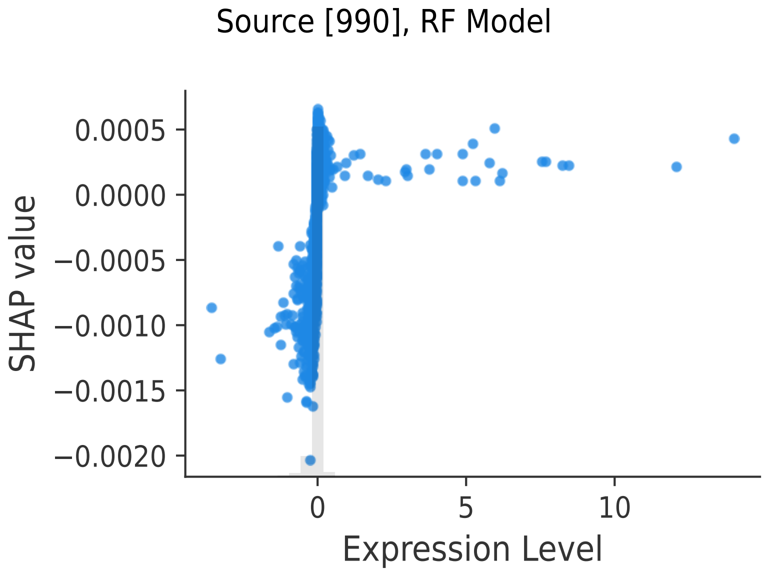
Source [990], RF Model
SHAP value
0.0005
0.0000
−0.0005
−0.0010
−0.0015
−0.0020
0
5
10
Expression Level

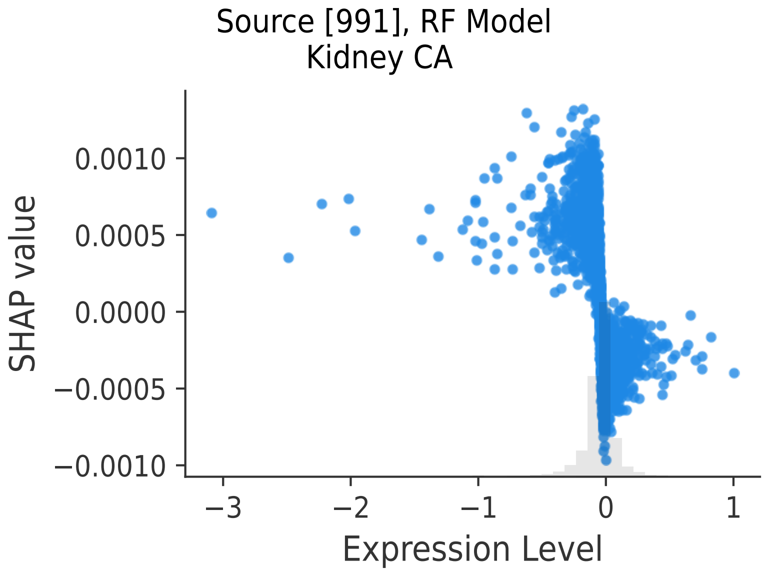

Source [991], RF Model
Kidney CA
0.0010
0.0005
0.0000
−0.0005
−0.0010
SHAP value
−3
−2
−1
0
1
Expression Level

Source [992], RF Model

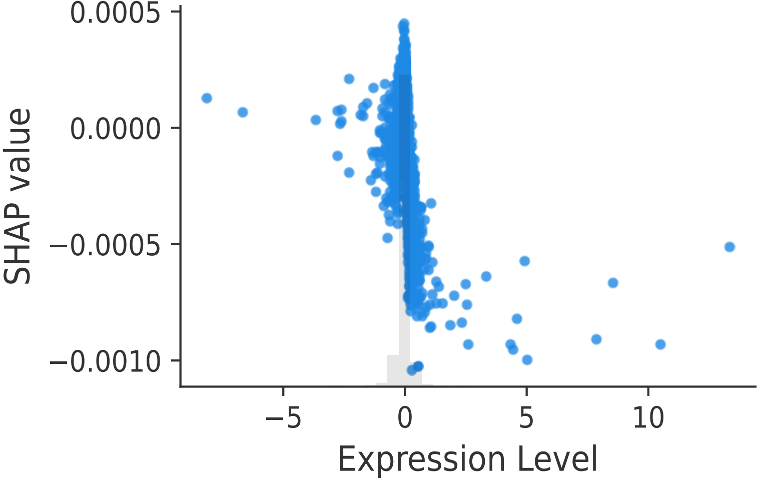

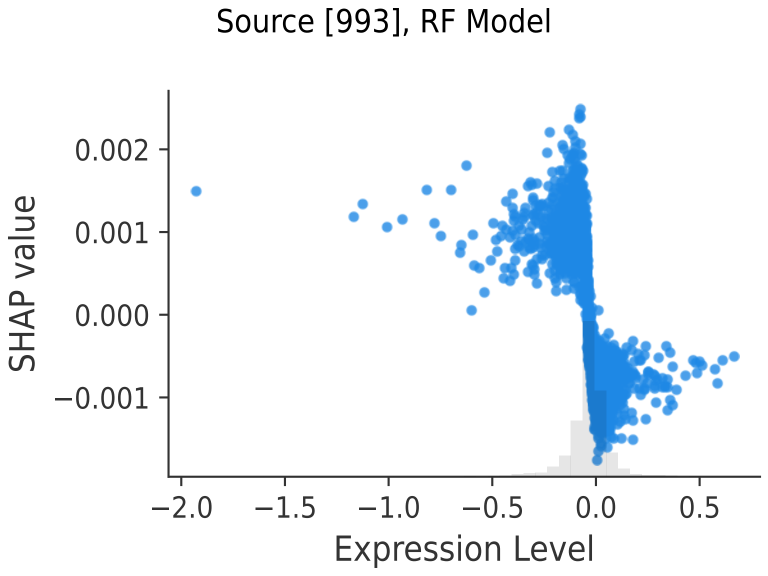
Source [993], RF Model
SHAP value
0.002
0.001
0.000
−0.001
−2.0
−1.5
−1.0
−0.5
0.0
0.5
Expression Level

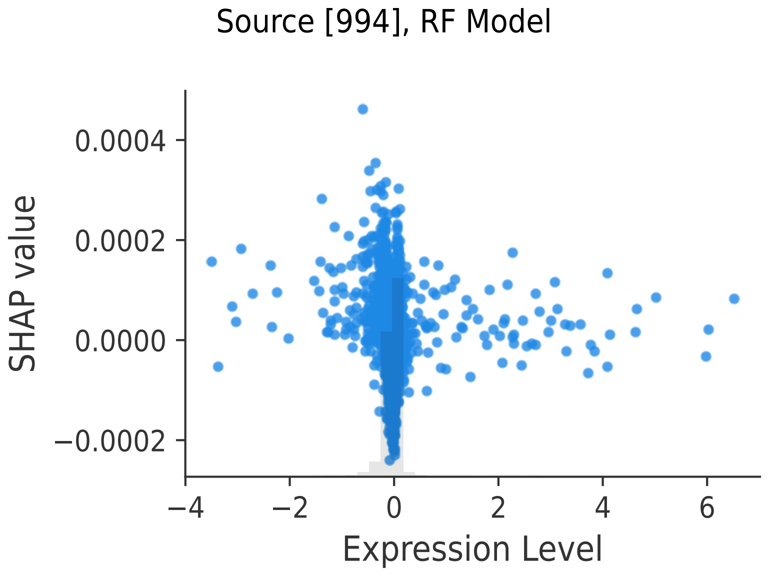

Source [994], RF Model
SHAP value
0.0004
0.0002
0.0000
−0.0002
−4
−2
0
2
4
6
Expression Level

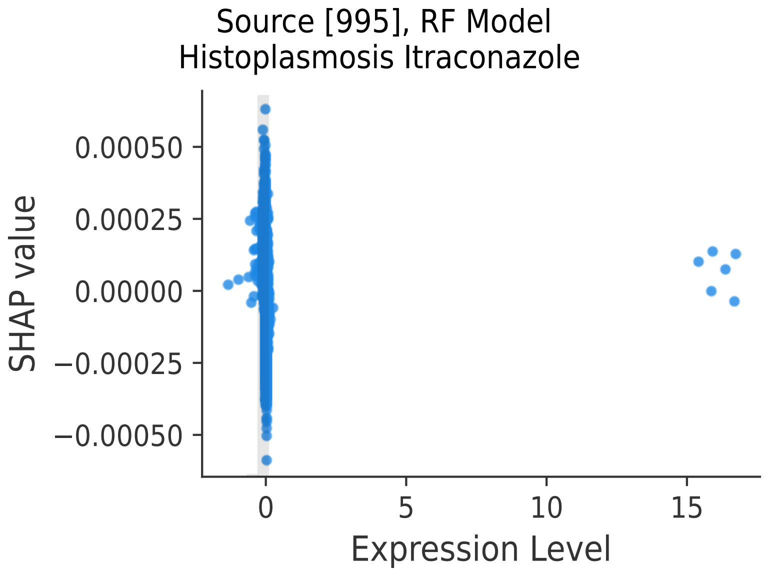
Source [995], RF Model
Histoplasmosis Itraconazole
SHAP value
0.00050
0.00025
0.00000
−0.00025
−0.00050
0
5
10
15
Expression Level

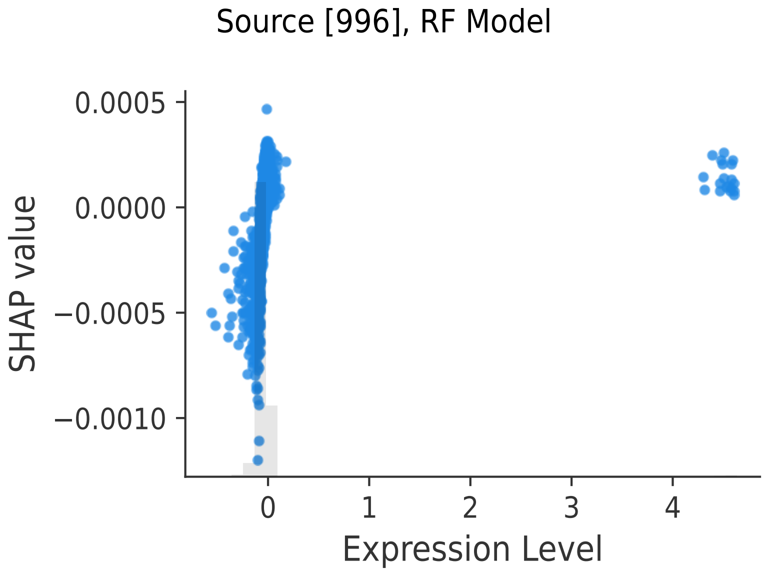

Source [996], RF Model
0.0005
0.0000
−0.0005
−0.0010
SHAP value
0
1
2
3
4
Expression Level

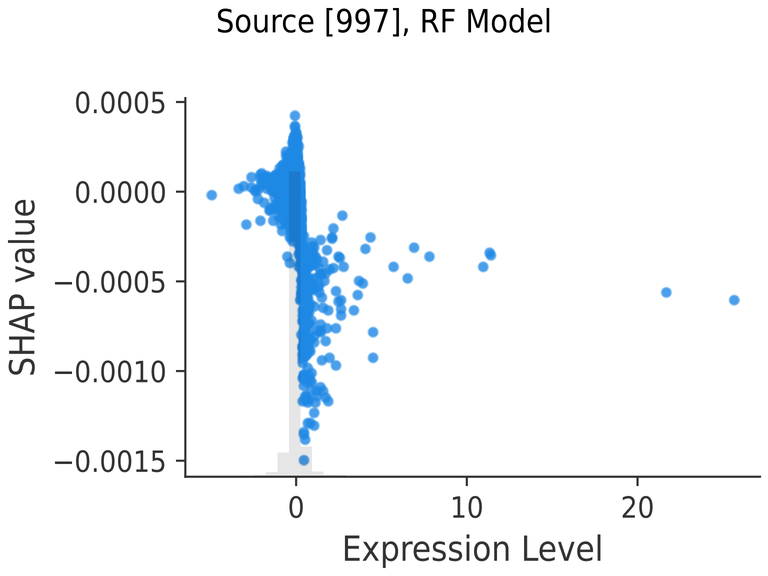
Source [997], RF Model
0.0005
0.0000
−0.0005
−0.0010
−0.0015
SHAP value
0
10
20
Expression Level

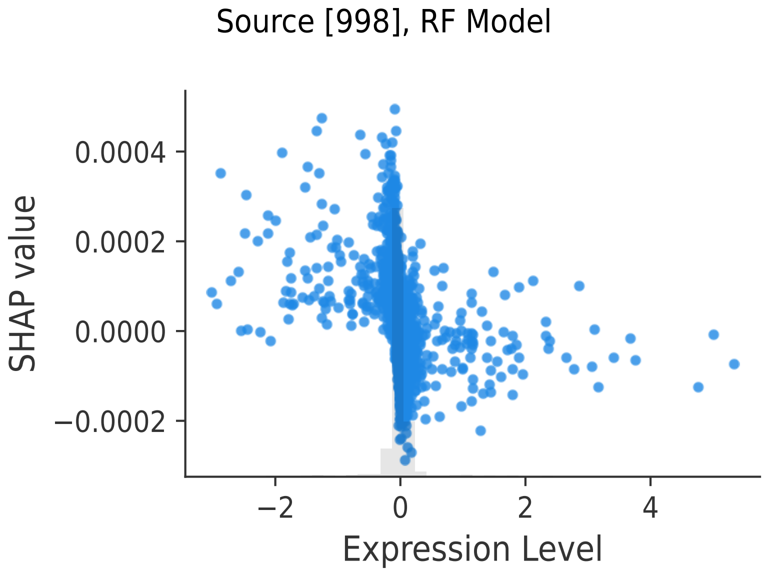

Source [998], RF Model
0.0004
0.0002
0.0000
−0.0002
SHAP value
−2
0
2
4
Expression Level

Source [999], RF Model

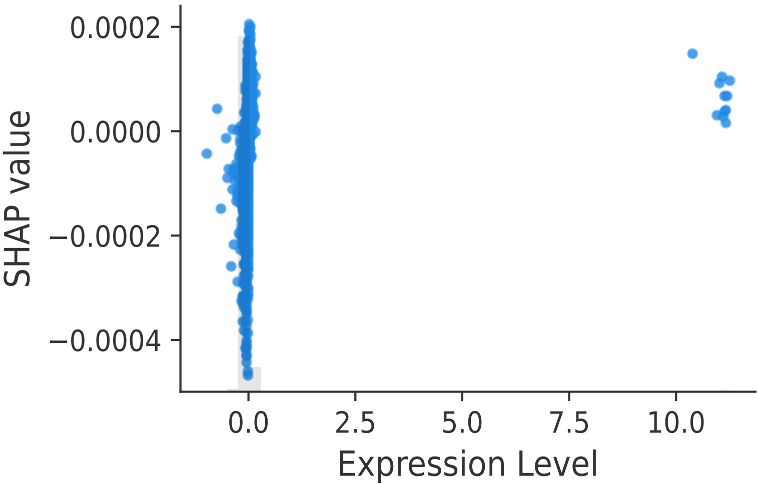

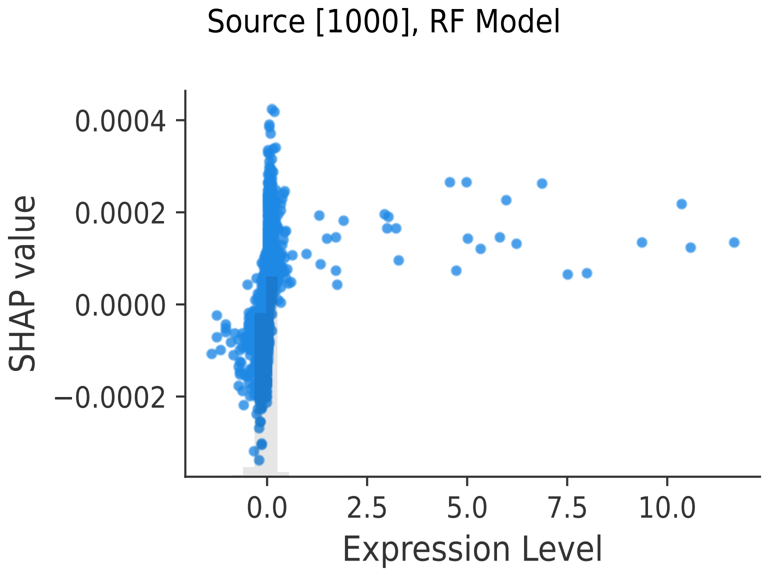
Source [1000], RF Model
0.0004
0.0002
0.0000
−0.0002
SHAP value
0.0
2.5
5.0
7.5
10.0
Expression Level

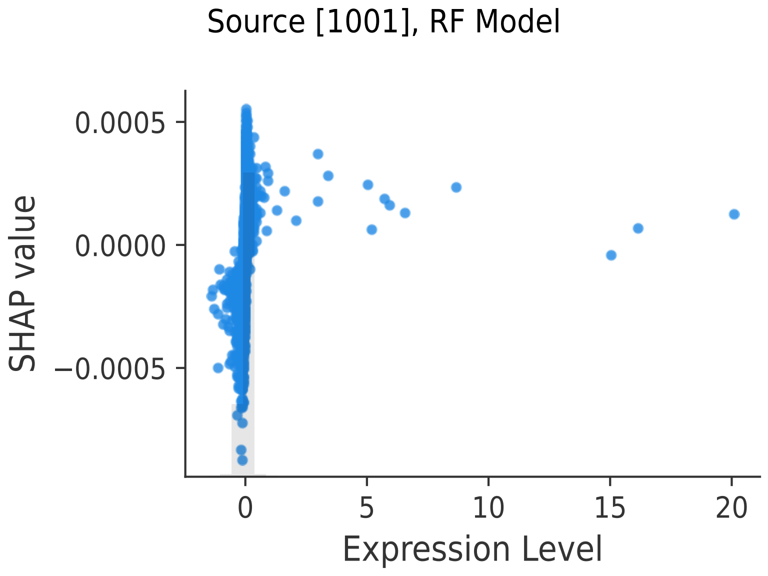

Source [1001], RF Model
0.0005
0.0000
−0.0005
SHAP value
0
5
10
15
20
Expression Level

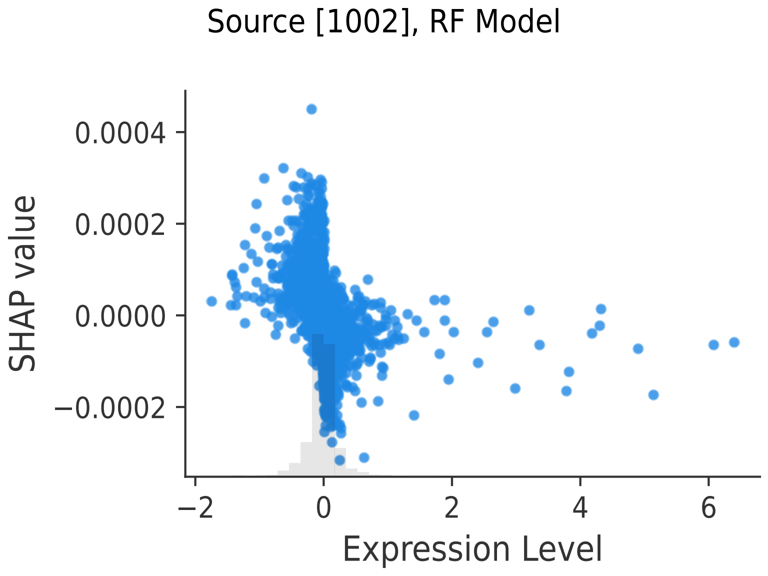

Source [1002], RF Model
SHAP value
0.0004
0.0002
0.0000
−0.0002
−2
0
2
4
6
Expression Level

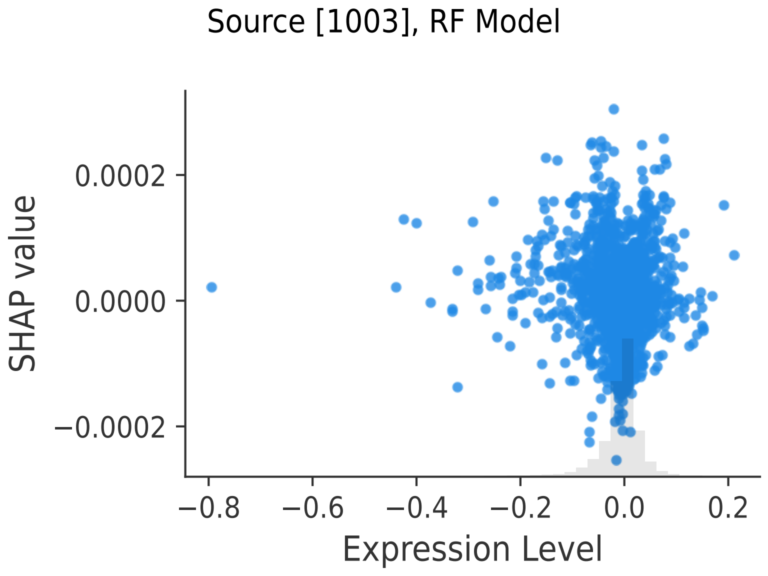

Source [1003], RF Model
SHAP value
0.0002
0.0000
−0.0002
−0.8
−0.6
−0.4
−0.2
0.0
0.2
Expression Level

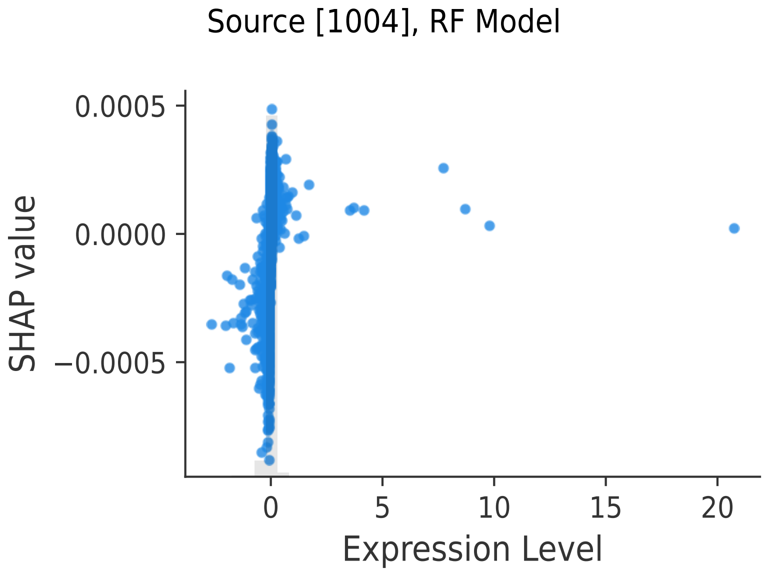

Source [1004], RF Model
0.0005
0.0000
−0.0005
SHAP value
0
5
10
15
20
Expression Level

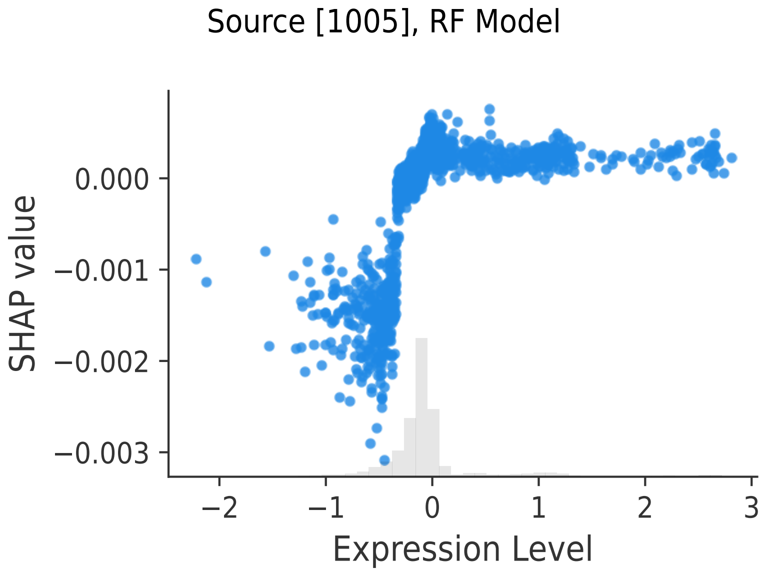
Source [1005], RF Model
SHAP value
0.000
−0.001
−0.002
−0.003
−2
−1
0
1
2
3
Expression Level

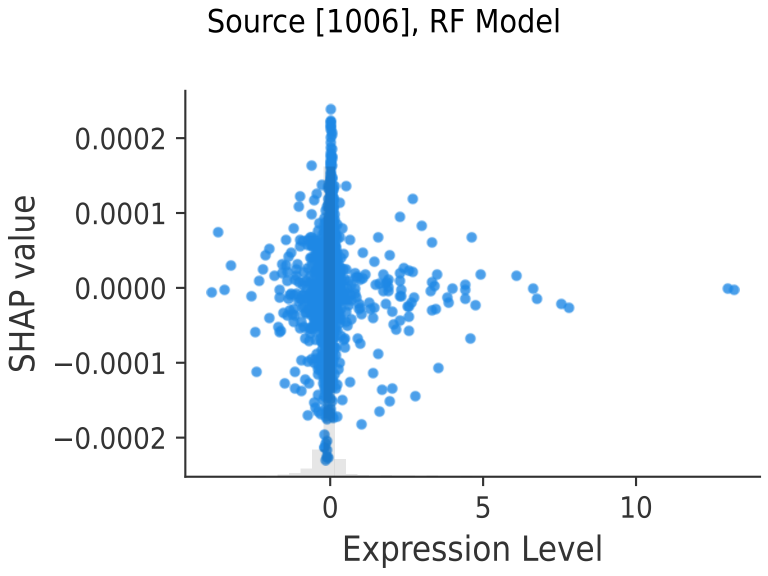
Source [1006], RF Model
0.0002
0.0001
0.0000
−0.0001
−0.0002
SHAP value
0
5
10
Expression Level

Source [1007], RF Model

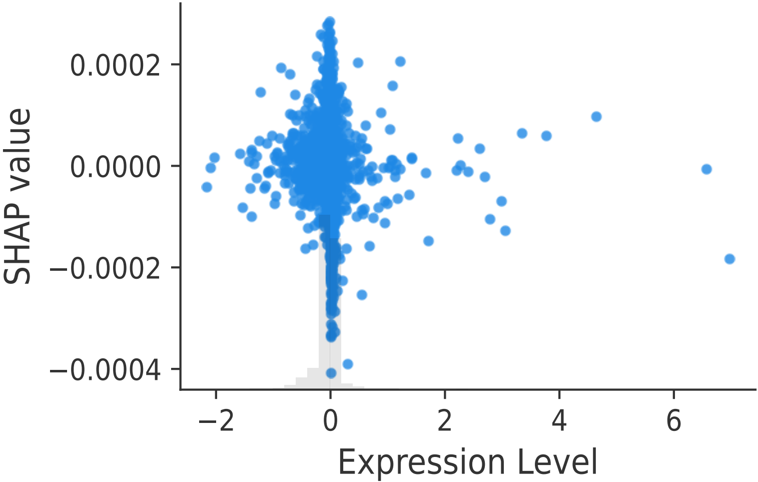

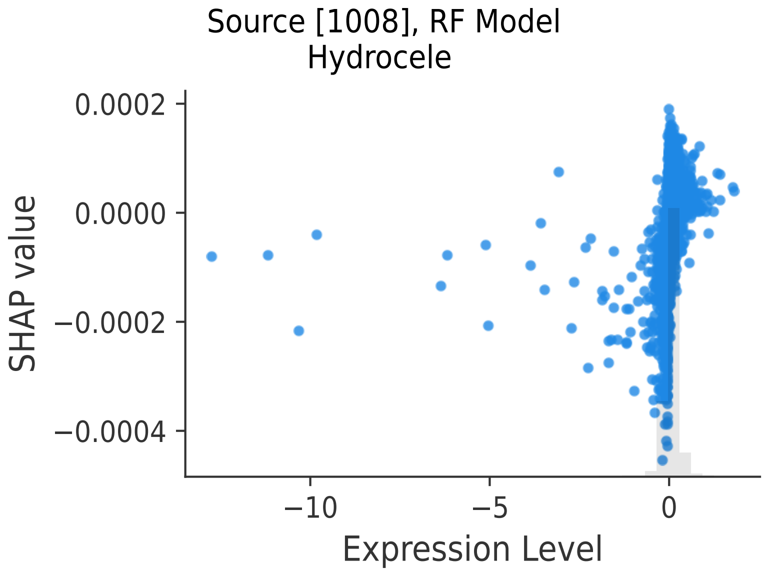

Source [1008], RF Model
Hydrocele
0.0002
0.0000
−0.0002
−0.0004
SHAP value
−10
−5
0
Expression Level

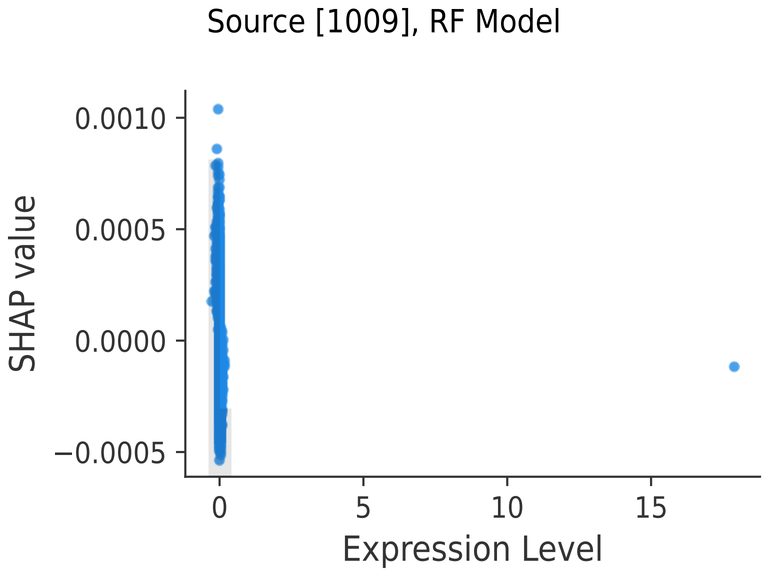

Source [1009], RF Model
SHAP value
0.0010
0.0005
0.0000
−0.0005
0
5
10
15
Expression Level

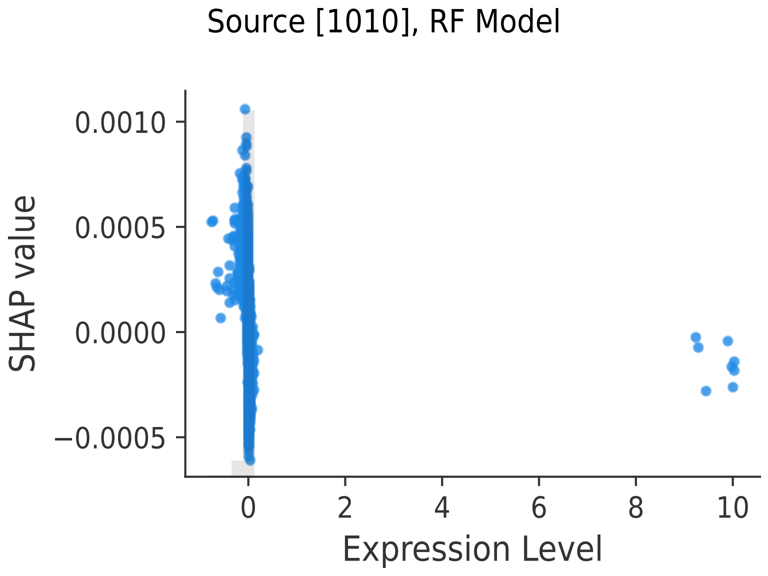

Source [1010], RF Model
0.0010
0.0005
0.0000
−0.0005
SHAP value
0
2
4
6
8
10
Expression Level

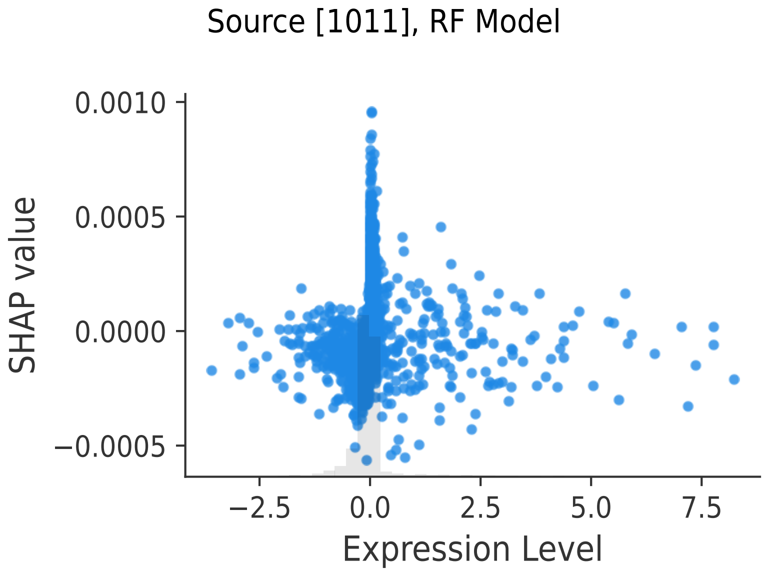

Source [1011], RF Model
0.0010
0.0005
0.0000
−0.0005
SHAP value
−2.5
0.0
2.5
5.0
7.5
Expression Level

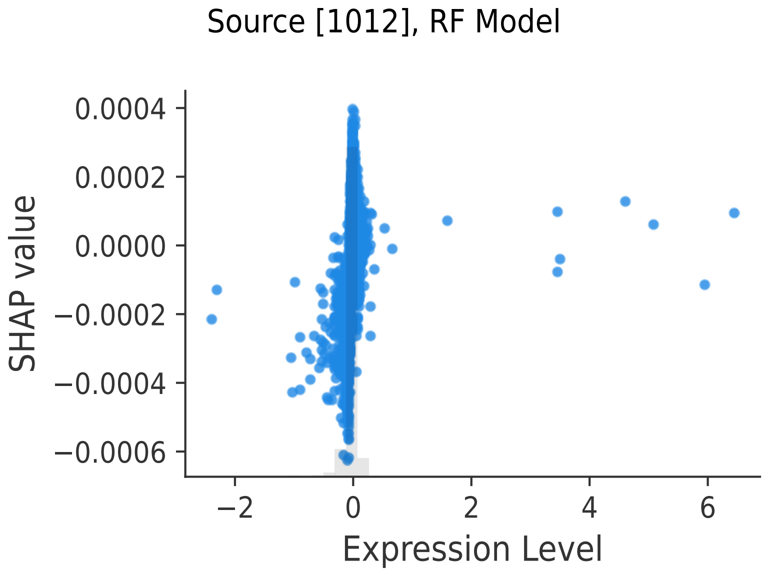

Source [1012], RF Model
SHAP value
0.0004
0.0002
0.0000
−0.0002
−0.0004
−0.0006
−2
0
2
4
6
Expression Level

Source [1013], RF Model

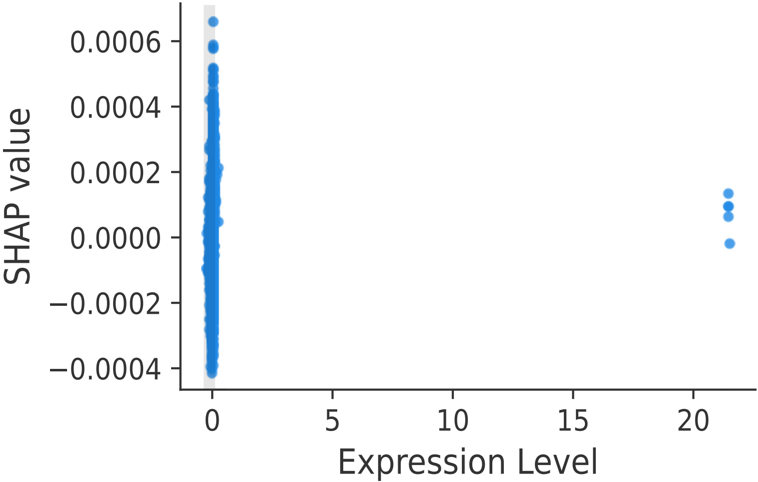

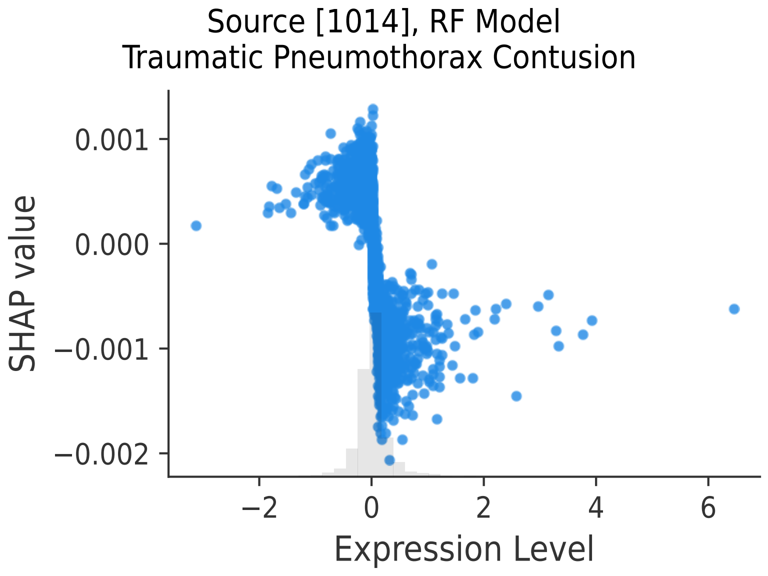

Source [1014], RF Model
Traumatic Pneumothorax Contusion
0.001
0.000
−0.001
−0.002
SHAP value
−2
0
2
4
6
Expression Level

Source [1015], RF Model

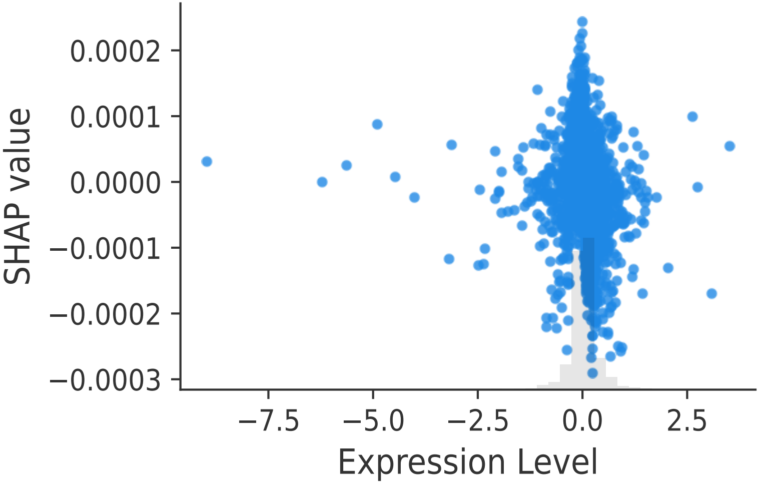

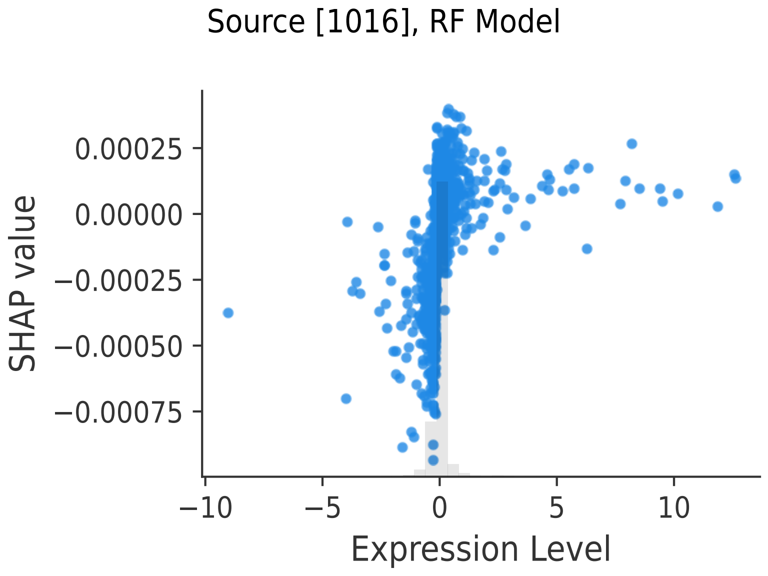
Source [1016], RF Model
SHAP value
0.00025
0.00000
−0.00025
−0.00050
−0.00075
−10
−5
0
5
10
Expression Level

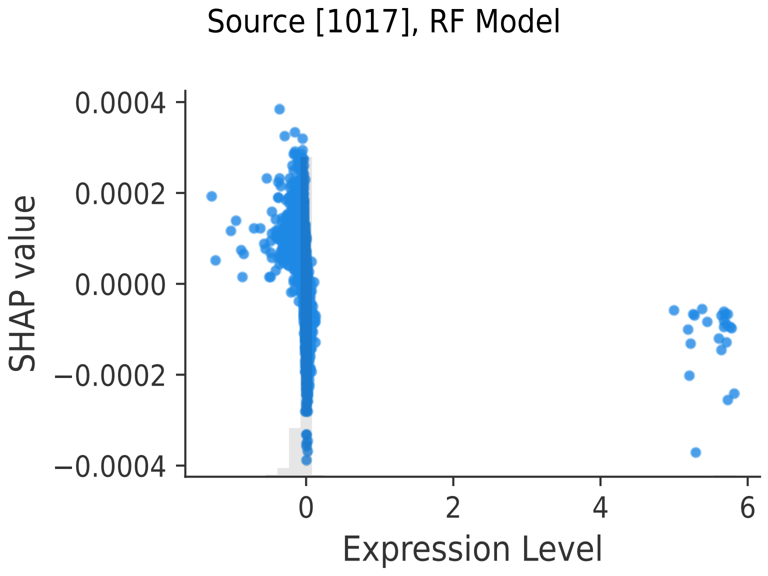
Source [1017], RF Model
0.0004
0.0002
0.0000
−0.0002
−0.0004
SHAP value
0
2
4
6
Expression Level

Source [1018], RF Model

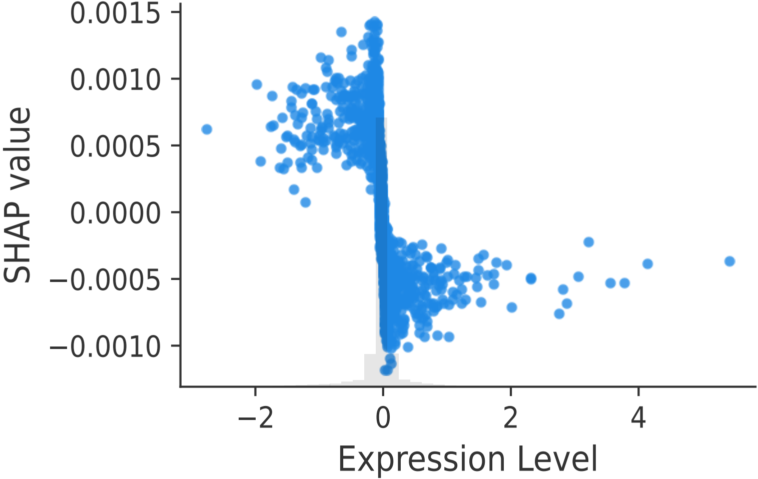

Source [1019], RF Model

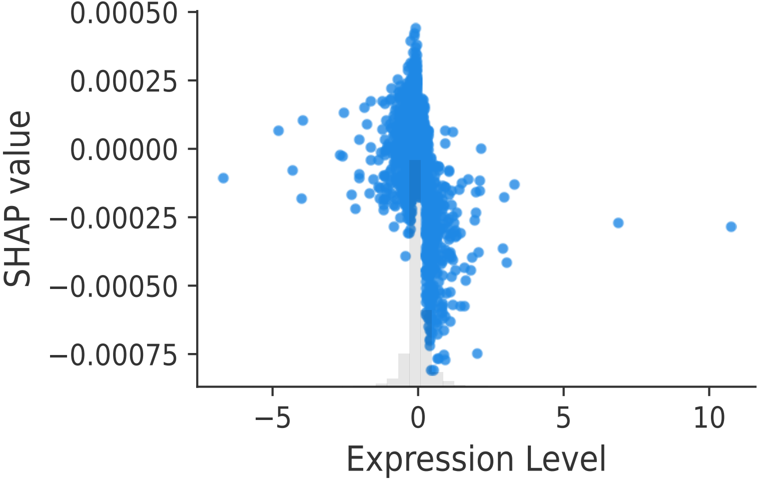

Source [1020], RF Model

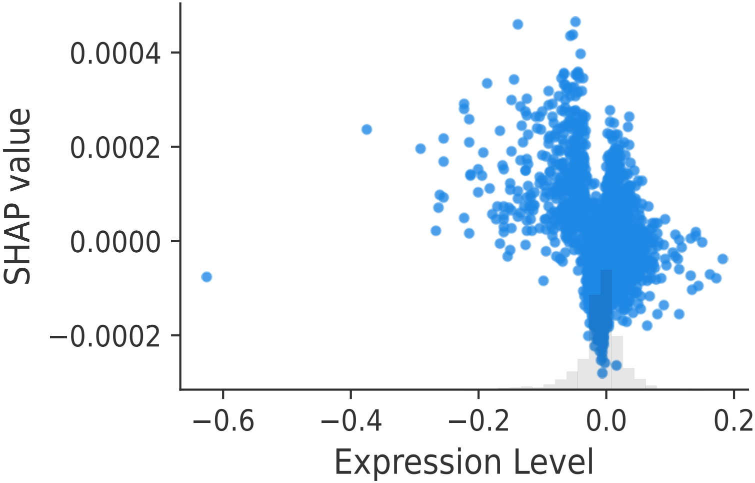

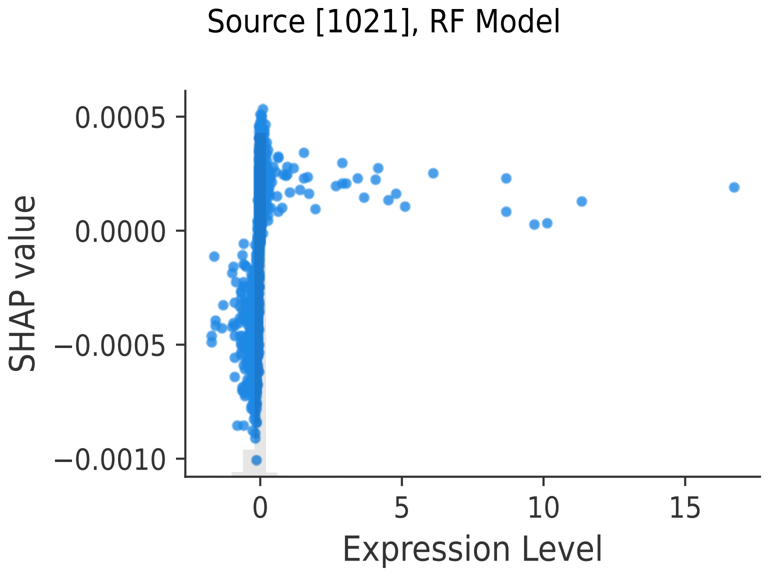

Source [1021], RF Model
0.0005
0.0000
−0.0005
−0.0010
SHAP value
0
5
10
15
Expression Level

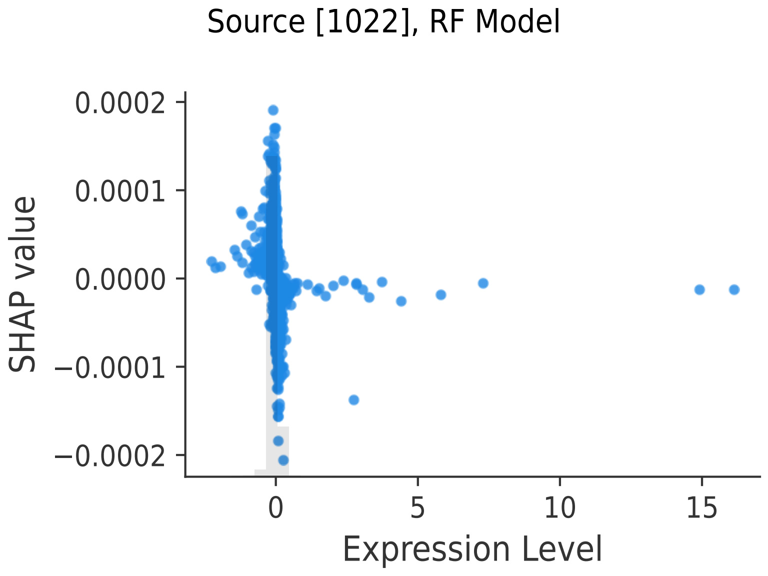
Source [1022], RF Model
SHAP value
0.0002
0.0001
0.0000
−0.0001
−0.0002
0
5
10
15
Expression Level

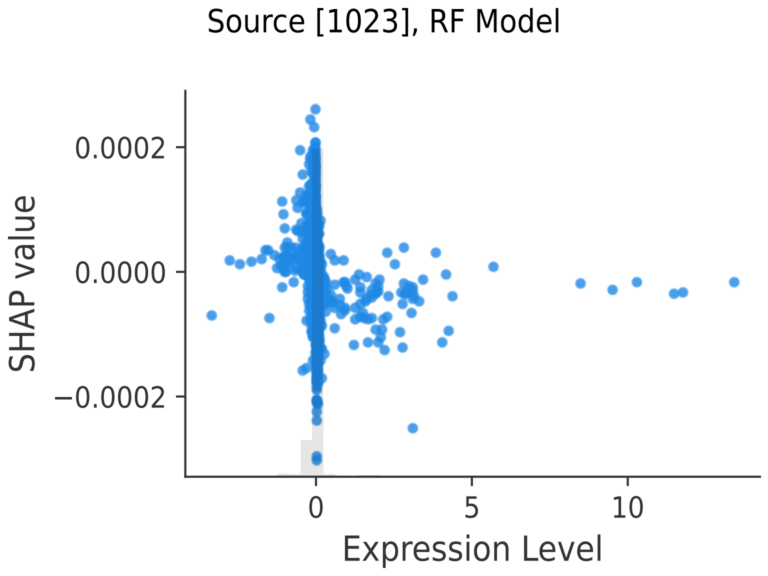

Source [1023], RF Model
0.0002
0.0000
−0.0002
SHAP value
0
5
10
Expression Level

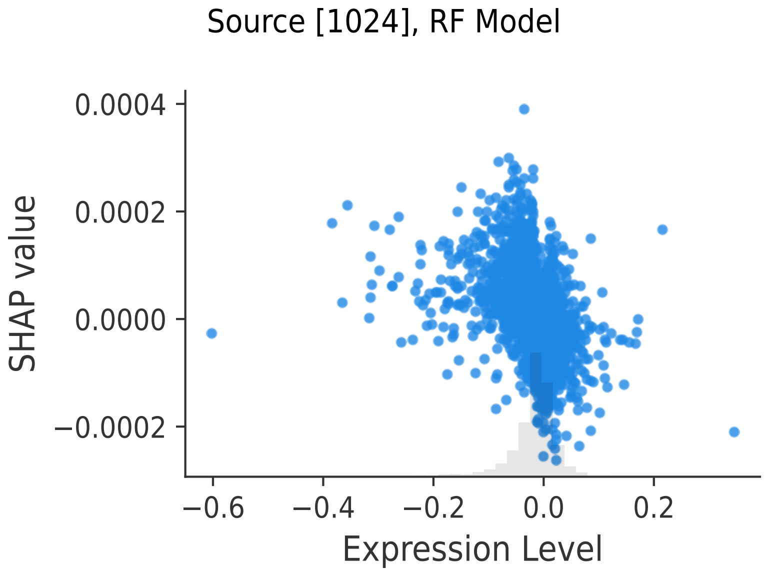

Source [1024], RF Model
0.0004
0.0002
0.0000
−0.0002
SHAP value
−0.6
−0.4
−0.2
0.0
0.2
Expression Level

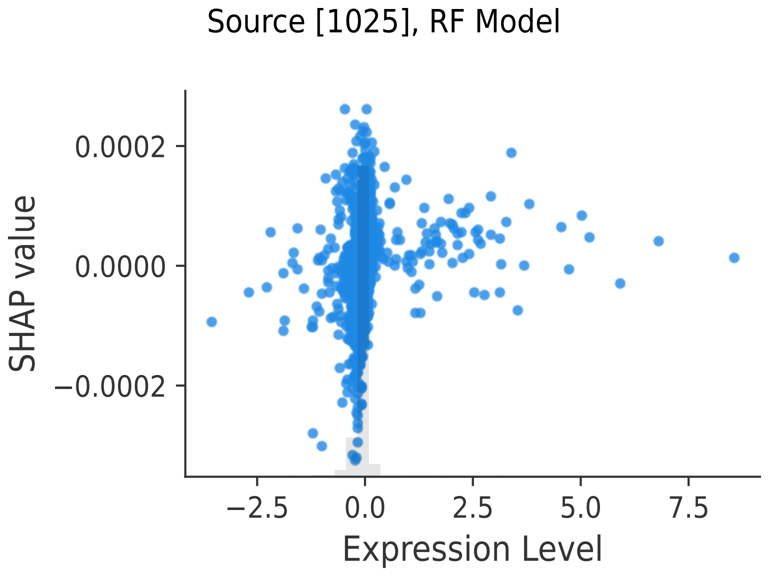

Source [1025], RF Model
0.0002
0.0000
−0.0002
SHAP value
−2.5
0.0
2.5
5.0
7.5
Expression Level

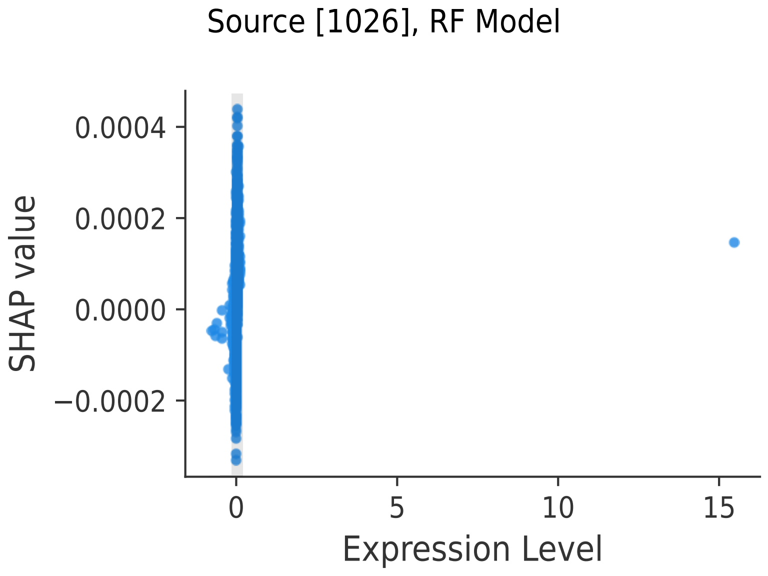

Source [1026], RF Model
0.0004
0.0002
0.0000
−0.0002
SHAP value
0
5
10
15
Expression Level

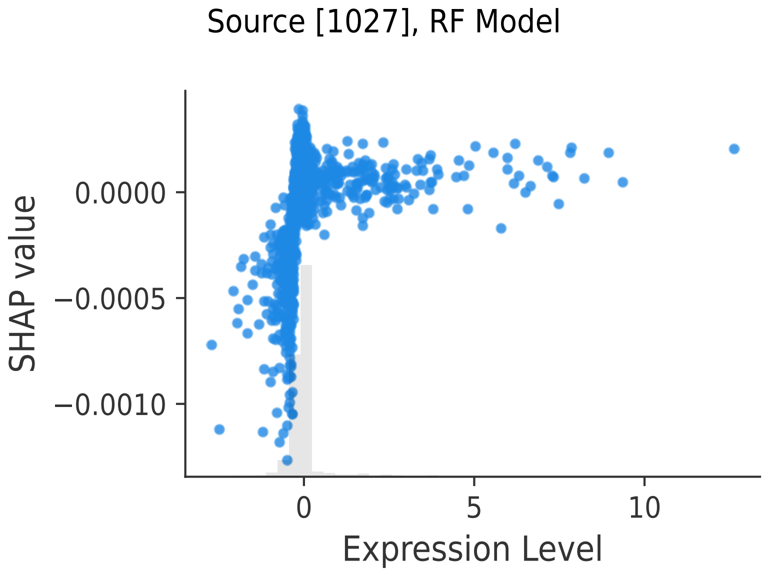

Source [1027], RF Model
SHAP value
0.0000
−0.0005
−0.0010
0
5
10
Expression Level

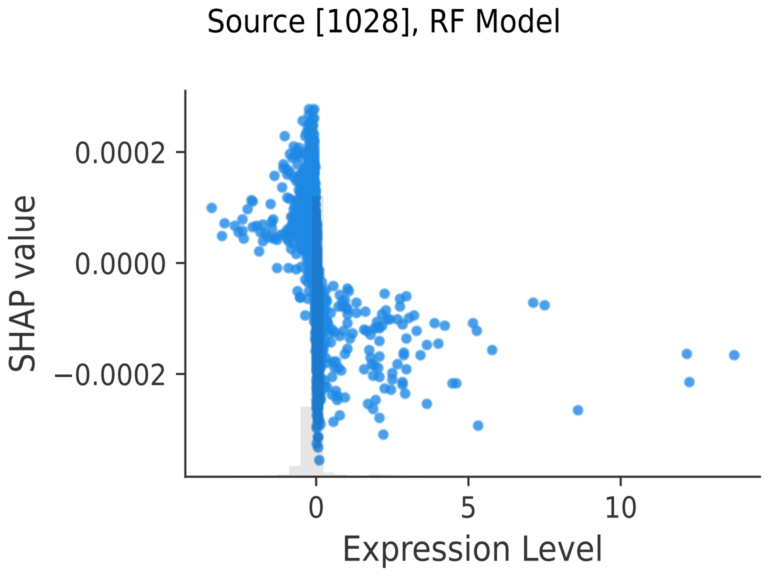
Source [1028], RF Model
SHAP value
0.0002
0.0000
−0.0002
0
5
10
Expression Level

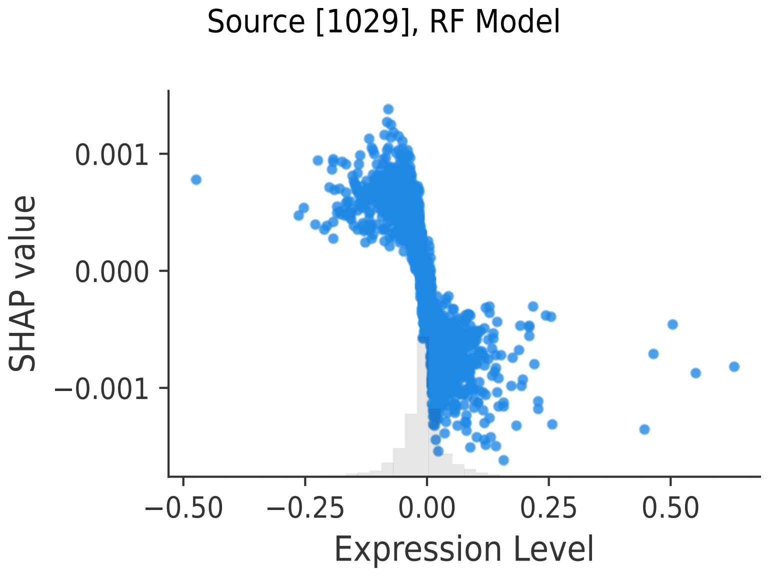

Source [1029], RF Model
SHAP value
0.001
0.000
−0.001
−0.50
−0.25
0.00
0.25
0.50
Expression Level

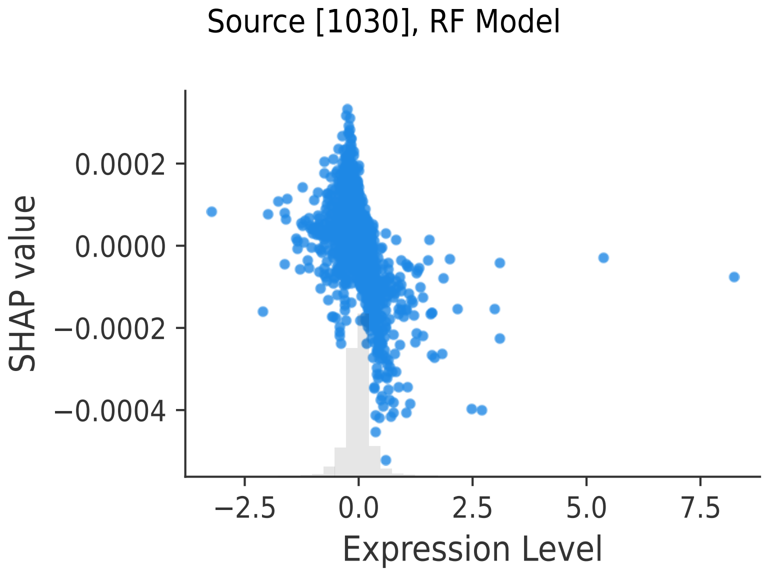

Source [1030], RF Model
0.0002
0.0000
−0.0002
−0.0004
SHAP value
−2.5
0.0
2.5
5.0
7.5
Expression Level

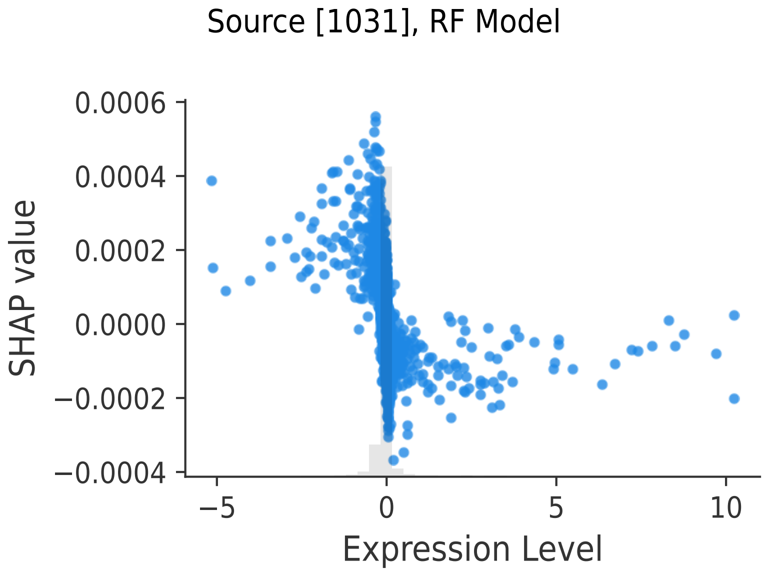
Source [1031], RF Model
0.0006
0.0004
0.0002
0.0000
−0.0002
−0.0004
SHAP value
−5
0
5
10
Expression Level

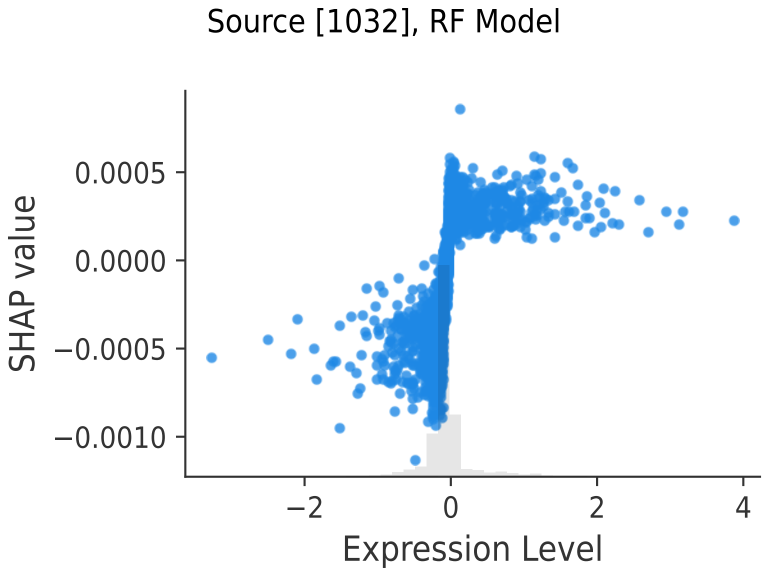

Source [1032], RF Model
SHAP value
0.0005
0.0000
−0.0005
−0.0010
−2
0
2
4
Expression Level

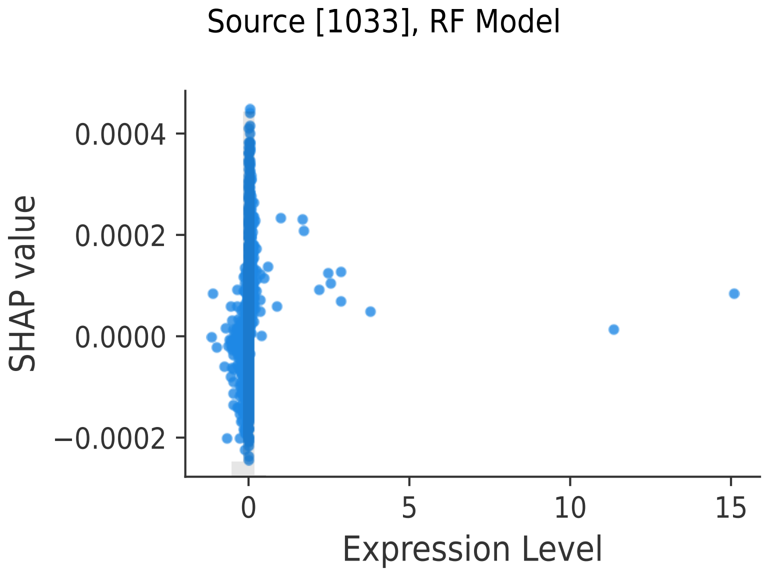
Source [1033], RF Model
SHAP value
0.0004
0.0002
0.0000
−0.0002
0
5
10
15
Expression Level

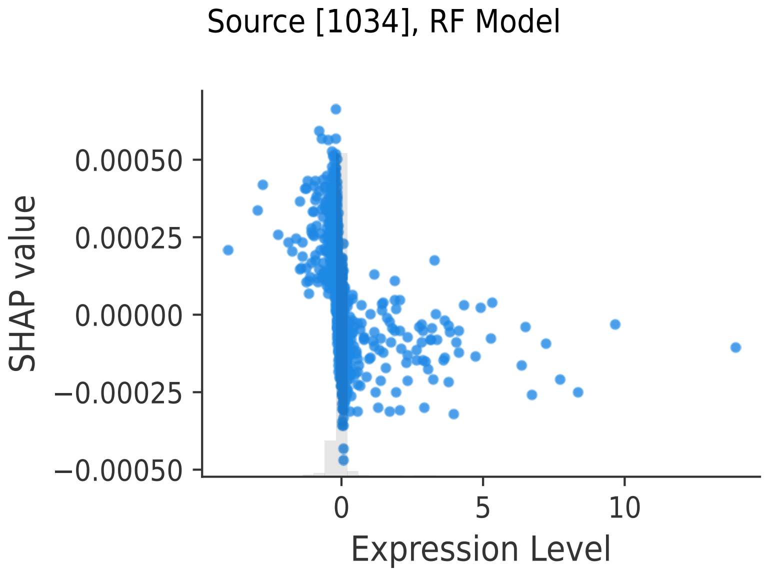
Source [1034], RF Model
SHAP value
0.00050
0.00025
0.00000
−0.00025
−0.00050
0
5
10
Expression Level

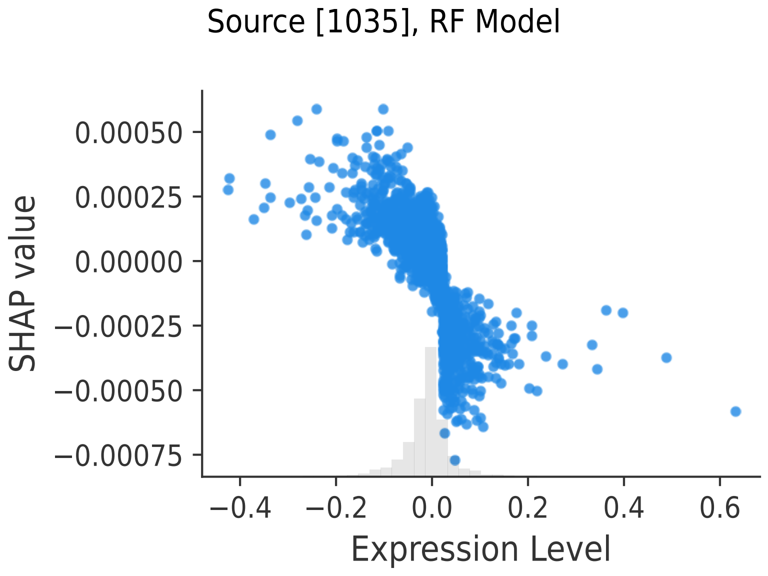
Source [1035], RF Model
SHAP value
0.00050
0.00025
0.00000
−0.00025
−0.00050
−0.00075
−0.4
−0.2
0.0
0.2
0.4
0.6
Expression Level

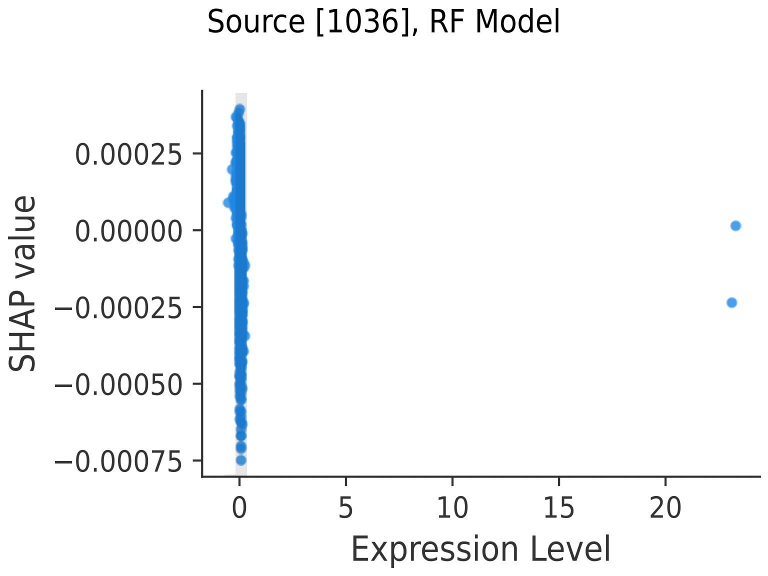

Source [1036], RF Model
SHAP value
0.00025
0.00000
−0.00025
−0.00050
−0.00075
0
5
10
15
20
Expression Level

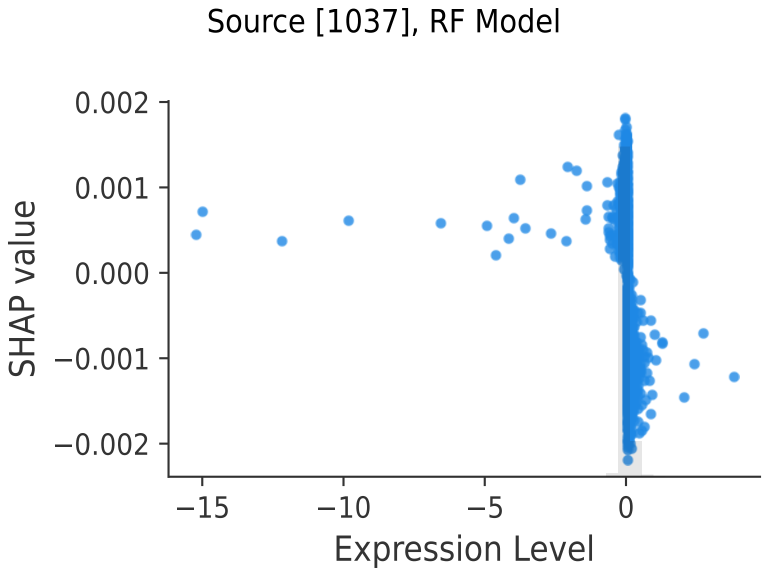

Source [1037], RF Model
SHAP value
0.002
0.001
0.000
−0.001
−0.002
−15
−10
−5
0
Expression Level

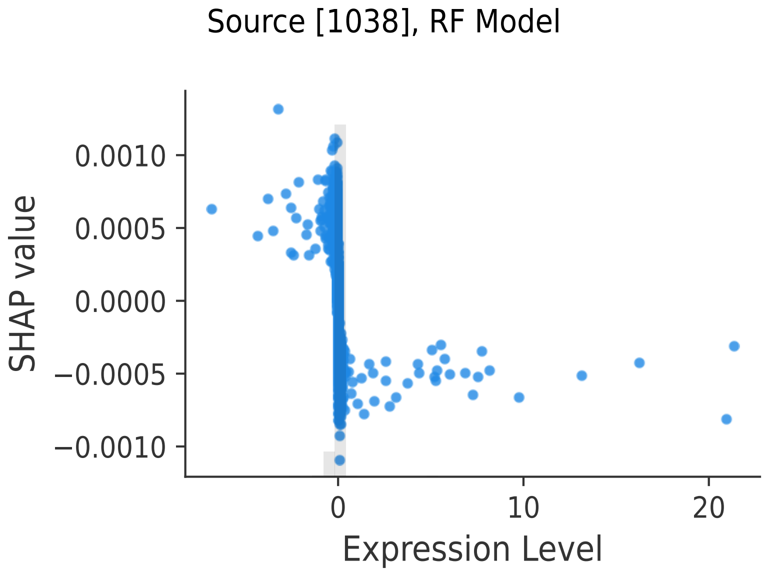

Source [1038], RF Model
0.0010
0.0005
0.0000
−0.0005
−0.0010
SHAP value
0
10
20
Expression Level

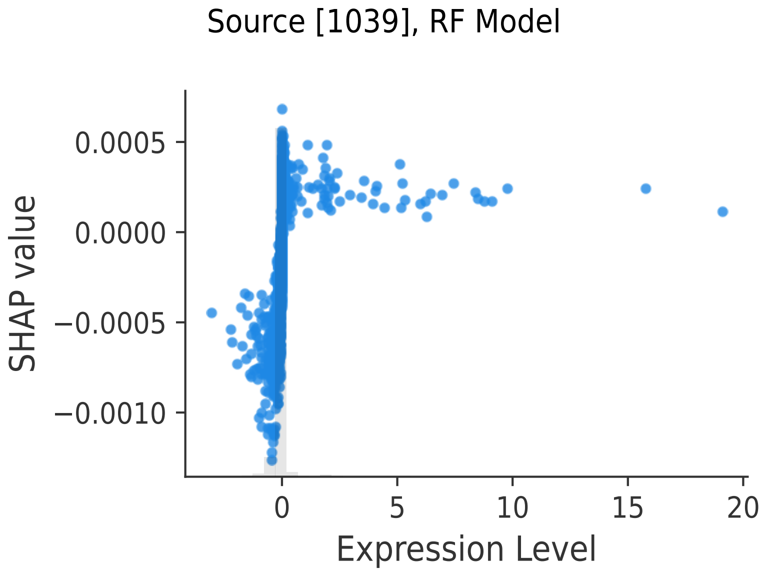
Source [1039], RF Model
0.0005
0.0000
−0.0005
−0.0010
SHAP value
0
5
10
15
20
Expression Level

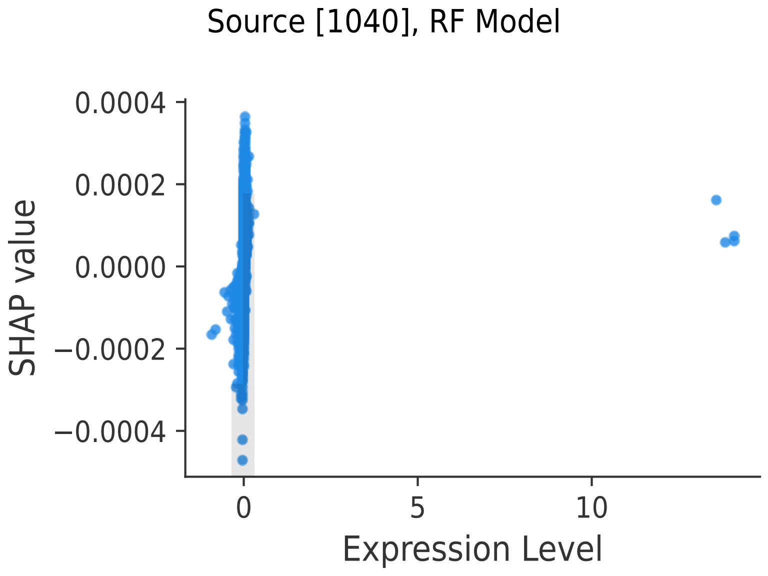

Source [1040], RF Model
0.0004
0.0002
0.0000
−0.0002
−0.0004
SHAP value
0
5
10
Expression Level

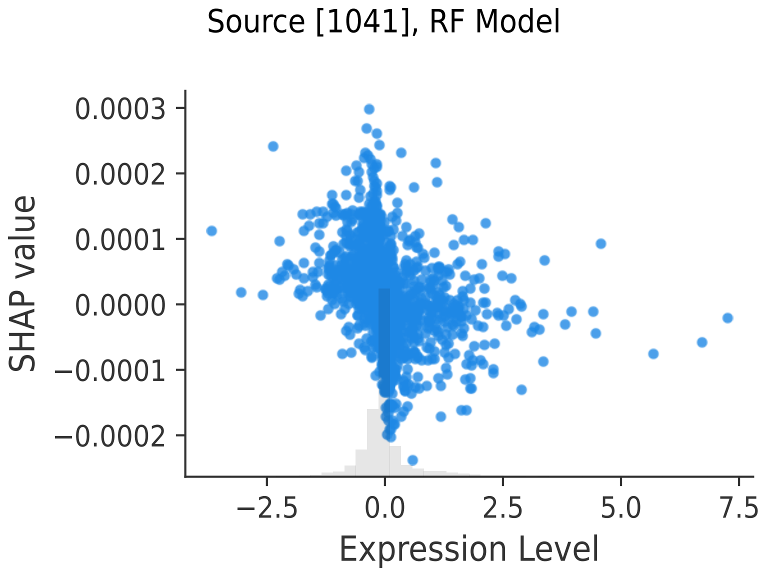
Source [1041], RF Model
SHAP value
0.0003
0.0002
0.0001
0.0000
−0.0001
−0.0002
−2.5
0.0
2.5
5.0
7.5
Expression Level

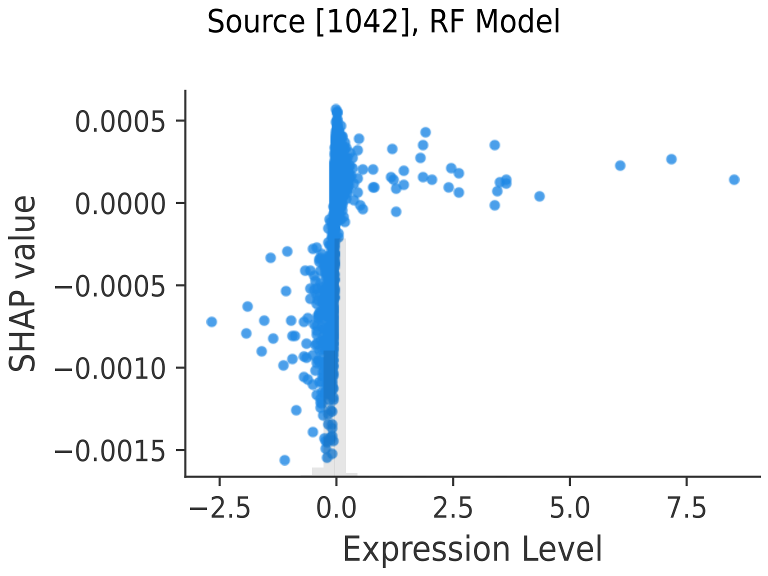
Source [1042], RF Model
SHAP value
0.0005
0.0000
−0.0005
−0.0010
−0.0015
−2.5
0.0
2.5
5.0
7.5
Expression Level

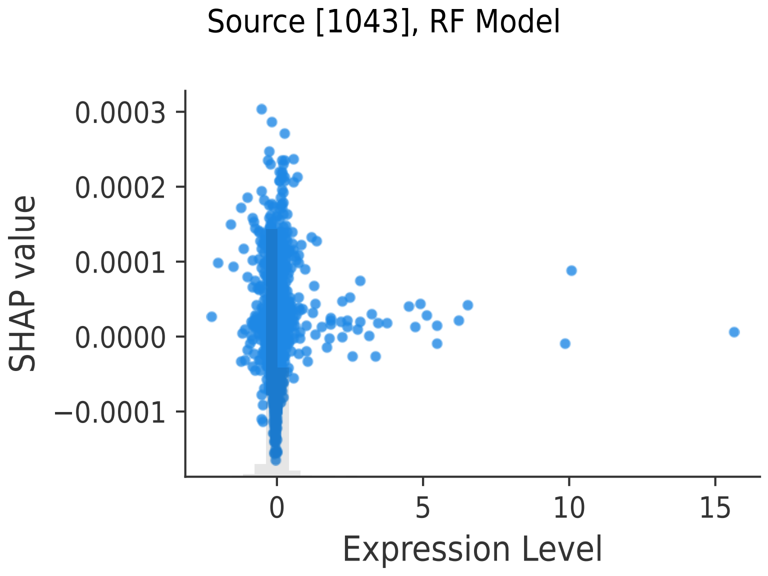
Source [1043], RF Model
SHAP value
0.0003
0.0002
0.0001
0.0000
−0.0001
0
5
10
15
Expression Level

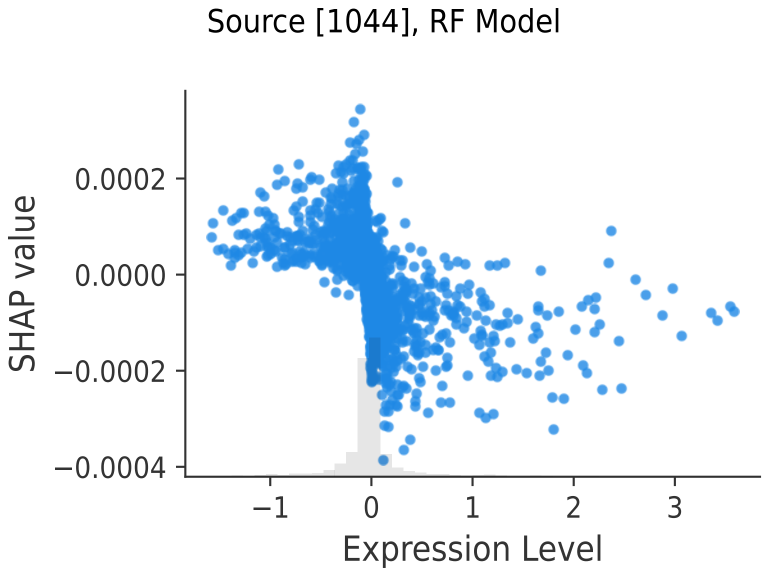
Source [1044], RF Model
SHAP value
0.0002
0.0000
−0.0002
−0.0004
−1
0
1
2
3
Expression Level

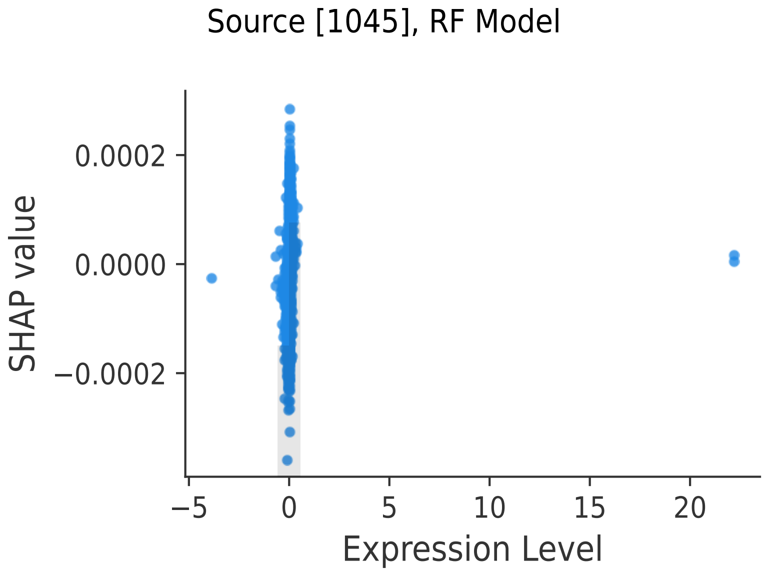

Source [1045], RF Model
SHAP value
0.0002
0.0000
−0.0002
−5
0
5
10
15
20
Expression Level

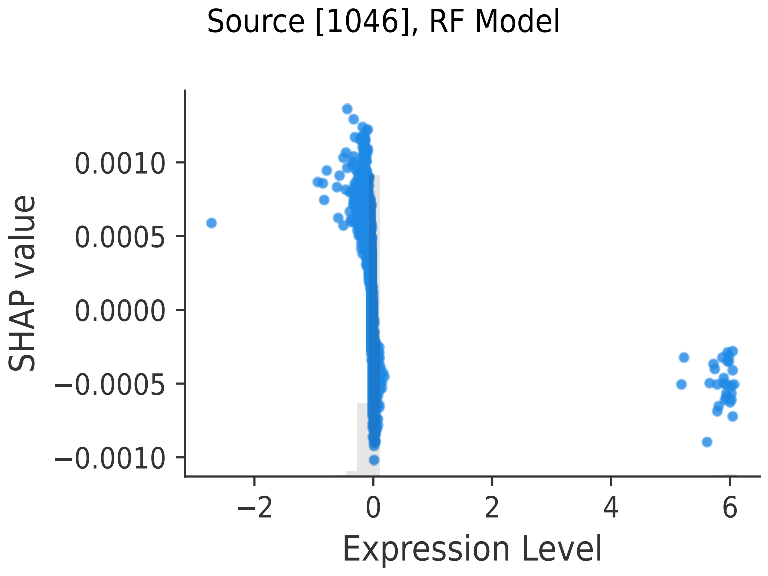
Source [1046], RF Model
SHAP value
0.0010
0.0005
0.0000
−0.0005
−0.0010
−2
0
2
4
6
Expression Level

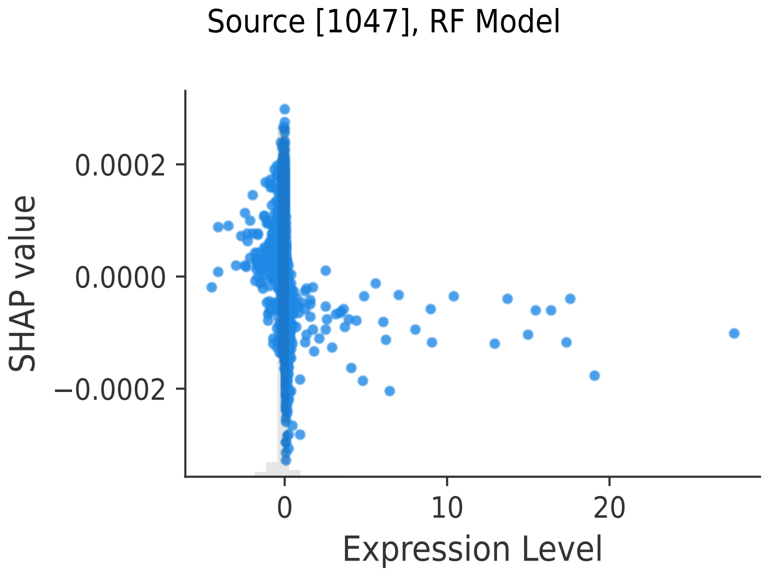

Source [1047], RF Model
0.0002
0.0000
−0.0002
SHAP value
0
10
20
Expression Level

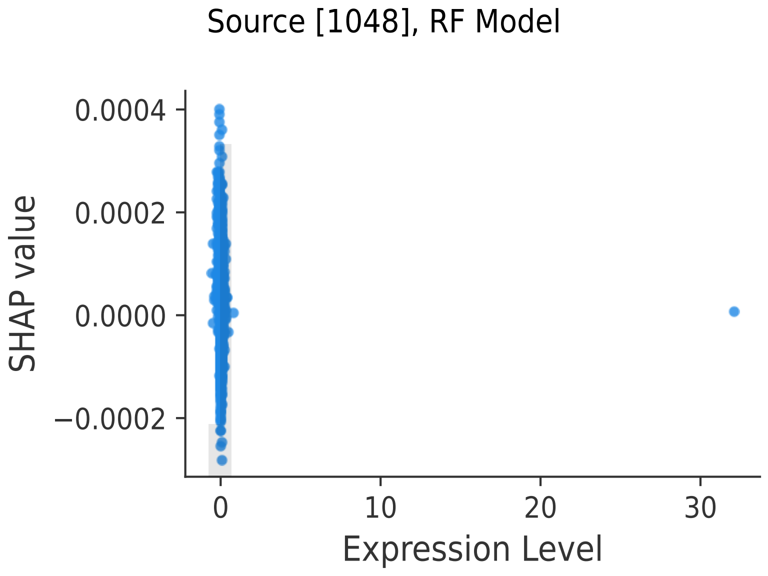

Source [1048], RF Model
SHAP value
0.0004
0.0002
0.0000
−0.0002
0
10
20
30
Expression Level

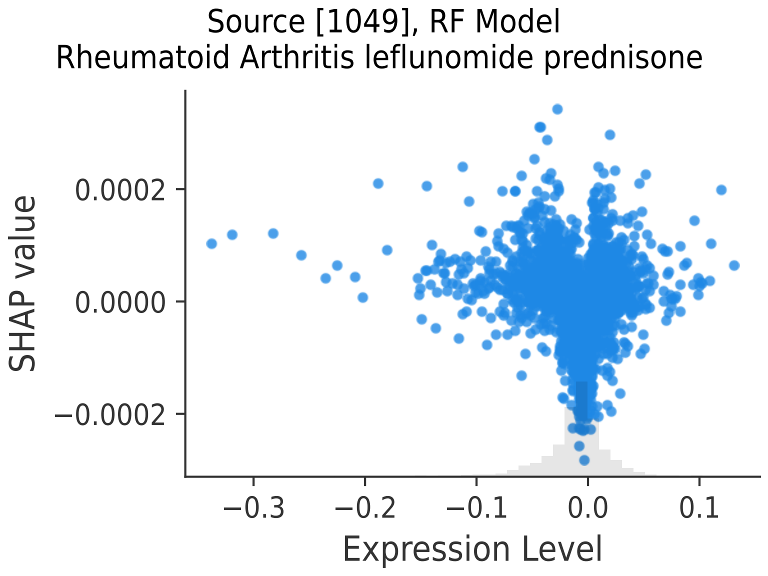
Source [1049], RF Model
Rheumatoid Arthritis leflunomide prednisone
SHAP value
0.0002
0.0000
−0.0002
−0.3
−0.2
−0.1
0.0
0.1
Expression Level

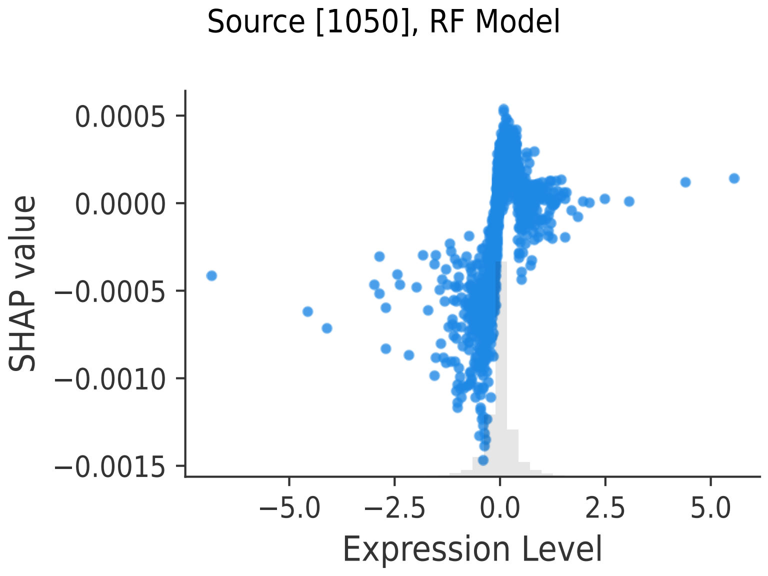

Source [1050], RF Model
0.0005
0.0000
−0.0005
−0.0010
−0.0015
SHAP value
−5.0
−2.5
0.0
2.5
5.0
Expression Level

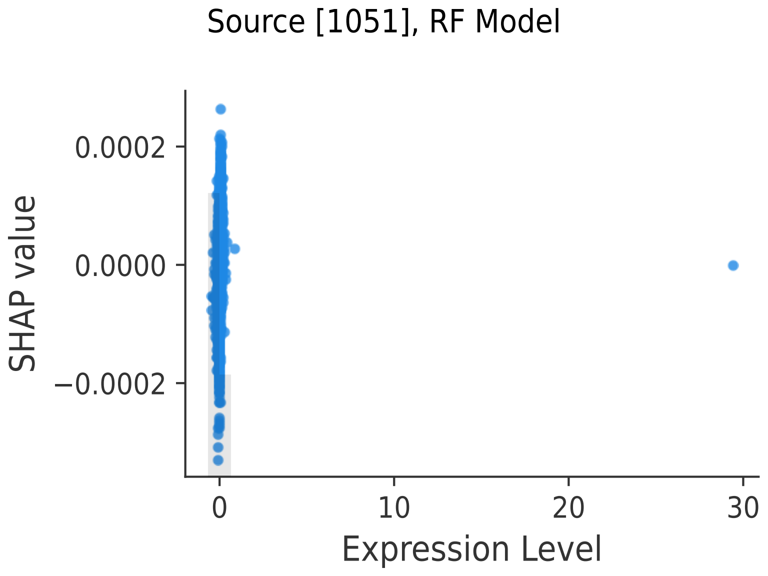

Source [1051], RF Model
SHAP value
0.0002
0.0000
−0.0002
0
10
20
30
Expression Level

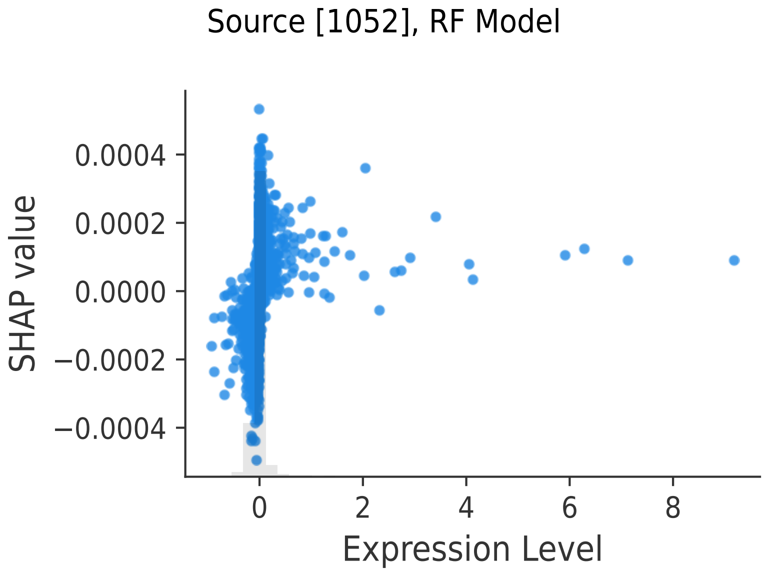
Source [1052], RF Model
SHAP value
0.0004
0.0002
0.0000
−0.0002
−0.0004
0
2
4
6
8
Expression Level

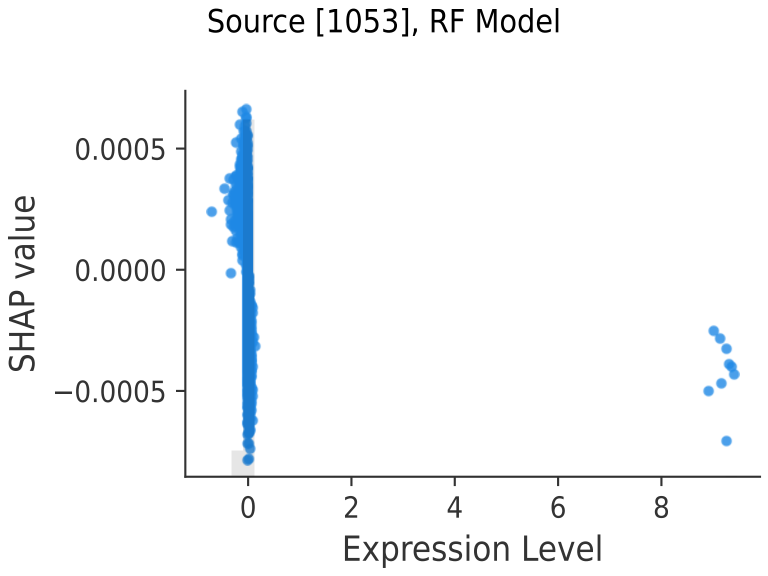

Source [1053], RF Model
SHAP value
0.0005
0.0000
−0.0005
0
2
4
6
8
Expression Level

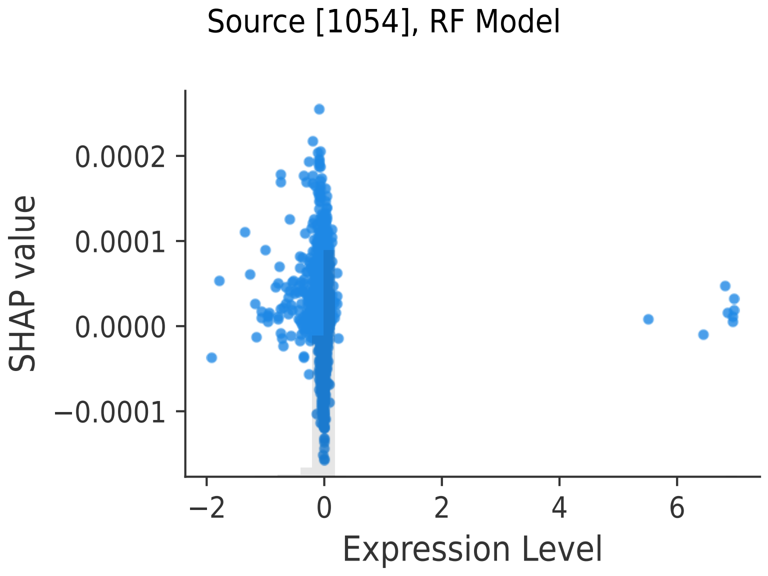
Source [1054], RF Model
SHAP value
0.0002
0.0001
0.0000
−0.0001
−2
0
2
4
6
Expression Level

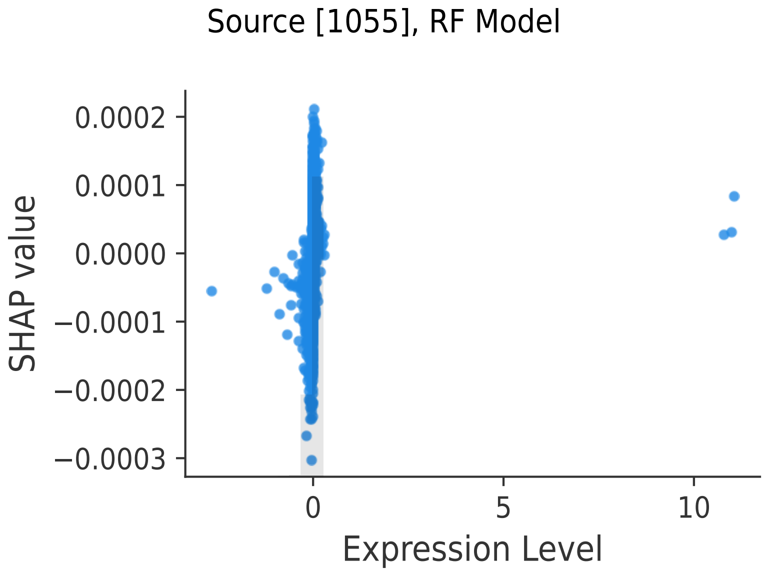
Source [1055], RF Model
SHAP value
0.0002
0.0001
0.0000
−0.0001
−0.0002
−0.0003
0
5
10
Expression Level

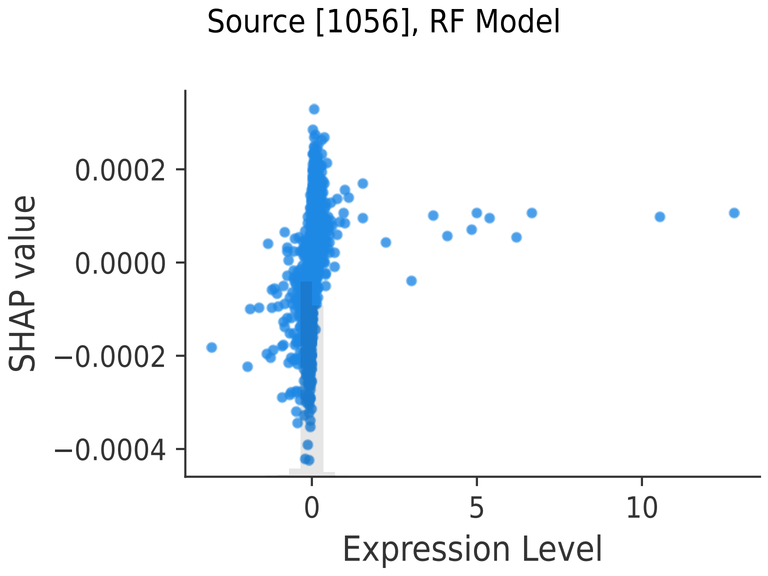
Source [1056], RF Model
SHAP value
0.0002
0.0000
−0.0002
−0.0004
0
5
10
Expression Level

Source [1057], RF Model

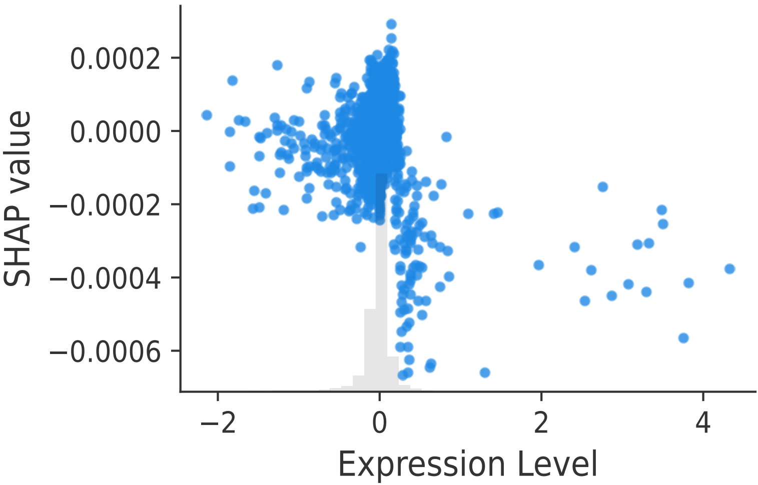

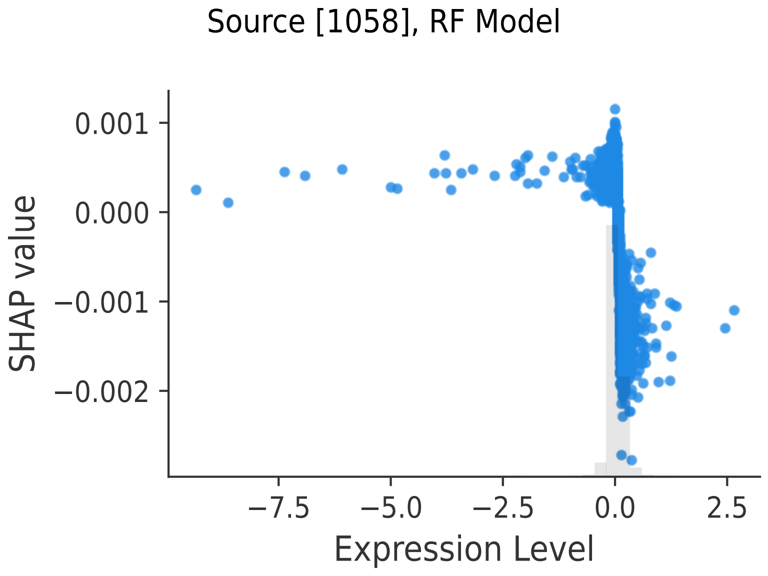

Source [1058], RF Model
0.001
0.000
−0.001
−0.002
SHAP value
−7.5
−5.0
−2.5
0.0
2.5
Expression Level

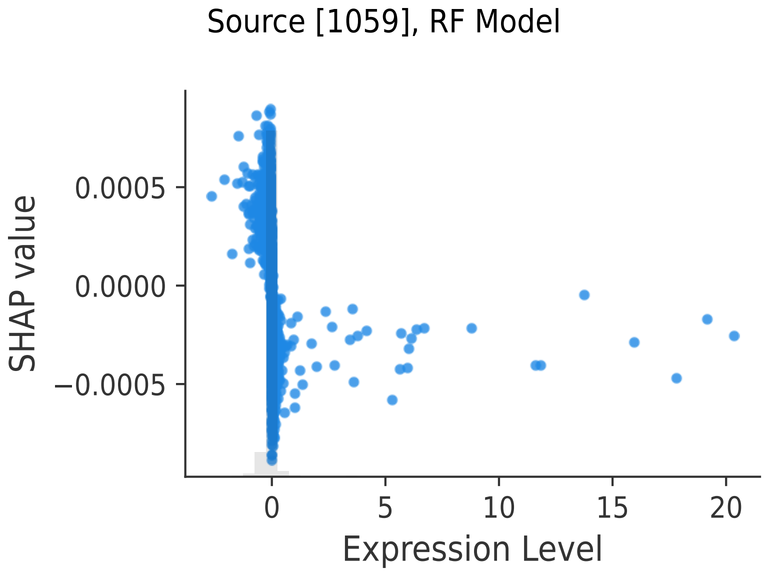

Source [1059], RF Model
SHAP value
0.0005
0.0000
−0.0005
0
5
10
15
20
Expression Level

Source [1060], RF Model

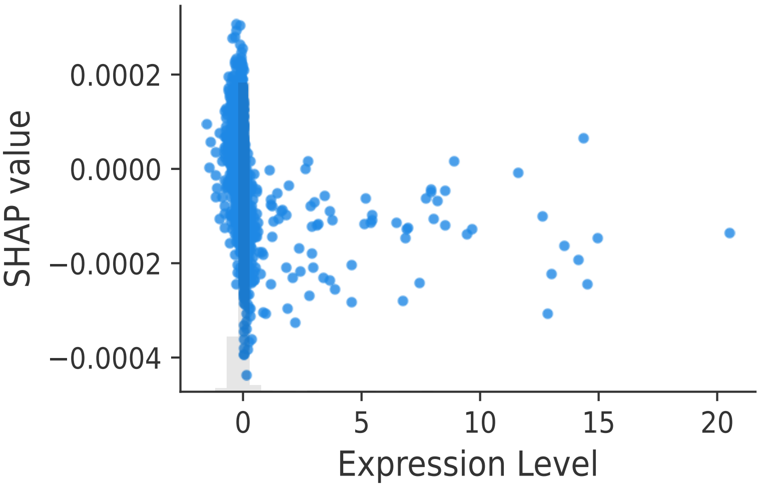

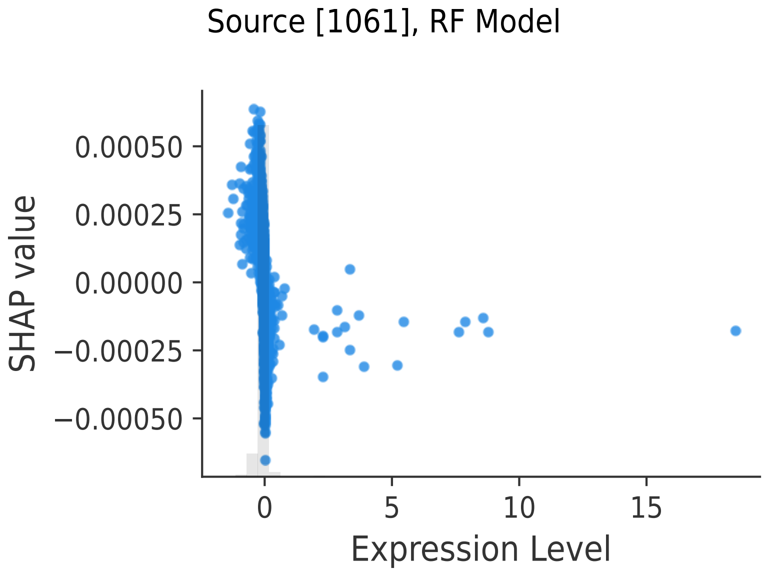

Source [1061], RF Model
SHAP value
0.00050
0.00025
0.00000
−0.00025
−0.00050
0
5
10
15
Expression Level

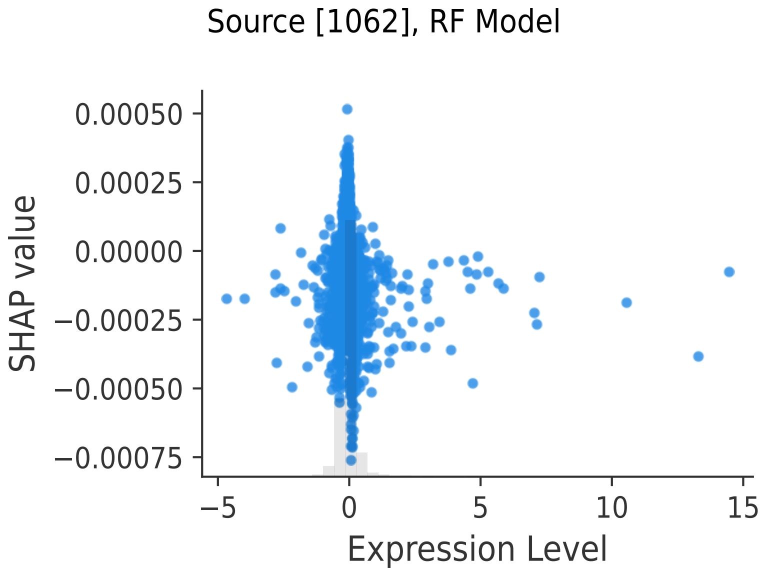
Source [1062], RF Model
SHAP value
0.00050
0.00025
0.00000
−0.00025
−0.00050
−0.00075
−5
0
5
10
15
Expression Level

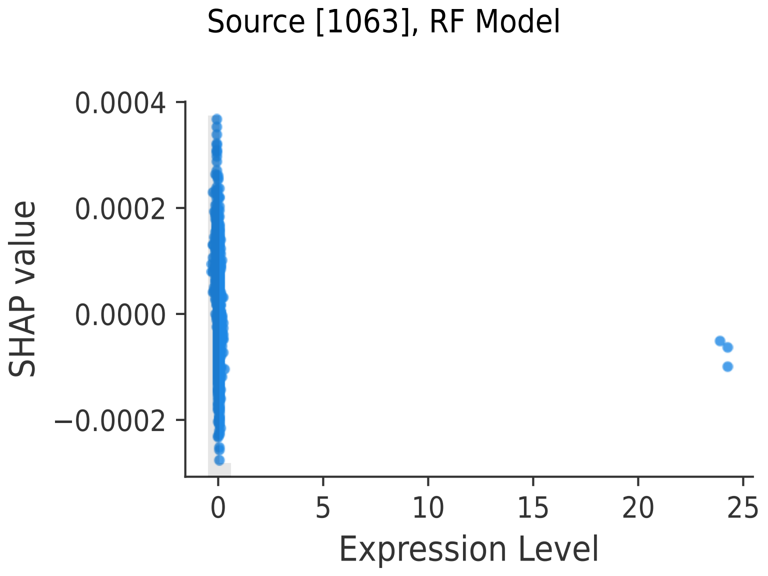
Source [1063], RF Model
SHAP value
0.0004
0.0002
0.0000
−0.0002
0
5
10
15
20
25
Expression Level

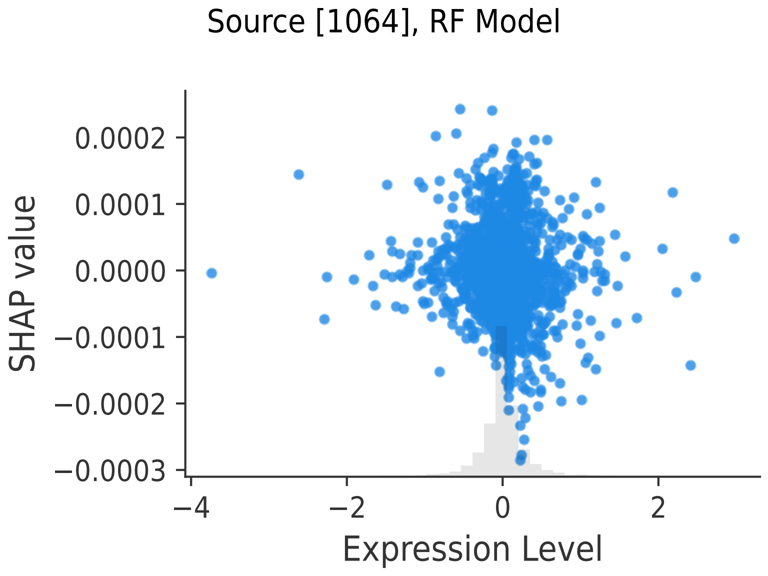
Source [1064], RF Model
SHAP value
0.0002
0.0001
0.0000
−0.0001
−0.0002
−0.0003
−4
−2
0
2
Expression Level

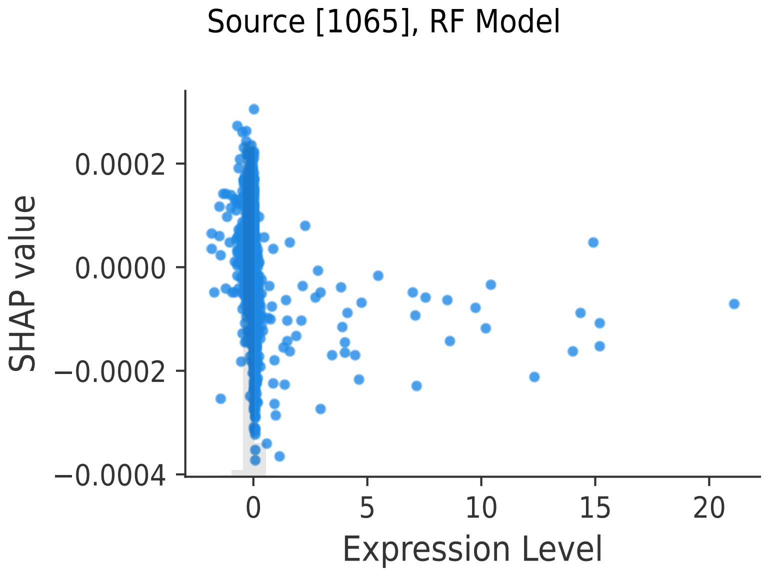

Source [1065], RF Model
SHAP value
0.0002
0.0000
−0.0002
−0.0004
0
5
10
15
20
Expression Level

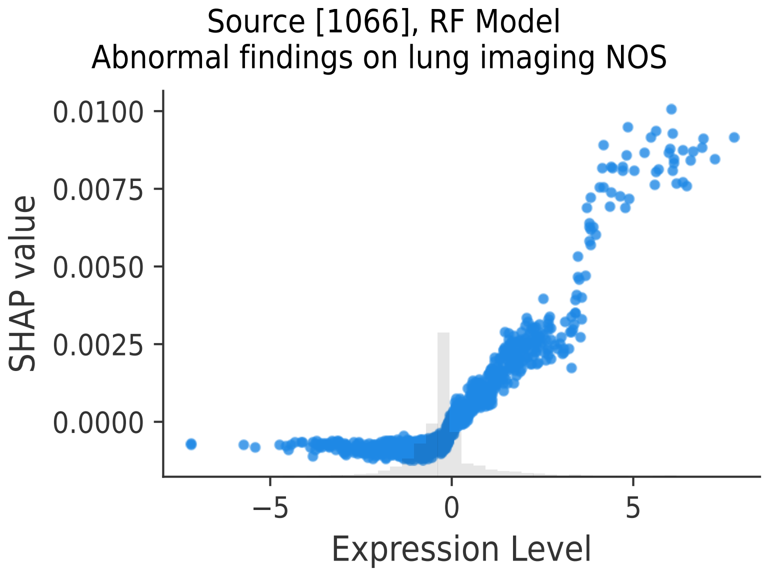
Source [1066], RF Model
Abnormal findings on lung imaging NOS
SHAP value
0.0100
0.0075
0.0050
0.0025
0.0000
−5
0
5
Expression Level

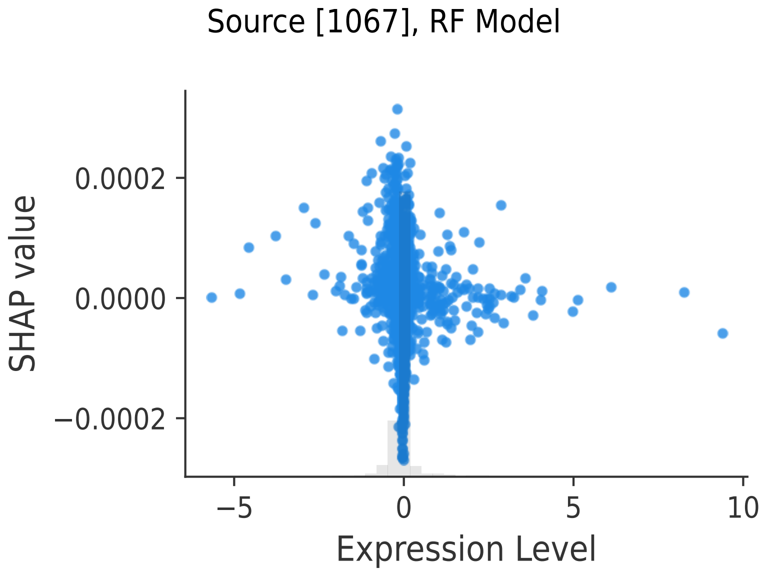

Source [1067], RF Model
SHAP value
0.0002
0.0000
−0.0002
−5
0
5
10
Expression Level

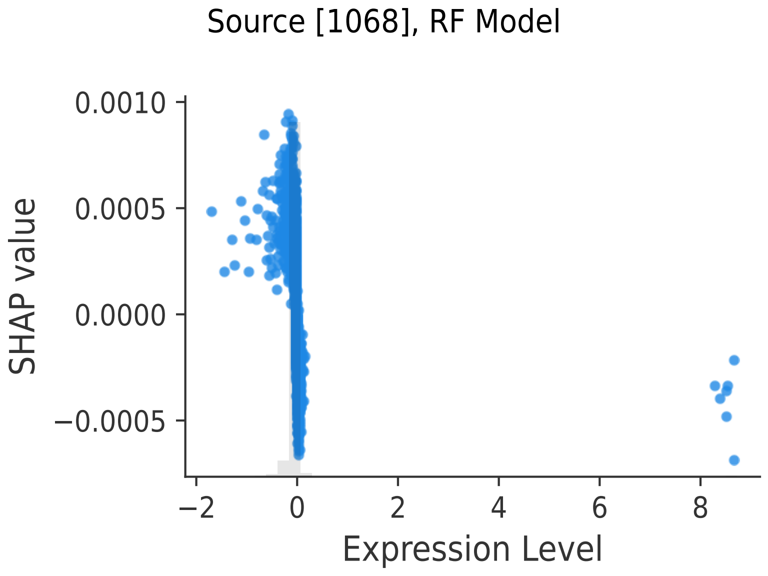
Source [1068], RF Model
SHAP value
0.0010
0.0005
0.0000
−0.0005
−2
0
2
4
6
8
Expression Level

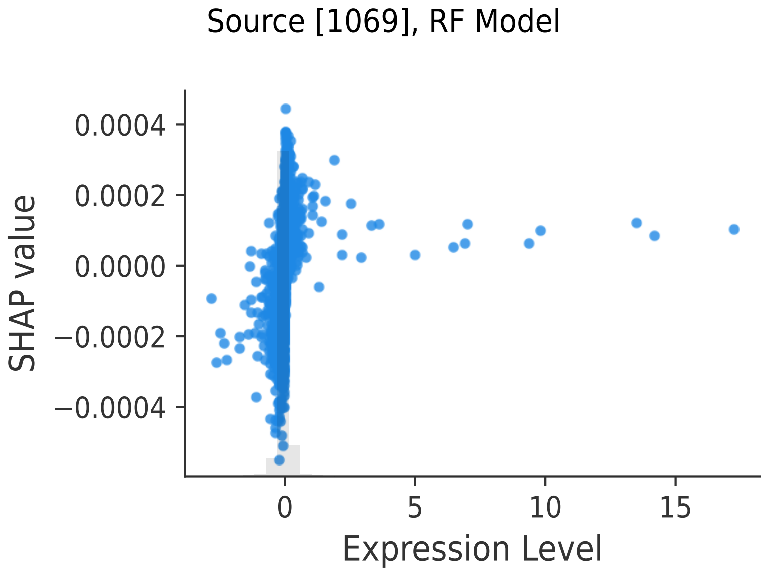

Source [1069], RF Model
0.0004
0.0002
0.0000
−0.0002
−0.0004
SHAP value
0
5
10
15
Expression Level

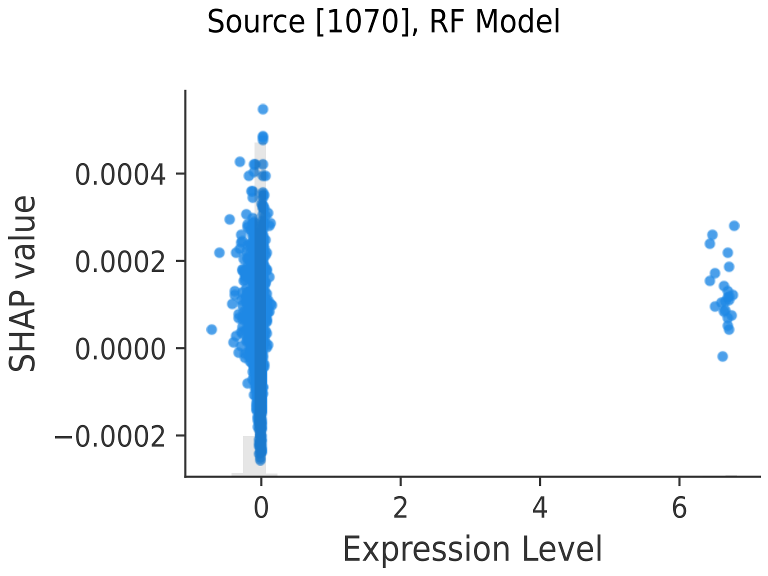

Source [1070], RF Model
SHAP value
0.0004
0.0002
0.0000
−0.0002
0
2
4
6
Expression Level

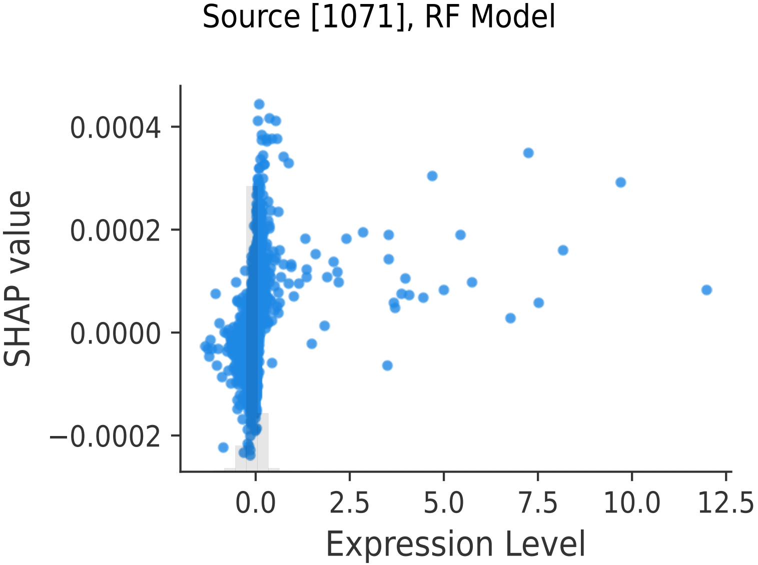

Source [1071], RF Model
SHAP value
0.0004
0.0002
0.0000
−0.0002
0.0
2.5
5.0
7.5
10.0
12.5
Expression Level

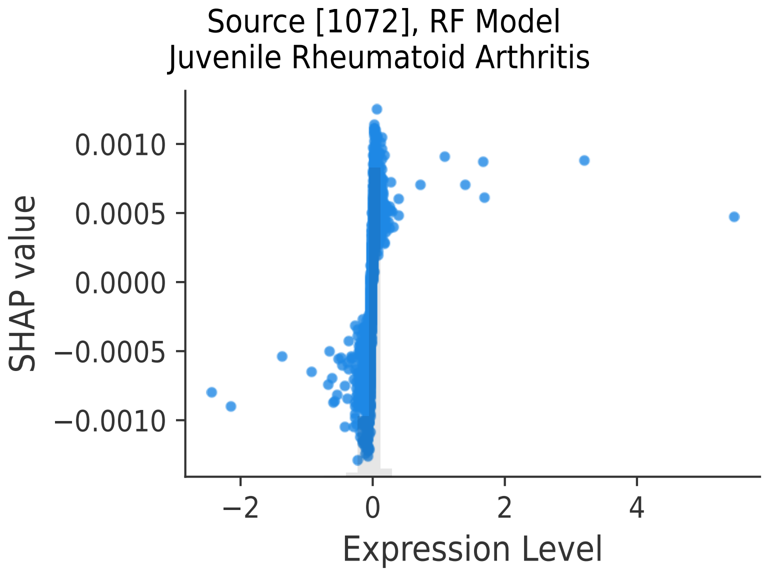

Source [1072], RF Model
Juvenile Rheumatoid Arthritis
SHAP value
0.0010
0.0005
0.0000
−0.0005
−0.0010
−2
0
2
4
Expression Level

Source [1073], RF Model

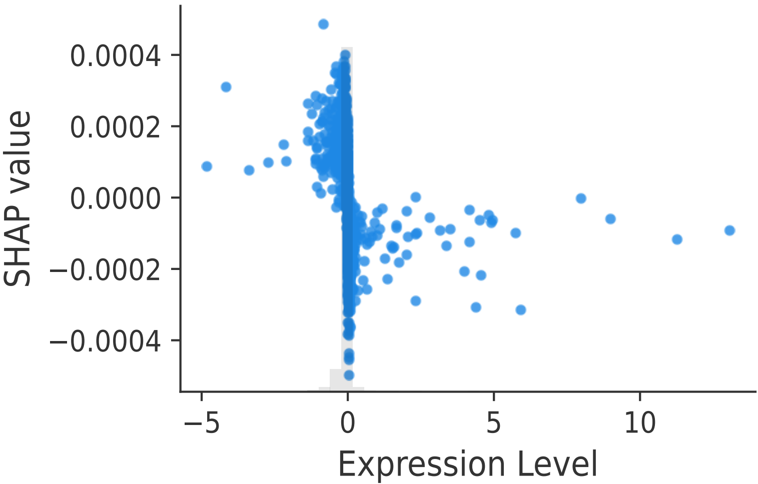

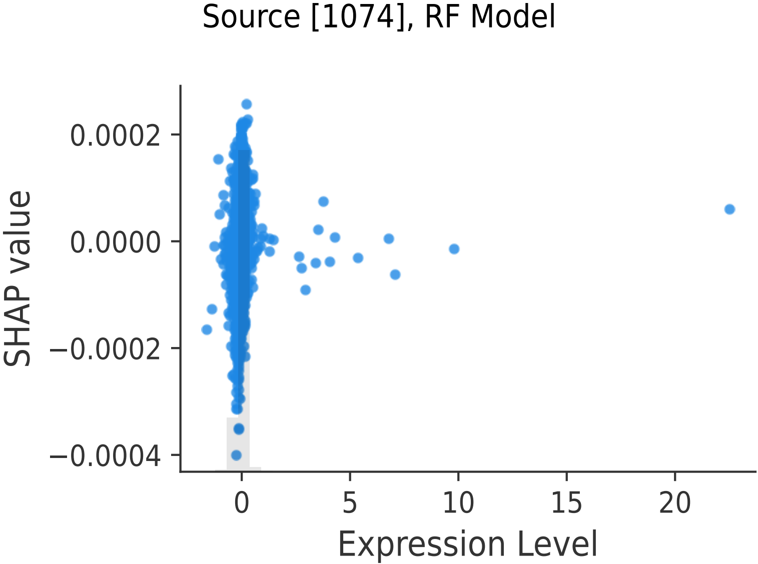
Source [1074], RF Model
0.0002
0.0000
−0.0002
−0.0004
SHAP value
0
5
10
15
20
Expression Level

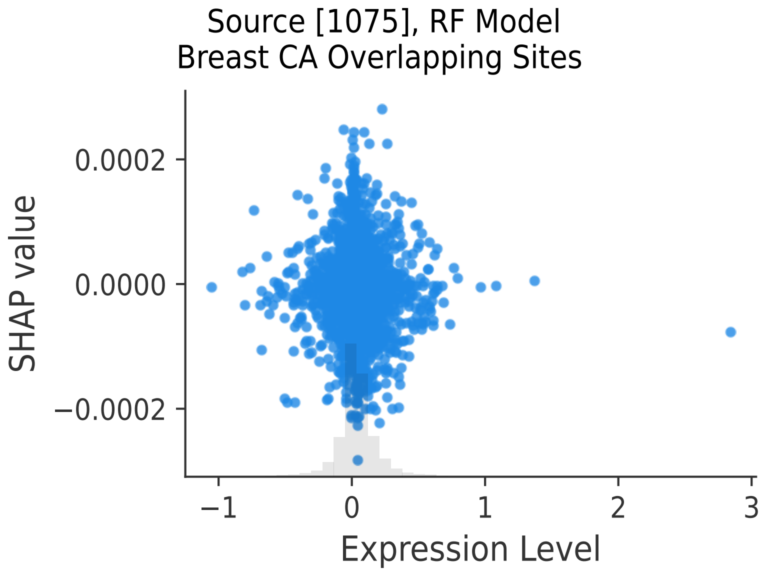

Source [1075], RF Model
Breast CA Overlapping Sites
0.0002
0.0000
−0.0002
SHAP value
−1
0
1
2
3
Expression Level

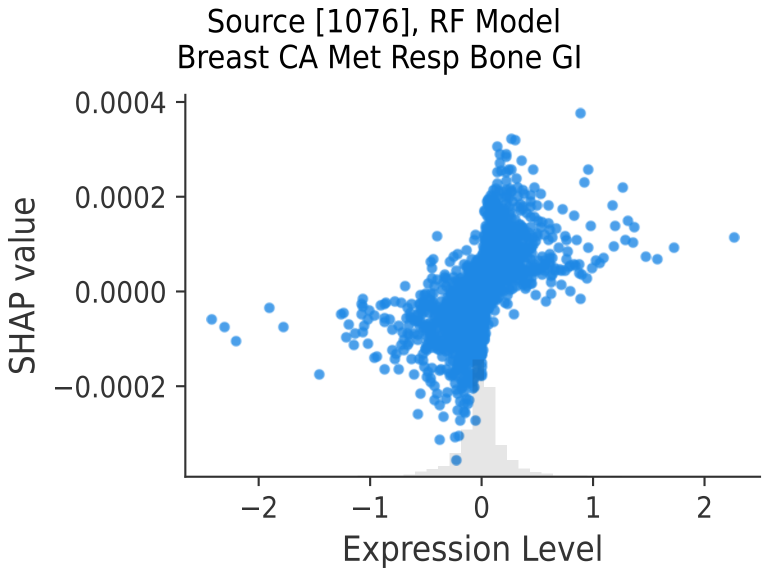

Source [1076], RF Model
Breast CA Met Resp Bone GI
0.0004
0.0002
0.0000
−0.0002
SHAP value
−2
−1
0
1
2
Expression Level

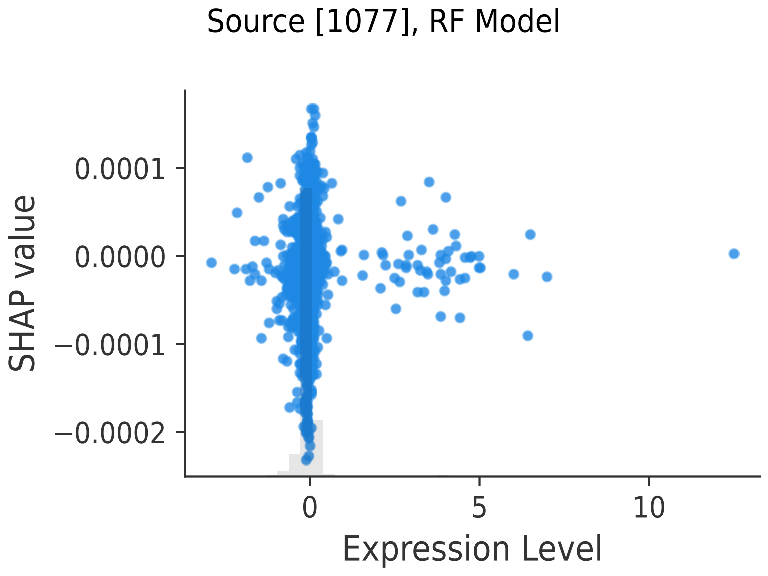
Source [1077], RF Model
0.0001
0.0000
−0.0001
−0.0002
SHAP value
0
5
10
Expression Level

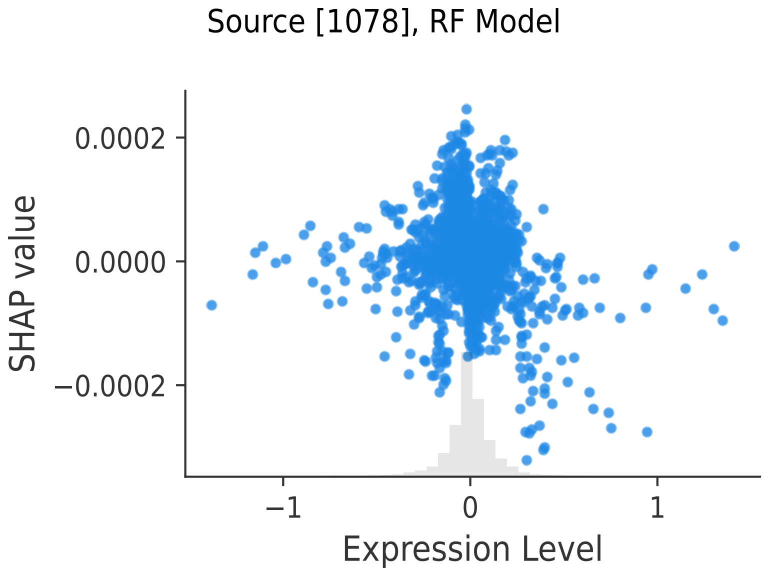
Source [1078], RF Model
0.0002
0.0000
−0.0002
SHAP value
−1
0
1
Expression Level

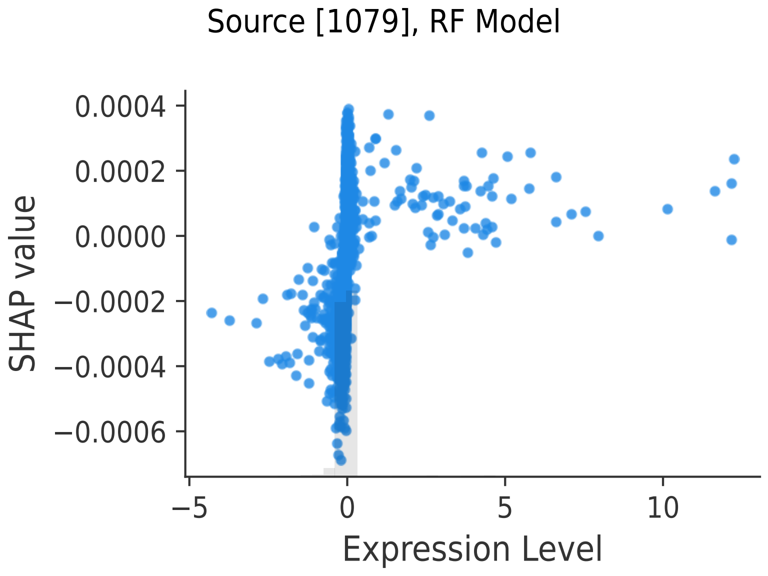
Source [1079], RF Model
SHAP value
0.0004
0.0002
0.0000
−0.0002
−0.0004
−0.0006
−5
0
5
10
Expression Level

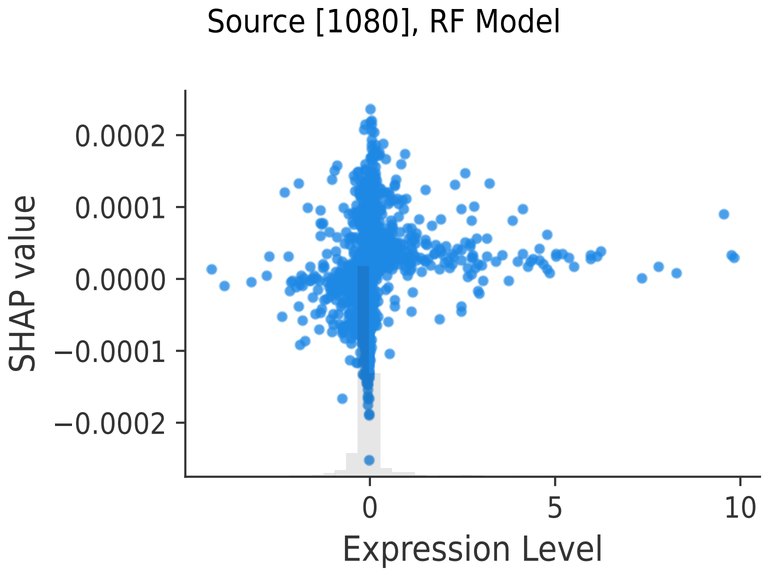

Source [1080], RF Model
SHAP value
0.0002
0.0001
0.0000
−0.0001
−0.0002
Expression Level
0
5
10

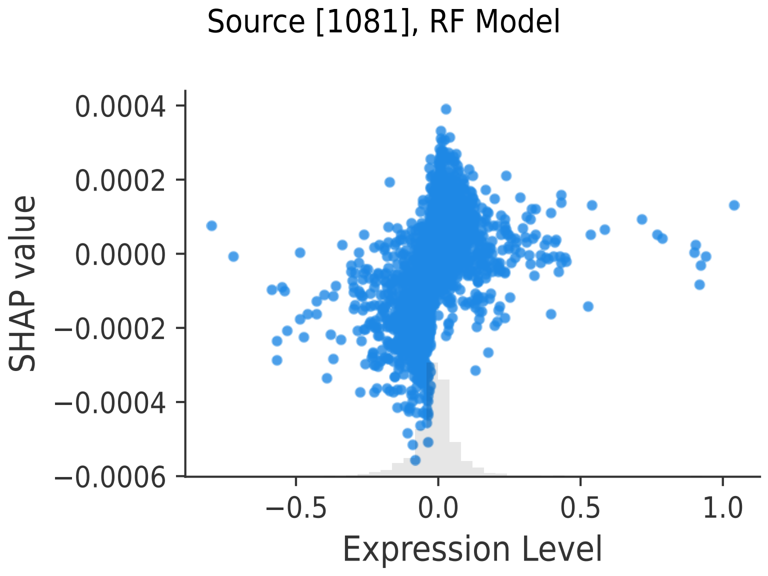
Source [1081], RF Model
0.0004
0.0002
0.0000
−0.0002
−0.0004
−0.0006
SHAP value
−0.5
0.0
0.5
1.0
Expression Level

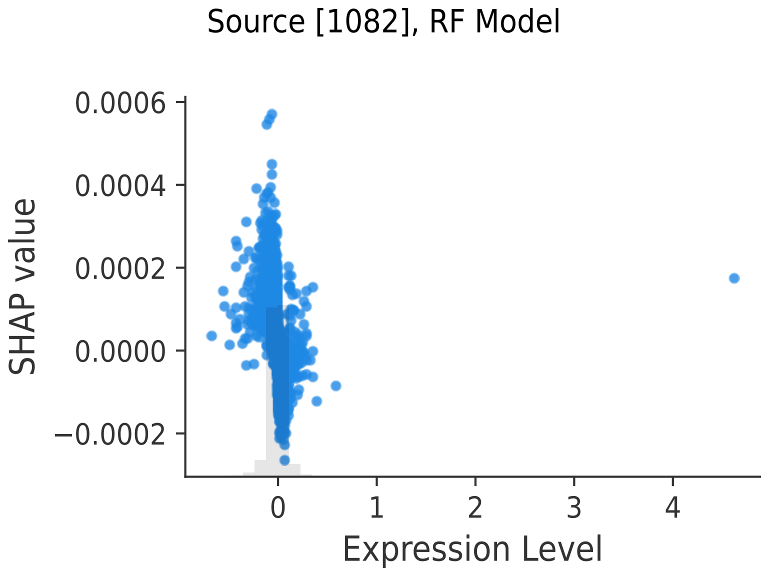
Source [1082], RF Model
SHAP value
0.0006
0.0004
0.0002
0.0000
−0.0002
0
1
2
3
4
Expression Level

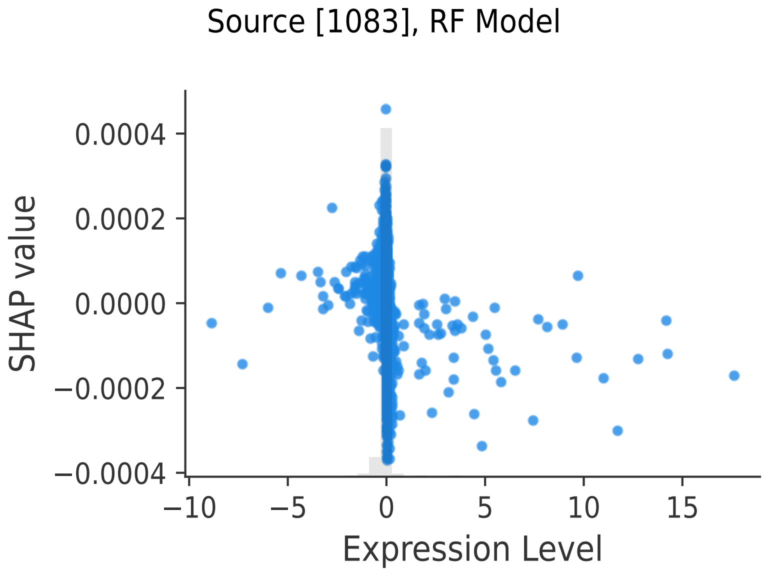
Source [1083], RF Model
SHAP value
0.0004
0.0002
0.0000
−0.0002
−0.0004
−10
−5
0
5
10
15
Expression Level

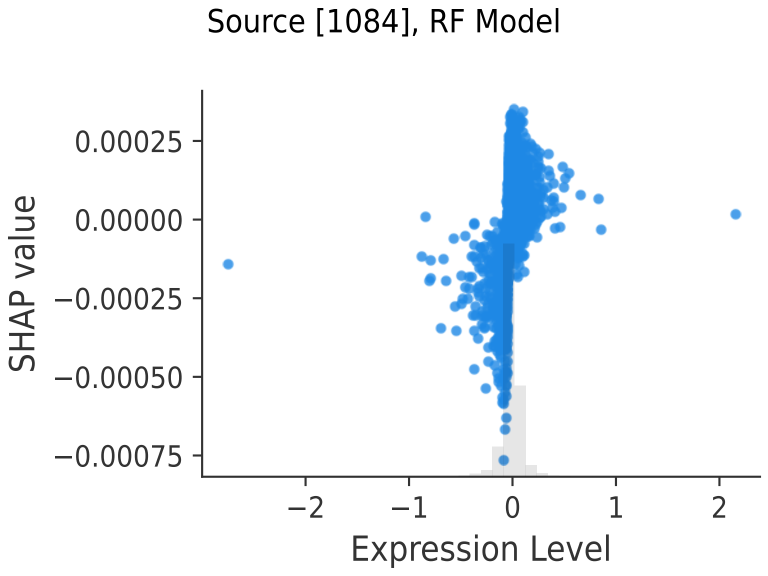

Source [1084], RF Model
0.00025
0.00000
−0.00025
−0.00050
−0.00075
SHAP value
−2
−1
0
1
2
Expression Level

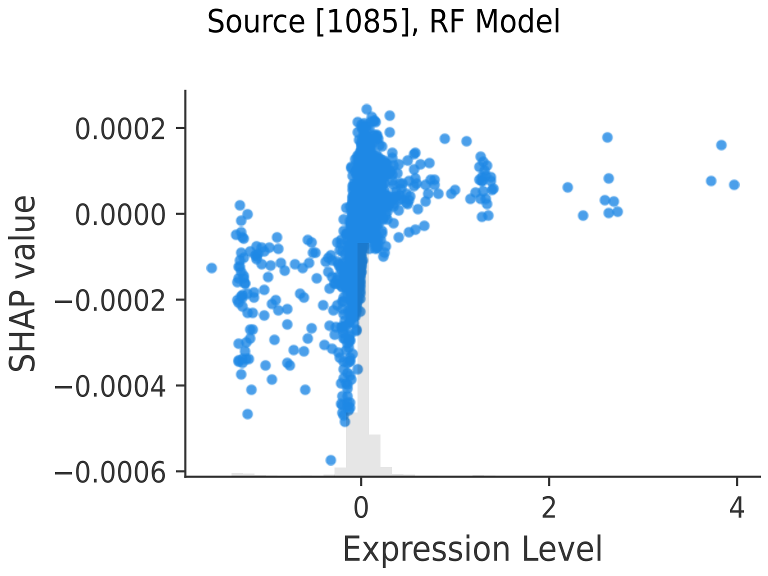
Source [1085], RF Model
0.0002
0.0000
−0.0002
−0.0004
−0.0006
SHAP value
0
2
4
Expression Level

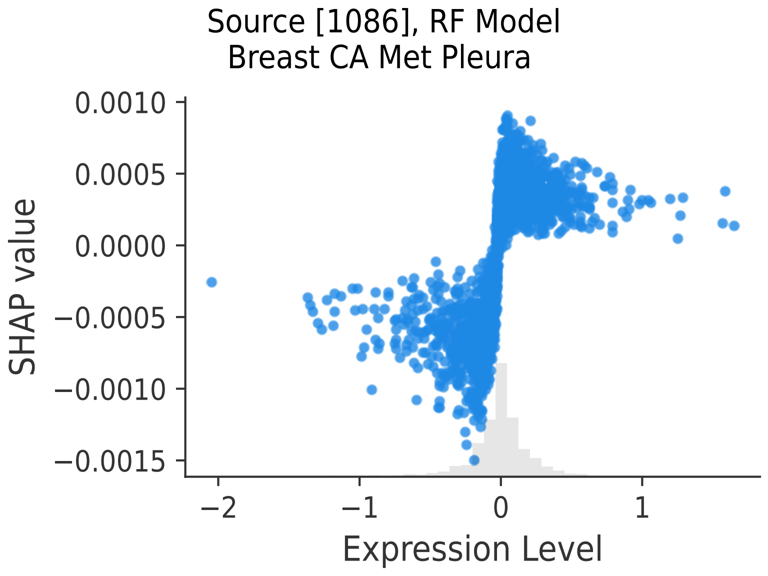
Source [1086], RF Model
Breast CA Met Pleura
SHAP value
0.0010
0.0005
0.0000
−0.0005
−0.0010
−0.0015
−2
−1
0
1
Expression Level

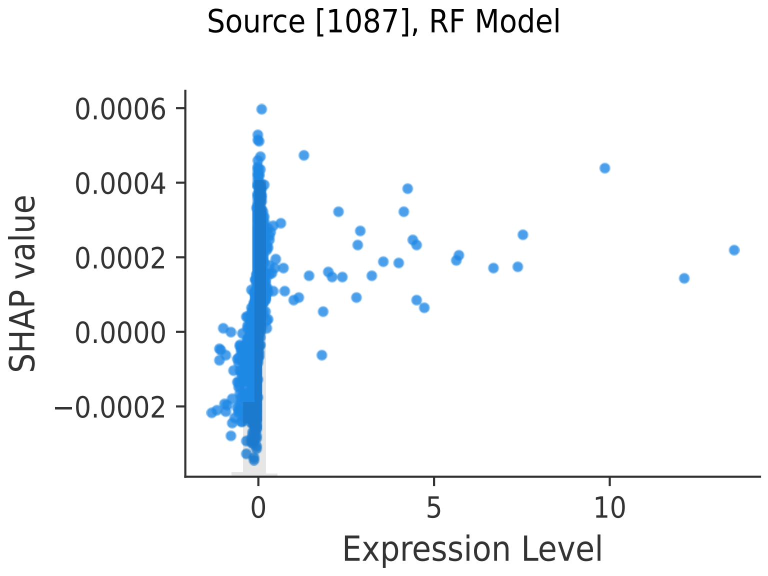

Source [1087], RF Model
0.0006
0.0004
0.0002
0.0000
−0.0002
SHAP value
0
5
10
Expression Level

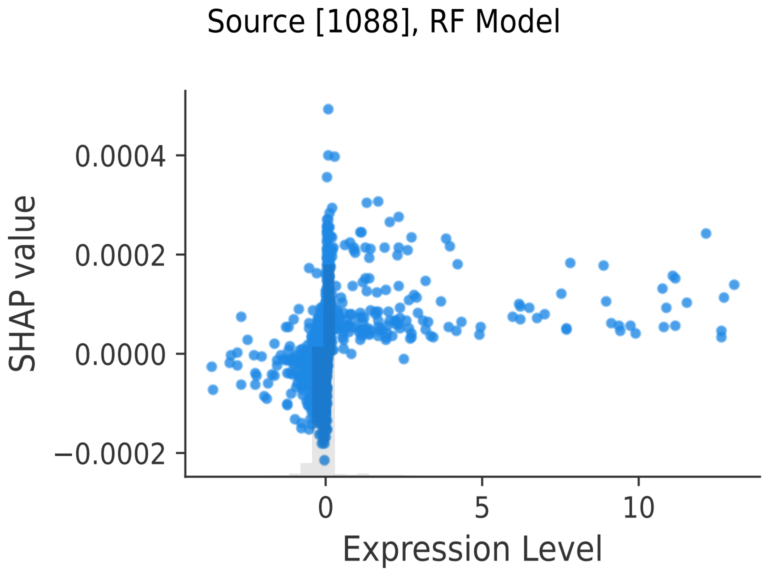
Source [1088], RF Model
0.0004
0.0002
0.0000
−0.0002
SHAP value
0
5
10
Expression Level

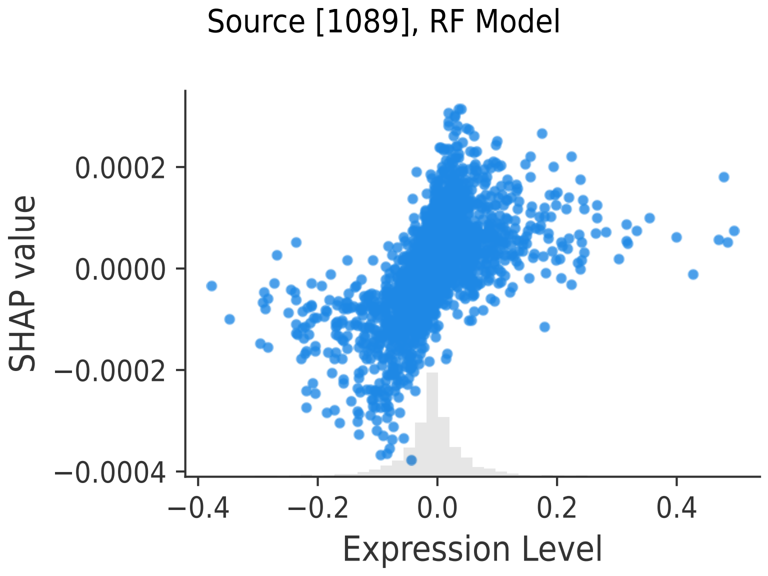
Source [1089], RF Model
SHAP value
0.0002
0.0000
−0.0002
−0.0004
−0.4
−0.2
0.0
0.2
0.4
Expression Level

Source [1090], RF Model

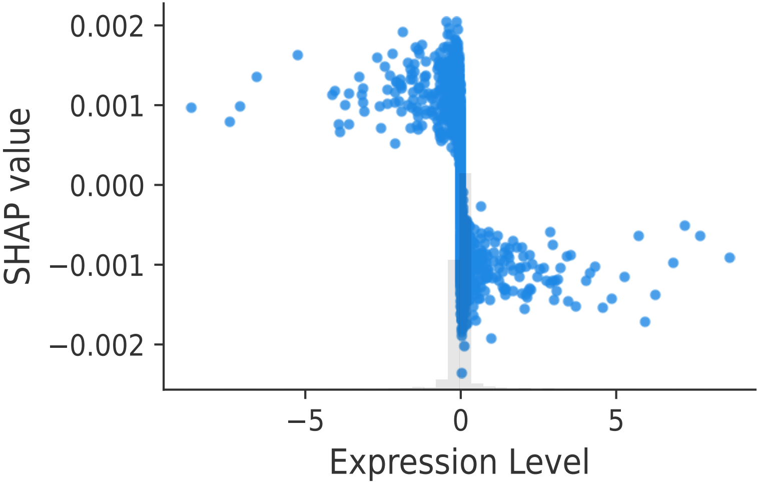

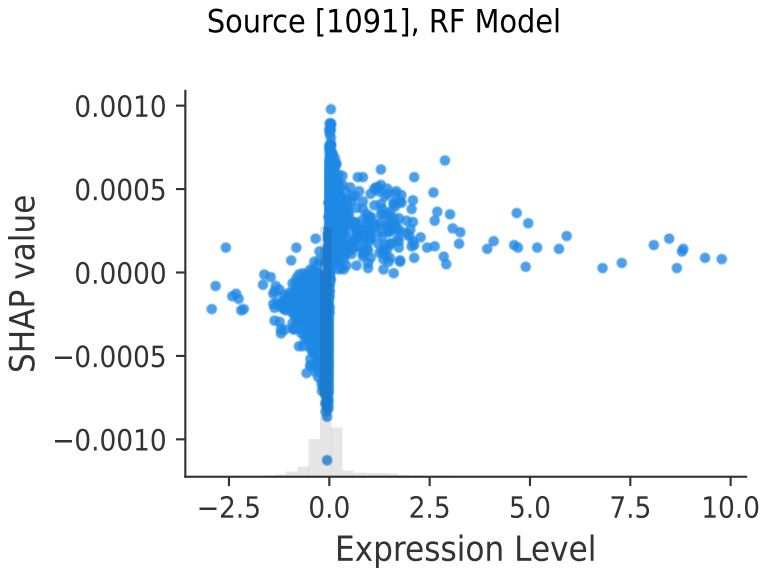

Source [1091], RF Model
SHAP value
0.0010
0.0005
0.0000
−0.0005
−0.0010
−2.5
0.0
2.5
5.0
7.5
10.0
Expression Level

Source [1092], RF Model

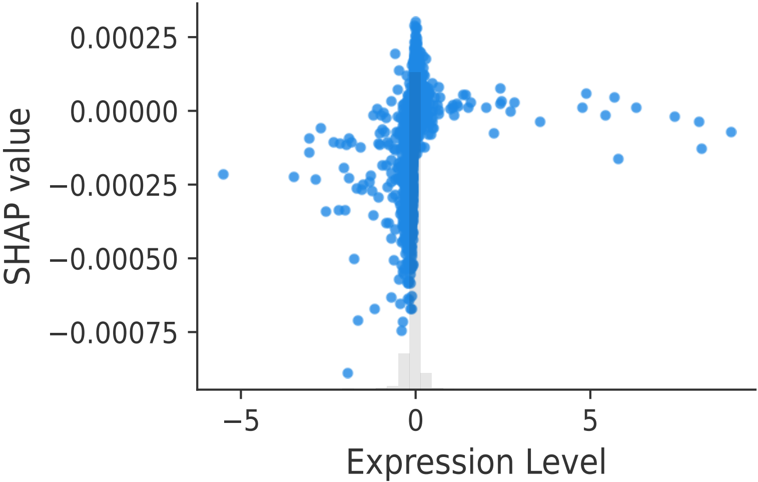

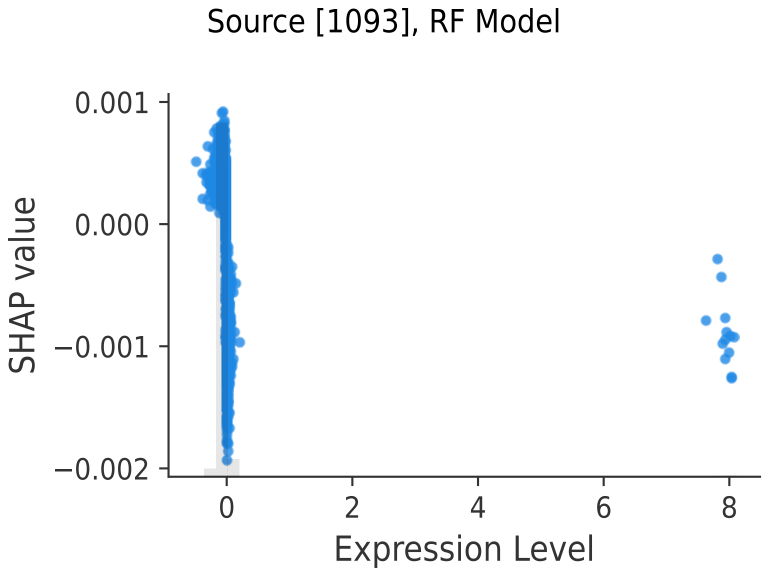
Source [1093], RF Model
SHAP value
0.001
0.000
−0.001
−0.002
0
2
4
6
8
Expression Level

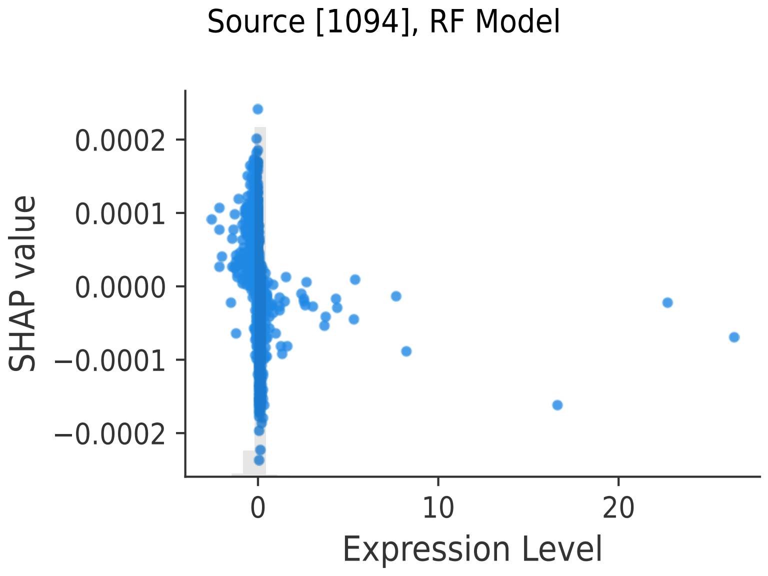

Source [1094], RF Model
0.0002
0.0001
0.0000
−0.0001
−0.0002
SHAP value
0
10
20
Expression Level

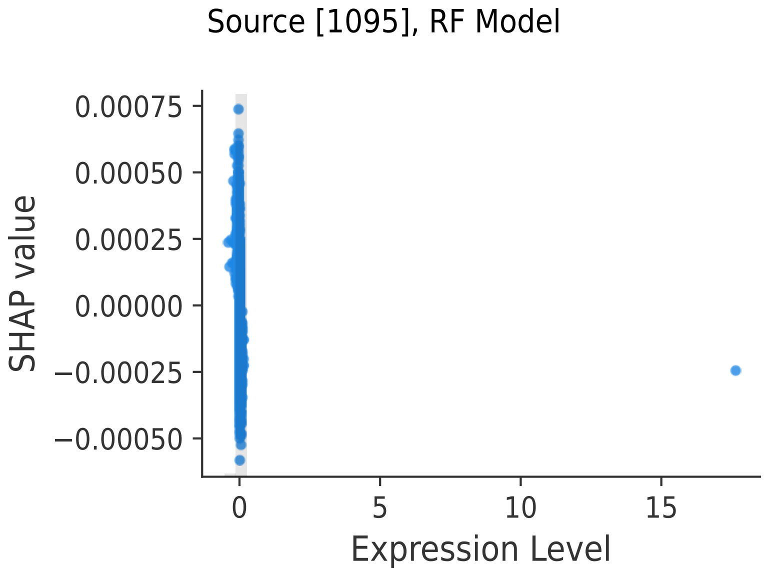

Source [1095], RF Model
0.00075
0.00050
0.00025
0.00000
−0.00025
−0.00050
SHAP value
0
5
10
15
Expression Level

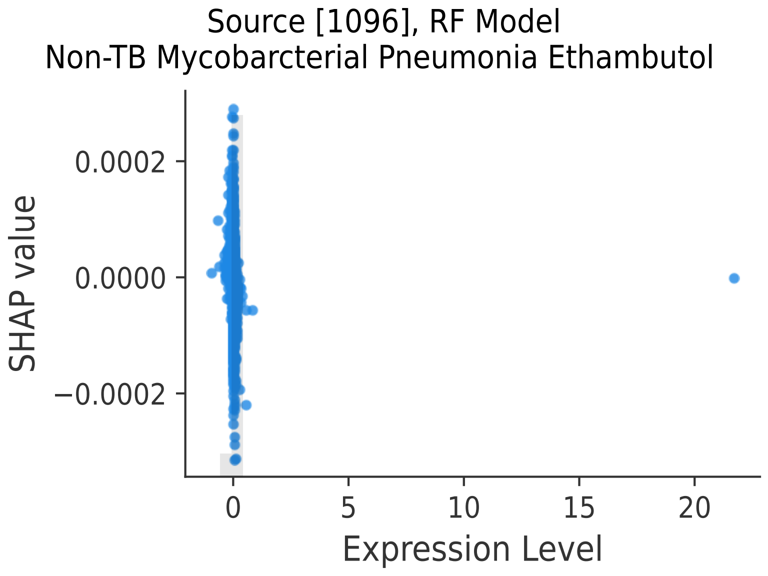
Source [1096], RF Model
Non-TB Mycobarcterial Pneumonia Ethambutol
0.0002
0.0000
−0.0002
SHAP value
0
5
10
15
20
Expression Level

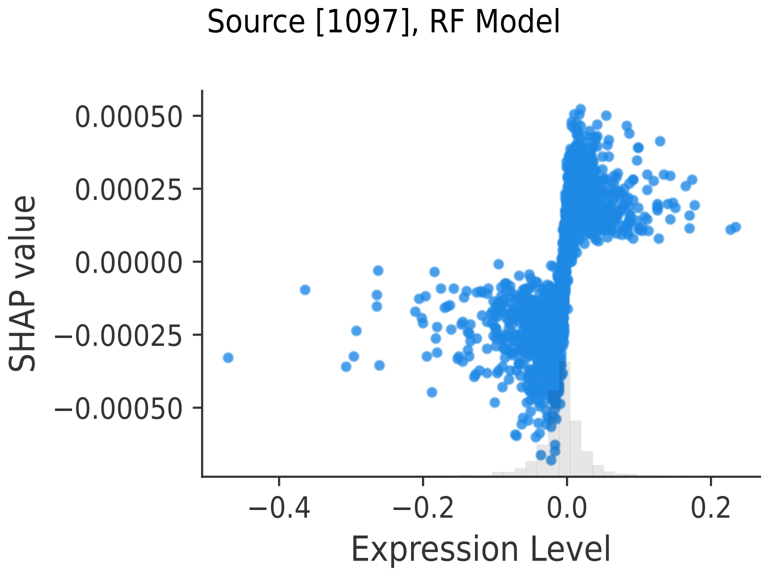
Source [1097], RF Model
SHAP value
0.00050
0.00025
0.00000
−0.00025
−0.00050
−0.4
−0.2
0.0
0.2
Expression Level

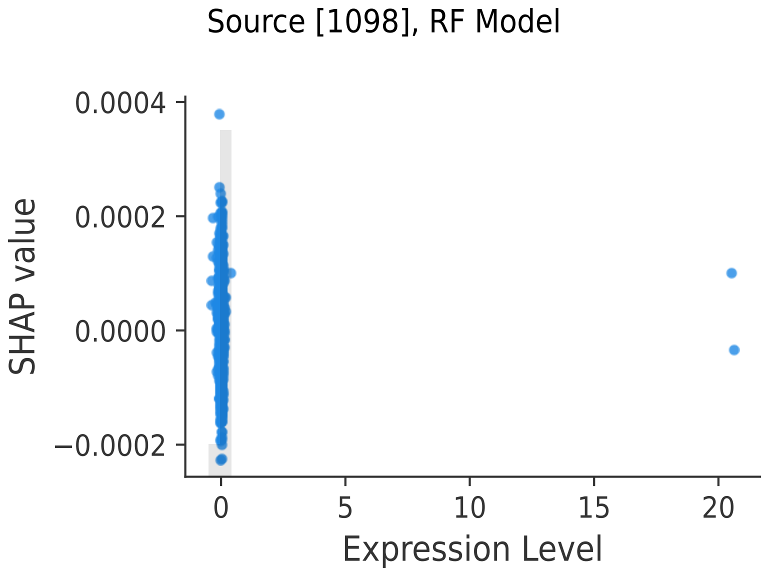

Source [1098], RF Model
0.0004
0.0002
0.0000
−0.0002
SHAP value
0
5
10
15
20
Expression Level

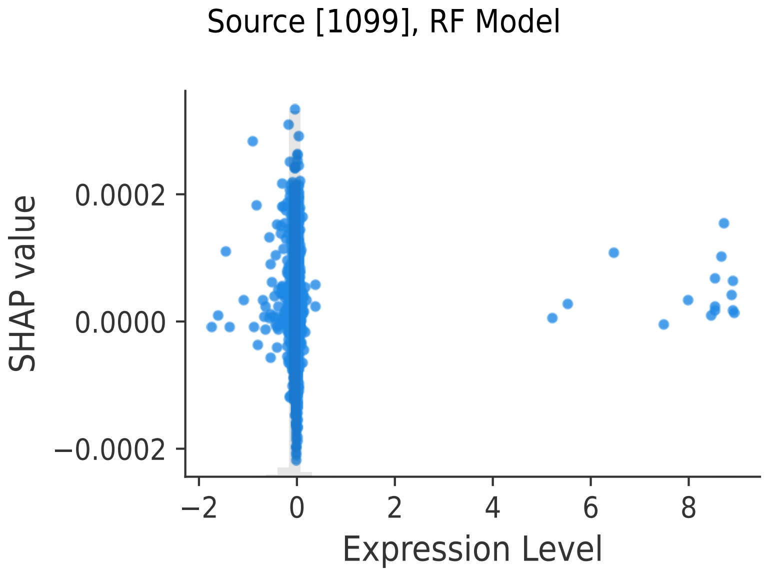
Source [1099], RF Model
SHAP value
0.0002
0.0000
−0.0002
−2
0
2
4
6
8
Expression Level

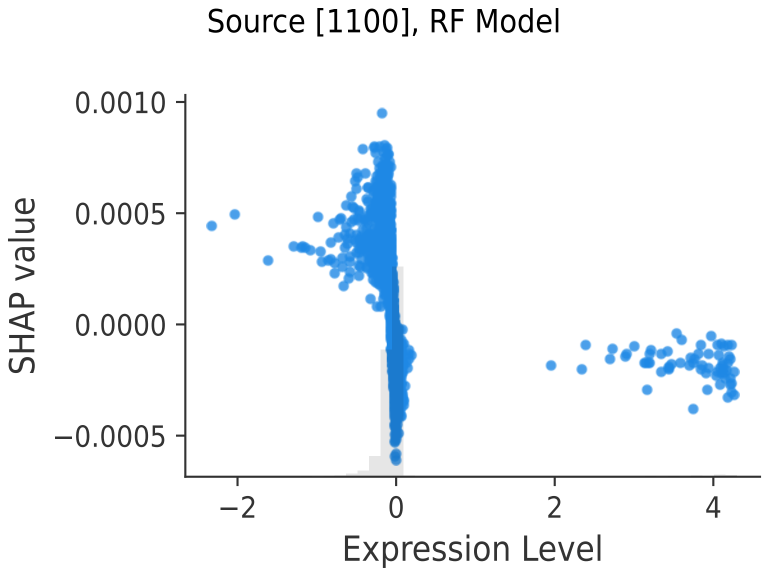

Source [1100], RF Model
0.0010
0.0005
0.0000
−0.0005
SHAP value
−2
0
2
4
Expression Level

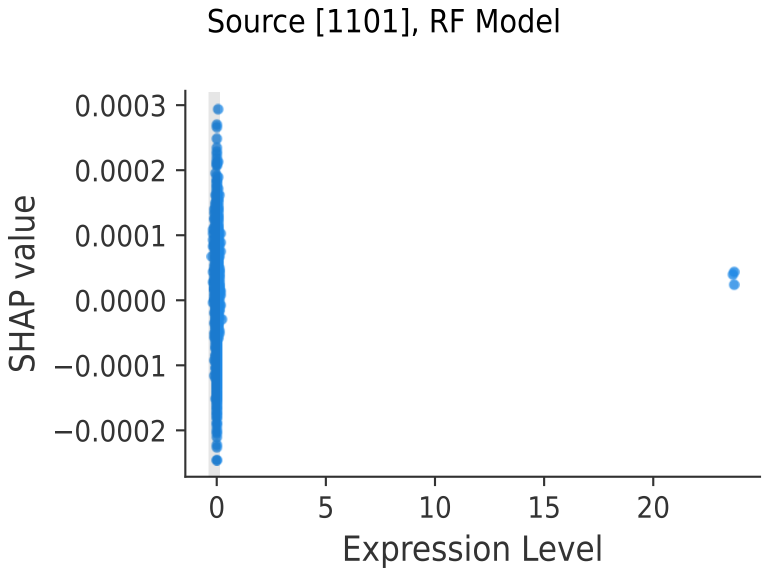
Source [1101], RF Model
SHAP value
0.0003
0.0002
0.0001
0.0000
−0.0001
−0.0002
0
5
10
15
20
Expression Level

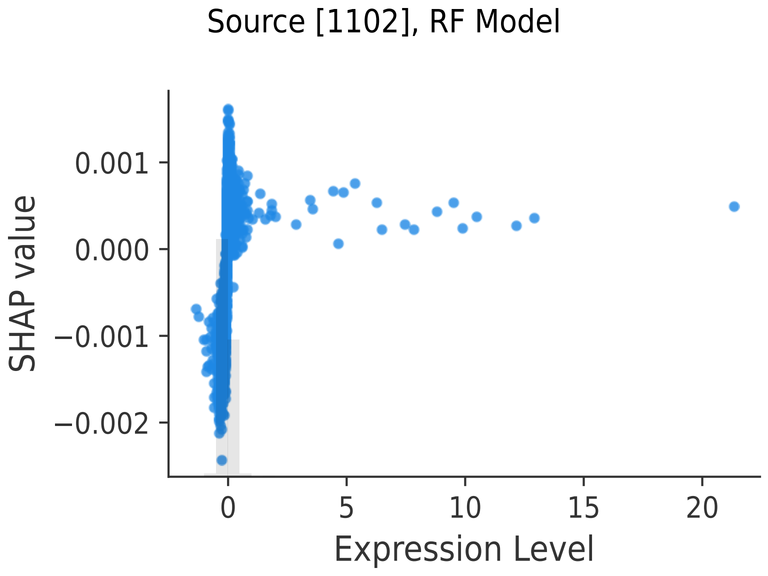
Source [1102], RF Model
SHAP value
0.001
0.000
−0.001
−0.002
0
5
10
15
20
Expression Level

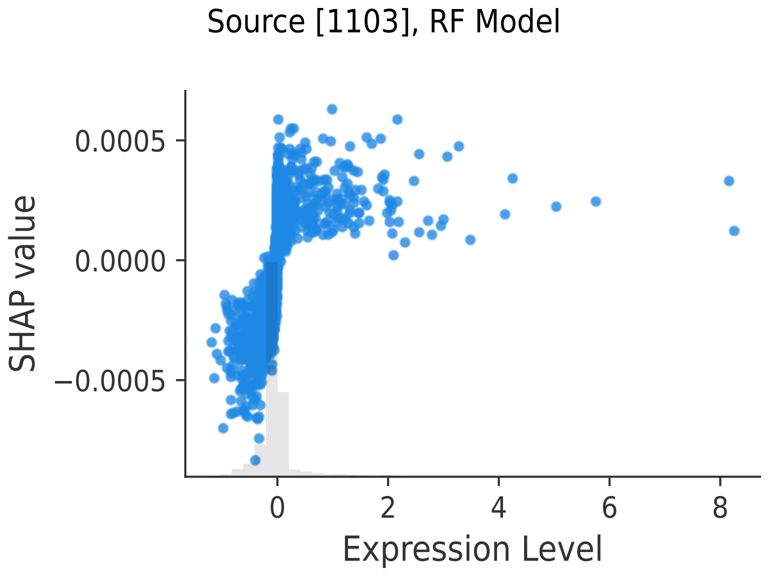
Source [1103], RF Model
SHAP value
0.0005
0.0000
−0.0005
0
2
4
6
8
Expression Level

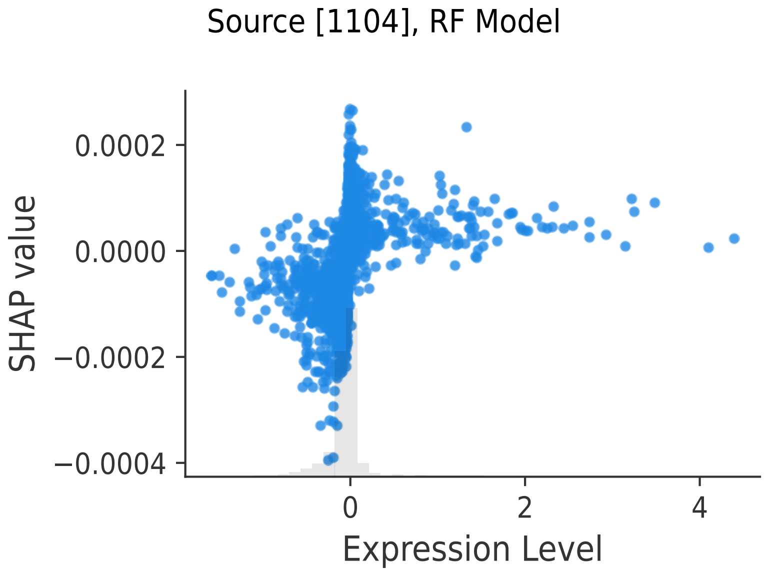
Source [1104], RF Model
0.0002
0.0000
−0.0002
−0.0004
SHAP value
0
2
4
Expression Level

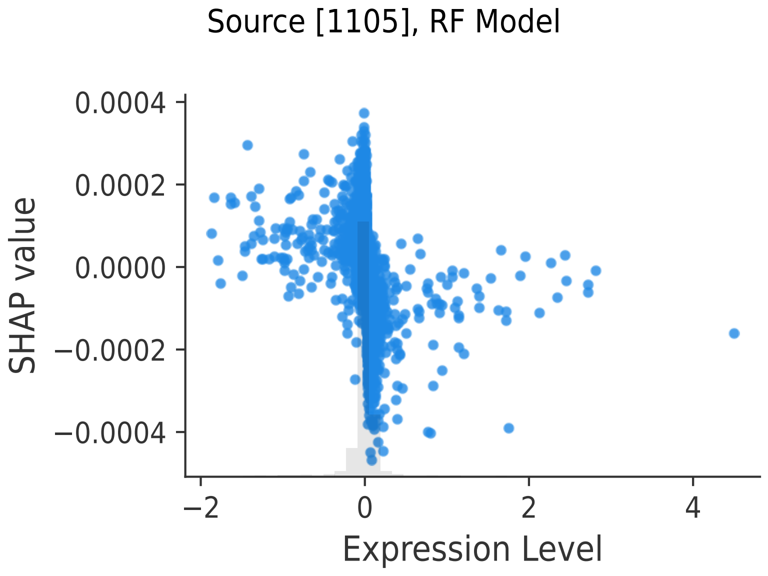
Source [1105], RF Model
SHAP value
0.0004
0.0002
0.0000
−0.0002
−0.0004
−2
0
2
4
Expression Level

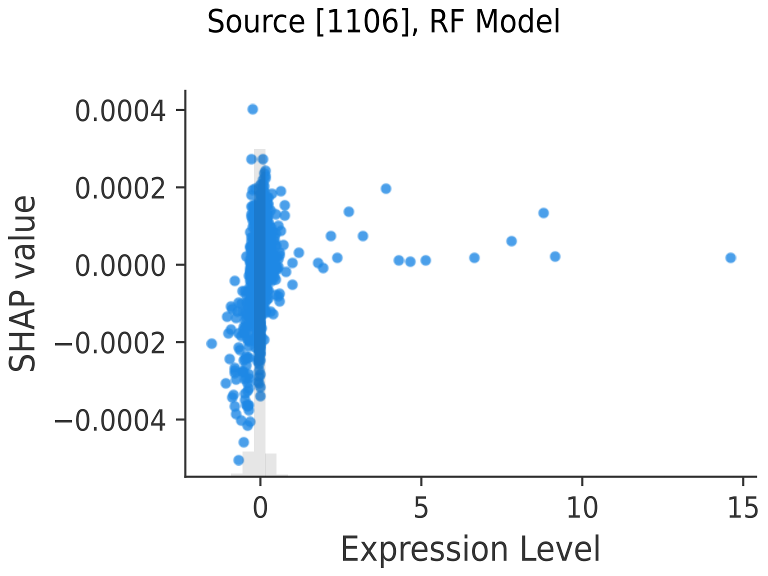

Source [1106], RF Model
0.0004
0.0002
0.0000
−0.0002
−0.0004
SHAP value
0
5
10
15
Expression Level

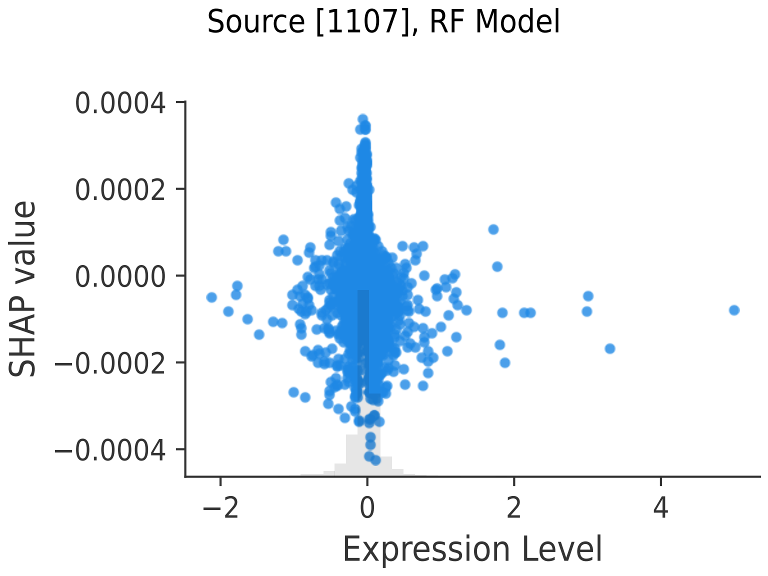
Source [1107], RF Model
0.0004
0.0002
0.0000
−0.0002
−0.0004
SHAP value
−2
0
2
4
Expression Level

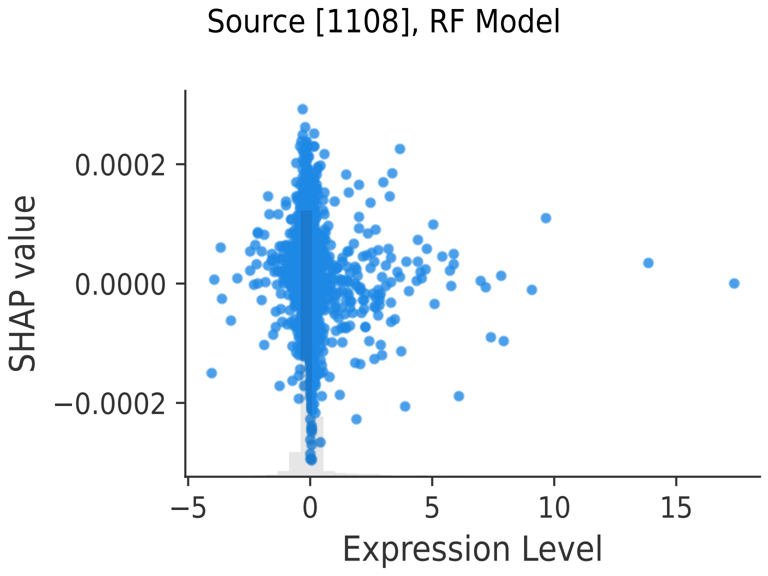

Source [1108], RF Model
SHAP value
0.0002
0.0000
−0.0002
−5
0
5
10
15
Expression Level

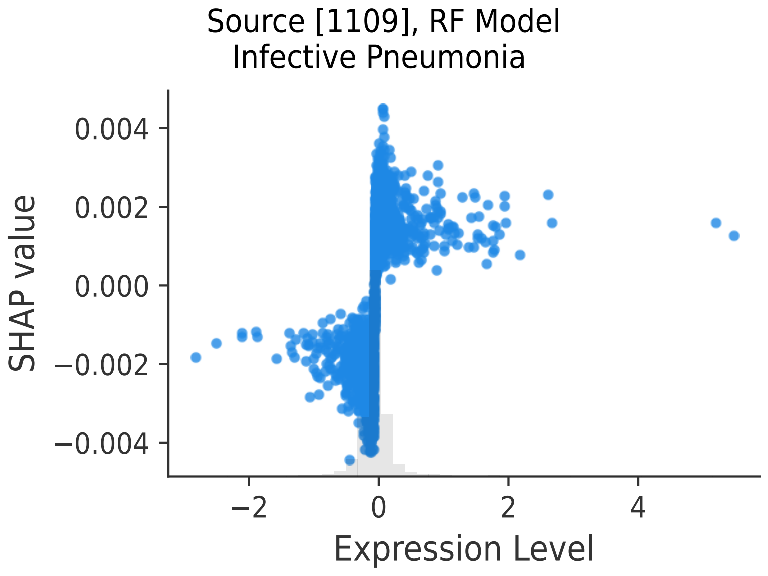

Source [1109], RF Model
Infective Pneumonia
SHAP value
0.004
0.002
0.000
−0.002
−0.004
−2
0
2
4
Expression Level

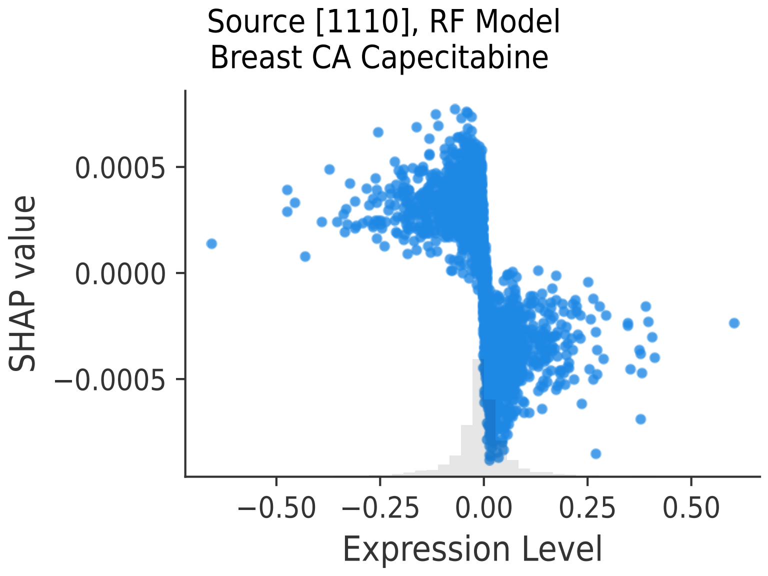
Source [1110], RF Model
Breast CA Capecitabine
0.0005
0.0000
−0.0005
SHAP value
−0.50
−0.25
0.00
0.25
0.50
Expression Level

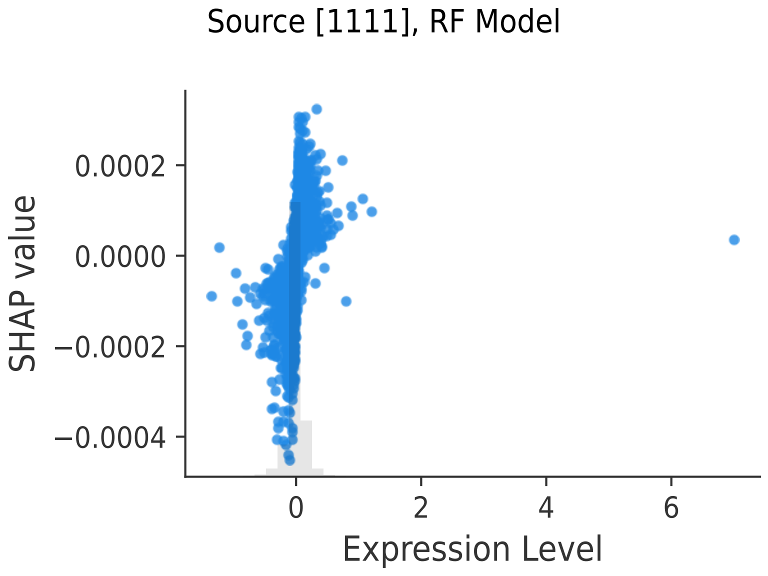

Source [1111], RF Model
SHAP value
0.0002
0.0000
−0.0002
−0.0004
0
2
4
6
Expression Level

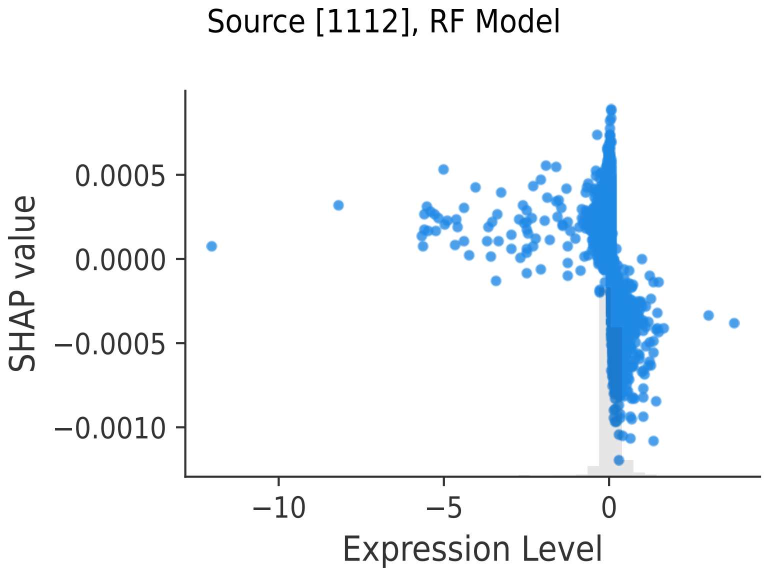

Source [1112], RF Model
SHAP value
0.0005
0.0000
−0.0005
−0.0010
−10
−5
0
Expression Level

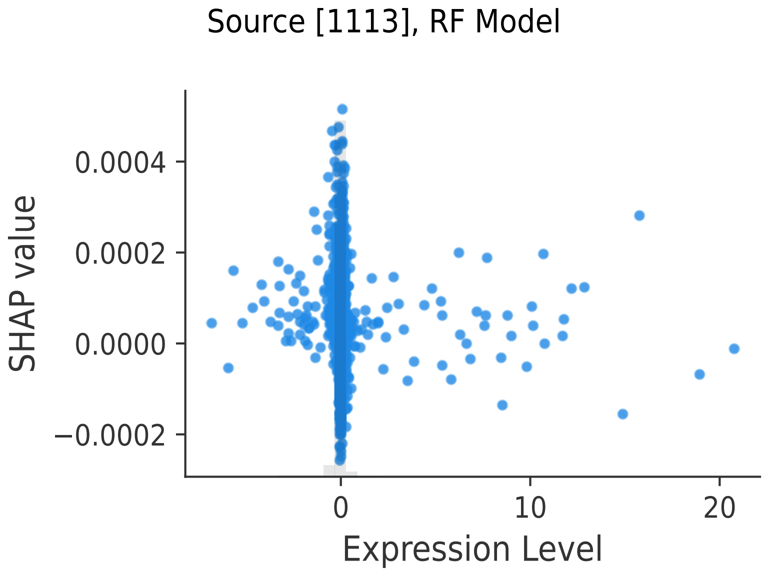
Source [1113], RF Model
0.0004
0.0002
0.0000
−0.0002
SHAP value
0
10
20
Expression Level

Source [1114], RF Model

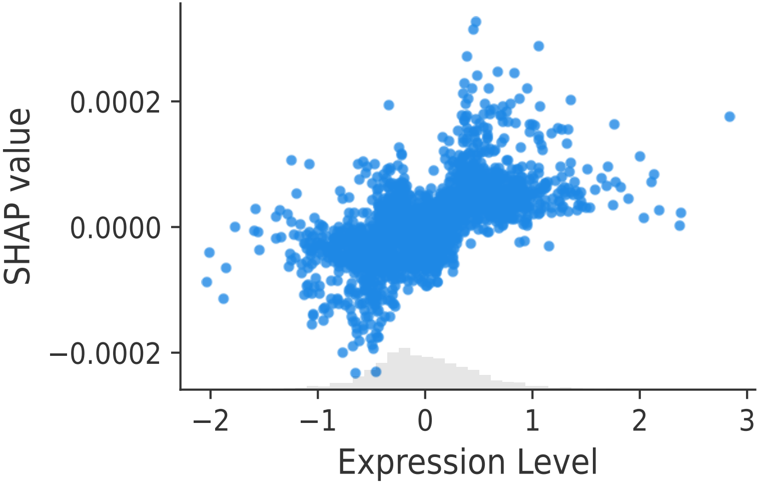

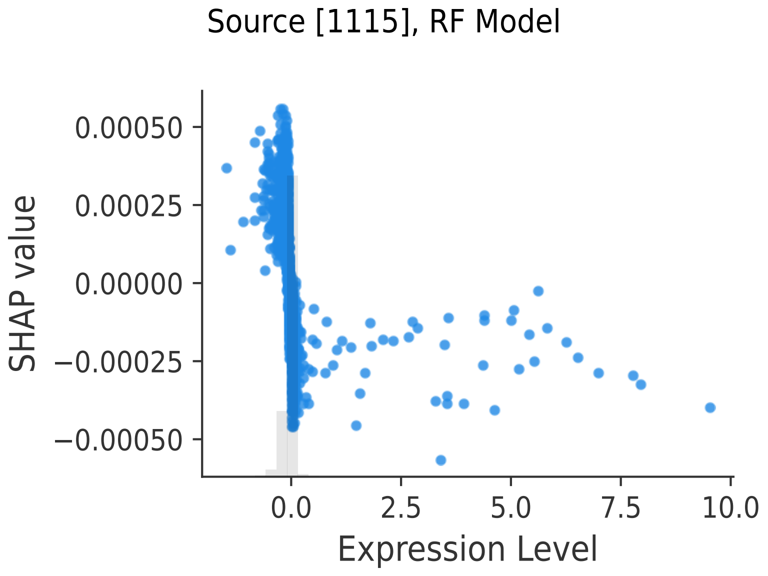
Source [1115], RF Model
SHAP value
0.00050
0.00025
0.00000
−0.00025
−0.00050
0.0
2.5
5.0
7.5
10.0
Expression Level

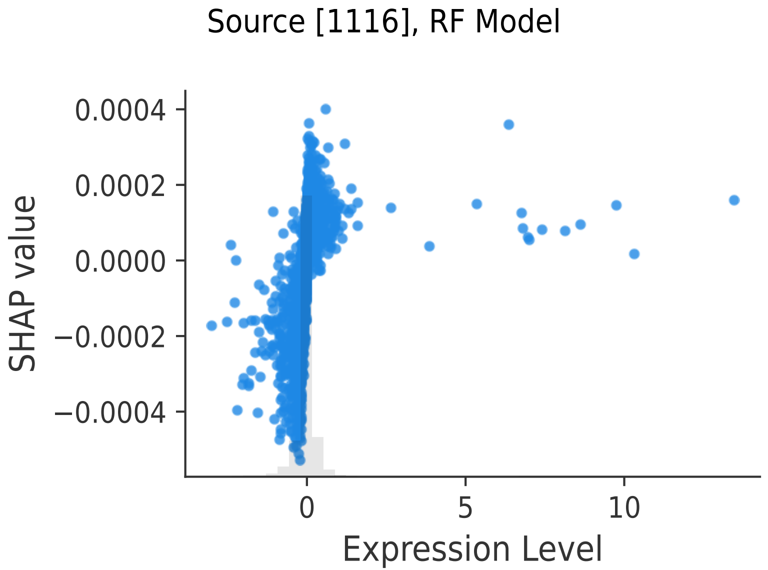

Source [1116], RF Model
0.0004
0.0002
0.0000
−0.0002
−0.0004
SHAP value
0
5
10
Expression Level

Source [1117], RF Model

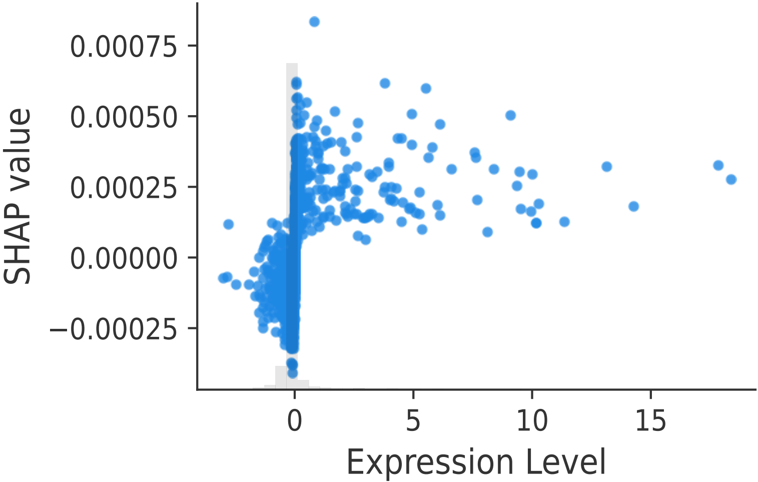

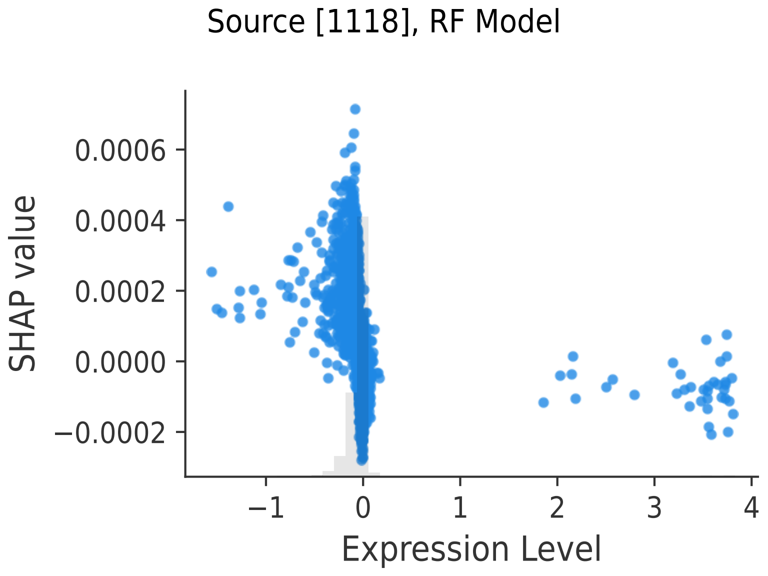

Source [1118], RF Model
SHAP value
0.0006
0.0004
0.0002
0.0000
−0.0002
−1
0
1
2
3
4
Expression Level

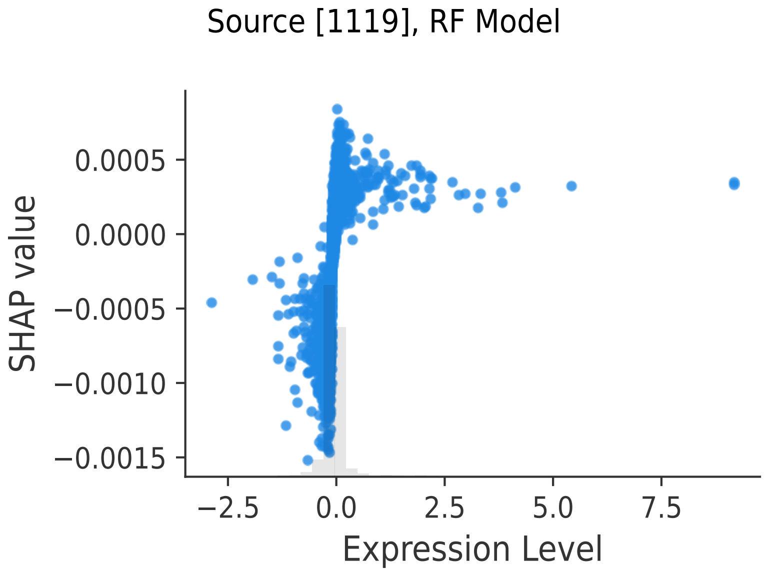
Source [1119], RF Model
SHAP value
0.0005
0.0000
−0.0005
−0.0010
−0.0015
−2.5
0.0
2.5
5.0
7.5
Expression Level

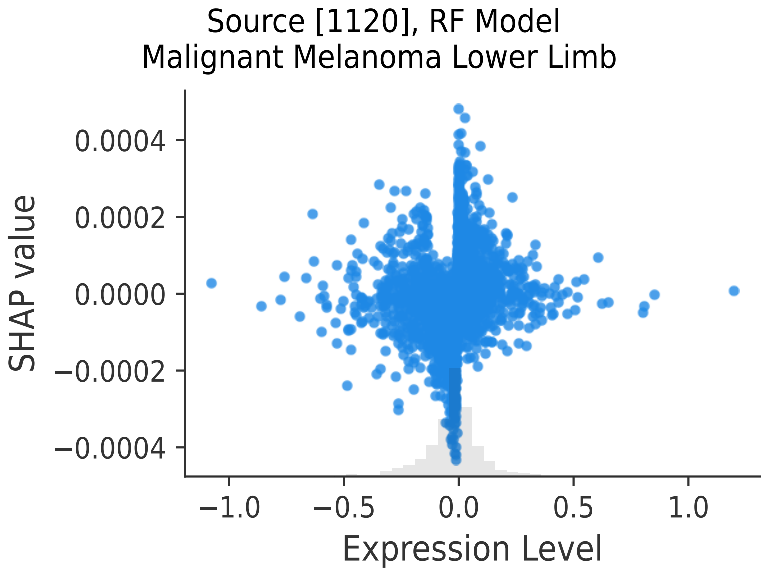
Source [1120], RF Model
Malignant Melanoma Lower Limb
0.0004
0.0002
0.0000
−0.0002
−0.0004
SHAP value
−1.0
−0.5
0.0
0.5
1.0
Expression Level

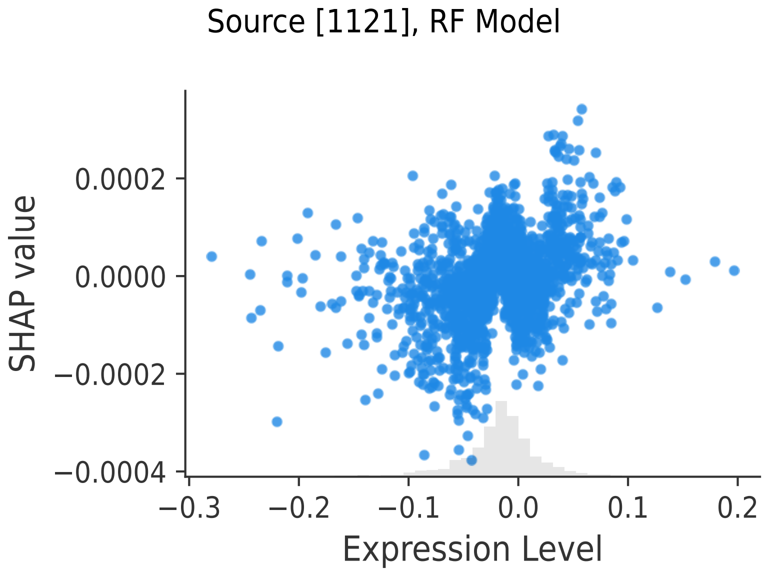

Source [1121], RF Model
SHAP value
0.0002
0.0000
−0.0002
−0.0004
−0.3
−0.2
−0.1
0.0
0.1
0.2
Expression Level

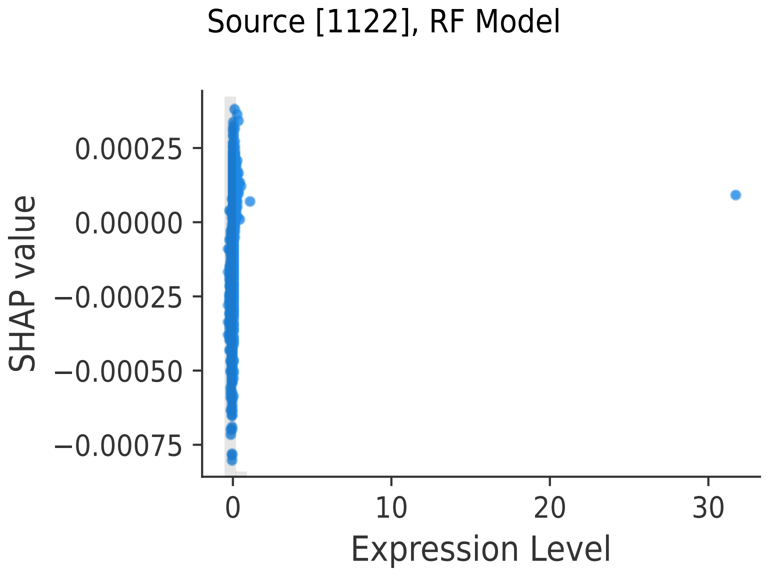
Source [1122], RF Model
SHAP value
0.00025
0.00000
−0.00025
−0.00050
−0.00075
0
10
20
30
Expression Level

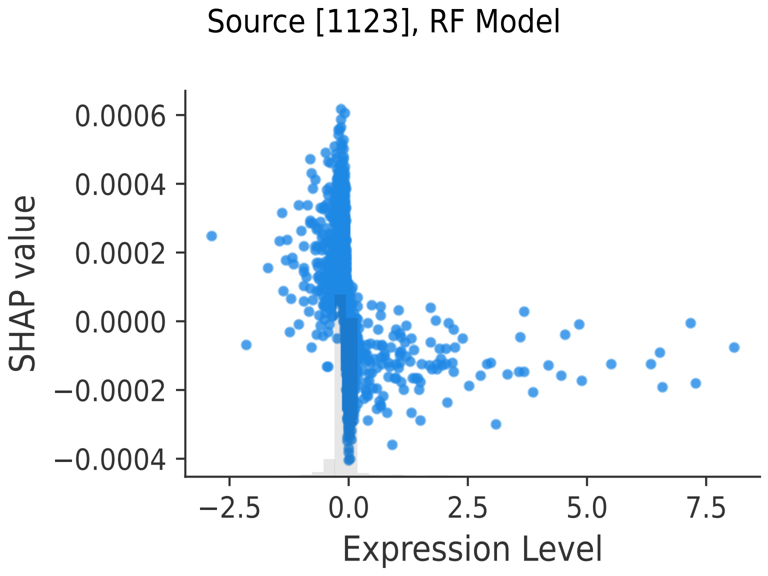

Source [1123], RF Model
SHAP value
0.0006
0.0004
0.0002
0.0000
−0.0002
−0.0004
−2.5
0.0
2.5
5.0
7.5
Expression Level

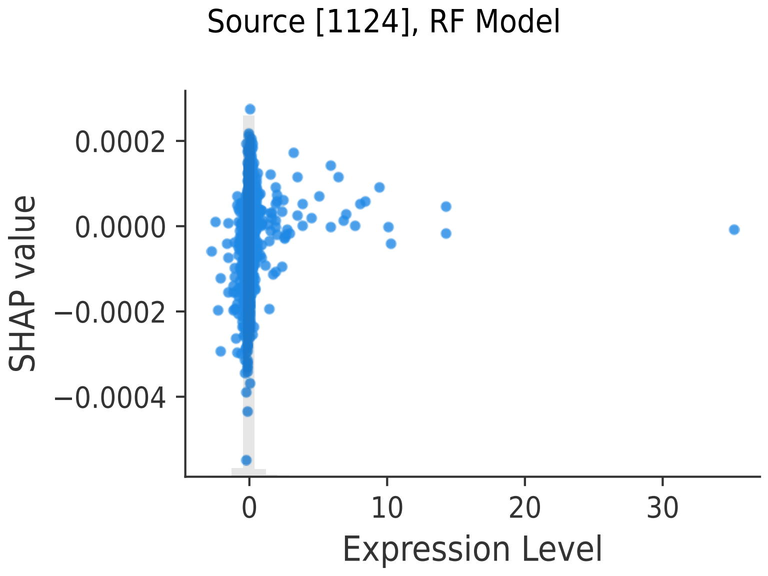
Source [1124], RF Model
SHAP value
0.0002
0.0000
−0.0002
−0.0004
0
10
20
30
Expression Level

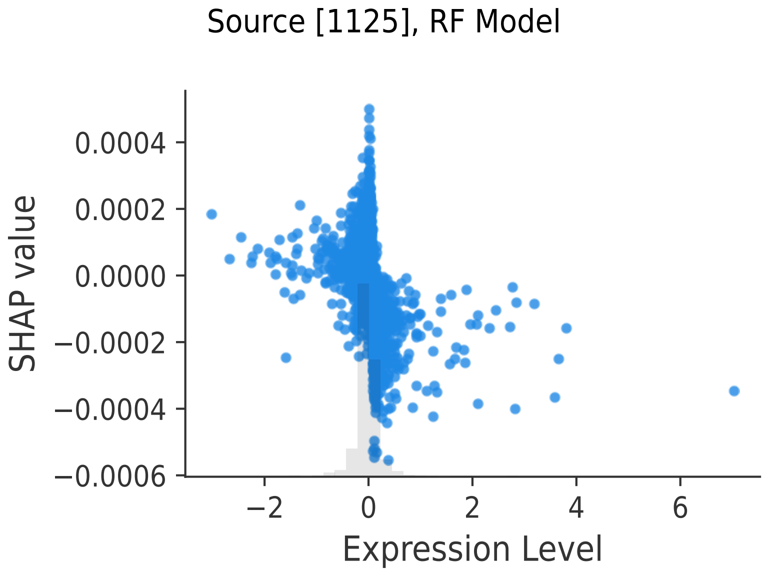

Source [1125], RF Model
SHAP value
0.0004
0.0002
0.0000
−0.0002
−0.0004
−0.0006
−2
0
2
4
6
Expression Level

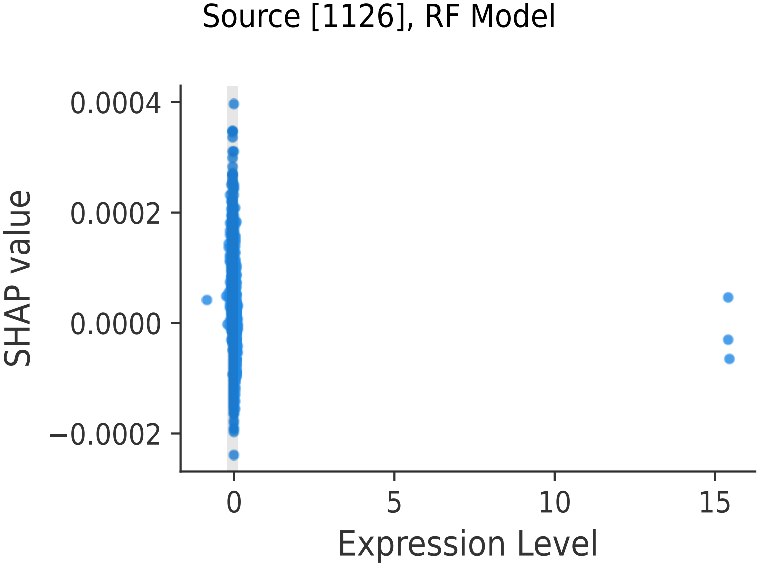
Source [1126], RF Model
SHAP value
0.0004
0.0002
0.0000
−0.0002
0
5
10
15
Expression Level

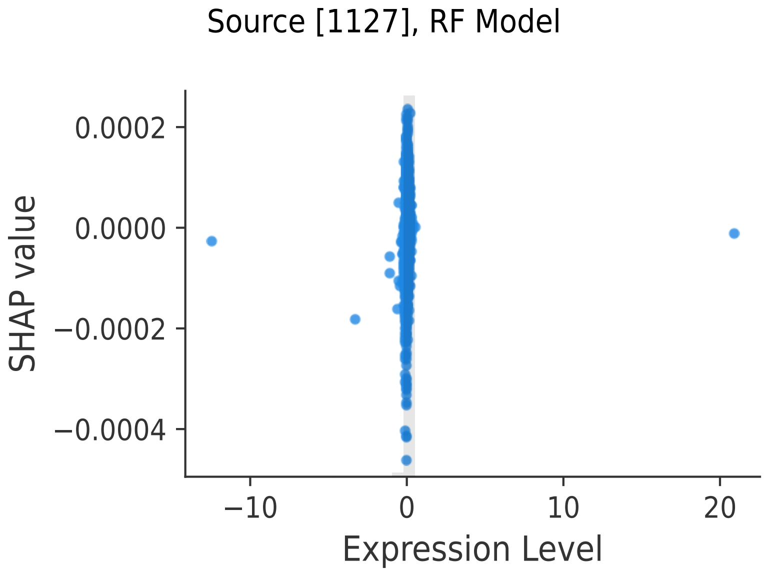
Source [1127], RF Model
SHAP value
0.0002
0.0000
−0.0002
−0.0004
−10
0
10
20
Expression Level

Source [1128], RF Model

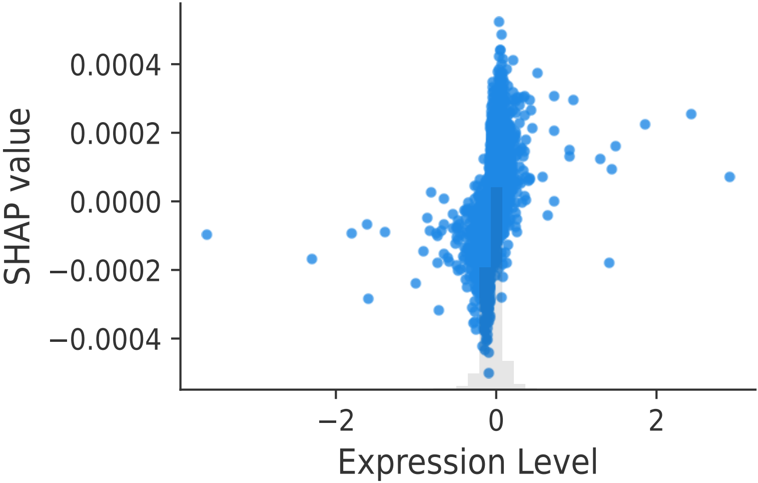

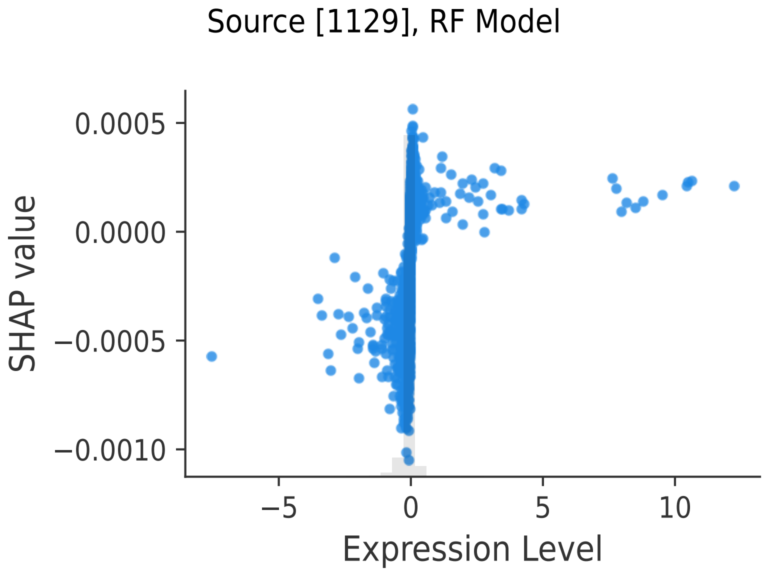

Source [1129], RF Model
0.0005
0.0000
−0.0005
−0.0010
SHAP value
−5
0
5
10
Expression Level

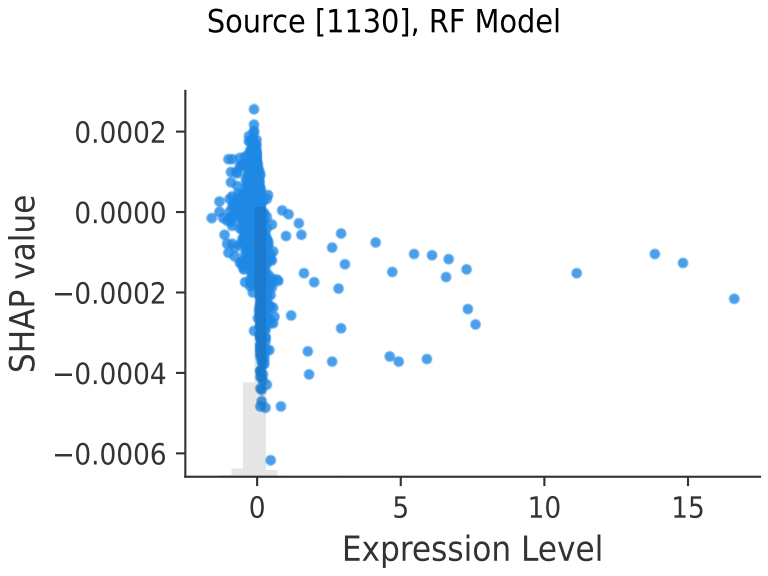
Source [1130], RF Model
SHAP value
0.0002
0.0000
−0.0002
−0.0004
−0.0006
0
5
10
15
Expression Level

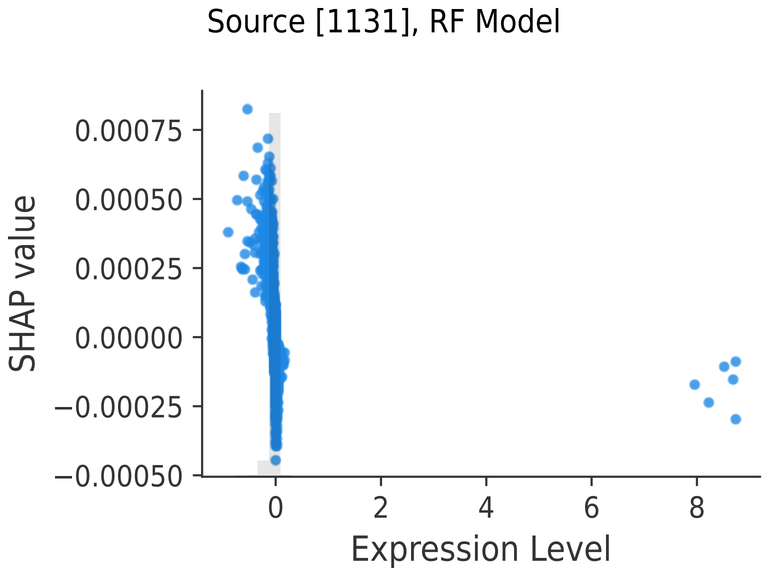

Source [1131], RF Model
SHAP value
0.00075
0.00050
0.00025
0.00000
−0.00025
−0.00050
0
2
4
6
8
Expression Level

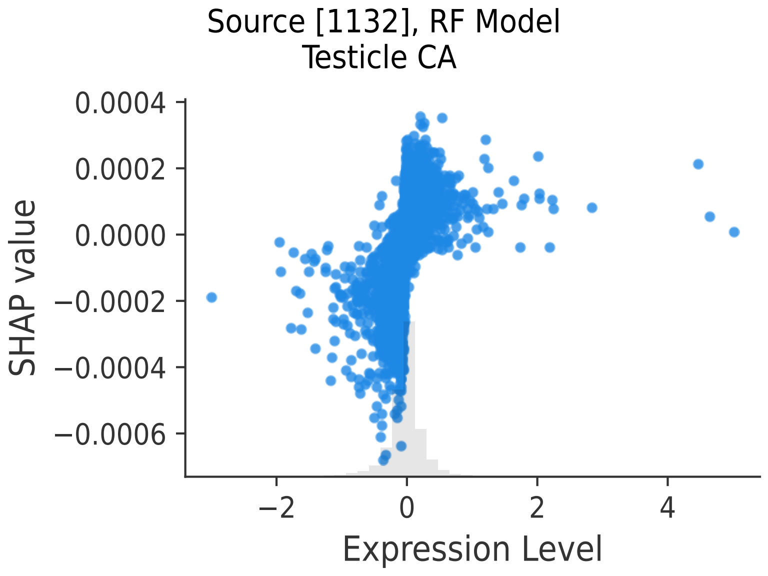

Source [1132], RF Model
Testicle CA
0.0004
0.0002
0.0000
−0.0002
−0.0004
−0.0006
SHAP value
−2
0
2
4
Expression Level

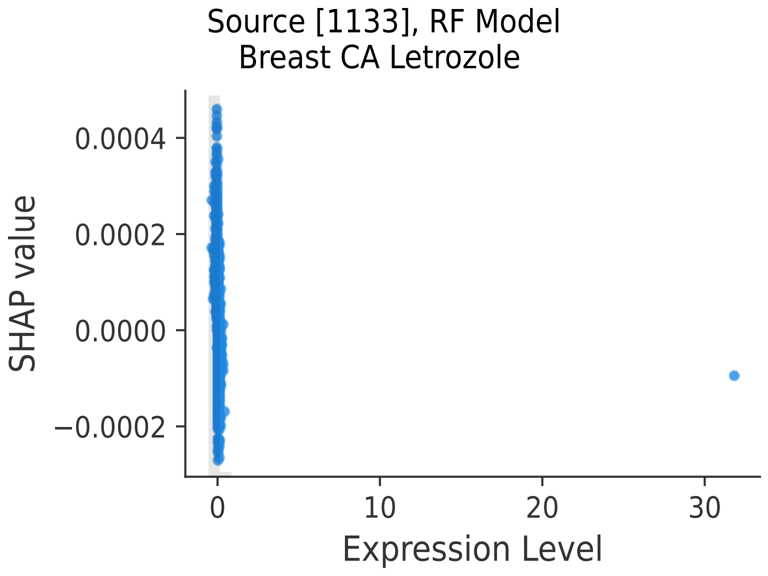
Source [1133], RF Model
Breast CA Letrozole
SHAP value
0.0004
0.0002
0.0000
−0.0002
0
10
20
30
Expression Level

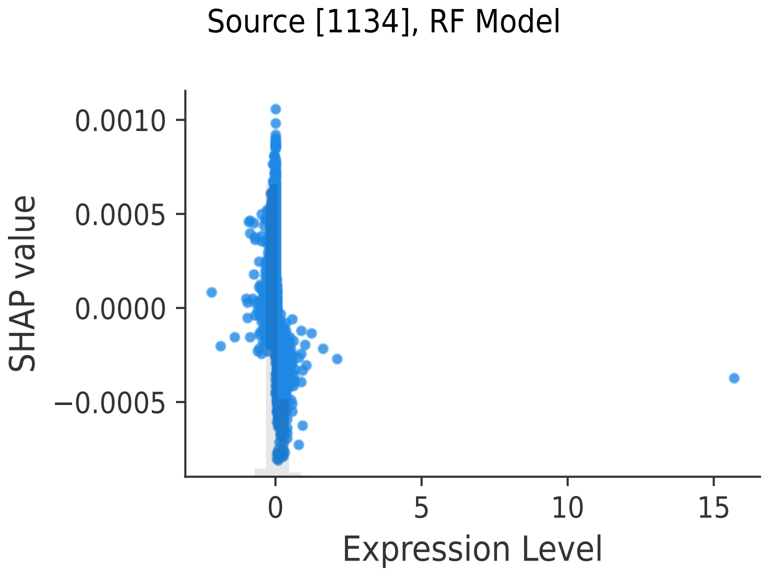
Source [1134], RF Model
SHAP value
0.0010
0.0005
0.0000
−0.0005
0
5
10
15
Expression Level

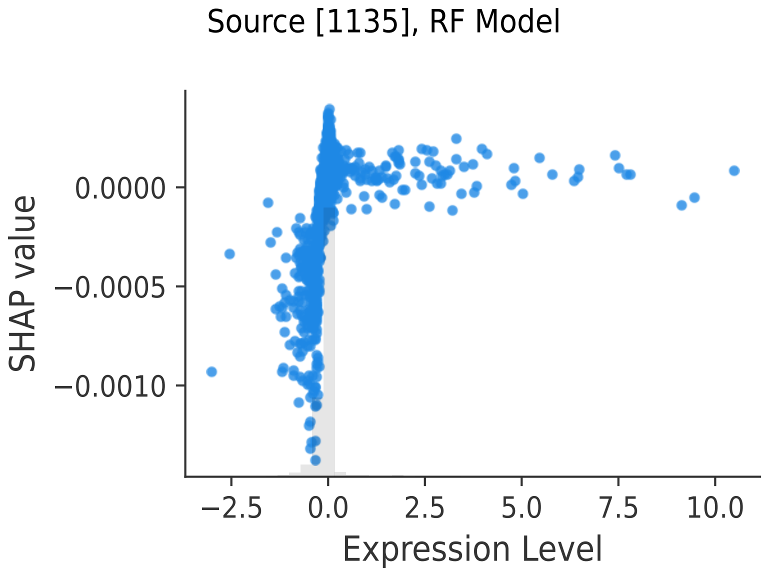

Source [1135], RF Model
SHAP value
0.0000
−0.0005
−0.0010
−2.5
0.0
2.5
5.0
7.5
10.0
Expression Level

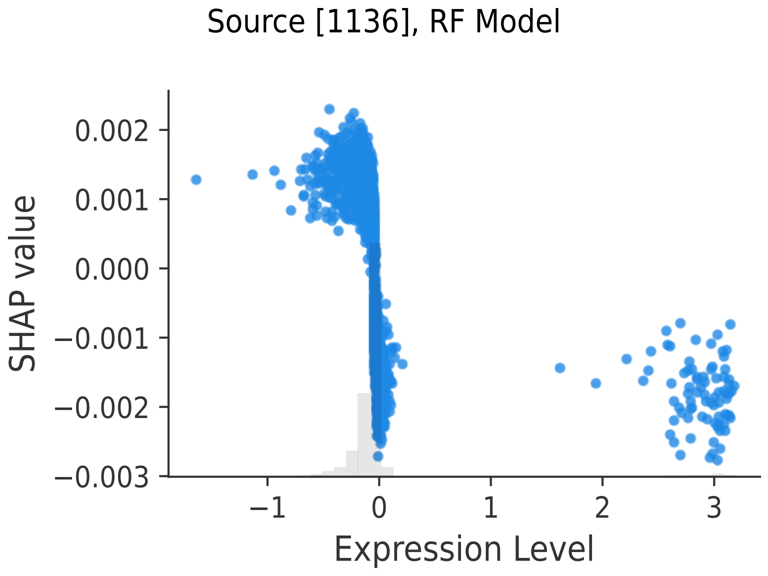

Source [1136], RF Model
SHAP value
0.002
0.001
0.000
−0.001
−0.002
−0.003
−1
0
1
2
3
Expression Level

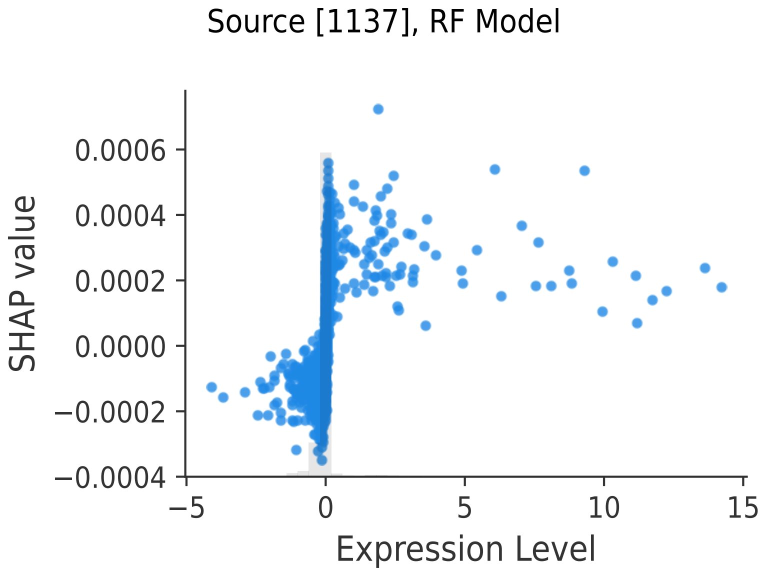
Source [1137], RF Model
SHAP value
0.0006
0.0004
0.0002
0.0000
−0.0002
−0.0004
−5
0
5
10
15
Expression Level

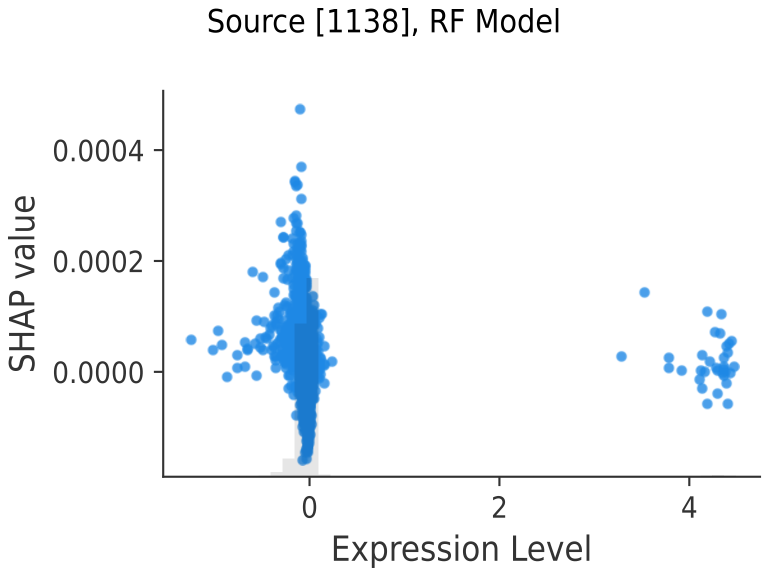
Source [1138], RF Model
0.0004
0.0002
0.0000
SHAP value
0
2
4
Expression Level

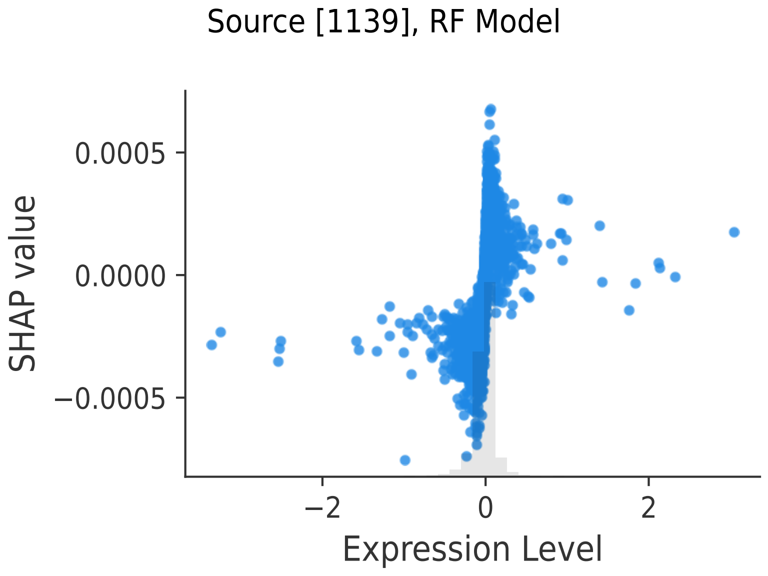

Source [1139], RF Model
0.0005
0.0000
−0.0005
SHAP value
−2
0
2
Expression Level

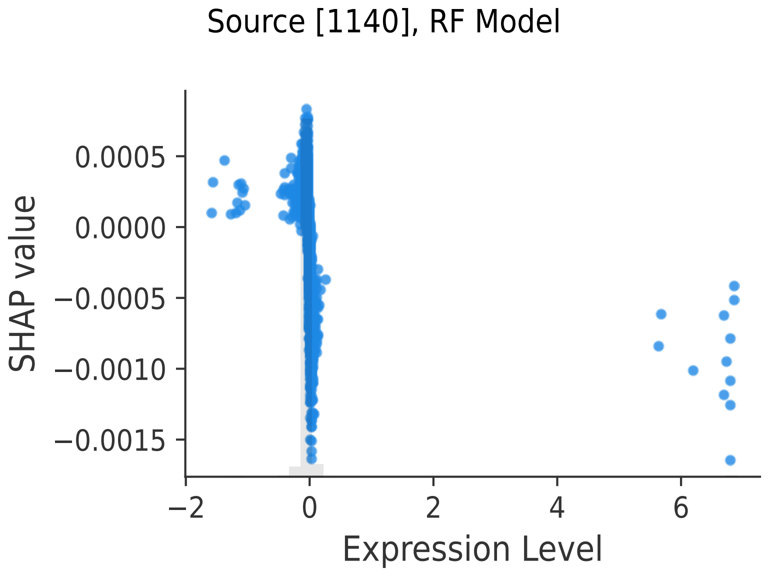

Source [1140], RF Model
0.0005
0.0000
−0.0005
−0.0010
−0.0015
SHAP value
−2
0
2
4
6
Expression Level

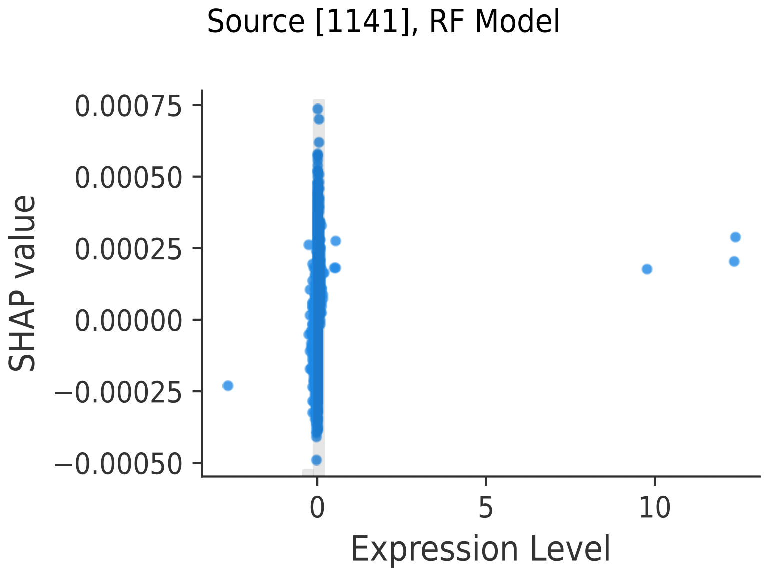

Source [1141], RF Model
0.00075
0.00050
0.00025
0.00000
−0.00025
−0.00050
SHAP value
0
5
10
Expression Level

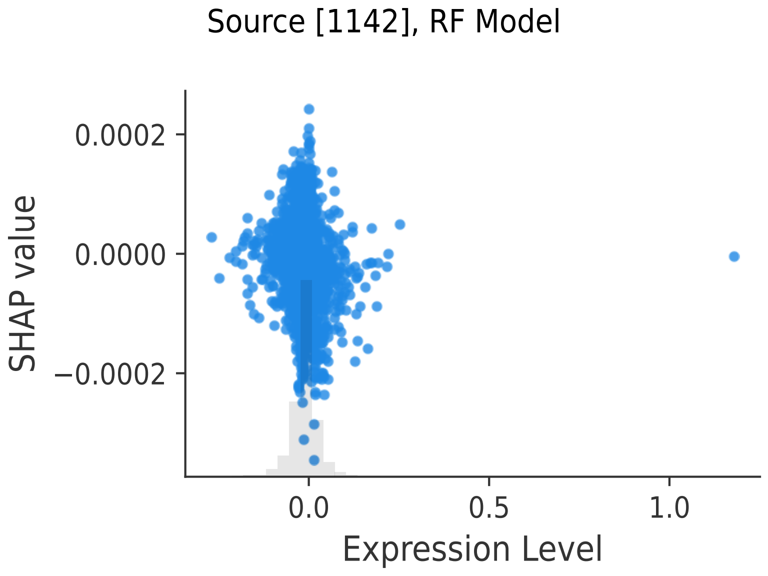
Source [1142], RF Model
0.0002
0.0000
−0.0002
SHAP value
0.0
0.5
1.0
Expression Level

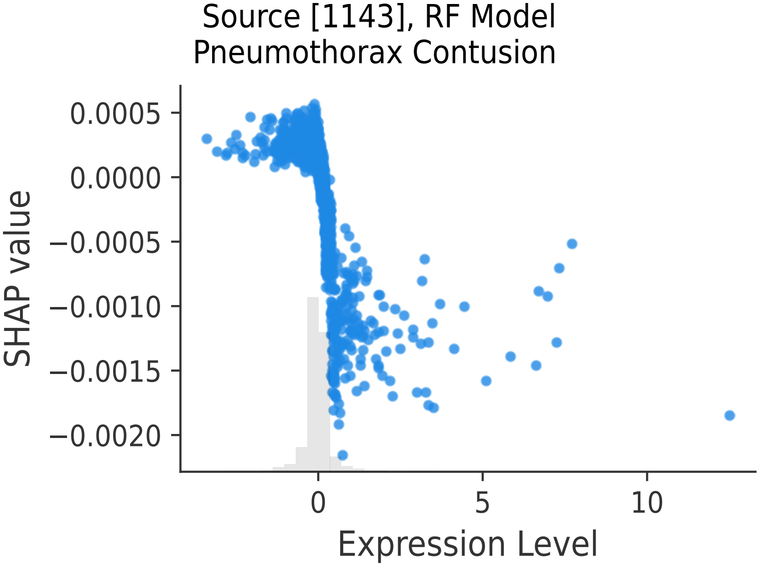
Source [1143], RF Model
Pneumothorax Contusion
SHAP value
0.0005
0.0000
−0.0005
−0.0010
−0.0015
−0.0020
0
5
10
Expression Level

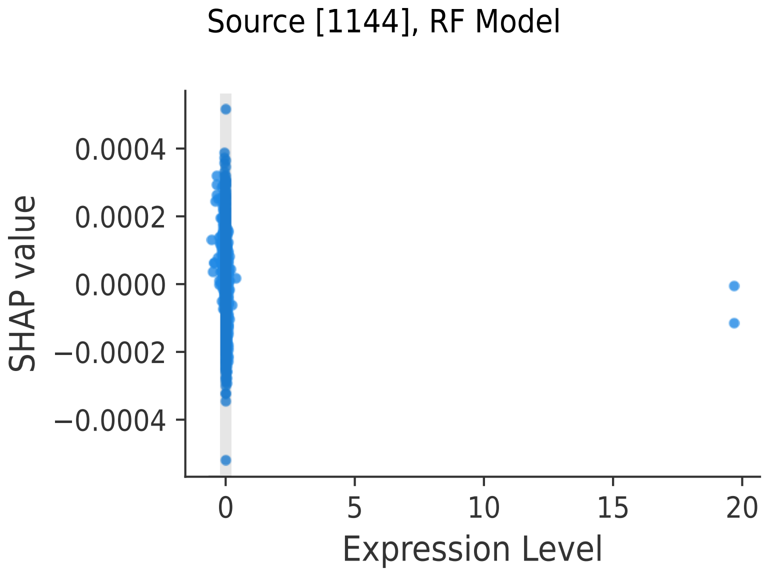

Source [1144], RF Model
0.0004
0.0002
0.0000
−0.0002
−0.0004
SHAP value
0
5
10
15
20
Expression Level

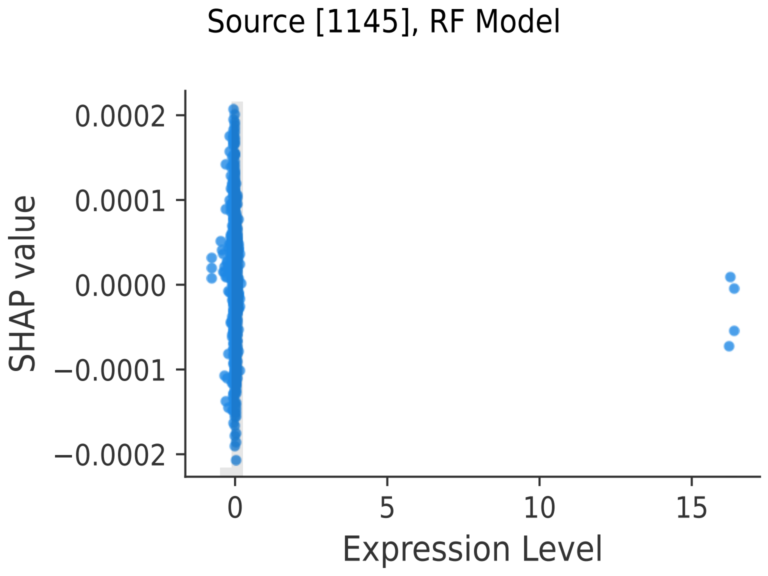

Source [1145], RF Model
0.0002
0.0001
0.0000
−0.0001
−0.0002
SHAP value
0
5
10
15
Expression Level

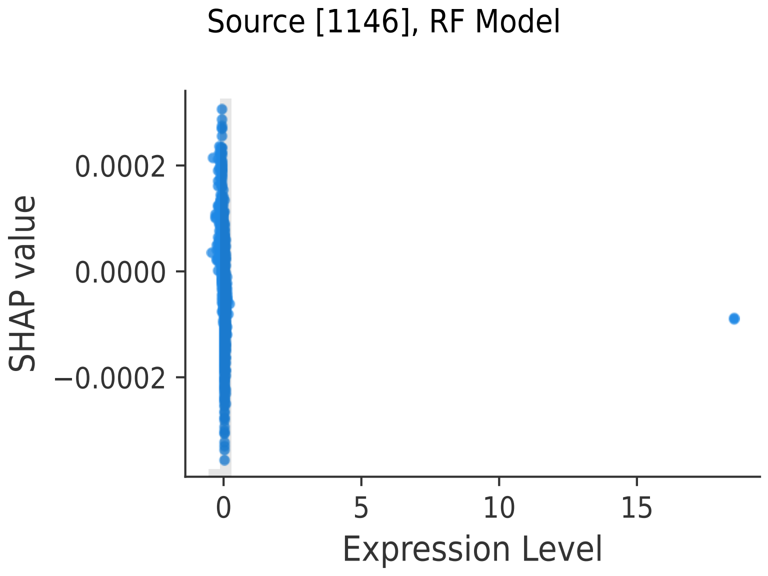
Source [1146], RF Model
0.0002
0.0000
−0.0002
SHAP value
0
5
10
15
Expression Level

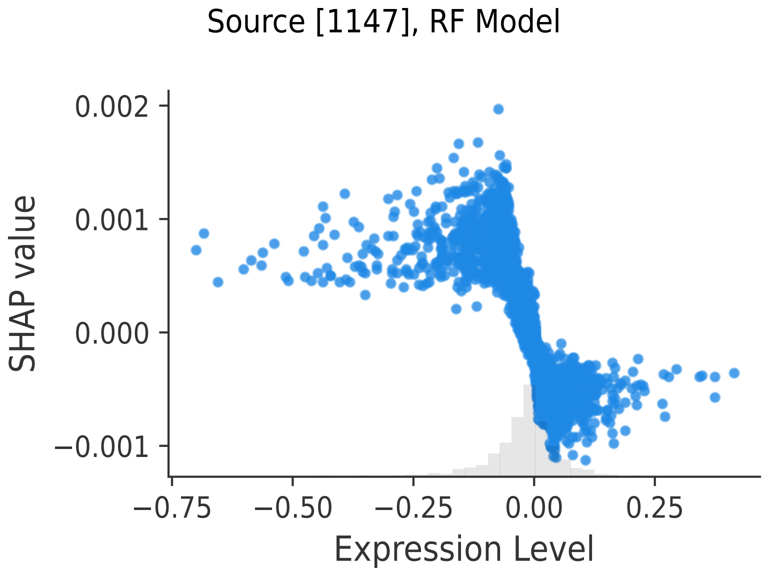
Source [1147], RF Model
0.002
0.001
0.000
−0.001
SHAP value
−0.75
−0.50
−0.25
0.00
0.25
Expression Level

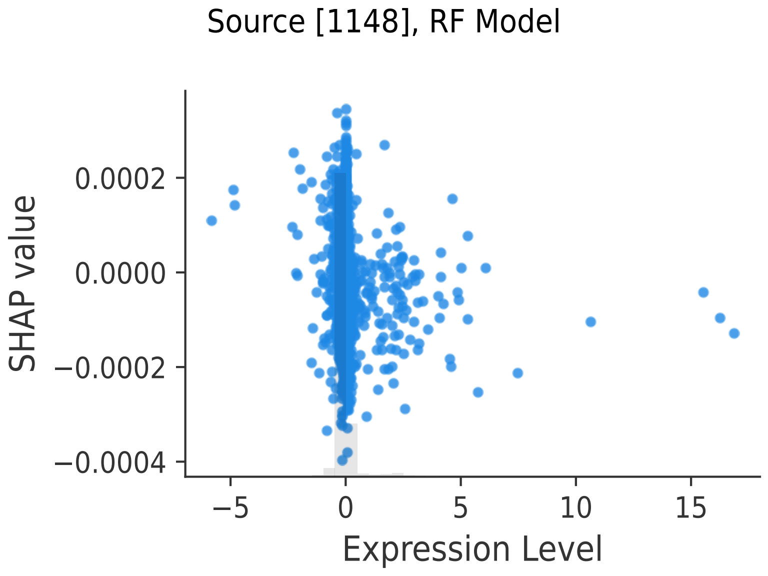
Source [1148], RF Model
SHAP value
0.0002
0.0000
−0.0002
−0.0004
−5
0
5
10
15
Expression Level

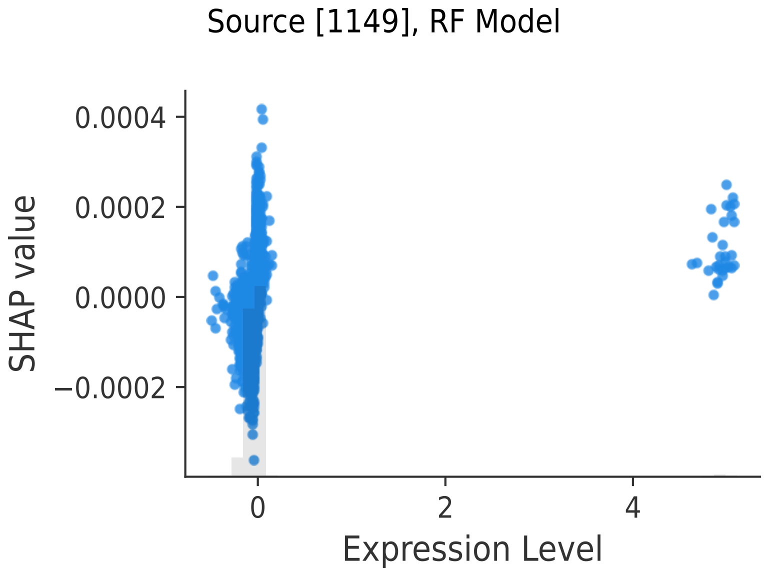
Source [1149], RF Model
SHAP value
0.0004
0.0002
0.0000
−0.0002
0
2
4
Expression Level

Source [1150], RF Model

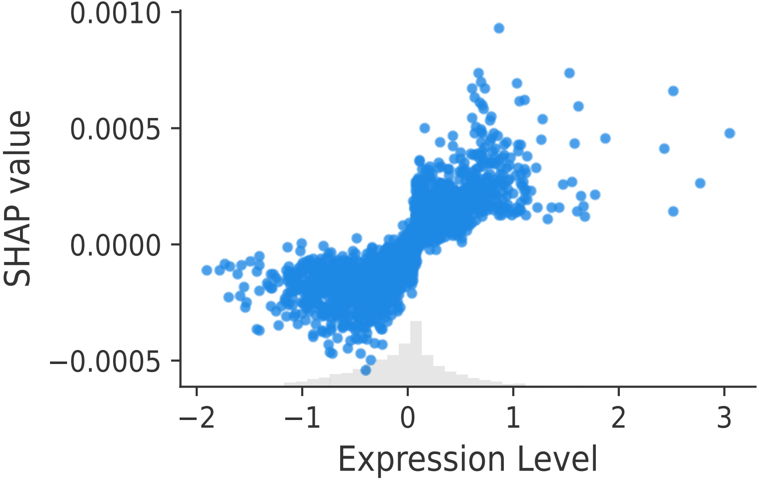

Source [1151], RF Model

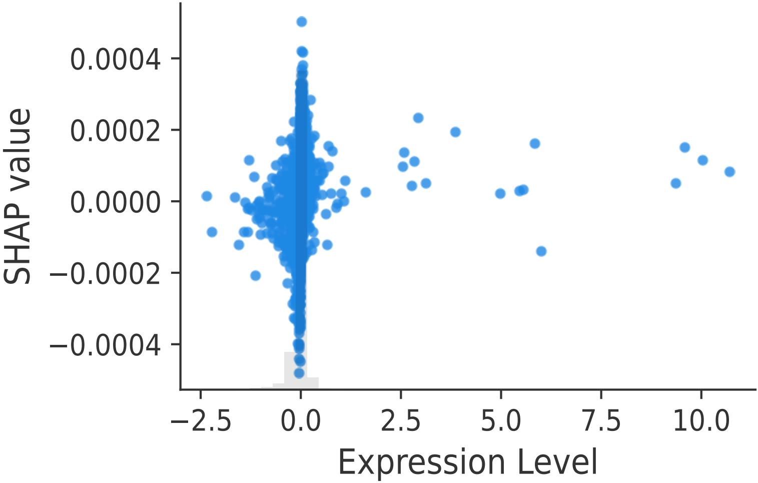

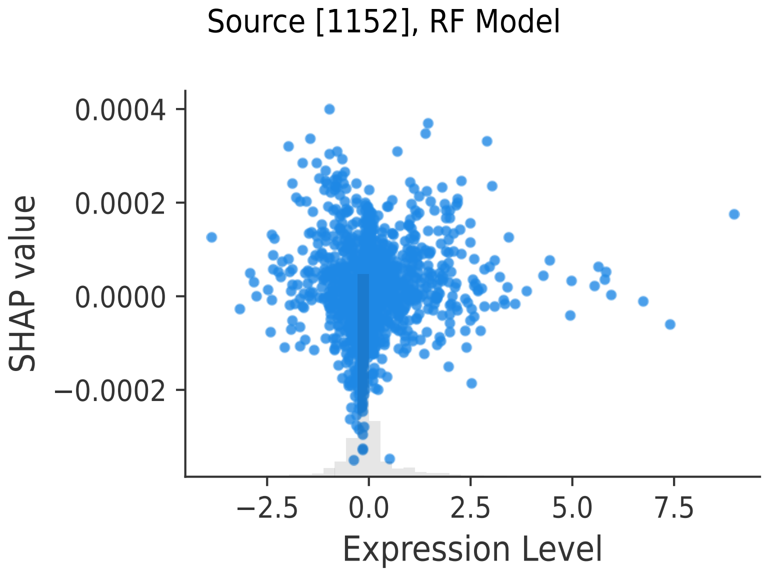

Source [1152], RF Model
0.0004
0.0002
0.0000
−0.0002
SHAP value
−2.5
0.0
2.5
5.0
7.5
Expression Level

Source [1153], RF Model

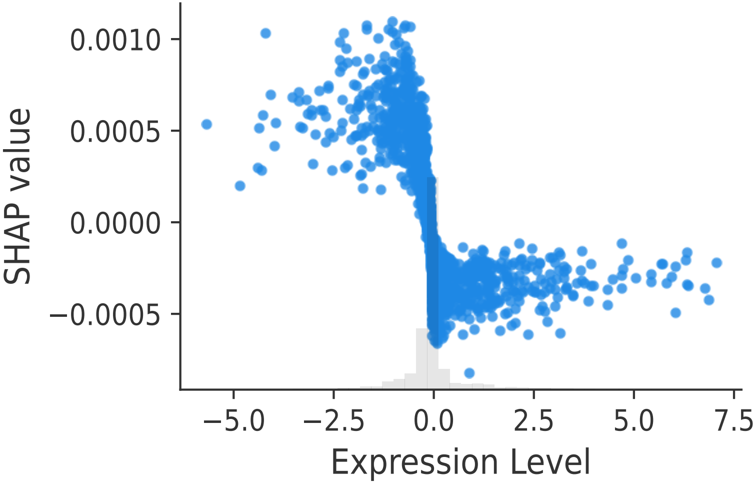

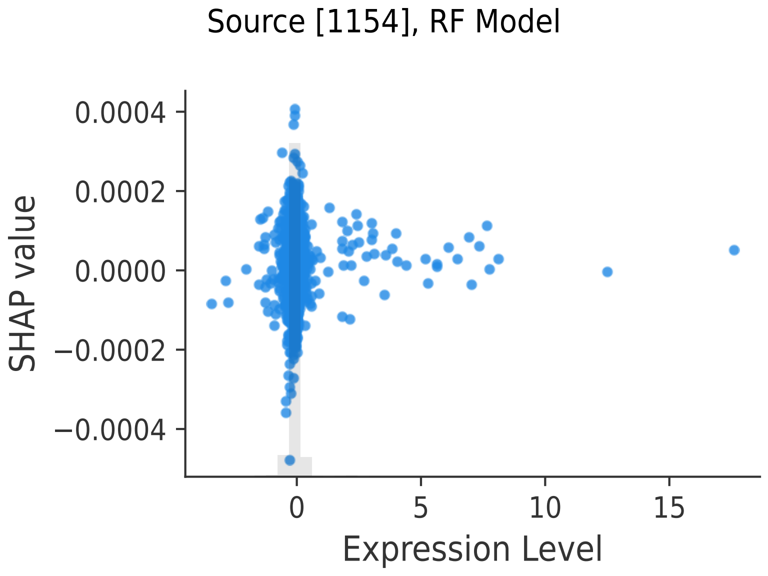

Source [1154], RF Model
SHAP value
0.0004
0.0002
0.0000
−0.0002
−0.0004
0
5
10
15
Expression Level

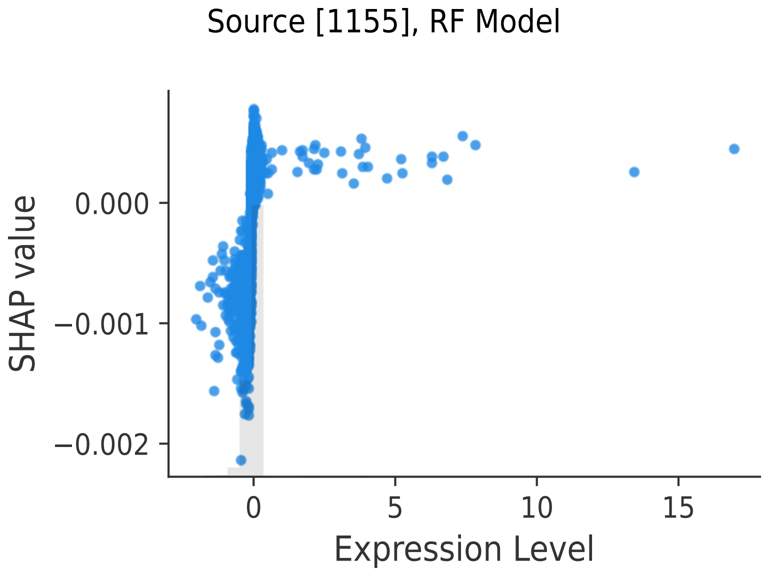

Source [1155], RF Model
SHAP value
0.000
−0.001
−0.002
0
5
10
15
Expression Level

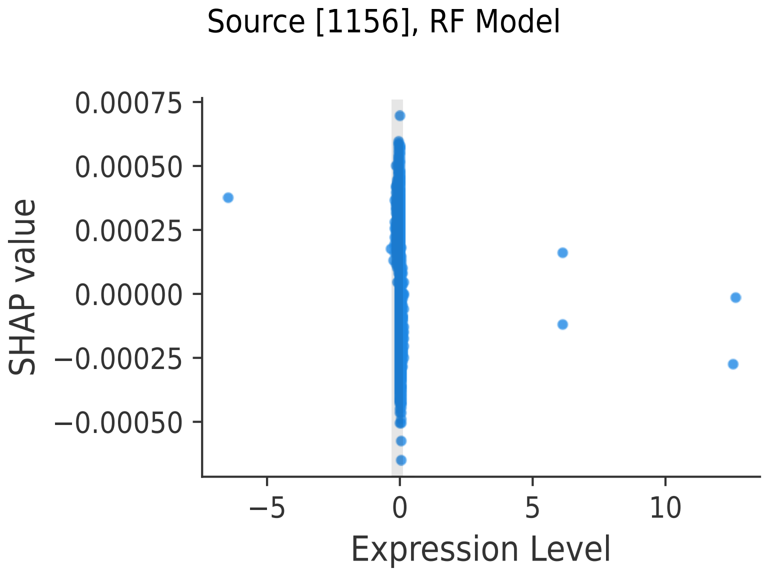

Source [1156], RF Model
SHAP value
0.00075
0.00050
0.00025
0.00000
−0.00025
−0.00050
−5
0
5
10
Expression Level

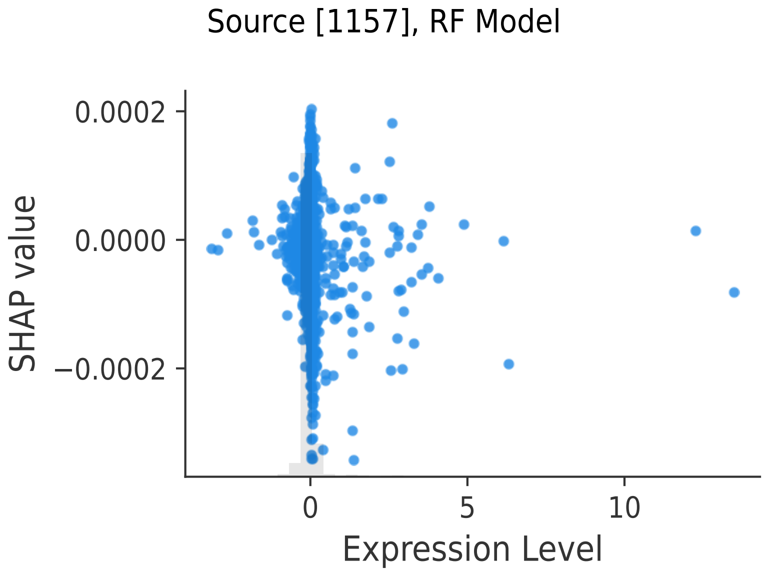

Source [1157], RF Model
0.0002
0.0000
−0.0002
SHAP value
0
5
10
Expression Level

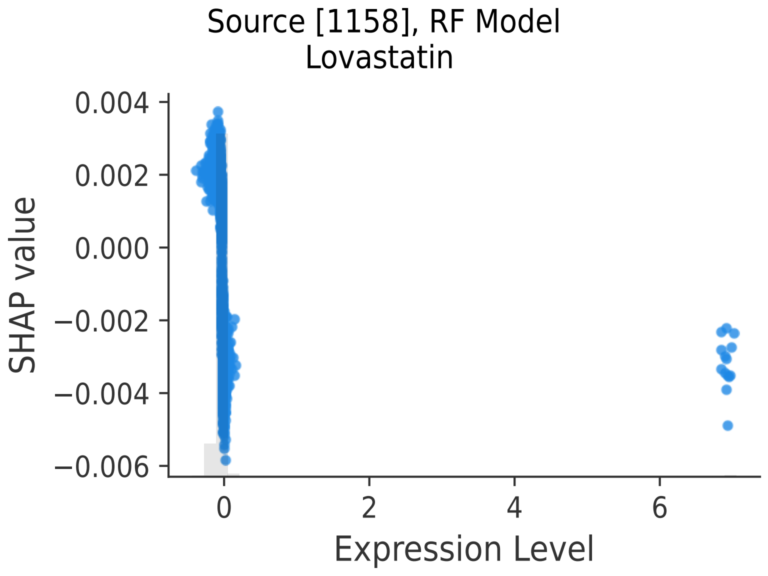

Source [1158], RF Model
Lovastatin
SHAP value
0.004
0.002
0.000
−0.002
−0.004
−0.006
0
2
4
6
Expression Level

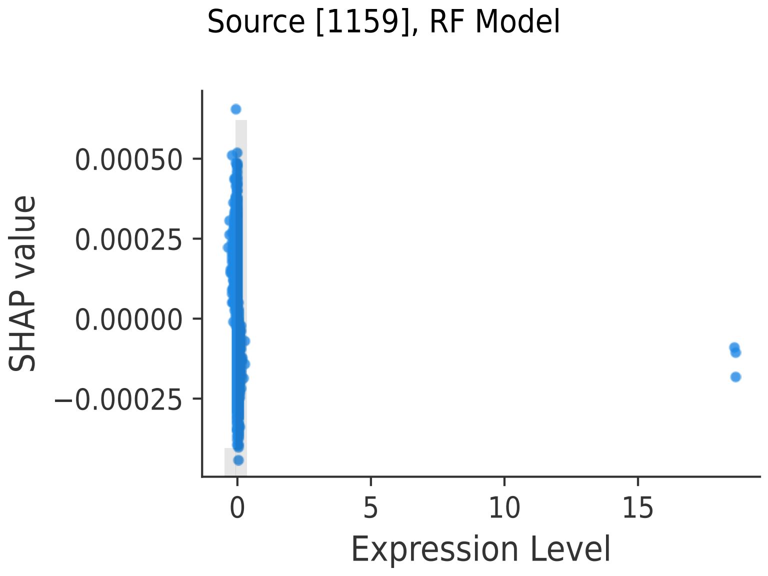
Source [1159], RF Model
SHAP value
0.00050
0.00025
0.00000
−0.00025
0
5
10
15
Expression Level

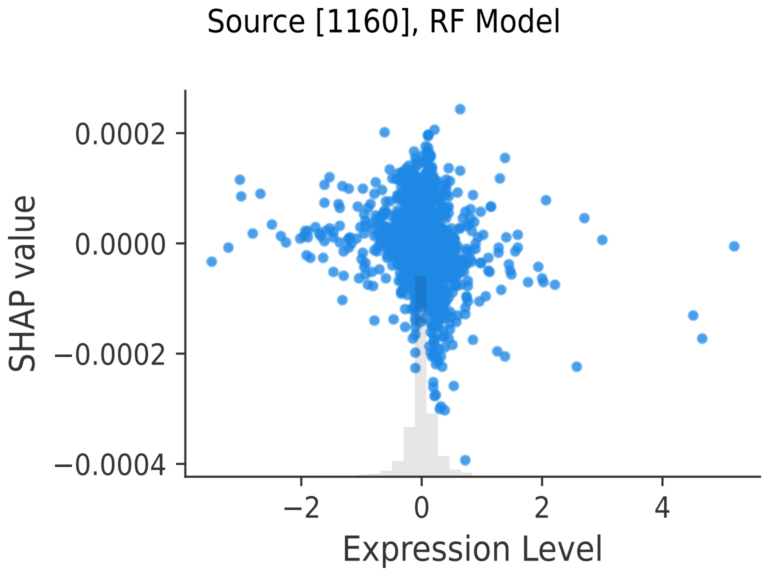

Source [1160], RF Model
SHAP value
0.0002
0.0000
−0.0002
−0.0004
−2
0
2
4
Expression Level

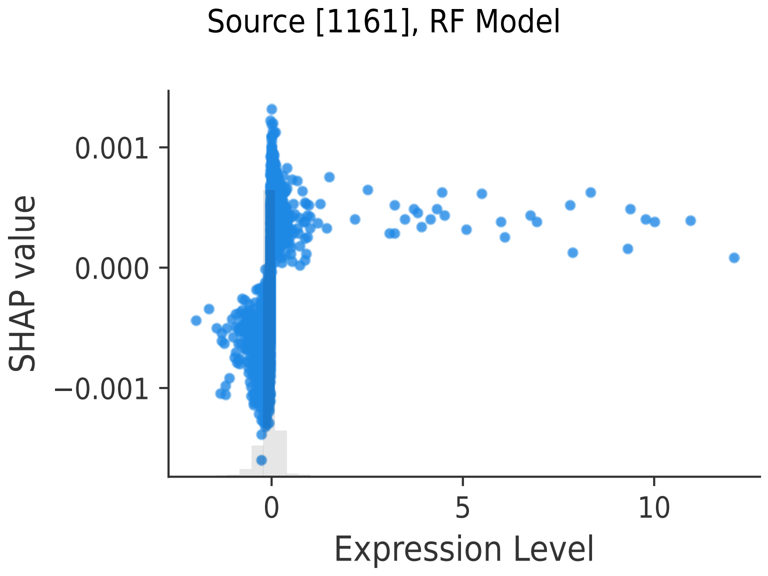
Source [1161], RF Model
0.001
0.000
−0.001
SHAP value
0
5
10
Expression Level

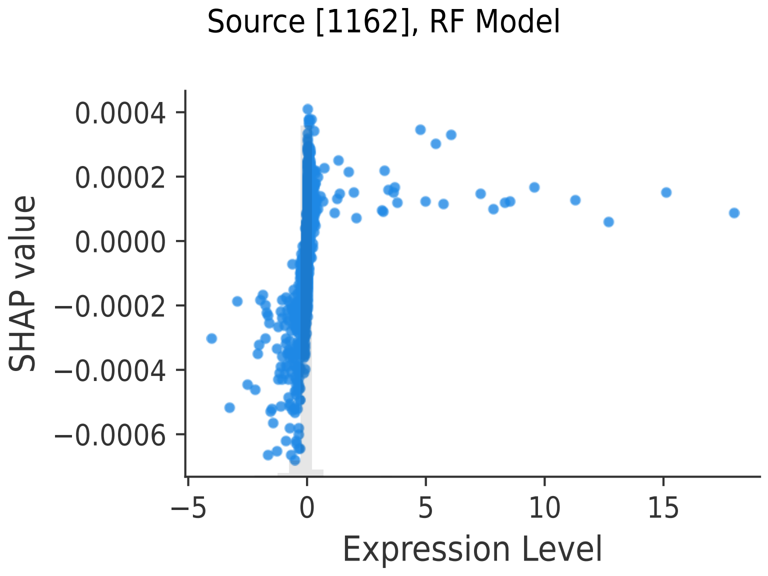

Source [1162], RF Model
SHAP value
0.0004
0.0002
0.0000
−0.0002
−0.0004
−0.0006
−5
0
5
10
15
Expression Level

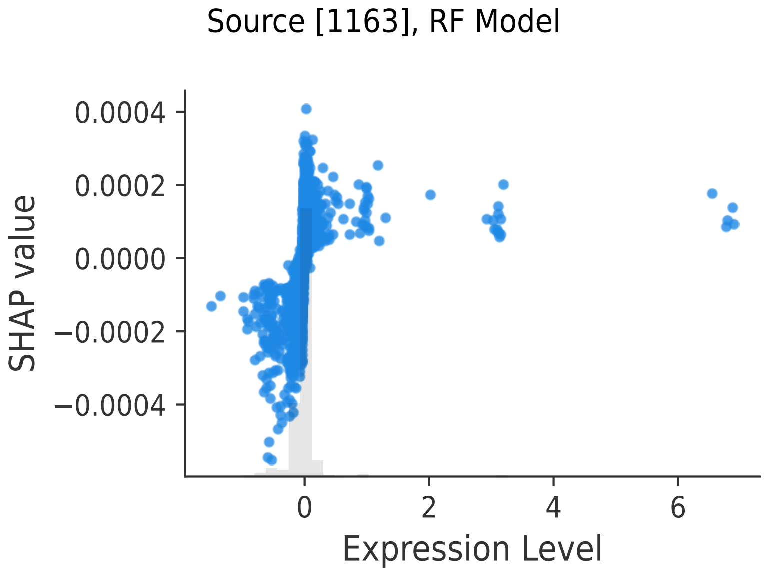
Source [1163], RF Model
SHAP value
0.0004
0.0002
0.0000
−0.0002
−0.0004
0
2
4
6
Expression Level

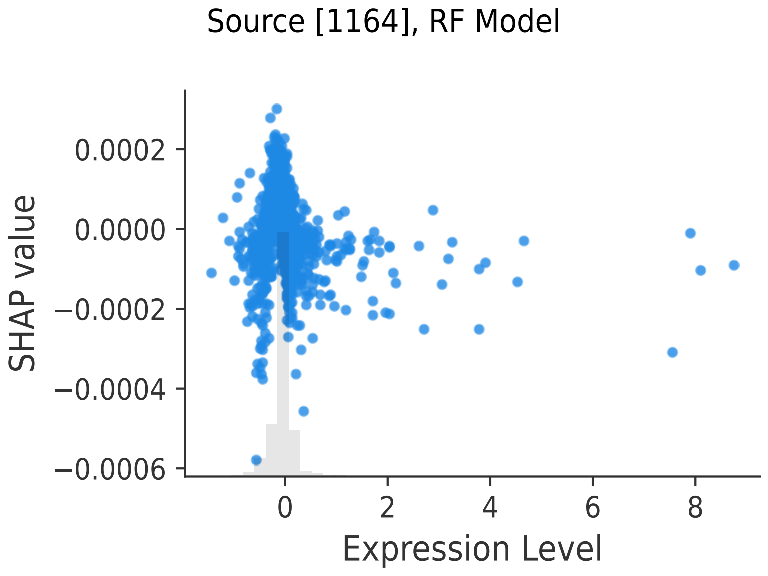

Source [1164], RF Model
SHAP value
0.0002
0.0000
−0.0002
−0.0004
−0.0006
0
2
4
6
8
Expression Level

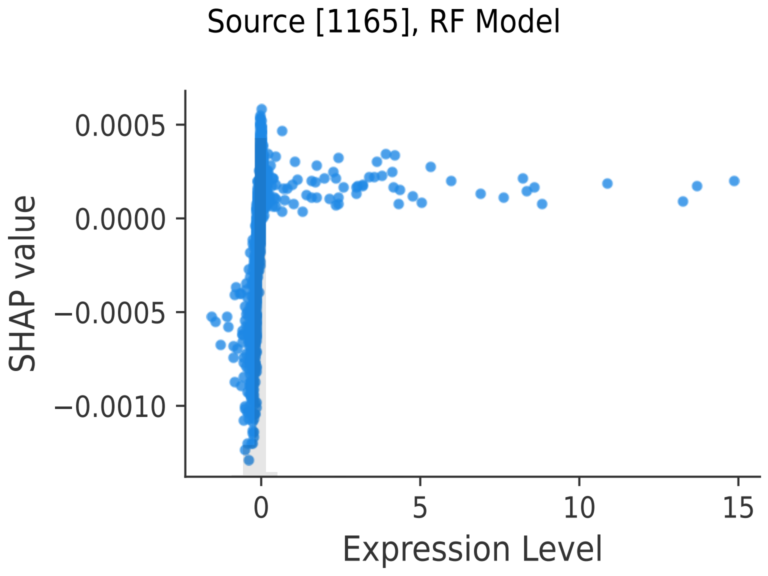
Source [1165], RF Model
SHAP value
0.0005
0.0000
−0.0005
−0.0010
0
5
10
15
Expression Level

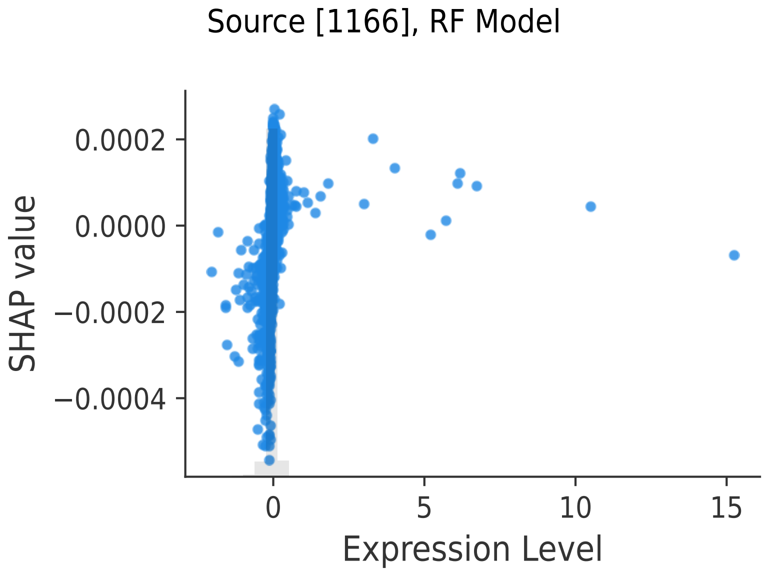
Source [1166], RF Model
0.0002
0.0000
−0.0002
−0.0004
SHAP value
0
5
10
15
Expression Level

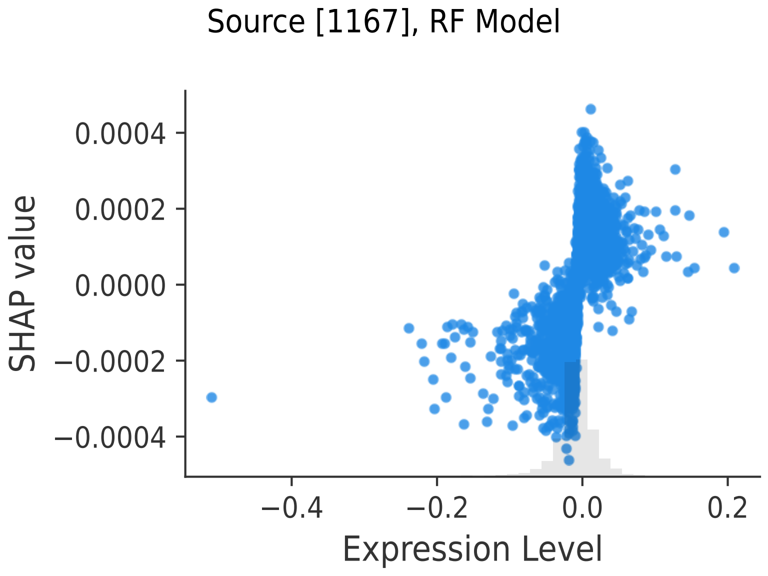
Source [1167], RF Model
SHAP value
0.0004
0.0002
0.0000
−0.0002
−0.0004
−0.4
−0.2
0.0
0.2
Expression Level

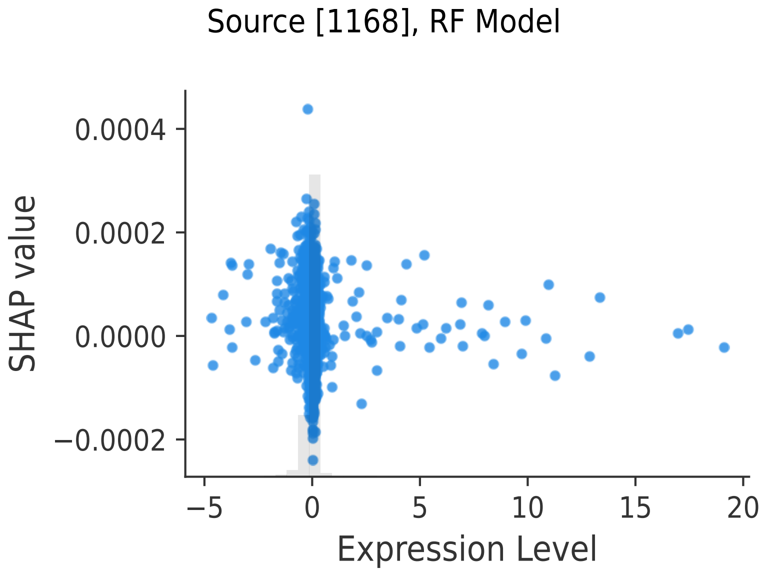

Source [1168], RF Model
SHAP value
0.0004
0.0002
0.0000
−0.0002
−5
0
5
10
15
20
Expression Level

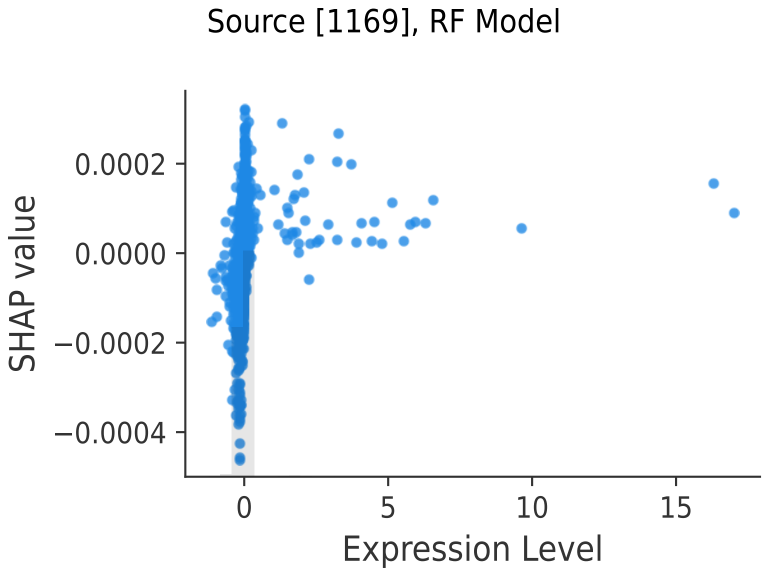

Source [1169], RF Model
SHAP value
0.0002
0.0000
−0.0002
−0.0004
0
5
10
15
Expression Level

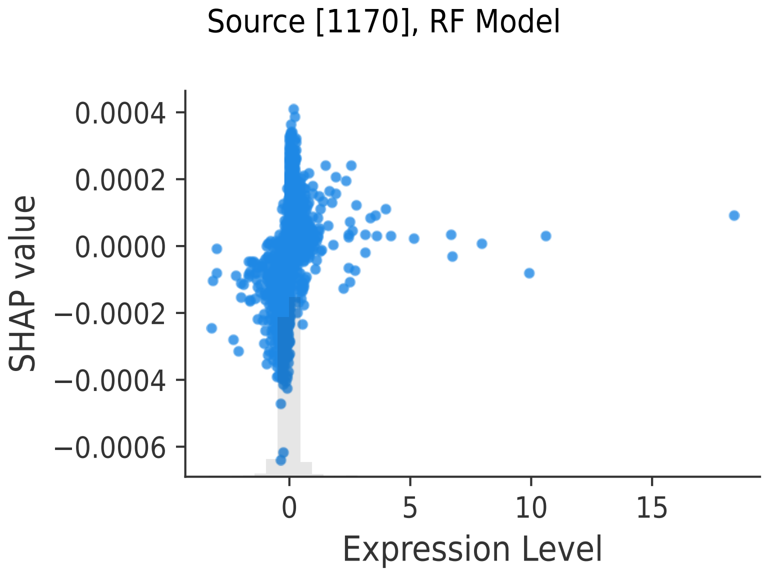

Source [1170], RF Model
0.0004
0.0002
0.0000
−0.0002
−0.0004
−0.0006
SHAP value
0
5
10
15
Expression Level

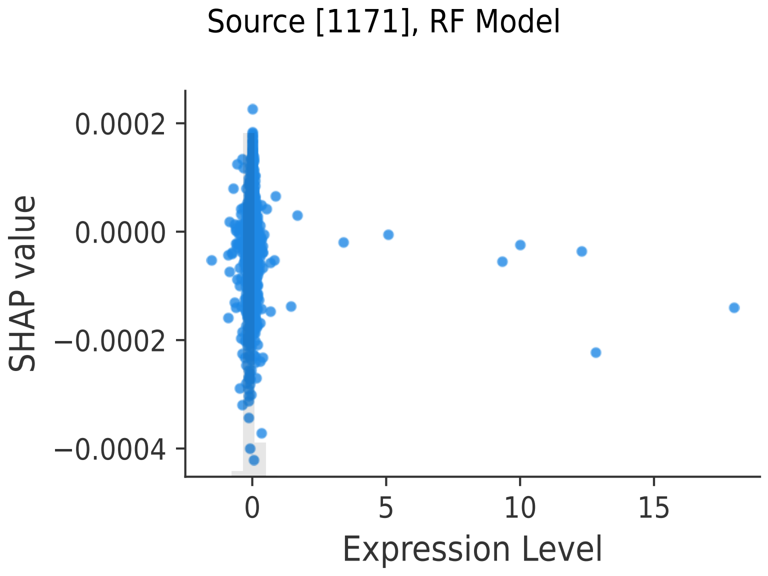

Source [1171], RF Model
SHAP value
0.0002
0.0000
−0.0002
−0.0004
0
5
10
15
Expression Level

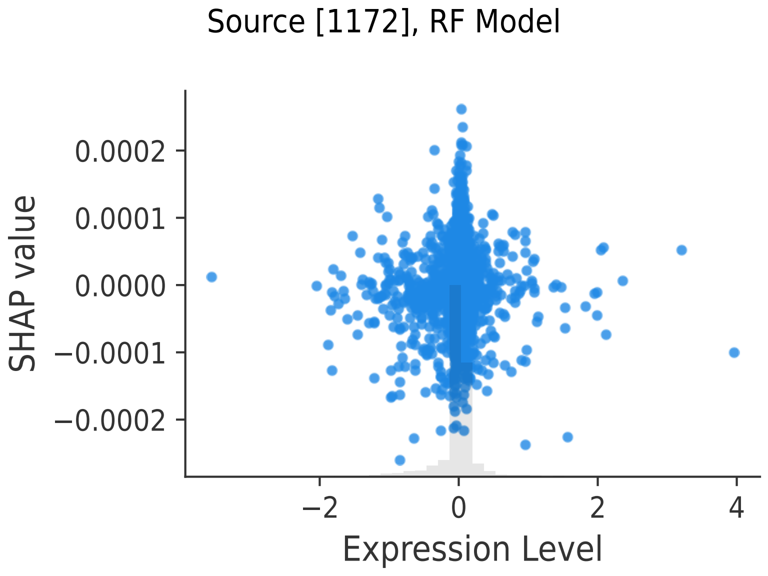

Source [1172], RF Model
SHAP value
0.0002
0.0001
0.0000
−0.0001
−0.0002
−2
0
2
4
Expression Level

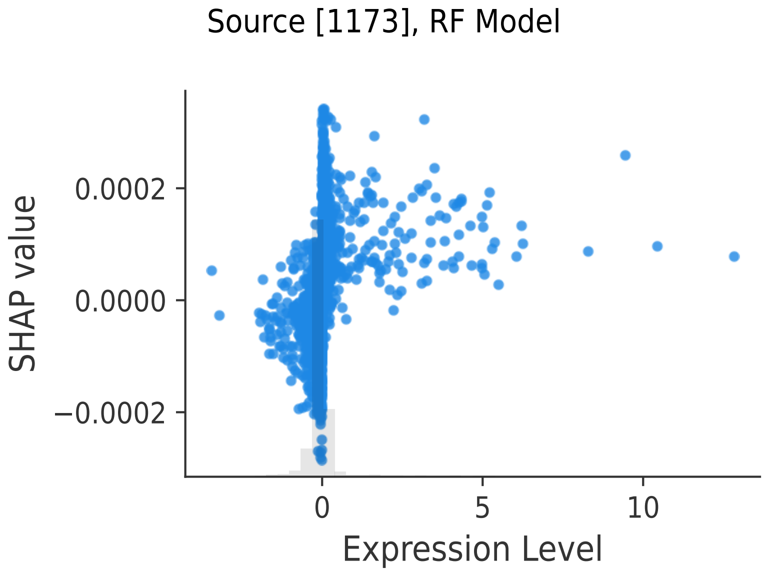
Source [1173], RF Model
SHAP value
0.0002
0.0000
−0.0002
0
5
10
Expression Level

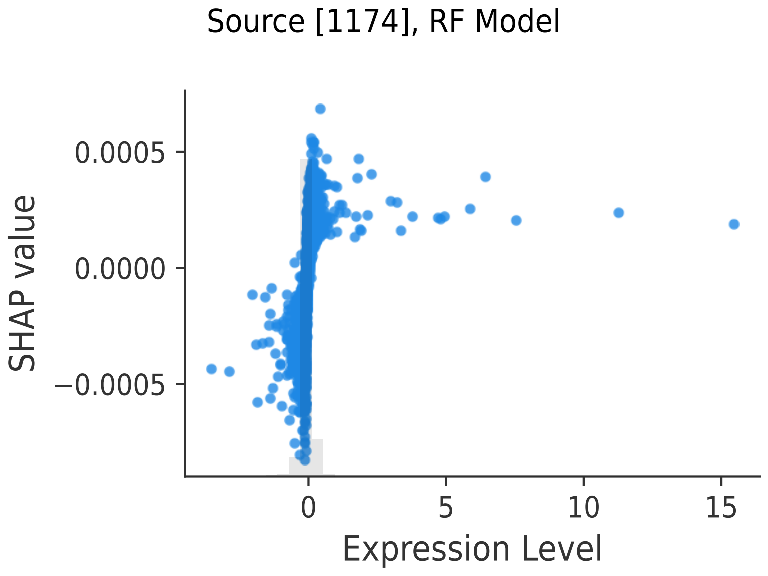

Source [1174], RF Model
0.0005
0.0000
−0.0005
SHAP value
0
5
10
15
Expression Level

Source [1175], RF Model

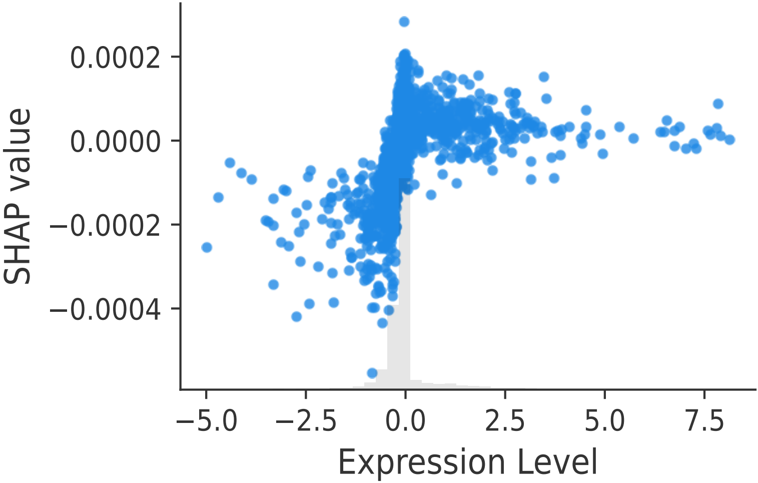

Source [1176], RF Model

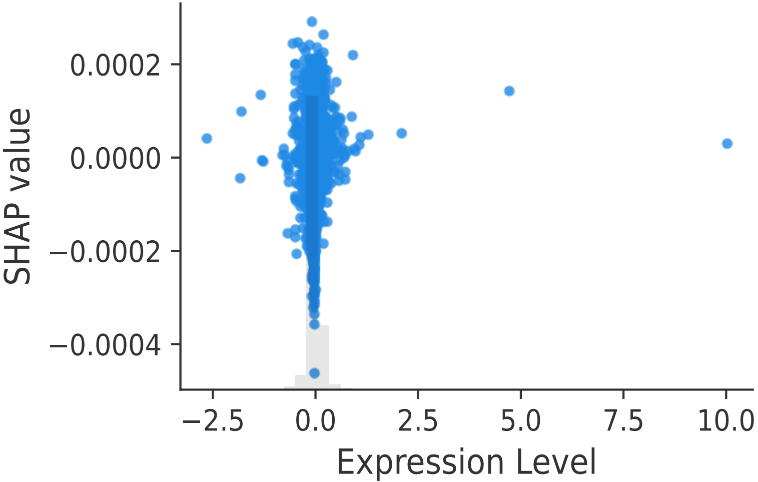

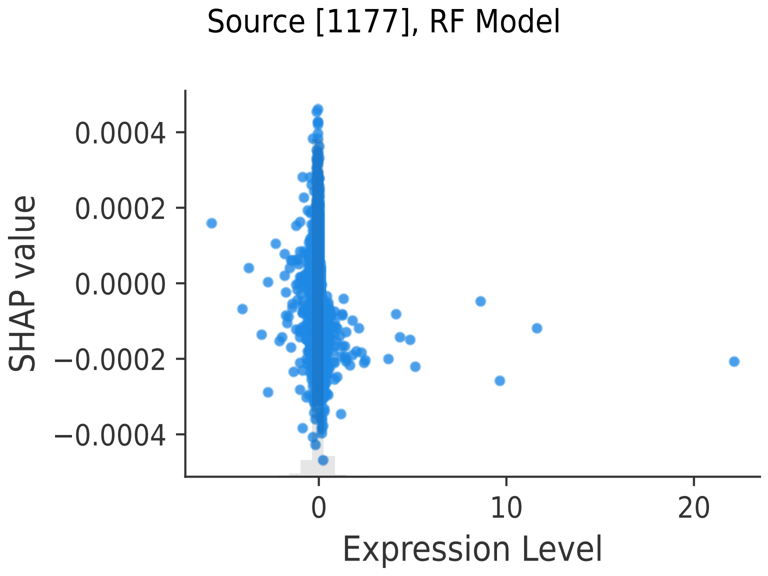
Source [1177], RF Model
SHAP value
0.0004
0.0002
0.0000
−0.0002
−0.0004
0
10
20
Expression Level

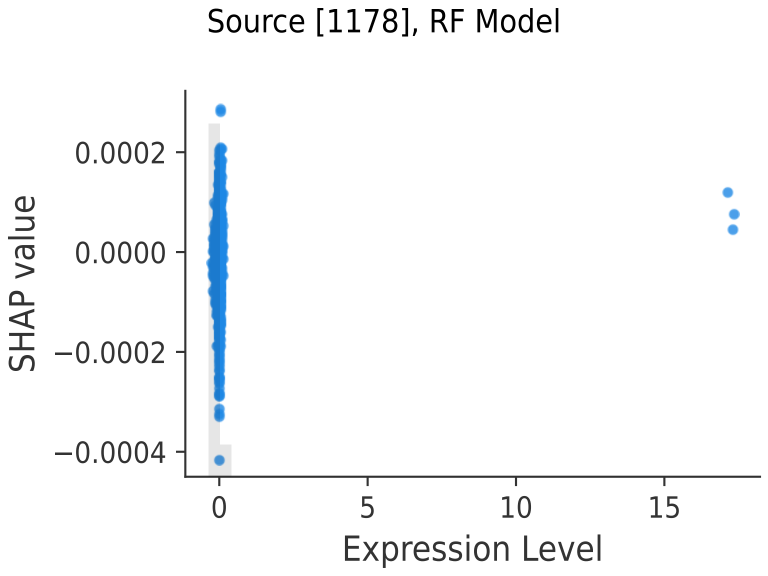

Source [1178], RF Model
0.0002
0.0000
−0.0002
−0.0004
SHAP value
0
5
10
15
Expression Level

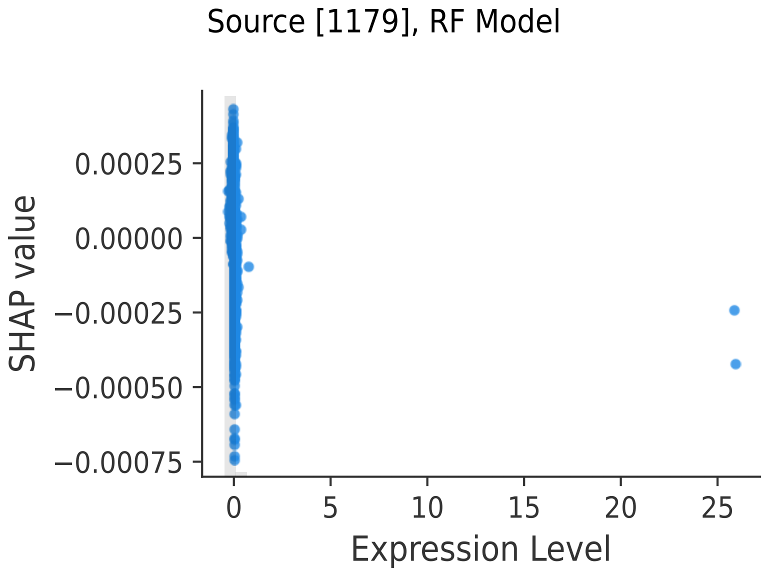
Source [1179], RF Model
SHAP value
0.00025
0.00000
−0.00025
−0.00050
−0.00075
0
5
10
15
20
25
Expression Level

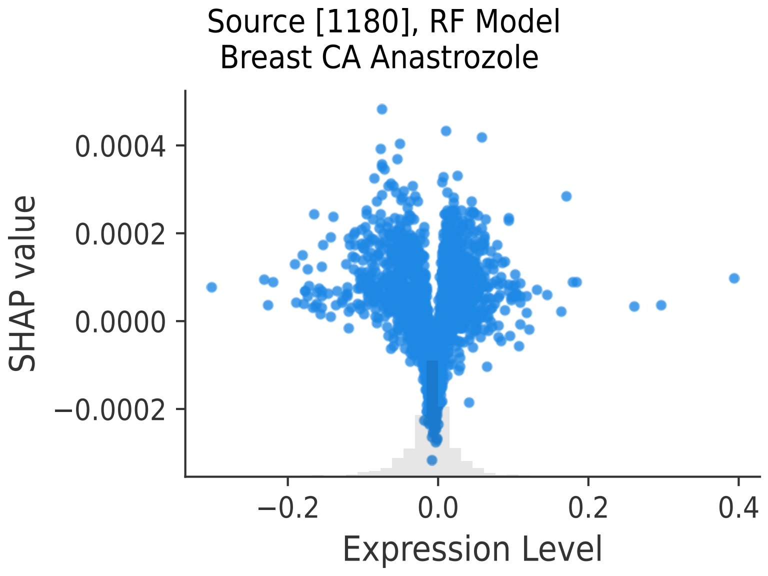

Source [1180], RF Model
Breast CA Anastrozole
SHAP value
0.0004
0.0002
0.0000
−0.0002
−0.2
0.0
0.2
0.4
Expression Level

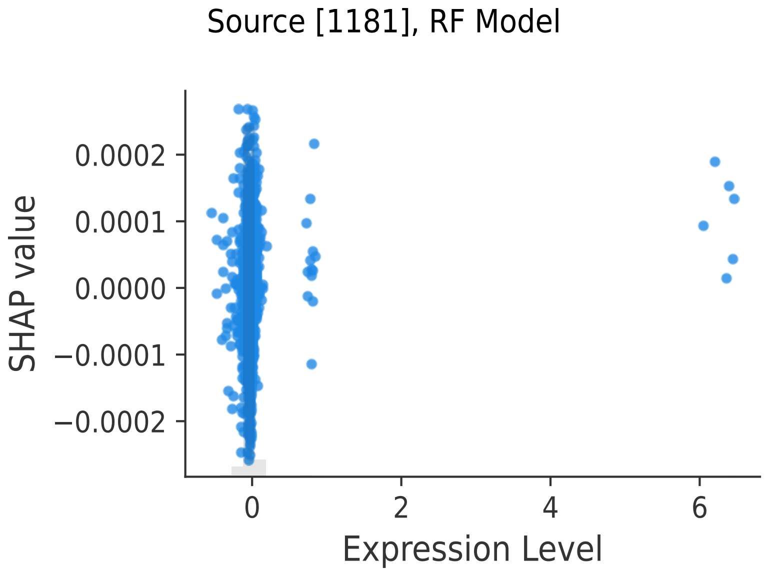
Source [1181], RF Model
SHAP value
0.0002
0.0001
0.0000
−0.0001
−0.0002
0
2
4
6
Expression Level

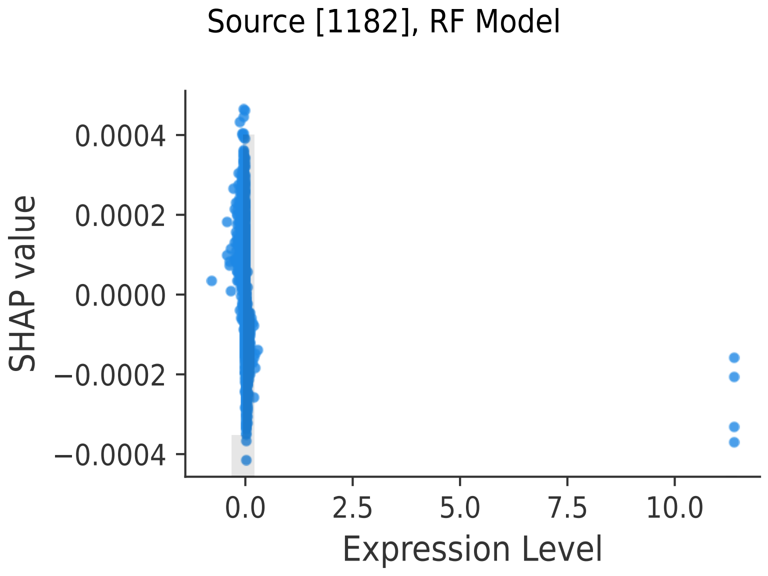

Source [1182], RF Model
0.0004
0.0002
0.0000
−0.0002
−0.0004
SHAP value
0.0
2.5
5.0
7.5
10.0
Expression Level

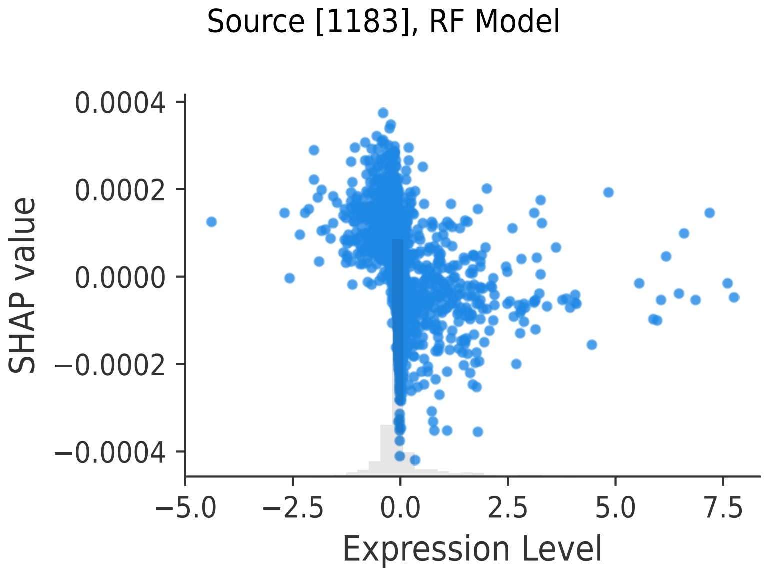
Source [1183], RF Model
0.0004
0.0002
0.0000
−0.0002
−0.0004
SHAP value
−5.0
−2.5
0.0
2.5
5.0
7.5
Expression Level

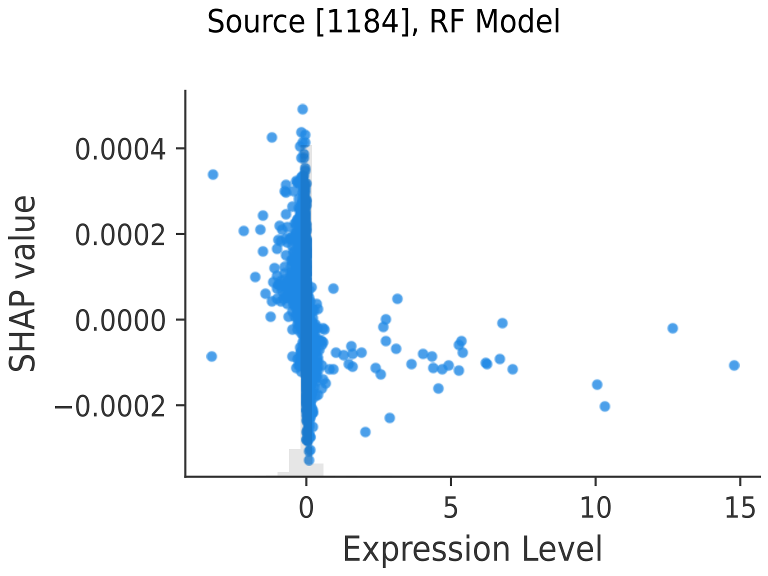
Source [1184], RF Model
SHAP value
0.0004
0.0002
0.0000
−0.0002
0
5
10
15
Expression Level

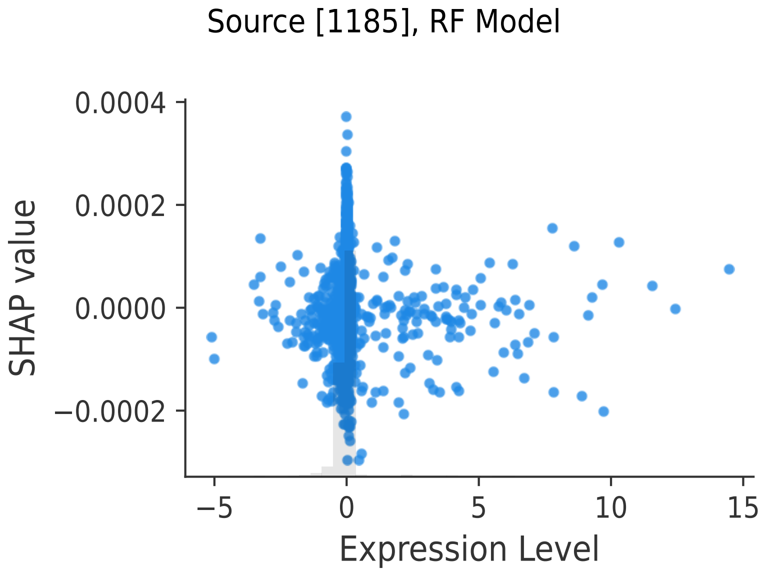
Source [1185], RF Model
0.0004
0.0002
0.0000
−0.0002
SHAP value
−5
0
5
10
15
Expression Level

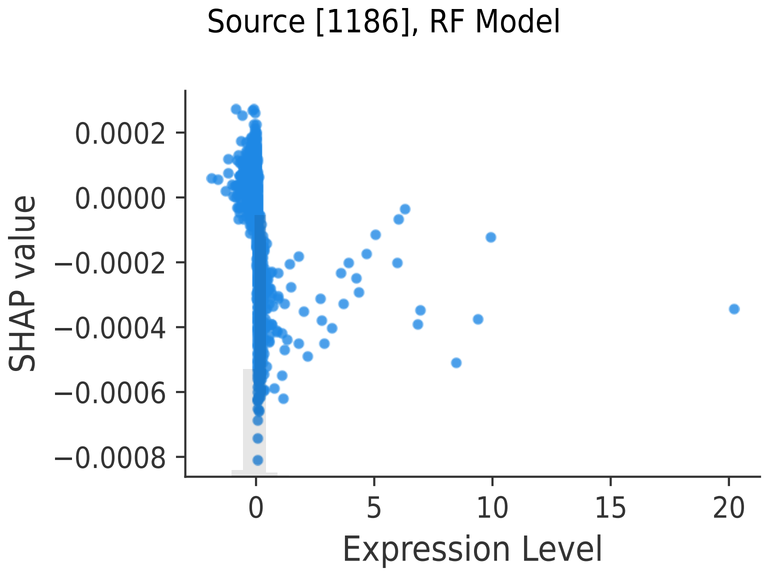

Source [1186], RF Model
SHAP value
0.0002
0.0000
−0.0002
−0.0004
−0.0006
−0.0008
0
5
10
15
20
Expression Level

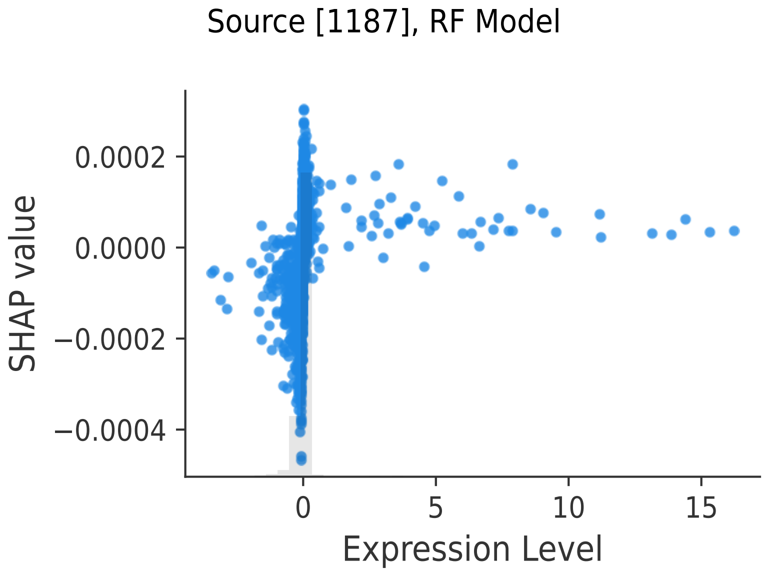

Source [1187], RF Model
0.0002
0.0000
−0.0002
−0.0004
SHAP value
0
5
10
15
Expression Level

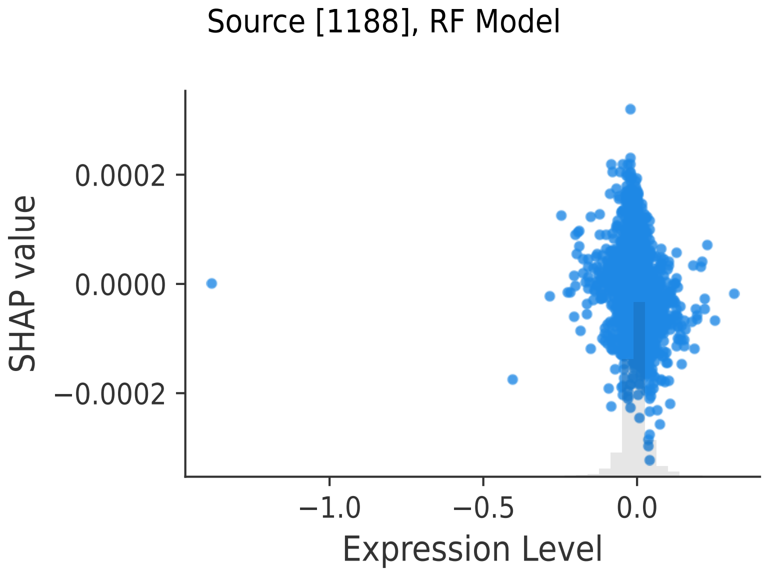
Source [1188], RF Model
SHAP value
0.0002
0.0000
−0.0002
−1.0
−0.5
0.0
Expression Level

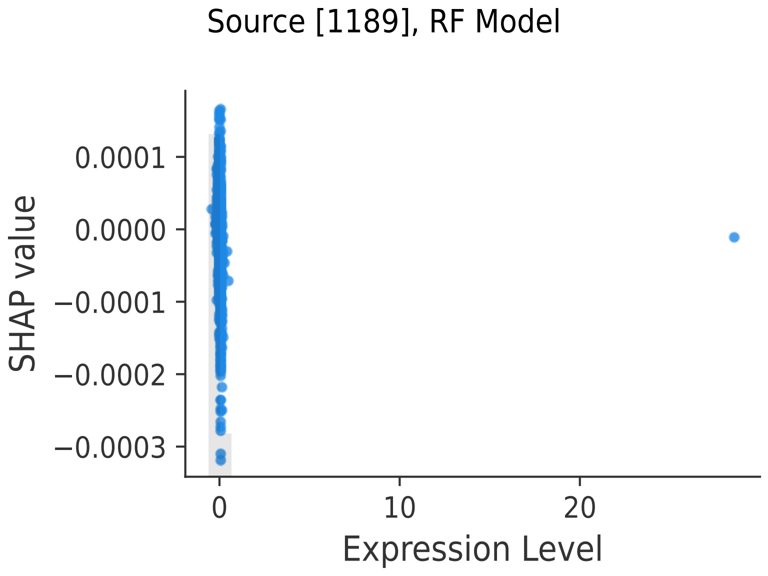
Source [1189], RF Model
SHAP value
0.0001
0.0000
−0.0001
−0.0002
−0.0003
0
10
20
Expression Level

Source [1190], RF Model
Breast Pain

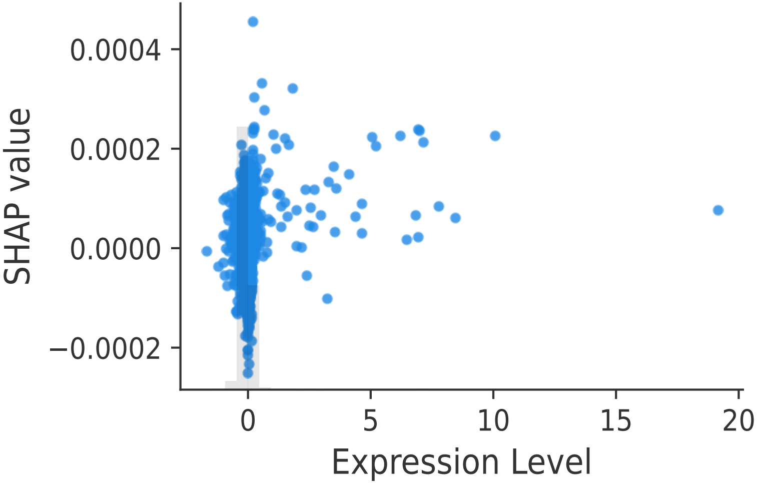

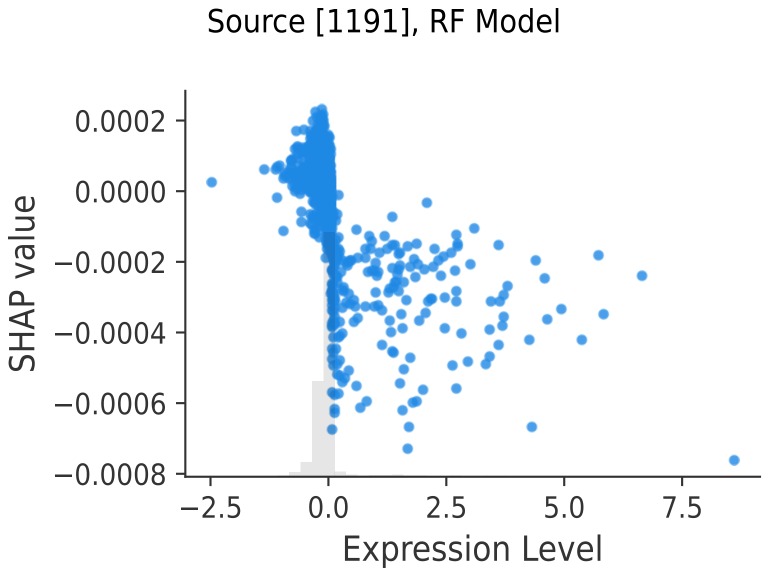
Source [1191], RF Model
SHAP value
0.0002
0.0000
−0.0002
−0.0004
−0.0006
−0.0008
−2.5
0.0
2.5
5.0
7.5
Expression Level

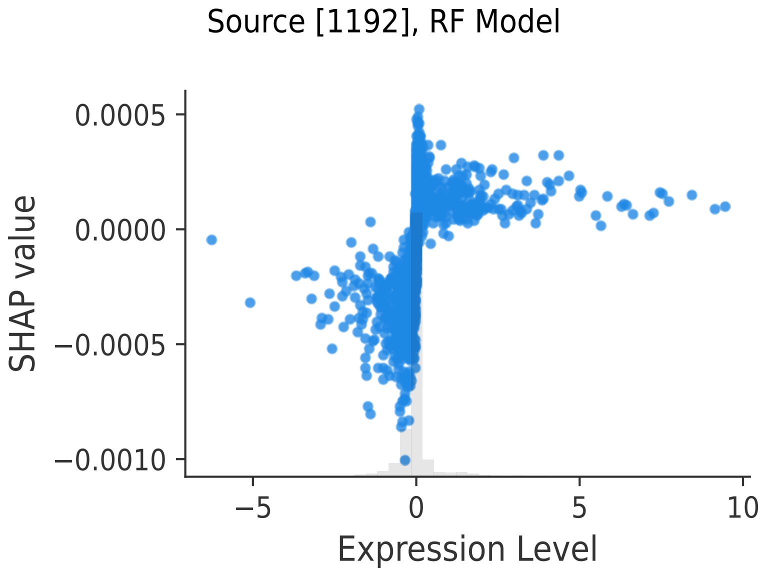

Source [1192], RF Model
0.0005
0.0000
−0.0005
−0.0010
SHAP value
−5
0
5
10
Expression Level

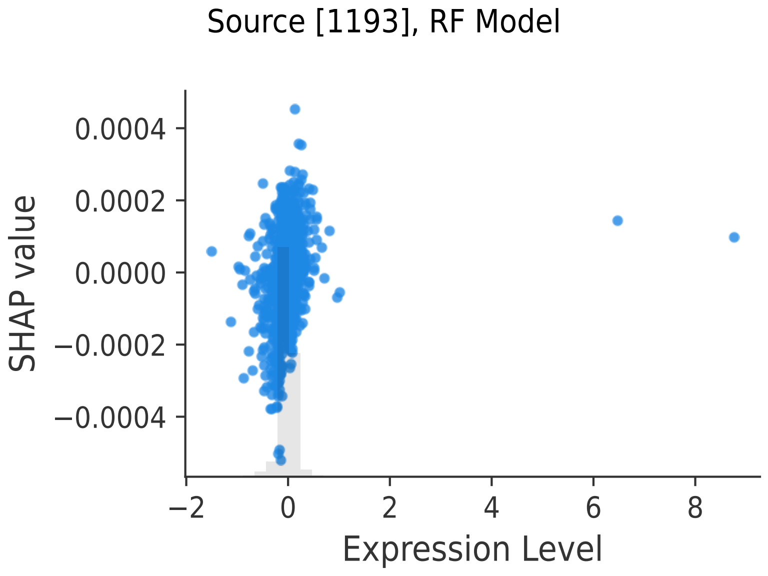

Source [1193], RF Model
SHAP value
0.0004
0.0002
0.0000
−0.0002
−0.0004
−2
0
2
4
6
8
Expression Level

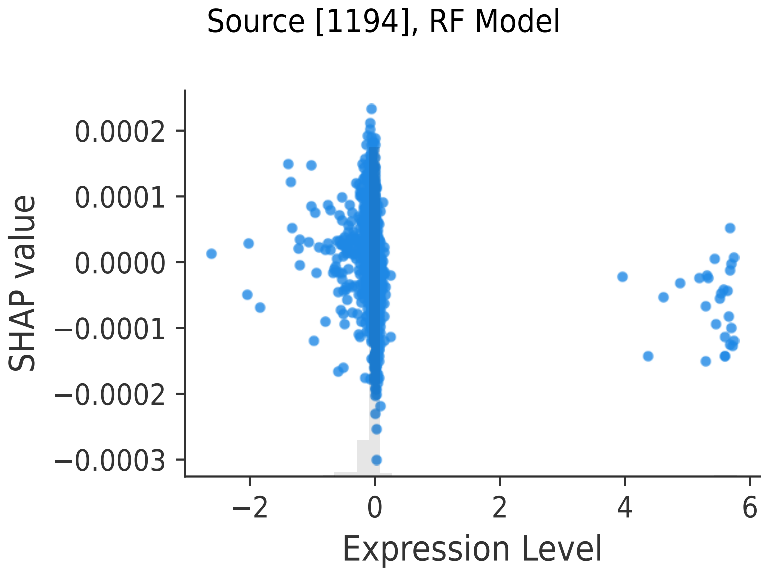
Source [1194], RF Model
SHAP value
0.0002
0.0001
0.0000
−0.0001
−0.0002
−0.0003
−2
0
2
4
6
Expression Level

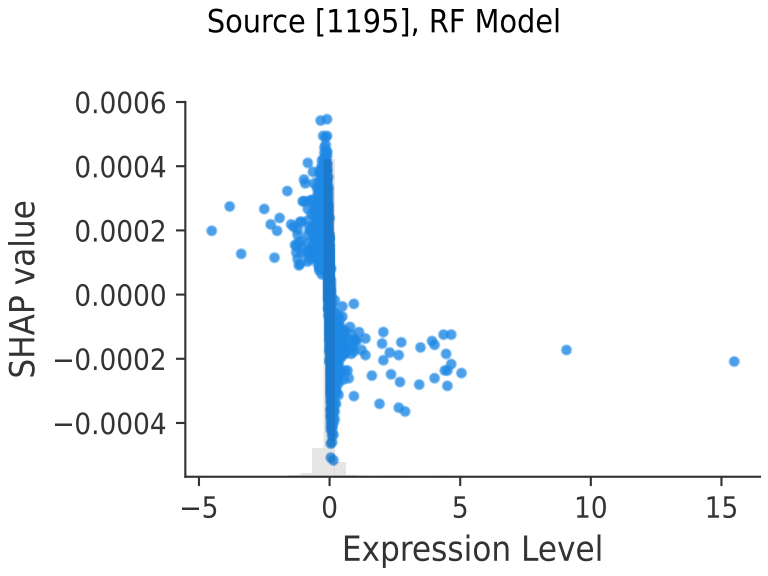
Source [1195], RF Model
SHAP value
0.0006
0.0004
0.0002
0.0000
−0.0002
−0.0004
Expression Level
−5
0
5
10
15

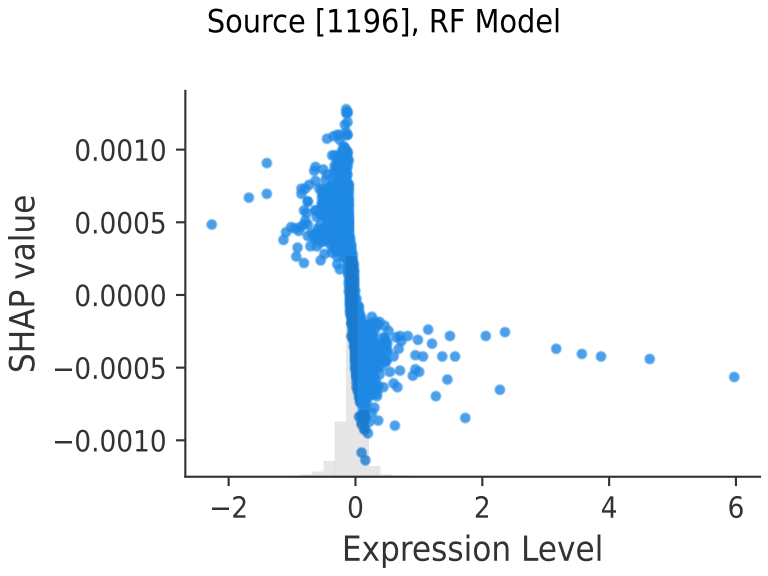

Source [1196], RF Model
SHAP value
0.0010
0.0005
0.0000
−0.0005
−0.0010
−2
0
2
4
6
Expression Level

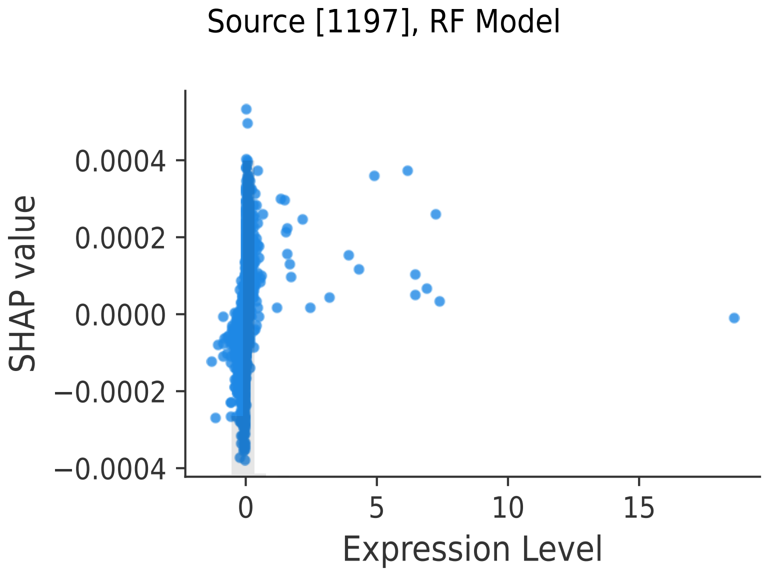

Source [1197], RF Model
SHAP value
0.0004
0.0002
0.0000
−0.0002
−0.0004
0
5
10
15
Expression Level

Source [1198], RF Model
Lower lobe primary malignancy

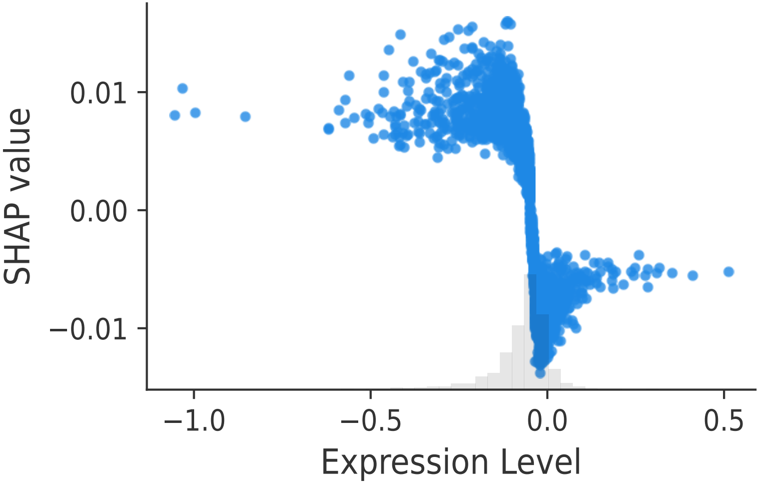

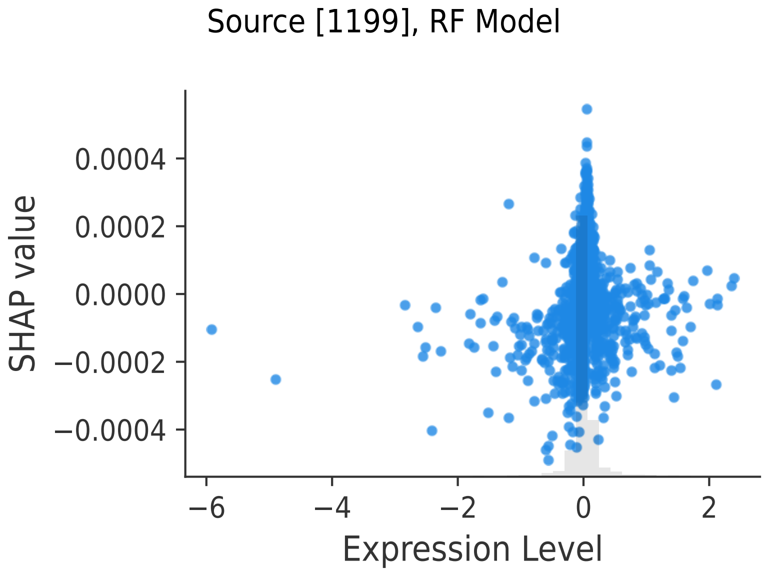

Source [1199], RF Model
SHAP value
0.0004
0.0002
0.0000
−0.0002
−0.0004
−6
−4
−2
0
2
Expression Level

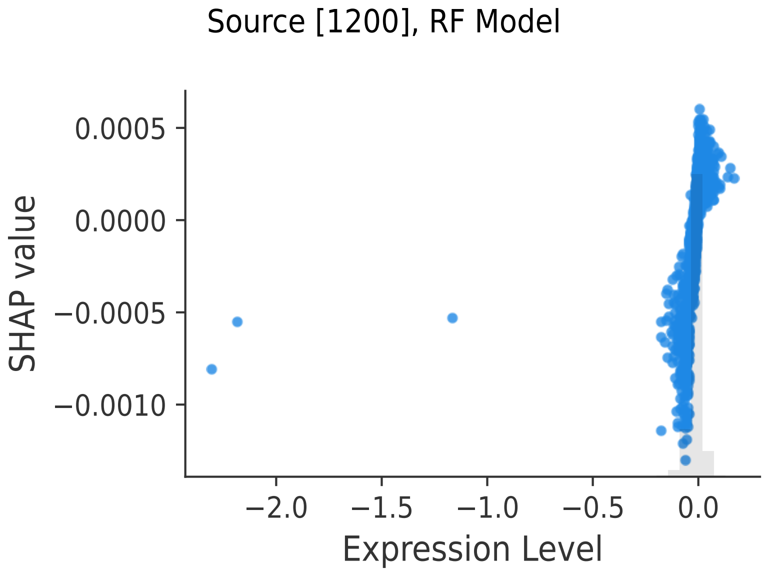

Source [1200], RF Model
0.0005
0.0000
−0.0005
−0.0010
SHAP value
−2.0
−1.5
−1.0
−0.5
0.0
Expression Level

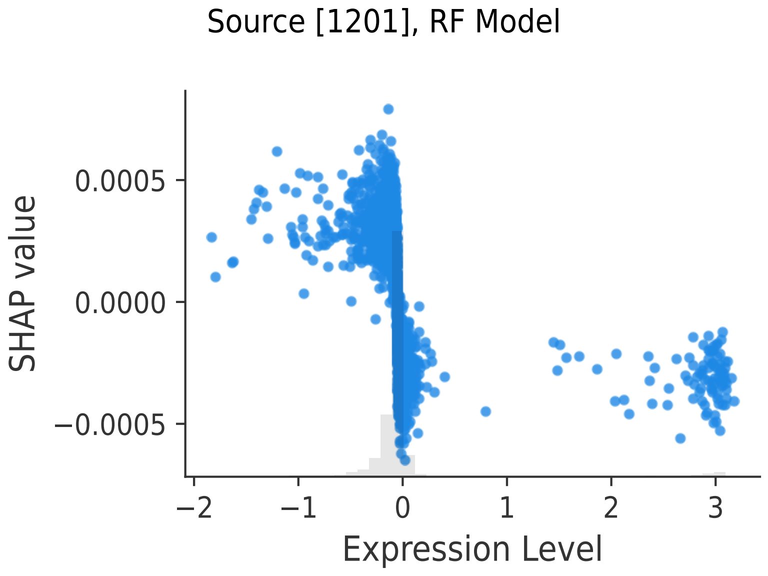

Source [1201], RF Model
0.0005
0.0000
−0.0005
SHAP value
−2
−1
0
1
2
3
Expression Level

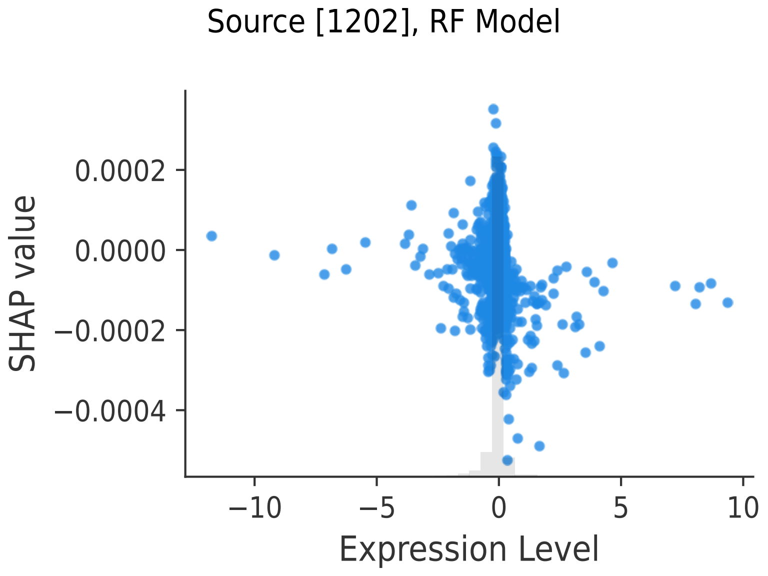
Source [1202], RF Model
SHAP value
0.0002
0.0000
−0.0002
−0.0004
Expression Level
−10
−5
0
5
10

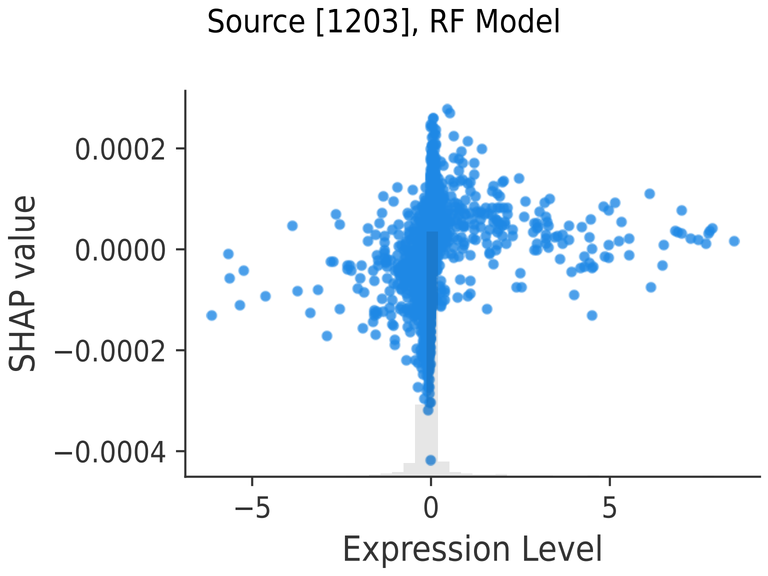
Source [1203], RF Model
0.0002
0.0000
−0.0002
−0.0004
SHAP value
−5
0
5
Expression Level

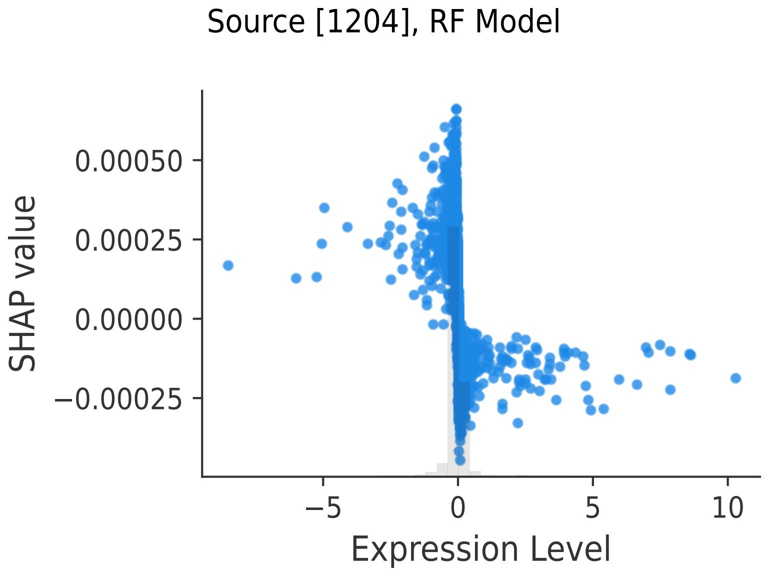
Source [1204], RF Model
0.00050
0.00025
0.00000
−0.00025
SHAP value
−5
0
5
10
Expression Level

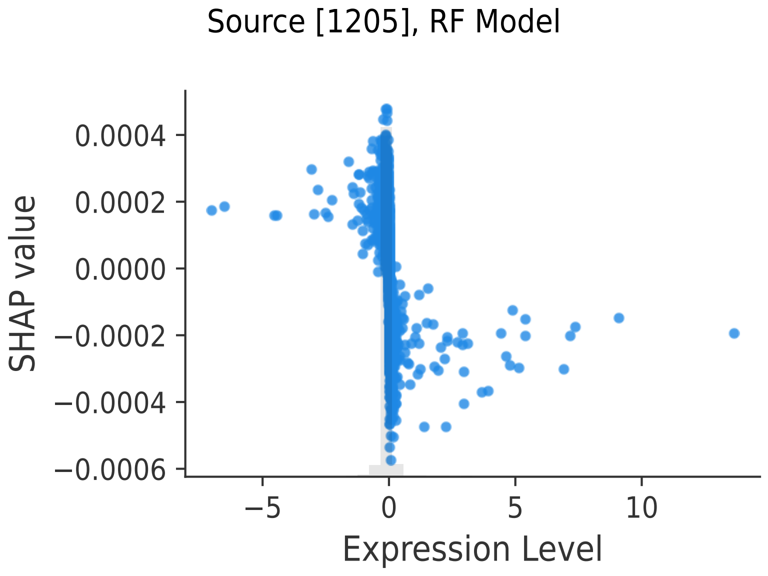

Source [1205], RF Model
0.0004
0.0002
0.0000
−0.0002
−0.0004
−0.0006
SHAP value
−5
0
5
10
Expression Level

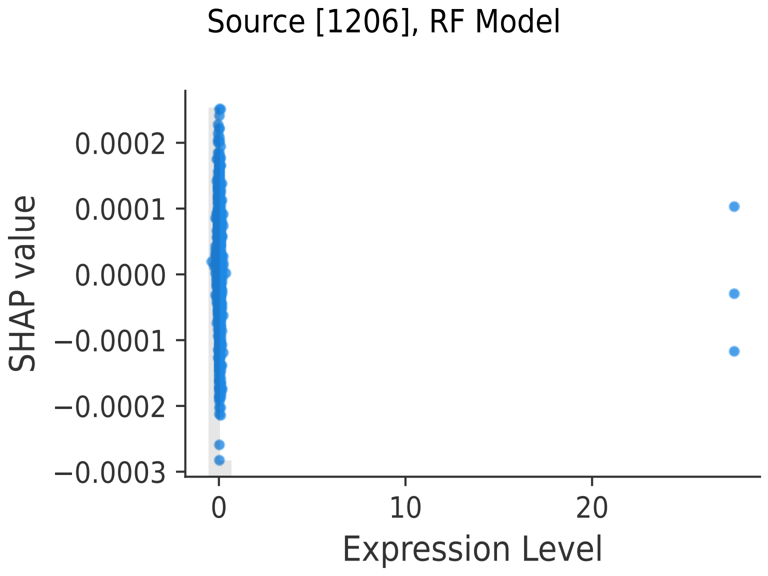
Source [1206], RF Model
SHAP value
0.0002
0.0001
0.0000
−0.0001
−0.0002
−0.0003
0
10
20
Expression Level

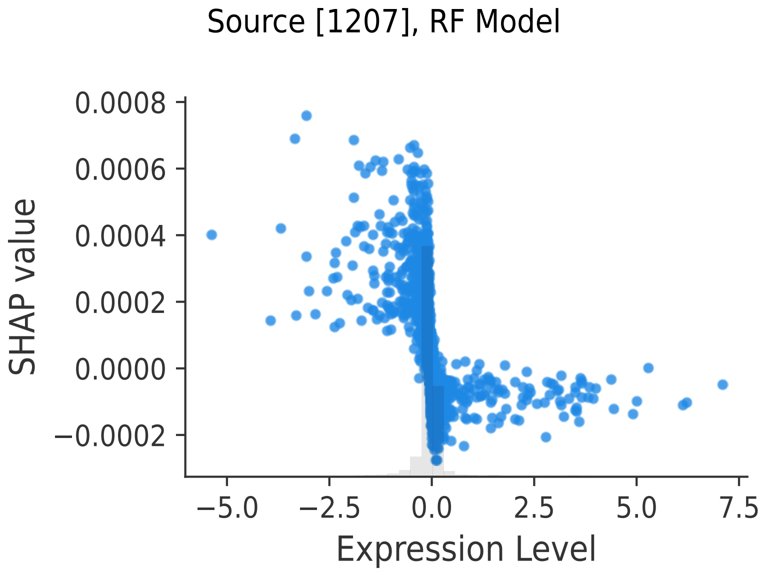

Source [1207], RF Model
0.0008
0.0006
0.0004
0.0002
0.0000
−0.0002
SHAP value
−5.0
−2.5
0.0
2.5
5.0
7.5
Expression Level

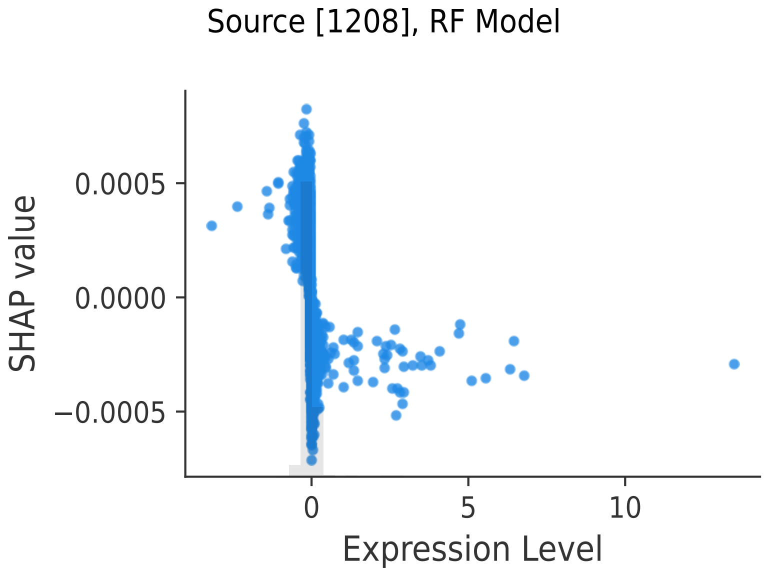
Source [1208], RF Model
SHAP value
0.0005
0.0000
−0.0005
0
5
10
Expression Level

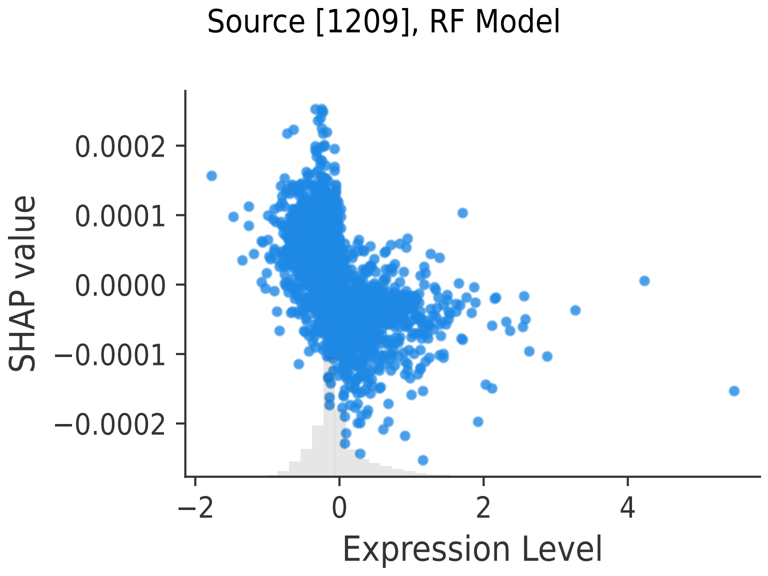
Source [1209], RF Model
0.0002
0.0001
0.0000
−0.0001
−0.0002
SHAP value
−2
0
2
4
Expression Level

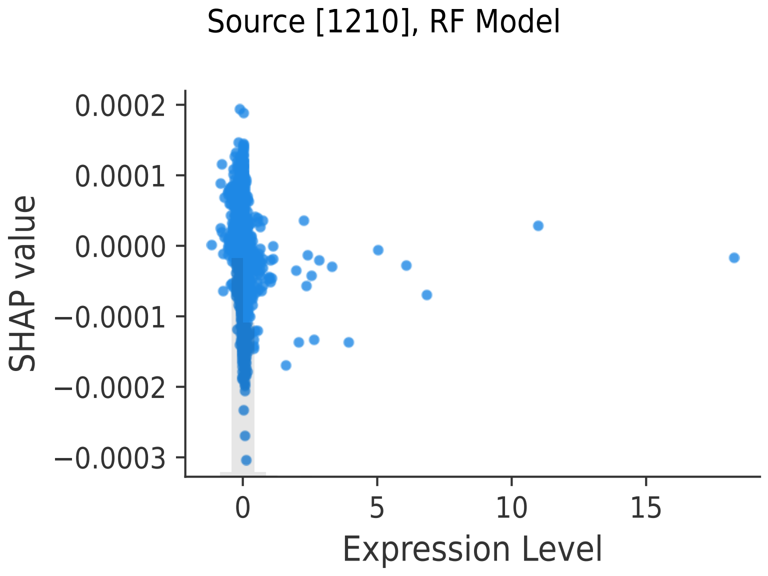

Source [1210], RF Model
0.0002
0.0001
0.0000
−0.0001
−0.0002
−0.0003
SHAP value
0
5
10
15
Expression Level

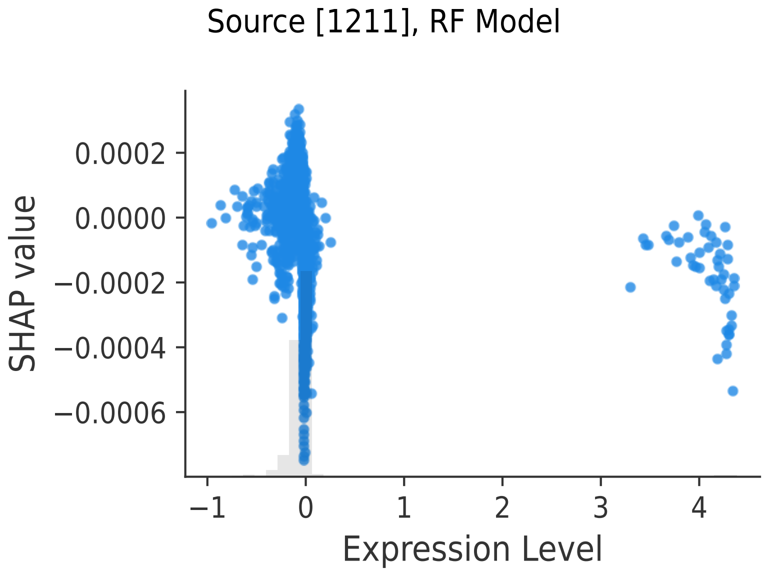

Source [1211], RF Model
SHAP value
0.0002
0.0000
−0.0002
−0.0004
−0.0006
−1
0
1
2
3
4
Expression Level

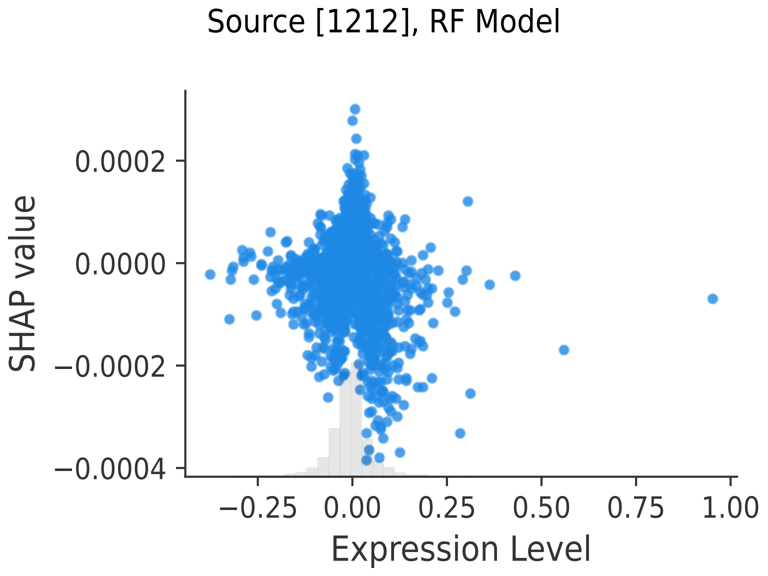

Source [1212], RF Model
SHAP value
0.0002
0.0000
−0.0002
−0.0004
−0.25
0.00
0.25
0.50
0.75
1.00
Expression Level

Source [1213], RF Model

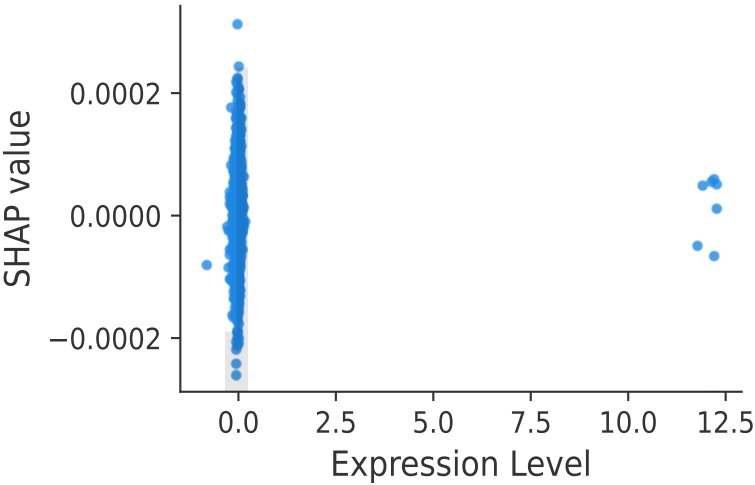

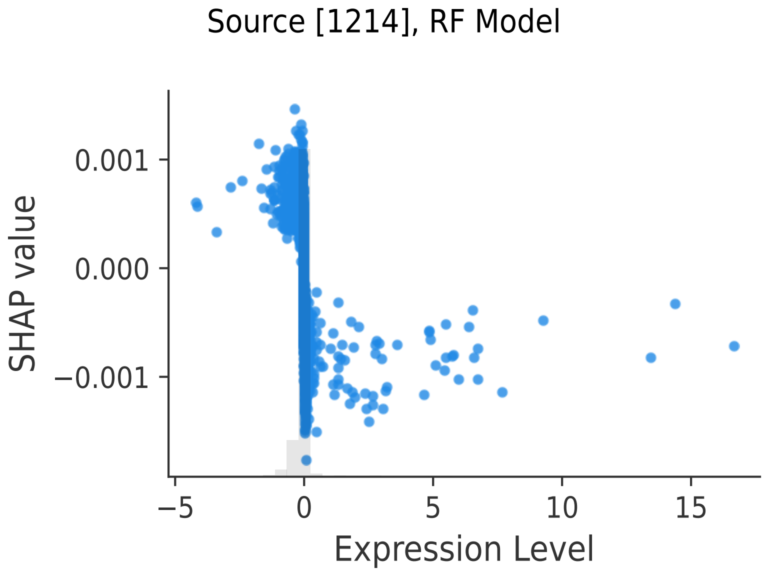

Source [1214], RF Model
SHAP value
0.001
0.000
−0.001
−5
0
5
10
15
Expression Level

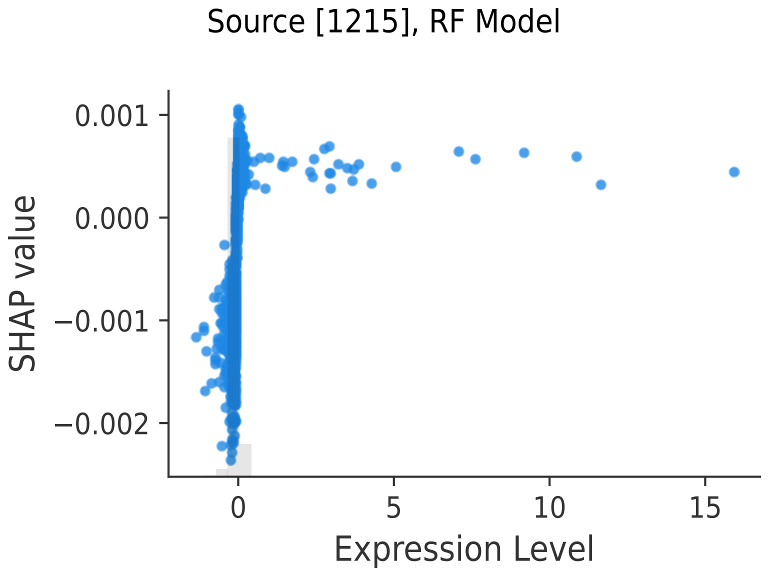
Source [1215], RF Model
SHAP value
0.001
0.000
−0.001
−0.002
0
5
10
15
Expression Level

Source [1216], RF Model

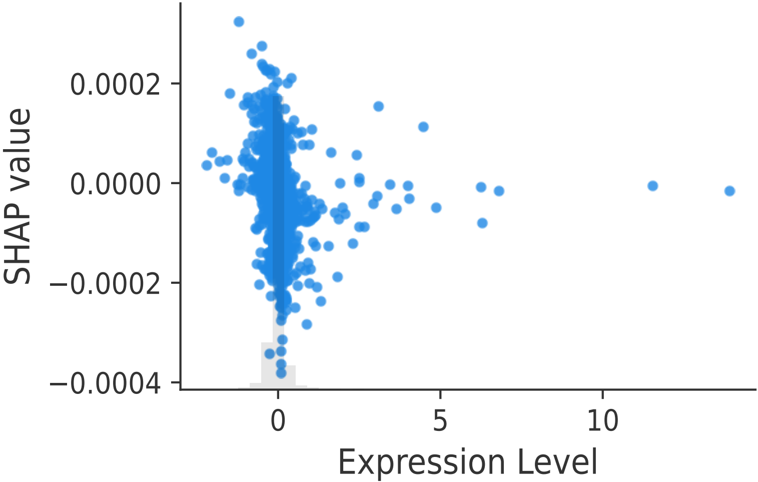

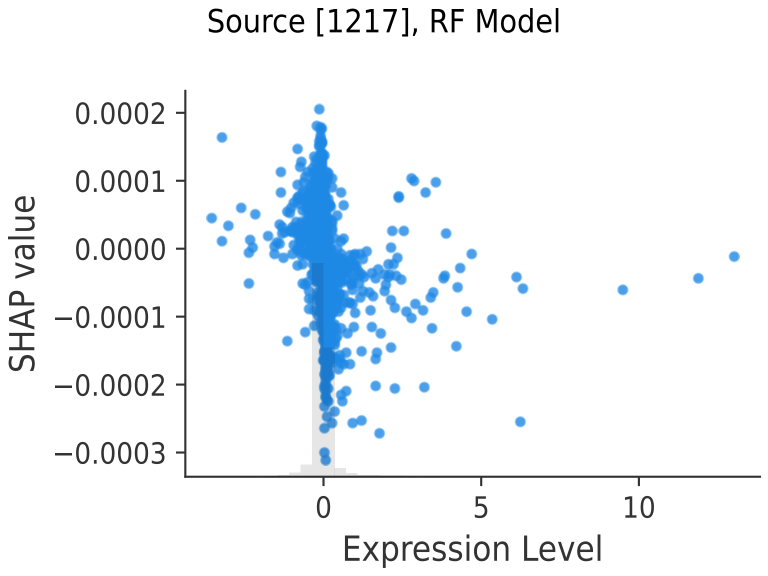
Source [1217], RF Model
SHAP value
0.0002
0.0001
0.0000
−0.0001
−0.0002
−0.0003
0
5
10
Expression Level

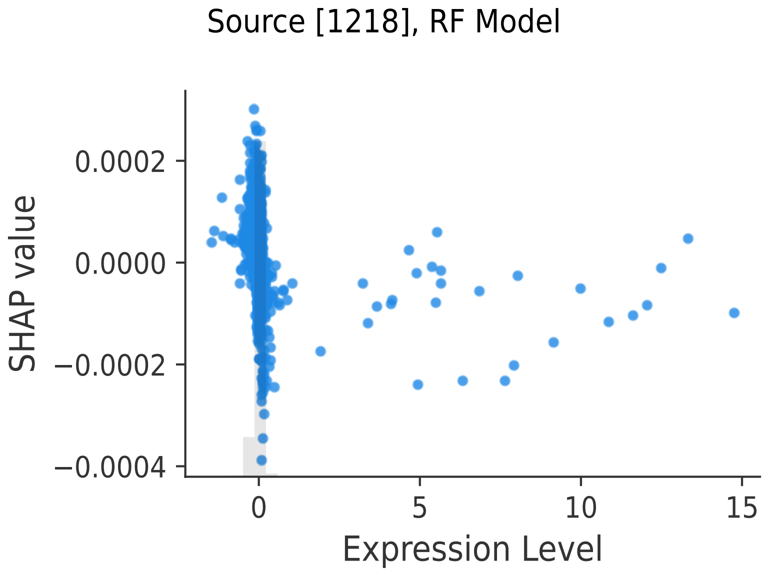
Source [1218], RF Model
SHAP value
0.0002
0.0000
−0.0002
−0.0004
0
5
10
15
Expression Level

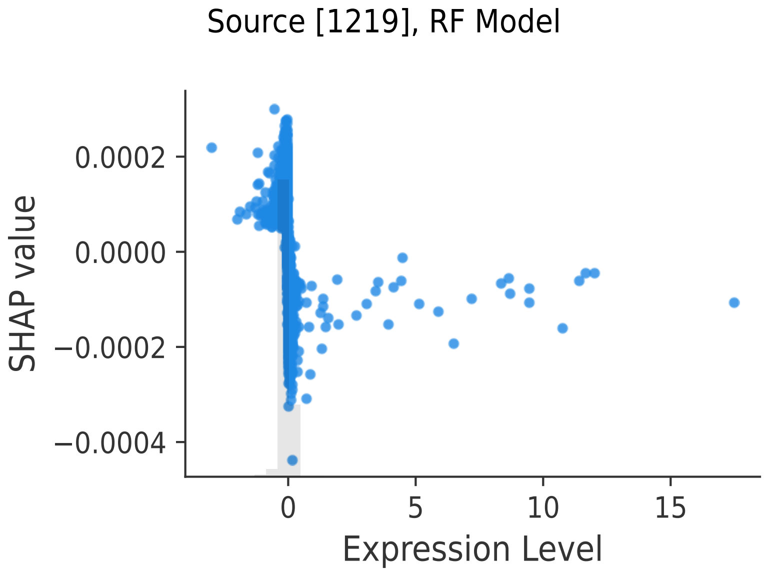
Source [1219], RF Model
SHAP value
0.0002
0.0000
−0.0002
−0.0004
0
5
10
15
Expression Level

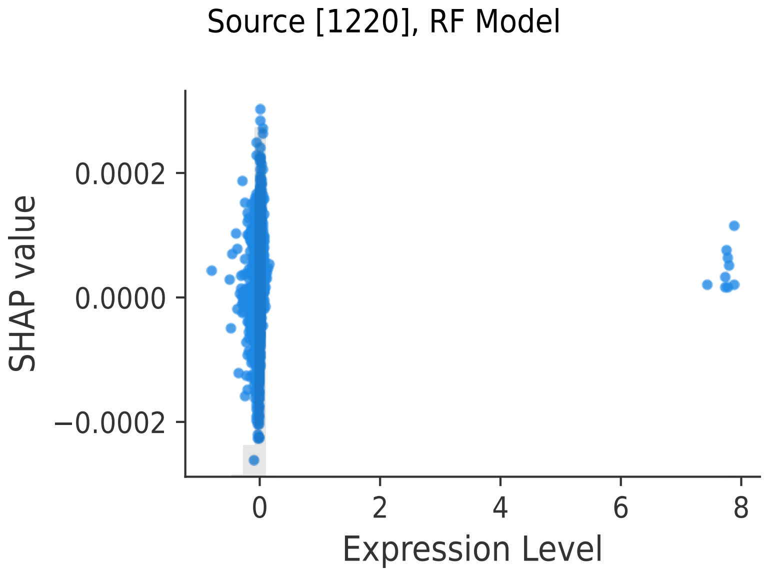

Source [1220], RF Model
SHAP value
0.0002
0.0000
−0.0002
0
2
4
6
8
Expression Level

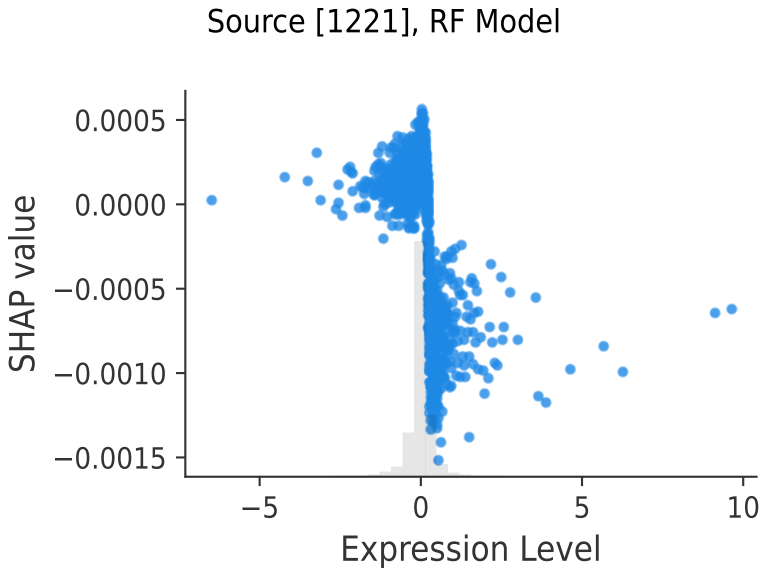

Source [1221], RF Model
0.0005
0.0000
−0.0005
−0.0010
−0.0015
SHAP value
−5
0
5
10
Expression Level

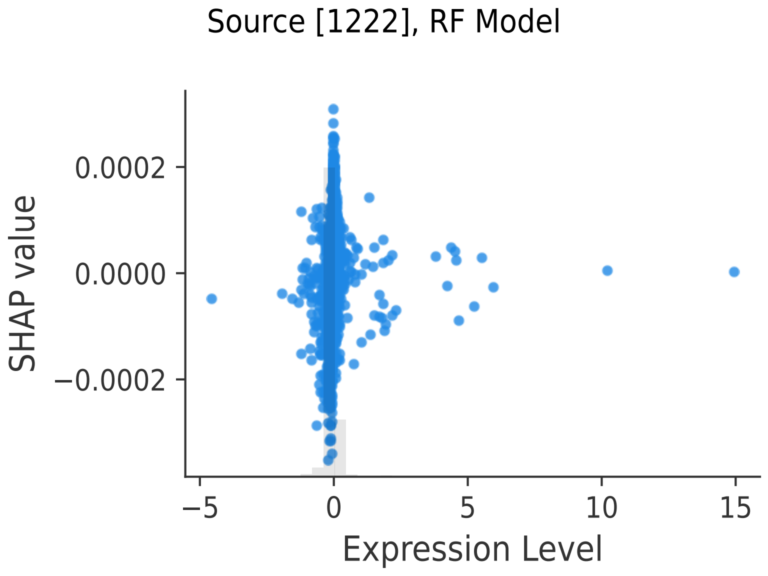

Source [1222], RF Model
SHAP value
0.0002
0.0000
−0.0002
−5
0
5
10
15
Expression Level

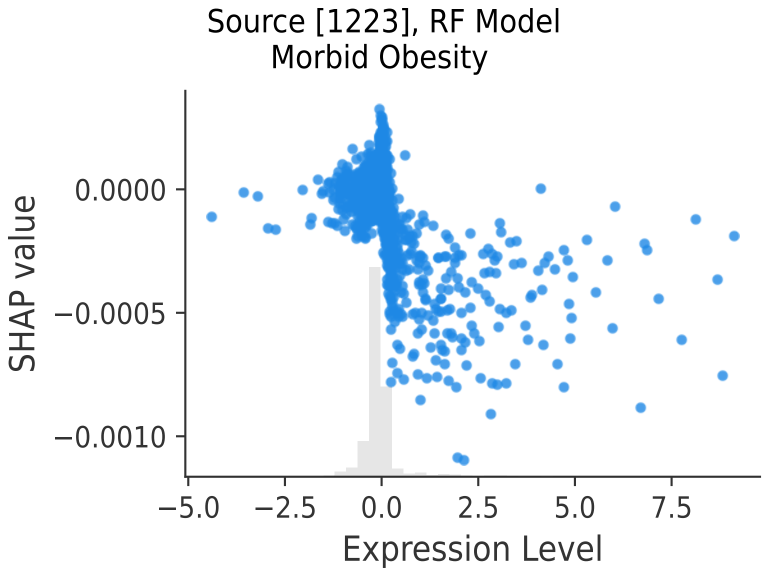
Source [1223], RF Model
Morbid Obesity
SHAP value
0.0000
−0.0005
−0.0010
−5.0
−2.5
0.0
2.5
5.0
7.5
Expression Level

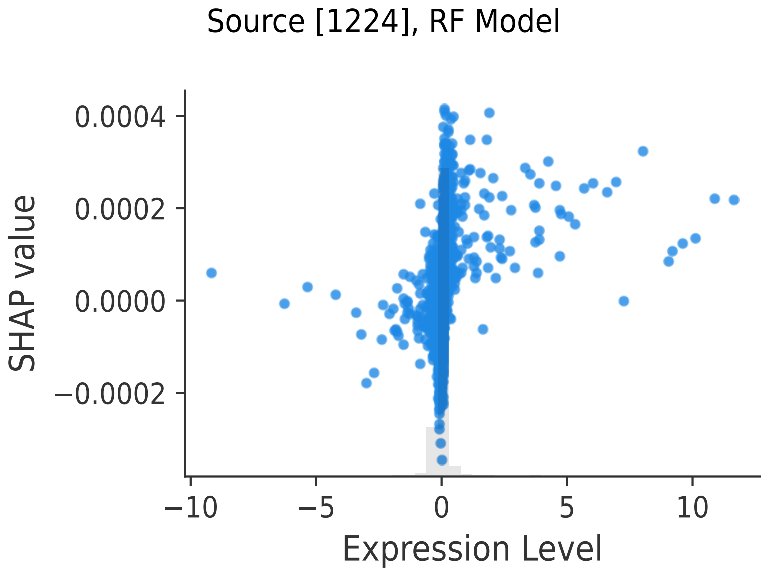
Source [1224], RF Model
SHAP value
0.0004
0.0002
0.0000
−0.0002
−10
−5
0
5
10
Expression Level

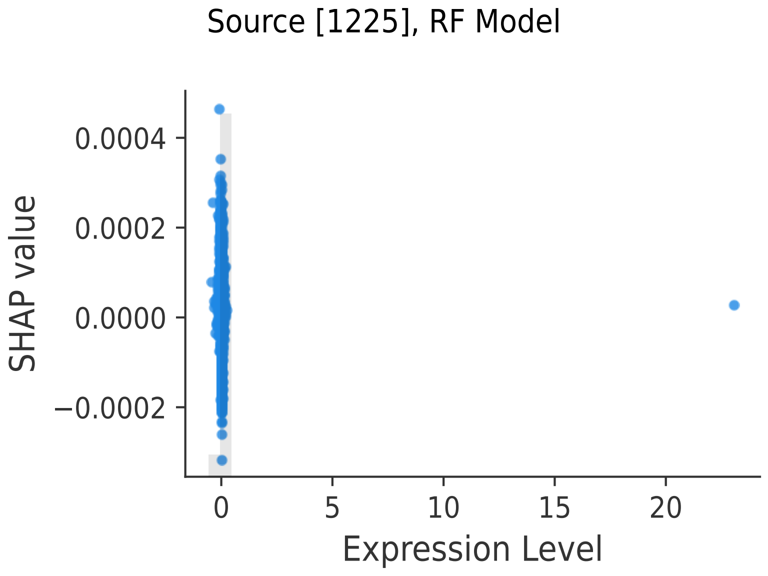
Source [1225], RF Model
0.0004
0.0002
0.0000
−0.0002
SHAP value
0
5
10
15
20
Expression Level

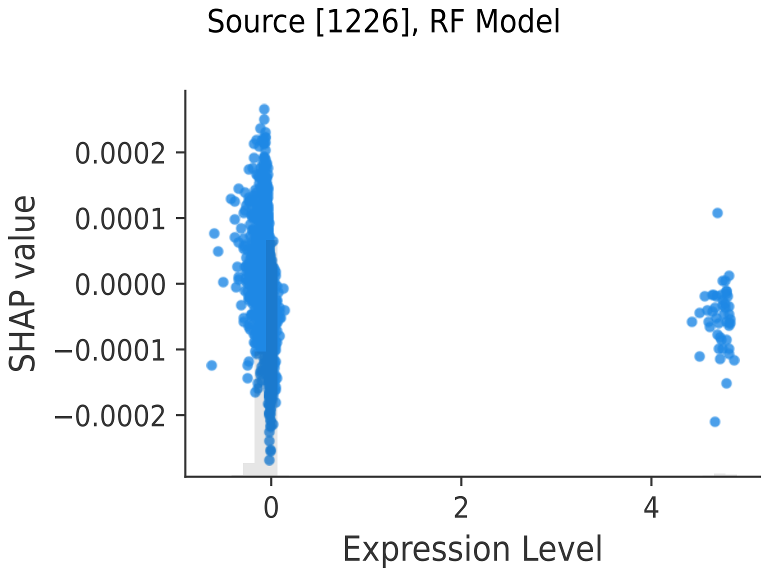

Source [1226], RF Model
0.0002
0.0001
0.0000
−0.0001
−0.0002
SHAP value
0
2
4
Expression Level

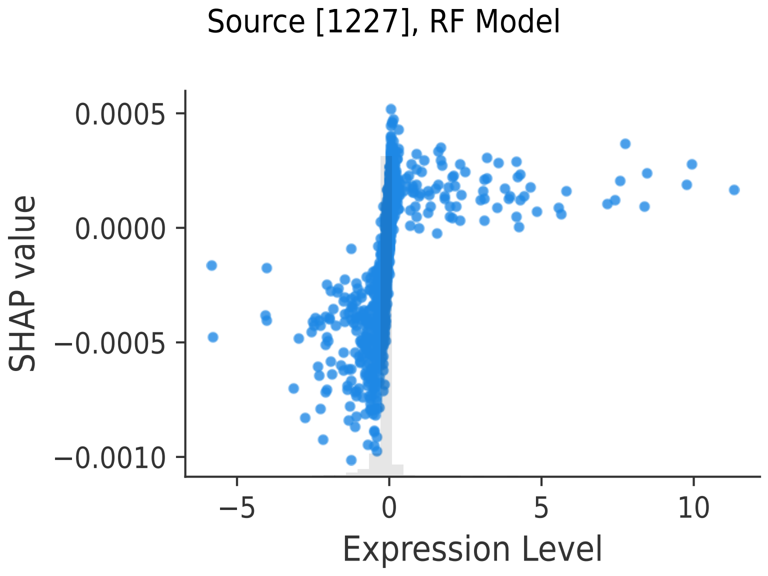

Source [1227], RF Model
0.0005
0.0000
−0.0005
−0.0010
SHAP value
−5
0
5
10
Expression Level

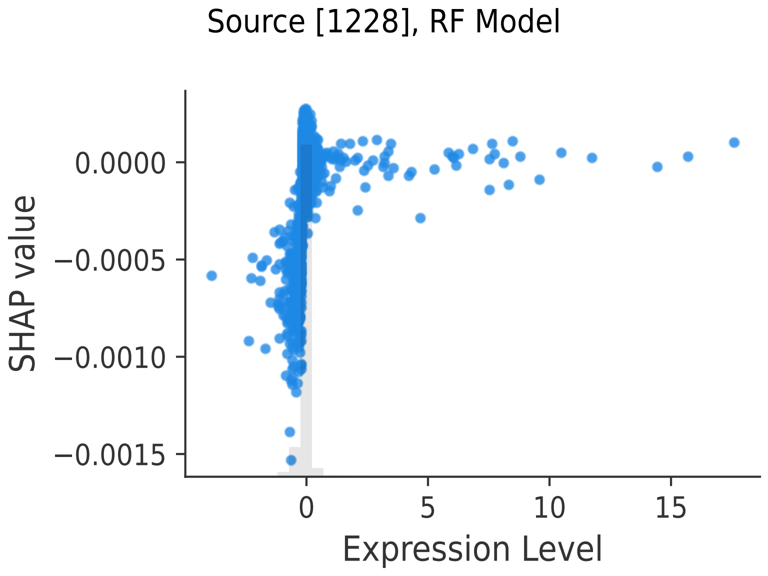
Source [1228], RF Model
SHAP value
0.0000
−0.0005
−0.0010
−0.0015
0
5
10
15
Expression Level

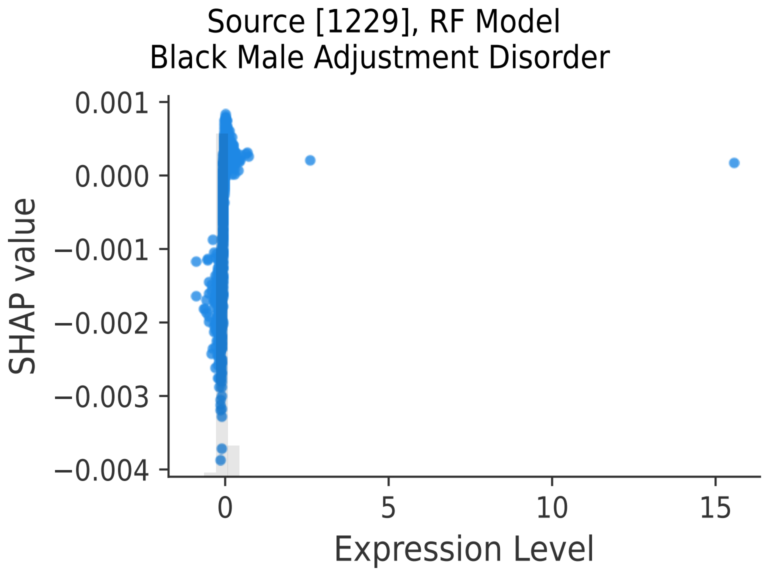

Source [1229], RF Model
Black Male Adjustment Disorder
SHAP value
0.001
0.000
−0.001
−0.002
−0.003
−0.004
0
5
10
15
Expression Level

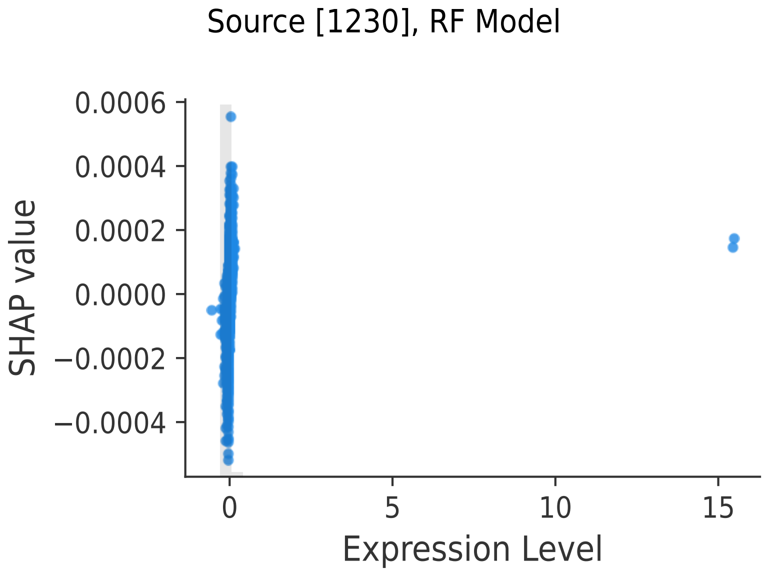

Source [1230], RF Model
0.0006
0.0004
0.0002
0.0000
−0.0002
−0.0004
SHAP value
0
5
10
15
Expression Level

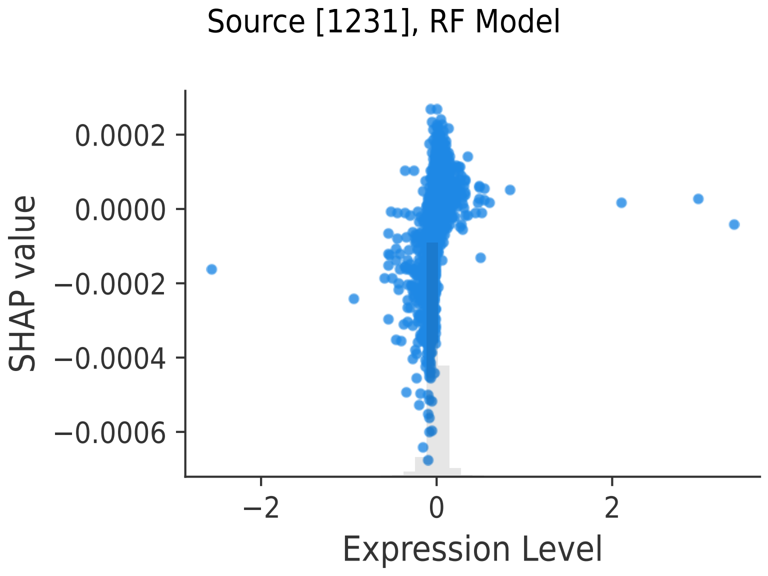

Source [1231], RF Model
0.0002
0.0000
−0.0002
−0.0004
−0.0006
SHAP value
−2
0
2
Expression Level

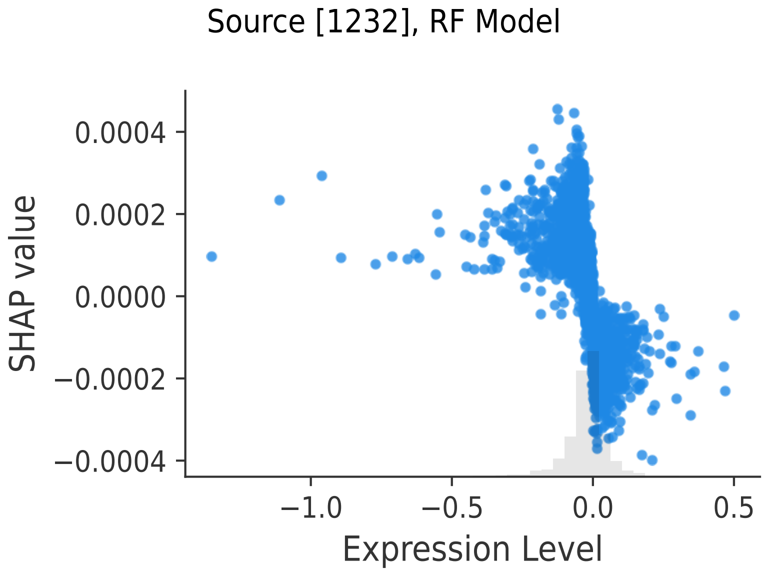
Source [1232], RF Model
SHAP value
0.0004
0.0002
0.0000
−0.0002
−0.0004
−1.0
−0.5
0.0
0.5
Expression Level

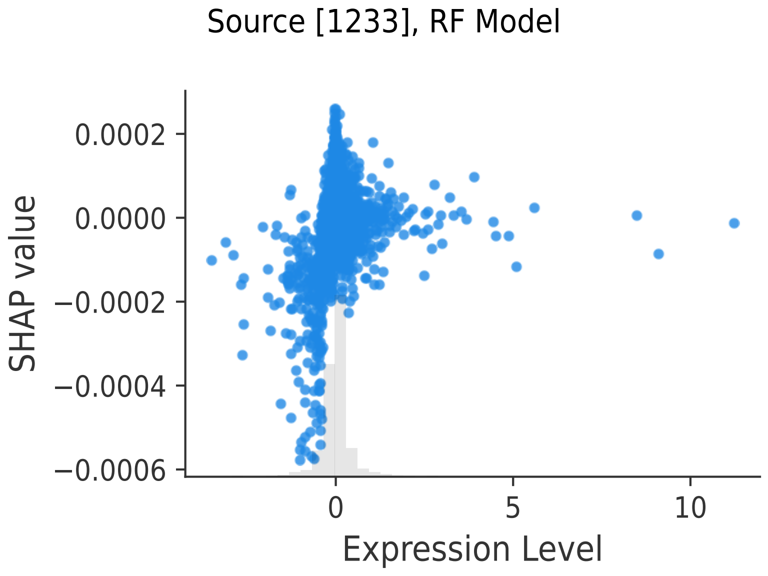

Source [1233], RF Model
0.0002
0.0000
−0.0002
−0.0004
−0.0006
SHAP value
0
5
10
Expression Level

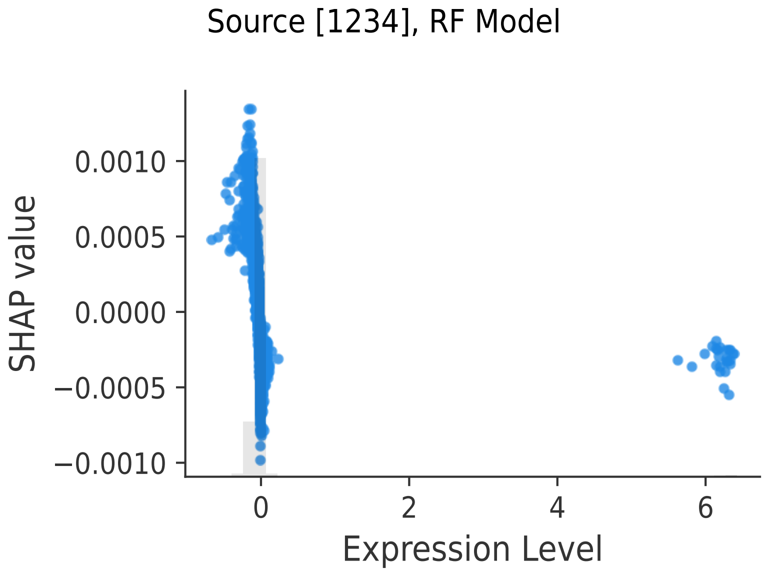
Source [1234], RF Model
SHAP value
0.0010
0.0005
0.0000
−0.0005
−0.0010
0
2
4
6
Expression Level

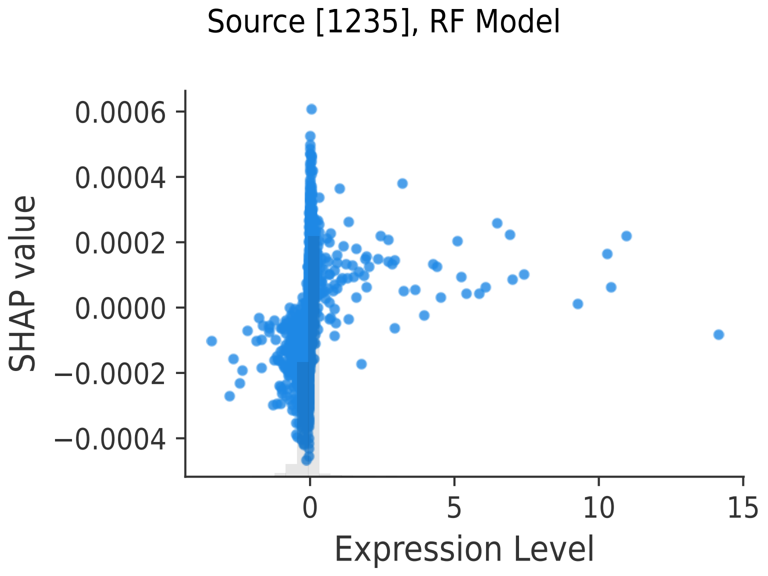

Source [1235], RF Model
SHAP value
0.0006
0.0004
0.0002
0.0000
−0.0002
−0.0004
Expression Level
0
5
10
15

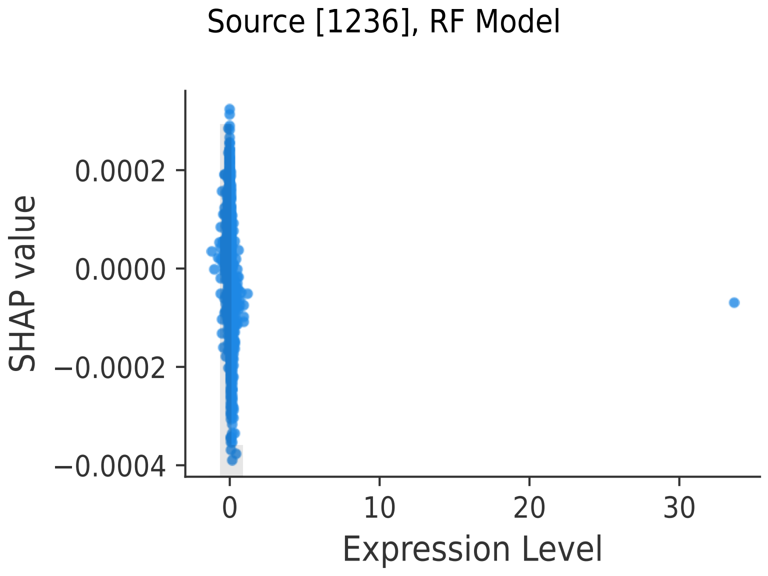

Source [1236], RF Model
SHAP value
0.0002
0.0000
−0.0002
−0.0004
0
10
20
30
Expression Level

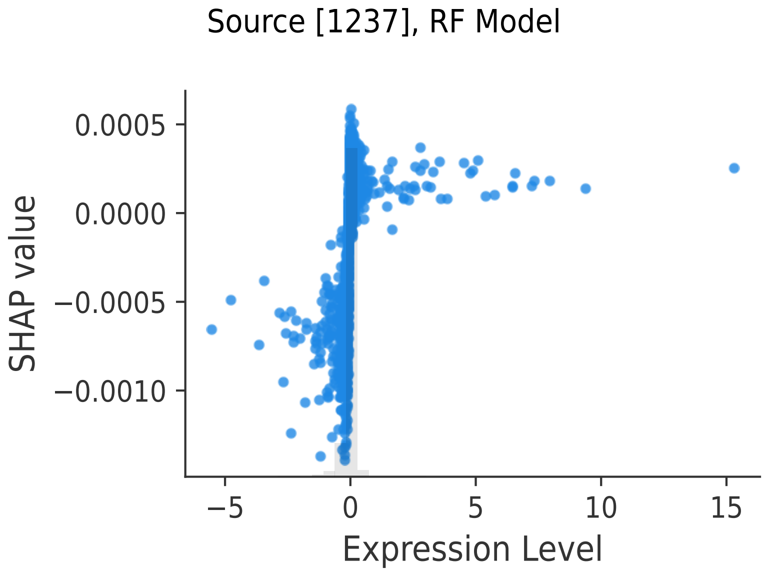
Source [1237], RF Model
SHAP value
0.0005
0.0000
−0.0005
−0.0010
−5
0
5
10
15
Expression Level

Source [1238], RF Model

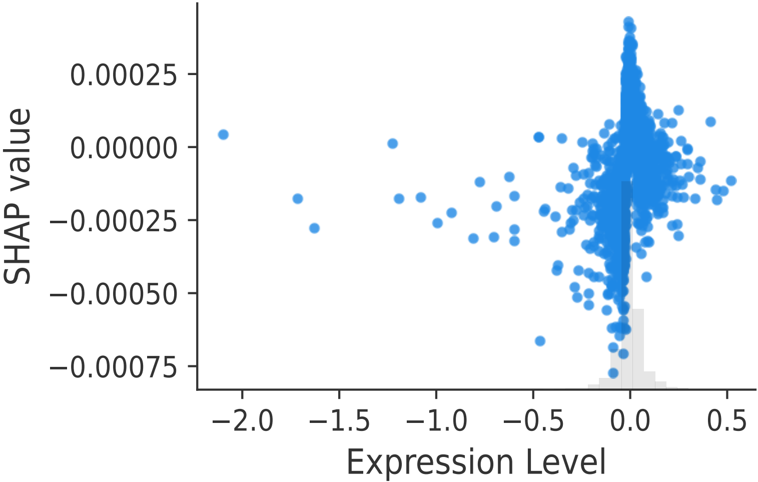

Source [1239], RF Model

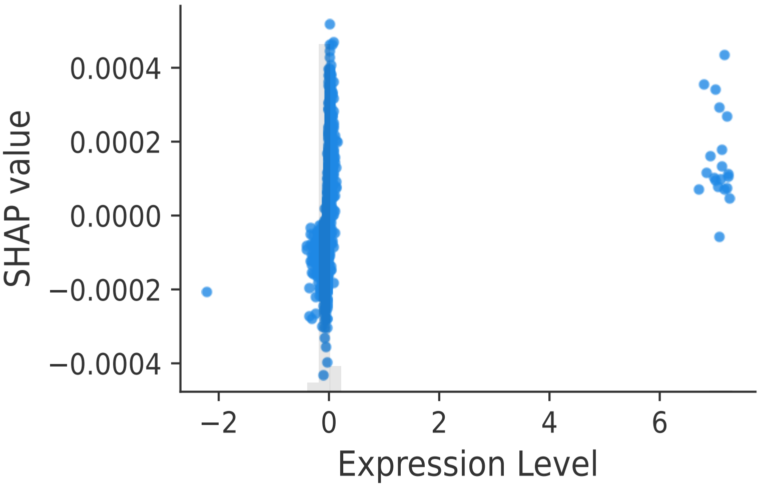

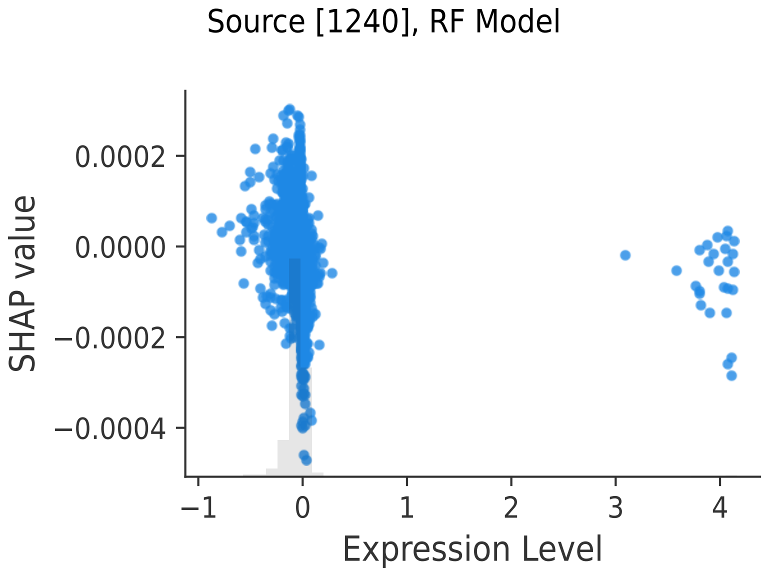

Source [1240], RF Model
SHAP value
0.0002
0.0000
−0.0002
−0.0004
−1
0
1
2
3
4
Expression Level

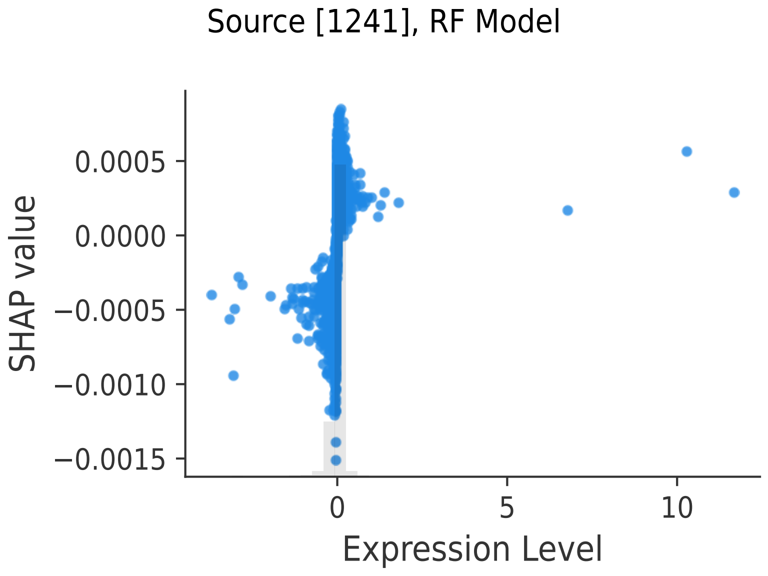
Source [1241], RF Model
SHAP value
0.0005
0.0000
−0.0005
−0.0010
−0.0015
0
5
10
Expression Level

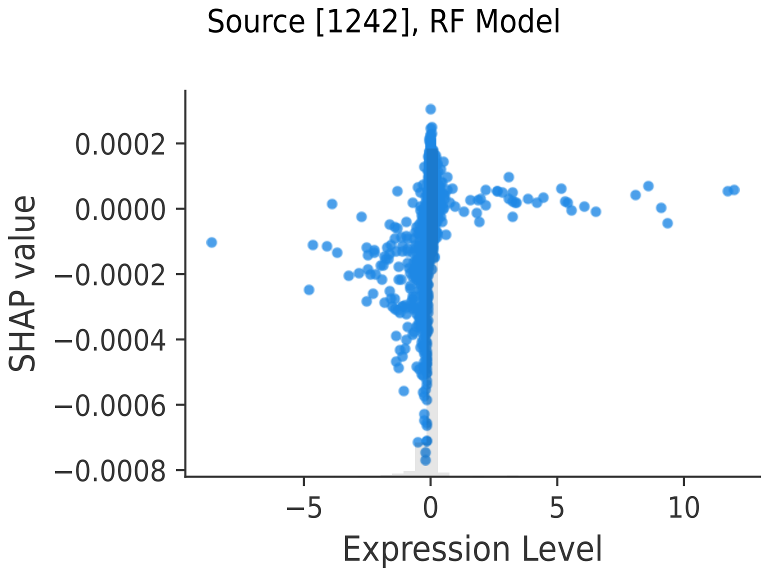

Source [1242], RF Model
SHAP value
0.0002
0.0000
−0.0002
−0.0004
−0.0006
−0.0008
−5
0
5
10
Expression Level

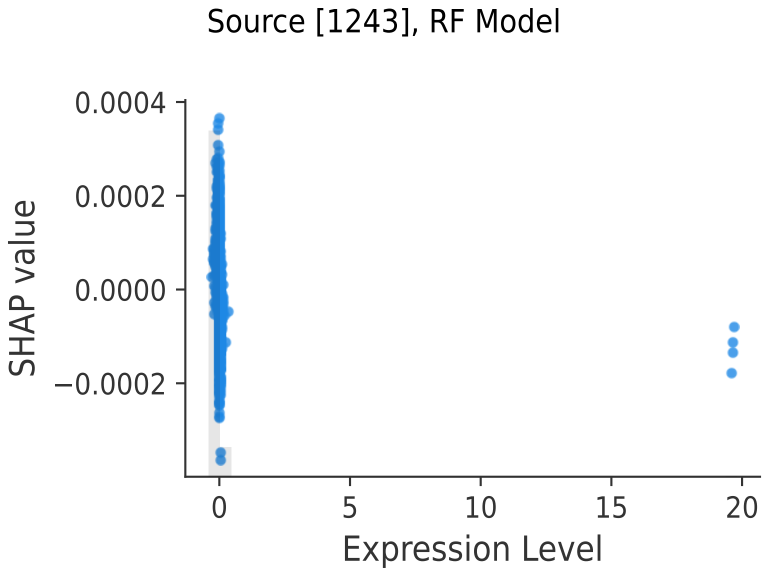

Source [1243], RF Model
0.0004
0.0002
0.0000
−0.0002
SHAP value
0
5
10
15
20
Expression Level

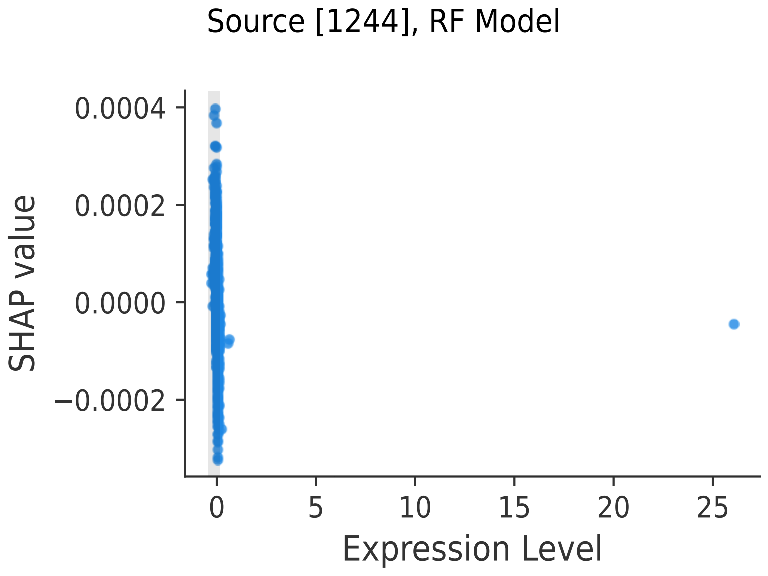
Source [1244], RF Model
SHAP value
0.0004
0.0002
0.0000
−0.0002
Expression Level
0
5
10
15
20
25

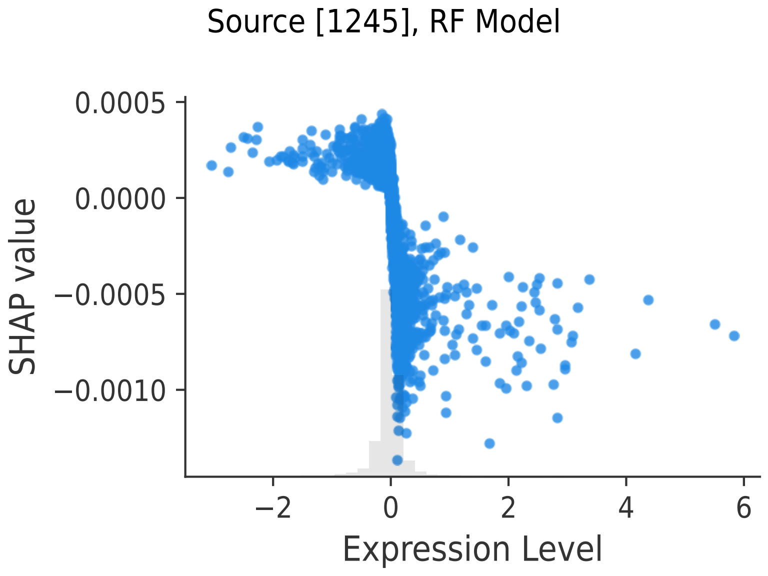
Source [1245], RF Model
0.0005
0.0000
−0.0005
−0.0010
SHAP value
−2
0
2
4
6
Expression Level

Source [1246], RF Model

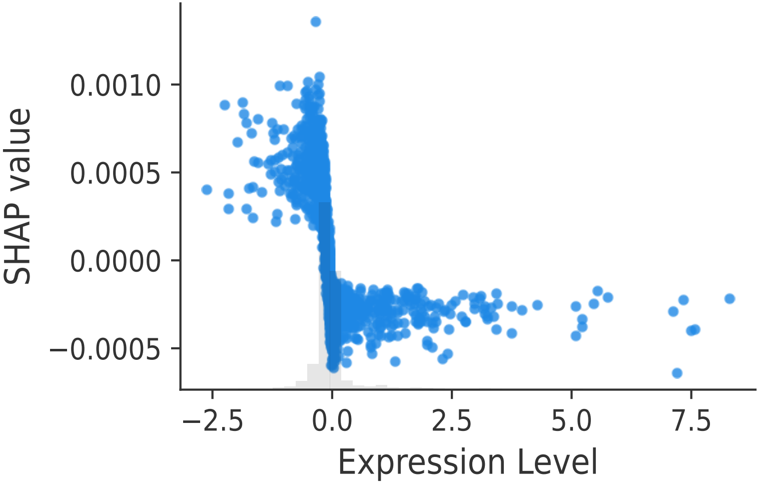

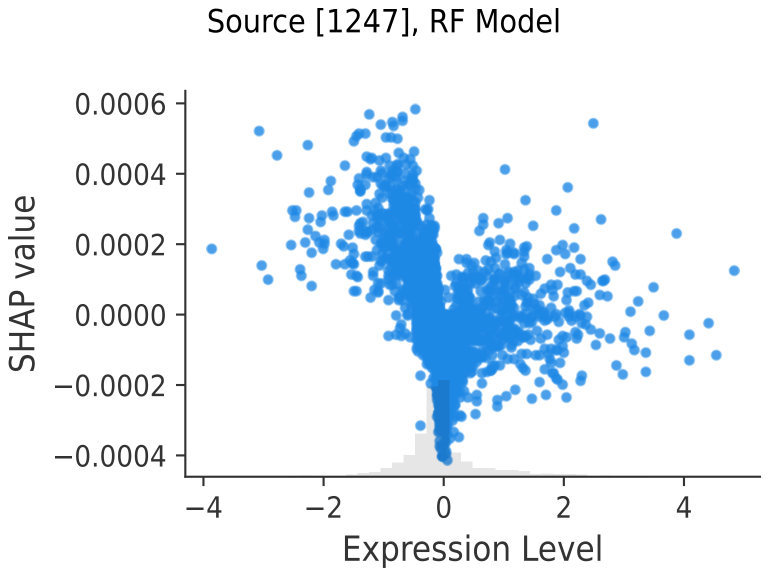
Source [1247], RF Model
0.0006
0.0004
0.0002
0.0000
−0.0002
−0.0004
SHAP value
−4
−2
0
2
4
Expression Level

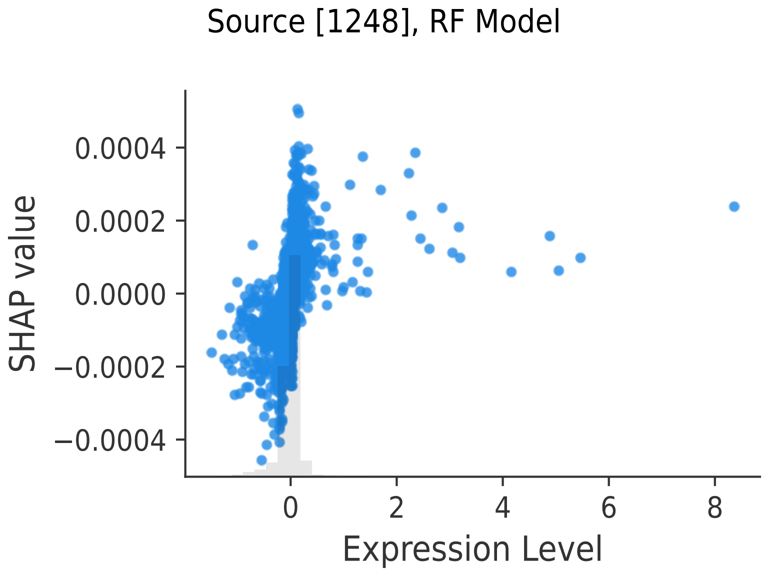

Source [1248], RF Model
SHAP value
0.0004
0.0002
0.0000
−0.0002
−0.0004
0
2
4
6
8
Expression Level

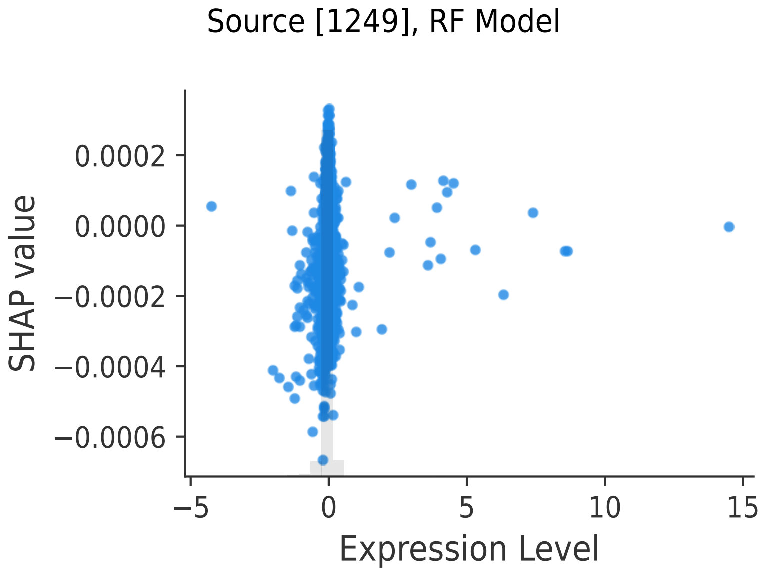

Source [1249], RF Model
SHAP value
0.0002
0.0000
−0.0002
−0.0004
−0.0006
−5
0
5
10
15
Expression Level

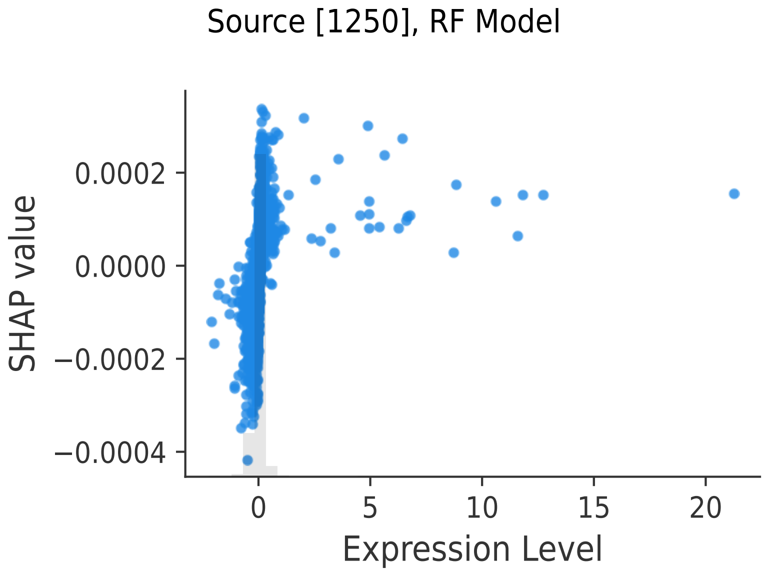

Source [1250], RF Model
SHAP value
0.0002
0.0000
−0.0002
−0.0004
0
5
10
15
20
Expression Level

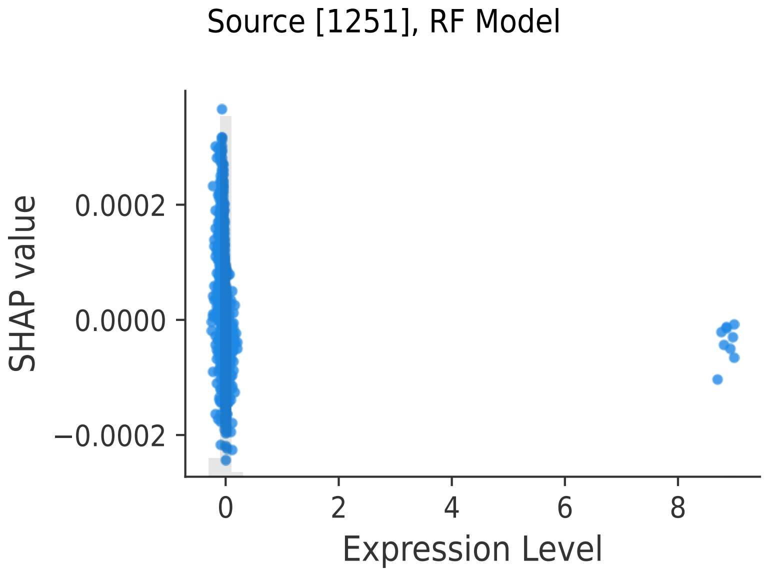
Source [1251], RF Model
SHAP value
0.0002
0.0000
−0.0002
0
2
4
6
8
Expression Level

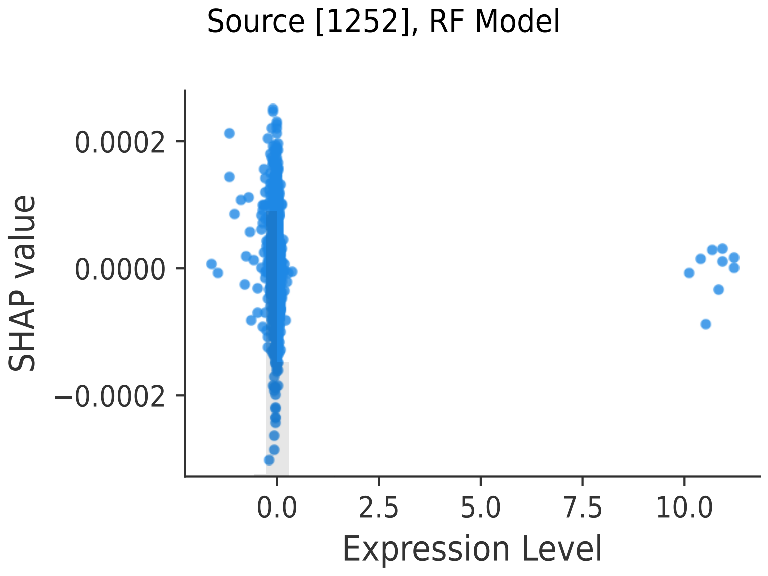
Source [1252], RF Model
SHAP value
0.0002
0.0000
−0.0002
0.0
2.5
5.0
7.5
10.0
Expression Level

Source [1253], RF Model

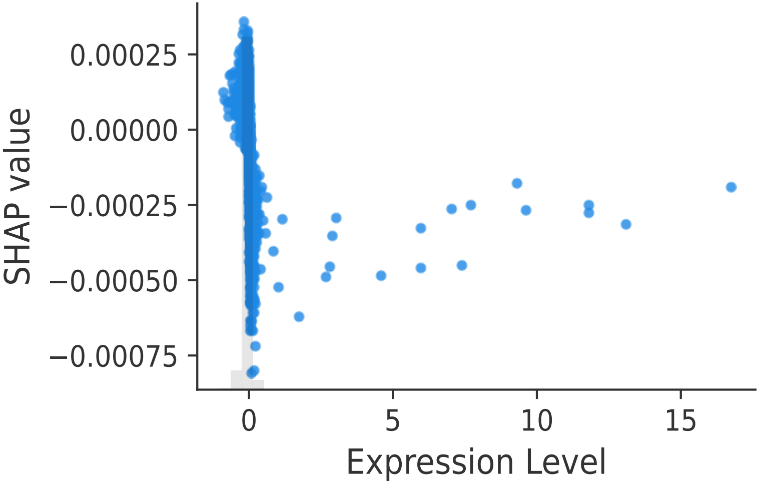

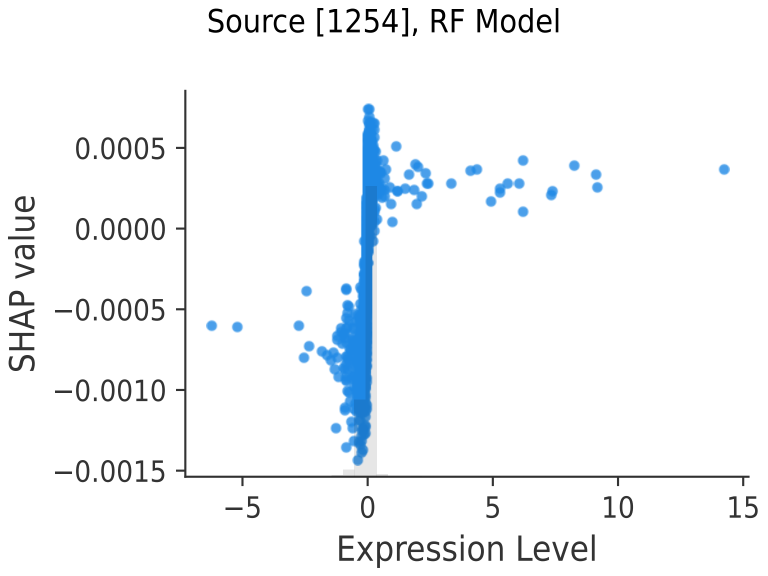
Source [1254], RF Model
SHAP value
0.0005
0.0000
−0.0005
−0.0010
−0.0015
−5
0
5
10
15
Expression Level

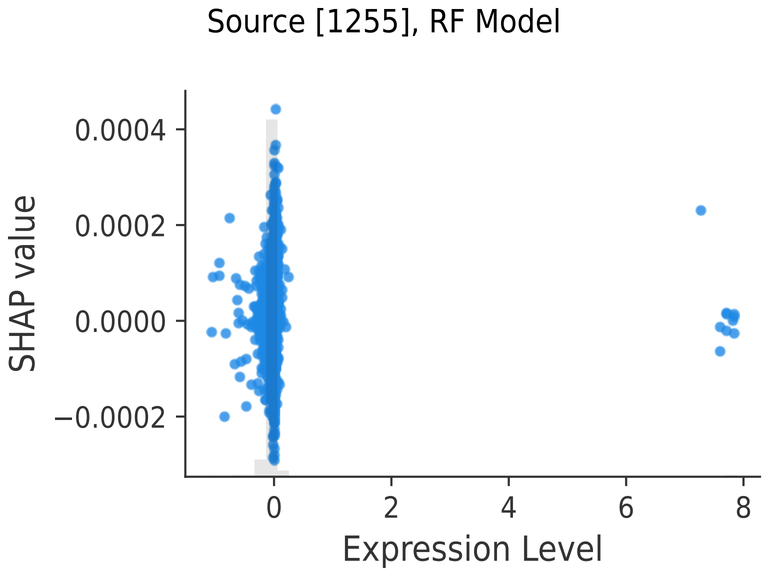

Source [1255], RF Model
SHAP value
0.0004
0.0002
0.0000
−0.0002
0
2
4
6
8
Expression Level

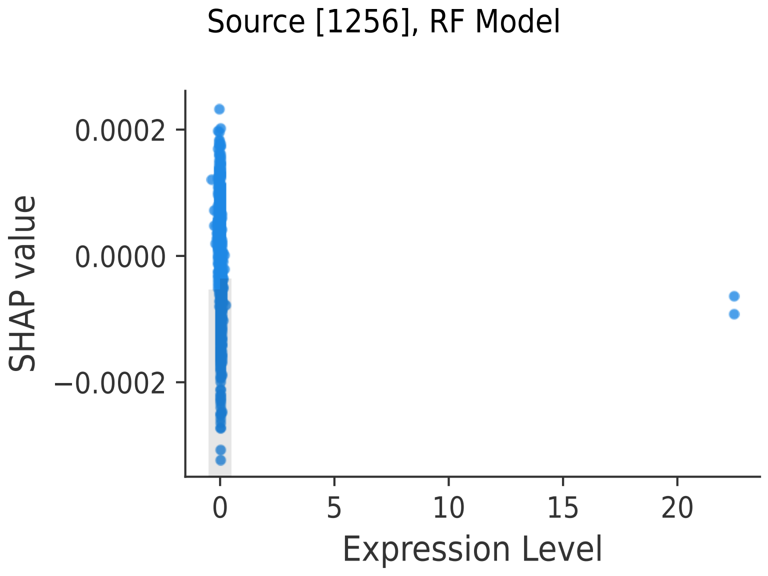
Source [1256], RF Model
SHAP value
0.0002
0.0000
−0.0002
0
5
10
15
20
Expression Level

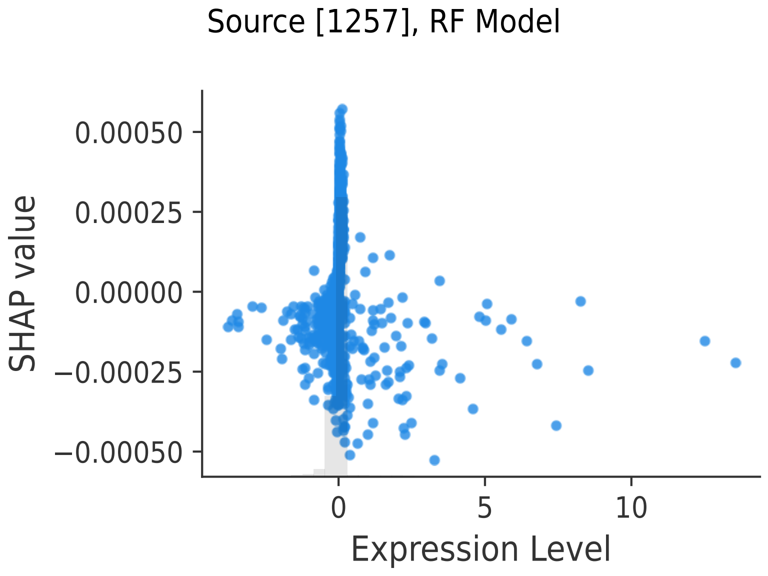
Source [1257], RF Model
0.00050
0.00025
0.00000
−0.00025
−0.00050
SHAP value
0
5
10
Expression Level

Source [1258], RF Model

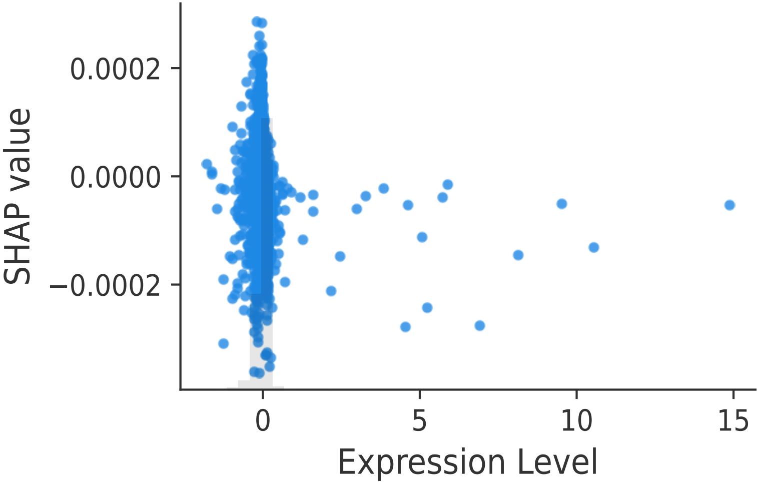

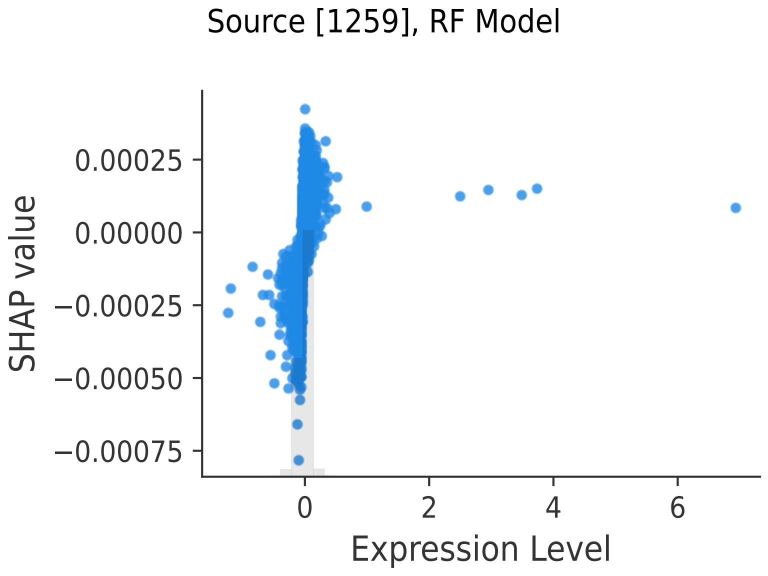
Source [1259], RF Model
0.00025
0.00000
−0.00025
−0.00050
−0.00075
SHAP value
0
2
4
6
Expression Level

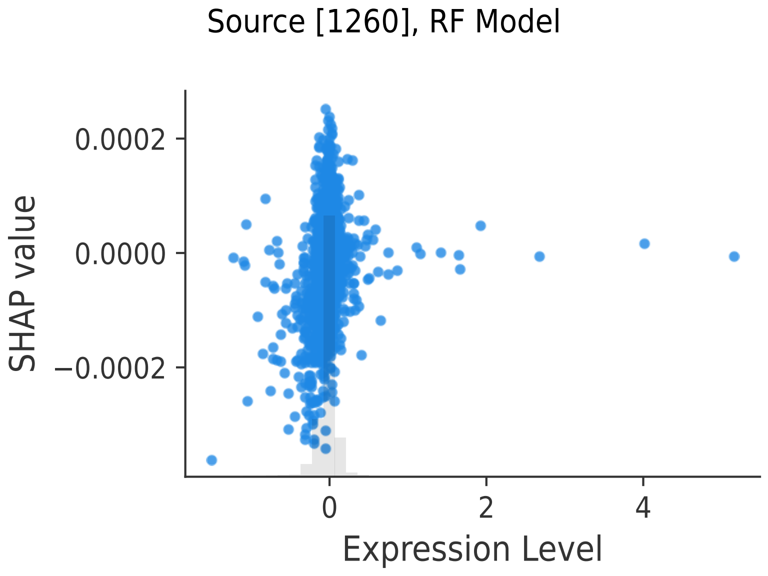
Source [1260], RF Model
SHAP value
0.0002
0.0000
−0.0002
0
2
4
Expression Level

Source [1261], RF Model

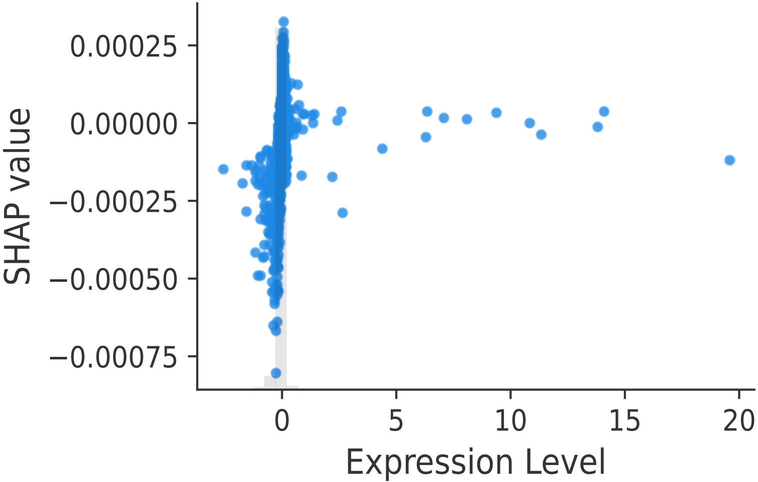

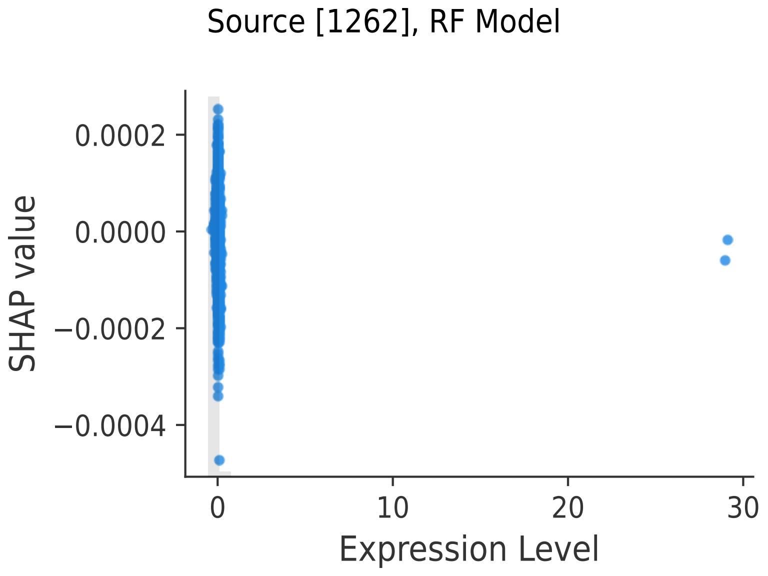
Source [1262], RF Model
0.0002
0.0000
−0.0002
−0.0004
SHAP value
0
10
20
30
Expression Level

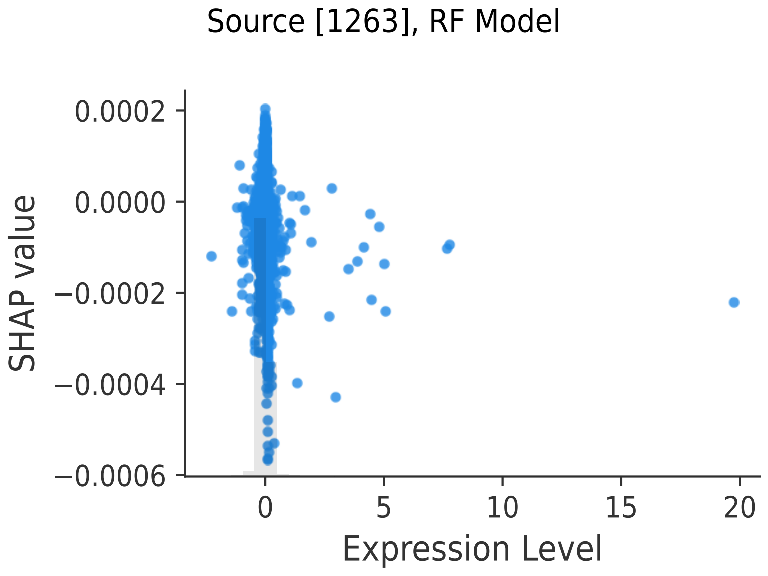
Source [1263], RF Model
0.0002
0.0000
−0.0002
−0.0004
−0.0006
SHAP value
0
5
10
15
20
Expression Level

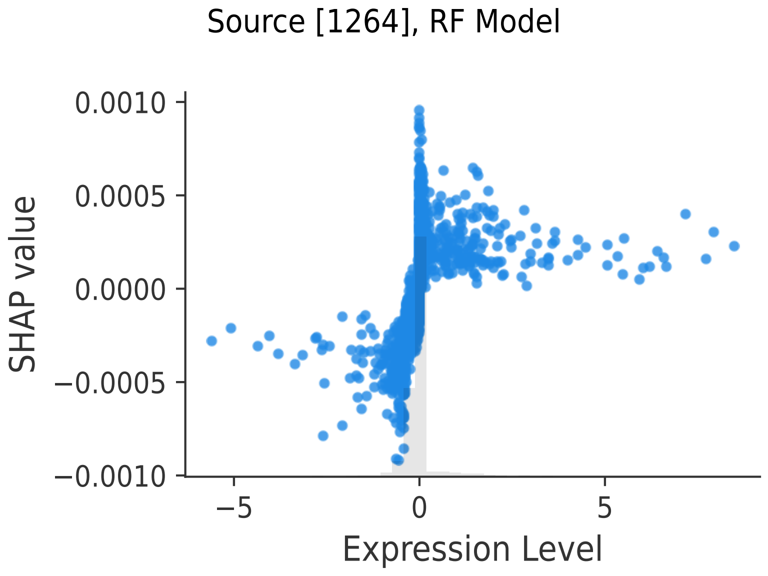

Source [1264], RF Model
0.0010
0.0005
0.0000
−0.0005
−0.0010
SHAP value
−5
0
5
Expression Level

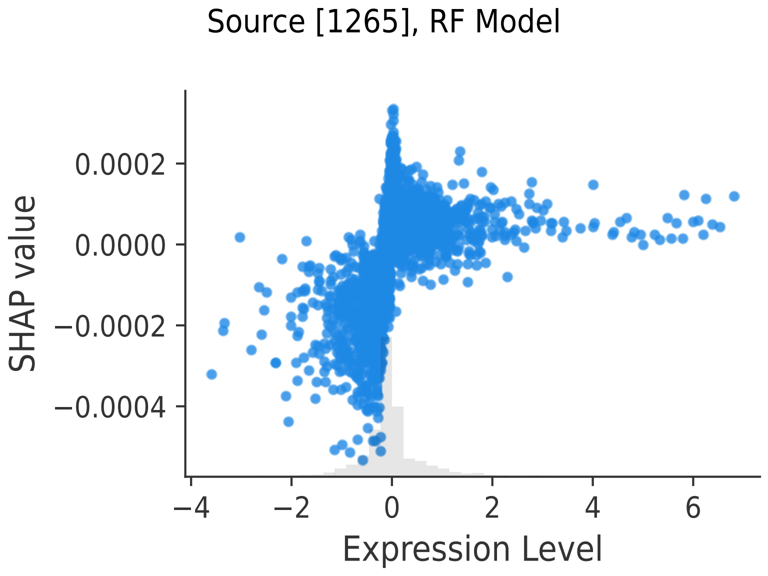
Source [1265], RF Model
SHAP value
0.0002
0.0000
−0.0002
−0.0004
−4
−2
0
2
4
6
Expression Level

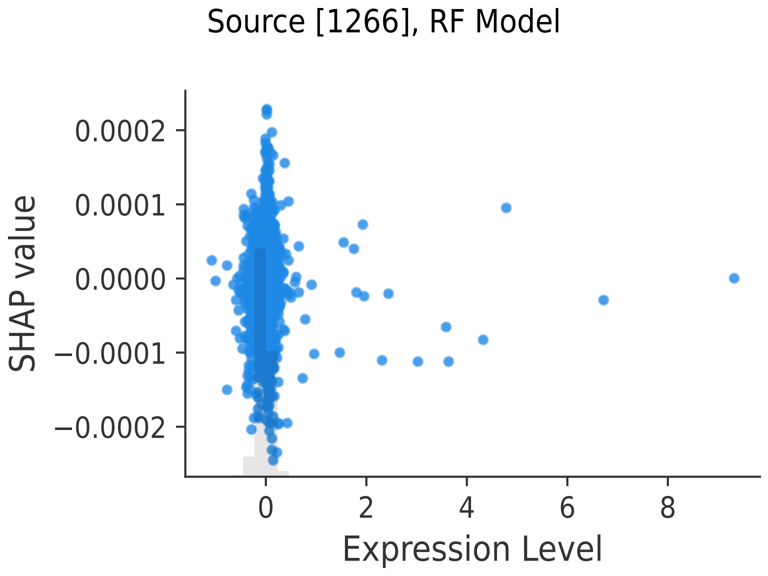

Source [1266], RF Model
SHAP value
0.0002
0.0001
0.0000
−0.0001
−0.0002
0
2
4
6
8
Expression Level

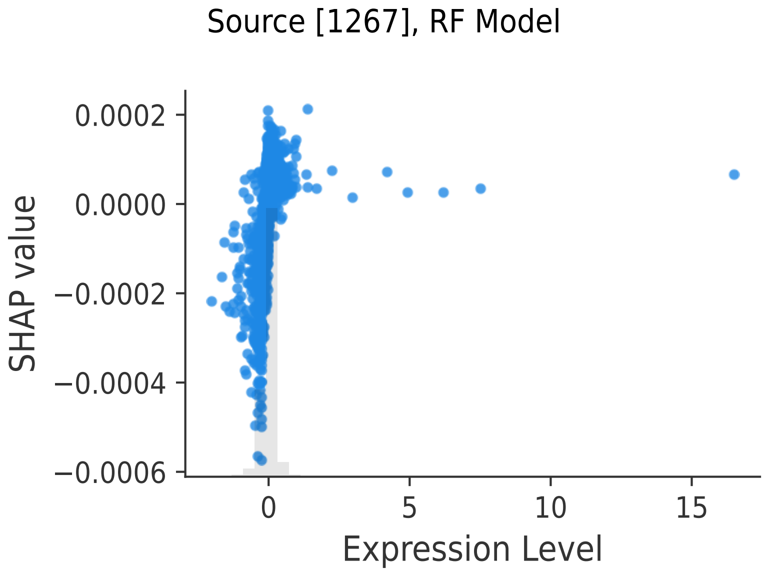
Source [1267], RF Model
0.0002
0.0000
−0.0002
−0.0004
−0.0006
SHAP value
0
5
10
15
Expression Level

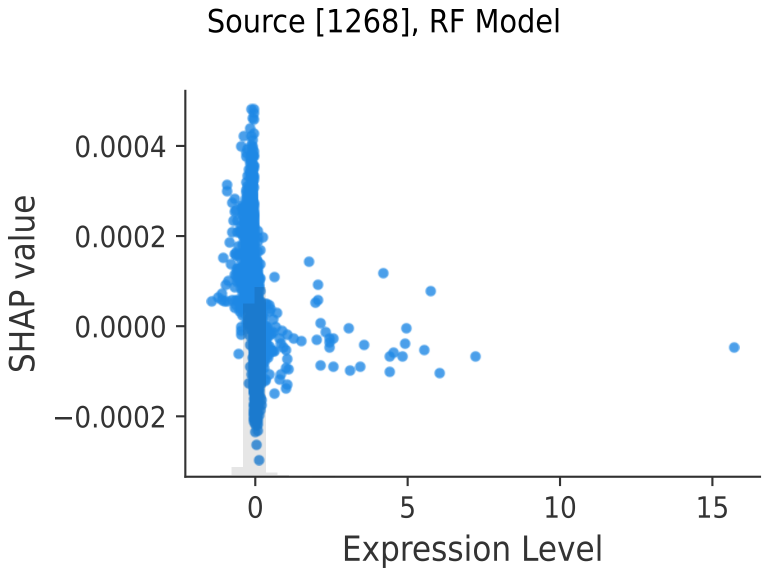

Source [1268], RF Model
0.0004
0.0002
0.0000
−0.0002
SHAP value
0
5
10
15
Expression Level

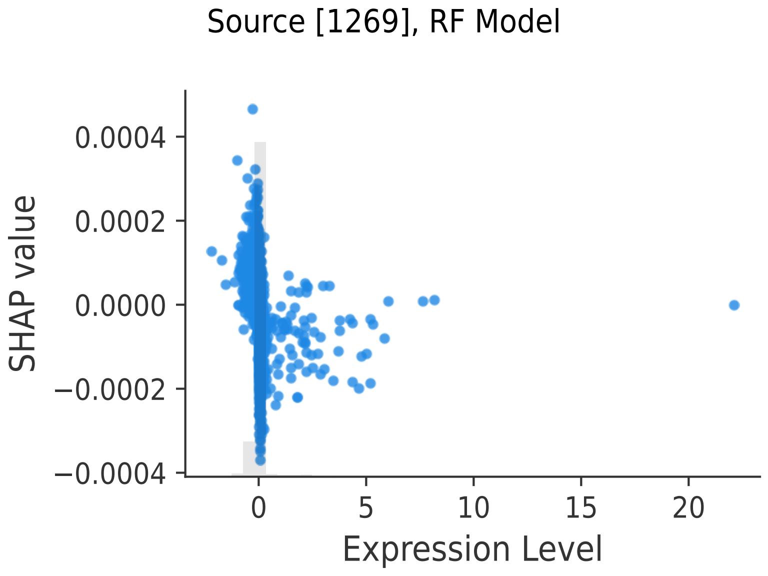

Source [1269], RF Model
SHAP value
0.0004
0.0002
0.0000
−0.0002
−0.0004
0
5
10
15
20
Expression Level

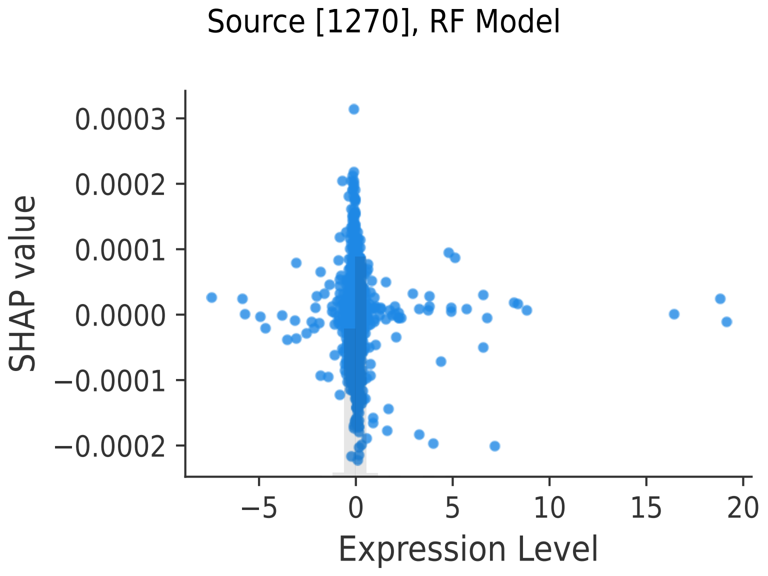
Source [1270], RF Model
SHAP value
0.0003
0.0002
0.0001
0.0000
−0.0001
−0.0002
−5
0
5
10
15
20
Expression Level

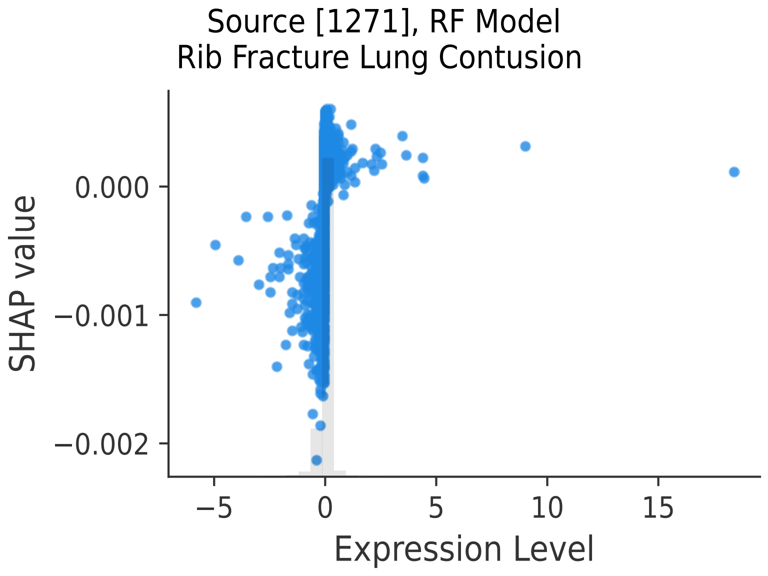

Source [1271], RF Model
Rib Fracture Lung Contusion
SHAP value
0.000
−0.001
−0.002
−5
0
5
10
15
Expression Level

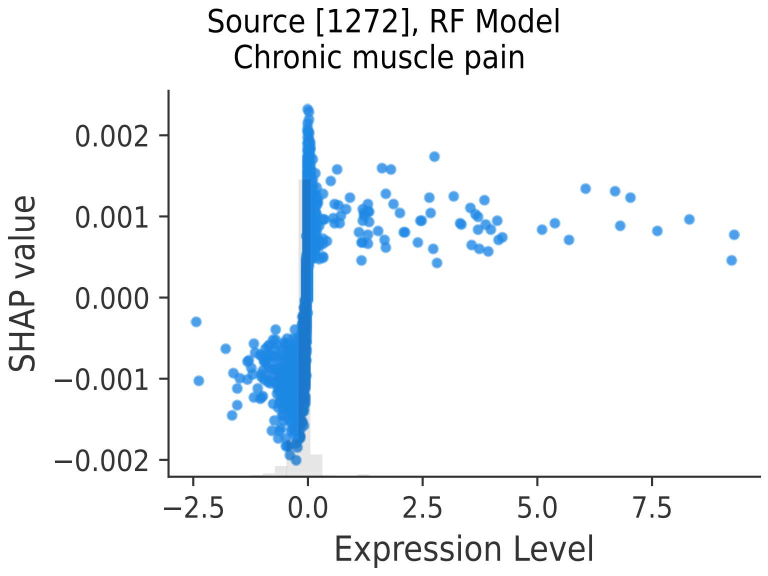
Source [1272], RF Model
Chronic muscle pain
SHAP value
0.002
0.001
0.000
−0.001
−0.002
−2.5
0.0
2.5
5.0
7.5
Expression Level

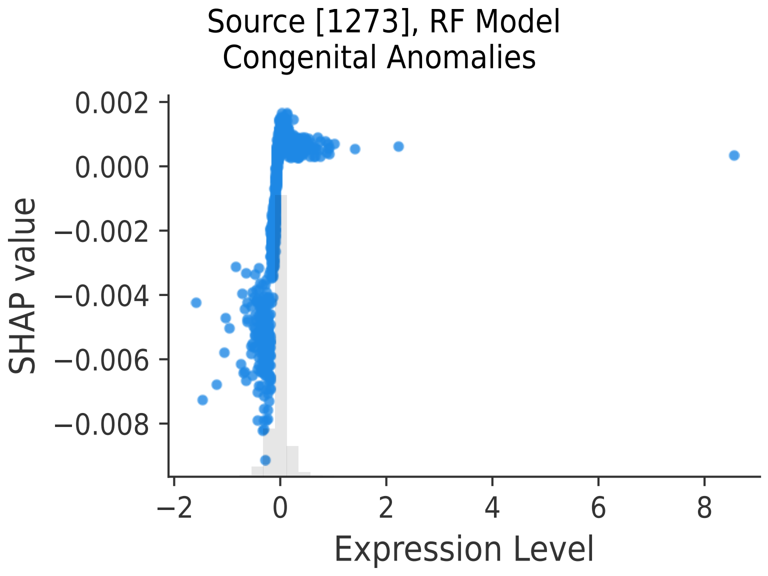

Source [1273], RF Model
Congenital Anomalies
0.002
0.000
−0.002
−0.004
−0.006
−0.008
SHAP value
−2
0
2
4
6
8
Expression Level

Source [1274], RF Model

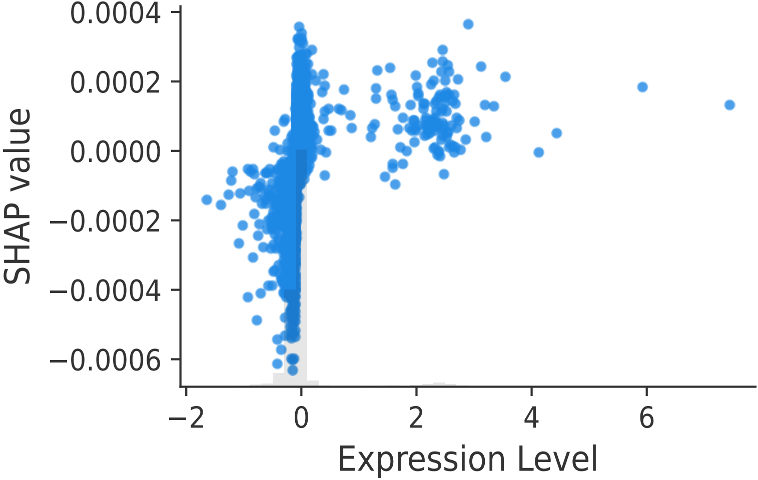

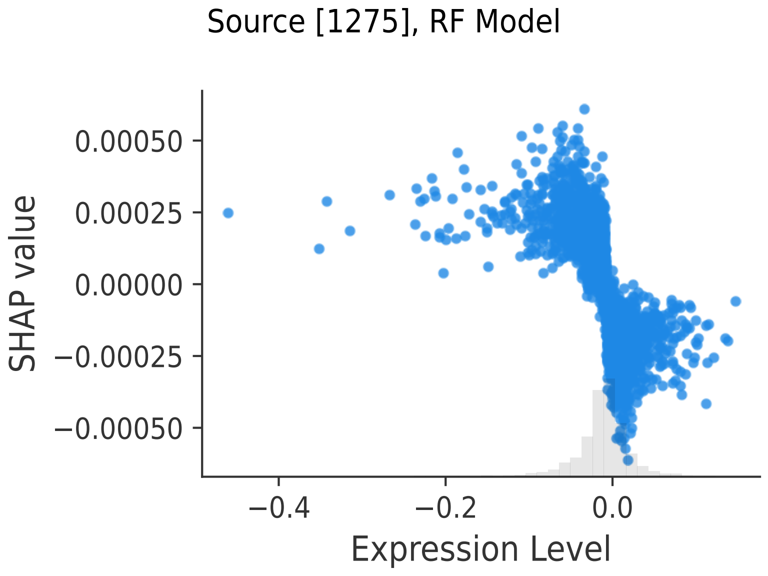
Source [1275], RF Model
0.00050
0.00025
0.00000
−0.00025
−0.00050
SHAP value
−0.4
−0.2
0.0
Expression Level

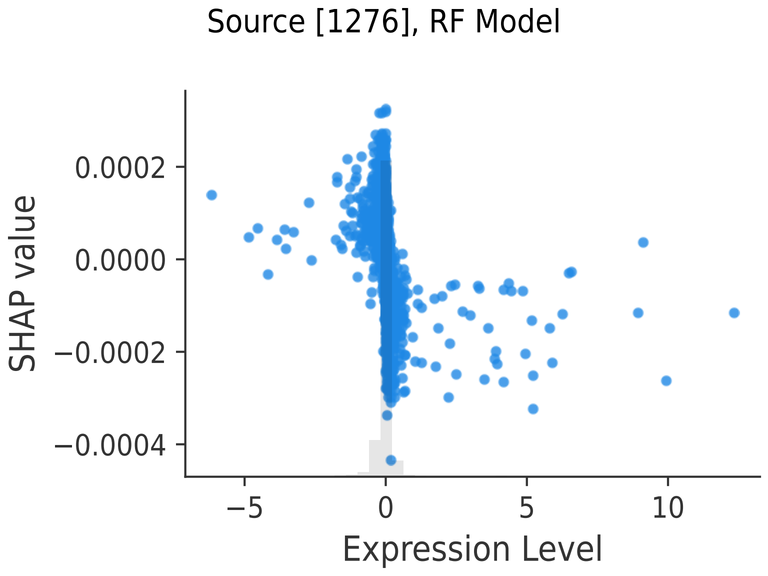

Source [1276], RF Model
SHAP value
0.0002
0.0000
−0.0002
−0.0004
−5
0
5
10
Expression Level

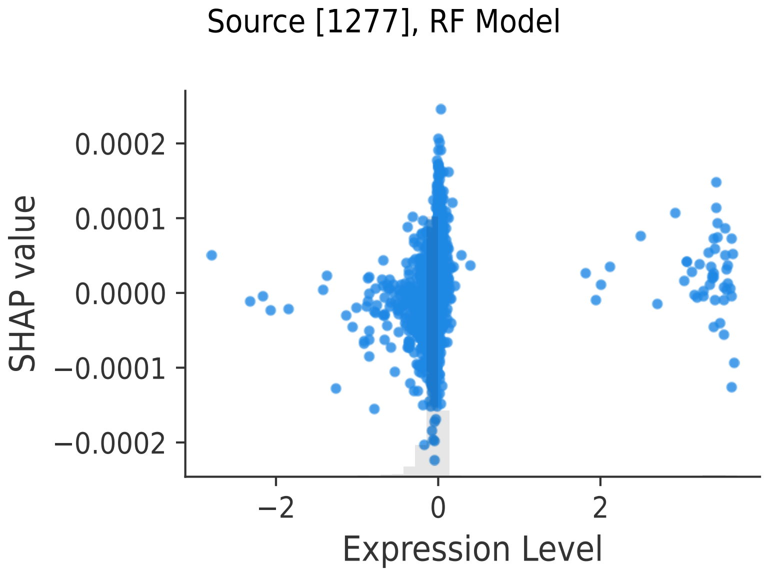
Source [1277], RF Model
SHAP value
0.0002
0.0001
0.0000
−0.0001
−0.0002
−2
0
2
Expression Level

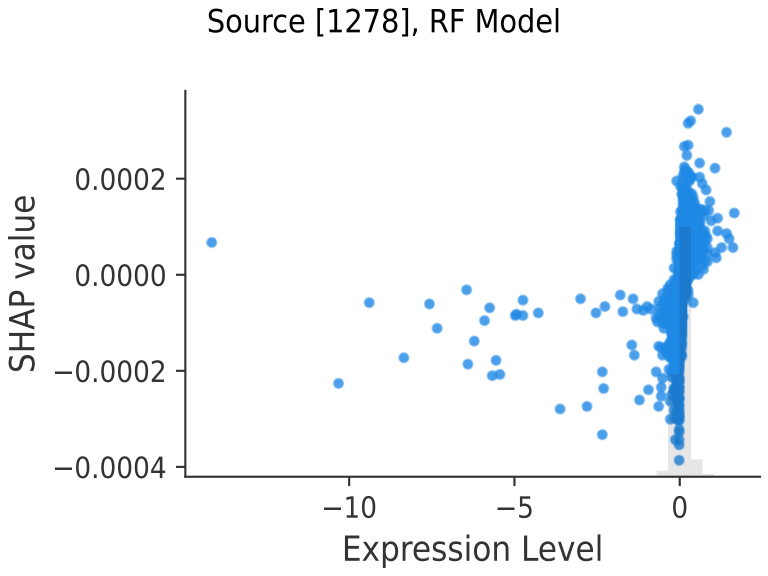
Source [1278], RF Model
SHAP value
0.0002
0.0000
−0.0002
−0.0004
−10
−5
0
Expression Level

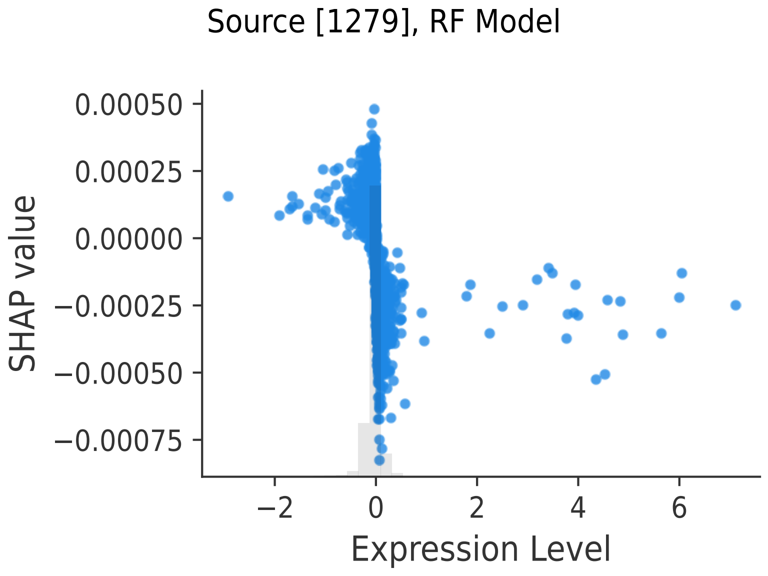

Source [1279], RF Model
0.00050
0.00025
0.00000
−0.00025
−0.00050
−0.00075
SHAP value
−2
0
2
4
6
Expression Level

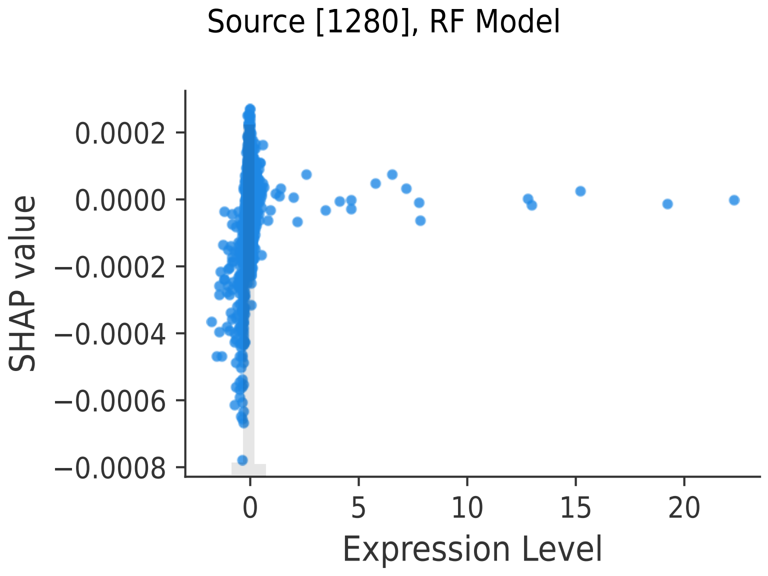
Source [1280], RF Model
SHAP value
0.0002
0.0000
−0.0002
−0.0004
−0.0006
−0.0008
0
5
10
15
20
Expression Level

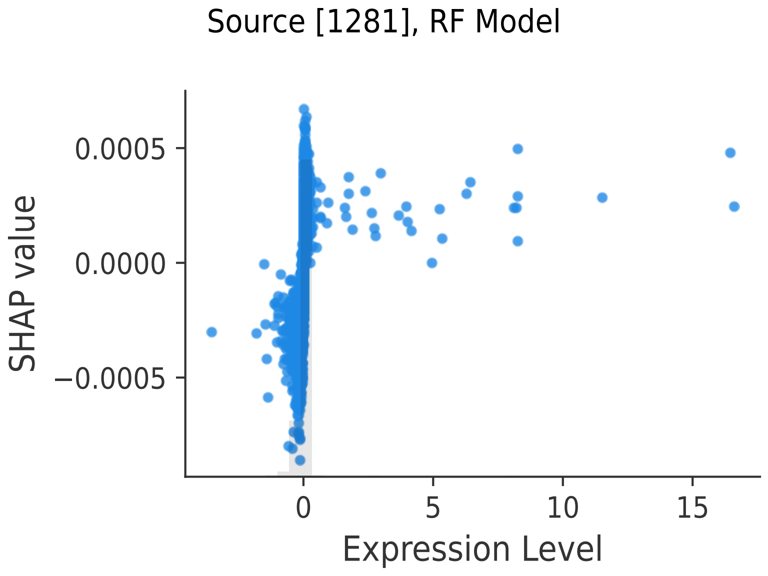

Source [1281], RF Model
0.0005
0.0000
−0.0005
SHAP value
0
5
10
15
Expression Level

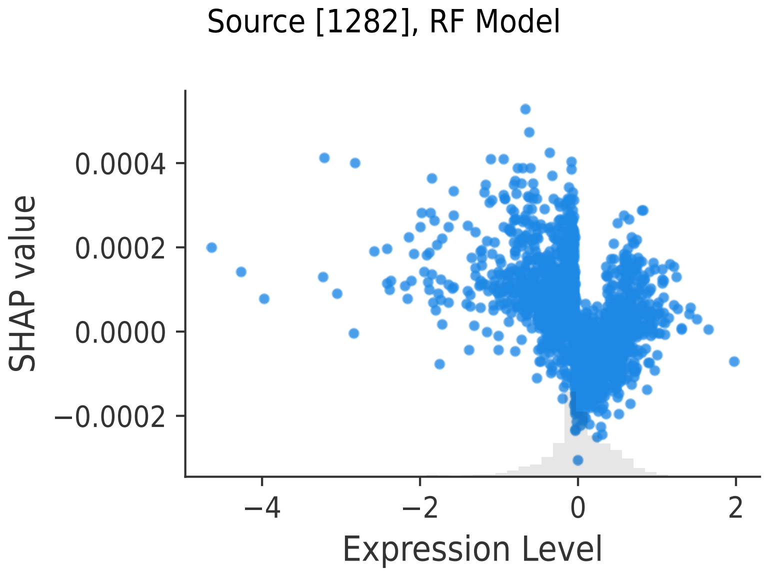

Source [1282], RF Model
SHAP value
0.0004
0.0002
0.0000
−0.0002
−4
−2
0
2
Expression Level

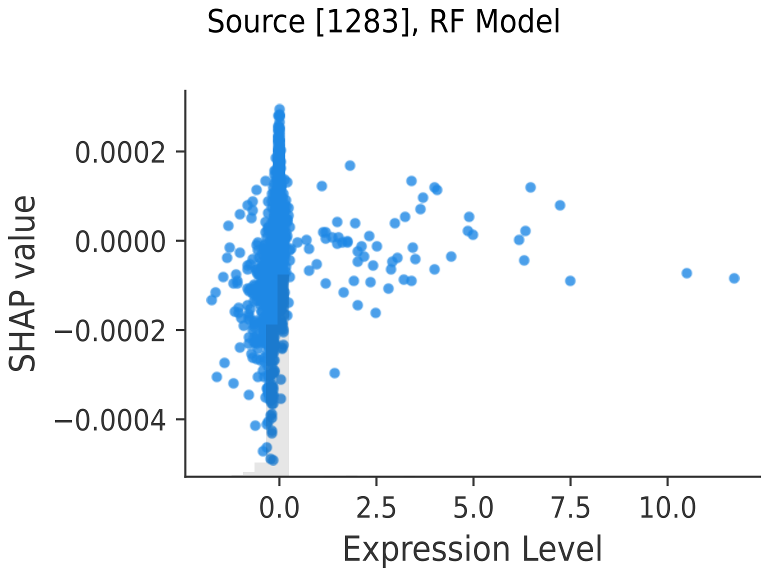
Source [1283], RF Model
SHAP value
0.0002
0.0000
−0.0002
−0.0004
0.0
2.5
5.0
7.5
10.0
Expression Level

Source [1284], RF Model

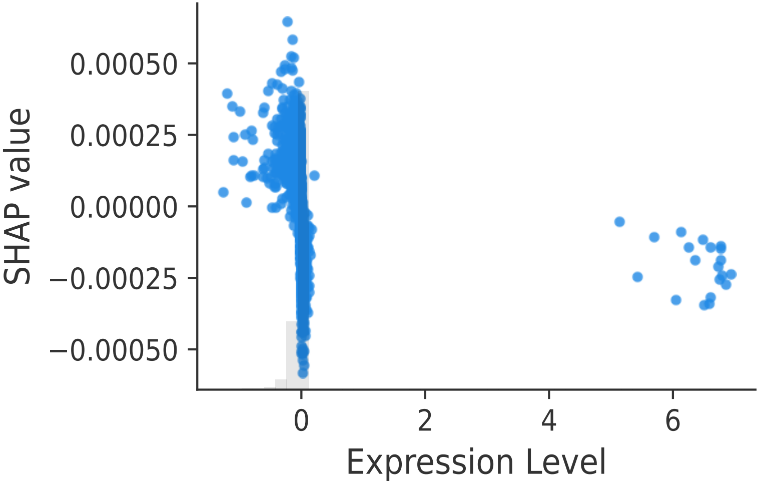

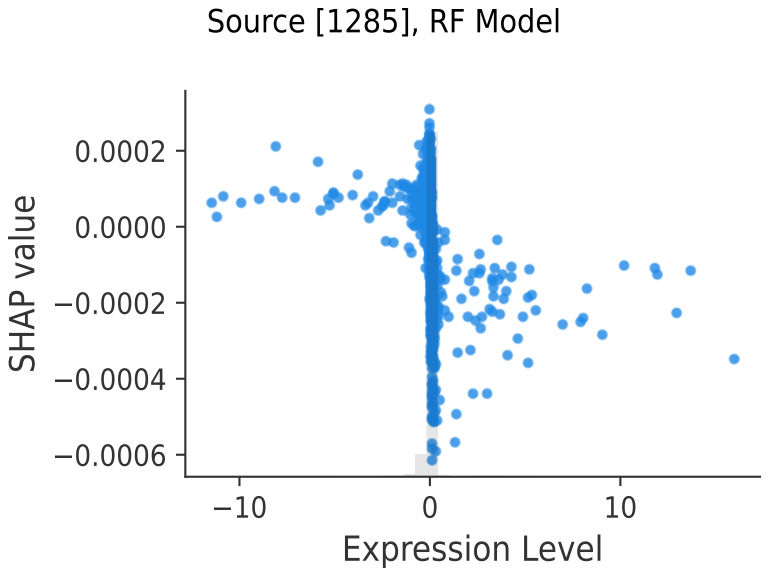
Source [1285], RF Model
SHAP value
0.0002
0.0000
−0.0002
−0.0004
−0.0006
−10
0
10
Expression Level

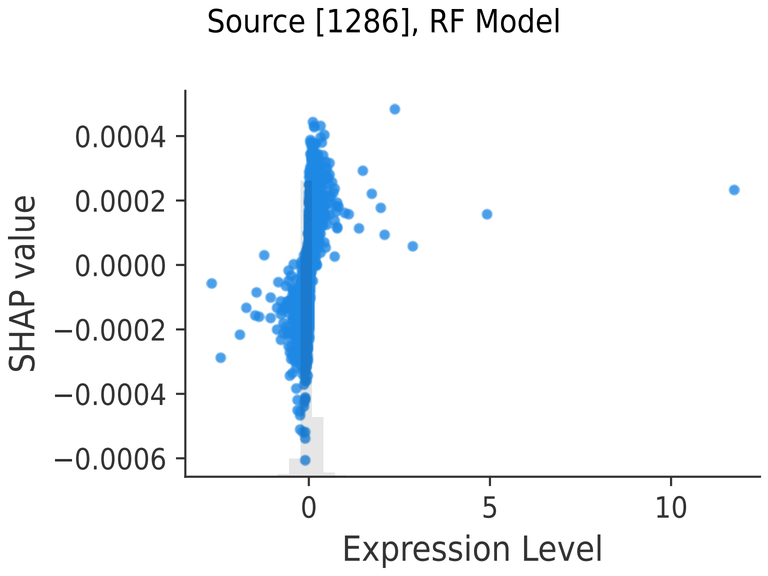

Source [1286], RF Model
SHAP value
0.0004
0.0002
0.0000
−0.0002
−0.0004
−0.0006
0
5
10
Expression Level

Source [1287], RF Model

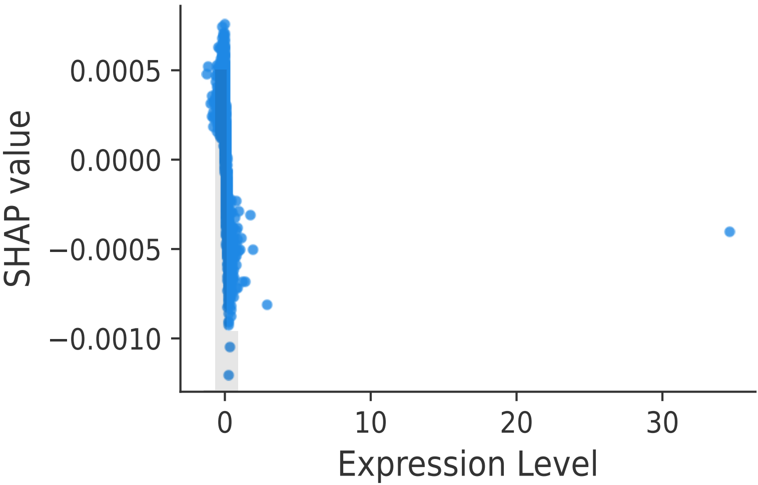

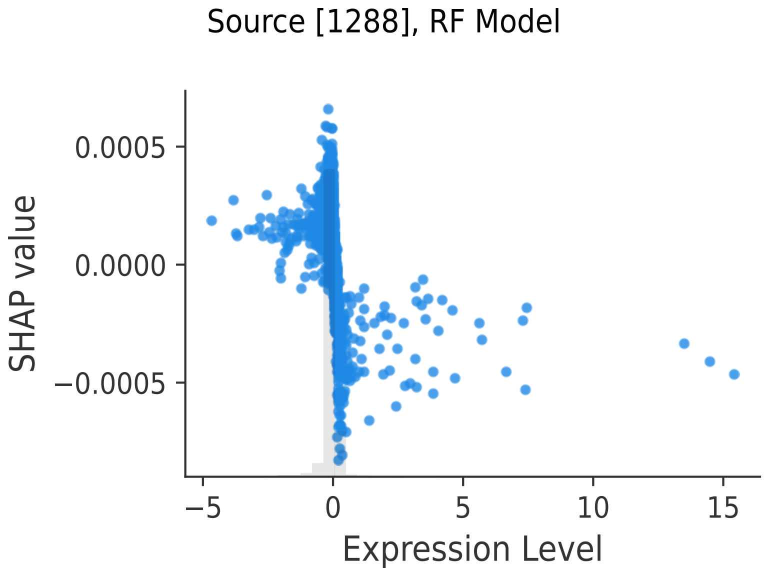

Source [1288], RF Model
0.0005
0.0000
−0.0005
SHAP value
−5
0
5
10
15
Expression Level

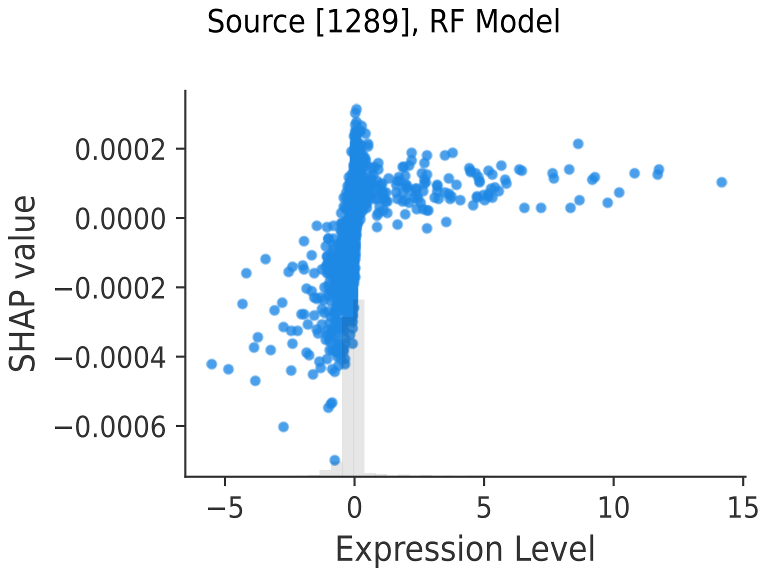

Source [1289], RF Model
SHAP value
0.0002
0.0000
−0.0002
−0.0004
−0.0006
−5
0
5
10
15
Expression Level

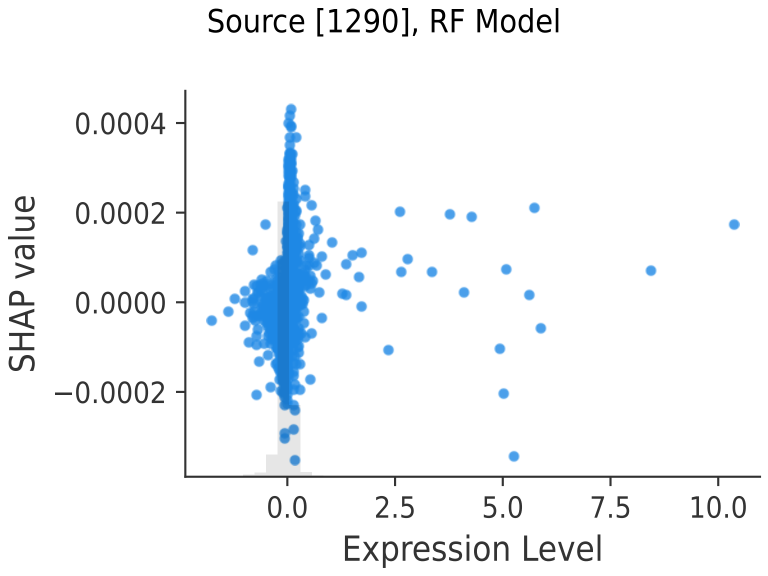
Source [1290], RF Model
SHAP value
0.0004
0.0002
0.0000
−0.0002
0.0
2.5
5.0
7.5
10.0
Expression Level

Source [1291], RF Model

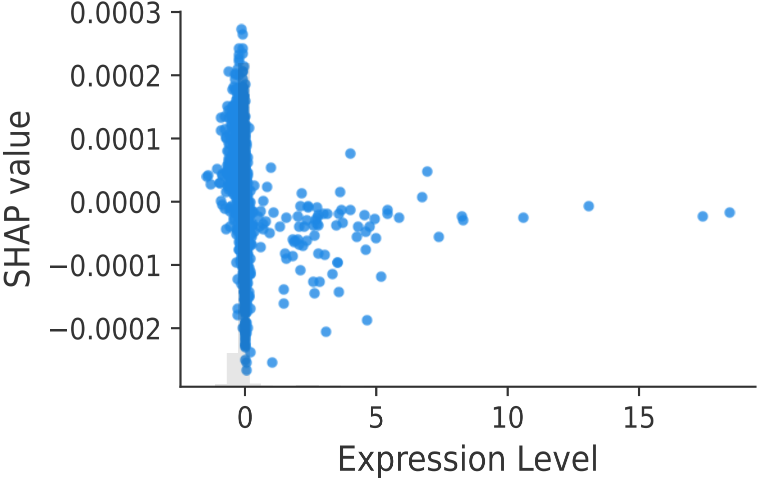

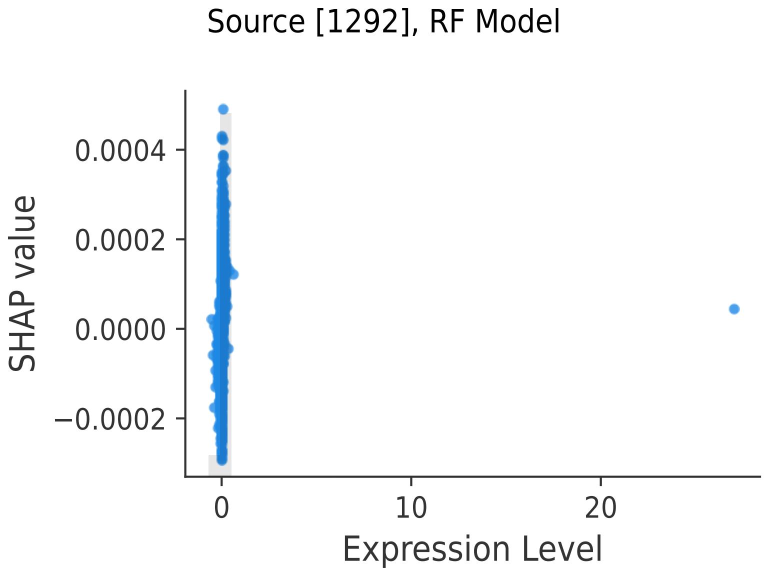

Source [1292], RF Model
SHAP value
0.0004
0.0002
0.0000
−0.0002
0
10
20
Expression Level

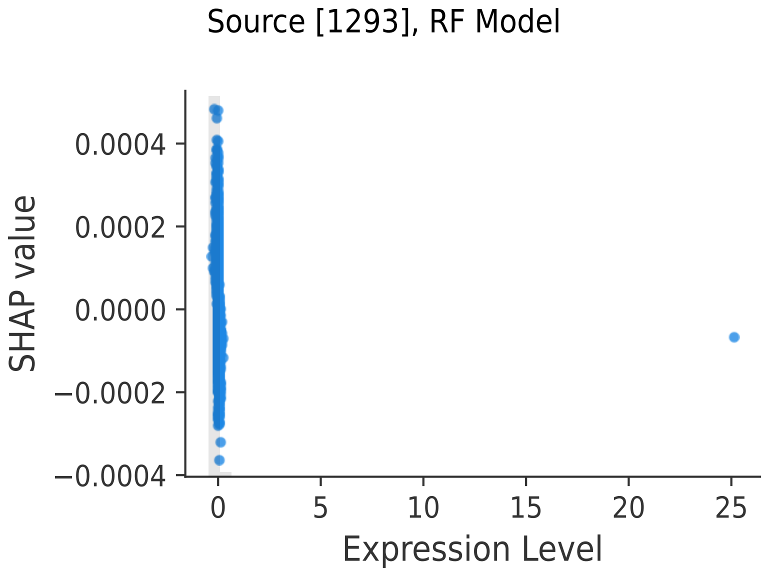
Source [1293], RF Model
SHAP value
0.0004
0.0002
0.0000
−0.0002
−0.0004
0
5
10
15
20
25
Expression Level

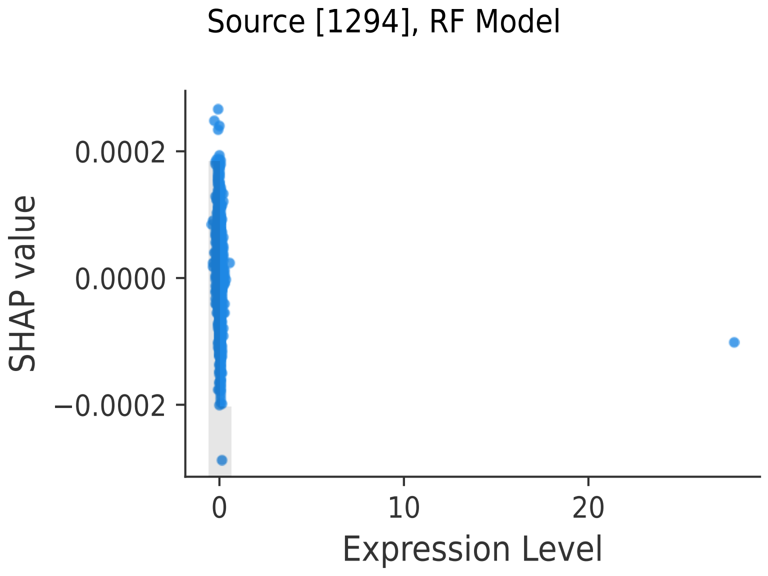
Source [1294], RF Model
SHAP value
0.0002
0.0000
−0.0002
Expression Level
0
10
20

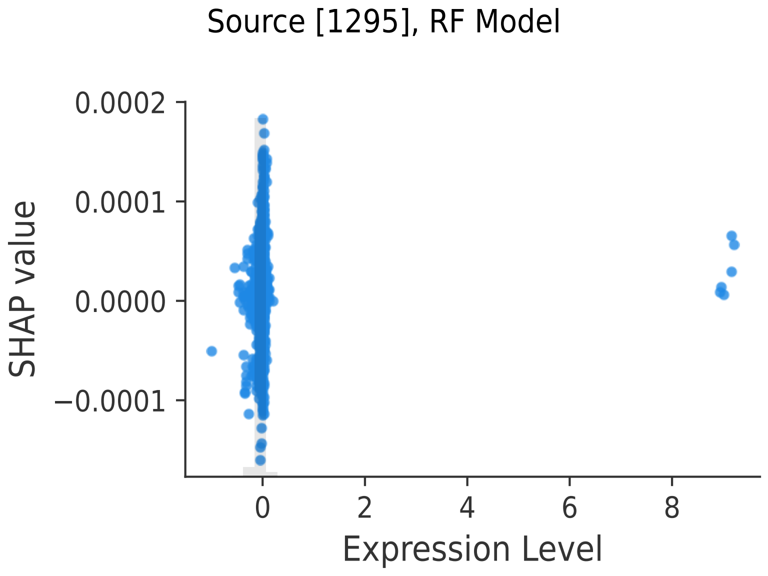
Source [1295], RF Model
0.0002
0.0001
0.0000
−0.0001
SHAP value
0
2
4
6
8
Expression Level

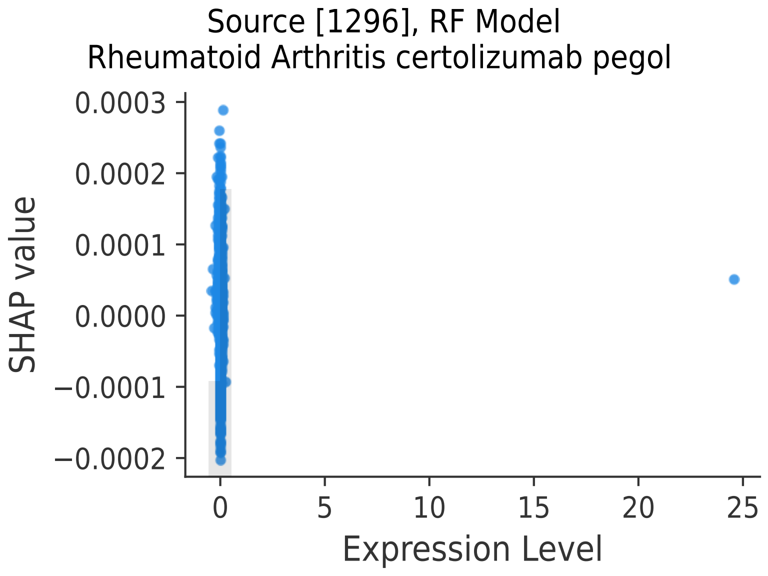

Source [1296], RF Model
Rheumatoid Arthritis certolizumab pegol
SHAP value
0.0003
0.0002
0.0001
0.0000
−0.0001
−0.0002
0
5
10
15
20
25
Expression Level

Source [1297], RF Model

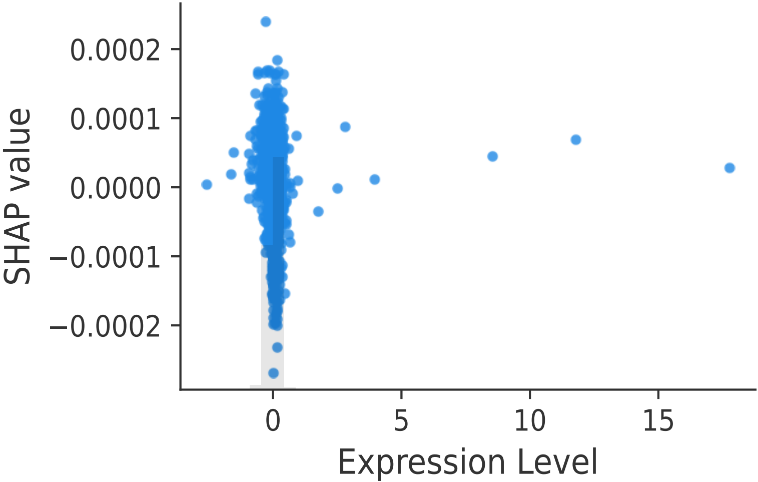

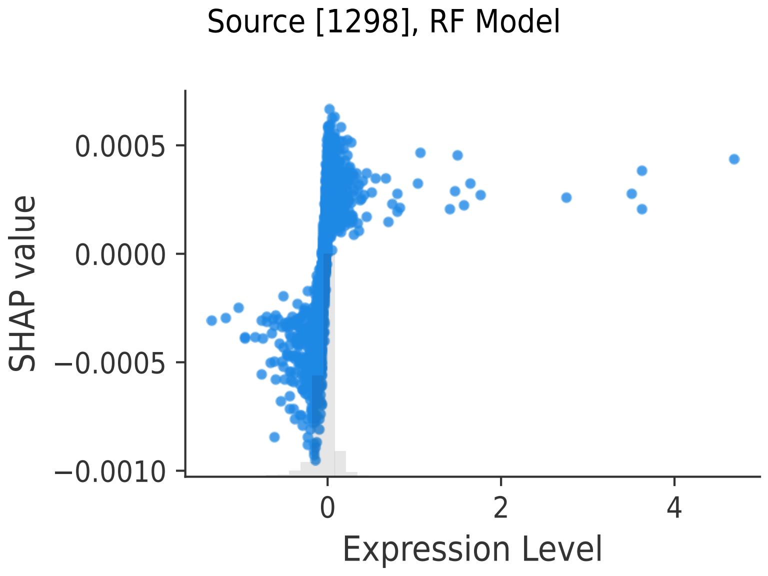
Source [1298], RF Model
SHAP value
0.0005
0.0000
−0.0005
−0.0010
0
2
4
Expression Level

Source [1299], RF Model

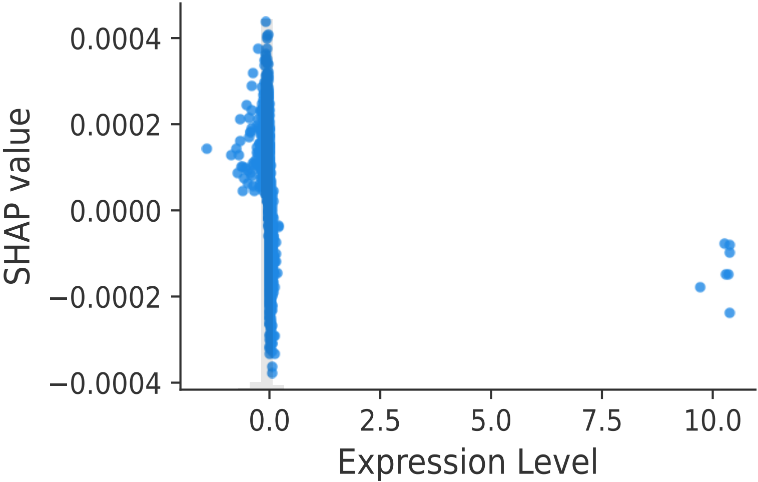

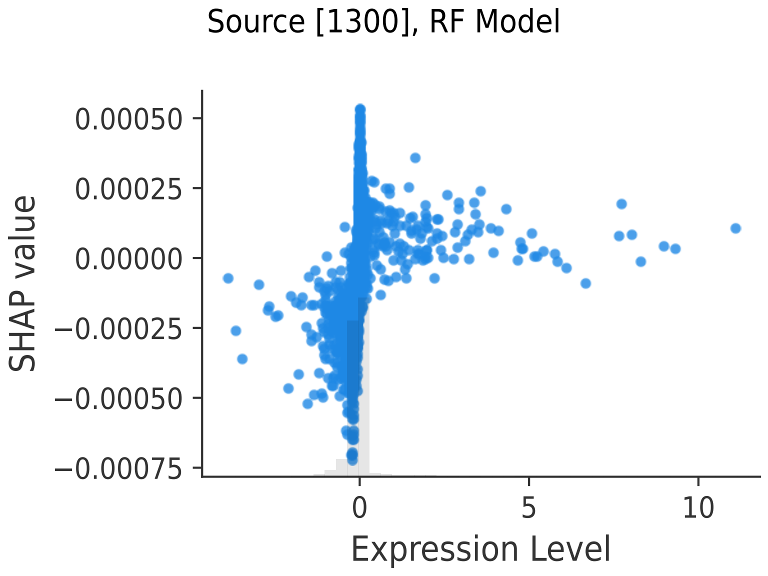
Source [1300], RF Model
SHAP value
0.00050
0.00025
0.00000
−0.00025
−0.00050
−0.00075
0
5
10
Expression Level

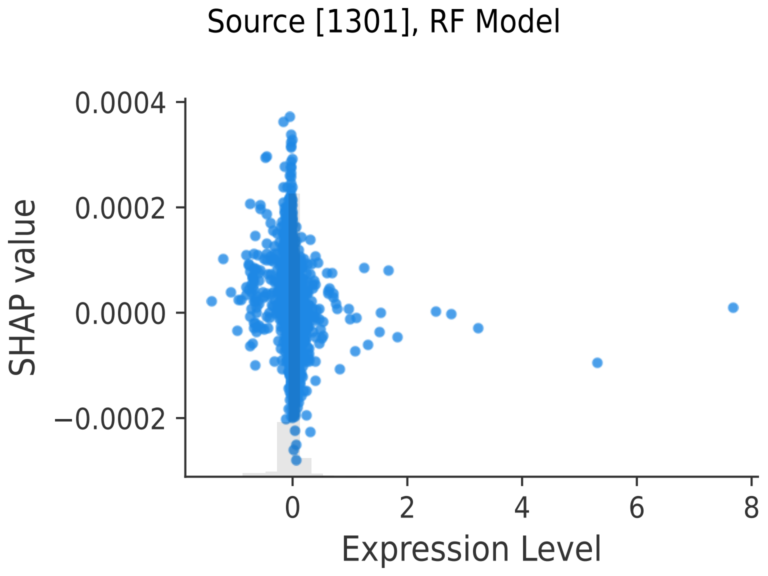
Source [1301], RF Model
0.0004
0.0002
0.0000
−0.0002
SHAP value
0
2
4
6
8
Expression Level

Source [1302], RF Model

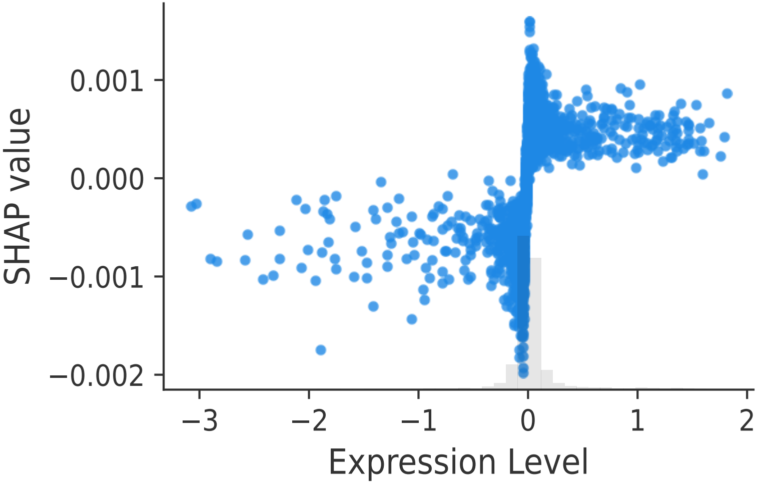

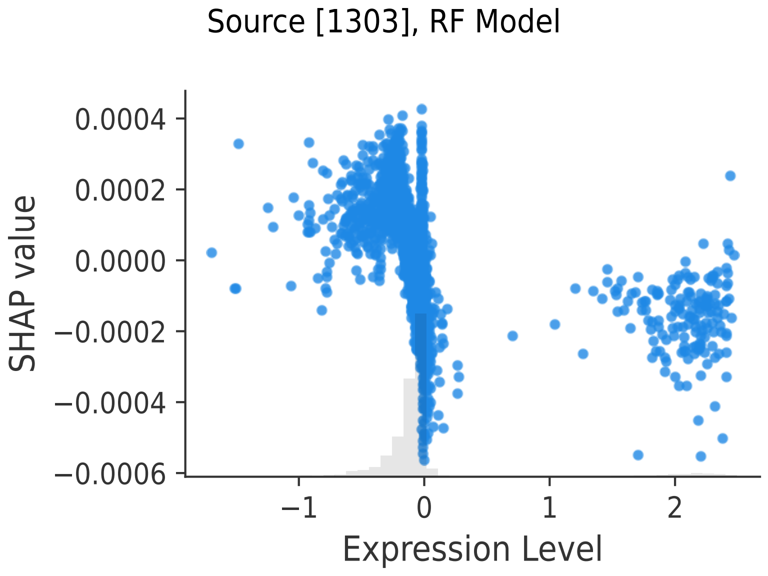

Source [1303], RF Model
SHAP value
0.0004
0.0002
0.0000
−0.0002
−0.0004
−0.0006
−1
0
1
2
Expression Level

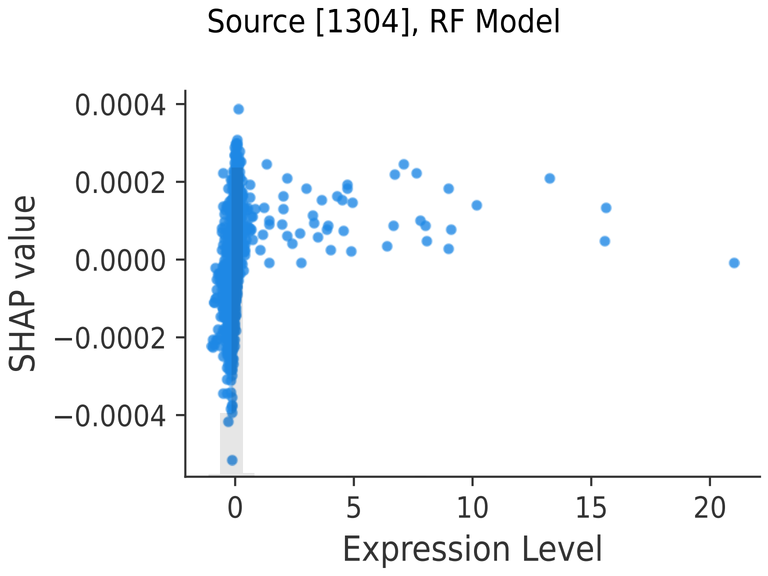
Source [1304], RF Model
0.0004
0.0002
0.0000
−0.0002
−0.0004
SHAP value
0
5
10
15
20
Expression Level

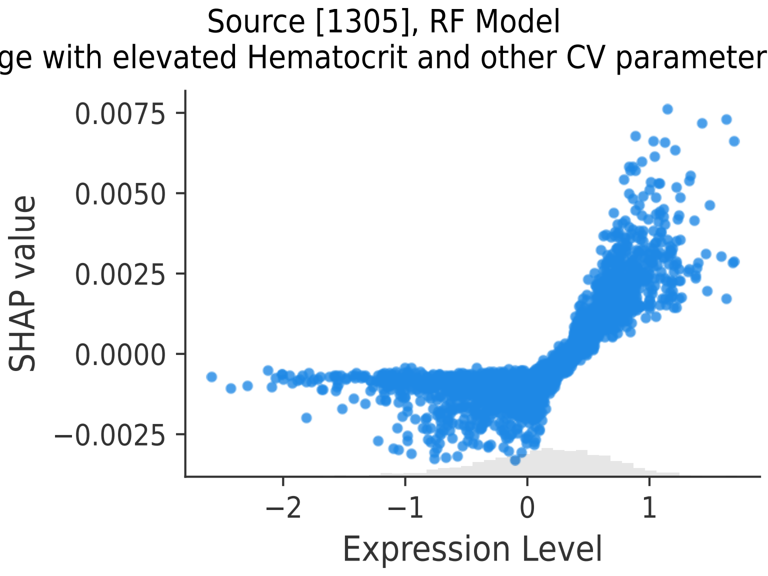
Source [1305], RF Model
ge with elevated Hematocrit and other CV parameter
SHAP value
0.0075
0.0050
0.0025
0.0000
−0.0025
−2
−1
0
1
Expression Level

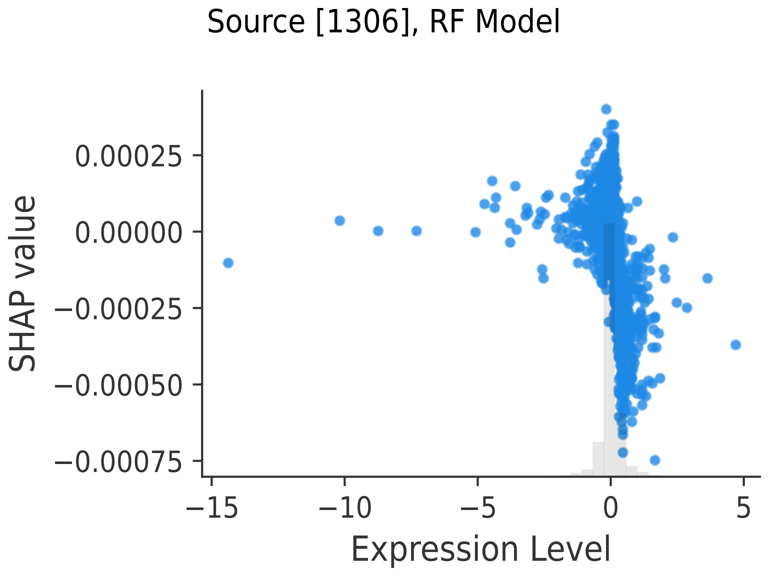
Source [1306], RF Model
SHAP value
0.00025
0.00000
−0.00025
−0.00050
−0.00075
−15
−10
−5
0
5
Expression Level

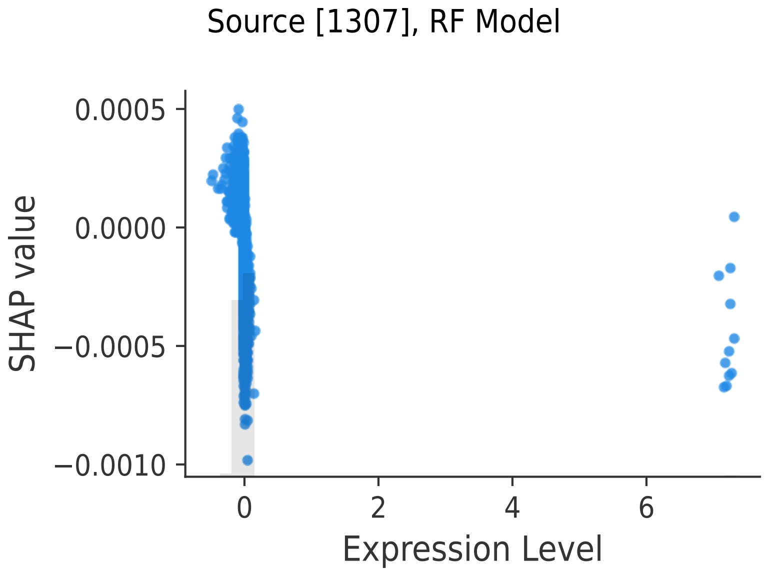

Source [1307], RF Model
0.0005
0.0000
−0.0005
−0.0010
SHAP value
0
2
4
6
Expression Level

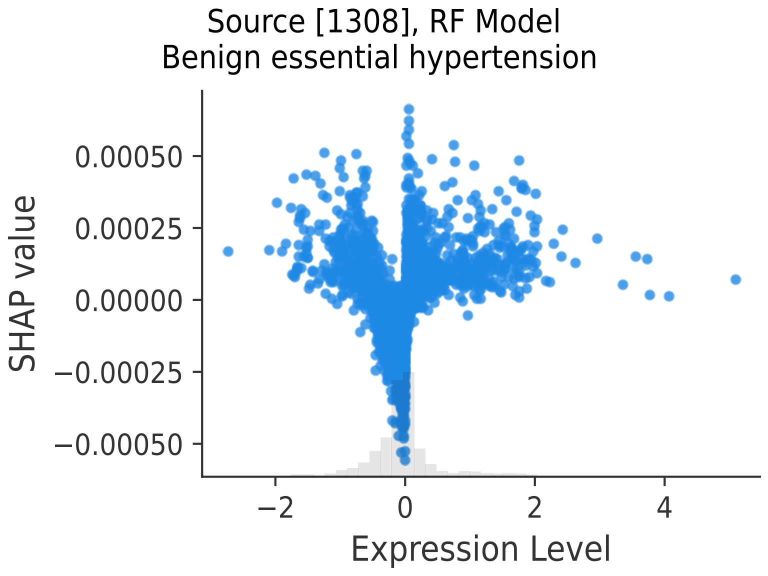
Source [1308], RF Model
Benign essential hypertension
SHAP value
0.00050
0.00025
0.00000
−0.00025
−0.00050
−2
0
2
4
Expression Level

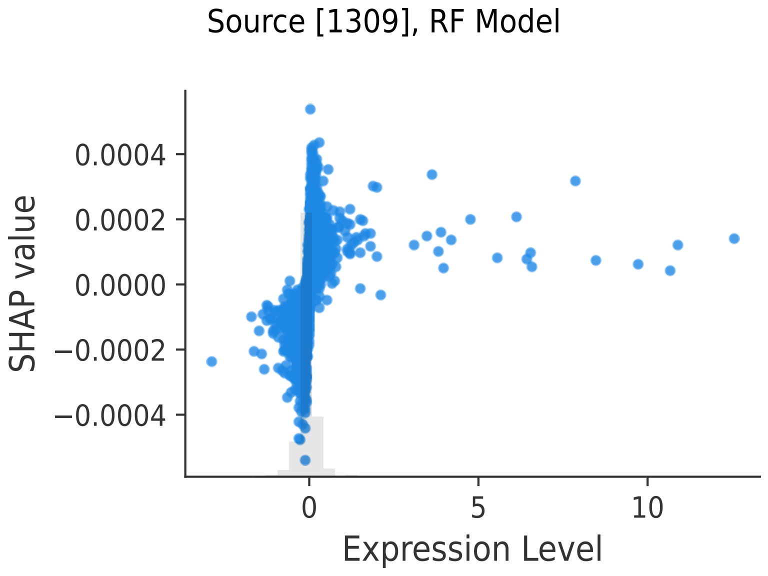

Source [1309], RF Model
SHAP value
0.0004
0.0002
0.0000
−0.0002
−0.0004
0
5
10
Expression Level

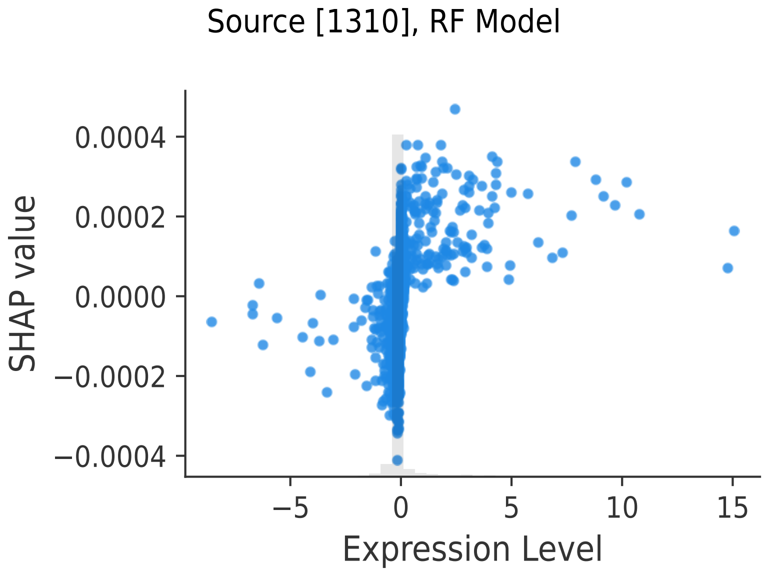

Source [1310], RF Model
SHAP value
0.0004
0.0002
0.0000
−0.0002
−0.0004
−5
0
5
10
15
Expression Level

Source [1311], RF Model

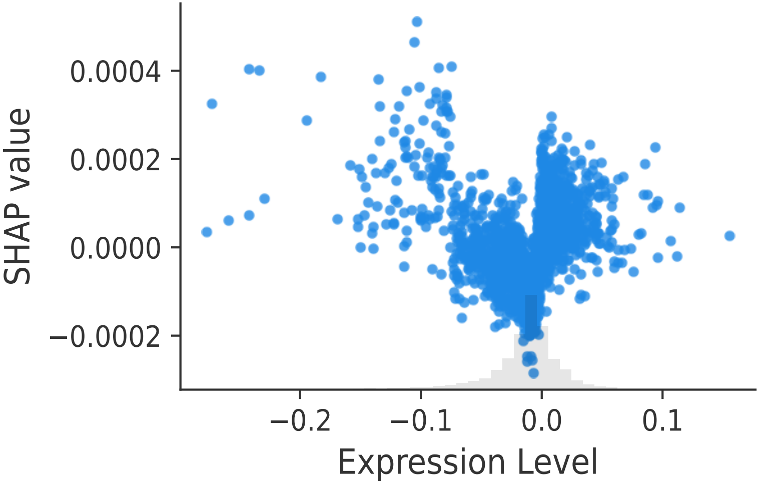

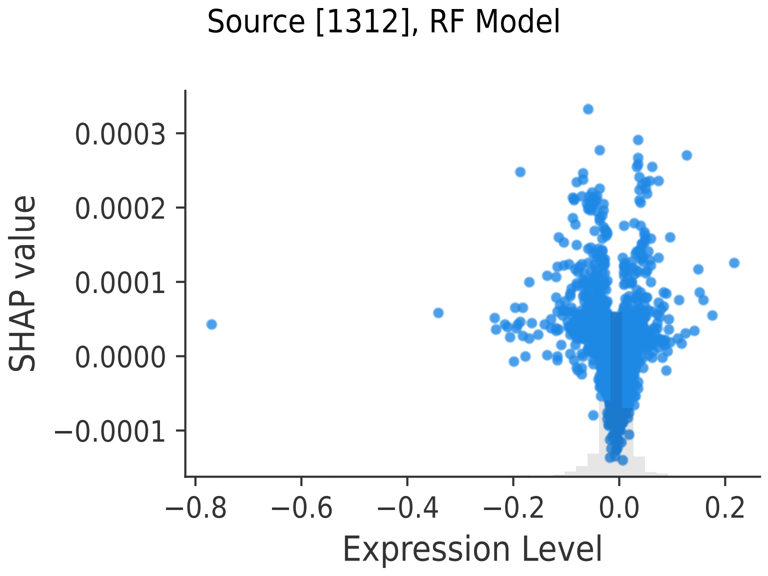
Source [1312], RF Model
SHAP value
0.0003
0.0002
0.0001
0.0000
−0.0001
−0.8
−0.6
−0.4
−0.2
0.0
0.2
Expression Level

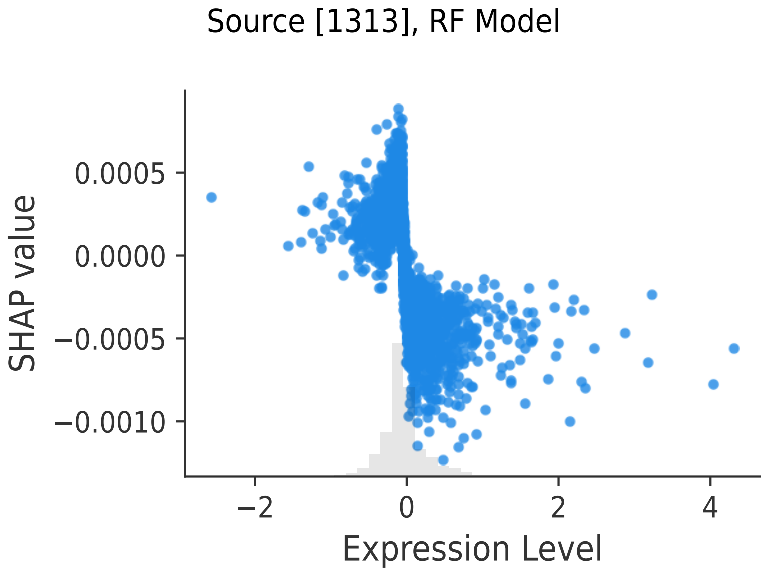

Source [1313], RF Model
SHAP value
0.0005
0.0000
−0.0005
−0.0010
−2
0
2
4
Expression Level

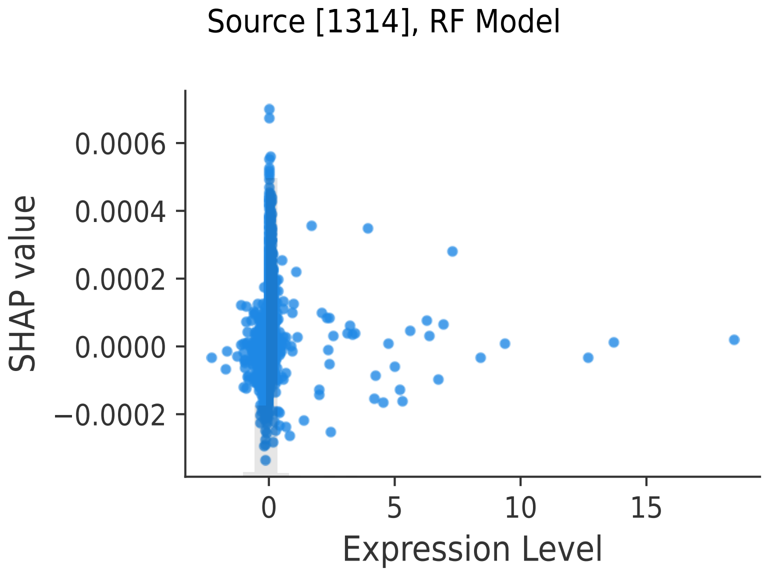
Source [1314], RF Model
SHAP value
0.0006
0.0004
0.0002
0.0000
−0.0002
0
5
10
15
Expression Level

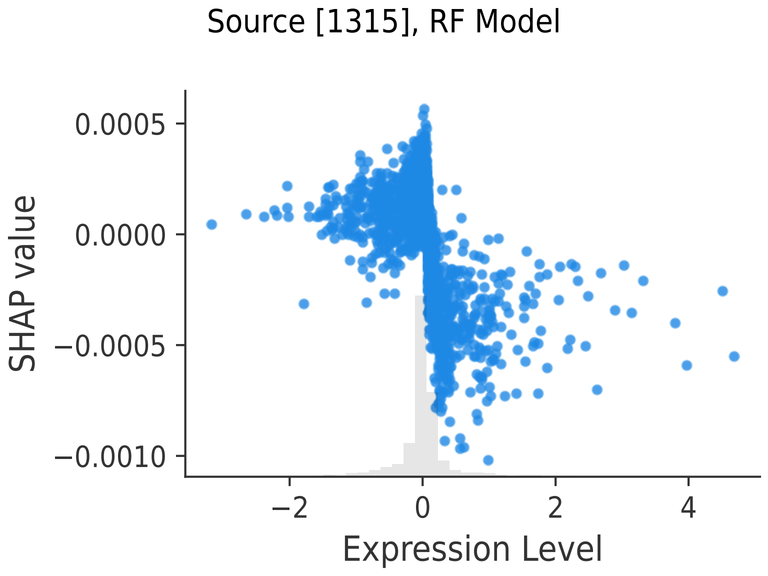
Source [1315], RF Model
0.0005
0.0000
−0.0005
−0.0010
SHAP value
−2
0
2
4
Expression Level

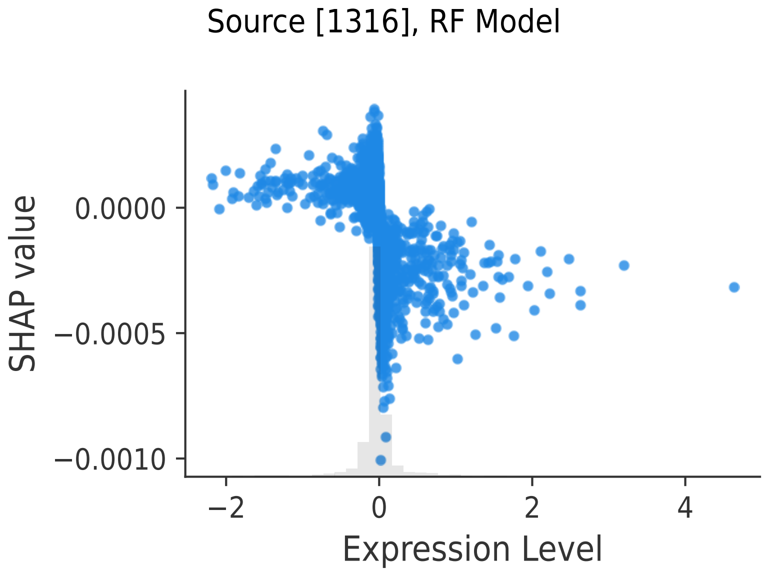
Source [1316], RF Model
SHAP value
0.0000
−0.0005
−0.0010
−2
0
2
4
Expression Level

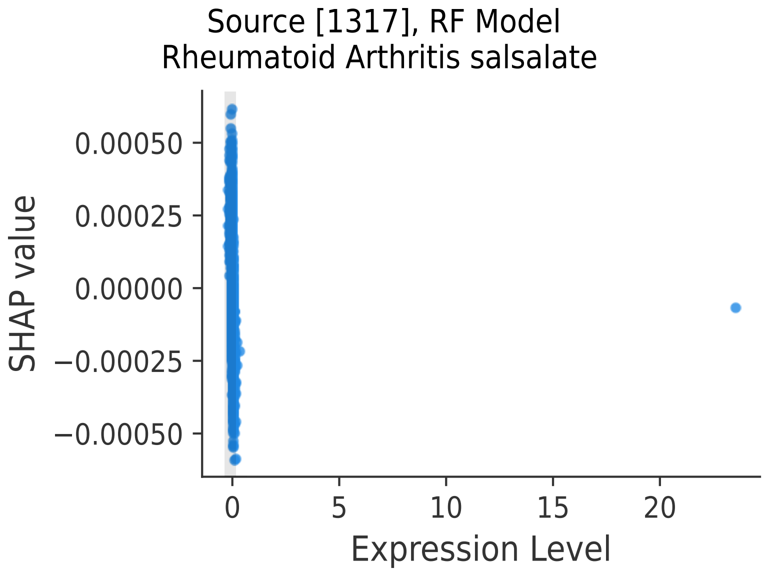

Source [1317], RF Model
Rheumatoid Arthritis salsalate
SHAP value
0.00050
0.00025
0.00000
−0.00025
−0.00050
0
5
10
15
20
Expression Level

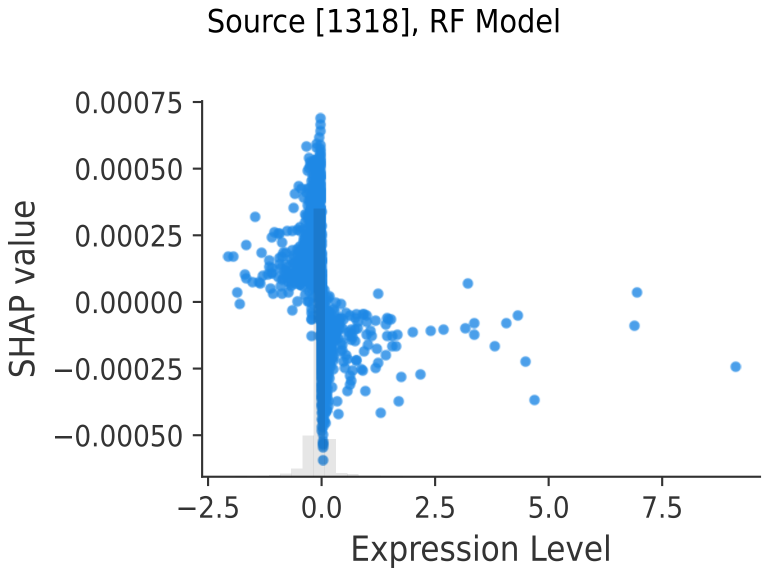
Source [1318], RF Model
0.00075
0.00050
0.00025
0.00000
−0.00025
−0.00050
SHAP value
−2.5
0.0
2.5
5.0
7.5
Expression Level

Source [1319], RF Model

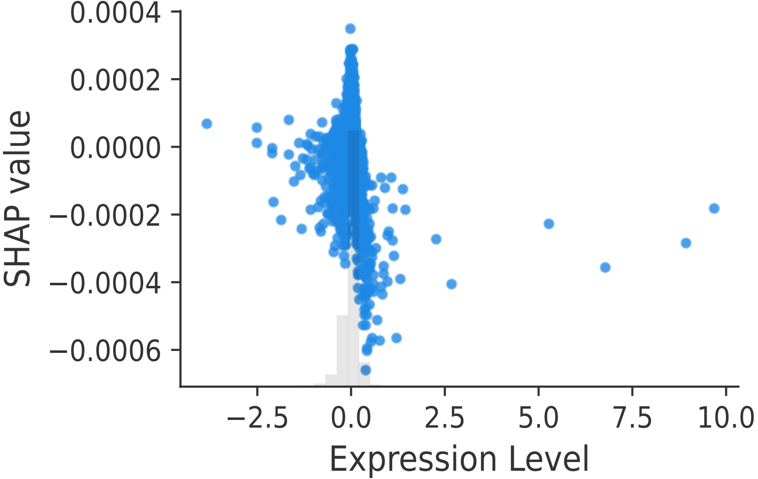

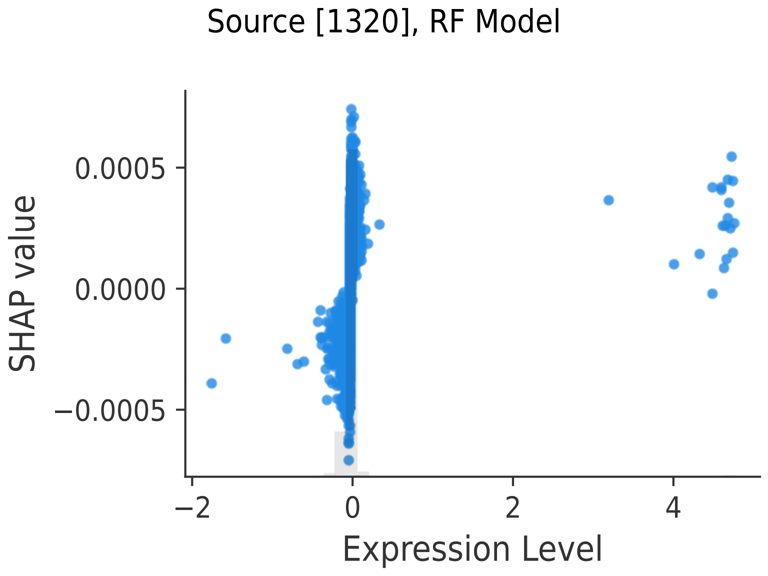

Source [1320], RF Model
SHAP value
0.0005
0.0000
−0.0005
−2
0
2
4
Expression Level

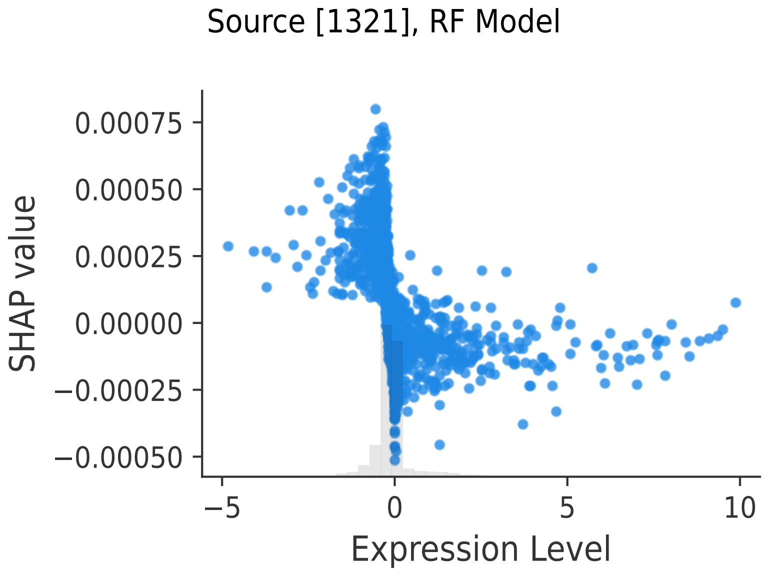

Source [1321], RF Model
0.00075
0.00050
0.00025
0.00000
−0.00025
−0.00050
SHAP value
−5
0
5
10
Expression Level

Source [1322], RF Model

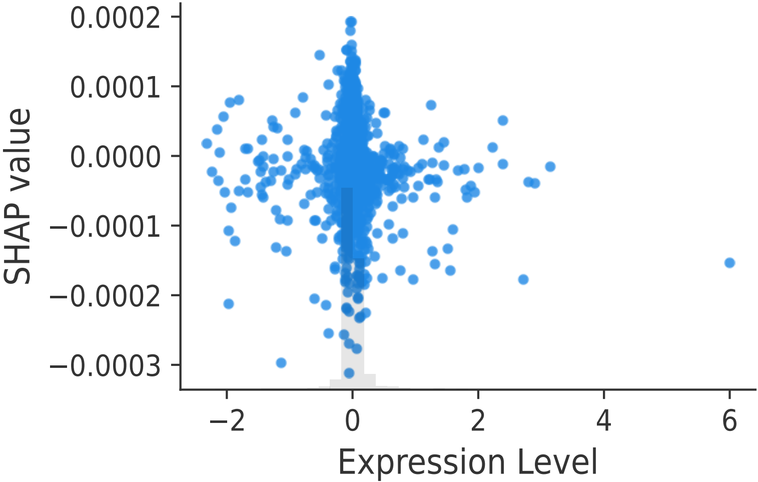

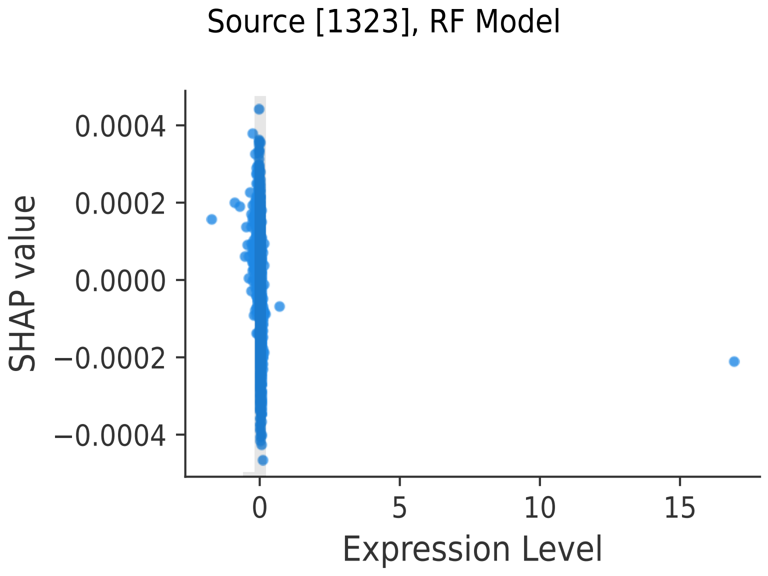
Source [1323], RF Model
0.0004
0.0002
0.0000
−0.0002
−0.0004
SHAP value
0
5
10
15
Expression Level

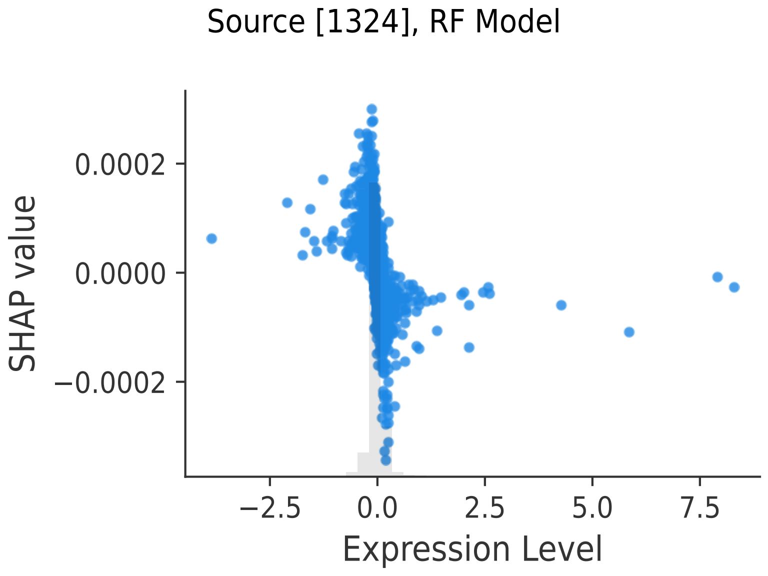
Source [1324], RF Model
0.0002
0.0000
−0.0002
SHAP value
−2.5
0.0
2.5
5.0
7.5
Expression Level

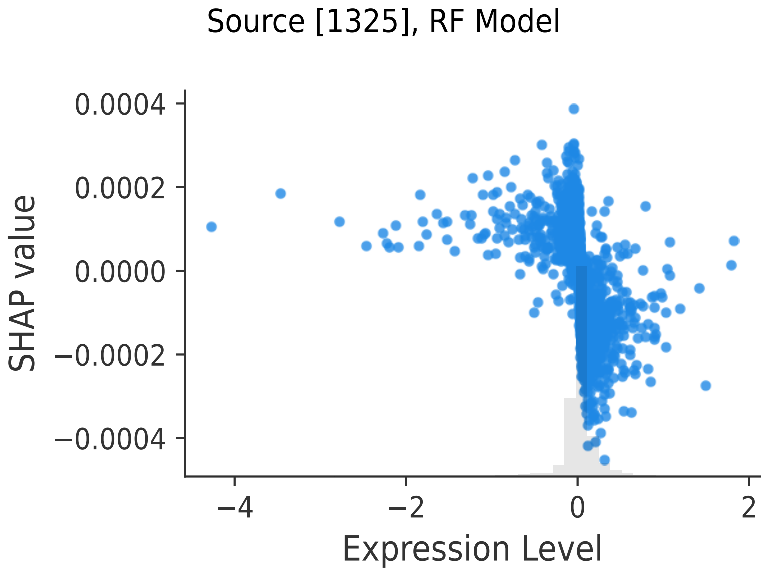

Source [1325], RF Model
0.0004
0.0002
0.0000
−0.0002
−0.0004
SHAP value
−4
−2
0
2
Expression Level

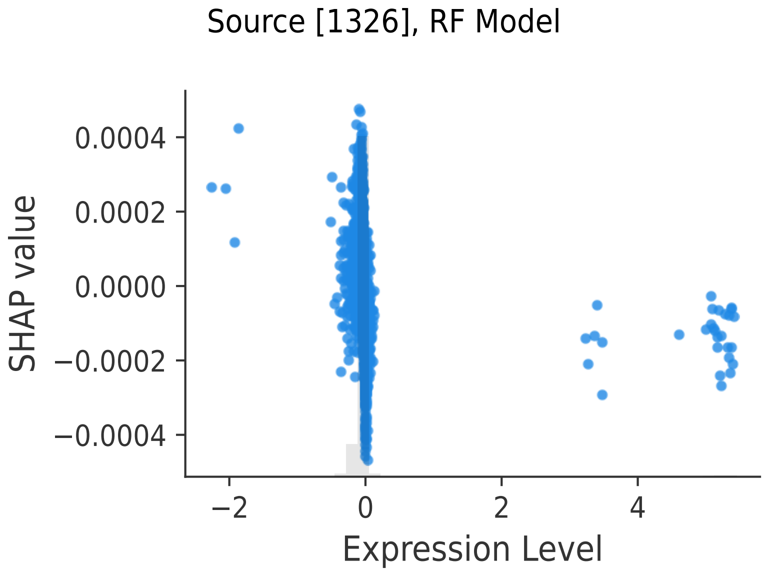
Source [1326], RF Model
SHAP value
0.0004
0.0002
0.0000
−0.0002
−0.0004
−2
0
2
4
Expression Level

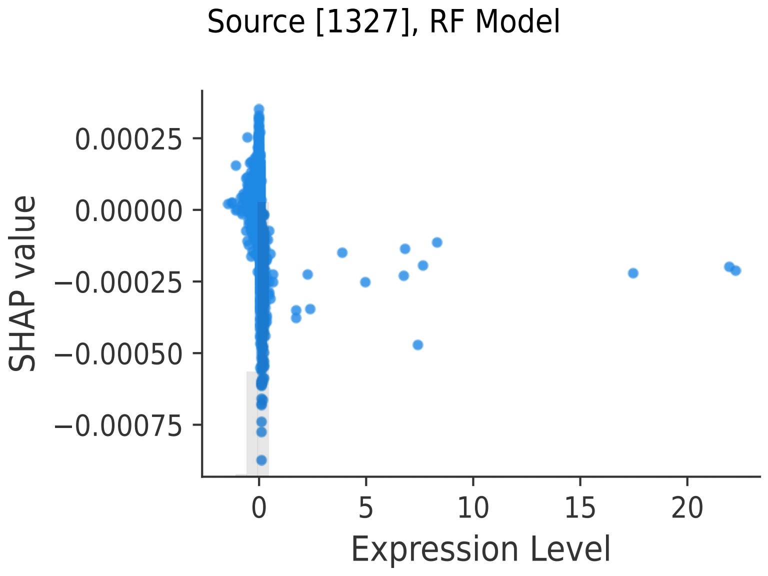
Source [1327], RF Model
SHAP value
0.00025
0.00000
−0.00025
−0.00050
−0.00075
0
5
10
15
20
Expression Level

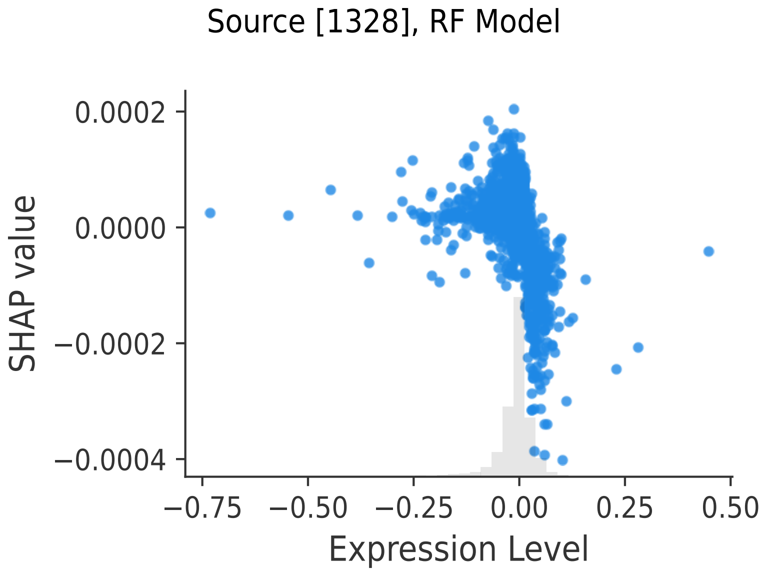
Source [1328], RF Model
SHAP value
0.0002
0.0000
−0.0002
−0.0004
Expression Level
−0.75
−0.50
−0.25
0.00
0.25
0.50

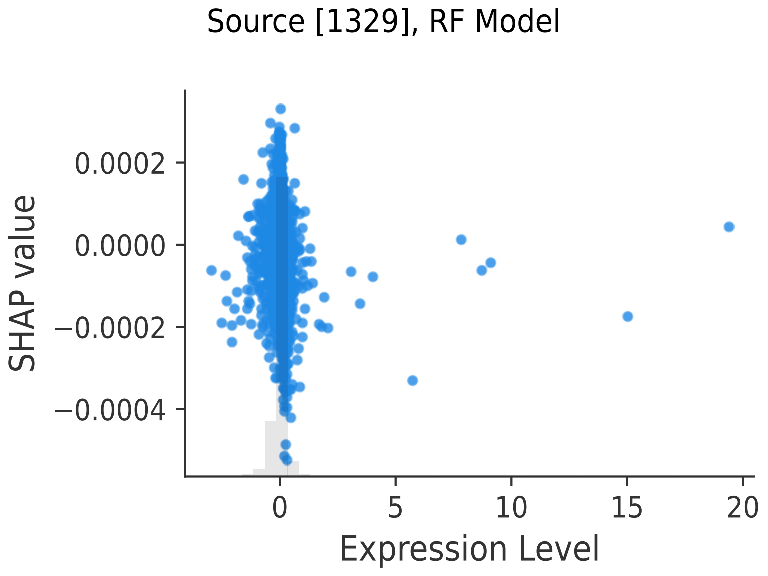
Source [1329], RF Model
0.0002
0.0000
−0.0002
−0.0004
SHAP value
0
5
10
15
20
Expression Level

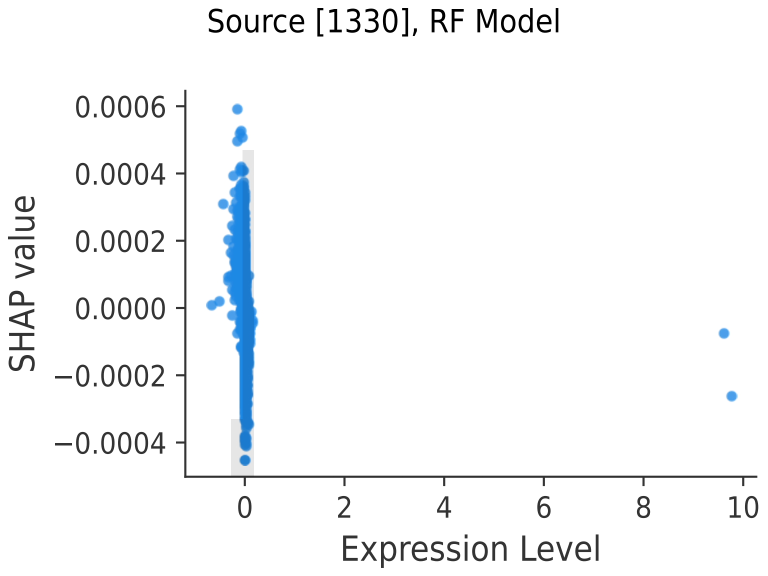

Source [1330], RF Model
0.0006
0.0004
0.0002
0.0000
−0.0002
−0.0004
SHAP value
0
2
4
6
8
10
Expression Level

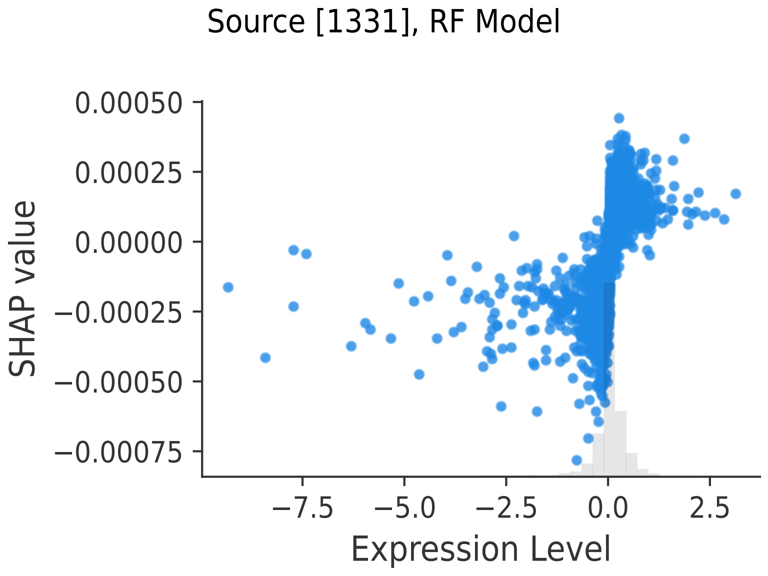

Source [1331], RF Model
0.00050
0.00025
0.00000
−0.00025
−0.00050
−0.00075
SHAP value
−7.5
−5.0
−2.5
0.0
2.5
Expression Level

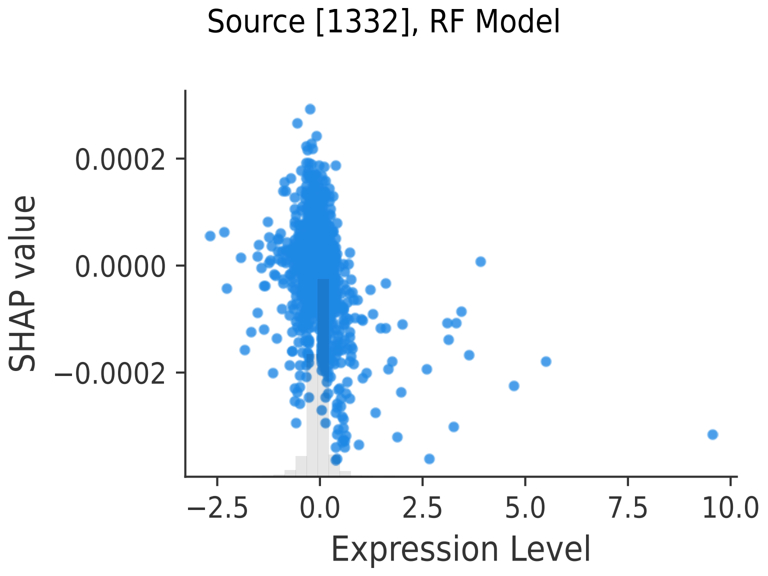
Source [1332], RF Model
0.0002
0.0000
−0.0002
SHAP value
−2.5
0.0
2.5
5.0
7.5
10.0
Expression Level

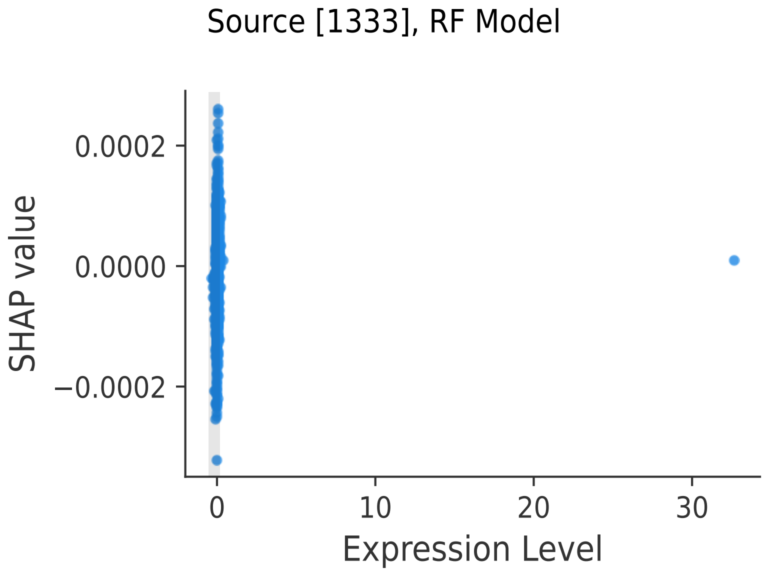
Source [1333], RF Model
SHAP value
0.0002
0.0000
−0.0002
0
10
20
30
Expression Level

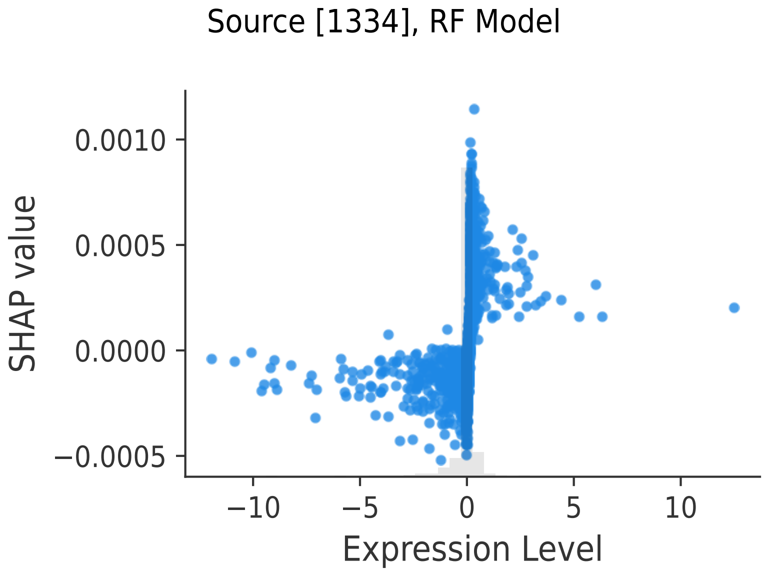

Source [1334], RF Model
SHAP value
0.0010
0.0005
0.0000
−0.0005
−10
−5
0
5
10
Expression Level

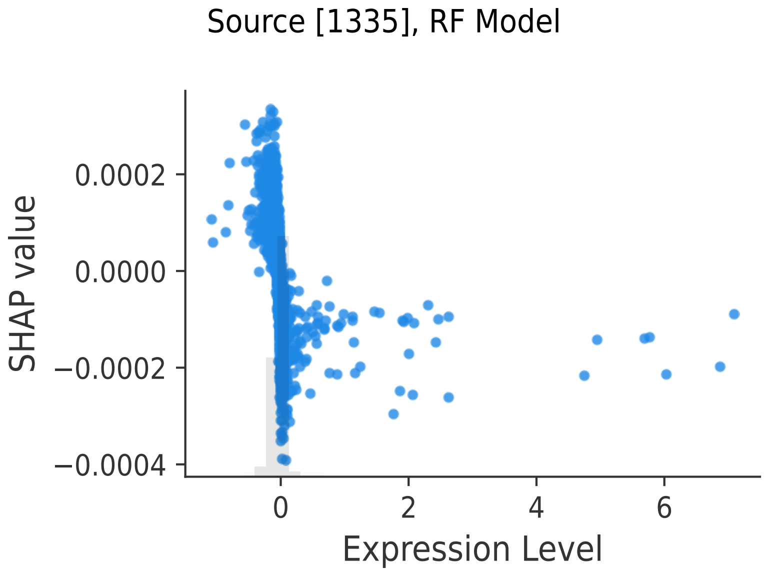

Source [1335], RF Model
SHAP value
0.0002
0.0000
−0.0002
−0.0004
0
2
4
6
Expression Level

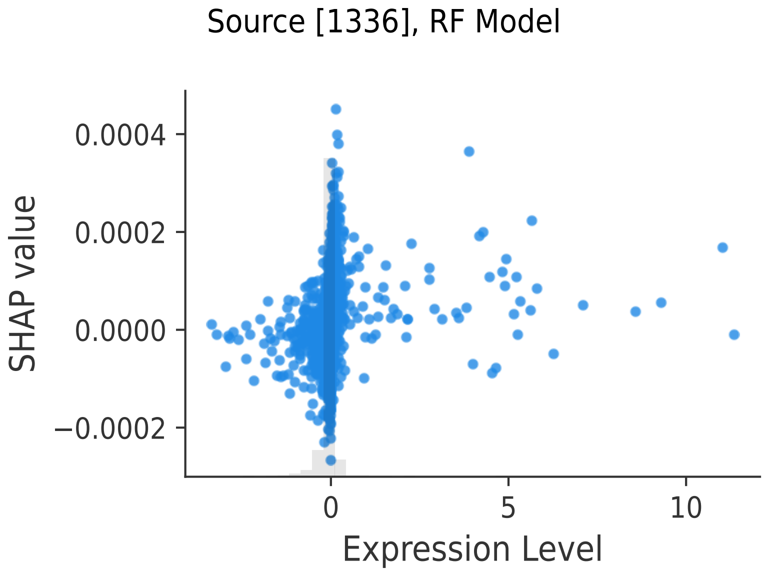

Source [1336], RF Model
0.0004
0.0002
0.0000
−0.0002
SHAP value
0
5
10
Expression Level

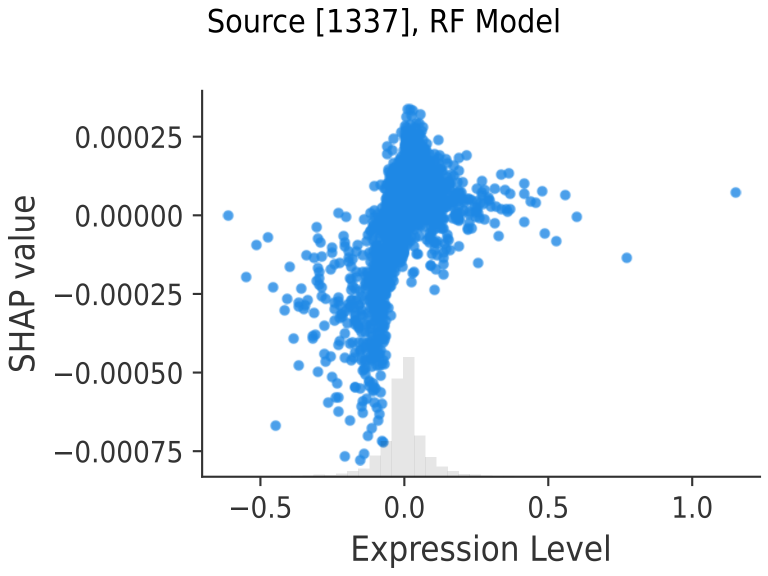
Source [1337], RF Model
SHAP value
0.00025
0.00000
−0.00025
−0.00050
−0.00075
−0.5
0.0
0.5
1.0
Expression Level

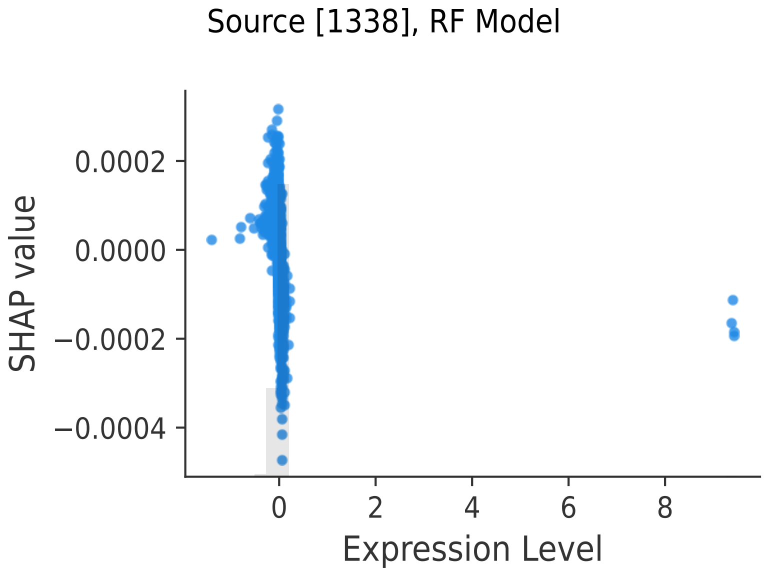
Source [1338], RF Model
SHAP value
0.0002
0.0000
−0.0002
−0.0004
0
2
4
6
8
Expression Level

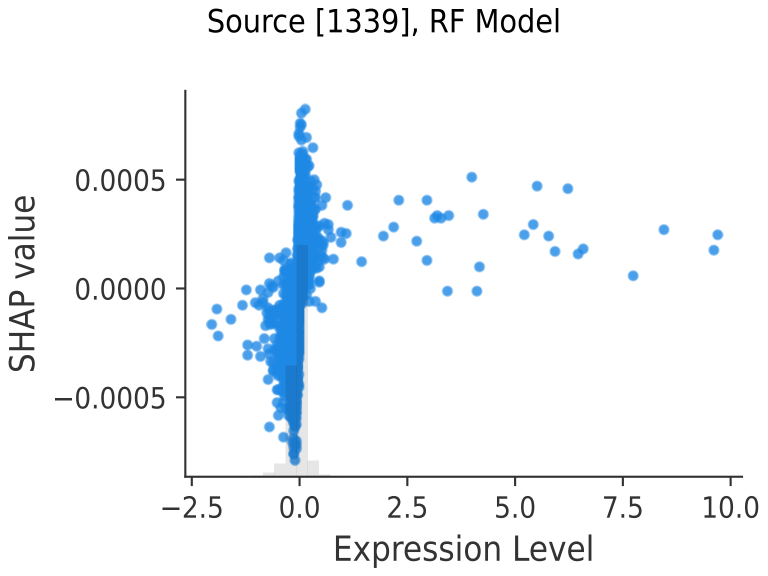

Source [1339], RF Model
SHAP value
0.0005
0.0000
−0.0005
−2.5
0.0
2.5
5.0
7.5
10.0
Expression Level

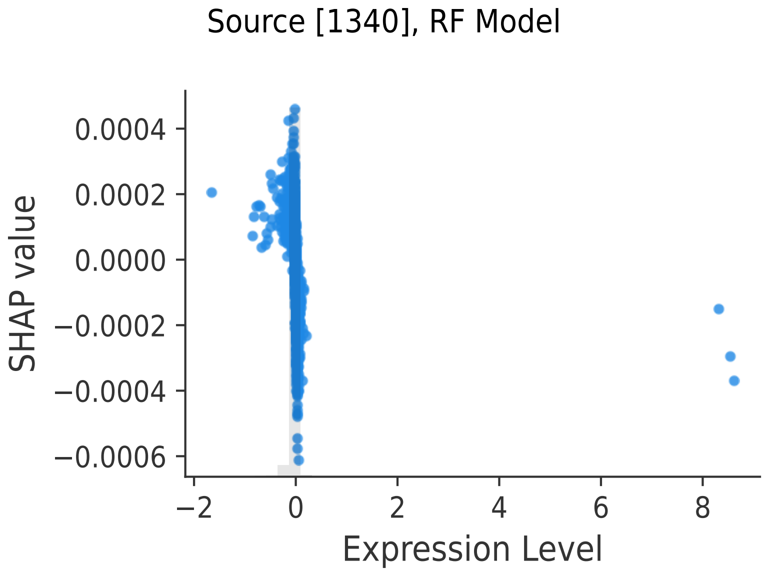
Source [1340], RF Model
SHAP value
0.0004
0.0002
0.0000
−0.0002
−0.0004
−0.0006
−2
0
2
4
6
8
Expression Level

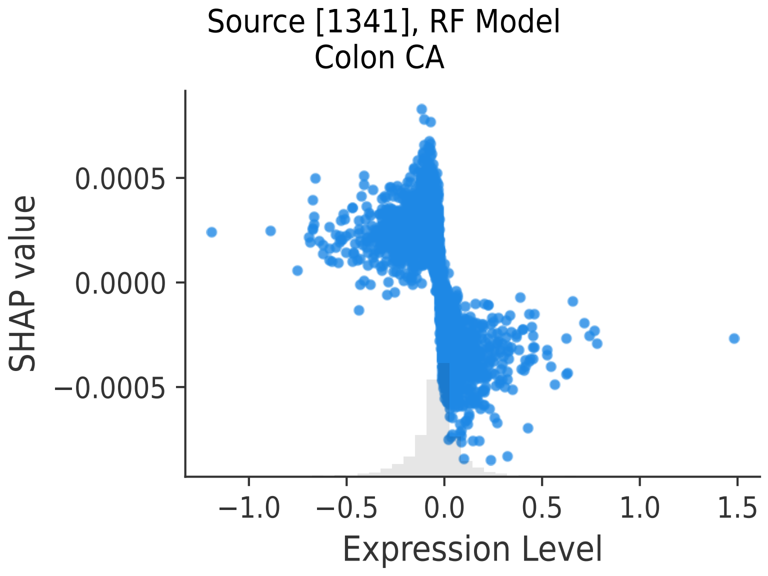
Source [1341], RF Model
Colon CA
0.0005
0.0000
−0.0005
SHAP value
−1.0
−0.5
0.0
0.5
1.0
1.5
Expression Level

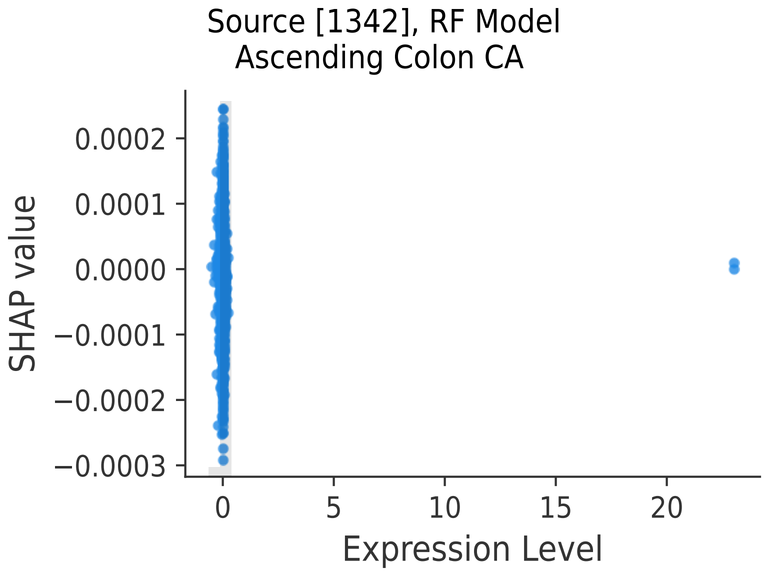

Source [1342], RF Model
Ascending Colon CA
SHAP value
0.0002
0.0001
0.0000
−0.0001
−0.0002
−0.0003
0
5
10
15
20
Expression Level

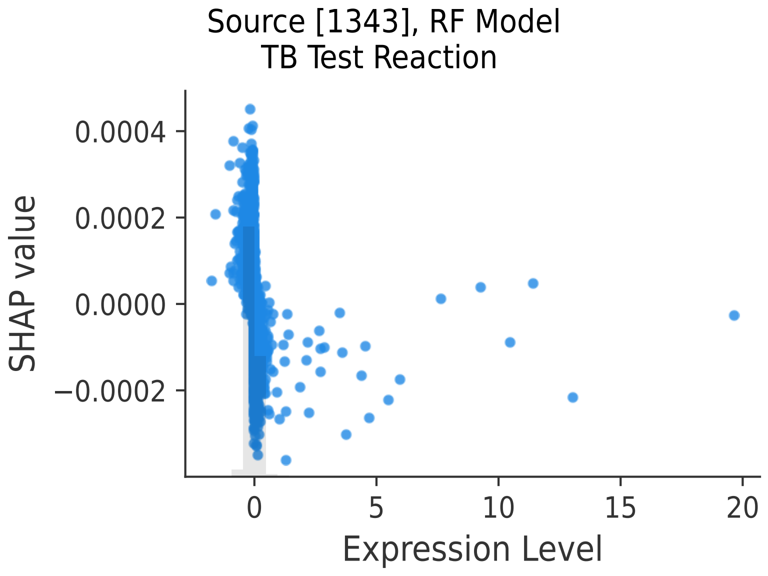

Source [1343], RF Model
TB Test Reaction
0.0004
0.0002
0.0000
−0.0002
SHAP value
0
5
10
15
20
Expression Level

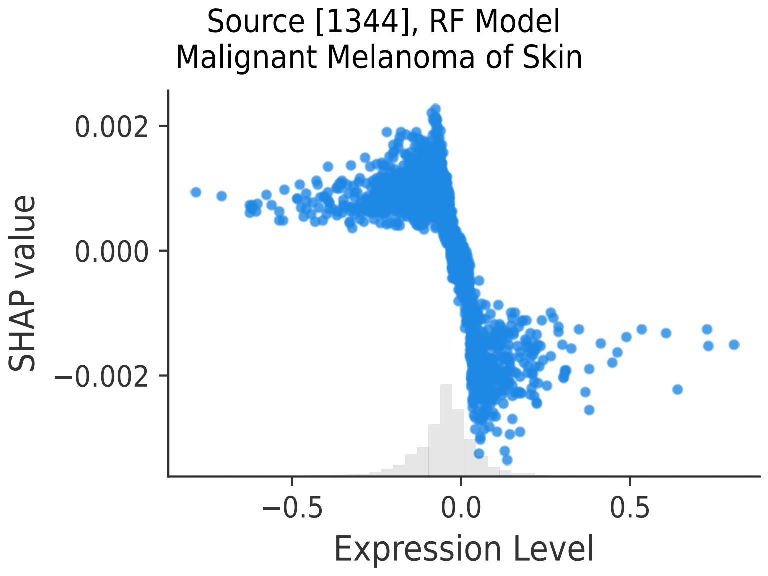
Source [1344], RF Model
Malignant Melanoma of Skin
SHAP value
0.002
0.000
−0.002
−0.5
0.0
0.5
Expression Level

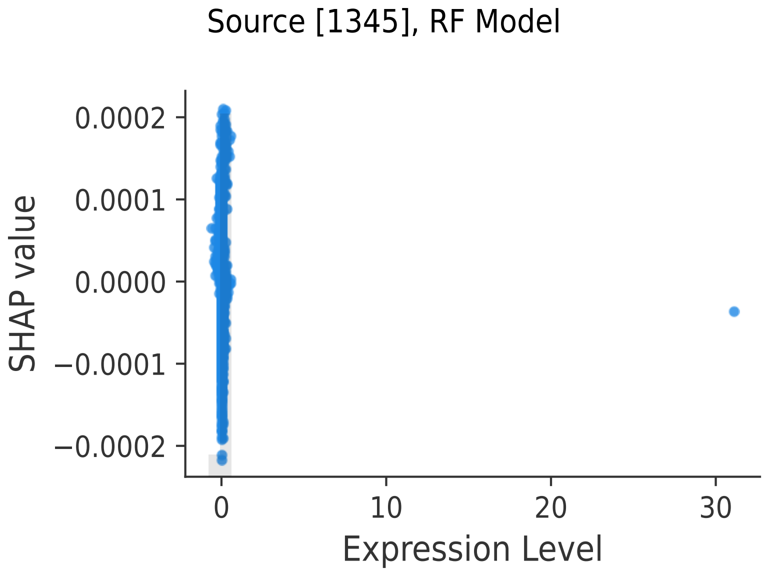
Source [1345], RF Model
SHAP value
0.0002
0.0001
0.0000
−0.0001
−0.0002
0
10
20
30
Expression Level

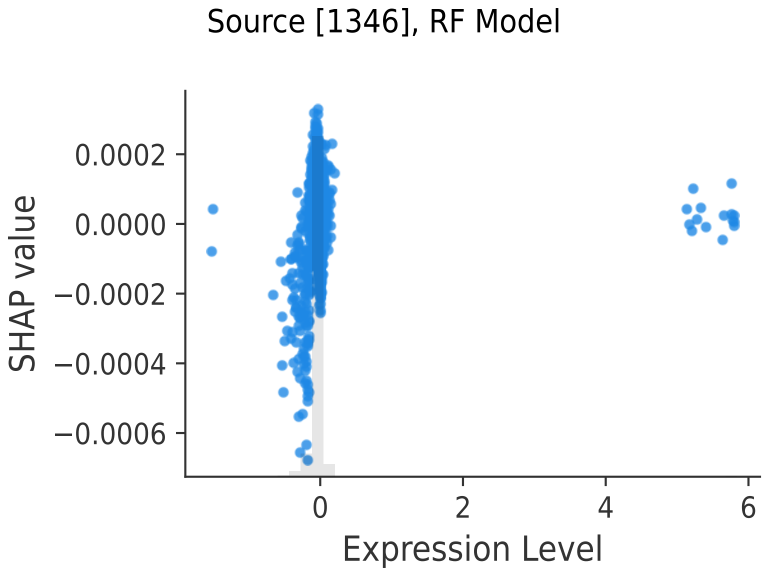
Source [1346], RF Model
SHAP value
0.0002
0.0000
−0.0002
−0.0004
−0.0006
0
2
4
6
Expression Level

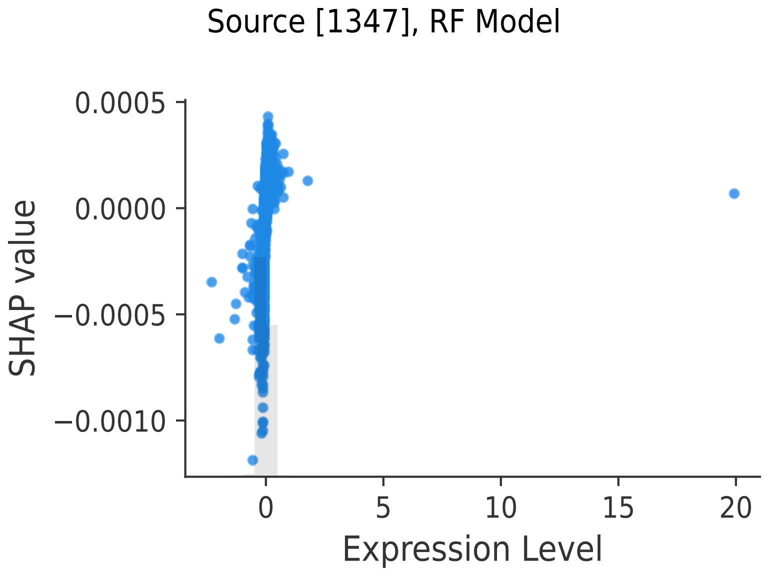
Source [1347], RF Model
SHAP value
0.0005
0.0000
−0.0005
−0.0010
0
5
10
15
20
Expression Level

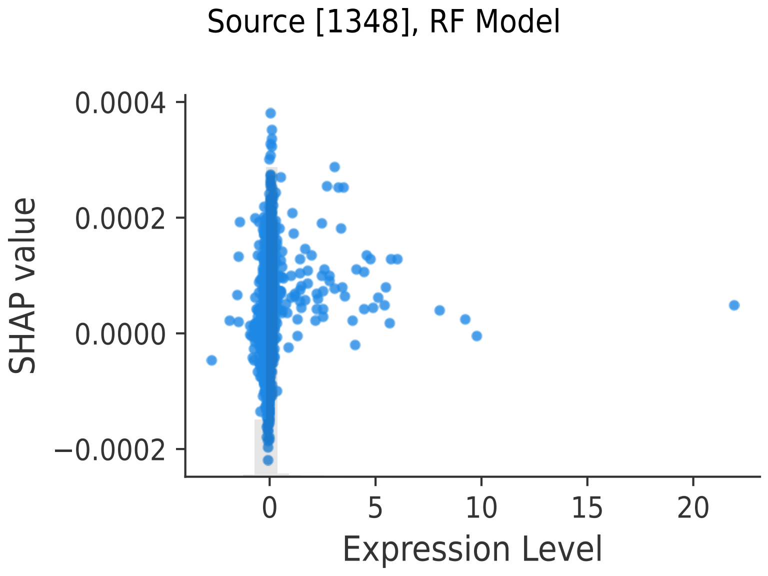

Source [1348], RF Model
SHAP value
0.0004
0.0002
0.0000
−0.0002
0
5
10
15
20
Expression Level

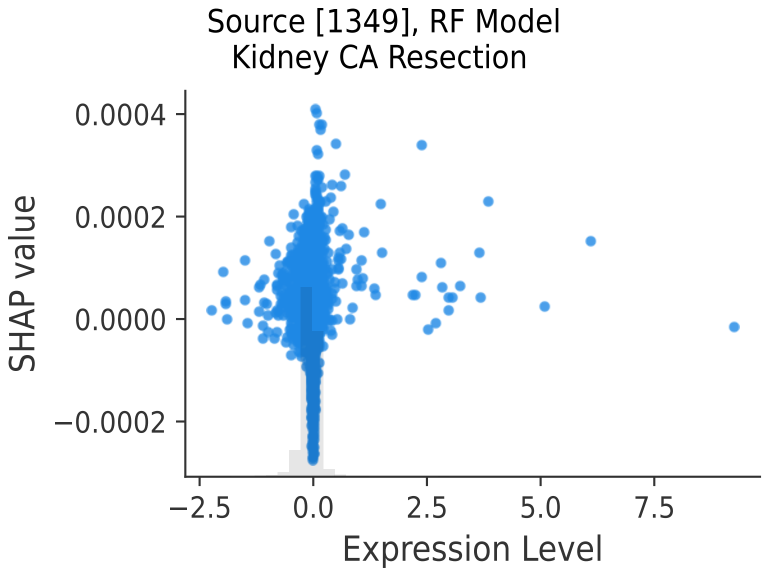

Source [1349], RF Model
Kidney CA Resection
SHAP value
0.0004
0.0002
0.0000
−0.0002
−2.5
0.0
2.5
5.0
7.5
Expression Level

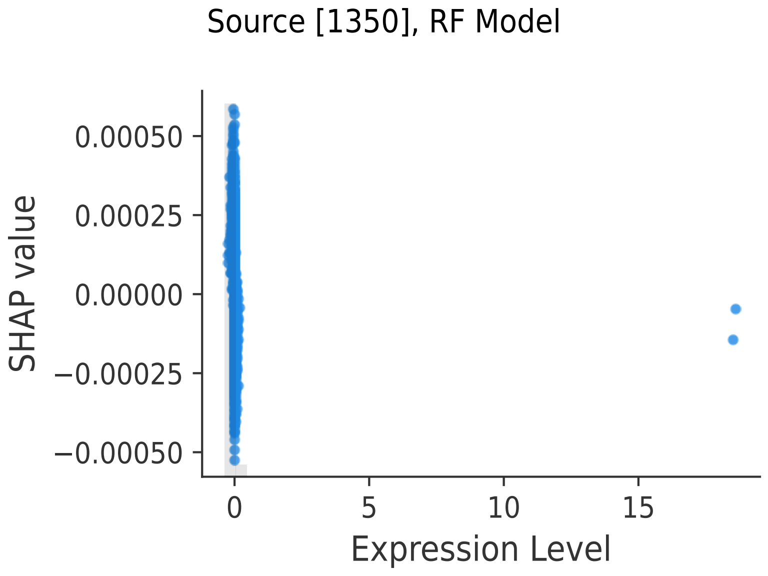

Source [1350], RF Model
0.00050
0.00025
0.00000
−0.00025
−0.00050
SHAP value
0
5
10
15
Expression Level

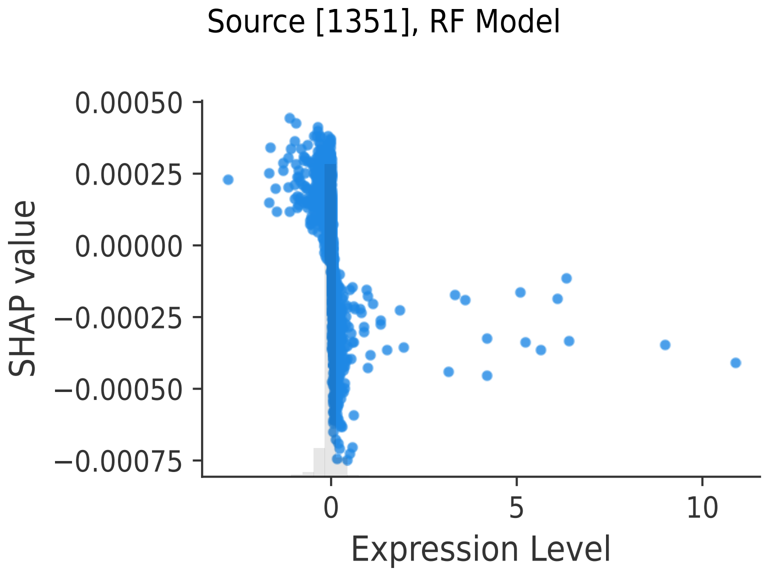

Source [1351], RF Model
0.00050
0.00025
0.00000
−0.00025
−0.00050
−0.00075
SHAP value
0
5
10
Expression Level

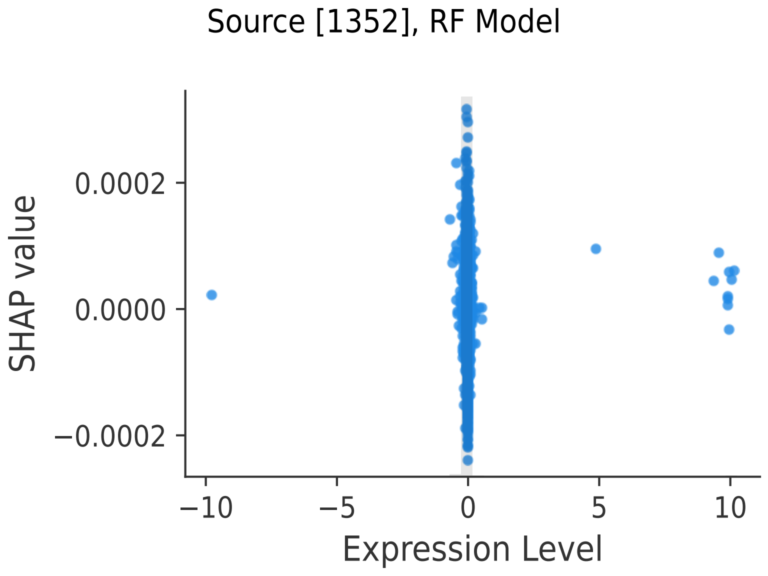

Source [1352], RF Model
SHAP value
0.0002
0.0000
−0.0002
−10
−5
0
5
10
Expression Level

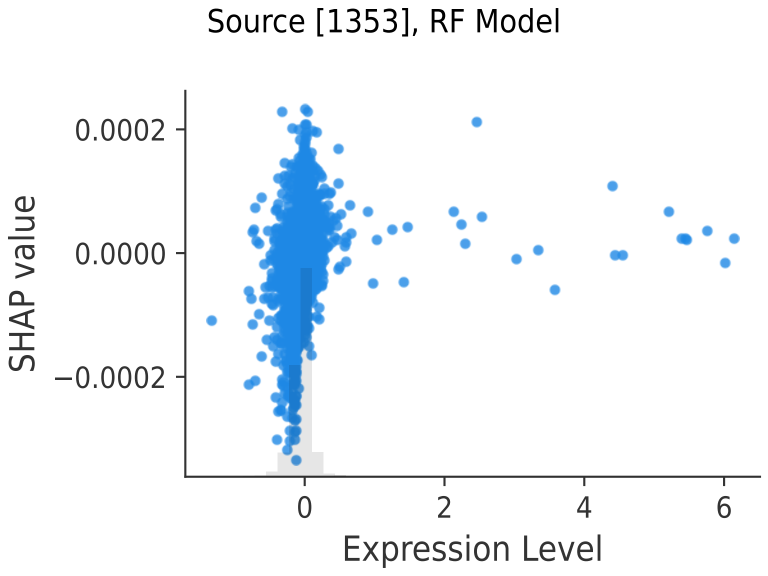

Source [1353], RF Model
0.0002
0.0000
−0.0002
SHAP value
0
2
4
6
Expression Level

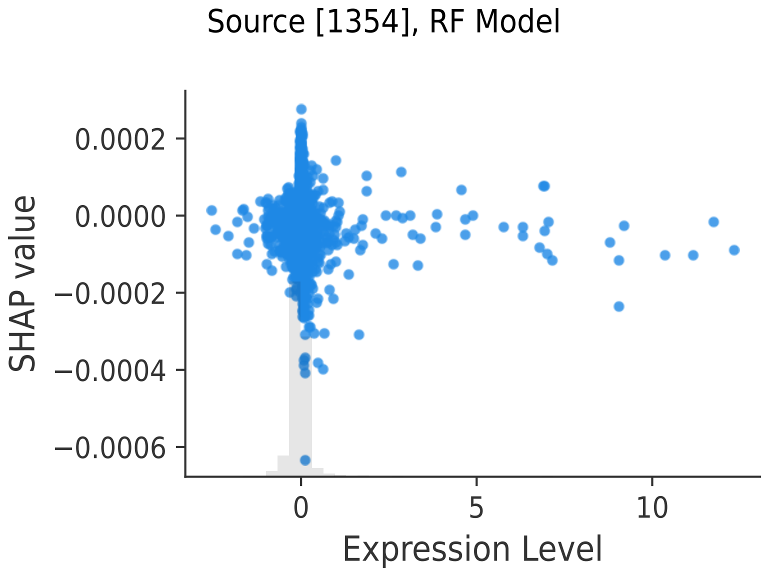

Source [1354], RF Model
0.0002
0.0000
−0.0002
−0.0004
−0.0006
SHAP value
0
5
10
Expression Level

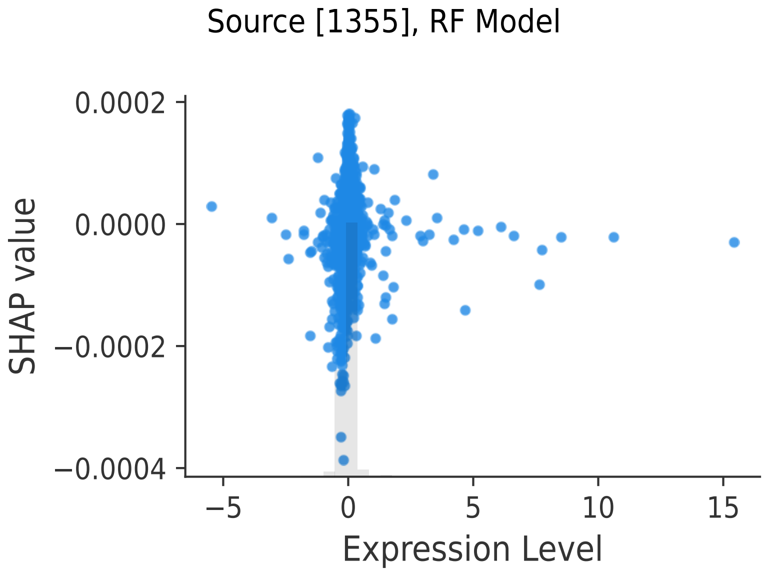
Source [1355], RF Model
0.0002
0.0000
−0.0002
−0.0004
SHAP value
−5
0
5
10
15
Expression Level

Source [1356], RF Model

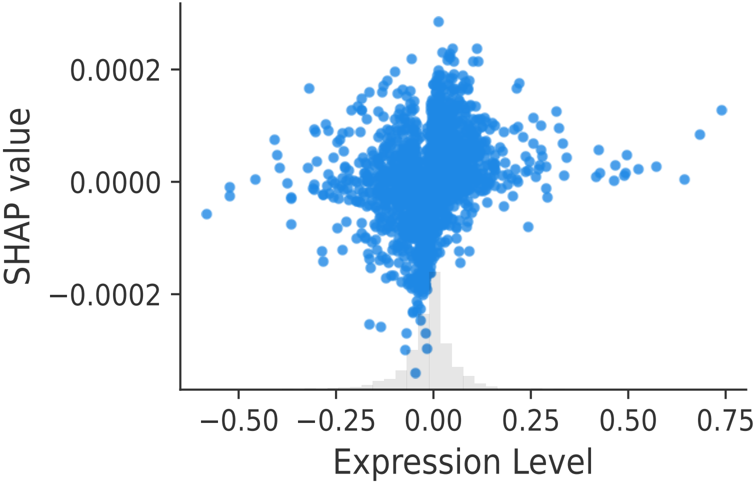

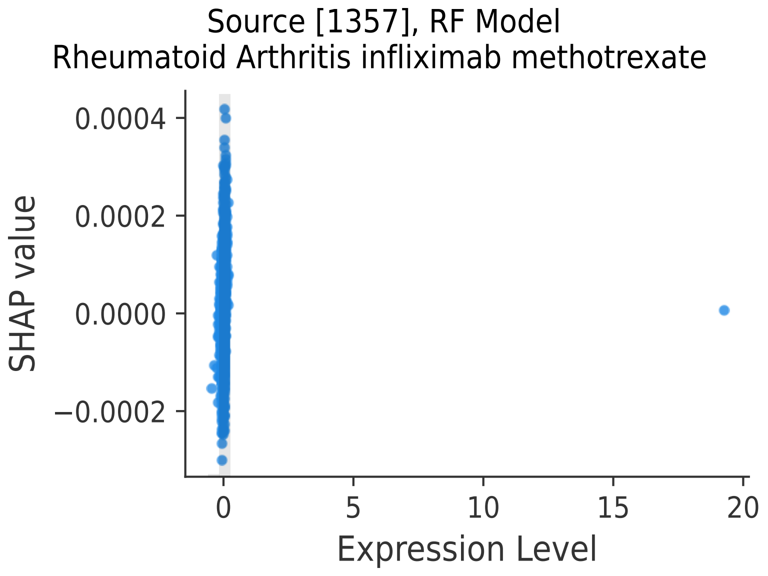
Source [1357], RF Model
Rheumatoid Arthritis infliximab methotrexate
SHAP value
0.0004
0.0002
0.0000
−0.0002
0
5
10
15
20
Expression Level

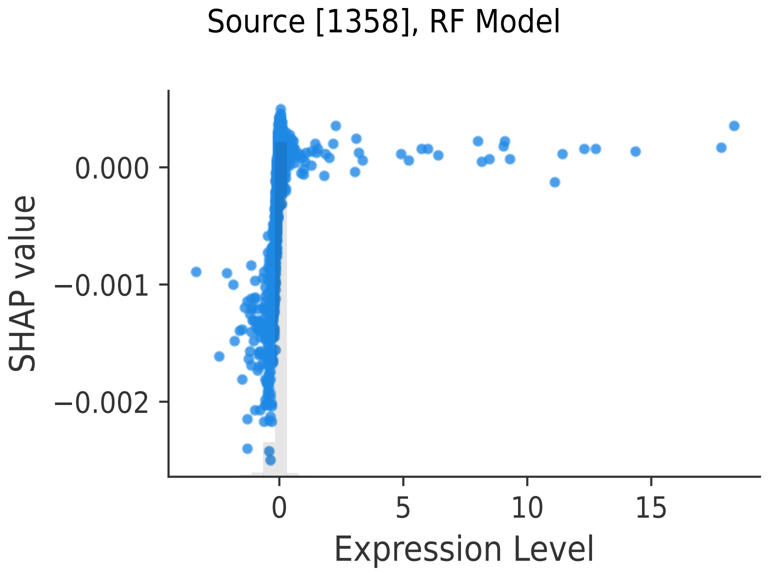
Source [1358], RF Model
SHAP value
0.000
−0.001
−0.002
0
5
10
15
Expression Level

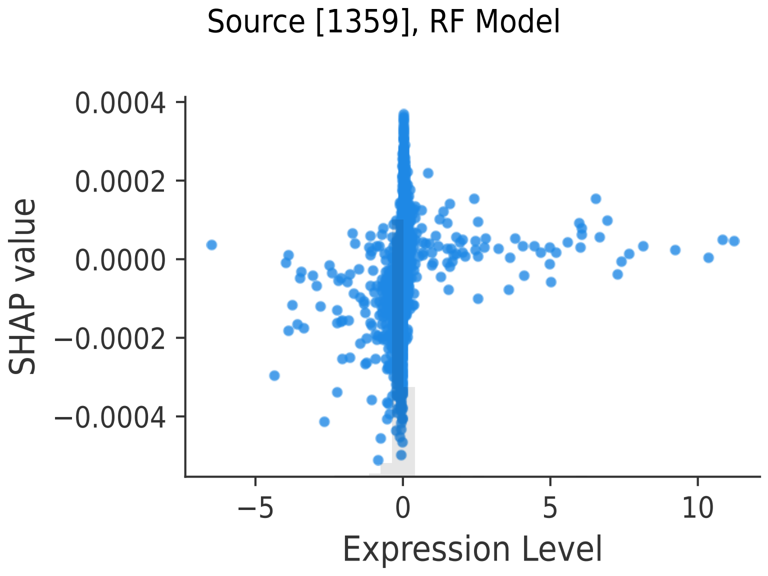
Source [1359], RF Model
0.0004
0.0002
0.0000
−0.0002
−0.0004
SHAP value
−5
0
5
10
Expression Level

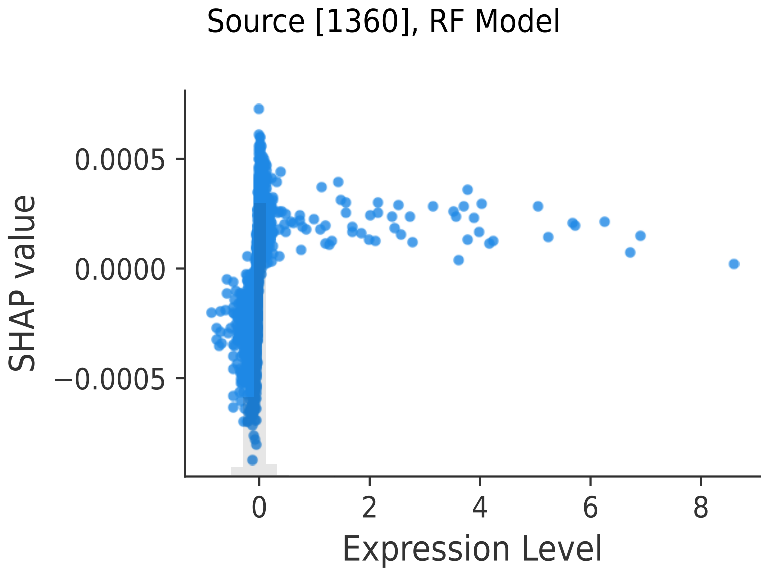
Source [1360], RF Model
0.0005
0.0000
−0.0005
SHAP value
0
2
4
6
8
Expression Level

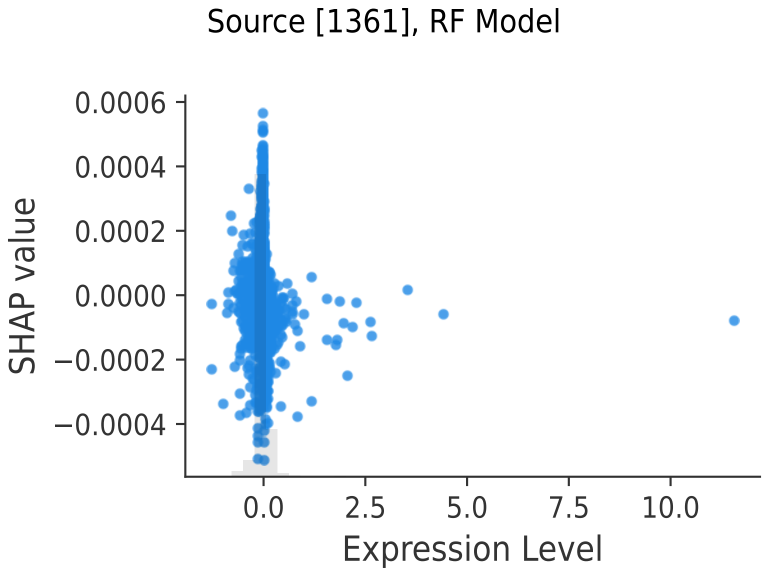

Source [1361], RF Model
SHAP value
0.0006
0.0004
0.0002
0.0000
−0.0002
−0.0004
0.0
2.5
5.0
7.5
10.0
Expression Level

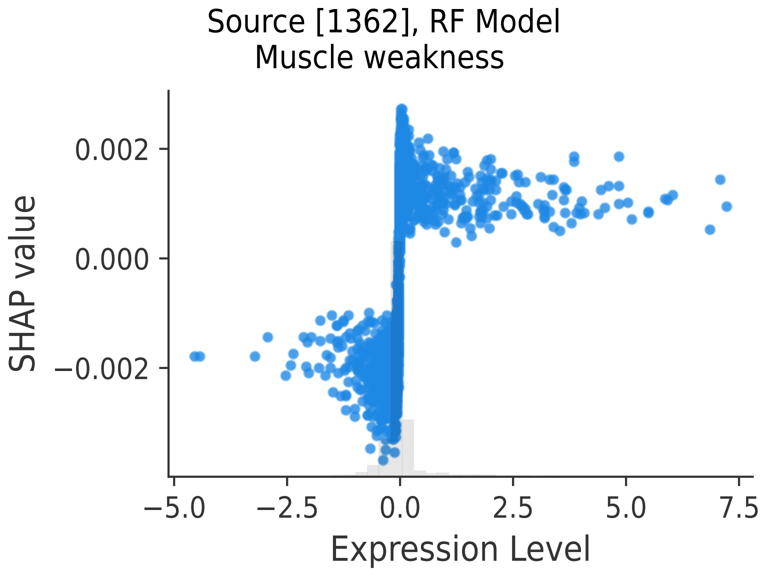

Source [1362], RF Model
Muscle weakness
SHAP value
0.002
0.000
−0.002
−5.0
−2.5
0.0
2.5
5.0
7.5
Expression Level

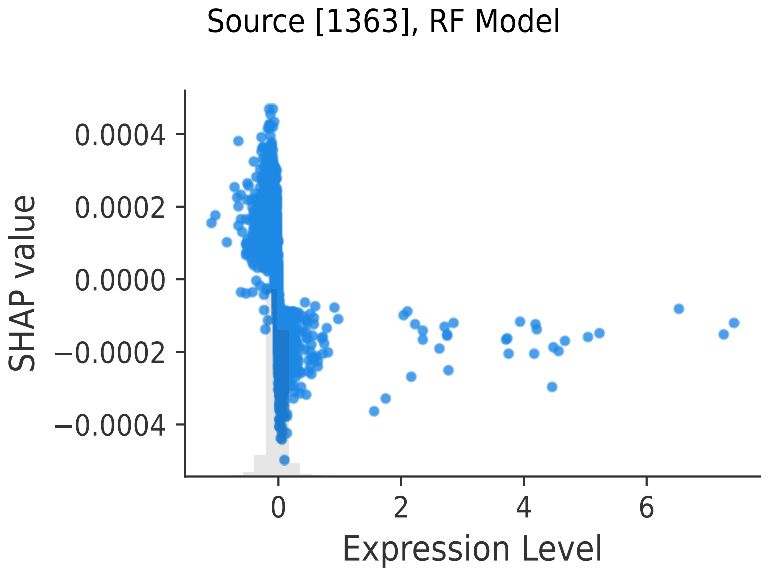

Source [1363], RF Model
SHAP value
0.0004
0.0002
0.0000
−0.0002
−0.0004
0
2
4
6
Expression Level

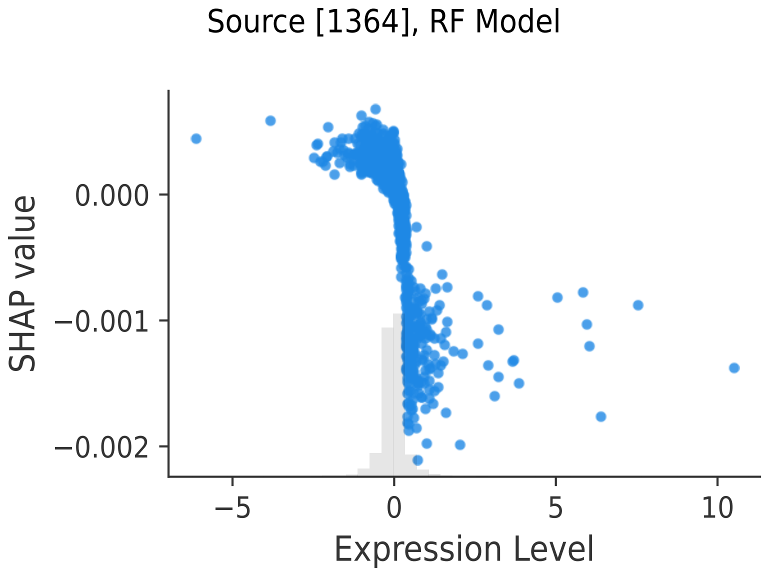
Source [1364], RF Model
SHAP value
0.000
−0.001
−0.002
−5
0
5
10
Expression Level

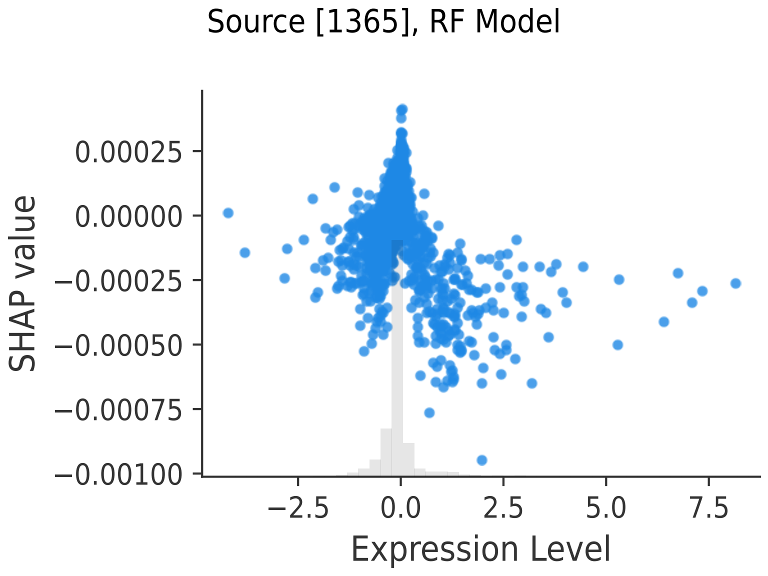
Source [1365], RF Model
0.00025
0.00000
−0.00025
−0.00050
−0.00075
−0.00100
SHAP value
−2.5
0.0
2.5
5.0
7.5
Expression Level

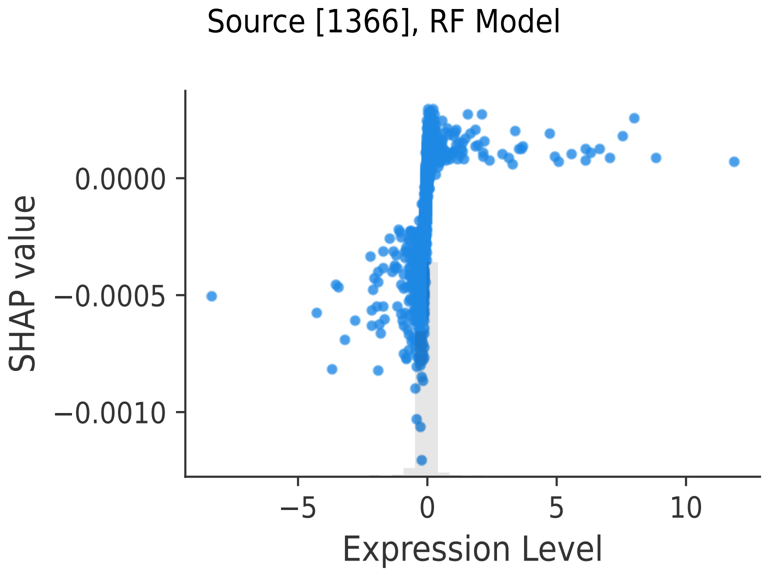

Source [1366], RF Model
0.0000
−0.0005
−0.0010
SHAP value
−5
0
5
10
Expression Level

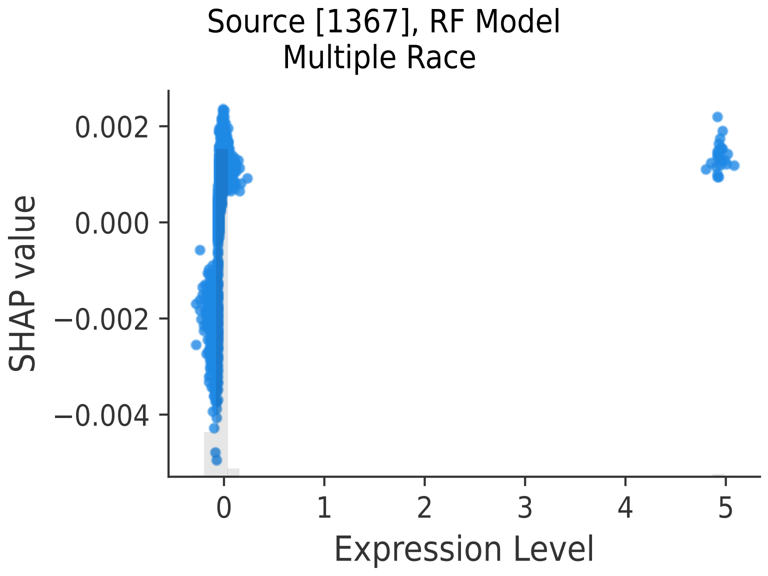

Source [1367], RF Model
Multiple Race
SHAP value
0.002
0.000
−0.002
−0.004
0
1
2
3
4
5
Expression Level

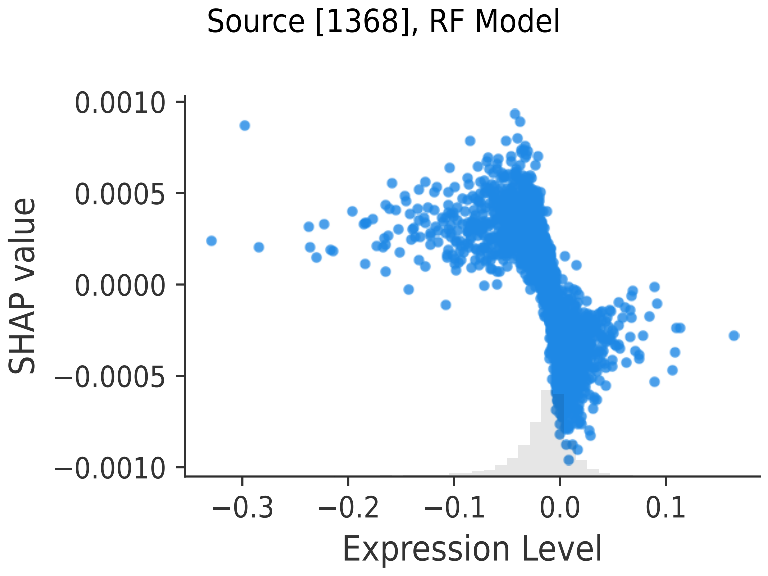

Source [1368], RF Model
0.0010
0.0005
0.0000
−0.0005
−0.0010
SHAP value
−0.3
−0.2
−0.1
0.0
0.1
Expression Level

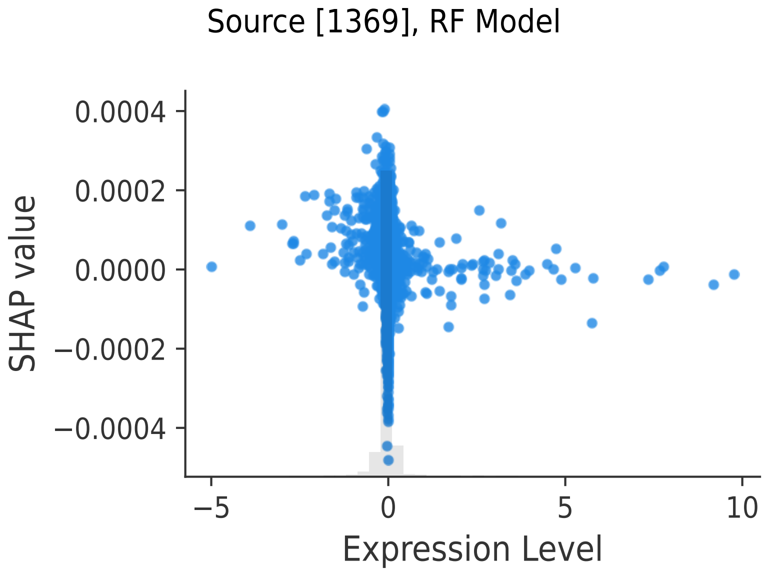
Source [1369], RF Model
SHAP value
0.0004
0.0002
0.0000
−0.0002
−0.0004
−5
0
5
10
Expression Level

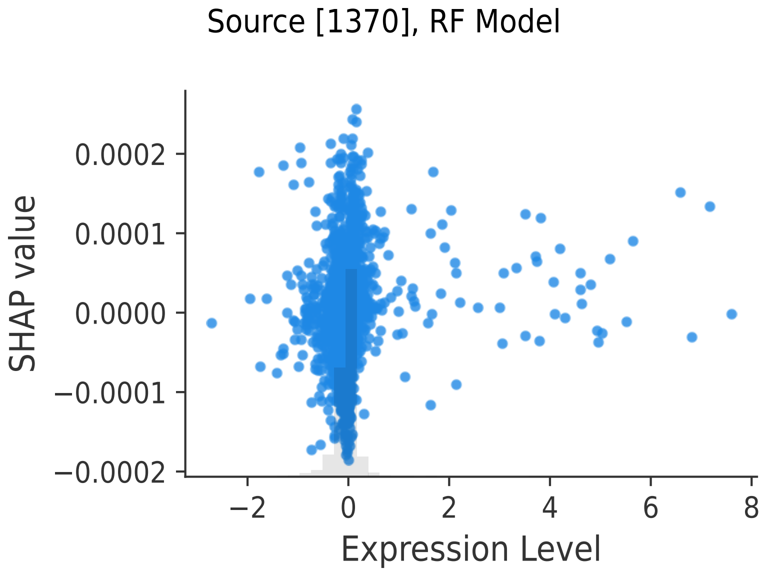
Source [1370], RF Model
SHAP value
0.0002
0.0001
0.0000
−0.0001
−0.0002
−2
0
2
4
6
8
Expression Level

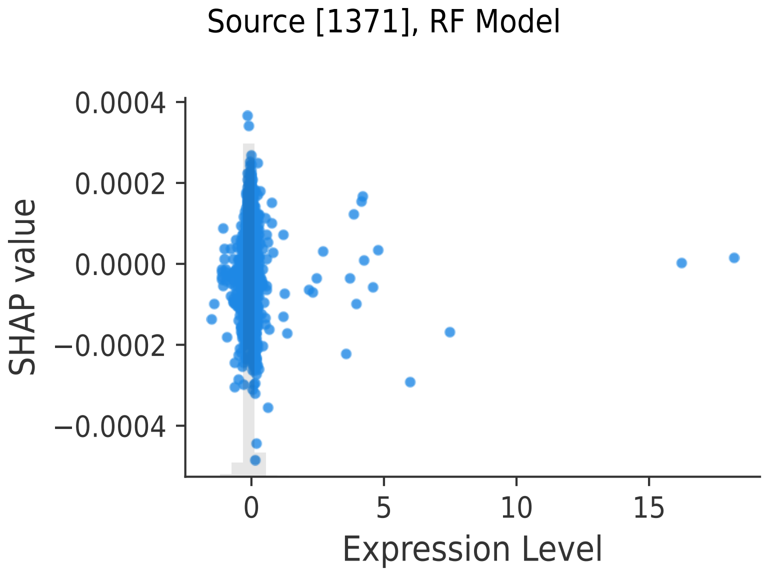
Source [1371], RF Model
0.0004
0.0002
0.0000
−0.0002
−0.0004
SHAP value
0
5
10
15
Expression Level

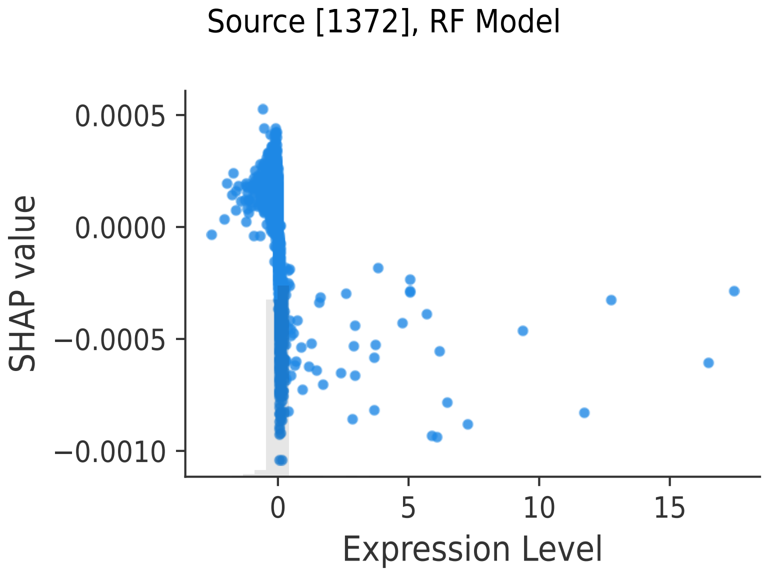
Source [1372], RF Model
SHAP value
0.0005
0.0000
−0.0005
−0.0010
0
5
10
15
Expression Level

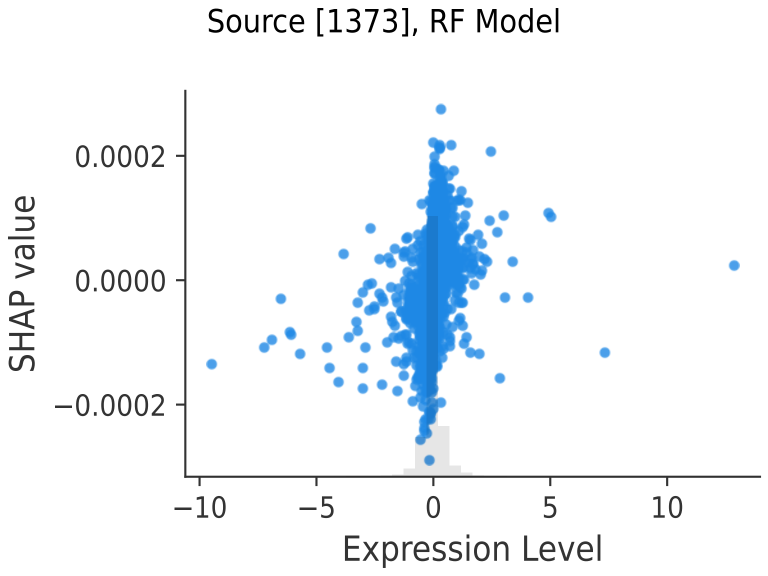

Source [1373], RF Model
SHAP value
0.0002
0.0000
−0.0002
−10
−5
0
5
10
Expression Level

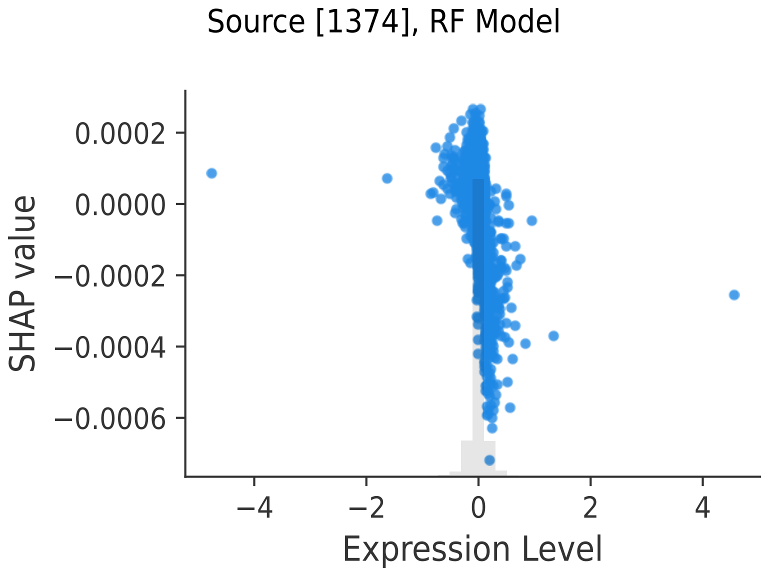
Source [1374], RF Model
SHAP value
0.0002
0.0000
−0.0002
−0.0004
−0.0006
Expression Level
−4
−2
0
2
4

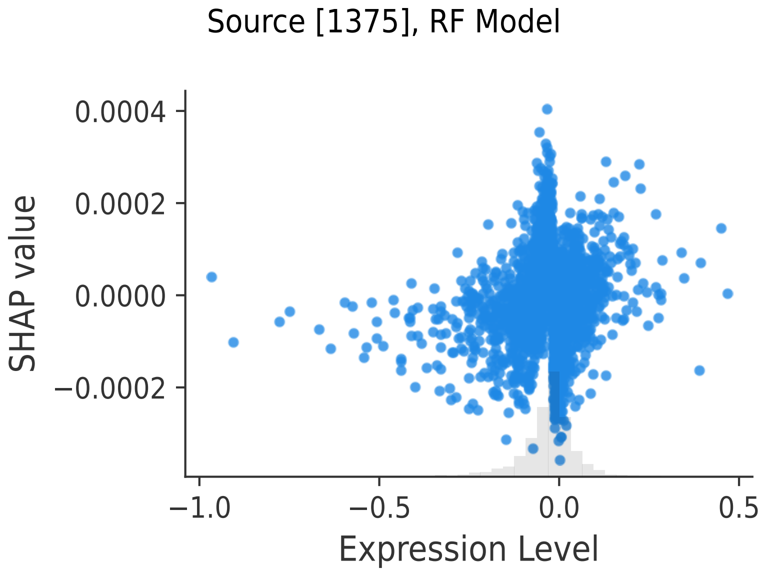
Source [1375], RF Model
0.0004
0.0002
0.0000
−0.0002
SHAP value
−1.0
−0.5
0.0
0.5
Expression Level

Source [1376], RF Model

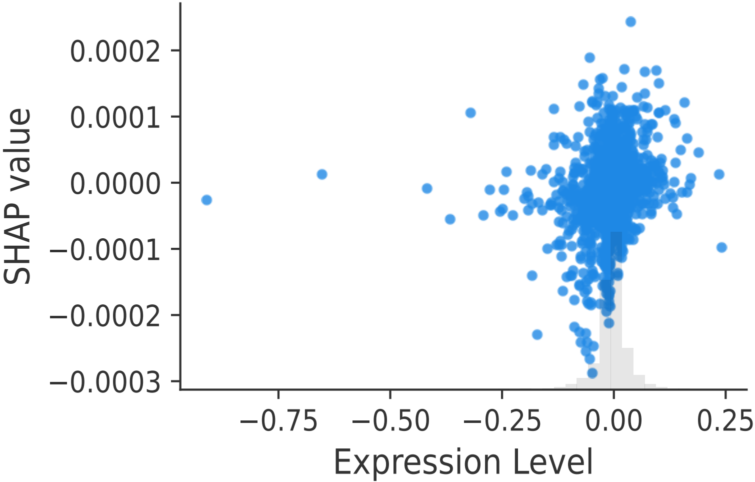

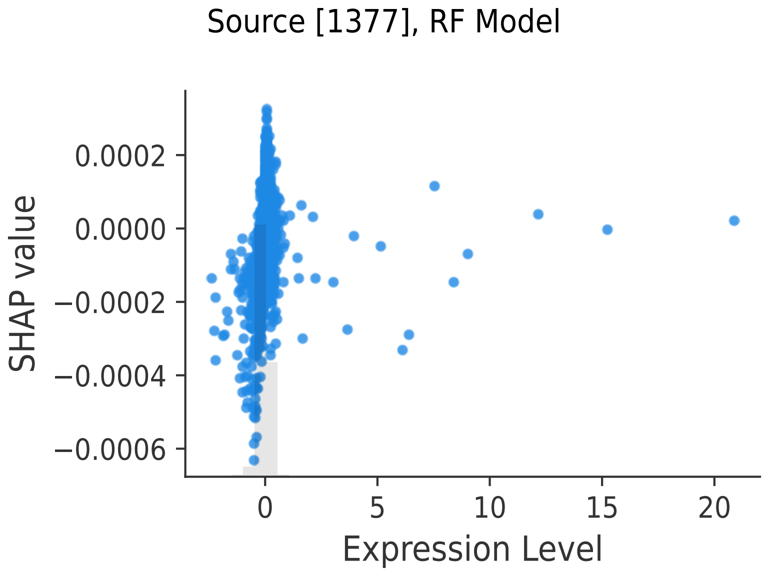

Source [1377], RF Model
SHAP value
0.0002
0.0000
−0.0002
−0.0004
−0.0006
0
5
10
15
20
Expression Level

Source [1378], RF Model

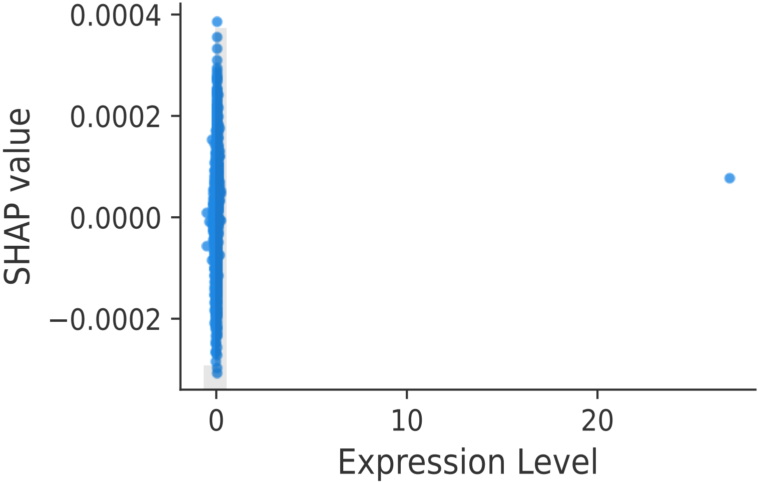

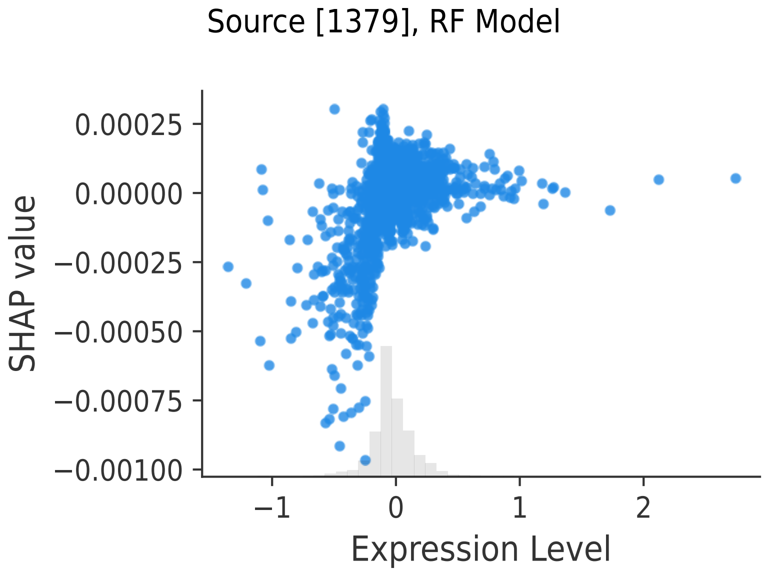

Source [1379], RF Model
0.00025
0.00000
−0.00025
−0.00050
−0.00075
−0.00100
SHAP value
−1
0
1
2
Expression Level

Source [1380], RF Model

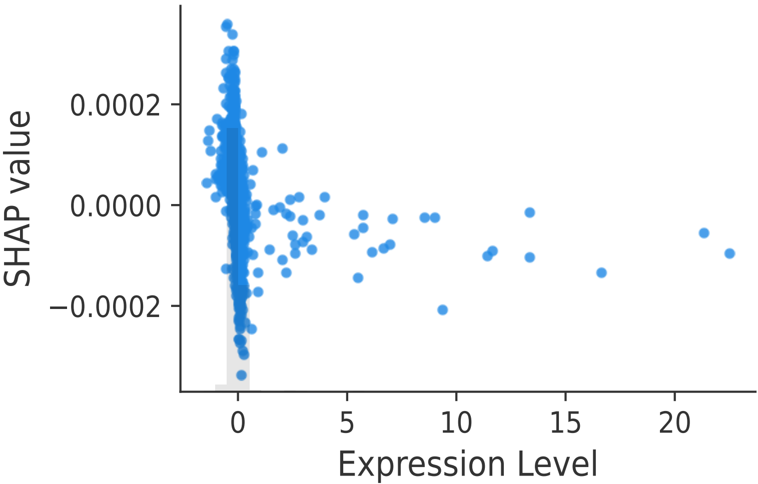

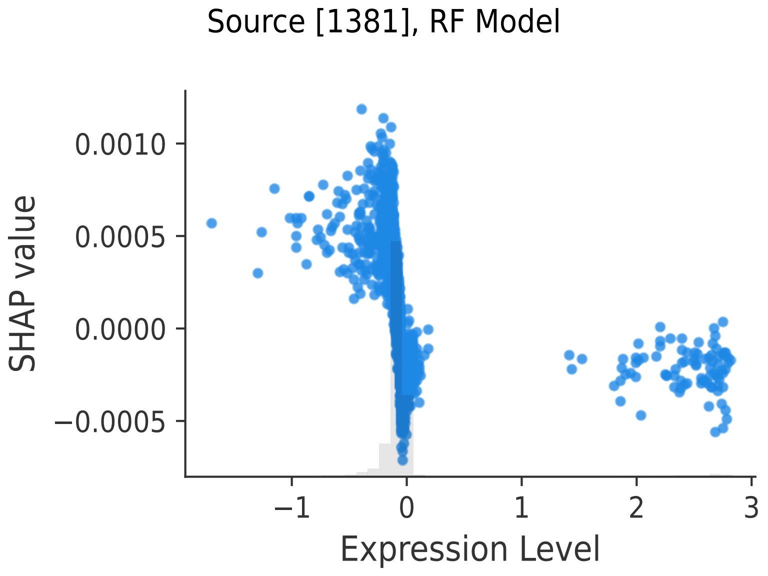
Source [1381], RF Model
SHAP value
0.0010
0.0005
0.0000
−0.0005
−1
0
1
2
3
Expression Level

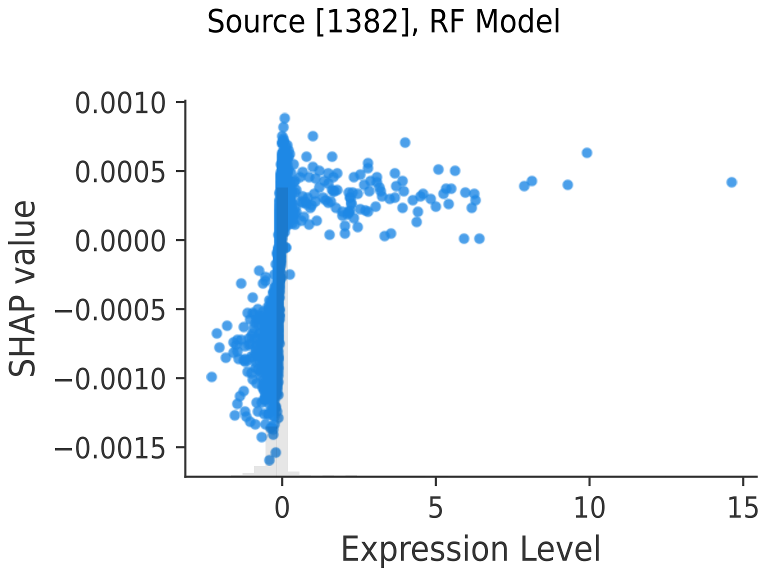
Source [1382], RF Model
SHAP value
0.0010
0.0005
0.0000
−0.0005
−0.0010
−0.0015
0
5
10
15
Expression Level

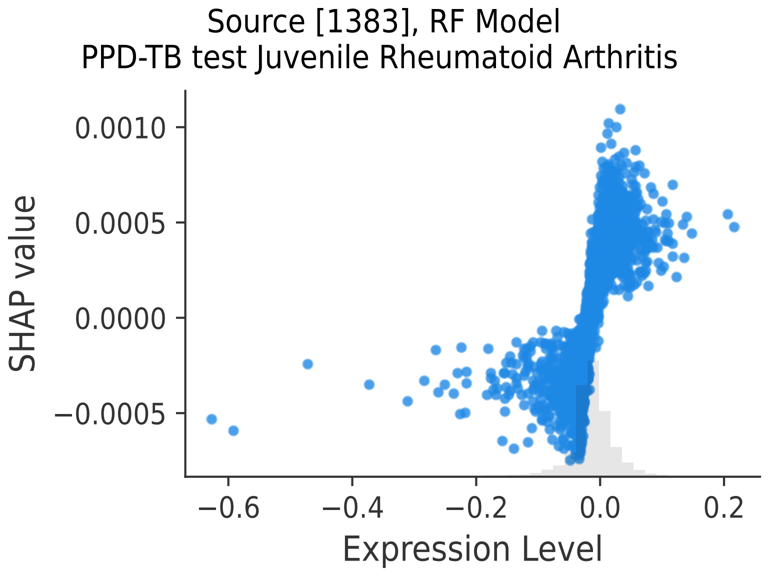

Source [1383], RF Model
PPD-TB test Juvenile Rheumatoid Arthritis
SHAP value
0.0010
0.0005
0.0000
−0.0005
−0.6
−0.4
−0.2
0.0
0.2
Expression Level

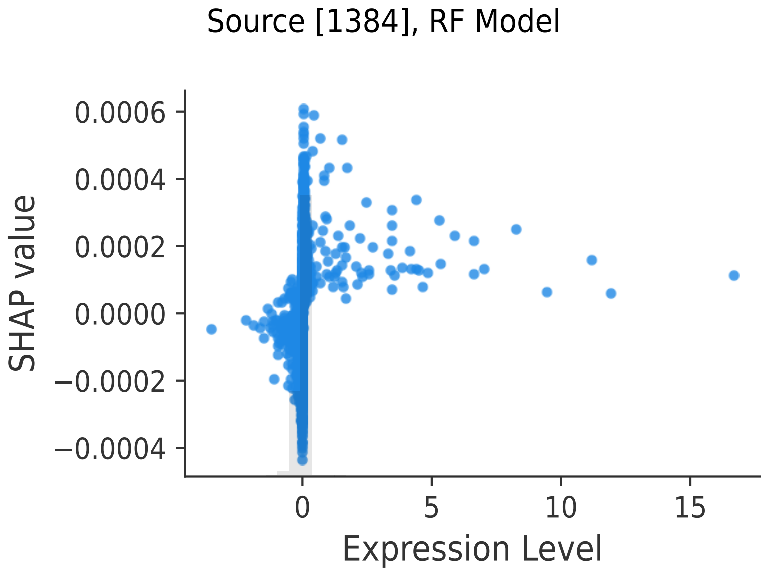

Source [1384], RF Model
0.0006
0.0004
0.0002
0.0000
−0.0002
−0.0004
SHAP value
0
5
10
15
Expression Level

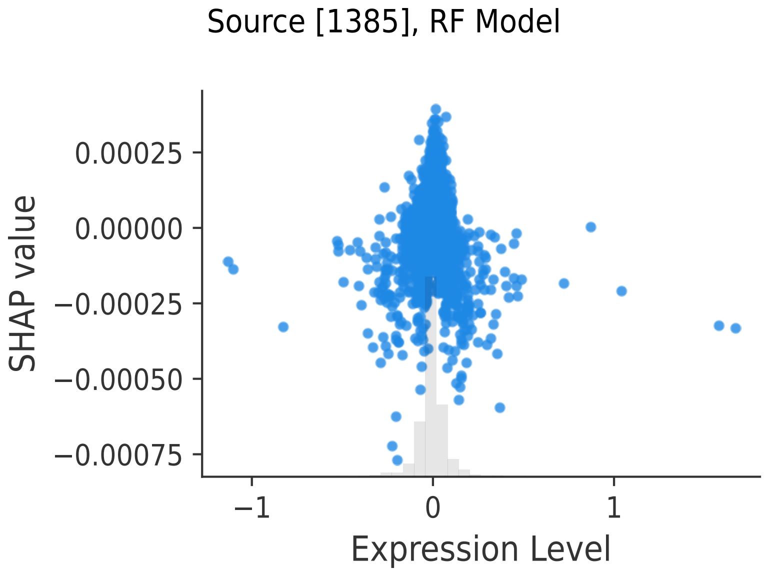
Source [1385], RF Model
0.00025
0.00000
−0.00025
−0.00050
−0.00075
SHAP value
−1
0
1
Expression Level

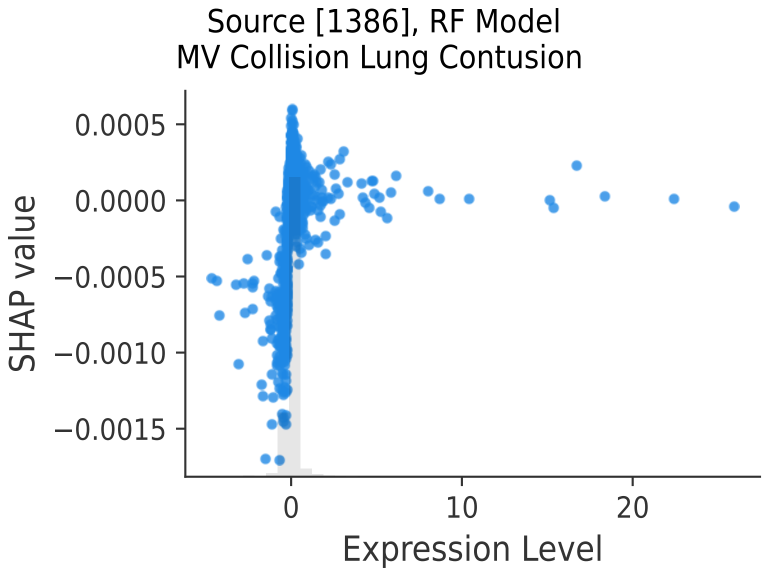
Source [1386], RF Model
MV Collision Lung Contusion
0.0005
0.0000
−0.0005
−0.0010
−0.0015
SHAP value
0
10
20
Expression Level

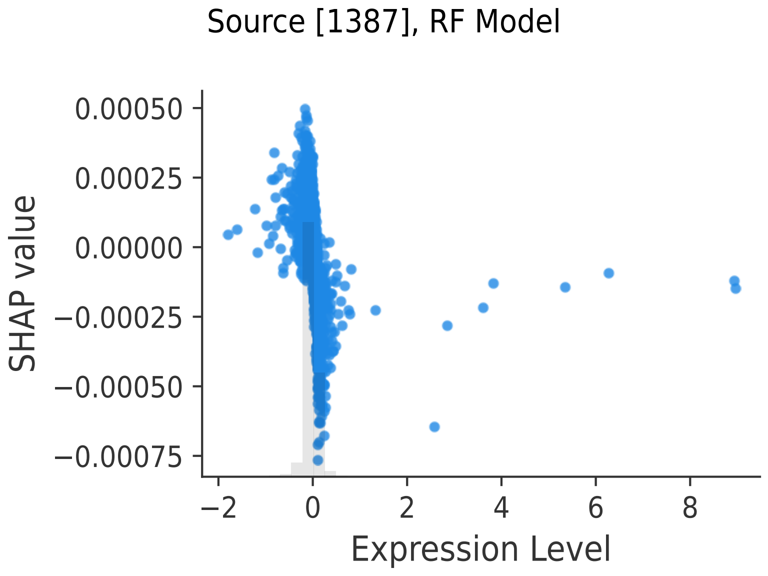

Source [1387], RF Model
SHAP value
0.00050
0.00025
0.00000
−0.00025
−0.00050
−0.00075
−2
0
2
4
6
8
Expression Level

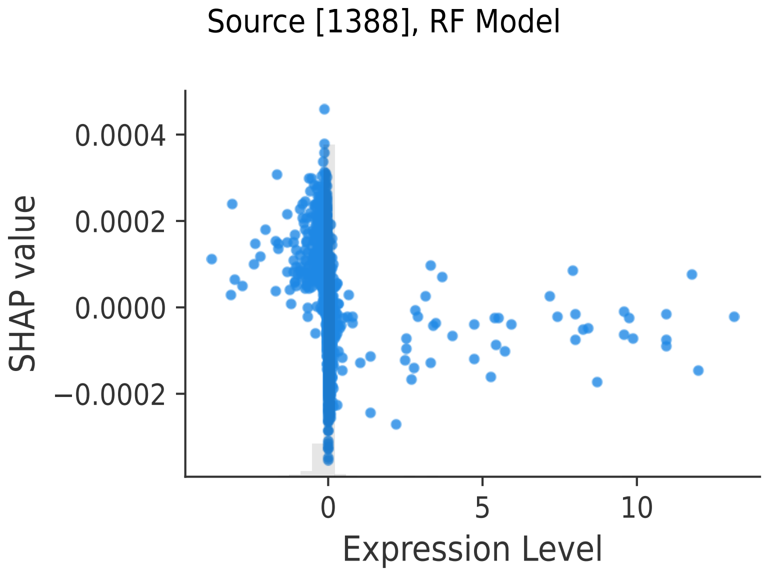
Source [1388], RF Model
SHAP value
0.0004
0.0002
0.0000
−0.0002
0
5
10
Expression Level

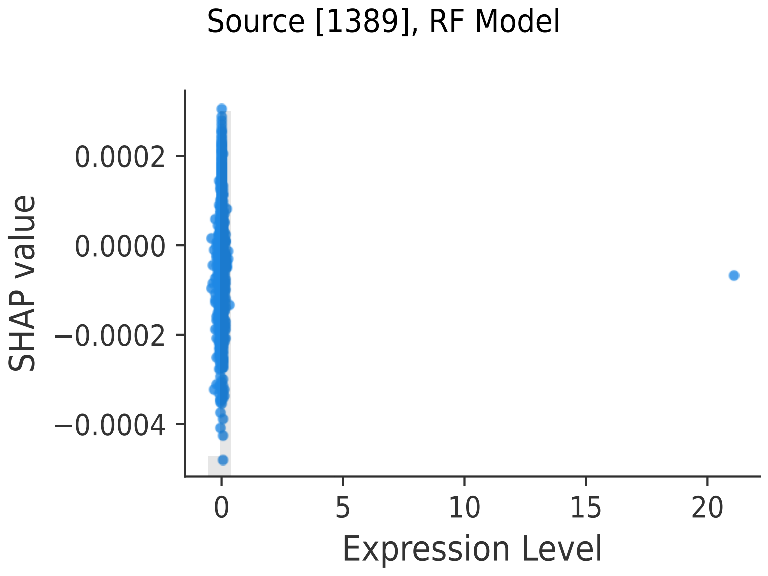
Source [1389], RF Model
SHAP value
0.0002
0.0000
−0.0002
−0.0004
0
5
10
15
20
Expression Level

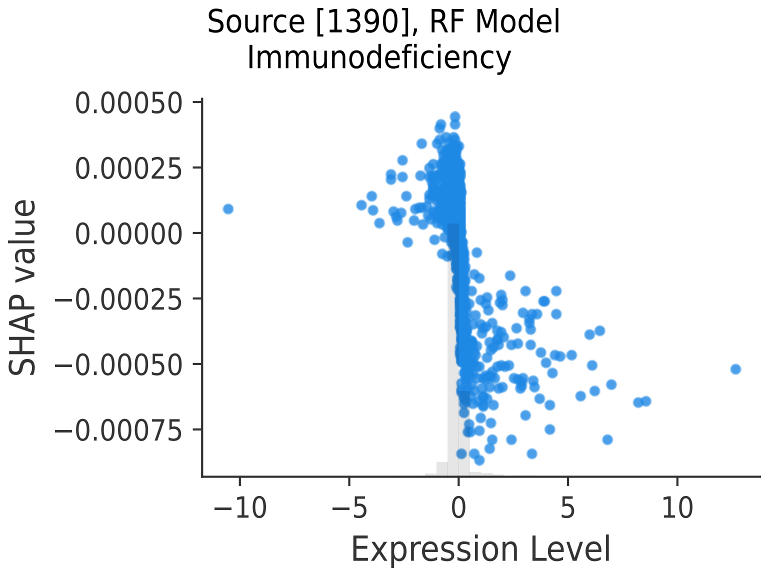
Source [1390], RF Model
Immunodeficiency
SHAP value
0.00050
0.00025
0.00000
−0.00025
−0.00050
−0.00075
−10
−5
0
5
10
Expression Level

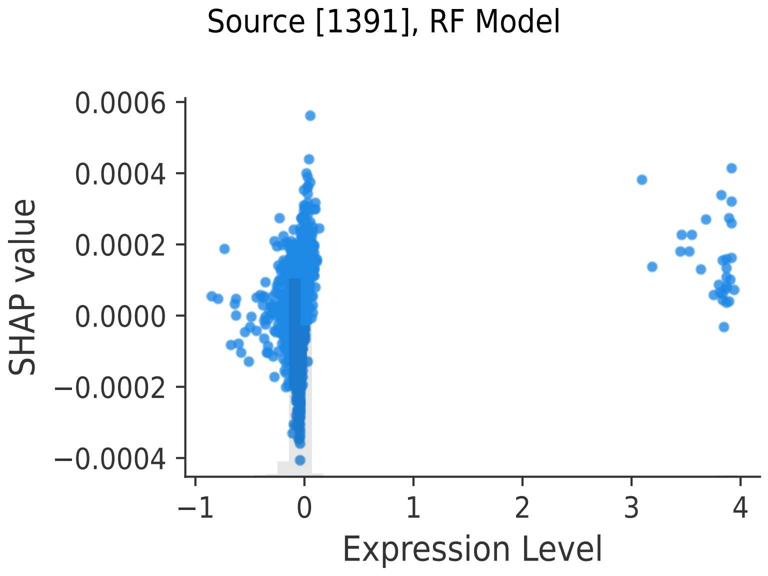
Source [1391], RF Model
SHAP value
0.0006
0.0004
0.0002
0.0000
−0.0002
−0.0004
−1
0
1
2
3
4
Expression Level

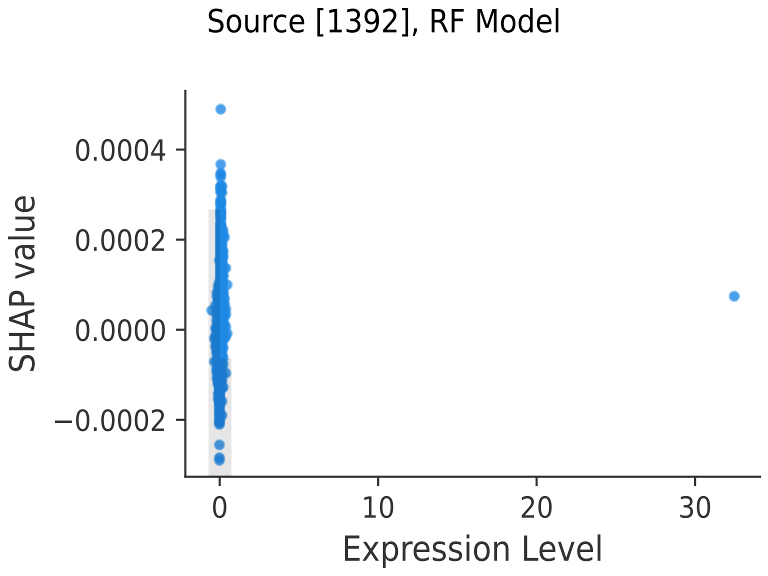
Source [1392], RF Model
0.0004
0.0002
0.0000
−0.0002
SHAP value
0
10
20
30
Expression Level

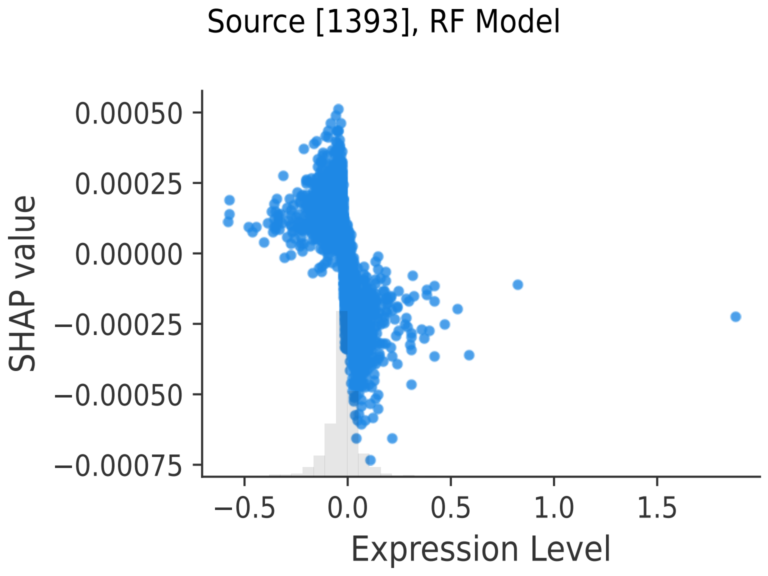

Source [1393], RF Model
SHAP value
0.00050
0.00025
0.00000
−0.00025
−0.00050
−0.00075
−0.5
0.0
0.5
1.0
1.5
Expression Level

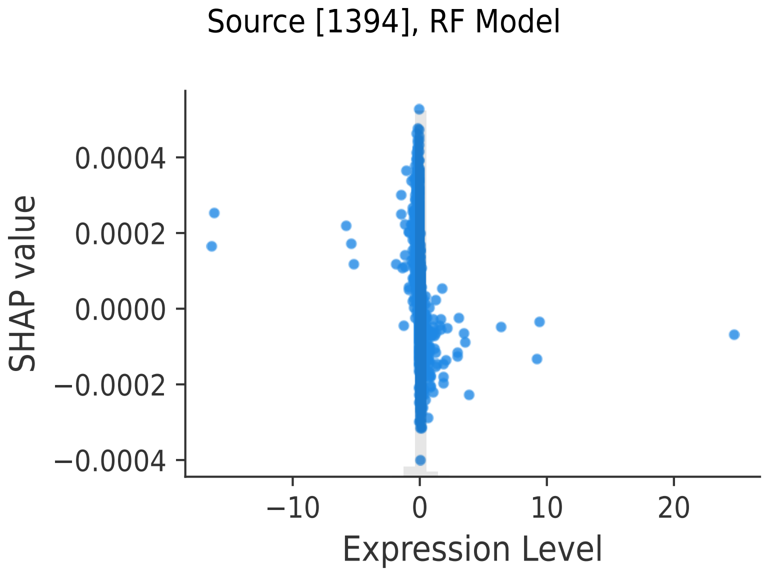
Source [1394], RF Model
SHAP value
0.0004
0.0002
0.0000
−0.0002
−0.0004
−10
0
10
20
Expression Level

Source [1395], RF Model

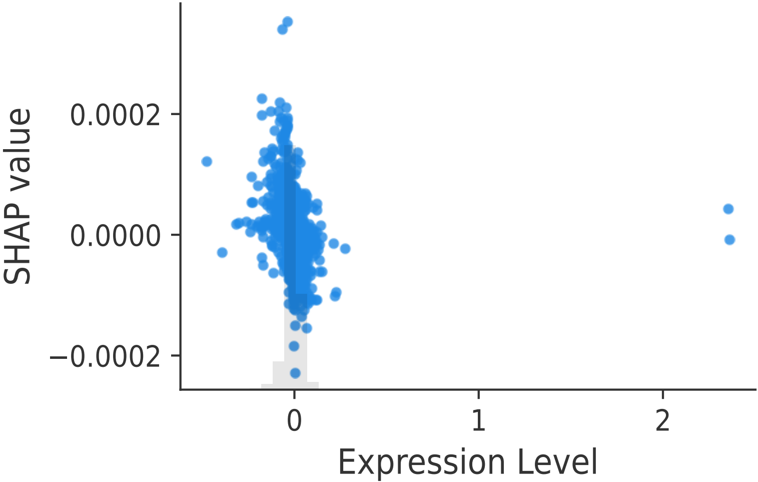

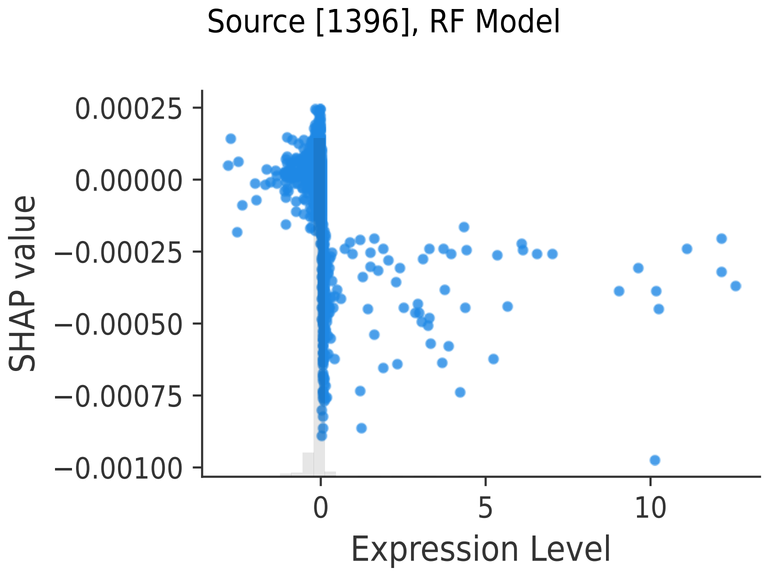
Source [1396], RF Model
SHAP value
0.00025
0.00000
−0.00025
−0.00050
−0.00075
−0.00100
0
5
10
Expression Level

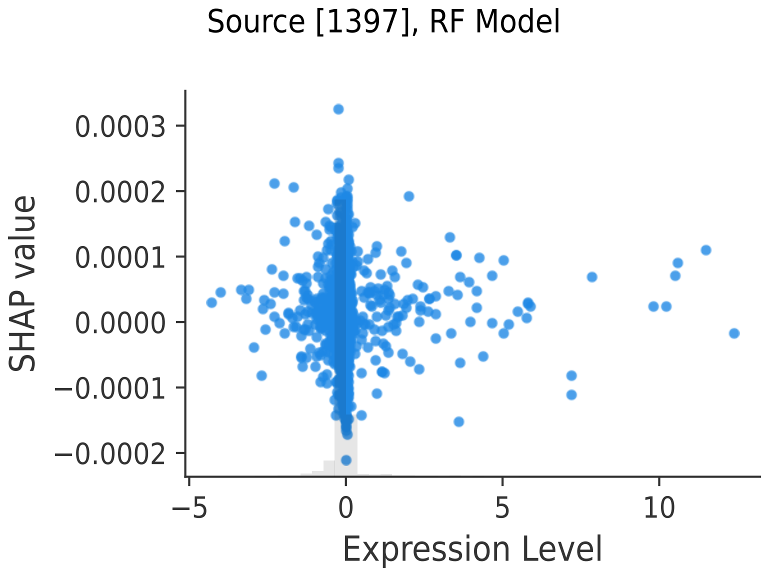
Source [1397], RF Model
SHAP value
0.0003
0.0002
0.0001
0.0000
−0.0001
−0.0002
−5
0
5
10
Expression Level

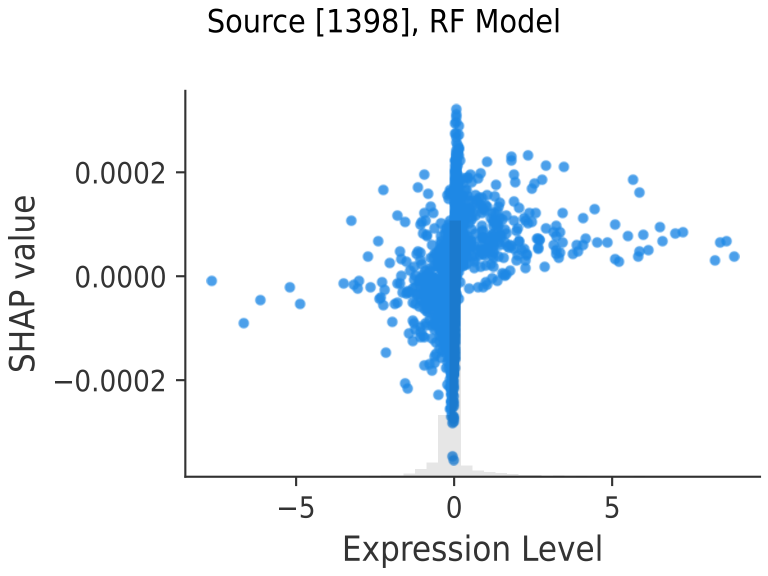
Source [1398], RF Model
SHAP value
0.0002
0.0000
−0.0002
−5
0
5
Expression Level

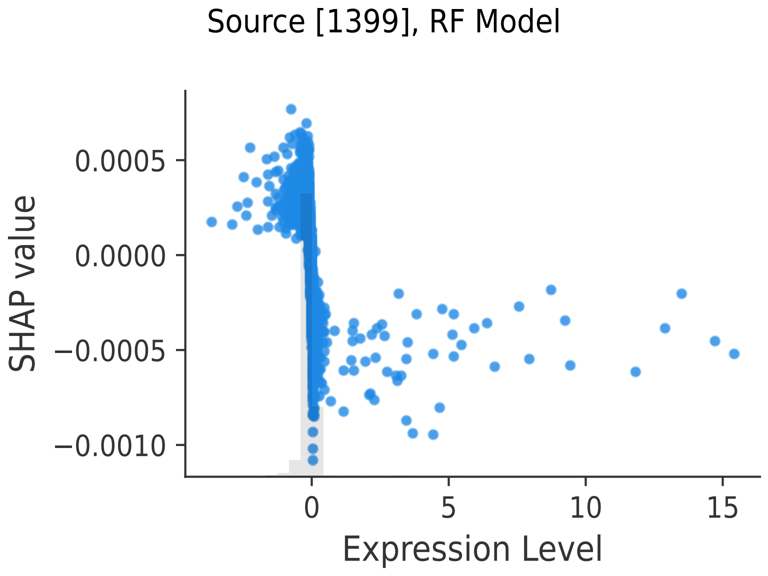

Source [1399], RF Model
SHAP value
0.0005
0.0000
−0.0005
−0.0010
0
5
10
15
Expression Level

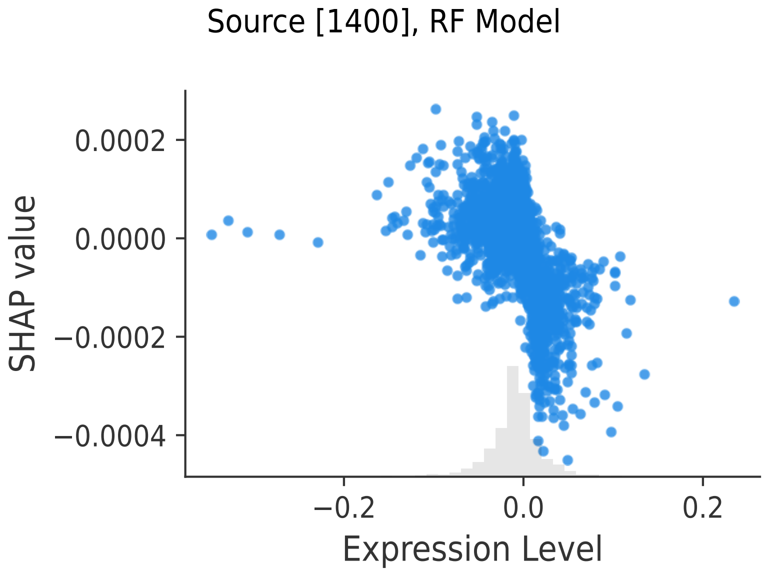

Source [1400], RF Model
0.0002
0.0000
−0.0002
−0.0004
SHAP value
−0.2
0.0
0.2
Expression Level

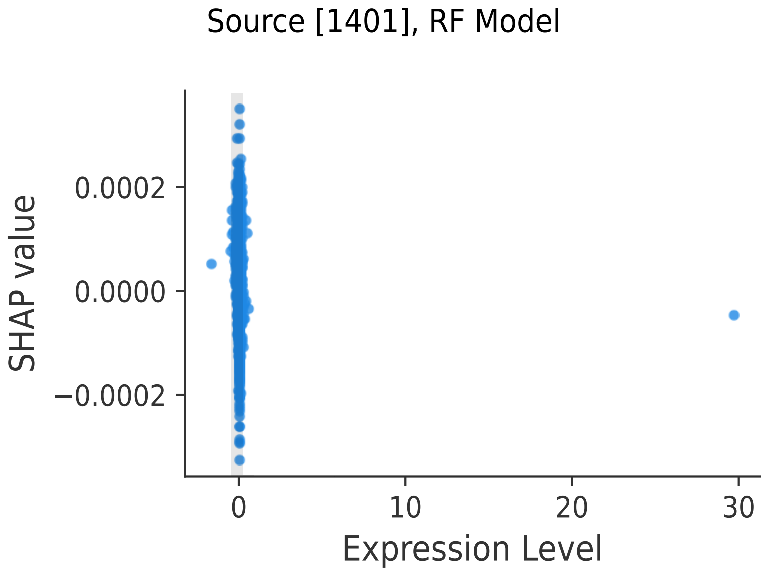

Source [1401], RF Model
SHAP value
0.0002
0.0000
−0.0002
0
10
20
30
Expression Level

Source [1402], RF Model

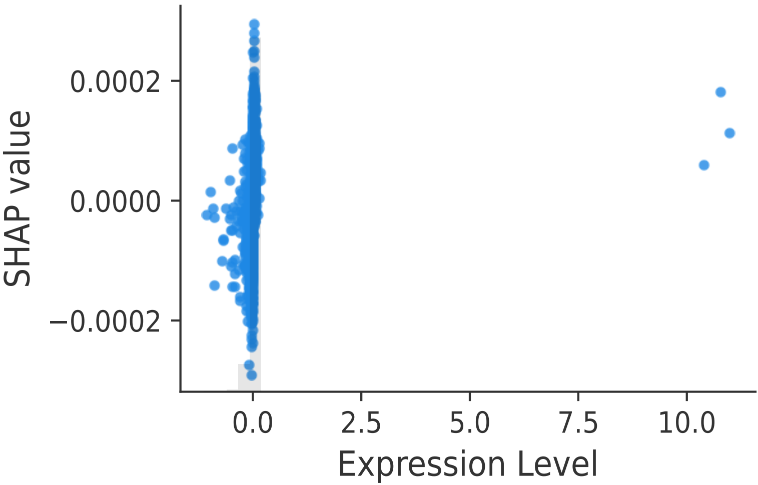

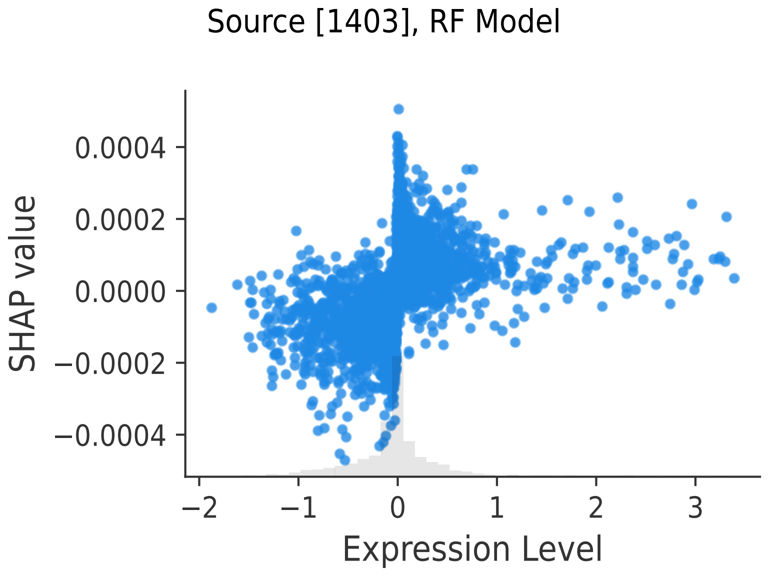
Source [1403], RF Model
SHAP value
0.0004
0.0002
0.0000
−0.0002
−0.0004
−2
−1
0
1
2
3
Expression Level

Source [1404], RF Model

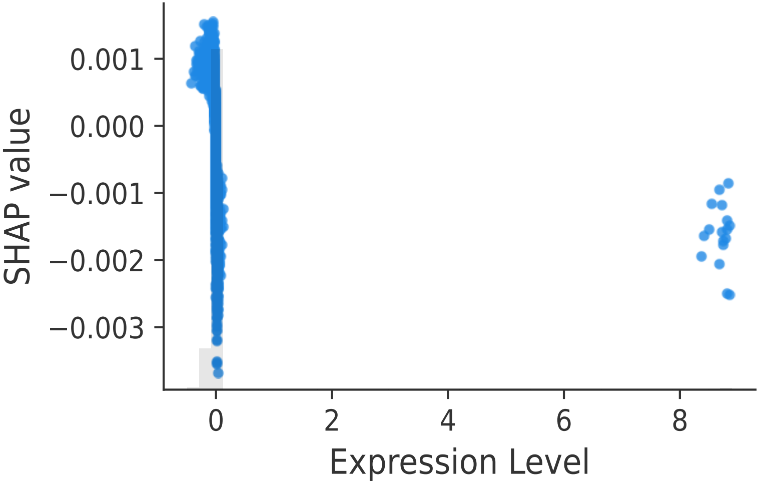

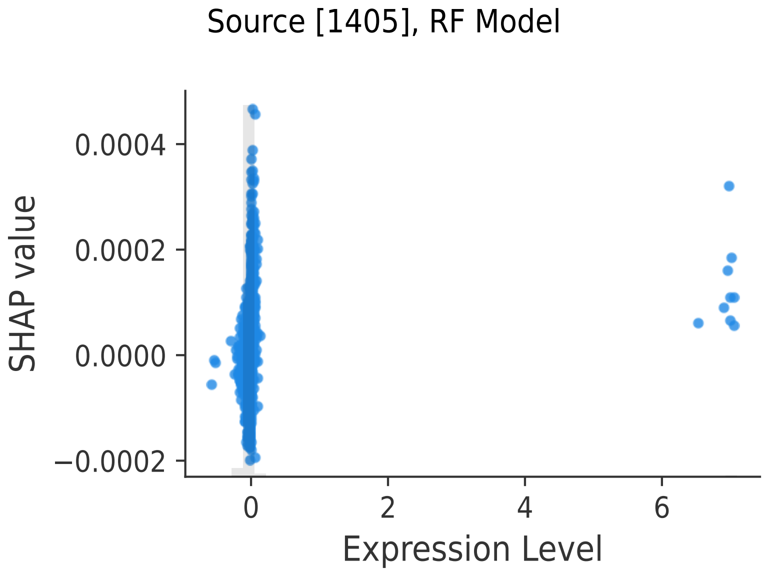

Source [1405], RF Model
SHAP value
0.0004
0.0002
0.0000
−0.0002
0
2
4
6
Expression Level

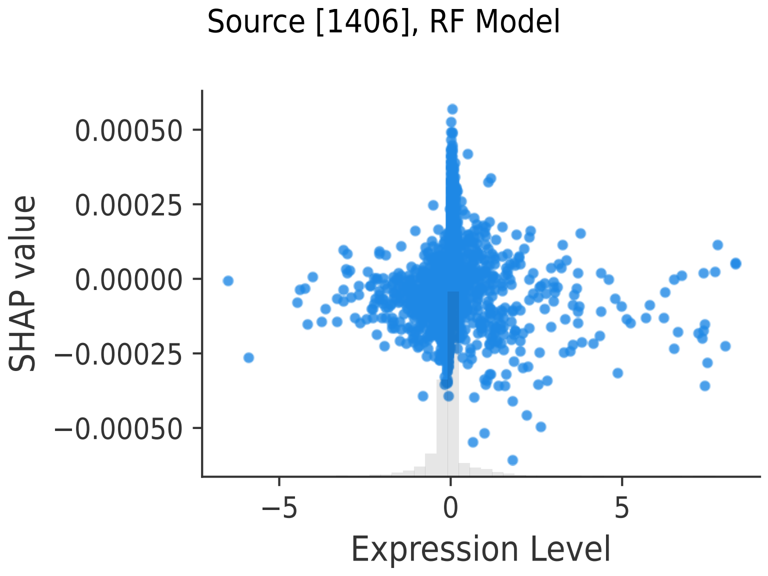

Source [1406], RF Model
0.00050
0.00025
0.00000
−0.00025
−0.00050
SHAP value
−5
0
5
Expression Level

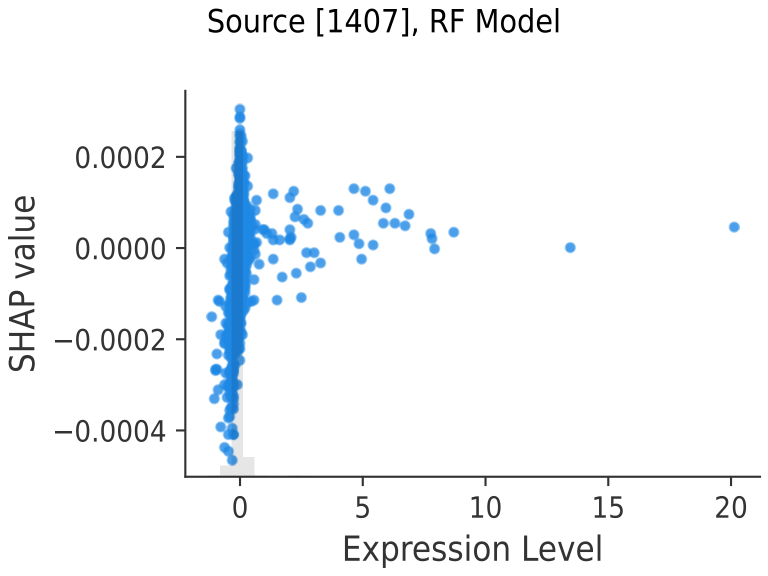

Source [1407], RF Model
0.0002
0.0000
−0.0002
−0.0004
SHAP value
0
5
10
15
20
Expression Level

Source [1408], RF Model

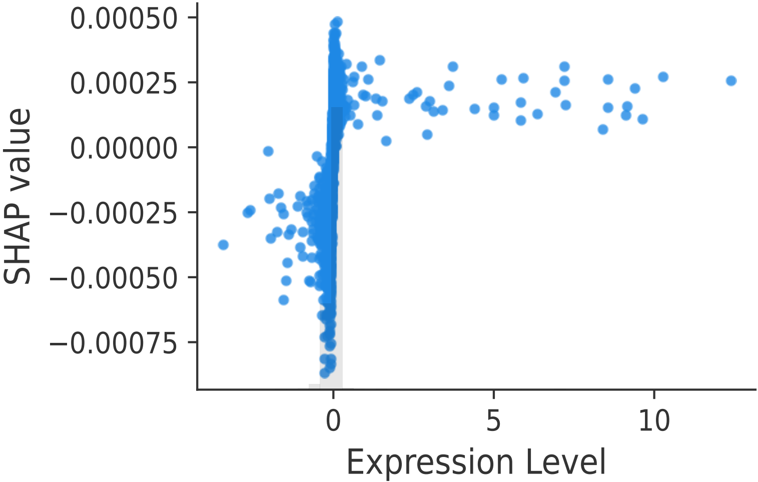

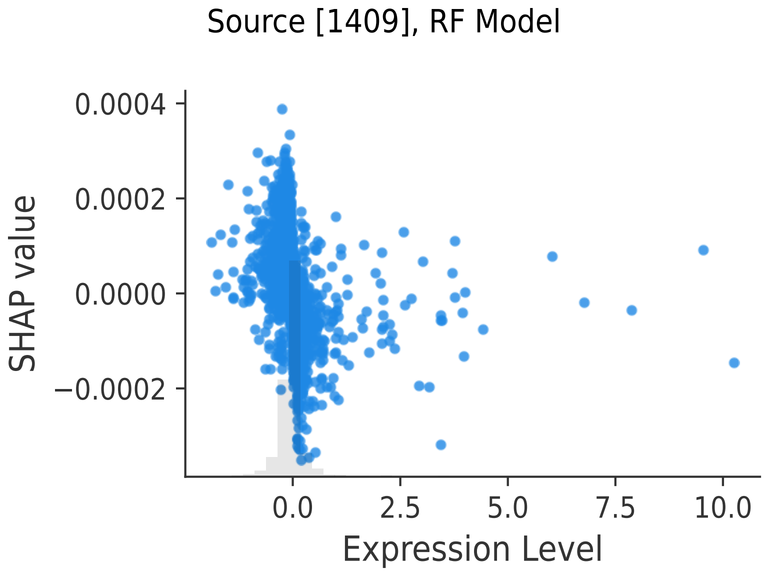

Source [1409], RF Model
0.0004
0.0002
0.0000
−0.0002
SHAP value
0.0
2.5
5.0
7.5
10.0
Expression Level

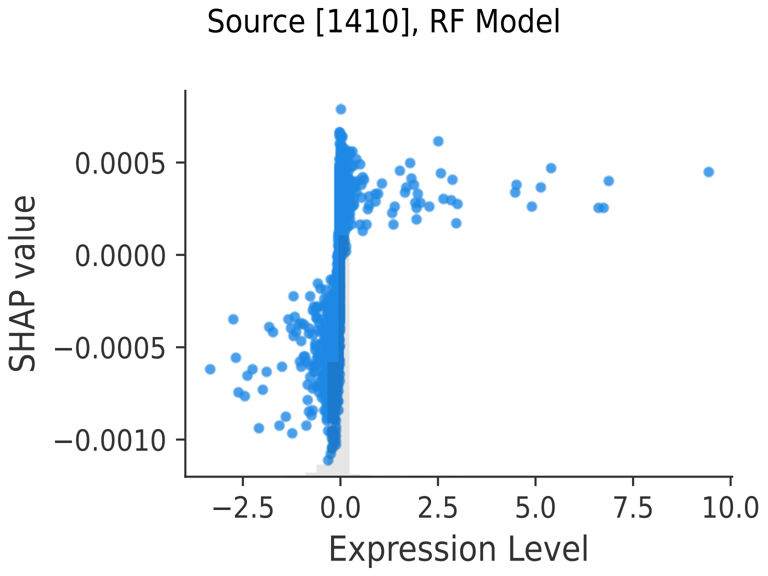

Source [1410], RF Model
SHAP value
0.0005
0.0000
−0.0005
−0.0010
−2.5
0.0
2.5
5.0
7.5
10.0
Expression Level

Source [1411], RF Model

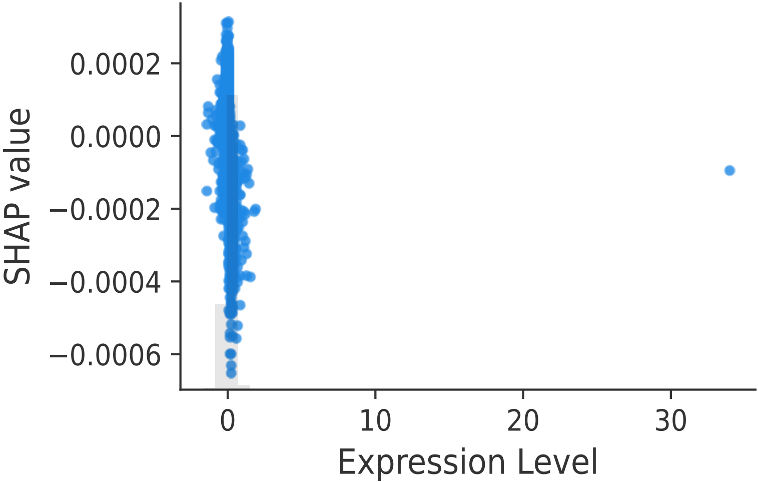

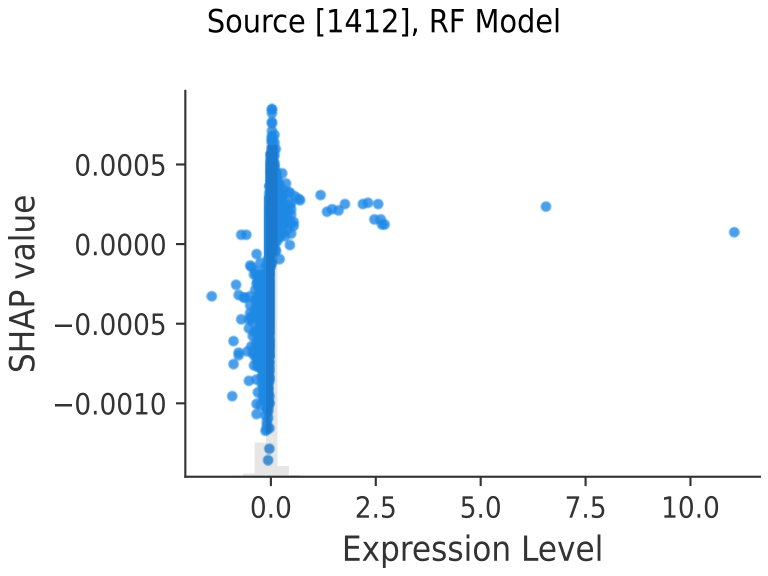
Source [1412], RF Model
SHAP value
0.0005
0.0000
−0.0005
−0.0010
0.0
2.5
5.0
7.5
10.0
Expression Level

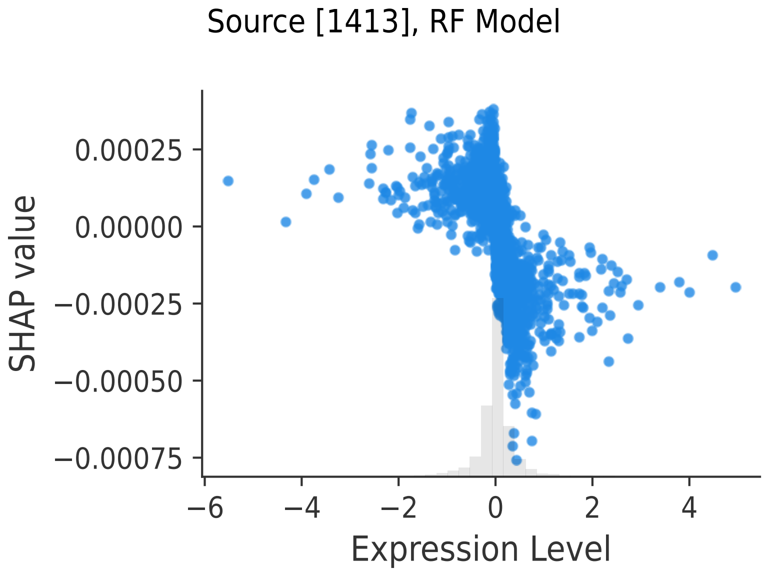
Source [1413], RF Model
0.00025
0.00000
−0.00025
−0.00050
−0.00075
SHAP value
−6
−4
−2
0
2
4
Expression Level

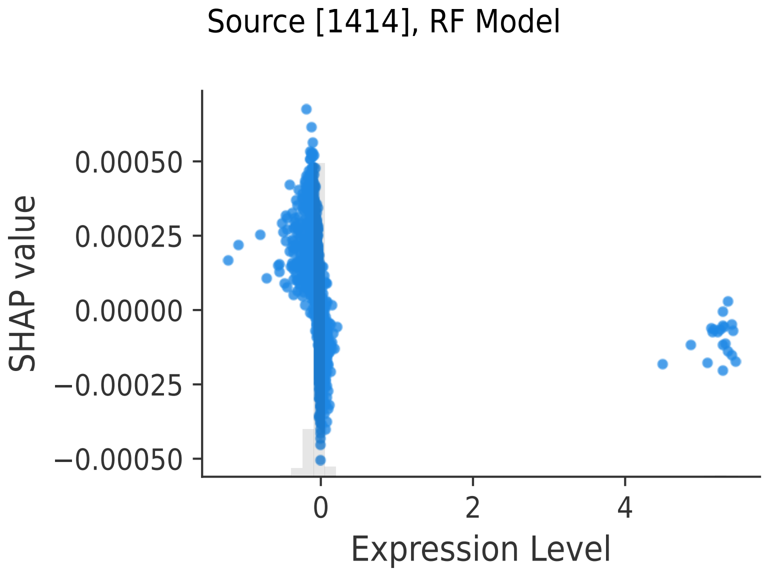
Source [1414], RF Model
SHAP value
0.00050
0.00025
0.00000
−0.00025
−0.00050
0
2
4
Expression Level

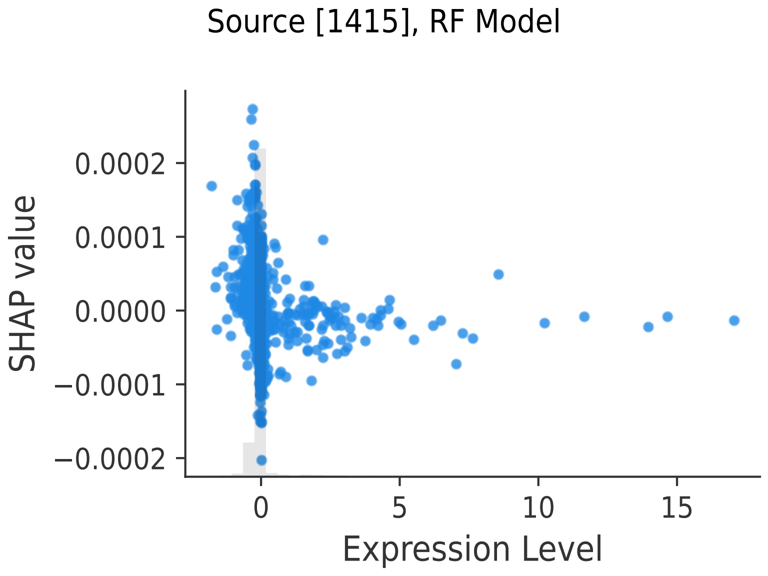

Source [1415], RF Model
SHAP value
0.0002
0.0001
0.0000
−0.0001
−0.0002
0
5
10
15
Expression Level

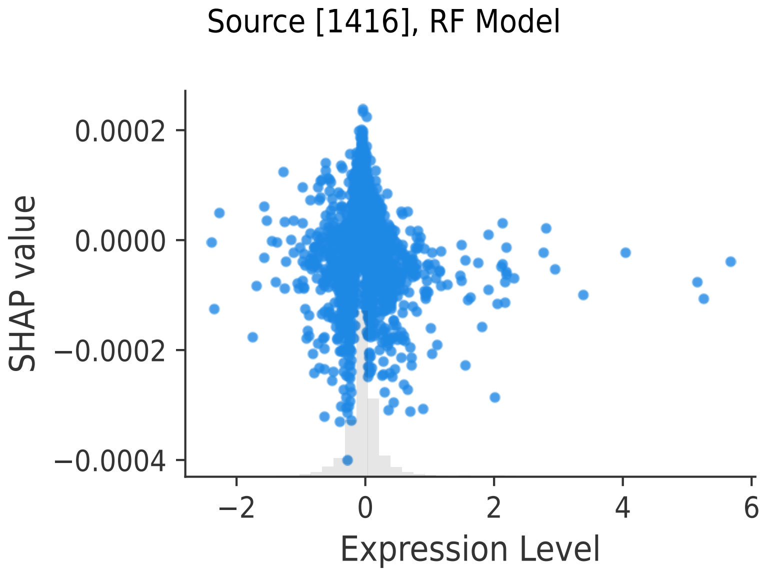
Source [1416], RF Model
SHAP value
0.0002
0.0000
−0.0002
−0.0004
−2
0
2
4
6
Expression Level

Source [1417], RF Model

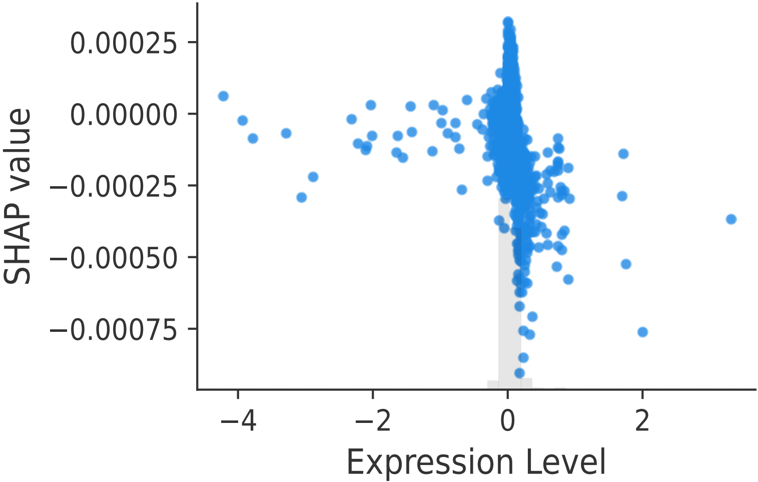

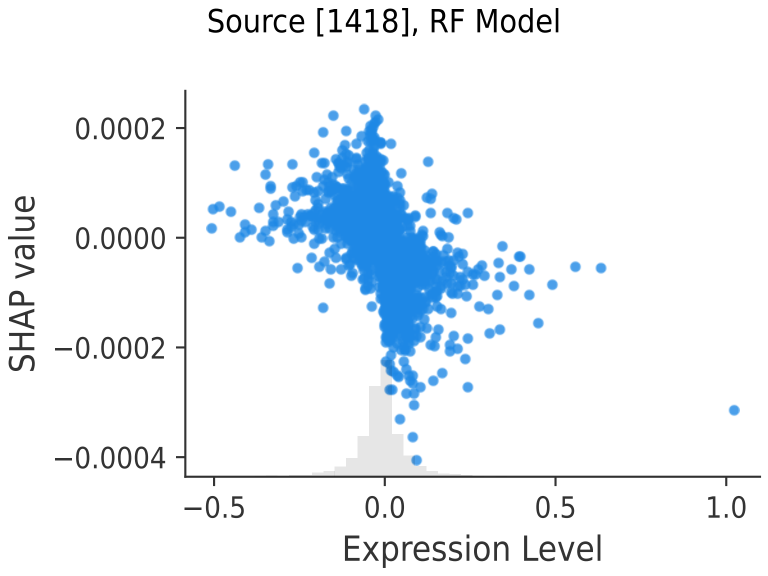
Source [1418], RF Model
0.0002
0.0000
−0.0002
−0.0004
SHAP value
−0.5
0.0
0.5
1.0
Expression Level

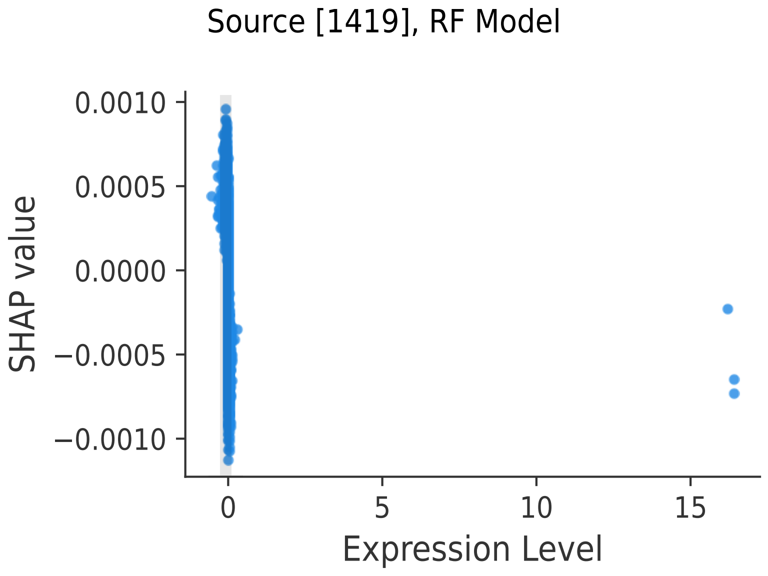
Source [1419], RF Model
SHAP value
0.0010
0.0005
0.0000
−0.0005
−0.0010
0
5
10
15
Expression Level

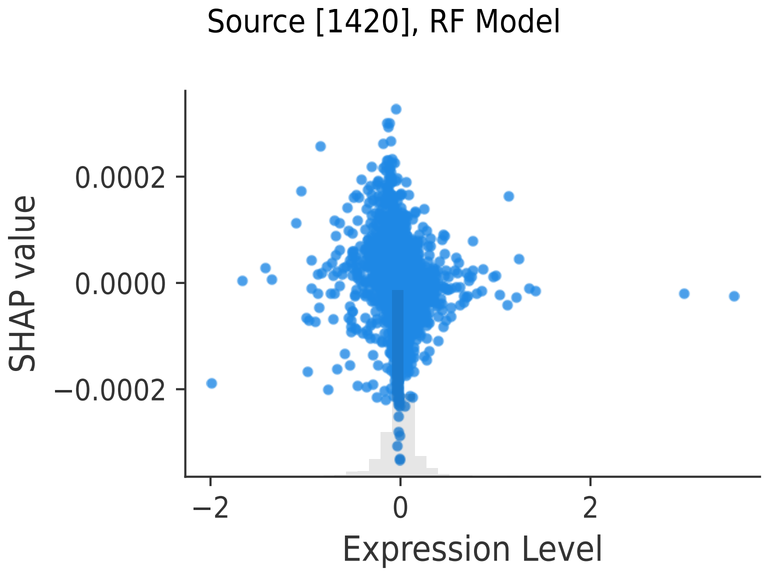
Source [1420], RF Model
0.0002
0.0000
−0.0002
SHAP value
−2
0
2
Expression Level

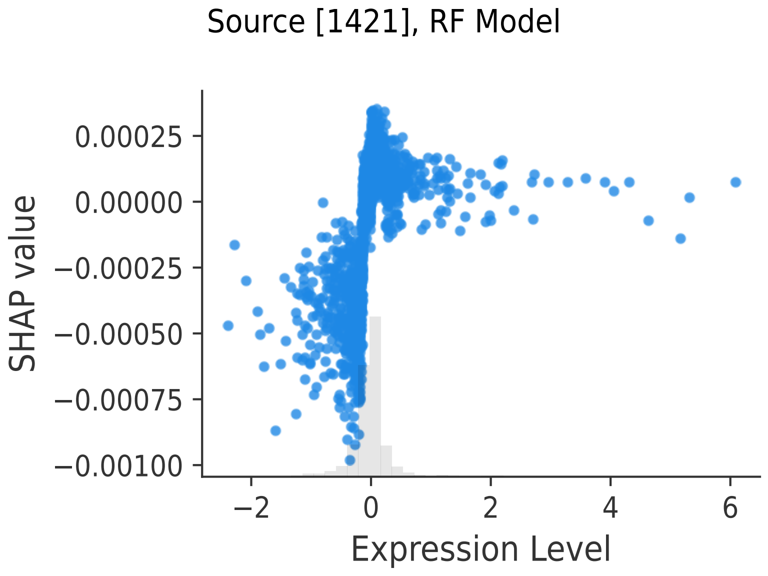

Source [1421], RF Model
0.00025
0.00000
−0.00025
−0.00050
−0.00075
−0.00100
SHAP value
−2
0
2
4
6
Expression Level

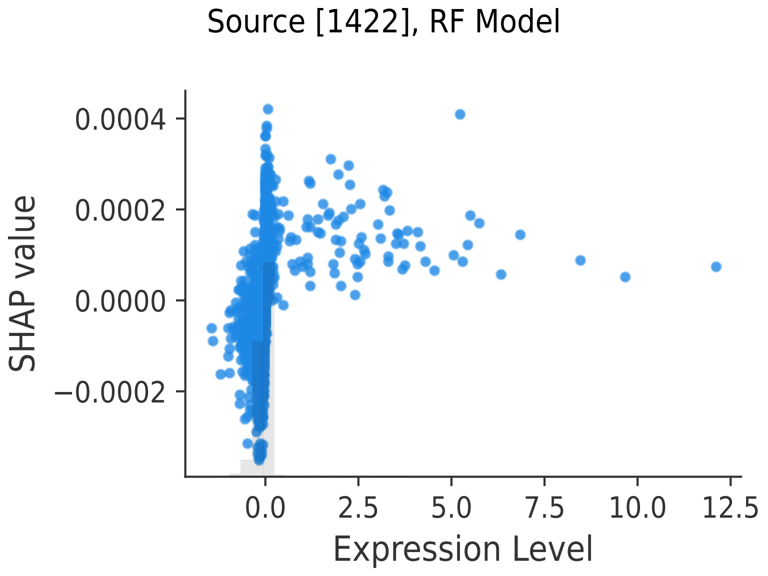
Source [1422], RF Model
SHAP value
0.0004
0.0002
0.0000
−0.0002
0.0
2.5
5.0
7.5
10.0
12.5
Expression Level

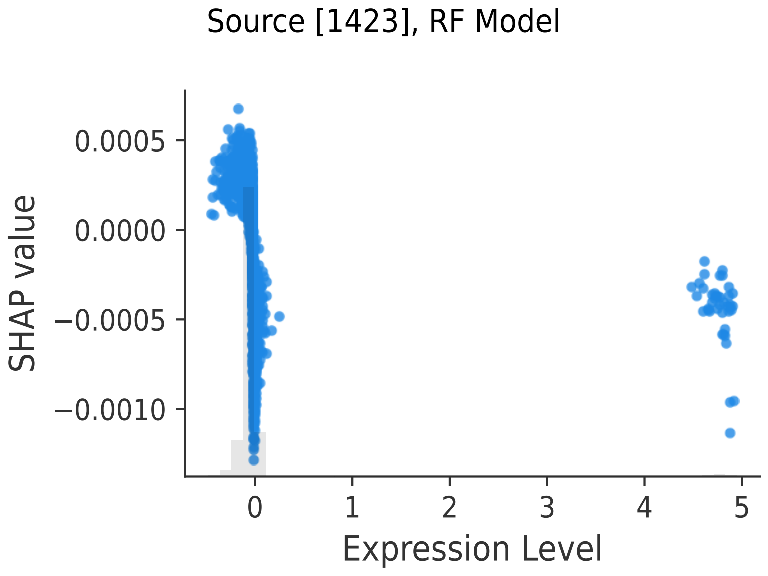

Source [1423], RF Model
0.0005
0.0000
−0.0005
−0.0010
SHAP value
0
1
2
3
4
5
Expression Level

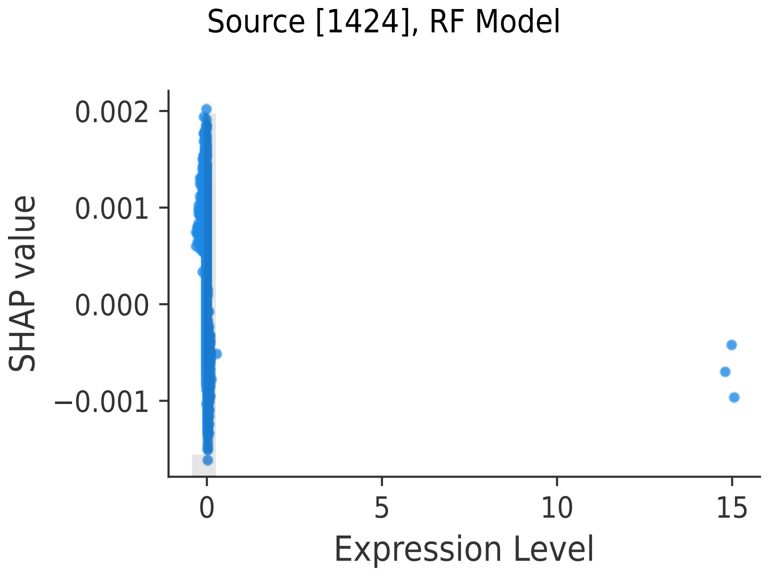

Source [1424], RF Model
SHAP value
0.002
0.001
0.000
−0.001
0
5
10
15
Expression Level

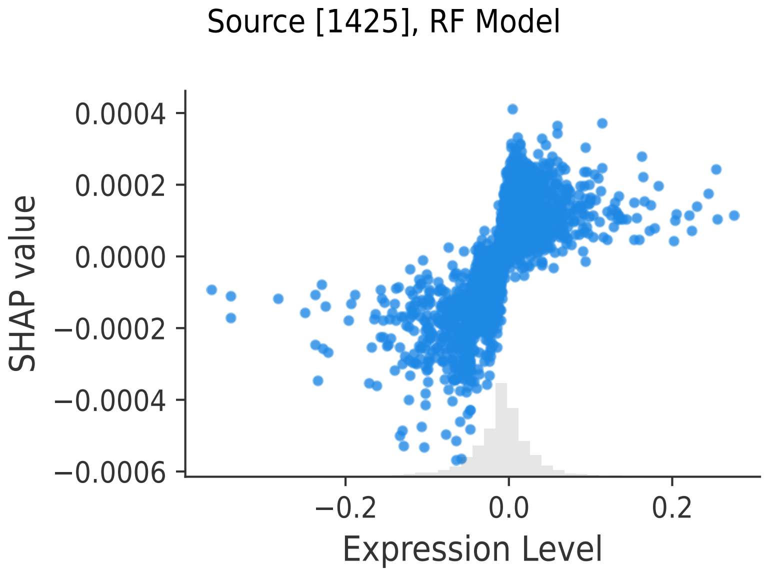

Source [1425], RF Model
0.0004
0.0002
0.0000
−0.0002
−0.0004
−0.0006
SHAP value
−0.2
0.0
0.2
Expression Level

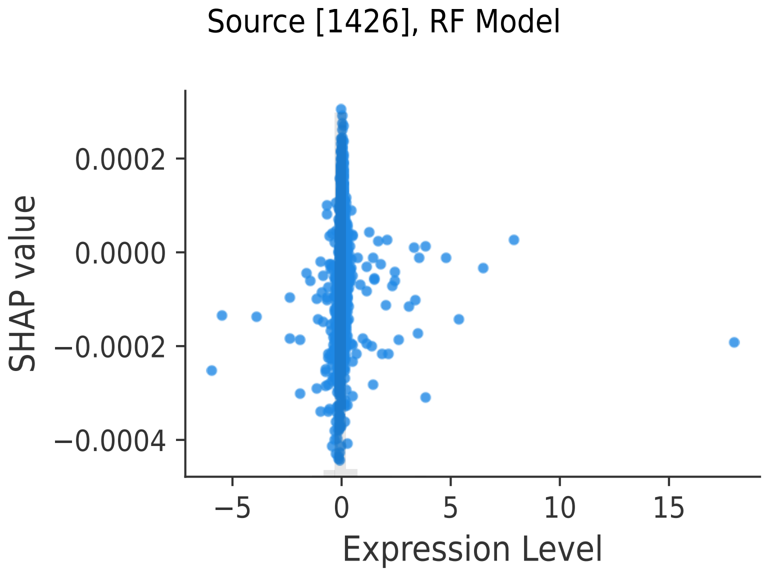

Source [1426], RF Model
0.0002
0.0000
−0.0002
−0.0004
SHAP value
−5
0
5
10
15
Expression Level

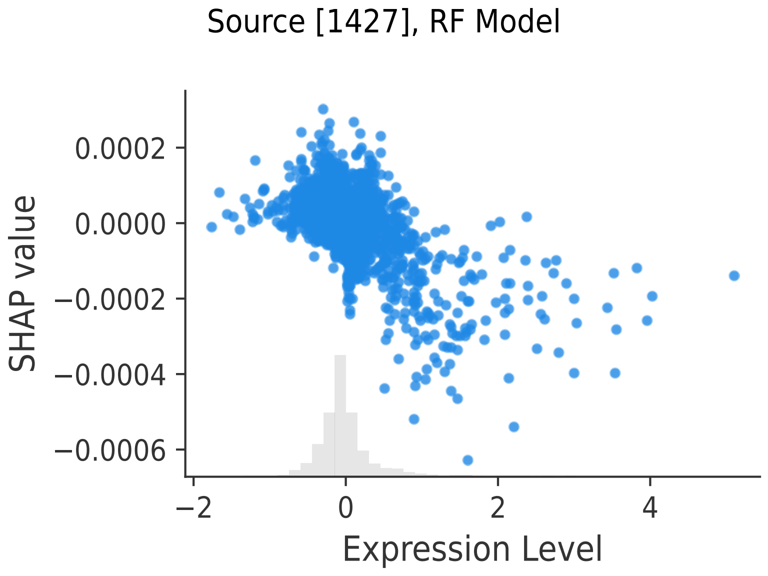

Source [1427], RF Model
SHAP value
0.0002
0.0000
−0.0002
−0.0004
−0.0006
−2
0
2
4
Expression Level

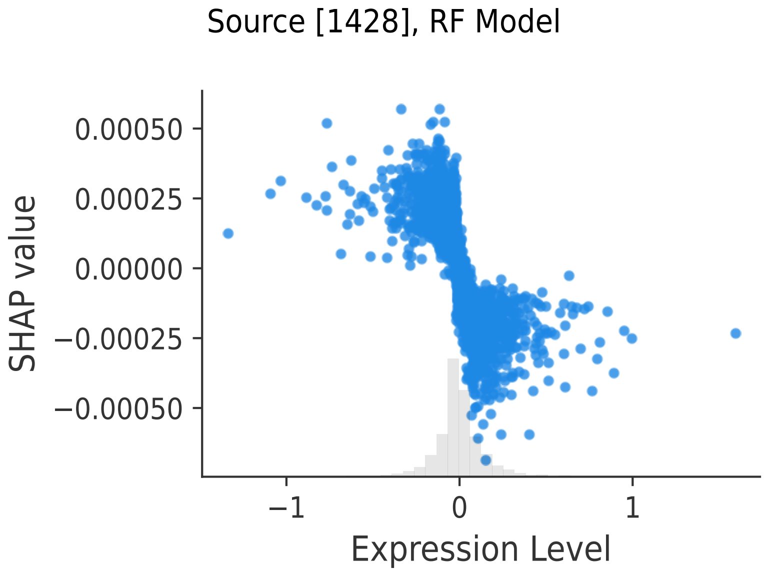
Source [1428], RF Model
SHAP value
0.00050
0.00025
0.00000
−0.00025
−0.00050
−1
0
1
Expression Level

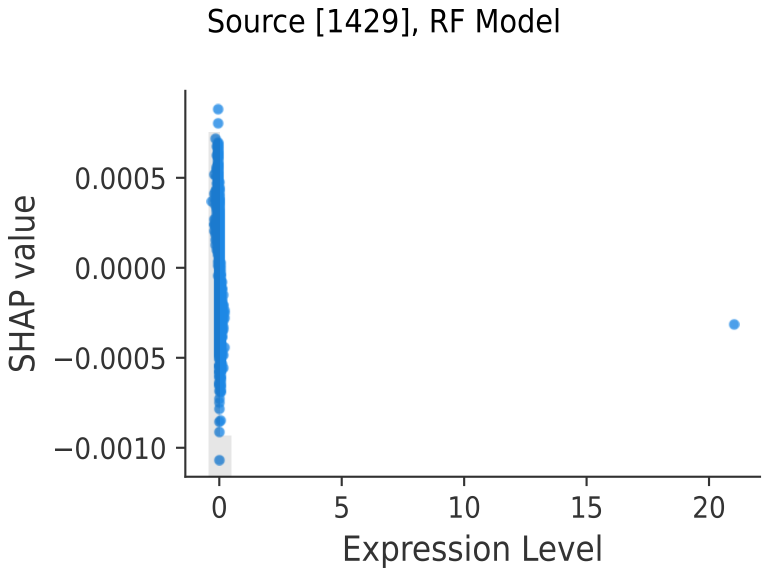

Source [1429], RF Model
SHAP value
0.0005
0.0000
−0.0005
−0.0010
0
5
10
15
20
Expression Level

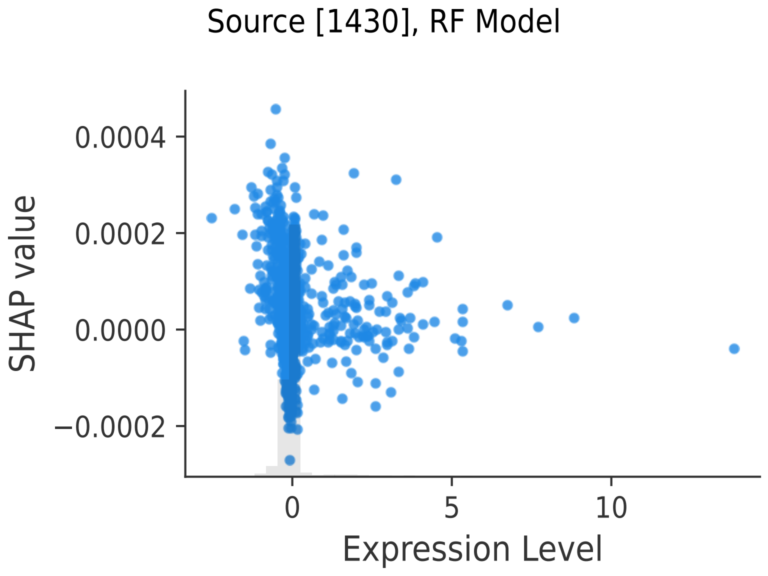

Source [1430], RF Model
SHAP value
0.0004
0.0002
0.0000
−0.0002
0
5
10
Expression Level

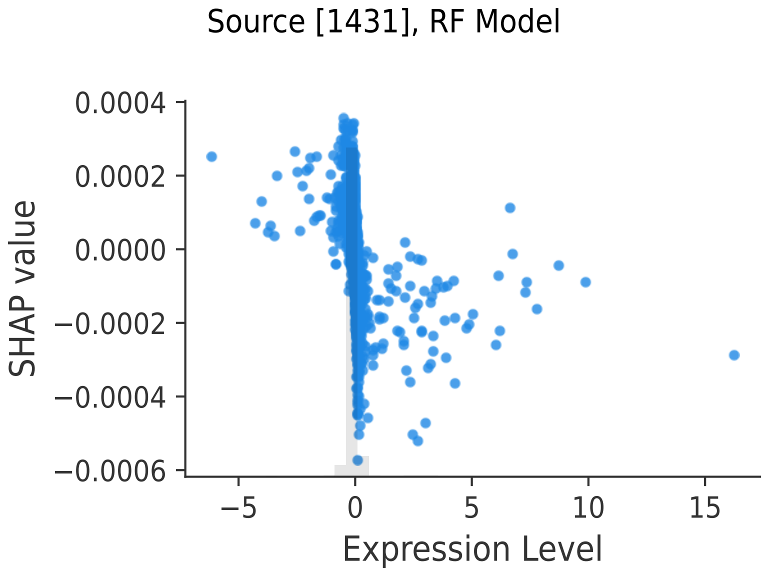
Source [1431], RF Model
SHAP value
Expression Level
0.0004
0.0002
0.0000
−0.0002
−0.0004
−0.0006
−5
0
5
10
15

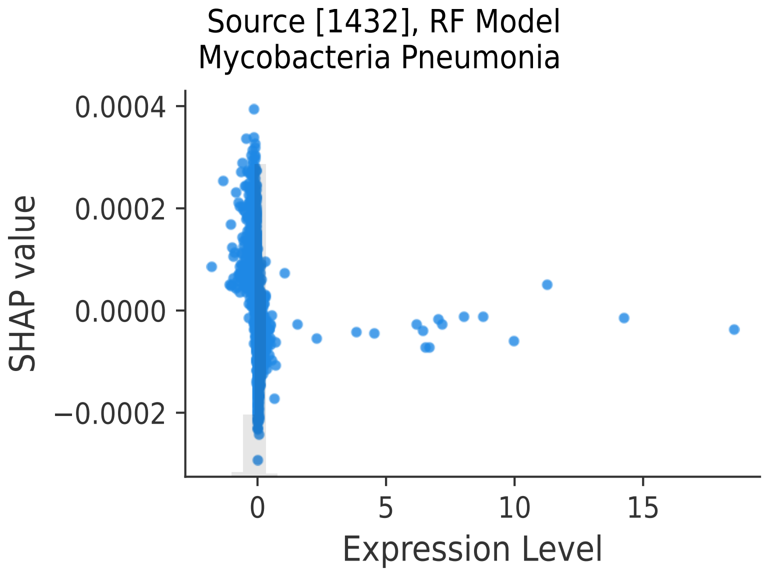
Source [1432], RF Model
Mycobacteria Pneumonia
SHAP value
0.0004
0.0002
0.0000
−0.0002
0
5
10
15
Expression Level

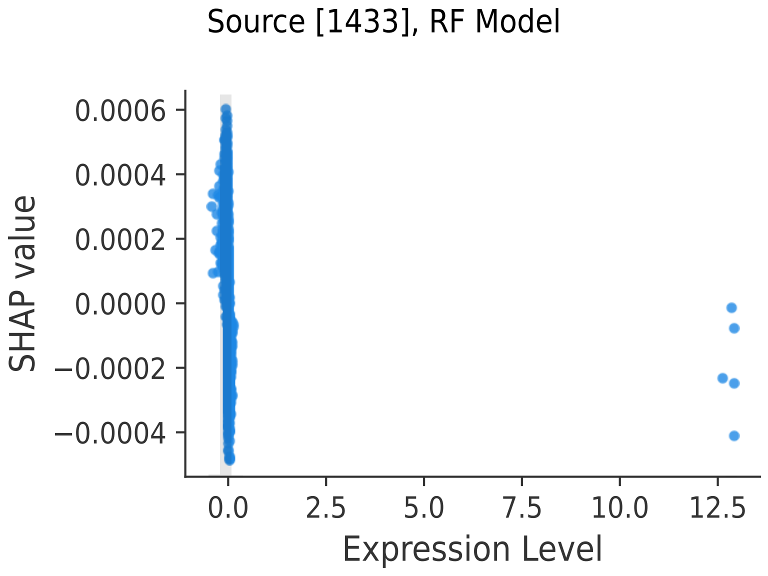
Source [1433], RF Model
SHAP value
0.0006
0.0004
0.0002
0.0000
−0.0002
−0.0004
0.0
2.5
5.0
7.5
10.0
12.5
Expression Level

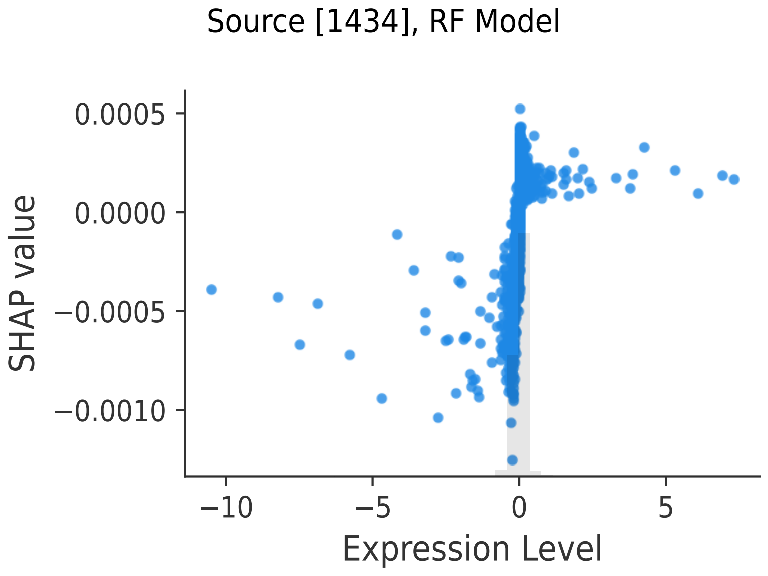
Source [1434], RF Model
SHAP value
0.0005
0.0000
−0.0005
−0.0010
−10
−5
0
5
Expression Level

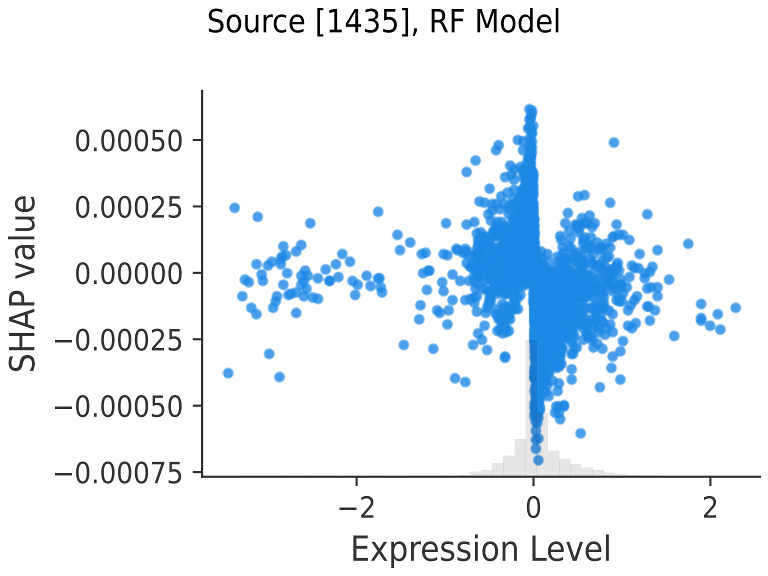

Source [1435], RF Model
0.00050
0.00025
0.00000
−0.00025
−0.00050
−0.00075
SHAP value
−2
0
2
Expression Level

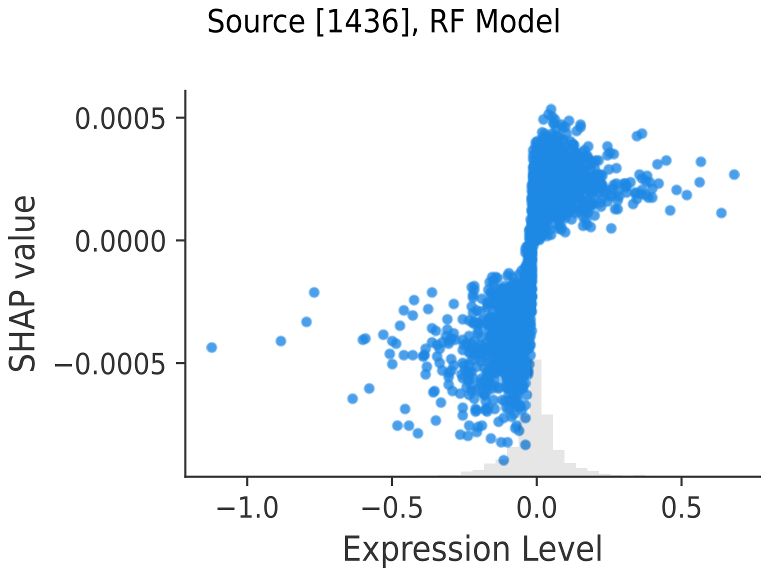

Source [1436], RF Model
0.0005
0.0000
−0.0005
SHAP value
−1.0
−0.5
0.0
0.5
Expression Level

Source [1437], RF Model

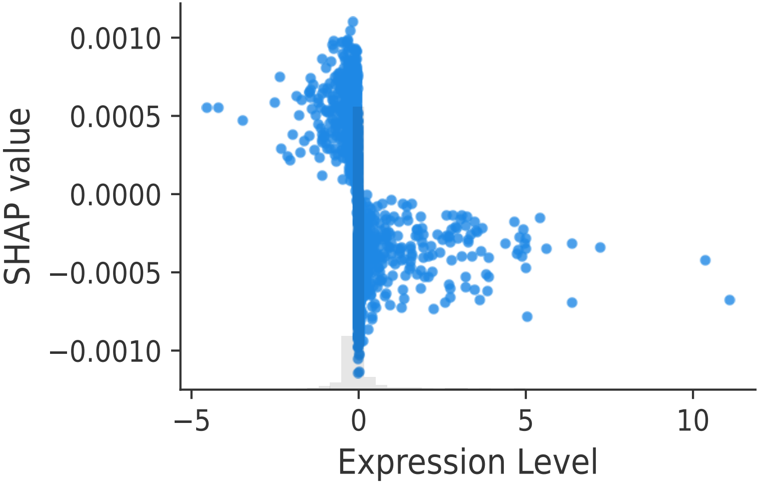

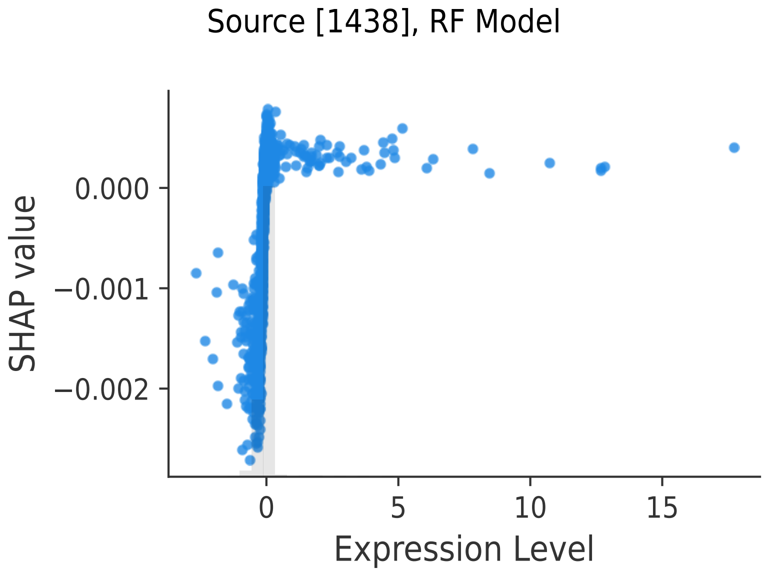

Source [1438], RF Model
SHAP value
0.000
−0.001
−0.002
0
5
10
15
Expression Level

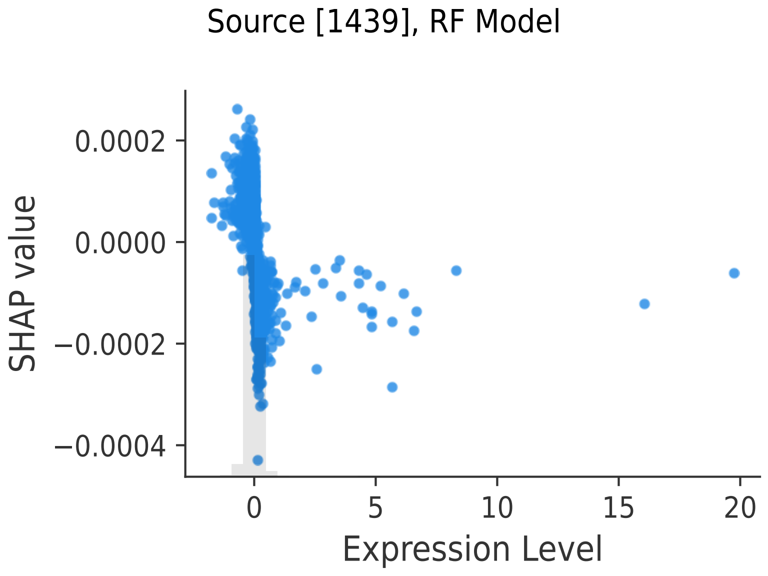
Source [1439], RF Model
SHAP value
0.0002
0.0000
−0.0002
−0.0004
0
5
10
15
20
Expression Level

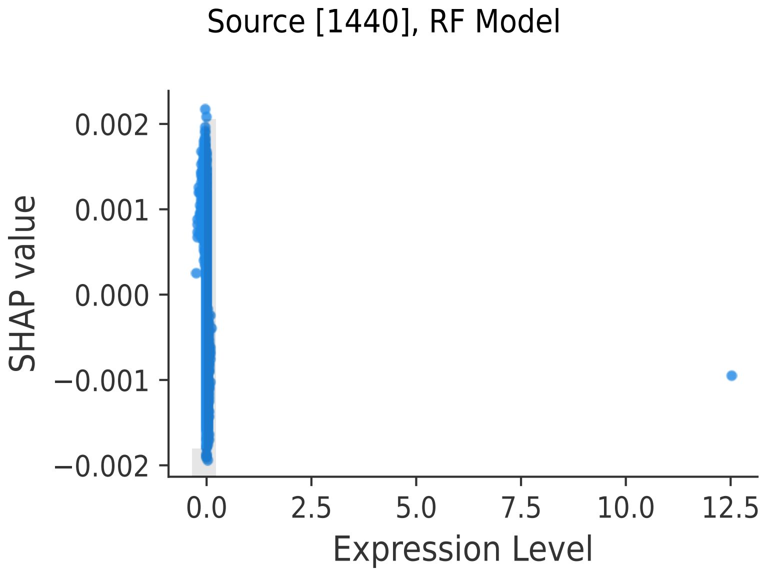
Source [1440], RF Model
SHAP value
0.002
0.001
0.000
−0.001
−0.002
0.0
2.5
5.0
7.5
10.0
12.5
Expression Level

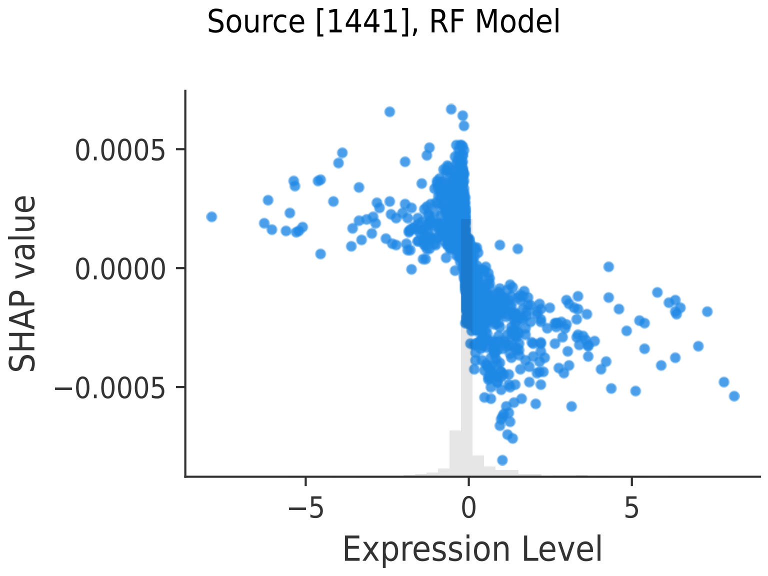

Source [1441], RF Model
0.0005
0.0000
−0.0005
SHAP value
−5
0
5
Expression Level

Source [1442], RF Model

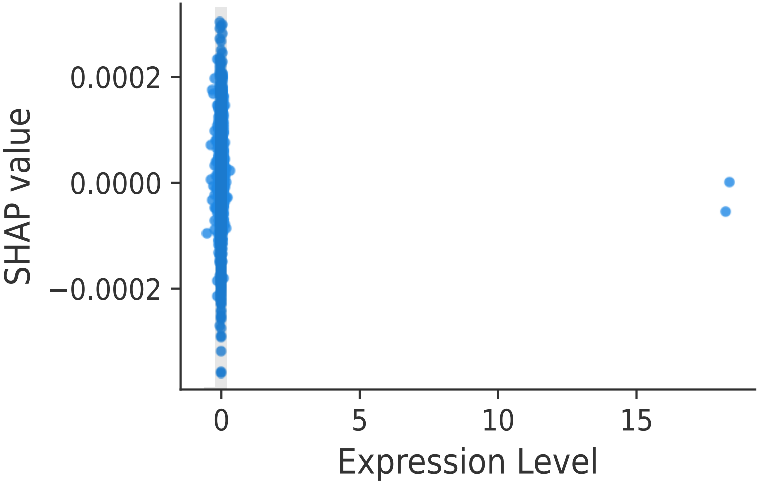

Source [1443], RF Model

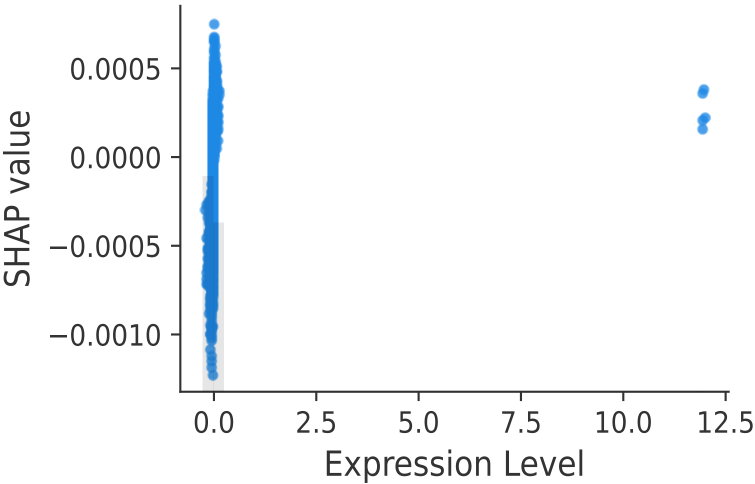

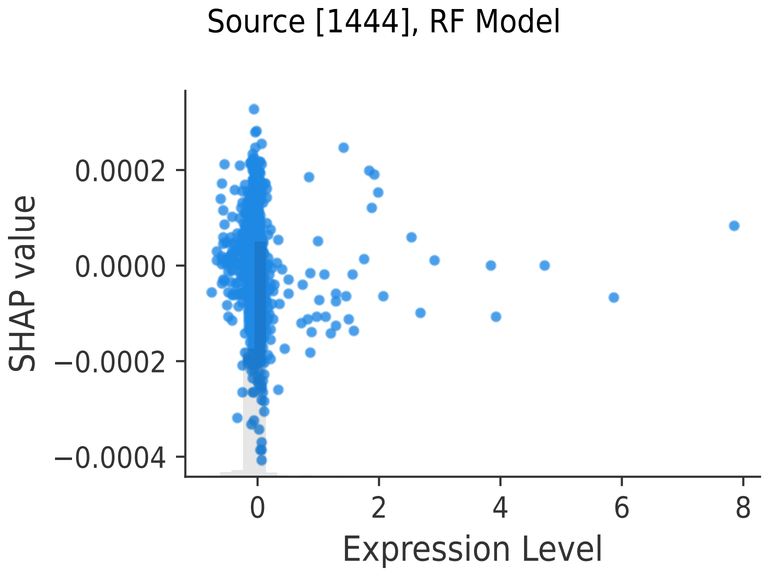

Source [1444], RF Model
SHAP value
0.0002
0.0000
−0.0002
−0.0004
0
2
4
6
8
Expression Level

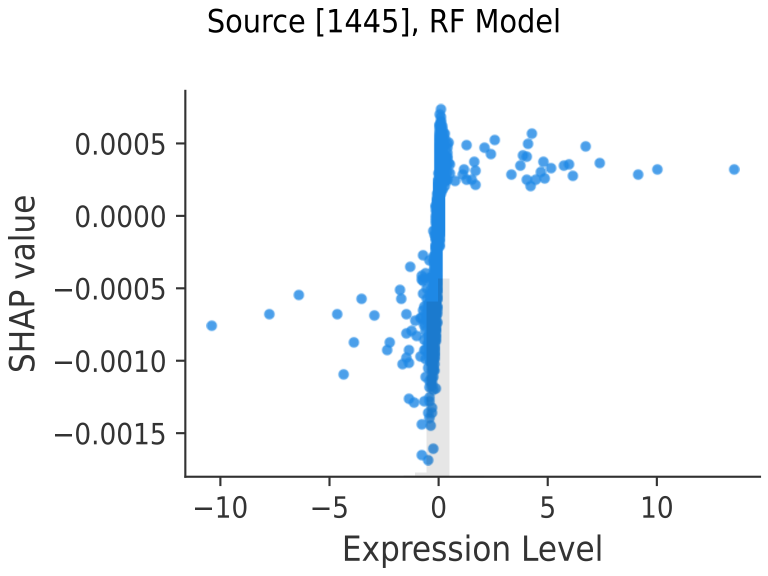
Source [1445], RF Model
SHAP value
0.0005
0.0000
−0.0005
−0.0010
−0.0015
−10
−5
0
5
10
Expression Level

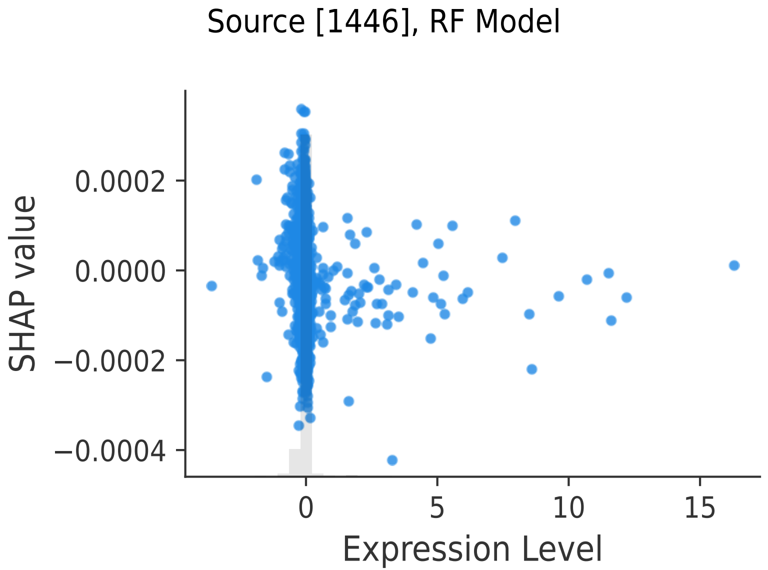
Source [1446], RF Model
0.0002
0.0000
−0.0002
−0.0004
SHAP value
0
5
10
15
Expression Level

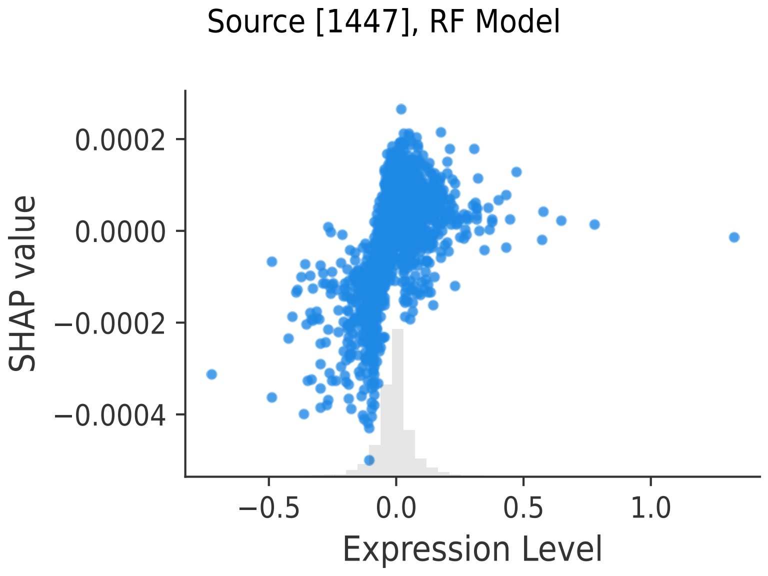
Source [1447], RF Model
0.0002
0.0000
−0.0002
−0.0004
SHAP value
−0.5
0.0
0.5
1.0
Expression Level

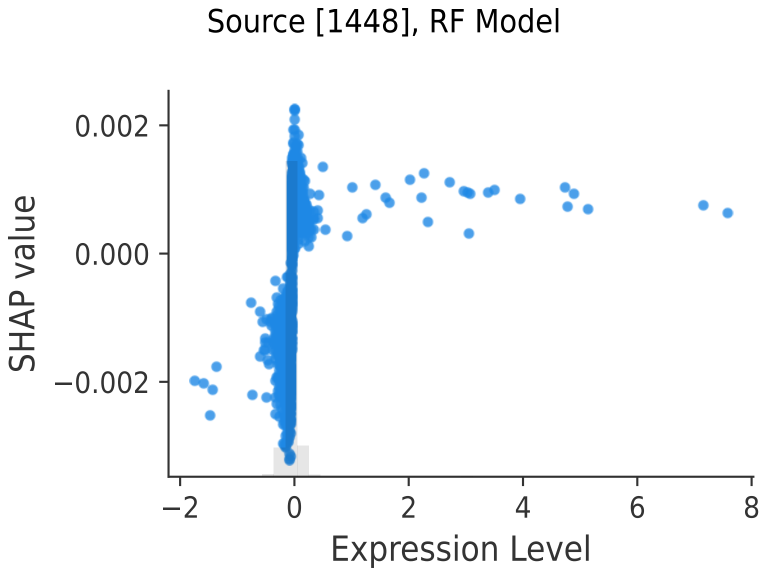

Source [1448], RF Model
0.002
0.000
−0.002
SHAP value
−2
0
2
4
6
8
Expression Level

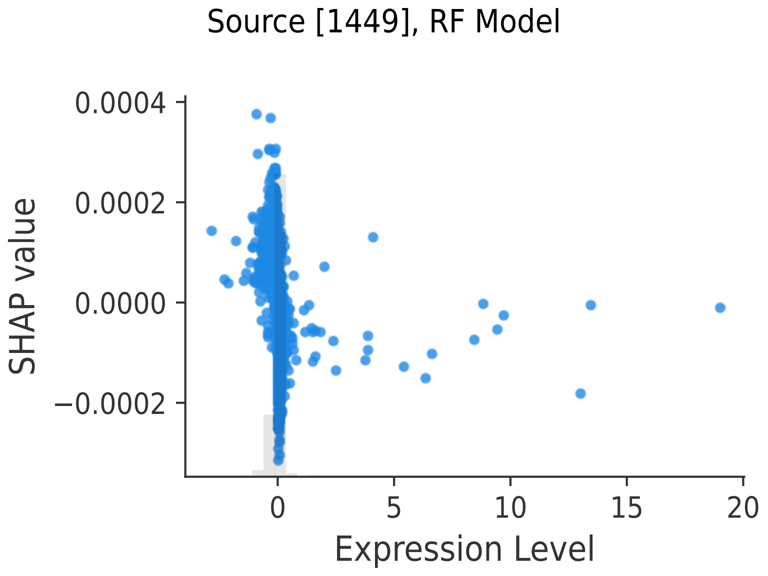
Source [1449], RF Model
0.0004
0.0002
0.0000
−0.0002
SHAP value
0
5
10
15
20
Expression Level

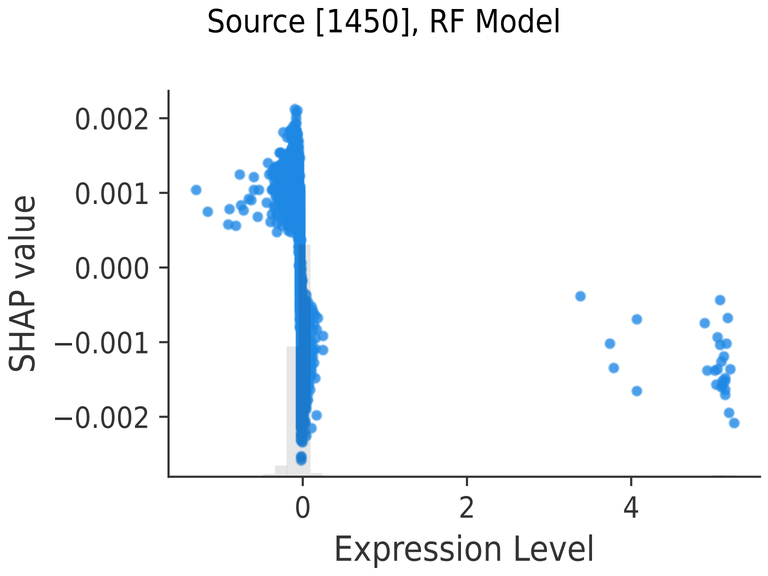

Source [1450], RF Model
SHAP value
0.002
0.001
0.000
−0.001
−0.002
0
2
4
Expression Level

Source [1451], RF Model

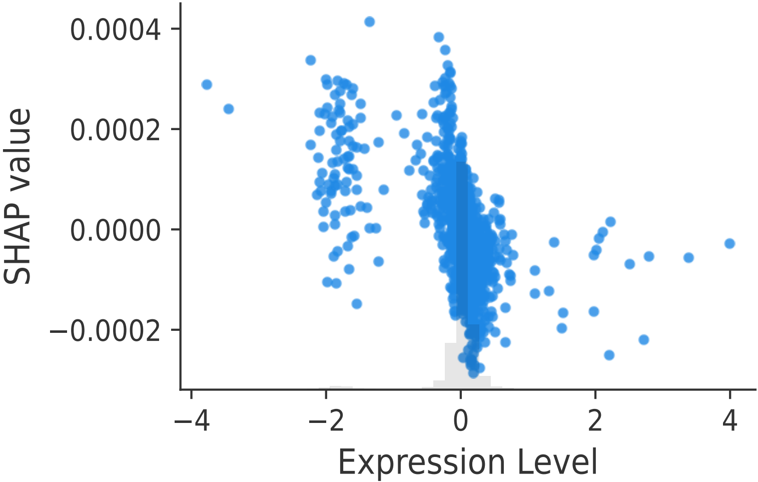

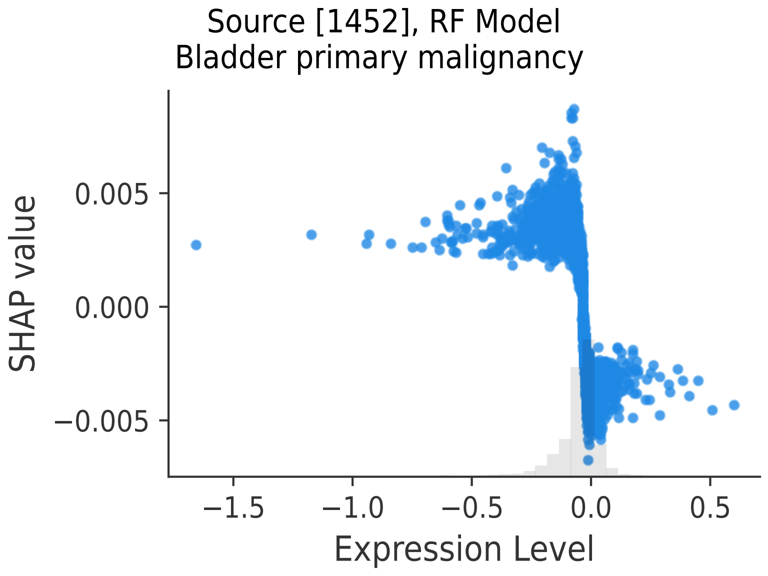

Source [1452], RF Model
Bladder primary malignancy
0.005
0.000
−0.005
SHAP value
−1.5
−1.0
−0.5
0.0
0.5
Expression Level

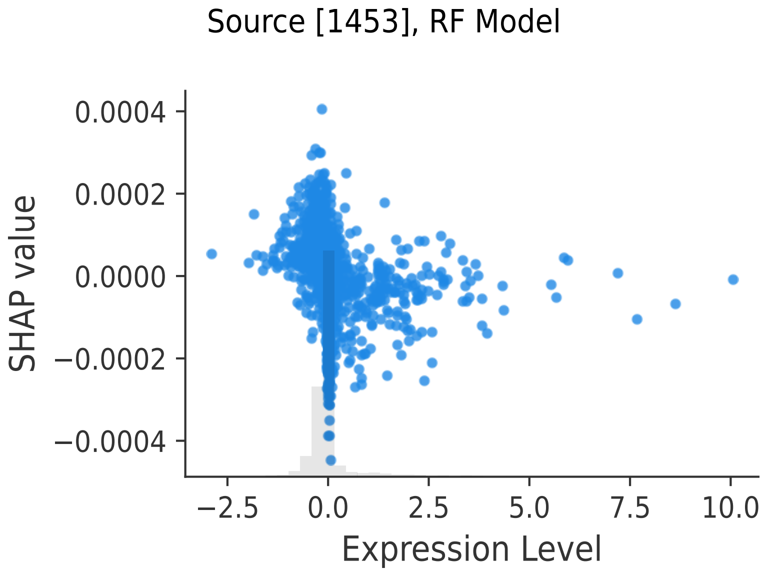
Source [1453], RF Model
0.0004
0.0002
0.0000
−0.0002
−0.0004
SHAP value
−2.5
0.0
2.5
5.0
7.5
10.0
Expression Level

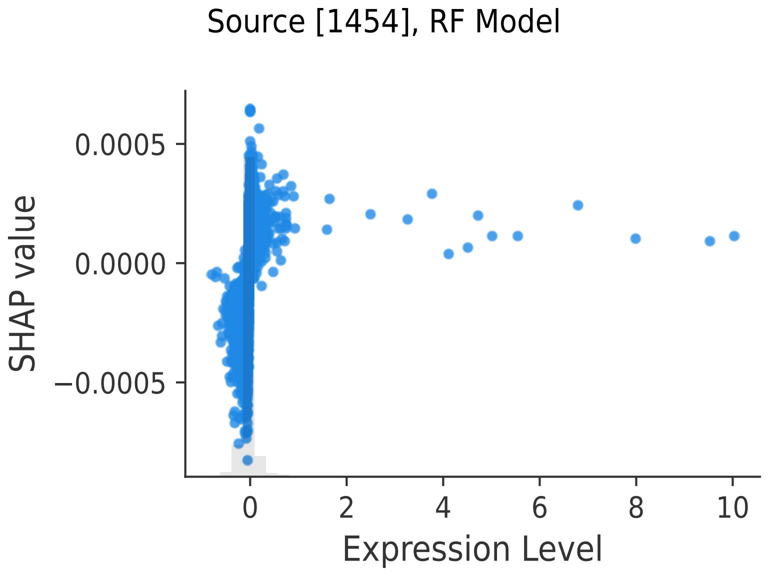
Source [1454], RF Model
0.0005
0.0000
−0.0005
SHAP value
0
2
4
6
8
10
Expression Level

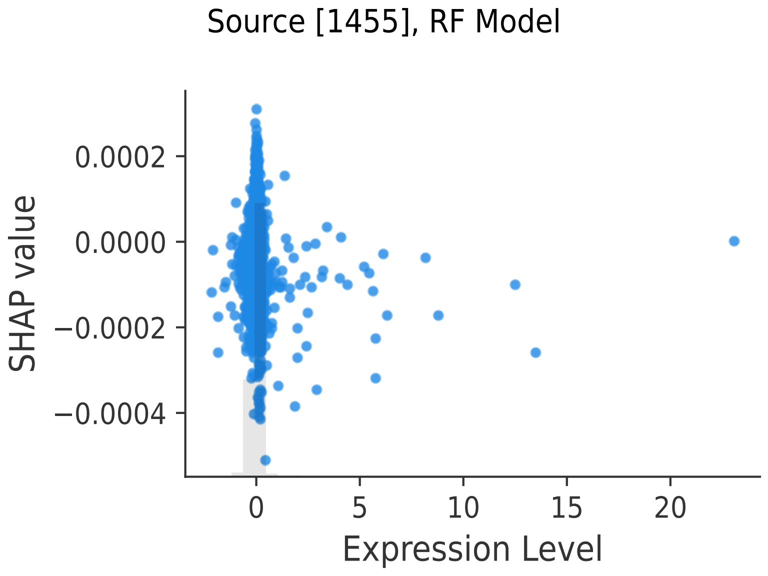
Source [1455], RF Model
0.0002
0.0000
−0.0002
−0.0004
SHAP value
0
5
10
15
20
Expression Level

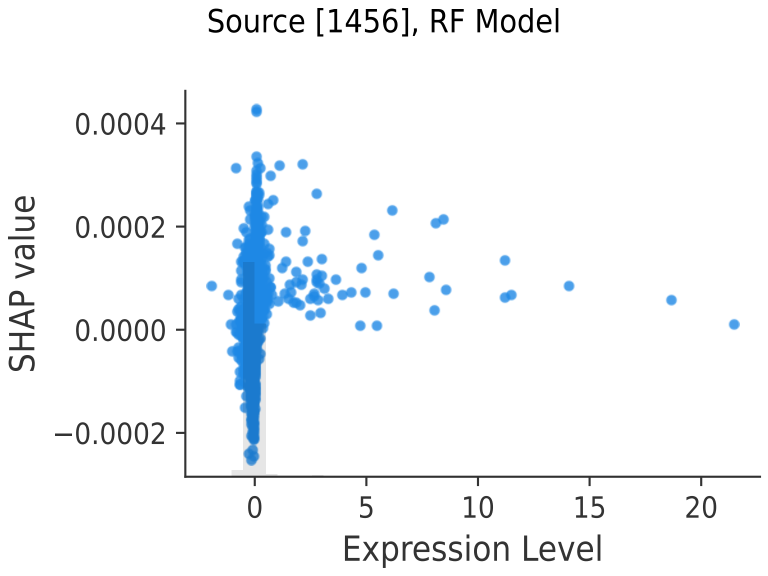

Source [1456], RF Model
0.0004
0.0002
0.0000
−0.0002
SHAP value
0
5
10
15
20
Expression Level

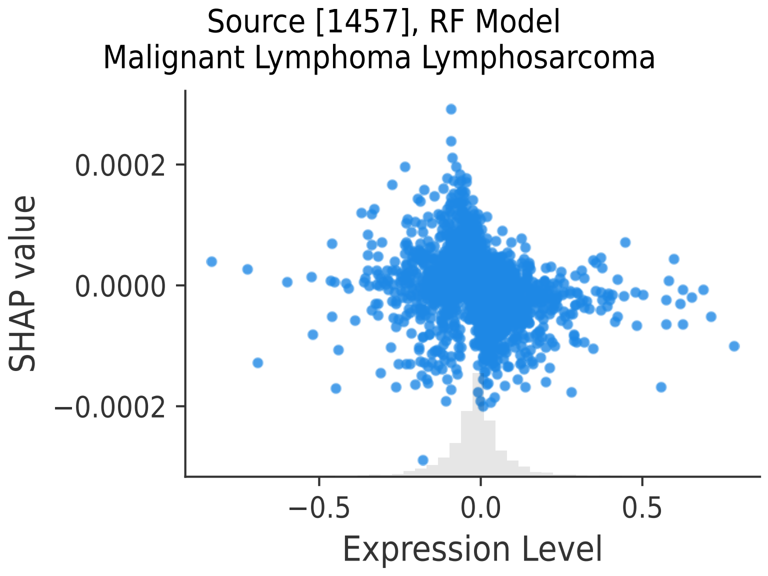
Source [1457], RF Model
Malignant Lymphoma Lymphosarcoma
SHAP value
0.0002
0.0000
−0.0002
−0.5
0.0
0.5
Expression Level

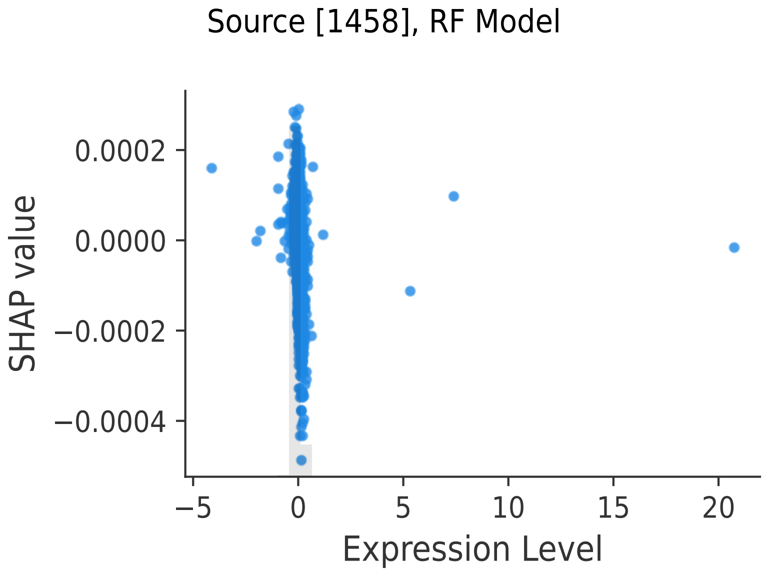

Source [1458], RF Model
SHAP value
0.0002
0.0000
−0.0002
−0.0004
−5
0
5
10
15
20
Expression Level

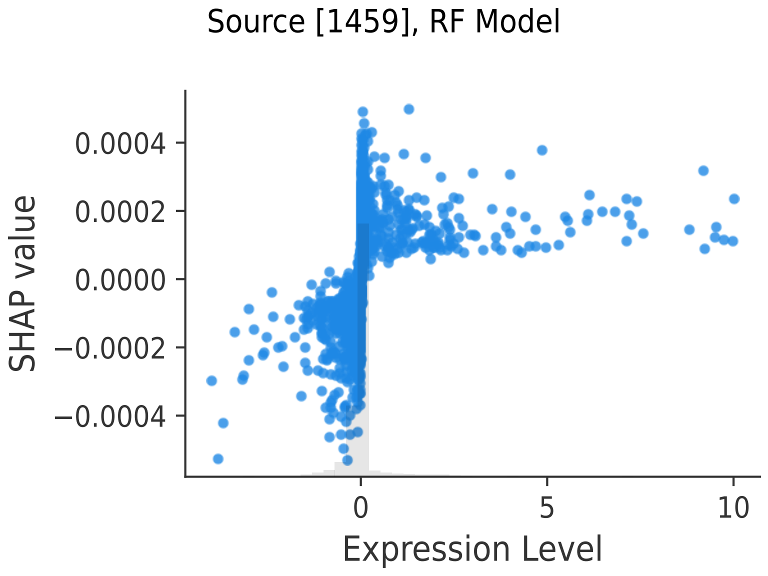
Source [1459], RF Model
SHAP value
0.0004
0.0002
0.0000
−0.0002
−0.0004
0
5
10
Expression Level

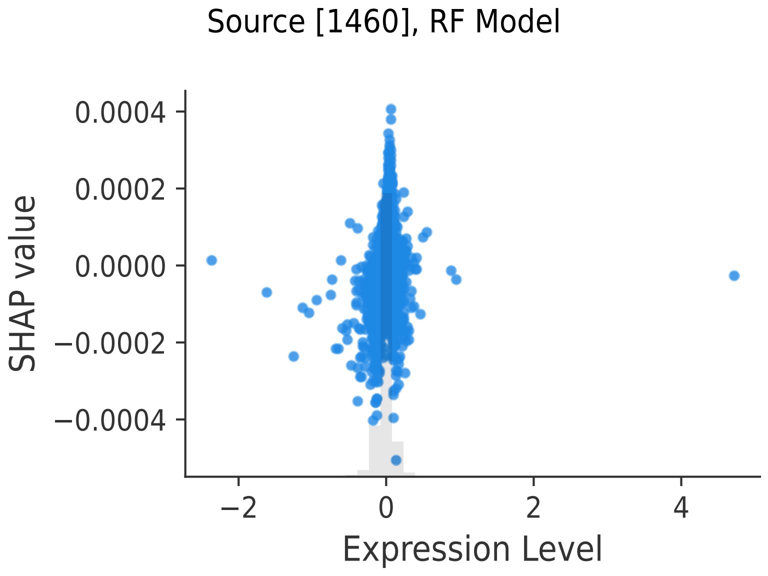
Source [1460], RF Model
0.0004
0.0002
0.0000
−0.0002
−0.0004
SHAP value
−2
0
2
4
Expression Level

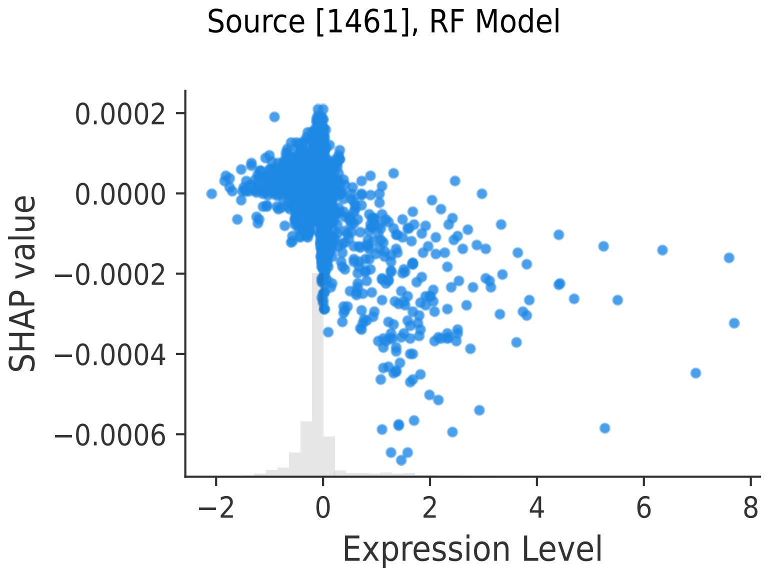
Source [1461], RF Model
0.0002
0.0000
−0.0002
−0.0004
−0.0006
SHAP value
−2
0
2
4
6
8
Expression Level

Source [1462], RF Model

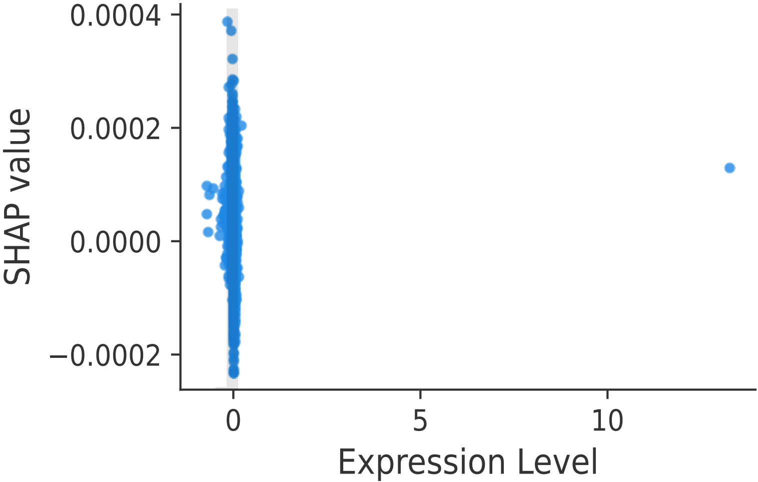

Source [1463], RF Model

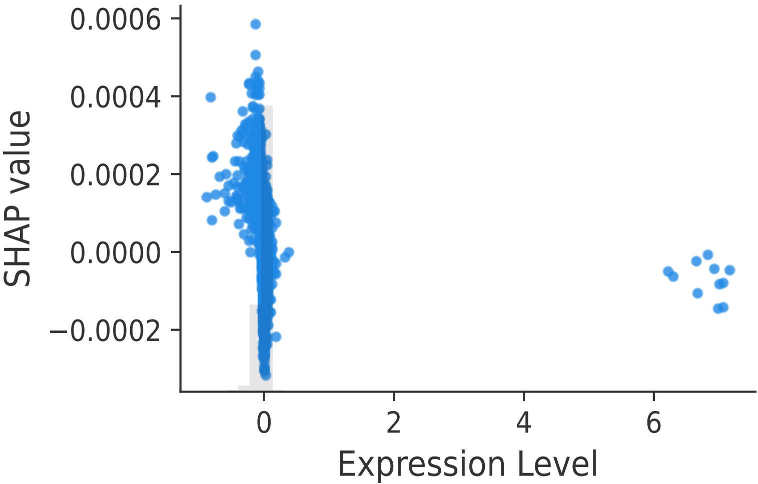

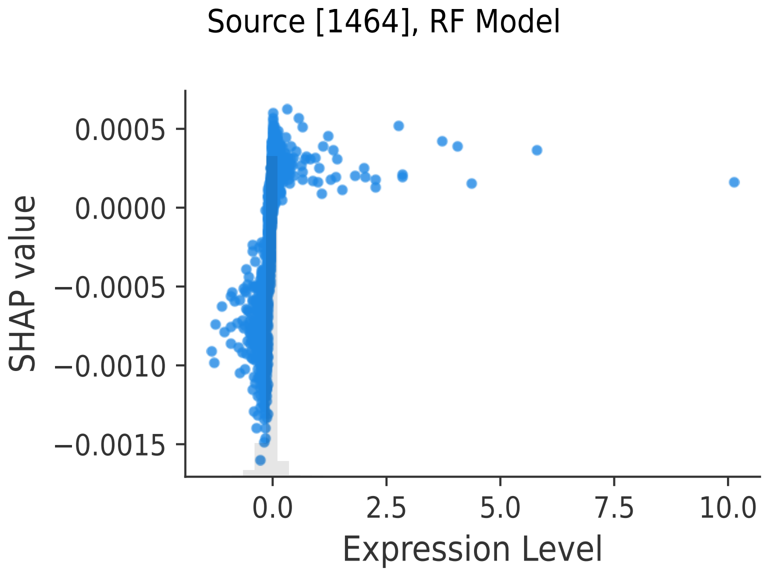
Source [1464], RF Model
0.0005
0.0000
−0.0005
−0.0010
−0.0015
SHAP value
0.0
2.5
5.0
7.5
10.0
Expression Level

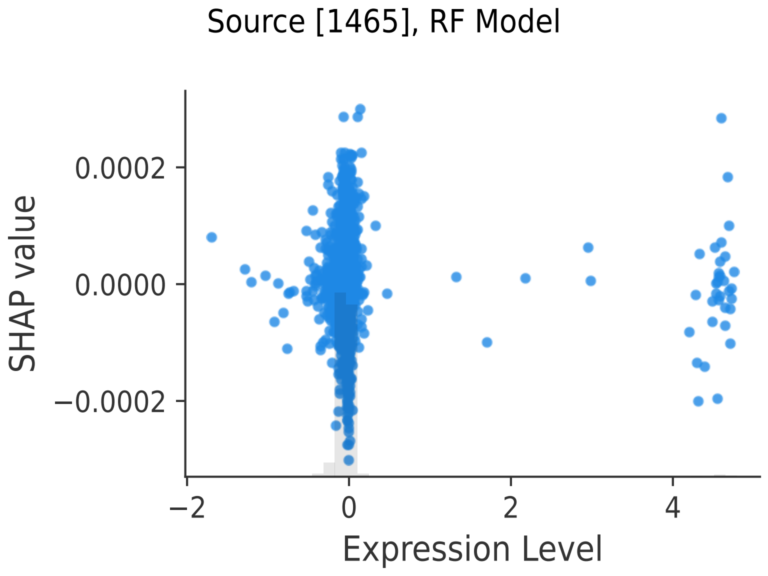
Source [1465], RF Model
SHAP value
0.0002
0.0000
−0.0002
−2
0
2
4
Expression Level

Source [1466], RF Model

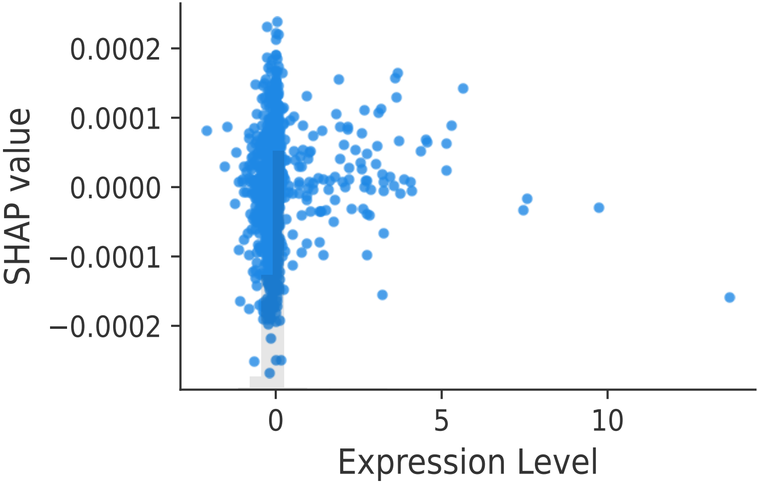

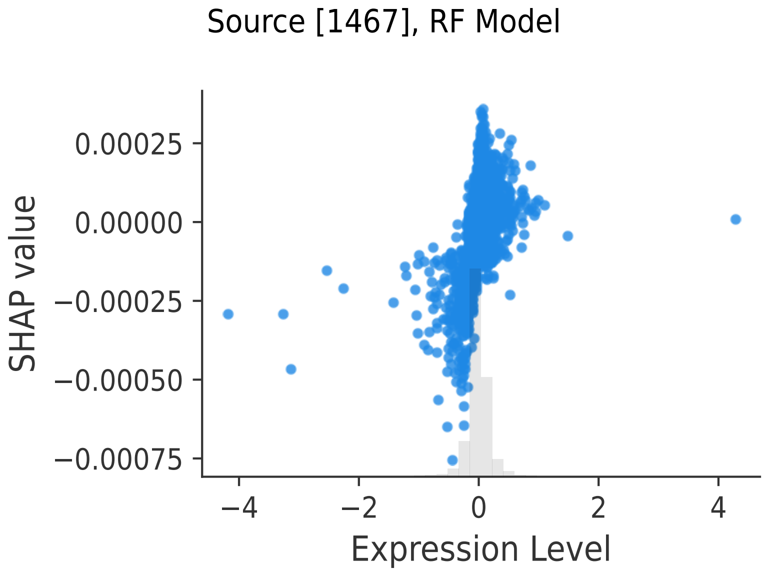

Source [1467], RF Model
SHAP value
0.00025
0.00000
−0.00025
−0.00050
−0.00075
−4
−2
0
2
4
Expression Level

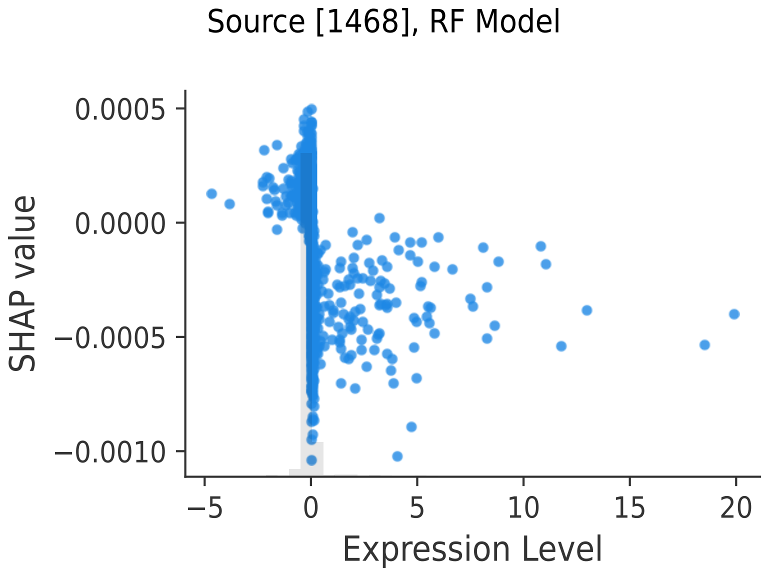

Source [1468], RF Model
0.0005
0.0000
−0.0005
−0.0010
SHAP value
−5
0
5
10
15
20
Expression Level

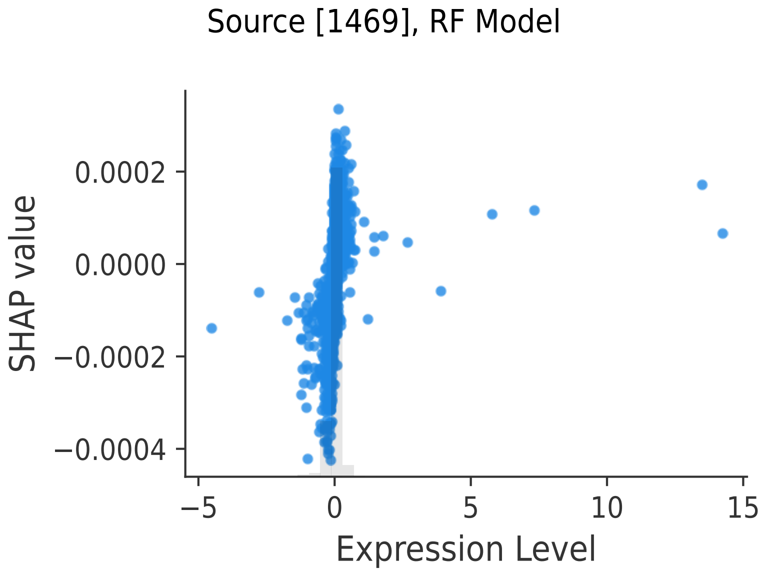

Source [1469], RF Model
SHAP value
0.0002
0.0000
−0.0002
−0.0004
−5
0
5
10
15
Expression Level

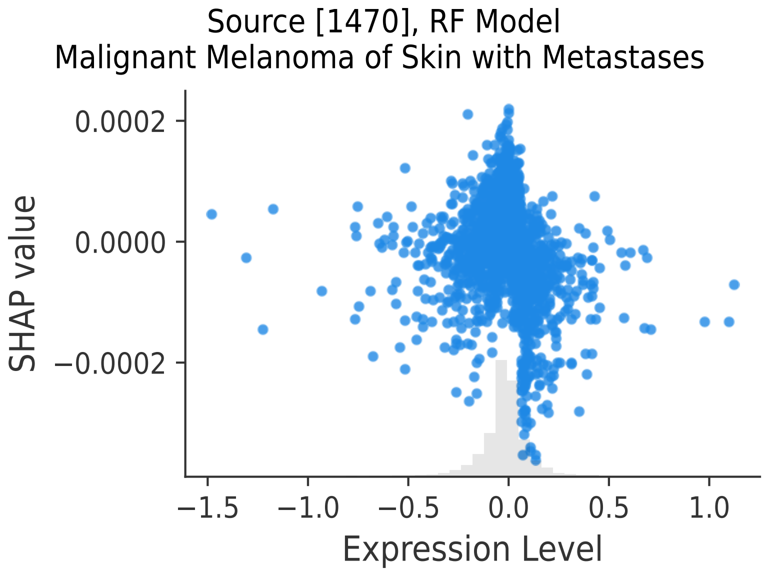

Source [1470], RF Model
Malignant Melanoma of Skin with Metastases
0.0002
0.0000
−0.0002
SHAP value
−1.5
−1.0
−0.5
0.0
0.5
1.0
Expression Level

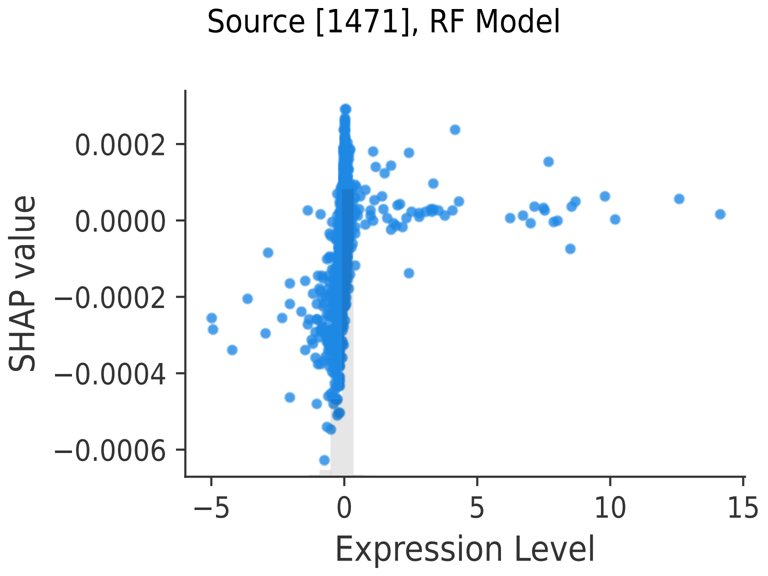
Source [1471], RF Model
SHAP value
0.0002
0.0000
−0.0002
−0.0004
−0.0006
−5
0
5
10
15
Expression Level

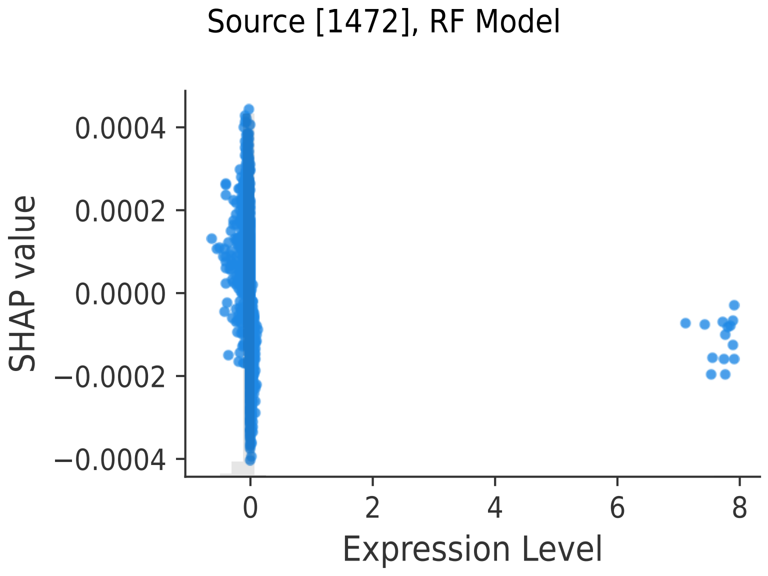

Source [1472], RF Model
SHAP value
0.0004
0.0002
0.0000
−0.0002
−0.0004
0
2
4
6
8
Expression Level

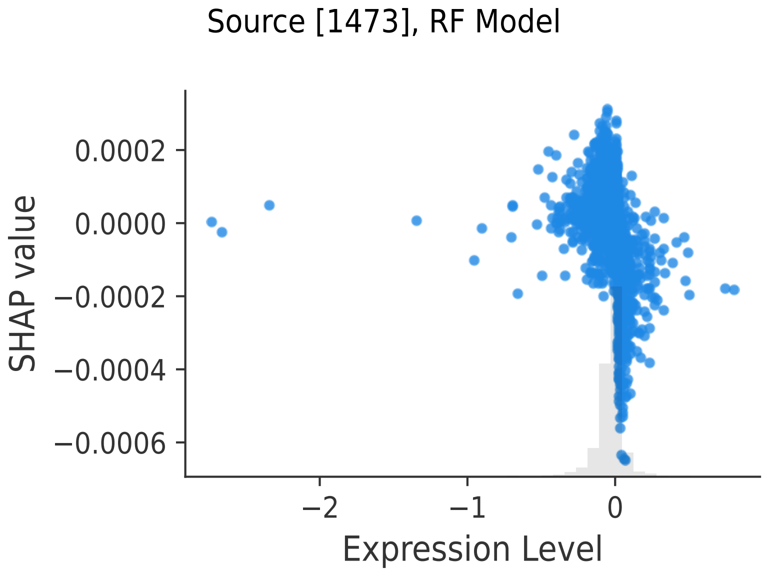
Source [1473], RF Model
SHAP value
0.0002
0.0000
−0.0002
−0.0004
−0.0006
−2
−1
0
Expression Level

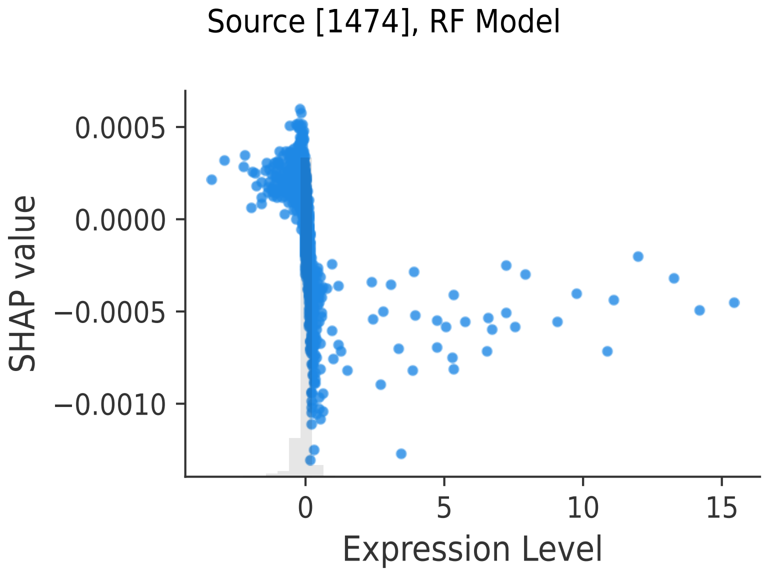

Source [1474], RF Model
0.0005
0.0000
−0.0005
−0.0010
SHAP value
0
5
10
15
Expression Level

Source [1475], RF Model

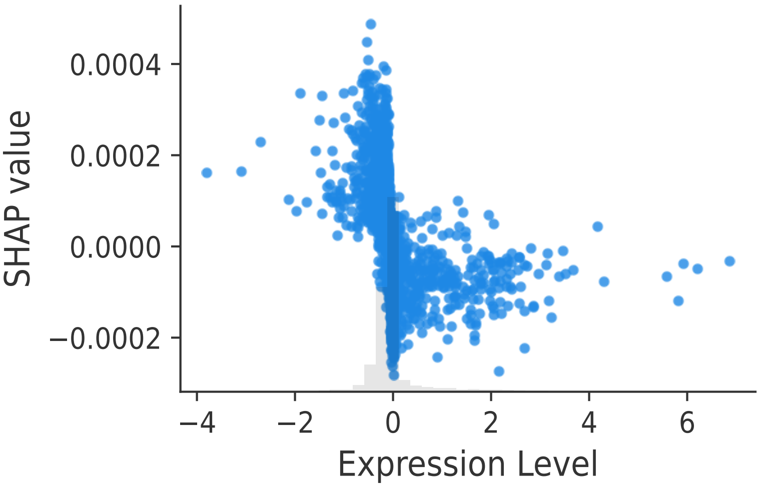

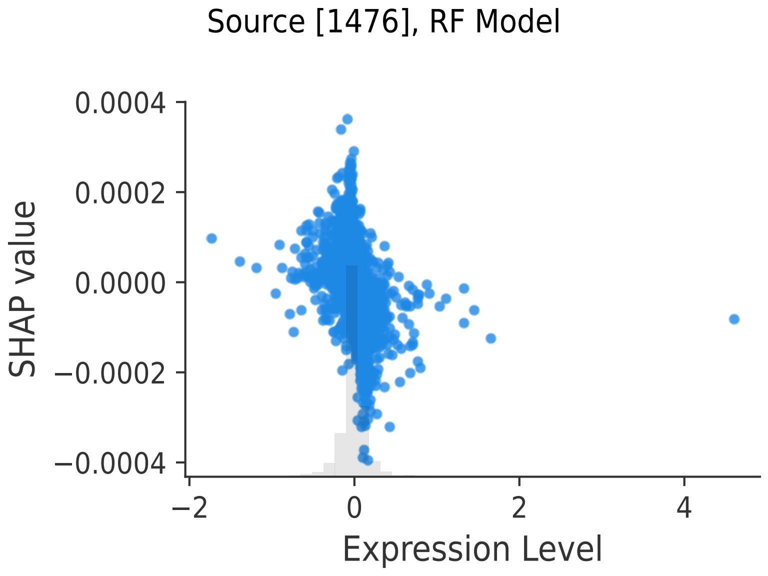

Source [1476], RF Model
0.0004
0.0002
0.0000
−0.0002
−0.0004
SHAP value
−2
0
2
4
Expression Level

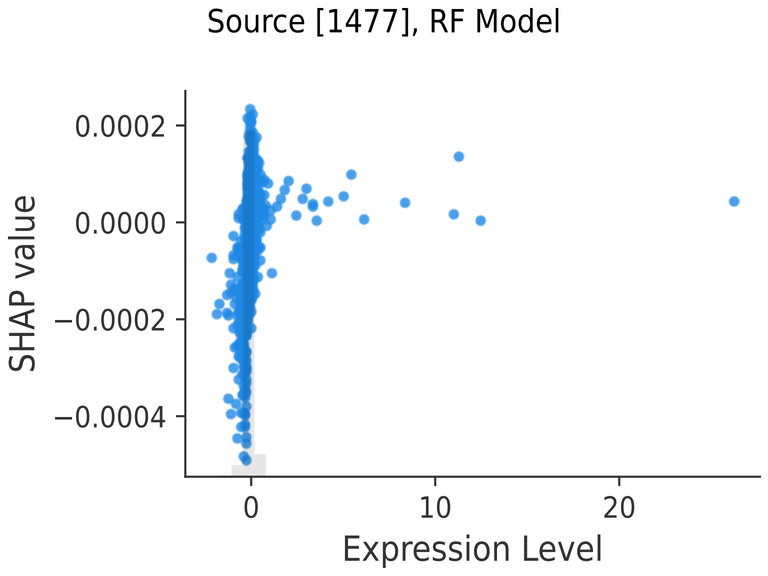
Source [1477], RF Model
0.0002
0.0000
−0.0002
−0.0004
SHAP value
0
10
20
Expression Level

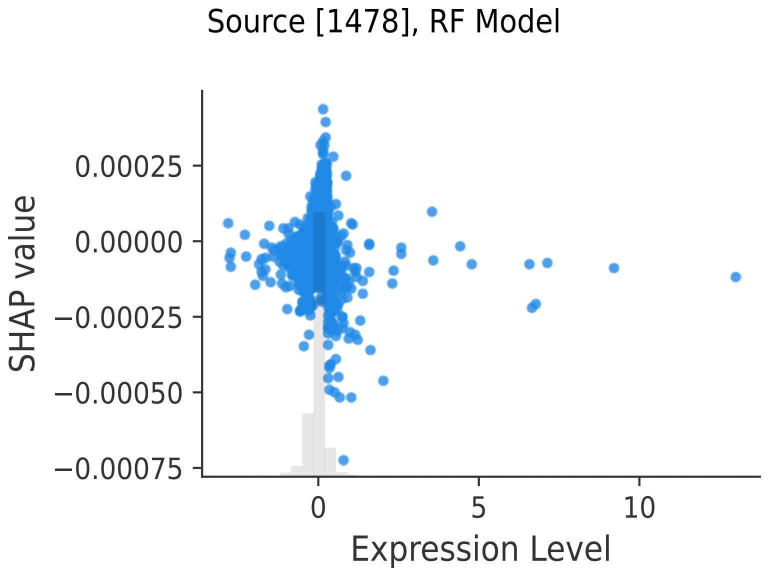
Source [1478], RF Model
0.00025
0.00000
−0.00025
−0.00050
−0.00075
SHAP value
0
5
10
Expression Level

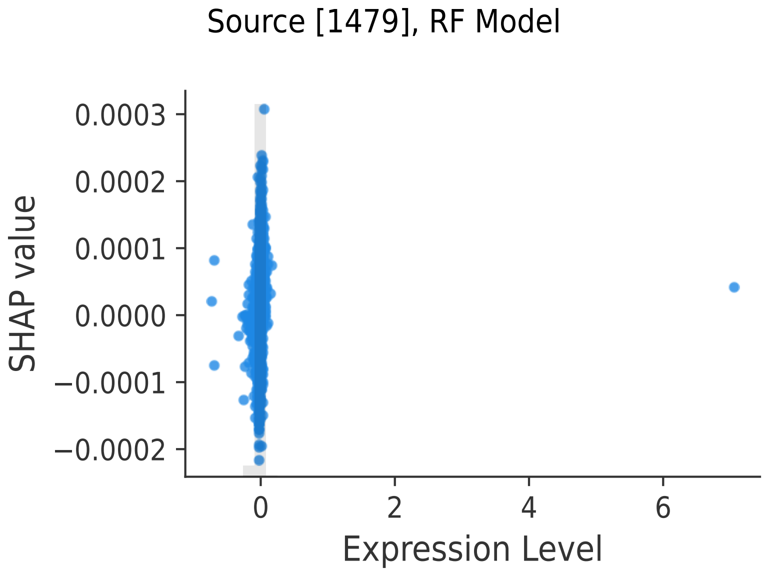
Source [1479], RF Model
SHAP value
0.0003
0.0002
0.0001
0.0000
−0.0001
−0.0002
0
2
4
6
Expression Level

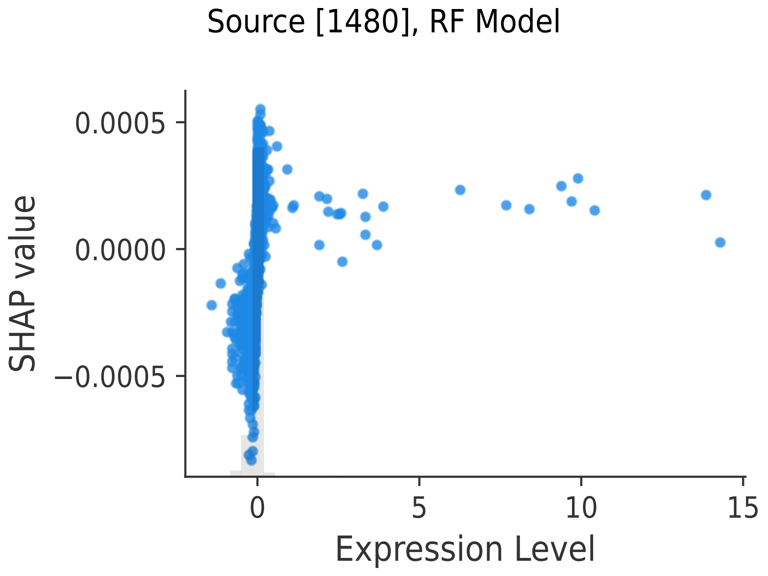
Source [1480], RF Model
SHAP value
0.0005
0.0000
−0.0005
0
5
10
15
Expression Level

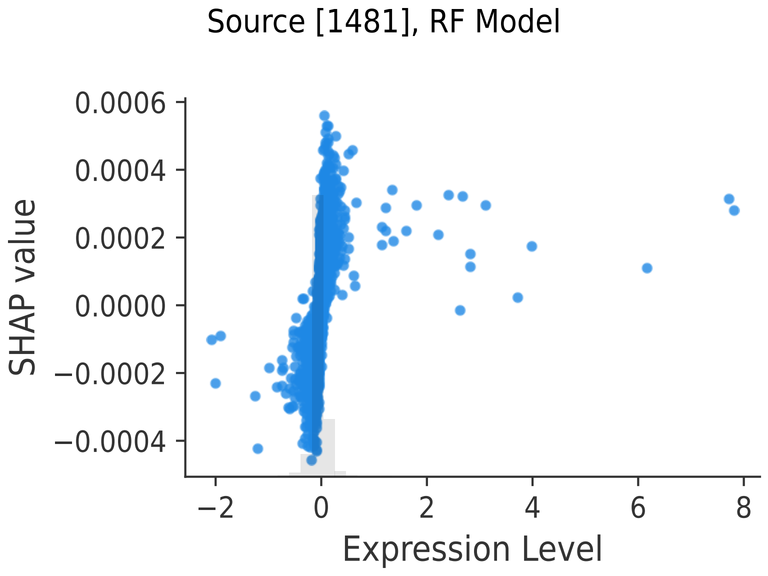
Source [1481], RF Model
SHAP value
0.0006
0.0004
0.0002
0.0000
−0.0002
−0.0004
−2
0
2
4
6
8
Expression Level

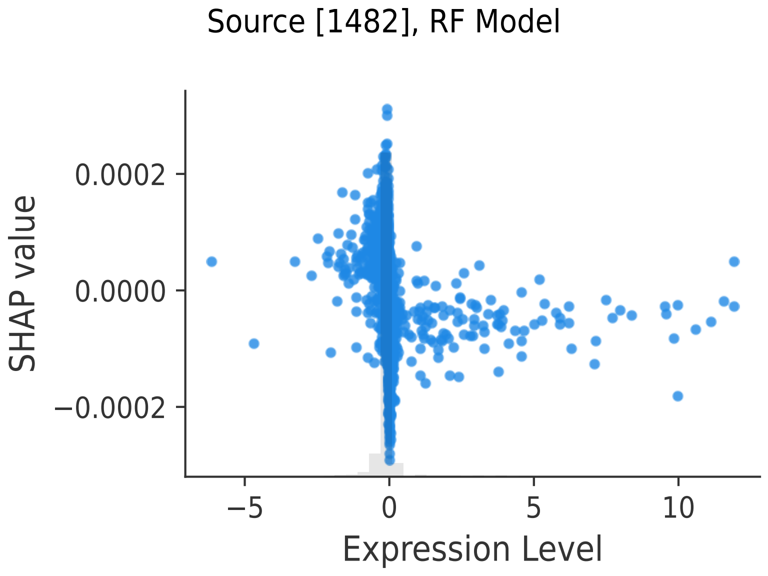

Source [1482], RF Model
SHAP value
0.0002
0.0000
−0.0002
−5
0
5
10
Expression Level

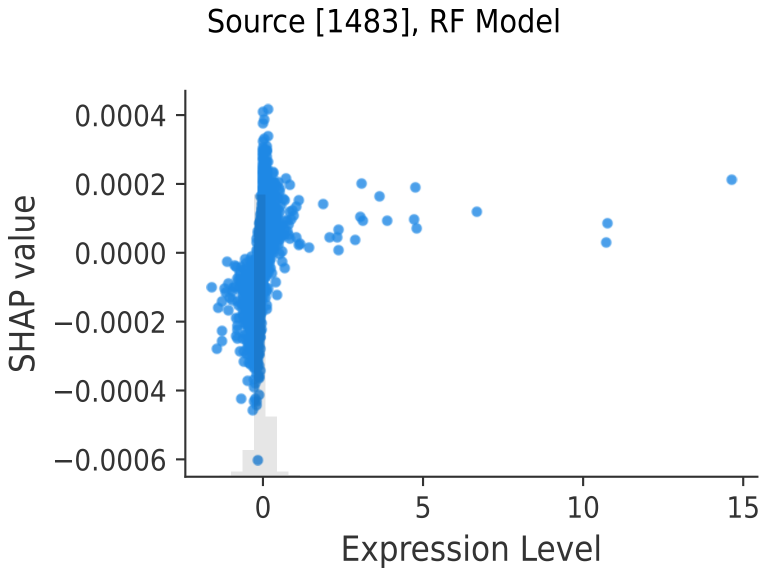
Source [1483], RF Model
SHAP value
0.0004
0.0002
0.0000
−0.0002
−0.0004
−0.0006
0
5
10
15
Expression Level

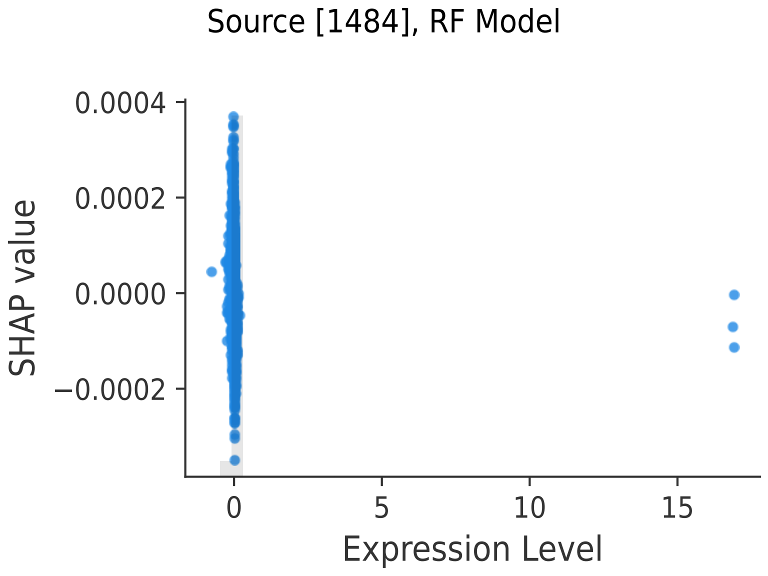

Source [1484], RF Model
0.0004
0.0002
0.0000
−0.0002
SHAP value
0
5
10
15
Expression Level

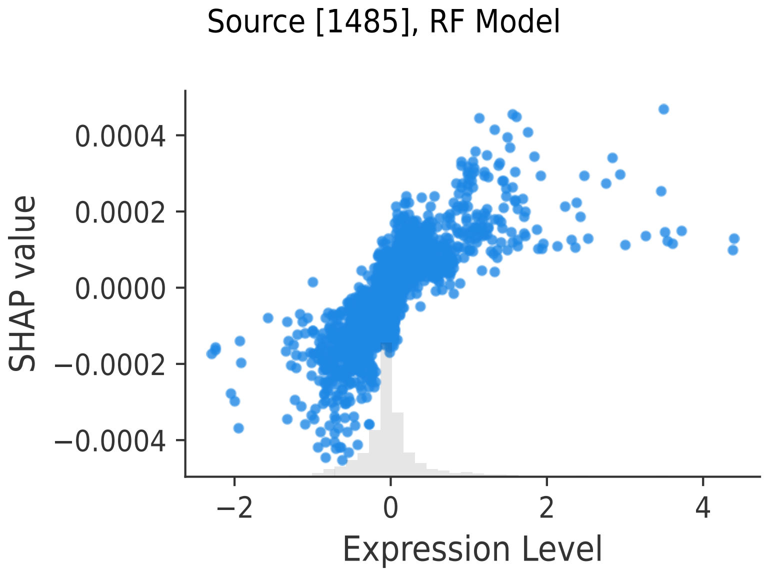

Source [1485], RF Model
SHAP value
0.0004
0.0002
0.0000
−0.0002
−0.0004
−2
0
2
4
Expression Level

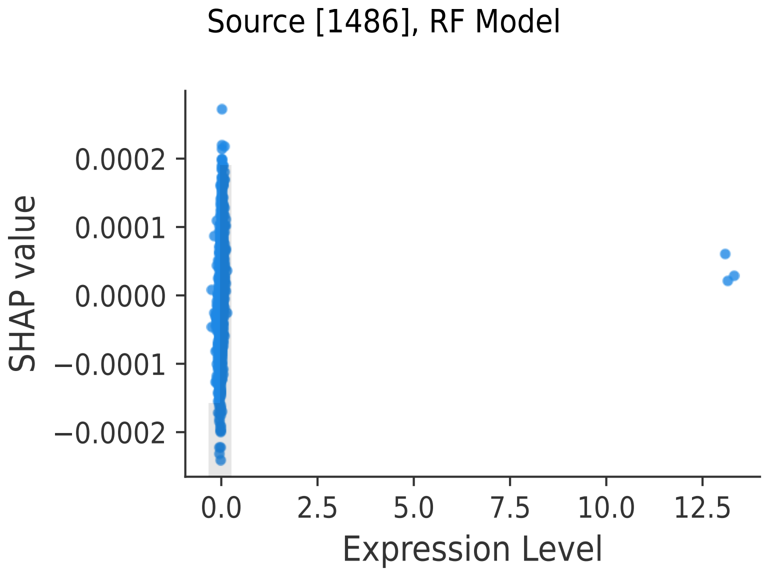

Source [1486], RF Model
SHAP value
0.0002
0.0001
0.0000
−0.0001
−0.0002
0.0
2.5
5.0
7.5
10.0
12.5
Expression Level

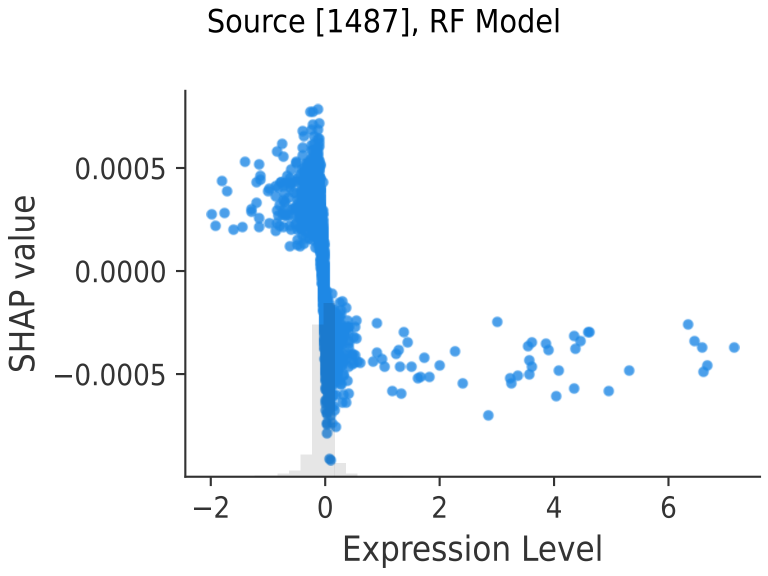
Source [1487], RF Model
0.0005
0.0000
−0.0005
SHAP value
−2
0
2
4
6
Expression Level

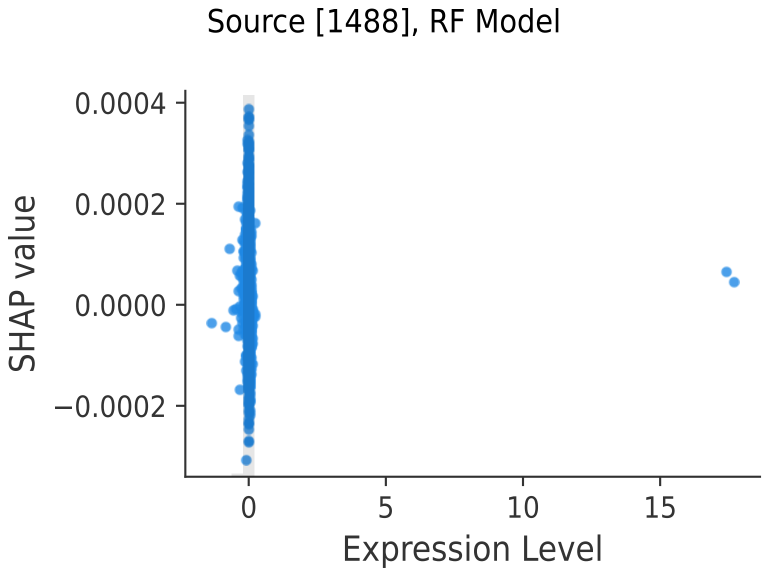
Source [1488], RF Model
0.0004
0.0002
0.0000
−0.0002
SHAP value
0
5
10
15
Expression Level

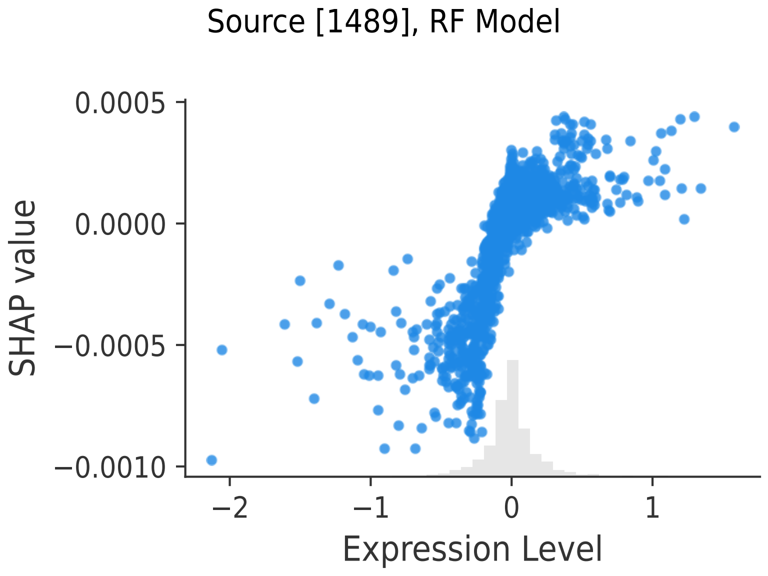

Source [1489], RF Model
0.0005
0.0000
−0.0005
−0.0010
SHAP value
−2
−1
0
1
Expression Level

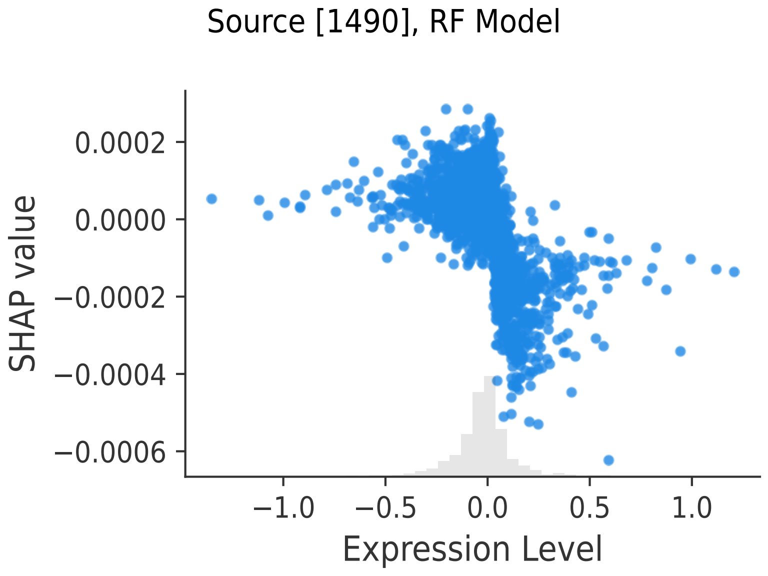
Source [1490], RF Model
0.0002
0.0000
−0.0002
−0.0004
−0.0006
SHAP value
−1.0
−0.5
0.0
0.5
1.0
Expression Level

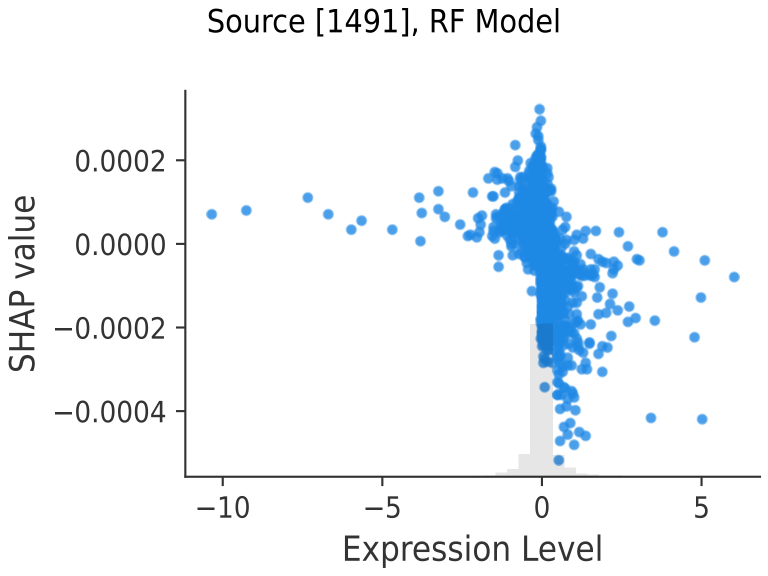
Source [1491], RF Model
0.0002
0.0000
−0.0002
−0.0004
SHAP value
−10
−5
0
5
Expression Level

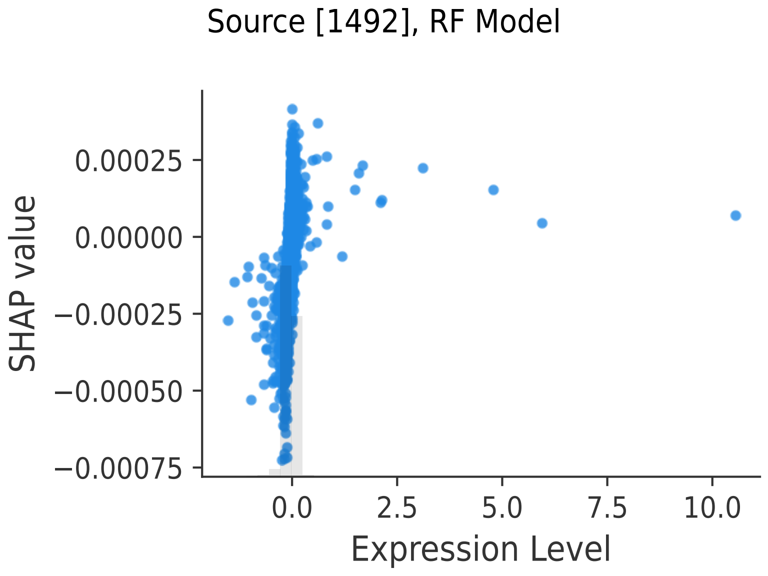
Source [1492], RF Model
SHAP value
0.00025
0.00000
−0.00025
−0.00050
−0.00075
0.0
2.5
5.0
7.5
10.0
Expression Level

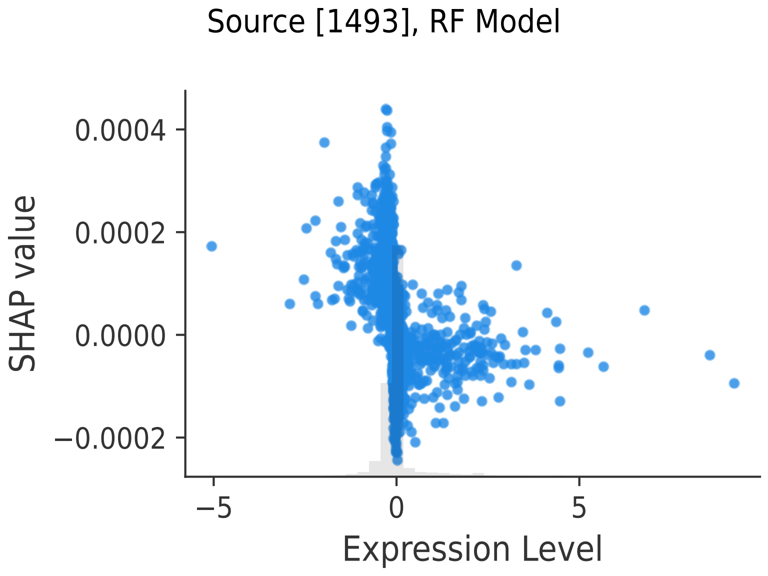

Source [1493], RF Model
SHAP value
0.0004
0.0002
0.0000
−0.0002
−5
0
5
Expression Level

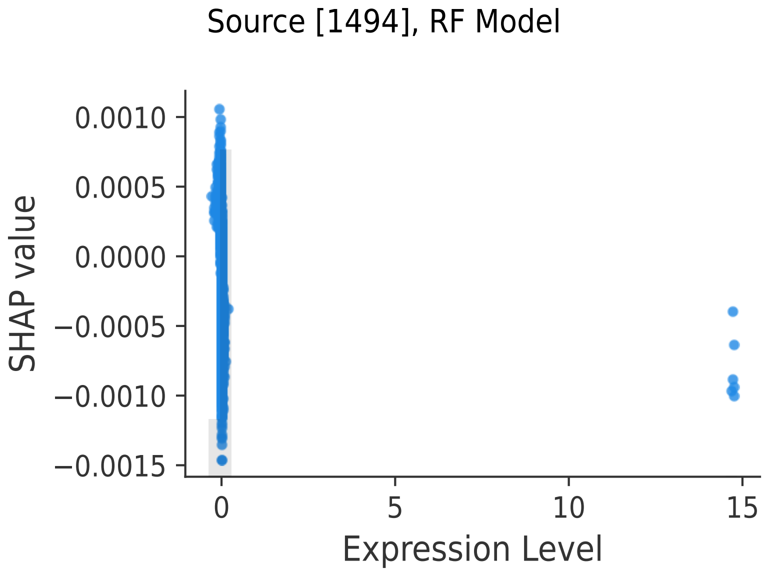
Source [1494], RF Model
SHAP value
0.0010
0.0005
0.0000
−0.0005
−0.0010
−0.0015
0
5
10
15
Expression Level

Source [1495], RF Model

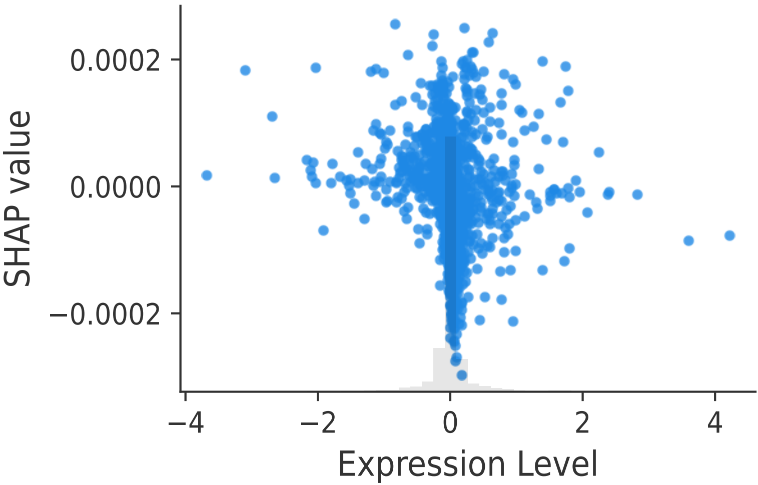

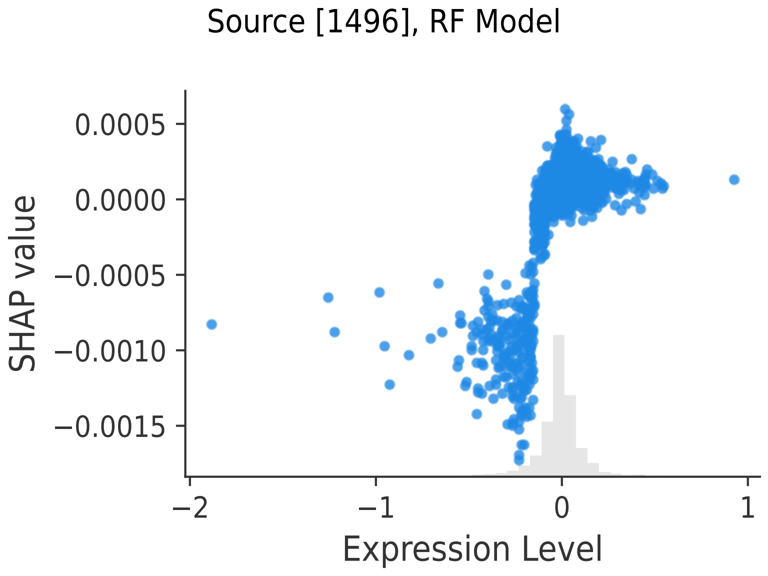
Source [1496], RF Model
SHAP value
0.0005
0.0000
−0.0005
−0.0010
−0.0015
−2
−1
0
1
Expression Level

Source [1497], RF Model

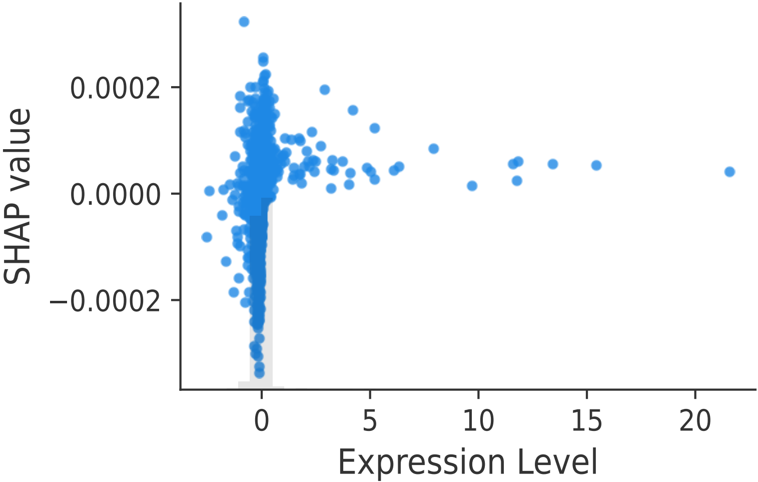

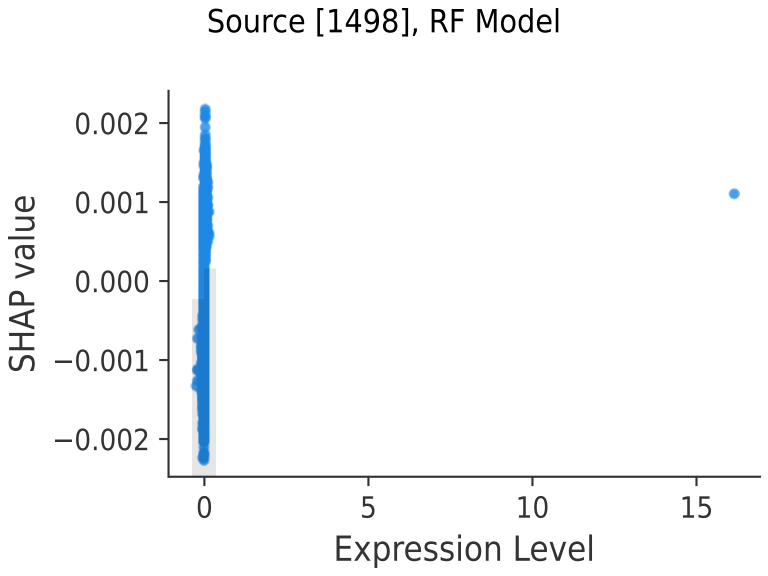
Source [1498], RF Model
SHAP value
0.002
0.001
0.000
−0.001
−0.002
0
5
10
15
Expression Level

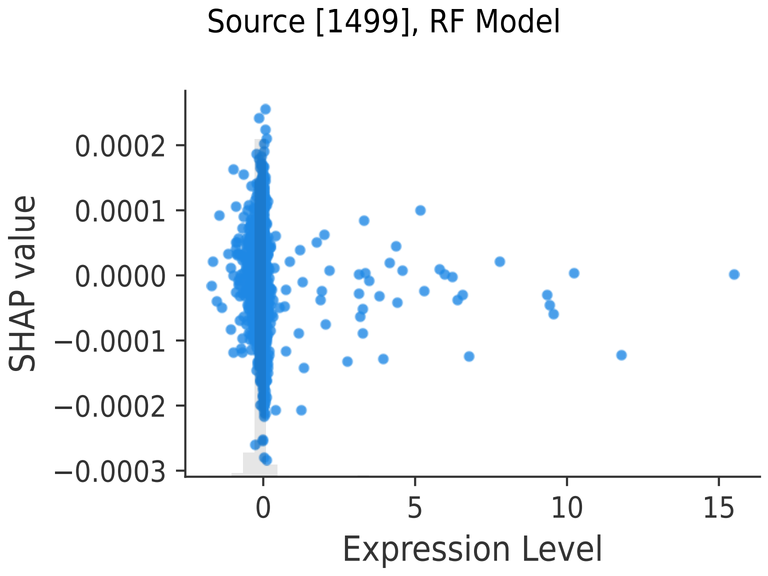
Source [1499], RF Model
SHAP value
0.0002
0.0001
0.0000
−0.0001
−0.0002
−0.0003
0
5
10
15
Expression Level

Source [1500], RF Model

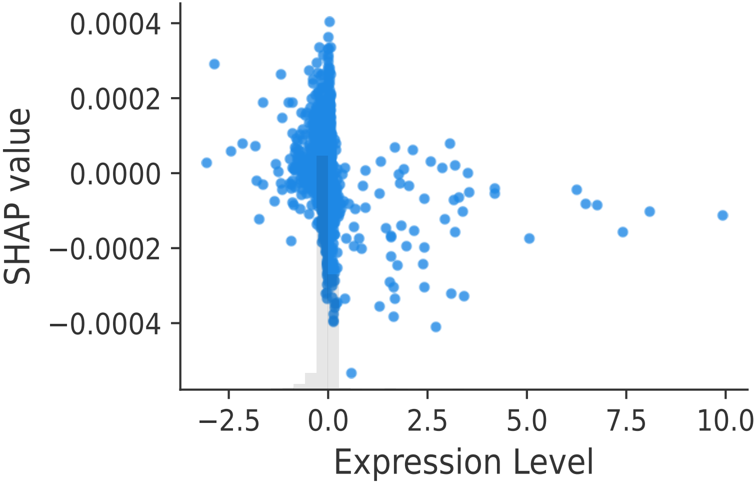

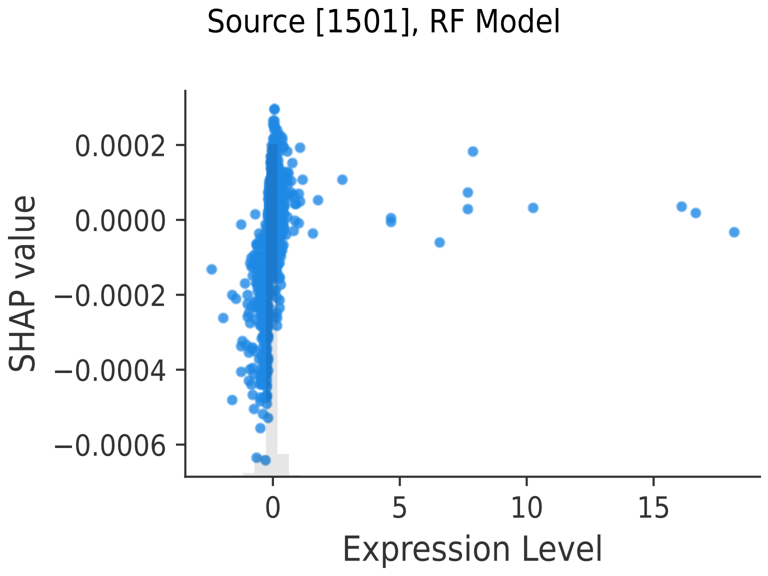

Source [1501], RF Model
SHAP value
0.0002
0.0000
−0.0002
−0.0004
−0.0006
0
5
10
15
Expression Level

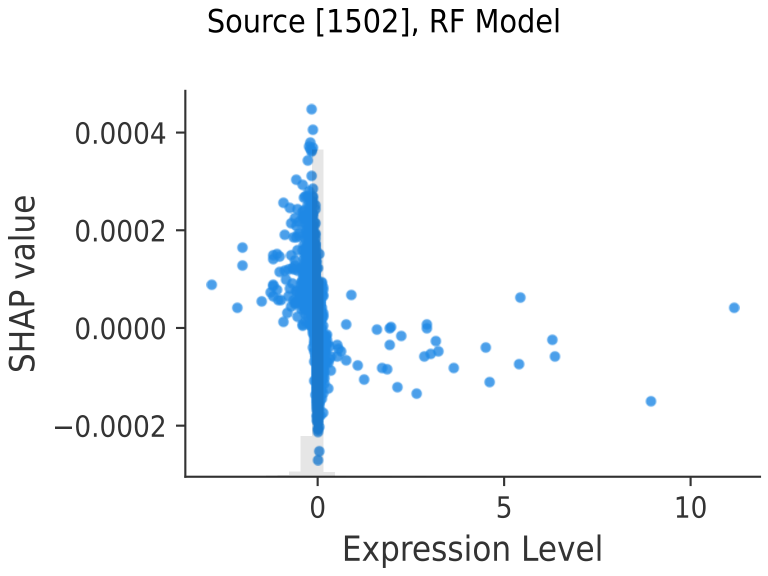

Source [1502], RF Model
0.0004
0.0002
0.0000
−0.0002
SHAP value
0
5
10
Expression Level

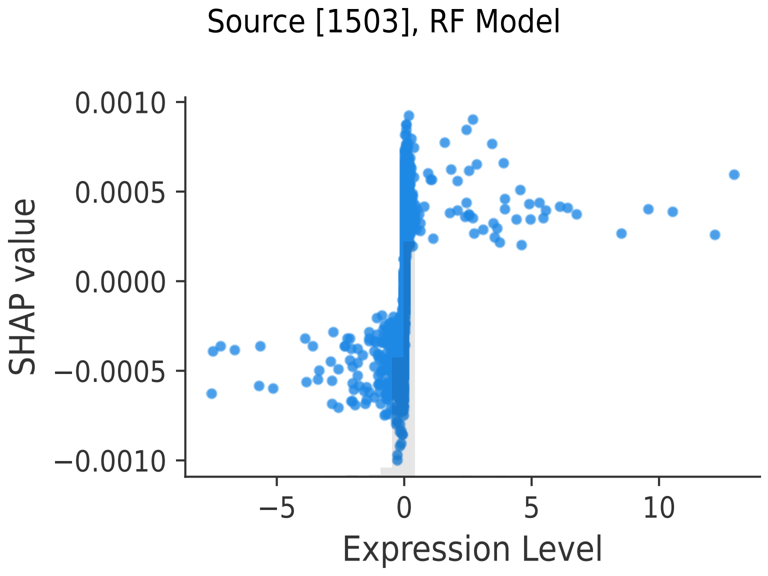
Source [1503], RF Model
SHAP value
0.0010
0.0005
0.0000
−0.0005
−0.0010
−5
0
5
10
Expression Level

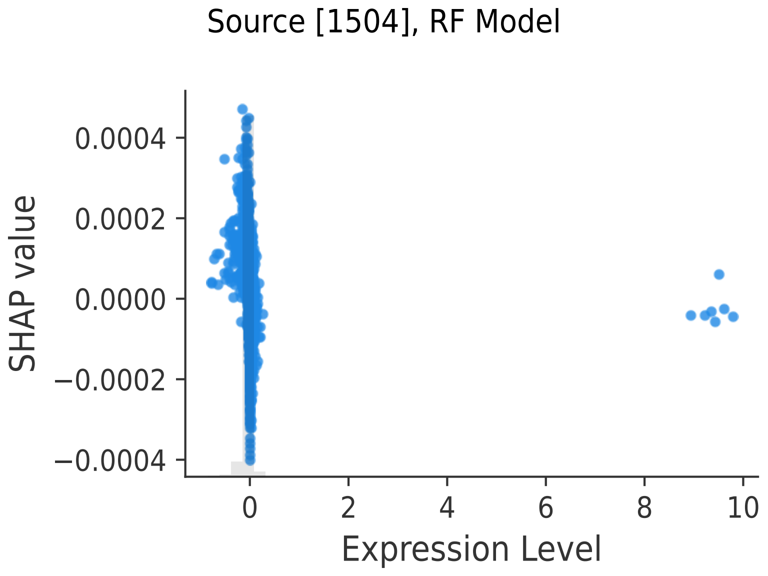
Source [1504], RF Model
SHAP value
0.0004
0.0002
0.0000
−0.0002
−0.0004
0
2
4
6
8
10
Expression Level

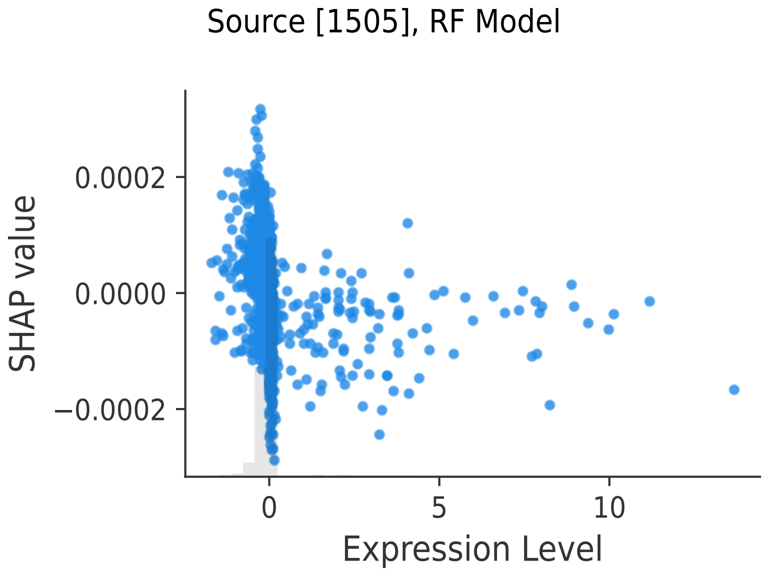

Source [1505], RF Model
0.0002
0.0000
−0.0002
SHAP value
0
5
10
Expression Level

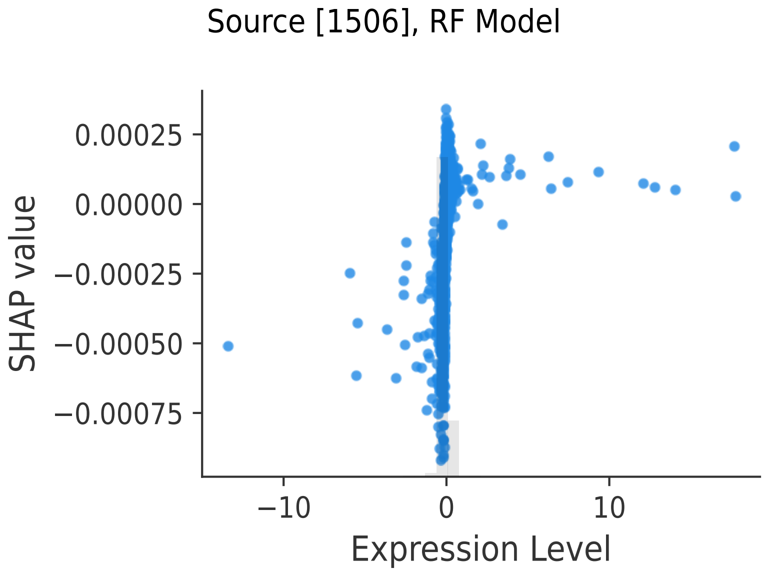

Source [1506], RF Model
SHAP value
0.00025
0.00000
−0.00025
−0.00050
−0.00075
−10
0
10
Expression Level

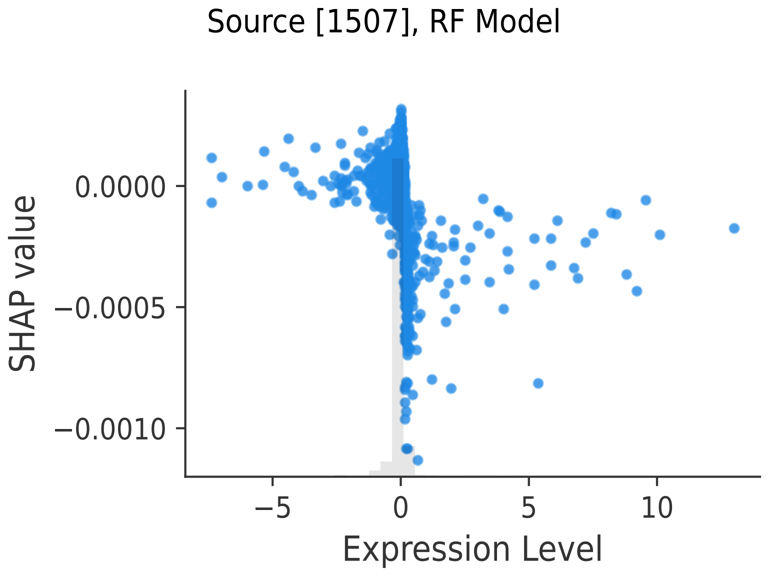
Source [1507], RF Model
SHAP value
0.0000
−0.0005
−0.0010
−5
0
5
10
Expression Level

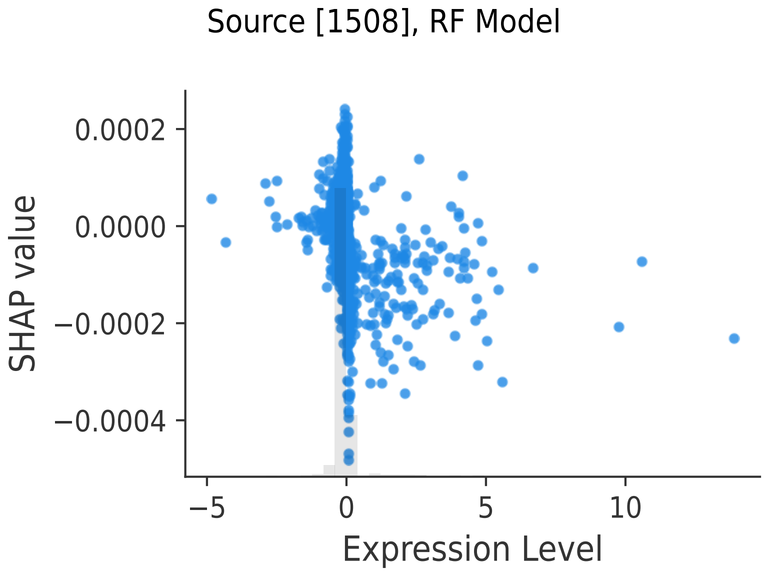
Source [1508], RF Model
0.0002
0.0000
−0.0002
−0.0004
SHAP value
−5
0
5
10
Expression Level

Source [1509], RF Model

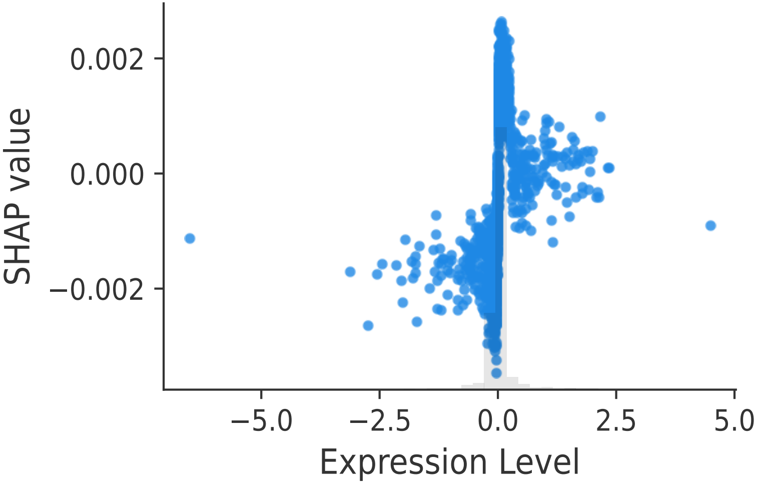

Source [1510], RF Model

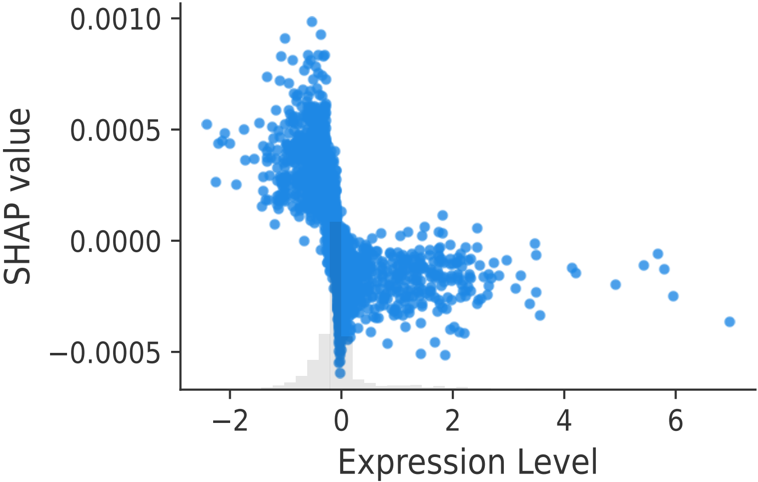

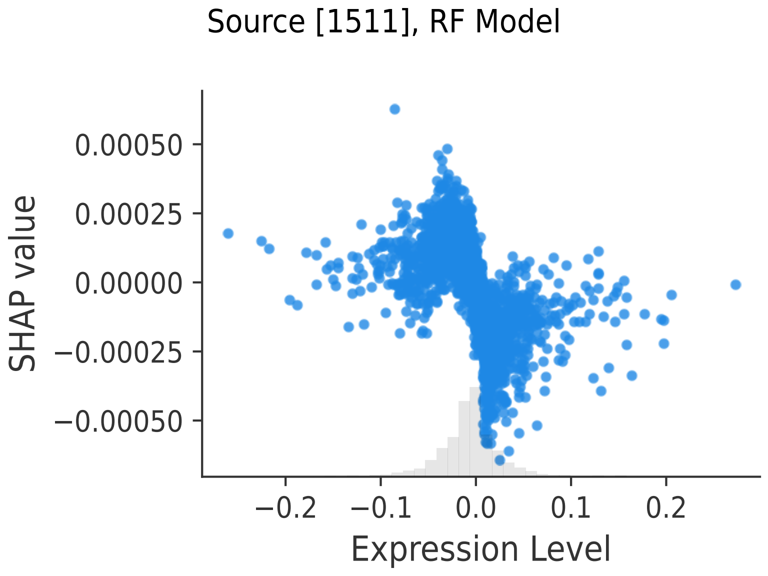

Source [1511], RF Model
0.00050
0.00025
0.00000
−0.00025
−0.00050
SHAP value
−0.2
−0.1
0.0
0.1
0.2
Expression Level

Source [1512], RF Model

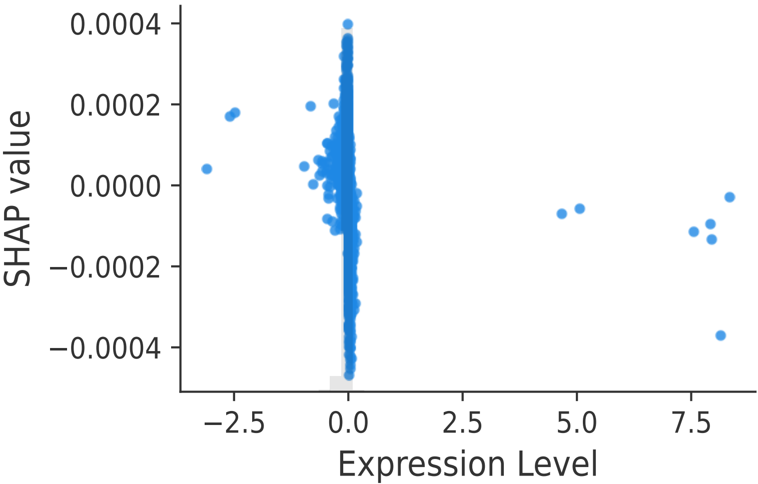

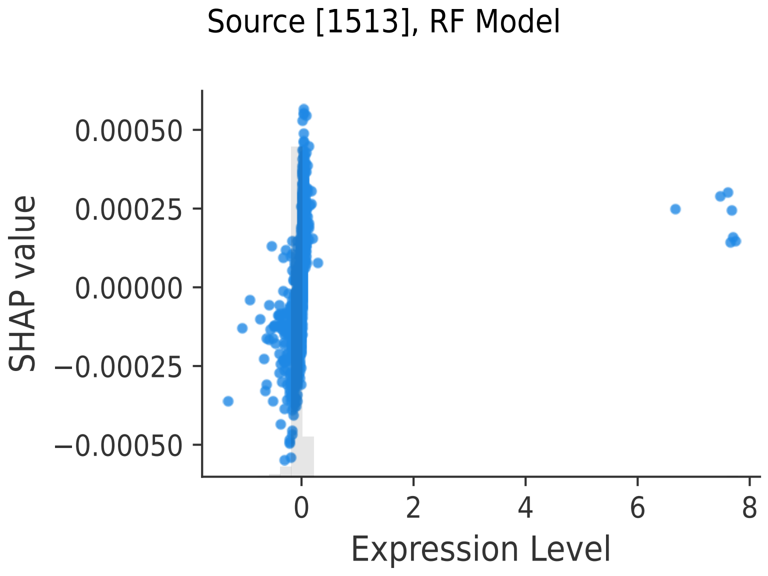
Source [1513], RF Model
SHAP value
0.00050
0.00025
0.00000
−0.00025
−0.00050
0
2
4
6
8
Expression Level

Source [1514], RF Model

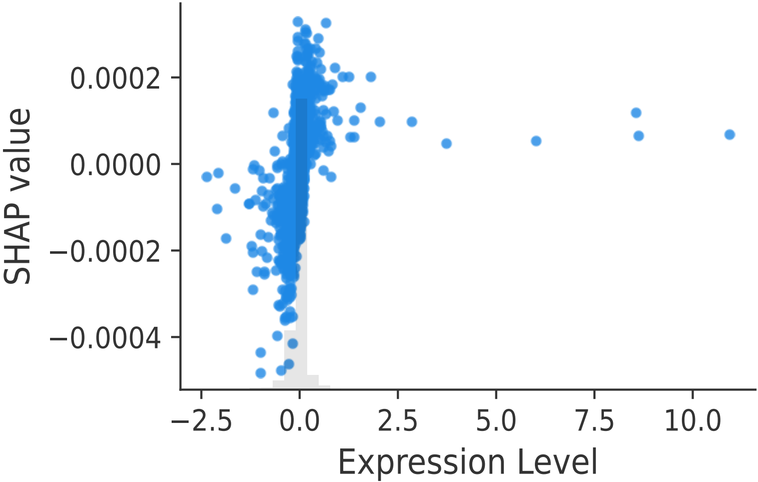

Source [1515], RF Model

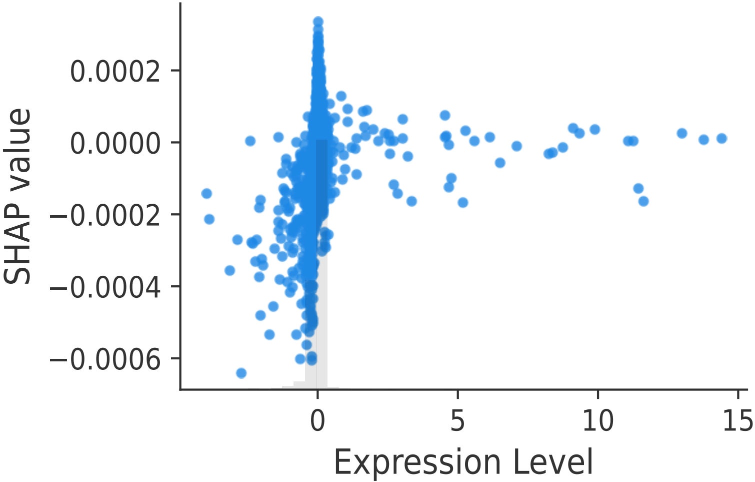

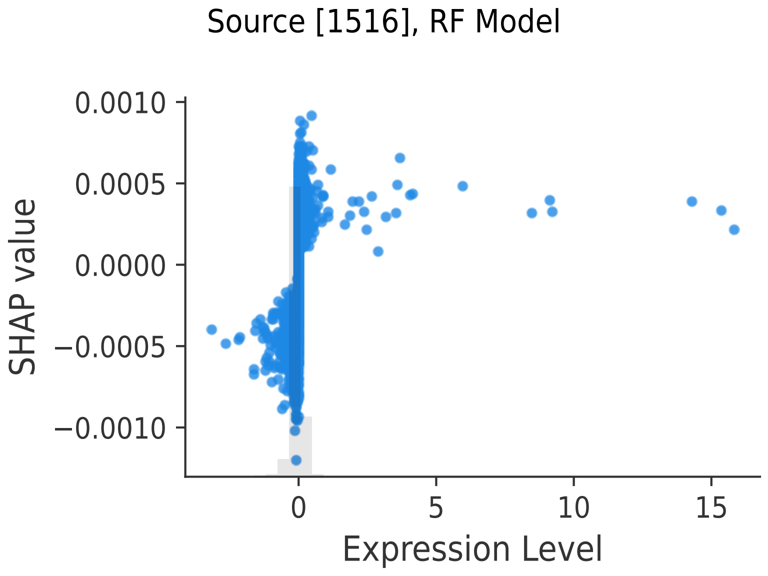

Source [1516], RF Model
0.0010
0.0005
0.0000
−0.0005
−0.0010
SHAP value
0
5
10
15
Expression Level

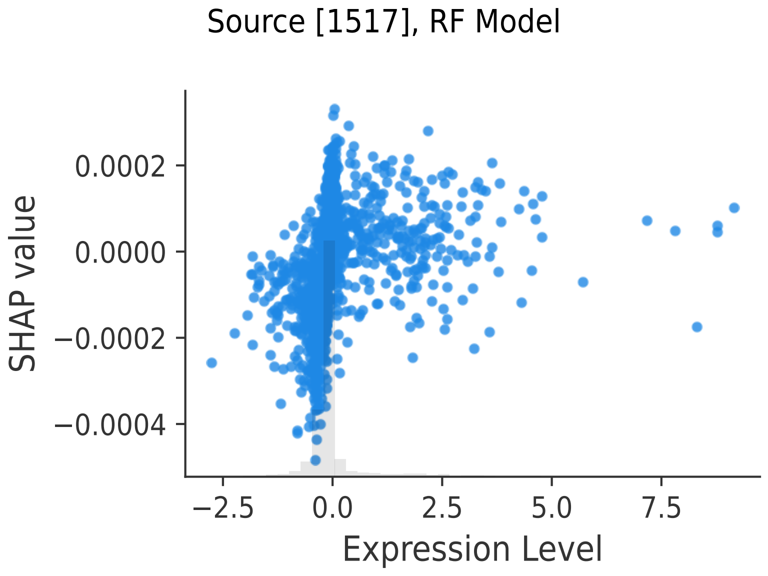

Source [1517], RF Model
SHAP value
0.0002
0.0000
−0.0002
−0.0004
−2.5
0.0
2.5
5.0
7.5
Expression Level

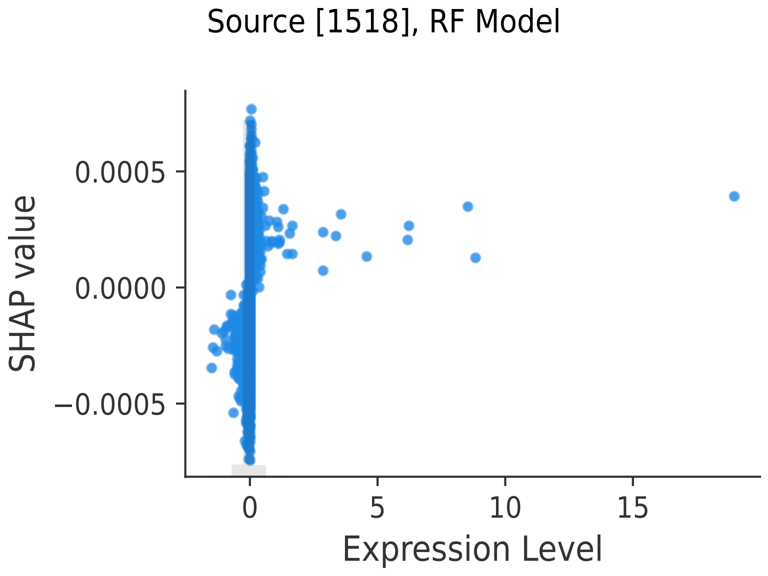

Source [1518], RF Model
0.0005
0.0000
−0.0005
SHAP value
0
5
10
15
Expression Level

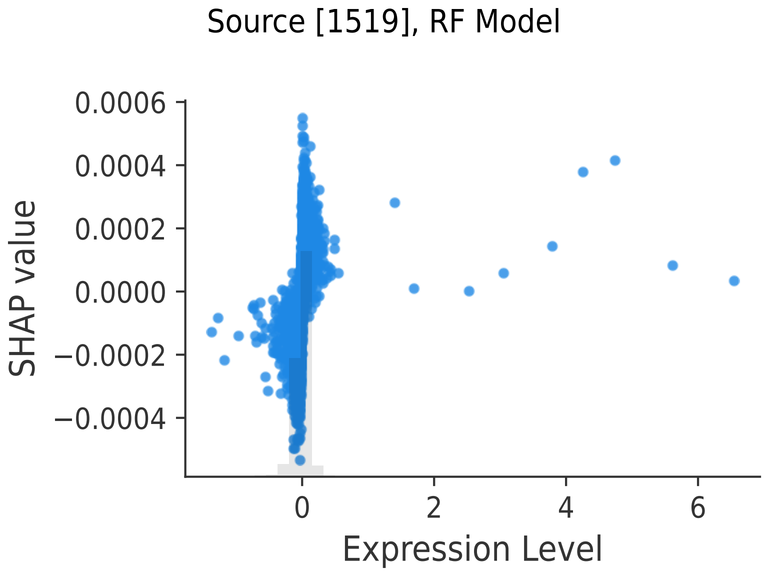
Source [1519], RF Model
0.0006
0.0004
0.0002
0.0000
−0.0002
−0.0004
SHAP value
0
2
4
6
Expression Level

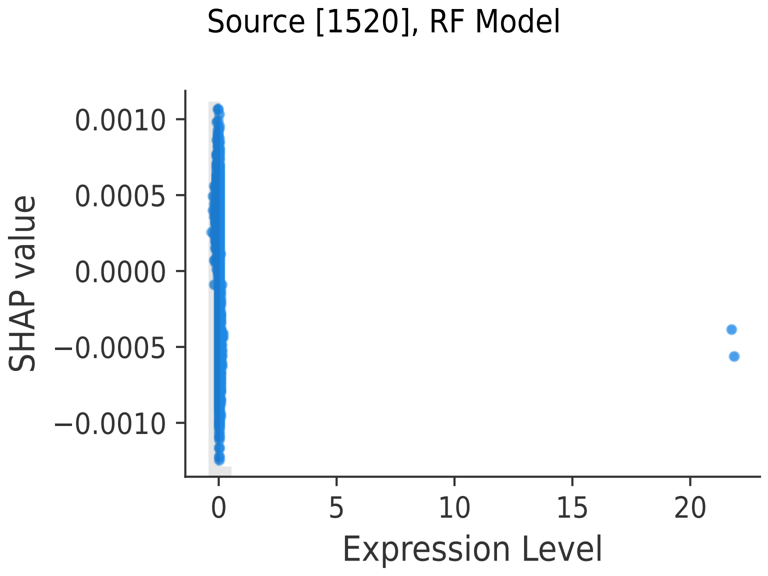
Source [1520], RF Model
SHAP value
0.0010
0.0005
0.0000
−0.0005
−0.0010
Expression Level
0
5
10
15
20

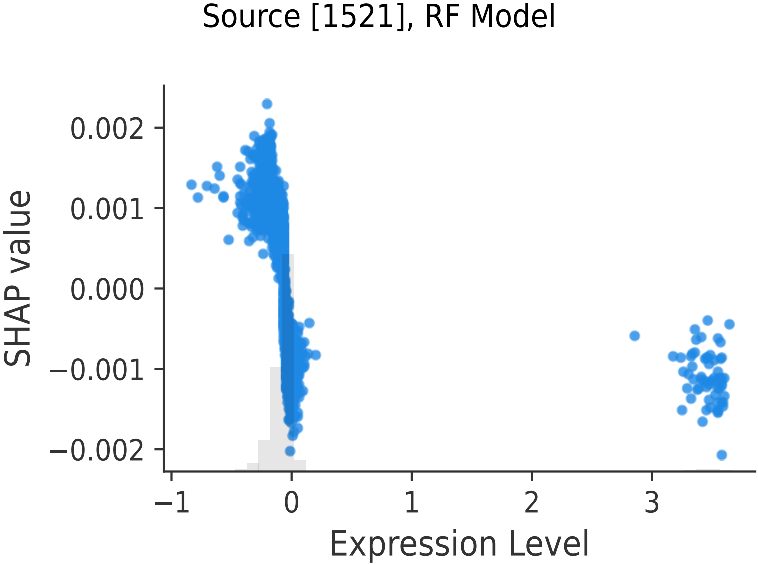
Source [1521], RF Model
SHAP value
0.002
0.001
0.000
−0.001
−0.002
−1
0
1
2
3
Expression Level

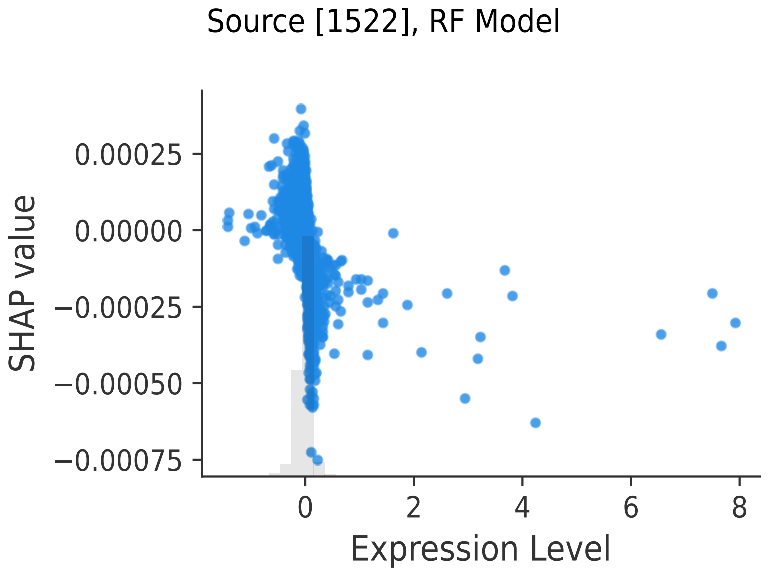
Source [1522], RF Model
SHAP value
0.00025
0.00000
−0.00025
−0.00050
−0.00075
Expression Level
0
2
4
6
8

Source [1523], RF Model

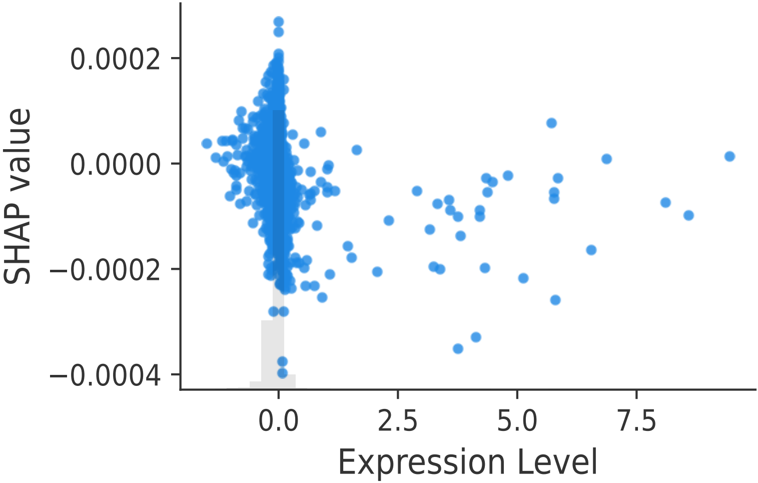

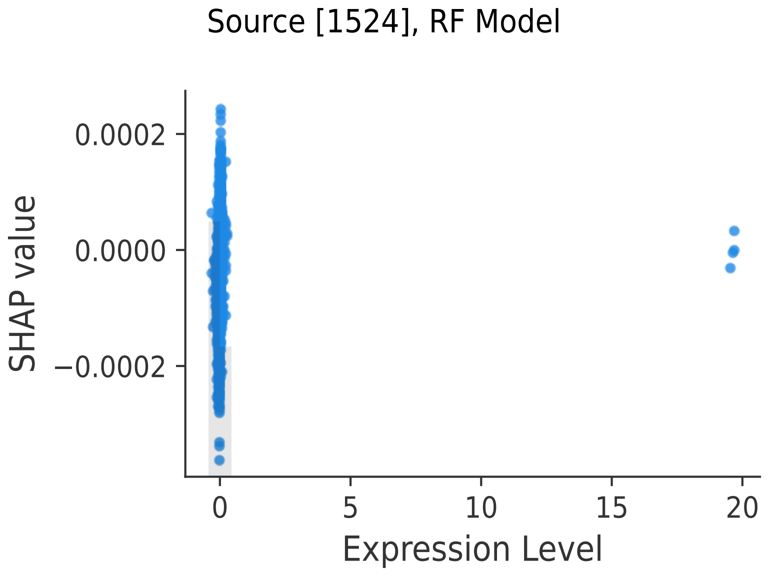

Source [1524], RF Model
0.0002
0.0000
−0.0002
SHAP value
0
5
10
15
20
Expression Level

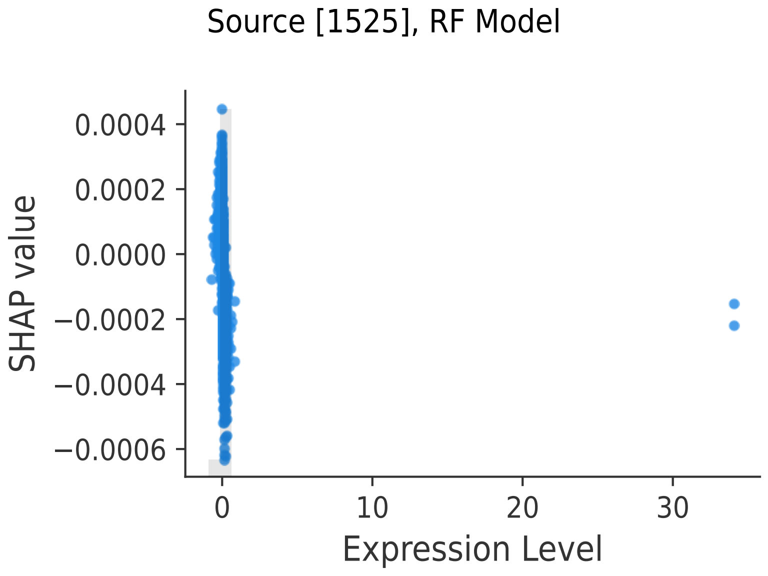

Source [1525], RF Model
0.0004
0.0002
0.0000
−0.0002
−0.0004
−0.0006
SHAP value
0
10
20
30
Expression Level

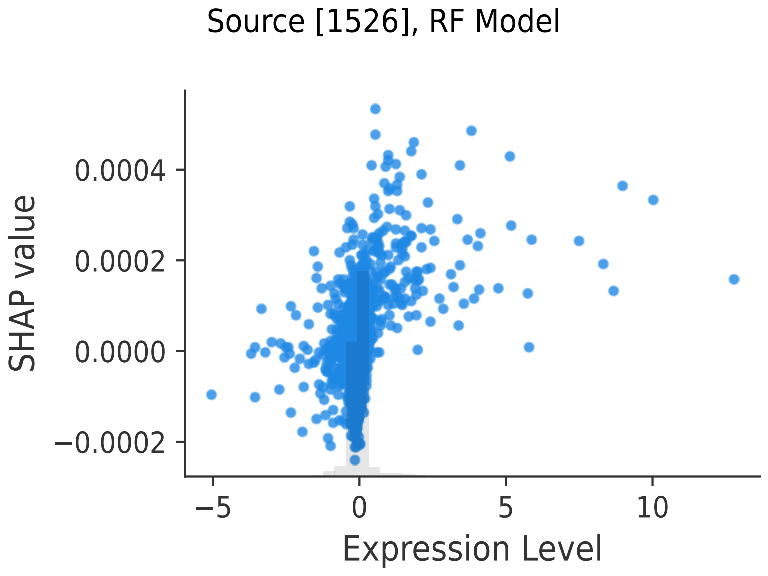
Source [1526], RF Model
SHAP value
0.0004
0.0002
0.0000
−0.0002
−5
0
5
10
Expression Level

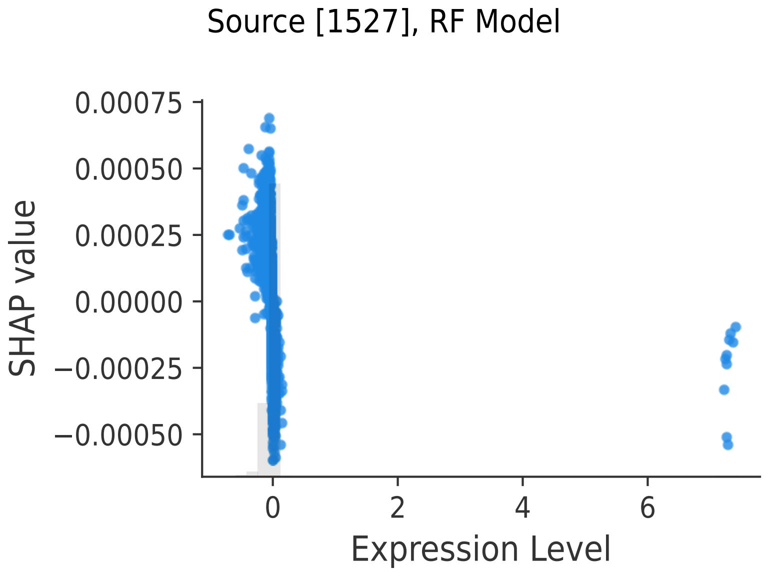
Source [1527], RF Model
SHAP value
0.00075
0.00050
0.00025
0.00000
−0.00025
−0.00050
0
2
4
6
Expression Level

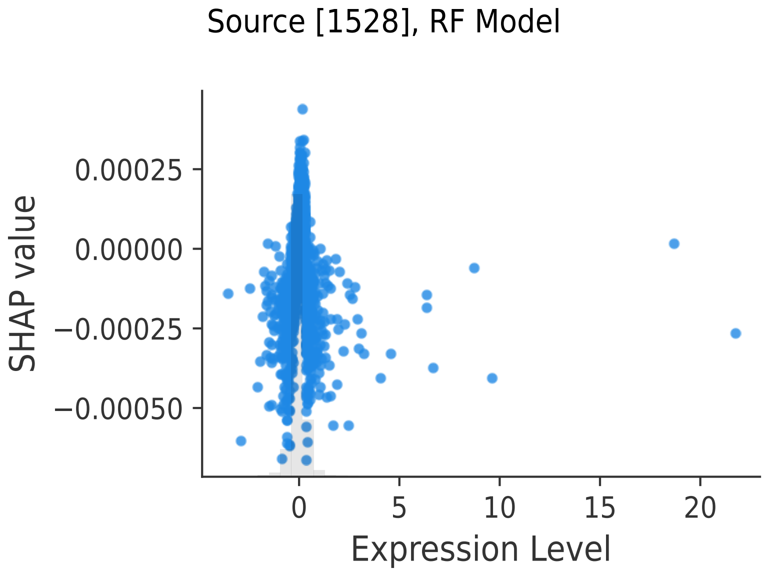

Source [1528], RF Model
SHAP value
0.00025
0.00000
−0.00025
−0.00050
0
5
10
15
20
Expression Level

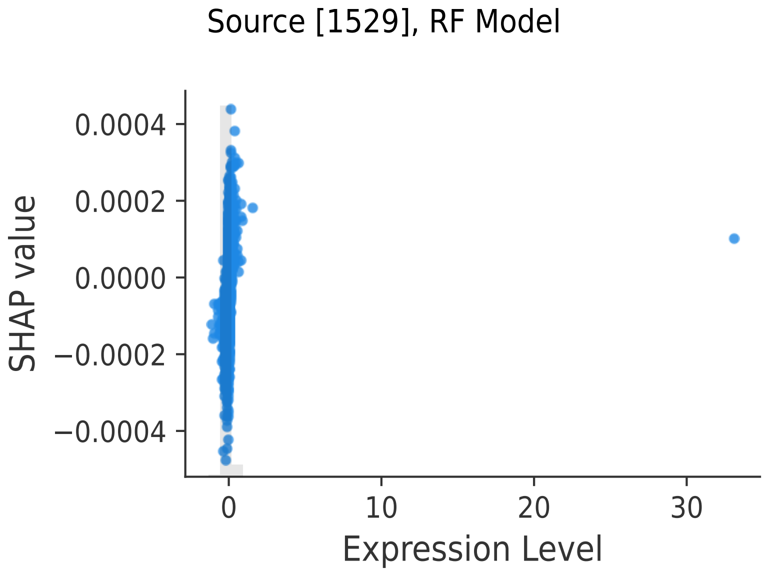
Source [1529], RF Model
0.0004
0.0002
0.0000
−0.0002
−0.0004
SHAP value
0
10
20
30
Expression Level

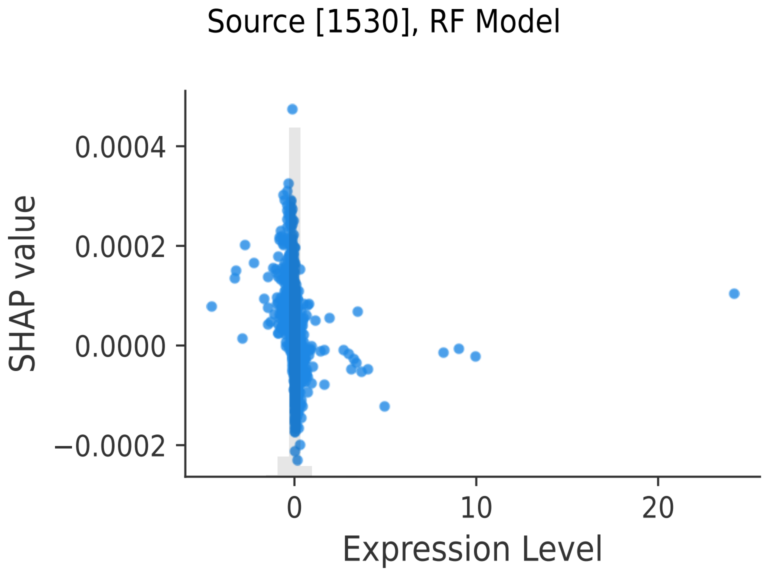

Source [1530], RF Model
0.0004
0.0002
0.0000
−0.0002
SHAP value
0
10
20
Expression Level

Source [1531], RF Model

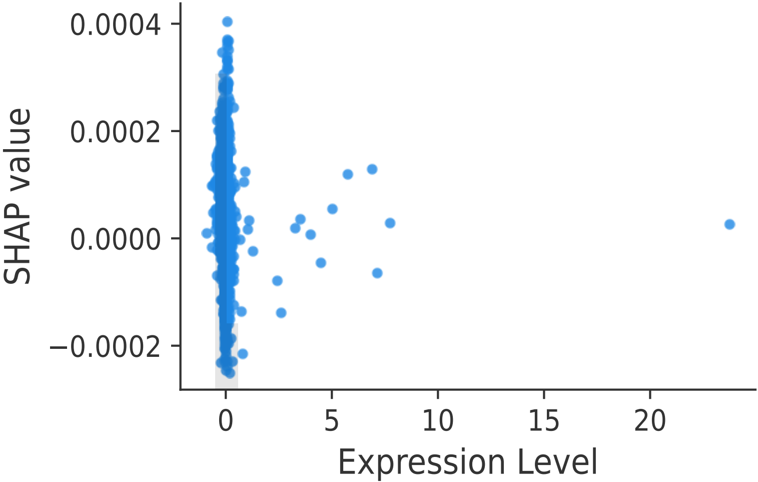

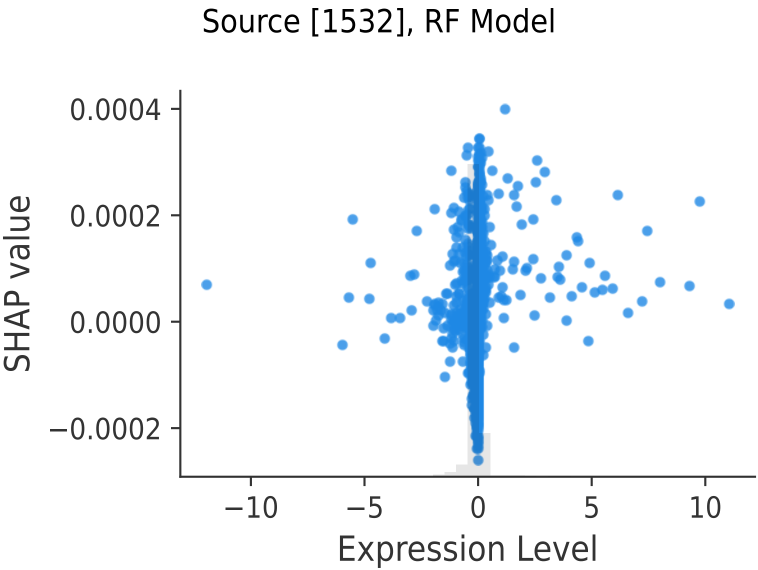

Source [1532], RF Model
SHAP value
0.0004
0.0002
0.0000
−0.0002
−10
−5
0
5
10
Expression Level

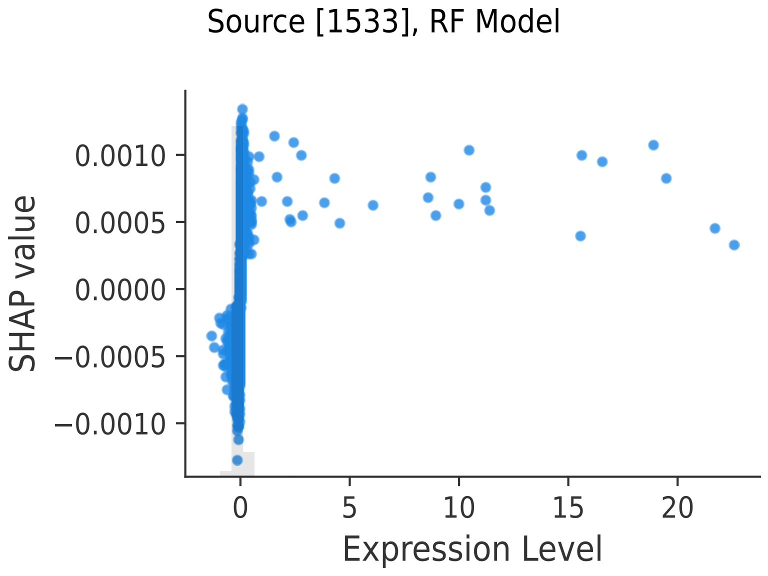
Source [1533], RF Model
SHAP value
0.0010
0.0005
0.0000
−0.0005
−0.0010
0
5
10
15
20
Expression Level

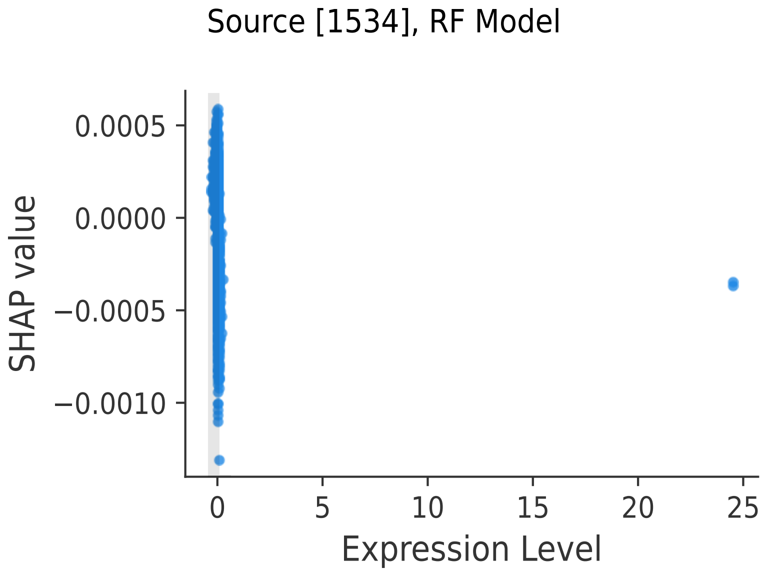
Source [1534], RF Model
SHAP value
0.0005
0.0000
−0.0005
−0.0010
0
5
10
15
20
25
Expression Level

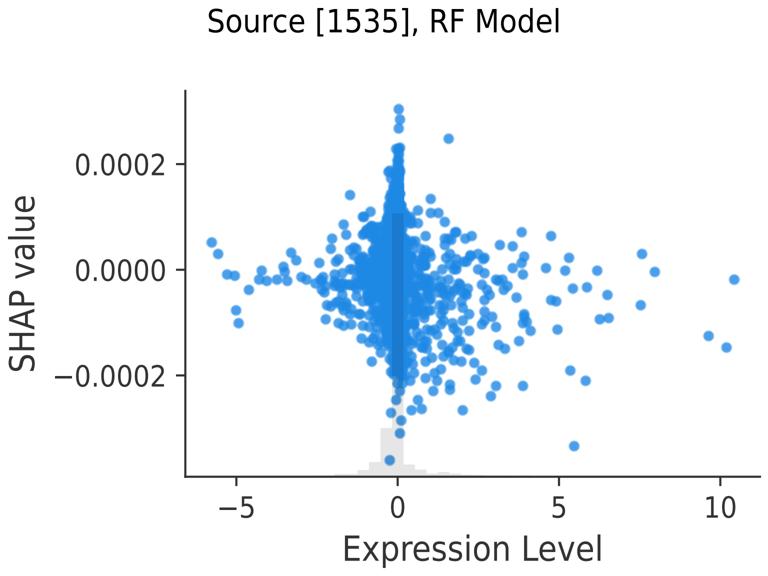
Source [1535], RF Model
0.0002
0.0000
−0.0002
SHAP value
−5
0
5
10
Expression Level

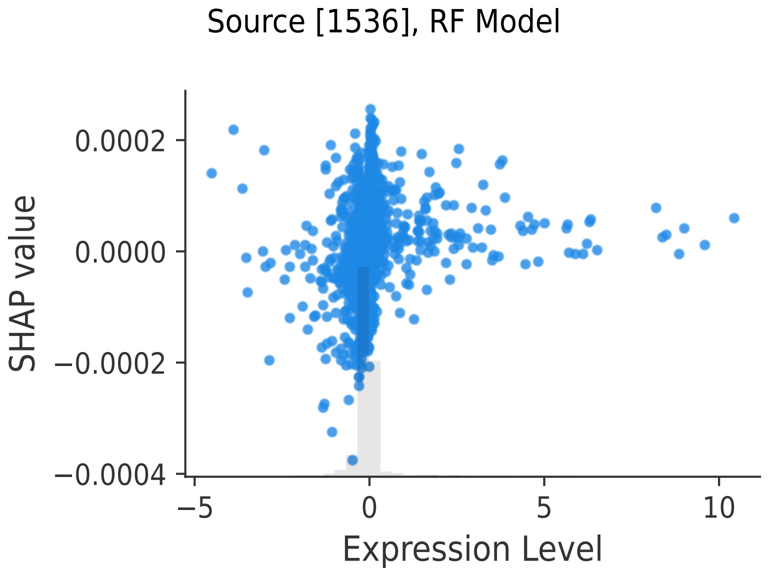

Source [1536], RF Model
SHAP value
0.0002
0.0000
−0.0002
−0.0004
Expression Level
−5
0
5
10

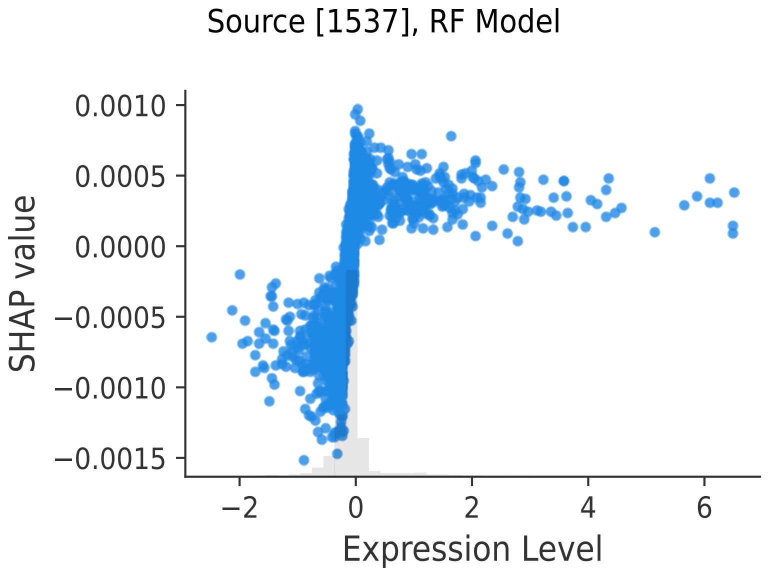

Source [1537], RF Model
SHAP value
0.0010
0.0005
0.0000
−0.0005
−0.0010
−0.0015
−2
0
2
4
6
Expression Level

Source [1538], RF Model

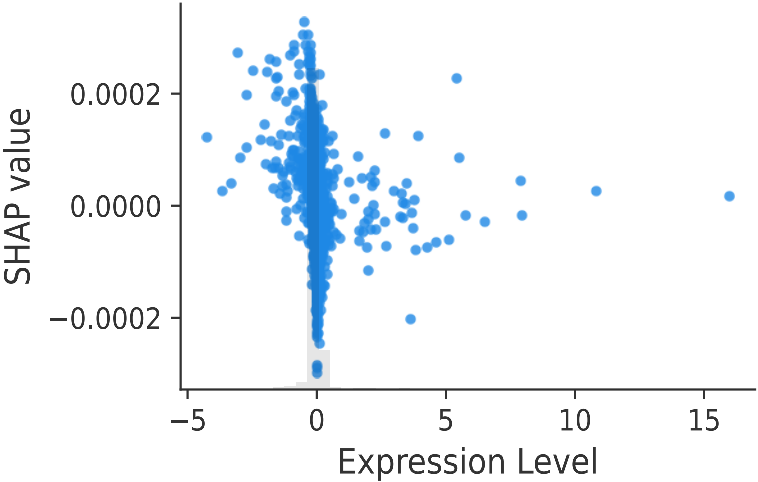

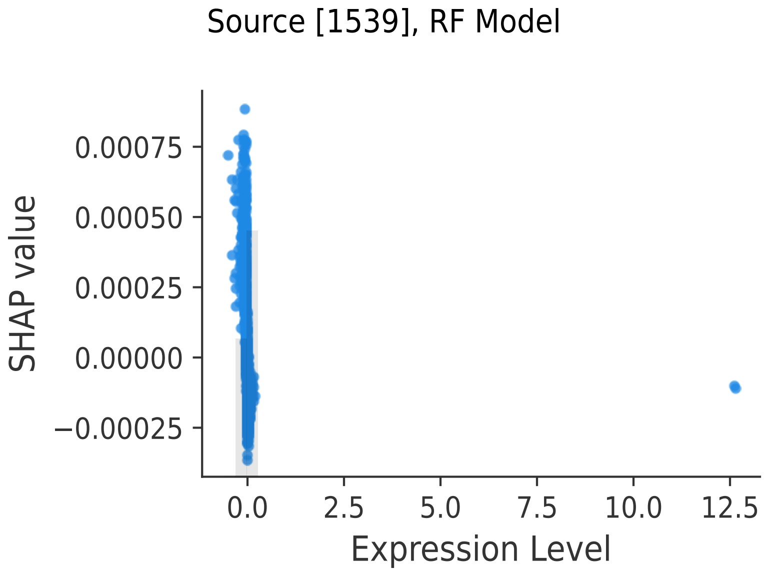
Source [1539], RF Model
SHAP value
0.00075
0.00050
0.00025
0.00000
−0.00025
0.0
2.5
5.0
7.5
10.0
12.5
Expression Level

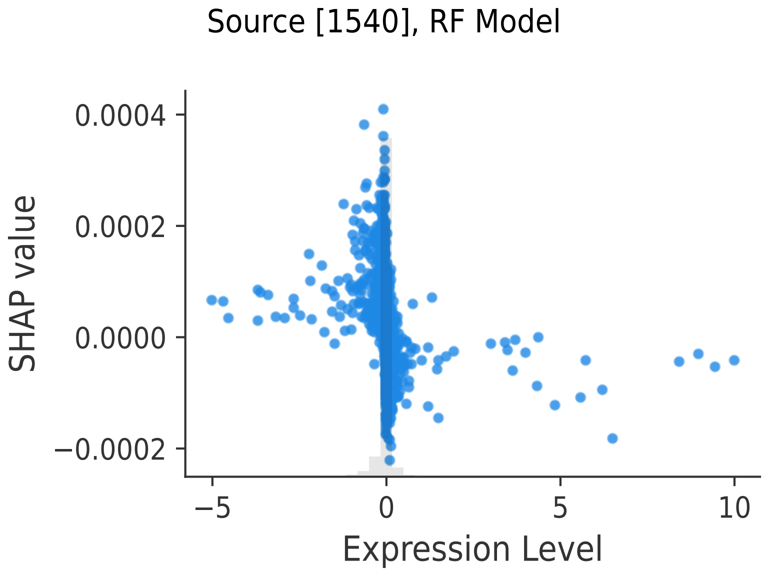
Source [1540], RF Model
SHAP value
0.0004
0.0002
0.0000
−0.0002
−5
0
5
10
Expression Level

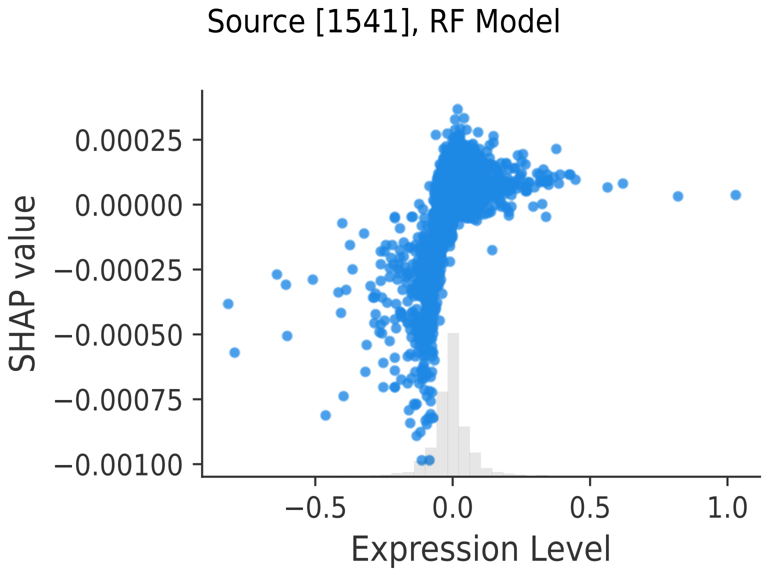

Source [1541], RF Model
0.00025
0.00000
−0.00025
−0.00050
−0.00075
−0.00100
SHAP value
−0.5
0.0
0.5
1.0
Expression Level

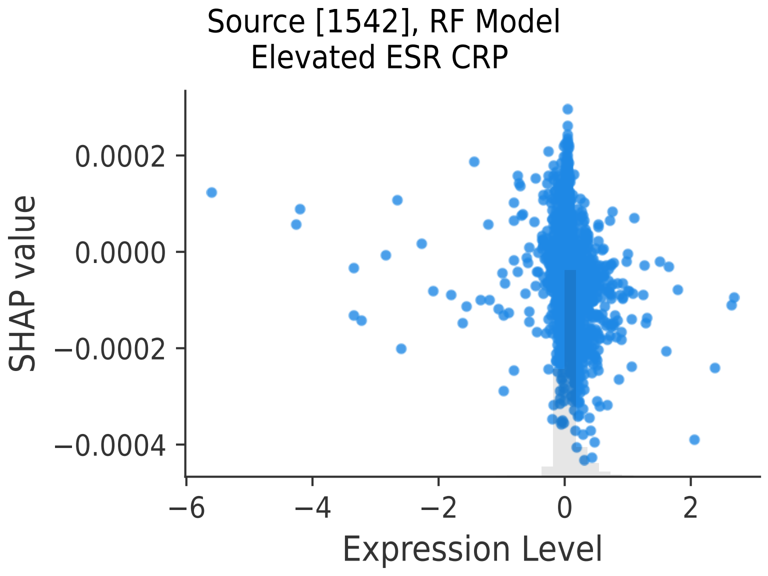

Source [1542], RF Model
Elevated ESR CRP
SHAP value
0.0002
0.0000
−0.0002
−0.0004
−6
−4
−2
0
2
Expression Level

Source [1543], RF Model

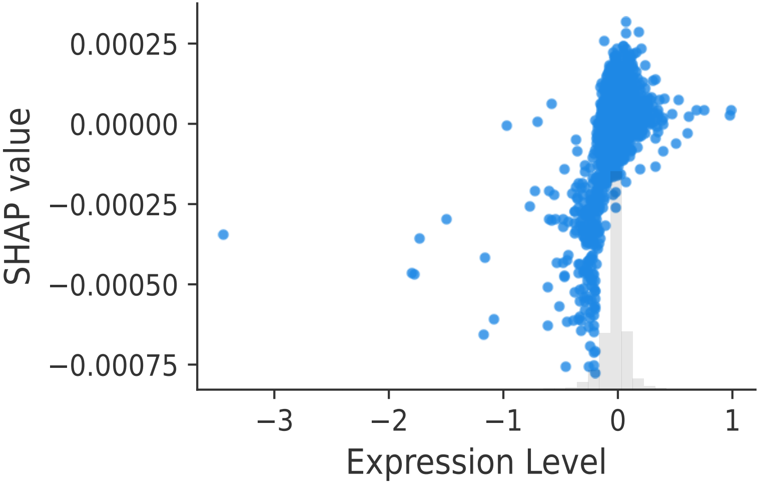

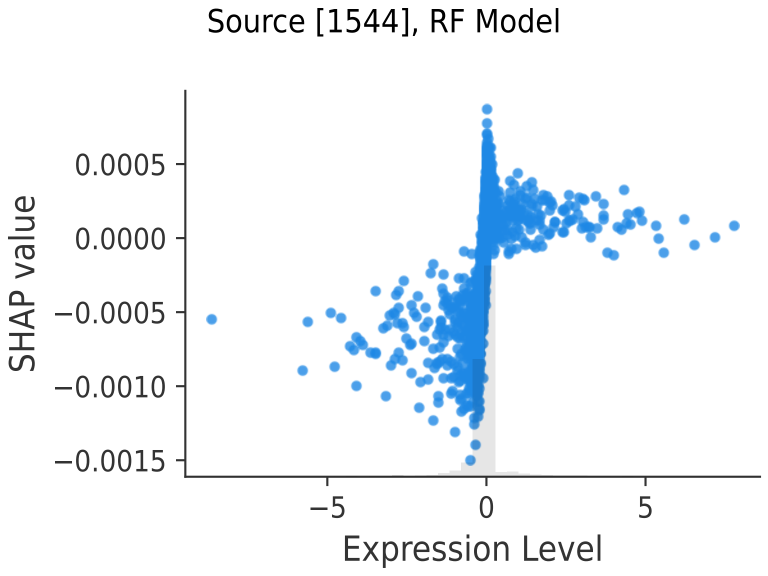
Source [1544], RF Model
SHAP value
0.0005
0.0000
−0.0005
−0.0010
−0.0015
−5
0
5
Expression Level

Source [1545], RF Model

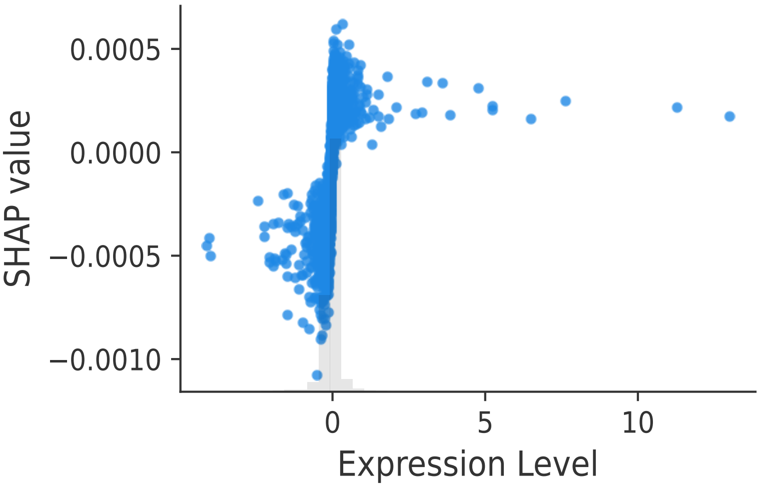

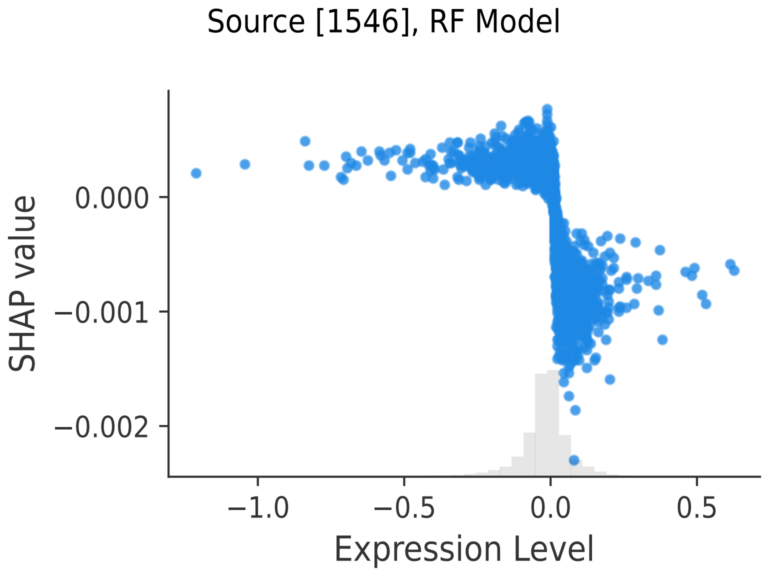
Source [1546], RF Model
SHAP value
0.000
−0.001
−0.002
−1.0
−0.5
0.0
0.5
Expression Level

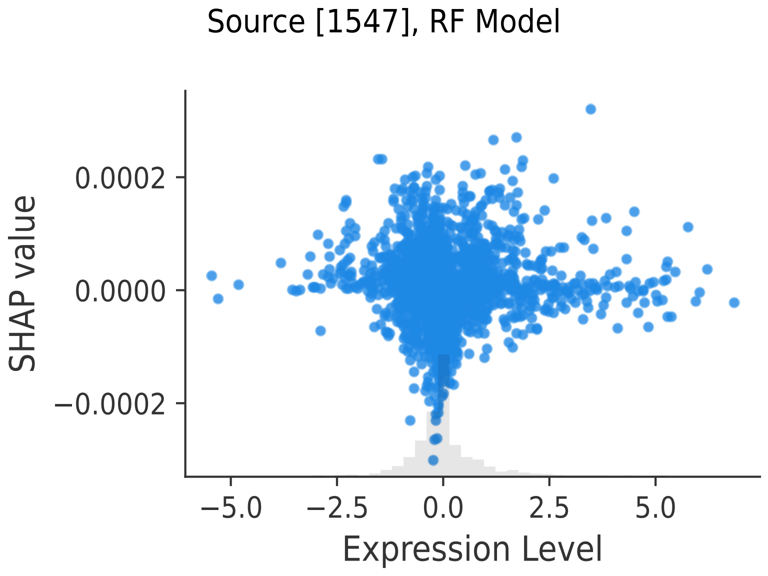

Source [1547], RF Model
0.0002
0.0000
−0.0002
SHAP value
−5.0
−2.5
0.0
2.5
5.0
Expression Level

Source [1548], RF Model

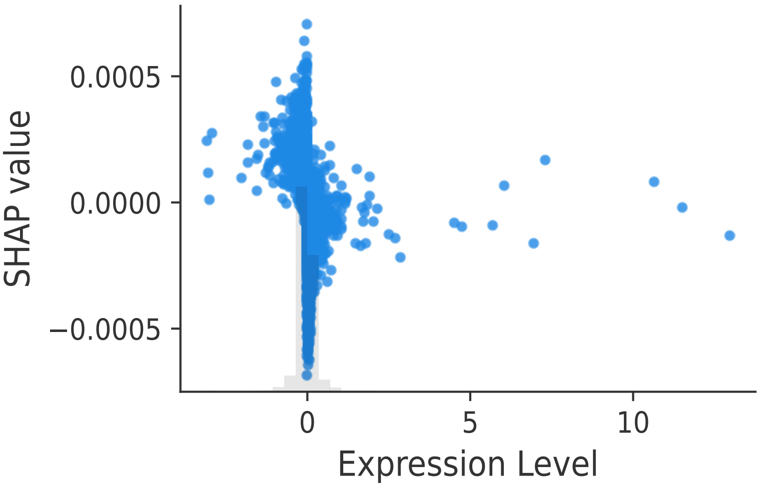

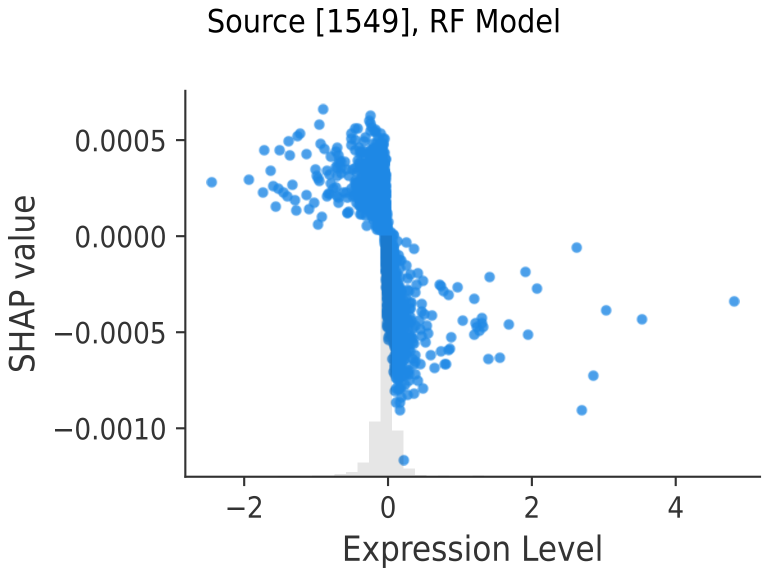
Source [1549], RF Model
0.0005
0.0000
−0.0005
−0.0010
SHAP value
−2
0
2
4
Expression Level

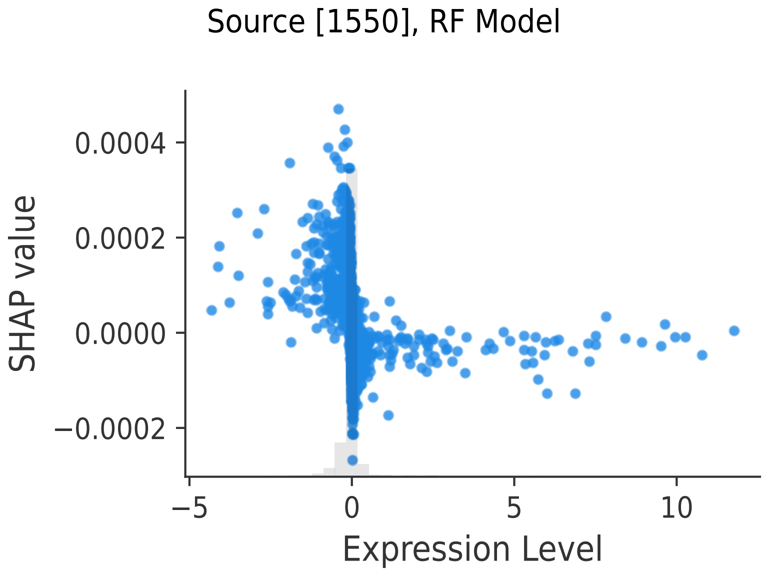

Source [1550], RF Model
0.0004
0.0002
0.0000
−0.0002
SHAP value
−5
0
5
10
Expression Level

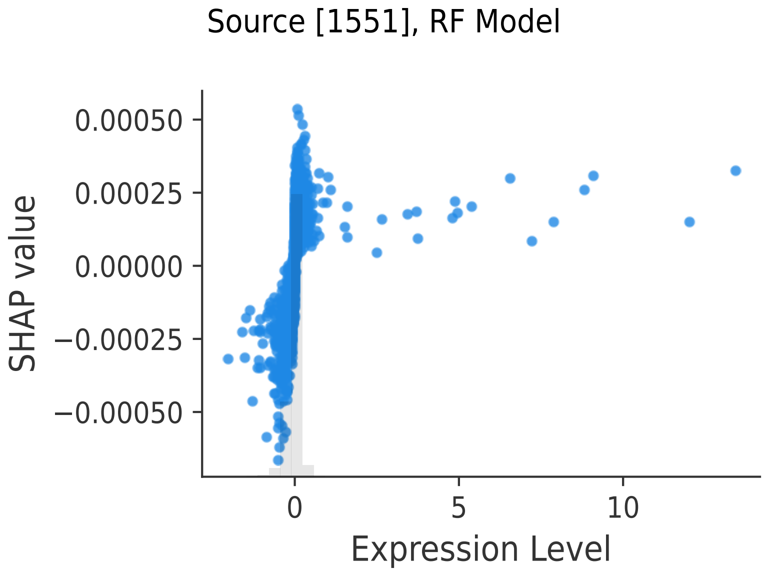
Source [1551], RF Model
0.00050
0.00025
0.00000
−0.00025
−0.00050
SHAP value
0
5
10
Expression Level

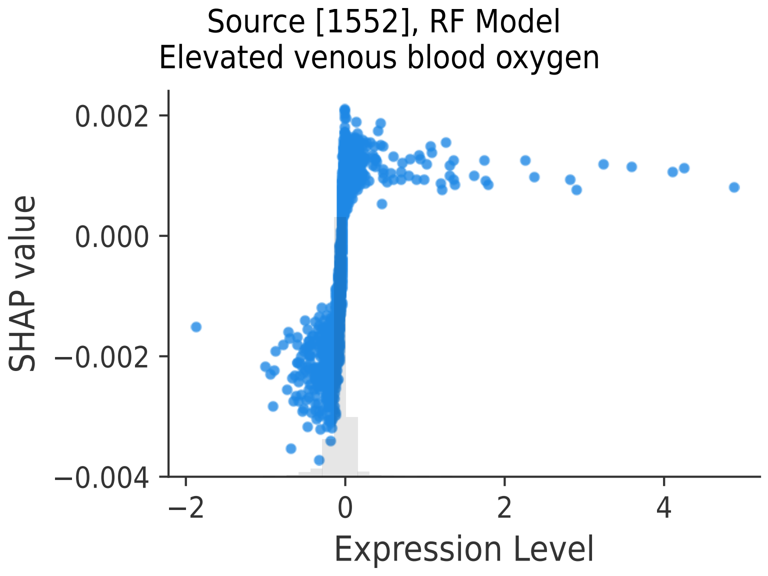

Source [1552], RF Model
Elevated venous blood oxygen
0.002
0.000
−0.002
−0.004
SHAP value
−2
0
2
4
Expression Level

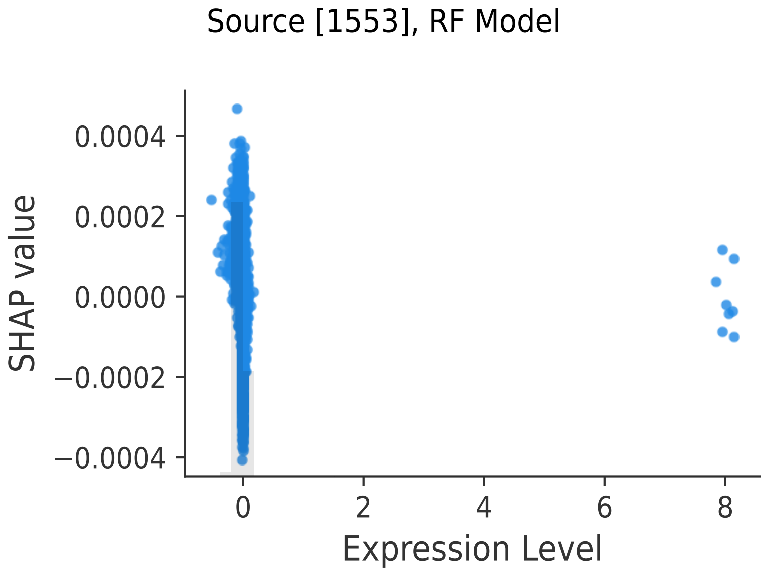
Source [1553], RF Model
SHAP value
0.0004
0.0002
0.0000
−0.0002
−0.0004
0
2
4
6
8
Expression Level

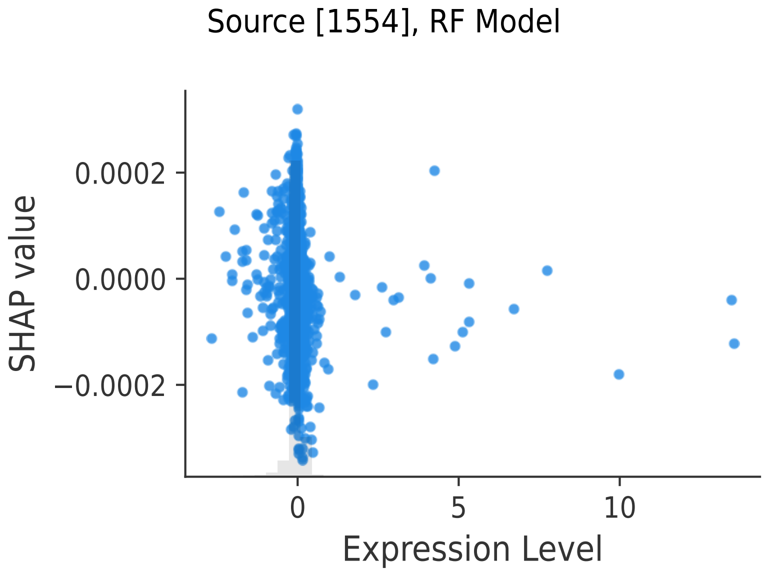

Source [1554], RF Model
SHAP value
0.0002
0.0000
−0.0002
0
5
10
Expression Level

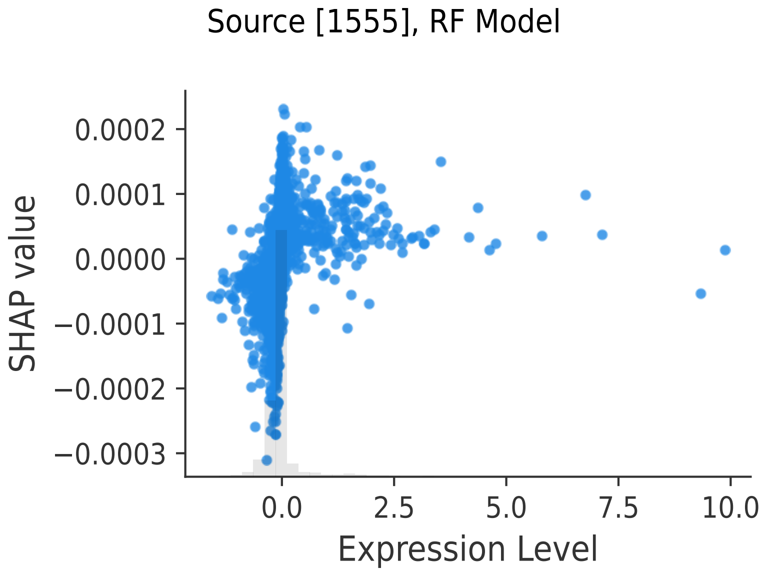

Source [1555], RF Model
SHAP value
0.0002
0.0001
0.0000
−0.0001
−0.0002
−0.0003
0.0
2.5
5.0
7.5
10.0
Expression Level

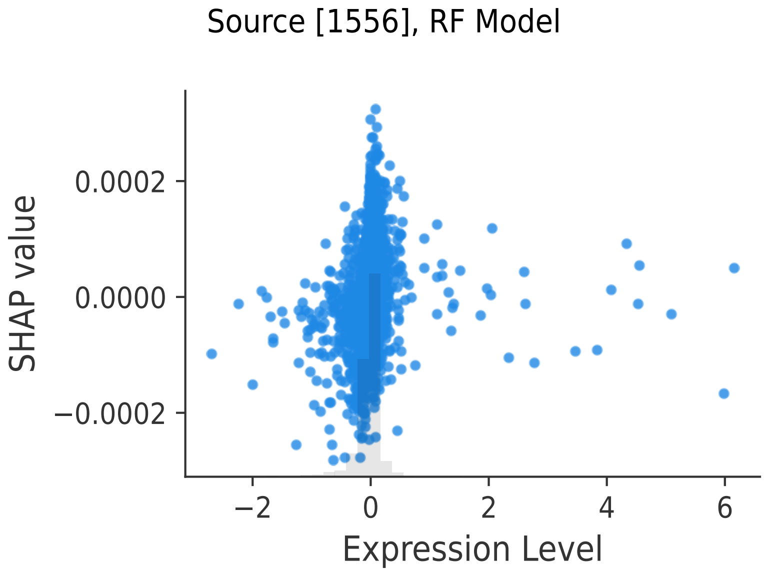
Source [1556], RF Model
SHAP value
0.0002
0.0000
−0.0002
−2
0
2
4
6
Expression Level

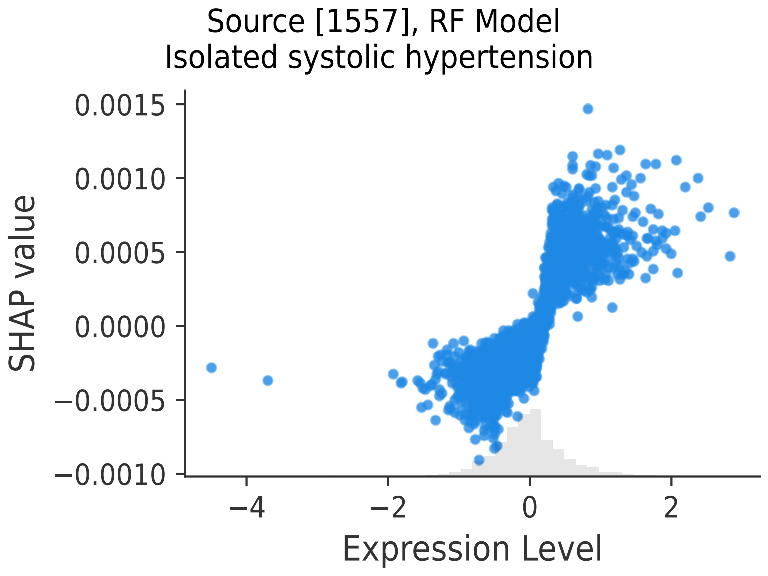
Source [1557], RF Model
Isolated systolic hypertension
SHAP value
0.0015
0.0010
0.0005
0.0000
−0.0005
−0.0010
−4
−2
0
2
Expression Level

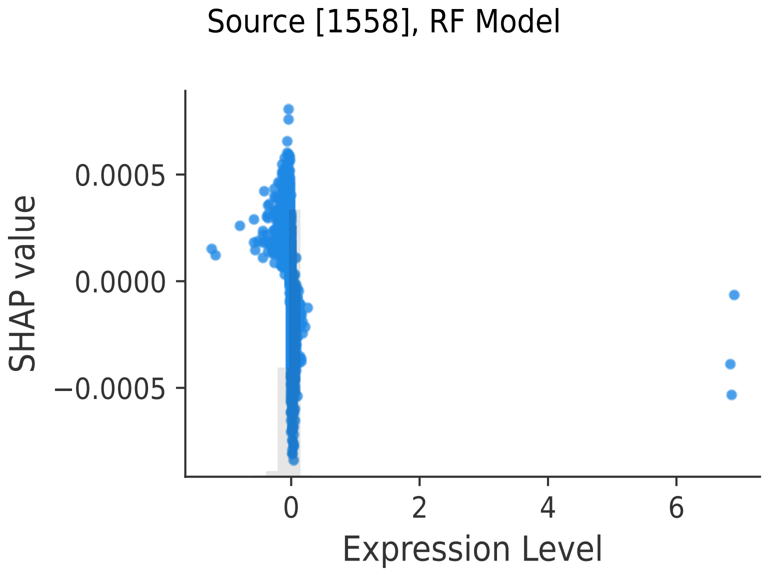

Source [1558], RF Model
SHAP value
0.0005
0.0000
−0.0005
0
2
4
6
Expression Level

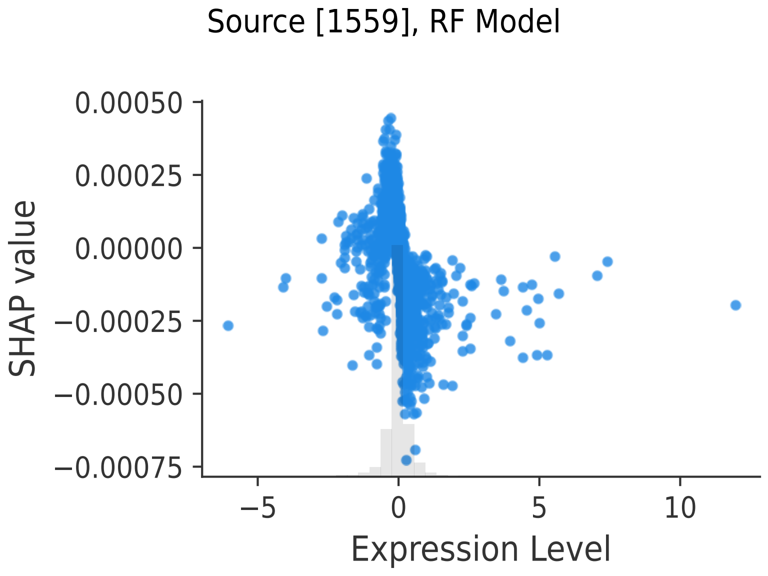

Source [1559], RF Model
0.00050
0.00025
0.00000
−0.00025
−0.00050
−0.00075
SHAP value
−5
0
5
10
Expression Level

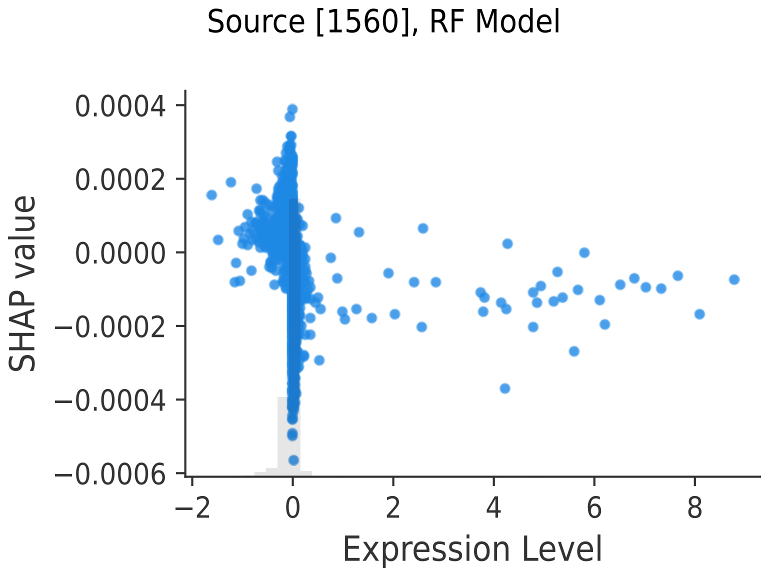
Source [1560], RF Model
SHAP value
0.0004
0.0002
0.0000
−0.0002
−0.0004
−0.0006
−2
0
2
4
6
8
Expression Level

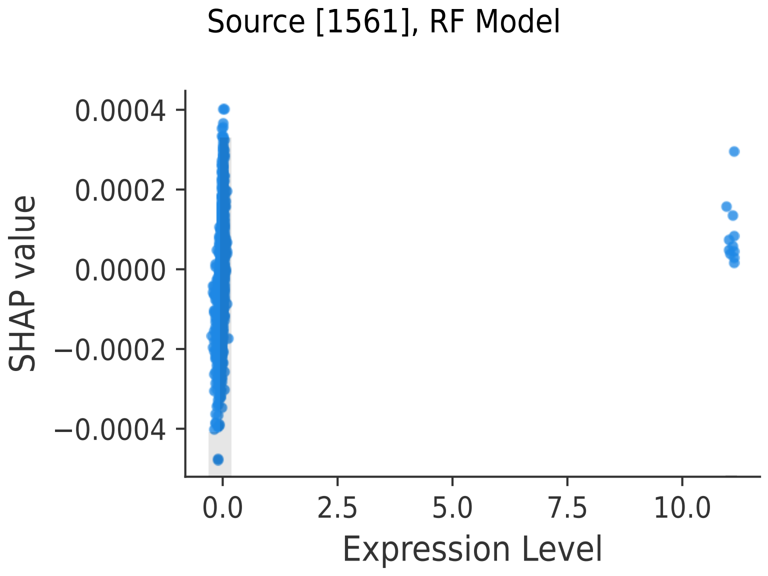

Source [1561], RF Model
0.0004
0.0002
0.0000
−0.0002
−0.0004
SHAP value
0.0
2.5
5.0
7.5
10.0
Expression Level

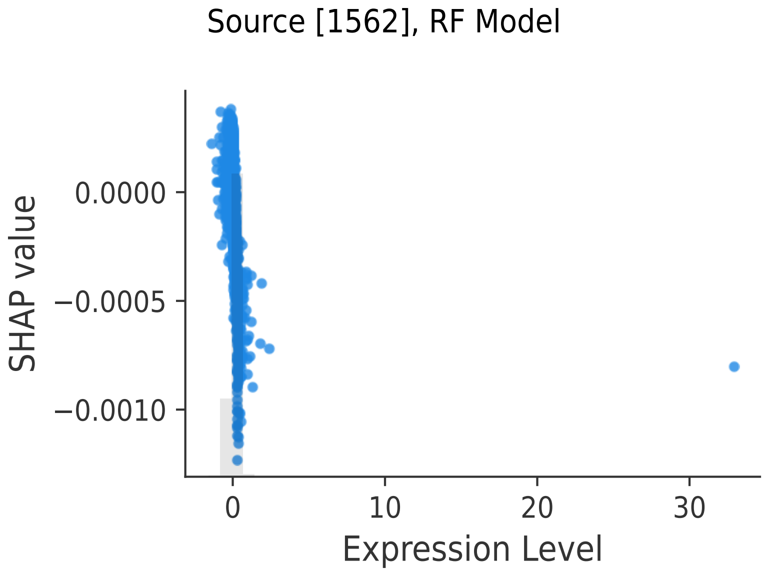
Source [1562], RF Model
SHAP value
0.0000
−0.0005
−0.0010
0
10
20
30
Expression Level

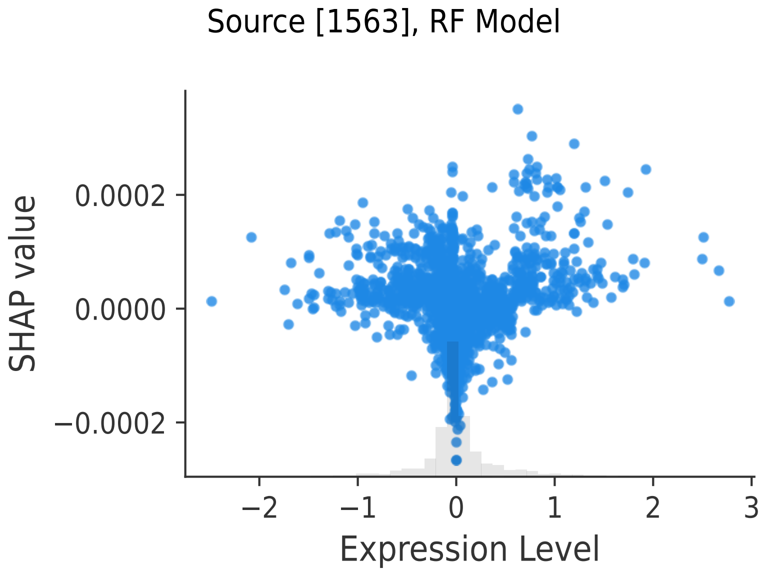

Source [1563], RF Model
SHAP value
0.0002
0.0000
−0.0002
−2
−1
0
1
2
3
Expression Level

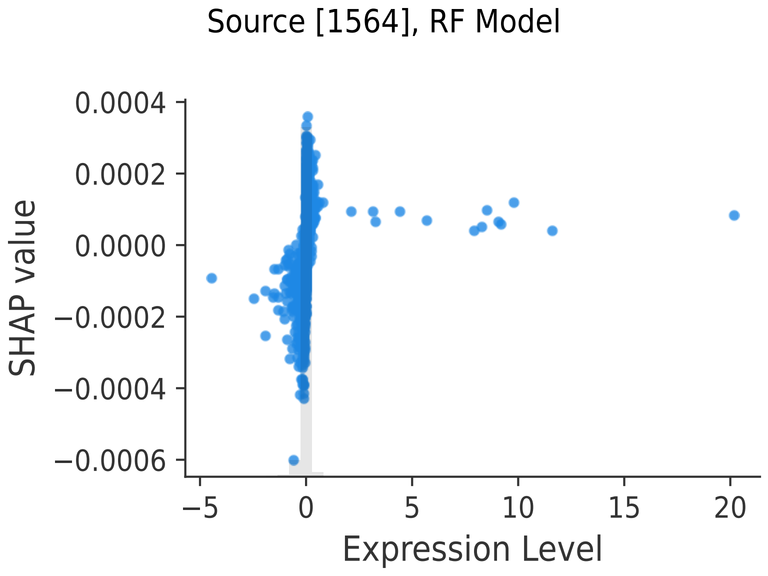

Source [1564], RF Model
0.0004
0.0002
0.0000
−0.0002
−0.0004
−0.0006
SHAP value
−5
0
5
10
15
20
Expression Level

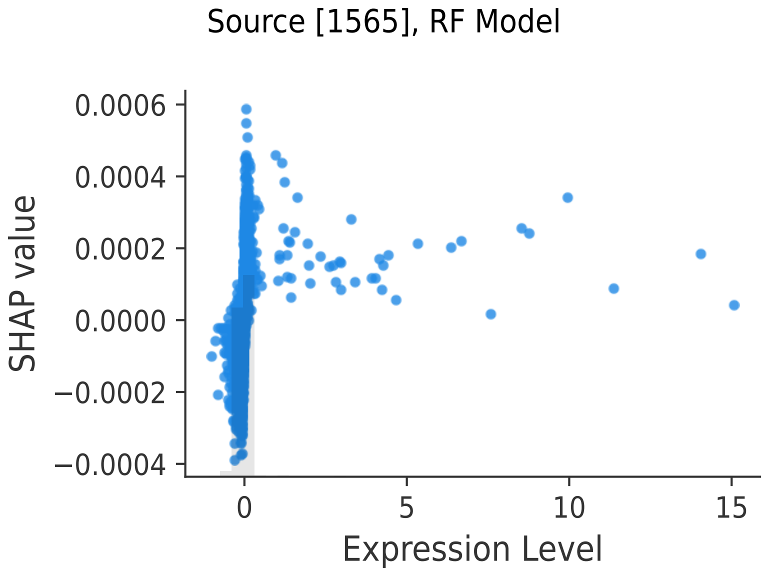
Source [1565], RF Model
SHAP value
0.0006
0.0004
0.0002
0.0000
−0.0002
−0.0004
0
5
10
15
Expression Level

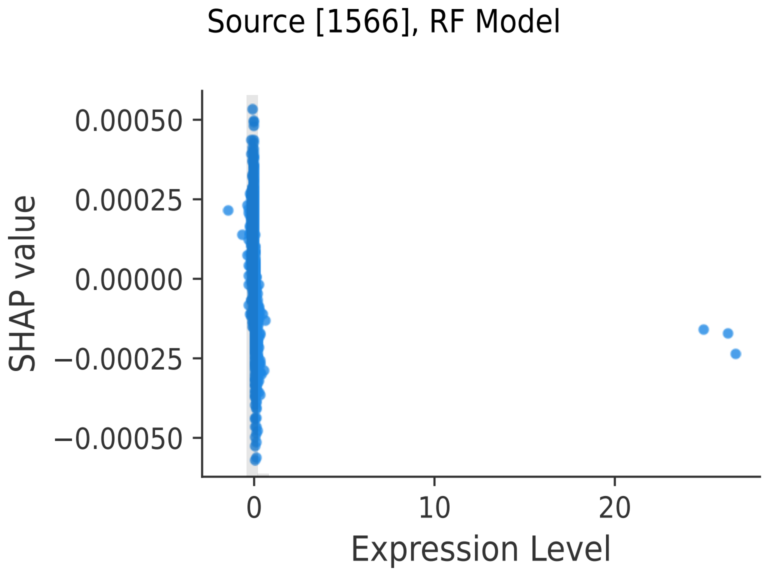

Source [1566], RF Model
0.00050
0.00025
0.00000
−0.00025
−0.00050
SHAP value
0
10
20
Expression Level

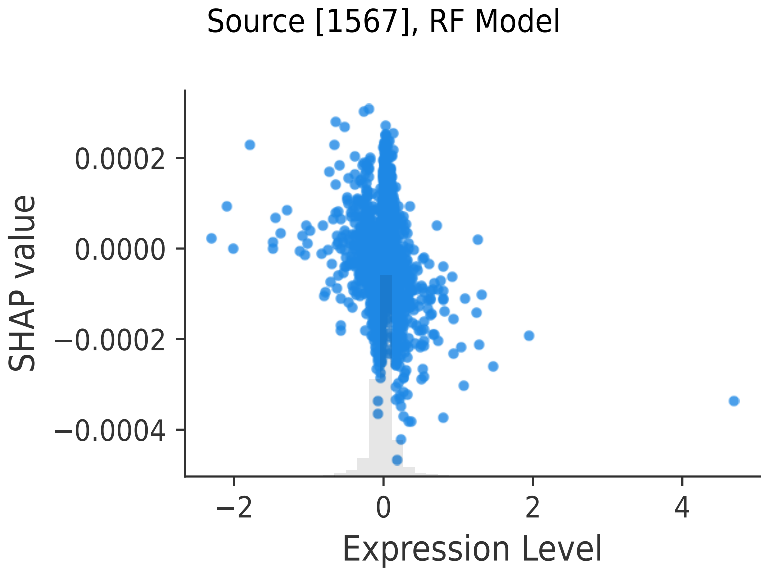
Source [1567], RF Model
0.0002
0.0000
−0.0002
−0.0004
SHAP value
−2
0
2
4
Expression Level

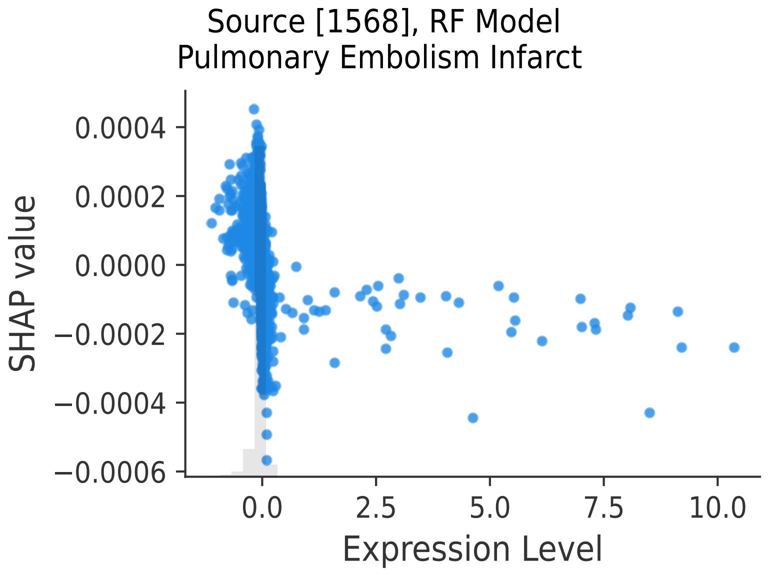
Source [1568], RF Model
Pulmonary Embolism Infarct
SHAP value
0.0004
0.0002
0.0000
−0.0002
−0.0004
−0.0006
0.0
2.5
5.0
7.5
10.0
Expression Level

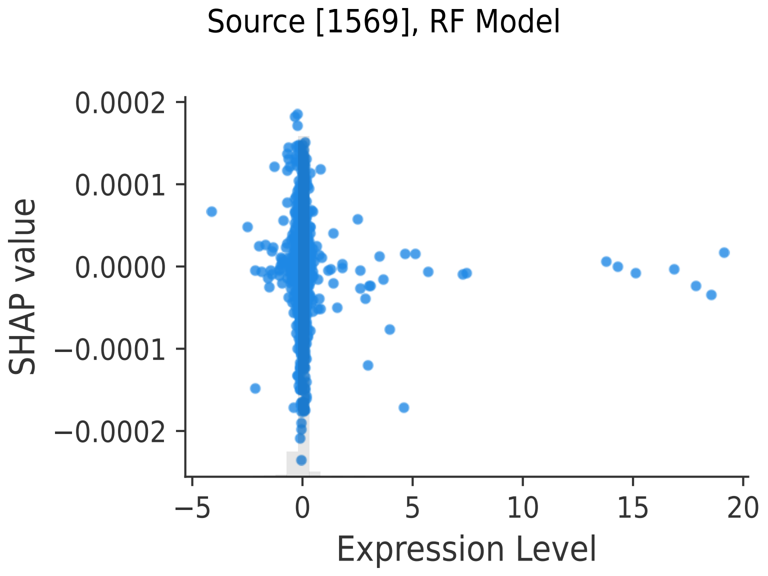
Source [1569], RF Model
SHAP value
0.0002
0.0001
0.0000
−0.0001
−0.0002
−5
0
5
10
15
20
Expression Level

Source [1570], RF Model

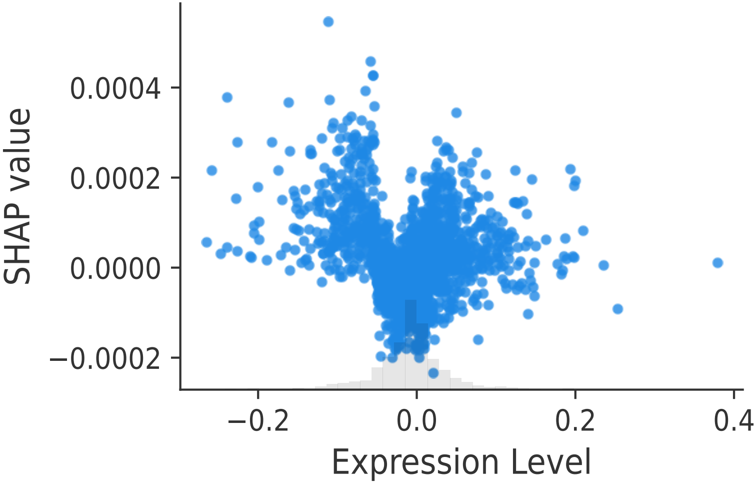

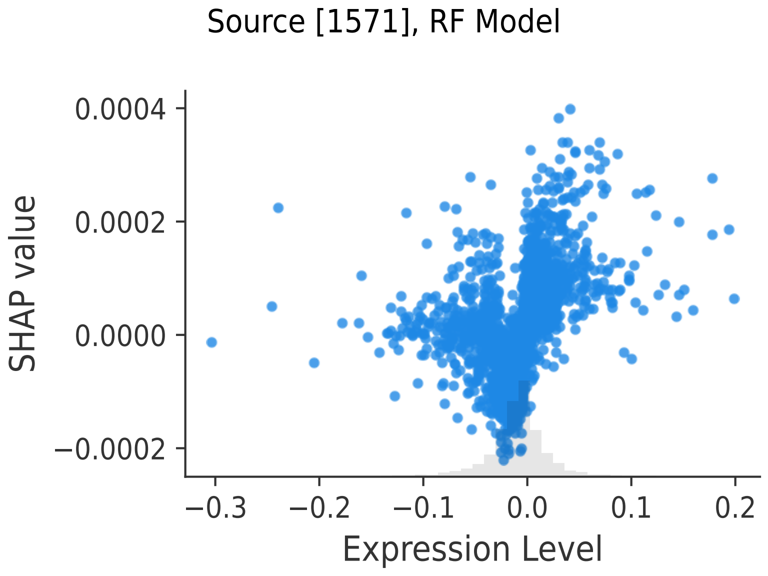
Source [1571], RF Model
SHAP value
0.0004
0.0002
0.0000
−0.0002
−0.3
−0.2
−0.1
0.0
0.1
0.2
Expression Level

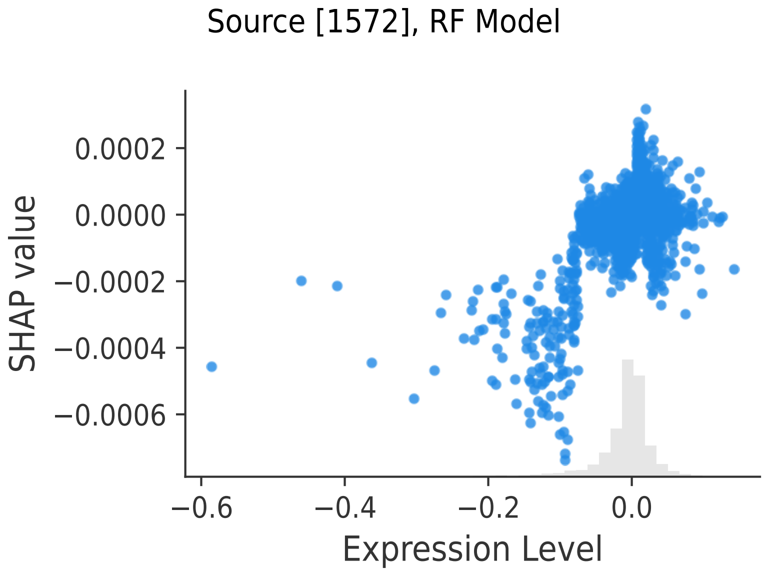
Source [1572], RF Model
SHAP value
0.0002
0.0000
−0.0002
−0.0004
−0.0006
−0.6
−0.4
−0.2
0.0
Expression Level

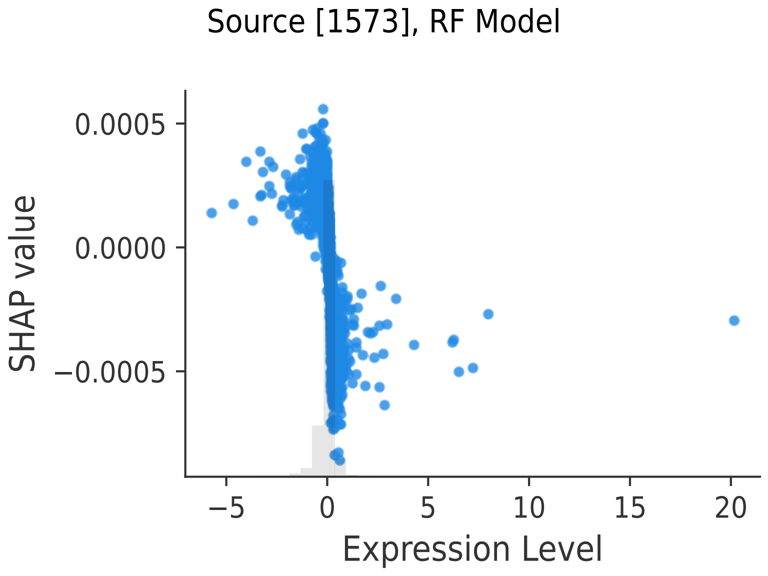

Source [1573], RF Model
SHAP value
0.0005
0.0000
−0.0005
−5
0
5
10
15
20
Expression Level

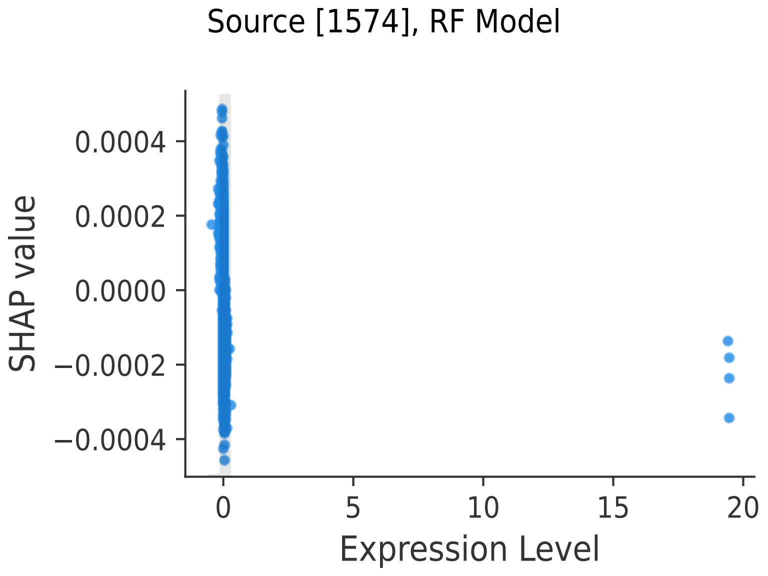
Source [1574], RF Model
SHAP value
0.0004
0.0002
0.0000
−0.0002
−0.0004
0
5
10
15
20
Expression Level

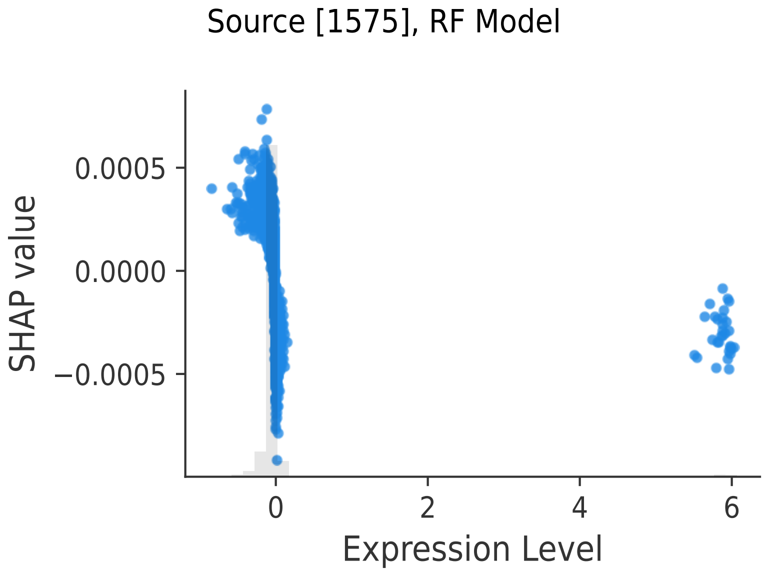
Source [1575], RF Model
SHAP value
0.0005
0.0000
−0.0005
0
2
4
6
Expression Level

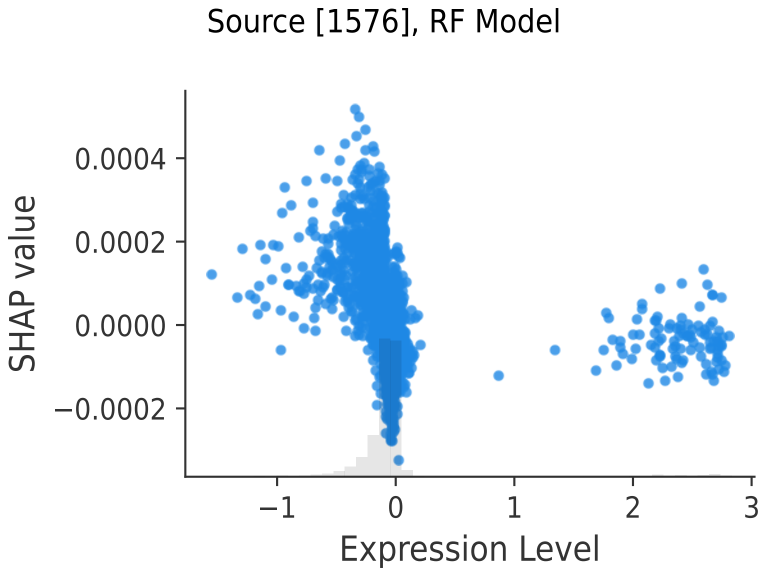

Source [1576], RF Model
0.0004
0.0002
0.0000
−0.0002
SHAP value
−1
0
1
2
3
Expression Level

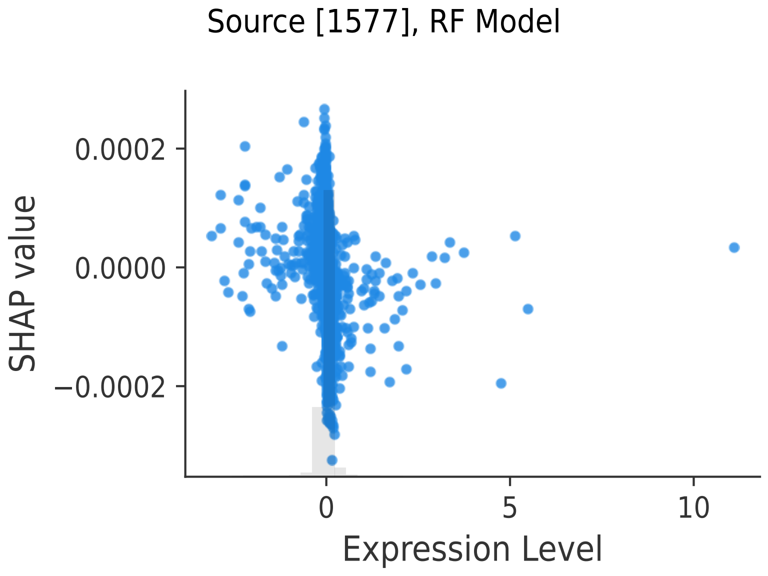

Source [1577], RF Model
0.0002
0.0000
−0.0002
SHAP value
0
5
10
Expression Level

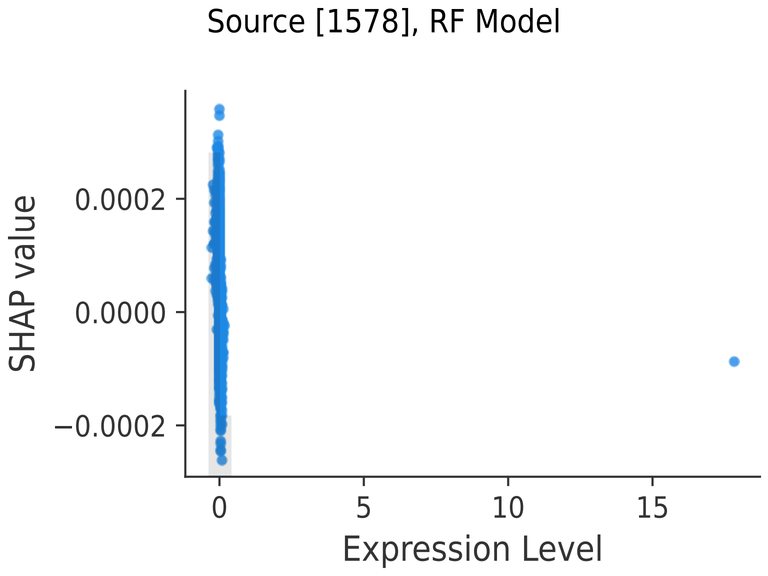
Source [1578], RF Model
SHAP value
0.0002
0.0000
−0.0002
0
5
10
15
Expression Level

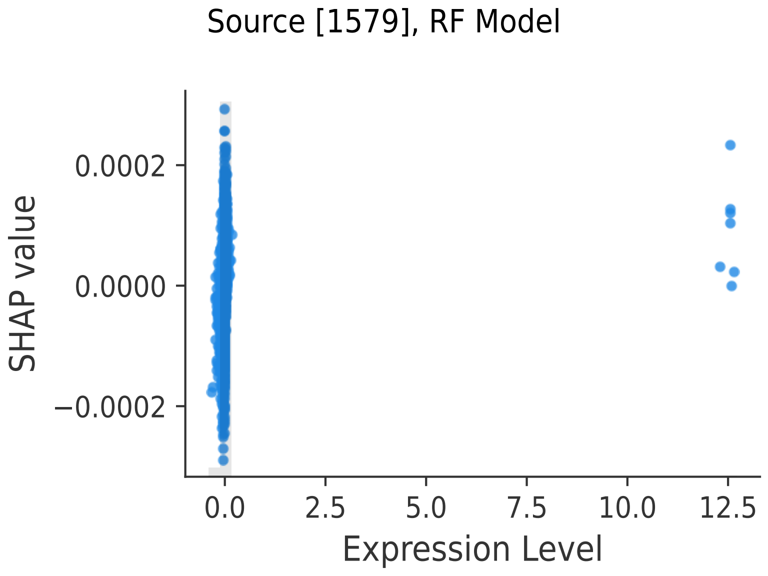
Source [1579], RF Model
SHAP value
0.0002
0.0000
−0.0002
0.0
2.5
5.0
7.5
10.0
12.5
Expression Level

Source [1580], RF Model

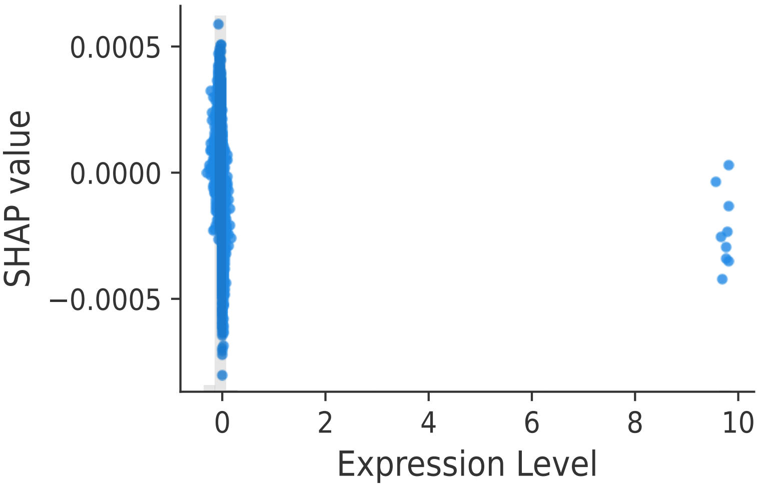

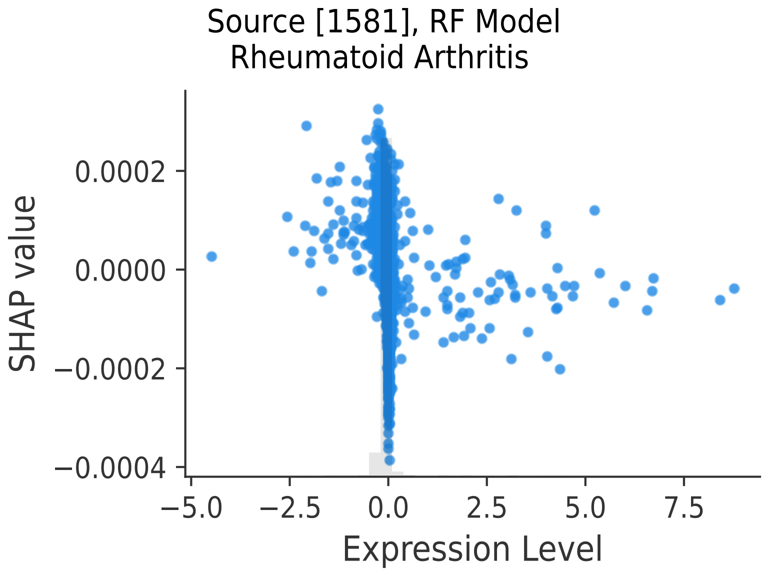

Source [1581], RF Model
Rheumatoid Arthritis
SHAP value
0.0002
0.0000
−0.0002
−0.0004
−5.0
−2.5
0.0
2.5
5.0
7.5
Expression Level

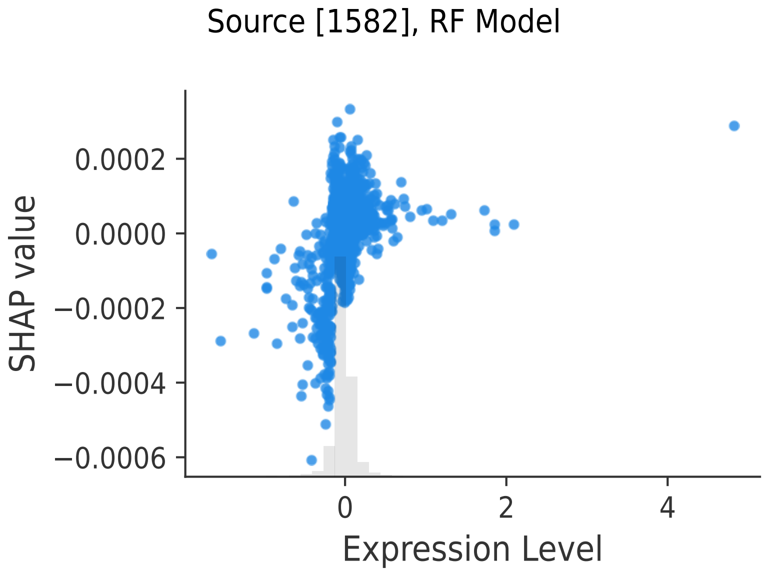

Source [1582], RF Model
0.0002
0.0000
−0.0002
−0.0004
−0.0006
SHAP value
0
2
4
Expression Level

Source [1583], RF Model

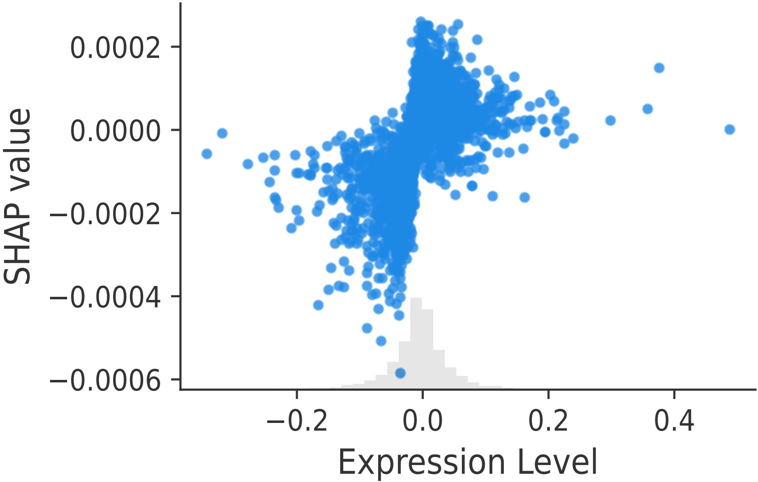

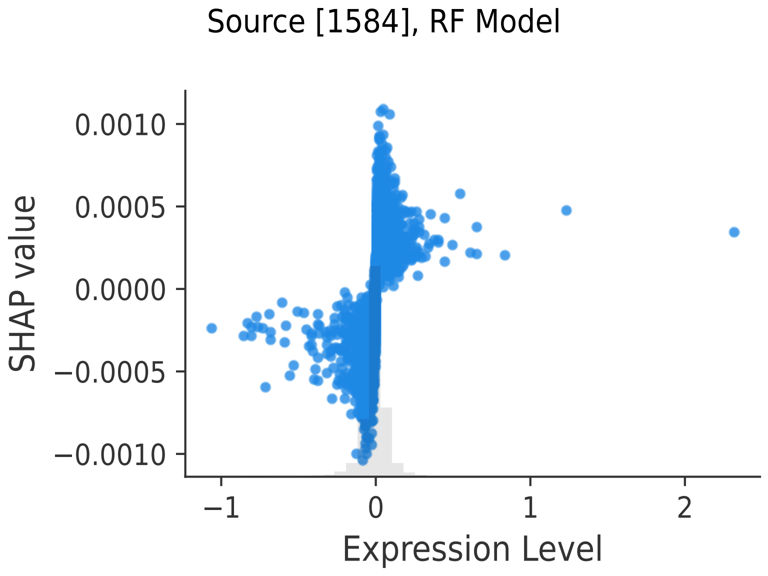

Source [1584], RF Model
0.0010
0.0005
0.0000
−0.0005
−0.0010
SHAP value
−1
0
1
2
Expression Level

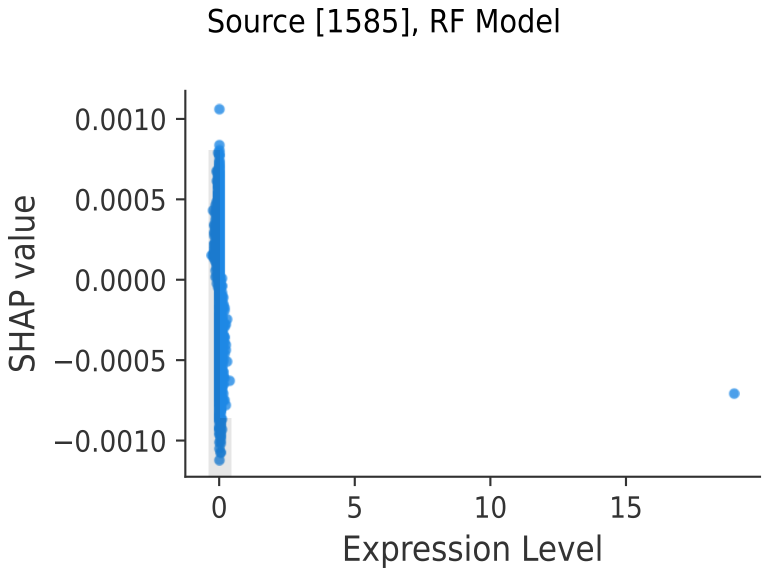
Source [1585], RF Model
SHAP value
0.0010
0.0005
0.0000
−0.0005
−0.0010
0
5
10
15
Expression Level

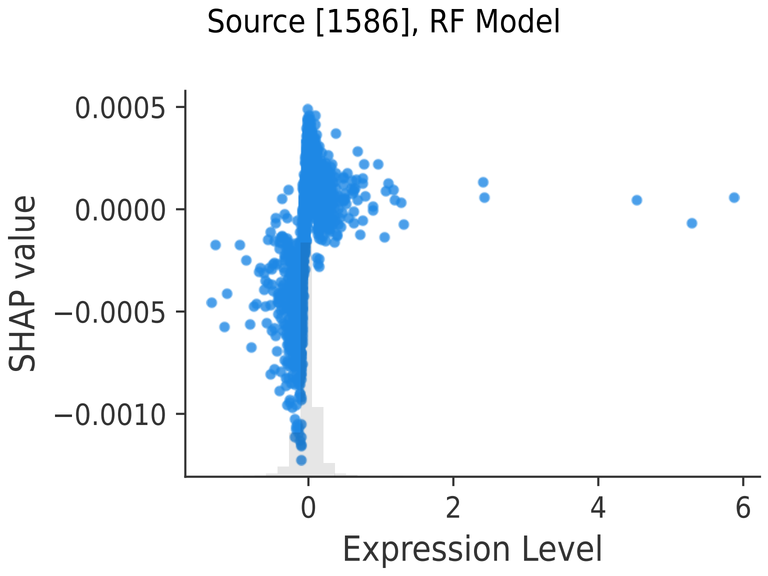

Source [1586], RF Model
SHAP value
0.0005
0.0000
−0.0005
−0.0010
0
2
4
6
Expression Level

Source [1587], RF Model

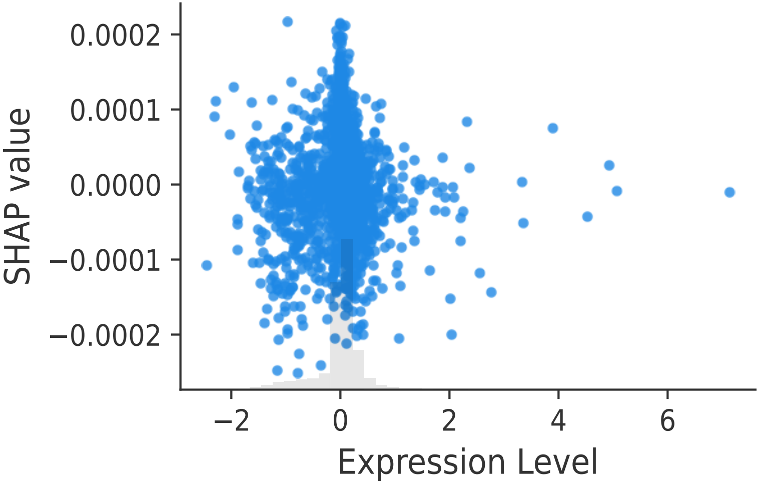

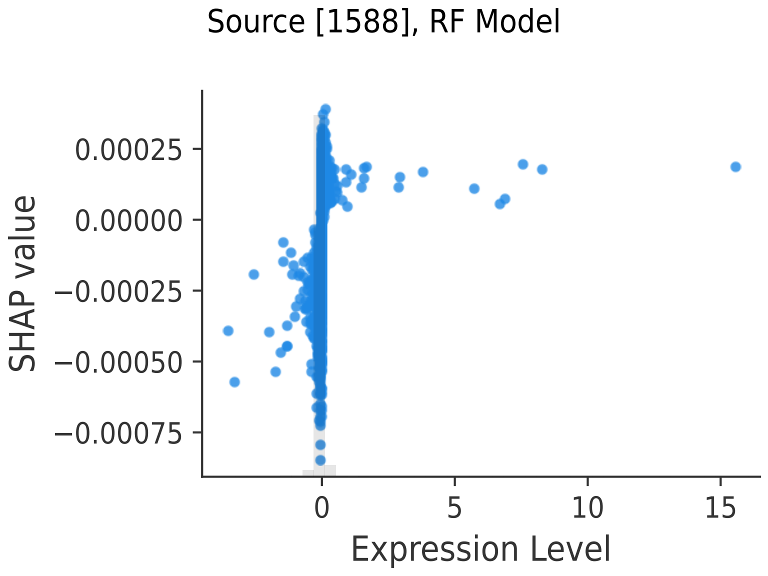
Source [1588], RF Model
SHAP value
0.00025
0.00000
−0.00025
−0.00050
−0.00075
0
5
10
15
Expression Level

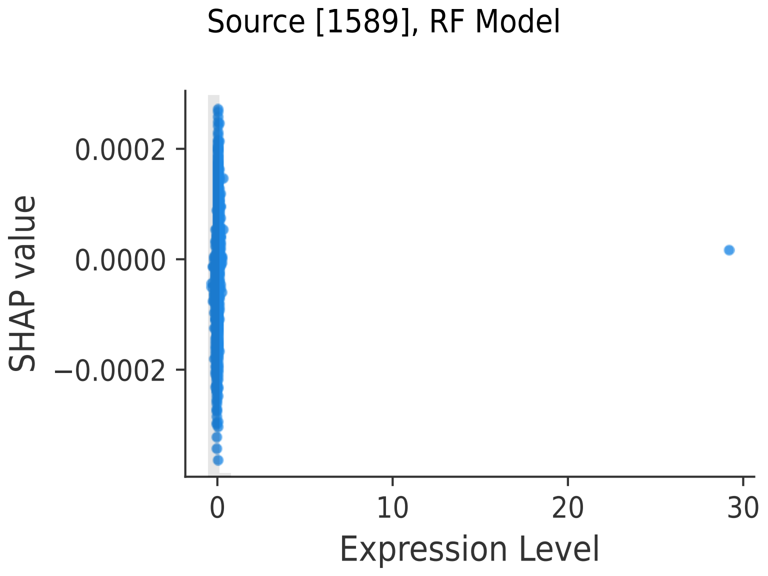

Source [1589], RF Model
SHAP value
0.0002
0.0000
−0.0002
0
10
20
30
Expression Level

Source [1590], RF Model

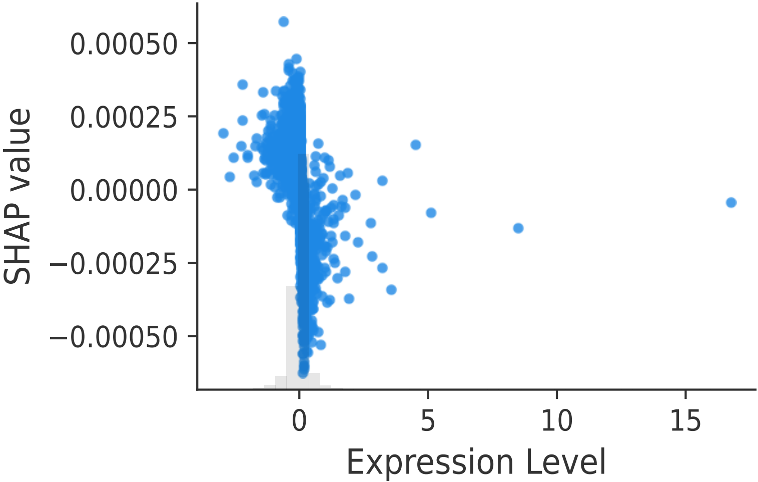

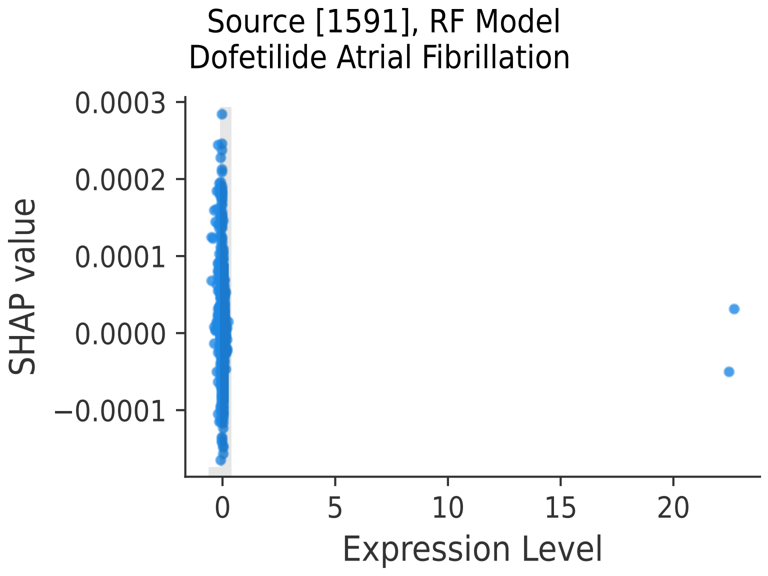

Source [1591], RF Model
Dofetilide Atrial Fibrillation
0.0003
0.0002
0.0001
0.0000
−0.0001
SHAP value
0
5
10
15
20
Expression Level

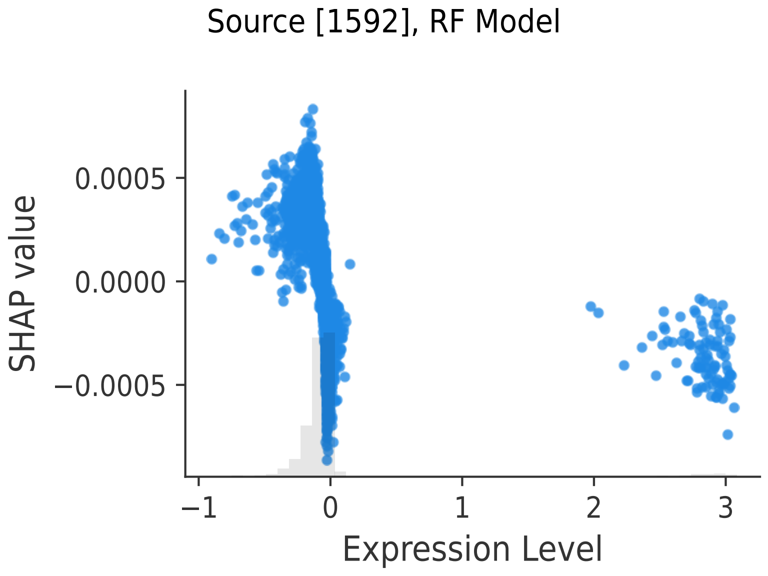

Source [1592], RF Model
0.0005
0.0000
−0.0005
SHAP value
−1
0
1
2
3
Expression Level

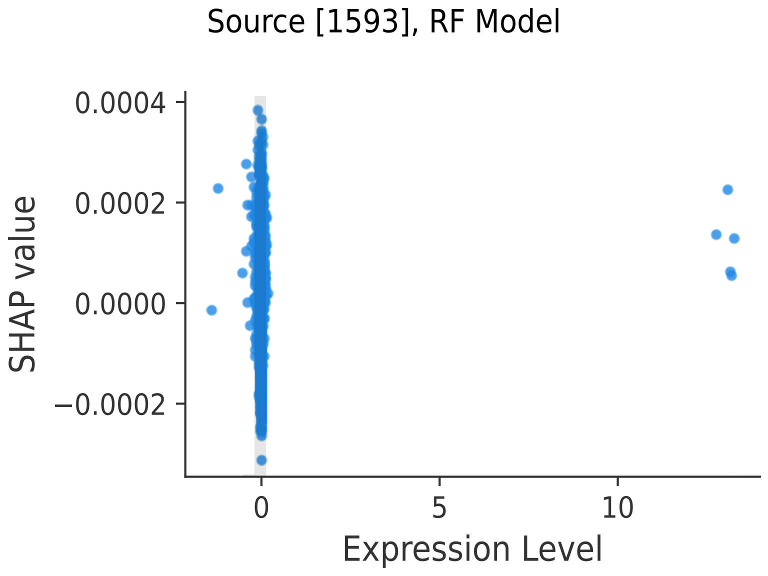
Source [1593], RF Model
SHAP value
0.0004
0.0002
0.0000
−0.0002
0
5
10
Expression Level

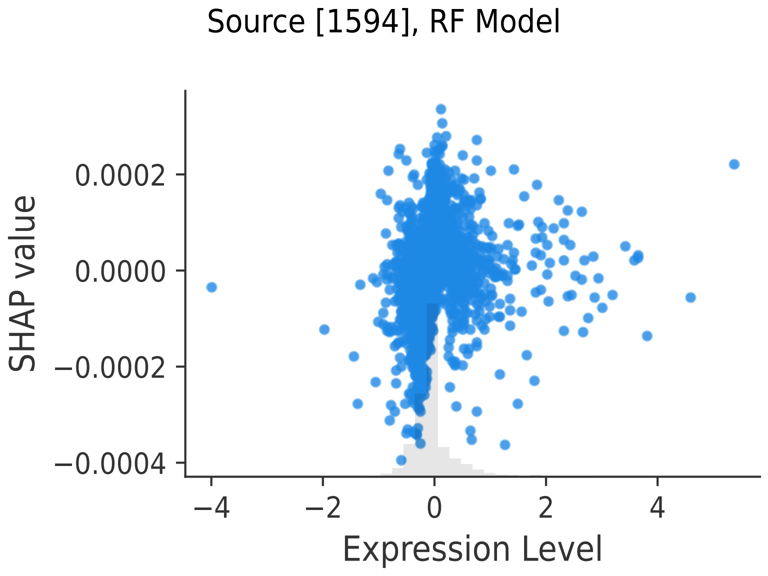
Source [1594], RF Model
SHAP value
0.0002
0.0000
−0.0002
−0.0004
−4
−2
0
2
4
Expression Level

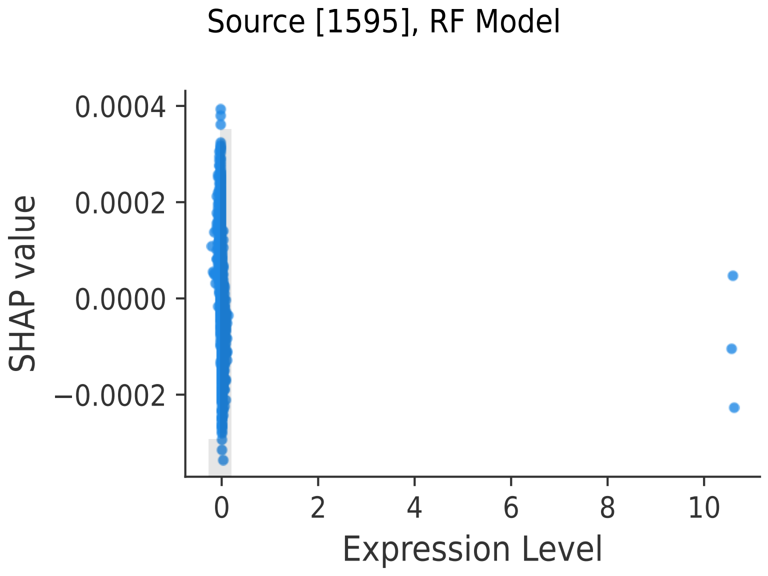
Source [1595], RF Model
0.0004
0.0002
0.0000
−0.0002
SHAP value
0
2
4
6
8
10
Expression Level

Source [1596], RF Model

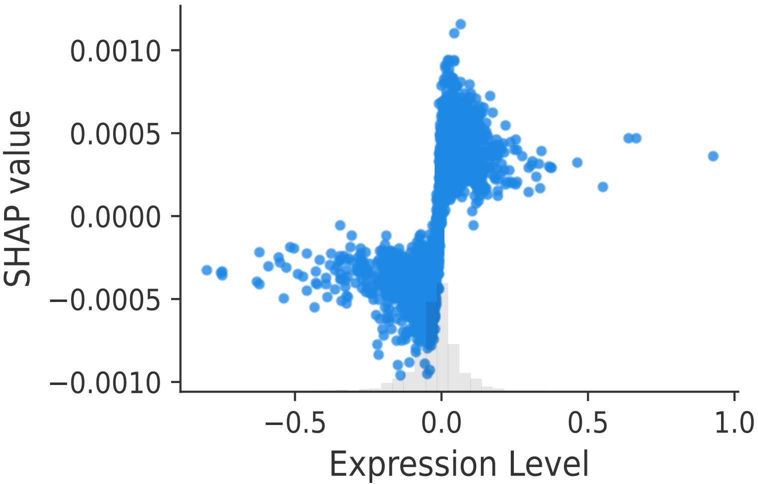

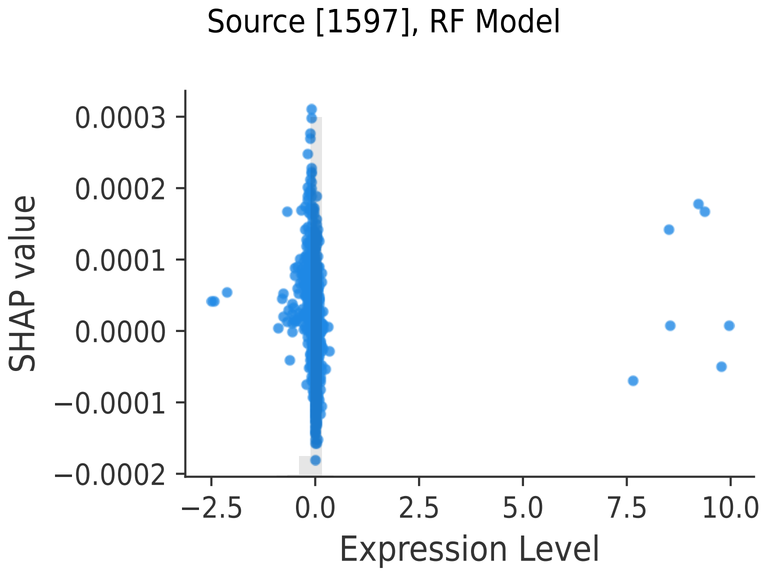

Source [1597], RF Model
SHAP value
0.0003
0.0002
0.0001
0.0000
−0.0001
−0.0002
−2.5
0.0
2.5
5.0
7.5
10.0
Expression Level

Source [1598], RF Model

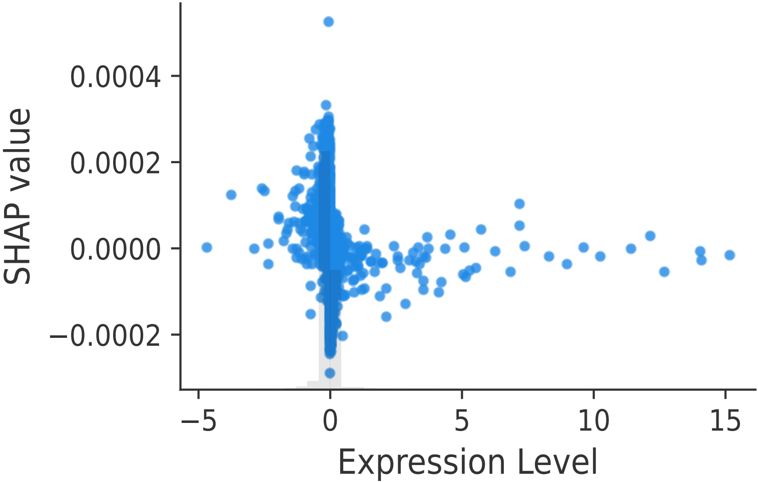

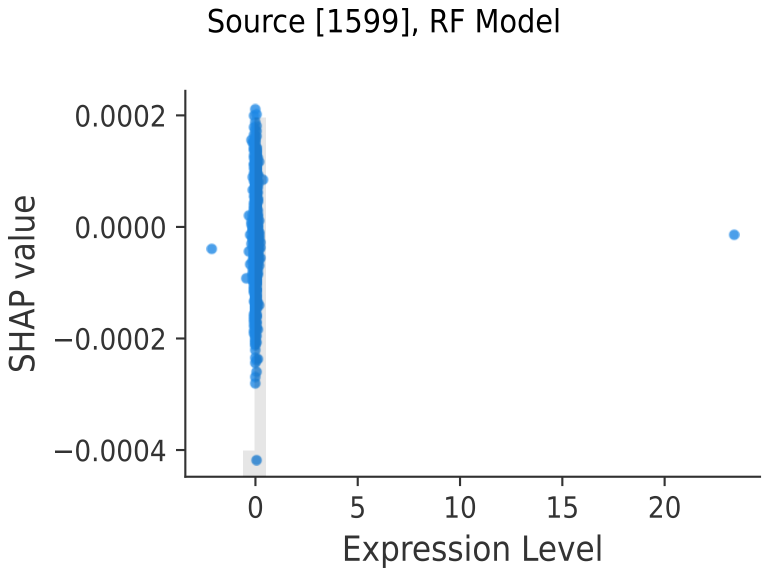
Source [1599], RF Model
0.0002
0.0000
−0.0002
−0.0004
SHAP value
0
5
10
15
20
Expression Level

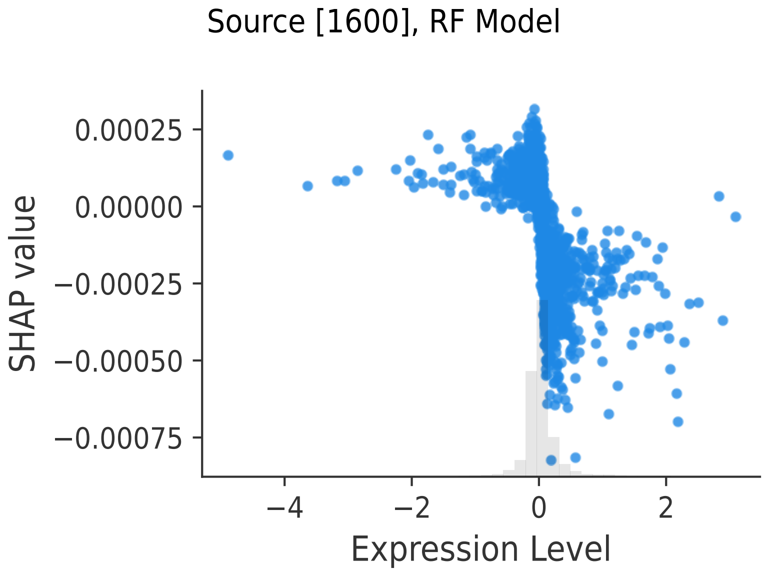
Source [1600], RF Model
0.00025
0.00000
−0.00025
−0.00050
−0.00075
SHAP value
−4
−2
0
2
Expression Level

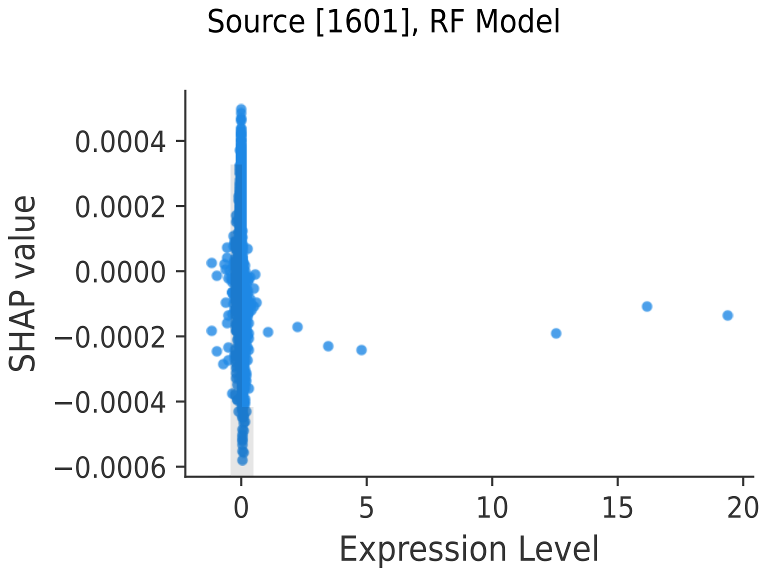

Source [1601], RF Model
SHAP value
0.0004
0.0002
0.0000
−0.0002
−0.0004
−0.0006
0
5
10
15
20
Expression Level

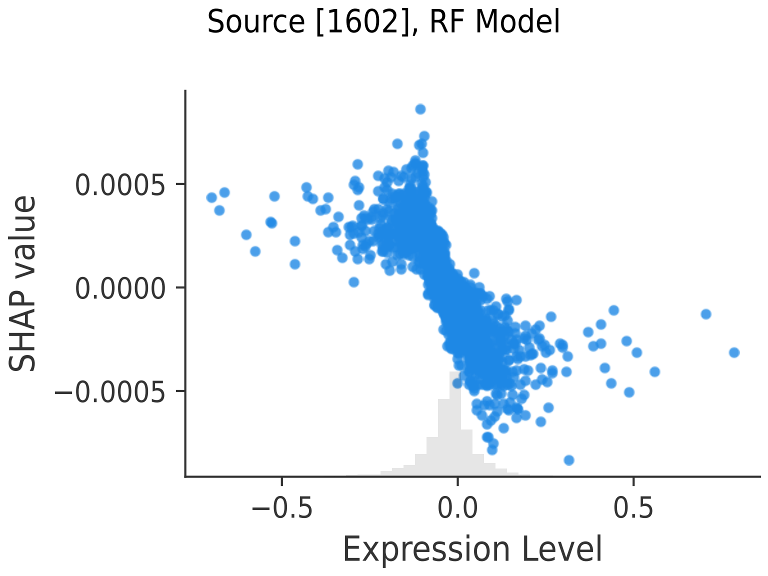

Source [1602], RF Model
SHAP value
0.0005
0.0000
−0.0005
−0.5
0.0
0.5
Expression Level

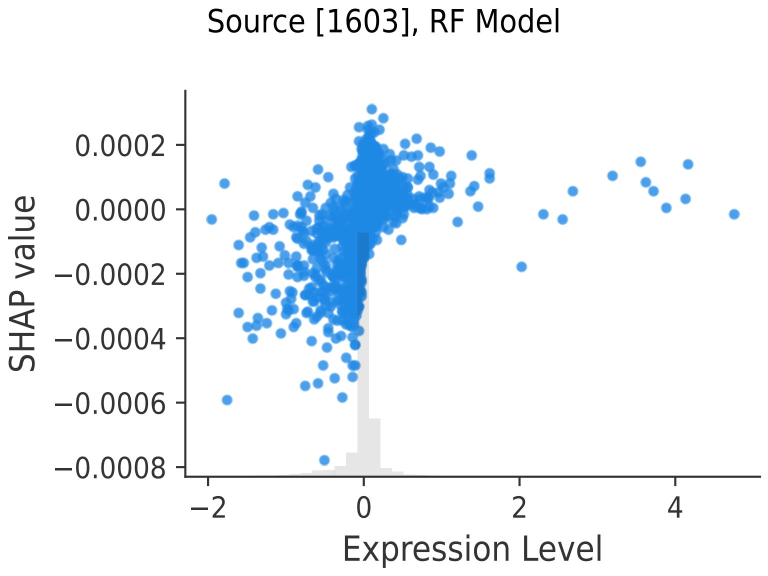
Source [1603], RF Model
SHAP value
0.0002
0.0000
−0.0002
−0.0004
−0.0006
−0.0008
−2
0
2
4
Expression Level

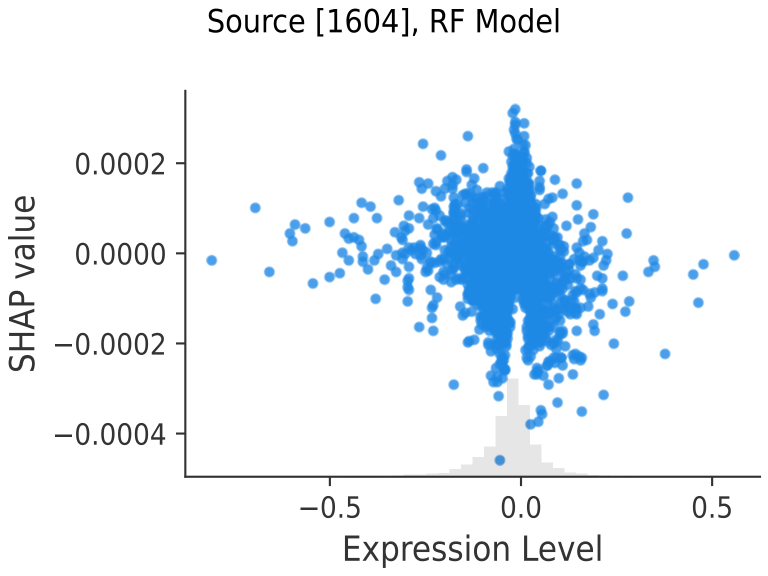

Source [1604], RF Model
0.0002
0.0000
−0.0002
−0.0004
SHAP value
−0.5
0.0
0.5
Expression Level

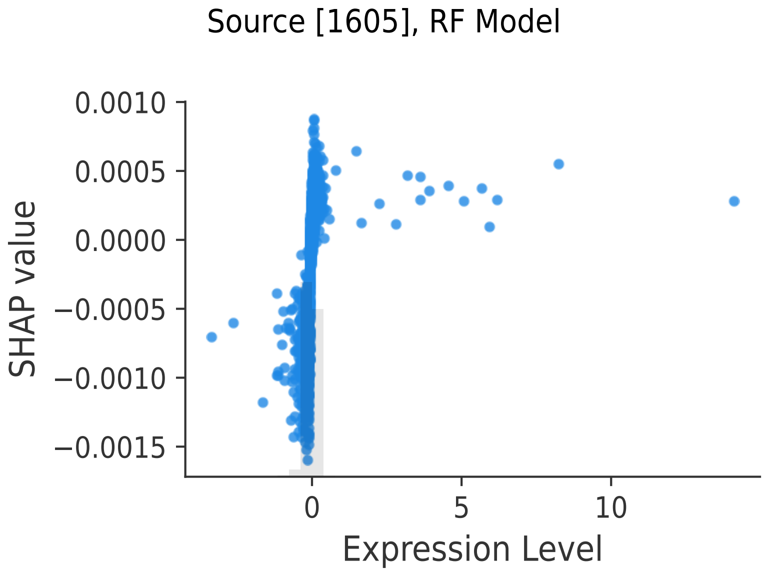

Source [1605], RF Model
0.0010
0.0005
0.0000
−0.0005
−0.0010
−0.0015
SHAP value
0
5
10
Expression Level

Source [1606], RF Model

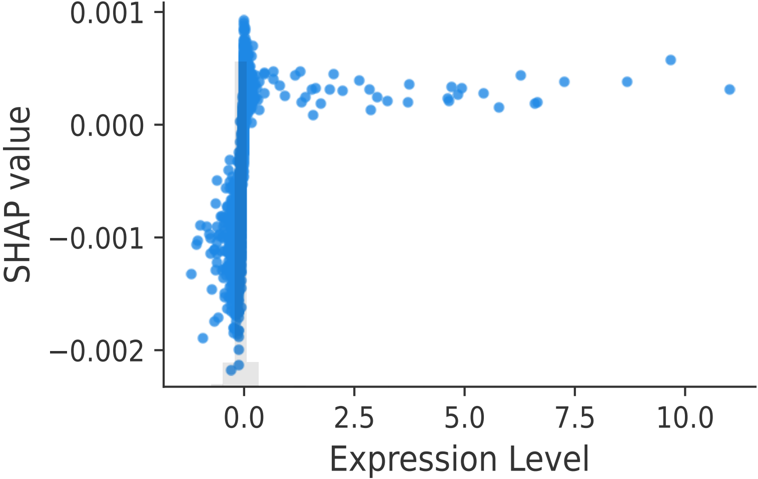

Source [1607], RF Model
Primary Malignant Neoplasm of Larynx or Supraglottis

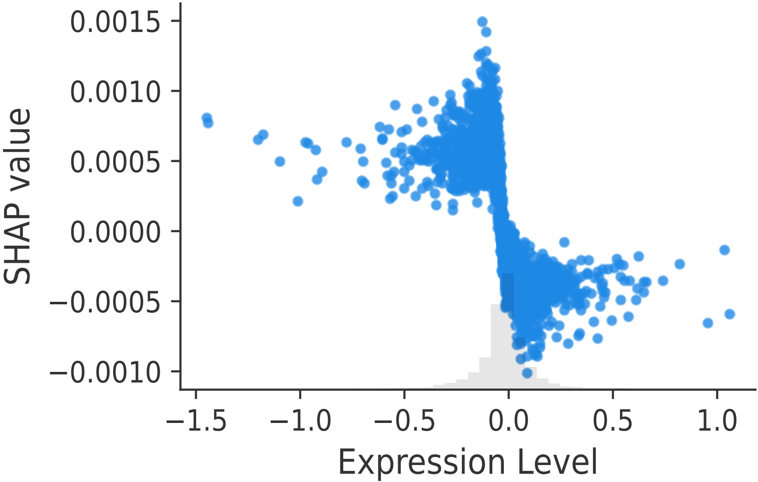

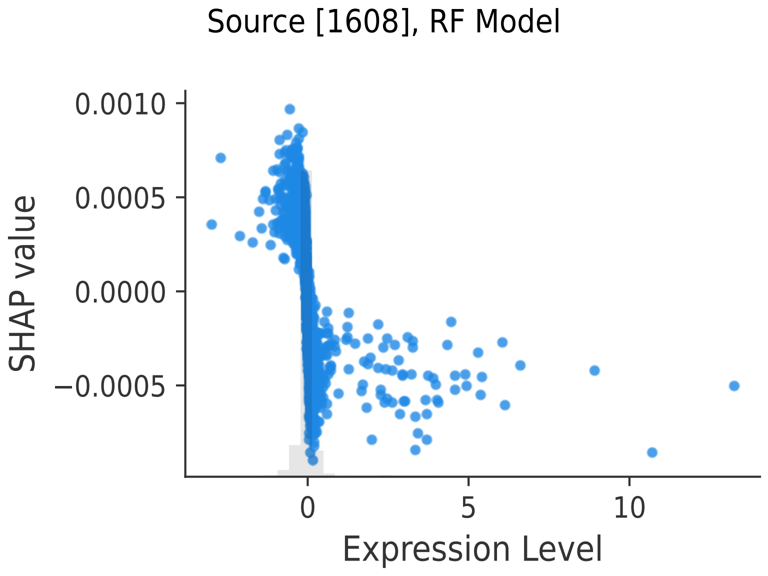

Source [1608], RF Model
0.0010
0.0005
0.0000
−0.0005
SHAP value
0
5
10
Expression Level

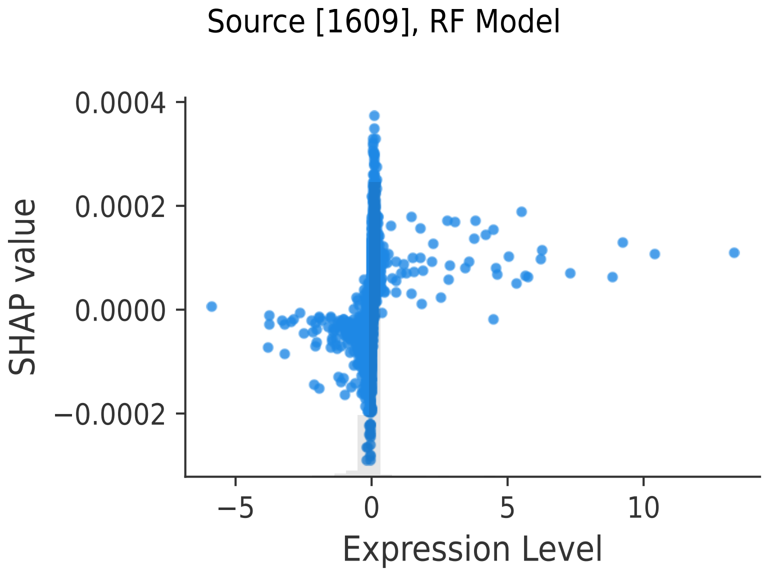

Source [1609], RF Model
0.0004
0.0002
0.0000
−0.0002
SHAP value
−5
0
5
10
Expression Level

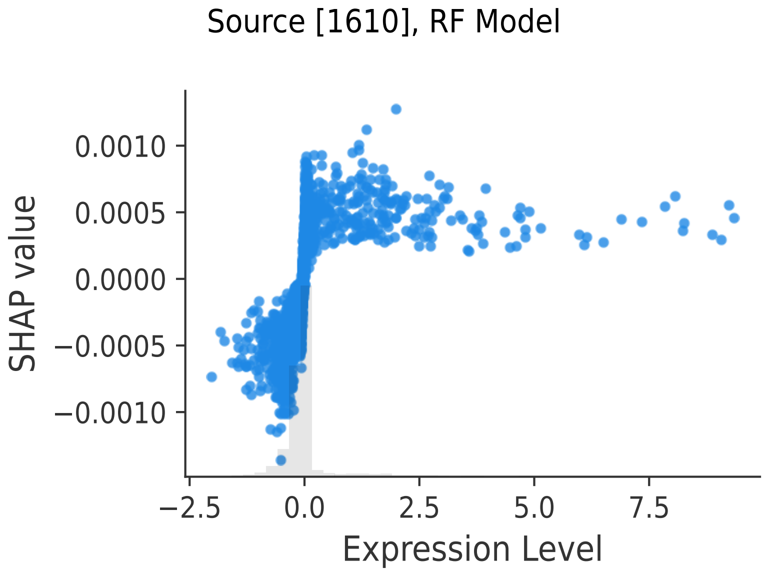
Source [1610], RF Model
SHAP value
0.0010
0.0005
0.0000
−0.0005
−0.0010
−2.5
0.0
2.5
5.0
7.5
Expression Level

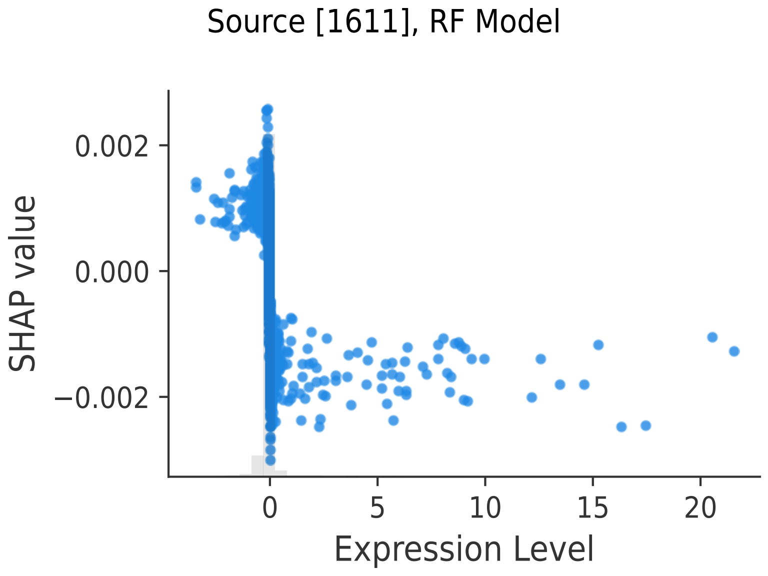

Source [1611], RF Model
SHAP value
0.002
0.000
−0.002
0
5
10
15
20
Expression Level

Source [1612], RF Model

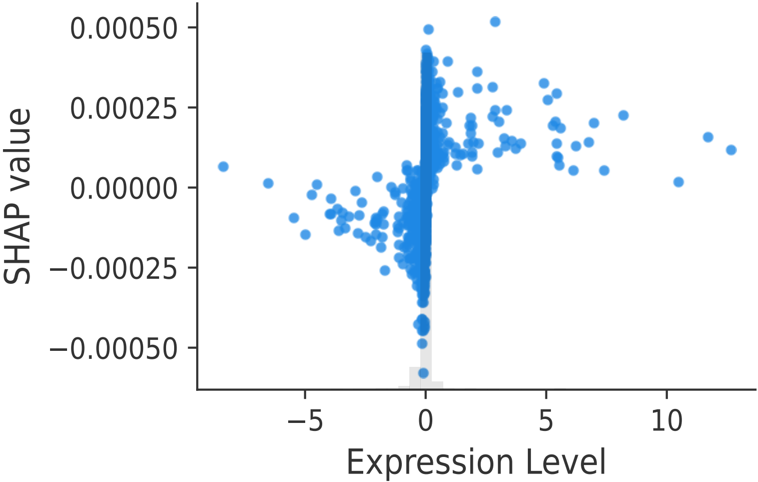

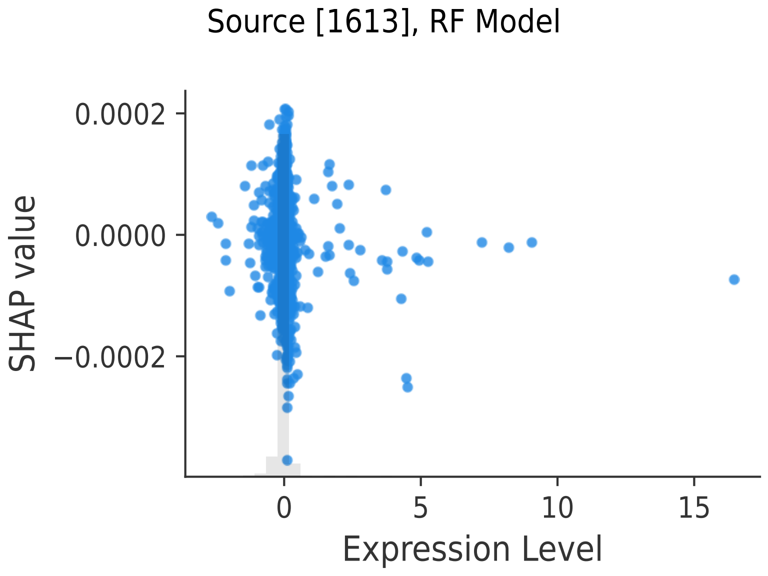

Source [1613], RF Model
0.0002
0.0000
−0.0002
SHAP value
0
5
10
15
Expression Level

Source [1614], RF Model

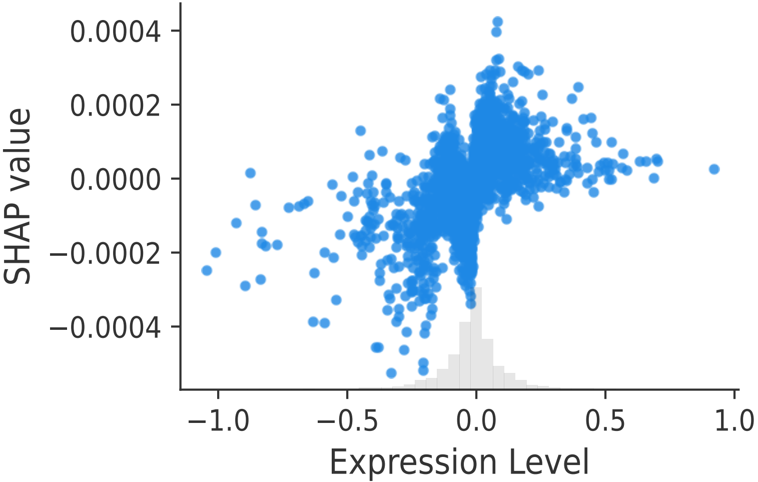

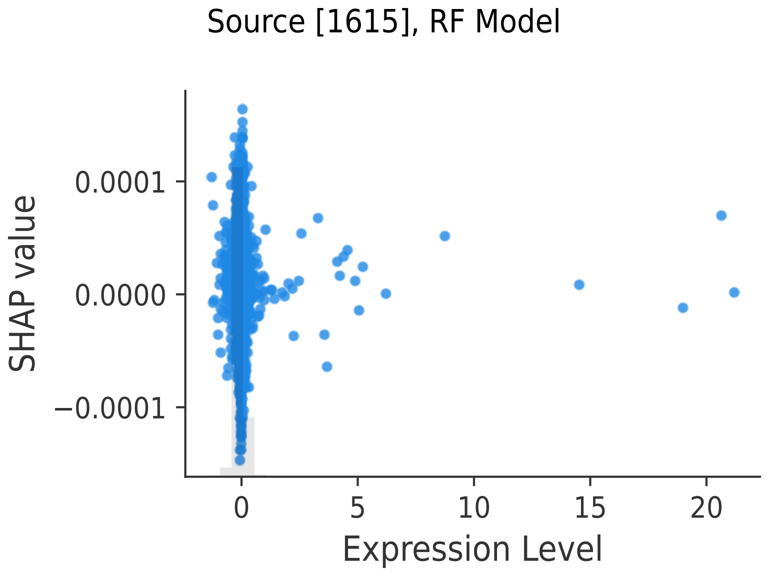

Source [1615], RF Model
SHAP value
0.0001
0.0000
−0.0001
0
5
10
15
20
Expression Level

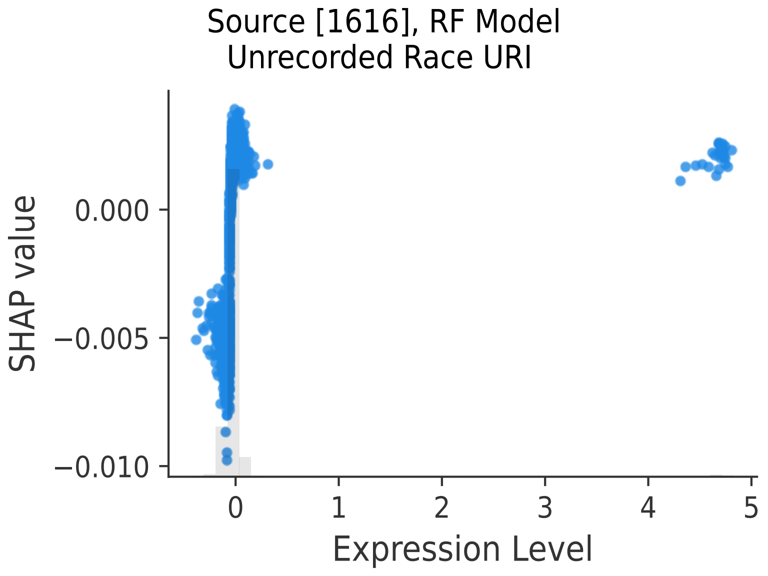

Source [1616], RF Model
Unrecorded Race URI
SHAP value
0.000
−0.005
−0.010
0
1
2
3
4
5
Expression Level

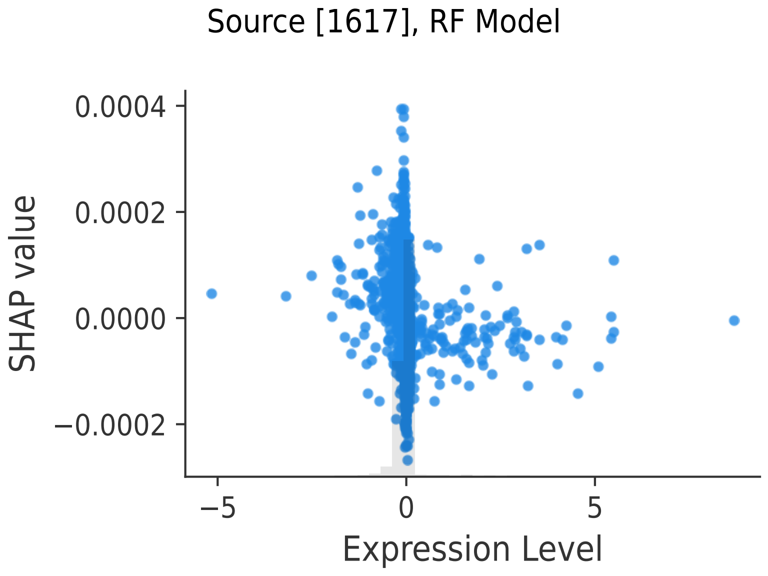

Source [1617], RF Model
0.0004
0.0002
0.0000
−0.0002
SHAP value
−5
0
5
Expression Level

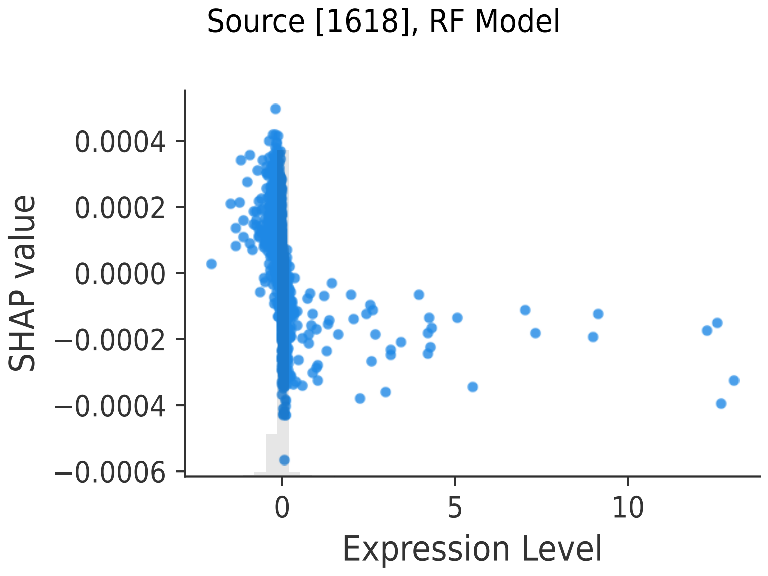
Source [1618], RF Model
0.0004
0.0002
0.0000
−0.0002
−0.0004
−0.0006
SHAP value
0
5
10
Expression Level

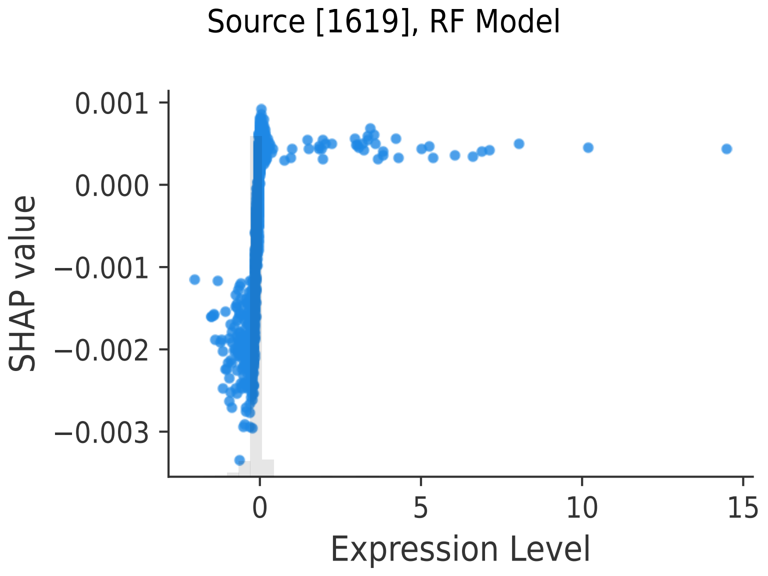
Source [1619], RF Model
0.001
0.000
−0.001
−0.002
−0.003
SHAP value
0
5
10
15
Expression Level

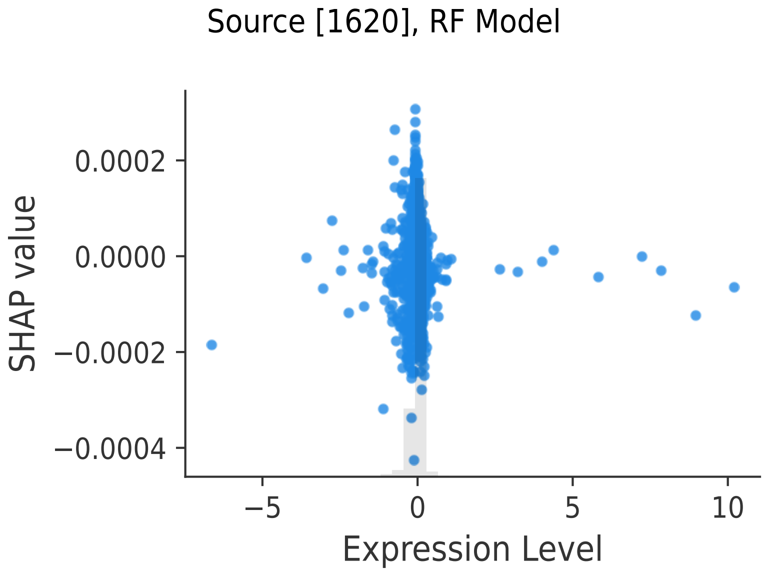

Source [1620], RF Model
SHAP value
0.0002
0.0000
−0.0002
−0.0004
−5
0
5
10
Expression Level

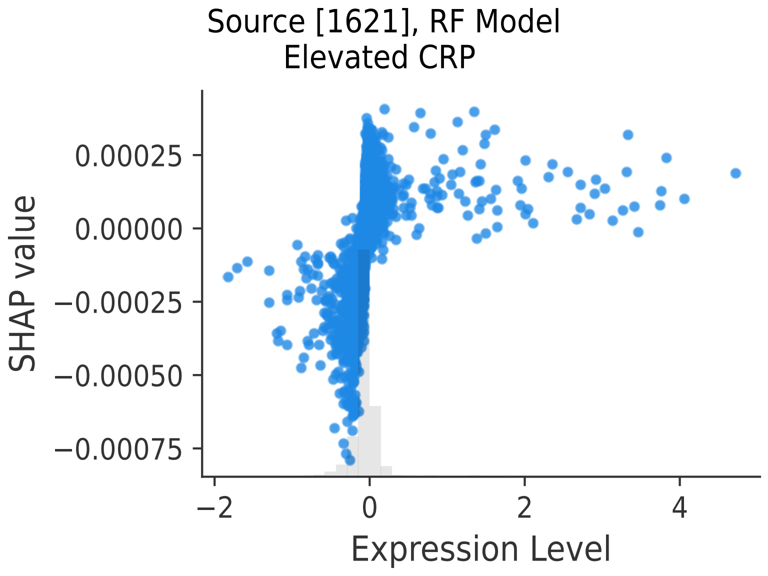
Source [1621], RF Model
Elevated CRP
SHAP value
0.00025
0.00000
−0.00025
−0.00050
−0.00075
−2
0
2
4
Expression Level

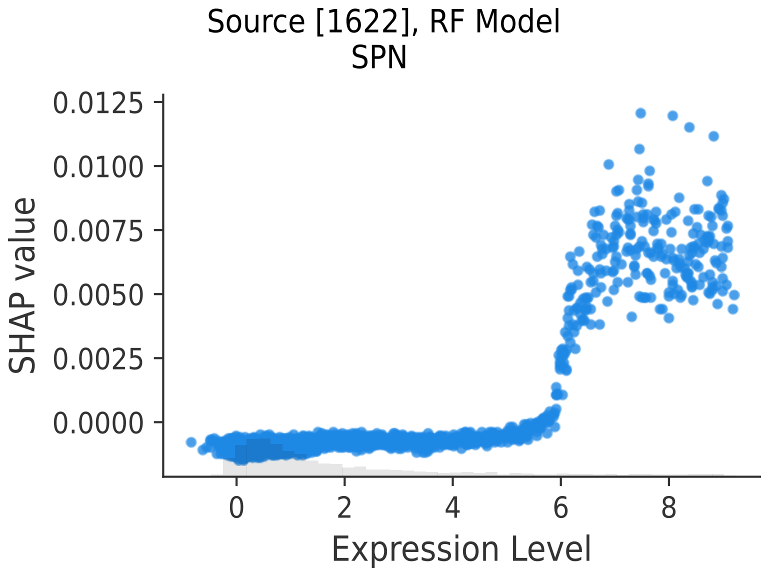

Source [1622], RF Model
SPN
SHAP value
0.0125
0.0100
0.0075
0.0050
0.0025
0.0000
0
2
4
6
8
Expression Level

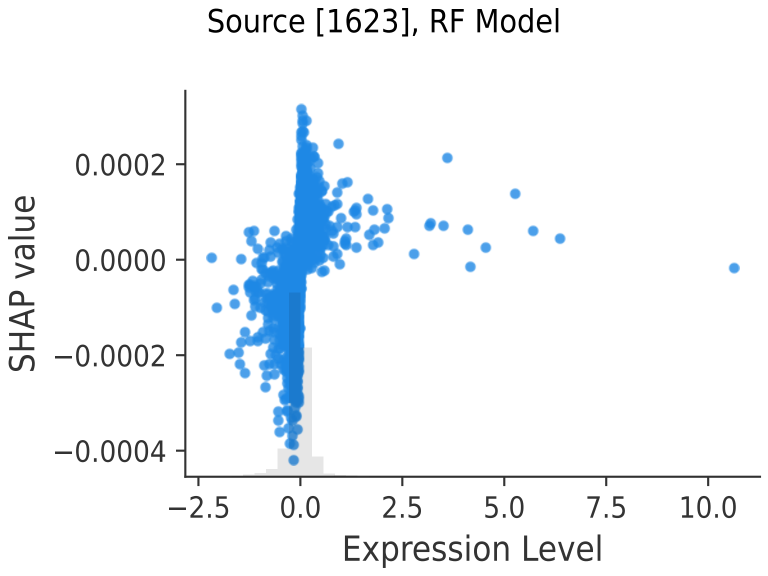
Source [1623], RF Model
SHAP value
0.0002
0.0000
−0.0002
−0.0004
−2.5
0.0
2.5
5.0
7.5
10.0
Expression Level

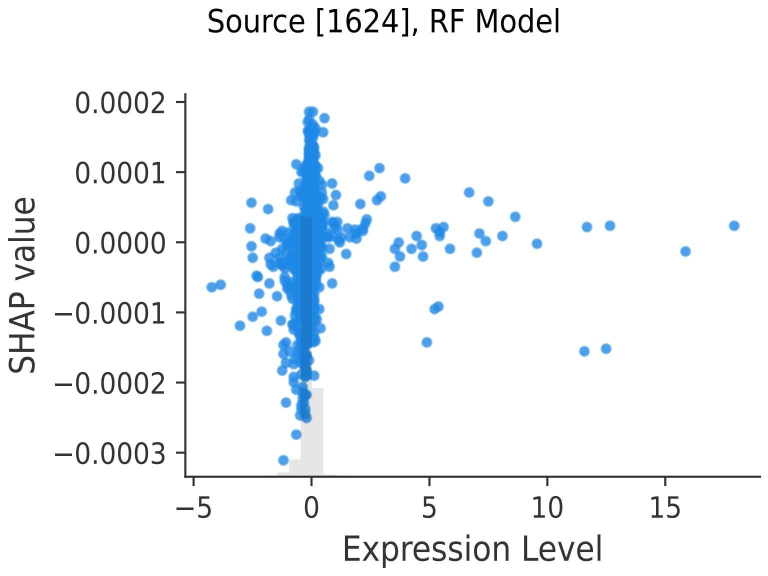

Source [1624], RF Model
0.0002
0.0001
0.0000
−0.0001
−0.0002
−0.0003
SHAP value
−5
0
5
10
15
Expression Level

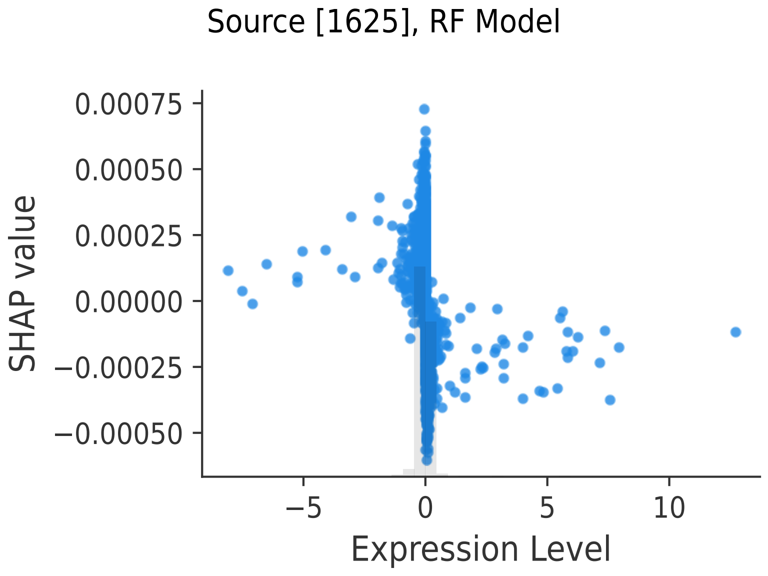

Source [1625], RF Model
0.00075
0.00050
0.00025
0.00000
−0.00025
−0.00050
SHAP value
−5
0
5
10
Expression Level

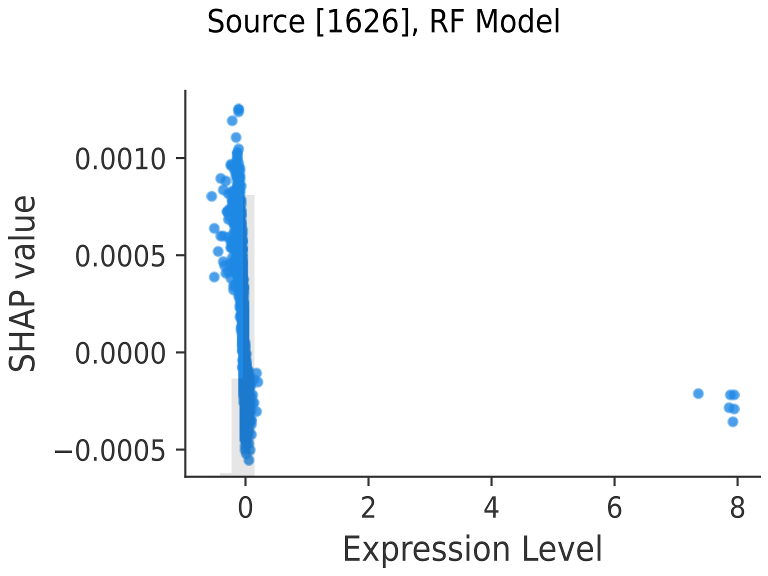

Source [1626], RF Model
SHAP value
0.0010
0.0005
0.0000
−0.0005
0
2
4
6
8
Expression Level

Source [1627], RF Model

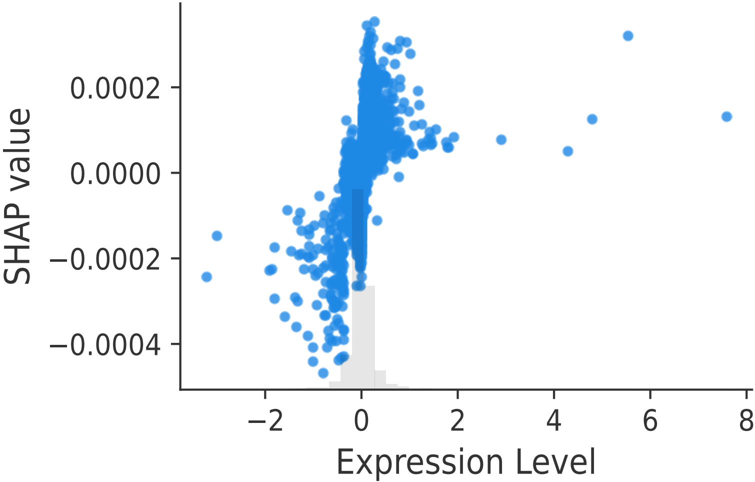

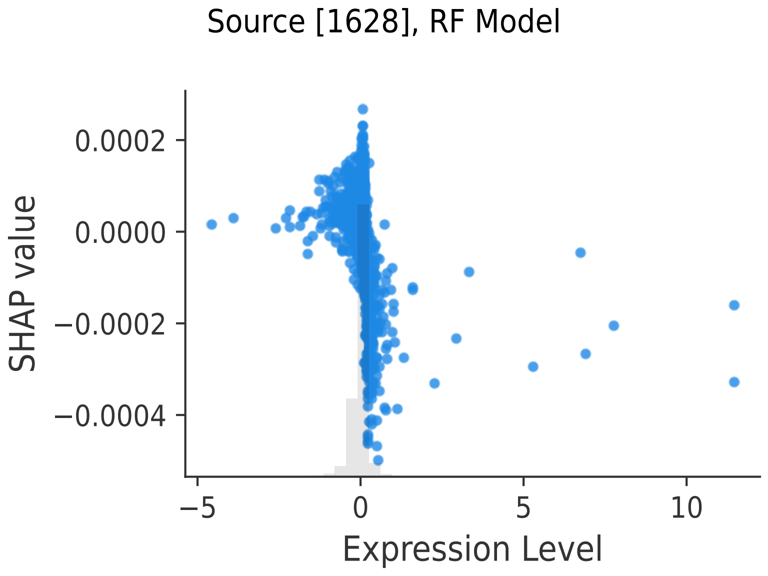

Source [1628], RF Model
SHAP value
0.0002
0.0000
−0.0002
−0.0004
−5
0
5
10
Expression Level

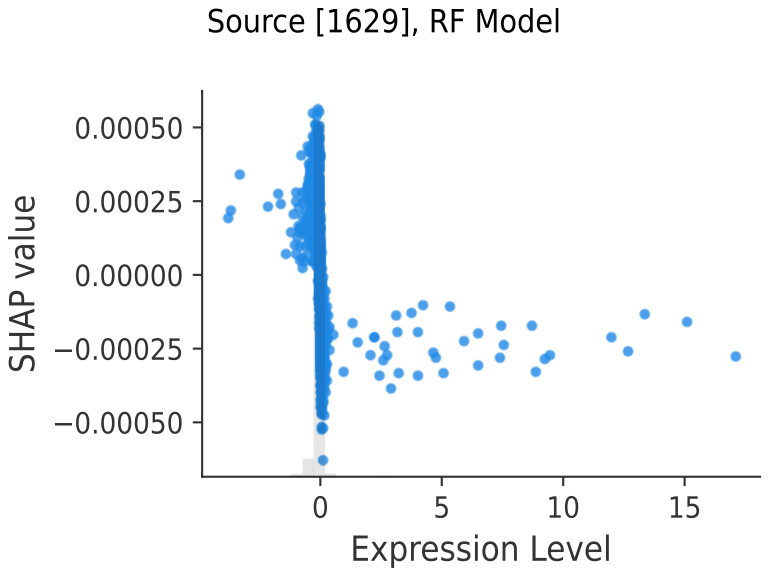

Source [1629], RF Model
SHAP value
0.00050
0.00025
0.00000
−0.00025
−0.00050
0
5
10
15
Expression Level

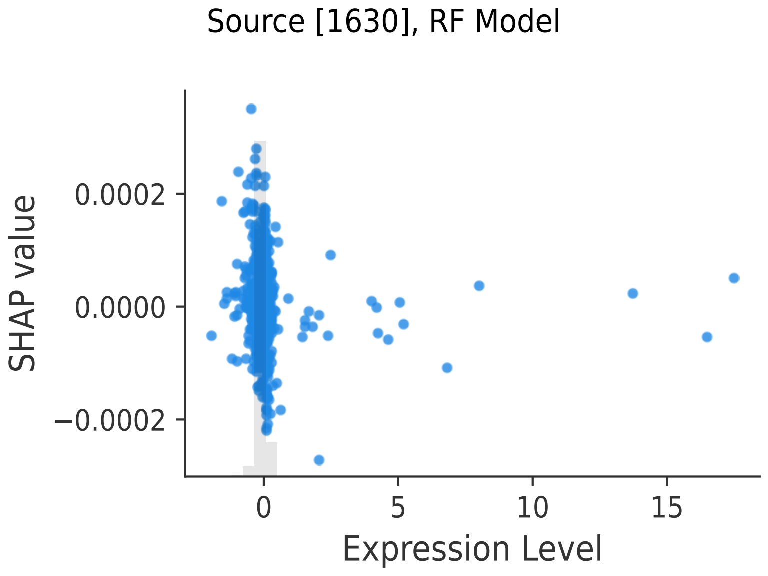
Source [1630], RF Model
SHAP value
0.0002
0.0000
−0.0002
0
5
10
15
Expression Level

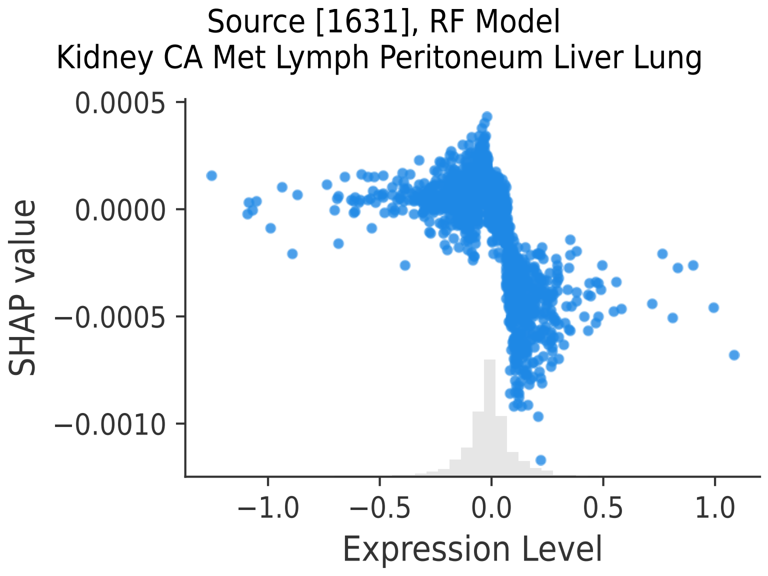

Source [1631], RF Model
Kidney CA Met Lymph Peritoneum Liver Lung
0.0005
0.0000
−0.0005
−0.0010
SHAP value
−1.0
−0.5
0.0
0.5
1.0
Expression Level

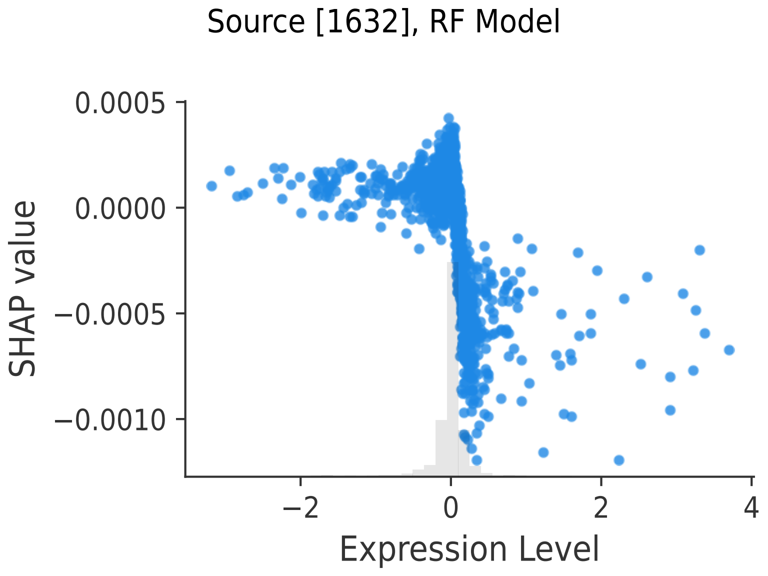

Source [1632], RF Model
0.0005
0.0000
−0.0005
−0.0010
SHAP value
−2
0
2
4
Expression Level

Source [1633], RF Model

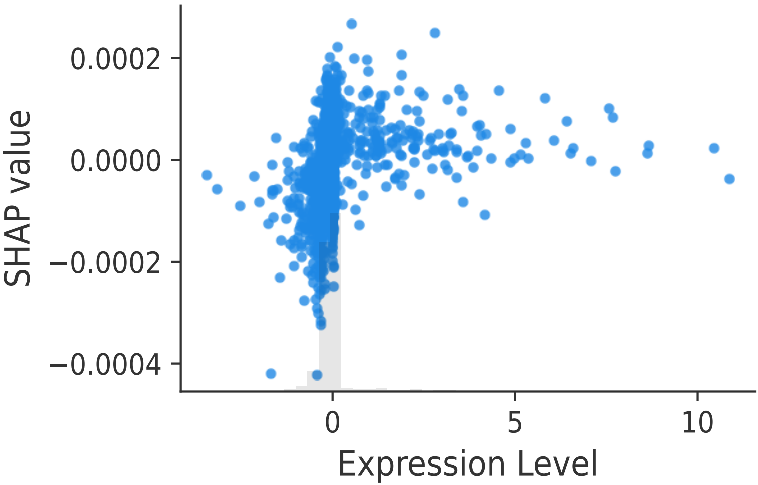

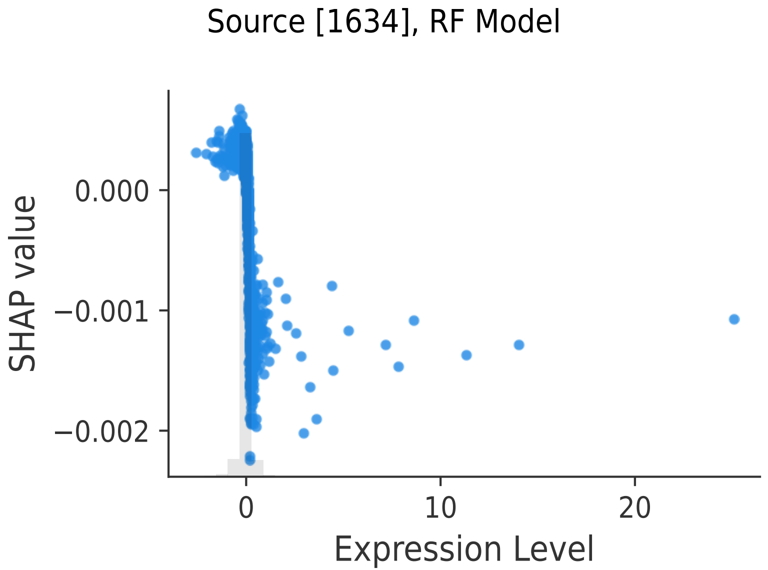

Source [1634], RF Model
SHAP value
0.000
−0.001
−0.002
0
10
20
Expression Level

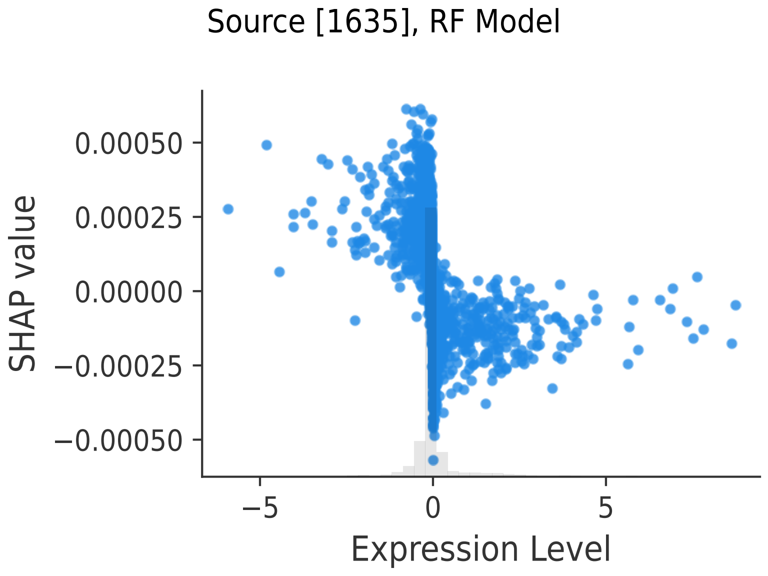
Source [1635], RF Model
SHAP value
0.00050
0.00025
0.00000
−0.00025
−0.00050
−5
0
5
Expression Level

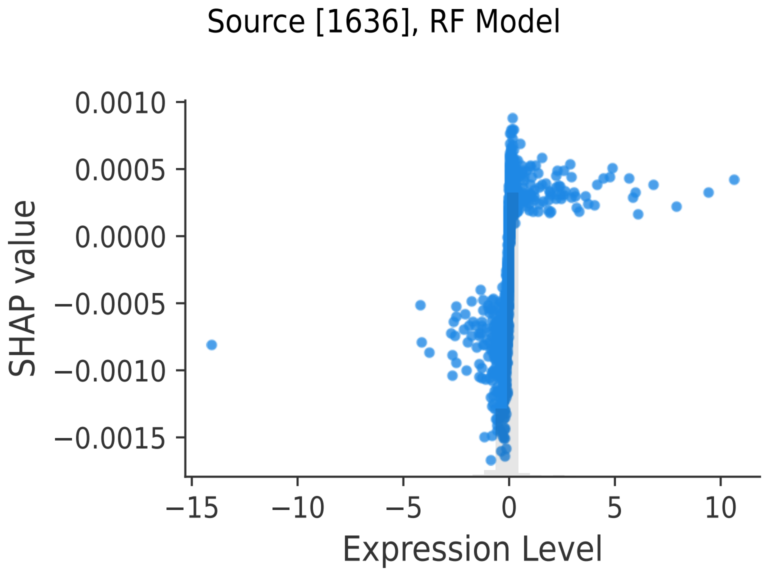
Source [1636], RF Model
0.0010
0.0005
0.0000
−0.0005
−0.0010
−0.0015
SHAP value
−15
−10
−5
0
5
10
Expression Level

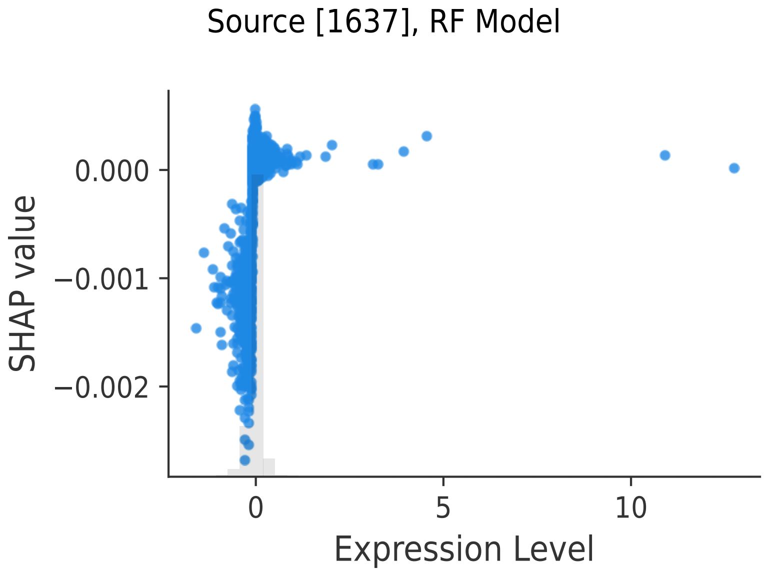
Source [1637], RF Model
SHAP value
0.000
−0.001
−0.002
0
5
10
Expression Level

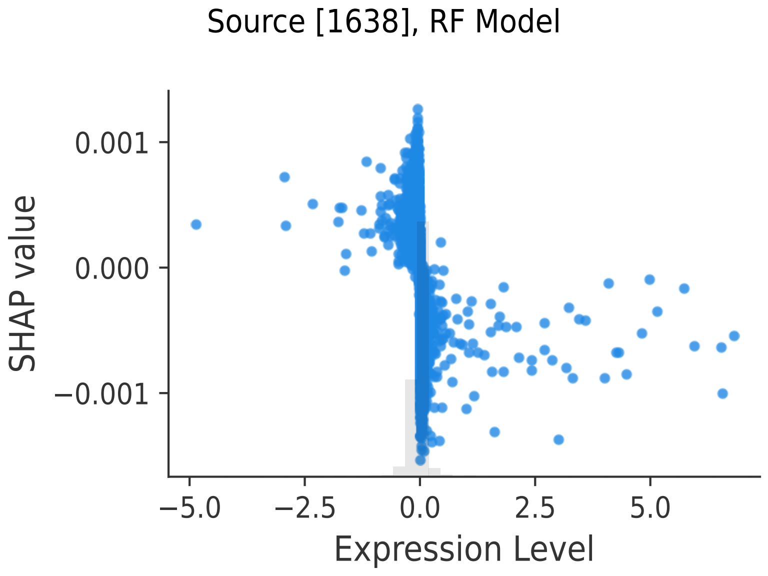
Source [1638], RF Model
0.001
0.000
−0.001
SHAP value
−5.0
−2.5
0.0
2.5
5.0
Expression Level

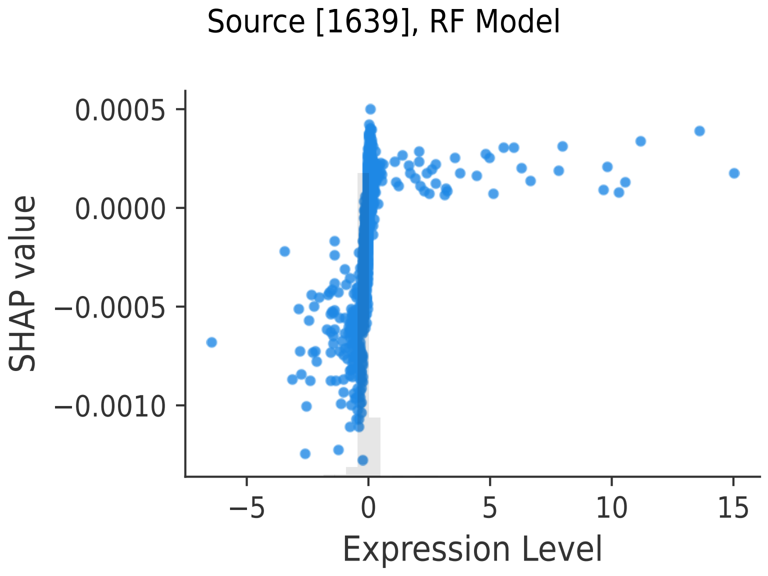
Source [1639], RF Model
SHAP value
0.0005
0.0000
−0.0005
−0.0010
−5
0
5
10
15
Expression Level

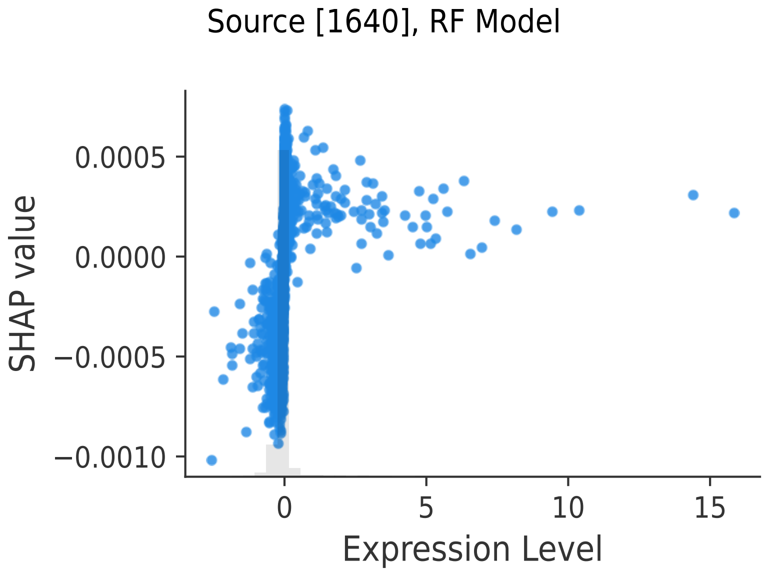

Source [1640], RF Model
SHAP value
0.0005
0.0000
−0.0005
−0.0010
0
5
10
15
Expression Level

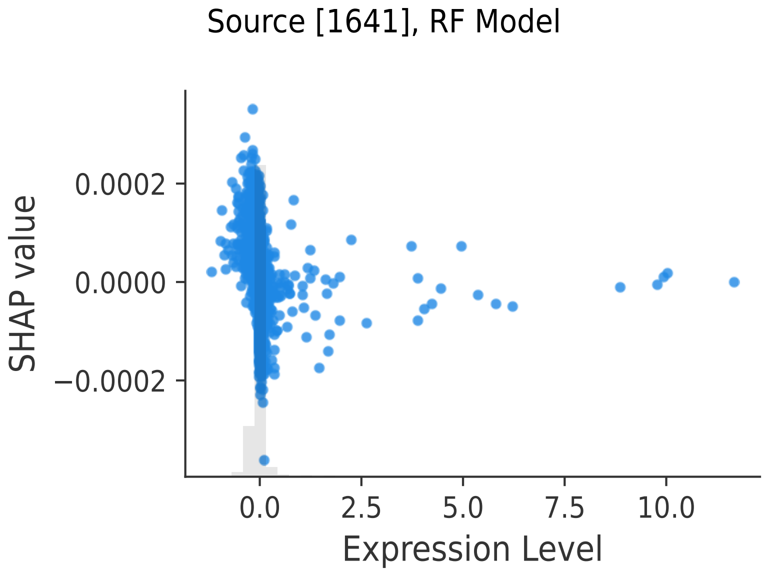

Source [1641], RF Model
0.0002
0.0000
−0.0002
SHAP value
0.0
2.5
5.0
7.5
10.0
Expression Level

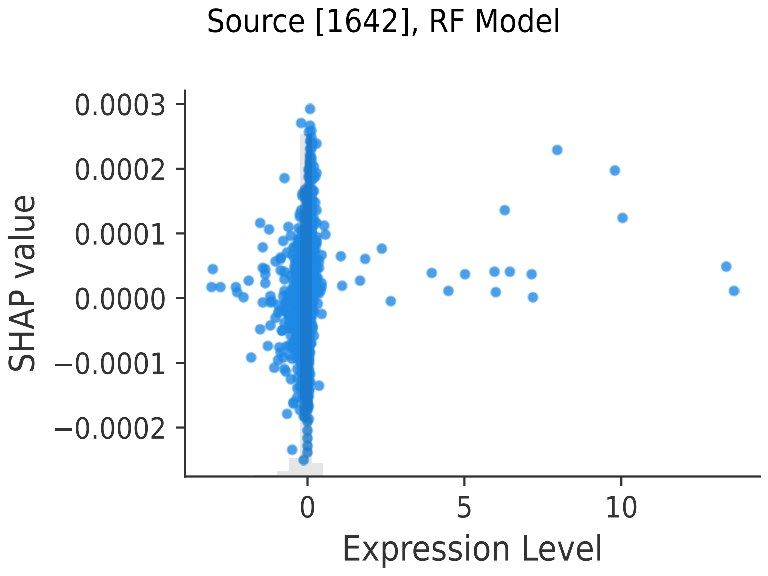
Source [1642], RF Model
0.0003
0.0002
0.0001
0.0000
−0.0001
−0.0002
SHAP value
0
5
10
Expression Level

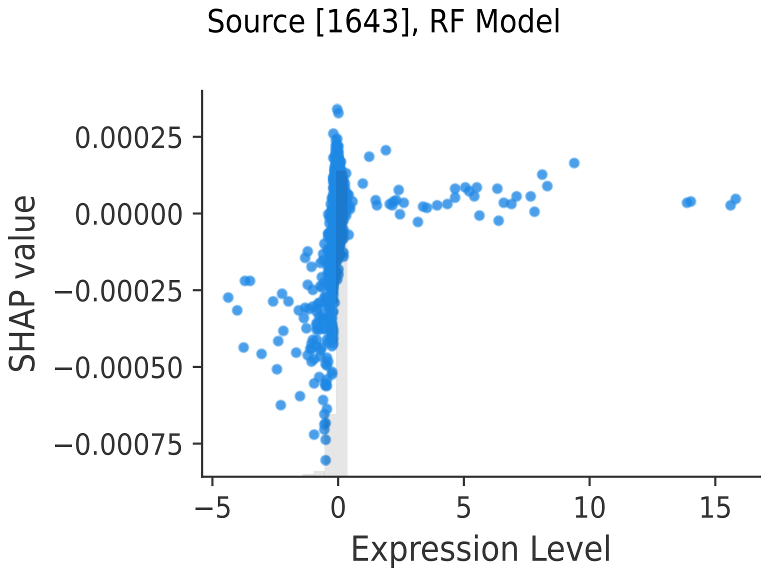

Source [1643], RF Model
0.00025
0.00000
−0.00025
−0.00050
−0.00075
SHAP value
−5
0
5
10
15
Expression Level

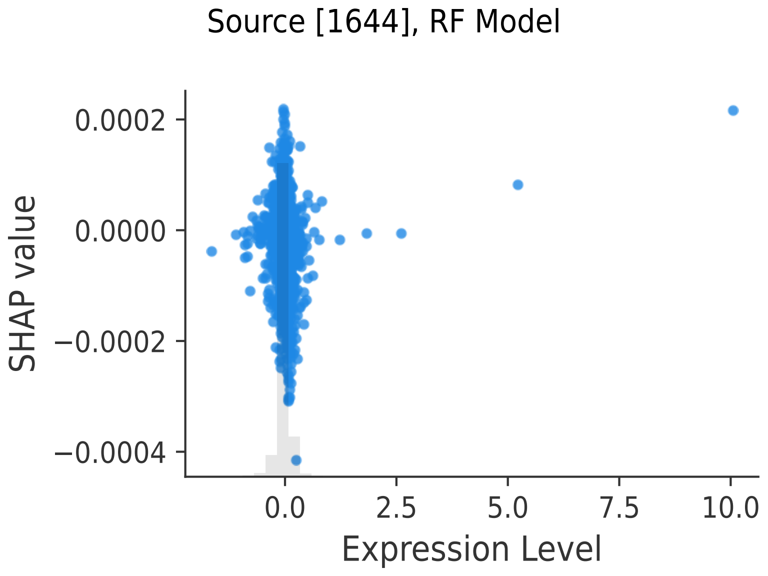

Source [1644], RF Model
0.0002
0.0000
−0.0002
−0.0004
SHAP value
0.0
2.5
5.0
7.5
10.0
Expression Level

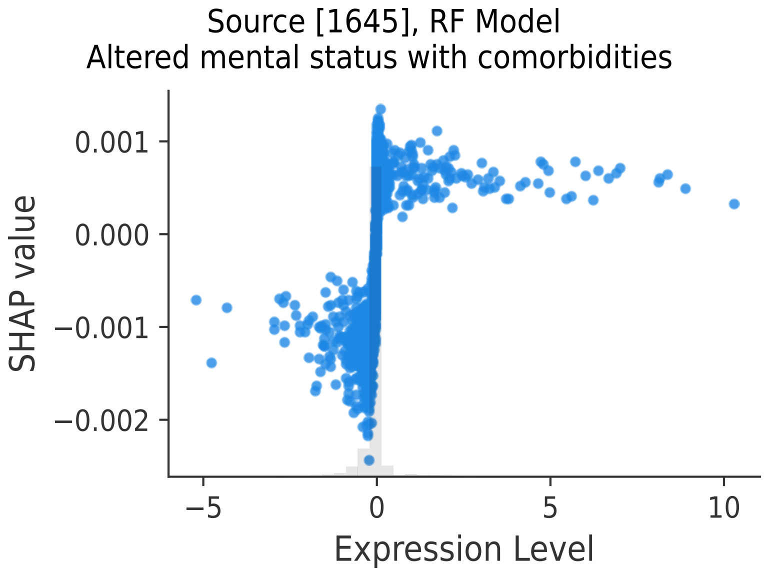

Source [1645], RF Model
Altered mental status with comorbidities
0.001
0.000
−0.001
−0.002
SHAP value
−5
0
5
10
Expression Level

Source [1646], RF Model

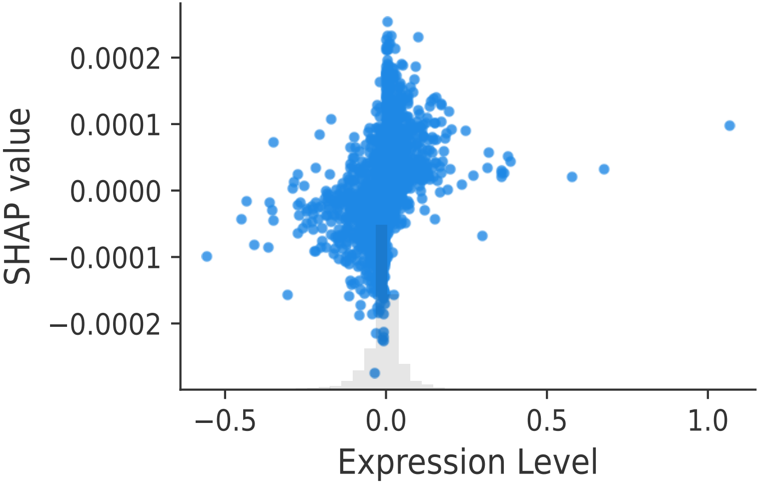

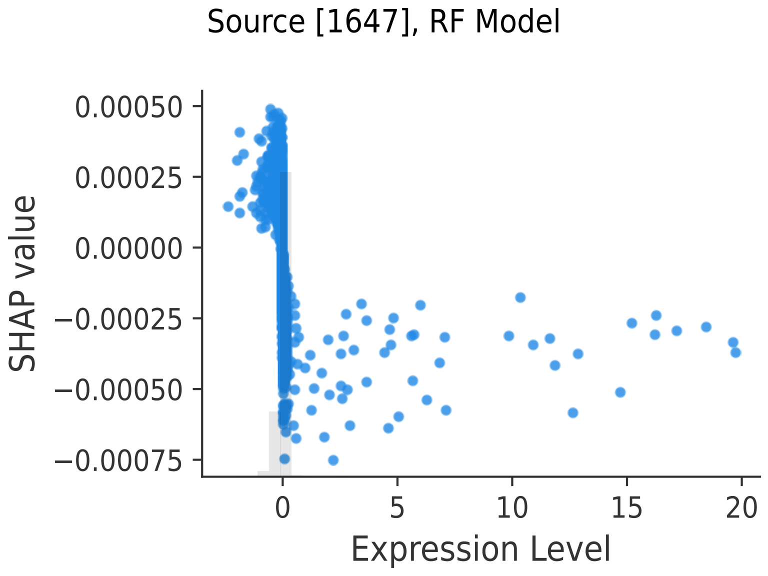
Source [1647], RF Model
SHAP value
0.00050
0.00025
0.00000
−0.00025
−0.00050
−0.00075
0
5
10
15
20
Expression Level

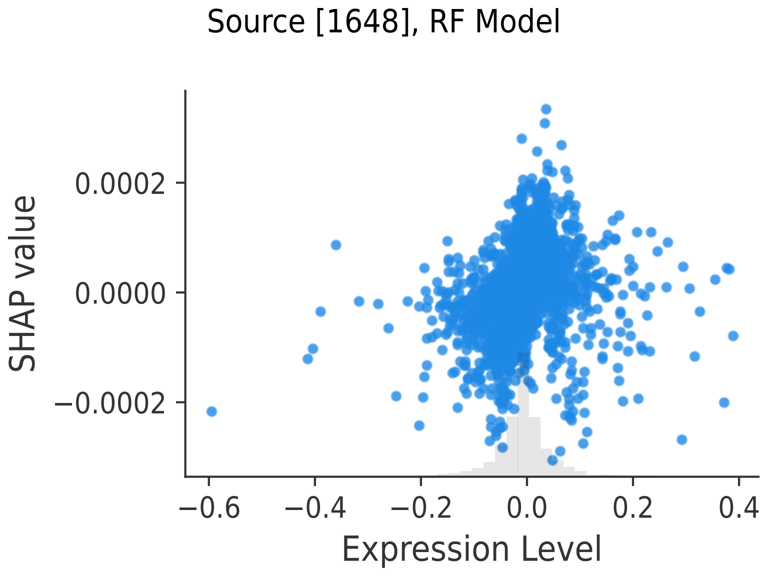
Source [1648], RF Model
SHAP value
0.0002
0.0000
−0.0002
−0.6
−0.4
−0.2
0.0
0.2
0.4
Expression Level

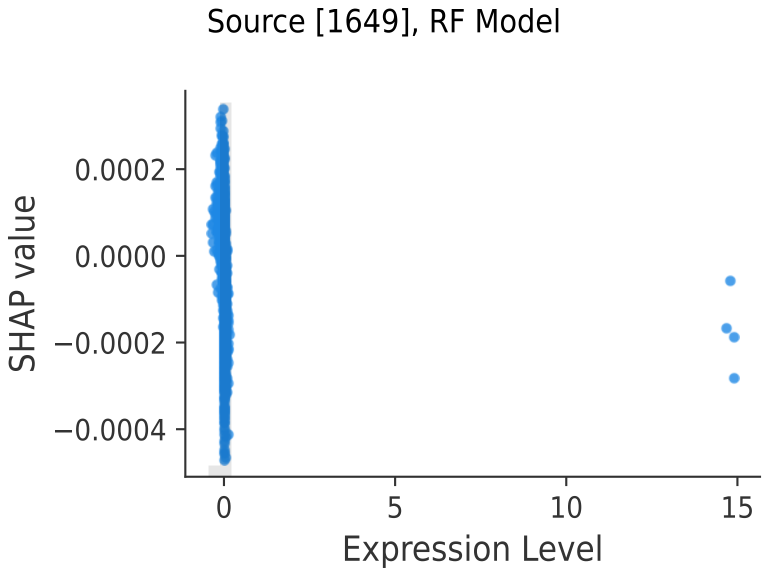
Source [1649], RF Model
0.0002
0.0000
−0.0002
−0.0004
SHAP value
0
5
10
15
Expression Level

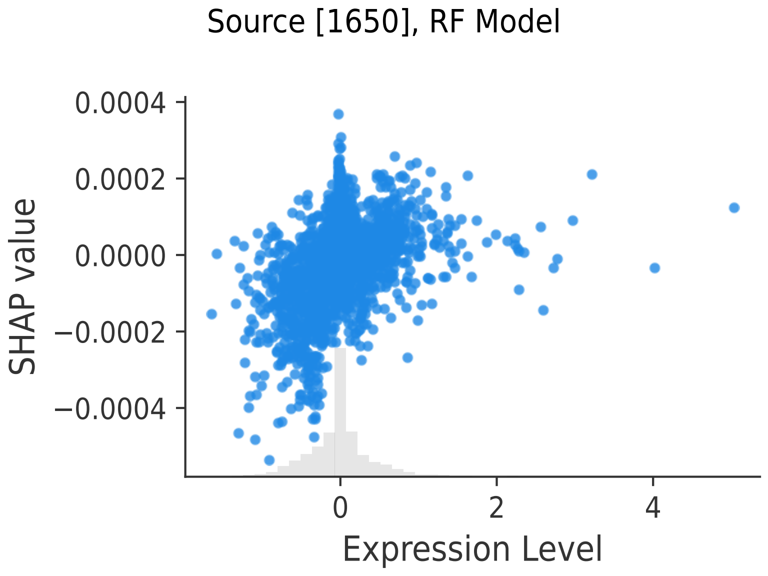

Source [1650], RF Model
0.0004
0.0002
0.0000
−0.0002
−0.0004
SHAP value
0
2
4
Expression Level

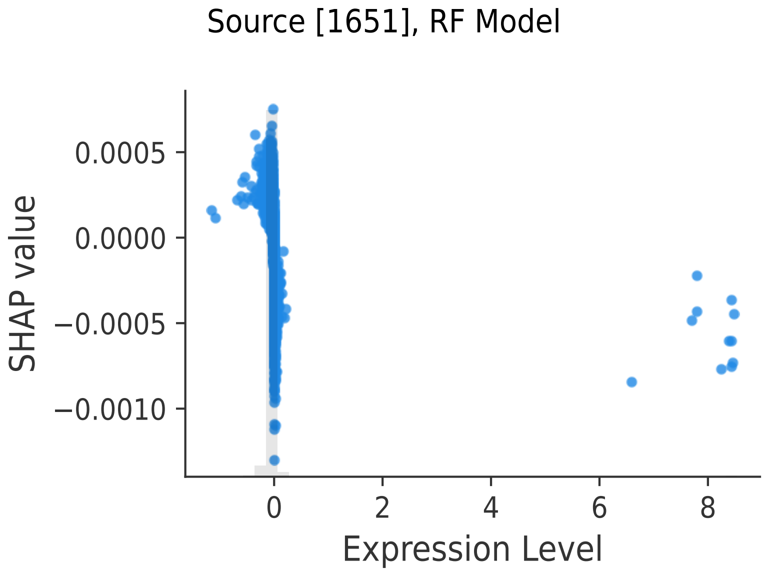

Source [1651], RF Model
SHAP value
0.0005
0.0000
−0.0005
−0.0010
0
2
4
6
8
Expression Level

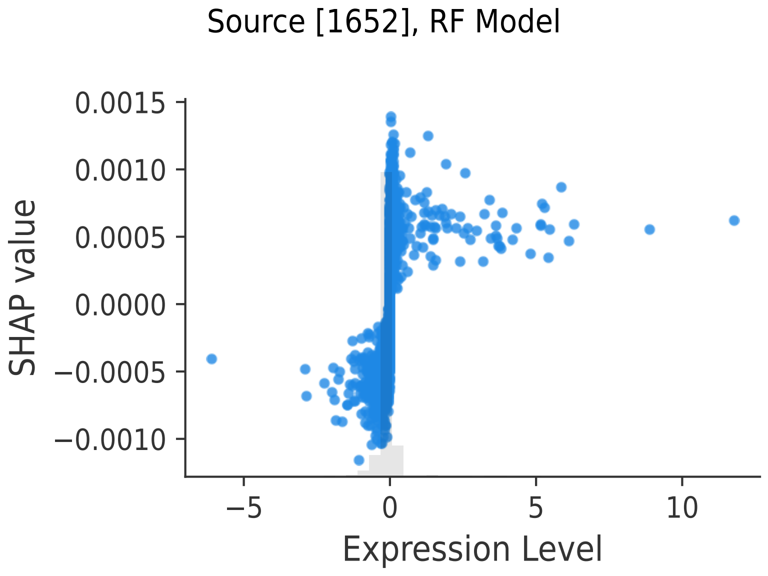

Source [1652], RF Model
0.0015
0.0010
0.0005
0.0000
−0.0005
−0.0010
SHAP value
−5
0
5
10
Expression Level

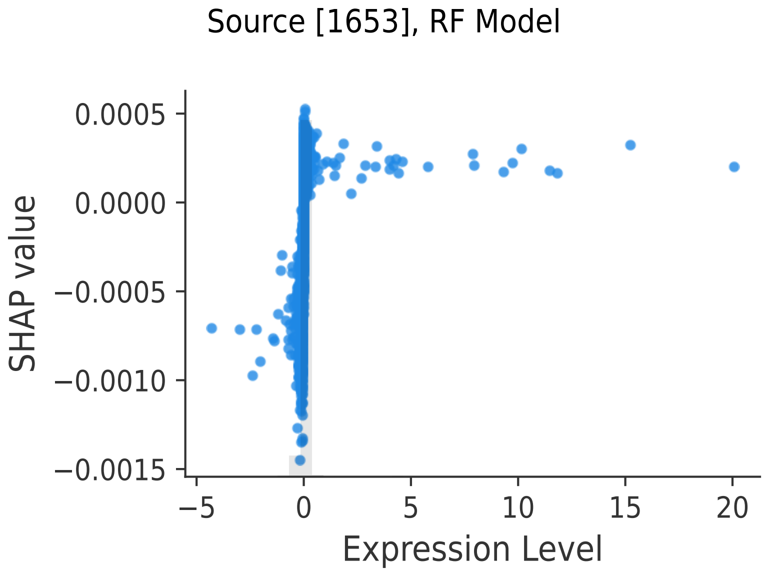
Source [1653], RF Model
SHAP value
0.0005
0.0000
−0.0005
−0.0010
−0.0015
−5
0
5
10
15
20
Expression Level

Source [1654], RF Model

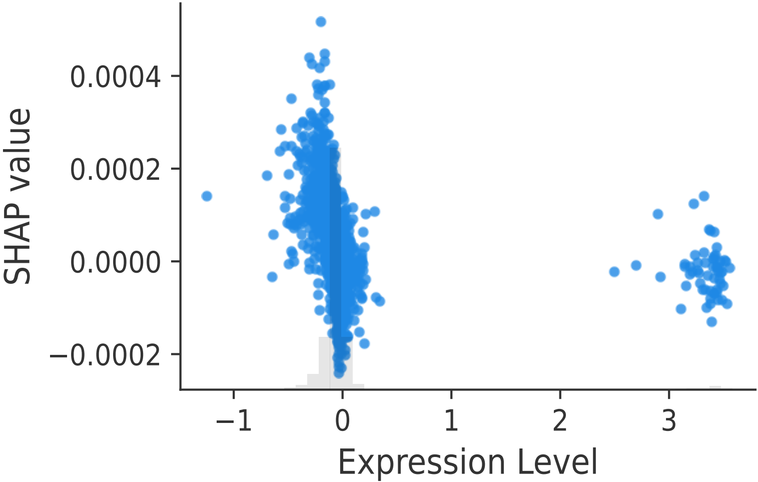

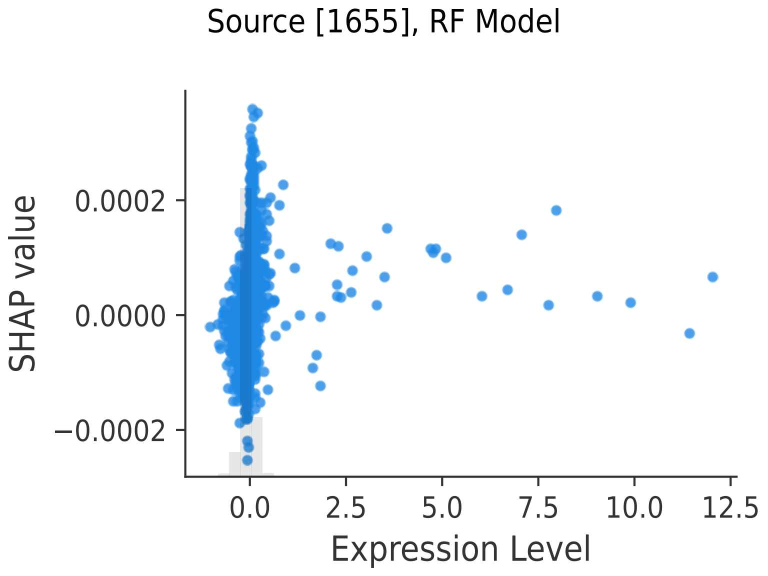
Source [1655], RF Model
SHAP value
0.0002
0.0000
−0.0002
0.0
2.5
5.0
7.5
10.0
12.5
Expression Level

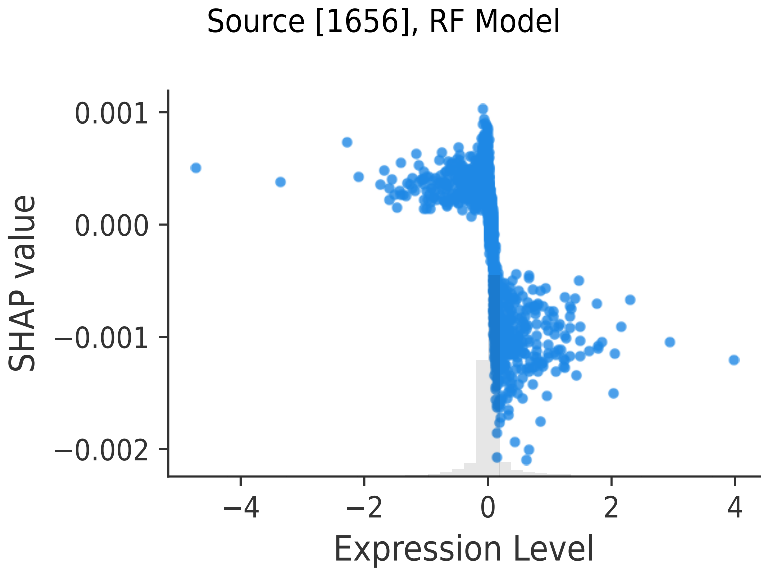
Source [1656], RF Model
SHAP value
0.001
0.000
−0.001
−0.002
−4
−2
0
2
4
Expression Level

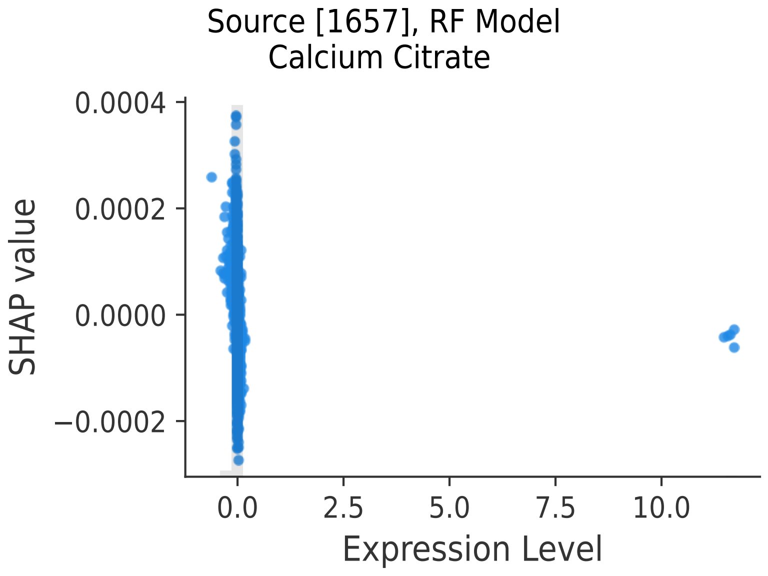
Source [1657], RF Model
Calcium Citrate
SHAP value
0.0004
0.0002
0.0000
−0.0002
0.0
2.5
5.0
7.5
10.0
Expression Level

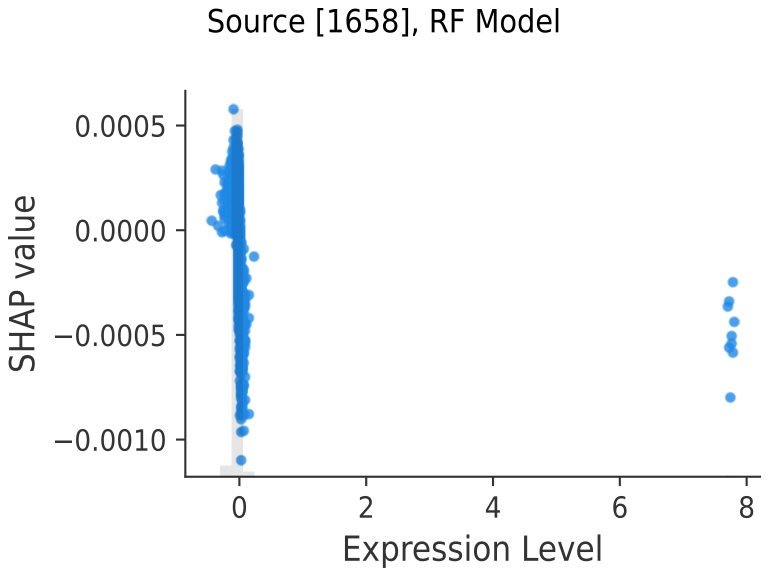
Source [1658], RF Model
SHAP value
0.0005
0.0000
−0.0005
−0.0010
0
2
4
6
8
Expression Level

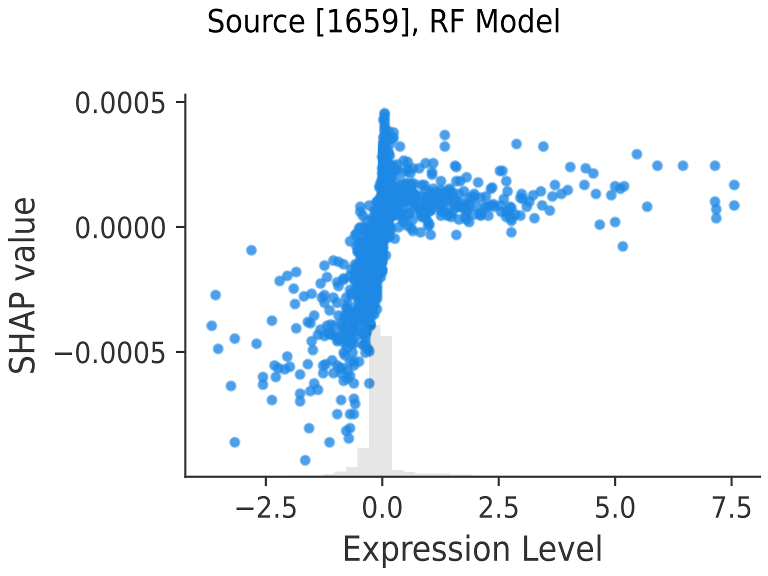

Source [1659], RF Model
0.0005
0.0000
−0.0005
SHAP value
−2.5
0.0
2.5
5.0
7.5
Expression Level

Source [1660], RF Model

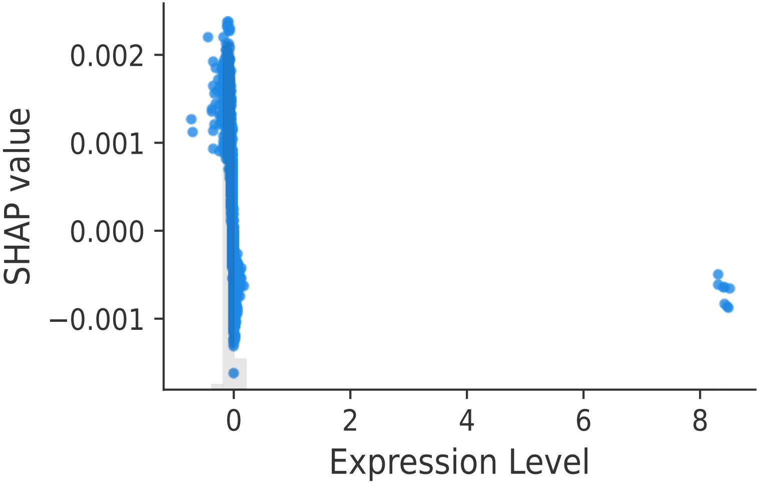

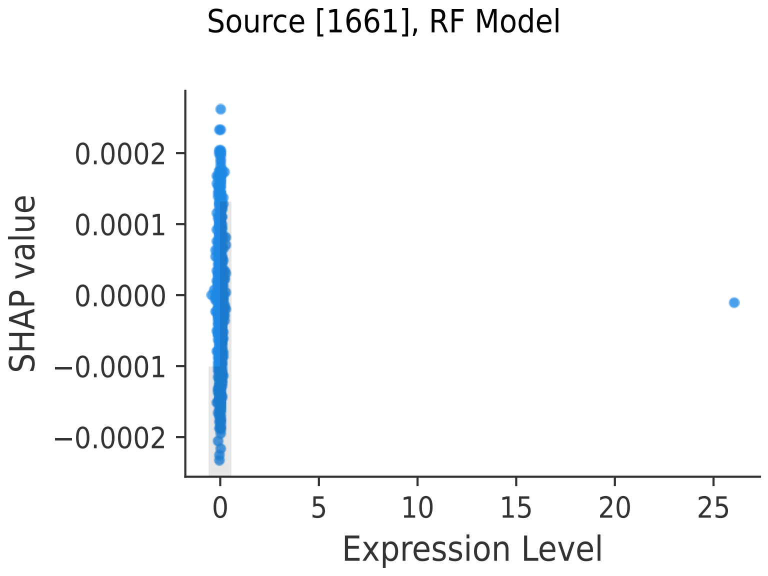

Source [1661], RF Model
SHAP value
0.0002
0.0001
0.0000
−0.0001
−0.0002
0
5
10
15
20
25
Expression Level

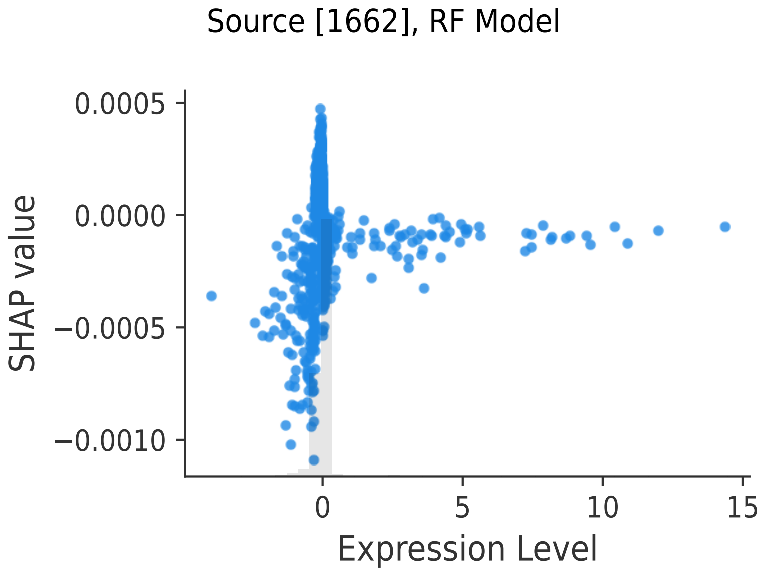

Source [1662], RF Model
0.0005
0.0000
−0.0005
−0.0010
SHAP value
0
5
10
15
Expression Level

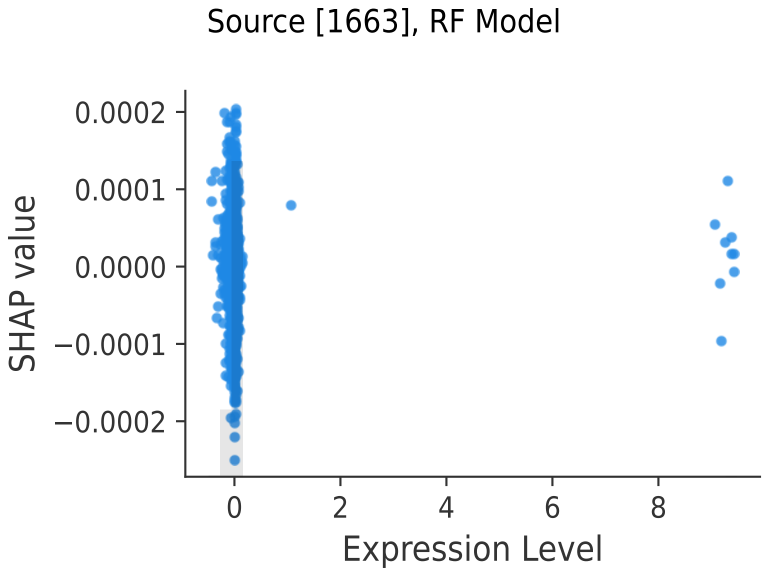

Source [1663], RF Model
SHAP value
0.0002
0.0001
0.0000
−0.0001
−0.0002
0
2
4
6
8
Expression Level

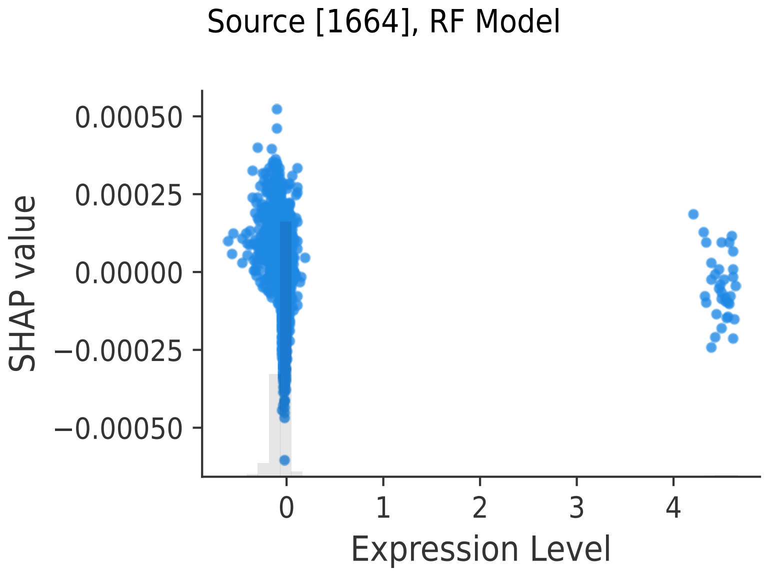

Source [1664], RF Model
SHAP value
0.00050
0.00025
0.00000
−0.00025
−0.00050
0
1
2
3
4
Expression Level

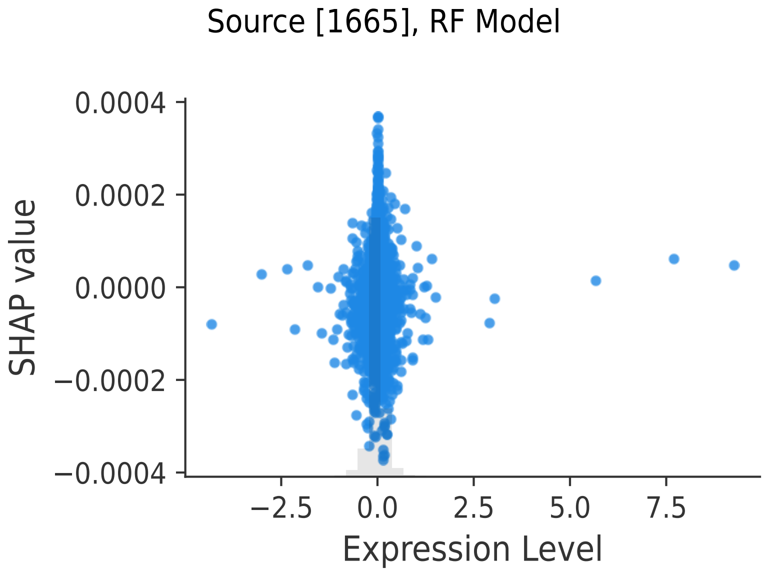
Source [1665], RF Model
0.0004
0.0002
0.0000
−0.0002
−0.0004
SHAP value
−2.5
0.0
2.5
5.0
7.5
Expression Level

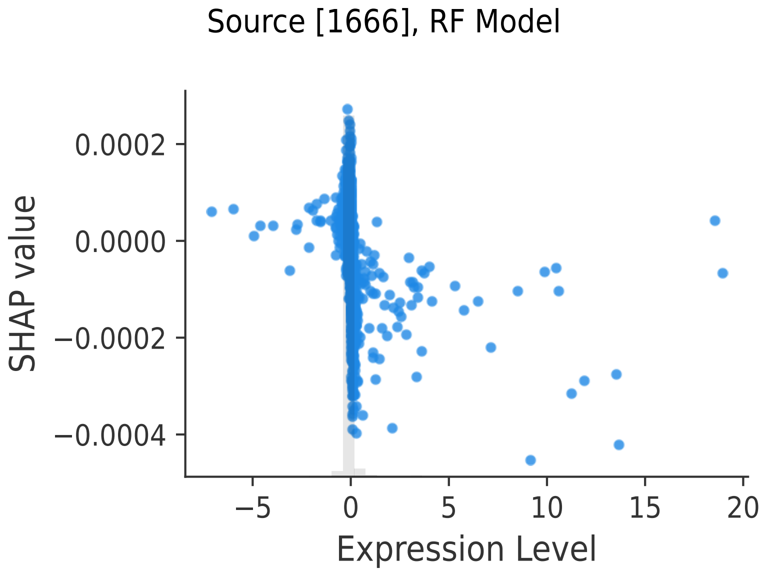

Source [1666], RF Model
0.0002
0.0000
−0.0002
−0.0004
SHAP value
−5
0
5
10
15
20
Expression Level

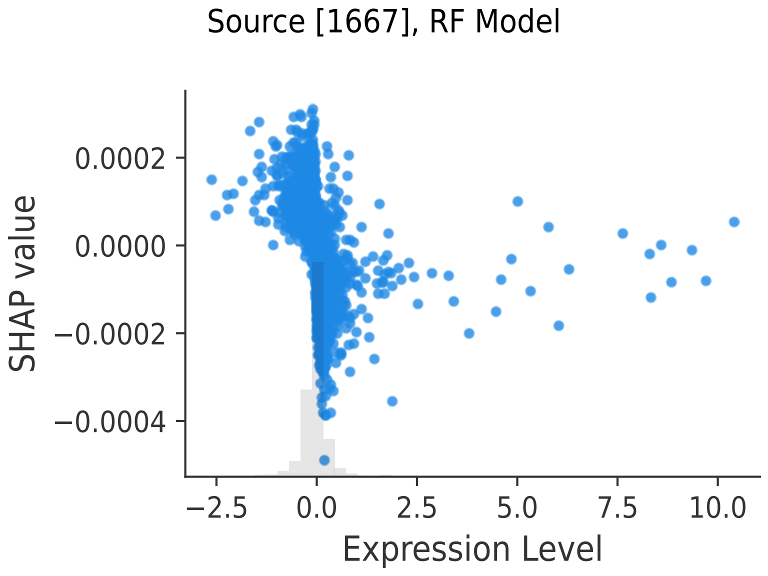

Source [1667], RF Model
SHAP value
0.0002
0.0000
−0.0002
−0.0004
−2.5
0.0
2.5
5.0
7.5
10.0
Expression Level

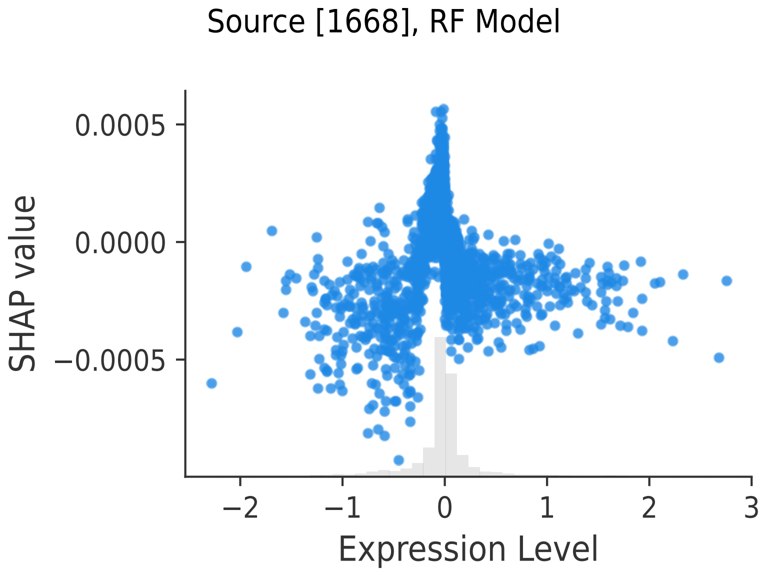
Source [1668], RF Model
SHAP value
0.0005
0.0000
−0.0005
Expression Level
−2
−1
0
1
2
3

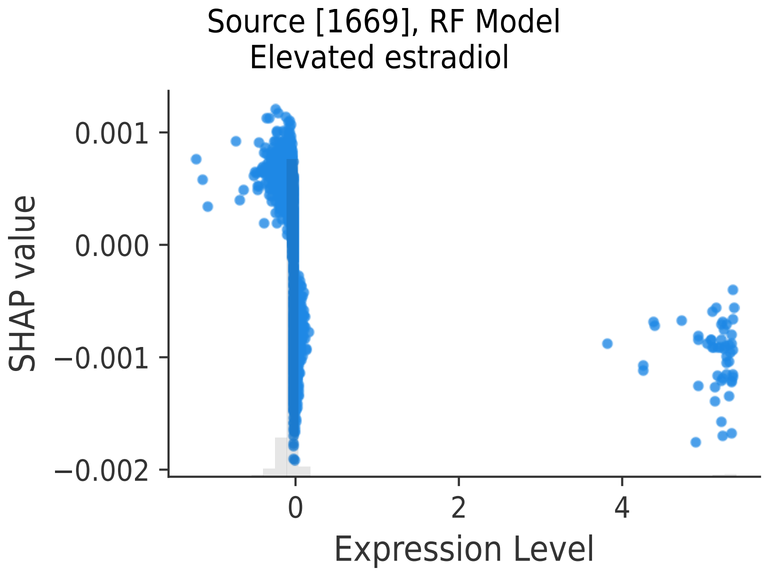
Source [1669], RF Model
Elevated estradiol
SHAP value
0.001
0.000
−0.001
−0.002
0
2
4
Expression Level

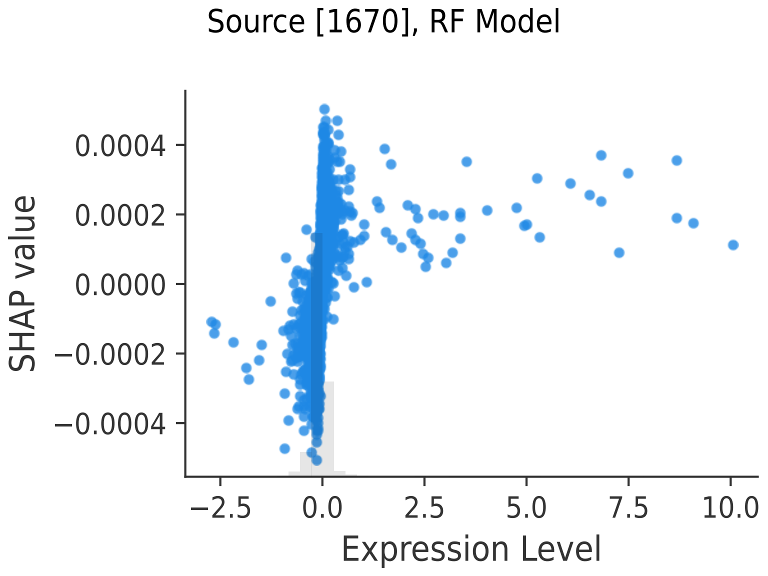
Source [1670], RF Model
SHAP value
0.0004
0.0002
0.0000
−0.0002
−0.0004
−2.5
0.0
2.5
5.0
7.5
10.0
Expression Level

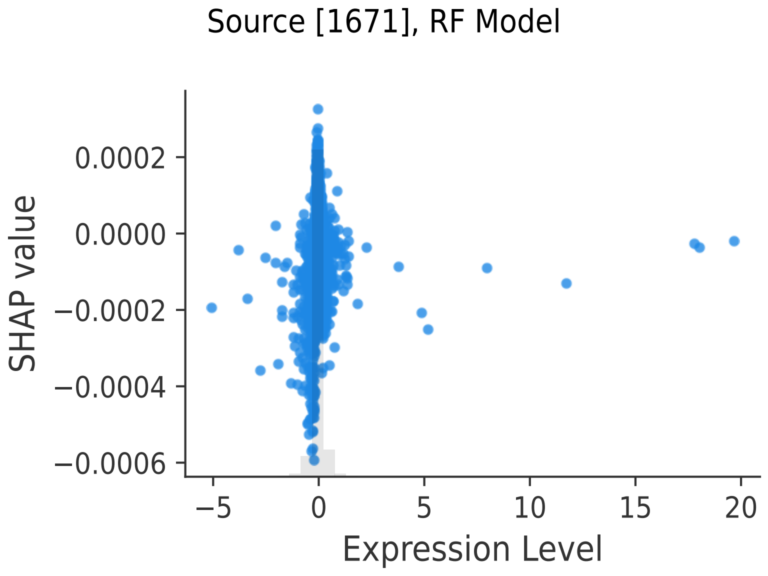
Source [1671], RF Model
SHAP value
0.0002
0.0000
−0.0002
−0.0004
−0.0006
−5
0
5
10
15
20
Expression Level

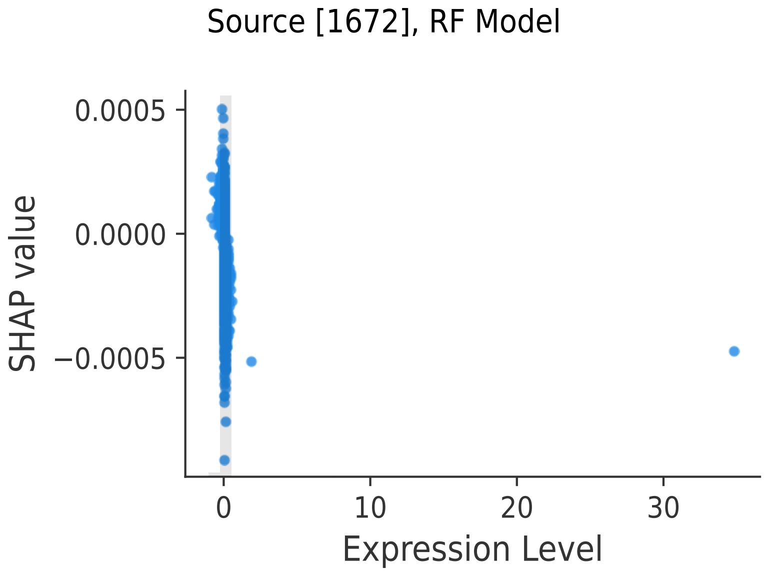
Source [1672], RF Model
SHAP value
0.0005
0.0000
−0.0005
0
10
20
30
Expression Level

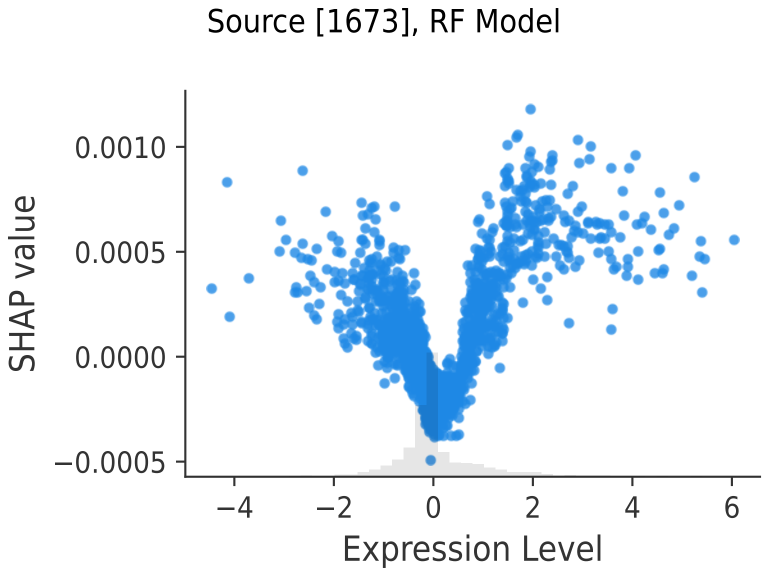

Source [1673], RF Model
SHAP value
0.0010
0.0005
0.0000
−0.0005
−4
−2
0
2
4
6
Expression Level

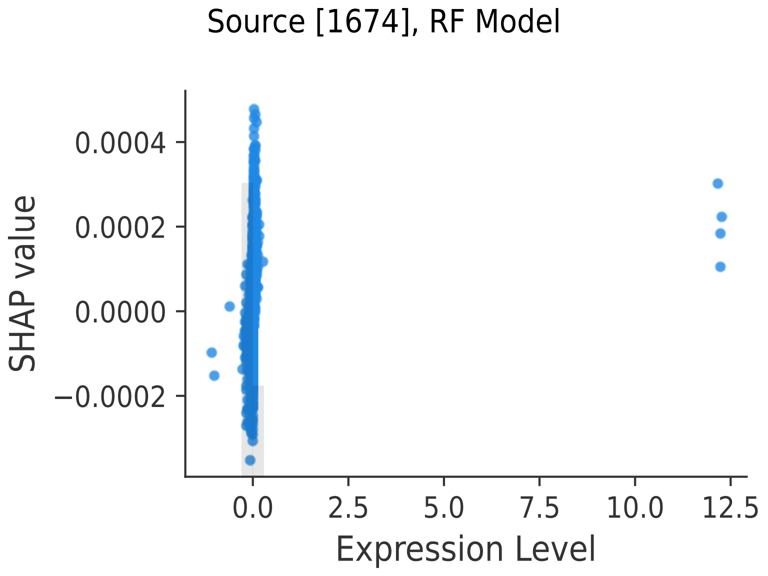

Source [1674], RF Model
SHAP value
0.0004
0.0002
0.0000
−0.0002
0.0
2.5
5.0
7.5
10.0
12.5
Expression Level

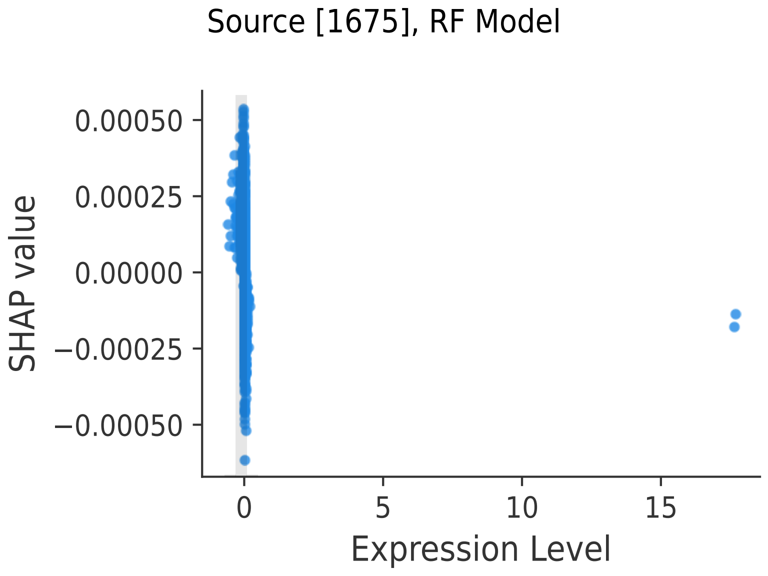
Source [1675], RF Model
0.00050
0.00025
0.00000
−0.00025
−0.00050
SHAP value
0
5
10
15
Expression Level

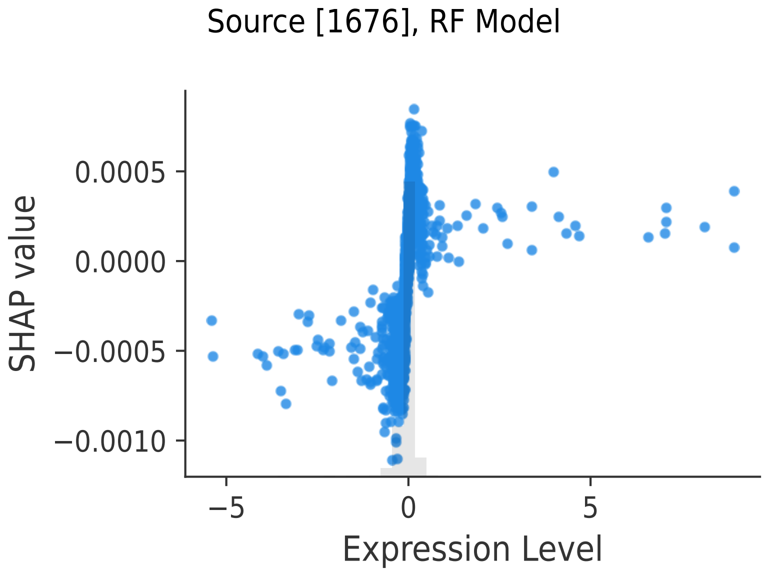

Source [1676], RF Model
0.0005
0.0000
−0.0005
−0.0010
SHAP value
−5
0
5
Expression Level

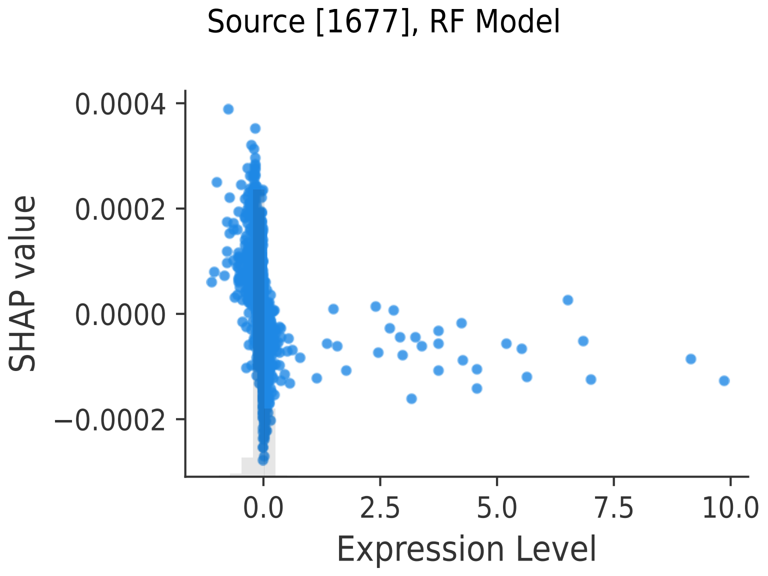

Source [1677], RF Model
0.0004
0.0002
0.0000
−0.0002
SHAP value
0.0
2.5
5.0
7.5
10.0
Expression Level

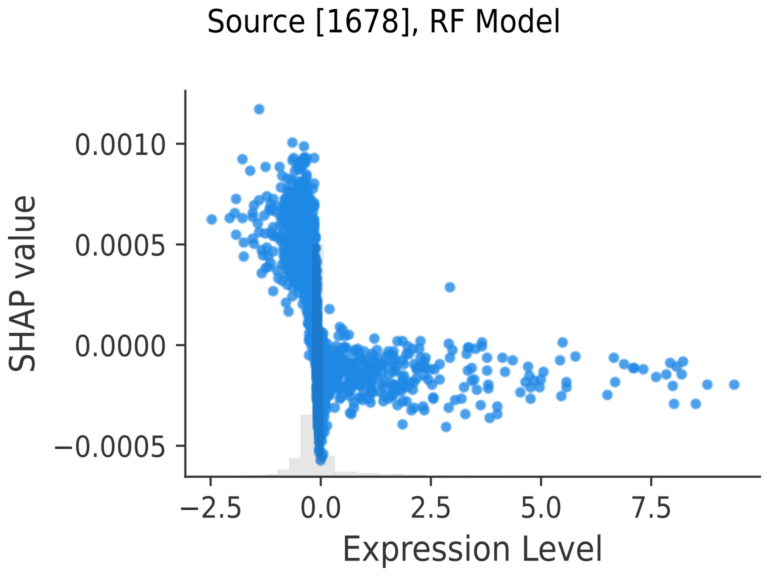

Source [1678], RF Model
SHAP value
0.0010
0.0005
0.0000
−0.0005
−2.5
0.0
2.5
5.0
7.5
Expression Level

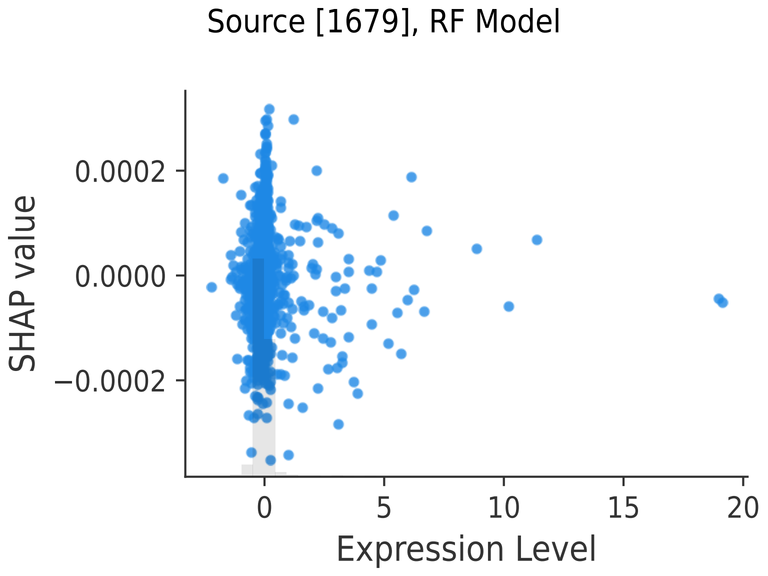
Source [1679], RF Model
SHAP value
0.0002
0.0000
−0.0002
0
5
10
15
20
Expression Level

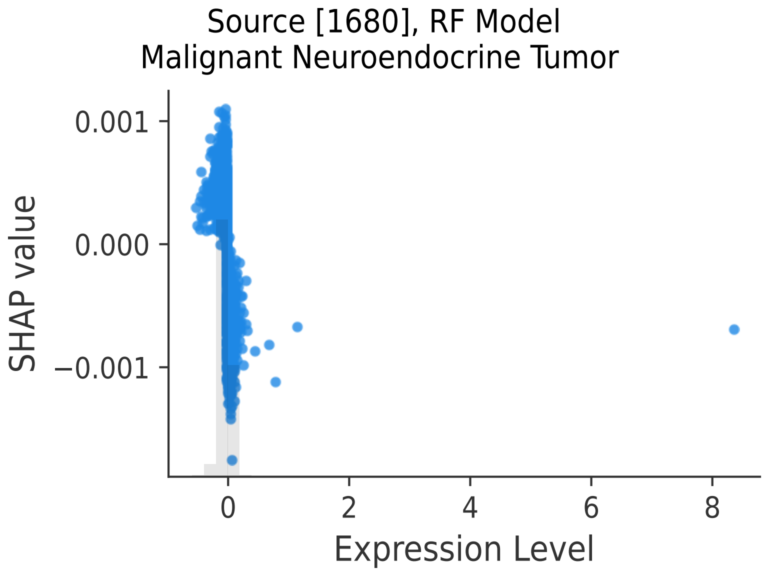
Source [1680], RF Model
Malignant Neuroendocrine Tumor
SHAP value
0.001
0.000
−0.001
0
2
4
6
8
Expression Level

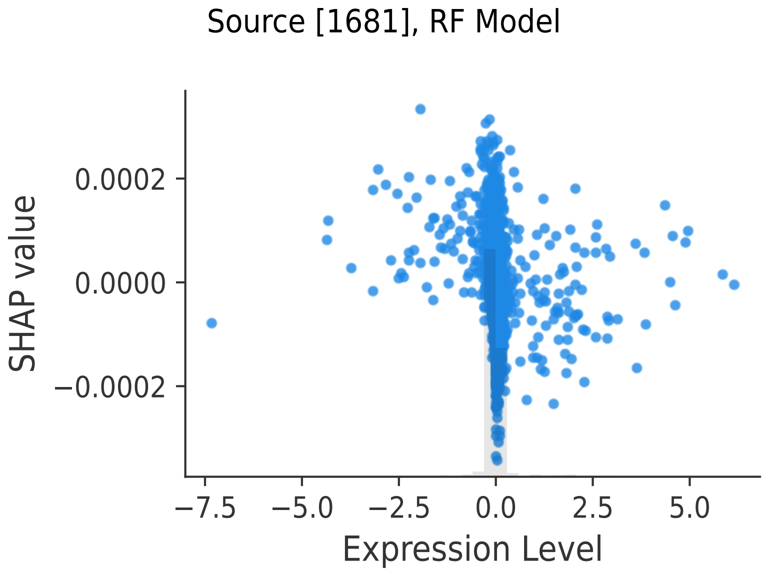

Source [1681], RF Model
SHAP value
0.0002
0.0000
−0.0002
−7.5
−5.0
−2.5
0.0
2.5
5.0
Expression Level

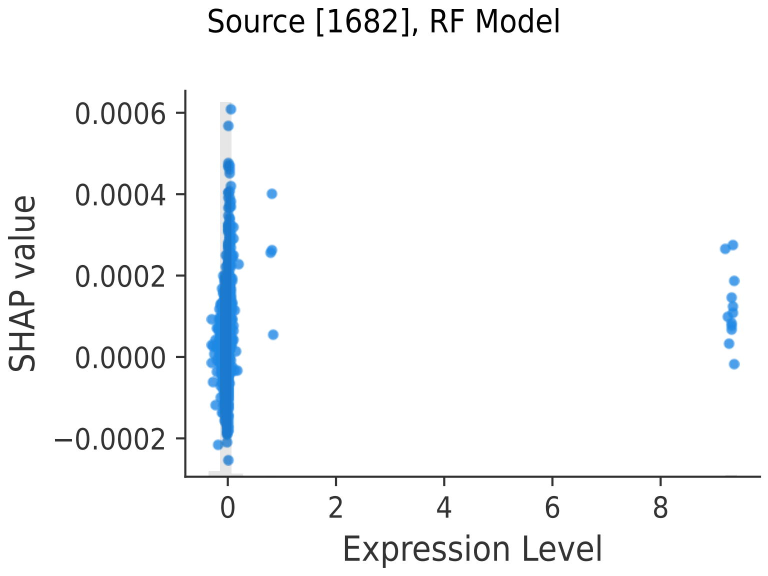
Source [1682], RF Model
SHAP value
0.0006
0.0004
0.0002
0.0000
−0.0002
0
2
4
6
8
Expression Level

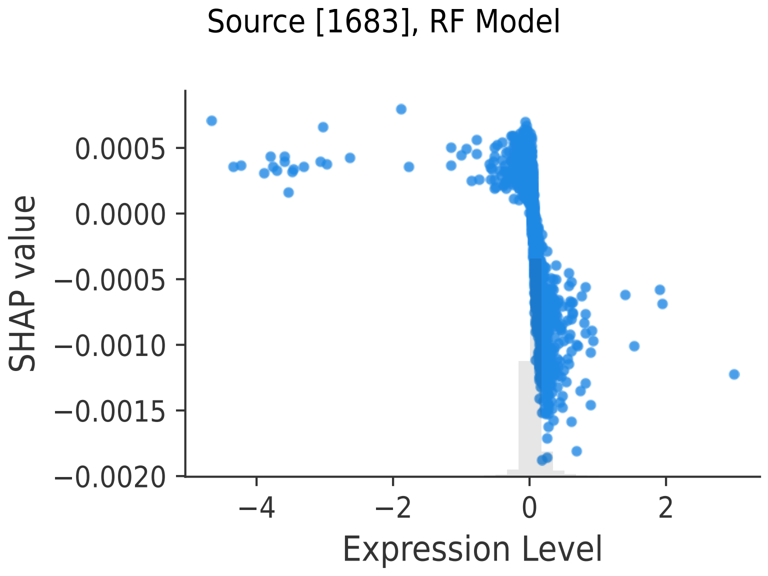

Source [1683], RF Model
SHAP value
0.0005
0.0000
−0.0005
−0.0010
−0.0015
−0.0020
−4
−2
0
2
Expression Level

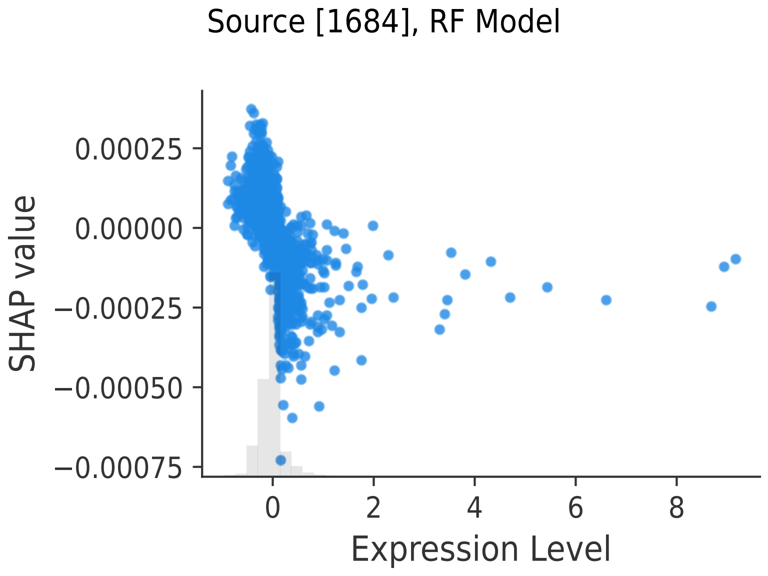

Source [1684], RF Model
0.00025
0.00000
−0.00025
−0.00050
−0.00075
SHAP value
0
2
4
6
8
Expression Level

Source [1685], RF Model

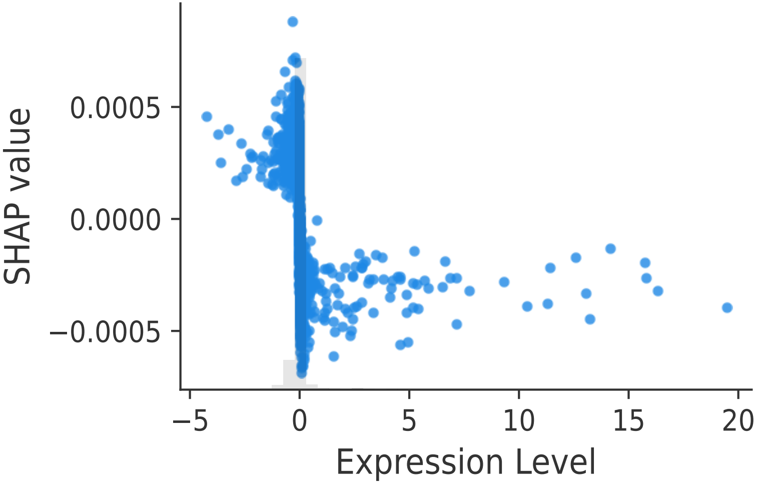

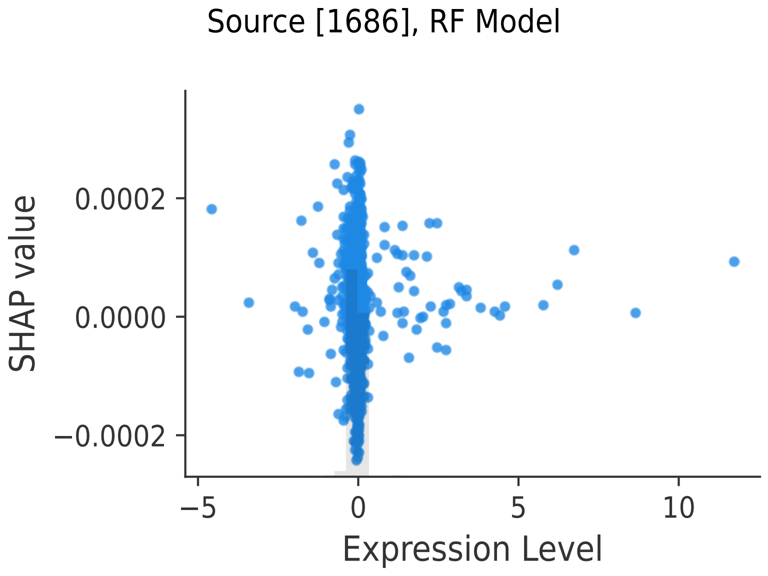
Source [1686], RF Model
0.0002
0.0000
−0.0002
SHAP value
−5
0
5
10
Expression Level

Source [1687], RF Model

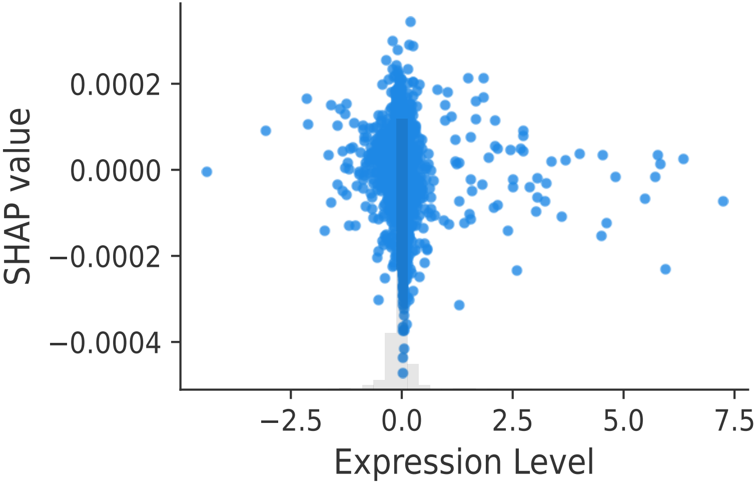

Source [1688], RF Model

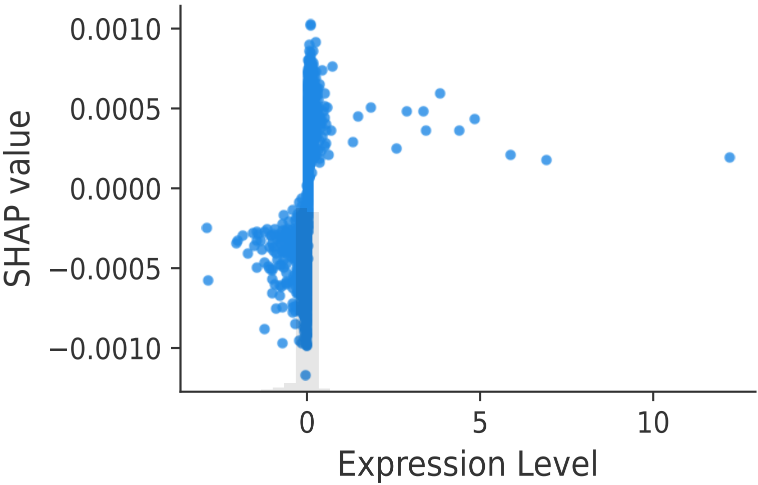

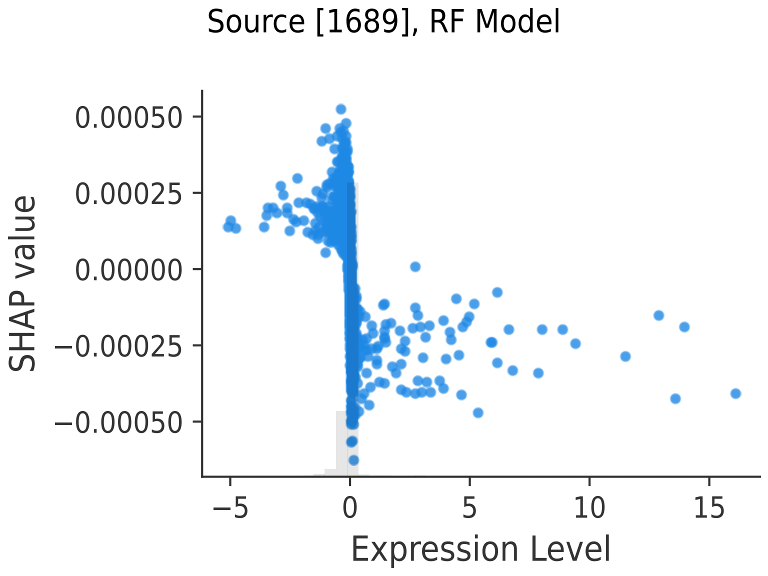

Source [1689], RF Model
SHAP value
0.00050
0.00025
0.00000
−0.00025
−0.00050
−5
0
5
10
15
Expression Level

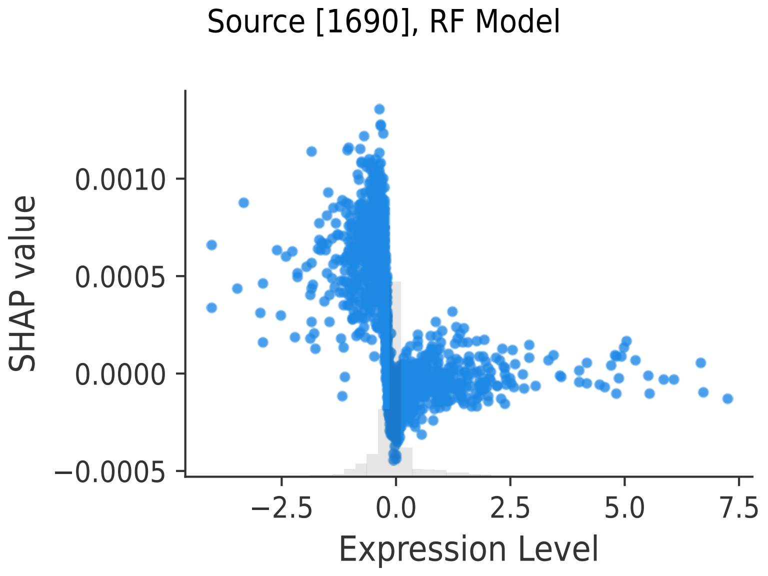
Source [1690], RF Model
SHAP value
0.0010
0.0005
0.0000
−0.0005
−2.5
0.0
2.5
5.0
7.5
Expression Level

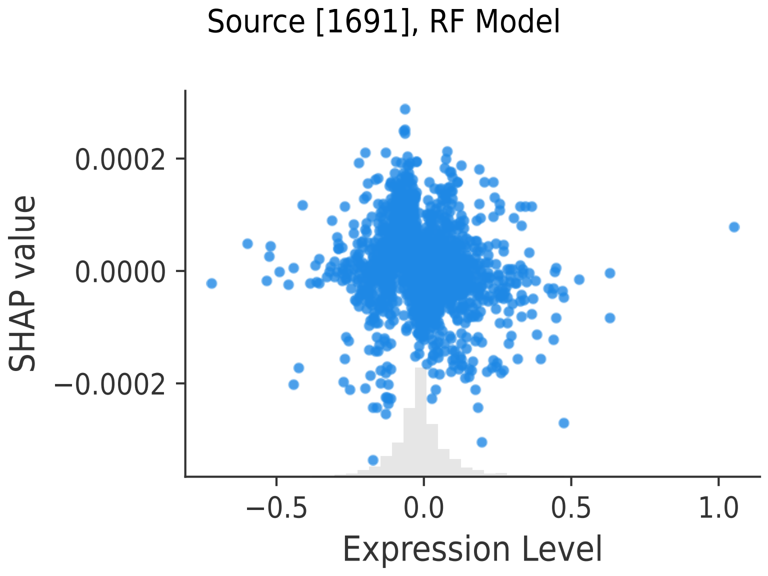

Source [1691], RF Model
0.0002
0.0000
−0.0002
SHAP value
−0.5
0.0
0.5
1.0
Expression Level

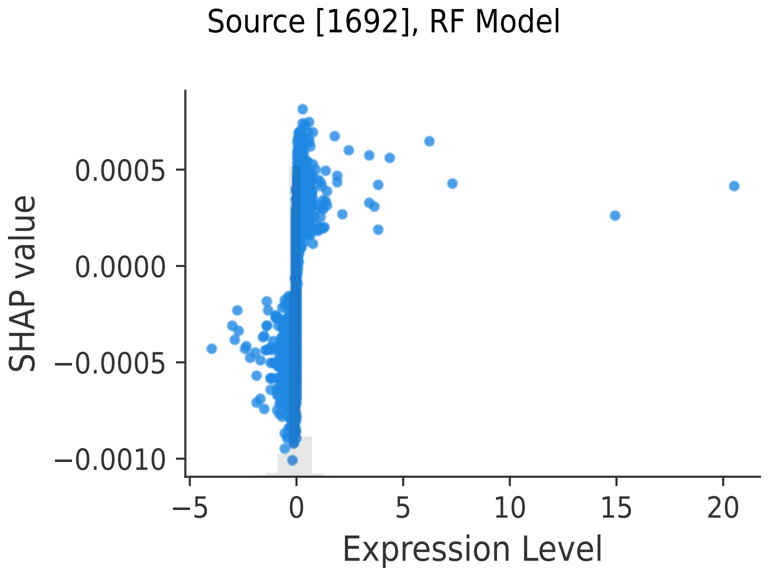

Source [1692], RF Model
SHAP value
0.0005
0.0000
−0.0005
−0.0010
−5
0
5
10
15
20
Expression Level

Source [1693], RF Model

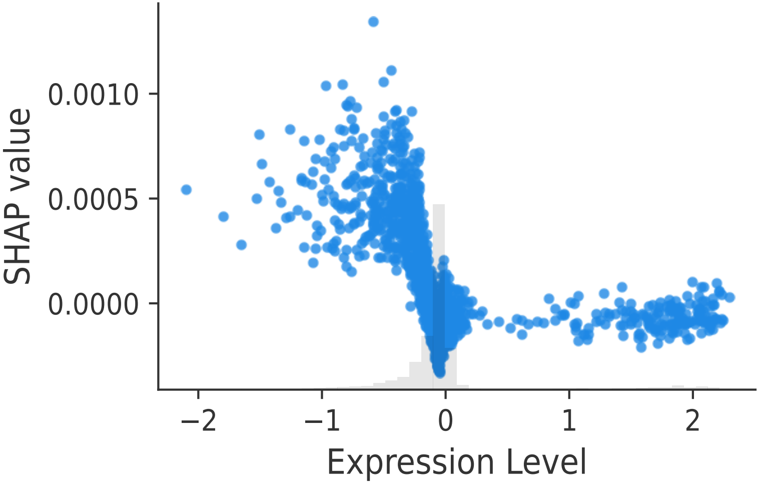

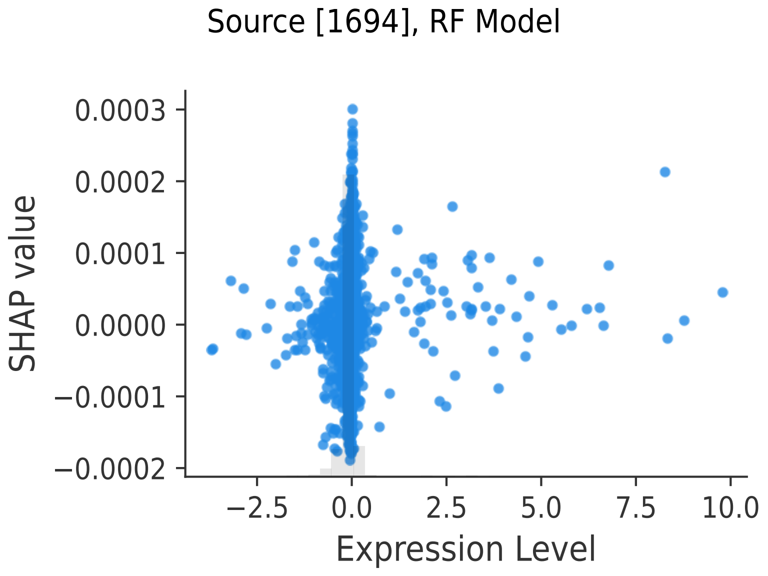
Source [1694], RF Model
SHAP value
0.0003
0.0002
0.0001
0.0000
−0.0001
−0.0002
−2.5
0.0
2.5
5.0
7.5
10.0
Expression Level

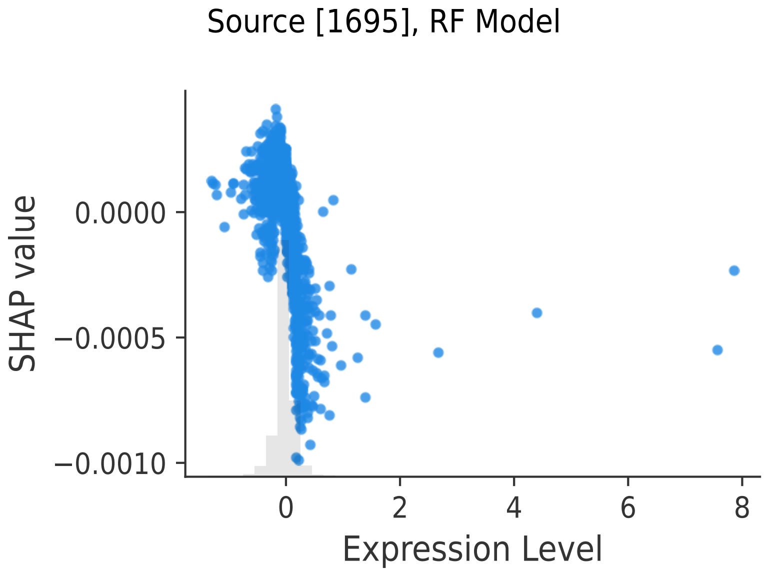
Source [1695], RF Model
SHAP value
0.0000
−0.0005
−0.0010
0
2
4
6
8
Expression Level

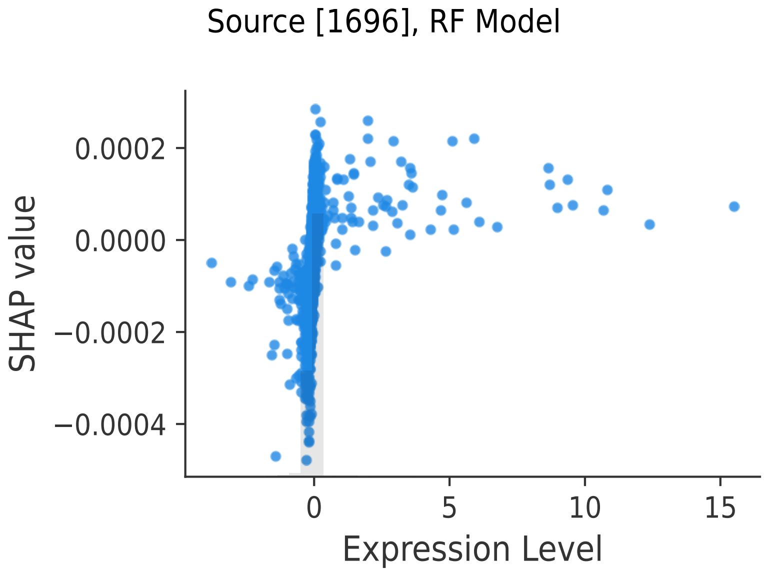

Source [1696], RF Model
0.0002
0.0000
−0.0002
−0.0004
SHAP value
0
5
10
15
Expression Level

Source [1697], RF Model

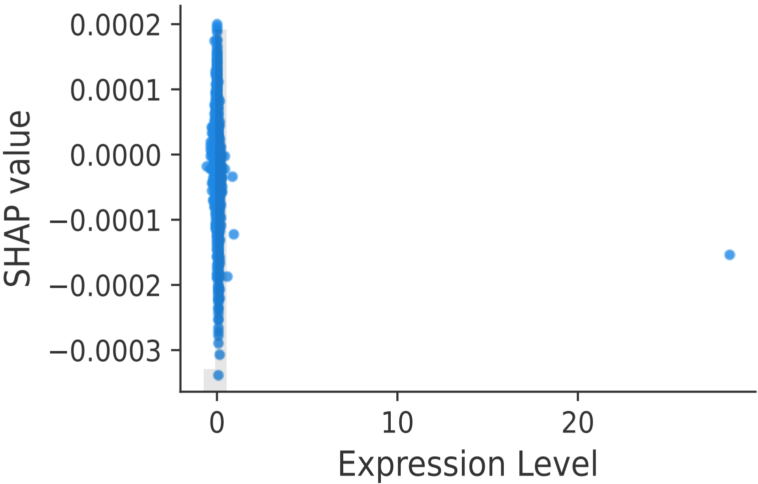

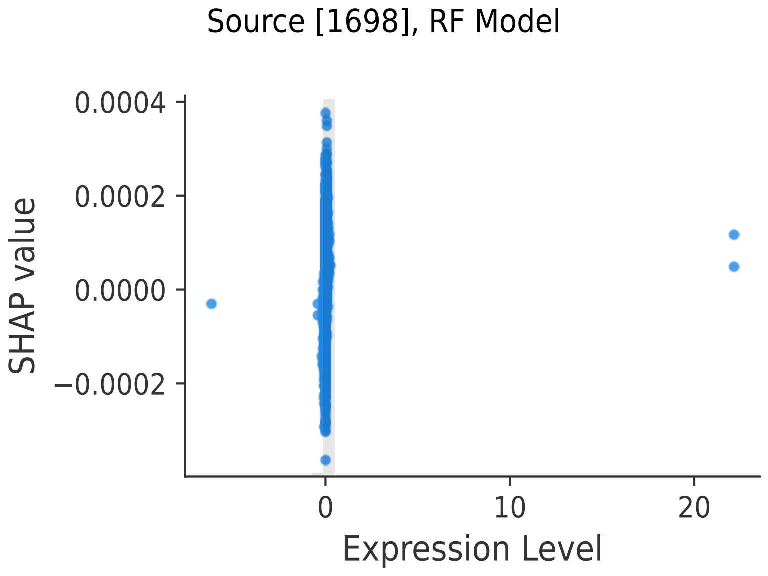
Source [1698], RF Model
0.0004
0.0002
0.0000
−0.0002
SHAP value
0
10
20
Expression Level

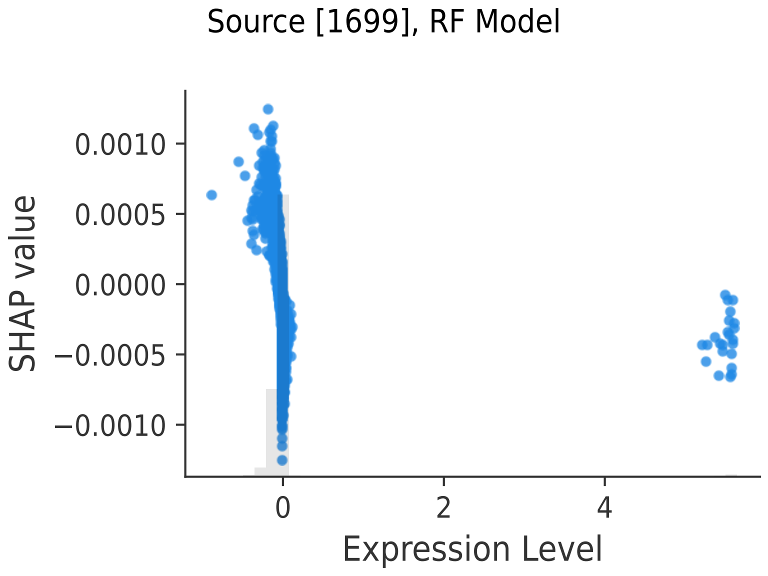
Source [1699], RF Model
0.0010
0.0005
0.0000
−0.0005
−0.0010
SHAP value
0
2
4
Expression Level

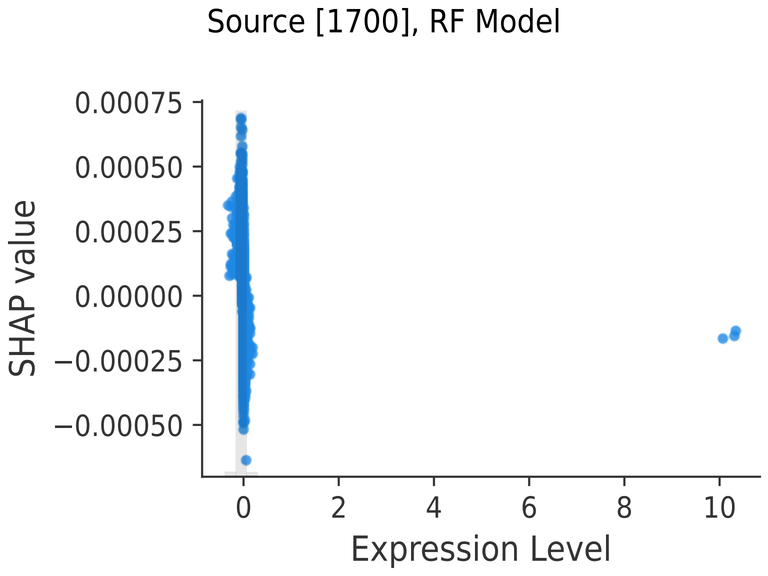
Source [1700], RF Model
SHAP value
0.00075
0.00050
0.00025
0.00000
−0.00025
−0.00050
0
2
4
6
8
10
Expression Level

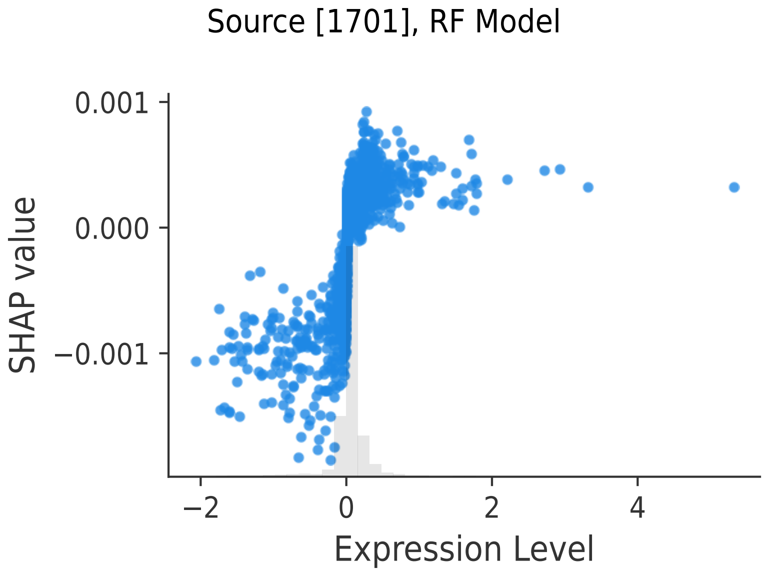

Source [1701], RF Model
SHAP value
0.001
0.000
−0.001
−2
0
2
4
Expression Level

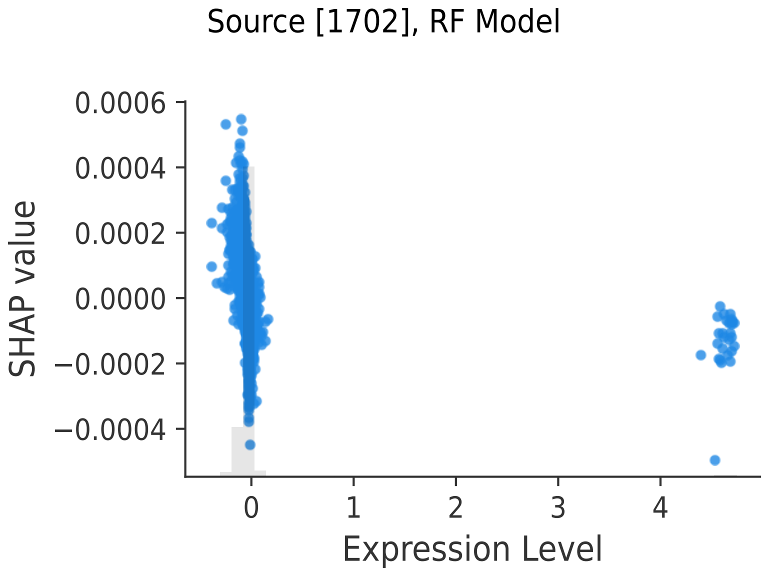

Source [1702], RF Model
SHAP value
0.0006
0.0004
0.0002
0.0000
−0.0002
−0.0004
0
1
2
3
4
Expression Level

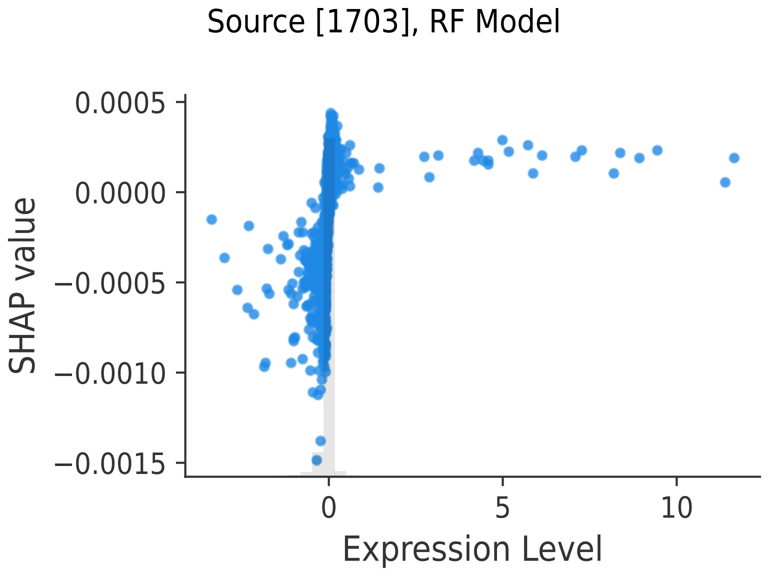

Source [1703], RF Model
0.0005
0.0000
−0.0005
−0.0010
−0.0015
SHAP value
0
5
10
Expression Level

Source [1704], RF Model

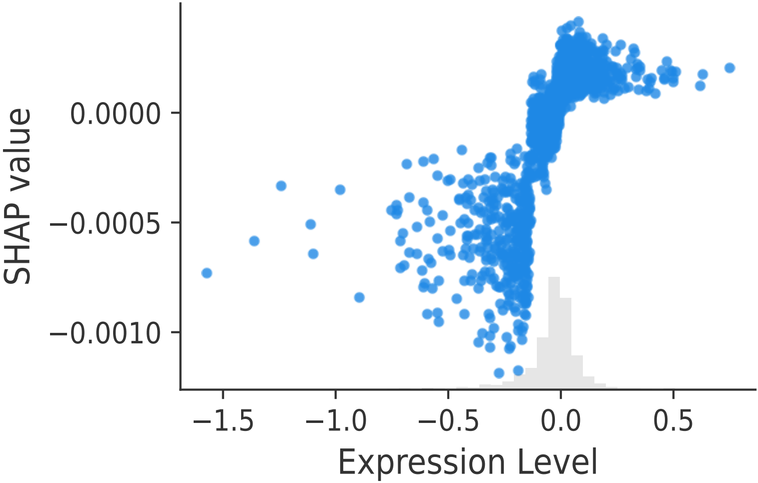

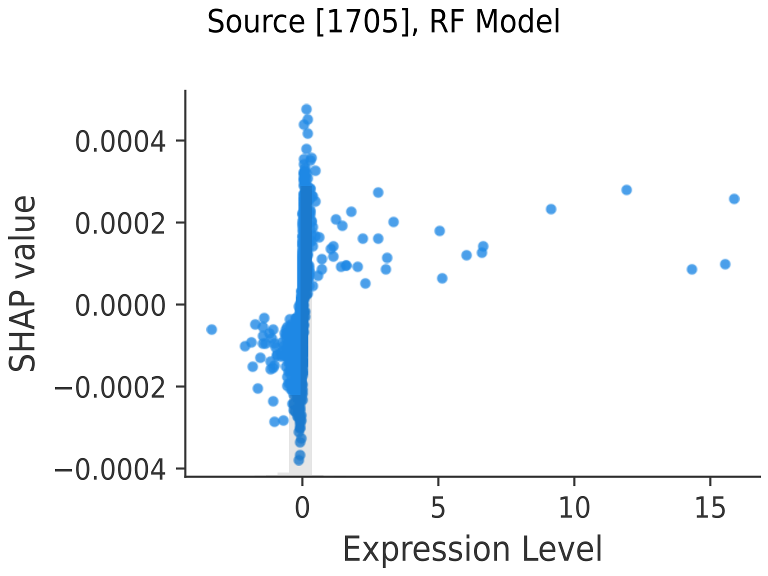

Source [1705], RF Model
SHAP value
0.0004
0.0002
0.0000
−0.0002
−0.0004
0
5
10
15
Expression Level

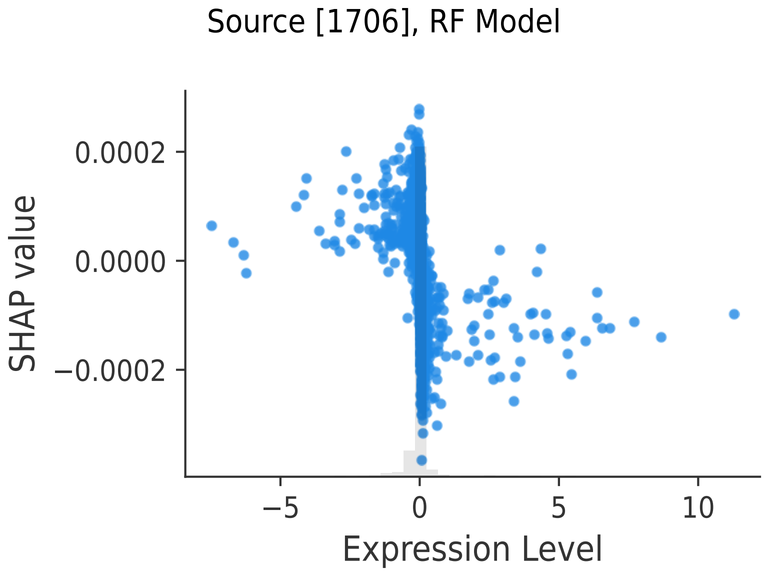

Source [1706], RF Model
SHAP value
0.0002
0.0000
−0.0002
−5
0
5
10
Expression Level

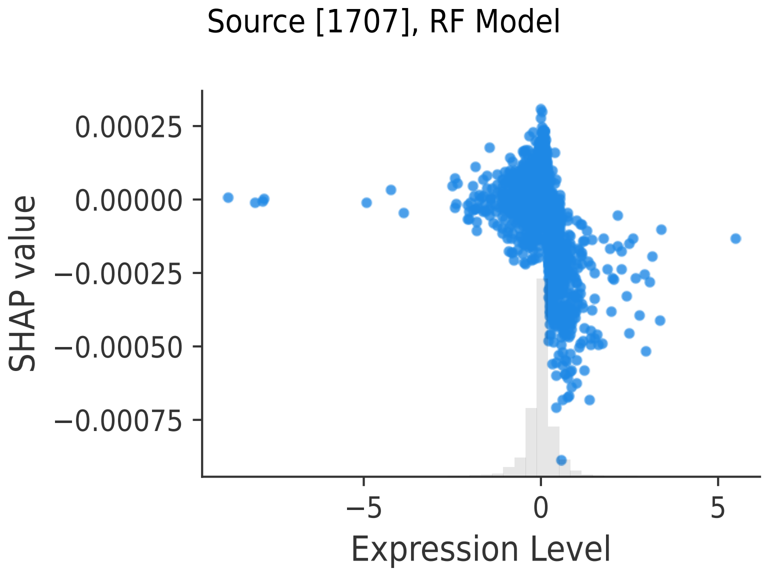
Source [1707], RF Model
SHAP value
0.00025
0.00000
−0.00025
−0.00050
−0.00075
−5
0
5
Expression Level

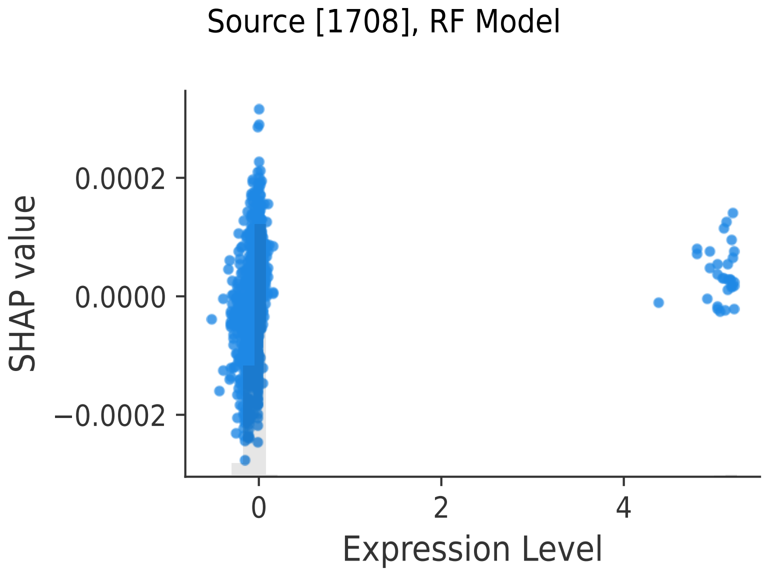
Source [1708], RF Model
SHAP value
0.0002
0.0000
−0.0002
0
2
4
Expression Level

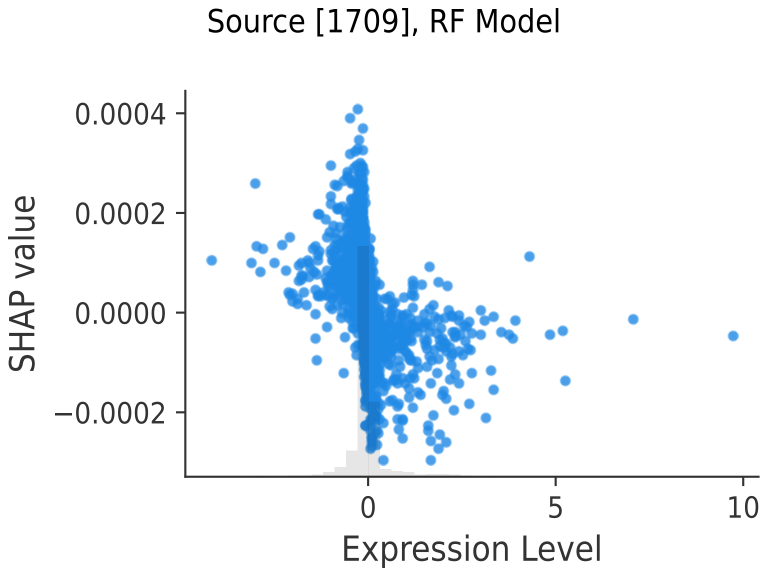
Source [1709], RF Model
0.0004
0.0002
0.0000
−0.0002
SHAP value
0
5
10
Expression Level

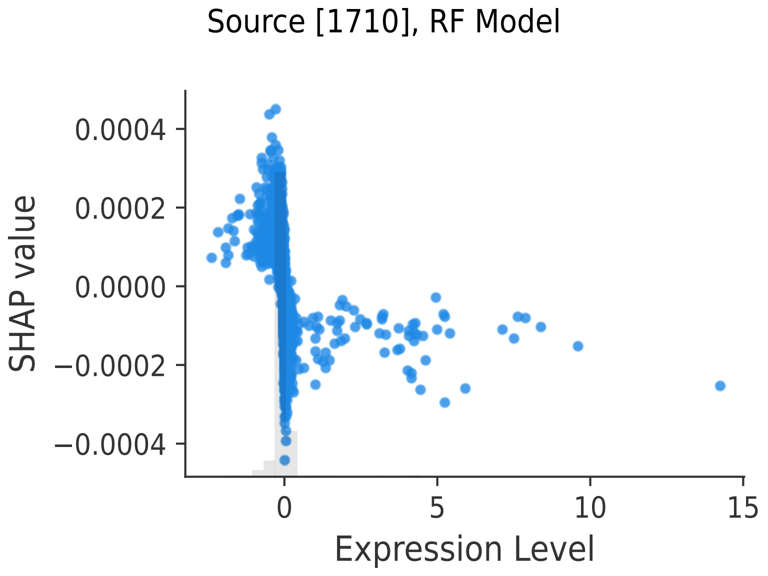
Source [1710], RF Model
SHAP value
0.0004
0.0002
0.0000
−0.0002
−0.0004
0
5
10
15
Expression Level

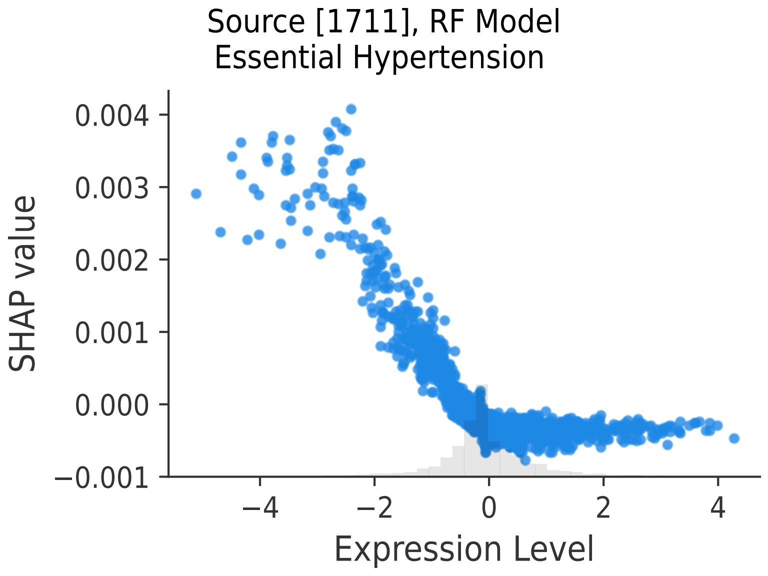
Source [1711], RF Model
Essential Hypertension
SHAP value
0.004
0.003
0.002
0.001
0.000
−0.001
−4
−2
0
2
4
Expression Level

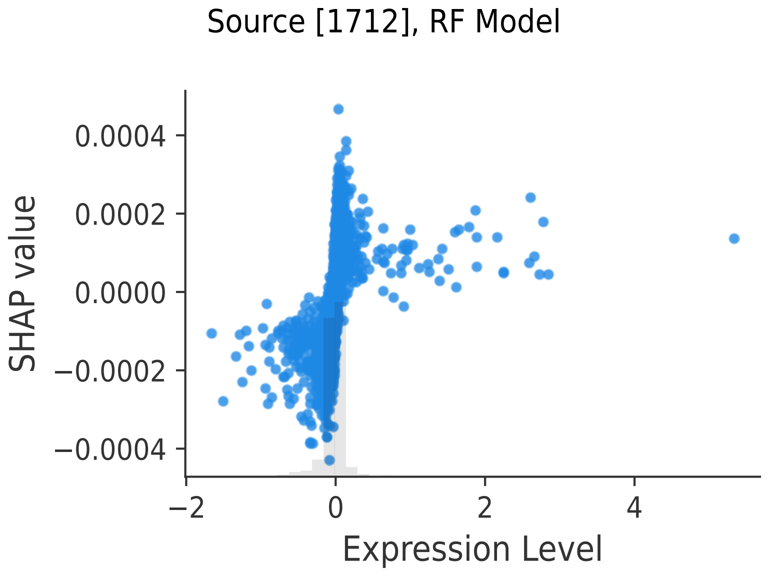
Source [1712], RF Model
SHAP value
Expression Level
0.0004
0.0002
0.0000
−0.0002
−0.0004
−2
0
2
4

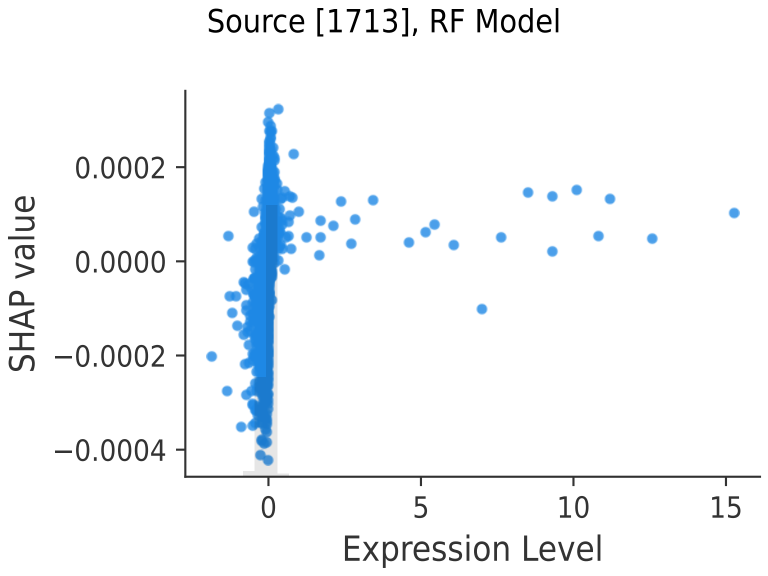
Source [1713], RF Model
0.0002
0.0000
−0.0002
−0.0004
SHAP value
0
5
10
15
Expression Level

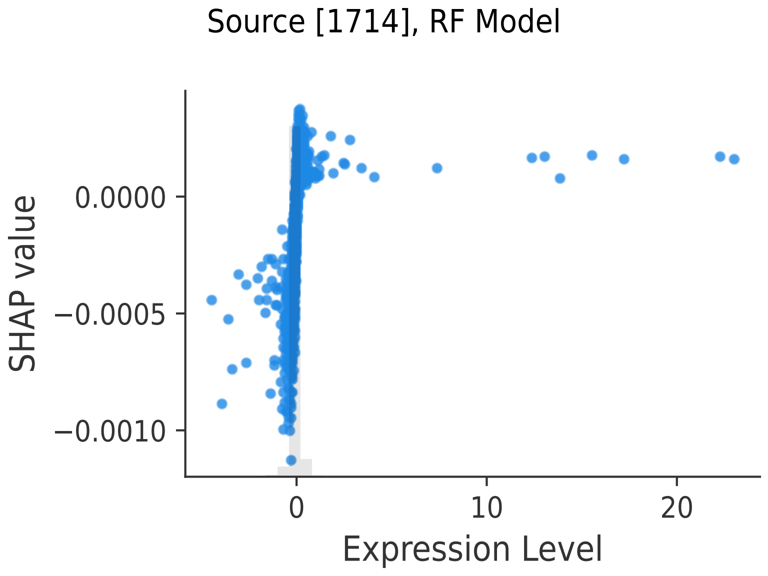

Source [1714], RF Model
SHAP value
0.0000
−0.0005
−0.0010
0
10
20
Expression Level

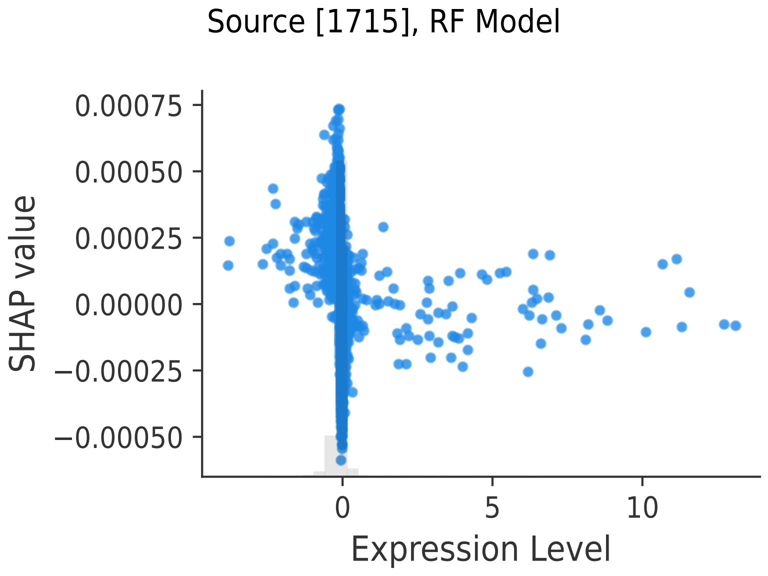

Source [1715], RF Model
SHAP value
0.00075
0.00050
0.00025
0.00000
−0.00025
−0.00050
0
5
10
Expression Level

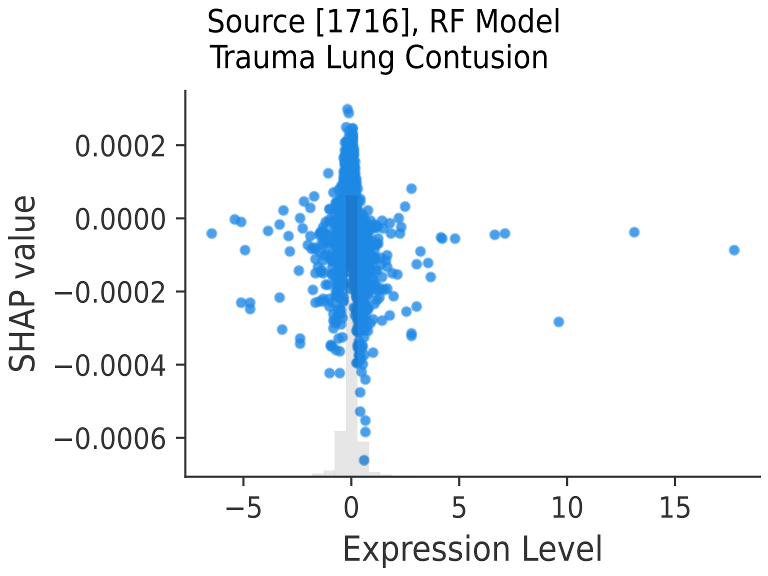

Source [1716], RF Model
Trauma Lung Contusion
0.0002
0.0000
−0.0002
−0.0004
−0.0006
SHAP value
−5
0
5
10
15
Expression Level

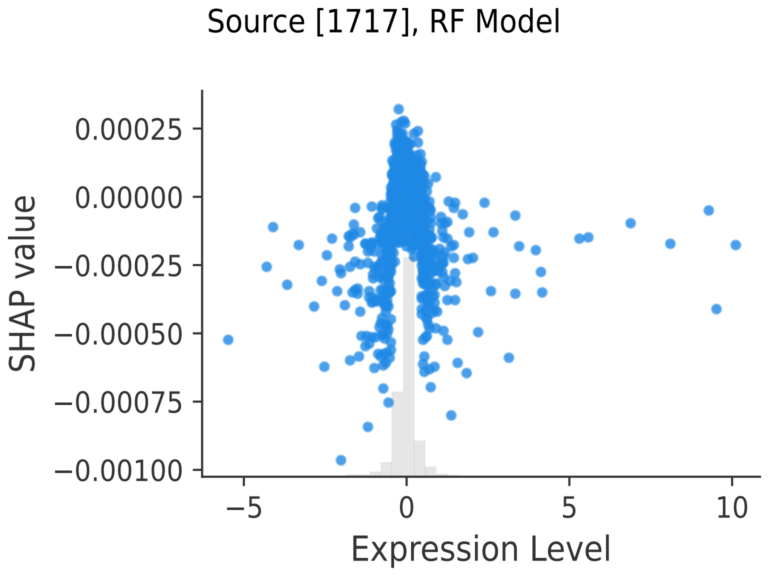
Source [1717], RF Model
0.00025
0.00000
−0.00025
−0.00050
−0.00075
−0.00100
SHAP value
−5
0
5
10
Expression Level

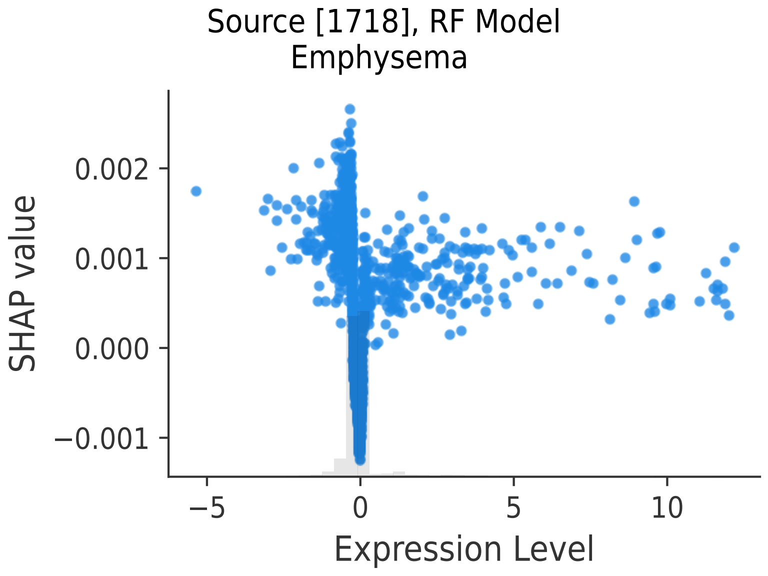
Source [1718], RF Model
Emphysema
SHAP value
0.002
0.001
0.000
−0.001
−5
0
5
10
Expression Level

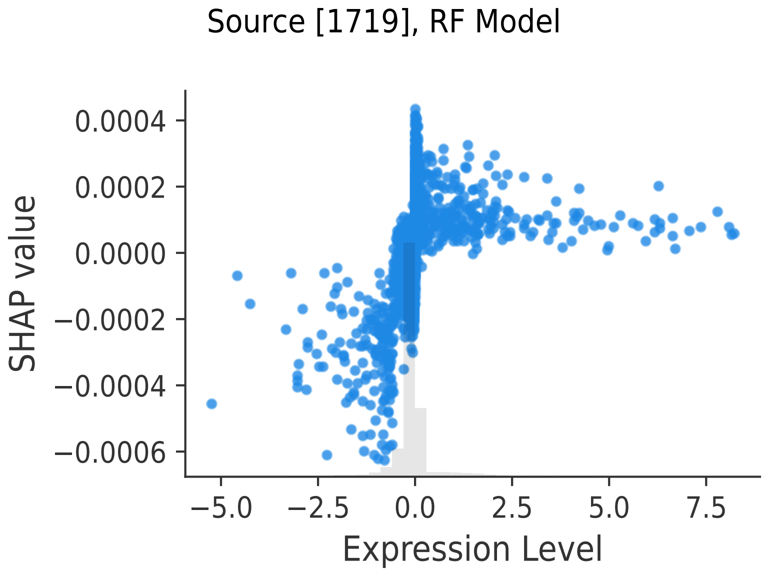
Source [1719], RF Model
0.0004
0.0002
0.0000
−0.0002
−0.0004
−0.0006
SHAP value
−5.0
−2.5
0.0
2.5
5.0
7.5
Expression Level

Source [1720], RF Model

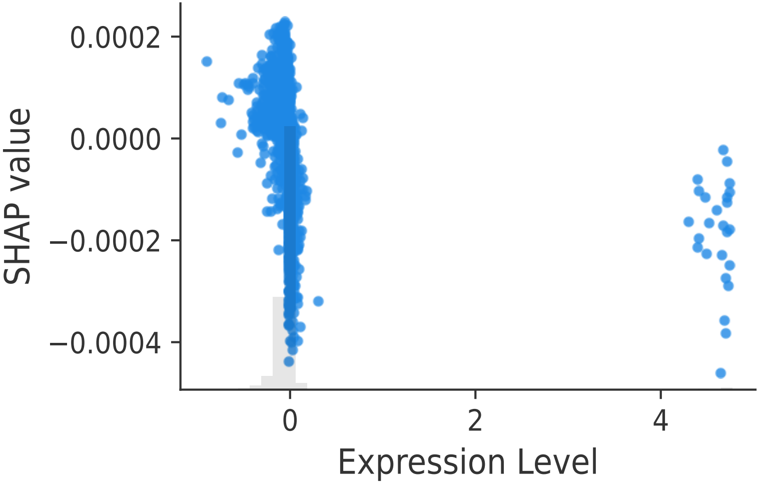

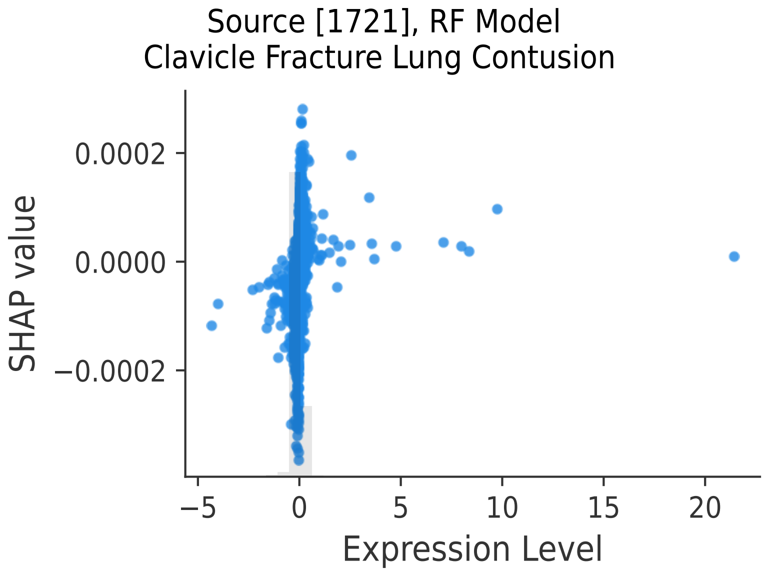

Source [1721], RF Model
Clavicle Fracture Lung Contusion
SHAP value
0.0002
0.0000
−0.0002
−5
0
5
10
15
20
Expression Level

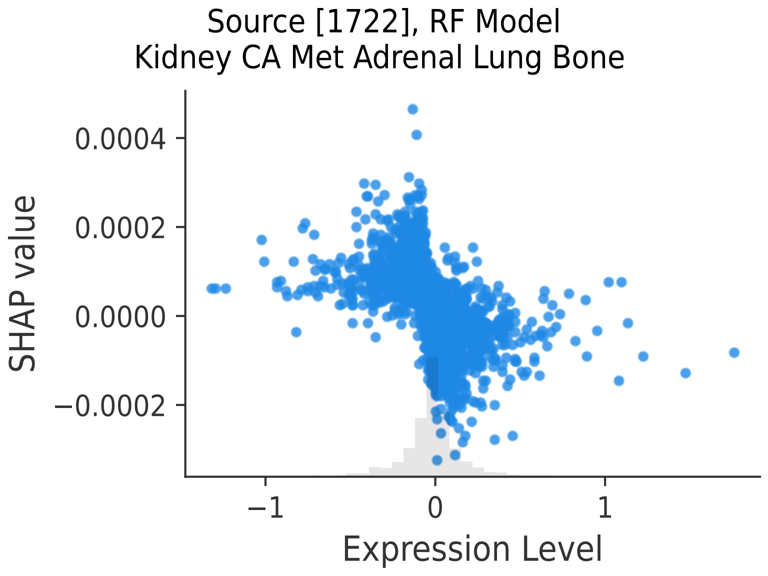
Source [1722], RF Model
Kidney CA Met Adrenal Lung Bone
SHAP value
0.0004
0.0002
0.0000
−0.0002
−1
0
1
Expression Level

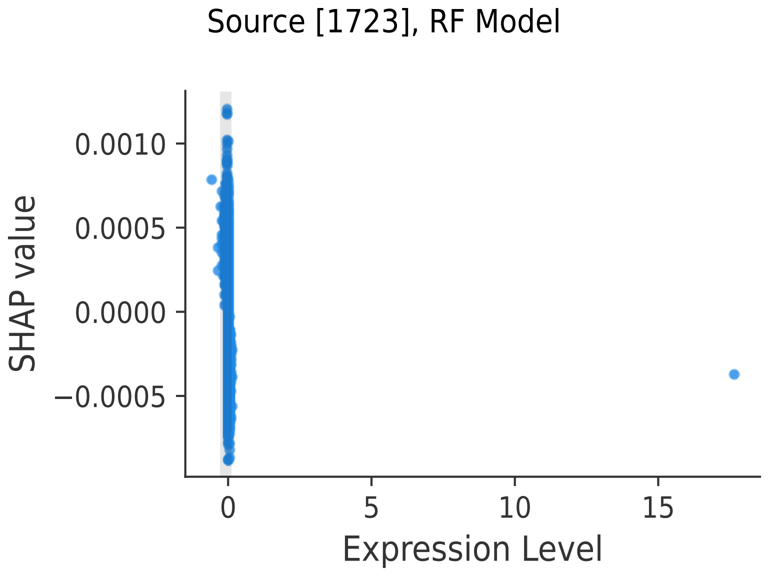
Source [1723], RF Model
SHAP value
0.0010
0.0005
0.0000
−0.0005
0
5
10
15
Expression Level

Source [1724], RF Model

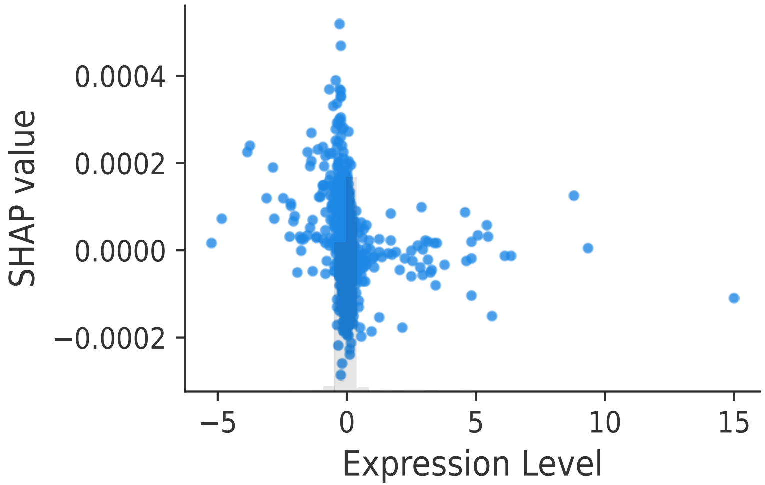

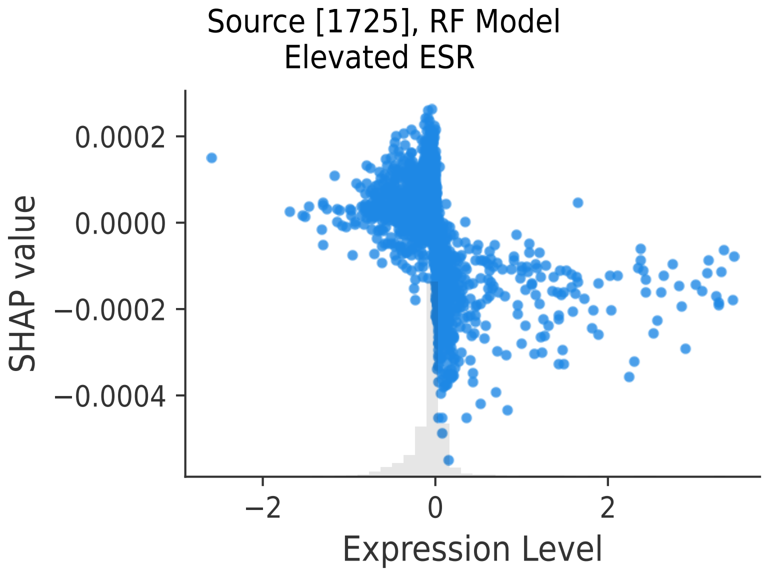
Source [1725], RF Model
Elevated ESR
SHAP value
0.0002
0.0000
−0.0002
−0.0004
−2
0
2
Expression Level

Source [1726], RF Model

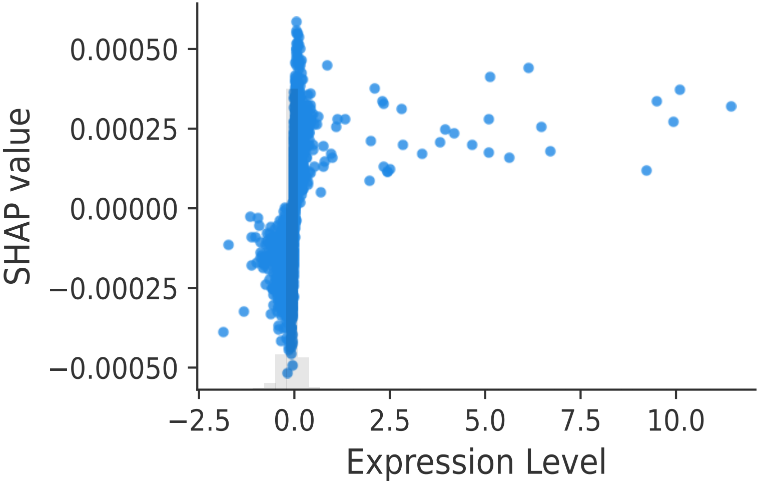

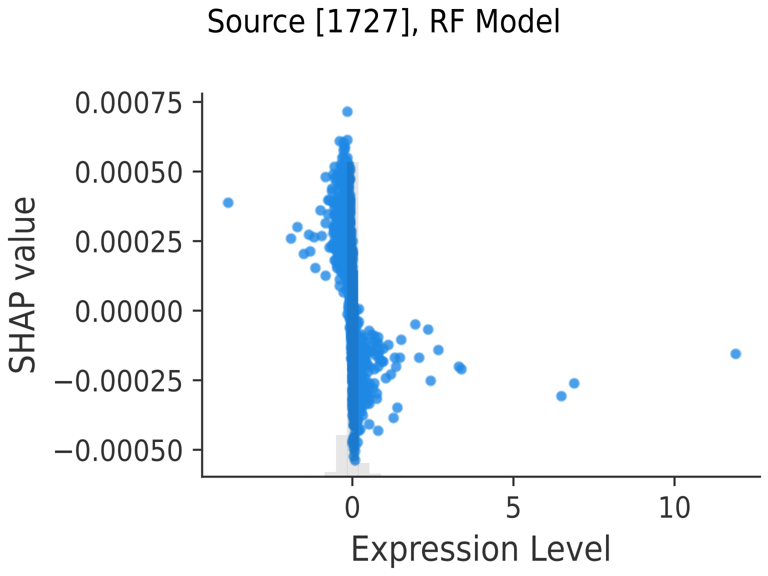
Source [1727], RF Model
SHAP value
0.00075
0.00050
0.00025
0.00000
−0.00025
−0.00050
0
5
10
Expression Level

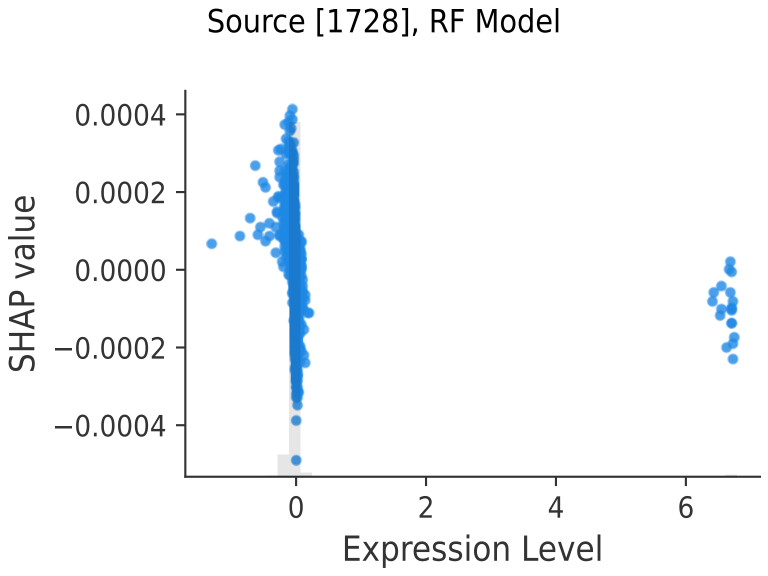

Source [1728], RF Model
SHAP value
0.0004
0.0002
0.0000
−0.0002
−0.0004
0
2
4
6
Expression Level

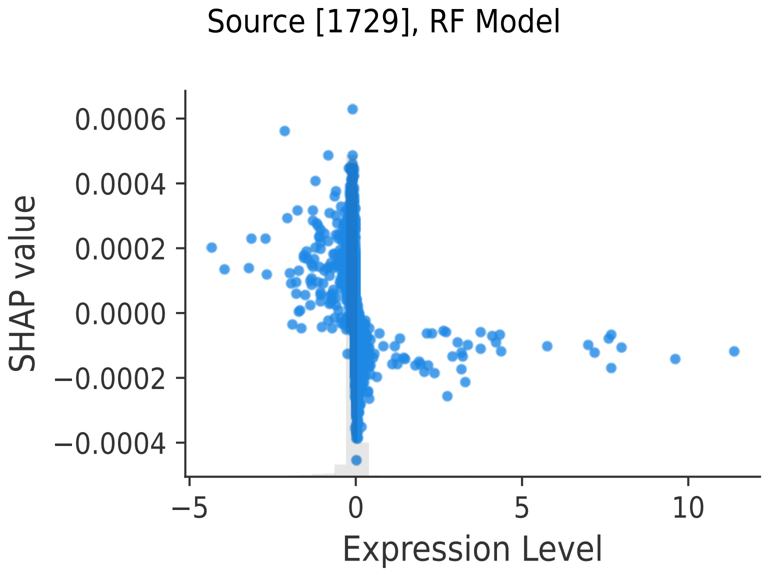
Source [1729], RF Model
SHAP value
0.0006
0.0004
0.0002
0.0000
−0.0002
−0.0004
−5
0
5
10
Expression Level

Source [1730], RF Model

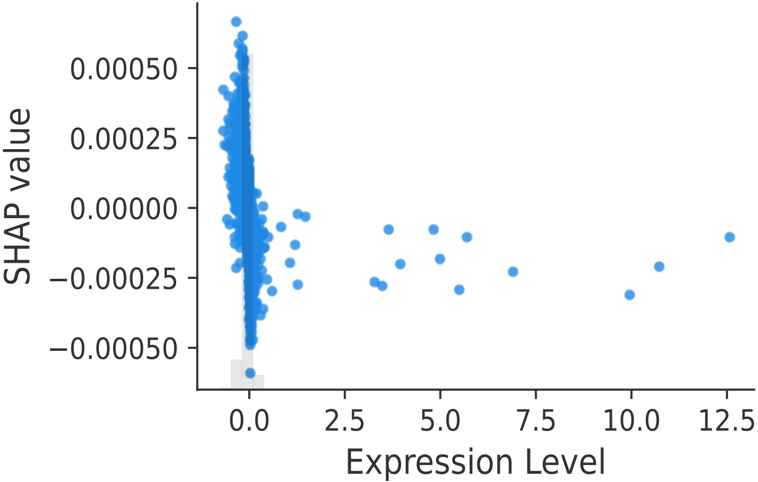

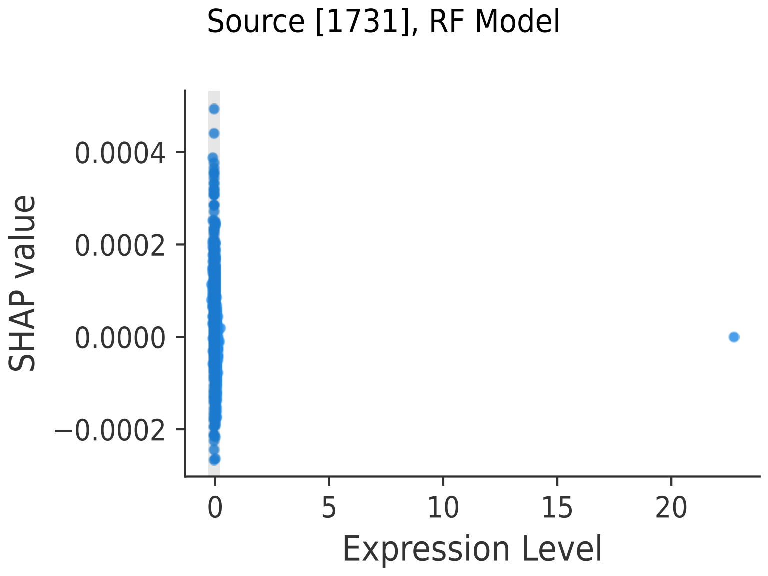
Source [1731], RF Model
SHAP value
0.0004
0.0002
0.0000
−0.0002
0
5
10
15
20
Expression Level

Source [1732], RF Model

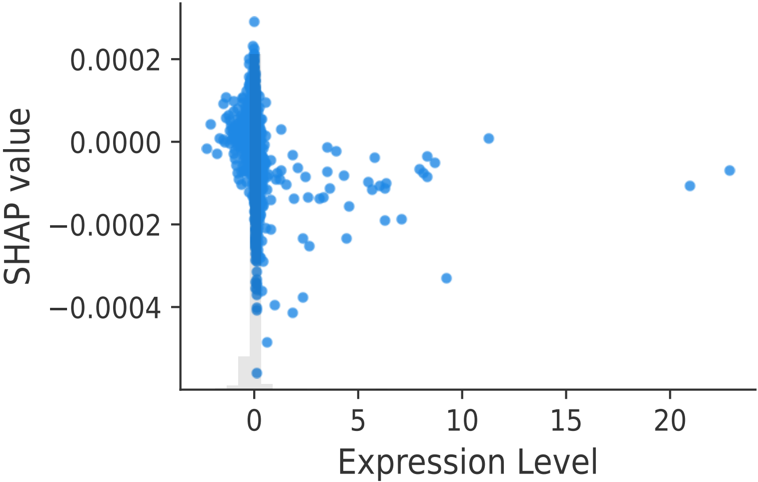

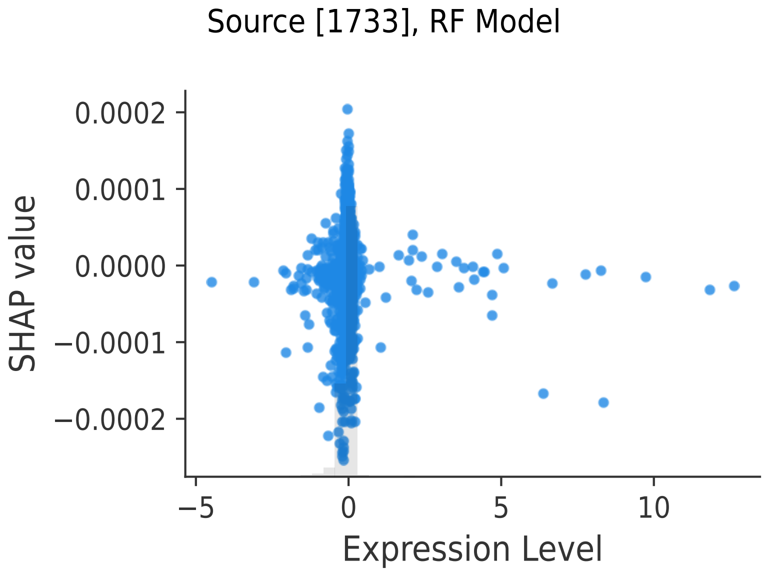
Source [1733], RF Model
SHAP value
0.0002
0.0001
0.0000
−0.0001
−0.0002
−5
0
5
10
Expression Level

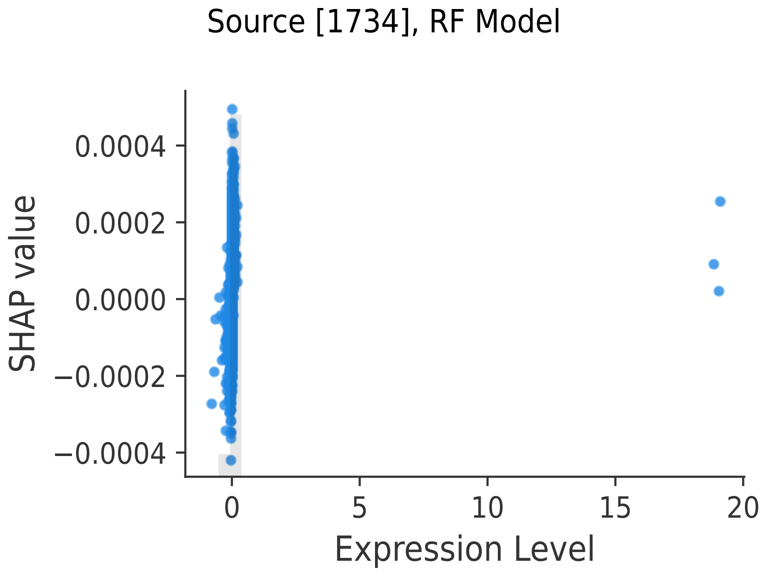
Source [1734], RF Model
SHAP value
0.0004
0.0002
0.0000
−0.0002
−0.0004
0
5
10
15
20
Expression Level

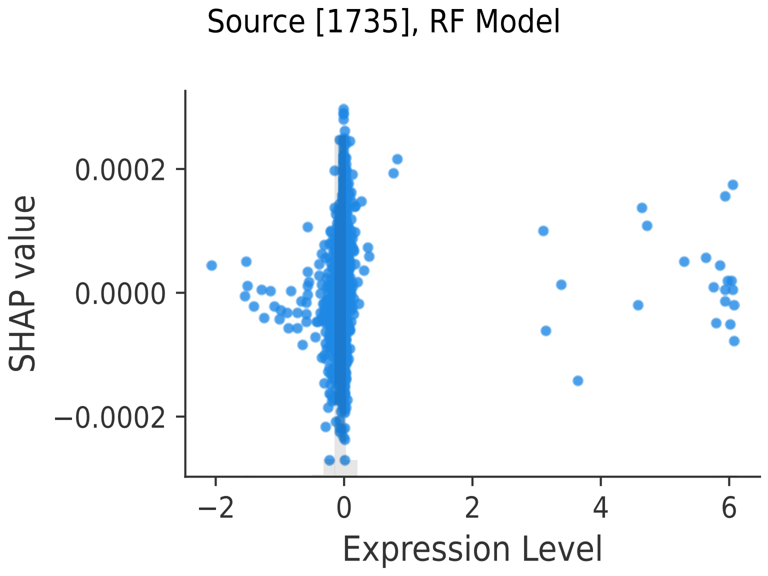

Source [1735], RF Model
SHAP value
0.0002
0.0000
−0.0002
−2
0
2
4
6
Expression Level

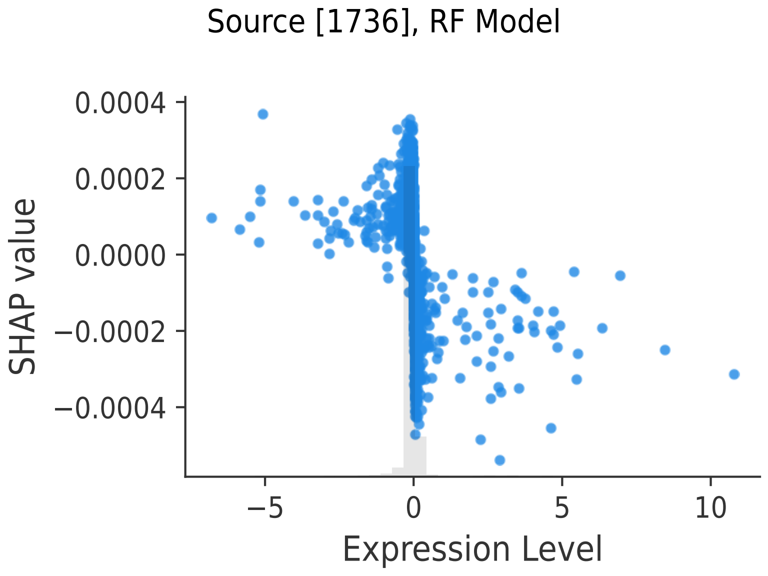

Source [1736], RF Model
SHAP value
0.0004
0.0002
0.0000
−0.0002
−0.0004
−5
0
5
10
Expression Level

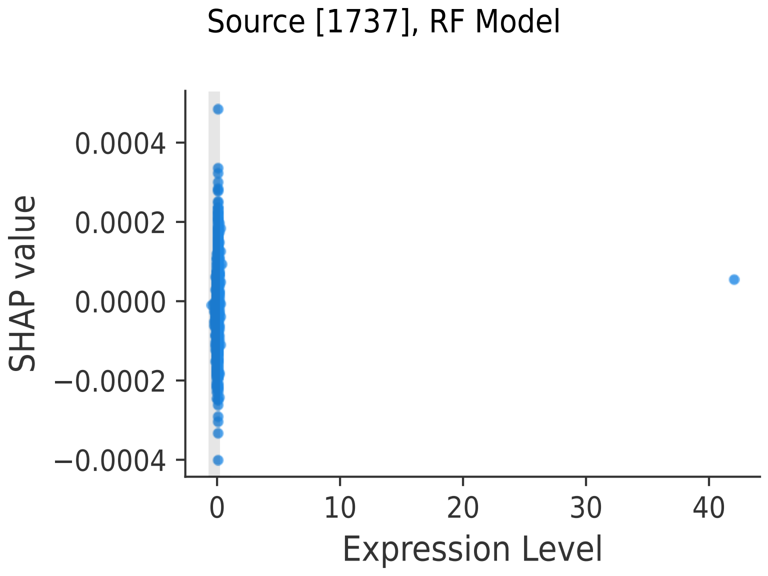

Source [1737], RF Model
0.0004
0.0002
0.0000
−0.0002
−0.0004
SHAP value
0
10
20
30
40
Expression Level

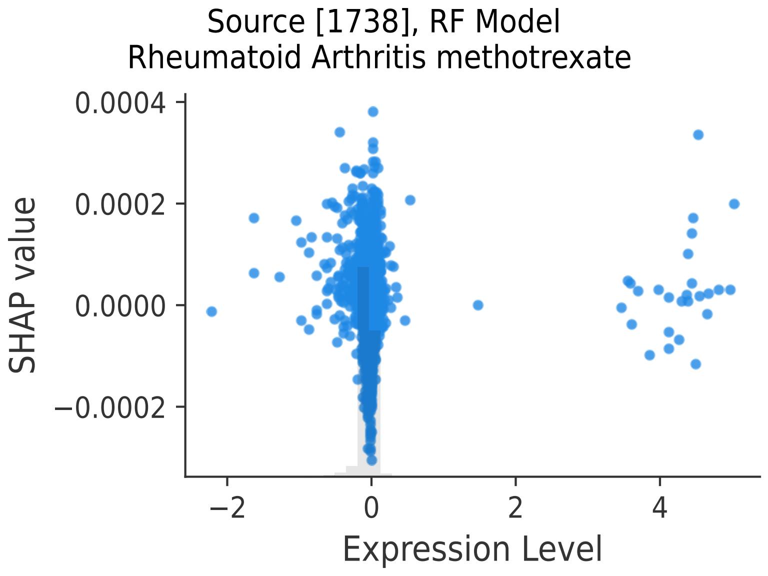
Source [1738], RF Model
Rheumatoid Arthritis methotrexate
0.0004
0.0002
0.0000
−0.0002
SHAP value
−2
0
2
4
Expression Level

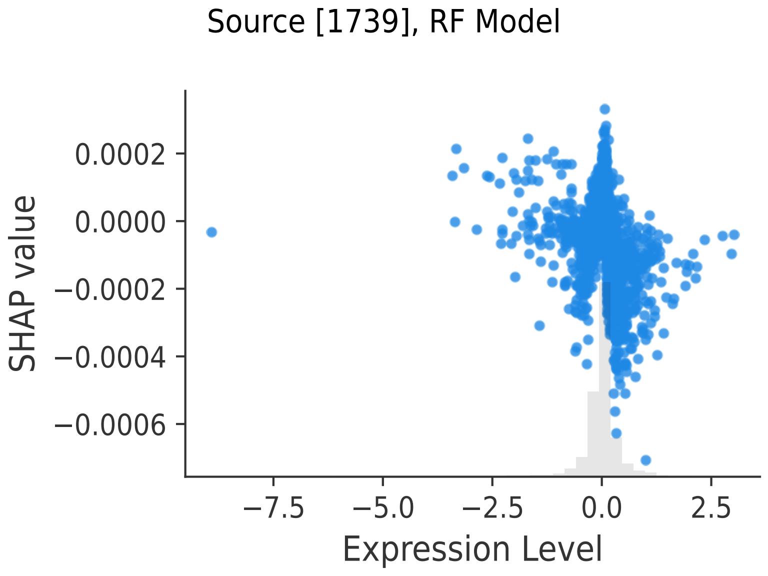
Source [1739], RF Model
SHAP value
0.0002
0.0000
−0.0002
−0.0004
−0.0006
−7.5
−5.0
−2.5
0.0
2.5
Expression Level

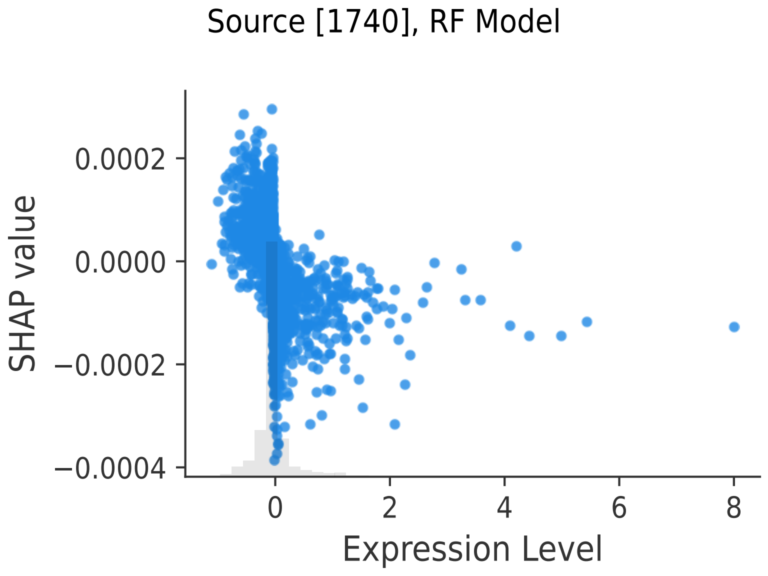
Source [1740], RF Model
SHAP value
0.0002
0.0000
−0.0002
−0.0004
0
2
4
6
8
Expression Level

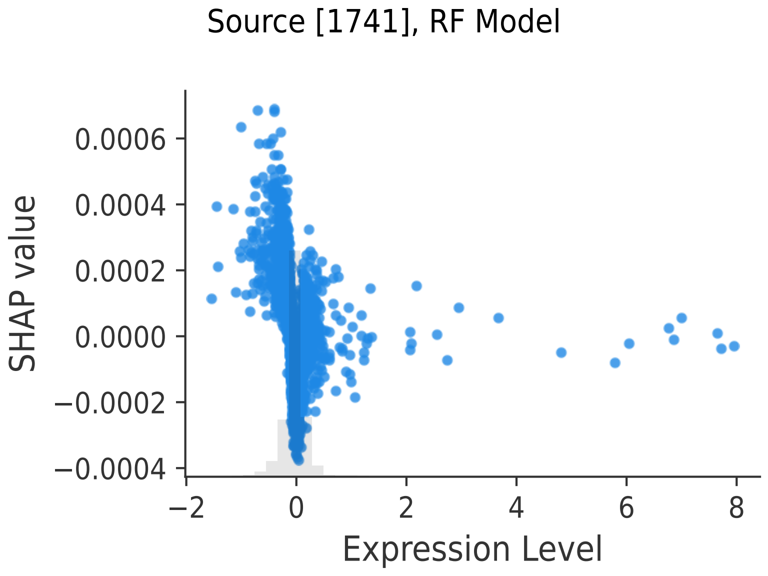
Source [1741], RF Model
SHAP value
0.0006
0.0004
0.0002
0.0000
−0.0002
−0.0004
−2
0
2
4
6
8
Expression Level

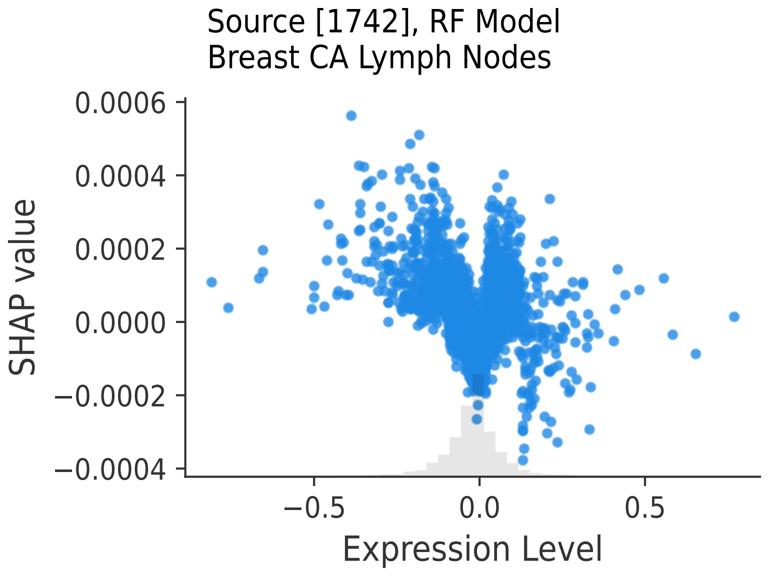
Source [1742], RF Model
Breast CA Lymph Nodes
SHAP value
0.0006
0.0004
0.0002
0.0000
−0.0002
−0.0004
−0.5
0.0
0.5
Expression Level

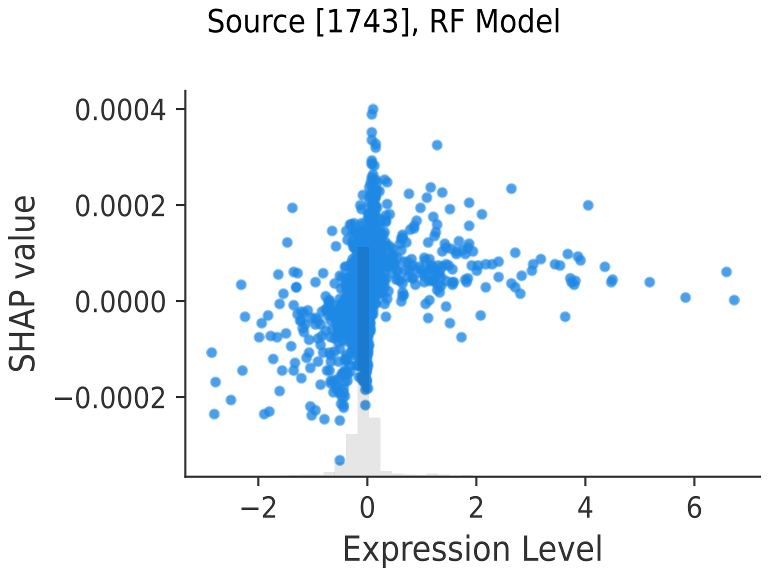
Source [1743], RF Model
SHAP value
0.0004
0.0002
0.0000
−0.0002
−2
0
2
4
6
Expression Level

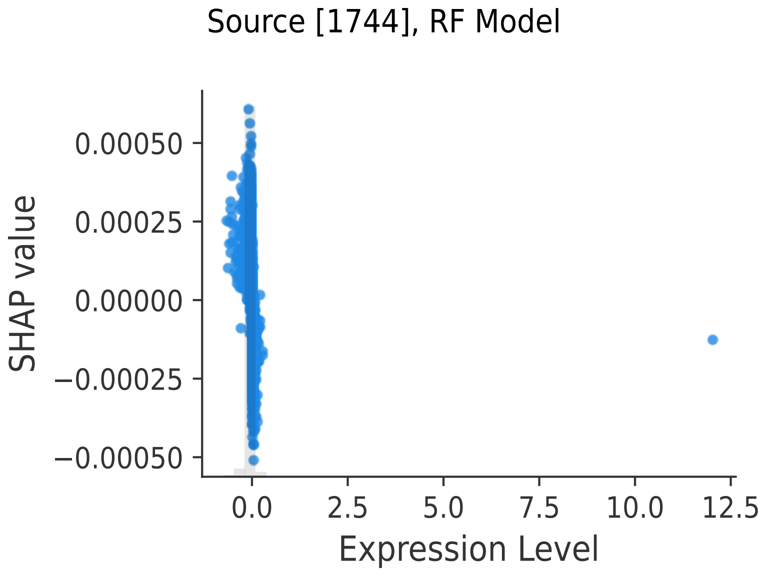

Source [1744], RF Model
SHAP value
0.00050
0.00025
0.00000
−0.00025
−0.00050
0.0
2.5
5.0
7.5
10.0
12.5
Expression Level

Source [1745], RF Model

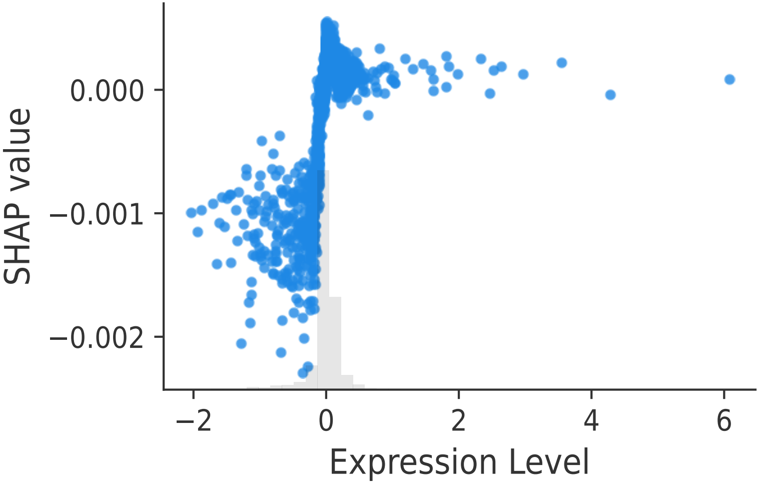

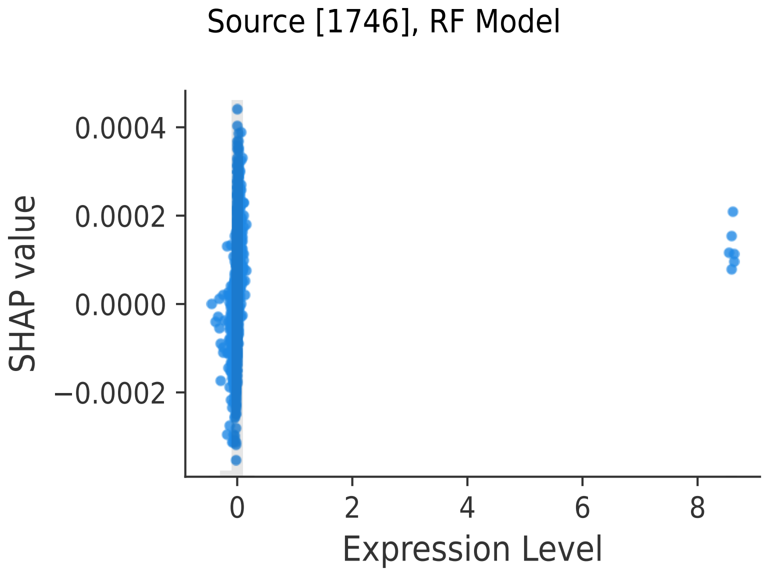

Source [1746], RF Model
0.0004
0.0002
0.0000
−0.0002
SHAP value
0
2
4
6
8
Expression Level

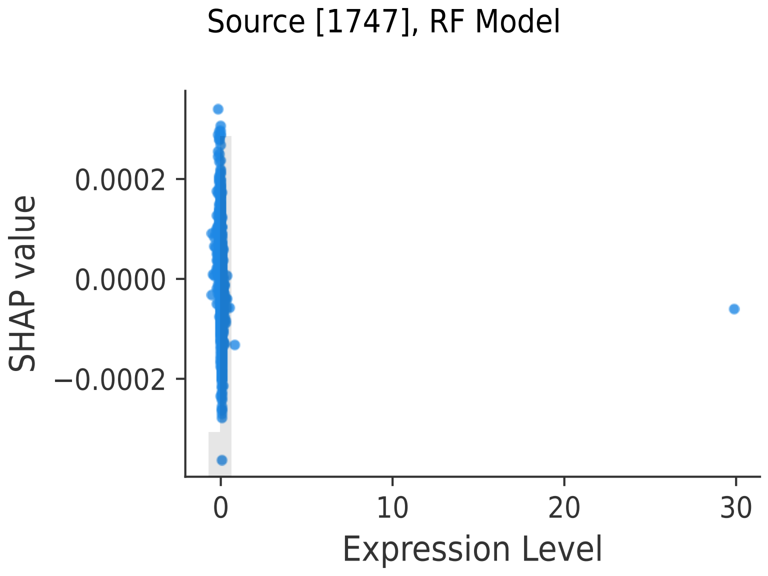

Source [1747], RF Model
SHAP value
0.0002
0.0000
−0.0002
0
10
20
30
Expression Level

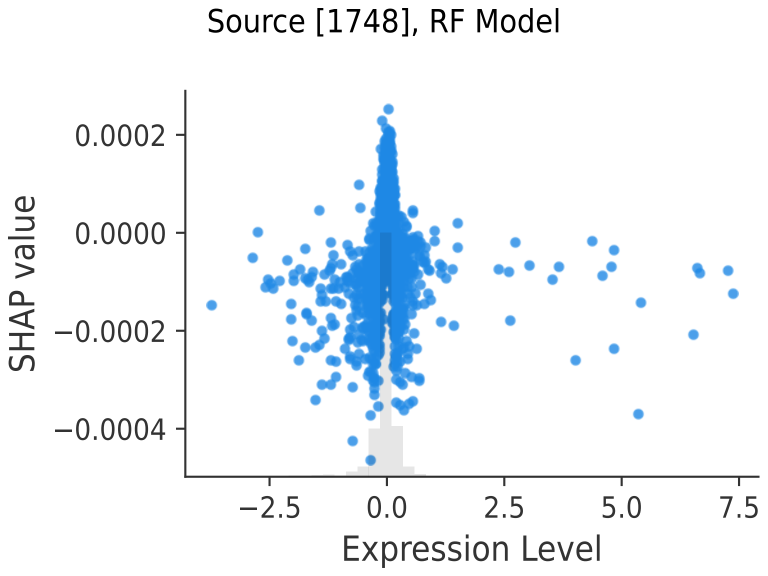

Source [1748], RF Model
SHAP value
0.0002
0.0000
−0.0002
−0.0004
−2.5
0.0
2.5
5.0
7.5
Expression Level

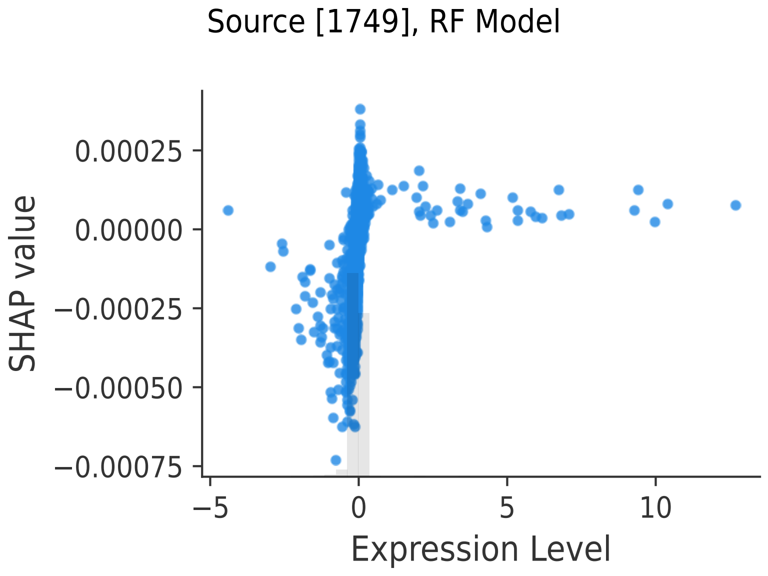

Source [1749], RF Model
SHAP value
0.00025
0.00000
−0.00025
−0.00050
−0.00075
−5
0
5
10
Expression Level

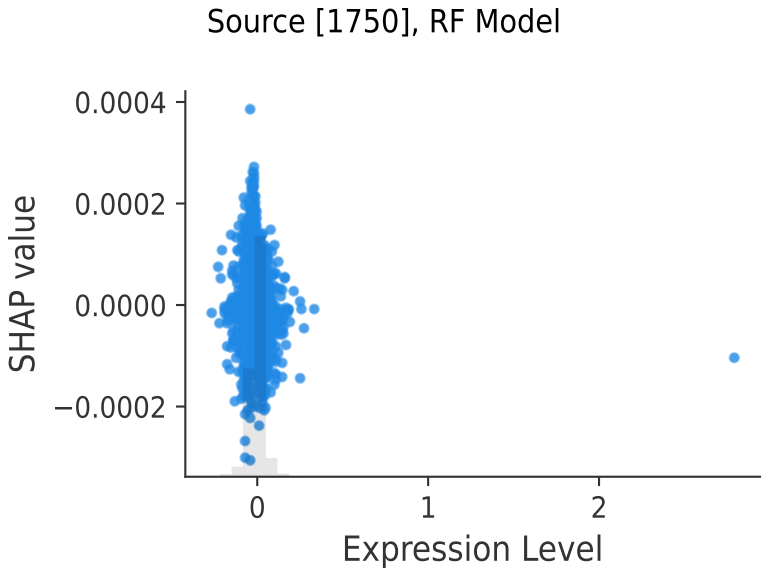

Source [1750], RF Model
SHAP value
0.0004
0.0002
0.0000
−0.0002
0
1
2
Expression Level

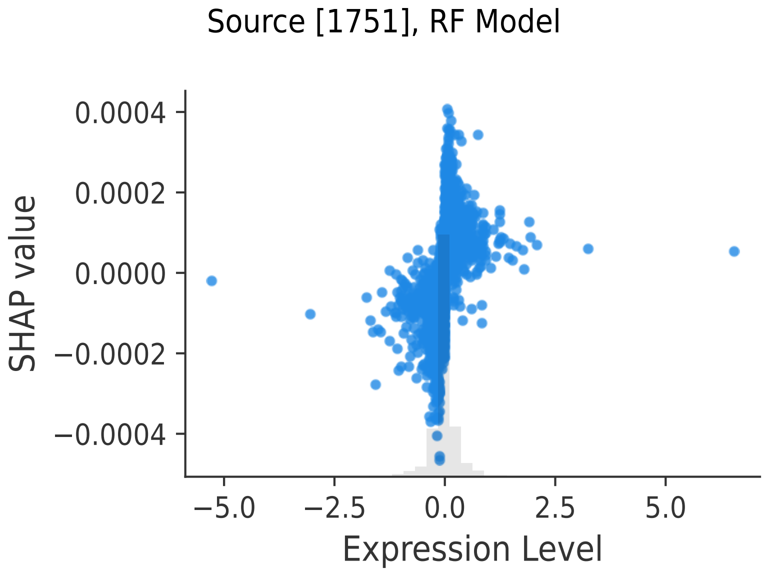

Source [1751], RF Model
SHAP value
0.0004
0.0002
0.0000
−0.0002
−0.0004
−5.0
−2.5
0.0
2.5
5.0
Expression Level

Source [1752], RF Model

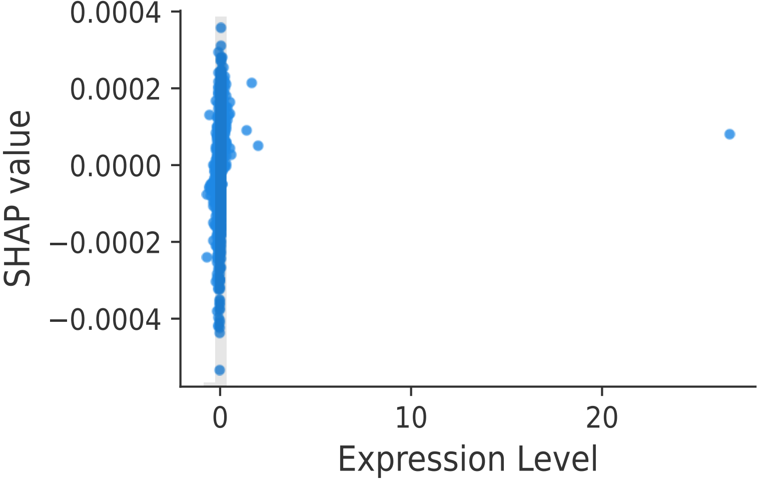

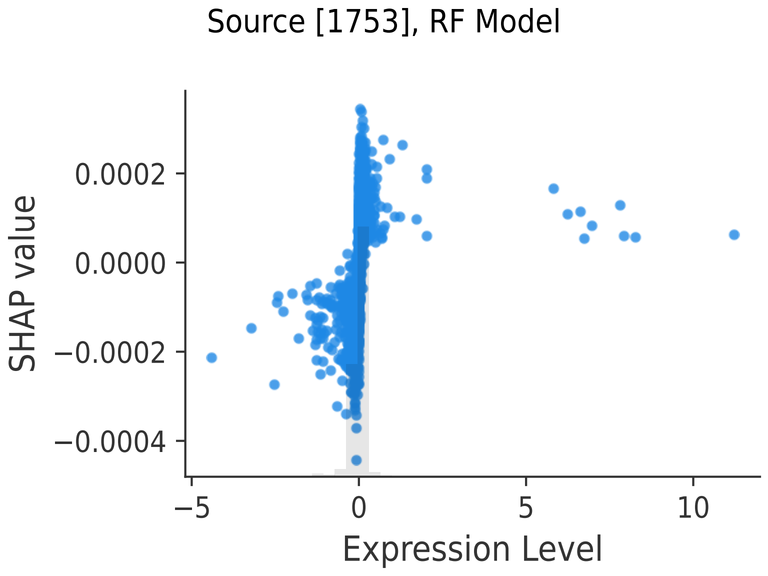

Source [1753], RF Model
SHAP value
0.0002
0.0000
−0.0002
−0.0004
−5
0
5
10
Expression Level

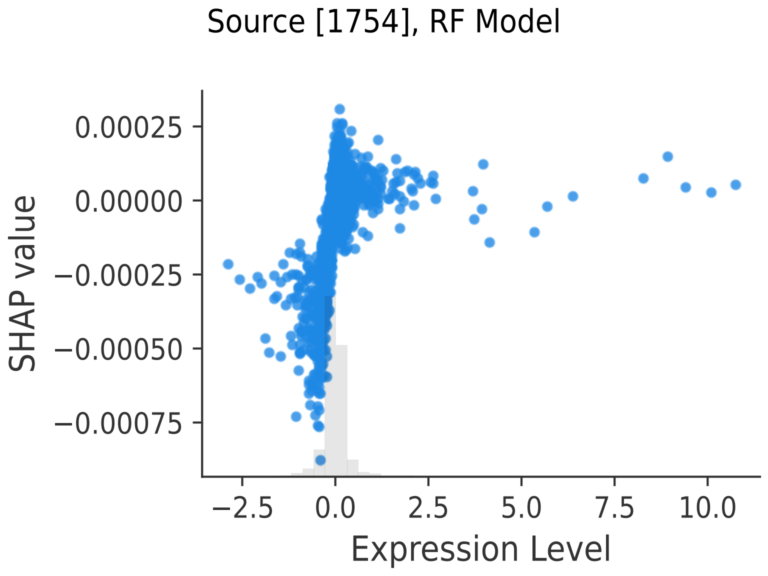
Source [1754], RF Model
0.00025
0.00000
−0.00025
−0.00050
−0.00075
SHAP value
−2.5
0.0
2.5
5.0
7.5
10.0
Expression Level

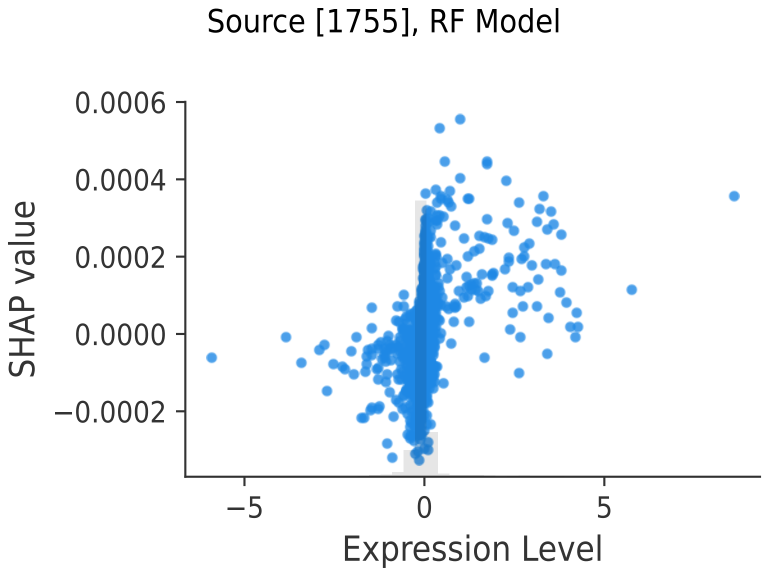

Source [1755], RF Model
0.0006
0.0004
0.0002
0.0000
−0.0002
SHAP value
−5
0
5
Expression Level

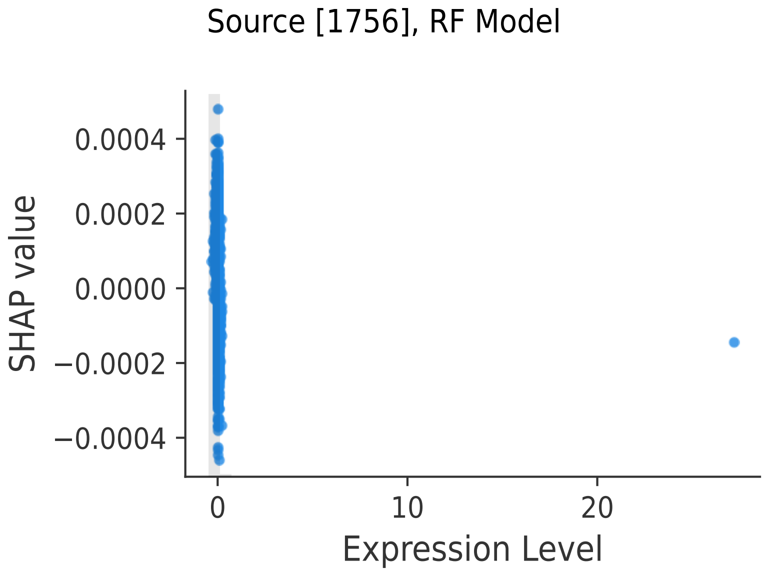
Source [1756], RF Model
SHAP value
0.0004
0.0002
0.0000
−0.0002
−0.0004
0
10
20
Expression Level

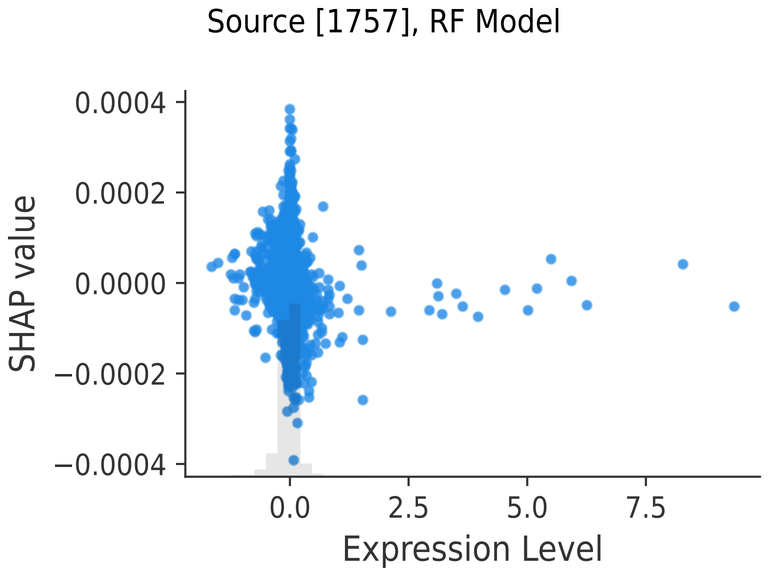

Source [1757], RF Model
SHAP value
0.0004
0.0002
0.0000
−0.0002
−0.0004
0.0
2.5
5.0
7.5
Expression Level

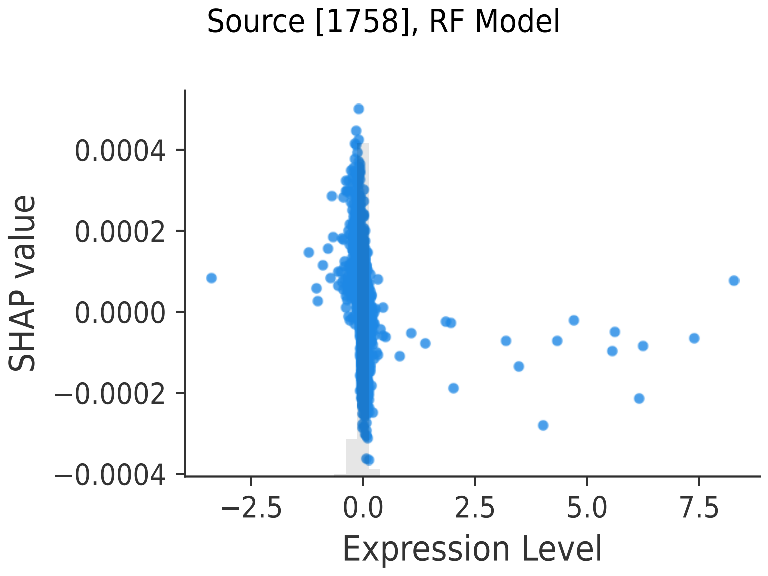

Source [1758], RF Model
SHAP value
0.0004
0.0002
0.0000
−0.0002
−0.0004
−2.5
0.0
2.5
5.0
7.5
Expression Level

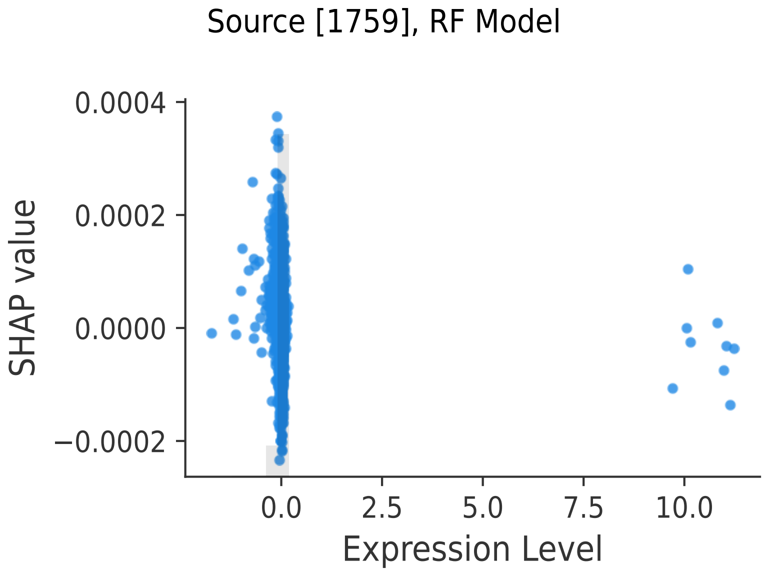

Source [1759], RF Model
0.0004
0.0002
0.0000
−0.0002
SHAP value
0.0
2.5
5.0
7.5
10.0
Expression Level

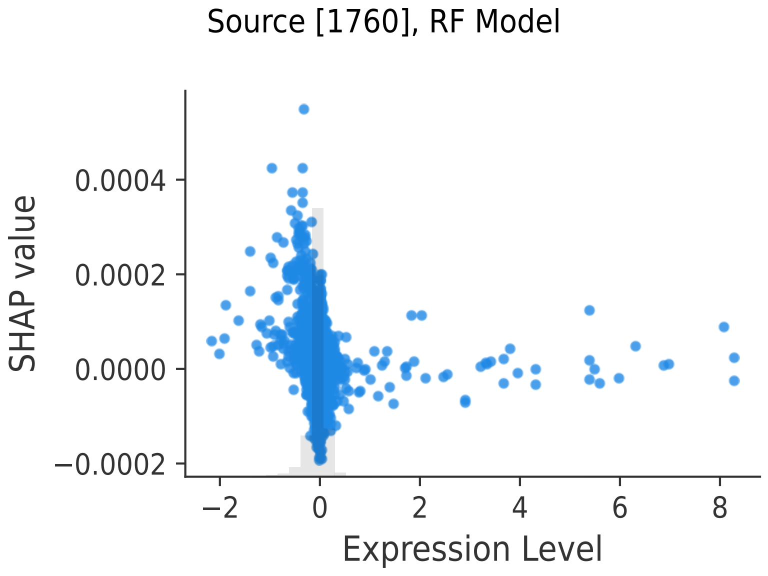
Source [1760], RF Model
SHAP value
0.0004
0.0002
0.0000
−0.0002
−2
0
2
4
6
8
Expression Level

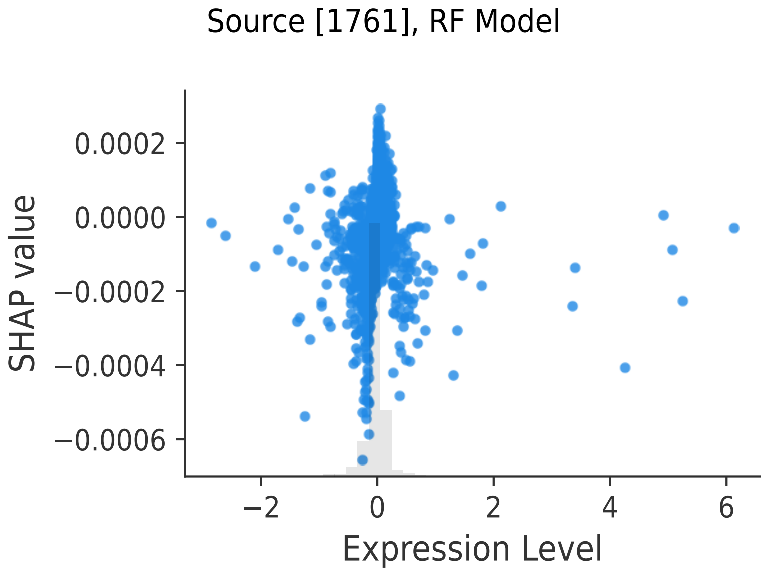
Source [1761], RF Model
SHAP value
0.0002
0.0000
−0.0002
−0.0004
−0.0006
−2
0
2
4
6
Expression Level

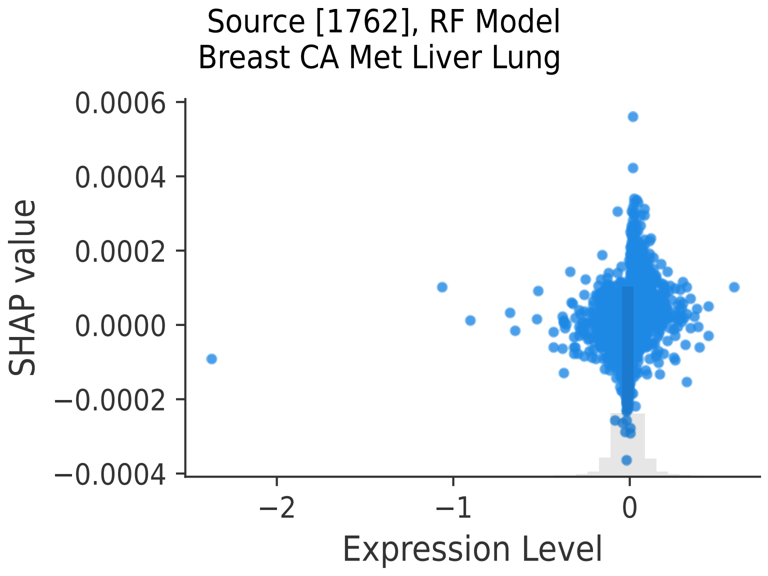

Source [1762], RF Model
Breast CA Met Liver Lung
SHAP value
0.0006
0.0004
0.0002
0.0000
−0.0002
−0.0004
−2
−1
0
Expression Level

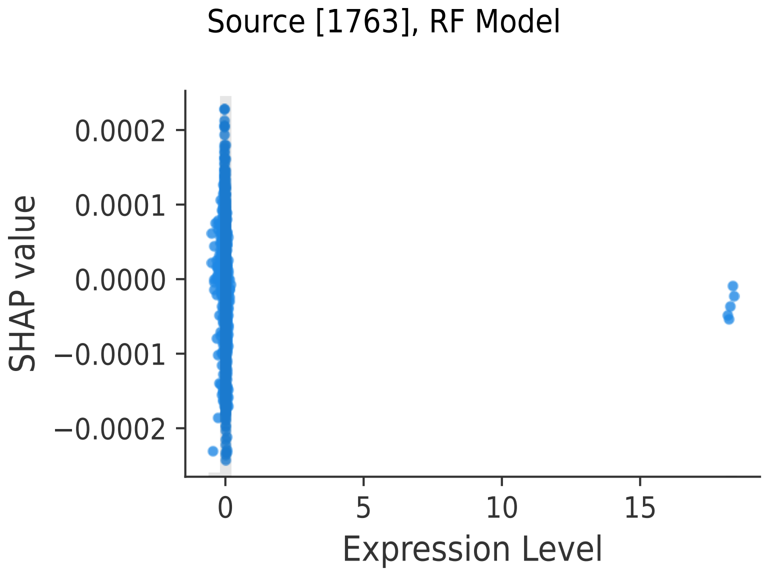

Source [1763], RF Model
SHAP value
0.0002
0.0001
0.0000
−0.0001
−0.0002
0
5
10
15
Expression Level

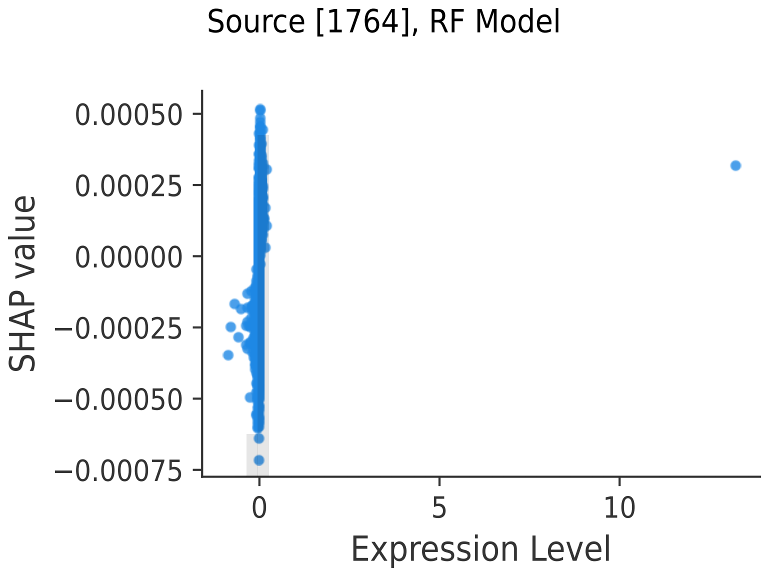
Source [1764], RF Model
SHAP value
0.00050
0.00025
0.00000
−0.00025
−0.00050
−0.00075
0
5
10
Expression Level

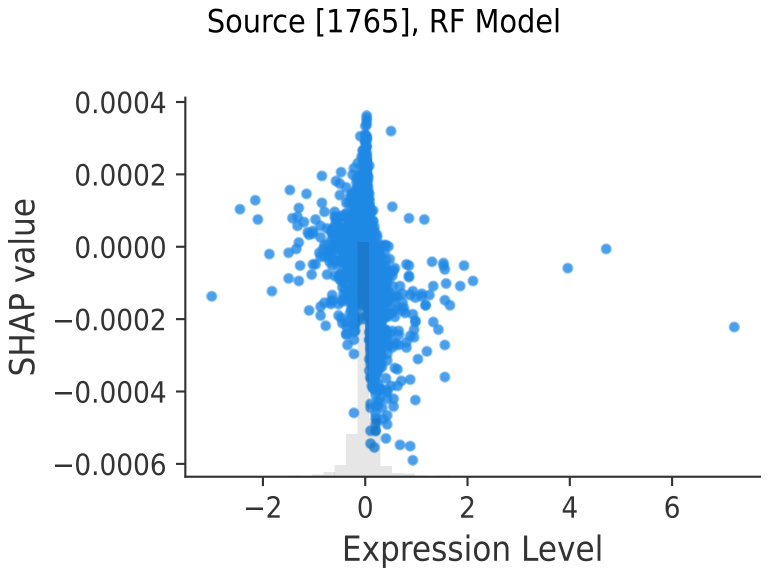

Source [1765], RF Model
0.0004
0.0002
0.0000
−0.0002
−0.0004
−0.0006
SHAP value
−2
0
2
4
6
Expression Level

Source [1766], RF Model

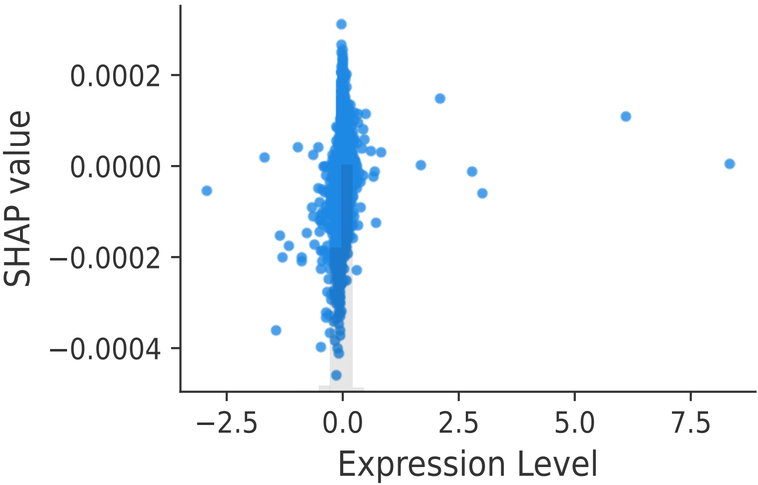

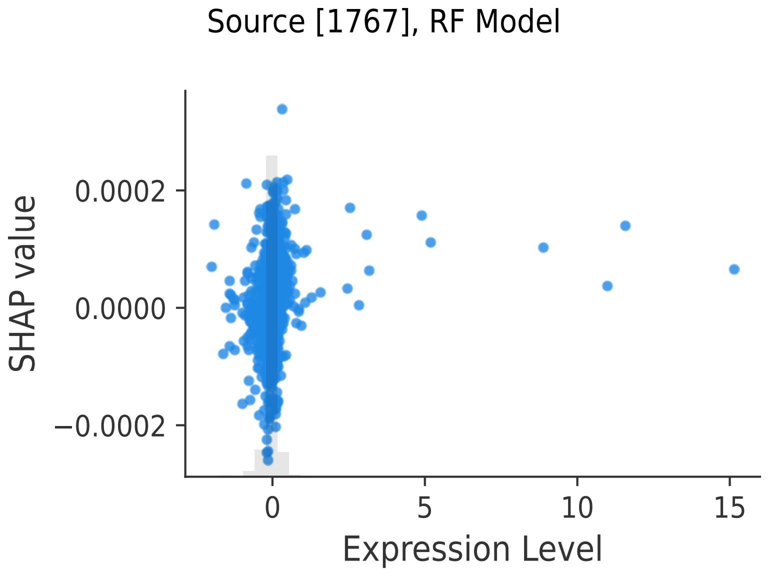

Source [1767], RF Model
SHAP value
0.0002
0.0000
−0.0002
0
5
10
15
Expression Level

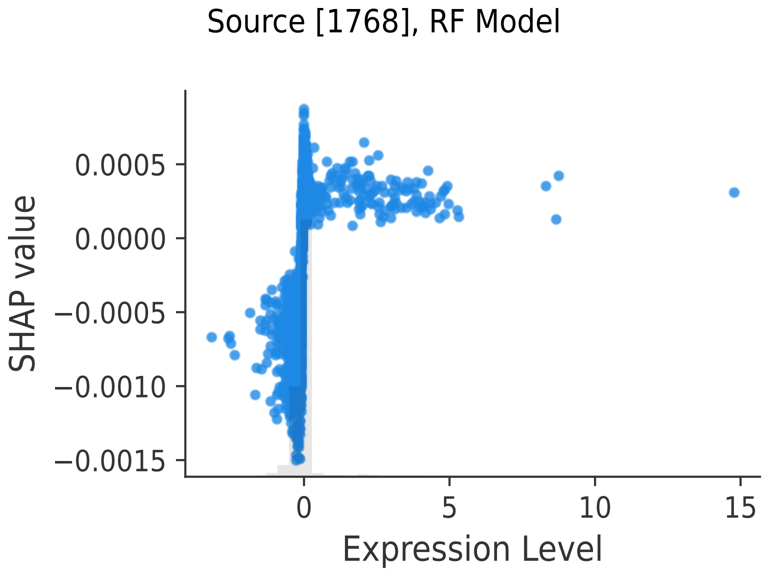

Source [1768], RF Model
0.0005
0.0000
−0.0005
−0.0010
−0.0015
SHAP value
0
5
10
15
Expression Level

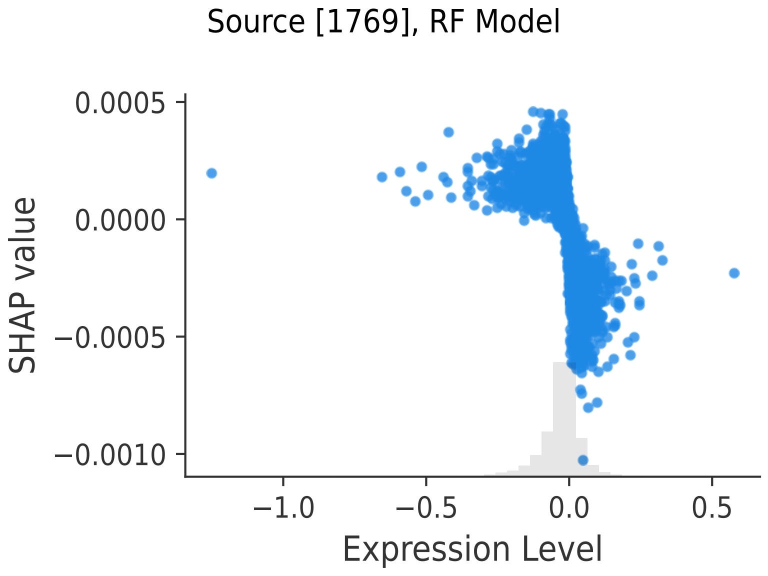
Source [1769], RF Model
0.0005
0.0000
−0.0005
−0.0010
SHAP value
−1.0
−0.5
0.0
0.5
Expression Level

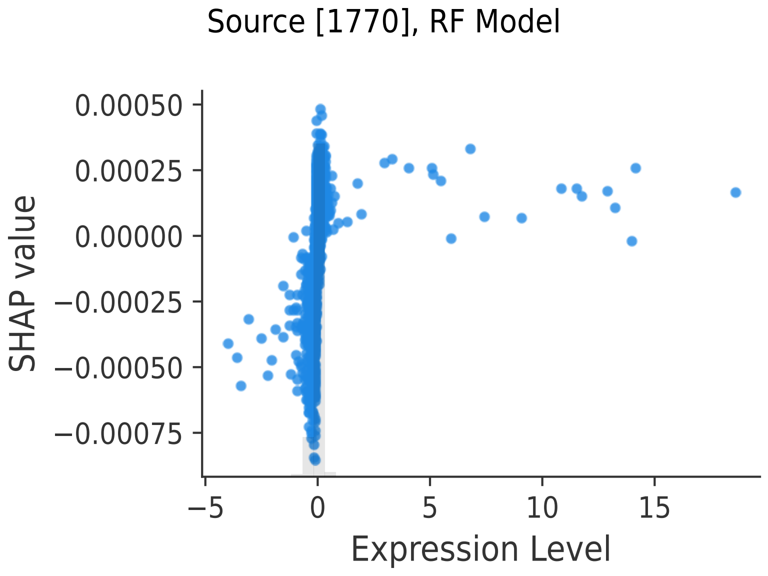

Source [1770], RF Model
0.00050
0.00025
0.00000
−0.00025
−0.00050
−0.00075
SHAP value
−5
0
5
10
15
Expression Level

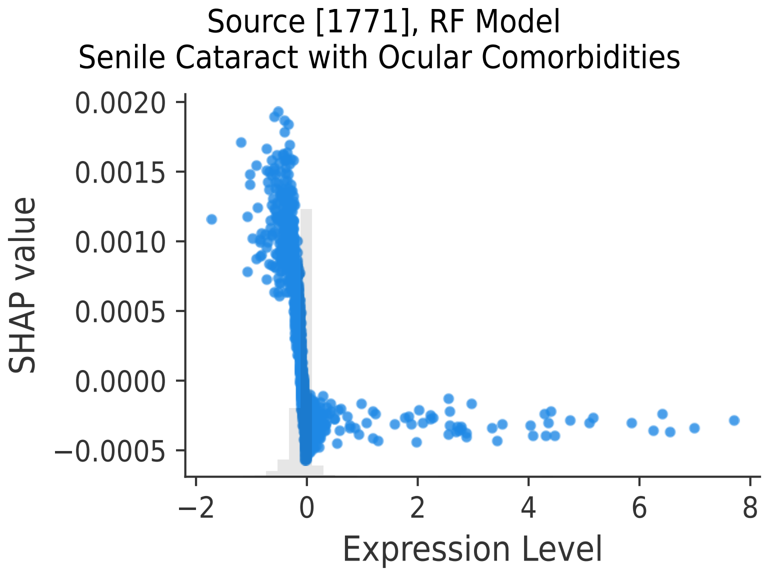

Source [1771], RF Model
Senile Cataract with Ocular Comorbidities
0.0020
0.0015
0.0010
0.0005
0.0000
−0.0005
SHAP value
−2
0
2
4
6
8
Expression Level

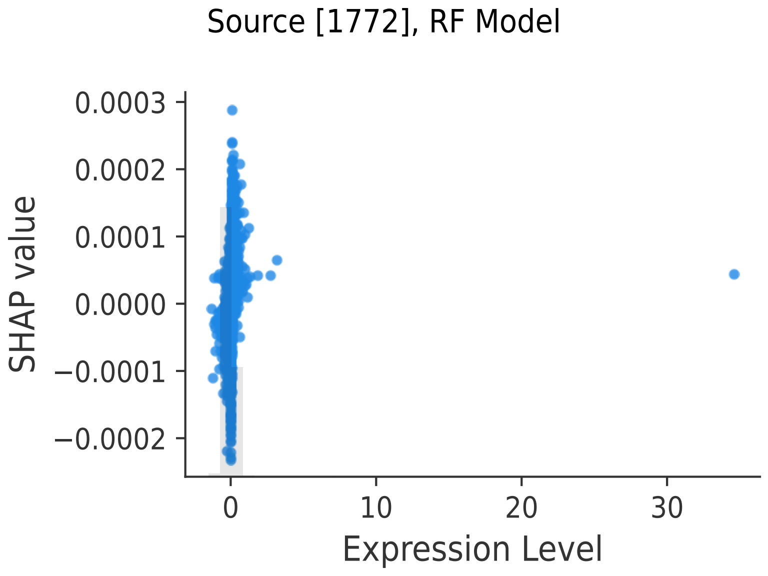
Source [1772], RF Model
SHAP value
0.0003
0.0002
0.0001
0.0000
−0.0001
−0.0002
0
10
20
30
Expression Level

Source [1773], RF Model

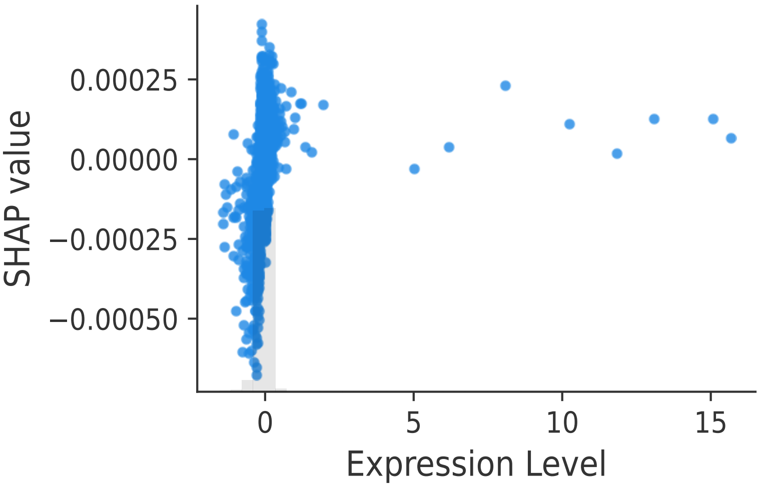

Source [1774], RF Model

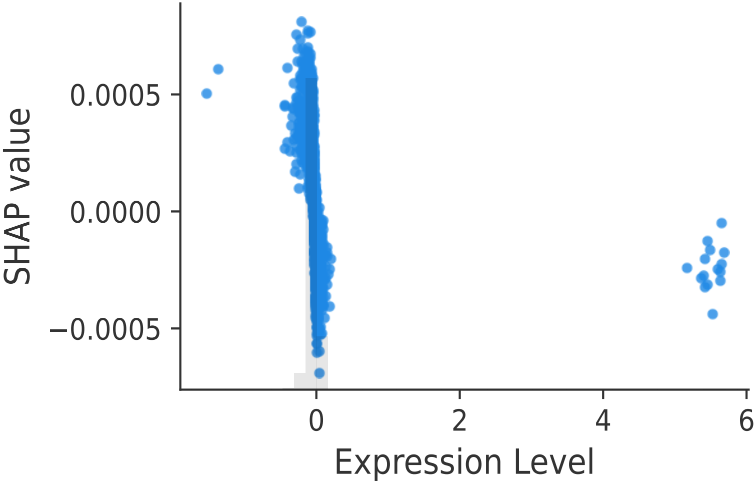

Source [1775], RF Model

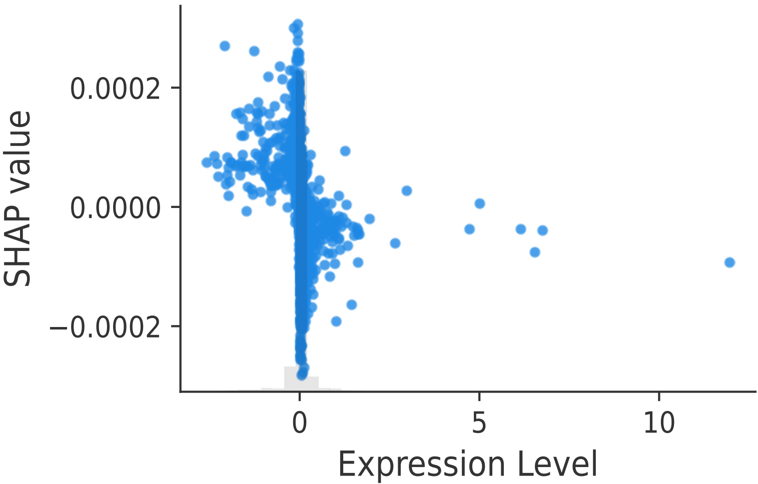

Source [1776], RF Model

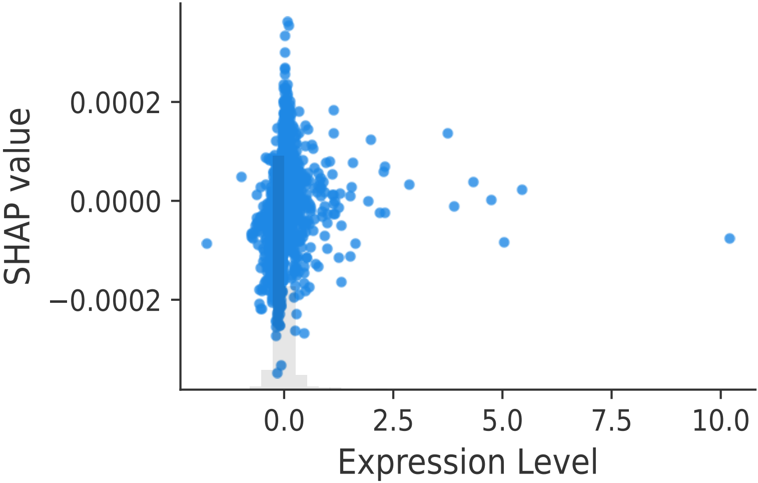

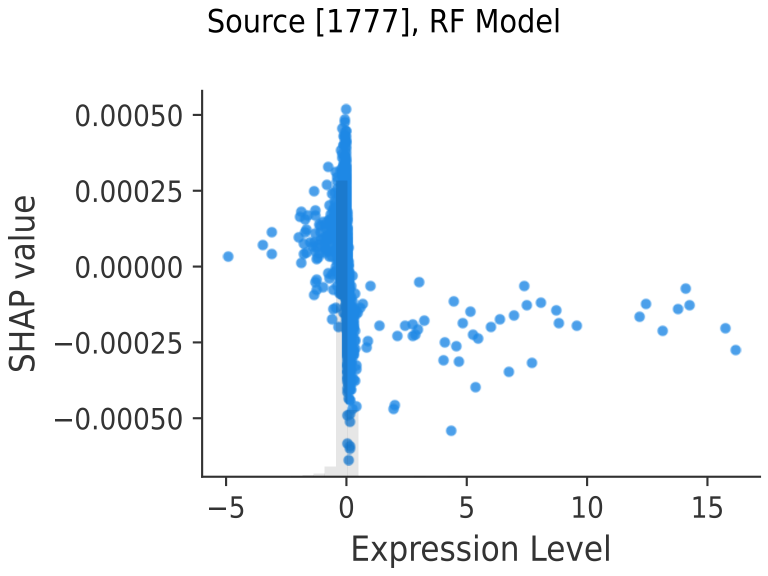
Source [1777], RF Model
SHAP value
0.00050
0.00025
0.00000
−0.00025
−0.00050
−5
0
5
10
15
Expression Level

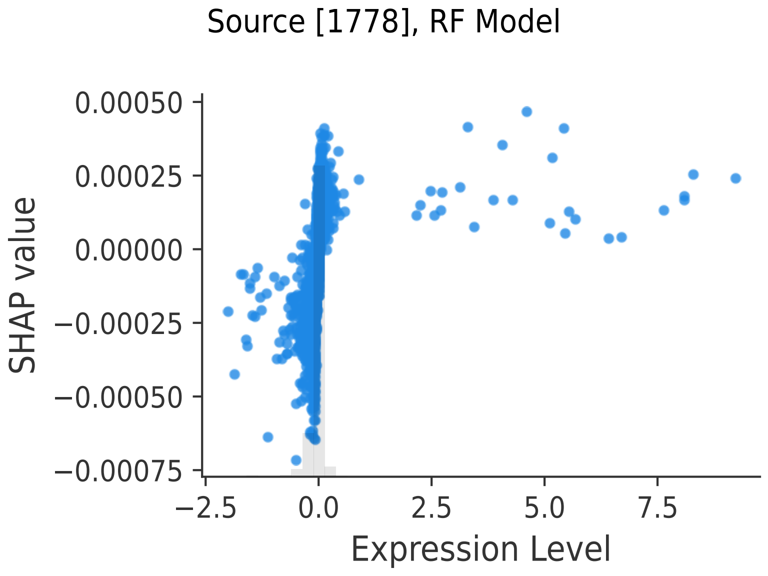
Source [1778], RF Model
0.00050
0.00025
0.00000
−0.00025
−0.00050
−0.00075
SHAP value
−2.5
0.0
2.5
5.0
7.5
Expression Level

Source [1779], RF Model

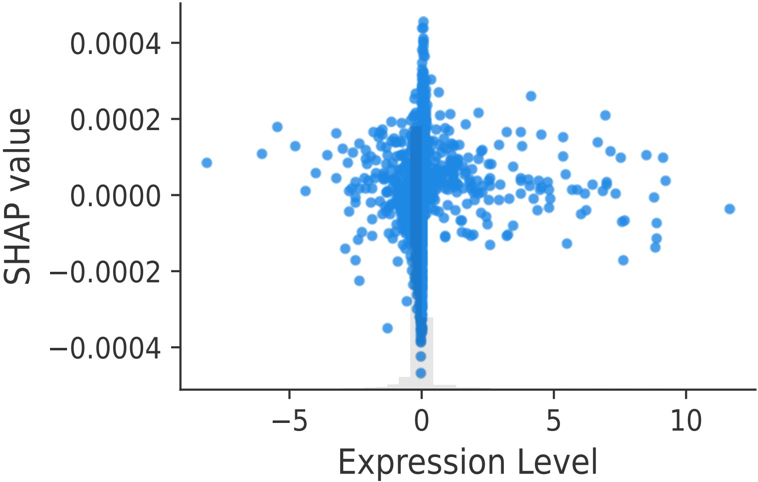

Source [1780], RF Model

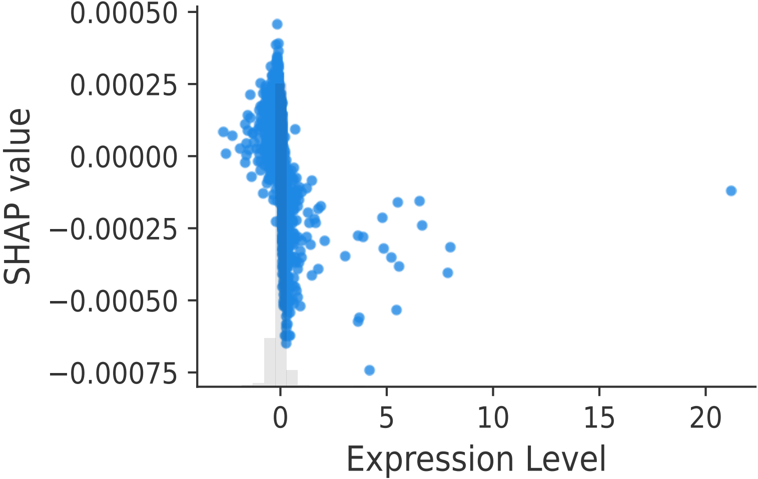

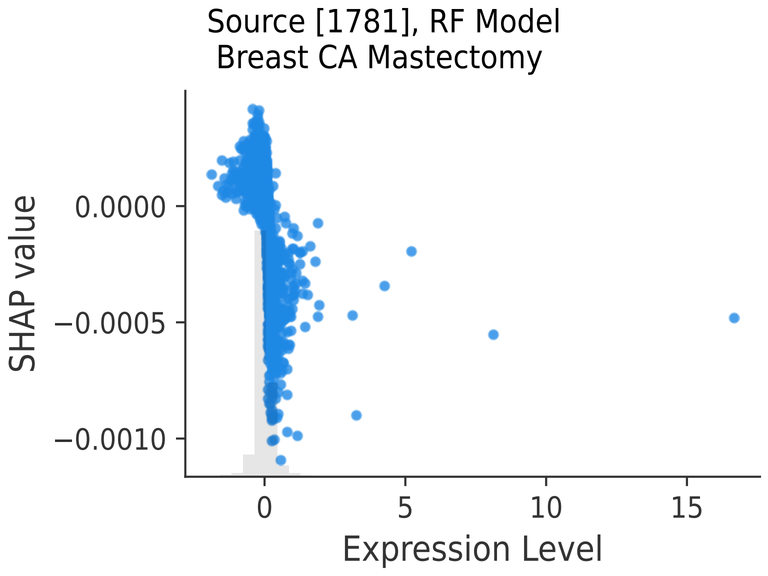

Source [1781], RF Model
Breast CA Mastectomy
SHAP value
0.0000
−0.0005
−0.0010
0
5
10
15
Expression Level

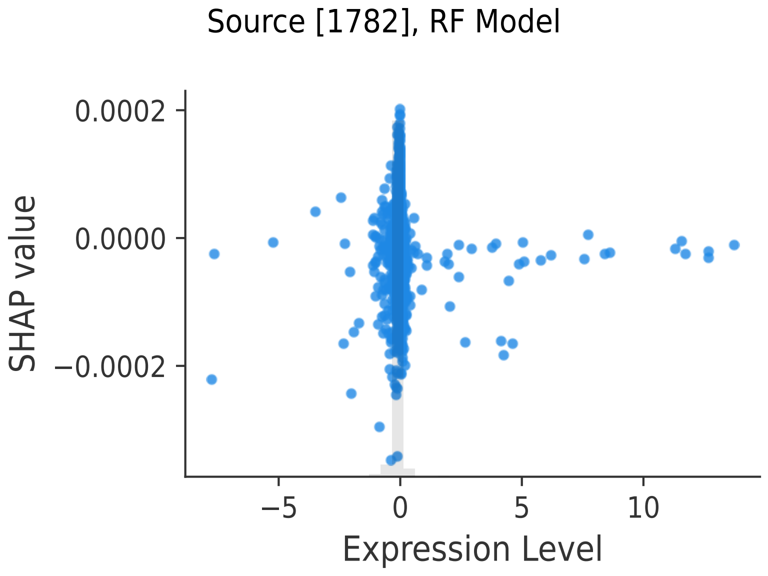
Source [1782], RF Model
SHAP value
0.0002
0.0000
−0.0002
−5
0
5
10
Expression Level

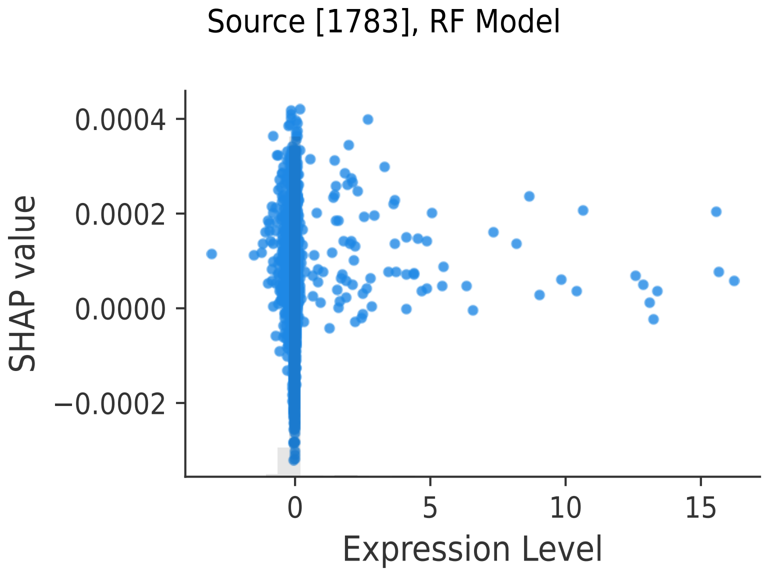
Source [1783], RF Model
SHAP value
0.0004
0.0002
0.0000
−0.0002
Expression Level
0
5
10
15

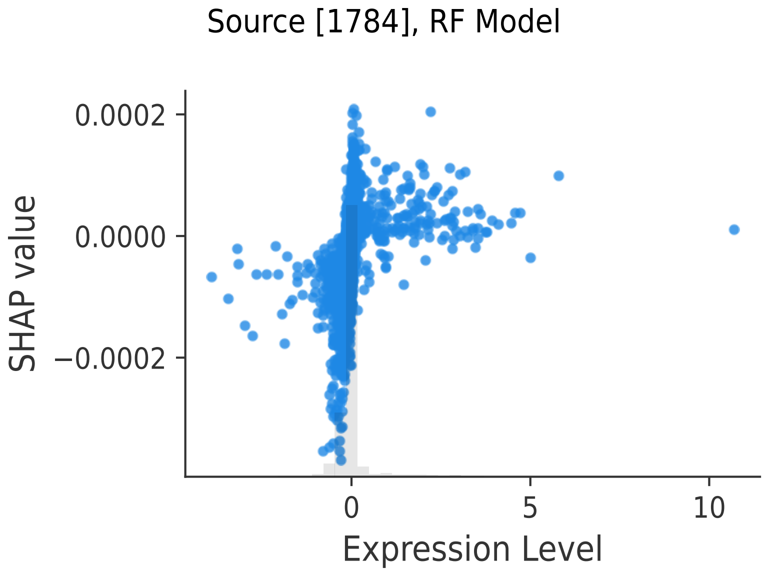
Source [1784], RF Model
0.0002
0.0000
−0.0002
SHAP value
0
5
10
Expression Level

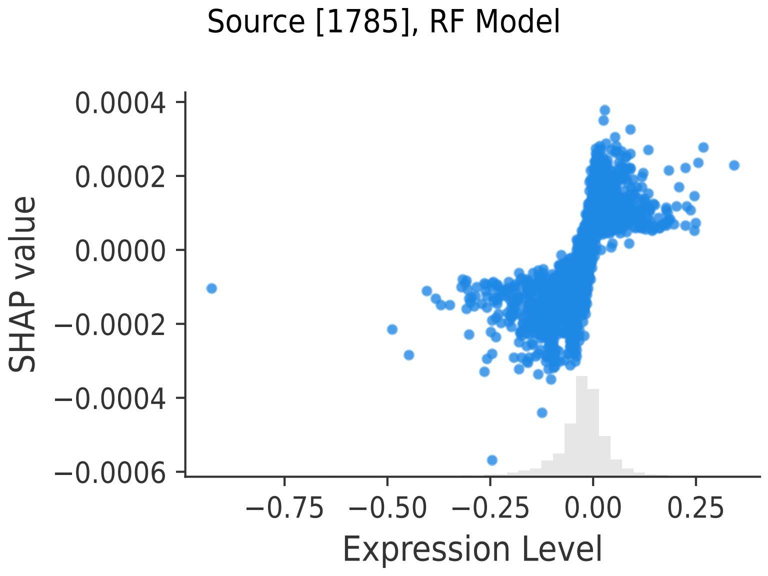

Source [1785], RF Model
0.0004
0.0002
0.0000
−0.0002
−0.0004
−0.0006
SHAP value
−0.75
−0.50
−0.25
0.00
0.25
Expression Level

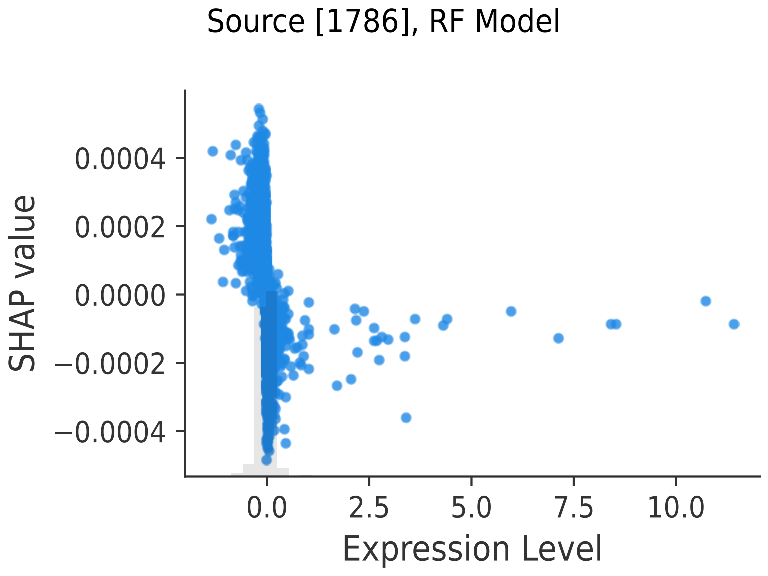

Source [1786], RF Model
SHAP value
0.0004
0.0002
0.0000
−0.0002
−0.0004
0.0
2.5
5.0
7.5
10.0
Expression Level

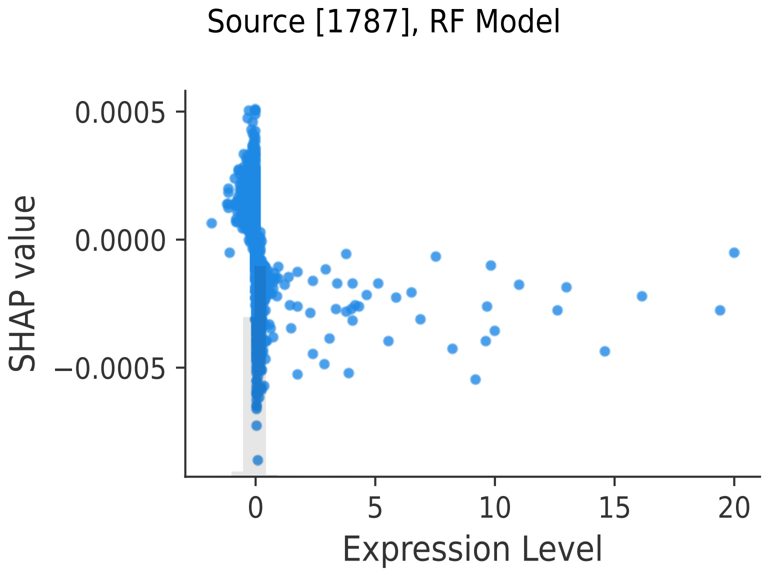

Source [1787], RF Model
SHAP value
0.0005
0.0000
−0.0005
0
5
10
15
20
Expression Level

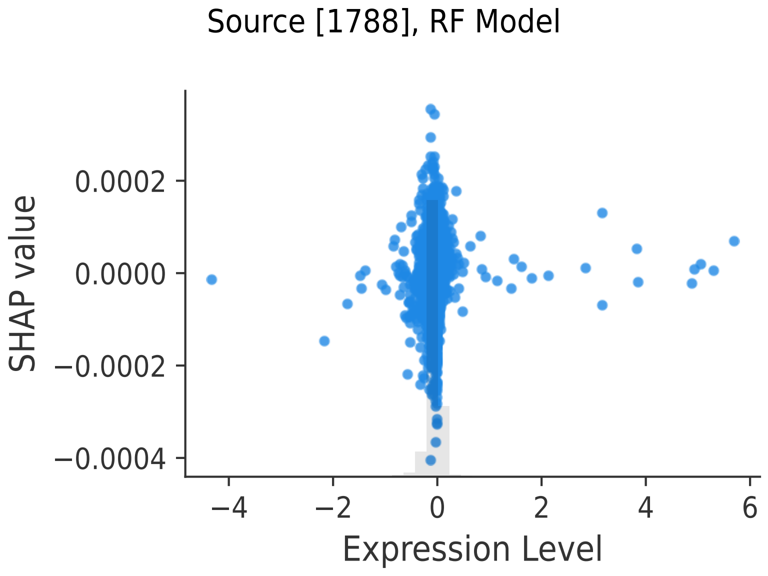

Source [1788], RF Model
SHAP value
0.0002
0.0000
−0.0002
−0.0004
−4
−2
0
2
4
6
Expression Level

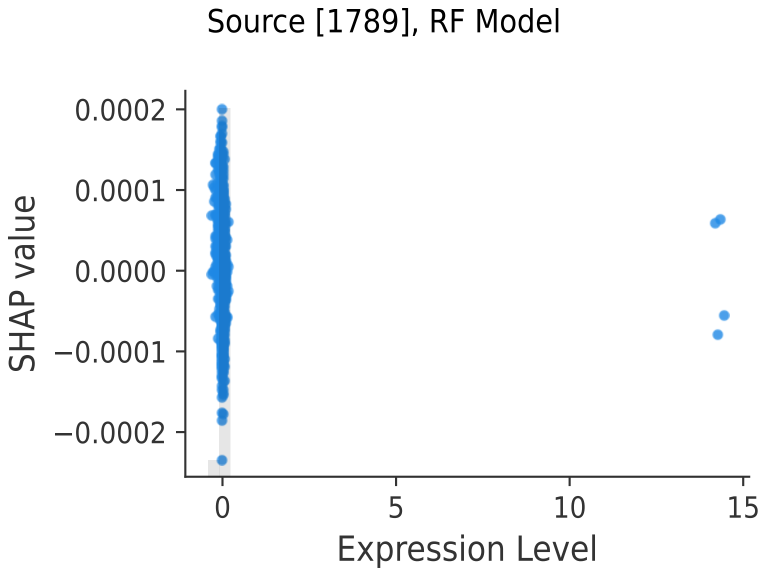

Source [1789], RF Model
SHAP value
0.0002
0.0001
0.0000
−0.0001
−0.0002
0
5
10
15
Expression Level

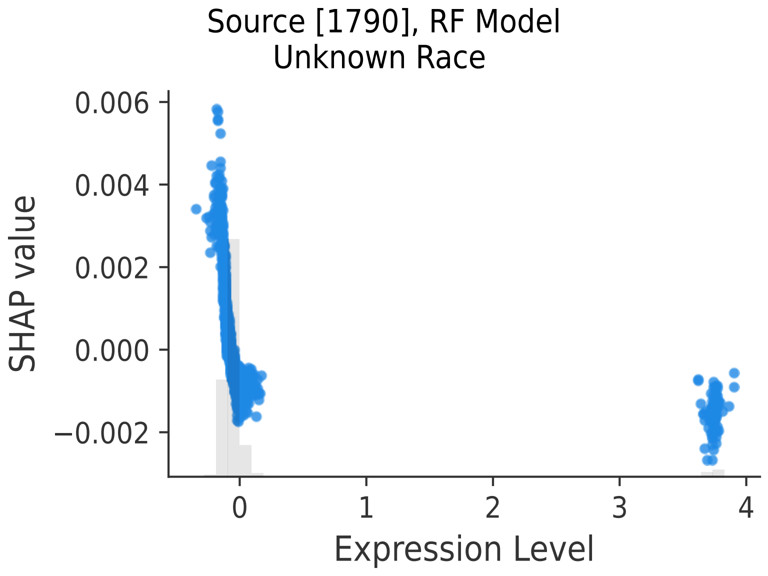
Source [1790], RF Model
Unknown Race
SHAP value
0.006
0.004
0.002
0.000
−0.002
0
1
2
3
4
Expression Level

Source [1791], RF Model

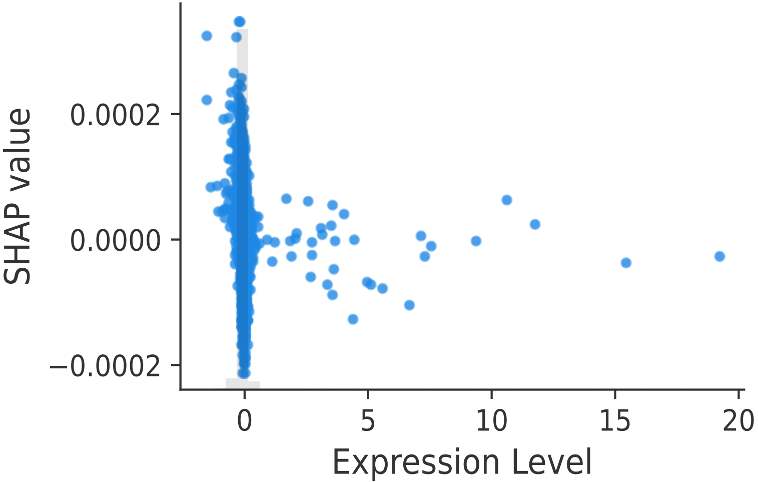

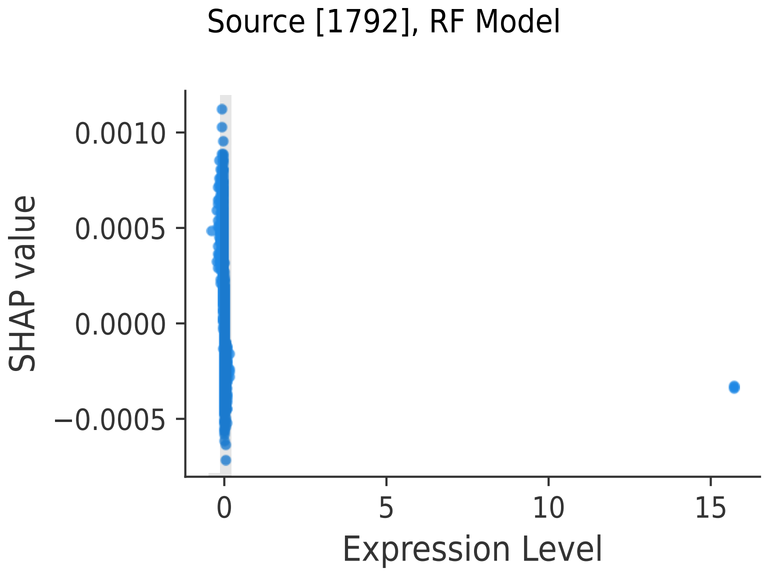

Source [1792], RF Model
0.0010
0.0005
0.0000
−0.0005
SHAP value
0
5
10
15
Expression Level

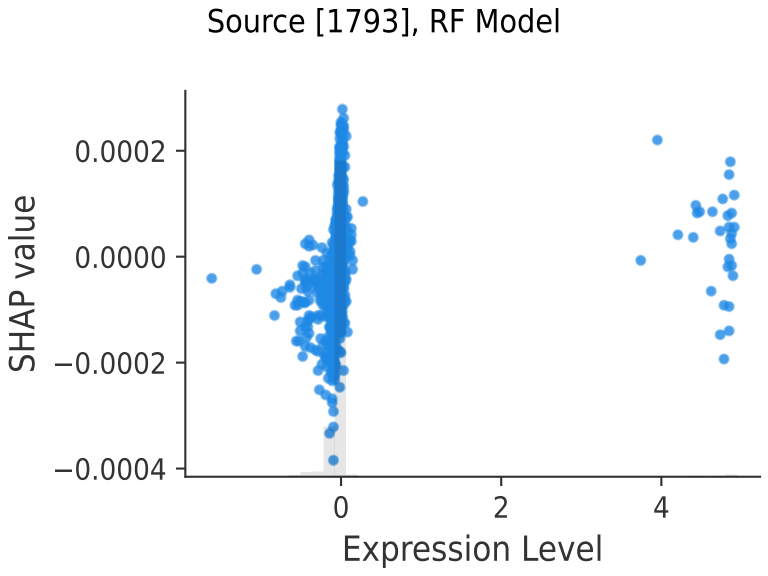
Source [1793], RF Model
0.0002
0.0000
−0.0002
−0.0004
SHAP value
0
2
4
Expression Level

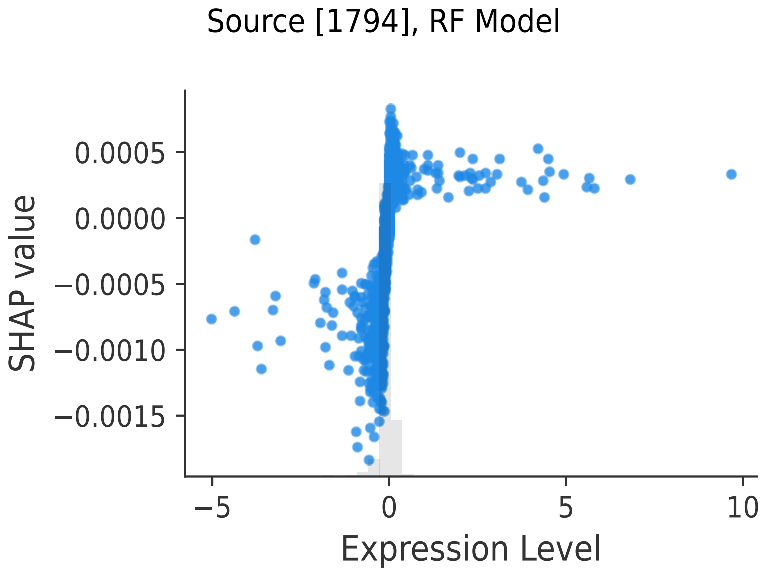

Source [1794], RF Model
0.0005
0.0000
−0.0005
−0.0010
−0.0015
SHAP value
−5
0
5
10
Expression Level

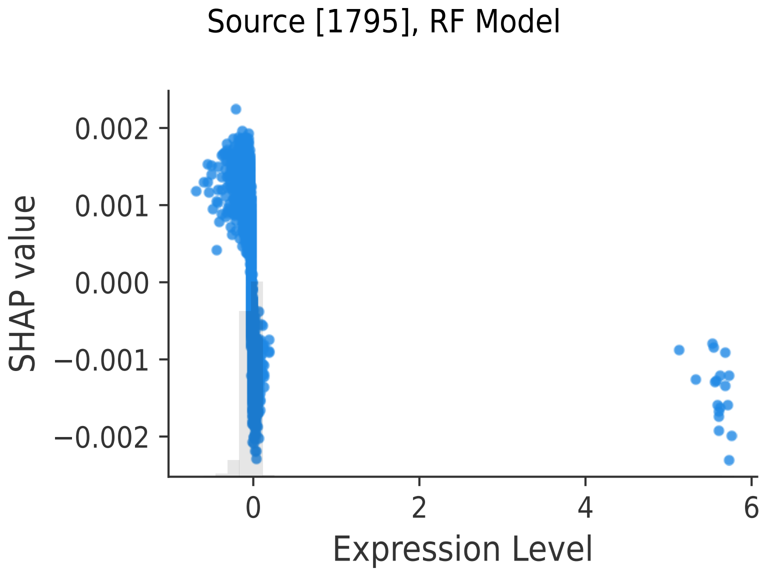

Source [1795], RF Model
SHAP value
0.002
0.001
0.000
−0.001
−0.002
0
2
4
6
Expression Level

Source [1796], RF Model

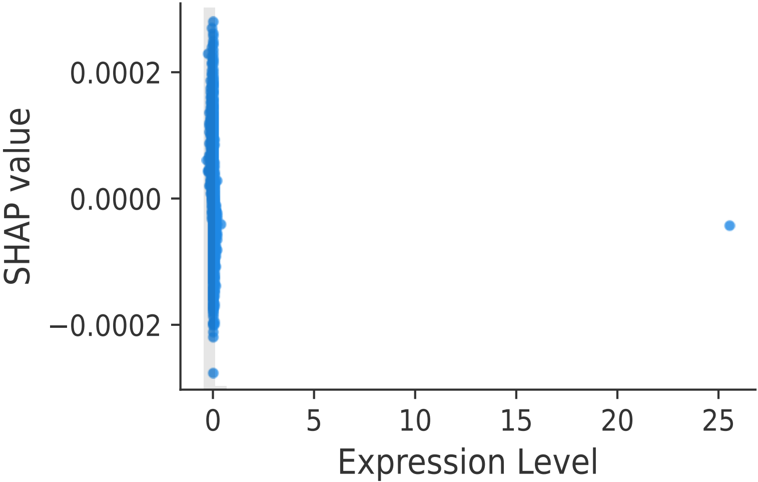

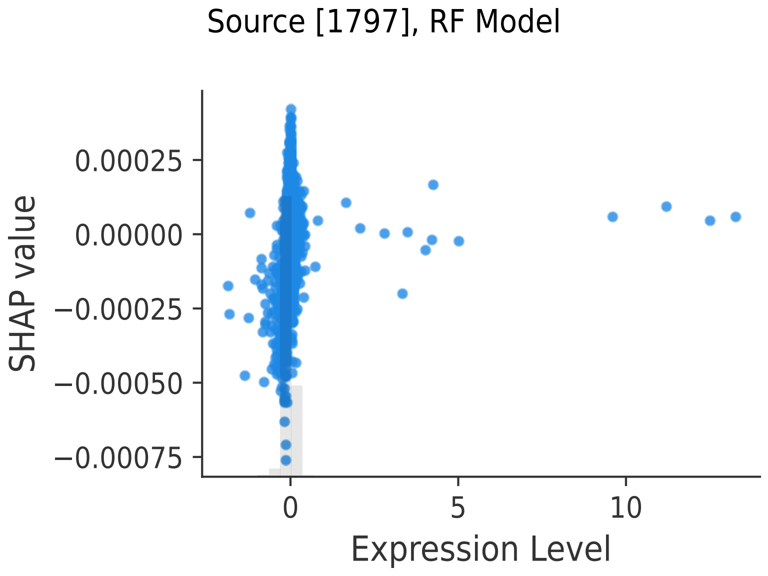

Source [1797], RF Model
SHAP value
0.00025
0.00000
−0.00025
−0.00050
−0.00075
0
5
10
Expression Level

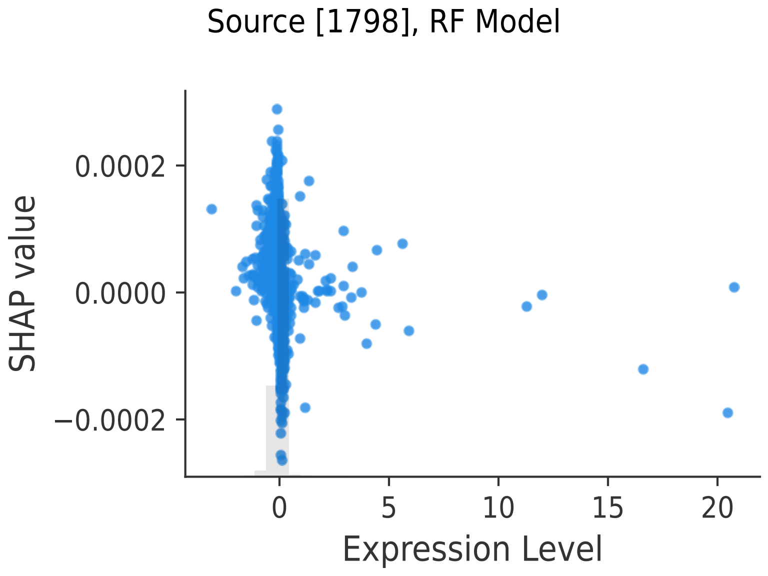
Source [1798], RF Model
SHAP value
0.0002
0.0000
−0.0002
Expression Level
0
5
10
15
20

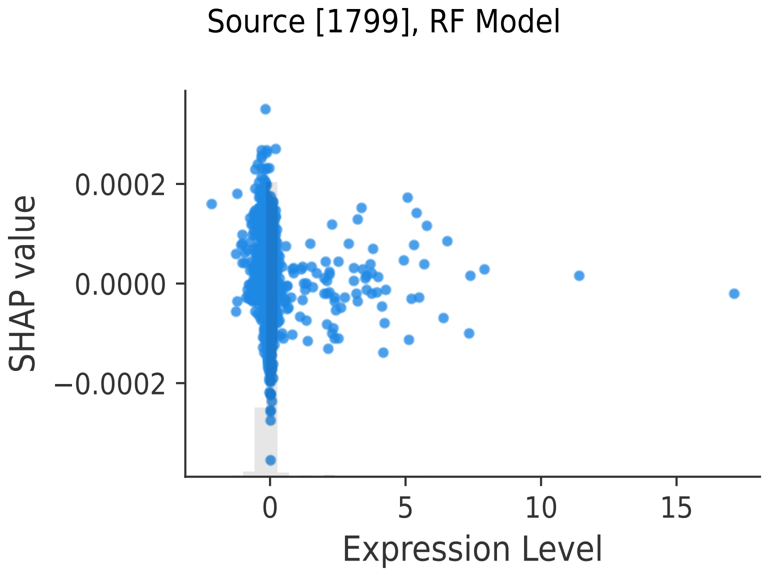
Source [1799], RF Model
SHAP value
0.0002
0.0000
−0.0002
0
5
10
15
Expression Level

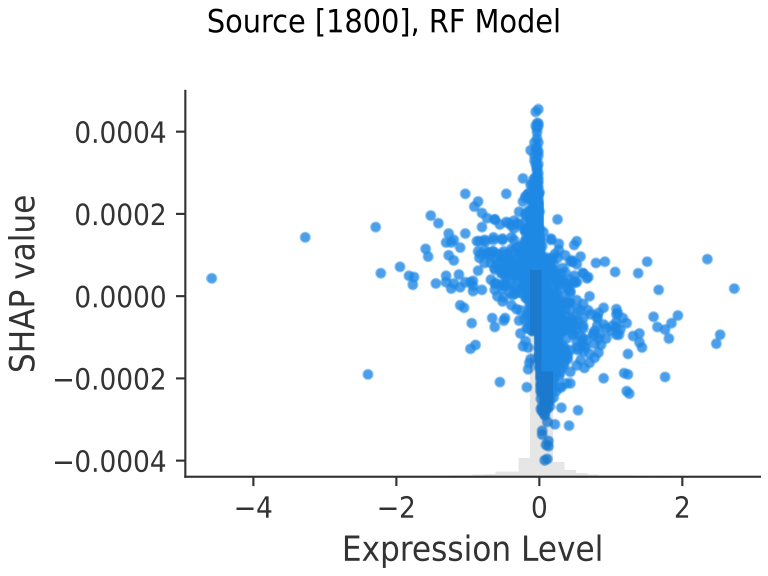

Source [1800], RF Model
SHAP value
0.0004
0.0002
0.0000
−0.0002
−0.0004
−4
−2
0
2
Expression Level

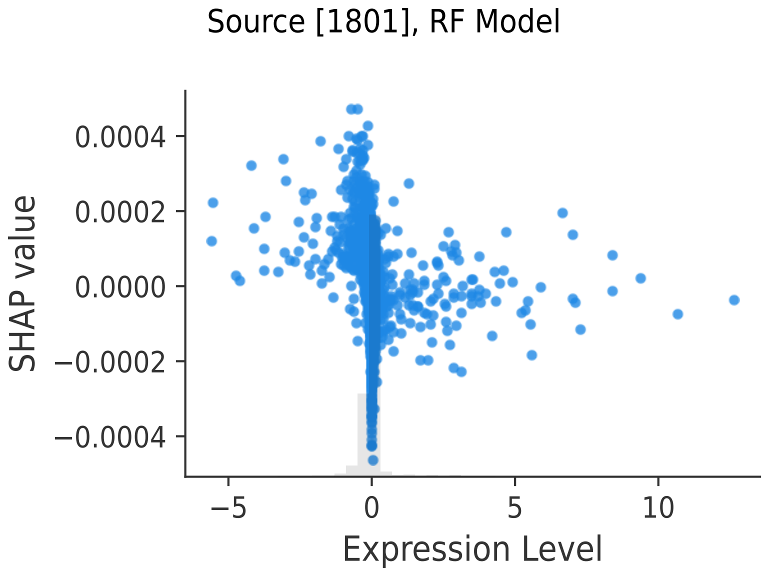
Source [1801], RF Model
SHAP value
0.0004
0.0002
0.0000
−0.0002
−0.0004
−5
0
5
10
Expression Level

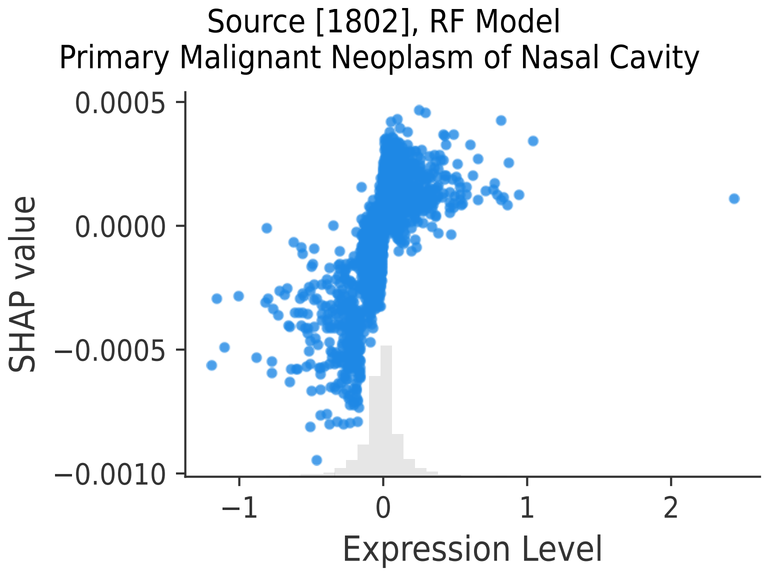

Source [1802], RF Model
Primary Malignant Neoplasm of Nasal Cavity
SHAP value
0.0005
0.0000
−0.0005
−0.0010
−1
0
1
2
Expression Level

Source [1803], RF Model

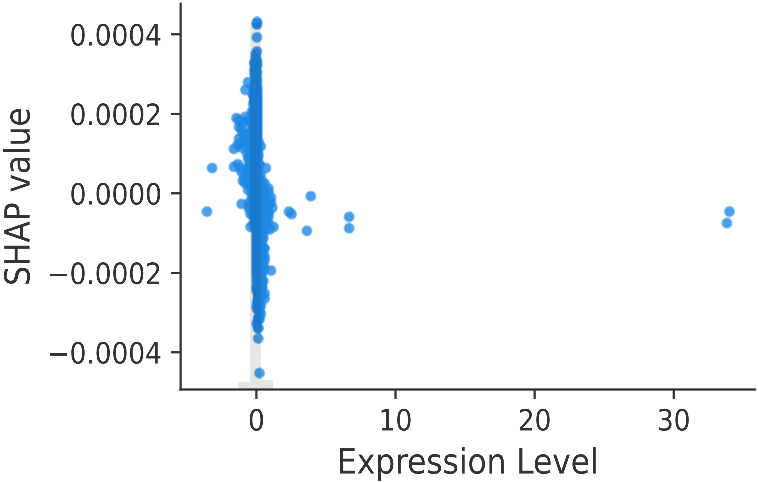

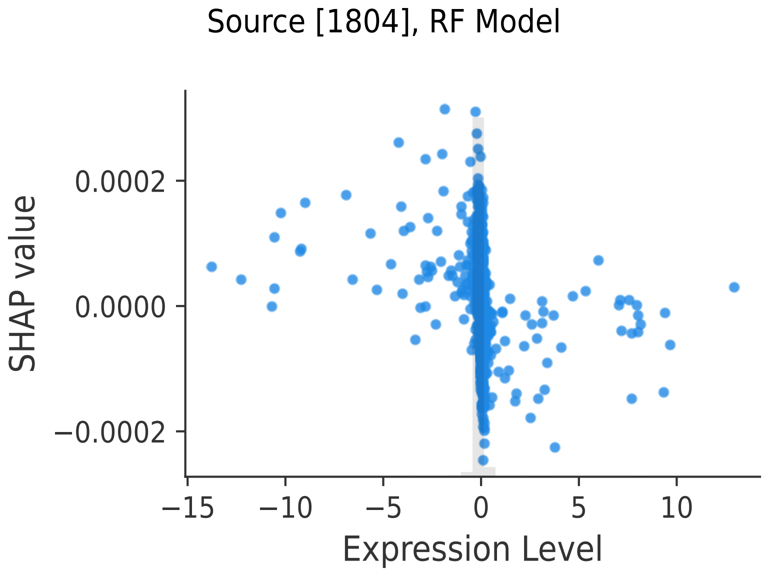

Source [1804], RF Model
SHAP value
0.0002
0.0000
−0.0002
−15
−10
−5
0
5
10
Expression Level

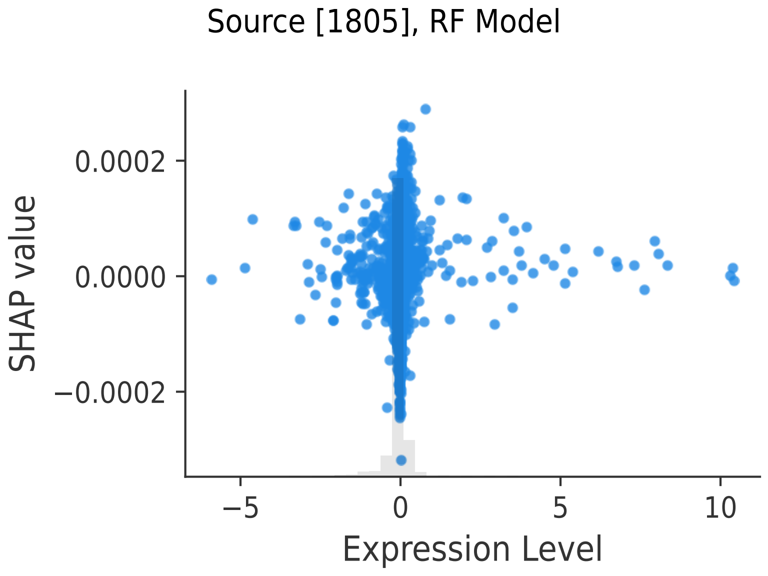
Source [1805], RF Model
SHAP value
0.0002
0.0000
−0.0002
−5
0
5
10
Expression Level

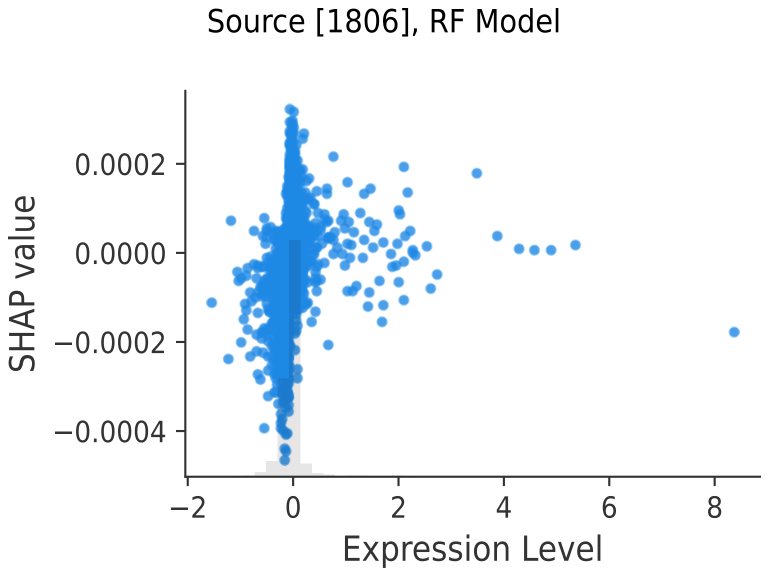

Source [1806], RF Model
0.0002
0.0000
−0.0002
−0.0004
SHAP value
−2
0
2
4
6
8
Expression Level

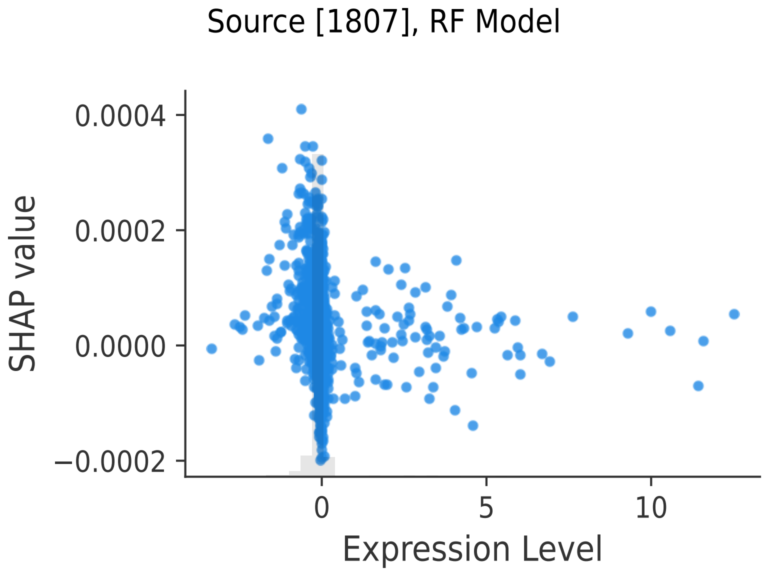

Source [1807], RF Model
SHAP value
0.0004
0.0002
0.0000
−0.0002
0
5
10
Expression Level

Source [1808], RF Model

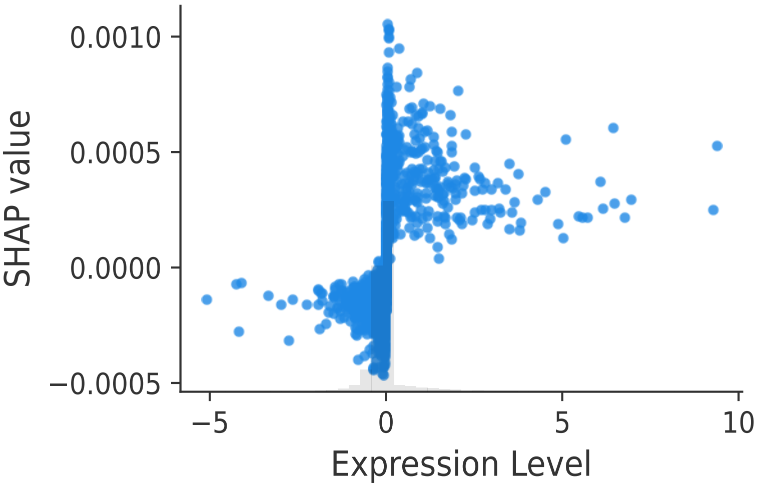

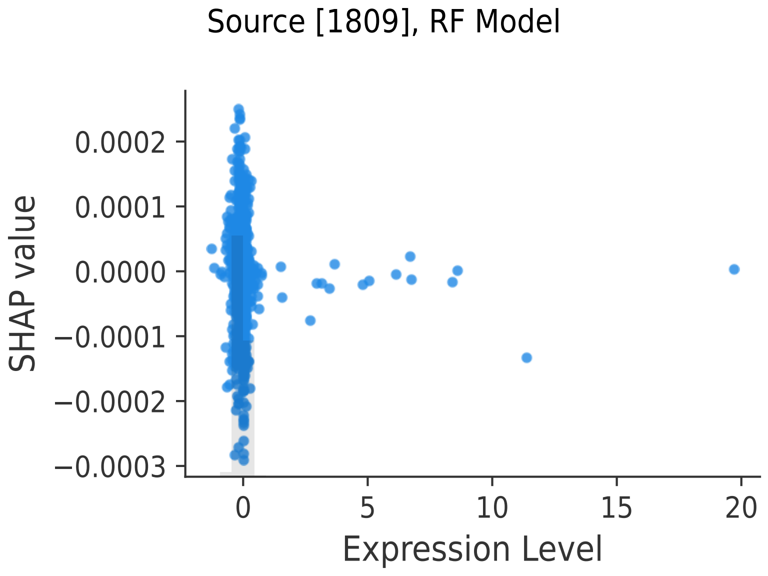
Source [1809], RF Model
SHAP value
0.0002
0.0001
0.0000
−0.0001
−0.0002
−0.0003
0
5
10
15
20
Expression Level

Source [1810], RF Model

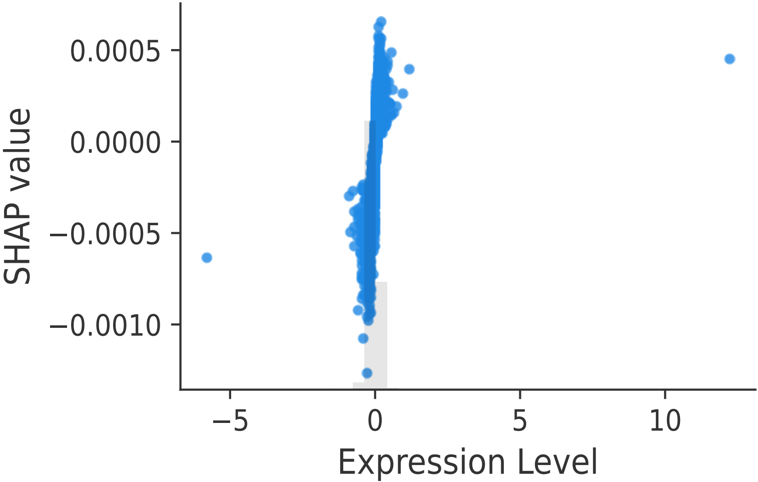

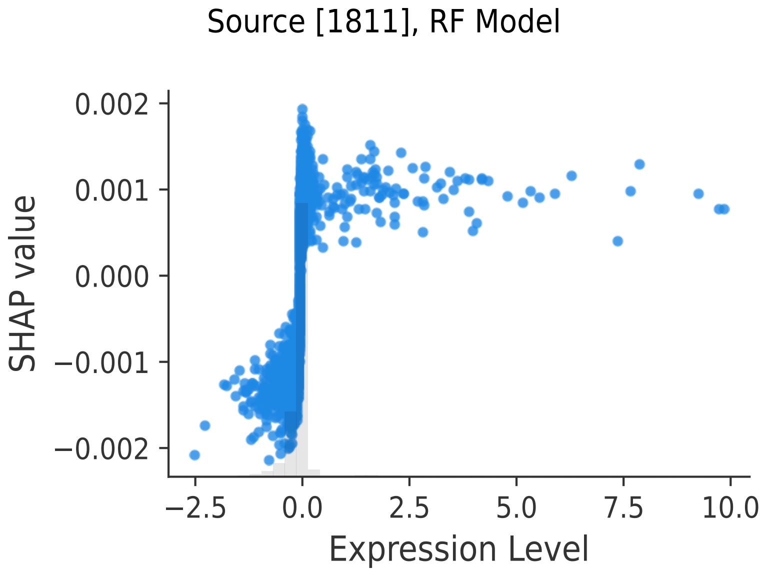
Source [1811], RF Model
SHAP value
0.002
0.001
0.000
−0.001
−0.002
−2.5
0.0
2.5
5.0
7.5
10.0
Expression Level

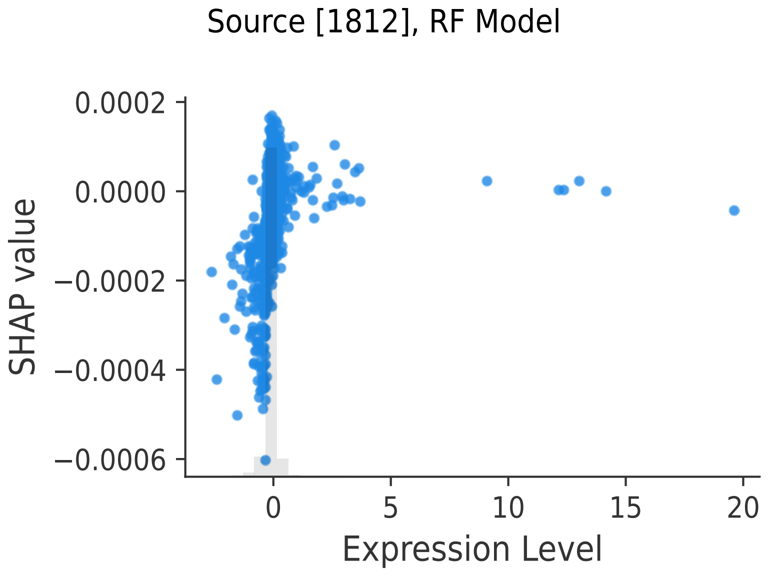

Source [1812], RF Model
SHAP value
0.0002
0.0000
−0.0002
−0.0004
−0.0006
0
5
10
15
20
Expression Level

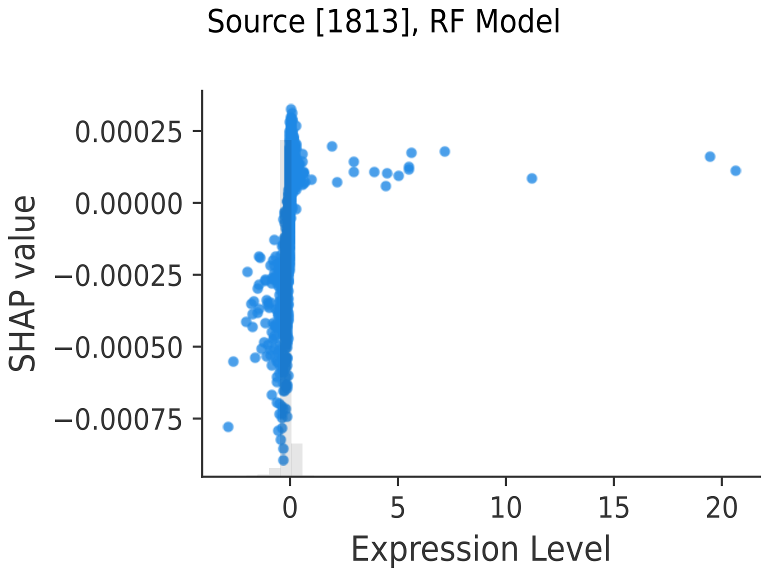

Source [1813], RF Model
SHAP value
0.00025
0.00000
−0.00025
−0.00050
−0.00075
0
5
10
15
20
Expression Level

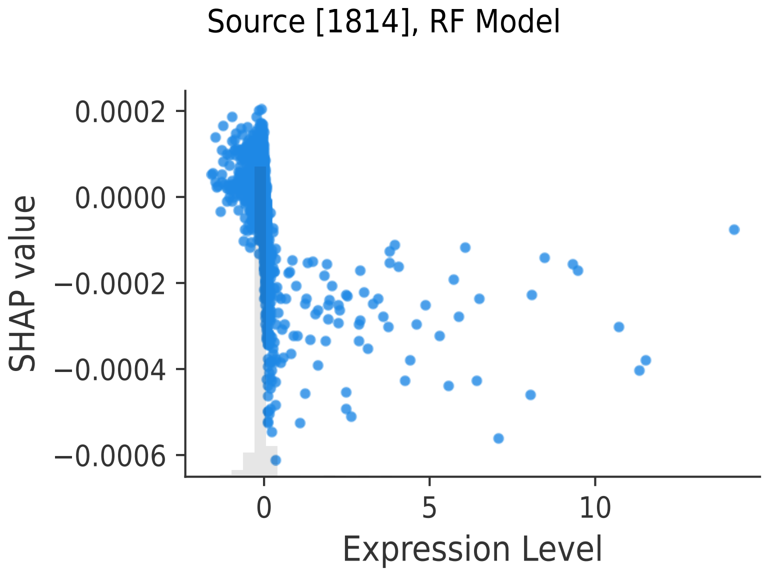
Source [1814], RF Model
0.0002
0.0000
−0.0002
−0.0004
−0.0006
SHAP value
0
5
10
Expression Level

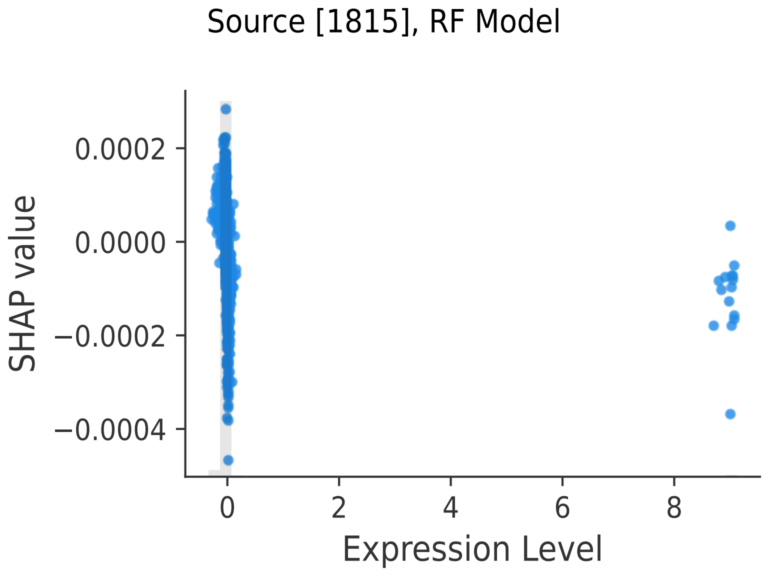
Source [1815], RF Model
0.0002
0.0000
−0.0002
−0.0004
SHAP value
0
2
4
6
8
Expression Level

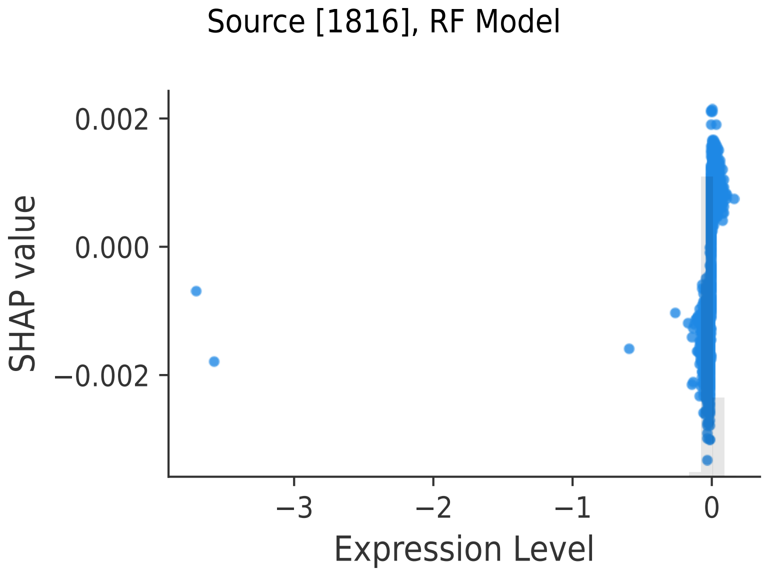
Source [1816], RF Model
SHAP value
0.002
0.000
−0.002
−3
−2
−1
0
Expression Level

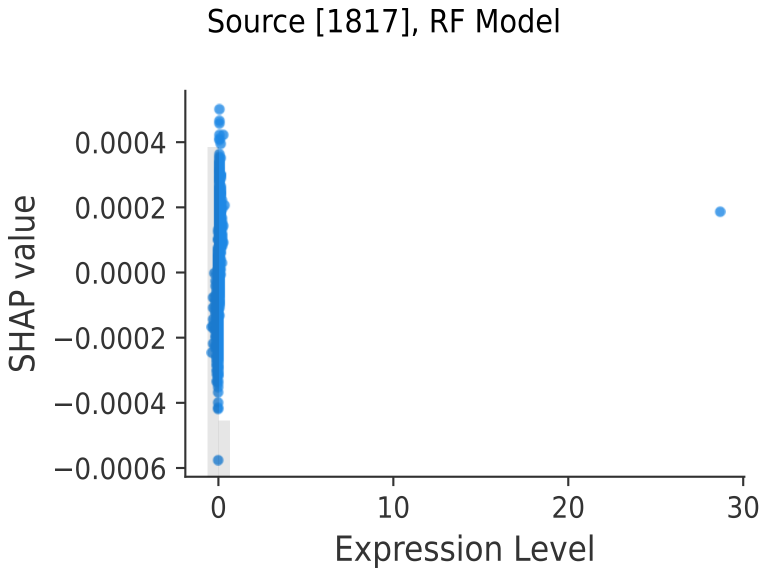
Source [1817], RF Model
SHAP value
0.0004
0.0002
0.0000
−0.0002
−0.0004
−0.0006
0
10
20
30
Expression Level

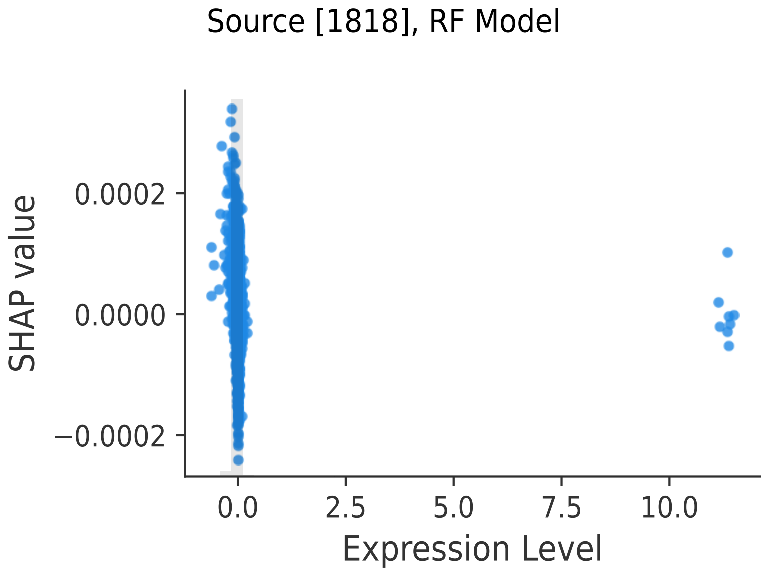
Source [1818], RF Model
SHAP value
0.0002
0.0000
−0.0002
0.0
2.5
5.0
7.5
10.0
Expression Level

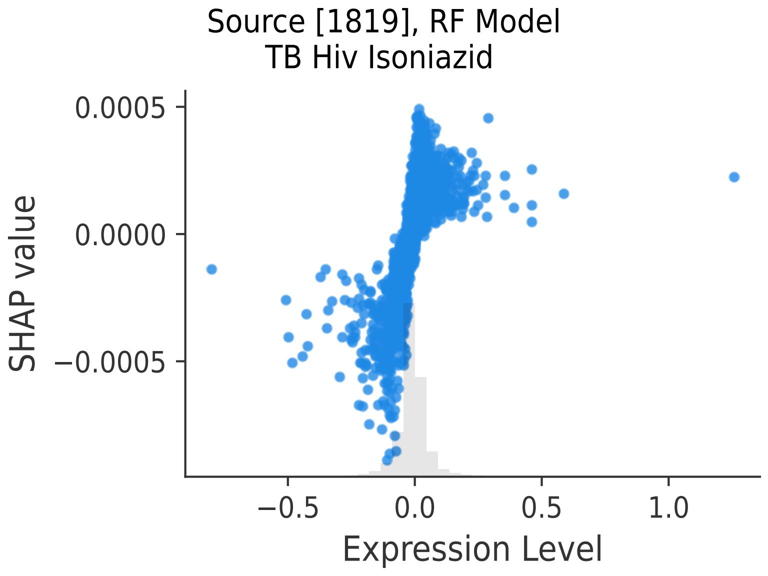
Source [1819], RF Model
TB Hiv Isoniazid
0.0005
0.0000
−0.0005
SHAP value
−0.5
0.0
0.5
1.0
Expression Level

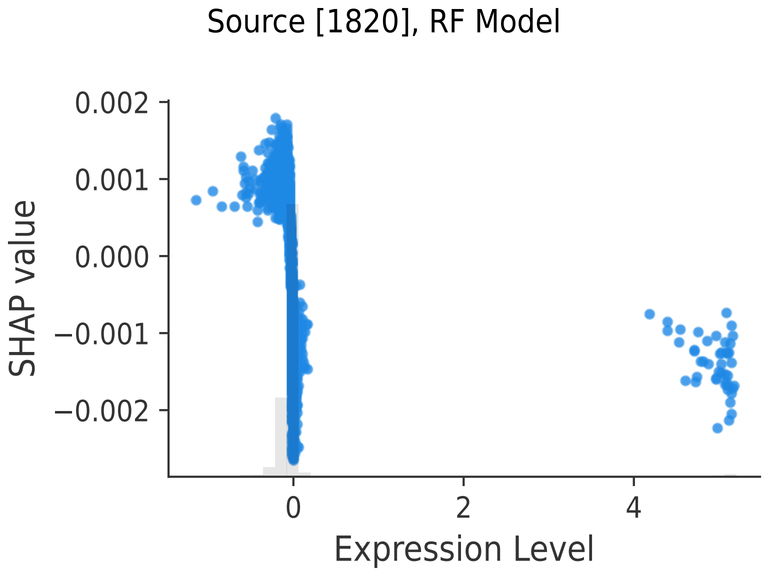

Source [1820], RF Model
0.002
0.001
0.000
−0.001
−0.002
SHAP value
0
2
4
Expression Level

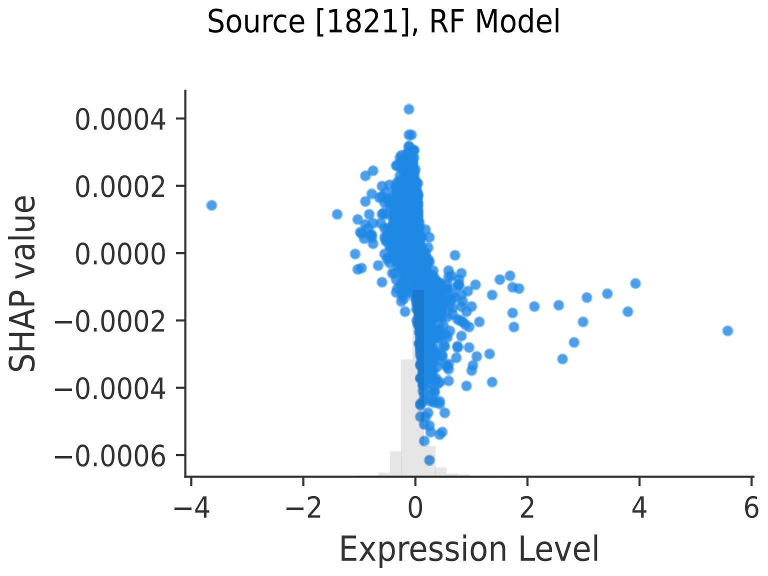

Source [1821], RF Model
SHAP value
0.0004
0.0002
0.0000
−0.0002
−0.0004
−0.0006
−4
−2
0
2
4
6
Expression Level

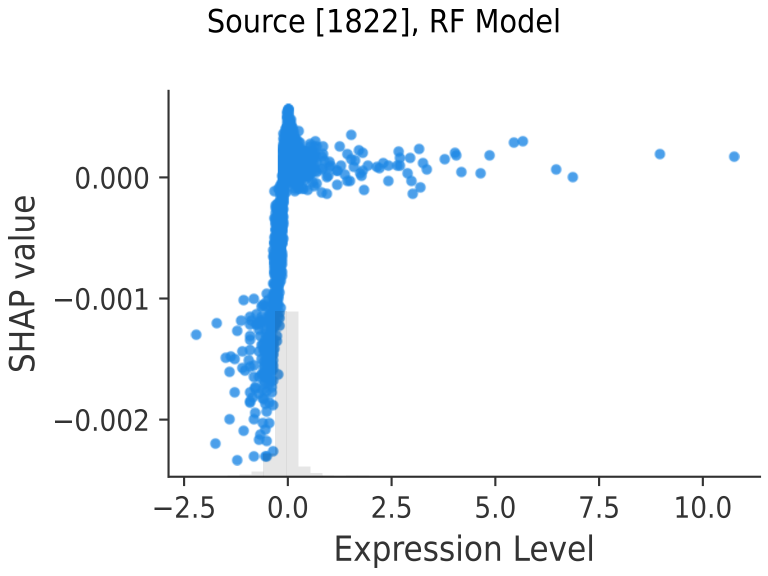

Source [1822], RF Model
SHAP value
0.000
−0.001
−0.002
−2.5
0.0
2.5
5.0
7.5
10.0
Expression Level

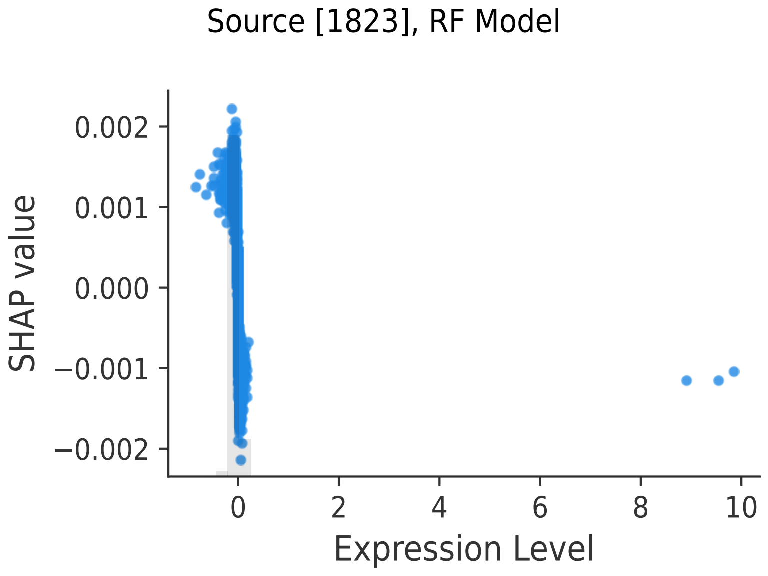
Source [1823], RF Model
SHAP value
0.002
0.001
0.000
−0.001
−0.002
0
2
4
6
8
10
Expression Level

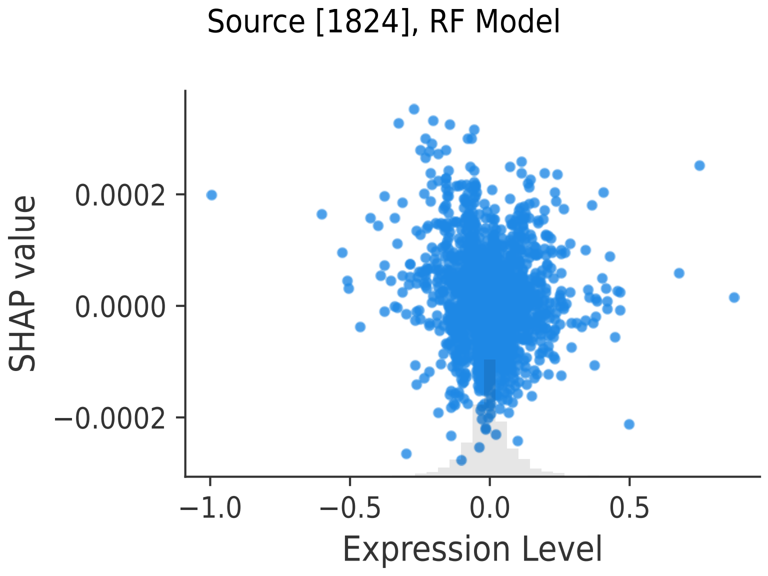

Source [1824], RF Model
SHAP value
0.0002
0.0000
−0.0002
−1.0
−0.5
0.0
0.5
Expression Level

Source [1825], RF Model

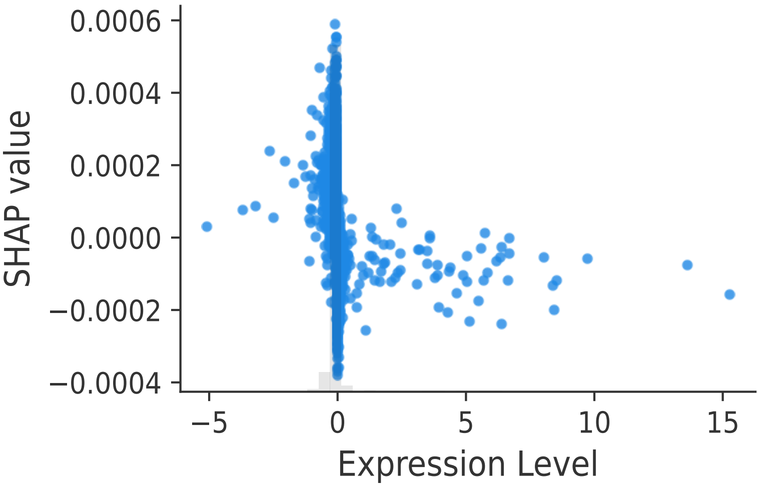

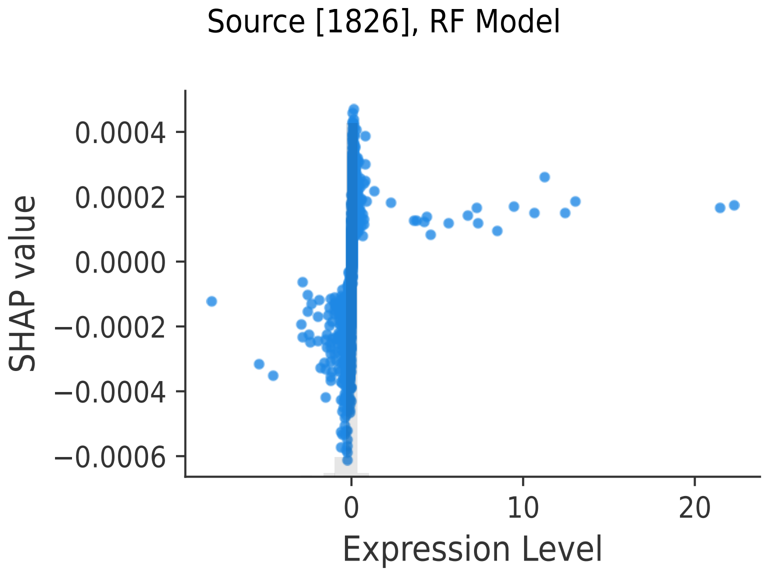

Source [1826], RF Model
SHAP value
0.0004
0.0002
0.0000
−0.0002
−0.0004
−0.0006
0
10
20
Expression Level

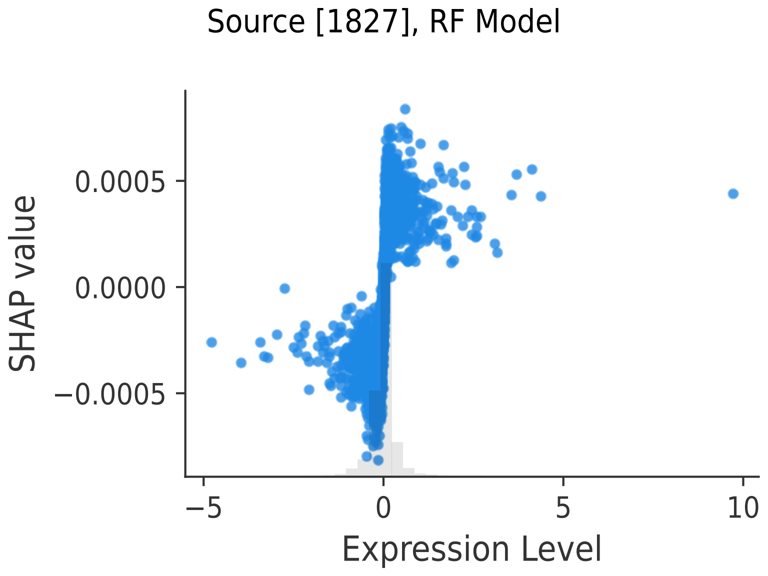

Source [1827], RF Model
SHAP value
0.0005
0.0000
−0.0005
−5
0
5
10
Expression Level

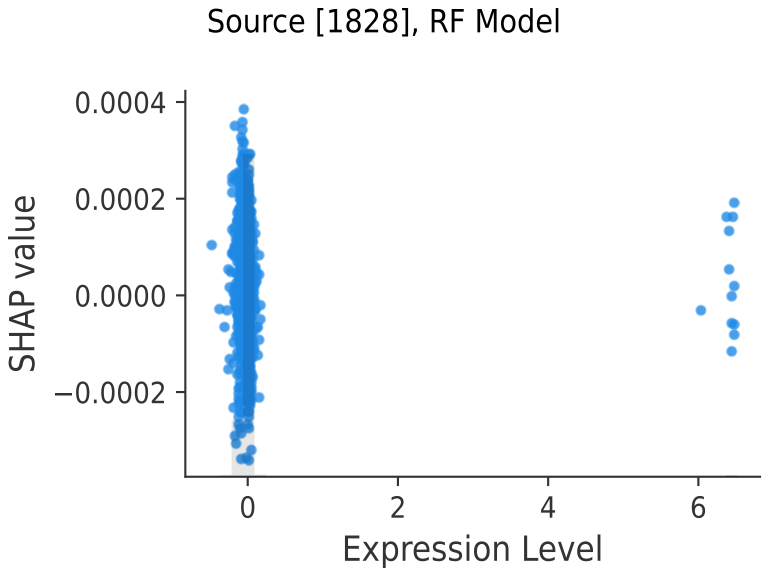
Source [1828], RF Model
SHAP value
0.0004
0.0002
0.0000
−0.0002
0
2
4
6
Expression Level

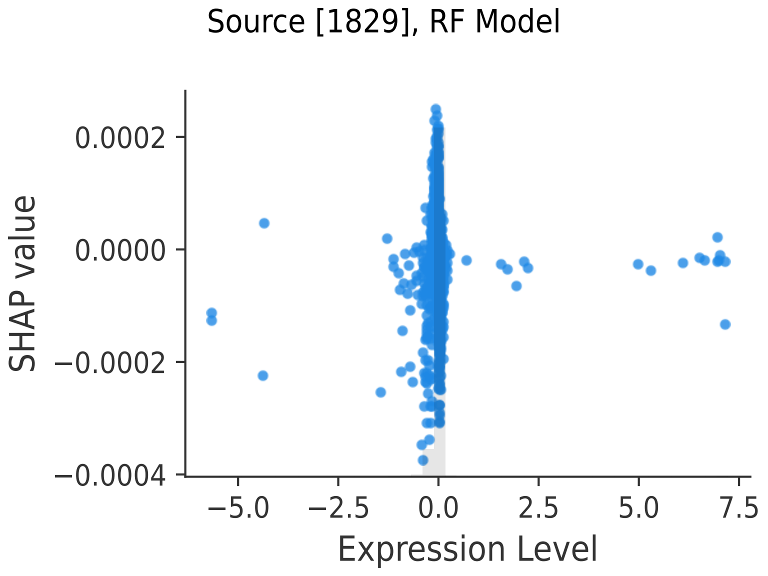

Source [1829], RF Model
0.0002
0.0000
−0.0002
−0.0004
SHAP value
−5.0
−2.5
0.0
2.5
5.0
7.5
Expression Level

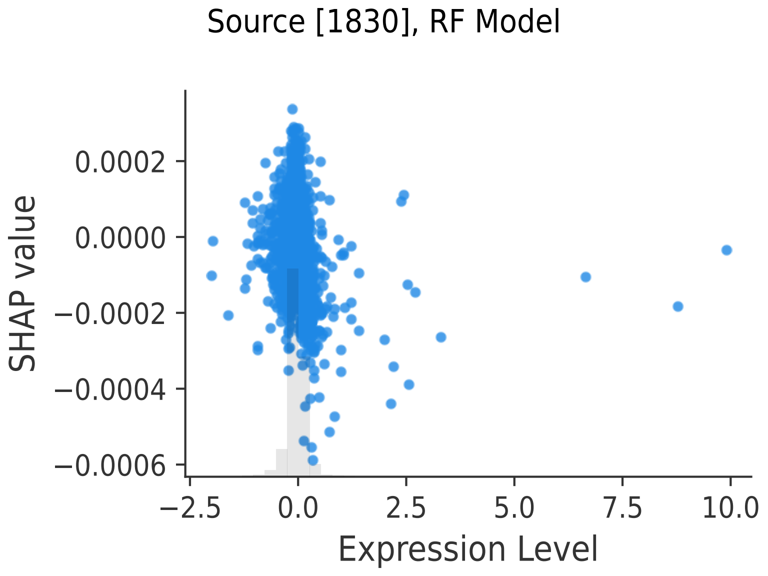
Source [1830], RF Model
SHAP value
0.0002
0.0000
−0.0002
−0.0004
−0.0006
−2.5
0.0
2.5
5.0
7.5
10.0
Expression Level

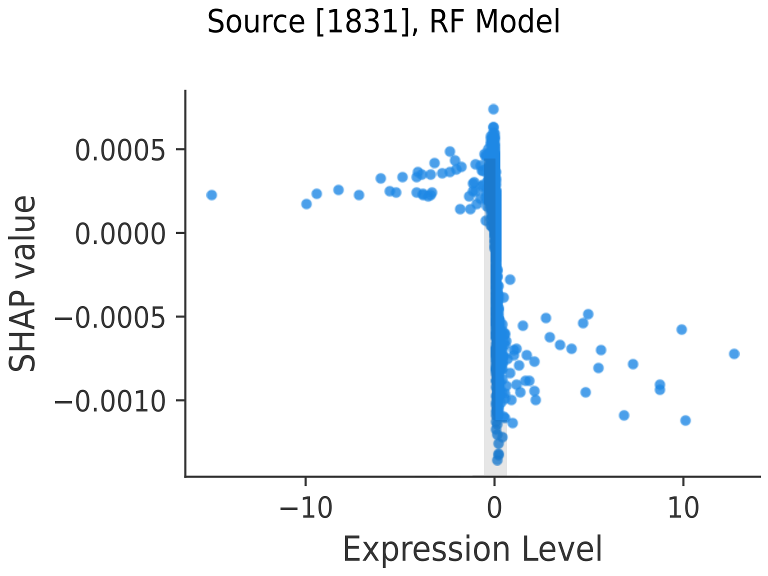
Source [1831], RF Model
SHAP value
0.0005
0.0000
−0.0005
−0.0010
−10
0
10
Expression Level

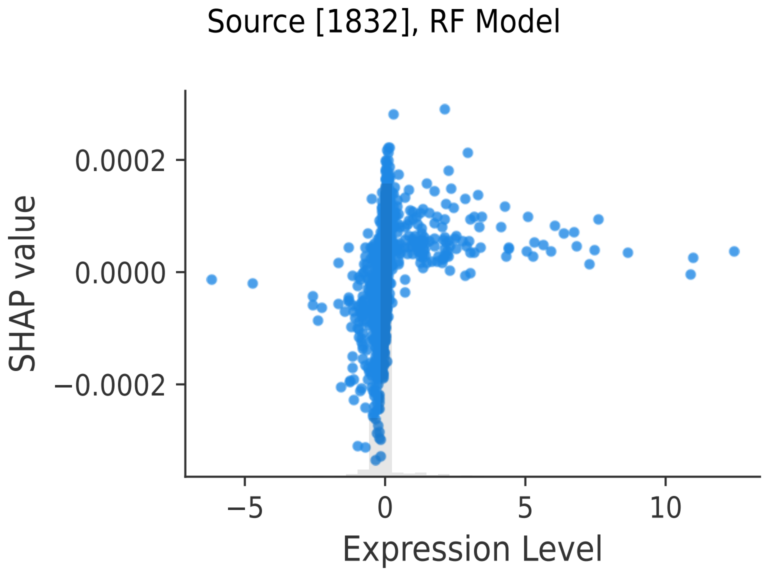

Source [1832], RF Model
SHAP value
0.0002
0.0000
−0.0002
−5
0
5
10
Expression Level

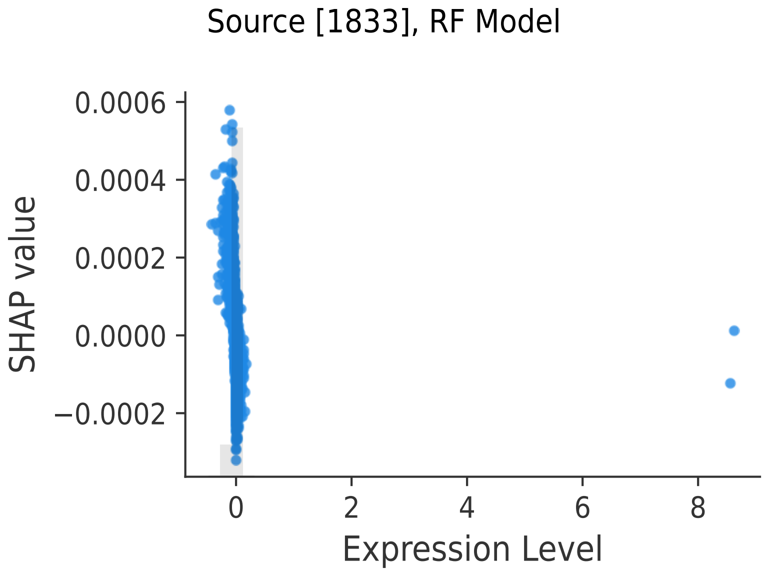

Source [1833], RF Model
SHAP value
0.0006
0.0004
0.0002
0.0000
−0.0002
0
2
4
6
8
Expression Level

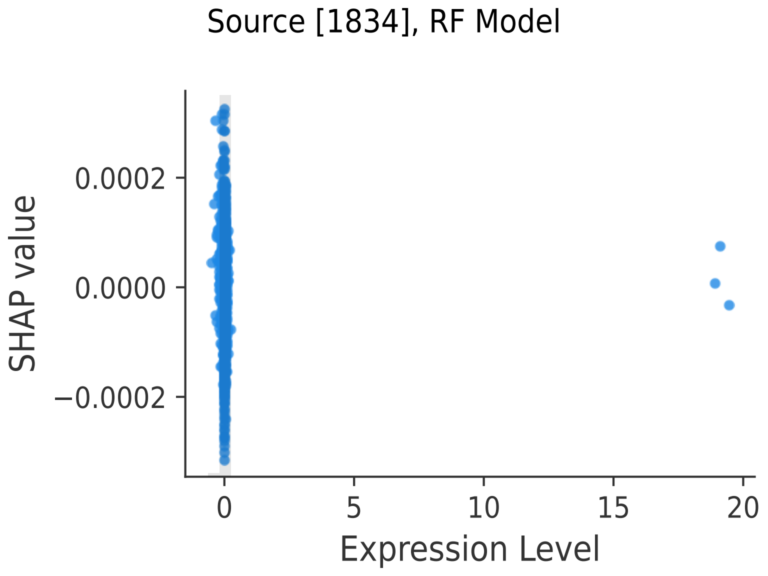

Source [1834], RF Model
SHAP value
0.0002
0.0000
−0.0002
0
5
10
15
20
Expression Level

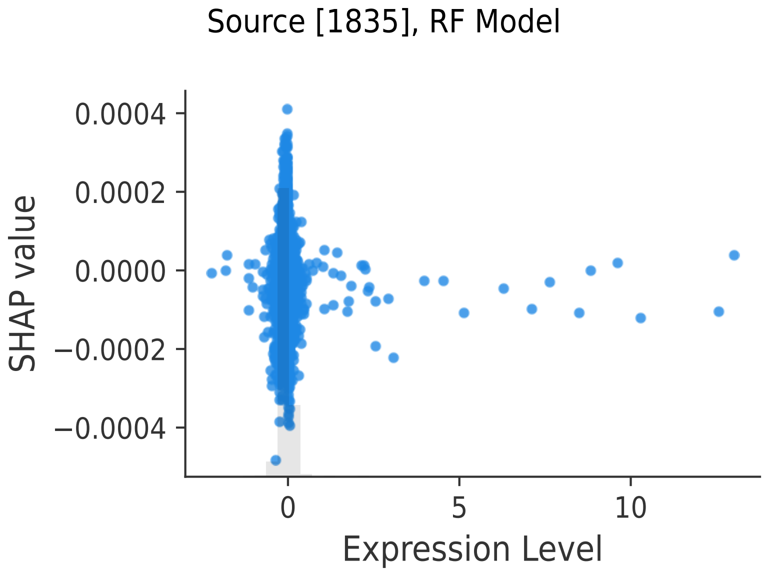
Source [1835], RF Model
0.0004
0.0002
0.0000
−0.0002
−0.0004
SHAP value
0
5
10
Expression Level

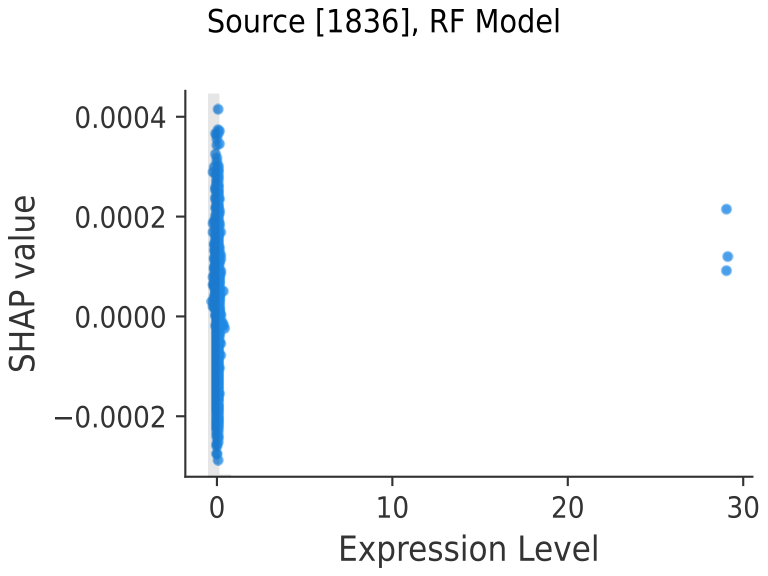
Source [1836], RF Model
SHAP value
0.0004
0.0002
0.0000
−0.0002
0
10
20
30
Expression Level

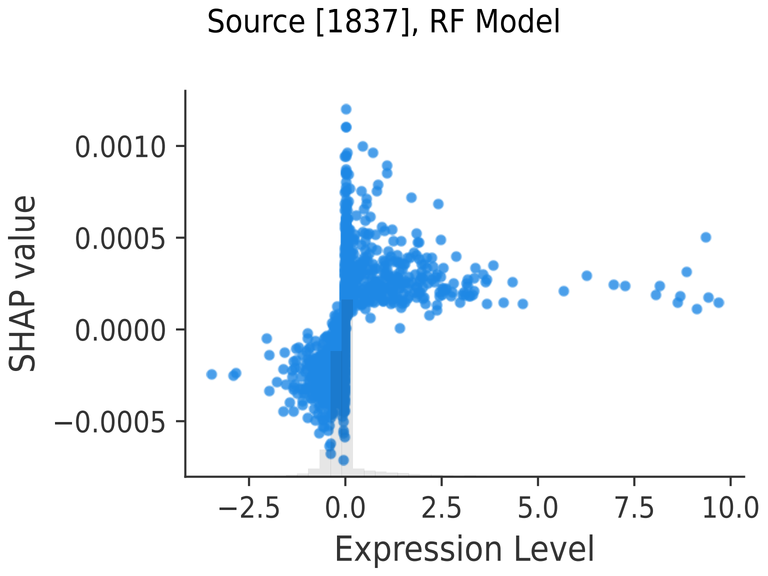
Source [1837], RF Model
SHAP value
0.0010
0.0005
0.0000
−0.0005
−2.5
0.0
2.5
5.0
7.5
10.0
Expression Level

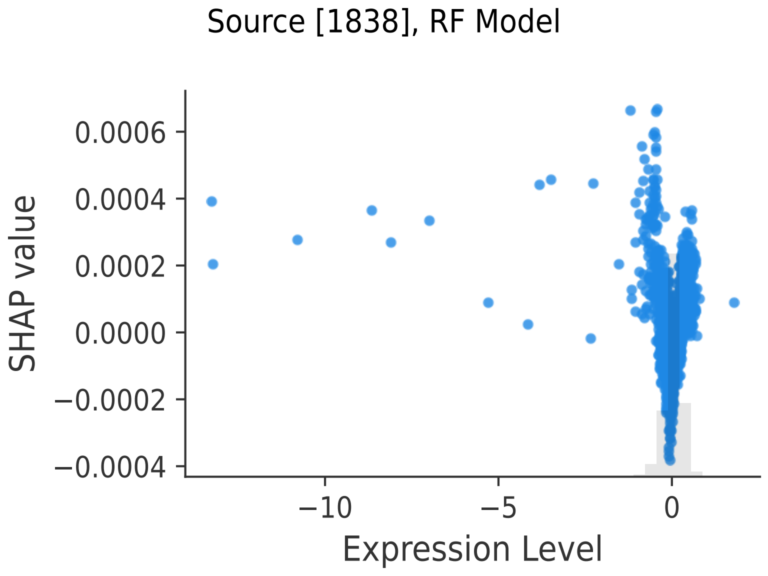
Source [1838], RF Model
SHAP value
0.0006
0.0004
0.0002
0.0000
−0.0002
−0.0004
−10
−5
0
Expression Level

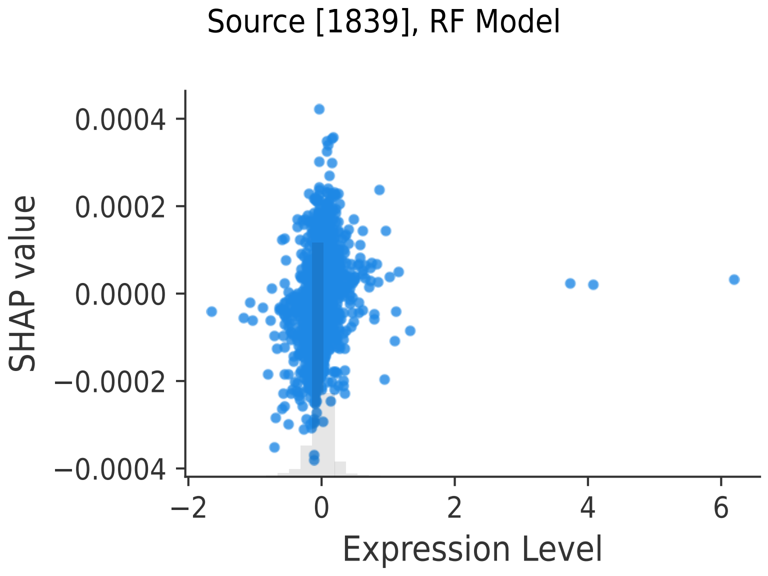
Source [1839], RF Model
SHAP value
0.0004
0.0002
0.0000
−0.0002
−0.0004
−2
0
2
4
6
Expression Level

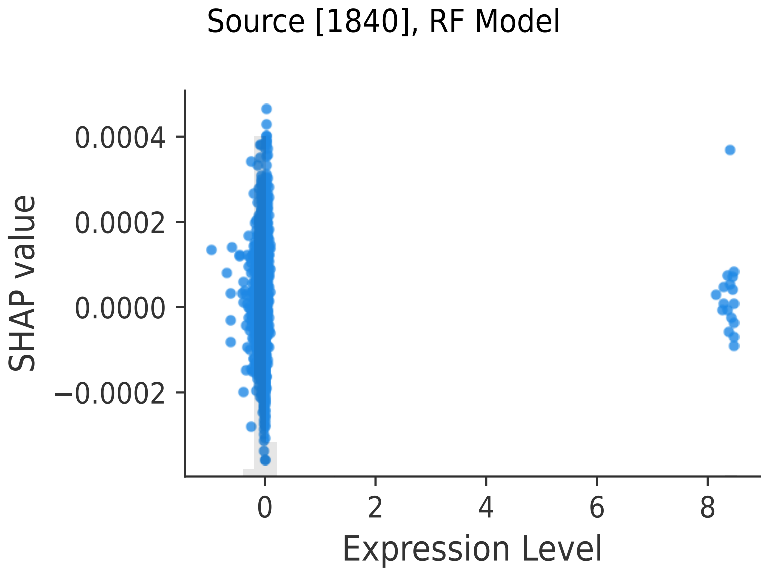
Source [1840], RF Model
SHAP value
0.0004
0.0002
0.0000
−0.0002
0
2
4
6
8
Expression Level

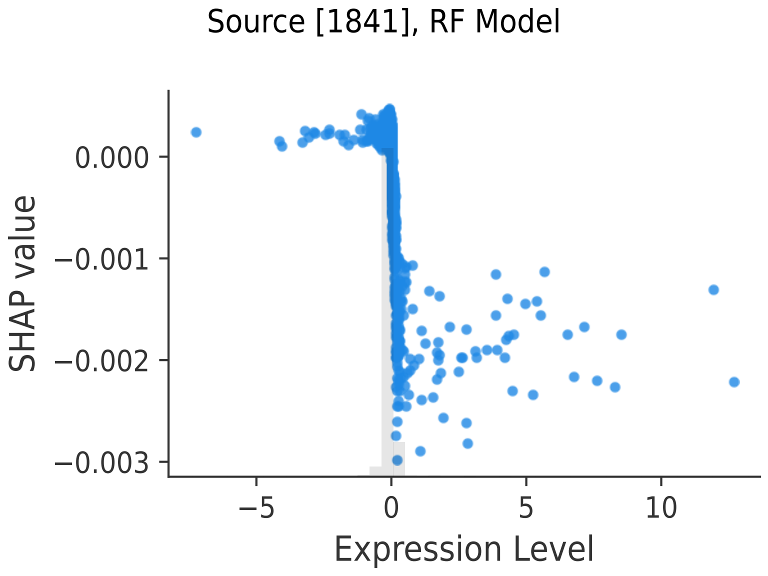
Source [1841], RF Model
SHAP value
0.000
−0.001
−0.002
−0.003
−5
0
5
10
Expression Level

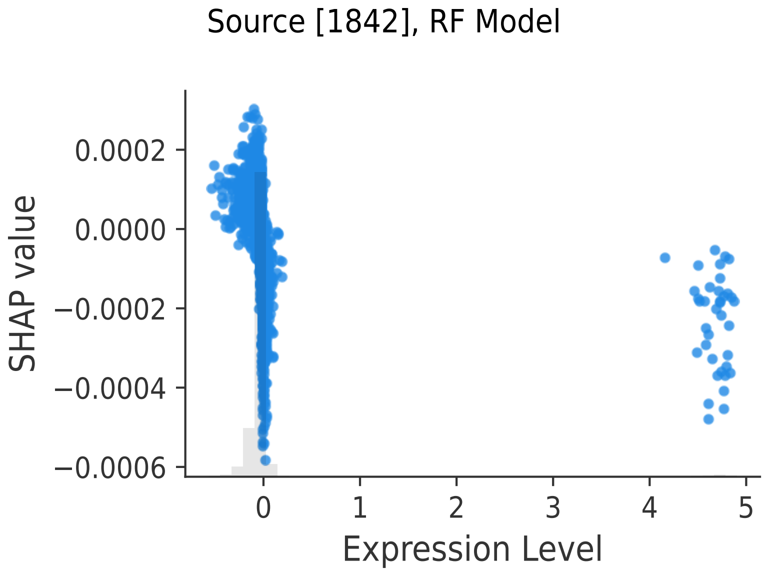

Source [1842], RF Model
0.0002
0.0000
−0.0002
−0.0004
−0.0006
SHAP value
0
1
2
3
4
5
Expression Level

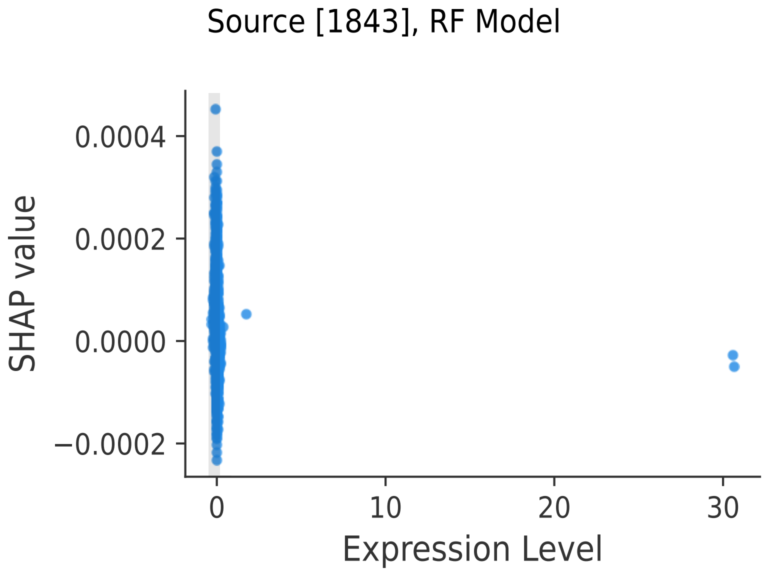

Source [1843], RF Model
0.0004
0.0002
0.0000
−0.0002
SHAP value
0
10
20
30
Expression Level

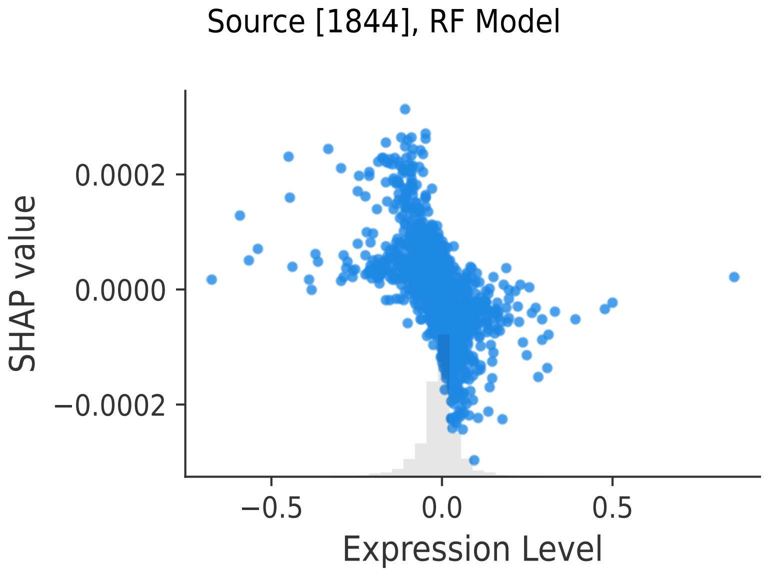
Source [1844], RF Model
SHAP value
0.0002
0.0000
−0.0002
−0.5
0.0
0.5
Expression Level

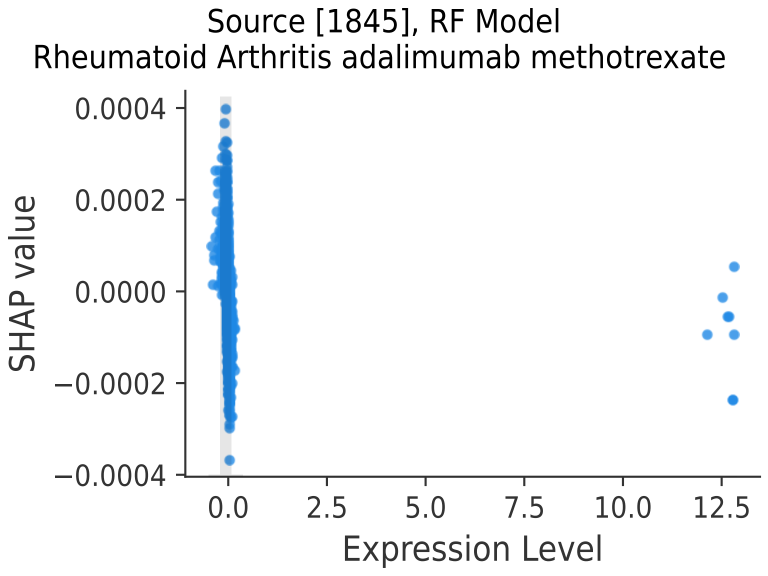
Source [1845], RF Model
Rheumatoid Arthritis adalimumab methotrexate
SHAP value
0.0004
0.0002
0.0000
−0.0002
−0.0004
0.0
2.5
5.0
7.5
10.0
12.5
Expression Level

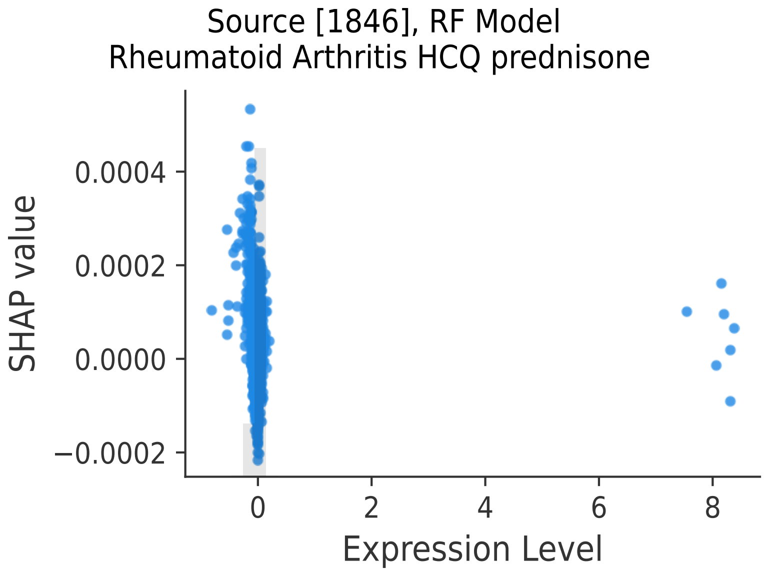

Source [1846], RF Model
Rheumatoid Arthritis HCQ prednisone
SHAP value
0.0004
0.0002
0.0000
−0.0002
0
2
4
6
8
Expression Level

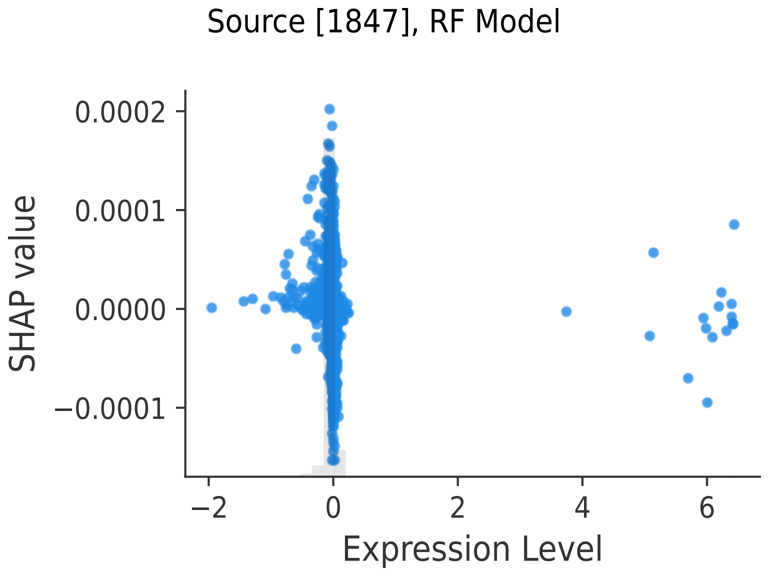
Source [1847], RF Model
0.0002
0.0001
0.0000
−0.0001
SHAP value
−2
0
2
4
6
Expression Level

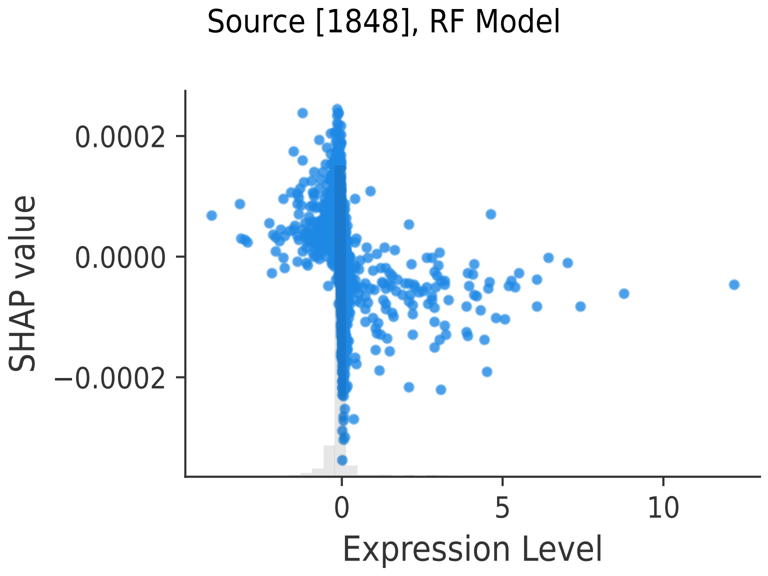
Source [1848], RF Model
0.0002
0.0000
−0.0002
SHAP value
0
5
10
Expression Level

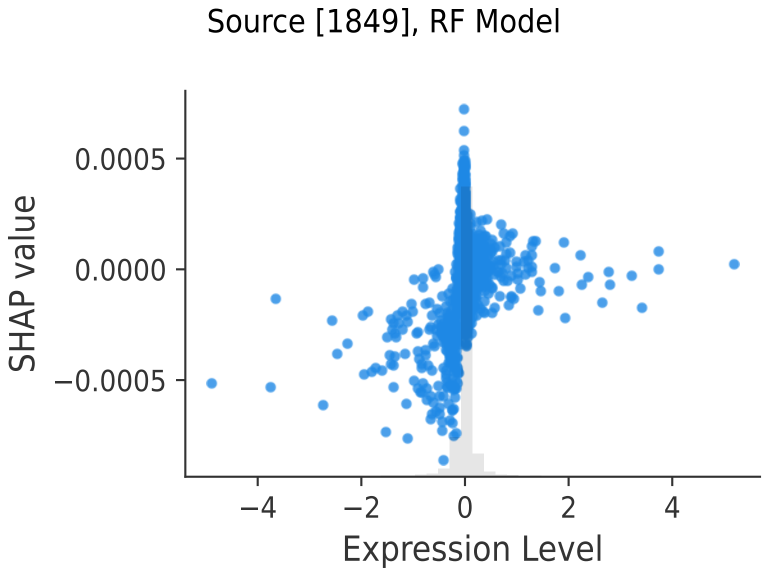
Source [1849], RF Model
SHAP value
0.0005
0.0000
−0.0005
−4
−2
0
2
4
Expression Level

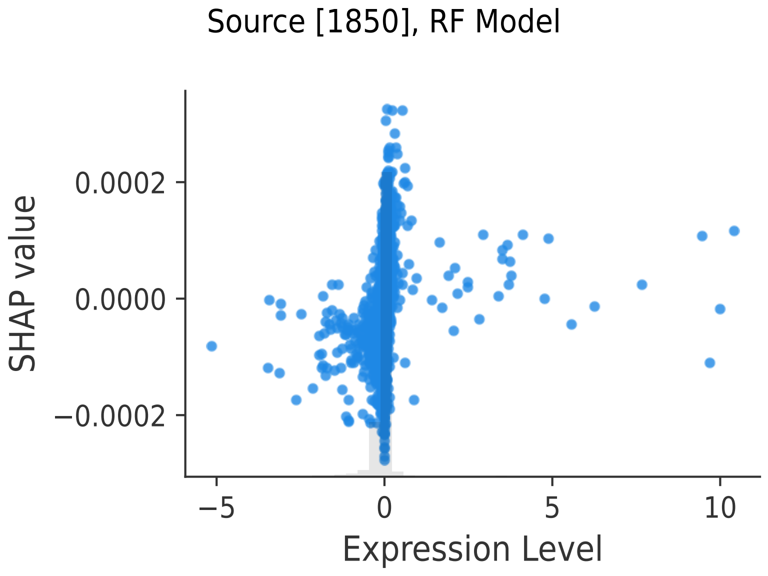
Source [1850], RF Model
SHAP value
0.0002
0.0000
−0.0002
−5
0
5
10
Expression Level

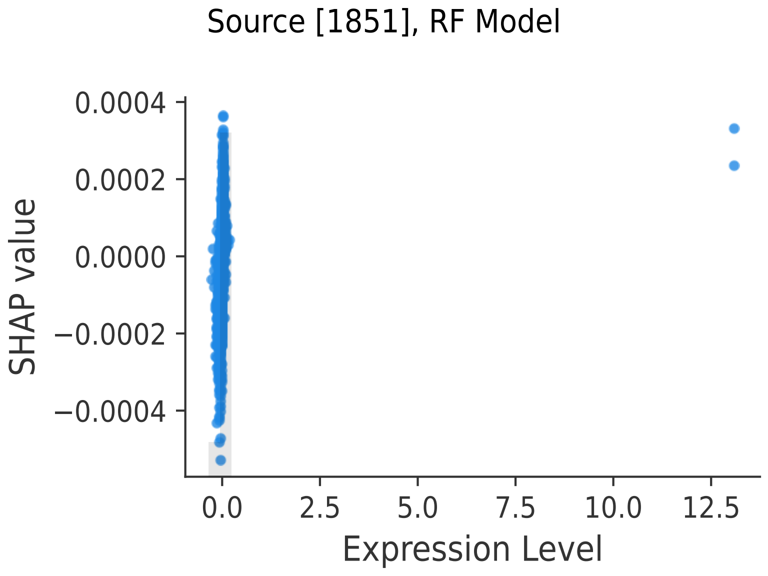
Source [1851], RF Model
0.0004
0.0002
0.0000
−0.0002
−0.0004
SHAP value
0.0
2.5
5.0
7.5
10.0
12.5
Expression Level

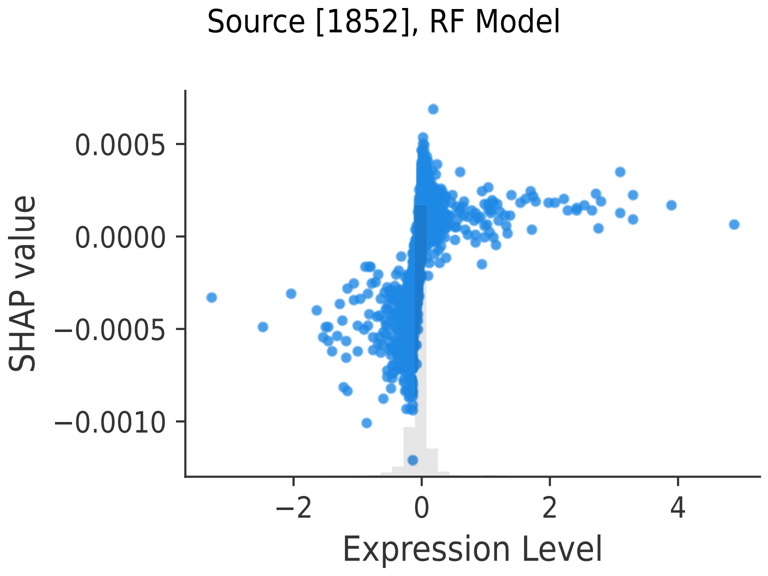

Source [1852], RF Model
0.0005
0.0000
−0.0005
−0.0010
SHAP value
−2
0
2
4
Expression Level

Source [1853], RF Model

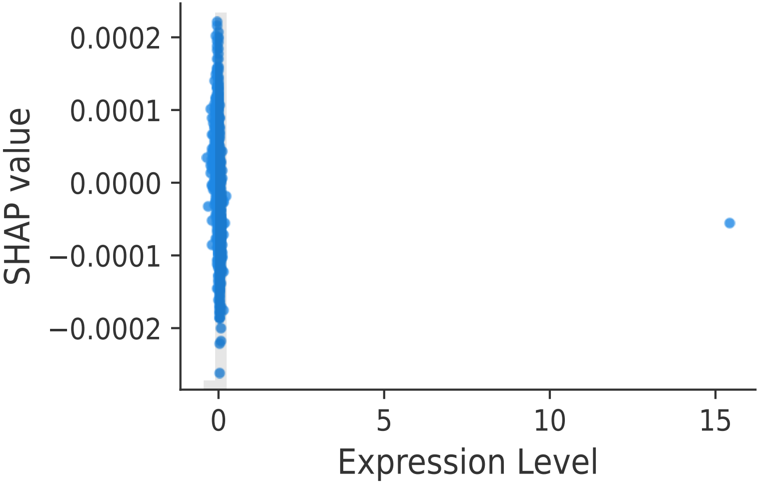

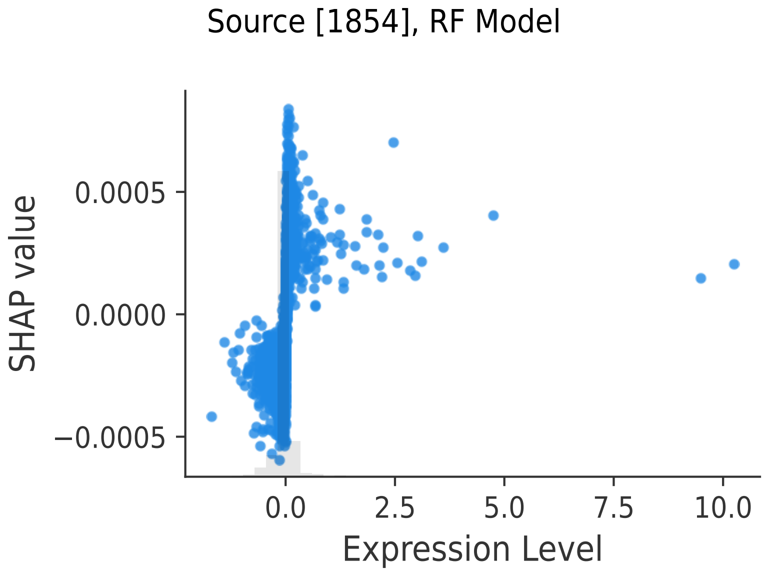
Source [1854], RF Model
SHAP value
0.0005
0.0000
−0.0005
0.0
2.5
5.0
7.5
10.0
Expression Level

Source [1855], RF Model

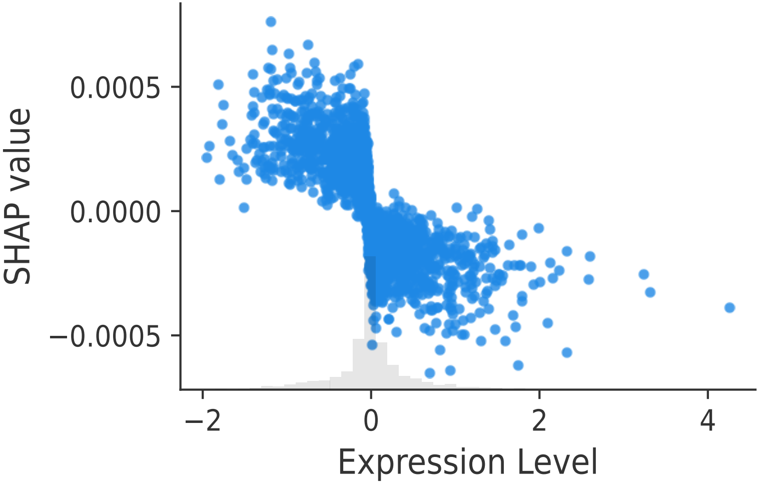

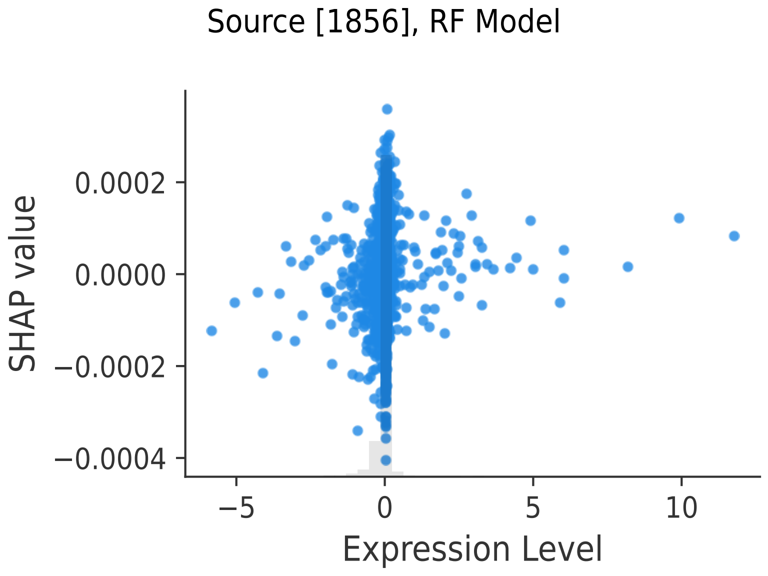

Source [1856], RF Model
SHAP value
0.0002
0.0000
−0.0002
−0.0004
−5
0
5
10
Expression Level

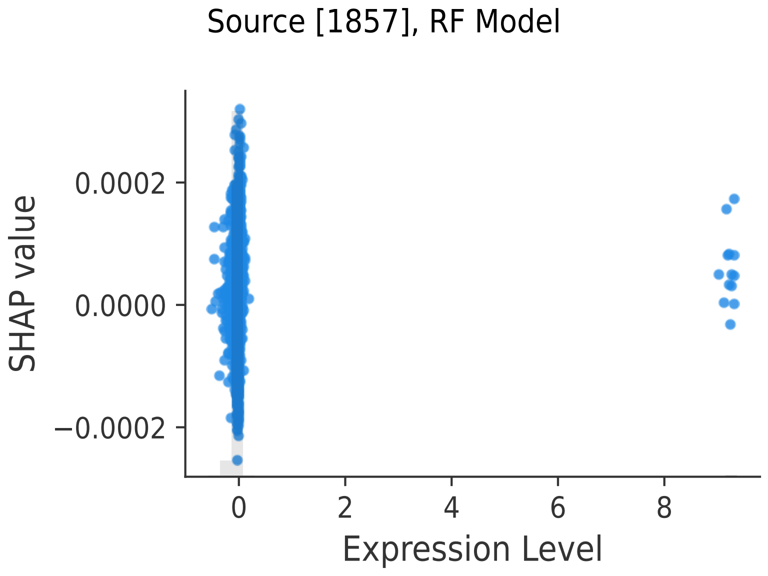
Source [1857], RF Model
0.0002
0.0000
−0.0002
SHAP value
0
2
4
6
8
Expression Level

Source [1858], RF Model

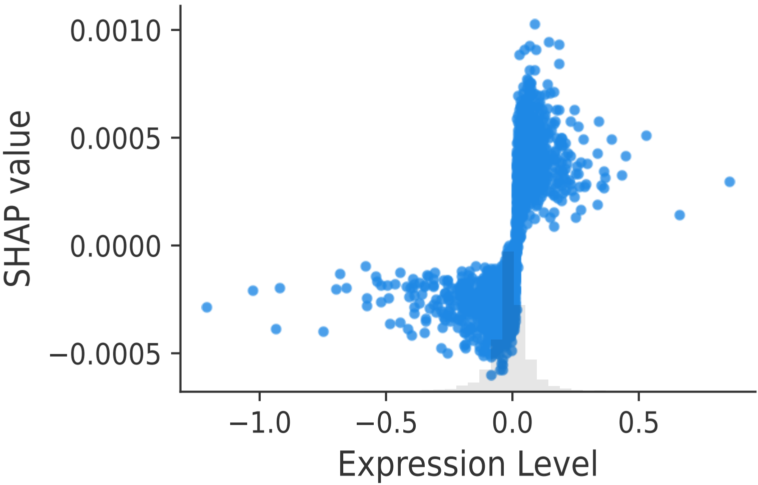

Source [1859], RF Model

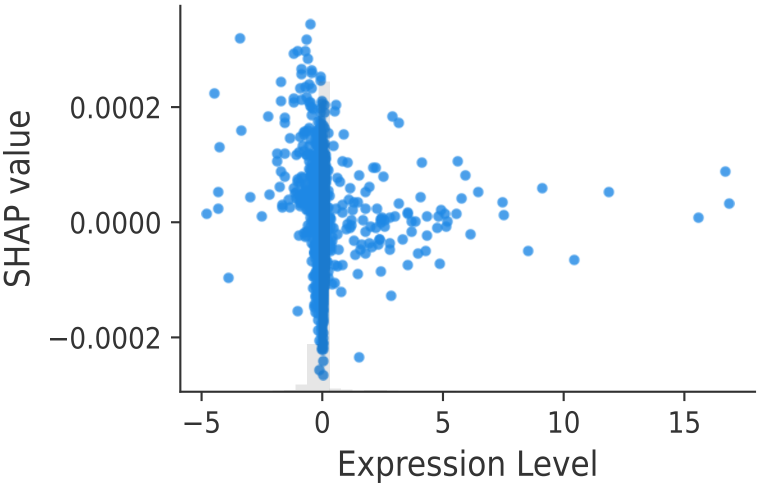

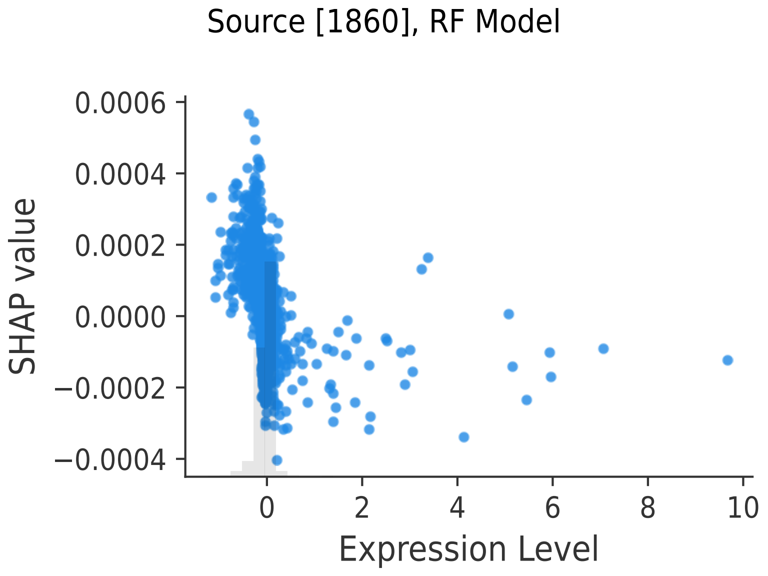
Source [1860], RF Model
0.0006
0.0004
0.0002
0.0000
−0.0002
−0.0004
SHAP value
0
2
4
6
8
10
Expression Level

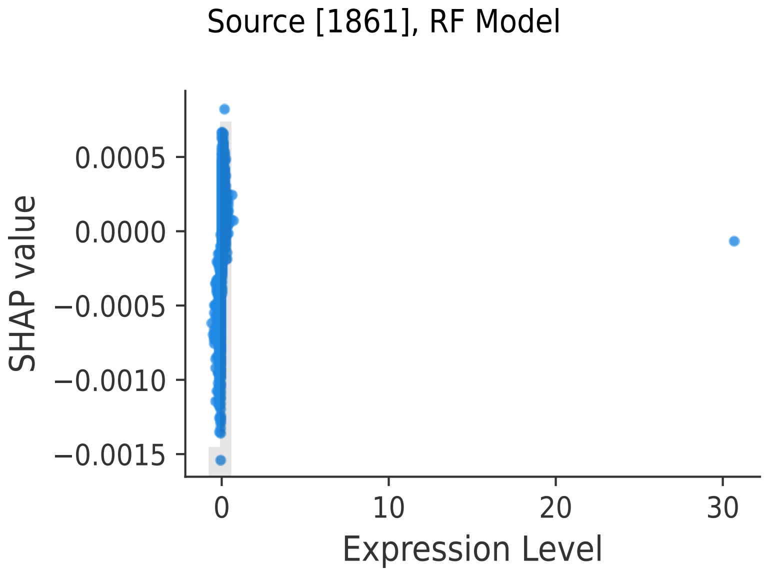

Source [1861], RF Model
0.0005
0.0000
−0.0005
−0.0010
−0.0015
SHAP value
0
10
20
30
Expression Level

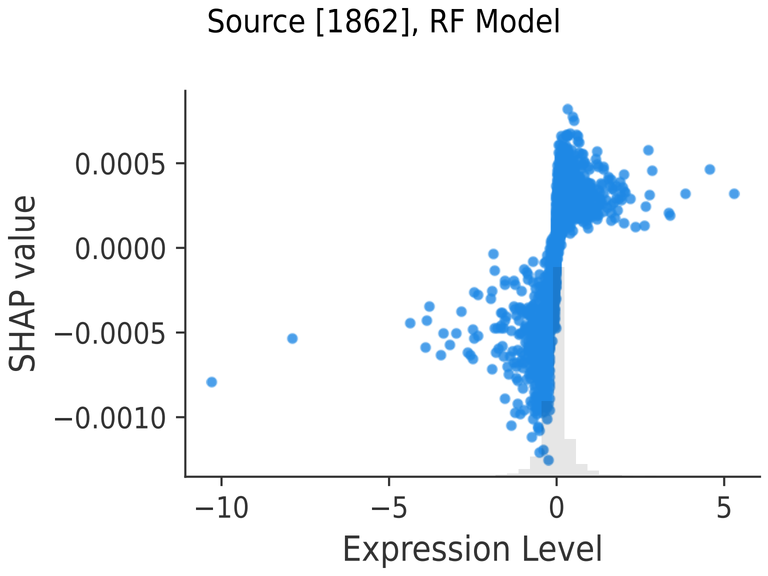
Source [1862], RF Model
SHAP value
0.0005
0.0000
−0.0005
−0.0010
−10
−5
0
5
Expression Level

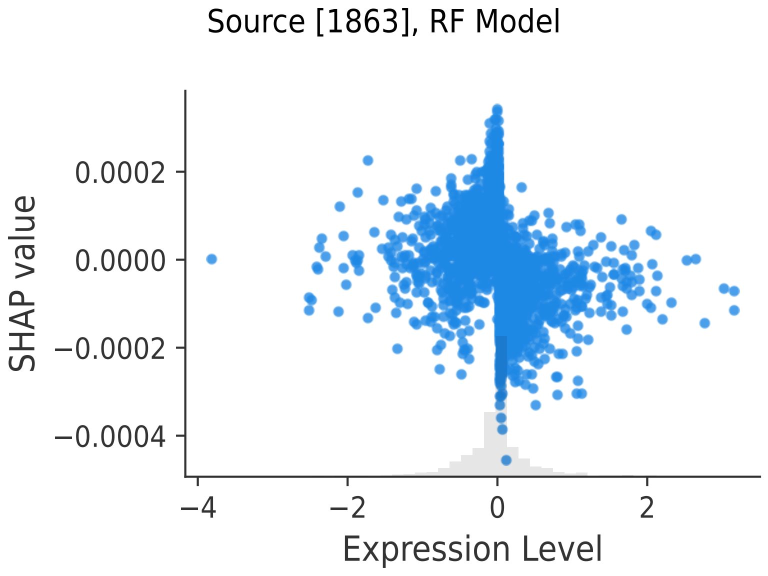

Source [1863], RF Model
0.0002
0.0000
−0.0002
−0.0004
SHAP value
−4
−2
0
2
Expression Level

Source [1864], RF Model

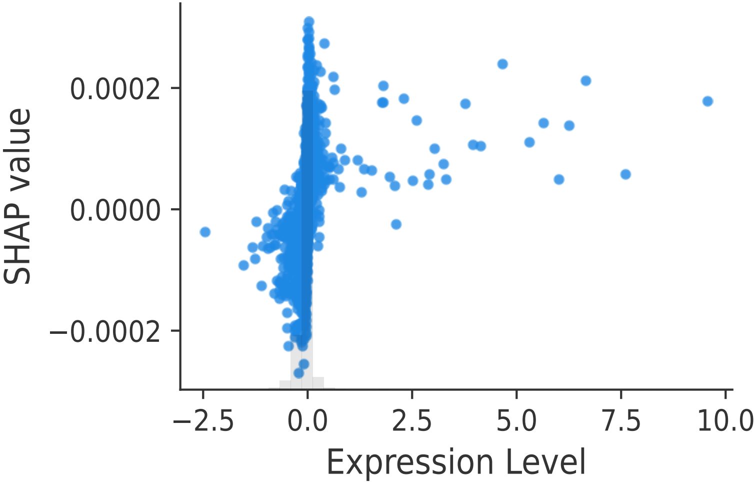

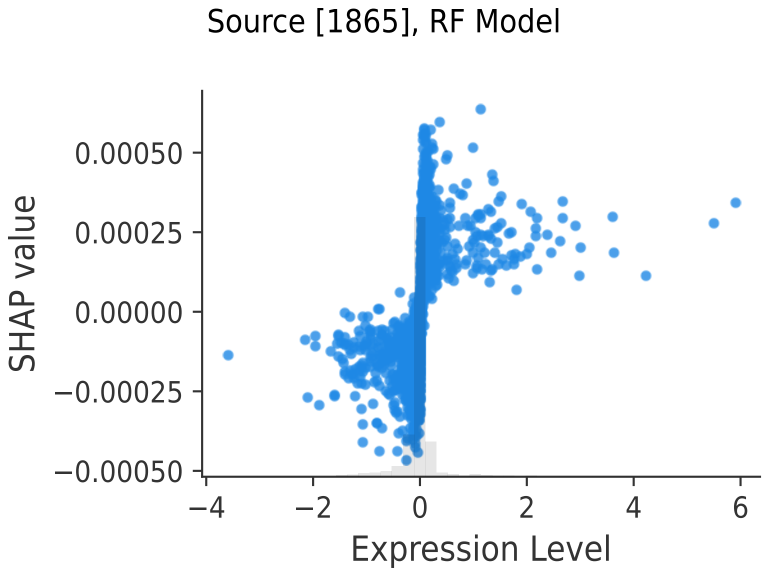

Source [1865], RF Model
0.00050
0.00025
0.00000
−0.00025
−0.00050
SHAP value
−4
−2
0
2
4
6
Expression Level

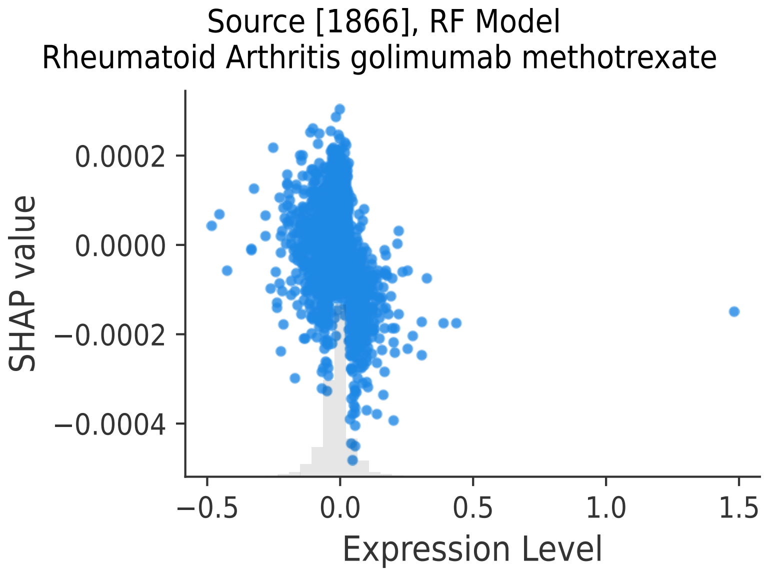
Source [1866], RF Model
Rheumatoid Arthritis golimumab methotrexate
0.0002
0.0000
−0.0002
−0.0004
SHAP value
−0.5
0.0
0.5
1.0
1.5
Expression Level

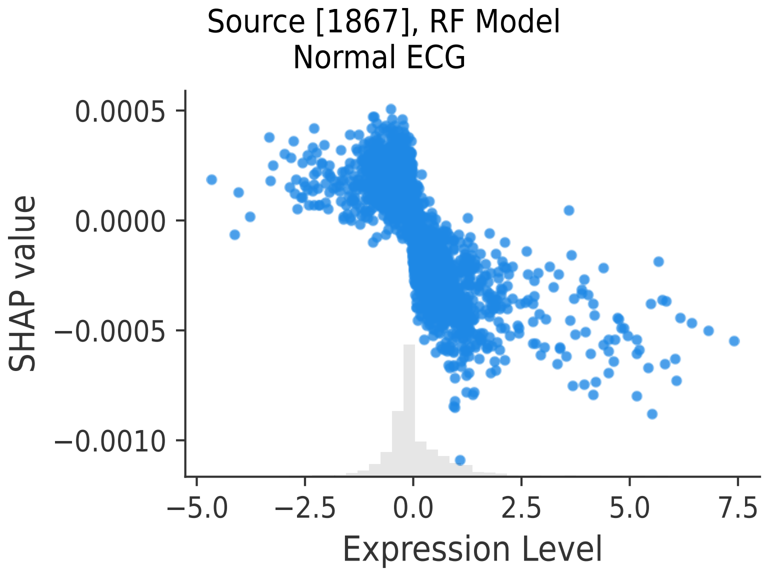
Source [1867], RF Model
Normal ECG
0.0005
0.0000
−0.0005
−0.0010
SHAP value
−5.0
−2.5
0.0
2.5
5.0
7.5
Expression Level

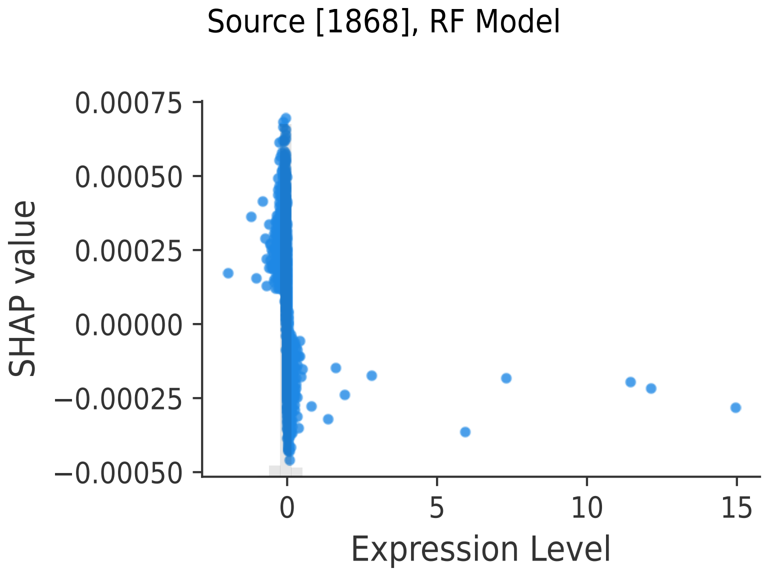
Source [1868], RF Model
SHAP value
0.00075
0.00050
0.00025
0.00000
−0.00025
−0.00050
0
5
10
15
Expression Level

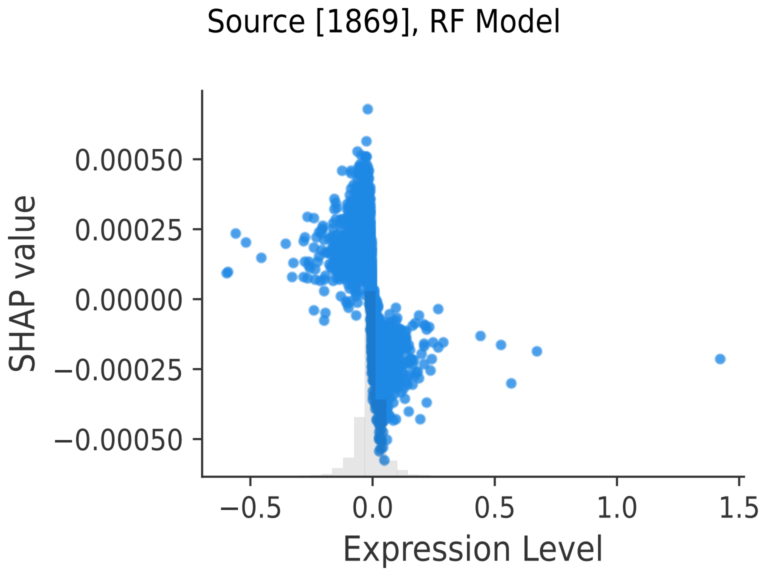

Source [1869], RF Model
SHAP value
0.00050
0.00025
0.00000
−0.00025
−0.00050
−0.5
0.0
0.5
1.0
1.5
Expression Level

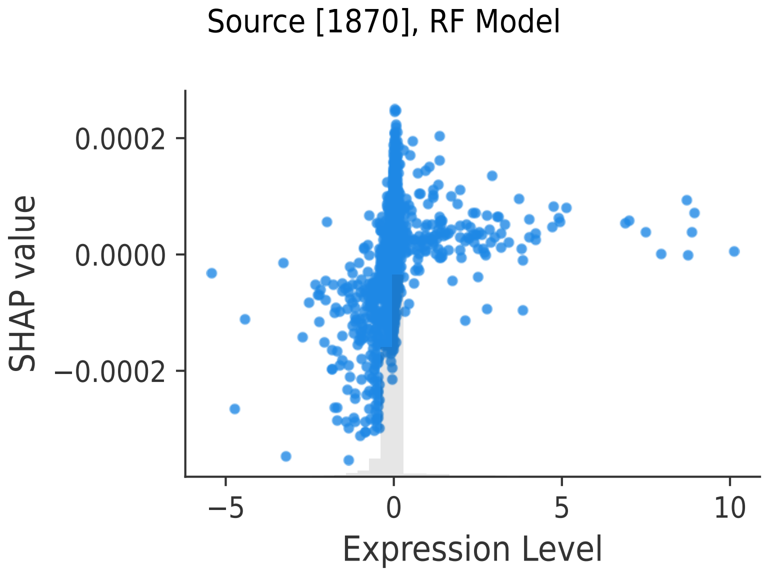

Source [1870], RF Model
SHAP value
0.0002
0.0000
−0.0002
−5
0
5
10
Expression Level

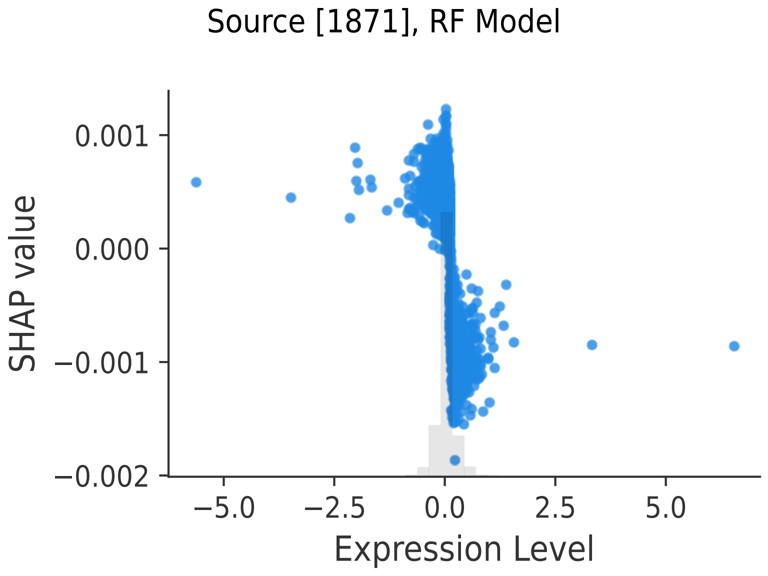
Source [1871], RF Model
SHAP value
0.001
0.000
−0.001
−0.002
−5.0
−2.5
0.0
2.5
5.0
Expression Level

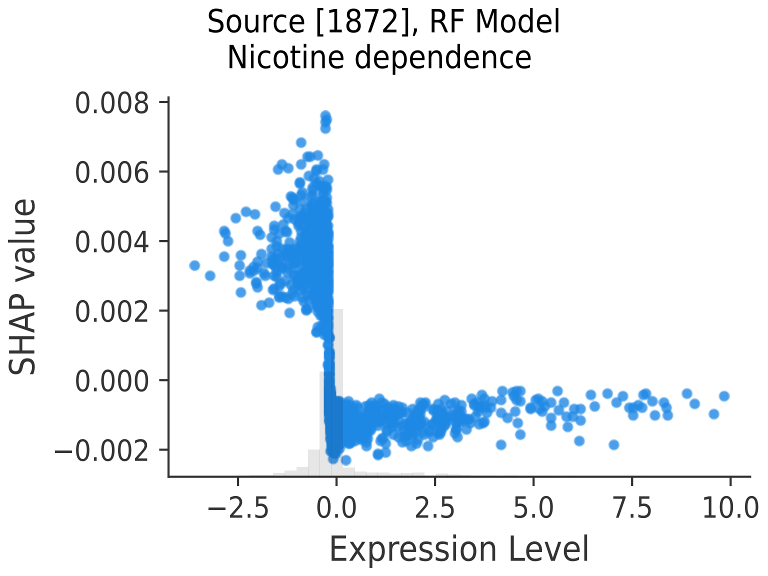
Source [1872], RF Model
Nicotine dependence
SHAP value
0.008
0.006
0.004
0.002
0.000
−0.002
−2.5
0.0
2.5
5.0
7.5
10.0
Expression Level

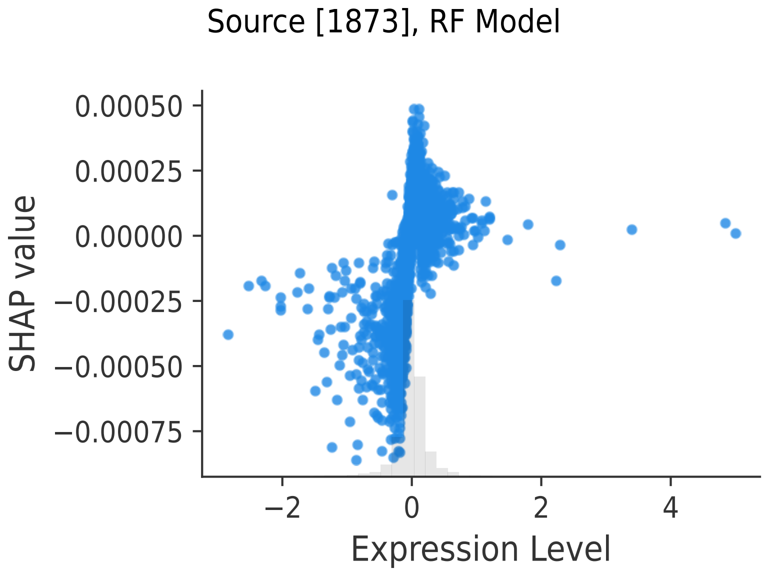

Source [1873], RF Model
0.00050
0.00025
0.00000
−0.00025
−0.00050
−0.00075
SHAP value
−2
0
2
4
Expression Level

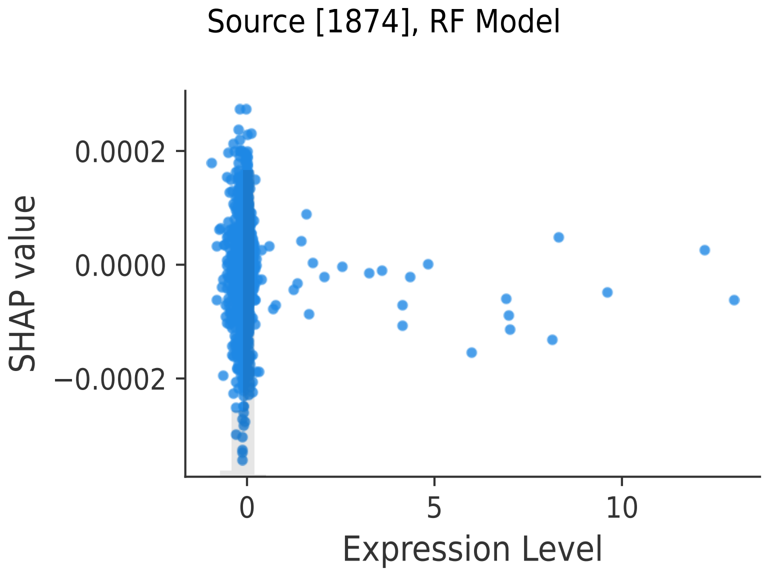

Source [1874], RF Model
0.0002
0.0000
−0.0002
SHAP value
0
5
10
Expression Level

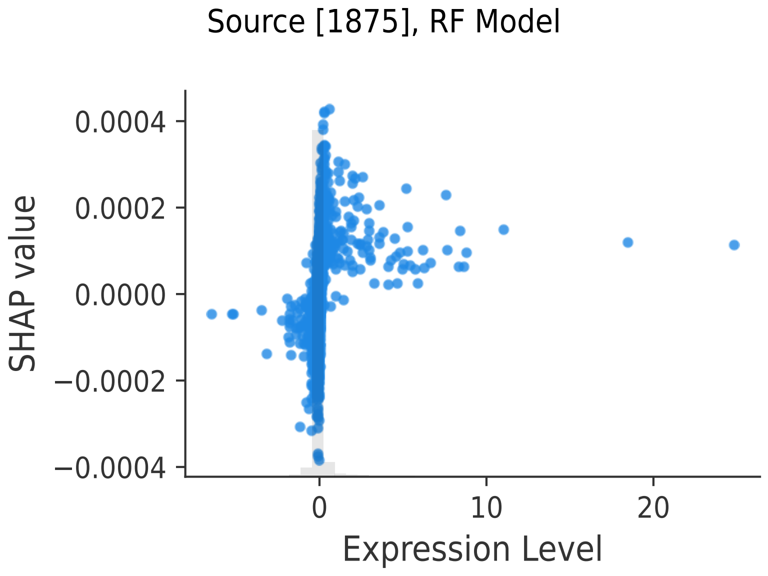
Source [1875], RF Model
SHAP value
0.0004
0.0002
0.0000
−0.0002
−0.0004
0
10
20
Expression Level

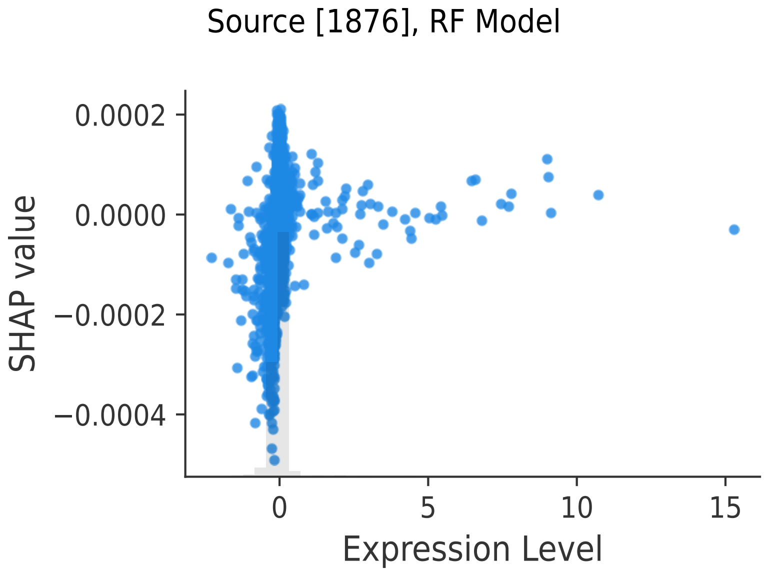

Source [1876], RF Model
SHAP value
0.0002
0.0000
−0.0002
−0.0004
0
5
10
15
Expression Level

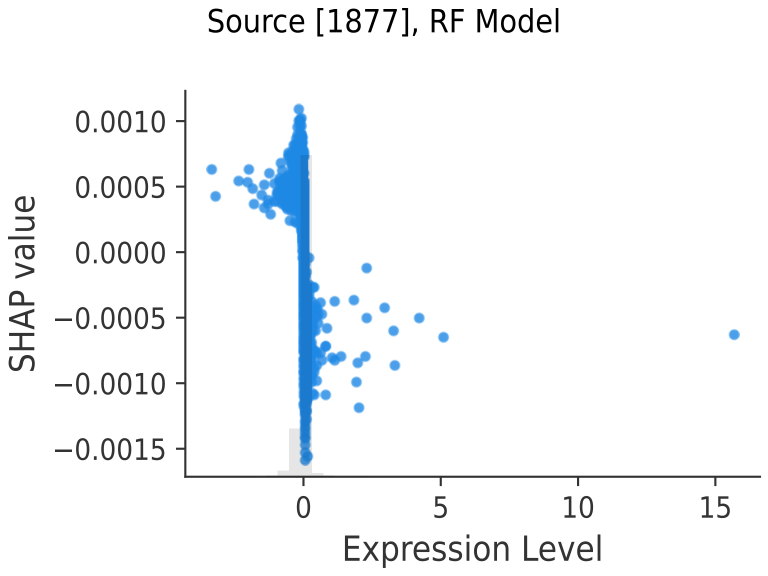
Source [1877], RF Model
SHAP value
0.0010
0.0005
0.0000
−0.0005
−0.0010
−0.0015
0
5
10
15
Expression Level

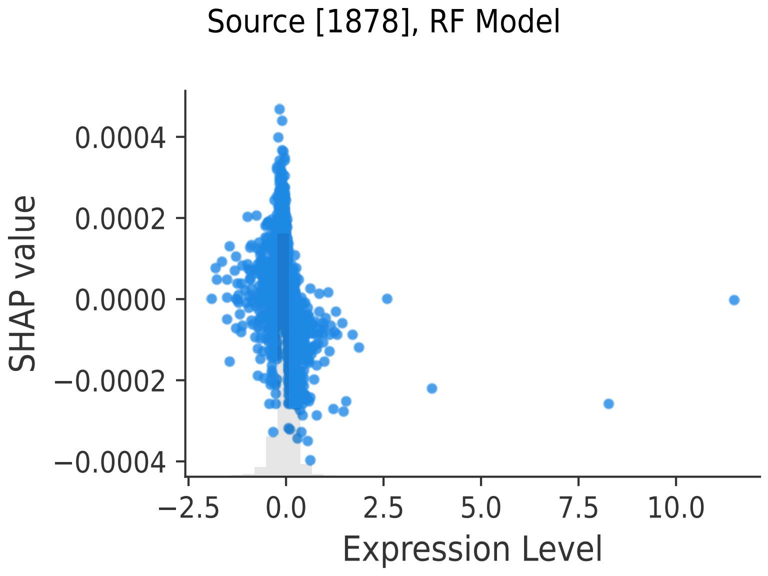
Source [1878], RF Model
0.0004
0.0002
0.0000
−0.0002
−0.0004
SHAP value
−2.5
0.0
2.5
5.0
7.5
10.0
Expression Level

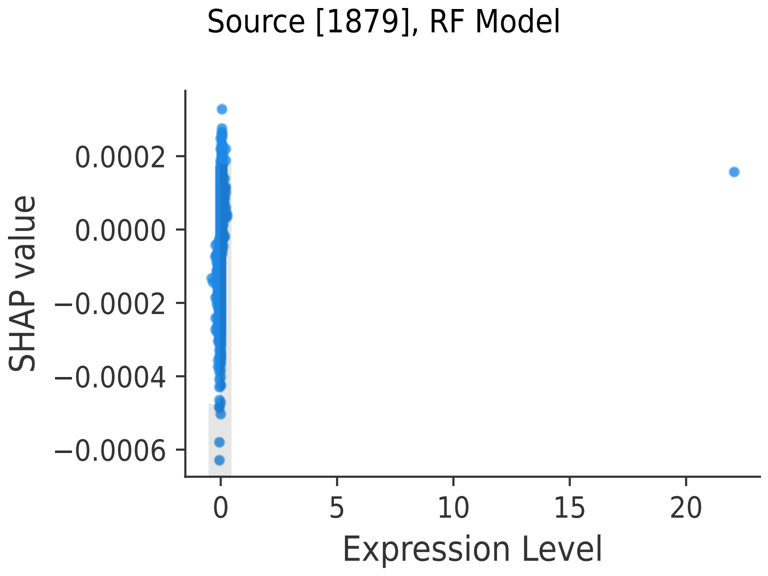

Source [1879], RF Model
SHAP value
0.0002
0.0000
−0.0002
−0.0004
−0.0006
0
5
10
15
20
Expression Level

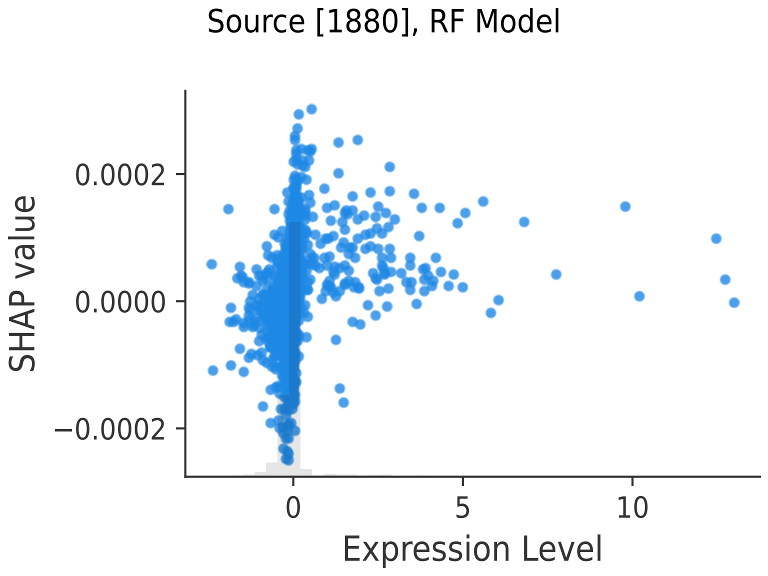

Source [1880], RF Model
SHAP value
0.0002
0.0000
−0.0002
0
5
10
Expression Level

Source [1881], RF Model

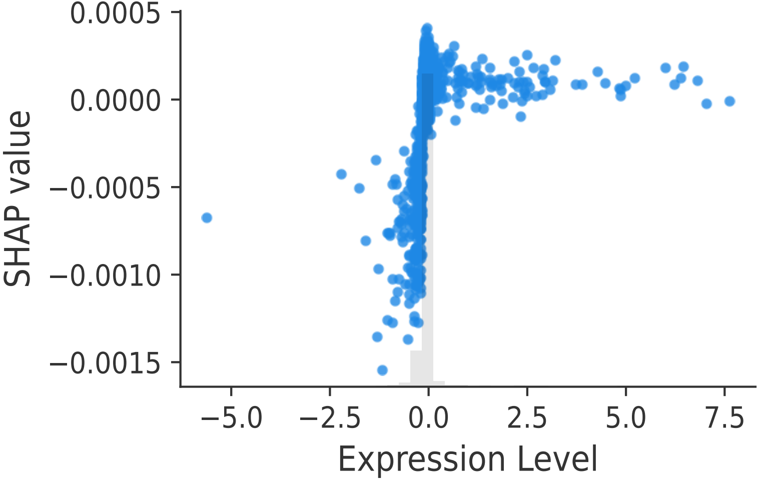

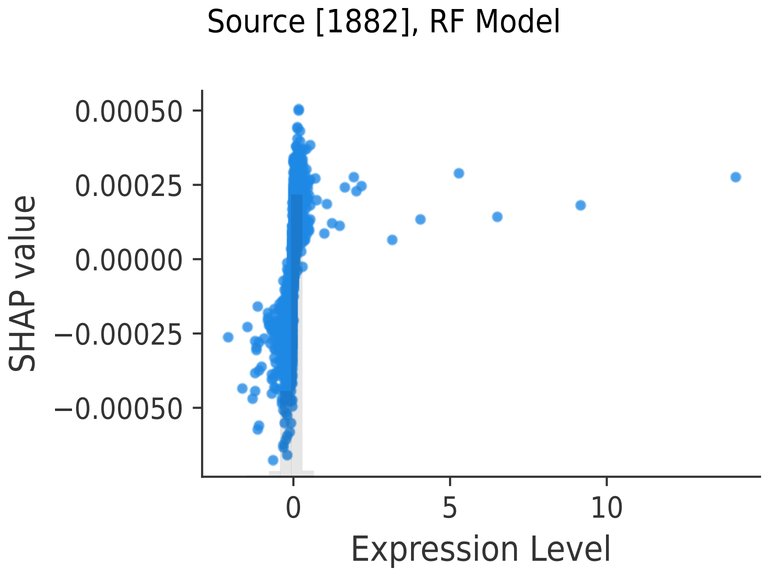

Source [1882], RF Model
0.00050
0.00025
0.00000
−0.00025
−0.00050
SHAP value
0
5
10
Expression Level

Source [1883], RF Model

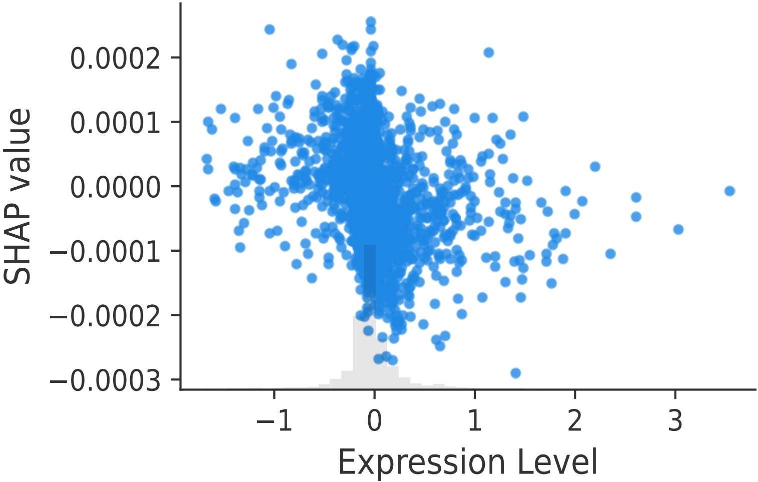

Source [1884], RF Model

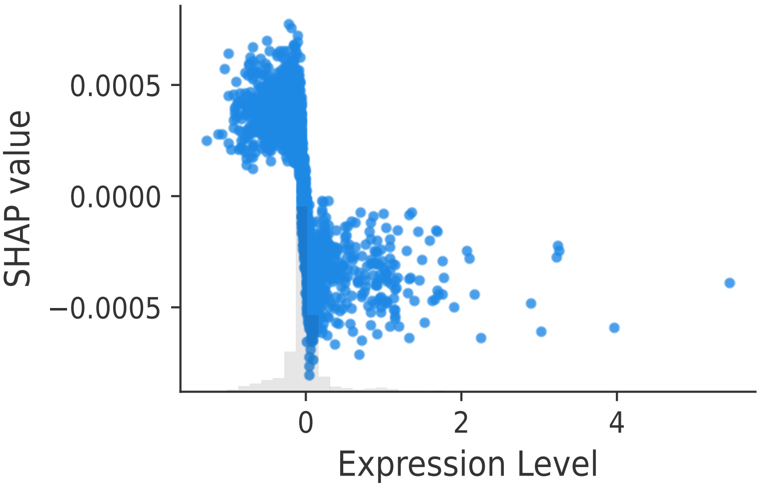

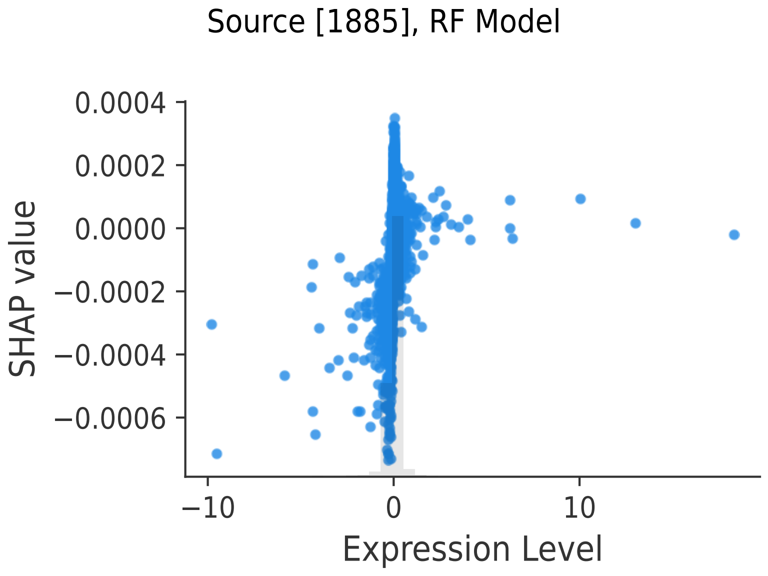

Source [1885], RF Model
0.0004
0.0002
0.0000
−0.0002
−0.0004
−0.0006
SHAP value
−10
0
10
Expression Level

Source [1886], RF Model
Diffuse large B-Cell Lymphoma Reticulosarcoma

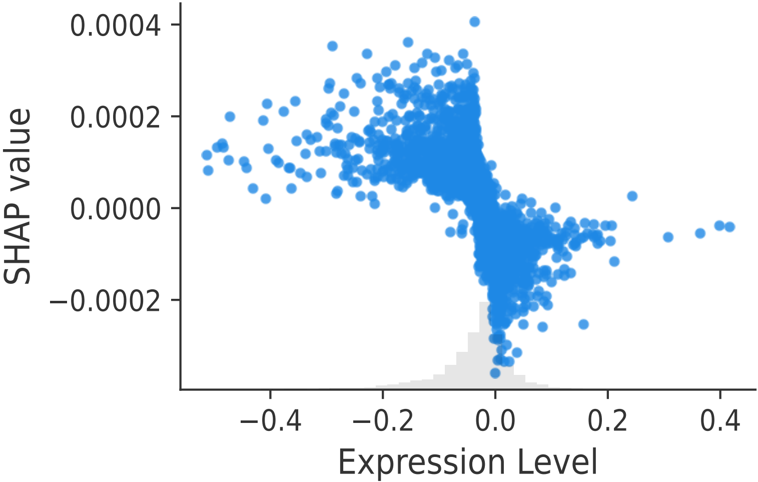

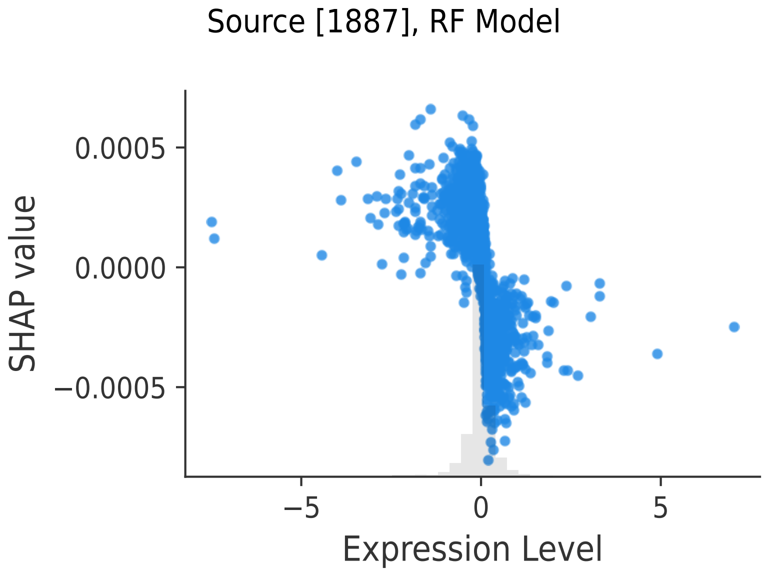
Source [1887], RF Model
SHAP value
0.0005
0.0000
−0.0005
−5
0
5
Expression Level

Source [1888], RF Model

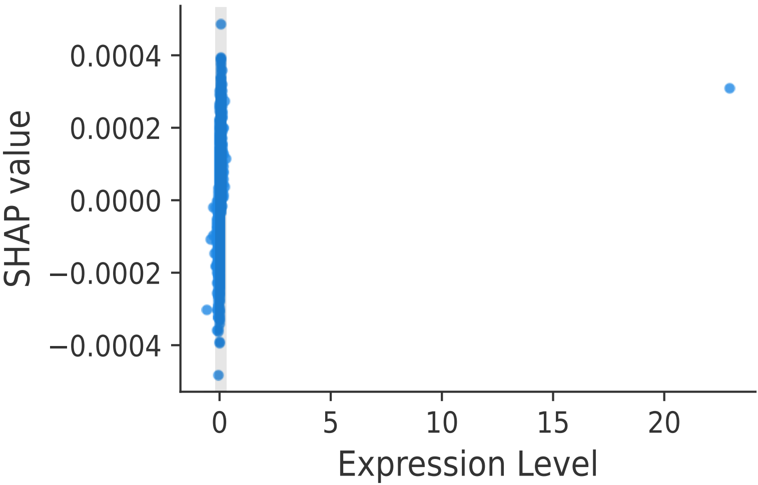

Source [1889], RF Model

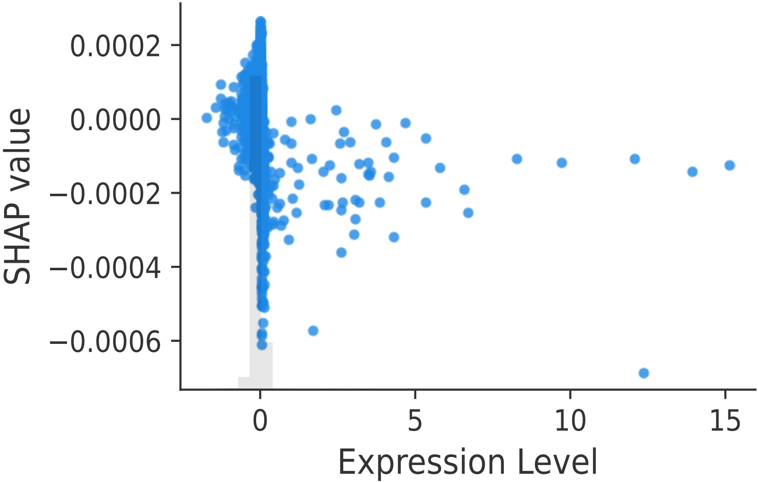

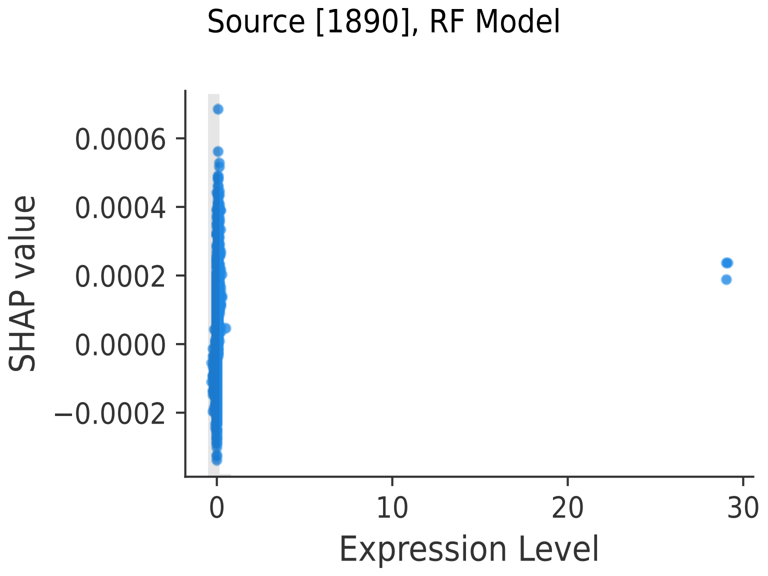
Source [1890], RF Model
SHAP value
0.0006
0.0004
0.0002
0.0000
−0.0002
0
10
20
30
Expression Level

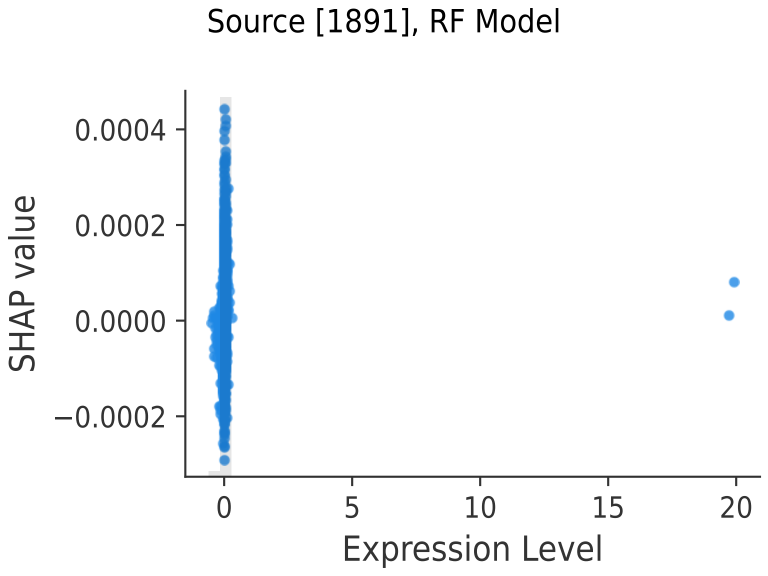
Source [1891], RF Model
SHAP value
0.0004
0.0002
0.0000
−0.0002
Expression Level
0
5
10
15
20

Source [1892], RF Model

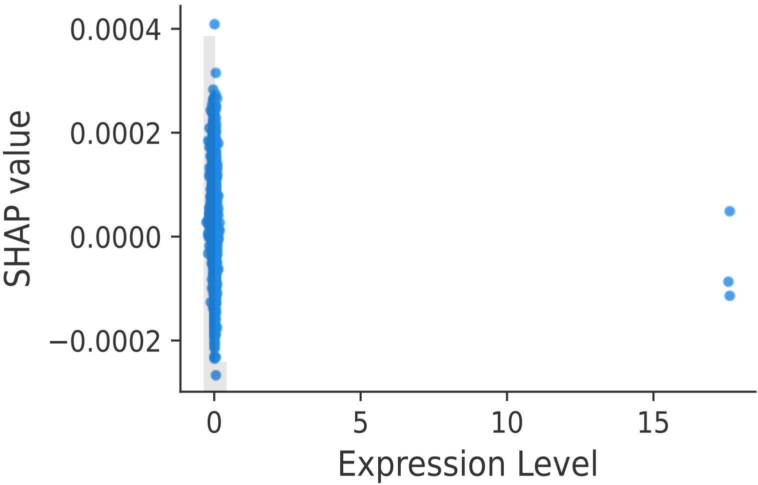

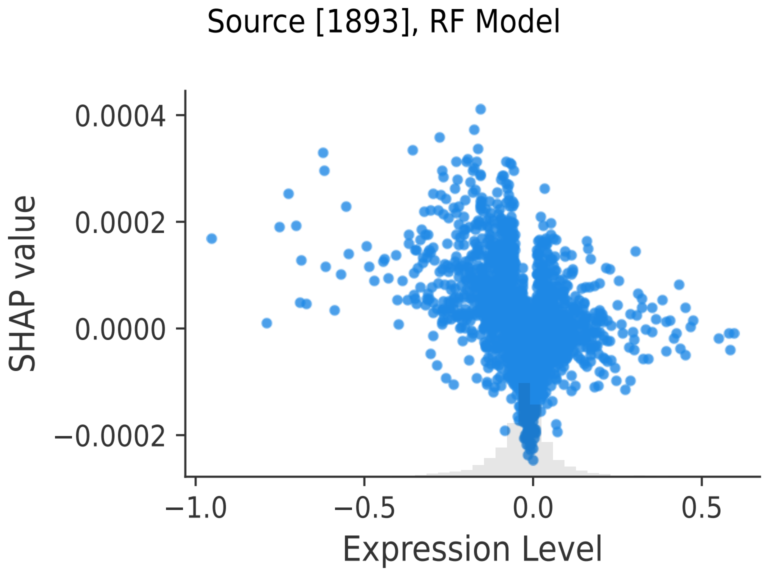

Source [1893], RF Model
0.0004
0.0002
0.0000
−0.0002
SHAP value
−1.0
−0.5
0.0
0.5
Expression Level

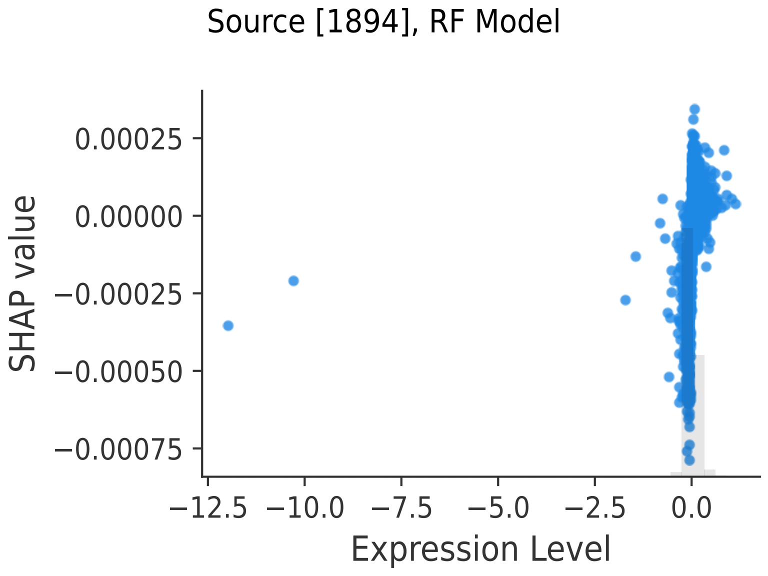
Source [1894], RF Model
0.00025
0.00000
−0.00025
−0.00050
−0.00075
SHAP value
−12.5
−10.0
−7.5
−5.0
−2.5
0.0
Expression Level

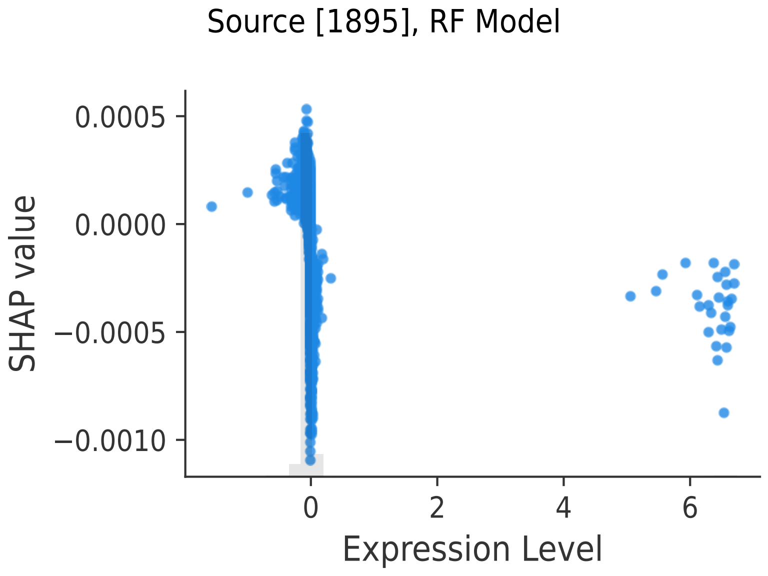
Source [1895], RF Model
0.0005
0.0000
−0.0005
−0.0010
SHAP value
0
2
4
6
Expression Level

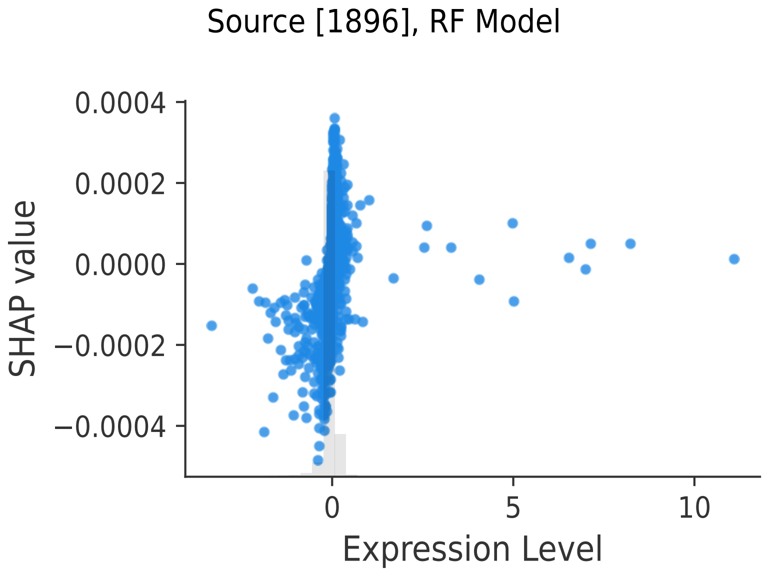
Source [1896], RF Model
0.0004
0.0002
0.0000
−0.0002
−0.0004
SHAP value
0
5
10
Expression Level

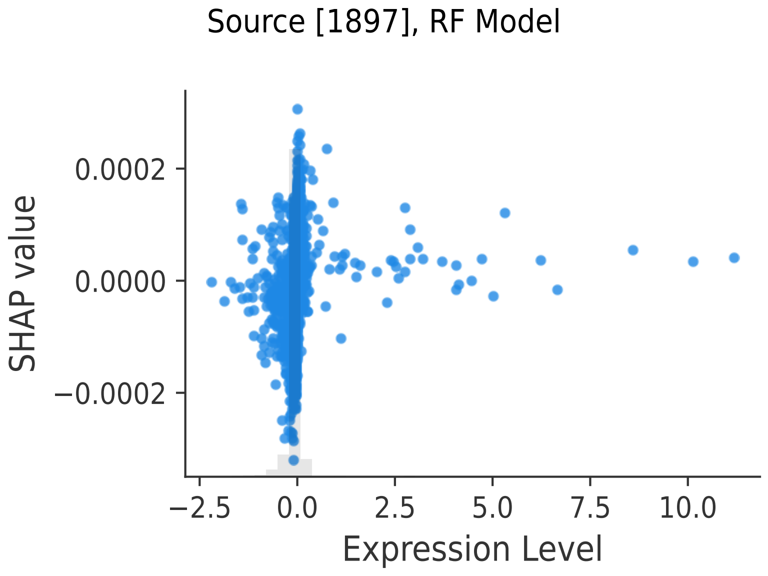
Source [1897], RF Model
SHAP value
0.0002
0.0000
−0.0002
−2.5
0.0
2.5
5.0
7.5
10.0
Expression Level

Source [1898], RF Model

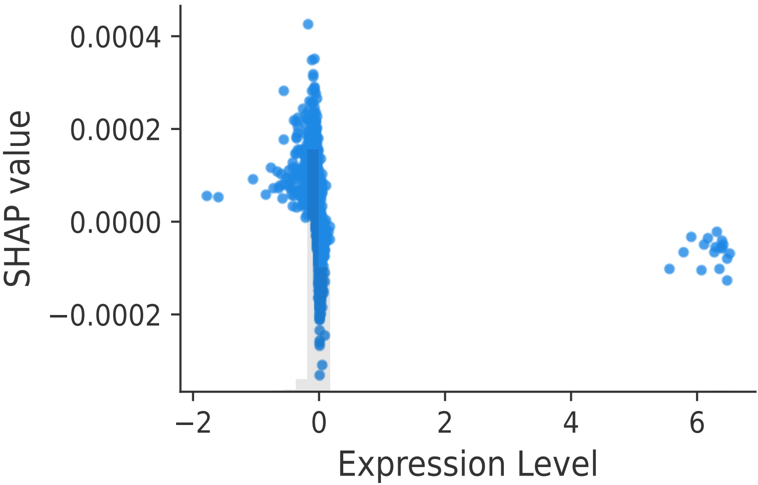

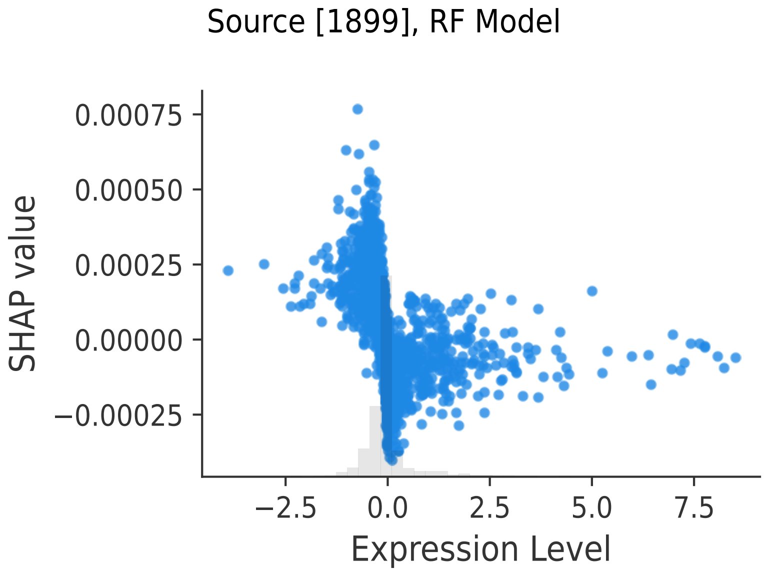

Source [1899], RF Model
SHAP value
0.00075
0.00050
0.00025
0.00000
−0.00025
−2.5
0.0
2.5
5.0
7.5
Expression Level

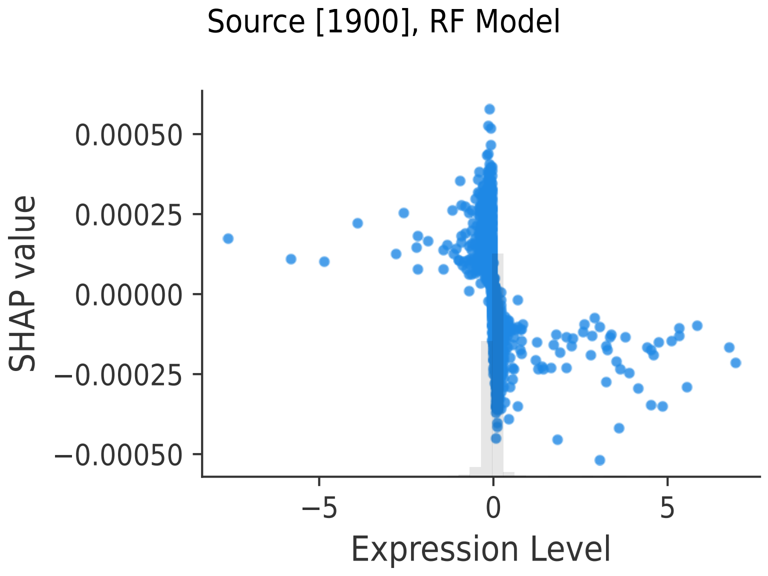
Source [1900], RF Model
0.00050
0.00025
0.00000
−0.00025
−0.00050
SHAP value
−5
0
5
Expression Level

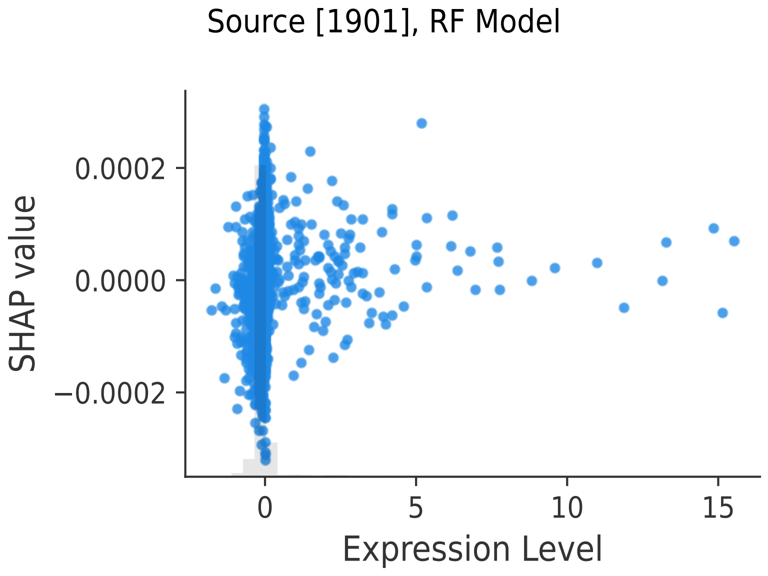
Source [1901], RF Model
SHAP value
0.0002
0.0000
−0.0002
0
5
10
15
Expression Level

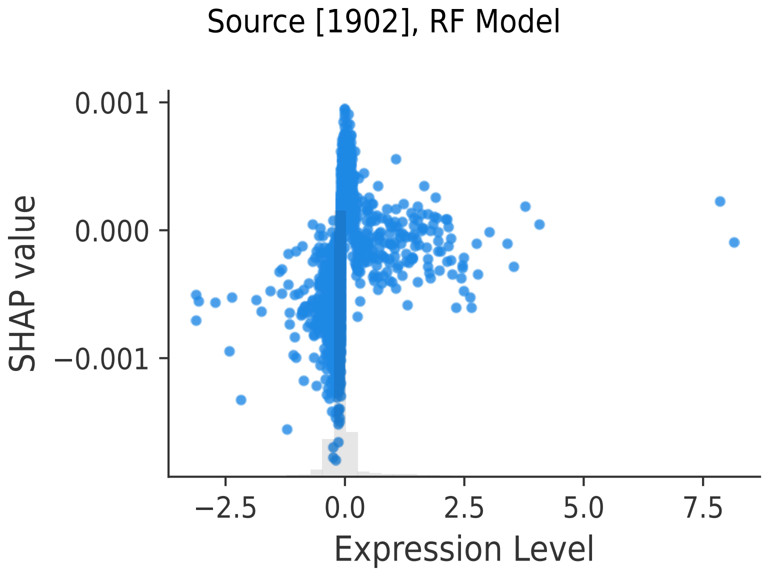

Source [1902], RF Model
0.001
0.000
−0.001
SHAP value
−2.5
0.0
2.5
5.0
7.5
Expression Level

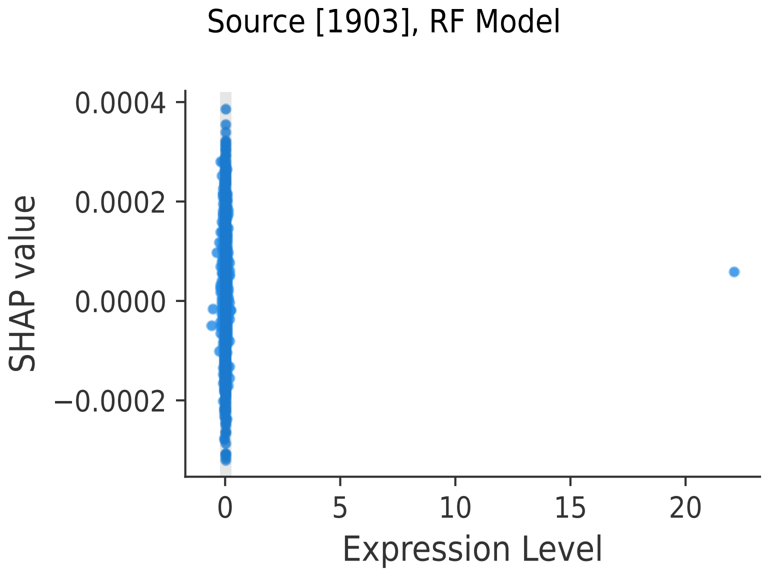

Source [1903], RF Model
0.0004
0.0002
0.0000
−0.0002
SHAP value
0
5
10
15
20
Expression Level

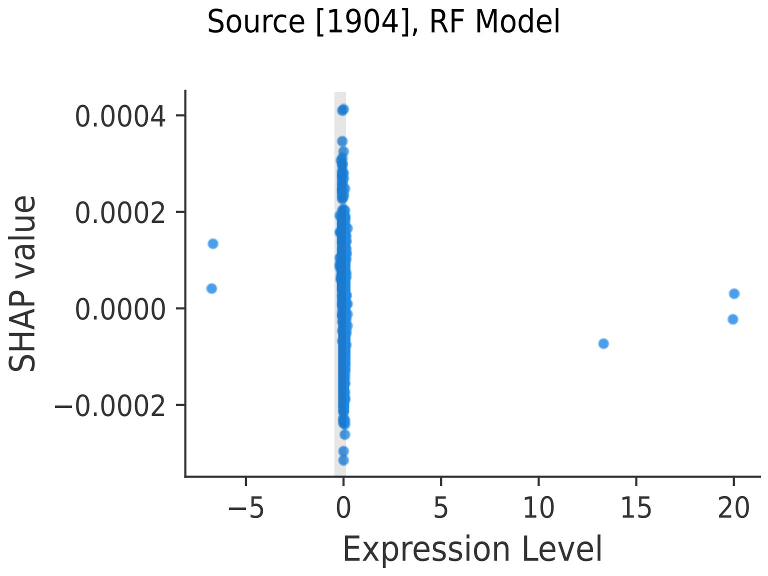

Source [1904], RF Model
0.0004
0.0002
0.0000
−0.0002
SHAP value
−5
0
5
10
15
20
Expression Level

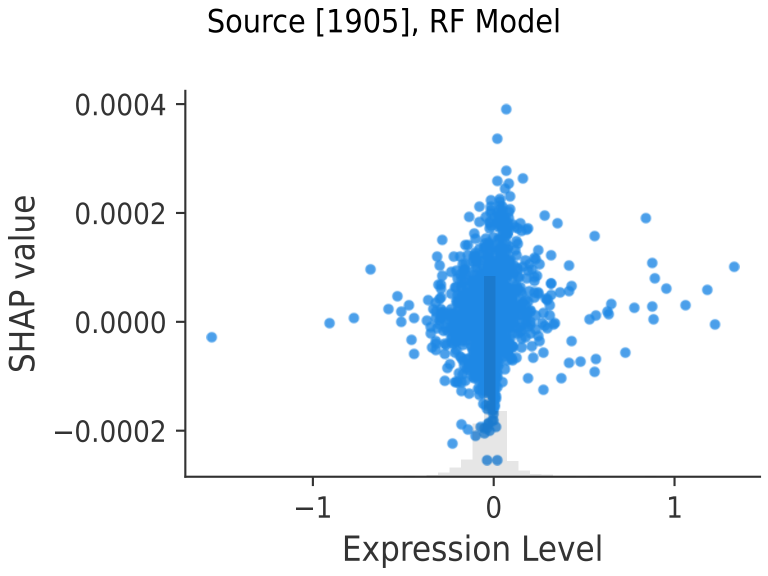
Source [1905], RF Model
0.0004
0.0002
0.0000
−0.0002
SHAP value
−1
0
1
Expression Level

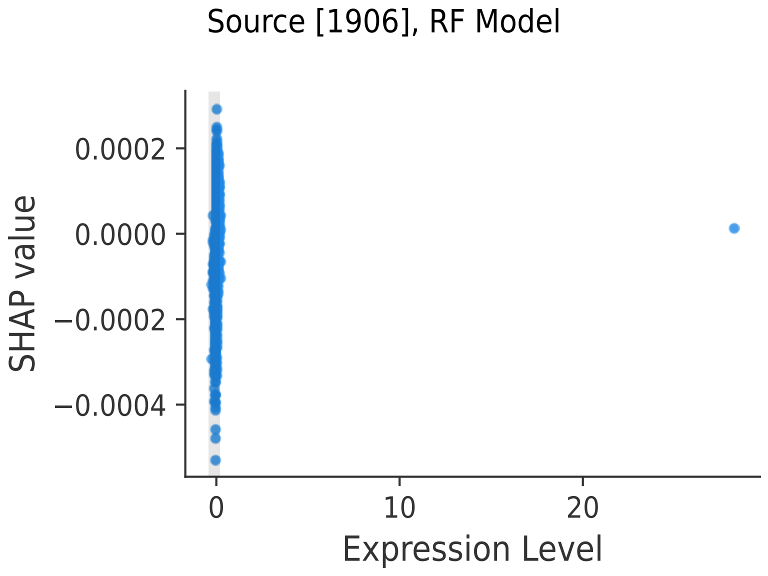

Source [1906], RF Model
0.0002
0.0000
−0.0002
−0.0004
SHAP value
0
10
20
Expression Level

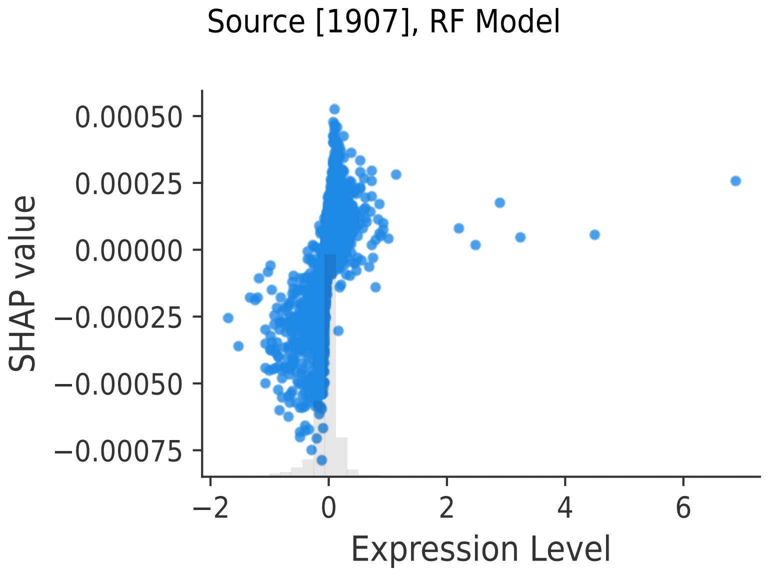
Source [1907], RF Model
SHAP value
0.00050
0.00025
0.00000
−0.00025
−0.00050
−0.00075
−2
0
2
4
6
Expression Level

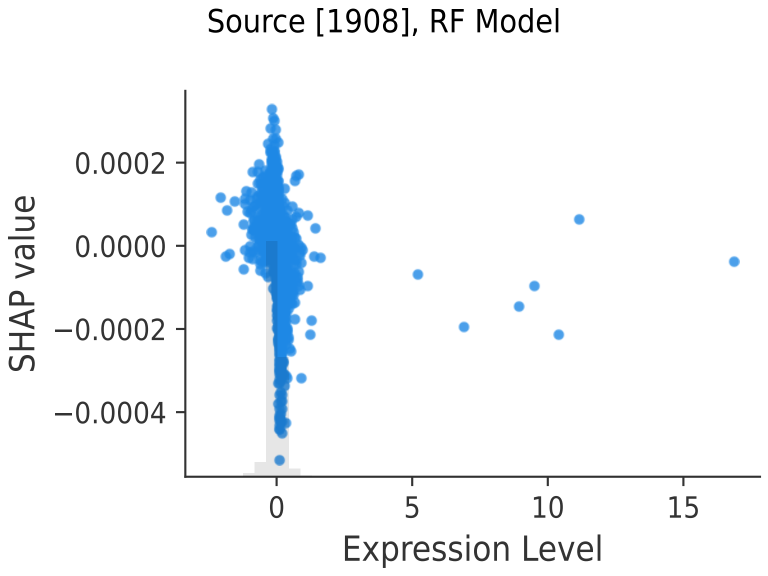

Source [1908], RF Model
SHAP value
0.0002
0.0000
−0.0002
−0.0004
0
5
10
15
Expression Level

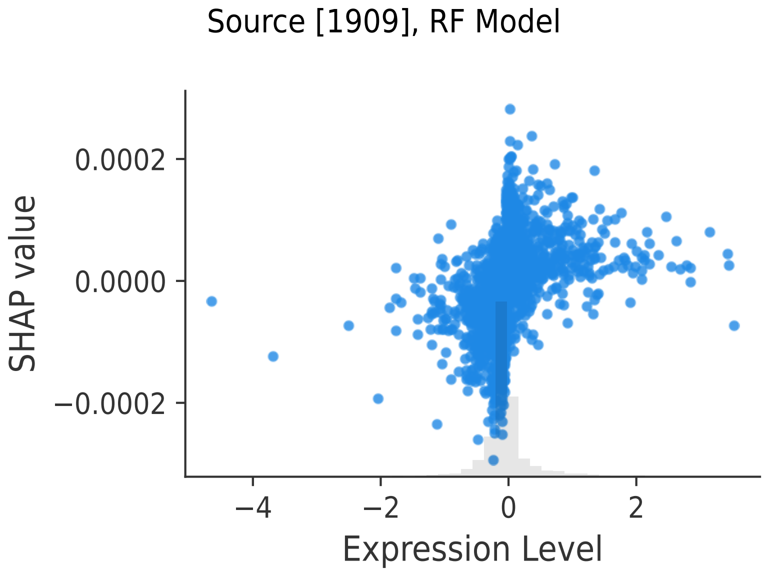
Source [1909], RF Model
0.0002
0.0000
−0.0002
SHAP value
−4
−2
0
2
Expression Level

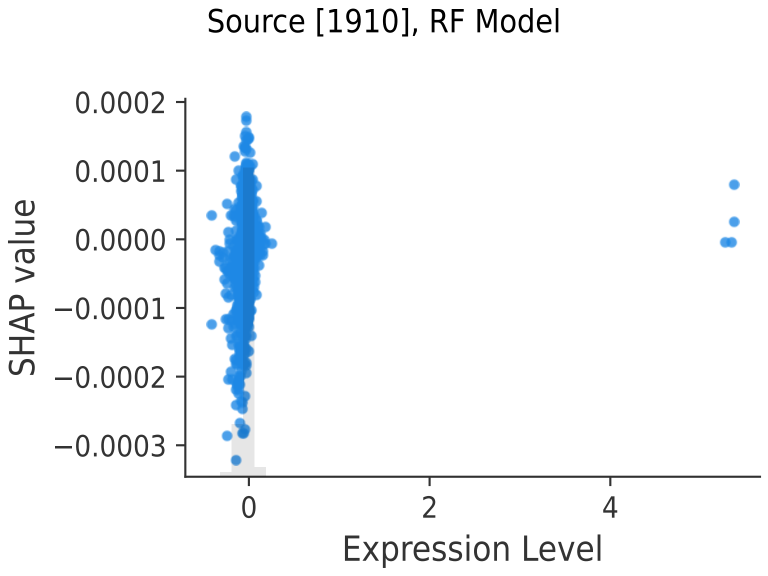
Source [1910], RF Model
0.0002
0.0001
0.0000
−0.0001
−0.0002
−0.0003
SHAP value
0
2
4
Expression Level

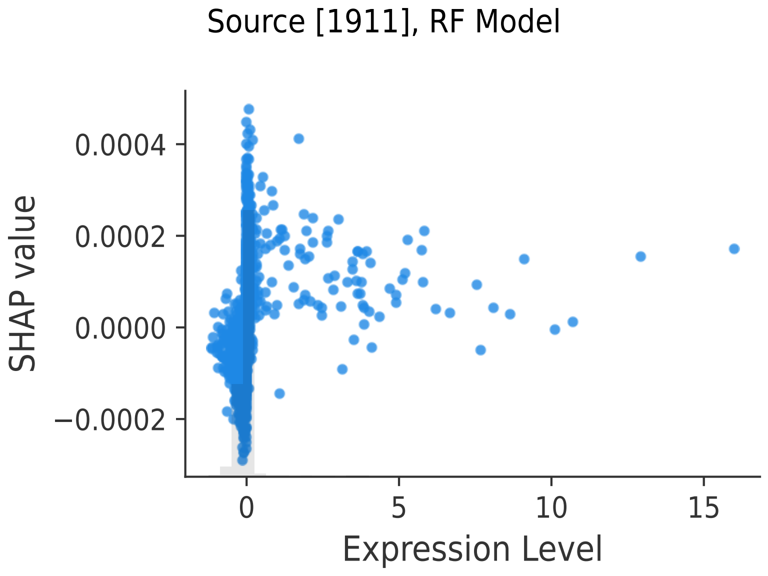
Source [1911], RF Model
0.0004
0.0002
0.0000
−0.0002
SHAP value
0
5
10
15
Expression Level

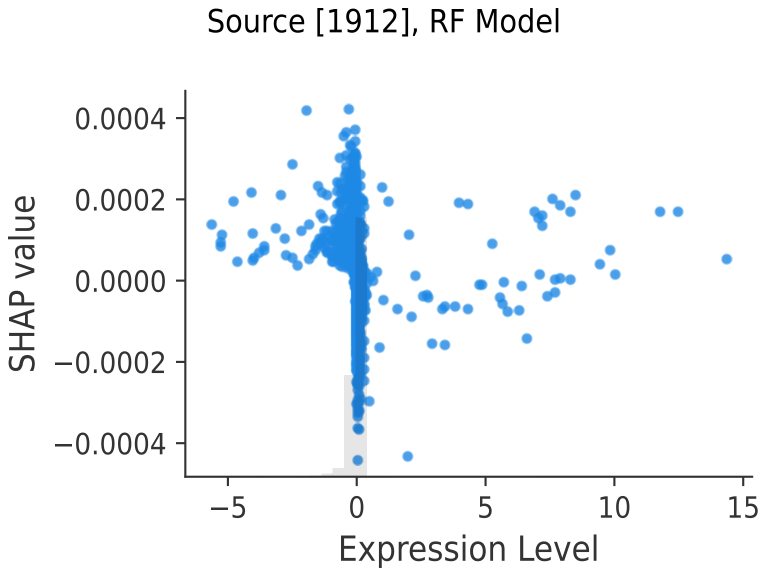

Source [1912], RF Model
SHAP value
0.0004
0.0002
0.0000
−0.0002
−0.0004
−5
0
5
10
15
Expression Level

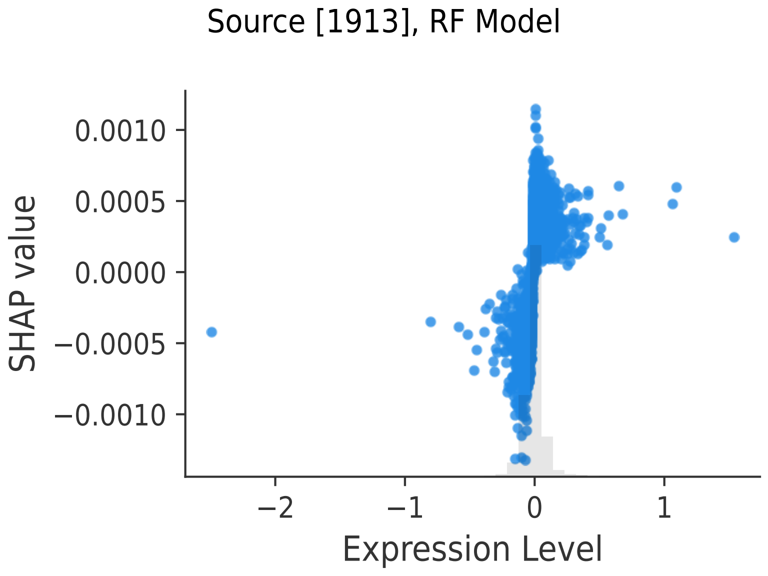
Source [1913], RF Model
0.0010
0.0005
0.0000
−0.0005
−0.0010
SHAP value
−2
−1
0
1
Expression Level

Source [1914], RF Model

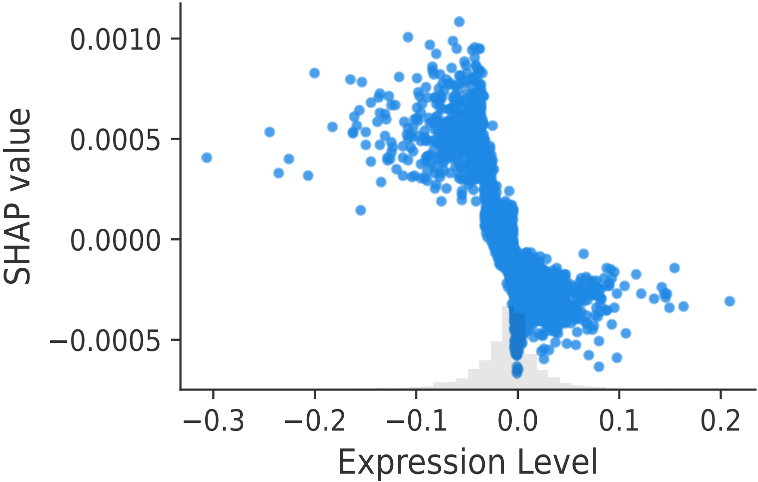

Source [1915], RF Model

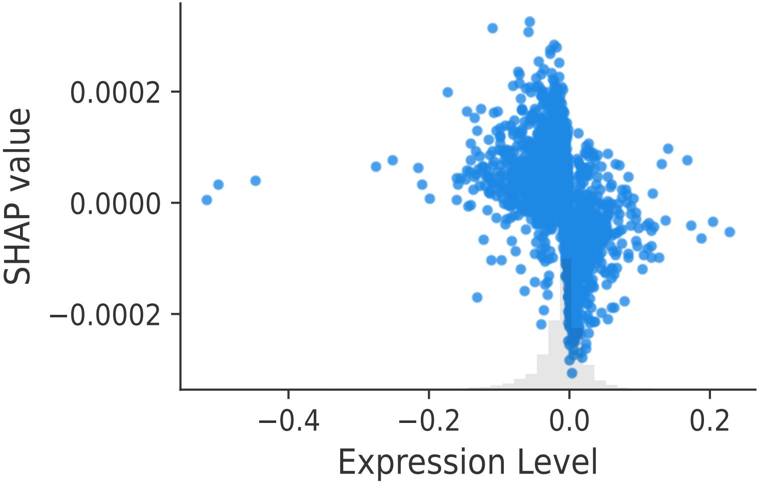

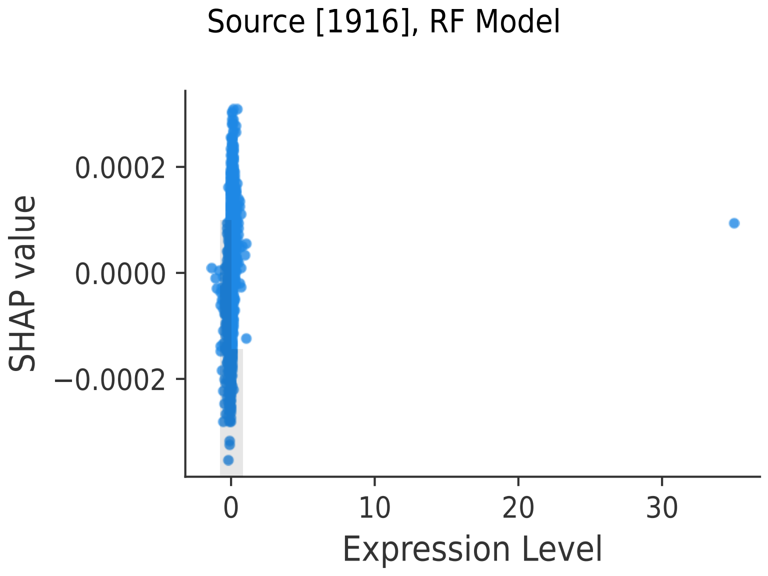
Source [1916], RF Model
SHAP value
0.0002
0.0000
−0.0002
0
10
20
30
Expression Level

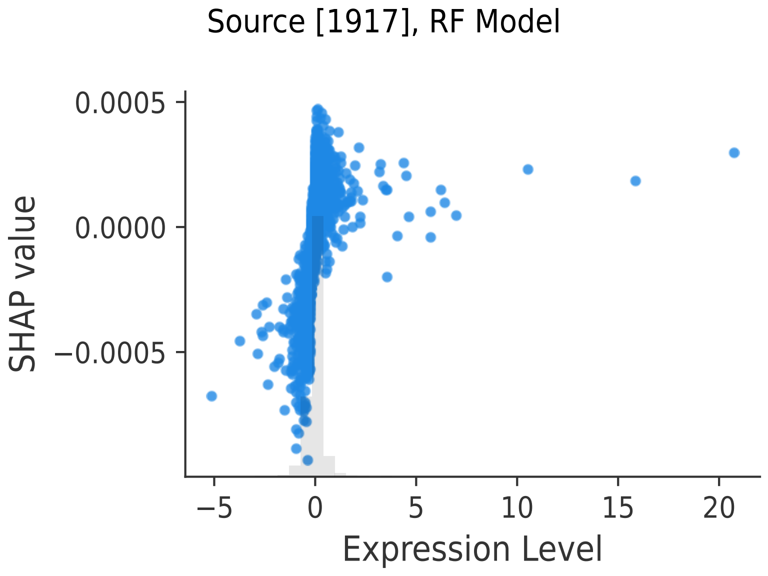
Source [1917], RF Model
SHAP value
0.0005
0.0000
−0.0005
Expression Level
−5
0
5
10
15
20

Source [1918], RF Model

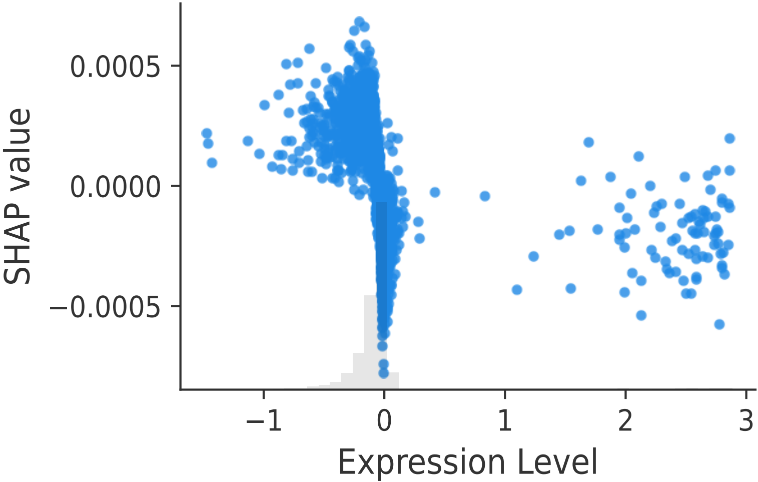

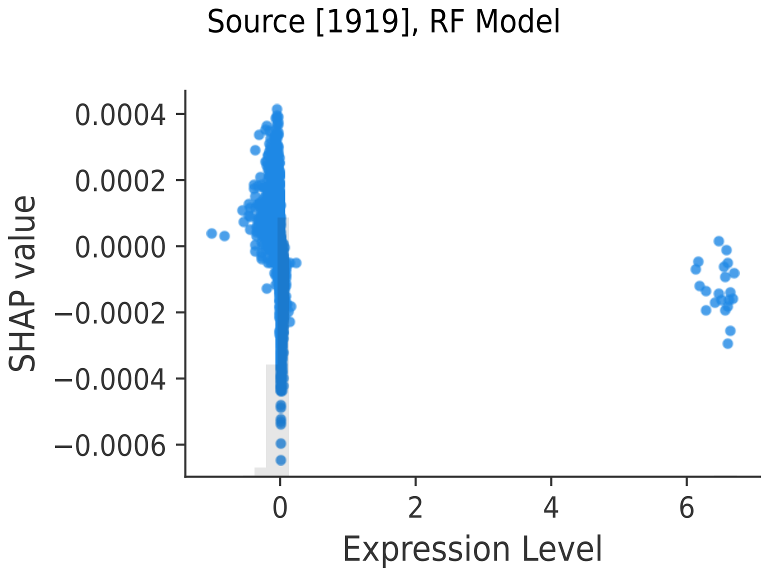

Source [1919], RF Model
SHAP value
0.0004
0.0002
0.0000
−0.0002
−0.0004
−0.0006
0
2
4
6
Expression Level

Source [1920], RF Model

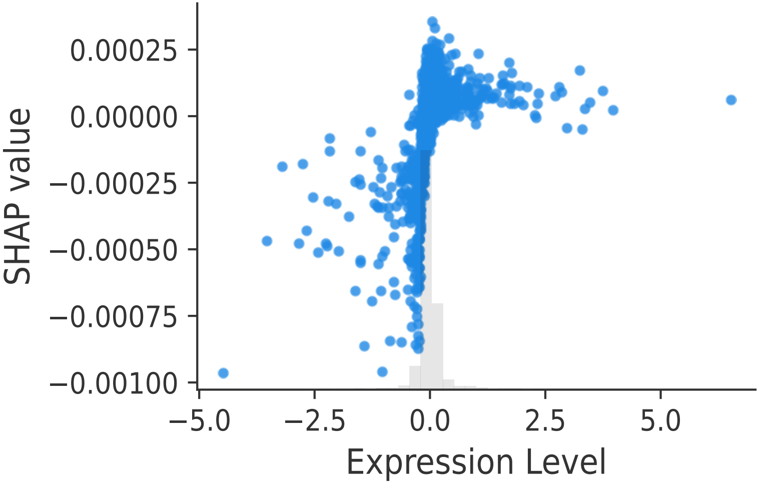

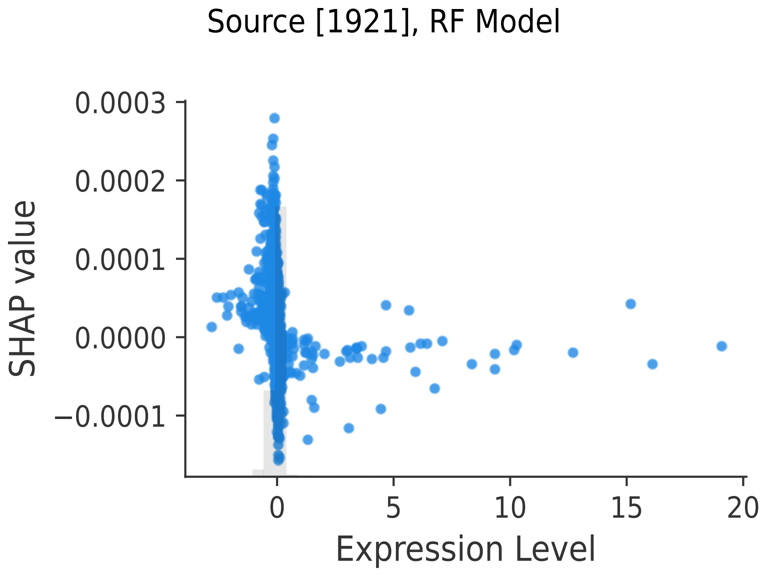

Source [1921], RF Model
SHAP value
0.0003
0.0002
0.0001
0.0000
−0.0001
0
5
10
15
20
Expression Level

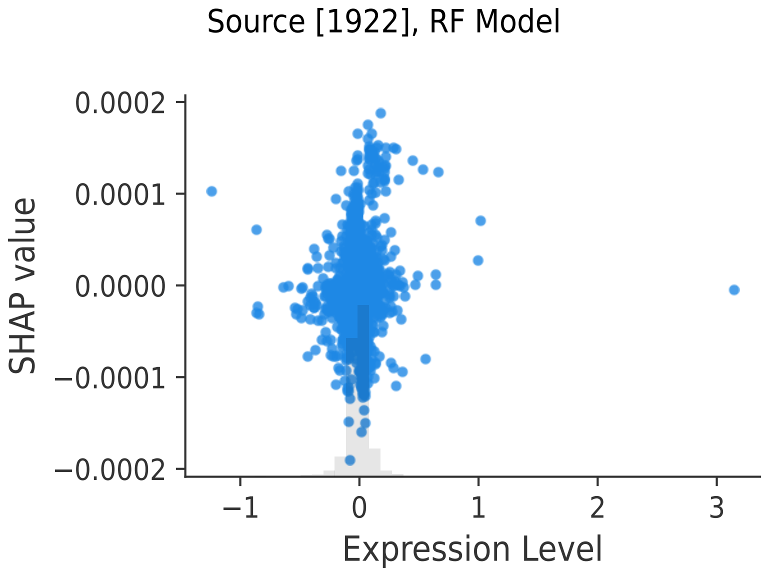

Source [1922], RF Model
SHAP value
0.0002
0.0001
0.0000
−0.0001
−0.0002
−1
0
1
2
3
Expression Level

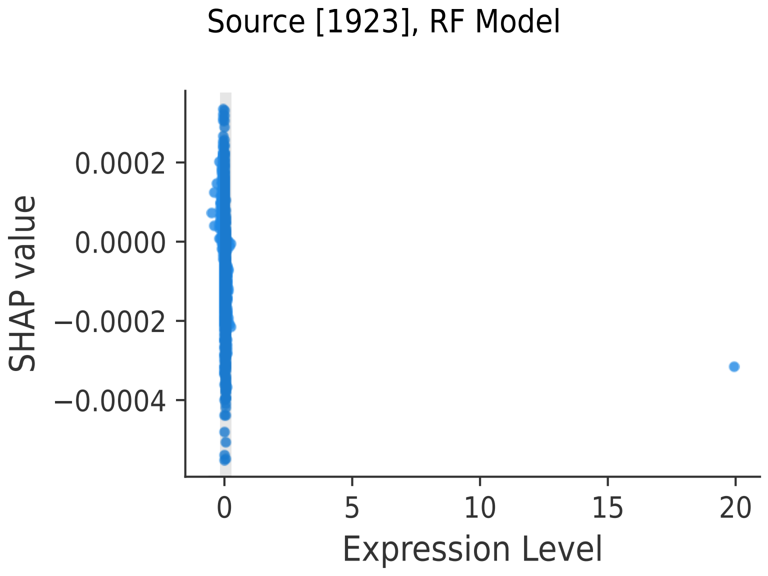
Source [1923], RF Model
SHAP value
0.0002
0.0000
−0.0002
−0.0004
0
5
10
15
20
Expression Level

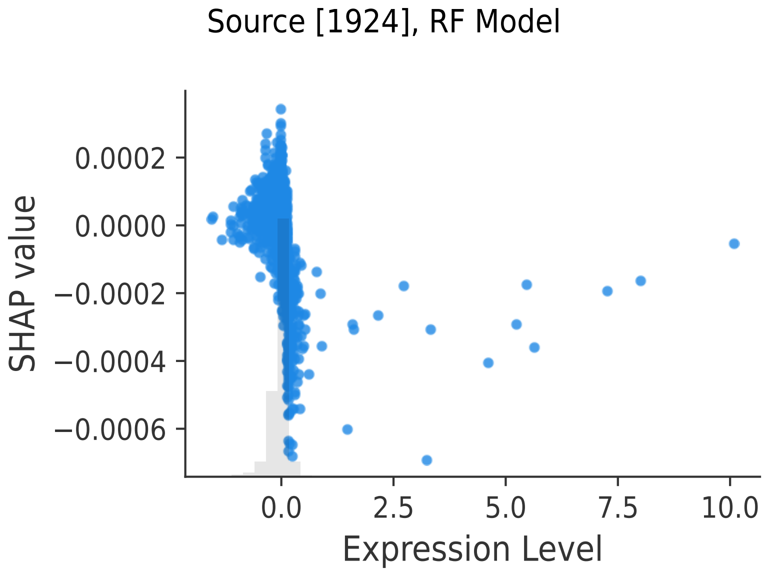

Source [1924], RF Model
0.0002
0.0000
−0.0002
−0.0004
−0.0006
SHAP value
0.0
2.5
5.0
7.5
10.0
Expression Level

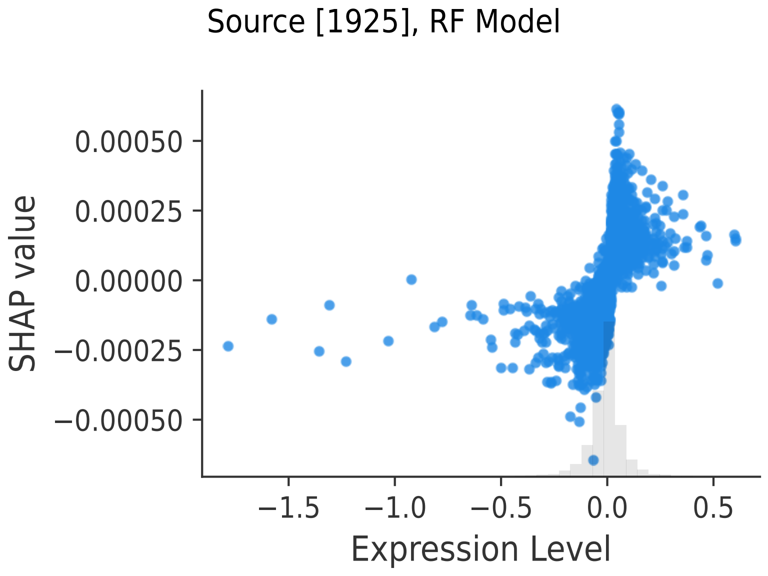

Source [1925], RF Model
SHAP value
0.00050
0.00025
0.00000
−0.00025
−0.00050
−1.5
−1.0
−0.5
0.0
0.5
Expression Level

Source [1926], RF Model

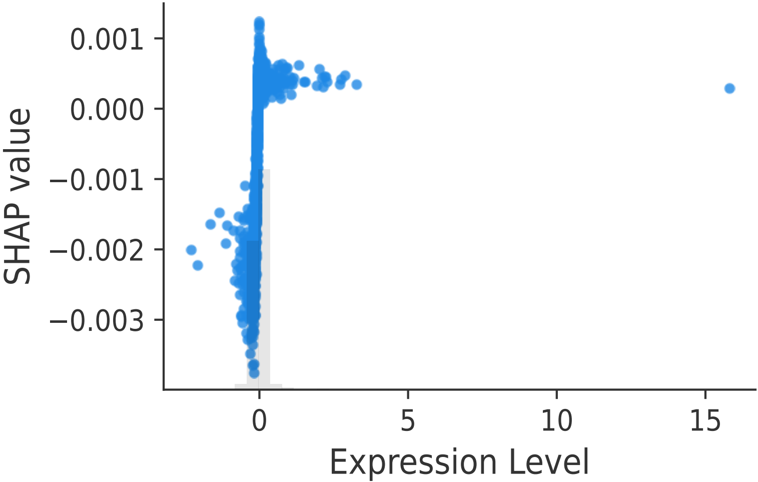

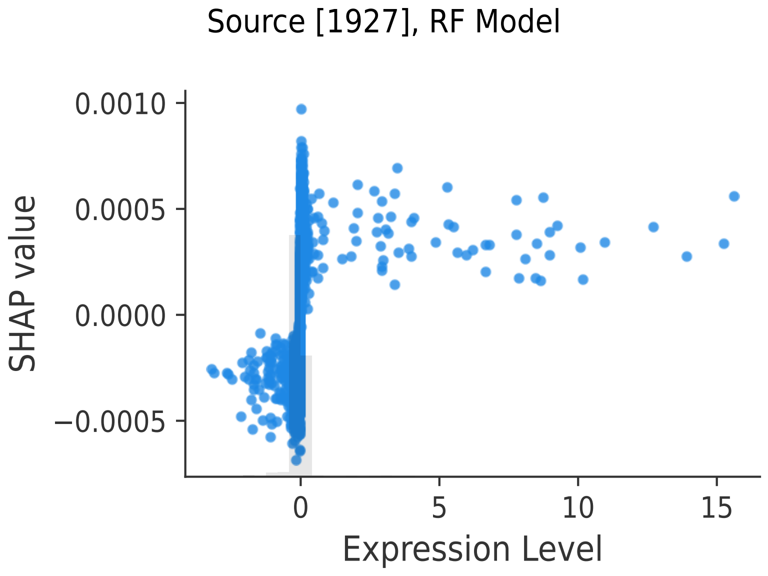

Source [1927], RF Model
0.0010
0.0005
0.0000
−0.0005
SHAP value
0
5
10
15
Expression Level

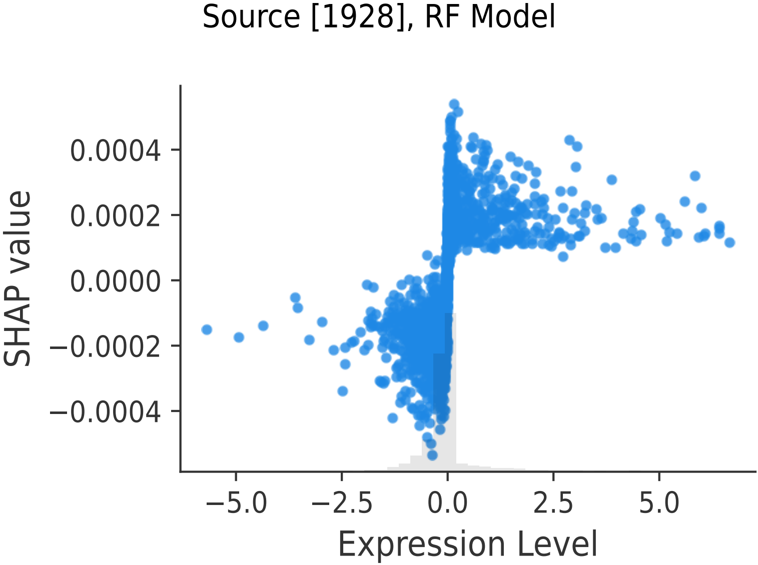

Source [1928], RF Model
SHAP value
0.0004
0.0002
0.0000
−0.0002
−0.0004
−5.0
−2.5
0.0
2.5
5.0
Expression Level

Source [1929], RF Model

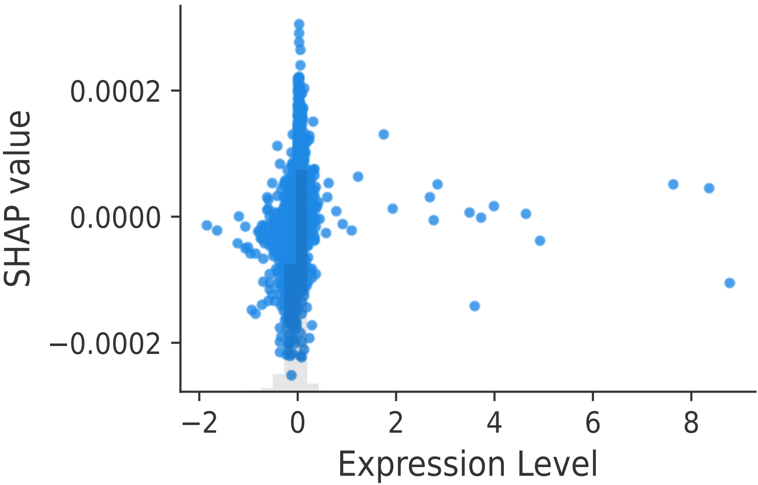

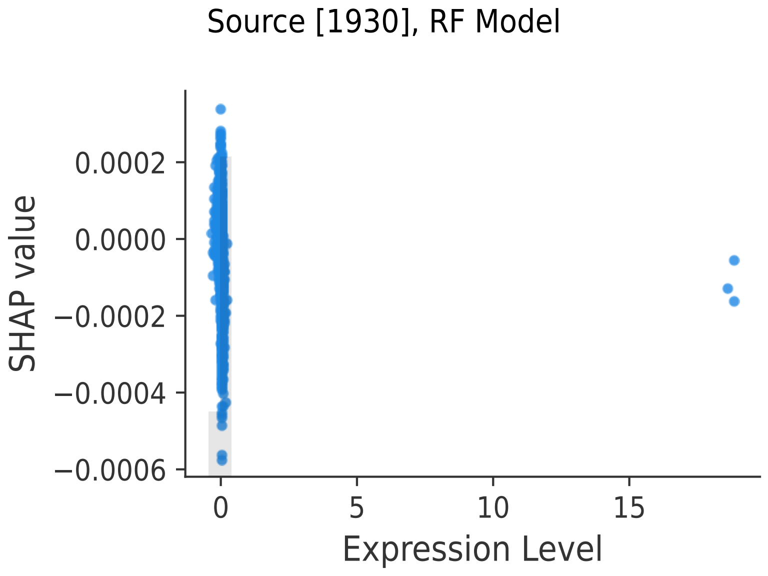
Source [1930], RF Model
SHAP value
0.0002
0.0000
−0.0002
−0.0004
−0.0006
0
5
10
15
Expression Level

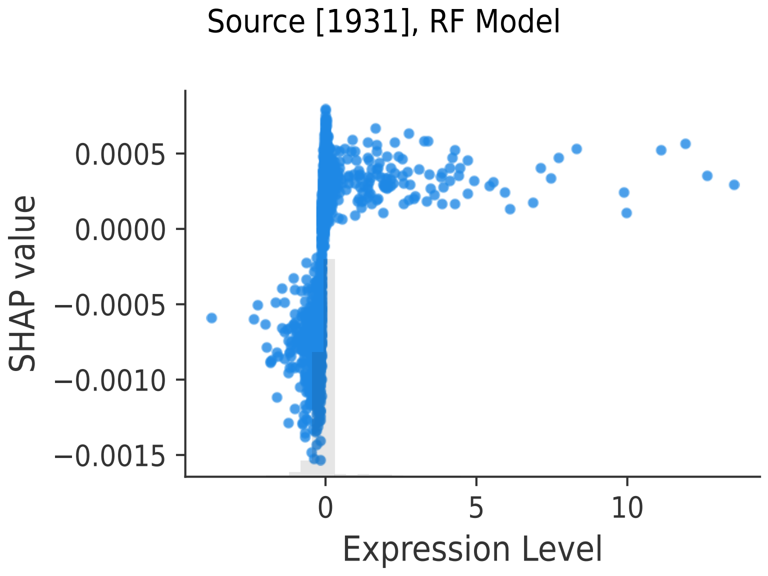
Source [1931], RF Model
SHAP value
0.0005
0.0000
−0.0005
−0.0010
−0.0015
0
5
10
Expression Level

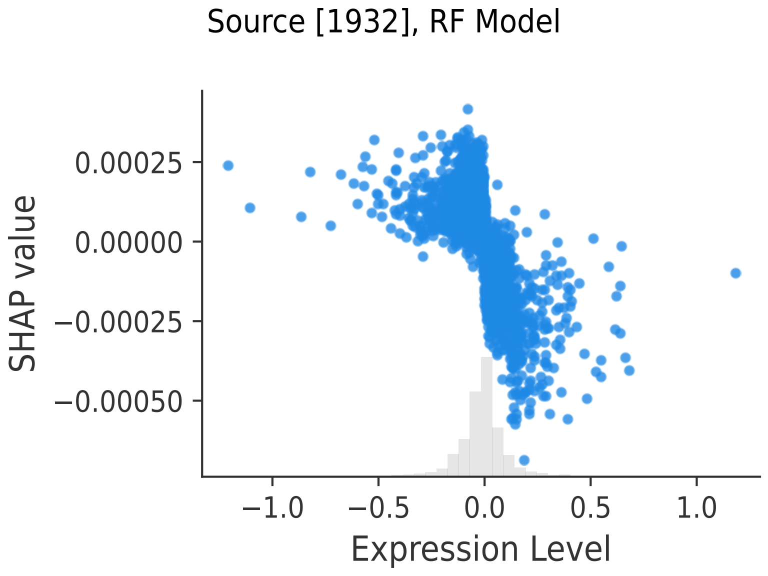

Source [1932], RF Model
SHAP value
0.00025
0.00000
−0.00025
−0.00050
−1.0
−0.5
0.0
0.5
1.0
Expression Level

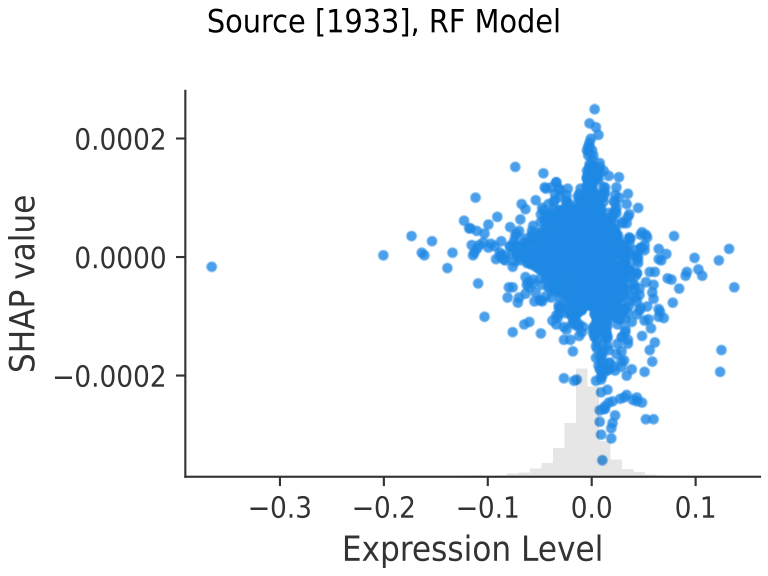

Source [1933], RF Model
0.0002
0.0000
−0.0002
SHAP value
−0.3
−0.2
−0.1
0.0
0.1
Expression Level

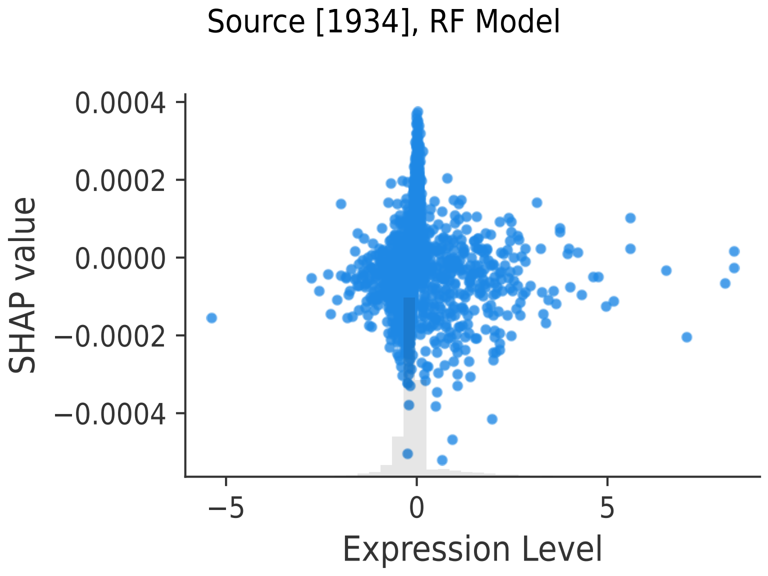

Source [1934], RF Model
SHAP value
0.0004
0.0002
0.0000
−0.0002
−0.0004
−5
0
5
Expression Level

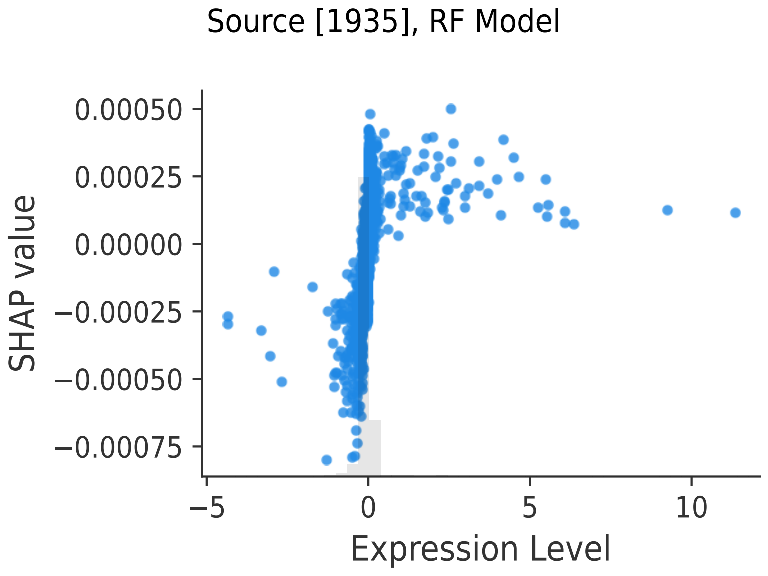
Source [1935], RF Model
SHAP value
0.00050
0.00025
0.00000
−0.00025
−0.00050
−0.00075
Expression Level
−5
0
5
10

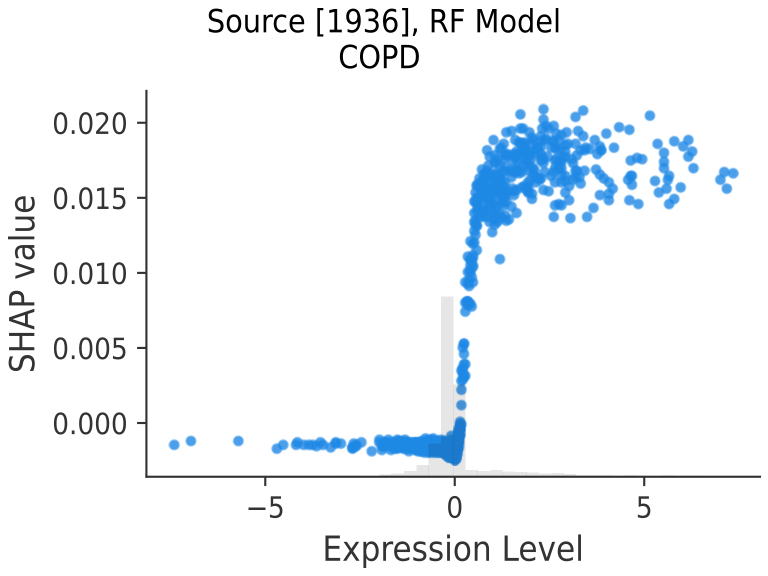
Source [1936], RF Model
COPD
SHAP value
0.020
0.015
0.010
0.005
0.000
−5
0
5
Expression Level

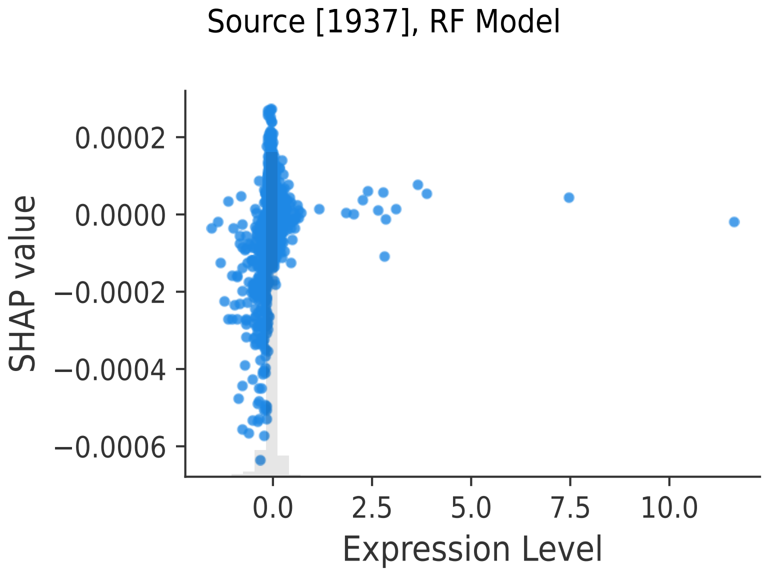

Source [1937], RF Model
0.0002
0.0000
−0.0002
−0.0004
−0.0006
SHAP value
0.0
2.5
5.0
7.5
10.0
Expression Level

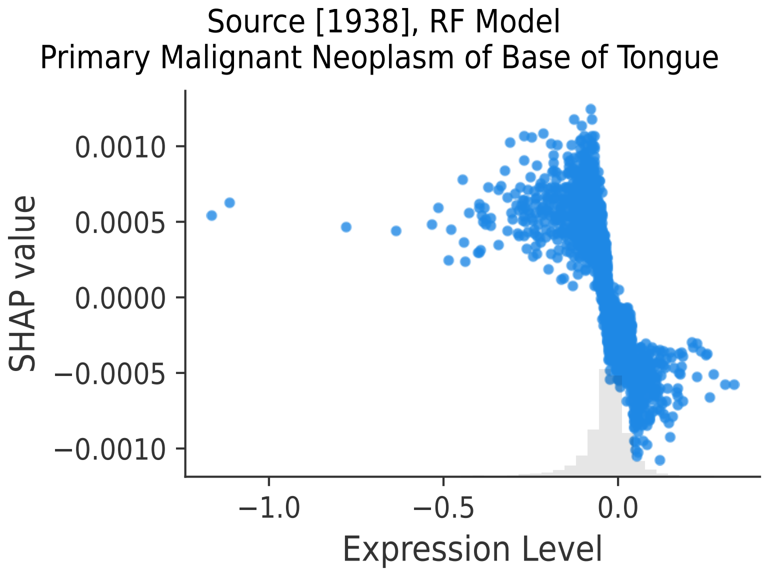
Source [1938], RF Model
Primary Malignant Neoplasm of Base of Tongue
SHAP value
0.0010
0.0005
0.0000
−0.0005
−0.0010
−1.0
−0.5
0.0
Expression Level

Source [1939], RF Model

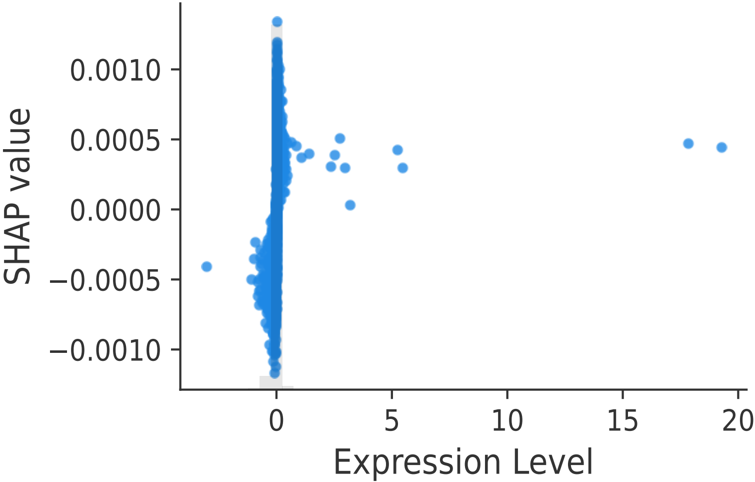

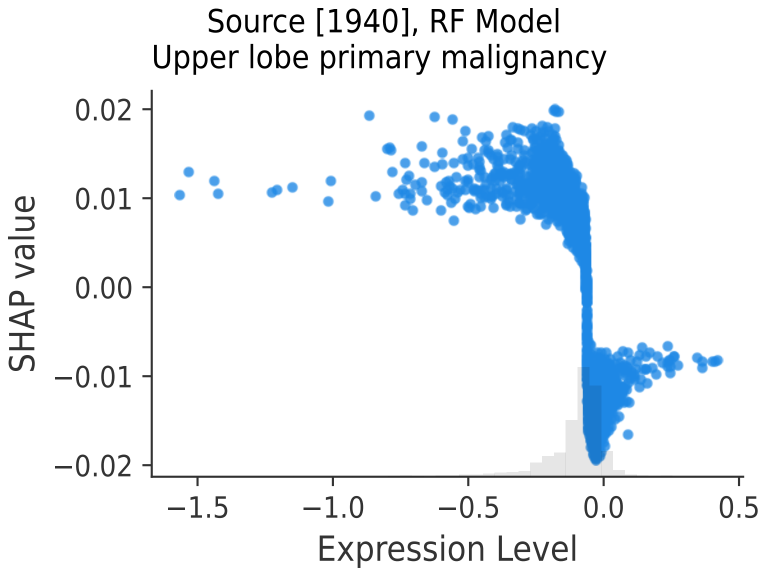
Source [1940], RF Model
Upper lobe primary malignancy
0.02
0.01
0.00
−0.01
−0.02
SHAP value
−1.5
−1.0
−0.5
0.0
0.5
Expression Level

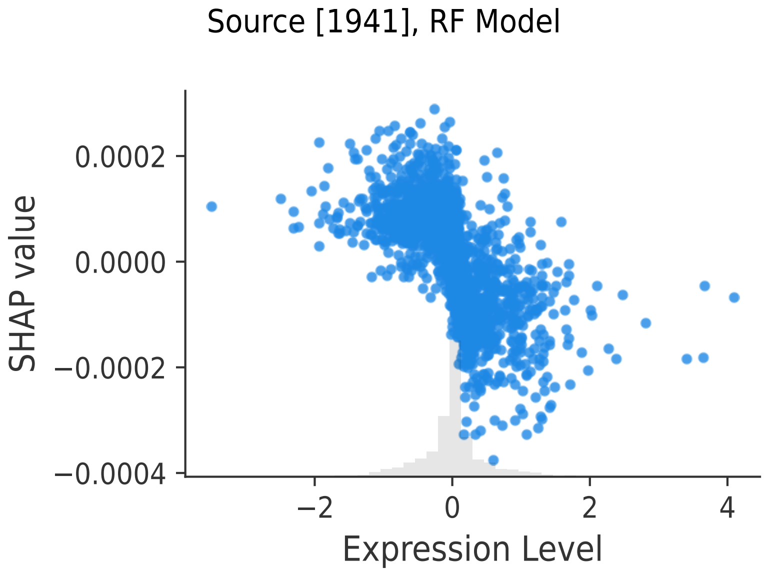
Source [1941], RF Model
0.0002
0.0000
−0.0002
−0.0004
SHAP value
−2
0
2
4
Expression Level

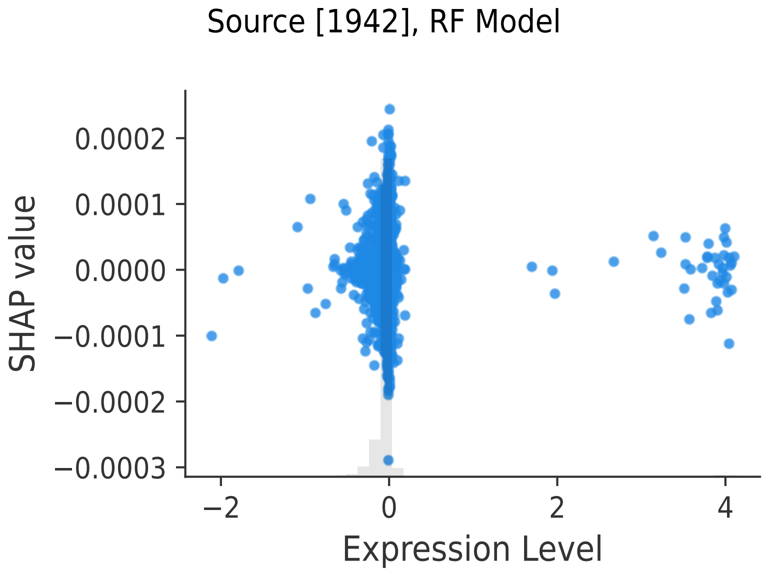

Source [1942], RF Model
SHAP value
0.0002
0.0001
0.0000
−0.0001
−0.0002
−0.0003
−2
0
2
4
Expression Level

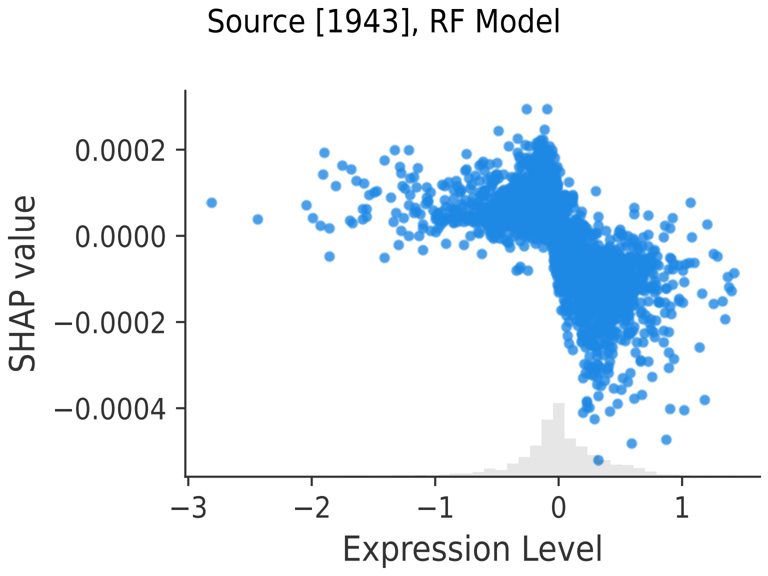

Source [1943], RF Model
0.0002
0.0000
−0.0002
−0.0004
SHAP value
−3
−2
−1
0
1
Expression Level

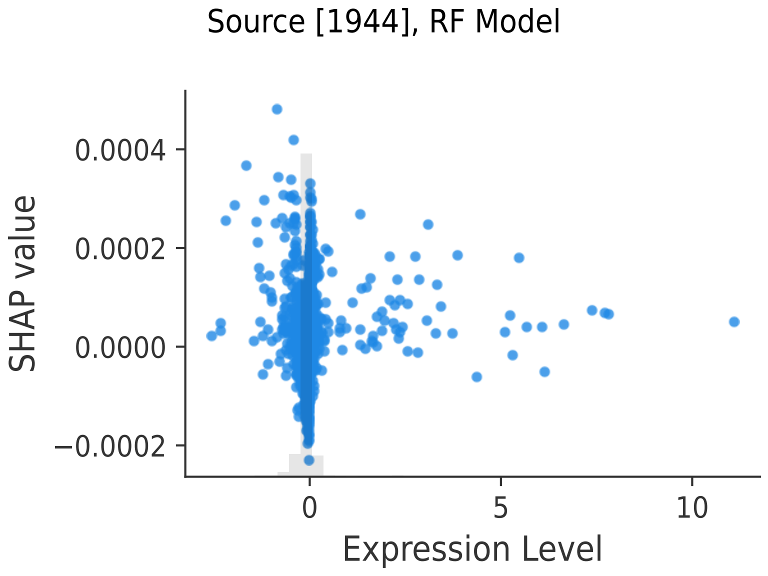
Source [1944], RF Model
SHAP value
0.0004
0.0002
0.0000
−0.0002
0
5
10
Expression Level

Source [1945], RF Model
Sigmoid Colon CA

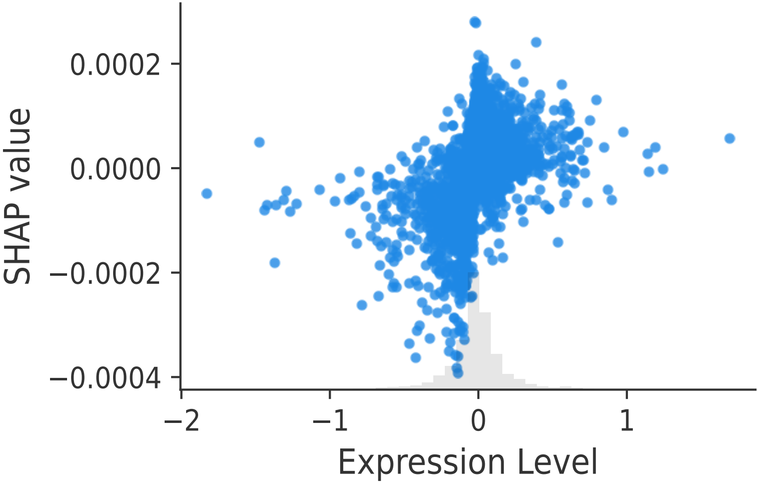

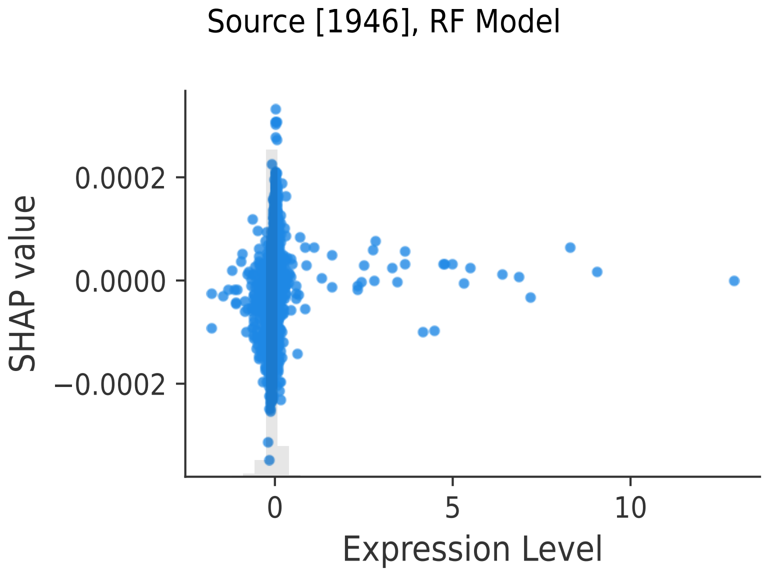

Source [1946], RF Model
0.0002
0.0000
−0.0002
SHAP value
0
5
10
Expression Level

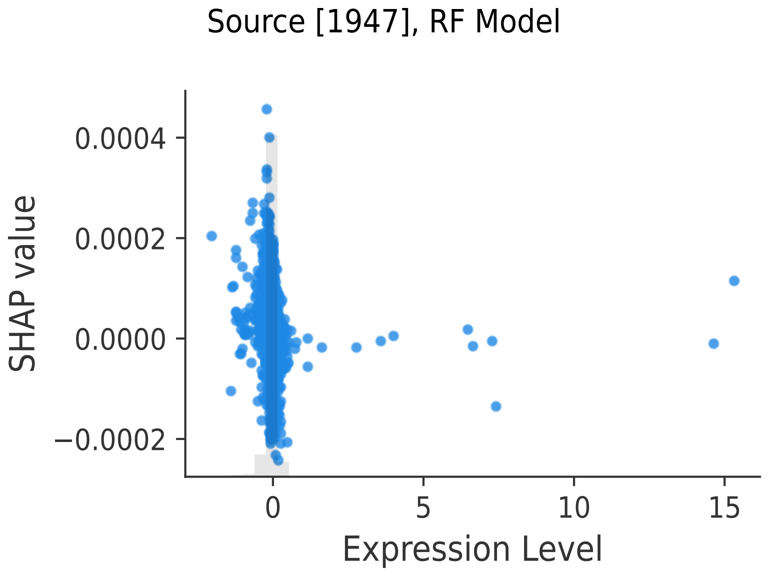
Source [1947], RF Model
0.0004
0.0002
0.0000
−0.0002
SHAP value
0
5
10
15
Expression Level

Source [1948], RF Model

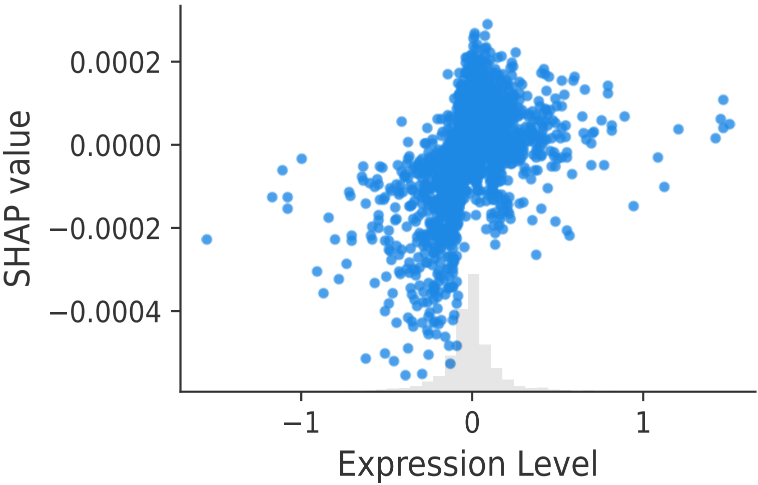

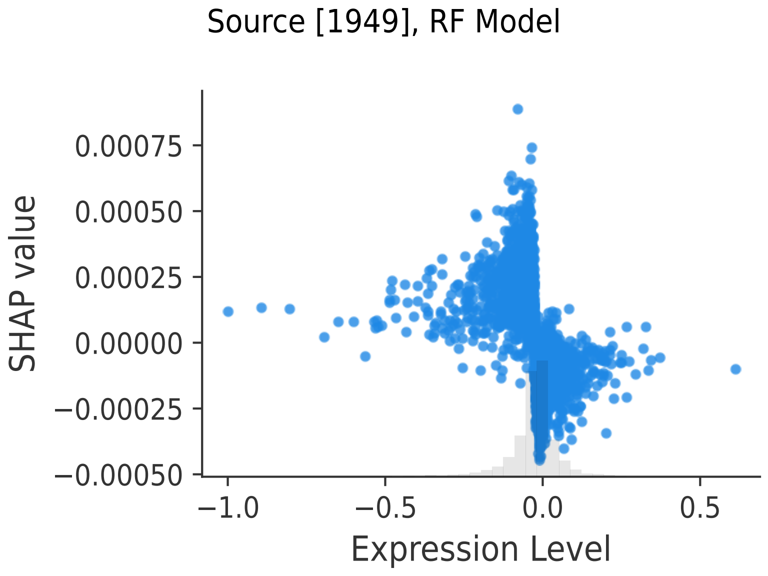
Source [1949], RF Model
0.00075
0.00050
0.00025
0.00000
−0.00025
−0.00050
SHAP value
−1.0
−0.5
0.0
0.5
Expression Level

Source [1950], RF Model
Metronidazole

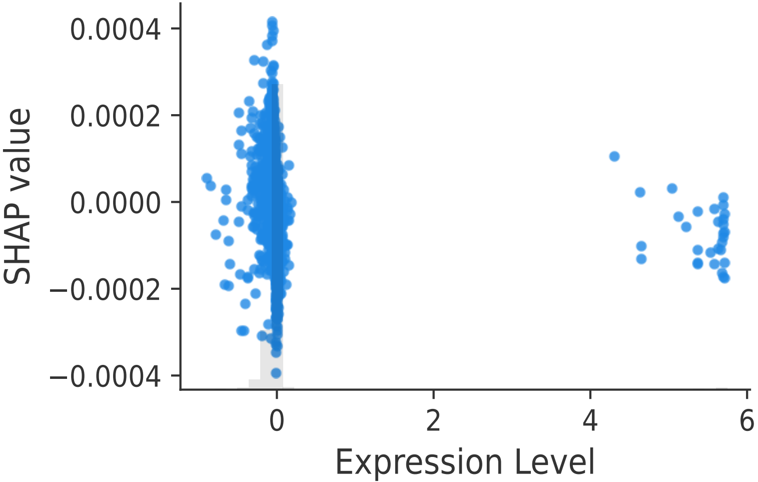

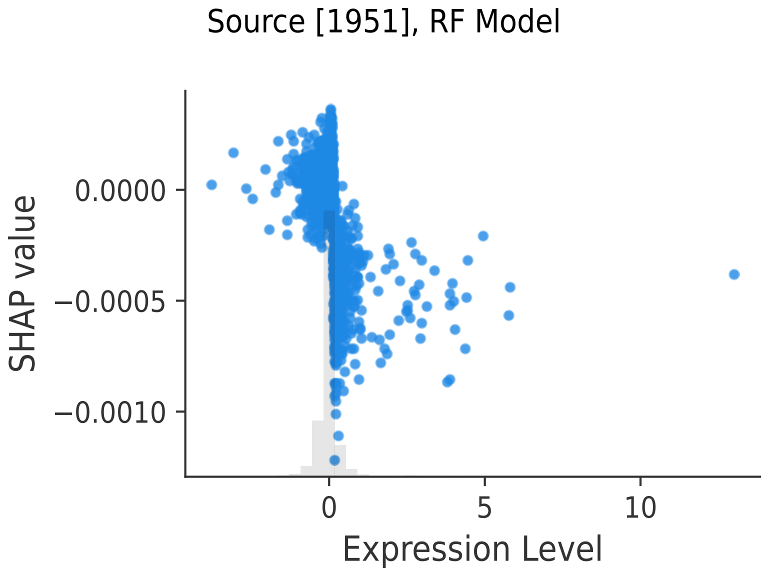
Source [1951], RF Model
SHAP value
0.0000
−0.0005
−0.0010
0
5
10
Expression Level

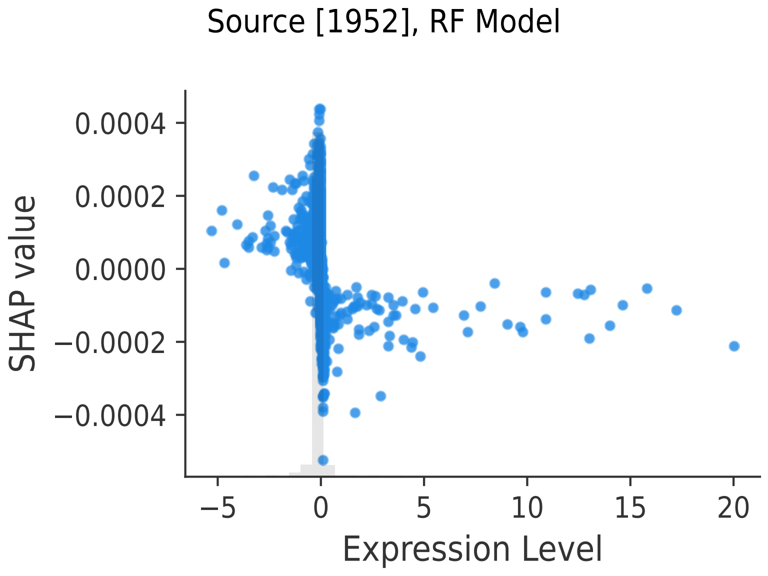
Source [1952], RF Model
SHAP value
0.0004
0.0002
0.0000
−0.0002
−0.0004
−5
0
5
10
15
20
Expression Level

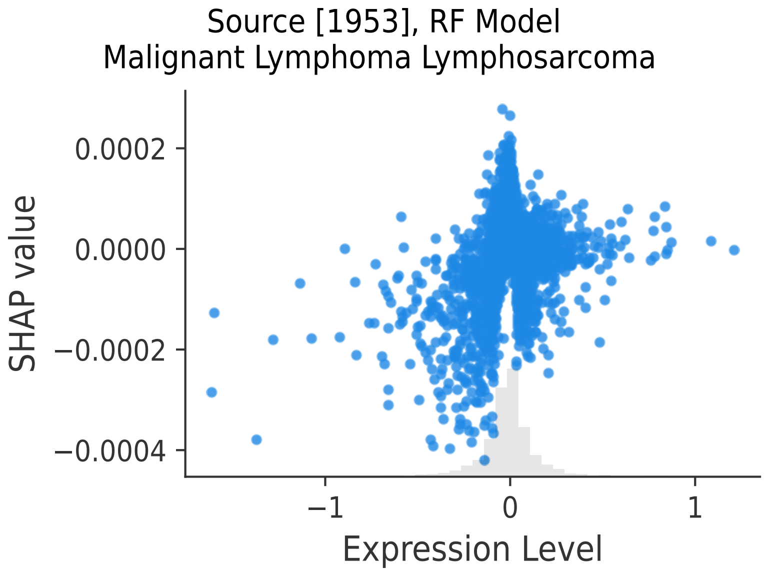

Source [1953], RF Model
Malignant Lymphoma Lymphosarcoma
SHAP value
0.0002
0.0000
−0.0002
−0.0004
−1
0
1
Expression Level

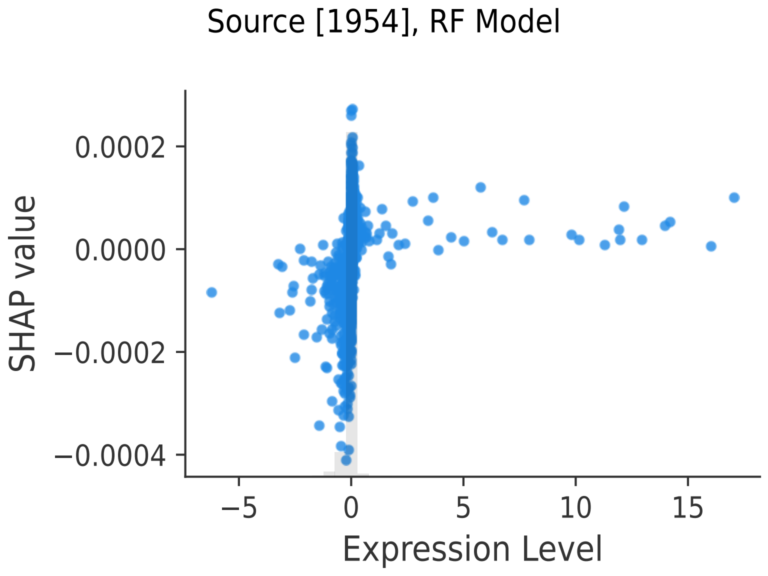

Source [1954], RF Model
SHAP value
0.0002
0.0000
−0.0002
−0.0004
−5
0
5
10
15
Expression Level

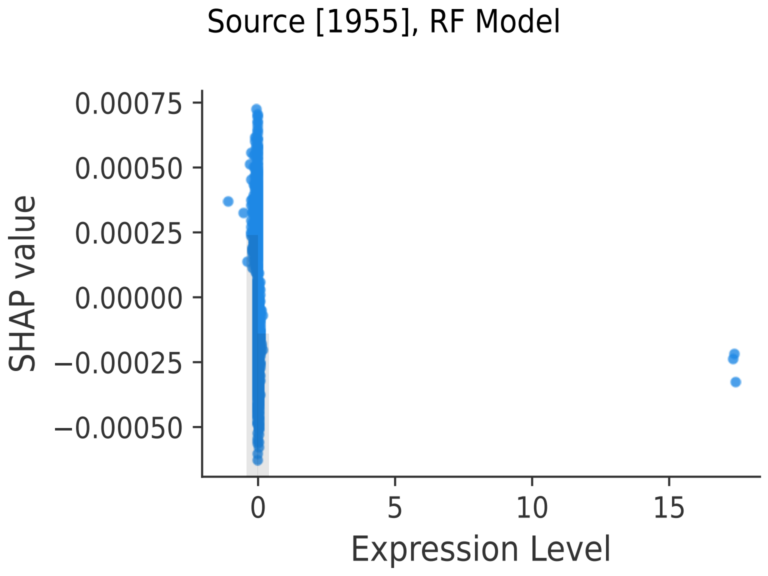
Source [1955], RF Model
0.00075
0.00050
0.00025
0.00000
−0.00025
−0.00050
SHAP value
0
5
10
15
Expression Level

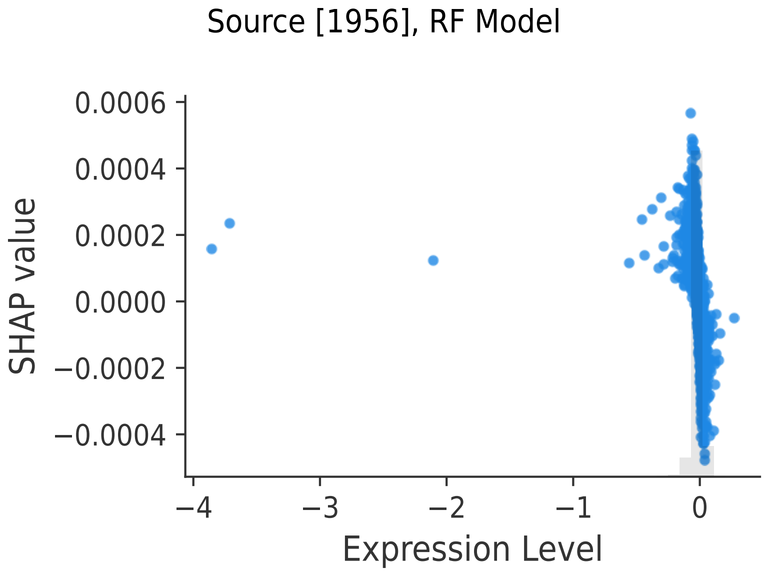
Source [1956], RF Model
SHAP value
0.0006
0.0004
0.0002
0.0000
−0.0002
−0.0004
−4
−3
−2
−1
0
Expression Level

Source [1957], RF Model

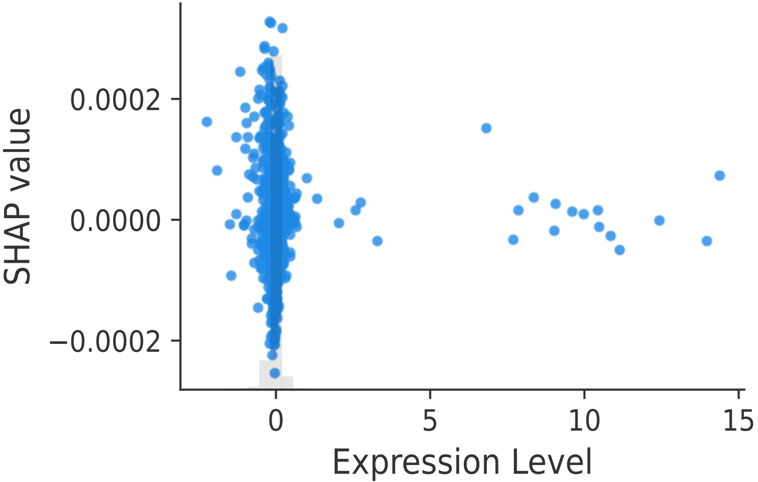

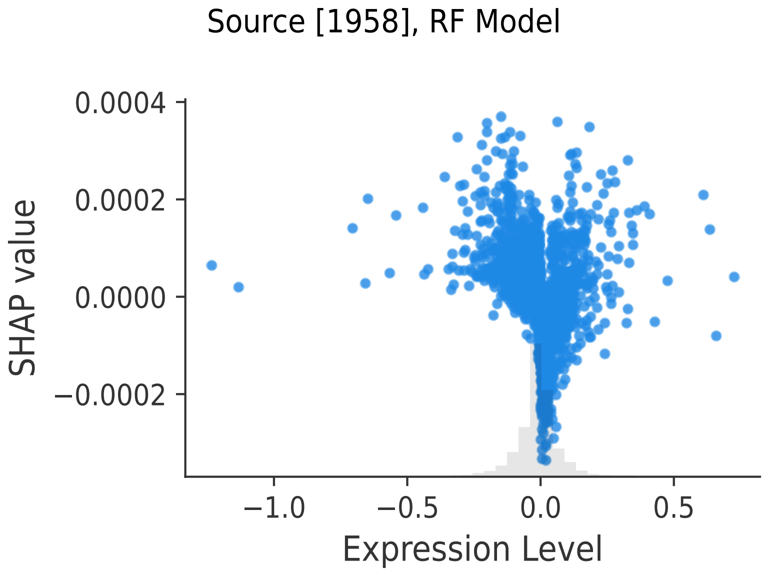

Source [1958], RF Model
SHAP value
0.0004
0.0002
0.0000
−0.0002
−1.0
−0.5
0.0
0.5
Expression Level

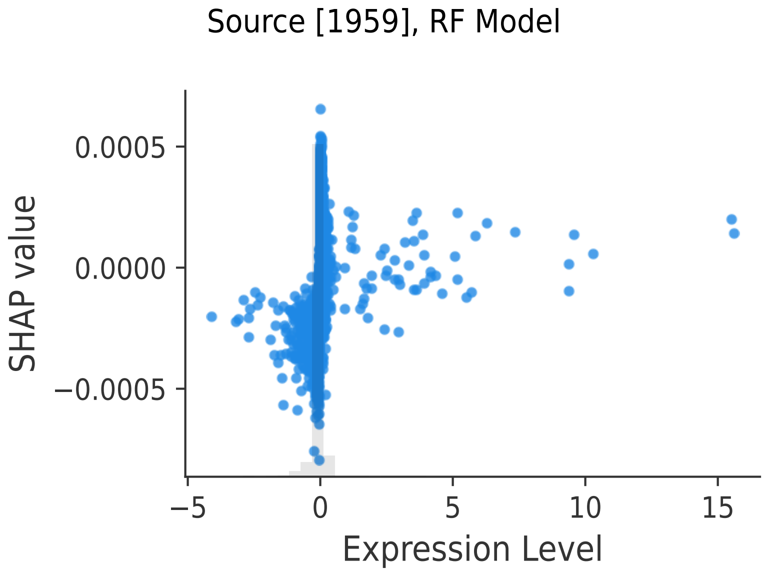

Source [1959], RF Model
SHAP value
0.0005
0.0000
−0.0005
−5
0
5
10
15
Expression Level

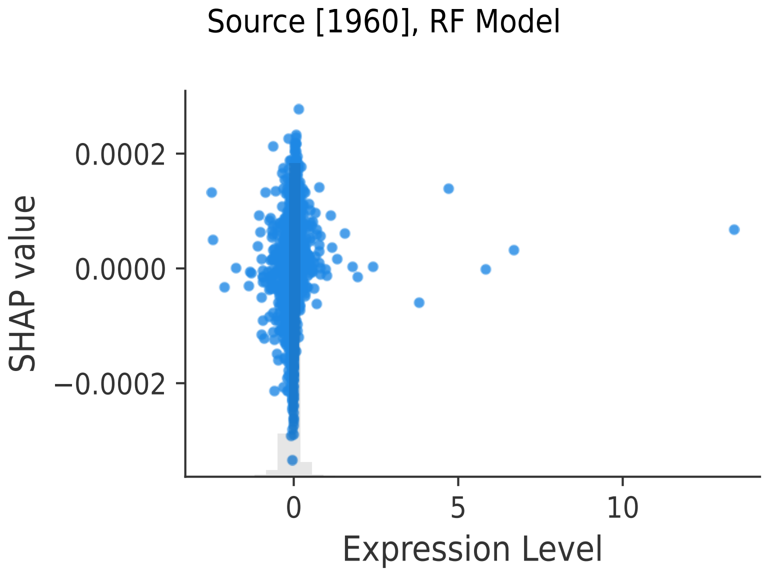
Source [1960], RF Model
0.0002
0.0000
−0.0002
SHAP value
0
5
10
Expression Level

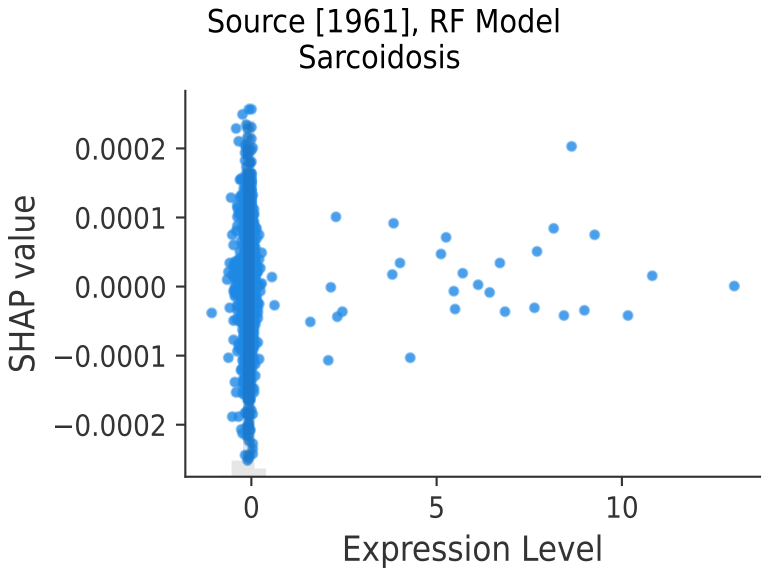
Source [1961], RF Model
Sarcoidosis
0.0002
0.0001
0.0000
−0.0001
−0.0002
SHAP value
0
5
10
Expression Level

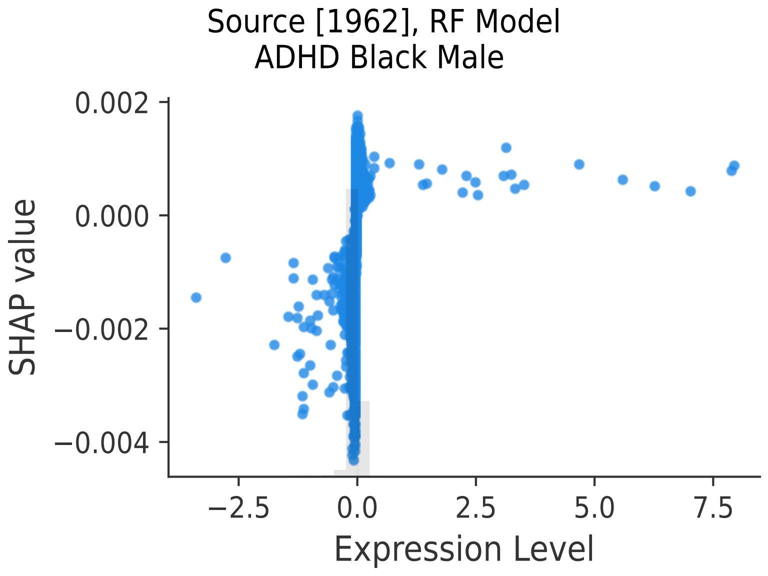

Source [1962], RF Model
ADHD Black Male
SHAP value
0.002
0.000
−0.002
−0.004
−2.5
0.0
2.5
5.0
7.5
Expression Level

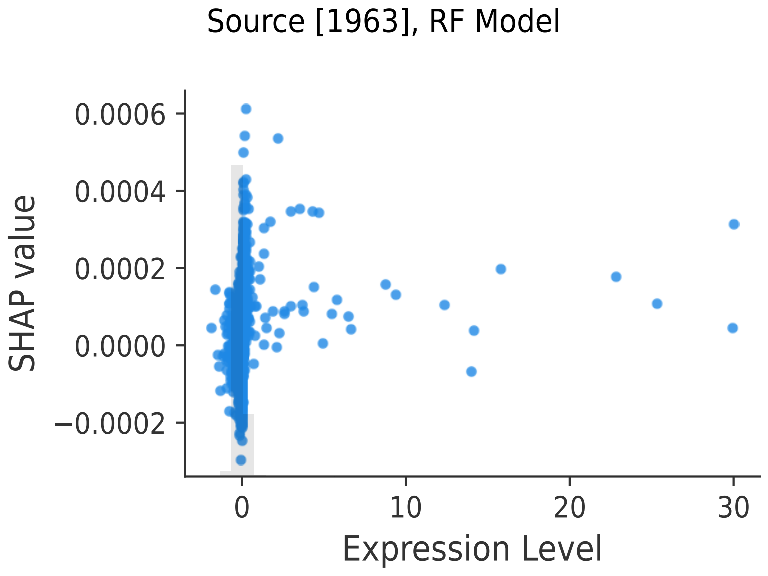

Source [1963], RF Model
0.0006
0.0004
0.0002
0.0000
−0.0002
SHAP value
0
10
20
30
Expression Level

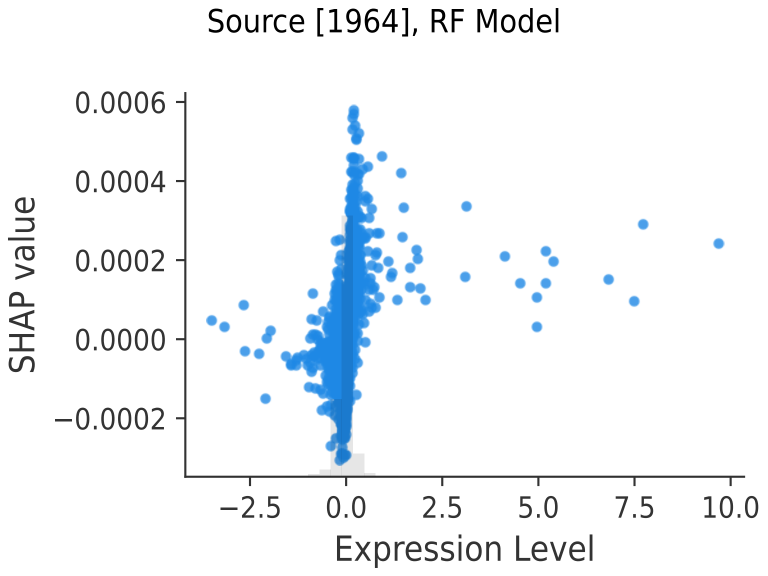
Source [1964], RF Model
0.0006
0.0004
0.0002
0.0000
−0.0002
SHAP value
−2.5
0.0
2.5
5.0
7.5
10.0
Expression Level

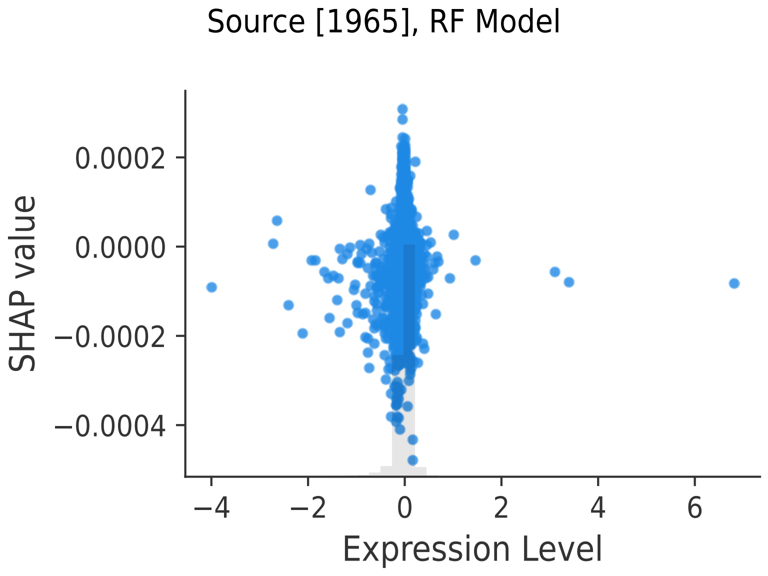

Source [1965], RF Model
SHAP value
0.0002
0.0000
−0.0002
−0.0004
−4
−2
0
2
4
6
Expression Level

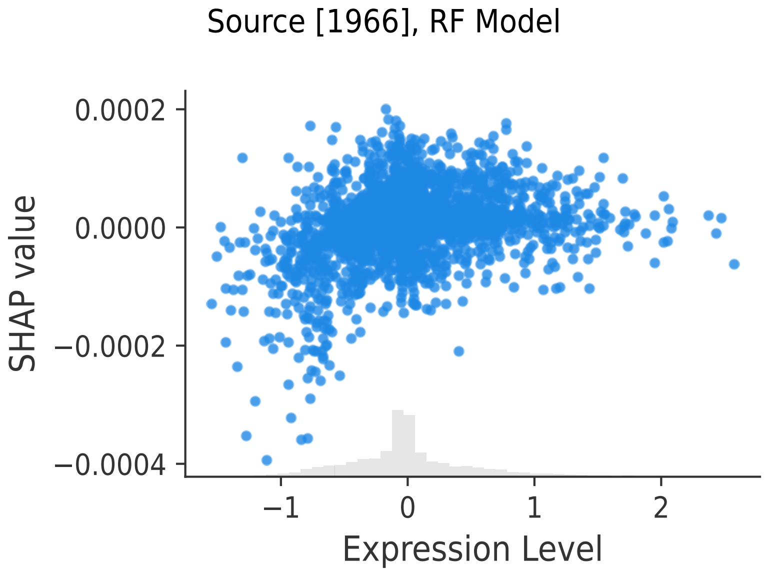

Source [1966], RF Model
0.0002
0.0000
−0.0002
−0.0004
SHAP value
−1
0
1
2
Expression Level

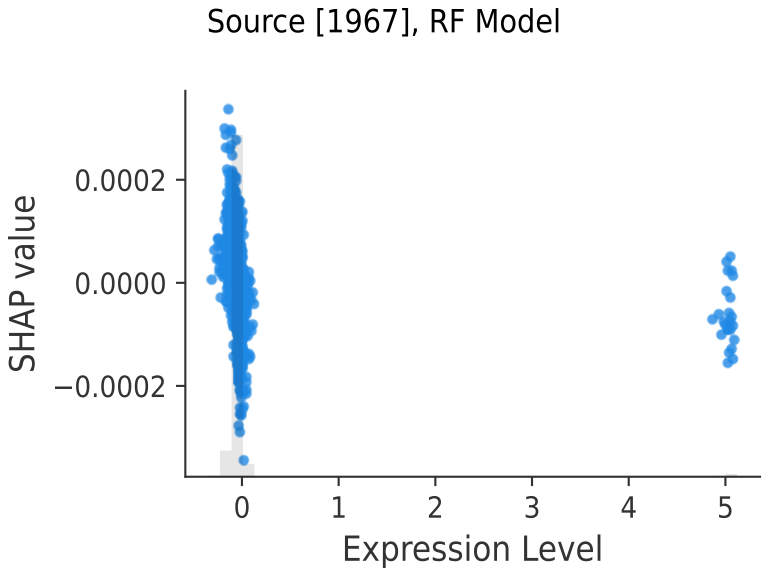
Source [1967], RF Model
SHAP value
0.0002
0.0000
−0.0002
0
1
2
3
4
5
Expression Level

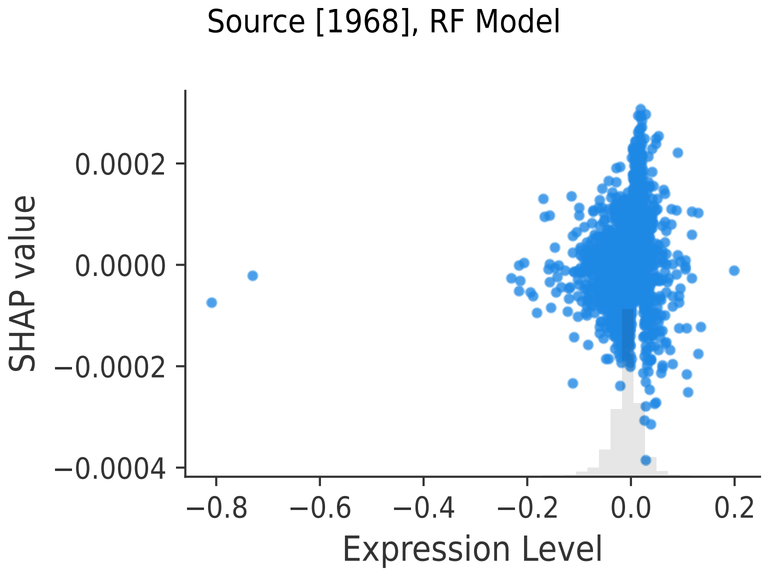
Source [1968], RF Model
SHAP value
0.0002
0.0000
−0.0002
−0.0004
−0.8
−0.6
−0.4
−0.2
0.0
0.2
Expression Level

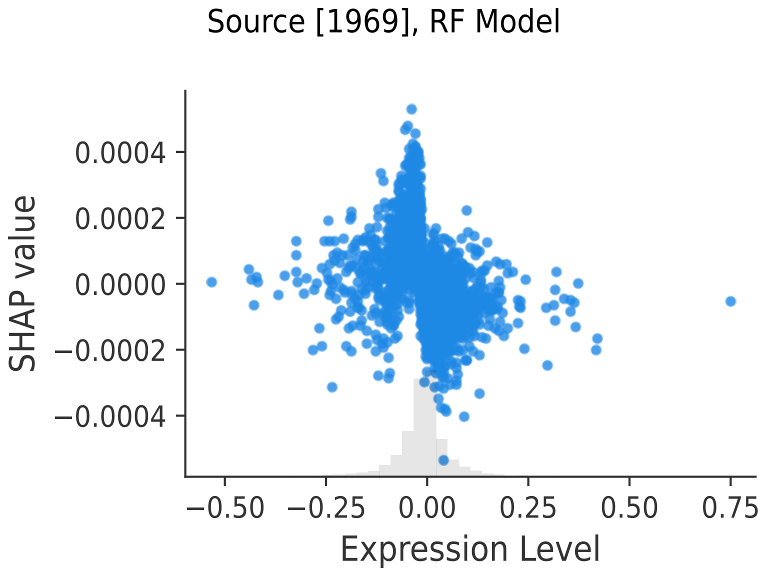
Source [1969], RF Model
SHAP value
0.0004
0.0002
0.0000
−0.0002
−0.0004
−0.50
−0.25
0.00
0.25
0.50
0.75
Expression Level

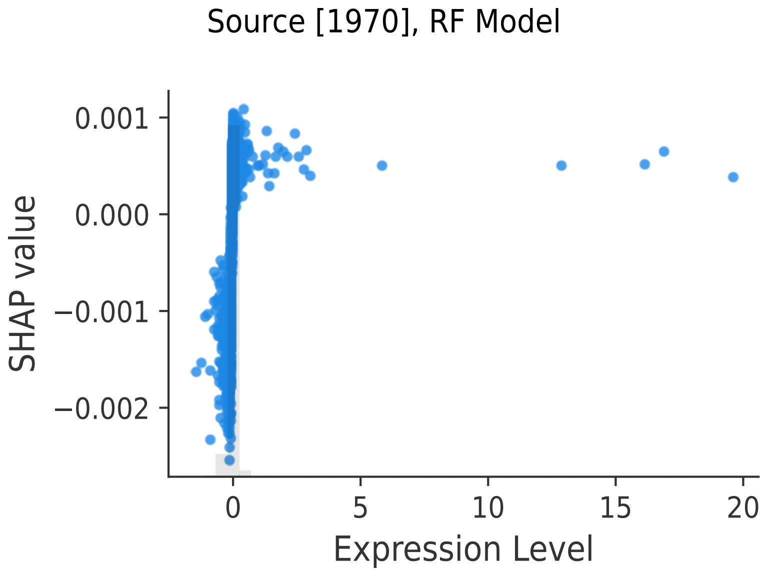

Source [1970], RF Model
SHAP value
0.001
0.000
−0.001
−0.002
0
5
10
15
20
Expression Level

Source [1971], RF Model

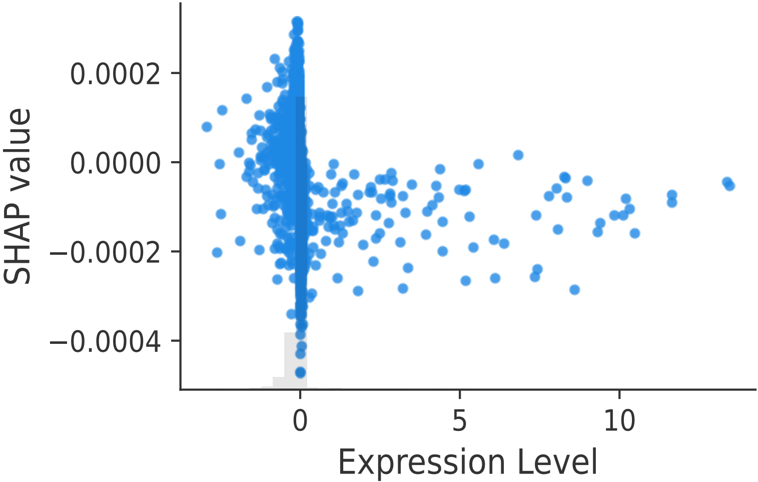

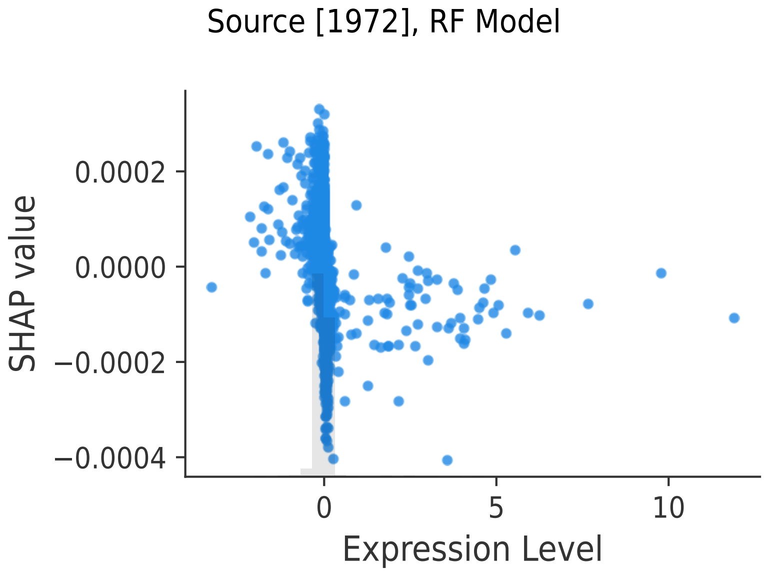

Source [1972], RF Model
SHAP value
0.0002
0.0000
−0.0002
−0.0004
0
5
10
Expression Level

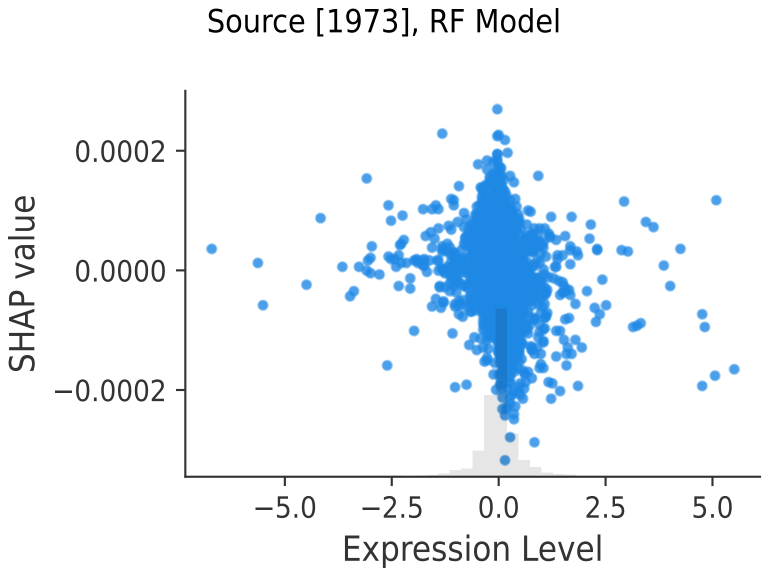
Source [1973], RF Model
0.0002
0.0000
−0.0002
SHAP value
−5.0
−2.5
0.0
2.5
5.0
Expression Level

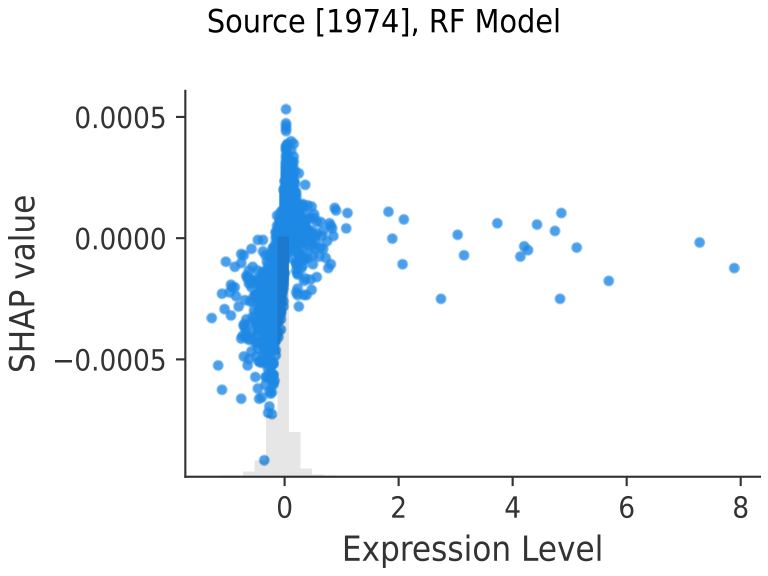

Source [1974], RF Model
0.0005
0.0000
−0.0005
SHAP value
0
2
4
6
8
Expression Level

Source [1975], RF Model

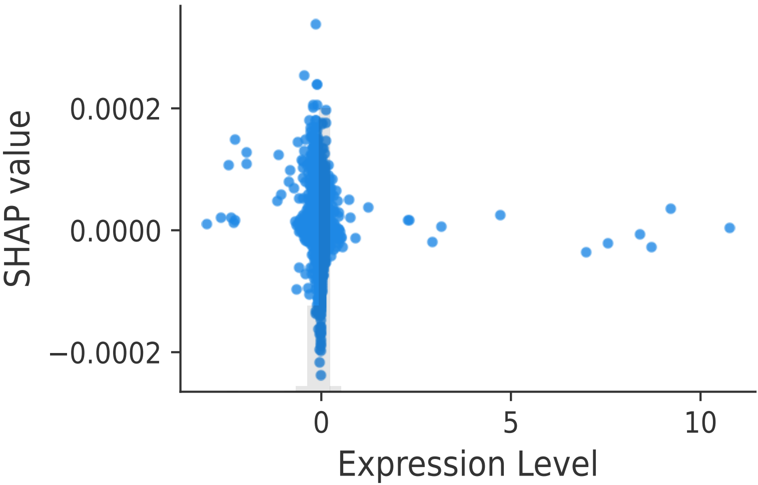

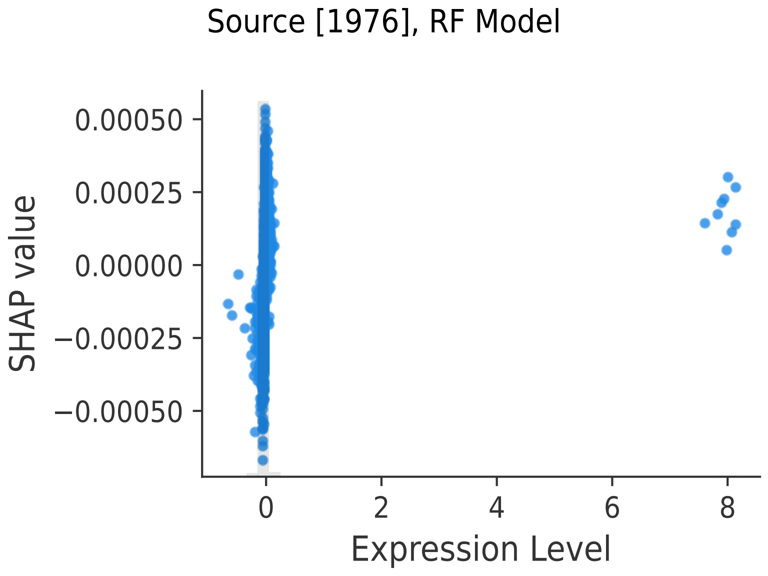

Source [1976], RF Model
SHAP value
0.00050
0.00025
0.00000
−0.00025
−0.00050
0
2
4
6
8
Expression Level

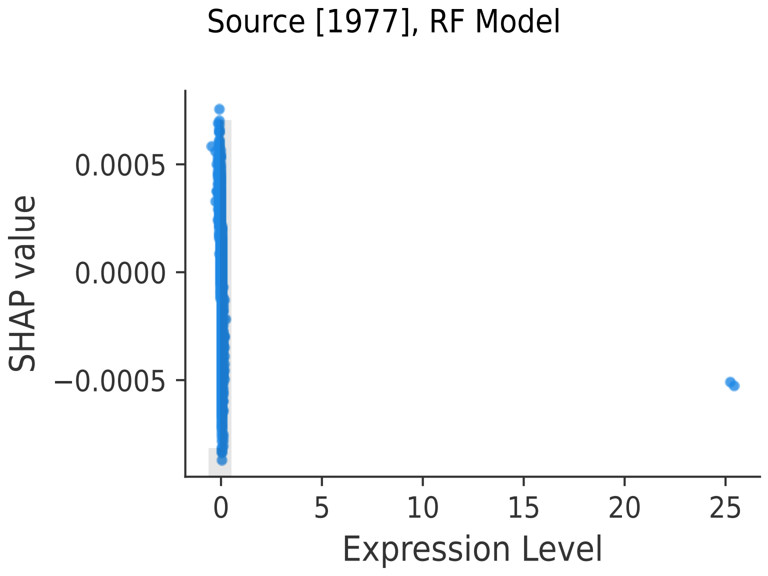
Source [1977], RF Model
SHAP value
0.0005
0.0000
−0.0005
0
5
10
15
20
25
Expression Level

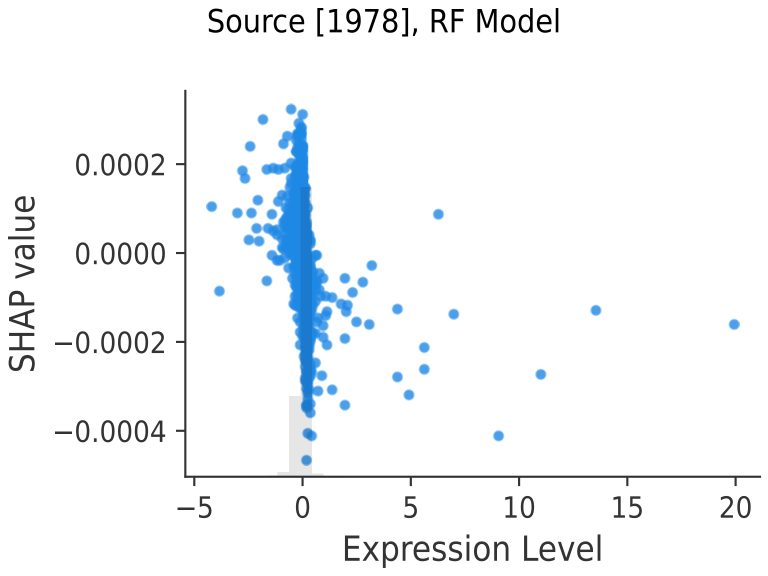
Source [1978], RF Model
SHAP value
0.0002
0.0000
−0.0002
−0.0004
Expression Level
−5
0
5
10
15
20

Source [1979], RF Model

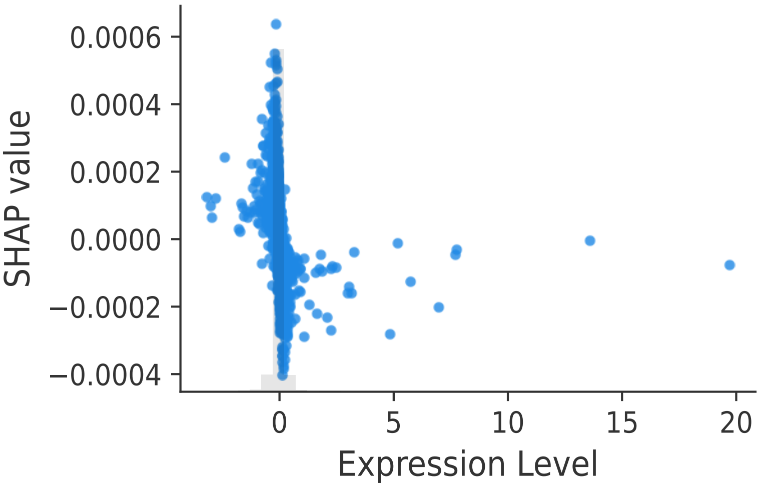

Source [1980], RF Model

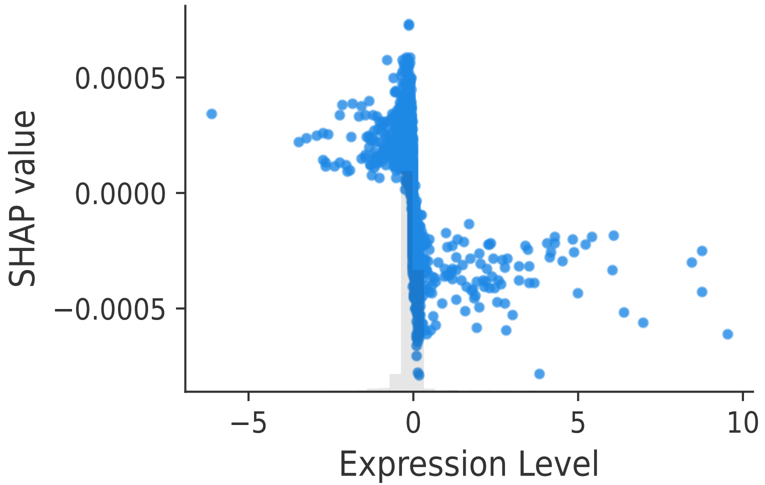

Source [1981], RF Model

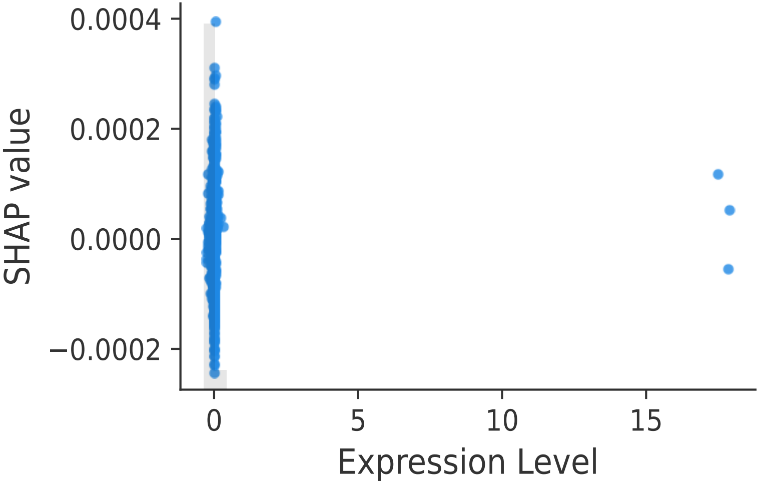

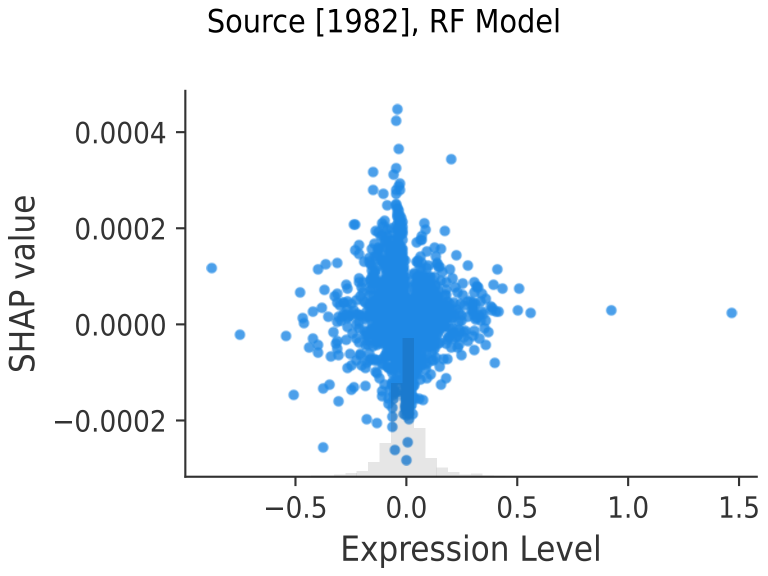

Source [1982], RF Model
SHAP value
0.0004
0.0002
0.0000
−0.0002
−0.5
0.0
0.5
1.0
1.5
Expression Level

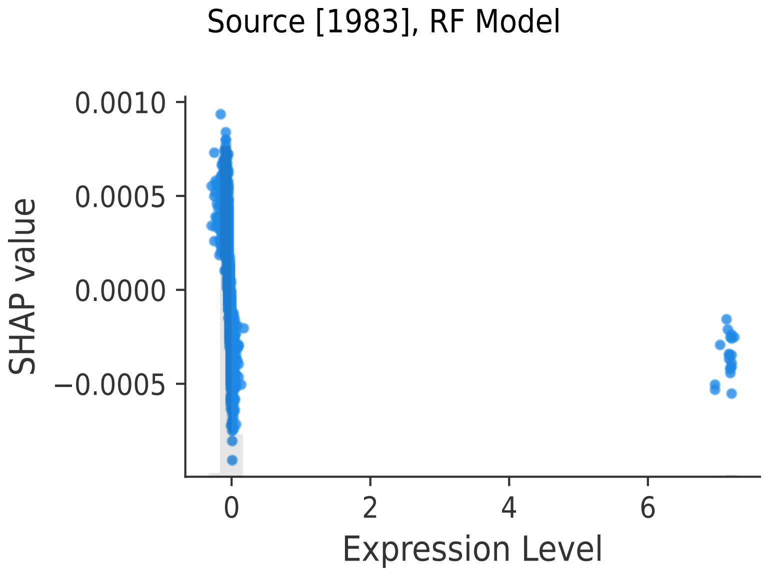

Source [1983], RF Model
SHAP value
0.0010
0.0005
0.0000
−0.0005
0
2
4
6
Expression Level

Source [1984], RF Model

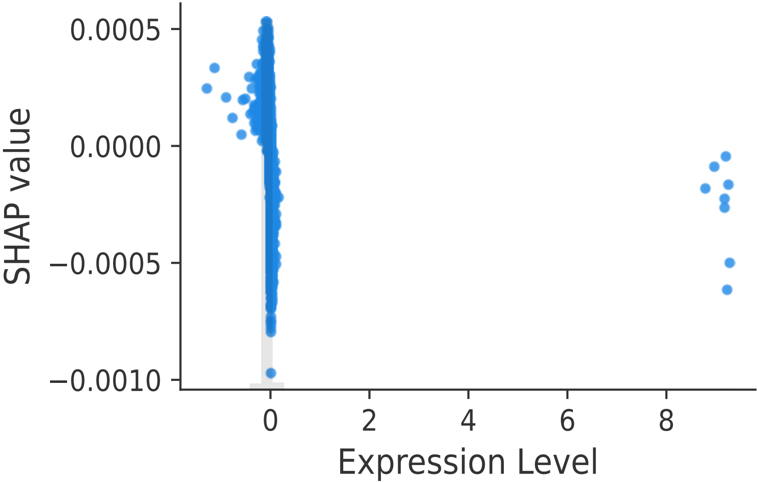

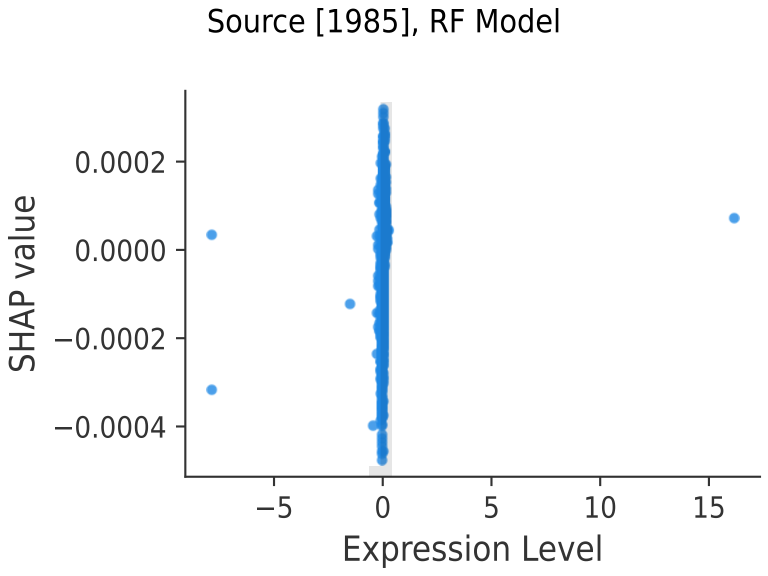

Source [1985], RF Model
SHAP value
0.0002
0.0000
−0.0002
−0.0004
−5
0
5
10
15
Expression Level

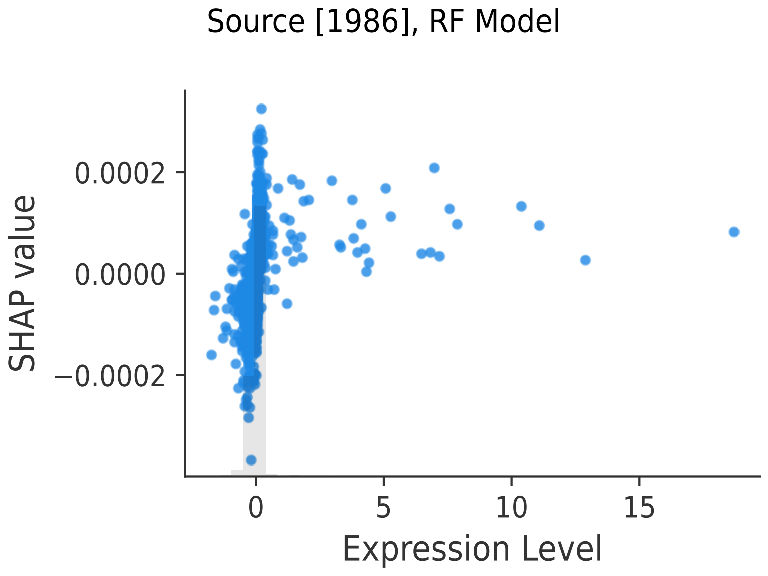
Source [1986], RF Model
SHAP value
0.0002
0.0000
−0.0002
0
5
10
15
Expression Level

Source [1987], RF Model

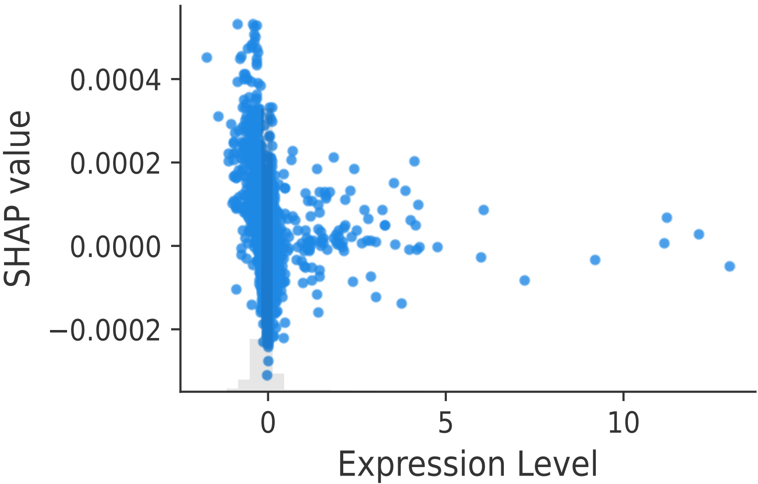

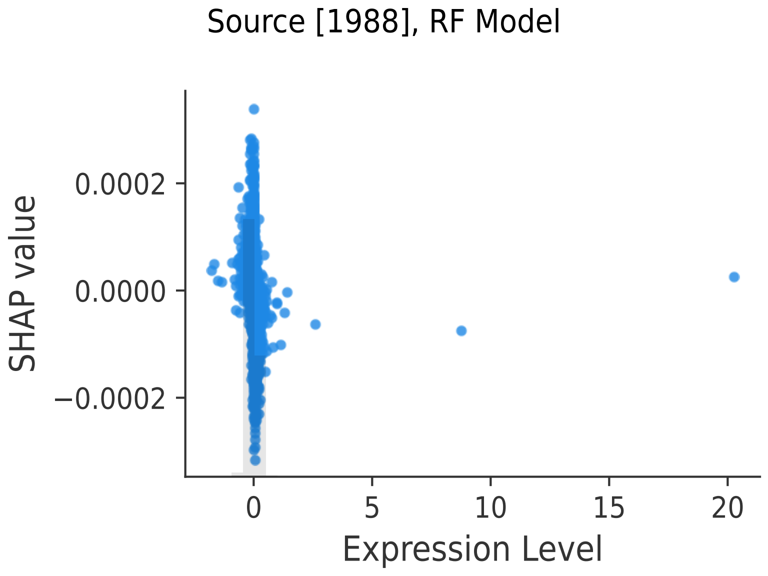

Source [1988], RF Model
SHAP value
0.0002
0.0000
−0.0002
0
5
10
15
20
Expression Level

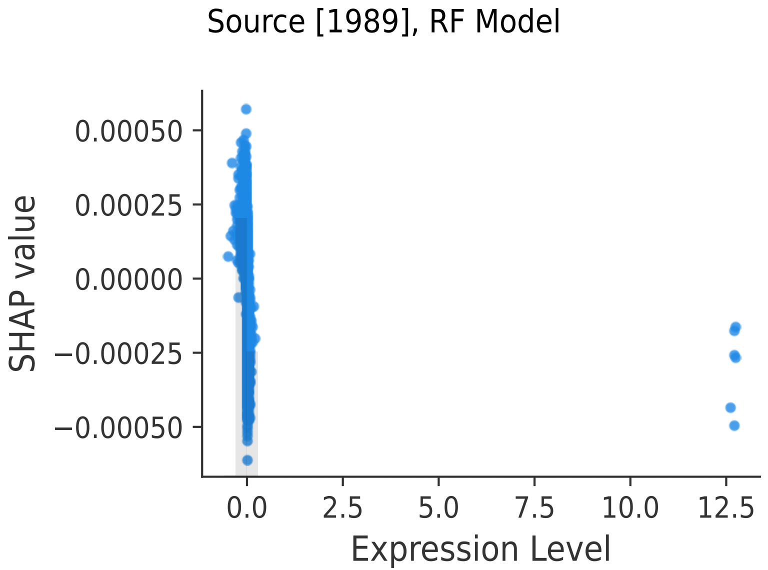
Source [1989], RF Model
SHAP value
0.00050
0.00025
0.00000
−0.00025
−0.00050
0.0
2.5
5.0
7.5
10.0
12.5
Expression Level

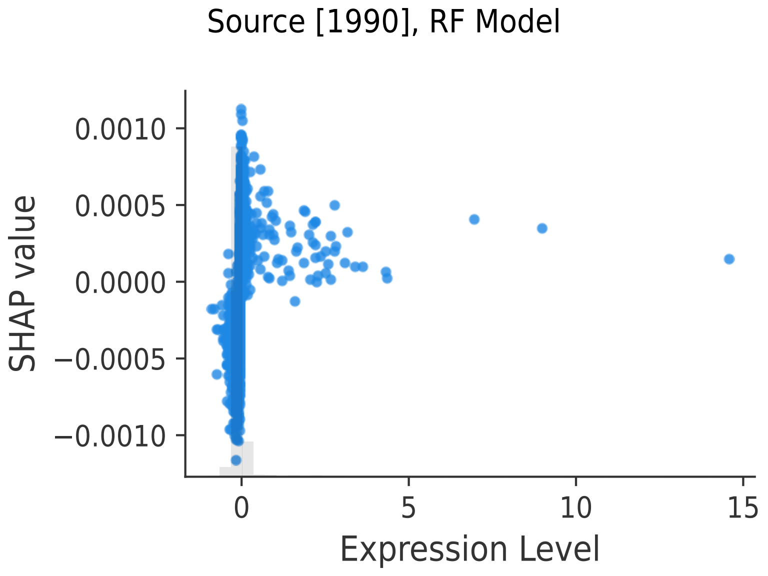

Source [1990], RF Model
SHAP value
0.0010
0.0005
0.0000
−0.0005
−0.0010
0
5
10
15
Expression Level

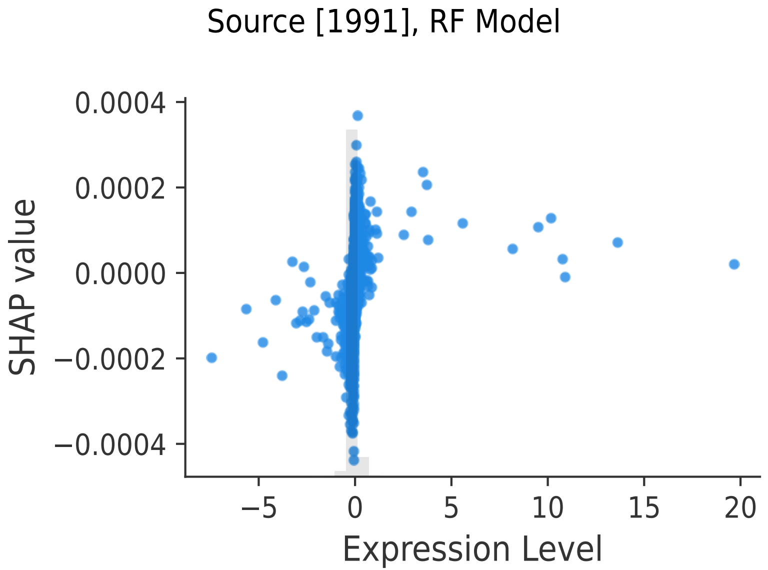
Source [1991], RF Model
0.0004
0.0002
0.0000
−0.0002
−0.0004
SHAP value
−5
0
5
10
15
20
Expression Level

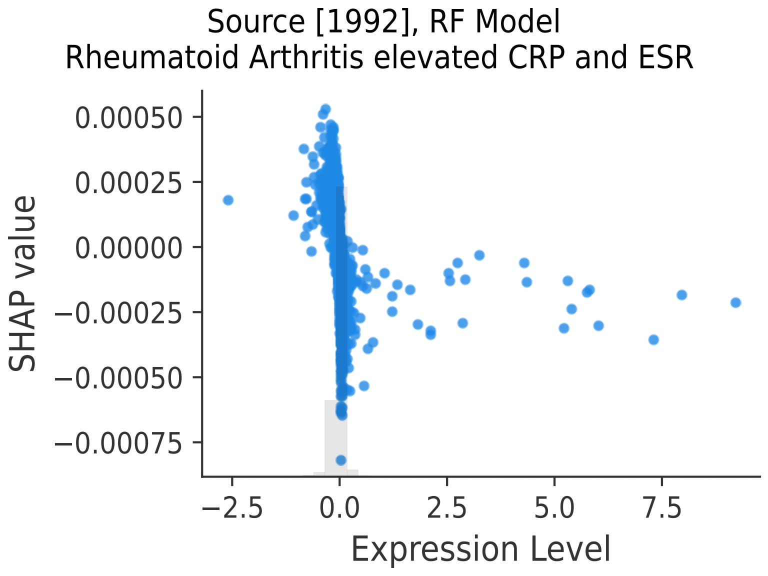
Source [1992], RF Model
Rheumatoid Arthritis elevated CRP and ESR
0.00050
0.00025
0.00000
−0.00025
−0.00050
−0.00075
SHAP value
−2.5
0.0
2.5
5.0
7.5
Expression Level

Source [1993], RF Model

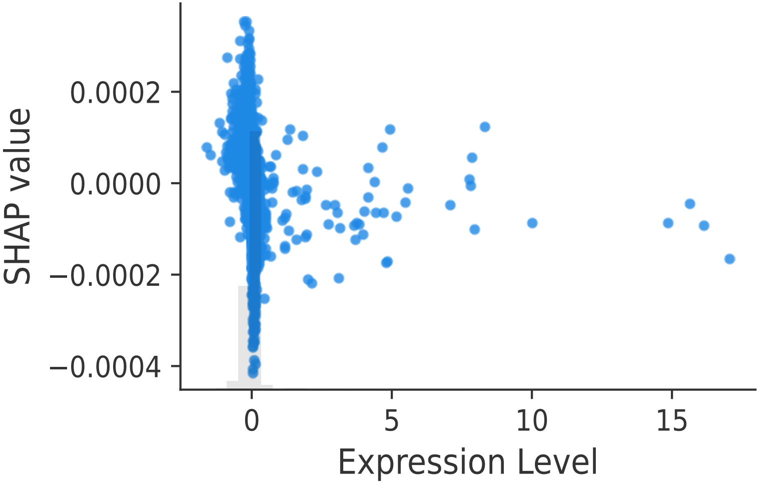

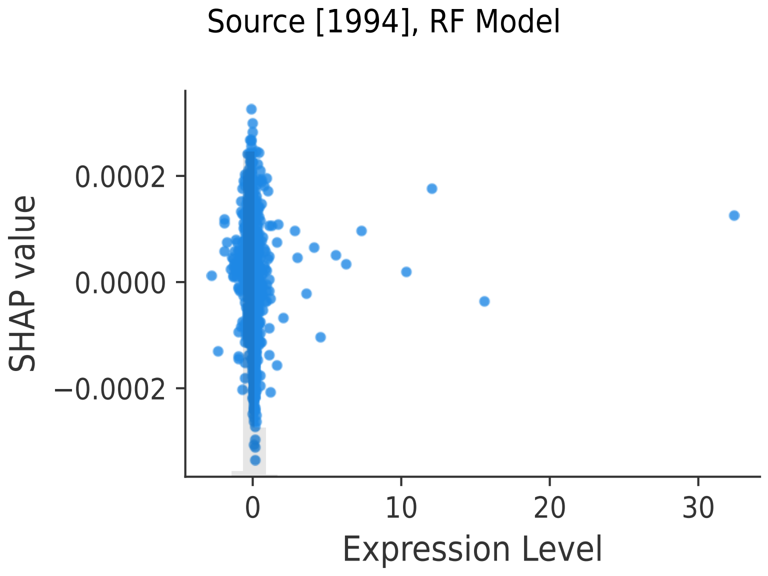
Source [1994], RF Model
SHAP value
0.0002
0.0000
−0.0002
0
10
20
30
Expression Level

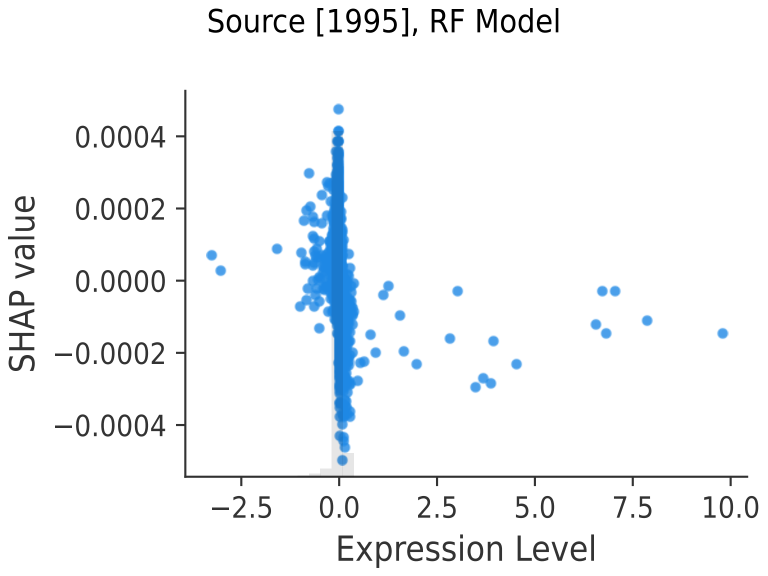
Source [1995], RF Model
0.0004
0.0002
0.0000
−0.0002
−0.0004
SHAP value
−2.5
0.0
2.5
5.0
7.5
10.0
Expression Level

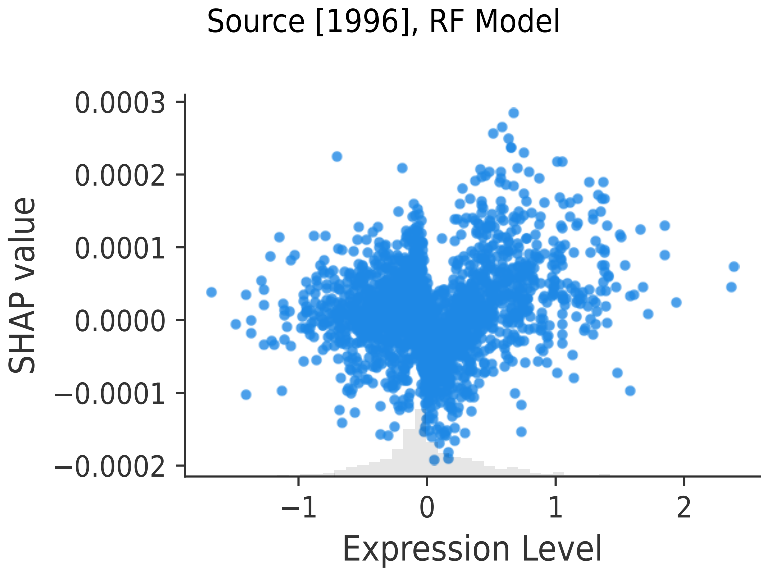
Source [1996], RF Model
0.0003
0.0002
0.0001
0.0000
−0.0001
−0.0002
SHAP value
−1
0
1
2
Expression Level

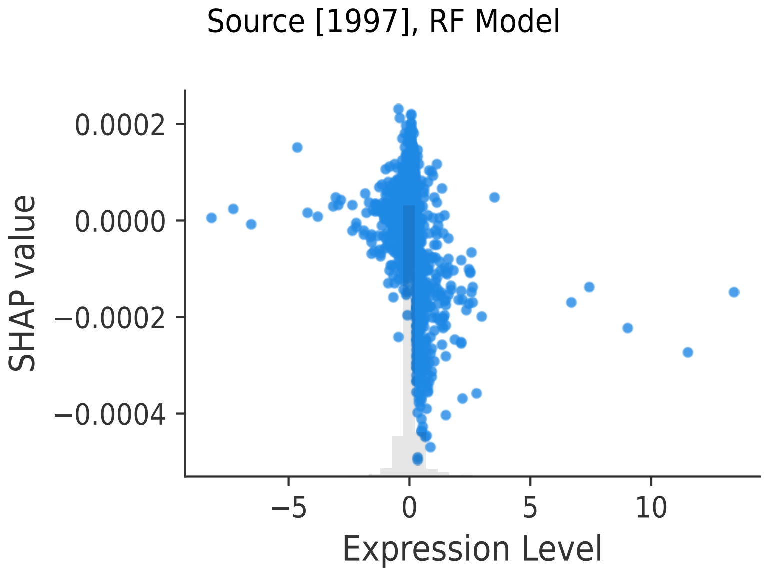
Source [1997], RF Model
0.0002
0.0000
−0.0002
−0.0004
SHAP value
−5
0
5
10
Expression Level

Source [1998], RF Model

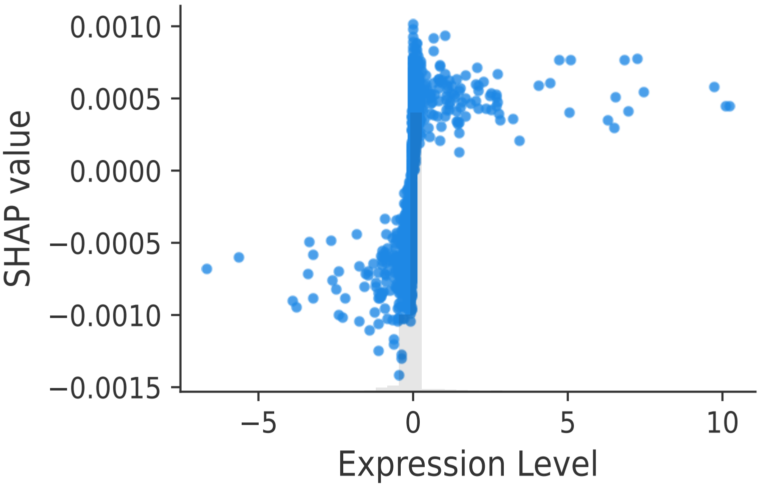

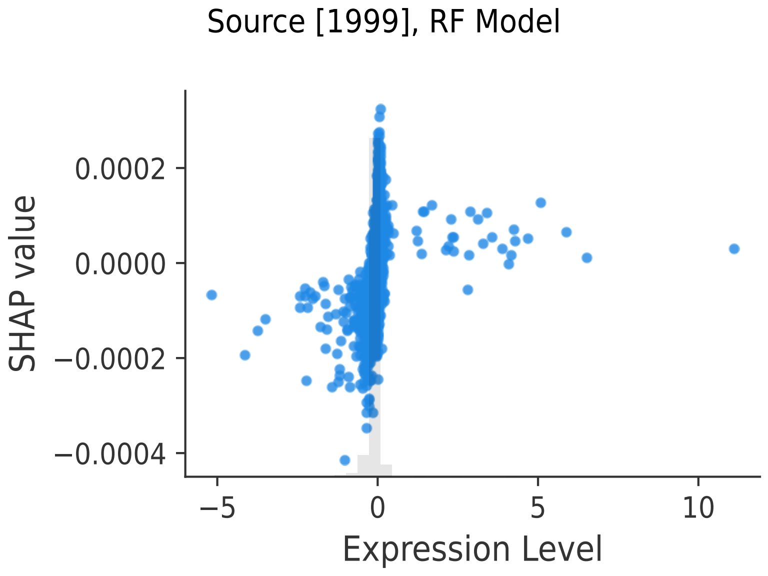
Source [1999], RF Model
SHAP value
0.0002
0.0000
−0.0002
−0.0004
−5
0
5
10
Expression Level
