# Supplementary material for: Unsupervised discovery of clinical disease signatures using probabilistic independence
Source: J Biomed Inform. Author manuscript; Available in PMC 2026 Jan 5. (PMC12767692; doi:10.1016/j.jbi.2025.104837)
Supplement: Supplement 3 [file NIHMS2115438-supplement-Supplement_3.pdf]

Signature [0]; 82 To Reach 0.975, (82 Plotted)

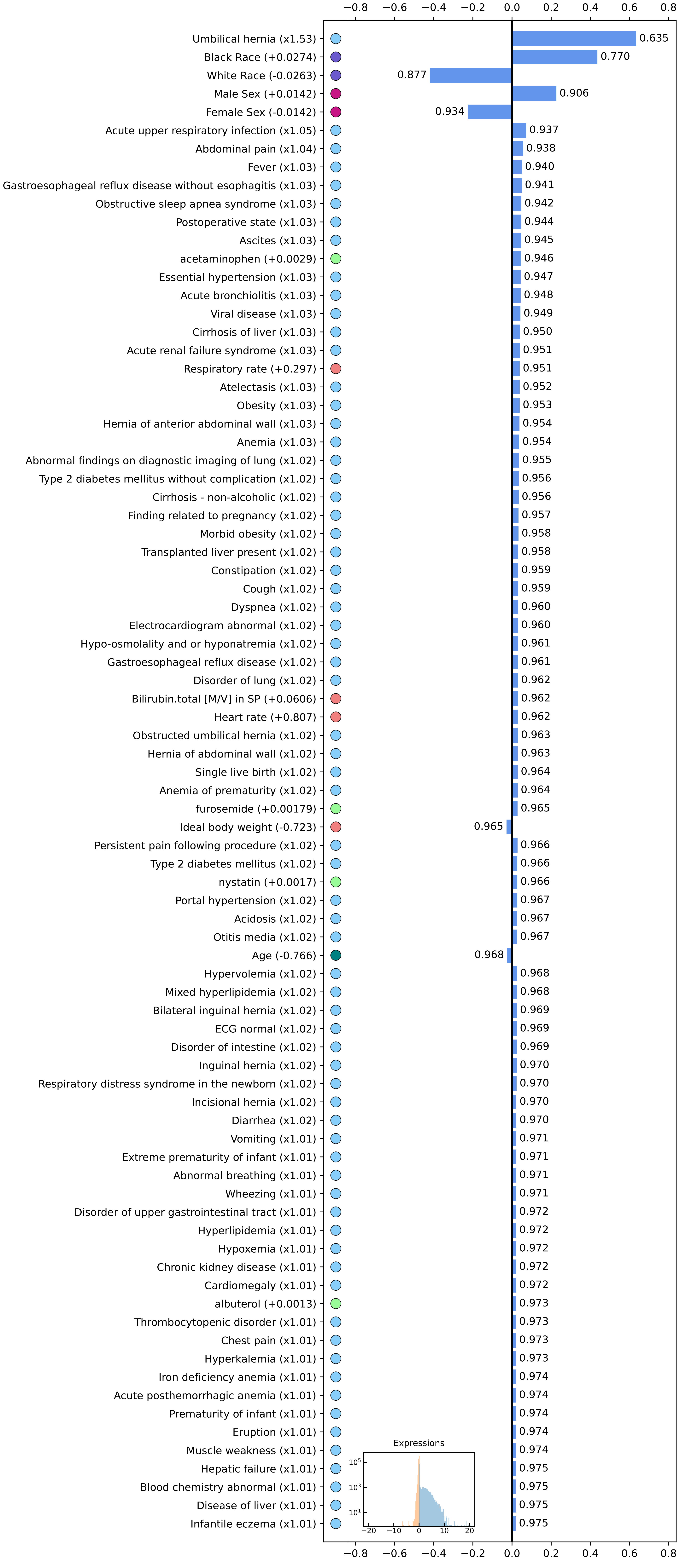

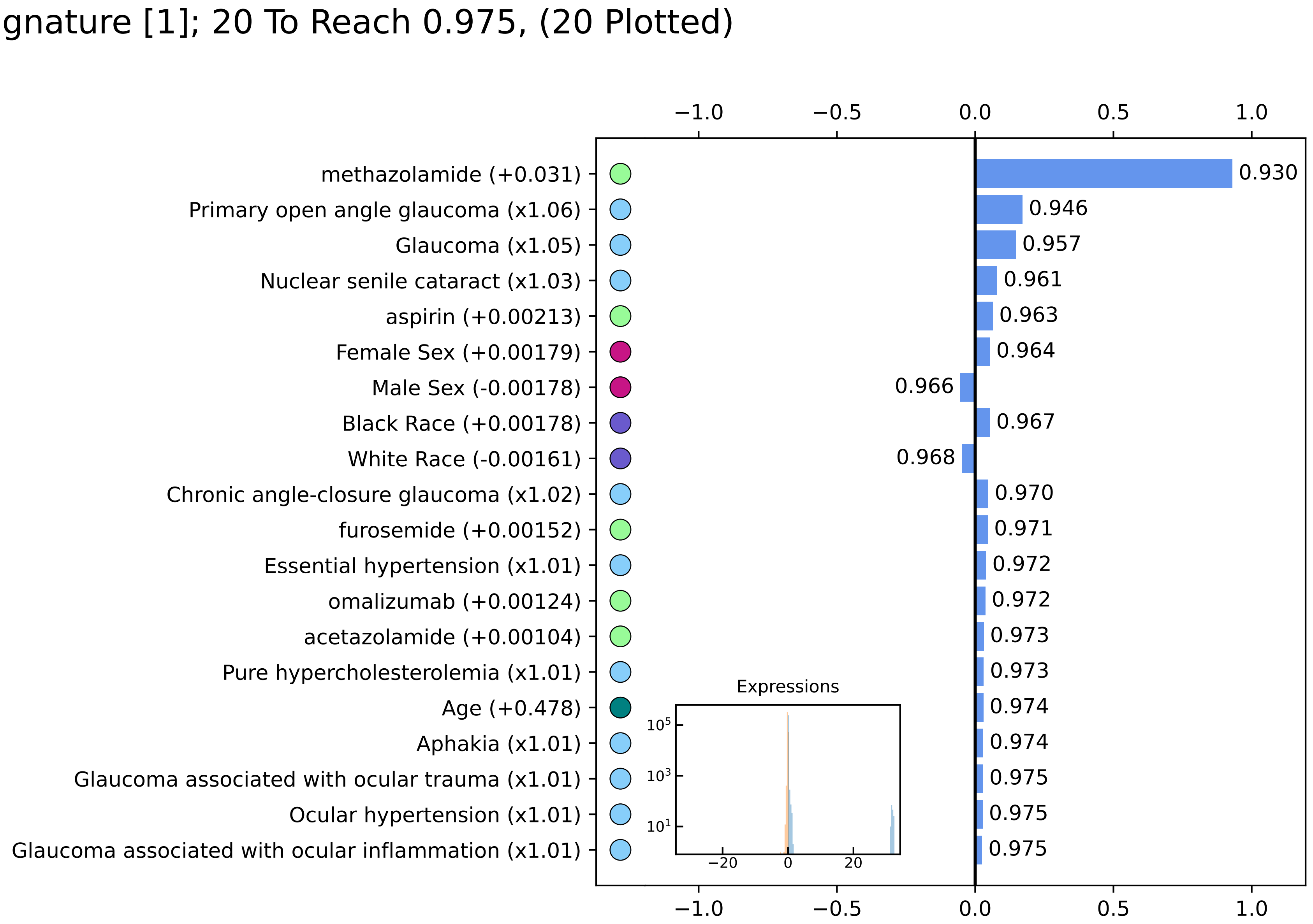
Signature [1]; 20 To Reach 0.975, (20 Plotted)
methazolamide (+0.031)
Primary open angle glaucoma (x1.06)
Glaucoma (x1.05)
Nuclear senile cataract (x1.03)
aspirin (+0.00213)
Female Sex (+0.00179)
Male Sex (-0.00178)
Black Race (+0.00178)
White Race (-0.00161)
Chronic angle-closure glaucoma (x1.02)
furosemide (+0.00152)
Essential hypertension (x1.01)
omalizumab (+0.00124)
acetazolamide (+0.00104)
Pure hypercholesterolemia (x1.01)
Age (+0.478)
Aphakia (x1.01)
Glaucoma associated with ocular trauma (x1.01)
Ocular hypertension (x1.01)
Glaucoma associated with ocular inflammation (x1.01)
0.930
0.946
0.957
0.961
0.963
0.964
0.966
0.967
0.968
0.970
0.971
0.972
0.972
0.973
0.973
0.974
0.974
0.975
0.975
0.975
Expressions
−1.0
−0.5
0.0
0.5
1.0

Signature [2]; 2 To Reach 0.975, (10 Plotted)

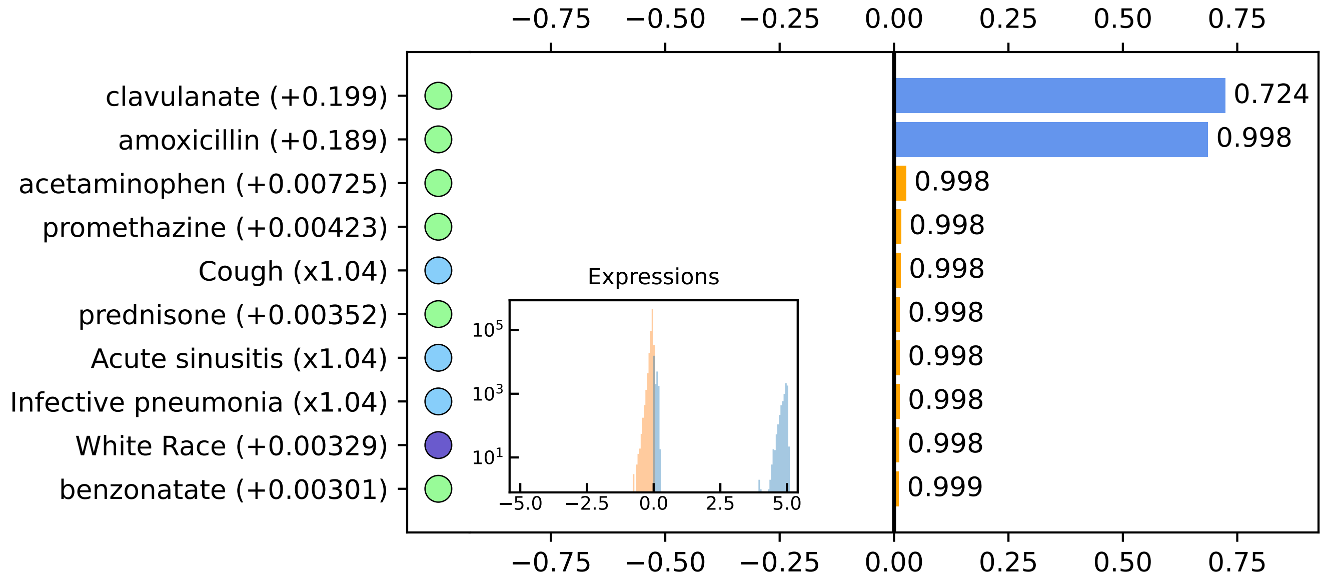

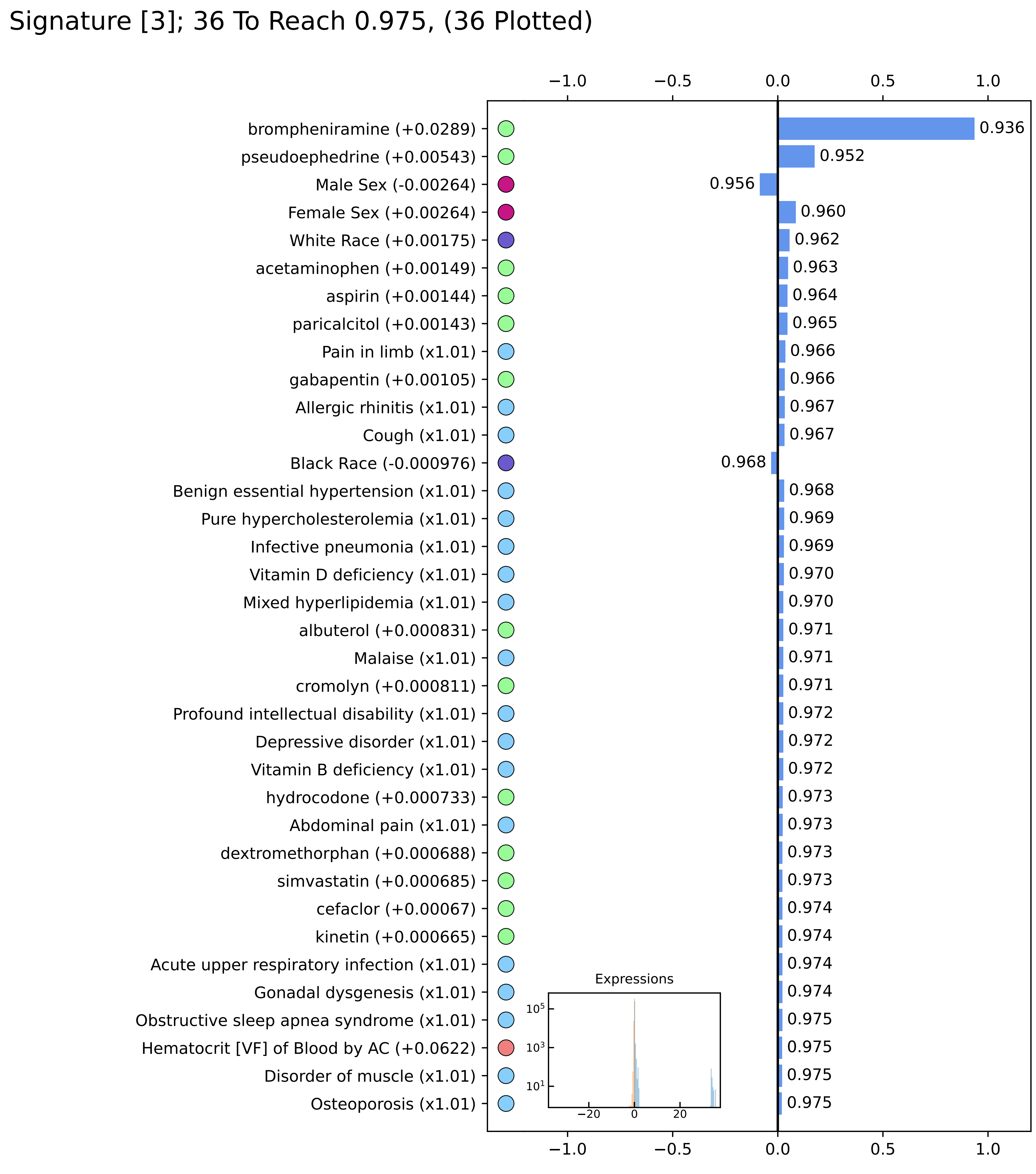

Signature [3]; 36 To Reach 0.975, (36 Plotted)
−1.0
−0.5
0.0
0.5
1.0
brompheniramine (+0.0289)
pseudoephedrine (+0.00543)
Male Sex (-0.00264)
Female Sex (+0.00264)
White Race (+0.00175)
acetaminophen (+0.00149)
aspirin (+0.00144)
paricalcitol (+0.00143)
Pain in limb (x1.01)
gabapentin (+0.00105)
Allergic rhinitis (x1.01)
Cough (x1.01)
Black Race (-0.000976)
Benign essential hypertension (x1.01)
Pure hypercholesterolemia (x1.01)
Infective pneumonia (x1.01)
Vitamin D deficiency (x1.01)
Mixed hyperlipidemia (x1.01)
albuterol (+0.000831)
Malaise (x1.01)
cromolyn (+0.000811)
Profound intellectual disability (x1.01)
Depressive disorder (x1.01)
Vitamin B deficiency (x1.01)
hydrocodone (+0.000733)
Abdominal pain (x1.01)
dextromethorphan (+0.000688)
simvastatin (+0.000685)
cefaclor (+0.00067)
kinetin (+0.000665)
Acute upper respiratory infection (x1.01)
Gonadal dysgenesis (x1.01)
Obstructive sleep apnea syndrome (x1.01)
Hematocrit [VF] of Blood by AC (+0.0622)
Disorder of muscle (x1.01)
Osteoporosis (x1.01)
0.936
0.952
0.956
0.960
0.962
0.963
0.964
0.965
0.966
0.966
0.967
0.967
0.968
0.968
0.969
0.969
0.970
0.970
0.971
0.971
0.971
0.972
0.972
0.972
0.973
0.973
0.973
0.973
0.974
0.974
0.974
0.974
0.975
0.975
0.975
0.975
Expressions
10^5
10^3
10^1
−20
0
20

Signature [4]; 1896 To Reach 0.975, (100 Plotted)

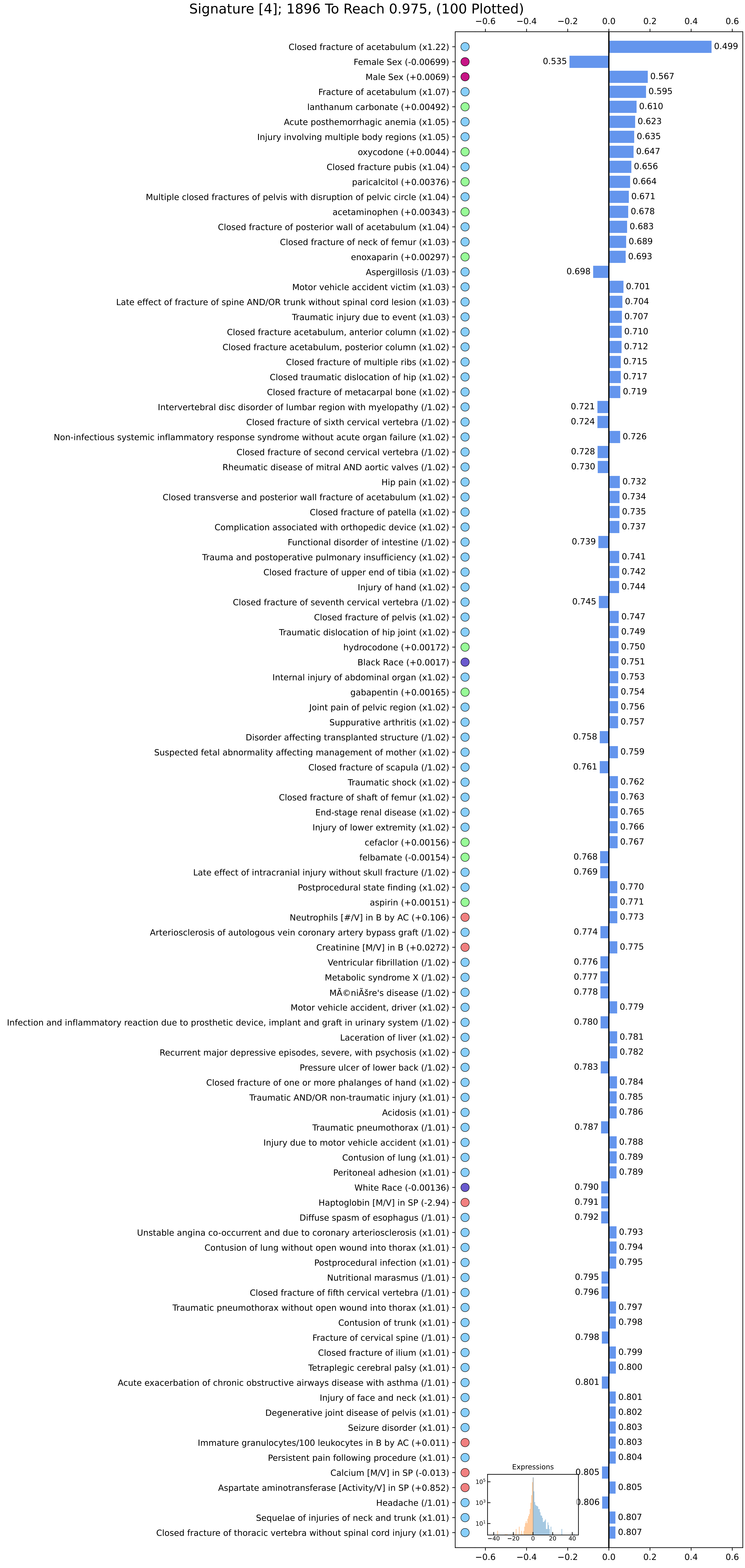

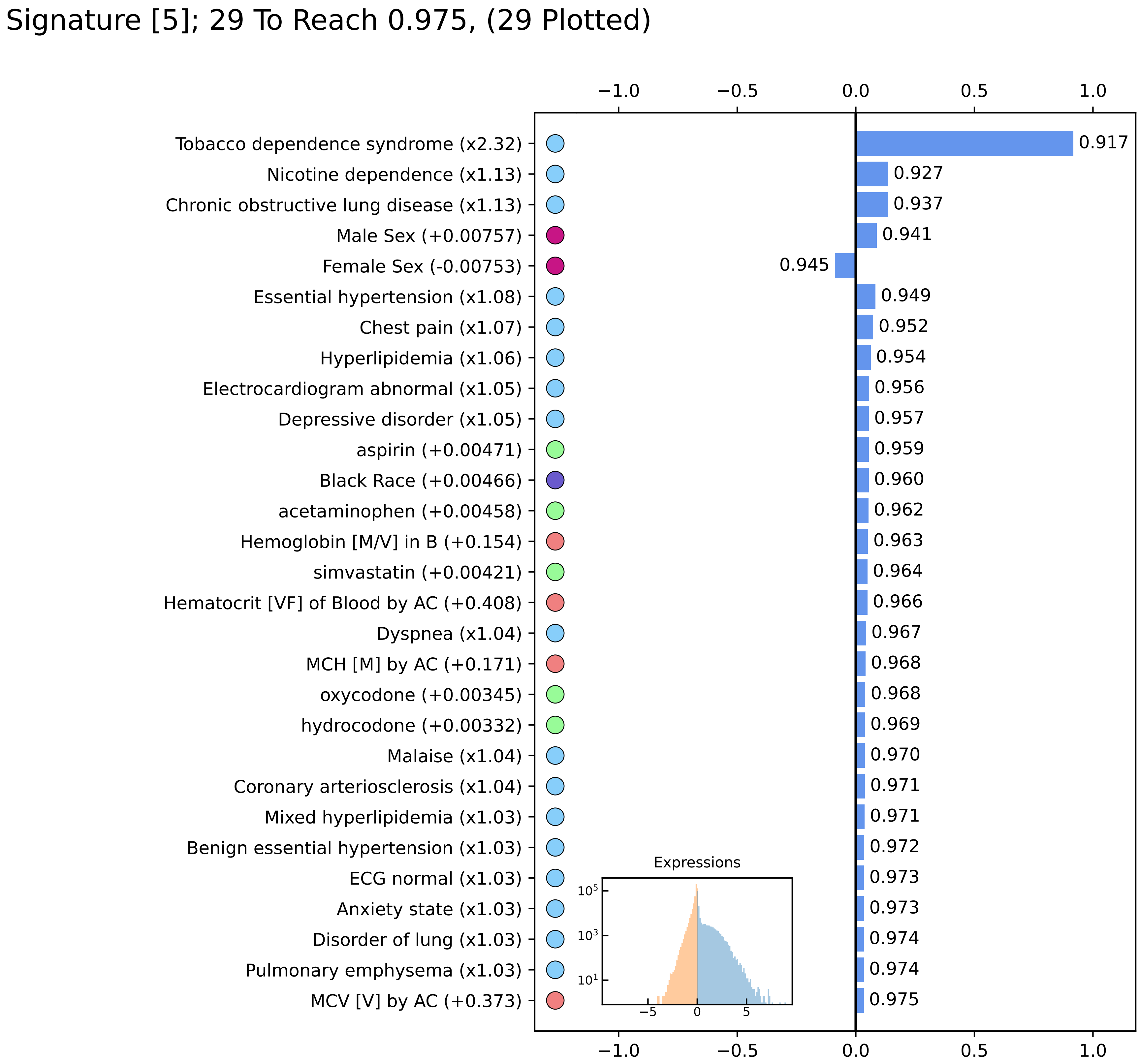
Signature [5]; 29 To Reach 0.975, (29 Plotted)
Tobacco dependence syndrome (x2.32) 0.917
Nicotine dependence (x1.13) 0.927
Chronic obstructive lung disease (x1.13) 0.937
Male Sex (+0.00757) 0.941
Female Sex (-0.00753) 0.945
Essential hypertension (x1.08) 0.949
Chest pain (x1.07) 0.952
Hyperlipidemia (x1.06) 0.954
Electrocardiogram abnormal (x1.05) 0.956
Depressive disorder (x1.05) 0.957
aspirin (+0.00471) 0.959
Black Race (+0.00466) 0.960
acetaminophen (+0.00458) 0.962
Hemoglobin [M/V] in B (+0.154) 0.963
simvastatin (+0.00421) 0.964
Hematocrit [VF] of Blood by AC (+0.408) 0.966
Dyspnea (x1.04) 0.967
MCH [M] by AC (+0.171) 0.968
oxycodone (+0.00345) 0.968
hydrocodone (+0.00332) 0.969
Malaise (x1.04) 0.970
Coronary arteriosclerosis (x1.04) 0.971
Mixed hyperlipidemia (x1.03) 0.971
Benign essential hypertension (x1.03) 0.972
ECG normal (x1.03) 0.973
Anxiety state (x1.03) 0.973
Disorder of lung (x1.03) 0.974
Pulmonary emphysema (x1.03) 0.974
MCV [V] by AC (+0.373) 0.975
−1.0 −0.5 0.0 0.5 1.0
Expressions
10^5
10^3
10^1
−5 0 5

Signature [6]; 12 To Reach 0.975, (12 Plotted)

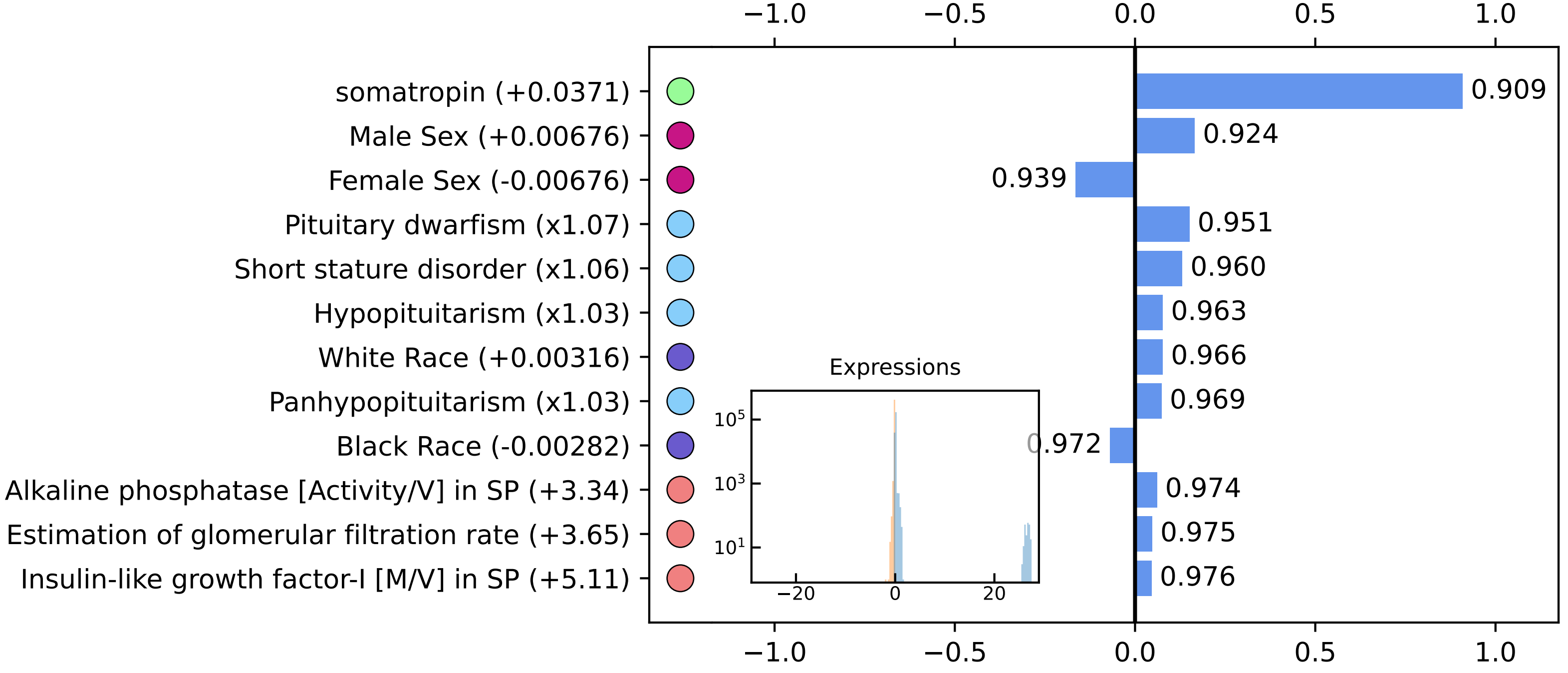

Signature [7]; 8 To Reach 0.975, (10 Plotted)

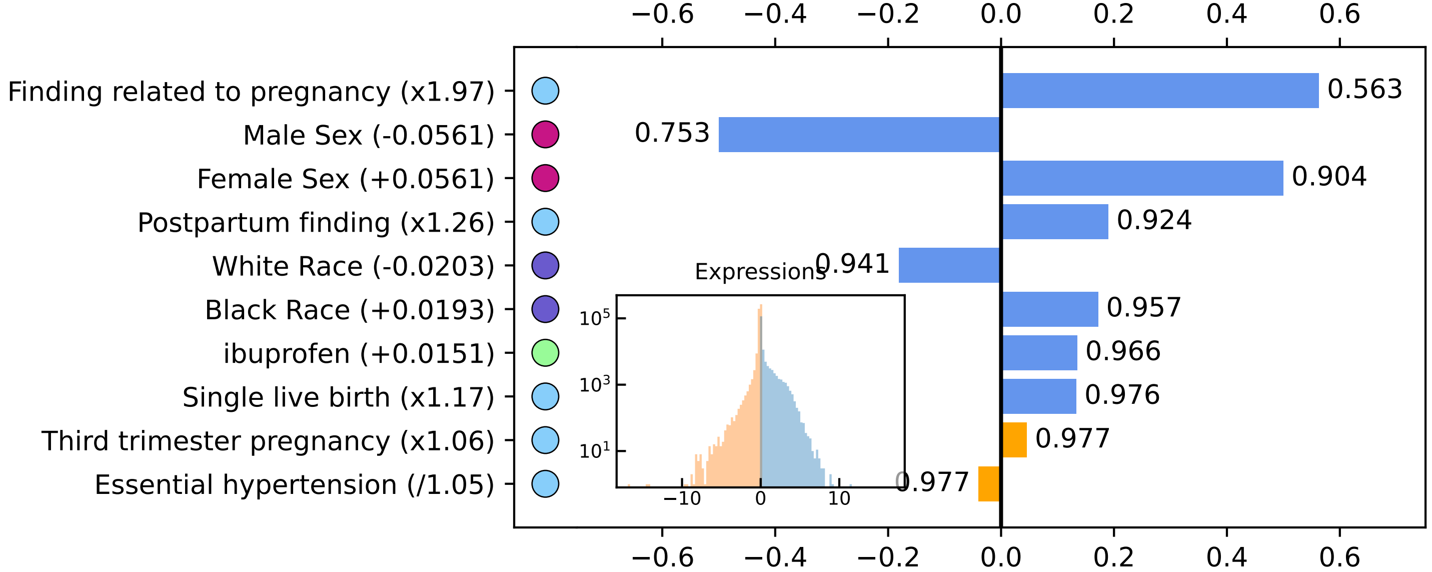

Signature [8]; 7 To Reach 0.975, (10 Plotted)

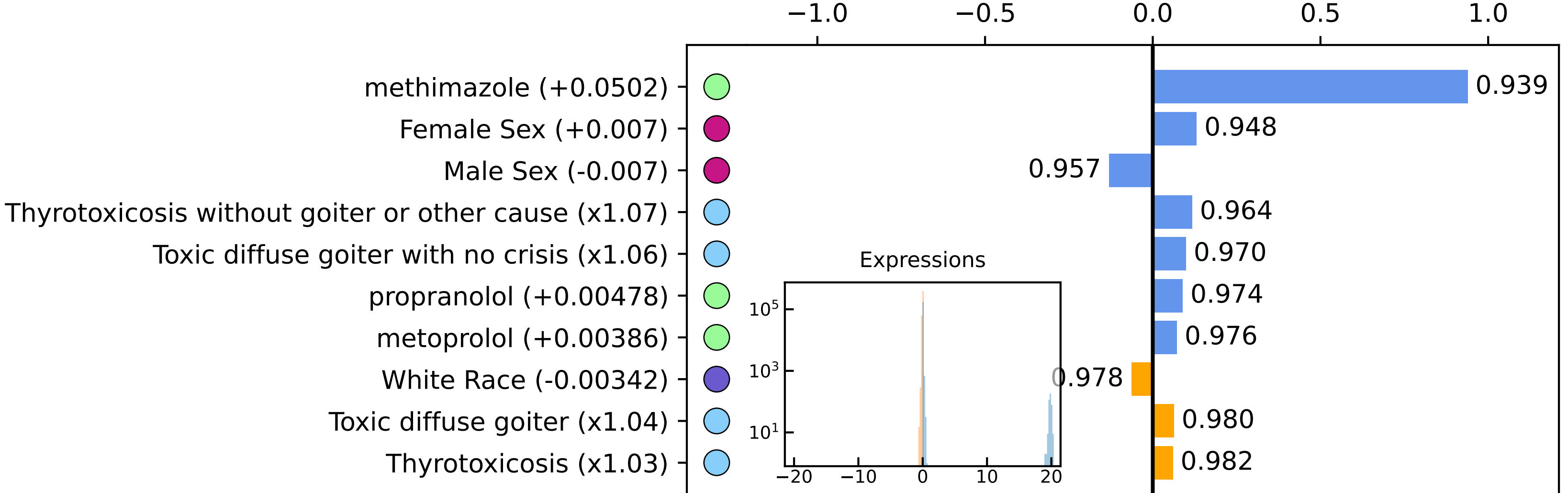

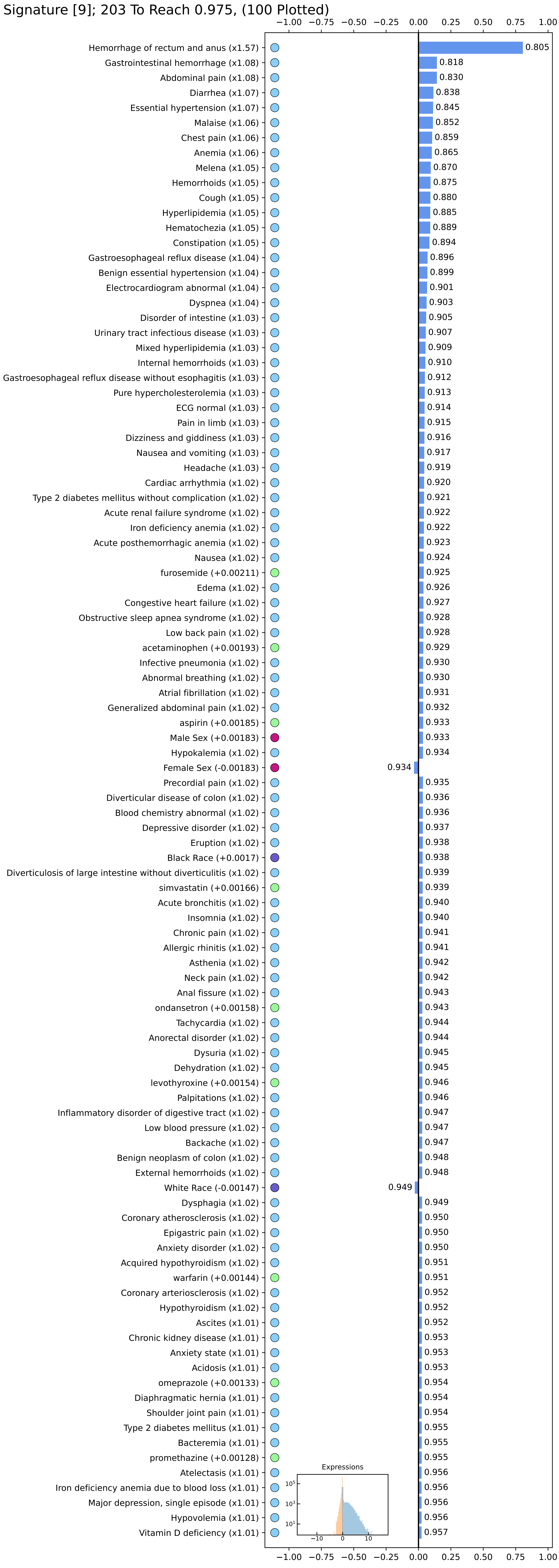

Signature [9]; 203 To Reach 0.975, (100 Plotted)
−1.00 −0.75 −0.50 −0.25 0.00 0.25 0.50 0.75 1.00
Hemorrhage of rectum and anus (x1.57) 0.805
Gastrointestinal hemorrhage (x1.08) 0.818
Abdominal pain (x1.08) 0.830
Diarrhea (x1.07) 0.838
Essential hypertension (x1.07) 0.845
Malaise (x1.06) 0.852
Chest pain (x1.06) 0.859
Anemia (x1.06) 0.865
Melena (x1.05) 0.870
Hemorrhoids (x1.05) 0.875
Cough (x1.05) 0.880
Hyperlipidemia (x1.05) 0.885
Hematochezia (x1.05) 0.889
Constipation (x1.05) 0.894
Gastroesophageal reflux disease (x1.04) 0.896
Benign essential hypertension (x1.04) 0.899
Electrocardiogram abnormal (x1.04) 0.901
Dyspnea (x1.04) 0.903
Disorder of intestine (x1.03) 0.905
Urinary tract infectious disease (x1.03) 0.907
Mixed hyperlipidemia (x1.03) 0.909
Internal hemorrhoids (x1.03) 0.910
Gastroesophageal reflux disease without esophagitis (x1.03) 0.912
Pure hypercholesterolemia (x1.03) 0.913
ECG normal (x1.03) 0.914
Pain in limb (x1.03) 0.915
Dizziness and giddiness (x1.03) 0.916
Nausea and vomiting (x1.03) 0.917
Headache (x1.03) 0.919
Cardiac arrhythmia (x1.02) 0.920
Type 2 diabetes mellitus without complication (x1.02) 0.921
Acute renal failure syndrome (x1.02) 0.922
Iron deficiency anemia (x1.02) 0.922
Acute posthemorrhagic anemia (x1.02) 0.923
Nausea (x1.02) 0.924
furosemide (+0.00211) 0.925
Edema (x1.02) 0.926
Congestive heart failure (x1.02) 0.927
Obstructive sleep apnea syndrome (x1.02) 0.928
Low back pain (x1.02) 0.928
acetaminophen (+0.00193) 0.929
Infective pneumonia (x1.02) 0.930
Abnormal breathing (x1.02) 0.930
Atrial fibrillation (x1.02) 0.931
Generalized abdominal pain (x1.02) 0.932
aspirin (+0.00185) 0.933
Male Sex (+0.00183) 0.933
Hypokalemia (x1.02) 0.934
Female Sex (-0.00183) 0.934
Precordial pain (x1.02) 0.935
Diverticular disease of colon (x1.02) 0.936
Blood chemistry abnormal (x1.02) 0.936
Depressive disorder (x1.02) 0.937
Eruption (x1.02) 0.938
Black Race (+0.0017) 0.938
Diverticulosis of large intestine without diverticulitis (x1.02) 0.939
simvastatin (+0.00166) 0.939
Acute bronchitis (x1.02) 0.940
Insomnia (x1.02) 0.940
Chronic pain (x1.02) 0.941
Allergic rhinitis (x1.02) 0.941
Asthenia (x1.02) 0.942
Neck pain (x1.02) 0.942
Anal fissure (x1.02) 0.943
ondansetron (+0.00158) 0.943
Tachycardia (x1.02) 0.944
Anorectal disorder (x1.02) 0.944
Dysuria (x1.02) 0.945
Dehydration (x1.02) 0.945
levothyroxine (+0.00154) 0.946
Palpitations (x1.02) 0.946
Inflammatory disorder of digestive tract (x1.02) 0.947
Low blood pressure (x1.02) 0.947
Backache (x1.02) 0.947
Benign neoplasm of colon (x1.02) 0.948
External hemorrhoids (x1.02) 0.948
White Race (-0.00147) 0.949
Dysphagia (x1.02) 0.949
Coronary atherosclerosis (x1.02) 0.950
Epigastric pain (x1.02) 0.950
Anxiety disorder (x1.02) 0.950
Acquired hypothyroidism (x1.02) 0.951
warfarin (+0.00144) 0.951
Coronary arteriosclerosis (x1.02) 0.952
Hypothyroidism (x1.02) 0.952
Ascites (x1.01) 0.952
Chronic kidney disease (x1.01) 0.953
Anxiety state (x1.01) 0.953
Acidosis (x1.01) 0.953
omeprazole (+0.00133) 0.954
Diaphragmatic hernia (x1.01) 0.954
Shoulder joint pain (x1.01) 0.954
Type 2 diabetes mellitus (x1.01) 0.955
Bacteremia (x1.01) 0.955
promethazine (+0.00128) 0.955
Atelectasis (x1.01) 0.956
Iron deficiency anemia due to blood loss (x1.01) 0.956
Major depression, single episode (x1.01) 0.956
Hypovolemia (x1.01) 0.956
Vitamin D deficiency (x1.01) 0.957
Expressions
−10 0 10

Signature [10]; 419 To Reach 0.975, (100 Plotted)

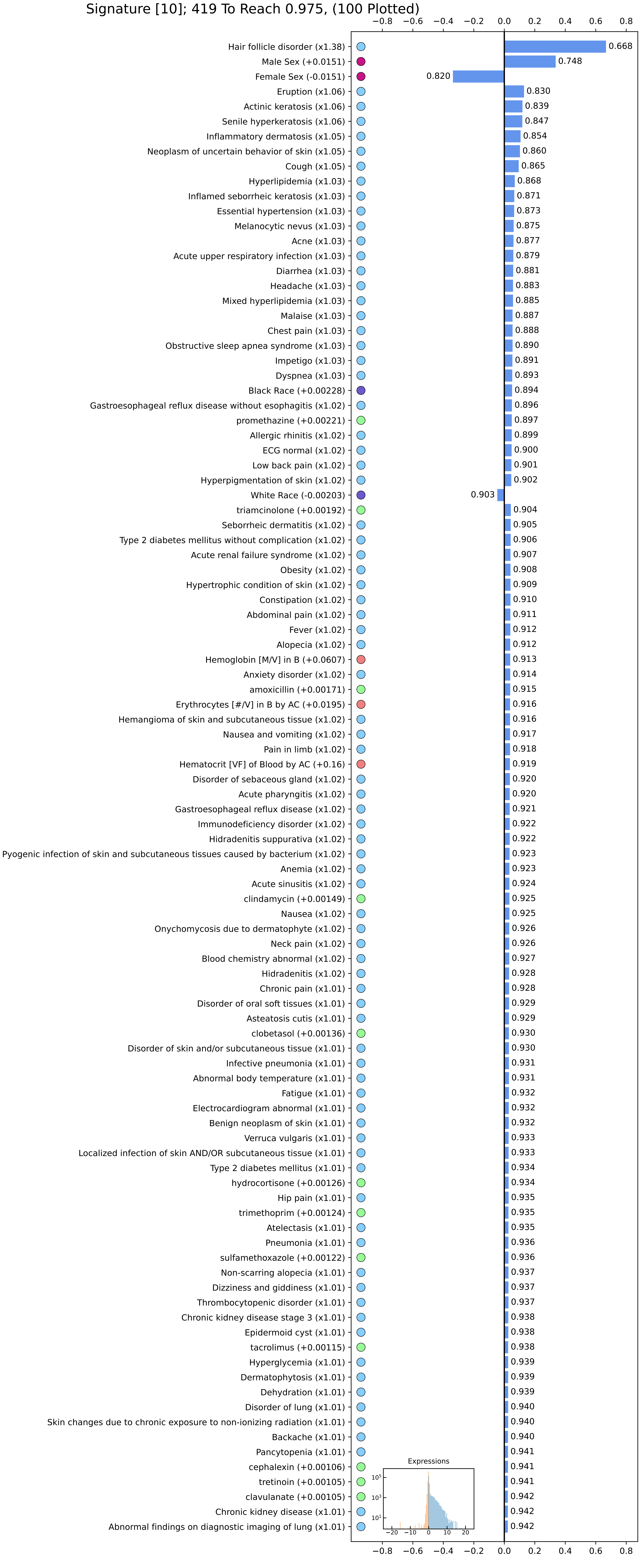

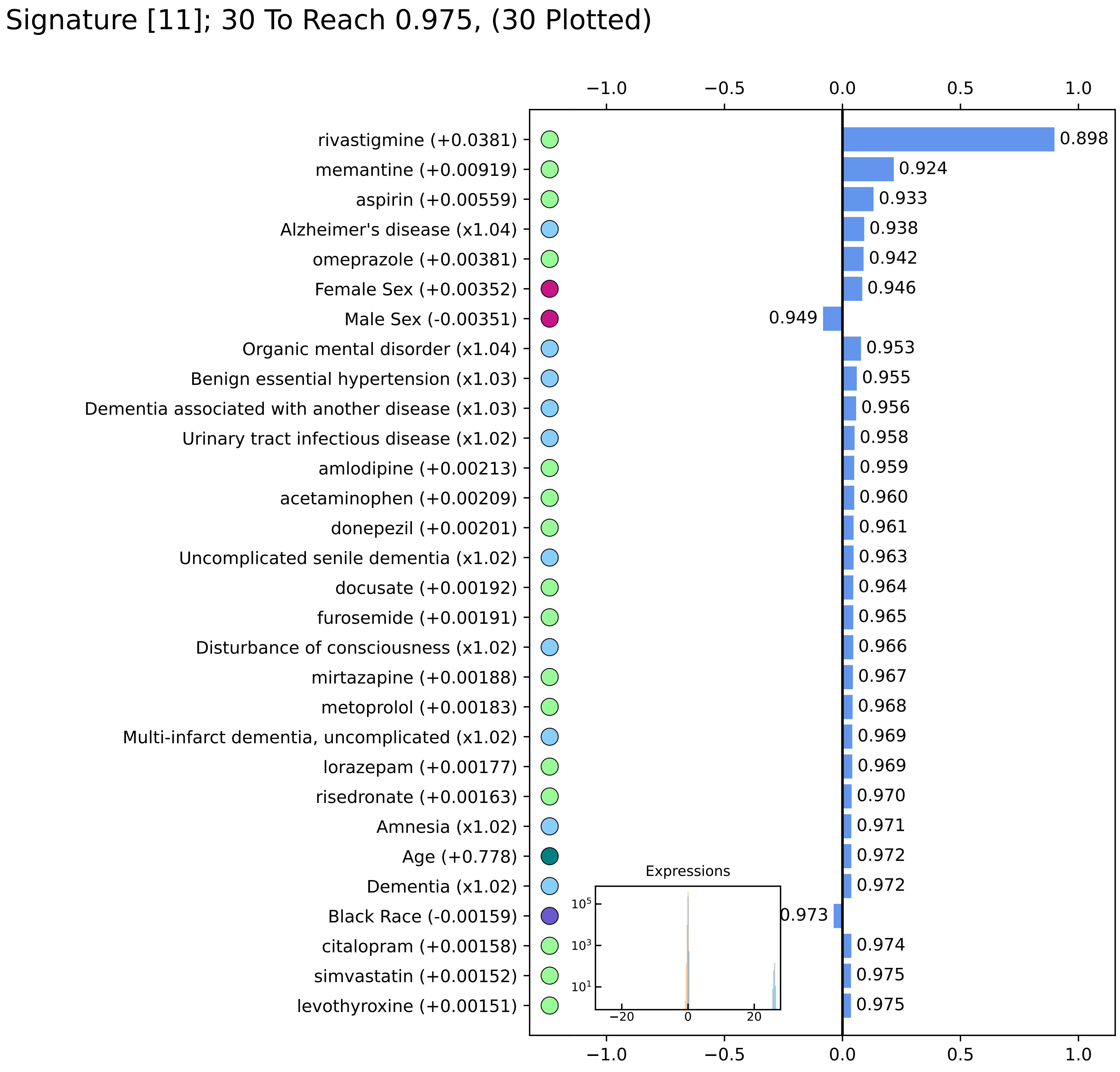

Signature [11]; 30 To Reach 0.975, (30 Plotted)
rivastigmine (+0.0381)
memantine (+0.00919)
aspirin (+0.00559)
Alzheimer's disease (x1.04)
omeprazole (+0.00381)
Female Sex (+0.00352)
Male Sex (-0.00351)
Organic mental disorder (x1.04)
Benign essential hypertension (x1.03)
Dementia associated with another disease (x1.03)
Urinary tract infectious disease (x1.02)
amlodipine (+0.00213)
acetaminophen (+0.00209)
donepezil (+0.00201)
Uncomplicated senile dementia (x1.02)
docusate (+0.00192)
furosemide (+0.00191)
Disturbance of consciousness (x1.02)
mirtazapine (+0.00188)
metoprolol (+0.00183)
Multi-infarct dementia, uncomplicated (x1.02)
lorazepam (+0.00177)
risedronate (+0.00163)
Amnesia (x1.02)
Age (+0.778)
Dementia (x1.02)
Black Race (-0.00159)
citalopram (+0.00158)
simvastatin (+0.00152)
levothyroxine (+0.00151)
0.898
0.924
0.933
0.938
0.942
0.946
0.949
0.953
0.955
0.956
0.958
0.959
0.960
0.961
0.963
0.964
0.965
0.966
0.967
0.968
0.969
0.969
0.970
0.971
0.972
0.972
0.973
0.974
0.975
0.975
−1.0
−0.5
0.0
0.5
1.0
Expressions
10^5
10^3
10^1
−20
0
20

Signature [12]; 4 To Reach 0.975, (10 Plotted)

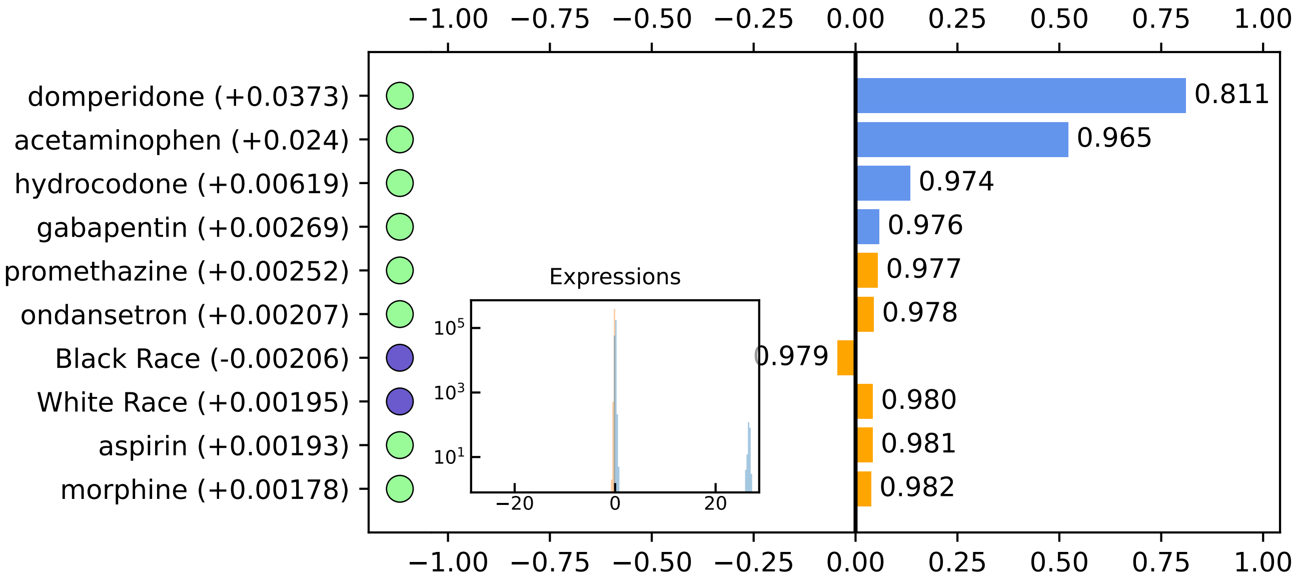

Signature [13]; 151 To Reach 0.975, (100 Plotted)

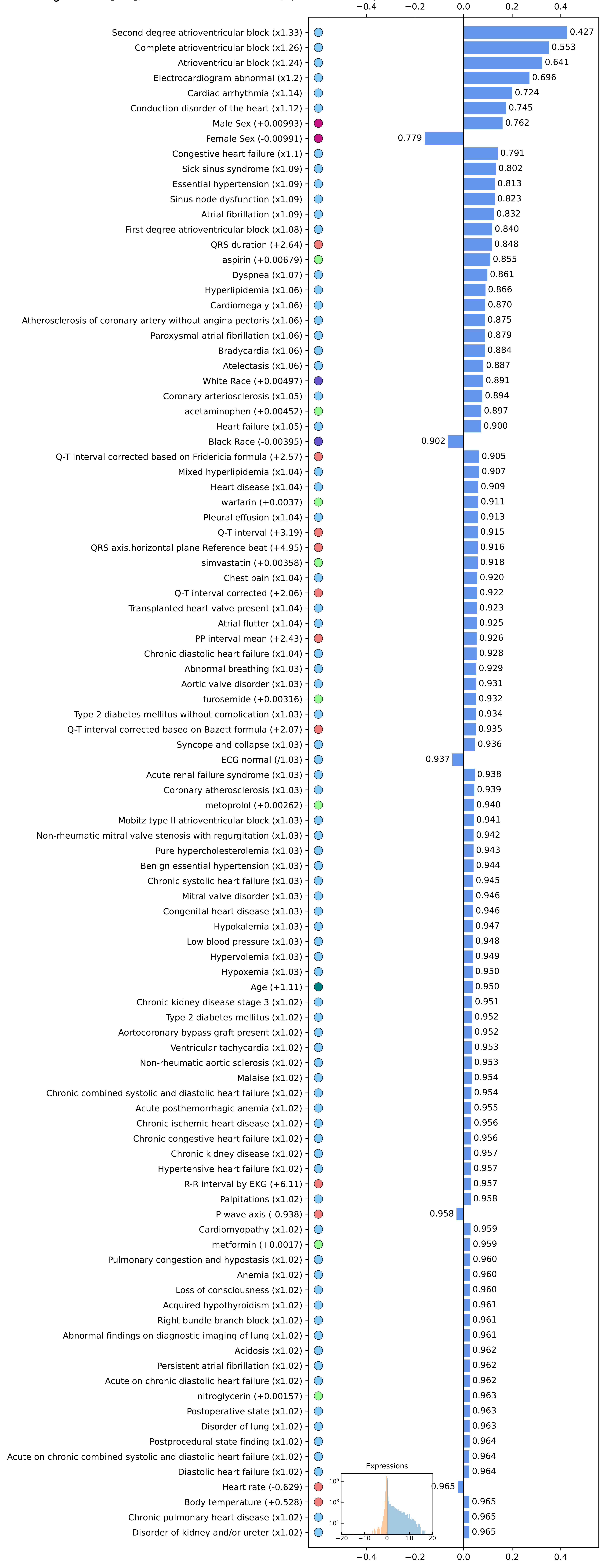

Signature [14]; 3 To Reach 0.975, (10 Plotted)

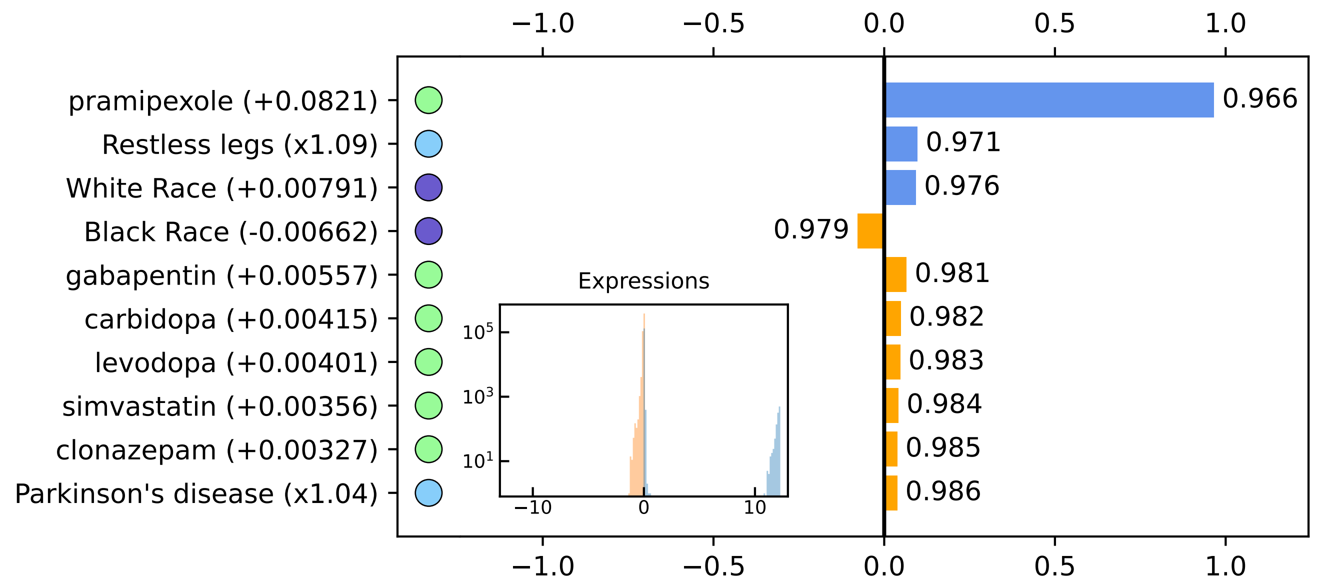

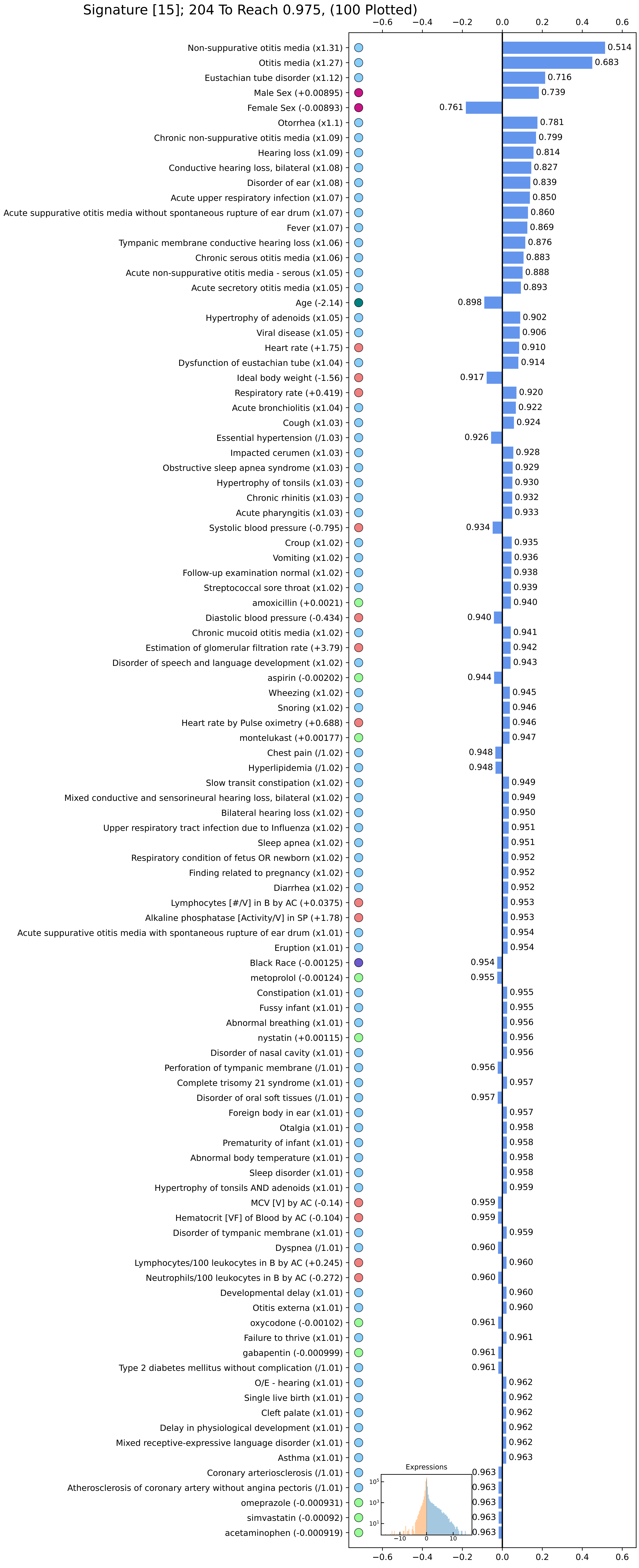
Signature [15]; 204 To Reach 0.975, (100 Plotted)
−0.6 −0.4 −0.2 0.0 0.2 0.4 0.6
Non-suppurative otitis media (x1.31) 0.514
Otitis media (x1.27) 0.683
Eustachian tube disorder (x1.12) 0.716
Male Sex (+0.00895) 0.739
Female Sex (-0.00893) 0.761
Otorrhea (x1.1) 0.781
Chronic non-suppurative otitis media (x1.09) 0.799
Hearing loss (x1.09) 0.814
Conductive hearing loss, bilateral (x1.08) 0.827
Disorder of ear (x1.08) 0.839
Acute upper respiratory infection (x1.07) 0.850
Acute suppurative otitis media without spontaneous rupture of ear drum (x1.07) 0.860
Fever (x1.07) 0.869
Tympanic membrane conductive hearing loss (x1.06) 0.876
Chronic serous otitis media (x1.06) 0.883
Acute non-suppurative otitis media - serous (x1.05) 0.888
Acute secretory otitis media (x1.05) 0.893
Age (-2.14) 0.898
Hypertrophy of adenoids (x1.05) 0.902
Viral disease (x1.05) 0.906
Heart rate (+1.75) 0.910
Dysfunction of eustachian tube (x1.04) 0.914
Ideal body weight (-1.56) 0.917
Respiratory rate (+0.419) 0.920
Acute bronchiolitis (x1.04) 0.922
Cough (x1.03) 0.924
Essential hypertension (/1.03) 0.926
Impacted cerumen (x1.03) 0.928
Obstructive sleep apnea syndrome (x1.03) 0.929
Hypertrophy of tonsils (x1.03) 0.930
Chronic rhinitis (x1.03) 0.932
Acute pharyngitis (x1.03) 0.933
Systolic blood pressure (-0.795) 0.934
Croup (x1.02) 0.935
Vomiting (x1.02) 0.936
Follow-up examination normal (x1.02) 0.938
Streptococcal sore throat (x1.02) 0.939
amoxicillin (+0.0021) 0.940
Diastolic blood pressure (-0.434) 0.940
Chronic mucoid otitis media (x1.02) 0.941
Estimation of glomerular filtration rate (+3.79) 0.942
Disorder of speech and language development (x1.02) 0.943
aspirin (-0.00202) 0.944
Wheezing (x1.02) 0.945
Snoring (x1.02) 0.946
Heart rate by Pulse oximetry (+0.688) 0.946
montelukast (+0.00177) 0.947
Chest pain (/1.02) 0.948
Hyperlipidemia (/1.02) 0.948
Slow transit constipation (x1.02) 0.949
Mixed conductive and sensorineural hearing loss, bilateral (x1.02) 0.949
Bilateral hearing loss (x1.02) 0.950
Upper respiratory tract infection due to Influenza (x1.02) 0.951
Sleep apnea (x1.02) 0.951
Respiratory condition of fetus OR newborn (x1.02) 0.952
Finding related to pregnancy (x1.02) 0.952
Diarrhea (x1.02) 0.952
Lymphocytes [#/V] in B by AC (+0.0375) 0.953
Alkaline phosphatase [Activity/V] in SP (+1.78) 0.953
Acute suppurative otitis media with spontaneous rupture of ear drum (x1.01) 0.954
Eruption (x1.01) 0.954
Black Race (-0.00125) 0.954
metoprolol (-0.00124) 0.955
Constipation (x1.01) 0.955
Fussy infant (x1.01) 0.955
Abnormal breathing (x1.01) 0.956
nystatin (+0.00115) 0.956
Disorder of nasal cavity (x1.01) 0.956
Perforation of tympanic membrane (/1.01) 0.956
Complete trisomy 21 syndrome (x1.01) 0.957
Disorder of oral soft tissues (/1.01) 0.957
Foreign body in ear (x1.01) 0.957
Otalgia (x1.01) 0.958
Prematurity of infant (x1.01) 0.958
Abnormal body temperature (x1.01) 0.958
Sleep disorder (x1.01) 0.958
Hypertrophy of tonsils AND adenoids (x1.01) 0.959
MCV [V] by AC (-0.14) 0.959
Hematocrit [VF] of Blood by AC (-0.104) 0.959
Disorder of tympanic membrane (x1.01) 0.959
Dyspnea (/1.01) 0.960
Lymphocytes/100 leukocytes in B by AC (+0.245) 0.960
Neutrophils/100 leukocytes in B by AC (-0.272) 0.960
Developmental delay (x1.01) 0.960
Otitis externa (x1.01) 0.960
oxycodone (-0.00102) 0.961
Failure to thrive (x1.01) 0.961
gabapentin (-0.000999) 0.961
Type 2 diabetes mellitus without complication (/1.01) 0.961
O/E - hearing (x1.01) 0.962
Single live birth (x1.01) 0.962
Cleft palate (x1.01) 0.962
Delay in physiological development (x1.01) 0.962
Mixed receptive-expressive language disorder (x1.01) 0.962
Asthma (x1.01) 0.963
Coronary arteriosclerosis (/1.01) 0.963
Atherosclerosis of coronary artery without angina pectoris (/1.01) 0.963
omeprazole (-0.000931) 0.963
simvastatin (-0.00092) 0.963
acetaminophen (-0.000919) 0.963
Expressions
10^5
10^3
10^1
−10 0 10

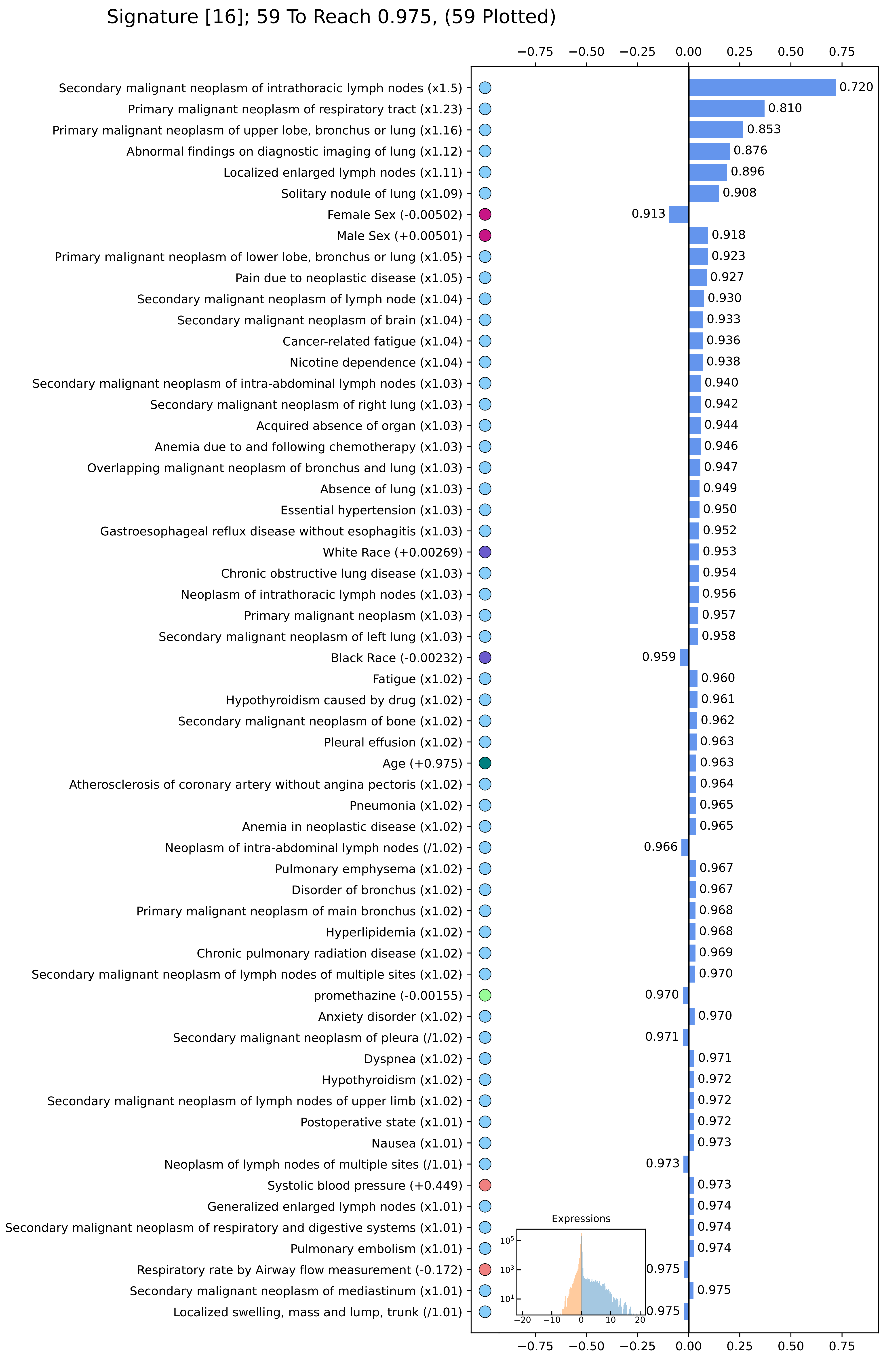
Signature [16]; 59 To Reach 0.975, (59 Plotted)
Secondary malignant neoplasm of intrathoracic lymph nodes (x1.5) 0.720
Primary malignant neoplasm of respiratory tract (x1.23) 0.810
Primary malignant neoplasm of upper lobe, bronchus or lung (x1.16) 0.853
Abnormal findings on diagnostic imaging of lung (x1.12) 0.876
Localized enlarged lymph nodes (x1.11) 0.896
Solitary nodule of lung (x1.09) 0.908
Female Sex (-0.00502) 0.913
Male Sex (+0.00501) 0.918
Primary malignant neoplasm of lower lobe, bronchus or lung (x1.05) 0.923
Pain due to neoplastic disease (x1.05) 0.927
Secondary malignant neoplasm of lymph node (x1.04) 0.930
Secondary malignant neoplasm of brain (x1.04) 0.933
Cancer-related fatigue (x1.04) 0.936
Nicotine dependence (x1.04) 0.938
Secondary malignant neoplasm of intra-abdominal lymph nodes (x1.03) 0.940
Secondary malignant neoplasm of right lung (x1.03) 0.942
Acquired absence of organ (x1.03) 0.944
Anemia due to and following chemotherapy (x1.03) 0.946
Overlapping malignant neoplasm of bronchus and lung (x1.03) 0.947
Absence of lung (x1.03) 0.949
Essential hypertension (x1.03) 0.950
Gastroesophageal reflux disease without esophagitis (x1.03) 0.952
White Race (+0.00269) 0.953
Chronic obstructive lung disease (x1.03) 0.954
Neoplasm of intrathoracic lymph nodes (x1.03) 0.956
Primary malignant neoplasm (x1.03) 0.957
Secondary malignant neoplasm of left lung (x1.03) 0.958
Black Race (-0.00232) 0.959
Fatigue (x1.02) 0.960
Hypothyroidism caused by drug (x1.02) 0.961
Secondary malignant neoplasm of bone (x1.02) 0.962
Pleural effusion (x1.02) 0.963
Age (+0.975) 0.963
Atherosclerosis of coronary artery without angina pectoris (x1.02) 0.964
Pneumonia (x1.02) 0.965
Anemia in neoplastic disease (x1.02) 0.965
Neoplasm of intra-abdominal lymph nodes (/1.02) 0.966
Pulmonary emphysema (x1.02) 0.967
Disorder of bronchus (x1.02) 0.967
Primary malignant neoplasm of main bronchus (x1.02) 0.968
Hyperlipidemia (x1.02) 0.968
Chronic pulmonary radiation disease (x1.02) 0.969
Secondary malignant neoplasm of lymph nodes of multiple sites (x1.02) 0.970
promethazine (-0.00155) 0.970
Anxiety disorder (x1.02) 0.970
Secondary malignant neoplasm of pleura (/1.02) 0.971
Dyspnea (x1.02) 0.971
Hypothyroidism (x1.02) 0.972
Secondary malignant neoplasm of lymph nodes of upper limb (x1.02) 0.972
Postoperative state (x1.01) 0.972
Nausea (x1.01) 0.973
Neoplasm of lymph nodes of multiple sites (/1.01) 0.973
Systolic blood pressure (+0.449) 0.973
Generalized enlarged lymph nodes (x1.01) 0.974
Secondary malignant neoplasm of respiratory and digestive systems (x1.01) 0.974
Pulmonary embolism (x1.01) 0.974
Respiratory rate by Airway flow measurement (-0.172) 0.975
Secondary malignant neoplasm of mediastinum (x1.01) 0.975
Localized swelling, mass and lump, trunk (/1.01) 0.975
−0.75 −0.50 −0.25 0.00 0.25 0.50 0.75
Expressions
10^5
10^3
10^1
−20 −10 0 10 20

Signature [17]; 5 To Reach 0.975, (10 Plotted)

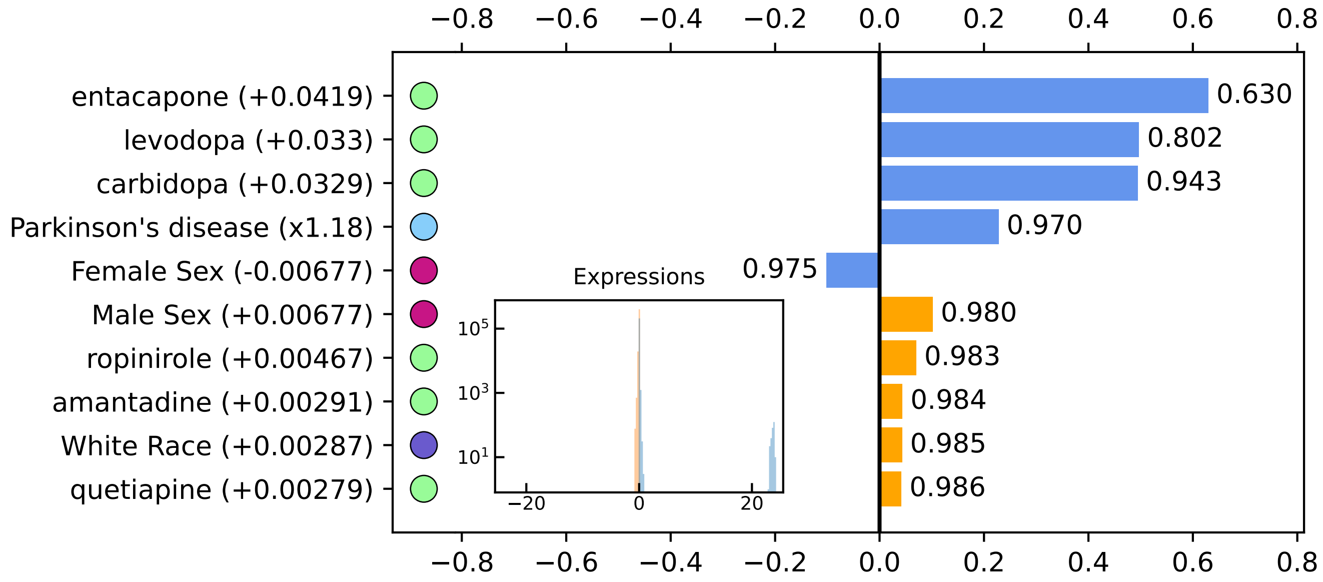

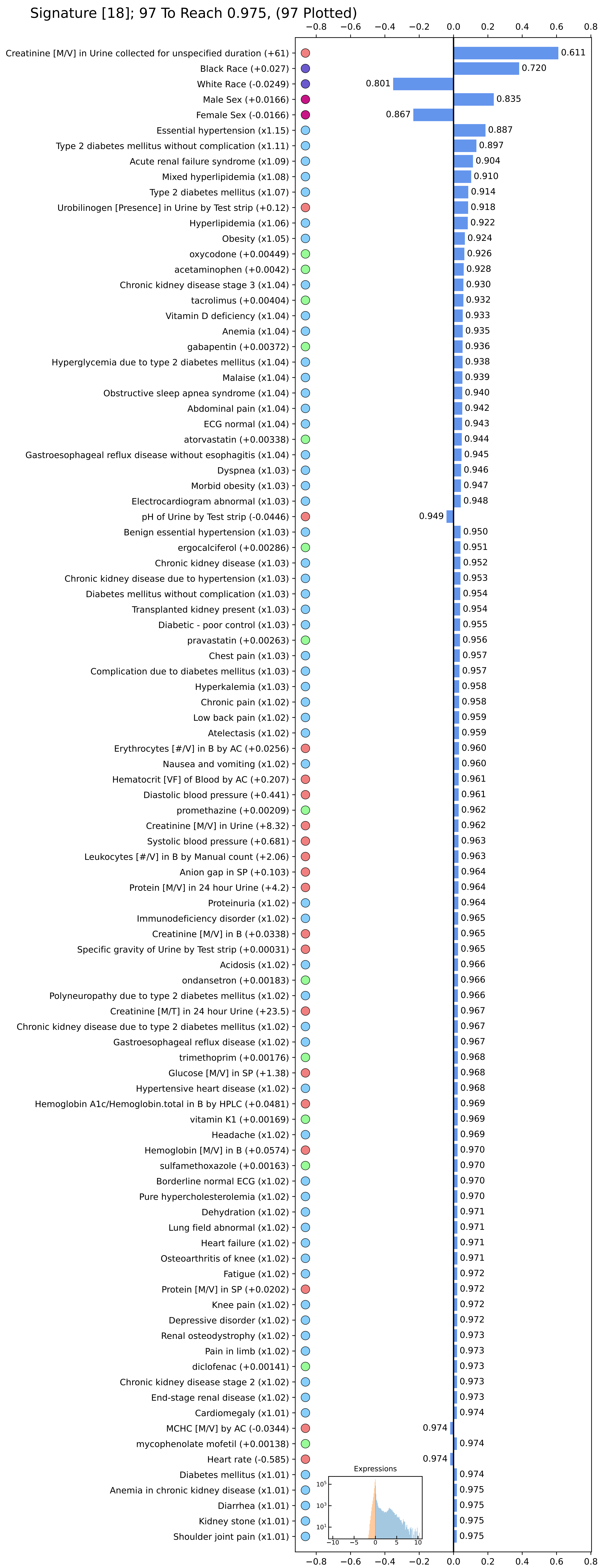

Signature [18]; 97 To Reach 0.975, (97 Plotted)
Creatinine [M/V] in Urine collected for unspecified duration (+61) 0.611
Black Race (+0.027) 0.720
White Race (-0.0249) 0.801
Male Sex (+0.0166) 0.835
Female Sex (-0.0166) 0.867
Essential hypertension (x1.15) 0.887
Type 2 diabetes mellitus without complication (x1.11) 0.897
Acute renal failure syndrome (x1.09) 0.904
Mixed hyperlipidemia (x1.08) 0.910
Type 2 diabetes mellitus (x1.07) 0.914
Urobilinogen [Presence] in Urine by Test strip (+0.12) 0.918
Hyperlipidemia (x1.06) 0.922
Obesity (x1.05) 0.924
oxycodone (+0.00449) 0.926
acetaminophen (+0.0042) 0.928
Chronic kidney disease stage 3 (x1.04) 0.930
tacrolimus (+0.00404) 0.932
Vitamin D deficiency (x1.04) 0.933
Anemia (x1.04) 0.935
gabapentin (+0.00372) 0.936
Hyperglycemia due to type 2 diabetes mellitus (x1.04) 0.938
Malaise (x1.04) 0.939
Obstructive sleep apnea syndrome (x1.04) 0.940
Abdominal pain (x1.04) 0.942
ECG normal (x1.04) 0.943
atorvastatin (+0.00338) 0.944
Gastroesophageal reflux disease without esophagitis (x1.04) 0.945
Dyspnea (x1.03) 0.946
Morbid obesity (x1.03) 0.947
Electrocardiogram abnormal (x1.03) 0.948
pH of Urine by Test strip (-0.0446) 0.949
Benign essential hypertension (x1.03) 0.950
ergocalciferol (+0.00286) 0.951
Chronic kidney disease (x1.03) 0.952
Chronic kidney disease due to hypertension (x1.03) 0.953
Diabetes mellitus without complication (x1.03) 0.954
Transplanted kidney present (x1.03) 0.954
Diabetic - poor control (x1.03) 0.955
pravastatin (+0.00263) 0.956
Chest pain (x1.03) 0.957
Complication due to diabetes mellitus (x1.03) 0.957
Hyperkalemia (x1.03) 0.958
Chronic pain (x1.02) 0.958
Low back pain (x1.02) 0.959
Atelectasis (x1.02) 0.959
Erythrocytes [#/V] in B by AC (+0.0256) 0.960
Nausea and vomiting (x1.02) 0.960
Hematocrit [VF] of Blood by AC (+0.207) 0.961
Diastolic blood pressure (+0.441) 0.961
promethazine (+0.00209) 0.962
Creatinine [M/V] in Urine (+8.32) 0.962
Systolic blood pressure (+0.681) 0.963
Leukocytes [#/V] in B by Manual count (+2.06) 0.963
Anion gap in SP (+0.103) 0.964
Protein [M/V] in 24 hour Urine (+4.2) 0.964
Proteinuria (x1.02) 0.964
Immunodeficiency disorder (x1.02) 0.965
Creatinine [M/V] in B (+0.0338) 0.965
Specific gravity of Urine by Test strip (+0.00031) 0.965
Acidosis (x1.02) 0.966
ondansetron (+0.00183) 0.966
Polyneuropathy due to type 2 diabetes mellitus (x1.02) 0.966
Creatinine [M/T] in 24 hour Urine (+23.5) 0.967
Chronic kidney disease due to type 2 diabetes mellitus (x1.02) 0.967
Gastroesophageal reflux disease (x1.02) 0.967
trimethoprim (+0.00176) 0.968
Glucose [M/V] in SP (+1.38) 0.968
Hypertensive heart disease (x1.02) 0.968
Hemoglobin A1c/Hemoglobin.total in B by HPLC (+0.0481) 0.969
vitamin K1 (+0.00169) 0.969
Headache (x1.02) 0.969
Hemoglobin [M/V] in B (+0.0574) 0.970
sulfamethoxazole (+0.00163) 0.970
Borderline normal ECG (x1.02) 0.970
Pure hypercholesterolemia (x1.02) 0.970
Dehydration (x1.02) 0.971
Lung field abnormal (x1.02) 0.971
Heart failure (x1.02) 0.971
Osteoarthritis of knee (x1.02) 0.971
Fatigue (x1.02) 0.972
Protein [M/V] in SP (+0.0202) 0.972
Knee pain (x1.02) 0.972
Depressive disorder (x1.02) 0.972
Renal osteodystrophy (x1.02) 0.973
Pain in limb (x1.02) 0.973
diclofenac (+0.00141) 0.973
Chronic kidney disease stage 2 (x1.02) 0.973
End-stage renal disease (x1.02) 0.973
Cardiomegaly (x1.01) 0.974
MCHC [M/V] by AC (-0.0344) 0.974
mycophenolate mofetil (+0.00138) 0.974
Heart rate (-0.585) 0.974
Diabetes mellitus (x1.01) 0.974
Anemia in chronic kidney disease (x1.01) 0.975
Diarrhea (x1.01) 0.975
Kidney stone (x1.01) 0.975
Shoulder joint pain (x1.01) 0.975
Expressions
−0.8 −0.6 −0.4 −0.2 0.0 0.2 0.4 0.6 0.8

Signature [19]; 243 To Reach 0.975, (100 Plotted)

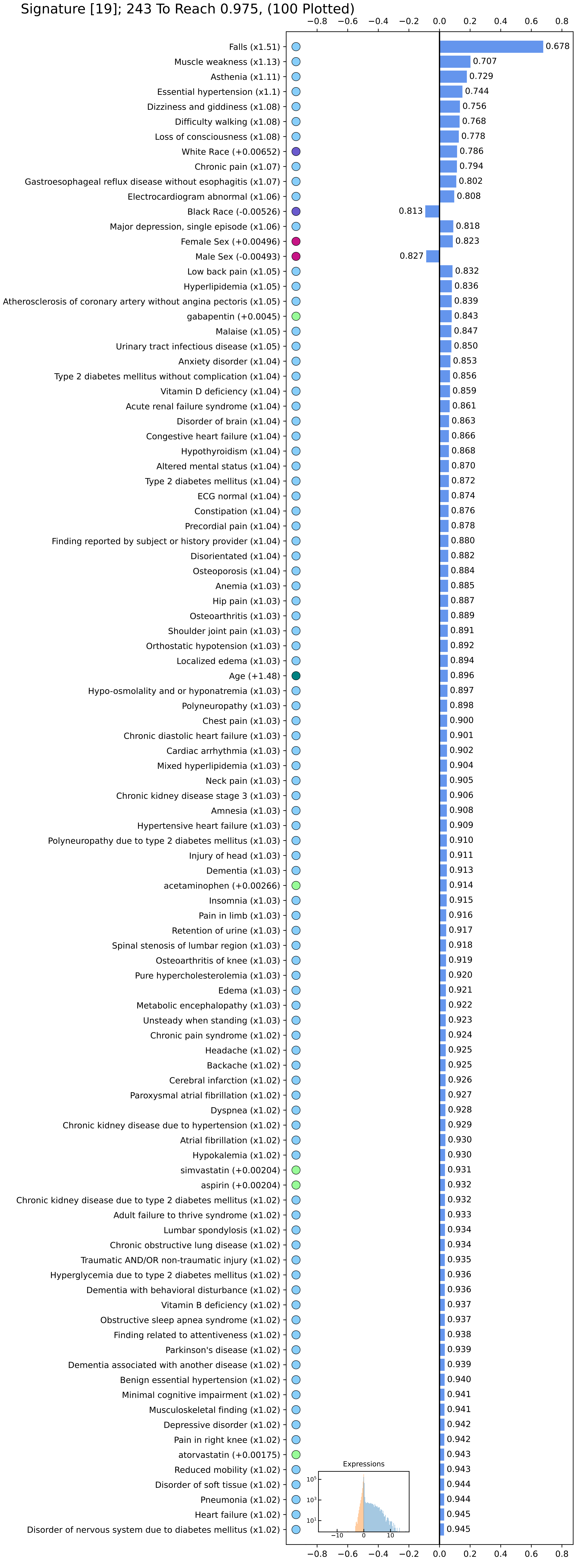

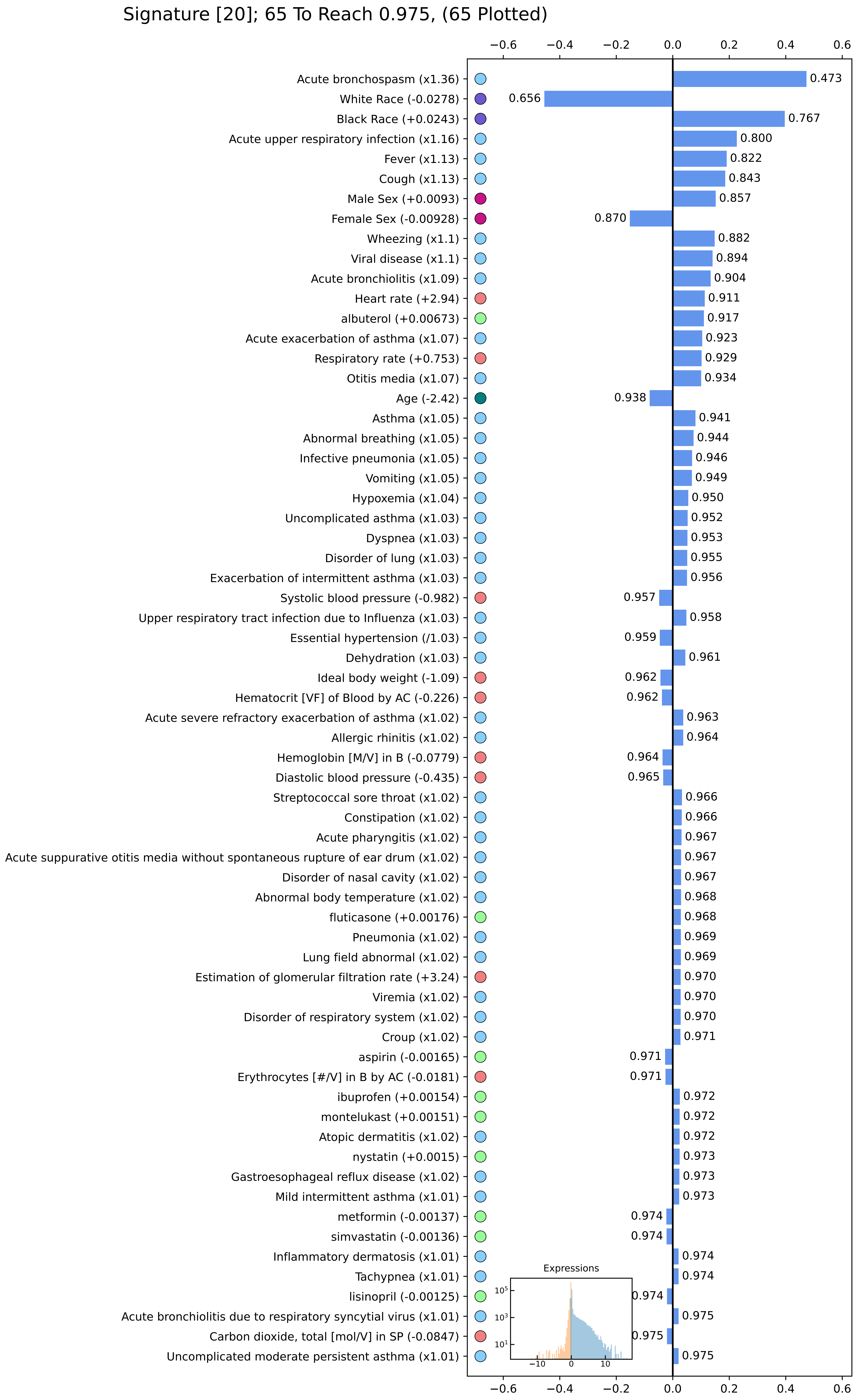
Signature [20]; 65 To Reach 0.975, (65 Plotted)
−0.6
−0.4
−0.2
0.0
0.2
0.4
0.6
Acute bronchospasm (x1.36) 0.473
White Race (-0.0278) 0.656
Black Race (+0.0243) 0.767
Acute upper respiratory infection (x1.16) 0.800
Fever (x1.13) 0.822
Cough (x1.13) 0.843
Male Sex (+0.0093) 0.857
Female Sex (-0.00928) 0.870
Wheezing (x1.1) 0.882
Viral disease (x1.1) 0.894
Acute bronchiolitis (x1.09) 0.904
Heart rate (+2.94) 0.911
albuterol (+0.00673) 0.917
Acute exacerbation of asthma (x1.07) 0.923
Respiratory rate (+0.753) 0.929
Otitis media (x1.07) 0.934
Age (-2.42) 0.938
Asthma (x1.05) 0.941
Abnormal breathing (x1.05) 0.944
Infective pneumonia (x1.05) 0.946
Vomiting (x1.05) 0.949
Hypoxemia (x1.04) 0.950
Uncomplicated asthma (x1.03) 0.952
Dyspnea (x1.03) 0.953
Disorder of lung (x1.03) 0.955
Exacerbation of intermittent asthma (x1.03) 0.956
Systolic blood pressure (-0.982) 0.957
Upper respiratory tract infection due to Influenza (x1.03) 0.958
Essential hypertension (/1.03) 0.959
Dehydration (x1.03) 0.961
Ideal body weight (-1.09) 0.962
Hematocrit [VF] of Blood by AC (-0.226) 0.962
Acute severe refractory exacerbation of asthma (x1.02) 0.963
Allergic rhinitis (x1.02) 0.964
Hemoglobin [M/V] in B (-0.0779) 0.964
Diastolic blood pressure (-0.435) 0.965
Streptococcal sore throat (x1.02) 0.966
Constipation (x1.02) 0.966
Acute pharyngitis (x1.02) 0.967
Acute suppurative otitis media without spontaneous rupture of ear drum (x1.02) 0.967
Disorder of nasal cavity (x1.02) 0.967
Abnormal body temperature (x1.02) 0.968
fluticasone (+0.00176) 0.968
Pneumonia (x1.02) 0.969
Lung field abnormal (x1.02) 0.969
Estimation of glomerular filtration rate (+3.24) 0.970
Viremia (x1.02) 0.970
Disorder of respiratory system (x1.02) 0.970
Croup (x1.02) 0.971
aspirin (-0.00165) 0.971
Erythrocytes [#/V] in B by AC (-0.0181) 0.971
ibuprofen (+0.00154) 0.972
montelukast (+0.00151) 0.972
Atopic dermatitis (x1.02) 0.972
nystatin (+0.0015) 0.973
Gastroesophageal reflux disease (x1.02) 0.973
Mild intermittent asthma (x1.01) 0.973
metformin (-0.00137) 0.974
simvastatin (-0.00136) 0.974
Inflammatory dermatosis (x1.01) 0.974
Tachypnea (x1.01) 0.974
lisinopril (-0.00125) 0.974
Acute bronchiolitis due to respiratory syncytial virus (x1.01) 0.975
Carbon dioxide, total [mol/V] in SP (-0.0847) 0.975
Uncomplicated moderate persistent asthma (x1.01) 0.975
Expressions
10^5
10^3
10^1
−10
0
10

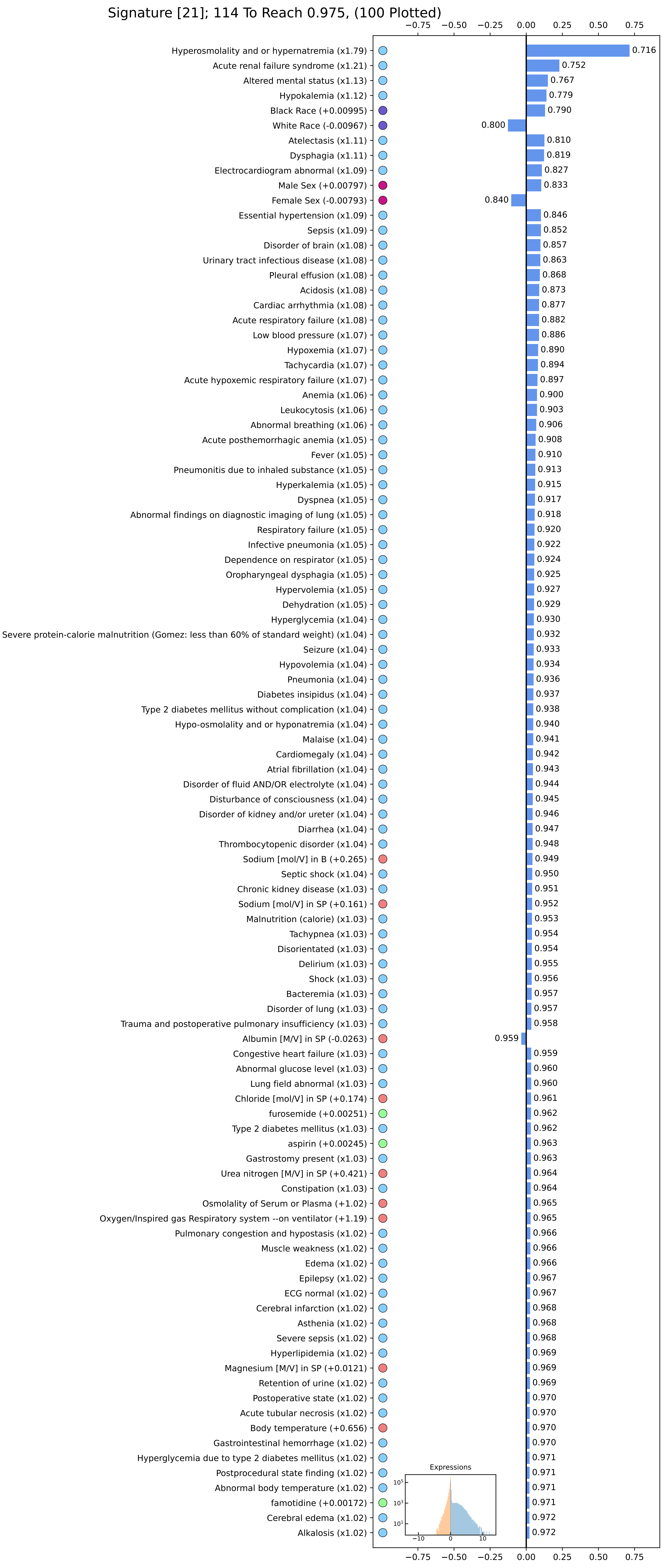
Signature [21]; 114 To Reach 0.975, (100 Plotted)
Hyperosmolality and or hypernatremia (x1.79) 0.716
Acute renal failure syndrome (x1.21) 0.752
Altered mental status (x1.13) 0.767
Hypokalemia (x1.12) 0.779
Black Race (+0.00995) 0.790
White Race (-0.00967) 0.800
Atelectasis (x1.11) 0.810
Dysphagia (x1.11) 0.819
Electrocardiogram abnormal (x1.09) 0.827
Male Sex (+0.00797) 0.833
Female Sex (-0.00793) 0.840
Essential hypertension (x1.09) 0.846
Sepsis (x1.09) 0.852
Disorder of brain (x1.08) 0.857
Urinary tract infectious disease (x1.08) 0.863
Pleural effusion (x1.08) 0.868
Acidosis (x1.08) 0.873
Cardiac arrhythmia (x1.08) 0.877
Acute respiratory failure (x1.08) 0.882
Low blood pressure (x1.07) 0.886
Hypoxemia (x1.07) 0.890
Tachycardia (x1.07) 0.894
Acute hypoxemic respiratory failure (x1.07) 0.897
Anemia (x1.06) 0.900
Leukocytosis (x1.06) 0.903
Abnormal breathing (x1.06) 0.906
Acute posthemorrhagic anemia (x1.05) 0.908
Fever (x1.05) 0.910
Pneumonitis due to inhaled substance (x1.05) 0.913
Hyperkalemia (x1.05) 0.915
Dyspnea (x1.05) 0.917
Abnormal findings on diagnostic imaging of lung (x1.05) 0.918
Respiratory failure (x1.05) 0.920
Infective pneumonia (x1.05) 0.922
Dependence on respirator (x1.05) 0.924
Oropharyngeal dysphagia (x1.05) 0.925
Hypervolemia (x1.05) 0.927
Dehydration (x1.05) 0.929
Hyperglycemia (x1.04) 0.930
Severe protein-calorie malnutrition (Gomez: less than 60% of standard weight) (x1.04) 0.932
Seizure (x1.04) 0.933
Hypovolemia (x1.04) 0.934
Pneumonia (x1.04) 0.936
Diabetes insipidus (x1.04) 0.937
Type 2 diabetes mellitus without complication (x1.04) 0.938
Hypo-osmolality and or hyponatremia (x1.04) 0.940
Malaise (x1.04) 0.941
Cardiomegaly (x1.04) 0.942
Atrial fibrillation (x1.04) 0.943
Disorder of fluid AND/OR electrolyte (x1.04) 0.944
Disturbance of consciousness (x1.04) 0.945
Disorder of kidney and/or ureter (x1.04) 0.946
Diarrhea (x1.04) 0.947
Thrombocytopenic disorder (x1.04) 0.948
Sodium [mol/V] in B (+0.265) 0.949
Septic shock (x1.04) 0.950
Chronic kidney disease (x1.03) 0.951
Sodium [mol/V] in SP (+0.161) 0.952
Malnutrition (calorie) (x1.03) 0.953
Tachypnea (x1.03) 0.954
Disorientated (x1.03) 0.954
Delirium (x1.03) 0.955
Shock (x1.03) 0.956
Bacteremia (x1.03) 0.957
Disorder of lung (x1.03) 0.957
Trauma and postoperative pulmonary insufficiency (x1.03) 0.958
Albumin [M/V] in SP (-0.0263) 0.959
Congestive heart failure (x1.03) 0.959
Abnormal glucose level (x1.03) 0.960
Lung field abnormal (x1.03) 0.960
Chloride [mol/V] in SP (+0.174) 0.961
furosemide (+0.00251) 0.962
Type 2 diabetes mellitus (x1.03) 0.962
aspirin (+0.00245) 0.963
Gastrostomy present (x1.03) 0.963
Urea nitrogen [M/V] in SP (+0.421) 0.964
Constipation (x1.03) 0.964
Osmolality of Serum or Plasma (+1.02) 0.965
Oxygen/Inspired gas Respiratory system --on ventilator (+1.19) 0.965
Pulmonary congestion and hypostasis (x1.02) 0.966
Muscle weakness (x1.02) 0.966
Edema (x1.02) 0.966
Epilepsy (x1.02) 0.967
ECG normal (x1.02) 0.967
Cerebral infarction (x1.02) 0.968
Asthenia (x1.02) 0.968
Severe sepsis (x1.02) 0.968
Hyperlipidemia (x1.02) 0.969
Magnesium [M/V] in SP (+0.0121) 0.969
Retention of urine (x1.02) 0.969
Postoperative state (x1.02) 0.970
Acute tubular necrosis (x1.02) 0.970
Body temperature (+0.656) 0.970
Gastrointestinal hemorrhage (x1.02) 0.970
Hyperglycemia due to type 2 diabetes mellitus (x1.02) 0.971
Postprocedural state finding (x1.02) 0.971
Abnormal body temperature (x1.02) 0.971
famotidine (+0.00172) 0.971
Cerebral edema (x1.02) 0.972
Alkalosis (x1.02) 0.972
Expressions
−0.75 −0.50 −0.25 0.00 0.25 0.50 0.75

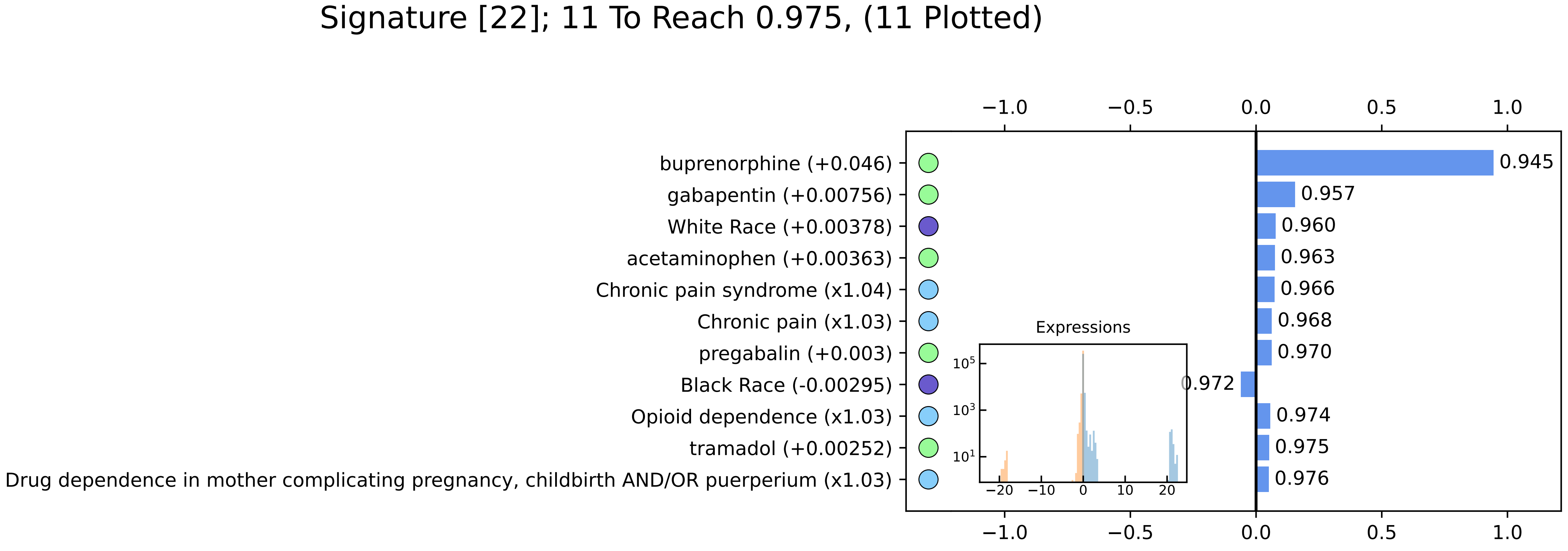
Signature [22]; 11 To Reach 0.975, (11 Plotted)
buprenorphine (+0.046)
gabapentin (+0.00756)
White Race (+0.00378)
acetaminophen (+0.00363)
Chronic pain syndrome (x1.04)
Chronic pain (x1.03)
pregabalin (+0.003)
Black Race (-0.00295)
Opioid dependence (x1.03)
tramadol (+0.00252)
Drug dependence in mother complicating pregnancy, childbirth AND/OR puerperium (x1.03)
0.945
0.957
0.960
0.963
0.966
0.968
0.970
0.972
0.974
0.975
0.976
−1.0
−0.5
0.0
0.5
1.0
Expressions
10^5
10^3
10^1
−20
−10
0
10
20

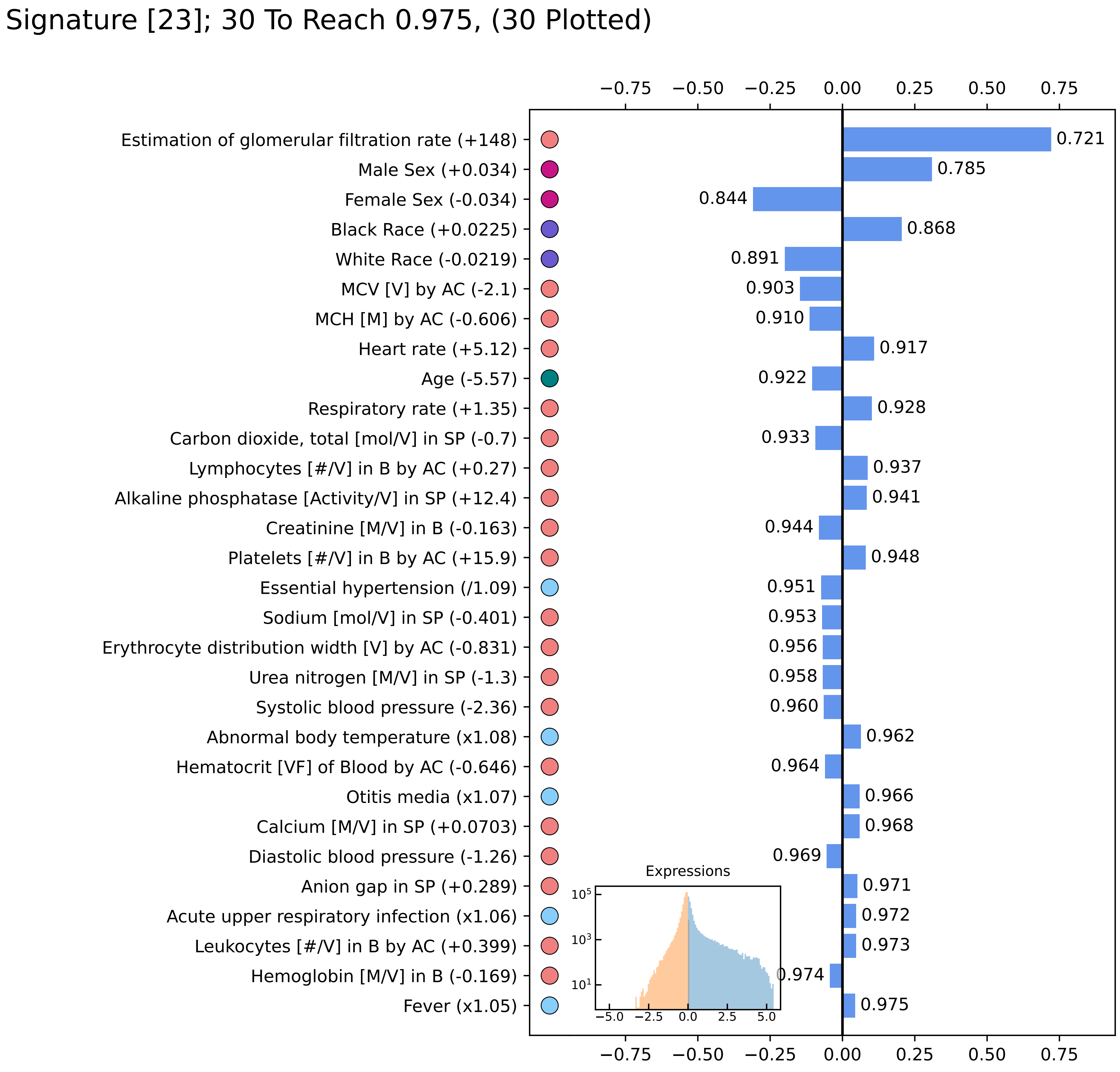

Signature [23]; 30 To Reach 0.975, (30 Plotted)
Estimation of glomerular filtration rate (+148)
Male Sex (+0.034)
Female Sex (-0.034)
Black Race (+0.0225)
White Race (-0.0219)
MCV [V] by AC (-2.1)
MCH [M] by AC (-0.606)
Heart rate (+5.12)
Age (-5.57)
Respiratory rate (+1.35)
Carbon dioxide, total [mol/V] in SP (-0.7)
Lymphocytes [#/V] in B by AC (+0.27)
Alkaline phosphatase [Activity/V] in SP (+12.4)
Creatinine [M/V] in B (-0.163)
Platelets [#/V] in B by AC (+15.9)
Essential hypertension (/1.09)
Sodium [mol/V] in SP (-0.401)
Erythrocyte distribution width [V] by AC (-0.831)
Urea nitrogen [M/V] in SP (-1.3)
Systolic blood pressure (-2.36)
Abnormal body temperature (x1.08)
Hematocrit [VF] of Blood by AC (-0.646)
Otitis media (x1.07)
Calcium [M/V] in SP (+0.0703)
Diastolic blood pressure (-1.26)
Anion gap in SP (+0.289)
Acute upper respiratory infection (x1.06)
Leukocytes [#/V] in B by AC (+0.399)
Hemoglobin [M/V] in B (-0.169)
Fever (x1.05)
0.721
0.785
0.844
0.868
0.891
0.903
0.910
0.917
0.922
0.928
0.933
0.937
0.941
0.944
0.948
0.951
0.953
0.956
0.958
0.960
0.962
0.964
0.966
0.968
0.969
0.971
0.972
0.973
0.974
0.975
−0.75 −0.50 −0.25 0.00 0.25 0.50 0.75
Expressions
10^5
10^3
10^1
−5.0 −2.5 0.0 2.5 5.0

Signature [24]; 170 To Reach 0.975, (100 Plotted)

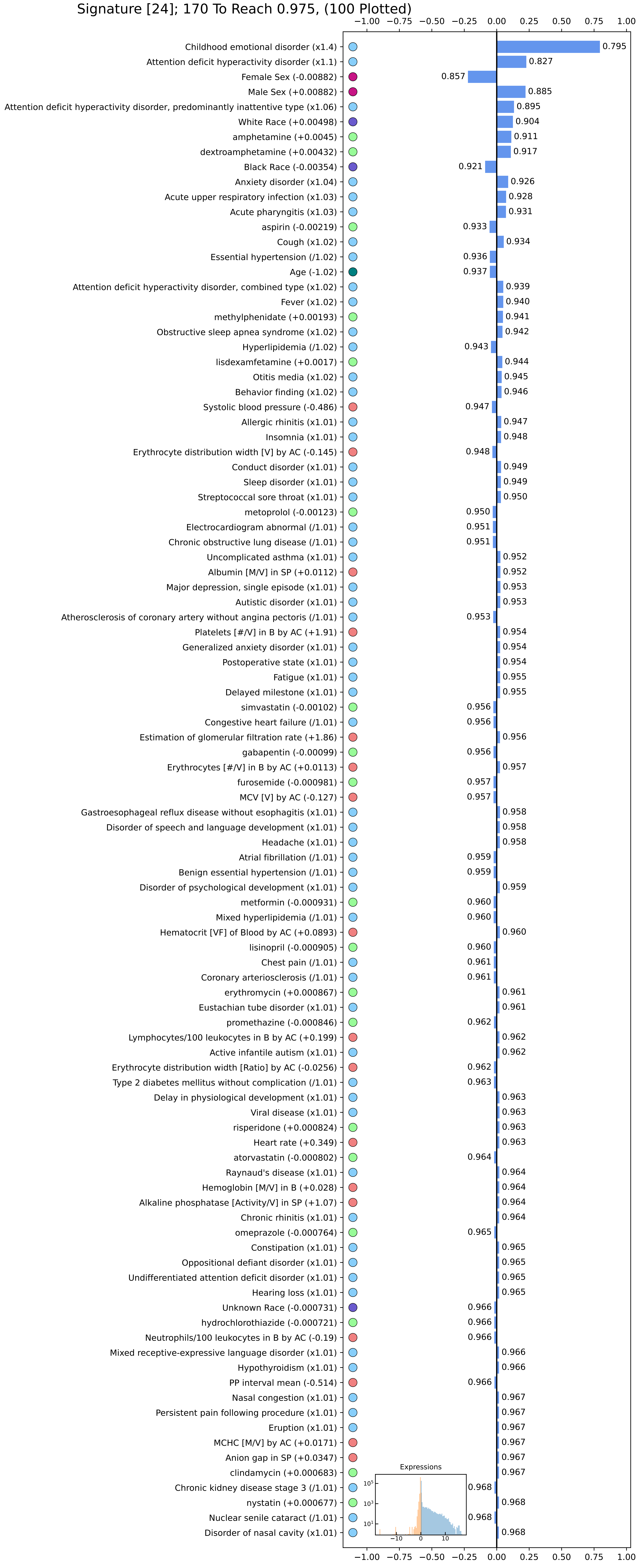

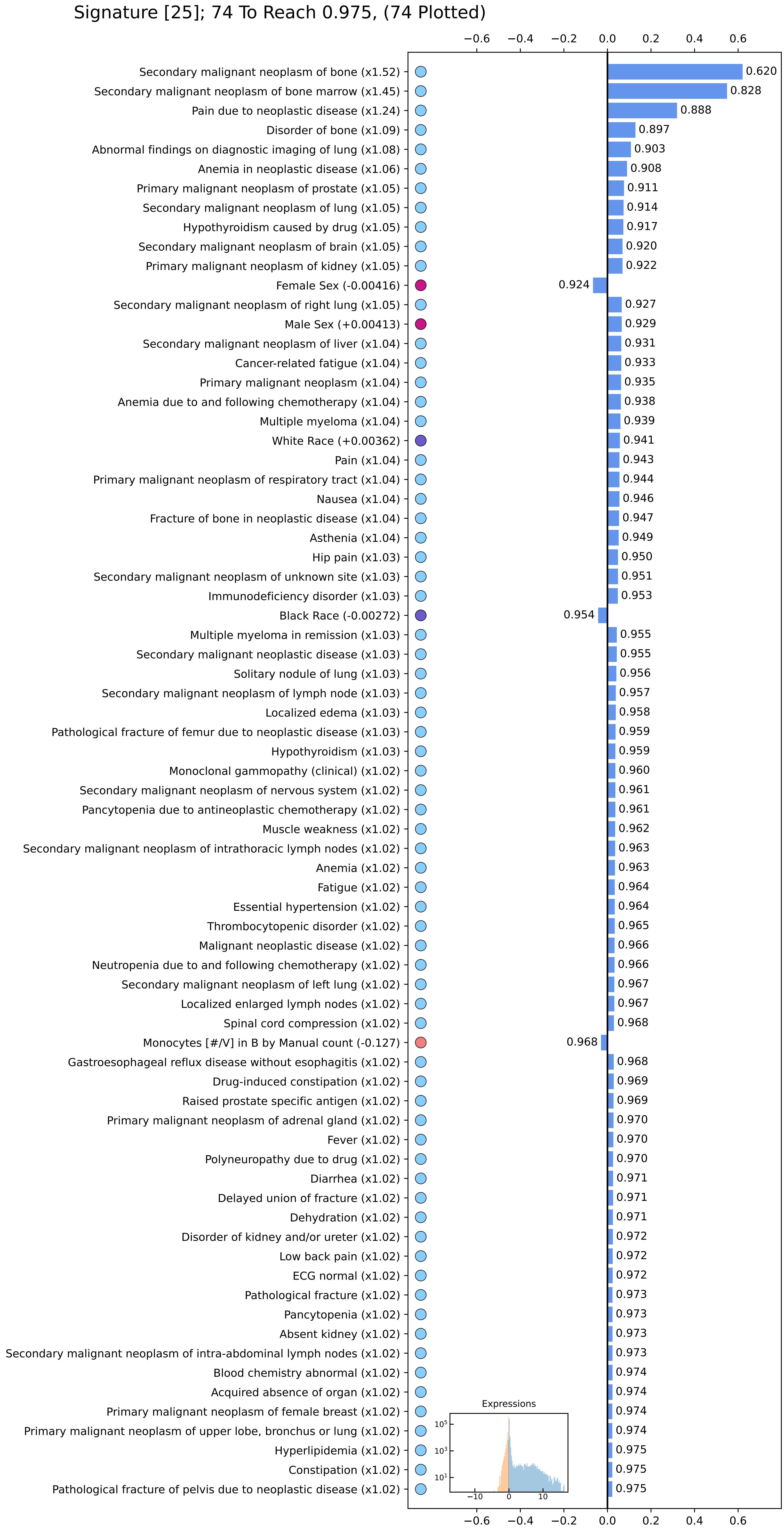
Signature [25]; 74 To Reach 0.975, (74 Plotted)
Secondary malignant neoplasm of bone (x1.52) 0.620
Secondary malignant neoplasm of bone marrow (x1.45) 0.828
Pain due to neoplastic disease (x1.24) 0.888
Disorder of bone (x1.09) 0.897
Abnormal findings on diagnostic imaging of lung (x1.08) 0.903
Anemia in neoplastic disease (x1.06) 0.908
Primary malignant neoplasm of prostate (x1.05) 0.911
Secondary malignant neoplasm of lung (x1.05) 0.914
Hypothyroidism caused by drug (x1.05) 0.917
Secondary malignant neoplasm of brain (x1.05) 0.920
Primary malignant neoplasm of kidney (x1.05) 0.922
Female Sex (-0.00416) 0.924
Secondary malignant neoplasm of right lung (x1.05) 0.927
Male Sex (+0.00413) 0.929
Secondary malignant neoplasm of liver (x1.04) 0.931
Cancer-related fatigue (x1.04) 0.933
Primary malignant neoplasm (x1.04) 0.935
Anemia due to and following chemotherapy (x1.04) 0.938
Multiple myeloma (x1.04) 0.939
White Race (+0.00362) 0.941
Pain (x1.04) 0.943
Primary malignant neoplasm of respiratory tract (x1.04) 0.944
Nausea (x1.04) 0.946
Fracture of bone in neoplastic disease (x1.04) 0.947
Asthenia (x1.04) 0.949
Hip pain (x1.03) 0.950
Secondary malignant neoplasm of unknown site (x1.03) 0.951
Immunodeficiency disorder (x1.03) 0.953
Black Race (-0.00272) 0.954
Multiple myeloma in remission (x1.03) 0.955
Secondary malignant neoplastic disease (x1.03) 0.955
Solitary nodule of lung (x1.03) 0.956
Secondary malignant neoplasm of lymph node (x1.03) 0.957
Localized edema (x1.03) 0.958
Pathological fracture of femur due to neoplastic disease (x1.03) 0.959
Hypothyroidism (x1.03) 0.959
Monoclonal gammopathy (clinical) (x1.02) 0.960
Secondary malignant neoplasm of nervous system (x1.02) 0.961
Pancytopenia due to antineoplastic chemotherapy (x1.02) 0.961
Muscle weakness (x1.02) 0.962
Secondary malignant neoplasm of intrathoracic lymph nodes (x1.02) 0.963
Anemia (x1.02) 0.963
Fatigue (x1.02) 0.964
Essential hypertension (x1.02) 0.964
Thrombocytopenic disorder (x1.02) 0.965
Malignant neoplastic disease (x1.02) 0.966
Neutropenia due to and following chemotherapy (x1.02) 0.966
Secondary malignant neoplasm of left lung (x1.02) 0.967
Localized enlarged lymph nodes (x1.02) 0.967
Spinal cord compression (x1.02) 0.968
Monocytes [#/V] in B by Manual count (-0.127) 0.968
Gastroesophageal reflux disease without esophagitis (x1.02) 0.968
Drug-induced constipation (x1.02) 0.969
Raised prostate specific antigen (x1.02) 0.969
Primary malignant neoplasm of adrenal gland (x1.02) 0.970
Fever (x1.02) 0.970
Polyneuropathy due to drug (x1.02) 0.970
Diarrhea (x1.02) 0.971
Delayed union of fracture (x1.02) 0.971
Dehydration (x1.02) 0.971
Disorder of kidney and/or ureter (x1.02) 0.972
Low back pain (x1.02) 0.972
ECG normal (x1.02) 0.972
Pathological fracture (x1.02) 0.973
Pancytopenia (x1.02) 0.973
Absent kidney (x1.02) 0.973
Secondary malignant neoplasm of intra-abdominal lymph nodes (x1.02) 0.973
Blood chemistry abnormal (x1.02) 0.974
Acquired absence of organ (x1.02) 0.974
Primary malignant neoplasm of female breast (x1.02) 0.974
Primary malignant neoplasm of upper lobe, bronchus or lung (x1.02) 0.974
Hyperlipidemia (x1.02) 0.975
Constipation (x1.02) 0.975
Pathological fracture of pelvis due to neoplastic disease (x1.02) 0.975
−0.6 −0.4 −0.2 0.0 0.2 0.4 0.6
Expressions

Signature [26]; 183 To Reach 0.975, (100 Plotted)

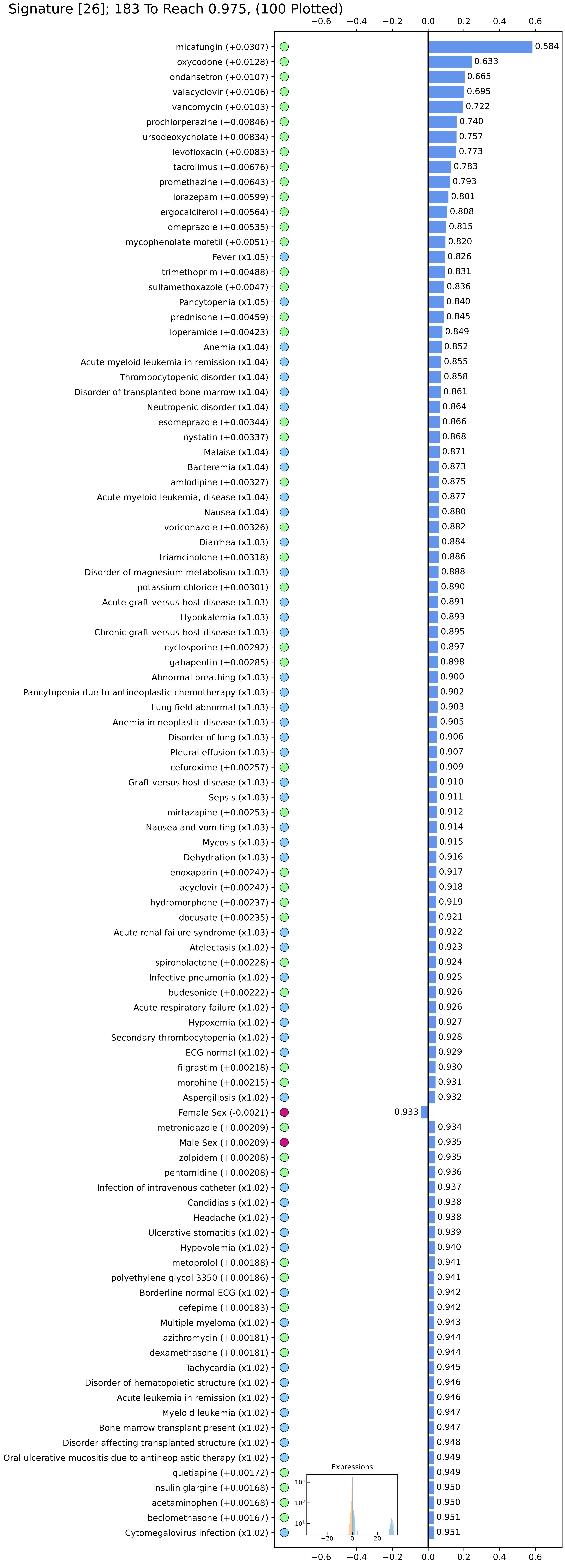

Signature [27]; 272 To Reach 0.975, (100 Plotted)

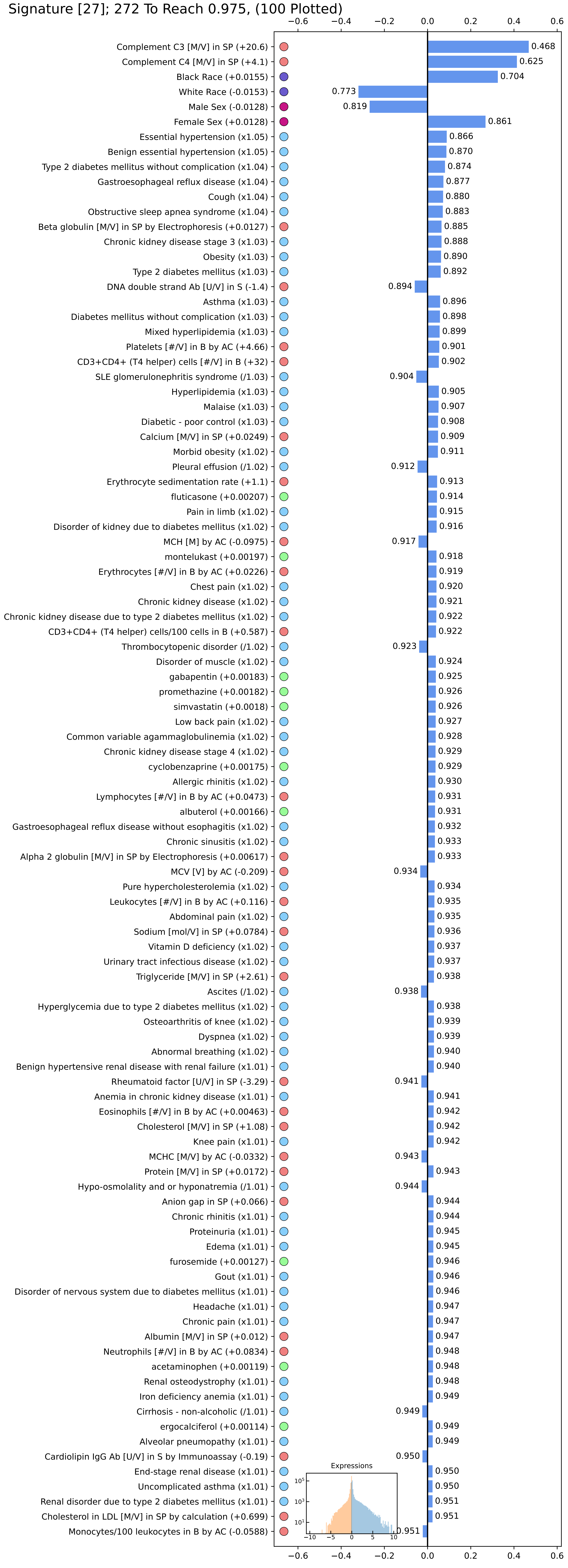

Signature [28]; 124 To Reach 0.975, (100 Plotted)

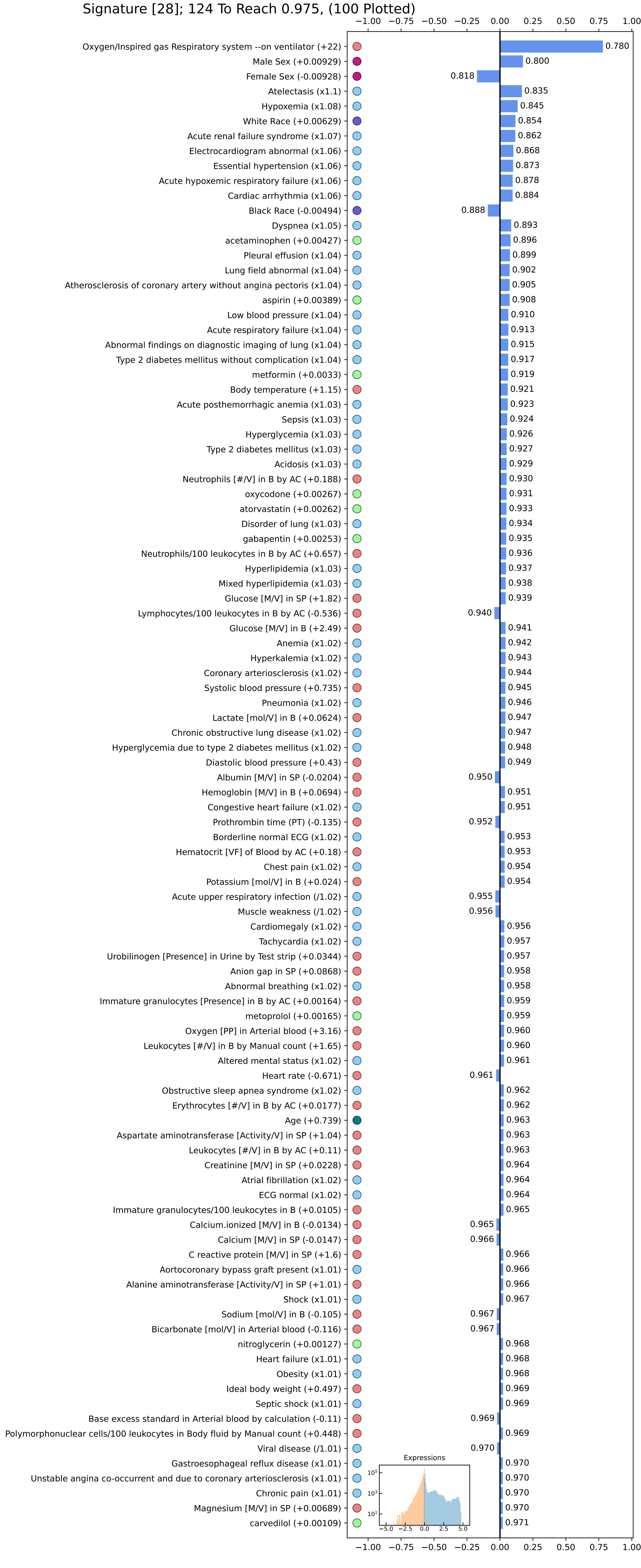

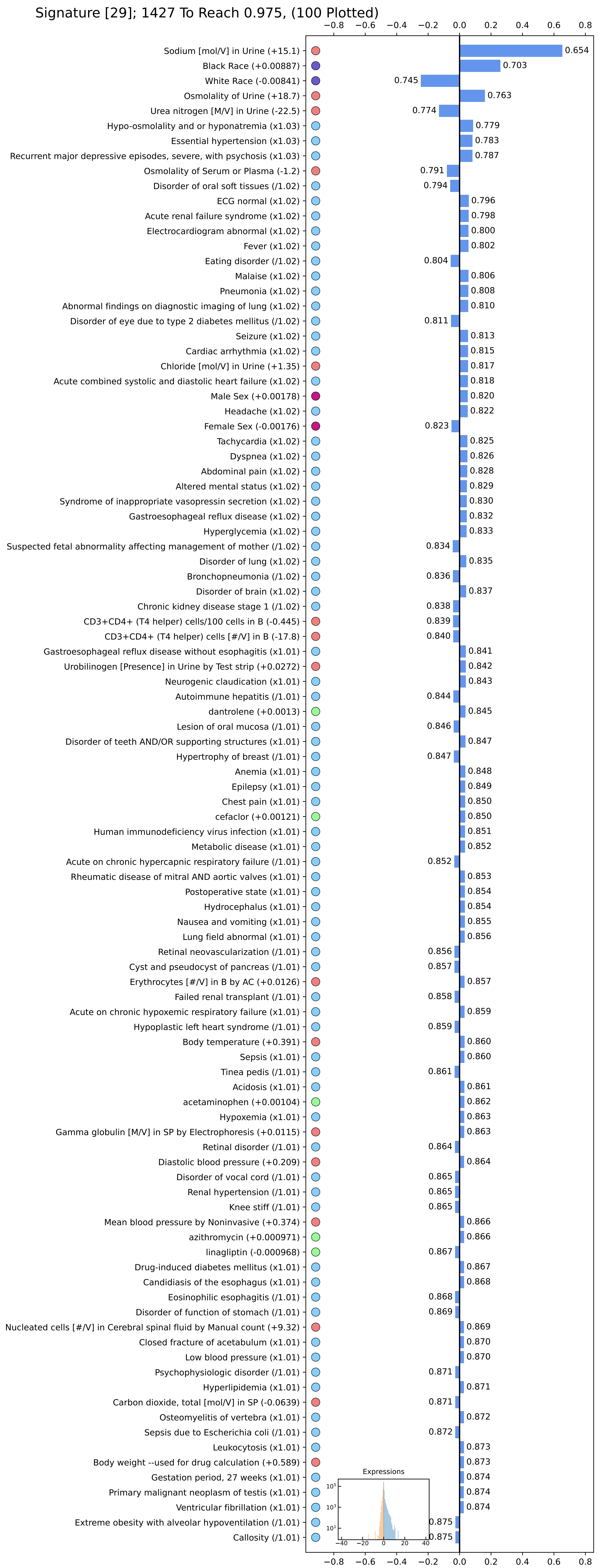

Signature [29]; 1427 To Reach 0.975, (100 Plotted)
Sodium [mol/V] in Urine (+15.1) 0.654
Black Race (+0.00887) 0.703
White Race (-0.00841) 0.745
Osmolality of Urine (+18.7) 0.763
Urea nitrogen [M/V] in Urine (-22.5) 0.774
Hypo-osmolality and or hyponatremia (x1.03) 0.779
Essential hypertension (x1.03) 0.783
Recurrent major depressive episodes, severe, with psychosis (x1.03) 0.787
Osmolality of Serum or Plasma (-1.2) 0.791
Disorder of oral soft tissues (/1.02) 0.794
ECG normal (x1.02) 0.796
Acute renal failure syndrome (x1.02) 0.798
Electrocardiogram abnormal (x1.02) 0.800
Fever (x1.02) 0.802
Eating disorder (/1.02) 0.804
Malaise (x1.02) 0.806
Pneumonia (x1.02) 0.808
Abnormal findings on diagnostic imaging of lung (x1.02) 0.810
Disorder of eye due to type 2 diabetes mellitus (/1.02) 0.811
Seizure (x1.02) 0.813
Cardiac arrhythmia (x1.02) 0.815
Chloride [mol/V] in Urine (+1.35) 0.817
Acute combined systolic and diastolic heart failure (x1.02) 0.818
Male Sex (+0.00178) 0.820
Headache (x1.02) 0.822
Female Sex (-0.00176) 0.823
Tachycardia (x1.02) 0.825
Dyspnea (x1.02) 0.826
Abdominal pain (x1.02) 0.828
Altered mental status (x1.02) 0.829
Syndrome of inappropriate vasopressin secretion (x1.02) 0.830
Gastroesophageal reflux disease (x1.02) 0.832
Hyperglycemia (x1.02) 0.833
Suspected fetal abnormality affecting management of mother (/1.02) 0.834
Disorder of lung (x1.02) 0.835
Bronchopneumonia (/1.02) 0.836
Disorder of brain (x1.02) 0.837
Chronic kidney disease stage 1 (/1.02) 0.838
CD3+CD4+ (T4 helper) cells/100 cells in B (-0.445) 0.839
CD3+CD4+ (T4 helper) cells [#/V] in B (-17.8) 0.840
Gastroesophageal reflux disease without esophagitis (x1.01) 0.841
Urobilinogen [Presence] in Urine by Test strip (+0.0272) 0.842
Neurogenic claudication (x1.01) 0.843
Autoimmune hepatitis (/1.01) 0.844
dantrolene (+0.0013) 0.845
Lesion of oral mucosa (/1.01) 0.846
Disorder of teeth AND/OR supporting structures (x1.01) 0.847
Hypertrophy of breast (/1.01) 0.847
Anemia (x1.01) 0.848
Epilepsy (x1.01) 0.849
Chest pain (x1.01) 0.850
cefaclor (+0.00121) 0.850
Human immunodeficiency virus infection (x1.01) 0.851
Metabolic disease (x1.01) 0.852
Acute on chronic hypercapnic respiratory failure (/1.01) 0.852
Rheumatic disease of mitral AND aortic valves (x1.01) 0.853
Postoperative state (x1.01) 0.854
Hydrocephalus (x1.01) 0.854
Nausea and vomiting (x1.01) 0.855
Lung field abnormal (x1.01) 0.856
Retinal neovascularization (/1.01) 0.856
Cyst and pseudocyst of pancreas (/1.01) 0.857
Erythrocytes [#/V] in B by AC (+0.0126) 0.857
Failed renal transplant (/1.01) 0.858
Acute on chronic hypoxemic respiratory failure (x1.01) 0.859
Hypoplastic left heart syndrome (/1.01) 0.859
Body temperature (+0.391) 0.860
Sepsis (x1.01) 0.860
Tinea pedis (/1.01) 0.861
Acidosis (x1.01) 0.861
acetaminophen (+0.00104) 0.862
Hypoxemia (x1.01) 0.863
Gamma globulin [M/V] in SP by Electrophoresis (+0.0115) 0.863
Retinal disorder (/1.01) 0.864
Diastolic blood pressure (+0.209) 0.864
Disorder of vocal cord (/1.01) 0.865
Renal hypertension (/1.01) 0.865
Knee stiff (/1.01) 0.865
Mean blood pressure by Noninvasive (+0.374) 0.866
azithromycin (+0.000971) 0.866
linagliptin (-0.000968) 0.867
Drug-induced diabetes mellitus (x1.01) 0.867
Candidiasis of the esophagus (x1.01) 0.868
Eosinophilic esophagitis (/1.01) 0.868
Disorder of function of stomach (/1.01) 0.869
Nucleated cells [#/V] in Cerebral spinal fluid by Manual count (+9.32) 0.869
Closed fracture of acetabulum (x1.01) 0.870
Low blood pressure (x1.01) 0.870
Psychophysiologic disorder (/1.01) 0.871
Hyperlipidemia (x1.01) 0.871
Carbon dioxide, total [mol/V] in SP (-0.0639) 0.871
Osteomyelitis of vertebra (x1.01) 0.872
Sepsis due to Escherichia coli (/1.01) 0.872
Leukocytosis (x1.01) 0.873
Body weight --used for drug calculation (+0.589) 0.873
Gestation period, 27 weeks (x1.01) 0.874
Primary malignant neoplasm of testis (x1.01) 0.874
Ventricular fibrillation (x1.01) 0.874
Extreme obesity with alveolar hypoventilation (/1.01) 0.875
Callosity (/1.01) 0.875
−0.8 −0.6 −0.4 −0.2 0.0 0.2 0.4 0.6 0.8
Expressions
10^5
10^3
10^1
−40 −20 0 20 40

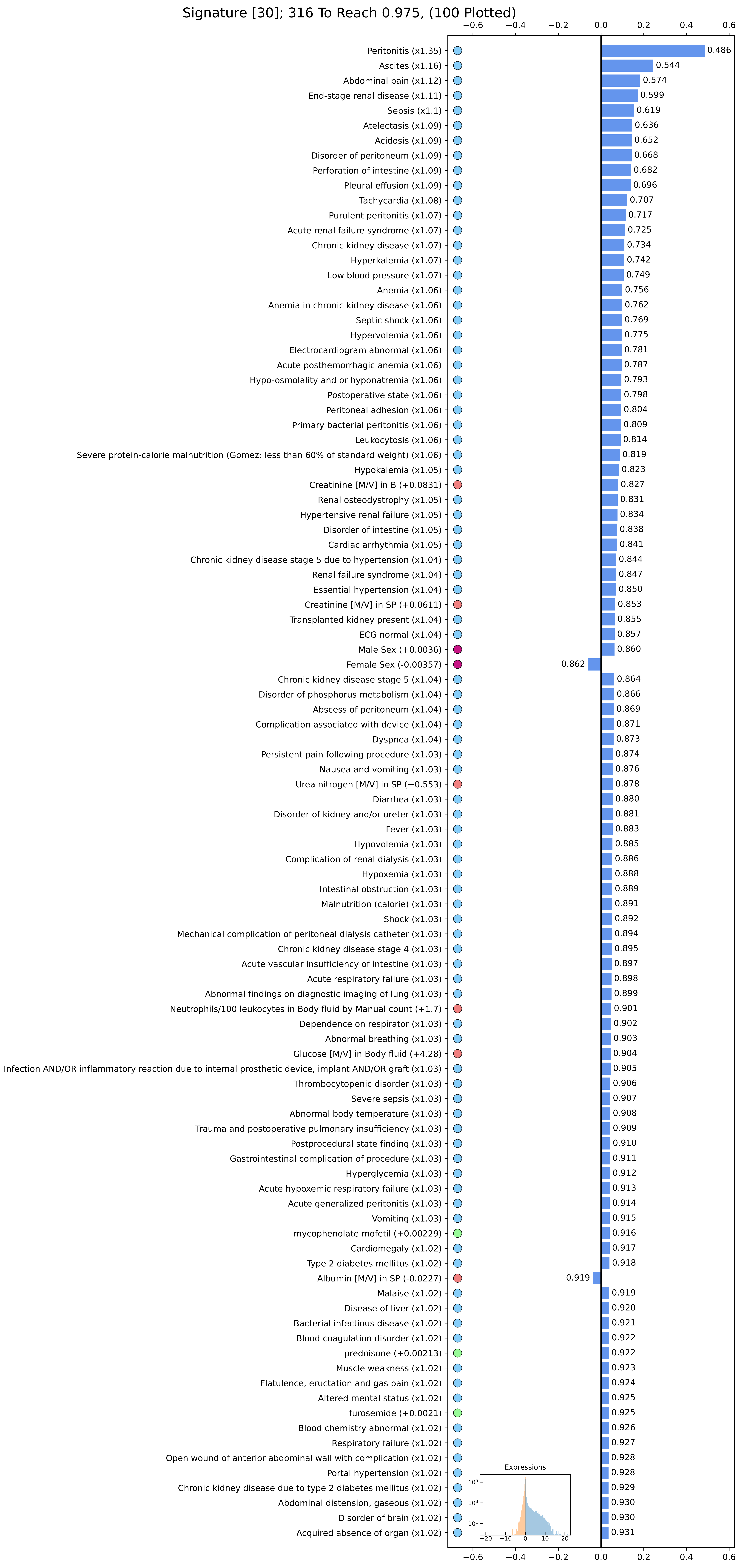

Signature [30]; 316 To Reach 0.975, (100 Plotted)
Peritonitis (x1.35) 0.486
Ascites (x1.16) 0.544
Abdominal pain (x1.12) 0.574
End-stage renal disease (x1.11) 0.599
Sepsis (x1.1) 0.619
Atelectasis (x1.09) 0.636
Acidosis (x1.09) 0.652
Disorder of peritoneum (x1.09) 0.668
Perforation of intestine (x1.09) 0.682
Pleural effusion (x1.09) 0.696
Tachycardia (x1.08) 0.707
Purulent peritonitis (x1.07) 0.717
Acute renal failure syndrome (x1.07) 0.725
Chronic kidney disease (x1.07) 0.734
Hyperkalemia (x1.07) 0.742
Low blood pressure (x1.07) 0.749
Anemia (x1.06) 0.756
Anemia in chronic kidney disease (x1.06) 0.762
Septic shock (x1.06) 0.769
Hypervolemia (x1.06) 0.775
Electrocardiogram abnormal (x1.06) 0.781
Acute posthemorrhagic anemia (x1.06) 0.787
Hypo-osmolality and or hyponatremia (x1.06) 0.793
Postoperative state (x1.06) 0.798
Peritoneal adhesion (x1.06) 0.804
Primary bacterial peritonitis (x1.06) 0.809
Leukocytosis (x1.06) 0.814
Severe protein-calorie malnutrition (Gomez: less than 60% of standard weight) (x1.06) 0.819
Hypokalemia (x1.05) 0.823
Creatinine [M/V] in B (+0.0831) 0.827
Renal osteodystrophy (x1.05) 0.831
Hypertensive renal failure (x1.05) 0.834
Disorder of intestine (x1.05) 0.838
Cardiac arrhythmia (x1.05) 0.841
Chronic kidney disease stage 5 due to hypertension (x1.04) 0.844
Renal failure syndrome (x1.04) 0.847
Essential hypertension (x1.04) 0.850
Creatinine [M/V] in SP (+0.0611) 0.853
Transplanted kidney present (x1.04) 0.855
ECG normal (x1.04) 0.857
Male Sex (+0.0036) 0.860
Female Sex (-0.00357) 0.862
Chronic kidney disease stage 5 (x1.04) 0.864
Disorder of phosphorus metabolism (x1.04) 0.866
Abscess of peritoneum (x1.04) 0.869
Complication associated with device (x1.04) 0.871
Dyspnea (x1.04) 0.873
Persistent pain following procedure (x1.03) 0.874
Nausea and vomiting (x1.03) 0.876
Urea nitrogen [M/V] in SP (+0.553) 0.878
Diarrhea (x1.03) 0.880
Disorder of kidney and/or ureter (x1.03) 0.881
Fever (x1.03) 0.883
Hypovolemia (x1.03) 0.885
Complication of renal dialysis (x1.03) 0.886
Hypoxemia (x1.03) 0.888
Intestinal obstruction (x1.03) 0.889
Malnutrition (calorie) (x1.03) 0.891
Shock (x1.03) 0.892
Mechanical complication of peritoneal dialysis catheter (x1.03) 0.894
Chronic kidney disease stage 4 (x1.03) 0.895
Acute vascular insufficiency of intestine (x1.03) 0.897
Acute respiratory failure (x1.03) 0.898
Abnormal findings on diagnostic imaging of lung (x1.03) 0.899
Neutrophils/100 leukocytes in Body fluid by Manual count (+1.7) 0.901
Dependence on respirator (x1.03) 0.902
Abnormal breathing (x1.03) 0.903
Glucose [M/V] in Body fluid (+4.28) 0.904
Infection AND/OR inflammatory reaction due to internal prosthetic device, implant AND/OR graft (x1.03) 0.905
Thrombocytopenic disorder (x1.03) 0.906
Severe sepsis (x1.03) 0.907
Abnormal body temperature (x1.03) 0.908
Trauma and postoperative pulmonary insufficiency (x1.03) 0.909
Postprocedural state finding (x1.03) 0.910
Gastrointestinal complication of procedure (x1.03) 0.911
Hyperglycemia (x1.03) 0.912
Acute hypoxemic respiratory failure (x1.03) 0.913
Acute generalized peritonitis (x1.03) 0.914
Vomiting (x1.03) 0.915
mycophenolate mofetil (+0.00229) 0.916
Cardiomegaly (x1.02) 0.917
Type 2 diabetes mellitus (x1.02) 0.918
Albumin [M/V] in SP (-0.0227) 0.919
Malaise (x1.02) 0.919
Disease of liver (x1.02) 0.920
Bacterial infectious disease (x1.02) 0.921
Blood coagulation disorder (x1.02) 0.922
prednisone (+0.00213) 0.922
Muscle weakness (x1.02) 0.923
Flatulence, eructation and gas pain (x1.02) 0.924
Altered mental status (x1.02) 0.925
furosemide (+0.0021) 0.925
Blood chemistry abnormal (x1.02) 0.926
Respiratory failure (x1.02) 0.927
Open wound of anterior abdominal wall with complication (x1.02) 0.928
Portal hypertension (x1.02) 0.928
Chronic kidney disease due to type 2 diabetes mellitus (x1.02) 0.929
Abdominal distension, gaseous (x1.02) 0.930
Disorder of brain (x1.02) 0.930
Acquired absence of organ (x1.02) 0.931
Expressions
−0.6 −0.4 −0.2 0.0 0.2 0.4 0.6

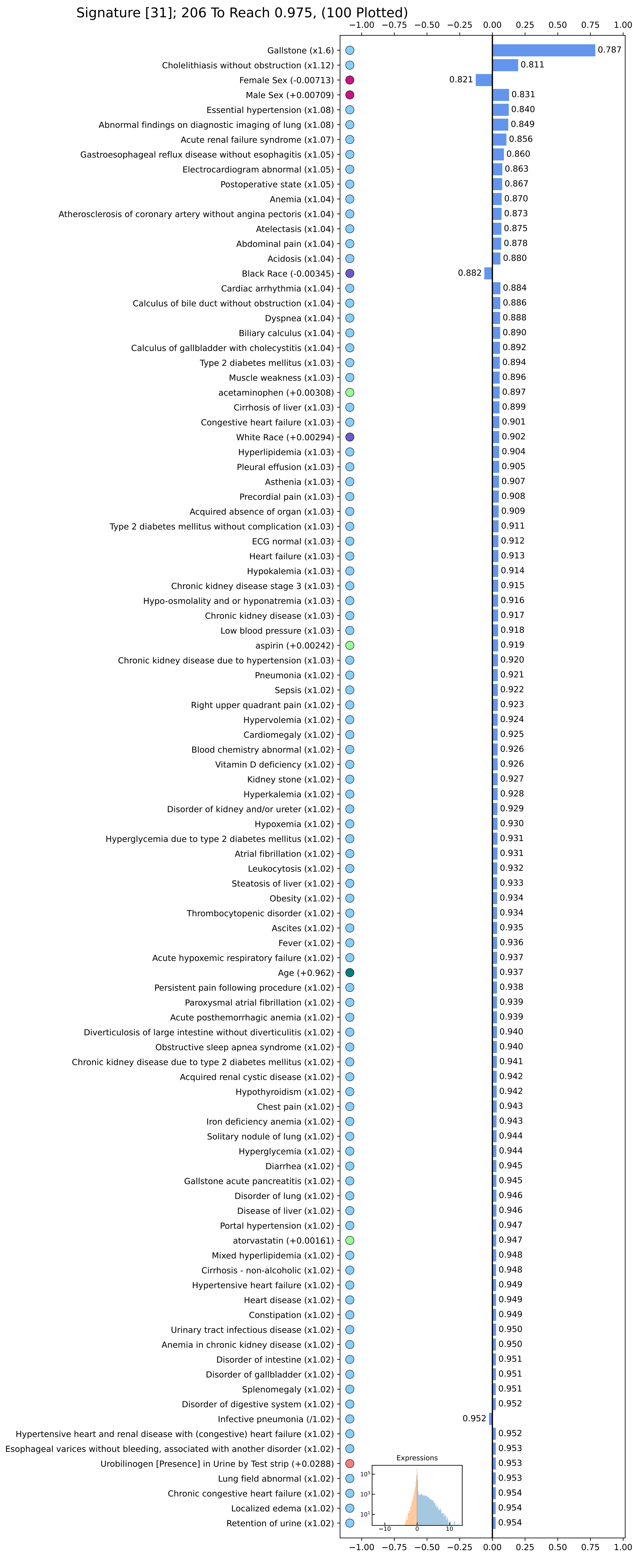

Signature [31]; 206 To Reach 0.975, (100 Plotted)
Gallstone (x1.6) 0.787
Cholelithiasis without obstruction (x1.12) 0.811
Female Sex (-0.00713) 0.821
Male Sex (+0.00709) 0.831
Essential hypertension (x1.08) 0.840
Abnormal findings on diagnostic imaging of lung (x1.08) 0.849
Acute renal failure syndrome (x1.07) 0.856
Gastroesophageal reflux disease without esophagitis (x1.05) 0.860
Electrocardiogram abnormal (x1.05) 0.863
Postoperative state (x1.05) 0.867
Anemia (x1.04) 0.870
Atherosclerosis of coronary artery without angina pectoris (x1.04) 0.873
Atelectasis (x1.04) 0.875
Abdominal pain (x1.04) 0.878
Acidosis (x1.04) 0.880
Black Race (-0.00345) 0.882
Cardiac arrhythmia (x1.04) 0.884
Calculus of bile duct without obstruction (x1.04) 0.886
Dyspnea (x1.04) 0.888
Biliary calculus (x1.04) 0.890
Calculus of gallbladder with cholecystitis (x1.04) 0.892
Type 2 diabetes mellitus (x1.03) 0.894
Muscle weakness (x1.03) 0.896
acetaminophen (+0.00308) 0.897
Cirrhosis of liver (x1.03) 0.899
Congestive heart failure (x1.03) 0.901
White Race (+0.00294) 0.902
Hyperlipidemia (x1.03) 0.904
Pleural effusion (x1.03) 0.905
Asthenia (x1.03) 0.907
Precordial pain (x1.03) 0.908
Acquired absence of organ (x1.03) 0.909
Type 2 diabetes mellitus without complication (x1.03) 0.911
ECG normal (x1.03) 0.912
Heart failure (x1.03) 0.913
Hypokalemia (x1.03) 0.914
Chronic kidney disease stage 3 (x1.03) 0.915
Hypo-osmolality and or hyponatremia (x1.03) 0.916
Chronic kidney disease (x1.03) 0.917
Low blood pressure (x1.03) 0.918
aspirin (+0.00242) 0.919
Chronic kidney disease due to hypertension (x1.03) 0.920
Pneumonia (x1.02) 0.921
Sepsis (x1.02) 0.922
Right upper quadrant pain (x1.02) 0.923
Hypervolemia (x1.02) 0.924
Cardiomegaly (x1.02) 0.925
Blood chemistry abnormal (x1.02) 0.926
Vitamin D deficiency (x1.02) 0.926
Kidney stone (x1.02) 0.927
Hyperkalemia (x1.02) 0.928
Disorder of kidney and/or ureter (x1.02) 0.929
Hypoxemia (x1.02) 0.930
Hyperglycemia due to type 2 diabetes mellitus (x1.02) 0.931
Atrial fibrillation (x1.02) 0.931
Leukocytosis (x1.02) 0.932
Steatosis of liver (x1.02) 0.933
Obesity (x1.02) 0.934
Thrombocytopenic disorder (x1.02) 0.934
Ascites (x1.02) 0.935
Fever (x1.02) 0.936
Acute hypoxemic respiratory failure (x1.02) 0.937
Age (+0.962) 0.937
Persistent pain following procedure (x1.02) 0.938
Paroxysmal atrial fibrillation (x1.02) 0.939
Acute posthemorrhagic anemia (x1.02) 0.939
Diverticulosis of large intestine without diverticulitis (x1.02) 0.940
Obstructive sleep apnea syndrome (x1.02) 0.940
Chronic kidney disease due to type 2 diabetes mellitus (x1.02) 0.941
Acquired renal cystic disease (x1.02) 0.942
Hypothyroidism (x1.02) 0.942
Chest pain (x1.02) 0.943
Iron deficiency anemia (x1.02) 0.943
Solitary nodule of lung (x1.02) 0.944
Hyperglycemia (x1.02) 0.944
Diarrhea (x1.02) 0.945
Gallstone acute pancreatitis (x1.02) 0.945
Disorder of lung (x1.02) 0.946
Disease of liver (x1.02) 0.946
Portal hypertension (x1.02) 0.947
atorvastatin (+0.00161) 0.947
Mixed hyperlipidemia (x1.02) 0.948
Cirrhosis - non-alcoholic (x1.02) 0.948
Hypertensive heart failure (x1.02) 0.949
Heart disease (x1.02) 0.949
Constipation (x1.02) 0.949
Urinary tract infectious disease (x1.02) 0.950
Anemia in chronic kidney disease (x1.02) 0.950
Disorder of intestine (x1.02) 0.951
Disorder of gallbladder (x1.02) 0.951
Splenomegaly (x1.02) 0.951
Disorder of digestive system (x1.02) 0.952
Infective pneumonia (/1.02) 0.952
Hypertensive heart and renal disease with (congestive) heart failure (x1.02) 0.952
Esophageal varices without bleeding, associated with another disorder (x1.02) 0.953
Urobilinogen [Presence] in Urine by Test strip (+0.0288) 0.953
Lung field abnormal (x1.02) 0.953
Chronic congestive heart failure (x1.02) 0.954
Localized edema (x1.02) 0.954
Retention of urine (x1.02) 0.954
Expressions
−1.00 −0.75 −0.50 −0.25 0.00 0.25 0.50 0.75 1.00

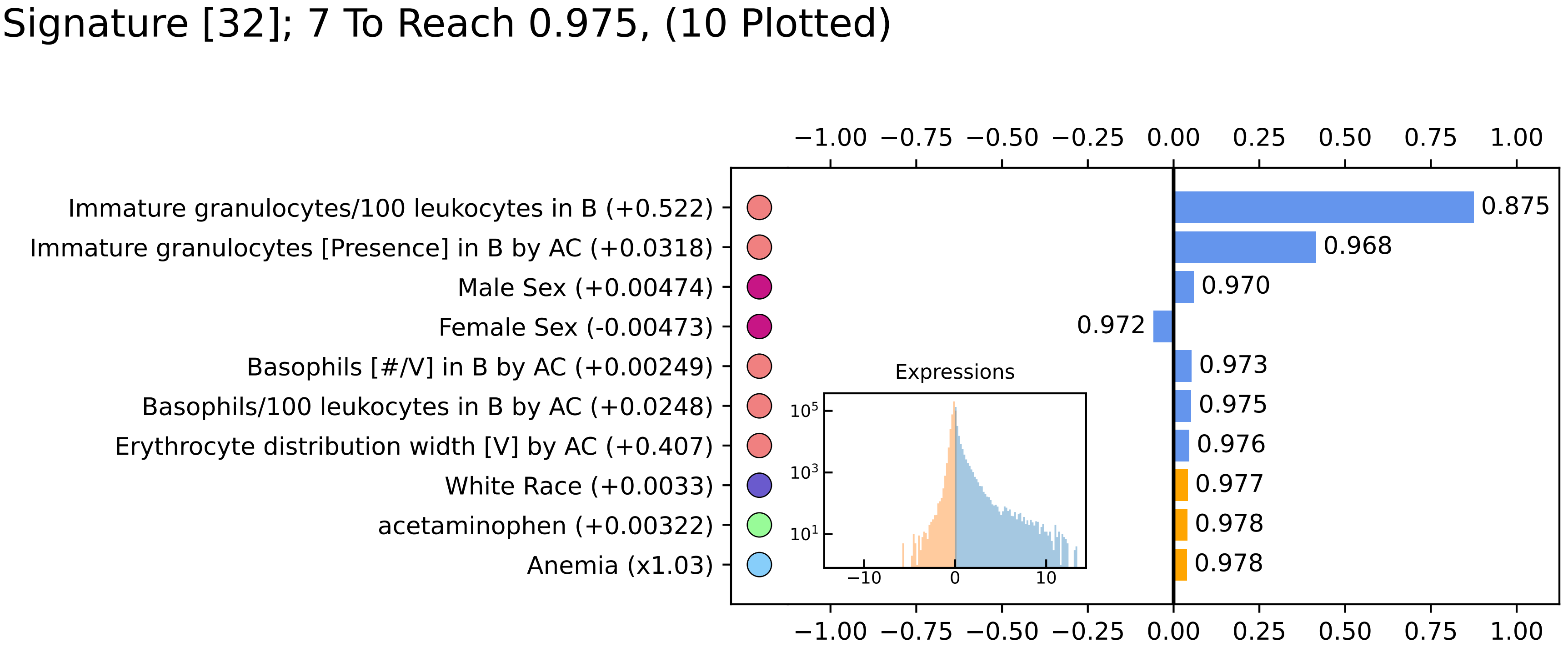
Signature [32]; 7 To Reach 0.975, (10 Plotted)
Immature granulocytes/100 leukocytes in B (+0.522)
Immature granulocytes [Presence] in B by AC (+0.0318)
Male Sex (+0.00474)
Female Sex (-0.00473)
Basophils [#/V] in B by AC (+0.00249)
Basophils/100 leukocytes in B by AC (+0.0248)
Erythrocyte distribution width [V] by AC (+0.407)
White Race (+0.0033)
acetaminophen (+0.00322)
Anemia (x1.03)
0.875
0.968
0.970
0.972
0.973
0.975
0.976
0.977
0.978
0.978
−1.00 −0.75 −0.50 −0.25 0.00 0.25 0.50 0.75 1.00
Expressions
10^5
10^3
10^1
−10 0 10

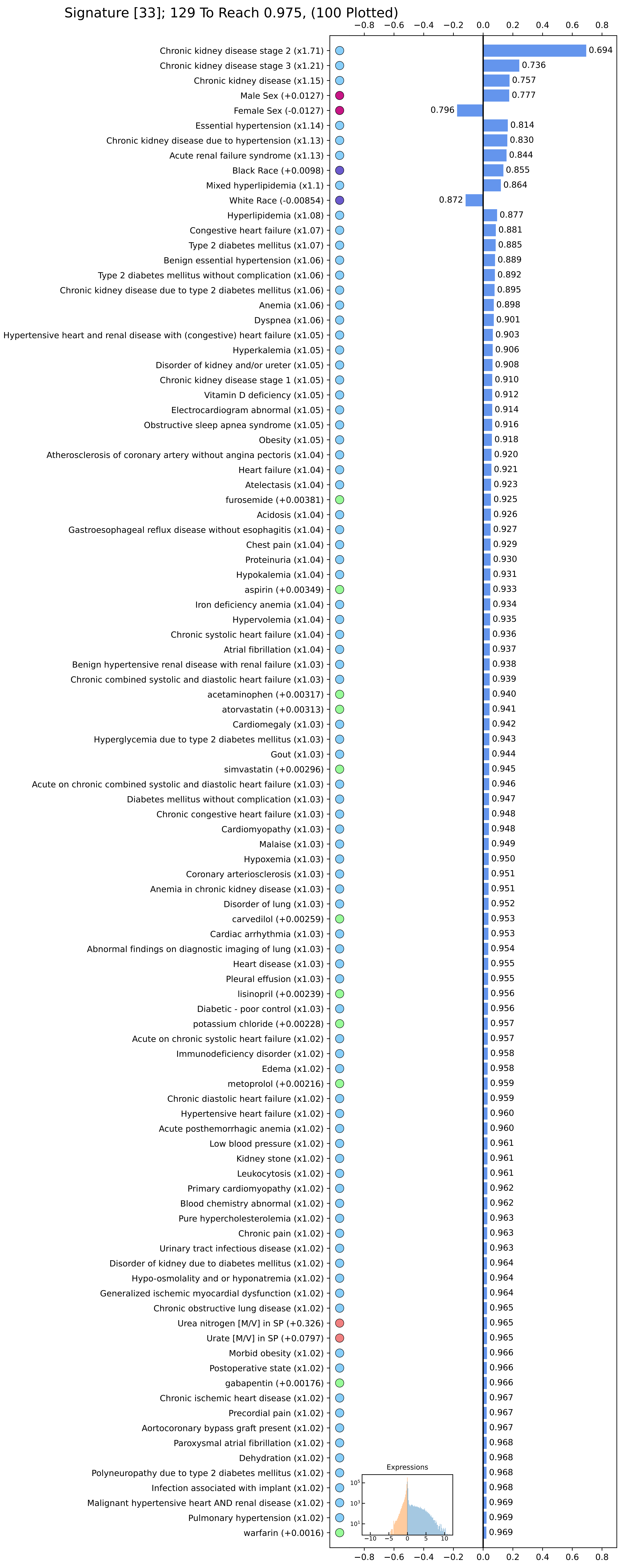

Signature [33]; 129 To Reach 0.975, (100 Plotted)
Chronic kidney disease stage 2 (x1.71) 0.694
Chronic kidney disease stage 3 (x1.21) 0.736
Chronic kidney disease (x1.15) 0.757
Male Sex (+0.0127) 0.777
Female Sex (-0.0127) 0.796
Essential hypertension (x1.14) 0.814
Chronic kidney disease due to hypertension (x1.13) 0.830
Acute renal failure syndrome (x1.13) 0.844
Black Race (+0.0098) 0.855
Mixed hyperlipidemia (x1.1) 0.864
White Race (-0.00854) 0.872
Hyperlipidemia (x1.08) 0.877
Congestive heart failure (x1.07) 0.881
Type 2 diabetes mellitus (x1.07) 0.885
Benign essential hypertension (x1.06) 0.889
Type 2 diabetes mellitus without complication (x1.06) 0.892
Chronic kidney disease due to type 2 diabetes mellitus (x1.06) 0.895
Anemia (x1.06) 0.898
Dyspnea (x1.06) 0.901
Hypertensive heart and renal disease with (congestive) heart failure (x1.05) 0.903
Hyperkalemia (x1.05) 0.906
Disorder of kidney and/or ureter (x1.05) 0.908
Chronic kidney disease stage 1 (x1.05) 0.910
Vitamin D deficiency (x1.05) 0.912
Electrocardiogram abnormal (x1.05) 0.914
Obstructive sleep apnea syndrome (x1.05) 0.916
Obesity (x1.05) 0.918
Atherosclerosis of coronary artery without angina pectoris (x1.04) 0.920
Heart failure (x1.04) 0.921
Atelectasis (x1.04) 0.923
furosemide (+0.00381) 0.925
Acidosis (x1.04) 0.926
Gastroesophageal reflux disease without esophagitis (x1.04) 0.927
Chest pain (x1.04) 0.929
Proteinuria (x1.04) 0.930
Hypokalemia (x1.04) 0.931
aspirin (+0.00349) 0.933
Iron deficiency anemia (x1.04) 0.934
Hypervolemia (x1.04) 0.935
Chronic systolic heart failure (x1.04) 0.936
Atrial fibrillation (x1.04) 0.937
Benign hypertensive renal disease with renal failure (x1.03) 0.938
Chronic combined systolic and diastolic heart failure (x1.03) 0.939
acetaminophen (+0.00317) 0.940
atorvastatin (+0.00313) 0.941
Cardiomegaly (x1.03) 0.942
Hyperglycemia due to type 2 diabetes mellitus (x1.03) 0.943
Gout (x1.03) 0.944
simvastatin (+0.00296) 0.945
Acute on chronic combined systolic and diastolic heart failure (x1.03) 0.946
Diabetes mellitus without complication (x1.03) 0.947
Chronic congestive heart failure (x1.03) 0.948
Cardiomyopathy (x1.03) 0.948
Malaise (x1.03) 0.949
Hypoxemia (x1.03) 0.950
Coronary arteriosclerosis (x1.03) 0.951
Anemia in chronic kidney disease (x1.03) 0.951
Disorder of lung (x1.03) 0.952
carvedilol (+0.00259) 0.953
Cardiac arrhythmia (x1.03) 0.953
Abnormal findings on diagnostic imaging of lung (x1.03) 0.954
Heart disease (x1.03) 0.955
Pleural effusion (x1.03) 0.955
lisinopril (+0.00239) 0.956
Diabetic - poor control (x1.03) 0.956
potassium chloride (+0.00228) 0.957
Acute on chronic systolic heart failure (x1.02) 0.957
Immunodeficiency disorder (x1.02) 0.958
Edema (x1.02) 0.958
metoprolol (+0.00216) 0.959
Chronic diastolic heart failure (x1.02) 0.959
Hypertensive heart failure (x1.02) 0.960
Acute posthemorrhagic anemia (x1.02) 0.960
Low blood pressure (x1.02) 0.961
Kidney stone (x1.02) 0.961
Leukocytosis (x1.02) 0.961
Primary cardiomyopathy (x1.02) 0.962
Blood chemistry abnormal (x1.02) 0.962
Pure hypercholesterolemia (x1.02) 0.963
Chronic pain (x1.02) 0.963
Urinary tract infectious disease (x1.02) 0.963
Disorder of kidney due to diabetes mellitus (x1.02) 0.964
Hypo-osmolality and or hyponatremia (x1.02) 0.964
Generalized ischemic myocardial dysfunction (x1.02) 0.964
Chronic obstructive lung disease (x1.02) 0.965
Urea nitrogen [M/V] in SP (+0.326) 0.965
Urate [M/V] in SP (+0.0797) 0.965
Morbid obesity (x1.02) 0.966
Postoperative state (x1.02) 0.966
gabapentin (+0.00176) 0.966
Chronic ischemic heart disease (x1.02) 0.967
Precordial pain (x1.02) 0.967
Aortocoronary bypass graft present (x1.02) 0.967
Paroxysmal atrial fibrillation (x1.02) 0.968
Dehydration (x1.02) 0.968
Polyneuropathy due to type 2 diabetes mellitus (x1.02) 0.968
Infection associated with implant (x1.02) 0.968
Malignant hypertensive heart AND renal disease (x1.02) 0.969
Pulmonary hypertension (x1.02) 0.969
warfarin (+0.0016) 0.969
Expressions
−0.8 −0.6 −0.4 −0.2 0.0 0.2 0.4 0.6 0.8

Signature [34]; 28 To Reach 0.975, (28 Plotted)

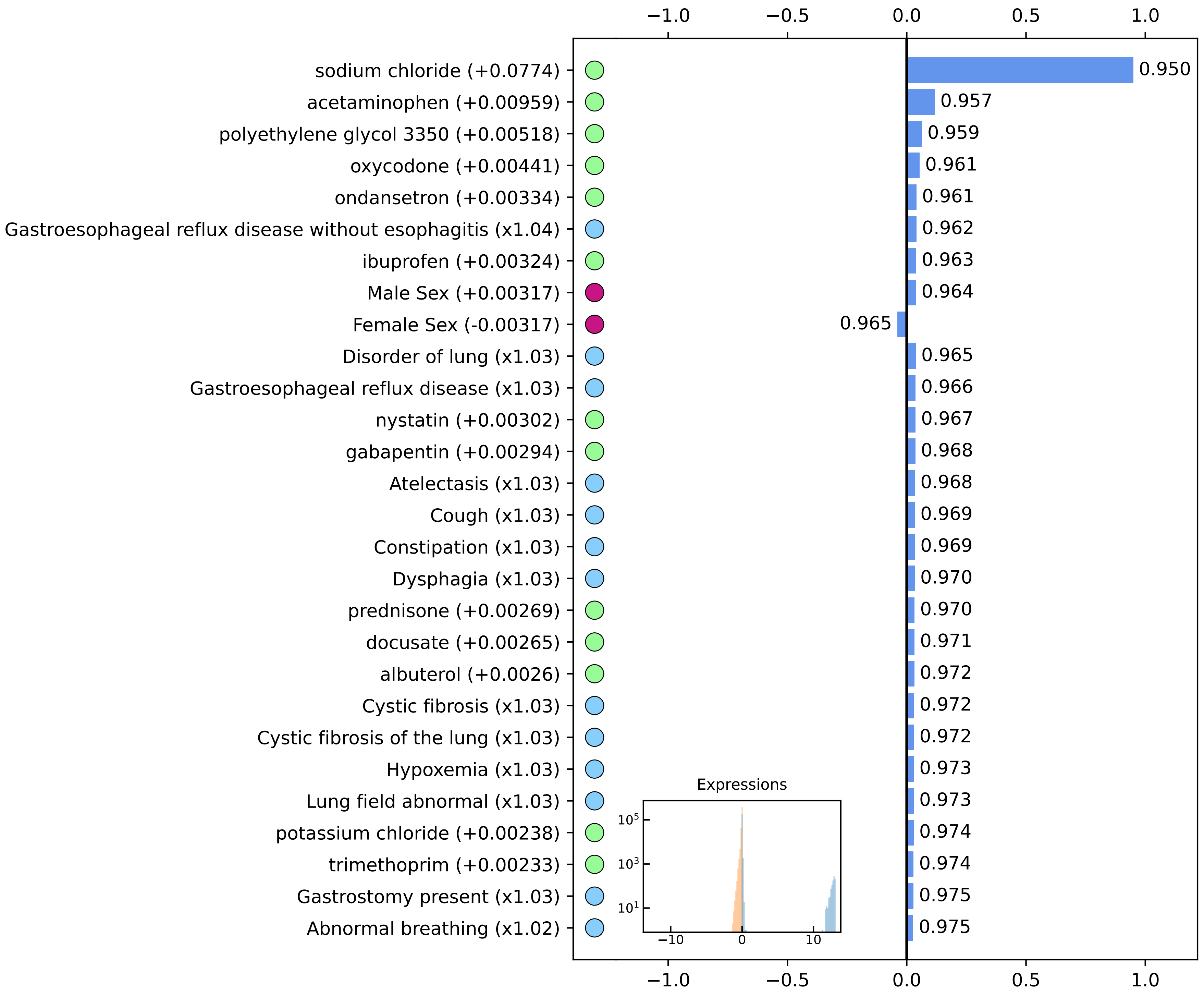

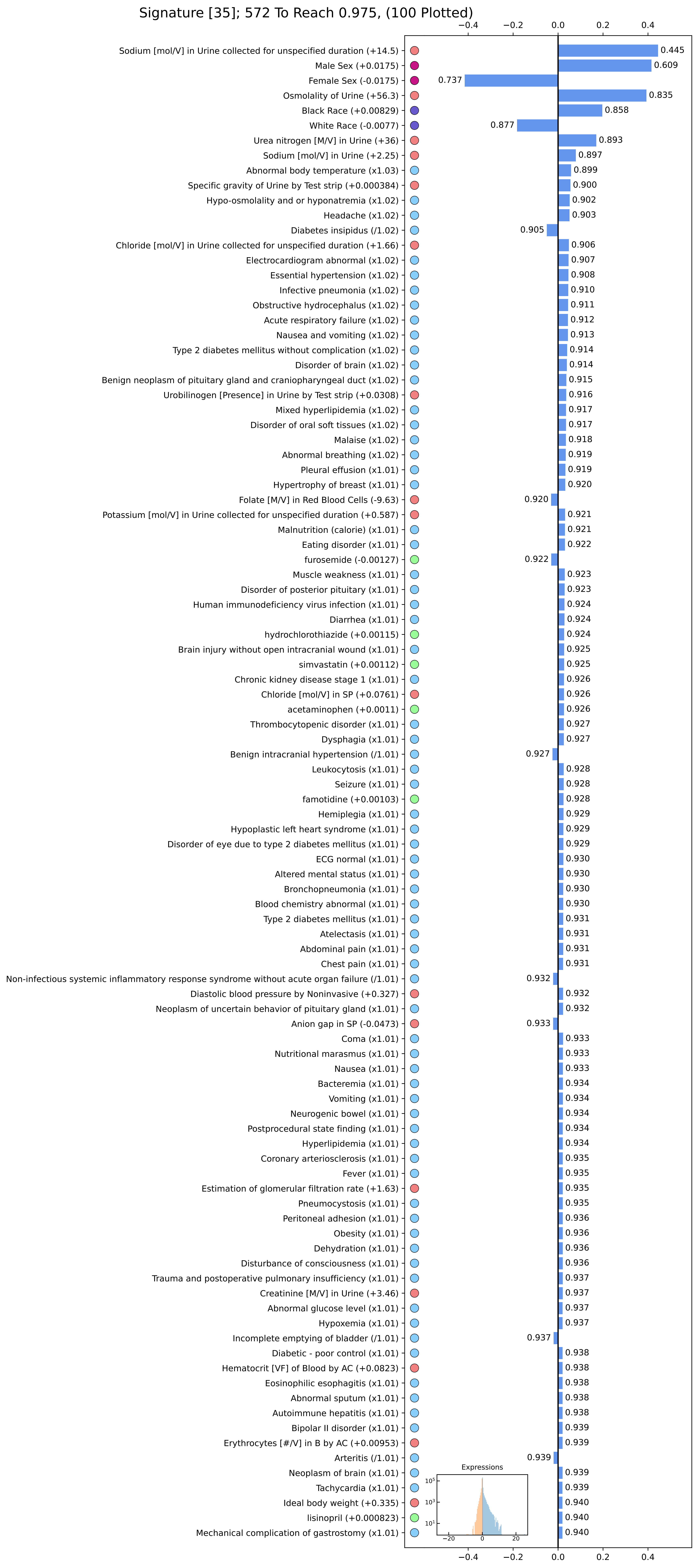

Signature [35]; 572 To Reach 0.975, (100 Plotted)
−0.4 −0.2 0.0 0.2 0.4
Sodium [mol/V] in Urine collected for unspecified duration (+14.5) 0.445
Male Sex (+0.0175) 0.609
Female Sex (-0.0175) 0.737
Osmolality of Urine (+56.3) 0.835
Black Race (+0.00829) 0.858
White Race (-0.0077) 0.877
Urea nitrogen [M/V] in Urine (+36) 0.893
Sodium [mol/V] in Urine (+2.25) 0.897
Abnormal body temperature (x1.03) 0.899
Specific gravity of Urine by Test strip (+0.000384) 0.900
Hypo-osmolality and or hyponatremia (x1.02) 0.902
Headache (x1.02) 0.903
Diabetes insipidus (/1.02) 0.905
Chloride [mol/V] in Urine collected for unspecified duration (+1.66) 0.906
Electrocardiogram abnormal (x1.02) 0.907
Essential hypertension (x1.02) 0.908
Infective pneumonia (x1.02) 0.910
Obstructive hydrocephalus (x1.02) 0.911
Acute respiratory failure (x1.02) 0.912
Nausea and vomiting (x1.02) 0.913
Type 2 diabetes mellitus without complication (x1.02) 0.914
Disorder of brain (x1.02) 0.914
Benign neoplasm of pituitary gland and craniopharyngeal duct (x1.02) 0.915
Urobilinogen [Presence] in Urine by Test strip (+0.0308) 0.916
Mixed hyperlipidemia (x1.02) 0.917
Disorder of oral soft tissues (x1.02) 0.917
Malaise (x1.02) 0.918
Abnormal breathing (x1.02) 0.919
Pleural effusion (x1.01) 0.919
Hypertrophy of breast (x1.01) 0.920
Folate [M/V] in Red Blood Cells (-9.63) 0.920
Potassium [mol/V] in Urine collected for unspecified duration (+0.587) 0.921
Malnutrition (calorie) (x1.01) 0.921
Eating disorder (x1.01) 0.922
furosemide (-0.00127) 0.922
Muscle weakness (x1.01) 0.923
Disorder of posterior pituitary (x1.01) 0.923
Human immunodeficiency virus infection (x1.01) 0.924
Diarrhea (x1.01) 0.924
hydrochlorothiazide (+0.00115) 0.924
Brain injury without open intracranial wound (x1.01) 0.925
simvastatin (+0.00112) 0.925
Chronic kidney disease stage 1 (x1.01) 0.926
Chloride [mol/V] in SP (+0.0761) 0.926
acetaminophen (+0.0011) 0.926
Thrombocytopenic disorder (x1.01) 0.927
Dysphagia (x1.01) 0.927
Benign intracranial hypertension (/1.01) 0.927
Leukocytosis (x1.01) 0.928
Seizure (x1.01) 0.928
famotidine (+0.00103) 0.928
Hemiplegia (x1.01) 0.929
Hypoplastic left heart syndrome (x1.01) 0.929
Disorder of eye due to type 2 diabetes mellitus (x1.01) 0.929
ECG normal (x1.01) 0.930
Altered mental status (x1.01) 0.930
Bronchopneumonia (x1.01) 0.930
Blood chemistry abnormal (x1.01) 0.930
Type 2 diabetes mellitus (x1.01) 0.931
Atelectasis (x1.01) 0.931
Abdominal pain (x1.01) 0.931
Chest pain (x1.01) 0.931
Non-infectious systemic inflammatory response syndrome without acute organ failure (/1.01) 0.932
Diastolic blood pressure by Noninvasive (+0.327) 0.932
Neoplasm of uncertain behavior of pituitary gland (x1.01) 0.932
Anion gap in SP (-0.0473) 0.933
Coma (x1.01) 0.933
Nutritional marasmus (x1.01) 0.933
Nausea (x1.01) 0.933
Bacteremia (x1.01) 0.934
Vomiting (x1.01) 0.934
Neurogenic bowel (x1.01) 0.934
Postprocedural state finding (x1.01) 0.934
Hyperlipidemia (x1.01) 0.934
Coronary arteriosclerosis (x1.01) 0.935
Fever (x1.01) 0.935
Estimation of glomerular filtration rate (+1.63) 0.935
Pneumocystosis (x1.01) 0.935
Peritoneal adhesion (x1.01) 0.936
Obesity (x1.01) 0.936
Dehydration (x1.01) 0.936
Disturbance of consciousness (x1.01) 0.936
Trauma and postoperative pulmonary insufficiency (x1.01) 0.937
Creatinine [M/V] in Urine (+3.46) 0.937
Abnormal glucose level (x1.01) 0.937
Hypoxemia (x1.01) 0.937
Incomplete emptying of bladder (/1.01) 0.937
Diabetic - poor control (x1.01) 0.938
Hematocrit [VF] of Blood by AC (+0.0823) 0.938
Eosinophilic esophagitis (x1.01) 0.938
Abnormal sputum (x1.01) 0.938
Autoimmune hepatitis (x1.01) 0.938
Bipolar II disorder (x1.01) 0.939
Erythrocytes [#/V] in B by AC (+0.00953) 0.939
Arteritis (/1.01) 0.939
Neoplasm of brain (x1.01) 0.939
Tachycardia (x1.01) 0.939
Ideal body weight (+0.335) 0.940
lisinopril (+0.000823) 0.940
Mechanical complication of gastrostomy (x1.01) 0.940
Expressions
10⁵ 10³ 10¹
−20 0 20
−0.4 −0.2 0.0 0.2 0.4

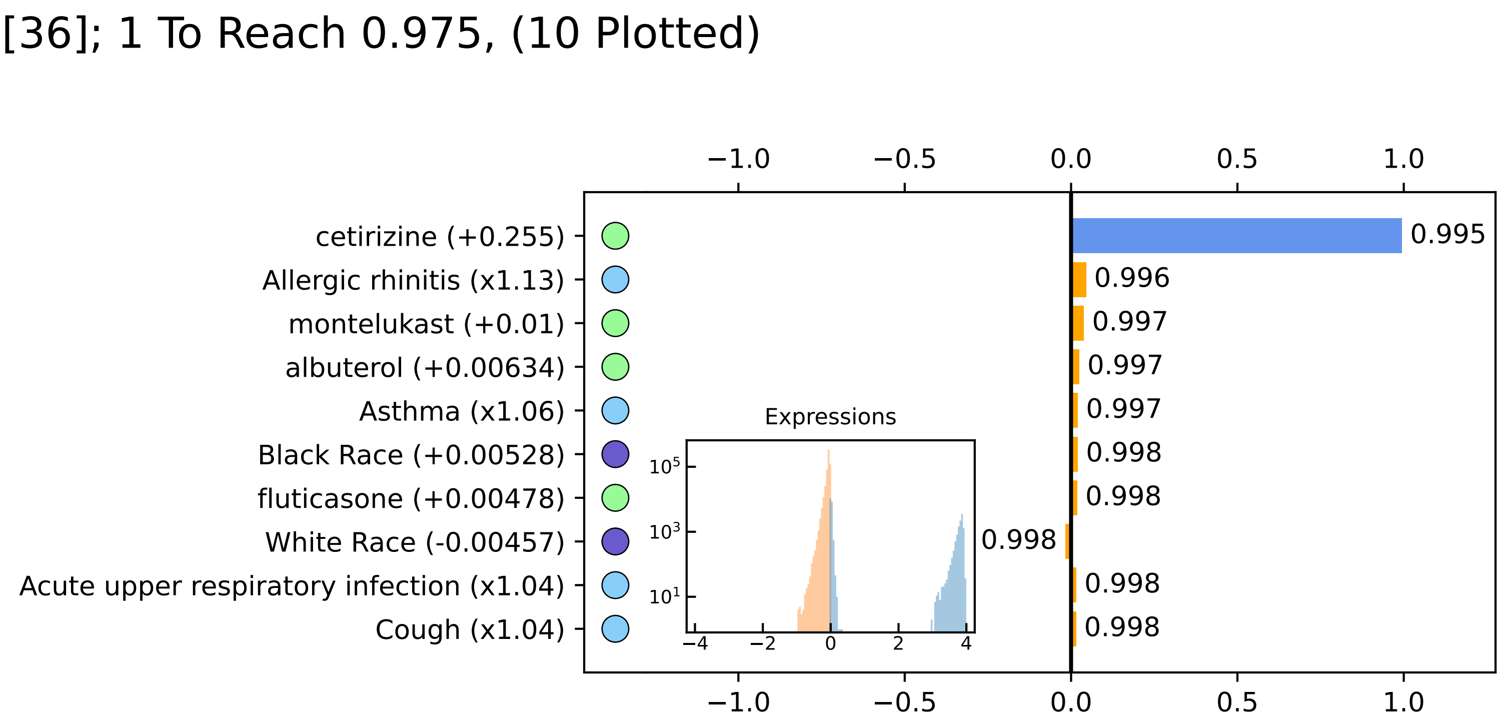
Signature [36]; 1 To Reach 0.975, (10 Plotted)
cetirizine (+0.255)
Allergic rhinitis (x1.13)
montelukast (+0.01)
albuterol (+0.00634)
Asthma (x1.06)
Black Race (+0.00528)
fluticasone (+0.00478)
White Race (-0.00457)
Acute upper respiratory infection (x1.04)
Cough (x1.04)
0.995
0.996
0.997
0.997
0.997
0.998
0.998
0.998
0.998
0.998
−1.0
−0.5
0.0
0.5
1.0
Expressions
10^5
10^3
10^1
−4
−2
0
2
4

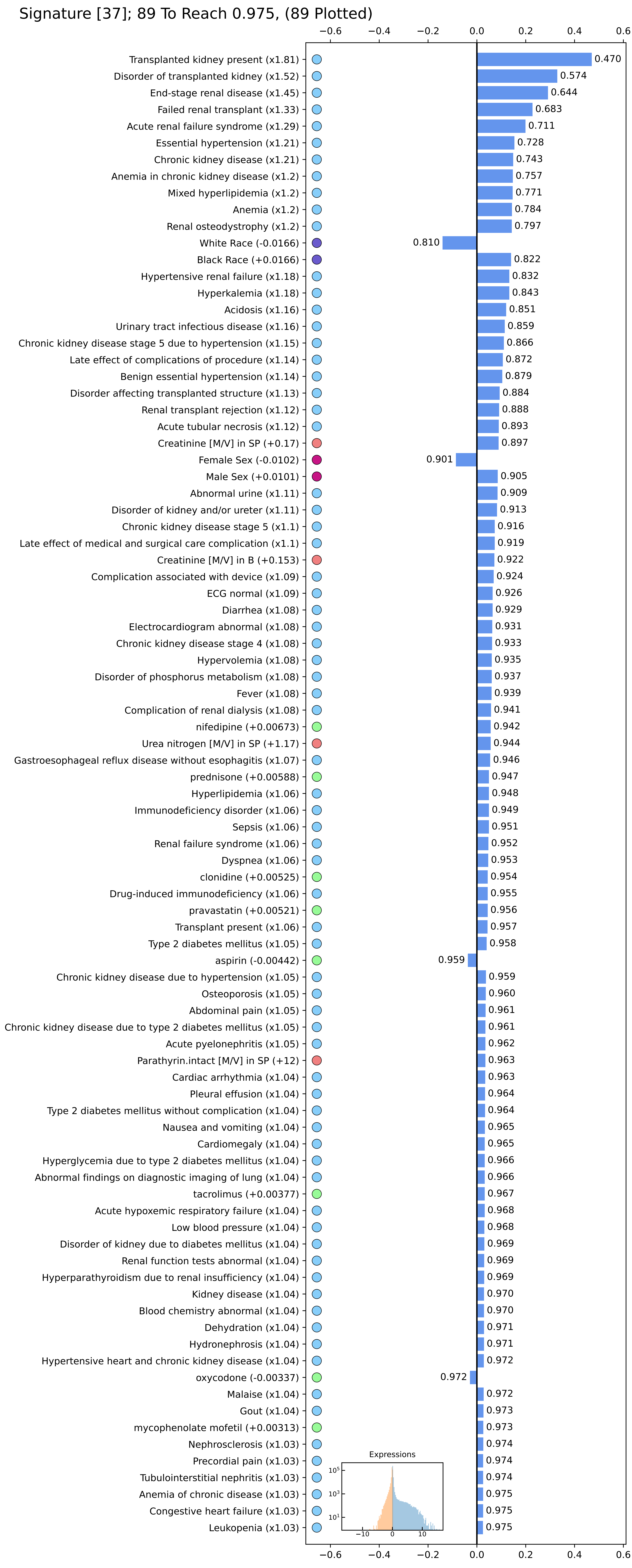
Signature [37]; 89 To Reach 0.975, (89 Plotted)
−0.6 −0.4 −0.2 0.0 0.2 0.4 0.6
Transplanted kidney present (x1.81) 0.470
Disorder of transplanted kidney (x1.52) 0.574
End-stage renal disease (x1.45) 0.644
Failed renal transplant (x1.33) 0.683
Acute renal failure syndrome (x1.29) 0.711
Essential hypertension (x1.21) 0.728
Chronic kidney disease (x1.21) 0.743
Anemia in chronic kidney disease (x1.2) 0.757
Mixed hyperlipidemia (x1.2) 0.771
Anemia (x1.2) 0.784
Renal osteodystrophy (x1.2) 0.797
White Race (-0.0166) 0.810
Black Race (+0.0166) 0.822
Hypertensive renal failure (x1.18) 0.832
Hyperkalemia (x1.18) 0.843
Acidosis (x1.16) 0.851
Urinary tract infectious disease (x1.16) 0.859
Chronic kidney disease stage 5 due to hypertension (x1.15) 0.866
Late effect of complications of procedure (x1.14) 0.872
Benign essential hypertension (x1.14) 0.879
Disorder affecting transplanted structure (x1.13) 0.884
Renal transplant rejection (x1.12) 0.888
Acute tubular necrosis (x1.12) 0.893
Creatinine [M/V] in SP (+0.17) 0.897
Female Sex (-0.0102) 0.901
Male Sex (+0.0101) 0.905
Abnormal urine (x1.11) 0.909
Disorder of kidney and/or ureter (x1.11) 0.913
Chronic kidney disease stage 5 (x1.1) 0.916
Late effect of medical and surgical care complication (x1.1) 0.919
Creatinine [M/V] in B (+0.153) 0.922
Complication associated with device (x1.09) 0.924
ECG normal (x1.09) 0.926
Diarrhea (x1.08) 0.929
Electrocardiogram abnormal (x1.08) 0.931
Chronic kidney disease stage 4 (x1.08) 0.933
Hypervolemia (x1.08) 0.935
Disorder of phosphorus metabolism (x1.08) 0.937
Fever (x1.08) 0.939
Complication of renal dialysis (x1.08) 0.941
nifedipine (+0.00673) 0.942
Urea nitrogen [M/V] in SP (+1.17) 0.944
Gastroesophageal reflux disease without esophagitis (x1.07) 0.946
prednisone (+0.00588) 0.947
Hyperlipidemia (x1.06) 0.948
Immunodeficiency disorder (x1.06) 0.949
Sepsis (x1.06) 0.951
Renal failure syndrome (x1.06) 0.952
Dyspnea (x1.06) 0.953
clonidine (+0.00525) 0.954
Drug-induced immunodeficiency (x1.06) 0.955
pravastatin (+0.00521) 0.956
Transplant present (x1.06) 0.957
Type 2 diabetes mellitus (x1.05) 0.958
aspirin (-0.00442) 0.959
Chronic kidney disease due to hypertension (x1.05) 0.959
Osteoporosis (x1.05) 0.960
Abdominal pain (x1.05) 0.961
Chronic kidney disease due to type 2 diabetes mellitus (x1.05) 0.961
Acute pyelonephritis (x1.05) 0.962
Parathyrin.intact [M/V] in SP (+12) 0.963
Cardiac arrhythmia (x1.04) 0.963
Pleural effusion (x1.04) 0.964
Type 2 diabetes mellitus without complication (x1.04) 0.964
Nausea and vomiting (x1.04) 0.965
Cardiomegaly (x1.04) 0.965
Hyperglycemia due to type 2 diabetes mellitus (x1.04) 0.966
Abnormal findings on diagnostic imaging of lung (x1.04) 0.966
tacrolimus (+0.00377) 0.967
Acute hypoxemic respiratory failure (x1.04) 0.968
Low blood pressure (x1.04) 0.968
Disorder of kidney due to diabetes mellitus (x1.04) 0.969
Renal function tests abnormal (x1.04) 0.969
Hyperparathyroidism due to renal insufficiency (x1.04) 0.969
Kidney disease (x1.04) 0.970
Blood chemistry abnormal (x1.04) 0.970
Dehydration (x1.04) 0.971
Hydronephrosis (x1.04) 0.971
Hypertensive heart and chronic kidney disease (x1.04) 0.972
oxycodone (-0.00337) 0.972
Malaise (x1.04) 0.972
Gout (x1.04) 0.973
mycophenolate mofetil (+0.00313) 0.973
Nephrosclerosis (x1.03) 0.974
Precordial pain (x1.03) 0.974
Tubulointerstitial nephritis (x1.03) 0.974
Anemia of chronic disease (x1.03) 0.975
Congestive heart failure (x1.03) 0.975
Leukopenia (x1.03) 0.975
Expressions
−10 0 10
−0.6 −0.4 −0.2 0.0 0.2 0.4 0.6

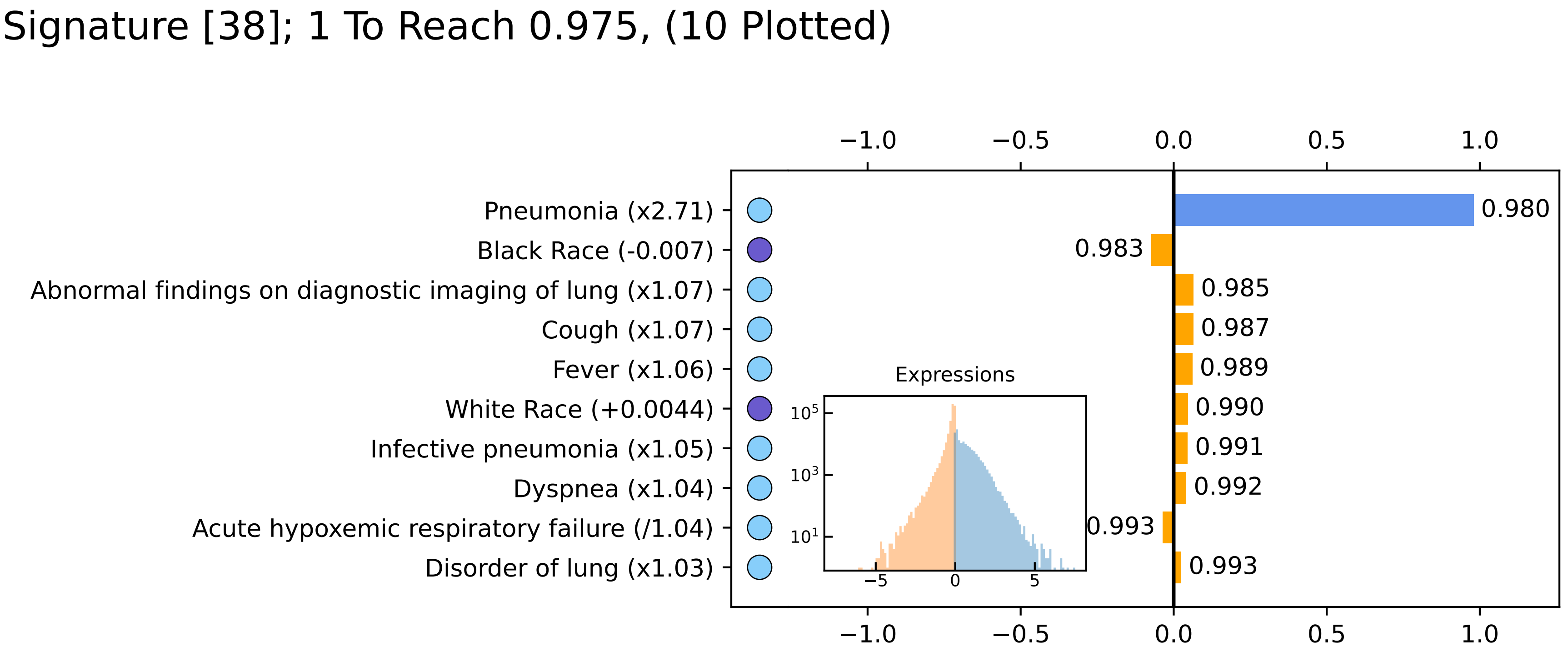
Signature [38]; 1 To Reach 0.975, (10 Plotted)
Pneumonia (x2.71)
Black Race (-0.007)
Abnormal findings on diagnostic imaging of lung (x1.07)
Cough (x1.07)
Fever (x1.06)
White Race (+0.0044)
Infective pneumonia (x1.05)
Dyspnea (x1.04)
Acute hypoxemic respiratory failure (/1.04)
Disorder of lung (x1.03)
0.980
0.983
0.985
0.987
0.989
0.990
0.991
0.992
0.993
0.993
Expressions
−1.0
−0.5
0.0
0.5
1.0

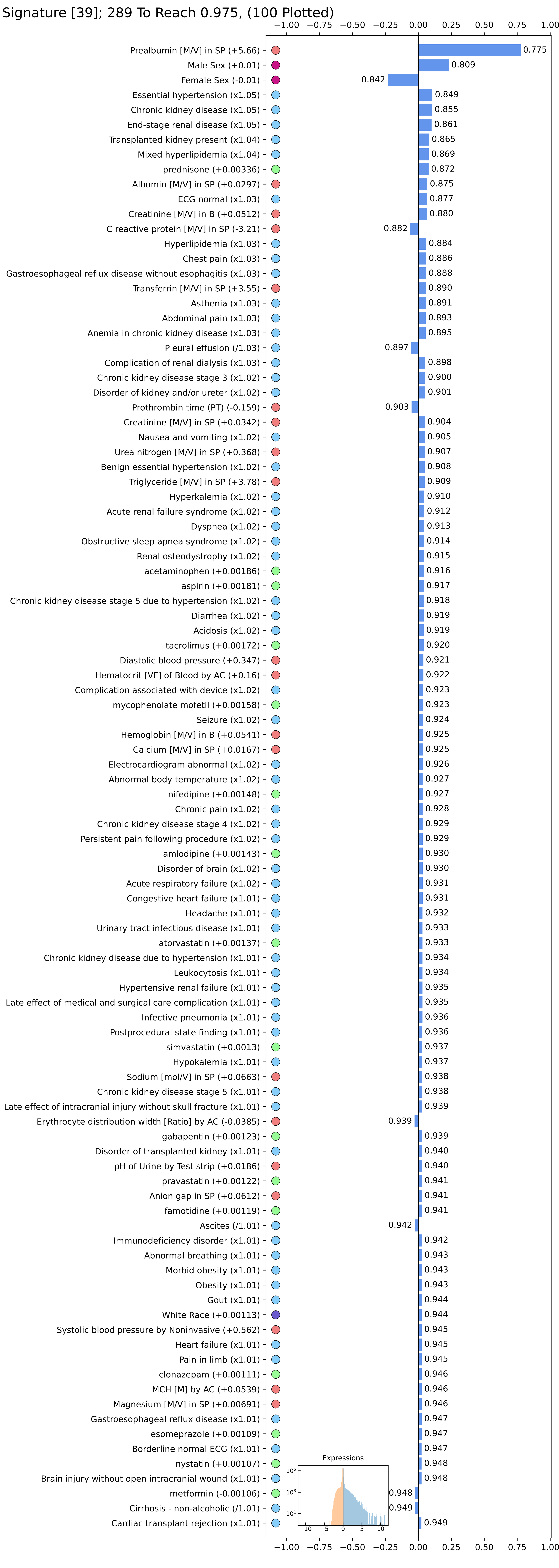

Signature [39]; 289 To Reach 0.975, (100 Plotted)
−1.00 −0.75 −0.50 −0.25 0.00 0.25 0.50 0.75 1.00
Prealbumin [M/V] in SP (+5.66) 0.775
Male Sex (+0.01) 0.809
Female Sex (-0.01) 0.842
Essential hypertension (x1.05) 0.849
Chronic kidney disease (x1.05) 0.855
End-stage renal disease (x1.05) 0.861
Transplanted kidney present (x1.04) 0.865
Mixed hyperlipidemia (x1.04) 0.869
prednisone (+0.00336) 0.872
Albumin [M/V] in SP (+0.0297) 0.875
ECG normal (x1.03) 0.877
Creatinine [M/V] in B (+0.0512) 0.880
C reactive protein [M/V] in SP (-3.21) 0.882
Hyperlipidemia (x1.03) 0.884
Chest pain (x1.03) 0.886
Gastroesophageal reflux disease without esophagitis (x1.03) 0.888
Transferrin [M/V] in SP (+3.55) 0.890
Asthenia (x1.03) 0.891
Abdominal pain (x1.03) 0.893
Anemia in chronic kidney disease (x1.03) 0.895
Pleural effusion (/1.03) 0.897
Complication of renal dialysis (x1.03) 0.898
Chronic kidney disease stage 3 (x1.02) 0.900
Disorder of kidney and/or ureter (x1.02) 0.901
Prothrombin time (PT) (-0.159) 0.903
Creatinine [M/V] in SP (+0.0342) 0.904
Nausea and vomiting (x1.02) 0.905
Urea nitrogen [M/V] in SP (+0.368) 0.907
Benign essential hypertension (x1.02) 0.908
Triglyceride [M/V] in SP (+3.78) 0.909
Hyperkalemia (x1.02) 0.910
Acute renal failure syndrome (x1.02) 0.912
Dyspnea (x1.02) 0.913
Obstructive sleep apnea syndrome (x1.02) 0.914
Renal osteodystrophy (x1.02) 0.915
acetaminophen (+0.00186) 0.916
aspirin (+0.00181) 0.917
Chronic kidney disease stage 5 due to hypertension (x1.02) 0.918
Diarrhea (x1.02) 0.919
Acidosis (x1.02) 0.919
tacrolimus (+0.00172) 0.920
Diastolic blood pressure (+0.347) 0.921
Hematocrit [VF] of Blood by AC (+0.16) 0.922
Complication associated with device (x1.02) 0.923
mycophenolate mofetil (+0.00158) 0.923
Seizure (x1.02) 0.924
Hemoglobin [M/V] in B (+0.0541) 0.925
Calcium [M/V] in SP (+0.0167) 0.925
Electrocardiogram abnormal (x1.02) 0.926
Abnormal body temperature (x1.02) 0.927
nifedipine (+0.00148) 0.927
Chronic pain (x1.02) 0.928
Chronic kidney disease stage 4 (x1.02) 0.929
Persistent pain following procedure (x1.02) 0.929
amlodipine (+0.00143) 0.930
Disorder of brain (x1.02) 0.930
Acute respiratory failure (x1.02) 0.931
Congestive heart failure (x1.01) 0.931
Headache (x1.01) 0.932
Urinary tract infectious disease (x1.01) 0.933
atorvastatin (+0.00137) 0.933
Chronic kidney disease due to hypertension (x1.01) 0.934
Leukocytosis (x1.01) 0.934
Hypertensive renal failure (x1.01) 0.935
Late effect of medical and surgical care complication (x1.01) 0.935
Infective pneumonia (x1.01) 0.936
Postprocedural state finding (x1.01) 0.936
simvastatin (+0.0013) 0.937
Hypokalemia (x1.01) 0.937
Sodium [mol/V] in SP (+0.0663) 0.938
Chronic kidney disease stage 5 (x1.01) 0.938
Late effect of intracranial injury without skull fracture (x1.01) 0.939
Erythrocyte distribution width [Ratio] by AC (-0.0385) 0.939
gabapentin (+0.00123) 0.939
Disorder of transplanted kidney (x1.01) 0.940
pH of Urine by Test strip (+0.0186) 0.940
pravastatin (+0.00122) 0.941
Anion gap in SP (+0.0612) 0.941
famotidine (+0.00119) 0.941
Ascites (/1.01) 0.942
Immunodeficiency disorder (x1.01) 0.942
Abnormal breathing (x1.01) 0.943
Morbid obesity (x1.01) 0.943
Obesity (x1.01) 0.943
Gout (x1.01) 0.944
White Race (+0.00113) 0.944
Systolic blood pressure by Noninvasive (+0.562) 0.945
Heart failure (x1.01) 0.945
Pain in limb (x1.01) 0.945
clonazepam (+0.00111) 0.946
MCH [M] by AC (+0.0539) 0.946
Magnesium [M/V] in SP (+0.00691) 0.946
Gastroesophageal reflux disease (x1.01) 0.947
esomeprazole (+0.00109) 0.947
Borderline normal ECG (x1.01) 0.947
nystatin (+0.00107) 0.948
Brain injury without open intracranial wound (x1.01) 0.948
metformin (-0.00106) 0.948
Cirrhosis - non-alcoholic (/1.01) 0.949
Cardiac transplant rejection (x1.01) 0.949
Expressions
10^5
10^3
10^1
−10 −5 0 5 10

Signature [40]; 103 To Reach 0.975, (100 Plotted)

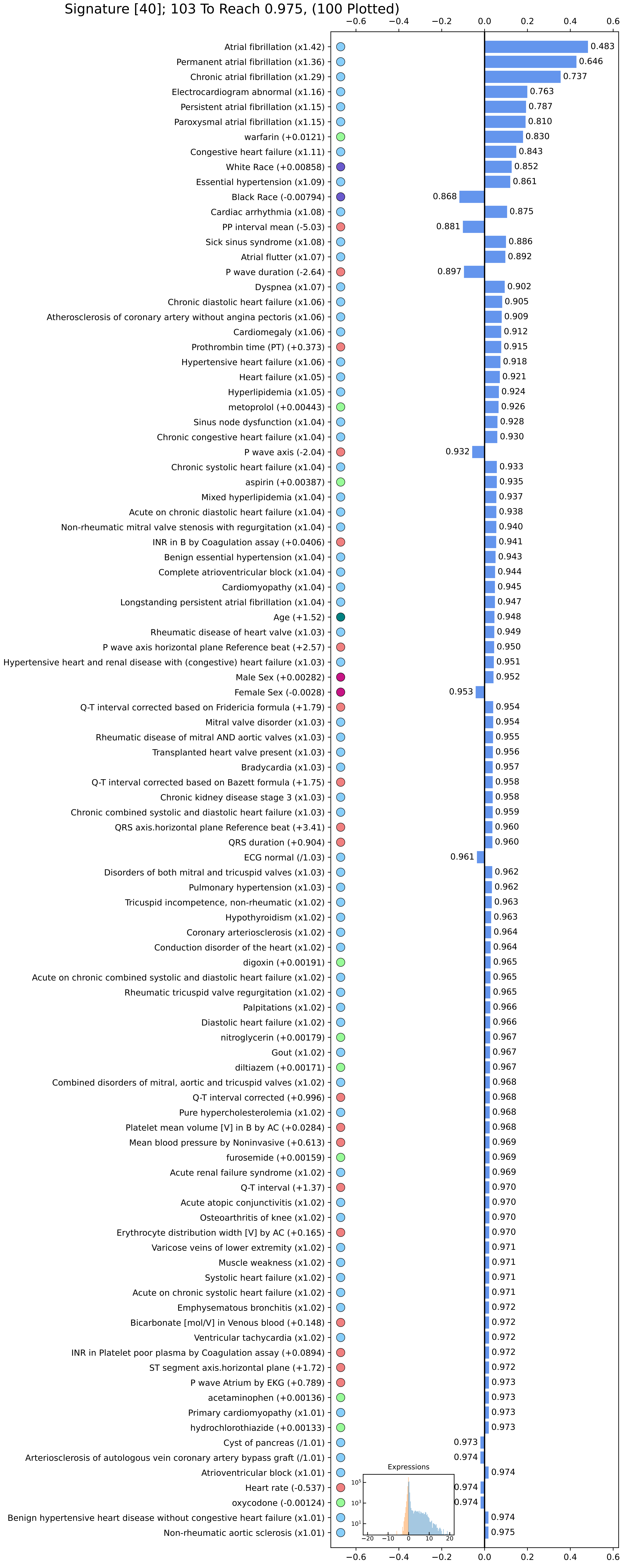

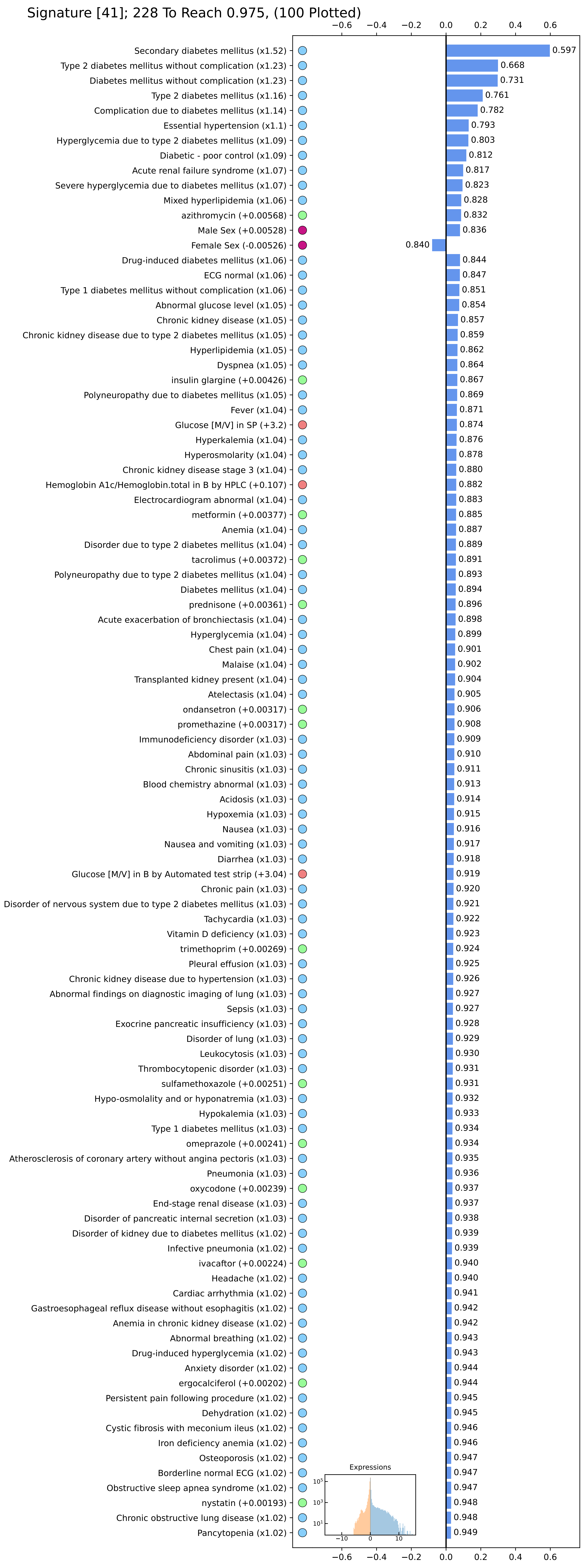
Signature [41]; 228 To Reach 0.975, (100 Plotted)
Secondary diabetes mellitus (x1.52) 0.597
Type 2 diabetes mellitus without complication (x1.23) 0.668
Diabetes mellitus without complication (x1.23) 0.731
Type 2 diabetes mellitus (x1.16) 0.761
Complication due to diabetes mellitus (x1.14) 0.782
Essential hypertension (x1.1) 0.793
Hyperglycemia due to type 2 diabetes mellitus (x1.09) 0.803
Diabetic - poor control (x1.09) 0.812
Acute renal failure syndrome (x1.07) 0.817
Severe hyperglycemia due to diabetes mellitus (x1.07) 0.823
Mixed hyperlipidemia (x1.06) 0.828
azithromycin (+0.00568) 0.832
Male Sex (+0.00528) 0.836
Female Sex (-0.00526) 0.840
Drug-induced diabetes mellitus (x1.06) 0.844
ECG normal (x1.06) 0.847
Type 1 diabetes mellitus without complication (x1.06) 0.851
Abnormal glucose level (x1.05) 0.854
Chronic kidney disease (x1.05) 0.857
Chronic kidney disease due to type 2 diabetes mellitus (x1.05) 0.859
Hyperlipidemia (x1.05) 0.862
Dyspnea (x1.05) 0.864
insulin glargine (+0.00426) 0.867
Polyneuropathy due to diabetes mellitus (x1.05) 0.869
Fever (x1.04) 0.871
Glucose [M/V] in SP (+3.2) 0.874
Hyperkalemia (x1.04) 0.876
Hyperosmolarity (x1.04) 0.878
Chronic kidney disease stage 3 (x1.04) 0.880
Hemoglobin A1c/Hemoglobin.total in B by HPLC (+0.107) 0.882
Electrocardiogram abnormal (x1.04) 0.883
metformin (+0.00377) 0.885
Anemia (x1.04) 0.887
Disorder due to type 2 diabetes mellitus (x1.04) 0.889
tacrolimus (+0.00372) 0.891
Polyneuropathy due to type 2 diabetes mellitus (x1.04) 0.893
Diabetes mellitus (x1.04) 0.894
prednisone (+0.00361) 0.896
Acute exacerbation of bronchiectasis (x1.04) 0.898
Hyperglycemia (x1.04) 0.899
Chest pain (x1.04) 0.901
Malaise (x1.04) 0.902
Transplanted kidney present (x1.04) 0.904
Atelectasis (x1.04) 0.905
ondansetron (+0.00317) 0.906
promethazine (+0.00317) 0.908
Immunodeficiency disorder (x1.03) 0.909
Abdominal pain (x1.03) 0.910
Chronic sinusitis (x1.03) 0.911
Blood chemistry abnormal (x1.03) 0.913
Acidosis (x1.03) 0.914
Hypoxemia (x1.03) 0.915
Nausea (x1.03) 0.916
Nausea and vomiting (x1.03) 0.917
Diarrhea (x1.03) 0.918
Glucose [M/V] in B by Automated test strip (+3.04) 0.919
Chronic pain (x1.03) 0.920
Disorder of nervous system due to type 2 diabetes mellitus (x1.03) 0.921
Tachycardia (x1.03) 0.922
Vitamin D deficiency (x1.03) 0.923
trimethoprim (+0.00269) 0.924
Pleural effusion (x1.03) 0.925
Chronic kidney disease due to hypertension (x1.03) 0.926
Abnormal findings on diagnostic imaging of lung (x1.03) 0.927
Sepsis (x1.03) 0.927
Exocrine pancreatic insufficiency (x1.03) 0.928
Disorder of lung (x1.03) 0.929
Leukocytosis (x1.03) 0.930
Thrombocytopenic disorder (x1.03) 0.931
sulfamethoxazole (+0.00251) 0.931
Hypo-osmolality and or hyponatremia (x1.03) 0.932
Hypokalemia (x1.03) 0.933
Type 1 diabetes mellitus (x1.03) 0.934
omeprazole (+0.00241) 0.934
Atherosclerosis of coronary artery without angina pectoris (x1.03) 0.935
Pneumonia (x1.03) 0.936
oxycodone (+0.00239) 0.937
End-stage renal disease (x1.03) 0.937
Disorder of pancreatic internal secretion (x1.03) 0.938
Disorder of kidney due to diabetes mellitus (x1.02) 0.939
Infective pneumonia (x1.02) 0.939
ivacaftor (+0.00224) 0.940
Headache (x1.02) 0.940
Cardiac arrhythmia (x1.02) 0.941
Gastroesophageal reflux disease without esophagitis (x1.02) 0.942
Anemia in chronic kidney disease (x1.02) 0.942
Abnormal breathing (x1.02) 0.943
Drug-induced hyperglycemia (x1.02) 0.943
Anxiety disorder (x1.02) 0.944
ergocalciferol (+0.00202) 0.944
Persistent pain following procedure (x1.02) 0.945
Dehydration (x1.02) 0.945
Cystic fibrosis with meconium ileus (x1.02) 0.946
Iron deficiency anemia (x1.02) 0.946
Osteoporosis (x1.02) 0.947
Borderline normal ECG (x1.02) 0.947
Obstructive sleep apnea syndrome (x1.02) 0.947
nystatin (+0.00193) 0.948
Chronic obstructive lung disease (x1.02) 0.948
Pancytopenia (x1.02) 0.949
Expressions
−0.6 −0.4 −0.2 0.0 0.2 0.4 0.6

Signature [42]; 215 To Reach 0.975, (100 Plotted)

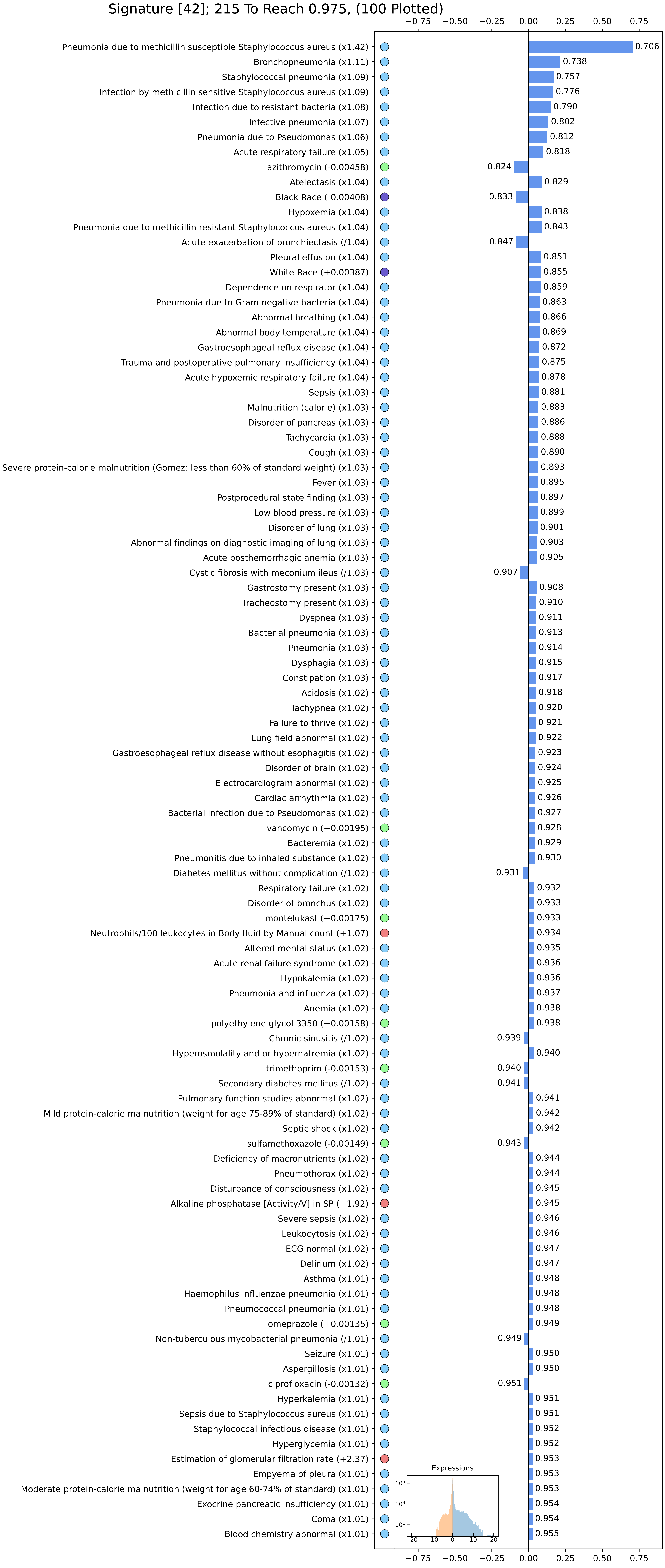

Signature [43]; 36 To Reach 0.975, (36 Plotted)

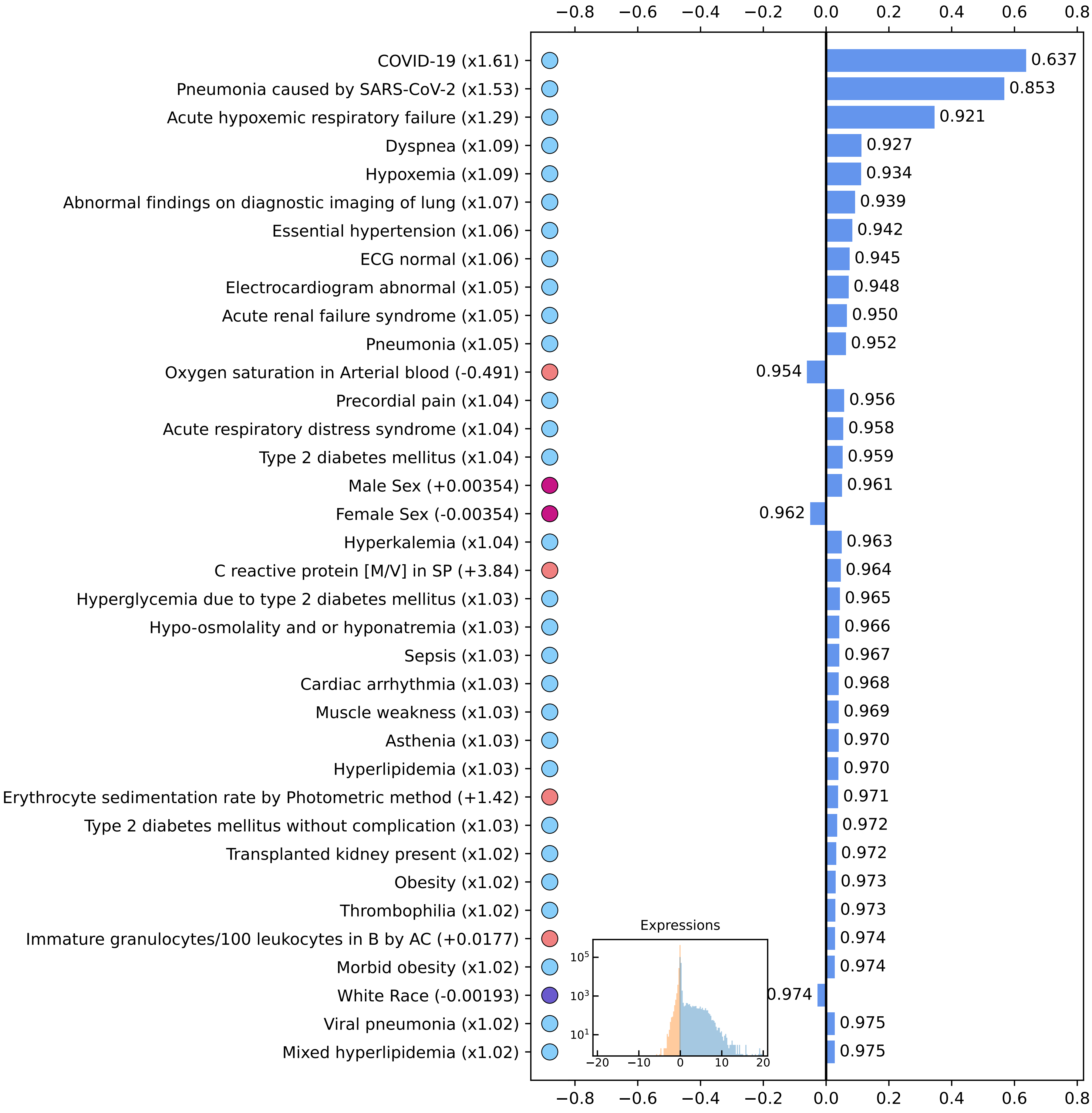

Signature [44]; 7 To Reach 0.975, (10 Plotted)

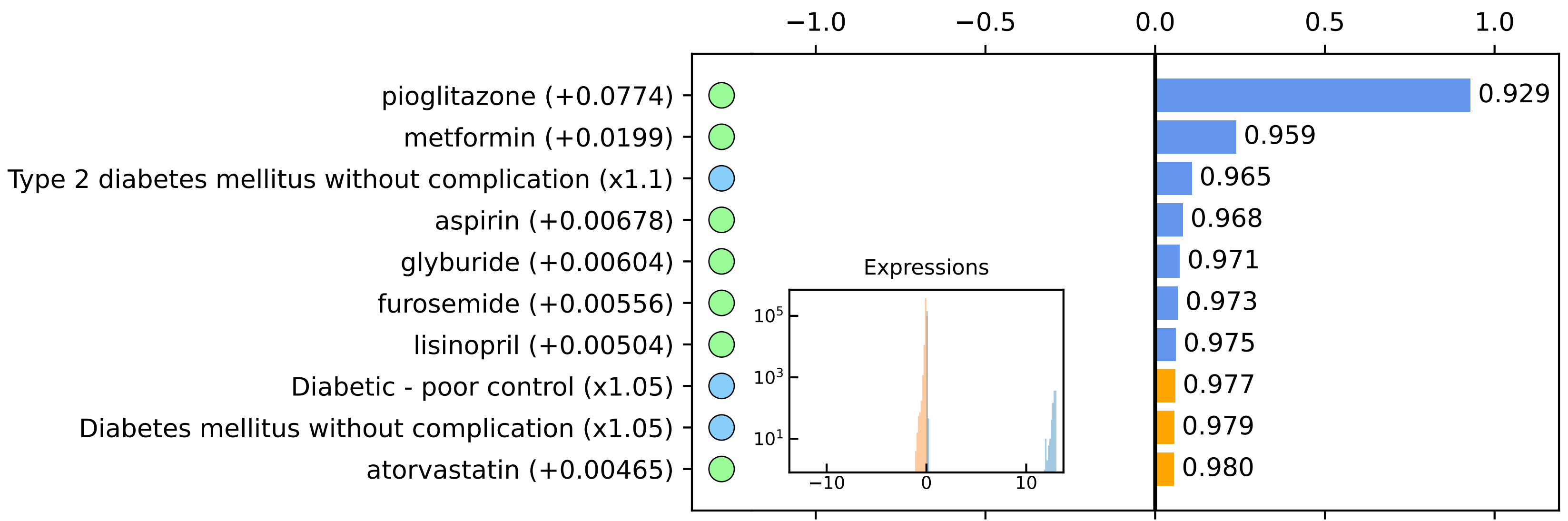

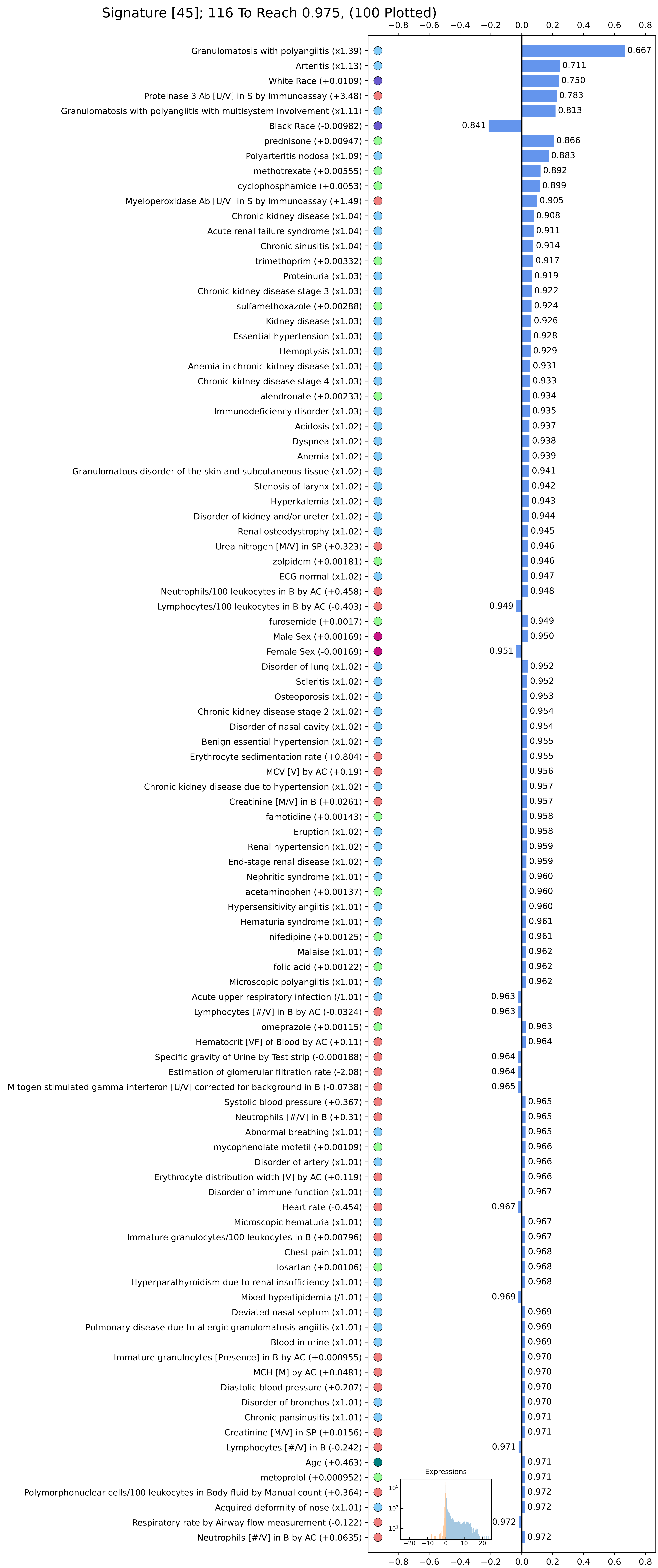

Signature [45]; 116 To Reach 0.975, (100 Plotted)
Granulomatosis with polyangiitis (x1.39) 0.667
Arteritis (x1.13) 0.711
White Race (+0.0109) 0.750
Proteinase 3 Ab [U/V] in S by Immunoassay (+3.48) 0.783
Granulomatosis with polyangiitis with multisystem involvement (x1.11) 0.813
Black Race (-0.00982) 0.841
prednisone (+0.00947) 0.866
Polyarteritis nodosa (x1.09) 0.883
methotrexate (+0.00555) 0.892
cyclophosphamide (+0.0053) 0.899
Myeloperoxidase Ab [U/V] in S by Immunoassay (+1.49) 0.905
Chronic kidney disease (x1.04) 0.908
Acute renal failure syndrome (x1.04) 0.911
Chronic sinusitis (x1.04) 0.914
trimethoprim (+0.00332) 0.917
Proteinuria (x1.03) 0.919
Chronic kidney disease stage 3 (x1.03) 0.922
sulfamethoxazole (+0.00288) 0.924
Kidney disease (x1.03) 0.926
Essential hypertension (x1.03) 0.928
Hemoptysis (x1.03) 0.929
Anemia in chronic kidney disease (x1.03) 0.931
Chronic kidney disease stage 4 (x1.03) 0.933
alendronate (+0.00233) 0.934
Immunodeficiency disorder (x1.03) 0.935
Acidosis (x1.02) 0.937
Dyspnea (x1.02) 0.938
Anemia (x1.02) 0.939
Granulomatous disorder of the skin and subcutaneous tissue (x1.02) 0.941
Stenosis of larynx (x1.02) 0.942
Hyperkalemia (x1.02) 0.943
Disorder of kidney and/or ureter (x1.02) 0.944
Renal osteodystrophy (x1.02) 0.945
Urea nitrogen [M/V] in SP (+0.323) 0.946
zolpidem (+0.00181) 0.946
ECG normal (x1.02) 0.947
Neutrophils/100 leukocytes in B by AC (+0.458) 0.948
Lymphocytes/100 leukocytes in B by AC (-0.403) 0.949
furosemide (+0.0017) 0.949
Male Sex (+0.00169) 0.950
Female Sex (-0.00169) 0.951
Disorder of lung (x1.02) 0.952
Scleritis (x1.02) 0.952
Osteoporosis (x1.02) 0.953
Chronic kidney disease stage 2 (x1.02) 0.954
Disorder of nasal cavity (x1.02) 0.954
Benign essential hypertension (x1.02) 0.955
Erythrocyte sedimentation rate (+0.804) 0.955
MCV [V] by AC (+0.19) 0.956
Chronic kidney disease due to hypertension (x1.02) 0.957
Creatinine [M/V] in B (+0.0261) 0.957
famotidine (+0.00143) 0.958
Eruption (x1.02) 0.958
Renal hypertension (x1.02) 0.959
End-stage renal disease (x1.02) 0.959
Nephritic syndrome (x1.01) 0.960
acetaminophen (+0.00137) 0.960
Hypersensitivity angiitis (x1.01) 0.960
Hematuria syndrome (x1.01) 0.961
nifedipine (+0.00125) 0.961
Malaise (x1.01) 0.962
folic acid (+0.00122) 0.962
Microscopic polyangiitis (x1.01) 0.962
Acute upper respiratory infection (/1.01) 0.963
Lymphocytes [#/V] in B by AC (-0.0324) 0.963
omeprazole (+0.00115) 0.963
Hematocrit [VF] of Blood by AC (+0.11) 0.964
Specific gravity of Urine by Test strip (-0.000188) 0.964
Estimation of glomerular filtration rate (-2.08) 0.964
Mitogen stimulated gamma interferon [U/V] corrected for background in B (-0.0738) 0.965
Systolic blood pressure (+0.367) 0.965
Neutrophils [#/V] in B (+0.31) 0.965
Abnormal breathing (x1.01) 0.965
mycophenolate mofetil (+0.00109) 0.966
Disorder of artery (x1.01) 0.966
Erythrocyte distribution width [V] by AC (+0.119) 0.966
Disorder of immune function (x1.01) 0.967
Heart rate (-0.454) 0.967
Microscopic hematuria (x1.01) 0.967
Immature granulocytes/100 leukocytes in B (+0.00796) 0.967
Chest pain (x1.01) 0.968
losartan (+0.00106) 0.968
Hyperparathyroidism due to renal insufficiency (x1.01) 0.968
Mixed hyperlipidemia (/1.01) 0.969
Deviated nasal septum (x1.01) 0.969
Pulmonary disease due to allergic granulomatosis angiitis (x1.01) 0.969
Blood in urine (x1.01) 0.969
Immature granulocytes [Presence] in B by AC (+0.000955) 0.970
MCH [M] by AC (+0.0481) 0.970
Diastolic blood pressure (+0.207) 0.970
Disorder of bronchus (x1.01) 0.970
Chronic pansinusitis (x1.01) 0.971
Creatinine [M/V] in SP (+0.0156) 0.971
Lymphocytes [#/V] in B (-0.242) 0.971
Age (+0.463) 0.971
metoprolol (+0.000952) 0.971
Polymorphonuclear cells/100 leukocytes in Body fluid by Manual count (+0.364) 0.972
Acquired deformity of nose (x1.01) 0.972
Respiratory rate by Airway flow measurement (-0.122) 0.972
Neutrophils [#/V] in B by AC (+0.0635) 0.972
−0.8 −0.6 −0.4 −0.2 0.0 0.2 0.4 0.6 0.8
Expressions

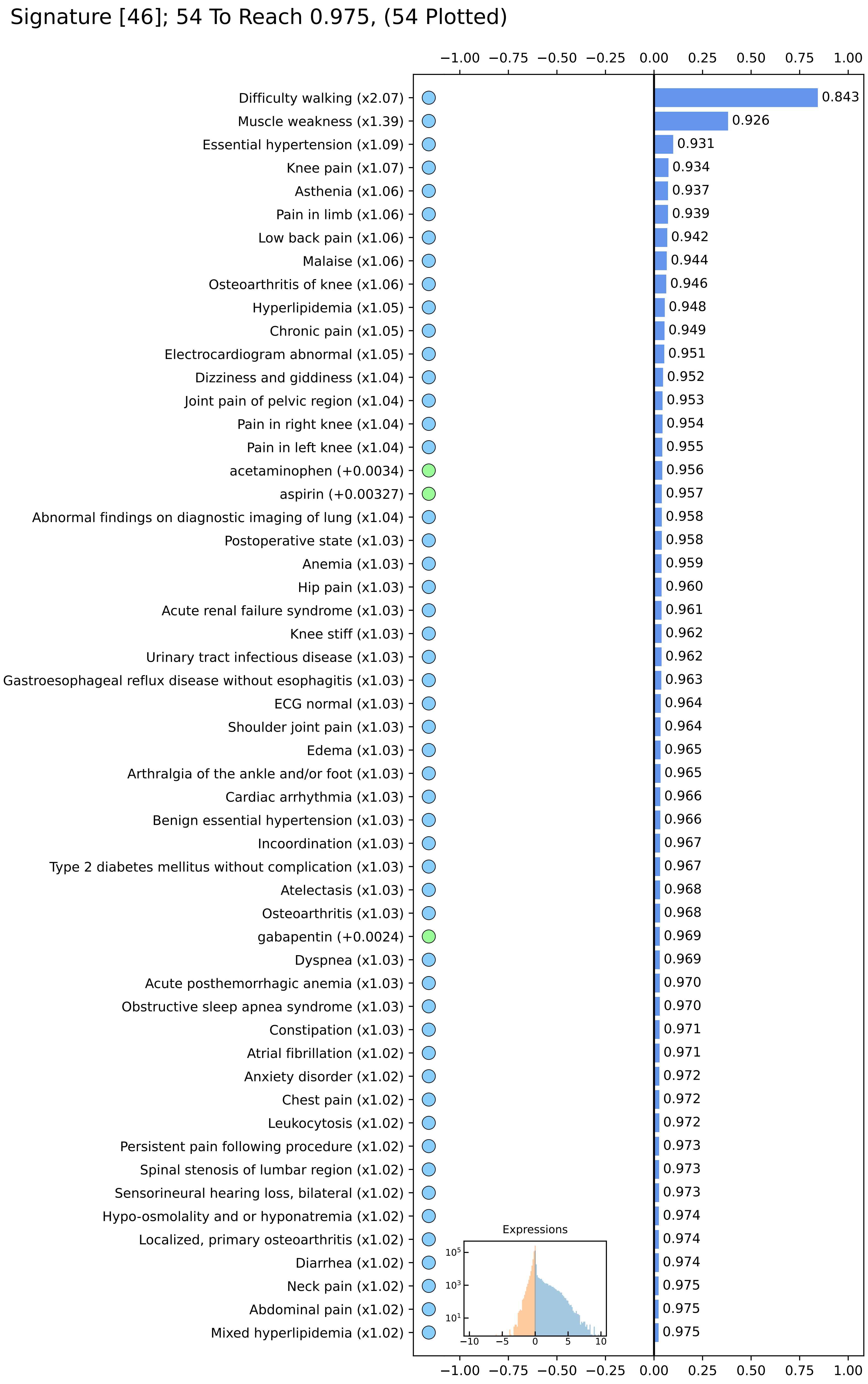
Signature [46]; 54 To Reach 0.975, (54 Plotted)
Difficulty walking (x2.07) 0.843
Muscle weakness (x1.39) 0.926
Essential hypertension (x1.09) 0.931
Knee pain (x1.07) 0.934
Asthenia (x1.06) 0.937
Pain in limb (x1.06) 0.939
Low back pain (x1.06) 0.942
Malaise (x1.06) 0.944
Osteoarthritis of knee (x1.06) 0.946
Hyperlipidemia (x1.05) 0.948
Chronic pain (x1.05) 0.949
Electrocardiogram abnormal (x1.05) 0.951
Dizziness and giddiness (x1.04) 0.952
Joint pain of pelvic region (x1.04) 0.953
Pain in right knee (x1.04) 0.954
Pain in left knee (x1.04) 0.955
acetaminophen (+0.0034) 0.956
aspirin (+0.00327) 0.957
Abnormal findings on diagnostic imaging of lung (x1.04) 0.958
Postoperative state (x1.03) 0.958
Anemia (x1.03) 0.959
Hip pain (x1.03) 0.960
Acute renal failure syndrome (x1.03) 0.961
Knee stiff (x1.03) 0.962
Urinary tract infectious disease (x1.03) 0.962
Gastroesophageal reflux disease without esophagitis (x1.03) 0.963
ECG normal (x1.03) 0.964
Shoulder joint pain (x1.03) 0.964
Edema (x1.03) 0.965
Arthralgia of the ankle and/or foot (x1.03) 0.965
Cardiac arrhythmia (x1.03) 0.966
Benign essential hypertension (x1.03) 0.966
Incoordination (x1.03) 0.967
Type 2 diabetes mellitus without complication (x1.03) 0.967
Atelectasis (x1.03) 0.968
Osteoarthritis (x1.03) 0.968
gabapentin (+0.0024) 0.969
Dyspnea (x1.03) 0.969
Acute posthemorrhagic anemia (x1.03) 0.970
Obstructive sleep apnea syndrome (x1.03) 0.970
Constipation (x1.03) 0.971
Atrial fibrillation (x1.02) 0.971
Anxiety disorder (x1.02) 0.972
Chest pain (x1.02) 0.972
Leukocytosis (x1.02) 0.972
Persistent pain following procedure (x1.02) 0.973
Spinal stenosis of lumbar region (x1.02) 0.973
Sensorineural hearing loss, bilateral (x1.02) 0.973
Hypo-osmolality and or hyponatremia (x1.02) 0.974
Localized, primary osteoarthritis (x1.02) 0.974
Diarrhea (x1.02) 0.974
Neck pain (x1.02) 0.975
Abdominal pain (x1.02) 0.975
Mixed hyperlipidemia (x1.02) 0.975
−1.00 −0.75 −0.50 −0.25 0.00 0.25 0.50 0.75 1.00
Expressions

Signature [47]; 55 To Reach 0.975, (55 Plotted)

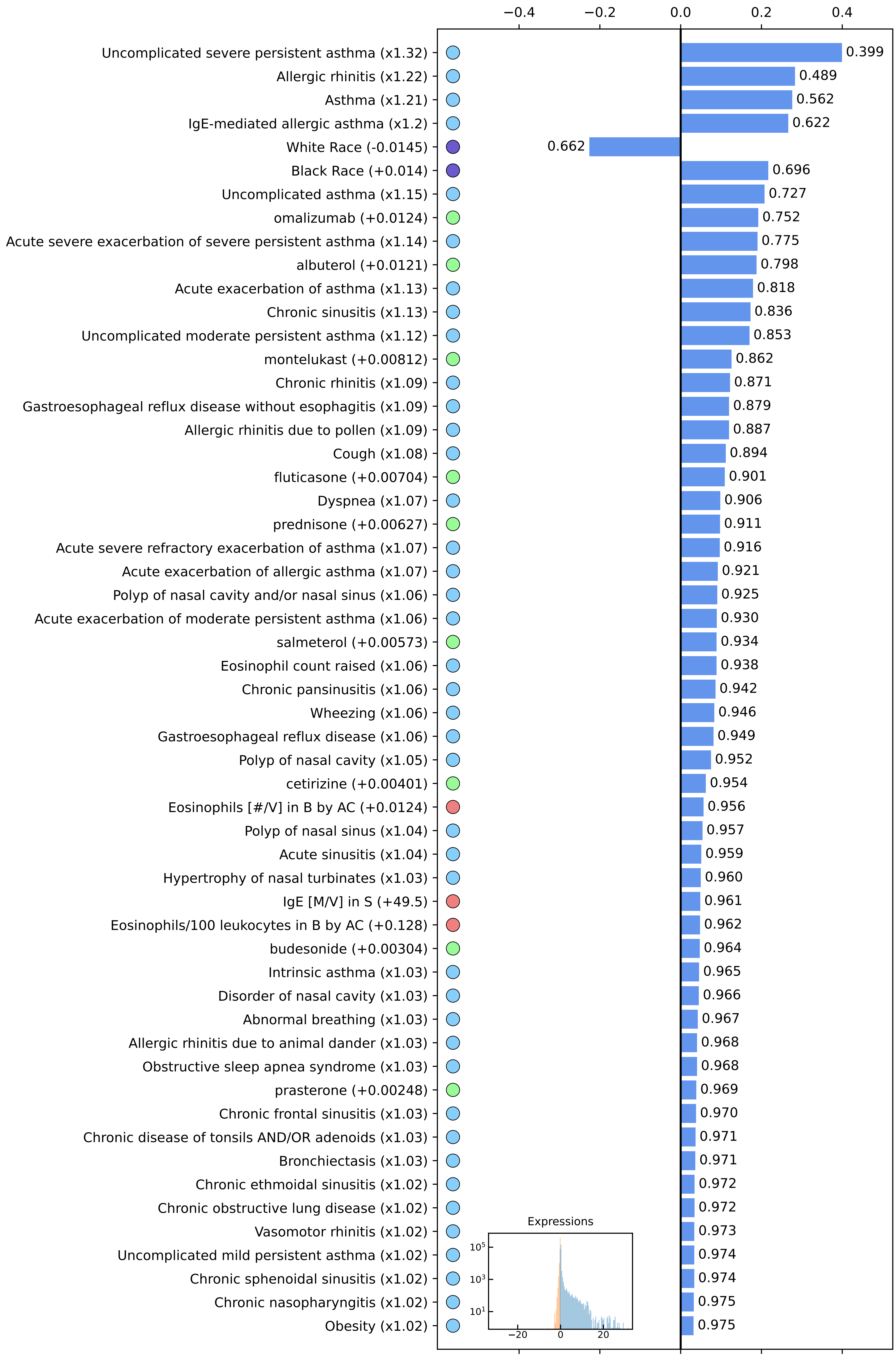

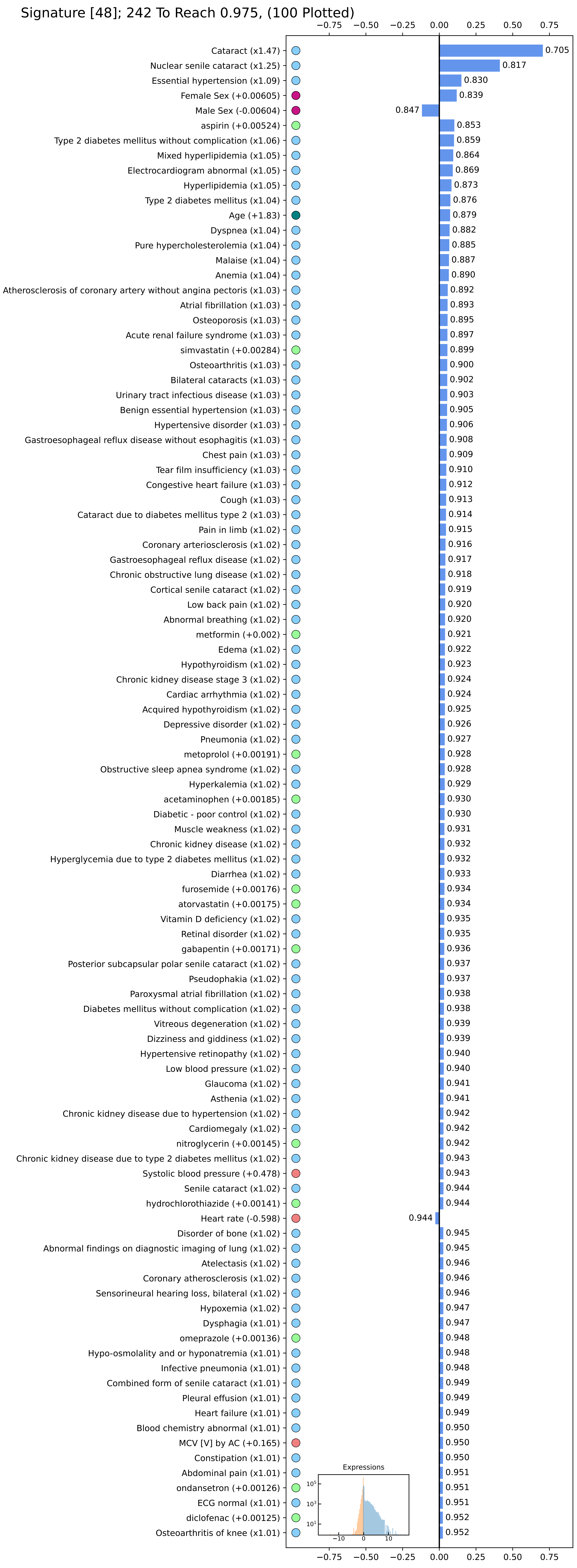
Signature [48]; 242 To Reach 0.975, (100 Plotted)
−0.75 −0.50 −0.25 0.00 0.25 0.50 0.75
Cataract (x1.47) 0.705
Nuclear senile cataract (x1.25) 0.817
Essential hypertension (x1.09) 0.830
Female Sex (+0.00605) 0.839
Male Sex (-0.00604) 0.847
aspirin (+0.00524) 0.853
Type 2 diabetes mellitus without complication (x1.06) 0.859
Mixed hyperlipidemia (x1.05) 0.864
Electrocardiogram abnormal (x1.05) 0.869
Hyperlipidemia (x1.05) 0.873
Type 2 diabetes mellitus (x1.04) 0.876
Age (+1.83) 0.879
Dyspnea (x1.04) 0.882
Pure hypercholesterolemia (x1.04) 0.885
Malaise (x1.04) 0.887
Anemia (x1.04) 0.890
Atherosclerosis of coronary artery without angina pectoris (x1.03) 0.892
Atrial fibrillation (x1.03) 0.893
Osteoporosis (x1.03) 0.895
Acute renal failure syndrome (x1.03) 0.897
simvastatin (+0.00284) 0.899
Osteoarthritis (x1.03) 0.900
Bilateral cataracts (x1.03) 0.902
Urinary tract infectious disease (x1.03) 0.903
Benign essential hypertension (x1.03) 0.905
Hypertensive disorder (x1.03) 0.906
Gastroesophageal reflux disease without esophagitis (x1.03) 0.908
Chest pain (x1.03) 0.909
Tear film insufficiency (x1.03) 0.910
Congestive heart failure (x1.03) 0.912
Cough (x1.03) 0.913
Cataract due to diabetes mellitus type 2 (x1.03) 0.914
Pain in limb (x1.02) 0.915
Coronary arteriosclerosis (x1.02) 0.916
Gastroesophageal reflux disease (x1.02) 0.917
Chronic obstructive lung disease (x1.02) 0.918
Cortical senile cataract (x1.02) 0.919
Low back pain (x1.02) 0.920
Abnormal breathing (x1.02) 0.920
metformin (+0.002) 0.921
Edema (x1.02) 0.922
Hypothyroidism (x1.02) 0.923
Chronic kidney disease stage 3 (x1.02) 0.924
Cardiac arrhythmia (x1.02) 0.924
Acquired hypothyroidism (x1.02) 0.925
Depressive disorder (x1.02) 0.926
Pneumonia (x1.02) 0.927
metoprolol (+0.00191) 0.928
Obstructive sleep apnea syndrome (x1.02) 0.928
Hyperkalemia (x1.02) 0.929
acetaminophen (+0.00185) 0.930
Diabetic - poor control (x1.02) 0.930
Muscle weakness (x1.02) 0.931
Chronic kidney disease (x1.02) 0.932
Hyperglycemia due to type 2 diabetes mellitus (x1.02) 0.932
Diarrhea (x1.02) 0.933
furosemide (+0.00176) 0.934
atorvastatin (+0.00175) 0.934
Vitamin D deficiency (x1.02) 0.935
Retinal disorder (x1.02) 0.935
gabapentin (+0.00171) 0.936
Posterior subcapsular polar senile cataract (x1.02) 0.937
Pseudophakia (x1.02) 0.937
Paroxysmal atrial fibrillation (x1.02) 0.938
Diabetes mellitus without complication (x1.02) 0.938
Vitreous degeneration (x1.02) 0.939
Dizziness and giddiness (x1.02) 0.939
Hypertensive retinopathy (x1.02) 0.940
Low blood pressure (x1.02) 0.940
Glaucoma (x1.02) 0.941
Asthenia (x1.02) 0.941
Chronic kidney disease due to hypertension (x1.02) 0.942
Cardiomegaly (x1.02) 0.942
nitroglycerin (+0.00145) 0.942
Chronic kidney disease due to type 2 diabetes mellitus (x1.02) 0.943
Systolic blood pressure (+0.478) 0.943
Senile cataract (x1.02) 0.944
hydrochlorothiazide (+0.00141) 0.944
Heart rate (-0.598) 0.944
Disorder of bone (x1.02) 0.945
Abnormal findings on diagnostic imaging of lung (x1.02) 0.945
Atelectasis (x1.02) 0.946
Coronary atherosclerosis (x1.02) 0.946
Sensorineural hearing loss, bilateral (x1.02) 0.946
Hypoxemia (x1.02) 0.947
Dysphagia (x1.01) 0.947
omeprazole (+0.00136) 0.948
Hypo-osmolality and or hyponatremia (x1.01) 0.948
Infective pneumonia (x1.01) 0.948
Combined form of senile cataract (x1.01) 0.949
Pleural effusion (x1.01) 0.949
Heart failure (x1.01) 0.949
Blood chemistry abnormal (x1.01) 0.950
MCV [V] by AC (+0.165) 0.950
Constipation (x1.01) 0.950
Abdominal pain (x1.01) 0.951
ondansetron (+0.00126) 0.951
ECG normal (x1.01) 0.951
diclofenac (+0.00125) 0.952
Osteoarthritis of knee (x1.01) 0.952
Expressions
10^5 10^3 10^1
−10 0 10
−0.75 −0.50 −0.25 0.00 0.25 0.50 0.75

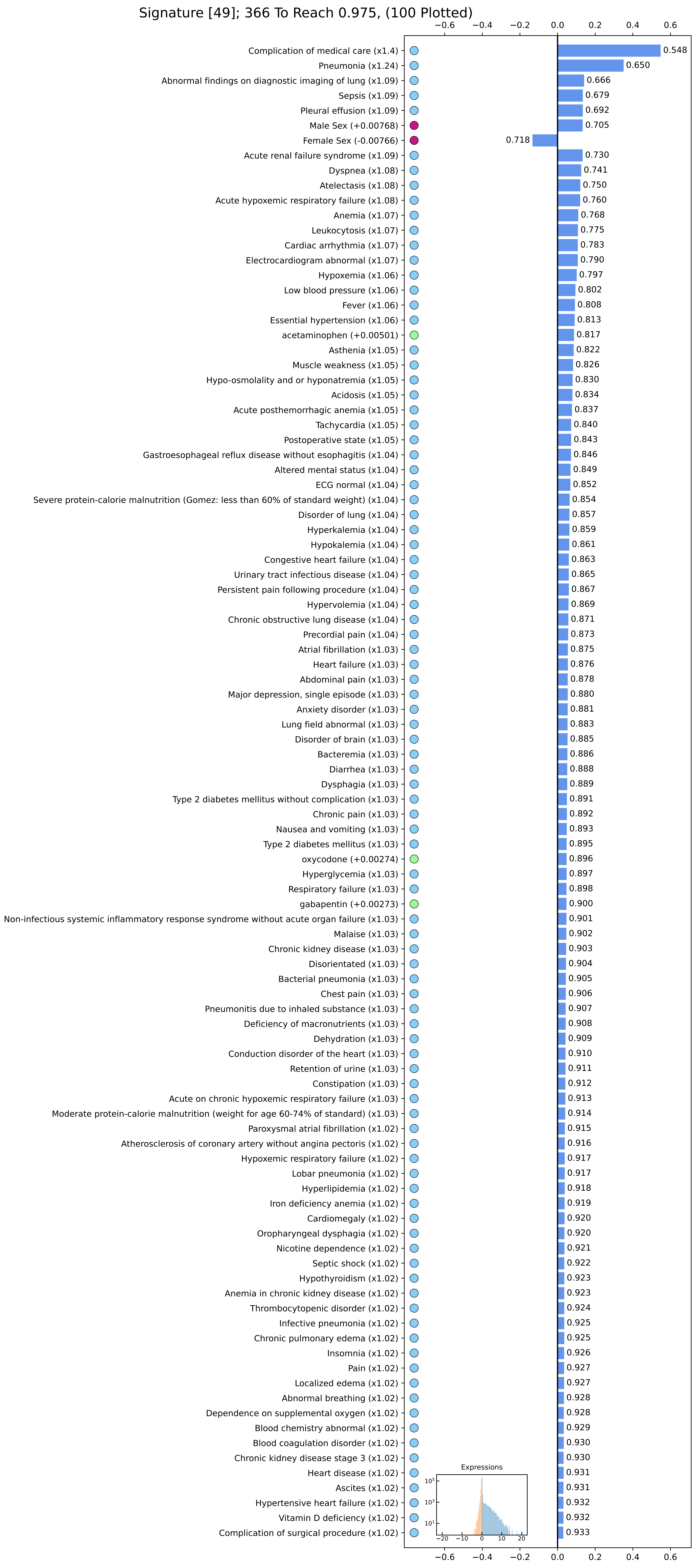
Signature [49]; 366 To Reach 0.975, (100 Plotted)
−0.6 −0.4 −0.2 0.0 0.2 0.4 0.6
Complication of medical care (x1.4) 0.548
Pneumonia (x1.24) 0.650
Abnormal findings on diagnostic imaging of lung (x1.09) 0.666
Sepsis (x1.09) 0.679
Pleural effusion (x1.09) 0.692
Male Sex (+0.00768) 0.705
Female Sex (-0.00766) 0.718
Acute renal failure syndrome (x1.09) 0.730
Dyspnea (x1.08) 0.741
Atelectasis (x1.08) 0.750
Acute hypoxemic respiratory failure (x1.08) 0.760
Anemia (x1.07) 0.768
Leukocytosis (x1.07) 0.775
Cardiac arrhythmia (x1.07) 0.783
Electrocardiogram abnormal (x1.07) 0.790
Hypoxemia (x1.06) 0.797
Low blood pressure (x1.06) 0.802
Fever (x1.06) 0.808
Essential hypertension (x1.06) 0.813
acetaminophen (+0.00501) 0.817
Asthenia (x1.05) 0.822
Muscle weakness (x1.05) 0.826
Hypo-osmolality and or hyponatremia (x1.05) 0.830
Acidosis (x1.05) 0.834
Acute posthemorrhagic anemia (x1.05) 0.837
Tachycardia (x1.05) 0.840
Postoperative state (x1.05) 0.843
Gastroesophageal reflux disease without esophagitis (x1.04) 0.846
Altered mental status (x1.04) 0.849
ECG normal (x1.04) 0.852
Severe protein-calorie malnutrition (Gomez: less than 60% of standard weight) (x1.04) 0.854
Disorder of lung (x1.04) 0.857
Hyperkalemia (x1.04) 0.859
Hypokalemia (x1.04) 0.861
Congestive heart failure (x1.04) 0.863
Urinary tract infectious disease (x1.04) 0.865
Persistent pain following procedure (x1.04) 0.867
Hypervolemia (x1.04) 0.869
Chronic obstructive lung disease (x1.04) 0.871
Precordial pain (x1.04) 0.873
Atrial fibrillation (x1.03) 0.875
Heart failure (x1.03) 0.876
Abdominal pain (x1.03) 0.878
Major depression, single episode (x1.03) 0.880
Anxiety disorder (x1.03) 0.881
Lung field abnormal (x1.03) 0.883
Disorder of brain (x1.03) 0.885
Bacteremia (x1.03) 0.886
Diarrhea (x1.03) 0.888
Dysphagia (x1.03) 0.889
Type 2 diabetes mellitus without complication (x1.03) 0.891
Chronic pain (x1.03) 0.892
Nausea and vomiting (x1.03) 0.893
Type 2 diabetes mellitus (x1.03) 0.895
oxycodone (+0.00274) 0.896
Hyperglycemia (x1.03) 0.897
Respiratory failure (x1.03) 0.898
gabapentin (+0.00273) 0.900
Non-infectious systemic inflammatory response syndrome without acute organ failure (x1.03) 0.901
Malaise (x1.03) 0.902
Chronic kidney disease (x1.03) 0.903
Disorientated (x1.03) 0.904
Bacterial pneumonia (x1.03) 0.905
Chest pain (x1.03) 0.906
Pneumonitis due to inhaled substance (x1.03) 0.907
Deficiency of macronutrients (x1.03) 0.908
Dehydration (x1.03) 0.909
Conduction disorder of the heart (x1.03) 0.910
Retention of urine (x1.03) 0.911
Constipation (x1.03) 0.912
Acute on chronic hypoxemic respiratory failure (x1.03) 0.913
Moderate protein-calorie malnutrition (weight for age 60-74% of standard) (x1.03) 0.914
Paroxysmal atrial fibrillation (x1.02) 0.915
Atherosclerosis of coronary artery without angina pectoris (x1.02) 0.916
Hypoxemic respiratory failure (x1.02) 0.917
Lobar pneumonia (x1.02) 0.917
Hyperlipidemia (x1.02) 0.918
Iron deficiency anemia (x1.02) 0.919
Cardiomegaly (x1.02) 0.920
Oropharyngeal dysphagia (x1.02) 0.920
Nicotine dependence (x1.02) 0.921
Septic shock (x1.02) 0.922
Hypothyroidism (x1.02) 0.923
Anemia in chronic kidney disease (x1.02) 0.923
Thrombocytopenic disorder (x1.02) 0.924
Infective pneumonia (x1.02) 0.925
Chronic pulmonary edema (x1.02) 0.925
Insomnia (x1.02) 0.926
Pain (x1.02) 0.927
Localized edema (x1.02) 0.927
Abnormal breathing (x1.02) 0.928
Dependence on supplemental oxygen (x1.02) 0.928
Blood chemistry abnormal (x1.02) 0.929
Blood coagulation disorder (x1.02) 0.930
Chronic kidney disease stage 3 (x1.02) 0.930
Heart disease (x1.02) 0.931
Ascites (x1.02) 0.931
Hypertensive heart failure (x1.02) 0.932
Vitamin D deficiency (x1.02) 0.932
Complication of surgical procedure (x1.02) 0.933
Expressions
−20 −10 0 10 20
−0.6 −0.4 −0.2 0.0 0.2 0.4 0.6

Signature [50]; 14 To Reach 0.975, (14 Plotted)

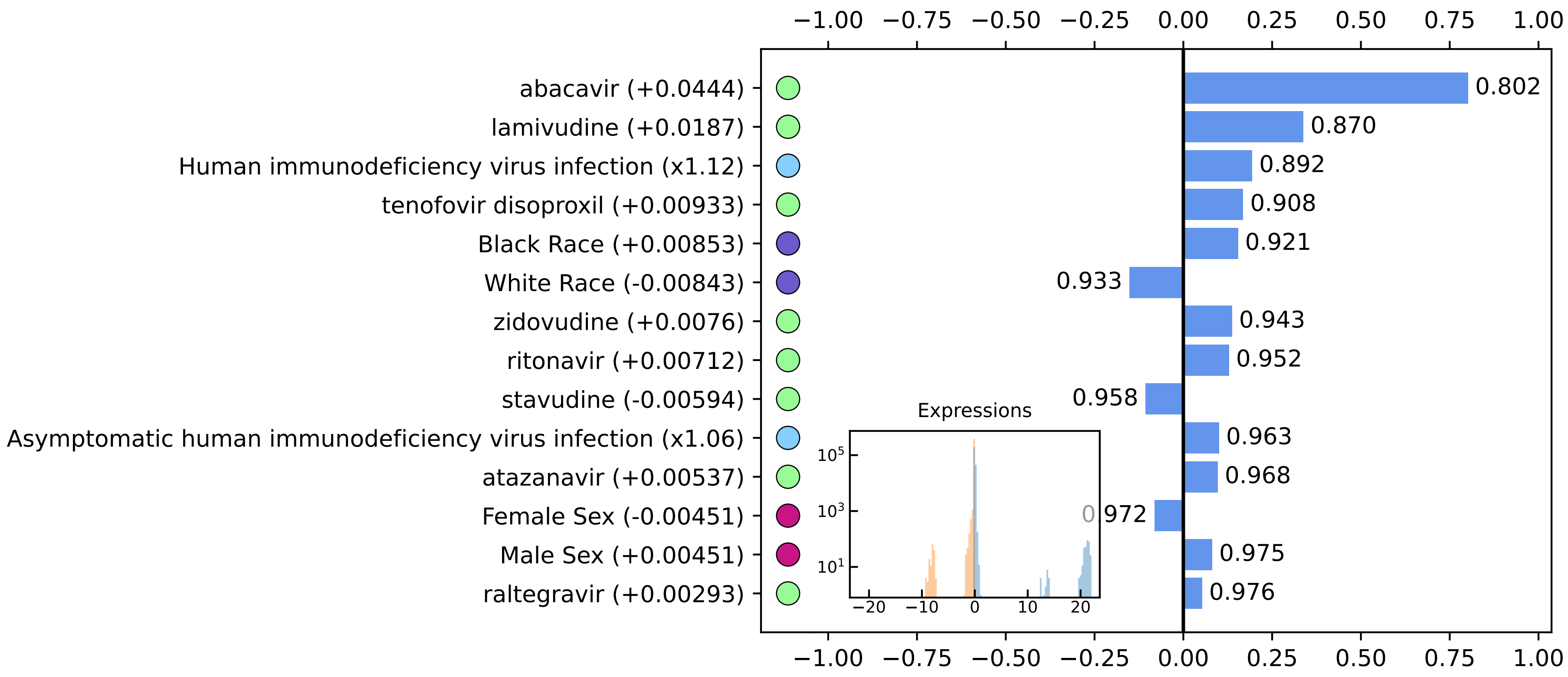

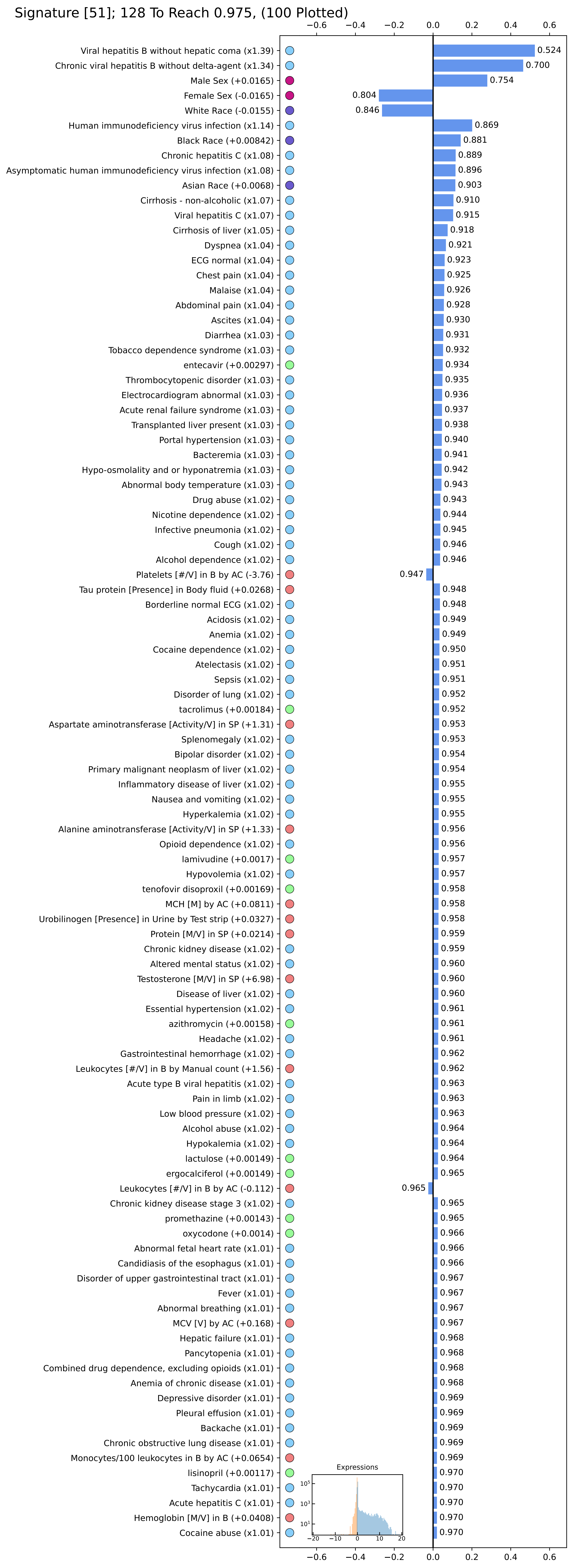

Signature [51]; 128 To Reach 0.975, (100 Plotted)
Viral hepatitis B without hepatic coma (x1.39) 0.524
Chronic viral hepatitis B without delta-agent (x1.34) 0.700
Male Sex (+0.0165) 0.754
Female Sex (-0.0165) 0.804
White Race (-0.0155) 0.846
Human immunodeficiency virus infection (x1.14) 0.869
Black Race (+0.00842) 0.881
Chronic hepatitis C (x1.08) 0.889
Asymptomatic human immunodeficiency virus infection (x1.08) 0.896
Asian Race (+0.0068) 0.903
Cirrhosis - non-alcoholic (x1.07) 0.910
Viral hepatitis C (x1.07) 0.915
Cirrhosis of liver (x1.05) 0.918
Dyspnea (x1.04) 0.921
ECG normal (x1.04) 0.923
Chest pain (x1.04) 0.925
Malaise (x1.04) 0.926
Abdominal pain (x1.04) 0.928
Ascites (x1.04) 0.930
Diarrhea (x1.03) 0.931
Tobacco dependence syndrome (x1.03) 0.932
entecavir (+0.00297) 0.934
Thrombocytopenic disorder (x1.03) 0.935
Electrocardiogram abnormal (x1.03) 0.936
Acute renal failure syndrome (x1.03) 0.937
Transplanted liver present (x1.03) 0.938
Portal hypertension (x1.03) 0.940
Bacteremia (x1.03) 0.941
Hypo-osmolality and or hyponatremia (x1.03) 0.942
Abnormal body temperature (x1.03) 0.943
Drug abuse (x1.02) 0.943
Nicotine dependence (x1.02) 0.944
Infective pneumonia (x1.02) 0.945
Cough (x1.02) 0.946
Alcohol dependence (x1.02) 0.946
Platelets [#/V] in B by AC (-3.76) 0.947
Tau protein [Presence] in Body fluid (+0.0268) 0.948
Borderline normal ECG (x1.02) 0.948
Acidosis (x1.02) 0.949
Anemia (x1.02) 0.949
Cocaine dependence (x1.02) 0.950
Atelectasis (x1.02) 0.951
Sepsis (x1.02) 0.951
Disorder of lung (x1.02) 0.952
tacrolimus (+0.00184) 0.952
Aspartate aminotransferase [Activity/V] in SP (+1.31) 0.953
Splenomegaly (x1.02) 0.953
Bipolar disorder (x1.02) 0.954
Primary malignant neoplasm of liver (x1.02) 0.954
Inflammatory disease of liver (x1.02) 0.955
Nausea and vomiting (x1.02) 0.955
Hyperkalemia (x1.02) 0.955
Alanine aminotransferase [Activity/V] in SP (+1.33) 0.956
Opioid dependence (x1.02) 0.956
lamivudine (+0.0017) 0.957
Hypovolemia (x1.02) 0.957
tenofovir disoproxil (+0.00169) 0.958
MCH [M] by AC (+0.0811) 0.958
Urobilinogen [Presence] in Urine by Test strip (+0.0327) 0.958
Protein [M/V] in SP (+0.0214) 0.959
Chronic kidney disease (x1.02) 0.959
Altered mental status (x1.02) 0.960
Testosterone [M/V] in SP (+6.98) 0.960
Disease of liver (x1.02) 0.960
Essential hypertension (x1.02) 0.961
azithromycin (+0.00158) 0.961
Headache (x1.02) 0.961
Gastrointestinal hemorrhage (x1.02) 0.962
Leukocytes [#/V] in B by Manual count (+1.56) 0.962
Acute type B viral hepatitis (x1.02) 0.963
Pain in limb (x1.02) 0.963
Low blood pressure (x1.02) 0.963
Alcohol abuse (x1.02) 0.964
Hypokalemia (x1.02) 0.964
lactulose (+0.00149) 0.964
ergocalciferol (+0.00149) 0.965
Leukocytes [#/V] in B by AC (-0.112) 0.965
Chronic kidney disease stage 3 (x1.02) 0.965
promethazine (+0.00143) 0.965
oxycodone (+0.0014) 0.966
Abnormal fetal heart rate (x1.01) 0.966
Candidiasis of the esophagus (x1.01) 0.966
Disorder of upper gastrointestinal tract (x1.01) 0.967
Fever (x1.01) 0.967
Abnormal breathing (x1.01) 0.967
MCV [V] by AC (+0.168) 0.967
Hepatic failure (x1.01) 0.968
Pancytopenia (x1.01) 0.968
Combined drug dependence, excluding opioids (x1.01) 0.968
Anemia of chronic disease (x1.01) 0.968
Depressive disorder (x1.01) 0.969
Pleural effusion (x1.01) 0.969
Backache (x1.01) 0.969
Chronic obstructive lung disease (x1.01) 0.969
Monocytes/100 leukocytes in B by AC (+0.0654) 0.969
lisinopril (+0.00117) 0.970
Tachycardia (x1.01) 0.970
Acute hepatitis C (x1.01) 0.970
Hemoglobin [M/V] in B (+0.0408) 0.970
Cocaine abuse (x1.01) 0.970
−0.6 −0.4 −0.2 0.0 0.2 0.4 0.6
Expressions
10^5 10^3 10^1
−20 −10 0 10 20

Signature [52]; 4 To Reach 0.975, (10 Plotted)

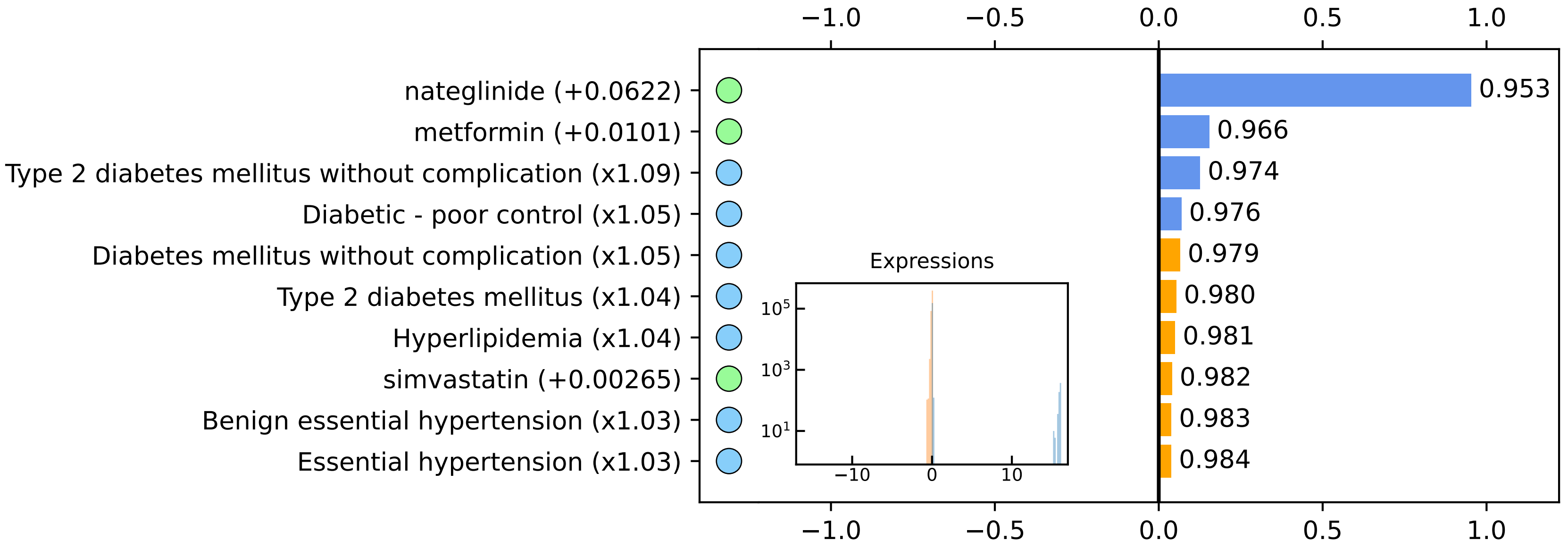

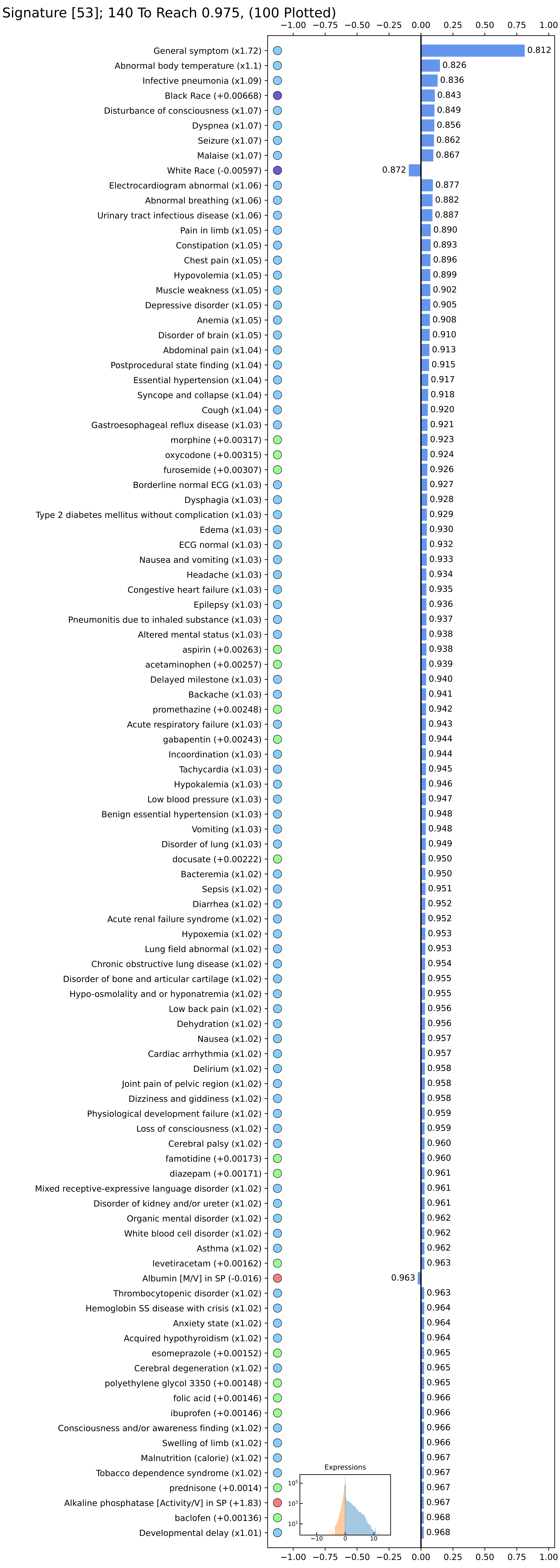

Signature [53]; 140 To Reach 0.975, (100 Plotted)
General symptom (x1.72) 0.812
Abnormal body temperature (x1.1) 0.826
Infective pneumonia (x1.09) 0.836
Black Race (+0.00668) 0.843
Disturbance of consciousness (x1.07) 0.849
Dyspnea (x1.07) 0.856
Seizure (x1.07) 0.862
Malaise (x1.07) 0.867
White Race (-0.00597) 0.872
Electrocardiogram abnormal (x1.06) 0.877
Abnormal breathing (x1.06) 0.882
Urinary tract infectious disease (x1.06) 0.887
Pain in limb (x1.05) 0.890
Constipation (x1.05) 0.893
Chest pain (x1.05) 0.896
Hypovolemia (x1.05) 0.899
Muscle weakness (x1.05) 0.902
Depressive disorder (x1.05) 0.905
Anemia (x1.05) 0.908
Disorder of brain (x1.05) 0.910
Abdominal pain (x1.04) 0.913
Postprocedural state finding (x1.04) 0.915
Essential hypertension (x1.04) 0.917
Syncope and collapse (x1.04) 0.918
Cough (x1.04) 0.920
Gastroesophageal reflux disease (x1.03) 0.921
morphine (+0.00317) 0.923
oxycodone (+0.00315) 0.924
furosemide (+0.00307) 0.926
Borderline normal ECG (x1.03) 0.927
Dysphagia (x1.03) 0.928
Type 2 diabetes mellitus without complication (x1.03) 0.929
Edema (x1.03) 0.930
ECG normal (x1.03) 0.932
Nausea and vomiting (x1.03) 0.933
Headache (x1.03) 0.934
Congestive heart failure (x1.03) 0.935
Epilepsy (x1.03) 0.936
Pneumonitis due to inhaled substance (x1.03) 0.937
Altered mental status (x1.03) 0.938
aspirin (+0.00263) 0.938
acetaminophen (+0.00257) 0.939
Delayed milestone (x1.03) 0.940
Backache (x1.03) 0.941
promethazine (+0.00248) 0.942
Acute respiratory failure (x1.03) 0.943
gabapentin (+0.00243) 0.944
Incoordination (x1.03) 0.944
Tachycardia (x1.03) 0.945
Hypokalemia (x1.03) 0.946
Low blood pressure (x1.03) 0.947
Benign essential hypertension (x1.03) 0.948
Vomiting (x1.03) 0.948
Disorder of lung (x1.03) 0.949
docusate (+0.00222) 0.950
Bacteremia (x1.02) 0.950
Sepsis (x1.02) 0.951
Diarrhea (x1.02) 0.952
Acute renal failure syndrome (x1.02) 0.952
Hypoxemia (x1.02) 0.953
Lung field abnormal (x1.02) 0.953
Chronic obstructive lung disease (x1.02) 0.954
Disorder of bone and articular cartilage (x1.02) 0.955
Hypo-osmolality and or hyponatremia (x1.02) 0.955
Low back pain (x1.02) 0.956
Dehydration (x1.02) 0.956
Nausea (x1.02) 0.957
Cardiac arrhythmia (x1.02) 0.957
Delirium (x1.02) 0.958
Joint pain of pelvic region (x1.02) 0.958
Dizziness and giddiness (x1.02) 0.958
Physiological development failure (x1.02) 0.959
Loss of consciousness (x1.02) 0.959
Cerebral palsy (x1.02) 0.960
famotidine (+0.00173) 0.960
diazepam (+0.00171) 0.961
Mixed receptive-expressive language disorder (x1.02) 0.961
Disorder of kidney and/or ureter (x1.02) 0.961
Organic mental disorder (x1.02) 0.962
White blood cell disorder (x1.02) 0.962
Asthma (x1.02) 0.962
levetiracetam (+0.00162) 0.963
Albumin [M/V] in SP (-0.016) 0.963
Thrombocytopenic disorder (x1.02) 0.963
Hemoglobin SS disease with crisis (x1.02) 0.964
Anxiety state (x1.02) 0.964
Acquired hypothyroidism (x1.02) 0.964
esomeprazole (+0.00152) 0.965
Cerebral degeneration (x1.02) 0.965
polyethylene glycol 3350 (+0.00148) 0.965
folic acid (+0.00146) 0.966
ibuprofen (+0.00146) 0.966
Consciousness and/or awareness finding (x1.02) 0.966
Swelling of limb (x1.02) 0.966
Malnutrition (calorie) (x1.02) 0.967
Tobacco dependence syndrome (x1.02) 0.967
prednisone (+0.0014) 0.967
Alkaline phosphatase [Activity/V] in SP (+1.83) 0.967
baclofen (+0.00136) 0.968
Developmental delay (x1.01) 0.968
Expressions
−1.00 −0.75 −0.50 −0.25 0.00 0.25 0.50 0.75 1.00

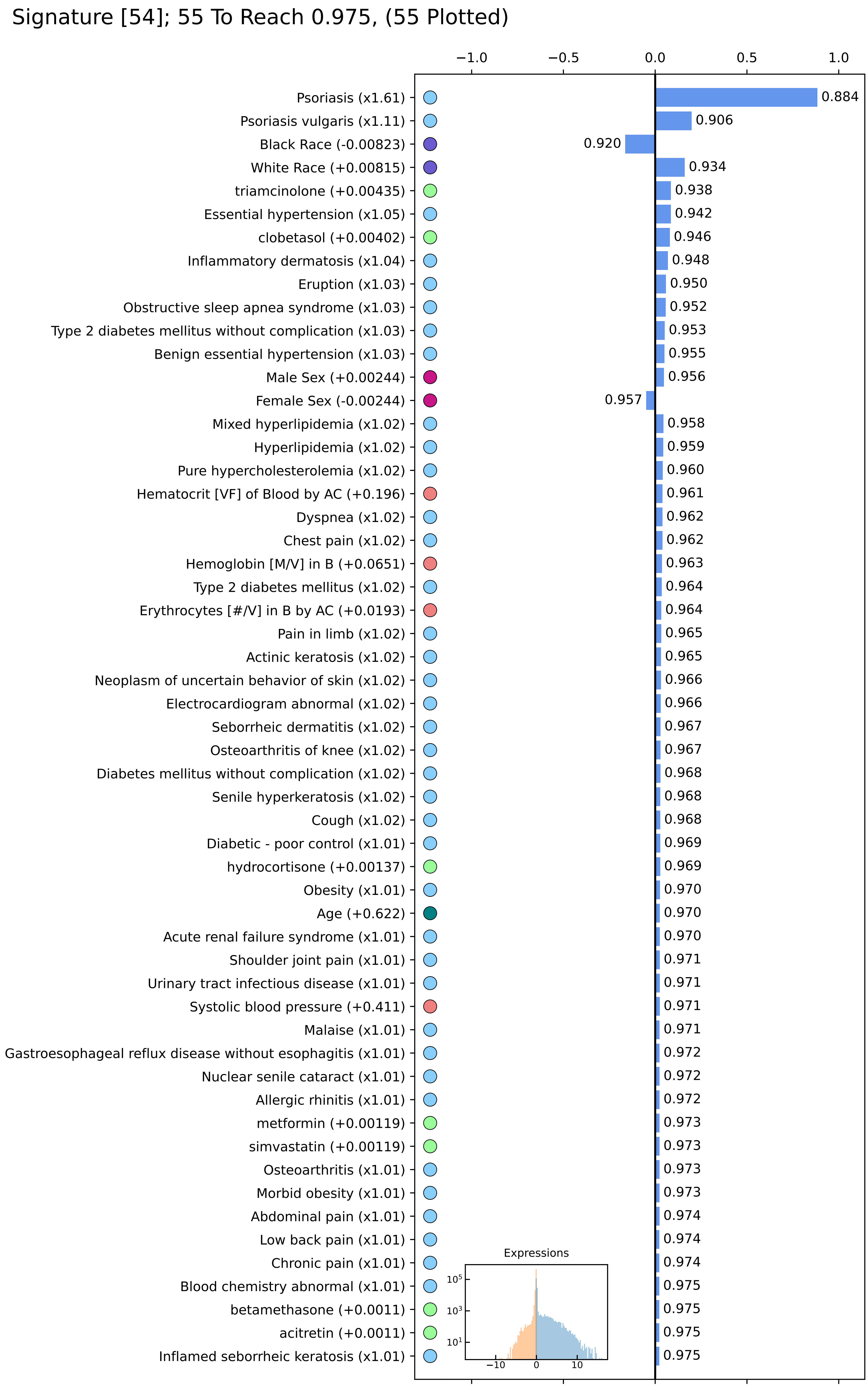

Signature [54]; 55 To Reach 0.975, (55 Plotted)
−1.0 −0.5 0.0 0.5 1.0
Psoriasis (x1.61) 0.884
Psoriasis vulgaris (x1.11) 0.906
Black Race (-0.00823) 0.920
White Race (+0.00815) 0.934
triamcinolone (+0.00435) 0.938
Essential hypertension (x1.05) 0.942
clobetasol (+0.00402) 0.946
Inflammatory dermatosis (x1.04) 0.948
Eruption (x1.03) 0.950
Obstructive sleep apnea syndrome (x1.03) 0.952
Type 2 diabetes mellitus without complication (x1.03) 0.953
Benign essential hypertension (x1.03) 0.955
Male Sex (+0.00244) 0.956
Female Sex (-0.00244) 0.957
Mixed hyperlipidemia (x1.02) 0.958
Hyperlipidemia (x1.02) 0.959
Pure hypercholesterolemia (x1.02) 0.960
Hematocrit [VF] of Blood by AC (+0.196) 0.961
Dyspnea (x1.02) 0.962
Chest pain (x1.02) 0.962
Hemoglobin [M/V] in B (+0.0651) 0.963
Type 2 diabetes mellitus (x1.02) 0.964
Erythrocytes [#/V] in B by AC (+0.0193) 0.964
Pain in limb (x1.02) 0.965
Actinic keratosis (x1.02) 0.965
Neoplasm of uncertain behavior of skin (x1.02) 0.966
Electrocardiogram abnormal (x1.02) 0.966
Seborrheic dermatitis (x1.02) 0.967
Osteoarthritis of knee (x1.02) 0.967
Diabetes mellitus without complication (x1.02) 0.968
Senile hyperkeratosis (x1.02) 0.968
Cough (x1.02) 0.968
Diabetic - poor control (x1.01) 0.969
hydrocortisone (+0.00137) 0.969
Obesity (x1.01) 0.970
Age (+0.622) 0.970
Acute renal failure syndrome (x1.01) 0.970
Shoulder joint pain (x1.01) 0.971
Urinary tract infectious disease (x1.01) 0.971
Systolic blood pressure (+0.411) 0.971
Malaise (x1.01) 0.971
Gastroesophageal reflux disease without esophagitis (x1.01) 0.972
Nuclear senile cataract (x1.01) 0.972
Allergic rhinitis (x1.01) 0.972
metformin (+0.00119) 0.973
simvastatin (+0.00119) 0.973
Osteoarthritis (x1.01) 0.973
Morbid obesity (x1.01) 0.973
Abdominal pain (x1.01) 0.974
Low back pain (x1.01) 0.974
Chronic pain (x1.01) 0.974
Blood chemistry abnormal (x1.01) 0.975
betamethasone (+0.0011) 0.975
acitretin (+0.0011) 0.975
Inflamed seborrheic keratosis (x1.01) 0.975
Expressions
10^5
10^3
10^1
−10 0 10
−1.0 −0.5 0.0 0.5 1.0

Signature [55]; 13 To Reach 0.975, (13 Plotted)

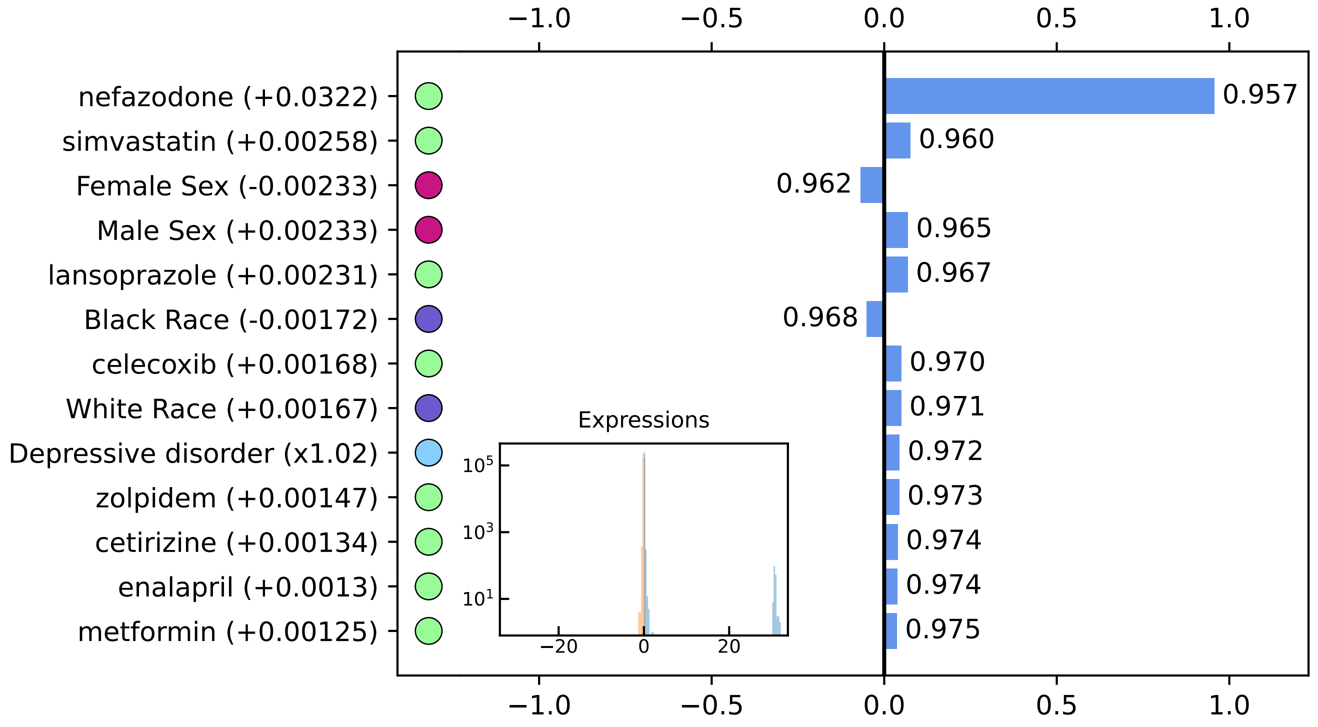

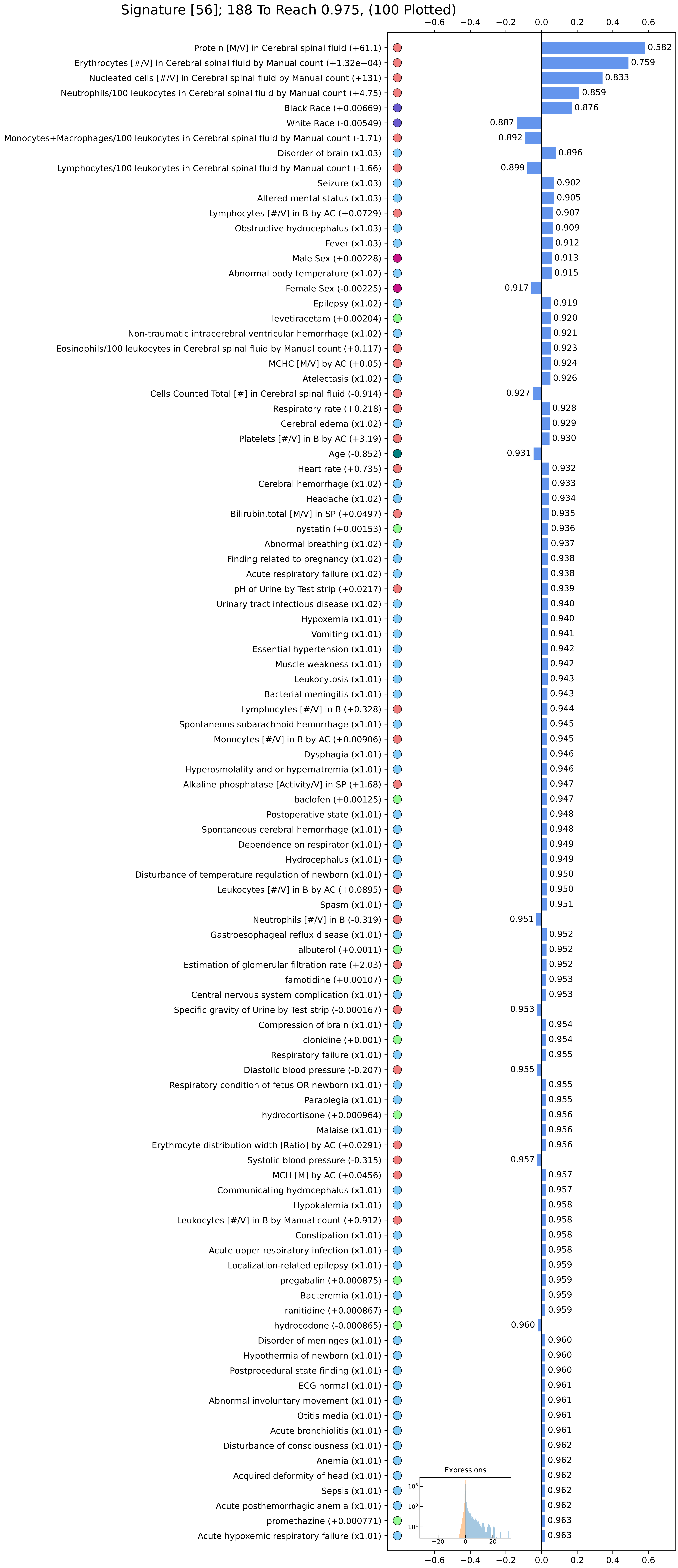
Signature [56]; 188 To Reach 0.975, (100 Plotted)
−0.6 −0.4 −0.2 0.0 0.2 0.4 0.6
Protein [M/V] in Cerebral spinal fluid (+61.1) 0.582
Erythrocytes [#/V] in Cerebral spinal fluid by Manual count (+1.32e+04) 0.759
Nucleated cells [#/V] in Cerebral spinal fluid by Manual count (+131) 0.833
Neutrophils/100 leukocytes in Cerebral spinal fluid by Manual count (+4.75) 0.859
Black Race (+0.00669) 0.876
White Race (-0.00549) 0.887
Monocytes+Macrophages/100 leukocytes in Cerebral spinal fluid by Manual count (-1.71) 0.892
Disorder of brain (x1.03) 0.896
Lymphocytes/100 leukocytes in Cerebral spinal fluid by Manual count (-1.66) 0.899
Seizure (x1.03) 0.902
Altered mental status (x1.03) 0.905
Lymphocytes [#/V] in B by AC (+0.0729) 0.907
Obstructive hydrocephalus (x1.03) 0.909
Fever (x1.03) 0.912
Male Sex (+0.00228) 0.913
Abnormal body temperature (x1.02) 0.915
Female Sex (-0.00225) 0.917
Epilepsy (x1.02) 0.919
levetiracetam (+0.00204) 0.920
Non-traumatic intracerebral ventricular hemorrhage (x1.02) 0.921
Eosinophils/100 leukocytes in Cerebral spinal fluid by Manual count (+0.117) 0.923
MCHC [M/V] by AC (+0.05) 0.924
Atelectasis (x1.02) 0.926
Cells Counted Total [#] in Cerebral spinal fluid (-0.914) 0.927
Respiratory rate (+0.218) 0.928
Cerebral edema (x1.02) 0.929
Platelets [#/V] in B by AC (+3.19) 0.930
Age (-0.852) 0.931
Heart rate (+0.735) 0.932
Cerebral hemorrhage (x1.02) 0.933
Headache (x1.02) 0.934
Bilirubin.total [M/V] in SP (+0.0497) 0.935
nystatin (+0.00153) 0.936
Abnormal breathing (x1.02) 0.937
Finding related to pregnancy (x1.02) 0.938
Acute respiratory failure (x1.02) 0.938
pH of Urine by Test strip (+0.0217) 0.939
Urinary tract infectious disease (x1.02) 0.940
Hypoxemia (x1.01) 0.940
Vomiting (x1.01) 0.941
Essential hypertension (x1.01) 0.942
Muscle weakness (x1.01) 0.942
Leukocytosis (x1.01) 0.943
Bacterial meningitis (x1.01) 0.943
Lymphocytes [#/V] in B (+0.328) 0.944
Spontaneous subarachnoid hemorrhage (x1.01) 0.945
Monocytes [#/V] in B by AC (+0.00906) 0.945
Dysphagia (x1.01) 0.946
Hyperosmolality and or hypernatremia (x1.01) 0.946
Alkaline phosphatase [Activity/V] in SP (+1.68) 0.947
baclofen (+0.00125) 0.947
Postoperative state (x1.01) 0.948
Spontaneous cerebral hemorrhage (x1.01) 0.948
Dependence on respirator (x1.01) 0.949
Hydrocephalus (x1.01) 0.949
Disturbance of temperature regulation of newborn (x1.01) 0.950
Leukocytes [#/V] in B by AC (+0.0895) 0.950
Spasm (x1.01) 0.951
Neutrophils [#/V] in B (-0.319) 0.951
Gastroesophageal reflux disease (x1.01) 0.952
albuterol (+0.0011) 0.952
Estimation of glomerular filtration rate (+2.03) 0.952
famotidine (+0.00107) 0.953
Central nervous system complication (x1.01) 0.953
Specific gravity of Urine by Test strip (-0.000167) 0.953
Compression of brain (x1.01) 0.954
clonidine (+0.001) 0.954
Respiratory failure (x1.01) 0.955
Diastolic blood pressure (-0.207) 0.955
Respiratory condition of fetus OR newborn (x1.01) 0.955
Paraplegia (x1.01) 0.955
hydrocortisone (+0.000964) 0.956
Malaise (x1.01) 0.956
Erythrocyte distribution width [Ratio] by AC (+0.0291) 0.956
Systolic blood pressure (-0.315) 0.957
MCH [M] by AC (+0.0456) 0.957
Communicating hydrocephalus (x1.01) 0.957
Hypokalemia (x1.01) 0.958
Leukocytes [#/V] in B by Manual count (+0.912) 0.958
Constipation (x1.01) 0.958
Acute upper respiratory infection (x1.01) 0.958
Localization-related epilepsy (x1.01) 0.959
pregabalin (+0.000875) 0.959
Bacteremia (x1.01) 0.959
ranitidine (+0.000867) 0.959
hydrocodone (-0.000865) 0.960
Disorder of meninges (x1.01) 0.960
Hypothermia of newborn (x1.01) 0.960
Postprocedural state finding (x1.01) 0.960
ECG normal (x1.01) 0.961
Abnormal involuntary movement (x1.01) 0.961
Otitis media (x1.01) 0.961
Acute bronchiolitis (x1.01) 0.961
Disturbance of consciousness (x1.01) 0.962
Anemia (x1.01) 0.962
Acquired deformity of head (x1.01) 0.962
Sepsis (x1.01) 0.962
Acute posthemorrhagic anemia (x1.01) 0.962
promethazine (+0.000771) 0.963
Acute hypoxemic respiratory failure (x1.01) 0.963
Expressions
−20 0 20
−0.6 −0.4 −0.2 0.0 0.2 0.4 0.6

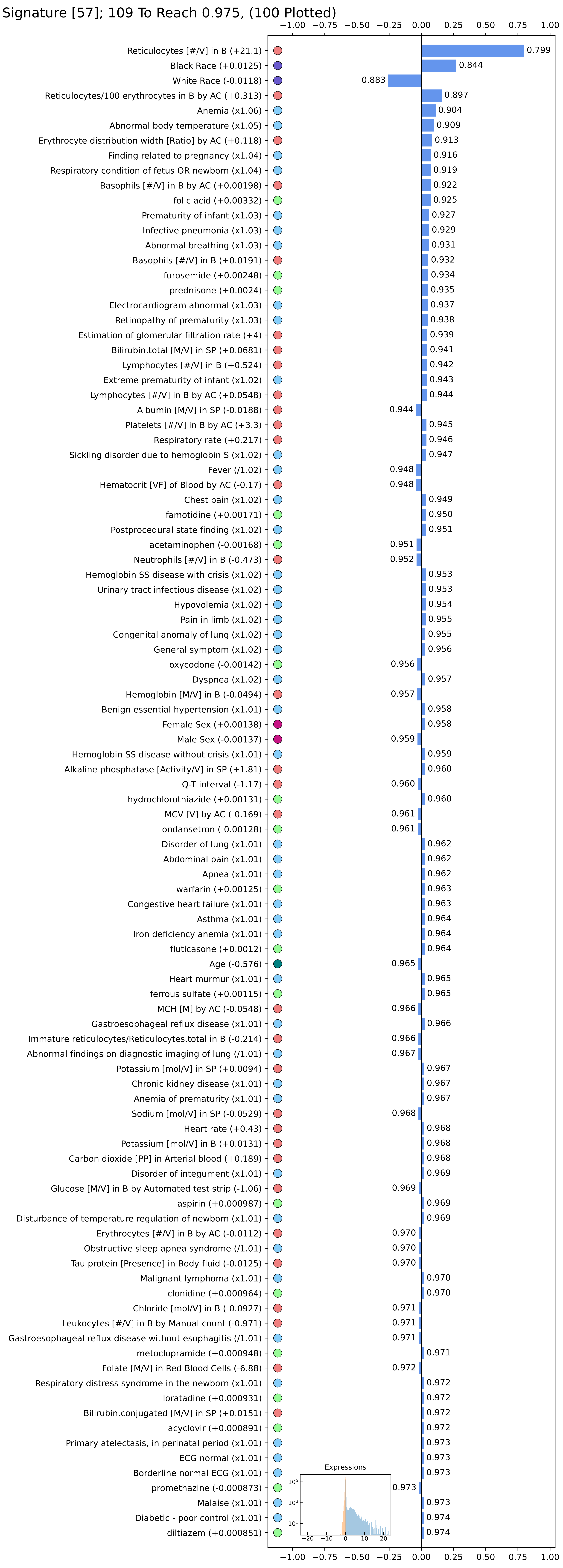
Signature [57]; 109 To Reach 0.975, (100 Plotted)
−1.00 −0.75 −0.50 −0.25 0.00 0.25 0.50 0.75 1.00
Reticulocytes [#/V] in B (+21.1) 0.799
Black Race (+0.0125) 0.844
White Race (-0.0118) 0.883
Reticulocytes/100 erythrocytes in B by AC (+0.313) 0.897
Anemia (x1.06) 0.904
Abnormal body temperature (x1.05) 0.909
Erythrocyte distribution width [Ratio] by AC (+0.118) 0.913
Finding related to pregnancy (x1.04) 0.916
Respiratory condition of fetus OR newborn (x1.04) 0.919
Basophils [#/V] in B by AC (+0.00198) 0.922
folic acid (+0.00332) 0.925
Prematurity of infant (x1.03) 0.927
Infective pneumonia (x1.03) 0.929
Abnormal breathing (x1.03) 0.931
Basophils [#/V] in B (+0.0191) 0.932
furosemide (+0.00248) 0.934
prednisone (+0.0024) 0.935
Electrocardiogram abnormal (x1.03) 0.937
Retinopathy of prematurity (x1.03) 0.938
Estimation of glomerular filtration rate (+4) 0.939
Bilirubin.total [M/V] in SP (+0.0681) 0.941
Lymphocytes [#/V] in B (+0.524) 0.942
Extreme prematurity of infant (x1.02) 0.943
Lymphocytes [#/V] in B by AC (+0.0548) 0.944
Albumin [M/V] in SP (-0.0188) 0.944
Platelets [#/V] in B by AC (+3.3) 0.945
Respiratory rate (+0.217) 0.946
Sickling disorder due to hemoglobin S (x1.02) 0.947
Fever (/1.02) 0.948
Hematocrit [VF] of Blood by AC (-0.17) 0.948
Chest pain (x1.02) 0.949
famotidine (+0.00171) 0.950
Postprocedural state finding (x1.02) 0.951
acetaminophen (-0.00168) 0.951
Neutrophils [#/V] in B (-0.473) 0.952
Hemoglobin SS disease with crisis (x1.02) 0.953
Urinary tract infectious disease (x1.02) 0.953
Hypovolemia (x1.02) 0.954
Pain in limb (x1.02) 0.955
Congenital anomaly of lung (x1.02) 0.955
General symptom (x1.02) 0.956
oxycodone (-0.00142) 0.956
Dyspnea (x1.02) 0.957
Hemoglobin [M/V] in B (-0.0494) 0.957
Benign essential hypertension (x1.01) 0.958
Female Sex (+0.00138) 0.958
Male Sex (-0.00137) 0.959
Hemoglobin SS disease without crisis (x1.01) 0.959
Alkaline phosphatase [Activity/V] in SP (+1.81) 0.960
Q-T interval (-1.17) 0.960
hydrochlorothiazide (+0.00131) 0.960
MCV [V] by AC (-0.169) 0.961
ondansetron (-0.00128) 0.961
Disorder of lung (x1.01) 0.962
Abdominal pain (x1.01) 0.962
Apnea (x1.01) 0.962
warfarin (+0.00125) 0.963
Congestive heart failure (x1.01) 0.963
Asthma (x1.01) 0.964
Iron deficiency anemia (x1.01) 0.964
fluticasone (+0.0012) 0.964
Age (-0.576) 0.965
Heart murmur (x1.01) 0.965
ferrous sulfate (+0.00115) 0.965
MCH [M] by AC (-0.0548) 0.966
Gastroesophageal reflux disease (x1.01) 0.966
Immature reticulocytes/Reticulocytes.total in B (-0.214) 0.966
Abnormal findings on diagnostic imaging of lung (/1.01) 0.967
Potassium [mol/V] in SP (+0.0094) 0.967
Chronic kidney disease (x1.01) 0.967
Anemia of prematurity (x1.01) 0.967
Sodium [mol/V] in SP (-0.0529) 0.968
Heart rate (+0.43) 0.968
Potassium [mol/V] in B (+0.0131) 0.968
Carbon dioxide [PP] in Arterial blood (+0.189) 0.968
Disorder of integument (x1.01) 0.969
Glucose [M/V] in B by Automated test strip (-1.06) 0.969
aspirin (+0.000987) 0.969
Disturbance of temperature regulation of newborn (x1.01) 0.969
Erythrocytes [#/V] in B by AC (-0.0112) 0.970
Obstructive sleep apnea syndrome (/1.01) 0.970
Tau protein [Presence] in Body fluid (-0.0125) 0.970
Malignant lymphoma (x1.01) 0.970
clonidine (+0.000964) 0.970
Chloride [mol/V] in B (-0.0927) 0.971
Leukocytes [#/V] in B by Manual count (-0.971) 0.971
Gastroesophageal reflux disease without esophagitis (/1.01) 0.971
metoclopramide (+0.000948) 0.971
Folate [M/V] in Red Blood Cells (-6.88) 0.972
Respiratory distress syndrome in the newborn (x1.01) 0.972
loratadine (+0.000931) 0.972
Bilirubin.conjugated [M/V] in SP (+0.0151) 0.972
acyclovir (+0.000891) 0.972
Primary atelectasis, in perinatal period (x1.01) 0.973
ECG normal (x1.01) 0.973
Borderline normal ECG (x1.01) 0.973
promethazine (-0.000873) 0.973
Malaise (x1.01) 0.973
Diabetic - poor control (x1.01) 0.974
diltiazem (+0.000851) 0.974
Expressions
10^5 10^3 10^1
−20 −10 0 10 20

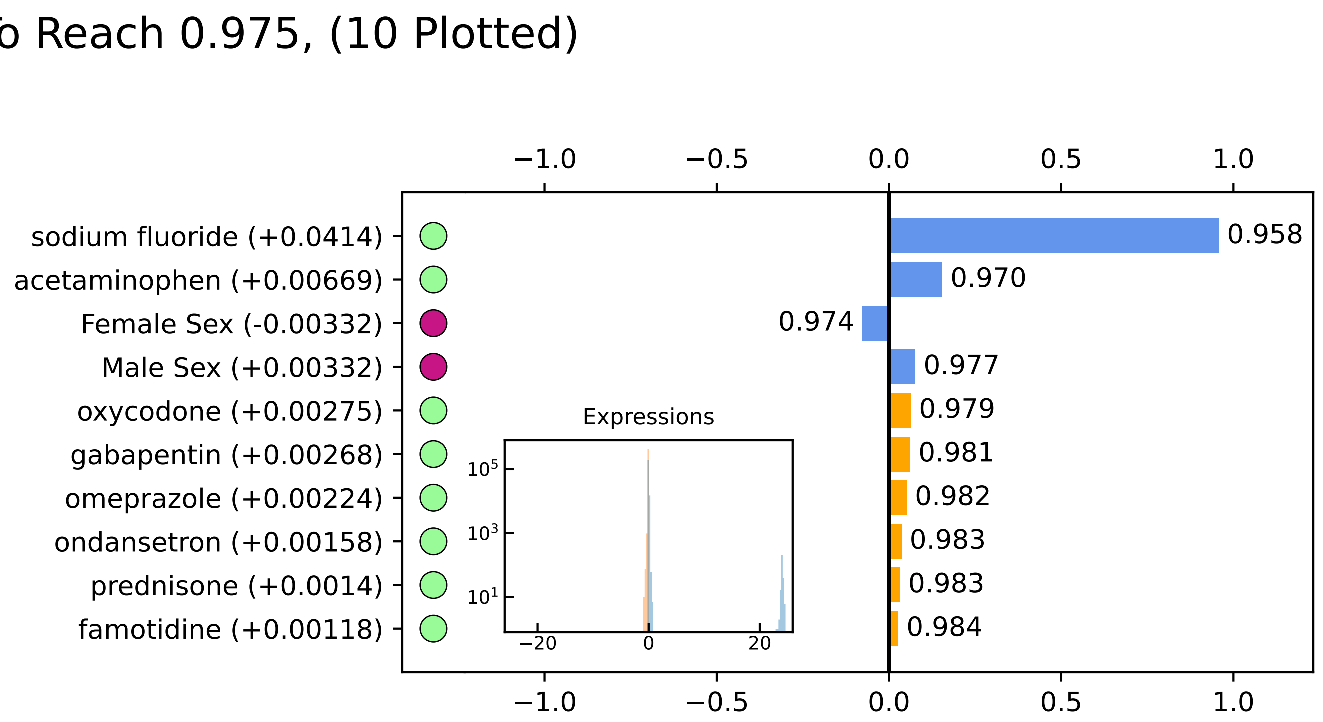
Signature [58]; 4 To Reach 0.975, (10 Plotted)
sodium fluoride (+0.0414)
acetaminophen (+0.00669)
Female Sex (-0.00332)
Male Sex (+0.00332)
oxycodone (+0.00275)
gabapentin (+0.00268)
omeprazole (+0.00224)
ondansetron (+0.00158)
prednisone (+0.0014)
famotidine (+0.00118)
0.958
0.970
0.974
0.977
0.979
0.981
0.982
0.983
0.983
0.984
Expressions
−1.0
−0.5
0.0
0.5
1.0

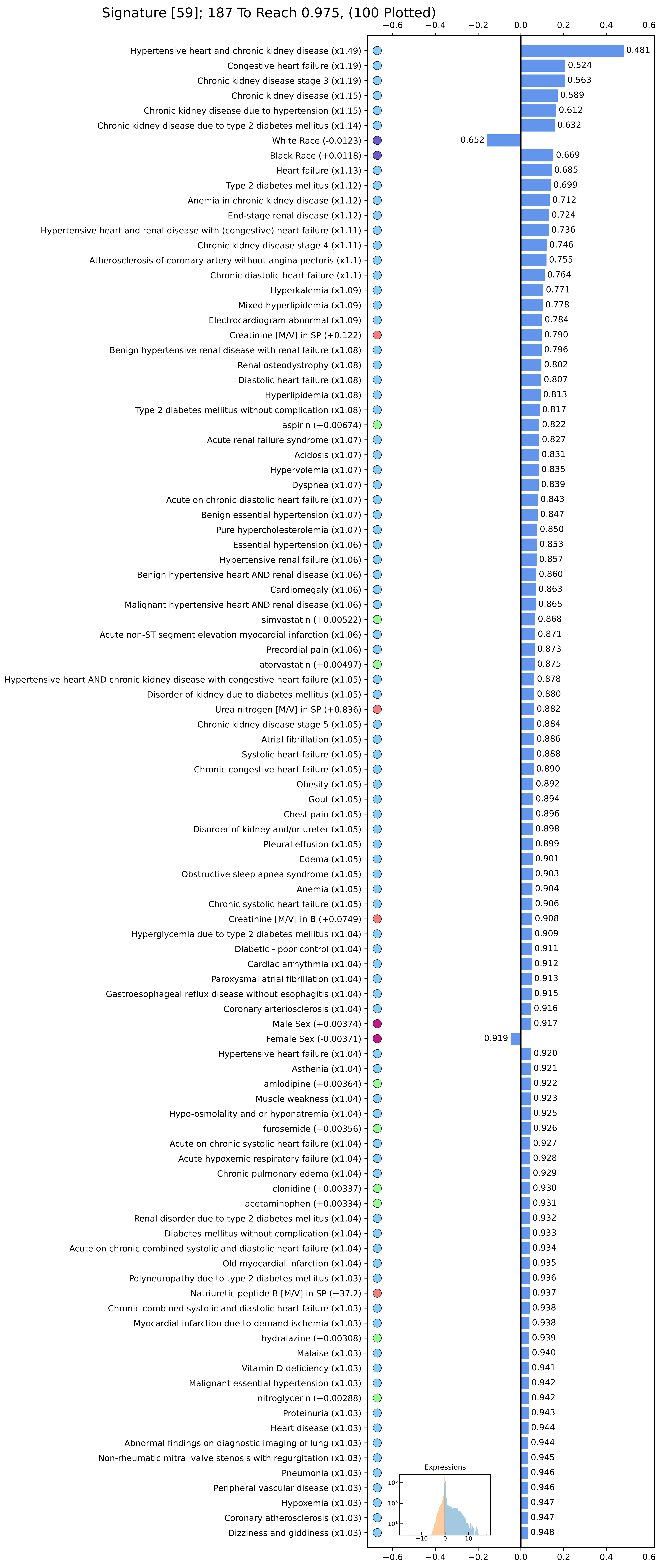
Signature [59]; 187 To Reach 0.975, (100 Plotted)
Hypertensive heart and chronic kidney disease (x1.49) 0.481
Congestive heart failure (x1.19) 0.524
Chronic kidney disease stage 3 (x1.19) 0.563
Chronic kidney disease (x1.15) 0.589
Chronic kidney disease due to hypertension (x1.15) 0.612
Chronic kidney disease due to type 2 diabetes mellitus (x1.14) 0.632
White Race (-0.0123) 0.652
Black Race (+0.0118) 0.669
Heart failure (x1.13) 0.685
Type 2 diabetes mellitus (x1.12) 0.699
Anemia in chronic kidney disease (x1.12) 0.712
End-stage renal disease (x1.12) 0.724
Hypertensive heart and renal disease with (congestive) heart failure (x1.11) 0.736
Chronic kidney disease stage 4 (x1.11) 0.746
Atherosclerosis of coronary artery without angina pectoris (x1.1) 0.755
Chronic diastolic heart failure (x1.1) 0.764
Hyperkalemia (x1.09) 0.771
Mixed hyperlipidemia (x1.09) 0.778
Electrocardiogram abnormal (x1.09) 0.784
Creatinine [M/V] in SP (+0.122) 0.790
Benign hypertensive renal disease with renal failure (x1.08) 0.796
Renal osteodystrophy (x1.08) 0.802
Diastolic heart failure (x1.08) 0.807
Hyperlipidemia (x1.08) 0.813
Type 2 diabetes mellitus without complication (x1.08) 0.817
aspirin (+0.00674) 0.822
Acute renal failure syndrome (x1.07) 0.827
Acidosis (x1.07) 0.831
Hypervolemia (x1.07) 0.835
Dyspnea (x1.07) 0.839
Acute on chronic diastolic heart failure (x1.07) 0.843
Benign essential hypertension (x1.07) 0.847
Pure hypercholesterolemia (x1.07) 0.850
Essential hypertension (x1.06) 0.853
Hypertensive renal failure (x1.06) 0.857
Benign hypertensive heart AND renal disease (x1.06) 0.860
Cardiomegaly (x1.06) 0.863
Malignant hypertensive heart AND renal disease (x1.06) 0.865
simvastatin (+0.00522) 0.868
Acute non-ST segment elevation myocardial infarction (x1.06) 0.871
Precordial pain (x1.06) 0.873
atorvastatin (+0.00497) 0.875
Hypertensive heart AND chronic kidney disease with congestive heart failure (x1.05) 0.878
Disorder of kidney due to diabetes mellitus (x1.05) 0.880
Urea nitrogen [M/V] in SP (+0.836) 0.882
Chronic kidney disease stage 5 (x1.05) 0.884
Atrial fibrillation (x1.05) 0.886
Systolic heart failure (x1.05) 0.888
Chronic congestive heart failure (x1.05) 0.890
Obesity (x1.05) 0.892
Gout (x1.05) 0.894
Chest pain (x1.05) 0.896
Disorder of kidney and/or ureter (x1.05) 0.898
Pleural effusion (x1.05) 0.899
Edema (x1.05) 0.901
Obstructive sleep apnea syndrome (x1.05) 0.903
Anemia (x1.05) 0.904
Chronic systolic heart failure (x1.05) 0.906
Creatinine [M/V] in B (+0.0749) 0.908
Hyperglycemia due to type 2 diabetes mellitus (x1.04) 0.909
Diabetic - poor control (x1.04) 0.911
Cardiac arrhythmia (x1.04) 0.912
Paroxysmal atrial fibrillation (x1.04) 0.913
Gastroesophageal reflux disease without esophagitis (x1.04) 0.915
Coronary arteriosclerosis (x1.04) 0.916
Male Sex (+0.00374) 0.917
Female Sex (-0.00371) 0.919
Hypertensive heart failure (x1.04) 0.920
Asthenia (x1.04) 0.921
amlodipine (+0.00364) 0.922
Muscle weakness (x1.04) 0.923
Hypo-osmolality and or hyponatremia (x1.04) 0.925
furosemide (+0.00356) 0.926
Acute on chronic systolic heart failure (x1.04) 0.927
Acute hypoxemic respiratory failure (x1.04) 0.928
Chronic pulmonary edema (x1.04) 0.929
clonidine (+0.00337) 0.930
acetaminophen (+0.00334) 0.931
Renal disorder due to type 2 diabetes mellitus (x1.04) 0.932
Diabetes mellitus without complication (x1.04) 0.933
Acute on chronic combined systolic and diastolic heart failure (x1.04) 0.934
Old myocardial infarction (x1.04) 0.935
Polyneuropathy due to type 2 diabetes mellitus (x1.03) 0.936
Natriuretic peptide B [M/V] in SP (+37.2) 0.937
Chronic combined systolic and diastolic heart failure (x1.03) 0.938
Myocardial infarction due to demand ischemia (x1.03) 0.938
hydralazine (+0.00308) 0.939
Malaise (x1.03) 0.940
Vitamin D deficiency (x1.03) 0.941
Malignant essential hypertension (x1.03) 0.942
nitroglycerin (+0.00288) 0.942
Proteinuria (x1.03) 0.943
Heart disease (x1.03) 0.944
Abnormal findings on diagnostic imaging of lung (x1.03) 0.944
Non-rheumatic mitral valve stenosis with regurgitation (x1.03) 0.945
Pneumonia (x1.03) 0.946
Peripheral vascular disease (x1.03) 0.946
Hypoxemia (x1.03) 0.947
Coronary atherosclerosis (x1.03) 0.947
Dizziness and giddiness (x1.03) 0.948
Expressions
−0.6 −0.4 −0.2 0.0 0.2 0.4 0.6

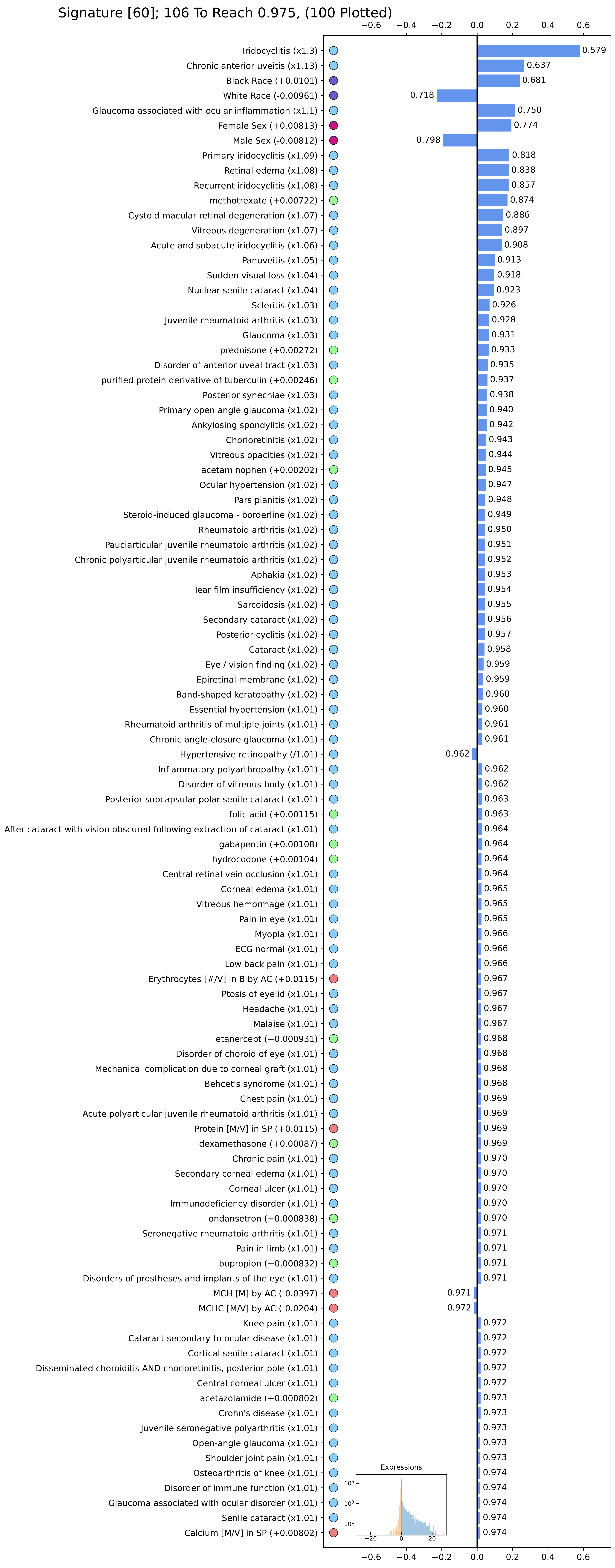
Signature [60]; 106 To Reach 0.975, (100 Plotted)
−0.6
−0.4
−0.2
0.0
0.2
0.4
0.6
Iridocyclitis (x1.3)
0.579
Chronic anterior uveitis (x1.13)
0.637
Black Race (+0.0101)
0.681
White Race (-0.00961)
0.718
Glaucoma associated with ocular inflammation (x1.1)
0.750
Female Sex (+0.00813)
0.774
Male Sex (-0.00812)
0.798
Primary iridocyclitis (x1.09)
0.818
Retinal edema (x1.08)
0.838
Recurrent iridocyclitis (x1.08)
0.857
methotrexate (+0.00722)
0.874
Cystoid macular retinal degeneration (x1.07)
0.886
Vitreous degeneration (x1.07)
0.897
Acute and subacute iridocyclitis (x1.06)
0.908
Panuveitis (x1.05)
0.913
Sudden visual loss (x1.04)
0.918
Nuclear senile cataract (x1.04)
0.923
Scleritis (x1.03)
0.926
Juvenile rheumatoid arthritis (x1.03)
0.928
Glaucoma (x1.03)
0.931
prednisone (+0.00272)
0.933
Disorder of anterior uveal tract (x1.03)
0.935
purified protein derivative of tuberculin (+0.00246)
0.937
Posterior synechiae (x1.03)
0.938
Primary open angle glaucoma (x1.02)
0.940
Ankylosing spondylitis (x1.02)
0.942
Chorioretinitis (x1.02)
0.943
Vitreous opacities (x1.02)
0.944
acetaminophen (+0.00202)
0.945
Ocular hypertension (x1.02)
0.947
Pars planitis (x1.02)
0.948
Steroid-induced glaucoma - borderline (x1.02)
0.949
Rheumatoid arthritis (x1.02)
0.950
Pauciarticular juvenile rheumatoid arthritis (x1.02)
0.951
Chronic polyarticular juvenile rheumatoid arthritis (x1.02)
0.952
Aphakia (x1.02)
0.953
Tear film insufficiency (x1.02)
0.954
Sarcoidosis (x1.02)
0.955
Secondary cataract (x1.02)
0.956
Posterior cyclitis (x1.02)
0.957
Cataract (x1.02)
0.958
Eye / vision finding (x1.02)
0.959
Epiretinal membrane (x1.02)
0.959
Band-shaped keratopathy (x1.02)
0.960
Essential hypertension (x1.01)
0.960
Rheumatoid arthritis of multiple joints (x1.01)
0.961
Chronic angle-closure glaucoma (x1.01)
0.961
Hypertensive retinopathy (/1.01)
0.962
Inflammatory polyarthropathy (x1.01)
0.962
Disorder of vitreous body (x1.01)
0.962
Posterior subcapsular polar senile cataract (x1.01)
0.963
folic acid (+0.00115)
0.963
After-cataract with vision obscured following extraction of cataract (x1.01)
0.964
gabapentin (+0.00108)
0.964
hydrocodone (+0.00104)
0.964
Central retinal vein occlusion (x1.01)
0.964
Corneal edema (x1.01)
0.965
Vitreous hemorrhage (x1.01)
0.965
Pain in eye (x1.01)
0.965
Myopia (x1.01)
0.966
ECG normal (x1.01)
0.966
Low back pain (x1.01)
0.966
Erythrocytes [#/V] in B by AC (+0.0115)
0.967
Ptosis of eyelid (x1.01)
0.967
Headache (x1.01)
0.967
Malaise (x1.01)
0.967
etanercept (+0.000931)
0.968
Disorder of choroid of eye (x1.01)
0.968
Mechanical complication due to corneal graft (x1.01)
0.968
Behcet's syndrome (x1.01)
0.968
Chest pain (x1.01)
0.969
Acute polyarticular juvenile rheumatoid arthritis (x1.01)
0.969
Protein [M/V] in SP (+0.0115)
0.969
dexamethasone (+0.00087)
0.969
Chronic pain (x1.01)
0.970
Secondary corneal edema (x1.01)
0.970
Corneal ulcer (x1.01)
0.970
Immunodeficiency disorder (x1.01)
0.970
ondansetron (+0.000838)
0.970
Seronegative rheumatoid arthritis (x1.01)
0.971
Pain in limb (x1.01)
0.971
bupropion (+0.000832)
0.971
Disorders of prostheses and implants of the eye (x1.01)
0.971
MCH [M] by AC (-0.0397)
0.971
MCHC [M/V] by AC (-0.0204)
0.972
Knee pain (x1.01)
0.972
Cataract secondary to ocular disease (x1.01)
0.972
Cortical senile cataract (x1.01)
0.972
Disseminated choroiditis AND chorioretinitis, posterior pole (x1.01)
0.972
Central corneal ulcer (x1.01)
0.972
acetazolamide (+0.000802)
0.973
Crohn's disease (x1.01)
0.973
Juvenile seronegative polyarthritis (x1.01)
0.973
Open-angle glaucoma (x1.01)
0.973
Shoulder joint pain (x1.01)
0.973
Osteoarthritis of knee (x1.01)
0.974
Disorder of immune function (x1.01)
0.974
Glaucoma associated with ocular disorder (x1.01)
0.974
Senile cataract (x1.01)
0.974
Calcium [M/V] in SP (+0.00802)
0.974
Expressions
10^5
10^3
10^1
−20
0
20

Signature [61]; 3 To Reach 0.975, (10 Plotted)

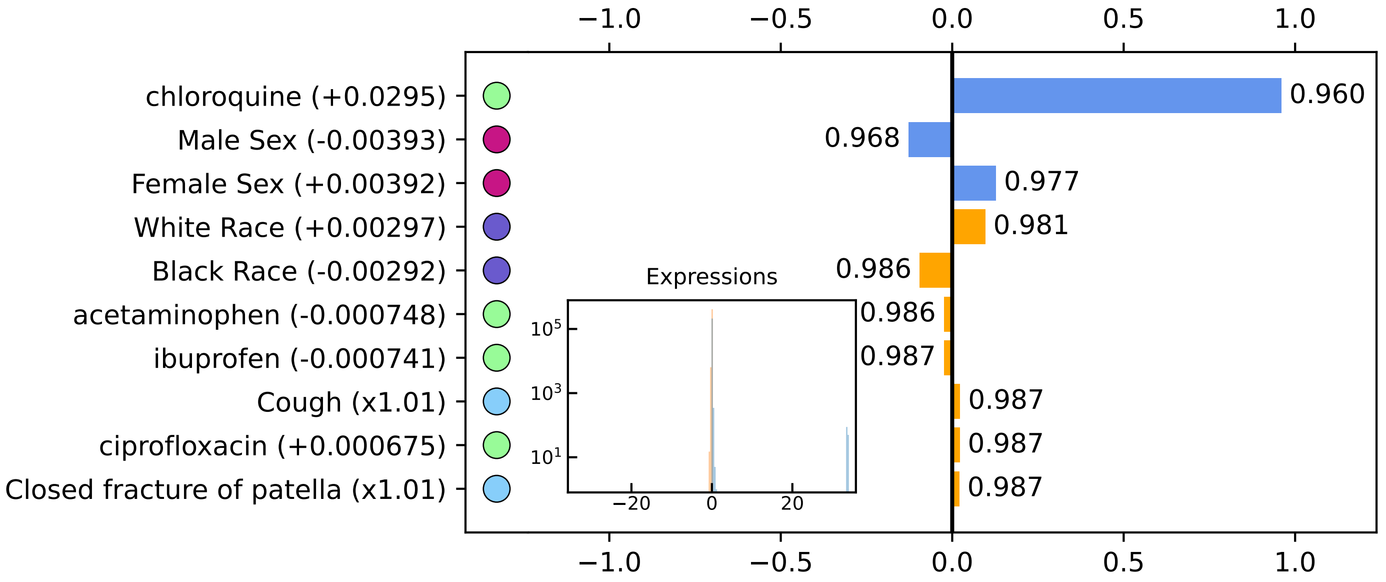

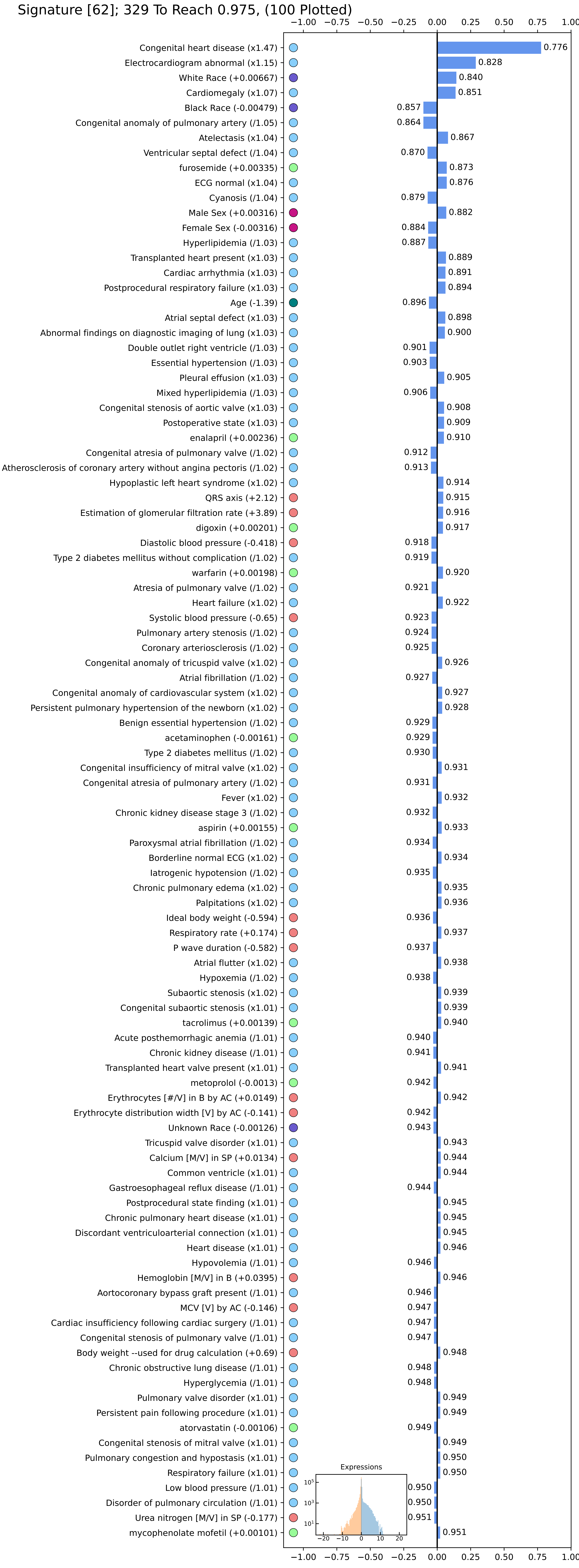

Signature [62]; 329 To Reach 0.975, (100 Plotted)
−1.00
−0.75
−0.50
−0.25
0.00
0.25
0.50
0.75
1.00
Congenital heart disease (x1.47)
0.776
Electrocardiogram abnormal (x1.15)
0.828
White Race (+0.00667)
0.840
Cardiomegaly (x1.07)
0.851
Black Race (-0.00479)
0.857
Congenital anomaly of pulmonary artery (/1.05)
0.864
Atelectasis (x1.04)
0.867
Ventricular septal defect (/1.04)
0.870
furosemide (+0.00335)
0.873
ECG normal (x1.04)
0.876
Cyanosis (/1.04)
0.879
Male Sex (+0.00316)
0.882
Female Sex (-0.00316)
0.884
Hyperlipidemia (/1.03)
0.887
Transplanted heart present (x1.03)
0.889
Cardiac arrhythmia (x1.03)
0.891
Postprocedural respiratory failure (x1.03)
0.894
Age (-1.39)
0.896
Atrial septal defect (x1.03)
0.898
Abnormal findings on diagnostic imaging of lung (x1.03)
0.900
Double outlet right ventricle (/1.03)
0.901
Essential hypertension (/1.03)
0.903
Pleural effusion (x1.03)
0.905
Mixed hyperlipidemia (/1.03)
0.906
Congenital stenosis of aortic valve (x1.03)
0.908
Postoperative state (x1.03)
0.909
enalapril (+0.00236)
0.910
Congenital atresia of pulmonary valve (/1.02)
0.912
Atherosclerosis of coronary artery without angina pectoris (/1.02)
0.913
Hypoplastic left heart syndrome (x1.02)
0.914
QRS axis (+2.12)
0.915
Estimation of glomerular filtration rate (+3.89)
0.916
digoxin (+0.00201)
0.917
Diastolic blood pressure (-0.418)
0.918
Type 2 diabetes mellitus without complication (/1.02)
0.919
warfarin (+0.00198)
0.920
Atresia of pulmonary valve (/1.02)
0.921
Heart failure (x1.02)
0.922
Systolic blood pressure (-0.65)
0.923
Pulmonary artery stenosis (/1.02)
0.924
Coronary arteriosclerosis (/1.02)
0.925
Congenital anomaly of tricuspid valve (x1.02)
0.926
Atrial fibrillation (/1.02)
0.927
Congenital anomaly of cardiovascular system (x1.02)
0.927
Persistent pulmonary hypertension of the newborn (x1.02)
0.928
Benign essential hypertension (/1.02)
0.929
acetaminophen (-0.00161)
0.929
Type 2 diabetes mellitus (/1.02)
0.930
Congenital insufficiency of mitral valve (x1.02)
0.931
Congenital atresia of pulmonary artery (/1.02)
0.931
Fever (x1.02)
0.932
Chronic kidney disease stage 3 (/1.02)
0.932
aspirin (+0.00155)
0.933
Paroxysmal atrial fibrillation (/1.02)
0.934
Borderline normal ECG (x1.02)
0.934
Iatrogenic hypotension (/1.02)
0.935
Chronic pulmonary edema (x1.02)
0.935
Palpitations (x1.02)
0.936
Ideal body weight (-0.594)
0.936
Respiratory rate (+0.174)
0.937
P wave duration (-0.582)
0.937
Atrial flutter (x1.02)
0.938
Hypoxemia (/1.02)
0.938
Subaortic stenosis (x1.02)
0.939
Congenital subaortic stenosis (x1.01)
0.939
tacrolimus (+0.00139)
0.940
Acute posthemorrhagic anemia (/1.01)
0.940
Chronic kidney disease (/1.01)
0.941
Transplanted heart valve present (x1.01)
0.941
metoprolol (-0.0013)
0.942
Erythrocytes [#/V] in B by AC (+0.0149)
0.942
Erythrocyte distribution width [V] by AC (-0.141)
0.942
Unknown Race (-0.00126)
0.943
Tricuspid valve disorder (x1.01)
0.943
Calcium [M/V] in SP (+0.0134)
0.944
Common ventricle (x1.01)
0.944
Gastroesophageal reflux disease (/1.01)
0.944
Postprocedural state finding (x1.01)
0.945
Chronic pulmonary heart disease (x1.01)
0.945
Discordant ventriculoarterial connection (x1.01)
0.945
Heart disease (x1.01)
0.946
Hypovolemia (/1.01)
0.946
Hemoglobin [M/V] in B (+0.0395)
0.946
Aortocoronary bypass graft present (/1.01)
0.946
MCV [V] by AC (-0.146)
0.947
Cardiac insufficiency following cardiac surgery (/1.01)
0.947
Congenital stenosis of pulmonary valve (/1.01)
0.947
Body weight --used for drug calculation (+0.69)
0.948
Chronic obstructive lung disease (/1.01)
0.948
Hyperglycemia (/1.01)
0.948
Pulmonary valve disorder (x1.01)
0.949
Persistent pain following procedure (x1.01)
0.949
atorvastatin (-0.00106)
0.949
Congenital stenosis of mitral valve (x1.01)
0.949
Pulmonary congestion and hypostasis (x1.01)
0.950
Respiratory failure (x1.01)
0.950
Low blood pressure (/1.01)
0.950
Disorder of pulmonary circulation (/1.01)
0.950
Urea nitrogen [M/V] in SP (-0.177)
0.951
mycophenolate mofetil (+0.00101)
0.951
Expressions
10^5
10^3
10^1
−20
−10
0
10
20

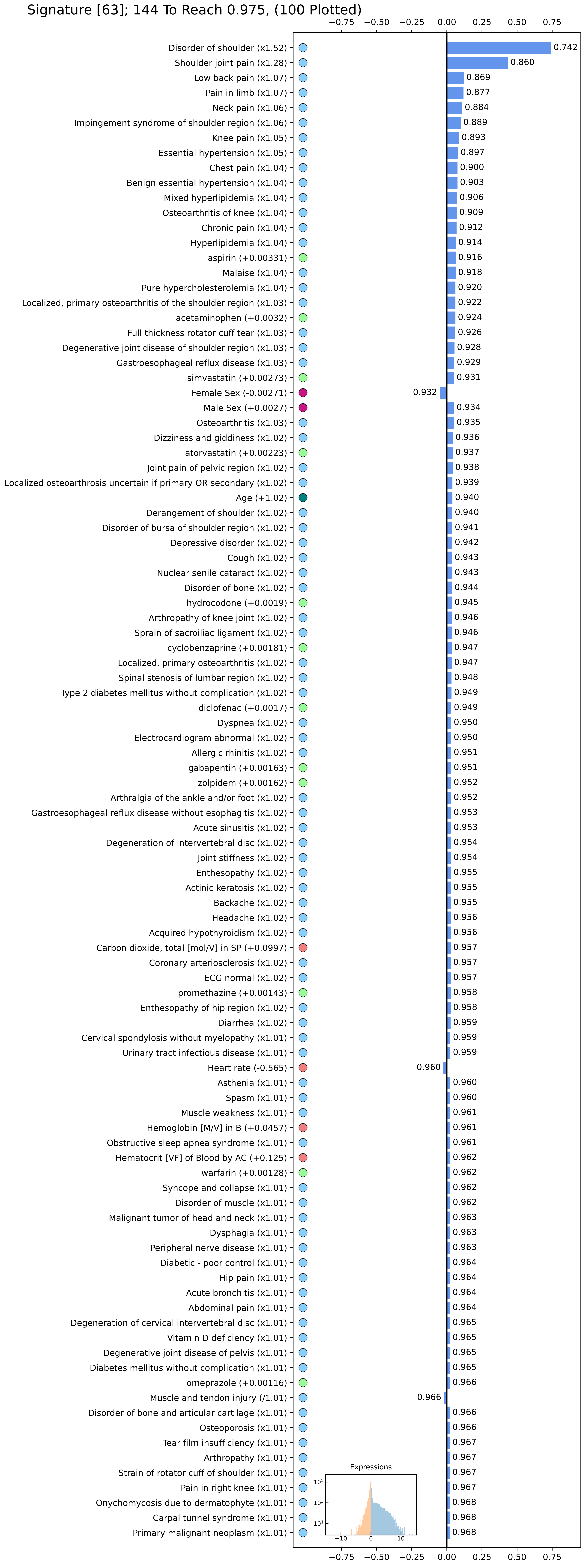
Signature [63]; 144 To Reach 0.975, (100 Plotted)
Disorder of shoulder (x1.52) 0.742
Shoulder joint pain (x1.28) 0.860
Low back pain (x1.07) 0.869
Pain in limb (x1.07) 0.877
Neck pain (x1.06) 0.884
Impingement syndrome of shoulder region (x1.06) 0.889
Knee pain (x1.05) 0.893
Essential hypertension (x1.05) 0.897
Chest pain (x1.04) 0.900
Benign essential hypertension (x1.04) 0.903
Mixed hyperlipidemia (x1.04) 0.906
Osteoarthritis of knee (x1.04) 0.909
Chronic pain (x1.04) 0.912
Hyperlipidemia (x1.04) 0.914
aspirin (+0.00331) 0.916
Malaise (x1.04) 0.918
Pure hypercholesterolemia (x1.04) 0.920
Localized, primary osteoarthritis of the shoulder region (x1.03) 0.922
acetaminophen (+0.0032) 0.924
Full thickness rotator cuff tear (x1.03) 0.926
Degenerative joint disease of shoulder region (x1.03) 0.928
Gastroesophageal reflux disease (x1.03) 0.929
simvastatin (+0.00273) 0.931
Female Sex (-0.00271) 0.932
Male Sex (+0.0027) 0.934
Osteoarthritis (x1.03) 0.935
Dizziness and giddiness (x1.02) 0.936
atorvastatin (+0.00223) 0.937
Joint pain of pelvic region (x1.02) 0.938
Localized osteoarthrosis uncertain if primary OR secondary (x1.02) 0.939
Age (+1.02) 0.940
Derangement of shoulder (x1.02) 0.940
Disorder of bursa of shoulder region (x1.02) 0.941
Depressive disorder (x1.02) 0.942
Cough (x1.02) 0.943
Nuclear senile cataract (x1.02) 0.943
Disorder of bone (x1.02) 0.944
hydrocodone (+0.0019) 0.945
Arthropathy of knee joint (x1.02) 0.946
Sprain of sacroiliac ligament (x1.02) 0.946
cyclobenzaprine (+0.00181) 0.947
Localized, primary osteoarthritis (x1.02) 0.947
Spinal stenosis of lumbar region (x1.02) 0.948
Type 2 diabetes mellitus without complication (x1.02) 0.949
diclofenac (+0.0017) 0.949
Dyspnea (x1.02) 0.950
Electrocardiogram abnormal (x1.02) 0.950
Allergic rhinitis (x1.02) 0.951
gabapentin (+0.00163) 0.951
zolpidem (+0.00162) 0.952
Arthralgia of the ankle and/or foot (x1.02) 0.952
Gastroesophageal reflux disease without esophagitis (x1.02) 0.953
Acute sinusitis (x1.02) 0.953
Degeneration of intervertebral disc (x1.02) 0.954
Joint stiffness (x1.02) 0.954
Enthesopathy (x1.02) 0.955
Actinic keratosis (x1.02) 0.955
Backache (x1.02) 0.955
Headache (x1.02) 0.956
Acquired hypothyroidism (x1.02) 0.956
Carbon dioxide, total [mol/V] in SP (+0.0997) 0.957
Coronary arteriosclerosis (x1.02) 0.957
ECG normal (x1.02) 0.957
promethazine (+0.00143) 0.958
Enthesopathy of hip region (x1.02) 0.958
Diarrhea (x1.02) 0.959
Cervical spondylosis without myelopathy (x1.01) 0.959
Urinary tract infectious disease (x1.01) 0.959
Heart rate (-0.565) 0.960
Asthenia (x1.01) 0.960
Spasm (x1.01) 0.960
Muscle weakness (x1.01) 0.961
Hemoglobin [M/V] in B (+0.0457) 0.961
Obstructive sleep apnea syndrome (x1.01) 0.961
Hematocrit [VF] of Blood by AC (+0.125) 0.962
warfarin (+0.00128) 0.962
Syncope and collapse (x1.01) 0.962
Disorder of muscle (x1.01) 0.962
Malignant tumor of head and neck (x1.01) 0.963
Dysphagia (x1.01) 0.963
Peripheral nerve disease (x1.01) 0.963
Diabetic - poor control (x1.01) 0.964
Hip pain (x1.01) 0.964
Acute bronchitis (x1.01) 0.964
Abdominal pain (x1.01) 0.964
Degeneration of cervical intervertebral disc (x1.01) 0.965
Vitamin D deficiency (x1.01) 0.965
Degenerative joint disease of pelvis (x1.01) 0.965
Diabetes mellitus without complication (x1.01) 0.965
omeprazole (+0.00116) 0.966
Muscle and tendon injury (/1.01) 0.966
Disorder of bone and articular cartilage (x1.01) 0.966
Osteoporosis (x1.01) 0.966
Tear film insufficiency (x1.01) 0.967
Arthropathy (x1.01) 0.967
Strain of rotator cuff of shoulder (x1.01) 0.967
Pain in right knee (x1.01) 0.967
Onychomycosis due to dermatophyte (x1.01) 0.968
Carpal tunnel syndrome (x1.01) 0.968
Primary malignant neoplasm (x1.01) 0.968
−0.75 −0.50 −0.25 0.00 0.25 0.50 0.75
Expressions
−10 0 10

Signature [64]; 210 To Reach 0.975, (100 Plotted)

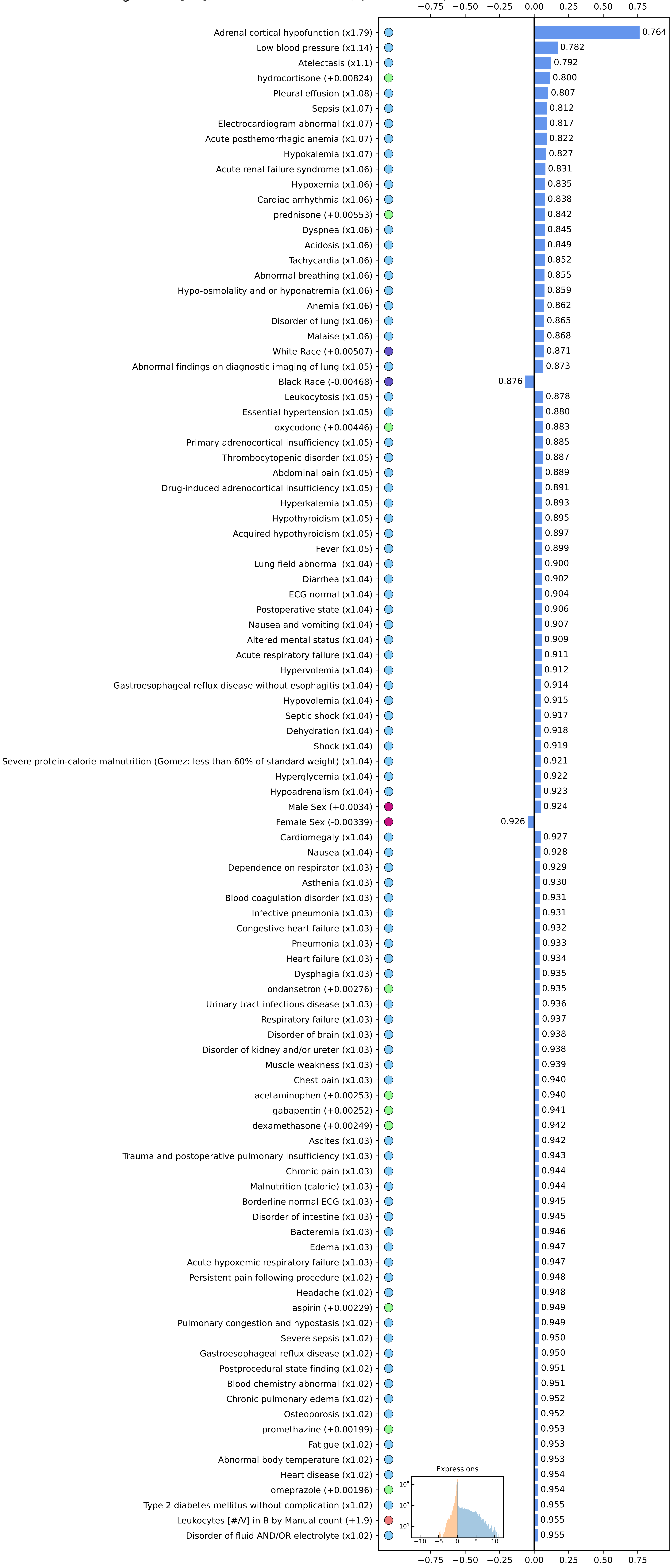

Signature [65]; 17 To Reach 0.975, (17 Plotted)

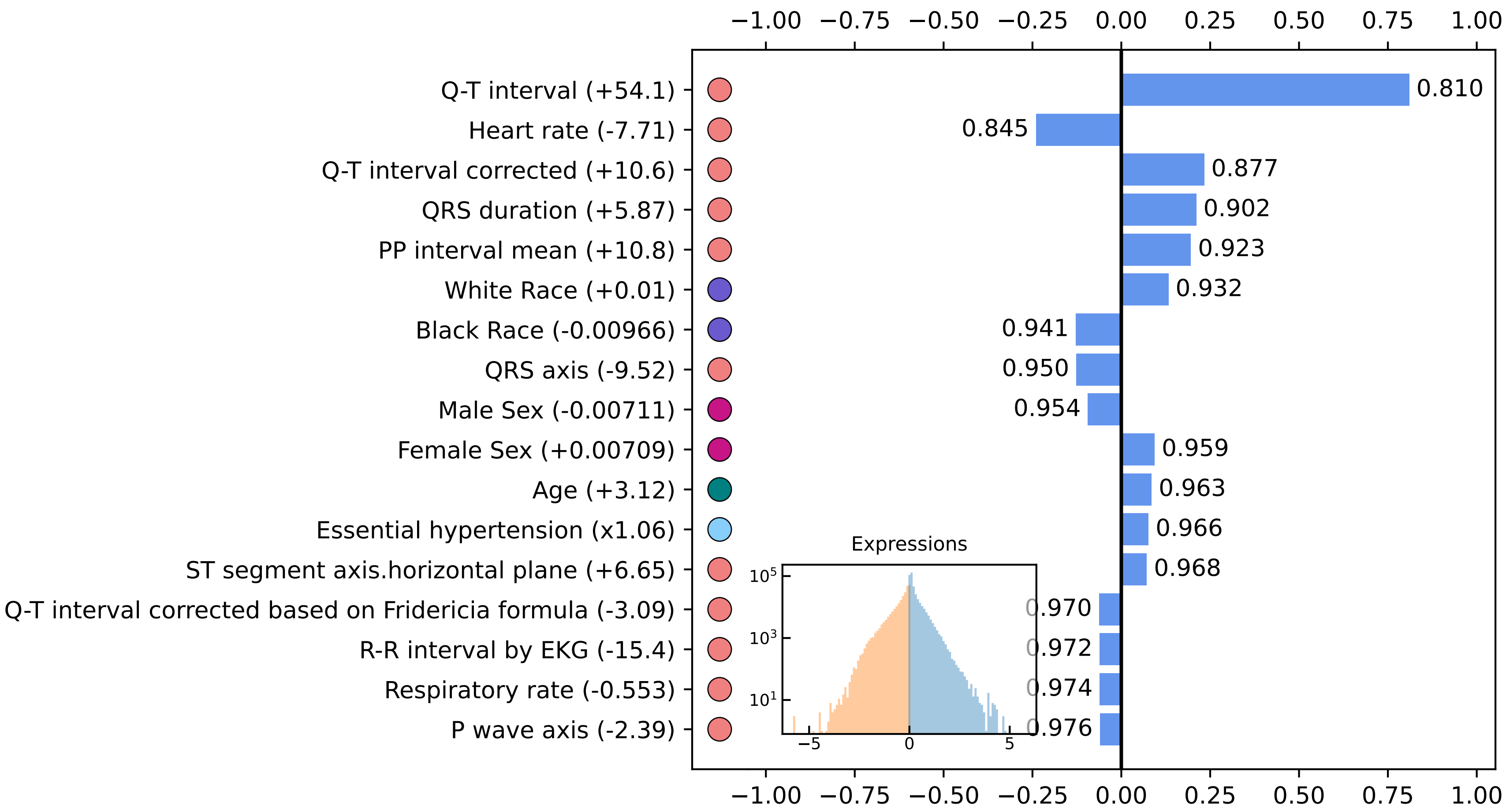

Signature [66]; 3 To Reach 0.975, (10 Plotted)

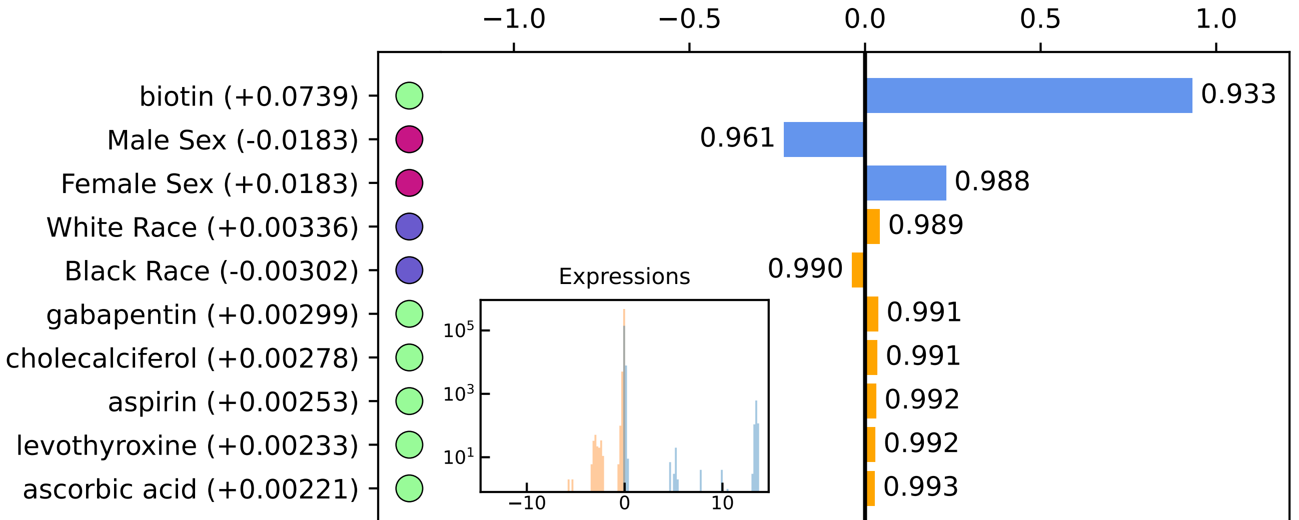

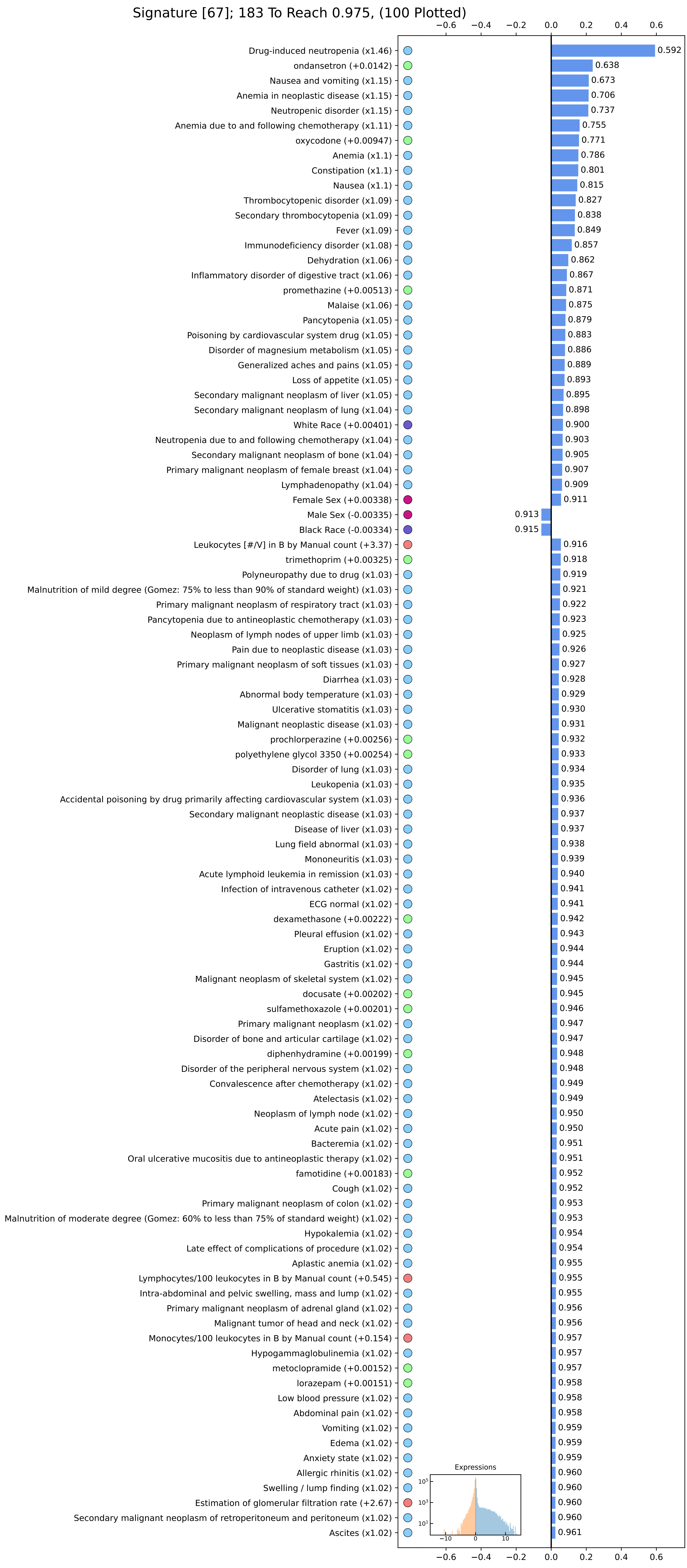

Signature [67]; 183 To Reach 0.975, (100 Plotted)
Drug-induced neutropenia (x1.46)
ondansetron (+0.0142)
Nausea and vomiting (x1.15)
Anemia in neoplastic disease (x1.15)
Neutropenic disorder (x1.15)
Anemia due to and following chemotherapy (x1.11)
oxycodone (+0.00947)
Anemia (x1.1)
Constipation (x1.1)
Nausea (x1.1)
Thrombocytopenic disorder (x1.09)
Secondary thrombocytopenia (x1.09)
Fever (x1.09)
Immunodeficiency disorder (x1.08)
Dehydration (x1.06)
Inflammatory disorder of digestive tract (x1.06)
promethazine (+0.00513)
Malaise (x1.06)
Pancytopenia (x1.05)
Poisoning by cardiovascular system drug (x1.05)
Disorder of magnesium metabolism (x1.05)
Generalized aches and pains (x1.05)
Loss of appetite (x1.05)
Secondary malignant neoplasm of liver (x1.05)
Secondary malignant neoplasm of lung (x1.04)
White Race (+0.00401)
Neutropenia due to and following chemotherapy (x1.04)
Secondary malignant neoplasm of bone (x1.04)
Primary malignant neoplasm of female breast (x1.04)
Lymphadenopathy (x1.04)
Female Sex (+0.00338)
Male Sex (-0.00335)
Black Race (-0.00334)
Leukocytes [#/V] in B by Manual count (+3.37)
trimethoprim (+0.00325)
Polyneuropathy due to drug (x1.03)
Malnutrition of mild degree (Gomez: 75% to less than 90% of standard weight) (x1.03)
Primary malignant neoplasm of respiratory tract (x1.03)
Pancytopenia due to antineoplastic chemotherapy (x1.03)
Neoplasm of lymph nodes of upper limb (x1.03)
Pain due to neoplastic disease (x1.03)
Primary malignant neoplasm of soft tissues (x1.03)
Diarrhea (x1.03)
Abnormal body temperature (x1.03)
Ulcerative stomatitis (x1.03)
Malignant neoplastic disease (x1.03)
prochlorperazine (+0.00256)
polyethylene glycol 3350 (+0.00254)
Disorder of lung (x1.03)
Leukopenia (x1.03)
Accidental poisoning by drug primarily affecting cardiovascular system (x1.03)
Secondary malignant neoplastic disease (x1.03)
Disease of liver (x1.03)
Lung field abnormal (x1.03)
Mononeuritis (x1.03)
Acute lymphoid leukemia in remission (x1.03)
Infection of intravenous catheter (x1.02)
ECG normal (x1.02)
dexamethasone (+0.00222)
Pleural effusion (x1.02)
Eruption (x1.02)
Gastritis (x1.02)
Malignant neoplasm of skeletal system (x1.02)
docusate (+0.00202)
sulfamethoxazole (+0.00201)
Primary malignant neoplasm (x1.02)
Disorder of bone and articular cartilage (x1.02)
diphenhydramine (+0.00199)
Disorder of the peripheral nervous system (x1.02)
Convalescence after chemotherapy (x1.02)
Atelectasis (x1.02)
Neoplasm of lymph node (x1.02)
Acute pain (x1.02)
Bacteremia (x1.02)
Oral ulcerative mucositis due to antineoplastic therapy (x1.02)
famotidine (+0.00183)
Cough (x1.02)
Primary malignant neoplasm of colon (x1.02)
Malnutrition of moderate degree (Gomez: 60% to less than 75% of standard weight) (x1.02)
Hypokalemia (x1.02)
Late effect of complications of procedure (x1.02)
Aplastic anemia (x1.02)
Lymphocytes/100 leukocytes in B by Manual count (+0.545)
Intra-abdominal and pelvic swelling, mass and lump (x1.02)
Primary malignant neoplasm of adrenal gland (x1.02)
Malignant tumor of head and neck (x1.02)
Monocytes/100 leukocytes in B by Manual count (+0.154)
Hypogammaglobulinemia (x1.02)
metoclopramide (+0.00152)
lorazepam (+0.00151)
Low blood pressure (x1.02)
Abdominal pain (x1.02)
Vomiting (x1.02)
Edema (x1.02)
Anxiety state (x1.02)
Allergic rhinitis (x1.02)
Swelling / lump finding (x1.02)
Estimation of glomerular filtration rate (+2.67)
Secondary malignant neoplasm of retroperitoneum and peritoneum (x1.02)
Ascites (x1.02)
0.592
0.638
0.673
0.706
0.737
0.755
0.771
0.786
0.801
0.815
0.827
0.838
0.849
0.857
0.862
0.867
0.871
0.875
0.879
0.883
0.886
0.889
0.893
0.895
0.898
0.900
0.903
0.905
0.907
0.909
0.911
0.913
0.915
0.916
0.918
0.919
0.921
0.922
0.923
0.925
0.926
0.927
0.928
0.929
0.930
0.931
0.932
0.933
0.934
0.935
0.936
0.937
0.937
0.938
0.939
0.940
0.941
0.941
0.942
0.943
0.944
0.944
0.945
0.945
0.946
0.947
0.947
0.948
0.948
0.949
0.949
0.950
0.950
0.951
0.951
0.952
0.952
0.953
0.953
0.954
0.954
0.955
0.955
0.955
0.956
0.956
0.957
0.957
0.957
0.958
0.958
0.958
0.959
0.959
0.959
0.960
0.960
0.960
0.960
0.961
Expressions
−0.6 −0.4 −0.2 0.0 0.2 0.4 0.6

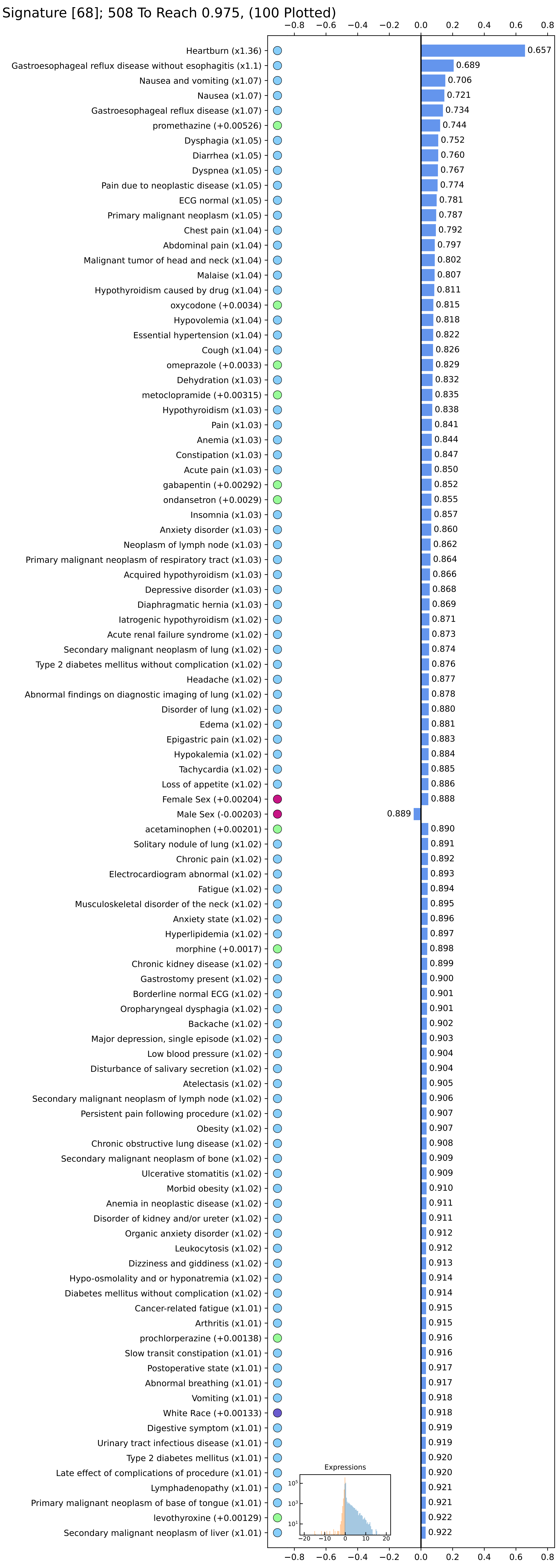
Signature [68]; 508 To Reach 0.975, (100 Plotted)
Heartburn (x1.36) 0.657
Gastroesophageal reflux disease without esophagitis (x1.1) 0.689
Nausea and vomiting (x1.07) 0.706
Nausea (x1.07) 0.721
Gastroesophageal reflux disease (x1.07) 0.734
promethazine (+0.00526) 0.744
Dysphagia (x1.05) 0.752
Diarrhea (x1.05) 0.760
Dyspnea (x1.05) 0.767
Pain due to neoplastic disease (x1.05) 0.774
ECG normal (x1.05) 0.781
Primary malignant neoplasm (x1.05) 0.787
Chest pain (x1.04) 0.792
Abdominal pain (x1.04) 0.797
Malignant tumor of head and neck (x1.04) 0.802
Malaise (x1.04) 0.807
Hypothyroidism caused by drug (x1.04) 0.811
oxycodone (+0.0034) 0.815
Hypovolemia (x1.04) 0.818
Essential hypertension (x1.04) 0.822
Cough (x1.04) 0.826
omeprazole (+0.0033) 0.829
Dehydration (x1.03) 0.832
metoclopramide (+0.00315) 0.835
Hypothyroidism (x1.03) 0.838
Pain (x1.03) 0.841
Anemia (x1.03) 0.844
Constipation (x1.03) 0.847
Acute pain (x1.03) 0.850
gabapentin (+0.00292) 0.852
ondansetron (+0.0029) 0.855
Insomnia (x1.03) 0.857
Anxiety disorder (x1.03) 0.860
Neoplasm of lymph node (x1.03) 0.862
Primary malignant neoplasm of respiratory tract (x1.03) 0.864
Acquired hypothyroidism (x1.03) 0.866
Depressive disorder (x1.03) 0.868
Diaphragmatic hernia (x1.03) 0.869
Iatrogenic hypothyroidism (x1.02) 0.871
Acute renal failure syndrome (x1.02) 0.873
Secondary malignant neoplasm of lung (x1.02) 0.874
Type 2 diabetes mellitus without complication (x1.02) 0.876
Headache (x1.02) 0.877
Abnormal findings on diagnostic imaging of lung (x1.02) 0.878
Disorder of lung (x1.02) 0.880
Edema (x1.02) 0.881
Epigastric pain (x1.02) 0.883
Hypokalemia (x1.02) 0.884
Tachycardia (x1.02) 0.885
Loss of appetite (x1.02) 0.886
Female Sex (+0.00204) 0.888
Male Sex (-0.00203) 0.889
acetaminophen (+0.00201) 0.890
Solitary nodule of lung (x1.02) 0.891
Chronic pain (x1.02) 0.892
Electrocardiogram abnormal (x1.02) 0.893
Fatigue (x1.02) 0.894
Musculoskeletal disorder of the neck (x1.02) 0.895
Anxiety state (x1.02) 0.896
Hyperlipidemia (x1.02) 0.897
morphine (+0.0017) 0.898
Chronic kidney disease (x1.02) 0.899
Gastrostomy present (x1.02) 0.900
Borderline normal ECG (x1.02) 0.901
Oropharyngeal dysphagia (x1.02) 0.901
Backache (x1.02) 0.902
Major depression, single episode (x1.02) 0.903
Low blood pressure (x1.02) 0.904
Disturbance of salivary secretion (x1.02) 0.904
Atelectasis (x1.02) 0.905
Secondary malignant neoplasm of lymph node (x1.02) 0.906
Persistent pain following procedure (x1.02) 0.907
Obesity (x1.02) 0.907
Chronic obstructive lung disease (x1.02) 0.908
Secondary malignant neoplasm of bone (x1.02) 0.909
Ulcerative stomatitis (x1.02) 0.909
Morbid obesity (x1.02) 0.910
Anemia in neoplastic disease (x1.02) 0.911
Disorder of kidney and/or ureter (x1.02) 0.911
Organic anxiety disorder (x1.02) 0.912
Leukocytosis (x1.02) 0.912
Dizziness and giddiness (x1.02) 0.913
Hypo-osmolality and or hyponatremia (x1.02) 0.914
Diabetes mellitus without complication (x1.02) 0.914
Cancer-related fatigue (x1.01) 0.915
Arthritis (x1.01) 0.915
prochlorperazine (+0.00138) 0.916
Slow transit constipation (x1.01) 0.916
Postoperative state (x1.01) 0.917
Abnormal breathing (x1.01) 0.917
Vomiting (x1.01) 0.918
White Race (+0.00133) 0.918
Digestive symptom (x1.01) 0.919
Urinary tract infectious disease (x1.01) 0.919
Type 2 diabetes mellitus (x1.01) 0.920
Late effect of complications of procedure (x1.01) 0.920
Lymphadenopathy (x1.01) 0.921
Primary malignant neoplasm of base of tongue (x1.01) 0.921
levothyroxine (+0.00129) 0.922
Secondary malignant neoplasm of liver (x1.01) 0.922
Expressions
−0.8 −0.6 −0.4 −0.2 0.0 0.2 0.4 0.6 0.8

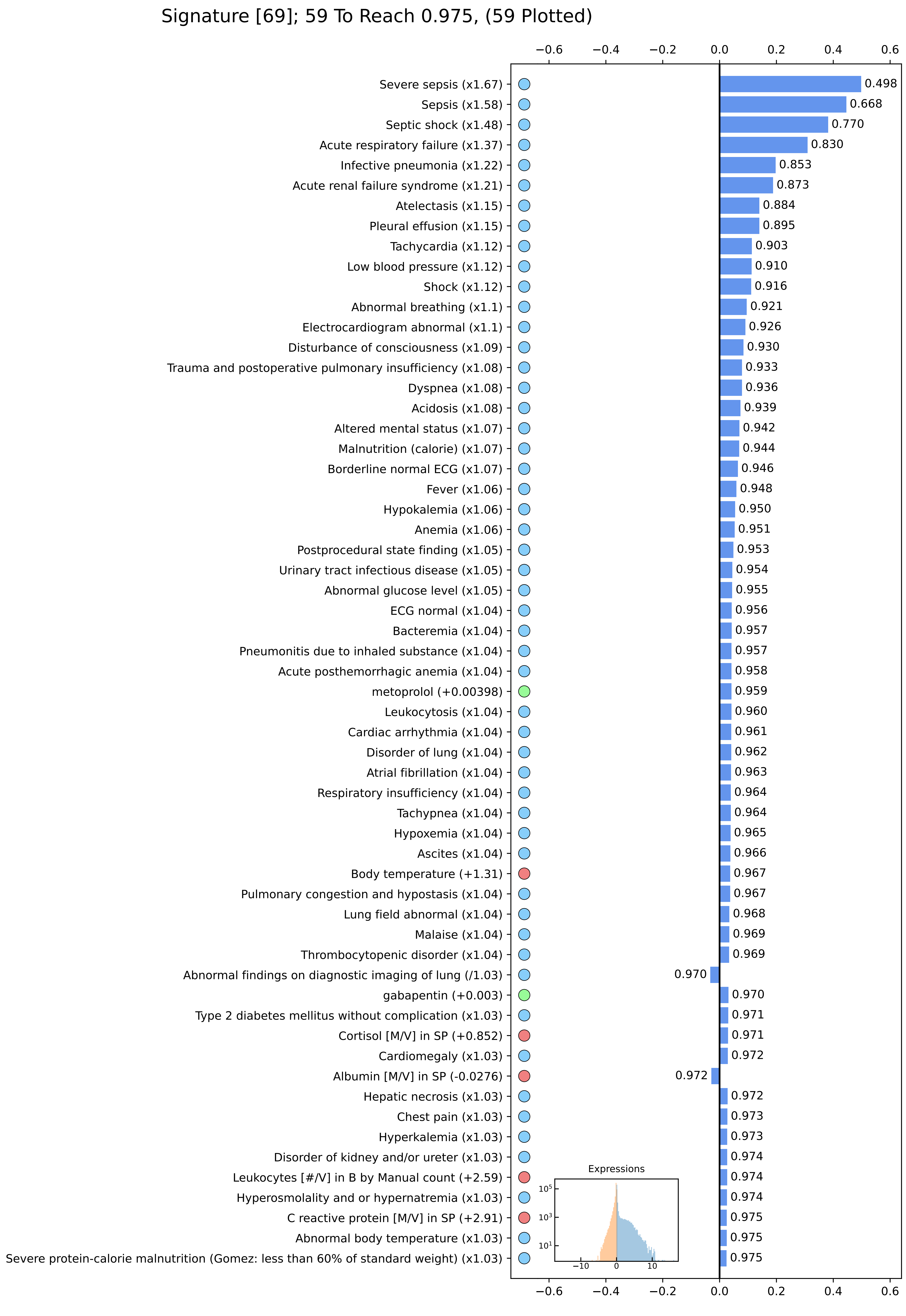
Signature [69]; 59 To Reach 0.975, (59 Plotted)
−0.6 −0.4 −0.2 0.0 0.2 0.4 0.6
Severe sepsis (x1.67) 0.498
Sepsis (x1.58) 0.668
Septic shock (x1.48) 0.770
Acute respiratory failure (x1.37) 0.830
Infective pneumonia (x1.22) 0.853
Acute renal failure syndrome (x1.21) 0.873
Atelectasis (x1.15) 0.884
Pleural effusion (x1.15) 0.895
Tachycardia (x1.12) 0.903
Low blood pressure (x1.12) 0.910
Shock (x1.12) 0.916
Abnormal breathing (x1.1) 0.921
Electrocardiogram abnormal (x1.1) 0.926
Disturbance of consciousness (x1.09) 0.930
Trauma and postoperative pulmonary insufficiency (x1.08) 0.933
Dyspnea (x1.08) 0.936
Acidosis (x1.08) 0.939
Altered mental status (x1.07) 0.942
Malnutrition (calorie) (x1.07) 0.944
Borderline normal ECG (x1.07) 0.946
Fever (x1.06) 0.948
Hypokalemia (x1.06) 0.950
Anemia (x1.06) 0.951
Postprocedural state finding (x1.05) 0.953
Urinary tract infectious disease (x1.05) 0.954
Abnormal glucose level (x1.05) 0.955
ECG normal (x1.04) 0.956
Bacteremia (x1.04) 0.957
Pneumonitis due to inhaled substance (x1.04) 0.957
Acute posthemorrhagic anemia (x1.04) 0.958
metoprolol (+0.00398) 0.959
Leukocytosis (x1.04) 0.960
Cardiac arrhythmia (x1.04) 0.961
Disorder of lung (x1.04) 0.962
Atrial fibrillation (x1.04) 0.963
Respiratory insufficiency (x1.04) 0.964
Tachypnea (x1.04) 0.964
Hypoxemia (x1.04) 0.965
Ascites (x1.04) 0.966
Body temperature (+1.31) 0.967
Pulmonary congestion and hypostasis (x1.04) 0.967
Lung field abnormal (x1.04) 0.968
Malaise (x1.04) 0.969
Thrombocytopenic disorder (x1.04) 0.969
Abnormal findings on diagnostic imaging of lung (/1.03) 0.970
gabapentin (+0.003) 0.970
Type 2 diabetes mellitus without complication (x1.03) 0.971
Cortisol [M/V] in SP (+0.852) 0.971
Cardiomegaly (x1.03) 0.972
Albumin [M/V] in SP (-0.0276) 0.972
Hepatic necrosis (x1.03) 0.972
Chest pain (x1.03) 0.973
Hyperkalemia (x1.03) 0.973
Disorder of kidney and/or ureter (x1.03) 0.974
Leukocytes [#/V] in B by Manual count (+2.59) 0.974
Hyperosmolality and or hypernatremia (x1.03) 0.974
C reactive protein [M/V] in SP (+2.91) 0.975
Abnormal body temperature (x1.03) 0.975
Severe protein-calorie malnutrition (Gomez: less than 60% of standard weight) (x1.03) 0.975
Expressions
10^5
10^3
10^1
−10 0 10
−0.6 −0.4 −0.2 0.0 0.2 0.4 0.6

Signature [70]; 8 To Reach 0.975, (10 Plotted)

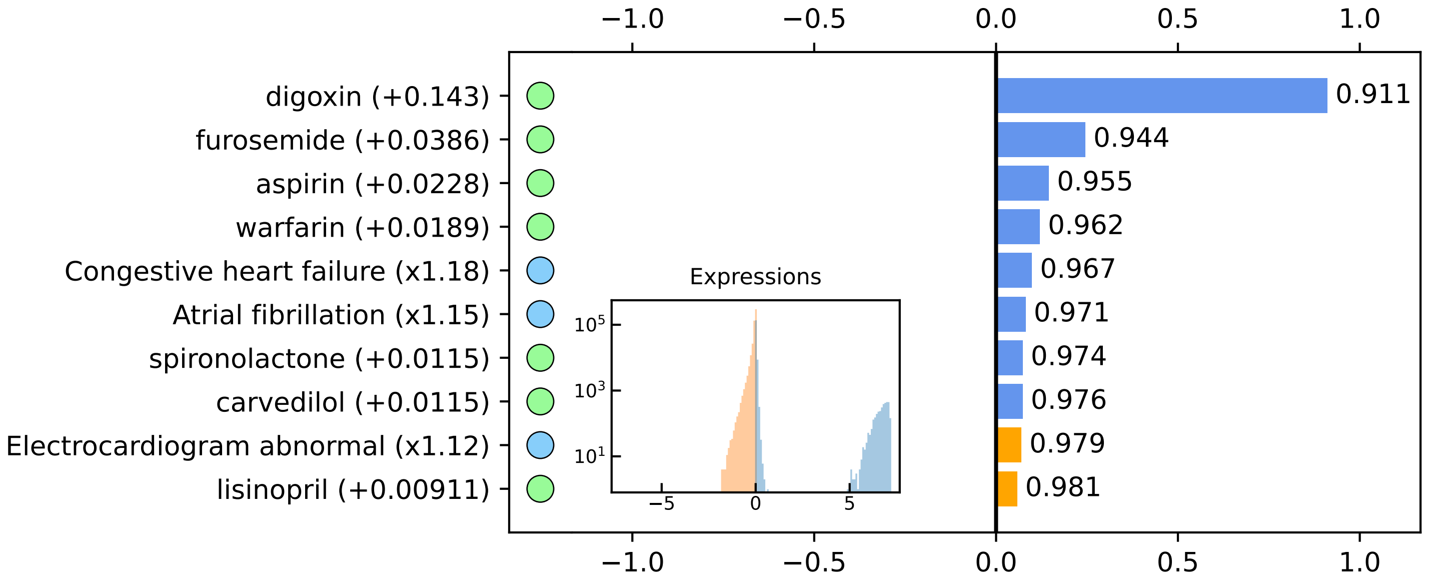

Signature [71]; 12 To Reach 0.975, (12 Plotted)

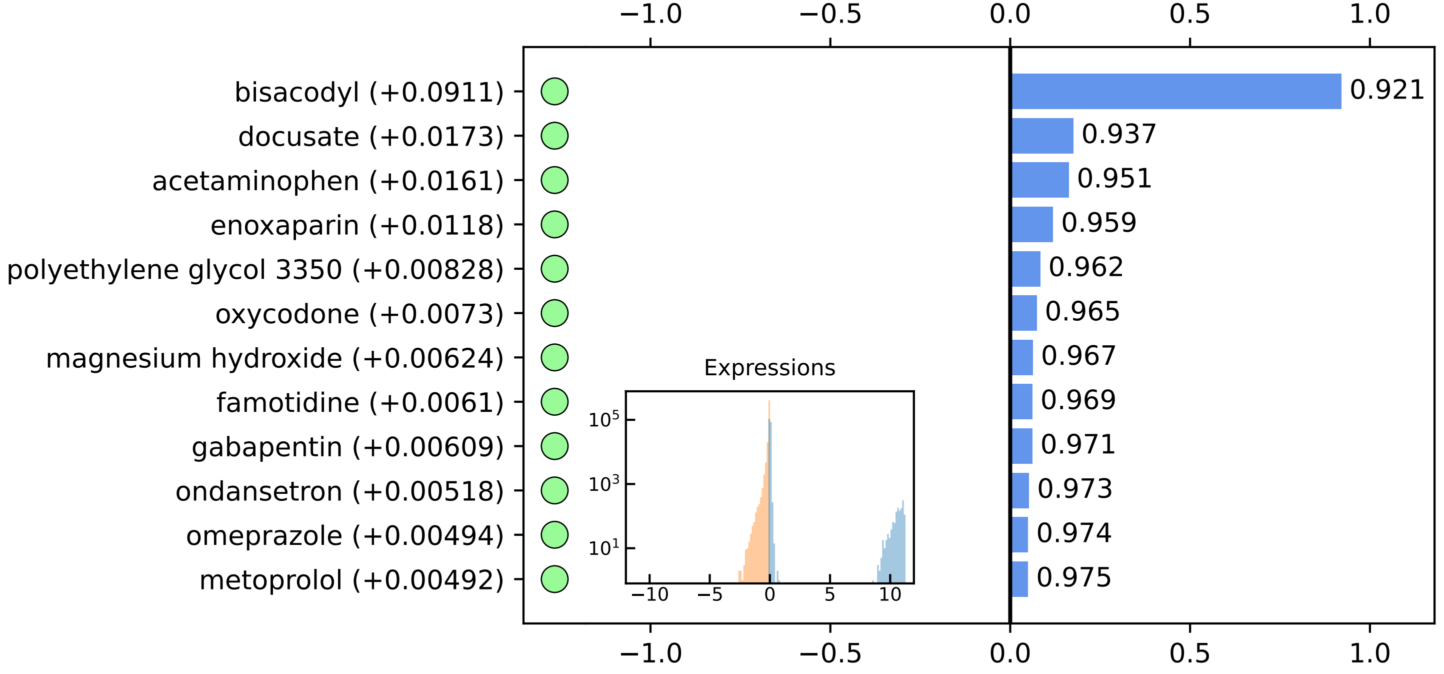

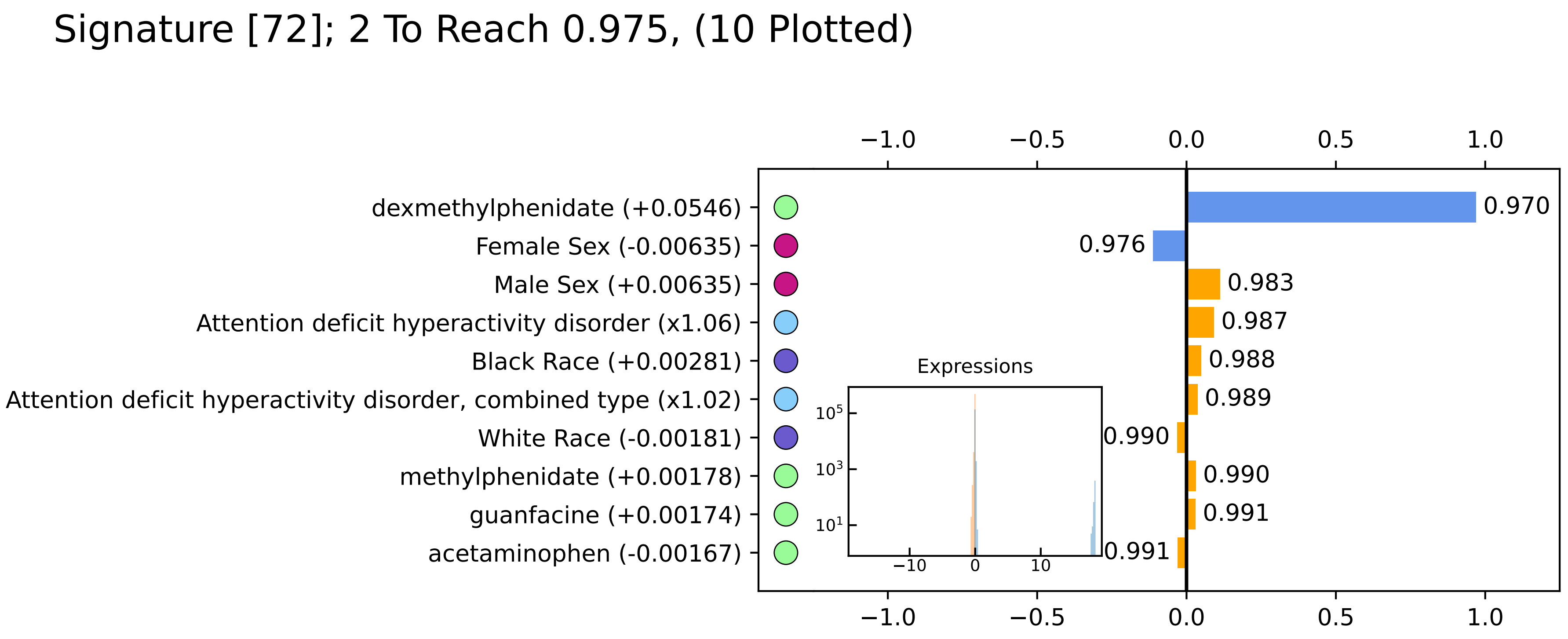
Signature [72]; 2 To Reach 0.975, (10 Plotted)
dexmethylphenidate (+0.0546)
Female Sex (-0.00635)
Male Sex (+0.00635)
Attention deficit hyperactivity disorder (x1.06)
Black Race (+0.00281)
Attention deficit hyperactivity disorder, combined type (x1.02)
White Race (-0.00181)
methylphenidate (+0.00178)
guanfacine (+0.00174)
acetaminophen (-0.00167)
0.970
0.976
0.983
0.987
0.988
0.989
0.990
0.990
0.991
0.991
Expressions
−1.0
−0.5
0.0
0.5
1.0
−10
0
10

Signature [73]; 77 To Reach 0.975, (77 Plotted)

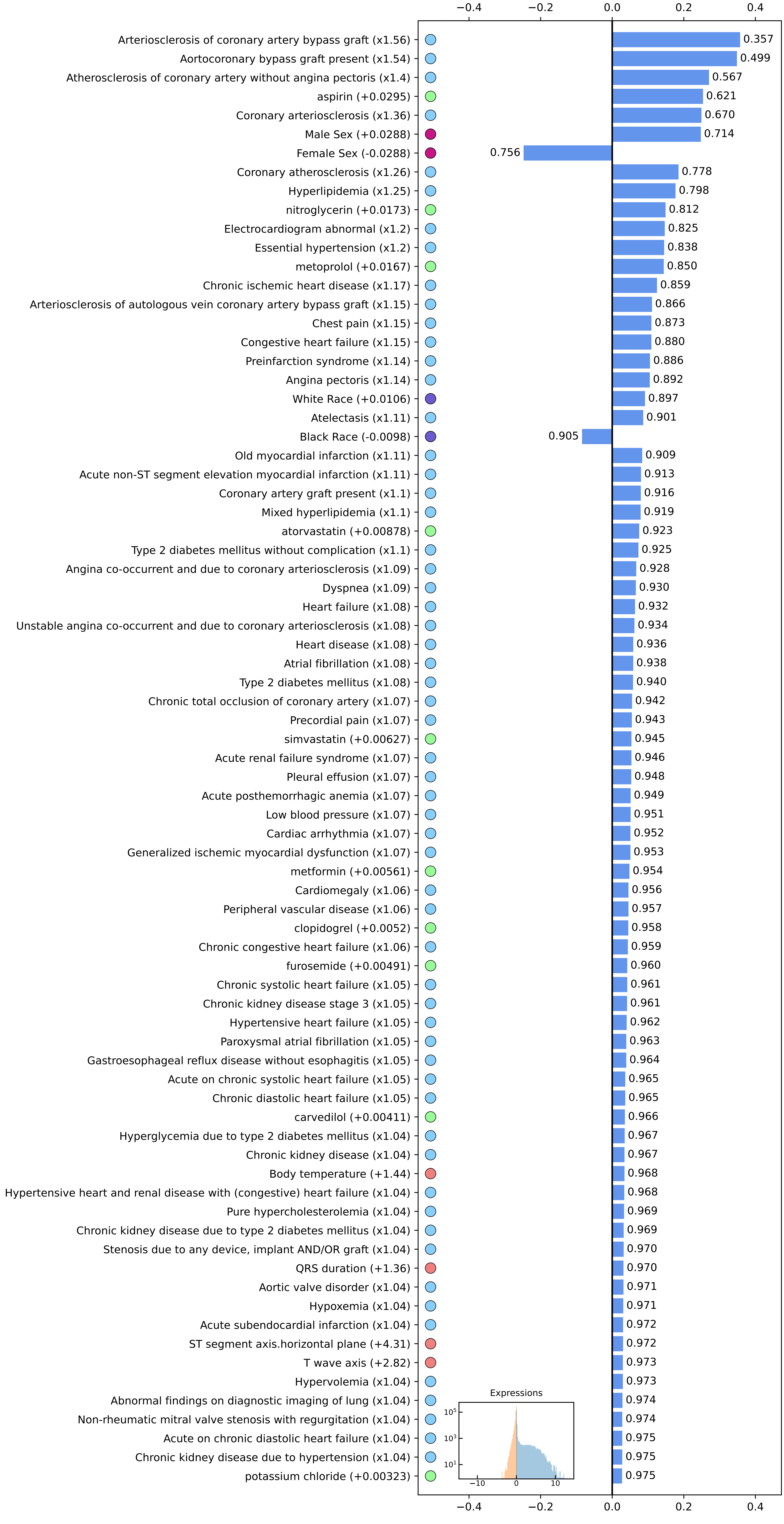

Signature [74]; 7 To Reach 0.975, (10 Plotted)

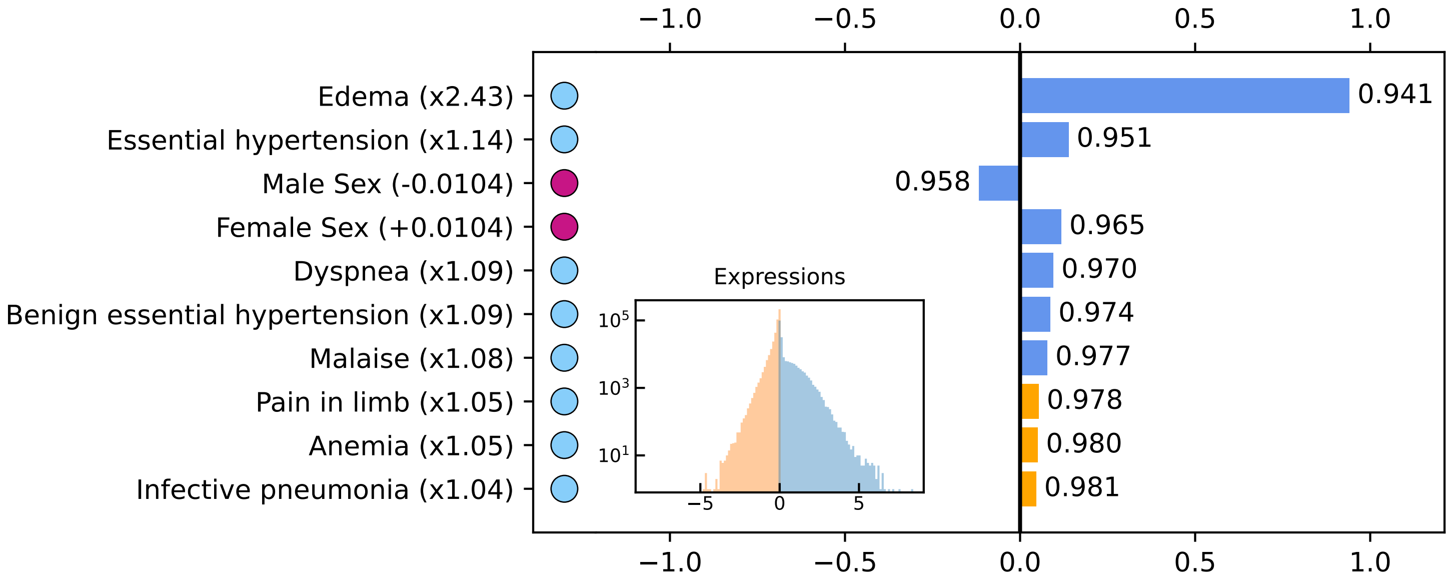

Signature [75]; 1 To Reach 0.975, (10 Plotted)

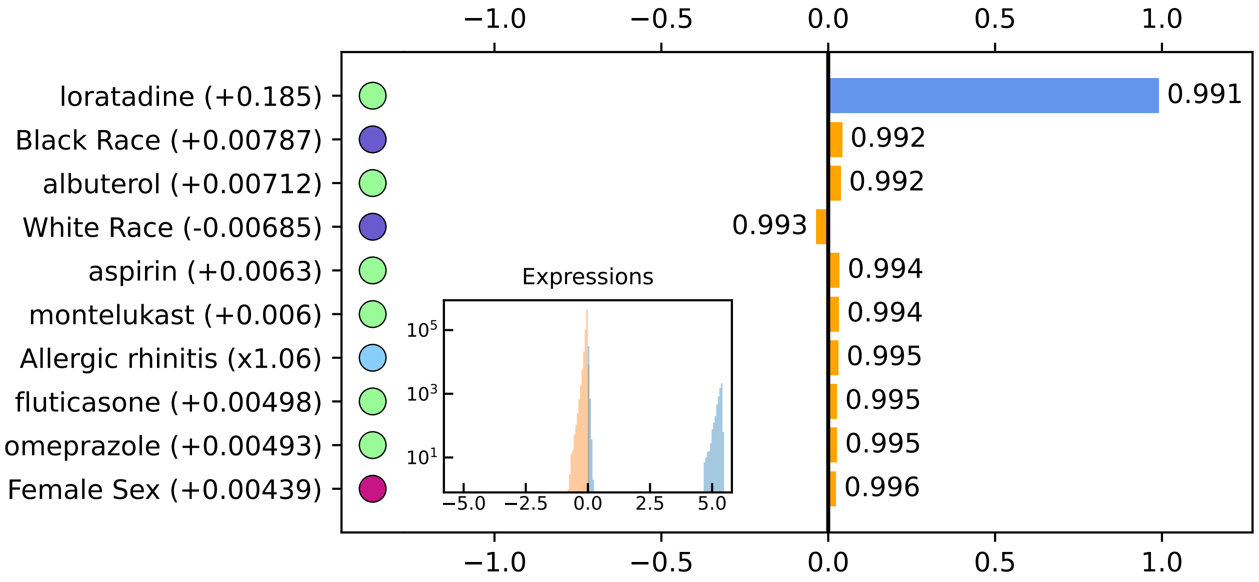

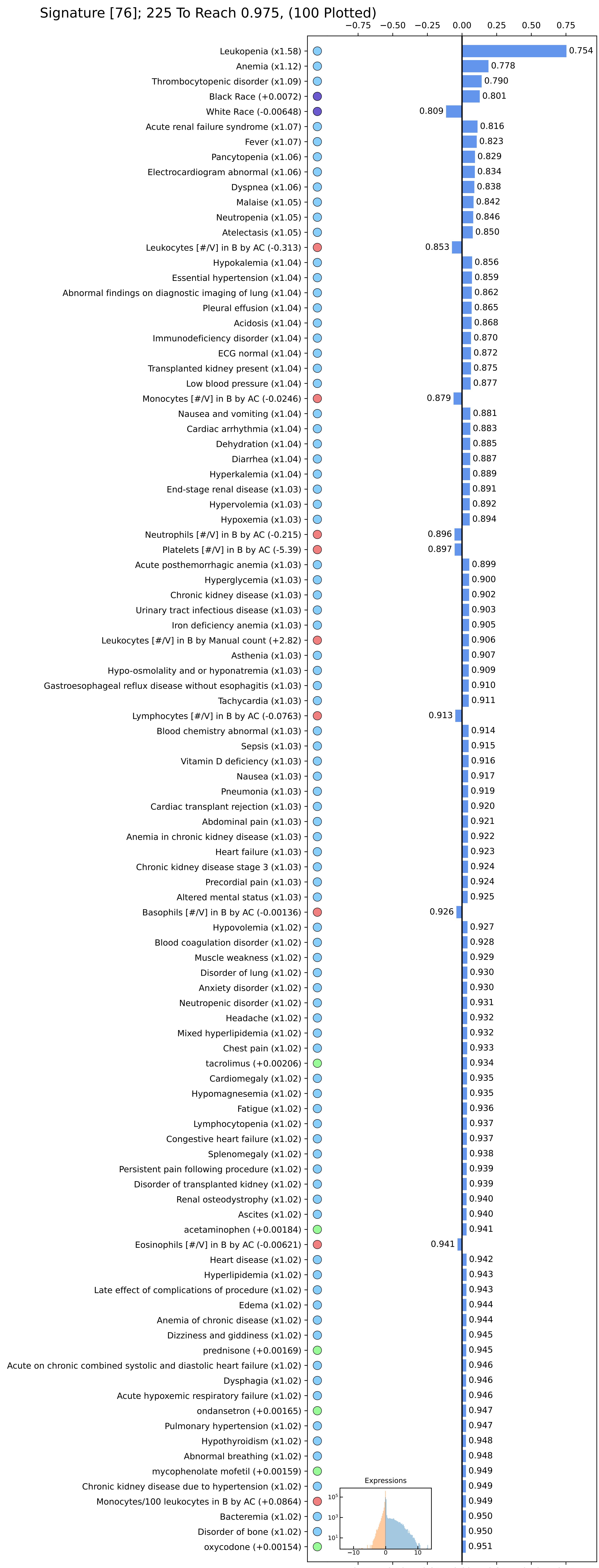

Signature [76]; 225 To Reach 0.975, (100 Plotted)
Leukopenia (x1.58) 0.754
Anemia (x1.12) 0.778
Thrombocytopenic disorder (x1.09) 0.790
Black Race (+0.0072) 0.801
White Race (-0.00648) 0.809
Acute renal failure syndrome (x1.07) 0.816
Fever (x1.07) 0.823
Pancytopenia (x1.06) 0.829
Electrocardiogram abnormal (x1.06) 0.834
Dyspnea (x1.06) 0.838
Malaise (x1.05) 0.842
Neutropenia (x1.05) 0.846
Atelectasis (x1.05) 0.850
Leukocytes [#/V] in B by AC (-0.313) 0.853
Hypokalemia (x1.04) 0.856
Essential hypertension (x1.04) 0.859
Abnormal findings on diagnostic imaging of lung (x1.04) 0.862
Pleural effusion (x1.04) 0.865
Acidosis (x1.04) 0.868
Immunodeficiency disorder (x1.04) 0.870
ECG normal (x1.04) 0.872
Transplanted kidney present (x1.04) 0.875
Low blood pressure (x1.04) 0.877
Monocytes [#/V] in B by AC (-0.0246) 0.879
Nausea and vomiting (x1.04) 0.881
Cardiac arrhythmia (x1.04) 0.883
Dehydration (x1.04) 0.885
Diarrhea (x1.04) 0.887
Hyperkalemia (x1.04) 0.889
End-stage renal disease (x1.03) 0.891
Hypervolemia (x1.03) 0.892
Hypoxemia (x1.03) 0.894
Neutrophils [#/V] in B by AC (-0.215) 0.896
Platelets [#/V] in B by AC (-5.39) 0.897
Acute posthemorrhagic anemia (x1.03) 0.899
Hyperglycemia (x1.03) 0.900
Chronic kidney disease (x1.03) 0.902
Urinary tract infectious disease (x1.03) 0.903
Iron deficiency anemia (x1.03) 0.905
Leukocytes [#/V] in B by Manual count (+2.82) 0.906
Asthenia (x1.03) 0.907
Hypo-osmolality and or hyponatremia (x1.03) 0.909
Gastroesophageal reflux disease without esophagitis (x1.03) 0.910
Tachycardia (x1.03) 0.911
Lymphocytes [#/V] in B by AC (-0.0763) 0.913
Blood chemistry abnormal (x1.03) 0.914
Sepsis (x1.03) 0.915
Vitamin D deficiency (x1.03) 0.916
Nausea (x1.03) 0.917
Pneumonia (x1.03) 0.919
Cardiac transplant rejection (x1.03) 0.920
Abdominal pain (x1.03) 0.921
Anemia in chronic kidney disease (x1.03) 0.922
Heart failure (x1.03) 0.923
Chronic kidney disease stage 3 (x1.03) 0.924
Precordial pain (x1.03) 0.924
Altered mental status (x1.03) 0.925
Basophils [#/V] in B by AC (-0.00136) 0.926
Hypovolemia (x1.02) 0.927
Blood coagulation disorder (x1.02) 0.928
Muscle weakness (x1.02) 0.929
Disorder of lung (x1.02) 0.930
Anxiety disorder (x1.02) 0.930
Neutropenic disorder (x1.02) 0.931
Headache (x1.02) 0.932
Mixed hyperlipidemia (x1.02) 0.932
Chest pain (x1.02) 0.933
tacrolimus (+0.00206) 0.934
Cardiomegaly (x1.02) 0.935
Hypomagnesemia (x1.02) 0.935
Fatigue (x1.02) 0.936
Lymphocytopenia (x1.02) 0.937
Congestive heart failure (x1.02) 0.937
Splenomegaly (x1.02) 0.938
Persistent pain following procedure (x1.02) 0.939
Disorder of transplanted kidney (x1.02) 0.939
Renal osteodystrophy (x1.02) 0.940
Ascites (x1.02) 0.940
acetaminophen (+0.00184) 0.941
Eosinophils [#/V] in B by AC (-0.00621) 0.941
Heart disease (x1.02) 0.942
Hyperlipidemia (x1.02) 0.943
Late effect of complications of procedure (x1.02) 0.943
Edema (x1.02) 0.944
Anemia of chronic disease (x1.02) 0.944
Dizziness and giddiness (x1.02) 0.945
prednisone (+0.00169) 0.945
Acute on chronic combined systolic and diastolic heart failure (x1.02) 0.946
Dysphagia (x1.02) 0.946
Acute hypoxemic respiratory failure (x1.02) 0.946
ondansetron (+0.00165) 0.947
Pulmonary hypertension (x1.02) 0.947
Hypothyroidism (x1.02) 0.948
Abnormal breathing (x1.02) 0.948
mycophenolate mofetil (+0.00159) 0.949
Chronic kidney disease due to hypertension (x1.02) 0.949
Monocytes/100 leukocytes in B by AC (+0.0864) 0.949
Bacteremia (x1.02) 0.950
Disorder of bone (x1.02) 0.950
oxycodone (+0.00154) 0.951
−0.75 −0.50 −0.25 0.00 0.25 0.50 0.75
Expressions

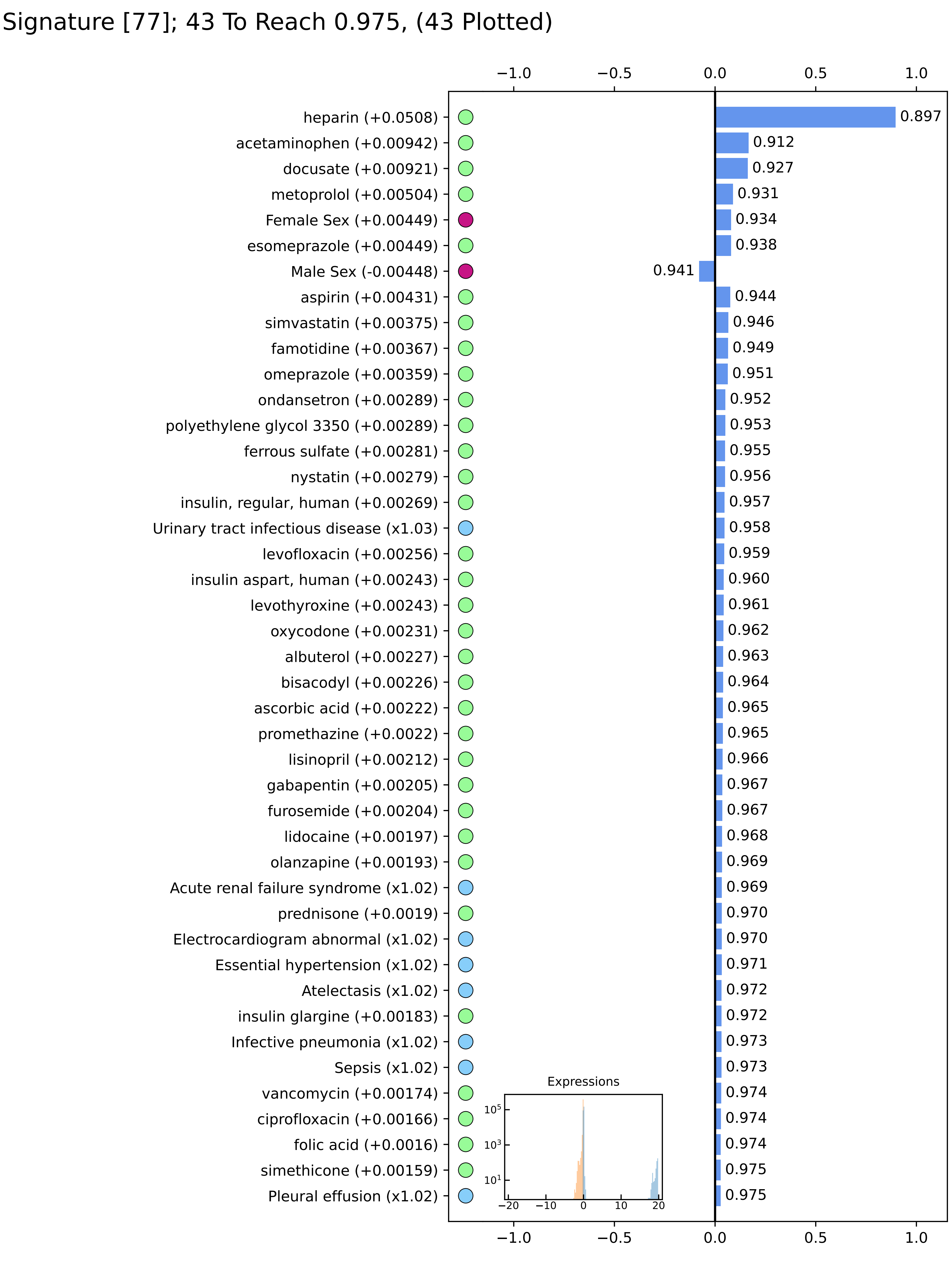
Signature [77]; 43 To Reach 0.975, (43 Plotted)
heparin (+0.0508) 0.897
acetaminophen (+0.00942) 0.912
docusate (+0.00921) 0.927
metoprolol (+0.00504) 0.931
Female Sex (+0.00449) 0.934
esomeprazole (+0.00449) 0.938
Male Sex (-0.00448) 0.941
aspirin (+0.00431) 0.944
simvastatin (+0.00375) 0.946
famotidine (+0.00367) 0.949
omeprazole (+0.00359) 0.951
ondansetron (+0.00289) 0.952
polyethylene glycol 3350 (+0.00289) 0.953
ferrous sulfate (+0.00281) 0.955
nystatin (+0.00279) 0.956
insulin, regular, human (+0.00269) 0.957
Urinary tract infectious disease (x1.03) 0.958
levofloxacin (+0.00256) 0.959
insulin aspart, human (+0.00243) 0.960
levothyroxine (+0.00243) 0.961
oxycodone (+0.00231) 0.962
albuterol (+0.00227) 0.963
bisacodyl (+0.00226) 0.964
ascorbic acid (+0.00222) 0.965
promethazine (+0.0022) 0.965
lisinopril (+0.00212) 0.966
gabapentin (+0.00205) 0.967
furosemide (+0.00204) 0.967
lidocaine (+0.00197) 0.968
olanzapine (+0.00193) 0.969
Acute renal failure syndrome (x1.02) 0.969
prednisone (+0.0019) 0.970
Electrocardiogram abnormal (x1.02) 0.970
Essential hypertension (x1.02) 0.971
Atelectasis (x1.02) 0.972
insulin glargine (+0.00183) 0.972
Infective pneumonia (x1.02) 0.973
Sepsis (x1.02) 0.973
vancomycin (+0.00174) 0.974
ciprofloxacin (+0.00166) 0.974
folic acid (+0.0016) 0.974
simethicone (+0.00159) 0.975
Pleural effusion (x1.02) 0.975
−1.0 −0.5 0.0 0.5 1.0
Expressions
10^5
10^3
10^1
−20 −10 0 10 20

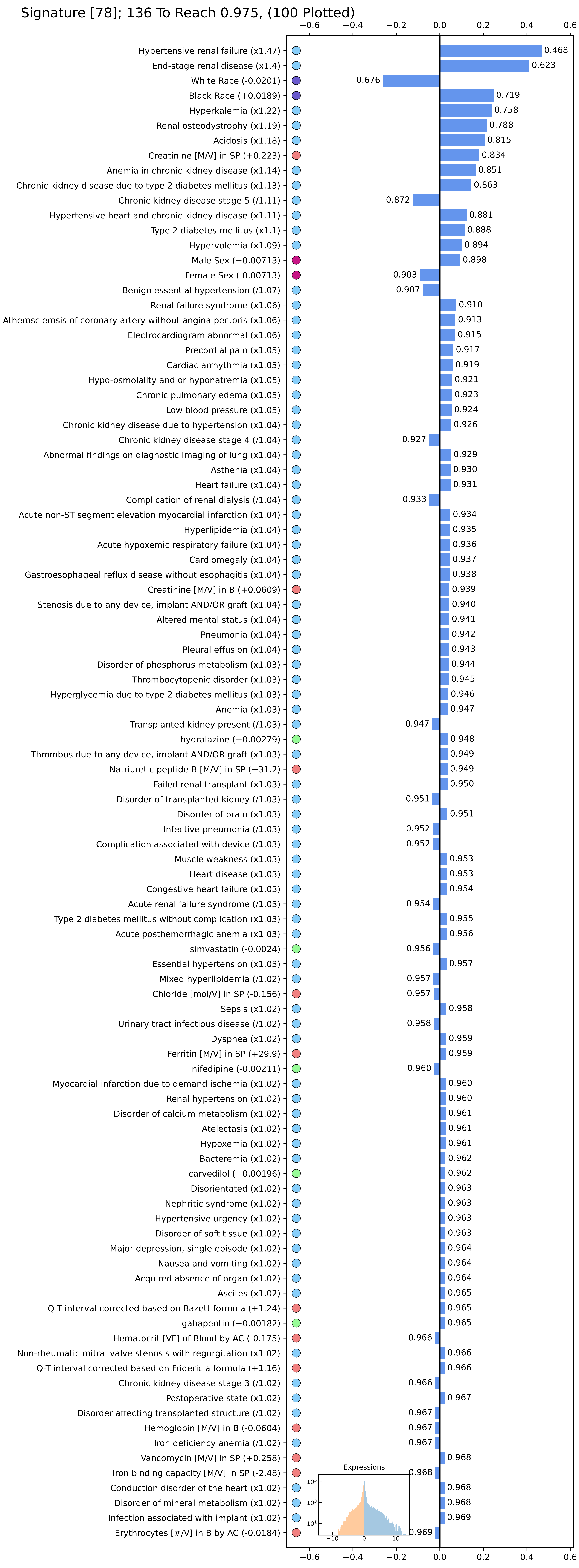

Signature [78]; 136 To Reach 0.975, (100 Plotted)
Hypertensive renal failure (x1.47) 0.468
End-stage renal disease (x1.4) 0.623
White Race (-0.0201) 0.676
Black Race (+0.0189) 0.719
Hyperkalemia (x1.22) 0.758
Renal osteodystrophy (x1.19) 0.788
Acidosis (x1.18) 0.815
Creatinine [M/V] in SP (+0.223) 0.834
Anemia in chronic kidney disease (x1.14) 0.851
Chronic kidney disease due to type 2 diabetes mellitus (x1.13) 0.863
Chronic kidney disease stage 5 (/1.11) 0.872
Hypertensive heart and chronic kidney disease (x1.11) 0.881
Type 2 diabetes mellitus (x1.1) 0.888
Hypervolemia (x1.09) 0.894
Male Sex (+0.00713) 0.898
Female Sex (-0.00713) 0.903
Benign essential hypertension (/1.07) 0.907
Renal failure syndrome (x1.06) 0.910
Atherosclerosis of coronary artery without angina pectoris (x1.06) 0.913
Electrocardiogram abnormal (x1.06) 0.915
Precordial pain (x1.05) 0.917
Cardiac arrhythmia (x1.05) 0.919
Hypo-osmolality and or hyponatremia (x1.05) 0.921
Chronic pulmonary edema (x1.05) 0.923
Low blood pressure (x1.05) 0.924
Chronic kidney disease due to hypertension (x1.04) 0.926
Chronic kidney disease stage 4 (/1.04) 0.927
Abnormal findings on diagnostic imaging of lung (x1.04) 0.929
Asthenia (x1.04) 0.930
Heart failure (x1.04) 0.931
Complication of renal dialysis (/1.04) 0.933
Acute non-ST segment elevation myocardial infarction (x1.04) 0.934
Hyperlipidemia (x1.04) 0.935
Acute hypoxemic respiratory failure (x1.04) 0.936
Cardiomegaly (x1.04) 0.937
Gastroesophageal reflux disease without esophagitis (x1.04) 0.938
Creatinine [M/V] in B (+0.0609) 0.939
Stenosis due to any device, implant AND/OR graft (x1.04) 0.940
Altered mental status (x1.04) 0.941
Pneumonia (x1.04) 0.942
Pleural effusion (x1.04) 0.943
Disorder of phosphorus metabolism (x1.03) 0.944
Thrombocytopenic disorder (x1.03) 0.945
Hyperglycemia due to type 2 diabetes mellitus (x1.03) 0.946
Anemia (x1.03) 0.947
Transplanted kidney present (/1.03) 0.947
hydralazine (+0.00279) 0.948
Thrombus due to any device, implant AND/OR graft (x1.03) 0.949
Natriuretic peptide B [M/V] in SP (+31.2) 0.949
Failed renal transplant (x1.03) 0.950
Disorder of transplanted kidney (/1.03) 0.951
Disorder of brain (x1.03) 0.951
Infective pneumonia (/1.03) 0.952
Complication associated with device (/1.03) 0.952
Muscle weakness (x1.03) 0.953
Heart disease (x1.03) 0.953
Congestive heart failure (x1.03) 0.954
Acute renal failure syndrome (/1.03) 0.954
Type 2 diabetes mellitus without complication (x1.03) 0.955
Acute posthemorrhagic anemia (x1.03) 0.956
simvastatin (-0.0024) 0.956
Essential hypertension (x1.03) 0.957
Mixed hyperlipidemia (/1.02) 0.957
Chloride [mol/V] in SP (-0.156) 0.957
Sepsis (x1.02) 0.958
Urinary tract infectious disease (/1.02) 0.958
Dyspnea (x1.02) 0.959
Ferritin [M/V] in SP (+29.9) 0.959
nifedipine (-0.00211) 0.960
Myocardial infarction due to demand ischemia (x1.02) 0.960
Renal hypertension (x1.02) 0.960
Disorder of calcium metabolism (x1.02) 0.961
Atelectasis (x1.02) 0.961
Hypoxemia (x1.02) 0.961
Bacteremia (x1.02) 0.962
carvedilol (+0.00196) 0.962
Disorientated (x1.02) 0.963
Nephritic syndrome (x1.02) 0.963
Hypertensive urgency (x1.02) 0.963
Disorder of soft tissue (x1.02) 0.963
Major depression, single episode (x1.02) 0.964
Nausea and vomiting (x1.02) 0.964
Acquired absence of organ (x1.02) 0.964
Ascites (x1.02) 0.965
Q-T interval corrected based on Bazett formula (+1.24) 0.965
gabapentin (+0.00182) 0.965
Hematocrit [VF] of Blood by AC (-0.175) 0.966
Non-rheumatic mitral valve stenosis with regurgitation (x1.02) 0.966
Q-T interval corrected based on Fridericia formula (+1.16) 0.966
Chronic kidney disease stage 3 (/1.02) 0.966
Postoperative state (x1.02) 0.967
Disorder affecting transplanted structure (/1.02) 0.967
Hemoglobin [M/V] in B (-0.0604) 0.967
Iron deficiency anemia (/1.02) 0.967
Vancomycin [M/V] in SP (+0.258) 0.968
Iron binding capacity [M/V] in SP (-2.48) 0.968
Conduction disorder of the heart (x1.02) 0.968
Disorder of mineral metabolism (x1.02) 0.968
Infection associated with implant (x1.02) 0.969
Erythrocytes [#/V] in B by AC (-0.0184) 0.969
Expressions

Signature [79]; 74 To Reach 0.975, (74 Plotted)

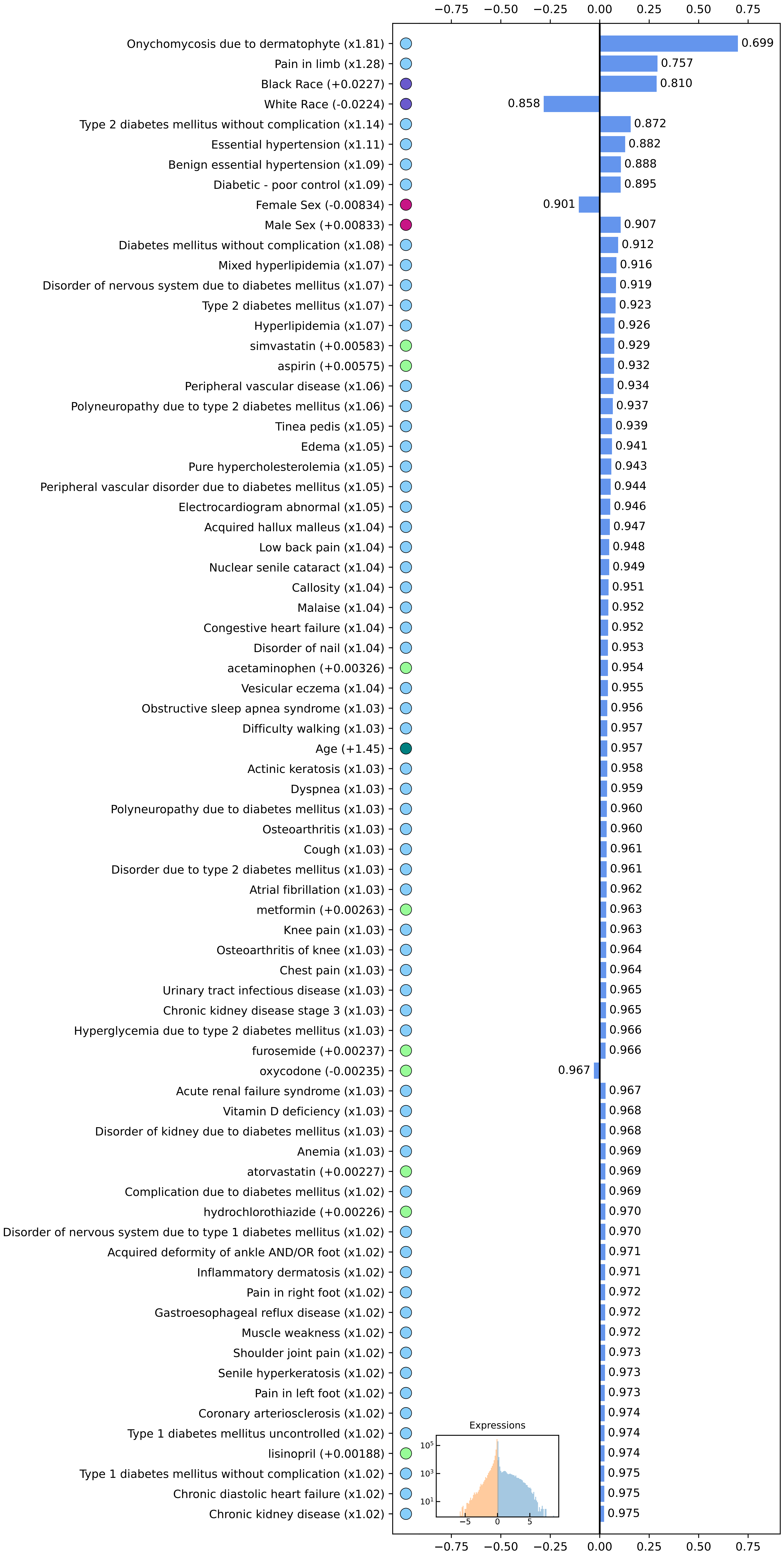

Signature [80]; 169 To Reach 0.975, (100 Plotted)

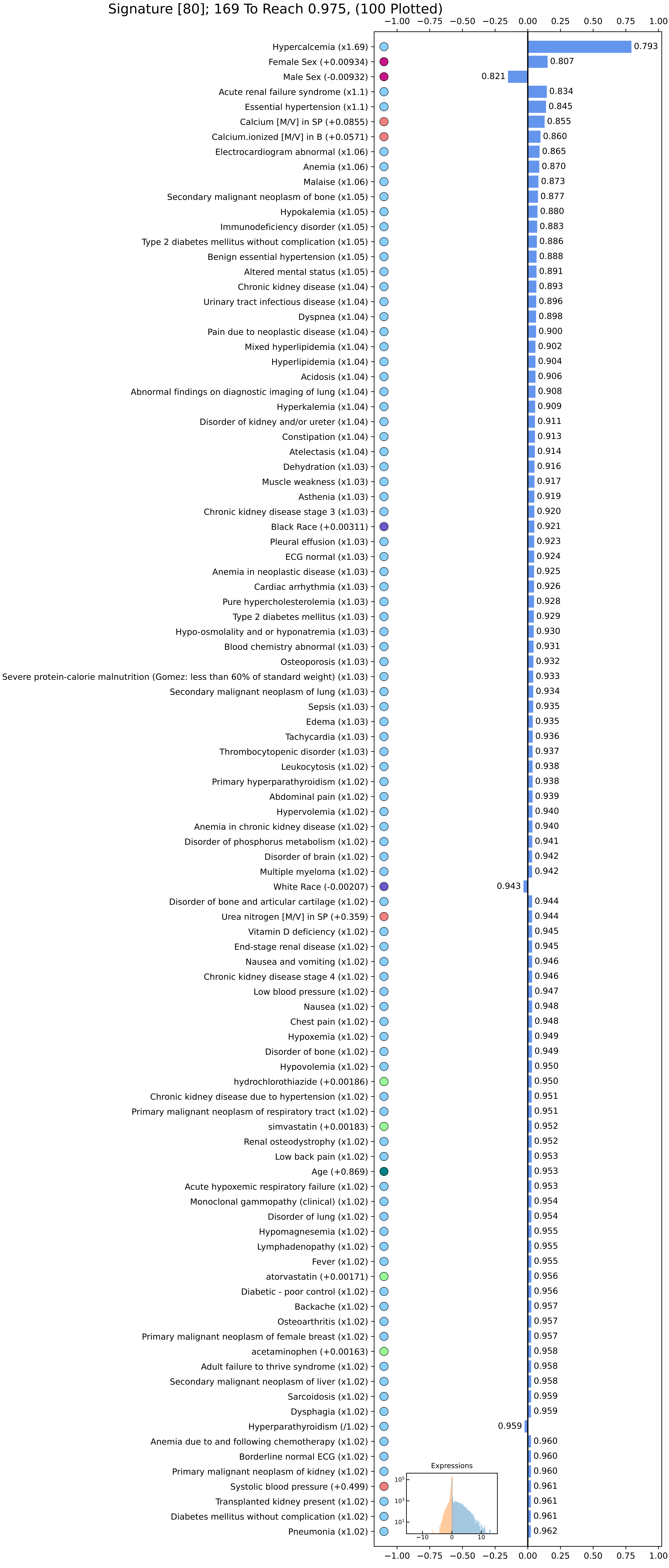

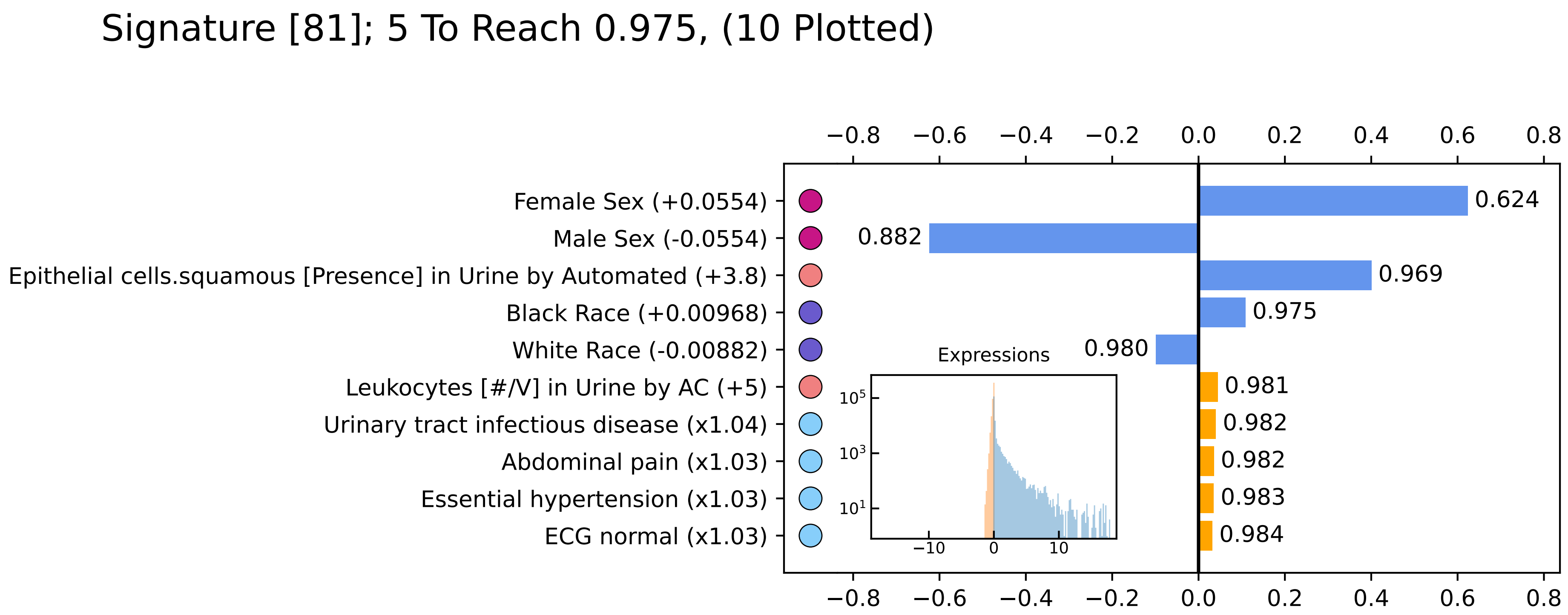
Signature [81]; 5 To Reach 0.975, (10 Plotted)
Female Sex (+0.0554)
Male Sex (-0.0554)
Epithelial cells.squamous [Presence] in Urine by Automated (+3.8)
Black Race (+0.00968)
White Race (-0.00882)
Leukocytes [#/V] in Urine by AC (+5)
Urinary tract infectious disease (x1.04)
Abdominal pain (x1.03)
Essential hypertension (x1.03)
ECG normal (x1.03)
0.624
0.882
0.969
0.975
0.980
0.981
0.982
0.982
0.983
0.984
Expressions
−0.8 −0.6 −0.4 −0.2 0.0 0.2 0.4 0.6 0.8

Signature [82]; 5 To Reach 0.975, (10 Plotted)

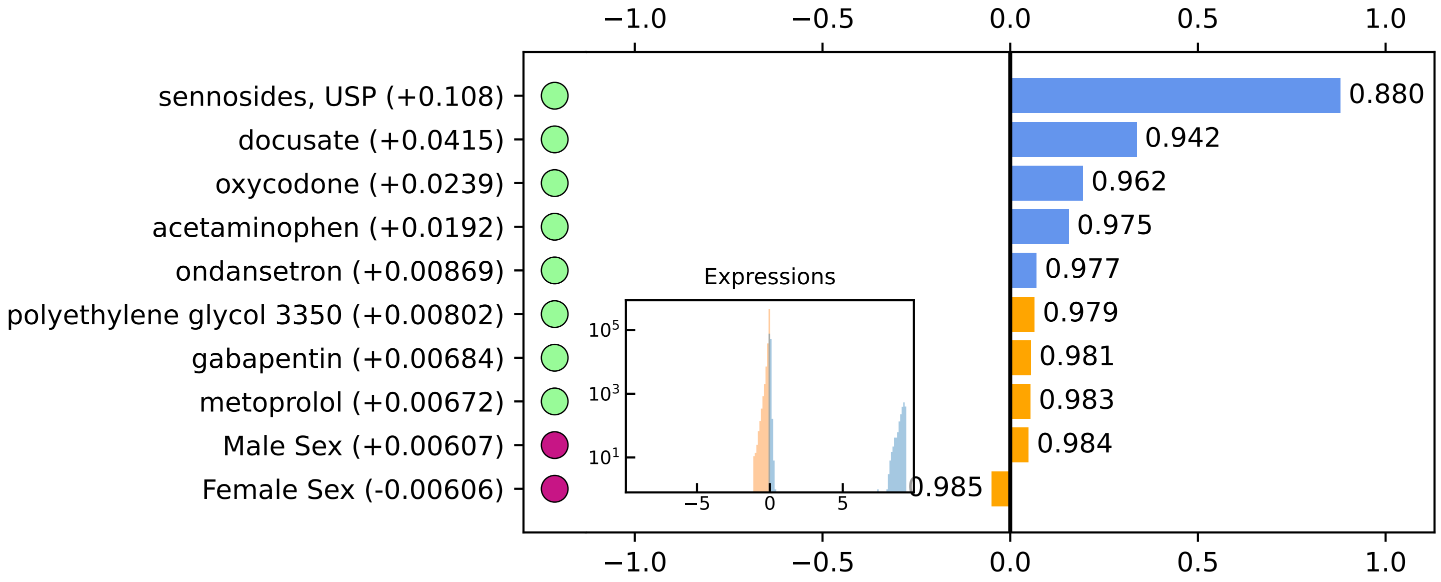

Signature [83]; 2 To Reach 0.975, (10 Plotted)

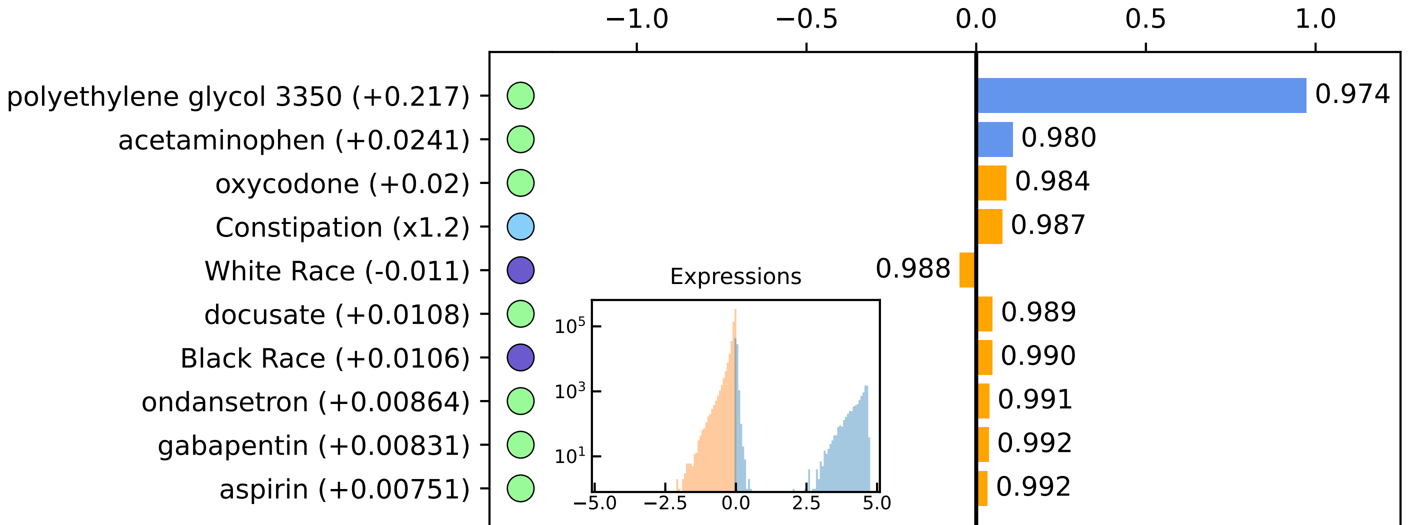

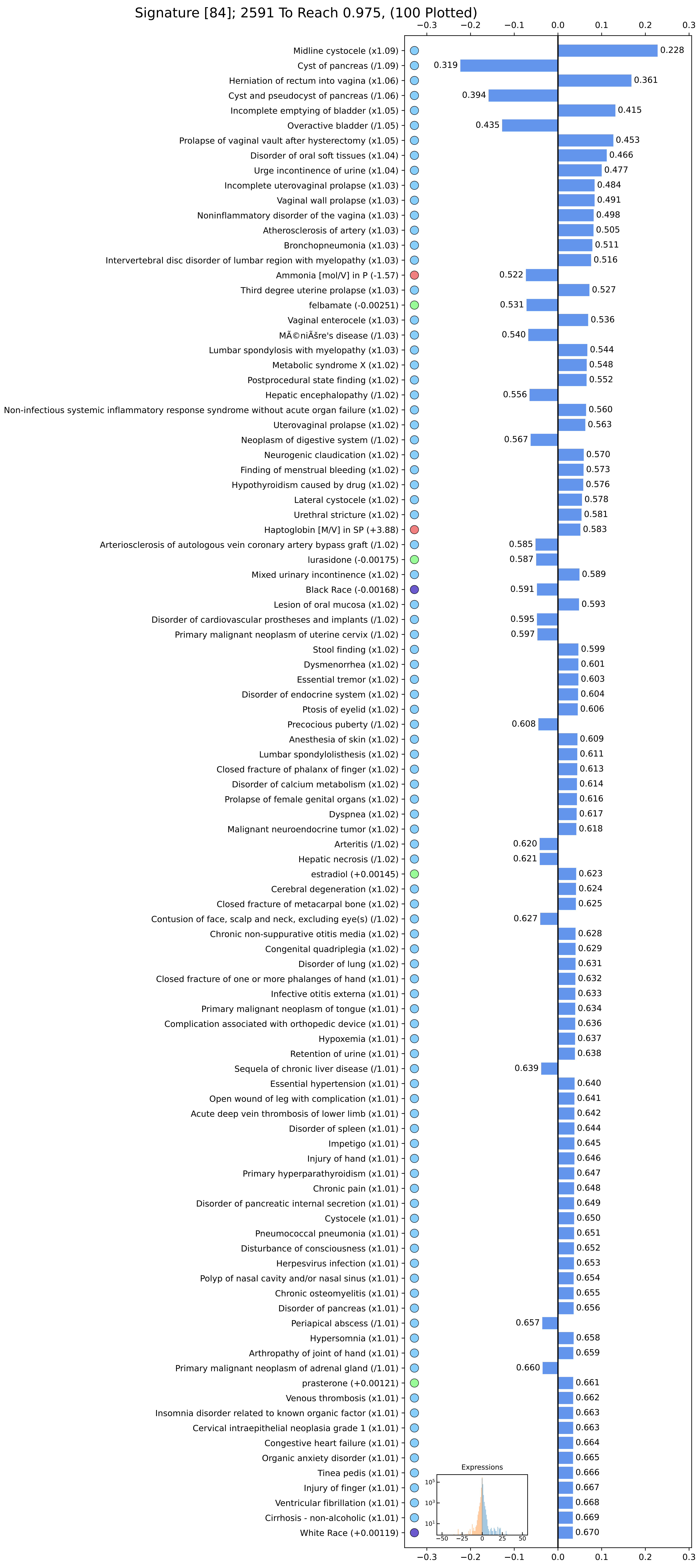

Signature [84]; 2591 To Reach 0.975, (100 Plotted)
Midline cystocele (x1.09) 0.228
Cyst of pancreas (/1.09) 0.319
Herniation of rectum into vagina (x1.06) 0.361
Cyst and pseudocyst of pancreas (/1.06) 0.394
Incomplete emptying of bladder (x1.05) 0.415
Overactive bladder (/1.05) 0.435
Prolapse of vaginal vault after hysterectomy (x1.05) 0.453
Disorder of oral soft tissues (x1.04) 0.466
Urge incontinence of urine (x1.04) 0.477
Incomplete uterovaginal prolapse (x1.03) 0.484
Vaginal wall prolapse (x1.03) 0.491
Noninflammatory disorder of the vagina (x1.03) 0.498
Atherosclerosis of artery (x1.03) 0.505
Bronchopneumonia (x1.03) 0.511
Intervertebral disc disorder of lumbar region with myelopathy (x1.03) 0.516
Ammonia [mol/V] in P (-1.57) 0.522
Third degree uterine prolapse (x1.03) 0.527
felbamate (-0.00251) 0.531
Vaginal enterocele (x1.03) 0.536
MÃ©niÃšre's disease (/1.03) 0.540
Lumbar spondylosis with myelopathy (x1.03) 0.544
Metabolic syndrome X (x1.02) 0.548
Postprocedural state finding (x1.02) 0.552
Hepatic encephalopathy (/1.02) 0.556
Non-infectious systemic inflammatory response syndrome without acute organ failure (x1.02) 0.560
Uterovaginal prolapse (x1.02) 0.563
Neoplasm of digestive system (/1.02) 0.567
Neurogenic claudication (x1.02) 0.570
Finding of menstrual bleeding (x1.02) 0.573
Hypothyroidism caused by drug (x1.02) 0.576
Lateral cystocele (x1.02) 0.578
Urethral stricture (x1.02) 0.581
Haptoglobin [M/V] in SP (+3.88) 0.583
Arteriosclerosis of autologous vein coronary artery bypass graft (/1.02) 0.585
lurasidone (-0.00175) 0.587
Mixed urinary incontinence (x1.02) 0.589
Black Race (-0.00168) 0.591
Lesion of oral mucosa (x1.02) 0.593
Disorder of cardiovascular prostheses and implants (/1.02) 0.595
Primary malignant neoplasm of uterine cervix (/1.02) 0.597
Stool finding (x1.02) 0.599
Dysmenorrhea (x1.02) 0.601
Essential tremor (x1.02) 0.603
Disorder of endocrine system (x1.02) 0.604
Ptosis of eyelid (x1.02) 0.606
Precocious puberty (/1.02) 0.608
Anesthesia of skin (x1.02) 0.609
Lumbar spondylolisthesis (x1.02) 0.611
Closed fracture of phalanx of finger (x1.02) 0.613
Disorder of calcium metabolism (x1.02) 0.614
Prolapse of female genital organs (x1.02) 0.616
Dyspnea (x1.02) 0.617
Malignant neuroendocrine tumor (x1.02) 0.618
Arteritis (/1.02) 0.620
Hepatic necrosis (/1.02) 0.621
estradiol (+0.00145) 0.623
Cerebral degeneration (x1.02) 0.624
Closed fracture of metacarpal bone (x1.02) 0.625
Contusion of face, scalp and neck, excluding eye(s) (/1.02) 0.627
Chronic non-suppurative otitis media (x1.02) 0.628
Congenital quadriplegia (x1.02) 0.629
Disorder of lung (x1.02) 0.631
Closed fracture of one or more phalanges of hand (x1.01) 0.632
Infective otitis externa (x1.01) 0.633
Primary malignant neoplasm of tongue (x1.01) 0.634
Complication associated with orthopedic device (x1.01) 0.636
Hypoxemia (x1.01) 0.637
Retention of urine (x1.01) 0.638
Sequela of chronic liver disease (/1.01) 0.639
Essential hypertension (x1.01) 0.640
Open wound of leg with complication (x1.01) 0.641
Acute deep vein thrombosis of lower limb (x1.01) 0.642
Disorder of spleen (x1.01) 0.644
Impetigo (x1.01) 0.645
Injury of hand (x1.01) 0.646
Primary hyperparathyroidism (x1.01) 0.647
Chronic pain (x1.01) 0.648
Disorder of pancreatic internal secretion (x1.01) 0.649
Cystocele (x1.01) 0.650
Pneumococcal pneumonia (x1.01) 0.651
Disturbance of consciousness (x1.01) 0.652
Herpesvirus infection (x1.01) 0.653
Polyp of nasal cavity and/or nasal sinus (x1.01) 0.654
Chronic osteomyelitis (x1.01) 0.655
Disorder of pancreas (x1.01) 0.656
Periapical abscess (/1.01) 0.657
Hypersomnia (x1.01) 0.658
Arthropathy of joint of hand (x1.01) 0.659
Primary malignant neoplasm of adrenal gland (/1.01) 0.660
prasterone (+0.00121) 0.661
Venous thrombosis (x1.01) 0.662
Insomnia disorder related to known organic factor (x1.01) 0.663
Cervical intraepithelial neoplasia grade 1 (x1.01) 0.663
Congestive heart failure (x1.01) 0.664
Organic anxiety disorder (x1.01) 0.665
Tinea pedis (x1.01) 0.666
Injury of finger (x1.01) 0.667
Ventricular fibrillation (x1.01) 0.668
Cirrhosis - non-alcoholic (x1.01) 0.669
White Race (+0.00119) 0.670
Expressions
−0.3 −0.2 −0.1 0.0 0.1 0.2 0.3

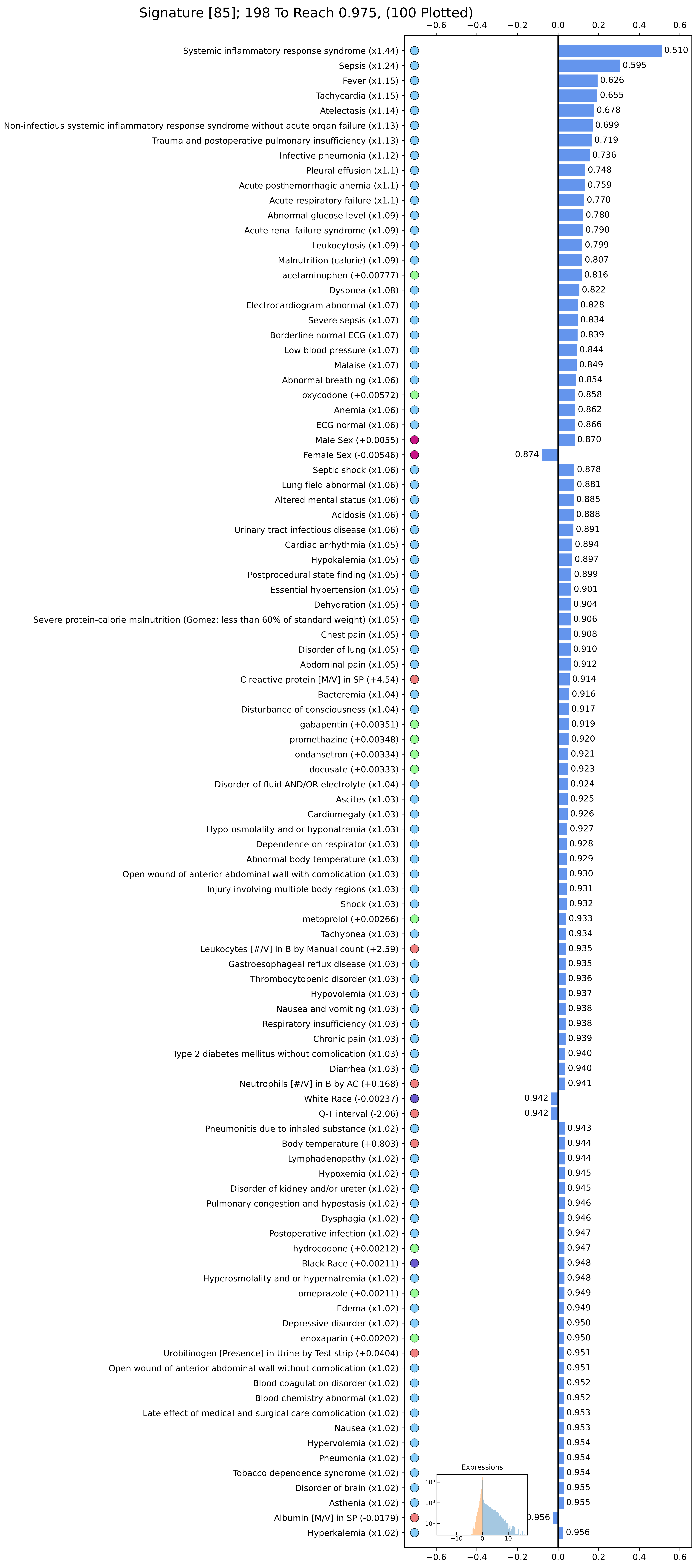

Signature [85]; 198 To Reach 0.975, (100 Plotted)
Systemic inflammatory response syndrome (x1.44) 0.510
Sepsis (x1.24) 0.595
Fever (x1.15) 0.626
Tachycardia (x1.15) 0.655
Atelectasis (x1.14) 0.678
Non-infectious systemic inflammatory response syndrome without acute organ failure (x1.13) 0.699
Trauma and postoperative pulmonary insufficiency (x1.13) 0.719
Infective pneumonia (x1.12) 0.736
Pleural effusion (x1.1) 0.748
Acute posthemorrhagic anemia (x1.1) 0.759
Acute respiratory failure (x1.1) 0.770
Abnormal glucose level (x1.09) 0.780
Acute renal failure syndrome (x1.09) 0.790
Leukocytosis (x1.09) 0.799
Malnutrition (calorie) (x1.09) 0.807
acetaminophen (+0.00777) 0.816
Dyspnea (x1.08) 0.822
Electrocardiogram abnormal (x1.07) 0.828
Severe sepsis (x1.07) 0.834
Borderline normal ECG (x1.07) 0.839
Low blood pressure (x1.07) 0.844
Malaise (x1.07) 0.849
Abnormal breathing (x1.06) 0.854
oxycodone (+0.00572) 0.858
Anemia (x1.06) 0.862
ECG normal (x1.06) 0.866
Male Sex (+0.0055) 0.870
Female Sex (-0.00546) 0.874
Septic shock (x1.06) 0.878
Lung field abnormal (x1.06) 0.881
Altered mental status (x1.06) 0.885
Acidosis (x1.06) 0.888
Urinary tract infectious disease (x1.06) 0.891
Cardiac arrhythmia (x1.05) 0.894
Hypokalemia (x1.05) 0.897
Postprocedural state finding (x1.05) 0.899
Essential hypertension (x1.05) 0.901
Dehydration (x1.05) 0.904
Severe protein-calorie malnutrition (Gomez: less than 60% of standard weight) (x1.05) 0.906
Chest pain (x1.05) 0.908
Disorder of lung (x1.05) 0.910
Abdominal pain (x1.05) 0.912
C reactive protein [M/V] in SP (+4.54) 0.914
Bacteremia (x1.04) 0.916
Disturbance of consciousness (x1.04) 0.917
gabapentin (+0.00351) 0.919
promethazine (+0.00348) 0.920
ondansetron (+0.00334) 0.921
docusate (+0.00333) 0.923
Disorder of fluid AND/OR electrolyte (x1.04) 0.924
Ascites (x1.03) 0.925
Cardiomegaly (x1.03) 0.926
Hypo-osmolality and or hyponatremia (x1.03) 0.927
Dependence on respirator (x1.03) 0.928
Abnormal body temperature (x1.03) 0.929
Open wound of anterior abdominal wall with complication (x1.03) 0.930
Injury involving multiple body regions (x1.03) 0.931
Shock (x1.03) 0.932
metoprolol (+0.00266) 0.933
Tachypnea (x1.03) 0.934
Leukocytes [#/V] in B by Manual count (+2.59) 0.935
Gastroesophageal reflux disease (x1.03) 0.935
Thrombocytopenic disorder (x1.03) 0.936
Hypovolemia (x1.03) 0.937
Nausea and vomiting (x1.03) 0.938
Respiratory insufficiency (x1.03) 0.938
Chronic pain (x1.03) 0.939
Type 2 diabetes mellitus without complication (x1.03) 0.940
Diarrhea (x1.03) 0.940
Neutrophils [#/V] in B by AC (+0.168) 0.941
White Race (-0.00237) 0.942
Q-T interval (-2.06) 0.942
Pneumonitis due to inhaled substance (x1.02) 0.943
Body temperature (+0.803) 0.944
Lymphadenopathy (x1.02) 0.944
Hypoxemia (x1.02) 0.945
Disorder of kidney and/or ureter (x1.02) 0.945
Pulmonary congestion and hypostasis (x1.02) 0.946
Dysphagia (x1.02) 0.946
Postoperative infection (x1.02) 0.947
hydrocodone (+0.00212) 0.947
Black Race (+0.00211) 0.948
Hyperosmolality and or hypernatremia (x1.02) 0.948
omeprazole (+0.00211) 0.949
Edema (x1.02) 0.949
Depressive disorder (x1.02) 0.950
enoxaparin (+0.00202) 0.950
Urobilinogen [Presence] in Urine by Test strip (+0.0404) 0.951
Open wound of anterior abdominal wall without complication (x1.02) 0.951
Blood coagulation disorder (x1.02) 0.952
Blood chemistry abnormal (x1.02) 0.952
Late effect of medical and surgical care complication (x1.02) 0.953
Nausea (x1.02) 0.953
Hypervolemia (x1.02) 0.954
Pneumonia (x1.02) 0.954
Tobacco dependence syndrome (x1.02) 0.954
Disorder of brain (x1.02) 0.955
Asthenia (x1.02) 0.955
Albumin [M/V] in SP (-0.0179) 0.956
Hyperkalemia (x1.02) 0.956
Expressions
−0.6 −0.4 −0.2 0.0 0.2 0.4 0.6

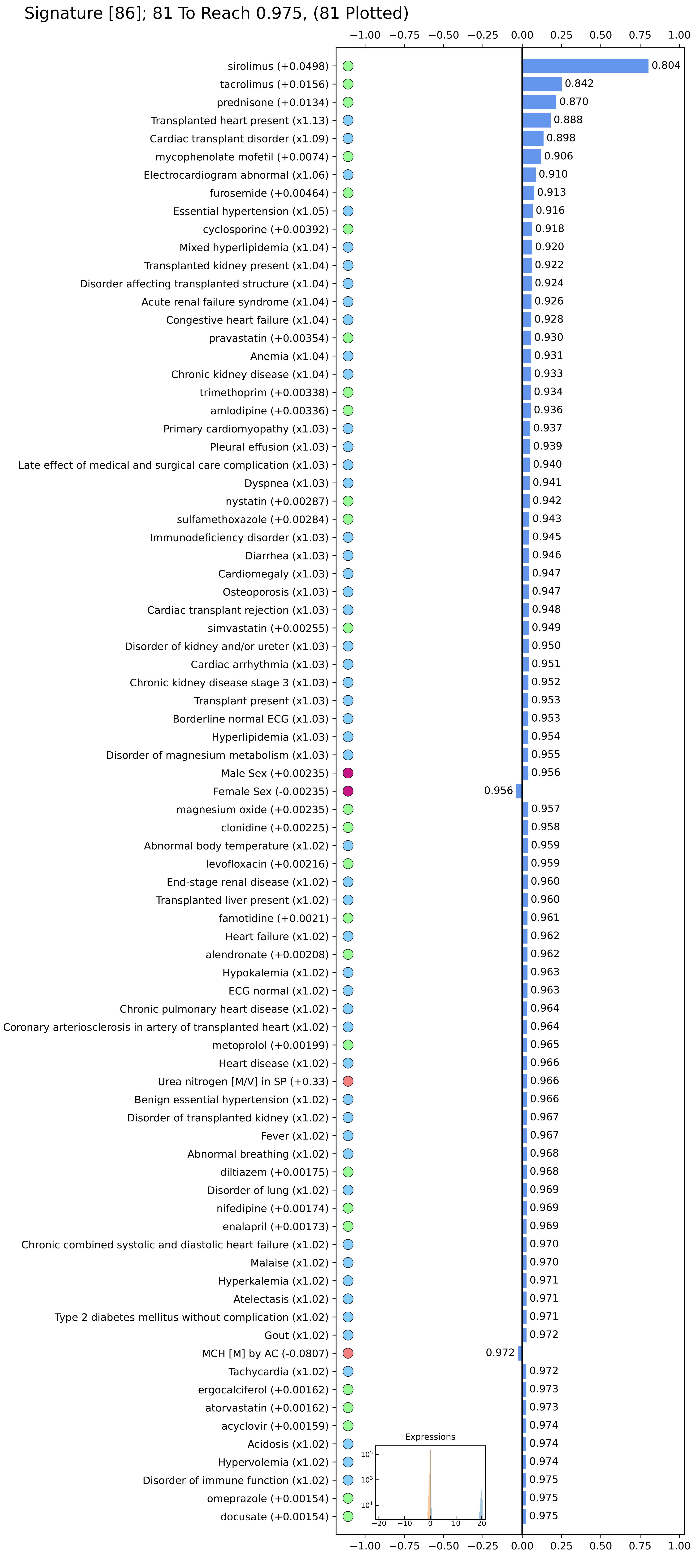
Signature [86]; 81 To Reach 0.975, (81 Plotted)
−1.00 −0.75 −0.50 −0.25 0.00 0.25 0.50 0.75 1.00
sirolimus (+0.0498) 0.804
tacrolimus (+0.0156) 0.842
prednisone (+0.0134) 0.870
Transplanted heart present (x1.13) 0.888
Cardiac transplant disorder (x1.09) 0.898
mycophenolate mofetil (+0.0074) 0.906
Electrocardiogram abnormal (x1.06) 0.910
furosemide (+0.00464) 0.913
Essential hypertension (x1.05) 0.916
cyclosporine (+0.00392) 0.918
Mixed hyperlipidemia (x1.04) 0.920
Transplanted kidney present (x1.04) 0.922
Disorder affecting transplanted structure (x1.04) 0.924
Acute renal failure syndrome (x1.04) 0.926
Congestive heart failure (x1.04) 0.928
pravastatin (+0.00354) 0.930
Anemia (x1.04) 0.931
Chronic kidney disease (x1.04) 0.933
trimethoprim (+0.00338) 0.934
amlodipine (+0.00336) 0.936
Primary cardiomyopathy (x1.03) 0.937
Pleural effusion (x1.03) 0.939
Late effect of medical and surgical care complication (x1.03) 0.940
Dyspnea (x1.03) 0.941
nystatin (+0.00287) 0.942
sulfamethoxazole (+0.00284) 0.943
Immunodeficiency disorder (x1.03) 0.945
Diarrhea (x1.03) 0.946
Cardiomegaly (x1.03) 0.947
Osteoporosis (x1.03) 0.947
Cardiac transplant rejection (x1.03) 0.948
simvastatin (+0.00255) 0.949
Disorder of kidney and/or ureter (x1.03) 0.950
Cardiac arrhythmia (x1.03) 0.951
Chronic kidney disease stage 3 (x1.03) 0.952
Transplant present (x1.03) 0.953
Borderline normal ECG (x1.03) 0.953
Hyperlipidemia (x1.03) 0.954
Disorder of magnesium metabolism (x1.03) 0.955
Male Sex (+0.00235) 0.956
Female Sex (-0.00235) 0.956
magnesium oxide (+0.00235) 0.957
clonidine (+0.00225) 0.958
Abnormal body temperature (x1.02) 0.959
levofloxacin (+0.00216) 0.959
End-stage renal disease (x1.02) 0.960
Transplanted liver present (x1.02) 0.960
famotidine (+0.0021) 0.961
Heart failure (x1.02) 0.962
alendronate (+0.00208) 0.962
Hypokalemia (x1.02) 0.963
ECG normal (x1.02) 0.963
Chronic pulmonary heart disease (x1.02) 0.964
Coronary arteriosclerosis in artery of transplanted heart (x1.02) 0.964
metoprolol (+0.00199) 0.965
Heart disease (x1.02) 0.966
Urea nitrogen [M/V] in SP (+0.33) 0.966
Benign essential hypertension (x1.02) 0.966
Disorder of transplanted kidney (x1.02) 0.967
Fever (x1.02) 0.967
Abnormal breathing (x1.02) 0.968
diltiazem (+0.00175) 0.968
Disorder of lung (x1.02) 0.969
nifedipine (+0.00174) 0.969
enalapril (+0.00173) 0.969
Chronic combined systolic and diastolic heart failure (x1.02) 0.970
Malaise (x1.02) 0.970
Hyperkalemia (x1.02) 0.971
Atelectasis (x1.02) 0.971
Type 2 diabetes mellitus without complication (x1.02) 0.971
Gout (x1.02) 0.972
MCH [M] by AC (-0.0807) 0.972
Tachycardia (x1.02) 0.972
ergocalciferol (+0.00162) 0.973
atorvastatin (+0.00162) 0.973
acyclovir (+0.00159) 0.974
Acidosis (x1.02) 0.974
Hypervolemia (x1.02) 0.974
Disorder of immune function (x1.02) 0.975
omeprazole (+0.00154) 0.975
docusate (+0.00154) 0.975
Expressions
10^5
10^3
10^1
−20 −10 0 10 20
−1.00 −0.75 −0.50 −0.25 0.00 0.25 0.50 0.75 1.00

Signature [87]; 87 To Reach 0.975, (87 Plotted)

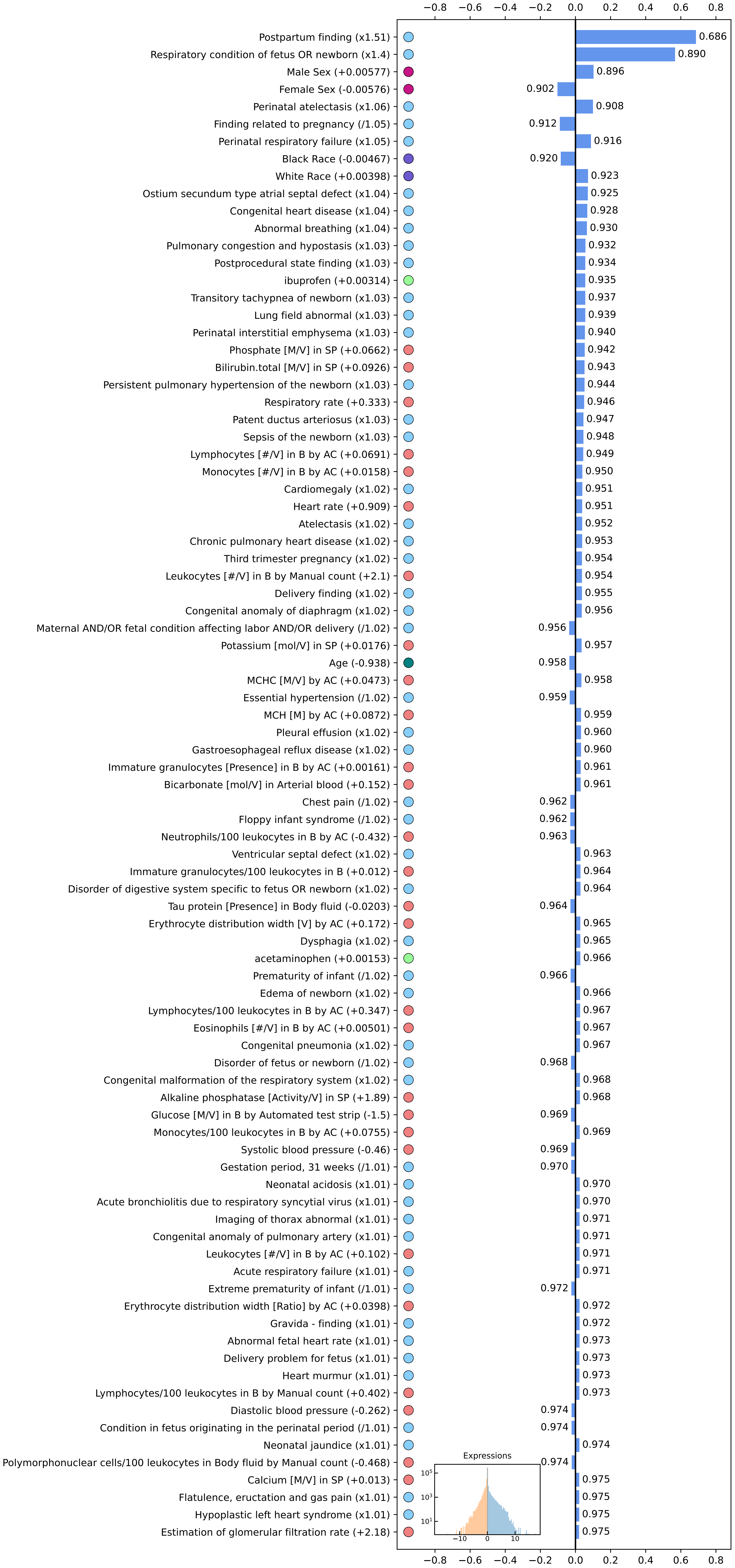

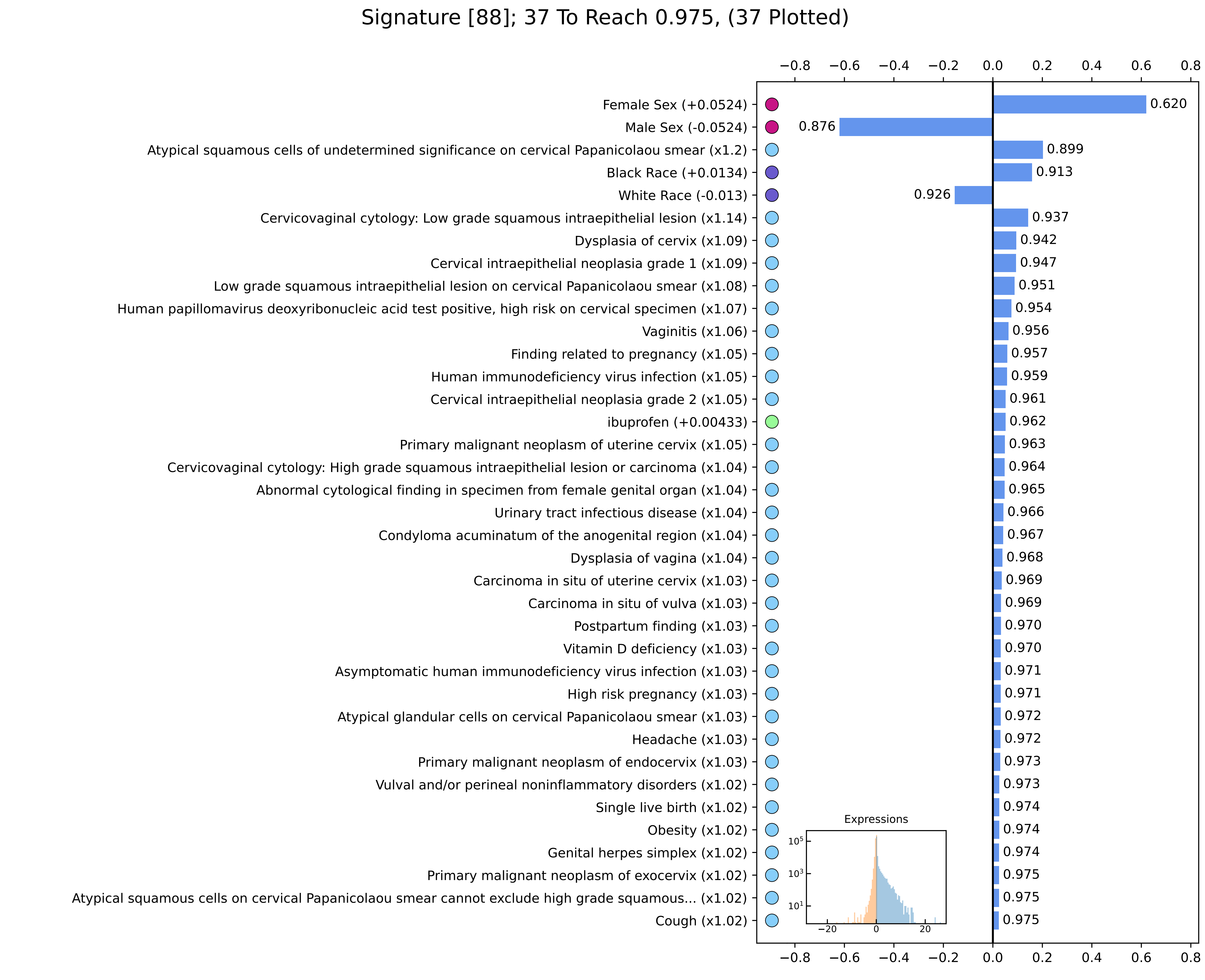

Signature [88]; 37 To Reach 0.975, (37 Plotted)
Female Sex (+0.0524) 0.620
Male Sex (-0.0524) 0.876
Atypical squamous cells of undetermined significance on cervical Papanicolaou smear (x1.2) 0.899
Black Race (+0.0134) 0.913
White Race (-0.013) 0.926
Cervicovaginal cytology: Low grade squamous intraepithelial lesion (x1.14) 0.937
Dysplasia of cervix (x1.09) 0.942
Cervical intraepithelial neoplasia grade 1 (x1.09) 0.947
Low grade squamous intraepithelial lesion on cervical Papanicolaou smear (x1.08) 0.951
Human papillomavirus deoxyribonucleic acid test positive, high risk on cervical specimen (x1.07) 0.954
Vaginitis (x1.06) 0.956
Finding related to pregnancy (x1.05) 0.957
Human immunodeficiency virus infection (x1.05) 0.959
Cervical intraepithelial neoplasia grade 2 (x1.05) 0.961
ibuprofen (+0.00433) 0.962
Primary malignant neoplasm of uterine cervix (x1.05) 0.963
Cervicovaginal cytology: High grade squamous intraepithelial lesion or carcinoma (x1.04) 0.964
Abnormal cytological finding in specimen from female genital organ (x1.04) 0.965
Urinary tract infectious disease (x1.04) 0.966
Condyloma acuminatum of the anogenital region (x1.04) 0.967
Dysplasia of vagina (x1.04) 0.968
Carcinoma in situ of uterine cervix (x1.03) 0.969
Carcinoma in situ of vulva (x1.03) 0.969
Postpartum finding (x1.03) 0.970
Vitamin D deficiency (x1.03) 0.970
Asymptomatic human immunodeficiency virus infection (x1.03) 0.971
High risk pregnancy (x1.03) 0.971
Atypical glandular cells on cervical Papanicolaou smear (x1.03) 0.972
Headache (x1.03) 0.972
Primary malignant neoplasm of endocervix (x1.03) 0.973
Vulval and/or perineal noninflammatory disorders (x1.02) 0.973
Single live birth (x1.02) 0.974
Obesity (x1.02) 0.974
Genital herpes simplex (x1.02) 0.974
Primary malignant neoplasm of exocervix (x1.02) 0.975
Atypical squamous cells on cervical Papanicolaou smear cannot exclude high grade squamous... (x1.02) 0.975
Cough (x1.02) 0.975
−0.8 −0.6 −0.4 −0.2 0.0 0.2 0.4 0.6 0.8
Expressions
10^5
10^3
10^1
−20 0 20

Signature [89]; 279 To Reach 0.975, (100 Plotted)

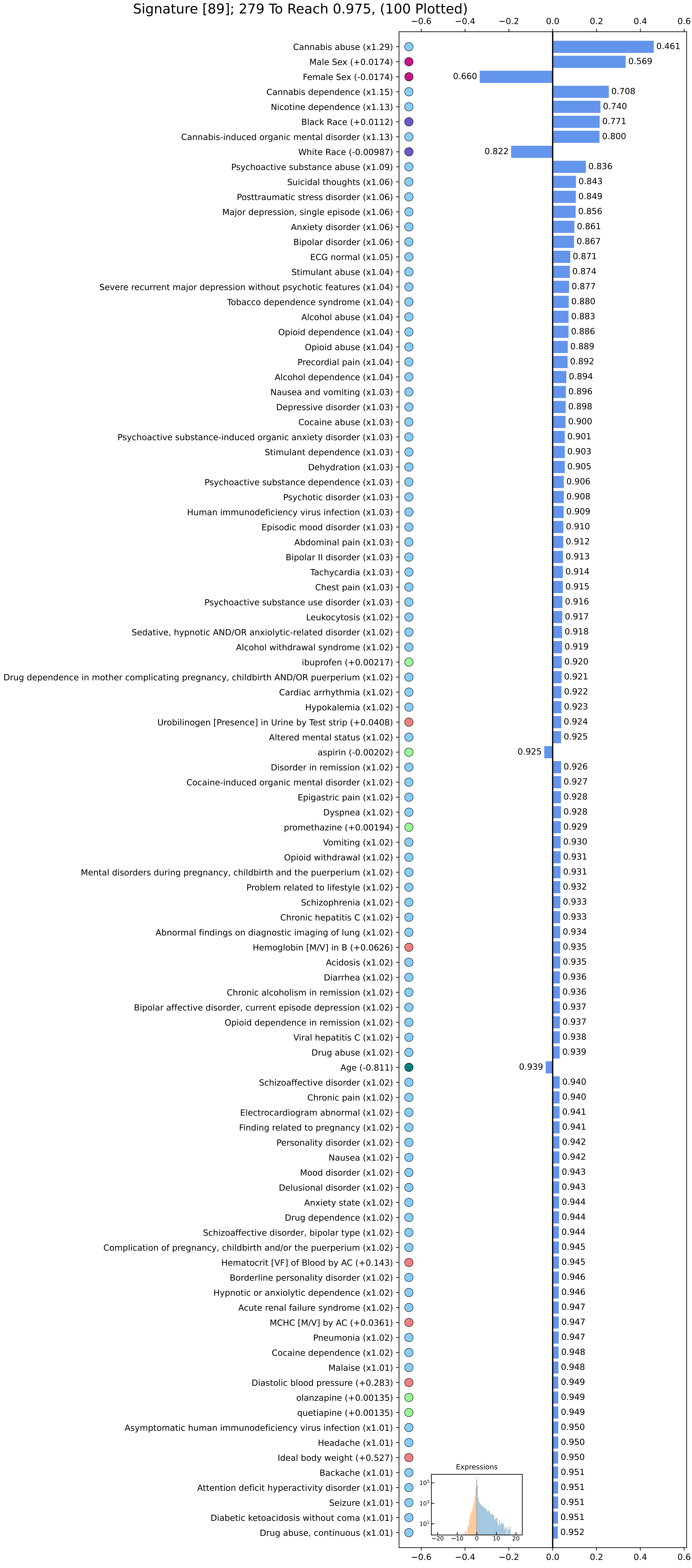

Signature [90]; 1 To Reach 0.975, (10 Plotted)

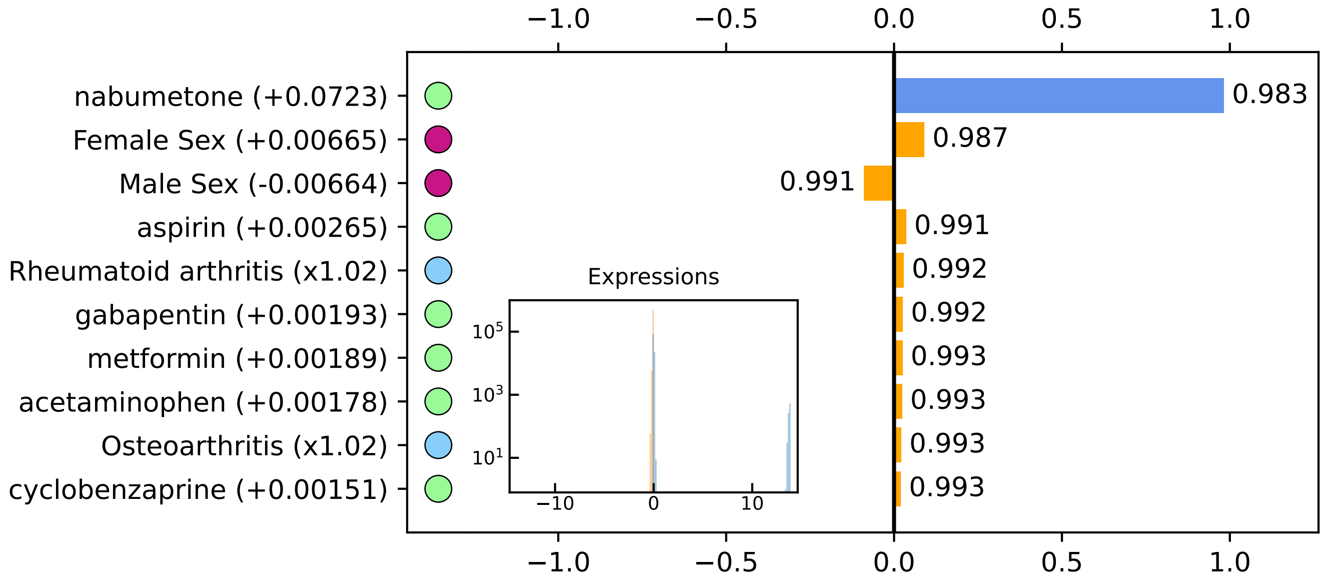

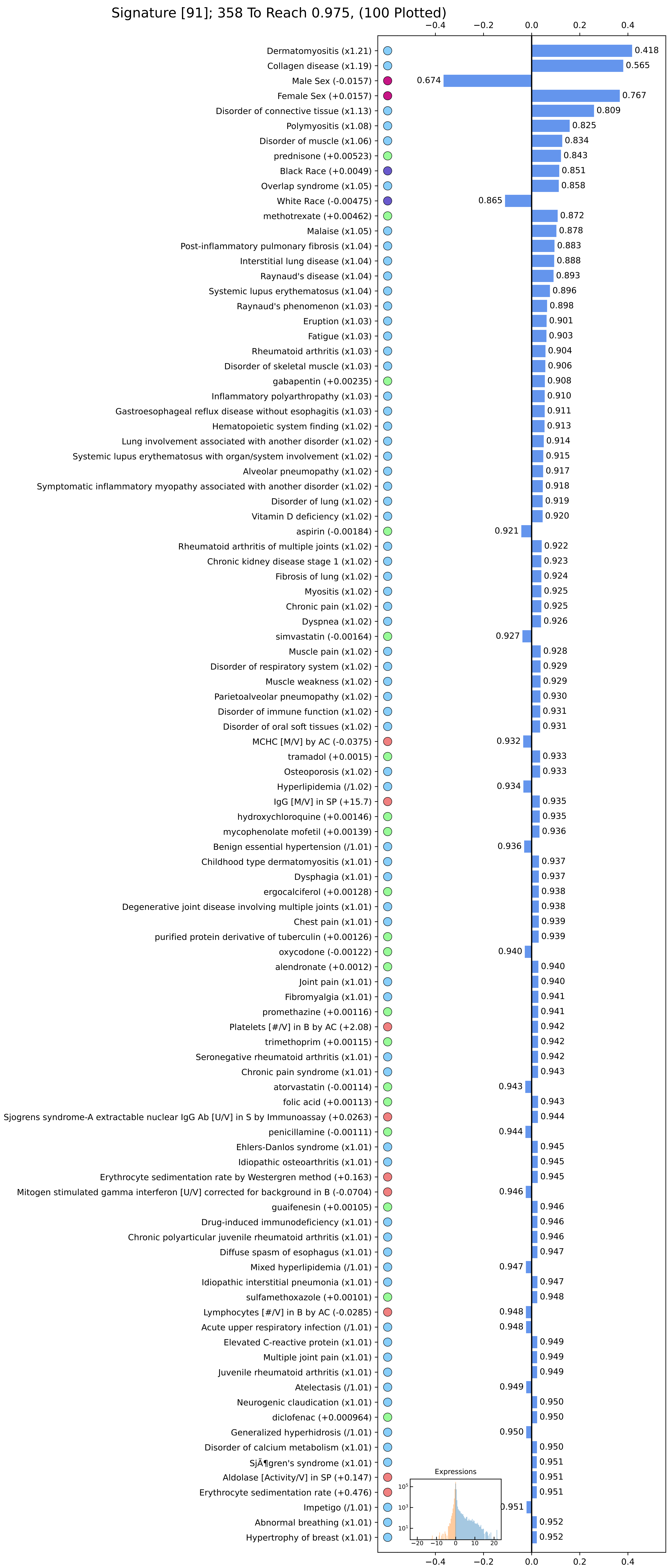

Signature [91]; 358 To Reach 0.975, (100 Plotted)
Dermatomyositis (x1.21) 0.418
Collagen disease (x1.19) 0.565
Male Sex (-0.0157) 0.674
Female Sex (+0.0157) 0.767
Disorder of connective tissue (x1.13) 0.809
Polymyositis (x1.08) 0.825
Disorder of muscle (x1.06) 0.834
prednisone (+0.00523) 0.843
Black Race (+0.0049) 0.851
Overlap syndrome (x1.05) 0.858
White Race (-0.00475) 0.865
methotrexate (+0.00462) 0.872
Malaise (x1.05) 0.878
Post-inflammatory pulmonary fibrosis (x1.04) 0.883
Interstitial lung disease (x1.04) 0.888
Raynaud's disease (x1.04) 0.893
Systemic lupus erythematosus (x1.04) 0.896
Raynaud's phenomenon (x1.03) 0.898
Eruption (x1.03) 0.901
Fatigue (x1.03) 0.903
Rheumatoid arthritis (x1.03) 0.904
Disorder of skeletal muscle (x1.03) 0.906
gabapentin (+0.00235) 0.908
Inflammatory polyarthropathy (x1.03) 0.910
Gastroesophageal reflux disease without esophagitis (x1.03) 0.911
Hematopoietic system finding (x1.02) 0.913
Lung involvement associated with another disorder (x1.02) 0.914
Systemic lupus erythematosus with organ/system involvement (x1.02) 0.915
Alveolar pneumopathy (x1.02) 0.917
Symptomatic inflammatory myopathy associated with another disorder (x1.02) 0.918
Disorder of lung (x1.02) 0.919
Vitamin D deficiency (x1.02) 0.920
aspirin (-0.00184) 0.921
Rheumatoid arthritis of multiple joints (x1.02) 0.922
Chronic kidney disease stage 1 (x1.02) 0.923
Fibrosis of lung (x1.02) 0.924
Myositis (x1.02) 0.925
Chronic pain (x1.02) 0.925
Dyspnea (x1.02) 0.926
simvastatin (-0.00164) 0.927
Muscle pain (x1.02) 0.928
Disorder of respiratory system (x1.02) 0.929
Muscle weakness (x1.02) 0.929
Parietoalveolar pneumopathy (x1.02) 0.930
Disorder of immune function (x1.02) 0.931
Disorder of oral soft tissues (x1.02) 0.931
MCHC [M/V] by AC (-0.0375) 0.932
tramadol (+0.0015) 0.933
Osteoporosis (x1.02) 0.933
Hyperlipidemia (/1.02) 0.934
IgG [M/V] in SP (+15.7) 0.935
hydroxychloroquine (+0.00146) 0.935
mycophenolate mofetil (+0.00139) 0.936
Benign essential hypertension (/1.01) 0.936
Childhood type dermatomyositis (x1.01) 0.937
Dysphagia (x1.01) 0.937
ergocalciferol (+0.00128) 0.938
Degenerative joint disease involving multiple joints (x1.01) 0.938
Chest pain (x1.01) 0.939
purified protein derivative of tuberculin (+0.00126) 0.939
oxycodone (-0.00122) 0.940
alendronate (+0.0012) 0.940
Joint pain (x1.01) 0.940
Fibromyalgia (x1.01) 0.941
promethazine (+0.00116) 0.941
Platelets [#/V] in B by AC (+2.08) 0.942
trimethoprim (+0.00115) 0.942
Seronegative rheumatoid arthritis (x1.01) 0.942
Chronic pain syndrome (x1.01) 0.943
atorvastatin (-0.00114) 0.943
folic acid (+0.00113) 0.943
Sjogrens syndrome-A extractable nuclear IgG Ab [U/V] in S by Immunoassay (+0.0263) 0.944
penicillamine (-0.00111) 0.944
Ehlers-Danlos syndrome (x1.01) 0.945
Idiopathic osteoarthritis (x1.01) 0.945
Erythrocyte sedimentation rate by Westergren method (+0.163) 0.945
Mitogen stimulated gamma interferon [U/V] corrected for background in B (-0.0704) 0.946
guaifenesin (+0.00105) 0.946
Drug-induced immunodeficiency (x1.01) 0.946
Chronic polyarticular juvenile rheumatoid arthritis (x1.01) 0.946
Diffuse spasm of esophagus (x1.01) 0.947
Mixed hyperlipidemia (/1.01) 0.947
Idiopathic interstitial pneumonia (x1.01) 0.947
sulfamethoxazole (+0.00101) 0.948
Lymphocytes [#/V] in B by AC (-0.0285) 0.948
Acute upper respiratory infection (/1.01) 0.948
Elevated C-reactive protein (x1.01) 0.949
Multiple joint pain (x1.01) 0.949
Juvenile rheumatoid arthritis (x1.01) 0.949
Atelectasis (/1.01) 0.949
Neurogenic claudication (x1.01) 0.950
diclofenac (+0.000964) 0.950
Generalized hyperhidrosis (/1.01) 0.950
Disorder of calcium metabolism (x1.01) 0.950
SjÃ¶gren's syndrome (x1.01) 0.951
Aldolase [Activity/V] in SP (+0.147) 0.951
Erythrocyte sedimentation rate (+0.476) 0.951
Impetigo (/1.01) 0.951
Abnormal breathing (x1.01) 0.952
Hypertrophy of breast (x1.01) 0.952
Expressions
−0.4 −0.2 0.0 0.2 0.4

Signature [92]; 105 To Reach 0.975, (100 Plotted)

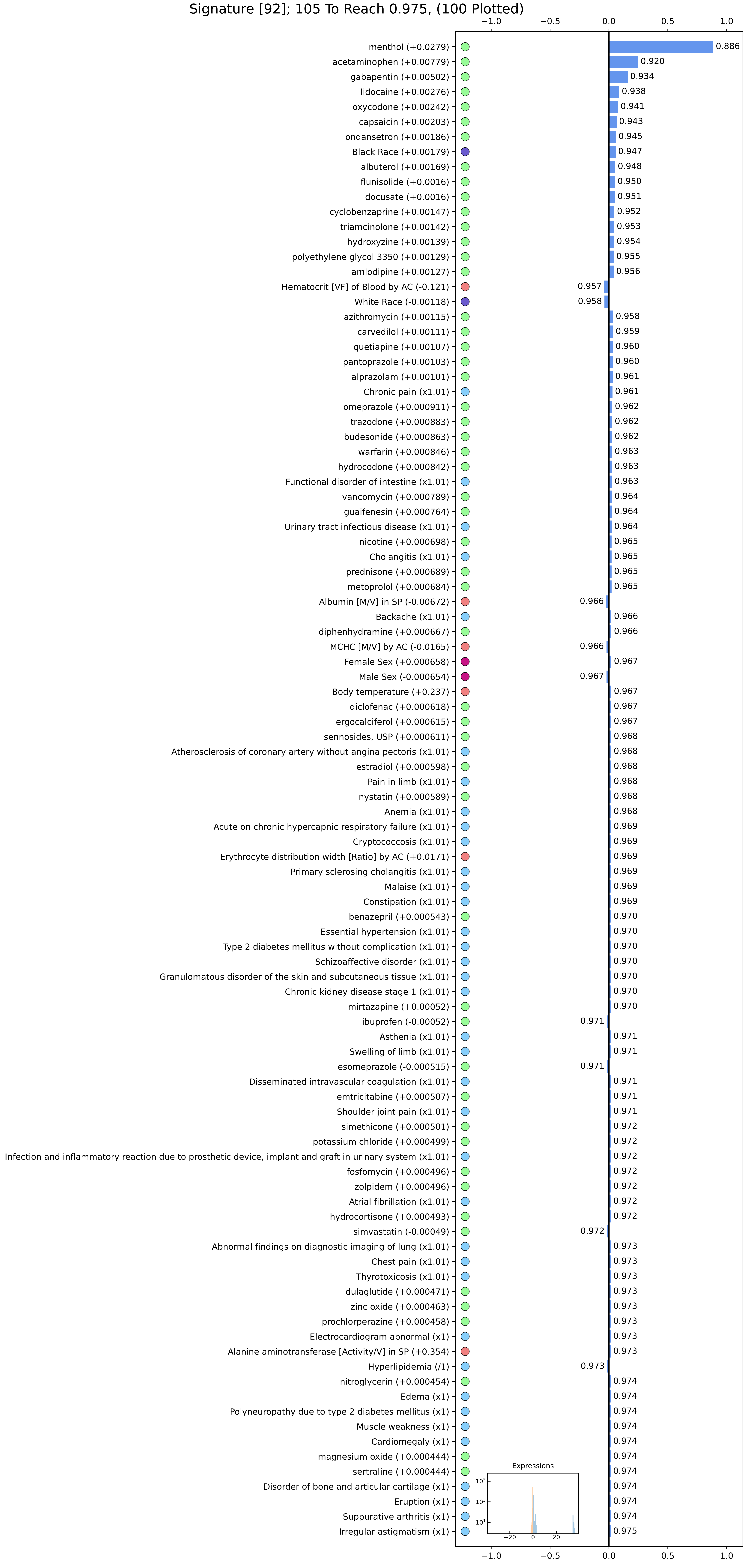

Signature [93]; 55 To Reach 0.975, (55 Plotted)

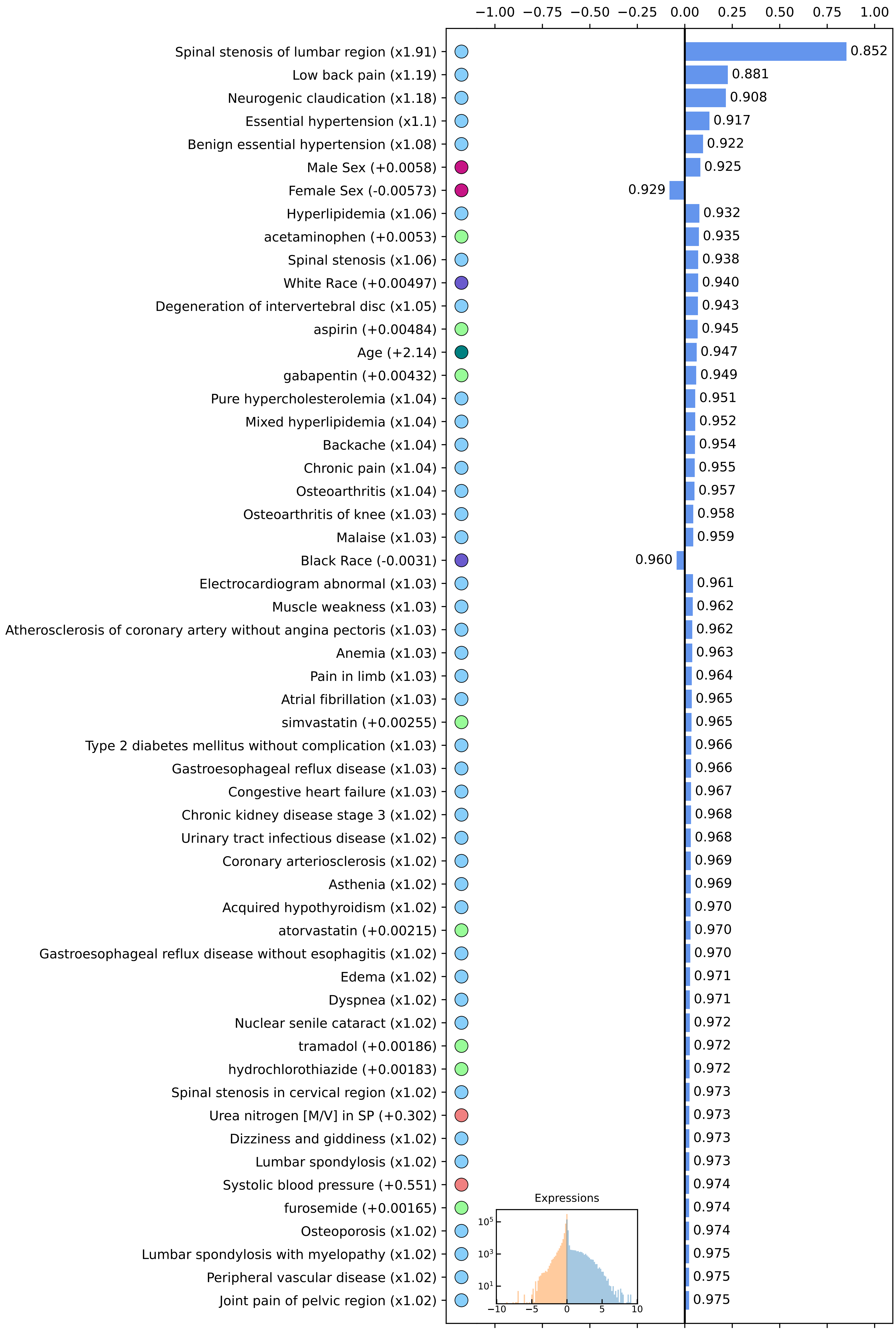

Signature [94]; 125 To Reach 0.975, (100 Plotted)

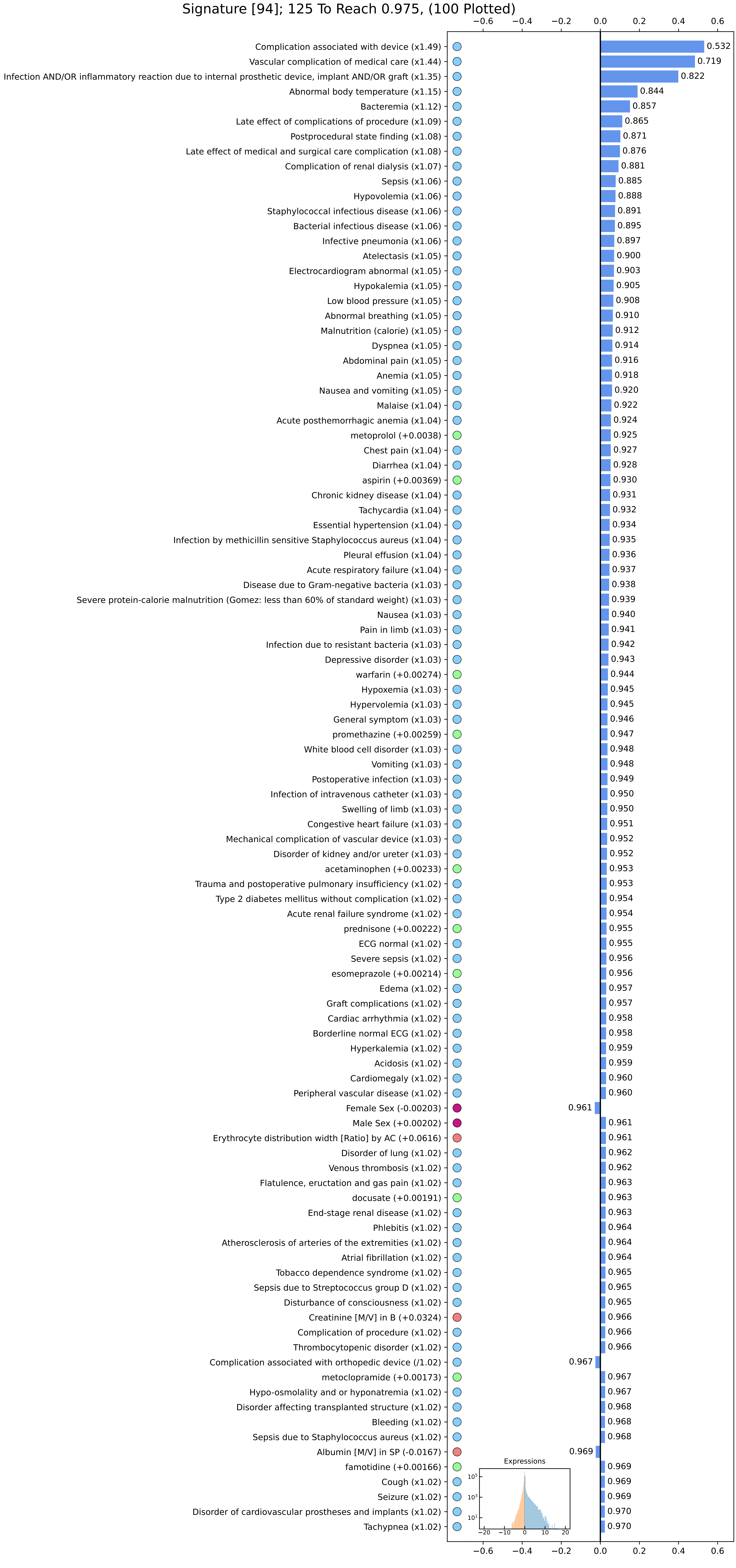

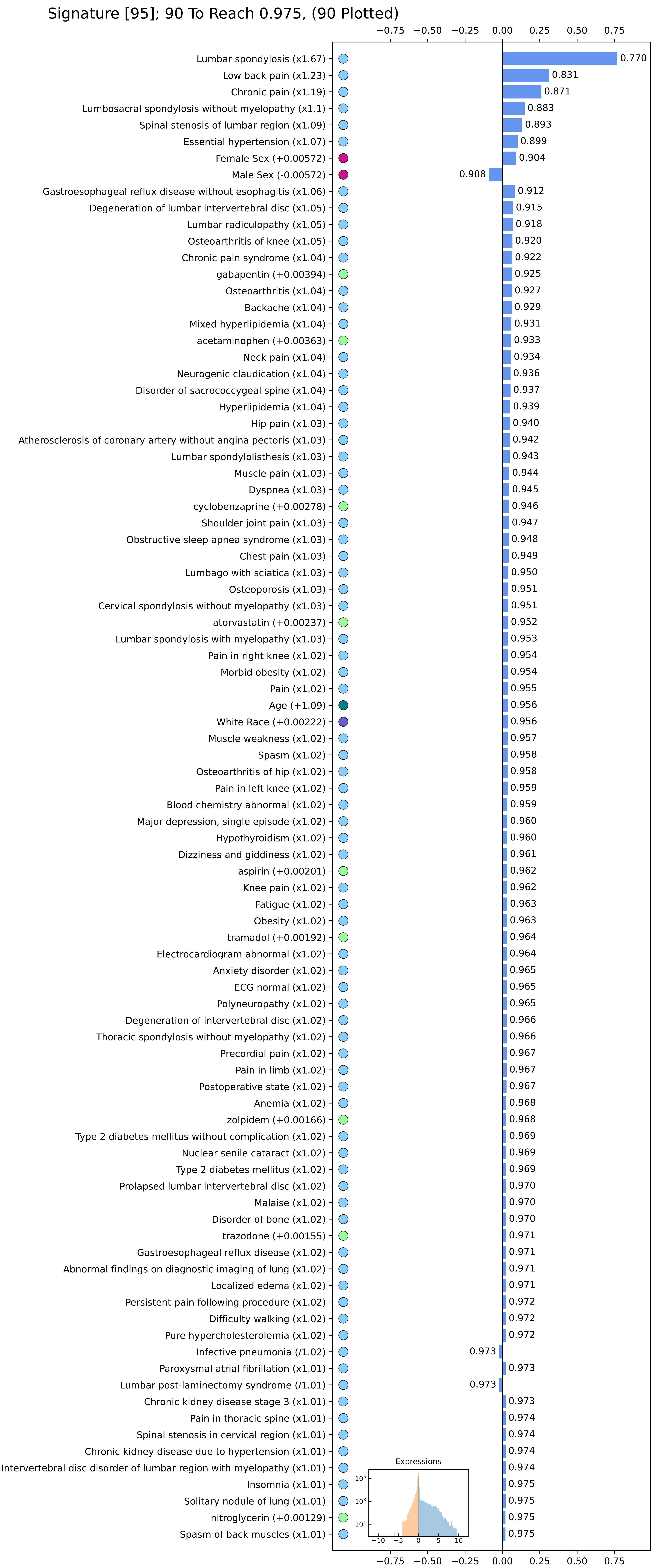

Signature [95]; 90 To Reach 0.975, (90 Plotted)
Lumbar spondylosis (x1.67) 0.770
Low back pain (x1.23) 0.831
Chronic pain (x1.19) 0.871
Lumbosacral spondylosis without myelopathy (x1.1) 0.883
Spinal stenosis of lumbar region (x1.09) 0.893
Essential hypertension (x1.07) 0.899
Female Sex (+0.00572) 0.904
Male Sex (-0.00572) 0.908
Gastroesophageal reflux disease without esophagitis (x1.06) 0.912
Degeneration of lumbar intervertebral disc (x1.05) 0.915
Lumbar radiculopathy (x1.05) 0.918
Osteoarthritis of knee (x1.05) 0.920
Chronic pain syndrome (x1.04) 0.922
gabapentin (+0.00394) 0.925
Osteoarthritis (x1.04) 0.927
Backache (x1.04) 0.929
Mixed hyperlipidemia (x1.04) 0.931
acetaminophen (+0.00363) 0.933
Neck pain (x1.04) 0.934
Neurogenic claudication (x1.04) 0.936
Disorder of sacrococcygeal spine (x1.04) 0.937
Hyperlipidemia (x1.04) 0.939
Hip pain (x1.03) 0.940
Atherosclerosis of coronary artery without angina pectoris (x1.03) 0.942
Lumbar spondylolisthesis (x1.03) 0.943
Muscle pain (x1.03) 0.944
Dyspnea (x1.03) 0.945
cyclobenzaprine (+0.00278) 0.946
Shoulder joint pain (x1.03) 0.947
Obstructive sleep apnea syndrome (x1.03) 0.948
Chest pain (x1.03) 0.949
Lumbago with sciatica (x1.03) 0.950
Osteoporosis (x1.03) 0.951
Cervical spondylosis without myelopathy (x1.03) 0.951
atorvastatin (+0.00237) 0.952
Lumbar spondylosis with myelopathy (x1.03) 0.953
Pain in right knee (x1.02) 0.954
Morbid obesity (x1.02) 0.954
Pain (x1.02) 0.955
Age (+1.09) 0.956
White Race (+0.00222) 0.956
Muscle weakness (x1.02) 0.957
Spasm (x1.02) 0.958
Osteoarthritis of hip (x1.02) 0.958
Pain in left knee (x1.02) 0.959
Blood chemistry abnormal (x1.02) 0.959
Major depression, single episode (x1.02) 0.960
Hypothyroidism (x1.02) 0.960
Dizziness and giddiness (x1.02) 0.961
aspirin (+0.00201) 0.962
Knee pain (x1.02) 0.962
Fatigue (x1.02) 0.963
Obesity (x1.02) 0.963
tramadol (+0.00192) 0.964
Electrocardiogram abnormal (x1.02) 0.964
Anxiety disorder (x1.02) 0.965
ECG normal (x1.02) 0.965
Polyneuropathy (x1.02) 0.965
Degeneration of intervertebral disc (x1.02) 0.966
Thoracic spondylosis without myelopathy (x1.02) 0.966
Precordial pain (x1.02) 0.967
Pain in limb (x1.02) 0.967
Postoperative state (x1.02) 0.967
Anemia (x1.02) 0.968
zolpidem (+0.00166) 0.968
Type 2 diabetes mellitus without complication (x1.02) 0.969
Nuclear senile cataract (x1.02) 0.969
Type 2 diabetes mellitus (x1.02) 0.969
Prolapsed lumbar intervertebral disc (x1.02) 0.970
Malaise (x1.02) 0.970
Disorder of bone (x1.02) 0.970
trazodone (+0.00155) 0.971
Gastroesophageal reflux disease (x1.02) 0.971
Abnormal findings on diagnostic imaging of lung (x1.02) 0.971
Localized edema (x1.02) 0.971
Persistent pain following procedure (x1.02) 0.972
Difficulty walking (x1.02) 0.972
Pure hypercholesterolemia (x1.02) 0.972
Infective pneumonia (/1.02) 0.973
Paroxysmal atrial fibrillation (x1.01) 0.973
Lumbar post-laminectomy syndrome (/1.01) 0.973
Chronic kidney disease stage 3 (x1.01) 0.973
Pain in thoracic spine (x1.01) 0.974
Spinal stenosis in cervical region (x1.01) 0.974
Chronic kidney disease due to hypertension (x1.01) 0.974
Intervertebral disc disorder of lumbar region with myelopathy (x1.01) 0.974
Insomnia (x1.01) 0.975
Solitary nodule of lung (x1.01) 0.975
nitroglycerin (+0.00129) 0.975
Spasm of back muscles (x1.01) 0.975
−0.75 −0.50 −0.25 0.00 0.25 0.50 0.75
Expressions

Signature [96]; 10 To Reach 0.975, (10 Plotted)

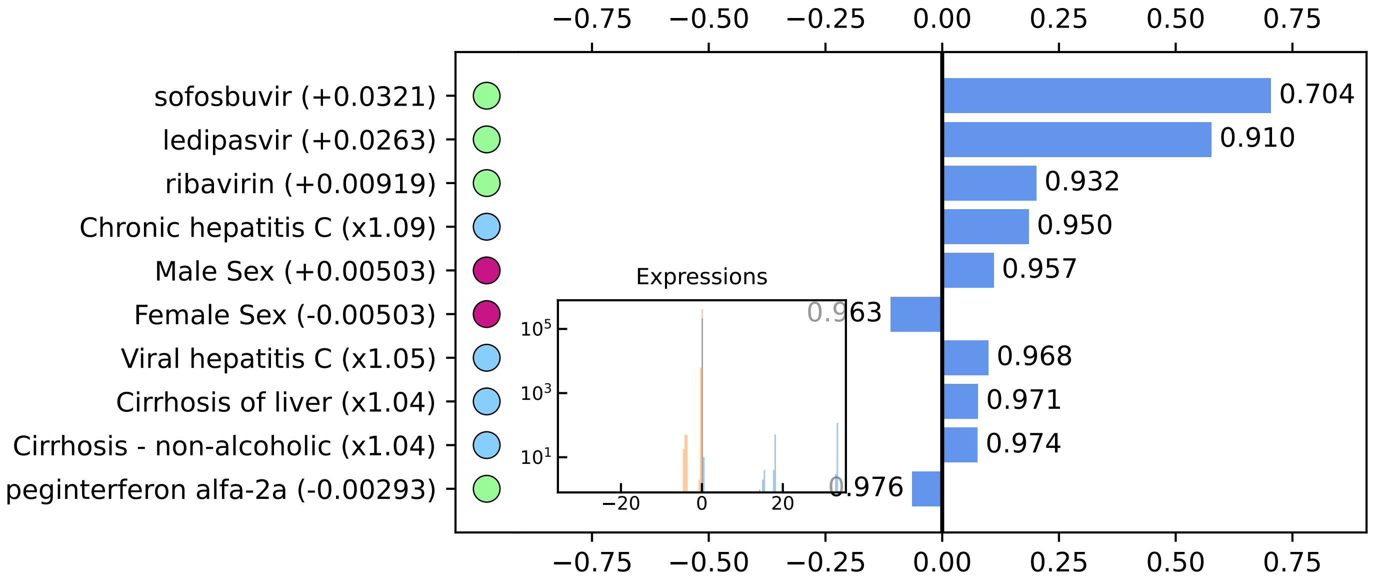

Signature [97]; 211 To Reach 0.975, (100 Plotted)

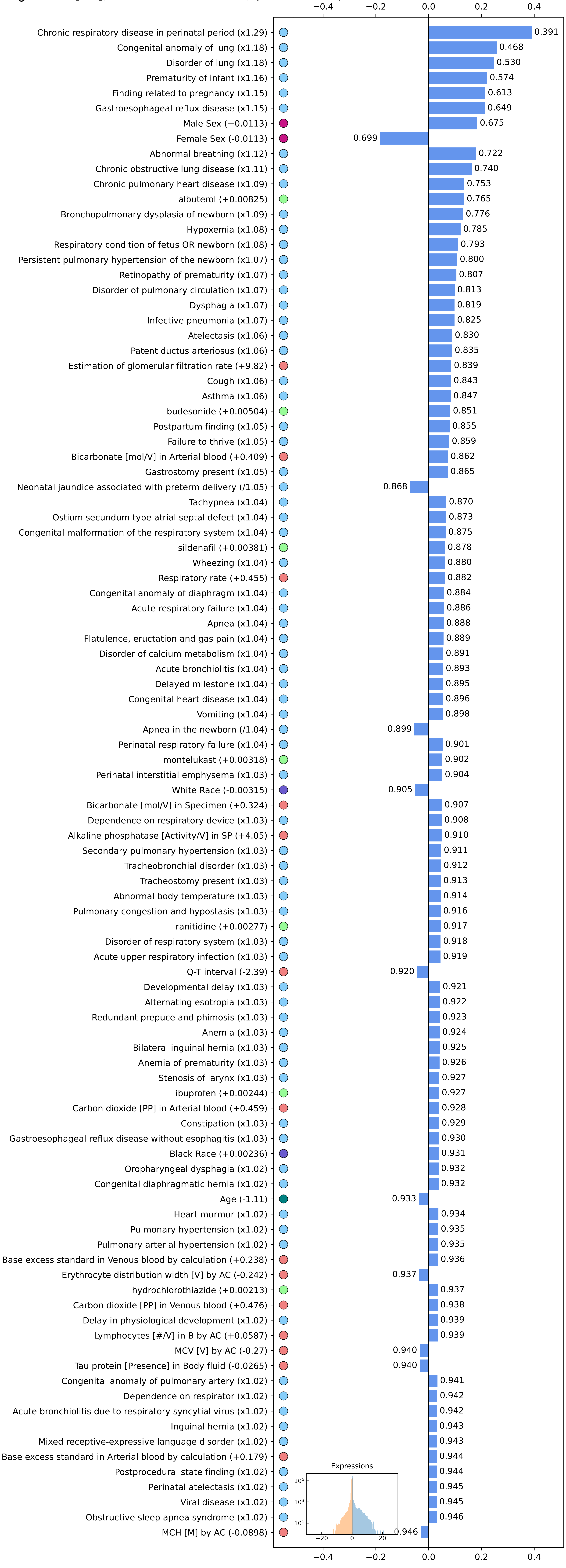

Signature [98]; 1 To Reach 0.975, (10 Plotted)

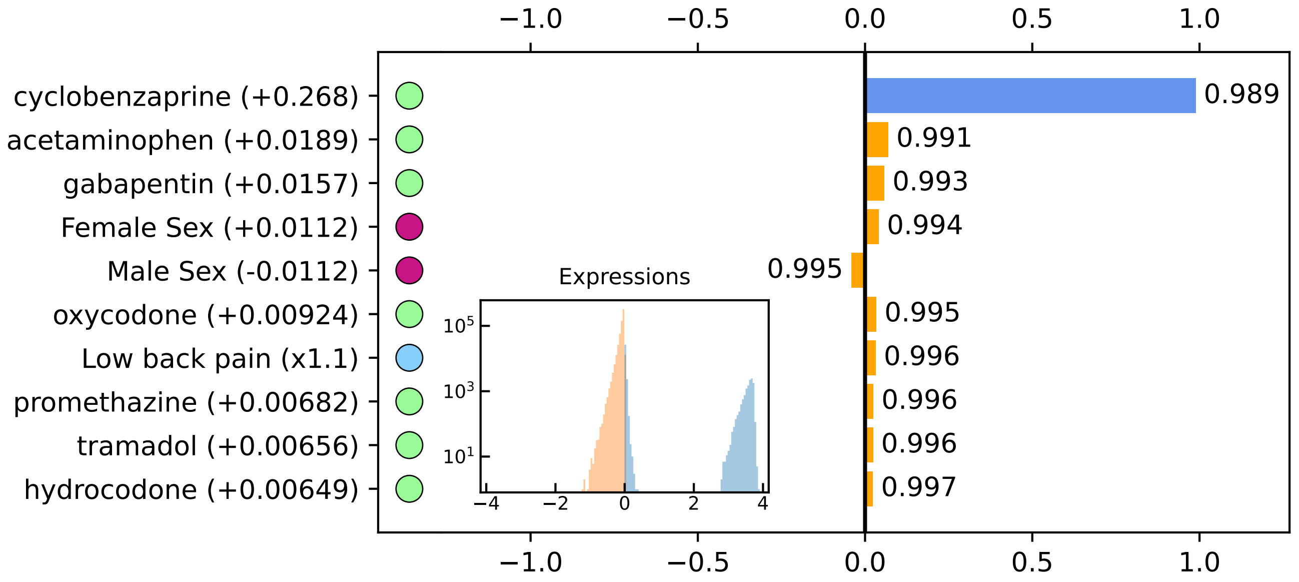

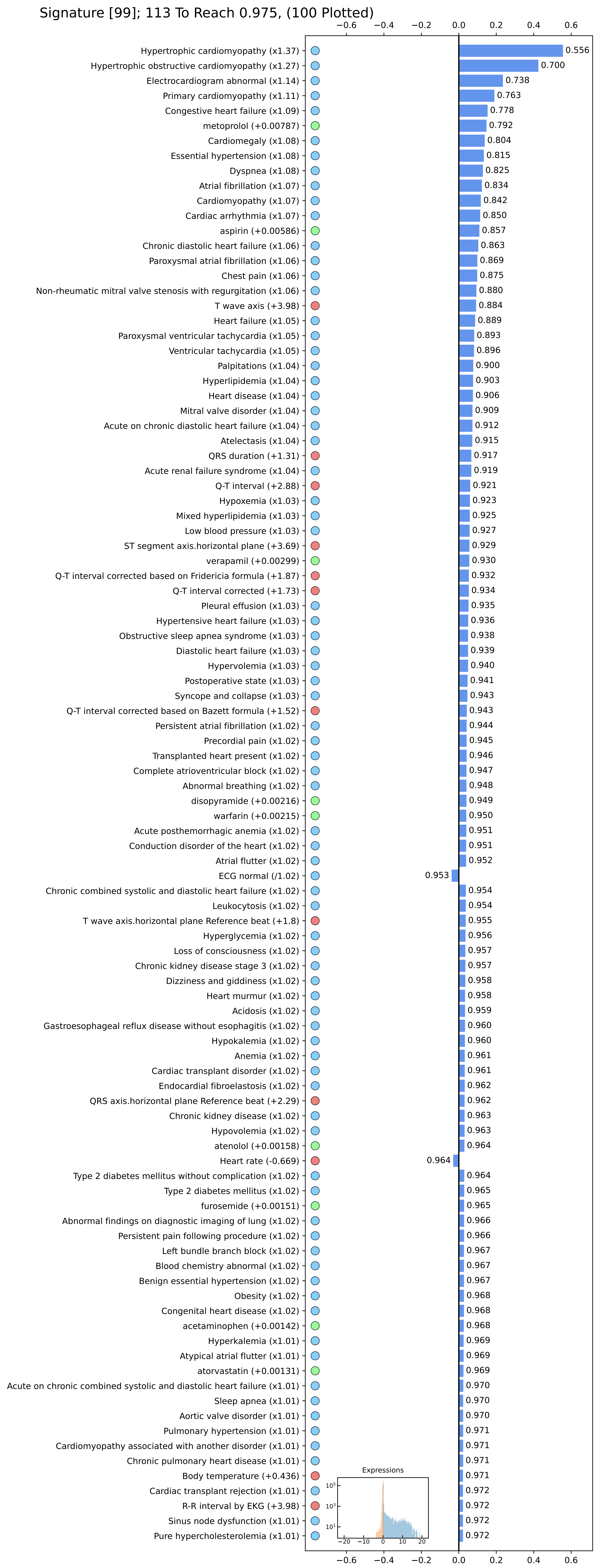

Signature [99]; 113 To Reach 0.975, (100 Plotted)
−0.6 −0.4 −0.2 0.0 0.2 0.4 0.6
Hypertrophic cardiomyopathy (x1.37) 0.556
Hypertrophic obstructive cardiomyopathy (x1.27) 0.700
Electrocardiogram abnormal (x1.14) 0.738
Primary cardiomyopathy (x1.11) 0.763
Congestive heart failure (x1.09) 0.778
metoprolol (+0.00787) 0.792
Cardiomegaly (x1.08) 0.804
Essential hypertension (x1.08) 0.815
Dyspnea (x1.08) 0.825
Atrial fibrillation (x1.07) 0.834
Cardiomyopathy (x1.07) 0.842
Cardiac arrhythmia (x1.07) 0.850
aspirin (+0.00586) 0.857
Chronic diastolic heart failure (x1.06) 0.863
Paroxysmal atrial fibrillation (x1.06) 0.869
Chest pain (x1.06) 0.875
Non-rheumatic mitral valve stenosis with regurgitation (x1.06) 0.880
T wave axis (+3.98) 0.884
Heart failure (x1.05) 0.889
Paroxysmal ventricular tachycardia (x1.05) 0.893
Ventricular tachycardia (x1.05) 0.896
Palpitations (x1.04) 0.900
Hyperlipidemia (x1.04) 0.903
Heart disease (x1.04) 0.906
Mitral valve disorder (x1.04) 0.909
Acute on chronic diastolic heart failure (x1.04) 0.912
Atelectasis (x1.04) 0.915
QRS duration (+1.31) 0.917
Acute renal failure syndrome (x1.04) 0.919
Q-T interval (+2.88) 0.921
Hypoxemia (x1.03) 0.923
Mixed hyperlipidemia (x1.03) 0.925
Low blood pressure (x1.03) 0.927
ST segment axis.horizontal plane (+3.69) 0.929
verapamil (+0.00299) 0.930
Q-T interval corrected based on Fridericia formula (+1.87) 0.932
Q-T interval corrected (+1.73) 0.934
Pleural effusion (x1.03) 0.935
Hypertensive heart failure (x1.03) 0.936
Obstructive sleep apnea syndrome (x1.03) 0.938
Diastolic heart failure (x1.03) 0.939
Hypervolemia (x1.03) 0.940
Postoperative state (x1.03) 0.941
Syncope and collapse (x1.03) 0.943
Q-T interval corrected based on Bazett formula (+1.52) 0.943
Persistent atrial fibrillation (x1.02) 0.944
Precordial pain (x1.02) 0.945
Transplanted heart present (x1.02) 0.946
Complete atrioventricular block (x1.02) 0.947
Abnormal breathing (x1.02) 0.948
disopyramide (+0.00216) 0.949
warfarin (+0.00215) 0.950
Acute posthemorrhagic anemia (x1.02) 0.951
Conduction disorder of the heart (x1.02) 0.951
Atrial flutter (x1.02) 0.952
ECG normal (/1.02) 0.953
Chronic combined systolic and diastolic heart failure (x1.02) 0.954
Leukocytosis (x1.02) 0.954
T wave axis.horizontal plane Reference beat (+1.8) 0.955
Hyperglycemia (x1.02) 0.956
Loss of consciousness (x1.02) 0.957
Chronic kidney disease stage 3 (x1.02) 0.957
Dizziness and giddiness (x1.02) 0.958
Heart murmur (x1.02) 0.958
Acidosis (x1.02) 0.959
Gastroesophageal reflux disease without esophagitis (x1.02) 0.960
Hypokalemia (x1.02) 0.960
Anemia (x1.02) 0.961
Cardiac transplant disorder (x1.02) 0.961
Endocardial fibroelastosis (x1.02) 0.962
QRS axis.horizontal plane Reference beat (+2.29) 0.962
Chronic kidney disease (x1.02) 0.963
Hypovolemia (x1.02) 0.963
atenolol (+0.00158) 0.964
Heart rate (-0.669) 0.964
Type 2 diabetes mellitus without complication (x1.02) 0.964
Type 2 diabetes mellitus (x1.02) 0.965
furosemide (+0.00151) 0.965
Abnormal findings on diagnostic imaging of lung (x1.02) 0.966
Persistent pain following procedure (x1.02) 0.966
Left bundle branch block (x1.02) 0.967
Blood chemistry abnormal (x1.02) 0.967
Benign essential hypertension (x1.02) 0.967
Obesity (x1.02) 0.968
Congenital heart disease (x1.02) 0.968
acetaminophen (+0.00142) 0.968
Hyperkalemia (x1.01) 0.969
Atypical atrial flutter (x1.01) 0.969
atorvastatin (+0.00131) 0.969
Acute on chronic combined systolic and diastolic heart failure (x1.01) 0.970
Sleep apnea (x1.01) 0.970
Aortic valve disorder (x1.01) 0.970
Pulmonary hypertension (x1.01) 0.971
Cardiomyopathy associated with another disorder (x1.01) 0.971
Chronic pulmonary heart disease (x1.01) 0.971
Body temperature (+0.436) 0.971
Cardiac transplant rejection (x1.01) 0.972
R-R interval by EKG (+3.98) 0.972
Sinus node dysfunction (x1.01) 0.972
Pure hypercholesterolemia (x1.01) 0.972
Expressions

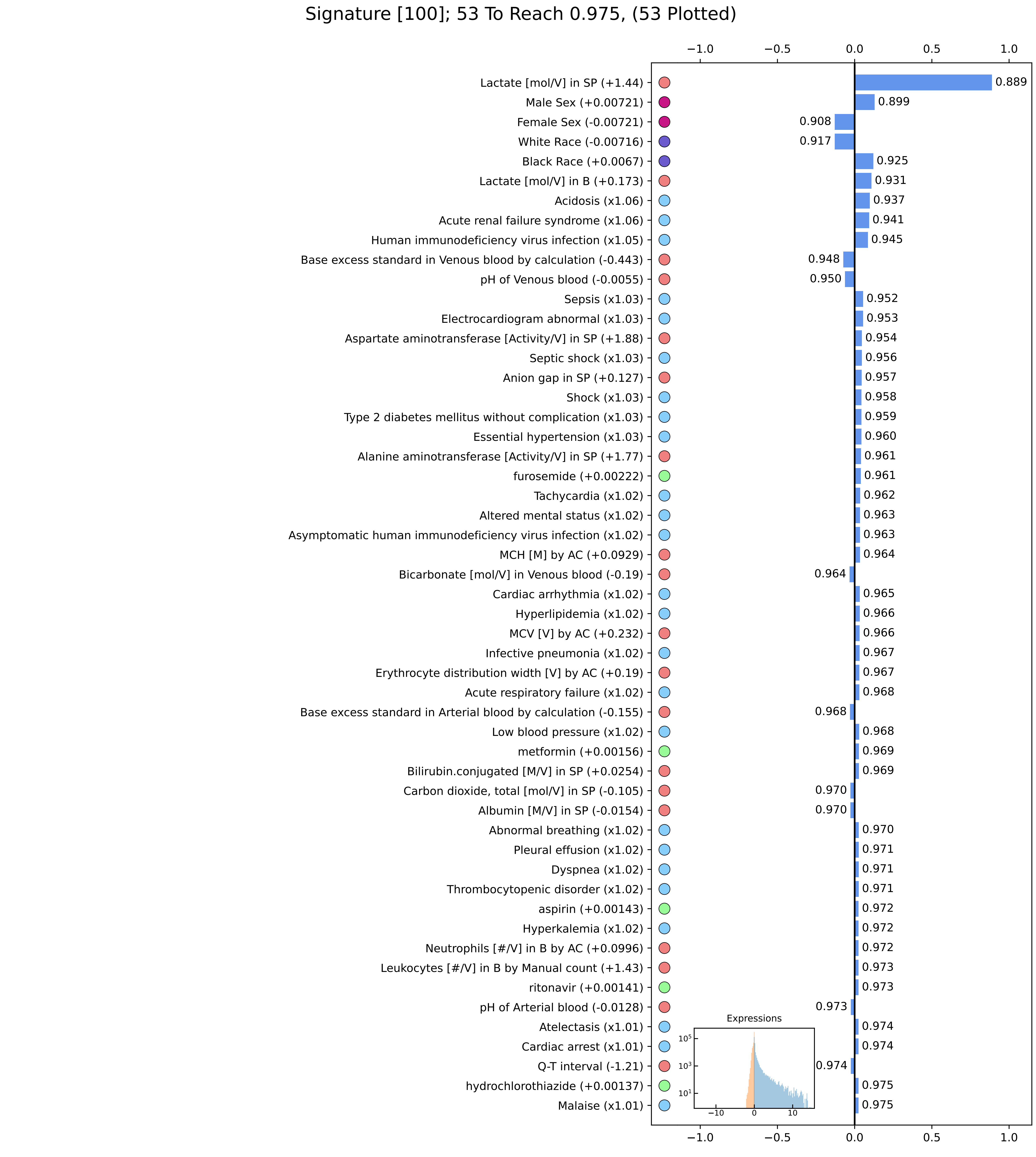

Signature [100]; 53 To Reach 0.975, (53 Plotted)
Lactate [mol/V] in SP (+1.44) 0.889
Male Sex (+0.00721) 0.899
Female Sex (-0.00721) 0.908
White Race (-0.00716) 0.917
Black Race (+0.0067) 0.925
Lactate [mol/V] in B (+0.173) 0.931
Acidosis (x1.06) 0.937
Acute renal failure syndrome (x1.06) 0.941
Human immunodeficiency virus infection (x1.05) 0.945
Base excess standard in Venous blood by calculation (-0.443) 0.948
pH of Venous blood (-0.0055) 0.950
Sepsis (x1.03) 0.952
Electrocardiogram abnormal (x1.03) 0.953
Aspartate aminotransferase [Activity/V] in SP (+1.88) 0.954
Septic shock (x1.03) 0.956
Anion gap in SP (+0.127) 0.957
Shock (x1.03) 0.958
Type 2 diabetes mellitus without complication (x1.03) 0.959
Essential hypertension (x1.03) 0.960
Alanine aminotransferase [Activity/V] in SP (+1.77) 0.961
furosemide (+0.00222) 0.961
Tachycardia (x1.02) 0.962
Altered mental status (x1.02) 0.963
Asymptomatic human immunodeficiency virus infection (x1.02) 0.963
MCH [M] by AC (+0.0929) 0.964
Bicarbonate [mol/V] in Venous blood (-0.19) 0.964
Cardiac arrhythmia (x1.02) 0.965
Hyperlipidemia (x1.02) 0.966
MCV [V] by AC (+0.232) 0.966
Infective pneumonia (x1.02) 0.967
Erythrocyte distribution width [V] by AC (+0.19) 0.967
Acute respiratory failure (x1.02) 0.968
Base excess standard in Arterial blood by calculation (-0.155) 0.968
Low blood pressure (x1.02) 0.968
metformin (+0.00156) 0.969
Bilirubin.conjugated [M/V] in SP (+0.0254) 0.969
Carbon dioxide, total [mol/V] in SP (-0.105) 0.970
Albumin [M/V] in SP (-0.0154) 0.970
Abnormal breathing (x1.02) 0.970
Pleural effusion (x1.02) 0.971
Dyspnea (x1.02) 0.971
Thrombocytopenic disorder (x1.02) 0.971
aspirin (+0.00143) 0.972
Hyperkalemia (x1.02) 0.972
Neutrophils [#/V] in B by AC (+0.0996) 0.972
Leukocytes [#/V] in B by Manual count (+1.43) 0.973
ritonavir (+0.00141) 0.973
pH of Arterial blood (-0.0128) 0.973
Atelectasis (x1.01) 0.974
Cardiac arrest (x1.01) 0.974
Q-T interval (-1.21) 0.974
hydrochlorothiazide (+0.00137) 0.975
Malaise (x1.01) 0.975
−1.0 −0.5 0.0 0.5 1.0
Expressions

Signature [101]; 80 To Reach 0.975, (80 Plotted)

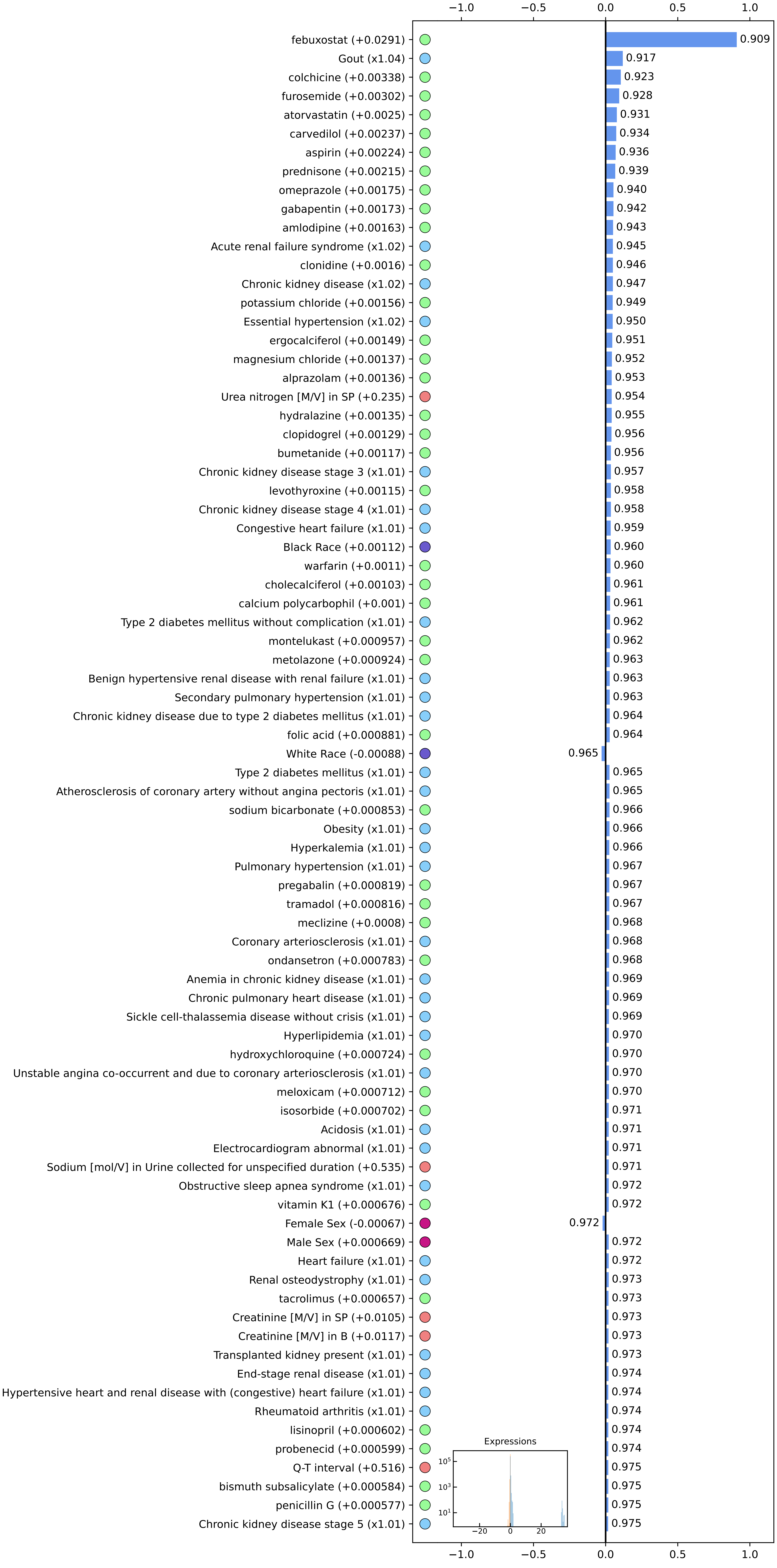

Signature [102]; 200 To Reach 0.975, (100 Plotted)

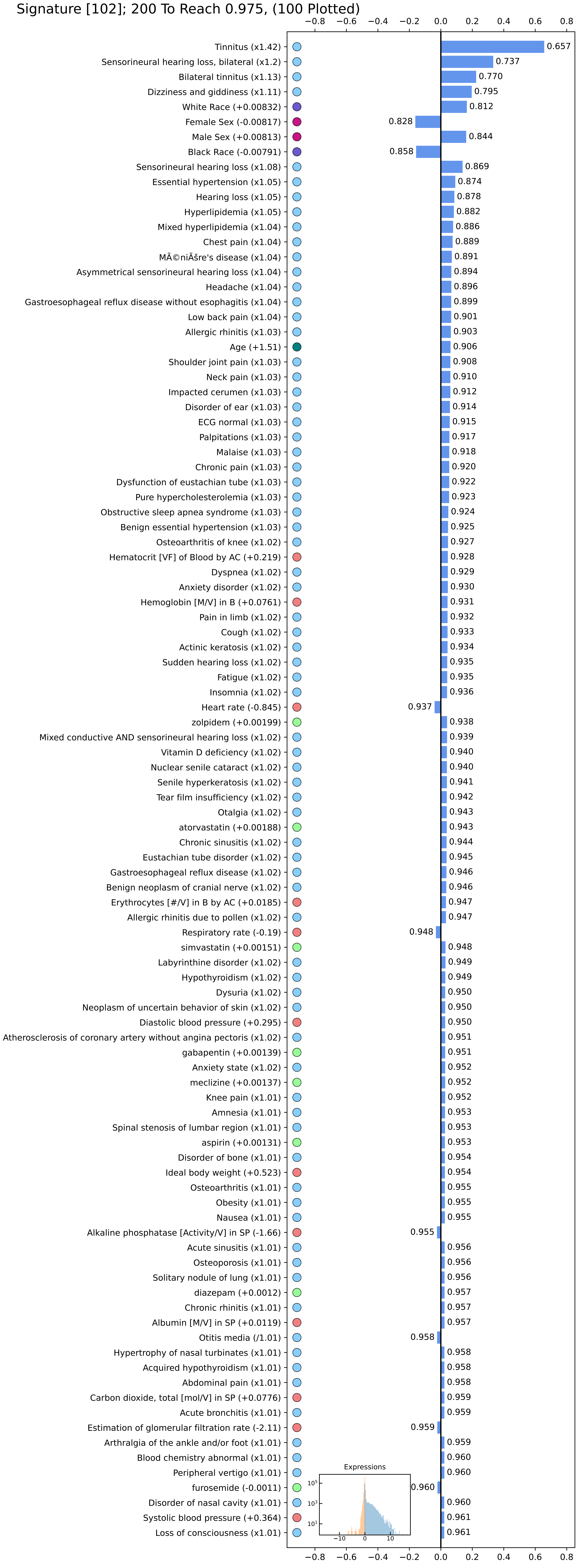

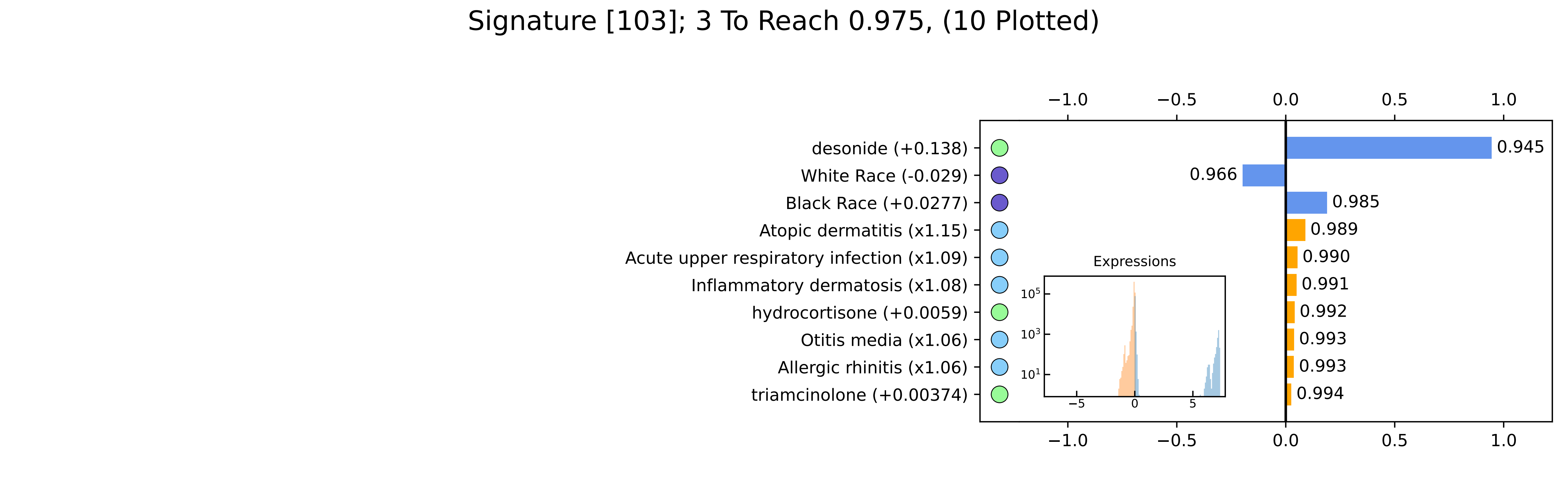

Signature [103]; 3 To Reach 0.975, (10 Plotted)
desonide (+0.138)
White Race (-0.029)
Black Race (+0.0277)
Atopic dermatitis (x1.15)
Acute upper respiratory infection (x1.09)
Inflammatory dermatosis (x1.08)
hydrocortisone (+0.0059)
Otitis media (x1.06)
Allergic rhinitis (x1.06)
triamcinolone (+0.00374)
0.945
0.966
0.985
0.989
0.990
0.991
0.992
0.993
0.993
0.994
Expressions
−1.0
−0.5
0.0
0.5
1.0

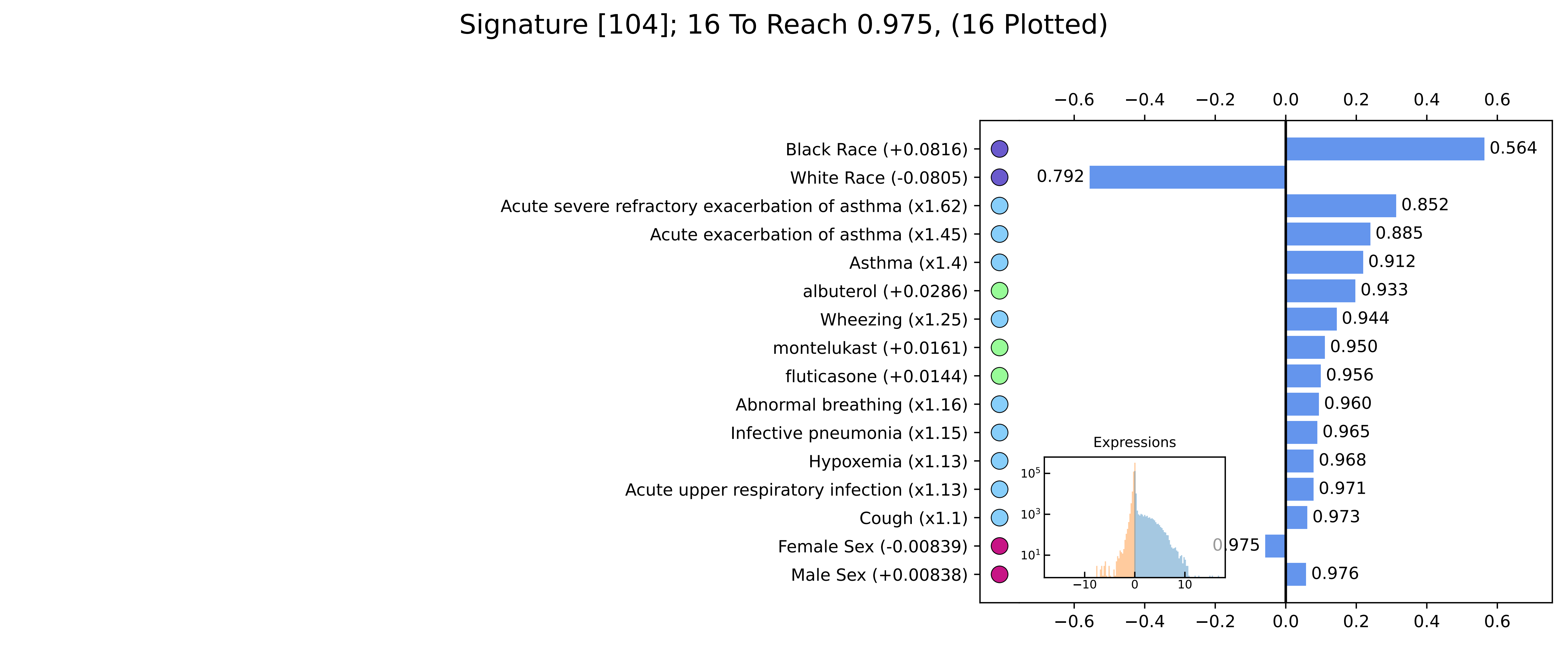

Signature [104]; 16 To Reach 0.975, (16 Plotted)
Black Race (+0.0816)
White Race (-0.0805)
Acute severe refractory exacerbation of asthma (x1.62)
Acute exacerbation of asthma (x1.45)
Asthma (x1.4)
albuterol (+0.0286)
Wheezing (x1.25)
montelukast (+0.0161)
fluticasone (+0.0144)
Abnormal breathing (x1.16)
Infective pneumonia (x1.15)
Hypoxemia (x1.13)
Acute upper respiratory infection (x1.13)
Cough (x1.1)
Female Sex (-0.00839)
Male Sex (+0.00838)
0.564
0.792
0.852
0.885
0.912
0.933
0.944
0.950
0.956
0.960
0.965
0.968
0.971
0.973
0.975
0.976
Expressions
−0.6
−0.4
−0.2
0.0
0.2
0.4
0.6

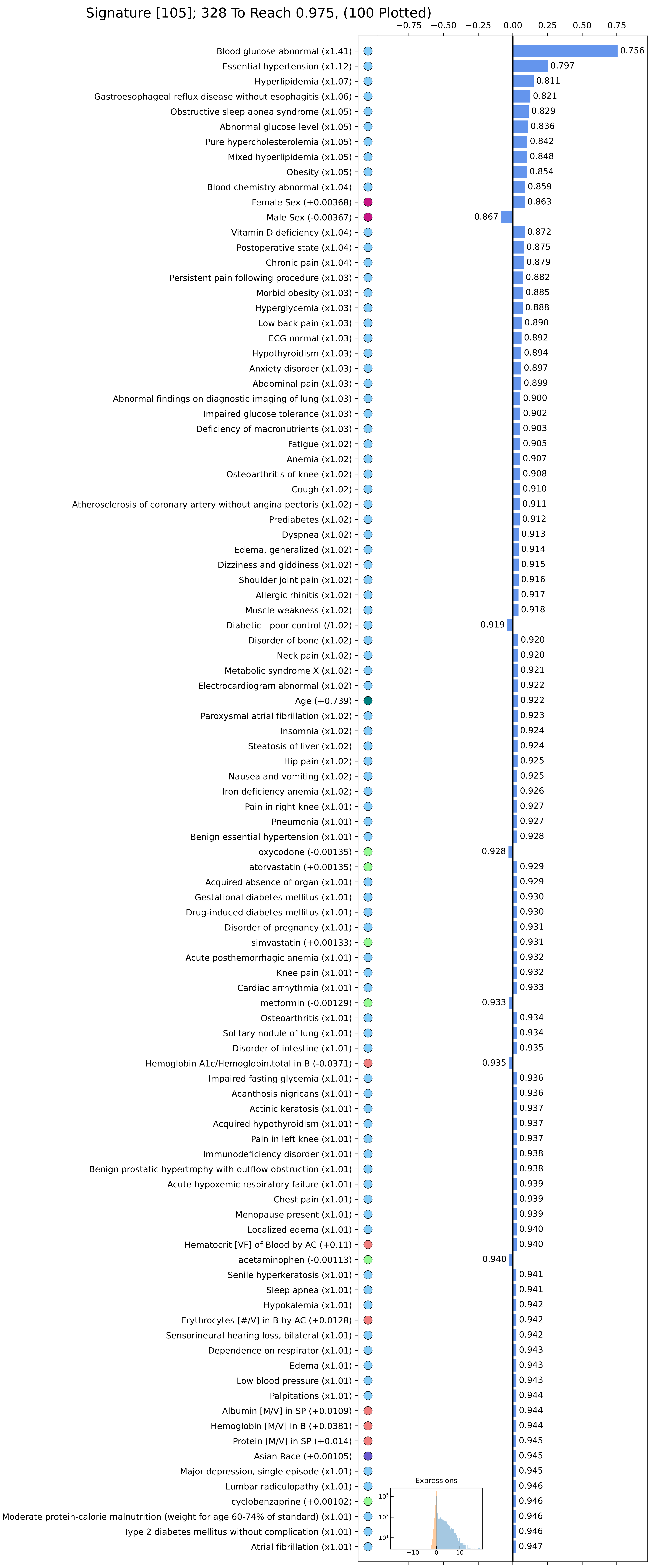

Signature [105]; 328 To Reach 0.975, (100 Plotted)
Blood glucose abnormal (x1.41) 0.756
Essential hypertension (x1.12) 0.797
Hyperlipidemia (x1.07) 0.811
Gastroesophageal reflux disease without esophagitis (x1.06) 0.821
Obstructive sleep apnea syndrome (x1.05) 0.829
Abnormal glucose level (x1.05) 0.836
Pure hypercholesterolemia (x1.05) 0.842
Mixed hyperlipidemia (x1.05) 0.848
Obesity (x1.05) 0.854
Blood chemistry abnormal (x1.04) 0.859
Female Sex (+0.00368) 0.863
Male Sex (-0.00367) 0.867
Vitamin D deficiency (x1.04) 0.872
Postoperative state (x1.04) 0.875
Chronic pain (x1.04) 0.879
Persistent pain following procedure (x1.03) 0.882
Morbid obesity (x1.03) 0.885
Hyperglycemia (x1.03) 0.888
Low back pain (x1.03) 0.890
ECG normal (x1.03) 0.892
Hypothyroidism (x1.03) 0.894
Anxiety disorder (x1.03) 0.897
Abdominal pain (x1.03) 0.899
Abnormal findings on diagnostic imaging of lung (x1.03) 0.900
Impaired glucose tolerance (x1.03) 0.902
Deficiency of macronutrients (x1.03) 0.903
Fatigue (x1.02) 0.905
Anemia (x1.02) 0.907
Osteoarthritis of knee (x1.02) 0.908
Cough (x1.02) 0.910
Atherosclerosis of coronary artery without angina pectoris (x1.02) 0.911
Prediabetes (x1.02) 0.912
Dyspnea (x1.02) 0.913
Edema, generalized (x1.02) 0.914
Dizziness and giddiness (x1.02) 0.915
Shoulder joint pain (x1.02) 0.916
Allergic rhinitis (x1.02) 0.917
Muscle weakness (x1.02) 0.918
Diabetic - poor control (/1.02) 0.919
Disorder of bone (x1.02) 0.920
Neck pain (x1.02) 0.920
Metabolic syndrome X (x1.02) 0.921
Electrocardiogram abnormal (x1.02) 0.922
Age (+0.739) 0.922
Paroxysmal atrial fibrillation (x1.02) 0.923
Insomnia (x1.02) 0.924
Steatosis of liver (x1.02) 0.924
Hip pain (x1.02) 0.925
Nausea and vomiting (x1.02) 0.925
Iron deficiency anemia (x1.02) 0.926
Pain in right knee (x1.01) 0.927
Pneumonia (x1.01) 0.927
Benign essential hypertension (x1.01) 0.928
oxycodone (-0.00135) 0.928
atorvastatin (+0.00135) 0.929
Acquired absence of organ (x1.01) 0.929
Gestational diabetes mellitus (x1.01) 0.930
Drug-induced diabetes mellitus (x1.01) 0.930
Disorder of pregnancy (x1.01) 0.931
simvastatin (+0.00133) 0.931
Acute posthemorrhagic anemia (x1.01) 0.932
Knee pain (x1.01) 0.932
Cardiac arrhythmia (x1.01) 0.933
metformin (-0.00129) 0.933
Osteoarthritis (x1.01) 0.934
Solitary nodule of lung (x1.01) 0.934
Disorder of intestine (x1.01) 0.935
Hemoglobin A1c/Hemoglobin.total in B (-0.0371) 0.935
Impaired fasting glycemia (x1.01) 0.936
Acanthosis nigricans (x1.01) 0.936
Actinic keratosis (x1.01) 0.937
Acquired hypothyroidism (x1.01) 0.937
Pain in left knee (x1.01) 0.937
Immunodeficiency disorder (x1.01) 0.938
Benign prostatic hypertrophy with outflow obstruction (x1.01) 0.938
Acute hypoxemic respiratory failure (x1.01) 0.939
Chest pain (x1.01) 0.939
Menopause present (x1.01) 0.939
Localized edema (x1.01) 0.940
Hematocrit [VF] of Blood by AC (+0.11) 0.940
acetaminophen (-0.00113) 0.940
Senile hyperkeratosis (x1.01) 0.941
Sleep apnea (x1.01) 0.941
Hypokalemia (x1.01) 0.942
Erythrocytes [#/V] in B by AC (+0.0128) 0.942
Sensorineural hearing loss, bilateral (x1.01) 0.942
Dependence on respirator (x1.01) 0.943
Edema (x1.01) 0.943
Low blood pressure (x1.01) 0.943
Palpitations (x1.01) 0.944
Albumin [M/V] in SP (+0.0109) 0.944
Hemoglobin [M/V] in B (+0.0381) 0.944
Protein [M/V] in SP (+0.014) 0.945
Asian Race (+0.00105) 0.945
Major depression, single episode (x1.01) 0.945
Lumbar radiculopathy (x1.01) 0.946
cyclobenzaprine (+0.00102) 0.946
Moderate protein-calorie malnutrition (weight for age 60-74% of standard) (x1.01) 0.946
Type 2 diabetes mellitus without complication (x1.01) 0.946
Atrial fibrillation (x1.01) 0.947
−0.75 −0.50 −0.25 0.00 0.25 0.50 0.75
Expressions

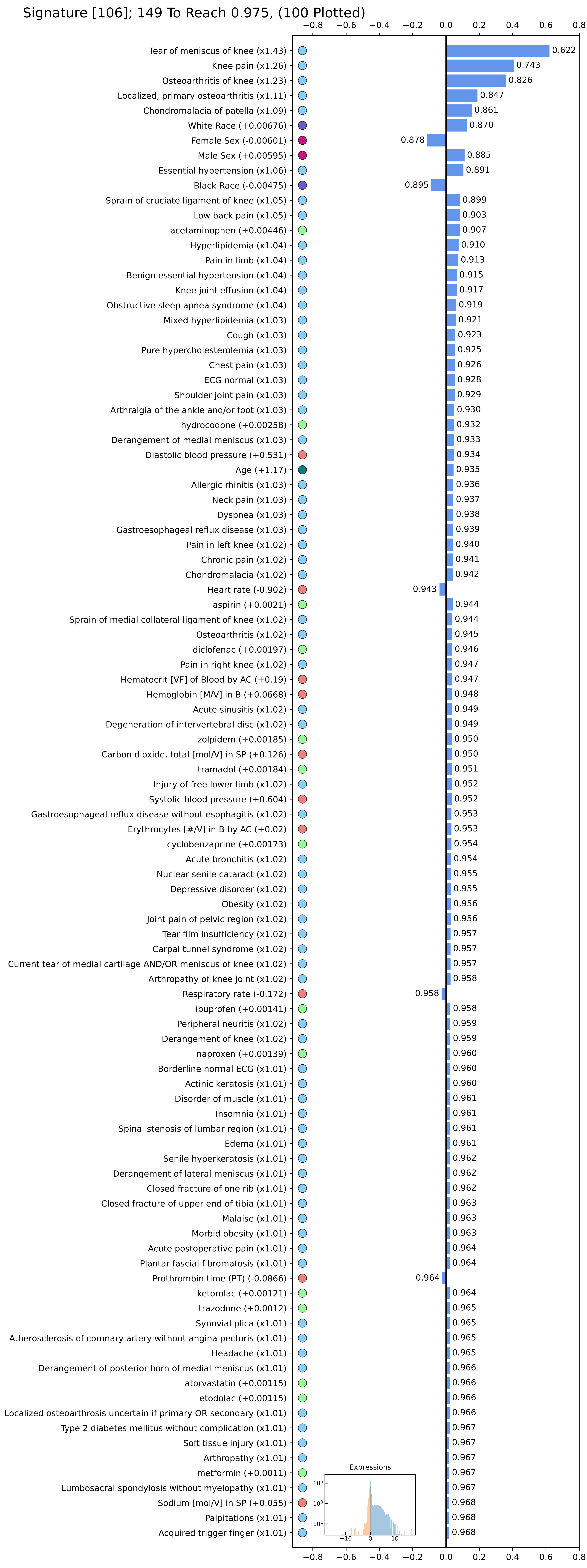

Signature [106]; 149 To Reach 0.975, (100 Plotted)
Tear of meniscus of knee (x1.43) 0.622
Knee pain (x1.26) 0.743
Osteoarthritis of knee (x1.23) 0.826
Localized, primary osteoarthritis (x1.11) 0.847
Chondromalacia of patella (x1.09) 0.861
White Race (+0.00676) 0.870
Female Sex (-0.00601) 0.878
Male Sex (+0.00595) 0.885
Essential hypertension (x1.06) 0.891
Black Race (-0.00475) 0.895
Sprain of cruciate ligament of knee (x1.05) 0.899
Low back pain (x1.05) 0.903
acetaminophen (+0.00446) 0.907
Hyperlipidemia (x1.04) 0.910
Pain in limb (x1.04) 0.913
Benign essential hypertension (x1.04) 0.915
Knee joint effusion (x1.04) 0.917
Obstructive sleep apnea syndrome (x1.04) 0.919
Mixed hyperlipidemia (x1.03) 0.921
Cough (x1.03) 0.923
Pure hypercholesterolemia (x1.03) 0.925
Chest pain (x1.03) 0.926
ECG normal (x1.03) 0.928
Shoulder joint pain (x1.03) 0.929
Arthralgia of the ankle and/or foot (x1.03) 0.930
hydrocodone (+0.00258) 0.932
Derangement of medial meniscus (x1.03) 0.933
Diastolic blood pressure (+0.531) 0.934
Age (+1.17) 0.935
Allergic rhinitis (x1.03) 0.936
Neck pain (x1.03) 0.937
Dyspnea (x1.03) 0.938
Gastroesophageal reflux disease (x1.03) 0.939
Pain in left knee (x1.02) 0.940
Chronic pain (x1.02) 0.941
Chondromalacia (x1.02) 0.942
Heart rate (-0.902) 0.943
aspirin (+0.0021) 0.944
Sprain of medial collateral ligament of knee (x1.02) 0.944
Osteoarthritis (x1.02) 0.945
diclofenac (+0.00197) 0.946
Pain in right knee (x1.02) 0.947
Hematocrit [VF] of Blood by AC (+0.19) 0.947
Hemoglobin [M/V] in B (+0.0668) 0.948
Acute sinusitis (x1.02) 0.949
Degeneration of intervertebral disc (x1.02) 0.949
zolpidem (+0.00185) 0.950
Carbon dioxide, total [mol/V] in SP (+0.126) 0.950
tramadol (+0.00184) 0.951
Injury of free lower limb (x1.02) 0.952
Systolic blood pressure (+0.604) 0.952
Gastroesophageal reflux disease without esophagitis (x1.02) 0.953
Erythrocytes [#/V] in B by AC (+0.02) 0.953
cyclobenzaprine (+0.00173) 0.954
Acute bronchitis (x1.02) 0.954
Nuclear senile cataract (x1.02) 0.955
Depressive disorder (x1.02) 0.955
Obesity (x1.02) 0.956
Joint pain of pelvic region (x1.02) 0.956
Tear film insufficiency (x1.02) 0.957
Carpal tunnel syndrome (x1.02) 0.957
Current tear of medial cartilage AND/OR meniscus of knee (x1.02) 0.957
Arthropathy of knee joint (x1.02) 0.958
Respiratory rate (-0.172) 0.958
ibuprofen (+0.00141) 0.958
Peripheral neuritis (x1.02) 0.959
Derangement of knee (x1.02) 0.959
naproxen (+0.00139) 0.960
Borderline normal ECG (x1.01) 0.960
Actinic keratosis (x1.01) 0.960
Disorder of muscle (x1.01) 0.961
Insomnia (x1.01) 0.961
Spinal stenosis of lumbar region (x1.01) 0.961
Edema (x1.01) 0.961
Senile hyperkeratosis (x1.01) 0.962
Derangement of lateral meniscus (x1.01) 0.962
Closed fracture of one rib (x1.01) 0.962
Closed fracture of upper end of tibia (x1.01) 0.963
Malaise (x1.01) 0.963
Morbid obesity (x1.01) 0.963
Acute postoperative pain (x1.01) 0.964
Plantar fascial fibromatosis (x1.01) 0.964
Prothrombin time (PT) (-0.0866) 0.964
ketorolac (+0.00121) 0.964
trazodone (+0.0012) 0.965
Synovial plica (x1.01) 0.965
Atherosclerosis of coronary artery without angina pectoris (x1.01) 0.965
Headache (x1.01) 0.965
Derangement of posterior horn of medial meniscus (x1.01) 0.966
atorvastatin (+0.00115) 0.966
etodolac (+0.00115) 0.966
Localized osteoarthrosis uncertain if primary OR secondary (x1.01) 0.966
Type 2 diabetes mellitus without complication (x1.01) 0.967
Soft tissue injury (x1.01) 0.967
Arthropathy (x1.01) 0.967
metformin (+0.0011) 0.967
Lumbosacral spondylosis without myelopathy (x1.01) 0.967
Sodium [mol/V] in SP (+0.055) 0.968
Palpitations (x1.01) 0.968
Acquired trigger finger (x1.01) 0.968
Expressions
−0.8 −0.6 −0.4 −0.2 0.0 0.2 0.4 0.6 0.8

Signature [107]; 320 To Reach 0.975, (100 Plotted)

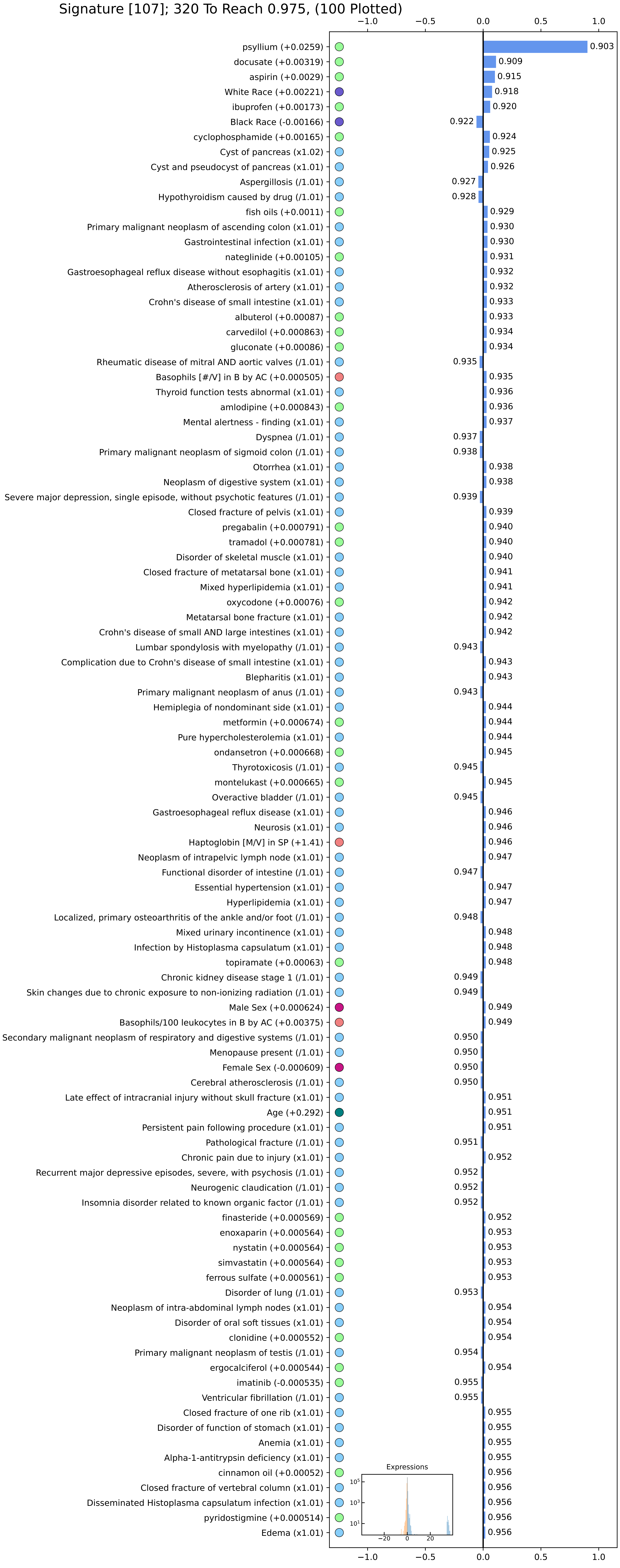

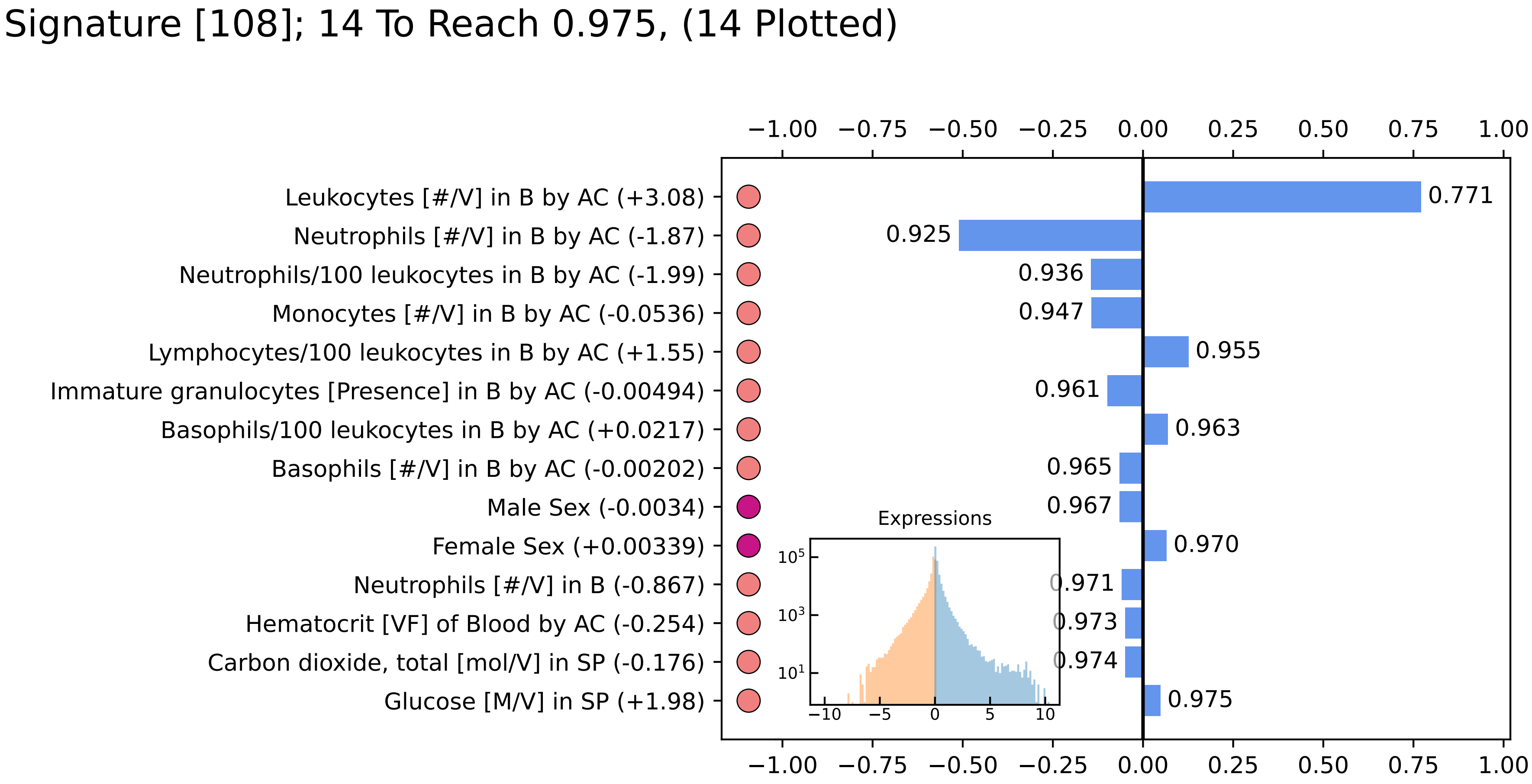

Signature [108]; 14 To Reach 0.975, (14 Plotted)
Leukocytes [#/V] in B by AC (+3.08)
Neutrophils [#/V] in B by AC (-1.87)
Neutrophils/100 leukocytes in B by AC (-1.99)
Monocytes [#/V] in B by AC (-0.0536)
Lymphocytes/100 leukocytes in B by AC (+1.55)
Immature granulocytes [Presence] in B by AC (-0.00494)
Basophils/100 leukocytes in B by AC (+0.0217)
Basophils [#/V] in B by AC (-0.00202)
Male Sex (-0.0034)
Female Sex (+0.00339)
Neutrophils [#/V] in B (-0.867)
Hematocrit [VF] of Blood by AC (-0.254)
Carbon dioxide, total [mol/V] in SP (-0.176)
Glucose [M/V] in SP (+1.98)
0.771
0.925
0.936
0.947
0.955
0.961
0.963
0.965
0.967
0.970
0.971
0.973
0.974
0.975
−1.00 −0.75 −0.50 −0.25 0.00 0.25 0.50 0.75 1.00
Expressions

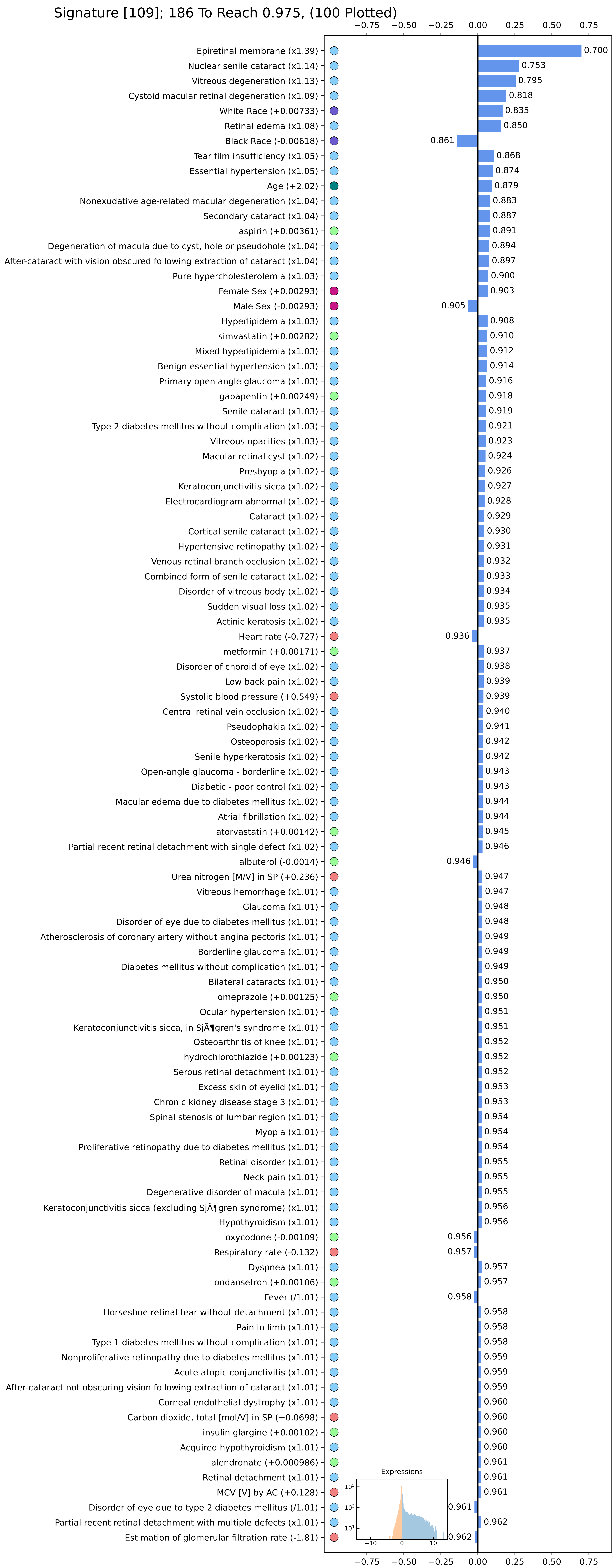

Signature [109]; 186 To Reach 0.975, (100 Plotted)
Epiretinal membrane (x1.39) 0.700
Nuclear senile cataract (x1.14) 0.753
Vitreous degeneration (x1.13) 0.795
Cystoid macular retinal degeneration (x1.09) 0.818
White Race (+0.00733) 0.835
Retinal edema (x1.08) 0.850
Black Race (-0.00618) 0.861
Tear film insufficiency (x1.05) 0.868
Essential hypertension (x1.05) 0.874
Age (+2.02) 0.879
Nonexudative age-related macular degeneration (x1.04) 0.883
Secondary cataract (x1.04) 0.887
aspirin (+0.00361) 0.891
Degeneration of macula due to cyst, hole or pseudohole (x1.04) 0.894
After-cataract with vision obscured following extraction of cataract (x1.04) 0.897
Pure hypercholesterolemia (x1.03) 0.900
Female Sex (+0.00293) 0.903
Male Sex (-0.00293) 0.905
Hyperlipidemia (x1.03) 0.908
simvastatin (+0.00282) 0.910
Mixed hyperlipidemia (x1.03) 0.912
Benign essential hypertension (x1.03) 0.914
Primary open angle glaucoma (x1.03) 0.916
gabapentin (+0.00249) 0.918
Senile cataract (x1.03) 0.919
Type 2 diabetes mellitus without complication (x1.03) 0.921
Vitreous opacities (x1.03) 0.923
Macular retinal cyst (x1.02) 0.924
Presbyopia (x1.02) 0.926
Keratoconjunctivitis sicca (x1.02) 0.927
Electrocardiogram abnormal (x1.02) 0.928
Cataract (x1.02) 0.929
Cortical senile cataract (x1.02) 0.930
Hypertensive retinopathy (x1.02) 0.931
Venous retinal branch occlusion (x1.02) 0.932
Combined form of senile cataract (x1.02) 0.933
Disorder of vitreous body (x1.02) 0.934
Sudden visual loss (x1.02) 0.935
Actinic keratosis (x1.02) 0.935
Heart rate (-0.727) 0.936
metformin (+0.00171) 0.937
Disorder of choroid of eye (x1.02) 0.938
Low back pain (x1.02) 0.939
Systolic blood pressure (+0.549) 0.939
Central retinal vein occlusion (x1.02) 0.940
Pseudophakia (x1.02) 0.941
Osteoporosis (x1.02) 0.942
Senile hyperkeratosis (x1.02) 0.942
Open-angle glaucoma - borderline (x1.02) 0.943
Diabetic - poor control (x1.02) 0.943
Macular edema due to diabetes mellitus (x1.02) 0.944
Atrial fibrillation (x1.02) 0.944
atorvastatin (+0.00142) 0.945
Partial recent retinal detachment with single defect (x1.02) 0.946
albuterol (-0.0014) 0.946
Urea nitrogen [M/V] in SP (+0.236) 0.947
Vitreous hemorrhage (x1.01) 0.947
Glaucoma (x1.01) 0.948
Disorder of eye due to diabetes mellitus (x1.01) 0.948
Atherosclerosis of coronary artery without angina pectoris (x1.01) 0.949
Borderline glaucoma (x1.01) 0.949
Diabetes mellitus without complication (x1.01) 0.949
Bilateral cataracts (x1.01) 0.950
omeprazole (+0.00125) 0.950
Ocular hypertension (x1.01) 0.951
Keratoconjunctivitis sicca, in SjÃ¶gren's syndrome (x1.01) 0.951
Osteoarthritis of knee (x1.01) 0.952
hydrochlorothiazide (+0.00123) 0.952
Serous retinal detachment (x1.01) 0.952
Excess skin of eyelid (x1.01) 0.953
Chronic kidney disease stage 3 (x1.01) 0.953
Spinal stenosis of lumbar region (x1.01) 0.954
Myopia (x1.01) 0.954
Proliferative retinopathy due to diabetes mellitus (x1.01) 0.954
Retinal disorder (x1.01) 0.955
Neck pain (x1.01) 0.955
Degenerative disorder of macula (x1.01) 0.955
Keratoconjunctivitis sicca (excluding SjÃ¶gren syndrome) (x1.01) 0.956
Hypothyroidism (x1.01) 0.956
oxycodone (-0.00109) 0.956
Respiratory rate (-0.132) 0.957
Dyspnea (x1.01) 0.957
ondansetron (+0.00106) 0.957
Fever (/1.01) 0.958
Horseshoe retinal tear without detachment (x1.01) 0.958
Pain in limb (x1.01) 0.958
Type 1 diabetes mellitus without complication (x1.01) 0.958
Nonproliferative retinopathy due to diabetes mellitus (x1.01) 0.959
Acute atopic conjunctivitis (x1.01) 0.959
After-cataract not obscuring vision following extraction of cataract (x1.01) 0.959
Corneal endothelial dystrophy (x1.01) 0.960
Carbon dioxide, total [mol/V] in SP (+0.0698) 0.960
insulin glargine (+0.00102) 0.960
Acquired hypothyroidism (x1.01) 0.960
alendronate (+0.000986) 0.961
Retinal detachment (x1.01) 0.961
MCV [V] by AC (+0.128) 0.961
Disorder of eye due to type 2 diabetes mellitus (/1.01) 0.961
Partial recent retinal detachment with multiple defects (x1.01) 0.962
Estimation of glomerular filtration rate (-1.81) 0.962
−0.75 −0.50 −0.25 0.00 0.25 0.50 0.75
Expressions

Signature [110]; 1 To Reach 0.975, (10 Plotted)

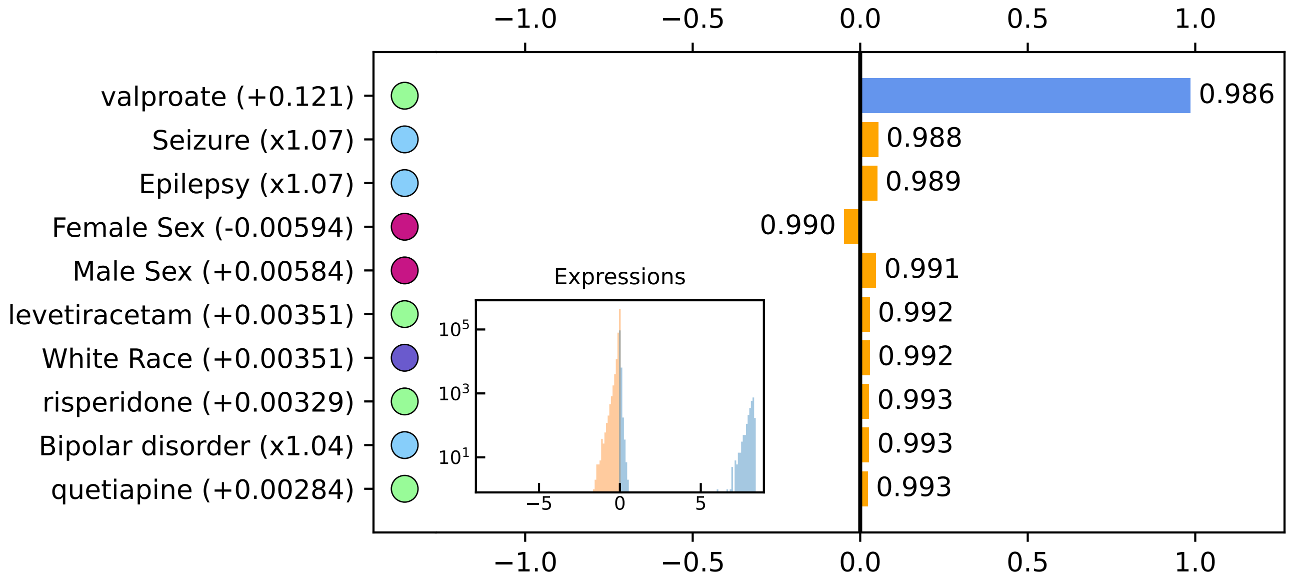

Signature [111]; 1 To Reach 0.975, (10 Plotted)

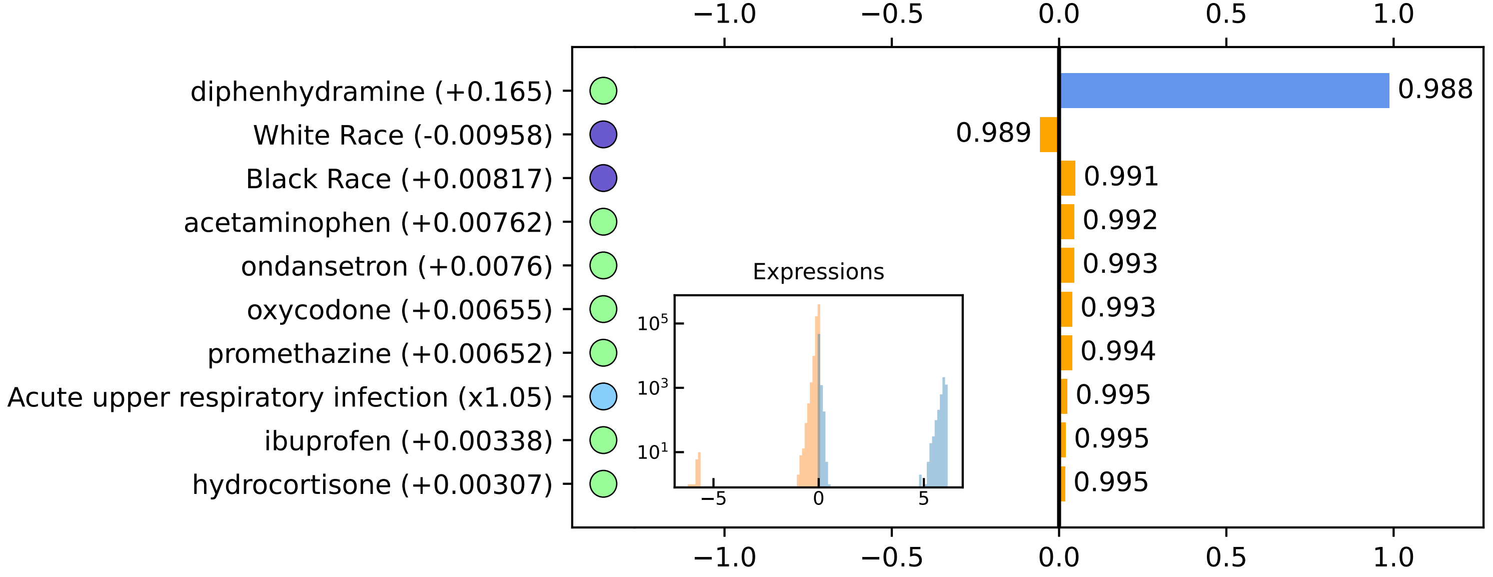

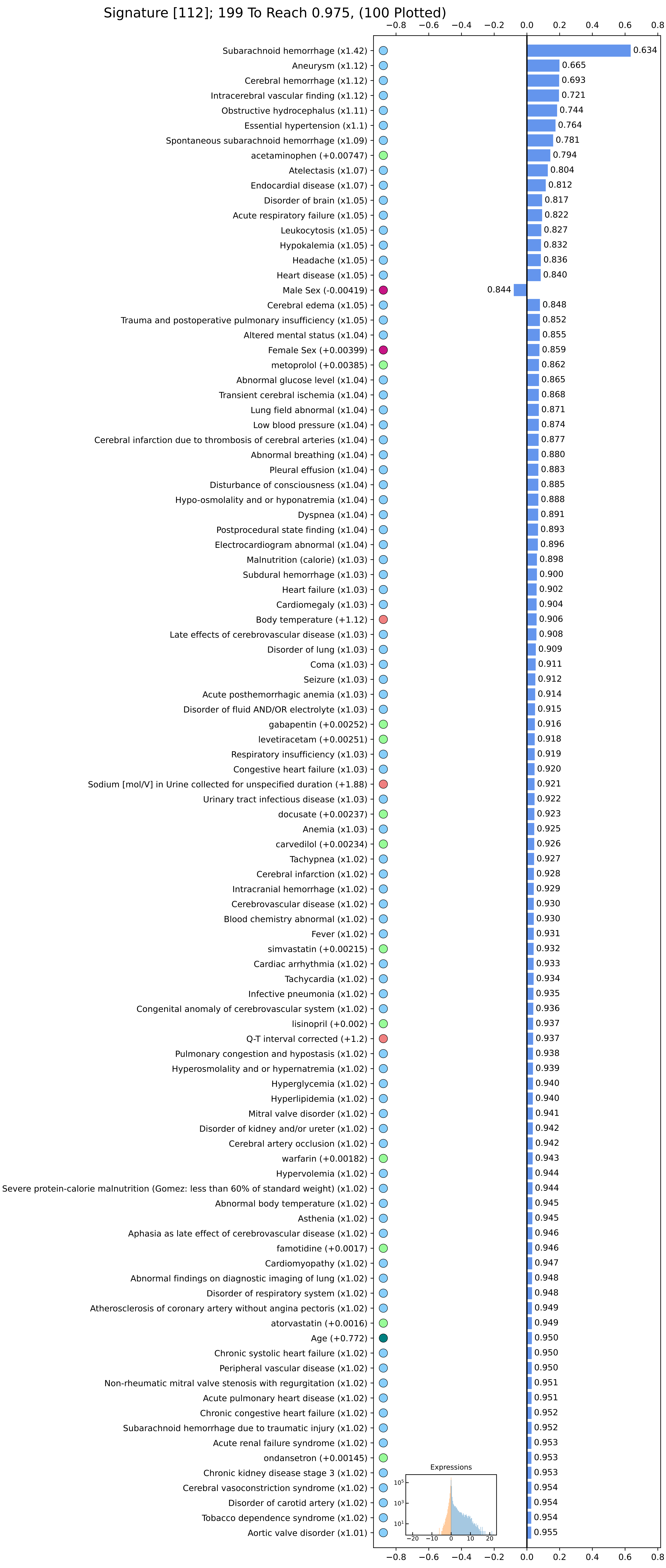

Signature [112]; 199 To Reach 0.975, (100 Plotted)
Subarachnoid hemorrhage (x1.42) 0.634
Aneurysm (x1.12) 0.665
Cerebral hemorrhage (x1.12) 0.693
Intracerebral vascular finding (x1.12) 0.721
Obstructive hydrocephalus (x1.11) 0.744
Essential hypertension (x1.1) 0.764
Spontaneous subarachnoid hemorrhage (x1.09) 0.781
acetaminophen (+0.00747) 0.794
Atelectasis (x1.07) 0.804
Endocardial disease (x1.07) 0.812
Disorder of brain (x1.05) 0.817
Acute respiratory failure (x1.05) 0.822
Leukocytosis (x1.05) 0.827
Hypokalemia (x1.05) 0.832
Headache (x1.05) 0.836
Heart disease (x1.05) 0.840
Male Sex (-0.00419) 0.844
Cerebral edema (x1.05) 0.848
Trauma and postoperative pulmonary insufficiency (x1.05) 0.852
Altered mental status (x1.04) 0.855
Female Sex (+0.00399) 0.859
metoprolol (+0.00385) 0.862
Abnormal glucose level (x1.04) 0.865
Transient cerebral ischemia (x1.04) 0.868
Lung field abnormal (x1.04) 0.871
Low blood pressure (x1.04) 0.874
Cerebral infarction due to thrombosis of cerebral arteries (x1.04) 0.877
Abnormal breathing (x1.04) 0.880
Pleural effusion (x1.04) 0.883
Disturbance of consciousness (x1.04) 0.885
Hypo-osmolality and or hyponatremia (x1.04) 0.888
Dyspnea (x1.04) 0.891
Postprocedural state finding (x1.04) 0.893
Electrocardiogram abnormal (x1.04) 0.896
Malnutrition (calorie) (x1.03) 0.898
Subdural hemorrhage (x1.03) 0.900
Heart failure (x1.03) 0.902
Cardiomegaly (x1.03) 0.904
Body temperature (+1.12) 0.906
Late effects of cerebrovascular disease (x1.03) 0.908
Disorder of lung (x1.03) 0.909
Coma (x1.03) 0.911
Seizure (x1.03) 0.912
Acute posthemorrhagic anemia (x1.03) 0.914
Disorder of fluid AND/OR electrolyte (x1.03) 0.915
gabapentin (+0.00252) 0.916
levetiracetam (+0.00251) 0.918
Respiratory insufficiency (x1.03) 0.919
Congestive heart failure (x1.03) 0.920
Sodium [mol/V] in Urine collected for unspecified duration (+1.88) 0.921
Urinary tract infectious disease (x1.03) 0.922
docusate (+0.00237) 0.923
Anemia (x1.03) 0.925
carvedilol (+0.00234) 0.926
Tachypnea (x1.02) 0.927
Cerebral infarction (x1.02) 0.928
Intracranial hemorrhage (x1.02) 0.929
Cerebrovascular disease (x1.02) 0.930
Blood chemistry abnormal (x1.02) 0.930
Fever (x1.02) 0.931
simvastatin (+0.00215) 0.932
Cardiac arrhythmia (x1.02) 0.933
Tachycardia (x1.02) 0.934
Infective pneumonia (x1.02) 0.935
Congenital anomaly of cerebrovascular system (x1.02) 0.936
lisinopril (+0.002) 0.937
Q-T interval corrected (+1.2) 0.937
Pulmonary congestion and hypostasis (x1.02) 0.938
Hyperosmolality and or hypernatremia (x1.02) 0.939
Hyperglycemia (x1.02) 0.940
Hyperlipidemia (x1.02) 0.940
Mitral valve disorder (x1.02) 0.941
Disorder of kidney and/or ureter (x1.02) 0.942
Cerebral artery occlusion (x1.02) 0.942
warfarin (+0.00182) 0.943
Hypervolemia (x1.02) 0.944
Severe protein-calorie malnutrition (Gomez: less than 60% of standard weight) (x1.02) 0.944
Abnormal body temperature (x1.02) 0.945
Asthenia (x1.02) 0.945
Aphasia as late effect of cerebrovascular disease (x1.02) 0.946
famotidine (+0.0017) 0.946
Cardiomyopathy (x1.02) 0.947
Abnormal findings on diagnostic imaging of lung (x1.02) 0.948
Disorder of respiratory system (x1.02) 0.948
Atherosclerosis of coronary artery without angina pectoris (x1.02) 0.949
atorvastatin (+0.0016) 0.949
Age (+0.772) 0.950
Chronic systolic heart failure (x1.02) 0.950
Peripheral vascular disease (x1.02) 0.950
Non-rheumatic mitral valve stenosis with regurgitation (x1.02) 0.951
Acute pulmonary heart disease (x1.02) 0.951
Chronic congestive heart failure (x1.02) 0.952
Subarachnoid hemorrhage due to traumatic injury (x1.02) 0.952
Acute renal failure syndrome (x1.02) 0.953
ondansetron (+0.00145) 0.953
Chronic kidney disease stage 3 (x1.02) 0.953
Cerebral vasoconstriction syndrome (x1.02) 0.954
Disorder of carotid artery (x1.02) 0.954
Tobacco dependence syndrome (x1.02) 0.954
Aortic valve disorder (x1.01) 0.955
−0.8 −0.6 −0.4 −0.2 0.0 0.2 0.4 0.6 0.8
Expressions
−20 −10 0 10 20

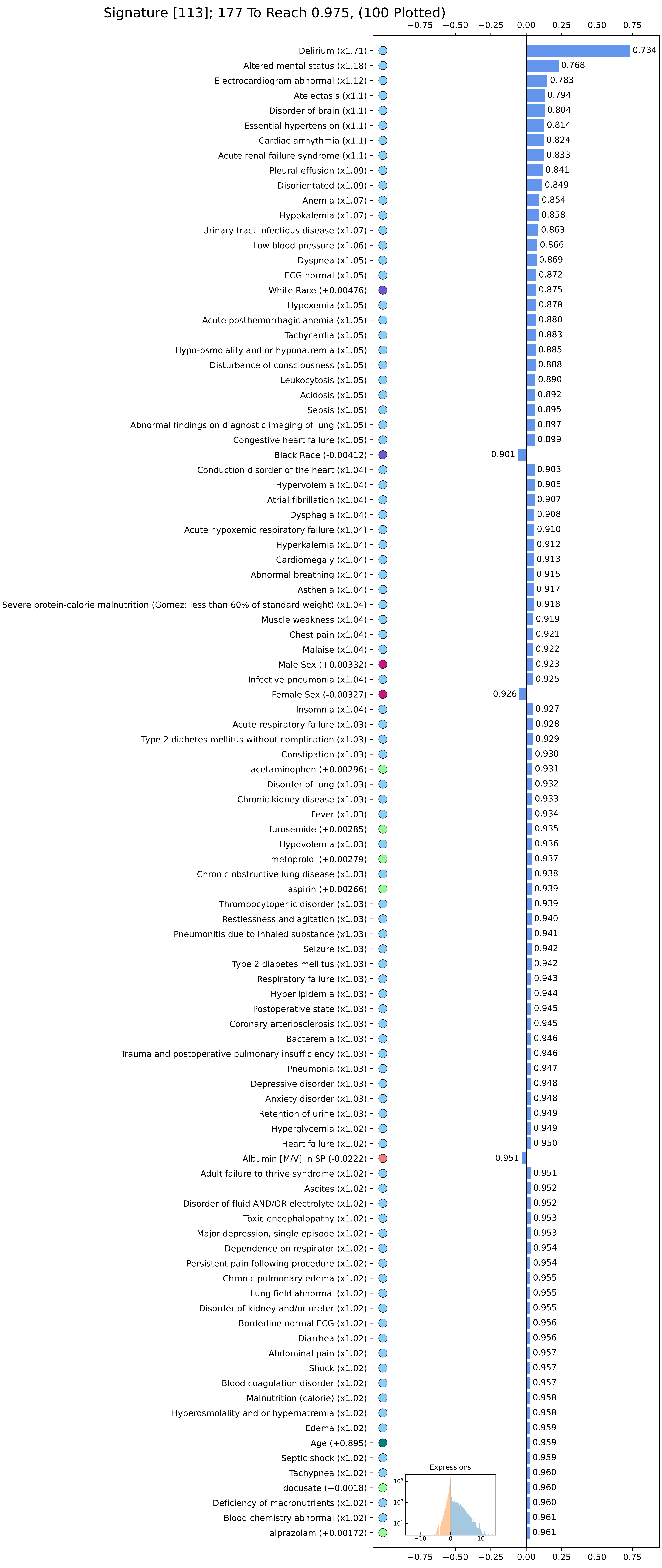
Signature [113]; 177 To Reach 0.975, (100 Plotted)
Delirium (x1.71) 0.734
Altered mental status (x1.18) 0.768
Electrocardiogram abnormal (x1.12) 0.783
Atelectasis (x1.1) 0.794
Disorder of brain (x1.1) 0.804
Essential hypertension (x1.1) 0.814
Cardiac arrhythmia (x1.1) 0.824
Acute renal failure syndrome (x1.1) 0.833
Pleural effusion (x1.09) 0.841
Disorientated (x1.09) 0.849
Anemia (x1.07) 0.854
Hypokalemia (x1.07) 0.858
Urinary tract infectious disease (x1.07) 0.863
Low blood pressure (x1.06) 0.866
Dyspnea (x1.05) 0.869
ECG normal (x1.05) 0.872
White Race (+0.00476) 0.875
Hypoxemia (x1.05) 0.878
Acute posthemorrhagic anemia (x1.05) 0.880
Tachycardia (x1.05) 0.883
Hypo-osmolality and or hyponatremia (x1.05) 0.885
Disturbance of consciousness (x1.05) 0.888
Leukocytosis (x1.05) 0.890
Acidosis (x1.05) 0.892
Sepsis (x1.05) 0.895
Abnormal findings on diagnostic imaging of lung (x1.05) 0.897
Congestive heart failure (x1.05) 0.899
Black Race (-0.00412) 0.901
Conduction disorder of the heart (x1.04) 0.903
Hypervolemia (x1.04) 0.905
Atrial fibrillation (x1.04) 0.907
Dysphagia (x1.04) 0.908
Acute hypoxemic respiratory failure (x1.04) 0.910
Hyperkalemia (x1.04) 0.912
Cardiomegaly (x1.04) 0.913
Abnormal breathing (x1.04) 0.915
Asthenia (x1.04) 0.917
Severe protein-calorie malnutrition (Gomez: less than 60% of standard weight) (x1.04) 0.918
Muscle weakness (x1.04) 0.919
Chest pain (x1.04) 0.921
Malaise (x1.04) 0.922
Male Sex (+0.00332) 0.923
Infective pneumonia (x1.04) 0.925
Female Sex (-0.00327) 0.926
Insomnia (x1.04) 0.927
Acute respiratory failure (x1.03) 0.928
Type 2 diabetes mellitus without complication (x1.03) 0.929
Constipation (x1.03) 0.930
acetaminophen (+0.00296) 0.931
Disorder of lung (x1.03) 0.932
Chronic kidney disease (x1.03) 0.933
Fever (x1.03) 0.934
furosemide (+0.00285) 0.935
Hypovolemia (x1.03) 0.936
metoprolol (+0.00279) 0.937
Chronic obstructive lung disease (x1.03) 0.938
aspirin (+0.00266) 0.939
Thrombocytopenic disorder (x1.03) 0.939
Restlessness and agitation (x1.03) 0.940
Pneumonitis due to inhaled substance (x1.03) 0.941
Seizure (x1.03) 0.942
Type 2 diabetes mellitus (x1.03) 0.942
Respiratory failure (x1.03) 0.943
Hyperlipidemia (x1.03) 0.944
Postoperative state (x1.03) 0.945
Coronary arteriosclerosis (x1.03) 0.945
Bacteremia (x1.03) 0.946
Trauma and postoperative pulmonary insufficiency (x1.03) 0.946
Pneumonia (x1.03) 0.947
Depressive disorder (x1.03) 0.948
Anxiety disorder (x1.03) 0.948
Retention of urine (x1.03) 0.949
Hyperglycemia (x1.02) 0.949
Heart failure (x1.02) 0.950
Albumin [M/V] in SP (-0.0222) 0.951
Adult failure to thrive syndrome (x1.02) 0.951
Ascites (x1.02) 0.952
Disorder of fluid AND/OR electrolyte (x1.02) 0.952
Toxic encephalopathy (x1.02) 0.953
Major depression, single episode (x1.02) 0.953
Dependence on respirator (x1.02) 0.954
Persistent pain following procedure (x1.02) 0.954
Chronic pulmonary edema (x1.02) 0.955
Lung field abnormal (x1.02) 0.955
Disorder of kidney and/or ureter (x1.02) 0.955
Borderline normal ECG (x1.02) 0.956
Diarrhea (x1.02) 0.956
Abdominal pain (x1.02) 0.957
Shock (x1.02) 0.957
Blood coagulation disorder (x1.02) 0.957
Malnutrition (calorie) (x1.02) 0.958
Hyperosmolality and or hypernatremia (x1.02) 0.958
Edema (x1.02) 0.959
Age (+0.895) 0.959
Septic shock (x1.02) 0.959
Tachypnea (x1.02) 0.960
docusate (+0.0018) 0.960
Deficiency of macronutrients (x1.02) 0.960
Blood chemistry abnormal (x1.02) 0.961
alprazolam (+0.00172) 0.961
−0.75 −0.50 −0.25 0.00 0.25 0.50 0.75
Expressions
10⁵
10³
10¹
−10 0 10

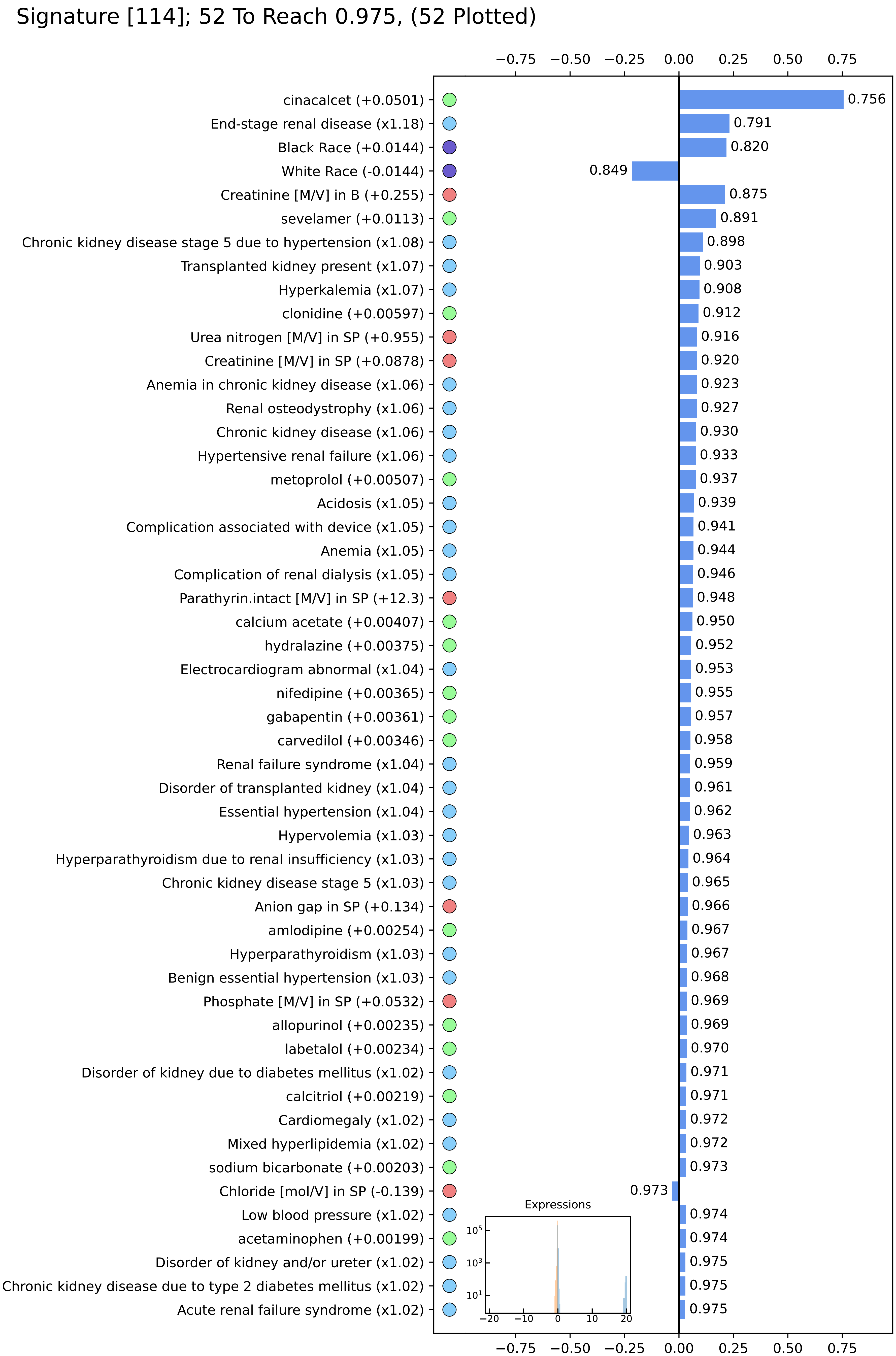
Signature [114]; 52 To Reach 0.975, (52 Plotted)
−0.75 −0.50 −0.25 0.00 0.25 0.50 0.75
cinacalcet (+0.0501) 0.756
End-stage renal disease (x1.18) 0.791
Black Race (+0.0144) 0.820
White Race (-0.0144) 0.849
Creatinine [M/V] in B (+0.255) 0.875
sevelamer (+0.0113) 0.891
Chronic kidney disease stage 5 due to hypertension (x1.08) 0.898
Transplanted kidney present (x1.07) 0.903
Hyperkalemia (x1.07) 0.908
clonidine (+0.00597) 0.912
Urea nitrogen [M/V] in SP (+0.955) 0.916
Creatinine [M/V] in SP (+0.0878) 0.920
Anemia in chronic kidney disease (x1.06) 0.923
Renal osteodystrophy (x1.06) 0.927
Chronic kidney disease (x1.06) 0.930
Hypertensive renal failure (x1.06) 0.933
metoprolol (+0.00507) 0.937
Acidosis (x1.05) 0.939
Complication associated with device (x1.05) 0.941
Anemia (x1.05) 0.944
Complication of renal dialysis (x1.05) 0.946
Parathyrin.intact [M/V] in SP (+12.3) 0.948
calcium acetate (+0.00407) 0.950
hydralazine (+0.00375) 0.952
Electrocardiogram abnormal (x1.04) 0.953
nifedipine (+0.00365) 0.955
gabapentin (+0.00361) 0.957
carvedilol (+0.00346) 0.958
Renal failure syndrome (x1.04) 0.959
Disorder of transplanted kidney (x1.04) 0.961
Essential hypertension (x1.04) 0.962
Hypervolemia (x1.03) 0.963
Hyperparathyroidism due to renal insufficiency (x1.03) 0.964
Chronic kidney disease stage 5 (x1.03) 0.965
Anion gap in SP (+0.134) 0.966
amlodipine (+0.00254) 0.967
Hyperparathyroidism (x1.03) 0.967
Benign essential hypertension (x1.03) 0.968
Phosphate [M/V] in SP (+0.0532) 0.969
allopurinol (+0.00235) 0.969
labetalol (+0.00234) 0.970
Disorder of kidney due to diabetes mellitus (x1.02) 0.971
calcitriol (+0.00219) 0.971
Cardiomegaly (x1.02) 0.972
Mixed hyperlipidemia (x1.02) 0.972
sodium bicarbonate (+0.00203) 0.973
Chloride [mol/V] in SP (-0.139) 0.973
Low blood pressure (x1.02) 0.974
acetaminophen (+0.00199) 0.974
Disorder of kidney and/or ureter (x1.02) 0.975
Chronic kidney disease due to type 2 diabetes mellitus (x1.02) 0.975
Acute renal failure syndrome (x1.02) 0.975
Expressions
10^5
10^3
10^1
−20 −10 0 10 20

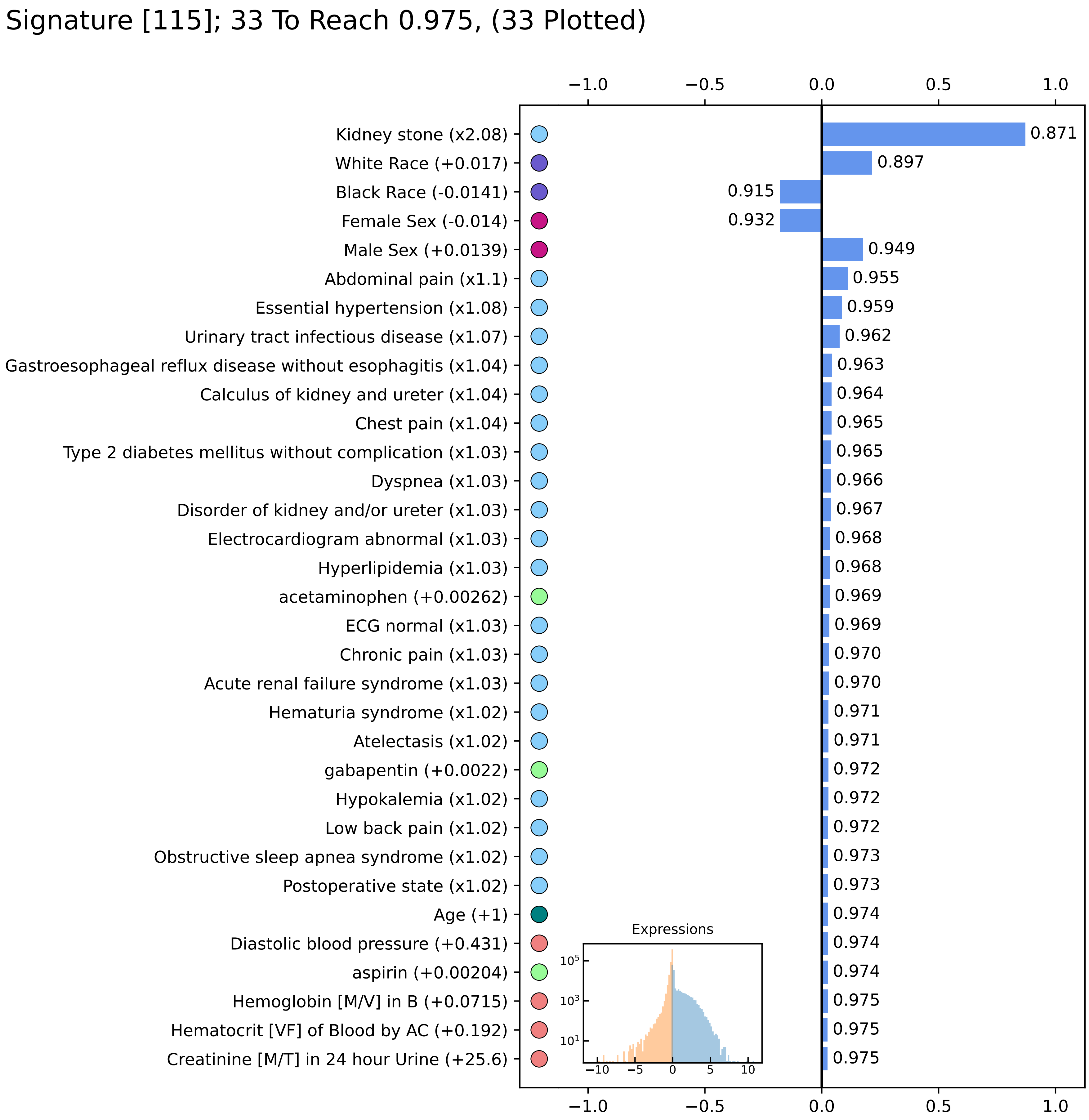

Signature [115]; 33 To Reach 0.975, (33 Plotted)
Kidney stone (x2.08) 0.871
White Race (+0.017) 0.897
Black Race (-0.0141) 0.915
Female Sex (-0.014) 0.932
Male Sex (+0.0139) 0.949
Abdominal pain (x1.1) 0.955
Essential hypertension (x1.08) 0.959
Urinary tract infectious disease (x1.07) 0.962
Gastroesophageal reflux disease without esophagitis (x1.04) 0.963
Calculus of kidney and ureter (x1.04) 0.964
Chest pain (x1.04) 0.965
Type 2 diabetes mellitus without complication (x1.03) 0.965
Dyspnea (x1.03) 0.966
Disorder of kidney and/or ureter (x1.03) 0.967
Electrocardiogram abnormal (x1.03) 0.968
Hyperlipidemia (x1.03) 0.968
acetaminophen (+0.00262) 0.969
ECG normal (x1.03) 0.969
Chronic pain (x1.03) 0.970
Acute renal failure syndrome (x1.03) 0.970
Hematuria syndrome (x1.02) 0.971
Atelectasis (x1.02) 0.971
gabapentin (+0.0022) 0.972
Hypokalemia (x1.02) 0.972
Low back pain (x1.02) 0.972
Obstructive sleep apnea syndrome (x1.02) 0.973
Postoperative state (x1.02) 0.973
Age (+1) 0.974
Diastolic blood pressure (+0.431) 0.974
aspirin (+0.00204) 0.974
Hemoglobin [M/V] in B (+0.0715) 0.975
Hematocrit [VF] of Blood by AC (+0.192) 0.975
Creatinine [M/T] in 24 hour Urine (+25.6) 0.975
−1.0 −0.5 0.0 0.5 1.0
Expressions

Signature [116]; 3 To Reach 0.975, (10 Plotted)

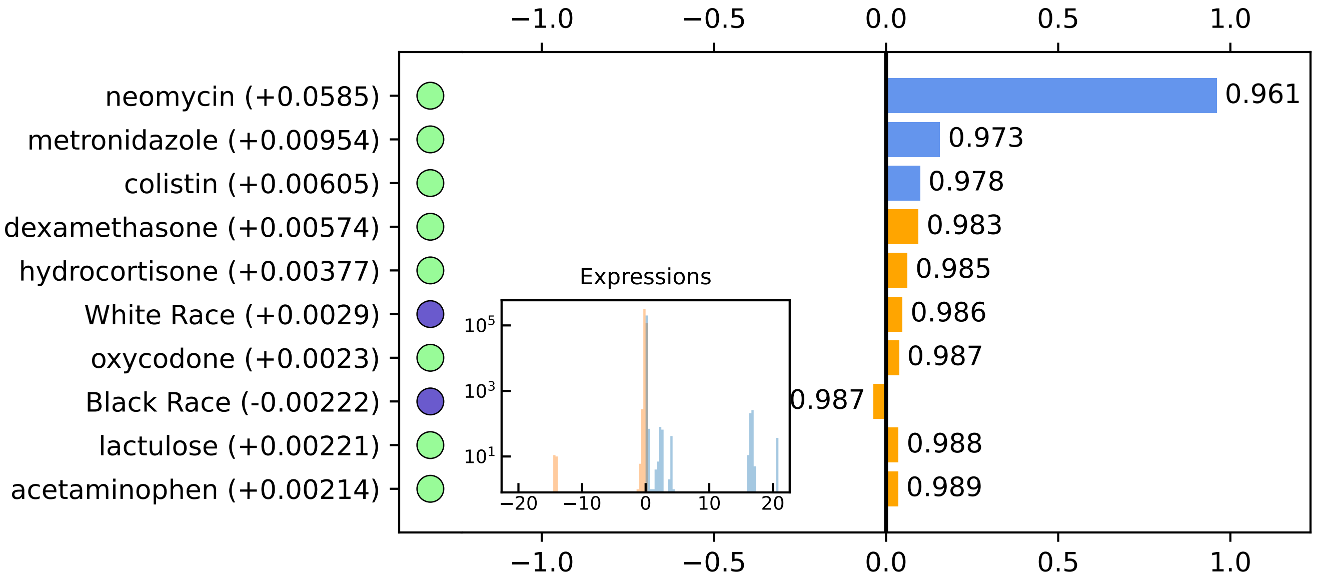

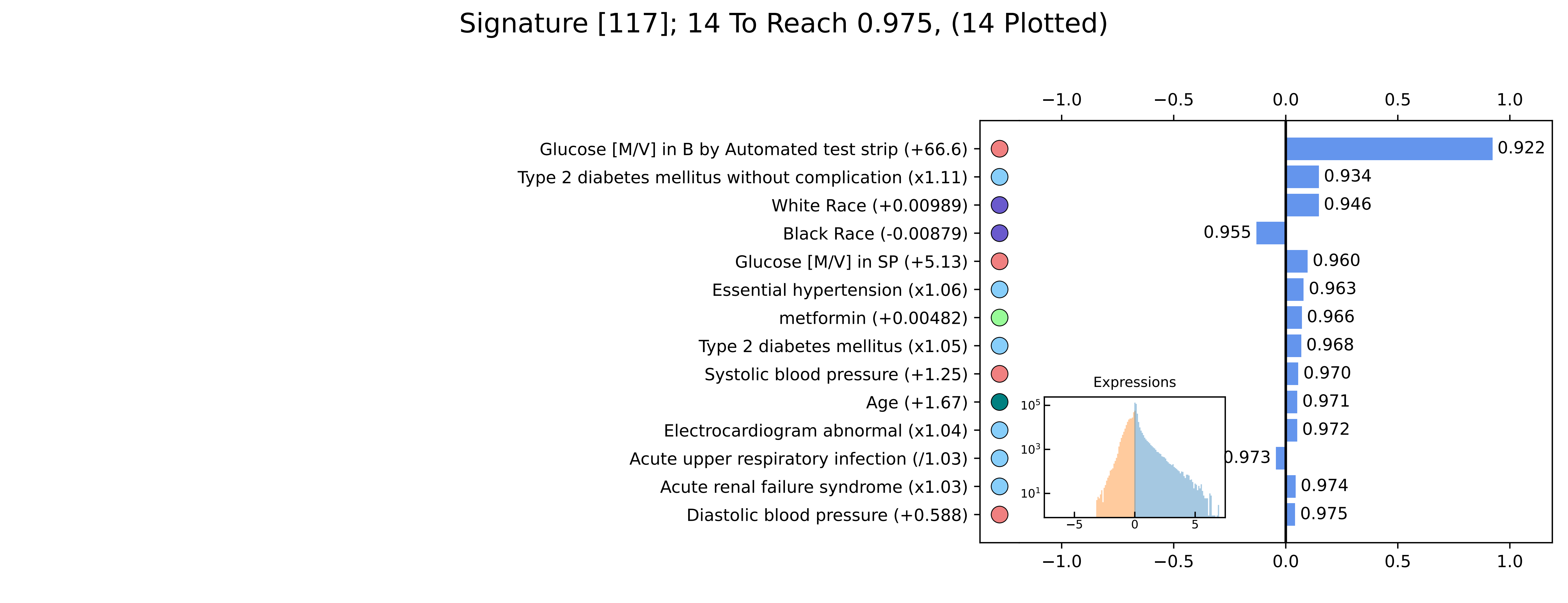

Signature [117]; 14 To Reach 0.975, (14 Plotted)
Glucose [M/V] in B by Automated test strip (+66.6)
Type 2 diabetes mellitus without complication (x1.11)
White Race (+0.00989)
Black Race (-0.00879)
Glucose [M/V] in SP (+5.13)
Essential hypertension (x1.06)
metformin (+0.00482)
Type 2 diabetes mellitus (x1.05)
Systolic blood pressure (+1.25)
Age (+1.67)
Electrocardiogram abnormal (x1.04)
Acute upper respiratory infection (/1.03)
Acute renal failure syndrome (x1.03)
Diastolic blood pressure (+0.588)
0.922
0.934
0.946
0.955
0.960
0.963
0.966
0.968
0.970
0.971
0.972
0.973
0.974
0.975
−1.0
−0.5
0.0
0.5
1.0
Expressions

Signature [118]; 3 To Reach 0.975, (10 Plotted)

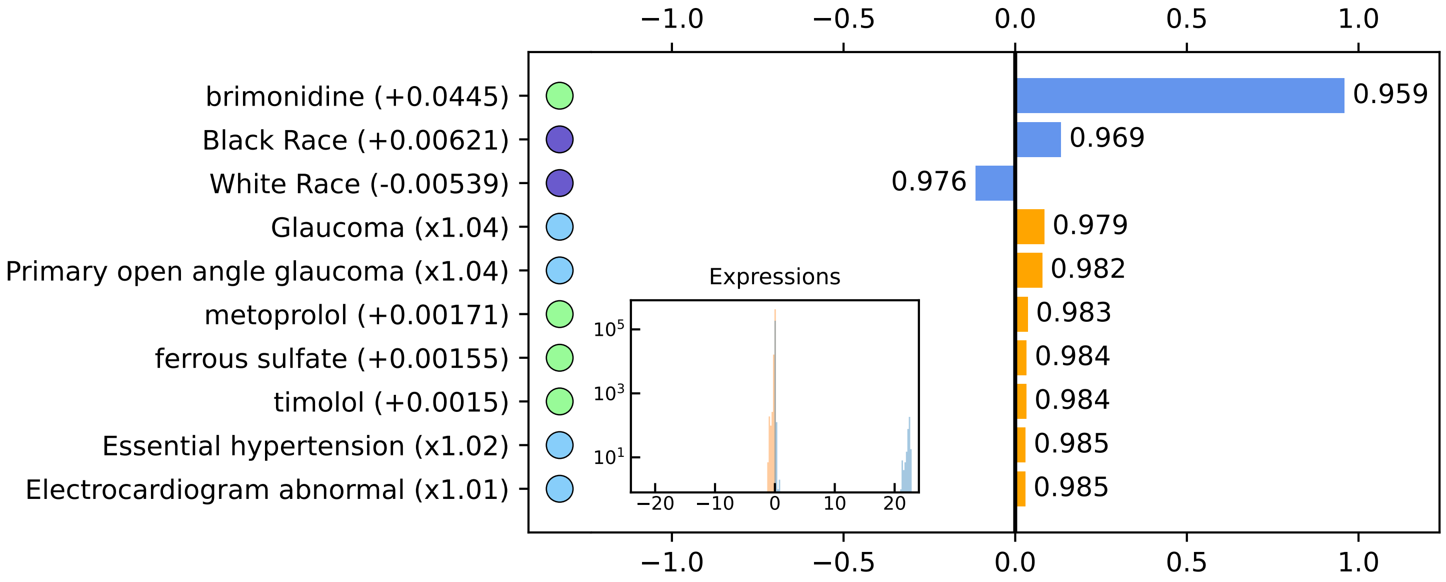

Signature [119]; 6 To Reach 0.975, (10 Plotted)

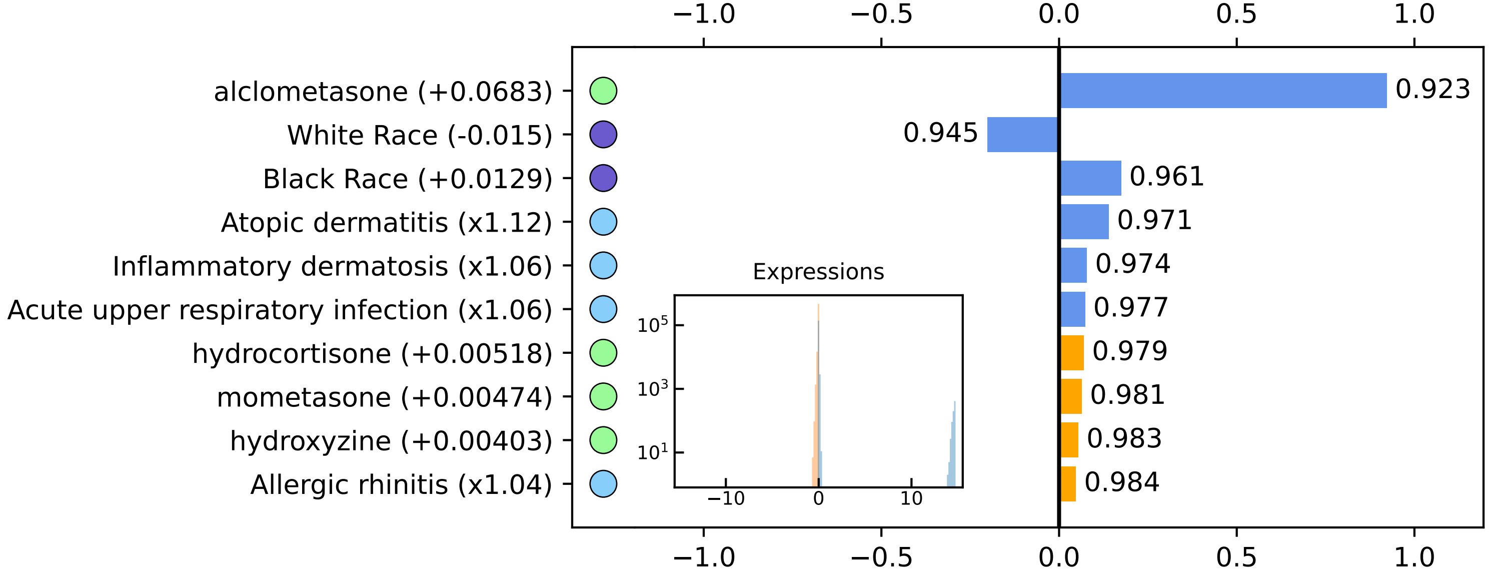

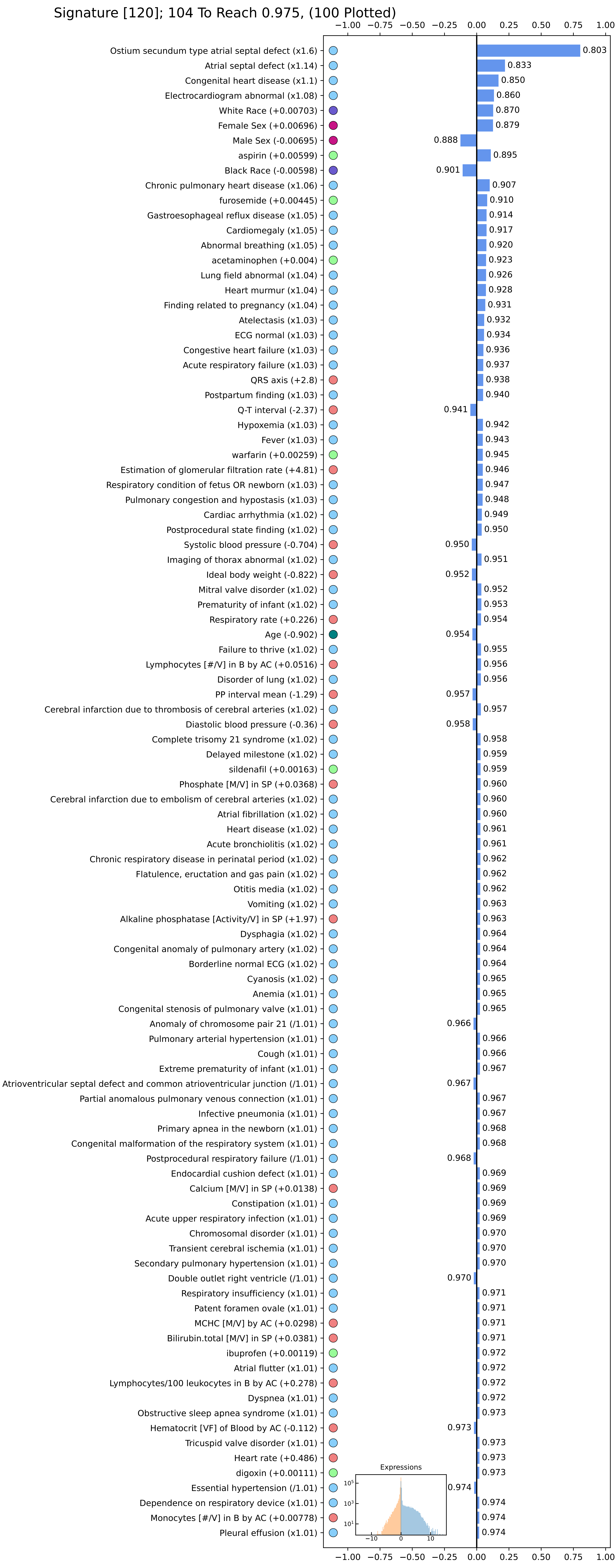
Signature [120]; 104 To Reach 0.975, (100 Plotted)
−1.00 −0.75 −0.50 −0.25 0.00 0.25 0.50 0.75 1.00
Ostium secundum type atrial septal defect (x1.6)
0.803
Atrial septal defect (x1.14)
0.833
Congenital heart disease (x1.1)
0.850
Electrocardiogram abnormal (x1.08)
0.860
White Race (+0.00703)
0.870
Female Sex (+0.00696)
0.879
Male Sex (-0.00695)
0.888
aspirin (+0.00599)
0.895
Black Race (-0.00598)
0.901
Chronic pulmonary heart disease (x1.06)
0.907
furosemide (+0.00445)
0.910
Gastroesophageal reflux disease (x1.05)
0.914
Cardiomegaly (x1.05)
0.917
Abnormal breathing (x1.05)
0.920
acetaminophen (+0.004)
0.923
Lung field abnormal (x1.04)
0.926
Heart murmur (x1.04)
0.928
Finding related to pregnancy (x1.04)
0.931
Atelectasis (x1.03)
0.932
ECG normal (x1.03)
0.934
Congestive heart failure (x1.03)
0.936
Acute respiratory failure (x1.03)
0.937
QRS axis (+2.8)
0.938
Postpartum finding (x1.03)
0.940
Q-T interval (-2.37)
0.941
Hypoxemia (x1.03)
0.942
Fever (x1.03)
0.943
warfarin (+0.00259)
0.945
Estimation of glomerular filtration rate (+4.81)
0.946
Respiratory condition of fetus OR newborn (x1.03)
0.947
Pulmonary congestion and hypostasis (x1.03)
0.948
Cardiac arrhythmia (x1.02)
0.949
Postprocedural state finding (x1.02)
0.950
Systolic blood pressure (-0.704)
0.950
Imaging of thorax abnormal (x1.02)
0.951
Ideal body weight (-0.822)
0.952
Mitral valve disorder (x1.02)
0.952
Prematurity of infant (x1.02)
0.953
Respiratory rate (+0.226)
0.954
Age (-0.902)
0.954
Failure to thrive (x1.02)
0.955
Lymphocytes [#/V] in B by AC (+0.0516)
0.956
Disorder of lung (x1.02)
0.956
PP interval mean (-1.29)
0.957
Cerebral infarction due to thrombosis of cerebral arteries (x1.02)
0.957
Diastolic blood pressure (-0.36)
0.958
Complete trisomy 21 syndrome (x1.02)
0.958
Delayed milestone (x1.02)
0.959
sildenafil (+0.00163)
0.959
Phosphate [M/V] in SP (+0.0368)
0.960
Cerebral infarction due to embolism of cerebral arteries (x1.02)
0.960
Atrial fibrillation (x1.02)
0.960
Heart disease (x1.02)
0.961
Acute bronchiolitis (x1.02)
0.961
Chronic respiratory disease in perinatal period (x1.02)
0.962
Flatulence, eructation and gas pain (x1.02)
0.962
Otitis media (x1.02)
0.962
Vomiting (x1.02)
0.963
Alkaline phosphatase [Activity/V] in SP (+1.97)
0.963
Dysphagia (x1.02)
0.964
Congenital anomaly of pulmonary artery (x1.02)
0.964
Borderline normal ECG (x1.02)
0.964
Cyanosis (x1.02)
0.965
Anemia (x1.01)
0.965
Congenital stenosis of pulmonary valve (x1.01)
0.965
Anomaly of chromosome pair 21 (/1.01)
0.966
Pulmonary arterial hypertension (x1.01)
0.966
Cough (x1.01)
0.966
Extreme prematurity of infant (x1.01)
0.967
Atrioventricular septal defect and common atrioventricular junction (/1.01)
0.967
Partial anomalous pulmonary venous connection (x1.01)
0.967
Infective pneumonia (x1.01)
0.967
Primary apnea in the newborn (x1.01)
0.968
Congenital malformation of the respiratory system (x1.01)
0.968
Postprocedural respiratory failure (/1.01)
0.968
Endocardial cushion defect (x1.01)
0.969
Calcium [M/V] in SP (+0.0138)
0.969
Constipation (x1.01)
0.969
Acute upper respiratory infection (x1.01)
0.969
Chromosomal disorder (x1.01)
0.970
Transient cerebral ischemia (x1.01)
0.970
Secondary pulmonary hypertension (x1.01)
0.970
Double outlet right ventricle (/1.01)
0.970
Respiratory insufficiency (x1.01)
0.971
Patent foramen ovale (x1.01)
0.971
MCHC [M/V] by AC (+0.0298)
0.971
Bilirubin.total [M/V] in SP (+0.0381)
0.971
ibuprofen (+0.00119)
0.972
Atrial flutter (x1.01)
0.972
Lymphocytes/100 leukocytes in B by AC (+0.278)
0.972
Dyspnea (x1.01)
0.972
Obstructive sleep apnea syndrome (x1.01)
0.973
Hematocrit [VF] of Blood by AC (-0.112)
0.973
Tricuspid valve disorder (x1.01)
0.973
Heart rate (+0.486)
0.973
digoxin (+0.00111)
0.973
Essential hypertension (/1.01)
0.974
Dependence on respiratory device (x1.01)
0.974
Monocytes [#/V] in B by AC (+0.00778)
0.974
Pleural effusion (x1.01)
0.974
Expressions
−10 0 10
−1.00 −0.75 −0.50 −0.25 0.00 0.25 0.50 0.75 1.00

Signature [121]; 1 To Reach 0.975, (10 Plotted)

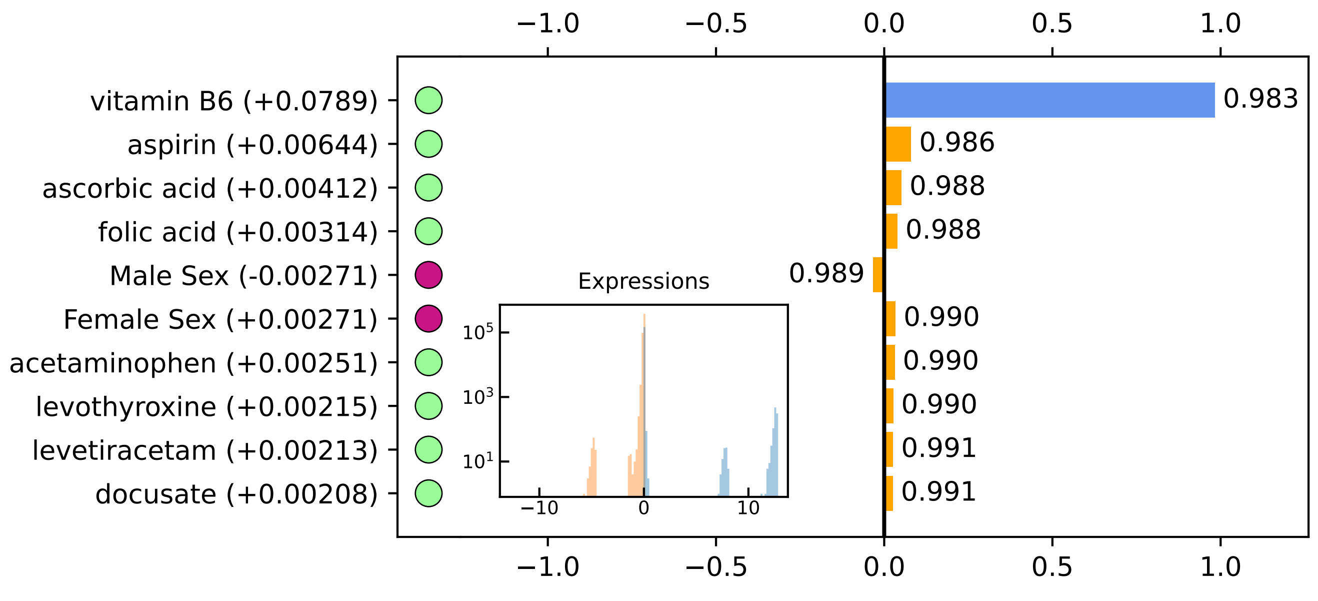

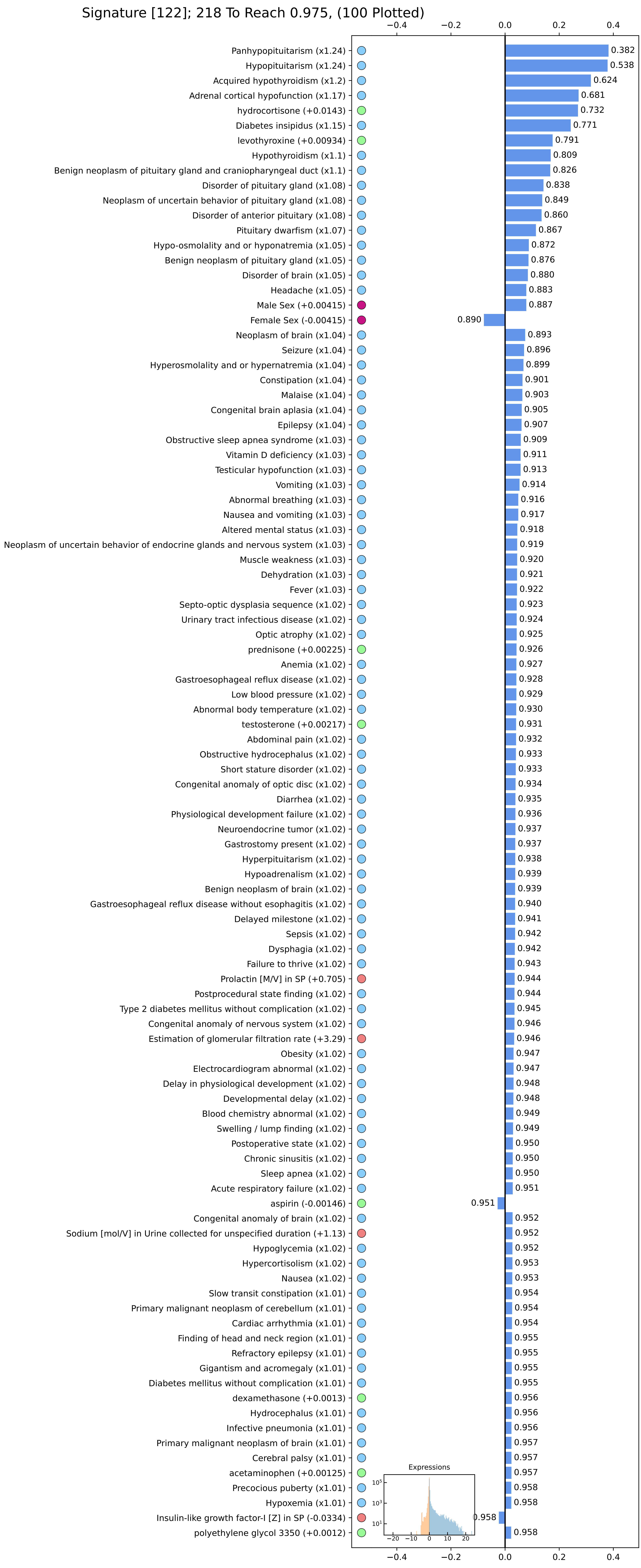

Signature [122]; 218 To Reach 0.975, (100 Plotted)
−0.4
−0.2
0.0
0.2
0.4
Panhypopituitarism (x1.24) 0.382
Hypopituitarism (x1.24) 0.538
Acquired hypothyroidism (x1.2) 0.624
Adrenal cortical hypofunction (x1.17) 0.681
hydrocortisone (+0.0143) 0.732
Diabetes insipidus (x1.15) 0.771
levothyroxine (+0.00934) 0.791
Hypothyroidism (x1.1) 0.809
Benign neoplasm of pituitary gland and craniopharyngeal duct (x1.1) 0.826
Disorder of pituitary gland (x1.08) 0.838
Neoplasm of uncertain behavior of pituitary gland (x1.08) 0.849
Disorder of anterior pituitary (x1.08) 0.860
Pituitary dwarfism (x1.07) 0.867
Hypo-osmolality and or hyponatremia (x1.05) 0.872
Benign neoplasm of pituitary gland (x1.05) 0.876
Disorder of brain (x1.05) 0.880
Headache (x1.05) 0.883
Male Sex (+0.00415) 0.887
Female Sex (-0.00415) 0.890
Neoplasm of brain (x1.04) 0.893
Seizure (x1.04) 0.896
Hyperosmolality and or hypernatremia (x1.04) 0.899
Constipation (x1.04) 0.901
Malaise (x1.04) 0.903
Congenital brain aplasia (x1.04) 0.905
Epilepsy (x1.04) 0.907
Obstructive sleep apnea syndrome (x1.03) 0.909
Vitamin D deficiency (x1.03) 0.911
Testicular hypofunction (x1.03) 0.913
Vomiting (x1.03) 0.914
Abnormal breathing (x1.03) 0.916
Nausea and vomiting (x1.03) 0.917
Altered mental status (x1.03) 0.918
Neoplasm of uncertain behavior of endocrine glands and nervous system (x1.03) 0.919
Muscle weakness (x1.03) 0.920
Dehydration (x1.03) 0.921
Fever (x1.03) 0.922
Septo-optic dysplasia sequence (x1.02) 0.923
Urinary tract infectious disease (x1.02) 0.924
Optic atrophy (x1.02) 0.925
prednisone (+0.00225) 0.926
Anemia (x1.02) 0.927
Gastroesophageal reflux disease (x1.02) 0.928
Low blood pressure (x1.02) 0.929
Abnormal body temperature (x1.02) 0.930
testosterone (+0.00217) 0.931
Abdominal pain (x1.02) 0.932
Obstructive hydrocephalus (x1.02) 0.933
Short stature disorder (x1.02) 0.933
Congenital anomaly of optic disc (x1.02) 0.934
Diarrhea (x1.02) 0.935
Physiological development failure (x1.02) 0.936
Neuroendocrine tumor (x1.02) 0.937
Gastrostomy present (x1.02) 0.937
Hyperpituitarism (x1.02) 0.938
Hypoadrenalism (x1.02) 0.939
Benign neoplasm of brain (x1.02) 0.939
Gastroesophageal reflux disease without esophagitis (x1.02) 0.940
Delayed milestone (x1.02) 0.941
Sepsis (x1.02) 0.942
Dysphagia (x1.02) 0.942
Failure to thrive (x1.02) 0.943
Prolactin [M/V] in SP (+0.705) 0.944
Postprocedural state finding (x1.02) 0.944
Type 2 diabetes mellitus without complication (x1.02) 0.945
Congenital anomaly of nervous system (x1.02) 0.946
Estimation of glomerular filtration rate (+3.29) 0.946
Obesity (x1.02) 0.947
Electrocardiogram abnormal (x1.02) 0.947
Delay in physiological development (x1.02) 0.948
Developmental delay (x1.02) 0.948
Blood chemistry abnormal (x1.02) 0.949
Swelling / lump finding (x1.02) 0.949
Postoperative state (x1.02) 0.950
Chronic sinusitis (x1.02) 0.950
Sleep apnea (x1.02) 0.950
Acute respiratory failure (x1.02) 0.951
aspirin (-0.00146) 0.951
Congenital anomaly of brain (x1.02) 0.952
Sodium [mol/V] in Urine collected for unspecified duration (+1.13) 0.952
Hypoglycemia (x1.02) 0.952
Hypercortisolism (x1.02) 0.953
Nausea (x1.02) 0.953
Slow transit constipation (x1.01) 0.954
Primary malignant neoplasm of cerebellum (x1.01) 0.954
Cardiac arrhythmia (x1.01) 0.954
Finding of head and neck region (x1.01) 0.955
Refractory epilepsy (x1.01) 0.955
Gigantism and acromegaly (x1.01) 0.955
Diabetes mellitus without complication (x1.01) 0.955
dexamethasone (+0.0013) 0.956
Hydrocephalus (x1.01) 0.956
Infective pneumonia (x1.01) 0.956
Primary malignant neoplasm of brain (x1.01) 0.957
Cerebral palsy (x1.01) 0.957
acetaminophen (+0.00125) 0.957
Precocious puberty (x1.01) 0.958
Hypoxemia (x1.01) 0.958
Insulin-like growth factor-I [Z] in SP (-0.0334) 0.958
polyethylene glycol 3350 (+0.0012) 0.958
Expressions
10^5
10^3
10^1
−20
−10
0
10
20

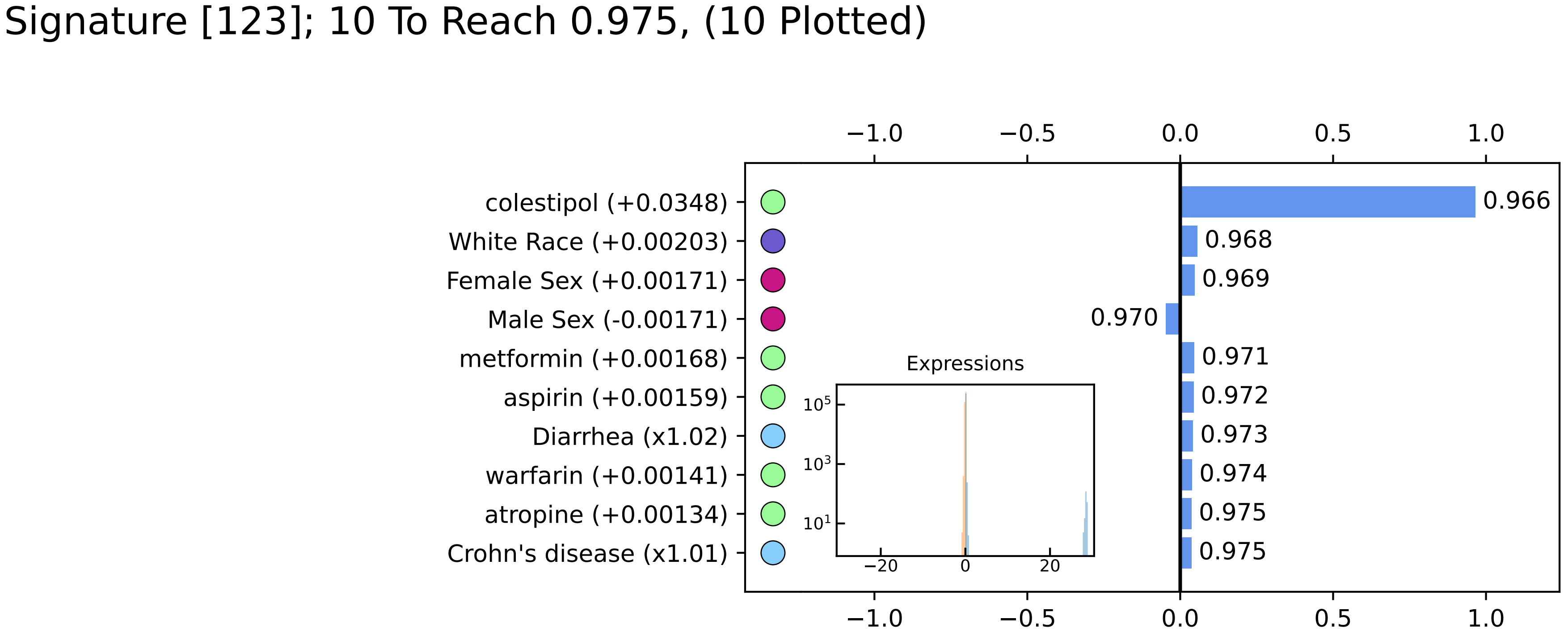

Signature [123]; 10 To Reach 0.975, (10 Plotted)
colestipol (+0.0348)
White Race (+0.00203)
Female Sex (+0.00171)
Male Sex (-0.00171)
metformin (+0.00168)
aspirin (+0.00159)
Diarrhea (x1.02)
warfarin (+0.00141)
atropine (+0.00134)
Crohn's disease (x1.01)
0.966
0.968
0.969
0.970
0.971
0.972
0.973
0.974
0.975
0.975
−1.0
−0.5
0.0
0.5
1.0
Expressions
10^5
10^3
10^1
−20
0
20

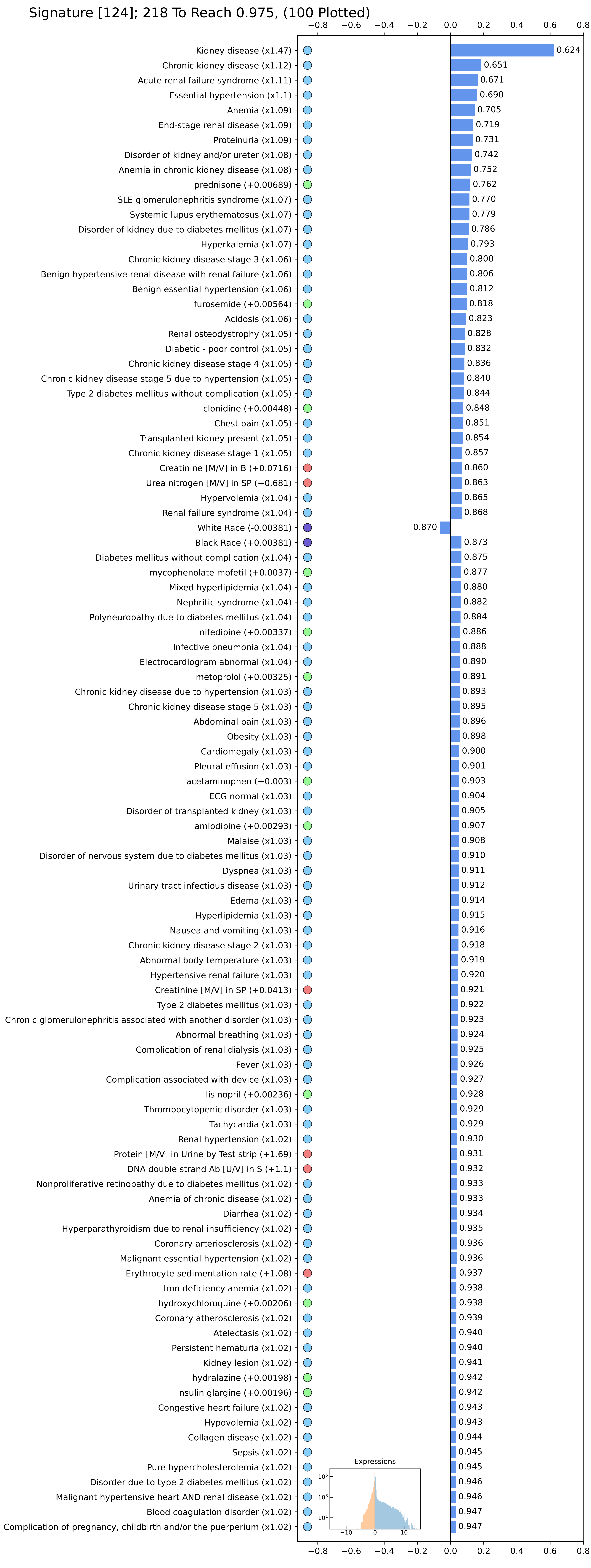
Signature [124]; 218 To Reach 0.975, (100 Plotted)
−0.8 −0.6 −0.4 −0.2 0.0 0.2 0.4 0.6 0.8
Kidney disease (x1.47) 0.624
Chronic kidney disease (x1.12) 0.651
Acute renal failure syndrome (x1.11) 0.671
Essential hypertension (x1.1) 0.690
Anemia (x1.09) 0.705
End-stage renal disease (x1.09) 0.719
Proteinuria (x1.09) 0.731
Disorder of kidney and/or ureter (x1.08) 0.742
Anemia in chronic kidney disease (x1.08) 0.752
prednisone (+0.00689) 0.762
SLE glomerulonephritis syndrome (x1.07) 0.770
Systemic lupus erythematosus (x1.07) 0.779
Disorder of kidney due to diabetes mellitus (x1.07) 0.786
Hyperkalemia (x1.07) 0.793
Chronic kidney disease stage 3 (x1.06) 0.800
Benign hypertensive renal disease with renal failure (x1.06) 0.806
Benign essential hypertension (x1.06) 0.812
furosemide (+0.00564) 0.818
Acidosis (x1.06) 0.823
Renal osteodystrophy (x1.05) 0.828
Diabetic - poor control (x1.05) 0.832
Chronic kidney disease stage 4 (x1.05) 0.836
Chronic kidney disease stage 5 due to hypertension (x1.05) 0.840
Type 2 diabetes mellitus without complication (x1.05) 0.844
clonidine (+0.00448) 0.848
Chest pain (x1.05) 0.851
Transplanted kidney present (x1.05) 0.854
Chronic kidney disease stage 1 (x1.05) 0.857
Creatinine [M/V] in B (+0.0716) 0.860
Urea nitrogen [M/V] in SP (+0.681) 0.863
Hypervolemia (x1.04) 0.865
Renal failure syndrome (x1.04) 0.868
White Race (-0.00381) 0.870
Black Race (+0.00381) 0.873
Diabetes mellitus without complication (x1.04) 0.875
mycophenolate mofetil (+0.0037) 0.877
Mixed hyperlipidemia (x1.04) 0.880
Nephritic syndrome (x1.04) 0.882
Polyneuropathy due to diabetes mellitus (x1.04) 0.884
nifedipine (+0.00337) 0.886
Infective pneumonia (x1.04) 0.888
Electrocardiogram abnormal (x1.04) 0.890
metoprolol (+0.00325) 0.891
Chronic kidney disease due to hypertension (x1.03) 0.893
Chronic kidney disease stage 5 (x1.03) 0.895
Abdominal pain (x1.03) 0.896
Obesity (x1.03) 0.898
Cardiomegaly (x1.03) 0.900
Pleural effusion (x1.03) 0.901
acetaminophen (+0.003) 0.903
ECG normal (x1.03) 0.904
Disorder of transplanted kidney (x1.03) 0.905
amlodipine (+0.00293) 0.907
Malaise (x1.03) 0.908
Disorder of nervous system due to diabetes mellitus (x1.03) 0.910
Dyspnea (x1.03) 0.911
Urinary tract infectious disease (x1.03) 0.912
Edema (x1.03) 0.914
Hyperlipidemia (x1.03) 0.915
Nausea and vomiting (x1.03) 0.916
Chronic kidney disease stage 2 (x1.03) 0.918
Abnormal body temperature (x1.03) 0.919
Hypertensive renal failure (x1.03) 0.920
Creatinine [M/V] in SP (+0.0413) 0.921
Type 2 diabetes mellitus (x1.03) 0.922
Chronic glomerulonephritis associated with another disorder (x1.03) 0.923
Abnormal breathing (x1.03) 0.924
Complication of renal dialysis (x1.03) 0.925
Fever (x1.03) 0.926
Complication associated with device (x1.03) 0.927
lisinopril (+0.00236) 0.928
Thrombocytopenic disorder (x1.03) 0.929
Tachycardia (x1.03) 0.929
Renal hypertension (x1.02) 0.930
Protein [M/V] in Urine by Test strip (+1.69) 0.931
DNA double strand Ab [U/V] in S (+1.1) 0.932
Nonproliferative retinopathy due to diabetes mellitus (x1.02) 0.933
Anemia of chronic disease (x1.02) 0.933
Diarrhea (x1.02) 0.934
Hyperparathyroidism due to renal insufficiency (x1.02) 0.935
Coronary arteriosclerosis (x1.02) 0.936
Malignant essential hypertension (x1.02) 0.936
Erythrocyte sedimentation rate (+1.08) 0.937
Iron deficiency anemia (x1.02) 0.938
hydroxychloroquine (+0.00206) 0.938
Coronary atherosclerosis (x1.02) 0.939
Atelectasis (x1.02) 0.940
Persistent hematuria (x1.02) 0.940
Kidney lesion (x1.02) 0.941
hydralazine (+0.00198) 0.942
insulin glargine (+0.00196) 0.942
Congestive heart failure (x1.02) 0.943
Hypovolemia (x1.02) 0.943
Collagen disease (x1.02) 0.944
Sepsis (x1.02) 0.945
Pure hypercholesterolemia (x1.02) 0.945
Disorder due to type 2 diabetes mellitus (x1.02) 0.946
Malignant hypertensive heart AND renal disease (x1.02) 0.946
Blood coagulation disorder (x1.02) 0.947
Complication of pregnancy, childbirth and/or the puerperium (x1.02) 0.947
Expressions
10^5
10^3
10^1
−10 0 10
−0.8 −0.6 −0.4 −0.2 0.0 0.2 0.4 0.6 0.8

Signature [125]; 3 To Reach 0.975, (10 Plotted)

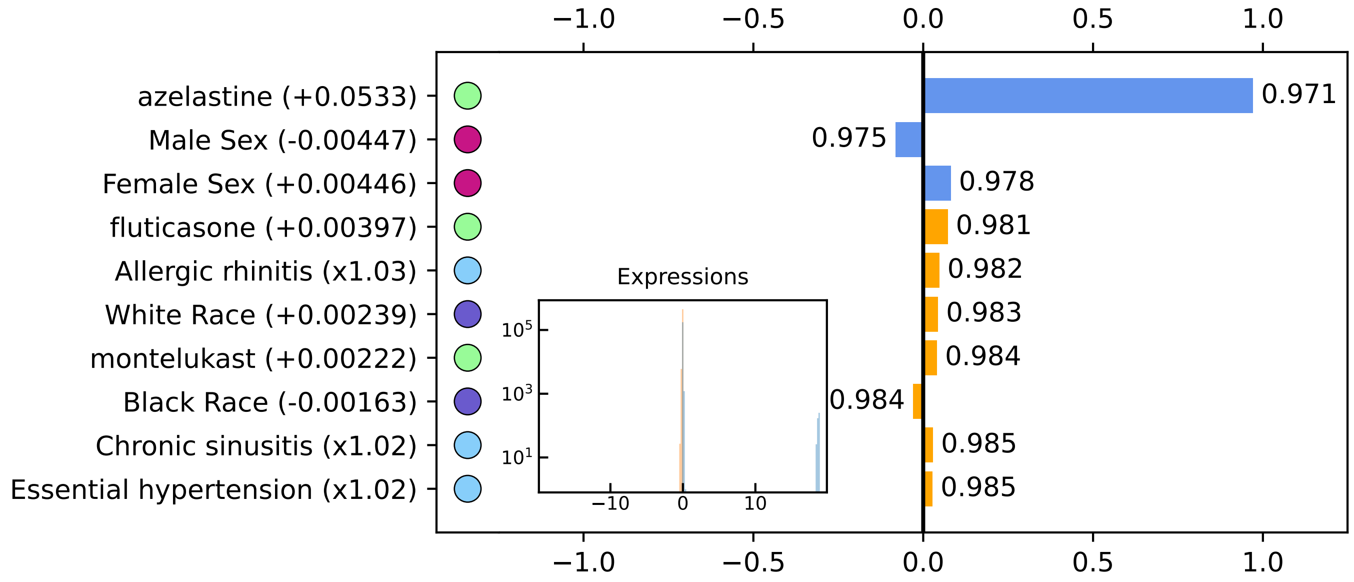

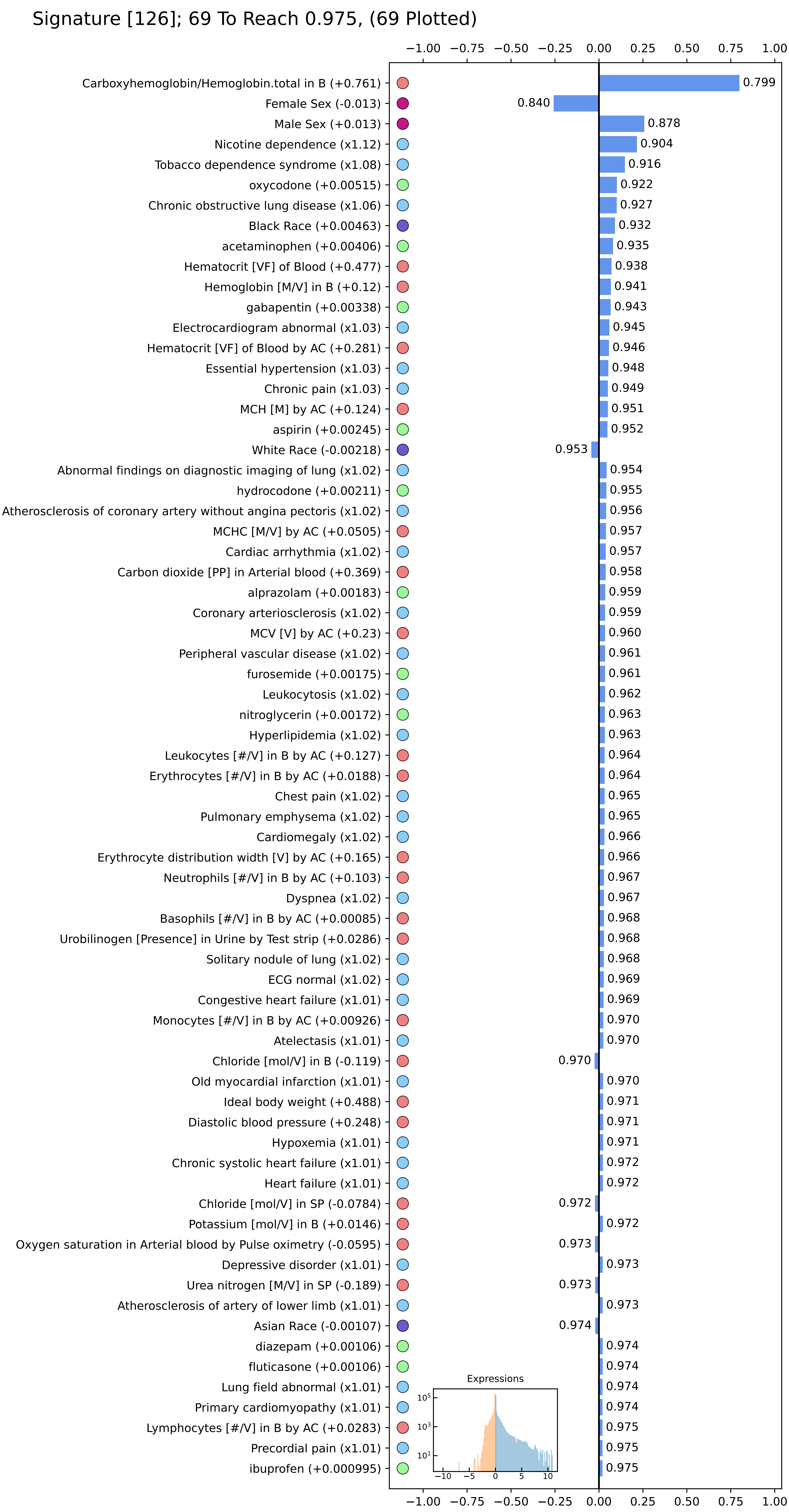
Signature [126]; 69 To Reach 0.975, (69 Plotted)
Carboxyhemoglobin/Hemoglobin.total in B (+0.761)
Female Sex (-0.013)
Male Sex (+0.013)
Nicotine dependence (x1.12)
Tobacco dependence syndrome (x1.08)
oxycodone (+0.00515)
Chronic obstructive lung disease (x1.06)
Black Race (+0.00463)
acetaminophen (+0.00406)
Hematocrit [VF] of Blood (+0.477)
Hemoglobin [M/V] in B (+0.12)
gabapentin (+0.00338)
Electrocardiogram abnormal (x1.03)
Hematocrit [VF] of Blood by AC (+0.281)
Essential hypertension (x1.03)
Chronic pain (x1.03)
MCH [M] by AC (+0.124)
aspirin (+0.00245)
White Race (-0.00218)
Abnormal findings on diagnostic imaging of lung (x1.02)
hydrocodone (+0.00211)
Atherosclerosis of coronary artery without angina pectoris (x1.02)
MCHC [M/V] by AC (+0.0505)
Cardiac arrhythmia (x1.02)
Carbon dioxide [PP] in Arterial blood (+0.369)
alprazolam (+0.00183)
Coronary arteriosclerosis (x1.02)
MCV [V] by AC (+0.23)
Peripheral vascular disease (x1.02)
furosemide (+0.00175)
Leukocytosis (x1.02)
nitroglycerin (+0.00172)
Hyperlipidemia (x1.02)
Leukocytes [#/V] in B by AC (+0.127)
Erythrocytes [#/V] in B by AC (+0.0188)
Chest pain (x1.02)
Pulmonary emphysema (x1.02)
Cardiomegaly (x1.02)
Erythrocyte distribution width [V] by AC (+0.165)
Neutrophils [#/V] in B by AC (+0.103)
Dyspnea (x1.02)
Basophils [#/V] in B by AC (+0.00085)
Urobilinogen [Presence] in Urine by Test strip (+0.0286)
Solitary nodule of lung (x1.02)
ECG normal (x1.02)
Congestive heart failure (x1.01)
Monocytes [#/V] in B by AC (+0.00926)
Atelectasis (x1.01)
Chloride [mol/V] in B (-0.119)
Old myocardial infarction (x1.01)
Ideal body weight (+0.488)
Diastolic blood pressure (+0.248)
Hypoxemia (x1.01)
Chronic systolic heart failure (x1.01)
Heart failure (x1.01)
Chloride [mol/V] in SP (-0.0784)
Potassium [mol/V] in B (+0.0146)
Oxygen saturation in Arterial blood by Pulse oximetry (-0.0595)
Depressive disorder (x1.01)
Urea nitrogen [M/V] in SP (-0.189)
Atherosclerosis of artery of lower limb (x1.01)
Asian Race (-0.00107)
diazepam (+0.00106)
fluticasone (+0.00106)
Lung field abnormal (x1.01)
Primary cardiomyopathy (x1.01)
Lymphocytes [#/V] in B by AC (+0.0283)
Precordial pain (x1.01)
ibuprofen (+0.000995)
0.799
0.840
0.878
0.904
0.916
0.922
0.927
0.932
0.935
0.938
0.941
0.943
0.945
0.946
0.948
0.949
0.951
0.952
0.953
0.954
0.955
0.956
0.957
0.957
0.958
0.959
0.959
0.960
0.961
0.961
0.962
0.963
0.963
0.964
0.964
0.965
0.965
0.966
0.966
0.967
0.967
0.968
0.968
0.968
0.969
0.969
0.970
0.970
0.970
0.970
0.971
0.971
0.971
0.972
0.972
0.972
0.972
0.973
0.973
0.973
0.973
0.974
0.974
0.974
0.974
0.974
0.975
0.975
0.975
−1.00 −0.75 −0.50 −0.25 0.00 0.25 0.50 0.75 1.00
Expressions
10^5
10^3
10^1
−10 −5 0 5 10

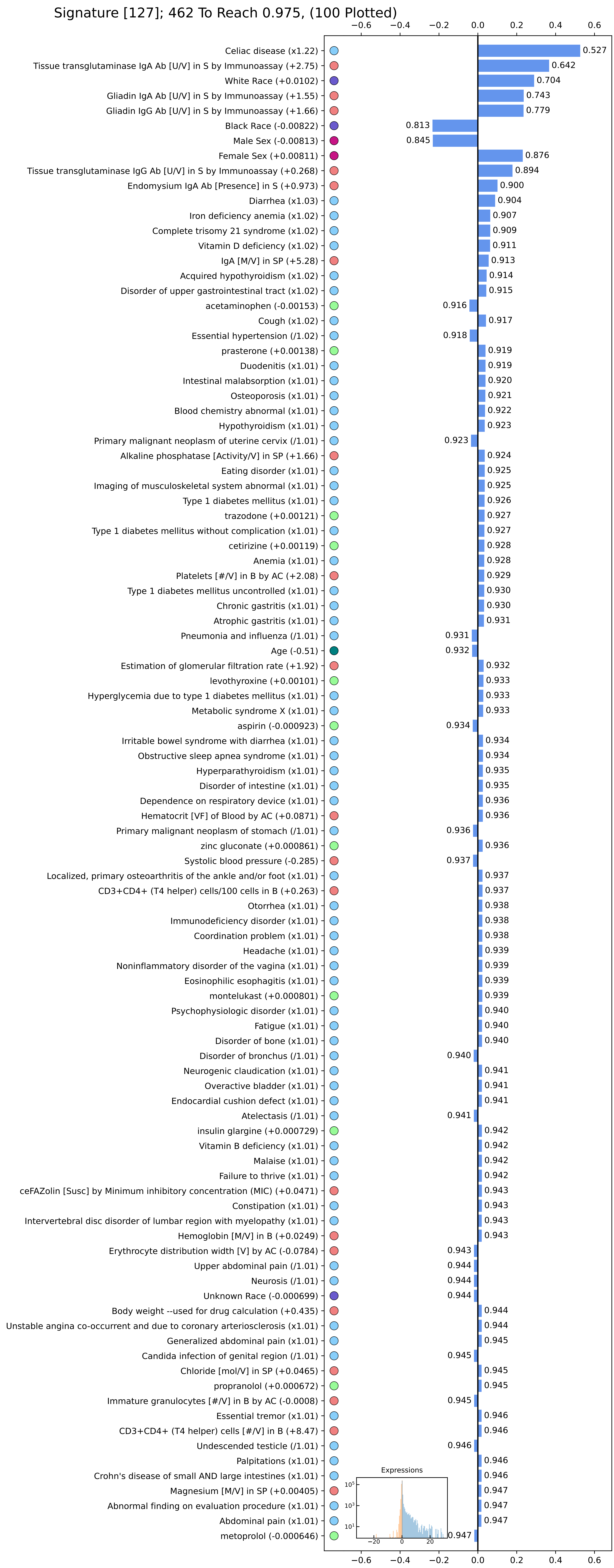

Signature [127]; 462 To Reach 0.975, (100 Plotted)
−0.6 −0.4 −0.2 0.0 0.2 0.4 0.6
Celiac disease (x1.22) 0.527
Tissue transglutaminase IgA Ab [U/V] in S by Immunoassay (+2.75) 0.642
White Race (+0.0102) 0.704
Gliadin IgA Ab [U/V] in S by Immunoassay (+1.55) 0.743
Gliadin IgG Ab [U/V] in S by Immunoassay (+1.66) 0.779
Black Race (-0.00822) 0.813
Male Sex (-0.00813) 0.845
Female Sex (+0.00811) 0.876
Tissue transglutaminase IgG Ab [U/V] in S by Immunoassay (+0.268) 0.894
Endomysium IgA Ab [Presence] in S (+0.973) 0.900
Diarrhea (x1.03) 0.904
Iron deficiency anemia (x1.02) 0.907
Complete trisomy 21 syndrome (x1.02) 0.909
Vitamin D deficiency (x1.02) 0.911
IgA [M/V] in SP (+5.28) 0.913
Acquired hypothyroidism (x1.02) 0.914
Disorder of upper gastrointestinal tract (x1.02) 0.915
acetaminophen (-0.00153) 0.916
Cough (x1.02) 0.917
Essential hypertension (/1.02) 0.918
prasterone (+0.00138) 0.919
Duodenitis (x1.01) 0.919
Intestinal malabsorption (x1.01) 0.920
Osteoporosis (x1.01) 0.921
Blood chemistry abnormal (x1.01) 0.922
Hypothyroidism (x1.01) 0.923
Primary malignant neoplasm of uterine cervix (/1.01) 0.923
Alkaline phosphatase [Activity/V] in SP (+1.66) 0.924
Eating disorder (x1.01) 0.925
Imaging of musculoskeletal system abnormal (x1.01) 0.925
Type 1 diabetes mellitus (x1.01) 0.926
trazodone (+0.00121) 0.927
Type 1 diabetes mellitus without complication (x1.01) 0.927
cetirizine (+0.00119) 0.928
Anemia (x1.01) 0.928
Platelets [#/V] in B by AC (+2.08) 0.929
Type 1 diabetes mellitus uncontrolled (x1.01) 0.930
Chronic gastritis (x1.01) 0.930
Atrophic gastritis (x1.01) 0.931
Pneumonia and influenza (/1.01) 0.931
Age (-0.51) 0.932
Estimation of glomerular filtration rate (+1.92) 0.932
levothyroxine (+0.00101) 0.933
Hyperglycemia due to type 1 diabetes mellitus (x1.01) 0.933
Metabolic syndrome X (x1.01) 0.933
aspirin (-0.000923) 0.934
Irritable bowel syndrome with diarrhea (x1.01) 0.934
Obstructive sleep apnea syndrome (x1.01) 0.934
Hyperparathyroidism (x1.01) 0.935
Disorder of intestine (x1.01) 0.935
Dependence on respiratory device (x1.01) 0.936
Hematocrit [VF] of Blood by AC (+0.0871) 0.936
Primary malignant neoplasm of stomach (/1.01) 0.936
zinc gluconate (+0.000861) 0.936
Systolic blood pressure (-0.285) 0.937
Localized, primary osteoarthritis of the ankle and/or foot (x1.01) 0.937
CD3+CD4+ (T4 helper) cells/100 cells in B (+0.263) 0.937
Otorrhea (x1.01) 0.938
Immunodeficiency disorder (x1.01) 0.938
Coordination problem (x1.01) 0.938
Headache (x1.01) 0.939
Noninflammatory disorder of the vagina (x1.01) 0.939
Eosinophilic esophagitis (x1.01) 0.939
montelukast (+0.000801) 0.939
Psychophysiologic disorder (x1.01) 0.940
Fatigue (x1.01) 0.940
Disorder of bone (x1.01) 0.940
Disorder of bronchus (/1.01) 0.940
Neurogenic claudication (x1.01) 0.941
Overactive bladder (x1.01) 0.941
Endocardial cushion defect (x1.01) 0.941
Atelectasis (/1.01) 0.941
insulin glargine (+0.000729) 0.942
Vitamin B deficiency (x1.01) 0.942
Malaise (x1.01) 0.942
Failure to thrive (x1.01) 0.942
ceFAZolin [Susc] by Minimum inhibitory concentration (MIC) (+0.0471) 0.943
Constipation (x1.01) 0.943
Intervertebral disc disorder of lumbar region with myelopathy (x1.01) 0.943
Hemoglobin [M/V] in B (+0.0249) 0.943
Erythrocyte distribution width [V] by AC (-0.0784) 0.943
Upper abdominal pain (/1.01) 0.944
Neurosis (/1.01) 0.944
Unknown Race (-0.000699) 0.944
Body weight --used for drug calculation (+0.435) 0.944
Unstable angina co-occurrent and due to coronary arteriosclerosis (x1.01) 0.944
Generalized abdominal pain (x1.01) 0.945
Candida infection of genital region (/1.01) 0.945
Chloride [mol/V] in SP (+0.0465) 0.945
propranolol (+0.000672) 0.945
Immature granulocytes [#/V] in B by AC (-0.0008) 0.945
Essential tremor (x1.01) 0.946
CD3+CD4+ (T4 helper) cells [#/V] in B (+8.47) 0.946
Undescended testicle (/1.01) 0.946
Palpitations (x1.01) 0.946
Crohn's disease of small AND large intestines (x1.01) 0.946
Magnesium [M/V] in SP (+0.00405) 0.947
Abnormal finding on evaluation procedure (x1.01) 0.947
Abdominal pain (x1.01) 0.947
metoprolol (-0.000646) 0.947
Expressions
10^5
10^3
10^1
−20 0 20
−0.6 −0.4 −0.2 0.0 0.2 0.4 0.6

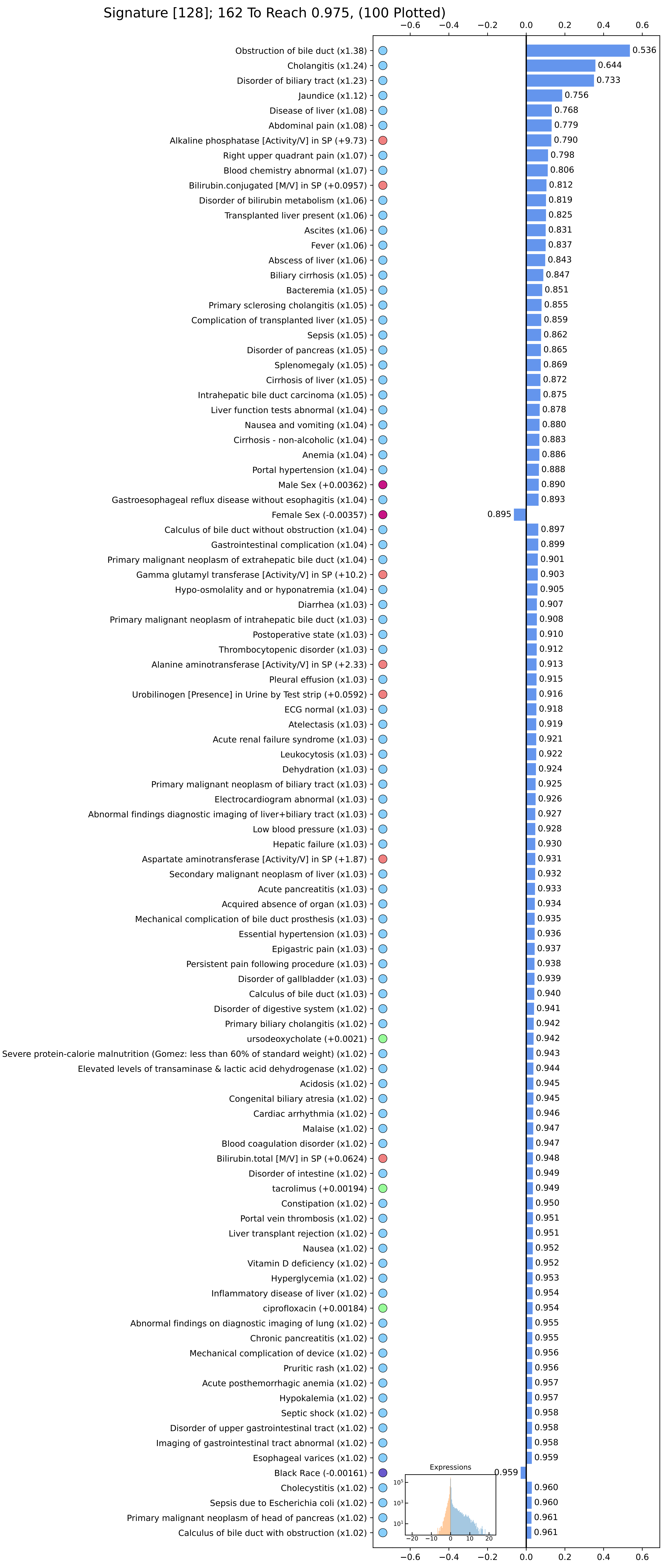

Signature [128]; 162 To Reach 0.975, (100 Plotted)
Obstruction of bile duct (x1.38) 0.536
Cholangitis (x1.24) 0.644
Disorder of biliary tract (x1.23) 0.733
Jaundice (x1.12) 0.756
Disease of liver (x1.08) 0.768
Abdominal pain (x1.08) 0.779
Alkaline phosphatase [Activity/V] in SP (+9.73) 0.790
Right upper quadrant pain (x1.07) 0.798
Blood chemistry abnormal (x1.07) 0.806
Bilirubin.conjugated [M/V] in SP (+0.0957) 0.812
Disorder of bilirubin metabolism (x1.06) 0.819
Transplanted liver present (x1.06) 0.825
Ascites (x1.06) 0.831
Fever (x1.06) 0.837
Abscess of liver (x1.06) 0.843
Biliary cirrhosis (x1.05) 0.847
Bacteremia (x1.05) 0.851
Primary sclerosing cholangitis (x1.05) 0.855
Complication of transplanted liver (x1.05) 0.859
Sepsis (x1.05) 0.862
Disorder of pancreas (x1.05) 0.865
Splenomegaly (x1.05) 0.869
Cirrhosis of liver (x1.05) 0.872
Intrahepatic bile duct carcinoma (x1.05) 0.875
Liver function tests abnormal (x1.04) 0.878
Nausea and vomiting (x1.04) 0.880
Cirrhosis - non-alcoholic (x1.04) 0.883
Anemia (x1.04) 0.886
Portal hypertension (x1.04) 0.888
Male Sex (+0.00362) 0.890
Gastroesophageal reflux disease without esophagitis (x1.04) 0.893
Female Sex (-0.00357) 0.895
Calculus of bile duct without obstruction (x1.04) 0.897
Gastrointestinal complication (x1.04) 0.899
Primary malignant neoplasm of extrahepatic bile duct (x1.04) 0.901
Gamma glutamyl transferase [Activity/V] in SP (+10.2) 0.903
Hypo-osmolality and or hyponatremia (x1.04) 0.905
Diarrhea (x1.03) 0.907
Primary malignant neoplasm of intrahepatic bile duct (x1.03) 0.908
Postoperative state (x1.03) 0.910
Thrombocytopenic disorder (x1.03) 0.912
Alanine aminotransferase [Activity/V] in SP (+2.33) 0.913
Pleural effusion (x1.03) 0.915
Urobilinogen [Presence] in Urine by Test strip (+0.0592) 0.916
ECG normal (x1.03) 0.918
Atelectasis (x1.03) 0.919
Acute renal failure syndrome (x1.03) 0.921
Leukocytosis (x1.03) 0.922
Dehydration (x1.03) 0.924
Primary malignant neoplasm of biliary tract (x1.03) 0.925
Electrocardiogram abnormal (x1.03) 0.926
Abnormal findings diagnostic imaging of liver+biliary tract (x1.03) 0.927
Low blood pressure (x1.03) 0.928
Hepatic failure (x1.03) 0.930
Aspartate aminotransferase [Activity/V] in SP (+1.87) 0.931
Secondary malignant neoplasm of liver (x1.03) 0.932
Acute pancreatitis (x1.03) 0.933
Acquired absence of organ (x1.03) 0.934
Mechanical complication of bile duct prosthesis (x1.03) 0.935
Essential hypertension (x1.03) 0.936
Epigastric pain (x1.03) 0.937
Persistent pain following procedure (x1.03) 0.938
Disorder of gallbladder (x1.03) 0.939
Calculus of bile duct (x1.03) 0.940
Disorder of digestive system (x1.02) 0.941
Primary biliary cholangitis (x1.02) 0.942
ursodeoxycholate (+0.0021) 0.942
Severe protein-calorie malnutrition (Gomez: less than 60% of standard weight) (x1.02) 0.943
Elevated levels of transaminase & lactic acid dehydrogenase (x1.02) 0.944
Acidosis (x1.02) 0.945
Congenital biliary atresia (x1.02) 0.945
Cardiac arrhythmia (x1.02) 0.946
Malaise (x1.02) 0.947
Blood coagulation disorder (x1.02) 0.947
Bilirubin.total [M/V] in SP (+0.0624) 0.948
Disorder of intestine (x1.02) 0.949
tacrolimus (+0.00194) 0.949
Constipation (x1.02) 0.950
Portal vein thrombosis (x1.02) 0.951
Liver transplant rejection (x1.02) 0.951
Nausea (x1.02) 0.952
Vitamin D deficiency (x1.02) 0.952
Hyperglycemia (x1.02) 0.953
Inflammatory disease of liver (x1.02) 0.954
ciprofloxacin (+0.00184) 0.954
Abnormal findings on diagnostic imaging of lung (x1.02) 0.955
Chronic pancreatitis (x1.02) 0.955
Mechanical complication of device (x1.02) 0.956
Pruritic rash (x1.02) 0.956
Acute posthemorrhagic anemia (x1.02) 0.957
Hypokalemia (x1.02) 0.957
Septic shock (x1.02) 0.958
Disorder of upper gastrointestinal tract (x1.02) 0.958
Imaging of gastrointestinal tract abnormal (x1.02) 0.958
Esophageal varices (x1.02) 0.959
Black Race (-0.00161) 0.959
Cholecystitis (x1.02) 0.960
Sepsis due to Escherichia coli (x1.02) 0.960
Primary malignant neoplasm of head of pancreas (x1.02) 0.961
Calculus of bile duct with obstruction (x1.02) 0.961
Expressions
−0.6 −0.4 −0.2 0.0 0.2 0.4 0.6

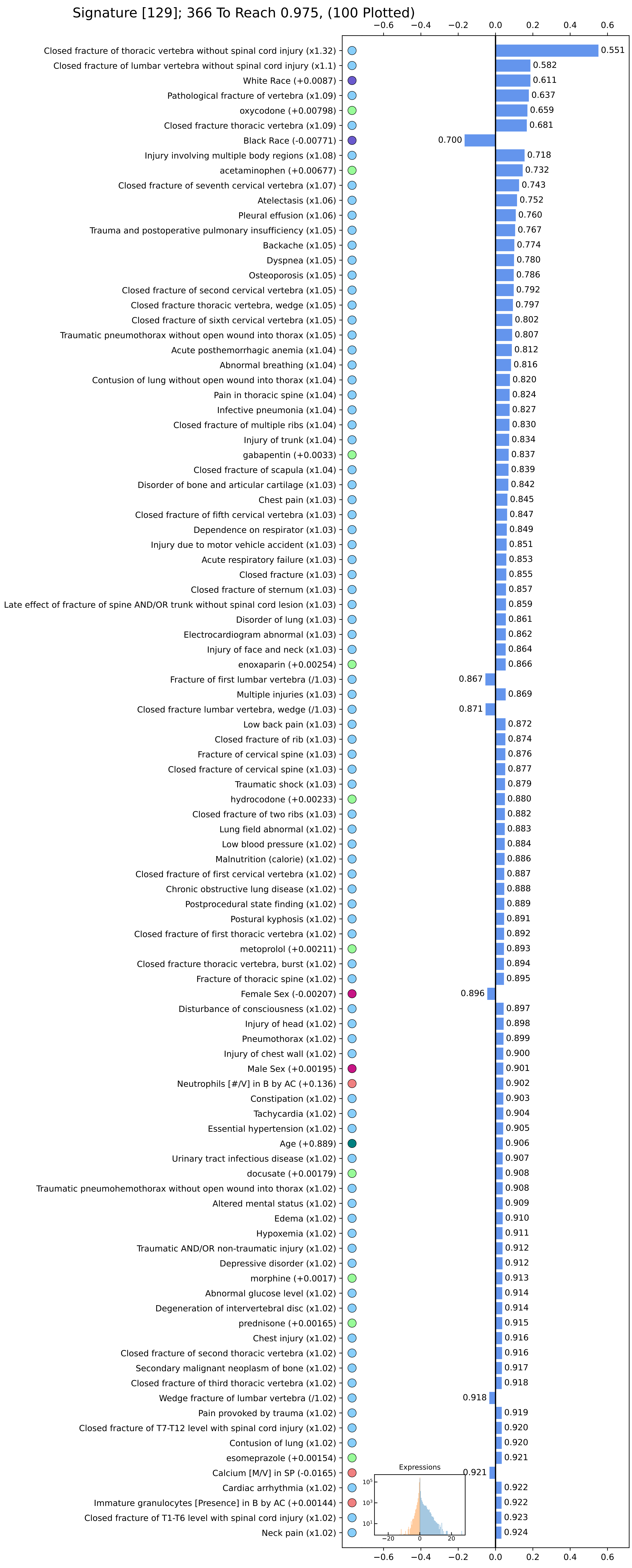

Signature [129]; 366 To Reach 0.975, (100 Plotted)
Closed fracture of thoracic vertebra without spinal cord injury (x1.32) 0.551
Closed fracture of lumbar vertebra without spinal cord injury (x1.1) 0.582
White Race (+0.0087) 0.611
Pathological fracture of vertebra (x1.09) 0.637
oxycodone (+0.00798) 0.659
Closed fracture thoracic vertebra (x1.09) 0.681
Black Race (-0.00771) 0.700
Injury involving multiple body regions (x1.08) 0.718
acetaminophen (+0.00677) 0.732
Closed fracture of seventh cervical vertebra (x1.07) 0.743
Atelectasis (x1.06) 0.752
Pleural effusion (x1.06) 0.760
Trauma and postoperative pulmonary insufficiency (x1.05) 0.767
Backache (x1.05) 0.774
Dyspnea (x1.05) 0.780
Osteoporosis (x1.05) 0.786
Closed fracture of second cervical vertebra (x1.05) 0.792
Closed fracture thoracic vertebra, wedge (x1.05) 0.797
Closed fracture of sixth cervical vertebra (x1.05) 0.802
Traumatic pneumothorax without open wound into thorax (x1.05) 0.807
Acute posthemorrhagic anemia (x1.04) 0.812
Abnormal breathing (x1.04) 0.816
Contusion of lung without open wound into thorax (x1.04) 0.820
Pain in thoracic spine (x1.04) 0.824
Infective pneumonia (x1.04) 0.827
Closed fracture of multiple ribs (x1.04) 0.830
Injury of trunk (x1.04) 0.834
gabapentin (+0.0033) 0.837
Closed fracture of scapula (x1.04) 0.839
Disorder of bone and articular cartilage (x1.03) 0.842
Chest pain (x1.03) 0.845
Closed fracture of fifth cervical vertebra (x1.03) 0.847
Dependence on respirator (x1.03) 0.849
Injury due to motor vehicle accident (x1.03) 0.851
Acute respiratory failure (x1.03) 0.853
Closed fracture (x1.03) 0.855
Closed fracture of sternum (x1.03) 0.857
Late effect of fracture of spine AND/OR trunk without spinal cord lesion (x1.03) 0.859
Disorder of lung (x1.03) 0.861
Electrocardiogram abnormal (x1.03) 0.862
Injury of face and neck (x1.03) 0.864
enoxaparin (+0.00254) 0.866
Fracture of first lumbar vertebra (/1.03) 0.867
Multiple injuries (x1.03) 0.869
Closed fracture lumbar vertebra, wedge (/1.03) 0.871
Low back pain (x1.03) 0.872
Closed fracture of rib (x1.03) 0.874
Fracture of cervical spine (x1.03) 0.876
Closed fracture of cervical spine (x1.03) 0.877
Traumatic shock (x1.03) 0.879
hydrocodone (+0.00233) 0.880
Closed fracture of two ribs (x1.03) 0.882
Lung field abnormal (x1.02) 0.883
Low blood pressure (x1.02) 0.884
Malnutrition (calorie) (x1.02) 0.886
Closed fracture of first cervical vertebra (x1.02) 0.887
Chronic obstructive lung disease (x1.02) 0.888
Postprocedural state finding (x1.02) 0.889
Postural kyphosis (x1.02) 0.891
Closed fracture of first thoracic vertebra (x1.02) 0.892
metoprolol (+0.00211) 0.893
Closed fracture thoracic vertebra, burst (x1.02) 0.894
Fracture of thoracic spine (x1.02) 0.895
Female Sex (-0.00207) 0.896
Disturbance of consciousness (x1.02) 0.897
Injury of head (x1.02) 0.898
Pneumothorax (x1.02) 0.899
Injury of chest wall (x1.02) 0.900
Male Sex (+0.00195) 0.901
Neutrophils [#/V] in B by AC (+0.136) 0.902
Constipation (x1.02) 0.903
Tachycardia (x1.02) 0.904
Essential hypertension (x1.02) 0.905
Age (+0.889) 0.906
Urinary tract infectious disease (x1.02) 0.907
docusate (+0.00179) 0.908
Traumatic pneumohemothorax without open wound into thorax (x1.02) 0.908
Altered mental status (x1.02) 0.909
Edema (x1.02) 0.910
Hypoxemia (x1.02) 0.911
Traumatic AND/OR non-traumatic injury (x1.02) 0.912
Depressive disorder (x1.02) 0.912
morphine (+0.0017) 0.913
Abnormal glucose level (x1.02) 0.914
Degeneration of intervertebral disc (x1.02) 0.914
prednisone (+0.00165) 0.915
Chest injury (x1.02) 0.916
Closed fracture of second thoracic vertebra (x1.02) 0.916
Secondary malignant neoplasm of bone (x1.02) 0.917
Closed fracture of third thoracic vertebra (x1.02) 0.918
Wedge fracture of lumbar vertebra (/1.02) 0.918
Pain provoked by trauma (x1.02) 0.919
Closed fracture of T7-T12 level with spinal cord injury (x1.02) 0.920
Contusion of lung (x1.02) 0.920
esomeprazole (+0.00154) 0.921
Calcium [M/V] in SP (-0.0165) 0.921
Cardiac arrhythmia (x1.02) 0.922
Immature granulocytes [Presence] in B by AC (+0.00144) 0.922
Closed fracture of T1-T6 level with spinal cord injury (x1.02) 0.923
Neck pain (x1.02) 0.924
−0.6 −0.4 −0.2 0.0 0.2 0.4 0.6
Expressions

Signature [130]; 15 To Reach 0.975, (15 Plotted)

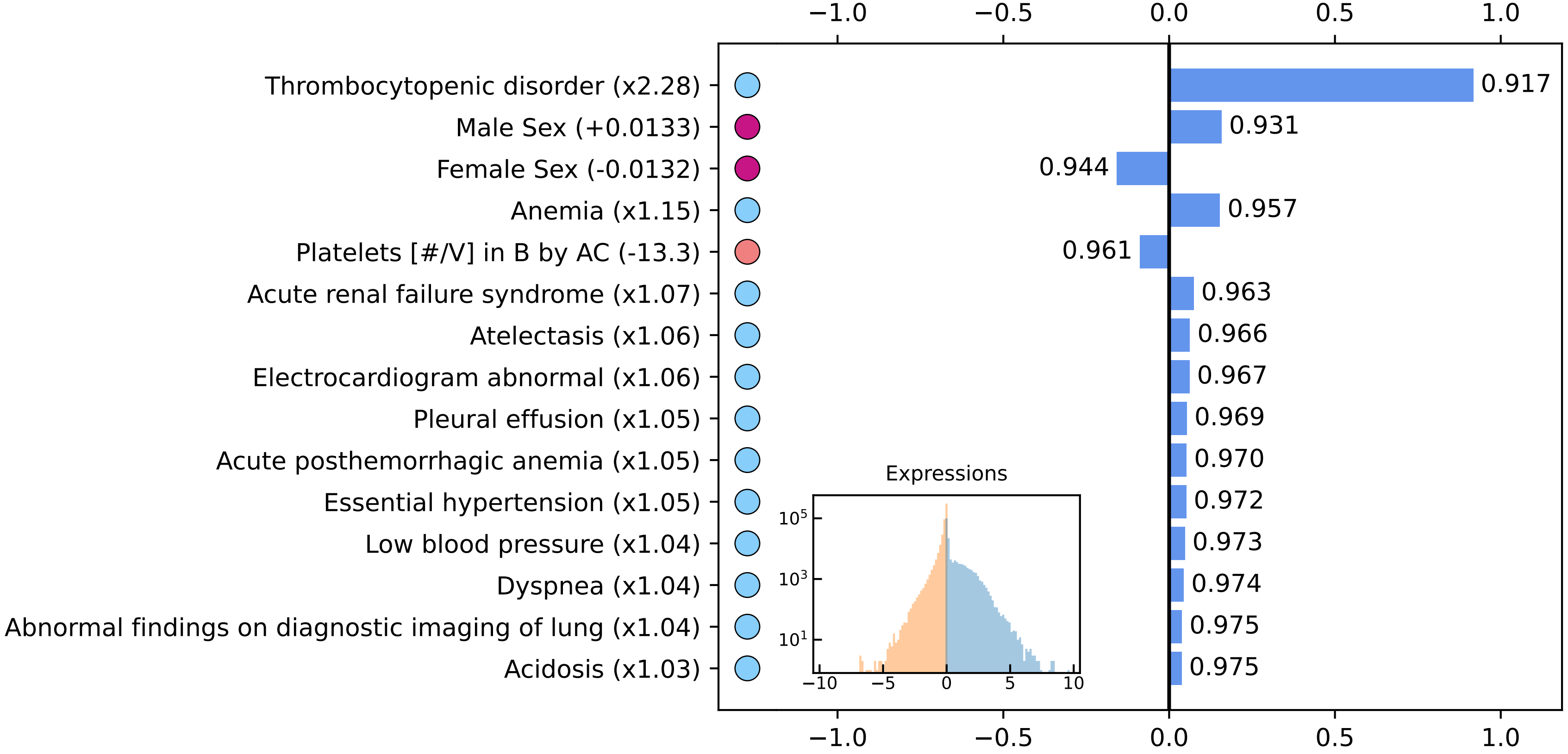

Signature [131]; 1 To Reach 0.975, (10 Plotted)

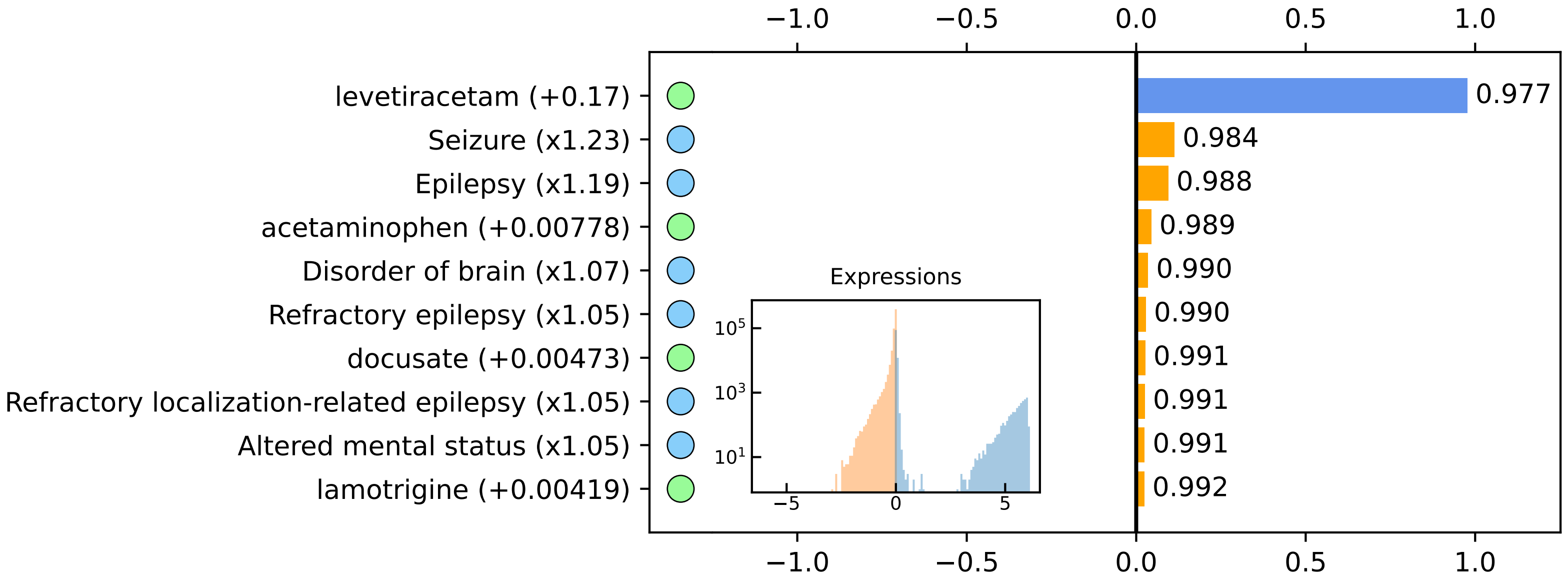

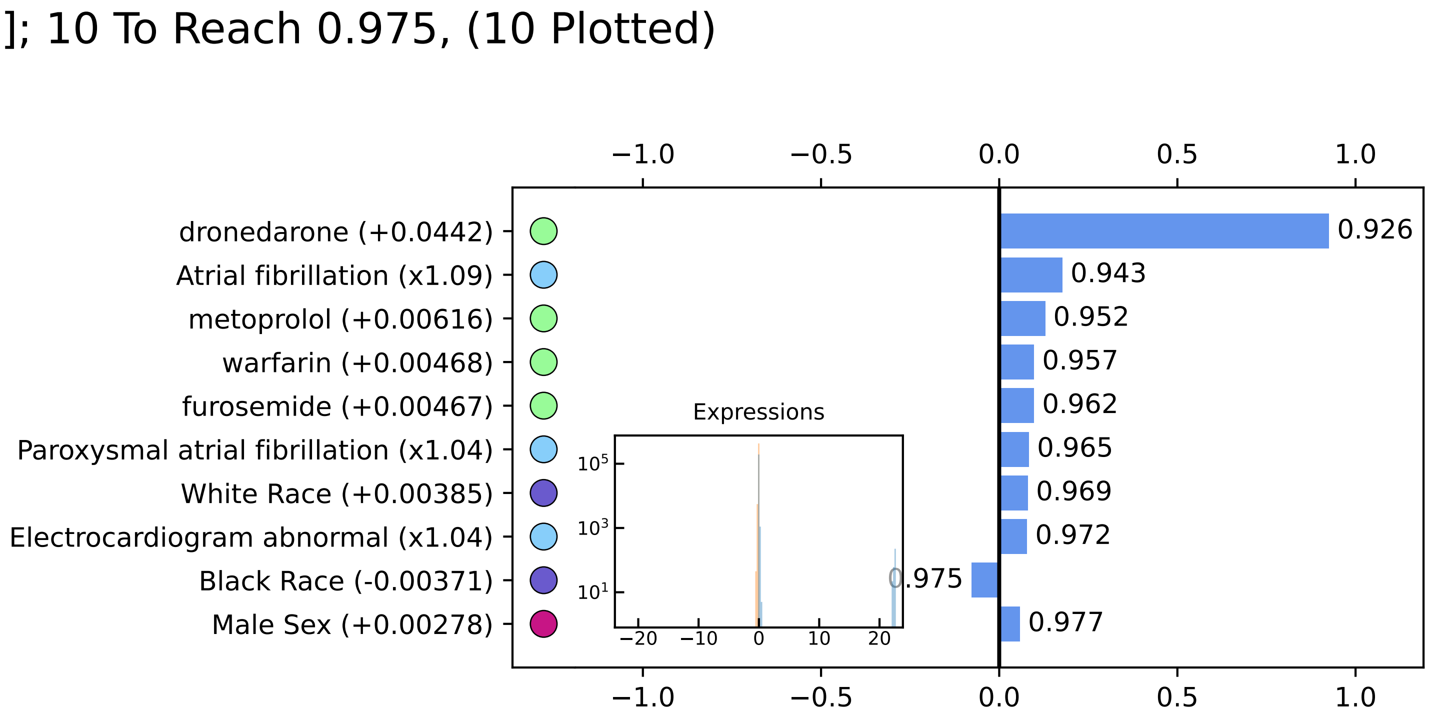
Signature [132]; 10 To Reach 0.975, (10 Plotted)
dronedarone (+0.0442)
Atrial fibrillation (x1.09)
metoprolol (+0.00616)
warfarin (+0.00468)
furosemide (+0.00467)
Paroxysmal atrial fibrillation (x1.04)
White Race (+0.00385)
Electrocardiogram abnormal (x1.04)
Black Race (-0.00371)
Male Sex (+0.00278)
0.926
0.943
0.952
0.957
0.962
0.965
0.969
0.972
0.975
0.977
−1.0
−0.5
0.0
0.5
1.0
Expressions
10^5
10^3
10^1
−20
−10
0
10
20

Signature [133]; 1 To Reach 0.975, (10 Plotted)

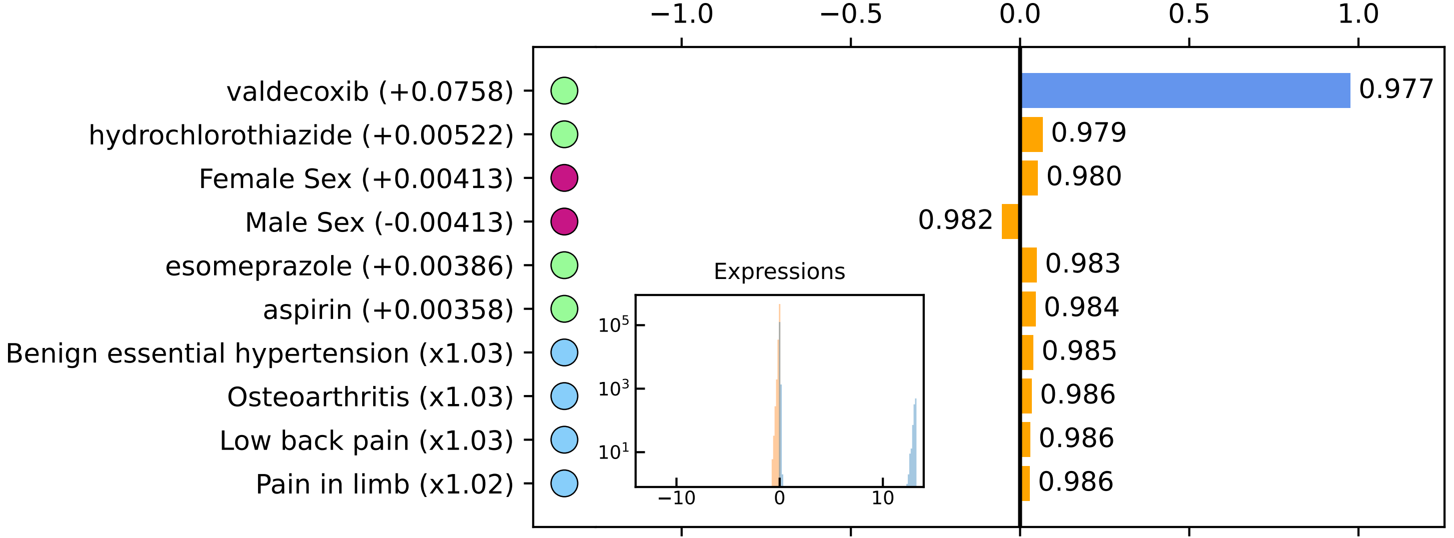

Signature [134]; 2 To Reach 0.975, (10 Plotted)

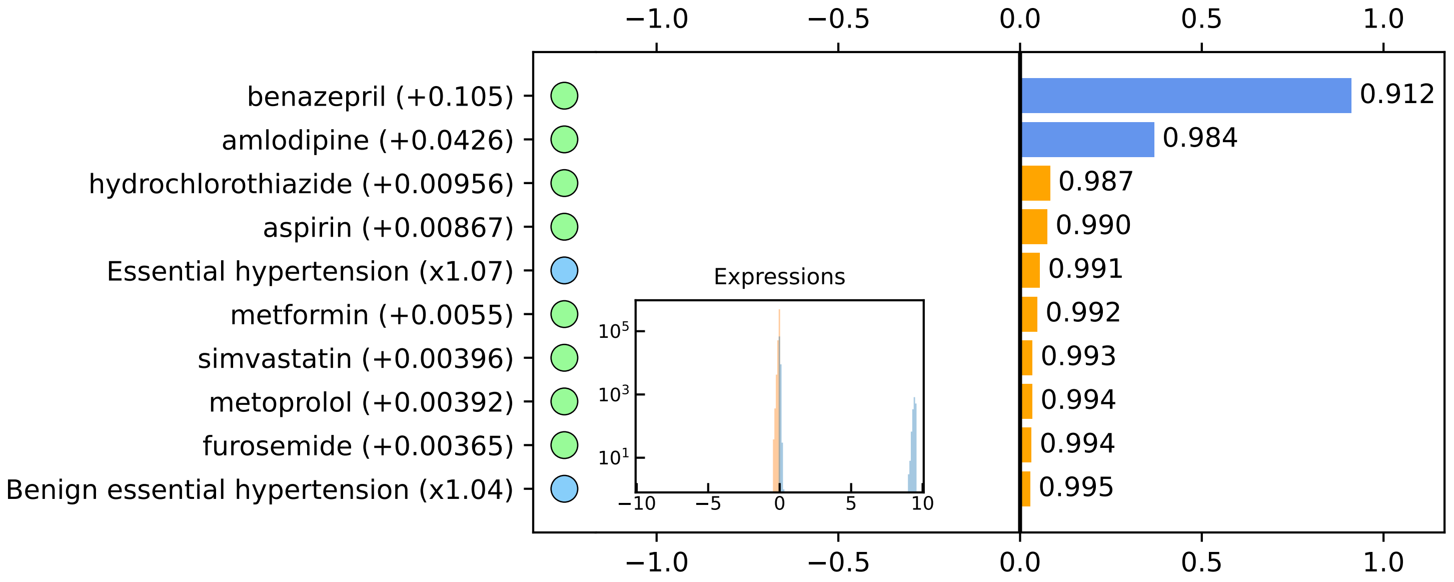

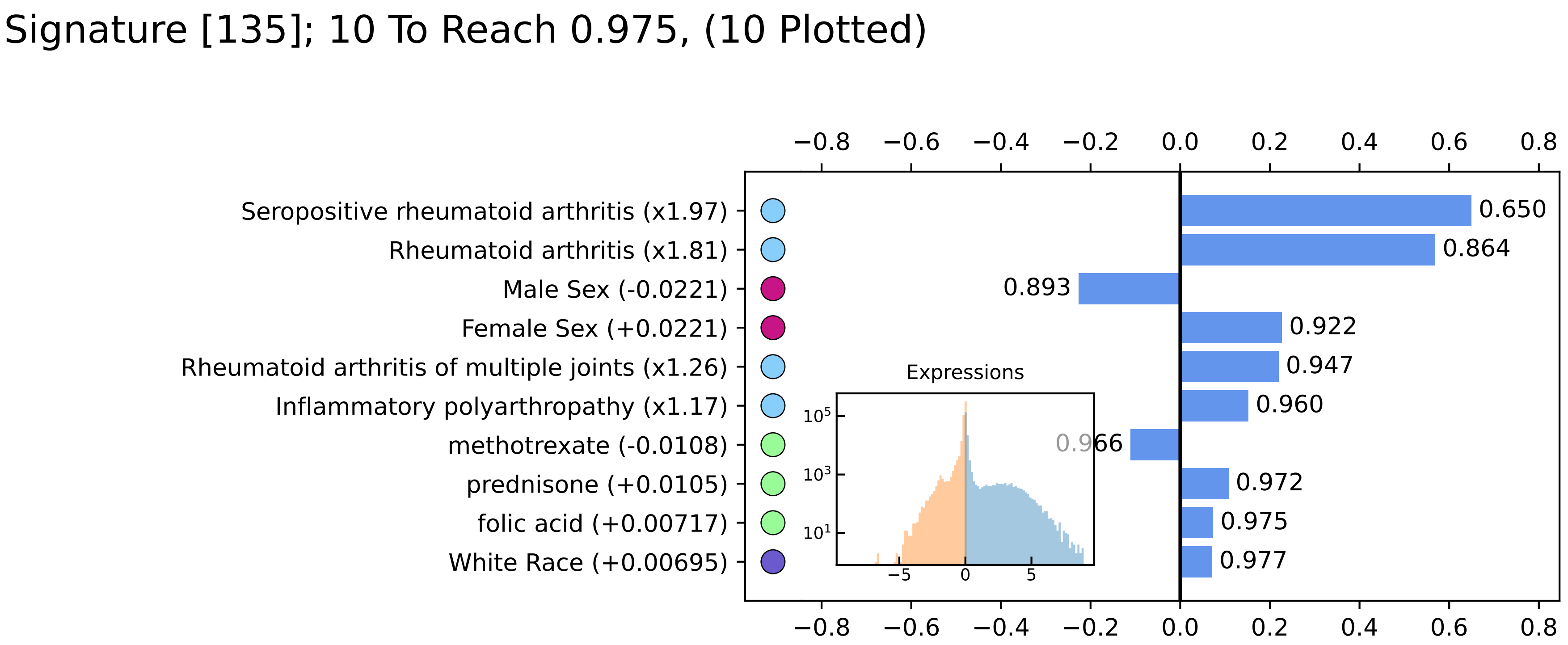

Signature [135]; 10 To Reach 0.975, (10 Plotted)
Seropositive rheumatoid arthritis (x1.97)
Rheumatoid arthritis (x1.81)
Male Sex (-0.0221)
Female Sex (+0.0221)
Rheumatoid arthritis of multiple joints (x1.26)
Inflammatory polyarthropathy (x1.17)
methotrexate (-0.0108)
prednisone (+0.0105)
folic acid (+0.00717)
White Race (+0.00695)
0.650
0.864
0.893
0.922
0.947
0.960
0.966
0.972
0.975
0.977
−0.8 −0.6 −0.4 −0.2 0.0 0.2 0.4 0.6 0.8
Expressions

Signature [136]; 19 To Reach 0.975, (19 Plotted)

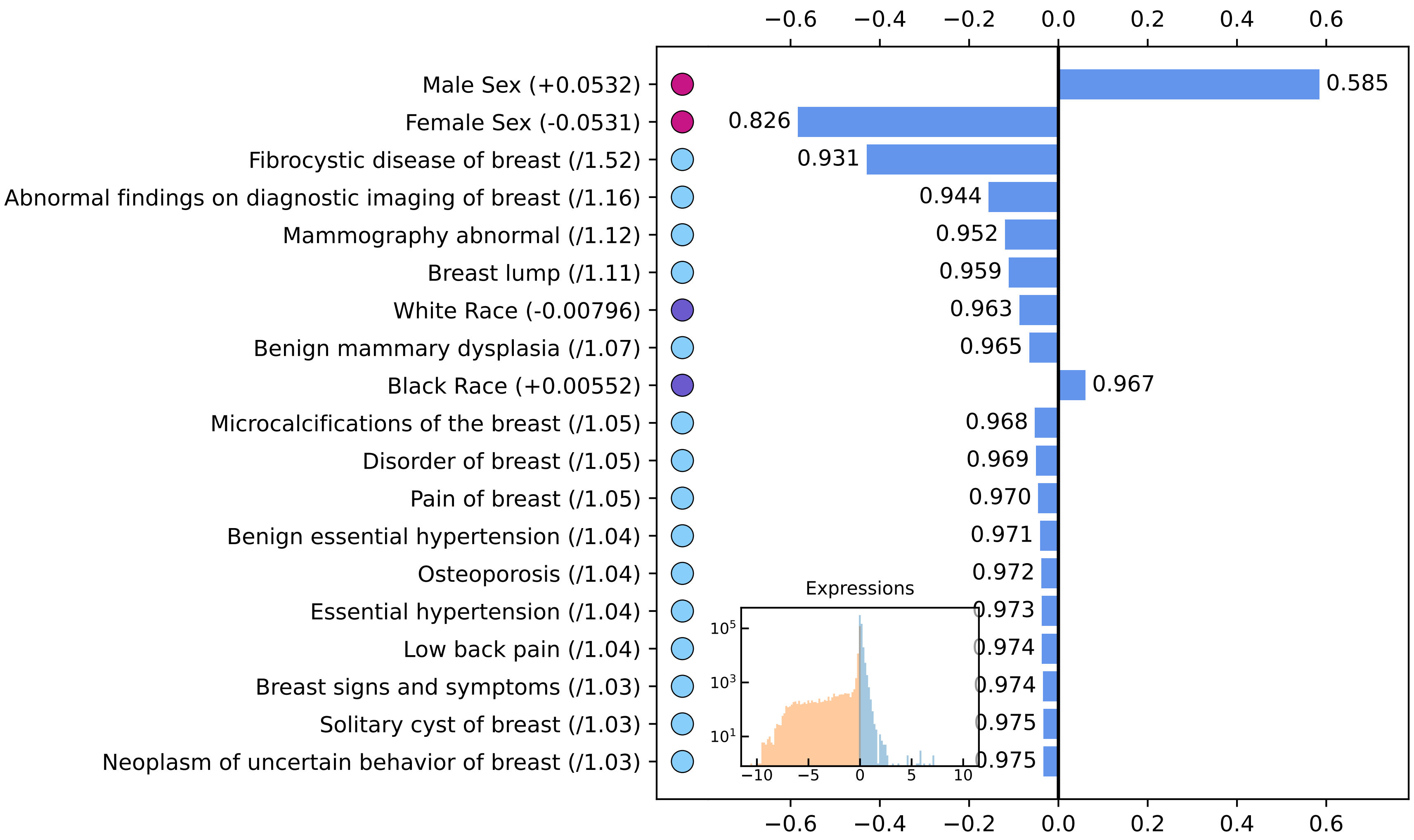

Signature [137]; 198 To Reach 0.975, (100 Plotted)

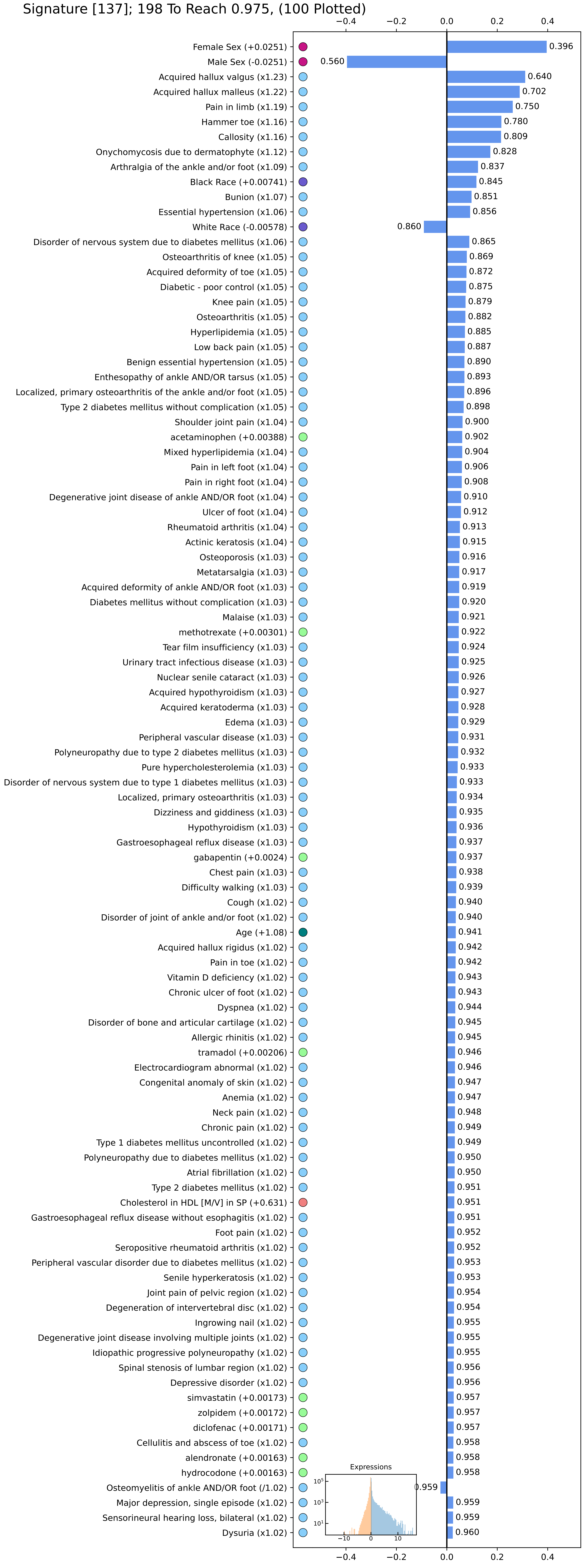

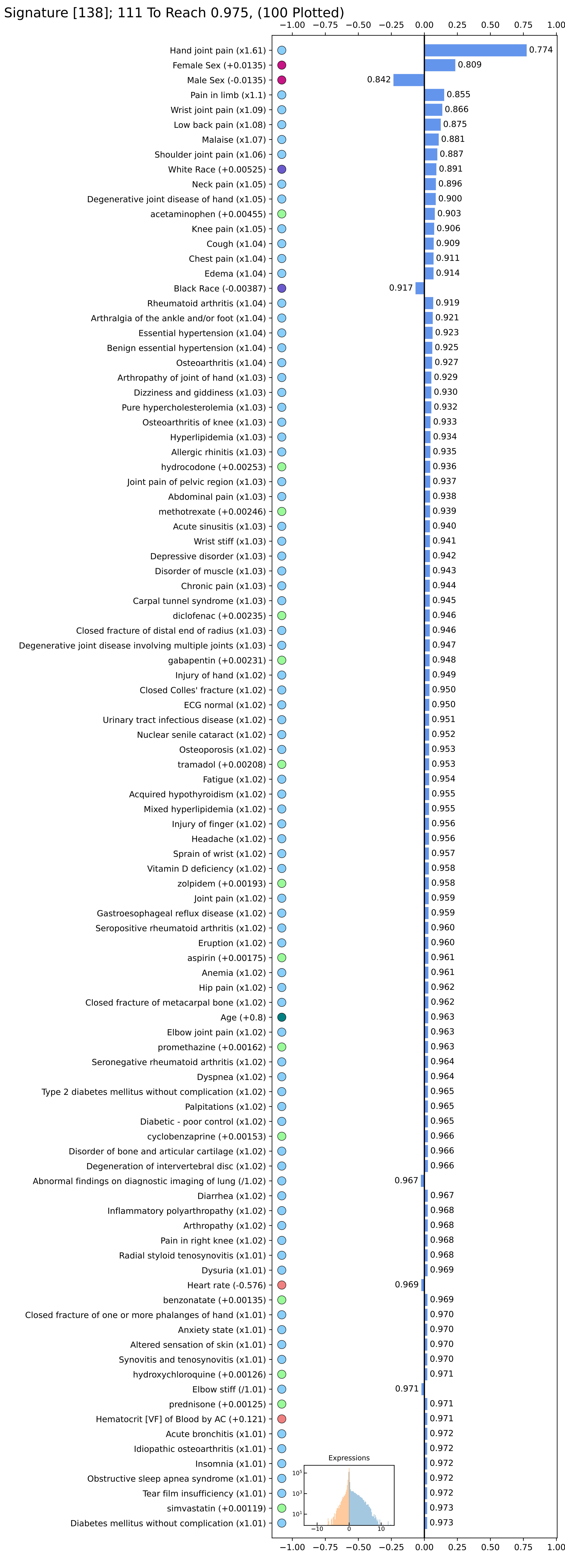

Signature [138]; 111 To Reach 0.975, (100 Plotted)
Hand joint pain (x1.61) 0.774
Female Sex (+0.0135) 0.809
Male Sex (-0.0135) 0.842
Pain in limb (x1.1) 0.855
Wrist joint pain (x1.09) 0.866
Low back pain (x1.08) 0.875
Malaise (x1.07) 0.881
Shoulder joint pain (x1.06) 0.887
White Race (+0.00525) 0.891
Neck pain (x1.05) 0.896
Degenerative joint disease of hand (x1.05) 0.900
acetaminophen (+0.00455) 0.903
Knee pain (x1.05) 0.906
Cough (x1.04) 0.909
Chest pain (x1.04) 0.911
Edema (x1.04) 0.914
Black Race (-0.00387) 0.917
Rheumatoid arthritis (x1.04) 0.919
Arthralgia of the ankle and/or foot (x1.04) 0.921
Essential hypertension (x1.04) 0.923
Benign essential hypertension (x1.04) 0.925
Osteoarthritis (x1.04) 0.927
Arthropathy of joint of hand (x1.03) 0.929
Dizziness and giddiness (x1.03) 0.930
Pure hypercholesterolemia (x1.03) 0.932
Osteoarthritis of knee (x1.03) 0.933
Hyperlipidemia (x1.03) 0.934
Allergic rhinitis (x1.03) 0.935
hydrocodone (+0.00253) 0.936
Joint pain of pelvic region (x1.03) 0.937
Abdominal pain (x1.03) 0.938
methotrexate (+0.00246) 0.939
Acute sinusitis (x1.03) 0.940
Wrist stiff (x1.03) 0.941
Depressive disorder (x1.03) 0.942
Disorder of muscle (x1.03) 0.943
Chronic pain (x1.03) 0.944
Carpal tunnel syndrome (x1.03) 0.945
diclofenac (+0.00235) 0.946
Closed fracture of distal end of radius (x1.03) 0.946
Degenerative joint disease involving multiple joints (x1.03) 0.947
gabapentin (+0.00231) 0.948
Injury of hand (x1.02) 0.949
Closed Colles' fracture (x1.02) 0.950
ECG normal (x1.02) 0.950
Urinary tract infectious disease (x1.02) 0.951
Nuclear senile cataract (x1.02) 0.952
Osteoporosis (x1.02) 0.953
tramadol (+0.00208) 0.953
Fatigue (x1.02) 0.954
Acquired hypothyroidism (x1.02) 0.955
Mixed hyperlipidemia (x1.02) 0.955
Injury of finger (x1.02) 0.956
Headache (x1.02) 0.956
Sprain of wrist (x1.02) 0.957
Vitamin D deficiency (x1.02) 0.958
zolpidem (+0.00193) 0.958
Joint pain (x1.02) 0.959
Gastroesophageal reflux disease (x1.02) 0.959
Seropositive rheumatoid arthritis (x1.02) 0.960
Eruption (x1.02) 0.960
aspirin (+0.00175) 0.961
Anemia (x1.02) 0.961
Hip pain (x1.02) 0.962
Closed fracture of metacarpal bone (x1.02) 0.962
Age (+0.8) 0.963
Elbow joint pain (x1.02) 0.963
promethazine (+0.00162) 0.963
Seronegative rheumatoid arthritis (x1.02) 0.964
Dyspnea (x1.02) 0.964
Type 2 diabetes mellitus without complication (x1.02) 0.965
Palpitations (x1.02) 0.965
Diabetic - poor control (x1.02) 0.965
cyclobenzaprine (+0.00153) 0.966
Disorder of bone and articular cartilage (x1.02) 0.966
Degeneration of intervertebral disc (x1.02) 0.966
Abnormal findings on diagnostic imaging of lung (/1.02) 0.967
Diarrhea (x1.02) 0.967
Inflammatory polyarthropathy (x1.02) 0.968
Arthropathy (x1.02) 0.968
Pain in right knee (x1.02) 0.968
Radial styloid tenosynovitis (x1.01) 0.968
Dysuria (x1.01) 0.969
Heart rate (-0.576) 0.969
benzonatate (+0.00135) 0.969
Closed fracture of one or more phalanges of hand (x1.01) 0.970
Anxiety state (x1.01) 0.970
Altered sensation of skin (x1.01) 0.970
Synovitis and tenosynovitis (x1.01) 0.970
hydroxychloroquine (+0.00126) 0.971
Elbow stiff (/1.01) 0.971
prednisone (+0.00125) 0.971
Hematocrit [VF] of Blood by AC (+0.121) 0.971
Acute bronchitis (x1.01) 0.972
Idiopathic osteoarthritis (x1.01) 0.972
Insomnia (x1.01) 0.972
Obstructive sleep apnea syndrome (x1.01) 0.972
Tear film insufficiency (x1.01) 0.972
simvastatin (+0.00119) 0.973
Diabetes mellitus without complication (x1.01) 0.973
Expressions
−1.00 −0.75 −0.50 −0.25 0.00 0.25 0.50 0.75 1.00

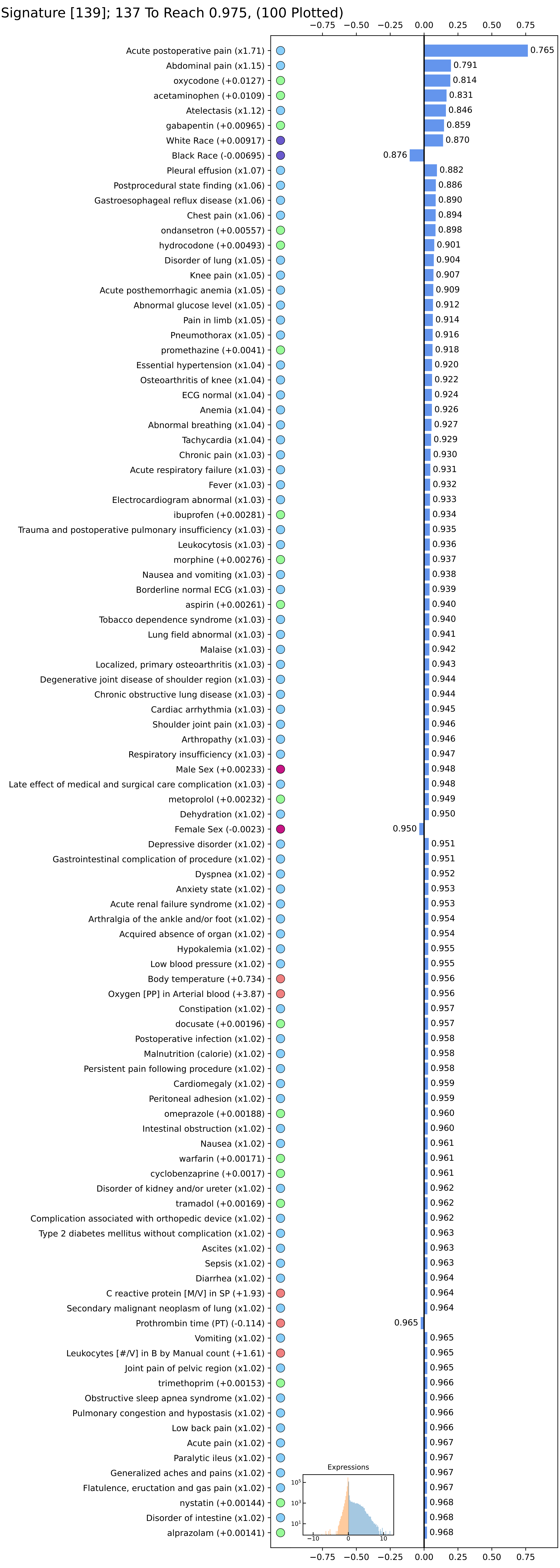
Signature [139]; 137 To Reach 0.975, (100 Plotted)
Acute postoperative pain (x1.71) 0.765
Abdominal pain (x1.15) 0.791
oxycodone (+0.0127) 0.814
acetaminophen (+0.0109) 0.831
Atelectasis (x1.12) 0.846
gabapentin (+0.00965) 0.859
White Race (+0.00917) 0.870
Black Race (-0.00695) 0.876
Pleural effusion (x1.07) 0.882
Postprocedural state finding (x1.06) 0.886
Gastroesophageal reflux disease (x1.06) 0.890
Chest pain (x1.06) 0.894
ondansetron (+0.00557) 0.898
hydrocodone (+0.00493) 0.901
Disorder of lung (x1.05) 0.904
Knee pain (x1.05) 0.907
Acute posthemorrhagic anemia (x1.05) 0.909
Abnormal glucose level (x1.05) 0.912
Pain in limb (x1.05) 0.914
Pneumothorax (x1.05) 0.916
promethazine (+0.0041) 0.918
Essential hypertension (x1.04) 0.920
Osteoarthritis of knee (x1.04) 0.922
ECG normal (x1.04) 0.924
Anemia (x1.04) 0.926
Abnormal breathing (x1.04) 0.927
Tachycardia (x1.04) 0.929
Chronic pain (x1.03) 0.930
Acute respiratory failure (x1.03) 0.931
Fever (x1.03) 0.932
Electrocardiogram abnormal (x1.03) 0.933
ibuprofen (+0.00281) 0.934
Trauma and postoperative pulmonary insufficiency (x1.03) 0.935
Leukocytosis (x1.03) 0.936
morphine (+0.00276) 0.937
Nausea and vomiting (x1.03) 0.938
Borderline normal ECG (x1.03) 0.939
aspirin (+0.00261) 0.940
Tobacco dependence syndrome (x1.03) 0.940
Lung field abnormal (x1.03) 0.941
Malaise (x1.03) 0.942
Localized, primary osteoarthritis (x1.03) 0.943
Degenerative joint disease of shoulder region (x1.03) 0.944
Chronic obstructive lung disease (x1.03) 0.944
Cardiac arrhythmia (x1.03) 0.945
Shoulder joint pain (x1.03) 0.946
Arthropathy (x1.03) 0.946
Respiratory insufficiency (x1.03) 0.947
Male Sex (+0.00233) 0.948
Late effect of medical and surgical care complication (x1.03) 0.948
metoprolol (+0.00232) 0.949
Dehydration (x1.02) 0.950
Female Sex (-0.0023) 0.950
Depressive disorder (x1.02) 0.951
Gastrointestinal complication of procedure (x1.02) 0.951
Dyspnea (x1.02) 0.952
Anxiety state (x1.02) 0.953
Acute renal failure syndrome (x1.02) 0.953
Arthralgia of the ankle and/or foot (x1.02) 0.954
Acquired absence of organ (x1.02) 0.954
Hypokalemia (x1.02) 0.955
Low blood pressure (x1.02) 0.955
Body temperature (+0.734) 0.956
Oxygen [PP] in Arterial blood (+3.87) 0.956
Constipation (x1.02) 0.957
docusate (+0.00196) 0.957
Postoperative infection (x1.02) 0.958
Malnutrition (calorie) (x1.02) 0.958
Persistent pain following procedure (x1.02) 0.958
Cardiomegaly (x1.02) 0.959
Peritoneal adhesion (x1.02) 0.959
omeprazole (+0.00188) 0.960
Intestinal obstruction (x1.02) 0.960
Nausea (x1.02) 0.961
warfarin (+0.00171) 0.961
cyclobenzaprine (+0.0017) 0.961
Disorder of kidney and/or ureter (x1.02) 0.962
tramadol (+0.00169) 0.962
Complication associated with orthopedic device (x1.02) 0.962
Type 2 diabetes mellitus without complication (x1.02) 0.963
Ascites (x1.02) 0.963
Sepsis (x1.02) 0.963
Diarrhea (x1.02) 0.964
C reactive protein [M/V] in SP (+1.93) 0.964
Secondary malignant neoplasm of lung (x1.02) 0.964
Prothrombin time (PT) (-0.114) 0.965
Vomiting (x1.02) 0.965
Leukocytes [#/V] in B by Manual count (+1.61) 0.965
Joint pain of pelvic region (x1.02) 0.965
trimethoprim (+0.00153) 0.966
Obstructive sleep apnea syndrome (x1.02) 0.966
Pulmonary congestion and hypostasis (x1.02) 0.966
Low back pain (x1.02) 0.966
Acute pain (x1.02) 0.967
Paralytic ileus (x1.02) 0.967
Generalized aches and pains (x1.02) 0.967
Flatulence, eructation and gas pain (x1.02) 0.967
nystatin (+0.00144) 0.968
Disorder of intestine (x1.02) 0.968
alprazolam (+0.00141) 0.968
−0.75 −0.50 −0.25 0.00 0.25 0.50 0.75
Expressions
10⁵ 10³ 10¹
−10 0 10

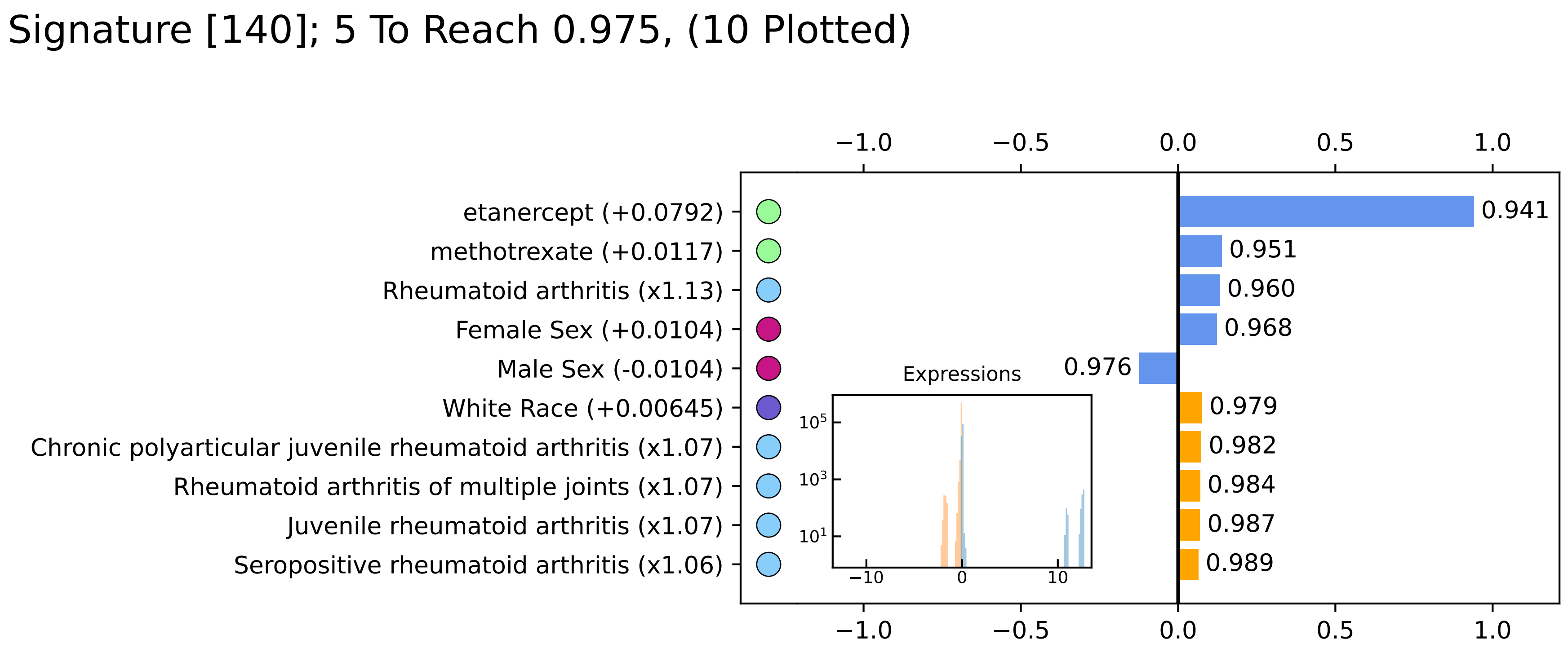
Signature [140]; 5 To Reach 0.975, (10 Plotted)
etanercept (+0.0792)
methotrexate (+0.0117)
Rheumatoid arthritis (x1.13)
Female Sex (+0.0104)
Male Sex (-0.0104)
White Race (+0.00645)
Chronic polyarticular juvenile rheumatoid arthritis (x1.07)
Rheumatoid arthritis of multiple joints (x1.07)
Juvenile rheumatoid arthritis (x1.07)
Seropositive rheumatoid arthritis (x1.06)
0.941
0.951
0.960
0.968
0.976
0.979
0.982
0.984
0.987
0.989
Expressions
−1.0
−0.5
0.0
0.5
1.0

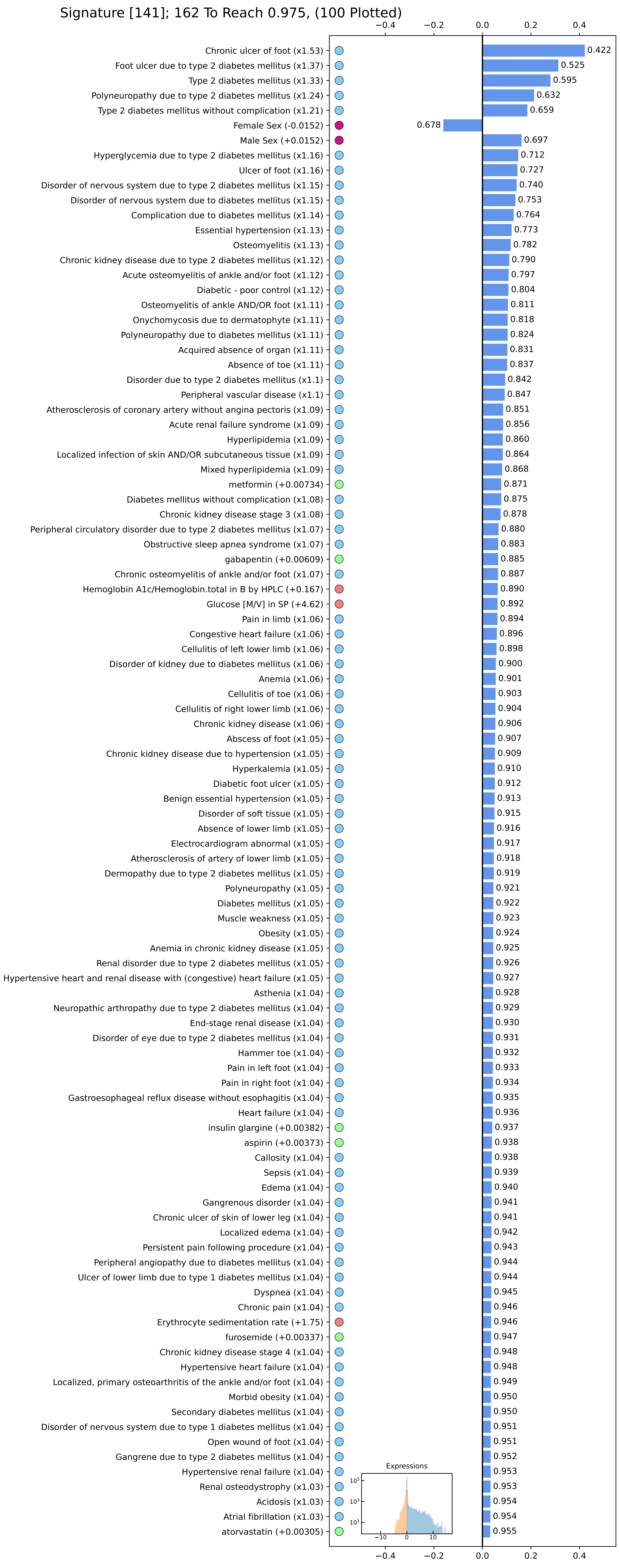

Signature [141]; 162 To Reach 0.975, (100 Plotted)
−0.4 −0.2 0.0 0.2 0.4
Chronic ulcer of foot (x1.53) 0.422
Foot ulcer due to type 2 diabetes mellitus (x1.37) 0.525
Type 2 diabetes mellitus (x1.33) 0.595
Polyneuropathy due to type 2 diabetes mellitus (x1.24) 0.632
Type 2 diabetes mellitus without complication (x1.21) 0.659
Female Sex (-0.0152) 0.678
Male Sex (+0.0152) 0.697
Hyperglycemia due to type 2 diabetes mellitus (x1.16) 0.712
Ulcer of foot (x1.16) 0.727
Disorder of nervous system due to type 2 diabetes mellitus (x1.15) 0.740
Disorder of nervous system due to diabetes mellitus (x1.15) 0.753
Complication due to diabetes mellitus (x1.14) 0.764
Essential hypertension (x1.13) 0.773
Osteomyelitis (x1.13) 0.782
Chronic kidney disease due to type 2 diabetes mellitus (x1.12) 0.790
Acute osteomyelitis of ankle and/or foot (x1.12) 0.797
Diabetic - poor control (x1.12) 0.804
Osteomyelitis of ankle AND/OR foot (x1.11) 0.811
Onychomycosis due to dermatophyte (x1.11) 0.818
Polyneuropathy due to diabetes mellitus (x1.11) 0.824
Acquired absence of organ (x1.11) 0.831
Absence of toe (x1.11) 0.837
Disorder due to type 2 diabetes mellitus (x1.1) 0.842
Peripheral vascular disease (x1.1) 0.847
Atherosclerosis of coronary artery without angina pectoris (x1.09) 0.851
Acute renal failure syndrome (x1.09) 0.856
Hyperlipidemia (x1.09) 0.860
Localized infection of skin AND/OR subcutaneous tissue (x1.09) 0.864
Mixed hyperlipidemia (x1.09) 0.868
metformin (+0.00734) 0.871
Diabetes mellitus without complication (x1.08) 0.875
Chronic kidney disease stage 3 (x1.08) 0.878
Peripheral circulatory disorder due to type 2 diabetes mellitus (x1.07) 0.880
Obstructive sleep apnea syndrome (x1.07) 0.883
gabapentin (+0.00609) 0.885
Chronic osteomyelitis of ankle and/or foot (x1.07) 0.887
Hemoglobin A1c/Hemoglobin.total in B by HPLC (+0.167) 0.890
Glucose [M/V] in SP (+4.62) 0.892
Pain in limb (x1.06) 0.894
Congestive heart failure (x1.06) 0.896
Cellulitis of left lower limb (x1.06) 0.898
Disorder of kidney due to diabetes mellitus (x1.06) 0.900
Anemia (x1.06) 0.901
Cellulitis of toe (x1.06) 0.903
Cellulitis of right lower limb (x1.06) 0.904
Chronic kidney disease (x1.06) 0.906
Abscess of foot (x1.05) 0.907
Chronic kidney disease due to hypertension (x1.05) 0.909
Hyperkalemia (x1.05) 0.910
Diabetic foot ulcer (x1.05) 0.912
Benign essential hypertension (x1.05) 0.913
Disorder of soft tissue (x1.05) 0.915
Absence of lower limb (x1.05) 0.916
Electrocardiogram abnormal (x1.05) 0.917
Atherosclerosis of artery of lower limb (x1.05) 0.918
Dermopathy due to type 2 diabetes mellitus (x1.05) 0.919
Polyneuropathy (x1.05) 0.921
Diabetes mellitus (x1.05) 0.922
Muscle weakness (x1.05) 0.923
Obesity (x1.05) 0.924
Anemia in chronic kidney disease (x1.05) 0.925
Renal disorder due to type 2 diabetes mellitus (x1.05) 0.926
Hypertensive heart and renal disease with (congestive) heart failure (x1.05) 0.927
Asthenia (x1.04) 0.928
Neuropathic arthropathy due to type 2 diabetes mellitus (x1.04) 0.929
End-stage renal disease (x1.04) 0.930
Disorder of eye due to type 2 diabetes mellitus (x1.04) 0.931
Hammer toe (x1.04) 0.932
Pain in left foot (x1.04) 0.933
Pain in right foot (x1.04) 0.934
Gastroesophageal reflux disease without esophagitis (x1.04) 0.935
Heart failure (x1.04) 0.936
insulin glargine (+0.00382) 0.937
aspirin (+0.00373) 0.938
Callosity (x1.04) 0.938
Sepsis (x1.04) 0.939
Edema (x1.04) 0.940
Gangrenous disorder (x1.04) 0.941
Chronic ulcer of skin of lower leg (x1.04) 0.941
Localized edema (x1.04) 0.942
Persistent pain following procedure (x1.04) 0.943
Peripheral angiopathy due to diabetes mellitus (x1.04) 0.944
Ulcer of lower limb due to type 1 diabetes mellitus (x1.04) 0.944
Dyspnea (x1.04) 0.945
Chronic pain (x1.04) 0.946
Erythrocyte sedimentation rate (+1.75) 0.946
furosemide (+0.00337) 0.947
Chronic kidney disease stage 4 (x1.04) 0.948
Hypertensive heart failure (x1.04) 0.948
Localized, primary osteoarthritis of the ankle and/or foot (x1.04) 0.949
Morbid obesity (x1.04) 0.950
Secondary diabetes mellitus (x1.04) 0.950
Disorder of nervous system due to type 1 diabetes mellitus (x1.04) 0.951
Open wound of foot (x1.04) 0.951
Gangrene due to type 2 diabetes mellitus (x1.04) 0.952
Hypertensive renal failure (x1.04) 0.953
Renal osteodystrophy (x1.03) 0.953
Acidosis (x1.03) 0.954
Atrial fibrillation (x1.03) 0.954
atorvastatin (+0.00305) 0.955
Expressions
10^5 10^3 10^1
−10 0 10
−0.4 −0.2 0.0 0.2 0.4

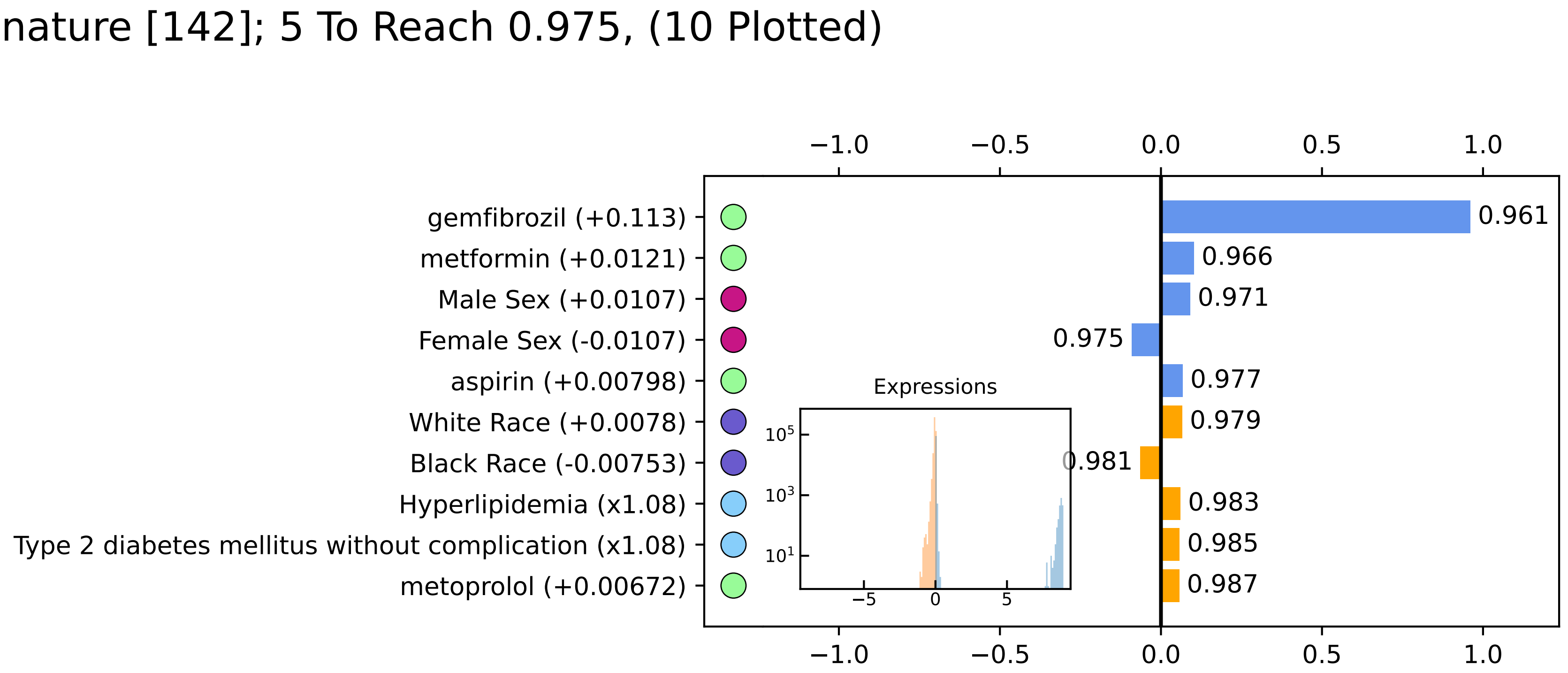

Signature [142]; 5 To Reach 0.975, (10 Plotted)
gemfibrozil (+0.113)
metformin (+0.0121)
Male Sex (+0.0107)
Female Sex (-0.0107)
aspirin (+0.00798)
White Race (+0.0078)
Black Race (-0.00753)
Hyperlipidemia (x1.08)
Type 2 diabetes mellitus without complication (x1.08)
metoprolol (+0.00672)
0.961
0.966
0.971
0.975
0.977
0.979
0.981
0.983
0.985
0.987
−1.0
−0.5
0.0
0.5
1.0
Expressions
10^5
10^3
10^1
−5
0
5

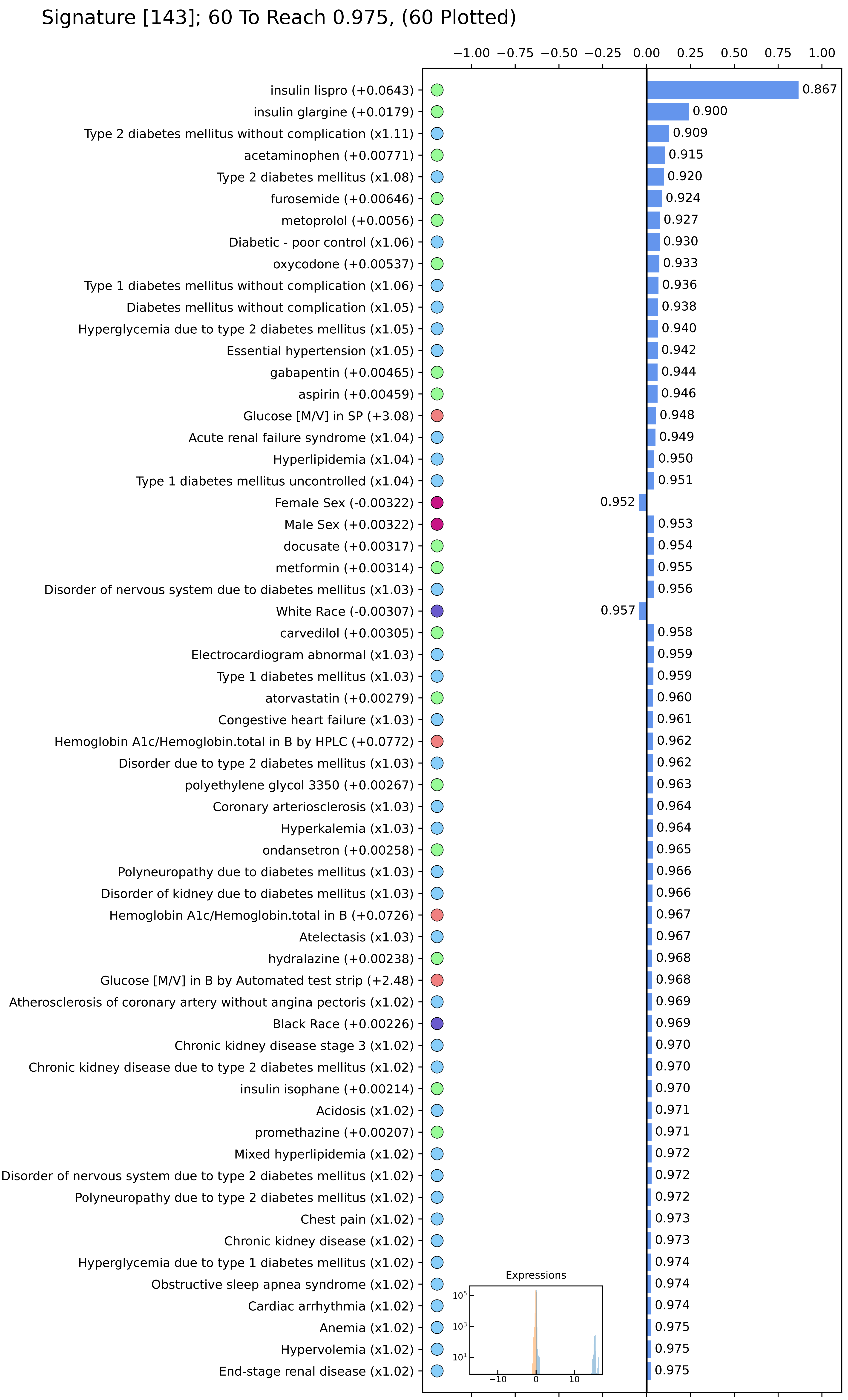

Signature [143]; 60 To Reach 0.975, (60 Plotted)
insulin lispro (+0.0643) 0.867
insulin glargine (+0.0179) 0.900
Type 2 diabetes mellitus without complication (x1.11) 0.909
acetaminophen (+0.00771) 0.915
Type 2 diabetes mellitus (x1.08) 0.920
furosemide (+0.00646) 0.924
metoprolol (+0.0056) 0.927
Diabetic - poor control (x1.06) 0.930
oxycodone (+0.00537) 0.933
Type 1 diabetes mellitus without complication (x1.06) 0.936
Diabetes mellitus without complication (x1.05) 0.938
Hyperglycemia due to type 2 diabetes mellitus (x1.05) 0.940
Essential hypertension (x1.05) 0.942
gabapentin (+0.00465) 0.944
aspirin (+0.00459) 0.946
Glucose [M/V] in SP (+3.08) 0.948
Acute renal failure syndrome (x1.04) 0.949
Hyperlipidemia (x1.04) 0.950
Type 1 diabetes mellitus uncontrolled (x1.04) 0.951
Female Sex (-0.00322) 0.952
Male Sex (+0.00322) 0.953
docusate (+0.00317) 0.954
metformin (+0.00314) 0.955
Disorder of nervous system due to diabetes mellitus (x1.03) 0.956
White Race (-0.00307) 0.957
carvedilol (+0.00305) 0.958
Electrocardiogram abnormal (x1.03) 0.959
Type 1 diabetes mellitus (x1.03) 0.959
atorvastatin (+0.00279) 0.960
Congestive heart failure (x1.03) 0.961
Hemoglobin A1c/Hemoglobin.total in B by HPLC (+0.0772) 0.962
Disorder due to type 2 diabetes mellitus (x1.03) 0.962
polyethylene glycol 3350 (+0.00267) 0.963
Coronary arteriosclerosis (x1.03) 0.964
Hyperkalemia (x1.03) 0.964
ondansetron (+0.00258) 0.965
Polyneuropathy due to diabetes mellitus (x1.03) 0.966
Disorder of kidney due to diabetes mellitus (x1.03) 0.966
Hemoglobin A1c/Hemoglobin.total in B (+0.0726) 0.967
Atelectasis (x1.03) 0.967
hydralazine (+0.00238) 0.968
Glucose [M/V] in B by Automated test strip (+2.48) 0.968
Atherosclerosis of coronary artery without angina pectoris (x1.02) 0.969
Black Race (+0.00226) 0.969
Chronic kidney disease stage 3 (x1.02) 0.970
Chronic kidney disease due to type 2 diabetes mellitus (x1.02) 0.970
insulin isophane (+0.00214) 0.970
Acidosis (x1.02) 0.971
promethazine (+0.00207) 0.971
Mixed hyperlipidemia (x1.02) 0.972
Disorder of nervous system due to type 2 diabetes mellitus (x1.02) 0.972
Polyneuropathy due to type 2 diabetes mellitus (x1.02) 0.972
Chest pain (x1.02) 0.973
Chronic kidney disease (x1.02) 0.973
Hyperglycemia due to type 1 diabetes mellitus (x1.02) 0.974
Obstructive sleep apnea syndrome (x1.02) 0.974
Cardiac arrhythmia (x1.02) 0.974
Anemia (x1.02) 0.975
Hypervolemia (x1.02) 0.975
End-stage renal disease (x1.02) 0.975
−1.00 −0.75 −0.50 −0.25 0.00 0.25 0.50 0.75 1.00
Expressions
10^5
10^3
10^1
−10
0
10

Signature [144]; 12 To Reach 0.975, (12 Plotted)

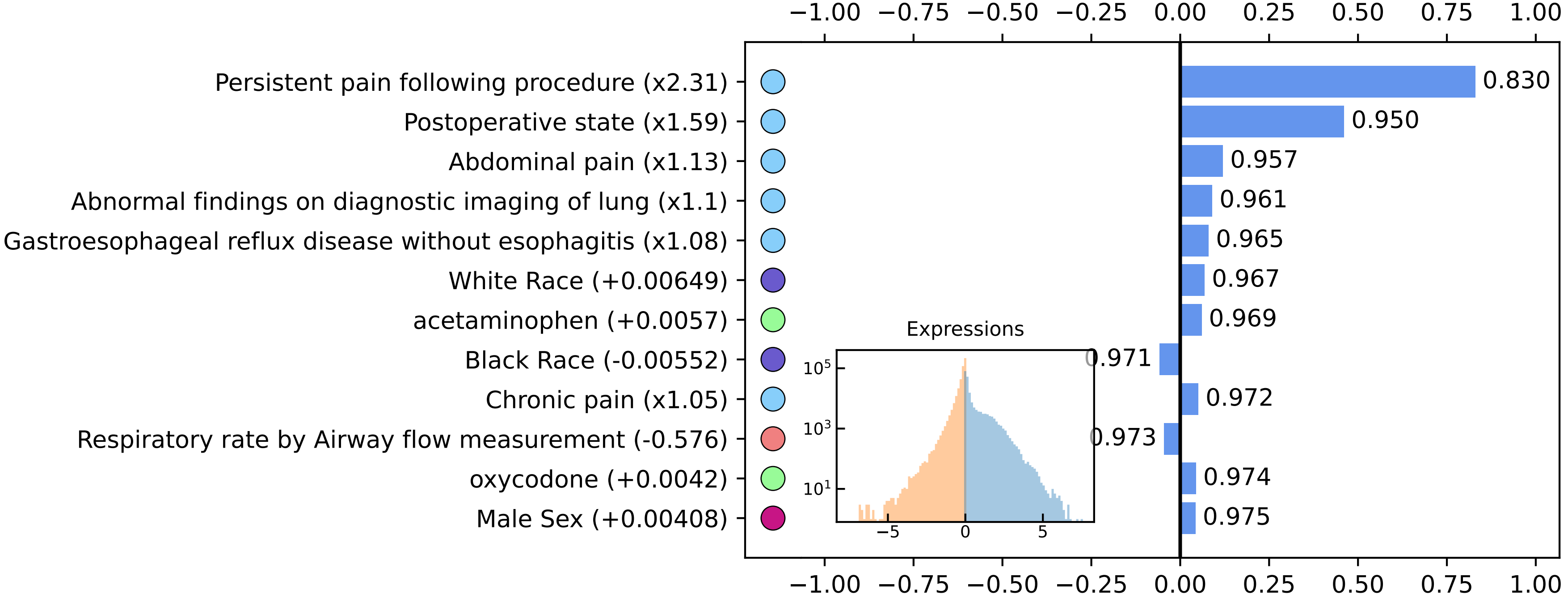

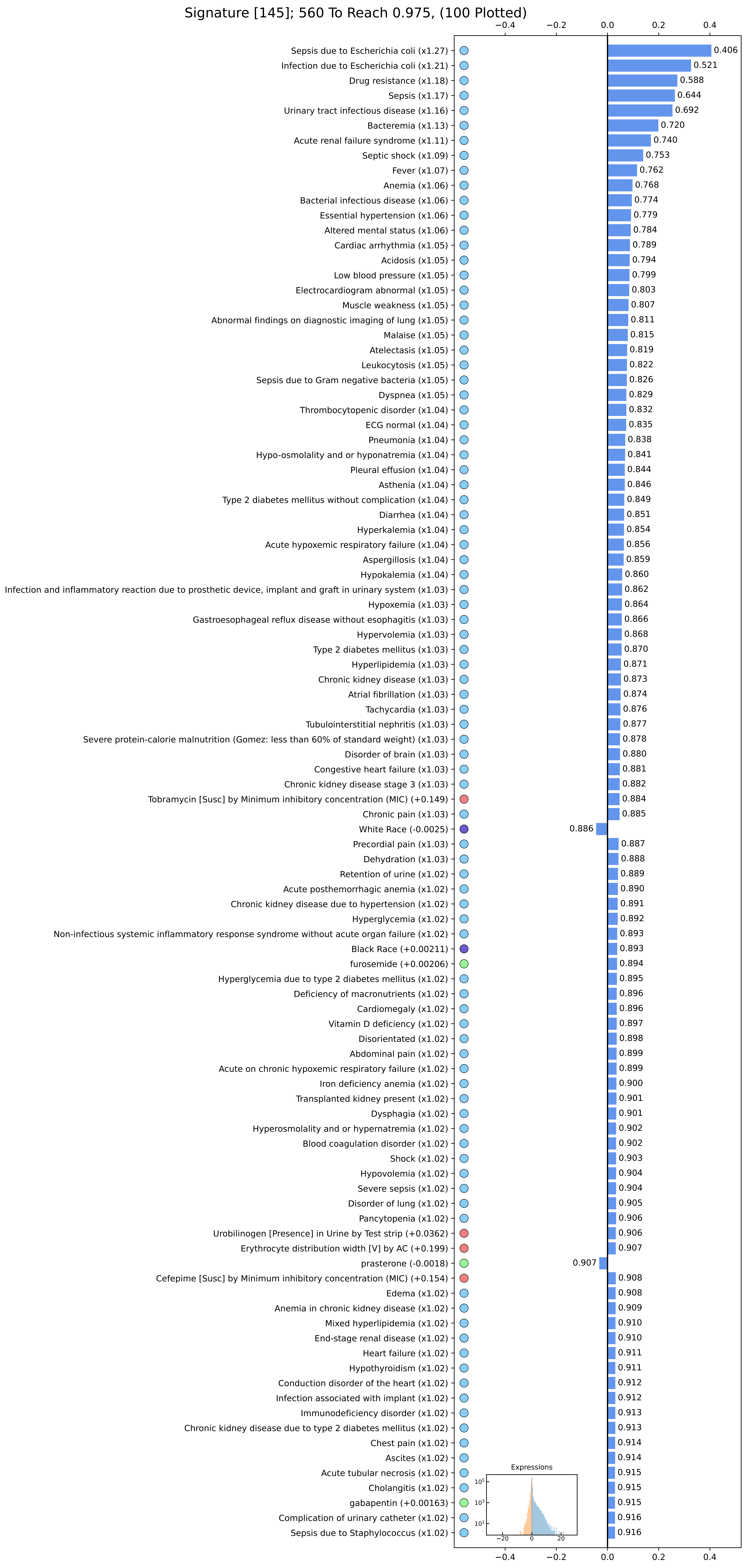
Signature [145]; 560 To Reach 0.975, (100 Plotted)
−0.4
−0.2
0.0
0.2
0.4
Sepsis due to Escherichia coli (x1.27)
Infection due to Escherichia coli (x1.21)
Drug resistance (x1.18)
Sepsis (x1.17)
Urinary tract infectious disease (x1.16)
Bacteremia (x1.13)
Acute renal failure syndrome (x1.11)
Septic shock (x1.09)
Fever (x1.07)
Anemia (x1.06)
Bacterial infectious disease (x1.06)
Essential hypertension (x1.06)
Altered mental status (x1.06)
Cardiac arrhythmia (x1.05)
Acidosis (x1.05)
Low blood pressure (x1.05)
Electrocardiogram abnormal (x1.05)
Muscle weakness (x1.05)
Abnormal findings on diagnostic imaging of lung (x1.05)
Malaise (x1.05)
Atelectasis (x1.05)
Leukocytosis (x1.05)
Sepsis due to Gram negative bacteria (x1.05)
Dyspnea (x1.05)
Thrombocytopenic disorder (x1.04)
ECG normal (x1.04)
Pneumonia (x1.04)
Hypo-osmolality and or hyponatremia (x1.04)
Pleural effusion (x1.04)
Asthenia (x1.04)
Type 2 diabetes mellitus without complication (x1.04)
Diarrhea (x1.04)
Hyperkalemia (x1.04)
Acute hypoxemic respiratory failure (x1.04)
Aspergillosis (x1.04)
Hypokalemia (x1.04)
Infection and inflammatory reaction due to prosthetic device, implant and graft in urinary system (x1.03)
Hypoxemia (x1.03)
Gastroesophageal reflux disease without esophagitis (x1.03)
Hypervolemia (x1.03)
Type 2 diabetes mellitus (x1.03)
Hyperlipidemia (x1.03)
Chronic kidney disease (x1.03)
Atrial fibrillation (x1.03)
Tachycardia (x1.03)
Tubulointerstitial nephritis (x1.03)
Severe protein-calorie malnutrition (Gomez: less than 60% of standard weight) (x1.03)
Disorder of brain (x1.03)
Congestive heart failure (x1.03)
Chronic kidney disease stage 3 (x1.03)
Tobramycin [Susc] by Minimum inhibitory concentration (MIC) (+0.149)
Chronic pain (x1.03)
White Race (-0.0025)
Precordial pain (x1.03)
Dehydration (x1.03)
Retention of urine (x1.02)
Acute posthemorrhagic anemia (x1.02)
Chronic kidney disease due to hypertension (x1.02)
Hyperglycemia (x1.02)
Non-infectious systemic inflammatory response syndrome without acute organ failure (x1.02)
Black Race (+0.00211)
furosemide (+0.00206)
Hyperglycemia due to type 2 diabetes mellitus (x1.02)
Deficiency of macronutrients (x1.02)
Cardiomegaly (x1.02)
Vitamin D deficiency (x1.02)
Disorientated (x1.02)
Abdominal pain (x1.02)
Acute on chronic hypoxemic respiratory failure (x1.02)
Iron deficiency anemia (x1.02)
Transplanted kidney present (x1.02)
Dysphagia (x1.02)
Hyperosmolality and or hypernatremia (x1.02)
Blood coagulation disorder (x1.02)
Shock (x1.02)
Hypovolemia (x1.02)
Severe sepsis (x1.02)
Disorder of lung (x1.02)
Pancytopenia (x1.02)
Urobilinogen [Presence] in Urine by Test strip (+0.0362)
Erythrocyte distribution width [V] by AC (+0.199)
prasterone (-0.0018)
Cefepime [Susc] by Minimum inhibitory concentration (MIC) (+0.154)
Edema (x1.02)
Anemia in chronic kidney disease (x1.02)
Mixed hyperlipidemia (x1.02)
End-stage renal disease (x1.02)
Heart failure (x1.02)
Hypothyroidism (x1.02)
Conduction disorder of the heart (x1.02)
Infection associated with implant (x1.02)
Immunodeficiency disorder (x1.02)
Chronic kidney disease due to type 2 diabetes mellitus (x1.02)
Chest pain (x1.02)
Ascites (x1.02)
Acute tubular necrosis (x1.02)
Cholangitis (x1.02)
gabapentin (+0.00163)
Complication of urinary catheter (x1.02)
Sepsis due to Staphylococcus (x1.02)
0.406
0.521
0.588
0.644
0.692
0.720
0.740
0.753
0.762
0.768
0.774
0.779
0.784
0.789
0.794
0.799
0.803
0.807
0.811
0.815
0.819
0.822
0.826
0.829
0.832
0.835
0.838
0.841
0.844
0.846
0.849
0.851
0.854
0.856
0.859
0.860
0.862
0.864
0.866
0.868
0.870
0.871
0.873
0.874
0.876
0.877
0.878
0.880
0.881
0.882
0.884
0.885
0.886
0.887
0.888
0.889
0.890
0.891
0.892
0.893
0.893
0.894
0.895
0.896
0.896
0.897
0.898
0.899
0.899
0.900
0.901
0.901
0.902
0.902
0.903
0.904
0.904
0.905
0.906
0.906
0.907
0.907
0.908
0.908
0.909
0.910
0.910
0.911
0.911
0.912
0.912
0.913
0.913
0.914
0.914
0.915
0.915
0.915
0.916
0.916
Expressions
−20
0
20

Signature [146]; 305 To Reach 0.975, (100 Plotted)

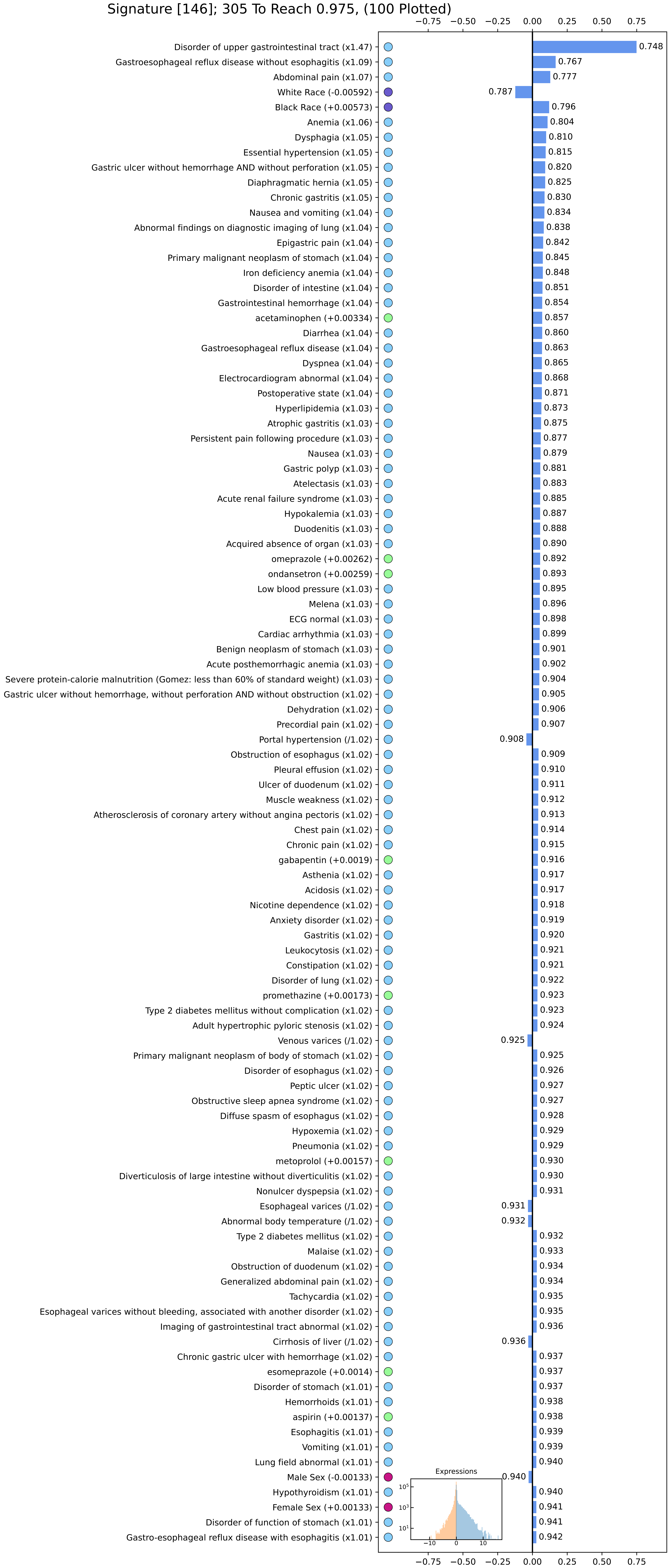

Signature [147]; 1 To Reach 0.975, (10 Plotted)

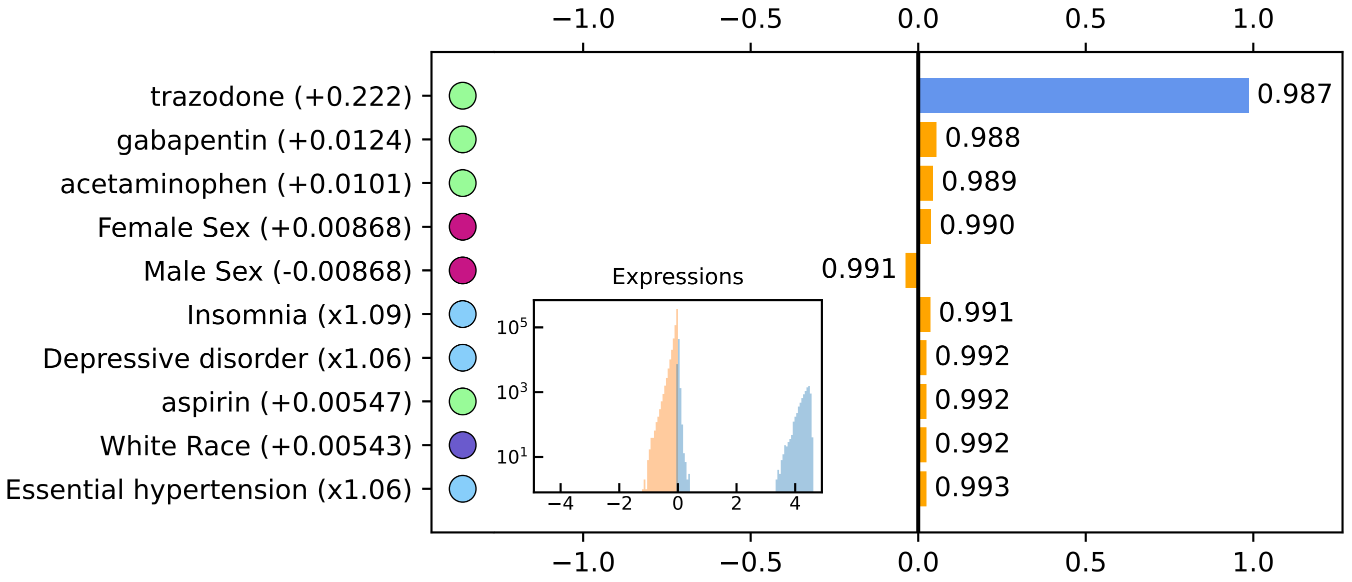

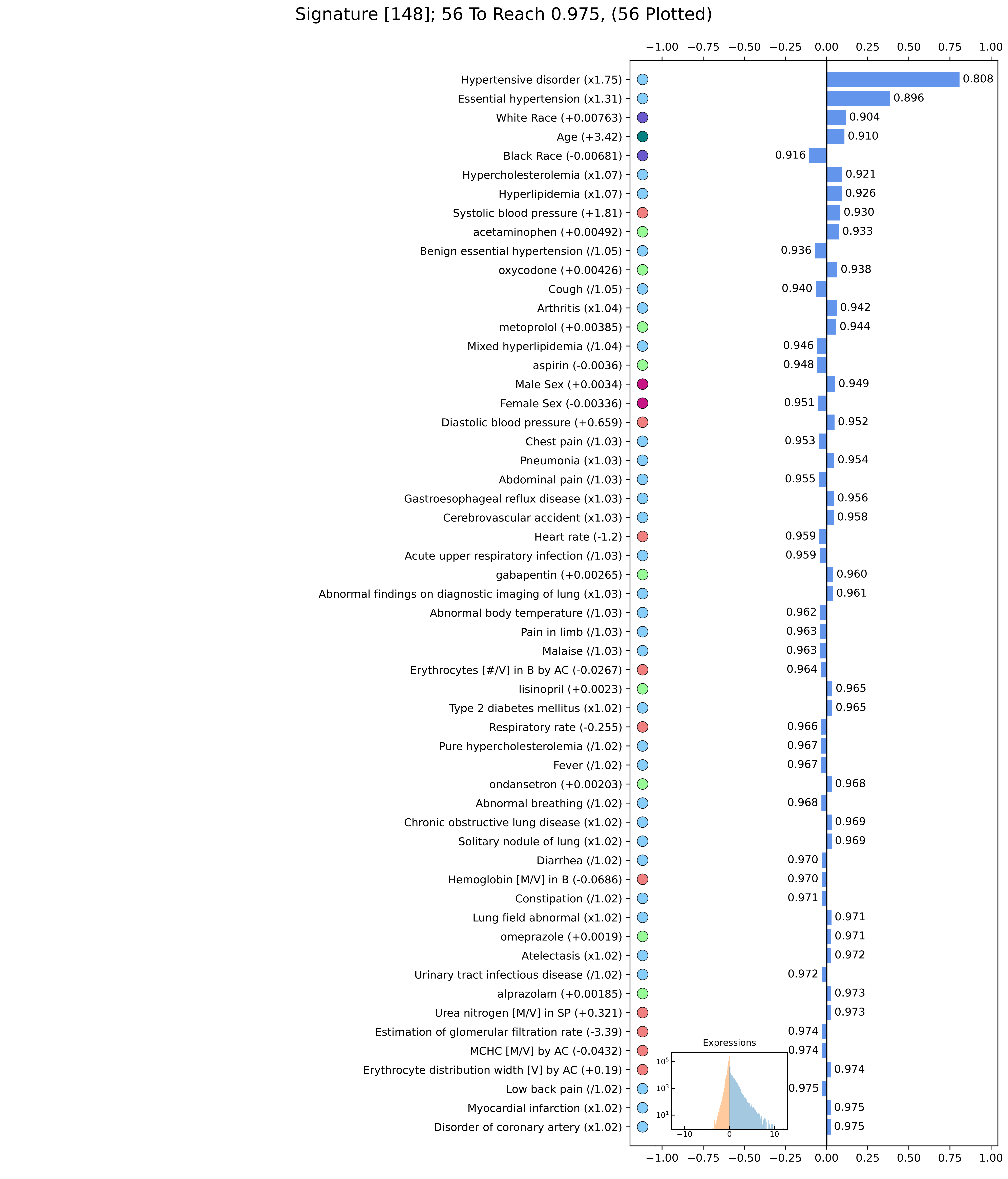
Signature [148]; 56 To Reach 0.975, (56 Plotted)
Hypertensive disorder (x1.75) 0.808
Essential hypertension (x1.31) 0.896
White Race (+0.00763) 0.904
Age (+3.42) 0.910
Black Race (-0.00681) 0.916
Hypercholesterolemia (x1.07) 0.921
Hyperlipidemia (x1.07) 0.926
Systolic blood pressure (+1.81) 0.930
acetaminophen (+0.00492) 0.933
Benign essential hypertension (/1.05) 0.936
oxycodone (+0.00426) 0.938
Cough (/1.05) 0.940
Arthritis (x1.04) 0.942
metoprolol (+0.00385) 0.944
Mixed hyperlipidemia (/1.04) 0.946
aspirin (-0.0036) 0.948
Male Sex (+0.0034) 0.949
Female Sex (-0.00336) 0.951
Diastolic blood pressure (+0.659) 0.952
Chest pain (/1.03) 0.953
Pneumonia (x1.03) 0.954
Abdominal pain (/1.03) 0.955
Gastroesophageal reflux disease (x1.03) 0.956
Cerebrovascular accident (x1.03) 0.958
Heart rate (-1.2) 0.959
Acute upper respiratory infection (/1.03) 0.959
gabapentin (+0.00265) 0.960
Abnormal findings on diagnostic imaging of lung (x1.03) 0.961
Abnormal body temperature (/1.03) 0.962
Pain in limb (/1.03) 0.963
Malaise (/1.03) 0.963
Erythrocytes [#/V] in B by AC (-0.0267) 0.964
lisinopril (+0.0023) 0.965
Type 2 diabetes mellitus (x1.02) 0.965
Respiratory rate (-0.255) 0.966
Pure hypercholesterolemia (/1.02) 0.967
Fever (/1.02) 0.967
ondansetron (+0.00203) 0.968
Abnormal breathing (/1.02) 0.968
Chronic obstructive lung disease (x1.02) 0.969
Solitary nodule of lung (x1.02) 0.969
Diarrhea (/1.02) 0.970
Hemoglobin [M/V] in B (-0.0686) 0.970
Constipation (/1.02) 0.971
Lung field abnormal (x1.02) 0.971
omeprazole (+0.0019) 0.971
Atelectasis (x1.02) 0.972
Urinary tract infectious disease (/1.02) 0.972
alprazolam (+0.00185) 0.973
Urea nitrogen [M/V] in SP (+0.321) 0.973
Estimation of glomerular filtration rate (-3.39) 0.974
MCHC [M/V] by AC (-0.0432) 0.974
Erythrocyte distribution width [V] by AC (+0.19) 0.974
Low back pain (/1.02) 0.975
Myocardial infarction (x1.02) 0.975
Disorder of coronary artery (x1.02) 0.975
−1.00 −0.75 −0.50 −0.25 0.00 0.25 0.50 0.75 1.00
Expressions
10⁵
10³
10¹
−10 0 10

Signature [149]; 1 To Reach 0.975, (10 Plotted)

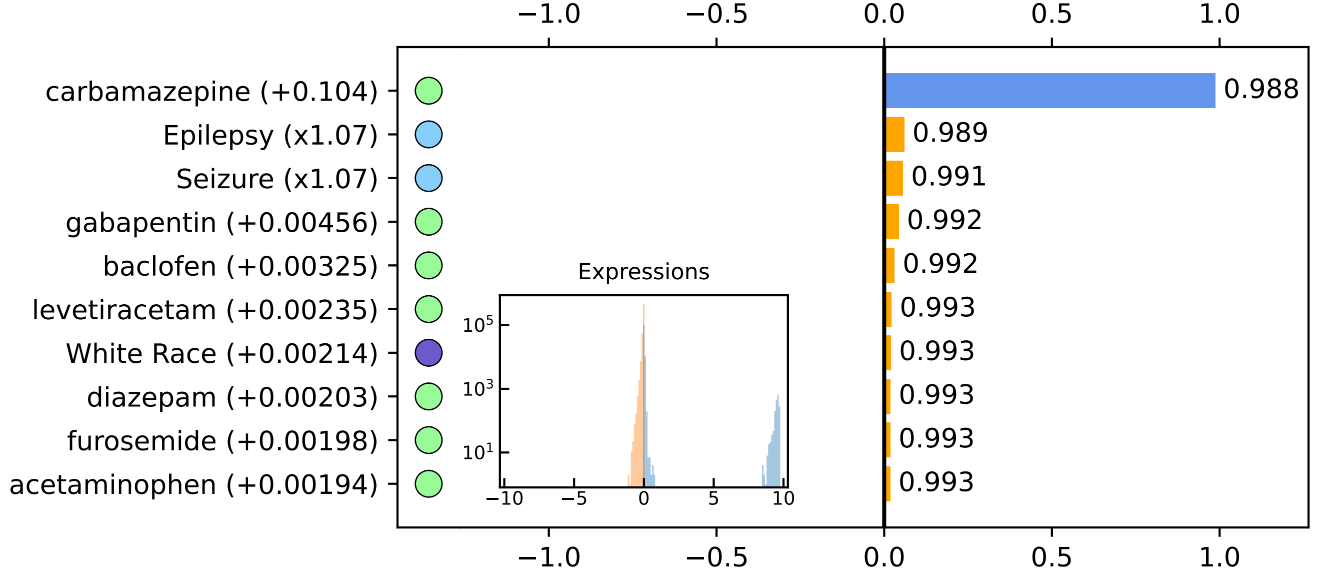

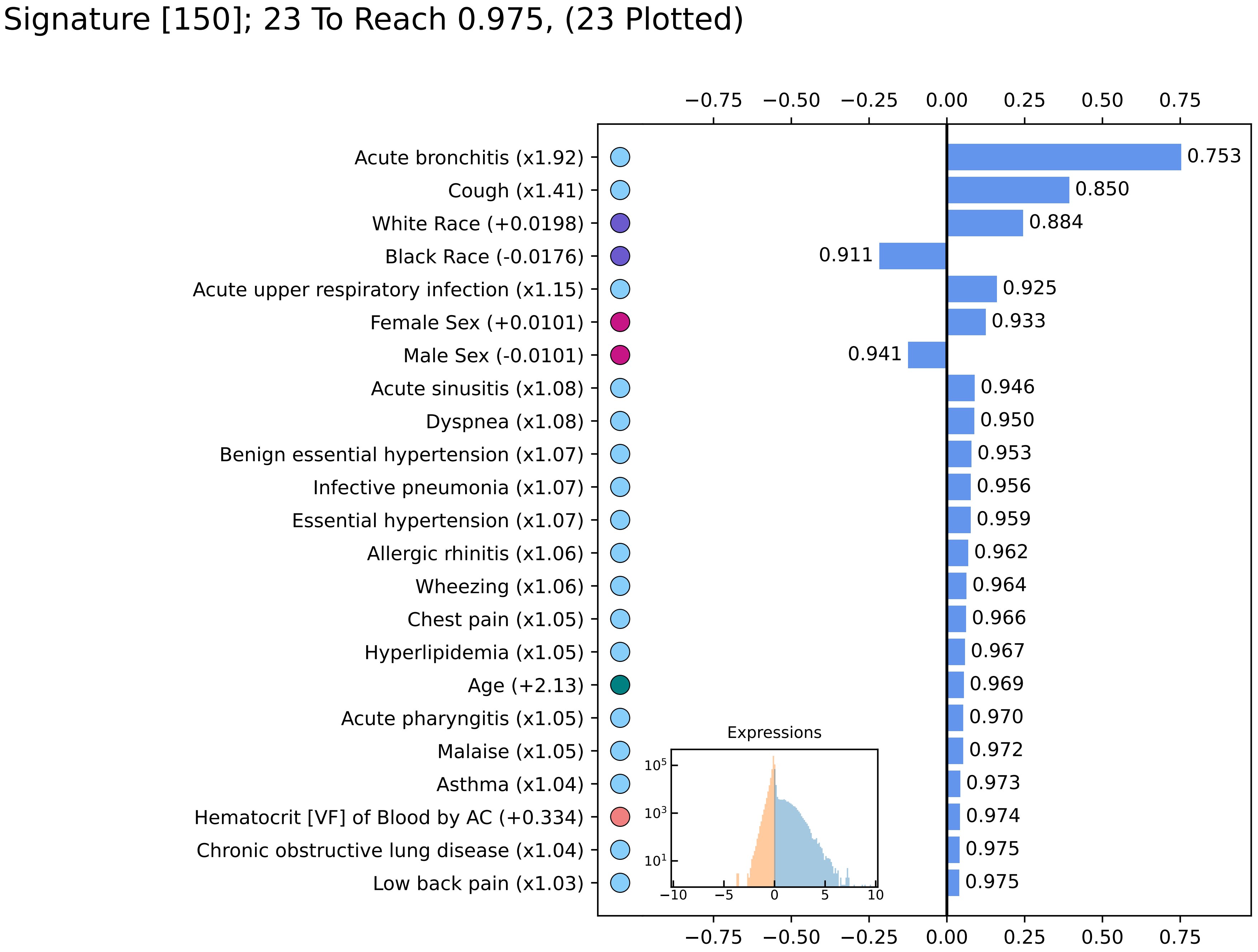
Signature [150]; 23 To Reach 0.975, (23 Plotted)
Acute bronchitis (x1.92) 0.753
Cough (x1.41) 0.850
White Race (+0.0198) 0.884
Black Race (-0.0176) 0.911
Acute upper respiratory infection (x1.15) 0.925
Female Sex (+0.0101) 0.933
Male Sex (-0.0101) 0.941
Acute sinusitis (x1.08) 0.946
Dyspnea (x1.08) 0.950
Benign essential hypertension (x1.07) 0.953
Infective pneumonia (x1.07) 0.956
Essential hypertension (x1.07) 0.959
Allergic rhinitis (x1.06) 0.962
Wheezing (x1.06) 0.964
Chest pain (x1.05) 0.966
Hyperlipidemia (x1.05) 0.967
Age (+2.13) 0.969
Acute pharyngitis (x1.05) 0.970
Malaise (x1.05) 0.972
Asthma (x1.04) 0.973
Hematocrit [VF] of Blood by AC (+0.334) 0.974
Chronic obstructive lung disease (x1.04) 0.975
Low back pain (x1.03) 0.975
−0.75 −0.50 −0.25 0.00 0.25 0.50 0.75
Expressions
−10 −5 0 5 10

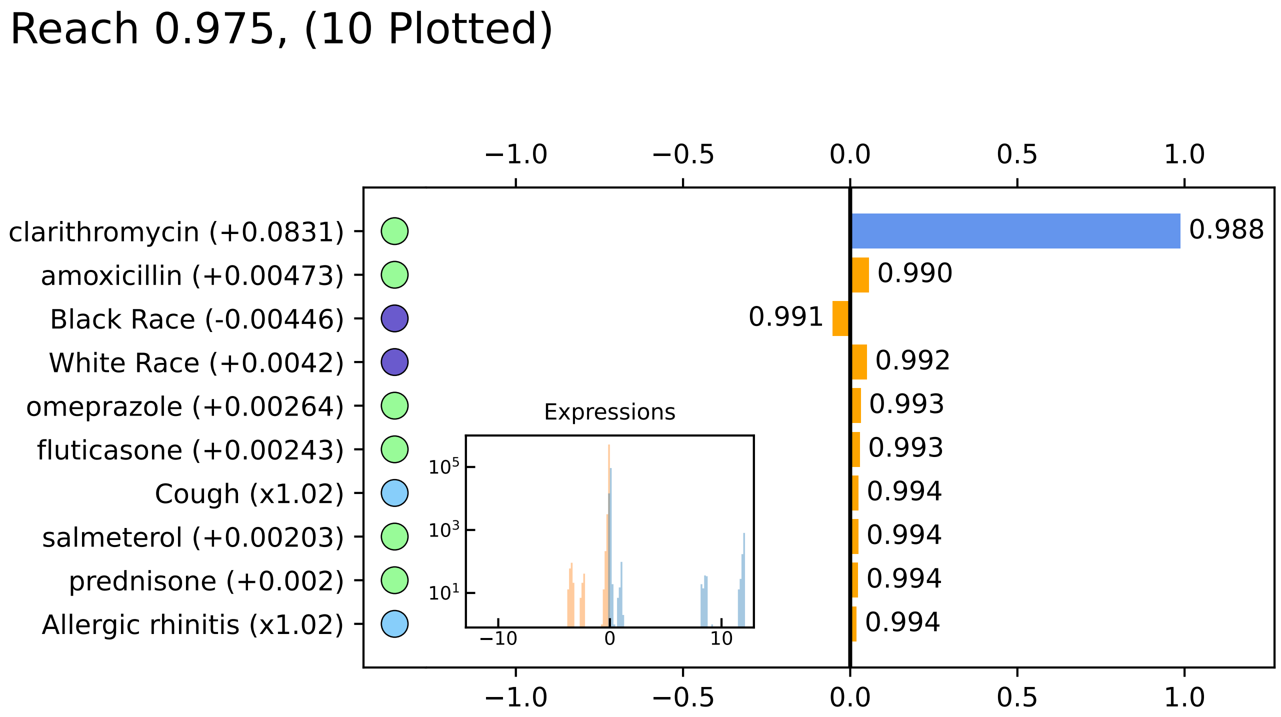
Signature [151]; 1 To Reach 0.975, (10 Plotted)
clarithromycin (+0.0831)
amoxicillin (+0.00473)
Black Race (-0.00446)
White Race (+0.0042)
omeprazole (+0.00264)
fluticasone (+0.00243)
Cough (x1.02)
salmeterol (+0.00203)
prednisone (+0.002)
Allergic rhinitis (x1.02)
0.988
0.990
0.991
0.992
0.993
0.993
0.994
0.994
0.994
0.994
Expressions
−1.0
−0.5
0.0
0.5
1.0

Signature [152]; 9 To Reach 0.975, (10 Plotted)

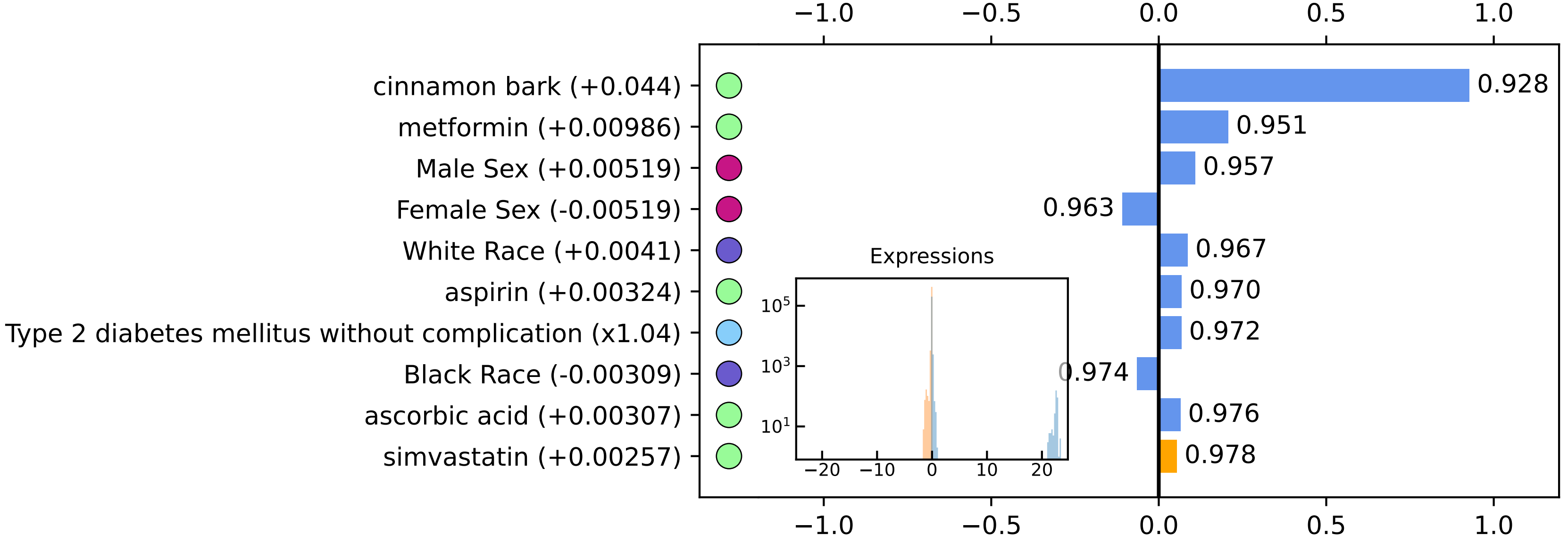

Signature [153]; 9 To Reach 0.975, (10 Plotted)

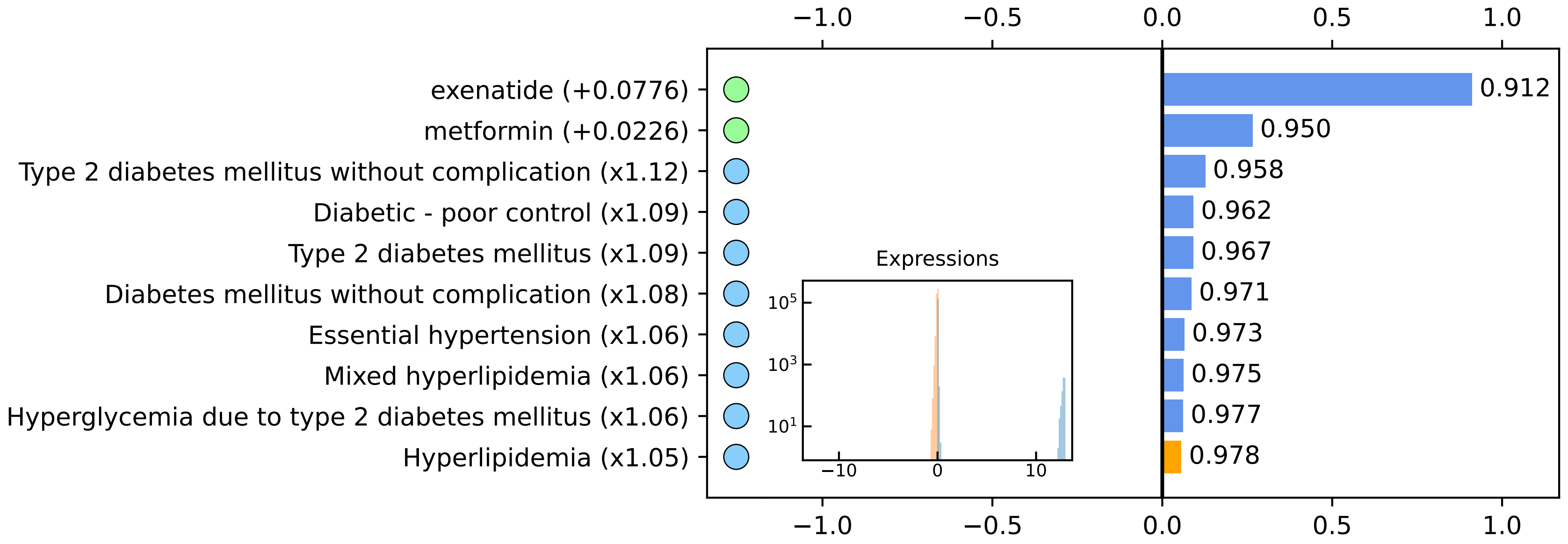

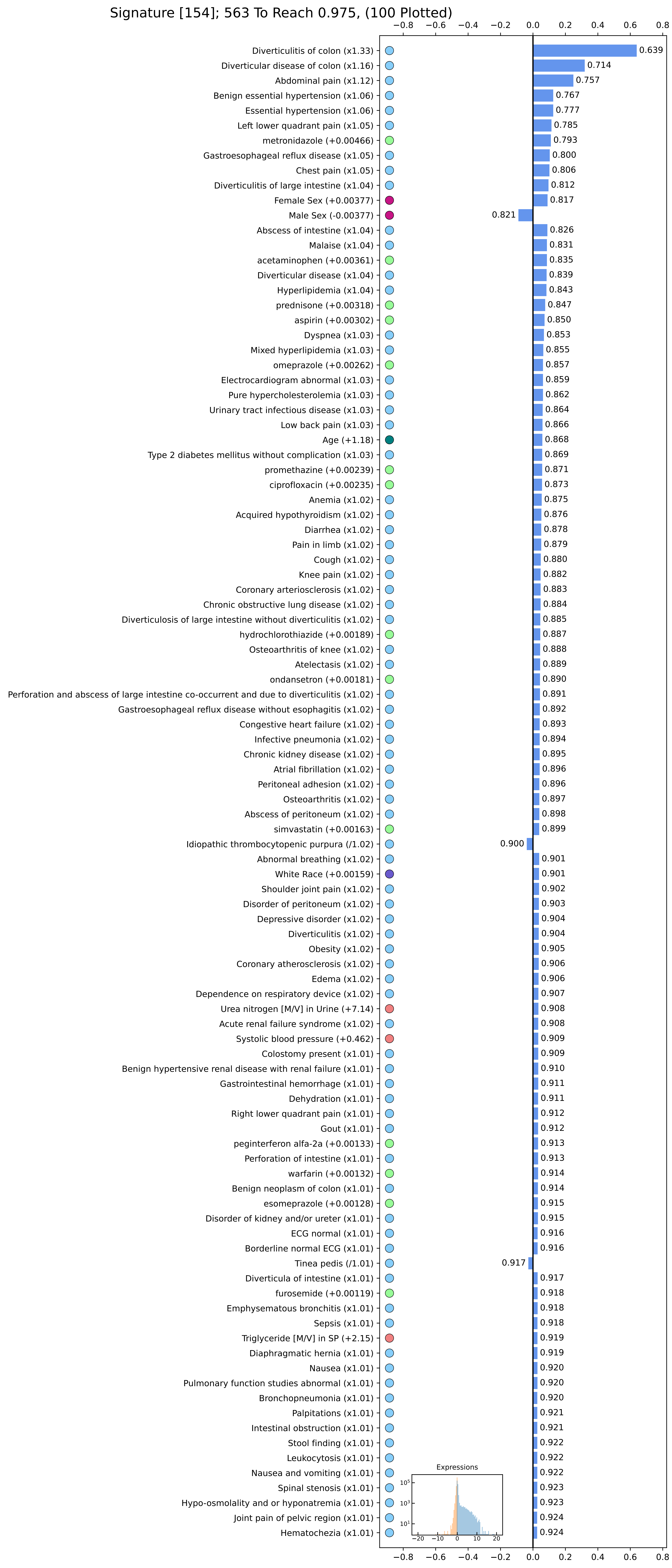

Signature [154]; 563 To Reach 0.975, (100 Plotted)
Diverticulitis of colon (x1.33) 0.639
Diverticular disease of colon (x1.16) 0.714
Abdominal pain (x1.12) 0.757
Benign essential hypertension (x1.06) 0.767
Essential hypertension (x1.06) 0.777
Left lower quadrant pain (x1.05) 0.785
metronidazole (+0.00466) 0.793
Gastroesophageal reflux disease (x1.05) 0.800
Chest pain (x1.05) 0.806
Diverticulitis of large intestine (x1.04) 0.812
Female Sex (+0.00377) 0.817
Male Sex (-0.00377) 0.821
Abscess of intestine (x1.04) 0.826
Malaise (x1.04) 0.831
acetaminophen (+0.00361) 0.835
Diverticular disease (x1.04) 0.839
Hyperlipidemia (x1.04) 0.843
prednisone (+0.00318) 0.847
aspirin (+0.00302) 0.850
Dyspnea (x1.03) 0.853
Mixed hyperlipidemia (x1.03) 0.855
omeprazole (+0.00262) 0.857
Electrocardiogram abnormal (x1.03) 0.859
Pure hypercholesterolemia (x1.03) 0.862
Urinary tract infectious disease (x1.03) 0.864
Low back pain (x1.03) 0.866
Age (+1.18) 0.868
Type 2 diabetes mellitus without complication (x1.03) 0.869
promethazine (+0.00239) 0.871
ciprofloxacin (+0.00235) 0.873
Anemia (x1.02) 0.875
Acquired hypothyroidism (x1.02) 0.876
Diarrhea (x1.02) 0.878
Pain in limb (x1.02) 0.879
Cough (x1.02) 0.880
Knee pain (x1.02) 0.882
Coronary arteriosclerosis (x1.02) 0.883
Chronic obstructive lung disease (x1.02) 0.884
Diverticulosis of large intestine without diverticulitis (x1.02) 0.885
hydrochlorothiazide (+0.00189) 0.887
Osteoarthritis of knee (x1.02) 0.888
Atelectasis (x1.02) 0.889
ondansetron (+0.00181) 0.890
Perforation and abscess of large intestine co-occurrent and due to diverticulitis (x1.02) 0.891
Gastroesophageal reflux disease without esophagitis (x1.02) 0.892
Congestive heart failure (x1.02) 0.893
Infective pneumonia (x1.02) 0.894
Chronic kidney disease (x1.02) 0.895
Atrial fibrillation (x1.02) 0.896
Peritoneal adhesion (x1.02) 0.896
Osteoarthritis (x1.02) 0.897
Abscess of peritoneum (x1.02) 0.898
simvastatin (+0.00163) 0.899
Idiopathic thrombocytopenic purpura (/1.02) 0.900
Abnormal breathing (x1.02) 0.901
White Race (+0.00159) 0.901
Shoulder joint pain (x1.02) 0.902
Disorder of peritoneum (x1.02) 0.903
Depressive disorder (x1.02) 0.904
Diverticulitis (x1.02) 0.904
Obesity (x1.02) 0.905
Coronary atherosclerosis (x1.02) 0.906
Edema (x1.02) 0.906
Dependence on respiratory device (x1.02) 0.907
Urea nitrogen [M/V] in Urine (+7.14) 0.908
Acute renal failure syndrome (x1.02) 0.908
Systolic blood pressure (+0.462) 0.909
Colostomy present (x1.01) 0.909
Benign hypertensive renal disease with renal failure (x1.01) 0.910
Gastrointestinal hemorrhage (x1.01) 0.911
Dehydration (x1.01) 0.911
Right lower quadrant pain (x1.01) 0.912
Gout (x1.01) 0.912
peginterferon alfa-2a (+0.00133) 0.913
Perforation of intestine (x1.01) 0.913
warfarin (+0.00132) 0.914
Benign neoplasm of colon (x1.01) 0.914
esomeprazole (+0.00128) 0.915
Disorder of kidney and/or ureter (x1.01) 0.915
ECG normal (x1.01) 0.916
Borderline normal ECG (x1.01) 0.916
Tinea pedis (/1.01) 0.917
Diverticula of intestine (x1.01) 0.917
furosemide (+0.00119) 0.918
Emphysematous bronchitis (x1.01) 0.918
Sepsis (x1.01) 0.918
Triglyceride [M/V] in SP (+2.15) 0.919
Diaphragmatic hernia (x1.01) 0.919
Nausea (x1.01) 0.920
Pulmonary function studies abnormal (x1.01) 0.920
Bronchopneumonia (x1.01) 0.920
Palpitations (x1.01) 0.921
Intestinal obstruction (x1.01) 0.921
Stool finding (x1.01) 0.922
Leukocytosis (x1.01) 0.922
Nausea and vomiting (x1.01) 0.922
Spinal stenosis (x1.01) 0.923
Hypo-osmolality and or hyponatremia (x1.01) 0.923
Joint pain of pelvic region (x1.01) 0.924
Hematochezia (x1.01) 0.924
−0.8 −0.6 −0.4 −0.2 0.0 0.2 0.4 0.6 0.8
Expressions

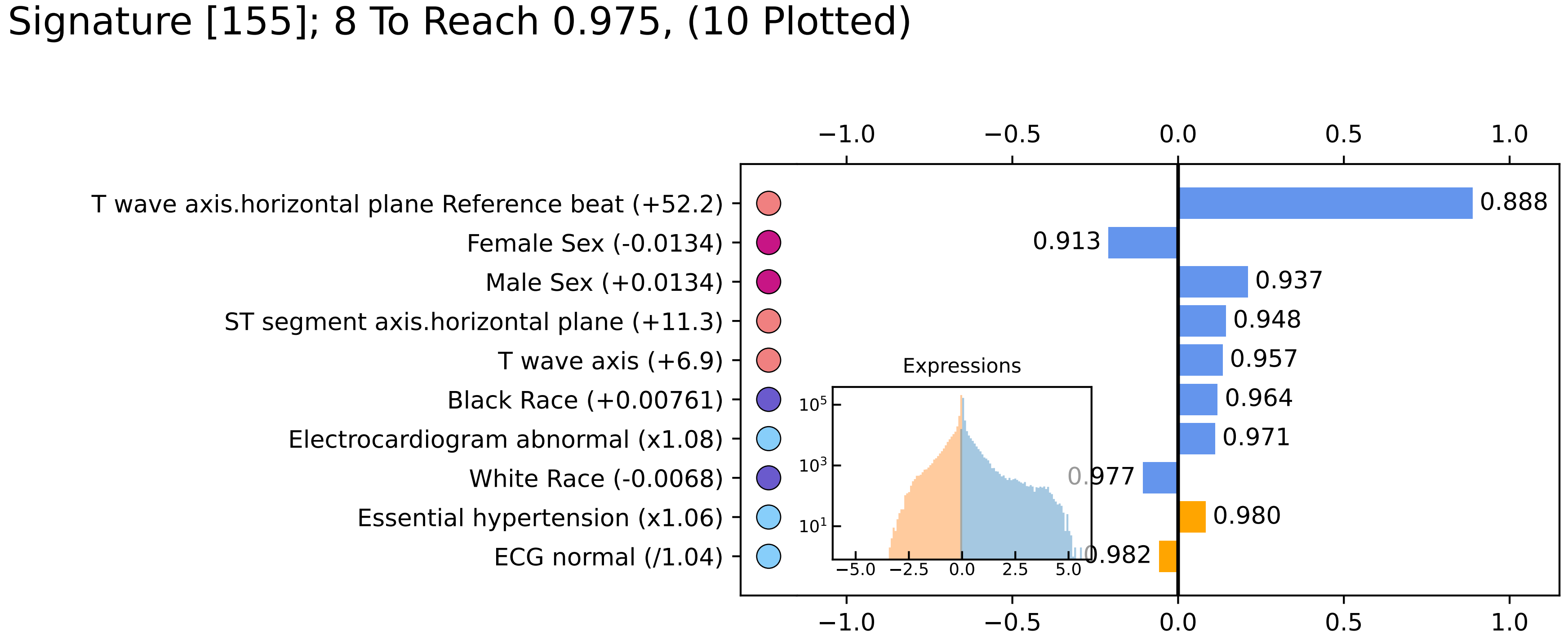
Signature [155]; 8 To Reach 0.975, (10 Plotted)
T wave axis.horizontal plane Reference beat (+52.2)
Female Sex (-0.0134)
Male Sex (+0.0134)
ST segment axis.horizontal plane (+11.3)
T wave axis (+6.9)
Black Race (+0.00761)
Electrocardiogram abnormal (x1.08)
White Race (-0.0068)
Essential hypertension (x1.06)
ECG normal (/1.04)
0.888
0.913
0.937
0.948
0.957
0.964
0.971
0.977
0.980
0.982
−1.0
−0.5
0.0
0.5
1.0
Expressions
10^5
10^3
10^1
−5.0
−2.5
0.0
2.5
5.0

Signature [156]; 121 To Reach 0.975, (100 Plotted)

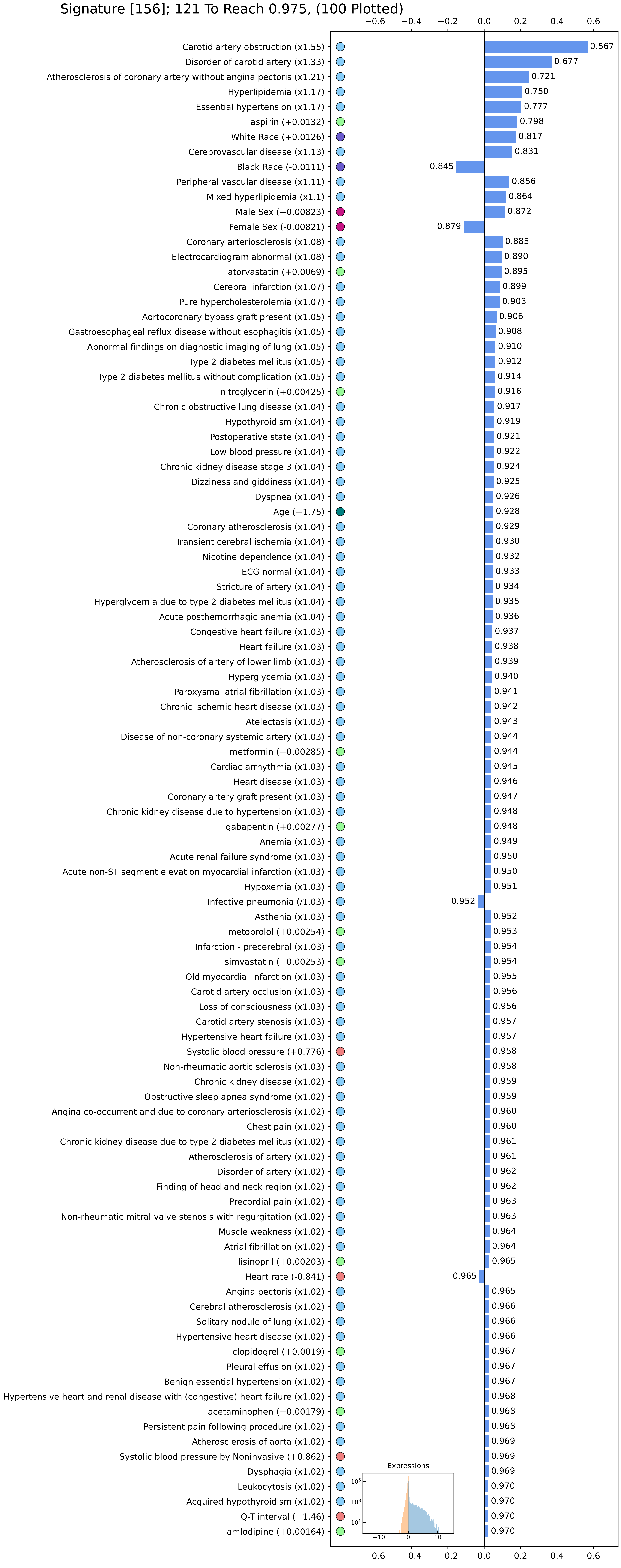

Signature [157]; 1 To Reach 0.975, (10 Plotted)

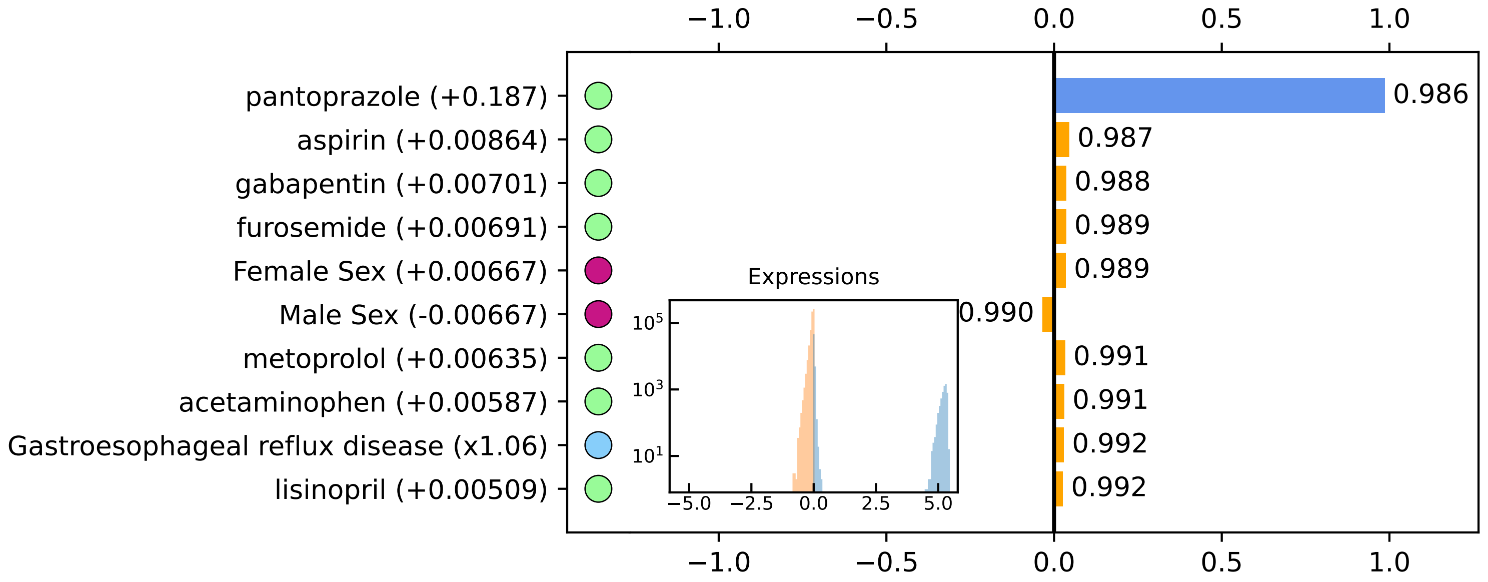

Signature [158]; 424 To Reach 0.975, (100 Plotted)

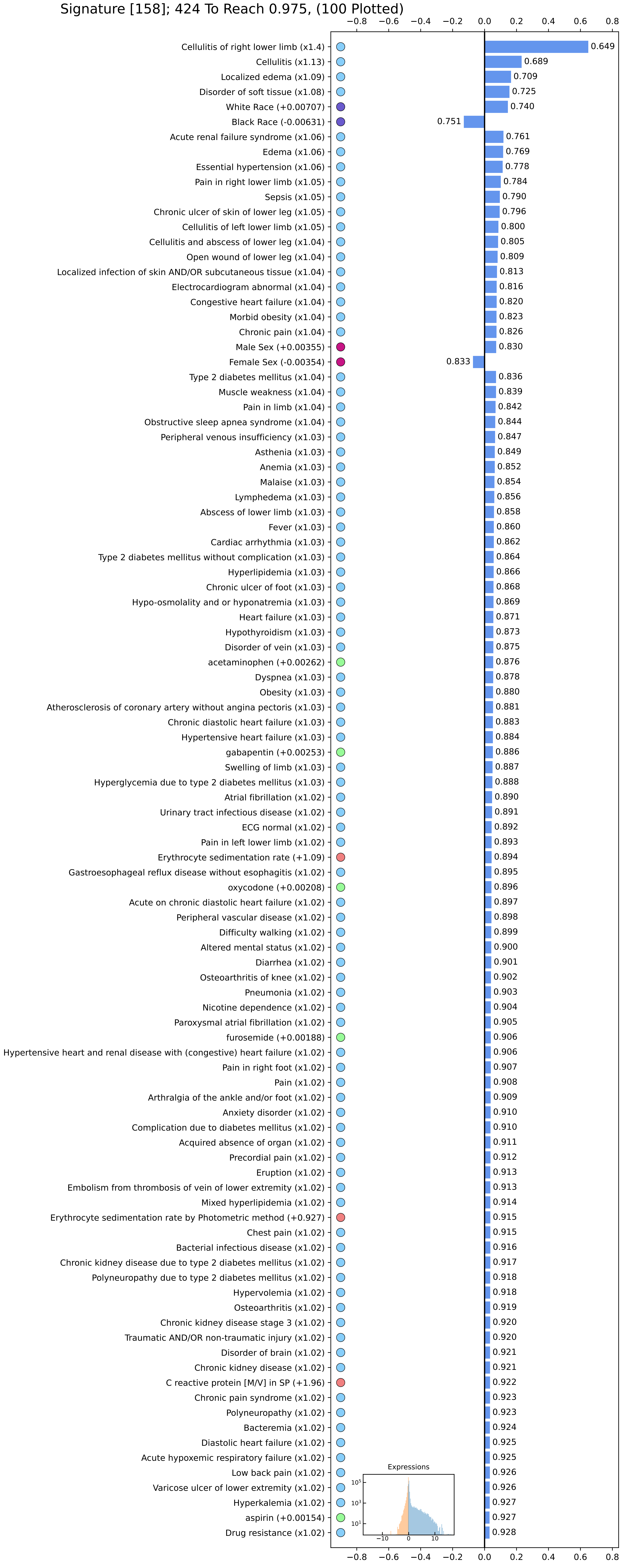

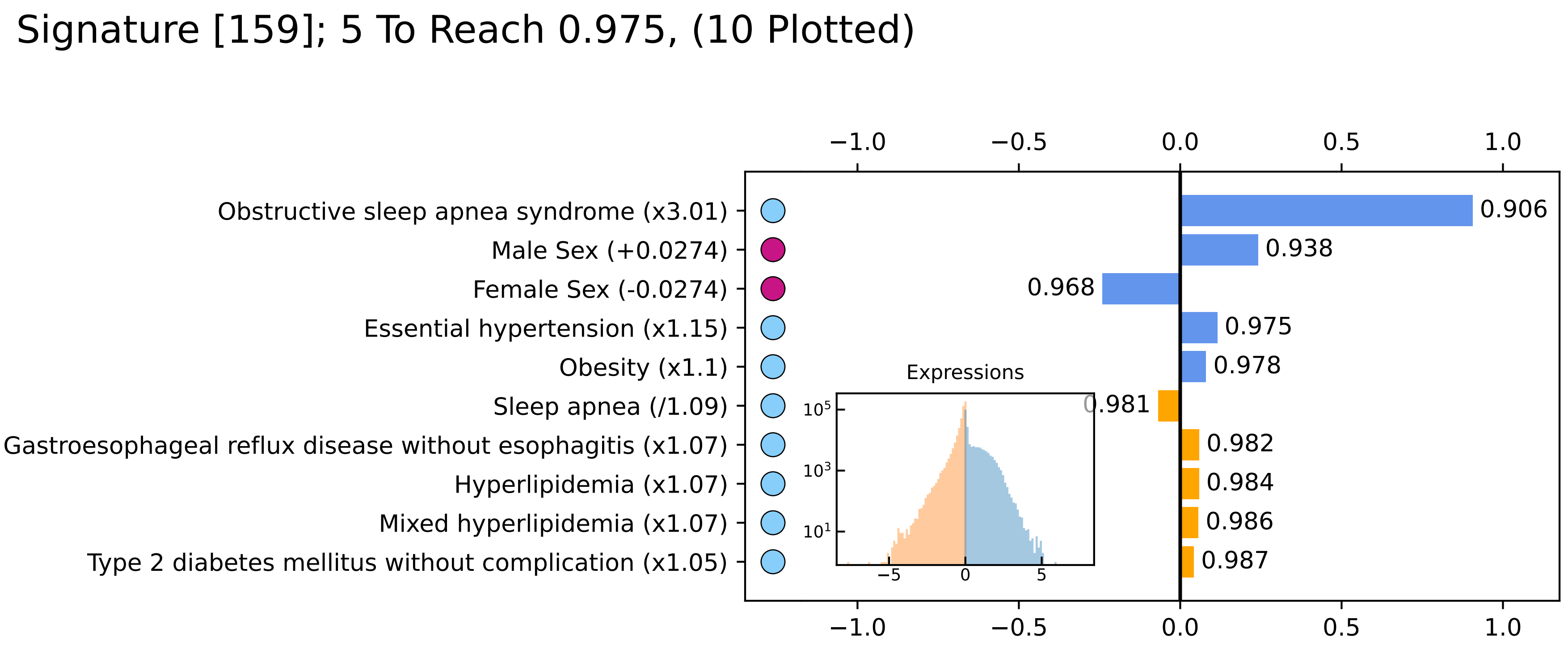
Signature [159]; 5 To Reach 0.975, (10 Plotted)
Obstructive sleep apnea syndrome (x3.01)
Male Sex (+0.0274)
Female Sex (-0.0274)
Essential hypertension (x1.15)
Obesity (x1.1)
Sleep apnea (/1.09)
Gastroesophageal reflux disease without esophagitis (x1.07)
Hyperlipidemia (x1.07)
Mixed hyperlipidemia (x1.07)
Type 2 diabetes mellitus without complication (x1.05)
0.906
0.938
0.968
0.975
0.978
0.981
0.982
0.984
0.986
0.987
Expressions
−1.0
−0.5
0.0
0.5
1.0

Signature [160]; 1 To Reach 0.975, (10 Plotted)

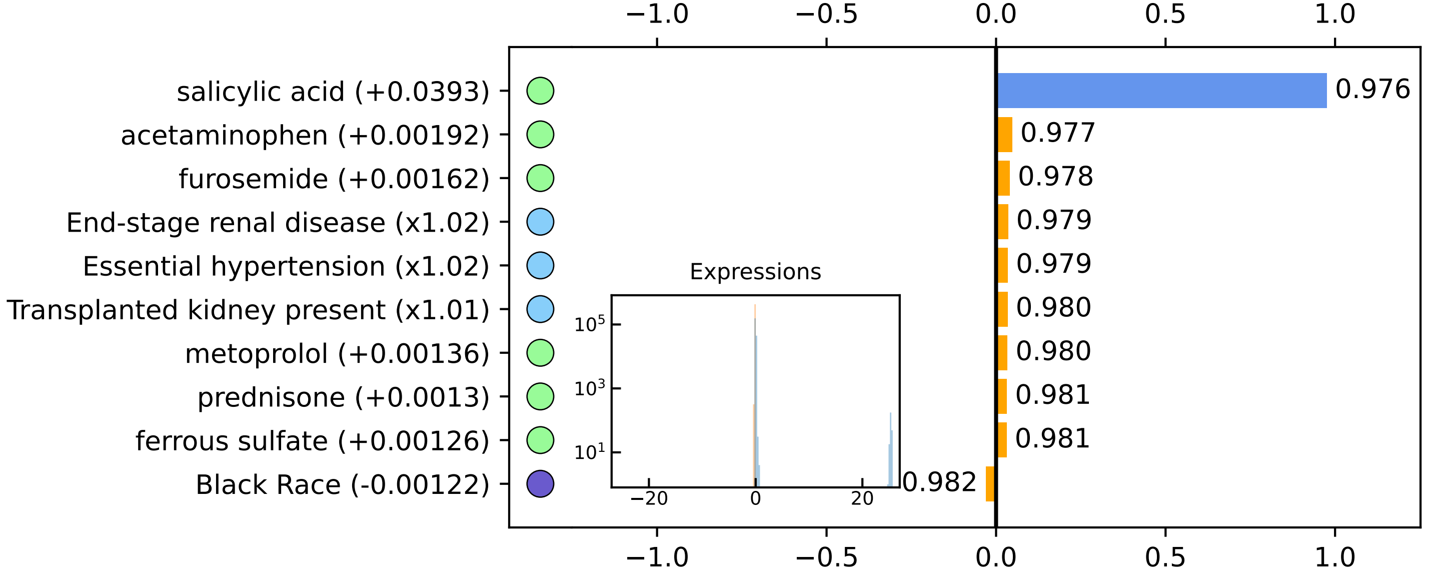

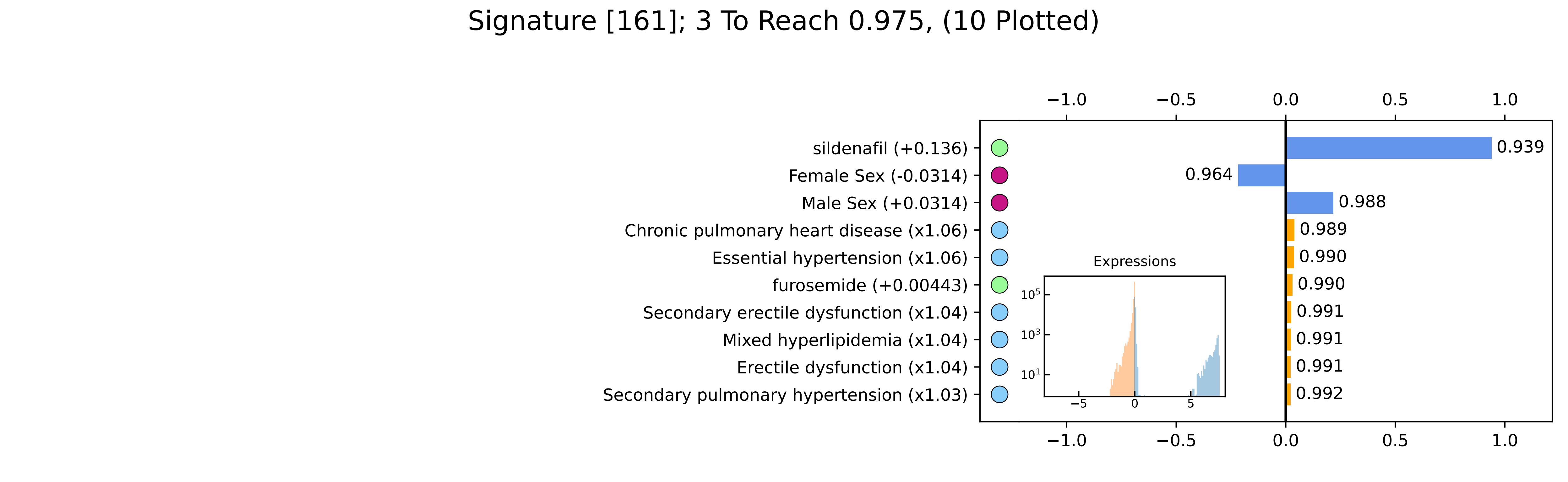
Signature [161]; 3 To Reach 0.975, (10 Plotted)
sildenafil (+0.136)
Female Sex (-0.0314)
Male Sex (+0.0314)
Chronic pulmonary heart disease (x1.06)
Essential hypertension (x1.06)
furosemide (+0.00443)
Secondary erectile dysfunction (x1.04)
Mixed hyperlipidemia (x1.04)
Erectile dysfunction (x1.04)
Secondary pulmonary hypertension (x1.03)
0.939
0.964
0.988
0.989
0.990
0.990
0.991
0.991
0.991
0.992
−1.0
−0.5
0.0
0.5
1.0
Expressions

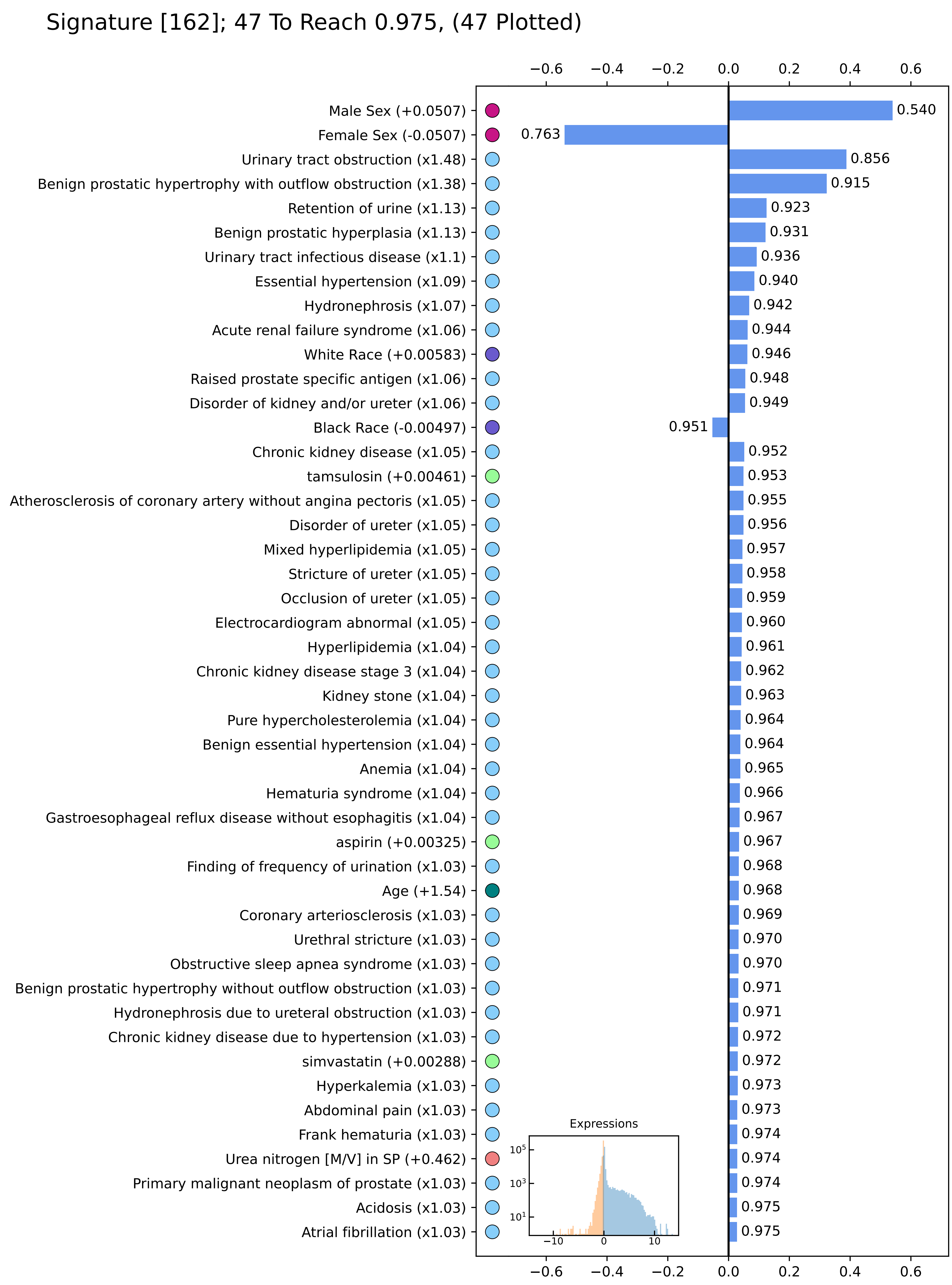
Signature [162]; 47 To Reach 0.975, (47 Plotted)
−0.6
−0.4
−0.2
0.0
0.2
0.4
0.6
Male Sex (+0.0507)
0.540
Female Sex (-0.0507)
0.763
Urinary tract obstruction (x1.48)
0.856
Benign prostatic hypertrophy with outflow obstruction (x1.38)
0.915
Retention of urine (x1.13)
0.923
Benign prostatic hyperplasia (x1.13)
0.931
Urinary tract infectious disease (x1.1)
0.936
Essential hypertension (x1.09)
0.940
Hydronephrosis (x1.07)
0.942
Acute renal failure syndrome (x1.06)
0.944
White Race (+0.00583)
0.946
Raised prostate specific antigen (x1.06)
0.948
Disorder of kidney and/or ureter (x1.06)
0.949
Black Race (-0.00497)
0.951
Chronic kidney disease (x1.05)
0.952
tamsulosin (+0.00461)
0.953
Atherosclerosis of coronary artery without angina pectoris (x1.05)
0.955
Disorder of ureter (x1.05)
0.956
Mixed hyperlipidemia (x1.05)
0.957
Stricture of ureter (x1.05)
0.958
Occlusion of ureter (x1.05)
0.959
Electrocardiogram abnormal (x1.05)
0.960
Hyperlipidemia (x1.04)
0.961
Chronic kidney disease stage 3 (x1.04)
0.962
Kidney stone (x1.04)
0.963
Pure hypercholesterolemia (x1.04)
0.964
Benign essential hypertension (x1.04)
0.964
Anemia (x1.04)
0.965
Hematuria syndrome (x1.04)
0.966
Gastroesophageal reflux disease without esophagitis (x1.04)
0.967
aspirin (+0.00325)
0.967
Finding of frequency of urination (x1.03)
0.968
Age (+1.54)
0.968
Coronary arteriosclerosis (x1.03)
0.969
Urethral stricture (x1.03)
0.970
Obstructive sleep apnea syndrome (x1.03)
0.970
Benign prostatic hypertrophy without outflow obstruction (x1.03)
0.971
Hydronephrosis due to ureteral obstruction (x1.03)
0.971
Chronic kidney disease due to hypertension (x1.03)
0.972
simvastatin (+0.00288)
0.972
Hyperkalemia (x1.03)
0.973
Abdominal pain (x1.03)
0.973
Frank hematuria (x1.03)
0.974
Urea nitrogen [M/V] in SP (+0.462)
0.974
Primary malignant neoplasm of prostate (x1.03)
0.974
Acidosis (x1.03)
0.975
Atrial fibrillation (x1.03)
0.975
Expressions
10^5
10^3
10^1
−10
0
10

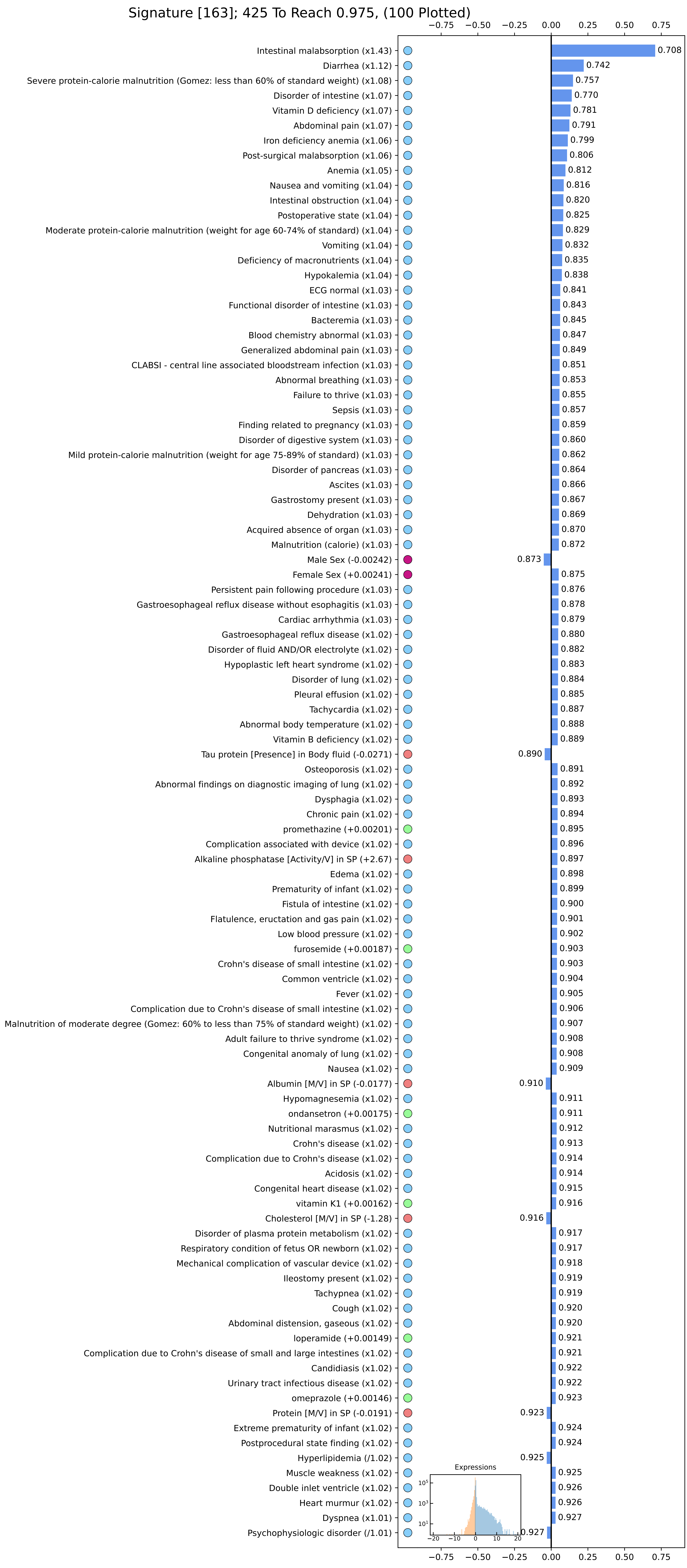

Signature [163]; 425 To Reach 0.975, (100 Plotted)
Intestinal malabsorption (x1.43) 0.708
Diarrhea (x1.12) 0.742
Severe protein-calorie malnutrition (Gomez: less than 60% of standard weight) (x1.08) 0.757
Disorder of intestine (x1.07) 0.770
Vitamin D deficiency (x1.07) 0.781
Abdominal pain (x1.07) 0.791
Iron deficiency anemia (x1.06) 0.799
Post-surgical malabsorption (x1.06) 0.806
Anemia (x1.05) 0.812
Nausea and vomiting (x1.04) 0.816
Intestinal obstruction (x1.04) 0.820
Postoperative state (x1.04) 0.825
Moderate protein-calorie malnutrition (weight for age 60-74% of standard) (x1.04) 0.829
Vomiting (x1.04) 0.832
Deficiency of macronutrients (x1.04) 0.835
Hypokalemia (x1.04) 0.838
ECG normal (x1.03) 0.841
Functional disorder of intestine (x1.03) 0.843
Bacteremia (x1.03) 0.845
Blood chemistry abnormal (x1.03) 0.847
Generalized abdominal pain (x1.03) 0.849
CLABSI - central line associated bloodstream infection (x1.03) 0.851
Abnormal breathing (x1.03) 0.853
Failure to thrive (x1.03) 0.855
Sepsis (x1.03) 0.857
Finding related to pregnancy (x1.03) 0.859
Disorder of digestive system (x1.03) 0.860
Mild protein-calorie malnutrition (weight for age 75-89% of standard) (x1.03) 0.862
Disorder of pancreas (x1.03) 0.864
Ascites (x1.03) 0.866
Gastrostomy present (x1.03) 0.867
Dehydration (x1.03) 0.869
Acquired absence of organ (x1.03) 0.870
Malnutrition (calorie) (x1.03) 0.872
Male Sex (-0.00242) 0.873
Female Sex (+0.00241) 0.875
Persistent pain following procedure (x1.03) 0.876
Gastroesophageal reflux disease without esophagitis (x1.03) 0.878
Cardiac arrhythmia (x1.03) 0.879
Gastroesophageal reflux disease (x1.02) 0.880
Disorder of fluid AND/OR electrolyte (x1.02) 0.882
Hypoplastic left heart syndrome (x1.02) 0.883
Disorder of lung (x1.02) 0.884
Pleural effusion (x1.02) 0.885
Tachycardia (x1.02) 0.887
Abnormal body temperature (x1.02) 0.888
Vitamin B deficiency (x1.02) 0.889
Tau protein [Presence] in Body fluid (-0.0271) 0.890
Osteoporosis (x1.02) 0.891
Abnormal findings on diagnostic imaging of lung (x1.02) 0.892
Dysphagia (x1.02) 0.893
Chronic pain (x1.02) 0.894
promethazine (+0.00201) 0.895
Complication associated with device (x1.02) 0.896
Alkaline phosphatase [Activity/V] in SP (+2.67) 0.897
Edema (x1.02) 0.898
Prematurity of infant (x1.02) 0.899
Fistula of intestine (x1.02) 0.900
Flatulence, eructation and gas pain (x1.02) 0.901
Low blood pressure (x1.02) 0.902
furosemide (+0.00187) 0.903
Crohn's disease of small intestine (x1.02) 0.903
Common ventricle (x1.02) 0.904
Fever (x1.02) 0.905
Complication due to Crohn's disease of small intestine (x1.02) 0.906
Malnutrition of moderate degree (Gomez: 60% to less than 75% of standard weight) (x1.02) 0.907
Adult failure to thrive syndrome (x1.02) 0.908
Congenital anomaly of lung (x1.02) 0.908
Nausea (x1.02) 0.909
Albumin [M/V] in SP (-0.0177) 0.910
Hypomagnesemia (x1.02) 0.911
ondansetron (+0.00175) 0.911
Nutritional marasmus (x1.02) 0.912
Crohn's disease (x1.02) 0.913
Complication due to Crohn's disease (x1.02) 0.914
Acidosis (x1.02) 0.914
Congenital heart disease (x1.02) 0.915
vitamin K1 (+0.00162) 0.916
Cholesterol [M/V] in SP (-1.28) 0.916
Disorder of plasma protein metabolism (x1.02) 0.917
Respiratory condition of fetus OR newborn (x1.02) 0.917
Mechanical complication of vascular device (x1.02) 0.918
Ileostomy present (x1.02) 0.919
Tachypnea (x1.02) 0.919
Cough (x1.02) 0.920
Abdominal distension, gaseous (x1.02) 0.920
loperamide (+0.00149) 0.921
Complication due to Crohn's disease of small and large intestines (x1.02) 0.921
Candidiasis (x1.02) 0.922
Urinary tract infectious disease (x1.02) 0.922
omeprazole (+0.00146) 0.923
Protein [M/V] in SP (-0.0191) 0.923
Extreme prematurity of infant (x1.02) 0.924
Postprocedural state finding (x1.02) 0.924
Hyperlipidemia (/1.02) 0.925
Muscle weakness (x1.02) 0.925
Double inlet ventricle (x1.02) 0.926
Heart murmur (x1.02) 0.926
Dyspnea (x1.01) 0.927
Psychophysiologic disorder (/1.01) 0.927
−0.75 −0.50 −0.25 0.00 0.25 0.50 0.75
Expressions

Signature [164]; 6 To Reach 0.975, (10 Plotted)

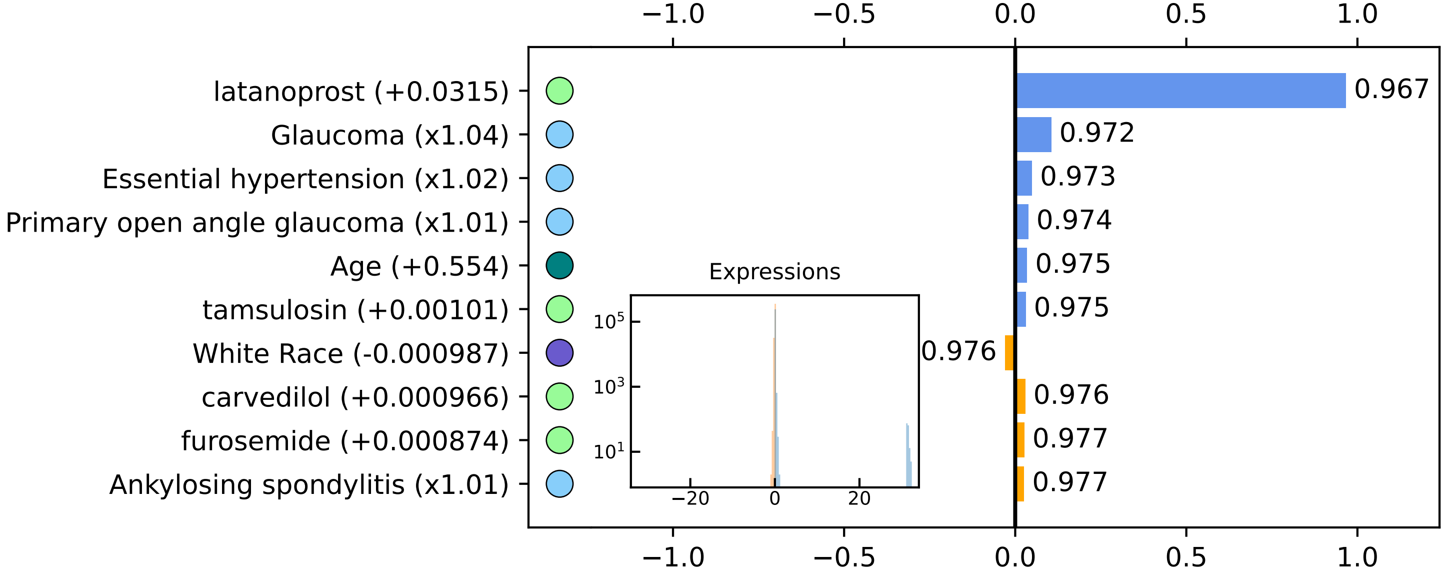

Signature [165]; 2 To Reach 0.975, (10 Plotted)

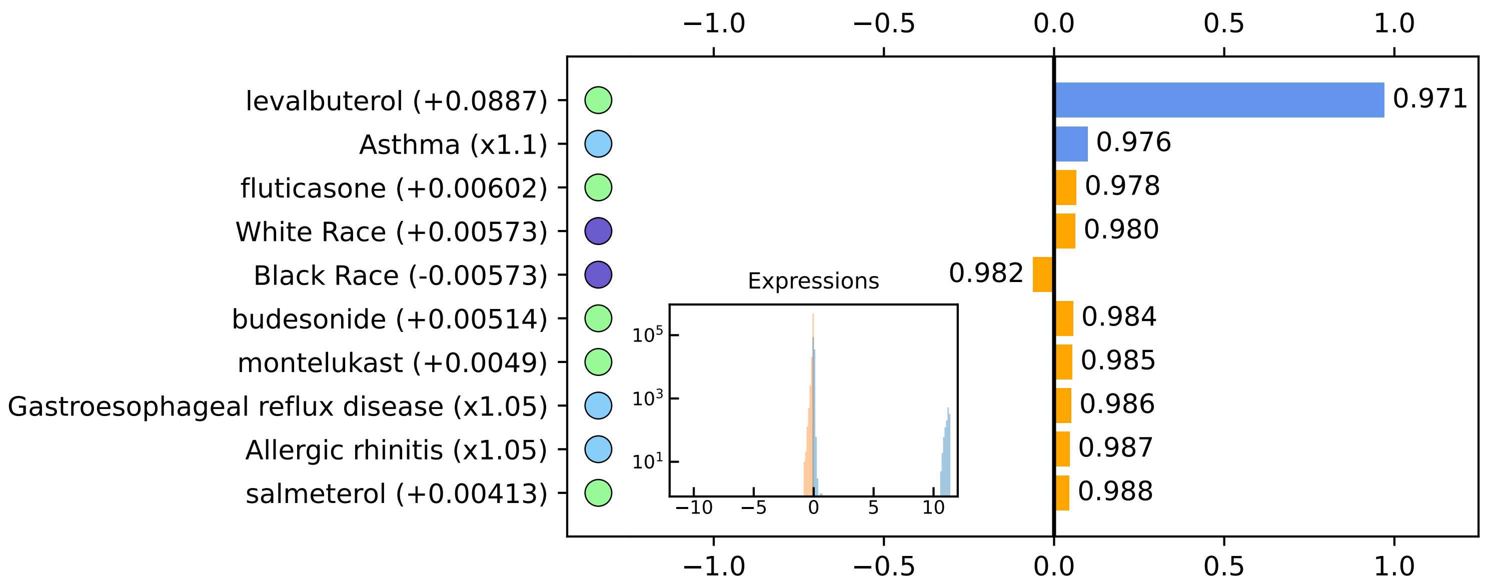

Signature [166]; 1 To Reach 0.975, (10 Plotted)

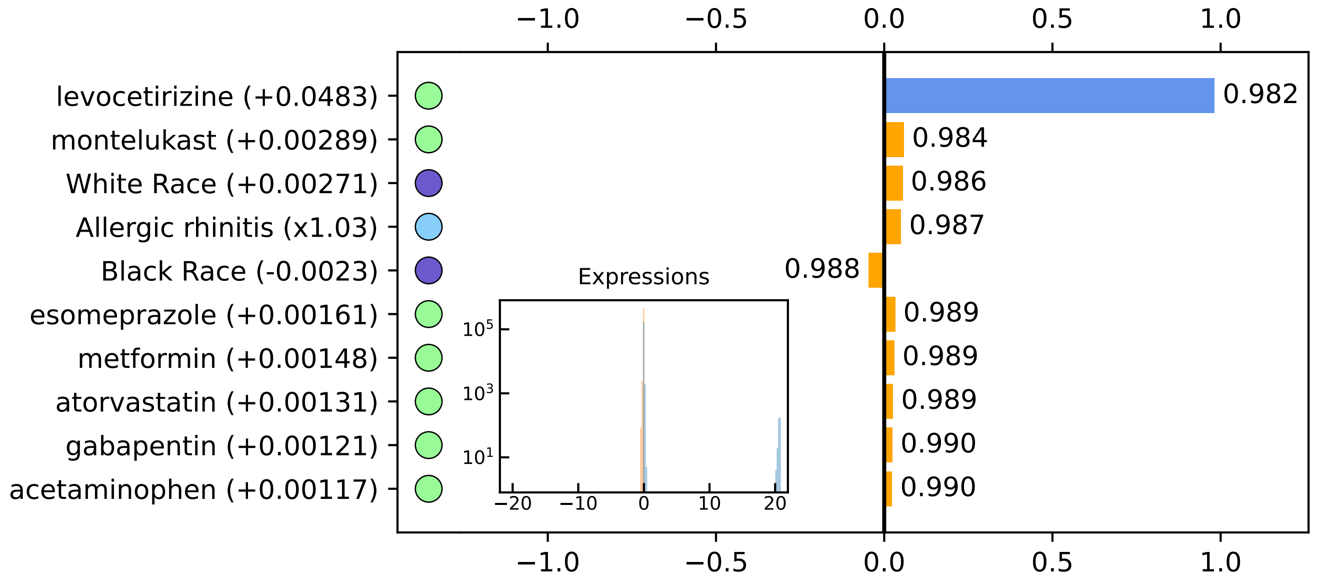

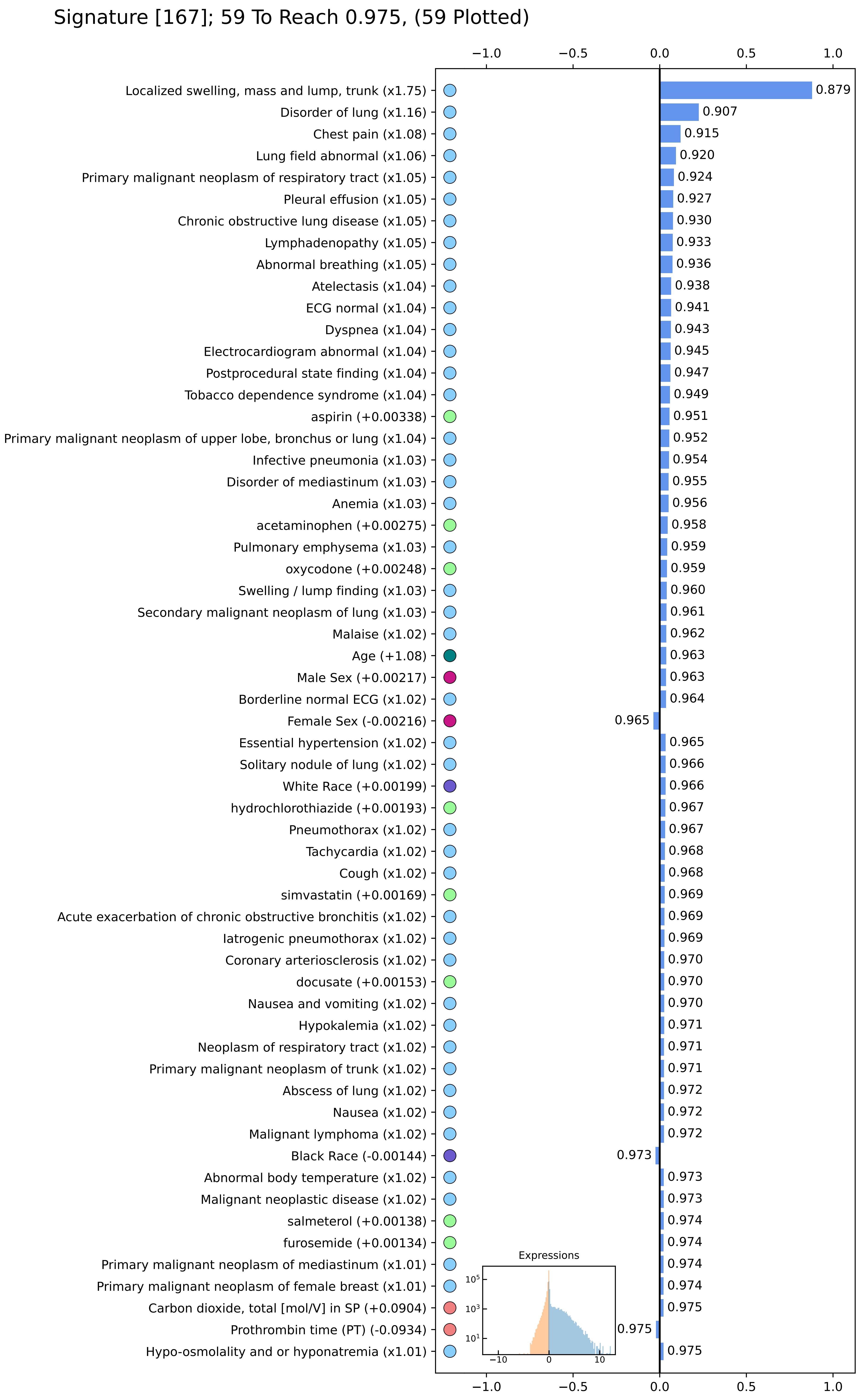

Signature [167]; 59 To Reach 0.975, (59 Plotted)
Localized swelling, mass and lump, trunk (x1.75) 0.879
Disorder of lung (x1.16) 0.907
Chest pain (x1.08) 0.915
Lung field abnormal (x1.06) 0.920
Primary malignant neoplasm of respiratory tract (x1.05) 0.924
Pleural effusion (x1.05) 0.927
Chronic obstructive lung disease (x1.05) 0.930
Lymphadenopathy (x1.05) 0.933
Abnormal breathing (x1.05) 0.936
Atelectasis (x1.04) 0.938
ECG normal (x1.04) 0.941
Dyspnea (x1.04) 0.943
Electrocardiogram abnormal (x1.04) 0.945
Postprocedural state finding (x1.04) 0.947
Tobacco dependence syndrome (x1.04) 0.949
aspirin (+0.00338) 0.951
Primary malignant neoplasm of upper lobe, bronchus or lung (x1.04) 0.952
Infective pneumonia (x1.03) 0.954
Disorder of mediastinum (x1.03) 0.955
Anemia (x1.03) 0.956
acetaminophen (+0.00275) 0.958
Pulmonary emphysema (x1.03) 0.959
oxycodone (+0.00248) 0.959
Swelling / lump finding (x1.03) 0.960
Secondary malignant neoplasm of lung (x1.03) 0.961
Malaise (x1.02) 0.962
Age (+1.08) 0.963
Male Sex (+0.00217) 0.963
Borderline normal ECG (x1.02) 0.964
Female Sex (-0.00216) 0.965
Essential hypertension (x1.02) 0.965
Solitary nodule of lung (x1.02) 0.966
White Race (+0.00199) 0.966
hydrochlorothiazide (+0.00193) 0.967
Pneumothorax (x1.02) 0.967
Tachycardia (x1.02) 0.968
Cough (x1.02) 0.968
simvastatin (+0.00169) 0.969
Acute exacerbation of chronic obstructive bronchitis (x1.02) 0.969
Iatrogenic pneumothorax (x1.02) 0.969
Coronary arteriosclerosis (x1.02) 0.970
docusate (+0.00153) 0.970
Nausea and vomiting (x1.02) 0.970
Hypokalemia (x1.02) 0.971
Neoplasm of respiratory tract (x1.02) 0.971
Primary malignant neoplasm of trunk (x1.02) 0.971
Abscess of lung (x1.02) 0.972
Nausea (x1.02) 0.972
Malignant lymphoma (x1.02) 0.972
Black Race (-0.00144) 0.973
Abnormal body temperature (x1.02) 0.973
Malignant neoplastic disease (x1.02) 0.973
salmeterol (+0.00138) 0.974
furosemide (+0.00134) 0.974
Primary malignant neoplasm of mediastinum (x1.01) 0.974
Primary malignant neoplasm of female breast (x1.01) 0.974
Carbon dioxide, total [mol/V] in SP (+0.0904) 0.975
Prothrombin time (PT) (-0.0934) 0.975
Hypo-osmolality and or hyponatremia (x1.01) 0.975
−1.0 −0.5 0.0 0.5 1.0
Expressions
10⁵ 10³ 10¹
−10 0 10

Signature [168]; 1 To Reach 0.975, (10 Plotted)

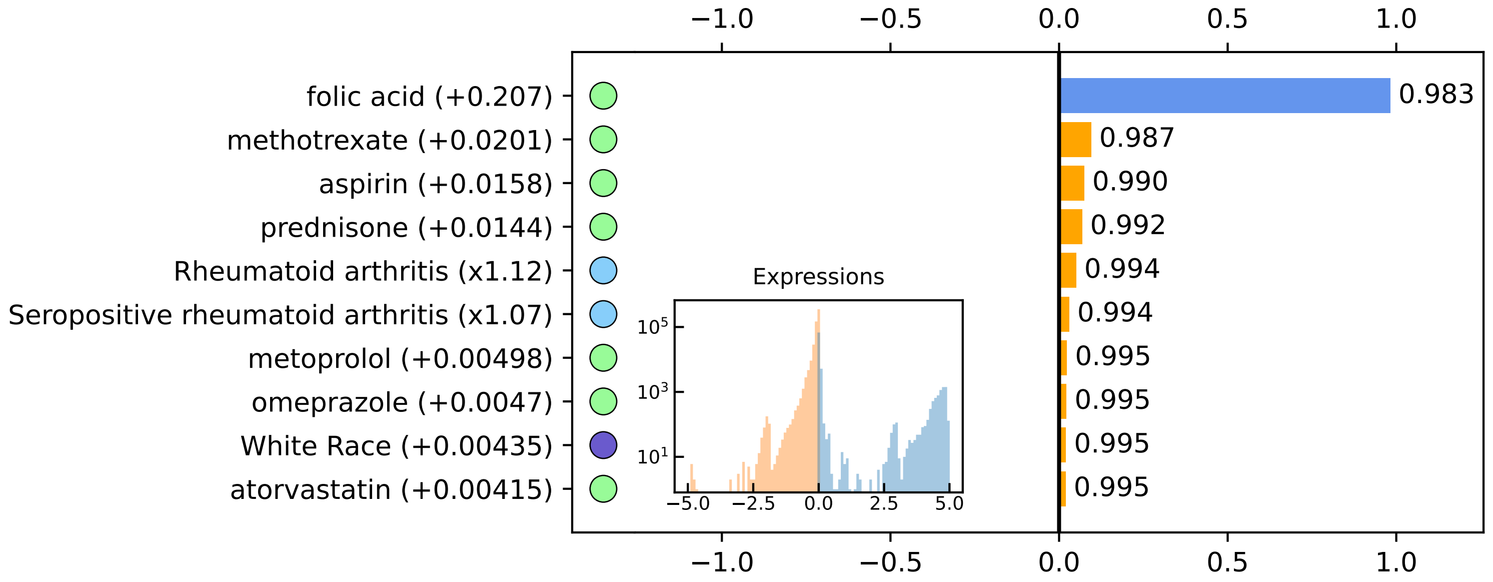

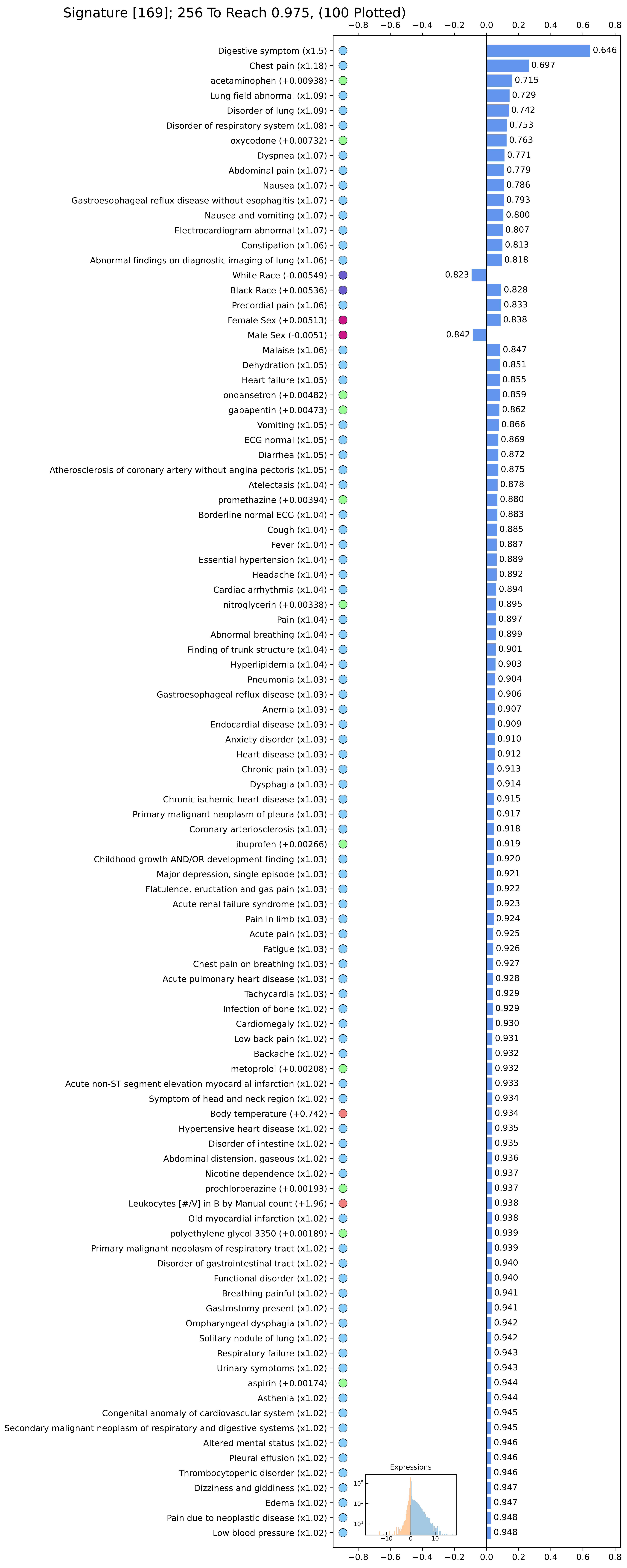
Signature [169]; 256 To Reach 0.975, (100 Plotted)
−0.8 −0.6 −0.4 −0.2 0.0 0.2 0.4 0.6 0.8
Digestive symptom (x1.5) 0.646
Chest pain (x1.18) 0.697
acetaminophen (+0.00938) 0.715
Lung field abnormal (x1.09) 0.729
Disorder of lung (x1.09) 0.742
Disorder of respiratory system (x1.08) 0.753
oxycodone (+0.00732) 0.763
Dyspnea (x1.07) 0.771
Abdominal pain (x1.07) 0.779
Nausea (x1.07) 0.786
Gastroesophageal reflux disease without esophagitis (x1.07) 0.793
Nausea and vomiting (x1.07) 0.800
Electrocardiogram abnormal (x1.07) 0.807
Constipation (x1.06) 0.813
Abnormal findings on diagnostic imaging of lung (x1.06) 0.818
White Race (-0.00549) 0.823
Black Race (+0.00536) 0.828
Precordial pain (x1.06) 0.833
Female Sex (+0.00513) 0.838
Male Sex (-0.0051) 0.842
Malaise (x1.06) 0.847
Dehydration (x1.05) 0.851
Heart failure (x1.05) 0.855
ondansetron (+0.00482) 0.859
gabapentin (+0.00473) 0.862
Vomiting (x1.05) 0.866
ECG normal (x1.05) 0.869
Diarrhea (x1.05) 0.872
Atherosclerosis of coronary artery without angina pectoris (x1.05) 0.875
Atelectasis (x1.04) 0.878
promethazine (+0.00394) 0.880
Borderline normal ECG (x1.04) 0.883
Cough (x1.04) 0.885
Fever (x1.04) 0.887
Essential hypertension (x1.04) 0.889
Headache (x1.04) 0.892
Cardiac arrhythmia (x1.04) 0.894
nitroglycerin (+0.00338) 0.895
Pain (x1.04) 0.897
Abnormal breathing (x1.04) 0.899
Finding of trunk structure (x1.04) 0.901
Hyperlipidemia (x1.04) 0.903
Pneumonia (x1.03) 0.904
Gastroesophageal reflux disease (x1.03) 0.906
Anemia (x1.03) 0.907
Endocardial disease (x1.03) 0.909
Anxiety disorder (x1.03) 0.910
Heart disease (x1.03) 0.912
Chronic pain (x1.03) 0.913
Dysphagia (x1.03) 0.914
Chronic ischemic heart disease (x1.03) 0.915
Primary malignant neoplasm of pleura (x1.03) 0.917
Coronary arteriosclerosis (x1.03) 0.918
ibuprofen (+0.00266) 0.919
Childhood growth AND/OR development finding (x1.03) 0.920
Major depression, single episode (x1.03) 0.921
Flatulence, eructation and gas pain (x1.03) 0.922
Acute renal failure syndrome (x1.03) 0.923
Pain in limb (x1.03) 0.924
Acute pain (x1.03) 0.925
Fatigue (x1.03) 0.926
Chest pain on breathing (x1.03) 0.927
Acute pulmonary heart disease (x1.03) 0.928
Tachycardia (x1.03) 0.929
Infection of bone (x1.02) 0.929
Cardiomegaly (x1.02) 0.930
Low back pain (x1.02) 0.931
Backache (x1.02) 0.932
metoprolol (+0.00208) 0.932
Acute non-ST segment elevation myocardial infarction (x1.02) 0.933
Symptom of head and neck region (x1.02) 0.934
Body temperature (+0.742) 0.934
Hypertensive heart disease (x1.02) 0.935
Disorder of intestine (x1.02) 0.935
Abdominal distension, gaseous (x1.02) 0.936
Nicotine dependence (x1.02) 0.937
prochlorperazine (+0.00193) 0.937
Leukocytes [#/V] in B by Manual count (+1.96) 0.938
Old myocardial infarction (x1.02) 0.938
polyethylene glycol 3350 (+0.00189) 0.939
Primary malignant neoplasm of respiratory tract (x1.02) 0.939
Disorder of gastrointestinal tract (x1.02) 0.940
Functional disorder (x1.02) 0.940
Breathing painful (x1.02) 0.941
Gastrostomy present (x1.02) 0.941
Oropharyngeal dysphagia (x1.02) 0.942
Solitary nodule of lung (x1.02) 0.942
Respiratory failure (x1.02) 0.943
Urinary symptoms (x1.02) 0.943
aspirin (+0.00174) 0.944
Asthenia (x1.02) 0.944
Congenital anomaly of cardiovascular system (x1.02) 0.945
Secondary malignant neoplasm of respiratory and digestive systems (x1.02) 0.945
Altered mental status (x1.02) 0.946
Pleural effusion (x1.02) 0.946
Thrombocytopenic disorder (x1.02) 0.946
Dizziness and giddiness (x1.02) 0.947
Edema (x1.02) 0.947
Pain due to neoplastic disease (x1.02) 0.948
Low blood pressure (x1.02) 0.948
Expressions
−10 0 10
−0.8 −0.6 −0.4 −0.2 0.0 0.2 0.4 0.6 0.8

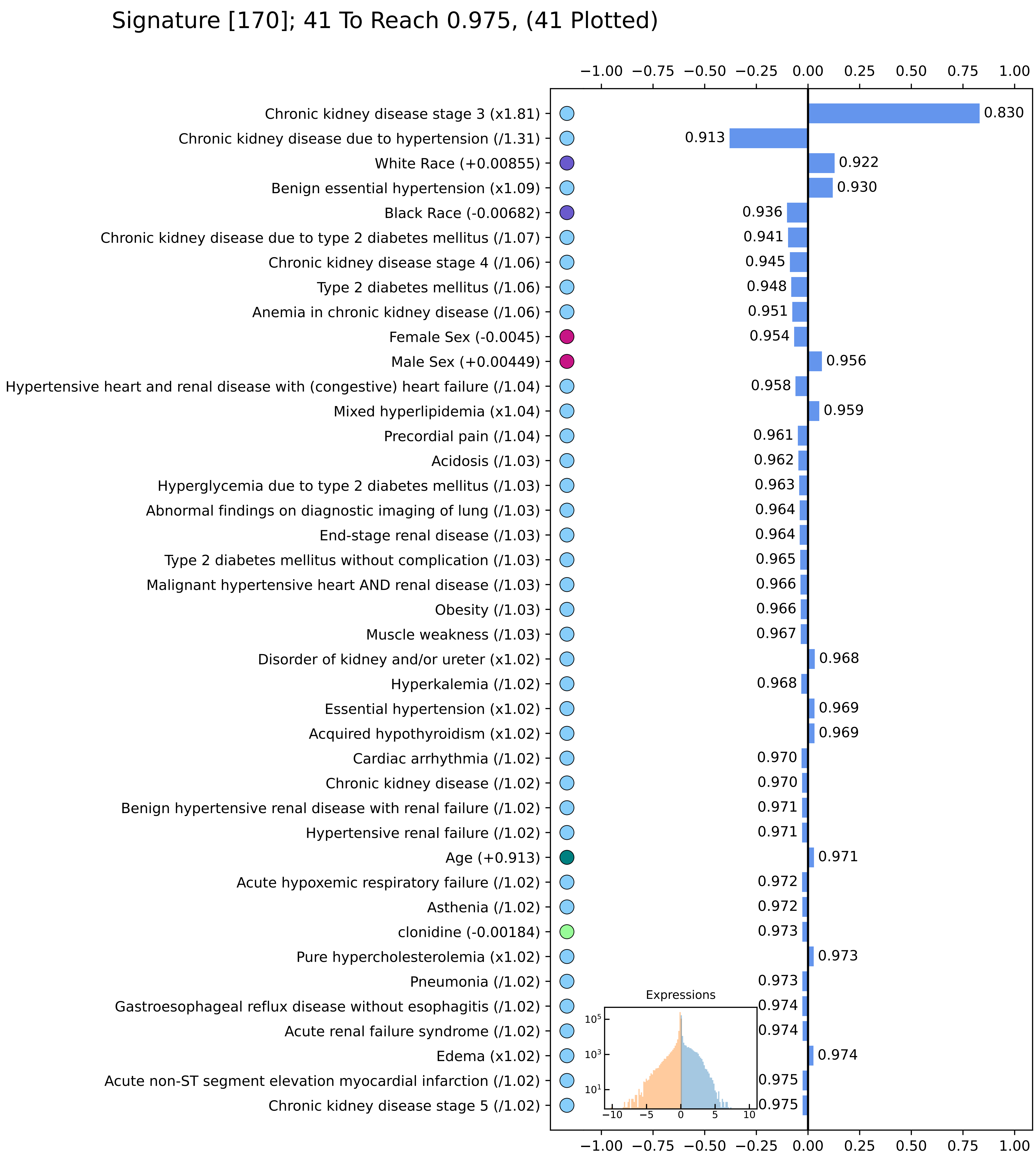

Signature [170]; 41 To Reach 0.975, (41 Plotted)
Chronic kidney disease stage 3 (x1.81) 0.830
Chronic kidney disease due to hypertension (/1.31) 0.913
White Race (+0.00855) 0.922
Benign essential hypertension (x1.09) 0.930
Black Race (-0.00682) 0.936
Chronic kidney disease due to type 2 diabetes mellitus (/1.07) 0.941
Chronic kidney disease stage 4 (/1.06) 0.945
Type 2 diabetes mellitus (/1.06) 0.948
Anemia in chronic kidney disease (/1.06) 0.951
Female Sex (-0.0045) 0.954
Male Sex (+0.00449) 0.956
Hypertensive heart and renal disease with (congestive) heart failure (/1.04) 0.958
Mixed hyperlipidemia (x1.04) 0.959
Precordial pain (/1.04) 0.961
Acidosis (/1.03) 0.962
Hyperglycemia due to type 2 diabetes mellitus (/1.03) 0.963
Abnormal findings on diagnostic imaging of lung (/1.03) 0.964
End-stage renal disease (/1.03) 0.964
Type 2 diabetes mellitus without complication (/1.03) 0.965
Malignant hypertensive heart AND renal disease (/1.03) 0.966
Obesity (/1.03) 0.966
Muscle weakness (/1.03) 0.967
Disorder of kidney and/or ureter (x1.02) 0.968
Hyperkalemia (/1.02) 0.968
Essential hypertension (x1.02) 0.969
Acquired hypothyroidism (x1.02) 0.969
Cardiac arrhythmia (/1.02) 0.970
Chronic kidney disease (/1.02) 0.970
Benign hypertensive renal disease with renal failure (/1.02) 0.971
Hypertensive renal failure (/1.02) 0.971
Age (+0.913) 0.971
Acute hypoxemic respiratory failure (/1.02) 0.972
Asthenia (/1.02) 0.972
clonidine (-0.00184) 0.973
Pure hypercholesterolemia (x1.02) 0.973
Pneumonia (/1.02) 0.973
Gastroesophageal reflux disease without esophagitis (/1.02) 0.974
Acute renal failure syndrome (/1.02) 0.974
Edema (x1.02) 0.974
Acute non-ST segment elevation myocardial infarction (/1.02) 0.975
Chronic kidney disease stage 5 (/1.02) 0.975
−1.00 −0.75 −0.50 −0.25 0.00 0.25 0.50 0.75 1.00
Expressions

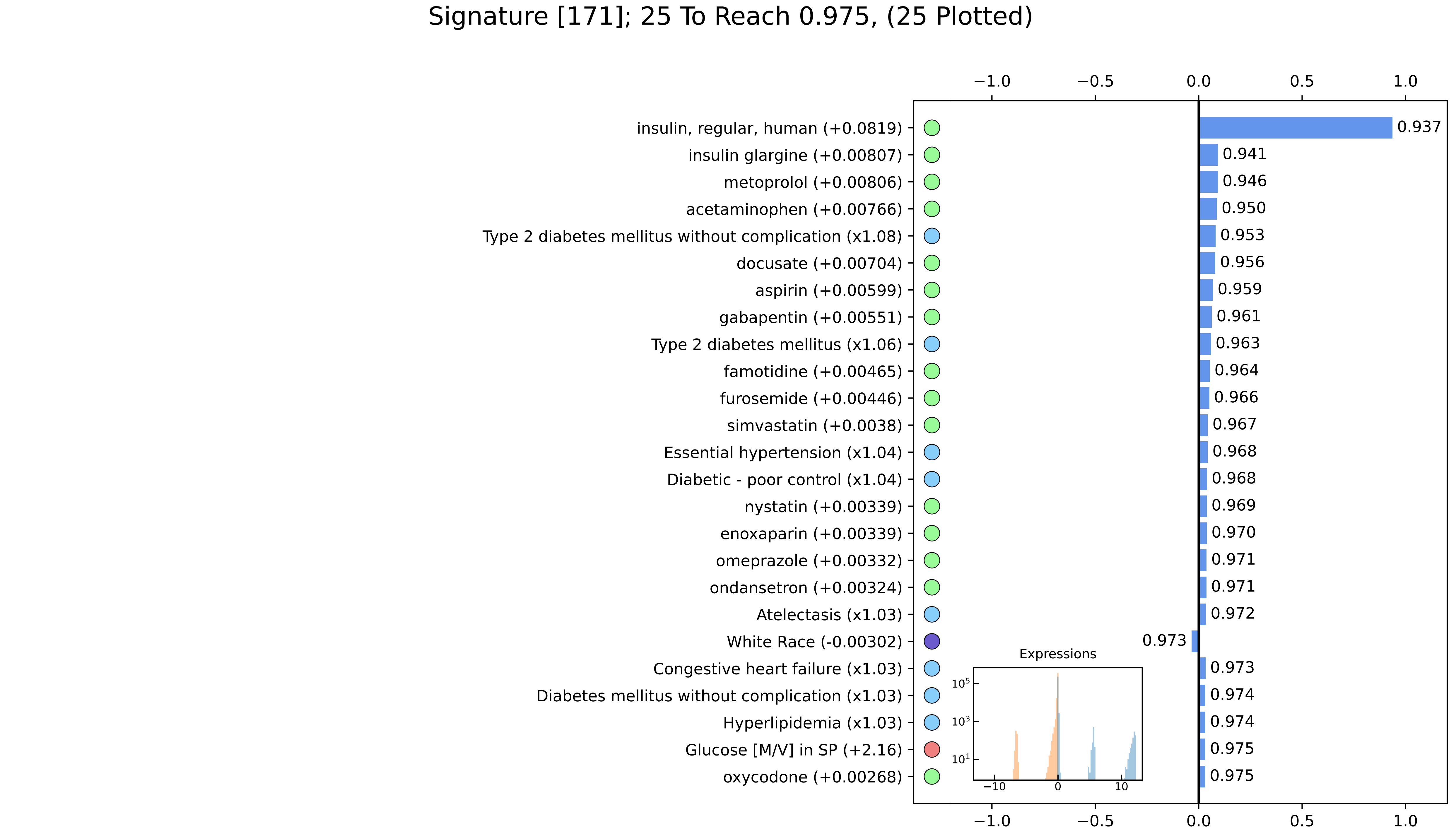

Signature [171]; 25 To Reach 0.975, (25 Plotted)
insulin, regular, human (+0.0819)
0.937
insulin glargine (+0.00807)
0.941
metoprolol (+0.00806)
0.946
acetaminophen (+0.00766)
0.950
Type 2 diabetes mellitus without complication (x1.08)
0.953
docusate (+0.00704)
0.956
aspirin (+0.00599)
0.959
gabapentin (+0.00551)
0.961
Type 2 diabetes mellitus (x1.06)
0.963
famotidine (+0.00465)
0.964
furosemide (+0.00446)
0.966
simvastatin (+0.0038)
0.967
Essential hypertension (x1.04)
0.968
Diabetic - poor control (x1.04)
0.968
nystatin (+0.00339)
0.969
enoxaparin (+0.00339)
0.970
omeprazole (+0.00332)
0.971
ondansetron (+0.00324)
0.971
Atelectasis (x1.03)
0.972
White Race (-0.00302)
0.973
Congestive heart failure (x1.03)
0.973
Diabetes mellitus without complication (x1.03)
0.974
Hyperlipidemia (x1.03)
0.974
Glucose [M/V] in SP (+2.16)
0.975
oxycodone (+0.00268)
0.975
−1.0
−0.5
0.0
0.5
1.0
Expressions
10^5
10^3
10^1
−10
0
10

Signature [172]; 6 To Reach 0.975, (10 Plotted)

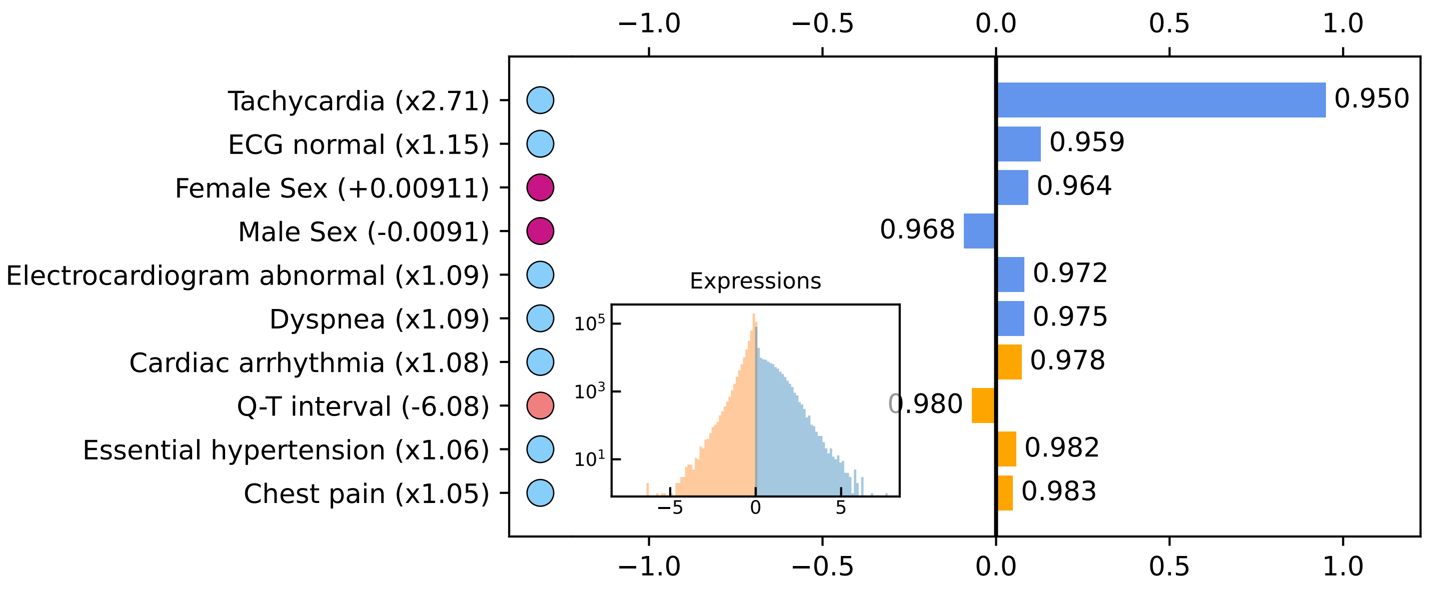

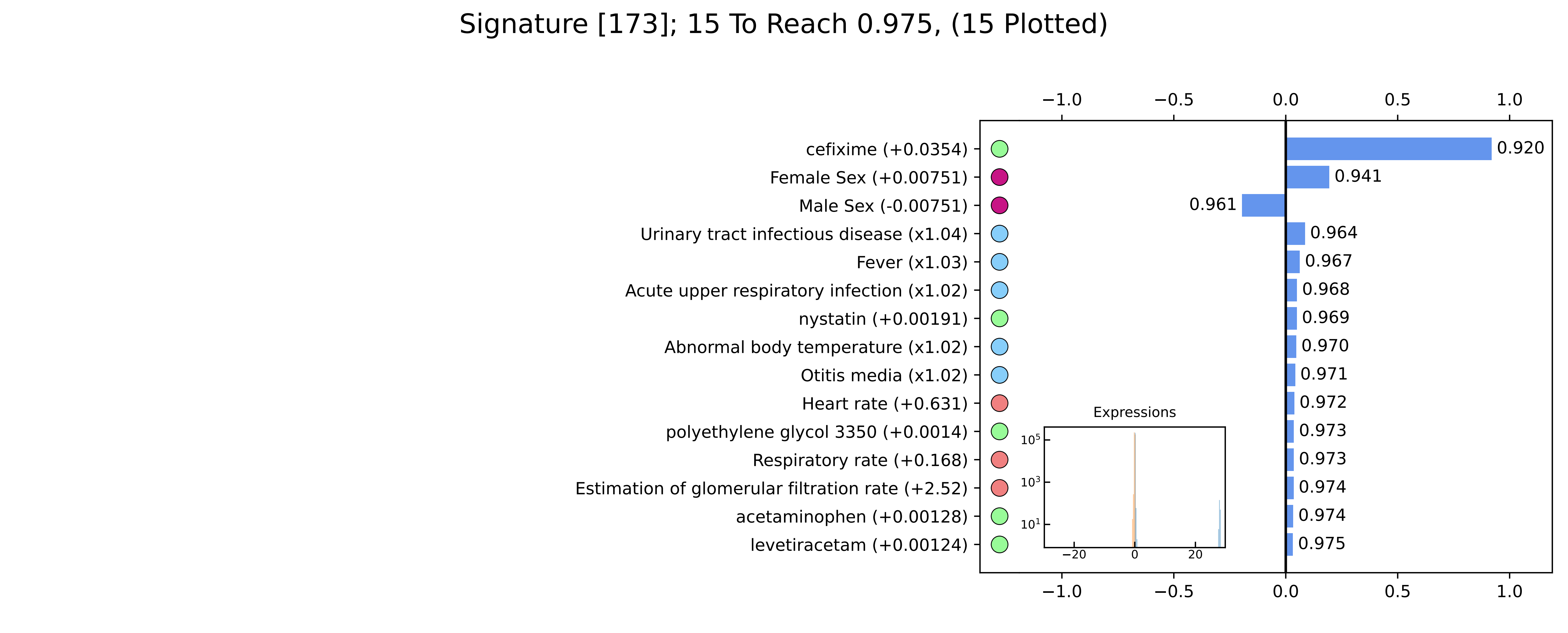

Signature [173]; 15 To Reach 0.975, (15 Plotted)
cefixime (+0.0354)
Female Sex (+0.00751)
Male Sex (-0.00751)
Urinary tract infectious disease (x1.04)
Fever (x1.03)
Acute upper respiratory infection (x1.02)
nystatin (+0.00191)
Abnormal body temperature (x1.02)
Otitis media (x1.02)
Heart rate (+0.631)
polyethylene glycol 3350 (+0.0014)
Respiratory rate (+0.168)
Estimation of glomerular filtration rate (+2.52)
acetaminophen (+0.00128)
levetiracetam (+0.00124)
0.920
0.941
0.961
0.964
0.967
0.968
0.969
0.970
0.971
0.972
0.973
0.973
0.974
0.974
0.975
−1.0
−0.5
0.0
0.5
1.0
Expressions
10^5
10^3
10^1
−20
0
20

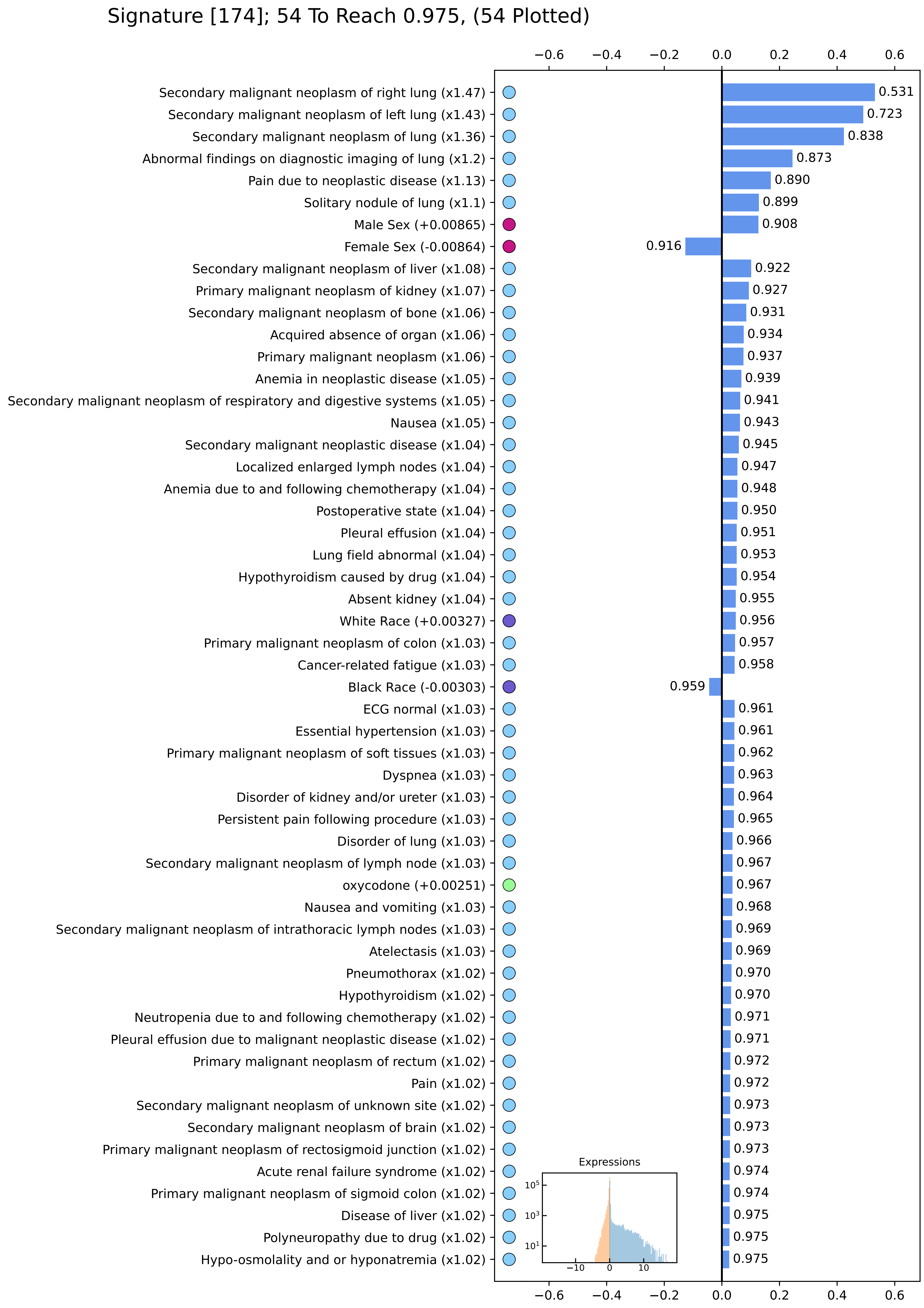

Signature [174]; 54 To Reach 0.975, (54 Plotted)
Secondary malignant neoplasm of right lung (x1.47) 0.531
Secondary malignant neoplasm of left lung (x1.43) 0.723
Secondary malignant neoplasm of lung (x1.36) 0.838
Abnormal findings on diagnostic imaging of lung (x1.2) 0.873
Pain due to neoplastic disease (x1.13) 0.890
Solitary nodule of lung (x1.1) 0.899
Male Sex (+0.00865) 0.908
Female Sex (-0.00864) 0.916
Secondary malignant neoplasm of liver (x1.08) 0.922
Primary malignant neoplasm of kidney (x1.07) 0.927
Secondary malignant neoplasm of bone (x1.06) 0.931
Acquired absence of organ (x1.06) 0.934
Primary malignant neoplasm (x1.06) 0.937
Anemia in neoplastic disease (x1.05) 0.939
Secondary malignant neoplasm of respiratory and digestive systems (x1.05) 0.941
Nausea (x1.05) 0.943
Secondary malignant neoplastic disease (x1.04) 0.945
Localized enlarged lymph nodes (x1.04) 0.947
Anemia due to and following chemotherapy (x1.04) 0.948
Postoperative state (x1.04) 0.950
Pleural effusion (x1.04) 0.951
Lung field abnormal (x1.04) 0.953
Hypothyroidism caused by drug (x1.04) 0.954
Absent kidney (x1.04) 0.955
White Race (+0.00327) 0.956
Primary malignant neoplasm of colon (x1.03) 0.957
Cancer-related fatigue (x1.03) 0.958
Black Race (-0.00303) 0.959
ECG normal (x1.03) 0.961
Essential hypertension (x1.03) 0.961
Primary malignant neoplasm of soft tissues (x1.03) 0.962
Dyspnea (x1.03) 0.963
Disorder of kidney and/or ureter (x1.03) 0.964
Persistent pain following procedure (x1.03) 0.965
Disorder of lung (x1.03) 0.966
Secondary malignant neoplasm of lymph node (x1.03) 0.967
oxycodone (+0.00251) 0.967
Nausea and vomiting (x1.03) 0.968
Secondary malignant neoplasm of intrathoracic lymph nodes (x1.03) 0.969
Atelectasis (x1.03) 0.969
Pneumothorax (x1.02) 0.970
Hypothyroidism (x1.02) 0.970
Neutropenia due to and following chemotherapy (x1.02) 0.971
Pleural effusion due to malignant neoplastic disease (x1.02) 0.971
Primary malignant neoplasm of rectum (x1.02) 0.972
Pain (x1.02) 0.972
Secondary malignant neoplasm of unknown site (x1.02) 0.973
Secondary malignant neoplasm of brain (x1.02) 0.973
Primary malignant neoplasm of rectosigmoid junction (x1.02) 0.973
Acute renal failure syndrome (x1.02) 0.974
Primary malignant neoplasm of sigmoid colon (x1.02) 0.974
Disease of liver (x1.02) 0.975
Polyneuropathy due to drug (x1.02) 0.975
Hypo-osmolality and or hyponatremia (x1.02) 0.975
−0.6 −0.4 −0.2 0.0 0.2 0.4 0.6
Expressions

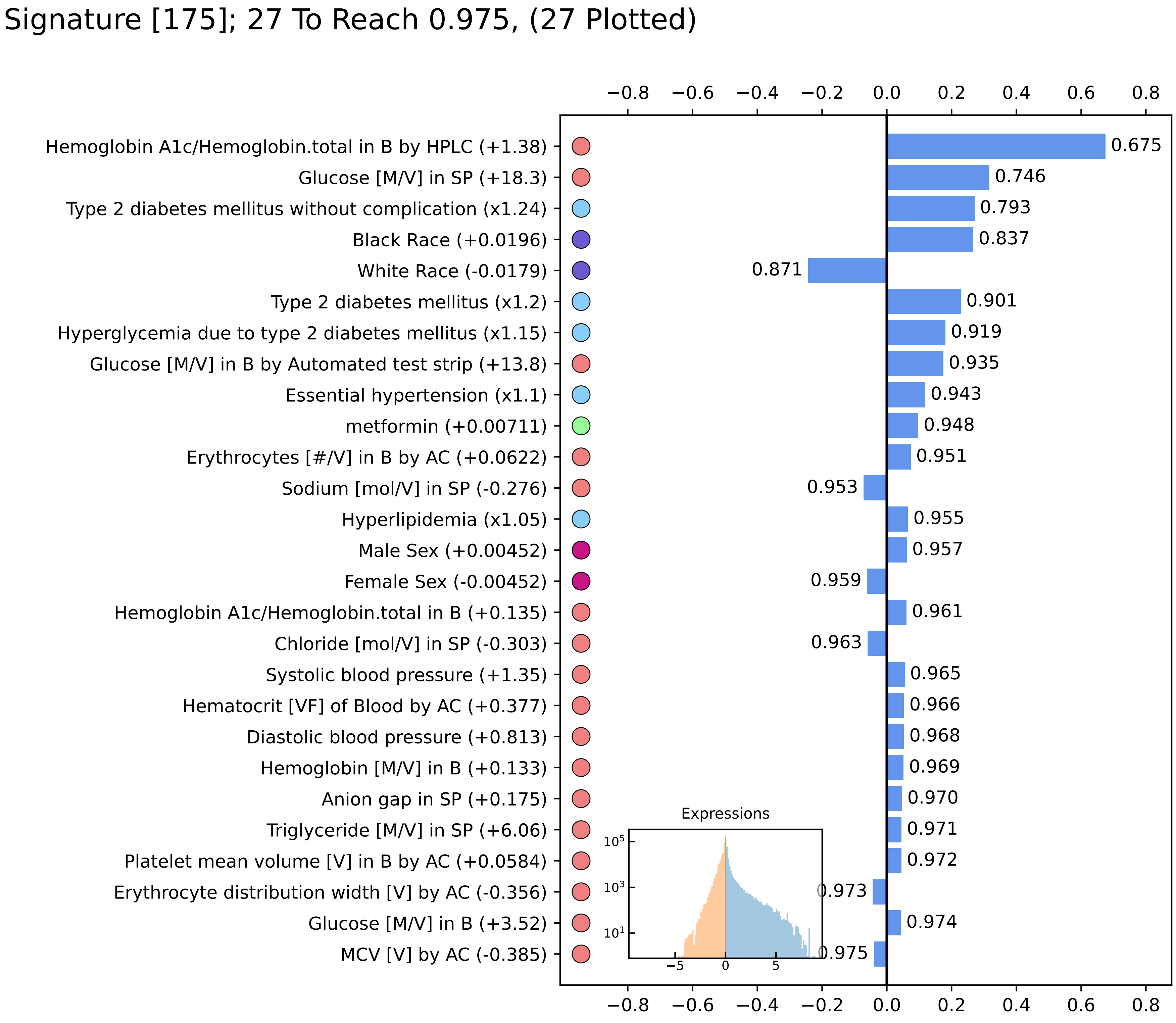
Signature [175]; 27 To Reach 0.975, (27 Plotted)
Hemoglobin A1c/Hemoglobin.total in B by HPLC (+1.38) 0.675
Glucose [M/V] in SP (+18.3) 0.746
Type 2 diabetes mellitus without complication (x1.24) 0.793
Black Race (+0.0196) 0.837
White Race (-0.0179) 0.871
Type 2 diabetes mellitus (x1.2) 0.901
Hyperglycemia due to type 2 diabetes mellitus (x1.15) 0.919
Glucose [M/V] in B by Automated test strip (+13.8) 0.935
Essential hypertension (x1.1) 0.943
metformin (+0.00711) 0.948
Erythrocytes [#/V] in B by AC (+0.0622) 0.951
Sodium [mol/V] in SP (-0.276) 0.953
Hyperlipidemia (x1.05) 0.955
Male Sex (+0.00452) 0.957
Female Sex (-0.00452) 0.959
Hemoglobin A1c/Hemoglobin.total in B (+0.135) 0.961
Chloride [mol/V] in SP (-0.303) 0.963
Systolic blood pressure (+1.35) 0.965
Hematocrit [VF] of Blood by AC (+0.377) 0.966
Diastolic blood pressure (+0.813) 0.968
Hemoglobin [M/V] in B (+0.133) 0.969
Anion gap in SP (+0.175) 0.970
Triglyceride [M/V] in SP (+6.06) 0.971
Platelet mean volume [V] in B by AC (+0.0584) 0.972
Erythrocyte distribution width [V] by AC (-0.356) 0.973
Glucose [M/V] in B (+3.52) 0.974
MCV [V] by AC (-0.385) 0.975
−0.8 −0.6 −0.4 −0.2 0.0 0.2 0.4 0.6 0.8
Expressions
−5 0 5

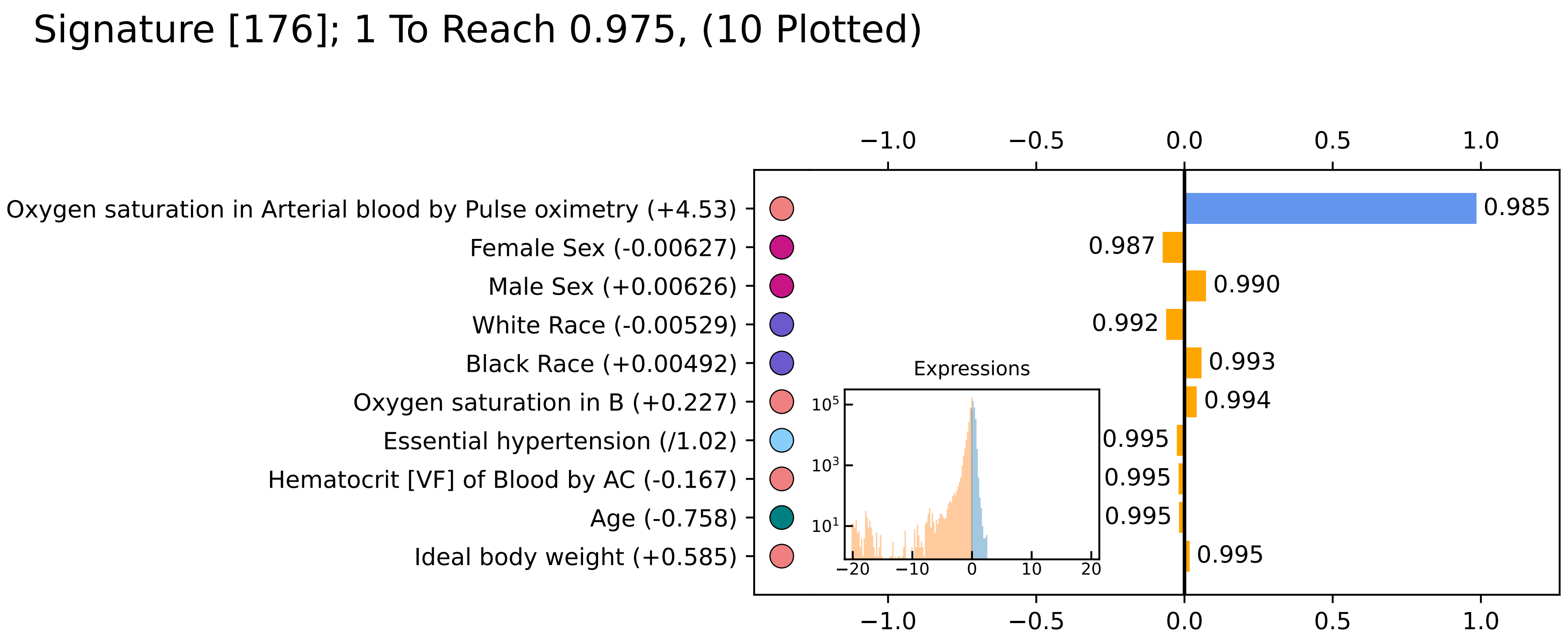

Signature [176]; 1 To Reach 0.975, (10 Plotted)
Oxygen saturation in Arterial blood by Pulse oximetry (+4.53)
Female Sex (-0.00627)
Male Sex (+0.00626)
White Race (-0.00529)
Black Race (+0.00492)
Oxygen saturation in B (+0.227)
Essential hypertension (/1.02)
Hematocrit [VF] of Blood by AC (-0.167)
Age (-0.758)
Ideal body weight (+0.585)
0.985
0.987
0.990
0.992
0.993
0.994
0.995
0.995
0.995
0.995
Expressions
−1.0
−0.5
0.0
0.5
1.0

Signature [177]; 242 To Reach 0.975, (100 Plotted)

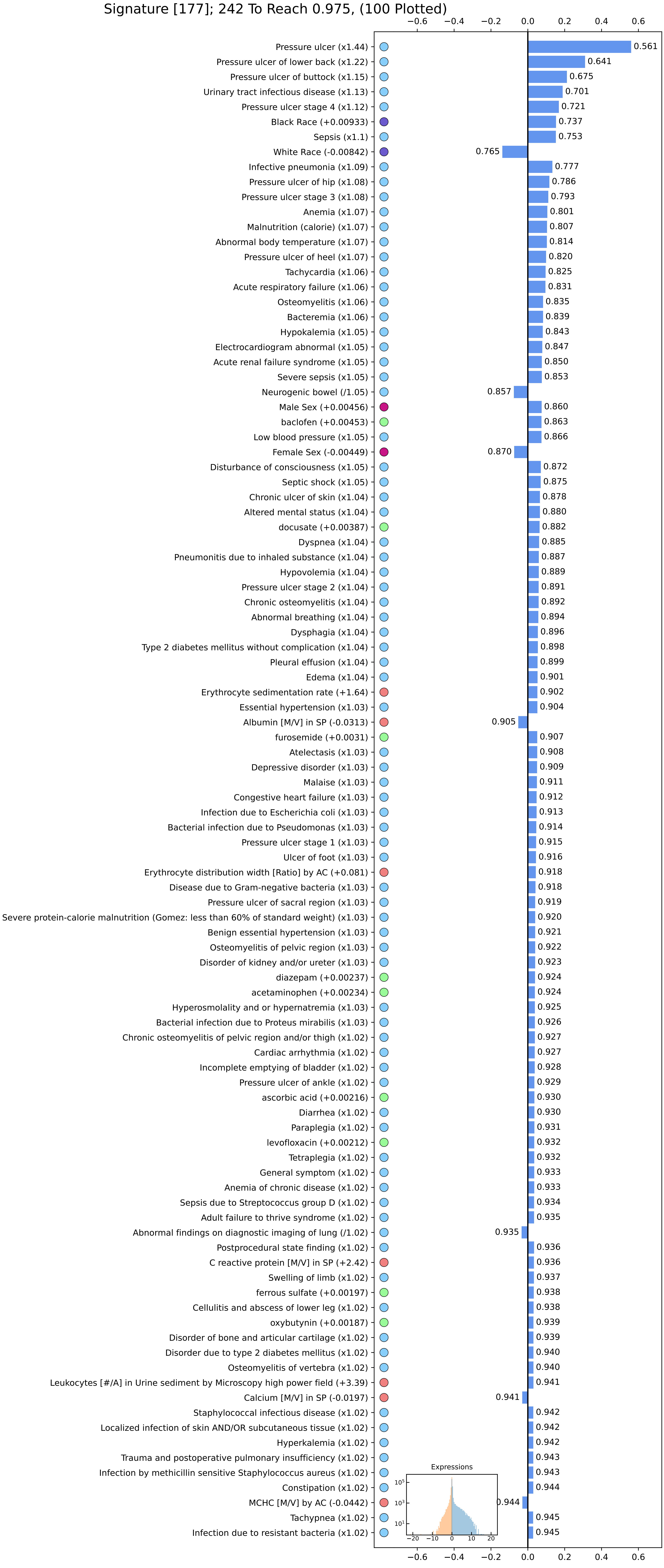

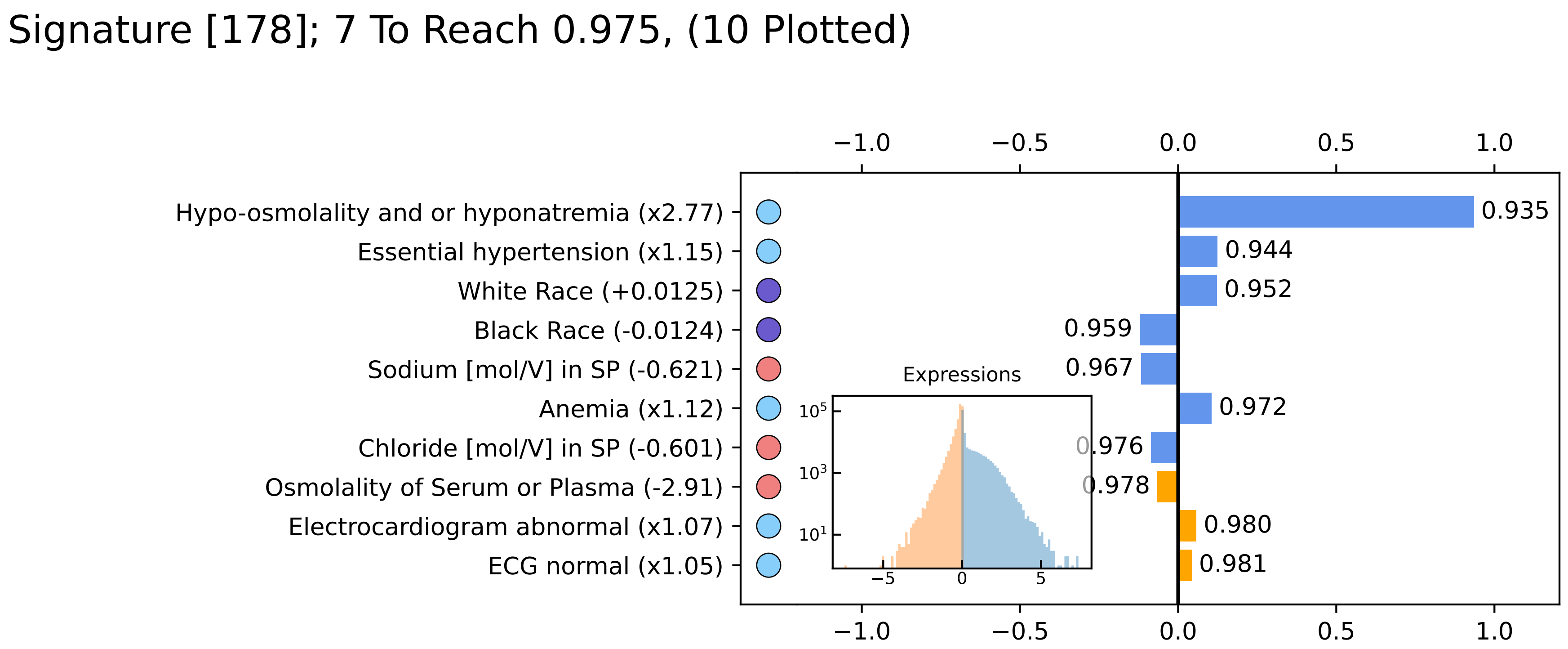

Signature [178]; 7 To Reach 0.975, (10 Plotted)
Hypo-osmolality and or hyponatremia (x2.77)
Essential hypertension (x1.15)
White Race (+0.0125)
Black Race (-0.0124)
Sodium [mol/V] in SP (-0.621)
Anemia (x1.12)
Chloride [mol/V] in SP (-0.601)
Osmolality of Serum or Plasma (-2.91)
Electrocardiogram abnormal (x1.07)
ECG normal (x1.05)
0.935
0.944
0.952
0.959
0.967
0.972
0.976
0.978
0.980
0.981
Expressions
−1.0
−0.5
0.0
0.5
1.0

Signature [179]; 64 To Reach 0.975, (64 Plotted)

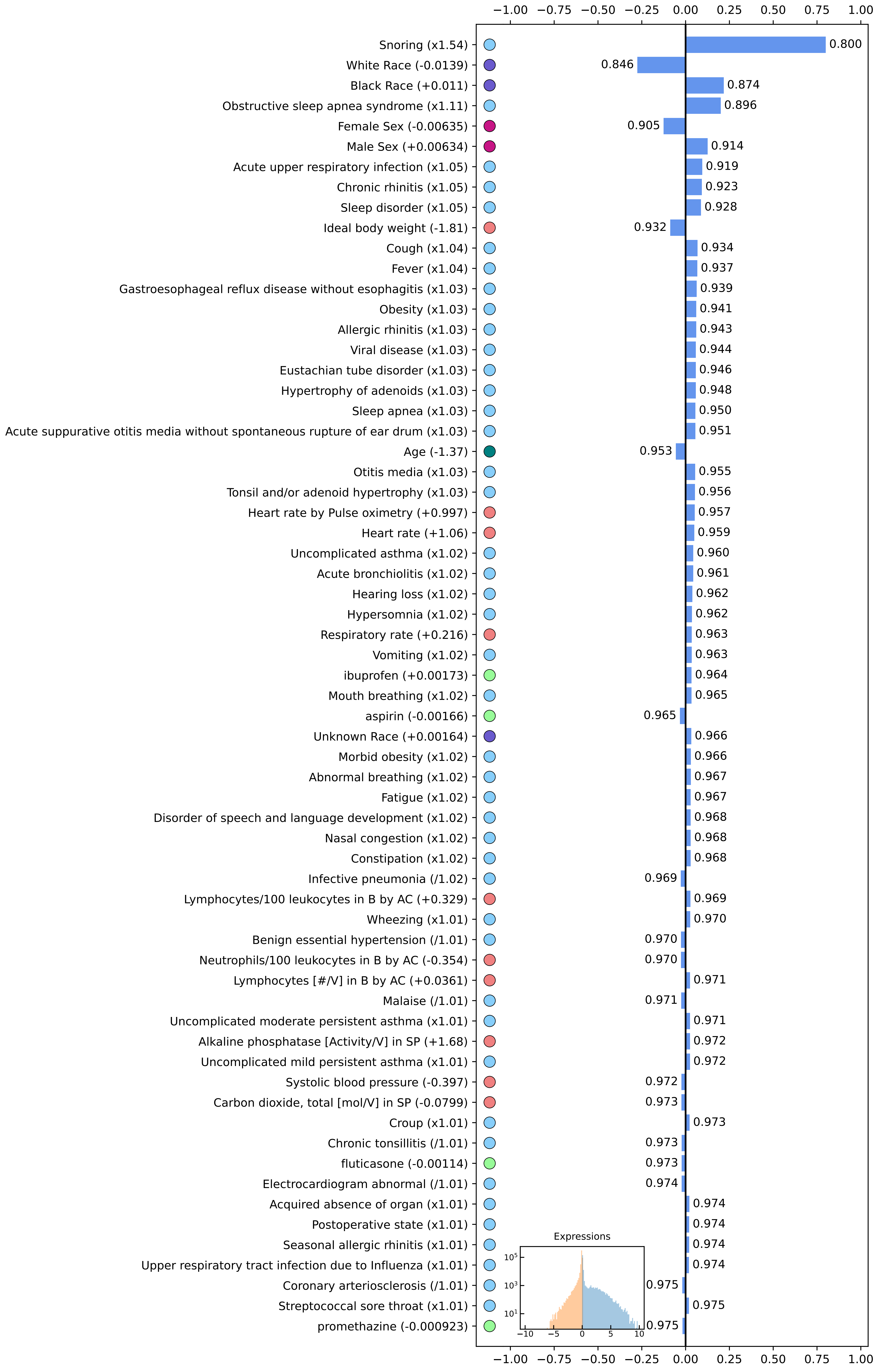

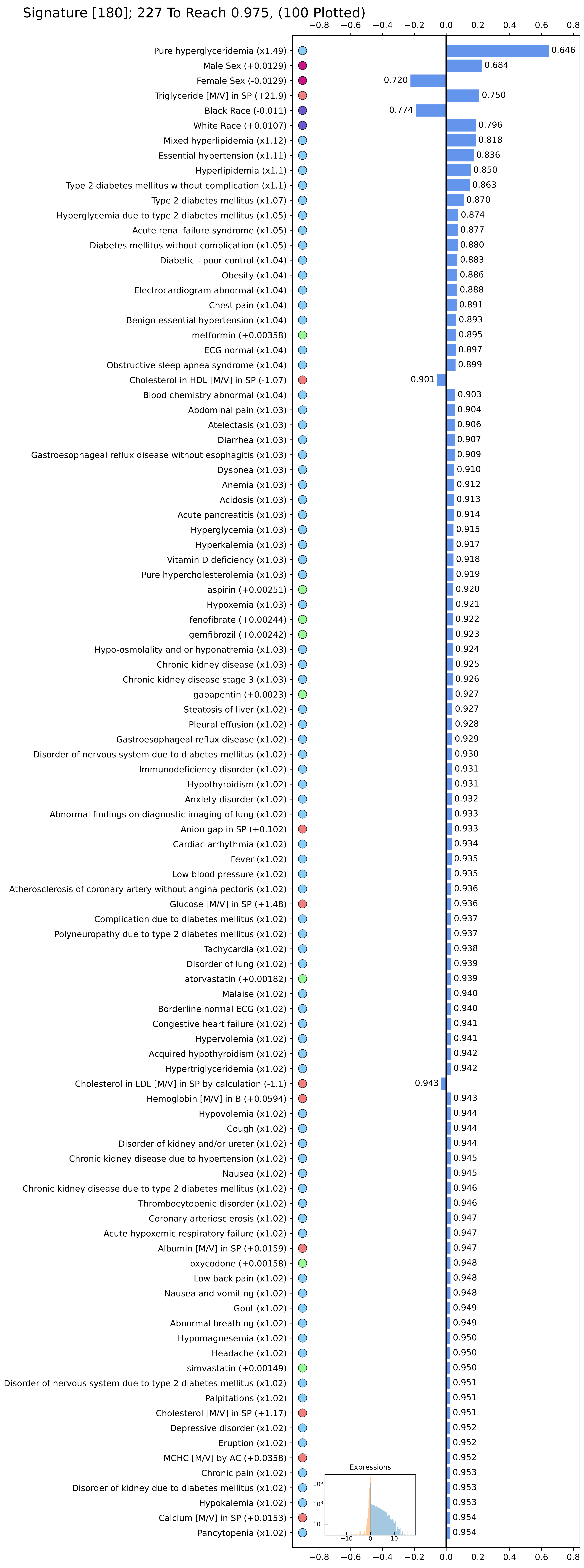
Signature [180]; 227 To Reach 0.975, (100 Plotted)
−0.8 −0.6 −0.4 −0.2 0.0 0.2 0.4 0.6 0.8
Pure hyperglyceridemia (x1.49) 0.646
Male Sex (+0.0129) 0.684
Female Sex (-0.0129) 0.720
Triglyceride [M/V] in SP (+21.9) 0.750
Black Race (-0.011) 0.774
White Race (+0.0107) 0.796
Mixed hyperlipidemia (x1.12) 0.818
Essential hypertension (x1.11) 0.836
Hyperlipidemia (x1.1) 0.850
Type 2 diabetes mellitus without complication (x1.1) 0.863
Type 2 diabetes mellitus (x1.07) 0.870
Hyperglycemia due to type 2 diabetes mellitus (x1.05) 0.874
Acute renal failure syndrome (x1.05) 0.877
Diabetes mellitus without complication (x1.05) 0.880
Diabetic - poor control (x1.04) 0.883
Obesity (x1.04) 0.886
Electrocardiogram abnormal (x1.04) 0.888
Chest pain (x1.04) 0.891
Benign essential hypertension (x1.04) 0.893
metformin (+0.00358) 0.895
ECG normal (x1.04) 0.897
Obstructive sleep apnea syndrome (x1.04) 0.899
Cholesterol in HDL [M/V] in SP (-1.07) 0.901
Blood chemistry abnormal (x1.04) 0.903
Abdominal pain (x1.03) 0.904
Atelectasis (x1.03) 0.906
Diarrhea (x1.03) 0.907
Gastroesophageal reflux disease without esophagitis (x1.03) 0.909
Dyspnea (x1.03) 0.910
Anemia (x1.03) 0.912
Acidosis (x1.03) 0.913
Acute pancreatitis (x1.03) 0.914
Hyperglycemia (x1.03) 0.915
Hyperkalemia (x1.03) 0.917
Vitamin D deficiency (x1.03) 0.918
Pure hypercholesterolemia (x1.03) 0.919
aspirin (+0.00251) 0.920
Hypoxemia (x1.03) 0.921
fenofibrate (+0.00244) 0.922
gemfibrozil (+0.00242) 0.923
Hypo-osmolality and or hyponatremia (x1.03) 0.924
Chronic kidney disease (x1.03) 0.925
Chronic kidney disease stage 3 (x1.03) 0.926
gabapentin (+0.0023) 0.927
Steatosis of liver (x1.02) 0.927
Pleural effusion (x1.02) 0.928
Gastroesophageal reflux disease (x1.02) 0.929
Disorder of nervous system due to diabetes mellitus (x1.02) 0.930
Immunodeficiency disorder (x1.02) 0.931
Hypothyroidism (x1.02) 0.931
Anxiety disorder (x1.02) 0.932
Abnormal findings on diagnostic imaging of lung (x1.02) 0.933
Anion gap in SP (+0.102) 0.933
Cardiac arrhythmia (x1.02) 0.934
Fever (x1.02) 0.935
Low blood pressure (x1.02) 0.935
Atherosclerosis of coronary artery without angina pectoris (x1.02) 0.936
Glucose [M/V] in SP (+1.48) 0.936
Complication due to diabetes mellitus (x1.02) 0.937
Polyneuropathy due to type 2 diabetes mellitus (x1.02) 0.937
Tachycardia (x1.02) 0.938
Disorder of lung (x1.02) 0.939
atorvastatin (+0.00182) 0.939
Malaise (x1.02) 0.940
Borderline normal ECG (x1.02) 0.940
Congestive heart failure (x1.02) 0.941
Hypervolemia (x1.02) 0.941
Acquired hypothyroidism (x1.02) 0.942
Hypertriglyceridemia (x1.02) 0.942
Cholesterol in LDL [M/V] in SP by calculation (-1.1) 0.943
Hemoglobin [M/V] in B (+0.0594) 0.943
Hypovolemia (x1.02) 0.944
Cough (x1.02) 0.944
Disorder of kidney and/or ureter (x1.02) 0.944
Chronic kidney disease due to hypertension (x1.02) 0.945
Nausea (x1.02) 0.945
Chronic kidney disease due to type 2 diabetes mellitus (x1.02) 0.946
Thrombocytopenic disorder (x1.02) 0.946
Coronary arteriosclerosis (x1.02) 0.947
Acute hypoxemic respiratory failure (x1.02) 0.947
Albumin [M/V] in SP (+0.0159) 0.947
oxycodone (+0.00158) 0.948
Low back pain (x1.02) 0.948
Nausea and vomiting (x1.02) 0.948
Gout (x1.02) 0.949
Abnormal breathing (x1.02) 0.949
Hypomagnesemia (x1.02) 0.950
Headache (x1.02) 0.950
simvastatin (+0.00149) 0.950
Disorder of nervous system due to type 2 diabetes mellitus (x1.02) 0.951
Palpitations (x1.02) 0.951
Cholesterol [M/V] in SP (+1.17) 0.951
Depressive disorder (x1.02) 0.952
Eruption (x1.02) 0.952
MCHC [M/V] by AC (+0.0358) 0.952
Chronic pain (x1.02) 0.953
Disorder of kidney due to diabetes mellitus (x1.02) 0.953
Hypokalemia (x1.02) 0.953
Calcium [M/V] in SP (+0.0153) 0.954
Pancytopenia (x1.02) 0.954
Expressions
10^5
10^3
10^1
−10 0 10

Signature [181]; 7 To Reach 0.975, (10 Plotted)

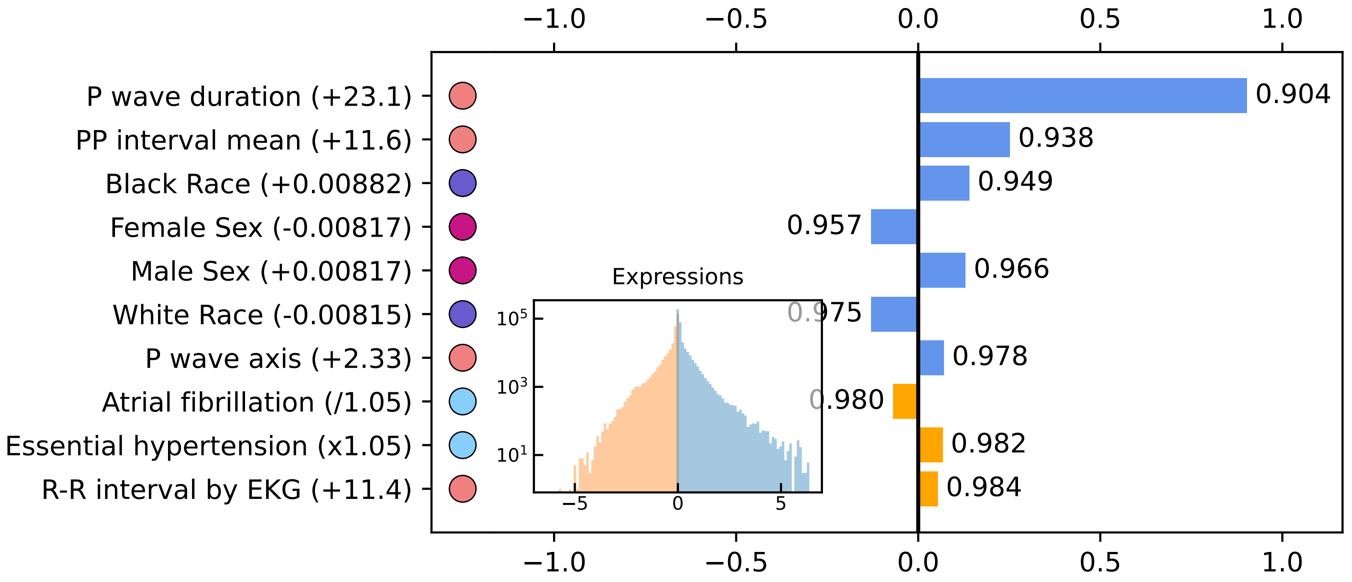

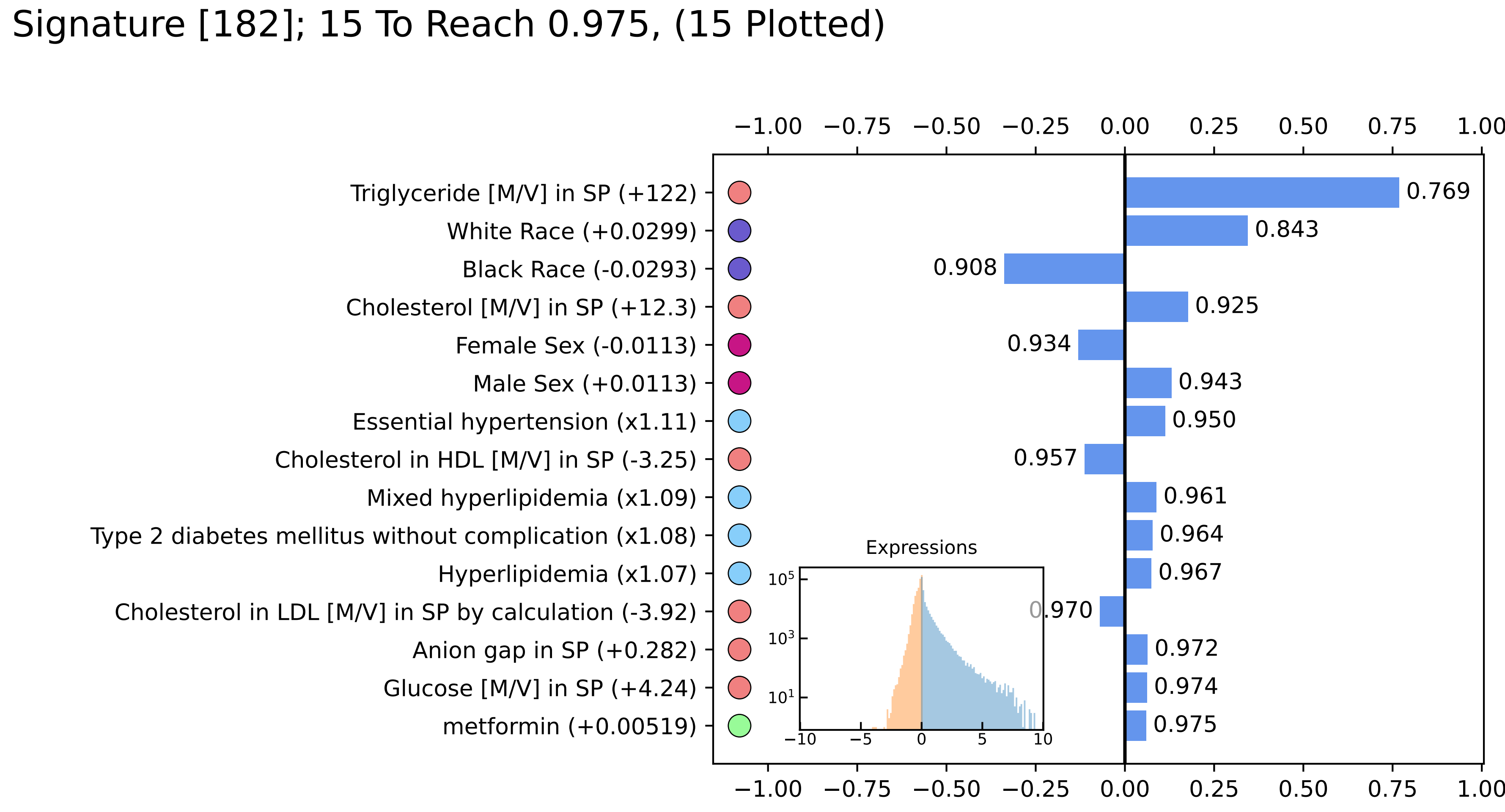

Signature [182]; 15 To Reach 0.975, (15 Plotted)
Triglyceride [M/V] in SP (+122)
White Race (+0.0299)
Black Race (-0.0293)
Cholesterol [M/V] in SP (+12.3)
Female Sex (-0.0113)
Male Sex (+0.0113)
Essential hypertension (x1.11)
Cholesterol in HDL [M/V] in SP (-3.25)
Mixed hyperlipidemia (x1.09)
Type 2 diabetes mellitus without complication (x1.08)
Hyperlipidemia (x1.07)
Cholesterol in LDL [M/V] in SP by calculation (-3.92)
Anion gap in SP (+0.282)
Glucose [M/V] in SP (+4.24)
metformin (+0.00519)
0.769
0.843
0.908
0.925
0.934
0.943
0.950
0.957
0.961
0.964
0.967
0.970
0.972
0.974
0.975
−1.00 −0.75 −0.50 −0.25 0.00 0.25 0.50 0.75 1.00
Expressions

Signature [183]; 7 To Reach 0.975, (10 Plotted)

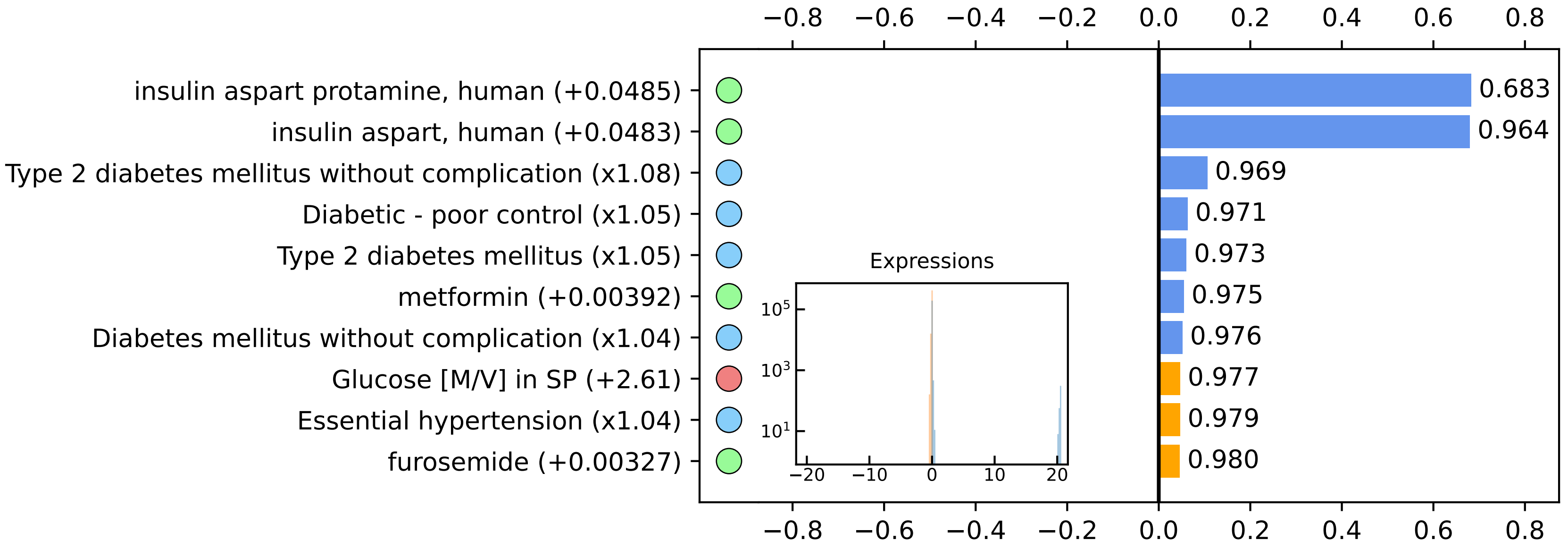

Signature [184]; 155 To Reach 0.975, (100 Plotted)

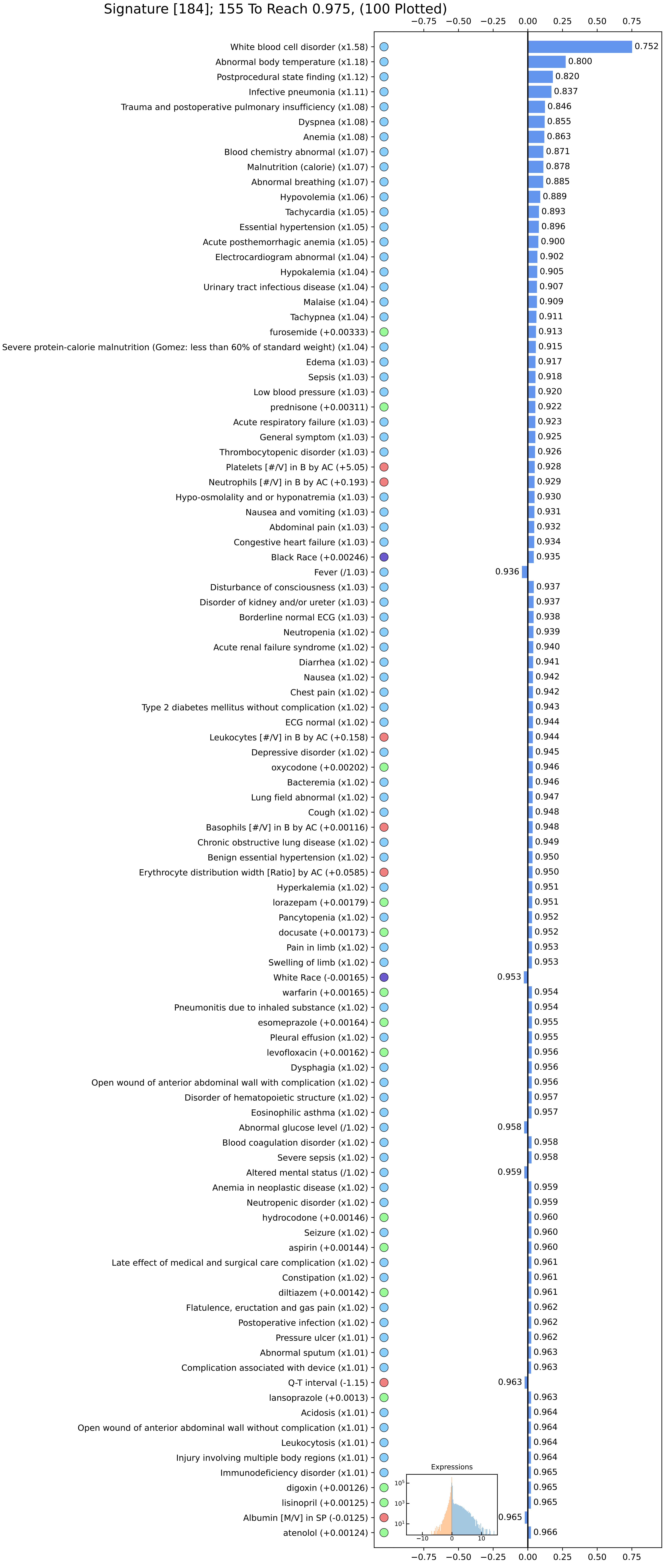

Signature [185]; 130 To Reach 0.975, (100 Plotted)

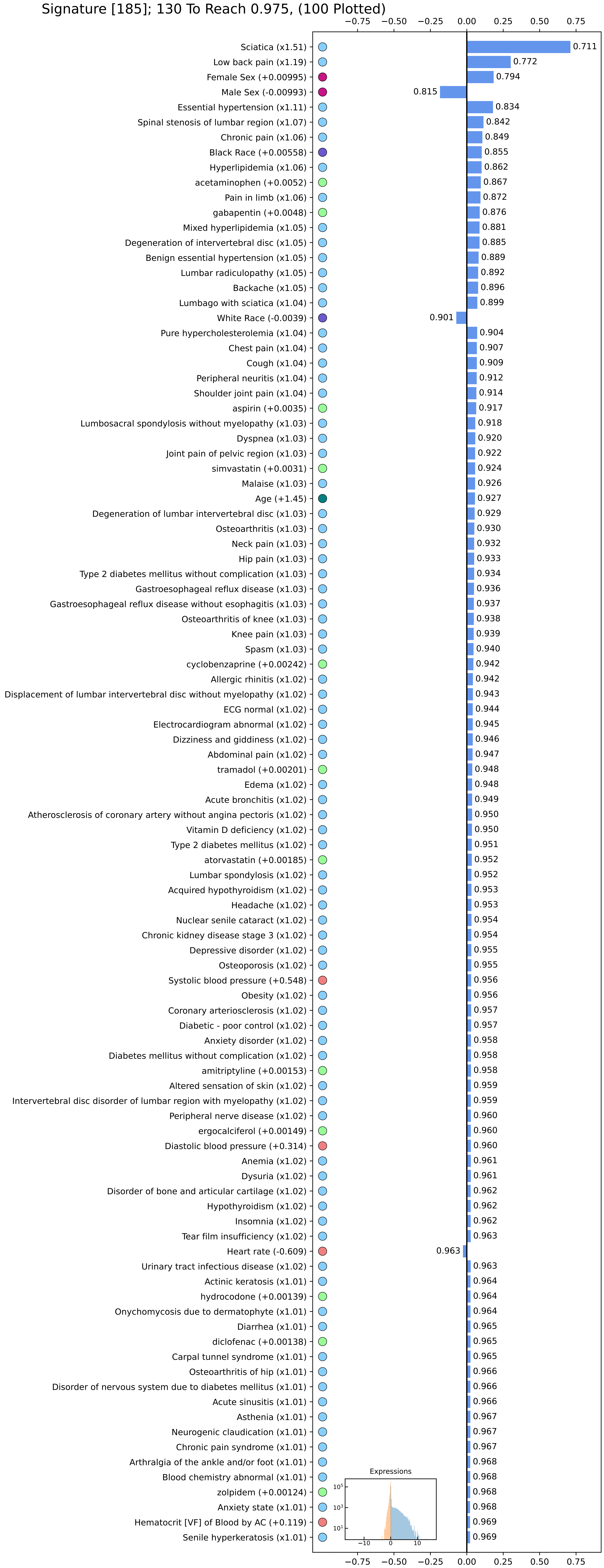

Signature [186]; 1162 To Reach 0.975, (100 Plotted)

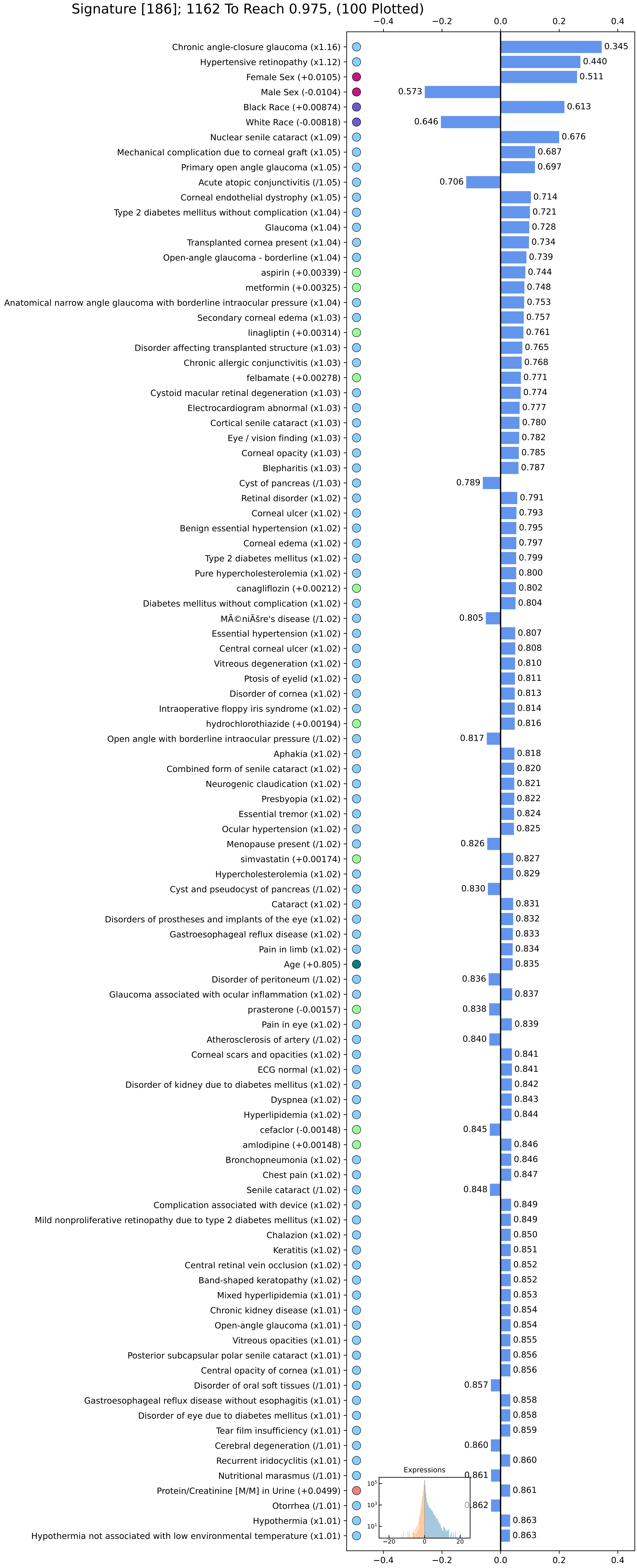

Signature [187]; 4 To Reach 0.975, (10 Plotted)

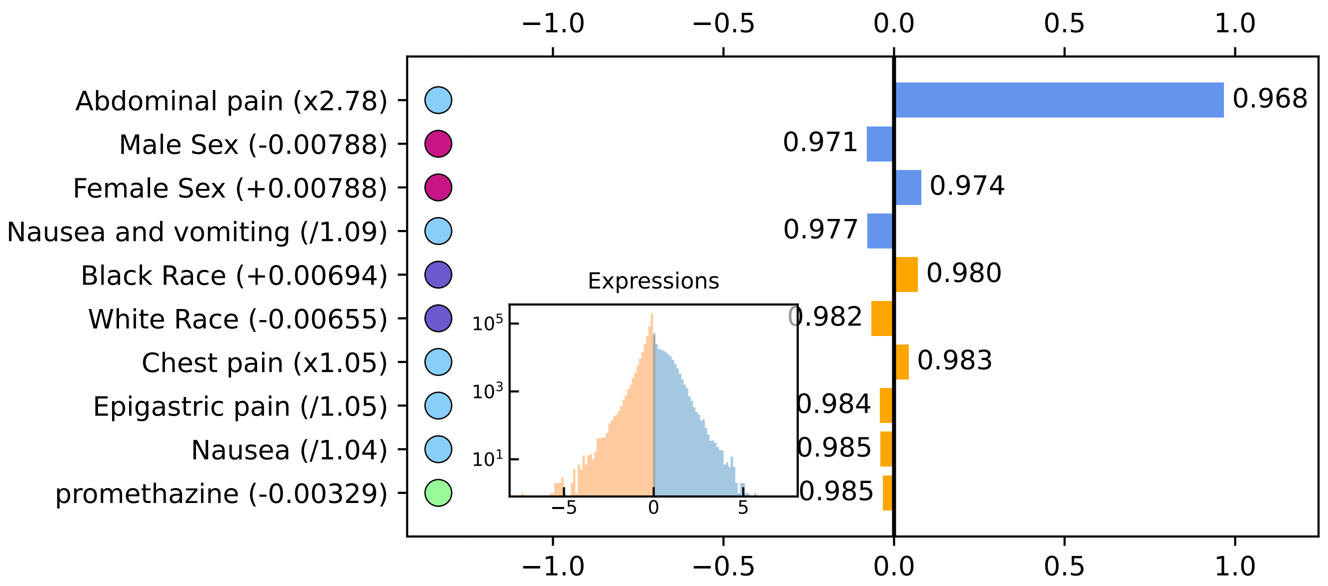

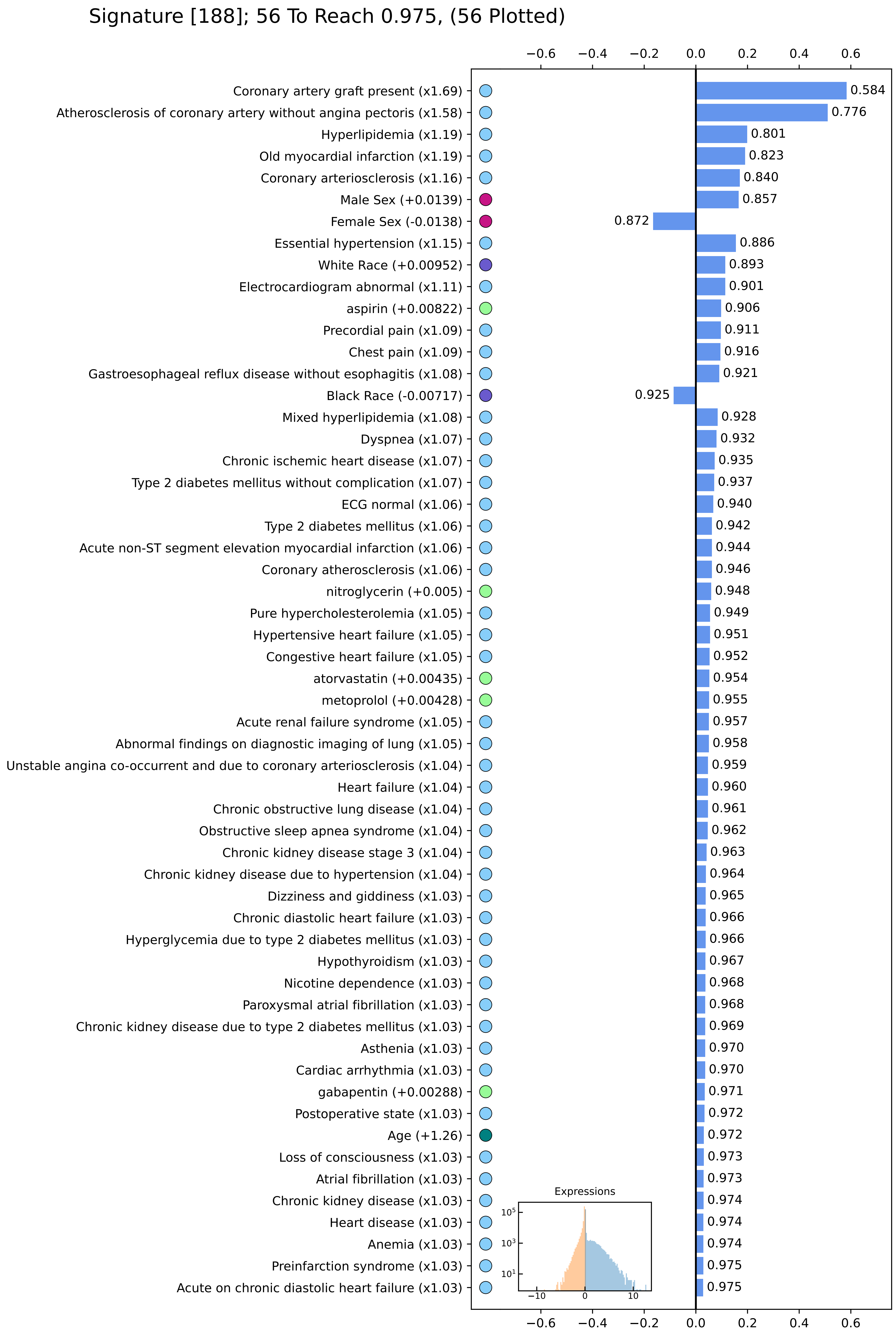
Signature [188]; 56 To Reach 0.975, (56 Plotted)
Coronary artery graft present (x1.69) 0.584
Atherosclerosis of coronary artery without angina pectoris (x1.58) 0.776
Hyperlipidemia (x1.19) 0.801
Old myocardial infarction (x1.19) 0.823
Coronary arteriosclerosis (x1.16) 0.840
Male Sex (+0.0139) 0.857
Female Sex (-0.0138) 0.872
Essential hypertension (x1.15) 0.886
White Race (+0.00952) 0.893
Electrocardiogram abnormal (x1.11) 0.901
aspirin (+0.00822) 0.906
Precordial pain (x1.09) 0.911
Chest pain (x1.09) 0.916
Gastroesophageal reflux disease without esophagitis (x1.08) 0.921
Black Race (-0.00717) 0.925
Mixed hyperlipidemia (x1.08) 0.928
Dyspnea (x1.07) 0.932
Chronic ischemic heart disease (x1.07) 0.935
Type 2 diabetes mellitus without complication (x1.07) 0.937
ECG normal (x1.06) 0.940
Type 2 diabetes mellitus (x1.06) 0.942
Acute non-ST segment elevation myocardial infarction (x1.06) 0.944
Coronary atherosclerosis (x1.06) 0.946
nitroglycerin (+0.005) 0.948
Pure hypercholesterolemia (x1.05) 0.949
Hypertensive heart failure (x1.05) 0.951
Congestive heart failure (x1.05) 0.952
atorvastatin (+0.00435) 0.954
metoprolol (+0.00428) 0.955
Acute renal failure syndrome (x1.05) 0.957
Abnormal findings on diagnostic imaging of lung (x1.05) 0.958
Unstable angina co-occurrent and due to coronary arteriosclerosis (x1.04) 0.959
Heart failure (x1.04) 0.960
Chronic obstructive lung disease (x1.04) 0.961
Obstructive sleep apnea syndrome (x1.04) 0.962
Chronic kidney disease stage 3 (x1.04) 0.963
Chronic kidney disease due to hypertension (x1.04) 0.964
Dizziness and giddiness (x1.03) 0.965
Chronic diastolic heart failure (x1.03) 0.966
Hyperglycemia due to type 2 diabetes mellitus (x1.03) 0.966
Hypothyroidism (x1.03) 0.967
Nicotine dependence (x1.03) 0.968
Paroxysmal atrial fibrillation (x1.03) 0.968
Chronic kidney disease due to type 2 diabetes mellitus (x1.03) 0.969
Asthenia (x1.03) 0.970
Cardiac arrhythmia (x1.03) 0.970
gabapentin (+0.00288) 0.971
Postoperative state (x1.03) 0.972
Age (+1.26) 0.972
Loss of consciousness (x1.03) 0.973
Atrial fibrillation (x1.03) 0.973
Chronic kidney disease (x1.03) 0.974
Heart disease (x1.03) 0.974
Anemia (x1.03) 0.974
Preinfarction syndrome (x1.03) 0.975
Acute on chronic diastolic heart failure (x1.03) 0.975
−0.6 −0.4 −0.2 0.0 0.2 0.4 0.6
Expressions
10^5
10^3
10^1
−10 0 10

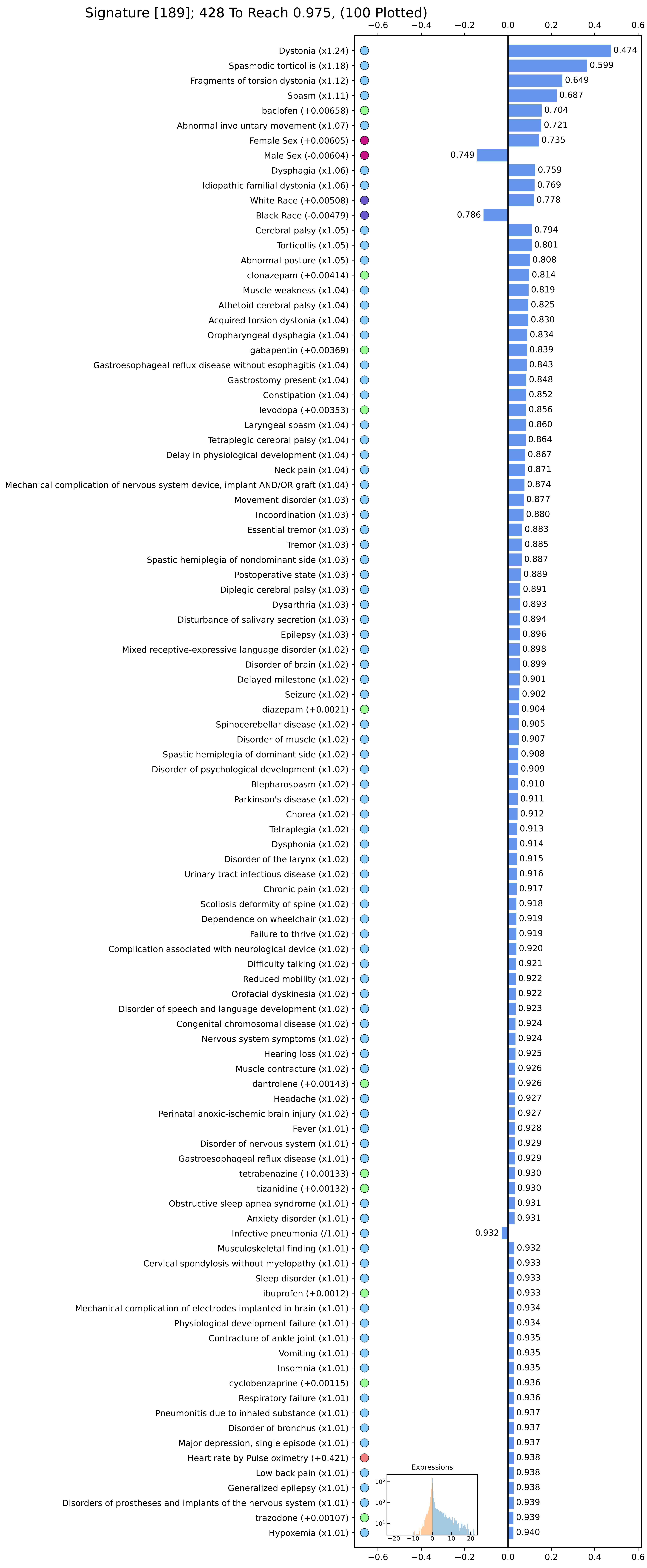

Signature [189]; 428 To Reach 0.975, (100 Plotted)
Dystonia (x1.24) 0.474
Spasmodic torticollis (x1.18) 0.599
Fragments of torsion dystonia (x1.12) 0.649
Spasm (x1.11) 0.687
baclofen (+0.00658) 0.704
Abnormal involuntary movement (x1.07) 0.721
Female Sex (+0.00605) 0.735
Male Sex (-0.00604) 0.749
Dysphagia (x1.06) 0.759
Idiopathic familial dystonia (x1.06) 0.769
White Race (+0.00508) 0.778
Black Race (-0.00479) 0.786
Cerebral palsy (x1.05) 0.794
Torticollis (x1.05) 0.801
Abnormal posture (x1.05) 0.808
clonazepam (+0.00414) 0.814
Muscle weakness (x1.04) 0.819
Athetoid cerebral palsy (x1.04) 0.825
Acquired torsion dystonia (x1.04) 0.830
Oropharyngeal dysphagia (x1.04) 0.834
gabapentin (+0.00369) 0.839
Gastroesophageal reflux disease without esophagitis (x1.04) 0.843
Gastrostomy present (x1.04) 0.848
Constipation (x1.04) 0.852
levodopa (+0.00353) 0.856
Laryngeal spasm (x1.04) 0.860
Tetraplegic cerebral palsy (x1.04) 0.864
Delay in physiological development (x1.04) 0.867
Neck pain (x1.04) 0.871
Mechanical complication of nervous system device, implant AND/OR graft (x1.04) 0.874
Movement disorder (x1.03) 0.877
Incoordination (x1.03) 0.880
Essential tremor (x1.03) 0.883
Tremor (x1.03) 0.885
Spastic hemiplegia of nondominant side (x1.03) 0.887
Postoperative state (x1.03) 0.889
Diplegic cerebral palsy (x1.03) 0.891
Dysarthria (x1.03) 0.893
Disturbance of salivary secretion (x1.03) 0.894
Epilepsy (x1.03) 0.896
Mixed receptive-expressive language disorder (x1.02) 0.898
Disorder of brain (x1.02) 0.899
Delayed milestone (x1.02) 0.901
Seizure (x1.02) 0.902
diazepam (+0.0021) 0.904
Spinocerebellar disease (x1.02) 0.905
Disorder of muscle (x1.02) 0.907
Spastic hemiplegia of dominant side (x1.02) 0.908
Disorder of psychological development (x1.02) 0.909
Blepharospasm (x1.02) 0.910
Parkinson's disease (x1.02) 0.911
Chorea (x1.02) 0.912
Tetraplegia (x1.02) 0.913
Dysphonia (x1.02) 0.914
Disorder of the larynx (x1.02) 0.915
Urinary tract infectious disease (x1.02) 0.916
Chronic pain (x1.02) 0.917
Scoliosis deformity of spine (x1.02) 0.918
Dependence on wheelchair (x1.02) 0.919
Failure to thrive (x1.02) 0.919
Complication associated with neurological device (x1.02) 0.920
Difficulty talking (x1.02) 0.921
Reduced mobility (x1.02) 0.922
Orofacial dyskinesia (x1.02) 0.922
Disorder of speech and language development (x1.02) 0.923
Congenital chromosomal disease (x1.02) 0.924
Nervous system symptoms (x1.02) 0.924
Hearing loss (x1.02) 0.925
Muscle contracture (x1.02) 0.926
dantrolene (+0.00143) 0.926
Headache (x1.02) 0.927
Perinatal anoxic-ischemic brain injury (x1.02) 0.927
Fever (x1.01) 0.928
Disorder of nervous system (x1.01) 0.929
Gastroesophageal reflux disease (x1.01) 0.929
tetrabenazine (+0.00133) 0.930
tizanidine (+0.00132) 0.930
Obstructive sleep apnea syndrome (x1.01) 0.931
Anxiety disorder (x1.01) 0.931
Infective pneumonia (/1.01) 0.932
Musculoskeletal finding (x1.01) 0.932
Cervical spondylosis without myelopathy (x1.01) 0.933
Sleep disorder (x1.01) 0.933
ibuprofen (+0.0012) 0.933
Mechanical complication of electrodes implanted in brain (x1.01) 0.934
Physiological development failure (x1.01) 0.934
Contracture of ankle joint (x1.01) 0.935
Vomiting (x1.01) 0.935
Insomnia (x1.01) 0.935
cyclobenzaprine (+0.00115) 0.936
Respiratory failure (x1.01) 0.936
Pneumonitis due to inhaled substance (x1.01) 0.937
Disorder of bronchus (x1.01) 0.937
Major depression, single episode (x1.01) 0.937
Heart rate by Pulse oximetry (+0.421) 0.938
Low back pain (x1.01) 0.938
Generalized epilepsy (x1.01) 0.938
Disorders of prostheses and implants of the nervous system (x1.01) 0.939
trazodone (+0.00107) 0.939
Hypoxemia (x1.01) 0.940
Expressions
−0.6 −0.4 −0.2 0.0 0.2 0.4 0.6

Signature [190]; 123 To Reach 0.975, (100 Plotted)

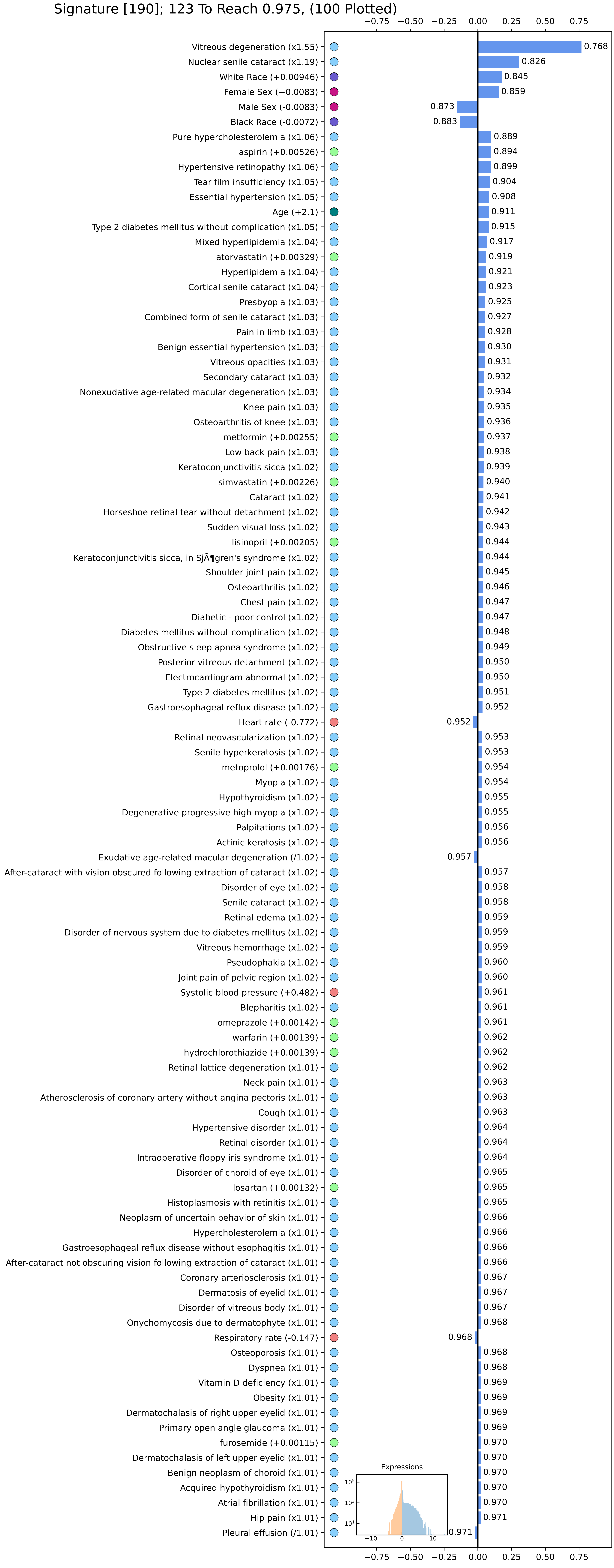

Signature [191]; 1 To Reach 0.975, (10 Plotted)

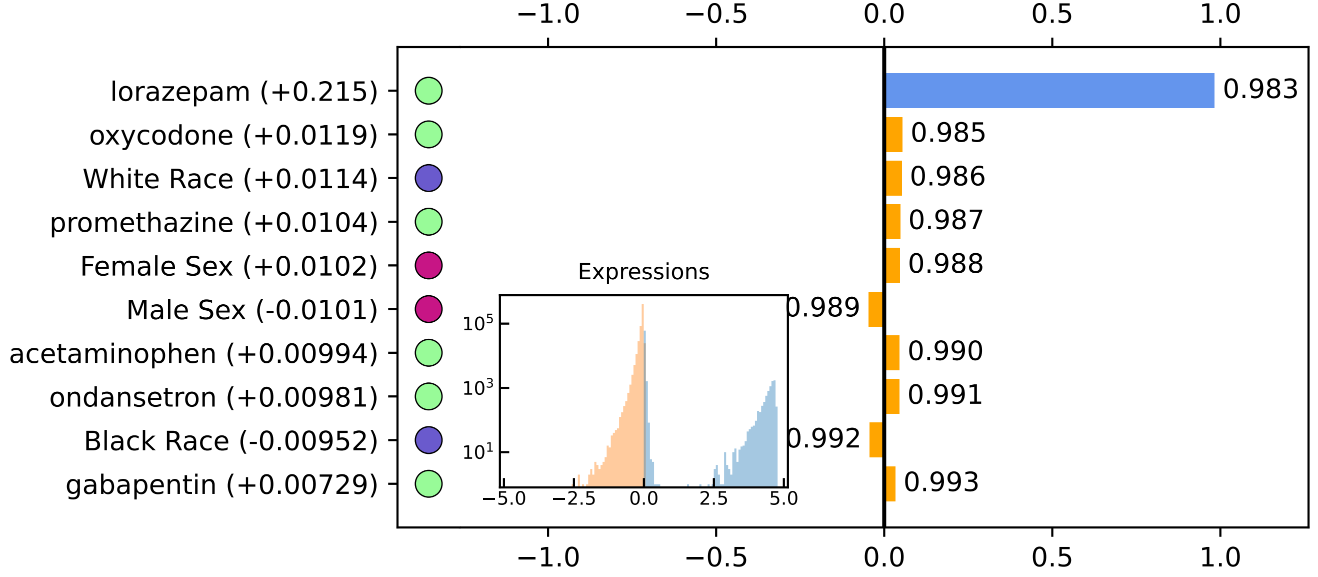

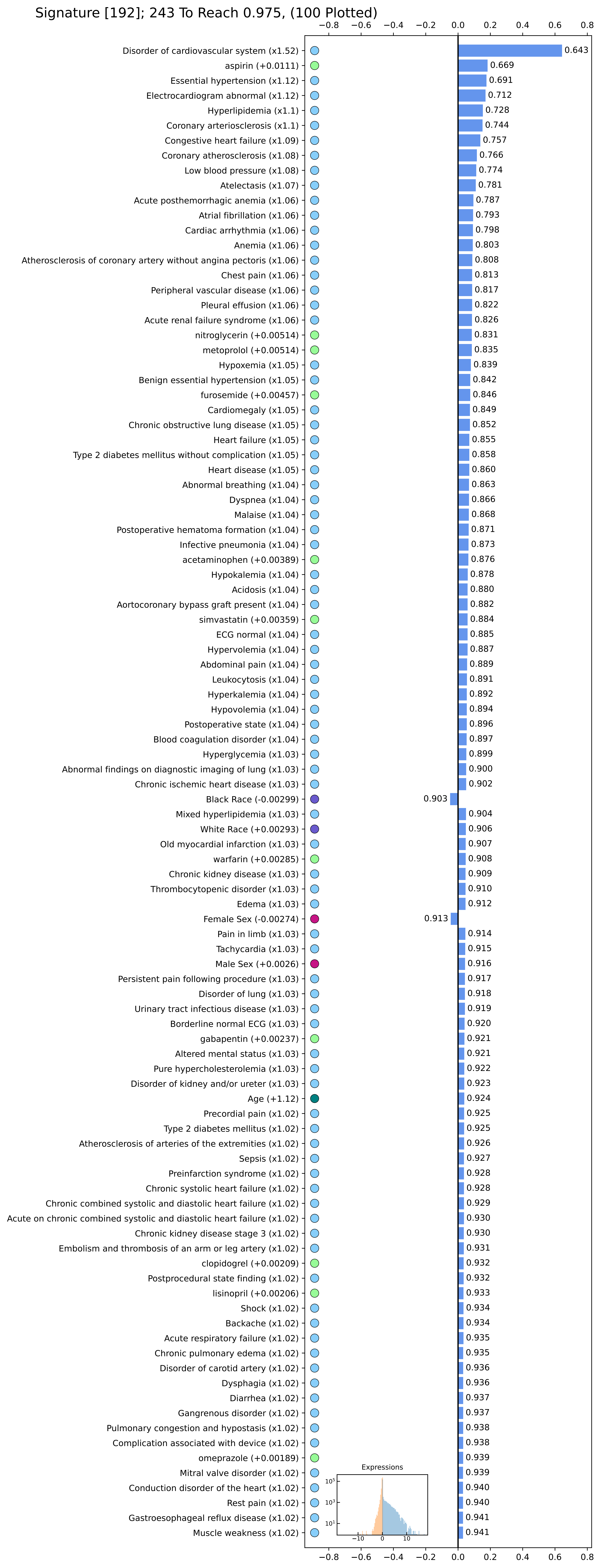
Signature [192]; 243 To Reach 0.975, (100 Plotted)
Disorder of cardiovascular system (x1.52) 0.643
aspirin (+0.0111) 0.669
Essential hypertension (x1.12) 0.691
Electrocardiogram abnormal (x1.12) 0.712
Hyperlipidemia (x1.1) 0.728
Coronary arteriosclerosis (x1.1) 0.744
Congestive heart failure (x1.09) 0.757
Coronary atherosclerosis (x1.08) 0.766
Low blood pressure (x1.08) 0.774
Atelectasis (x1.07) 0.781
Acute posthemorrhagic anemia (x1.06) 0.787
Atrial fibrillation (x1.06) 0.793
Cardiac arrhythmia (x1.06) 0.798
Anemia (x1.06) 0.803
Atherosclerosis of coronary artery without angina pectoris (x1.06) 0.808
Chest pain (x1.06) 0.813
Peripheral vascular disease (x1.06) 0.817
Pleural effusion (x1.06) 0.822
Acute renal failure syndrome (x1.06) 0.826
nitroglycerin (+0.00514) 0.831
metoprolol (+0.00514) 0.835
Hypoxemia (x1.05) 0.839
Benign essential hypertension (x1.05) 0.842
furosemide (+0.00457) 0.846
Cardiomegaly (x1.05) 0.849
Chronic obstructive lung disease (x1.05) 0.852
Heart failure (x1.05) 0.855
Type 2 diabetes mellitus without complication (x1.05) 0.858
Heart disease (x1.05) 0.860
Abnormal breathing (x1.04) 0.863
Dyspnea (x1.04) 0.866
Malaise (x1.04) 0.868
Postoperative hematoma formation (x1.04) 0.871
Infective pneumonia (x1.04) 0.873
acetaminophen (+0.00389) 0.876
Hypokalemia (x1.04) 0.878
Acidosis (x1.04) 0.880
Aortocoronary bypass graft present (x1.04) 0.882
simvastatin (+0.00359) 0.884
ECG normal (x1.04) 0.885
Hypervolemia (x1.04) 0.887
Abdominal pain (x1.04) 0.889
Leukocytosis (x1.04) 0.891
Hyperkalemia (x1.04) 0.892
Hypovolemia (x1.04) 0.894
Postoperative state (x1.04) 0.896
Blood coagulation disorder (x1.04) 0.897
Hyperglycemia (x1.03) 0.899
Abnormal findings on diagnostic imaging of lung (x1.03) 0.900
Chronic ischemic heart disease (x1.03) 0.902
Black Race (-0.00299) 0.903
Mixed hyperlipidemia (x1.03) 0.904
White Race (+0.00293) 0.906
Old myocardial infarction (x1.03) 0.907
warfarin (+0.00285) 0.908
Chronic kidney disease (x1.03) 0.909
Thrombocytopenic disorder (x1.03) 0.910
Edema (x1.03) 0.912
Female Sex (-0.00274) 0.913
Pain in limb (x1.03) 0.914
Tachycardia (x1.03) 0.915
Male Sex (+0.0026) 0.916
Persistent pain following procedure (x1.03) 0.917
Disorder of lung (x1.03) 0.918
Urinary tract infectious disease (x1.03) 0.919
Borderline normal ECG (x1.03) 0.920
gabapentin (+0.00237) 0.921
Altered mental status (x1.03) 0.921
Pure hypercholesterolemia (x1.03) 0.922
Disorder of kidney and/or ureter (x1.03) 0.923
Age (+1.12) 0.924
Precordial pain (x1.02) 0.925
Type 2 diabetes mellitus (x1.02) 0.925
Atherosclerosis of arteries of the extremities (x1.02) 0.926
Sepsis (x1.02) 0.927
Preinfarction syndrome (x1.02) 0.928
Chronic systolic heart failure (x1.02) 0.928
Chronic combined systolic and diastolic heart failure (x1.02) 0.929
Acute on chronic combined systolic and diastolic heart failure (x1.02) 0.930
Chronic kidney disease stage 3 (x1.02) 0.930
Embolism and thrombosis of an arm or leg artery (x1.02) 0.931
clopidogrel (+0.00209) 0.932
Postprocedural state finding (x1.02) 0.932
lisinopril (+0.00206) 0.933
Shock (x1.02) 0.934
Backache (x1.02) 0.934
Acute respiratory failure (x1.02) 0.935
Chronic pulmonary edema (x1.02) 0.935
Disorder of carotid artery (x1.02) 0.936
Dysphagia (x1.02) 0.936
Diarrhea (x1.02) 0.937
Gangrenous disorder (x1.02) 0.937
Pulmonary congestion and hypostasis (x1.02) 0.938
Complication associated with device (x1.02) 0.938
omeprazole (+0.00189) 0.939
Mitral valve disorder (x1.02) 0.939
Conduction disorder of the heart (x1.02) 0.940
Rest pain (x1.02) 0.940
Gastroesophageal reflux disease (x1.02) 0.941
Muscle weakness (x1.02) 0.941
Expressions
−0.8 −0.6 −0.4 −0.2 0.0 0.2 0.4 0.6 0.8

Signature [193]; 1 To Reach 0.975, (10 Plotted)

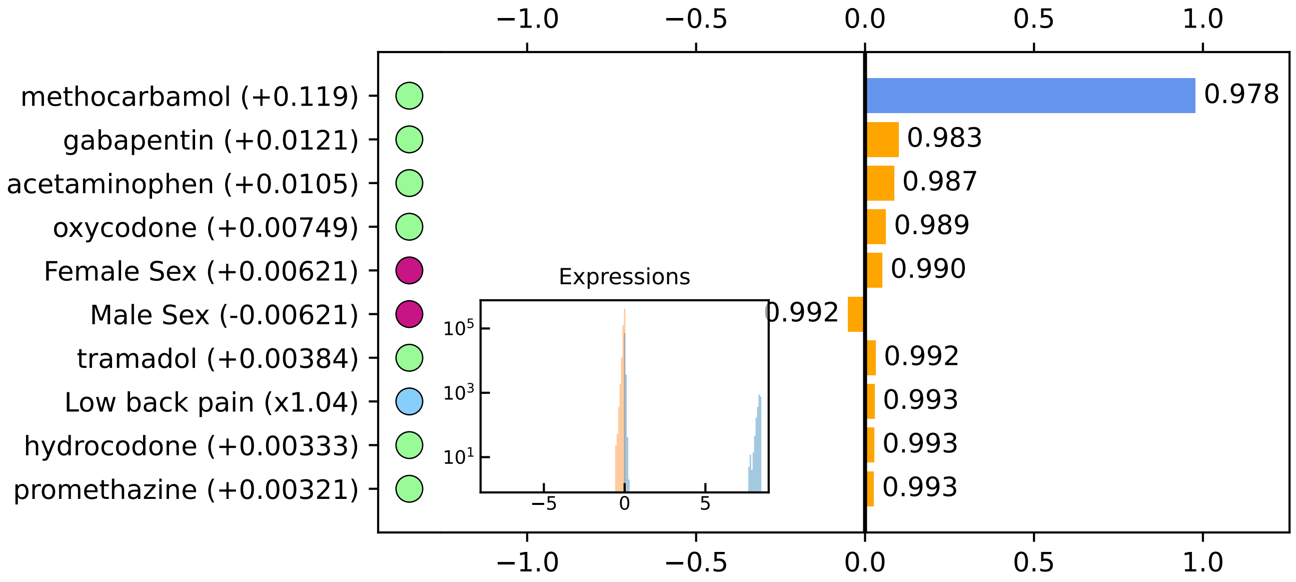

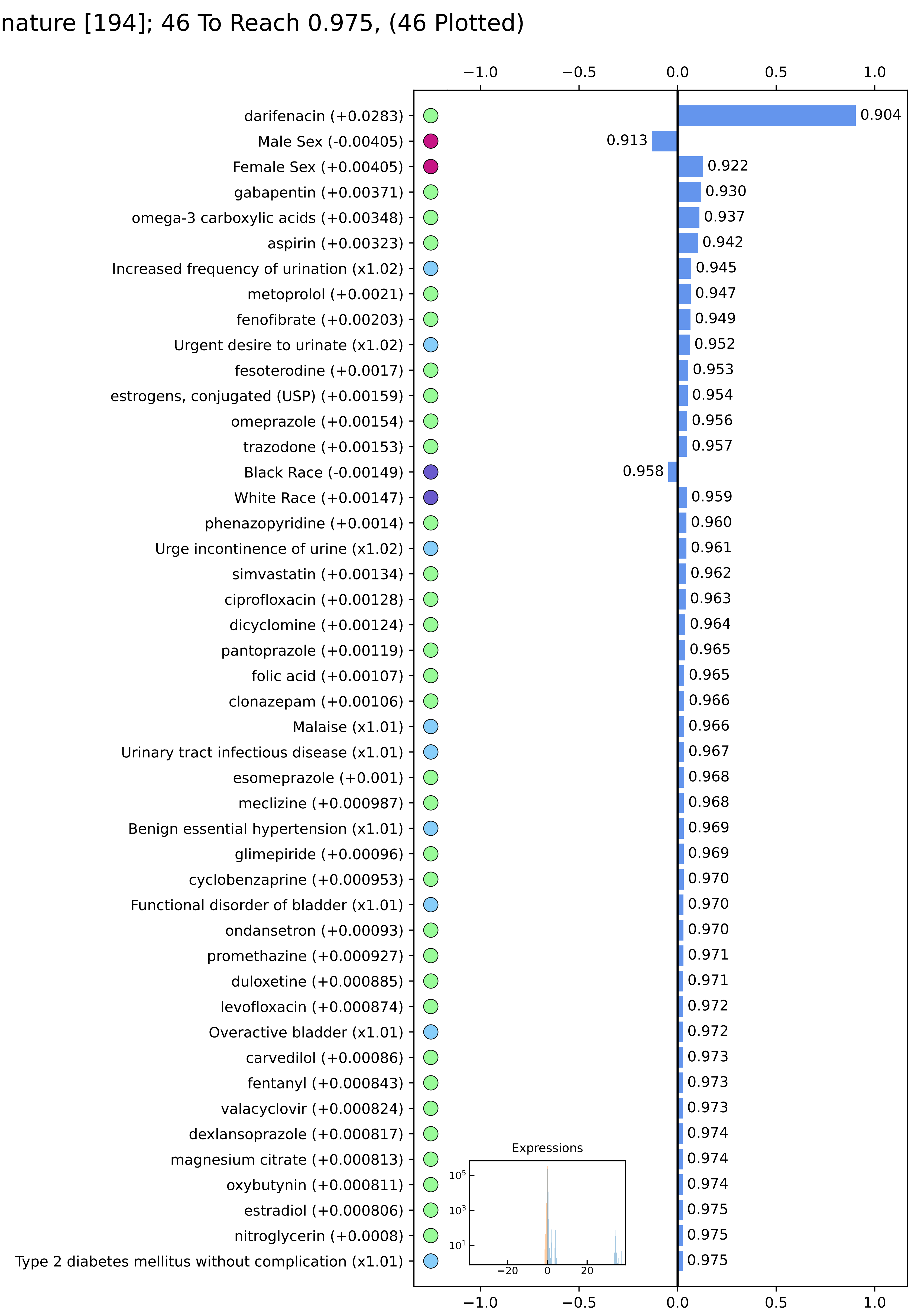
Signature [194]; 46 To Reach 0.975, (46 Plotted)
darifenacin (+0.0283) 0.904
Male Sex (-0.00405) 0.913
Female Sex (+0.00405) 0.922
gabapentin (+0.00371) 0.930
omega-3 carboxylic acids (+0.00348) 0.937
aspirin (+0.00323) 0.942
Increased frequency of urination (x1.02) 0.945
metoprolol (+0.0021) 0.947
fenofibrate (+0.00203) 0.949
Urgent desire to urinate (x1.02) 0.952
fesoterodine (+0.0017) 0.953
estrogens, conjugated (USP) (+0.00159) 0.954
omeprazole (+0.00154) 0.956
trazodone (+0.00153) 0.957
Black Race (-0.00149) 0.958
White Race (+0.00147) 0.959
phenazopyridine (+0.0014) 0.960
Urge incontinence of urine (x1.02) 0.961
simvastatin (+0.00134) 0.962
ciprofloxacin (+0.00128) 0.963
dicyclomine (+0.00124) 0.964
pantoprazole (+0.00119) 0.965
folic acid (+0.00107) 0.965
clonazepam (+0.00106) 0.966
Malaise (x1.01) 0.966
Urinary tract infectious disease (x1.01) 0.967
esomeprazole (+0.001) 0.968
meclizine (+0.000987) 0.968
Benign essential hypertension (x1.01) 0.969
glimepiride (+0.00096) 0.969
cyclobenzaprine (+0.000953) 0.970
Functional disorder of bladder (x1.01) 0.970
ondansetron (+0.00093) 0.970
promethazine (+0.000927) 0.971
duloxetine (+0.000885) 0.971
levofloxacin (+0.000874) 0.972
Overactive bladder (x1.01) 0.972
carvedilol (+0.00086) 0.973
fentanyl (+0.000843) 0.973
valacyclovir (+0.000824) 0.973
dexlansoprazole (+0.000817) 0.974
magnesium citrate (+0.000813) 0.974
oxybutynin (+0.000811) 0.974
estradiol (+0.000806) 0.975
nitroglycerin (+0.0008) 0.975
Type 2 diabetes mellitus without complication (x1.01) 0.975
−1.0
−0.5
0.0
0.5
1.0
Expressions
10^5
10^3
10^1
−20
0
20

Signature [195]; 1 To Reach 0.975, (10 Plotted)

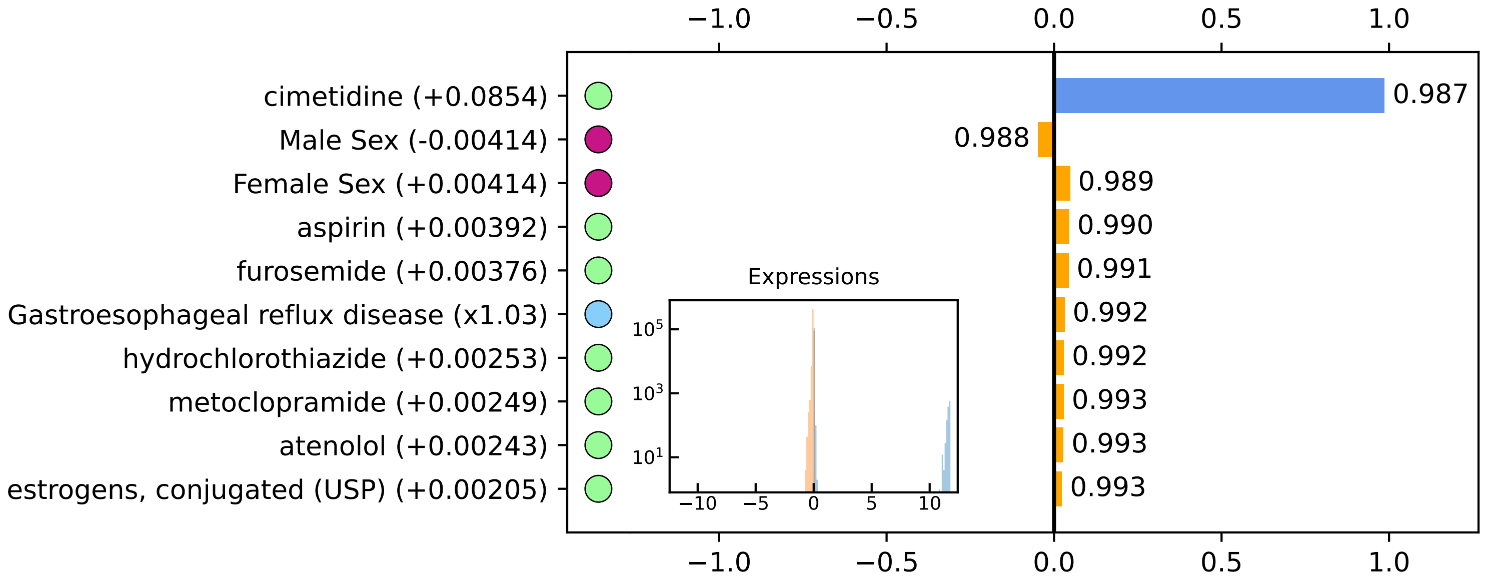

Signature [196]; 1 To Reach 0.975, (10 Plotted)

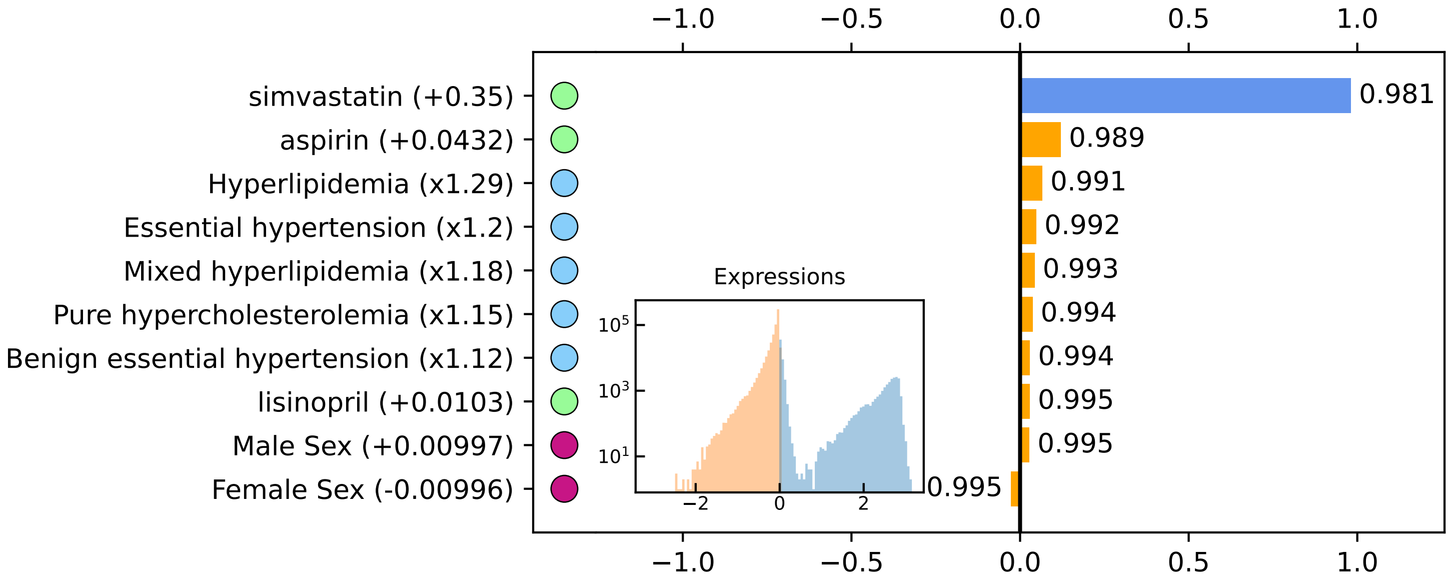

Signature [197]; 2 To Reach 0.975, (10 Plotted)

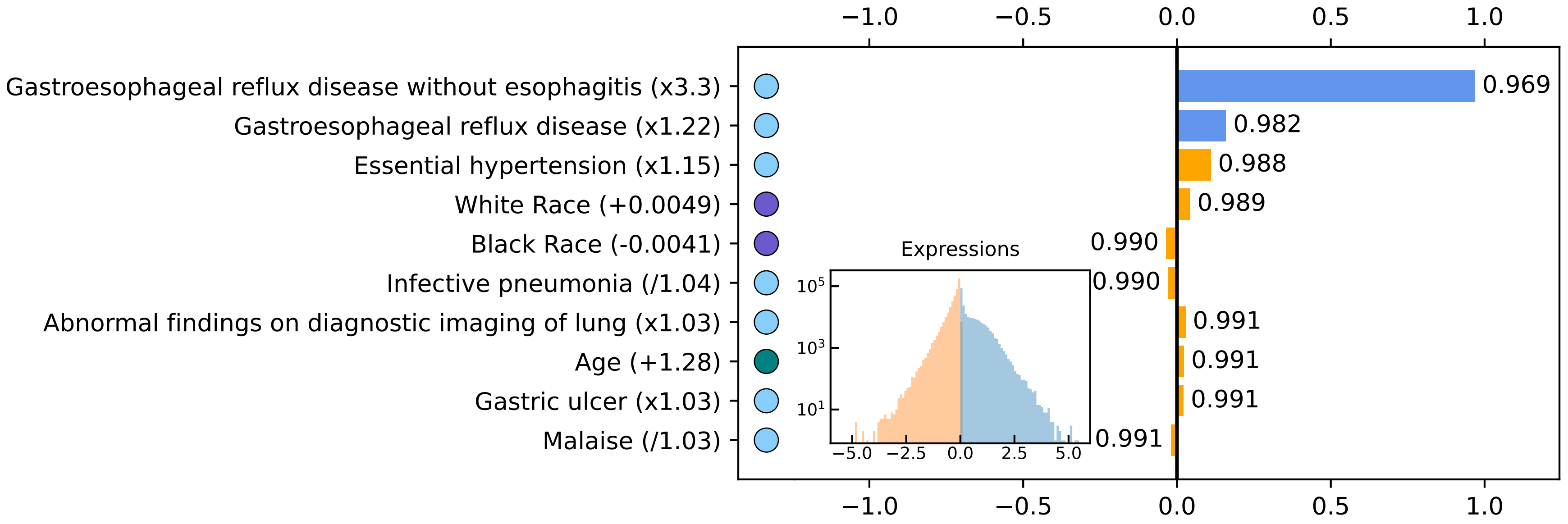

Signature [198]; 75 To Reach 0.975, (75 Plotted)

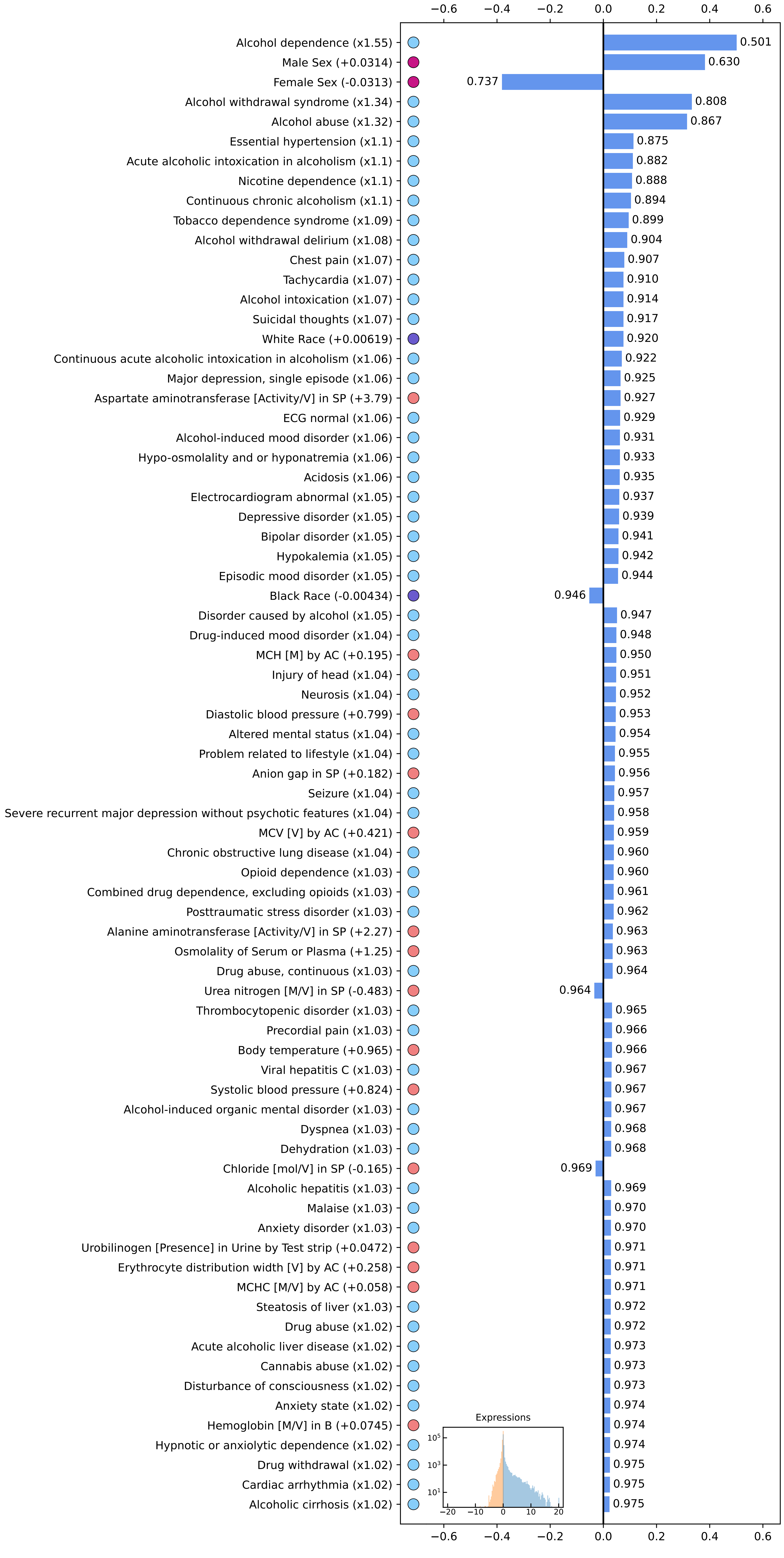

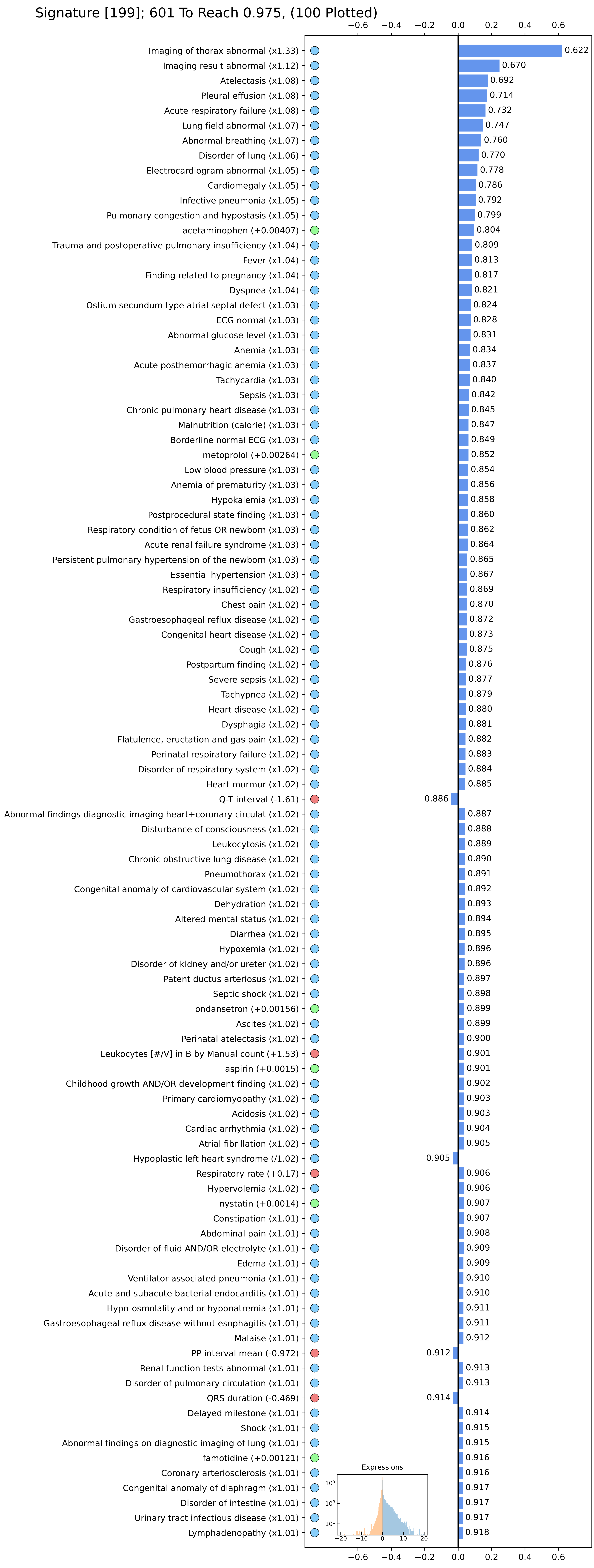
Signature [199]; 601 To Reach 0.975, (100 Plotted)
Imaging of thorax abnormal (x1.33) 0.622
Imaging result abnormal (x1.12) 0.670
Atelectasis (x1.08) 0.692
Pleural effusion (x1.08) 0.714
Acute respiratory failure (x1.08) 0.732
Lung field abnormal (x1.07) 0.747
Abnormal breathing (x1.07) 0.760
Disorder of lung (x1.06) 0.770
Electrocardiogram abnormal (x1.05) 0.778
Cardiomegaly (x1.05) 0.786
Infective pneumonia (x1.05) 0.792
Pulmonary congestion and hypostasis (x1.05) 0.799
acetaminophen (+0.00407) 0.804
Trauma and postoperative pulmonary insufficiency (x1.04) 0.809
Fever (x1.04) 0.813
Finding related to pregnancy (x1.04) 0.817
Dyspnea (x1.04) 0.821
Ostium secundum type atrial septal defect (x1.03) 0.824
ECG normal (x1.03) 0.828
Abnormal glucose level (x1.03) 0.831
Anemia (x1.03) 0.834
Acute posthemorrhagic anemia (x1.03) 0.837
Tachycardia (x1.03) 0.840
Sepsis (x1.03) 0.842
Chronic pulmonary heart disease (x1.03) 0.845
Malnutrition (calorie) (x1.03) 0.847
Borderline normal ECG (x1.03) 0.849
metoprolol (+0.00264) 0.852
Low blood pressure (x1.03) 0.854
Anemia of prematurity (x1.03) 0.856
Hypokalemia (x1.03) 0.858
Postprocedural state finding (x1.03) 0.860
Respiratory condition of fetus OR newborn (x1.03) 0.862
Acute renal failure syndrome (x1.03) 0.864
Persistent pulmonary hypertension of the newborn (x1.03) 0.865
Essential hypertension (x1.03) 0.867
Respiratory insufficiency (x1.02) 0.869
Chest pain (x1.02) 0.870
Gastroesophageal reflux disease (x1.02) 0.872
Congenital heart disease (x1.02) 0.873
Cough (x1.02) 0.875
Postpartum finding (x1.02) 0.876
Severe sepsis (x1.02) 0.877
Tachypnea (x1.02) 0.879
Heart disease (x1.02) 0.880
Dysphagia (x1.02) 0.881
Flatulence, eructation and gas pain (x1.02) 0.882
Perinatal respiratory failure (x1.02) 0.883
Disorder of respiratory system (x1.02) 0.884
Heart murmur (x1.02) 0.885
Q-T interval (-1.61) 0.886
Abnormal findings diagnostic imaging heart+coronary circulat (x1.02) 0.887
Disturbance of consciousness (x1.02) 0.888
Leukocytosis (x1.02) 0.889
Chronic obstructive lung disease (x1.02) 0.890
Pneumothorax (x1.02) 0.891
Congenital anomaly of cardiovascular system (x1.02) 0.892
Dehydration (x1.02) 0.893
Altered mental status (x1.02) 0.894
Diarrhea (x1.02) 0.895
Hypoxemia (x1.02) 0.896
Disorder of kidney and/or ureter (x1.02) 0.896
Patent ductus arteriosus (x1.02) 0.897
Septic shock (x1.02) 0.898
ondansetron (+0.00156) 0.899
Ascites (x1.02) 0.899
Perinatal atelectasis (x1.02) 0.900
Leukocytes [#/V] in B by Manual count (+1.53) 0.901
aspirin (+0.0015) 0.901
Childhood growth AND/OR development finding (x1.02) 0.902
Primary cardiomyopathy (x1.02) 0.903
Acidosis (x1.02) 0.903
Cardiac arrhythmia (x1.02) 0.904
Atrial fibrillation (x1.02) 0.905
Hypoplastic left heart syndrome (/1.02) 0.905
Respiratory rate (+0.17) 0.906
Hypervolemia (x1.02) 0.906
nystatin (+0.0014) 0.907
Constipation (x1.01) 0.907
Abdominal pain (x1.01) 0.908
Disorder of fluid AND/OR electrolyte (x1.01) 0.909
Edema (x1.01) 0.909
Ventilator associated pneumonia (x1.01) 0.910
Acute and subacute bacterial endocarditis (x1.01) 0.910
Hypo-osmolality and or hyponatremia (x1.01) 0.911
Gastroesophageal reflux disease without esophagitis (x1.01) 0.911
Malaise (x1.01) 0.912
PP interval mean (-0.972) 0.912
Renal function tests abnormal (x1.01) 0.913
Disorder of pulmonary circulation (x1.01) 0.913
QRS duration (-0.469) 0.914
Delayed milestone (x1.01) 0.914
Shock (x1.01) 0.915
Abnormal findings on diagnostic imaging of lung (x1.01) 0.915
famotidine (+0.00121) 0.916
Coronary arteriosclerosis (x1.01) 0.916
Congenital anomaly of diaphragm (x1.01) 0.917
Disorder of intestine (x1.01) 0.917
Urinary tract infectious disease (x1.01) 0.917
Lymphadenopathy (x1.01) 0.918
−0.6 −0.4 −0.2 0.0 0.2 0.4 0.6
Expressions
10^5 10^3 10^1
−20 −10 0 10 20

Signature [200]; 3 To Reach 0.975, (10 Plotted)

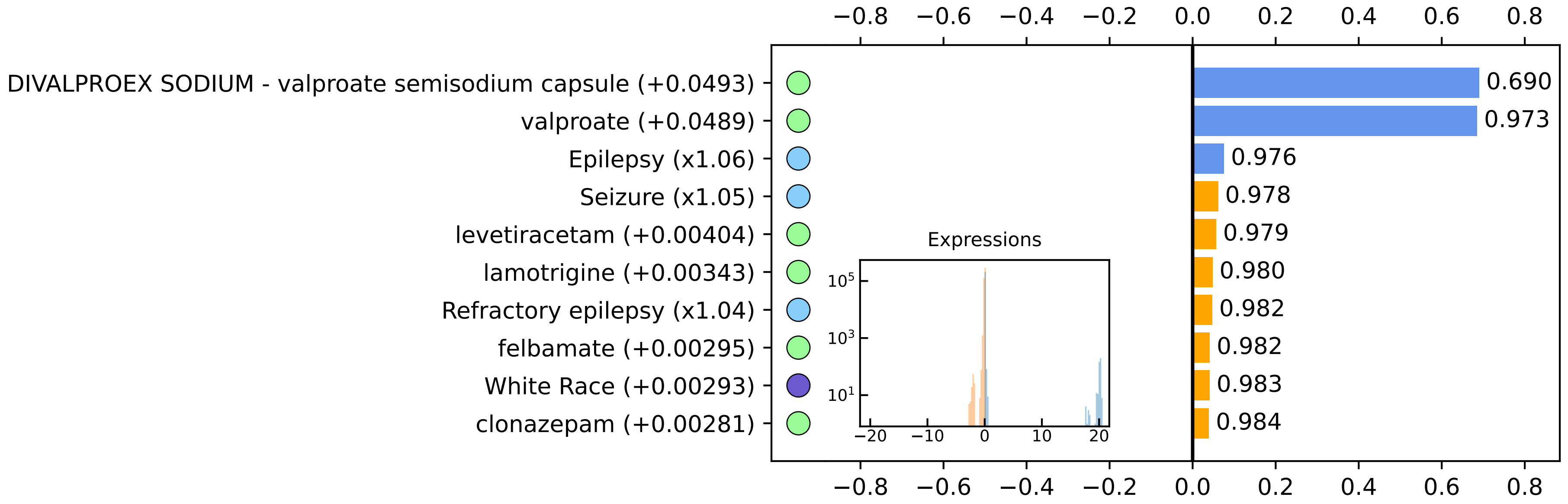

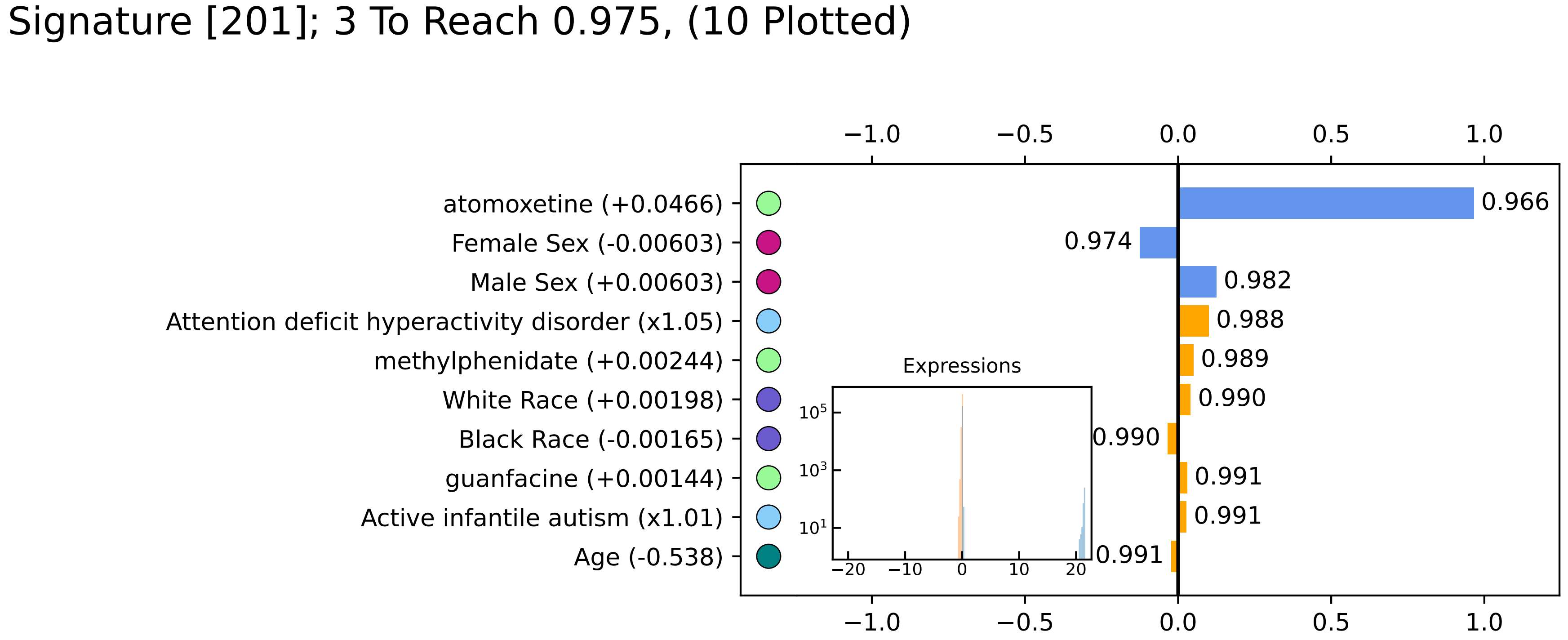

Signature [201]; 3 To Reach 0.975, (10 Plotted)
atomoxetine (+0.0466)
Female Sex (-0.00603)
Male Sex (+0.00603)
Attention deficit hyperactivity disorder (x1.05)
methylphenidate (+0.00244)
White Race (+0.00198)
Black Race (-0.00165)
guanfacine (+0.00144)
Active infantile autism (x1.01)
Age (-0.538)
0.966
0.974
0.982
0.988
0.989
0.990
0.990
0.991
0.991
0.991
−1.0
−0.5
0.0
0.5
1.0
Expressions
10^5
10^3
10^1
−20
−10
0
10
20

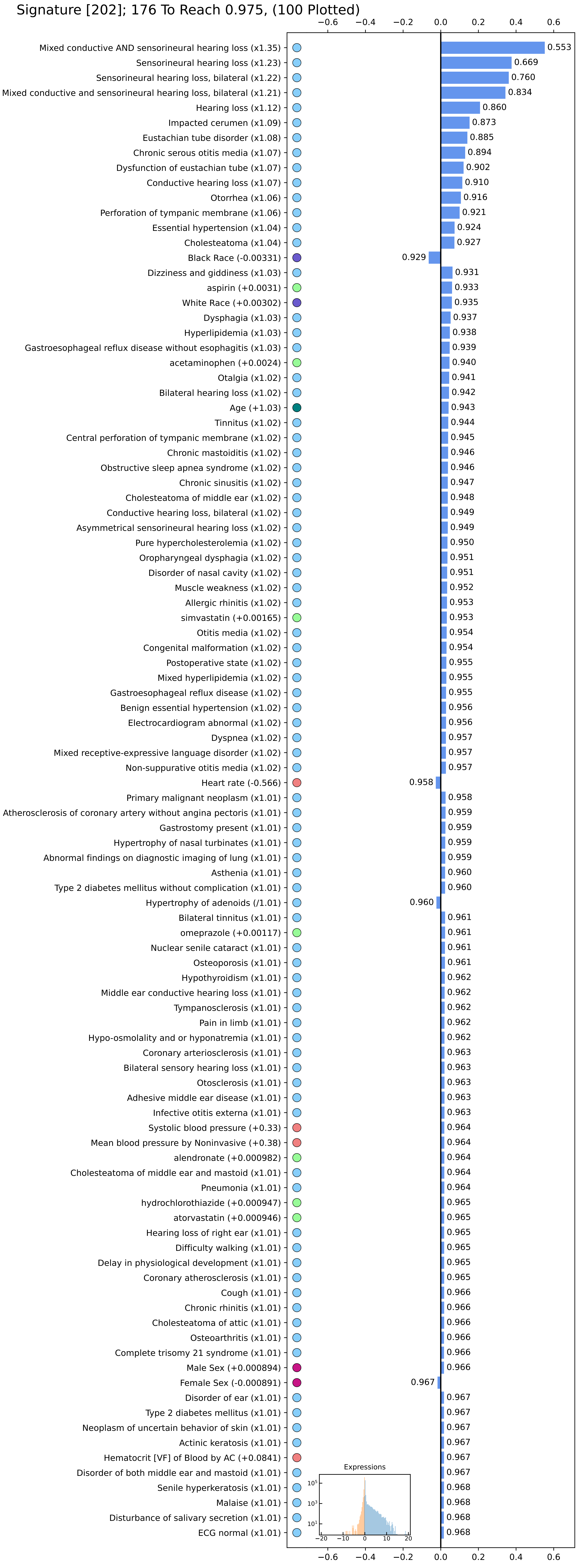
Signature [202]; 176 To Reach 0.975, (100 Plotted)
−0.6 −0.4 −0.2 0.0 0.2 0.4 0.6
Mixed conductive AND sensorineural hearing loss (x1.35) 0.553
Sensorineural hearing loss (x1.23) 0.669
Sensorineural hearing loss, bilateral (x1.22) 0.760
Mixed conductive and sensorineural hearing loss, bilateral (x1.21) 0.834
Hearing loss (x1.12) 0.860
Impacted cerumen (x1.09) 0.873
Eustachian tube disorder (x1.08) 0.885
Chronic serous otitis media (x1.07) 0.894
Dysfunction of eustachian tube (x1.07) 0.902
Conductive hearing loss (x1.07) 0.910
Otorrhea (x1.06) 0.916
Perforation of tympanic membrane (x1.06) 0.921
Essential hypertension (x1.04) 0.924
Cholesteatoma (x1.04) 0.927
Black Race (-0.00331) 0.929
Dizziness and giddiness (x1.03) 0.931
aspirin (+0.0031) 0.933
White Race (+0.00302) 0.935
Dysphagia (x1.03) 0.937
Hyperlipidemia (x1.03) 0.938
Gastroesophageal reflux disease without esophagitis (x1.03) 0.939
acetaminophen (+0.0024) 0.940
Otalgia (x1.02) 0.941
Bilateral hearing loss (x1.02) 0.942
Age (+1.03) 0.943
Tinnitus (x1.02) 0.944
Central perforation of tympanic membrane (x1.02) 0.945
Chronic mastoiditis (x1.02) 0.946
Obstructive sleep apnea syndrome (x1.02) 0.946
Chronic sinusitis (x1.02) 0.947
Cholesteatoma of middle ear (x1.02) 0.948
Conductive hearing loss, bilateral (x1.02) 0.949
Asymmetrical sensorineural hearing loss (x1.02) 0.949
Pure hypercholesterolemia (x1.02) 0.950
Oropharyngeal dysphagia (x1.02) 0.951
Disorder of nasal cavity (x1.02) 0.951
Muscle weakness (x1.02) 0.952
Allergic rhinitis (x1.02) 0.953
simvastatin (+0.00165) 0.953
Otitis media (x1.02) 0.954
Congenital malformation (x1.02) 0.954
Postoperative state (x1.02) 0.955
Mixed hyperlipidemia (x1.02) 0.955
Gastroesophageal reflux disease (x1.02) 0.955
Benign essential hypertension (x1.02) 0.956
Electrocardiogram abnormal (x1.02) 0.956
Dyspnea (x1.02) 0.957
Mixed receptive-expressive language disorder (x1.02) 0.957
Non-suppurative otitis media (x1.02) 0.957
Heart rate (-0.566) 0.958
Primary malignant neoplasm (x1.01) 0.958
Atherosclerosis of coronary artery without angina pectoris (x1.01) 0.959
Gastrostomy present (x1.01) 0.959
Hypertrophy of nasal turbinates (x1.01) 0.959
Abnormal findings on diagnostic imaging of lung (x1.01) 0.959
Asthenia (x1.01) 0.960
Type 2 diabetes mellitus without complication (x1.01) 0.960
Hypertrophy of adenoids (/1.01) 0.960
Bilateral tinnitus (x1.01) 0.961
omeprazole (+0.00117) 0.961
Nuclear senile cataract (x1.01) 0.961
Osteoporosis (x1.01) 0.961
Hypothyroidism (x1.01) 0.962
Middle ear conductive hearing loss (x1.01) 0.962
Tympanosclerosis (x1.01) 0.962
Pain in limb (x1.01) 0.962
Hypo-osmolality and or hyponatremia (x1.01) 0.962
Coronary arteriosclerosis (x1.01) 0.963
Bilateral sensory hearing loss (x1.01) 0.963
Otosclerosis (x1.01) 0.963
Adhesive middle ear disease (x1.01) 0.963
Infective otitis externa (x1.01) 0.963
Systolic blood pressure (+0.33) 0.964
Mean blood pressure by Noninvasive (+0.38) 0.964
alendronate (+0.000982) 0.964
Cholesteatoma of middle ear and mastoid (x1.01) 0.964
Pneumonia (x1.01) 0.964
hydrochlorothiazide (+0.000947) 0.965
atorvastatin (+0.000946) 0.965
Hearing loss of right ear (x1.01) 0.965
Difficulty walking (x1.01) 0.965
Delay in physiological development (x1.01) 0.965
Coronary atherosclerosis (x1.01) 0.965
Cough (x1.01) 0.966
Chronic rhinitis (x1.01) 0.966
Cholesteatoma of attic (x1.01) 0.966
Osteoarthritis (x1.01) 0.966
Complete trisomy 21 syndrome (x1.01) 0.966
Male Sex (+0.000894) 0.966
Female Sex (-0.000891) 0.967
Disorder of ear (x1.01) 0.967
Type 2 diabetes mellitus (x1.01) 0.967
Neoplasm of uncertain behavior of skin (x1.01) 0.967
Actinic keratosis (x1.01) 0.967
Hematocrit [VF] of Blood by AC (+0.0841) 0.967
Disorder of both middle ear and mastoid (x1.01) 0.967
Senile hyperkeratosis (x1.01) 0.968
Malaise (x1.01) 0.968
Disturbance of salivary secretion (x1.01) 0.968
ECG normal (x1.01) 0.968
Expressions
10^5
10^3
10^1
−20 −10 0 10 20
−0.6 −0.4 −0.2 0.0 0.2 0.4 0.6

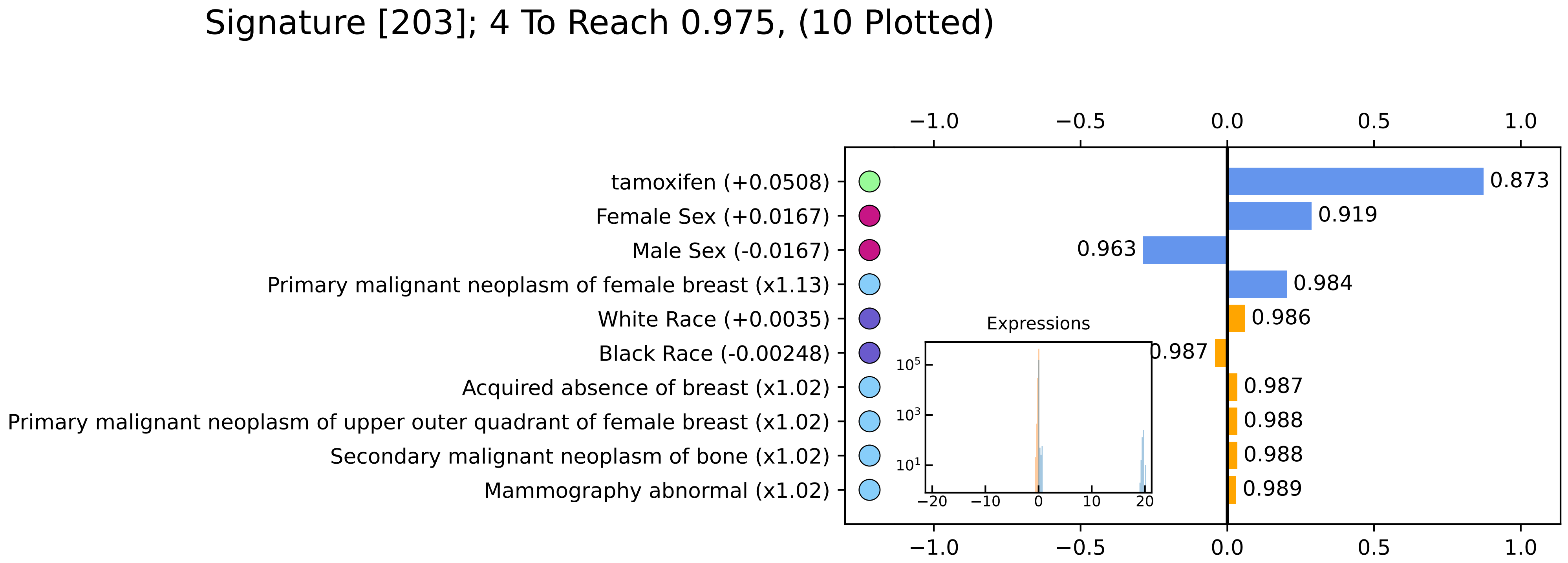
Signature [203]; 4 To Reach 0.975, (10 Plotted)
tamoxifen (+0.0508)
Female Sex (+0.0167)
Male Sex (-0.0167)
Primary malignant neoplasm of female breast (x1.13)
White Race (+0.0035)
Black Race (-0.00248)
Acquired absence of breast (x1.02)
Primary malignant neoplasm of upper outer quadrant of female breast (x1.02)
Secondary malignant neoplasm of bone (x1.02)
Mammography abnormal (x1.02)
0.873
0.919
0.963
0.984
0.986
0.987
0.987
0.988
0.988
0.989
−1.0
−0.5
0.0
0.5
1.0
Expressions
−20
−10
0
10
20

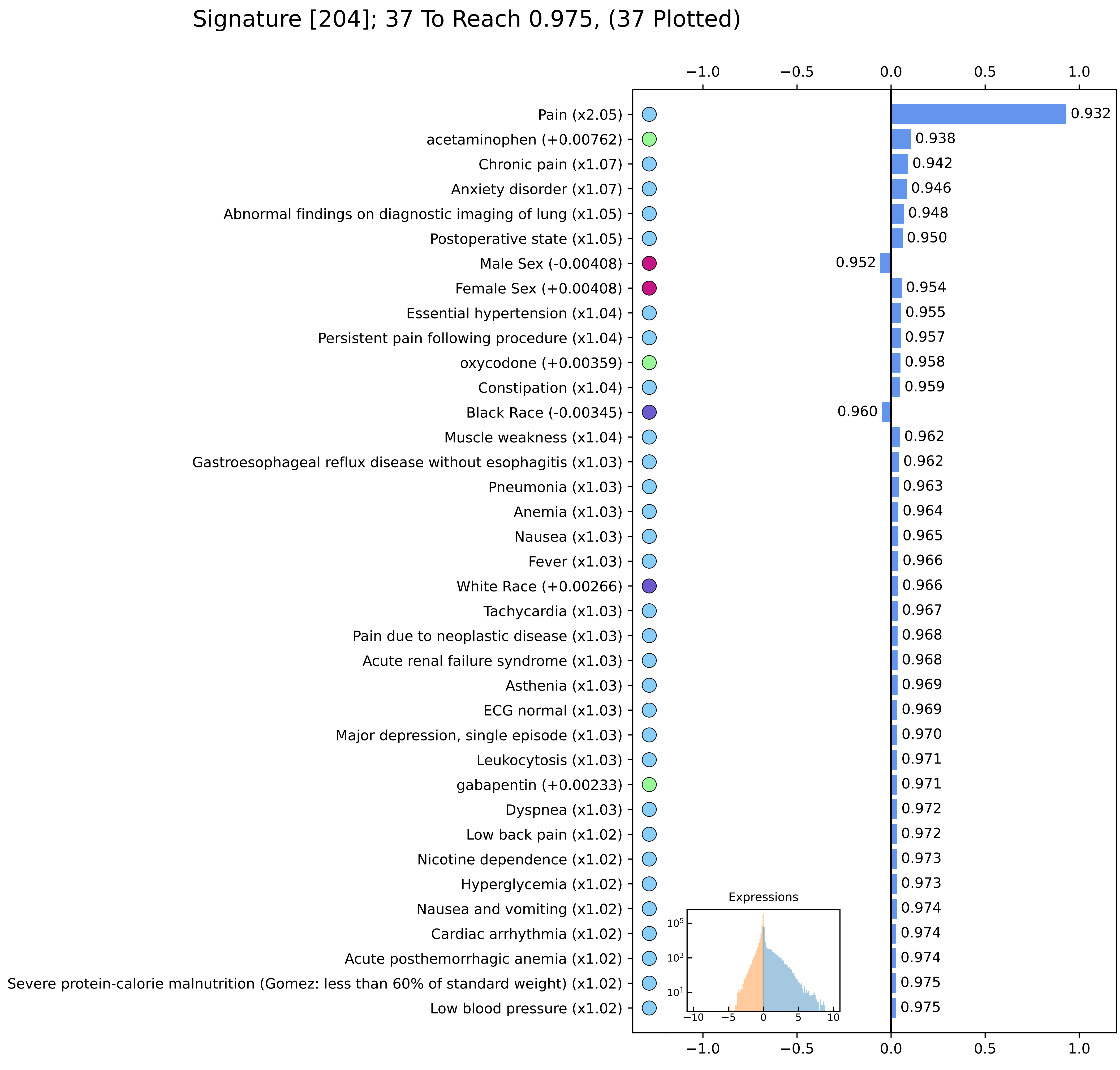

Signature [204]; 37 To Reach 0.975, (37 Plotted)
Pain (x2.05) 0.932
acetaminophen (+0.00762) 0.938
Chronic pain (x1.07) 0.942
Anxiety disorder (x1.07) 0.946
Abnormal findings on diagnostic imaging of lung (x1.05) 0.948
Postoperative state (x1.05) 0.950
Male Sex (-0.00408) 0.952
Female Sex (+0.00408) 0.954
Essential hypertension (x1.04) 0.955
Persistent pain following procedure (x1.04) 0.957
oxycodone (+0.00359) 0.958
Constipation (x1.04) 0.959
Black Race (-0.00345) 0.960
Muscle weakness (x1.04) 0.962
Gastroesophageal reflux disease without esophagitis (x1.03) 0.962
Pneumonia (x1.03) 0.963
Anemia (x1.03) 0.964
Nausea (x1.03) 0.965
Fever (x1.03) 0.966
White Race (+0.00266) 0.966
Tachycardia (x1.03) 0.967
Pain due to neoplastic disease (x1.03) 0.968
Acute renal failure syndrome (x1.03) 0.968
Asthenia (x1.03) 0.969
ECG normal (x1.03) 0.969
Major depression, single episode (x1.03) 0.970
Leukocytosis (x1.03) 0.971
gabapentin (+0.00233) 0.971
Dyspnea (x1.03) 0.972
Low back pain (x1.02) 0.972
Nicotine dependence (x1.02) 0.973
Hyperglycemia (x1.02) 0.973
Nausea and vomiting (x1.02) 0.974
Cardiac arrhythmia (x1.02) 0.974
Acute posthemorrhagic anemia (x1.02) 0.974
Severe protein-calorie malnutrition (Gomez: less than 60% of standard weight) (x1.02) 0.975
Low blood pressure (x1.02) 0.975
−1.0 −0.5 0.0 0.5 1.0
Expressions

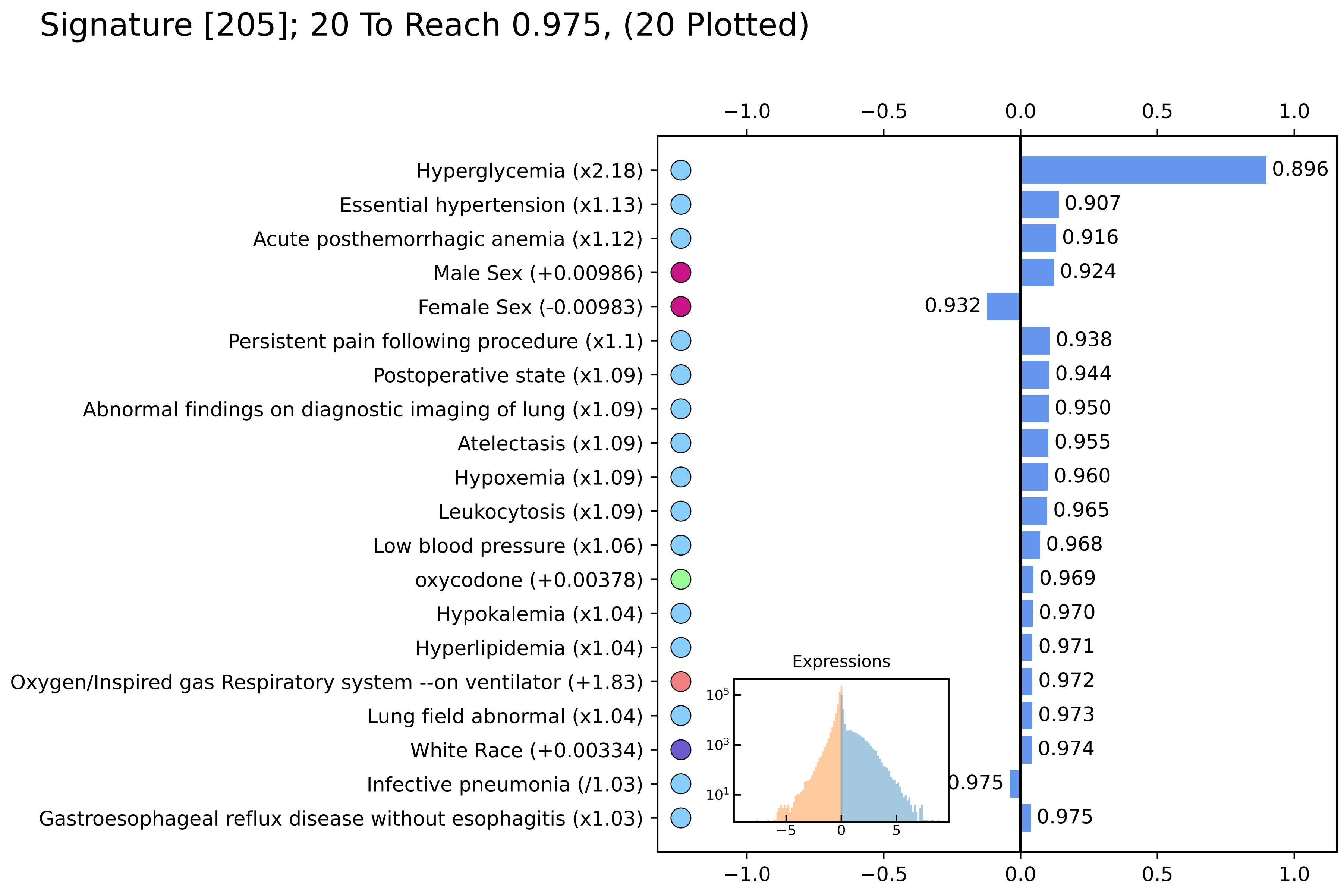

Signature [205]; 20 To Reach 0.975, (20 Plotted)
Hyperglycemia (x2.18) 0.896
Essential hypertension (x1.13) 0.907
Acute posthemorrhagic anemia (x1.12) 0.916
Male Sex (+0.00986) 0.924
Female Sex (-0.00983) 0.932
Persistent pain following procedure (x1.1) 0.938
Postoperative state (x1.09) 0.944
Abnormal findings on diagnostic imaging of lung (x1.09) 0.950
Atelectasis (x1.09) 0.955
Hypoxemia (x1.09) 0.960
Leukocytosis (x1.09) 0.965
Low blood pressure (x1.06) 0.968
oxycodone (+0.00378) 0.969
Hypokalemia (x1.04) 0.970
Hyperlipidemia (x1.04) 0.971
Oxygen/Inspired gas Respiratory system --on ventilator (+1.83) 0.972
Lung field abnormal (x1.04) 0.973
White Race (+0.00334) 0.974
Infective pneumonia (/1.03) 0.975
Gastroesophageal reflux disease without esophagitis (x1.03) 0.975
−1.0 −0.5 0.0 0.5 1.0
Expressions

Signature [206]; 76 To Reach 0.975, (76 Plotted)

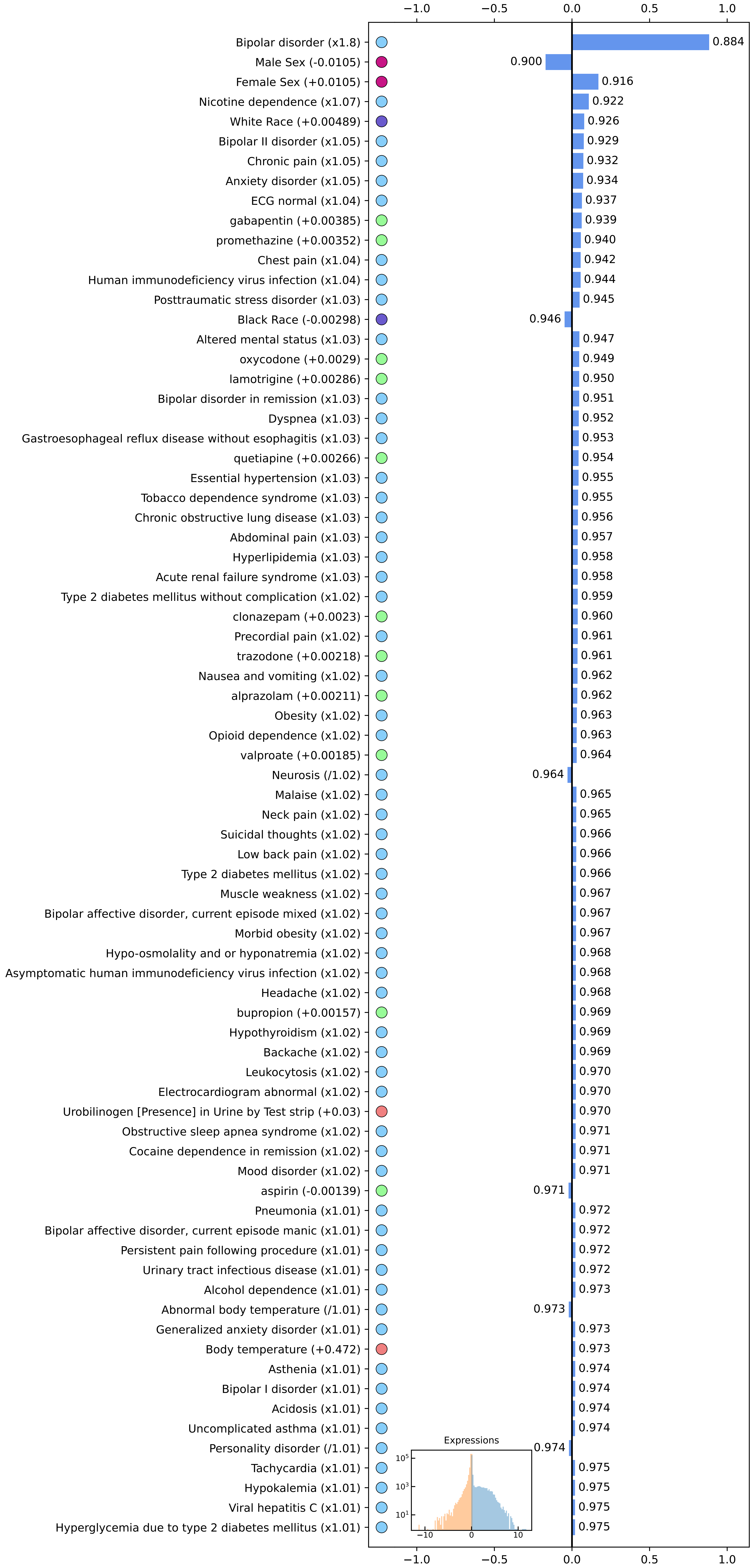

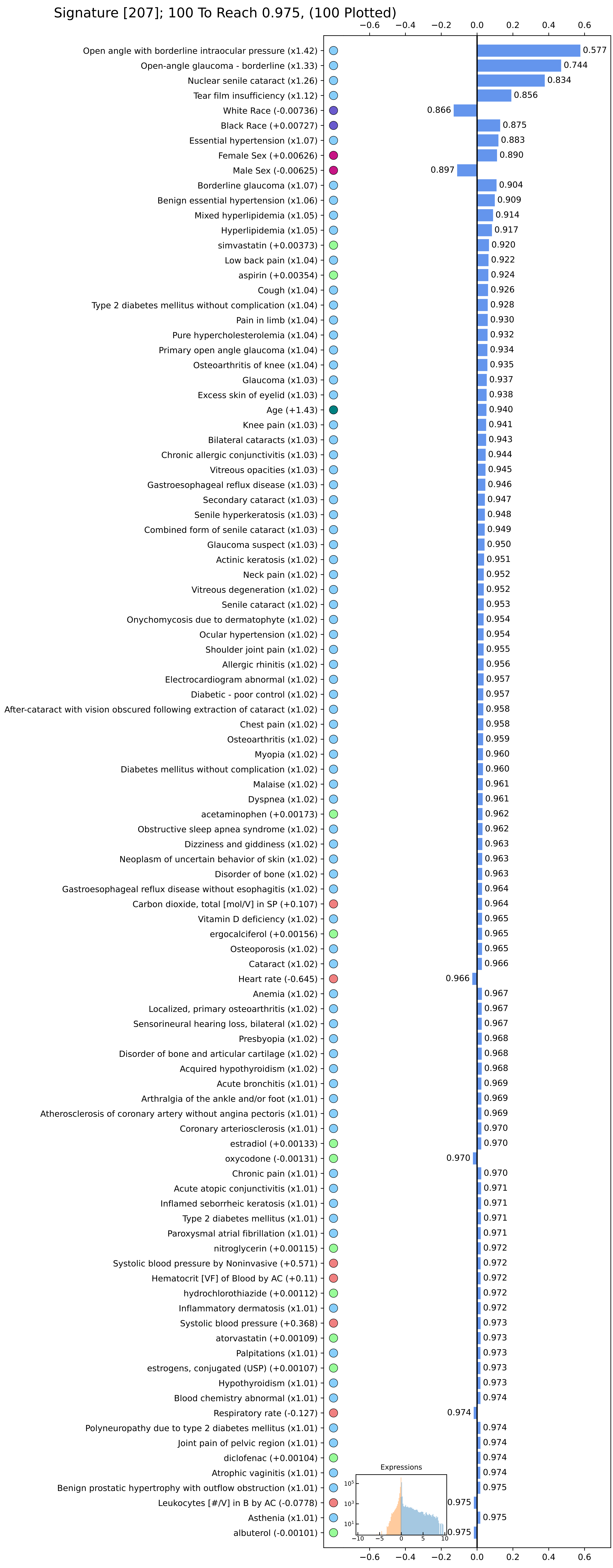

Signature [207]; 100 To Reach 0.975, (100 Plotted)
Open angle with borderline intraocular pressure (x1.42) 0.577
Open-angle glaucoma - borderline (x1.33) 0.744
Nuclear senile cataract (x1.26) 0.834
Tear film insufficiency (x1.12) 0.856
White Race (-0.00736) 0.866
Black Race (+0.00727) 0.875
Essential hypertension (x1.07) 0.883
Female Sex (+0.00626) 0.890
Male Sex (-0.00625) 0.897
Borderline glaucoma (x1.07) 0.904
Benign essential hypertension (x1.06) 0.909
Mixed hyperlipidemia (x1.05) 0.914
Hyperlipidemia (x1.05) 0.917
simvastatin (+0.00373) 0.920
Low back pain (x1.04) 0.922
aspirin (+0.00354) 0.924
Cough (x1.04) 0.926
Type 2 diabetes mellitus without complication (x1.04) 0.928
Pain in limb (x1.04) 0.930
Pure hypercholesterolemia (x1.04) 0.932
Primary open angle glaucoma (x1.04) 0.934
Osteoarthritis of knee (x1.04) 0.935
Glaucoma (x1.03) 0.937
Excess skin of eyelid (x1.03) 0.938
Age (+1.43) 0.940
Knee pain (x1.03) 0.941
Bilateral cataracts (x1.03) 0.943
Chronic allergic conjunctivitis (x1.03) 0.944
Vitreous opacities (x1.03) 0.945
Gastroesophageal reflux disease (x1.03) 0.946
Secondary cataract (x1.03) 0.947
Senile hyperkeratosis (x1.03) 0.948
Combined form of senile cataract (x1.03) 0.949
Glaucoma suspect (x1.03) 0.950
Actinic keratosis (x1.02) 0.951
Neck pain (x1.02) 0.952
Vitreous degeneration (x1.02) 0.952
Senile cataract (x1.02) 0.953
Onychomycosis due to dermatophyte (x1.02) 0.954
Ocular hypertension (x1.02) 0.954
Shoulder joint pain (x1.02) 0.955
Allergic rhinitis (x1.02) 0.956
Electrocardiogram abnormal (x1.02) 0.957
Diabetic - poor control (x1.02) 0.957
After-cataract with vision obscured following extraction of cataract (x1.02) 0.958
Chest pain (x1.02) 0.958
Osteoarthritis (x1.02) 0.959
Myopia (x1.02) 0.960
Diabetes mellitus without complication (x1.02) 0.960
Malaise (x1.02) 0.961
Dyspnea (x1.02) 0.961
acetaminophen (+0.00173) 0.962
Obstructive sleep apnea syndrome (x1.02) 0.962
Dizziness and giddiness (x1.02) 0.963
Neoplasm of uncertain behavior of skin (x1.02) 0.963
Disorder of bone (x1.02) 0.963
Gastroesophageal reflux disease without esophagitis (x1.02) 0.964
Carbon dioxide, total [mol/V] in SP (+0.107) 0.964
Vitamin D deficiency (x1.02) 0.965
ergocalciferol (+0.00156) 0.965
Osteoporosis (x1.02) 0.965
Cataract (x1.02) 0.966
Heart rate (-0.645) 0.966
Anemia (x1.02) 0.967
Localized, primary osteoarthritis (x1.02) 0.967
Sensorineural hearing loss, bilateral (x1.02) 0.967
Presbyopia (x1.02) 0.968
Disorder of bone and articular cartilage (x1.02) 0.968
Acquired hypothyroidism (x1.02) 0.968
Acute bronchitis (x1.01) 0.969
Arthralgia of the ankle and/or foot (x1.01) 0.969
Atherosclerosis of coronary artery without angina pectoris (x1.01) 0.969
Coronary arteriosclerosis (x1.01) 0.970
estradiol (+0.00133) 0.970
oxycodone (-0.00131) 0.970
Chronic pain (x1.01) 0.970
Acute atopic conjunctivitis (x1.01) 0.971
Inflamed seborrheic keratosis (x1.01) 0.971
Type 2 diabetes mellitus (x1.01) 0.971
Paroxysmal atrial fibrillation (x1.01) 0.971
nitroglycerin (+0.00115) 0.972
Systolic blood pressure by Noninvasive (+0.571) 0.972
Hematocrit [VF] of Blood by AC (+0.11) 0.972
hydrochlorothiazide (+0.00112) 0.972
Inflammatory dermatosis (x1.01) 0.972
Systolic blood pressure (+0.368) 0.973
atorvastatin (+0.00109) 0.973
Palpitations (x1.01) 0.973
estrogens, conjugated (USP) (+0.00107) 0.973
Hypothyroidism (x1.01) 0.973
Blood chemistry abnormal (x1.01) 0.974
Respiratory rate (-0.127) 0.974
Polyneuropathy due to type 2 diabetes mellitus (x1.01) 0.974
Joint pain of pelvic region (x1.01) 0.974
diclofenac (+0.00104) 0.974
Atrophic vaginitis (x1.01) 0.974
Benign prostatic hypertrophy with outflow obstruction (x1.01) 0.975
Leukocytes [#/V] in B by AC (-0.0778) 0.975
Asthenia (x1.01) 0.975
albuterol (-0.00101) 0.975
−0.6 −0.4 −0.2 0.0 0.2 0.4 0.6
Expressions

Signature [208]; 1 To Reach 0.975, (10 Plotted)

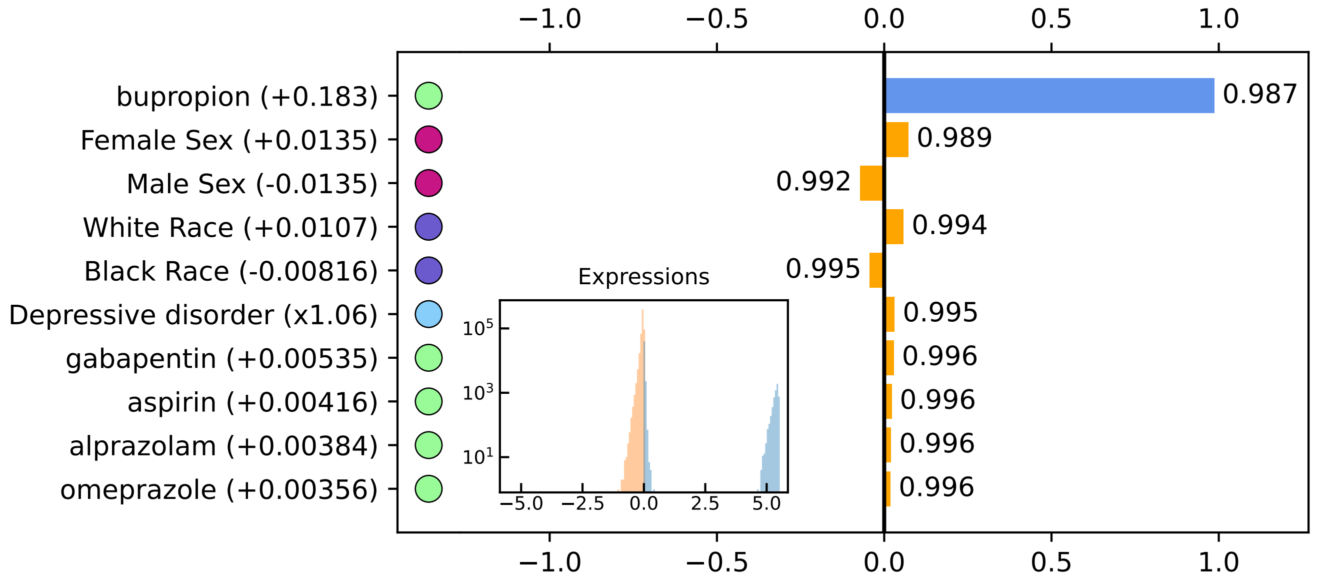

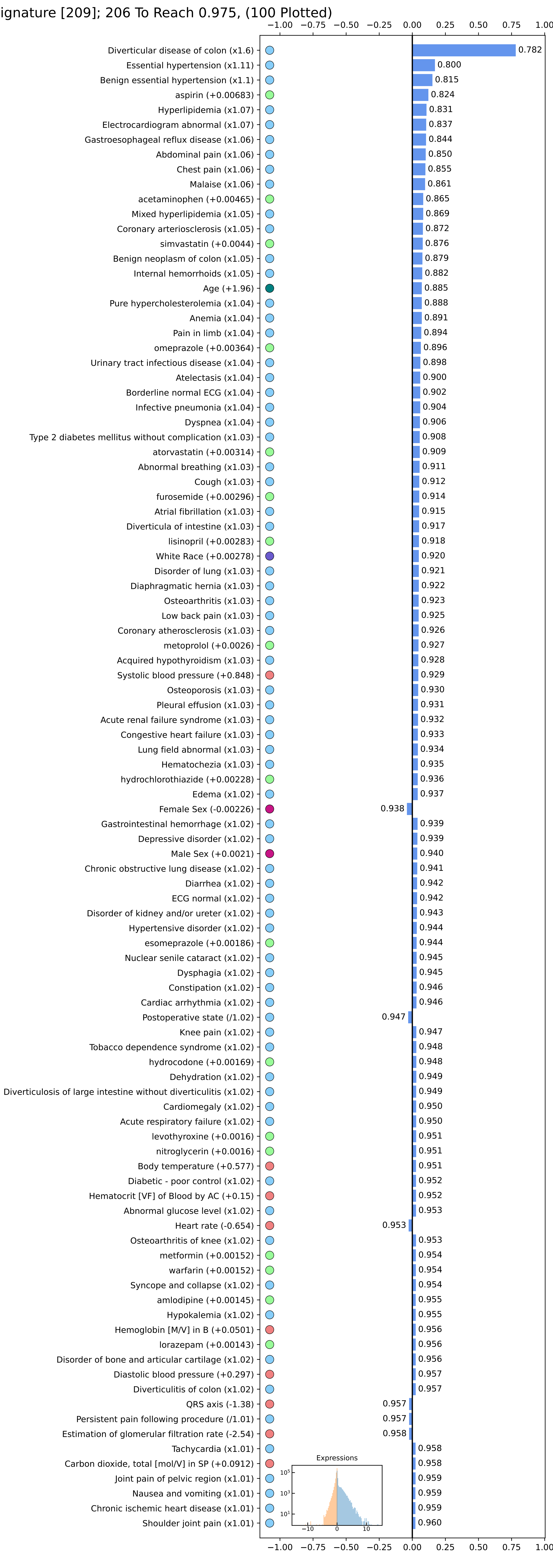

Signature [209]; 206 To Reach 0.975, (100 Plotted)
−1.00 −0.75 −0.50 −0.25 0.00 0.25 0.50 0.75 1.00
Diverticular disease of colon (x1.6) 0.782
Essential hypertension (x1.11) 0.800
Benign essential hypertension (x1.1) 0.815
aspirin (+0.00683) 0.824
Hyperlipidemia (x1.07) 0.831
Electrocardiogram abnormal (x1.07) 0.837
Gastroesophageal reflux disease (x1.06) 0.844
Abdominal pain (x1.06) 0.850
Chest pain (x1.06) 0.855
Malaise (x1.06) 0.861
acetaminophen (+0.00465) 0.865
Mixed hyperlipidemia (x1.05) 0.869
Coronary arteriosclerosis (x1.05) 0.872
simvastatin (+0.0044) 0.876
Benign neoplasm of colon (x1.05) 0.879
Internal hemorrhoids (x1.05) 0.882
Age (+1.96) 0.885
Pure hypercholesterolemia (x1.04) 0.888
Anemia (x1.04) 0.891
Pain in limb (x1.04) 0.894
omeprazole (+0.00364) 0.896
Urinary tract infectious disease (x1.04) 0.898
Atelectasis (x1.04) 0.900
Borderline normal ECG (x1.04) 0.902
Infective pneumonia (x1.04) 0.904
Dyspnea (x1.04) 0.906
Type 2 diabetes mellitus without complication (x1.03) 0.908
atorvastatin (+0.00314) 0.909
Abnormal breathing (x1.03) 0.911
Cough (x1.03) 0.912
furosemide (+0.00296) 0.914
Atrial fibrillation (x1.03) 0.915
Diverticula of intestine (x1.03) 0.917
lisinopril (+0.00283) 0.918
White Race (+0.00278) 0.920
Disorder of lung (x1.03) 0.921
Diaphragmatic hernia (x1.03) 0.922
Osteoarthritis (x1.03) 0.923
Low back pain (x1.03) 0.925
Coronary atherosclerosis (x1.03) 0.926
metoprolol (+0.0026) 0.927
Acquired hypothyroidism (x1.03) 0.928
Systolic blood pressure (+0.848) 0.929
Osteoporosis (x1.03) 0.930
Pleural effusion (x1.03) 0.931
Acute renal failure syndrome (x1.03) 0.932
Congestive heart failure (x1.03) 0.933
Lung field abnormal (x1.03) 0.934
Hematochezia (x1.03) 0.935
hydrochlorothiazide (+0.00228) 0.936
Edema (x1.02) 0.937
Female Sex (-0.00226) 0.938
Gastrointestinal hemorrhage (x1.02) 0.939
Depressive disorder (x1.02) 0.939
Male Sex (+0.0021) 0.940
Chronic obstructive lung disease (x1.02) 0.941
Diarrhea (x1.02) 0.942
ECG normal (x1.02) 0.942
Disorder of kidney and/or ureter (x1.02) 0.943
Hypertensive disorder (x1.02) 0.944
esomeprazole (+0.00186) 0.944
Nuclear senile cataract (x1.02) 0.945
Dysphagia (x1.02) 0.945
Constipation (x1.02) 0.946
Cardiac arrhythmia (x1.02) 0.946
Postoperative state (/1.02) 0.947
Knee pain (x1.02) 0.947
Tobacco dependence syndrome (x1.02) 0.948
hydrocodone (+0.00169) 0.948
Dehydration (x1.02) 0.949
Diverticulosis of large intestine without diverticulitis (x1.02) 0.949
Cardiomegaly (x1.02) 0.950
Acute respiratory failure (x1.02) 0.950
levothyroxine (+0.0016) 0.951
nitroglycerin (+0.0016) 0.951
Body temperature (+0.577) 0.951
Diabetic - poor control (x1.02) 0.952
Hematocrit [VF] of Blood by AC (+0.15) 0.952
Abnormal glucose level (x1.02) 0.953
Heart rate (-0.654) 0.953
Osteoarthritis of knee (x1.02) 0.953
metformin (+0.00152) 0.954
warfarin (+0.00152) 0.954
Syncope and collapse (x1.02) 0.954
amlodipine (+0.00145) 0.955
Hypokalemia (x1.02) 0.955
Hemoglobin [M/V] in B (+0.0501) 0.956
lorazepam (+0.00143) 0.956
Disorder of bone and articular cartilage (x1.02) 0.956
Diastolic blood pressure (+0.297) 0.957
Diverticulitis of colon (x1.02) 0.957
QRS axis (-1.38) 0.957
Persistent pain following procedure (/1.01) 0.957
Estimation of glomerular filtration rate (-2.54) 0.958
Tachycardia (x1.01) 0.958
Carbon dioxide, total [mol/V] in SP (+0.0912) 0.958
Joint pain of pelvic region (x1.01) 0.959
Nausea and vomiting (x1.01) 0.959
Chronic ischemic heart disease (x1.01) 0.959
Shoulder joint pain (x1.01) 0.960
Expressions
10^5
10^3
10^1
−10
0
10
−1.00 −0.75 −0.50 −0.25 0.00 0.25 0.50 0.75 1.00

Signature [210]; 8 To Reach 0.975, (10 Plotted)

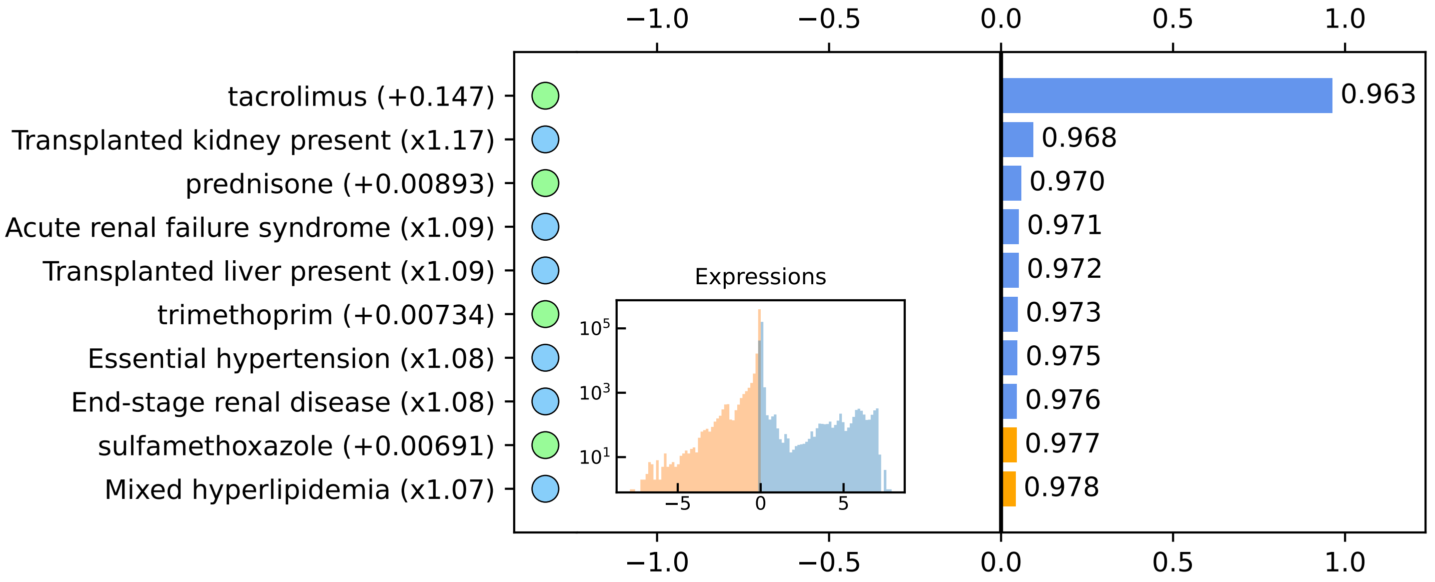

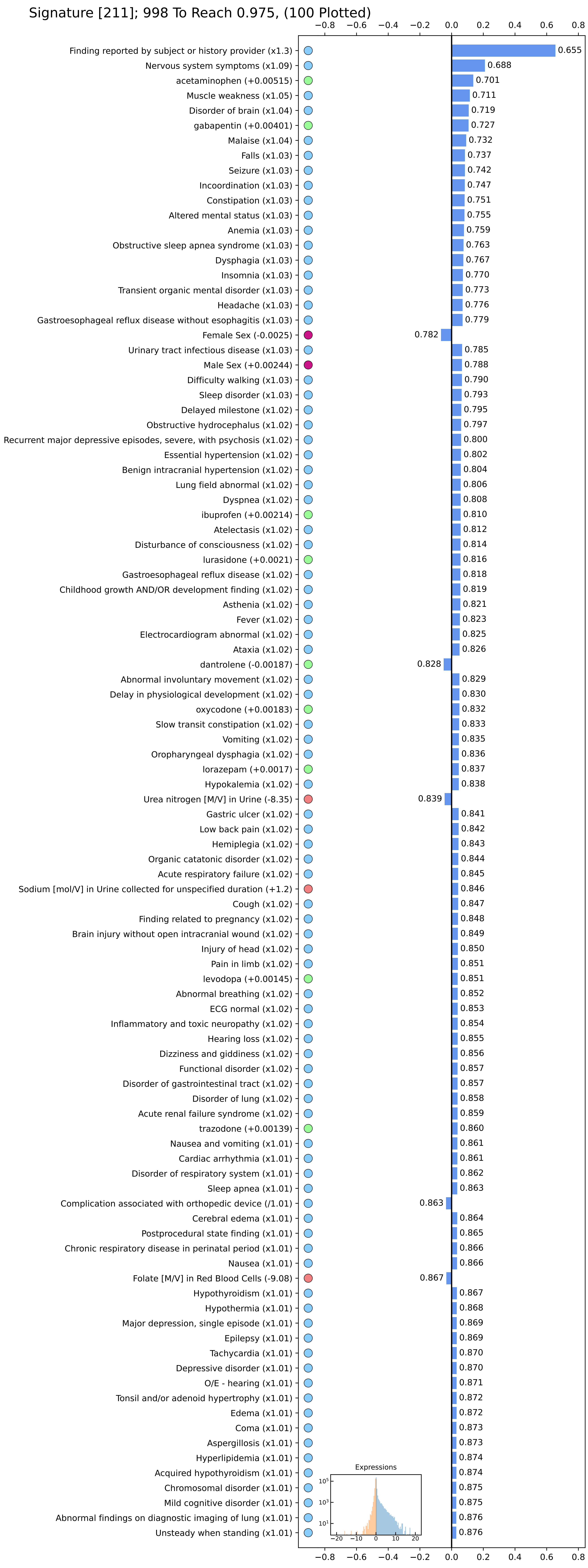

Signature [211]; 998 To Reach 0.975, (100 Plotted)
Finding reported by subject or history provider (x1.3) 0.655
Nervous system symptoms (x1.09) 0.688
acetaminophen (+0.00515) 0.701
Muscle weakness (x1.05) 0.711
Disorder of brain (x1.04) 0.719
gabapentin (+0.00401) 0.727
Malaise (x1.04) 0.732
Falls (x1.03) 0.737
Seizure (x1.03) 0.742
Incoordination (x1.03) 0.747
Constipation (x1.03) 0.751
Altered mental status (x1.03) 0.755
Anemia (x1.03) 0.759
Obstructive sleep apnea syndrome (x1.03) 0.763
Dysphagia (x1.03) 0.767
Insomnia (x1.03) 0.770
Transient organic mental disorder (x1.03) 0.773
Headache (x1.03) 0.776
Gastroesophageal reflux disease without esophagitis (x1.03) 0.779
Female Sex (-0.0025) 0.782
Urinary tract infectious disease (x1.03) 0.785
Male Sex (+0.00244) 0.788
Difficulty walking (x1.03) 0.790
Sleep disorder (x1.03) 0.793
Delayed milestone (x1.02) 0.795
Obstructive hydrocephalus (x1.02) 0.797
Recurrent major depressive episodes, severe, with psychosis (x1.02) 0.800
Essential hypertension (x1.02) 0.802
Benign intracranial hypertension (x1.02) 0.804
Lung field abnormal (x1.02) 0.806
Dyspnea (x1.02) 0.808
ibuprofen (+0.00214) 0.810
Atelectasis (x1.02) 0.812
Disturbance of consciousness (x1.02) 0.814
lurasidone (+0.0021) 0.816
Gastroesophageal reflux disease (x1.02) 0.818
Childhood growth AND/OR development finding (x1.02) 0.819
Asthenia (x1.02) 0.821
Fever (x1.02) 0.823
Electrocardiogram abnormal (x1.02) 0.825
Ataxia (x1.02) 0.826
dantrolene (-0.00187) 0.828
Abnormal involuntary movement (x1.02) 0.829
Delay in physiological development (x1.02) 0.830
oxycodone (+0.00183) 0.832
Slow transit constipation (x1.02) 0.833
Vomiting (x1.02) 0.835
Oropharyngeal dysphagia (x1.02) 0.836
lorazepam (+0.0017) 0.837
Hypokalemia (x1.02) 0.838
Urea nitrogen [M/V] in Urine (-8.35) 0.839
Gastric ulcer (x1.02) 0.841
Low back pain (x1.02) 0.842
Hemiplegia (x1.02) 0.843
Organic catatonic disorder (x1.02) 0.844
Acute respiratory failure (x1.02) 0.845
Sodium [mol/V] in Urine collected for unspecified duration (+1.2) 0.846
Cough (x1.02) 0.847
Finding related to pregnancy (x1.02) 0.848
Brain injury without open intracranial wound (x1.02) 0.849
Injury of head (x1.02) 0.850
Pain in limb (x1.02) 0.851
levodopa (+0.00145) 0.851
Abnormal breathing (x1.02) 0.852
ECG normal (x1.02) 0.853
Inflammatory and toxic neuropathy (x1.02) 0.854
Hearing loss (x1.02) 0.855
Dizziness and giddiness (x1.02) 0.856
Functional disorder (x1.02) 0.857
Disorder of gastrointestinal tract (x1.02) 0.857
Disorder of lung (x1.02) 0.858
Acute renal failure syndrome (x1.02) 0.859
trazodone (+0.00139) 0.860
Nausea and vomiting (x1.01) 0.861
Cardiac arrhythmia (x1.01) 0.861
Disorder of respiratory system (x1.01) 0.862
Sleep apnea (x1.01) 0.863
Complication associated with orthopedic device (/1.01) 0.863
Cerebral edema (x1.01) 0.864
Postprocedural state finding (x1.01) 0.865
Chronic respiratory disease in perinatal period (x1.01) 0.866
Nausea (x1.01) 0.866
Folate [M/V] in Red Blood Cells (-9.08) 0.867
Hypothyroidism (x1.01) 0.867
Hypothermia (x1.01) 0.868
Major depression, single episode (x1.01) 0.869
Epilepsy (x1.01) 0.869
Tachycardia (x1.01) 0.870
Depressive disorder (x1.01) 0.870
O/E - hearing (x1.01) 0.871
Tonsil and/or adenoid hypertrophy (x1.01) 0.872
Edema (x1.01) 0.872
Coma (x1.01) 0.873
Aspergillosis (x1.01) 0.873
Hyperlipidemia (x1.01) 0.874
Acquired hypothyroidism (x1.01) 0.874
Chromosomal disorder (x1.01) 0.875
Mild cognitive disorder (x1.01) 0.875
Abnormal findings on diagnostic imaging of lung (x1.01) 0.876
Unsteady when standing (x1.01) 0.876
−0.8 −0.6 −0.4 −0.2 0.0 0.2 0.4 0.6 0.8
Expressions

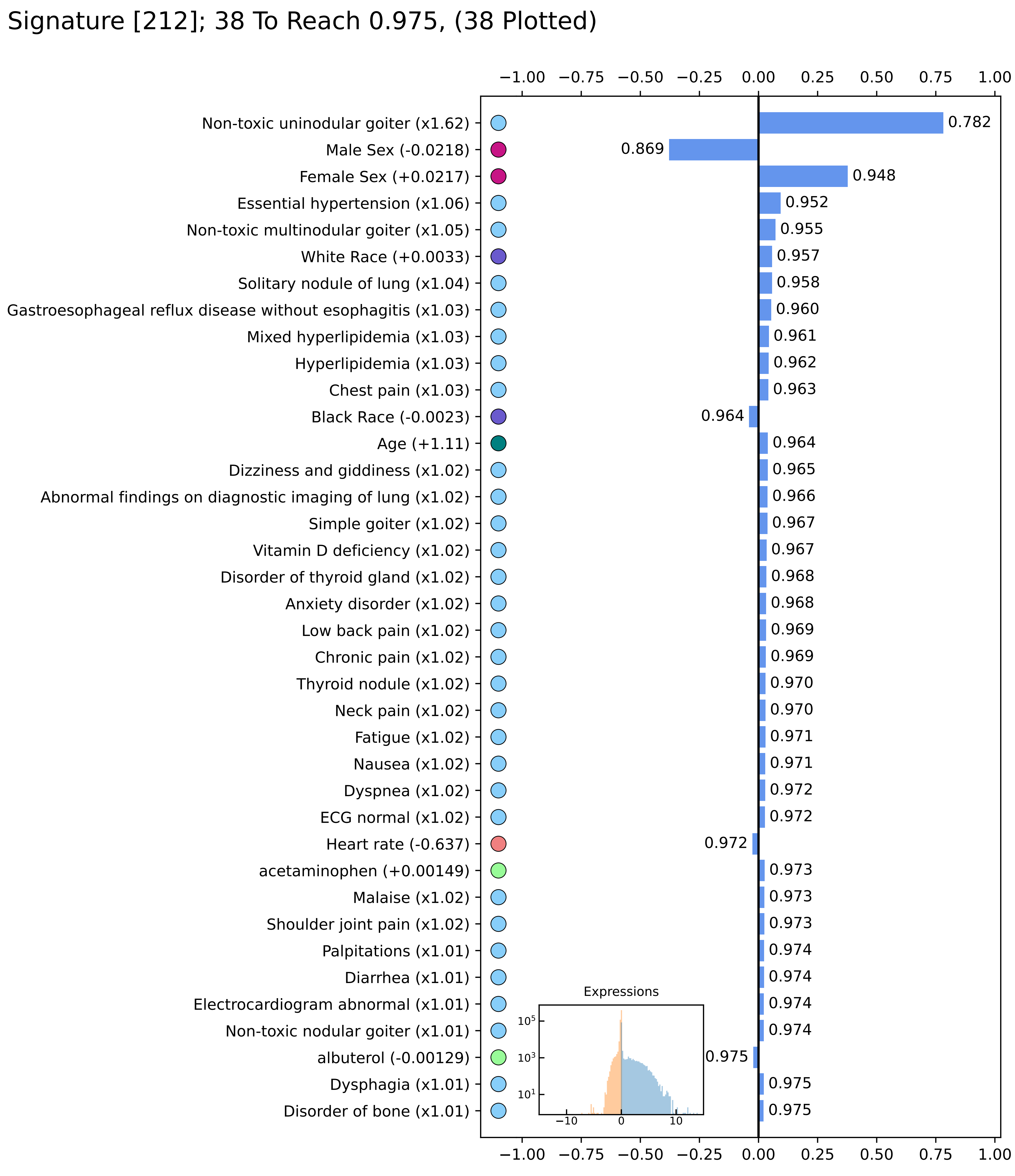

Signature [212]; 38 To Reach 0.975, (38 Plotted)
Non-toxic uninodular goiter (x1.62) 0.782
Male Sex (-0.0218) 0.869
Female Sex (+0.0217) 0.948
Essential hypertension (x1.06) 0.952
Non-toxic multinodular goiter (x1.05) 0.955
White Race (+0.0033) 0.957
Solitary nodule of lung (x1.04) 0.958
Gastroesophageal reflux disease without esophagitis (x1.03) 0.960
Mixed hyperlipidemia (x1.03) 0.961
Hyperlipidemia (x1.03) 0.962
Chest pain (x1.03) 0.963
Black Race (-0.0023) 0.964
Age (+1.11) 0.964
Dizziness and giddiness (x1.02) 0.965
Abnormal findings on diagnostic imaging of lung (x1.02) 0.966
Simple goiter (x1.02) 0.967
Vitamin D deficiency (x1.02) 0.967
Disorder of thyroid gland (x1.02) 0.968
Anxiety disorder (x1.02) 0.968
Low back pain (x1.02) 0.969
Chronic pain (x1.02) 0.969
Thyroid nodule (x1.02) 0.970
Neck pain (x1.02) 0.970
Fatigue (x1.02) 0.971
Nausea (x1.02) 0.971
Dyspnea (x1.02) 0.972
ECG normal (x1.02) 0.972
Heart rate (-0.637) 0.972
acetaminophen (+0.00149) 0.973
Malaise (x1.02) 0.973
Shoulder joint pain (x1.02) 0.973
Palpitations (x1.01) 0.974
Diarrhea (x1.01) 0.974
Electrocardiogram abnormal (x1.01) 0.974
Non-toxic nodular goiter (x1.01) 0.974
albuterol (-0.00129) 0.975
Dysphagia (x1.01) 0.975
Disorder of bone (x1.01) 0.975
−1.00 −0.75 −0.50 −0.25 0.00 0.25 0.50 0.75 1.00
Expressions
10^5
10^3
10^1
−10 0 10

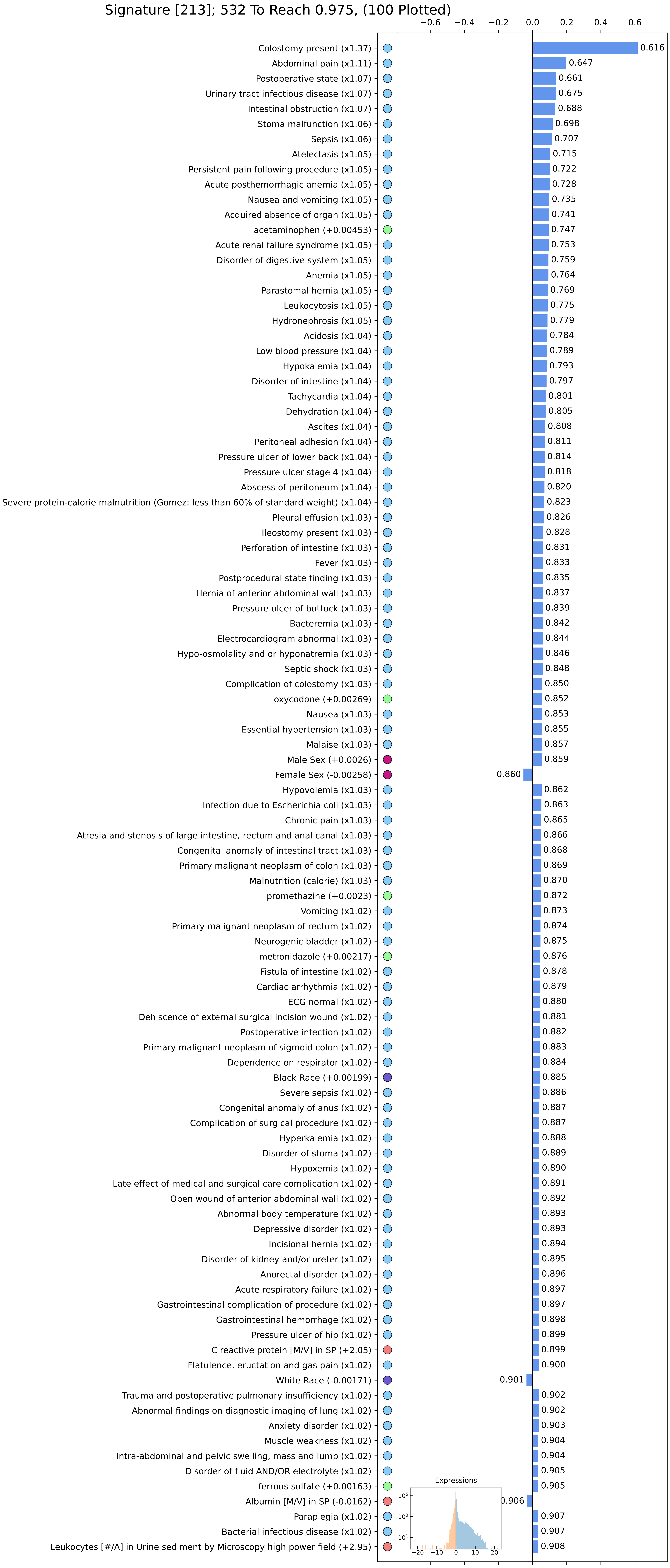

Signature [213]; 532 To Reach 0.975, (100 Plotted)
Colostomy present (x1.37) 0.616
Abdominal pain (x1.11) 0.647
Postoperative state (x1.07) 0.661
Urinary tract infectious disease (x1.07) 0.675
Intestinal obstruction (x1.07) 0.688
Stoma malfunction (x1.06) 0.698
Sepsis (x1.06) 0.707
Atelectasis (x1.05) 0.715
Persistent pain following procedure (x1.05) 0.722
Acute posthemorrhagic anemia (x1.05) 0.728
Nausea and vomiting (x1.05) 0.735
Acquired absence of organ (x1.05) 0.741
acetaminophen (+0.00453) 0.747
Acute renal failure syndrome (x1.05) 0.753
Disorder of digestive system (x1.05) 0.759
Anemia (x1.05) 0.764
Parastomal hernia (x1.05) 0.769
Leukocytosis (x1.05) 0.775
Hydronephrosis (x1.05) 0.779
Acidosis (x1.04) 0.784
Low blood pressure (x1.04) 0.789
Hypokalemia (x1.04) 0.793
Disorder of intestine (x1.04) 0.797
Tachycardia (x1.04) 0.801
Dehydration (x1.04) 0.805
Ascites (x1.04) 0.808
Peritoneal adhesion (x1.04) 0.811
Pressure ulcer of lower back (x1.04) 0.814
Pressure ulcer stage 4 (x1.04) 0.818
Abscess of peritoneum (x1.04) 0.820
Severe protein-calorie malnutrition (Gomez: less than 60% of standard weight) (x1.04) 0.823
Pleural effusion (x1.03) 0.826
Ileostomy present (x1.03) 0.828
Perforation of intestine (x1.03) 0.831
Fever (x1.03) 0.833
Postprocedural state finding (x1.03) 0.835
Hernia of anterior abdominal wall (x1.03) 0.837
Pressure ulcer of buttock (x1.03) 0.839
Bacteremia (x1.03) 0.842
Electrocardiogram abnormal (x1.03) 0.844
Hypo-osmolality and or hyponatremia (x1.03) 0.846
Septic shock (x1.03) 0.848
Complication of colostomy (x1.03) 0.850
oxycodone (+0.00269) 0.852
Nausea (x1.03) 0.853
Essential hypertension (x1.03) 0.855
Malaise (x1.03) 0.857
Male Sex (+0.0026) 0.859
Female Sex (-0.00258) 0.860
Hypovolemia (x1.03) 0.862
Infection due to Escherichia coli (x1.03) 0.863
Chronic pain (x1.03) 0.865
Atresia and stenosis of large intestine, rectum and anal canal (x1.03) 0.866
Congenital anomaly of intestinal tract (x1.03) 0.868
Primary malignant neoplasm of colon (x1.03) 0.869
Malnutrition (calorie) (x1.03) 0.870
promethazine (+0.0023) 0.872
Vomiting (x1.02) 0.873
Primary malignant neoplasm of rectum (x1.02) 0.874
Neurogenic bladder (x1.02) 0.875
metronidazole (+0.00217) 0.876
Fistula of intestine (x1.02) 0.878
Cardiac arrhythmia (x1.02) 0.879
ECG normal (x1.02) 0.880
Dehiscence of external surgical incision wound (x1.02) 0.881
Postoperative infection (x1.02) 0.882
Primary malignant neoplasm of sigmoid colon (x1.02) 0.883
Dependence on respirator (x1.02) 0.884
Black Race (+0.00199) 0.885
Severe sepsis (x1.02) 0.886
Congenital anomaly of anus (x1.02) 0.887
Complication of surgical procedure (x1.02) 0.887
Hyperkalemia (x1.02) 0.888
Disorder of stoma (x1.02) 0.889
Hypoxemia (x1.02) 0.890
Late effect of medical and surgical care complication (x1.02) 0.891
Open wound of anterior abdominal wall (x1.02) 0.892
Abnormal body temperature (x1.02) 0.893
Depressive disorder (x1.02) 0.893
Incisional hernia (x1.02) 0.894
Disorder of kidney and/or ureter (x1.02) 0.895
Anorectal disorder (x1.02) 0.896
Acute respiratory failure (x1.02) 0.897
Gastrointestinal complication of procedure (x1.02) 0.897
Gastrointestinal hemorrhage (x1.02) 0.898
Pressure ulcer of hip (x1.02) 0.899
C reactive protein [M/V] in SP (+2.05) 0.899
Flatulence, eructation and gas pain (x1.02) 0.900
White Race (-0.00171) 0.901
Trauma and postoperative pulmonary insufficiency (x1.02) 0.902
Abnormal findings on diagnostic imaging of lung (x1.02) 0.902
Anxiety disorder (x1.02) 0.903
Muscle weakness (x1.02) 0.904
Intra-abdominal and pelvic swelling, mass and lump (x1.02) 0.904
Disorder of fluid AND/OR electrolyte (x1.02) 0.905
ferrous sulfate (+0.00163) 0.905
Albumin [M/V] in SP (-0.0162) 0.906
Paraplegia (x1.02) 0.907
Bacterial infectious disease (x1.02) 0.907
Leukocytes [#/A] in Urine sediment by Microscopy high power field (+2.95) 0.908
−0.6 −0.4 −0.2 0.0 0.2 0.4 0.6
Expressions
−20 −10 0 10 20

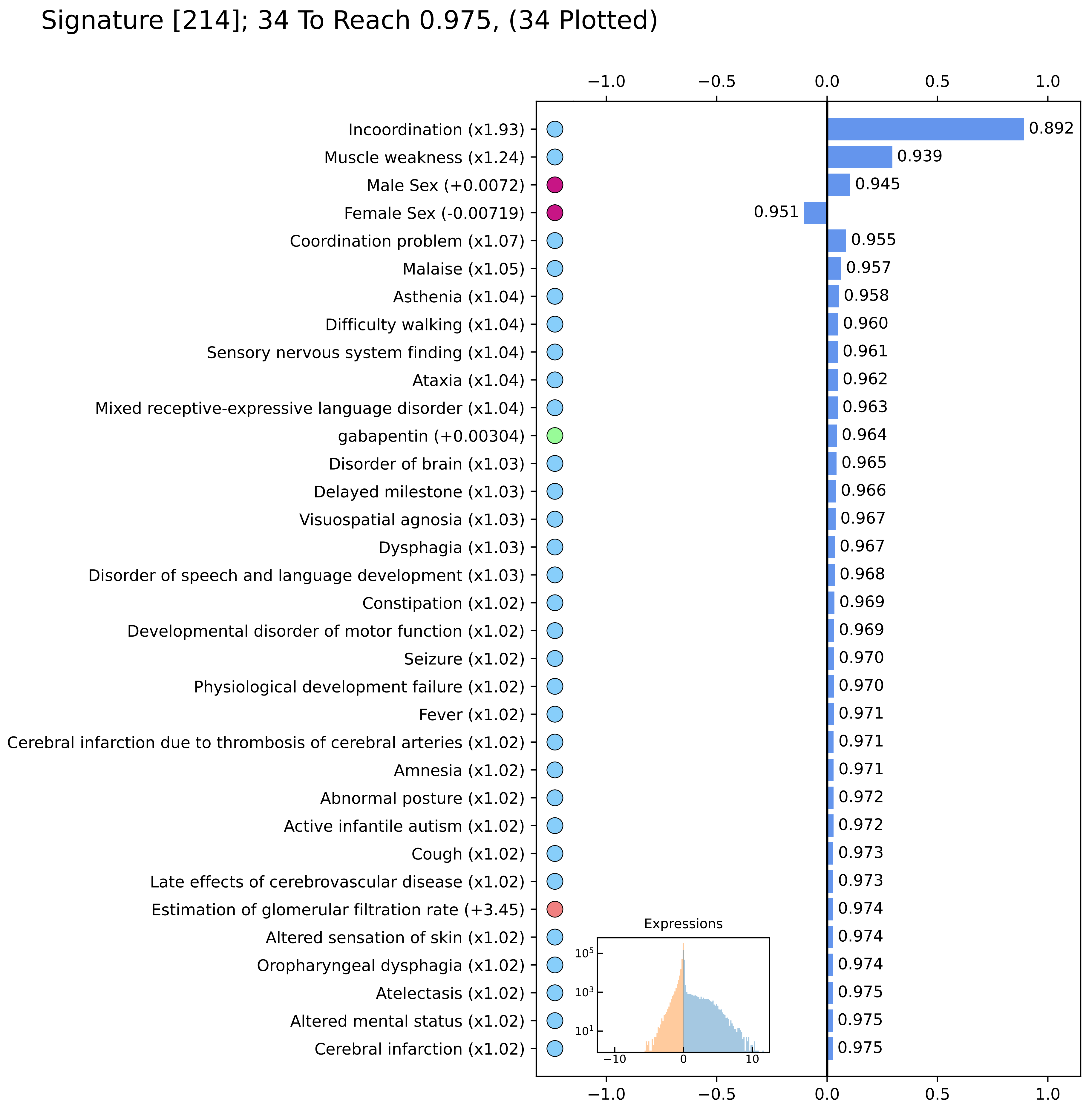

Signature [214]; 34 To Reach 0.975, (34 Plotted)
Incoordination (x1.93)
0.892
Muscle weakness (x1.24)
0.939
Male Sex (+0.0072)
0.945
Female Sex (-0.00719)
0.951
Coordination problem (x1.07)
0.955
Malaise (x1.05)
0.957
Asthenia (x1.04)
0.958
Difficulty walking (x1.04)
0.960
Sensory nervous system finding (x1.04)
0.961
Ataxia (x1.04)
0.962
Mixed receptive-expressive language disorder (x1.04)
0.963
gabapentin (+0.00304)
0.964
Disorder of brain (x1.03)
0.965
Delayed milestone (x1.03)
0.966
Visuospatial agnosia (x1.03)
0.967
Dysphagia (x1.03)
0.967
Disorder of speech and language development (x1.03)
0.968
Constipation (x1.02)
0.969
Developmental disorder of motor function (x1.02)
0.969
Seizure (x1.02)
0.970
Physiological development failure (x1.02)
0.970
Fever (x1.02)
0.971
Cerebral infarction due to thrombosis of cerebral arteries (x1.02)
0.971
Amnesia (x1.02)
0.971
Abnormal posture (x1.02)
0.972
Active infantile autism (x1.02)
0.972
Cough (x1.02)
0.973
Late effects of cerebrovascular disease (x1.02)
0.973
Estimation of glomerular filtration rate (+3.45)
0.974
Altered sensation of skin (x1.02)
0.974
Oropharyngeal dysphagia (x1.02)
0.974
Atelectasis (x1.02)
0.975
Altered mental status (x1.02)
0.975
Cerebral infarction (x1.02)
0.975
Expressions

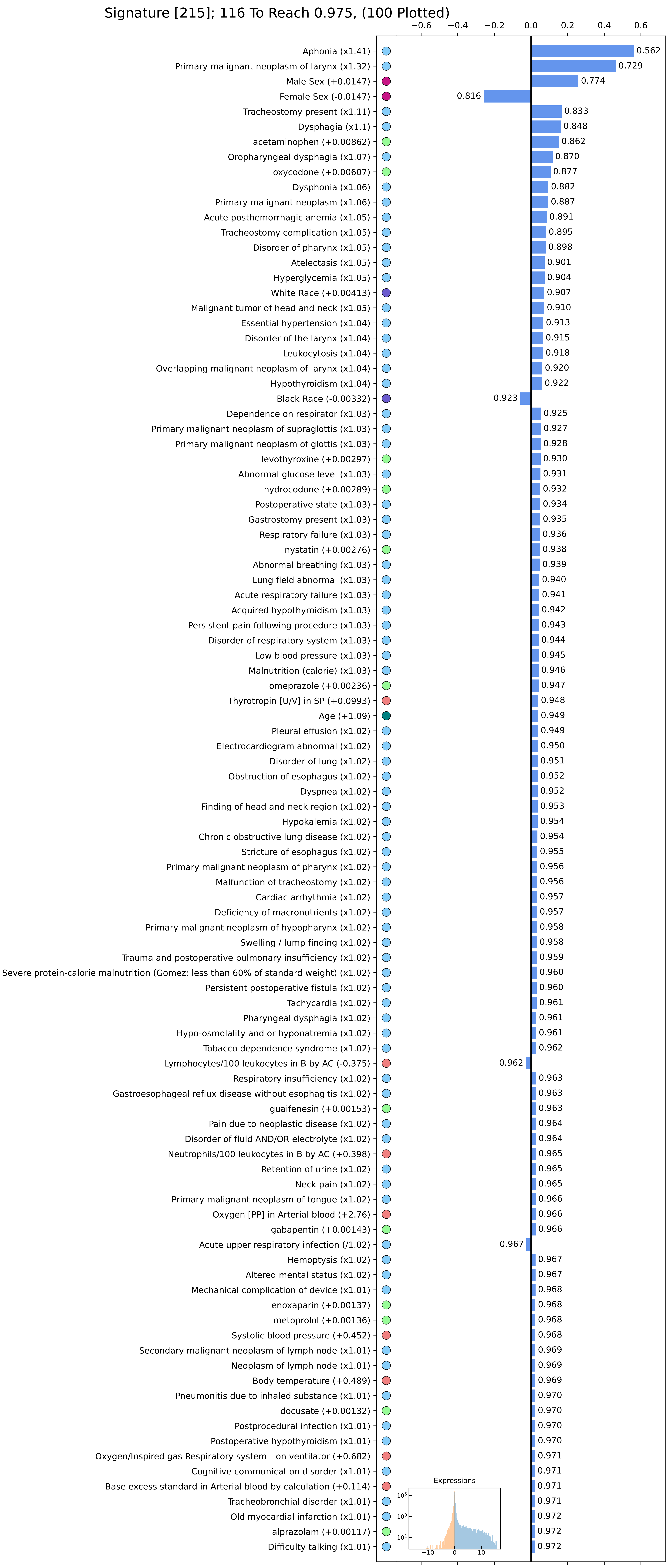
Signature [215]; 116 To Reach 0.975, (100 Plotted)
Aphonia (x1.41) 0.562
Primary malignant neoplasm of larynx (x1.32) 0.729
Male Sex (+0.0147) 0.774
Female Sex (-0.0147) 0.816
Tracheostomy present (x1.11) 0.833
Dysphagia (x1.1) 0.848
acetaminophen (+0.00862) 0.862
Oropharyngeal dysphagia (x1.07) 0.870
oxycodone (+0.00607) 0.877
Dysphonia (x1.06) 0.882
Primary malignant neoplasm (x1.06) 0.887
Acute posthemorrhagic anemia (x1.05) 0.891
Tracheostomy complication (x1.05) 0.895
Disorder of pharynx (x1.05) 0.898
Atelectasis (x1.05) 0.901
Hyperglycemia (x1.05) 0.904
White Race (+0.00413) 0.907
Malignant tumor of head and neck (x1.05) 0.910
Essential hypertension (x1.04) 0.913
Disorder of the larynx (x1.04) 0.915
Leukocytosis (x1.04) 0.918
Overlapping malignant neoplasm of larynx (x1.04) 0.920
Hypothyroidism (x1.04) 0.922
Black Race (-0.00332) 0.923
Dependence on respirator (x1.03) 0.925
Primary malignant neoplasm of supraglottis (x1.03) 0.927
Primary malignant neoplasm of glottis (x1.03) 0.928
levothyroxine (+0.00297) 0.930
Abnormal glucose level (x1.03) 0.931
hydrocodone (+0.00289) 0.932
Postoperative state (x1.03) 0.934
Gastrostomy present (x1.03) 0.935
Respiratory failure (x1.03) 0.936
nystatin (+0.00276) 0.938
Abnormal breathing (x1.03) 0.939
Lung field abnormal (x1.03) 0.940
Acute respiratory failure (x1.03) 0.941
Acquired hypothyroidism (x1.03) 0.942
Persistent pain following procedure (x1.03) 0.943
Disorder of respiratory system (x1.03) 0.944
Low blood pressure (x1.03) 0.945
Malnutrition (calorie) (x1.03) 0.946
omeprazole (+0.00236) 0.947
Thyrotropin [U/V] in SP (+0.0993) 0.948
Age (+1.09) 0.949
Pleural effusion (x1.02) 0.949
Electrocardiogram abnormal (x1.02) 0.950
Disorder of lung (x1.02) 0.951
Obstruction of esophagus (x1.02) 0.952
Dyspnea (x1.02) 0.952
Finding of head and neck region (x1.02) 0.953
Hypokalemia (x1.02) 0.954
Chronic obstructive lung disease (x1.02) 0.954
Stricture of esophagus (x1.02) 0.955
Primary malignant neoplasm of pharynx (x1.02) 0.956
Malfunction of tracheostomy (x1.02) 0.956
Cardiac arrhythmia (x1.02) 0.957
Deficiency of macronutrients (x1.02) 0.957
Primary malignant neoplasm of hypopharynx (x1.02) 0.958
Swelling / lump finding (x1.02) 0.958
Trauma and postoperative pulmonary insufficiency (x1.02) 0.959
Severe protein-calorie malnutrition (Gomez: less than 60% of standard weight) (x1.02) 0.960
Persistent postoperative fistula (x1.02) 0.960
Tachycardia (x1.02) 0.961
Pharyngeal dysphagia (x1.02) 0.961
Hypo-osmolality and or hyponatremia (x1.02) 0.961
Tobacco dependence syndrome (x1.02) 0.962
Lymphocytes/100 leukocytes in B by AC (-0.375) 0.962
Respiratory insufficiency (x1.02) 0.963
Gastroesophageal reflux disease without esophagitis (x1.02) 0.963
guaifenesin (+0.00153) 0.963
Pain due to neoplastic disease (x1.02) 0.964
Disorder of fluid AND/OR electrolyte (x1.02) 0.964
Neutrophils/100 leukocytes in B by AC (+0.398) 0.965
Retention of urine (x1.02) 0.965
Neck pain (x1.02) 0.965
Primary malignant neoplasm of tongue (x1.02) 0.966
Oxygen [PP] in Arterial blood (+2.76) 0.966
gabapentin (+0.00143) 0.966
Acute upper respiratory infection (/1.02) 0.967
Hemoptysis (x1.02) 0.967
Altered mental status (x1.02) 0.967
Mechanical complication of device (x1.01) 0.968
enoxaparin (+0.00137) 0.968
metoprolol (+0.00136) 0.968
Systolic blood pressure (+0.452) 0.968
Secondary malignant neoplasm of lymph node (x1.01) 0.969
Neoplasm of lymph node (x1.01) 0.969
Body temperature (+0.489) 0.969
Pneumonitis due to inhaled substance (x1.01) 0.970
docusate (+0.00132) 0.970
Postprocedural infection (x1.01) 0.970
Postoperative hypothyroidism (x1.01) 0.970
Oxygen/Inspired gas Respiratory system --on ventilator (+0.682) 0.971
Cognitive communication disorder (x1.01) 0.971
Base excess standard in Arterial blood by calculation (+0.114) 0.971
Tracheobronchial disorder (x1.01) 0.971
Old myocardial infarction (x1.01) 0.972
alprazolam (+0.00117) 0.972
Difficulty talking (x1.01) 0.972
Expressions
−0.6 −0.4 −0.2 0.0 0.2 0.4 0.6

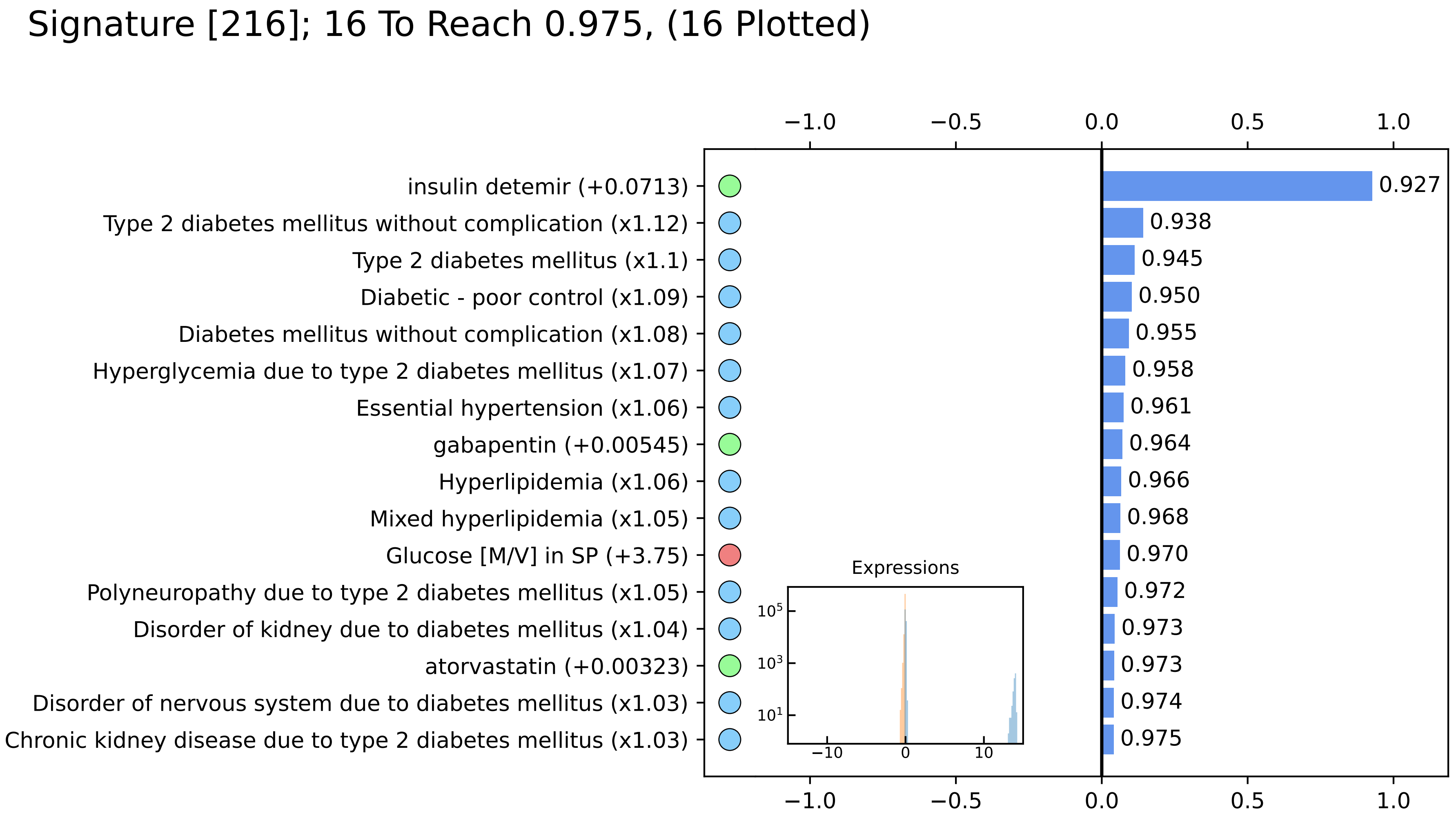

Signature [216]; 16 To Reach 0.975, (16 Plotted)
insulin detemir (+0.0713)
Type 2 diabetes mellitus without complication (x1.12)
Type 2 diabetes mellitus (x1.1)
Diabetic - poor control (x1.09)
Diabetes mellitus without complication (x1.08)
Hyperglycemia due to type 2 diabetes mellitus (x1.07)
Essential hypertension (x1.06)
gabapentin (+0.00545)
Hyperlipidemia (x1.06)
Mixed hyperlipidemia (x1.05)
Glucose [M/V] in SP (+3.75)
Polyneuropathy due to type 2 diabetes mellitus (x1.05)
Disorder of kidney due to diabetes mellitus (x1.04)
atorvastatin (+0.00323)
Disorder of nervous system due to diabetes mellitus (x1.03)
Chronic kidney disease due to type 2 diabetes mellitus (x1.03)
0.927
0.938
0.945
0.950
0.955
0.958
0.961
0.964
0.966
0.968
0.970
0.972
0.973
0.973
0.974
0.975
−1.0
−0.5
0.0
0.5
1.0
Expressions

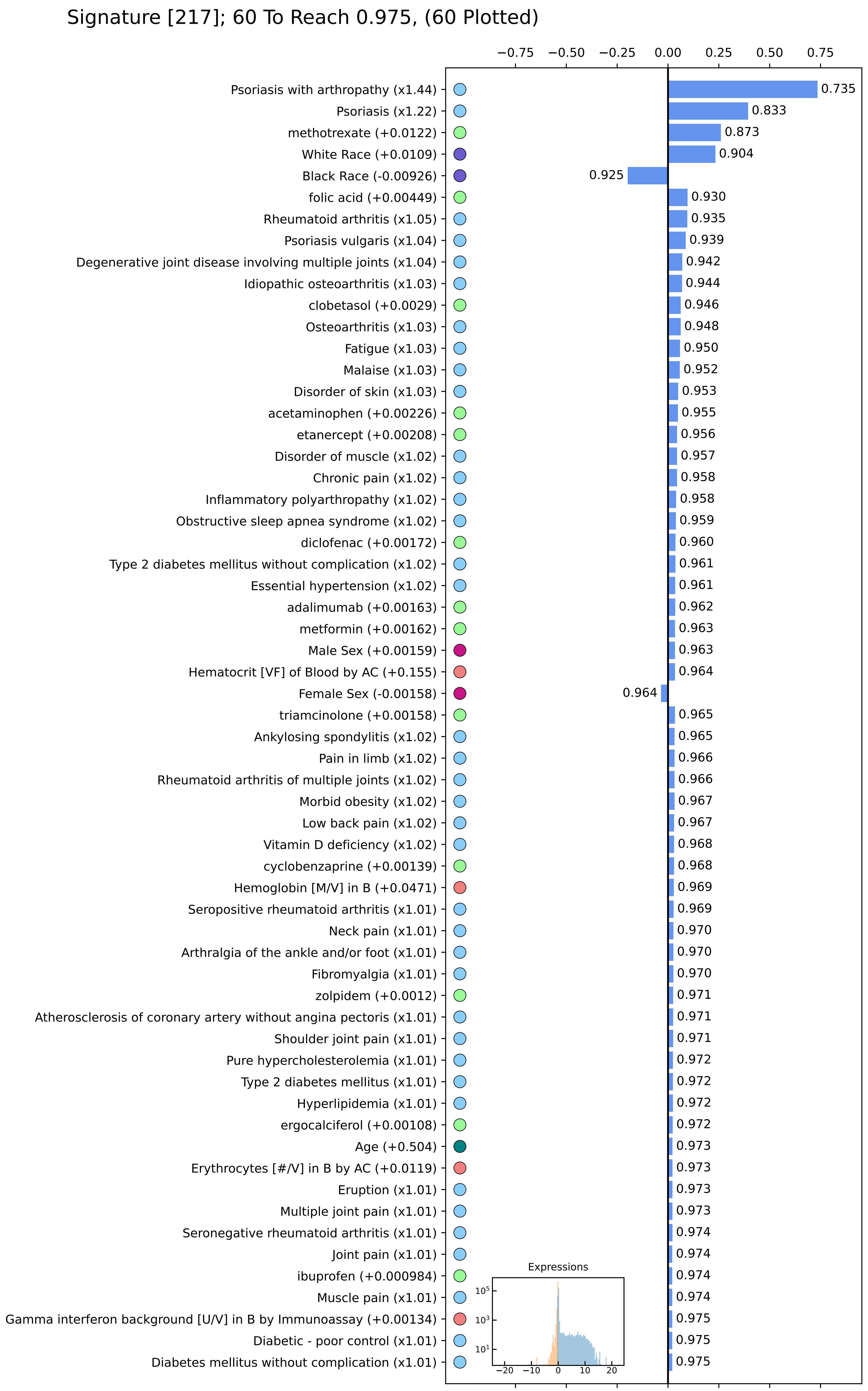

Signature [217]; 60 To Reach 0.975, (60 Plotted)
Psoriasis with arthropathy (x1.44)
Psoriasis (x1.22)
methotrexate (+0.0122)
White Race (+0.0109)
Black Race (-0.00926)
folic acid (+0.00449)
Rheumatoid arthritis (x1.05)
Psoriasis vulgaris (x1.04)
Degenerative joint disease involving multiple joints (x1.04)
Idiopathic osteoarthritis (x1.03)
clobetasol (+0.0029)
Osteoarthritis (x1.03)
Fatigue (x1.03)
Malaise (x1.03)
Disorder of skin (x1.03)
acetaminophen (+0.00226)
etanercept (+0.00208)
Disorder of muscle (x1.02)
Chronic pain (x1.02)
Inflammatory polyarthropathy (x1.02)
Obstructive sleep apnea syndrome (x1.02)
diclofenac (+0.00172)
Type 2 diabetes mellitus without complication (x1.02)
Essential hypertension (x1.02)
adalimumab (+0.00163)
metformin (+0.00162)
Male Sex (+0.00159)
Hematocrit [VF] of Blood by AC (+0.155)
Female Sex (-0.00158)
triamcinolone (+0.00158)
Ankylosing spondylitis (x1.02)
Pain in limb (x1.02)
Rheumatoid arthritis of multiple joints (x1.02)
Morbid obesity (x1.02)
Low back pain (x1.02)
Vitamin D deficiency (x1.02)
cyclobenzaprine (+0.00139)
Hemoglobin [M/V] in B (+0.0471)
Seropositive rheumatoid arthritis (x1.01)
Neck pain (x1.01)
Arthralgia of the ankle and/or foot (x1.01)
Fibromyalgia (x1.01)
zolpidem (+0.0012)
Atherosclerosis of coronary artery without angina pectoris (x1.01)
Shoulder joint pain (x1.01)
Pure hypercholesterolemia (x1.01)
Type 2 diabetes mellitus (x1.01)
Hyperlipidemia (x1.01)
ergocalciferol (+0.00108)
Age (+0.504)
Erythrocytes [#/V] in B by AC (+0.0119)
Eruption (x1.01)
Multiple joint pain (x1.01)
Seronegative rheumatoid arthritis (x1.01)
Joint pain (x1.01)
ibuprofen (+0.000984)
Muscle pain (x1.01)
Gamma interferon background [U/V] in B by Immunoassay (+0.00134)
Diabetic - poor control (x1.01)
Diabetes mellitus without complication (x1.01)
0.735
0.833
0.873
0.904
0.925
0.930
0.935
0.939
0.942
0.944
0.946
0.948
0.950
0.952
0.953
0.955
0.956
0.957
0.958
0.958
0.959
0.960
0.961
0.961
0.962
0.963
0.963
0.964
0.964
0.965
0.965
0.966
0.966
0.967
0.967
0.968
0.968
0.969
0.969
0.970
0.970
0.970
0.971
0.971
0.971
0.972
0.972
0.972
0.972
0.973
0.973
0.973
0.973
0.974
0.974
0.974
0.974
0.975
0.975
0.975
−0.75 −0.50 −0.25 0.00 0.25 0.50 0.75
Expressions

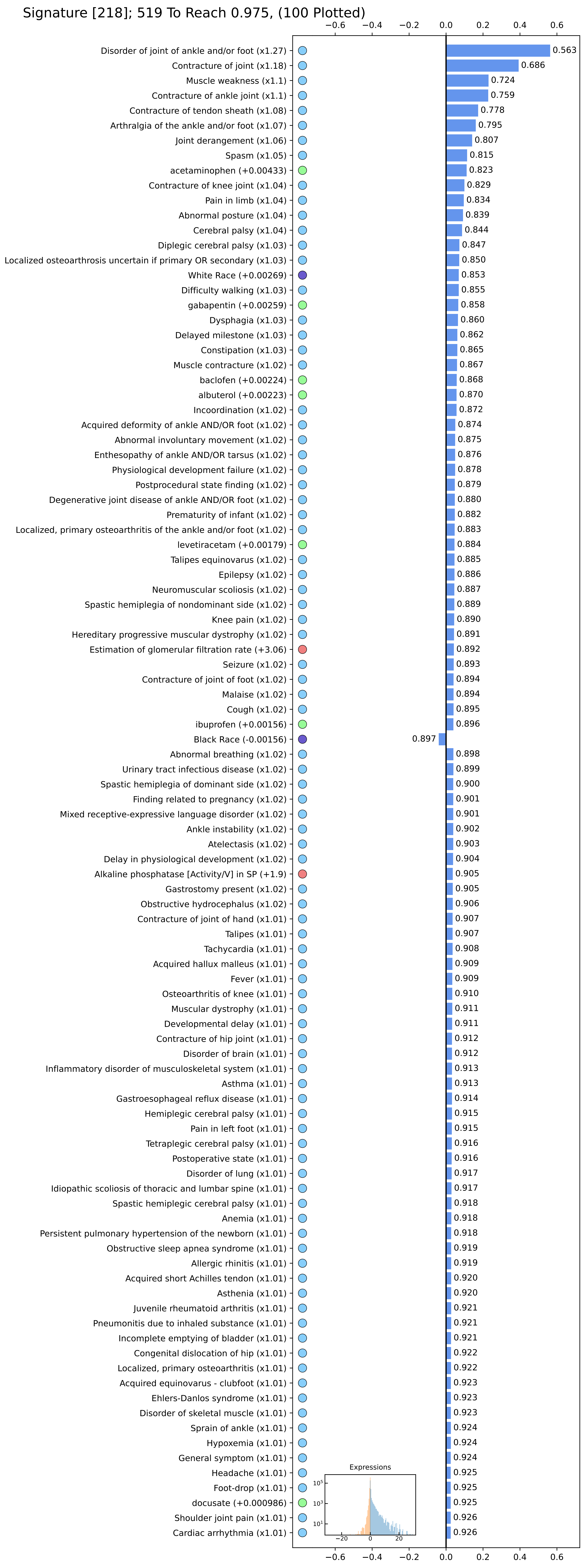

Signature [218]; 519 To Reach 0.975, (100 Plotted)
Disorder of joint of ankle and/or foot (x1.27)
Contracture of joint (x1.18)
Muscle weakness (x1.1)
Contracture of ankle joint (x1.1)
Contracture of tendon sheath (x1.08)
Arthralgia of the ankle and/or foot (x1.07)
Joint derangement (x1.06)
Spasm (x1.05)
acetaminophen (+0.00433)
Contracture of knee joint (x1.04)
Pain in limb (x1.04)
Abnormal posture (x1.04)
Cerebral palsy (x1.04)
Diplegic cerebral palsy (x1.03)
Localized osteoarthrosis uncertain if primary OR secondary (x1.03)
White Race (+0.00269)
Difficulty walking (x1.03)
gabapentin (+0.00259)
Dysphagia (x1.03)
Delayed milestone (x1.03)
Constipation (x1.03)
Muscle contracture (x1.02)
baclofen (+0.00224)
albuterol (+0.00223)
Incoordination (x1.02)
Acquired deformity of ankle AND/OR foot (x1.02)
Abnormal involuntary movement (x1.02)
Enthesopathy of ankle AND/OR tarsus (x1.02)
Physiological development failure (x1.02)
Postprocedural state finding (x1.02)
Degenerative joint disease of ankle AND/OR foot (x1.02)
Prematurity of infant (x1.02)
Localized, primary osteoarthritis of the ankle and/or foot (x1.02)
levetiracetam (+0.00179)
Talipes equinovarus (x1.02)
Epilepsy (x1.02)
Neuromuscular scoliosis (x1.02)
Spastic hemiplegia of nondominant side (x1.02)
Knee pain (x1.02)
Hereditary progressive muscular dystrophy (x1.02)
Estimation of glomerular filtration rate (+3.06)
Seizure (x1.02)
Contracture of joint of foot (x1.02)
Malaise (x1.02)
Cough (x1.02)
ibuprofen (+0.00156)
Black Race (-0.00156)
Abnormal breathing (x1.02)
Urinary tract infectious disease (x1.02)
Spastic hemiplegia of dominant side (x1.02)
Finding related to pregnancy (x1.02)
Mixed receptive-expressive language disorder (x1.02)
Ankle instability (x1.02)
Atelectasis (x1.02)
Delay in physiological development (x1.02)
Alkaline phosphatase [Activity/V] in SP (+1.9)
Gastrostomy present (x1.02)
Obstructive hydrocephalus (x1.02)
Contracture of joint of hand (x1.01)
Talipes (x1.01)
Tachycardia (x1.01)
Acquired hallux malleus (x1.01)
Fever (x1.01)
Osteoarthritis of knee (x1.01)
Muscular dystrophy (x1.01)
Developmental delay (x1.01)
Contracture of hip joint (x1.01)
Disorder of brain (x1.01)
Inflammatory disorder of musculoskeletal system (x1.01)
Asthma (x1.01)
Gastroesophageal reflux disease (x1.01)
Hemiplegic cerebral palsy (x1.01)
Pain in left foot (x1.01)
Tetraplegic cerebral palsy (x1.01)
Postoperative state (x1.01)
Disorder of lung (x1.01)
Idiopathic scoliosis of thoracic and lumbar spine (x1.01)
Spastic hemiplegic cerebral palsy (x1.01)
Anemia (x1.01)
Persistent pulmonary hypertension of the newborn (x1.01)
Obstructive sleep apnea syndrome (x1.01)
Allergic rhinitis (x1.01)
Acquired short Achilles tendon (x1.01)
Asthenia (x1.01)
Juvenile rheumatoid arthritis (x1.01)
Pneumonitis due to inhaled substance (x1.01)
Incomplete emptying of bladder (x1.01)
Congenital dislocation of hip (x1.01)
Localized, primary osteoarthritis (x1.01)
Acquired equinovarus - clubfoot (x1.01)
Ehlers-Danlos syndrome (x1.01)
Disorder of skeletal muscle (x1.01)
Sprain of ankle (x1.01)
Hypoxemia (x1.01)
General symptom (x1.01)
Headache (x1.01)
Foot-drop (x1.01)
docusate (+0.000986)
Shoulder joint pain (x1.01)
Cardiac arrhythmia (x1.01)
0.563
0.686
0.724
0.759
0.778
0.795
0.807
0.815
0.823
0.829
0.834
0.839
0.844
0.847
0.850
0.853
0.855
0.858
0.860
0.862
0.865
0.867
0.868
0.870
0.872
0.874
0.875
0.876
0.878
0.879
0.880
0.882
0.883
0.884
0.885
0.886
0.887
0.889
0.890
0.891
0.892
0.893
0.894
0.894
0.895
0.896
0.897
0.898
0.899
0.900
0.901
0.901
0.902
0.903
0.904
0.905
0.905
0.906
0.907
0.907
0.908
0.909
0.909
0.910
0.911
0.911
0.912
0.912
0.913
0.913
0.914
0.915
0.915
0.916
0.916
0.917
0.917
0.918
0.918
0.918
0.919
0.919
0.920
0.920
0.921
0.921
0.921
0.922
0.922
0.923
0.923
0.923
0.924
0.924
0.924
0.925
0.925
0.925
0.926
0.926
−0.6 −0.4 −0.2 0.0 0.2 0.4 0.6
Expressions
−20 0 20

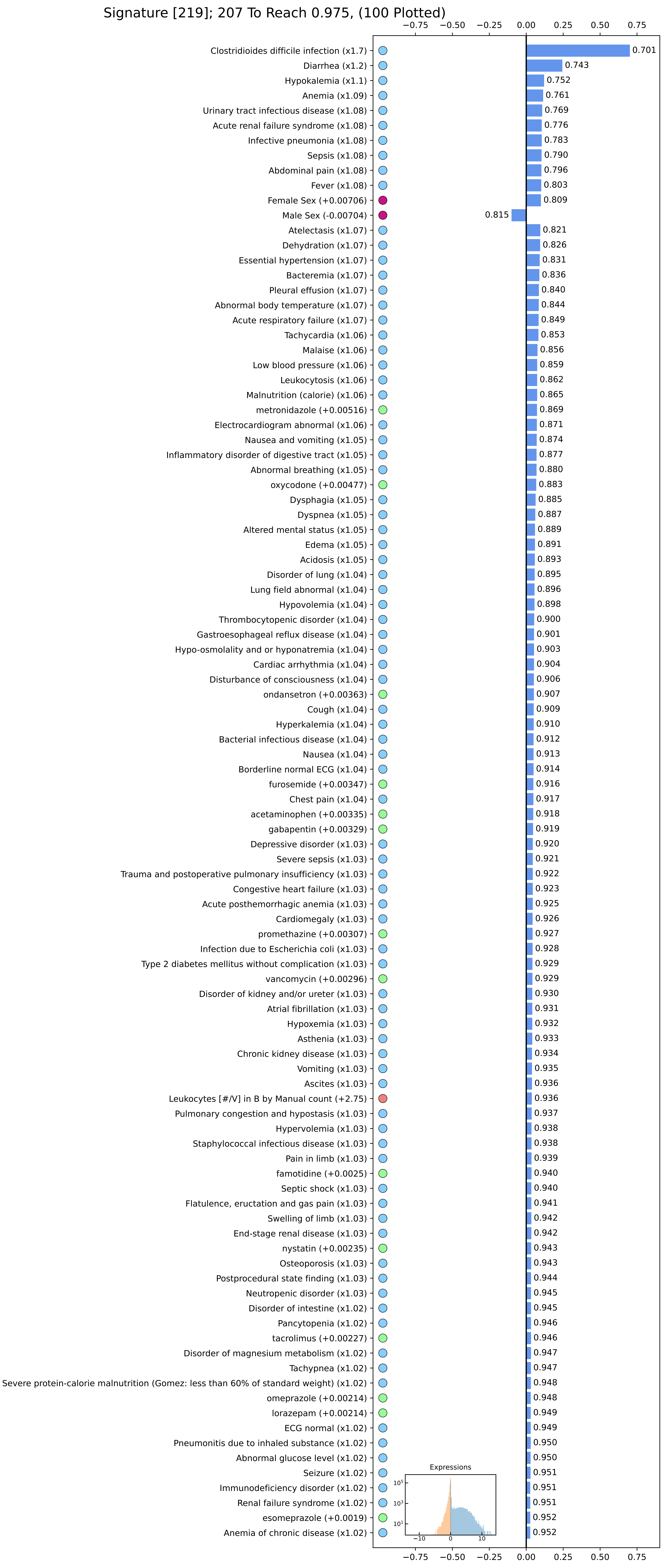

Signature [219]; 207 To Reach 0.975, (100 Plotted)
Clostridioides difficile infection (x1.7) 0.701
Diarrhea (x1.2) 0.743
Hypokalemia (x1.1) 0.752
Anemia (x1.09) 0.761
Urinary tract infectious disease (x1.08) 0.769
Acute renal failure syndrome (x1.08) 0.776
Infective pneumonia (x1.08) 0.783
Sepsis (x1.08) 0.790
Abdominal pain (x1.08) 0.796
Fever (x1.08) 0.803
Female Sex (+0.00706) 0.809
Male Sex (-0.00704) 0.815
Atelectasis (x1.07) 0.821
Dehydration (x1.07) 0.826
Essential hypertension (x1.07) 0.831
Bacteremia (x1.07) 0.836
Pleural effusion (x1.07) 0.840
Abnormal body temperature (x1.07) 0.844
Acute respiratory failure (x1.07) 0.849
Tachycardia (x1.06) 0.853
Malaise (x1.06) 0.856
Low blood pressure (x1.06) 0.859
Leukocytosis (x1.06) 0.862
Malnutrition (calorie) (x1.06) 0.865
metronidazole (+0.00516) 0.869
Electrocardiogram abnormal (x1.06) 0.871
Nausea and vomiting (x1.05) 0.874
Inflammatory disorder of digestive tract (x1.05) 0.877
Abnormal breathing (x1.05) 0.880
oxycodone (+0.00477) 0.883
Dysphagia (x1.05) 0.885
Dyspnea (x1.05) 0.887
Altered mental status (x1.05) 0.889
Edema (x1.05) 0.891
Acidosis (x1.05) 0.893
Disorder of lung (x1.04) 0.895
Lung field abnormal (x1.04) 0.896
Hypovolemia (x1.04) 0.898
Thrombocytopenic disorder (x1.04) 0.900
Gastroesophageal reflux disease (x1.04) 0.901
Hypo-osmolality and or hyponatremia (x1.04) 0.903
Cardiac arrhythmia (x1.04) 0.904
Disturbance of consciousness (x1.04) 0.906
ondansetron (+0.00363) 0.907
Cough (x1.04) 0.909
Hyperkalemia (x1.04) 0.910
Bacterial infectious disease (x1.04) 0.912
Nausea (x1.04) 0.913
Borderline normal ECG (x1.04) 0.914
furosemide (+0.00347) 0.916
Chest pain (x1.04) 0.917
acetaminophen (+0.00335) 0.918
gabapentin (+0.00329) 0.919
Depressive disorder (x1.03) 0.920
Severe sepsis (x1.03) 0.921
Trauma and postoperative pulmonary insufficiency (x1.03) 0.922
Congestive heart failure (x1.03) 0.923
Acute posthemorrhagic anemia (x1.03) 0.925
Cardiomegaly (x1.03) 0.926
promethazine (+0.00307) 0.927
Infection due to Escherichia coli (x1.03) 0.928
Type 2 diabetes mellitus without complication (x1.03) 0.929
vancomycin (+0.00296) 0.929
Disorder of kidney and/or ureter (x1.03) 0.930
Atrial fibrillation (x1.03) 0.931
Hypoxemia (x1.03) 0.932
Asthenia (x1.03) 0.933
Chronic kidney disease (x1.03) 0.934
Vomiting (x1.03) 0.935
Ascites (x1.03) 0.936
Leukocytes [#/V] in B by Manual count (+2.75) 0.936
Pulmonary congestion and hypostasis (x1.03) 0.937
Hypervolemia (x1.03) 0.938
Staphylococcal infectious disease (x1.03) 0.938
Pain in limb (x1.03) 0.939
famotidine (+0.0025) 0.940
Septic shock (x1.03) 0.940
Flatulence, eructation and gas pain (x1.03) 0.941
Swelling of limb (x1.03) 0.942
End-stage renal disease (x1.03) 0.942
nystatin (+0.00235) 0.943
Osteoporosis (x1.03) 0.943
Postprocedural state finding (x1.03) 0.944
Neutropenic disorder (x1.03) 0.945
Disorder of intestine (x1.02) 0.945
Pancytopenia (x1.02) 0.946
tacrolimus (+0.00227) 0.946
Disorder of magnesium metabolism (x1.02) 0.947
Tachypnea (x1.02) 0.947
Severe protein-calorie malnutrition (Gomez: less than 60% of standard weight) (x1.02) 0.948
omeprazole (+0.00214) 0.948
lorazepam (+0.00214) 0.949
ECG normal (x1.02) 0.949
Pneumonitis due to inhaled substance (x1.02) 0.950
Abnormal glucose level (x1.02) 0.950
Seizure (x1.02) 0.951
Immunodeficiency disorder (x1.02) 0.951
Renal failure syndrome (x1.02) 0.951
esomeprazole (+0.0019) 0.952
Anemia of chronic disease (x1.02) 0.952
Expressions
−0.75 −0.50 −0.25 0.00 0.25 0.50 0.75

Signature [220]; 84 To Reach 0.975, (84 Plotted)

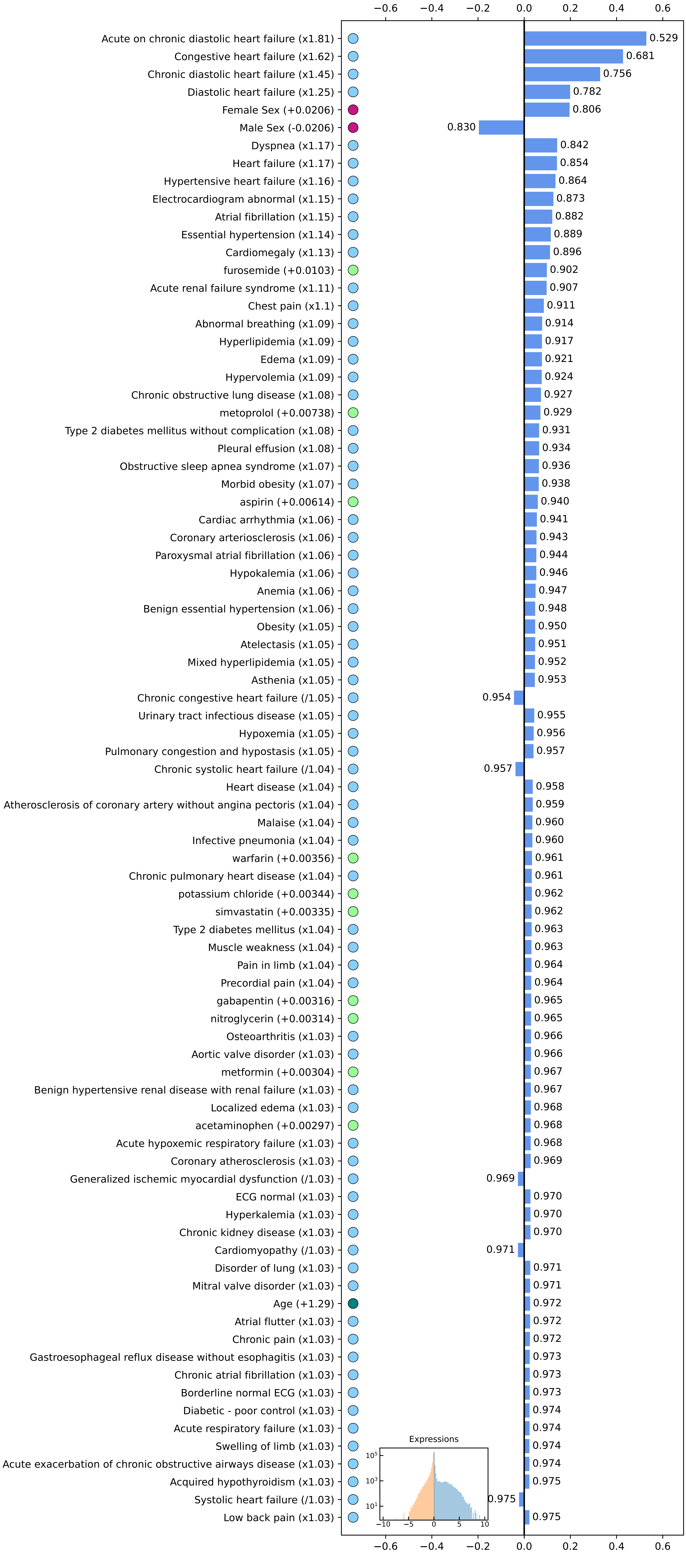

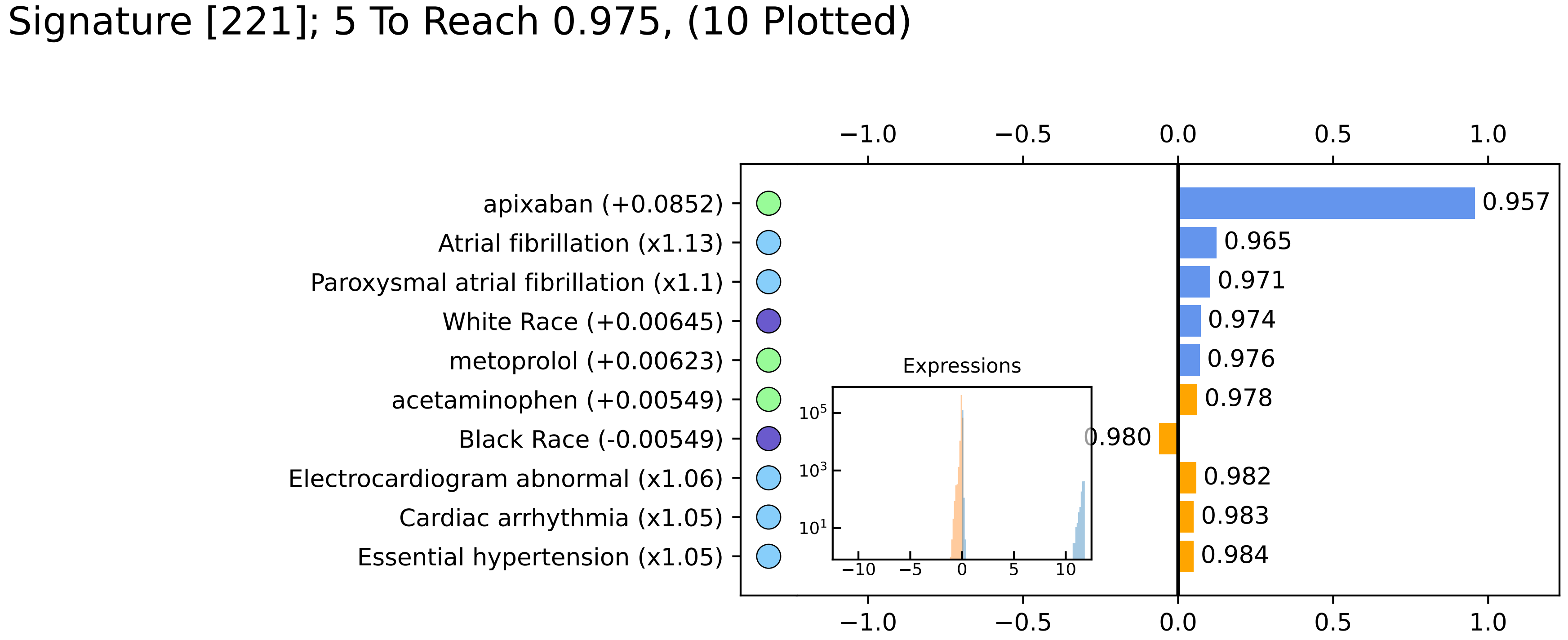
Signature [221]; 5 To Reach 0.975, (10 Plotted)
apixaban (+0.0852)
Atrial fibrillation (x1.13)
Paroxysmal atrial fibrillation (x1.1)
White Race (+0.00645)
metoprolol (+0.00623)
acetaminophen (+0.00549)
Black Race (-0.00549)
Electrocardiogram abnormal (x1.06)
Cardiac arrhythmia (x1.05)
Essential hypertension (x1.05)
0.957
0.965
0.971
0.974
0.976
0.978
0.980
0.982
0.983
0.984
Expressions
−1.0
−0.5
0.0
0.5
1.0

Signature [222]; 197 To Reach 0.975, (100 Plotted)

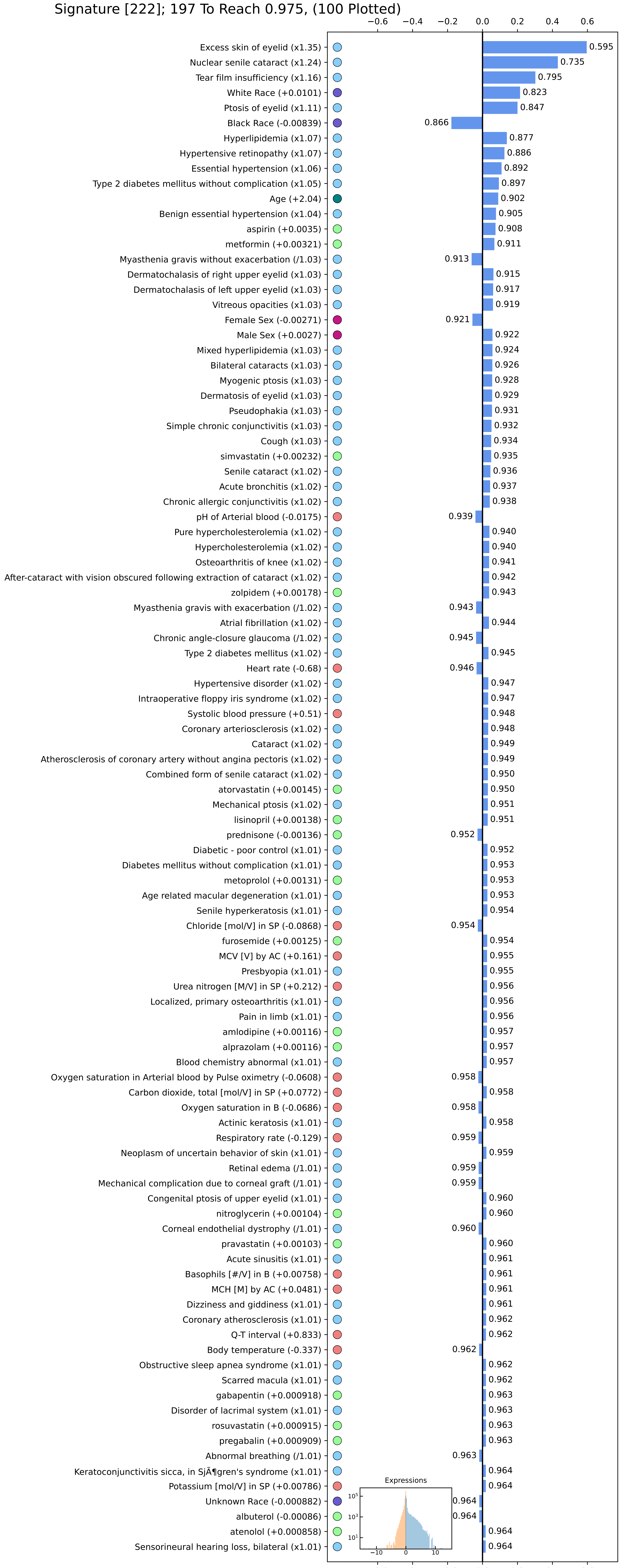

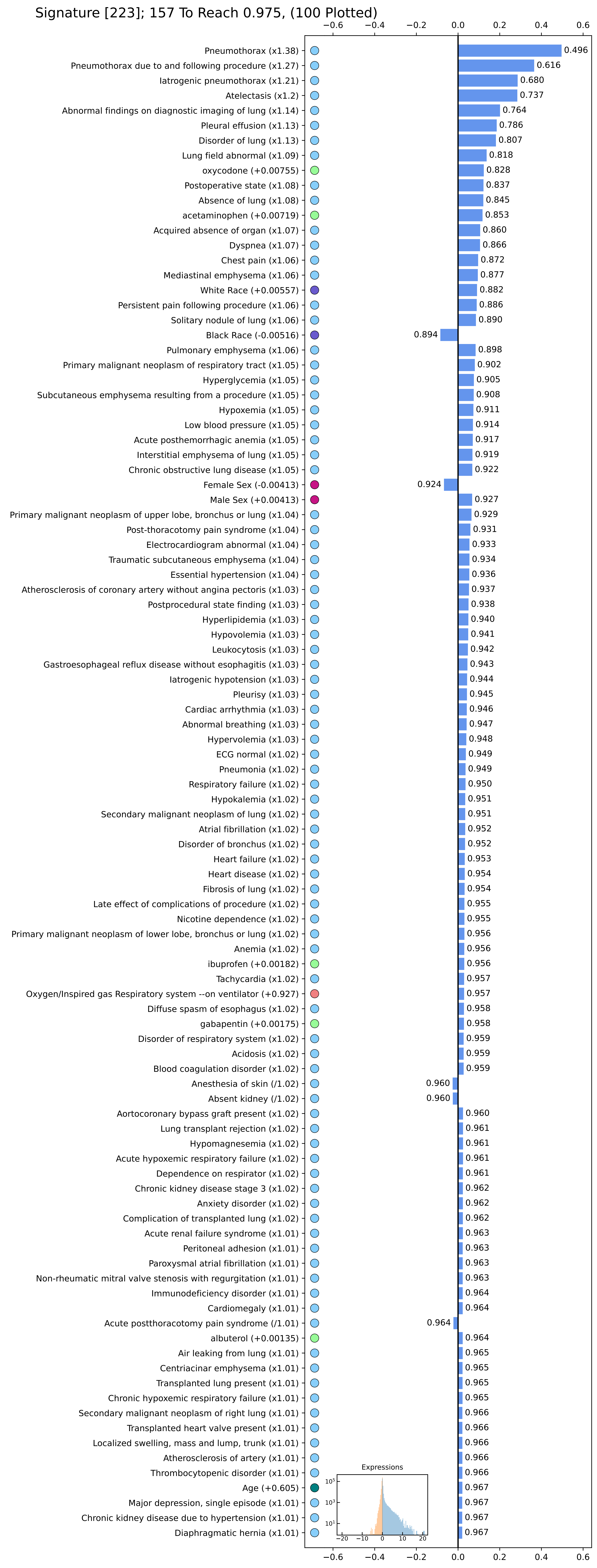

Signature [223]; 157 To Reach 0.975, (100 Plotted)
Pneumothorax (x1.38) 0.496
Pneumothorax due to and following procedure (x1.27) 0.616
Iatrogenic pneumothorax (x1.21) 0.680
Atelectasis (x1.2) 0.737
Abnormal findings on diagnostic imaging of lung (x1.14) 0.764
Pleural effusion (x1.13) 0.786
Disorder of lung (x1.13) 0.807
Lung field abnormal (x1.09) 0.818
oxycodone (+0.00755) 0.828
Postoperative state (x1.08) 0.837
Absence of lung (x1.08) 0.845
acetaminophen (+0.00719) 0.853
Acquired absence of organ (x1.07) 0.860
Dyspnea (x1.07) 0.866
Chest pain (x1.06) 0.872
Mediastinal emphysema (x1.06) 0.877
White Race (+0.00557) 0.882
Persistent pain following procedure (x1.06) 0.886
Solitary nodule of lung (x1.06) 0.890
Black Race (-0.00516) 0.894
Pulmonary emphysema (x1.06) 0.898
Primary malignant neoplasm of respiratory tract (x1.05) 0.902
Hyperglycemia (x1.05) 0.905
Subcutaneous emphysema resulting from a procedure (x1.05) 0.908
Hypoxemia (x1.05) 0.911
Low blood pressure (x1.05) 0.914
Acute posthemorrhagic anemia (x1.05) 0.917
Interstitial emphysema of lung (x1.05) 0.919
Chronic obstructive lung disease (x1.05) 0.922
Female Sex (-0.00413) 0.924
Male Sex (+0.00413) 0.927
Primary malignant neoplasm of upper lobe, bronchus or lung (x1.04) 0.929
Post-thoracotomy pain syndrome (x1.04) 0.931
Electrocardiogram abnormal (x1.04) 0.933
Traumatic subcutaneous emphysema (x1.04) 0.934
Essential hypertension (x1.04) 0.936
Atherosclerosis of coronary artery without angina pectoris (x1.03) 0.937
Postprocedural state finding (x1.03) 0.938
Hyperlipidemia (x1.03) 0.940
Hypovolemia (x1.03) 0.941
Leukocytosis (x1.03) 0.942
Gastroesophageal reflux disease without esophagitis (x1.03) 0.943
Iatrogenic hypotension (x1.03) 0.944
Pleurisy (x1.03) 0.945
Cardiac arrhythmia (x1.03) 0.946
Abnormal breathing (x1.03) 0.947
Hypervolemia (x1.03) 0.948
ECG normal (x1.02) 0.949
Pneumonia (x1.02) 0.949
Respiratory failure (x1.02) 0.950
Hypokalemia (x1.02) 0.951
Secondary malignant neoplasm of lung (x1.02) 0.951
Atrial fibrillation (x1.02) 0.952
Disorder of bronchus (x1.02) 0.952
Heart failure (x1.02) 0.953
Heart disease (x1.02) 0.954
Fibrosis of lung (x1.02) 0.954
Late effect of complications of procedure (x1.02) 0.955
Nicotine dependence (x1.02) 0.955
Primary malignant neoplasm of lower lobe, bronchus or lung (x1.02) 0.956
Anemia (x1.02) 0.956
ibuprofen (+0.00182) 0.956
Tachycardia (x1.02) 0.957
Oxygen/Inspired gas Respiratory system --on ventilator (+0.927) 0.957
Diffuse spasm of esophagus (x1.02) 0.958
gabapentin (+0.00175) 0.958
Disorder of respiratory system (x1.02) 0.959
Acidosis (x1.02) 0.959
Blood coagulation disorder (x1.02) 0.959
Anesthesia of skin (/1.02) 0.960
Absent kidney (/1.02) 0.960
Aortocoronary bypass graft present (x1.02) 0.960
Lung transplant rejection (x1.02) 0.961
Hypomagnesemia (x1.02) 0.961
Acute hypoxemic respiratory failure (x1.02) 0.961
Dependence on respirator (x1.02) 0.961
Chronic kidney disease stage 3 (x1.02) 0.962
Anxiety disorder (x1.02) 0.962
Complication of transplanted lung (x1.02) 0.962
Acute renal failure syndrome (x1.01) 0.963
Peritoneal adhesion (x1.01) 0.963
Paroxysmal atrial fibrillation (x1.01) 0.963
Non-rheumatic mitral valve stenosis with regurgitation (x1.01) 0.963
Immunodeficiency disorder (x1.01) 0.964
Cardiomegaly (x1.01) 0.964
Acute postthoracotomy pain syndrome (/1.01) 0.964
albuterol (+0.00135) 0.964
Air leaking from lung (x1.01) 0.965
Centriacinar emphysema (x1.01) 0.965
Transplanted lung present (x1.01) 0.965
Chronic hypoxemic respiratory failure (x1.01) 0.965
Secondary malignant neoplasm of right lung (x1.01) 0.966
Transplanted heart valve present (x1.01) 0.966
Localized swelling, mass and lump, trunk (x1.01) 0.966
Atherosclerosis of artery (x1.01) 0.966
Thrombocytopenic disorder (x1.01) 0.966
Age (+0.605) 0.967
Major depression, single episode (x1.01) 0.967
Chronic kidney disease due to hypertension (x1.01) 0.967
Diaphragmatic hernia (x1.01) 0.967
Expressions
−0.6 −0.4 −0.2 0.0 0.2 0.4 0.6

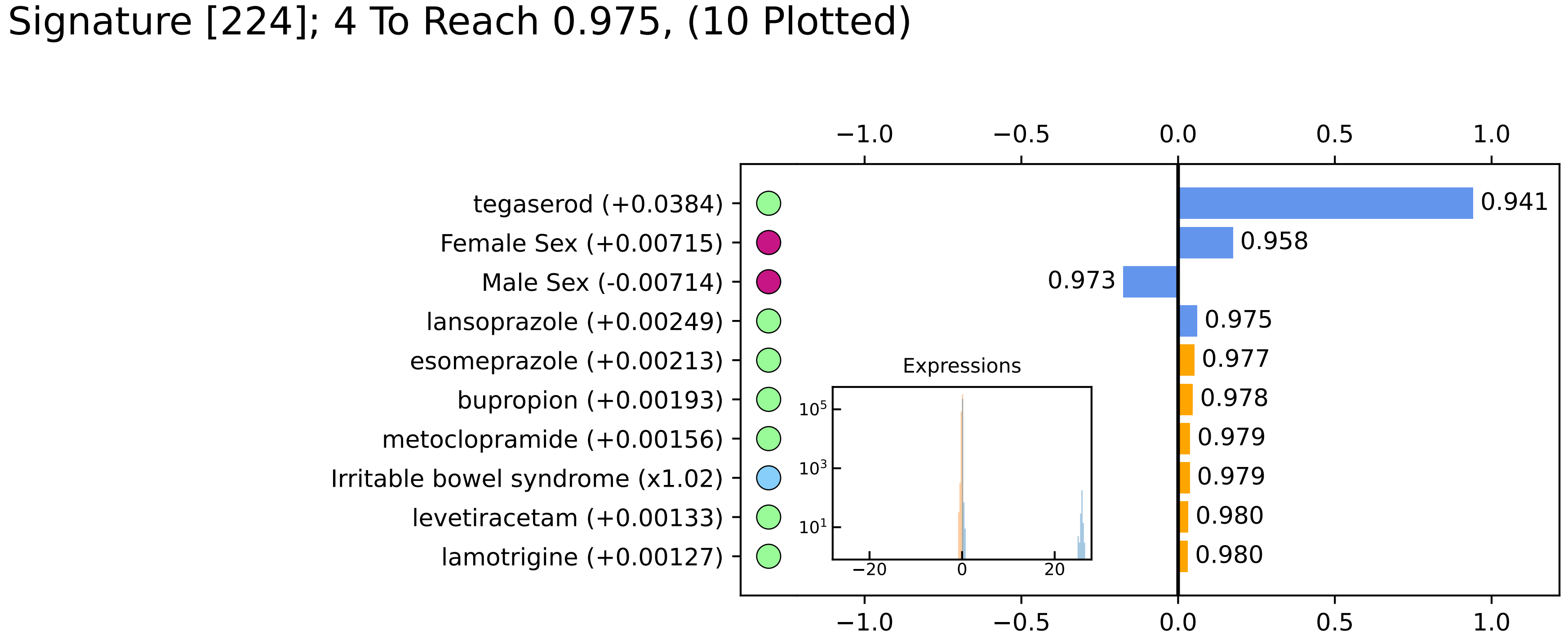

Signature [224]; 4 To Reach 0.975, (10 Plotted)
tegaserod (+0.0384)
Female Sex (+0.00715)
Male Sex (-0.00714)
lansoprazole (+0.00249)
esomeprazole (+0.00213)
bupropion (+0.00193)
metoclopramide (+0.00156)
Irritable bowel syndrome (x1.02)
levetiracetam (+0.00133)
lamotrigine (+0.00127)
0.941
0.958
0.973
0.975
0.977
0.978
0.979
0.979
0.980
0.980
−1.0
−0.5
0.0
0.5
1.0
Expressions
10^5
10^3
10^1
−20
0
20

Signature [225]; 2 To Reach 0.975, (10 Plotted)

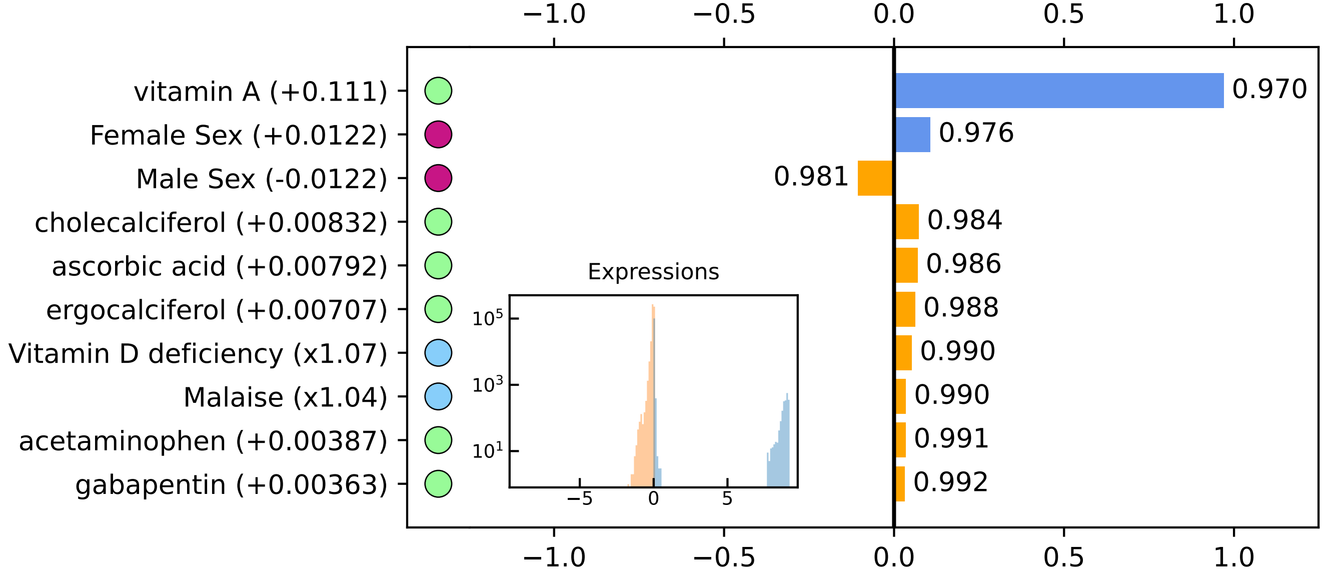

Signature [226]; 76 To Reach 0.975, (76 Plotted)

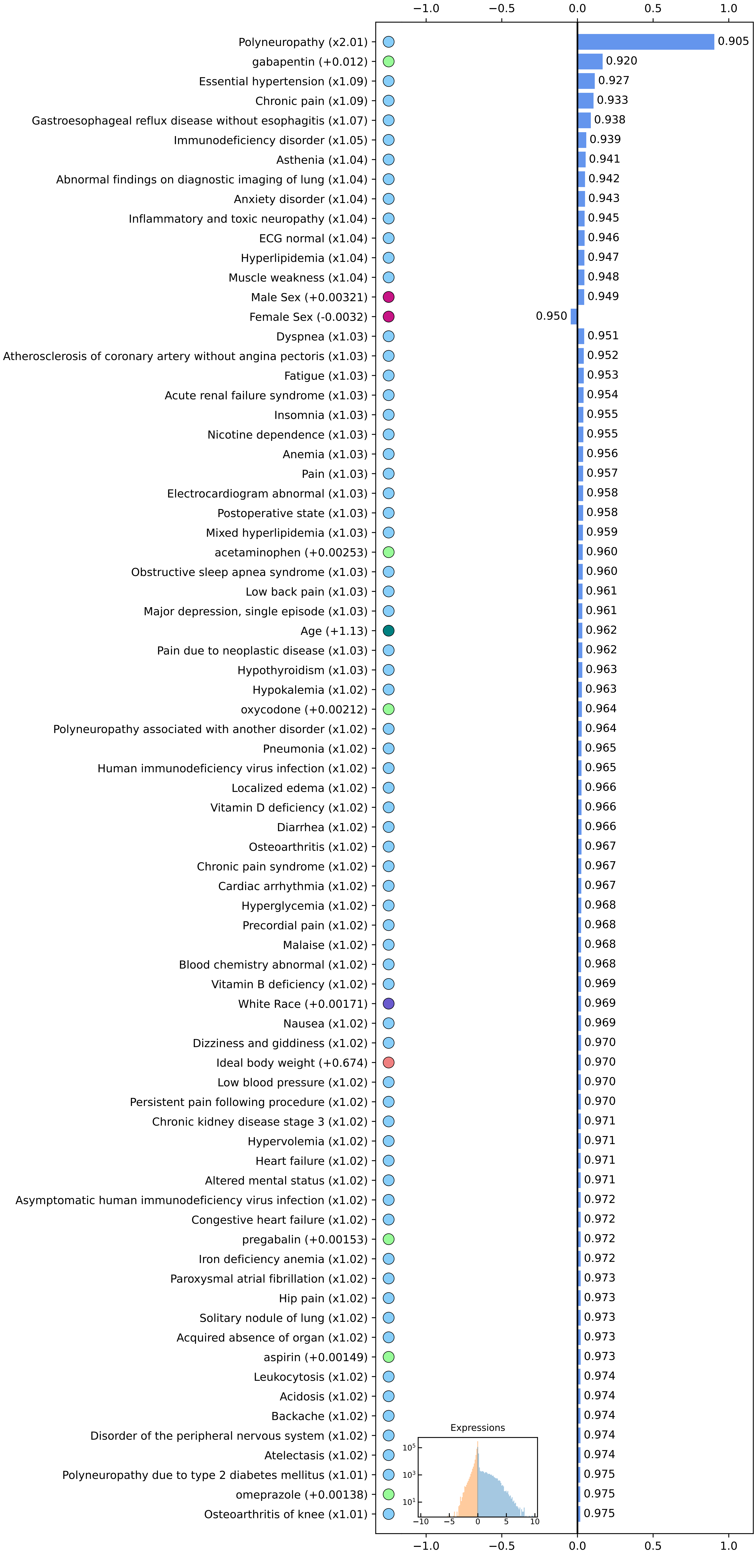

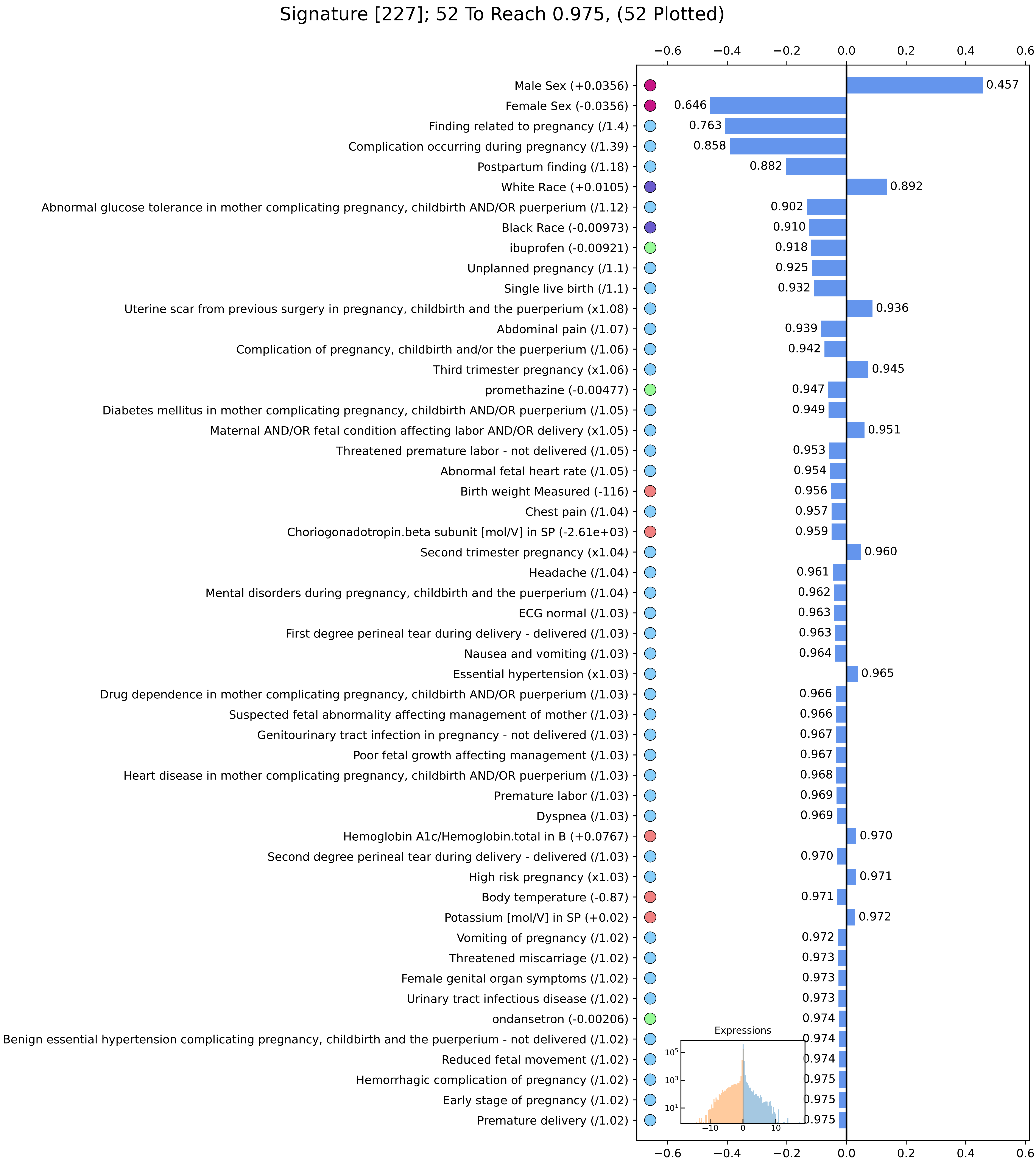
Signature [227]; 52 To Reach 0.975, (52 Plotted)
Male Sex (+0.0356) 0.457
Female Sex (-0.0356) 0.646
Finding related to pregnancy (/1.4) 0.763
Complication occurring during pregnancy (/1.39) 0.858
Postpartum finding (/1.18) 0.882
White Race (+0.0105) 0.892
Abnormal glucose tolerance in mother complicating pregnancy, childbirth AND/OR puerperium (/1.12) 0.902
Black Race (-0.00973) 0.910
ibuprofen (-0.00921) 0.918
Unplanned pregnancy (/1.1) 0.925
Single live birth (/1.1) 0.932
Uterine scar from previous surgery in pregnancy, childbirth and the puerperium (x1.08) 0.936
Abdominal pain (/1.07) 0.939
Complication of pregnancy, childbirth and/or the puerperium (/1.06) 0.942
Third trimester pregnancy (x1.06) 0.945
promethazine (-0.00477) 0.947
Diabetes mellitus in mother complicating pregnancy, childbirth AND/OR puerperium (/1.05) 0.949
Maternal AND/OR fetal condition affecting labor AND/OR delivery (x1.05) 0.951
Threatened premature labor - not delivered (/1.05) 0.953
Abnormal fetal heart rate (/1.05) 0.954
Birth weight Measured (-116) 0.956
Chest pain (/1.04) 0.957
Choriogonadotropin.beta subunit [mol/V] in SP (-2.61e+03) 0.959
Second trimester pregnancy (x1.04) 0.960
Headache (/1.04) 0.961
Mental disorders during pregnancy, childbirth and the puerperium (/1.04) 0.962
ECG normal (/1.03) 0.963
First degree perineal tear during delivery - delivered (/1.03) 0.963
Nausea and vomiting (/1.03) 0.964
Essential hypertension (x1.03) 0.965
Drug dependence in mother complicating pregnancy, childbirth AND/OR puerperium (/1.03) 0.966
Suspected fetal abnormality affecting management of mother (/1.03) 0.966
Genitourinary tract infection in pregnancy - not delivered (/1.03) 0.967
Poor fetal growth affecting management (/1.03) 0.967
Heart disease in mother complicating pregnancy, childbirth AND/OR puerperium (/1.03) 0.968
Premature labor (/1.03) 0.969
Dyspnea (/1.03) 0.969
Hemoglobin A1c/Hemoglobin.total in B (+0.0767) 0.970
Second degree perineal tear during delivery - delivered (/1.03) 0.970
High risk pregnancy (x1.03) 0.971
Body temperature (-0.87) 0.971
Potassium [mol/V] in SP (+0.02) 0.972
Vomiting of pregnancy (/1.02) 0.972
Threatened miscarriage (/1.02) 0.973
Female genital organ symptoms (/1.02) 0.973
Urinary tract infectious disease (/1.02) 0.973
ondansetron (-0.00206) 0.974
Benign essential hypertension complicating pregnancy, childbirth and the puerperium - not delivered (/1.02) 0.974
Reduced fetal movement (/1.02) 0.974
Hemorrhagic complication of pregnancy (/1.02) 0.975
Early stage of pregnancy (/1.02) 0.975
Premature delivery (/1.02) 0.975
−0.6 −0.4 −0.2 0.0 0.2 0.4 0.6
Expressions
−10 0 10

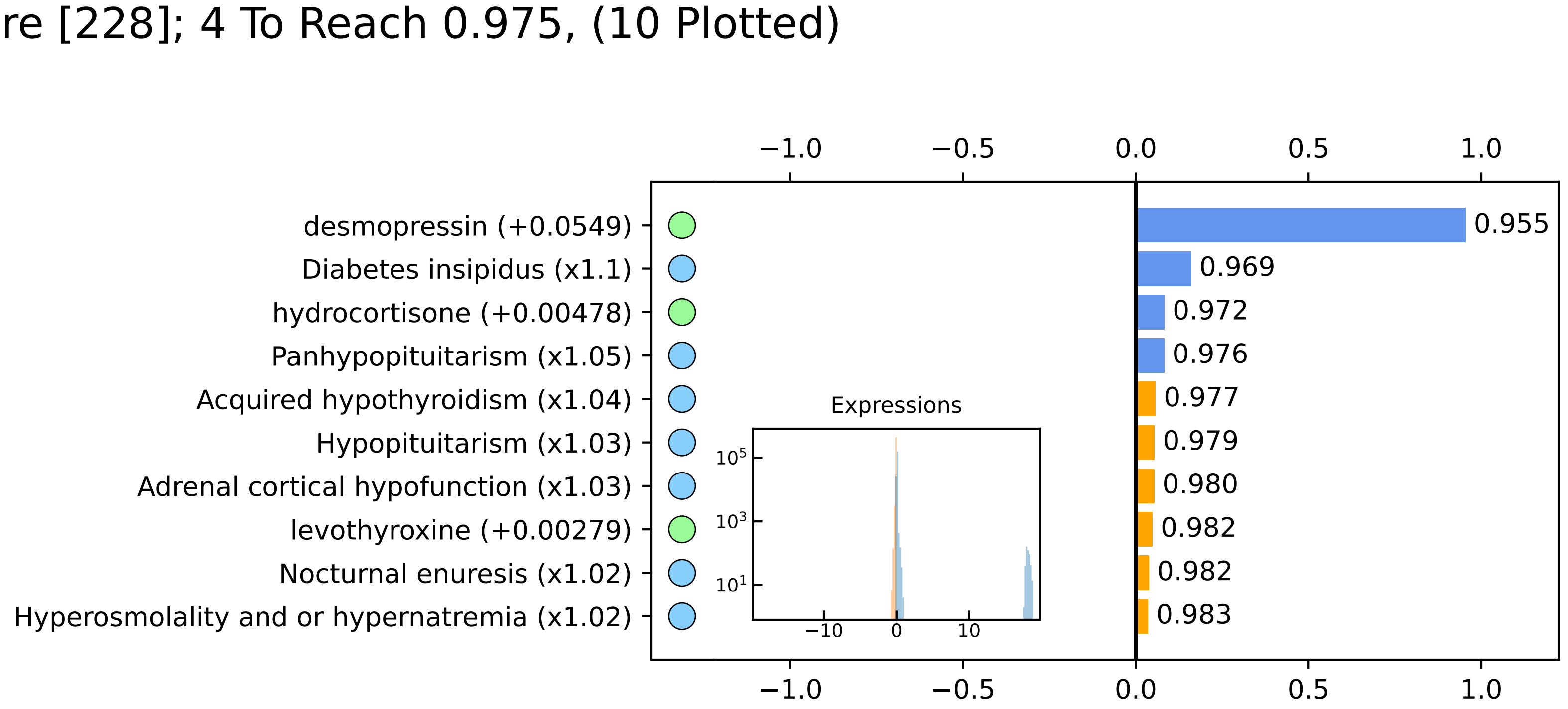
Signature [228]; 4 To Reach 0.975, (10 Plotted)
desmopressin (+0.0549)
Diabetes insipidus (x1.1)
hydrocortisone (+0.00478)
Panhypopituitarism (x1.05)
Acquired hypothyroidism (x1.04)
Hypopituitarism (x1.03)
Adrenal cortical hypofunction (x1.03)
levothyroxine (+0.00279)
Nocturnal enuresis (x1.02)
Hyperosmolality and or hypernatremia (x1.02)
0.955
0.969
0.972
0.976
0.977
0.979
0.980
0.982
0.982
0.983
−1.0
−0.5
0.0
0.5
1.0
Expressions
10^5
10^3
10^1
−10
0
10

Signature [229]; 345 To Reach 0.975, (100 Plotted)

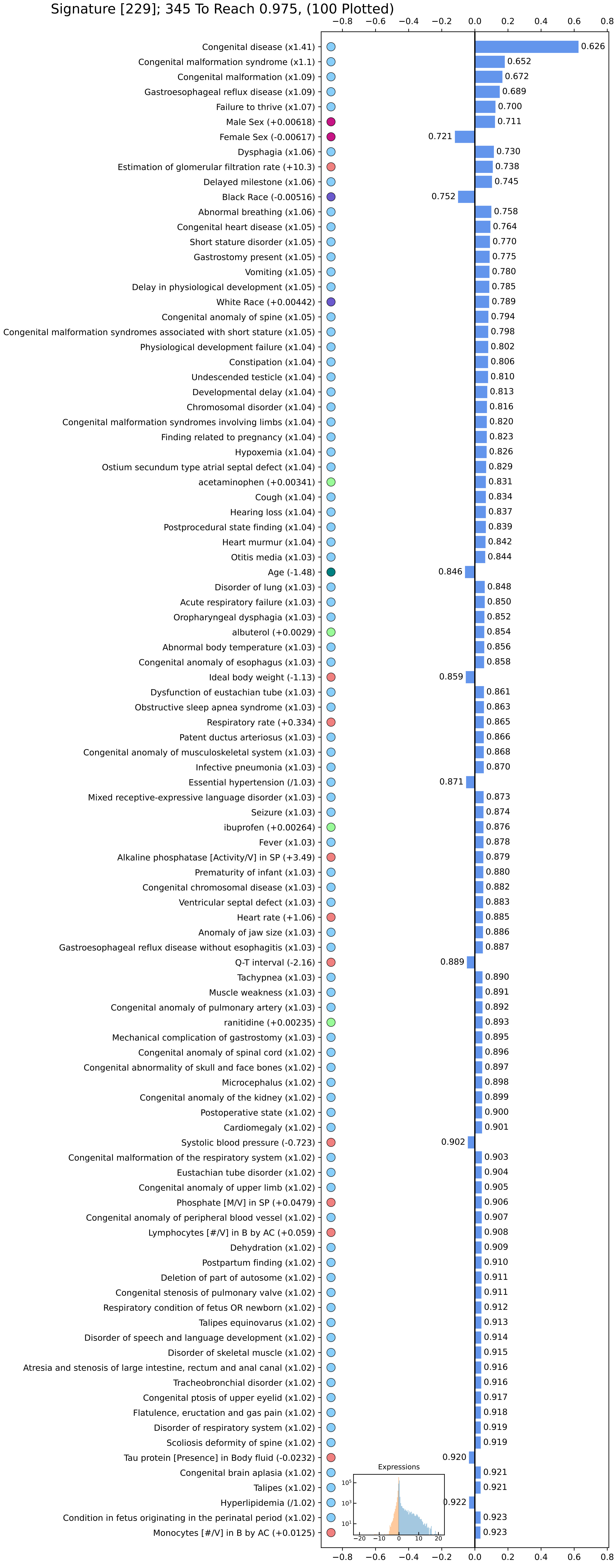

Signature [230]; 12 To Reach 0.975, (12 Plotted)

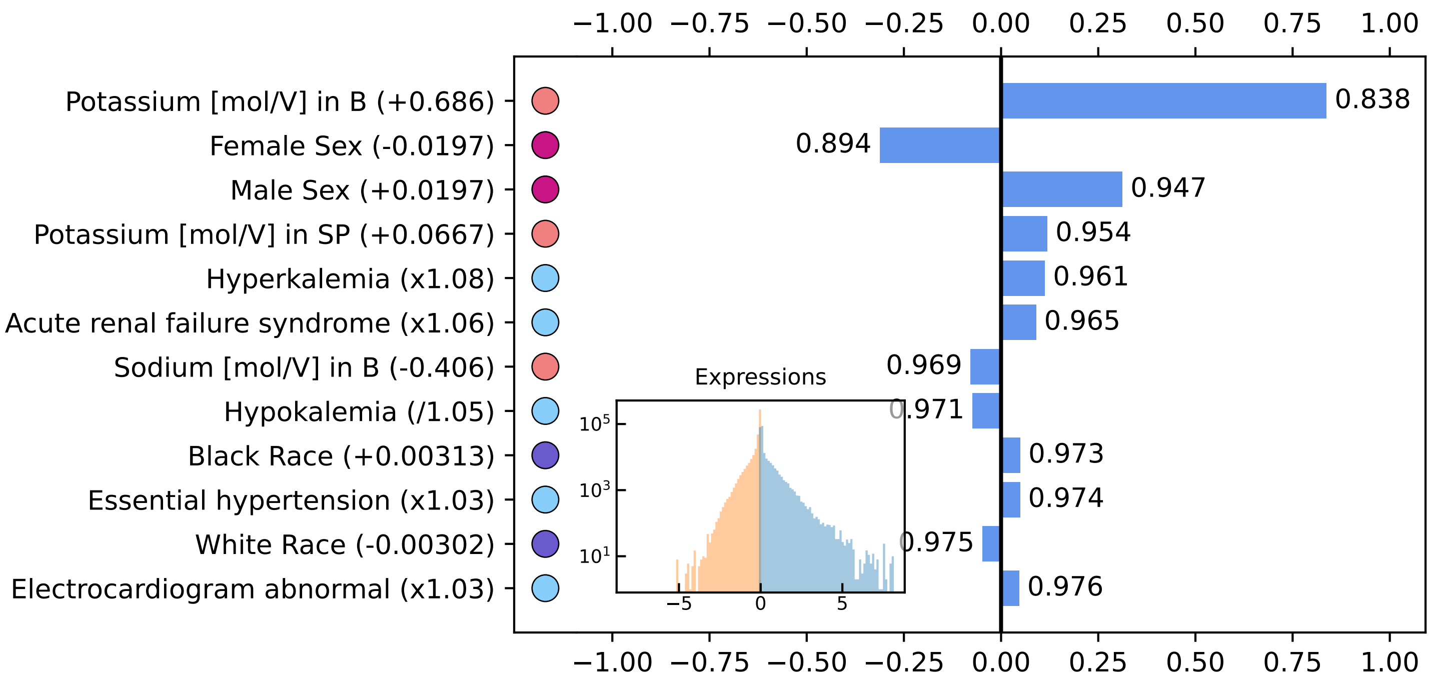

Signature [231]; 451 To Reach 0.975, (100 Plotted)

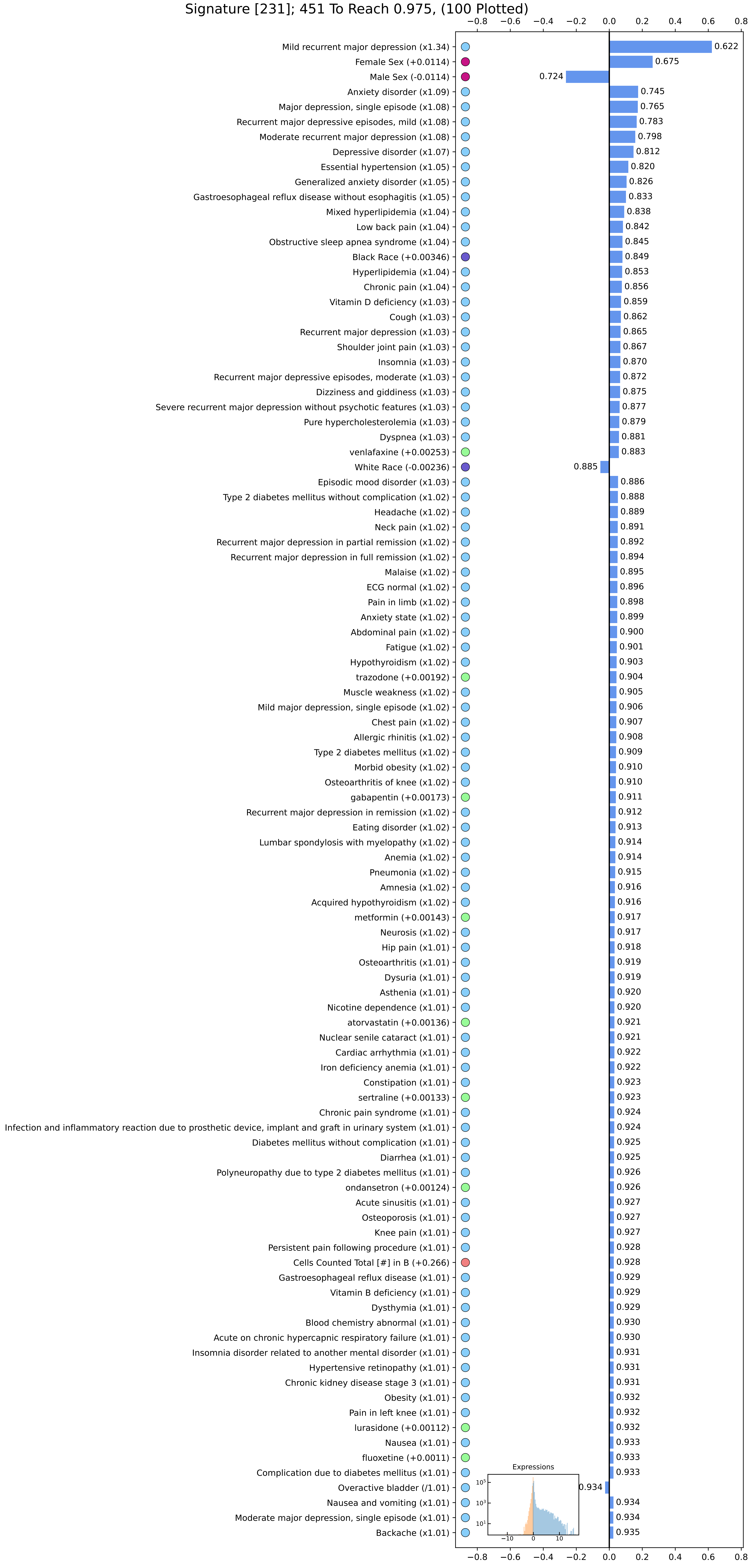

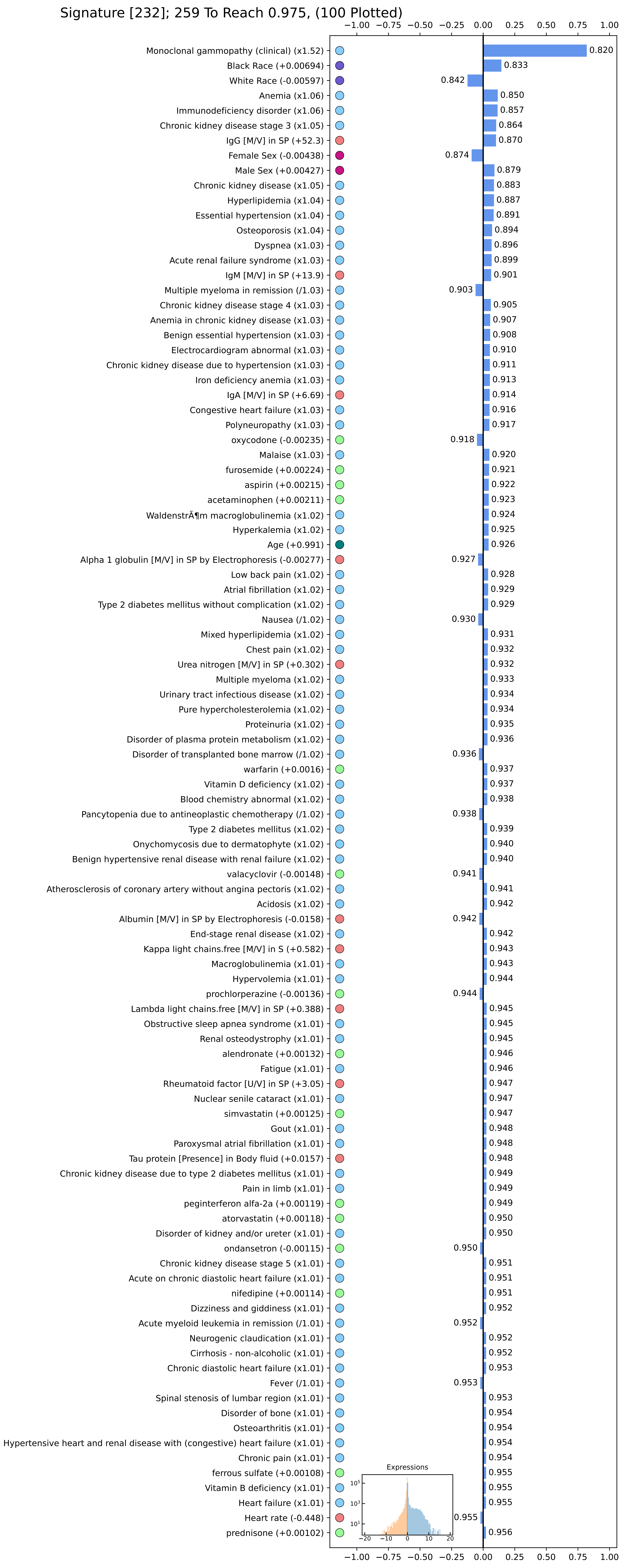

Signature [232]; 259 To Reach 0.975, (100 Plotted)
−1.00 −0.75 −0.50 −0.25 0.00 0.25 0.50 0.75 1.00
Monoclonal gammopathy (clinical) (x1.52) 0.820
Black Race (+0.00694) 0.833
White Race (-0.00597) 0.842
Anemia (x1.06) 0.850
Immunodeficiency disorder (x1.06) 0.857
Chronic kidney disease stage 3 (x1.05) 0.864
IgG [M/V] in SP (+52.3) 0.870
Female Sex (-0.00438) 0.874
Male Sex (+0.00427) 0.879
Chronic kidney disease (x1.05) 0.883
Hyperlipidemia (x1.04) 0.887
Essential hypertension (x1.04) 0.891
Osteoporosis (x1.04) 0.894
Dyspnea (x1.03) 0.896
Acute renal failure syndrome (x1.03) 0.899
IgM [M/V] in SP (+13.9) 0.901
Multiple myeloma in remission (/1.03) 0.903
Chronic kidney disease stage 4 (x1.03) 0.905
Anemia in chronic kidney disease (x1.03) 0.907
Benign essential hypertension (x1.03) 0.908
Electrocardiogram abnormal (x1.03) 0.910
Chronic kidney disease due to hypertension (x1.03) 0.911
Iron deficiency anemia (x1.03) 0.913
IgA [M/V] in SP (+6.69) 0.914
Congestive heart failure (x1.03) 0.916
Polyneuropathy (x1.03) 0.917
oxycodone (-0.00235) 0.918
Malaise (x1.03) 0.920
furosemide (+0.00224) 0.921
aspirin (+0.00215) 0.922
acetaminophen (+0.00211) 0.923
WaldenstrÃ¶m macroglobulinemia (x1.02) 0.924
Hyperkalemia (x1.02) 0.925
Age (+0.991) 0.926
Alpha 1 globulin [M/V] in SP by Electrophoresis (-0.00277) 0.927
Low back pain (x1.02) 0.928
Atrial fibrillation (x1.02) 0.929
Type 2 diabetes mellitus without complication (x1.02) 0.929
Nausea (/1.02) 0.930
Mixed hyperlipidemia (x1.02) 0.931
Chest pain (x1.02) 0.932
Urea nitrogen [M/V] in SP (+0.302) 0.932
Multiple myeloma (x1.02) 0.933
Urinary tract infectious disease (x1.02) 0.934
Pure hypercholesterolemia (x1.02) 0.934
Proteinuria (x1.02) 0.935
Disorder of plasma protein metabolism (x1.02) 0.936
Disorder of transplanted bone marrow (/1.02) 0.936
warfarin (+0.0016) 0.937
Vitamin D deficiency (x1.02) 0.937
Blood chemistry abnormal (x1.02) 0.938
Pancytopenia due to antineoplastic chemotherapy (/1.02) 0.938
Type 2 diabetes mellitus (x1.02) 0.939
Onychomycosis due to dermatophyte (x1.02) 0.940
Benign hypertensive renal disease with renal failure (x1.02) 0.940
valacyclovir (-0.00148) 0.941
Atherosclerosis of coronary artery without angina pectoris (x1.02) 0.941
Acidosis (x1.02) 0.942
Albumin [M/V] in SP by Electrophoresis (-0.0158) 0.942
End-stage renal disease (x1.02) 0.942
Kappa light chains.free [M/V] in S (+0.582) 0.943
Macroglobulinemia (x1.01) 0.943
Hypervolemia (x1.01) 0.944
prochlorperazine (-0.00136) 0.944
Lambda light chains.free [M/V] in SP (+0.388) 0.945
Obstructive sleep apnea syndrome (x1.01) 0.945
Renal osteodystrophy (x1.01) 0.945
alendronate (+0.00132) 0.946
Fatigue (x1.01) 0.946
Rheumatoid factor [U/V] in SP (+3.05) 0.947
Nuclear senile cataract (x1.01) 0.947
simvastatin (+0.00125) 0.947
Gout (x1.01) 0.948
Paroxysmal atrial fibrillation (x1.01) 0.948
Tau protein [Presence] in Body fluid (+0.0157) 0.948
Chronic kidney disease due to type 2 diabetes mellitus (x1.01) 0.949
Pain in limb (x1.01) 0.949
peginterferon alfa-2a (+0.00119) 0.949
atorvastatin (+0.00118) 0.950
Disorder of kidney and/or ureter (x1.01) 0.950
ondansetron (-0.00115) 0.950
Chronic kidney disease stage 5 (x1.01) 0.951
Acute on chronic diastolic heart failure (x1.01) 0.951
nifedipine (+0.00114) 0.951
Dizziness and giddiness (x1.01) 0.952
Acute myeloid leukemia in remission (/1.01) 0.952
Neurogenic claudication (x1.01) 0.952
Cirrhosis - non-alcoholic (x1.01) 0.952
Chronic diastolic heart failure (x1.01) 0.953
Fever (/1.01) 0.953
Spinal stenosis of lumbar region (x1.01) 0.953
Disorder of bone (x1.01) 0.954
Osteoarthritis (x1.01) 0.954
Hypertensive heart and renal disease with (congestive) heart failure (x1.01) 0.954
Chronic pain (x1.01) 0.954
ferrous sulfate (+0.00108) 0.955
Vitamin B deficiency (x1.01) 0.955
Heart failure (x1.01) 0.955
Heart rate (-0.448) 0.955
prednisone (+0.00102) 0.956
Expressions
10^5 10^3 10^1
−20 −10 0 10 20
−1.00 −0.75 −0.50 −0.25 0.00 0.25 0.50 0.75 1.00

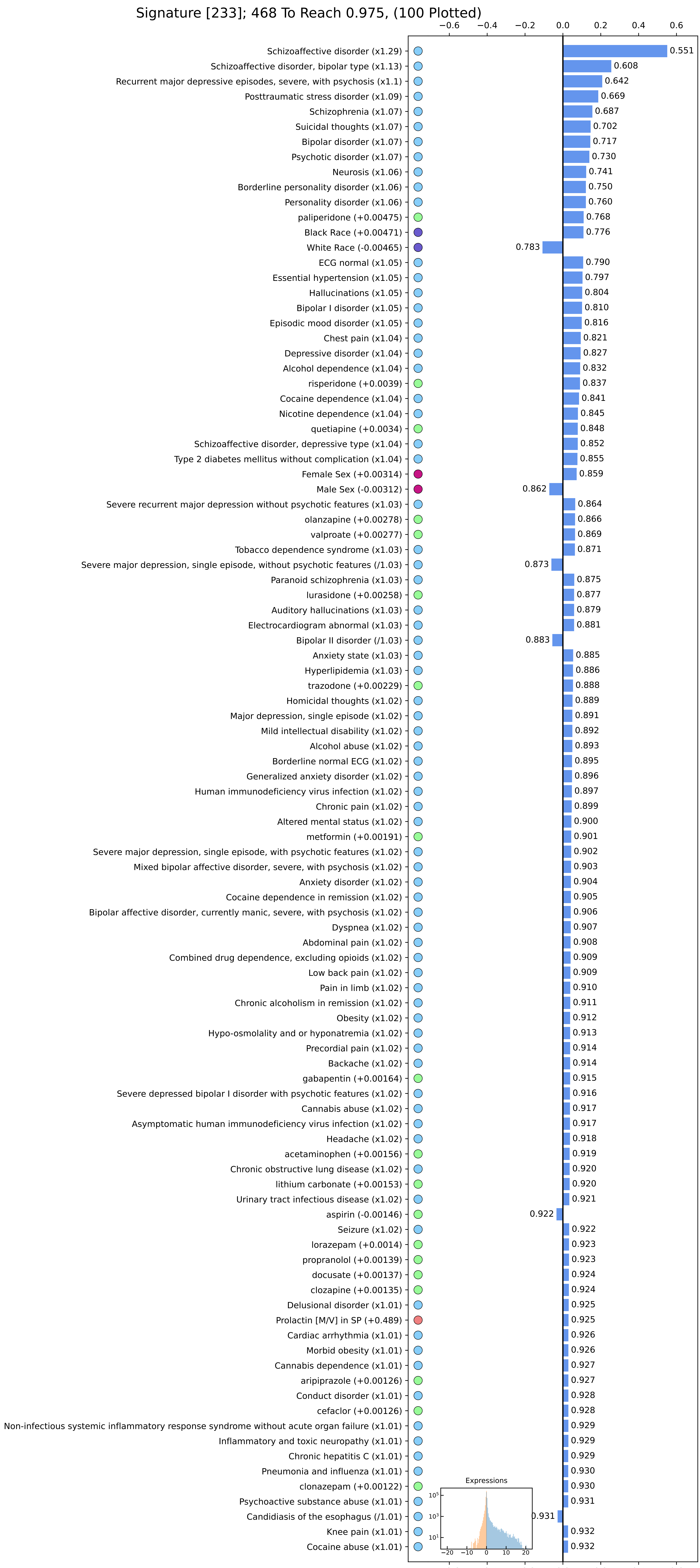

Signature [233]; 468 To Reach 0.975, (100 Plotted)
Schizoaffective disorder (x1.29) 0.551
Schizoaffective disorder, bipolar type (x1.13) 0.608
Recurrent major depressive episodes, severe, with psychosis (x1.1) 0.642
Posttraumatic stress disorder (x1.09) 0.669
Schizophrenia (x1.07) 0.687
Suicidal thoughts (x1.07) 0.702
Bipolar disorder (x1.07) 0.717
Psychotic disorder (x1.07) 0.730
Neurosis (x1.06) 0.741
Borderline personality disorder (x1.06) 0.750
Personality disorder (x1.06) 0.760
paliperidone (+0.00475) 0.768
Black Race (+0.00471) 0.776
White Race (-0.00465) 0.783
ECG normal (x1.05) 0.790
Essential hypertension (x1.05) 0.797
Hallucinations (x1.05) 0.804
Bipolar I disorder (x1.05) 0.810
Episodic mood disorder (x1.05) 0.816
Chest pain (x1.04) 0.821
Depressive disorder (x1.04) 0.827
Alcohol dependence (x1.04) 0.832
risperidone (+0.0039) 0.837
Cocaine dependence (x1.04) 0.841
Nicotine dependence (x1.04) 0.845
quetiapine (+0.0034) 0.848
Schizoaffective disorder, depressive type (x1.04) 0.852
Type 2 diabetes mellitus without complication (x1.04) 0.855
Female Sex (+0.00314) 0.859
Male Sex (-0.00312) 0.862
Severe recurrent major depression without psychotic features (x1.03) 0.864
olanzapine (+0.00278) 0.866
valproate (+0.00277) 0.869
Tobacco dependence syndrome (x1.03) 0.871
Severe major depression, single episode, without psychotic features (/1.03) 0.873
Paranoid schizophrenia (x1.03) 0.875
lurasidone (+0.00258) 0.877
Auditory hallucinations (x1.03) 0.879
Electrocardiogram abnormal (x1.03) 0.881
Bipolar II disorder (/1.03) 0.883
Anxiety state (x1.03) 0.885
Hyperlipidemia (x1.03) 0.886
trazodone (+0.00229) 0.888
Homicidal thoughts (x1.02) 0.889
Major depression, single episode (x1.02) 0.891
Mild intellectual disability (x1.02) 0.892
Alcohol abuse (x1.02) 0.893
Borderline normal ECG (x1.02) 0.895
Generalized anxiety disorder (x1.02) 0.896
Human immunodeficiency virus infection (x1.02) 0.897
Chronic pain (x1.02) 0.899
Altered mental status (x1.02) 0.900
metformin (+0.00191) 0.901
Severe major depression, single episode, with psychotic features (x1.02) 0.902
Mixed bipolar affective disorder, severe, with psychosis (x1.02) 0.903
Anxiety disorder (x1.02) 0.904
Cocaine dependence in remission (x1.02) 0.905
Bipolar affective disorder, currently manic, severe, with psychosis (x1.02) 0.906
Dyspnea (x1.02) 0.907
Abdominal pain (x1.02) 0.908
Combined drug dependence, excluding opioids (x1.02) 0.909
Low back pain (x1.02) 0.909
Pain in limb (x1.02) 0.910
Chronic alcoholism in remission (x1.02) 0.911
Obesity (x1.02) 0.912
Hypo-osmolality and or hyponatremia (x1.02) 0.913
Precordial pain (x1.02) 0.914
Backache (x1.02) 0.914
gabapentin (+0.00164) 0.915
Severe depressed bipolar I disorder with psychotic features (x1.02) 0.916
Cannabis abuse (x1.02) 0.917
Asymptomatic human immunodeficiency virus infection (x1.02) 0.917
Headache (x1.02) 0.918
acetaminophen (+0.00156) 0.919
Chronic obstructive lung disease (x1.02) 0.920
lithium carbonate (+0.00153) 0.920
Urinary tract infectious disease (x1.02) 0.921
aspirin (-0.00146) 0.922
Seizure (x1.02) 0.922
lorazepam (+0.0014) 0.923
propranolol (+0.00139) 0.923
docusate (+0.00137) 0.924
clozapine (+0.00135) 0.924
Delusional disorder (x1.01) 0.925
Prolactin [M/V] in SP (+0.489) 0.925
Cardiac arrhythmia (x1.01) 0.926
Morbid obesity (x1.01) 0.926
Cannabis dependence (x1.01) 0.927
aripiprazole (+0.00126) 0.927
Conduct disorder (x1.01) 0.928
cefaclor (+0.00126) 0.928
Non-infectious systemic inflammatory response syndrome without acute organ failure (x1.01) 0.929
Inflammatory and toxic neuropathy (x1.01) 0.929
Chronic hepatitis C (x1.01) 0.929
Pneumonia and influenza (x1.01) 0.930
clonazepam (+0.00122) 0.930
Psychoactive substance abuse (x1.01) 0.931
Candidiasis of the esophagus (/1.01) 0.931
Knee pain (x1.01) 0.932
Cocaine abuse (x1.01) 0.932
−0.6 −0.4 −0.2 0.0 0.2 0.4 0.6
Expressions

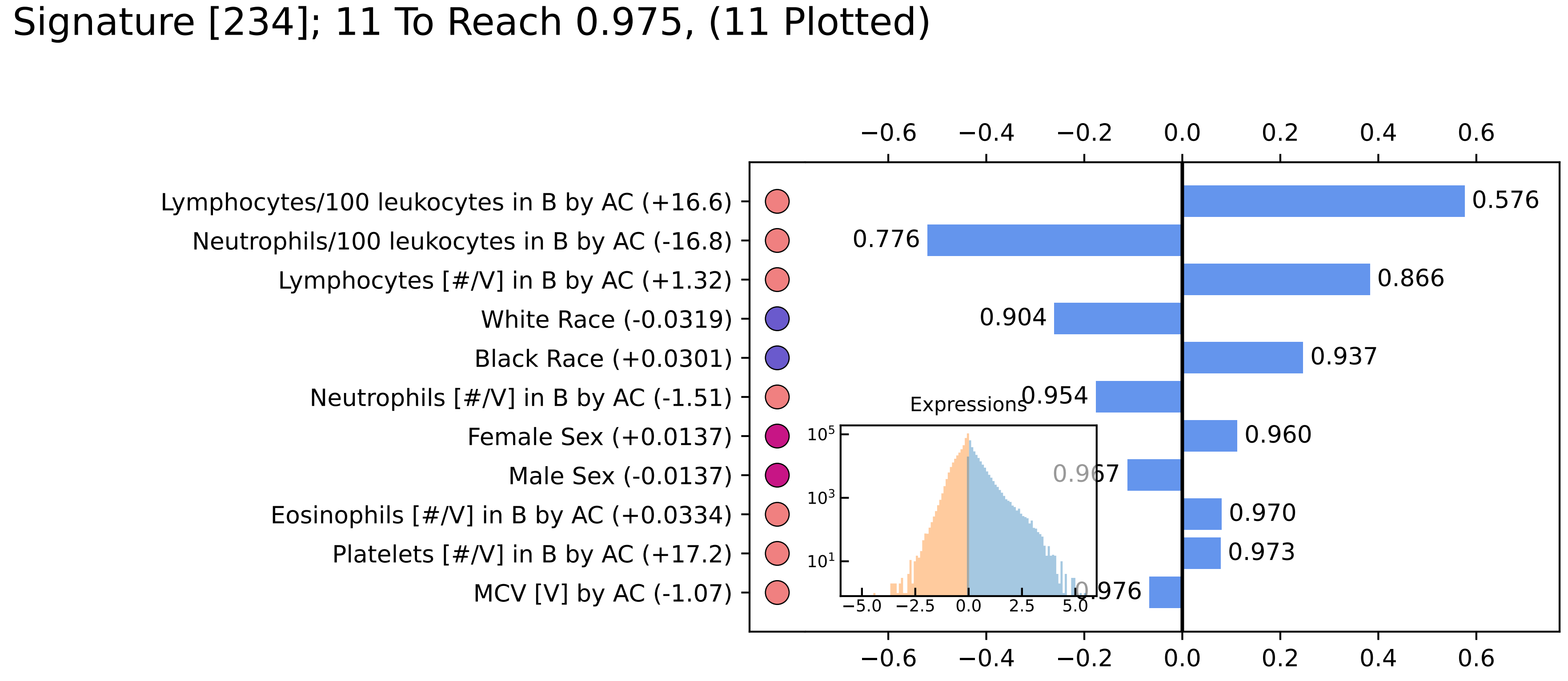
Signature [234]; 11 To Reach 0.975, (11 Plotted)
Lymphocytes/100 leukocytes in B by AC (+16.6)
Neutrophils/100 leukocytes in B by AC (-16.8)
Lymphocytes [#/V] in B by AC (+1.32)
White Race (-0.0319)
Black Race (+0.0301)
Neutrophils [#/V] in B by AC (-1.51)
Female Sex (+0.0137)
Male Sex (-0.0137)
Eosinophils [#/V] in B by AC (+0.0334)
Platelets [#/V] in B by AC (+17.2)
MCV [V] by AC (-1.07)
0.576
0.776
0.866
0.904
0.937
0.954
0.960
0.967
0.970
0.973
0.976
−0.6
−0.4
−0.2
0.0
0.2
0.4
0.6
Expressions
10^5
10^3
10^1
−5.0
−2.5
0.0
2.5
5.0

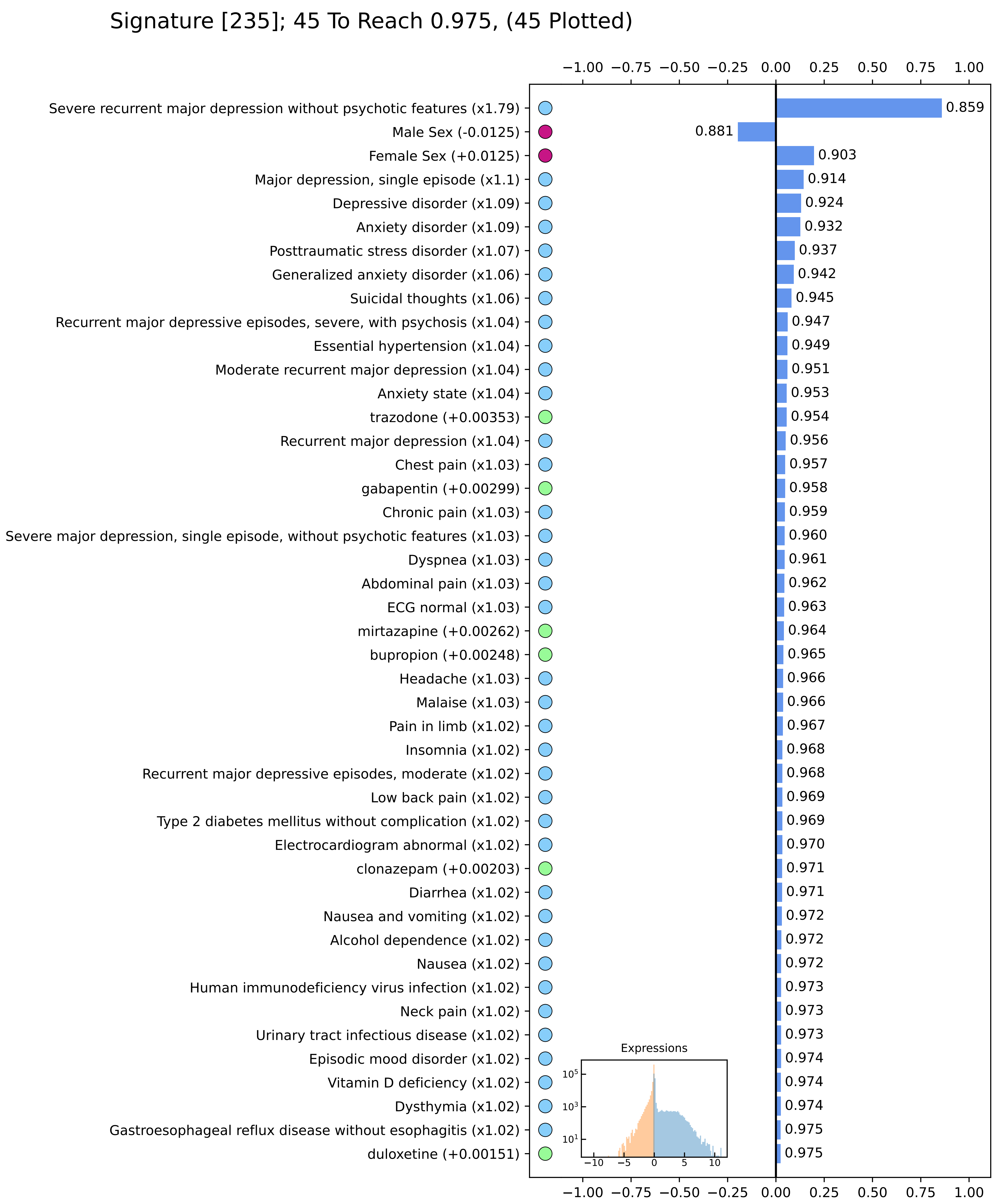

Signature [235]; 45 To Reach 0.975, (45 Plotted)
Severe recurrent major depression without psychotic features (x1.79) 0.859
Male Sex (-0.0125) 0.881
Female Sex (+0.0125) 0.903
Major depression, single episode (x1.1) 0.914
Depressive disorder (x1.09) 0.924
Anxiety disorder (x1.09) 0.932
Posttraumatic stress disorder (x1.07) 0.937
Generalized anxiety disorder (x1.06) 0.942
Suicidal thoughts (x1.06) 0.945
Recurrent major depressive episodes, severe, with psychosis (x1.04) 0.947
Essential hypertension (x1.04) 0.949
Moderate recurrent major depression (x1.04) 0.951
Anxiety state (x1.04) 0.953
trazodone (+0.00353) 0.954
Recurrent major depression (x1.04) 0.956
Chest pain (x1.03) 0.957
gabapentin (+0.00299) 0.958
Chronic pain (x1.03) 0.959
Severe major depression, single episode, without psychotic features (x1.03) 0.960
Dyspnea (x1.03) 0.961
Abdominal pain (x1.03) 0.962
ECG normal (x1.03) 0.963
mirtazapine (+0.00262) 0.964
bupropion (+0.00248) 0.965
Headache (x1.03) 0.966
Malaise (x1.03) 0.966
Pain in limb (x1.02) 0.967
Insomnia (x1.02) 0.968
Recurrent major depressive episodes, moderate (x1.02) 0.968
Low back pain (x1.02) 0.969
Type 2 diabetes mellitus without complication (x1.02) 0.969
Electrocardiogram abnormal (x1.02) 0.970
clonazepam (+0.00203) 0.971
Diarrhea (x1.02) 0.971
Nausea and vomiting (x1.02) 0.972
Alcohol dependence (x1.02) 0.972
Nausea (x1.02) 0.972
Human immunodeficiency virus infection (x1.02) 0.973
Neck pain (x1.02) 0.973
Urinary tract infectious disease (x1.02) 0.973
Episodic mood disorder (x1.02) 0.974
Vitamin D deficiency (x1.02) 0.974
Dysthymia (x1.02) 0.974
Gastroesophageal reflux disease without esophagitis (x1.02) 0.975
duloxetine (+0.00151) 0.975
Expressions
−1.00 −0.75 −0.50 −0.25 0.00 0.25 0.50 0.75 1.00

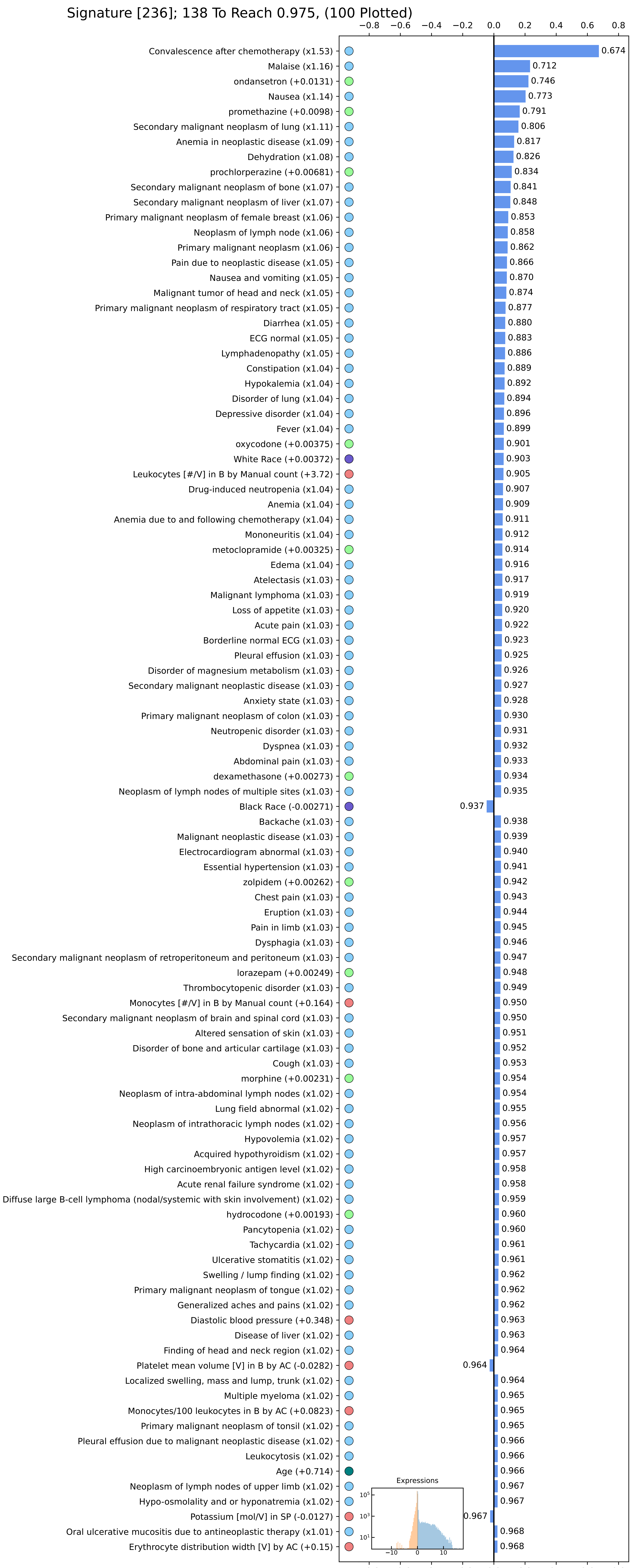

Signature [236]; 138 To Reach 0.975, (100 Plotted)
Convalescence after chemotherapy (x1.53) 0.674
Malaise (x1.16) 0.712
ondansetron (+0.0131) 0.746
Nausea (x1.14) 0.773
promethazine (+0.0098) 0.791
Secondary malignant neoplasm of lung (x1.11) 0.806
Anemia in neoplastic disease (x1.09) 0.817
Dehydration (x1.08) 0.826
prochlorperazine (+0.00681) 0.834
Secondary malignant neoplasm of bone (x1.07) 0.841
Secondary malignant neoplasm of liver (x1.07) 0.848
Primary malignant neoplasm of female breast (x1.06) 0.853
Neoplasm of lymph node (x1.06) 0.858
Primary malignant neoplasm (x1.06) 0.862
Pain due to neoplastic disease (x1.05) 0.866
Nausea and vomiting (x1.05) 0.870
Malignant tumor of head and neck (x1.05) 0.874
Primary malignant neoplasm of respiratory tract (x1.05) 0.877
Diarrhea (x1.05) 0.880
ECG normal (x1.05) 0.883
Lymphadenopathy (x1.05) 0.886
Constipation (x1.04) 0.889
Hypokalemia (x1.04) 0.892
Disorder of lung (x1.04) 0.894
Depressive disorder (x1.04) 0.896
Fever (x1.04) 0.899
oxycodone (+0.00375) 0.901
White Race (+0.00372) 0.903
Leukocytes [#/V] in B by Manual count (+3.72) 0.905
Drug-induced neutropenia (x1.04) 0.907
Anemia (x1.04) 0.909
Anemia due to and following chemotherapy (x1.04) 0.911
Mononeuritis (x1.04) 0.912
metoclopramide (+0.00325) 0.914
Edema (x1.04) 0.916
Atelectasis (x1.03) 0.917
Malignant lymphoma (x1.03) 0.919
Loss of appetite (x1.03) 0.920
Acute pain (x1.03) 0.922
Borderline normal ECG (x1.03) 0.923
Pleural effusion (x1.03) 0.925
Disorder of magnesium metabolism (x1.03) 0.926
Secondary malignant neoplastic disease (x1.03) 0.927
Anxiety state (x1.03) 0.928
Primary malignant neoplasm of colon (x1.03) 0.930
Neutropenic disorder (x1.03) 0.931
Dyspnea (x1.03) 0.932
Abdominal pain (x1.03) 0.933
dexamethasone (+0.00273) 0.934
Neoplasm of lymph nodes of multiple sites (x1.03) 0.935
Black Race (-0.00271) 0.937
Backache (x1.03) 0.938
Malignant neoplastic disease (x1.03) 0.939
Electrocardiogram abnormal (x1.03) 0.940
Essential hypertension (x1.03) 0.941
zolpidem (+0.00262) 0.942
Chest pain (x1.03) 0.943
Eruption (x1.03) 0.944
Pain in limb (x1.03) 0.945
Dysphagia (x1.03) 0.946
Secondary malignant neoplasm of retroperitoneum and peritoneum (x1.03) 0.947
lorazepam (+0.00249) 0.948
Thrombocytopenic disorder (x1.03) 0.949
Monocytes [#/V] in B by Manual count (+0.164) 0.950
Secondary malignant neoplasm of brain and spinal cord (x1.03) 0.950
Altered sensation of skin (x1.03) 0.951
Disorder of bone and articular cartilage (x1.03) 0.952
Cough (x1.03) 0.953
morphine (+0.00231) 0.954
Neoplasm of intra-abdominal lymph nodes (x1.02) 0.954
Lung field abnormal (x1.02) 0.955
Neoplasm of intrathoracic lymph nodes (x1.02) 0.956
Hypovolemia (x1.02) 0.957
Acquired hypothyroidism (x1.02) 0.957
High carcinoembryonic antigen level (x1.02) 0.958
Acute renal failure syndrome (x1.02) 0.958
Diffuse large B-cell lymphoma (nodal/systemic with skin involvement) (x1.02) 0.959
hydrocodone (+0.00193) 0.960
Pancytopenia (x1.02) 0.960
Tachycardia (x1.02) 0.961
Ulcerative stomatitis (x1.02) 0.961
Swelling / lump finding (x1.02) 0.962
Primary malignant neoplasm of tongue (x1.02) 0.962
Generalized aches and pains (x1.02) 0.962
Diastolic blood pressure (+0.348) 0.963
Disease of liver (x1.02) 0.963
Finding of head and neck region (x1.02) 0.964
Platelet mean volume [V] in B by AC (-0.0282) 0.964
Localized swelling, mass and lump, trunk (x1.02) 0.964
Multiple myeloma (x1.02) 0.965
Monocytes/100 leukocytes in B by AC (+0.0823) 0.965
Primary malignant neoplasm of tonsil (x1.02) 0.965
Pleural effusion due to malignant neoplastic disease (x1.02) 0.966
Leukocytosis (x1.02) 0.966
Age (+0.714) 0.966
Neoplasm of lymph nodes of upper limb (x1.02) 0.967
Hypo-osmolality and or hyponatremia (x1.02) 0.967
Potassium [mol/V] in SP (-0.0127) 0.967
Oral ulcerative mucositis due to antineoplastic therapy (x1.01) 0.968
Erythrocyte distribution width [V] by AC (+0.15) 0.968
−0.8 −0.6 −0.4 −0.2 0.0 0.2 0.4 0.6 0.8
Expressions
10^5 10^3 10^1
−10 0 10

Signature [237]; 3 To Reach 0.975, (10 Plotted)

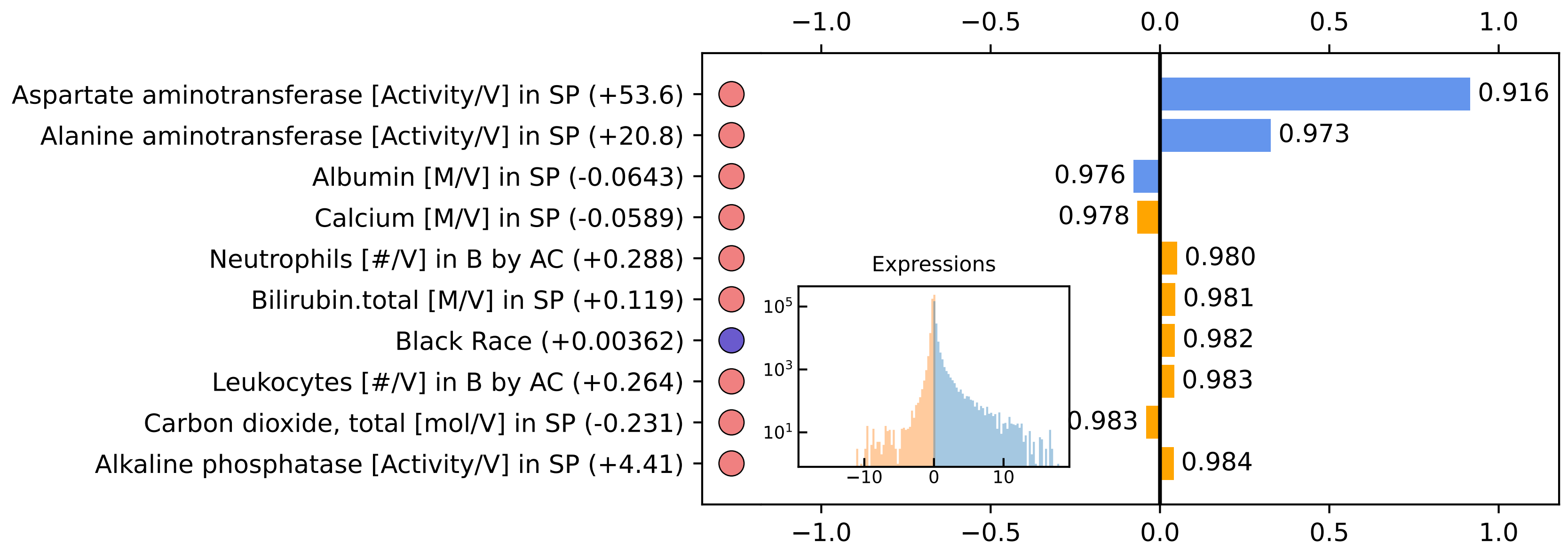

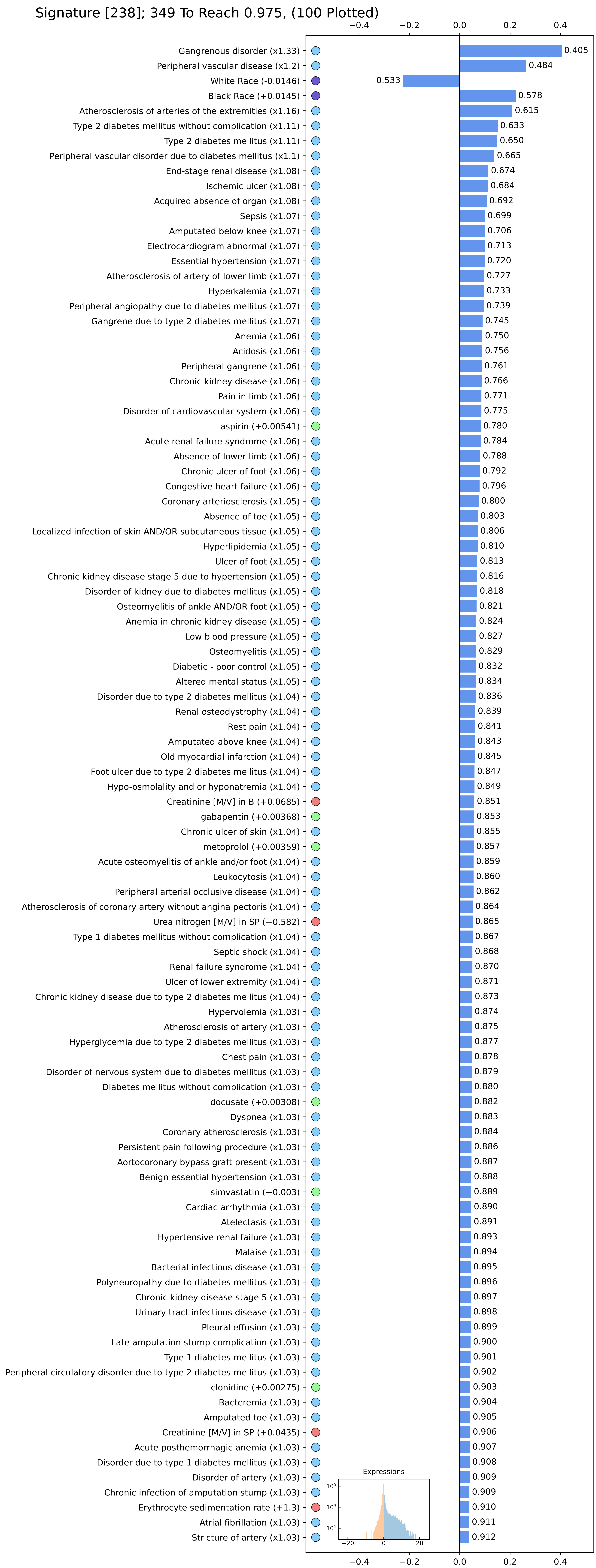

Signature [238]; 349 To Reach 0.975, (100 Plotted)
Gangrenous disorder (x1.33) 0.405
Peripheral vascular disease (x1.2) 0.484
White Race (-0.0146) 0.533
Black Race (+0.0145) 0.578
Atherosclerosis of arteries of the extremities (x1.16) 0.615
Type 2 diabetes mellitus without complication (x1.11) 0.633
Type 2 diabetes mellitus (x1.11) 0.650
Peripheral vascular disorder due to diabetes mellitus (x1.1) 0.665
End-stage renal disease (x1.08) 0.674
Ischemic ulcer (x1.08) 0.684
Acquired absence of organ (x1.08) 0.692
Sepsis (x1.07) 0.699
Amputated below knee (x1.07) 0.706
Electrocardiogram abnormal (x1.07) 0.713
Essential hypertension (x1.07) 0.720
Atherosclerosis of artery of lower limb (x1.07) 0.727
Hyperkalemia (x1.07) 0.733
Peripheral angiopathy due to diabetes mellitus (x1.07) 0.739
Gangrene due to type 2 diabetes mellitus (x1.07) 0.745
Anemia (x1.06) 0.750
Acidosis (x1.06) 0.756
Peripheral gangrene (x1.06) 0.761
Chronic kidney disease (x1.06) 0.766
Pain in limb (x1.06) 0.771
Disorder of cardiovascular system (x1.06) 0.775
aspirin (+0.00541) 0.780
Acute renal failure syndrome (x1.06) 0.784
Absence of lower limb (x1.06) 0.788
Chronic ulcer of foot (x1.06) 0.792
Congestive heart failure (x1.06) 0.796
Coronary arteriosclerosis (x1.05) 0.800
Absence of toe (x1.05) 0.803
Localized infection of skin AND/OR subcutaneous tissue (x1.05) 0.806
Hyperlipidemia (x1.05) 0.810
Ulcer of foot (x1.05) 0.813
Chronic kidney disease stage 5 due to hypertension (x1.05) 0.816
Disorder of kidney due to diabetes mellitus (x1.05) 0.818
Osteomyelitis of ankle AND/OR foot (x1.05) 0.821
Anemia in chronic kidney disease (x1.05) 0.824
Low blood pressure (x1.05) 0.827
Osteomyelitis (x1.05) 0.829
Diabetic - poor control (x1.05) 0.832
Altered mental status (x1.05) 0.834
Disorder due to type 2 diabetes mellitus (x1.04) 0.836
Renal osteodystrophy (x1.04) 0.839
Rest pain (x1.04) 0.841
Amputated above knee (x1.04) 0.843
Old myocardial infarction (x1.04) 0.845
Foot ulcer due to type 2 diabetes mellitus (x1.04) 0.847
Hypo-osmolality and or hyponatremia (x1.04) 0.849
Creatinine [M/V] in B (+0.0685) 0.851
gabapentin (+0.00368) 0.853
Chronic ulcer of skin (x1.04) 0.855
metoprolol (+0.00359) 0.857
Acute osteomyelitis of ankle and/or foot (x1.04) 0.859
Leukocytosis (x1.04) 0.860
Peripheral arterial occlusive disease (x1.04) 0.862
Atherosclerosis of coronary artery without angina pectoris (x1.04) 0.864
Urea nitrogen [M/V] in SP (+0.582) 0.865
Type 1 diabetes mellitus without complication (x1.04) 0.867
Septic shock (x1.04) 0.868
Renal failure syndrome (x1.04) 0.870
Ulcer of lower extremity (x1.04) 0.871
Chronic kidney disease due to type 2 diabetes mellitus (x1.04) 0.873
Hypervolemia (x1.03) 0.874
Atherosclerosis of artery (x1.03) 0.875
Hyperglycemia due to type 2 diabetes mellitus (x1.03) 0.877
Chest pain (x1.03) 0.878
Disorder of nervous system due to diabetes mellitus (x1.03) 0.879
Diabetes mellitus without complication (x1.03) 0.880
docusate (+0.00308) 0.882
Dyspnea (x1.03) 0.883
Coronary atherosclerosis (x1.03) 0.884
Persistent pain following procedure (x1.03) 0.886
Aortocoronary bypass graft present (x1.03) 0.887
Benign essential hypertension (x1.03) 0.888
simvastatin (+0.003) 0.889
Cardiac arrhythmia (x1.03) 0.890
Atelectasis (x1.03) 0.891
Hypertensive renal failure (x1.03) 0.893
Malaise (x1.03) 0.894
Bacterial infectious disease (x1.03) 0.895
Polyneuropathy due to diabetes mellitus (x1.03) 0.896
Chronic kidney disease stage 5 (x1.03) 0.897
Urinary tract infectious disease (x1.03) 0.898
Pleural effusion (x1.03) 0.899
Late amputation stump complication (x1.03) 0.900
Type 1 diabetes mellitus (x1.03) 0.901
Peripheral circulatory disorder due to type 2 diabetes mellitus (x1.03) 0.902
clonidine (+0.00275) 0.903
Bacteremia (x1.03) 0.904
Amputated toe (x1.03) 0.905
Creatinine [M/V] in SP (+0.0435) 0.906
Acute posthemorrhagic anemia (x1.03) 0.907
Disorder due to type 1 diabetes mellitus (x1.03) 0.908
Disorder of artery (x1.03) 0.909
Chronic infection of amputation stump (x1.03) 0.909
Erythrocyte sedimentation rate (+1.3) 0.910
Atrial fibrillation (x1.03) 0.911
Stricture of artery (x1.03) 0.912
Expressions
−0.4 −0.2 0.0 0.2 0.4

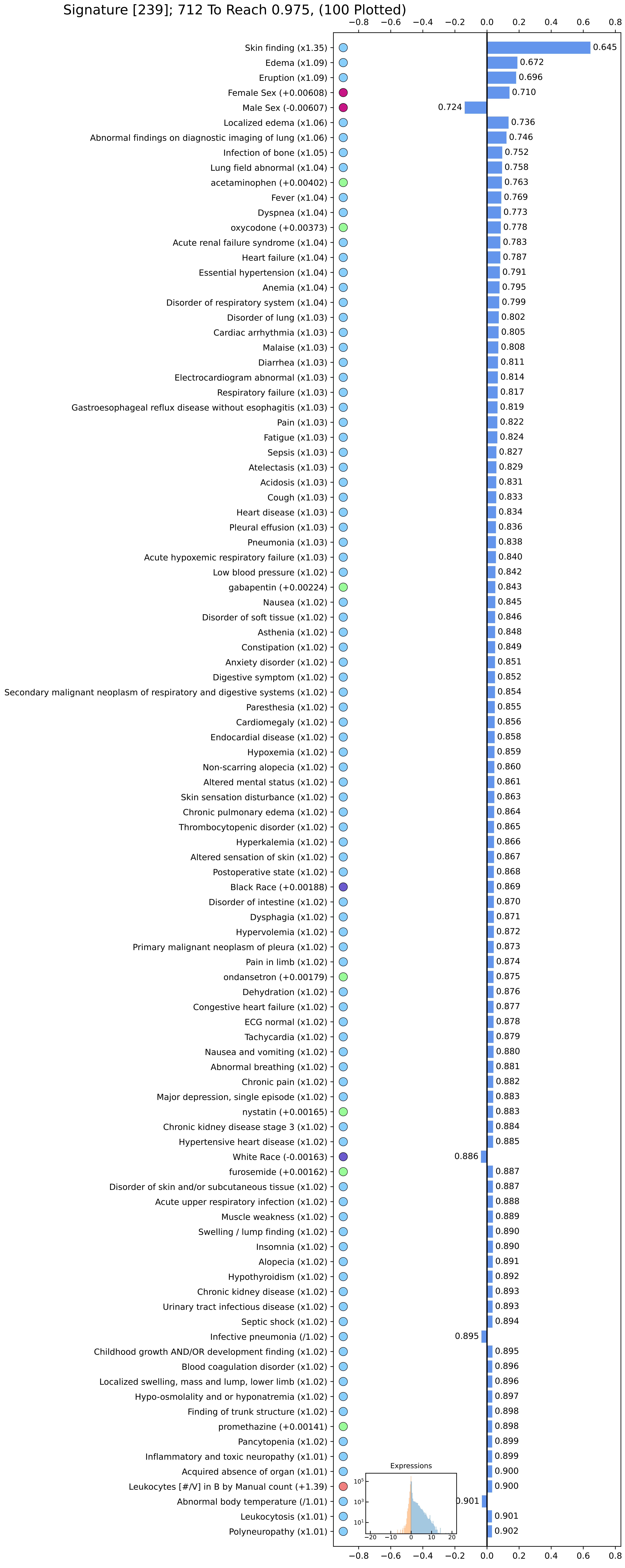

Signature [239]; 712 To Reach 0.975, (100 Plotted)
−0.8 −0.6 −0.4 −0.2 0.0 0.2 0.4 0.6 0.8
Skin finding (x1.35) 0.645
Edema (x1.09) 0.672
Eruption (x1.09) 0.696
Female Sex (+0.00608) 0.710
Male Sex (-0.00607) 0.724
Localized edema (x1.06) 0.736
Abnormal findings on diagnostic imaging of lung (x1.06) 0.746
Infection of bone (x1.05) 0.752
Lung field abnormal (x1.04) 0.758
acetaminophen (+0.00402) 0.763
Fever (x1.04) 0.769
Dyspnea (x1.04) 0.773
oxycodone (+0.00373) 0.778
Acute renal failure syndrome (x1.04) 0.783
Heart failure (x1.04) 0.787
Essential hypertension (x1.04) 0.791
Anemia (x1.04) 0.795
Disorder of respiratory system (x1.04) 0.799
Disorder of lung (x1.03) 0.802
Cardiac arrhythmia (x1.03) 0.805
Malaise (x1.03) 0.808
Diarrhea (x1.03) 0.811
Electrocardiogram abnormal (x1.03) 0.814
Respiratory failure (x1.03) 0.817
Gastroesophageal reflux disease without esophagitis (x1.03) 0.819
Pain (x1.03) 0.822
Fatigue (x1.03) 0.824
Sepsis (x1.03) 0.827
Atelectasis (x1.03) 0.829
Acidosis (x1.03) 0.831
Cough (x1.03) 0.833
Heart disease (x1.03) 0.834
Pleural effusion (x1.03) 0.836
Pneumonia (x1.03) 0.838
Acute hypoxemic respiratory failure (x1.03) 0.840
Low blood pressure (x1.02) 0.842
gabapentin (+0.00224) 0.843
Nausea (x1.02) 0.845
Disorder of soft tissue (x1.02) 0.846
Asthenia (x1.02) 0.848
Constipation (x1.02) 0.849
Anxiety disorder (x1.02) 0.851
Digestive symptom (x1.02) 0.852
Secondary malignant neoplasm of respiratory and digestive systems (x1.02) 0.854
Paresthesia (x1.02) 0.855
Cardiomegaly (x1.02) 0.856
Endocardial disease (x1.02) 0.858
Hypoxemia (x1.02) 0.859
Non-scarring alopecia (x1.02) 0.860
Altered mental status (x1.02) 0.861
Skin sensation disturbance (x1.02) 0.863
Chronic pulmonary edema (x1.02) 0.864
Thrombocytopenic disorder (x1.02) 0.865
Hyperkalemia (x1.02) 0.866
Altered sensation of skin (x1.02) 0.867
Postoperative state (x1.02) 0.868
Black Race (+0.00188) 0.869
Disorder of intestine (x1.02) 0.870
Dysphagia (x1.02) 0.871
Hypervolemia (x1.02) 0.872
Primary malignant neoplasm of pleura (x1.02) 0.873
Pain in limb (x1.02) 0.874
ondansetron (+0.00179) 0.875
Dehydration (x1.02) 0.876
Congestive heart failure (x1.02) 0.877
ECG normal (x1.02) 0.878
Tachycardia (x1.02) 0.879
Nausea and vomiting (x1.02) 0.880
Abnormal breathing (x1.02) 0.881
Chronic pain (x1.02) 0.882
Major depression, single episode (x1.02) 0.883
nystatin (+0.00165) 0.883
Chronic kidney disease stage 3 (x1.02) 0.884
Hypertensive heart disease (x1.02) 0.885
White Race (-0.00163) 0.886
furosemide (+0.00162) 0.887
Disorder of skin and/or subcutaneous tissue (x1.02) 0.887
Acute upper respiratory infection (x1.02) 0.888
Muscle weakness (x1.02) 0.889
Swelling / lump finding (x1.02) 0.890
Insomnia (x1.02) 0.890
Alopecia (x1.02) 0.891
Hypothyroidism (x1.02) 0.892
Chronic kidney disease (x1.02) 0.893
Urinary tract infectious disease (x1.02) 0.893
Septic shock (x1.02) 0.894
Infective pneumonia (/1.02) 0.895
Childhood growth AND/OR development finding (x1.02) 0.895
Blood coagulation disorder (x1.02) 0.896
Localized swelling, mass and lump, lower limb (x1.02) 0.896
Hypo-osmolality and or hyponatremia (x1.02) 0.897
Finding of trunk structure (x1.02) 0.898
promethazine (+0.00141) 0.898
Pancytopenia (x1.02) 0.899
Inflammatory and toxic neuropathy (x1.01) 0.899
Acquired absence of organ (x1.01) 0.900
Leukocytes [#/V] in B by Manual count (+1.39) 0.900
Abnormal body temperature (/1.01) 0.901
Leukocytosis (x1.01) 0.901
Polyneuropathy (x1.01) 0.902
Expressions
10^5 10^3 10^1
−20 −10 0 10 20

Signature [240]; 3 To Reach 0.975, (10 Plotted)

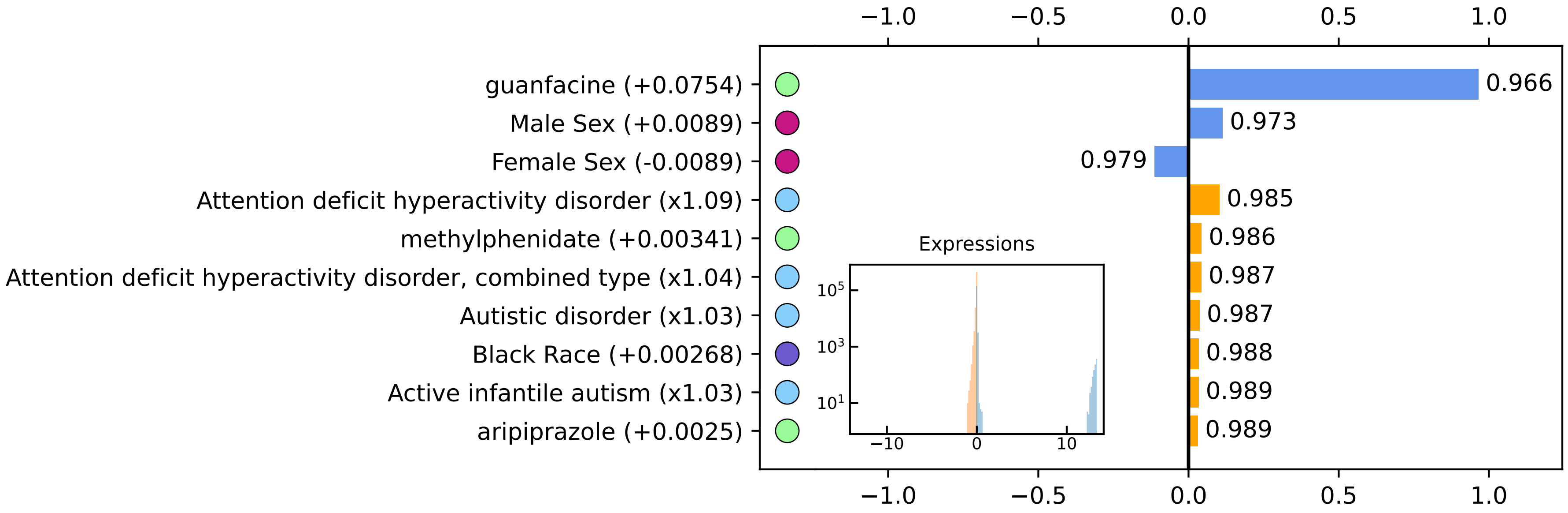

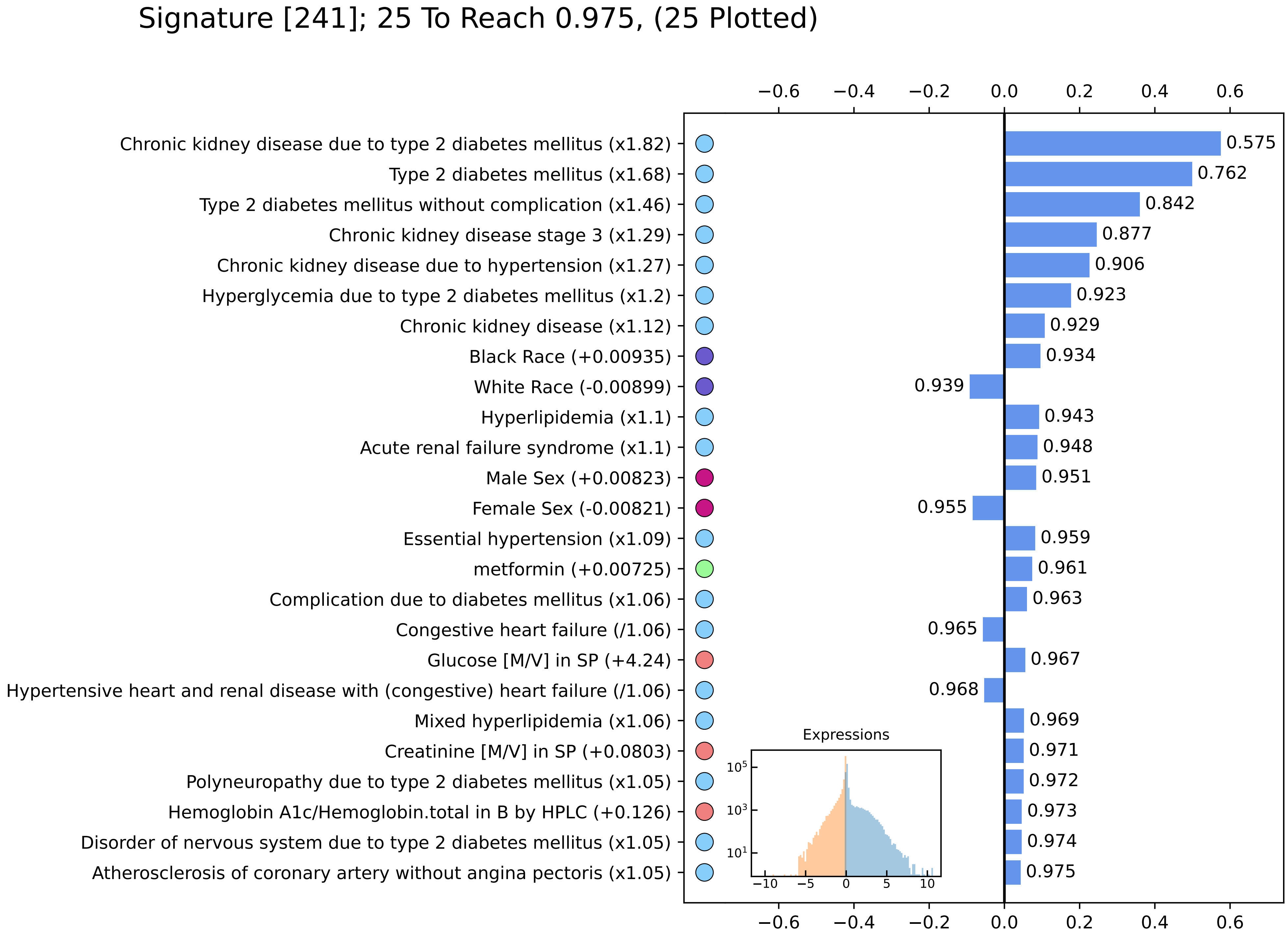

Signature [241]; 25 To Reach 0.975, (25 Plotted)
Chronic kidney disease due to type 2 diabetes mellitus (x1.82)
Type 2 diabetes mellitus (x1.68)
Type 2 diabetes mellitus without complication (x1.46)
Chronic kidney disease stage 3 (x1.29)
Chronic kidney disease due to hypertension (x1.27)
Hyperglycemia due to type 2 diabetes mellitus (x1.2)
Chronic kidney disease (x1.12)
Black Race (+0.00935)
White Race (-0.00899)
Hyperlipidemia (x1.1)
Acute renal failure syndrome (x1.1)
Male Sex (+0.00823)
Female Sex (-0.00821)
Essential hypertension (x1.09)
metformin (+0.00725)
Complication due to diabetes mellitus (x1.06)
Congestive heart failure (/1.06)
Glucose [M/V] in SP (+4.24)
Hypertensive heart and renal disease with (congestive) heart failure (/1.06)
Mixed hyperlipidemia (x1.06)
Creatinine [M/V] in SP (+0.0803)
Polyneuropathy due to type 2 diabetes mellitus (x1.05)
Hemoglobin A1c/Hemoglobin.total in B by HPLC (+0.126)
Disorder of nervous system due to type 2 diabetes mellitus (x1.05)
Atherosclerosis of coronary artery without angina pectoris (x1.05)
0.575
0.762
0.842
0.877
0.906
0.923
0.929
0.934
0.939
0.943
0.948
0.951
0.955
0.959
0.961
0.963
0.965
0.967
0.968
0.969
0.971
0.972
0.973
0.974
0.975
−0.6
−0.4
−0.2
0.0
0.2
0.4
0.6
Expressions
10^5
10^3
10^1
−10
−5
0
5
10

Signature [242]; 1 To Reach 0.975, (10 Plotted)

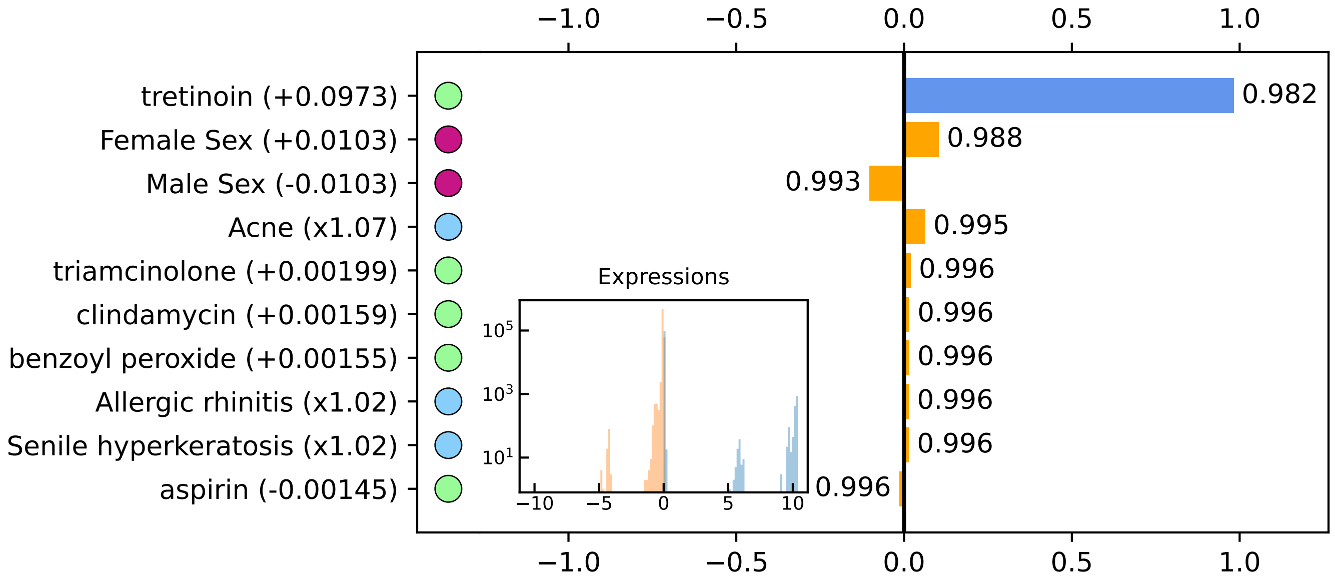

Signature [243]; 1 To Reach 0.975, (10 Plotted)

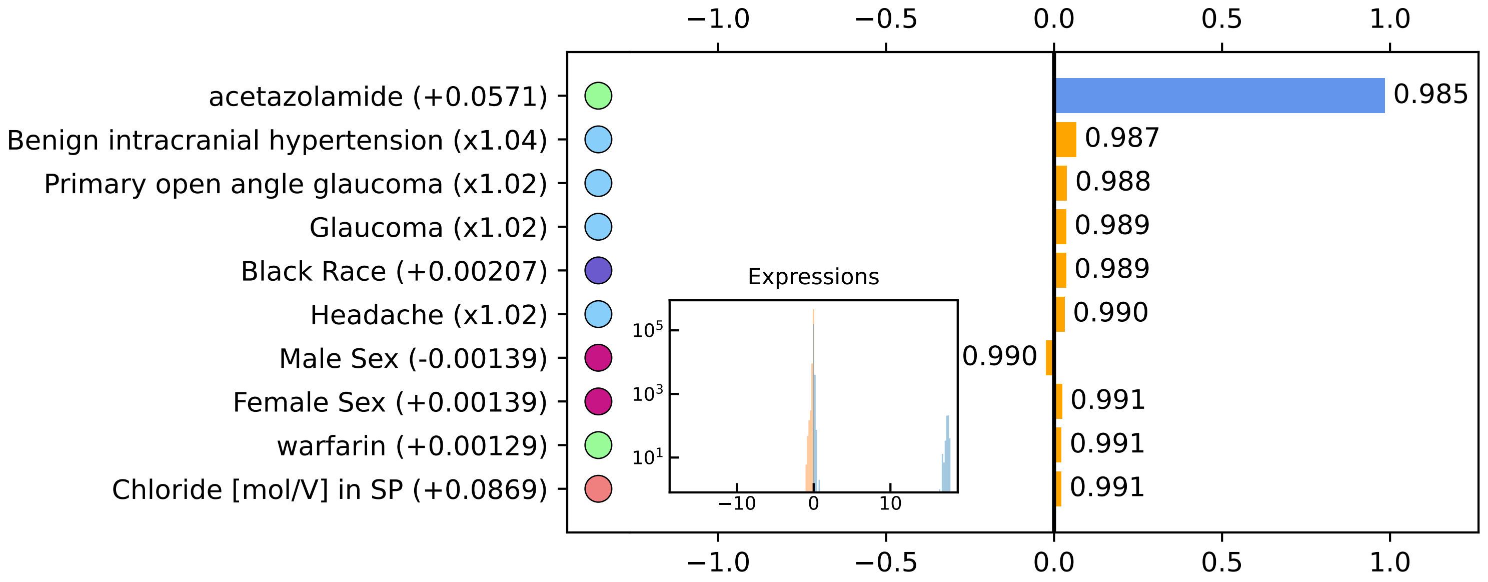

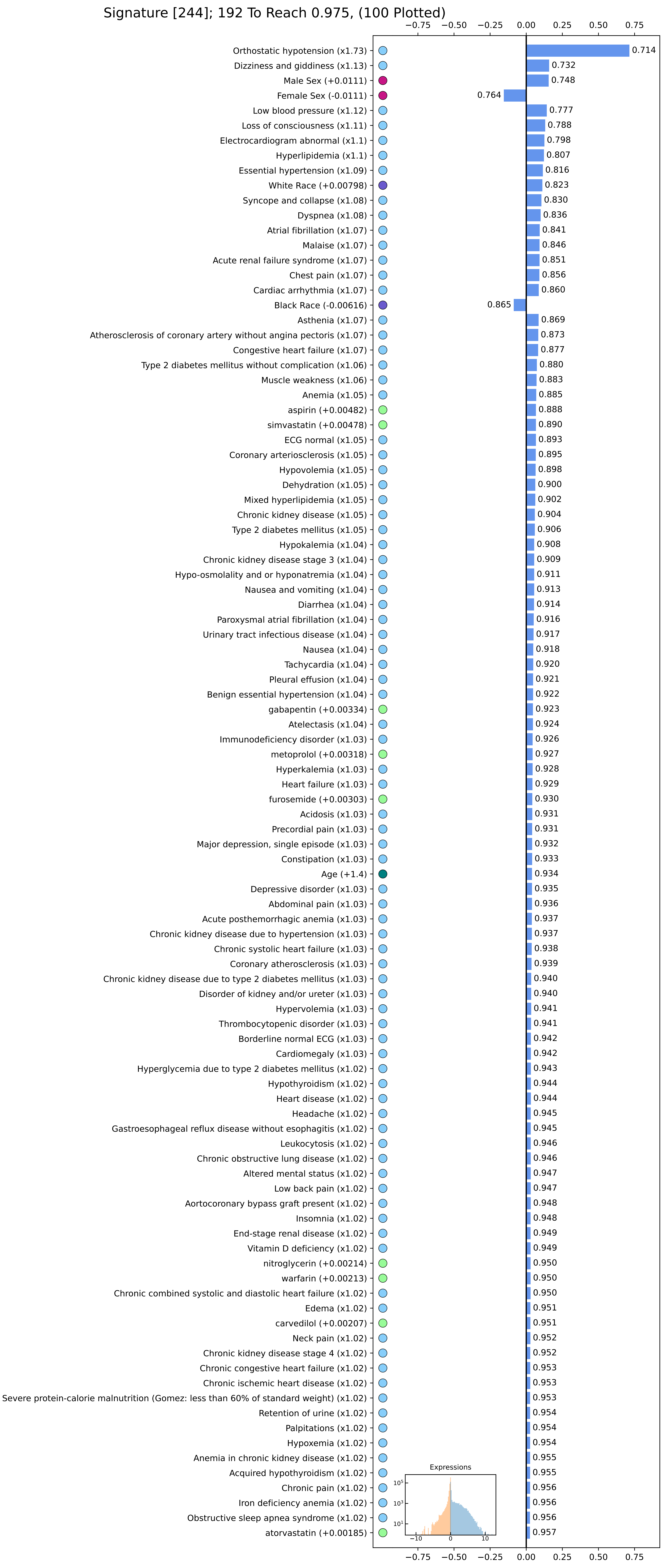

Signature [244]; 192 To Reach 0.975, (100 Plotted)
Orthostatic hypotension (x1.73) 0.714
Dizziness and giddiness (x1.13) 0.732
Male Sex (+0.0111) 0.748
Female Sex (-0.0111) 0.764
Low blood pressure (x1.12) 0.777
Loss of consciousness (x1.11) 0.788
Electrocardiogram abnormal (x1.1) 0.798
Hyperlipidemia (x1.1) 0.807
Essential hypertension (x1.09) 0.816
White Race (+0.00798) 0.823
Syncope and collapse (x1.08) 0.830
Dyspnea (x1.08) 0.836
Atrial fibrillation (x1.07) 0.841
Malaise (x1.07) 0.846
Acute renal failure syndrome (x1.07) 0.851
Chest pain (x1.07) 0.856
Cardiac arrhythmia (x1.07) 0.860
Black Race (-0.00616) 0.865
Asthenia (x1.07) 0.869
Atherosclerosis of coronary artery without angina pectoris (x1.07) 0.873
Congestive heart failure (x1.07) 0.877
Type 2 diabetes mellitus without complication (x1.06) 0.880
Muscle weakness (x1.06) 0.883
Anemia (x1.05) 0.885
aspirin (+0.00482) 0.888
simvastatin (+0.00478) 0.890
ECG normal (x1.05) 0.893
Coronary arteriosclerosis (x1.05) 0.895
Hypovolemia (x1.05) 0.898
Dehydration (x1.05) 0.900
Mixed hyperlipidemia (x1.05) 0.902
Chronic kidney disease (x1.05) 0.904
Type 2 diabetes mellitus (x1.05) 0.906
Hypokalemia (x1.04) 0.908
Chronic kidney disease stage 3 (x1.04) 0.909
Hypo-osmolality and or hyponatremia (x1.04) 0.911
Nausea and vomiting (x1.04) 0.913
Diarrhea (x1.04) 0.914
Paroxysmal atrial fibrillation (x1.04) 0.916
Urinary tract infectious disease (x1.04) 0.917
Nausea (x1.04) 0.918
Tachycardia (x1.04) 0.920
Pleural effusion (x1.04) 0.921
Benign essential hypertension (x1.04) 0.922
gabapentin (+0.00334) 0.923
Atelectasis (x1.04) 0.924
Immunodeficiency disorder (x1.03) 0.926
metoprolol (+0.00318) 0.927
Hyperkalemia (x1.03) 0.928
Heart failure (x1.03) 0.929
furosemide (+0.00303) 0.930
Acidosis (x1.03) 0.931
Precordial pain (x1.03) 0.931
Major depression, single episode (x1.03) 0.932
Constipation (x1.03) 0.933
Age (+1.4) 0.934
Depressive disorder (x1.03) 0.935
Abdominal pain (x1.03) 0.936
Acute posthemorrhagic anemia (x1.03) 0.937
Chronic kidney disease due to hypertension (x1.03) 0.937
Chronic systolic heart failure (x1.03) 0.938
Coronary atherosclerosis (x1.03) 0.939
Chronic kidney disease due to type 2 diabetes mellitus (x1.03) 0.940
Disorder of kidney and/or ureter (x1.03) 0.940
Hypervolemia (x1.03) 0.941
Thrombocytopenic disorder (x1.03) 0.941
Borderline normal ECG (x1.03) 0.942
Cardiomegaly (x1.03) 0.942
Hyperglycemia due to type 2 diabetes mellitus (x1.02) 0.943
Hypothyroidism (x1.02) 0.944
Heart disease (x1.02) 0.944
Headache (x1.02) 0.945
Gastroesophageal reflux disease without esophagitis (x1.02) 0.945
Leukocytosis (x1.02) 0.946
Chronic obstructive lung disease (x1.02) 0.946
Altered mental status (x1.02) 0.947
Low back pain (x1.02) 0.947
Aortocoronary bypass graft present (x1.02) 0.948
Insomnia (x1.02) 0.948
End-stage renal disease (x1.02) 0.949
Vitamin D deficiency (x1.02) 0.949
nitroglycerin (+0.00214) 0.950
warfarin (+0.00213) 0.950
Chronic combined systolic and diastolic heart failure (x1.02) 0.950
Edema (x1.02) 0.951
carvedilol (+0.00207) 0.951
Neck pain (x1.02) 0.952
Chronic kidney disease stage 4 (x1.02) 0.952
Chronic congestive heart failure (x1.02) 0.953
Chronic ischemic heart disease (x1.02) 0.953
Severe protein-calorie malnutrition (Gomez: less than 60% of standard weight) (x1.02) 0.953
Retention of urine (x1.02) 0.954
Palpitations (x1.02) 0.954
Hypoxemia (x1.02) 0.954
Anemia in chronic kidney disease (x1.02) 0.955
Acquired hypothyroidism (x1.02) 0.955
Chronic pain (x1.02) 0.956
Iron deficiency anemia (x1.02) 0.956
Obstructive sleep apnea syndrome (x1.02) 0.956
atorvastatin (+0.00185) 0.957
−0.75 −0.50 −0.25 0.00 0.25 0.50 0.75
Expressions

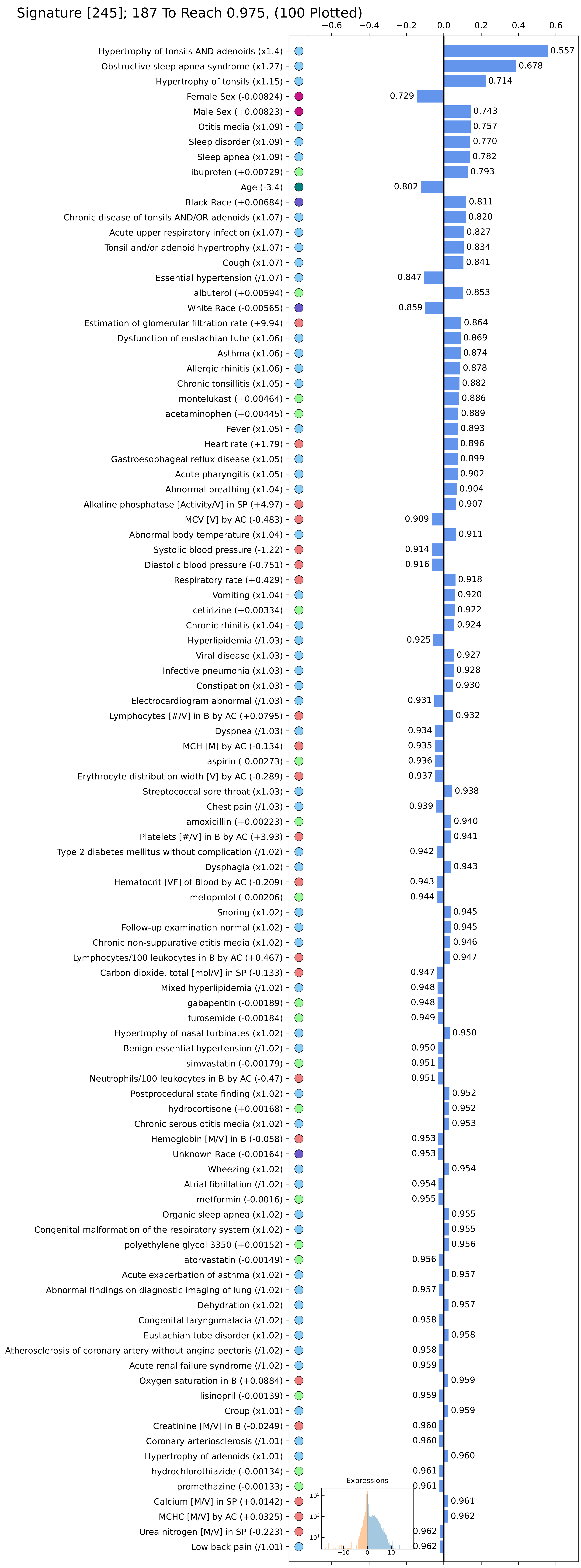

Signature [245]; 187 To Reach 0.975, (100 Plotted)
Hypertrophy of tonsils AND adenoids (x1.4)
Obstructive sleep apnea syndrome (x1.27)
Hypertrophy of tonsils (x1.15)
Female Sex (-0.00824)
Male Sex (+0.00823)
Otitis media (x1.09)
Sleep disorder (x1.09)
Sleep apnea (x1.09)
ibuprofen (+0.00729)
Age (-3.4)
Black Race (+0.00684)
Chronic disease of tonsils AND/OR adenoids (x1.07)
Acute upper respiratory infection (x1.07)
Tonsil and/or adenoid hypertrophy (x1.07)
Cough (x1.07)
Essential hypertension (/1.07)
albuterol (+0.00594)
White Race (-0.00565)
Estimation of glomerular filtration rate (+9.94)
Dysfunction of eustachian tube (x1.06)
Asthma (x1.06)
Allergic rhinitis (x1.06)
Chronic tonsillitis (x1.05)
montelukast (+0.00464)
acetaminophen (+0.00445)
Fever (x1.05)
Heart rate (+1.79)
Gastroesophageal reflux disease (x1.05)
Acute pharyngitis (x1.05)
Abnormal breathing (x1.04)
Alkaline phosphatase [Activity/V] in SP (+4.97)
MCV [V] by AC (-0.483)
Abnormal body temperature (x1.04)
Systolic blood pressure (-1.22)
Diastolic blood pressure (-0.751)
Respiratory rate (+0.429)
Vomiting (x1.04)
cetirizine (+0.00334)
Chronic rhinitis (x1.04)
Hyperlipidemia (/1.03)
Viral disease (x1.03)
Infective pneumonia (x1.03)
Constipation (x1.03)
Electrocardiogram abnormal (/1.03)
Lymphocytes [#/V] in B by AC (+0.0795)
Dyspnea (/1.03)
MCH [M] by AC (-0.134)
aspirin (-0.00273)
Erythrocyte distribution width [V] by AC (-0.289)
Streptococcal sore throat (x1.03)
Chest pain (/1.03)
amoxicillin (+0.00223)
Platelets [#/V] in B by AC (+3.93)
Type 2 diabetes mellitus without complication (/1.02)
Dysphagia (x1.02)
Hematocrit [VF] of Blood by AC (-0.209)
metoprolol (-0.00206)
Snoring (x1.02)
Follow-up examination normal (x1.02)
Chronic non-suppurative otitis media (x1.02)
Lymphocytes/100 leukocytes in B by AC (+0.467)
Carbon dioxide, total [mol/V] in SP (-0.133)
Mixed hyperlipidemia (/1.02)
gabapentin (-0.00189)
furosemide (-0.00184)
Hypertrophy of nasal turbinates (x1.02)
Benign essential hypertension (/1.02)
simvastatin (-0.00179)
Neutrophils/100 leukocytes in B by AC (-0.47)
Postprocedural state finding (x1.02)
hydrocortisone (+0.00168)
Chronic serous otitis media (x1.02)
Hemoglobin [M/V] in B (-0.058)
Unknown Race (-0.00164)
Wheezing (x1.02)
Atrial fibrillation (/1.02)
metformin (-0.0016)
Organic sleep apnea (x1.02)
Congenital malformation of the respiratory system (x1.02)
polyethylene glycol 3350 (+0.00152)
atorvastatin (-0.00149)
Acute exacerbation of asthma (x1.02)
Abnormal findings on diagnostic imaging of lung (/1.02)
Dehydration (x1.02)
Congenital laryngomalacia (/1.02)
Eustachian tube disorder (x1.02)
Atherosclerosis of coronary artery without angina pectoris (/1.02)
Acute renal failure syndrome (/1.02)
Oxygen saturation in B (+0.0884)
lisinopril (-0.00139)
Croup (x1.01)
Creatinine [M/V] in B (-0.0249)
Coronary arteriosclerosis (/1.01)
Hypertrophy of adenoids (x1.01)
hydrochlorothiazide (-0.00134)
promethazine (-0.00133)
Calcium [M/V] in SP (+0.0142)
MCHC [M/V] by AC (+0.0325)
Urea nitrogen [M/V] in SP (-0.223)
Low back pain (/1.01)
0.557
0.678
0.714
0.729
0.743
0.757
0.770
0.782
0.793
0.802
0.811
0.820
0.827
0.834
0.841
0.847
0.853
0.859
0.864
0.869
0.874
0.878
0.882
0.886
0.889
0.893
0.896
0.899
0.902
0.904
0.907
0.909
0.911
0.914
0.916
0.918
0.920
0.922
0.924
0.925
0.927
0.928
0.930
0.931
0.932
0.934
0.935
0.936
0.937
0.938
0.939
0.940
0.941
0.942
0.943
0.943
0.944
0.945
0.945
0.946
0.947
0.947
0.948
0.948
0.949
0.950
0.950
0.951
0.951
0.952
0.952
0.953
0.953
0.953
0.954
0.954
0.955
0.955
0.955
0.956
0.956
0.957
0.957
0.957
0.958
0.958
0.958
0.959
0.959
0.959
0.959
0.960
0.960
0.960
0.961
0.961
0.961
0.962
0.962
0.962
−0.6
−0.4
−0.2
0.0
0.2
0.4
0.6
Expressions
10^5
10^3
10^1
−10
0
10

Signature [246]; 3 To Reach 0.975, (10 Plotted)

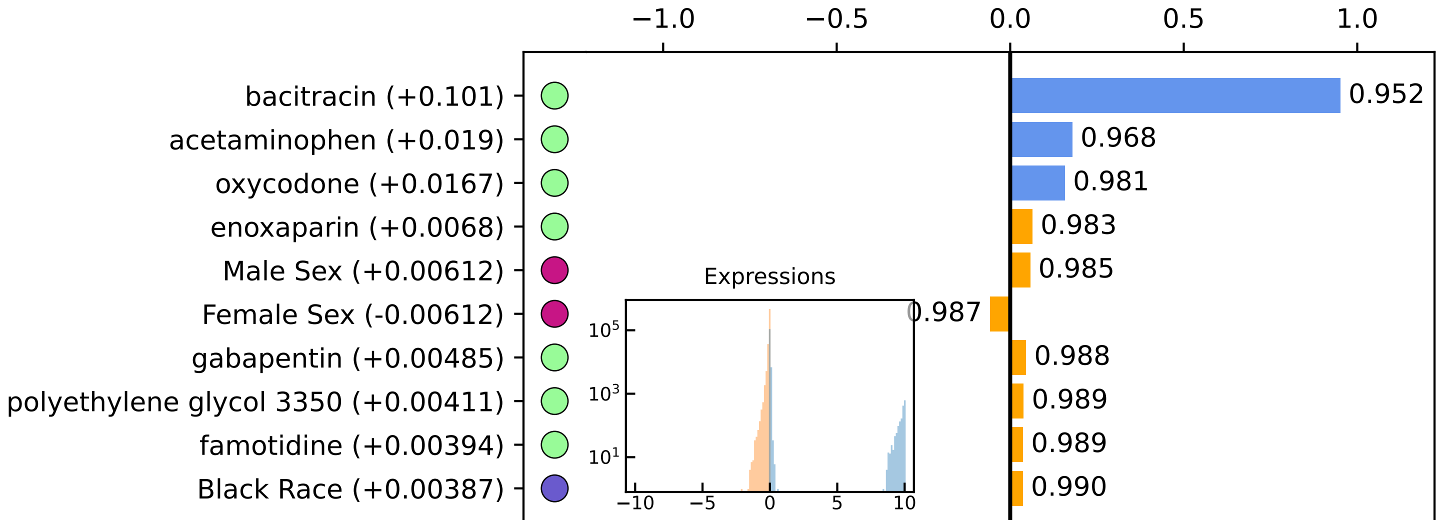

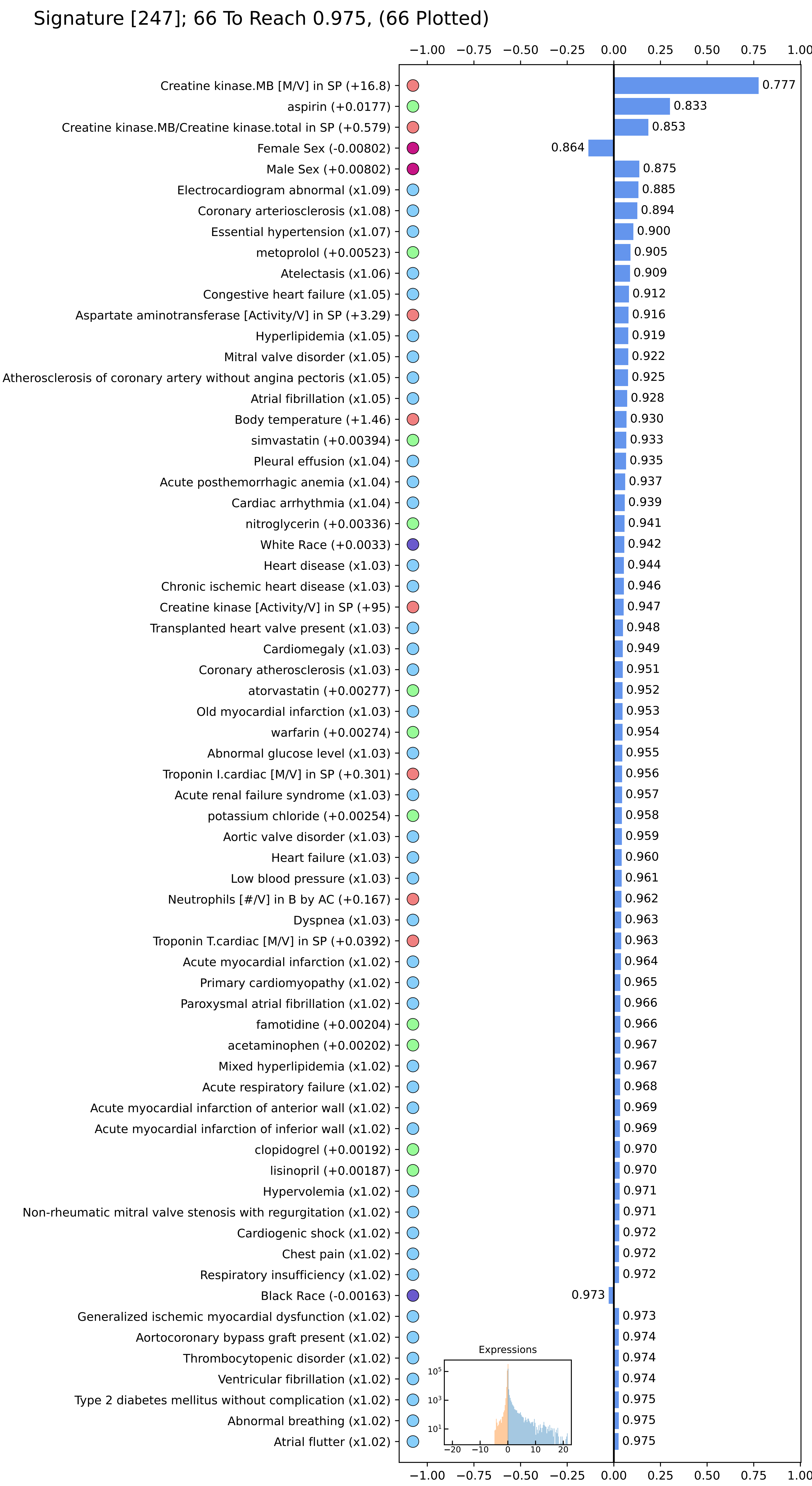

Signature [247]; 66 To Reach 0.975, (66 Plotted)
Creatine kinase.MB [M/V] in SP (+16.8) 0.777
aspirin (+0.0177) 0.833
Creatine kinase.MB/Creatine kinase.total in SP (+0.579) 0.853
Female Sex (-0.00802) 0.864
Male Sex (+0.00802) 0.875
Electrocardiogram abnormal (x1.09) 0.885
Coronary arteriosclerosis (x1.08) 0.894
Essential hypertension (x1.07) 0.900
metoprolol (+0.00523) 0.905
Atelectasis (x1.06) 0.909
Congestive heart failure (x1.05) 0.912
Aspartate aminotransferase [Activity/V] in SP (+3.29) 0.916
Hyperlipidemia (x1.05) 0.919
Mitral valve disorder (x1.05) 0.922
Atherosclerosis of coronary artery without angina pectoris (x1.05) 0.925
Atrial fibrillation (x1.05) 0.928
Body temperature (+1.46) 0.930
simvastatin (+0.00394) 0.933
Pleural effusion (x1.04) 0.935
Acute posthemorrhagic anemia (x1.04) 0.937
Cardiac arrhythmia (x1.04) 0.939
nitroglycerin (+0.00336) 0.941
White Race (+0.0033) 0.942
Heart disease (x1.03) 0.944
Chronic ischemic heart disease (x1.03) 0.946
Creatine kinase [Activity/V] in SP (+95) 0.947
Transplanted heart valve present (x1.03) 0.948
Cardiomegaly (x1.03) 0.949
Coronary atherosclerosis (x1.03) 0.951
atorvastatin (+0.00277) 0.952
Old myocardial infarction (x1.03) 0.953
warfarin (+0.00274) 0.954
Abnormal glucose level (x1.03) 0.955
Troponin I.cardiac [M/V] in SP (+0.301) 0.956
Acute renal failure syndrome (x1.03) 0.957
potassium chloride (+0.00254) 0.958
Aortic valve disorder (x1.03) 0.959
Heart failure (x1.03) 0.960
Low blood pressure (x1.03) 0.961
Neutrophils [#/V] in B by AC (+0.167) 0.962
Dyspnea (x1.03) 0.963
Troponin T.cardiac [M/V] in SP (+0.0392) 0.963
Acute myocardial infarction (x1.02) 0.964
Primary cardiomyopathy (x1.02) 0.965
Paroxysmal atrial fibrillation (x1.02) 0.966
famotidine (+0.00204) 0.966
acetaminophen (+0.00202) 0.967
Mixed hyperlipidemia (x1.02) 0.967
Acute respiratory failure (x1.02) 0.968
Acute myocardial infarction of anterior wall (x1.02) 0.969
Acute myocardial infarction of inferior wall (x1.02) 0.969
clopidogrel (+0.00192) 0.970
lisinopril (+0.00187) 0.970
Hypervolemia (x1.02) 0.971
Non-rheumatic mitral valve stenosis with regurgitation (x1.02) 0.971
Cardiogenic shock (x1.02) 0.972
Chest pain (x1.02) 0.972
Respiratory insufficiency (x1.02) 0.972
Black Race (-0.00163) 0.973
Generalized ischemic myocardial dysfunction (x1.02) 0.973
Aortocoronary bypass graft present (x1.02) 0.974
Thrombocytopenic disorder (x1.02) 0.974
Ventricular fibrillation (x1.02) 0.974
Type 2 diabetes mellitus without complication (x1.02) 0.975
Abnormal breathing (x1.02) 0.975
Atrial flutter (x1.02) 0.975
−1.00 −0.75 −0.50 −0.25 0.00 0.25 0.50 0.75 1.00
Expressions
10^5
10^3
10^1
−20 −10 0 10 20

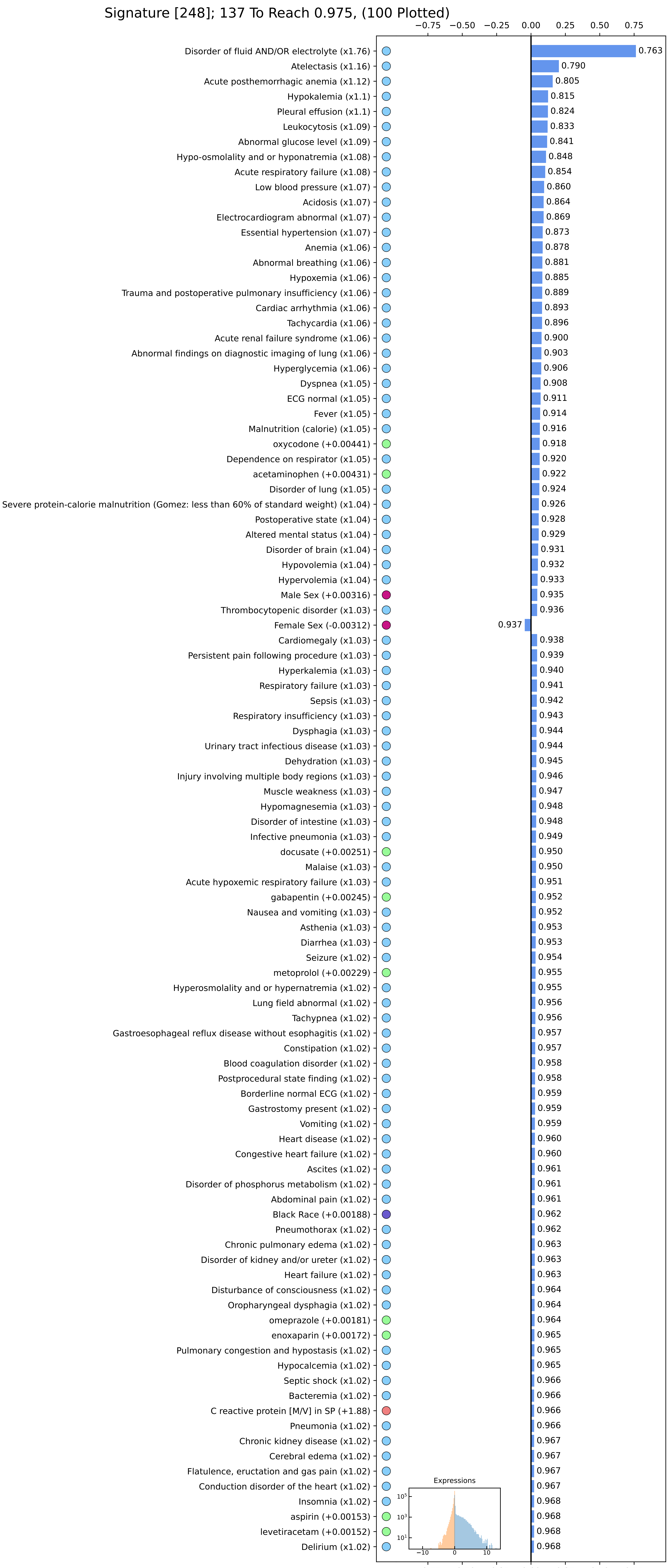

Signature [248]; 137 To Reach 0.975, (100 Plotted)
−0.75 −0.50 −0.25 0.00 0.25 0.50 0.75
Disorder of fluid AND/OR electrolyte (x1.76) 0.763
Atelectasis (x1.16) 0.790
Acute posthemorrhagic anemia (x1.12) 0.805
Hypokalemia (x1.1) 0.815
Pleural effusion (x1.1) 0.824
Leukocytosis (x1.09) 0.833
Abnormal glucose level (x1.09) 0.841
Hypo-osmolality and or hyponatremia (x1.08) 0.848
Acute respiratory failure (x1.08) 0.854
Low blood pressure (x1.07) 0.860
Acidosis (x1.07) 0.864
Electrocardiogram abnormal (x1.07) 0.869
Essential hypertension (x1.07) 0.873
Anemia (x1.06) 0.878
Abnormal breathing (x1.06) 0.881
Hypoxemia (x1.06) 0.885
Trauma and postoperative pulmonary insufficiency (x1.06) 0.889
Cardiac arrhythmia (x1.06) 0.893
Tachycardia (x1.06) 0.896
Acute renal failure syndrome (x1.06) 0.900
Abnormal findings on diagnostic imaging of lung (x1.06) 0.903
Hyperglycemia (x1.06) 0.906
Dyspnea (x1.05) 0.908
ECG normal (x1.05) 0.911
Fever (x1.05) 0.914
Malnutrition (calorie) (x1.05) 0.916
oxycodone (+0.00441) 0.918
Dependence on respirator (x1.05) 0.920
acetaminophen (+0.00431) 0.922
Disorder of lung (x1.05) 0.924
Severe protein-calorie malnutrition (Gomez: less than 60% of standard weight) (x1.04) 0.926
Postoperative state (x1.04) 0.928
Altered mental status (x1.04) 0.929
Disorder of brain (x1.04) 0.931
Hypovolemia (x1.04) 0.932
Hypervolemia (x1.04) 0.933
Male Sex (+0.00316) 0.935
Thrombocytopenic disorder (x1.03) 0.936
Female Sex (-0.00312) 0.937
Cardiomegaly (x1.03) 0.938
Persistent pain following procedure (x1.03) 0.939
Hyperkalemia (x1.03) 0.940
Respiratory failure (x1.03) 0.941
Sepsis (x1.03) 0.942
Respiratory insufficiency (x1.03) 0.943
Dysphagia (x1.03) 0.944
Urinary tract infectious disease (x1.03) 0.944
Dehydration (x1.03) 0.945
Injury involving multiple body regions (x1.03) 0.946
Muscle weakness (x1.03) 0.947
Hypomagnesemia (x1.03) 0.948
Disorder of intestine (x1.03) 0.948
Infective pneumonia (x1.03) 0.949
docusate (+0.00251) 0.950
Malaise (x1.03) 0.950
Acute hypoxemic respiratory failure (x1.03) 0.951
gabapentin (+0.00245) 0.952
Nausea and vomiting (x1.03) 0.952
Asthenia (x1.03) 0.953
Diarrhea (x1.03) 0.953
Seizure (x1.02) 0.954
metoprolol (+0.00229) 0.955
Hyperosmolality and or hypernatremia (x1.02) 0.955
Lung field abnormal (x1.02) 0.956
Tachypnea (x1.02) 0.956
Gastroesophageal reflux disease without esophagitis (x1.02) 0.957
Constipation (x1.02) 0.957
Blood coagulation disorder (x1.02) 0.958
Postprocedural state finding (x1.02) 0.958
Borderline normal ECG (x1.02) 0.959
Gastrostomy present (x1.02) 0.959
Vomiting (x1.02) 0.959
Heart disease (x1.02) 0.960
Congestive heart failure (x1.02) 0.960
Ascites (x1.02) 0.961
Disorder of phosphorus metabolism (x1.02) 0.961
Abdominal pain (x1.02) 0.961
Black Race (+0.00188) 0.962
Pneumothorax (x1.02) 0.962
Chronic pulmonary edema (x1.02) 0.963
Disorder of kidney and/or ureter (x1.02) 0.963
Heart failure (x1.02) 0.963
Disturbance of consciousness (x1.02) 0.964
Oropharyngeal dysphagia (x1.02) 0.964
omeprazole (+0.00181) 0.964
enoxaparin (+0.00172) 0.965
Pulmonary congestion and hypostasis (x1.02) 0.965
Hypocalcemia (x1.02) 0.965
Septic shock (x1.02) 0.966
Bacteremia (x1.02) 0.966
C reactive protein [M/V] in SP (+1.88) 0.966
Pneumonia (x1.02) 0.966
Chronic kidney disease (x1.02) 0.967
Cerebral edema (x1.02) 0.967
Flatulence, eructation and gas pain (x1.02) 0.967
Conduction disorder of the heart (x1.02) 0.967
Insomnia (x1.02) 0.968
aspirin (+0.00153) 0.968
levetiracetam (+0.00152) 0.968
Delirium (x1.02) 0.968
Expressions

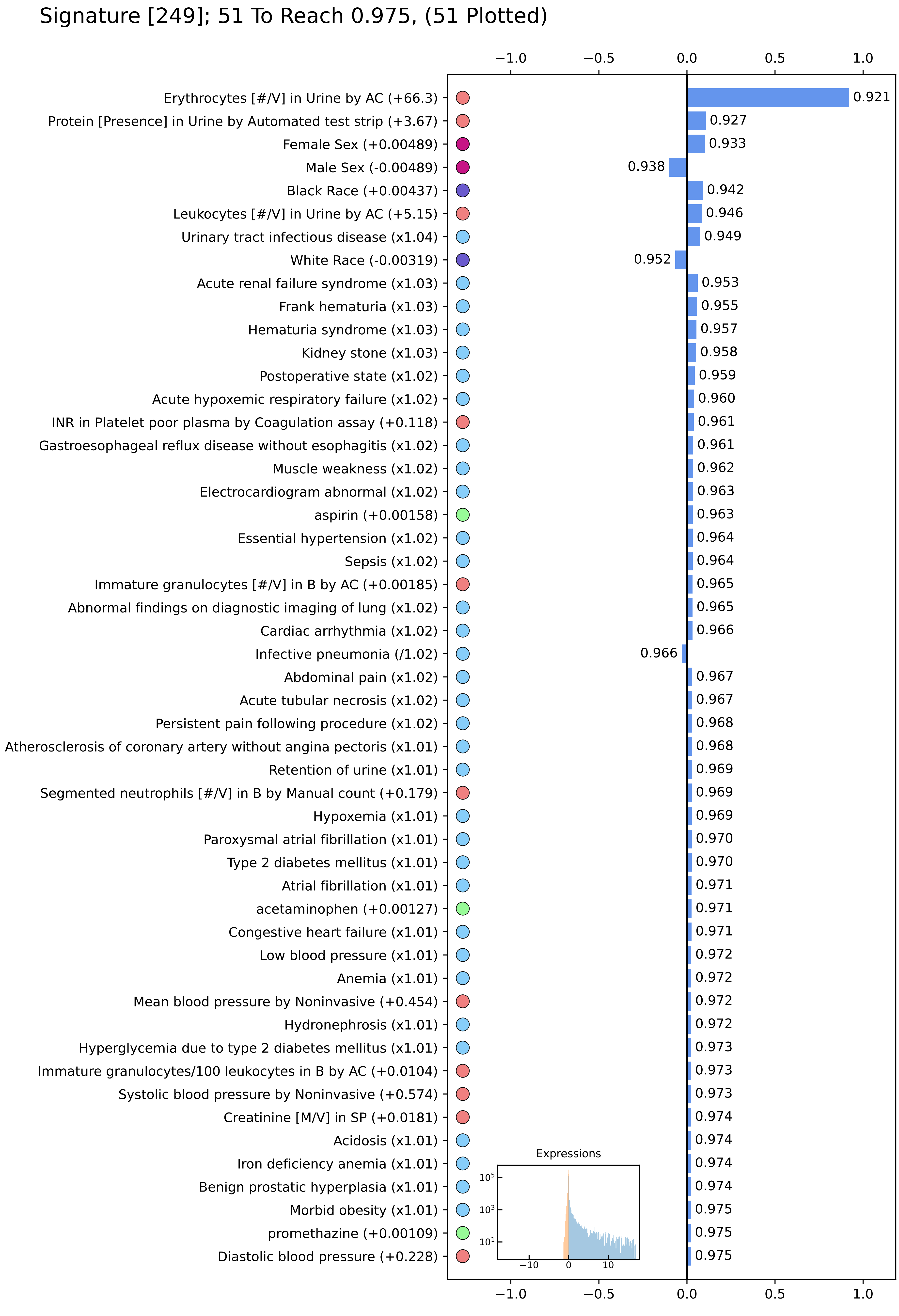

Signature [249]; 51 To Reach 0.975, (51 Plotted)
Erythrocytes [#/V] in Urine by AC (+66.3) 0.921
Protein [Presence] in Urine by Automated test strip (+3.67) 0.927
Female Sex (+0.00489) 0.933
Male Sex (-0.00489) 0.938
Black Race (+0.00437) 0.942
Leukocytes [#/V] in Urine by AC (+5.15) 0.946
Urinary tract infectious disease (x1.04) 0.949
White Race (-0.00319) 0.952
Acute renal failure syndrome (x1.03) 0.953
Frank hematuria (x1.03) 0.955
Hematuria syndrome (x1.03) 0.957
Kidney stone (x1.03) 0.958
Postoperative state (x1.02) 0.959
Acute hypoxemic respiratory failure (x1.02) 0.960
INR in Platelet poor plasma by Coagulation assay (+0.118) 0.961
Gastroesophageal reflux disease without esophagitis (x1.02) 0.961
Muscle weakness (x1.02) 0.962
Electrocardiogram abnormal (x1.02) 0.963
aspirin (+0.00158) 0.963
Essential hypertension (x1.02) 0.964
Sepsis (x1.02) 0.964
Immature granulocytes [#/V] in B by AC (+0.00185) 0.965
Abnormal findings on diagnostic imaging of lung (x1.02) 0.965
Cardiac arrhythmia (x1.02) 0.966
Infective pneumonia (/1.02) 0.966
Abdominal pain (x1.02) 0.967
Acute tubular necrosis (x1.02) 0.967
Persistent pain following procedure (x1.02) 0.968
Atherosclerosis of coronary artery without angina pectoris (x1.01) 0.968
Retention of urine (x1.01) 0.969
Segmented neutrophils [#/V] in B by Manual count (+0.179) 0.969
Hypoxemia (x1.01) 0.969
Paroxysmal atrial fibrillation (x1.01) 0.970
Type 2 diabetes mellitus (x1.01) 0.970
Atrial fibrillation (x1.01) 0.971
acetaminophen (+0.00127) 0.971
Congestive heart failure (x1.01) 0.971
Low blood pressure (x1.01) 0.972
Anemia (x1.01) 0.972
Mean blood pressure by Noninvasive (+0.454) 0.972
Hydronephrosis (x1.01) 0.972
Hyperglycemia due to type 2 diabetes mellitus (x1.01) 0.973
Immature granulocytes/100 leukocytes in B by AC (+0.0104) 0.973
Systolic blood pressure by Noninvasive (+0.574) 0.973
Creatinine [M/V] in SP (+0.0181) 0.974
Acidosis (x1.01) 0.974
Iron deficiency anemia (x1.01) 0.974
Benign prostatic hyperplasia (x1.01) 0.974
Morbid obesity (x1.01) 0.975
promethazine (+0.00109) 0.975
Diastolic blood pressure (+0.228) 0.975
−1.0 −0.5 0.0 0.5 1.0
Expressions

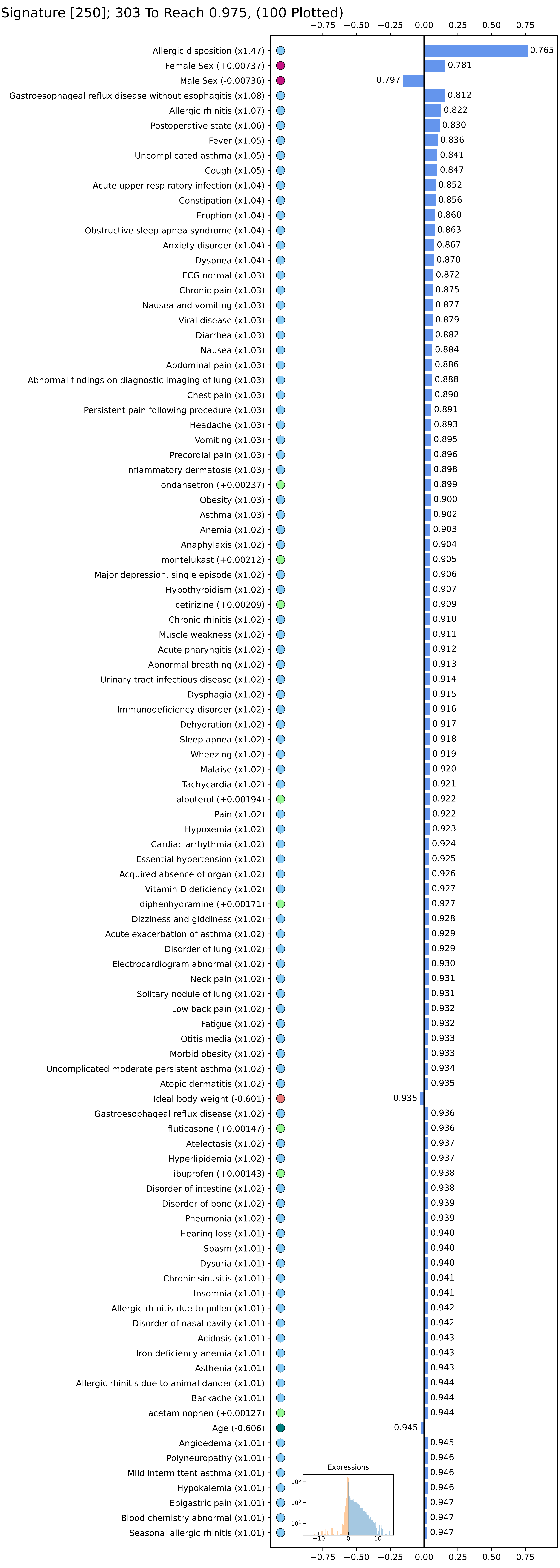

Signature [250]; 303 To Reach 0.975, (100 Plotted)
Allergic disposition (x1.47) 0.765
Female Sex (+0.00737) 0.781
Male Sex (-0.00736) 0.797
Gastroesophageal reflux disease without esophagitis (x1.08) 0.812
Allergic rhinitis (x1.07) 0.822
Postoperative state (x1.06) 0.830
Fever (x1.05) 0.836
Uncomplicated asthma (x1.05) 0.841
Cough (x1.05) 0.847
Acute upper respiratory infection (x1.04) 0.852
Constipation (x1.04) 0.856
Eruption (x1.04) 0.860
Obstructive sleep apnea syndrome (x1.04) 0.863
Anxiety disorder (x1.04) 0.867
Dyspnea (x1.04) 0.870
ECG normal (x1.03) 0.872
Chronic pain (x1.03) 0.875
Nausea and vomiting (x1.03) 0.877
Viral disease (x1.03) 0.879
Diarrhea (x1.03) 0.882
Nausea (x1.03) 0.884
Abdominal pain (x1.03) 0.886
Abnormal findings on diagnostic imaging of lung (x1.03) 0.888
Chest pain (x1.03) 0.890
Persistent pain following procedure (x1.03) 0.891
Headache (x1.03) 0.893
Vomiting (x1.03) 0.895
Precordial pain (x1.03) 0.896
Inflammatory dermatosis (x1.03) 0.898
ondansetron (+0.00237) 0.899
Obesity (x1.03) 0.900
Asthma (x1.03) 0.902
Anemia (x1.02) 0.903
Anaphylaxis (x1.02) 0.904
montelukast (+0.00212) 0.905
Major depression, single episode (x1.02) 0.906
Hypothyroidism (x1.02) 0.907
cetirizine (+0.00209) 0.909
Chronic rhinitis (x1.02) 0.910
Muscle weakness (x1.02) 0.911
Acute pharyngitis (x1.02) 0.912
Abnormal breathing (x1.02) 0.913
Urinary tract infectious disease (x1.02) 0.914
Dysphagia (x1.02) 0.915
Immunodeficiency disorder (x1.02) 0.916
Dehydration (x1.02) 0.917
Sleep apnea (x1.02) 0.918
Wheezing (x1.02) 0.919
Malaise (x1.02) 0.920
Tachycardia (x1.02) 0.921
albuterol (+0.00194) 0.922
Pain (x1.02) 0.922
Hypoxemia (x1.02) 0.923
Cardiac arrhythmia (x1.02) 0.924
Essential hypertension (x1.02) 0.925
Acquired absence of organ (x1.02) 0.926
Vitamin D deficiency (x1.02) 0.927
diphenhydramine (+0.00171) 0.927
Dizziness and giddiness (x1.02) 0.928
Acute exacerbation of asthma (x1.02) 0.929
Disorder of lung (x1.02) 0.929
Electrocardiogram abnormal (x1.02) 0.930
Neck pain (x1.02) 0.931
Solitary nodule of lung (x1.02) 0.931
Low back pain (x1.02) 0.932
Fatigue (x1.02) 0.932
Otitis media (x1.02) 0.933
Morbid obesity (x1.02) 0.933
Uncomplicated moderate persistent asthma (x1.02) 0.934
Atopic dermatitis (x1.02) 0.935
Ideal body weight (-0.601) 0.935
Gastroesophageal reflux disease (x1.02) 0.936
fluticasone (+0.00147) 0.936
Atelectasis (x1.02) 0.937
Hyperlipidemia (x1.02) 0.937
ibuprofen (+0.00143) 0.938
Disorder of intestine (x1.02) 0.938
Disorder of bone (x1.02) 0.939
Pneumonia (x1.02) 0.939
Hearing loss (x1.01) 0.940
Spasm (x1.01) 0.940
Dysuria (x1.01) 0.940
Chronic sinusitis (x1.01) 0.941
Insomnia (x1.01) 0.941
Allergic rhinitis due to pollen (x1.01) 0.942
Disorder of nasal cavity (x1.01) 0.942
Acidosis (x1.01) 0.943
Iron deficiency anemia (x1.01) 0.943
Asthenia (x1.01) 0.943
Allergic rhinitis due to animal dander (x1.01) 0.944
Backache (x1.01) 0.944
acetaminophen (+0.00127) 0.944
Age (-0.606) 0.945
Angioedema (x1.01) 0.945
Polyneuropathy (x1.01) 0.946
Mild intermittent asthma (x1.01) 0.946
Hypokalemia (x1.01) 0.946
Epigastric pain (x1.01) 0.947
Blood chemistry abnormal (x1.01) 0.947
Seasonal allergic rhinitis (x1.01) 0.947
−0.75 −0.50 −0.25 0.00 0.25 0.50 0.75
Expressions

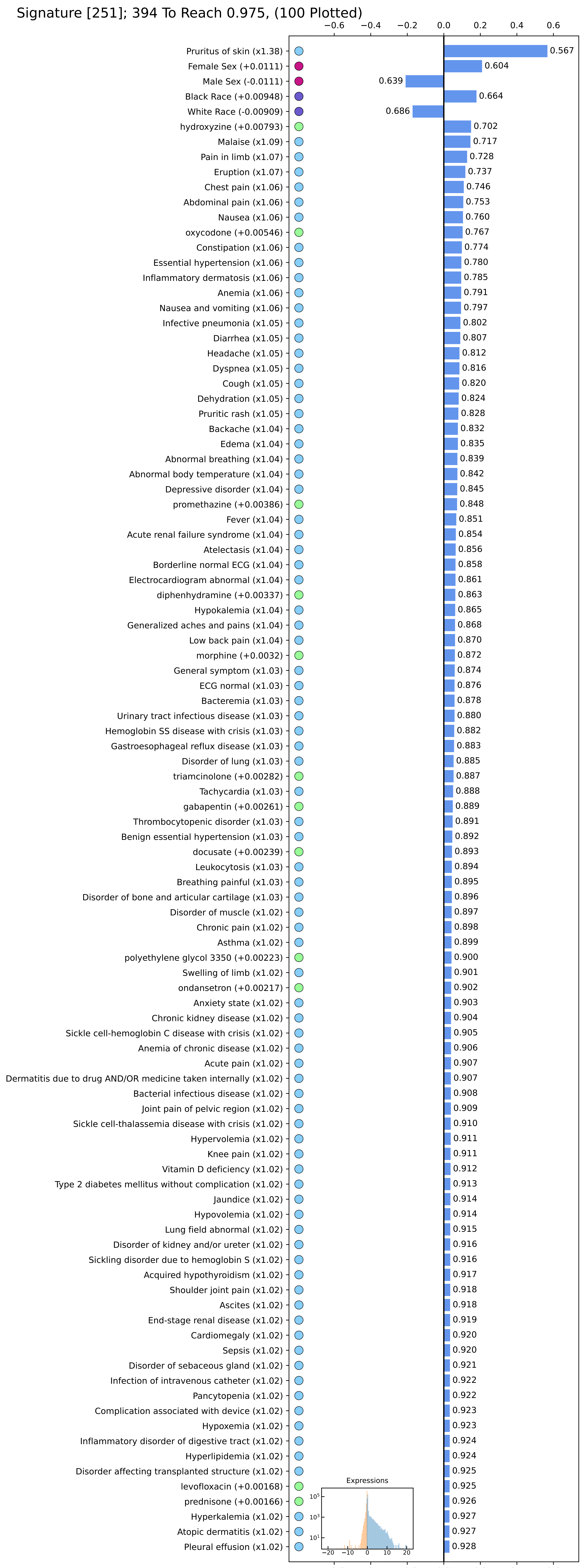

Signature [251]; 394 To Reach 0.975, (100 Plotted)
Pruritus of skin (x1.38) 0.567
Female Sex (+0.0111) 0.604
Male Sex (-0.0111) 0.639
Black Race (+0.00948) 0.664
White Race (-0.00909) 0.686
hydroxyzine (+0.00793) 0.702
Malaise (x1.09) 0.717
Pain in limb (x1.07) 0.728
Eruption (x1.07) 0.737
Chest pain (x1.06) 0.746
Abdominal pain (x1.06) 0.753
Nausea (x1.06) 0.760
oxycodone (+0.00546) 0.767
Constipation (x1.06) 0.774
Essential hypertension (x1.06) 0.780
Inflammatory dermatosis (x1.06) 0.785
Anemia (x1.06) 0.791
Nausea and vomiting (x1.06) 0.797
Infective pneumonia (x1.05) 0.802
Diarrhea (x1.05) 0.807
Headache (x1.05) 0.812
Dyspnea (x1.05) 0.816
Cough (x1.05) 0.820
Dehydration (x1.05) 0.824
Pruritic rash (x1.05) 0.828
Backache (x1.04) 0.832
Edema (x1.04) 0.835
Abnormal breathing (x1.04) 0.839
Abnormal body temperature (x1.04) 0.842
Depressive disorder (x1.04) 0.845
promethazine (+0.00386) 0.848
Fever (x1.04) 0.851
Acute renal failure syndrome (x1.04) 0.854
Atelectasis (x1.04) 0.856
Borderline normal ECG (x1.04) 0.858
Electrocardiogram abnormal (x1.04) 0.861
diphenhydramine (+0.00337) 0.863
Hypokalemia (x1.04) 0.865
Generalized aches and pains (x1.04) 0.868
Low back pain (x1.04) 0.870
morphine (+0.0032) 0.872
General symptom (x1.03) 0.874
ECG normal (x1.03) 0.876
Bacteremia (x1.03) 0.878
Urinary tract infectious disease (x1.03) 0.880
Hemoglobin SS disease with crisis (x1.03) 0.882
Gastroesophageal reflux disease (x1.03) 0.883
Disorder of lung (x1.03) 0.885
triamcinolone (+0.00282) 0.887
Tachycardia (x1.03) 0.888
gabapentin (+0.00261) 0.889
Thrombocytopenic disorder (x1.03) 0.891
Benign essential hypertension (x1.03) 0.892
docusate (+0.00239) 0.893
Leukocytosis (x1.03) 0.894
Breathing painful (x1.03) 0.895
Disorder of bone and articular cartilage (x1.03) 0.896
Disorder of muscle (x1.02) 0.897
Chronic pain (x1.02) 0.898
Asthma (x1.02) 0.899
polyethylene glycol 3350 (+0.00223) 0.900
Swelling of limb (x1.02) 0.901
ondansetron (+0.00217) 0.902
Anxiety state (x1.02) 0.903
Chronic kidney disease (x1.02) 0.904
Sickle cell-hemoglobin C disease with crisis (x1.02) 0.905
Anemia of chronic disease (x1.02) 0.906
Acute pain (x1.02) 0.907
Dermatitis due to drug AND/OR medicine taken internally (x1.02) 0.907
Bacterial infectious disease (x1.02) 0.908
Joint pain of pelvic region (x1.02) 0.909
Sickle cell-thalassemia disease with crisis (x1.02) 0.910
Hypervolemia (x1.02) 0.911
Knee pain (x1.02) 0.911
Vitamin D deficiency (x1.02) 0.912
Type 2 diabetes mellitus without complication (x1.02) 0.913
Jaundice (x1.02) 0.914
Hypovolemia (x1.02) 0.914
Lung field abnormal (x1.02) 0.915
Disorder of kidney and/or ureter (x1.02) 0.916
Sickling disorder due to hemoglobin S (x1.02) 0.916
Acquired hypothyroidism (x1.02) 0.917
Shoulder joint pain (x1.02) 0.918
Ascites (x1.02) 0.918
End-stage renal disease (x1.02) 0.919
Cardiomegaly (x1.02) 0.920
Sepsis (x1.02) 0.920
Disorder of sebaceous gland (x1.02) 0.921
Infection of intravenous catheter (x1.02) 0.922
Pancytopenia (x1.02) 0.922
Complication associated with device (x1.02) 0.923
Hypoxemia (x1.02) 0.923
Inflammatory disorder of digestive tract (x1.02) 0.924
Hyperlipidemia (x1.02) 0.924
Disorder affecting transplanted structure (x1.02) 0.925
levofloxacin (+0.00168) 0.925
prednisone (+0.00166) 0.926
Hyperkalemia (x1.02) 0.927
Atopic dermatitis (x1.02) 0.927
Pleural effusion (x1.02) 0.928
Expressions
−0.6 −0.4 −0.2 0.0 0.2 0.4 0.6

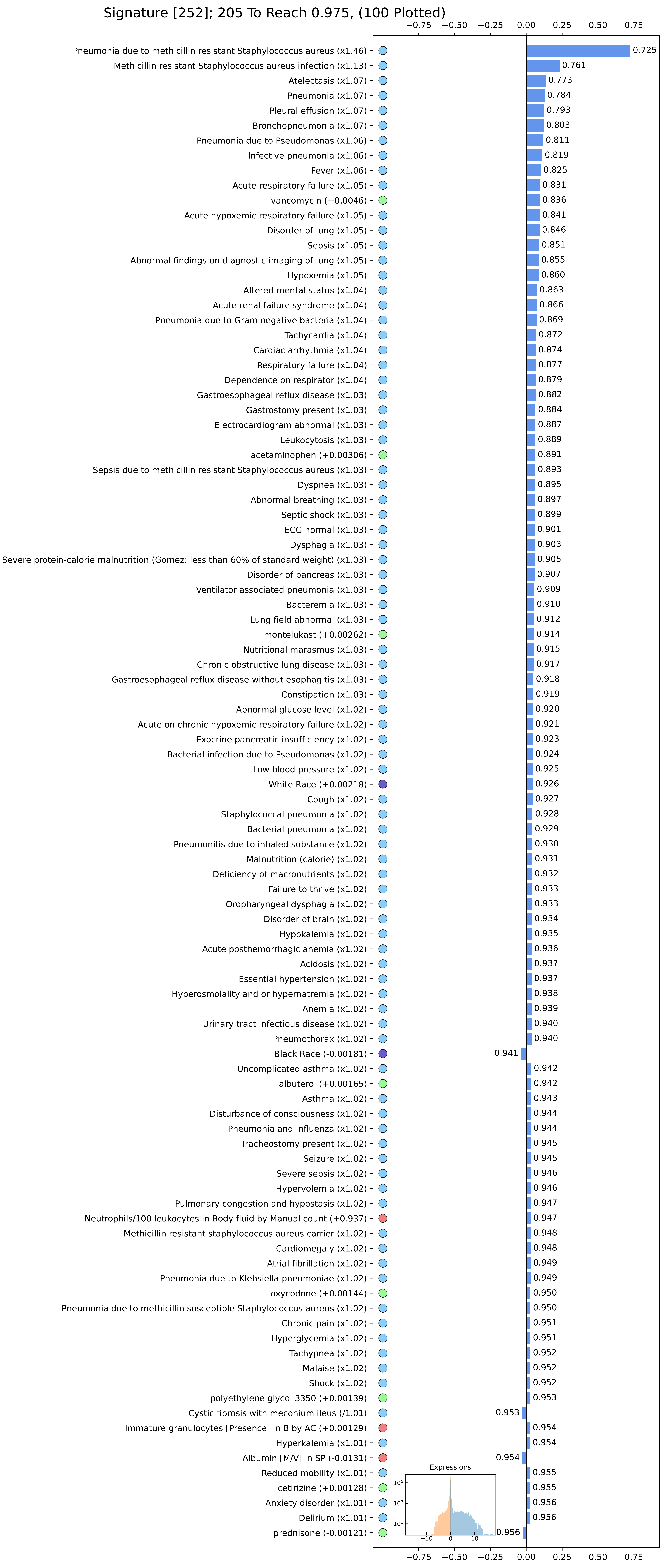

Signature [252]; 205 To Reach 0.975, (100 Plotted)
Pneumonia due to methicillin resistant Staphylococcus aureus (x1.46) 0.725
Methicillin resistant Staphylococcus aureus infection (x1.13) 0.761
Atelectasis (x1.07) 0.773
Pneumonia (x1.07) 0.784
Pleural effusion (x1.07) 0.793
Bronchopneumonia (x1.07) 0.803
Pneumonia due to Pseudomonas (x1.06) 0.811
Infective pneumonia (x1.06) 0.819
Fever (x1.06) 0.825
Acute respiratory failure (x1.05) 0.831
vancomycin (+0.0046) 0.836
Acute hypoxemic respiratory failure (x1.05) 0.841
Disorder of lung (x1.05) 0.846
Sepsis (x1.05) 0.851
Abnormal findings on diagnostic imaging of lung (x1.05) 0.855
Hypoxemia (x1.05) 0.860
Altered mental status (x1.04) 0.863
Acute renal failure syndrome (x1.04) 0.866
Pneumonia due to Gram negative bacteria (x1.04) 0.869
Tachycardia (x1.04) 0.872
Cardiac arrhythmia (x1.04) 0.874
Respiratory failure (x1.04) 0.877
Dependence on respirator (x1.04) 0.879
Gastroesophageal reflux disease (x1.03) 0.882
Gastrostomy present (x1.03) 0.884
Electrocardiogram abnormal (x1.03) 0.887
Leukocytosis (x1.03) 0.889
acetaminophen (+0.00306) 0.891
Sepsis due to methicillin resistant Staphylococcus aureus (x1.03) 0.893
Dyspnea (x1.03) 0.895
Abnormal breathing (x1.03) 0.897
Septic shock (x1.03) 0.899
ECG normal (x1.03) 0.901
Dysphagia (x1.03) 0.903
Severe protein-calorie malnutrition (Gomez: less than 60% of standard weight) (x1.03) 0.905
Disorder of pancreas (x1.03) 0.907
Ventilator associated pneumonia (x1.03) 0.909
Bacteremia (x1.03) 0.910
Lung field abnormal (x1.03) 0.912
montelukast (+0.00262) 0.914
Nutritional marasmus (x1.03) 0.915
Chronic obstructive lung disease (x1.03) 0.917
Gastroesophageal reflux disease without esophagitis (x1.03) 0.918
Constipation (x1.03) 0.919
Abnormal glucose level (x1.02) 0.920
Acute on chronic hypoxemic respiratory failure (x1.02) 0.921
Exocrine pancreatic insufficiency (x1.02) 0.923
Bacterial infection due to Pseudomonas (x1.02) 0.924
Low blood pressure (x1.02) 0.925
White Race (+0.00218) 0.926
Cough (x1.02) 0.927
Staphylococcal pneumonia (x1.02) 0.928
Bacterial pneumonia (x1.02) 0.929
Pneumonitis due to inhaled substance (x1.02) 0.930
Malnutrition (calorie) (x1.02) 0.931
Deficiency of macronutrients (x1.02) 0.932
Failure to thrive (x1.02) 0.933
Oropharyngeal dysphagia (x1.02) 0.933
Disorder of brain (x1.02) 0.934
Hypokalemia (x1.02) 0.935
Acute posthemorrhagic anemia (x1.02) 0.936
Acidosis (x1.02) 0.937
Essential hypertension (x1.02) 0.937
Hyperosmolality and or hypernatremia (x1.02) 0.938
Anemia (x1.02) 0.939
Urinary tract infectious disease (x1.02) 0.940
Pneumothorax (x1.02) 0.940
Black Race (-0.00181) 0.941
Uncomplicated asthma (x1.02) 0.942
albuterol (+0.00165) 0.942
Asthma (x1.02) 0.943
Disturbance of consciousness (x1.02) 0.944
Pneumonia and influenza (x1.02) 0.944
Tracheostomy present (x1.02) 0.945
Seizure (x1.02) 0.945
Severe sepsis (x1.02) 0.946
Hypervolemia (x1.02) 0.946
Pulmonary congestion and hypostasis (x1.02) 0.947
Neutrophils/100 leukocytes in Body fluid by Manual count (+0.937) 0.947
Methicillin resistant staphylococcus aureus carrier (x1.02) 0.948
Cardiomegaly (x1.02) 0.948
Atrial fibrillation (x1.02) 0.949
Pneumonia due to Klebsiella pneumoniae (x1.02) 0.949
oxycodone (+0.00144) 0.950
Pneumonia due to methicillin susceptible Staphylococcus aureus (x1.02) 0.950
Chronic pain (x1.02) 0.951
Hyperglycemia (x1.02) 0.951
Tachypnea (x1.02) 0.952
Malaise (x1.02) 0.952
Shock (x1.02) 0.952
polyethylene glycol 3350 (+0.00139) 0.953
Cystic fibrosis with meconium ileus (/1.01) 0.953
Immature granulocytes [Presence] in B by AC (+0.00129) 0.954
Hyperkalemia (x1.01) 0.954
Albumin [M/V] in SP (-0.0131) 0.954
Reduced mobility (x1.01) 0.955
cetirizine (+0.00128) 0.955
Anxiety disorder (x1.01) 0.956
Delirium (x1.01) 0.956
prednisone (-0.00121) 0.956
−0.75 −0.50 −0.25 0.00 0.25 0.50 0.75
Expressions

Signature [253]; 22 To Reach 0.975, (22 Plotted)

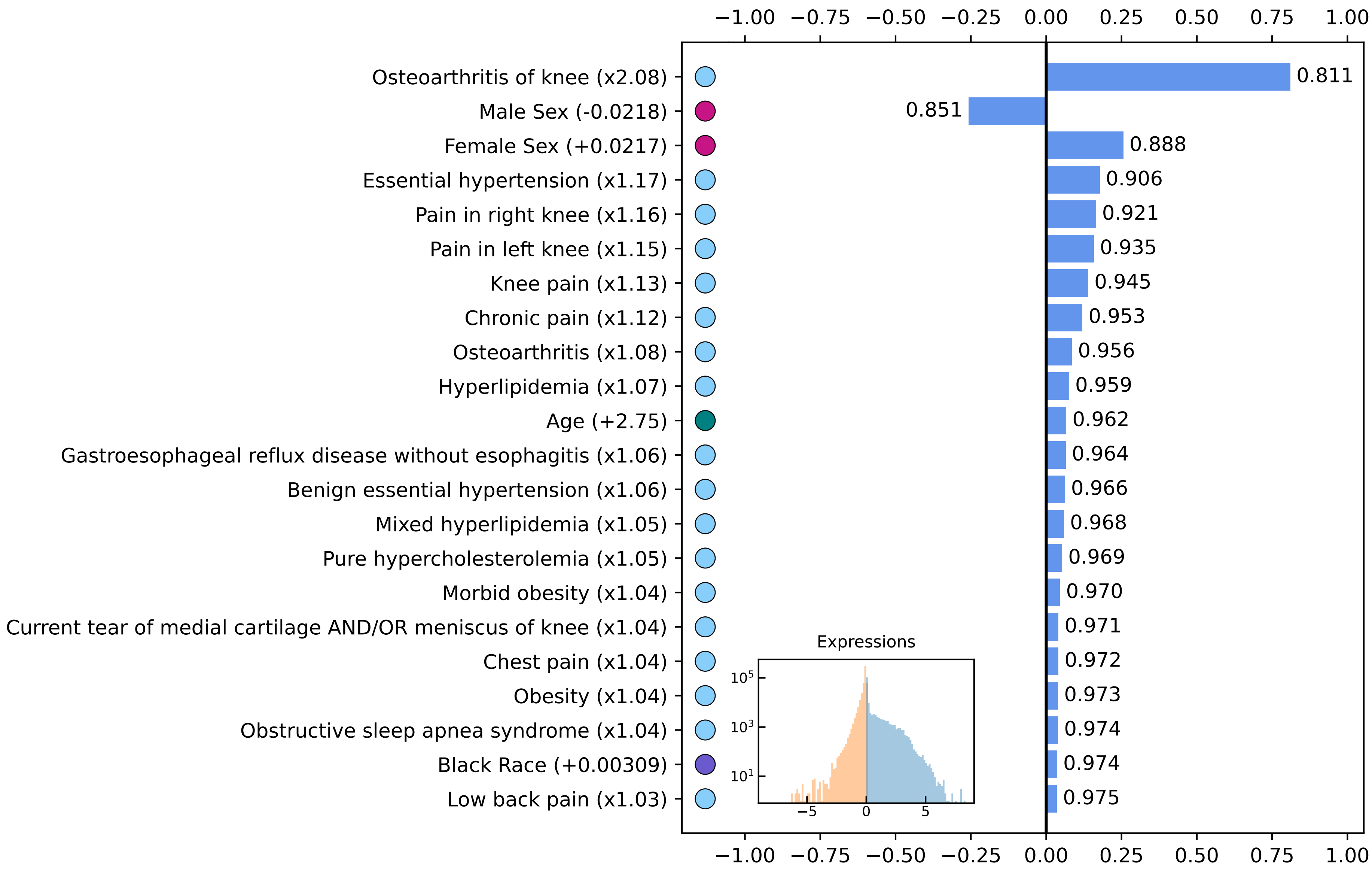

Signature [254]; 7 To Reach 0.975, (10 Plotted)

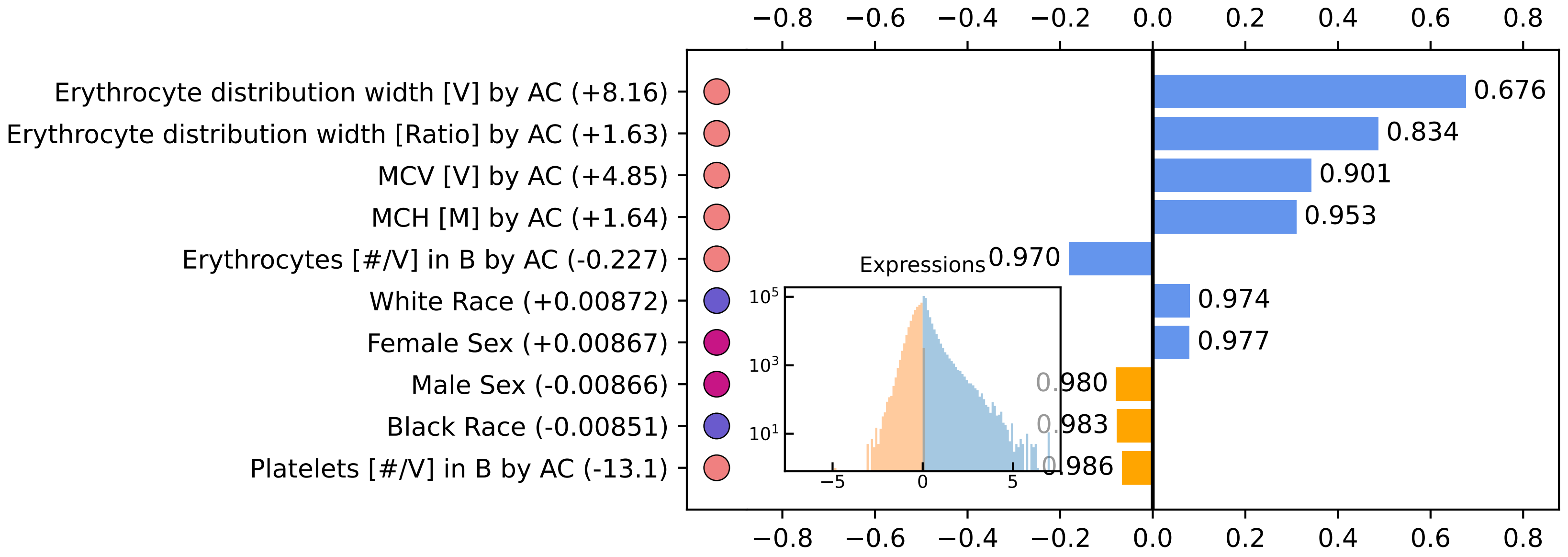

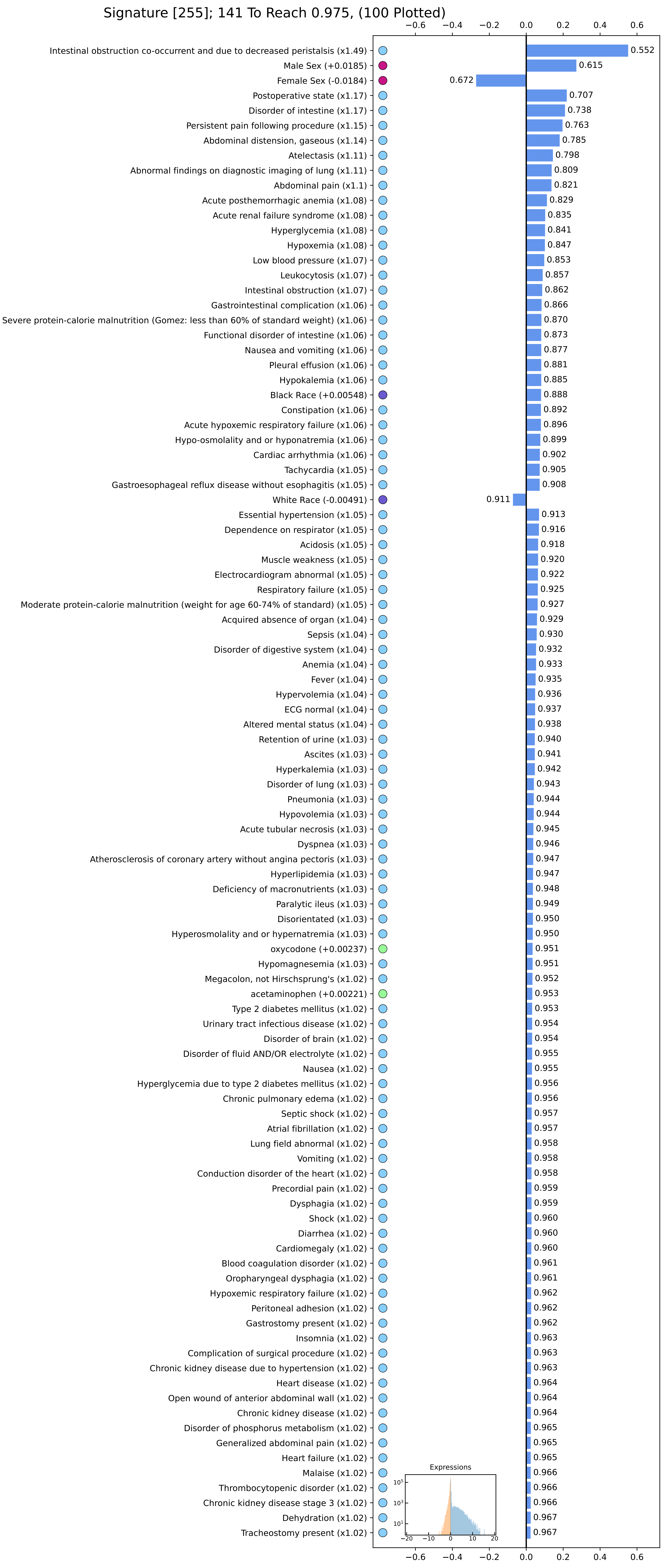

Signature [255]; 141 To Reach 0.975, (100 Plotted)
Intestinal obstruction co-occurrent and due to decreased peristalsis (x1.49) 0.552
Male Sex (+0.0185) 0.615
Female Sex (-0.0184) 0.672
Postoperative state (x1.17) 0.707
Disorder of intestine (x1.17) 0.738
Persistent pain following procedure (x1.15) 0.763
Abdominal distension, gaseous (x1.14) 0.785
Atelectasis (x1.11) 0.798
Abnormal findings on diagnostic imaging of lung (x1.11) 0.809
Abdominal pain (x1.1) 0.821
Acute posthemorrhagic anemia (x1.08) 0.829
Acute renal failure syndrome (x1.08) 0.835
Hyperglycemia (x1.08) 0.841
Hypoxemia (x1.08) 0.847
Low blood pressure (x1.07) 0.853
Leukocytosis (x1.07) 0.857
Intestinal obstruction (x1.07) 0.862
Gastrointestinal complication (x1.06) 0.866
Severe protein-calorie malnutrition (Gomez: less than 60% of standard weight) (x1.06) 0.870
Functional disorder of intestine (x1.06) 0.873
Nausea and vomiting (x1.06) 0.877
Pleural effusion (x1.06) 0.881
Hypokalemia (x1.06) 0.885
Black Race (+0.00548) 0.888
Constipation (x1.06) 0.892
Acute hypoxemic respiratory failure (x1.06) 0.896
Hypo-osmolality and or hyponatremia (x1.06) 0.899
Cardiac arrhythmia (x1.06) 0.902
Tachycardia (x1.05) 0.905
Gastroesophageal reflux disease without esophagitis (x1.05) 0.908
White Race (-0.00491) 0.911
Essential hypertension (x1.05) 0.913
Dependence on respirator (x1.05) 0.916
Acidosis (x1.05) 0.918
Muscle weakness (x1.05) 0.920
Electrocardiogram abnormal (x1.05) 0.922
Respiratory failure (x1.05) 0.925
Moderate protein-calorie malnutrition (weight for age 60-74% of standard) (x1.05) 0.927
Acquired absence of organ (x1.04) 0.929
Sepsis (x1.04) 0.930
Disorder of digestive system (x1.04) 0.932
Anemia (x1.04) 0.933
Fever (x1.04) 0.935
Hypervolemia (x1.04) 0.936
ECG normal (x1.04) 0.937
Altered mental status (x1.04) 0.938
Retention of urine (x1.03) 0.940
Ascites (x1.03) 0.941
Hyperkalemia (x1.03) 0.942
Disorder of lung (x1.03) 0.943
Pneumonia (x1.03) 0.944
Hypovolemia (x1.03) 0.944
Acute tubular necrosis (x1.03) 0.945
Dyspnea (x1.03) 0.946
Atherosclerosis of coronary artery without angina pectoris (x1.03) 0.947
Hyperlipidemia (x1.03) 0.947
Deficiency of macronutrients (x1.03) 0.948
Paralytic ileus (x1.03) 0.949
Disorientated (x1.03) 0.950
Hyperosmolality and or hypernatremia (x1.03) 0.950
oxycodone (+0.00237) 0.951
Hypomagnesemia (x1.03) 0.951
Megacolon, not Hirschsprung's (x1.02) 0.952
acetaminophen (+0.00221) 0.953
Type 2 diabetes mellitus (x1.02) 0.953
Urinary tract infectious disease (x1.02) 0.954
Disorder of brain (x1.02) 0.954
Disorder of fluid AND/OR electrolyte (x1.02) 0.955
Nausea (x1.02) 0.955
Hyperglycemia due to type 2 diabetes mellitus (x1.02) 0.956
Chronic pulmonary edema (x1.02) 0.956
Septic shock (x1.02) 0.957
Atrial fibrillation (x1.02) 0.957
Lung field abnormal (x1.02) 0.958
Vomiting (x1.02) 0.958
Conduction disorder of the heart (x1.02) 0.958
Precordial pain (x1.02) 0.959
Dysphagia (x1.02) 0.959
Shock (x1.02) 0.960
Diarrhea (x1.02) 0.960
Cardiomegaly (x1.02) 0.960
Blood coagulation disorder (x1.02) 0.961
Oropharyngeal dysphagia (x1.02) 0.961
Hypoxemic respiratory failure (x1.02) 0.962
Peritoneal adhesion (x1.02) 0.962
Gastrostomy present (x1.02) 0.962
Insomnia (x1.02) 0.963
Complication of surgical procedure (x1.02) 0.963
Chronic kidney disease due to hypertension (x1.02) 0.963
Heart disease (x1.02) 0.964
Open wound of anterior abdominal wall (x1.02) 0.964
Chronic kidney disease (x1.02) 0.964
Disorder of phosphorus metabolism (x1.02) 0.965
Generalized abdominal pain (x1.02) 0.965
Heart failure (x1.02) 0.965
Malaise (x1.02) 0.966
Thrombocytopenic disorder (x1.02) 0.966
Chronic kidney disease stage 3 (x1.02) 0.966
Dehydration (x1.02) 0.967
Tracheostomy present (x1.02) 0.967
Expressions
−0.6 −0.4 −0.2 0.0 0.2 0.4 0.6

Signature [256]; 12 To Reach 0.975, (12 Plotted)

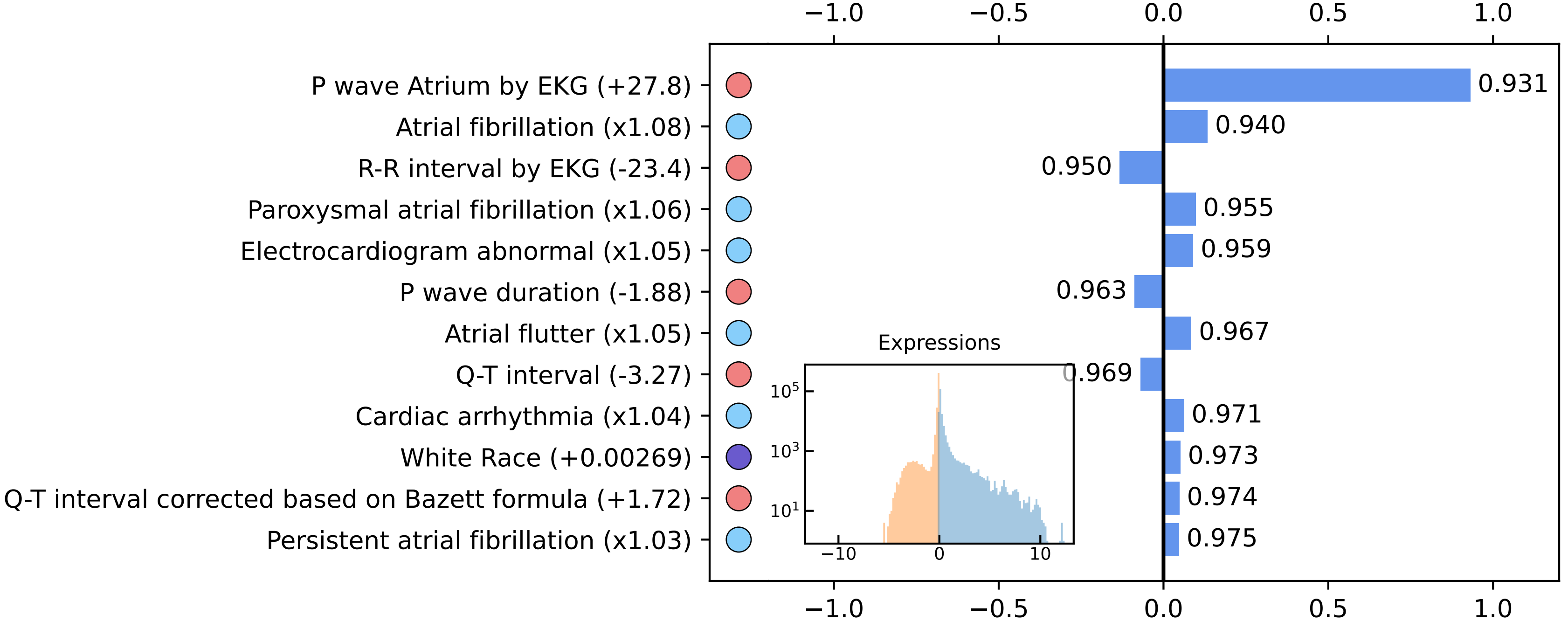

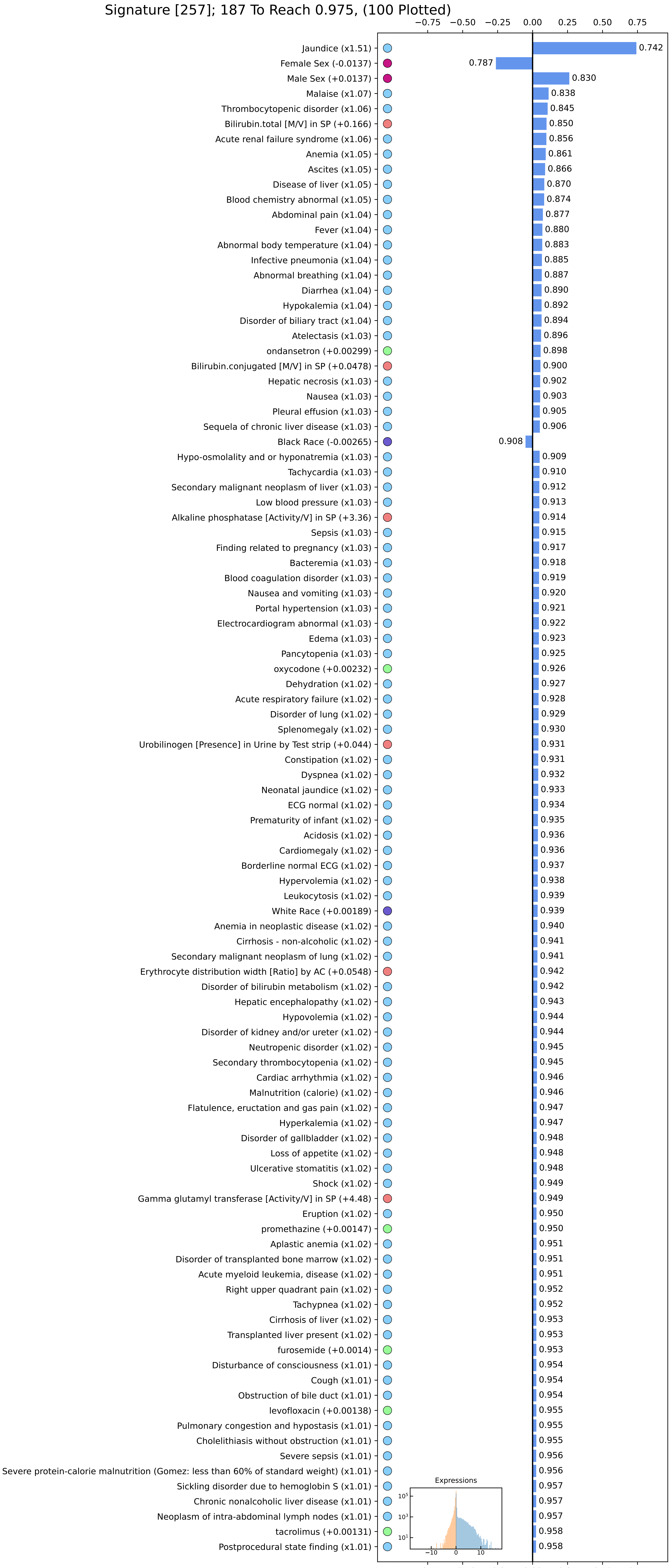

Signature [257]; 187 To Reach 0.975, (100 Plotted)
Jaundice (x1.51)
Female Sex (-0.0137)
Male Sex (+0.0137)
Malaise (x1.07)
Thrombocytopenic disorder (x1.06)
Bilirubin.total [M/V] in SP (+0.166)
Acute renal failure syndrome (x1.06)
Anemia (x1.05)
Ascites (x1.05)
Disease of liver (x1.05)
Blood chemistry abnormal (x1.05)
Abdominal pain (x1.04)
Fever (x1.04)
Abnormal body temperature (x1.04)
Infective pneumonia (x1.04)
Abnormal breathing (x1.04)
Diarrhea (x1.04)
Hypokalemia (x1.04)
Disorder of biliary tract (x1.04)
Atelectasis (x1.03)
ondansetron (+0.00299)
Bilirubin.conjugated [M/V] in SP (+0.0478)
Hepatic necrosis (x1.03)
Nausea (x1.03)
Pleural effusion (x1.03)
Sequela of chronic liver disease (x1.03)
Black Race (-0.00265)
Hypo-osmolality and or hyponatremia (x1.03)
Tachycardia (x1.03)
Secondary malignant neoplasm of liver (x1.03)
Low blood pressure (x1.03)
Alkaline phosphatase [Activity/V] in SP (+3.36)
Sepsis (x1.03)
Finding related to pregnancy (x1.03)
Bacteremia (x1.03)
Blood coagulation disorder (x1.03)
Nausea and vomiting (x1.03)
Portal hypertension (x1.03)
Electrocardiogram abnormal (x1.03)
Edema (x1.03)
Pancytopenia (x1.03)
oxycodone (+0.00232)
Dehydration (x1.02)
Acute respiratory failure (x1.02)
Disorder of lung (x1.02)
Splenomegaly (x1.02)
Urobilinogen [Presence] in Urine by Test strip (+0.044)
Constipation (x1.02)
Dyspnea (x1.02)
Neonatal jaundice (x1.02)
ECG normal (x1.02)
Prematurity of infant (x1.02)
Acidosis (x1.02)
Cardiomegaly (x1.02)
Borderline normal ECG (x1.02)
Hypervolemia (x1.02)
Leukocytosis (x1.02)
White Race (+0.00189)
Anemia in neoplastic disease (x1.02)
Cirrhosis - non-alcoholic (x1.02)
Secondary malignant neoplasm of lung (x1.02)
Erythrocyte distribution width [Ratio] by AC (+0.0548)
Disorder of bilirubin metabolism (x1.02)
Hepatic encephalopathy (x1.02)
Hypovolemia (x1.02)
Disorder of kidney and/or ureter (x1.02)
Neutropenic disorder (x1.02)
Secondary thrombocytopenia (x1.02)
Cardiac arrhythmia (x1.02)
Malnutrition (calorie) (x1.02)
Flatulence, eructation and gas pain (x1.02)
Hyperkalemia (x1.02)
Disorder of gallbladder (x1.02)
Loss of appetite (x1.02)
Ulcerative stomatitis (x1.02)
Shock (x1.02)
Gamma glutamyl transferase [Activity/V] in SP (+4.48)
Eruption (x1.02)
promethazine (+0.00147)
Aplastic anemia (x1.02)
Disorder of transplanted bone marrow (x1.02)
Acute myeloid leukemia, disease (x1.02)
Right upper quadrant pain (x1.02)
Tachypnea (x1.02)
Cirrhosis of liver (x1.02)
Transplanted liver present (x1.02)
furosemide (+0.0014)
Disturbance of consciousness (x1.01)
Cough (x1.01)
Obstruction of bile duct (x1.01)
levofloxacin (+0.00138)
Pulmonary congestion and hypostasis (x1.01)
Cholelithiasis without obstruction (x1.01)
Severe sepsis (x1.01)
Severe protein-calorie malnutrition (Gomez: less than 60% of standard weight) (x1.01)
Sickling disorder due to hemoglobin S (x1.01)
Chronic nonalcoholic liver disease (x1.01)
Neoplasm of intra-abdominal lymph nodes (x1.01)
tacrolimus (+0.00131)
Postprocedural state finding (x1.01)
0.742
0.787
0.830
0.838
0.845
0.850
0.856
0.861
0.866
0.870
0.874
0.877
0.880
0.883
0.885
0.887
0.890
0.892
0.894
0.896
0.898
0.900
0.902
0.903
0.905
0.906
0.908
0.909
0.910
0.912
0.913
0.914
0.915
0.917
0.918
0.919
0.920
0.921
0.922
0.923
0.925
0.926
0.927
0.928
0.929
0.930
0.931
0.931
0.932
0.933
0.934
0.935
0.936
0.936
0.937
0.938
0.939
0.939
0.940
0.941
0.941
0.942
0.942
0.943
0.944
0.944
0.945
0.945
0.946
0.946
0.947
0.947
0.948
0.948
0.948
0.949
0.949
0.950
0.950
0.951
0.951
0.951
0.952
0.952
0.953
0.953
0.953
0.954
0.954
0.954
0.955
0.955
0.955
0.956
0.956
0.957
0.957
0.957
0.958
0.958
−0.75 −0.50 −0.25 0.00 0.25 0.50 0.75
Expressions

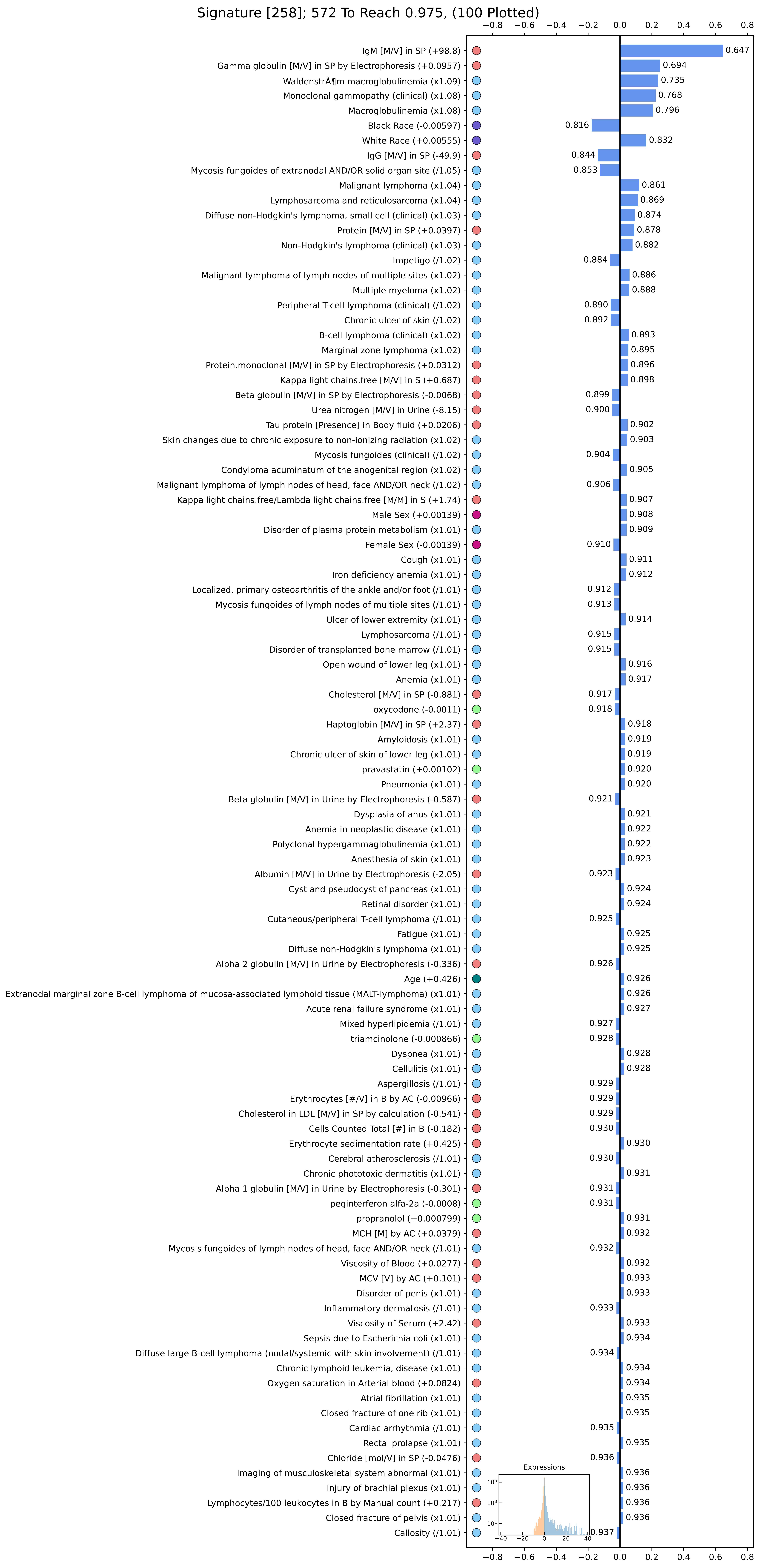

Signature [258]; 572 To Reach 0.975, (100 Plotted)
IgM [M/V] in SP (+98.8) 0.647
Gamma globulin [M/V] in SP by Electrophoresis (+0.0957) 0.694
WaldenstrÃ¶m macroglobulinemia (x1.09) 0.735
Monoclonal gammopathy (clinical) (x1.08) 0.768
Macroglobulinemia (x1.08) 0.796
Black Race (-0.00597) 0.816
White Race (+0.00555) 0.832
IgG [M/V] in SP (-49.9) 0.844
Mycosis fungoides of extranodal AND/OR solid organ site (/1.05) 0.853
Malignant lymphoma (x1.04) 0.861
Lymphosarcoma and reticulosarcoma (x1.04) 0.869
Diffuse non-Hodgkin's lymphoma, small cell (clinical) (x1.03) 0.874
Protein [M/V] in SP (+0.0397) 0.878
Non-Hodgkin's lymphoma (clinical) (x1.03) 0.882
Impetigo (/1.02) 0.884
Malignant lymphoma of lymph nodes of multiple sites (x1.02) 0.886
Multiple myeloma (x1.02) 0.888
Peripheral T-cell lymphoma (clinical) (/1.02) 0.890
Chronic ulcer of skin (/1.02) 0.892
B-cell lymphoma (clinical) (x1.02) 0.893
Marginal zone lymphoma (x1.02) 0.895
Protein.monoclonal [M/V] in SP by Electrophoresis (+0.0312) 0.896
Kappa light chains.free [M/V] in S (+0.687) 0.898
Beta globulin [M/V] in SP by Electrophoresis (-0.0068) 0.899
Urea nitrogen [M/V] in Urine (-8.15) 0.900
Tau protein [Presence] in Body fluid (+0.0206) 0.902
Skin changes due to chronic exposure to non-ionizing radiation (x1.02) 0.903
Mycosis fungoides (clinical) (/1.02) 0.904
Condyloma acuminatum of the anogenital region (x1.02) 0.905
Malignant lymphoma of lymph nodes of head, face AND/OR neck (/1.02) 0.906
Kappa light chains.free/Lambda light chains.free [M/M] in S (+1.74) 0.907
Male Sex (+0.00139) 0.908
Disorder of plasma protein metabolism (x1.01) 0.909
Female Sex (-0.00139) 0.910
Cough (x1.01) 0.911
Iron deficiency anemia (x1.01) 0.912
Localized, primary osteoarthritis of the ankle and/or foot (/1.01) 0.912
Mycosis fungoides of lymph nodes of multiple sites (/1.01) 0.913
Ulcer of lower extremity (x1.01) 0.914
Lymphosarcoma (/1.01) 0.915
Disorder of transplanted bone marrow (/1.01) 0.915
Open wound of lower leg (x1.01) 0.916
Anemia (x1.01) 0.917
Cholesterol [M/V] in SP (-0.881) 0.917
oxycodone (-0.0011) 0.918
Haptoglobin [M/V] in SP (+2.37) 0.918
Amyloidosis (x1.01) 0.919
Chronic ulcer of skin of lower leg (x1.01) 0.919
pravastatin (+0.00102) 0.920
Pneumonia (x1.01) 0.920
Beta globulin [M/V] in Urine by Electrophoresis (-0.587) 0.921
Dysplasia of anus (x1.01) 0.921
Anemia in neoplastic disease (x1.01) 0.922
Polyclonal hypergammaglobulinemia (x1.01) 0.922
Anesthesia of skin (x1.01) 0.923
Albumin [M/V] in Urine by Electrophoresis (-2.05) 0.923
Cyst and pseudocyst of pancreas (x1.01) 0.924
Retinal disorder (x1.01) 0.924
Cutaneous/peripheral T-cell lymphoma (/1.01) 0.925
Fatigue (x1.01) 0.925
Diffuse non-Hodgkin's lymphoma (x1.01) 0.925
Alpha 2 globulin [M/V] in Urine by Electrophoresis (-0.336) 0.926
Age (+0.426) 0.926
Extranodal marginal zone B-cell lymphoma of mucosa-associated lymphoid tissue (MALT-lymphoma) (x1.01) 0.926
Acute renal failure syndrome (x1.01) 0.927
Mixed hyperlipidemia (/1.01) 0.927
triamcinolone (-0.000866) 0.928
Dyspnea (x1.01) 0.928
Cellulitis (x1.01) 0.928
Aspergillosis (/1.01) 0.929
Erythrocytes [#/V] in B by AC (-0.00966) 0.929
Cholesterol in LDL [M/V] in SP by calculation (-0.541) 0.929
Cells Counted Total [#] in B (-0.182) 0.930
Erythrocyte sedimentation rate (+0.425) 0.930
Cerebral atherosclerosis (/1.01) 0.930
Chronic phototoxic dermatitis (x1.01) 0.931
Alpha 1 globulin [M/V] in Urine by Electrophoresis (-0.301) 0.931
peginterferon alfa-2a (-0.0008) 0.931
propranolol (+0.000799) 0.931
MCH [M] by AC (+0.0379) 0.932
Mycosis fungoides of lymph nodes of head, face AND/OR neck (/1.01) 0.932
Viscosity of Blood (+0.0277) 0.932
MCV [V] by AC (+0.101) 0.933
Disorder of penis (x1.01) 0.933
Inflammatory dermatosis (/1.01) 0.933
Viscosity of Serum (+2.42) 0.933
Sepsis due to Escherichia coli (x1.01) 0.934
Diffuse large B-cell lymphoma (nodal/systemic with skin involvement) (/1.01) 0.934
Chronic lymphoid leukemia, disease (x1.01) 0.934
Oxygen saturation in Arterial blood (+0.0824) 0.934
Atrial fibrillation (x1.01) 0.935
Closed fracture of one rib (x1.01) 0.935
Cardiac arrhythmia (/1.01) 0.935
Rectal prolapse (x1.01) 0.935
Chloride [mol/V] in SP (-0.0476) 0.936
Imaging of musculoskeletal system abnormal (x1.01) 0.936
Injury of brachial plexus (x1.01) 0.936
Lymphocytes/100 leukocytes in B by Manual count (+0.217) 0.936
Closed fracture of pelvis (x1.01) 0.936
Callosity (/1.01) 0.937
Expressions
−0.8 −0.6 −0.4 −0.2 0.0 0.2 0.4 0.6 0.8

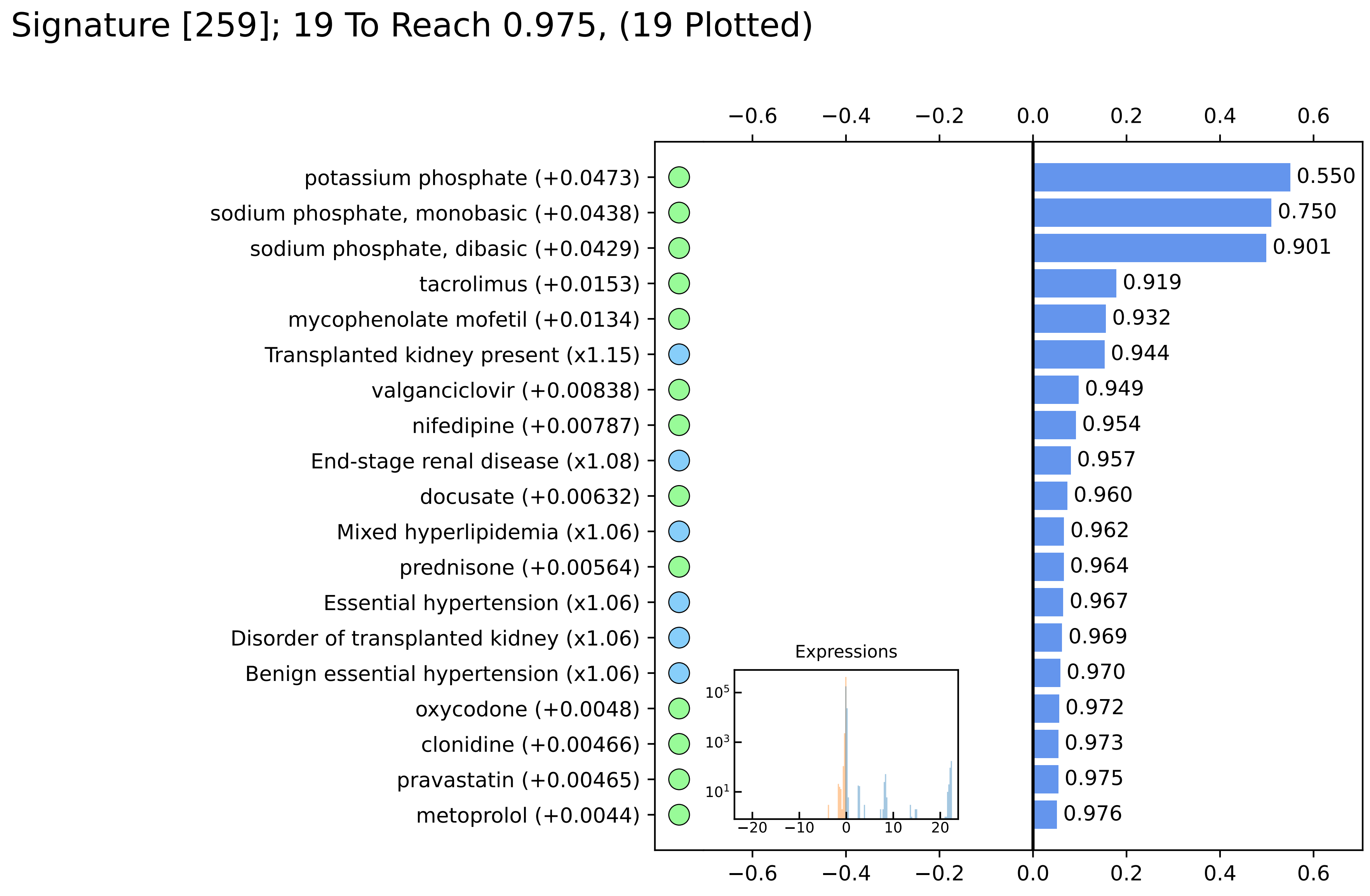
Signature [259]; 19 To Reach 0.975, (19 Plotted)
potassium phosphate (+0.0473)
sodium phosphate, monobasic (+0.0438)
sodium phosphate, dibasic (+0.0429)
tacrolimus (+0.0153)
mycophenolate mofetil (+0.0134)
Transplanted kidney present (x1.15)
valganciclovir (+0.00838)
nifedipine (+0.00787)
End-stage renal disease (x1.08)
docusate (+0.00632)
Mixed hyperlipidemia (x1.06)
prednisone (+0.00564)
Essential hypertension (x1.06)
Disorder of transplanted kidney (x1.06)
Benign essential hypertension (x1.06)
oxycodone (+0.0048)
clonidine (+0.00466)
pravastatin (+0.00465)
metoprolol (+0.0044)
0.550
0.750
0.901
0.919
0.932
0.944
0.949
0.954
0.957
0.960
0.962
0.964
0.967
0.969
0.970
0.972
0.973
0.975
0.976
−0.6
−0.4
−0.2
0.0
0.2
0.4
0.6
Expressions
−20
−10
0
10
20

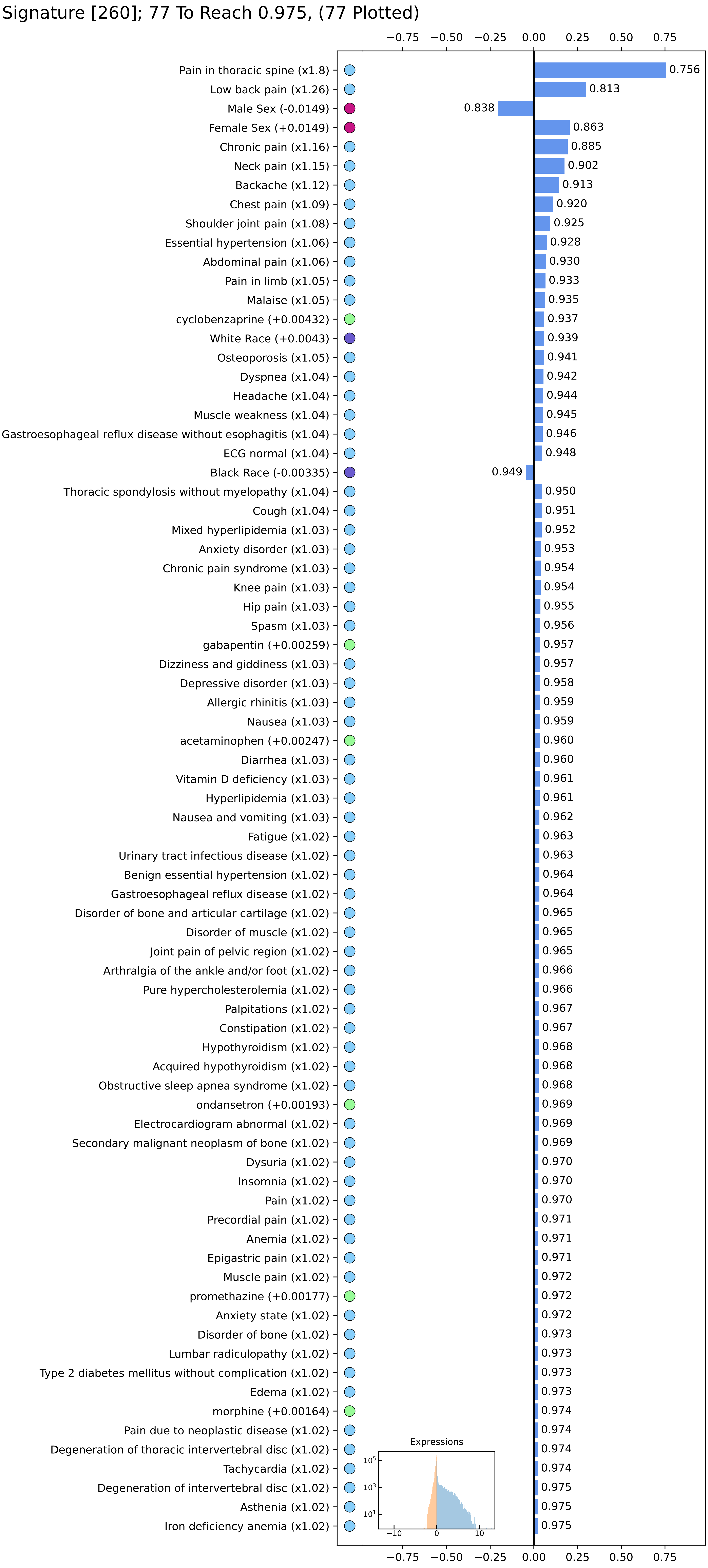
Signature [260]; 77 To Reach 0.975, (77 Plotted)
Pain in thoracic spine (x1.8) 0.756
Low back pain (x1.26) 0.813
Male Sex (-0.0149) 0.838
Female Sex (+0.0149) 0.863
Chronic pain (x1.16) 0.885
Neck pain (x1.15) 0.902
Backache (x1.12) 0.913
Chest pain (x1.09) 0.920
Shoulder joint pain (x1.08) 0.925
Essential hypertension (x1.06) 0.928
Abdominal pain (x1.06) 0.930
Pain in limb (x1.05) 0.933
Malaise (x1.05) 0.935
cyclobenzaprine (+0.00432) 0.937
White Race (+0.0043) 0.939
Osteoporosis (x1.05) 0.941
Dyspnea (x1.04) 0.942
Headache (x1.04) 0.944
Muscle weakness (x1.04) 0.945
Gastroesophageal reflux disease without esophagitis (x1.04) 0.946
ECG normal (x1.04) 0.948
Black Race (-0.00335) 0.949
Thoracic spondylosis without myelopathy (x1.04) 0.950
Cough (x1.04) 0.951
Mixed hyperlipidemia (x1.03) 0.952
Anxiety disorder (x1.03) 0.953
Chronic pain syndrome (x1.03) 0.954
Knee pain (x1.03) 0.954
Hip pain (x1.03) 0.955
Spasm (x1.03) 0.956
gabapentin (+0.00259) 0.957
Dizziness and giddiness (x1.03) 0.957
Depressive disorder (x1.03) 0.958
Allergic rhinitis (x1.03) 0.959
Nausea (x1.03) 0.959
acetaminophen (+0.00247) 0.960
Diarrhea (x1.03) 0.960
Vitamin D deficiency (x1.03) 0.961
Hyperlipidemia (x1.03) 0.961
Nausea and vomiting (x1.03) 0.962
Fatigue (x1.02) 0.963
Urinary tract infectious disease (x1.02) 0.963
Benign essential hypertension (x1.02) 0.964
Gastroesophageal reflux disease (x1.02) 0.964
Disorder of bone and articular cartilage (x1.02) 0.965
Disorder of muscle (x1.02) 0.965
Joint pain of pelvic region (x1.02) 0.965
Arthralgia of the ankle and/or foot (x1.02) 0.966
Pure hypercholesterolemia (x1.02) 0.966
Palpitations (x1.02) 0.967
Constipation (x1.02) 0.967
Hypothyroidism (x1.02) 0.968
Acquired hypothyroidism (x1.02) 0.968
Obstructive sleep apnea syndrome (x1.02) 0.968
ondansetron (+0.00193) 0.969
Electrocardiogram abnormal (x1.02) 0.969
Secondary malignant neoplasm of bone (x1.02) 0.969
Dysuria (x1.02) 0.970
Insomnia (x1.02) 0.970
Pain (x1.02) 0.970
Precordial pain (x1.02) 0.971
Anemia (x1.02) 0.971
Epigastric pain (x1.02) 0.971
Muscle pain (x1.02) 0.972
promethazine (+0.00177) 0.972
Anxiety state (x1.02) 0.972
Disorder of bone (x1.02) 0.973
Lumbar radiculopathy (x1.02) 0.973
Type 2 diabetes mellitus without complication (x1.02) 0.973
Edema (x1.02) 0.973
morphine (+0.00164) 0.974
Pain due to neoplastic disease (x1.02) 0.974
Degeneration of thoracic intervertebral disc (x1.02) 0.974
Tachycardia (x1.02) 0.974
Degeneration of intervertebral disc (x1.02) 0.975
Asthenia (x1.02) 0.975
Iron deficiency anemia (x1.02) 0.975
−0.75 −0.50 −0.25 0.00 0.25 0.50 0.75
Expressions
−10 0 10

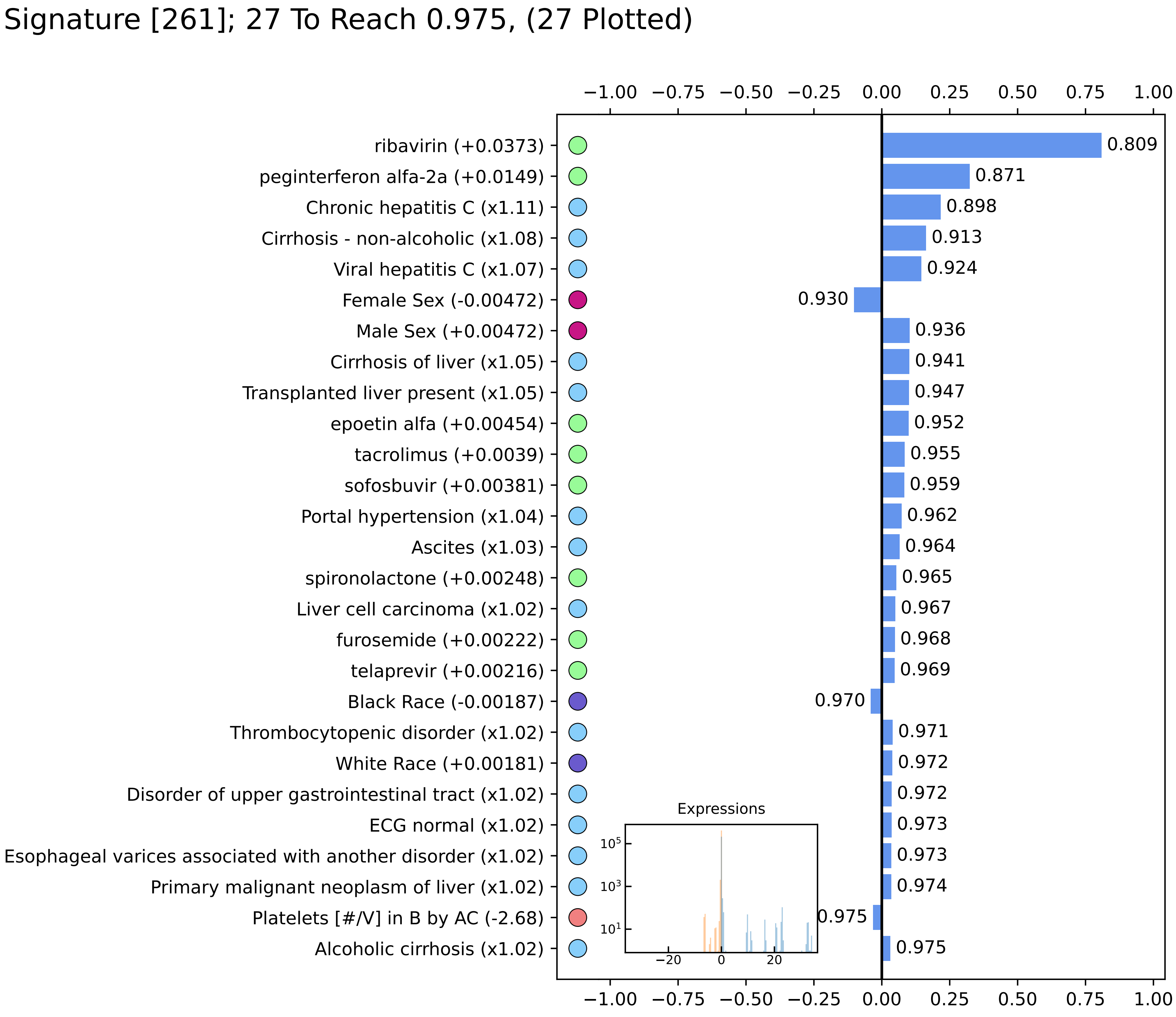
Signature [261]; 27 To Reach 0.975, (27 Plotted)
ribavirin (+0.0373) 0.809
peginterferon alfa-2a (+0.0149) 0.871
Chronic hepatitis C (x1.11) 0.898
Cirrhosis - non-alcoholic (x1.08) 0.913
Viral hepatitis C (x1.07) 0.924
Female Sex (-0.00472) 0.930
Male Sex (+0.00472) 0.936
Cirrhosis of liver (x1.05) 0.941
Transplanted liver present (x1.05) 0.947
epoetin alfa (+0.00454) 0.952
tacrolimus (+0.0039) 0.955
sofosbuvir (+0.00381) 0.959
Portal hypertension (x1.04) 0.962
Ascites (x1.03) 0.964
spironolactone (+0.00248) 0.965
Liver cell carcinoma (x1.02) 0.967
furosemide (+0.00222) 0.968
telaprevir (+0.00216) 0.969
Black Race (-0.00187) 0.970
Thrombocytopenic disorder (x1.02) 0.971
White Race (+0.00181) 0.972
Disorder of upper gastrointestinal tract (x1.02) 0.972
ECG normal (x1.02) 0.973
Esophageal varices associated with another disorder (x1.02) 0.973
Primary malignant neoplasm of liver (x1.02) 0.974
Platelets [#/V] in B by AC (-2.68) 0.975
Alcoholic cirrhosis (x1.02) 0.975
−1.00 −0.75 −0.50 −0.25 0.00 0.25 0.50 0.75 1.00
Expressions
10^5
10^3
10^1
−20 0 20

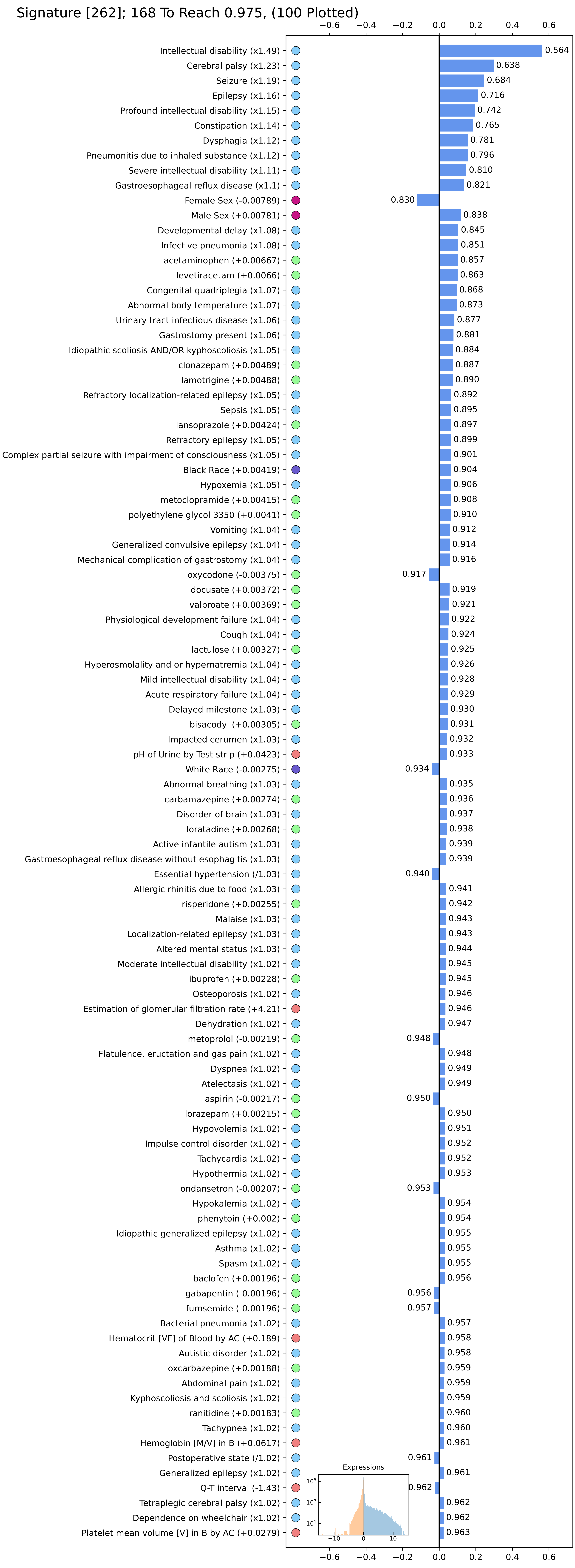

Signature [262]; 168 To Reach 0.975, (100 Plotted)
Intellectual disability (x1.49) 0.564
Cerebral palsy (x1.23) 0.638
Seizure (x1.19) 0.684
Epilepsy (x1.16) 0.716
Profound intellectual disability (x1.15) 0.742
Constipation (x1.14) 0.765
Dysphagia (x1.12) 0.781
Pneumonitis due to inhaled substance (x1.12) 0.796
Severe intellectual disability (x1.11) 0.810
Gastroesophageal reflux disease (x1.1) 0.821
Female Sex (-0.00789) 0.830
Male Sex (+0.00781) 0.838
Developmental delay (x1.08) 0.845
Infective pneumonia (x1.08) 0.851
acetaminophen (+0.00667) 0.857
levetiracetam (+0.0066) 0.863
Congenital quadriplegia (x1.07) 0.868
Abnormal body temperature (x1.07) 0.873
Urinary tract infectious disease (x1.06) 0.877
Gastrostomy present (x1.06) 0.881
Idiopathic scoliosis AND/OR kyphoscoliosis (x1.05) 0.884
clonazepam (+0.00489) 0.887
lamotrigine (+0.00488) 0.890
Refractory localization-related epilepsy (x1.05) 0.892
Sepsis (x1.05) 0.895
lansoprazole (+0.00424) 0.897
Refractory epilepsy (x1.05) 0.899
Complex partial seizure with impairment of consciousness (x1.05) 0.901
Black Race (+0.00419) 0.904
Hypoxemia (x1.05) 0.906
metoclopramide (+0.00415) 0.908
polyethylene glycol 3350 (+0.0041) 0.910
Vomiting (x1.04) 0.912
Generalized convulsive epilepsy (x1.04) 0.914
Mechanical complication of gastrostomy (x1.04) 0.916
oxycodone (-0.00375) 0.917
docusate (+0.00372) 0.919
valproate (+0.00369) 0.921
Physiological development failure (x1.04) 0.922
Cough (x1.04) 0.924
lactulose (+0.00327) 0.925
Hyperosmolality and or hypernatremia (x1.04) 0.926
Mild intellectual disability (x1.04) 0.928
Acute respiratory failure (x1.04) 0.929
Delayed milestone (x1.03) 0.930
bisacodyl (+0.00305) 0.931
Impacted cerumen (x1.03) 0.932
pH of Urine by Test strip (+0.0423) 0.933
White Race (-0.00275) 0.934
Abnormal breathing (x1.03) 0.935
carbamazepine (+0.00274) 0.936
Disorder of brain (x1.03) 0.937
loratadine (+0.00268) 0.938
Active infantile autism (x1.03) 0.939
Gastroesophageal reflux disease without esophagitis (x1.03) 0.939
Essential hypertension (/1.03) 0.940
Allergic rhinitis due to food (x1.03) 0.941
risperidone (+0.00255) 0.942
Malaise (x1.03) 0.943
Localization-related epilepsy (x1.03) 0.943
Altered mental status (x1.03) 0.944
Moderate intellectual disability (x1.02) 0.945
ibuprofen (+0.00228) 0.945
Osteoporosis (x1.02) 0.946
Estimation of glomerular filtration rate (+4.21) 0.946
Dehydration (x1.02) 0.947
metoprolol (-0.00219) 0.948
Flatulence, eructation and gas pain (x1.02) 0.948
Dyspnea (x1.02) 0.949
Atelectasis (x1.02) 0.949
aspirin (-0.00217) 0.950
lorazepam (+0.00215) 0.950
Hypovolemia (x1.02) 0.951
Impulse control disorder (x1.02) 0.952
Tachycardia (x1.02) 0.952
Hypothermia (x1.02) 0.953
ondansetron (-0.00207) 0.953
Hypokalemia (x1.02) 0.954
phenytoin (+0.002) 0.954
Idiopathic generalized epilepsy (x1.02) 0.955
Asthma (x1.02) 0.955
Spasm (x1.02) 0.955
baclofen (+0.00196) 0.956
gabapentin (-0.00196) 0.956
furosemide (-0.00196) 0.957
Bacterial pneumonia (x1.02) 0.957
Hematocrit [VF] of Blood by AC (+0.189) 0.958
Autistic disorder (x1.02) 0.958
oxcarbazepine (+0.00188) 0.959
Abdominal pain (x1.02) 0.959
Kyphoscoliosis and scoliosis (x1.02) 0.959
ranitidine (+0.00183) 0.960
Tachypnea (x1.02) 0.960
Hemoglobin [M/V] in B (+0.0617) 0.961
Postoperative state (/1.02) 0.961
Generalized epilepsy (x1.02) 0.961
Q-T interval (-1.43) 0.962
Tetraplegic cerebral palsy (x1.02) 0.962
Dependence on wheelchair (x1.02) 0.962
Platelet mean volume [V] in B by AC (+0.0279) 0.963
−0.6 −0.4 −0.2 0.0 0.2 0.4 0.6
Expressions

Signature [263]; 221 To Reach 0.975, (100 Plotted)

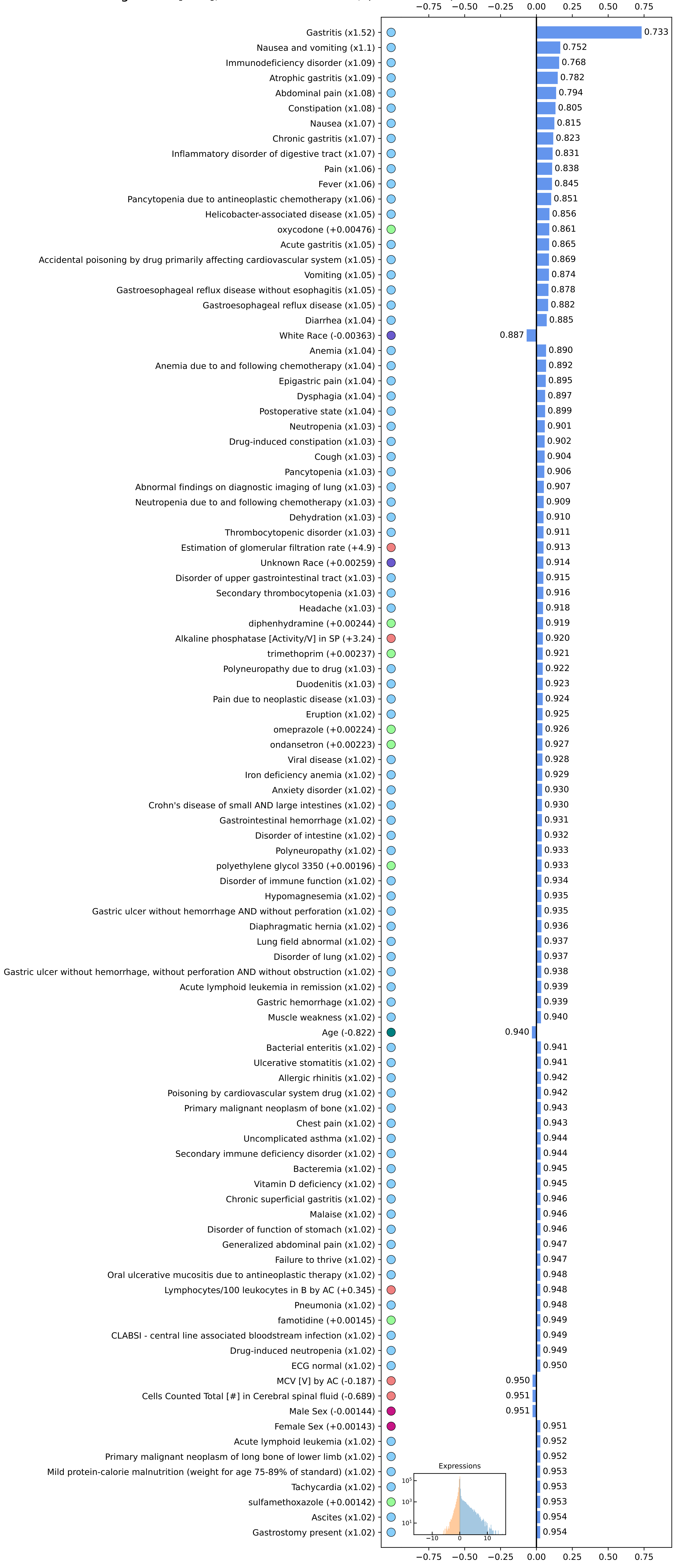

Signature [264]; 1 To Reach 0.975, (10 Plotted)

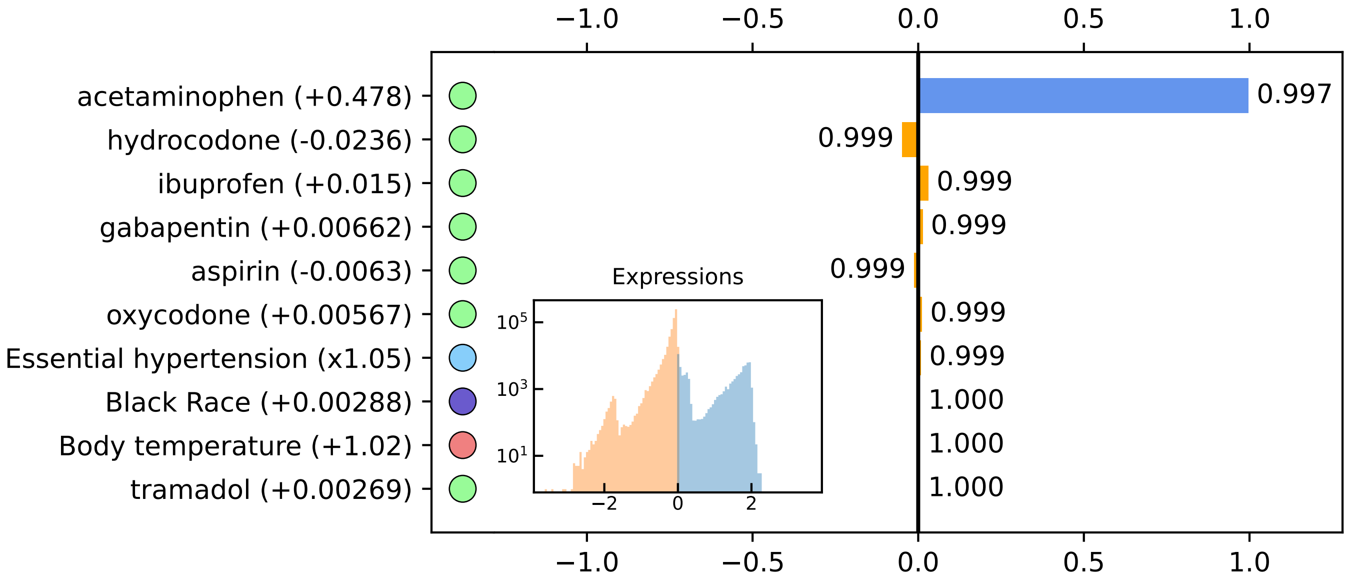

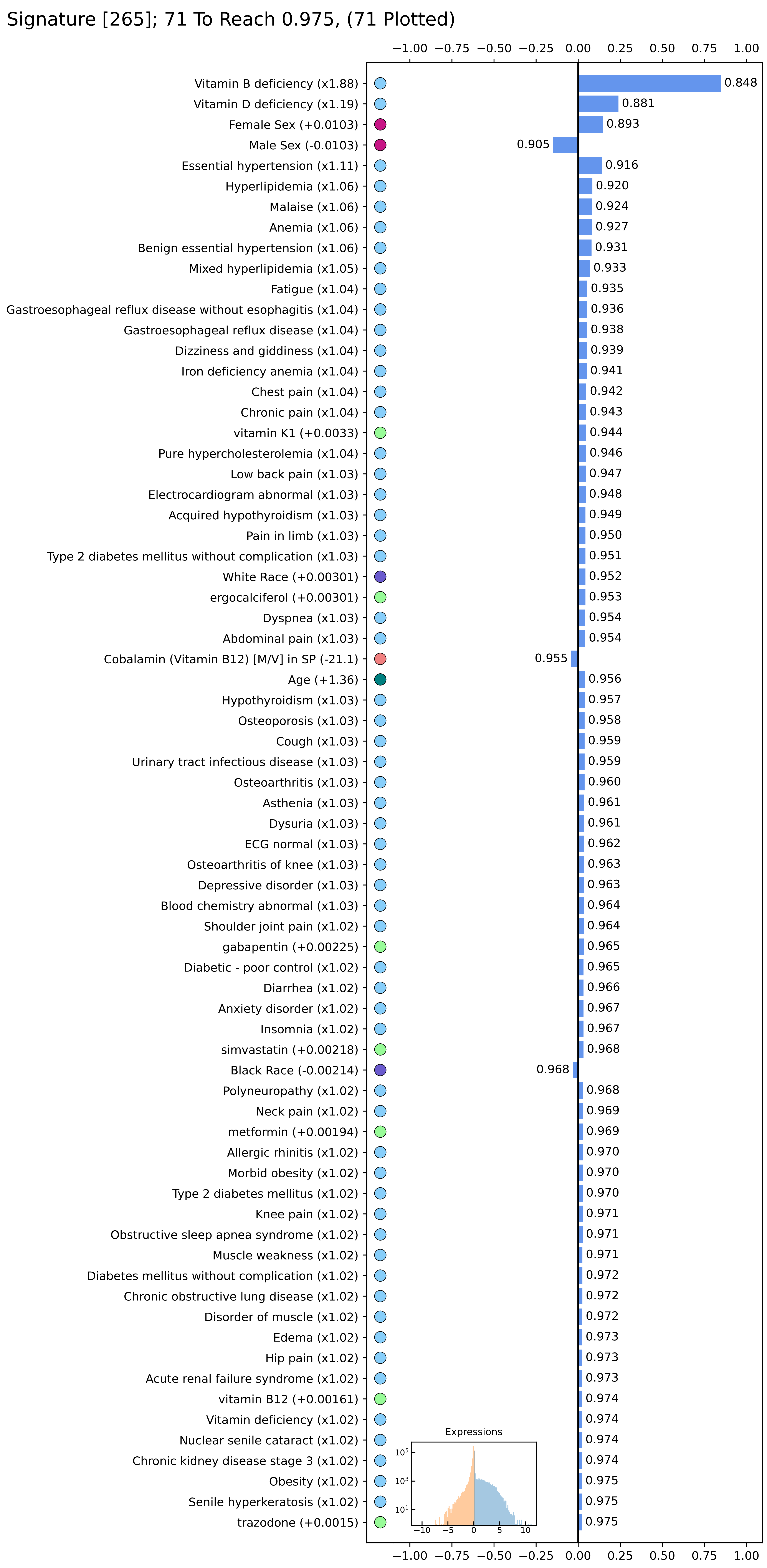

Signature [265]; 71 To Reach 0.975, (71 Plotted)
Vitamin B deficiency (x1.88) 0.848
Vitamin D deficiency (x1.19) 0.881
Female Sex (+0.0103) 0.893
Male Sex (-0.0103) 0.905
Essential hypertension (x1.11) 0.916
Hyperlipidemia (x1.06) 0.920
Malaise (x1.06) 0.924
Anemia (x1.06) 0.927
Benign essential hypertension (x1.06) 0.931
Mixed hyperlipidemia (x1.05) 0.933
Fatigue (x1.04) 0.935
Gastroesophageal reflux disease without esophagitis (x1.04) 0.936
Gastroesophageal reflux disease (x1.04) 0.938
Dizziness and giddiness (x1.04) 0.939
Iron deficiency anemia (x1.04) 0.941
Chest pain (x1.04) 0.942
Chronic pain (x1.04) 0.943
vitamin K1 (+0.0033) 0.944
Pure hypercholesterolemia (x1.04) 0.946
Low back pain (x1.03) 0.947
Electrocardiogram abnormal (x1.03) 0.948
Acquired hypothyroidism (x1.03) 0.949
Pain in limb (x1.03) 0.950
Type 2 diabetes mellitus without complication (x1.03) 0.951
White Race (+0.00301) 0.952
ergocalciferol (+0.00301) 0.953
Dyspnea (x1.03) 0.954
Abdominal pain (x1.03) 0.954
Cobalamin (Vitamin B12) [M/V] in SP (-21.1) 0.955
Age (+1.36) 0.956
Hypothyroidism (x1.03) 0.957
Osteoporosis (x1.03) 0.958
Cough (x1.03) 0.959
Urinary tract infectious disease (x1.03) 0.959
Osteoarthritis (x1.03) 0.960
Asthenia (x1.03) 0.961
Dysuria (x1.03) 0.961
ECG normal (x1.03) 0.962
Osteoarthritis of knee (x1.03) 0.963
Depressive disorder (x1.03) 0.963
Blood chemistry abnormal (x1.03) 0.964
Shoulder joint pain (x1.02) 0.964
gabapentin (+0.00225) 0.965
Diabetic - poor control (x1.02) 0.965
Diarrhea (x1.02) 0.966
Anxiety disorder (x1.02) 0.967
Insomnia (x1.02) 0.967
simvastatin (+0.00218) 0.968
Black Race (-0.00214) 0.968
Polyneuropathy (x1.02) 0.968
Neck pain (x1.02) 0.969
metformin (+0.00194) 0.969
Allergic rhinitis (x1.02) 0.970
Morbid obesity (x1.02) 0.970
Type 2 diabetes mellitus (x1.02) 0.970
Knee pain (x1.02) 0.971
Obstructive sleep apnea syndrome (x1.02) 0.971
Muscle weakness (x1.02) 0.971
Diabetes mellitus without complication (x1.02) 0.972
Chronic obstructive lung disease (x1.02) 0.972
Disorder of muscle (x1.02) 0.972
Edema (x1.02) 0.973
Hip pain (x1.02) 0.973
Acute renal failure syndrome (x1.02) 0.973
vitamin B12 (+0.00161) 0.974
Vitamin deficiency (x1.02) 0.974
Nuclear senile cataract (x1.02) 0.974
Chronic kidney disease stage 3 (x1.02) 0.974
Obesity (x1.02) 0.975
Senile hyperkeratosis (x1.02) 0.975
trazodone (+0.0015) 0.975
−1.00 −0.75 −0.50 −0.25 0.00 0.25 0.50 0.75 1.00
Expressions

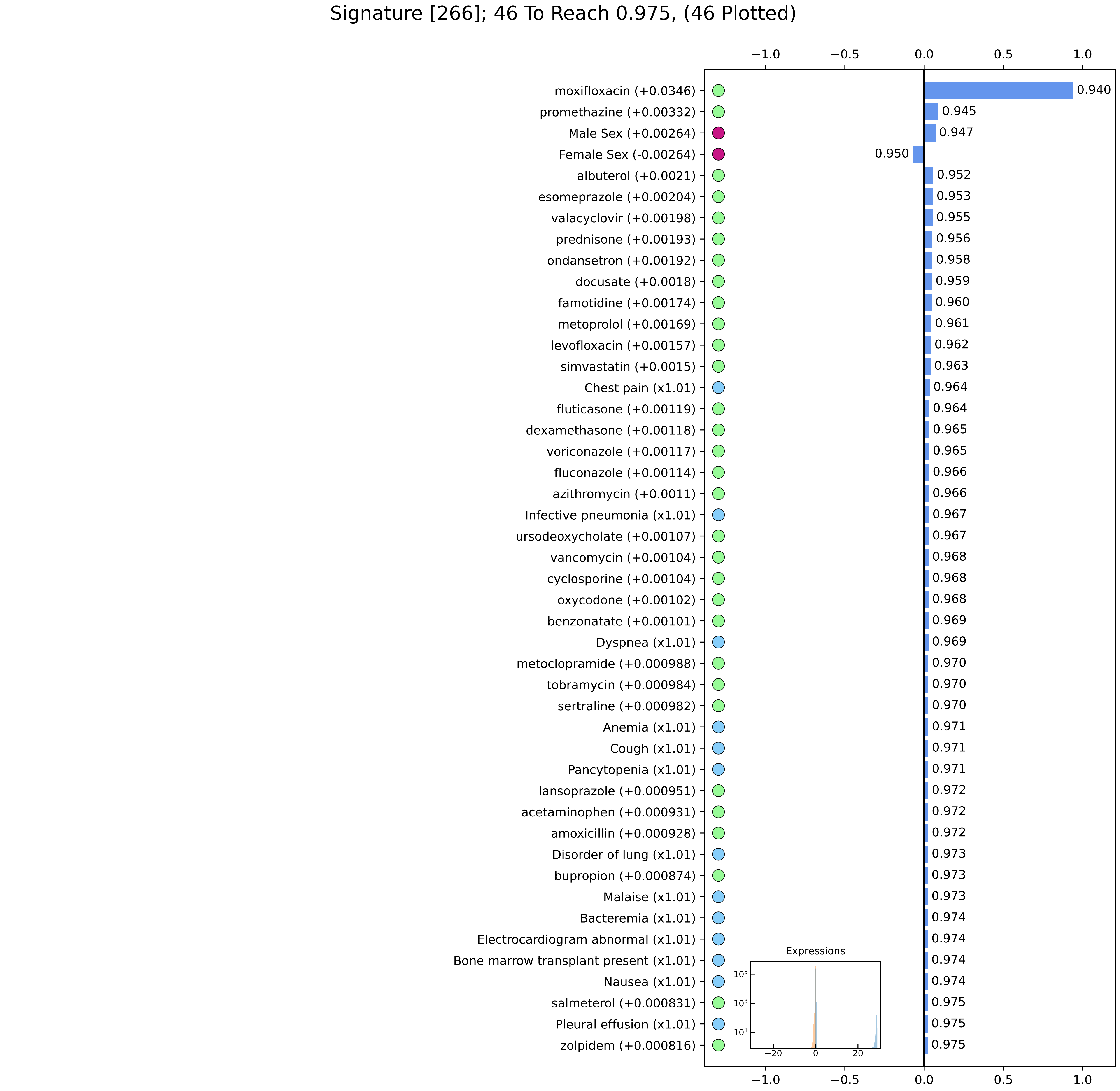

Signature [266]; 46 To Reach 0.975, (46 Plotted)
moxifloxacin (+0.0346) 0.940
promethazine (+0.00332) 0.945
Male Sex (+0.00264) 0.947
Female Sex (-0.00264) 0.950
albuterol (+0.0021) 0.952
esomeprazole (+0.00204) 0.953
valacyclovir (+0.00198) 0.955
prednisone (+0.00193) 0.956
ondansetron (+0.00192) 0.958
docusate (+0.0018) 0.959
famotidine (+0.00174) 0.960
metoprolol (+0.00169) 0.961
levofloxacin (+0.00157) 0.962
simvastatin (+0.0015) 0.963
Chest pain (x1.01) 0.964
fluticasone (+0.00119) 0.964
dexamethasone (+0.00118) 0.965
voriconazole (+0.00117) 0.965
fluconazole (+0.00114) 0.966
azithromycin (+0.0011) 0.966
Infective pneumonia (x1.01) 0.967
ursodeoxycholate (+0.00107) 0.967
vancomycin (+0.00104) 0.968
cyclosporine (+0.00104) 0.968
oxycodone (+0.00102) 0.968
benzonatate (+0.00101) 0.969
Dyspnea (x1.01) 0.969
metoclopramide (+0.000988) 0.970
tobramycin (+0.000984) 0.970
sertraline (+0.000982) 0.970
Anemia (x1.01) 0.971
Cough (x1.01) 0.971
Pancytopenia (x1.01) 0.971
lansoprazole (+0.000951) 0.972
acetaminophen (+0.000931) 0.972
amoxicillin (+0.000928) 0.972
Disorder of lung (x1.01) 0.973
bupropion (+0.000874) 0.973
Malaise (x1.01) 0.973
Bacteremia (x1.01) 0.974
Electrocardiogram abnormal (x1.01) 0.974
Bone marrow transplant present (x1.01) 0.974
Nausea (x1.01) 0.974
salmeterol (+0.000831) 0.975
Pleural effusion (x1.01) 0.975
zolpidem (+0.000816) 0.975
−1.0 −0.5 0.0 0.5 1.0
Expressions
10^5 10^3 10^1
−20 0 20

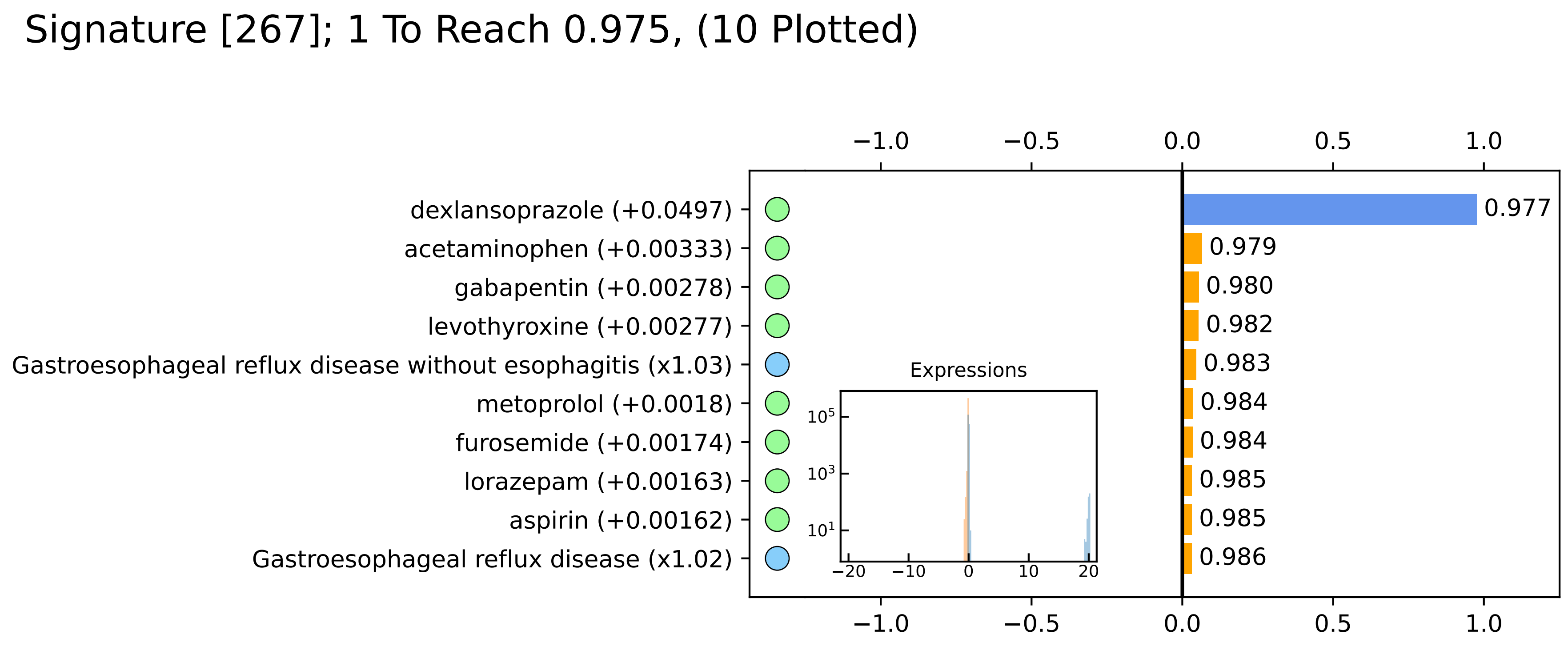

Signature [267]; 1 To Reach 0.975, (10 Plotted)
dexlansoprazole (+0.0497)
acetaminophen (+0.00333)
gabapentin (+0.00278)
levothyroxine (+0.00277)
Gastroesophageal reflux disease without esophagitis (x1.03)
metoprolol (+0.0018)
furosemide (+0.00174)
lorazepam (+0.00163)
aspirin (+0.00162)
Gastroesophageal reflux disease (x1.02)
0.977
0.979
0.980
0.982
0.983
0.984
0.984
0.985
0.985
0.986
Expressions
−1.0 −0.5 0.0 0.5 1.0

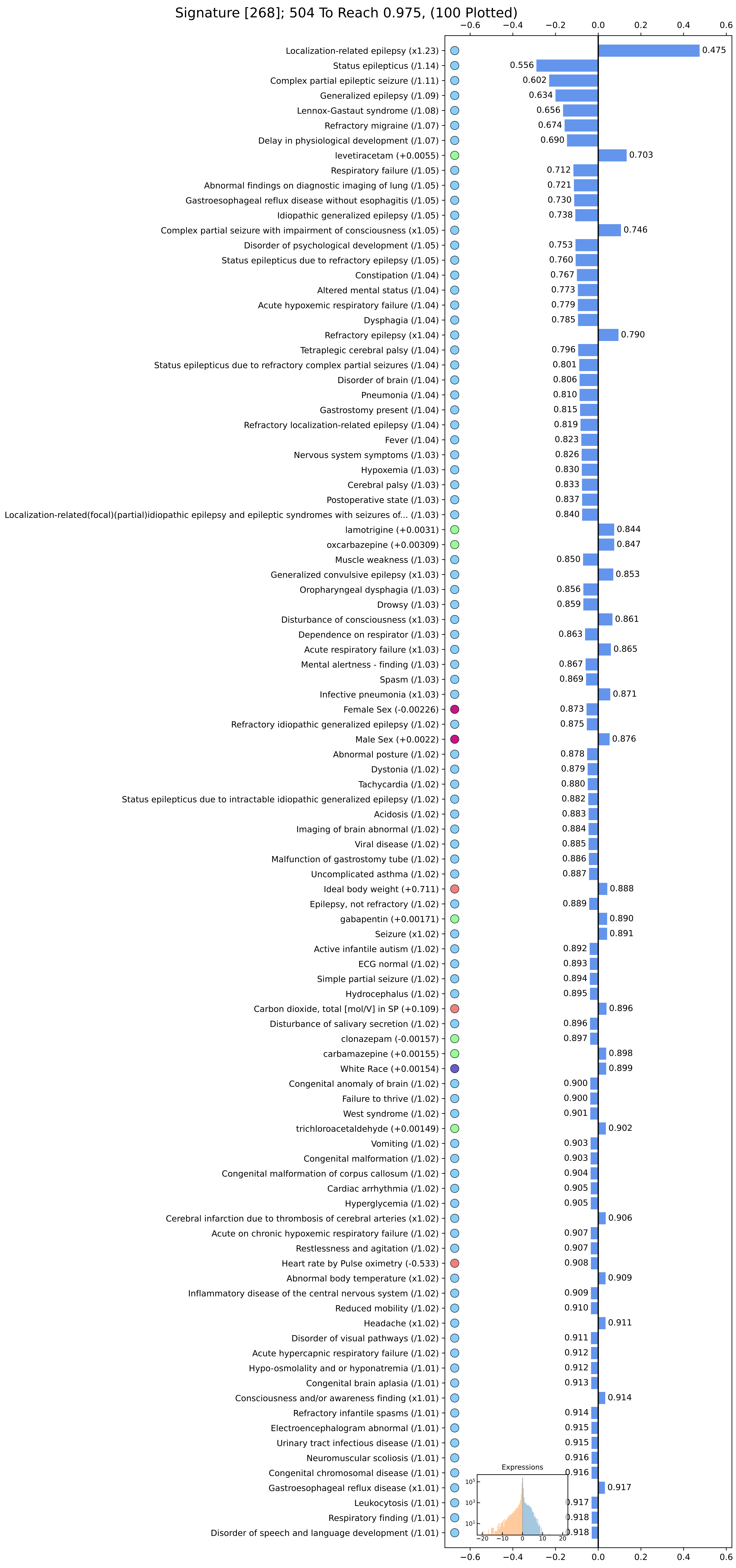
Signature [268]; 504 To Reach 0.975, (100 Plotted)
−0.6 −0.4 −0.2 0.0 0.2 0.4 0.6
Localization-related epilepsy (x1.23) 0.475
Status epilepticus (/1.14) 0.556
Complex partial epileptic seizure (/1.11) 0.602
Generalized epilepsy (/1.09) 0.634
Lennox-Gastaut syndrome (/1.08) 0.656
Refractory migraine (/1.07) 0.674
Delay in physiological development (/1.07) 0.690
levetiracetam (+0.0055) 0.703
Respiratory failure (/1.05) 0.712
Abnormal findings on diagnostic imaging of lung (/1.05) 0.721
Gastroesophageal reflux disease without esophagitis (/1.05) 0.730
Idiopathic generalized epilepsy (/1.05) 0.738
Complex partial seizure with impairment of consciousness (x1.05) 0.746
Disorder of psychological development (/1.05) 0.753
Status epilepticus due to refractory epilepsy (/1.05) 0.760
Constipation (/1.04) 0.767
Altered mental status (/1.04) 0.773
Acute hypoxemic respiratory failure (/1.04) 0.779
Dysphagia (/1.04) 0.785
Refractory epilepsy (x1.04) 0.790
Tetraplegic cerebral palsy (/1.04) 0.796
Status epilepticus due to refractory complex partial seizures (/1.04) 0.801
Disorder of brain (/1.04) 0.806
Pneumonia (/1.04) 0.810
Gastrostomy present (/1.04) 0.815
Refractory localization-related epilepsy (/1.04) 0.819
Fever (/1.04) 0.823
Nervous system symptoms (/1.03) 0.826
Hypoxemia (/1.03) 0.830
Cerebral palsy (/1.03) 0.833
Postoperative state (/1.03) 0.837
Localization-related(focal)(partial)idiopathic epilepsy and epileptic syndromes with seizures of... (/1.03) 0.840
lamotrigine (+0.0031) 0.844
oxcarbazepine (+0.00309) 0.847
Muscle weakness (/1.03) 0.850
Generalized convulsive epilepsy (x1.03) 0.853
Oropharyngeal dysphagia (/1.03) 0.856
Drowsy (/1.03) 0.859
Disturbance of consciousness (x1.03) 0.861
Dependence on respirator (/1.03) 0.863
Acute respiratory failure (x1.03) 0.865
Mental alertness - finding (/1.03) 0.867
Spasm (/1.03) 0.869
Infective pneumonia (x1.03) 0.871
Female Sex (-0.00226) 0.873
Refractory idiopathic generalized epilepsy (/1.02) 0.875
Male Sex (+0.0022) 0.876
Abnormal posture (/1.02) 0.878
Dystonia (/1.02) 0.879
Tachycardia (/1.02) 0.880
Status epilepticus due to intractable idiopathic generalized epilepsy (/1.02) 0.882
Acidosis (/1.02) 0.883
Imaging of brain abnormal (/1.02) 0.884
Viral disease (/1.02) 0.885
Malfunction of gastrostomy tube (/1.02) 0.886
Uncomplicated asthma (/1.02) 0.887
Ideal body weight (+0.711) 0.888
Epilepsy, not refractory (/1.02) 0.889
gabapentin (+0.00171) 0.890
Seizure (x1.02) 0.891
Active infantile autism (/1.02) 0.892
ECG normal (/1.02) 0.893
Simple partial seizure (/1.02) 0.894
Hydrocephalus (/1.02) 0.895
Carbon dioxide, total [mol/V] in SP (+0.109) 0.896
Disturbance of salivary secretion (/1.02) 0.896
clonazepam (-0.00157) 0.897
carbamazepine (+0.00155) 0.898
White Race (+0.00154) 0.899
Congenital anomaly of brain (/1.02) 0.900
Failure to thrive (/1.02) 0.900
West syndrome (/1.02) 0.901
trichloroacetaldehyde (+0.00149) 0.902
Vomiting (/1.02) 0.903
Congenital malformation (/1.02) 0.903
Congenital malformation of corpus callosum (/1.02) 0.904
Cardiac arrhythmia (/1.02) 0.905
Hyperglycemia (/1.02) 0.905
Cerebral infarction due to thrombosis of cerebral arteries (x1.02) 0.906
Acute on chronic hypoxemic respiratory failure (/1.02) 0.907
Restlessness and agitation (/1.02) 0.907
Heart rate by Pulse oximetry (-0.533) 0.908
Abnormal body temperature (x1.02) 0.909
Inflammatory disease of the central nervous system (/1.02) 0.909
Reduced mobility (/1.02) 0.910
Headache (x1.02) 0.911
Disorder of visual pathways (/1.02) 0.911
Acute hypercapnic respiratory failure (/1.02) 0.912
Hypo-osmolality and or hyponatremia (/1.01) 0.912
Congenital brain aplasia (/1.01) 0.913
Consciousness and/or awareness finding (x1.01) 0.914
Refractory infantile spasms (/1.01) 0.914
Electroencephalogram abnormal (/1.01) 0.915
Urinary tract infectious disease (/1.01) 0.915
Neuromuscular scoliosis (/1.01) 0.916
Congenital chromosomal disease (/1.01) 0.916
Gastroesophageal reflux disease (x1.01) 0.917
Leukocytosis (/1.01) 0.917
Respiratory finding (/1.01) 0.918
Disorder of speech and language development (/1.01) 0.918
Expressions
−20 −10 0 10 20
−0.6 −0.4 −0.2 0.0 0.2 0.4 0.6

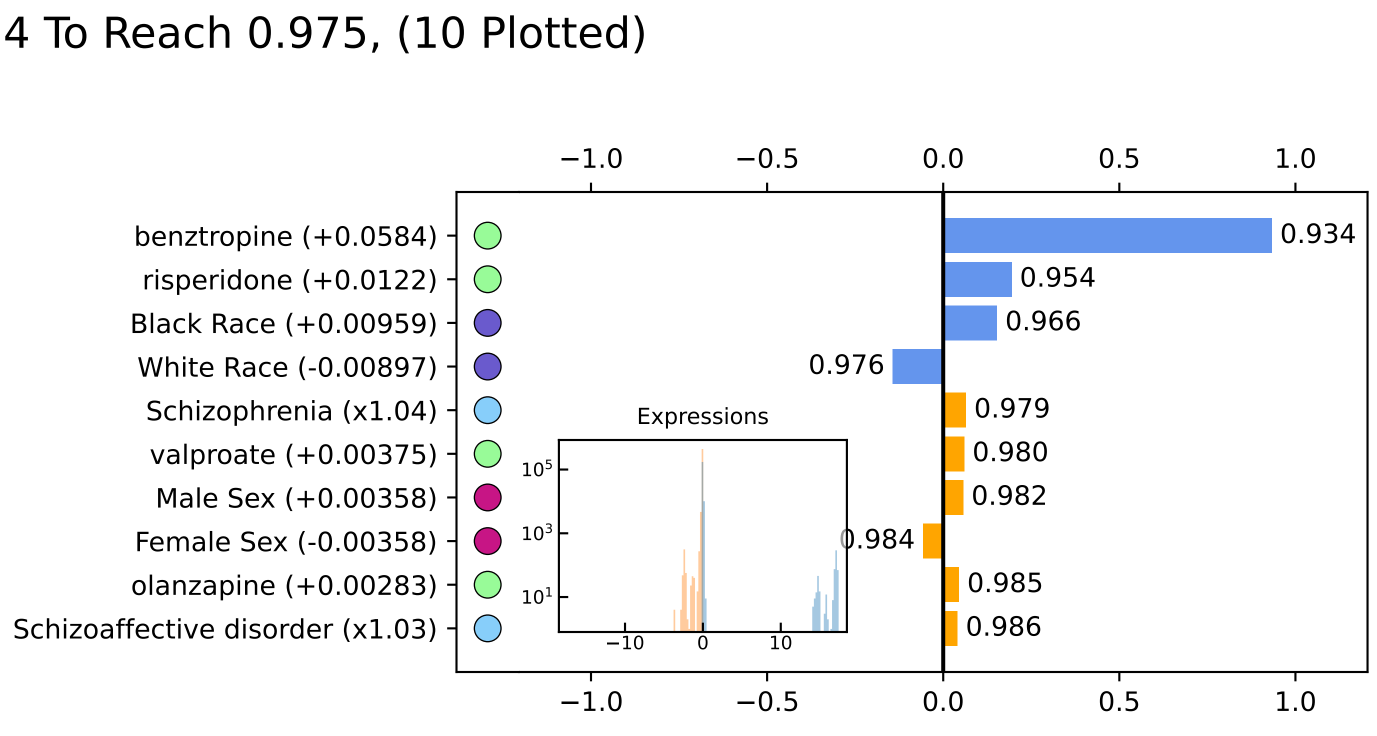

Signature [269]; 4 To Reach 0.975, (10 Plotted)
benztropine (+0.0584)
risperidone (+0.0122)
Black Race (+0.00959)
White Race (-0.00897)
Schizophrenia (x1.04)
valproate (+0.00375)
Male Sex (+0.00358)
Female Sex (-0.00358)
olanzapine (+0.00283)
Schizoaffective disorder (x1.03)
0.934
0.954
0.966
0.976
0.979
0.980
0.982
0.984
0.985
0.986
Expressions
−1.0
−0.5
0.0
0.5
1.0

Signature [270]; 209 To Reach 0.975, (100 Plotted)

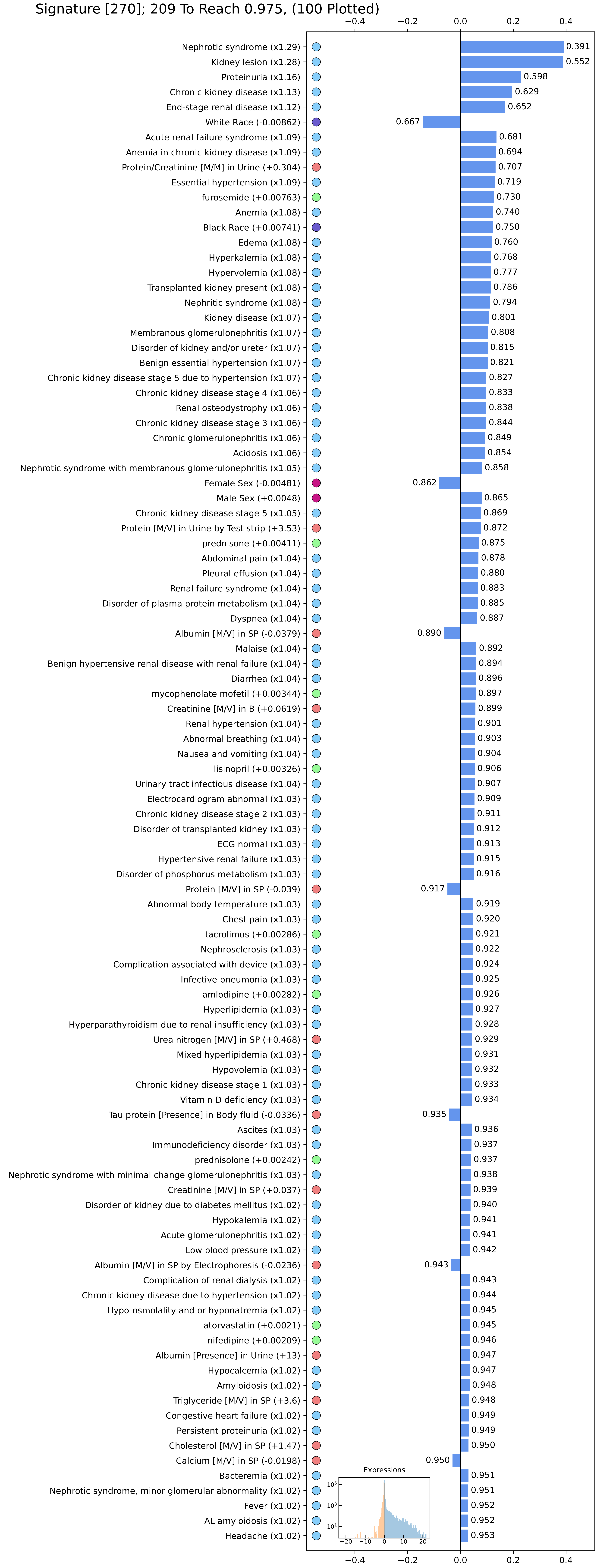

Signature [271]; 1 To Reach 0.975, (10 Plotted)

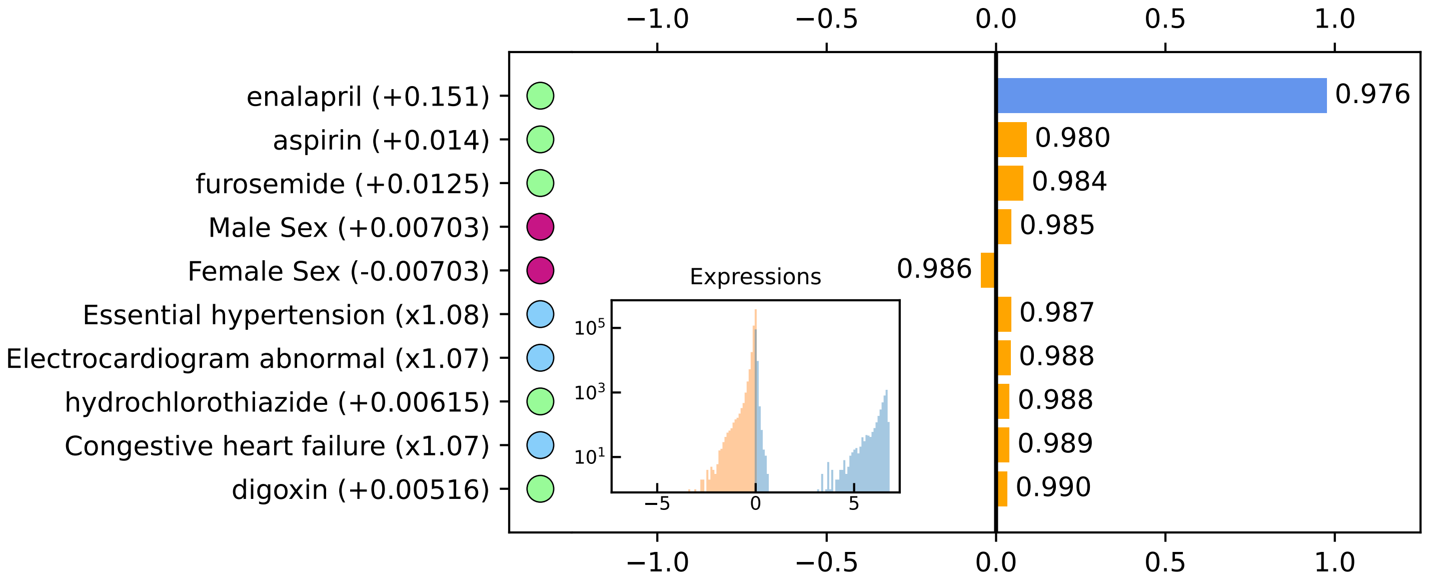

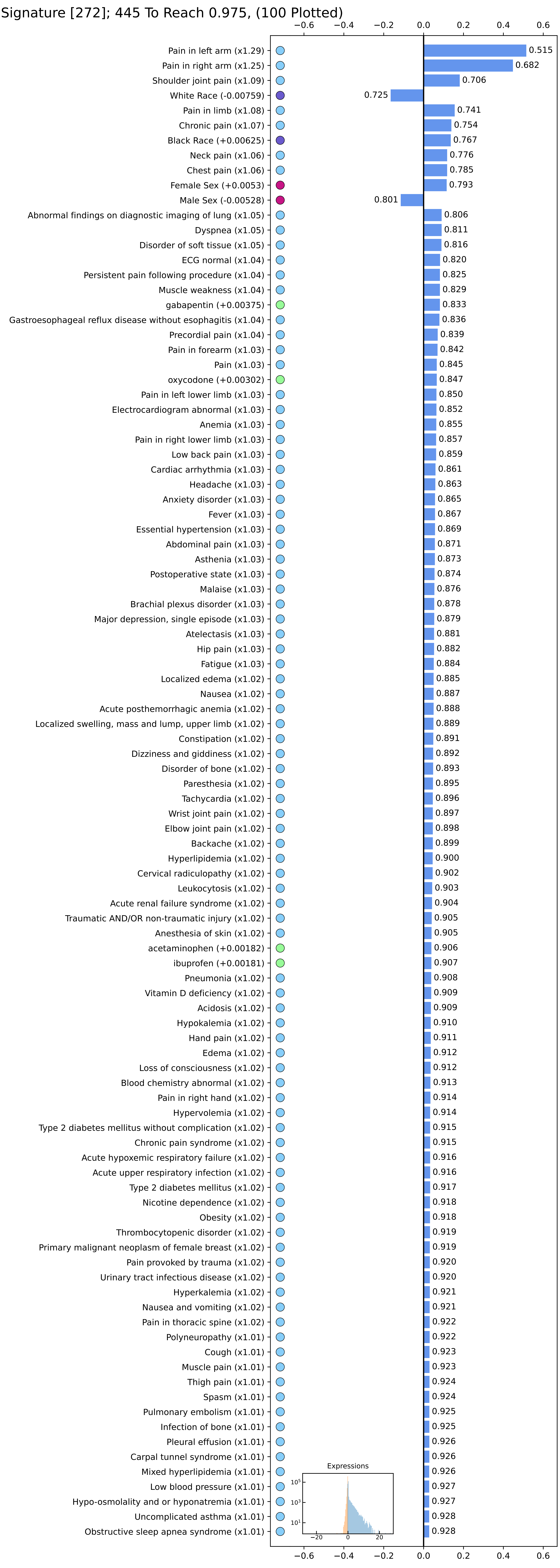
Signature [272]; 445 To Reach 0.975, (100 Plotted)
Pain in left arm (x1.29) 0.515
Pain in right arm (x1.25) 0.682
Shoulder joint pain (x1.09) 0.706
White Race (-0.00759) 0.725
Pain in limb (x1.08) 0.741
Chronic pain (x1.07) 0.754
Black Race (+0.00625) 0.767
Neck pain (x1.06) 0.776
Chest pain (x1.06) 0.785
Female Sex (+0.0053) 0.793
Male Sex (-0.00528) 0.801
Abnormal findings on diagnostic imaging of lung (x1.05) 0.806
Dyspnea (x1.05) 0.811
Disorder of soft tissue (x1.05) 0.816
ECG normal (x1.04) 0.820
Persistent pain following procedure (x1.04) 0.825
Muscle weakness (x1.04) 0.829
gabapentin (+0.00375) 0.833
Gastroesophageal reflux disease without esophagitis (x1.04) 0.836
Precordial pain (x1.04) 0.839
Pain in forearm (x1.03) 0.842
Pain (x1.03) 0.845
oxycodone (+0.00302) 0.847
Pain in left lower limb (x1.03) 0.850
Electrocardiogram abnormal (x1.03) 0.852
Anemia (x1.03) 0.855
Pain in right lower limb (x1.03) 0.857
Low back pain (x1.03) 0.859
Cardiac arrhythmia (x1.03) 0.861
Headache (x1.03) 0.863
Anxiety disorder (x1.03) 0.865
Fever (x1.03) 0.867
Essential hypertension (x1.03) 0.869
Abdominal pain (x1.03) 0.871
Asthenia (x1.03) 0.873
Postoperative state (x1.03) 0.874
Malaise (x1.03) 0.876
Brachial plexus disorder (x1.03) 0.878
Major depression, single episode (x1.03) 0.879
Atelectasis (x1.03) 0.881
Hip pain (x1.03) 0.882
Fatigue (x1.03) 0.884
Localized edema (x1.02) 0.885
Nausea (x1.02) 0.887
Acute posthemorrhagic anemia (x1.02) 0.888
Localized swelling, mass and lump, upper limb (x1.02) 0.889
Constipation (x1.02) 0.891
Dizziness and giddiness (x1.02) 0.892
Disorder of bone (x1.02) 0.893
Paresthesia (x1.02) 0.895
Tachycardia (x1.02) 0.896
Wrist joint pain (x1.02) 0.897
Elbow joint pain (x1.02) 0.898
Backache (x1.02) 0.899
Hyperlipidemia (x1.02) 0.900
Cervical radiculopathy (x1.02) 0.902
Leukocytosis (x1.02) 0.903
Acute renal failure syndrome (x1.02) 0.904
Traumatic AND/OR non-traumatic injury (x1.02) 0.905
Anesthesia of skin (x1.02) 0.905
acetaminophen (+0.00182) 0.906
ibuprofen (+0.00181) 0.907
Pneumonia (x1.02) 0.908
Vitamin D deficiency (x1.02) 0.909
Acidosis (x1.02) 0.909
Hypokalemia (x1.02) 0.910
Hand pain (x1.02) 0.911
Edema (x1.02) 0.912
Loss of consciousness (x1.02) 0.912
Blood chemistry abnormal (x1.02) 0.913
Pain in right hand (x1.02) 0.914
Hypervolemia (x1.02) 0.914
Type 2 diabetes mellitus without complication (x1.02) 0.915
Chronic pain syndrome (x1.02) 0.915
Acute hypoxemic respiratory failure (x1.02) 0.916
Acute upper respiratory infection (x1.02) 0.916
Type 2 diabetes mellitus (x1.02) 0.917
Nicotine dependence (x1.02) 0.918
Obesity (x1.02) 0.918
Thrombocytopenic disorder (x1.02) 0.919
Primary malignant neoplasm of female breast (x1.02) 0.919
Pain provoked by trauma (x1.02) 0.920
Urinary tract infectious disease (x1.02) 0.920
Hyperkalemia (x1.02) 0.921
Nausea and vomiting (x1.02) 0.921
Pain in thoracic spine (x1.02) 0.922
Polyneuropathy (x1.01) 0.922
Cough (x1.01) 0.923
Muscle pain (x1.01) 0.923
Thigh pain (x1.01) 0.924
Spasm (x1.01) 0.924
Pulmonary embolism (x1.01) 0.925
Infection of bone (x1.01) 0.925
Pleural effusion (x1.01) 0.926
Carpal tunnel syndrome (x1.01) 0.926
Mixed hyperlipidemia (x1.01) 0.926
Low blood pressure (x1.01) 0.927
Hypo-osmolality and or hyponatremia (x1.01) 0.927
Uncomplicated asthma (x1.01) 0.928
Obstructive sleep apnea syndrome (x1.01) 0.928
−0.6 −0.4 −0.2 0.0 0.2 0.4 0.6
Expressions
−20 0 20

Signature [273]; 57 To Reach 0.975, (57 Plotted)

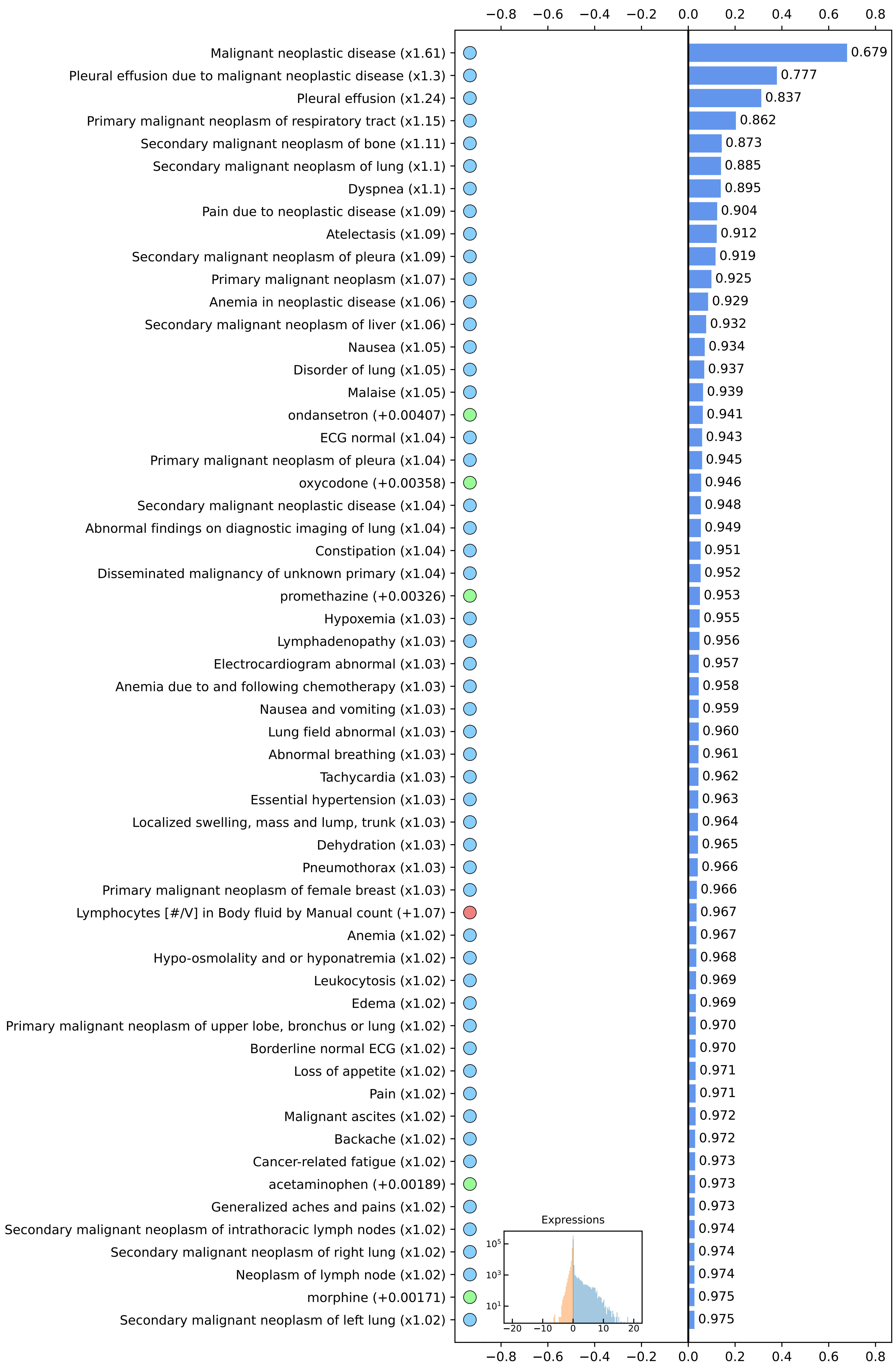

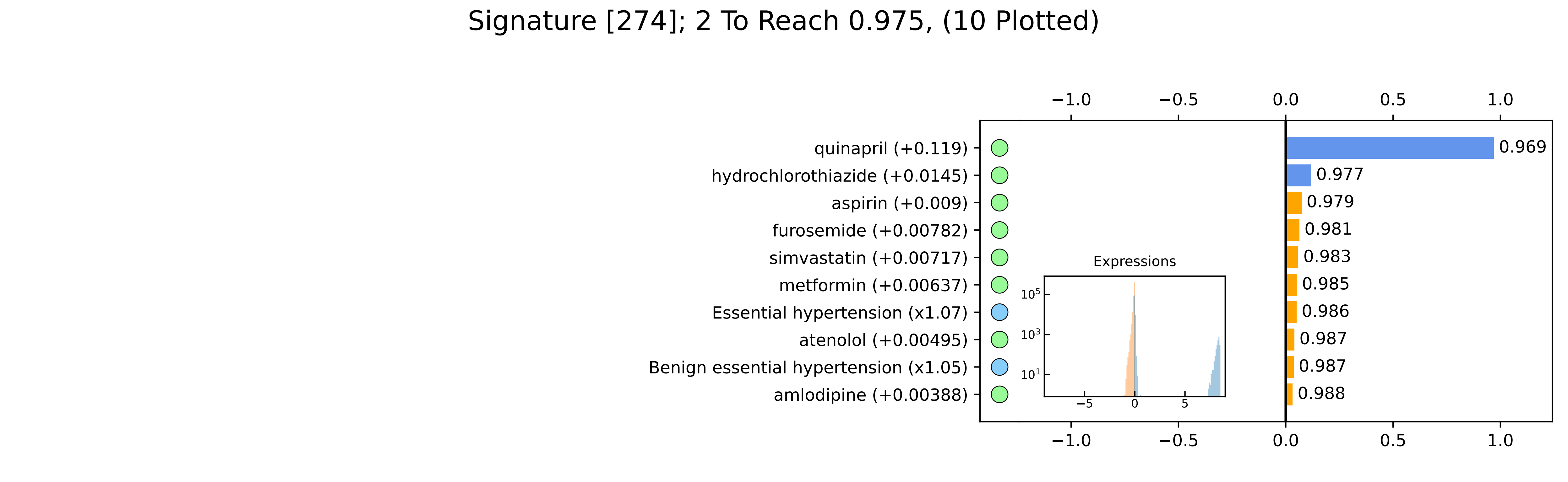

Signature [274]; 2 To Reach 0.975, (10 Plotted)
quinapril (+0.119)
hydrochlorothiazide (+0.0145)
aspirin (+0.009)
furosemide (+0.00782)
simvastatin (+0.00717)
metformin (+0.00637)
Essential hypertension (x1.07)
atenolol (+0.00495)
Benign essential hypertension (x1.05)
amlodipine (+0.00388)
0.969
0.977
0.979
0.981
0.983
0.985
0.986
0.987
0.987
0.988
Expressions
−1.0
−0.5
0.0
0.5
1.0

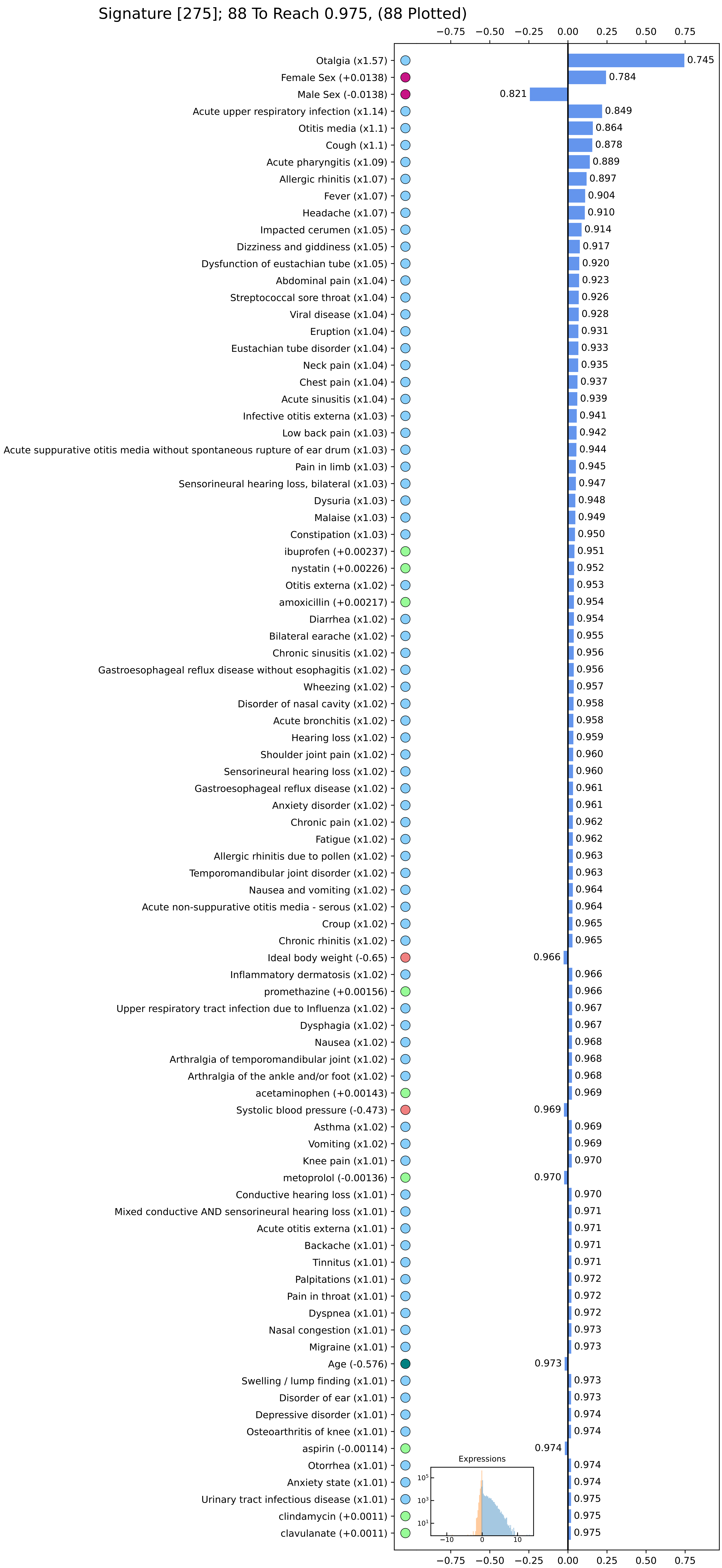

Signature [275]; 88 To Reach 0.975, (88 Plotted)
Otalgia (x1.57) 0.745
Female Sex (+0.0138) 0.784
Male Sex (-0.0138) 0.821
Acute upper respiratory infection (x1.14) 0.849
Otitis media (x1.1) 0.864
Cough (x1.1) 0.878
Acute pharyngitis (x1.09) 0.889
Allergic rhinitis (x1.07) 0.897
Fever (x1.07) 0.904
Headache (x1.07) 0.910
Impacted cerumen (x1.05) 0.914
Dizziness and giddiness (x1.05) 0.917
Dysfunction of eustachian tube (x1.05) 0.920
Abdominal pain (x1.04) 0.923
Streptococcal sore throat (x1.04) 0.926
Viral disease (x1.04) 0.928
Eruption (x1.04) 0.931
Eustachian tube disorder (x1.04) 0.933
Neck pain (x1.04) 0.935
Chest pain (x1.04) 0.937
Acute sinusitis (x1.04) 0.939
Infective otitis externa (x1.03) 0.941
Low back pain (x1.03) 0.942
Acute suppurative otitis media without spontaneous rupture of ear drum (x1.03) 0.944
Pain in limb (x1.03) 0.945
Sensorineural hearing loss, bilateral (x1.03) 0.947
Dysuria (x1.03) 0.948
Malaise (x1.03) 0.949
Constipation (x1.03) 0.950
ibuprofen (+0.00237) 0.951
nystatin (+0.00226) 0.952
Otitis externa (x1.02) 0.953
amoxicillin (+0.00217) 0.954
Diarrhea (x1.02) 0.954
Bilateral earache (x1.02) 0.955
Chronic sinusitis (x1.02) 0.956
Gastroesophageal reflux disease without esophagitis (x1.02) 0.956
Wheezing (x1.02) 0.957
Disorder of nasal cavity (x1.02) 0.958
Acute bronchitis (x1.02) 0.958
Hearing loss (x1.02) 0.959
Shoulder joint pain (x1.02) 0.960
Sensorineural hearing loss (x1.02) 0.960
Gastroesophageal reflux disease (x1.02) 0.961
Anxiety disorder (x1.02) 0.961
Chronic pain (x1.02) 0.962
Fatigue (x1.02) 0.962
Allergic rhinitis due to pollen (x1.02) 0.963
Temporomandibular joint disorder (x1.02) 0.963
Nausea and vomiting (x1.02) 0.964
Acute non-suppurative otitis media - serous (x1.02) 0.964
Croup (x1.02) 0.965
Chronic rhinitis (x1.02) 0.965
Ideal body weight (-0.65) 0.966
Inflammatory dermatosis (x1.02) 0.966
promethazine (+0.00156) 0.966
Upper respiratory tract infection due to Influenza (x1.02) 0.967
Dysphagia (x1.02) 0.967
Nausea (x1.02) 0.968
Arthralgia of temporomandibular joint (x1.02) 0.968
Arthralgia of the ankle and/or foot (x1.02) 0.968
acetaminophen (+0.00143) 0.969
Systolic blood pressure (-0.473) 0.969
Asthma (x1.02) 0.969
Vomiting (x1.02) 0.969
Knee pain (x1.01) 0.970
metoprolol (-0.00136) 0.970
Conductive hearing loss (x1.01) 0.970
Mixed conductive AND sensorineural hearing loss (x1.01) 0.971
Acute otitis externa (x1.01) 0.971
Backache (x1.01) 0.971
Tinnitus (x1.01) 0.971
Palpitations (x1.01) 0.972
Pain in throat (x1.01) 0.972
Dyspnea (x1.01) 0.972
Nasal congestion (x1.01) 0.973
Migraine (x1.01) 0.973
Age (-0.576) 0.973
Swelling / lump finding (x1.01) 0.973
Disorder of ear (x1.01) 0.973
Depressive disorder (x1.01) 0.974
Osteoarthritis of knee (x1.01) 0.974
aspirin (-0.00114) 0.974
Otorrhea (x1.01) 0.974
Anxiety state (x1.01) 0.974
Urinary tract infectious disease (x1.01) 0.975
clindamycin (+0.0011) 0.975
clavulanate (+0.0011) 0.975
Expressions
−0.75 −0.50 −0.25 0.00 0.25 0.50 0.75

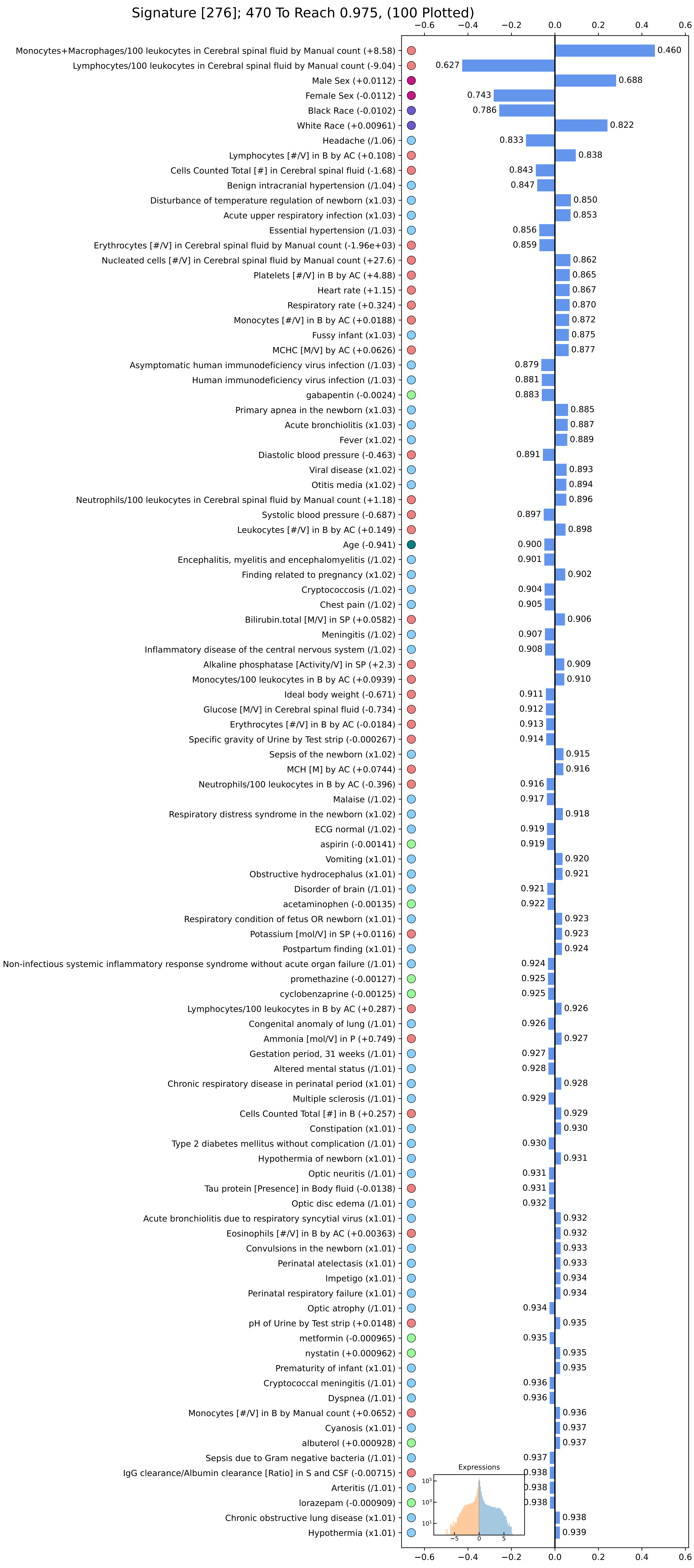

Signature [276]; 470 To Reach 0.975, (100 Plotted)
Monocytes+Macrophages/100 leukocytes in Cerebral spinal fluid by Manual count (+8.58)
Lymphocytes/100 leukocytes in Cerebral spinal fluid by Manual count (-9.04)
Male Sex (+0.0112)
Female Sex (-0.0112)
Black Race (-0.0102)
White Race (+0.00961)
Headache (/1.06)
Lymphocytes [#/V] in B by AC (+0.108)
Cells Counted Total [#] in Cerebral spinal fluid (-1.68)
Benign intracranial hypertension (/1.04)
Disturbance of temperature regulation of newborn (x1.03)
Acute upper respiratory infection (x1.03)
Essential hypertension (/1.03)
Erythrocytes [#/V] in Cerebral spinal fluid by Manual count (-1.96e+03)
Nucleated cells [#/V] in Cerebral spinal fluid by Manual count (+27.6)
Platelets [#/V] in B by AC (+4.88)
Heart rate (+1.15)
Respiratory rate (+0.324)
Monocytes [#/V] in B by AC (+0.0188)
Fussy infant (x1.03)
MCHC [M/V] by AC (+0.0626)
Asymptomatic human immunodeficiency virus infection (/1.03)
Human immunodeficiency virus infection (/1.03)
gabapentin (-0.0024)
Primary apnea in the newborn (x1.03)
Acute bronchiolitis (x1.03)
Fever (x1.02)
Diastolic blood pressure (-0.463)
Viral disease (x1.02)
Otitis media (x1.02)
Neutrophils/100 leukocytes in Cerebral spinal fluid by Manual count (+1.18)
Systolic blood pressure (-0.687)
Leukocytes [#/V] in B by AC (+0.149)
Age (-0.941)
Encephalitis, myelitis and encephalomyelitis (/1.02)
Finding related to pregnancy (x1.02)
Cryptococcosis (/1.02)
Chest pain (/1.02)
Bilirubin.total [M/V] in SP (+0.0582)
Meningitis (/1.02)
Inflammatory disease of the central nervous system (/1.02)
Alkaline phosphatase [Activity/V] in SP (+2.3)
Monocytes/100 leukocytes in B by AC (+0.0939)
Ideal body weight (-0.671)
Glucose [M/V] in Cerebral spinal fluid (-0.734)
Erythrocytes [#/V] in B by AC (-0.0184)
Specific gravity of Urine by Test strip (-0.000267)
Sepsis of the newborn (x1.02)
MCH [M] by AC (+0.0744)
Neutrophils/100 leukocytes in B by AC (-0.396)
Malaise (/1.02)
Respiratory distress syndrome in the newborn (x1.02)
ECG normal (/1.02)
aspirin (-0.00141)
Vomiting (x1.01)
Obstructive hydrocephalus (x1.01)
Disorder of brain (/1.01)
acetaminophen (-0.00135)
Respiratory condition of fetus OR newborn (x1.01)
Potassium [mol/V] in SP (+0.0116)
Postpartum finding (x1.01)
Non-infectious systemic inflammatory response syndrome without acute organ failure (/1.01)
promethazine (-0.00127)
cyclobenzaprine (-0.00125)
Lymphocytes/100 leukocytes in B by AC (+0.287)
Congenital anomaly of lung (/1.01)
Ammonia [mol/V] in P (+0.749)
Gestation period, 31 weeks (/1.01)
Altered mental status (/1.01)
Chronic respiratory disease in perinatal period (x1.01)
Multiple sclerosis (/1.01)
Cells Counted Total [#] in B (+0.257)
Constipation (x1.01)
Type 2 diabetes mellitus without complication (/1.01)
Hypothermia of newborn (x1.01)
Optic neuritis (/1.01)
Tau protein [Presence] in Body fluid (-0.0138)
Optic disc edema (/1.01)
Acute bronchiolitis due to respiratory syncytial virus (x1.01)
Eosinophils [#/V] in B by AC (+0.00363)
Convulsions in the newborn (x1.01)
Perinatal atelectasis (x1.01)
Impetigo (x1.01)
Perinatal respiratory failure (x1.01)
Optic atrophy (/1.01)
pH of Urine by Test strip (+0.0148)
metformin (-0.000965)
nystatin (+0.000962)
Prematurity of infant (x1.01)
Cryptococcal meningitis (/1.01)
Dyspnea (/1.01)
Monocytes [#/V] in B by Manual count (+0.0652)
Cyanosis (x1.01)
albuterol (+0.000928)
Sepsis due to Gram negative bacteria (/1.01)
IgG clearance/Albumin clearance [Ratio] in S and CSF (-0.00715)
Arteritis (/1.01)
lorazepam (-0.000909)
Chronic obstructive lung disease (x1.01)
Hypothermia (x1.01)
0.460
0.627
0.688
0.743
0.786
0.822
0.833
0.838
0.843
0.847
0.850
0.853
0.856
0.859
0.862
0.865
0.867
0.870
0.872
0.875
0.877
0.879
0.881
0.883
0.885
0.887
0.889
0.891
0.893
0.894
0.896
0.897
0.898
0.900
0.901
0.902
0.904
0.905
0.906
0.907
0.908
0.909
0.910
0.911
0.912
0.913
0.914
0.915
0.916
0.916
0.917
0.918
0.919
0.919
0.920
0.921
0.921
0.922
0.923
0.923
0.924
0.924
0.925
0.925
0.926
0.926
0.927
0.927
0.928
0.928
0.929
0.929
0.930
0.930
0.931
0.931
0.931
0.932
0.932
0.932
0.933
0.933
0.934
0.934
0.934
0.935
0.935
0.935
0.935
0.936
0.936
0.936
0.937
0.937
0.937
0.938
0.938
0.938
0.938
0.939
−0.6
−0.4
−0.2
0.0
0.2
0.4
0.6
Expressions

Signature [277]; 1065 To Reach 0.975, (100 Plotted)

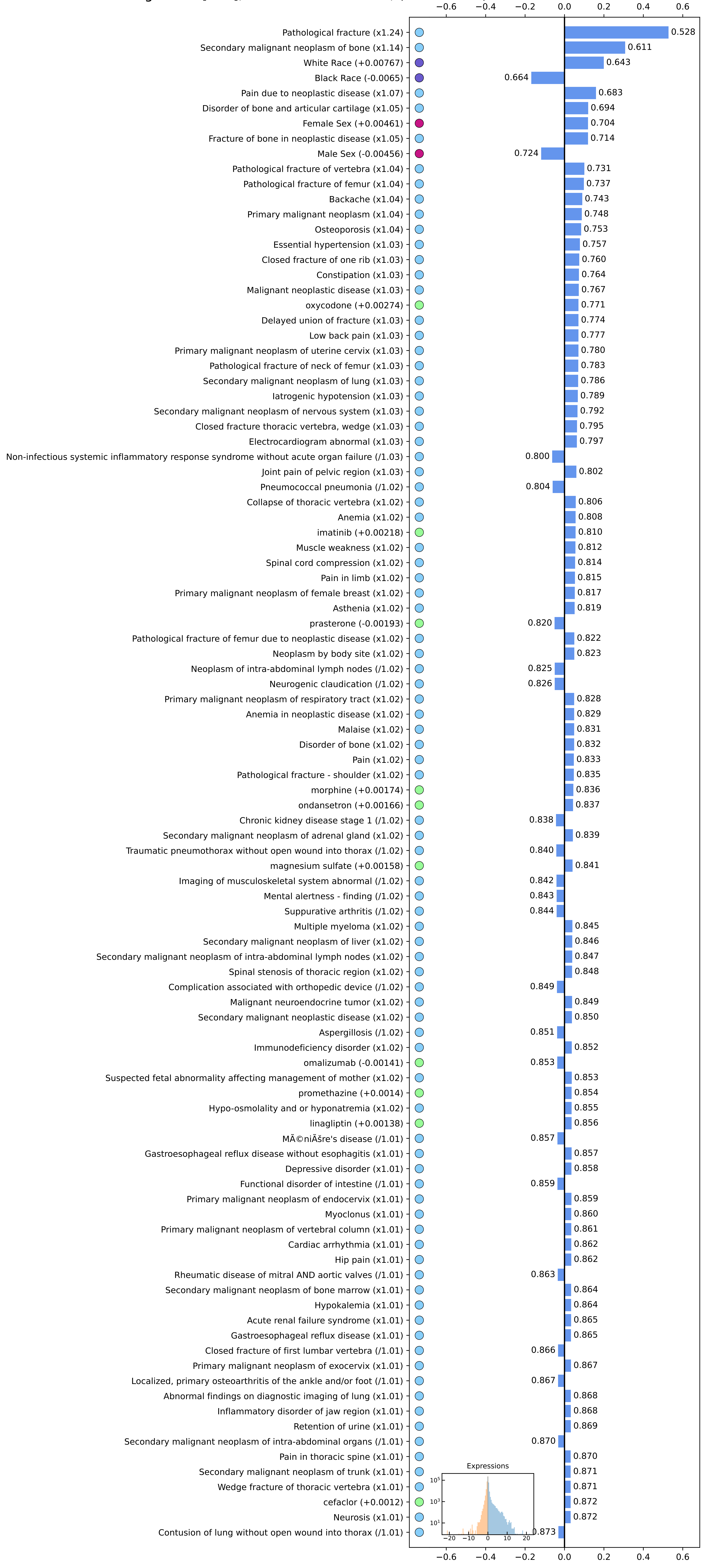

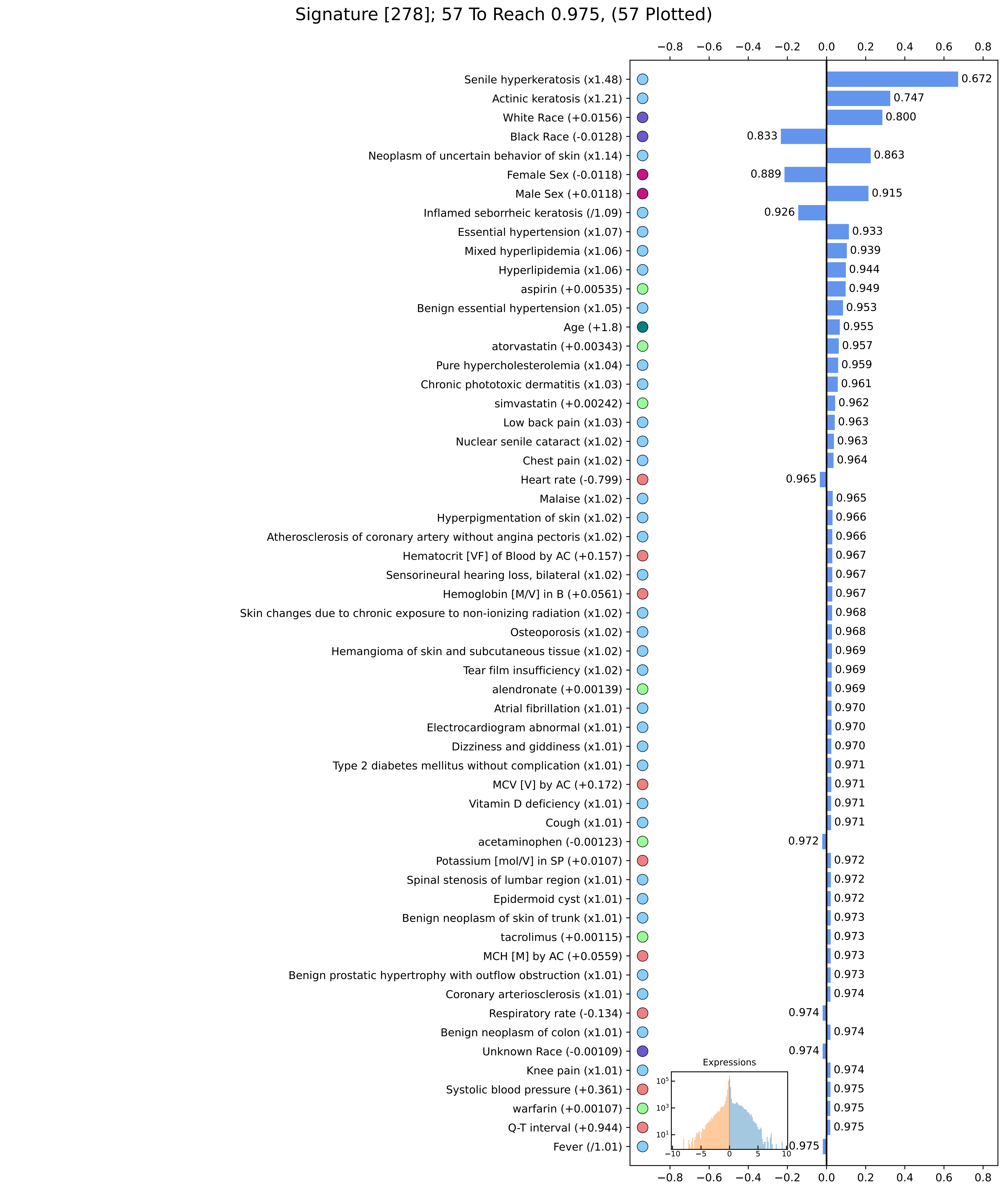

Signature [278]; 57 To Reach 0.975, (57 Plotted)
Senile hyperkeratosis (x1.48) 0.672
Actinic keratosis (x1.21) 0.747
White Race (+0.0156) 0.800
Black Race (-0.0128) 0.833
Neoplasm of uncertain behavior of skin (x1.14) 0.863
Female Sex (-0.0118) 0.889
Male Sex (+0.0118) 0.915
Inflamed seborrheic keratosis (/1.09) 0.926
Essential hypertension (x1.07) 0.933
Mixed hyperlipidemia (x1.06) 0.939
Hyperlipidemia (x1.06) 0.944
aspirin (+0.00535) 0.949
Benign essential hypertension (x1.05) 0.953
Age (+1.8) 0.955
atorvastatin (+0.00343) 0.957
Pure hypercholesterolemia (x1.04) 0.959
Chronic phototoxic dermatitis (x1.03) 0.961
simvastatin (+0.00242) 0.962
Low back pain (x1.03) 0.963
Nuclear senile cataract (x1.02) 0.963
Chest pain (x1.02) 0.964
Heart rate (-0.799) 0.965
Malaise (x1.02) 0.965
Hyperpigmentation of skin (x1.02) 0.966
Atherosclerosis of coronary artery without angina pectoris (x1.02) 0.966
Hematocrit [VF] of Blood by AC (+0.157) 0.967
Sensorineural hearing loss, bilateral (x1.02) 0.967
Hemoglobin [M/V] in B (+0.0561) 0.967
Skin changes due to chronic exposure to non-ionizing radiation (x1.02) 0.968
Osteoporosis (x1.02) 0.968
Hemangioma of skin and subcutaneous tissue (x1.02) 0.969
Tear film insufficiency (x1.02) 0.969
alendronate (+0.00139) 0.969
Atrial fibrillation (x1.01) 0.970
Electrocardiogram abnormal (x1.01) 0.970
Dizziness and giddiness (x1.01) 0.970
Type 2 diabetes mellitus without complication (x1.01) 0.971
MCV [V] by AC (+0.172) 0.971
Vitamin D deficiency (x1.01) 0.971
Cough (x1.01) 0.971
acetaminophen (-0.00123) 0.972
Potassium [mol/V] in SP (+0.0107) 0.972
Spinal stenosis of lumbar region (x1.01) 0.972
Epidermoid cyst (x1.01) 0.972
Benign neoplasm of skin of trunk (x1.01) 0.973
tacrolimus (+0.00115) 0.973
MCH [M] by AC (+0.0559) 0.973
Benign prostatic hypertrophy with outflow obstruction (x1.01) 0.973
Coronary arteriosclerosis (x1.01) 0.974
Respiratory rate (-0.134) 0.974
Benign neoplasm of colon (x1.01) 0.974
Unknown Race (-0.00109) 0.974
Knee pain (x1.01) 0.974
Systolic blood pressure (+0.361) 0.975
warfarin (+0.00107) 0.975
Q-T interval (+0.944) 0.975
Fever (/1.01) 0.975
−0.8 −0.6 −0.4 −0.2 0.0 0.2 0.4 0.6 0.8
Expressions

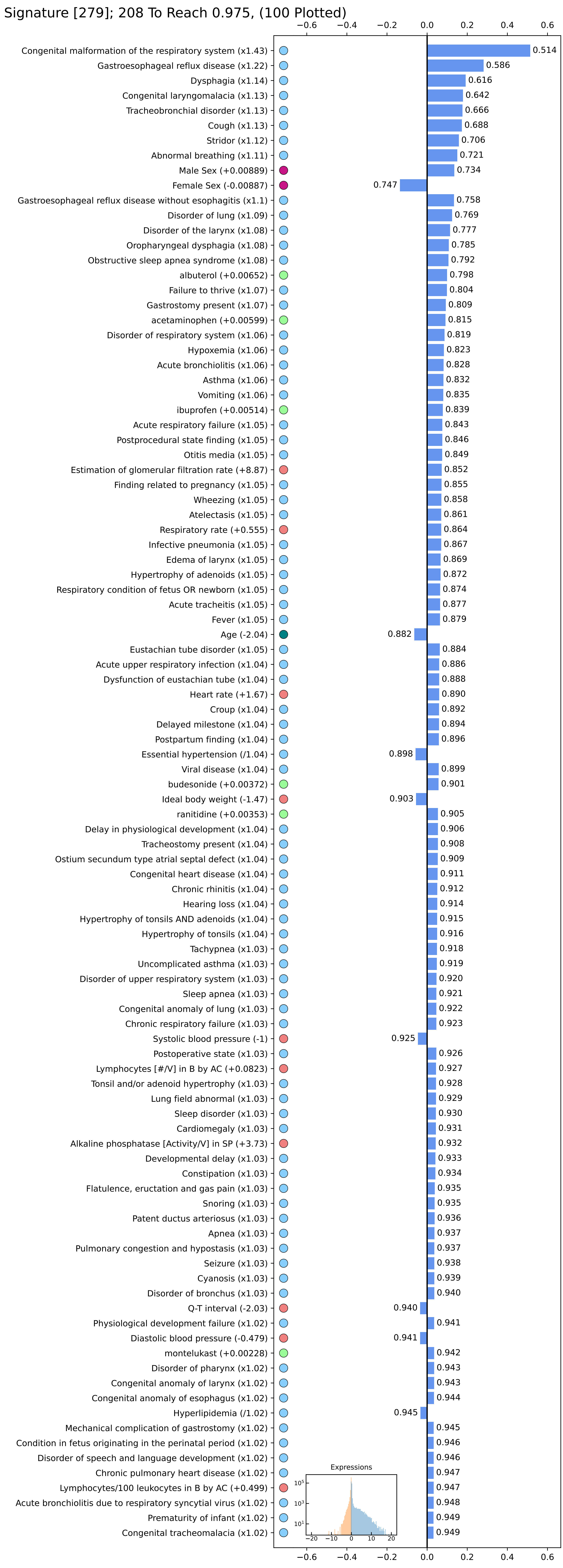

Signature [279]; 208 To Reach 0.975, (100 Plotted)
Congenital malformation of the respiratory system (x1.43) 0.514
Gastroesophageal reflux disease (x1.22) 0.586
Dysphagia (x1.14) 0.616
Congenital laryngomalacia (x1.13) 0.642
Tracheobronchial disorder (x1.13) 0.666
Cough (x1.13) 0.688
Stridor (x1.12) 0.706
Abnormal breathing (x1.11) 0.721
Male Sex (+0.00889) 0.734
Female Sex (-0.00887) 0.747
Gastroesophageal reflux disease without esophagitis (x1.1) 0.758
Disorder of lung (x1.09) 0.769
Disorder of the larynx (x1.08) 0.777
Oropharyngeal dysphagia (x1.08) 0.785
Obstructive sleep apnea syndrome (x1.08) 0.792
albuterol (+0.00652) 0.798
Failure to thrive (x1.07) 0.804
Gastrostomy present (x1.07) 0.809
acetaminophen (+0.00599) 0.815
Disorder of respiratory system (x1.06) 0.819
Hypoxemia (x1.06) 0.823
Acute bronchiolitis (x1.06) 0.828
Asthma (x1.06) 0.832
Vomiting (x1.06) 0.835
ibuprofen (+0.00514) 0.839
Acute respiratory failure (x1.05) 0.843
Postprocedural state finding (x1.05) 0.846
Otitis media (x1.05) 0.849
Estimation of glomerular filtration rate (+8.87) 0.852
Finding related to pregnancy (x1.05) 0.855
Wheezing (x1.05) 0.858
Atelectasis (x1.05) 0.861
Respiratory rate (+0.555) 0.864
Infective pneumonia (x1.05) 0.867
Edema of larynx (x1.05) 0.869
Hypertrophy of adenoids (x1.05) 0.872
Respiratory condition of fetus OR newborn (x1.05) 0.874
Acute tracheitis (x1.05) 0.877
Fever (x1.05) 0.879
Age (-2.04) 0.882
Eustachian tube disorder (x1.05) 0.884
Acute upper respiratory infection (x1.04) 0.886
Dysfunction of eustachian tube (x1.04) 0.888
Heart rate (+1.67) 0.890
Croup (x1.04) 0.892
Delayed milestone (x1.04) 0.894
Postpartum finding (x1.04) 0.896
Essential hypertension (/1.04) 0.898
Viral disease (x1.04) 0.899
budesonide (+0.00372) 0.901
Ideal body weight (-1.47) 0.903
ranitidine (+0.00353) 0.905
Delay in physiological development (x1.04) 0.906
Tracheostomy present (x1.04) 0.908
Ostium secundum type atrial septal defect (x1.04) 0.909
Congenital heart disease (x1.04) 0.911
Chronic rhinitis (x1.04) 0.912
Hearing loss (x1.04) 0.914
Hypertrophy of tonsils AND adenoids (x1.04) 0.915
Hypertrophy of tonsils (x1.04) 0.916
Tachypnea (x1.03) 0.918
Uncomplicated asthma (x1.03) 0.919
Disorder of upper respiratory system (x1.03) 0.920
Sleep apnea (x1.03) 0.921
Congenital anomaly of lung (x1.03) 0.922
Chronic respiratory failure (x1.03) 0.923
Systolic blood pressure (-1) 0.925
Postoperative state (x1.03) 0.926
Lymphocytes [#/V] in B by AC (+0.0823) 0.927
Tonsil and/or adenoid hypertrophy (x1.03) 0.928
Lung field abnormal (x1.03) 0.929
Sleep disorder (x1.03) 0.930
Cardiomegaly (x1.03) 0.931
Alkaline phosphatase [Activity/V] in SP (+3.73) 0.932
Developmental delay (x1.03) 0.933
Constipation (x1.03) 0.934
Flatulence, eructation and gas pain (x1.03) 0.935
Snoring (x1.03) 0.935
Patent ductus arteriosus (x1.03) 0.936
Apnea (x1.03) 0.937
Pulmonary congestion and hypostasis (x1.03) 0.937
Seizure (x1.03) 0.938
Cyanosis (x1.03) 0.939
Disorder of bronchus (x1.03) 0.940
Q-T interval (-2.03) 0.940
Physiological development failure (x1.02) 0.941
Diastolic blood pressure (-0.479) 0.941
montelukast (+0.00228) 0.942
Disorder of pharynx (x1.02) 0.943
Congenital anomaly of larynx (x1.02) 0.943
Congenital anomaly of esophagus (x1.02) 0.944
Hyperlipidemia (/1.02) 0.945
Mechanical complication of gastrostomy (x1.02) 0.945
Condition in fetus originating in the perinatal period (x1.02) 0.946
Disorder of speech and language development (x1.02) 0.946
Chronic pulmonary heart disease (x1.02) 0.947
Lymphocytes/100 leukocytes in B by AC (+0.499) 0.947
Acute bronchiolitis due to respiratory syncytial virus (x1.02) 0.948
Prematurity of infant (x1.02) 0.949
Congenital tracheomalacia (x1.02) 0.949
−0.6 −0.4 −0.2 0.0 0.2 0.4 0.6
Expressions

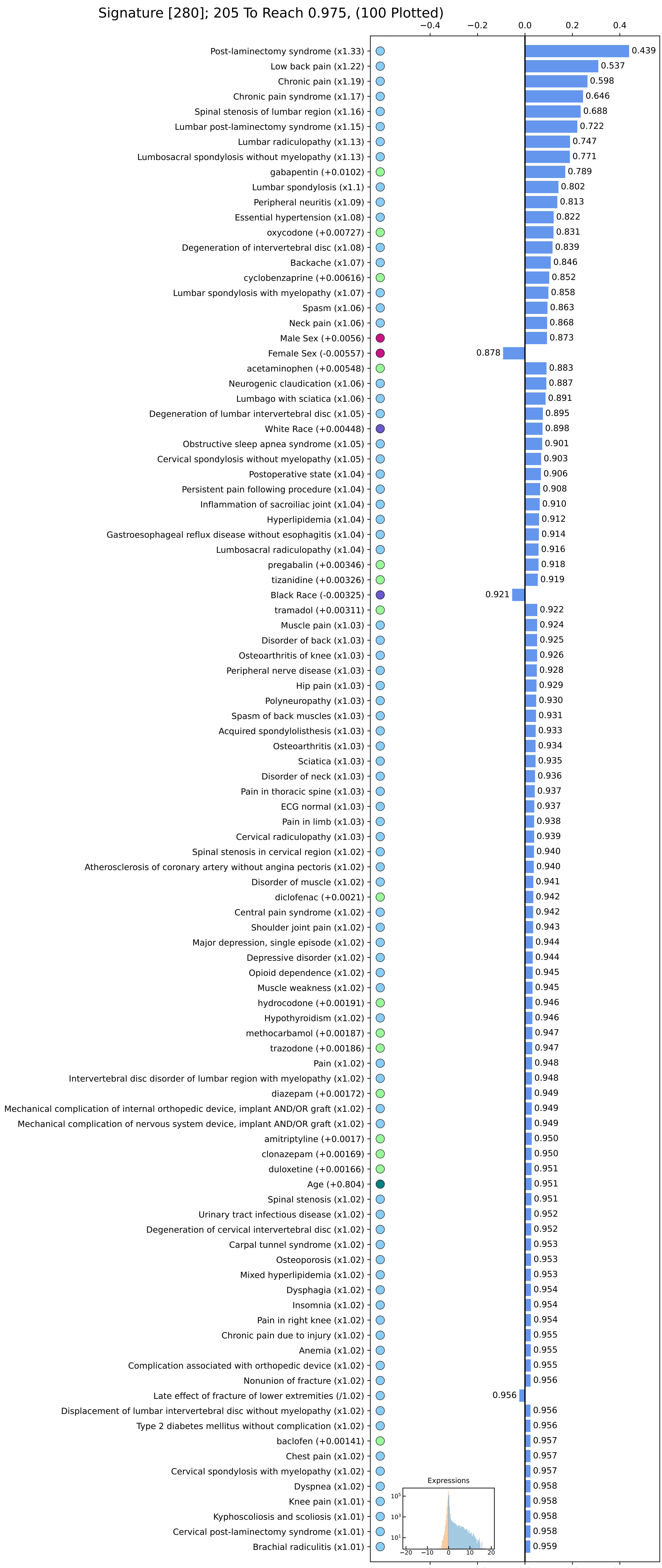

Signature [280]; 205 To Reach 0.975, (100 Plotted)
Post-laminectomy syndrome (x1.33) 0.439
Low back pain (x1.22) 0.537
Chronic pain (x1.19) 0.598
Chronic pain syndrome (x1.17) 0.646
Spinal stenosis of lumbar region (x1.16) 0.688
Lumbar post-laminectomy syndrome (x1.15) 0.722
Lumbar radiculopathy (x1.13) 0.747
Lumbosacral spondylosis without myelopathy (x1.13) 0.771
gabapentin (+0.0102) 0.789
Lumbar spondylosis (x1.1) 0.802
Peripheral neuritis (x1.09) 0.813
Essential hypertension (x1.08) 0.822
oxycodone (+0.00727) 0.831
Degeneration of intervertebral disc (x1.08) 0.839
Backache (x1.07) 0.846
cyclobenzaprine (+0.00616) 0.852
Lumbar spondylosis with myelopathy (x1.07) 0.858
Spasm (x1.06) 0.863
Neck pain (x1.06) 0.868
Male Sex (+0.0056) 0.873
Female Sex (-0.00557) 0.878
acetaminophen (+0.00548) 0.883
Neurogenic claudication (x1.06) 0.887
Lumbago with sciatica (x1.06) 0.891
Degeneration of lumbar intervertebral disc (x1.05) 0.895
White Race (+0.00448) 0.898
Obstructive sleep apnea syndrome (x1.05) 0.901
Cervical spondylosis without myelopathy (x1.05) 0.903
Postoperative state (x1.04) 0.906
Persistent pain following procedure (x1.04) 0.908
Inflammation of sacroiliac joint (x1.04) 0.910
Hyperlipidemia (x1.04) 0.912
Gastroesophageal reflux disease without esophagitis (x1.04) 0.914
Lumbosacral radiculopathy (x1.04) 0.916
pregabalin (+0.00346) 0.918
tizanidine (+0.00326) 0.919
Black Race (-0.00325) 0.921
tramadol (+0.00311) 0.922
Muscle pain (x1.03) 0.924
Disorder of back (x1.03) 0.925
Osteoarthritis of knee (x1.03) 0.926
Peripheral nerve disease (x1.03) 0.928
Hip pain (x1.03) 0.929
Polyneuropathy (x1.03) 0.930
Spasm of back muscles (x1.03) 0.931
Acquired spondylolisthesis (x1.03) 0.933
Osteoarthritis (x1.03) 0.934
Sciatica (x1.03) 0.935
Disorder of neck (x1.03) 0.936
Pain in thoracic spine (x1.03) 0.937
ECG normal (x1.03) 0.937
Pain in limb (x1.03) 0.938
Cervical radiculopathy (x1.03) 0.939
Spinal stenosis in cervical region (x1.02) 0.940
Atherosclerosis of coronary artery without angina pectoris (x1.02) 0.940
Disorder of muscle (x1.02) 0.941
diclofenac (+0.0021) 0.942
Central pain syndrome (x1.02) 0.942
Shoulder joint pain (x1.02) 0.943
Major depression, single episode (x1.02) 0.944
Depressive disorder (x1.02) 0.944
Opioid dependence (x1.02) 0.945
Muscle weakness (x1.02) 0.945
hydrocodone (+0.00191) 0.946
Hypothyroidism (x1.02) 0.946
methocarbamol (+0.00187) 0.947
trazodone (+0.00186) 0.947
Pain (x1.02) 0.948
Intervertebral disc disorder of lumbar region with myelopathy (x1.02) 0.948
diazepam (+0.00172) 0.949
Mechanical complication of internal orthopedic device, implant AND/OR graft (x1.02) 0.949
Mechanical complication of nervous system device, implant AND/OR graft (x1.02) 0.949
amitriptyline (+0.0017) 0.950
clonazepam (+0.00169) 0.950
duloxetine (+0.00166) 0.951
Age (+0.804) 0.951
Spinal stenosis (x1.02) 0.951
Urinary tract infectious disease (x1.02) 0.952
Degeneration of cervical intervertebral disc (x1.02) 0.952
Carpal tunnel syndrome (x1.02) 0.953
Osteoporosis (x1.02) 0.953
Mixed hyperlipidemia (x1.02) 0.953
Dysphagia (x1.02) 0.954
Insomnia (x1.02) 0.954
Pain in right knee (x1.02) 0.954
Chronic pain due to injury (x1.02) 0.955
Anemia (x1.02) 0.955
Complication associated with orthopedic device (x1.02) 0.955
Nonunion of fracture (x1.02) 0.956
Late effect of fracture of lower extremities (/1.02) 0.956
Displacement of lumbar intervertebral disc without myelopathy (x1.02) 0.956
Type 2 diabetes mellitus without complication (x1.02) 0.956
baclofen (+0.00141) 0.957
Chest pain (x1.02) 0.957
Cervical spondylosis with myelopathy (x1.02) 0.957
Dyspnea (x1.02) 0.958
Knee pain (x1.01) 0.958
Kyphoscoliosis and scoliosis (x1.01) 0.958
Cervical post-laminectomy syndrome (x1.01) 0.958
Brachial radiculitis (x1.01) 0.959
−0.4 −0.2 0.0 0.2 0.4
Expressions

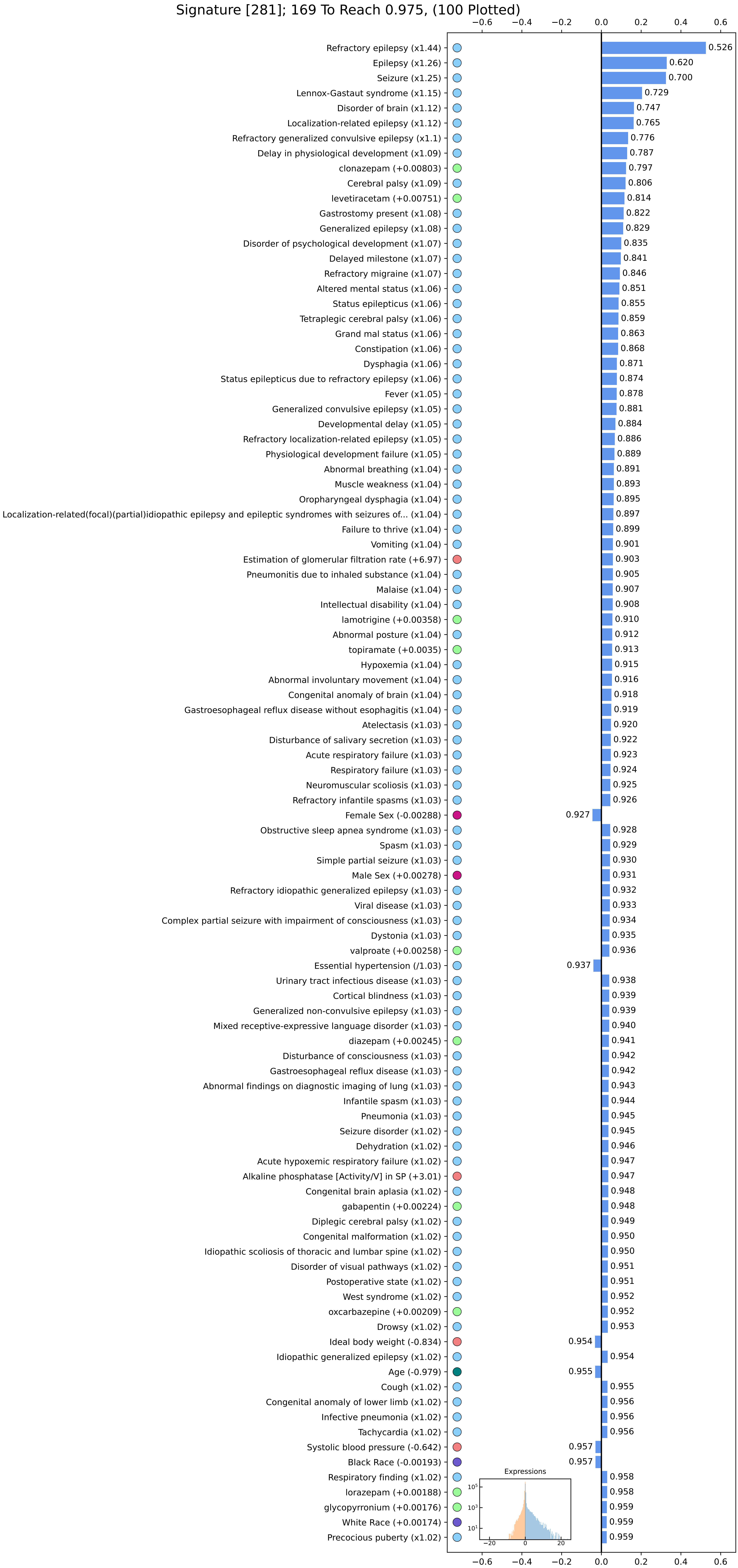
Signature [281]; 169 To Reach 0.975, (100 Plotted)
Refractory epilepsy (x1.44) 0.526
Epilepsy (x1.26) 0.620
Seizure (x1.25) 0.700
Lennox-Gastaut syndrome (x1.15) 0.729
Disorder of brain (x1.12) 0.747
Localization-related epilepsy (x1.12) 0.765
Refractory generalized convulsive epilepsy (x1.1) 0.776
Delay in physiological development (x1.09) 0.787
clonazepam (+0.00803) 0.797
Cerebral palsy (x1.09) 0.806
levetiracetam (+0.00751) 0.814
Gastrostomy present (x1.08) 0.822
Generalized epilepsy (x1.08) 0.829
Disorder of psychological development (x1.07) 0.835
Delayed milestone (x1.07) 0.841
Refractory migraine (x1.07) 0.846
Altered mental status (x1.06) 0.851
Status epilepticus (x1.06) 0.855
Tetraplegic cerebral palsy (x1.06) 0.859
Grand mal status (x1.06) 0.863
Constipation (x1.06) 0.868
Dysphagia (x1.06) 0.871
Status epilepticus due to refractory epilepsy (x1.06) 0.874
Fever (x1.05) 0.878
Generalized convulsive epilepsy (x1.05) 0.881
Developmental delay (x1.05) 0.884
Refractory localization-related epilepsy (x1.05) 0.886
Physiological development failure (x1.05) 0.889
Abnormal breathing (x1.04) 0.891
Muscle weakness (x1.04) 0.893
Oropharyngeal dysphagia (x1.04) 0.895
Localization-related(focal)(partial)idiopathic epilepsy and epileptic syndromes with seizures of... (x1.04) 0.897
Failure to thrive (x1.04) 0.899
Vomiting (x1.04) 0.901
Estimation of glomerular filtration rate (+6.97) 0.903
Pneumonitis due to inhaled substance (x1.04) 0.905
Malaise (x1.04) 0.907
Intellectual disability (x1.04) 0.908
lamotrigine (+0.00358) 0.910
Abnormal posture (x1.04) 0.912
topiramate (+0.0035) 0.913
Hypoxemia (x1.04) 0.915
Abnormal involuntary movement (x1.04) 0.916
Congenital anomaly of brain (x1.04) 0.918
Gastroesophageal reflux disease without esophagitis (x1.04) 0.919
Atelectasis (x1.03) 0.920
Disturbance of salivary secretion (x1.03) 0.922
Acute respiratory failure (x1.03) 0.923
Respiratory failure (x1.03) 0.924
Neuromuscular scoliosis (x1.03) 0.925
Refractory infantile spasms (x1.03) 0.926
Female Sex (-0.00288) 0.927
Obstructive sleep apnea syndrome (x1.03) 0.928
Spasm (x1.03) 0.929
Simple partial seizure (x1.03) 0.930
Male Sex (+0.00278) 0.931
Refractory idiopathic generalized epilepsy (x1.03) 0.932
Viral disease (x1.03) 0.933
Complex partial seizure with impairment of consciousness (x1.03) 0.934
Dystonia (x1.03) 0.935
valproate (+0.00258) 0.936
Essential hypertension (/1.03) 0.937
Urinary tract infectious disease (x1.03) 0.938
Cortical blindness (x1.03) 0.939
Generalized non-convulsive epilepsy (x1.03) 0.939
Mixed receptive-expressive language disorder (x1.03) 0.940
diazepam (+0.00245) 0.941
Disturbance of consciousness (x1.03) 0.942
Gastroesophageal reflux disease (x1.03) 0.942
Abnormal findings on diagnostic imaging of lung (x1.03) 0.943
Infantile spasm (x1.03) 0.944
Pneumonia (x1.03) 0.945
Seizure disorder (x1.02) 0.945
Dehydration (x1.02) 0.946
Acute hypoxemic respiratory failure (x1.02) 0.947
Alkaline phosphatase [Activity/V] in SP (+3.01) 0.947
Congenital brain aplasia (x1.02) 0.948
gabapentin (+0.00224) 0.948
Diplegic cerebral palsy (x1.02) 0.949
Congenital malformation (x1.02) 0.950
Idiopathic scoliosis of thoracic and lumbar spine (x1.02) 0.950
Disorder of visual pathways (x1.02) 0.951
Postoperative state (x1.02) 0.951
West syndrome (x1.02) 0.952
oxcarbazepine (+0.00209) 0.952
Drowsy (x1.02) 0.953
Ideal body weight (-0.834) 0.954
Idiopathic generalized epilepsy (x1.02) 0.954
Age (-0.979) 0.955
Cough (x1.02) 0.955
Congenital anomaly of lower limb (x1.02) 0.956
Infective pneumonia (x1.02) 0.956
Tachycardia (x1.02) 0.956
Systolic blood pressure (-0.642) 0.957
Black Race (-0.00193) 0.957
Respiratory finding (x1.02) 0.958
lorazepam (+0.00188) 0.958
glycopyrronium (+0.00176) 0.959
White Race (+0.00174) 0.959
Precocious puberty (x1.02) 0.959
−0.6 −0.4 −0.2 0.0 0.2 0.4 0.6
Expressions
10^5 10^3 10^1
−20 0 20

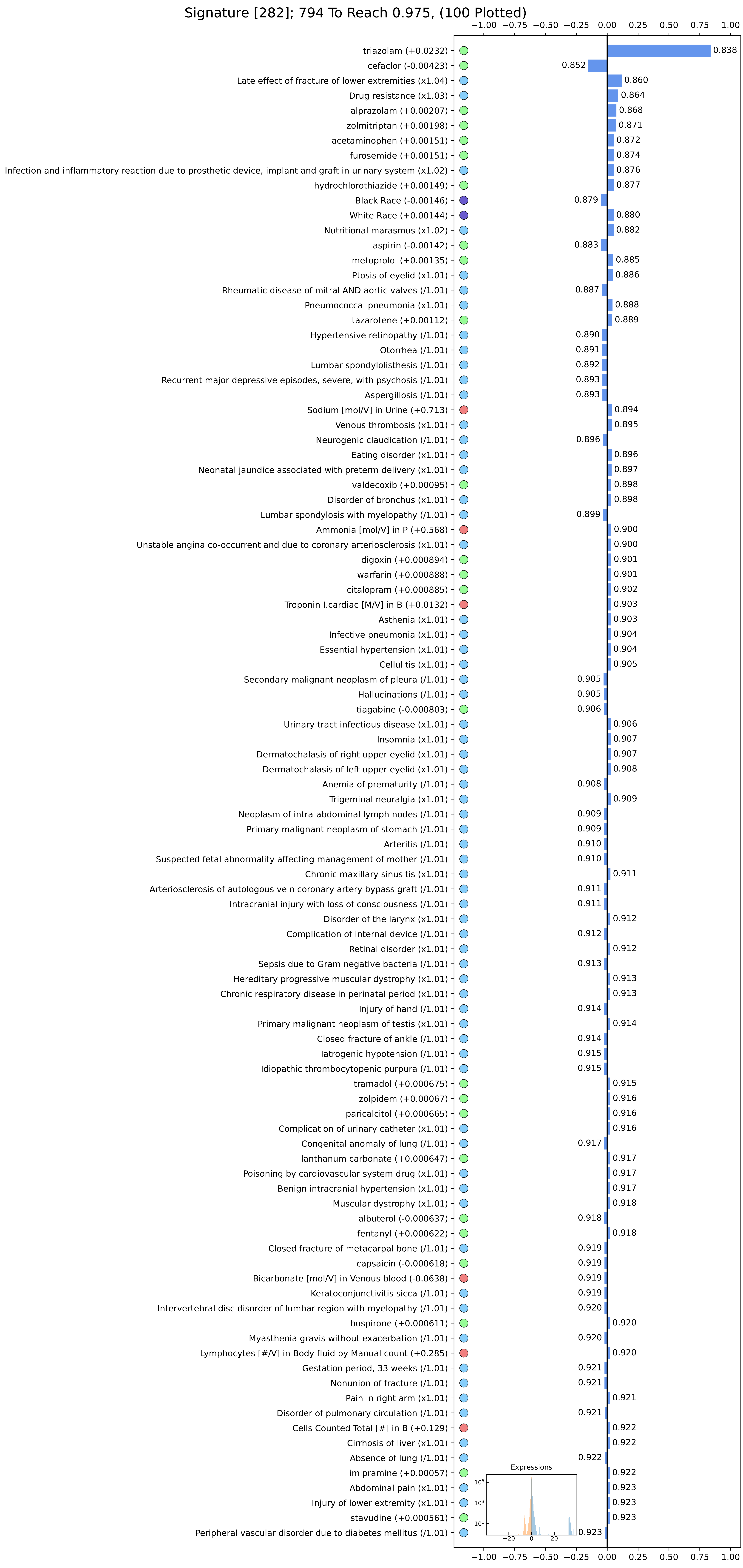

Signature [282]; 794 To Reach 0.975, (100 Plotted)
triazolam (+0.0232) 0.838
cefaclor (-0.00423) 0.852
Late effect of fracture of lower extremities (x1.04) 0.860
Drug resistance (x1.03) 0.864
alprazolam (+0.00207) 0.868
zolmitriptan (+0.00198) 0.871
acetaminophen (+0.00151) 0.872
furosemide (+0.00151) 0.874
Infection and inflammatory reaction due to prosthetic device, implant and graft in urinary system (x1.02) 0.876
hydrochlorothiazide (+0.00149) 0.877
Black Race (-0.00146) 0.879
White Race (+0.00144) 0.880
Nutritional marasmus (x1.02) 0.882
aspirin (-0.00142) 0.883
metoprolol (+0.00135) 0.885
Ptosis of eyelid (x1.01) 0.886
Rheumatic disease of mitral AND aortic valves (/1.01) 0.887
Pneumococcal pneumonia (x1.01) 0.888
tazarotene (+0.00112) 0.889
Hypertensive retinopathy (/1.01) 0.890
Otorrhea (/1.01) 0.891
Lumbar spondylolisthesis (/1.01) 0.892
Recurrent major depressive episodes, severe, with psychosis (/1.01) 0.893
Aspergillosis (/1.01) 0.893
Sodium [mol/V] in Urine (+0.713) 0.894
Venous thrombosis (x1.01) 0.895
Neurogenic claudication (/1.01) 0.896
Eating disorder (x1.01) 0.896
Neonatal jaundice associated with preterm delivery (x1.01) 0.897
valdecoxib (+0.00095) 0.898
Disorder of bronchus (x1.01) 0.898
Lumbar spondylosis with myelopathy (/1.01) 0.899
Ammonia [mol/V] in P (+0.568) 0.900
Unstable angina co-occurrent and due to coronary arteriosclerosis (x1.01) 0.900
digoxin (+0.000894) 0.901
warfarin (+0.000888) 0.901
citalopram (+0.000885) 0.902
Troponin I.cardiac [M/V] in B (+0.0132) 0.903
Asthenia (x1.01) 0.903
Infective pneumonia (x1.01) 0.904
Essential hypertension (x1.01) 0.904
Cellulitis (x1.01) 0.905
Secondary malignant neoplasm of pleura (/1.01) 0.905
Hallucinations (/1.01) 0.905
tiagabine (-0.000803) 0.906
Urinary tract infectious disease (x1.01) 0.906
Insomnia (x1.01) 0.907
Dermatochalasis of right upper eyelid (x1.01) 0.907
Dermatochalasis of left upper eyelid (x1.01) 0.908
Anemia of prematurity (/1.01) 0.908
Trigeminal neuralgia (x1.01) 0.909
Neoplasm of intra-abdominal lymph nodes (/1.01) 0.909
Primary malignant neoplasm of stomach (/1.01) 0.909
Arteritis (/1.01) 0.910
Suspected fetal abnormality affecting management of mother (/1.01) 0.910
Chronic maxillary sinusitis (x1.01) 0.911
Arteriosclerosis of autologous vein coronary artery bypass graft (/1.01) 0.911
Intracranial injury with loss of consciousness (/1.01) 0.911
Disorder of the larynx (x1.01) 0.912
Complication of internal device (/1.01) 0.912
Retinal disorder (x1.01) 0.912
Sepsis due to Gram negative bacteria (/1.01) 0.913
Hereditary progressive muscular dystrophy (x1.01) 0.913
Chronic respiratory disease in perinatal period (x1.01) 0.913
Injury of hand (/1.01) 0.914
Primary malignant neoplasm of testis (x1.01) 0.914
Closed fracture of ankle (/1.01) 0.914
Iatrogenic hypotension (/1.01) 0.915
Idiopathic thrombocytopenic purpura (/1.01) 0.915
tramadol (+0.000675) 0.915
zolpidem (+0.00067) 0.916
paricalcitol (+0.000665) 0.916
Complication of urinary catheter (x1.01) 0.916
Congenital anomaly of lung (/1.01) 0.917
lanthanum carbonate (+0.000647) 0.917
Poisoning by cardiovascular system drug (x1.01) 0.917
Benign intracranial hypertension (x1.01) 0.917
Muscular dystrophy (x1.01) 0.918
albuterol (-0.000637) 0.918
fentanyl (+0.000622) 0.918
Closed fracture of metacarpal bone (/1.01) 0.919
capsaicin (-0.000618) 0.919
Bicarbonate [mol/V] in Venous blood (-0.0638) 0.919
Keratoconjunctivitis sicca (/1.01) 0.919
Intervertebral disc disorder of lumbar region with myelopathy (/1.01) 0.920
buspirone (+0.000611) 0.920
Myasthenia gravis without exacerbation (/1.01) 0.920
Lymphocytes [#/V] in Body fluid by Manual count (+0.285) 0.920
Gestation period, 33 weeks (/1.01) 0.921
Nonunion of fracture (/1.01) 0.921
Pain in right arm (x1.01) 0.921
Disorder of pulmonary circulation (/1.01) 0.921
Cells Counted Total [#] in B (+0.129) 0.922
Cirrhosis of liver (x1.01) 0.922
Absence of lung (/1.01) 0.922
imipramine (+0.00057) 0.922
Abdominal pain (x1.01) 0.923
Injury of lower extremity (x1.01) 0.923
stavudine (+0.000561) 0.923
Peripheral vascular disorder due to diabetes mellitus (/1.01) 0.923
Expressions
−1.00 −0.75 −0.50 −0.25 0.00 0.25 0.50 0.75 1.00

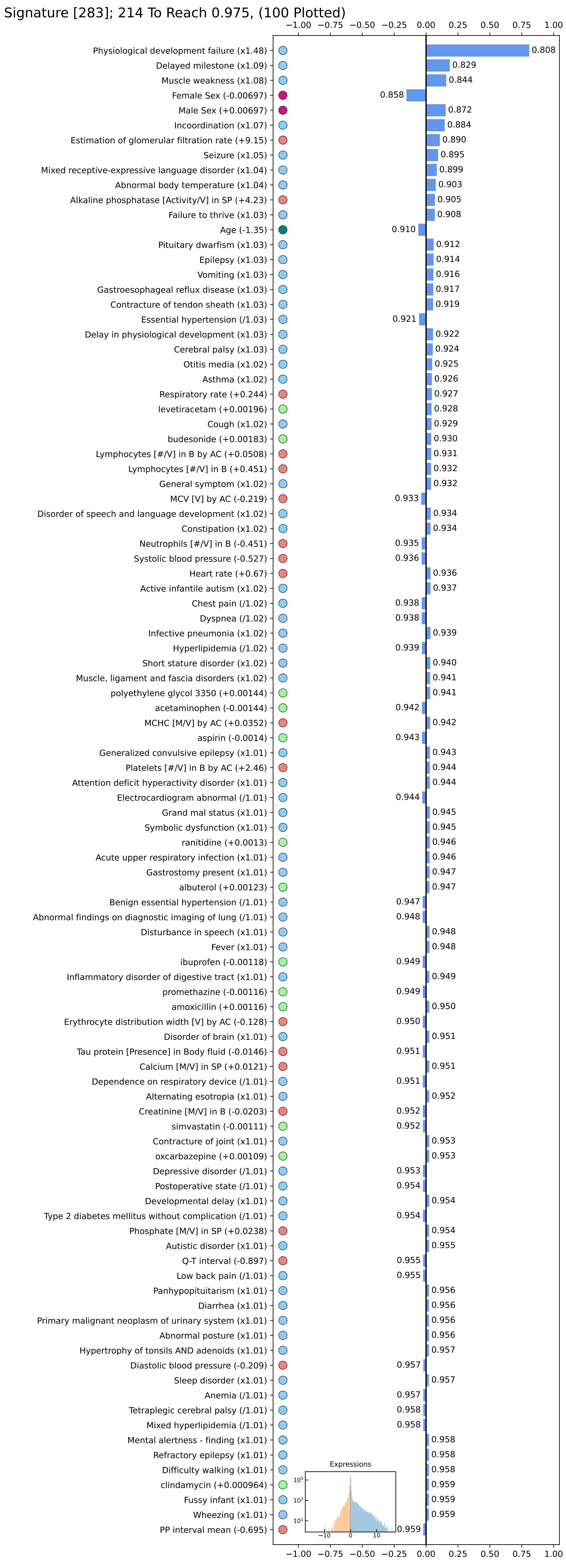

Signature [283]; 214 To Reach 0.975, (100 Plotted)
Physiological development failure (x1.48) 0.808
Delayed milestone (x1.09) 0.829
Muscle weakness (x1.08) 0.844
Female Sex (-0.00697) 0.858
Male Sex (+0.00697) 0.872
Incoordination (x1.07) 0.884
Estimation of glomerular filtration rate (+9.15) 0.890
Seizure (x1.05) 0.895
Mixed receptive-expressive language disorder (x1.04) 0.899
Abnormal body temperature (x1.04) 0.903
Alkaline phosphatase [Activity/V] in SP (+4.23) 0.905
Failure to thrive (x1.03) 0.908
Age (-1.35) 0.910
Pituitary dwarfism (x1.03) 0.912
Epilepsy (x1.03) 0.914
Vomiting (x1.03) 0.916
Gastroesophageal reflux disease (x1.03) 0.917
Contracture of tendon sheath (x1.03) 0.919
Essential hypertension (/1.03) 0.921
Delay in physiological development (x1.03) 0.922
Cerebral palsy (x1.03) 0.924
Otitis media (x1.02) 0.925
Asthma (x1.02) 0.926
Respiratory rate (+0.244) 0.927
levetiracetam (+0.00196) 0.928
Cough (x1.02) 0.929
budesonide (+0.00183) 0.930
Lymphocytes [#/V] in B by AC (+0.0508) 0.931
Lymphocytes [#/V] in B (+0.451) 0.932
General symptom (x1.02) 0.932
MCV [V] by AC (-0.219) 0.933
Disorder of speech and language development (x1.02) 0.934
Constipation (x1.02) 0.934
Neutrophils [#/V] in B (-0.451) 0.935
Systolic blood pressure (-0.527) 0.936
Heart rate (+0.67) 0.936
Active infantile autism (x1.02) 0.937
Chest pain (/1.02) 0.938
Dyspnea (/1.02) 0.938
Infective pneumonia (x1.02) 0.939
Hyperlipidemia (/1.02) 0.939
Short stature disorder (x1.02) 0.940
Muscle, ligament and fascia disorders (x1.02) 0.941
polyethylene glycol 3350 (+0.00144) 0.941
acetaminophen (-0.00144) 0.942
MCHC [M/V] by AC (+0.0352) 0.942
aspirin (-0.0014) 0.943
Generalized convulsive epilepsy (x1.01) 0.943
Platelets [#/V] in B by AC (+2.46) 0.944
Attention deficit hyperactivity disorder (x1.01) 0.944
Electrocardiogram abnormal (/1.01) 0.944
Grand mal status (x1.01) 0.945
Symbolic dysfunction (x1.01) 0.945
ranitidine (+0.0013) 0.946
Acute upper respiratory infection (x1.01) 0.946
Gastrostomy present (x1.01) 0.947
albuterol (+0.00123) 0.947
Benign essential hypertension (/1.01) 0.947
Abnormal findings on diagnostic imaging of lung (/1.01) 0.948
Disturbance in speech (x1.01) 0.948
Fever (x1.01) 0.948
ibuprofen (-0.00118) 0.949
Inflammatory disorder of digestive tract (x1.01) 0.949
promethazine (-0.00116) 0.949
amoxicillin (+0.00116) 0.950
Erythrocyte distribution width [V] by AC (-0.128) 0.950
Disorder of brain (x1.01) 0.951
Tau protein [Presence] in Body fluid (-0.0146) 0.951
Calcium [M/V] in SP (+0.0121) 0.951
Dependence on respiratory device (/1.01) 0.951
Alternating esotropia (x1.01) 0.952
Creatinine [M/V] in B (-0.0203) 0.952
simvastatin (-0.00111) 0.952
Contracture of joint (x1.01) 0.953
oxcarbazepine (+0.00109) 0.953
Depressive disorder (/1.01) 0.953
Postoperative state (/1.01) 0.954
Developmental delay (x1.01) 0.954
Type 2 diabetes mellitus without complication (/1.01) 0.954
Phosphate [M/V] in SP (+0.0238) 0.954
Autistic disorder (x1.01) 0.955
Q-T interval (-0.897) 0.955
Low back pain (/1.01) 0.955
Panhypopituitarism (x1.01) 0.956
Diarrhea (x1.01) 0.956
Primary malignant neoplasm of urinary system (x1.01) 0.956
Abnormal posture (x1.01) 0.956
Hypertrophy of tonsils AND adenoids (x1.01) 0.957
Diastolic blood pressure (-0.209) 0.957
Sleep disorder (x1.01) 0.957
Anemia (/1.01) 0.957
Tetraplegic cerebral palsy (/1.01) 0.958
Mixed hyperlipidemia (/1.01) 0.958
Mental alertness - finding (x1.01) 0.958
Refractory epilepsy (x1.01) 0.958
Difficulty walking (x1.01) 0.958
clindamycin (+0.000964) 0.959
Fussy infant (x1.01) 0.959
Wheezing (x1.01) 0.959
PP interval mean (-0.695) 0.959
Expressions
−1.00 −0.75 −0.50 −0.25 0.00 0.25 0.50 0.75 1.00

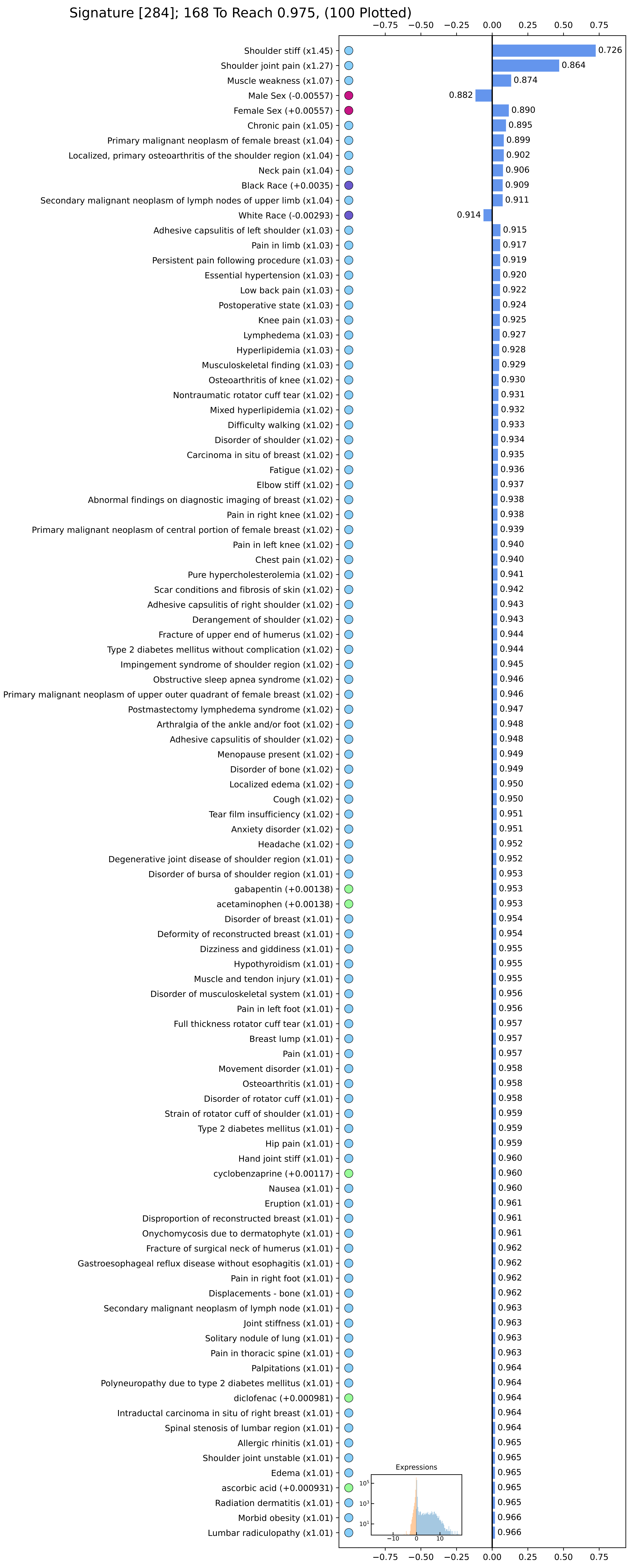

Signature [284]; 168 To Reach 0.975, (100 Plotted)
Shoulder stiff (x1.45) 0.726
Shoulder joint pain (x1.27) 0.864
Muscle weakness (x1.07) 0.874
Male Sex (-0.00557) 0.882
Female Sex (+0.00557) 0.890
Chronic pain (x1.05) 0.895
Primary malignant neoplasm of female breast (x1.04) 0.899
Localized, primary osteoarthritis of the shoulder region (x1.04) 0.902
Neck pain (x1.04) 0.906
Black Race (+0.0035) 0.909
Secondary malignant neoplasm of lymph nodes of upper limb (x1.04) 0.911
White Race (-0.00293) 0.914
Adhesive capsulitis of left shoulder (x1.03) 0.915
Pain in limb (x1.03) 0.917
Persistent pain following procedure (x1.03) 0.919
Essential hypertension (x1.03) 0.920
Low back pain (x1.03) 0.922
Postoperative state (x1.03) 0.924
Knee pain (x1.03) 0.925
Lymphedema (x1.03) 0.927
Hyperlipidemia (x1.03) 0.928
Musculoskeletal finding (x1.03) 0.929
Osteoarthritis of knee (x1.02) 0.930
Nontraumatic rotator cuff tear (x1.02) 0.931
Mixed hyperlipidemia (x1.02) 0.932
Difficulty walking (x1.02) 0.933
Disorder of shoulder (x1.02) 0.934
Carcinoma in situ of breast (x1.02) 0.935
Fatigue (x1.02) 0.936
Elbow stiff (x1.02) 0.937
Abnormal findings on diagnostic imaging of breast (x1.02) 0.938
Pain in right knee (x1.02) 0.938
Primary malignant neoplasm of central portion of female breast (x1.02) 0.939
Pain in left knee (x1.02) 0.940
Chest pain (x1.02) 0.940
Pure hypercholesterolemia (x1.02) 0.941
Scar conditions and fibrosis of skin (x1.02) 0.942
Adhesive capsulitis of right shoulder (x1.02) 0.943
Derangement of shoulder (x1.02) 0.943
Fracture of upper end of humerus (x1.02) 0.944
Type 2 diabetes mellitus without complication (x1.02) 0.944
Impingement syndrome of shoulder region (x1.02) 0.945
Obstructive sleep apnea syndrome (x1.02) 0.946
Primary malignant neoplasm of upper outer quadrant of female breast (x1.02) 0.946
Postmastectomy lymphedema syndrome (x1.02) 0.947
Arthralgia of the ankle and/or foot (x1.02) 0.948
Adhesive capsulitis of shoulder (x1.02) 0.948
Menopause present (x1.02) 0.949
Disorder of bone (x1.02) 0.949
Localized edema (x1.02) 0.950
Cough (x1.02) 0.950
Tear film insufficiency (x1.02) 0.951
Anxiety disorder (x1.02) 0.951
Headache (x1.02) 0.952
Degenerative joint disease of shoulder region (x1.01) 0.952
Disorder of bursa of shoulder region (x1.01) 0.953
gabapentin (+0.00138) 0.953
acetaminophen (+0.00138) 0.953
Disorder of breast (x1.01) 0.954
Deformity of reconstructed breast (x1.01) 0.954
Dizziness and giddiness (x1.01) 0.955
Hypothyroidism (x1.01) 0.955
Muscle and tendon injury (x1.01) 0.955
Disorder of musculoskeletal system (x1.01) 0.956
Pain in left foot (x1.01) 0.956
Full thickness rotator cuff tear (x1.01) 0.957
Breast lump (x1.01) 0.957
Pain (x1.01) 0.957
Movement disorder (x1.01) 0.958
Osteoarthritis (x1.01) 0.958
Disorder of rotator cuff (x1.01) 0.958
Strain of rotator cuff of shoulder (x1.01) 0.959
Type 2 diabetes mellitus (x1.01) 0.959
Hip pain (x1.01) 0.959
Hand joint stiff (x1.01) 0.960
cyclobenzaprine (+0.00117) 0.960
Nausea (x1.01) 0.960
Eruption (x1.01) 0.961
Disproportion of reconstructed breast (x1.01) 0.961
Onychomycosis due to dermatophyte (x1.01) 0.961
Fracture of surgical neck of humerus (x1.01) 0.962
Gastroesophageal reflux disease without esophagitis (x1.01) 0.962
Pain in right foot (x1.01) 0.962
Displacements - bone (x1.01) 0.962
Secondary malignant neoplasm of lymph node (x1.01) 0.963
Joint stiffness (x1.01) 0.963
Solitary nodule of lung (x1.01) 0.963
Pain in thoracic spine (x1.01) 0.963
Palpitations (x1.01) 0.964
Polyneuropathy due to type 2 diabetes mellitus (x1.01) 0.964
diclofenac (+0.000981) 0.964
Intraductal carcinoma in situ of right breast (x1.01) 0.964
Spinal stenosis of lumbar region (x1.01) 0.964
Allergic rhinitis (x1.01) 0.965
Shoulder joint unstable (x1.01) 0.965
Edema (x1.01) 0.965
ascorbic acid (+0.000931) 0.965
Radiation dermatitis (x1.01) 0.965
Morbid obesity (x1.01) 0.966
Lumbar radiculopathy (x1.01) 0.966
Expressions
−0.75 −0.50 −0.25 0.00 0.25 0.50 0.75

Signature [285]; 37 To Reach 0.975, (37 Plotted)

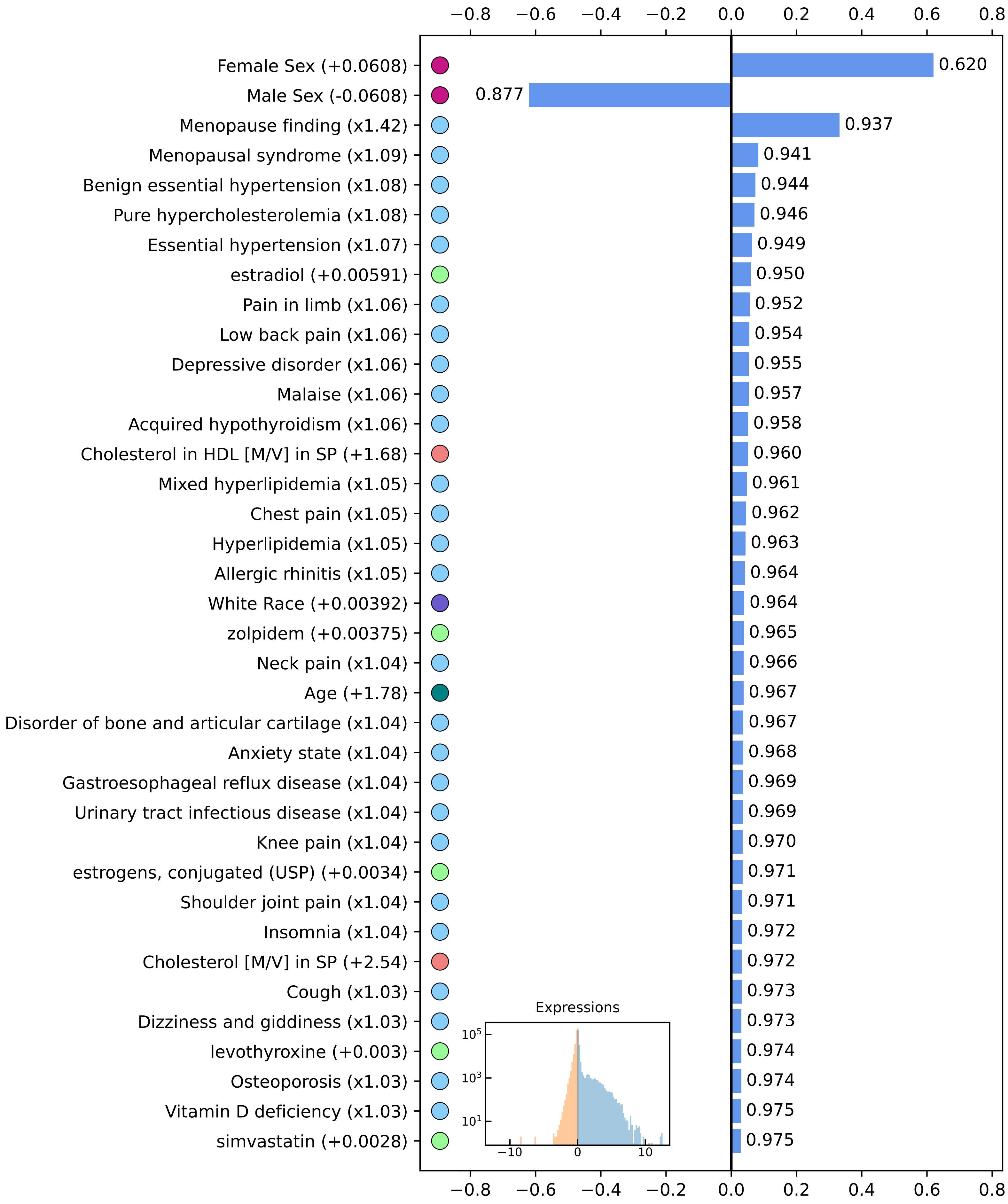

Signature [286]; 1 To Reach 0.975, (10 Plotted)

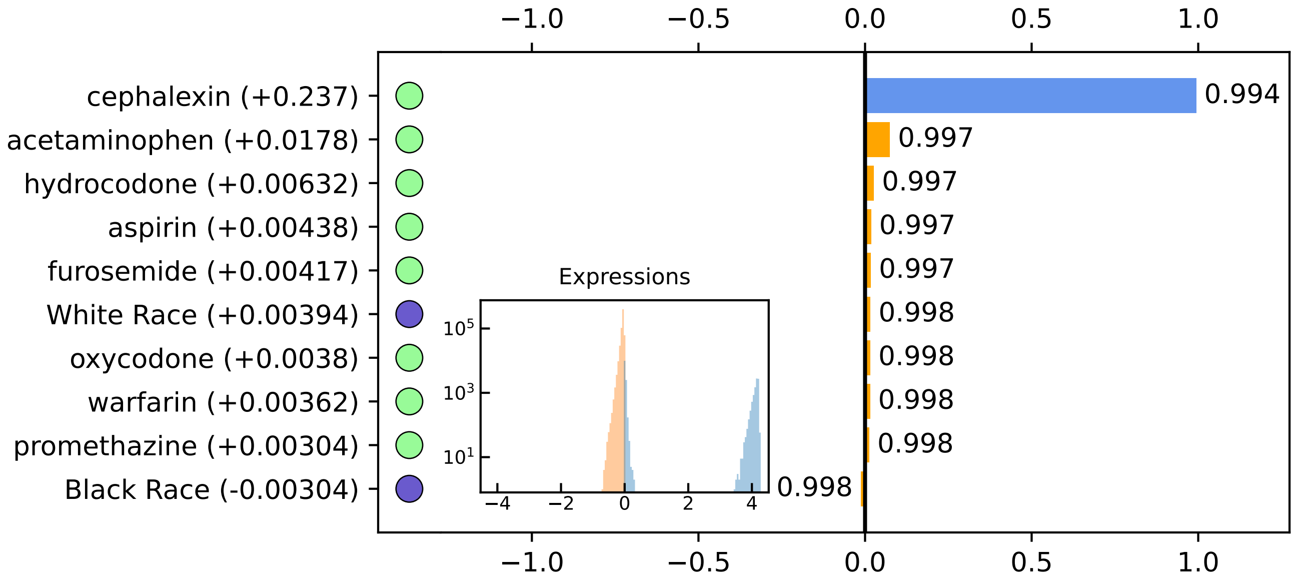

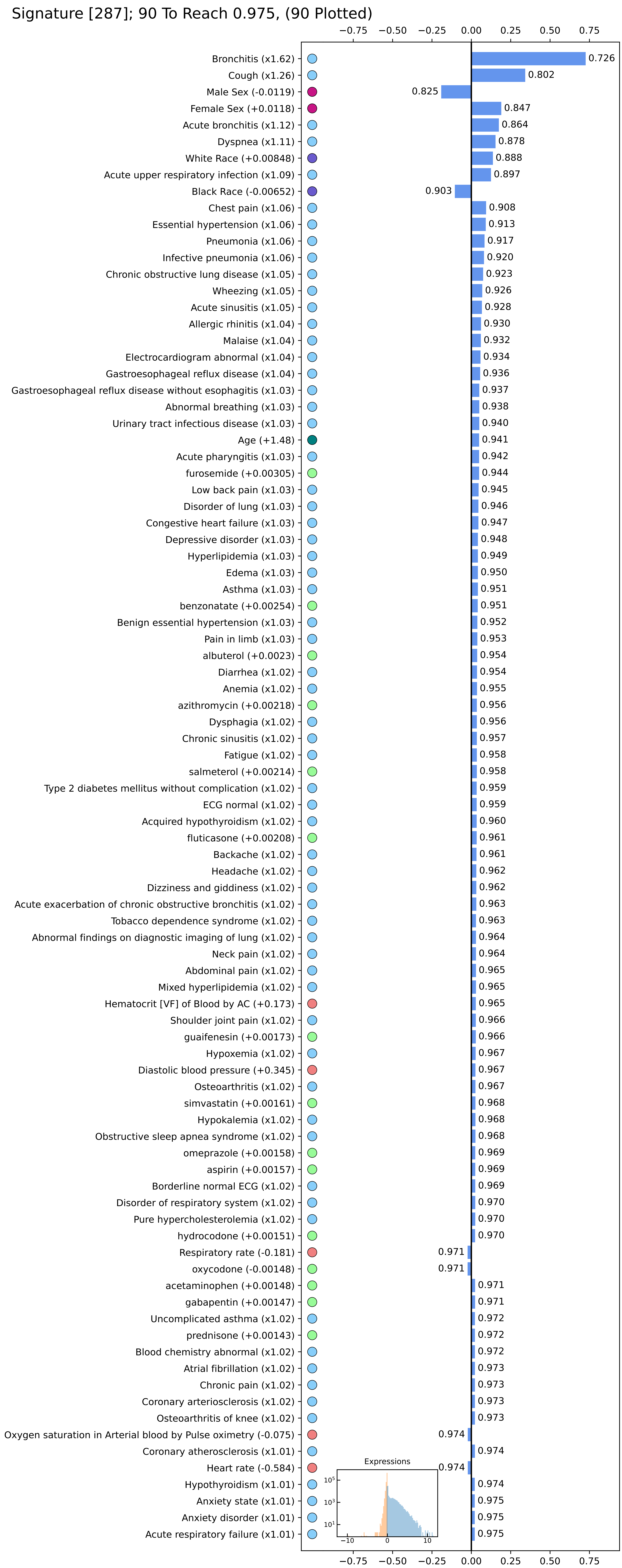

Signature [287]; 90 To Reach 0.975, (90 Plotted)
Bronchitis (x1.62) 0.726
Cough (x1.26) 0.802
Male Sex (-0.0119) 0.825
Female Sex (+0.0118) 0.847
Acute bronchitis (x1.12) 0.864
Dyspnea (x1.11) 0.878
White Race (+0.00848) 0.888
Acute upper respiratory infection (x1.09) 0.897
Black Race (-0.00652) 0.903
Chest pain (x1.06) 0.908
Essential hypertension (x1.06) 0.913
Pneumonia (x1.06) 0.917
Infective pneumonia (x1.06) 0.920
Chronic obstructive lung disease (x1.05) 0.923
Wheezing (x1.05) 0.926
Acute sinusitis (x1.05) 0.928
Allergic rhinitis (x1.04) 0.930
Malaise (x1.04) 0.932
Electrocardiogram abnormal (x1.04) 0.934
Gastroesophageal reflux disease (x1.04) 0.936
Gastroesophageal reflux disease without esophagitis (x1.03) 0.937
Abnormal breathing (x1.03) 0.938
Urinary tract infectious disease (x1.03) 0.940
Age (+1.48) 0.941
Acute pharyngitis (x1.03) 0.942
furosemide (+0.00305) 0.944
Low back pain (x1.03) 0.945
Disorder of lung (x1.03) 0.946
Congestive heart failure (x1.03) 0.947
Depressive disorder (x1.03) 0.948
Hyperlipidemia (x1.03) 0.949
Edema (x1.03) 0.950
Asthma (x1.03) 0.951
benzonatate (+0.00254) 0.951
Benign essential hypertension (x1.03) 0.952
Pain in limb (x1.03) 0.953
albuterol (+0.0023) 0.954
Diarrhea (x1.02) 0.954
Anemia (x1.02) 0.955
azithromycin (+0.00218) 0.956
Dysphagia (x1.02) 0.956
Chronic sinusitis (x1.02) 0.957
Fatigue (x1.02) 0.958
salmeterol (+0.00214) 0.958
Type 2 diabetes mellitus without complication (x1.02) 0.959
ECG normal (x1.02) 0.959
Acquired hypothyroidism (x1.02) 0.960
fluticasone (+0.00208) 0.961
Backache (x1.02) 0.961
Headache (x1.02) 0.962
Dizziness and giddiness (x1.02) 0.962
Acute exacerbation of chronic obstructive bronchitis (x1.02) 0.963
Tobacco dependence syndrome (x1.02) 0.963
Abnormal findings on diagnostic imaging of lung (x1.02) 0.964
Neck pain (x1.02) 0.964
Abdominal pain (x1.02) 0.965
Mixed hyperlipidemia (x1.02) 0.965
Hematocrit [VF] of Blood by AC (+0.173) 0.965
Shoulder joint pain (x1.02) 0.966
guaifenesin (+0.00173) 0.966
Hypoxemia (x1.02) 0.967
Diastolic blood pressure (+0.345) 0.967
Osteoarthritis (x1.02) 0.967
simvastatin (+0.00161) 0.968
Hypokalemia (x1.02) 0.968
Obstructive sleep apnea syndrome (x1.02) 0.968
omeprazole (+0.00158) 0.969
aspirin (+0.00157) 0.969
Borderline normal ECG (x1.02) 0.969
Disorder of respiratory system (x1.02) 0.970
Pure hypercholesterolemia (x1.02) 0.970
hydrocodone (+0.00151) 0.970
Respiratory rate (-0.181) 0.971
oxycodone (-0.00148) 0.971
acetaminophen (+0.00148) 0.971
gabapentin (+0.00147) 0.971
Uncomplicated asthma (x1.02) 0.972
prednisone (+0.00143) 0.972
Blood chemistry abnormal (x1.02) 0.972
Atrial fibrillation (x1.02) 0.973
Chronic pain (x1.02) 0.973
Coronary arteriosclerosis (x1.02) 0.973
Osteoarthritis of knee (x1.02) 0.973
Oxygen saturation in Arterial blood by Pulse oximetry (-0.075) 0.974
Coronary atherosclerosis (x1.01) 0.974
Heart rate (-0.584) 0.974
Hypothyroidism (x1.01) 0.974
Anxiety state (x1.01) 0.975
Anxiety disorder (x1.01) 0.975
Acute respiratory failure (x1.01) 0.975
Expressions
−0.75 −0.50 −0.25 0.00 0.25 0.50 0.75

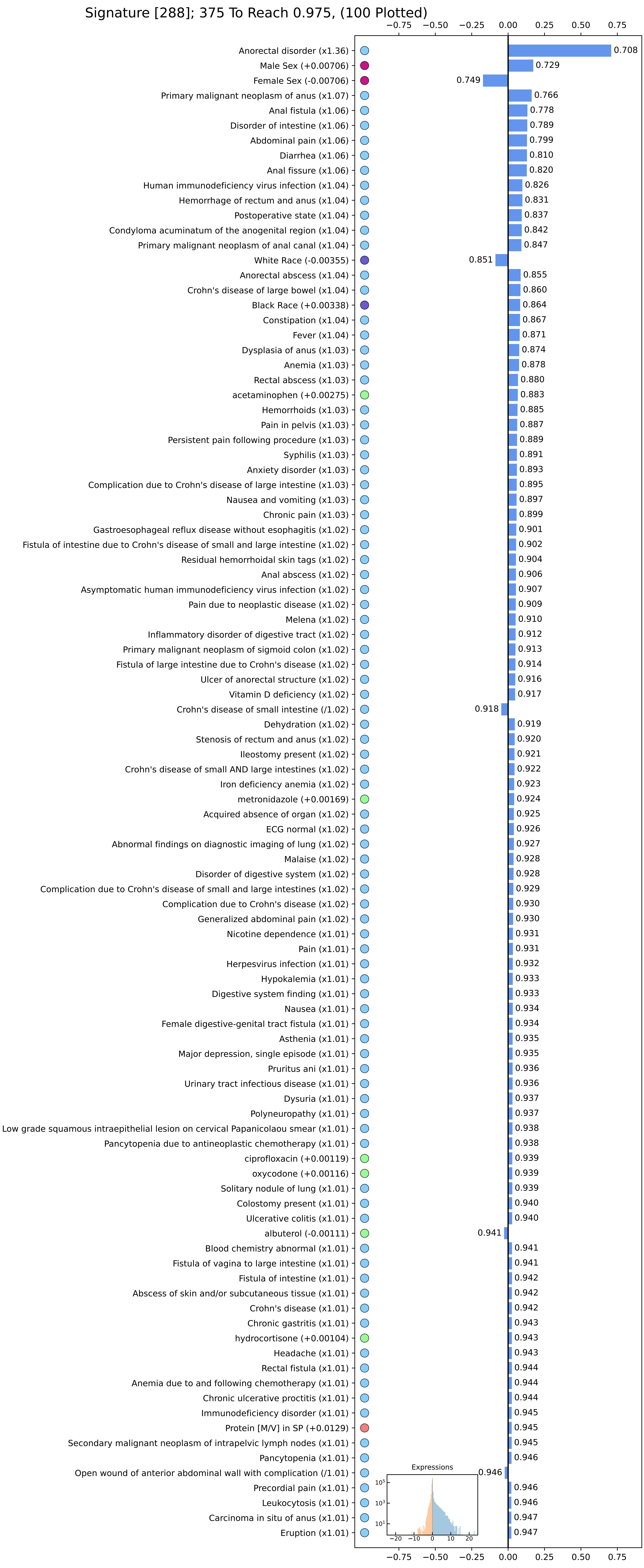

Signature [288]; 375 To Reach 0.975, (100 Plotted)
Anorectal disorder (x1.36) 0.708
Male Sex (+0.00706) 0.729
Female Sex (-0.00706) 0.749
Primary malignant neoplasm of anus (x1.07) 0.766
Anal fistula (x1.06) 0.778
Disorder of intestine (x1.06) 0.789
Abdominal pain (x1.06) 0.799
Diarrhea (x1.06) 0.810
Anal fissure (x1.06) 0.820
Human immunodeficiency virus infection (x1.04) 0.826
Hemorrhage of rectum and anus (x1.04) 0.831
Postoperative state (x1.04) 0.837
Condyloma acuminatum of the anogenital region (x1.04) 0.842
Primary malignant neoplasm of anal canal (x1.04) 0.847
White Race (-0.00355) 0.851
Anorectal abscess (x1.04) 0.855
Crohn's disease of large bowel (x1.04) 0.860
Black Race (+0.00338) 0.864
Constipation (x1.04) 0.867
Fever (x1.04) 0.871
Dysplasia of anus (x1.03) 0.874
Anemia (x1.03) 0.878
Rectal abscess (x1.03) 0.880
acetaminophen (+0.00275) 0.883
Hemorrhoids (x1.03) 0.885
Pain in pelvis (x1.03) 0.887
Persistent pain following procedure (x1.03) 0.889
Syphilis (x1.03) 0.891
Anxiety disorder (x1.03) 0.893
Complication due to Crohn's disease of large intestine (x1.03) 0.895
Nausea and vomiting (x1.03) 0.897
Chronic pain (x1.03) 0.899
Gastroesophageal reflux disease without esophagitis (x1.02) 0.901
Fistula of intestine due to Crohn's disease of small and large intestine (x1.02) 0.902
Residual hemorrhoidal skin tags (x1.02) 0.904
Anal abscess (x1.02) 0.906
Asymptomatic human immunodeficiency virus infection (x1.02) 0.907
Pain due to neoplastic disease (x1.02) 0.909
Melena (x1.02) 0.910
Inflammatory disorder of digestive tract (x1.02) 0.912
Primary malignant neoplasm of sigmoid colon (x1.02) 0.913
Fistula of large intestine due to Crohn's disease (x1.02) 0.914
Ulcer of anorectal structure (x1.02) 0.916
Vitamin D deficiency (x1.02) 0.917
Crohn's disease of small intestine (/1.02) 0.918
Dehydration (x1.02) 0.919
Stenosis of rectum and anus (x1.02) 0.920
Ileostomy present (x1.02) 0.921
Crohn's disease of small AND large intestines (x1.02) 0.922
Iron deficiency anemia (x1.02) 0.923
metronidazole (+0.00169) 0.924
Acquired absence of organ (x1.02) 0.925
ECG normal (x1.02) 0.926
Abnormal findings on diagnostic imaging of lung (x1.02) 0.927
Malaise (x1.02) 0.928
Disorder of digestive system (x1.02) 0.928
Complication due to Crohn's disease of small and large intestines (x1.02) 0.929
Complication due to Crohn's disease (x1.02) 0.930
Generalized abdominal pain (x1.02) 0.930
Nicotine dependence (x1.01) 0.931
Pain (x1.01) 0.931
Herpesvirus infection (x1.01) 0.932
Hypokalemia (x1.01) 0.933
Digestive system finding (x1.01) 0.933
Nausea (x1.01) 0.934
Female digestive-genital tract fistula (x1.01) 0.934
Asthenia (x1.01) 0.935
Major depression, single episode (x1.01) 0.935
Pruritus ani (x1.01) 0.936
Urinary tract infectious disease (x1.01) 0.936
Dysuria (x1.01) 0.937
Polyneuropathy (x1.01) 0.937
Low grade squamous intraepithelial lesion on cervical Papanicolaou smear (x1.01) 0.938
Pancytopenia due to antineoplastic chemotherapy (x1.01) 0.938
ciprofloxacin (+0.00119) 0.939
oxycodone (+0.00116) 0.939
Solitary nodule of lung (x1.01) 0.939
Colostomy present (x1.01) 0.940
Ulcerative colitis (x1.01) 0.940
albuterol (-0.00111) 0.941
Blood chemistry abnormal (x1.01) 0.941
Fistula of vagina to large intestine (x1.01) 0.941
Fistula of intestine (x1.01) 0.942
Abscess of skin and/or subcutaneous tissue (x1.01) 0.942
Crohn's disease (x1.01) 0.942
Chronic gastritis (x1.01) 0.943
hydrocortisone (+0.00104) 0.943
Headache (x1.01) 0.943
Rectal fistula (x1.01) 0.944
Anemia due to and following chemotherapy (x1.01) 0.944
Chronic ulcerative proctitis (x1.01) 0.944
Immunodeficiency disorder (x1.01) 0.945
Protein [M/V] in SP (+0.0129) 0.945
Secondary malignant neoplasm of intrapelvic lymph nodes (x1.01) 0.945
Pancytopenia (x1.01) 0.946
Open wound of anterior abdominal wall with complication (/1.01) 0.946
Precordial pain (x1.01) 0.946
Leukocytosis (x1.01) 0.946
Carcinoma in situ of anus (x1.01) 0.947
Eruption (x1.01) 0.947
−0.75 −0.50 −0.25 0.00 0.25 0.50 0.75
Expressions

Signature [289]; 12 To Reach 0.975, (12 Plotted)

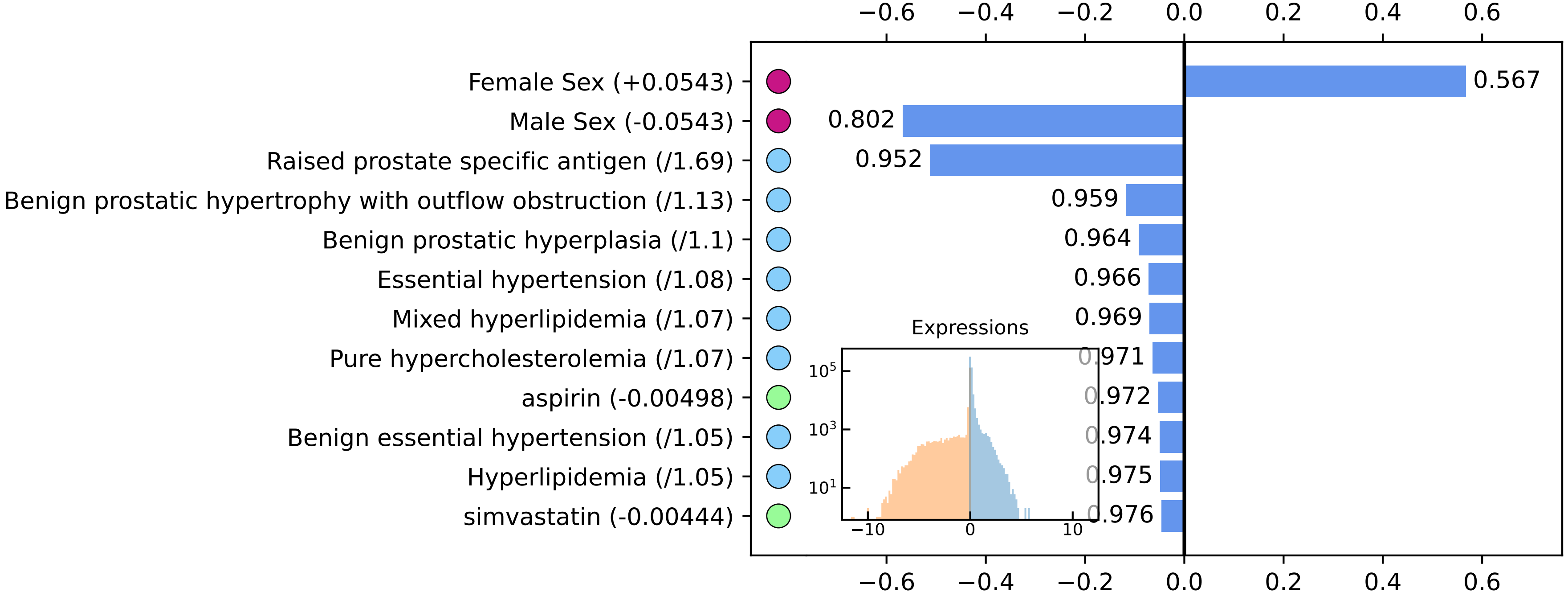

Signature [290]; 9 To Reach 0.975, (10 Plotted)

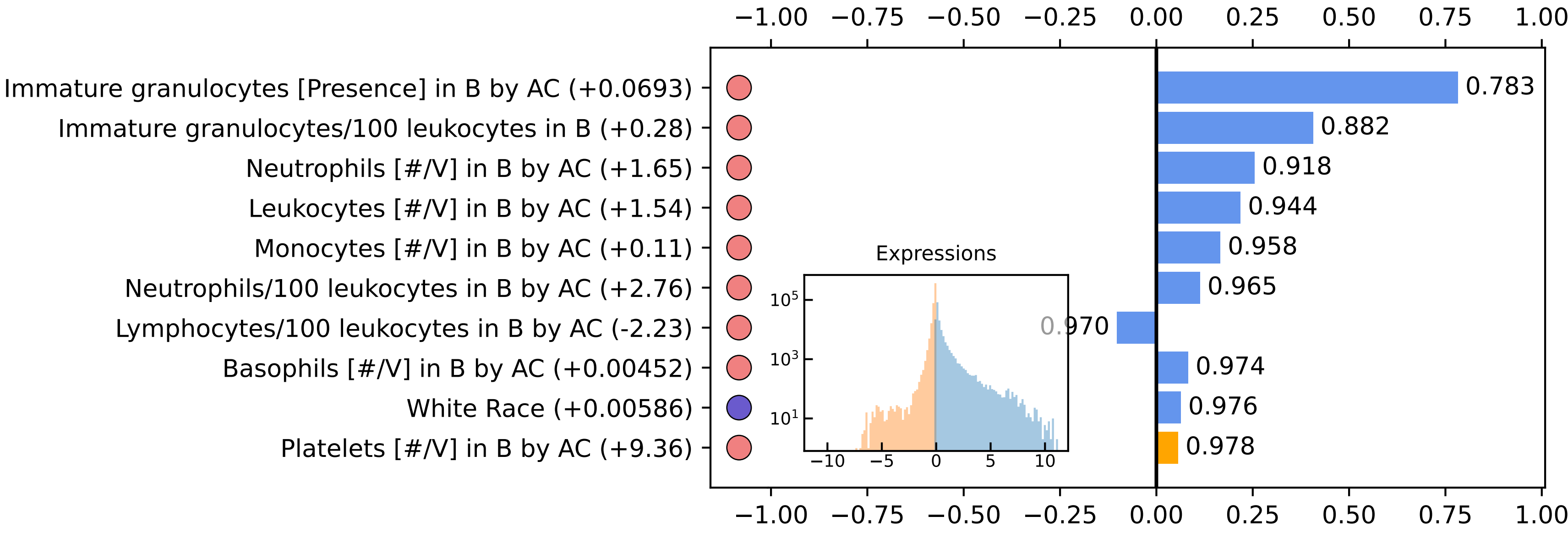

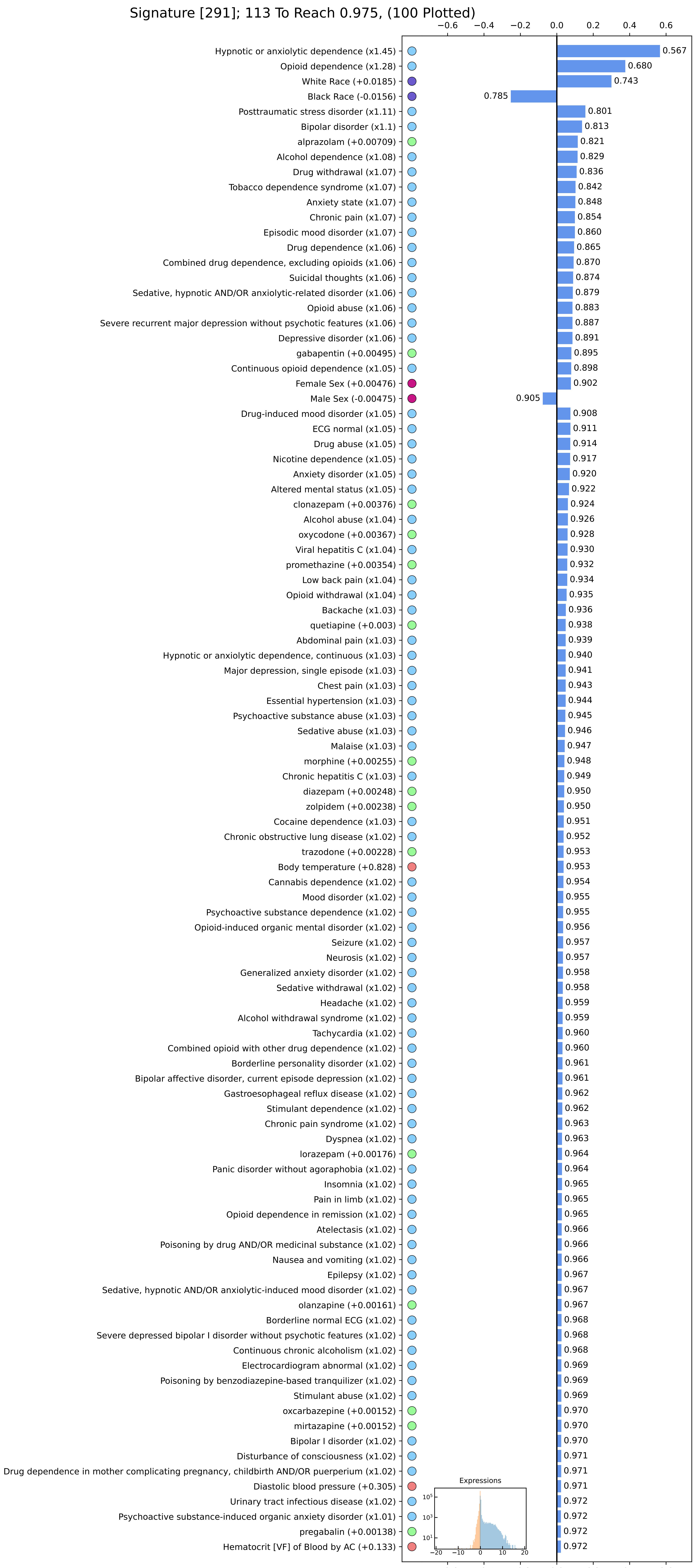
Signature [291]; 113 To Reach 0.975, (100 Plotted)
Hypnotic or anxiolytic dependence (x1.45) 0.567
Opioid dependence (x1.28) 0.680
White Race (+0.0185) 0.743
Black Race (-0.0156) 0.785
Posttraumatic stress disorder (x1.11) 0.801
Bipolar disorder (x1.1) 0.813
alprazolam (+0.00709) 0.821
Alcohol dependence (x1.08) 0.829
Drug withdrawal (x1.07) 0.836
Tobacco dependence syndrome (x1.07) 0.842
Anxiety state (x1.07) 0.848
Chronic pain (x1.07) 0.854
Episodic mood disorder (x1.07) 0.860
Drug dependence (x1.06) 0.865
Combined drug dependence, excluding opioids (x1.06) 0.870
Suicidal thoughts (x1.06) 0.874
Sedative, hypnotic AND/OR anxiolytic-related disorder (x1.06) 0.879
Opioid abuse (x1.06) 0.883
Severe recurrent major depression without psychotic features (x1.06) 0.887
Depressive disorder (x1.06) 0.891
gabapentin (+0.00495) 0.895
Continuous opioid dependence (x1.05) 0.898
Female Sex (+0.00476) 0.902
Male Sex (-0.00475) 0.905
Drug-induced mood disorder (x1.05) 0.908
ECG normal (x1.05) 0.911
Drug abuse (x1.05) 0.914
Nicotine dependence (x1.05) 0.917
Anxiety disorder (x1.05) 0.920
Altered mental status (x1.05) 0.922
clonazepam (+0.00376) 0.924
Alcohol abuse (x1.04) 0.926
oxycodone (+0.00367) 0.928
Viral hepatitis C (x1.04) 0.930
promethazine (+0.00354) 0.932
Low back pain (x1.04) 0.934
Opioid withdrawal (x1.04) 0.935
Backache (x1.03) 0.936
quetiapine (+0.003) 0.938
Abdominal pain (x1.03) 0.939
Hypnotic or anxiolytic dependence, continuous (x1.03) 0.940
Major depression, single episode (x1.03) 0.941
Chest pain (x1.03) 0.943
Essential hypertension (x1.03) 0.944
Psychoactive substance abuse (x1.03) 0.945
Sedative abuse (x1.03) 0.946
Malaise (x1.03) 0.947
morphine (+0.00255) 0.948
Chronic hepatitis C (x1.03) 0.949
diazepam (+0.00248) 0.950
zolpidem (+0.00238) 0.950
Cocaine dependence (x1.03) 0.951
Chronic obstructive lung disease (x1.02) 0.952
trazodone (+0.00228) 0.953
Body temperature (+0.828) 0.953
Cannabis dependence (x1.02) 0.954
Mood disorder (x1.02) 0.955
Psychoactive substance dependence (x1.02) 0.955
Opioid-induced organic mental disorder (x1.02) 0.956
Seizure (x1.02) 0.957
Neurosis (x1.02) 0.957
Generalized anxiety disorder (x1.02) 0.958
Sedative withdrawal (x1.02) 0.958
Headache (x1.02) 0.959
Alcohol withdrawal syndrome (x1.02) 0.959
Tachycardia (x1.02) 0.960
Combined opioid with other drug dependence (x1.02) 0.960
Borderline personality disorder (x1.02) 0.961
Bipolar affective disorder, current episode depression (x1.02) 0.961
Gastroesophageal reflux disease (x1.02) 0.962
Stimulant dependence (x1.02) 0.962
Chronic pain syndrome (x1.02) 0.963
Dyspnea (x1.02) 0.963
lorazepam (+0.00176) 0.964
Panic disorder without agoraphobia (x1.02) 0.964
Insomnia (x1.02) 0.965
Pain in limb (x1.02) 0.965
Opioid dependence in remission (x1.02) 0.965
Atelectasis (x1.02) 0.966
Poisoning by drug AND/OR medicinal substance (x1.02) 0.966
Nausea and vomiting (x1.02) 0.966
Epilepsy (x1.02) 0.967
Sedative, hypnotic AND/OR anxiolytic-induced mood disorder (x1.02) 0.967
olanzapine (+0.00161) 0.967
Borderline normal ECG (x1.02) 0.968
Severe depressed bipolar I disorder without psychotic features (x1.02) 0.968
Continuous chronic alcoholism (x1.02) 0.968
Electrocardiogram abnormal (x1.02) 0.969
Poisoning by benzodiazepine-based tranquilizer (x1.02) 0.969
Stimulant abuse (x1.02) 0.969
oxcarbazepine (+0.00152) 0.970
mirtazapine (+0.00152) 0.970
Bipolar I disorder (x1.02) 0.970
Disturbance of consciousness (x1.02) 0.971
Drug dependence in mother complicating pregnancy, childbirth AND/OR puerperium (x1.02) 0.971
Diastolic blood pressure (+0.305) 0.971
Urinary tract infectious disease (x1.02) 0.972
Psychoactive substance-induced organic anxiety disorder (x1.01) 0.972
pregabalin (+0.00138) 0.972
Hematocrit [VF] of Blood by AC (+0.133) 0.972
−0.6 −0.4 −0.2 0.0 0.2 0.4 0.6
Expressions

Signature [292]; 26 To Reach 0.975, (26 Plotted)

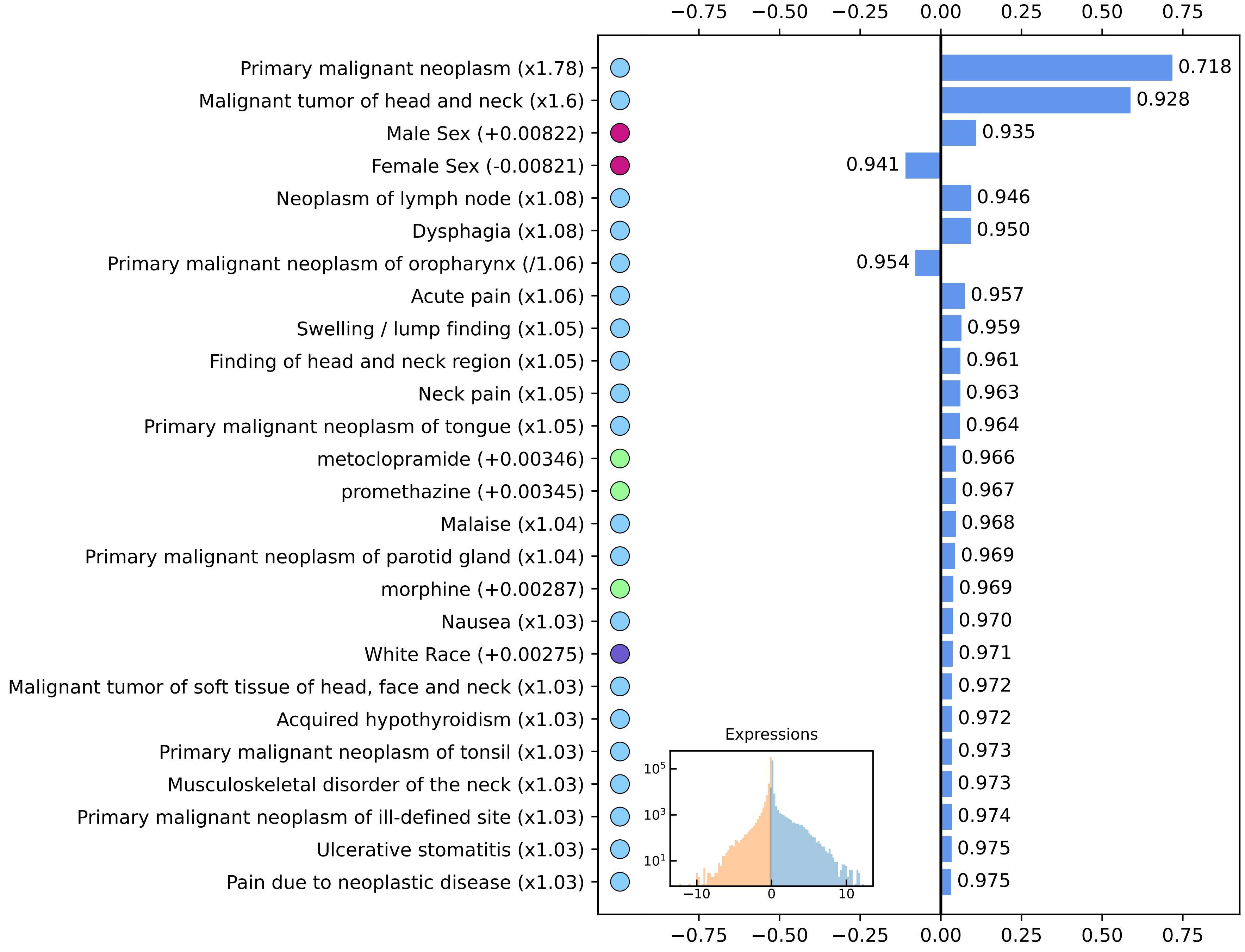

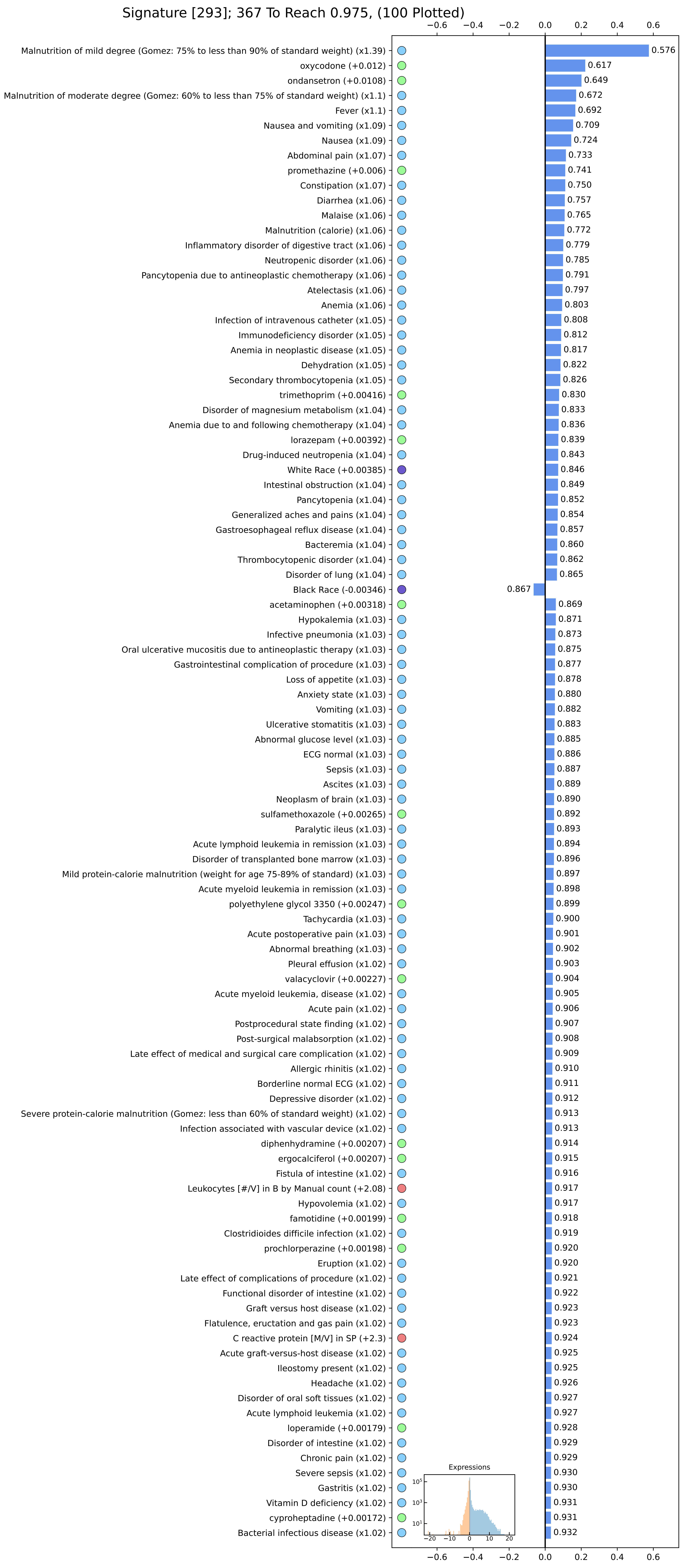

Signature [293]; 367 To Reach 0.975, (100 Plotted)
Malnutrition of mild degree (Gomez: 75% to less than 90% of standard weight) (x1.39)
oxycodone (+0.012)
ondansetron (+0.0108)
Malnutrition of moderate degree (Gomez: 60% to less than 75% of standard weight) (x1.1)
Fever (x1.1)
Nausea and vomiting (x1.09)
Nausea (x1.09)
Abdominal pain (x1.07)
promethazine (+0.006)
Constipation (x1.07)
Diarrhea (x1.06)
Malaise (x1.06)
Malnutrition (calorie) (x1.06)
Inflammatory disorder of digestive tract (x1.06)
Neutropenic disorder (x1.06)
Pancytopenia due to antineoplastic chemotherapy (x1.06)
Atelectasis (x1.06)
Anemia (x1.06)
Infection of intravenous catheter (x1.05)
Immunodeficiency disorder (x1.05)
Anemia in neoplastic disease (x1.05)
Dehydration (x1.05)
Secondary thrombocytopenia (x1.05)
trimethoprim (+0.00416)
Disorder of magnesium metabolism (x1.04)
Anemia due to and following chemotherapy (x1.04)
lorazepam (+0.00392)
Drug-induced neutropenia (x1.04)
White Race (+0.00385)
Intestinal obstruction (x1.04)
Pancytopenia (x1.04)
Generalized aches and pains (x1.04)
Gastroesophageal reflux disease (x1.04)
Bacteremia (x1.04)
Thrombocytopenic disorder (x1.04)
Disorder of lung (x1.04)
Black Race (-0.00346)
acetaminophen (+0.00318)
Hypokalemia (x1.03)
Infective pneumonia (x1.03)
Oral ulcerative mucositis due to antineoplastic therapy (x1.03)
Gastrointestinal complication of procedure (x1.03)
Loss of appetite (x1.03)
Anxiety state (x1.03)
Vomiting (x1.03)
Ulcerative stomatitis (x1.03)
Abnormal glucose level (x1.03)
ECG normal (x1.03)
Sepsis (x1.03)
Ascites (x1.03)
Neoplasm of brain (x1.03)
sulfamethoxazole (+0.00265)
Paralytic ileus (x1.03)
Acute lymphoid leukemia in remission (x1.03)
Disorder of transplanted bone marrow (x1.03)
Mild protein-calorie malnutrition (weight for age 75-89% of standard) (x1.03)
Acute myeloid leukemia in remission (x1.03)
polyethylene glycol 3350 (+0.00247)
Tachycardia (x1.03)
Acute postoperative pain (x1.03)
Abnormal breathing (x1.03)
Pleural effusion (x1.02)
valacyclovir (+0.00227)
Acute myeloid leukemia, disease (x1.02)
Acute pain (x1.02)
Postprocedural state finding (x1.02)
Post-surgical malabsorption (x1.02)
Late effect of medical and surgical care complication (x1.02)
Allergic rhinitis (x1.02)
Borderline normal ECG (x1.02)
Depressive disorder (x1.02)
Severe protein-calorie malnutrition (Gomez: less than 60% of standard weight) (x1.02)
Infection associated with vascular device (x1.02)
diphenhydramine (+0.00207)
ergocalciferol (+0.00207)
Fistula of intestine (x1.02)
Leukocytes [#/V] in B by Manual count (+2.08)
Hypovolemia (x1.02)
famotidine (+0.00199)
Clostridioides difficile infection (x1.02)
prochlorperazine (+0.00198)
Eruption (x1.02)
Late effect of complications of procedure (x1.02)
Functional disorder of intestine (x1.02)
Graft versus host disease (x1.02)
Flatulence, eructation and gas pain (x1.02)
C reactive protein [M/V] in SP (+2.3)
Acute graft-versus-host disease (x1.02)
Ileostomy present (x1.02)
Headache (x1.02)
Disorder of oral soft tissues (x1.02)
Acute lymphoid leukemia (x1.02)
loperamide (+0.00179)
Disorder of intestine (x1.02)
Chronic pain (x1.02)
Severe sepsis (x1.02)
Gastritis (x1.02)
Vitamin D deficiency (x1.02)
cyproheptadine (+0.00172)
Bacterial infectious disease (x1.02)
0.576 0.617 0.649 0.672 0.692 0.709 0.724 0.733 0.741 0.750 0.757 0.765 0.772 0.779 0.785 0.791 0.797 0.803 0.808 0.812 0.817 0.822 0.826 0.830 0.833 0.836 0.839 0.843 0.846 0.849 0.852 0.854 0.857 0.860 0.862 0.865 0.867 0.869 0.871 0.873 0.875 0.877 0.878 0.880 0.882 0.883 0.885 0.886 0.887 0.889 0.890 0.892 0.893 0.894 0.896 0.897 0.898 0.899 0.900 0.901 0.902 0.903 0.904 0.905 0.906 0.907 0.908 0.909 0.910 0.911 0.912 0.913 0.913 0.914 0.915 0.916 0.917 0.917 0.918 0.919 0.920 0.920 0.921 0.922 0.923 0.923 0.924 0.925 0.925 0.926 0.927 0.927 0.928 0.929 0.929 0.930 0.930 0.931 0.931 0.932
−0.6 −0.4 −0.2 0.0 0.2 0.4 0.6
Expressions
10^5 10^3 10^1
−20 −10 0 10 20

Signature [294]; 84 To Reach 0.975, (84 Plotted)

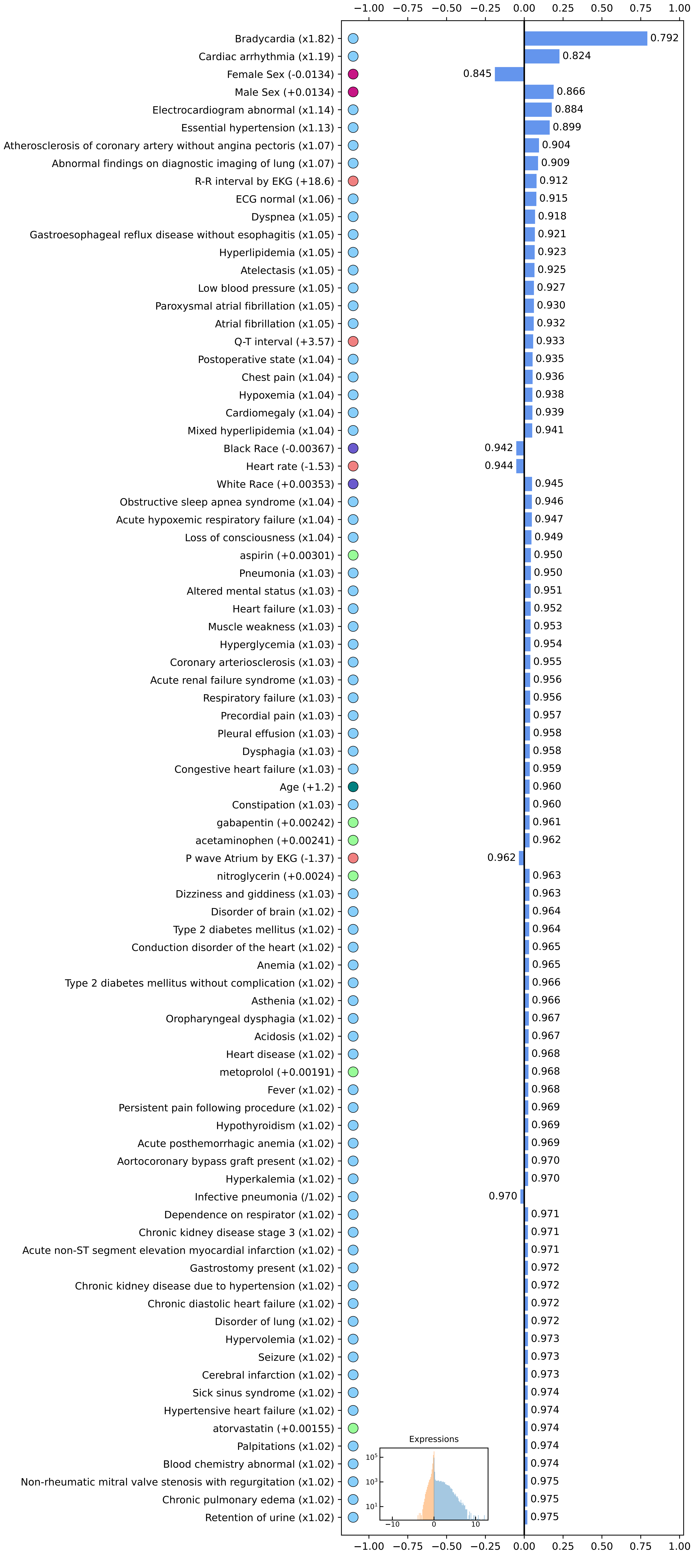

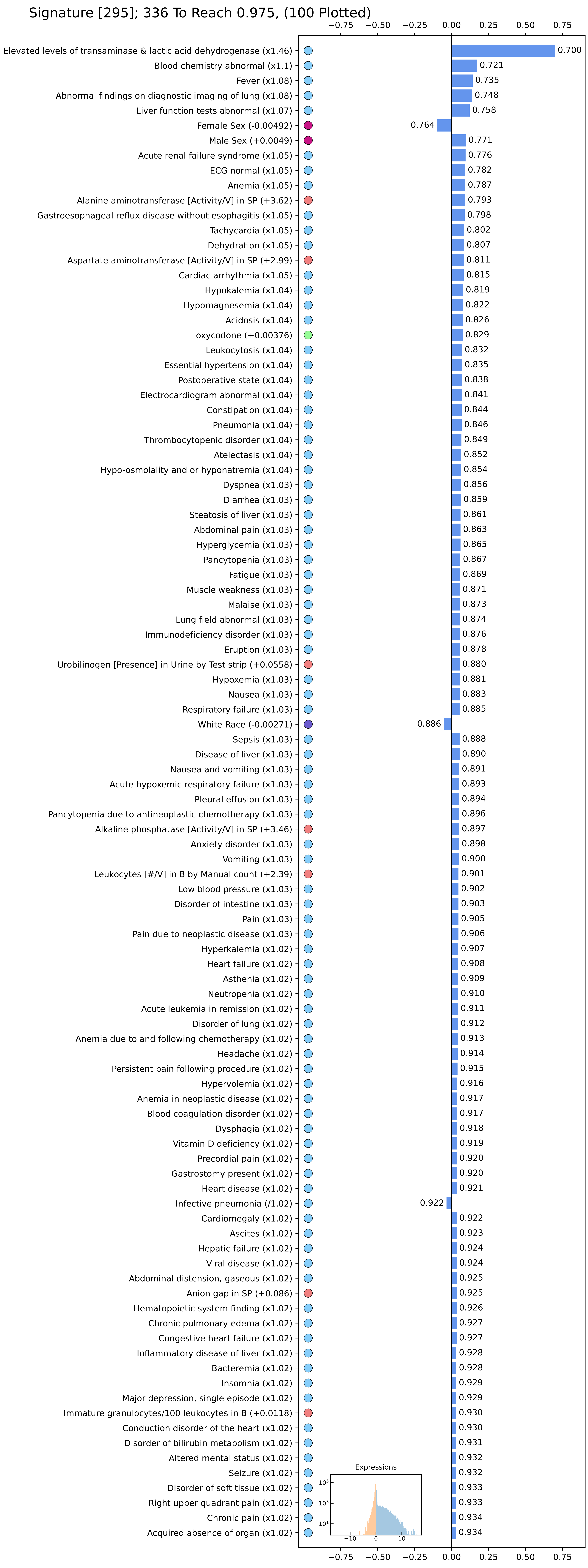

Signature [295]; 336 To Reach 0.975, (100 Plotted)
−0.75 −0.50 −0.25 0.00 0.25 0.50 0.75
Elevated levels of transaminase & lactic acid dehydrogenase (x1.46) 0.700
Blood chemistry abnormal (x1.1) 0.721
Fever (x1.08) 0.735
Abnormal findings on diagnostic imaging of lung (x1.08) 0.748
Liver function tests abnormal (x1.07) 0.758
Female Sex (-0.00492) 0.764
Male Sex (+0.0049) 0.771
Acute renal failure syndrome (x1.05) 0.776
ECG normal (x1.05) 0.782
Anemia (x1.05) 0.787
Alanine aminotransferase [Activity/V] in SP (+3.62) 0.793
Gastroesophageal reflux disease without esophagitis (x1.05) 0.798
Tachycardia (x1.05) 0.802
Dehydration (x1.05) 0.807
Aspartate aminotransferase [Activity/V] in SP (+2.99) 0.811
Cardiac arrhythmia (x1.05) 0.815
Hypokalemia (x1.04) 0.819
Hypomagnesemia (x1.04) 0.822
Acidosis (x1.04) 0.826
oxycodone (+0.00376) 0.829
Leukocytosis (x1.04) 0.832
Essential hypertension (x1.04) 0.835
Postoperative state (x1.04) 0.838
Electrocardiogram abnormal (x1.04) 0.841
Constipation (x1.04) 0.844
Pneumonia (x1.04) 0.846
Thrombocytopenic disorder (x1.04) 0.849
Atelectasis (x1.04) 0.852
Hypo-osmolality and or hyponatremia (x1.04) 0.854
Dyspnea (x1.03) 0.856
Diarrhea (x1.03) 0.859
Steatosis of liver (x1.03) 0.861
Abdominal pain (x1.03) 0.863
Hyperglycemia (x1.03) 0.865
Pancytopenia (x1.03) 0.867
Fatigue (x1.03) 0.869
Muscle weakness (x1.03) 0.871
Malaise (x1.03) 0.873
Lung field abnormal (x1.03) 0.874
Immunodeficiency disorder (x1.03) 0.876
Eruption (x1.03) 0.878
Urobilinogen [Presence] in Urine by Test strip (+0.0558) 0.880
Hypoxemia (x1.03) 0.881
Nausea (x1.03) 0.883
Respiratory failure (x1.03) 0.885
White Race (-0.00271) 0.886
Sepsis (x1.03) 0.888
Disease of liver (x1.03) 0.890
Nausea and vomiting (x1.03) 0.891
Acute hypoxemic respiratory failure (x1.03) 0.893
Pleural effusion (x1.03) 0.894
Pancytopenia due to antineoplastic chemotherapy (x1.03) 0.896
Alkaline phosphatase [Activity/V] in SP (+3.46) 0.897
Anxiety disorder (x1.03) 0.898
Vomiting (x1.03) 0.900
Leukocytes [#/V] in B by Manual count (+2.39) 0.901
Low blood pressure (x1.03) 0.902
Disorder of intestine (x1.03) 0.903
Pain (x1.03) 0.905
Pain due to neoplastic disease (x1.03) 0.906
Hyperkalemia (x1.02) 0.907
Heart failure (x1.02) 0.908
Asthenia (x1.02) 0.909
Neutropenia (x1.02) 0.910
Acute leukemia in remission (x1.02) 0.911
Disorder of lung (x1.02) 0.912
Anemia due to and following chemotherapy (x1.02) 0.913
Headache (x1.02) 0.914
Persistent pain following procedure (x1.02) 0.915
Hypervolemia (x1.02) 0.916
Anemia in neoplastic disease (x1.02) 0.917
Blood coagulation disorder (x1.02) 0.917
Dysphagia (x1.02) 0.918
Vitamin D deficiency (x1.02) 0.919
Precordial pain (x1.02) 0.920
Gastrostomy present (x1.02) 0.920
Heart disease (x1.02) 0.921
Infective pneumonia (/1.02) 0.922
Cardiomegaly (x1.02) 0.922
Ascites (x1.02) 0.923
Hepatic failure (x1.02) 0.924
Viral disease (x1.02) 0.924
Abdominal distension, gaseous (x1.02) 0.925
Anion gap in SP (+0.086) 0.925
Hematopoietic system finding (x1.02) 0.926
Chronic pulmonary edema (x1.02) 0.927
Congestive heart failure (x1.02) 0.927
Inflammatory disease of liver (x1.02) 0.928
Bacteremia (x1.02) 0.928
Insomnia (x1.02) 0.929
Major depression, single episode (x1.02) 0.929
Immature granulocytes/100 leukocytes in B (+0.0118) 0.930
Conduction disorder of the heart (x1.02) 0.930
Disorder of bilirubin metabolism (x1.02) 0.931
Altered mental status (x1.02) 0.932
Seizure (x1.02) 0.932
Disorder of soft tissue (x1.02) 0.933
Right upper quadrant pain (x1.02) 0.933
Chronic pain (x1.02) 0.934
Acquired absence of organ (x1.02) 0.934
Expressions

Signature [296]; 4 To Reach 0.975, (10 Plotted)

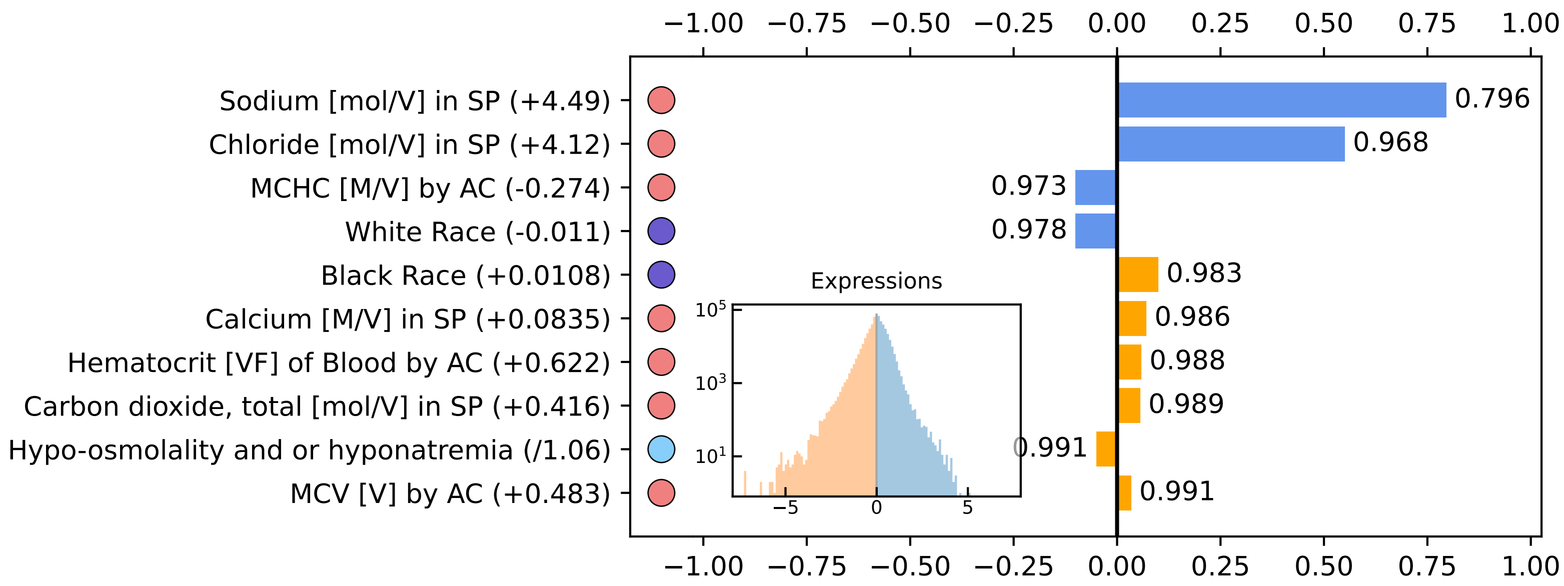

Signature [297]; 5 To Reach 0.975, (10 Plotted)

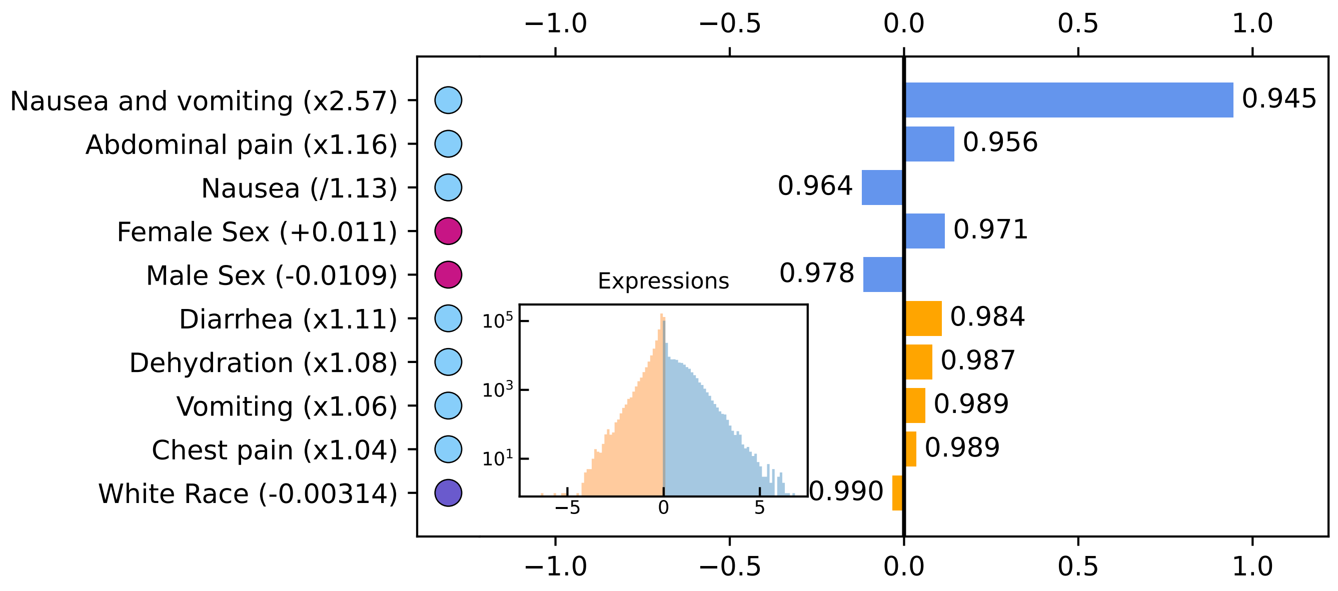

Signature [298]; 96 To Reach 0.975, (96 Plotted)

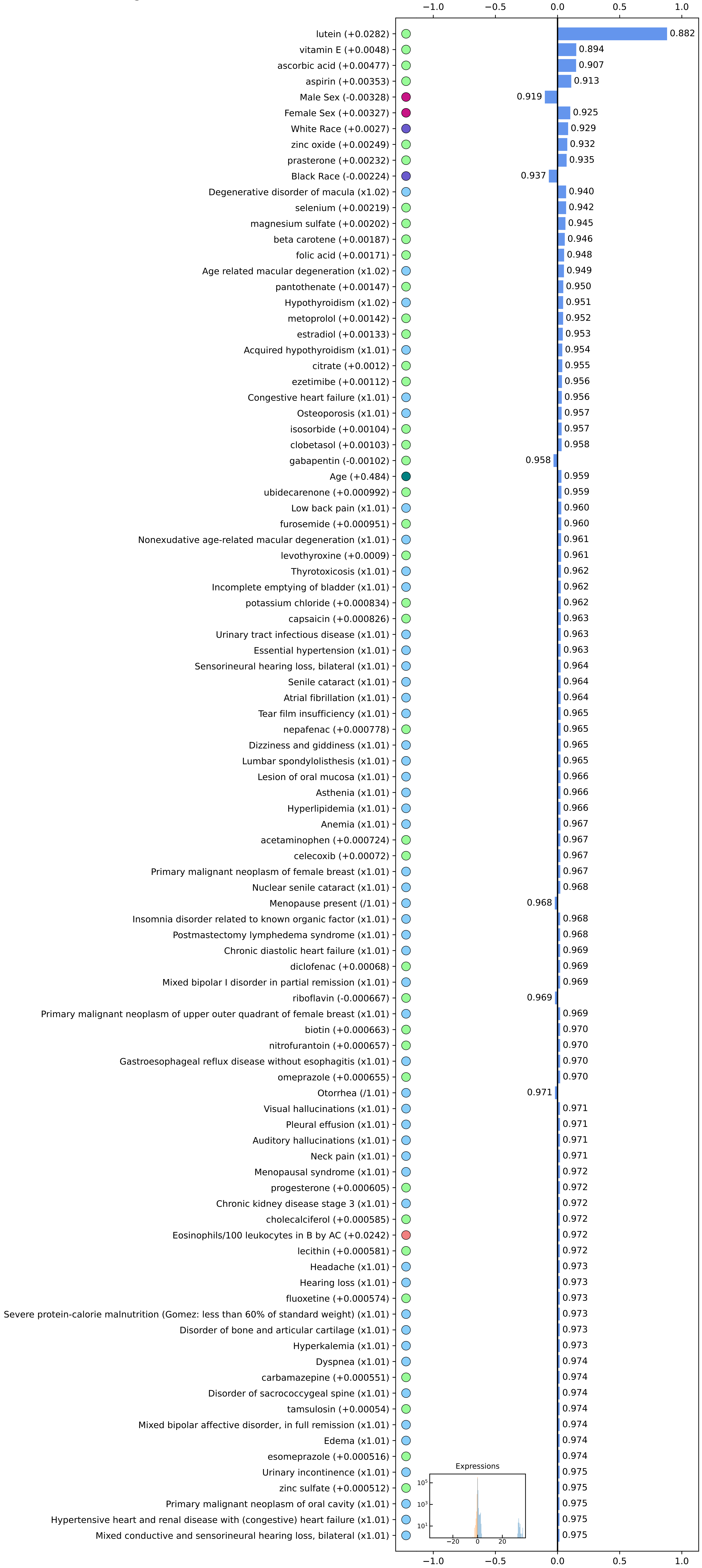

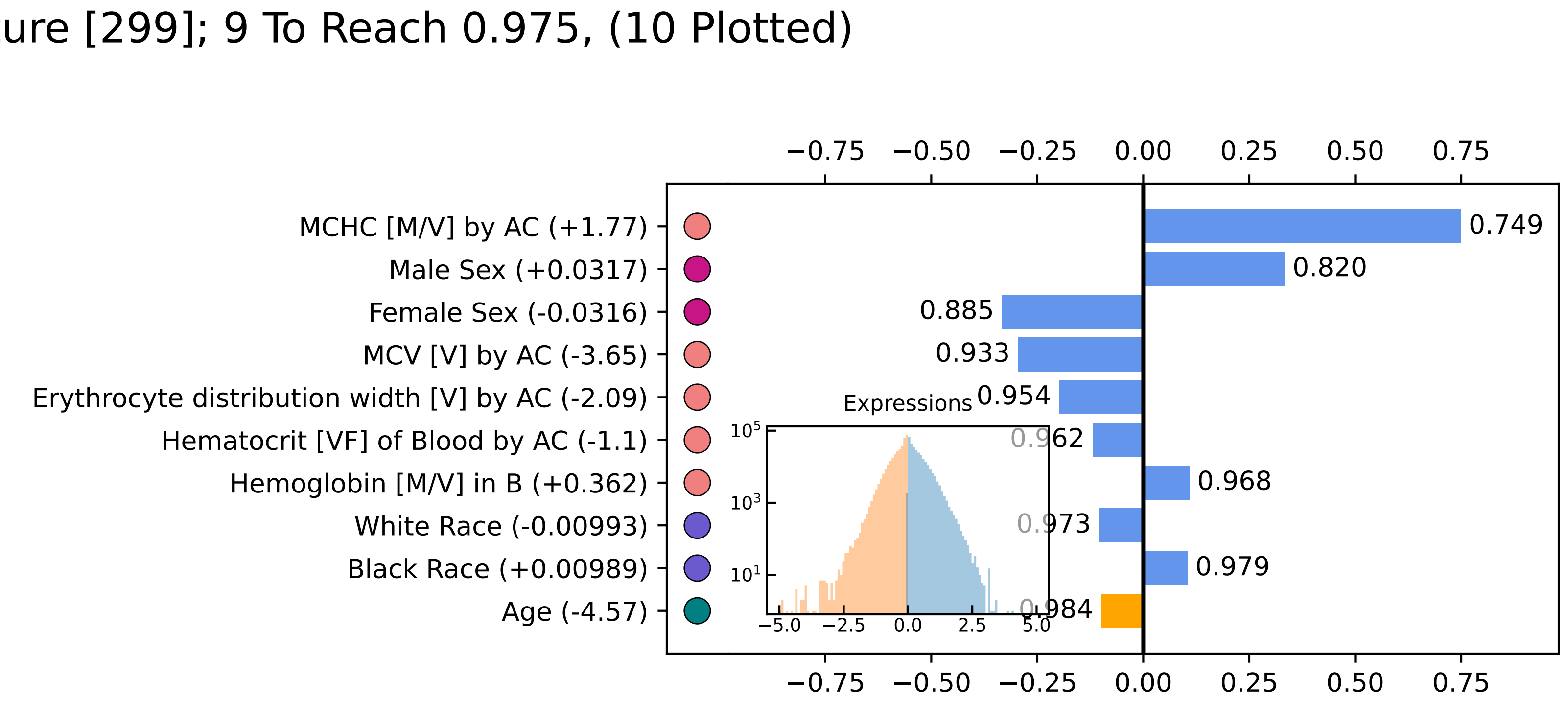

Signature [299]; 9 To Reach 0.975, (10 Plotted)
MCHC [M/V] by AC (+1.77)
Male Sex (+0.0317)
Female Sex (-0.0316)
MCV [V] by AC (-3.65)
Erythrocyte distribution width [V] by AC (-2.09)
Hematocrit [VF] of Blood by AC (-1.1)
Hemoglobin [M/V] in B (+0.362)
White Race (-0.00993)
Black Race (+0.00989)
Age (-4.57)
0.749
0.820
0.885
0.933
0.954
0.962
0.968
0.973
0.979
0.984
Expressions
−0.75 −0.50 −0.25 0.00 0.25 0.50 0.75

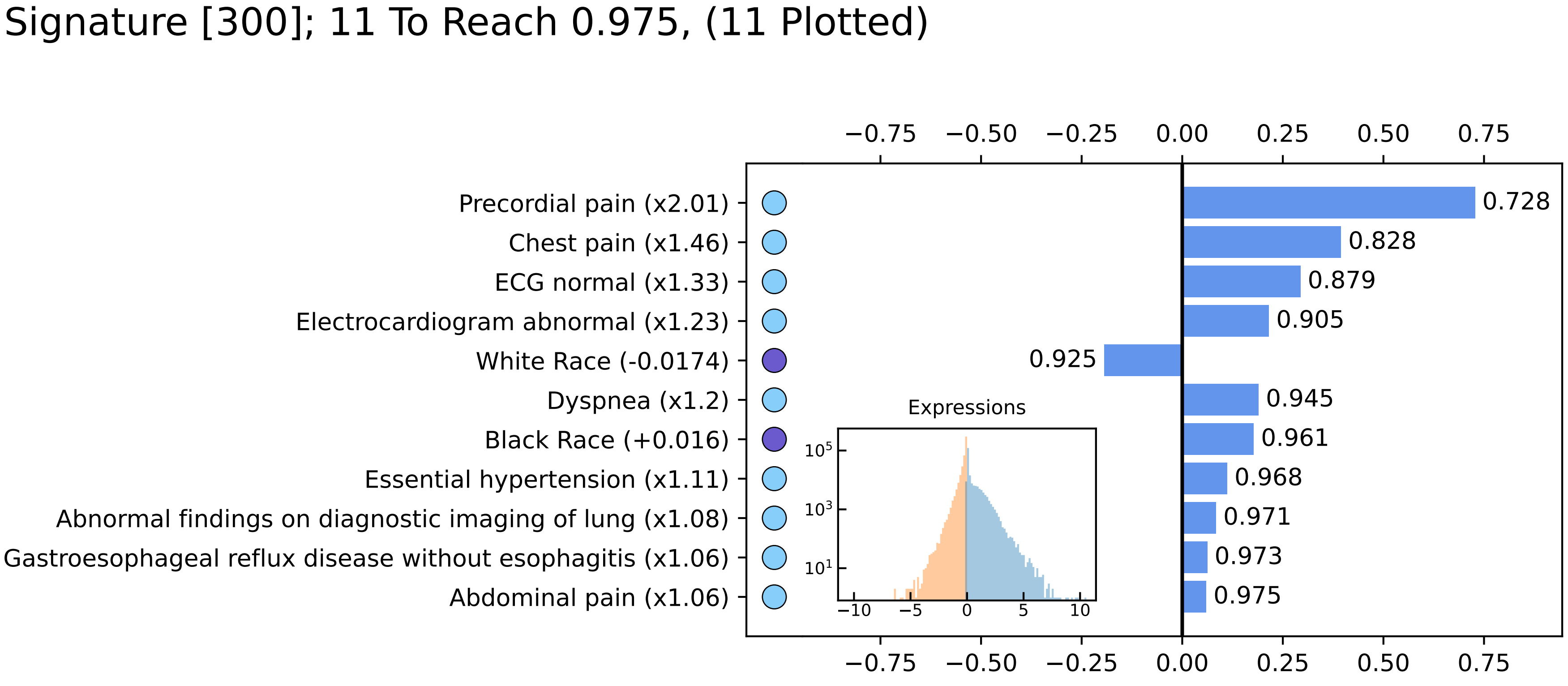

Signature [300]; 11 To Reach 0.975, (11 Plotted)
Precordial pain (x2.01)
Chest pain (x1.46)
ECG normal (x1.33)
Electrocardiogram abnormal (x1.23)
White Race (-0.0174)
Dyspnea (x1.2)
Black Race (+0.016)
Essential hypertension (x1.11)
Abnormal findings on diagnostic imaging of lung (x1.08)
Gastroesophageal reflux disease without esophagitis (x1.06)
Abdominal pain (x1.06)
0.728
0.828
0.879
0.905
0.925
0.945
0.961
0.968
0.971
0.973
0.975
−0.75 −0.50 −0.25 0.00 0.25 0.50 0.75
Expressions

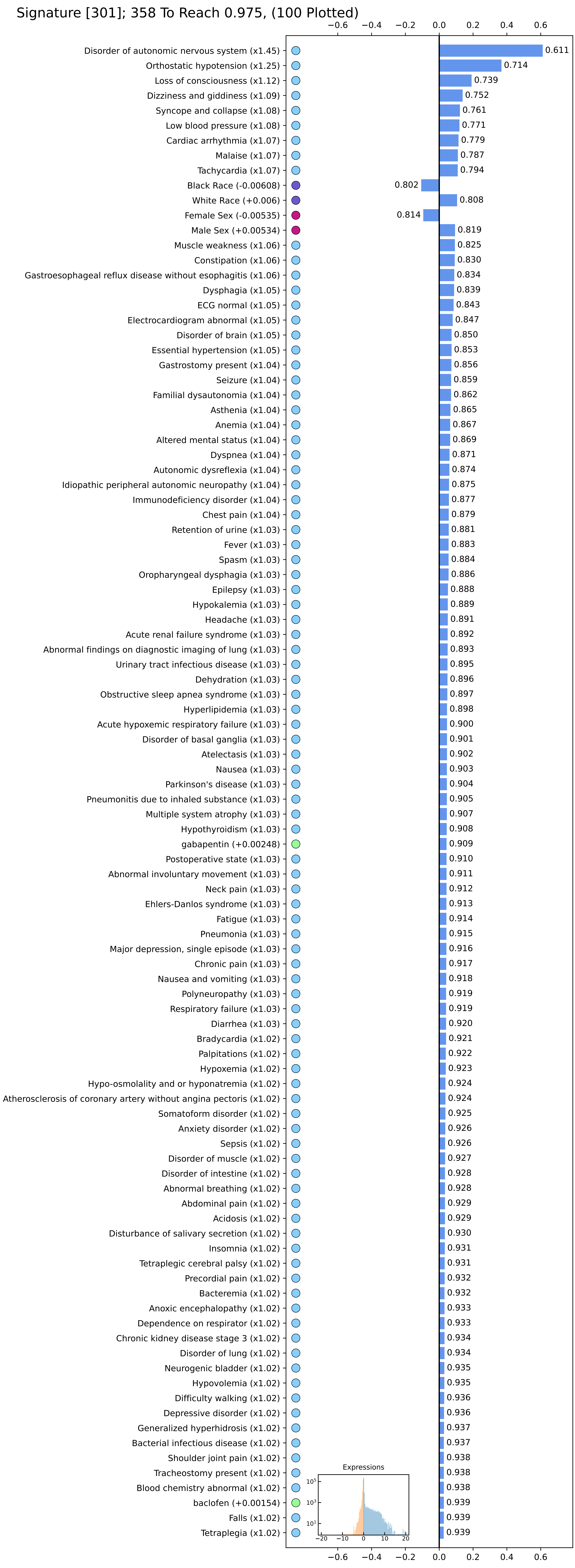

Signature [301]; 358 To Reach 0.975, (100 Plotted)
Disorder of autonomic nervous system (x1.45) 0.611
Orthostatic hypotension (x1.25) 0.714
Loss of consciousness (x1.12) 0.739
Dizziness and giddiness (x1.09) 0.752
Syncope and collapse (x1.08) 0.761
Low blood pressure (x1.08) 0.771
Cardiac arrhythmia (x1.07) 0.779
Malaise (x1.07) 0.787
Tachycardia (x1.07) 0.794
Black Race (-0.00608) 0.802
White Race (+0.006) 0.808
Female Sex (-0.00535) 0.814
Male Sex (+0.00534) 0.819
Muscle weakness (x1.06) 0.825
Constipation (x1.06) 0.830
Gastroesophageal reflux disease without esophagitis (x1.06) 0.834
Dysphagia (x1.05) 0.839
ECG normal (x1.05) 0.843
Electrocardiogram abnormal (x1.05) 0.847
Disorder of brain (x1.05) 0.850
Essential hypertension (x1.05) 0.853
Gastrostomy present (x1.04) 0.856
Seizure (x1.04) 0.859
Familial dysautonomia (x1.04) 0.862
Asthenia (x1.04) 0.865
Anemia (x1.04) 0.867
Altered mental status (x1.04) 0.869
Dyspnea (x1.04) 0.871
Autonomic dysreflexia (x1.04) 0.874
Idiopathic peripheral autonomic neuropathy (x1.04) 0.875
Immunodeficiency disorder (x1.04) 0.877
Chest pain (x1.04) 0.879
Retention of urine (x1.03) 0.881
Fever (x1.03) 0.883
Spasm (x1.03) 0.884
Oropharyngeal dysphagia (x1.03) 0.886
Epilepsy (x1.03) 0.888
Hypokalemia (x1.03) 0.889
Headache (x1.03) 0.891
Acute renal failure syndrome (x1.03) 0.892
Abnormal findings on diagnostic imaging of lung (x1.03) 0.893
Urinary tract infectious disease (x1.03) 0.895
Dehydration (x1.03) 0.896
Obstructive sleep apnea syndrome (x1.03) 0.897
Hyperlipidemia (x1.03) 0.898
Acute hypoxemic respiratory failure (x1.03) 0.900
Disorder of basal ganglia (x1.03) 0.901
Atelectasis (x1.03) 0.902
Nausea (x1.03) 0.903
Parkinson's disease (x1.03) 0.904
Pneumonitis due to inhaled substance (x1.03) 0.905
Multiple system atrophy (x1.03) 0.907
Hypothyroidism (x1.03) 0.908
gabapentin (+0.00248) 0.909
Postoperative state (x1.03) 0.910
Abnormal involuntary movement (x1.03) 0.911
Neck pain (x1.03) 0.912
Ehlers-Danlos syndrome (x1.03) 0.913
Fatigue (x1.03) 0.914
Pneumonia (x1.03) 0.915
Major depression, single episode (x1.03) 0.916
Chronic pain (x1.03) 0.917
Nausea and vomiting (x1.03) 0.918
Polyneuropathy (x1.03) 0.919
Respiratory failure (x1.03) 0.919
Diarrhea (x1.03) 0.920
Bradycardia (x1.02) 0.921
Palpitations (x1.02) 0.922
Hypoxemia (x1.02) 0.923
Hypo-osmolality and or hyponatremia (x1.02) 0.924
Atherosclerosis of coronary artery without angina pectoris (x1.02) 0.924
Somatoform disorder (x1.02) 0.925
Anxiety disorder (x1.02) 0.926
Sepsis (x1.02) 0.926
Disorder of muscle (x1.02) 0.927
Disorder of intestine (x1.02) 0.928
Abnormal breathing (x1.02) 0.928
Abdominal pain (x1.02) 0.929
Acidosis (x1.02) 0.929
Disturbance of salivary secretion (x1.02) 0.930
Insomnia (x1.02) 0.931
Tetraplegic cerebral palsy (x1.02) 0.931
Precordial pain (x1.02) 0.932
Bacteremia (x1.02) 0.932
Anoxic encephalopathy (x1.02) 0.933
Dependence on respirator (x1.02) 0.933
Chronic kidney disease stage 3 (x1.02) 0.934
Disorder of lung (x1.02) 0.934
Neurogenic bladder (x1.02) 0.935
Hypovolemia (x1.02) 0.935
Difficulty walking (x1.02) 0.936
Depressive disorder (x1.02) 0.936
Generalized hyperhidrosis (x1.02) 0.937
Bacterial infectious disease (x1.02) 0.937
Shoulder joint pain (x1.02) 0.938
Tracheostomy present (x1.02) 0.938
Blood chemistry abnormal (x1.02) 0.938
baclofen (+0.00154) 0.939
Falls (x1.02) 0.939
Tetraplegia (x1.02) 0.939
−0.6 −0.4 −0.2 0.0 0.2 0.4 0.6
Expressions

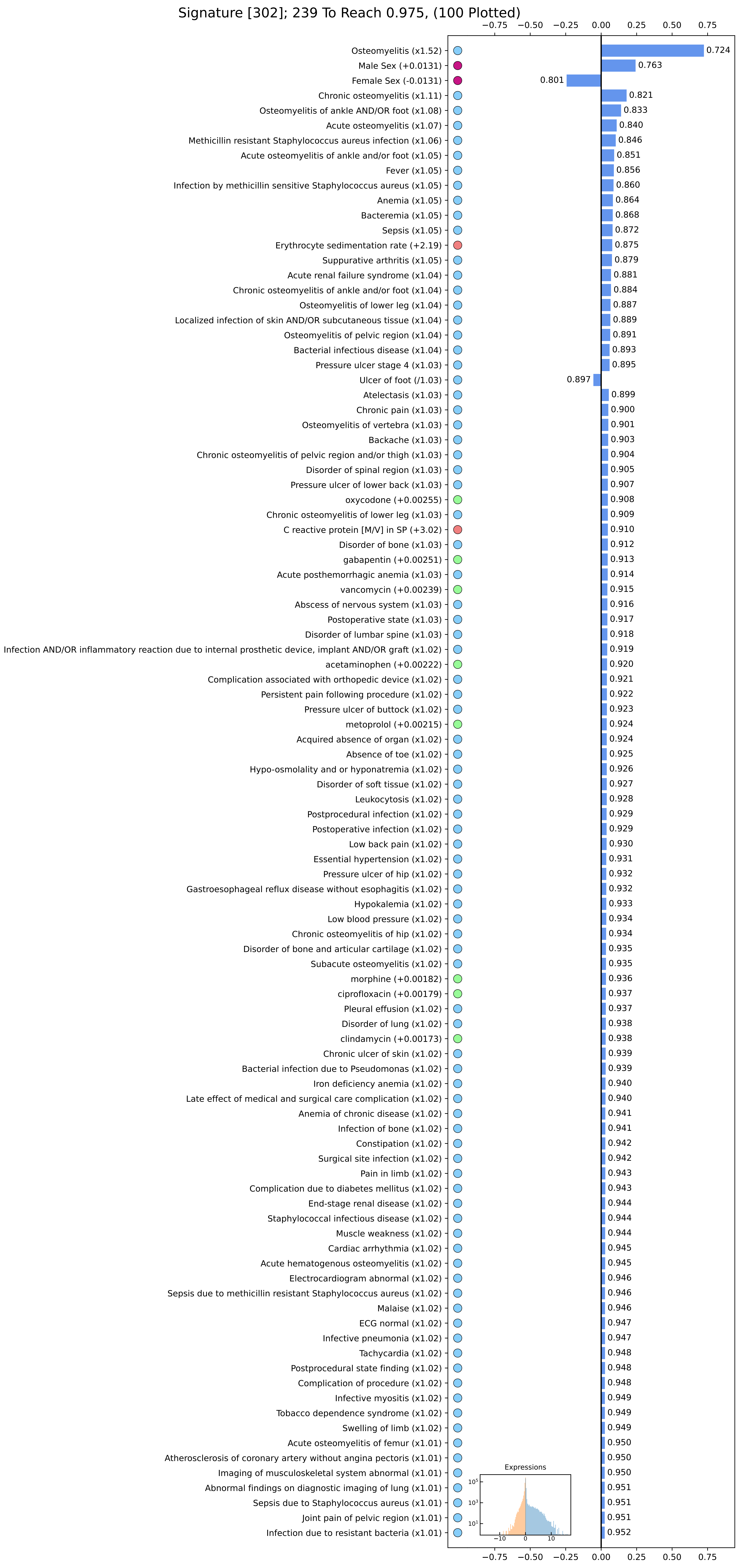

Signature [302]; 239 To Reach 0.975, (100 Plotted)
−0.75 −0.50 −0.25 0.00 0.25 0.50 0.75
Osteomyelitis (x1.52) 0.724
Male Sex (+0.0131) 0.763
Female Sex (-0.0131) 0.801
Chronic osteomyelitis (x1.11) 0.821
Osteomyelitis of ankle AND/OR foot (x1.08) 0.833
Acute osteomyelitis (x1.07) 0.840
Methicillin resistant Staphylococcus aureus infection (x1.06) 0.846
Acute osteomyelitis of ankle and/or foot (x1.05) 0.851
Fever (x1.05) 0.856
Infection by methicillin sensitive Staphylococcus aureus (x1.05) 0.860
Anemia (x1.05) 0.864
Bacteremia (x1.05) 0.868
Sepsis (x1.05) 0.872
Erythrocyte sedimentation rate (+2.19) 0.875
Suppurative arthritis (x1.05) 0.879
Acute renal failure syndrome (x1.04) 0.881
Chronic osteomyelitis of ankle and/or foot (x1.04) 0.884
Osteomyelitis of lower leg (x1.04) 0.887
Localized infection of skin AND/OR subcutaneous tissue (x1.04) 0.889
Osteomyelitis of pelvic region (x1.04) 0.891
Bacterial infectious disease (x1.04) 0.893
Pressure ulcer stage 4 (x1.03) 0.895
Ulcer of foot (/1.03) 0.897
Atelectasis (x1.03) 0.899
Chronic pain (x1.03) 0.900
Osteomyelitis of vertebra (x1.03) 0.901
Backache (x1.03) 0.903
Chronic osteomyelitis of pelvic region and/or thigh (x1.03) 0.904
Disorder of spinal region (x1.03) 0.905
Pressure ulcer of lower back (x1.03) 0.907
oxycodone (+0.00255) 0.908
Chronic osteomyelitis of lower leg (x1.03) 0.909
C reactive protein [M/V] in SP (+3.02) 0.910
Disorder of bone (x1.03) 0.912
gabapentin (+0.00251) 0.913
Acute posthemorrhagic anemia (x1.03) 0.914
vancomycin (+0.00239) 0.915
Abscess of nervous system (x1.03) 0.916
Postoperative state (x1.03) 0.917
Disorder of lumbar spine (x1.03) 0.918
Infection AND/OR inflammatory reaction due to internal prosthetic device, implant AND/OR graft (x1.02) 0.919
acetaminophen (+0.00222) 0.920
Complication associated with orthopedic device (x1.02) 0.921
Persistent pain following procedure (x1.02) 0.922
Pressure ulcer of buttock (x1.02) 0.923
metoprolol (+0.00215) 0.924
Acquired absence of organ (x1.02) 0.924
Absence of toe (x1.02) 0.925
Hypo-osmolality and or hyponatremia (x1.02) 0.926
Disorder of soft tissue (x1.02) 0.927
Leukocytosis (x1.02) 0.928
Postprocedural infection (x1.02) 0.929
Postoperative infection (x1.02) 0.929
Low back pain (x1.02) 0.930
Essential hypertension (x1.02) 0.931
Pressure ulcer of hip (x1.02) 0.932
Gastroesophageal reflux disease without esophagitis (x1.02) 0.932
Hypokalemia (x1.02) 0.933
Low blood pressure (x1.02) 0.934
Chronic osteomyelitis of hip (x1.02) 0.934
Disorder of bone and articular cartilage (x1.02) 0.935
Subacute osteomyelitis (x1.02) 0.935
morphine (+0.00182) 0.936
ciprofloxacin (+0.00179) 0.937
Pleural effusion (x1.02) 0.937
Disorder of lung (x1.02) 0.938
clindamycin (+0.00173) 0.938
Chronic ulcer of skin (x1.02) 0.939
Bacterial infection due to Pseudomonas (x1.02) 0.939
Iron deficiency anemia (x1.02) 0.940
Late effect of medical and surgical care complication (x1.02) 0.940
Anemia of chronic disease (x1.02) 0.941
Infection of bone (x1.02) 0.941
Constipation (x1.02) 0.942
Surgical site infection (x1.02) 0.942
Pain in limb (x1.02) 0.943
Complication due to diabetes mellitus (x1.02) 0.943
End-stage renal disease (x1.02) 0.944
Staphylococcal infectious disease (x1.02) 0.944
Muscle weakness (x1.02) 0.944
Cardiac arrhythmia (x1.02) 0.945
Acute hematogenous osteomyelitis (x1.02) 0.945
Electrocardiogram abnormal (x1.02) 0.946
Sepsis due to methicillin resistant Staphylococcus aureus (x1.02) 0.946
Malaise (x1.02) 0.946
ECG normal (x1.02) 0.947
Infective pneumonia (x1.02) 0.947
Tachycardia (x1.02) 0.948
Postprocedural state finding (x1.02) 0.948
Complication of procedure (x1.02) 0.948
Infective myositis (x1.02) 0.949
Tobacco dependence syndrome (x1.02) 0.949
Swelling of limb (x1.02) 0.949
Acute osteomyelitis of femur (x1.01) 0.950
Atherosclerosis of coronary artery without angina pectoris (x1.01) 0.950
Imaging of musculoskeletal system abnormal (x1.01) 0.950
Abnormal findings on diagnostic imaging of lung (x1.01) 0.951
Sepsis due to Staphylococcus aureus (x1.01) 0.951
Joint pain of pelvic region (x1.01) 0.951
Infection due to resistant bacteria (x1.01) 0.952
Expressions
10^5
10^3
10^1
−10 0 10
−0.75 −0.50 −0.25 0.00 0.25 0.50 0.75

Signature [303]; 196 To Reach 0.975, (100 Plotted)

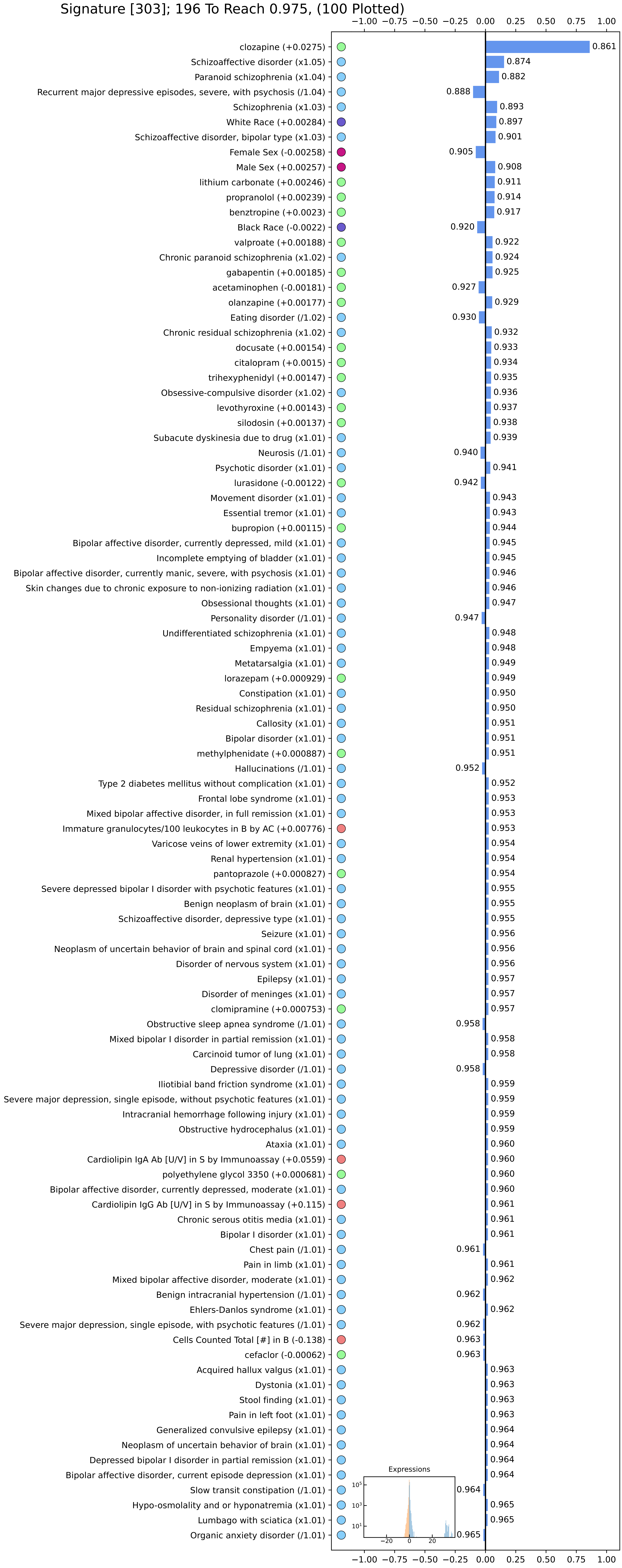

Signature [304]; 7 To Reach 0.975, (10 Plotted)

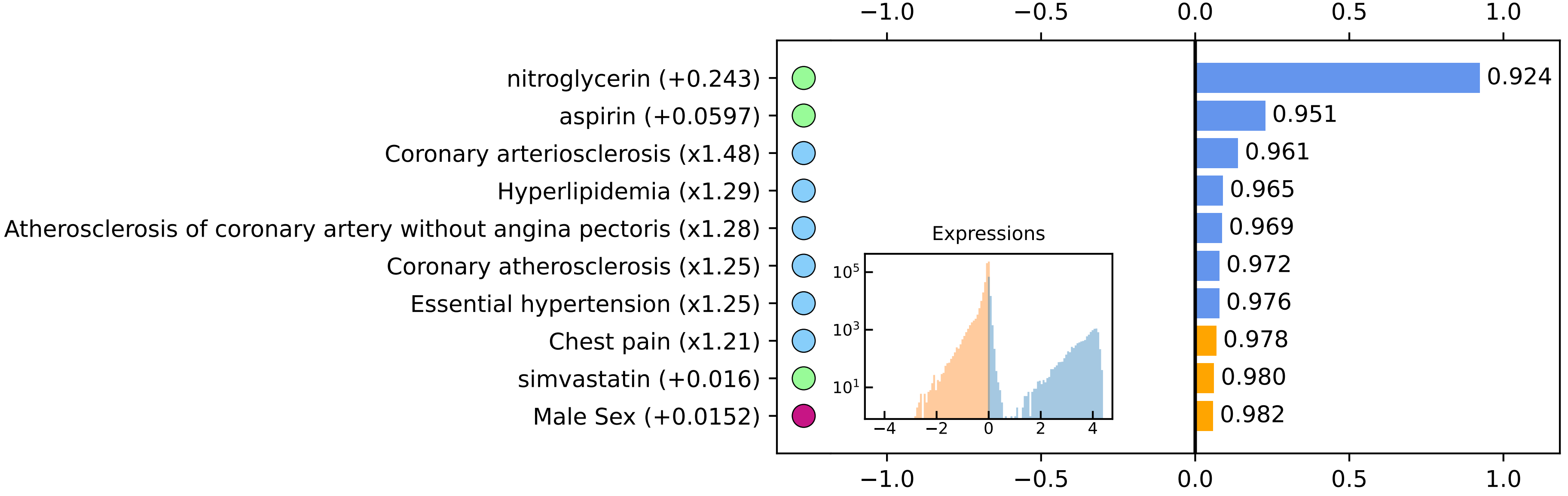

Signature [305]; 204 To Reach 0.975, (100 Plotted)

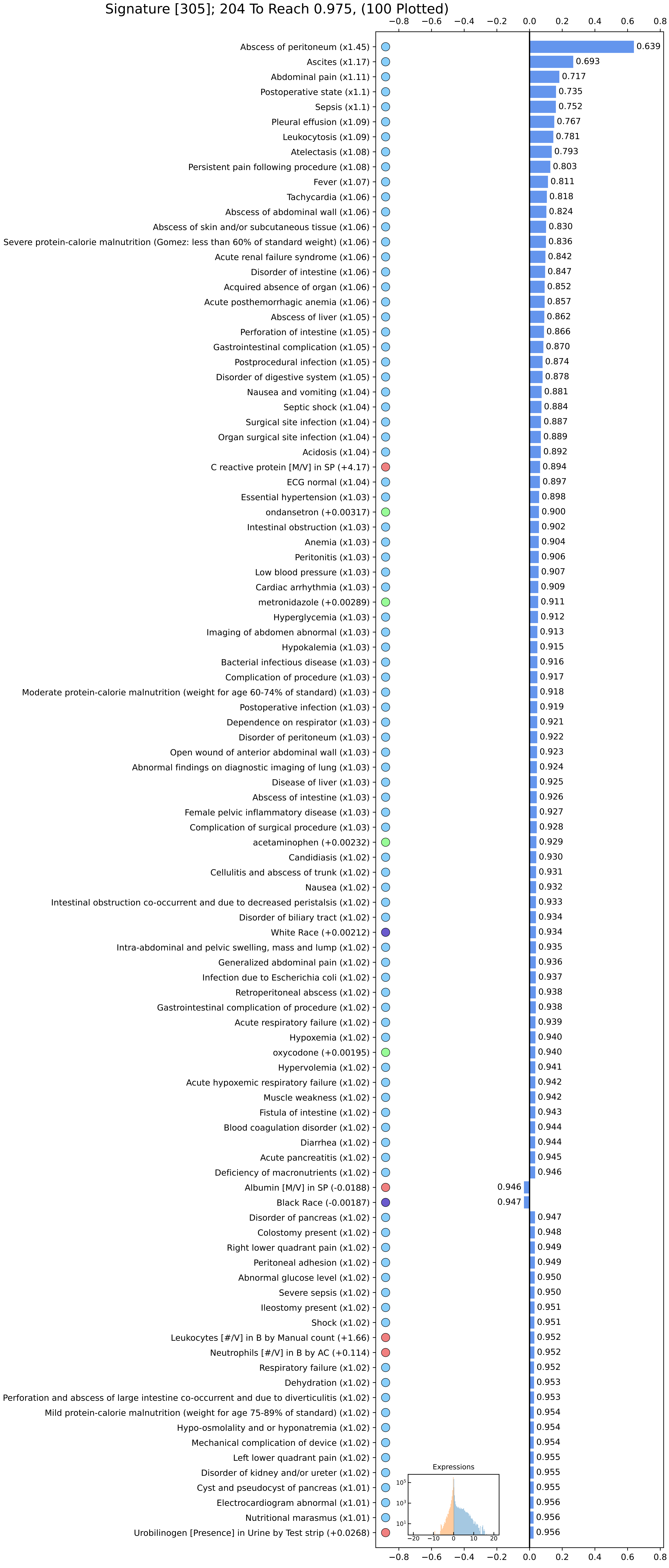

Signature [306]; 1 To Reach 0.975, (10 Plotted)

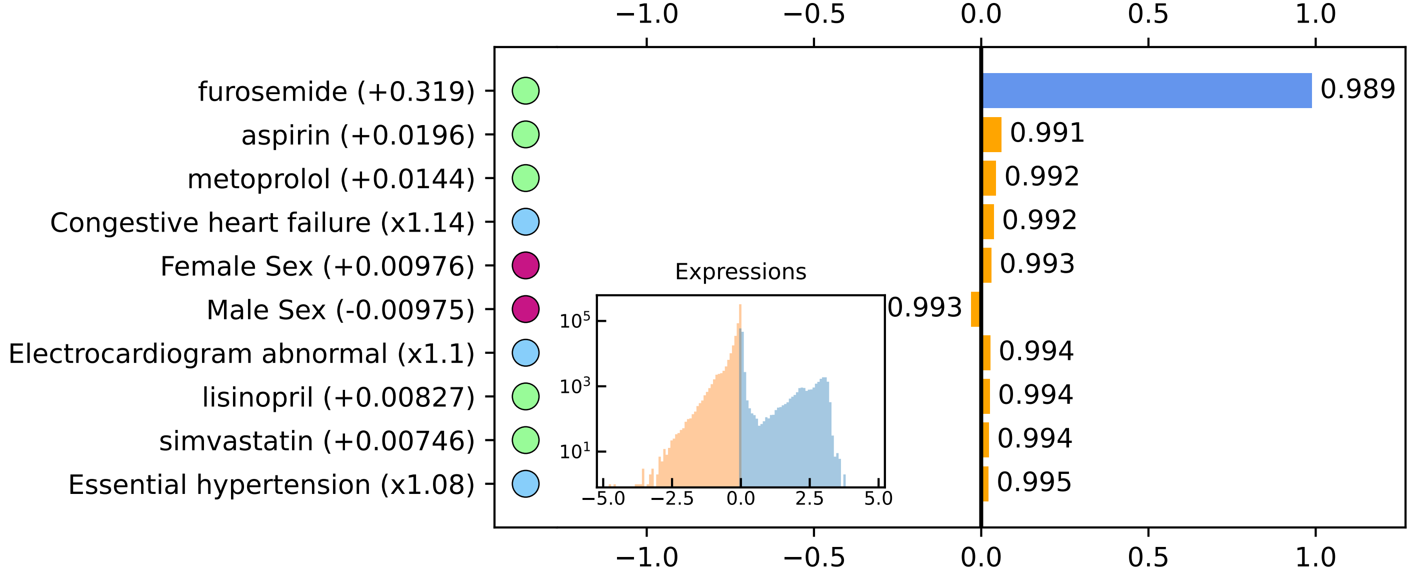

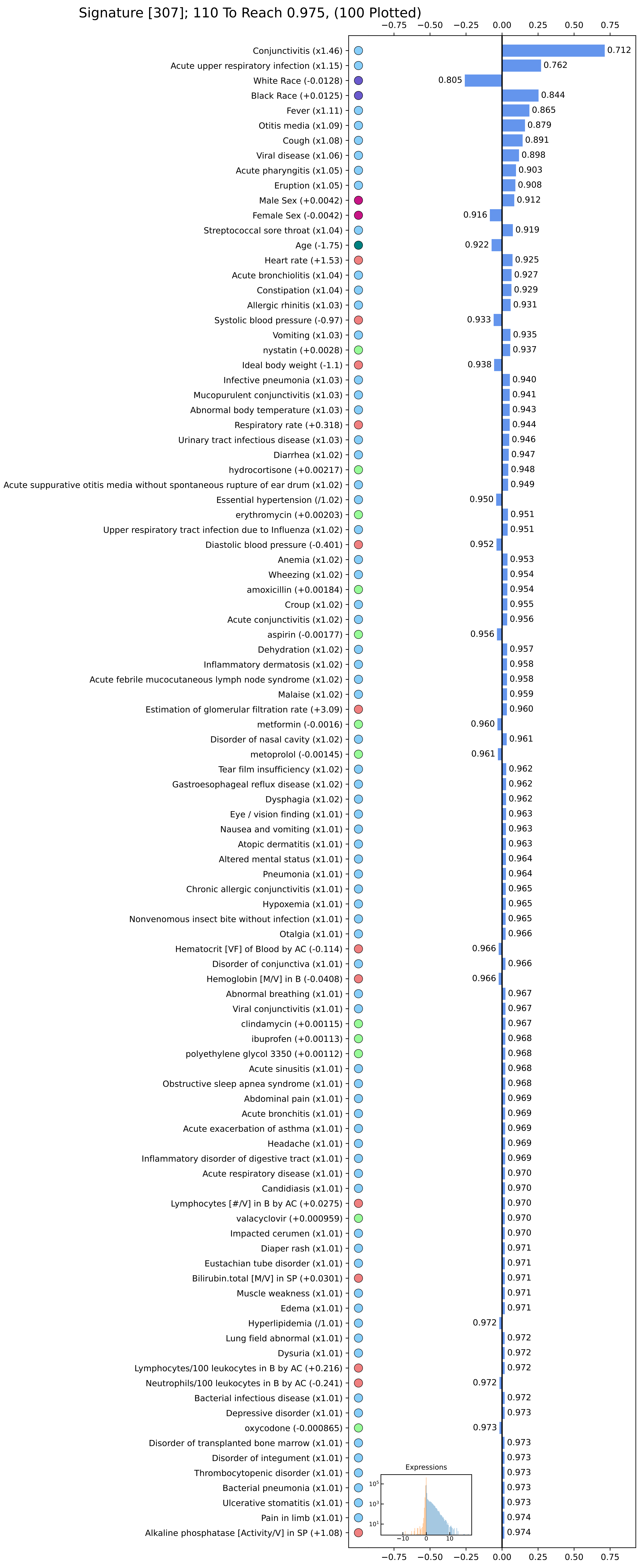

Signature [307]; 110 To Reach 0.975, (100 Plotted)
Conjunctivitis (x1.46) 0.712
Acute upper respiratory infection (x1.15) 0.762
White Race (-0.0128) 0.805
Black Race (+0.0125) 0.844
Fever (x1.11) 0.865
Otitis media (x1.09) 0.879
Cough (x1.08) 0.891
Viral disease (x1.06) 0.898
Acute pharyngitis (x1.05) 0.903
Eruption (x1.05) 0.908
Male Sex (+0.0042) 0.912
Female Sex (-0.0042) 0.916
Streptococcal sore throat (x1.04) 0.919
Age (-1.75) 0.922
Heart rate (+1.53) 0.925
Acute bronchiolitis (x1.04) 0.927
Constipation (x1.04) 0.929
Allergic rhinitis (x1.03) 0.931
Systolic blood pressure (-0.97) 0.933
Vomiting (x1.03) 0.935
nystatin (+0.0028) 0.937
Ideal body weight (-1.1) 0.938
Infective pneumonia (x1.03) 0.940
Mucopurulent conjunctivitis (x1.03) 0.941
Abnormal body temperature (x1.03) 0.943
Respiratory rate (+0.318) 0.944
Urinary tract infectious disease (x1.03) 0.946
Diarrhea (x1.02) 0.947
hydrocortisone (+0.00217) 0.948
Acute suppurative otitis media without spontaneous rupture of ear drum (x1.02) 0.949
Essential hypertension (/1.02) 0.950
erythromycin (+0.00203) 0.951
Upper respiratory tract infection due to Influenza (x1.02) 0.951
Diastolic blood pressure (-0.401) 0.952
Anemia (x1.02) 0.953
Wheezing (x1.02) 0.954
amoxicillin (+0.00184) 0.954
Croup (x1.02) 0.955
Acute conjunctivitis (x1.02) 0.956
aspirin (-0.00177) 0.956
Dehydration (x1.02) 0.957
Inflammatory dermatosis (x1.02) 0.958
Acute febrile mucocutaneous lymph node syndrome (x1.02) 0.958
Malaise (x1.02) 0.959
Estimation of glomerular filtration rate (+3.09) 0.960
metformin (-0.0016) 0.960
Disorder of nasal cavity (x1.02) 0.961
metoprolol (-0.00145) 0.961
Tear film insufficiency (x1.02) 0.962
Gastroesophageal reflux disease (x1.02) 0.962
Dysphagia (x1.02) 0.962
Eye / vision finding (x1.01) 0.963
Nausea and vomiting (x1.01) 0.963
Atopic dermatitis (x1.01) 0.963
Altered mental status (x1.01) 0.964
Pneumonia (x1.01) 0.964
Chronic allergic conjunctivitis (x1.01) 0.965
Hypoxemia (x1.01) 0.965
Nonvenomous insect bite without infection (x1.01) 0.965
Otalgia (x1.01) 0.966
Hematocrit [VF] of Blood by AC (-0.114) 0.966
Disorder of conjunctiva (x1.01) 0.966
Hemoglobin [M/V] in B (-0.0408) 0.966
Abnormal breathing (x1.01) 0.967
Viral conjunctivitis (x1.01) 0.967
clindamycin (+0.00115) 0.967
ibuprofen (+0.00113) 0.968
polyethylene glycol 3350 (+0.00112) 0.968
Acute sinusitis (x1.01) 0.968
Obstructive sleep apnea syndrome (x1.01) 0.968
Abdominal pain (x1.01) 0.969
Acute bronchitis (x1.01) 0.969
Acute exacerbation of asthma (x1.01) 0.969
Headache (x1.01) 0.969
Inflammatory disorder of digestive tract (x1.01) 0.969
Acute respiratory disease (x1.01) 0.970
Candidiasis (x1.01) 0.970
Lymphocytes [#/V] in B by AC (+0.0275) 0.970
valacyclovir (+0.000959) 0.970
Impacted cerumen (x1.01) 0.970
Diaper rash (x1.01) 0.971
Eustachian tube disorder (x1.01) 0.971
Bilirubin.total [M/V] in SP (+0.0301) 0.971
Muscle weakness (x1.01) 0.971
Edema (x1.01) 0.971
Hyperlipidemia (/1.01) 0.972
Lung field abnormal (x1.01) 0.972
Dysuria (x1.01) 0.972
Lymphocytes/100 leukocytes in B by AC (+0.216) 0.972
Neutrophils/100 leukocytes in B by AC (-0.241) 0.972
Bacterial infectious disease (x1.01) 0.972
Depressive disorder (x1.01) 0.973
oxycodone (-0.000865) 0.973
Disorder of transplanted bone marrow (x1.01) 0.973
Disorder of integument (x1.01) 0.973
Thrombocytopenic disorder (x1.01) 0.973
Bacterial pneumonia (x1.01) 0.973
Ulcerative stomatitis (x1.01) 0.973
Pain in limb (x1.01) 0.974
Alkaline phosphatase [Activity/V] in SP (+1.08) 0.974
−0.75 −0.50 −0.25 0.00 0.25 0.50 0.75
Expressions

Signature [308]; 1 To Reach 0.975, (10 Plotted)

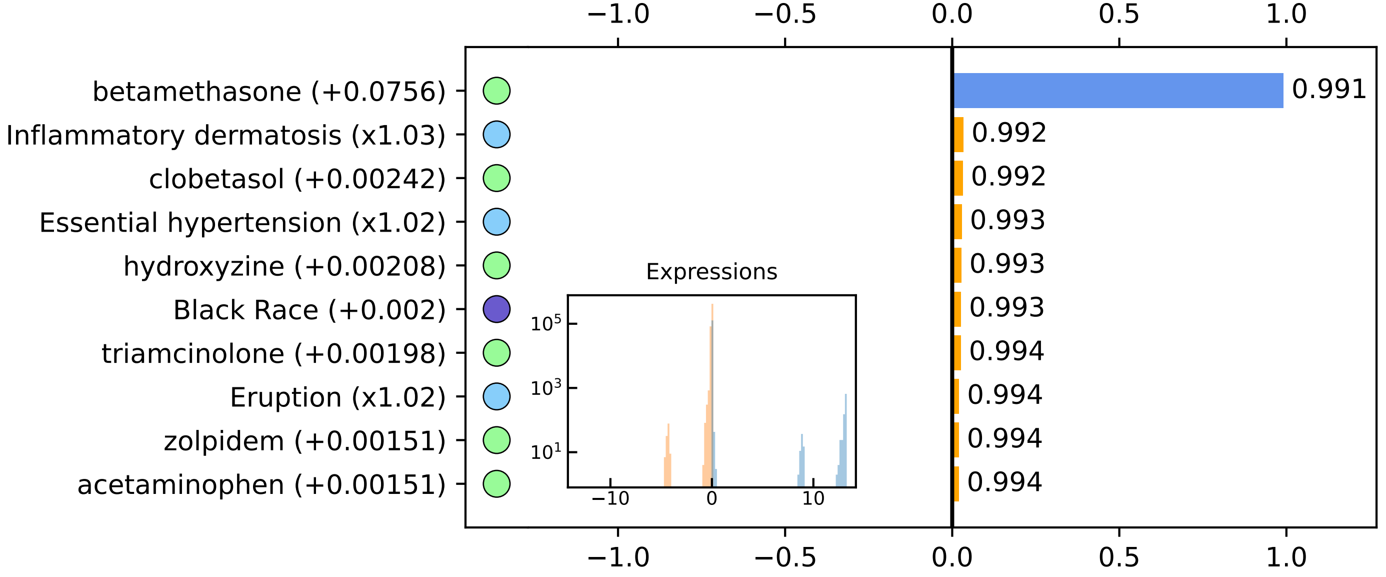

Signature [309]; 66 To Reach 0.975, (66 Plotted)

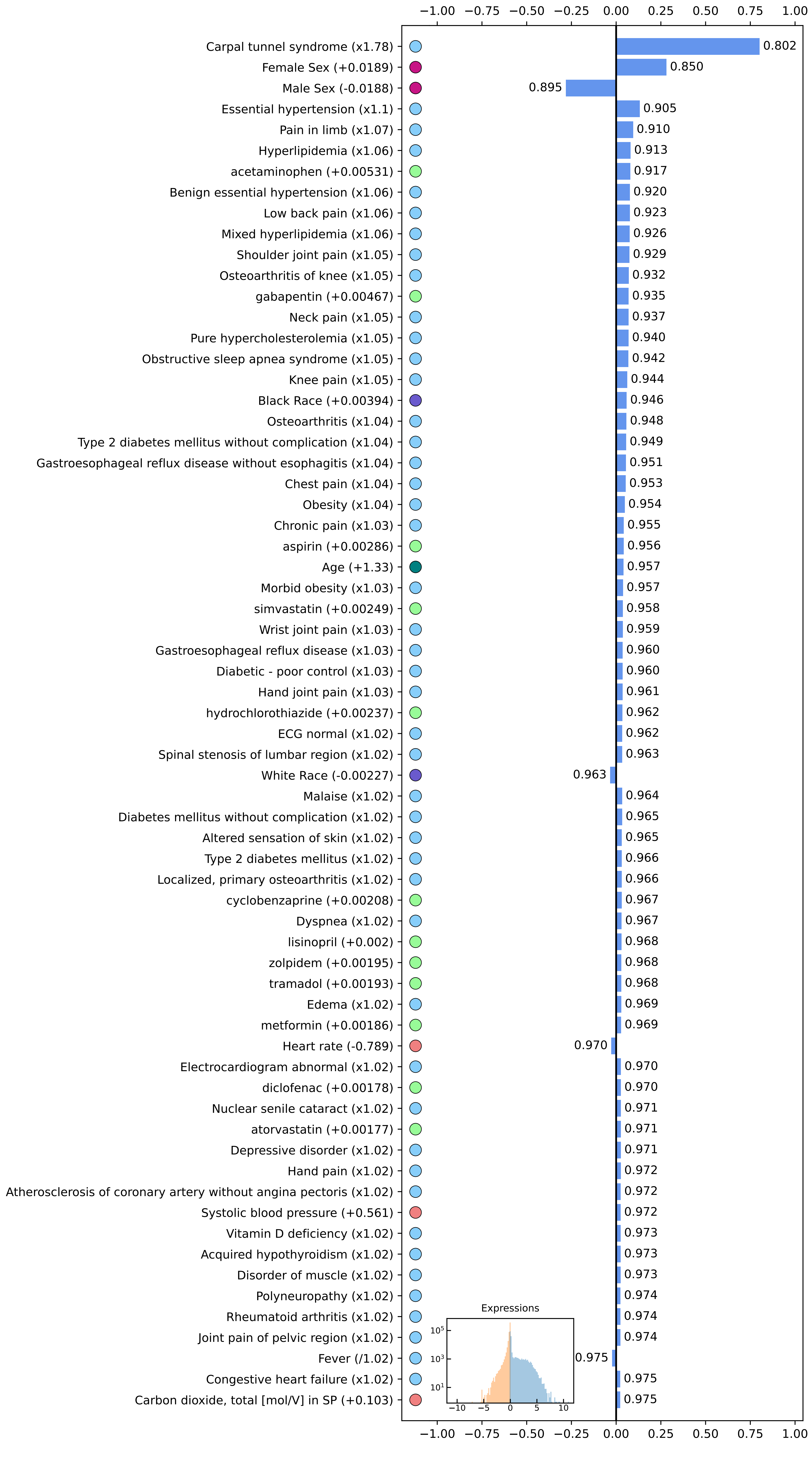

Signature [310]; 25 To Reach 0.975, (25 Plotted)

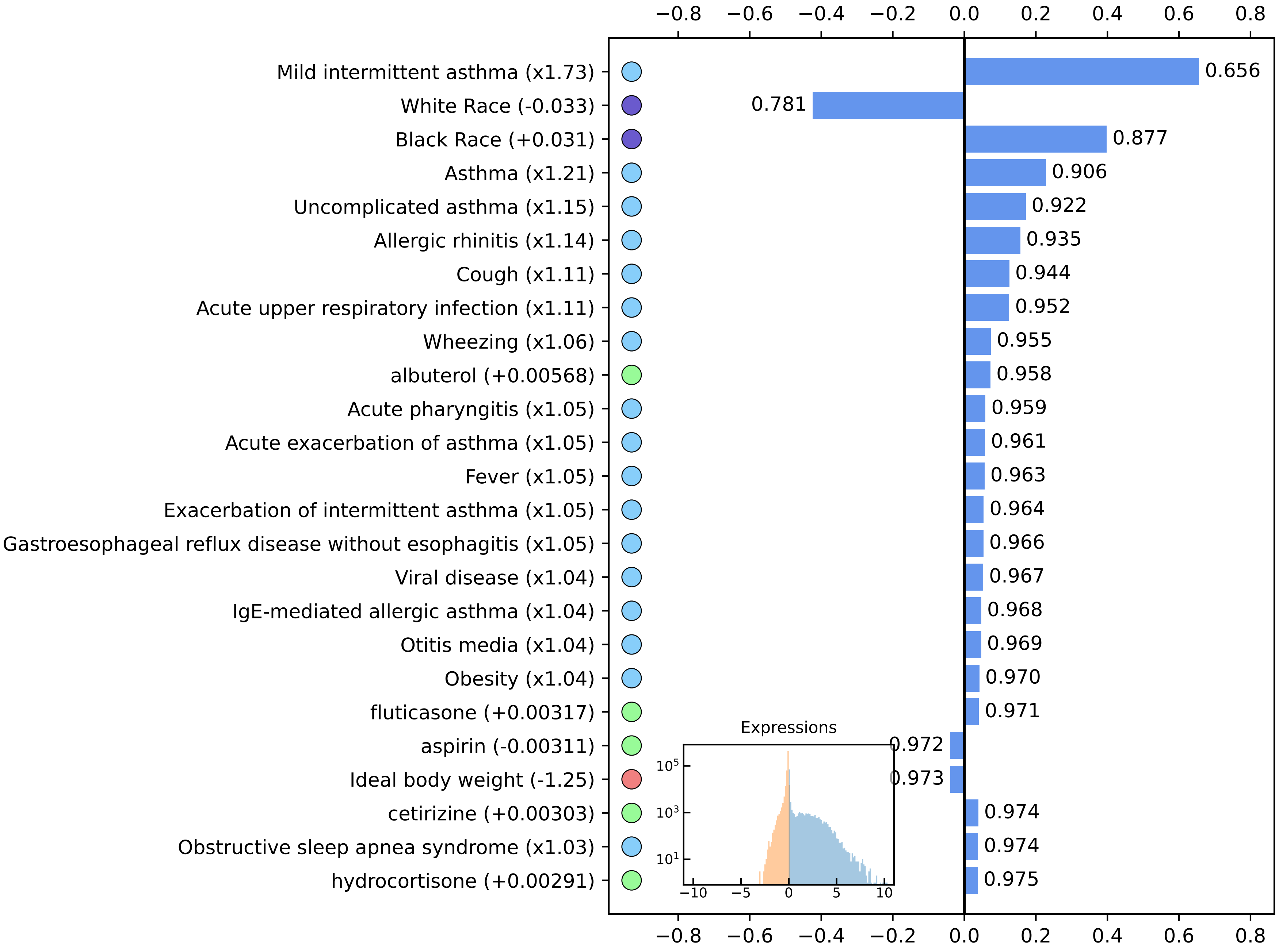

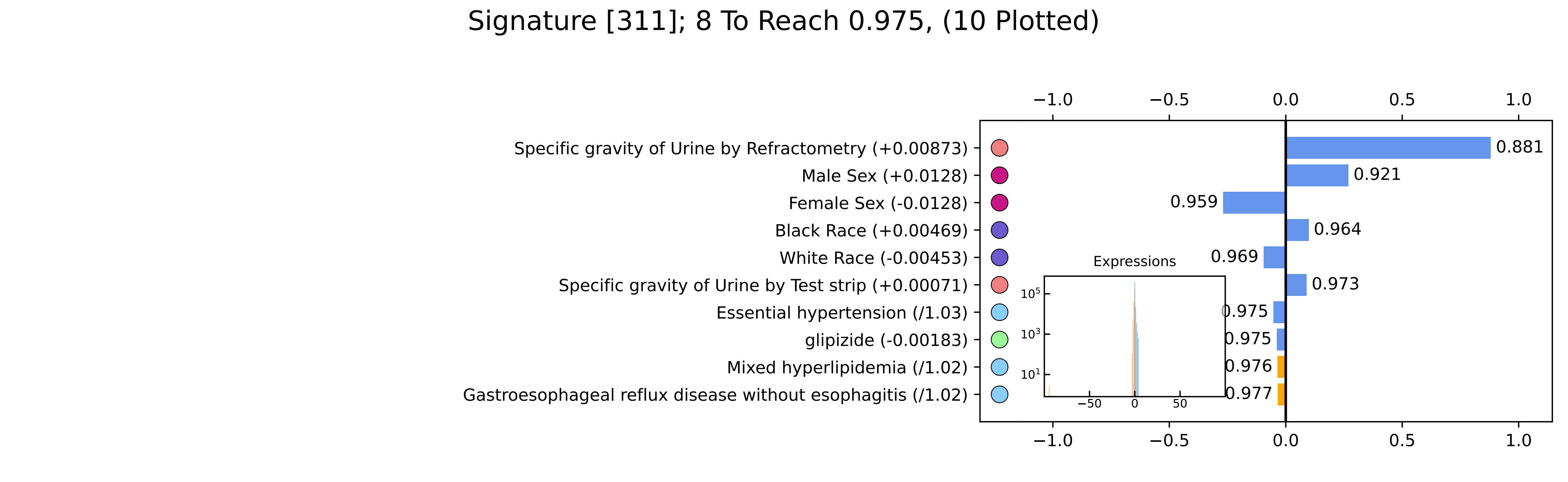

Signature [311]; 8 To Reach 0.975, (10 Plotted)
Specific gravity of Urine by Refractometry (+0.00873)
Male Sex (+0.0128)
Female Sex (-0.0128)
Black Race (+0.00469)
White Race (-0.00453)
Specific gravity of Urine by Test strip (+0.00071)
Essential hypertension (/1.03)
glipizide (-0.00183)
Mixed hyperlipidemia (/1.02)
Gastroesophageal reflux disease without esophagitis (/1.02)
0.881
0.921
0.959
0.964
0.969
0.973
0.975
0.975
0.976
0.977
Expressions
−1.0
−0.5
0.0
0.5
1.0
−50
0
50

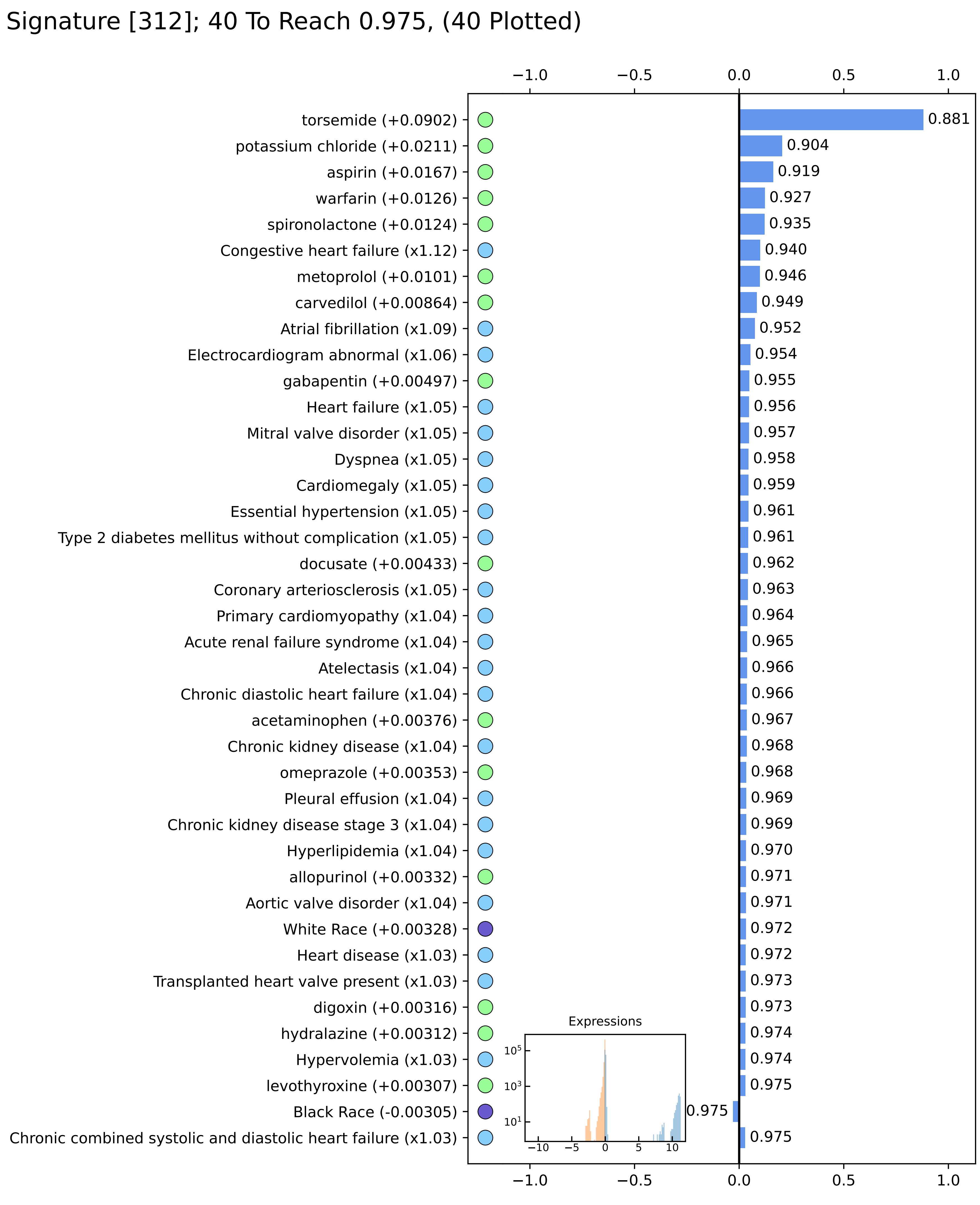

Signature [312]; 40 To Reach 0.975, (40 Plotted)
−1.0
−0.5
0.0
0.5
1.0
torsemide (+0.0902)
potassium chloride (+0.0211)
aspirin (+0.0167)
warfarin (+0.0126)
spironolactone (+0.0124)
Congestive heart failure (x1.12)
metoprolol (+0.0101)
carvedilol (+0.00864)
Atrial fibrillation (x1.09)
Electrocardiogram abnormal (x1.06)
gabapentin (+0.00497)
Heart failure (x1.05)
Mitral valve disorder (x1.05)
Dyspnea (x1.05)
Cardiomegaly (x1.05)
Essential hypertension (x1.05)
Type 2 diabetes mellitus without complication (x1.05)
docusate (+0.00433)
Coronary arteriosclerosis (x1.05)
Primary cardiomyopathy (x1.04)
Acute renal failure syndrome (x1.04)
Atelectasis (x1.04)
Chronic diastolic heart failure (x1.04)
acetaminophen (+0.00376)
Chronic kidney disease (x1.04)
omeprazole (+0.00353)
Pleural effusion (x1.04)
Chronic kidney disease stage 3 (x1.04)
Hyperlipidemia (x1.04)
allopurinol (+0.00332)
Aortic valve disorder (x1.04)
White Race (+0.00328)
Heart disease (x1.03)
Transplanted heart valve present (x1.03)
digoxin (+0.00316)
hydralazine (+0.00312)
Hypervolemia (x1.03)
levothyroxine (+0.00307)
Black Race (-0.00305)
Chronic combined systolic and diastolic heart failure (x1.03)
0.881
0.904
0.919
0.927
0.935
0.940
0.946
0.949
0.952
0.954
0.955
0.956
0.957
0.958
0.959
0.961
0.961
0.962
0.963
0.964
0.965
0.966
0.966
0.967
0.968
0.968
0.969
0.969
0.970
0.971
0.971
0.972
0.972
0.973
0.973
0.974
0.974
0.975
0.975
0.975
Expressions
10^5
10^3
10^1
−10
−5
0
5
10

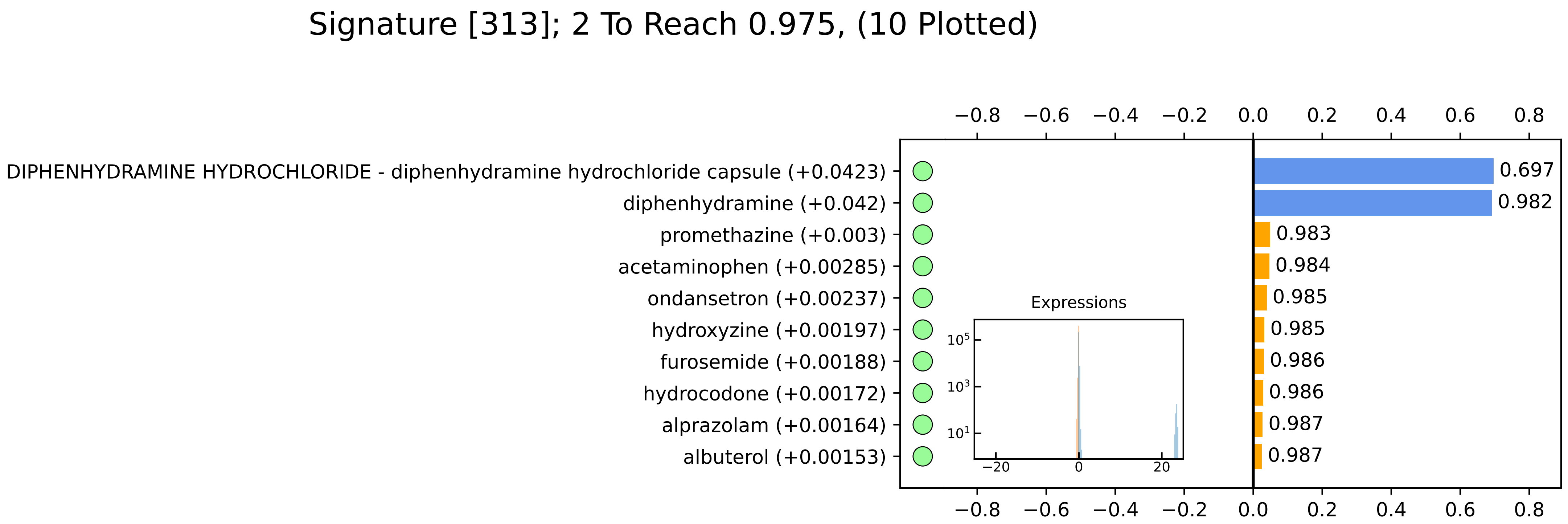
Signature [313]; 2 To Reach 0.975, (10 Plotted)
DIPHENHYDRAMINE HYDROCHLORIDE - diphenhydramine hydrochloride capsule (+0.0423)
diphenhydramine (+0.042)
promethazine (+0.003)
acetaminophen (+0.00285)
ondansetron (+0.00237)
hydroxyzine (+0.00197)
furosemide (+0.00188)
hydrocodone (+0.00172)
alprazolam (+0.00164)
albuterol (+0.00153)
0.697
0.982
0.983
0.984
0.985
0.985
0.986
0.986
0.987
0.987
Expressions
−0.8
−0.6
−0.4
−0.2
0.0
0.2
0.4
0.6
0.8

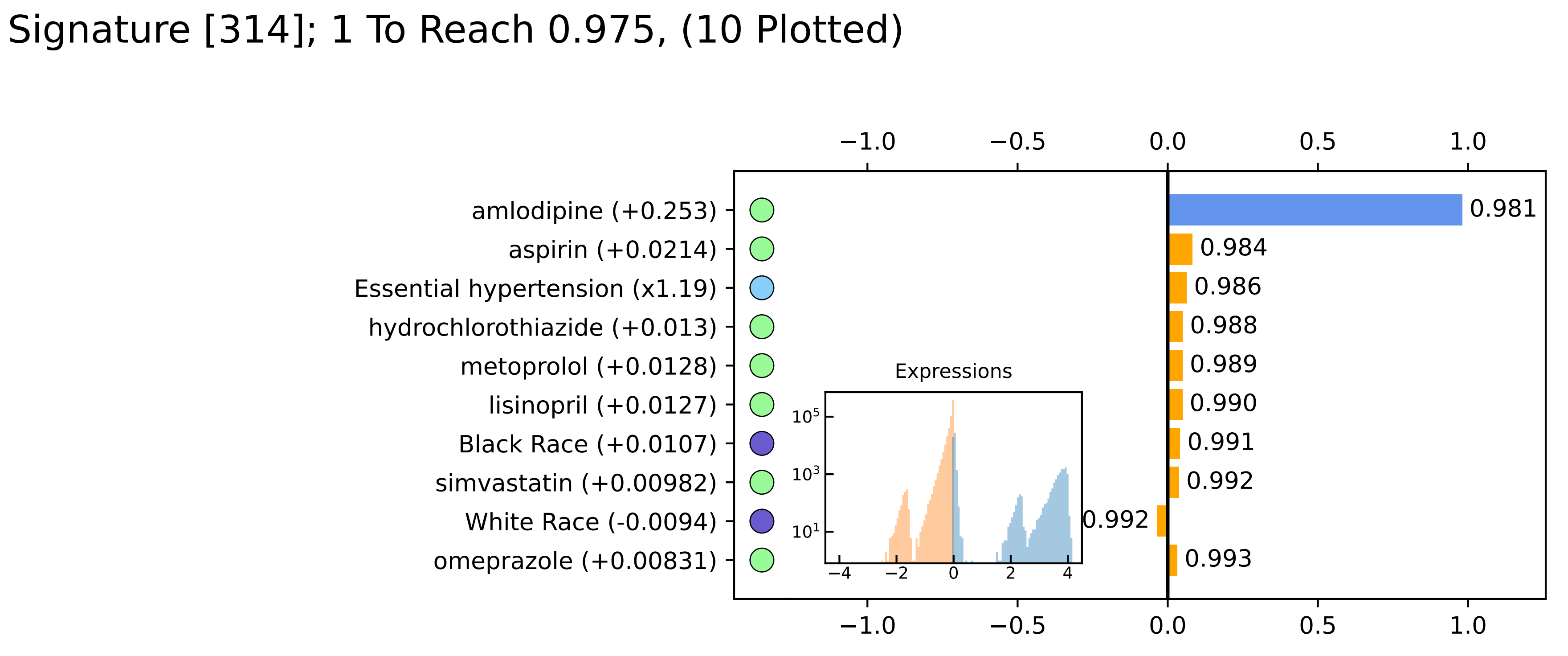

Signature [314]; 1 To Reach 0.975, (10 Plotted)
amlodipine (+0.253)
aspirin (+0.0214)
Essential hypertension (x1.19)
hydrochlorothiazide (+0.013)
metoprolol (+0.0128)
lisinopril (+0.0127)
Black Race (+0.0107)
simvastatin (+0.00982)
White Race (-0.0094)
omeprazole (+0.00831)
0.981
0.984
0.986
0.988
0.989
0.990
0.991
0.992
0.992
0.993
Expressions
−1.0
−0.5
0.0
0.5
1.0

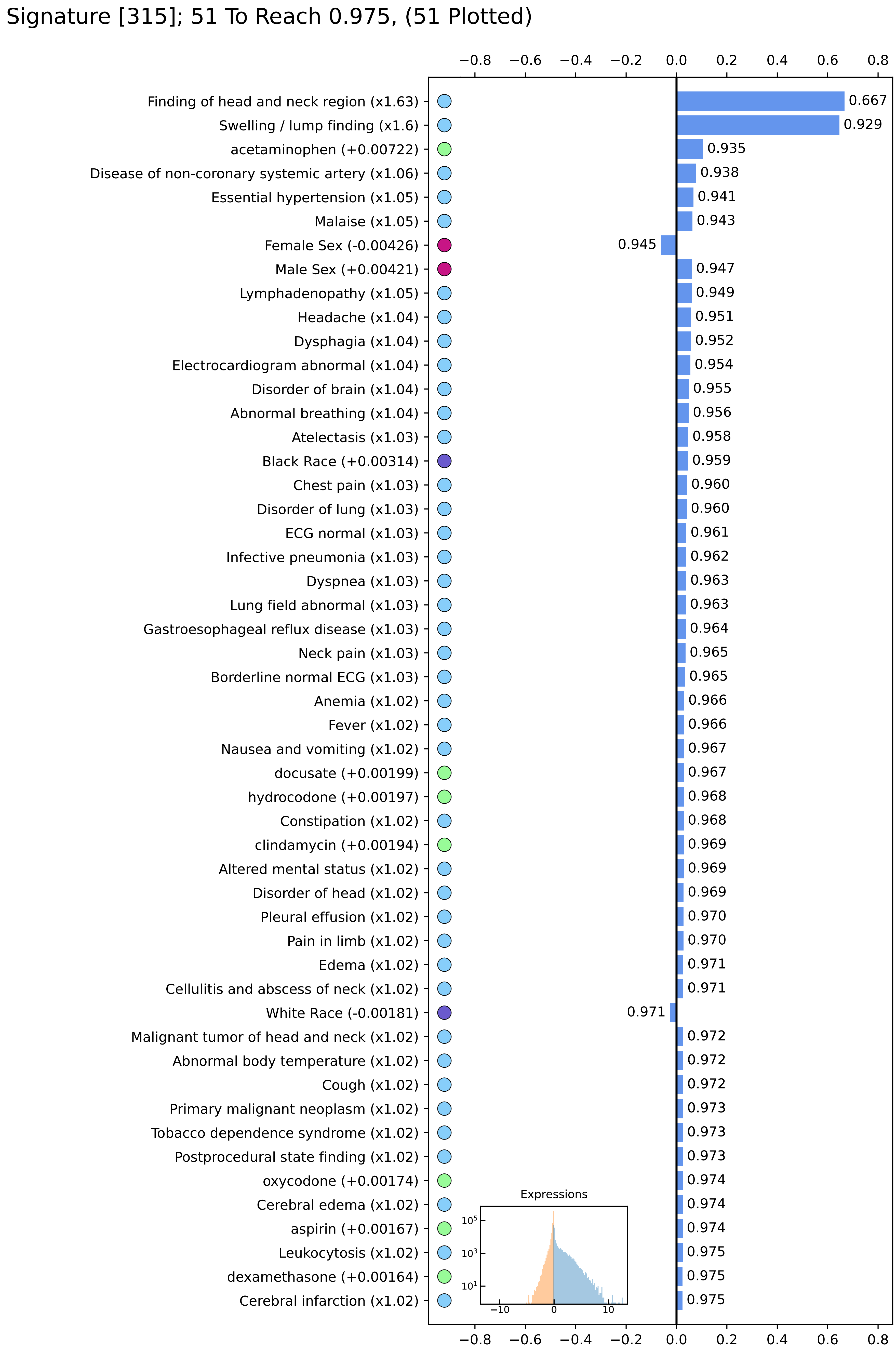

Signature [315]; 51 To Reach 0.975, (51 Plotted)
Finding of head and neck region (x1.63) 0.667
Swelling / lump finding (x1.6) 0.929
acetaminophen (+0.00722) 0.935
Disease of non-coronary systemic artery (x1.06) 0.938
Essential hypertension (x1.05) 0.941
Malaise (x1.05) 0.943
Female Sex (-0.00426) 0.945
Male Sex (+0.00421) 0.947
Lymphadenopathy (x1.05) 0.949
Headache (x1.04) 0.951
Dysphagia (x1.04) 0.952
Electrocardiogram abnormal (x1.04) 0.954
Disorder of brain (x1.04) 0.955
Abnormal breathing (x1.04) 0.956
Atelectasis (x1.03) 0.958
Black Race (+0.00314) 0.959
Chest pain (x1.03) 0.960
Disorder of lung (x1.03) 0.960
ECG normal (x1.03) 0.961
Infective pneumonia (x1.03) 0.962
Dyspnea (x1.03) 0.963
Lung field abnormal (x1.03) 0.963
Gastroesophageal reflux disease (x1.03) 0.964
Neck pain (x1.03) 0.965
Borderline normal ECG (x1.03) 0.965
Anemia (x1.02) 0.966
Fever (x1.02) 0.966
Nausea and vomiting (x1.02) 0.967
docusate (+0.00199) 0.967
hydrocodone (+0.00197) 0.968
Constipation (x1.02) 0.968
clindamycin (+0.00194) 0.969
Altered mental status (x1.02) 0.969
Disorder of head (x1.02) 0.969
Pleural effusion (x1.02) 0.970
Pain in limb (x1.02) 0.970
Edema (x1.02) 0.971
Cellulitis and abscess of neck (x1.02) 0.971
White Race (-0.00181) 0.971
Malignant tumor of head and neck (x1.02) 0.972
Abnormal body temperature (x1.02) 0.972
Cough (x1.02) 0.972
Primary malignant neoplasm (x1.02) 0.973
Tobacco dependence syndrome (x1.02) 0.973
Postprocedural state finding (x1.02) 0.973
oxycodone (+0.00174) 0.974
Cerebral edema (x1.02) 0.974
aspirin (+0.00167) 0.974
Leukocytosis (x1.02) 0.975
dexamethasone (+0.00164) 0.975
Cerebral infarction (x1.02) 0.975
−0.8 −0.6 −0.4 −0.2 0.0 0.2 0.4 0.6 0.8
Expressions
10^5
10^3
10^1
−10 0 10

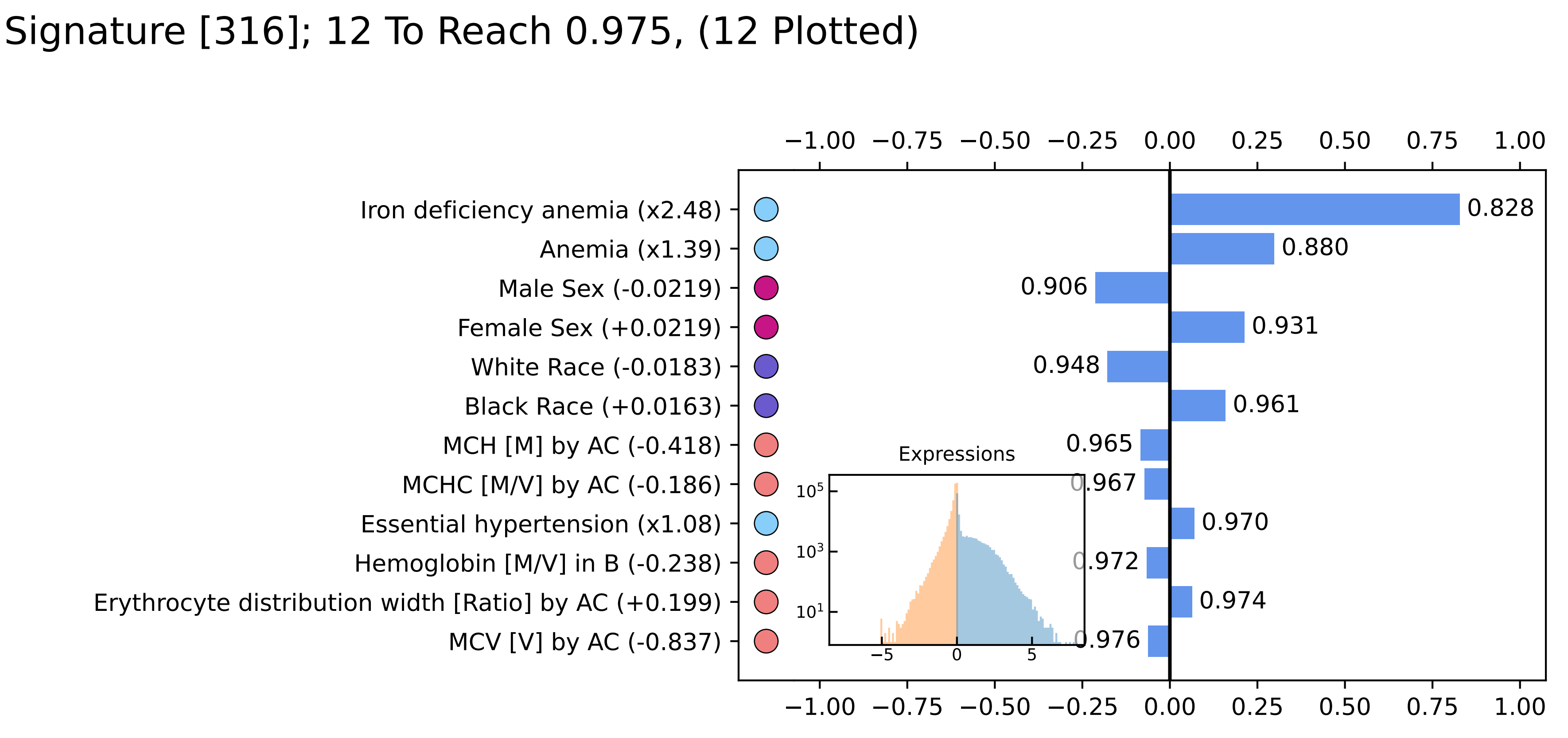
Signature [316]; 12 To Reach 0.975, (12 Plotted)
Iron deficiency anemia (x2.48)
Anemia (x1.39)
Male Sex (-0.0219)
Female Sex (+0.0219)
White Race (-0.0183)
Black Race (+0.0163)
MCH [M] by AC (-0.418)
MCHC [M/V] by AC (-0.186)
Essential hypertension (x1.08)
Hemoglobin [M/V] in B (-0.238)
Erythrocyte distribution width [Ratio] by AC (+0.199)
MCV [V] by AC (-0.837)
0.828
0.880
0.906
0.931
0.948
0.961
0.965
0.967
0.970
0.972
0.974
0.976
Expressions

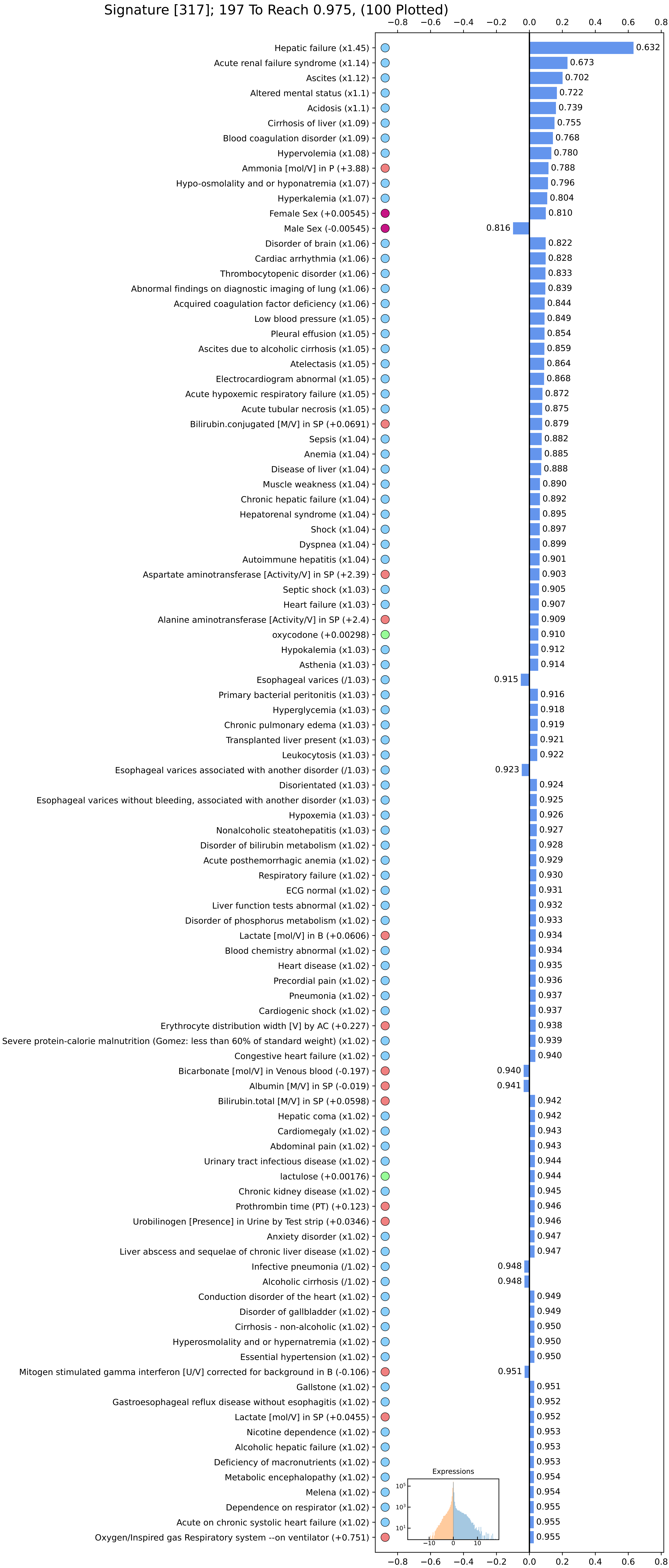

Signature [317]; 197 To Reach 0.975, (100 Plotted)
Hepatic failure (x1.45) 0.632
Acute renal failure syndrome (x1.14) 0.673
Ascites (x1.12) 0.702
Altered mental status (x1.1) 0.722
Acidosis (x1.1) 0.739
Cirrhosis of liver (x1.09) 0.755
Blood coagulation disorder (x1.09) 0.768
Hypervolemia (x1.08) 0.780
Ammonia [mol/V] in P (+3.88) 0.788
Hypo-osmolality and or hyponatremia (x1.07) 0.796
Hyperkalemia (x1.07) 0.804
Female Sex (+0.00545) 0.810
Male Sex (-0.00545) 0.816
Disorder of brain (x1.06) 0.822
Cardiac arrhythmia (x1.06) 0.828
Thrombocytopenic disorder (x1.06) 0.833
Abnormal findings on diagnostic imaging of lung (x1.06) 0.839
Acquired coagulation factor deficiency (x1.06) 0.844
Low blood pressure (x1.05) 0.849
Pleural effusion (x1.05) 0.854
Ascites due to alcoholic cirrhosis (x1.05) 0.859
Atelectasis (x1.05) 0.864
Electrocardiogram abnormal (x1.05) 0.868
Acute hypoxemic respiratory failure (x1.05) 0.872
Acute tubular necrosis (x1.05) 0.875
Bilirubin.conjugated [M/V] in SP (+0.0691) 0.879
Sepsis (x1.04) 0.882
Anemia (x1.04) 0.885
Disease of liver (x1.04) 0.888
Muscle weakness (x1.04) 0.890
Chronic hepatic failure (x1.04) 0.892
Hepatorenal syndrome (x1.04) 0.895
Shock (x1.04) 0.897
Dyspnea (x1.04) 0.899
Autoimmune hepatitis (x1.04) 0.901
Aspartate aminotransferase [Activity/V] in SP (+2.39) 0.903
Septic shock (x1.03) 0.905
Heart failure (x1.03) 0.907
Alanine aminotransferase [Activity/V] in SP (+2.4) 0.909
oxycodone (+0.00298) 0.910
Hypokalemia (x1.03) 0.912
Asthenia (x1.03) 0.914
Esophageal varices (/1.03) 0.915
Primary bacterial peritonitis (x1.03) 0.916
Hyperglycemia (x1.03) 0.918
Chronic pulmonary edema (x1.03) 0.919
Transplanted liver present (x1.03) 0.921
Leukocytosis (x1.03) 0.922
Esophageal varices associated with another disorder (/1.03) 0.923
Disorientated (x1.03) 0.924
Esophageal varices without bleeding, associated with another disorder (x1.03) 0.925
Hypoxemia (x1.03) 0.926
Nonalcoholic steatohepatitis (x1.03) 0.927
Disorder of bilirubin metabolism (x1.02) 0.928
Acute posthemorrhagic anemia (x1.02) 0.929
Respiratory failure (x1.02) 0.930
ECG normal (x1.02) 0.931
Liver function tests abnormal (x1.02) 0.932
Disorder of phosphorus metabolism (x1.02) 0.933
Lactate [mol/V] in B (+0.0606) 0.934
Blood chemistry abnormal (x1.02) 0.934
Heart disease (x1.02) 0.935
Precordial pain (x1.02) 0.936
Pneumonia (x1.02) 0.937
Cardiogenic shock (x1.02) 0.937
Erythrocyte distribution width [V] by AC (+0.227) 0.938
Severe protein-calorie malnutrition (Gomez: less than 60% of standard weight) (x1.02) 0.939
Congestive heart failure (x1.02) 0.940
Bicarbonate [mol/V] in Venous blood (-0.197) 0.940
Albumin [M/V] in SP (-0.019) 0.941
Bilirubin.total [M/V] in SP (+0.0598) 0.942
Hepatic coma (x1.02) 0.942
Cardiomegaly (x1.02) 0.943
Abdominal pain (x1.02) 0.943
Urinary tract infectious disease (x1.02) 0.944
lactulose (+0.00176) 0.944
Chronic kidney disease (x1.02) 0.945
Prothrombin time (PT) (+0.123) 0.946
Urobilinogen [Presence] in Urine by Test strip (+0.0346) 0.946
Anxiety disorder (x1.02) 0.947
Liver abscess and sequelae of chronic liver disease (x1.02) 0.947
Infective pneumonia (/1.02) 0.948
Alcoholic cirrhosis (/1.02) 0.948
Conduction disorder of the heart (x1.02) 0.949
Disorder of gallbladder (x1.02) 0.949
Cirrhosis - non-alcoholic (x1.02) 0.950
Hyperosmolality and or hypernatremia (x1.02) 0.950
Essential hypertension (x1.02) 0.950
Mitogen stimulated gamma interferon [U/V] corrected for background in B (-0.106) 0.951
Gallstone (x1.02) 0.951
Gastroesophageal reflux disease without esophagitis (x1.02) 0.952
Lactate [mol/V] in SP (+0.0455) 0.952
Nicotine dependence (x1.02) 0.953
Alcoholic hepatic failure (x1.02) 0.953
Deficiency of macronutrients (x1.02) 0.953
Metabolic encephalopathy (x1.02) 0.954
Melena (x1.02) 0.954
Dependence on respirator (x1.02) 0.955
Acute on chronic systolic heart failure (x1.02) 0.955
Oxygen/Inspired gas Respiratory system --on ventilator (+0.751) 0.955
Expressions
−0.8 −0.6 −0.4 −0.2 0.0 0.2 0.4 0.6 0.8

Signature [318]; 11 To Reach 0.975, (11 Plotted)

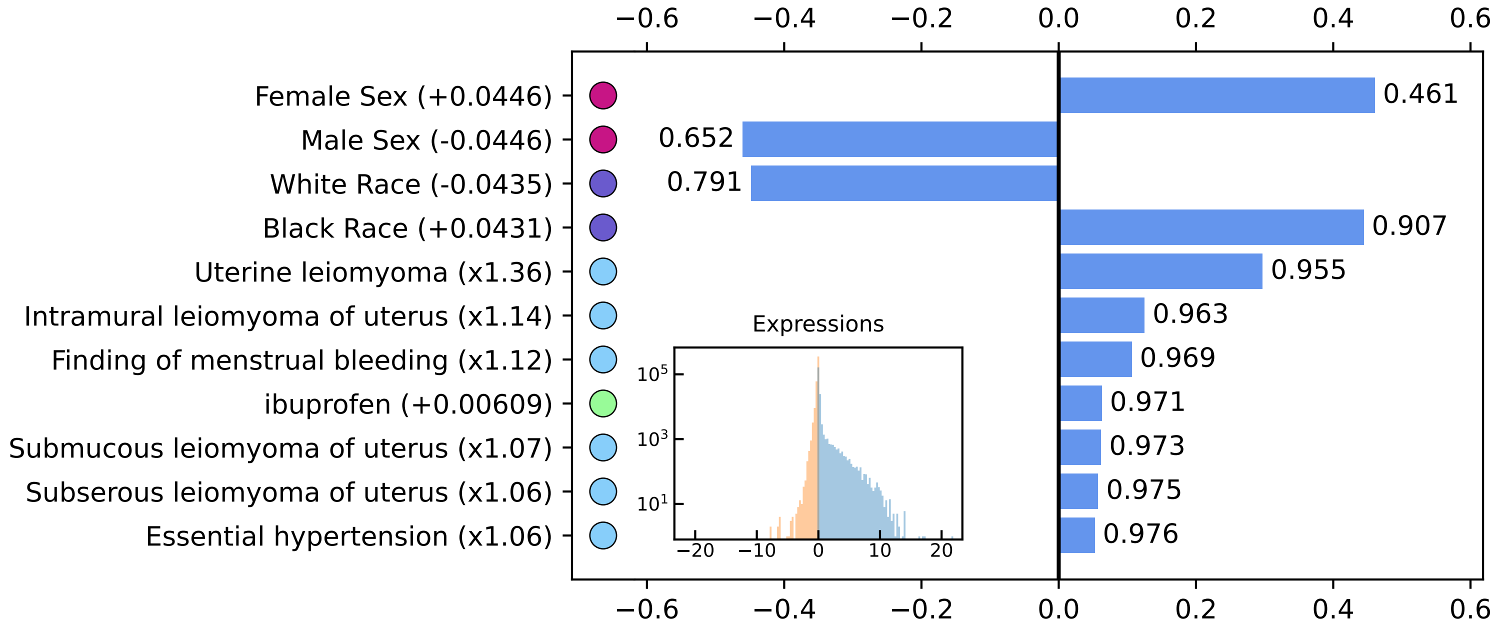

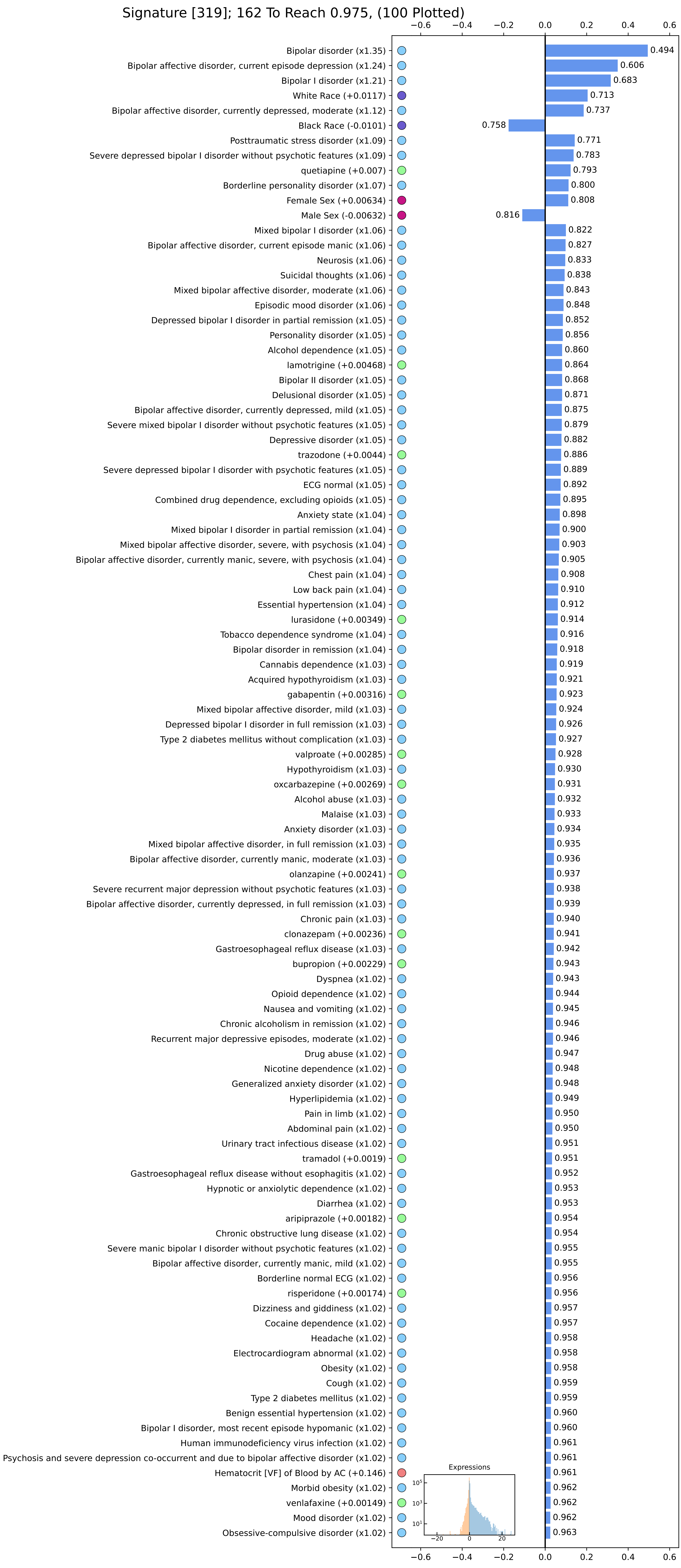

Signature [319]; 162 To Reach 0.975, (100 Plotted)
Bipolar disorder (x1.35) 0.494
Bipolar affective disorder, current episode depression (x1.24) 0.606
Bipolar I disorder (x1.21) 0.683
White Race (+0.0117) 0.713
Bipolar affective disorder, currently depressed, moderate (x1.12) 0.737
Black Race (-0.0101) 0.758
Posttraumatic stress disorder (x1.09) 0.771
Severe depressed bipolar I disorder without psychotic features (x1.09) 0.783
quetiapine (+0.007) 0.793
Borderline personality disorder (x1.07) 0.800
Female Sex (+0.00634) 0.808
Male Sex (-0.00632) 0.816
Mixed bipolar I disorder (x1.06) 0.822
Bipolar affective disorder, current episode manic (x1.06) 0.827
Neurosis (x1.06) 0.833
Suicidal thoughts (x1.06) 0.838
Mixed bipolar affective disorder, moderate (x1.06) 0.843
Episodic mood disorder (x1.06) 0.848
Depressed bipolar I disorder in partial remission (x1.05) 0.852
Personality disorder (x1.05) 0.856
Alcohol dependence (x1.05) 0.860
lamotrigine (+0.00468) 0.864
Bipolar II disorder (x1.05) 0.868
Delusional disorder (x1.05) 0.871
Bipolar affective disorder, currently depressed, mild (x1.05) 0.875
Severe mixed bipolar I disorder without psychotic features (x1.05) 0.879
Depressive disorder (x1.05) 0.882
trazodone (+0.0044) 0.886
Severe depressed bipolar I disorder with psychotic features (x1.05) 0.889
ECG normal (x1.05) 0.892
Combined drug dependence, excluding opioids (x1.05) 0.895
Anxiety state (x1.04) 0.898
Mixed bipolar I disorder in partial remission (x1.04) 0.900
Mixed bipolar affective disorder, severe, with psychosis (x1.04) 0.903
Bipolar affective disorder, currently manic, severe, with psychosis (x1.04) 0.905
Chest pain (x1.04) 0.908
Low back pain (x1.04) 0.910
Essential hypertension (x1.04) 0.912
lurasidone (+0.00349) 0.914
Tobacco dependence syndrome (x1.04) 0.916
Bipolar disorder in remission (x1.04) 0.918
Cannabis dependence (x1.03) 0.919
Acquired hypothyroidism (x1.03) 0.921
gabapentin (+0.00316) 0.923
Mixed bipolar affective disorder, mild (x1.03) 0.924
Depressed bipolar I disorder in full remission (x1.03) 0.926
Type 2 diabetes mellitus without complication (x1.03) 0.927
valproate (+0.00285) 0.928
Hypothyroidism (x1.03) 0.930
oxcarbazepine (+0.00269) 0.931
Alcohol abuse (x1.03) 0.932
Malaise (x1.03) 0.933
Anxiety disorder (x1.03) 0.934
Mixed bipolar affective disorder, in full remission (x1.03) 0.935
Bipolar affective disorder, currently manic, moderate (x1.03) 0.936
olanzapine (+0.00241) 0.937
Severe recurrent major depression without psychotic features (x1.03) 0.938
Bipolar affective disorder, currently depressed, in full remission (x1.03) 0.939
Chronic pain (x1.03) 0.940
clonazepam (+0.00236) 0.941
Gastroesophageal reflux disease (x1.03) 0.942
bupropion (+0.00229) 0.943
Dyspnea (x1.02) 0.943
Opioid dependence (x1.02) 0.944
Nausea and vomiting (x1.02) 0.945
Chronic alcoholism in remission (x1.02) 0.946
Recurrent major depressive episodes, moderate (x1.02) 0.946
Drug abuse (x1.02) 0.947
Nicotine dependence (x1.02) 0.948
Generalized anxiety disorder (x1.02) 0.948
Hyperlipidemia (x1.02) 0.949
Pain in limb (x1.02) 0.950
Abdominal pain (x1.02) 0.950
Urinary tract infectious disease (x1.02) 0.951
tramadol (+0.0019) 0.951
Gastroesophageal reflux disease without esophagitis (x1.02) 0.952
Hypnotic or anxiolytic dependence (x1.02) 0.953
Diarrhea (x1.02) 0.953
aripiprazole (+0.00182) 0.954
Chronic obstructive lung disease (x1.02) 0.954
Severe manic bipolar I disorder without psychotic features (x1.02) 0.955
Bipolar affective disorder, currently manic, mild (x1.02) 0.955
Borderline normal ECG (x1.02) 0.956
risperidone (+0.00174) 0.956
Dizziness and giddiness (x1.02) 0.957
Cocaine dependence (x1.02) 0.957
Headache (x1.02) 0.958
Electrocardiogram abnormal (x1.02) 0.958
Obesity (x1.02) 0.958
Cough (x1.02) 0.959
Type 2 diabetes mellitus (x1.02) 0.959
Benign essential hypertension (x1.02) 0.960
Bipolar I disorder, most recent episode hypomanic (x1.02) 0.960
Human immunodeficiency virus infection (x1.02) 0.961
Psychosis and severe depression co-occurrent and due to bipolar affective disorder (x1.02) 0.961
Hematocrit [VF] of Blood by AC (+0.146) 0.961
Morbid obesity (x1.02) 0.962
venlafaxine (+0.00149) 0.962
Mood disorder (x1.02) 0.962
Obsessive-compulsive disorder (x1.02) 0.963
Expressions
−0.6 −0.4 −0.2 0.0 0.2 0.4 0.6

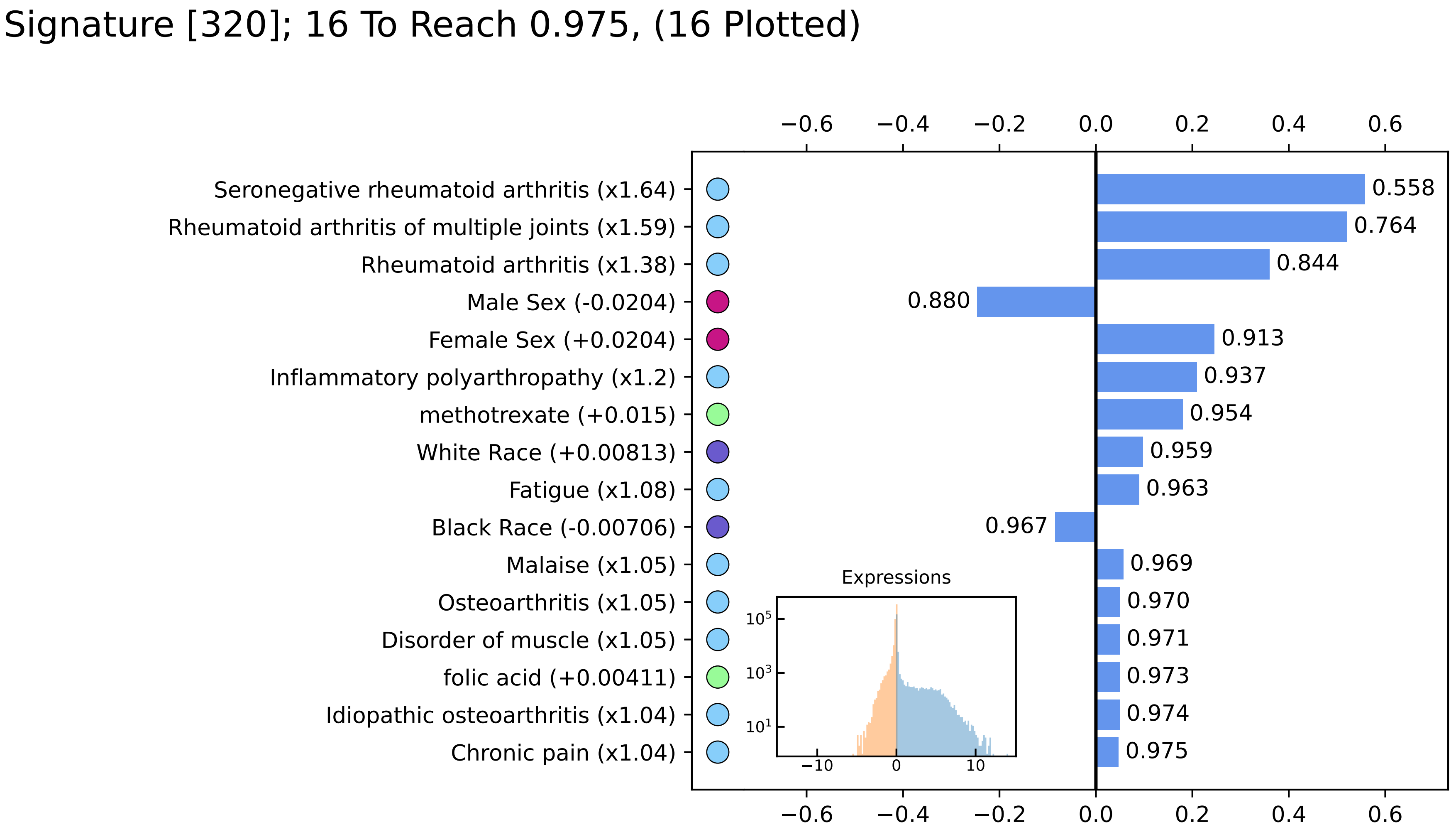

Signature [320]; 16 To Reach 0.975, (16 Plotted)
Seronegative rheumatoid arthritis (x1.64)
Rheumatoid arthritis of multiple joints (x1.59)
Rheumatoid arthritis (x1.38)
Male Sex (-0.0204)
Female Sex (+0.0204)
Inflammatory polyarthropathy (x1.2)
methotrexate (+0.015)
White Race (+0.00813)
Fatigue (x1.08)
Black Race (-0.00706)
Malaise (x1.05)
Osteoarthritis (x1.05)
Disorder of muscle (x1.05)
folic acid (+0.00411)
Idiopathic osteoarthritis (x1.04)
Chronic pain (x1.04)
0.558
0.764
0.844
0.880
0.913
0.937
0.954
0.959
0.963
0.967
0.969
0.970
0.971
0.973
0.974
0.975
−0.6
−0.4
−0.2
0.0
0.2
0.4
0.6
Expressions
10^5
10^3
10^1
−10
0
10

Signature [321]; 429 To Reach 0.975, (100 Plotted)

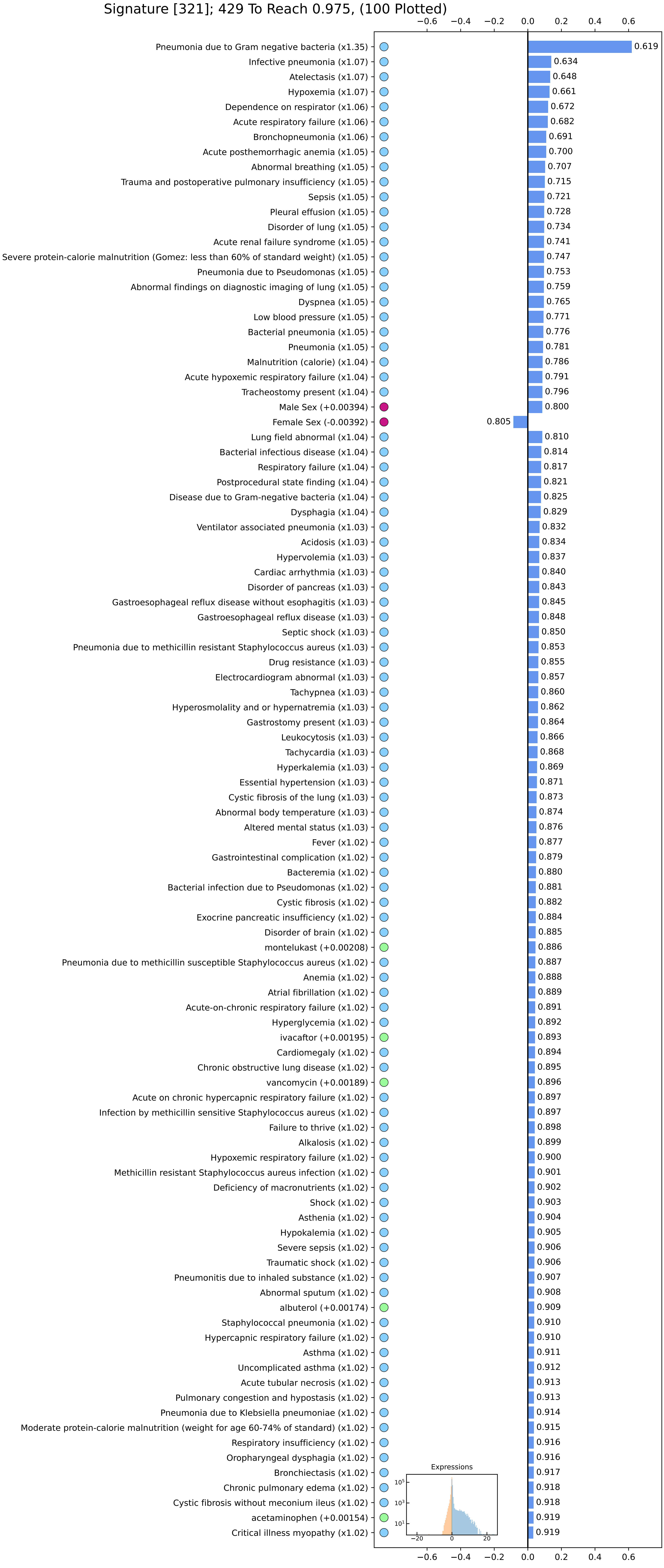

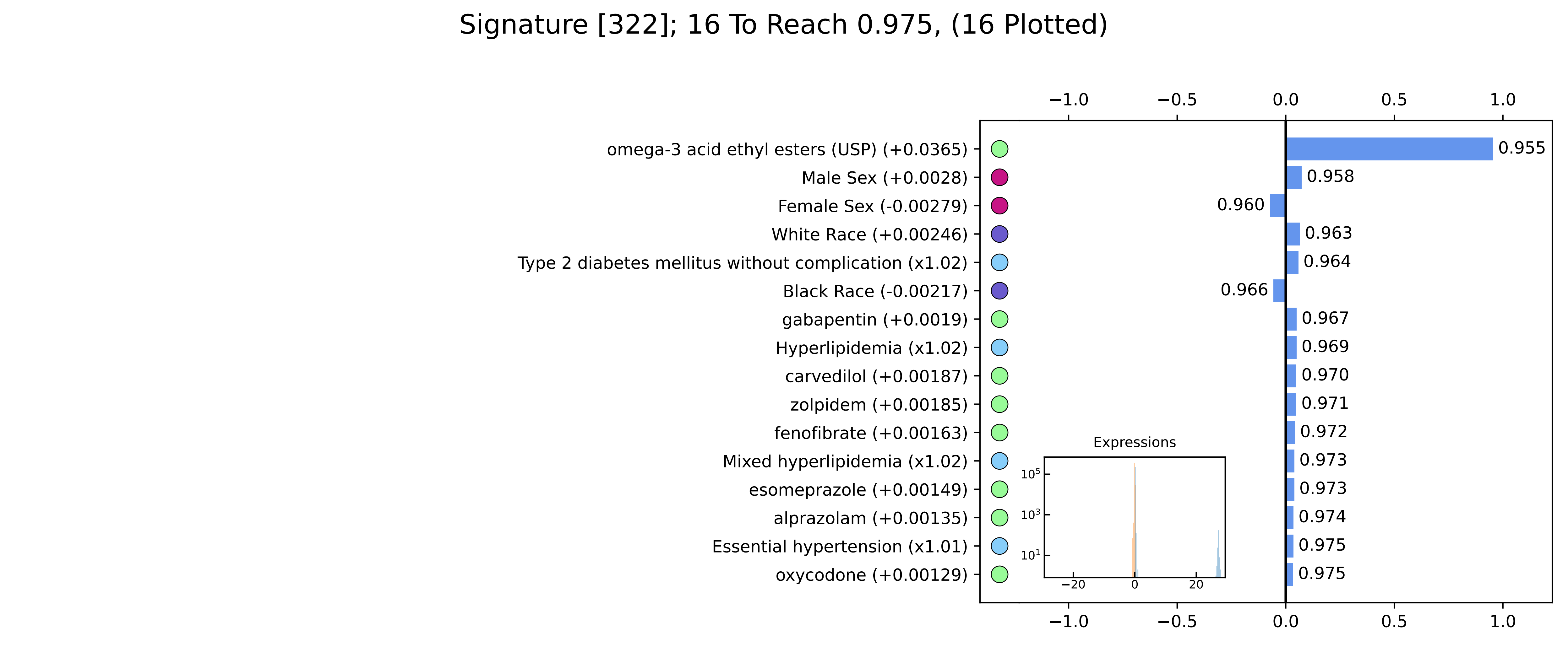

Signature [322]; 16 To Reach 0.975, (16 Plotted)
omega-3 acid ethyl esters (USP) (+0.0365)
Male Sex (+0.0028)
Female Sex (-0.00279)
White Race (+0.00246)
Type 2 diabetes mellitus without complication (x1.02)
Black Race (-0.00217)
gabapentin (+0.0019)
Hyperlipidemia (x1.02)
carvedilol (+0.00187)
zolpidem (+0.00185)
fenofibrate (+0.00163)
Mixed hyperlipidemia (x1.02)
esomeprazole (+0.00149)
alprazolam (+0.00135)
Essential hypertension (x1.01)
oxycodone (+0.00129)
0.955
0.958
0.960
0.963
0.964
0.966
0.967
0.969
0.970
0.971
0.972
0.973
0.973
0.974
0.975
0.975
−1.0
−0.5
0.0
0.5
1.0
Expressions
10^5
10^3
10^1
−20
0
20

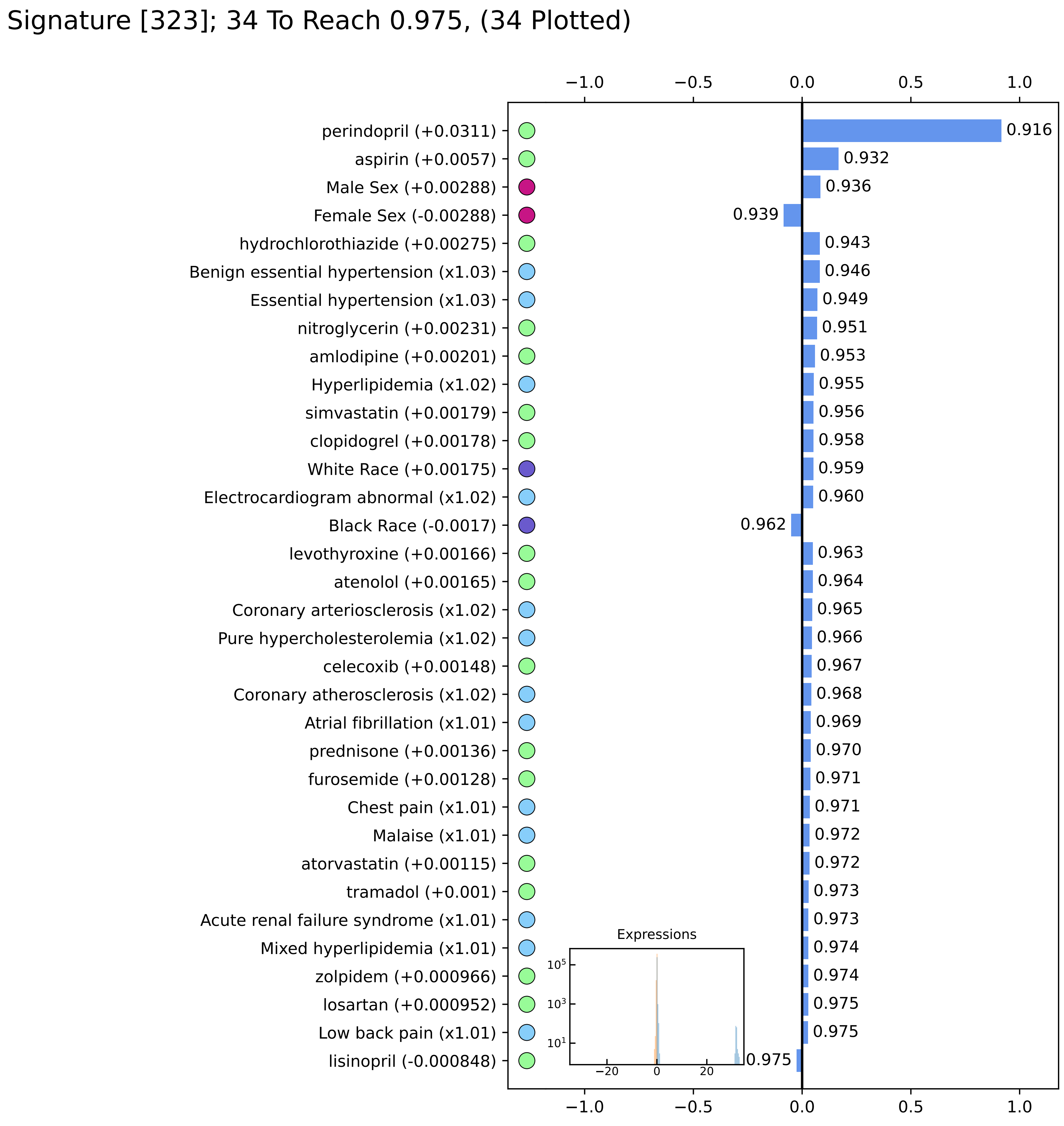

Signature [323]; 34 To Reach 0.975, (34 Plotted)
perindopril (+0.0311) 0.916
aspirin (+0.0057) 0.932
Male Sex (+0.00288) 0.936
Female Sex (-0.00288) 0.939
hydrochlorothiazide (+0.00275) 0.943
Benign essential hypertension (x1.03) 0.946
Essential hypertension (x1.03) 0.949
nitroglycerin (+0.00231) 0.951
amlodipine (+0.00201) 0.953
Hyperlipidemia (x1.02) 0.955
simvastatin (+0.00179) 0.956
clopidogrel (+0.00178) 0.958
White Race (+0.00175) 0.959
Electrocardiogram abnormal (x1.02) 0.960
Black Race (-0.0017) 0.962
levothyroxine (+0.00166) 0.963
atenolol (+0.00165) 0.964
Coronary arteriosclerosis (x1.02) 0.965
Pure hypercholesterolemia (x1.02) 0.966
celecoxib (+0.00148) 0.967
Coronary atherosclerosis (x1.02) 0.968
Atrial fibrillation (x1.01) 0.969
prednisone (+0.00136) 0.970
furosemide (+0.00128) 0.971
Chest pain (x1.01) 0.971
Malaise (x1.01) 0.972
atorvastatin (+0.00115) 0.972
tramadol (+0.001) 0.973
Acute renal failure syndrome (x1.01) 0.973
Mixed hyperlipidemia (x1.01) 0.974
zolpidem (+0.000966) 0.974
losartan (+0.000952) 0.975
Low back pain (x1.01) 0.975
lisinopril (-0.000848) 0.975
−1.0 −0.5 0.0 0.5 1.0
Expressions
−20 0 20

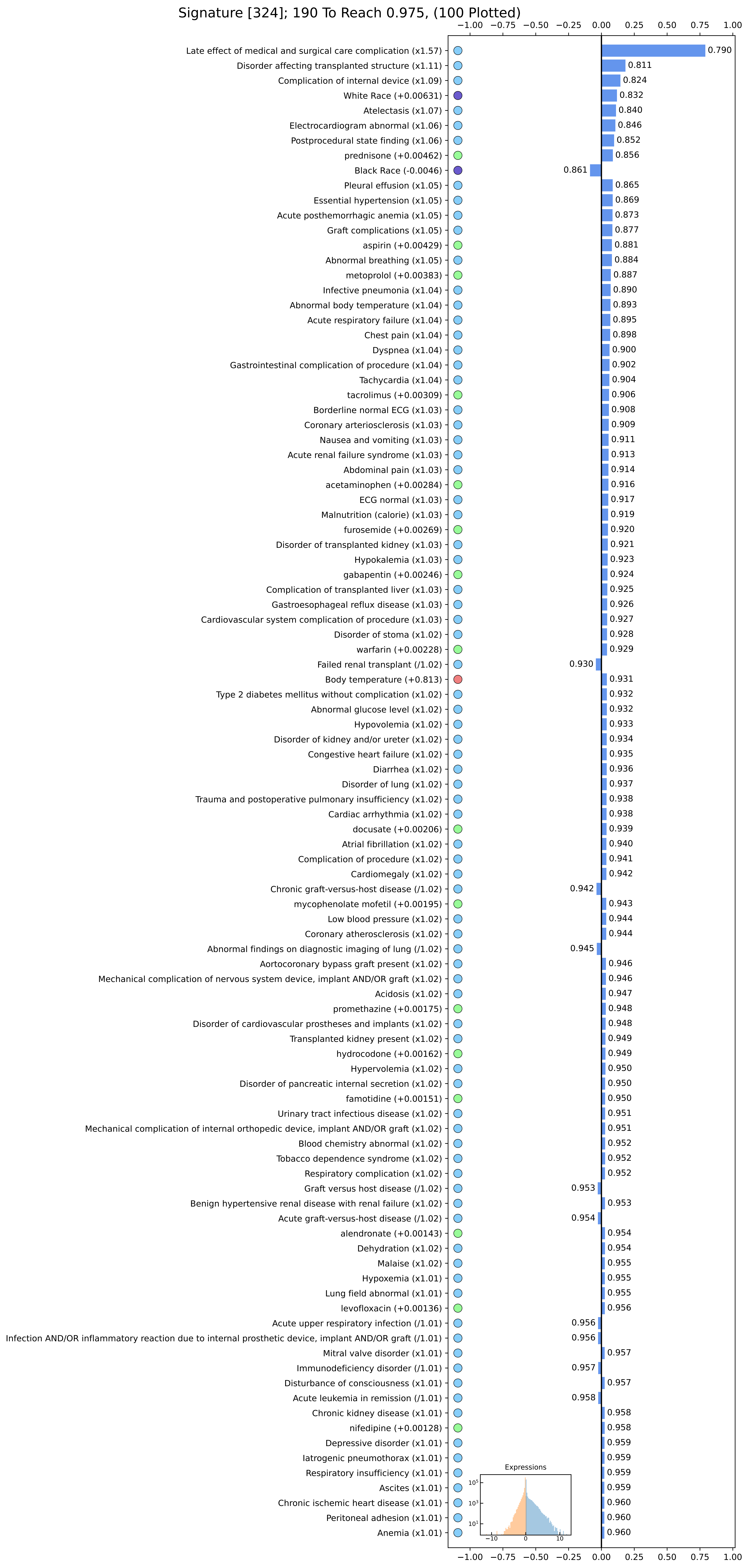

Signature [324]; 190 To Reach 0.975, (100 Plotted)
Late effect of medical and surgical care complication (x1.57) 0.790
Disorder affecting transplanted structure (x1.11) 0.811
Complication of internal device (x1.09) 0.824
White Race (+0.00631) 0.832
Atelectasis (x1.07) 0.840
Electrocardiogram abnormal (x1.06) 0.846
Postprocedural state finding (x1.06) 0.852
prednisone (+0.00462) 0.856
Black Race (-0.0046) 0.861
Pleural effusion (x1.05) 0.865
Essential hypertension (x1.05) 0.869
Acute posthemorrhagic anemia (x1.05) 0.873
Graft complications (x1.05) 0.877
aspirin (+0.00429) 0.881
Abnormal breathing (x1.05) 0.884
metoprolol (+0.00383) 0.887
Infective pneumonia (x1.04) 0.890
Abnormal body temperature (x1.04) 0.893
Acute respiratory failure (x1.04) 0.895
Chest pain (x1.04) 0.898
Dyspnea (x1.04) 0.900
Gastrointestinal complication of procedure (x1.04) 0.902
Tachycardia (x1.04) 0.904
tacrolimus (+0.00309) 0.906
Borderline normal ECG (x1.03) 0.908
Coronary arteriosclerosis (x1.03) 0.909
Nausea and vomiting (x1.03) 0.911
Acute renal failure syndrome (x1.03) 0.913
Abdominal pain (x1.03) 0.914
acetaminophen (+0.00284) 0.916
ECG normal (x1.03) 0.917
Malnutrition (calorie) (x1.03) 0.919
furosemide (+0.00269) 0.920
Disorder of transplanted kidney (x1.03) 0.921
Hypokalemia (x1.03) 0.923
gabapentin (+0.00246) 0.924
Complication of transplanted liver (x1.03) 0.925
Gastroesophageal reflux disease (x1.03) 0.926
Cardiovascular system complication of procedure (x1.03) 0.927
Disorder of stoma (x1.02) 0.928
warfarin (+0.00228) 0.929
Failed renal transplant (/1.02) 0.930
Body temperature (+0.813) 0.931
Type 2 diabetes mellitus without complication (x1.02) 0.932
Abnormal glucose level (x1.02) 0.932
Hypovolemia (x1.02) 0.933
Disorder of kidney and/or ureter (x1.02) 0.934
Congestive heart failure (x1.02) 0.935
Diarrhea (x1.02) 0.936
Disorder of lung (x1.02) 0.937
Trauma and postoperative pulmonary insufficiency (x1.02) 0.938
Cardiac arrhythmia (x1.02) 0.938
docusate (+0.00206) 0.939
Atrial fibrillation (x1.02) 0.940
Complication of procedure (x1.02) 0.941
Cardiomegaly (x1.02) 0.942
Chronic graft-versus-host disease (/1.02) 0.942
mycophenolate mofetil (+0.00195) 0.943
Low blood pressure (x1.02) 0.944
Coronary atherosclerosis (x1.02) 0.944
Abnormal findings on diagnostic imaging of lung (/1.02) 0.945
Aortocoronary bypass graft present (x1.02) 0.946
Mechanical complication of nervous system device, implant AND/OR graft (x1.02) 0.946
Acidosis (x1.02) 0.947
promethazine (+0.00175) 0.948
Disorder of cardiovascular prostheses and implants (x1.02) 0.948
Transplanted kidney present (x1.02) 0.949
hydrocodone (+0.00162) 0.949
Hypervolemia (x1.02) 0.950
Disorder of pancreatic internal secretion (x1.02) 0.950
famotidine (+0.00151) 0.950
Urinary tract infectious disease (x1.02) 0.951
Mechanical complication of internal orthopedic device, implant AND/OR graft (x1.02) 0.951
Blood chemistry abnormal (x1.02) 0.952
Tobacco dependence syndrome (x1.02) 0.952
Respiratory complication (x1.02) 0.952
Graft versus host disease (/1.02) 0.953
Benign hypertensive renal disease with renal failure (x1.02) 0.953
Acute graft-versus-host disease (/1.02) 0.954
alendronate (+0.00143) 0.954
Dehydration (x1.02) 0.954
Malaise (x1.02) 0.955
Hypoxemia (x1.01) 0.955
Lung field abnormal (x1.01) 0.955
levofloxacin (+0.00136) 0.956
Acute upper respiratory infection (/1.01) 0.956
Infection AND/OR inflammatory reaction due to internal prosthetic device, implant AND/OR graft (/1.01) 0.956
Mitral valve disorder (x1.01) 0.957
Immunodeficiency disorder (/1.01) 0.957
Disturbance of consciousness (x1.01) 0.957
Acute leukemia in remission (/1.01) 0.958
Chronic kidney disease (x1.01) 0.958
nifedipine (+0.00128) 0.958
Depressive disorder (x1.01) 0.959
Iatrogenic pneumothorax (x1.01) 0.959
Respiratory insufficiency (x1.01) 0.959
Ascites (x1.01) 0.959
Chronic ischemic heart disease (x1.01) 0.960
Peritoneal adhesion (x1.01) 0.960
Anemia (x1.01) 0.960
−1.00 −0.75 −0.50 −0.25 0.00 0.25 0.50 0.75 1.00
Expressions
−10 0 10

Signature [325]; 704 To Reach 0.975, (100 Plotted)

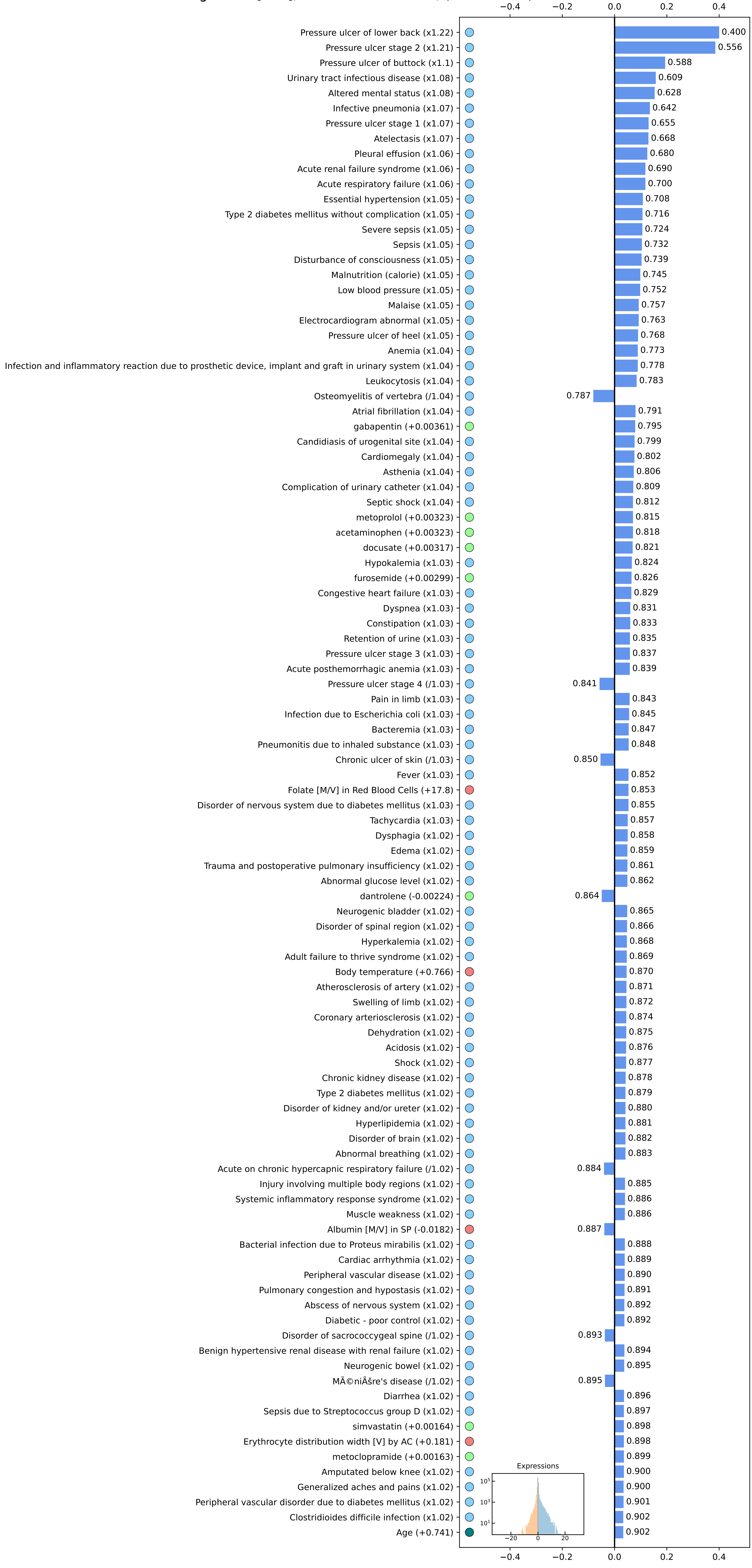

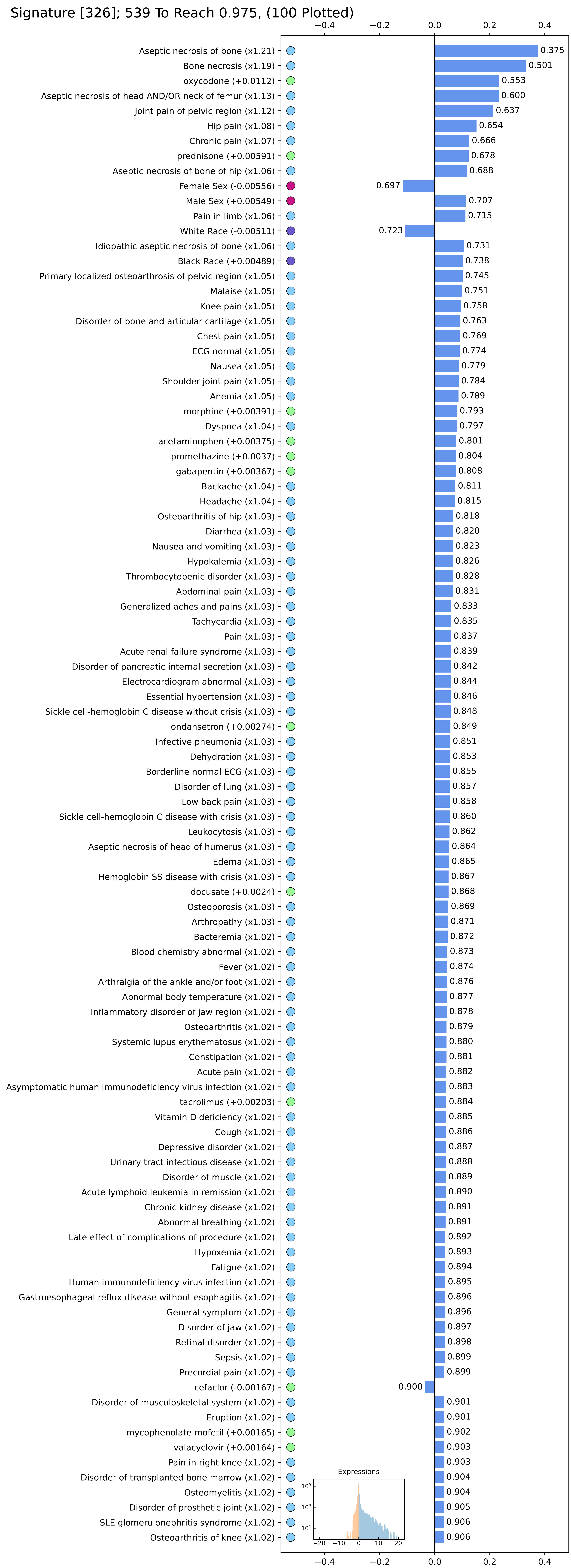

Signature [326]; 539 To Reach 0.975, (100 Plotted)
Aseptic necrosis of bone (x1.21) 0.375
Bone necrosis (x1.19) 0.501
oxycodone (+0.0112) 0.553
Aseptic necrosis of head AND/OR neck of femur (x1.13) 0.600
Joint pain of pelvic region (x1.12) 0.637
Hip pain (x1.08) 0.654
Chronic pain (x1.07) 0.666
prednisone (+0.00591) 0.678
Aseptic necrosis of bone of hip (x1.06) 0.688
Female Sex (-0.00556) 0.697
Male Sex (+0.00549) 0.707
Pain in limb (x1.06) 0.715
White Race (-0.00511) 0.723
Idiopathic aseptic necrosis of bone (x1.06) 0.731
Black Race (+0.00489) 0.738
Primary localized osteoarthrosis of pelvic region (x1.05) 0.745
Malaise (x1.05) 0.751
Knee pain (x1.05) 0.758
Disorder of bone and articular cartilage (x1.05) 0.763
Chest pain (x1.05) 0.769
ECG normal (x1.05) 0.774
Nausea (x1.05) 0.779
Shoulder joint pain (x1.05) 0.784
Anemia (x1.05) 0.789
morphine (+0.00391) 0.793
Dyspnea (x1.04) 0.797
acetaminophen (+0.00375) 0.801
promethazine (+0.0037) 0.804
gabapentin (+0.00367) 0.808
Backache (x1.04) 0.811
Headache (x1.04) 0.815
Osteoarthritis of hip (x1.03) 0.818
Diarrhea (x1.03) 0.820
Nausea and vomiting (x1.03) 0.823
Hypokalemia (x1.03) 0.826
Thrombocytopenic disorder (x1.03) 0.828
Abdominal pain (x1.03) 0.831
Generalized aches and pains (x1.03) 0.833
Tachycardia (x1.03) 0.835
Pain (x1.03) 0.837
Acute renal failure syndrome (x1.03) 0.839
Disorder of pancreatic internal secretion (x1.03) 0.842
Electrocardiogram abnormal (x1.03) 0.844
Essential hypertension (x1.03) 0.846
Sickle cell-hemoglobin C disease without crisis (x1.03) 0.848
ondansetron (+0.00274) 0.849
Infective pneumonia (x1.03) 0.851
Dehydration (x1.03) 0.853
Borderline normal ECG (x1.03) 0.855
Disorder of lung (x1.03) 0.857
Low back pain (x1.03) 0.858
Sickle cell-hemoglobin C disease with crisis (x1.03) 0.860
Leukocytosis (x1.03) 0.862
Aseptic necrosis of head of humerus (x1.03) 0.864
Edema (x1.03) 0.865
Hemoglobin SS disease with crisis (x1.03) 0.867
docusate (+0.0024) 0.868
Osteoporosis (x1.03) 0.869
Arthropathy (x1.03) 0.871
Bacteremia (x1.02) 0.872
Blood chemistry abnormal (x1.02) 0.873
Fever (x1.02) 0.874
Arthralgia of the ankle and/or foot (x1.02) 0.876
Abnormal body temperature (x1.02) 0.877
Inflammatory disorder of jaw region (x1.02) 0.878
Osteoarthritis (x1.02) 0.879
Systemic lupus erythematosus (x1.02) 0.880
Constipation (x1.02) 0.881
Acute pain (x1.02) 0.882
Asymptomatic human immunodeficiency virus infection (x1.02) 0.883
tacrolimus (+0.00203) 0.884
Vitamin D deficiency (x1.02) 0.885
Cough (x1.02) 0.886
Depressive disorder (x1.02) 0.887
Urinary tract infectious disease (x1.02) 0.888
Disorder of muscle (x1.02) 0.889
Acute lymphoid leukemia in remission (x1.02) 0.890
Chronic kidney disease (x1.02) 0.891
Abnormal breathing (x1.02) 0.891
Late effect of complications of procedure (x1.02) 0.892
Hypoxemia (x1.02) 0.893
Fatigue (x1.02) 0.894
Human immunodeficiency virus infection (x1.02) 0.895
Gastroesophageal reflux disease without esophagitis (x1.02) 0.896
General symptom (x1.02) 0.896
Disorder of jaw (x1.02) 0.897
Retinal disorder (x1.02) 0.898
Sepsis (x1.02) 0.899
Precordial pain (x1.02) 0.899
cefaclor (-0.00167) 0.900
Disorder of musculoskeletal system (x1.02) 0.901
Eruption (x1.02) 0.901
mycophenolate mofetil (+0.00165) 0.902
valacyclovir (+0.00164) 0.903
Pain in right knee (x1.02) 0.903
Disorder of transplanted bone marrow (x1.02) 0.904
Osteomyelitis (x1.02) 0.904
Disorder of prosthetic joint (x1.02) 0.905
SLE glomerulonephritis syndrome (x1.02) 0.906
Osteoarthritis of knee (x1.02) 0.906
Expressions
−0.4 −0.2 0.0 0.2 0.4

Signature [327]; 5 To Reach 0.975, (10 Plotted)

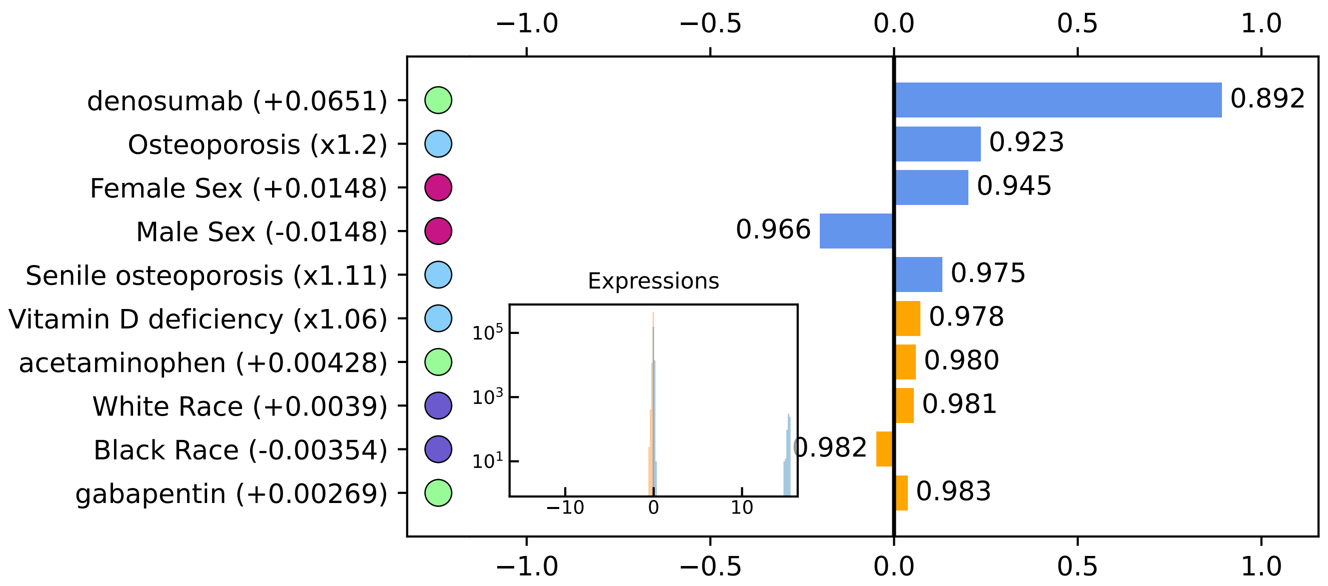

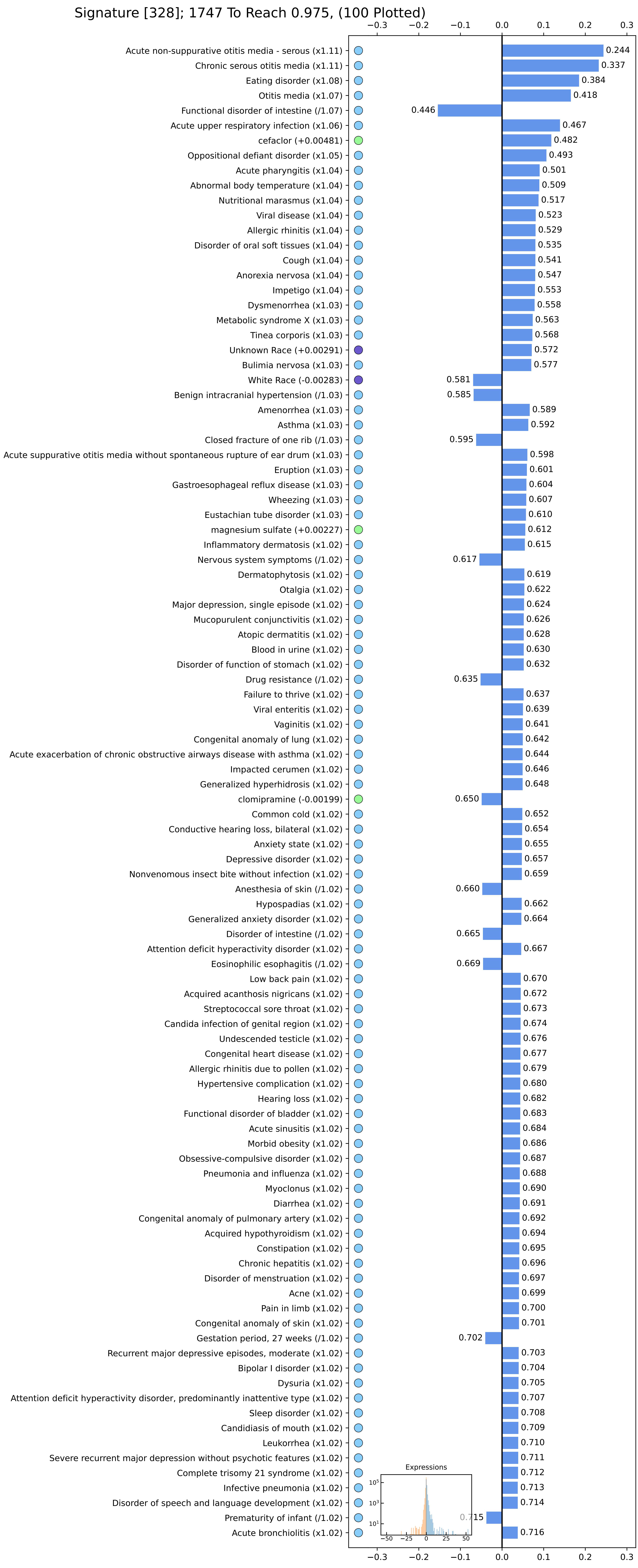
Signature [328]; 1747 To Reach 0.975, (100 Plotted)
Acute non-suppurative otitis media - serous (x1.11) 0.244
Chronic serous otitis media (x1.11) 0.337
Eating disorder (x1.08) 0.384
Otitis media (x1.07) 0.418
Functional disorder of intestine (/1.07) 0.446
Acute upper respiratory infection (x1.06) 0.467
cefaclor (+0.00481) 0.482
Oppositional defiant disorder (x1.05) 0.493
Acute pharyngitis (x1.04) 0.501
Abnormal body temperature (x1.04) 0.509
Nutritional marasmus (x1.04) 0.517
Viral disease (x1.04) 0.523
Allergic rhinitis (x1.04) 0.529
Disorder of oral soft tissues (x1.04) 0.535
Cough (x1.04) 0.541
Anorexia nervosa (x1.04) 0.547
Impetigo (x1.04) 0.553
Dysmenorrhea (x1.03) 0.558
Metabolic syndrome X (x1.03) 0.563
Tinea corporis (x1.03) 0.568
Unknown Race (+0.00291) 0.572
Bulimia nervosa (x1.03) 0.577
White Race (-0.00283) 0.581
Benign intracranial hypertension (/1.03) 0.585
Amenorrhea (x1.03) 0.589
Asthma (x1.03) 0.592
Closed fracture of one rib (/1.03) 0.595
Acute suppurative otitis media without spontaneous rupture of ear drum (x1.03) 0.598
Eruption (x1.03) 0.601
Gastroesophageal reflux disease (x1.03) 0.604
Wheezing (x1.03) 0.607
Eustachian tube disorder (x1.03) 0.610
magnesium sulfate (+0.00227) 0.612
Inflammatory dermatosis (x1.02) 0.615
Nervous system symptoms (/1.02) 0.617
Dermatophytosis (x1.02) 0.619
Otalgia (x1.02) 0.622
Major depression, single episode (x1.02) 0.624
Mucopurulent conjunctivitis (x1.02) 0.626
Atopic dermatitis (x1.02) 0.628
Blood in urine (x1.02) 0.630
Disorder of function of stomach (x1.02) 0.632
Drug resistance (/1.02) 0.635
Failure to thrive (x1.02) 0.637
Viral enteritis (x1.02) 0.639
Vaginitis (x1.02) 0.641
Congenital anomaly of lung (x1.02) 0.642
Acute exacerbation of chronic obstructive airways disease with asthma (x1.02) 0.644
Impacted cerumen (x1.02) 0.646
Generalized hyperhidrosis (x1.02) 0.648
clomipramine (-0.00199) 0.650
Common cold (x1.02) 0.652
Conductive hearing loss, bilateral (x1.02) 0.654
Anxiety state (x1.02) 0.655
Depressive disorder (x1.02) 0.657
Nonvenomous insect bite without infection (x1.02) 0.659
Anesthesia of skin (/1.02) 0.660
Hypospadias (x1.02) 0.662
Generalized anxiety disorder (x1.02) 0.664
Disorder of intestine (/1.02) 0.665
Attention deficit hyperactivity disorder (x1.02) 0.667
Eosinophilic esophagitis (/1.02) 0.669
Low back pain (x1.02) 0.670
Acquired acanthosis nigricans (x1.02) 0.672
Streptococcal sore throat (x1.02) 0.673
Candida infection of genital region (x1.02) 0.674
Undescended testicle (x1.02) 0.676
Congenital heart disease (x1.02) 0.677
Allergic rhinitis due to pollen (x1.02) 0.679
Hypertensive complication (x1.02) 0.680
Hearing loss (x1.02) 0.682
Functional disorder of bladder (x1.02) 0.683
Acute sinusitis (x1.02) 0.684
Morbid obesity (x1.02) 0.686
Obsessive-compulsive disorder (x1.02) 0.687
Pneumonia and influenza (x1.02) 0.688
Myoclonus (x1.02) 0.690
Diarrhea (x1.02) 0.691
Congenital anomaly of pulmonary artery (x1.02) 0.692
Acquired hypothyroidism (x1.02) 0.694
Constipation (x1.02) 0.695
Chronic hepatitis (x1.02) 0.696
Disorder of menstruation (x1.02) 0.697
Acne (x1.02) 0.699
Pain in limb (x1.02) 0.700
Congenital anomaly of skin (x1.02) 0.701
Gestation period, 27 weeks (/1.02) 0.702
Recurrent major depressive episodes, moderate (x1.02) 0.703
Bipolar I disorder (x1.02) 0.704
Dysuria (x1.02) 0.705
Attention deficit hyperactivity disorder, predominantly inattentive type (x1.02) 0.707
Sleep disorder (x1.02) 0.708
Candidiasis of mouth (x1.02) 0.709
Leukorrhea (x1.02) 0.710
Severe recurrent major depression without psychotic features (x1.02) 0.711
Complete trisomy 21 syndrome (x1.02) 0.712
Infective pneumonia (x1.02) 0.713
Disorder of speech and language development (x1.02) 0.714
Prematurity of infant (/1.02) 0.715
Acute bronchiolitis (x1.02) 0.716
−0.3 −0.2 −0.1 0.0 0.1 0.2 0.3
Expressions
−50 −25 0 25 50

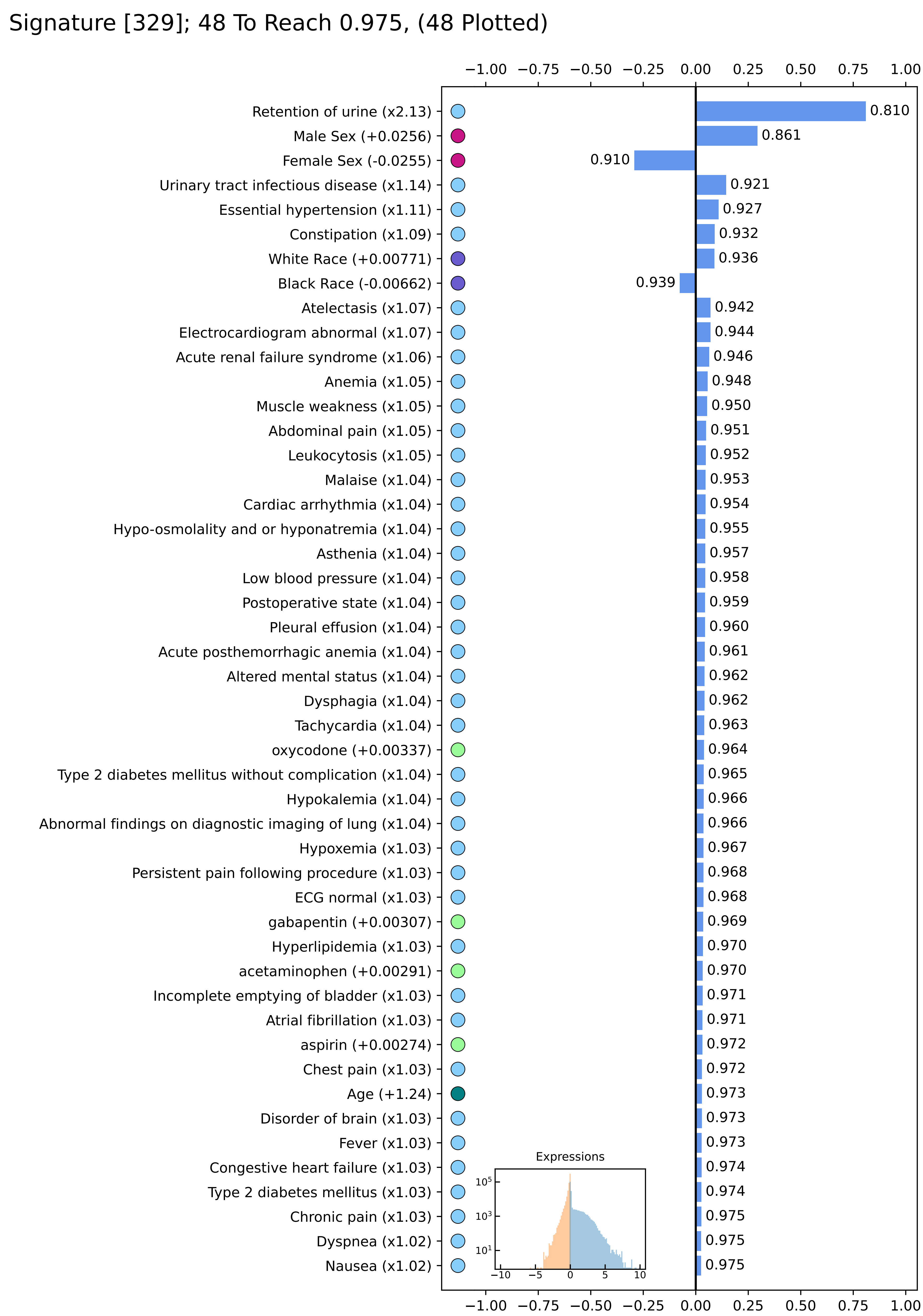

Signature [329]; 48 To Reach 0.975, (48 Plotted)
Retention of urine (x2.13) 0.810
Male Sex (+0.0256) 0.861
Female Sex (-0.0255) 0.910
Urinary tract infectious disease (x1.14) 0.921
Essential hypertension (x1.11) 0.927
Constipation (x1.09) 0.932
White Race (+0.00771) 0.936
Black Race (-0.00662) 0.939
Atelectasis (x1.07) 0.942
Electrocardiogram abnormal (x1.07) 0.944
Acute renal failure syndrome (x1.06) 0.946
Anemia (x1.05) 0.948
Muscle weakness (x1.05) 0.950
Abdominal pain (x1.05) 0.951
Leukocytosis (x1.05) 0.952
Malaise (x1.04) 0.953
Cardiac arrhythmia (x1.04) 0.954
Hypo-osmolality and or hyponatremia (x1.04) 0.955
Asthenia (x1.04) 0.957
Low blood pressure (x1.04) 0.958
Postoperative state (x1.04) 0.959
Pleural effusion (x1.04) 0.960
Acute posthemorrhagic anemia (x1.04) 0.961
Altered mental status (x1.04) 0.962
Dysphagia (x1.04) 0.962
Tachycardia (x1.04) 0.963
oxycodone (+0.00337) 0.964
Type 2 diabetes mellitus without complication (x1.04) 0.965
Hypokalemia (x1.04) 0.966
Abnormal findings on diagnostic imaging of lung (x1.04) 0.966
Hypoxemia (x1.03) 0.967
Persistent pain following procedure (x1.03) 0.968
ECG normal (x1.03) 0.968
gabapentin (+0.00307) 0.969
Hyperlipidemia (x1.03) 0.970
acetaminophen (+0.00291) 0.970
Incomplete emptying of bladder (x1.03) 0.971
Atrial fibrillation (x1.03) 0.971
aspirin (+0.00274) 0.972
Chest pain (x1.03) 0.972
Age (+1.24) 0.973
Disorder of brain (x1.03) 0.973
Fever (x1.03) 0.973
Congestive heart failure (x1.03) 0.974
Type 2 diabetes mellitus (x1.03) 0.974
Chronic pain (x1.03) 0.975
Dyspnea (x1.02) 0.975
Nausea (x1.02) 0.975
Expressions
−1.00 −0.75 −0.50 −0.25 0.00 0.25 0.50 0.75 1.00

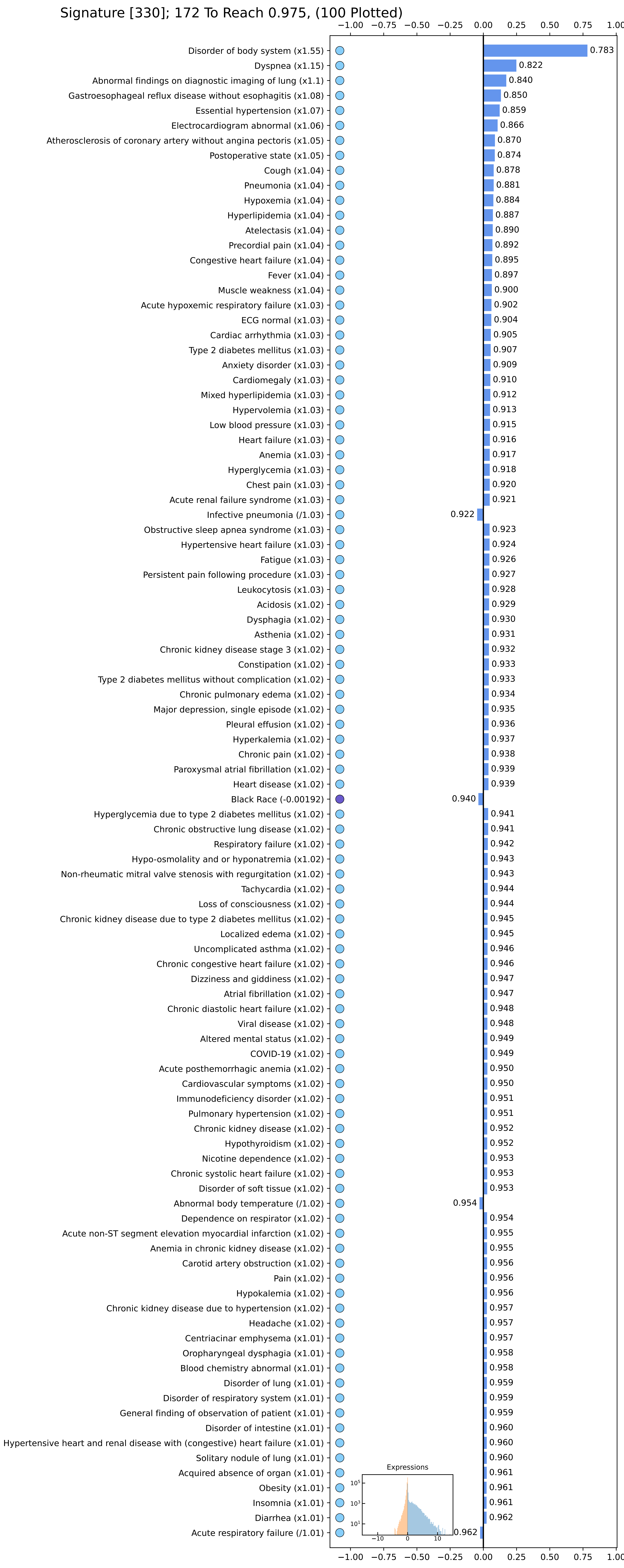

Signature [330]; 172 To Reach 0.975, (100 Plotted)
Disorder of body system (x1.55) 0.783
Dyspnea (x1.15) 0.822
Abnormal findings on diagnostic imaging of lung (x1.1) 0.840
Gastroesophageal reflux disease without esophagitis (x1.08) 0.850
Essential hypertension (x1.07) 0.859
Electrocardiogram abnormal (x1.06) 0.866
Atherosclerosis of coronary artery without angina pectoris (x1.05) 0.870
Postoperative state (x1.05) 0.874
Cough (x1.04) 0.878
Pneumonia (x1.04) 0.881
Hypoxemia (x1.04) 0.884
Hyperlipidemia (x1.04) 0.887
Atelectasis (x1.04) 0.890
Precordial pain (x1.04) 0.892
Congestive heart failure (x1.04) 0.895
Fever (x1.04) 0.897
Muscle weakness (x1.04) 0.900
Acute hypoxemic respiratory failure (x1.03) 0.902
ECG normal (x1.03) 0.904
Cardiac arrhythmia (x1.03) 0.905
Type 2 diabetes mellitus (x1.03) 0.907
Anxiety disorder (x1.03) 0.909
Cardiomegaly (x1.03) 0.910
Mixed hyperlipidemia (x1.03) 0.912
Hypervolemia (x1.03) 0.913
Low blood pressure (x1.03) 0.915
Heart failure (x1.03) 0.916
Anemia (x1.03) 0.917
Hyperglycemia (x1.03) 0.918
Chest pain (x1.03) 0.920
Acute renal failure syndrome (x1.03) 0.921
Infective pneumonia (/1.03) 0.922
Obstructive sleep apnea syndrome (x1.03) 0.923
Hypertensive heart failure (x1.03) 0.924
Fatigue (x1.03) 0.926
Persistent pain following procedure (x1.03) 0.927
Leukocytosis (x1.03) 0.928
Acidosis (x1.02) 0.929
Dysphagia (x1.02) 0.930
Asthenia (x1.02) 0.931
Chronic kidney disease stage 3 (x1.02) 0.932
Constipation (x1.02) 0.933
Type 2 diabetes mellitus without complication (x1.02) 0.933
Chronic pulmonary edema (x1.02) 0.934
Major depression, single episode (x1.02) 0.935
Pleural effusion (x1.02) 0.936
Hyperkalemia (x1.02) 0.937
Chronic pain (x1.02) 0.938
Paroxysmal atrial fibrillation (x1.02) 0.939
Heart disease (x1.02) 0.939
Black Race (-0.00192) 0.940
Hyperglycemia due to type 2 diabetes mellitus (x1.02) 0.941
Chronic obstructive lung disease (x1.02) 0.941
Respiratory failure (x1.02) 0.942
Hypo-osmolality and or hyponatremia (x1.02) 0.943
Non-rheumatic mitral valve stenosis with regurgitation (x1.02) 0.943
Tachycardia (x1.02) 0.944
Loss of consciousness (x1.02) 0.944
Chronic kidney disease due to type 2 diabetes mellitus (x1.02) 0.945
Localized edema (x1.02) 0.945
Uncomplicated asthma (x1.02) 0.946
Chronic congestive heart failure (x1.02) 0.946
Dizziness and giddiness (x1.02) 0.947
Atrial fibrillation (x1.02) 0.947
Chronic diastolic heart failure (x1.02) 0.948
Viral disease (x1.02) 0.948
Altered mental status (x1.02) 0.949
COVID-19 (x1.02) 0.949
Acute posthemorrhagic anemia (x1.02) 0.950
Cardiovascular symptoms (x1.02) 0.950
Immunodeficiency disorder (x1.02) 0.951
Pulmonary hypertension (x1.02) 0.951
Chronic kidney disease (x1.02) 0.952
Hypothyroidism (x1.02) 0.952
Nicotine dependence (x1.02) 0.953
Chronic systolic heart failure (x1.02) 0.953
Disorder of soft tissue (x1.02) 0.953
Abnormal body temperature (/1.02) 0.954
Dependence on respirator (x1.02) 0.954
Acute non-ST segment elevation myocardial infarction (x1.02) 0.955
Anemia in chronic kidney disease (x1.02) 0.955
Carotid artery obstruction (x1.02) 0.956
Pain (x1.02) 0.956
Hypokalemia (x1.02) 0.956
Chronic kidney disease due to hypertension (x1.02) 0.957
Headache (x1.02) 0.957
Centriacinar emphysema (x1.01) 0.957
Oropharyngeal dysphagia (x1.01) 0.958
Blood chemistry abnormal (x1.01) 0.958
Disorder of lung (x1.01) 0.959
Disorder of respiratory system (x1.01) 0.959
General finding of observation of patient (x1.01) 0.959
Disorder of intestine (x1.01) 0.960
Hypertensive heart and renal disease with (congestive) heart failure (x1.01) 0.960
Solitary nodule of lung (x1.01) 0.960
Acquired absence of organ (x1.01) 0.961
Obesity (x1.01) 0.961
Insomnia (x1.01) 0.961
Diarrhea (x1.01) 0.962
Acute respiratory failure (/1.01) 0.962
Expressions
−1.00 −0.75 −0.50 −0.25 0.00 0.25 0.50 0.75 1.00

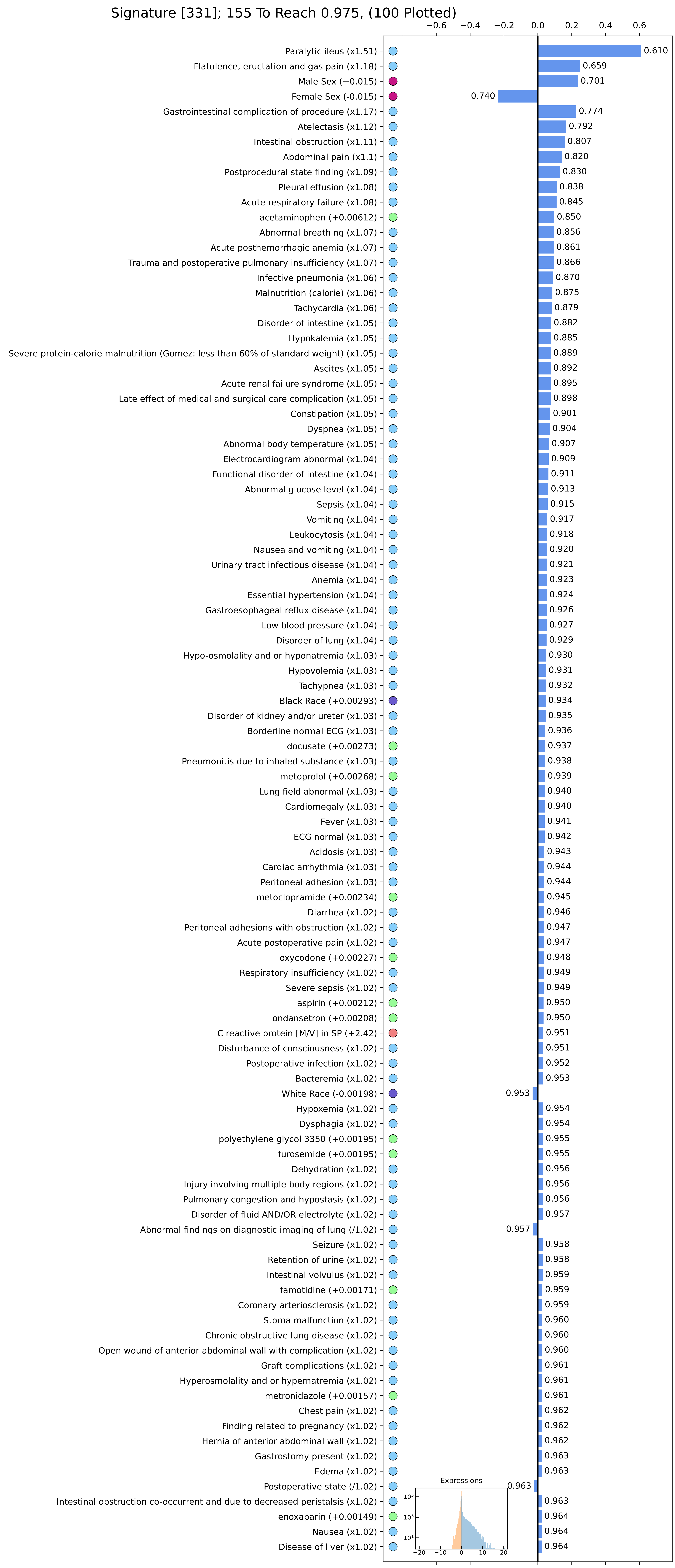

Signature [331]; 155 To Reach 0.975, (100 Plotted)
Paralytic ileus (x1.51) 0.610
Flatulence, eructation and gas pain (x1.18) 0.659
Male Sex (+0.015) 0.701
Female Sex (-0.015) 0.740
Gastrointestinal complication of procedure (x1.17) 0.774
Atelectasis (x1.12) 0.792
Intestinal obstruction (x1.11) 0.807
Abdominal pain (x1.1) 0.820
Postprocedural state finding (x1.09) 0.830
Pleural effusion (x1.08) 0.838
Acute respiratory failure (x1.08) 0.845
acetaminophen (+0.00612) 0.850
Abnormal breathing (x1.07) 0.856
Acute posthemorrhagic anemia (x1.07) 0.861
Trauma and postoperative pulmonary insufficiency (x1.07) 0.866
Infective pneumonia (x1.06) 0.870
Malnutrition (calorie) (x1.06) 0.875
Tachycardia (x1.06) 0.879
Disorder of intestine (x1.05) 0.882
Hypokalemia (x1.05) 0.885
Severe protein-calorie malnutrition (Gomez: less than 60% of standard weight) (x1.05) 0.889
Ascites (x1.05) 0.892
Acute renal failure syndrome (x1.05) 0.895
Late effect of medical and surgical care complication (x1.05) 0.898
Constipation (x1.05) 0.901
Dyspnea (x1.05) 0.904
Abnormal body temperature (x1.05) 0.907
Electrocardiogram abnormal (x1.04) 0.909
Functional disorder of intestine (x1.04) 0.911
Abnormal glucose level (x1.04) 0.913
Sepsis (x1.04) 0.915
Vomiting (x1.04) 0.917
Leukocytosis (x1.04) 0.918
Nausea and vomiting (x1.04) 0.920
Urinary tract infectious disease (x1.04) 0.921
Anemia (x1.04) 0.923
Essential hypertension (x1.04) 0.924
Gastroesophageal reflux disease (x1.04) 0.926
Low blood pressure (x1.04) 0.927
Disorder of lung (x1.04) 0.929
Hypo-osmolality and or hyponatremia (x1.03) 0.930
Hypovolemia (x1.03) 0.931
Tachypnea (x1.03) 0.932
Black Race (+0.00293) 0.934
Disorder of kidney and/or ureter (x1.03) 0.935
Borderline normal ECG (x1.03) 0.936
docusate (+0.00273) 0.937
Pneumonitis due to inhaled substance (x1.03) 0.938
metoprolol (+0.00268) 0.939
Lung field abnormal (x1.03) 0.940
Cardiomegaly (x1.03) 0.940
Fever (x1.03) 0.941
ECG normal (x1.03) 0.942
Acidosis (x1.03) 0.943
Cardiac arrhythmia (x1.03) 0.944
Peritoneal adhesion (x1.03) 0.944
metoclopramide (+0.00234) 0.945
Diarrhea (x1.02) 0.946
Peritoneal adhesions with obstruction (x1.02) 0.947
Acute postoperative pain (x1.02) 0.947
oxycodone (+0.00227) 0.948
Respiratory insufficiency (x1.02) 0.949
Severe sepsis (x1.02) 0.949
aspirin (+0.00212) 0.950
ondansetron (+0.00208) 0.950
C reactive protein [M/V] in SP (+2.42) 0.951
Disturbance of consciousness (x1.02) 0.951
Postoperative infection (x1.02) 0.952
Bacteremia (x1.02) 0.953
White Race (-0.00198) 0.953
Hypoxemia (x1.02) 0.954
Dysphagia (x1.02) 0.954
polyethylene glycol 3350 (+0.00195) 0.955
furosemide (+0.00195) 0.955
Dehydration (x1.02) 0.956
Injury involving multiple body regions (x1.02) 0.956
Pulmonary congestion and hypostasis (x1.02) 0.956
Disorder of fluid AND/OR electrolyte (x1.02) 0.957
Abnormal findings on diagnostic imaging of lung (/1.02) 0.957
Seizure (x1.02) 0.958
Retention of urine (x1.02) 0.958
Intestinal volvulus (x1.02) 0.959
famotidine (+0.00171) 0.959
Coronary arteriosclerosis (x1.02) 0.959
Stoma malfunction (x1.02) 0.960
Chronic obstructive lung disease (x1.02) 0.960
Open wound of anterior abdominal wall with complication (x1.02) 0.960
Graft complications (x1.02) 0.961
Hyperosmolality and or hypernatremia (x1.02) 0.961
metronidazole (+0.00157) 0.961
Chest pain (x1.02) 0.962
Finding related to pregnancy (x1.02) 0.962
Hernia of anterior abdominal wall (x1.02) 0.962
Gastrostomy present (x1.02) 0.963
Edema (x1.02) 0.963
Postoperative state (/1.02) 0.963
Intestinal obstruction co-occurrent and due to decreased peristalsis (x1.02) 0.963
enoxaparin (+0.00149) 0.964
Nausea (x1.02) 0.964
Disease of liver (x1.02) 0.964
−0.6 −0.4 −0.2 0.0 0.2 0.4 0.6
Expressions

Signature [332]; 4 To Reach 0.975, (10 Plotted)

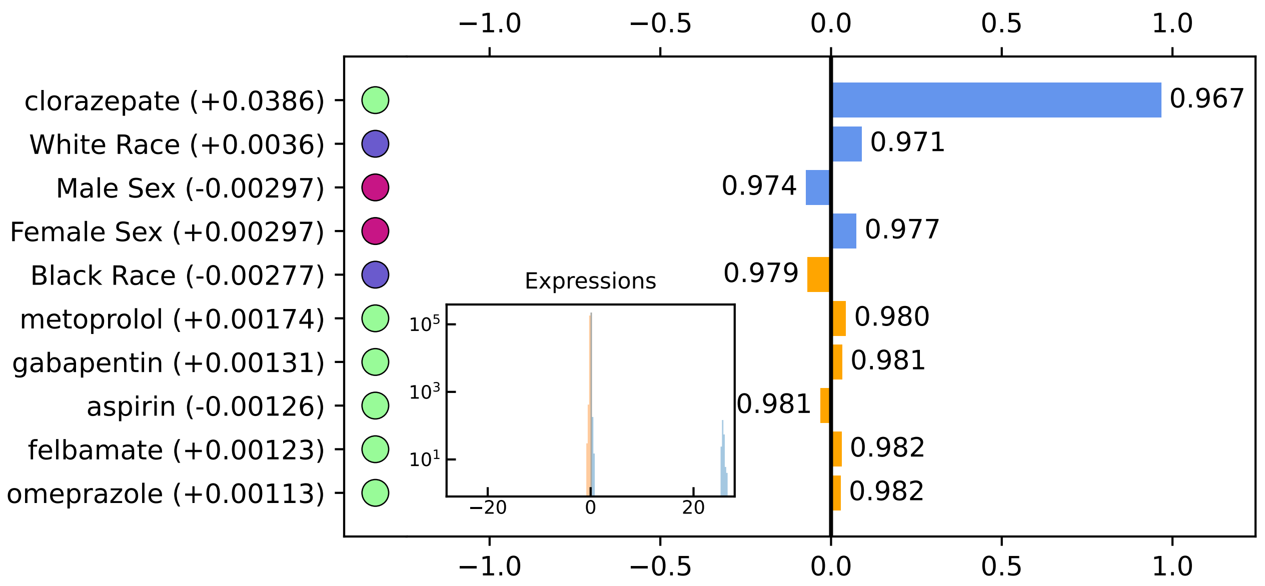

Signature [333]; 329 To Reach 0.975, (100 Plotted)

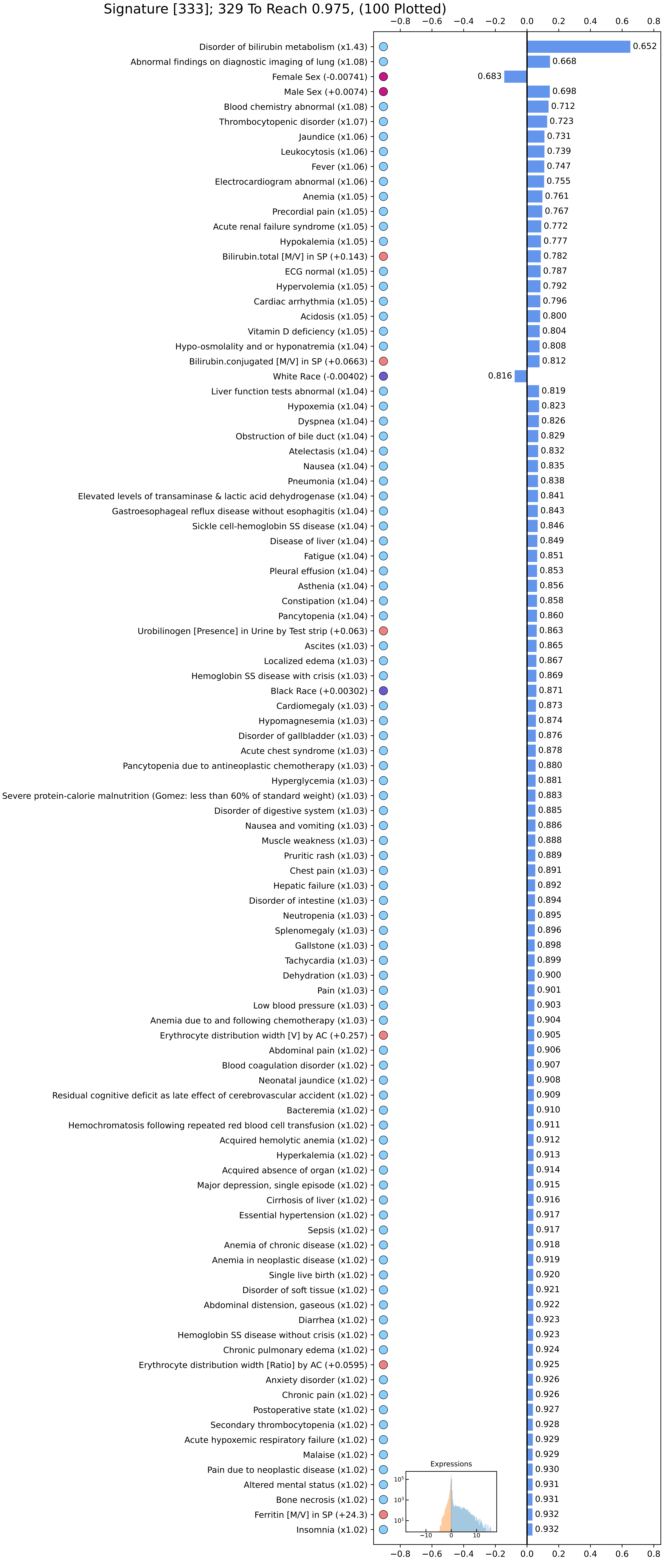

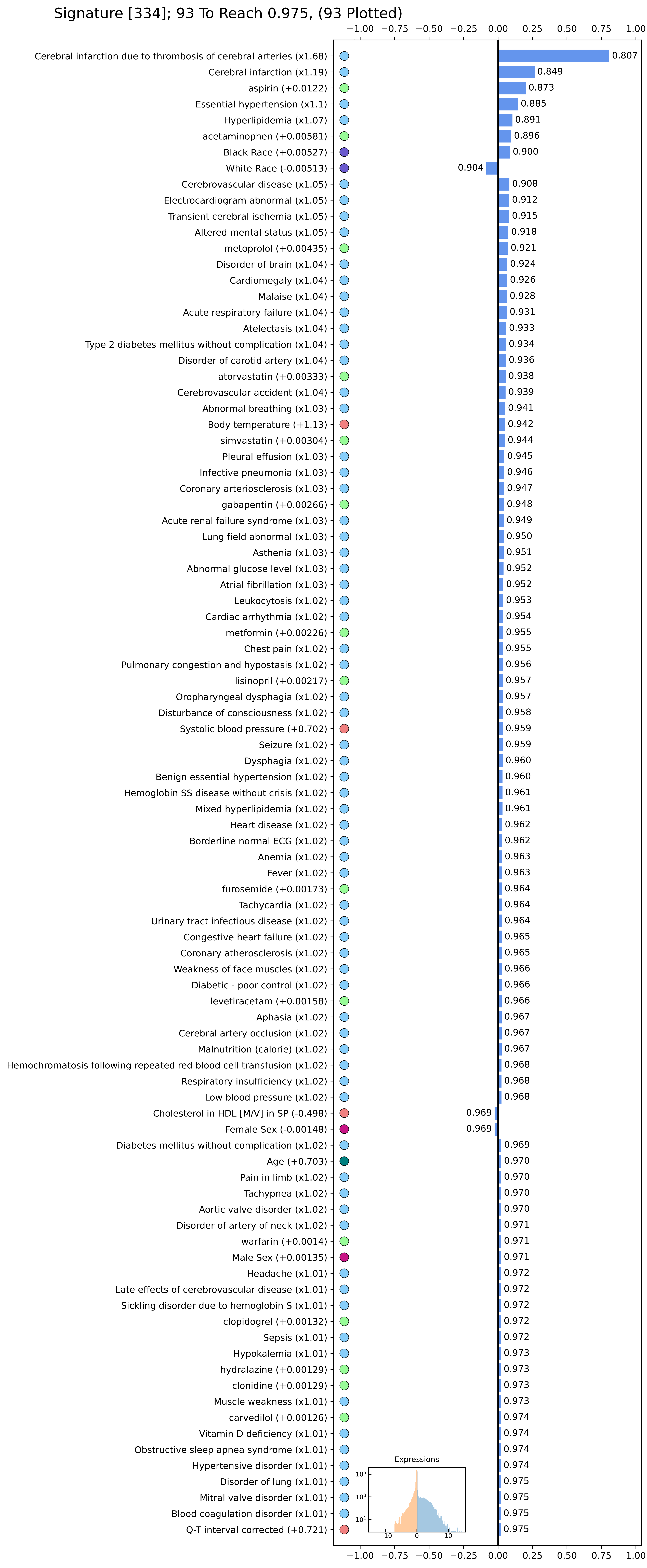

Signature [334]; 93 To Reach 0.975, (93 Plotted)
Cerebral infarction due to thrombosis of cerebral arteries (x1.68) 0.807
Cerebral infarction (x1.19) 0.849
aspirin (+0.0122) 0.873
Essential hypertension (x1.1) 0.885
Hyperlipidemia (x1.07) 0.891
acetaminophen (+0.00581) 0.896
Black Race (+0.00527) 0.900
White Race (-0.00513) 0.904
Cerebrovascular disease (x1.05) 0.908
Electrocardiogram abnormal (x1.05) 0.912
Transient cerebral ischemia (x1.05) 0.915
Altered mental status (x1.05) 0.918
metoprolol (+0.00435) 0.921
Disorder of brain (x1.04) 0.924
Cardiomegaly (x1.04) 0.926
Malaise (x1.04) 0.928
Acute respiratory failure (x1.04) 0.931
Atelectasis (x1.04) 0.933
Type 2 diabetes mellitus without complication (x1.04) 0.934
Disorder of carotid artery (x1.04) 0.936
atorvastatin (+0.00333) 0.938
Cerebrovascular accident (x1.04) 0.939
Abnormal breathing (x1.03) 0.941
Body temperature (+1.13) 0.942
simvastatin (+0.00304) 0.944
Pleural effusion (x1.03) 0.945
Infective pneumonia (x1.03) 0.946
Coronary arteriosclerosis (x1.03) 0.947
gabapentin (+0.00266) 0.948
Acute renal failure syndrome (x1.03) 0.949
Lung field abnormal (x1.03) 0.950
Asthenia (x1.03) 0.951
Abnormal glucose level (x1.03) 0.952
Atrial fibrillation (x1.03) 0.952
Leukocytosis (x1.02) 0.953
Cardiac arrhythmia (x1.02) 0.954
metformin (+0.00226) 0.955
Chest pain (x1.02) 0.955
Pulmonary congestion and hypostasis (x1.02) 0.956
lisinopril (+0.00217) 0.957
Oropharyngeal dysphagia (x1.02) 0.957
Disturbance of consciousness (x1.02) 0.958
Systolic blood pressure (+0.702) 0.959
Seizure (x1.02) 0.959
Dysphagia (x1.02) 0.960
Benign essential hypertension (x1.02) 0.960
Hemoglobin SS disease without crisis (x1.02) 0.961
Mixed hyperlipidemia (x1.02) 0.961
Heart disease (x1.02) 0.962
Borderline normal ECG (x1.02) 0.962
Anemia (x1.02) 0.963
Fever (x1.02) 0.963
furosemide (+0.00173) 0.964
Tachycardia (x1.02) 0.964
Urinary tract infectious disease (x1.02) 0.964
Congestive heart failure (x1.02) 0.965
Coronary atherosclerosis (x1.02) 0.965
Weakness of face muscles (x1.02) 0.966
Diabetic - poor control (x1.02) 0.966
levetiracetam (+0.00158) 0.966
Aphasia (x1.02) 0.967
Cerebral artery occlusion (x1.02) 0.967
Malnutrition (calorie) (x1.02) 0.967
Hemochromatosis following repeated red blood cell transfusion (x1.02) 0.968
Respiratory insufficiency (x1.02) 0.968
Low blood pressure (x1.02) 0.968
Cholesterol in HDL [M/V] in SP (-0.498) 0.969
Female Sex (-0.00148) 0.969
Diabetes mellitus without complication (x1.02) 0.969
Age (+0.703) 0.970
Pain in limb (x1.02) 0.970
Tachypnea (x1.02) 0.970
Aortic valve disorder (x1.02) 0.970
Disorder of artery of neck (x1.02) 0.971
warfarin (+0.0014) 0.971
Male Sex (+0.00135) 0.971
Headache (x1.01) 0.972
Late effects of cerebrovascular disease (x1.01) 0.972
Sickling disorder due to hemoglobin S (x1.01) 0.972
clopidogrel (+0.00132) 0.972
Sepsis (x1.01) 0.972
Hypokalemia (x1.01) 0.973
hydralazine (+0.00129) 0.973
clonidine (+0.00129) 0.973
Muscle weakness (x1.01) 0.973
carvedilol (+0.00126) 0.974
Vitamin D deficiency (x1.01) 0.974
Obstructive sleep apnea syndrome (x1.01) 0.974
Hypertensive disorder (x1.01) 0.974
Disorder of lung (x1.01) 0.975
Mitral valve disorder (x1.01) 0.975
Blood coagulation disorder (x1.01) 0.975
Q-T interval corrected (+0.721) 0.975
−1.00 −0.75 −0.50 −0.25 0.00 0.25 0.50 0.75 1.00
Expressions

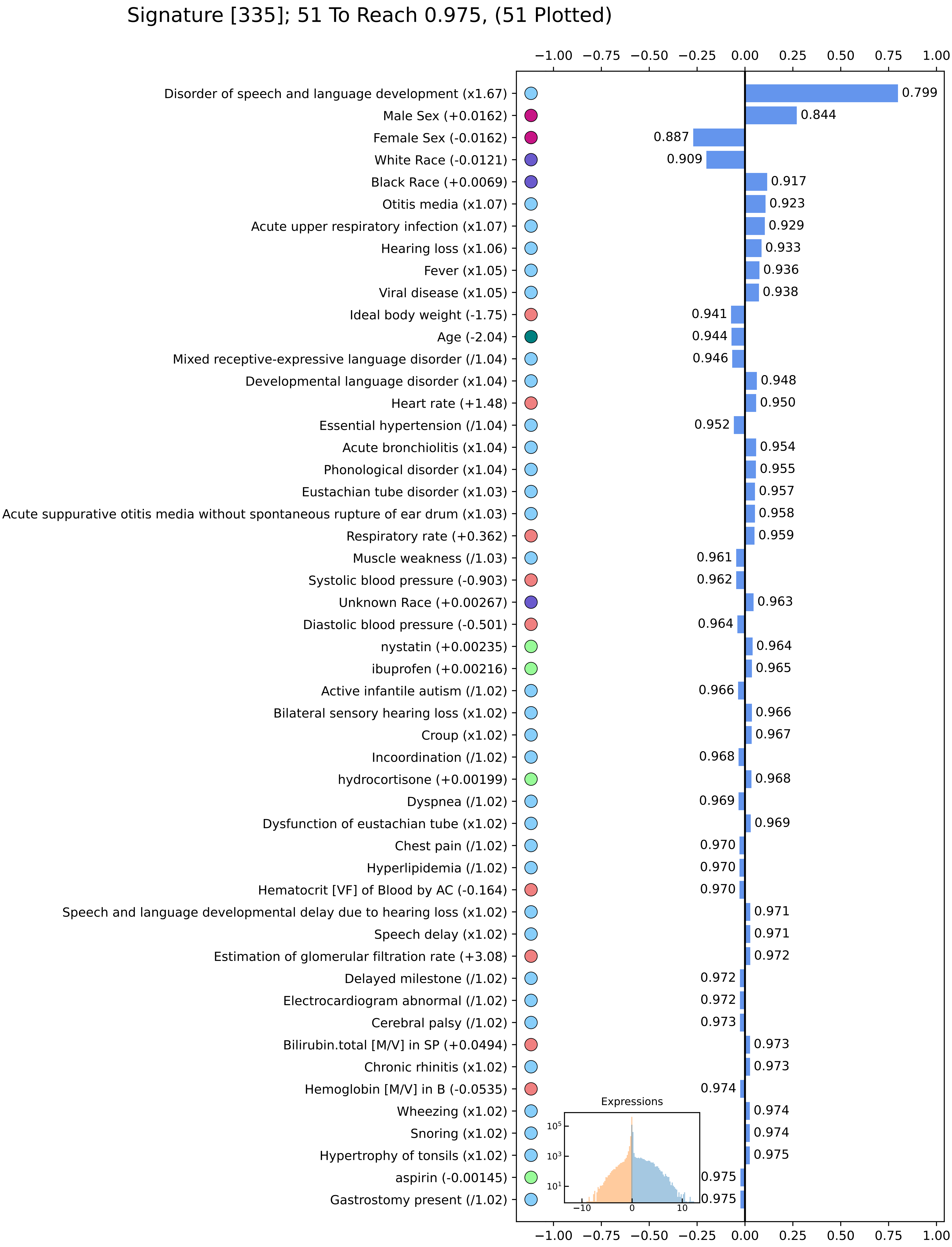

Signature [335]; 51 To Reach 0.975, (51 Plotted)
Disorder of speech and language development (x1.67) 0.799
Male Sex (+0.0162) 0.844
Female Sex (-0.0162) 0.887
White Race (-0.0121) 0.909
Black Race (+0.0069) 0.917
Otitis media (x1.07) 0.923
Acute upper respiratory infection (x1.07) 0.929
Hearing loss (x1.06) 0.933
Fever (x1.05) 0.936
Viral disease (x1.05) 0.938
Ideal body weight (-1.75) 0.941
Age (-2.04) 0.944
Mixed receptive-expressive language disorder (/1.04) 0.946
Developmental language disorder (x1.04) 0.948
Heart rate (+1.48) 0.950
Essential hypertension (/1.04) 0.952
Acute bronchiolitis (x1.04) 0.954
Phonological disorder (x1.04) 0.955
Eustachian tube disorder (x1.03) 0.957
Acute suppurative otitis media without spontaneous rupture of ear drum (x1.03) 0.958
Respiratory rate (+0.362) 0.959
Muscle weakness (/1.03) 0.961
Systolic blood pressure (-0.903) 0.962
Unknown Race (+0.00267) 0.963
Diastolic blood pressure (-0.501) 0.964
nystatin (+0.00235) 0.964
ibuprofen (+0.00216) 0.965
Active infantile autism (/1.02) 0.966
Bilateral sensory hearing loss (x1.02) 0.966
Croup (x1.02) 0.967
Incoordination (/1.02) 0.968
hydrocortisone (+0.00199) 0.968
Dyspnea (/1.02) 0.969
Dysfunction of eustachian tube (x1.02) 0.969
Chest pain (/1.02) 0.970
Hyperlipidemia (/1.02) 0.970
Hematocrit [VF] of Blood by AC (-0.164) 0.970
Speech and language developmental delay due to hearing loss (x1.02) 0.971
Speech delay (x1.02) 0.971
Estimation of glomerular filtration rate (+3.08) 0.972
Delayed milestone (/1.02) 0.972
Electrocardiogram abnormal (/1.02) 0.972
Cerebral palsy (/1.02) 0.973
Bilirubin.total [M/V] in SP (+0.0494) 0.973
Chronic rhinitis (x1.02) 0.973
Hemoglobin [M/V] in B (-0.0535) 0.974
Wheezing (x1.02) 0.974
Snoring (x1.02) 0.974
Hypertrophy of tonsils (x1.02) 0.975
aspirin (-0.00145) 0.975
Gastrostomy present (/1.02) 0.975
−1.00 −0.75 −0.50 −0.25 0.00 0.25 0.50 0.75 1.00
Expressions

Signature [336]; 4 To Reach 0.975, (10 Plotted)

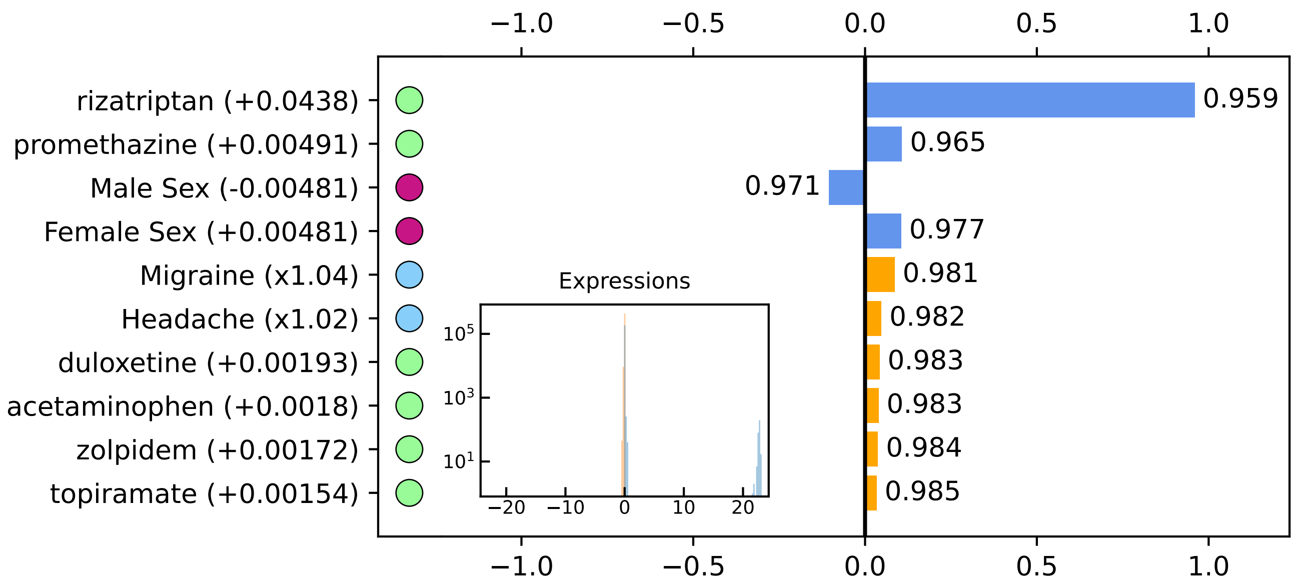

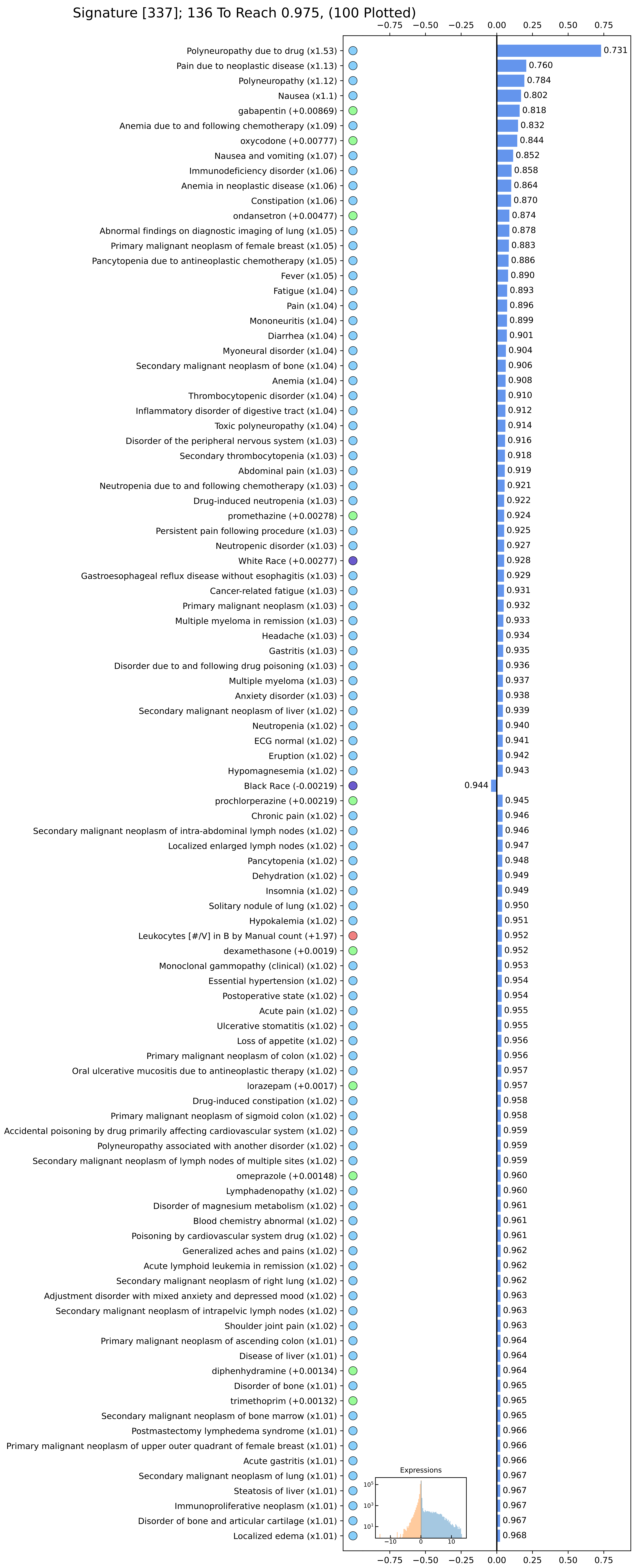

Signature [337]; 136 To Reach 0.975, (100 Plotted)
Polyneuropathy due to drug (x1.53) 0.731
Pain due to neoplastic disease (x1.13) 0.760
Polyneuropathy (x1.12) 0.784
Nausea (x1.1) 0.802
gabapentin (+0.00869) 0.818
Anemia due to and following chemotherapy (x1.09) 0.832
oxycodone (+0.00777) 0.844
Nausea and vomiting (x1.07) 0.852
Immunodeficiency disorder (x1.06) 0.858
Anemia in neoplastic disease (x1.06) 0.864
Constipation (x1.06) 0.870
ondansetron (+0.00477) 0.874
Abnormal findings on diagnostic imaging of lung (x1.05) 0.878
Primary malignant neoplasm of female breast (x1.05) 0.883
Pancytopenia due to antineoplastic chemotherapy (x1.05) 0.886
Fever (x1.05) 0.890
Fatigue (x1.04) 0.893
Pain (x1.04) 0.896
Mononeuritis (x1.04) 0.899
Diarrhea (x1.04) 0.901
Myoneural disorder (x1.04) 0.904
Secondary malignant neoplasm of bone (x1.04) 0.906
Anemia (x1.04) 0.908
Thrombocytopenic disorder (x1.04) 0.910
Inflammatory disorder of digestive tract (x1.04) 0.912
Toxic polyneuropathy (x1.04) 0.914
Disorder of the peripheral nervous system (x1.03) 0.916
Secondary thrombocytopenia (x1.03) 0.918
Abdominal pain (x1.03) 0.919
Neutropenia due to and following chemotherapy (x1.03) 0.921
Drug-induced neutropenia (x1.03) 0.922
promethazine (+0.00278) 0.924
Persistent pain following procedure (x1.03) 0.925
Neutropenic disorder (x1.03) 0.927
White Race (+0.00277) 0.928
Gastroesophageal reflux disease without esophagitis (x1.03) 0.929
Cancer-related fatigue (x1.03) 0.931
Primary malignant neoplasm (x1.03) 0.932
Multiple myeloma in remission (x1.03) 0.933
Headache (x1.03) 0.934
Gastritis (x1.03) 0.935
Disorder due to and following drug poisoning (x1.03) 0.936
Multiple myeloma (x1.03) 0.937
Anxiety disorder (x1.03) 0.938
Secondary malignant neoplasm of liver (x1.02) 0.939
Neutropenia (x1.02) 0.940
ECG normal (x1.02) 0.941
Eruption (x1.02) 0.942
Hypomagnesemia (x1.02) 0.943
Black Race (-0.00219) 0.944
prochlorperazine (+0.00219) 0.945
Chronic pain (x1.02) 0.946
Secondary malignant neoplasm of intra-abdominal lymph nodes (x1.02) 0.946
Localized enlarged lymph nodes (x1.02) 0.947
Pancytopenia (x1.02) 0.948
Dehydration (x1.02) 0.949
Insomnia (x1.02) 0.949
Solitary nodule of lung (x1.02) 0.950
Hypokalemia (x1.02) 0.951
Leukocytes [#/V] in B by Manual count (+1.97) 0.952
dexamethasone (+0.0019) 0.952
Monoclonal gammopathy (clinical) (x1.02) 0.953
Essential hypertension (x1.02) 0.954
Postoperative state (x1.02) 0.954
Acute pain (x1.02) 0.955
Ulcerative stomatitis (x1.02) 0.955
Loss of appetite (x1.02) 0.956
Primary malignant neoplasm of colon (x1.02) 0.956
Oral ulcerative mucositis due to antineoplastic therapy (x1.02) 0.957
lorazepam (+0.0017) 0.957
Drug-induced constipation (x1.02) 0.958
Primary malignant neoplasm of sigmoid colon (x1.02) 0.958
Accidental poisoning by drug primarily affecting cardiovascular system (x1.02) 0.959
Polyneuropathy associated with another disorder (x1.02) 0.959
Secondary malignant neoplasm of lymph nodes of multiple sites (x1.02) 0.959
omeprazole (+0.00148) 0.960
Lymphadenopathy (x1.02) 0.960
Disorder of magnesium metabolism (x1.02) 0.961
Blood chemistry abnormal (x1.02) 0.961
Poisoning by cardiovascular system drug (x1.02) 0.961
Generalized aches and pains (x1.02) 0.962
Acute lymphoid leukemia in remission (x1.02) 0.962
Secondary malignant neoplasm of right lung (x1.02) 0.962
Adjustment disorder with mixed anxiety and depressed mood (x1.02) 0.963
Secondary malignant neoplasm of intrapelvic lymph nodes (x1.02) 0.963
Shoulder joint pain (x1.02) 0.963
Primary malignant neoplasm of ascending colon (x1.01) 0.964
Disease of liver (x1.01) 0.964
diphenhydramine (+0.00134) 0.964
Disorder of bone (x1.01) 0.965
trimethoprim (+0.00132) 0.965
Secondary malignant neoplasm of bone marrow (x1.01) 0.965
Postmastectomy lymphedema syndrome (x1.01) 0.966
Primary malignant neoplasm of upper outer quadrant of female breast (x1.01) 0.966
Acute gastritis (x1.01) 0.966
Secondary malignant neoplasm of lung (x1.01) 0.967
Steatosis of liver (x1.01) 0.967
Immunoproliferative neoplasm (x1.01) 0.967
Disorder of bone and articular cartilage (x1.01) 0.967
Localized edema (x1.01) 0.968
−0.75 −0.50 −0.25 0.00 0.25 0.50 0.75
Expressions

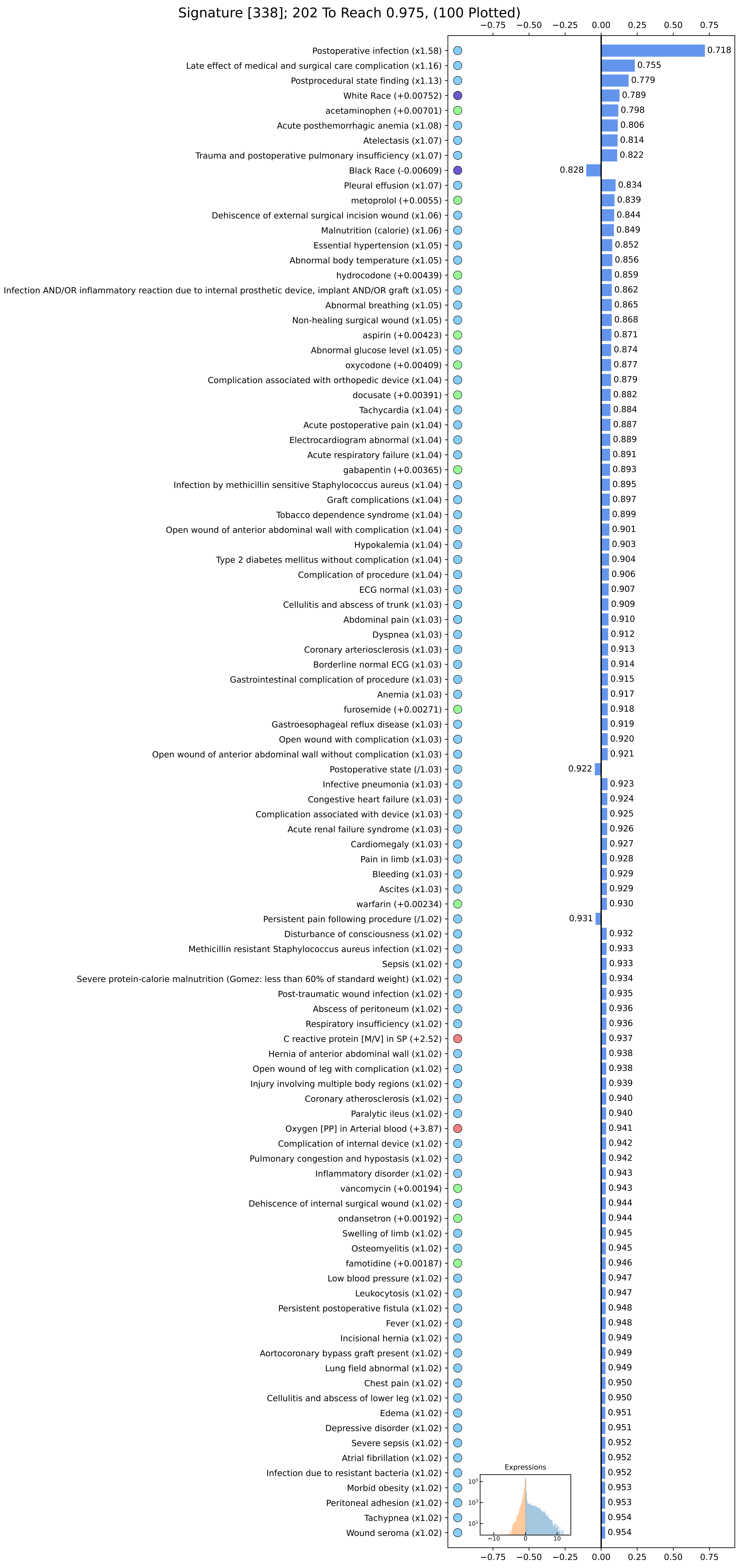

Signature [338]; 202 To Reach 0.975, (100 Plotted)
Postoperative infection (x1.58) 0.718
Late effect of medical and surgical care complication (x1.16) 0.755
Postprocedural state finding (x1.13) 0.779
White Race (+0.00752) 0.789
acetaminophen (+0.00701) 0.798
Acute posthemorrhagic anemia (x1.08) 0.806
Atelectasis (x1.07) 0.814
Trauma and postoperative pulmonary insufficiency (x1.07) 0.822
Black Race (-0.00609) 0.828
Pleural effusion (x1.07) 0.834
metoprolol (+0.0055) 0.839
Dehiscence of external surgical incision wound (x1.06) 0.844
Malnutrition (calorie) (x1.06) 0.849
Essential hypertension (x1.05) 0.852
Abnormal body temperature (x1.05) 0.856
hydrocodone (+0.00439) 0.859
Infection AND/OR inflammatory reaction due to internal prosthetic device, implant AND/OR graft (x1.05) 0.862
Abnormal breathing (x1.05) 0.865
Non-healing surgical wound (x1.05) 0.868
aspirin (+0.00423) 0.871
Abnormal glucose level (x1.05) 0.874
oxycodone (+0.00409) 0.877
Complication associated with orthopedic device (x1.04) 0.879
docusate (+0.00391) 0.882
Tachycardia (x1.04) 0.884
Acute postoperative pain (x1.04) 0.887
Electrocardiogram abnormal (x1.04) 0.889
Acute respiratory failure (x1.04) 0.891
gabapentin (+0.00365) 0.893
Infection by methicillin sensitive Staphylococcus aureus (x1.04) 0.895
Graft complications (x1.04) 0.897
Tobacco dependence syndrome (x1.04) 0.899
Open wound of anterior abdominal wall with complication (x1.04) 0.901
Hypokalemia (x1.04) 0.903
Type 2 diabetes mellitus without complication (x1.04) 0.904
Complication of procedure (x1.04) 0.906
ECG normal (x1.03) 0.907
Cellulitis and abscess of trunk (x1.03) 0.909
Abdominal pain (x1.03) 0.910
Dyspnea (x1.03) 0.912
Coronary arteriosclerosis (x1.03) 0.913
Borderline normal ECG (x1.03) 0.914
Gastrointestinal complication of procedure (x1.03) 0.915
Anemia (x1.03) 0.917
furosemide (+0.00271) 0.918
Gastroesophageal reflux disease (x1.03) 0.919
Open wound with complication (x1.03) 0.920
Open wound of anterior abdominal wall without complication (x1.03) 0.921
Postoperative state (/1.03) 0.922
Infective pneumonia (x1.03) 0.923
Congestive heart failure (x1.03) 0.924
Complication associated with device (x1.03) 0.925
Acute renal failure syndrome (x1.03) 0.926
Cardiomegaly (x1.03) 0.927
Pain in limb (x1.03) 0.928
Bleeding (x1.03) 0.929
Ascites (x1.03) 0.929
warfarin (+0.00234) 0.930
Persistent pain following procedure (/1.02) 0.931
Disturbance of consciousness (x1.02) 0.932
Methicillin resistant Staphylococcus aureus infection (x1.02) 0.933
Sepsis (x1.02) 0.933
Severe protein-calorie malnutrition (Gomez: less than 60% of standard weight) (x1.02) 0.934
Post-traumatic wound infection (x1.02) 0.935
Abscess of peritoneum (x1.02) 0.936
Respiratory insufficiency (x1.02) 0.936
C reactive protein [M/V] in SP (+2.52) 0.937
Hernia of anterior abdominal wall (x1.02) 0.938
Open wound of leg with complication (x1.02) 0.938
Injury involving multiple body regions (x1.02) 0.939
Coronary atherosclerosis (x1.02) 0.940
Paralytic ileus (x1.02) 0.940
Oxygen [PP] in Arterial blood (+3.87) 0.941
Complication of internal device (x1.02) 0.942
Pulmonary congestion and hypostasis (x1.02) 0.942
Inflammatory disorder (x1.02) 0.943
vancomycin (+0.00194) 0.943
Dehiscence of internal surgical wound (x1.02) 0.944
ondansetron (+0.00192) 0.944
Swelling of limb (x1.02) 0.945
Osteomyelitis (x1.02) 0.945
famotidine (+0.00187) 0.946
Low blood pressure (x1.02) 0.947
Leukocytosis (x1.02) 0.947
Persistent postoperative fistula (x1.02) 0.948
Fever (x1.02) 0.948
Incisional hernia (x1.02) 0.949
Aortocoronary bypass graft present (x1.02) 0.949
Lung field abnormal (x1.02) 0.949
Chest pain (x1.02) 0.950
Cellulitis and abscess of lower leg (x1.02) 0.950
Edema (x1.02) 0.951
Depressive disorder (x1.02) 0.951
Severe sepsis (x1.02) 0.952
Atrial fibrillation (x1.02) 0.952
Infection due to resistant bacteria (x1.02) 0.952
Morbid obesity (x1.02) 0.953
Peritoneal adhesion (x1.02) 0.953
Tachypnea (x1.02) 0.954
Wound seroma (x1.02) 0.954
−0.75 −0.50 −0.25 0.00 0.25 0.50 0.75
Expressions

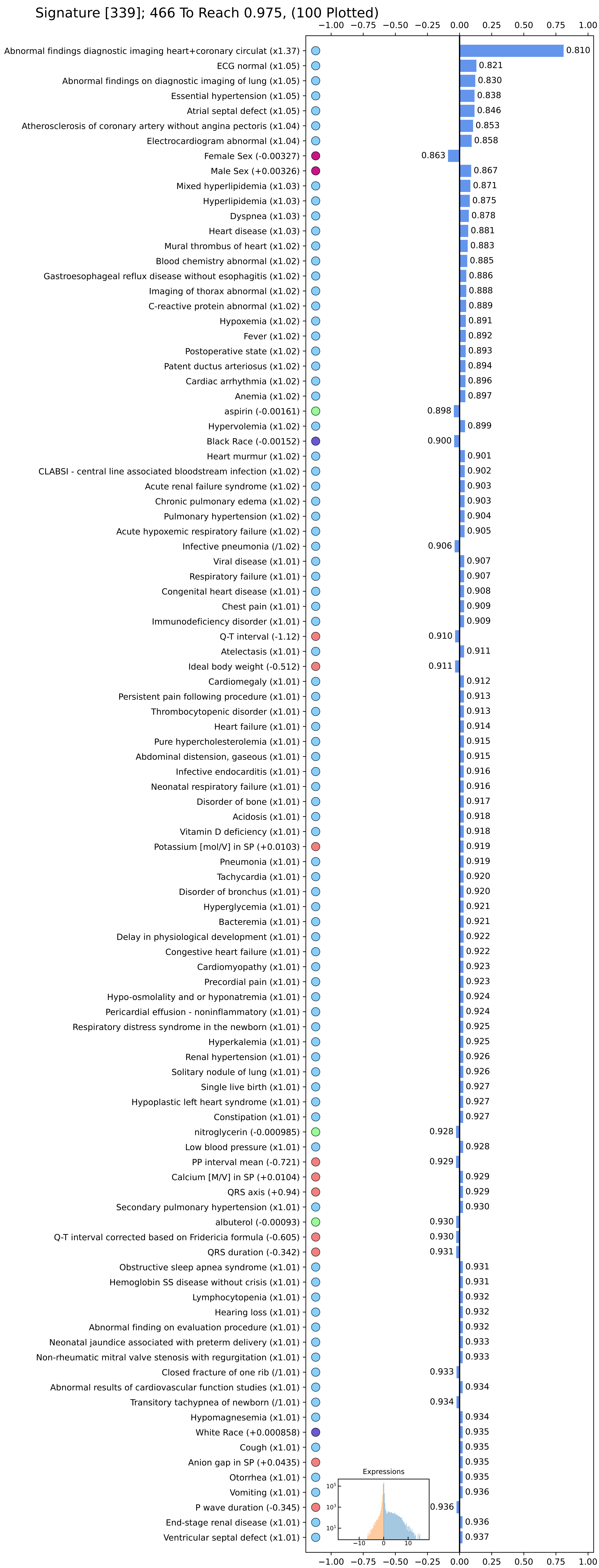

Signature [339]; 466 To Reach 0.975, (100 Plotted)
Abnormal findings diagnostic imaging heart+coronary circulat (x1.37) 0.810
ECG normal (x1.05) 0.821
Abnormal findings on diagnostic imaging of lung (x1.05) 0.830
Essential hypertension (x1.05) 0.838
Atrial septal defect (x1.05) 0.846
Atherosclerosis of coronary artery without angina pectoris (x1.04) 0.853
Electrocardiogram abnormal (x1.04) 0.858
Female Sex (-0.00327) 0.863
Male Sex (+0.00326) 0.867
Mixed hyperlipidemia (x1.03) 0.871
Hyperlipidemia (x1.03) 0.875
Dyspnea (x1.03) 0.878
Heart disease (x1.03) 0.881
Mural thrombus of heart (x1.02) 0.883
Blood chemistry abnormal (x1.02) 0.885
Gastroesophageal reflux disease without esophagitis (x1.02) 0.886
Imaging of thorax abnormal (x1.02) 0.888
C-reactive protein abnormal (x1.02) 0.889
Hypoxemia (x1.02) 0.891
Fever (x1.02) 0.892
Postoperative state (x1.02) 0.893
Patent ductus arteriosus (x1.02) 0.894
Cardiac arrhythmia (x1.02) 0.896
Anemia (x1.02) 0.897
aspirin (-0.00161) 0.898
Hypervolemia (x1.02) 0.899
Black Race (-0.00152) 0.900
Heart murmur (x1.02) 0.901
CLABSI - central line associated bloodstream infection (x1.02) 0.902
Acute renal failure syndrome (x1.02) 0.903
Chronic pulmonary edema (x1.02) 0.903
Pulmonary hypertension (x1.02) 0.904
Acute hypoxemic respiratory failure (x1.02) 0.905
Infective pneumonia (/1.02) 0.906
Viral disease (x1.01) 0.907
Respiratory failure (x1.01) 0.907
Congenital heart disease (x1.01) 0.908
Chest pain (x1.01) 0.909
Immunodeficiency disorder (x1.01) 0.909
Q-T interval (-1.12) 0.910
Atelectasis (x1.01) 0.911
Ideal body weight (-0.512) 0.911
Cardiomegaly (x1.01) 0.912
Persistent pain following procedure (x1.01) 0.913
Thrombocytopenic disorder (x1.01) 0.913
Heart failure (x1.01) 0.914
Pure hypercholesterolemia (x1.01) 0.915
Abdominal distension, gaseous (x1.01) 0.915
Infective endocarditis (x1.01) 0.916
Neonatal respiratory failure (x1.01) 0.916
Disorder of bone (x1.01) 0.917
Acidosis (x1.01) 0.918
Vitamin D deficiency (x1.01) 0.918
Potassium [mol/V] in SP (+0.0103) 0.919
Pneumonia (x1.01) 0.919
Tachycardia (x1.01) 0.920
Disorder of bronchus (x1.01) 0.920
Hyperglycemia (x1.01) 0.921
Bacteremia (x1.01) 0.921
Delay in physiological development (x1.01) 0.922
Congestive heart failure (x1.01) 0.922
Cardiomyopathy (x1.01) 0.923
Precordial pain (x1.01) 0.923
Hypo-osmolality and or hyponatremia (x1.01) 0.924
Pericardial effusion - noninflammatory (x1.01) 0.924
Respiratory distress syndrome in the newborn (x1.01) 0.925
Hyperkalemia (x1.01) 0.925
Renal hypertension (x1.01) 0.926
Solitary nodule of lung (x1.01) 0.926
Single live birth (x1.01) 0.927
Hypoplastic left heart syndrome (x1.01) 0.927
Constipation (x1.01) 0.927
nitroglycerin (-0.000985) 0.928
Low blood pressure (x1.01) 0.928
PP interval mean (-0.721) 0.929
Calcium [M/V] in SP (+0.0104) 0.929
QRS axis (+0.94) 0.929
Secondary pulmonary hypertension (x1.01) 0.930
albuterol (-0.00093) 0.930
Q-T interval corrected based on Fridericia formula (-0.605) 0.930
QRS duration (-0.342) 0.931
Obstructive sleep apnea syndrome (x1.01) 0.931
Hemoglobin SS disease without crisis (x1.01) 0.931
Lymphocytopenia (x1.01) 0.932
Hearing loss (x1.01) 0.932
Abnormal finding on evaluation procedure (x1.01) 0.932
Neonatal jaundice associated with preterm delivery (x1.01) 0.933
Non-rheumatic mitral valve stenosis with regurgitation (x1.01) 0.933
Closed fracture of one rib (/1.01) 0.933
Abnormal results of cardiovascular function studies (x1.01) 0.934
Transitory tachypnea of newborn (/1.01) 0.934
Hypomagnesemia (x1.01) 0.934
White Race (+0.000858) 0.935
Cough (x1.01) 0.935
Anion gap in SP (+0.0435) 0.935
Otorrhea (x1.01) 0.935
Vomiting (x1.01) 0.936
P wave duration (-0.345) 0.936
End-stage renal disease (x1.01) 0.936
Ventricular septal defect (x1.01) 0.937
−1.00 −0.75 −0.50 −0.25 0.00 0.25 0.50 0.75 1.00
Expressions

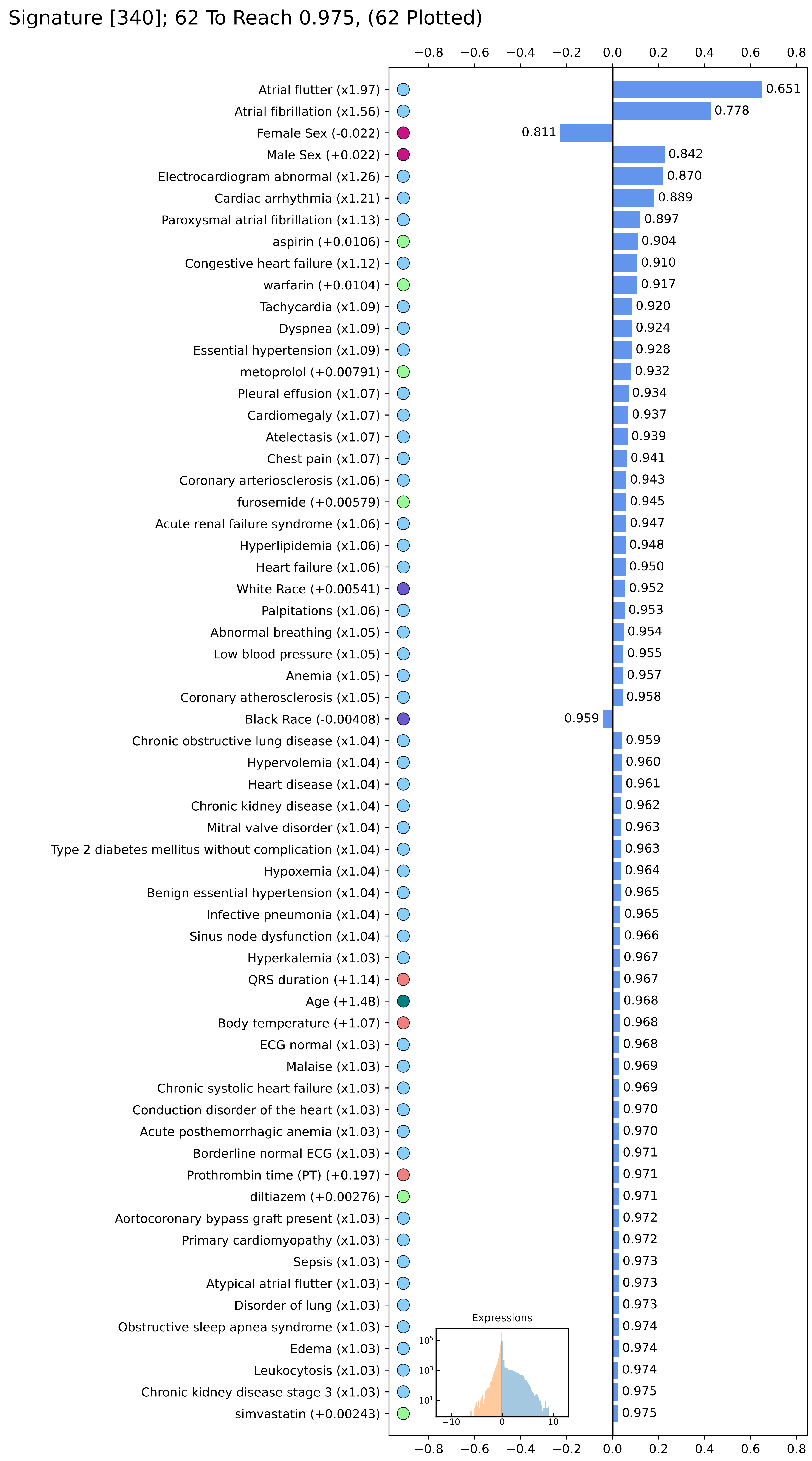

Signature [340]; 62 To Reach 0.975, (62 Plotted)
Atrial flutter (x1.97) 0.651
Atrial fibrillation (x1.56) 0.778
Female Sex (-0.022) 0.811
Male Sex (+0.022) 0.842
Electrocardiogram abnormal (x1.26) 0.870
Cardiac arrhythmia (x1.21) 0.889
Paroxysmal atrial fibrillation (x1.13) 0.897
aspirin (+0.0106) 0.904
Congestive heart failure (x1.12) 0.910
warfarin (+0.0104) 0.917
Tachycardia (x1.09) 0.920
Dyspnea (x1.09) 0.924
Essential hypertension (x1.09) 0.928
metoprolol (+0.00791) 0.932
Pleural effusion (x1.07) 0.934
Cardiomegaly (x1.07) 0.937
Atelectasis (x1.07) 0.939
Chest pain (x1.07) 0.941
Coronary arteriosclerosis (x1.06) 0.943
furosemide (+0.00579) 0.945
Acute renal failure syndrome (x1.06) 0.947
Hyperlipidemia (x1.06) 0.948
Heart failure (x1.06) 0.950
White Race (+0.00541) 0.952
Palpitations (x1.06) 0.953
Abnormal breathing (x1.05) 0.954
Low blood pressure (x1.05) 0.955
Anemia (x1.05) 0.957
Coronary atherosclerosis (x1.05) 0.958
Black Race (-0.00408) 0.959
Chronic obstructive lung disease (x1.04) 0.959
Hypervolemia (x1.04) 0.960
Heart disease (x1.04) 0.961
Chronic kidney disease (x1.04) 0.962
Mitral valve disorder (x1.04) 0.963
Type 2 diabetes mellitus without complication (x1.04) 0.963
Hypoxemia (x1.04) 0.964
Benign essential hypertension (x1.04) 0.965
Infective pneumonia (x1.04) 0.965
Sinus node dysfunction (x1.04) 0.966
Hyperkalemia (x1.03) 0.967
QRS duration (+1.14) 0.967
Age (+1.48) 0.968
Body temperature (+1.07) 0.968
ECG normal (x1.03) 0.968
Malaise (x1.03) 0.969
Chronic systolic heart failure (x1.03) 0.969
Conduction disorder of the heart (x1.03) 0.970
Acute posthemorrhagic anemia (x1.03) 0.970
Borderline normal ECG (x1.03) 0.971
Prothrombin time (PT) (+0.197) 0.971
diltiazem (+0.00276) 0.971
Aortocoronary bypass graft present (x1.03) 0.972
Primary cardiomyopathy (x1.03) 0.972
Sepsis (x1.03) 0.973
Atypical atrial flutter (x1.03) 0.973
Disorder of lung (x1.03) 0.973
Obstructive sleep apnea syndrome (x1.03) 0.974
Edema (x1.03) 0.974
Leukocytosis (x1.03) 0.974
Chronic kidney disease stage 3 (x1.03) 0.975
simvastatin (+0.00243) 0.975
−0.8 −0.6 −0.4 −0.2 0.0 0.2 0.4 0.6 0.8
Expressions
10^5
10^3
10^1
−10 0 10

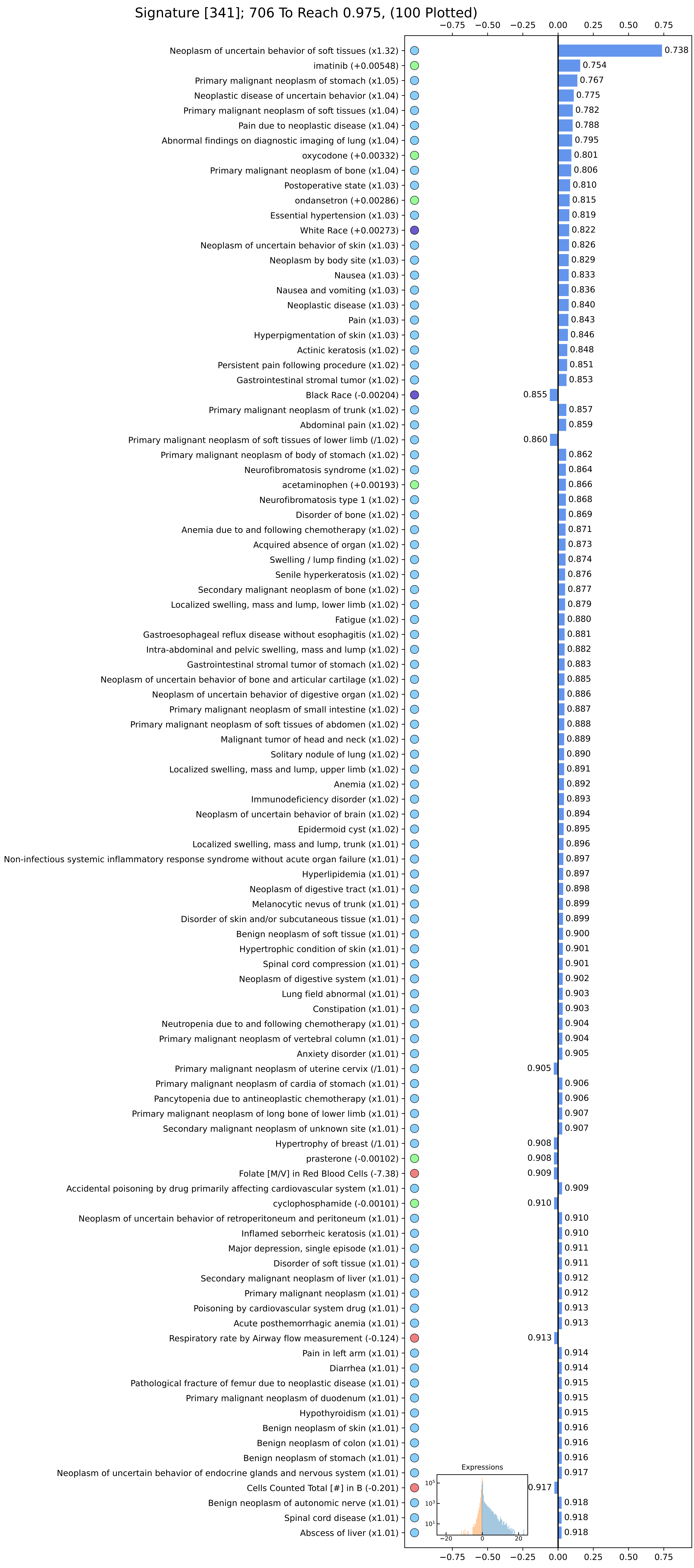

Signature [341]; 706 To Reach 0.975, (100 Plotted)
Neoplasm of uncertain behavior of soft tissues (x1.32) 0.738
imatinib (+0.00548) 0.754
Primary malignant neoplasm of stomach (x1.05) 0.767
Neoplastic disease of uncertain behavior (x1.04) 0.775
Primary malignant neoplasm of soft tissues (x1.04) 0.782
Pain due to neoplastic disease (x1.04) 0.788
Abnormal findings on diagnostic imaging of lung (x1.04) 0.795
oxycodone (+0.00332) 0.801
Primary malignant neoplasm of bone (x1.04) 0.806
Postoperative state (x1.03) 0.810
ondansetron (+0.00286) 0.815
Essential hypertension (x1.03) 0.819
White Race (+0.00273) 0.822
Neoplasm of uncertain behavior of skin (x1.03) 0.826
Neoplasm by body site (x1.03) 0.829
Nausea (x1.03) 0.833
Nausea and vomiting (x1.03) 0.836
Neoplastic disease (x1.03) 0.840
Pain (x1.03) 0.843
Hyperpigmentation of skin (x1.03) 0.846
Actinic keratosis (x1.02) 0.848
Persistent pain following procedure (x1.02) 0.851
Gastrointestinal stromal tumor (x1.02) 0.853
Black Race (-0.00204) 0.855
Primary malignant neoplasm of trunk (x1.02) 0.857
Abdominal pain (x1.02) 0.859
Primary malignant neoplasm of soft tissues of lower limb (/1.02) 0.860
Primary malignant neoplasm of body of stomach (x1.02) 0.862
Neurofibromatosis syndrome (x1.02) 0.864
acetaminophen (+0.00193) 0.866
Neurofibromatosis type 1 (x1.02) 0.868
Disorder of bone (x1.02) 0.869
Anemia due to and following chemotherapy (x1.02) 0.871
Acquired absence of organ (x1.02) 0.873
Swelling / lump finding (x1.02) 0.874
Senile hyperkeratosis (x1.02) 0.876
Secondary malignant neoplasm of bone (x1.02) 0.877
Localized swelling, mass and lump, lower limb (x1.02) 0.879
Fatigue (x1.02) 0.880
Gastroesophageal reflux disease without esophagitis (x1.02) 0.881
Intra-abdominal and pelvic swelling, mass and lump (x1.02) 0.882
Gastrointestinal stromal tumor of stomach (x1.02) 0.883
Neoplasm of uncertain behavior of bone and articular cartilage (x1.02) 0.885
Neoplasm of uncertain behavior of digestive organ (x1.02) 0.886
Primary malignant neoplasm of small intestine (x1.02) 0.887
Primary malignant neoplasm of soft tissues of abdomen (x1.02) 0.888
Malignant tumor of head and neck (x1.02) 0.889
Solitary nodule of lung (x1.02) 0.890
Localized swelling, mass and lump, upper limb (x1.02) 0.891
Anemia (x1.02) 0.892
Immunodeficiency disorder (x1.02) 0.893
Neoplasm of uncertain behavior of brain (x1.02) 0.894
Epidermoid cyst (x1.02) 0.895
Localized swelling, mass and lump, trunk (x1.01) 0.896
Non-infectious systemic inflammatory response syndrome without acute organ failure (x1.01) 0.897
Hyperlipidemia (x1.01) 0.897
Neoplasm of digestive tract (x1.01) 0.898
Melanocytic nevus of trunk (x1.01) 0.899
Disorder of skin and/or subcutaneous tissue (x1.01) 0.899
Benign neoplasm of soft tissue (x1.01) 0.900
Hypertrophic condition of skin (x1.01) 0.901
Spinal cord compression (x1.01) 0.901
Neoplasm of digestive system (x1.01) 0.902
Lung field abnormal (x1.01) 0.903
Constipation (x1.01) 0.903
Neutropenia due to and following chemotherapy (x1.01) 0.904
Primary malignant neoplasm of vertebral column (x1.01) 0.904
Anxiety disorder (x1.01) 0.905
Primary malignant neoplasm of uterine cervix (/1.01) 0.905
Primary malignant neoplasm of cardia of stomach (x1.01) 0.906
Pancytopenia due to antineoplastic chemotherapy (x1.01) 0.906
Primary malignant neoplasm of long bone of lower limb (x1.01) 0.907
Secondary malignant neoplasm of unknown site (x1.01) 0.907
Hypertrophy of breast (/1.01) 0.908
prasterone (-0.00102) 0.908
Folate [M/V] in Red Blood Cells (-7.38) 0.909
Accidental poisoning by drug primarily affecting cardiovascular system (x1.01) 0.909
cyclophosphamide (-0.00101) 0.910
Neoplasm of uncertain behavior of retroperitoneum and peritoneum (x1.01) 0.910
Inflamed seborrheic keratosis (x1.01) 0.910
Major depression, single episode (x1.01) 0.911
Disorder of soft tissue (x1.01) 0.911
Secondary malignant neoplasm of liver (x1.01) 0.912
Primary malignant neoplasm (x1.01) 0.912
Poisoning by cardiovascular system drug (x1.01) 0.913
Acute posthemorrhagic anemia (x1.01) 0.913
Respiratory rate by Airway flow measurement (-0.124) 0.913
Pain in left arm (x1.01) 0.914
Diarrhea (x1.01) 0.914
Pathological fracture of femur due to neoplastic disease (x1.01) 0.915
Primary malignant neoplasm of duodenum (x1.01) 0.915
Hypothyroidism (x1.01) 0.915
Benign neoplasm of skin (x1.01) 0.916
Benign neoplasm of colon (x1.01) 0.916
Benign neoplasm of stomach (x1.01) 0.916
Neoplasm of uncertain behavior of endocrine glands and nervous system (x1.01) 0.917
Cells Counted Total [#] in B (-0.201) 0.917
Benign neoplasm of autonomic nerve (x1.01) 0.918
Spinal cord disease (x1.01) 0.918
Abscess of liver (x1.01) 0.918
−0.75 −0.50 −0.25 0.00 0.25 0.50 0.75
Expressions

Signature [342]; 1 To Reach 0.975, (10 Plotted)

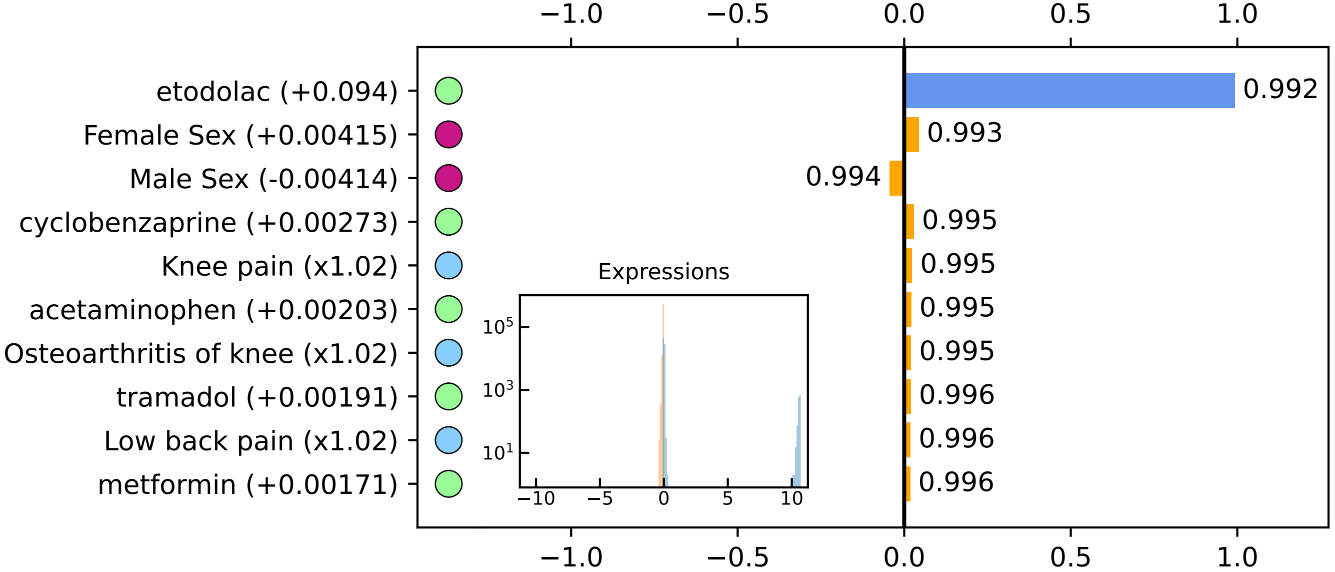

Signature [343]; 2 To Reach 0.975, (10 Plotted)

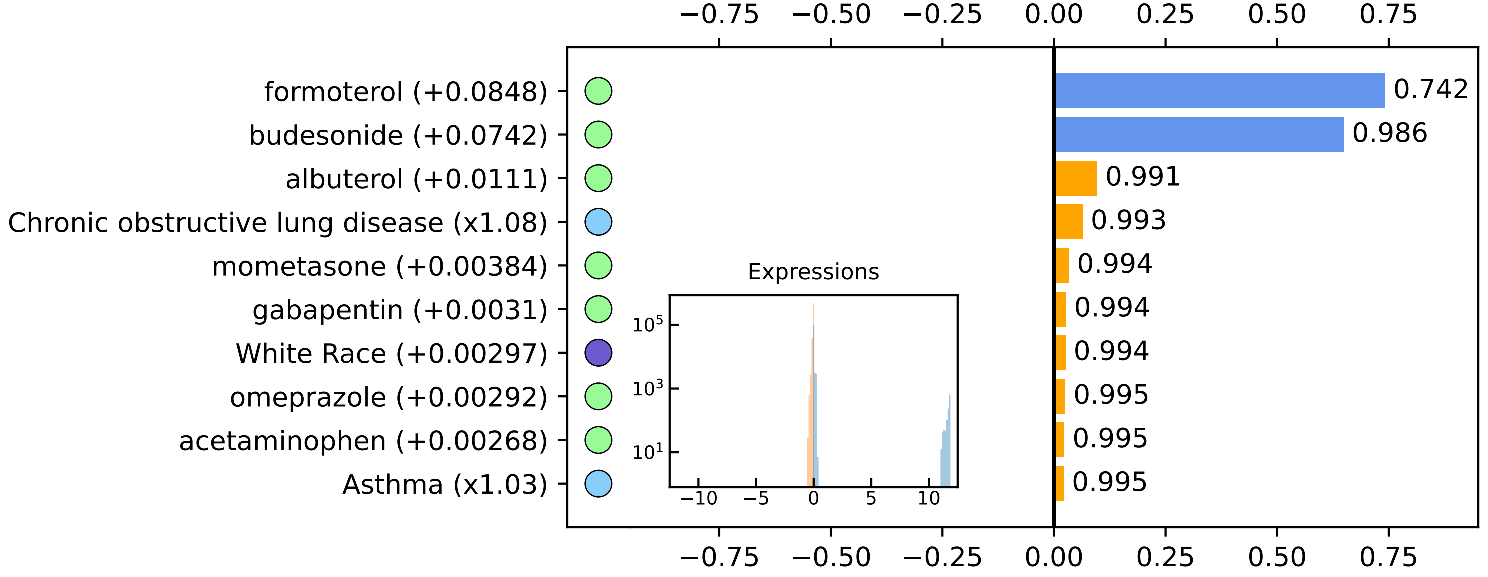

Signature [344]; 1 To Reach 0.975, (10 Plotted)

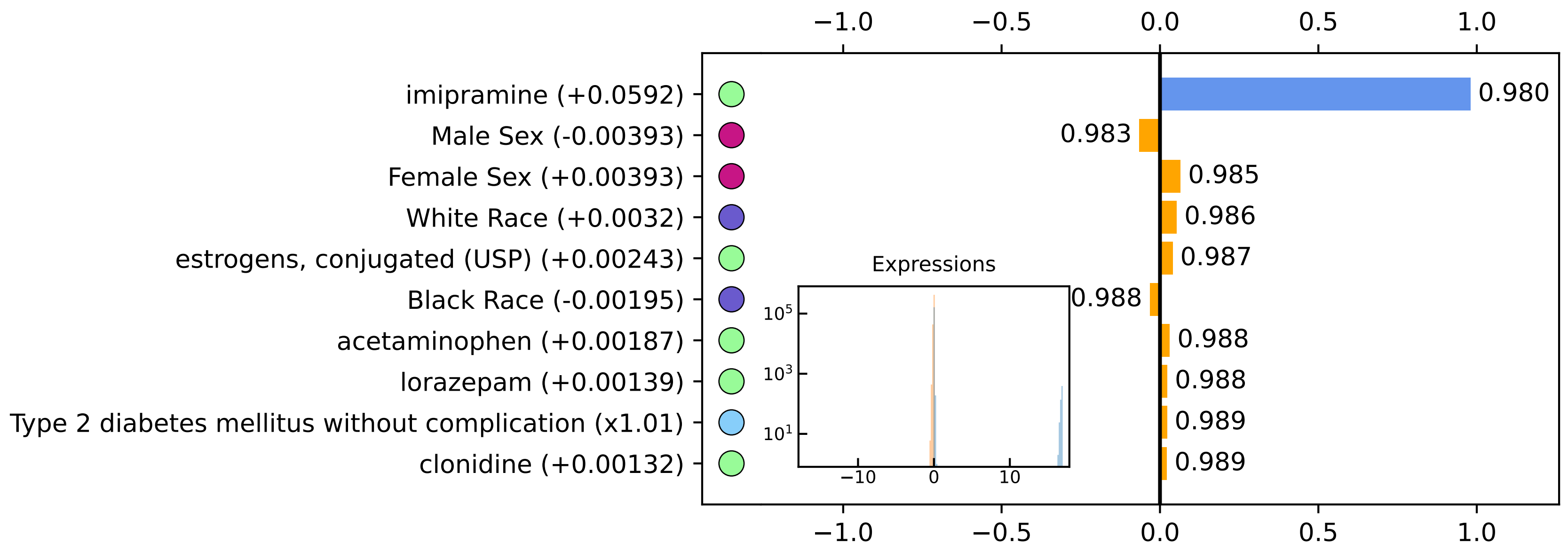

Signature [345]; 4 To Reach 0.975, (10 Plotted)

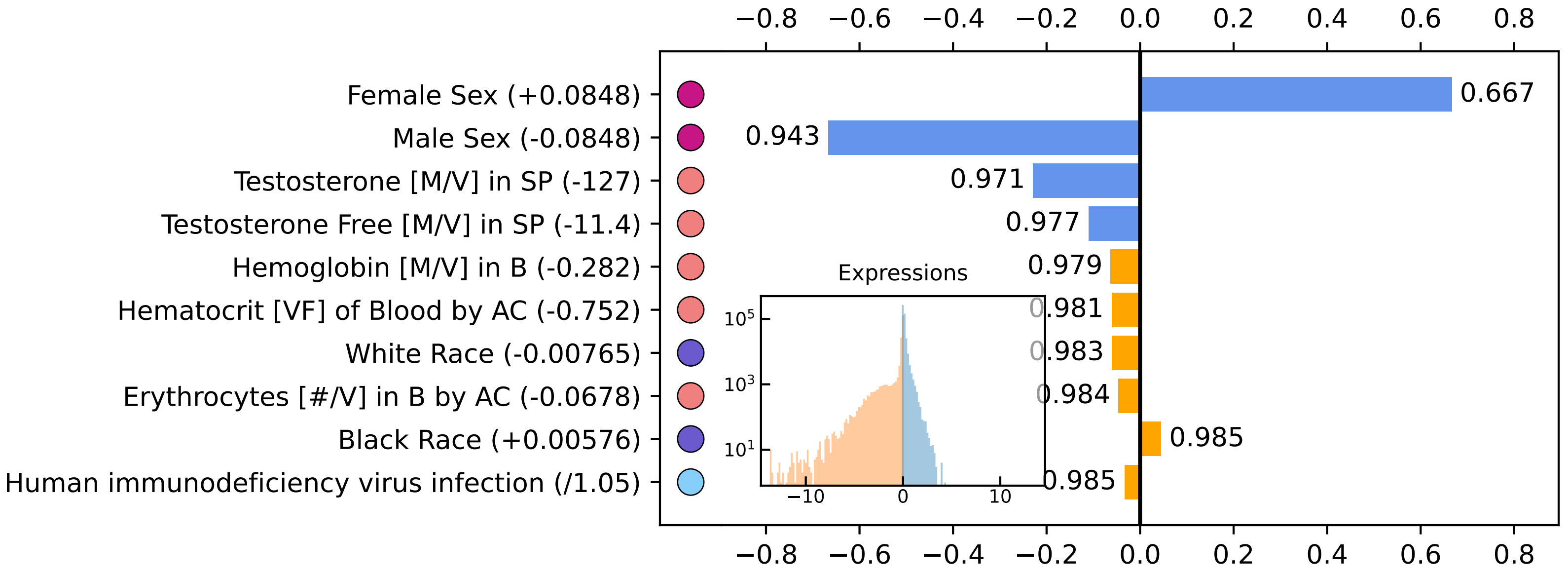

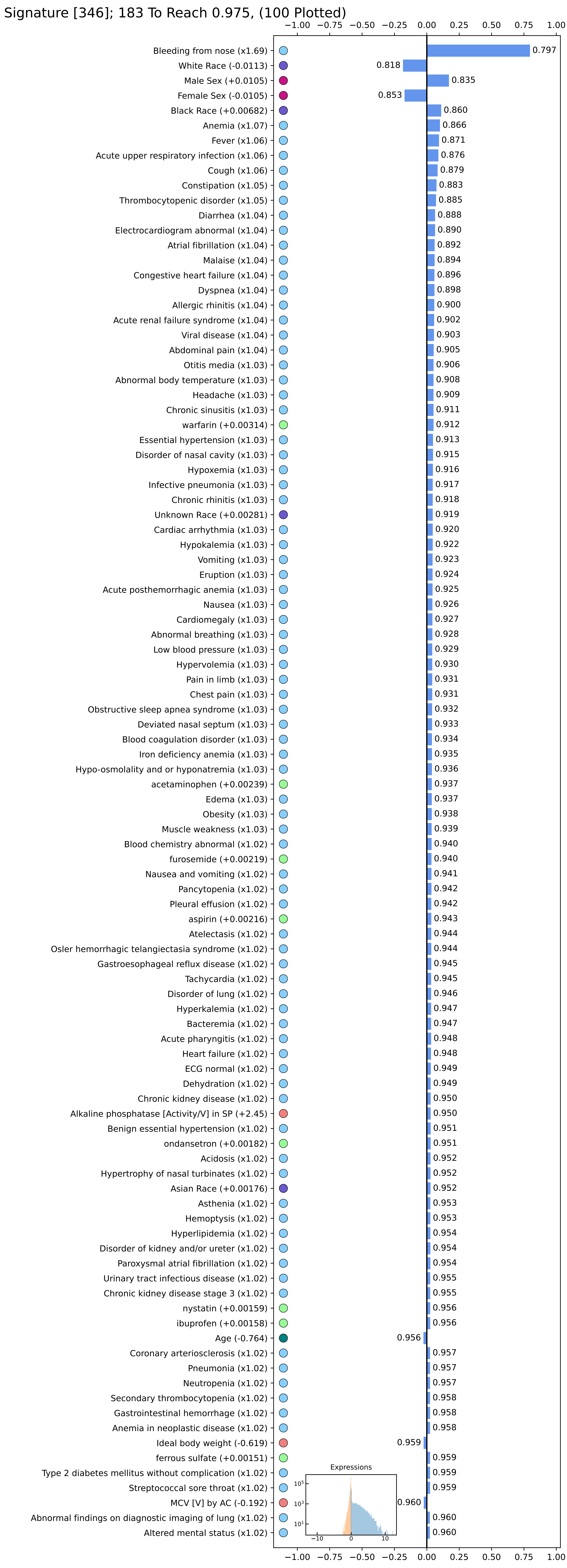

Signature [346]; 183 To Reach 0.975, (100 Plotted)
Bleeding from nose (x1.69) 0.797
White Race (-0.0113) 0.818
Male Sex (+0.0105) 0.835
Female Sex (-0.0105) 0.853
Black Race (+0.00682) 0.860
Anemia (x1.07) 0.866
Fever (x1.06) 0.871
Acute upper respiratory infection (x1.06) 0.876
Cough (x1.06) 0.879
Constipation (x1.05) 0.883
Thrombocytopenic disorder (x1.05) 0.885
Diarrhea (x1.04) 0.888
Electrocardiogram abnormal (x1.04) 0.890
Atrial fibrillation (x1.04) 0.892
Malaise (x1.04) 0.894
Congestive heart failure (x1.04) 0.896
Dyspnea (x1.04) 0.898
Allergic rhinitis (x1.04) 0.900
Acute renal failure syndrome (x1.04) 0.902
Viral disease (x1.04) 0.903
Abdominal pain (x1.04) 0.905
Otitis media (x1.03) 0.906
Abnormal body temperature (x1.03) 0.908
Headache (x1.03) 0.909
Chronic sinusitis (x1.03) 0.911
warfarin (+0.00314) 0.912
Essential hypertension (x1.03) 0.913
Disorder of nasal cavity (x1.03) 0.915
Hypoxemia (x1.03) 0.916
Infective pneumonia (x1.03) 0.917
Chronic rhinitis (x1.03) 0.918
Unknown Race (+0.00281) 0.919
Cardiac arrhythmia (x1.03) 0.920
Hypokalemia (x1.03) 0.922
Vomiting (x1.03) 0.923
Eruption (x1.03) 0.924
Acute posthemorrhagic anemia (x1.03) 0.925
Nausea (x1.03) 0.926
Cardiomegaly (x1.03) 0.927
Abnormal breathing (x1.03) 0.928
Low blood pressure (x1.03) 0.929
Hypervolemia (x1.03) 0.930
Pain in limb (x1.03) 0.931
Chest pain (x1.03) 0.931
Obstructive sleep apnea syndrome (x1.03) 0.932
Deviated nasal septum (x1.03) 0.933
Blood coagulation disorder (x1.03) 0.934
Iron deficiency anemia (x1.03) 0.935
Hypo-osmolality and or hyponatremia (x1.03) 0.936
acetaminophen (+0.00239) 0.937
Edema (x1.03) 0.937
Obesity (x1.03) 0.938
Muscle weakness (x1.03) 0.939
Blood chemistry abnormal (x1.02) 0.940
furosemide (+0.00219) 0.940
Nausea and vomiting (x1.02) 0.941
Pancytopenia (x1.02) 0.942
Pleural effusion (x1.02) 0.942
aspirin (+0.00216) 0.943
Atelectasis (x1.02) 0.944
Osler hemorrhagic telangiectasia syndrome (x1.02) 0.944
Gastroesophageal reflux disease (x1.02) 0.945
Tachycardia (x1.02) 0.945
Disorder of lung (x1.02) 0.946
Hyperkalemia (x1.02) 0.947
Bacteremia (x1.02) 0.947
Acute pharyngitis (x1.02) 0.948
Heart failure (x1.02) 0.948
ECG normal (x1.02) 0.949
Dehydration (x1.02) 0.949
Chronic kidney disease (x1.02) 0.950
Alkaline phosphatase [Activity/V] in SP (+2.45) 0.950
Benign essential hypertension (x1.02) 0.951
ondansetron (+0.00182) 0.951
Acidosis (x1.02) 0.952
Hypertrophy of nasal turbinates (x1.02) 0.952
Asian Race (+0.00176) 0.952
Asthenia (x1.02) 0.953
Hemoptysis (x1.02) 0.953
Hyperlipidemia (x1.02) 0.954
Disorder of kidney and/or ureter (x1.02) 0.954
Paroxysmal atrial fibrillation (x1.02) 0.954
Urinary tract infectious disease (x1.02) 0.955
Chronic kidney disease stage 3 (x1.02) 0.955
nystatin (+0.00159) 0.956
ibuprofen (+0.00158) 0.956
Age (-0.764) 0.956
Coronary arteriosclerosis (x1.02) 0.957
Pneumonia (x1.02) 0.957
Neutropenia (x1.02) 0.957
Secondary thrombocytopenia (x1.02) 0.958
Gastrointestinal hemorrhage (x1.02) 0.958
Anemia in neoplastic disease (x1.02) 0.958
Ideal body weight (-0.619) 0.959
ferrous sulfate (+0.00151) 0.959
Type 2 diabetes mellitus without complication (x1.02) 0.959
Streptococcal sore throat (x1.02) 0.959
MCV [V] by AC (-0.192) 0.960
Abnormal findings on diagnostic imaging of lung (x1.02) 0.960
Altered mental status (x1.02) 0.960
−1.00 −0.75 −0.50 −0.25 0.00 0.25 0.50 0.75 1.00
Expressions

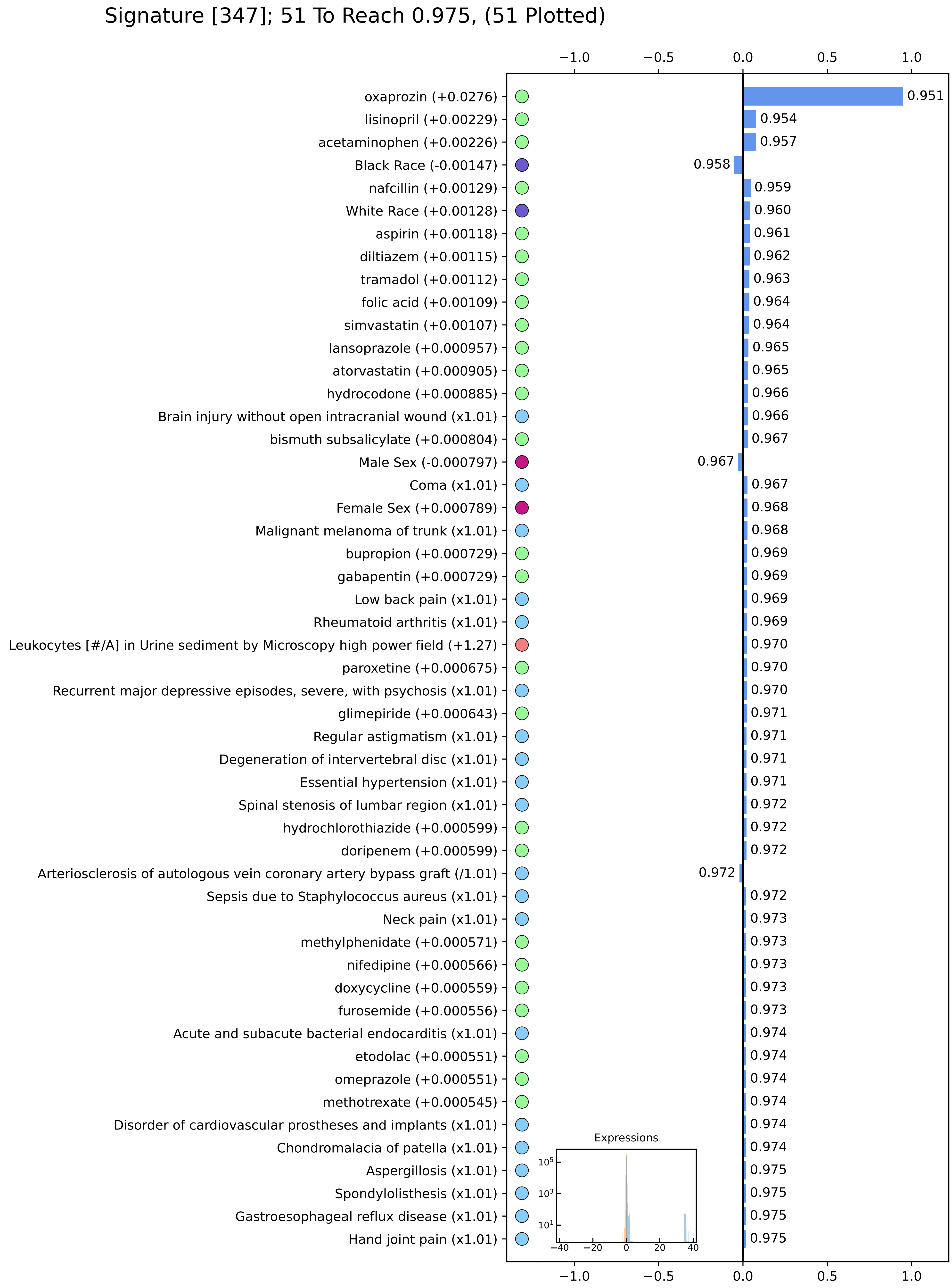

Signature [347]; 51 To Reach 0.975, (51 Plotted)
oxaprozin (+0.0276)
lisinopril (+0.00229)
acetaminophen (+0.00226)
Black Race (-0.00147)
nafcillin (+0.00129)
White Race (+0.00128)
aspirin (+0.00118)
diltiazem (+0.00115)
tramadol (+0.00112)
folic acid (+0.00109)
simvastatin (+0.00107)
lansoprazole (+0.000957)
atorvastatin (+0.000905)
hydrocodone (+0.000885)
Brain injury without open intracranial wound (x1.01)
bismuth subsalicylate (+0.000804)
Male Sex (-0.000797)
Coma (x1.01)
Female Sex (+0.000789)
Malignant melanoma of trunk (x1.01)
bupropion (+0.000729)
gabapentin (+0.000729)
Low back pain (x1.01)
Rheumatoid arthritis (x1.01)
Leukocytes [#/A] in Urine sediment by Microscopy high power field (+1.27)
paroxetine (+0.000675)
Recurrent major depressive episodes, severe, with psychosis (x1.01)
glimepiride (+0.000643)
Regular astigmatism (x1.01)
Degeneration of intervertebral disc (x1.01)
Essential hypertension (x1.01)
Spinal stenosis of lumbar region (x1.01)
hydrochlorothiazide (+0.000599)
doripenem (+0.000599)
Arteriosclerosis of autologous vein coronary artery bypass graft (/1.01)
Sepsis due to Staphylococcus aureus (x1.01)
Neck pain (x1.01)
methylphenidate (+0.000571)
nifedipine (+0.000566)
doxycycline (+0.000559)
furosemide (+0.000556)
Acute and subacute bacterial endocarditis (x1.01)
etodolac (+0.000551)
omeprazole (+0.000551)
methotrexate (+0.000545)
Disorder of cardiovascular prostheses and implants (x1.01)
Chondromalacia of patella (x1.01)
Aspergillosis (x1.01)
Spondylolisthesis (x1.01)
Gastroesophageal reflux disease (x1.01)
Hand joint pain (x1.01)
0.951
0.954
0.957
0.958
0.959
0.960
0.961
0.962
0.963
0.964
0.964
0.965
0.965
0.966
0.966
0.967
0.967
0.967
0.968
0.968
0.969
0.969
0.969
0.969
0.970
0.970
0.970
0.971
0.971
0.971
0.971
0.972
0.972
0.972
0.972
0.972
0.973
0.973
0.973
0.973
0.973
0.974
0.974
0.974
0.974
0.974
0.974
0.975
0.975
0.975
0.975
−1.0
−0.5
0.0
0.5
1.0
Expressions
−40
−20
0
20
40

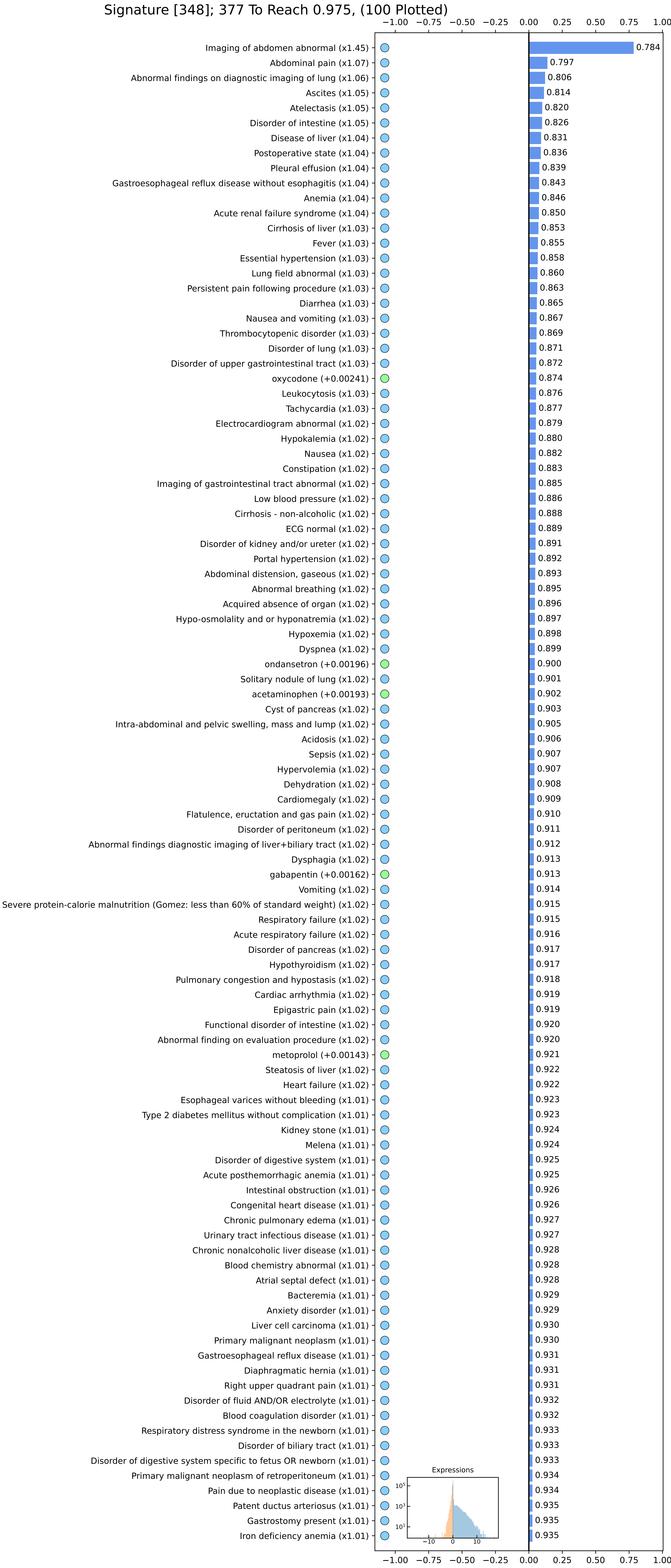
Signature [348]; 377 To Reach 0.975, (100 Plotted)
Imaging of abdomen abnormal (x1.45) 0.784
Abdominal pain (x1.07) 0.797
Abnormal findings on diagnostic imaging of lung (x1.06) 0.806
Ascites (x1.05) 0.814
Atelectasis (x1.05) 0.820
Disorder of intestine (x1.05) 0.826
Disease of liver (x1.04) 0.831
Postoperative state (x1.04) 0.836
Pleural effusion (x1.04) 0.839
Gastroesophageal reflux disease without esophagitis (x1.04) 0.843
Anemia (x1.04) 0.846
Acute renal failure syndrome (x1.04) 0.850
Cirrhosis of liver (x1.03) 0.853
Fever (x1.03) 0.855
Essential hypertension (x1.03) 0.858
Lung field abnormal (x1.03) 0.860
Persistent pain following procedure (x1.03) 0.863
Diarrhea (x1.03) 0.865
Nausea and vomiting (x1.03) 0.867
Thrombocytopenic disorder (x1.03) 0.869
Disorder of lung (x1.03) 0.871
Disorder of upper gastrointestinal tract (x1.03) 0.872
oxycodone (+0.00241) 0.874
Leukocytosis (x1.03) 0.876
Tachycardia (x1.03) 0.877
Electrocardiogram abnormal (x1.02) 0.879
Hypokalemia (x1.02) 0.880
Nausea (x1.02) 0.882
Constipation (x1.02) 0.883
Imaging of gastrointestinal tract abnormal (x1.02) 0.885
Low blood pressure (x1.02) 0.886
Cirrhosis - non-alcoholic (x1.02) 0.888
ECG normal (x1.02) 0.889
Disorder of kidney and/or ureter (x1.02) 0.891
Portal hypertension (x1.02) 0.892
Abdominal distension, gaseous (x1.02) 0.893
Abnormal breathing (x1.02) 0.895
Acquired absence of organ (x1.02) 0.896
Hypo-osmolality and or hyponatremia (x1.02) 0.897
Hypoxemia (x1.02) 0.898
Dyspnea (x1.02) 0.899
ondansetron (+0.00196) 0.900
Solitary nodule of lung (x1.02) 0.901
acetaminophen (+0.00193) 0.902
Cyst of pancreas (x1.02) 0.903
Intra-abdominal and pelvic swelling, mass and lump (x1.02) 0.905
Acidosis (x1.02) 0.906
Sepsis (x1.02) 0.907
Hypervolemia (x1.02) 0.907
Dehydration (x1.02) 0.908
Cardiomegaly (x1.02) 0.909
Flatulence, eructation and gas pain (x1.02) 0.910
Disorder of peritoneum (x1.02) 0.911
Abnormal findings diagnostic imaging of liver+biliary tract (x1.02) 0.912
Dysphagia (x1.02) 0.913
gabapentin (+0.00162) 0.913
Vomiting (x1.02) 0.914
Severe protein-calorie malnutrition (Gomez: less than 60% of standard weight) (x1.02) 0.915
Respiratory failure (x1.02) 0.915
Acute respiratory failure (x1.02) 0.916
Disorder of pancreas (x1.02) 0.917
Hypothyroidism (x1.02) 0.917
Pulmonary congestion and hypostasis (x1.02) 0.918
Cardiac arrhythmia (x1.02) 0.919
Epigastric pain (x1.02) 0.919
Functional disorder of intestine (x1.02) 0.920
Abnormal finding on evaluation procedure (x1.02) 0.920
metoprolol (+0.00143) 0.921
Steatosis of liver (x1.02) 0.922
Heart failure (x1.02) 0.922
Esophageal varices without bleeding (x1.01) 0.923
Type 2 diabetes mellitus without complication (x1.01) 0.923
Kidney stone (x1.01) 0.924
Melena (x1.01) 0.924
Disorder of digestive system (x1.01) 0.925
Acute posthemorrhagic anemia (x1.01) 0.925
Intestinal obstruction (x1.01) 0.926
Congenital heart disease (x1.01) 0.926
Chronic pulmonary edema (x1.01) 0.927
Urinary tract infectious disease (x1.01) 0.927
Chronic nonalcoholic liver disease (x1.01) 0.928
Blood chemistry abnormal (x1.01) 0.928
Atrial septal defect (x1.01) 0.928
Bacteremia (x1.01) 0.929
Anxiety disorder (x1.01) 0.929
Liver cell carcinoma (x1.01) 0.930
Primary malignant neoplasm (x1.01) 0.930
Gastroesophageal reflux disease (x1.01) 0.931
Diaphragmatic hernia (x1.01) 0.931
Right upper quadrant pain (x1.01) 0.931
Disorder of fluid AND/OR electrolyte (x1.01) 0.932
Blood coagulation disorder (x1.01) 0.932
Respiratory distress syndrome in the newborn (x1.01) 0.933
Disorder of biliary tract (x1.01) 0.933
Disorder of digestive system specific to fetus OR newborn (x1.01) 0.933
Primary malignant neoplasm of retroperitoneum (x1.01) 0.934
Pain due to neoplastic disease (x1.01) 0.934
Patent ductus arteriosus (x1.01) 0.935
Gastrostomy present (x1.01) 0.935
Iron deficiency anemia (x1.01) 0.935
Expressions
−1.00 −0.75 −0.50 −0.25 0.00 0.25 0.50 0.75 1.00

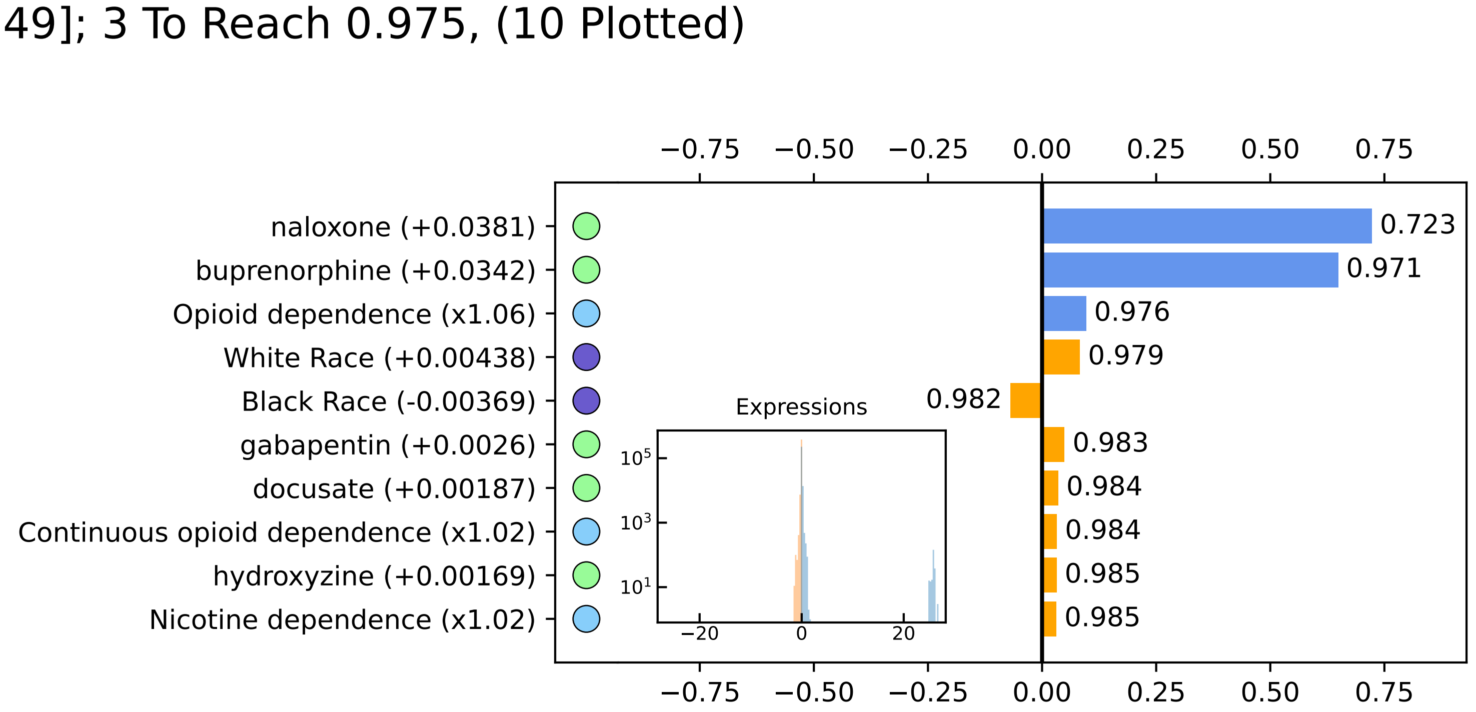
Signature [349]; 3 To Reach 0.975, (10 Plotted)
naloxone (+0.0381)
buprenorphine (+0.0342)
Opioid dependence (x1.06)
White Race (+0.00438)
Black Race (-0.00369)
gabapentin (+0.0026)
docusate (+0.00187)
Continuous opioid dependence (x1.02)
hydroxyzine (+0.00169)
Nicotine dependence (x1.02)
0.723
0.971
0.976
0.979
0.982
0.983
0.984
0.984
0.985
0.985
Expressions
−0.75
−0.50
−0.25
0.00
0.25
0.50
0.75

Signature [350]; 1423 To Reach 0.975, (100 Plotted)

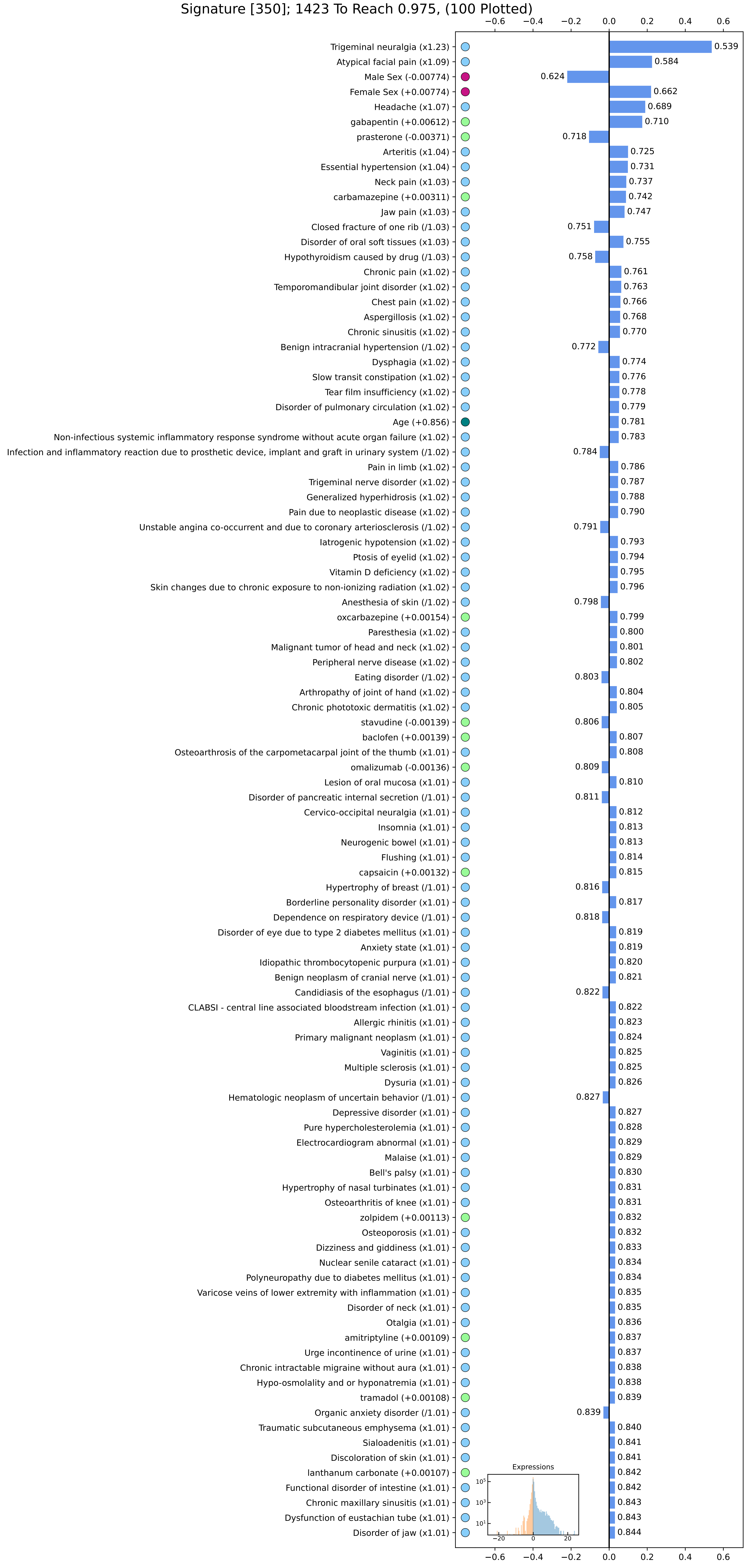

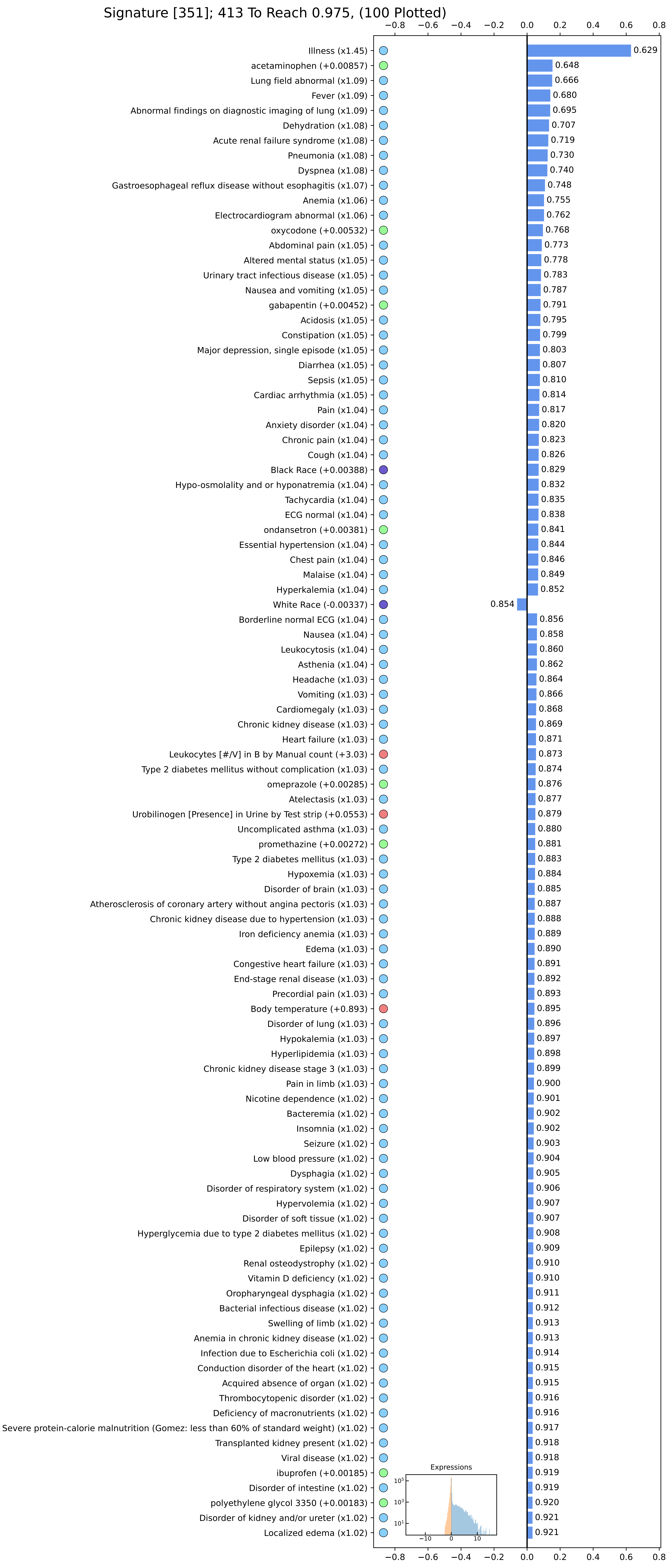

Signature [351]; 413 To Reach 0.975, (100 Plotted)
Illness (x1.45) 0.629
acetaminophen (+0.00857) 0.648
Lung field abnormal (x1.09) 0.666
Fever (x1.09) 0.680
Abnormal findings on diagnostic imaging of lung (x1.09) 0.695
Dehydration (x1.08) 0.707
Acute renal failure syndrome (x1.08) 0.719
Pneumonia (x1.08) 0.730
Dyspnea (x1.08) 0.740
Gastroesophageal reflux disease without esophagitis (x1.07) 0.748
Anemia (x1.06) 0.755
Electrocardiogram abnormal (x1.06) 0.762
oxycodone (+0.00532) 0.768
Abdominal pain (x1.05) 0.773
Altered mental status (x1.05) 0.778
Urinary tract infectious disease (x1.05) 0.783
Nausea and vomiting (x1.05) 0.787
gabapentin (+0.00452) 0.791
Acidosis (x1.05) 0.795
Constipation (x1.05) 0.799
Major depression, single episode (x1.05) 0.803
Diarrhea (x1.05) 0.807
Sepsis (x1.05) 0.810
Cardiac arrhythmia (x1.05) 0.814
Pain (x1.04) 0.817
Anxiety disorder (x1.04) 0.820
Chronic pain (x1.04) 0.823
Cough (x1.04) 0.826
Black Race (+0.00388) 0.829
Hypo-osmolality and or hyponatremia (x1.04) 0.832
Tachycardia (x1.04) 0.835
ECG normal (x1.04) 0.838
ondansetron (+0.00381) 0.841
Essential hypertension (x1.04) 0.844
Chest pain (x1.04) 0.846
Malaise (x1.04) 0.849
Hyperkalemia (x1.04) 0.852
White Race (-0.00337) 0.854
Borderline normal ECG (x1.04) 0.856
Nausea (x1.04) 0.858
Leukocytosis (x1.04) 0.860
Asthenia (x1.04) 0.862
Headache (x1.03) 0.864
Vomiting (x1.03) 0.866
Cardiomegaly (x1.03) 0.868
Chronic kidney disease (x1.03) 0.869
Heart failure (x1.03) 0.871
Leukocytes [#/V] in B by Manual count (+3.03) 0.873
Type 2 diabetes mellitus without complication (x1.03) 0.874
omeprazole (+0.00285) 0.876
Atelectasis (x1.03) 0.877
Urobilinogen [Presence] in Urine by Test strip (+0.0553) 0.879
Uncomplicated asthma (x1.03) 0.880
promethazine (+0.00272) 0.881
Type 2 diabetes mellitus (x1.03) 0.883
Hypoxemia (x1.03) 0.884
Disorder of brain (x1.03) 0.885
Atherosclerosis of coronary artery without angina pectoris (x1.03) 0.887
Chronic kidney disease due to hypertension (x1.03) 0.888
Iron deficiency anemia (x1.03) 0.889
Edema (x1.03) 0.890
Congestive heart failure (x1.03) 0.891
End-stage renal disease (x1.03) 0.892
Precordial pain (x1.03) 0.893
Body temperature (+0.893) 0.895
Disorder of lung (x1.03) 0.896
Hypokalemia (x1.03) 0.897
Hyperlipidemia (x1.03) 0.898
Chronic kidney disease stage 3 (x1.03) 0.899
Pain in limb (x1.03) 0.900
Nicotine dependence (x1.02) 0.901
Bacteremia (x1.02) 0.902
Insomnia (x1.02) 0.902
Seizure (x1.02) 0.903
Low blood pressure (x1.02) 0.904
Dysphagia (x1.02) 0.905
Disorder of respiratory system (x1.02) 0.906
Hypervolemia (x1.02) 0.907
Disorder of soft tissue (x1.02) 0.907
Hyperglycemia due to type 2 diabetes mellitus (x1.02) 0.908
Epilepsy (x1.02) 0.909
Renal osteodystrophy (x1.02) 0.910
Vitamin D deficiency (x1.02) 0.910
Oropharyngeal dysphagia (x1.02) 0.911
Bacterial infectious disease (x1.02) 0.912
Swelling of limb (x1.02) 0.913
Anemia in chronic kidney disease (x1.02) 0.913
Infection due to Escherichia coli (x1.02) 0.914
Conduction disorder of the heart (x1.02) 0.915
Acquired absence of organ (x1.02) 0.915
Thrombocytopenic disorder (x1.02) 0.916
Deficiency of macronutrients (x1.02) 0.916
Severe protein-calorie malnutrition (Gomez: less than 60% of standard weight) (x1.02) 0.917
Transplanted kidney present (x1.02) 0.918
Viral disease (x1.02) 0.918
ibuprofen (+0.00185) 0.919
Disorder of intestine (x1.02) 0.919
polyethylene glycol 3350 (+0.00183) 0.920
Disorder of kidney and/or ureter (x1.02) 0.921
Localized edema (x1.02) 0.921
Expressions
−0.8 −0.6 −0.4 −0.2 0.0 0.2 0.4 0.6 0.8

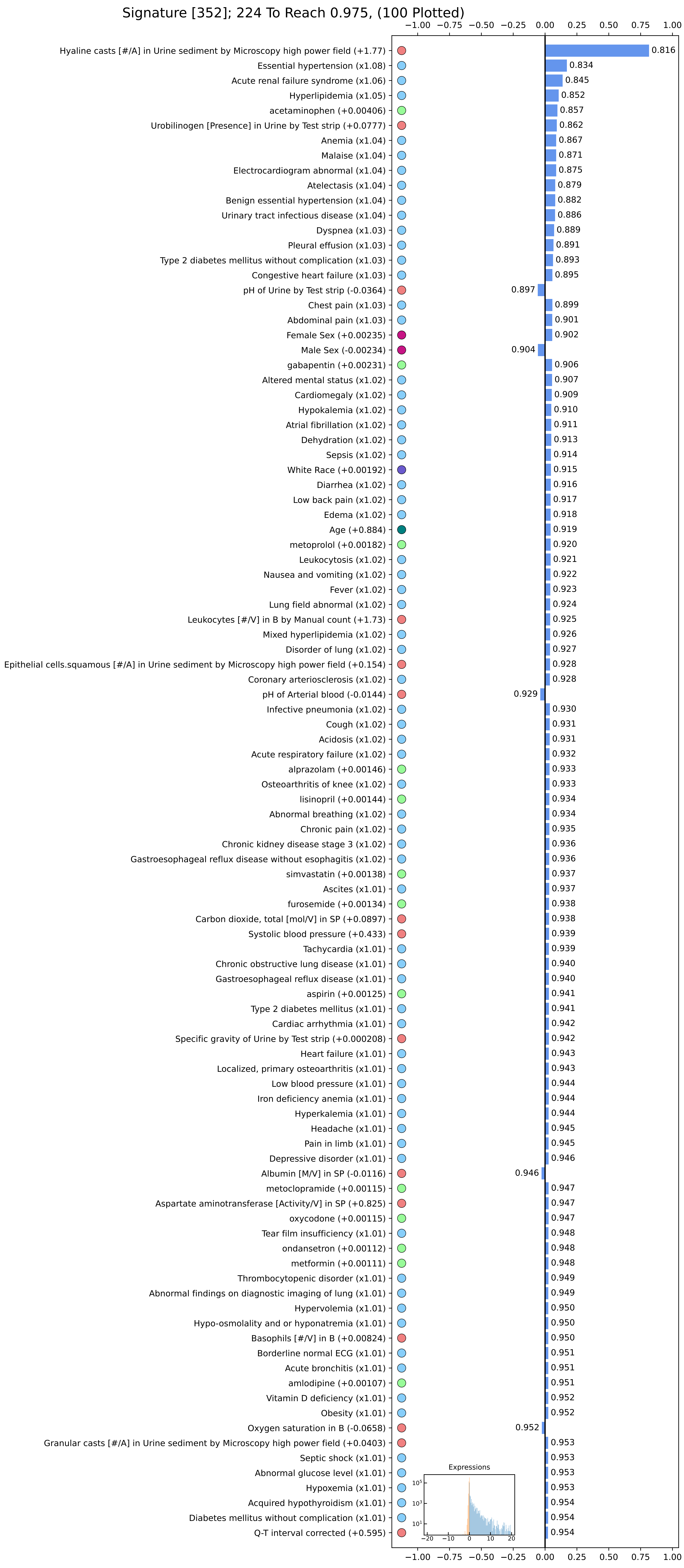

Signature [352]; 224 To Reach 0.975, (100 Plotted)
Hyaline casts [#/A] in Urine sediment by Microscopy high power field (+1.77) 0.816
Essential hypertension (x1.08) 0.834
Acute renal failure syndrome (x1.06) 0.845
Hyperlipidemia (x1.05) 0.852
acetaminophen (+0.00406) 0.857
Urobilinogen [Presence] in Urine by Test strip (+0.0777) 0.862
Anemia (x1.04) 0.867
Malaise (x1.04) 0.871
Electrocardiogram abnormal (x1.04) 0.875
Atelectasis (x1.04) 0.879
Benign essential hypertension (x1.04) 0.882
Urinary tract infectious disease (x1.04) 0.886
Dyspnea (x1.03) 0.889
Pleural effusion (x1.03) 0.891
Type 2 diabetes mellitus without complication (x1.03) 0.893
Congestive heart failure (x1.03) 0.895
pH of Urine by Test strip (-0.0364) 0.897
Chest pain (x1.03) 0.899
Abdominal pain (x1.03) 0.901
Female Sex (+0.00235) 0.902
Male Sex (-0.00234) 0.904
gabapentin (+0.00231) 0.906
Altered mental status (x1.02) 0.907
Cardiomegaly (x1.02) 0.909
Hypokalemia (x1.02) 0.910
Atrial fibrillation (x1.02) 0.911
Dehydration (x1.02) 0.913
Sepsis (x1.02) 0.914
White Race (+0.00192) 0.915
Diarrhea (x1.02) 0.916
Low back pain (x1.02) 0.917
Edema (x1.02) 0.918
Age (+0.884) 0.919
metoprolol (+0.00182) 0.920
Leukocytosis (x1.02) 0.921
Nausea and vomiting (x1.02) 0.922
Fever (x1.02) 0.923
Lung field abnormal (x1.02) 0.924
Leukocytes [#/V] in B by Manual count (+1.73) 0.925
Mixed hyperlipidemia (x1.02) 0.926
Disorder of lung (x1.02) 0.927
Epithelial cells.squamous [#/A] in Urine sediment by Microscopy high power field (+0.154) 0.928
Coronary arteriosclerosis (x1.02) 0.928
pH of Arterial blood (-0.0144) 0.929
Infective pneumonia (x1.02) 0.930
Cough (x1.02) 0.931
Acidosis (x1.02) 0.931
Acute respiratory failure (x1.02) 0.932
alprazolam (+0.00146) 0.933
Osteoarthritis of knee (x1.02) 0.933
lisinopril (+0.00144) 0.934
Abnormal breathing (x1.02) 0.934
Chronic pain (x1.02) 0.935
Chronic kidney disease stage 3 (x1.02) 0.936
Gastroesophageal reflux disease without esophagitis (x1.02) 0.936
simvastatin (+0.00138) 0.937
Ascites (x1.01) 0.937
furosemide (+0.00134) 0.938
Carbon dioxide, total [mol/V] in SP (+0.0897) 0.938
Systolic blood pressure (+0.433) 0.939
Tachycardia (x1.01) 0.939
Chronic obstructive lung disease (x1.01) 0.940
Gastroesophageal reflux disease (x1.01) 0.940
aspirin (+0.00125) 0.941
Type 2 diabetes mellitus (x1.01) 0.941
Cardiac arrhythmia (x1.01) 0.942
Specific gravity of Urine by Test strip (+0.000208) 0.942
Heart failure (x1.01) 0.943
Localized, primary osteoarthritis (x1.01) 0.943
Low blood pressure (x1.01) 0.944
Iron deficiency anemia (x1.01) 0.944
Hyperkalemia (x1.01) 0.944
Headache (x1.01) 0.945
Pain in limb (x1.01) 0.945
Depressive disorder (x1.01) 0.946
Albumin [M/V] in SP (-0.0116) 0.946
metoclopramide (+0.00115) 0.947
Aspartate aminotransferase [Activity/V] in SP (+0.825) 0.947
oxycodone (+0.00115) 0.947
Tear film insufficiency (x1.01) 0.948
ondansetron (+0.00112) 0.948
metformin (+0.00111) 0.948
Thrombocytopenic disorder (x1.01) 0.949
Abnormal findings on diagnostic imaging of lung (x1.01) 0.949
Hypervolemia (x1.01) 0.950
Hypo-osmolality and or hyponatremia (x1.01) 0.950
Basophils [#/V] in B (+0.00824) 0.950
Borderline normal ECG (x1.01) 0.951
Acute bronchitis (x1.01) 0.951
amlodipine (+0.00107) 0.951
Vitamin D deficiency (x1.01) 0.952
Obesity (x1.01) 0.952
Oxygen saturation in B (-0.0658) 0.952
Granular casts [#/A] in Urine sediment by Microscopy high power field (+0.0403) 0.953
Septic shock (x1.01) 0.953
Abnormal glucose level (x1.01) 0.953
Hypoxemia (x1.01) 0.953
Acquired hypothyroidism (x1.01) 0.954
Diabetes mellitus without complication (x1.01) 0.954
Q-T interval corrected (+0.595) 0.954
−1.00 −0.75 −0.50 −0.25 0.00 0.25 0.50 0.75 1.00
Expressions

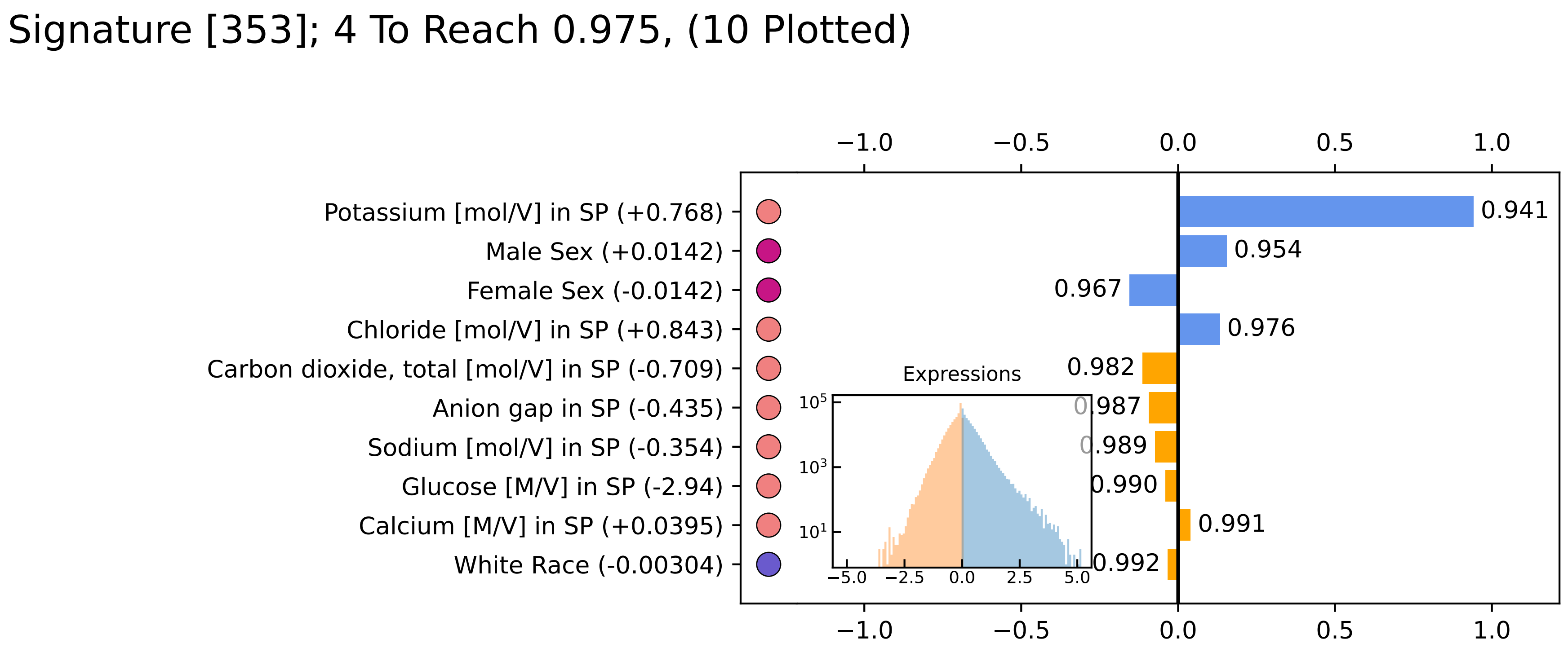

Signature [353]; 4 To Reach 0.975, (10 Plotted)
Potassium [mol/V] in SP (+0.768)
Male Sex (+0.0142)
Female Sex (-0.0142)
Chloride [mol/V] in SP (+0.843)
Carbon dioxide, total [mol/V] in SP (-0.709)
Anion gap in SP (-0.435)
Sodium [mol/V] in SP (-0.354)
Glucose [M/V] in SP (-2.94)
Calcium [M/V] in SP (+0.0395)
White Race (-0.00304)
0.941
0.954
0.967
0.976
0.982
0.987
0.989
0.990
0.991
0.992
Expressions
−1.0
−0.5
0.0
0.5
1.0

Signature [354]; 68 To Reach 0.975, (68 Plotted)

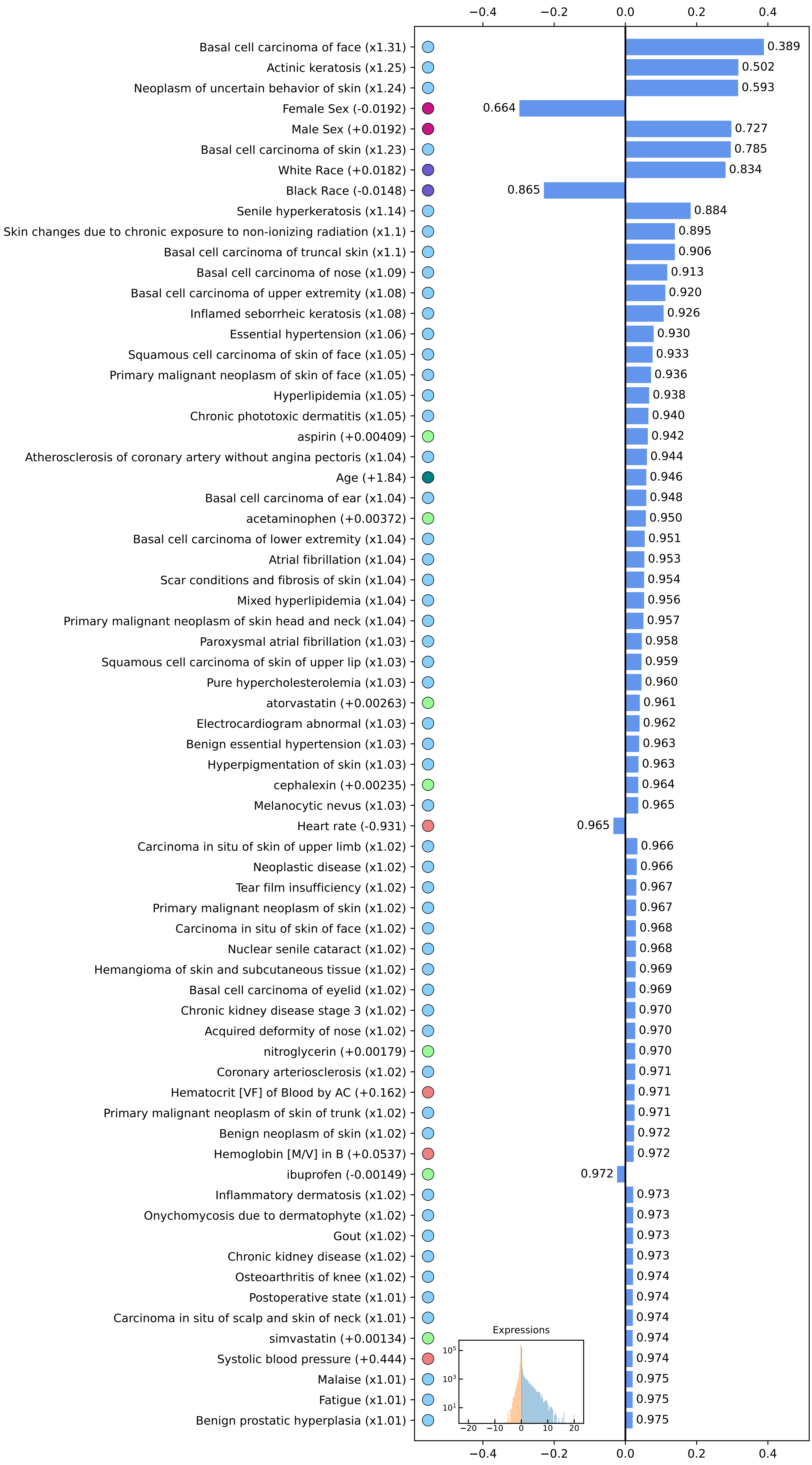

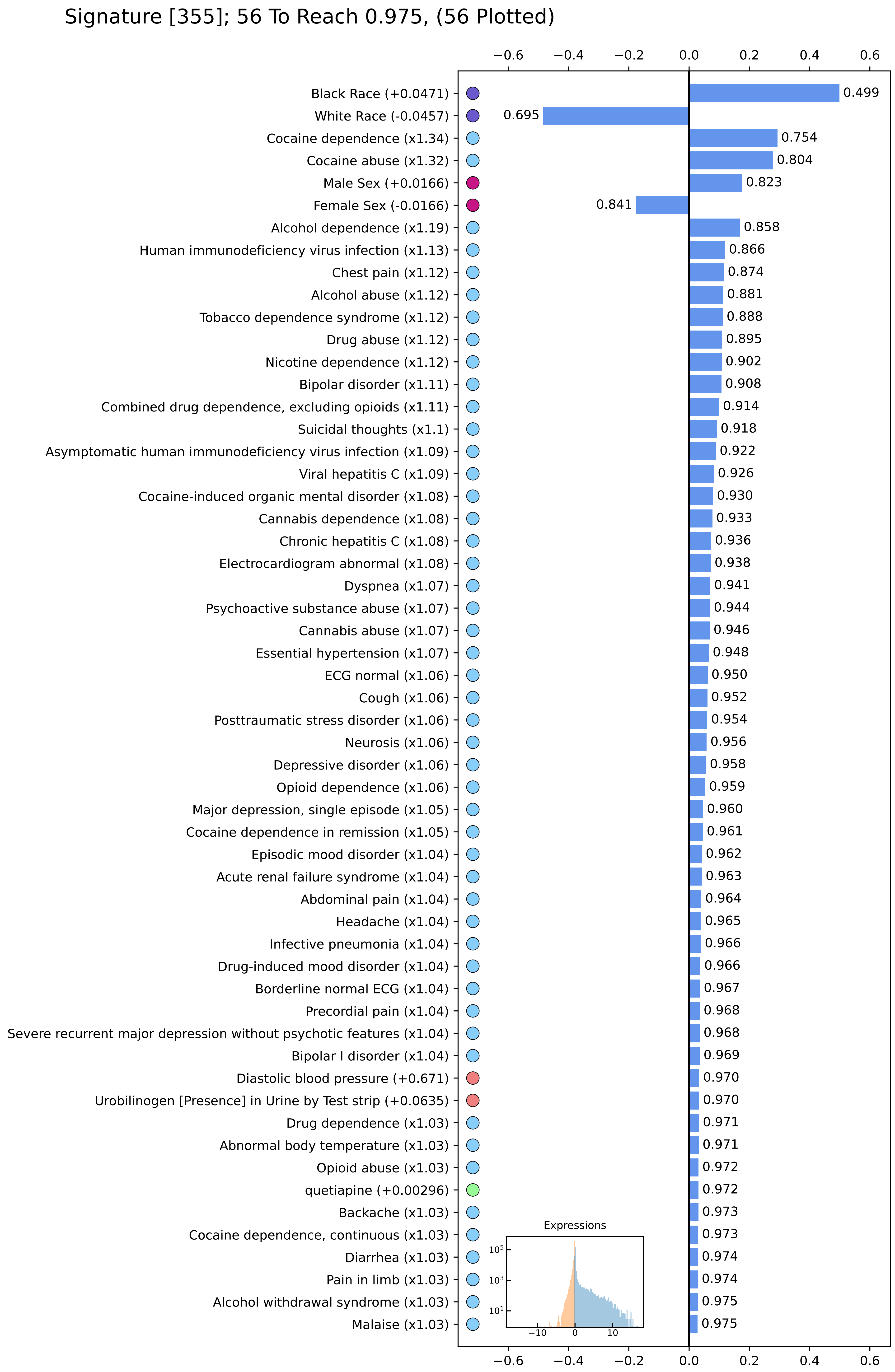

Signature [355]; 56 To Reach 0.975, (56 Plotted)
Black Race (+0.0471) 0.499
White Race (-0.0457) 0.695
Cocaine dependence (x1.34) 0.754
Cocaine abuse (x1.32) 0.804
Male Sex (+0.0166) 0.823
Female Sex (-0.0166) 0.841
Alcohol dependence (x1.19) 0.858
Human immunodeficiency virus infection (x1.13) 0.866
Chest pain (x1.12) 0.874
Alcohol abuse (x1.12) 0.881
Tobacco dependence syndrome (x1.12) 0.888
Drug abuse (x1.12) 0.895
Nicotine dependence (x1.12) 0.902
Bipolar disorder (x1.11) 0.908
Combined drug dependence, excluding opioids (x1.11) 0.914
Suicidal thoughts (x1.1) 0.918
Asymptomatic human immunodeficiency virus infection (x1.09) 0.922
Viral hepatitis C (x1.09) 0.926
Cocaine-induced organic mental disorder (x1.08) 0.930
Cannabis dependence (x1.08) 0.933
Chronic hepatitis C (x1.08) 0.936
Electrocardiogram abnormal (x1.08) 0.938
Dyspnea (x1.07) 0.941
Psychoactive substance abuse (x1.07) 0.944
Cannabis abuse (x1.07) 0.946
Essential hypertension (x1.07) 0.948
ECG normal (x1.06) 0.950
Cough (x1.06) 0.952
Posttraumatic stress disorder (x1.06) 0.954
Neurosis (x1.06) 0.956
Depressive disorder (x1.06) 0.958
Opioid dependence (x1.06) 0.959
Major depression, single episode (x1.05) 0.960
Cocaine dependence in remission (x1.05) 0.961
Episodic mood disorder (x1.04) 0.962
Acute renal failure syndrome (x1.04) 0.963
Abdominal pain (x1.04) 0.964
Headache (x1.04) 0.965
Infective pneumonia (x1.04) 0.966
Drug-induced mood disorder (x1.04) 0.966
Borderline normal ECG (x1.04) 0.967
Precordial pain (x1.04) 0.968
Severe recurrent major depression without psychotic features (x1.04) 0.968
Bipolar I disorder (x1.04) 0.969
Diastolic blood pressure (+0.671) 0.970
Urobilinogen [Presence] in Urine by Test strip (+0.0635) 0.970
Drug dependence (x1.03) 0.971
Abnormal body temperature (x1.03) 0.971
Opioid abuse (x1.03) 0.972
quetiapine (+0.00296) 0.972
Backache (x1.03) 0.973
Cocaine dependence, continuous (x1.03) 0.973
Diarrhea (x1.03) 0.974
Pain in limb (x1.03) 0.974
Alcohol withdrawal syndrome (x1.03) 0.975
Malaise (x1.03) 0.975
−0.6 −0.4 −0.2 0.0 0.2 0.4 0.6
Expressions

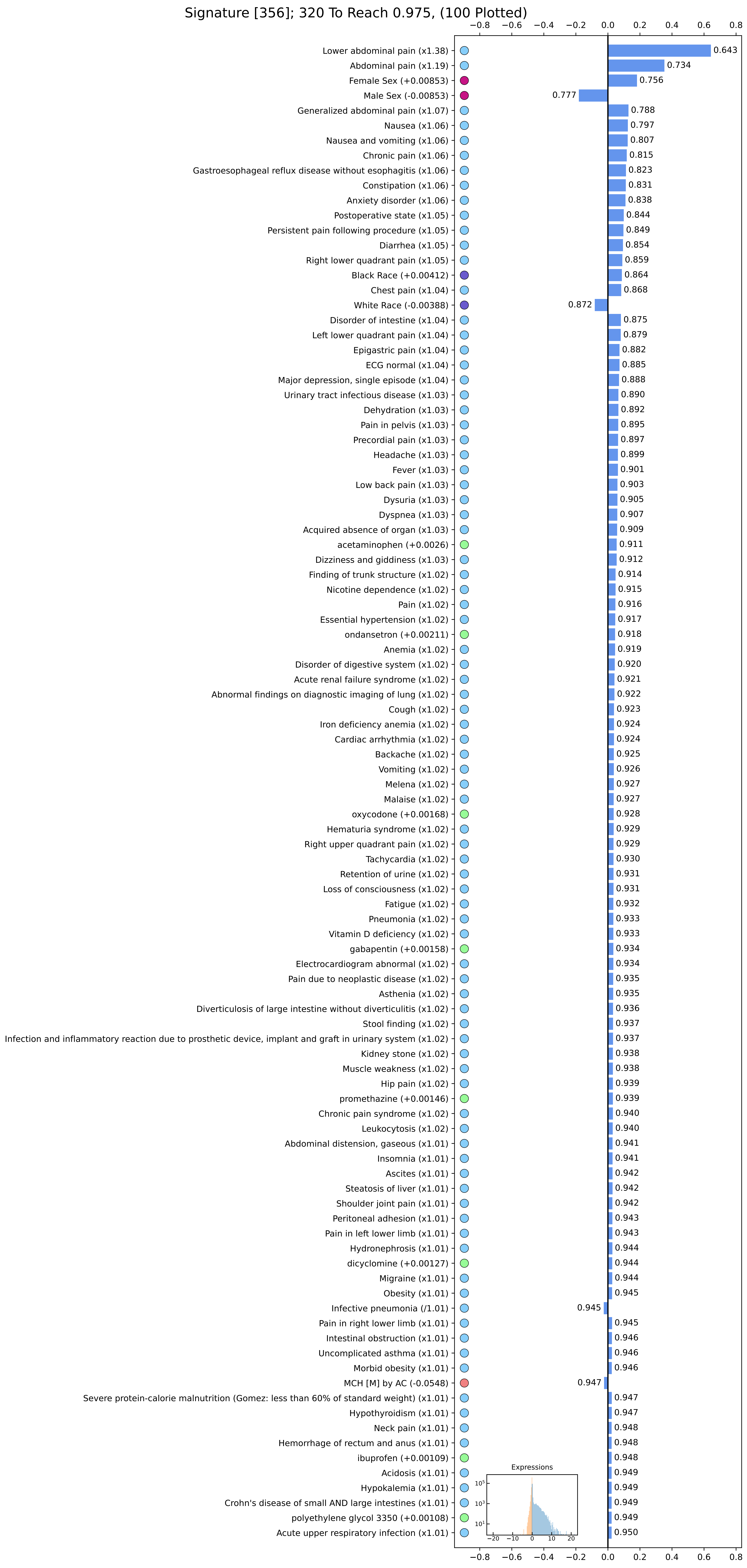

Signature [356]; 320 To Reach 0.975, (100 Plotted)
Lower abdominal pain (x1.38) 0.643
Abdominal pain (x1.19) 0.734
Female Sex (+0.00853) 0.756
Male Sex (-0.00853) 0.777
Generalized abdominal pain (x1.07) 0.788
Nausea (x1.06) 0.797
Nausea and vomiting (x1.06) 0.807
Chronic pain (x1.06) 0.815
Gastroesophageal reflux disease without esophagitis (x1.06) 0.823
Constipation (x1.06) 0.831
Anxiety disorder (x1.06) 0.838
Postoperative state (x1.05) 0.844
Persistent pain following procedure (x1.05) 0.849
Diarrhea (x1.05) 0.854
Right lower quadrant pain (x1.05) 0.859
Black Race (+0.00412) 0.864
Chest pain (x1.04) 0.868
White Race (-0.00388) 0.872
Disorder of intestine (x1.04) 0.875
Left lower quadrant pain (x1.04) 0.879
Epigastric pain (x1.04) 0.882
ECG normal (x1.04) 0.885
Major depression, single episode (x1.04) 0.888
Urinary tract infectious disease (x1.03) 0.890
Dehydration (x1.03) 0.892
Pain in pelvis (x1.03) 0.895
Precordial pain (x1.03) 0.897
Headache (x1.03) 0.899
Fever (x1.03) 0.901
Low back pain (x1.03) 0.903
Dysuria (x1.03) 0.905
Dyspnea (x1.03) 0.907
Acquired absence of organ (x1.03) 0.909
acetaminophen (+0.0026) 0.911
Dizziness and giddiness (x1.03) 0.912
Finding of trunk structure (x1.02) 0.914
Nicotine dependence (x1.02) 0.915
Pain (x1.02) 0.916
Essential hypertension (x1.02) 0.917
ondansetron (+0.00211) 0.918
Anemia (x1.02) 0.919
Disorder of digestive system (x1.02) 0.920
Acute renal failure syndrome (x1.02) 0.921
Abnormal findings on diagnostic imaging of lung (x1.02) 0.922
Cough (x1.02) 0.923
Iron deficiency anemia (x1.02) 0.924
Cardiac arrhythmia (x1.02) 0.924
Backache (x1.02) 0.925
Vomiting (x1.02) 0.926
Melena (x1.02) 0.927
Malaise (x1.02) 0.927
oxycodone (+0.00168) 0.928
Hematuria syndrome (x1.02) 0.929
Right upper quadrant pain (x1.02) 0.929
Tachycardia (x1.02) 0.930
Retention of urine (x1.02) 0.931
Loss of consciousness (x1.02) 0.931
Fatigue (x1.02) 0.932
Pneumonia (x1.02) 0.933
Vitamin D deficiency (x1.02) 0.933
gabapentin (+0.00158) 0.934
Electrocardiogram abnormal (x1.02) 0.934
Pain due to neoplastic disease (x1.02) 0.935
Asthenia (x1.02) 0.935
Diverticulosis of large intestine without diverticulitis (x1.02) 0.936
Stool finding (x1.02) 0.937
Infection and inflammatory reaction due to prosthetic device, implant and graft in urinary system (x1.02) 0.937
Kidney stone (x1.02) 0.938
Muscle weakness (x1.02) 0.938
Hip pain (x1.02) 0.939
promethazine (+0.00146) 0.939
Chronic pain syndrome (x1.02) 0.940
Leukocytosis (x1.02) 0.940
Abdominal distension, gaseous (x1.01) 0.941
Insomnia (x1.01) 0.941
Ascites (x1.01) 0.942
Steatosis of liver (x1.01) 0.942
Shoulder joint pain (x1.01) 0.942
Peritoneal adhesion (x1.01) 0.943
Pain in left lower limb (x1.01) 0.943
Hydronephrosis (x1.01) 0.944
dicyclomine (+0.00127) 0.944
Migraine (x1.01) 0.944
Obesity (x1.01) 0.945
Infective pneumonia (/1.01) 0.945
Pain in right lower limb (x1.01) 0.945
Intestinal obstruction (x1.01) 0.946
Uncomplicated asthma (x1.01) 0.946
Morbid obesity (x1.01) 0.946
MCH [M] by AC (-0.0548) 0.947
Severe protein-calorie malnutrition (Gomez: less than 60% of standard weight) (x1.01) 0.947
Hypothyroidism (x1.01) 0.947
Neck pain (x1.01) 0.948
Hemorrhage of rectum and anus (x1.01) 0.948
ibuprofen (+0.00109) 0.948
Acidosis (x1.01) 0.949
Hypokalemia (x1.01) 0.949
Crohn's disease of small AND large intestines (x1.01) 0.949
polyethylene glycol 3350 (+0.00108) 0.949
Acute upper respiratory infection (x1.01) 0.950
−0.8 −0.6 −0.4 −0.2 0.0 0.2 0.4 0.6 0.8
Expressions

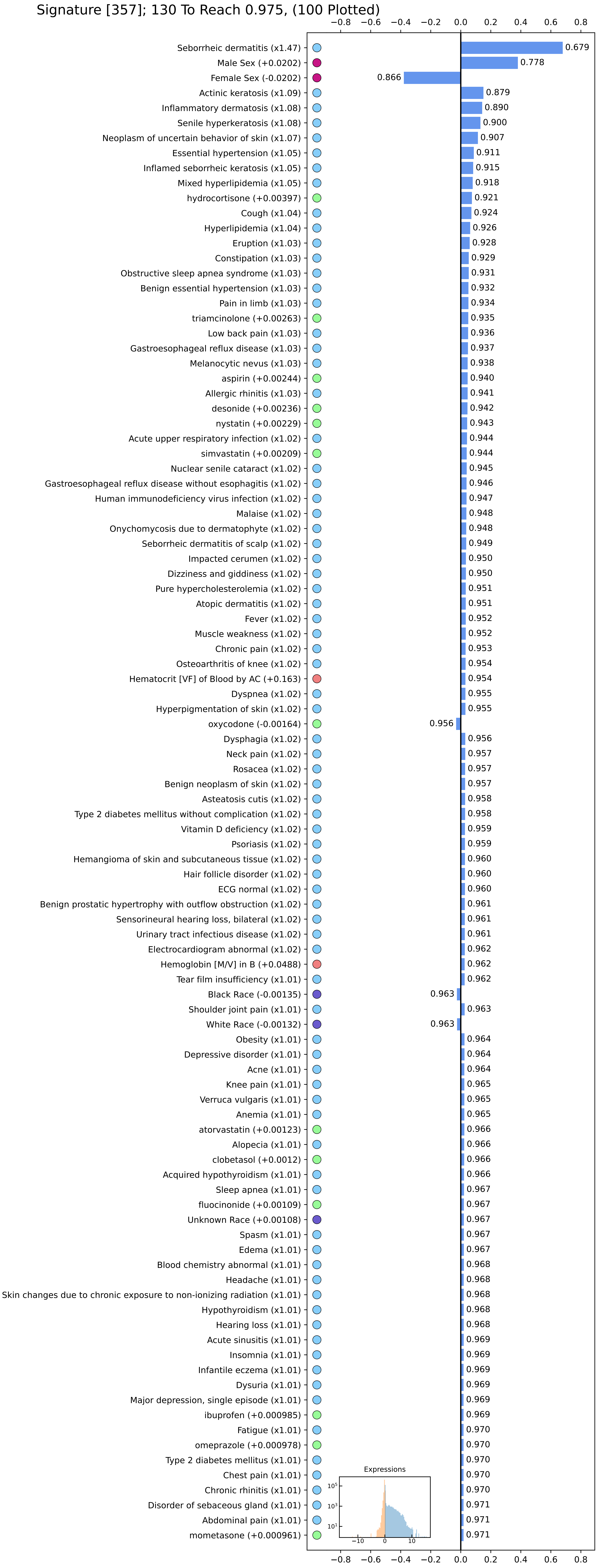

Signature [357]; 130 To Reach 0.975, (100 Plotted)
Seborrheic dermatitis (x1.47)
Male Sex (+0.0202)
Female Sex (-0.0202)
Actinic keratosis (x1.09)
Inflammatory dermatosis (x1.08)
Senile hyperkeratosis (x1.08)
Neoplasm of uncertain behavior of skin (x1.07)
Essential hypertension (x1.05)
Inflamed seborrheic keratosis (x1.05)
Mixed hyperlipidemia (x1.05)
hydrocortisone (+0.00397)
Cough (x1.04)
Hyperlipidemia (x1.04)
Eruption (x1.03)
Constipation (x1.03)
Obstructive sleep apnea syndrome (x1.03)
Benign essential hypertension (x1.03)
Pain in limb (x1.03)
triamcinolone (+0.00263)
Low back pain (x1.03)
Gastroesophageal reflux disease (x1.03)
Melanocytic nevus (x1.03)
aspirin (+0.00244)
Allergic rhinitis (x1.03)
desonide (+0.00236)
nystatin (+0.00229)
Acute upper respiratory infection (x1.02)
simvastatin (+0.00209)
Nuclear senile cataract (x1.02)
Gastroesophageal reflux disease without esophagitis (x1.02)
Human immunodeficiency virus infection (x1.02)
Malaise (x1.02)
Onychomycosis due to dermatophyte (x1.02)
Seborrheic dermatitis of scalp (x1.02)
Impacted cerumen (x1.02)
Dizziness and giddiness (x1.02)
Pure hypercholesterolemia (x1.02)
Atopic dermatitis (x1.02)
Fever (x1.02)
Muscle weakness (x1.02)
Chronic pain (x1.02)
Osteoarthritis of knee (x1.02)
Hematocrit [VF] of Blood by AC (+0.163)
Dyspnea (x1.02)
Hyperpigmentation of skin (x1.02)
oxycodone (-0.00164)
Dysphagia (x1.02)
Neck pain (x1.02)
Rosacea (x1.02)
Benign neoplasm of skin (x1.02)
Asteatosis cutis (x1.02)
Type 2 diabetes mellitus without complication (x1.02)
Vitamin D deficiency (x1.02)
Psoriasis (x1.02)
Hemangioma of skin and subcutaneous tissue (x1.02)
Hair follicle disorder (x1.02)
ECG normal (x1.02)
Benign prostatic hypertrophy with outflow obstruction (x1.02)
Sensorineural hearing loss, bilateral (x1.02)
Urinary tract infectious disease (x1.02)
Electrocardiogram abnormal (x1.02)
Hemoglobin [M/V] in B (+0.0488)
Tear film insufficiency (x1.01)
Black Race (-0.00135)
Shoulder joint pain (x1.01)
White Race (-0.00132)
Obesity (x1.01)
Depressive disorder (x1.01)
Acne (x1.01)
Knee pain (x1.01)
Verruca vulgaris (x1.01)
Anemia (x1.01)
atorvastatin (+0.00123)
Alopecia (x1.01)
clobetasol (+0.0012)
Acquired hypothyroidism (x1.01)
Sleep apnea (x1.01)
fluocinonide (+0.00109)
Unknown Race (+0.00108)
Spasm (x1.01)
Edema (x1.01)
Blood chemistry abnormal (x1.01)
Headache (x1.01)
Skin changes due to chronic exposure to non-ionizing radiation (x1.01)
Hypothyroidism (x1.01)
Hearing loss (x1.01)
Acute sinusitis (x1.01)
Insomnia (x1.01)
Infantile eczema (x1.01)
Dysuria (x1.01)
Major depression, single episode (x1.01)
ibuprofen (+0.000985)
Fatigue (x1.01)
omeprazole (+0.000978)
Type 2 diabetes mellitus (x1.01)
Chest pain (x1.01)
Chronic rhinitis (x1.01)
Disorder of sebaceous gland (x1.01)
Abdominal pain (x1.01)
mometasone (+0.000961)
0.679
0.778
0.866
0.879
0.890
0.900
0.907
0.911
0.915
0.918
0.921
0.924
0.926
0.928
0.929
0.931
0.932
0.934
0.935
0.936
0.937
0.938
0.940
0.941
0.942
0.943
0.944
0.944
0.945
0.946
0.947
0.948
0.948
0.949
0.950
0.950
0.951
0.951
0.952
0.952
0.953
0.954
0.954
0.955
0.955
0.956
0.956
0.957
0.957
0.957
0.958
0.958
0.959
0.959
0.960
0.960
0.960
0.961
0.961
0.961
0.962
0.962
0.962
0.963
0.963
0.963
0.964
0.964
0.964
0.965
0.965
0.965
0.966
0.966
0.966
0.966
0.967
0.967
0.967
0.967
0.967
0.968
0.968
0.968
0.968
0.968
0.969
0.969
0.969
0.969
0.969
0.969
0.970
0.970
0.970
0.970
0.970
0.971
0.971
0.971
−0.8 −0.6 −0.4 −0.2 0.0 0.2 0.4 0.6 0.8
Expressions
−10
0
10

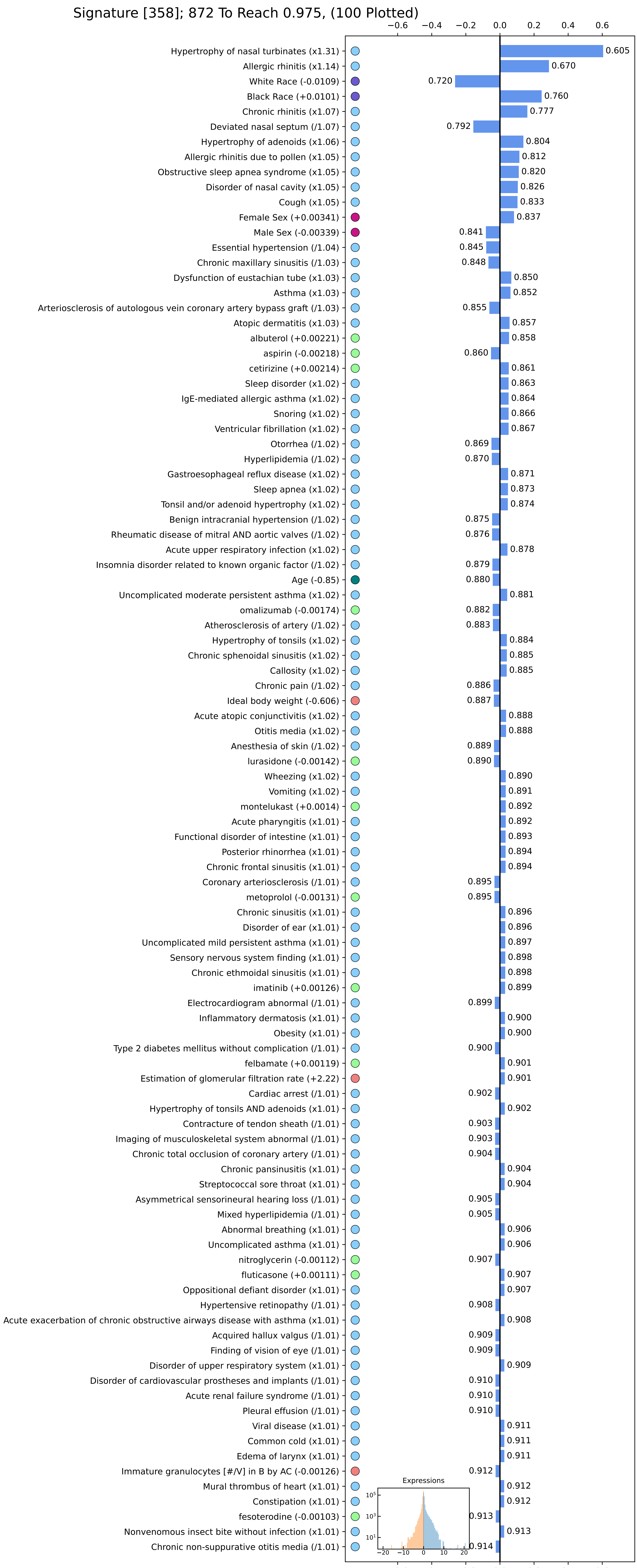

Signature [358]; 872 To Reach 0.975, (100 Plotted)
Hypertrophy of nasal turbinates (x1.31) 0.605
Allergic rhinitis (x1.14) 0.670
White Race (-0.0109) 0.720
Black Race (+0.0101) 0.760
Chronic rhinitis (x1.07) 0.777
Deviated nasal septum (/1.07) 0.792
Hypertrophy of adenoids (x1.06) 0.804
Allergic rhinitis due to pollen (x1.05) 0.812
Obstructive sleep apnea syndrome (x1.05) 0.820
Disorder of nasal cavity (x1.05) 0.826
Cough (x1.05) 0.833
Female Sex (+0.00341) 0.837
Male Sex (-0.00339) 0.841
Essential hypertension (/1.04) 0.845
Chronic maxillary sinusitis (/1.03) 0.848
Dysfunction of eustachian tube (x1.03) 0.850
Asthma (x1.03) 0.852
Arteriosclerosis of autologous vein coronary artery bypass graft (/1.03) 0.855
Atopic dermatitis (x1.03) 0.857
albuterol (+0.00221) 0.858
aspirin (-0.00218) 0.860
cetirizine (+0.00214) 0.861
Sleep disorder (x1.02) 0.863
IgE-mediated allergic asthma (x1.02) 0.864
Snoring (x1.02) 0.866
Ventricular fibrillation (x1.02) 0.867
Otorrhea (/1.02) 0.869
Hyperlipidemia (/1.02) 0.870
Gastroesophageal reflux disease (x1.02) 0.871
Sleep apnea (x1.02) 0.873
Tonsil and/or adenoid hypertrophy (x1.02) 0.874
Benign intracranial hypertension (/1.02) 0.875
Rheumatic disease of mitral AND aortic valves (/1.02) 0.876
Acute upper respiratory infection (x1.02) 0.878
Insomnia disorder related to known organic factor (/1.02) 0.879
Age (-0.85) 0.880
Uncomplicated moderate persistent asthma (x1.02) 0.881
omalizumab (-0.00174) 0.882
Atherosclerosis of artery (/1.02) 0.883
Hypertrophy of tonsils (x1.02) 0.884
Chronic sphenoidal sinusitis (x1.02) 0.885
Callosity (x1.02) 0.885
Chronic pain (/1.02) 0.886
Ideal body weight (-0.606) 0.887
Acute atopic conjunctivitis (x1.02) 0.888
Otitis media (x1.02) 0.888
Anesthesia of skin (/1.02) 0.889
lurasidone (-0.00142) 0.890
Wheezing (x1.02) 0.890
Vomiting (x1.02) 0.891
montelukast (+0.0014) 0.892
Acute pharyngitis (x1.01) 0.892
Functional disorder of intestine (x1.01) 0.893
Posterior rhinorrhea (x1.01) 0.894
Chronic frontal sinusitis (x1.01) 0.894
Coronary arteriosclerosis (/1.01) 0.895
metoprolol (-0.00131) 0.895
Chronic sinusitis (x1.01) 0.896
Disorder of ear (x1.01) 0.896
Uncomplicated mild persistent asthma (x1.01) 0.897
Sensory nervous system finding (x1.01) 0.898
Chronic ethmoidal sinusitis (x1.01) 0.898
imatinib (+0.00126) 0.899
Electrocardiogram abnormal (/1.01) 0.899
Inflammatory dermatosis (x1.01) 0.900
Obesity (x1.01) 0.900
Type 2 diabetes mellitus without complication (/1.01) 0.900
felbamate (+0.00119) 0.901
Estimation of glomerular filtration rate (+2.22) 0.901
Cardiac arrest (/1.01) 0.902
Hypertrophy of tonsils AND adenoids (x1.01) 0.902
Contracture of tendon sheath (/1.01) 0.903
Imaging of musculoskeletal system abnormal (/1.01) 0.903
Chronic total occlusion of coronary artery (/1.01) 0.904
Chronic pansinusitis (x1.01) 0.904
Streptococcal sore throat (x1.01) 0.904
Asymmetrical sensorineural hearing loss (/1.01) 0.905
Mixed hyperlipidemia (/1.01) 0.905
Abnormal breathing (x1.01) 0.906
Uncomplicated asthma (x1.01) 0.906
nitroglycerin (-0.00112) 0.907
fluticasone (+0.00111) 0.907
Oppositional defiant disorder (x1.01) 0.907
Hypertensive retinopathy (/1.01) 0.908
Acute exacerbation of chronic obstructive airways disease with asthma (x1.01) 0.908
Acquired hallux valgus (/1.01) 0.909
Finding of vision of eye (/1.01) 0.909
Disorder of upper respiratory system (x1.01) 0.909
Disorder of cardiovascular prostheses and implants (/1.01) 0.910
Acute renal failure syndrome (/1.01) 0.910
Pleural effusion (/1.01) 0.910
Viral disease (x1.01) 0.911
Common cold (x1.01) 0.911
Edema of larynx (x1.01) 0.911
Immature granulocytes [#/V] in B by AC (-0.00126) 0.912
Mural thrombus of heart (x1.01) 0.912
Constipation (x1.01) 0.912
fesoterodine (-0.00103) 0.913
Nonvenomous insect bite without infection (x1.01) 0.913
Chronic non-suppurative otitis media (/1.01) 0.914
Expressions
−0.6 −0.4 −0.2 0.0 0.2 0.4 0.6

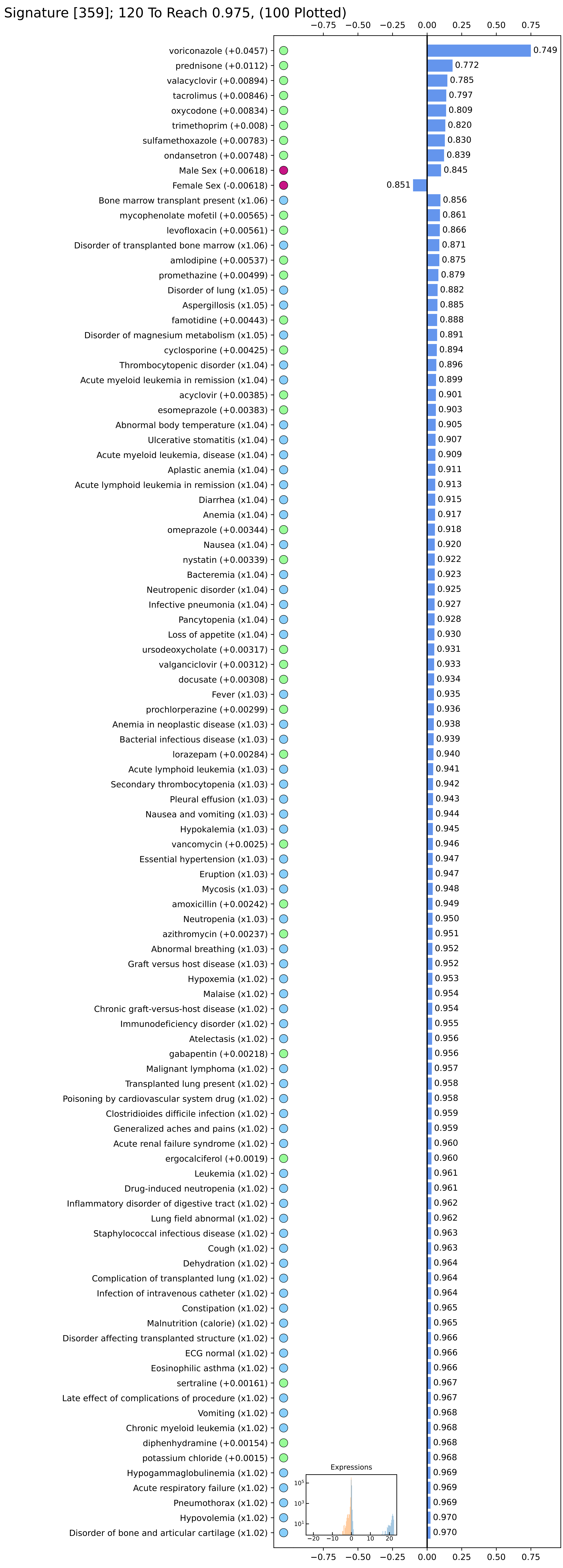

Signature [359]; 120 To Reach 0.975, (100 Plotted)
voriconazole (+0.0457) 0.749
prednisone (+0.0112) 0.772
valacyclovir (+0.00894) 0.785
tacrolimus (+0.00846) 0.797
oxycodone (+0.00834) 0.809
trimethoprim (+0.008) 0.820
sulfamethoxazole (+0.00783) 0.830
ondansetron (+0.00748) 0.839
Male Sex (+0.00618) 0.845
Female Sex (-0.00618) 0.851
Bone marrow transplant present (x1.06) 0.856
mycophenolate mofetil (+0.00565) 0.861
levofloxacin (+0.00561) 0.866
Disorder of transplanted bone marrow (x1.06) 0.871
amlodipine (+0.00537) 0.875
promethazine (+0.00499) 0.879
Disorder of lung (x1.05) 0.882
Aspergillosis (x1.05) 0.885
famotidine (+0.00443) 0.888
Disorder of magnesium metabolism (x1.05) 0.891
cyclosporine (+0.00425) 0.894
Thrombocytopenic disorder (x1.04) 0.896
Acute myeloid leukemia in remission (x1.04) 0.899
acyclovir (+0.00385) 0.901
esomeprazole (+0.00383) 0.903
Abnormal body temperature (x1.04) 0.905
Ulcerative stomatitis (x1.04) 0.907
Acute myeloid leukemia, disease (x1.04) 0.909
Aplastic anemia (x1.04) 0.911
Acute lymphoid leukemia in remission (x1.04) 0.913
Diarrhea (x1.04) 0.915
Anemia (x1.04) 0.917
omeprazole (+0.00344) 0.918
Nausea (x1.04) 0.920
nystatin (+0.00339) 0.922
Bacteremia (x1.04) 0.923
Neutropenic disorder (x1.04) 0.925
Infective pneumonia (x1.04) 0.927
Pancytopenia (x1.04) 0.928
Loss of appetite (x1.04) 0.930
ursodeoxycholate (+0.00317) 0.931
valganciclovir (+0.00312) 0.933
docusate (+0.00308) 0.934
Fever (x1.03) 0.935
prochlorperazine (+0.00299) 0.936
Anemia in neoplastic disease (x1.03) 0.938
Bacterial infectious disease (x1.03) 0.939
lorazepam (+0.00284) 0.940
Acute lymphoid leukemia (x1.03) 0.941
Secondary thrombocytopenia (x1.03) 0.942
Pleural effusion (x1.03) 0.943
Nausea and vomiting (x1.03) 0.944
Hypokalemia (x1.03) 0.945
vancomycin (+0.0025) 0.946
Essential hypertension (x1.03) 0.947
Eruption (x1.03) 0.947
Mycosis (x1.03) 0.948
amoxicillin (+0.00242) 0.949
Neutropenia (x1.03) 0.950
azithromycin (+0.00237) 0.951
Abnormal breathing (x1.03) 0.952
Graft versus host disease (x1.03) 0.952
Hypoxemia (x1.02) 0.953
Malaise (x1.02) 0.954
Chronic graft-versus-host disease (x1.02) 0.954
Immunodeficiency disorder (x1.02) 0.955
Atelectasis (x1.02) 0.956
gabapentin (+0.00218) 0.956
Malignant lymphoma (x1.02) 0.957
Transplanted lung present (x1.02) 0.958
Poisoning by cardiovascular system drug (x1.02) 0.958
Clostridioides difficile infection (x1.02) 0.959
Generalized aches and pains (x1.02) 0.959
Acute renal failure syndrome (x1.02) 0.960
ergocalciferol (+0.0019) 0.960
Leukemia (x1.02) 0.961
Drug-induced neutropenia (x1.02) 0.961
Inflammatory disorder of digestive tract (x1.02) 0.962
Lung field abnormal (x1.02) 0.962
Staphylococcal infectious disease (x1.02) 0.963
Cough (x1.02) 0.963
Dehydration (x1.02) 0.964
Complication of transplanted lung (x1.02) 0.964
Infection of intravenous catheter (x1.02) 0.964
Constipation (x1.02) 0.965
Malnutrition (calorie) (x1.02) 0.965
Disorder affecting transplanted structure (x1.02) 0.966
ECG normal (x1.02) 0.966
Eosinophilic asthma (x1.02) 0.966
sertraline (+0.00161) 0.967
Late effect of complications of procedure (x1.02) 0.967
Vomiting (x1.02) 0.968
Chronic myeloid leukemia (x1.02) 0.968
diphenhydramine (+0.00154) 0.968
potassium chloride (+0.0015) 0.968
Hypogammaglobulinemia (x1.02) 0.969
Acute respiratory failure (x1.02) 0.969
Pneumothorax (x1.02) 0.969
Hypovolemia (x1.02) 0.970
Disorder of bone and articular cartilage (x1.02) 0.970
−0.75 −0.50 −0.25 0.00 0.25 0.50 0.75
Expressions
−20 −10 0 10 20

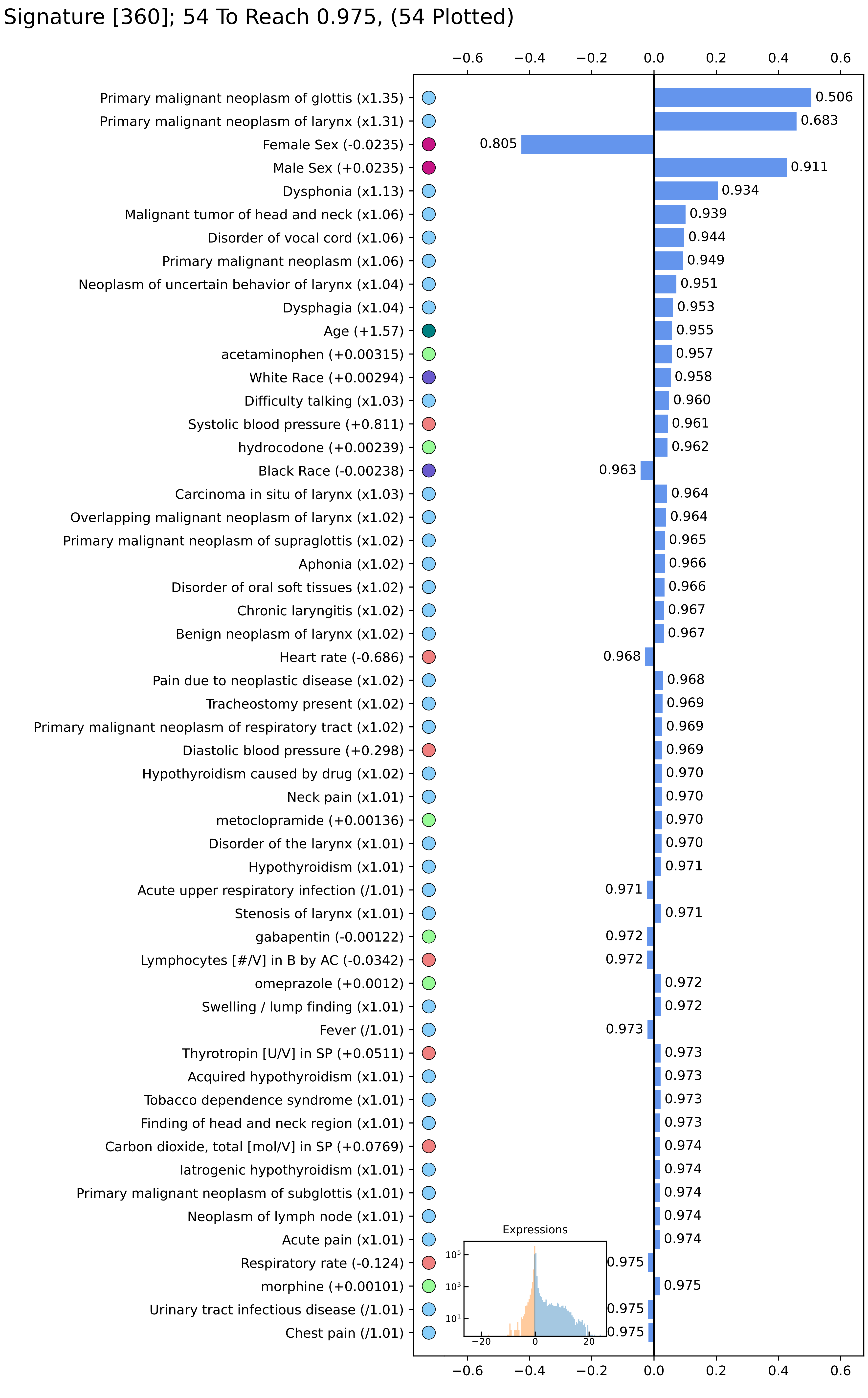

Signature [360]; 54 To Reach 0.975, (54 Plotted)
Primary malignant neoplasm of glottis (x1.35) 0.506
Primary malignant neoplasm of larynx (x1.31) 0.683
Female Sex (-0.0235) 0.805
Male Sex (+0.0235) 0.911
Dysphonia (x1.13) 0.934
Malignant tumor of head and neck (x1.06) 0.939
Disorder of vocal cord (x1.06) 0.944
Primary malignant neoplasm (x1.06) 0.949
Neoplasm of uncertain behavior of larynx (x1.04) 0.951
Dysphagia (x1.04) 0.953
Age (+1.57) 0.955
acetaminophen (+0.00315) 0.957
White Race (+0.00294) 0.958
Difficulty talking (x1.03) 0.960
Systolic blood pressure (+0.811) 0.961
hydrocodone (+0.00239) 0.962
Black Race (-0.00238) 0.963
Carcinoma in situ of larynx (x1.03) 0.964
Overlapping malignant neoplasm of larynx (x1.02) 0.964
Primary malignant neoplasm of supraglottis (x1.02) 0.965
Aphonia (x1.02) 0.966
Disorder of oral soft tissues (x1.02) 0.966
Chronic laryngitis (x1.02) 0.967
Benign neoplasm of larynx (x1.02) 0.967
Heart rate (-0.686) 0.968
Pain due to neoplastic disease (x1.02) 0.968
Tracheostomy present (x1.02) 0.969
Primary malignant neoplasm of respiratory tract (x1.02) 0.969
Diastolic blood pressure (+0.298) 0.969
Hypothyroidism caused by drug (x1.02) 0.970
Neck pain (x1.01) 0.970
metoclopramide (+0.00136) 0.970
Disorder of the larynx (x1.01) 0.970
Hypothyroidism (x1.01) 0.971
Acute upper respiratory infection (/1.01) 0.971
Stenosis of larynx (x1.01) 0.971
gabapentin (-0.00122) 0.972
Lymphocytes [#/V] in B by AC (-0.0342) 0.972
omeprazole (+0.0012) 0.972
Swelling / lump finding (x1.01) 0.972
Fever (/1.01) 0.973
Thyrotropin [U/V] in SP (+0.0511) 0.973
Acquired hypothyroidism (x1.01) 0.973
Tobacco dependence syndrome (x1.01) 0.973
Finding of head and neck region (x1.01) 0.973
Carbon dioxide, total [mol/V] in SP (+0.0769) 0.974
Iatrogenic hypothyroidism (x1.01) 0.974
Primary malignant neoplasm of subglottis (x1.01) 0.974
Neoplasm of lymph node (x1.01) 0.974
Acute pain (x1.01) 0.974
Respiratory rate (-0.124) 0.975
morphine (+0.00101) 0.975
Urinary tract infectious disease (/1.01) 0.975
Chest pain (/1.01) 0.975
−0.6 −0.4 −0.2 0.0 0.2 0.4 0.6
Expressions

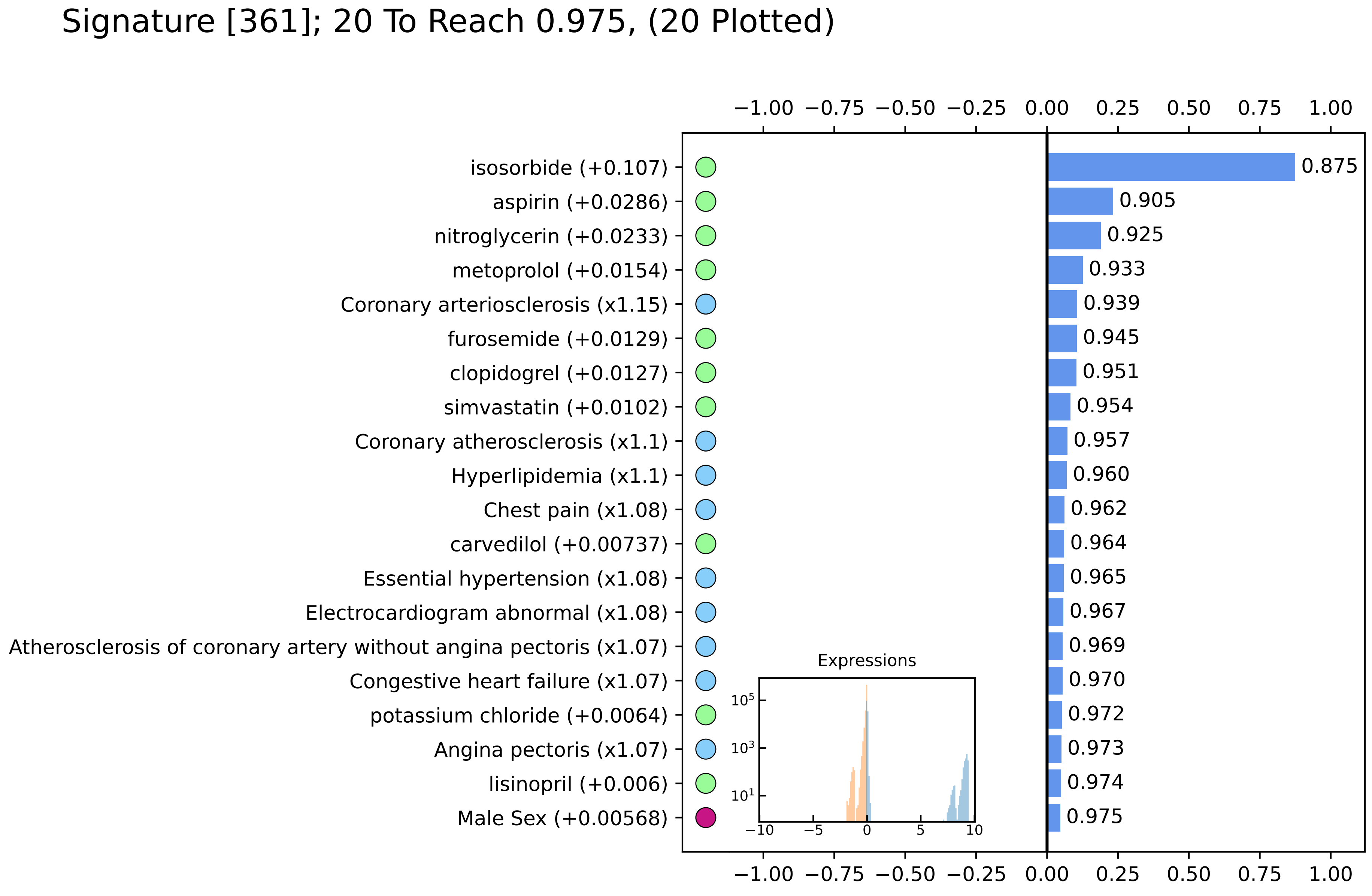
Signature [361]; 20 To Reach 0.975, (20 Plotted)
isosorbide (+0.107)
aspirin (+0.0286)
nitroglycerin (+0.0233)
metoprolol (+0.0154)
Coronary arteriosclerosis (x1.15)
furosemide (+0.0129)
clopidogrel (+0.0127)
simvastatin (+0.0102)
Coronary atherosclerosis (x1.1)
Hyperlipidemia (x1.1)
Chest pain (x1.08)
carvedilol (+0.00737)
Essential hypertension (x1.08)
Electrocardiogram abnormal (x1.08)
Atherosclerosis of coronary artery without angina pectoris (x1.07)
Congestive heart failure (x1.07)
potassium chloride (+0.0064)
Angina pectoris (x1.07)
lisinopril (+0.006)
Male Sex (+0.00568)
0.875
0.905
0.925
0.933
0.939
0.945
0.951
0.954
0.957
0.960
0.962
0.964
0.965
0.967
0.969
0.970
0.972
0.973
0.974
0.975
Expressions
−1.00 −0.75 −0.50 −0.25 0.00 0.25 0.50 0.75 1.00

Signature [362]; 33 To Reach 0.975, (33 Plotted)

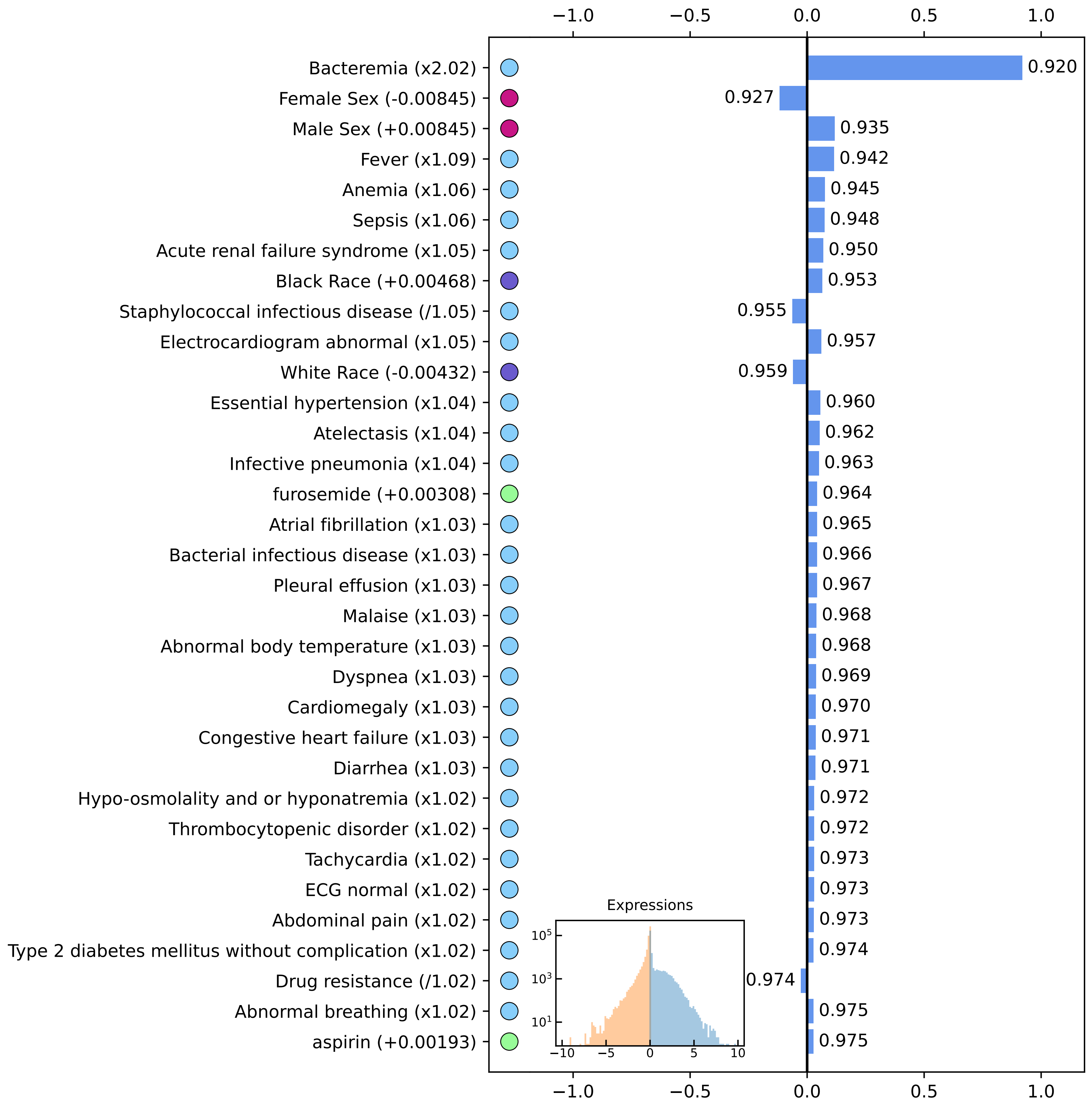

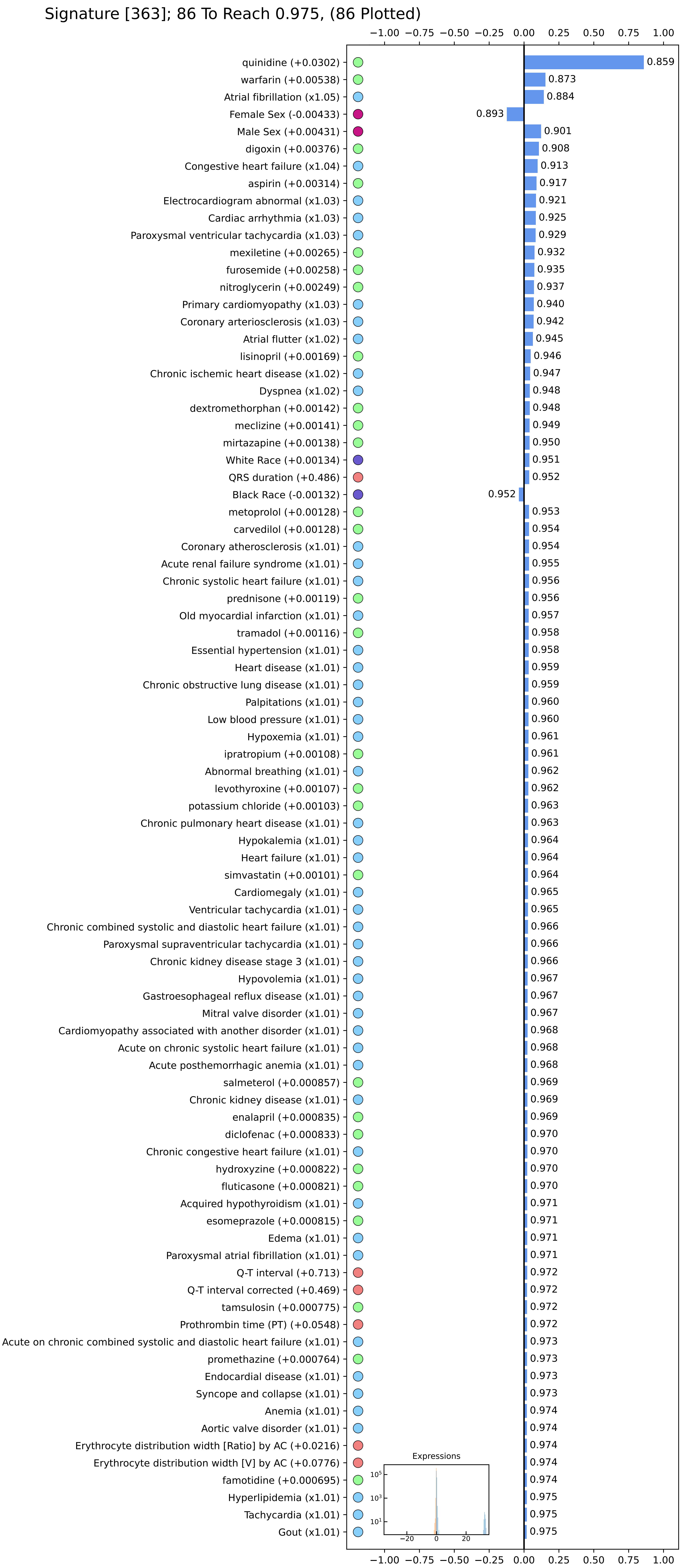

Signature [363]; 86 To Reach 0.975, (86 Plotted)
quinidine (+0.0302) 0.859
warfarin (+0.00538) 0.873
Atrial fibrillation (x1.05) 0.884
Female Sex (-0.00433) 0.893
Male Sex (+0.00431) 0.901
digoxin (+0.00376) 0.908
Congestive heart failure (x1.04) 0.913
aspirin (+0.00314) 0.917
Electrocardiogram abnormal (x1.03) 0.921
Cardiac arrhythmia (x1.03) 0.925
Paroxysmal ventricular tachycardia (x1.03) 0.929
mexiletine (+0.00265) 0.932
furosemide (+0.00258) 0.935
nitroglycerin (+0.00249) 0.937
Primary cardiomyopathy (x1.03) 0.940
Coronary arteriosclerosis (x1.03) 0.942
Atrial flutter (x1.02) 0.945
lisinopril (+0.00169) 0.946
Chronic ischemic heart disease (x1.02) 0.947
Dyspnea (x1.02) 0.948
dextromethorphan (+0.00142) 0.948
meclizine (+0.00141) 0.949
mirtazapine (+0.00138) 0.950
White Race (+0.00134) 0.951
QRS duration (+0.486) 0.952
Black Race (-0.00132) 0.952
metoprolol (+0.00128) 0.953
carvedilol (+0.00128) 0.954
Coronary atherosclerosis (x1.01) 0.954
Acute renal failure syndrome (x1.01) 0.955
Chronic systolic heart failure (x1.01) 0.956
prednisone (+0.00119) 0.956
Old myocardial infarction (x1.01) 0.957
tramadol (+0.00116) 0.958
Essential hypertension (x1.01) 0.958
Heart disease (x1.01) 0.959
Chronic obstructive lung disease (x1.01) 0.959
Palpitations (x1.01) 0.960
Low blood pressure (x1.01) 0.960
Hypoxemia (x1.01) 0.961
ipratropium (+0.00108) 0.961
Abnormal breathing (x1.01) 0.962
levothyroxine (+0.00107) 0.962
potassium chloride (+0.00103) 0.963
Chronic pulmonary heart disease (x1.01) 0.963
Hypokalemia (x1.01) 0.964
Heart failure (x1.01) 0.964
simvastatin (+0.00101) 0.964
Cardiomegaly (x1.01) 0.965
Ventricular tachycardia (x1.01) 0.965
Chronic combined systolic and diastolic heart failure (x1.01) 0.966
Paroxysmal supraventricular tachycardia (x1.01) 0.966
Chronic kidney disease stage 3 (x1.01) 0.966
Hypovolemia (x1.01) 0.967
Gastroesophageal reflux disease (x1.01) 0.967
Mitral valve disorder (x1.01) 0.967
Cardiomyopathy associated with another disorder (x1.01) 0.968
Acute on chronic systolic heart failure (x1.01) 0.968
Acute posthemorrhagic anemia (x1.01) 0.968
salmeterol (+0.000857) 0.969
Chronic kidney disease (x1.01) 0.969
enalapril (+0.000835) 0.969
diclofenac (+0.000833) 0.970
Chronic congestive heart failure (x1.01) 0.970
hydroxyzine (+0.000822) 0.970
fluticasone (+0.000821) 0.970
Acquired hypothyroidism (x1.01) 0.971
esomeprazole (+0.000815) 0.971
Edema (x1.01) 0.971
Paroxysmal atrial fibrillation (x1.01) 0.971
Q-T interval (+0.713) 0.972
Q-T interval corrected (+0.469) 0.972
tamsulosin (+0.000775) 0.972
Prothrombin time (PT) (+0.0548) 0.972
Acute on chronic combined systolic and diastolic heart failure (x1.01) 0.973
promethazine (+0.000764) 0.973
Endocardial disease (x1.01) 0.973
Syncope and collapse (x1.01) 0.973
Anemia (x1.01) 0.974
Aortic valve disorder (x1.01) 0.974
Erythrocyte distribution width [Ratio] by AC (+0.0216) 0.974
Erythrocyte distribution width [V] by AC (+0.0776) 0.974
famotidine (+0.000695) 0.974
Hyperlipidemia (x1.01) 0.975
Tachycardia (x1.01) 0.975
Gout (x1.01) 0.975
−1.00 −0.75 −0.50 −0.25 0.00 0.25 0.50 0.75 1.00
Expressions

Signature [364]; 14 To Reach 0.975, (14 Plotted)

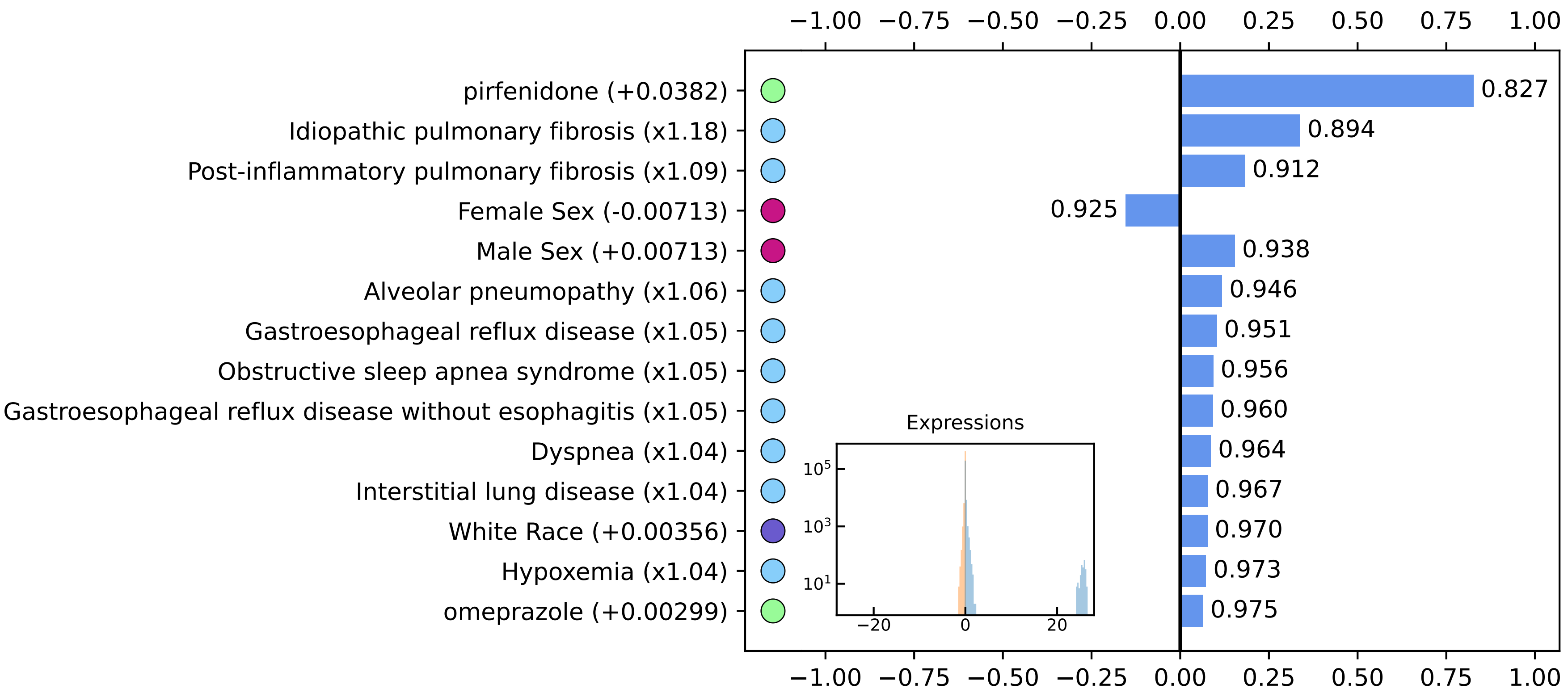

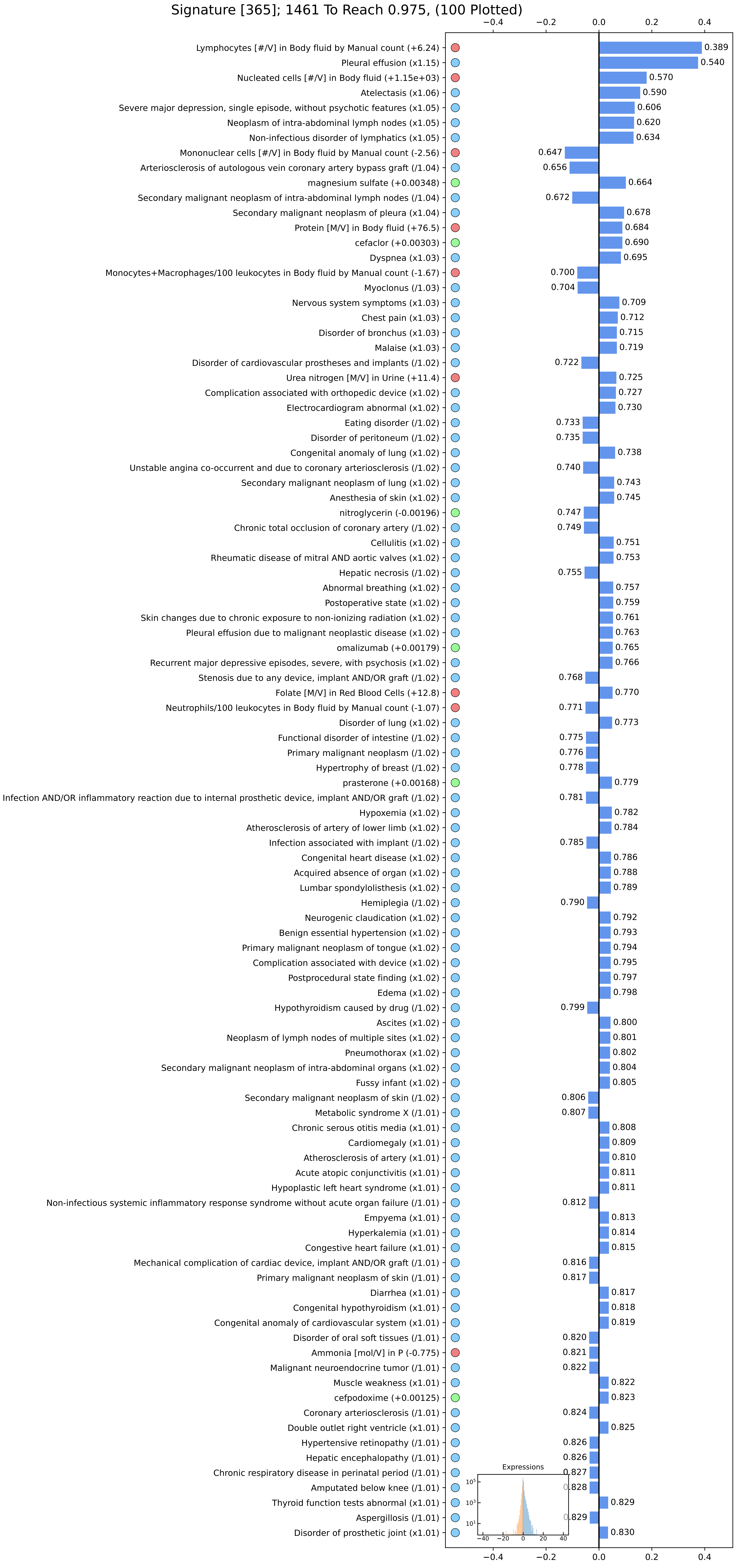

Signature [365]; 1461 To Reach 0.975, (100 Plotted)
Lymphocytes [#/V] in Body fluid by Manual count (+6.24) 0.389
Pleural effusion (x1.15) 0.540
Nucleated cells [#/V] in Body fluid (+1.15e+03) 0.570
Atelectasis (x1.06) 0.590
Severe major depression, single episode, without psychotic features (x1.05) 0.606
Neoplasm of intra-abdominal lymph nodes (x1.05) 0.620
Non-infectious disorder of lymphatics (x1.05) 0.634
Mononuclear cells [#/V] in Body fluid by Manual count (-2.56) 0.647
Arteriosclerosis of autologous vein coronary artery bypass graft (/1.04) 0.656
magnesium sulfate (+0.00348) 0.664
Secondary malignant neoplasm of intra-abdominal lymph nodes (/1.04) 0.672
Secondary malignant neoplasm of pleura (x1.04) 0.678
Protein [M/V] in Body fluid (+76.5) 0.684
cefaclor (+0.00303) 0.690
Dyspnea (x1.03) 0.695
Monocytes+Macrophages/100 leukocytes in Body fluid by Manual count (-1.67) 0.700
Myoclonus (/1.03) 0.704
Nervous system symptoms (x1.03) 0.709
Chest pain (x1.03) 0.712
Disorder of bronchus (x1.03) 0.715
Malaise (x1.03) 0.719
Disorder of cardiovascular prostheses and implants (/1.02) 0.722
Urea nitrogen [M/V] in Urine (+11.4) 0.725
Complication associated with orthopedic device (x1.02) 0.727
Electrocardiogram abnormal (x1.02) 0.730
Eating disorder (/1.02) 0.733
Disorder of peritoneum (/1.02) 0.735
Congenital anomaly of lung (x1.02) 0.738
Unstable angina co-occurrent and due to coronary arteriosclerosis (/1.02) 0.740
Secondary malignant neoplasm of lung (x1.02) 0.743
Anesthesia of skin (x1.02) 0.745
nitroglycerin (-0.00196) 0.747
Chronic total occlusion of coronary artery (/1.02) 0.749
Cellulitis (x1.02) 0.751
Rheumatic disease of mitral AND aortic valves (x1.02) 0.753
Hepatic necrosis (/1.02) 0.755
Abnormal breathing (x1.02) 0.757
Postoperative state (x1.02) 0.759
Skin changes due to chronic exposure to non-ionizing radiation (x1.02) 0.761
Pleural effusion due to malignant neoplastic disease (x1.02) 0.763
omalizumab (+0.00179) 0.765
Recurrent major depressive episodes, severe, with psychosis (x1.02) 0.766
Stenosis due to any device, implant AND/OR graft (/1.02) 0.768
Folate [M/V] in Red Blood Cells (+12.8) 0.770
Neutrophils/100 leukocytes in Body fluid by Manual count (-1.07) 0.771
Disorder of lung (x1.02) 0.773
Functional disorder of intestine (/1.02) 0.775
Primary malignant neoplasm (/1.02) 0.776
Hypertrophy of breast (/1.02) 0.778
prasterone (+0.00168) 0.779
Infection AND/OR inflammatory reaction due to internal prosthetic device, implant AND/OR graft (/1.02) 0.781
Hypoxemia (x1.02) 0.782
Atherosclerosis of artery of lower limb (x1.02) 0.784
Infection associated with implant (/1.02) 0.785
Congenital heart disease (x1.02) 0.786
Acquired absence of organ (x1.02) 0.788
Lumbar spondylolisthesis (x1.02) 0.789
Hemiplegia (/1.02) 0.790
Neurogenic claudication (x1.02) 0.792
Benign essential hypertension (x1.02) 0.793
Primary malignant neoplasm of tongue (x1.02) 0.794
Complication associated with device (x1.02) 0.795
Postprocedural state finding (x1.02) 0.797
Edema (x1.02) 0.798
Hypothyroidism caused by drug (/1.02) 0.799
Ascites (x1.02) 0.800
Neoplasm of lymph nodes of multiple sites (x1.02) 0.801
Pneumothorax (x1.02) 0.802
Secondary malignant neoplasm of intra-abdominal organs (x1.02) 0.804
Fussy infant (x1.02) 0.805
Secondary malignant neoplasm of skin (/1.02) 0.806
Metabolic syndrome X (/1.01) 0.807
Chronic serous otitis media (x1.01) 0.808
Cardiomegaly (x1.01) 0.809
Atherosclerosis of artery (x1.01) 0.810
Acute atopic conjunctivitis (x1.01) 0.811
Hypoplastic left heart syndrome (x1.01) 0.811
Non-infectious systemic inflammatory response syndrome without acute organ failure (/1.01) 0.812
Empyema (x1.01) 0.813
Hyperkalemia (x1.01) 0.814
Congestive heart failure (x1.01) 0.815
Mechanical complication of cardiac device, implant AND/OR graft (/1.01) 0.816
Primary malignant neoplasm of skin (/1.01) 0.817
Diarrhea (x1.01) 0.817
Congenital hypothyroidism (x1.01) 0.818
Congenital anomaly of cardiovascular system (x1.01) 0.819
Disorder of oral soft tissues (/1.01) 0.820
Ammonia [mol/V] in P (-0.775) 0.821
Malignant neuroendocrine tumor (/1.01) 0.822
Muscle weakness (x1.01) 0.822
cefpodoxime (+0.00125) 0.823
Coronary arteriosclerosis (/1.01) 0.824
Double outlet right ventricle (x1.01) 0.825
Hypertensive retinopathy (/1.01) 0.826
Hepatic encephalopathy (/1.01) 0.826
Chronic respiratory disease in perinatal period (/1.01) 0.827
Amputated below knee (/1.01) 0.828
Thyroid function tests abnormal (x1.01) 0.829
Aspergillosis (/1.01) 0.829
Disorder of prosthetic joint (x1.01) 0.830
−0.4 −0.2 0.0 0.2 0.4
Expressions

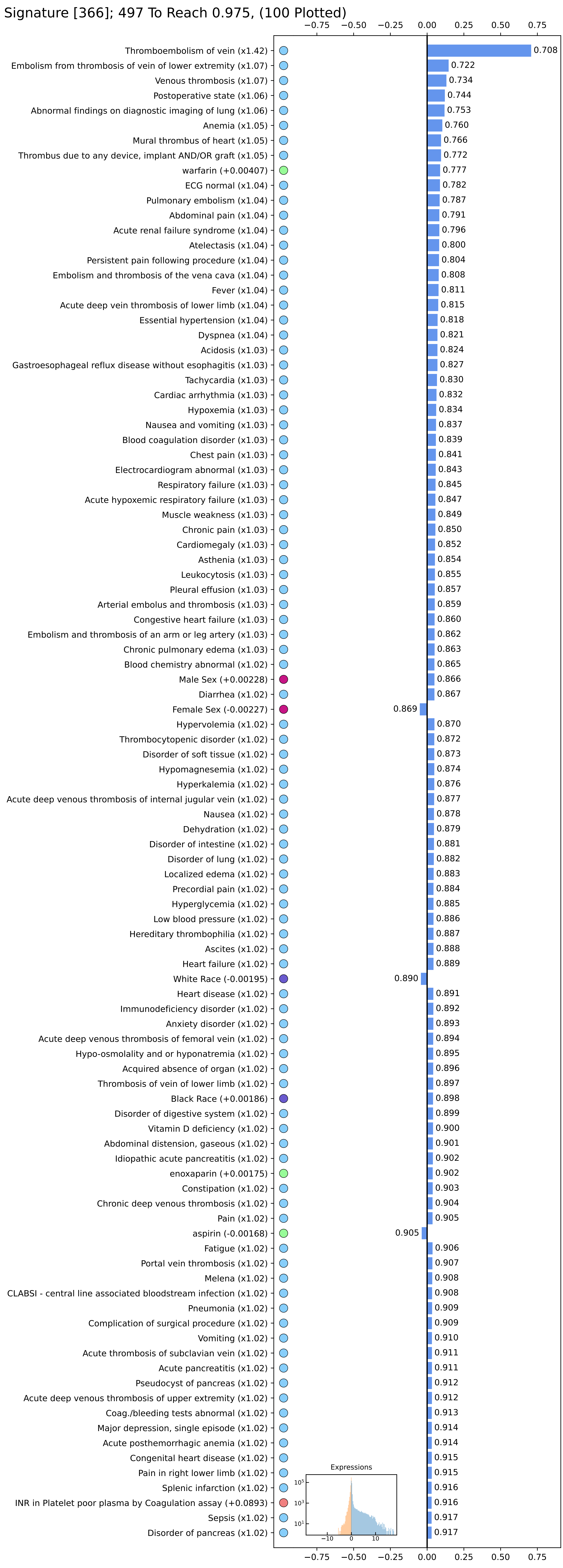
Signature [366]; 497 To Reach 0.975, (100 Plotted)
Thromboembolism of vein (x1.42) 0.708
Embolism from thrombosis of vein of lower extremity (x1.07) 0.722
Venous thrombosis (x1.07) 0.734
Postoperative state (x1.06) 0.744
Abnormal findings on diagnostic imaging of lung (x1.06) 0.753
Anemia (x1.05) 0.760
Mural thrombus of heart (x1.05) 0.766
Thrombus due to any device, implant AND/OR graft (x1.05) 0.772
warfarin (+0.00407) 0.777
ECG normal (x1.04) 0.782
Pulmonary embolism (x1.04) 0.787
Abdominal pain (x1.04) 0.791
Acute renal failure syndrome (x1.04) 0.796
Atelectasis (x1.04) 0.800
Persistent pain following procedure (x1.04) 0.804
Embolism and thrombosis of the vena cava (x1.04) 0.808
Fever (x1.04) 0.811
Acute deep vein thrombosis of lower limb (x1.04) 0.815
Essential hypertension (x1.04) 0.818
Dyspnea (x1.04) 0.821
Acidosis (x1.03) 0.824
Gastroesophageal reflux disease without esophagitis (x1.03) 0.827
Tachycardia (x1.03) 0.830
Cardiac arrhythmia (x1.03) 0.832
Hypoxemia (x1.03) 0.834
Nausea and vomiting (x1.03) 0.837
Blood coagulation disorder (x1.03) 0.839
Chest pain (x1.03) 0.841
Electrocardiogram abnormal (x1.03) 0.843
Respiratory failure (x1.03) 0.845
Acute hypoxemic respiratory failure (x1.03) 0.847
Muscle weakness (x1.03) 0.849
Chronic pain (x1.03) 0.850
Cardiomegaly (x1.03) 0.852
Asthenia (x1.03) 0.854
Leukocytosis (x1.03) 0.855
Pleural effusion (x1.03) 0.857
Arterial embolus and thrombosis (x1.03) 0.859
Congestive heart failure (x1.03) 0.860
Embolism and thrombosis of an arm or leg artery (x1.03) 0.862
Chronic pulmonary edema (x1.03) 0.863
Blood chemistry abnormal (x1.02) 0.865
Male Sex (+0.00228) 0.866
Diarrhea (x1.02) 0.867
Female Sex (-0.00227) 0.869
Hypervolemia (x1.02) 0.870
Thrombocytopenic disorder (x1.02) 0.872
Disorder of soft tissue (x1.02) 0.873
Hypomagnesemia (x1.02) 0.874
Hyperkalemia (x1.02) 0.876
Acute deep venous thrombosis of internal jugular vein (x1.02) 0.877
Nausea (x1.02) 0.878
Dehydration (x1.02) 0.879
Disorder of intestine (x1.02) 0.881
Disorder of lung (x1.02) 0.882
Localized edema (x1.02) 0.883
Precordial pain (x1.02) 0.884
Hyperglycemia (x1.02) 0.885
Low blood pressure (x1.02) 0.886
Hereditary thrombophilia (x1.02) 0.887
Ascites (x1.02) 0.888
Heart failure (x1.02) 0.889
White Race (-0.00195) 0.890
Heart disease (x1.02) 0.891
Immunodeficiency disorder (x1.02) 0.892
Anxiety disorder (x1.02) 0.893
Acute deep venous thrombosis of femoral vein (x1.02) 0.894
Hypo-osmolality and or hyponatremia (x1.02) 0.895
Acquired absence of organ (x1.02) 0.896
Thrombosis of vein of lower limb (x1.02) 0.897
Black Race (+0.00186) 0.898
Disorder of digestive system (x1.02) 0.899
Vitamin D deficiency (x1.02) 0.900
Abdominal distension, gaseous (x1.02) 0.901
Idiopathic acute pancreatitis (x1.02) 0.902
enoxaparin (+0.00175) 0.902
Constipation (x1.02) 0.903
Chronic deep venous thrombosis (x1.02) 0.904
Pain (x1.02) 0.905
aspirin (-0.00168) 0.905
Fatigue (x1.02) 0.906
Portal vein thrombosis (x1.02) 0.907
Melena (x1.02) 0.908
CLABSI - central line associated bloodstream infection (x1.02) 0.908
Pneumonia (x1.02) 0.909
Complication of surgical procedure (x1.02) 0.909
Vomiting (x1.02) 0.910
Acute thrombosis of subclavian vein (x1.02) 0.911
Acute pancreatitis (x1.02) 0.911
Pseudocyst of pancreas (x1.02) 0.912
Acute deep venous thrombosis of upper extremity (x1.02) 0.912
Coag./bleeding tests abnormal (x1.02) 0.913
Major depression, single episode (x1.02) 0.914
Acute posthemorrhagic anemia (x1.02) 0.914
Congenital heart disease (x1.02) 0.915
Pain in right lower limb (x1.02) 0.915
Splenic infarction (x1.02) 0.916
INR in Platelet poor plasma by Coagulation assay (+0.0893) 0.916
Sepsis (x1.02) 0.917
Disorder of pancreas (x1.02) 0.917
−0.75 −0.50 −0.25 0.00 0.25 0.50 0.75
Expressions
−10 0 10

Signature [367]; 269 To Reach 0.975, (100 Plotted)

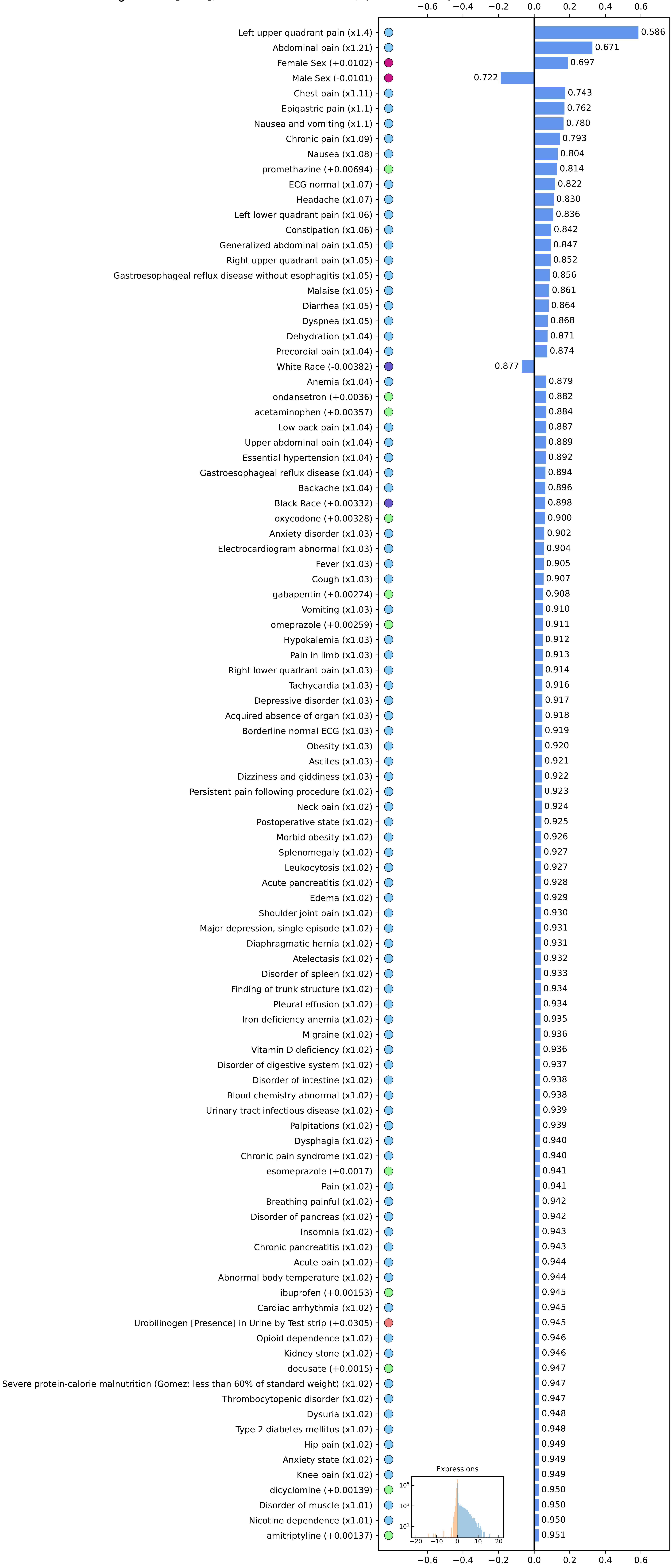

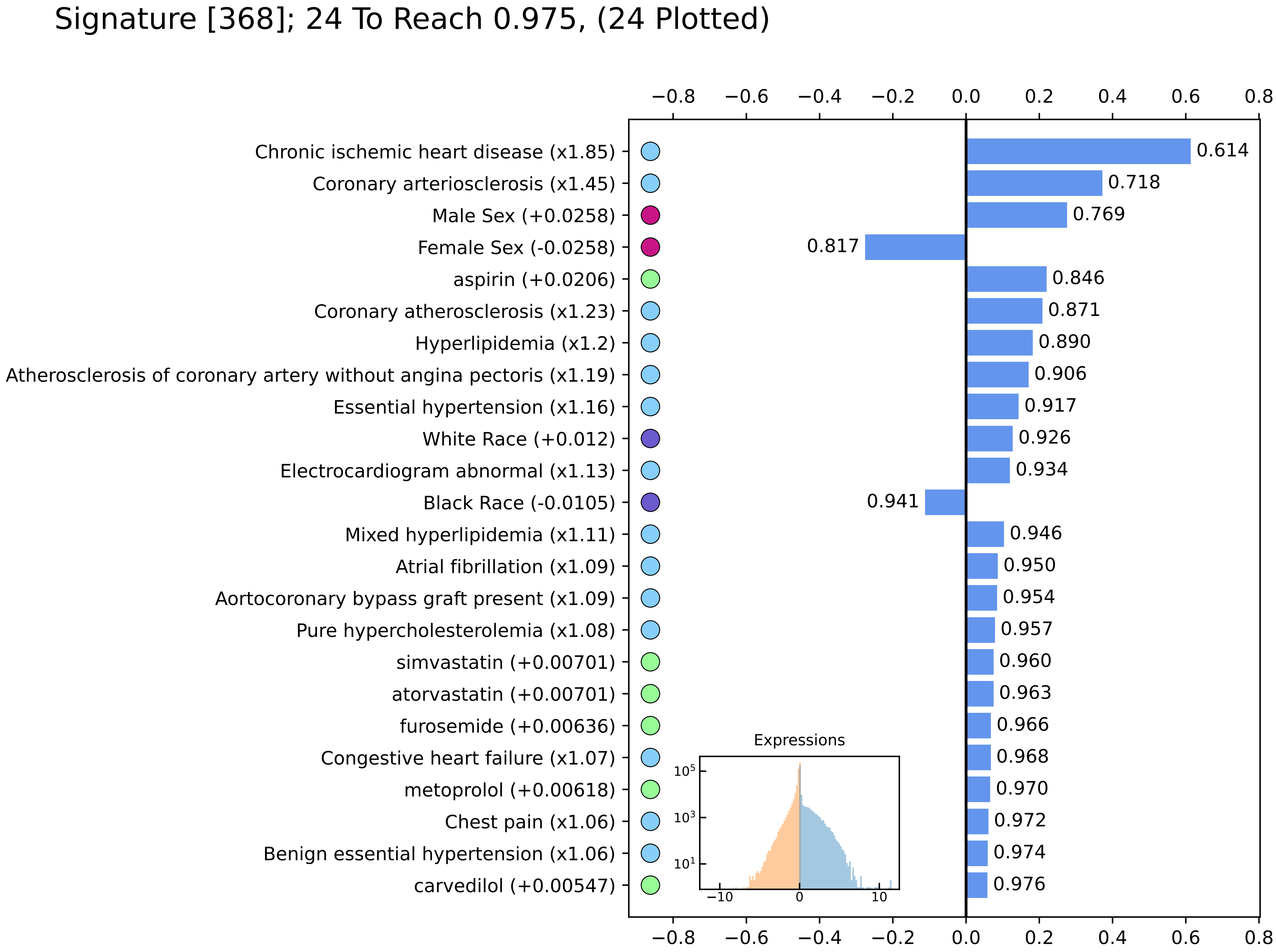

Signature [368]; 24 To Reach 0.975, (24 Plotted)
Chronic ischemic heart disease (x1.85)
0.614
Coronary arteriosclerosis (x1.45)
0.718
Male Sex (+0.0258)
0.769
Female Sex (-0.0258)
0.817
aspirin (+0.0206)
0.846
Coronary atherosclerosis (x1.23)
0.871
Hyperlipidemia (x1.2)
0.890
Atherosclerosis of coronary artery without angina pectoris (x1.19)
0.906
Essential hypertension (x1.16)
0.917
White Race (+0.012)
0.926
Electrocardiogram abnormal (x1.13)
0.934
Black Race (-0.0105)
0.941
Mixed hyperlipidemia (x1.11)
0.946
Atrial fibrillation (x1.09)
0.950
Aortocoronary bypass graft present (x1.09)
0.954
Pure hypercholesterolemia (x1.08)
0.957
simvastatin (+0.00701)
0.960
atorvastatin (+0.00701)
0.963
furosemide (+0.00636)
0.966
Congestive heart failure (x1.07)
0.968
metoprolol (+0.00618)
0.970
Chest pain (x1.06)
0.972
Benign essential hypertension (x1.06)
0.974
carvedilol (+0.00547)
0.976
Expressions
−0.8 −0.6 −0.4 −0.2 0.0 0.2 0.4 0.6 0.8

Signature [369]; 3 To Reach 0.975, (10 Plotted)

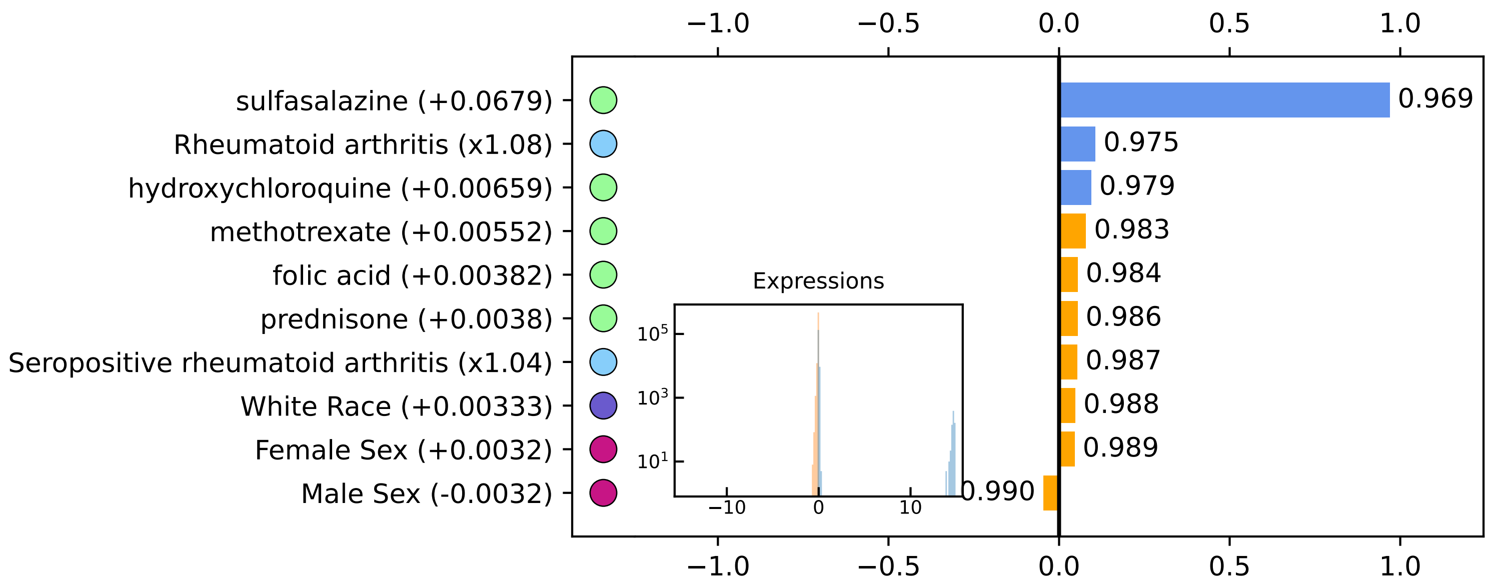

Signature [370]; 5 To Reach 0.975, (10 Plotted)

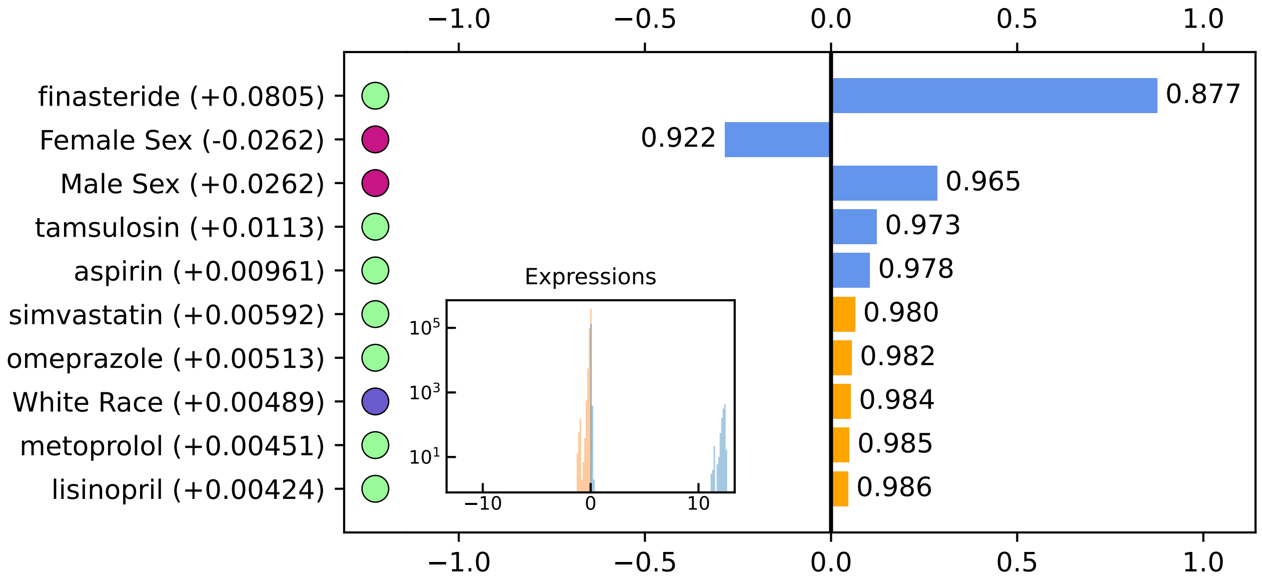

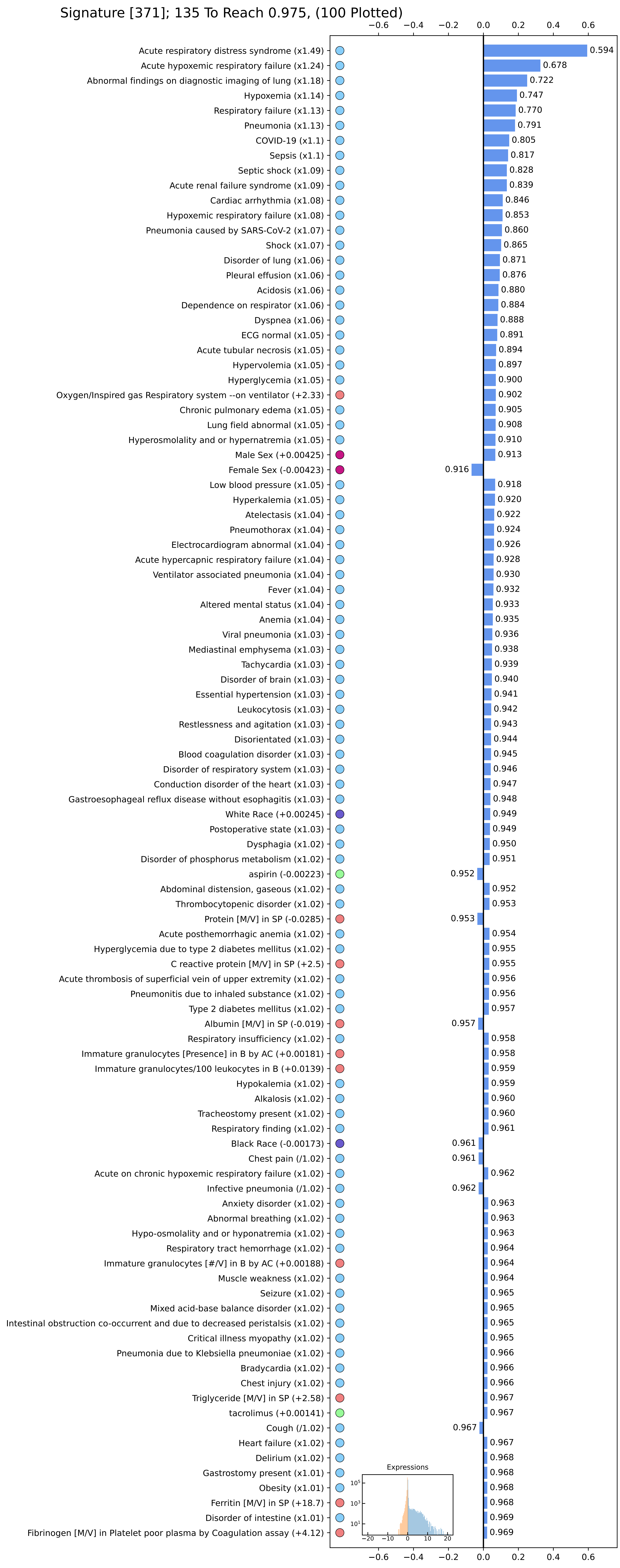

Signature [371]; 135 To Reach 0.975, (100 Plotted)
Acute respiratory distress syndrome (x1.49) 0.594
Acute hypoxemic respiratory failure (x1.24) 0.678
Abnormal findings on diagnostic imaging of lung (x1.18) 0.722
Hypoxemia (x1.14) 0.747
Respiratory failure (x1.13) 0.770
Pneumonia (x1.13) 0.791
COVID-19 (x1.1) 0.805
Sepsis (x1.1) 0.817
Septic shock (x1.09) 0.828
Acute renal failure syndrome (x1.09) 0.839
Cardiac arrhythmia (x1.08) 0.846
Hypoxemic respiratory failure (x1.08) 0.853
Pneumonia caused by SARS-CoV-2 (x1.07) 0.860
Shock (x1.07) 0.865
Disorder of lung (x1.06) 0.871
Pleural effusion (x1.06) 0.876
Acidosis (x1.06) 0.880
Dependence on respirator (x1.06) 0.884
Dyspnea (x1.06) 0.888
ECG normal (x1.05) 0.891
Acute tubular necrosis (x1.05) 0.894
Hypervolemia (x1.05) 0.897
Hyperglycemia (x1.05) 0.900
Oxygen/Inspired gas Respiratory system --on ventilator (+2.33) 0.902
Chronic pulmonary edema (x1.05) 0.905
Lung field abnormal (x1.05) 0.908
Hyperosmolality and or hypernatremia (x1.05) 0.910
Male Sex (+0.00425) 0.913
Female Sex (-0.00423) 0.916
Low blood pressure (x1.05) 0.918
Hyperkalemia (x1.05) 0.920
Atelectasis (x1.04) 0.922
Pneumothorax (x1.04) 0.924
Electrocardiogram abnormal (x1.04) 0.926
Acute hypercapnic respiratory failure (x1.04) 0.928
Ventilator associated pneumonia (x1.04) 0.930
Fever (x1.04) 0.932
Altered mental status (x1.04) 0.933
Anemia (x1.04) 0.935
Viral pneumonia (x1.03) 0.936
Mediastinal emphysema (x1.03) 0.938
Tachycardia (x1.03) 0.939
Disorder of brain (x1.03) 0.940
Essential hypertension (x1.03) 0.941
Leukocytosis (x1.03) 0.942
Restlessness and agitation (x1.03) 0.943
Disorientated (x1.03) 0.944
Blood coagulation disorder (x1.03) 0.945
Disorder of respiratory system (x1.03) 0.946
Conduction disorder of the heart (x1.03) 0.947
Gastroesophageal reflux disease without esophagitis (x1.03) 0.948
White Race (+0.00245) 0.949
Postoperative state (x1.03) 0.949
Dysphagia (x1.02) 0.950
Disorder of phosphorus metabolism (x1.02) 0.951
aspirin (-0.00223) 0.952
Abdominal distension, gaseous (x1.02) 0.952
Thrombocytopenic disorder (x1.02) 0.953
Protein [M/V] in SP (-0.0285) 0.953
Acute posthemorrhagic anemia (x1.02) 0.954
Hyperglycemia due to type 2 diabetes mellitus (x1.02) 0.955
C reactive protein [M/V] in SP (+2.5) 0.955
Acute thrombosis of superficial vein of upper extremity (x1.02) 0.956
Pneumonitis due to inhaled substance (x1.02) 0.956
Type 2 diabetes mellitus (x1.02) 0.957
Albumin [M/V] in SP (-0.019) 0.957
Respiratory insufficiency (x1.02) 0.958
Immature granulocytes [Presence] in B by AC (+0.00181) 0.958
Immature granulocytes/100 leukocytes in B (+0.0139) 0.959
Hypokalemia (x1.02) 0.959
Alkalosis (x1.02) 0.960
Tracheostomy present (x1.02) 0.960
Respiratory finding (x1.02) 0.961
Black Race (-0.00173) 0.961
Chest pain (/1.02) 0.961
Acute on chronic hypoxemic respiratory failure (x1.02) 0.962
Infective pneumonia (/1.02) 0.962
Anxiety disorder (x1.02) 0.963
Abnormal breathing (x1.02) 0.963
Hypo-osmolality and or hyponatremia (x1.02) 0.963
Respiratory tract hemorrhage (x1.02) 0.964
Immature granulocytes [#/V] in B by AC (+0.00188) 0.964
Muscle weakness (x1.02) 0.964
Seizure (x1.02) 0.965
Mixed acid-base balance disorder (x1.02) 0.965
Intestinal obstruction co-occurrent and due to decreased peristalsis (x1.02) 0.965
Critical illness myopathy (x1.02) 0.965
Pneumonia due to Klebsiella pneumoniae (x1.02) 0.966
Bradycardia (x1.02) 0.966
Chest injury (x1.02) 0.966
Triglyceride [M/V] in SP (+2.58) 0.967
tacrolimus (+0.00141) 0.967
Cough (/1.02) 0.967
Heart failure (x1.02) 0.967
Delirium (x1.02) 0.968
Gastrostomy present (x1.01) 0.968
Obesity (x1.01) 0.968
Ferritin [M/V] in SP (+18.7) 0.968
Disorder of intestine (x1.01) 0.969
Fibrinogen [M/V] in Platelet poor plasma by Coagulation assay (+4.12) 0.969
−0.6 −0.4 −0.2 0.0 0.2 0.4 0.6
Expressions

Signature [372]; 272 To Reach 0.975, (100 Plotted)

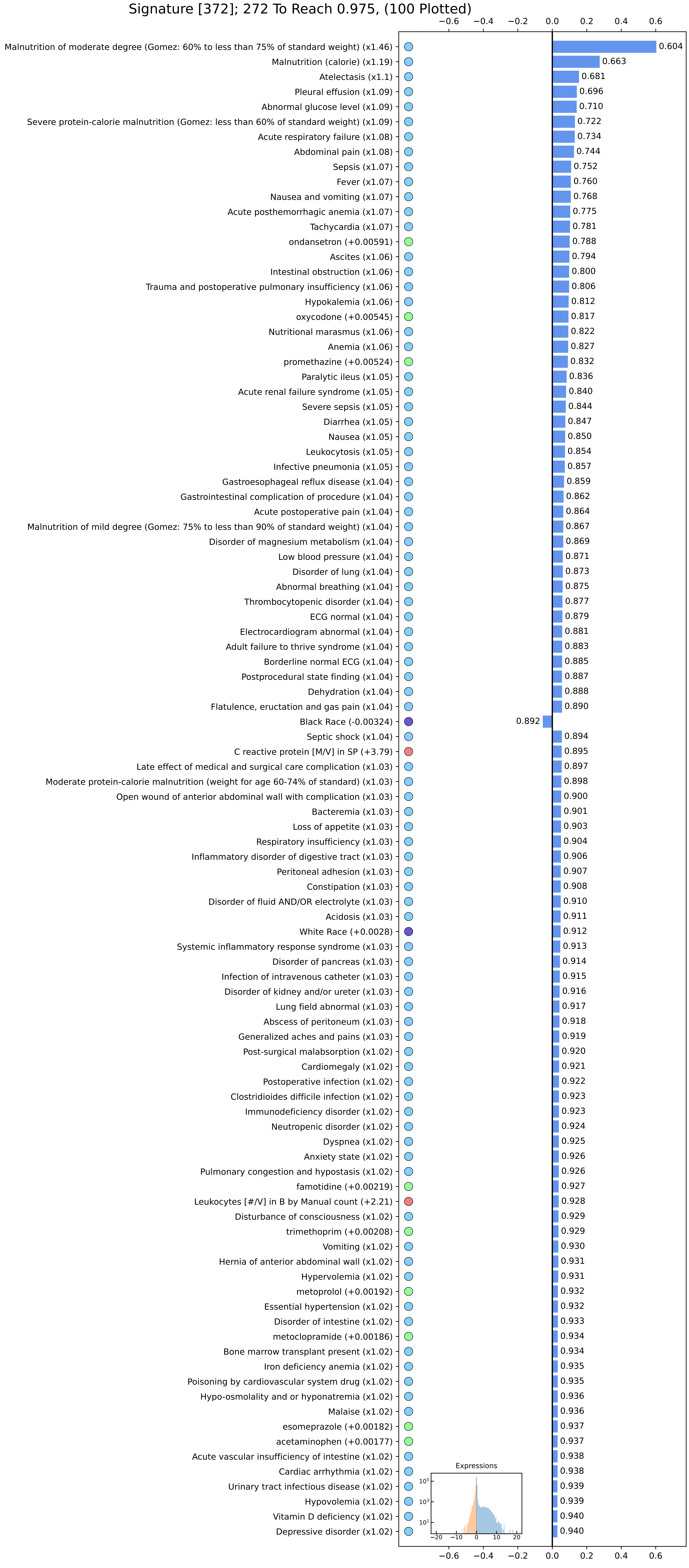

Signature [373]; 1 To Reach 0.975, (10 Plotted)

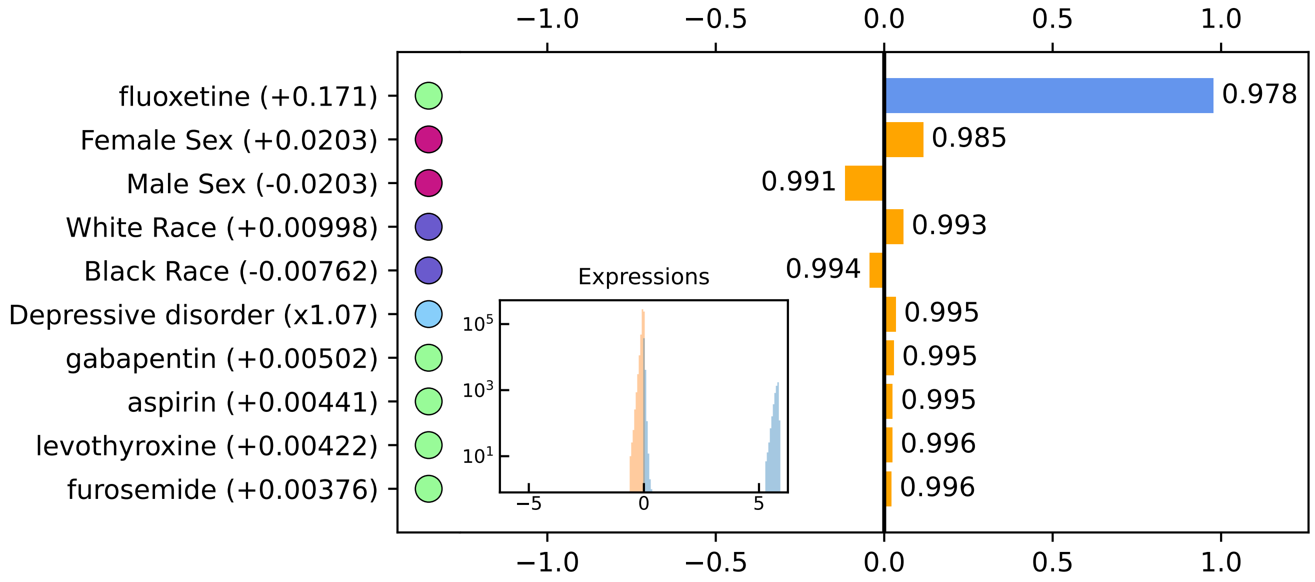

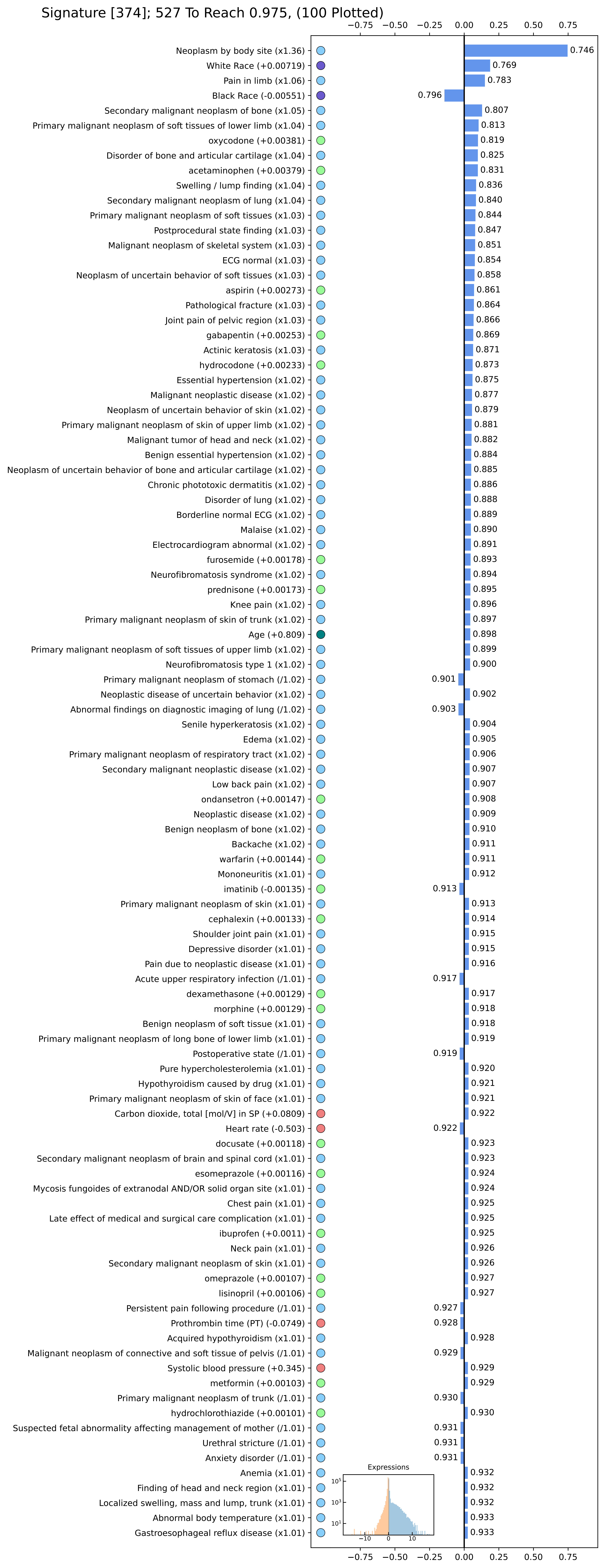

Signature [374]; 527 To Reach 0.975, (100 Plotted)
Neoplasm by body site (x1.36) 0.746
White Race (+0.00719) 0.769
Pain in limb (x1.06) 0.783
Black Race (-0.00551) 0.796
Secondary malignant neoplasm of bone (x1.05) 0.807
Primary malignant neoplasm of soft tissues of lower limb (x1.04) 0.813
oxycodone (+0.00381) 0.819
Disorder of bone and articular cartilage (x1.04) 0.825
acetaminophen (+0.00379) 0.831
Swelling / lump finding (x1.04) 0.836
Secondary malignant neoplasm of lung (x1.04) 0.840
Primary malignant neoplasm of soft tissues (x1.03) 0.844
Postprocedural state finding (x1.03) 0.847
Malignant neoplasm of skeletal system (x1.03) 0.851
ECG normal (x1.03) 0.854
Neoplasm of uncertain behavior of soft tissues (x1.03) 0.858
aspirin (+0.00273) 0.861
Pathological fracture (x1.03) 0.864
Joint pain of pelvic region (x1.03) 0.866
gabapentin (+0.00253) 0.869
Actinic keratosis (x1.03) 0.871
hydrocodone (+0.00233) 0.873
Essential hypertension (x1.02) 0.875
Malignant neoplastic disease (x1.02) 0.877
Neoplasm of uncertain behavior of skin (x1.02) 0.879
Primary malignant neoplasm of skin of upper limb (x1.02) 0.881
Malignant tumor of head and neck (x1.02) 0.882
Benign essential hypertension (x1.02) 0.884
Neoplasm of uncertain behavior of bone and articular cartilage (x1.02) 0.885
Chronic phototoxic dermatitis (x1.02) 0.886
Disorder of lung (x1.02) 0.888
Borderline normal ECG (x1.02) 0.889
Malaise (x1.02) 0.890
Electrocardiogram abnormal (x1.02) 0.891
furosemide (+0.00178) 0.893
Neurofibromatosis syndrome (x1.02) 0.894
prednisone (+0.00173) 0.895
Knee pain (x1.02) 0.896
Primary malignant neoplasm of skin of trunk (x1.02) 0.897
Age (+0.809) 0.898
Primary malignant neoplasm of soft tissues of upper limb (x1.02) 0.899
Neurofibromatosis type 1 (x1.02) 0.900
Primary malignant neoplasm of stomach (/1.02) 0.901
Neoplastic disease of uncertain behavior (x1.02) 0.902
Abnormal findings on diagnostic imaging of lung (/1.02) 0.903
Senile hyperkeratosis (x1.02) 0.904
Edema (x1.02) 0.905
Primary malignant neoplasm of respiratory tract (x1.02) 0.906
Secondary malignant neoplastic disease (x1.02) 0.907
Low back pain (x1.02) 0.907
ondansetron (+0.00147) 0.908
Neoplastic disease (x1.02) 0.909
Benign neoplasm of bone (x1.02) 0.910
Backache (x1.02) 0.911
warfarin (+0.00144) 0.911
Mononeuritis (x1.01) 0.912
imatinib (-0.00135) 0.913
Primary malignant neoplasm of skin (x1.01) 0.913
cephalexin (+0.00133) 0.914
Shoulder joint pain (x1.01) 0.915
Depressive disorder (x1.01) 0.915
Pain due to neoplastic disease (x1.01) 0.916
Acute upper respiratory infection (/1.01) 0.917
dexamethasone (+0.00129) 0.917
morphine (+0.00129) 0.918
Benign neoplasm of soft tissue (x1.01) 0.918
Primary malignant neoplasm of long bone of lower limb (x1.01) 0.919
Postoperative state (/1.01) 0.919
Pure hypercholesterolemia (x1.01) 0.920
Hypothyroidism caused by drug (x1.01) 0.921
Primary malignant neoplasm of skin of face (x1.01) 0.921
Carbon dioxide, total [mol/V] in SP (+0.0809) 0.922
Heart rate (-0.503) 0.922
docusate (+0.00118) 0.923
Secondary malignant neoplasm of brain and spinal cord (x1.01) 0.923
esomeprazole (+0.00116) 0.924
Mycosis fungoides of extranodal AND/OR solid organ site (x1.01) 0.924
Chest pain (x1.01) 0.925
Late effect of medical and surgical care complication (x1.01) 0.925
ibuprofen (+0.0011) 0.925
Neck pain (x1.01) 0.926
Secondary malignant neoplasm of skin (x1.01) 0.926
omeprazole (+0.00107) 0.927
lisinopril (+0.00106) 0.927
Persistent pain following procedure (/1.01) 0.927
Prothrombin time (PT) (-0.0749) 0.928
Acquired hypothyroidism (x1.01) 0.928
Malignant neoplasm of connective and soft tissue of pelvis (/1.01) 0.929
Systolic blood pressure (+0.345) 0.929
metformin (+0.00103) 0.929
Primary malignant neoplasm of trunk (/1.01) 0.930
hydrochlorothiazide (+0.00101) 0.930
Suspected fetal abnormality affecting management of mother (/1.01) 0.931
Urethral stricture (/1.01) 0.931
Anxiety disorder (/1.01) 0.931
Anemia (x1.01) 0.932
Finding of head and neck region (x1.01) 0.932
Localized swelling, mass and lump, trunk (x1.01) 0.932
Abnormal body temperature (x1.01) 0.933
Gastroesophageal reflux disease (x1.01) 0.933
−0.75 −0.50 −0.25 0.00 0.25 0.50 0.75
Expressions

Signature [375]; 123 To Reach 0.975, (100 Plotted)

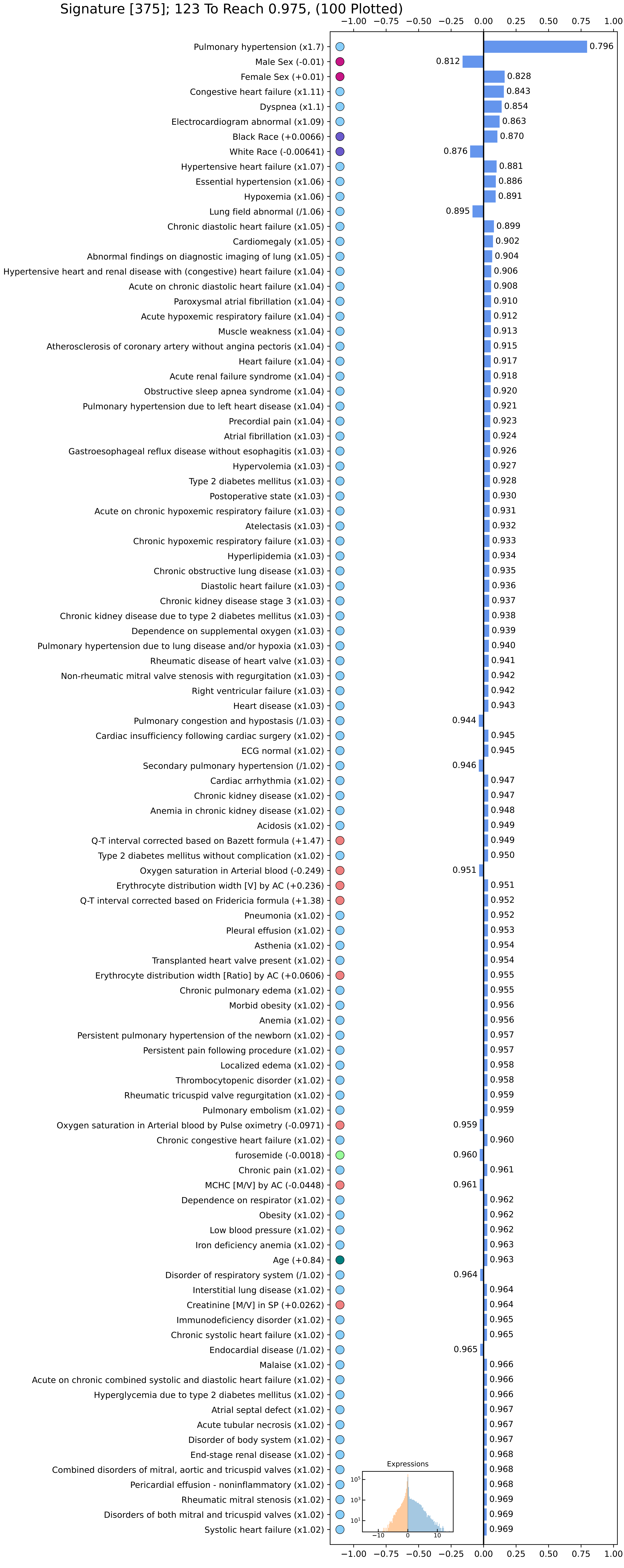

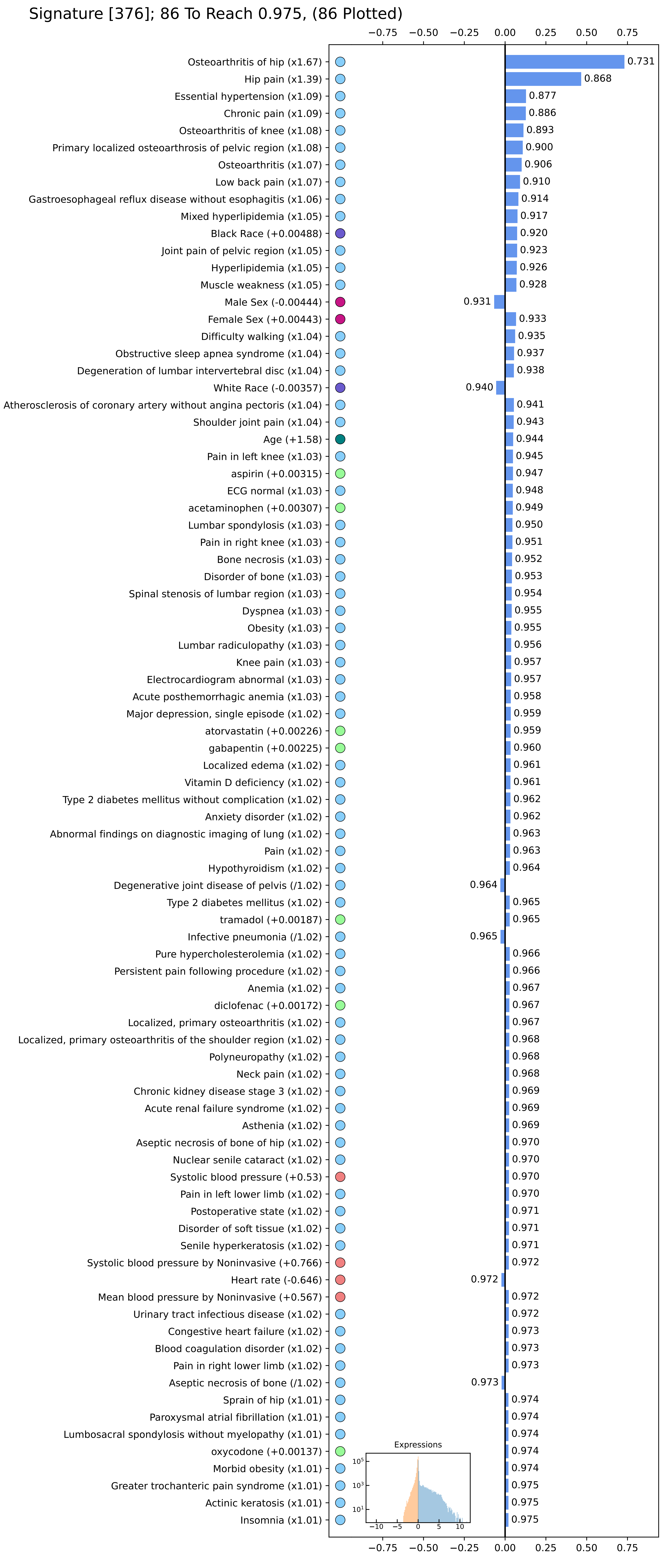

Signature [376]; 86 To Reach 0.975, (86 Plotted)
Osteoarthritis of hip (x1.67) 0.731
Hip pain (x1.39) 0.868
Essential hypertension (x1.09) 0.877
Chronic pain (x1.09) 0.886
Osteoarthritis of knee (x1.08) 0.893
Primary localized osteoarthrosis of pelvic region (x1.08) 0.900
Osteoarthritis (x1.07) 0.906
Low back pain (x1.07) 0.910
Gastroesophageal reflux disease without esophagitis (x1.06) 0.914
Mixed hyperlipidemia (x1.05) 0.917
Black Race (+0.00488) 0.920
Joint pain of pelvic region (x1.05) 0.923
Hyperlipidemia (x1.05) 0.926
Muscle weakness (x1.05) 0.928
Male Sex (-0.00444) 0.931
Female Sex (+0.00443) 0.933
Difficulty walking (x1.04) 0.935
Obstructive sleep apnea syndrome (x1.04) 0.937
Degeneration of lumbar intervertebral disc (x1.04) 0.938
White Race (-0.00357) 0.940
Atherosclerosis of coronary artery without angina pectoris (x1.04) 0.941
Shoulder joint pain (x1.04) 0.943
Age (+1.58) 0.944
Pain in left knee (x1.03) 0.945
aspirin (+0.00315) 0.947
ECG normal (x1.03) 0.948
acetaminophen (+0.00307) 0.949
Lumbar spondylosis (x1.03) 0.950
Pain in right knee (x1.03) 0.951
Bone necrosis (x1.03) 0.952
Disorder of bone (x1.03) 0.953
Spinal stenosis of lumbar region (x1.03) 0.954
Dyspnea (x1.03) 0.955
Obesity (x1.03) 0.955
Lumbar radiculopathy (x1.03) 0.956
Knee pain (x1.03) 0.957
Electrocardiogram abnormal (x1.03) 0.957
Acute posthemorrhagic anemia (x1.03) 0.958
Major depression, single episode (x1.02) 0.959
atorvastatin (+0.00226) 0.959
gabapentin (+0.00225) 0.960
Localized edema (x1.02) 0.961
Vitamin D deficiency (x1.02) 0.961
Type 2 diabetes mellitus without complication (x1.02) 0.962
Anxiety disorder (x1.02) 0.962
Abnormal findings on diagnostic imaging of lung (x1.02) 0.963
Pain (x1.02) 0.963
Hypothyroidism (x1.02) 0.964
Degenerative joint disease of pelvis (/1.02) 0.964
Type 2 diabetes mellitus (x1.02) 0.965
tramadol (+0.00187) 0.965
Infective pneumonia (/1.02) 0.965
Pure hypercholesterolemia (x1.02) 0.966
Persistent pain following procedure (x1.02) 0.966
Anemia (x1.02) 0.967
diclofenac (+0.00172) 0.967
Localized, primary osteoarthritis (x1.02) 0.967
Localized, primary osteoarthritis of the shoulder region (x1.02) 0.968
Polyneuropathy (x1.02) 0.968
Neck pain (x1.02) 0.968
Chronic kidney disease stage 3 (x1.02) 0.969
Acute renal failure syndrome (x1.02) 0.969
Asthenia (x1.02) 0.969
Aseptic necrosis of bone of hip (x1.02) 0.970
Nuclear senile cataract (x1.02) 0.970
Systolic blood pressure (+0.53) 0.970
Pain in left lower limb (x1.02) 0.970
Postoperative state (x1.02) 0.971
Disorder of soft tissue (x1.02) 0.971
Senile hyperkeratosis (x1.02) 0.971
Systolic blood pressure by Noninvasive (+0.766) 0.972
Heart rate (-0.646) 0.972
Mean blood pressure by Noninvasive (+0.567) 0.972
Urinary tract infectious disease (x1.02) 0.972
Congestive heart failure (x1.02) 0.973
Blood coagulation disorder (x1.02) 0.973
Pain in right lower limb (x1.02) 0.973
Aseptic necrosis of bone (/1.02) 0.973
Sprain of hip (x1.01) 0.974
Paroxysmal atrial fibrillation (x1.01) 0.974
Lumbosacral spondylosis without myelopathy (x1.01) 0.974
oxycodone (+0.00137) 0.974
Morbid obesity (x1.01) 0.974
Greater trochanteric pain syndrome (x1.01) 0.975
Actinic keratosis (x1.01) 0.975
Insomnia (x1.01) 0.975
−0.75 −0.50 −0.25 0.00 0.25 0.50 0.75
Expressions

Signature [377]; 187 To Reach 0.975, (100 Plotted)

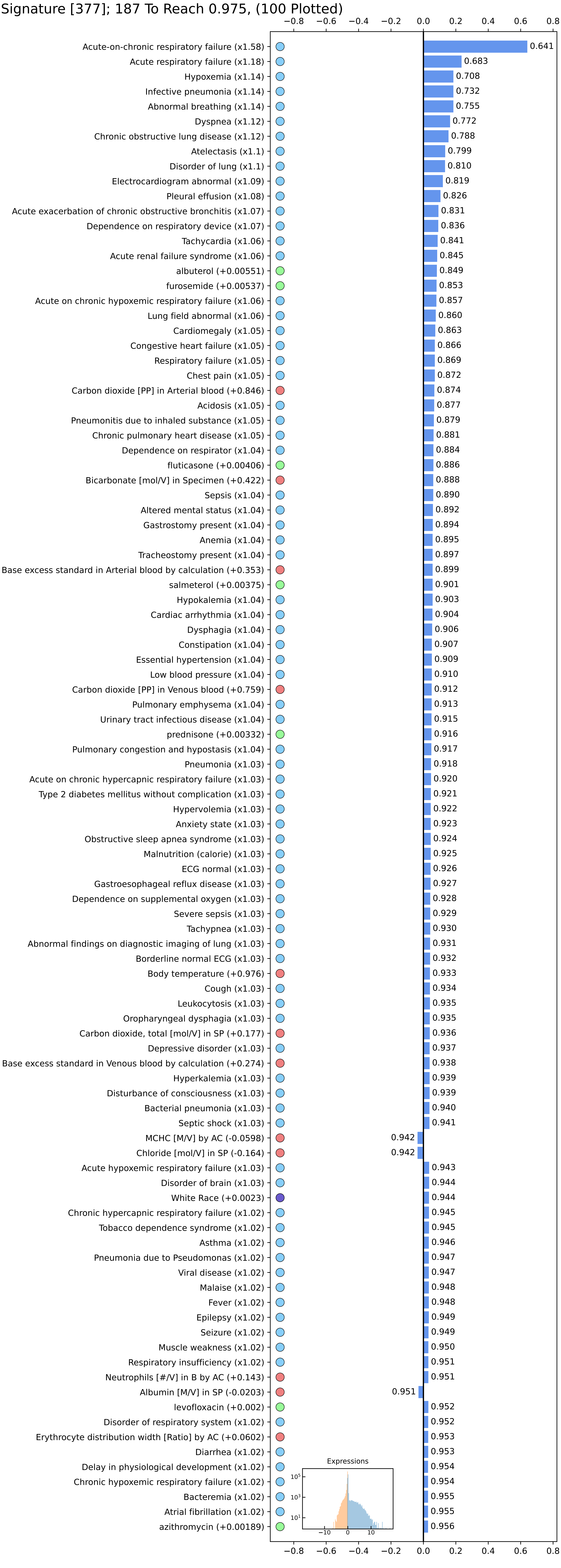

Signature [378]; 9 To Reach 0.975, (10 Plotted)

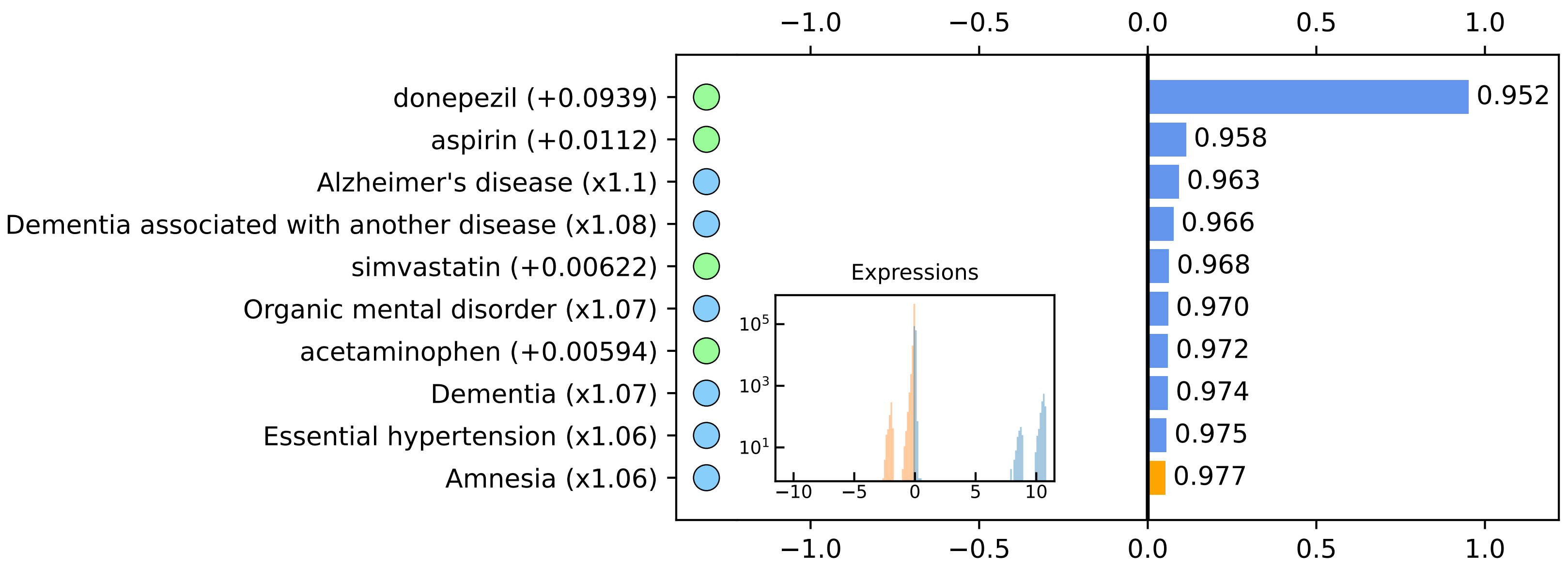

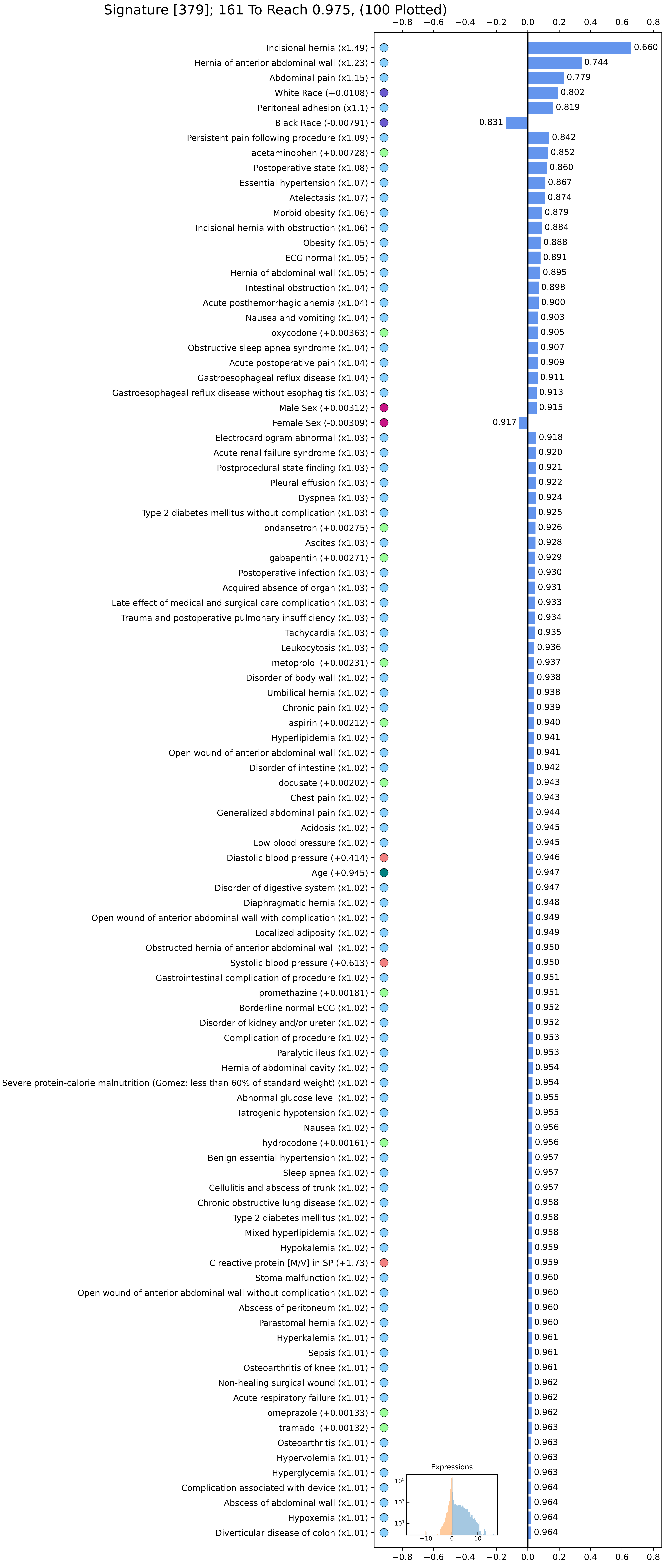
Signature [379]; 161 To Reach 0.975, (100 Plotted)
Incisional hernia (x1.49) 0.660
Hernia of anterior abdominal wall (x1.23) 0.744
Abdominal pain (x1.15) 0.779
White Race (+0.0108) 0.802
Peritoneal adhesion (x1.1) 0.819
Black Race (-0.00791) 0.831
Persistent pain following procedure (x1.09) 0.842
acetaminophen (+0.00728) 0.852
Postoperative state (x1.08) 0.860
Essential hypertension (x1.07) 0.867
Atelectasis (x1.07) 0.874
Morbid obesity (x1.06) 0.879
Incisional hernia with obstruction (x1.06) 0.884
Obesity (x1.05) 0.888
ECG normal (x1.05) 0.891
Hernia of abdominal wall (x1.05) 0.895
Intestinal obstruction (x1.04) 0.898
Acute posthemorrhagic anemia (x1.04) 0.900
Nausea and vomiting (x1.04) 0.903
oxycodone (+0.00363) 0.905
Obstructive sleep apnea syndrome (x1.04) 0.907
Acute postoperative pain (x1.04) 0.909
Gastroesophageal reflux disease (x1.04) 0.911
Gastroesophageal reflux disease without esophagitis (x1.03) 0.913
Male Sex (+0.00312) 0.915
Female Sex (-0.00309) 0.917
Electrocardiogram abnormal (x1.03) 0.918
Acute renal failure syndrome (x1.03) 0.920
Postprocedural state finding (x1.03) 0.921
Pleural effusion (x1.03) 0.922
Dyspnea (x1.03) 0.924
Type 2 diabetes mellitus without complication (x1.03) 0.925
ondansetron (+0.00275) 0.926
Ascites (x1.03) 0.928
gabapentin (+0.00271) 0.929
Postoperative infection (x1.03) 0.930
Acquired absence of organ (x1.03) 0.931
Late effect of medical and surgical care complication (x1.03) 0.933
Trauma and postoperative pulmonary insufficiency (x1.03) 0.934
Tachycardia (x1.03) 0.935
Leukocytosis (x1.03) 0.936
metoprolol (+0.00231) 0.937
Disorder of body wall (x1.02) 0.938
Umbilical hernia (x1.02) 0.938
Chronic pain (x1.02) 0.939
aspirin (+0.00212) 0.940
Hyperlipidemia (x1.02) 0.941
Open wound of anterior abdominal wall (x1.02) 0.941
Disorder of intestine (x1.02) 0.942
docusate (+0.00202) 0.943
Chest pain (x1.02) 0.943
Generalized abdominal pain (x1.02) 0.944
Acidosis (x1.02) 0.945
Low blood pressure (x1.02) 0.945
Diastolic blood pressure (+0.414) 0.946
Age (+0.945) 0.947
Disorder of digestive system (x1.02) 0.947
Diaphragmatic hernia (x1.02) 0.948
Open wound of anterior abdominal wall with complication (x1.02) 0.949
Localized adiposity (x1.02) 0.949
Obstructed hernia of anterior abdominal wall (x1.02) 0.950
Systolic blood pressure (+0.613) 0.950
Gastrointestinal complication of procedure (x1.02) 0.951
promethazine (+0.00181) 0.951
Borderline normal ECG (x1.02) 0.952
Disorder of kidney and/or ureter (x1.02) 0.952
Complication of procedure (x1.02) 0.953
Paralytic ileus (x1.02) 0.953
Hernia of abdominal cavity (x1.02) 0.954
Severe protein-calorie malnutrition (Gomez: less than 60% of standard weight) (x1.02) 0.954
Abnormal glucose level (x1.02) 0.955
Iatrogenic hypotension (x1.02) 0.955
Nausea (x1.02) 0.956
hydrocodone (+0.00161) 0.956
Benign essential hypertension (x1.02) 0.957
Sleep apnea (x1.02) 0.957
Cellulitis and abscess of trunk (x1.02) 0.957
Chronic obstructive lung disease (x1.02) 0.958
Type 2 diabetes mellitus (x1.02) 0.958
Mixed hyperlipidemia (x1.02) 0.958
Hypokalemia (x1.02) 0.959
C reactive protein [M/V] in SP (+1.73) 0.959
Stoma malfunction (x1.02) 0.960
Open wound of anterior abdominal wall without complication (x1.02) 0.960
Abscess of peritoneum (x1.02) 0.960
Parastomal hernia (x1.02) 0.960
Hyperkalemia (x1.01) 0.961
Sepsis (x1.01) 0.961
Osteoarthritis of knee (x1.01) 0.961
Non-healing surgical wound (x1.01) 0.962
Acute respiratory failure (x1.01) 0.962
omeprazole (+0.00133) 0.962
tramadol (+0.00132) 0.963
Osteoarthritis (x1.01) 0.963
Hypervolemia (x1.01) 0.963
Hyperglycemia (x1.01) 0.963
Complication associated with device (x1.01) 0.964
Abscess of abdominal wall (x1.01) 0.964
Hypoxemia (x1.01) 0.964
Diverticular disease of colon (x1.01) 0.964
−0.8 −0.6 −0.4 −0.2 0.0 0.2 0.4 0.6 0.8
Expressions
−10 0 10

Signature [380]; 133 To Reach 0.975, (100 Plotted)

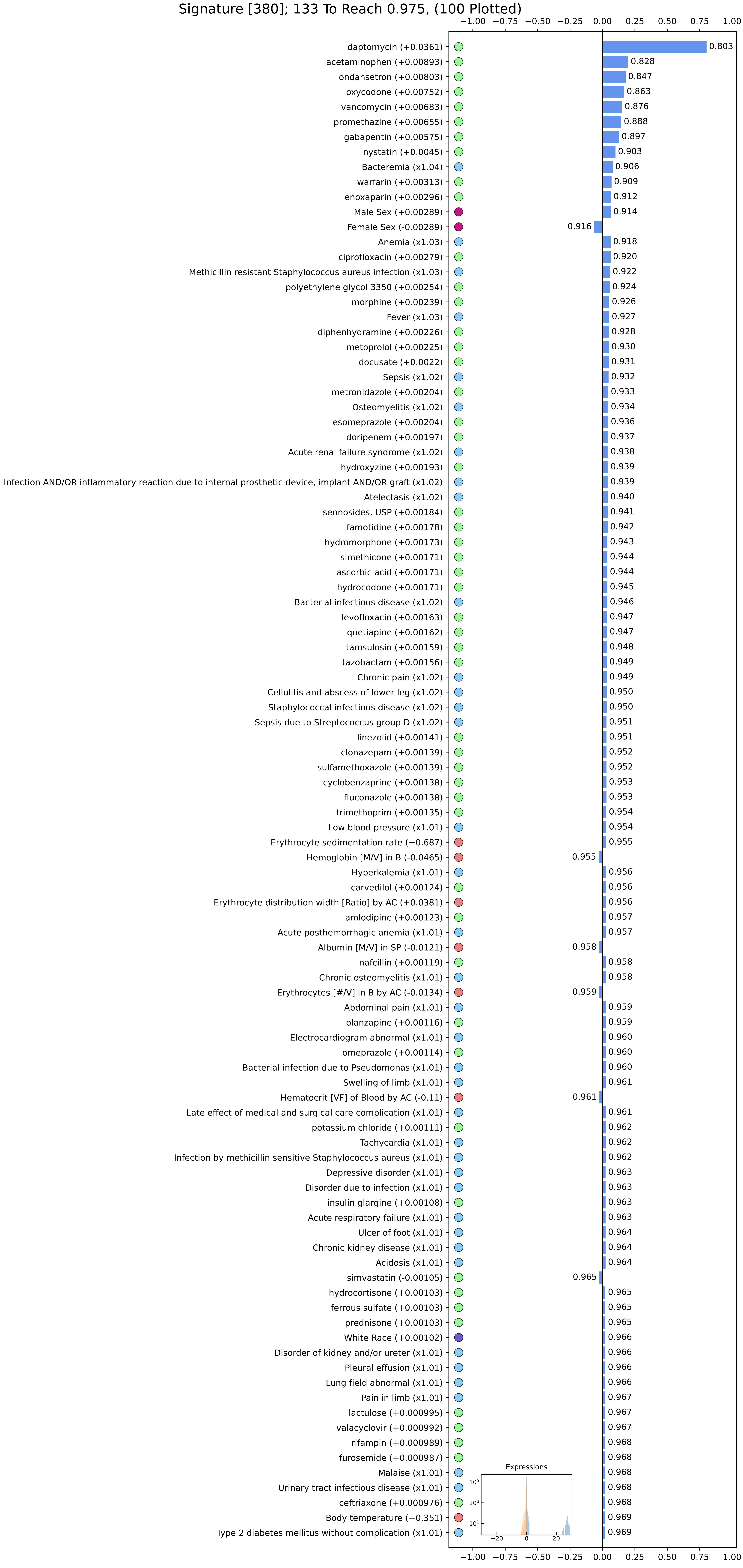

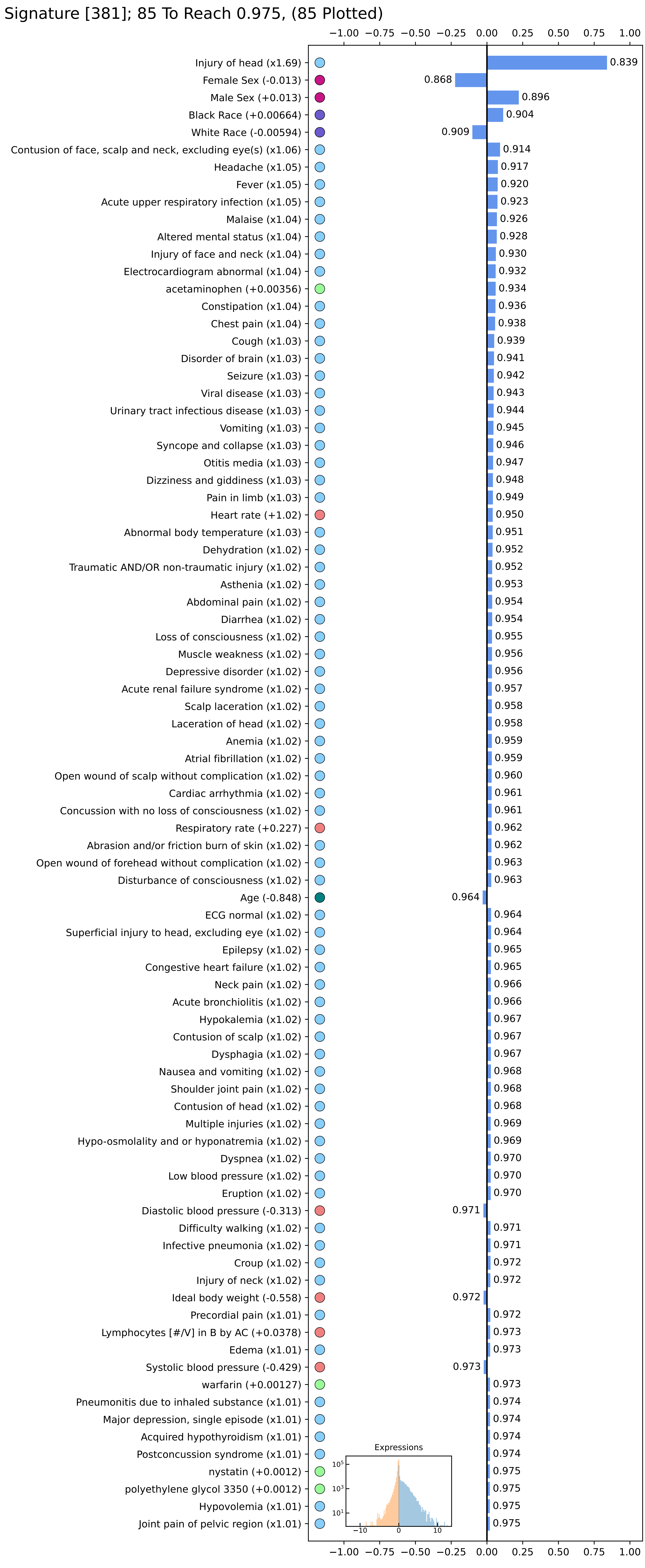

Signature [381]; 85 To Reach 0.975, (85 Plotted)
Injury of head (x1.69) 0.839
Female Sex (-0.013) 0.868
Male Sex (+0.013) 0.896
Black Race (+0.00664) 0.904
White Race (-0.00594) 0.909
Contusion of face, scalp and neck, excluding eye(s) (x1.06) 0.914
Headache (x1.05) 0.917
Fever (x1.05) 0.920
Acute upper respiratory infection (x1.05) 0.923
Malaise (x1.04) 0.926
Altered mental status (x1.04) 0.928
Injury of face and neck (x1.04) 0.930
Electrocardiogram abnormal (x1.04) 0.932
acetaminophen (+0.00356) 0.934
Constipation (x1.04) 0.936
Chest pain (x1.04) 0.938
Cough (x1.03) 0.939
Disorder of brain (x1.03) 0.941
Seizure (x1.03) 0.942
Viral disease (x1.03) 0.943
Urinary tract infectious disease (x1.03) 0.944
Vomiting (x1.03) 0.945
Syncope and collapse (x1.03) 0.946
Otitis media (x1.03) 0.947
Dizziness and giddiness (x1.03) 0.948
Pain in limb (x1.03) 0.949
Heart rate (+1.02) 0.950
Abnormal body temperature (x1.03) 0.951
Dehydration (x1.02) 0.952
Traumatic AND/OR non-traumatic injury (x1.02) 0.952
Asthenia (x1.02) 0.953
Abdominal pain (x1.02) 0.954
Diarrhea (x1.02) 0.954
Loss of consciousness (x1.02) 0.955
Muscle weakness (x1.02) 0.956
Depressive disorder (x1.02) 0.956
Acute renal failure syndrome (x1.02) 0.957
Scalp laceration (x1.02) 0.958
Laceration of head (x1.02) 0.958
Anemia (x1.02) 0.959
Atrial fibrillation (x1.02) 0.959
Open wound of scalp without complication (x1.02) 0.960
Cardiac arrhythmia (x1.02) 0.961
Concussion with no loss of consciousness (x1.02) 0.961
Respiratory rate (+0.227) 0.962
Abrasion and/or friction burn of skin (x1.02) 0.962
Open wound of forehead without complication (x1.02) 0.963
Disturbance of consciousness (x1.02) 0.963
Age (-0.848) 0.964
ECG normal (x1.02) 0.964
Superficial injury to head, excluding eye (x1.02) 0.964
Epilepsy (x1.02) 0.965
Congestive heart failure (x1.02) 0.965
Neck pain (x1.02) 0.966
Acute bronchiolitis (x1.02) 0.966
Hypokalemia (x1.02) 0.967
Contusion of scalp (x1.02) 0.967
Dysphagia (x1.02) 0.967
Nausea and vomiting (x1.02) 0.968
Shoulder joint pain (x1.02) 0.968
Contusion of head (x1.02) 0.968
Multiple injuries (x1.02) 0.969
Hypo-osmolality and or hyponatremia (x1.02) 0.969
Dyspnea (x1.02) 0.970
Low blood pressure (x1.02) 0.970
Eruption (x1.02) 0.970
Diastolic blood pressure (-0.313) 0.971
Difficulty walking (x1.02) 0.971
Infective pneumonia (x1.02) 0.971
Croup (x1.02) 0.972
Injury of neck (x1.02) 0.972
Ideal body weight (-0.558) 0.972
Precordial pain (x1.01) 0.972
Lymphocytes [#/V] in B by AC (+0.0378) 0.973
Edema (x1.01) 0.973
Systolic blood pressure (-0.429) 0.973
warfarin (+0.00127) 0.973
Pneumonitis due to inhaled substance (x1.01) 0.974
Major depression, single episode (x1.01) 0.974
Acquired hypothyroidism (x1.01) 0.974
Postconcussion syndrome (x1.01) 0.974
nystatin (+0.0012) 0.975
polyethylene glycol 3350 (+0.0012) 0.975
Hypovolemia (x1.01) 0.975
Joint pain of pelvic region (x1.01) 0.975
−1.00 −0.75 −0.50 −0.25 0.00 0.25 0.50 0.75 1.00
Expressions

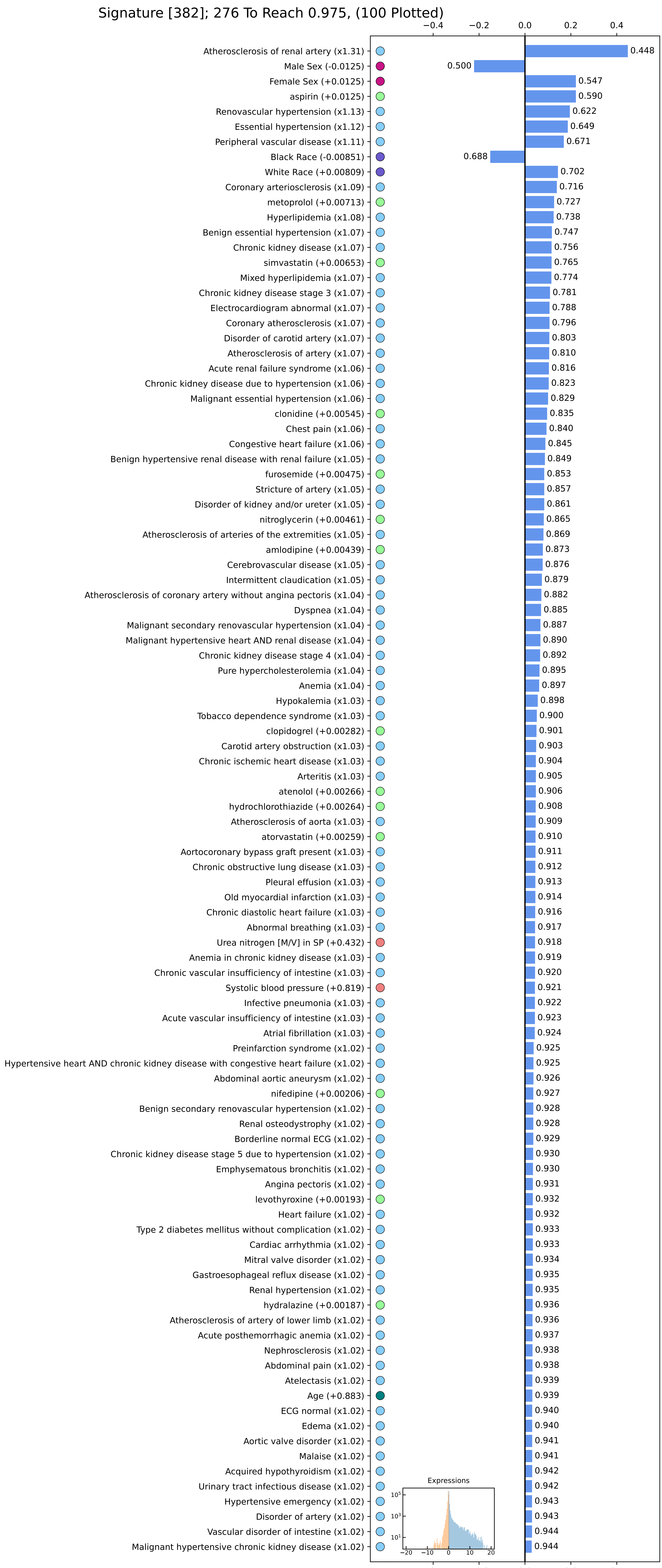

Signature [382]; 276 To Reach 0.975, (100 Plotted)
Atherosclerosis of renal artery (x1.31) 0.448
Male Sex (-0.0125) 0.500
Female Sex (+0.0125) 0.547
aspirin (+0.0125) 0.590
Renovascular hypertension (x1.13) 0.622
Essential hypertension (x1.12) 0.649
Peripheral vascular disease (x1.11) 0.671
Black Race (-0.00851) 0.688
White Race (+0.00809) 0.702
Coronary arteriosclerosis (x1.09) 0.716
metoprolol (+0.00713) 0.727
Hyperlipidemia (x1.08) 0.738
Benign essential hypertension (x1.07) 0.747
Chronic kidney disease (x1.07) 0.756
simvastatin (+0.00653) 0.765
Mixed hyperlipidemia (x1.07) 0.774
Chronic kidney disease stage 3 (x1.07) 0.781
Electrocardiogram abnormal (x1.07) 0.788
Coronary atherosclerosis (x1.07) 0.796
Disorder of carotid artery (x1.07) 0.803
Atherosclerosis of artery (x1.07) 0.810
Acute renal failure syndrome (x1.06) 0.816
Chronic kidney disease due to hypertension (x1.06) 0.823
Malignant essential hypertension (x1.06) 0.829
clonidine (+0.00545) 0.835
Chest pain (x1.06) 0.840
Congestive heart failure (x1.06) 0.845
Benign hypertensive renal disease with renal failure (x1.05) 0.849
furosemide (+0.00475) 0.853
Stricture of artery (x1.05) 0.857
Disorder of kidney and/or ureter (x1.05) 0.861
nitroglycerin (+0.00461) 0.865
Atherosclerosis of arteries of the extremities (x1.05) 0.869
amlodipine (+0.00439) 0.873
Cerebrovascular disease (x1.05) 0.876
Intermittent claudication (x1.05) 0.879
Atherosclerosis of coronary artery without angina pectoris (x1.04) 0.882
Dyspnea (x1.04) 0.885
Malignant secondary renovascular hypertension (x1.04) 0.887
Malignant hypertensive heart AND renal disease (x1.04) 0.890
Chronic kidney disease stage 4 (x1.04) 0.892
Pure hypercholesterolemia (x1.04) 0.895
Anemia (x1.04) 0.897
Hypokalemia (x1.03) 0.898
Tobacco dependence syndrome (x1.03) 0.900
clopidogrel (+0.00282) 0.901
Carotid artery obstruction (x1.03) 0.903
Chronic ischemic heart disease (x1.03) 0.904
Arteritis (x1.03) 0.905
atenolol (+0.00266) 0.906
hydrochlorothiazide (+0.00264) 0.908
Atherosclerosis of aorta (x1.03) 0.909
atorvastatin (+0.00259) 0.910
Aortocoronary bypass graft present (x1.03) 0.911
Chronic obstructive lung disease (x1.03) 0.912
Pleural effusion (x1.03) 0.913
Old myocardial infarction (x1.03) 0.914
Chronic diastolic heart failure (x1.03) 0.916
Abnormal breathing (x1.03) 0.917
Urea nitrogen [M/V] in SP (+0.432) 0.918
Anemia in chronic kidney disease (x1.03) 0.919
Chronic vascular insufficiency of intestine (x1.03) 0.920
Systolic blood pressure (+0.819) 0.921
Infective pneumonia (x1.03) 0.922
Acute vascular insufficiency of intestine (x1.03) 0.923
Atrial fibrillation (x1.03) 0.924
Preinfarction syndrome (x1.02) 0.925
Hypertensive heart AND chronic kidney disease with congestive heart failure (x1.02) 0.925
Abdominal aortic aneurysm (x1.02) 0.926
nifedipine (+0.00206) 0.927
Benign secondary renovascular hypertension (x1.02) 0.928
Renal osteodystrophy (x1.02) 0.928
Borderline normal ECG (x1.02) 0.929
Chronic kidney disease stage 5 due to hypertension (x1.02) 0.930
Emphysematous bronchitis (x1.02) 0.930
Angina pectoris (x1.02) 0.931
levothyroxine (+0.00193) 0.932
Heart failure (x1.02) 0.932
Type 2 diabetes mellitus without complication (x1.02) 0.933
Cardiac arrhythmia (x1.02) 0.933
Mitral valve disorder (x1.02) 0.934
Gastroesophageal reflux disease (x1.02) 0.935
Renal hypertension (x1.02) 0.935
hydralazine (+0.00187) 0.936
Atherosclerosis of artery of lower limb (x1.02) 0.936
Acute posthemorrhagic anemia (x1.02) 0.937
Nephrosclerosis (x1.02) 0.938
Abdominal pain (x1.02) 0.938
Atelectasis (x1.02) 0.939
Age (+0.883) 0.939
ECG normal (x1.02) 0.940
Edema (x1.02) 0.940
Aortic valve disorder (x1.02) 0.941
Malaise (x1.02) 0.941
Acquired hypothyroidism (x1.02) 0.942
Urinary tract infectious disease (x1.02) 0.942
Hypertensive emergency (x1.02) 0.943
Disorder of artery (x1.02) 0.943
Vascular disorder of intestine (x1.02) 0.944
Malignant hypertensive chronic kidney disease (x1.02) 0.944
−0.4 −0.2 0.0 0.2 0.4
Expressions

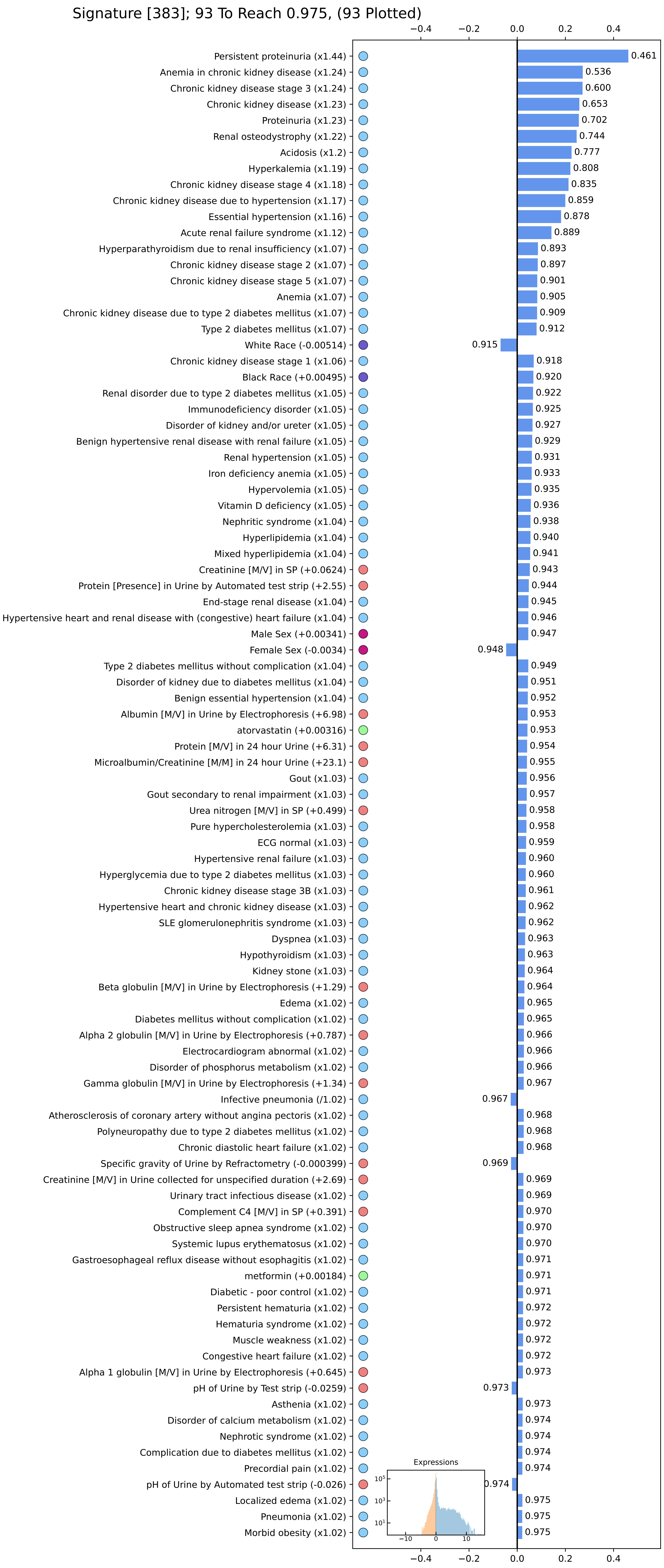
Signature [383]; 93 To Reach 0.975, (93 Plotted)
Persistent proteinuria (x1.44) 0.461
Anemia in chronic kidney disease (x1.24) 0.536
Chronic kidney disease stage 3 (x1.24) 0.600
Chronic kidney disease (x1.23) 0.653
Proteinuria (x1.23) 0.702
Renal osteodystrophy (x1.22) 0.744
Acidosis (x1.2) 0.777
Hyperkalemia (x1.19) 0.808
Chronic kidney disease stage 4 (x1.18) 0.835
Chronic kidney disease due to hypertension (x1.17) 0.859
Essential hypertension (x1.16) 0.878
Acute renal failure syndrome (x1.12) 0.889
Hyperparathyroidism due to renal insufficiency (x1.07) 0.893
Chronic kidney disease stage 2 (x1.07) 0.897
Chronic kidney disease stage 5 (x1.07) 0.901
Anemia (x1.07) 0.905
Chronic kidney disease due to type 2 diabetes mellitus (x1.07) 0.909
Type 2 diabetes mellitus (x1.07) 0.912
White Race (-0.00514) 0.915
Chronic kidney disease stage 1 (x1.06) 0.918
Black Race (+0.00495) 0.920
Renal disorder due to type 2 diabetes mellitus (x1.05) 0.922
Immunodeficiency disorder (x1.05) 0.925
Disorder of kidney and/or ureter (x1.05) 0.927
Benign hypertensive renal disease with renal failure (x1.05) 0.929
Renal hypertension (x1.05) 0.931
Iron deficiency anemia (x1.05) 0.933
Hypervolemia (x1.05) 0.935
Vitamin D deficiency (x1.05) 0.936
Nephritic syndrome (x1.04) 0.938
Hyperlipidemia (x1.04) 0.940
Mixed hyperlipidemia (x1.04) 0.941
Creatinine [M/V] in SP (+0.0624) 0.943
Protein [Presence] in Urine by Automated test strip (+2.55) 0.944
End-stage renal disease (x1.04) 0.945
Hypertensive heart and renal disease with (congestive) heart failure (x1.04) 0.946
Male Sex (+0.00341) 0.947
Female Sex (-0.0034) 0.948
Type 2 diabetes mellitus without complication (x1.04) 0.949
Disorder of kidney due to diabetes mellitus (x1.04) 0.951
Benign essential hypertension (x1.04) 0.952
Albumin [M/V] in Urine by Electrophoresis (+6.98) 0.953
atorvastatin (+0.00316) 0.953
Protein [M/V] in 24 hour Urine (+6.31) 0.954
Microalbumin/Creatinine [M/M] in 24 hour Urine (+23.1) 0.955
Gout (x1.03) 0.956
Gout secondary to renal impairment (x1.03) 0.957
Urea nitrogen [M/V] in SP (+0.499) 0.958
Pure hypercholesterolemia (x1.03) 0.958
ECG normal (x1.03) 0.959
Hypertensive renal failure (x1.03) 0.960
Hyperglycemia due to type 2 diabetes mellitus (x1.03) 0.960
Chronic kidney disease stage 3B (x1.03) 0.961
Hypertensive heart and chronic kidney disease (x1.03) 0.962
SLE glomerulonephritis syndrome (x1.03) 0.962
Dyspnea (x1.03) 0.963
Hypothyroidism (x1.03) 0.963
Kidney stone (x1.03) 0.964
Beta globulin [M/V] in Urine by Electrophoresis (+1.29) 0.964
Edema (x1.02) 0.965
Diabetes mellitus without complication (x1.02) 0.965
Alpha 2 globulin [M/V] in Urine by Electrophoresis (+0.787) 0.966
Electrocardiogram abnormal (x1.02) 0.966
Disorder of phosphorus metabolism (x1.02) 0.966
Gamma globulin [M/V] in Urine by Electrophoresis (+1.34) 0.967
Infective pneumonia (/1.02) 0.967
Atherosclerosis of coronary artery without angina pectoris (x1.02) 0.968
Polyneuropathy due to type 2 diabetes mellitus (x1.02) 0.968
Chronic diastolic heart failure (x1.02) 0.968
Specific gravity of Urine by Refractometry (-0.000399) 0.969
Creatinine [M/V] in Urine collected for unspecified duration (+2.69) 0.969
Urinary tract infectious disease (x1.02) 0.969
Complement C4 [M/V] in SP (+0.391) 0.970
Obstructive sleep apnea syndrome (x1.02) 0.970
Systemic lupus erythematosus (x1.02) 0.970
Gastroesophageal reflux disease without esophagitis (x1.02) 0.971
metformin (+0.00184) 0.971
Diabetic - poor control (x1.02) 0.971
Persistent hematuria (x1.02) 0.972
Hematuria syndrome (x1.02) 0.972
Muscle weakness (x1.02) 0.972
Congestive heart failure (x1.02) 0.972
Alpha 1 globulin [M/V] in Urine by Electrophoresis (+0.645) 0.973
pH of Urine by Test strip (-0.0259) 0.973
Asthenia (x1.02) 0.973
Disorder of calcium metabolism (x1.02) 0.974
Nephrotic syndrome (x1.02) 0.974
Complication due to diabetes mellitus (x1.02) 0.974
Precordial pain (x1.02) 0.974
pH of Urine by Automated test strip (-0.026) 0.974
Localized edema (x1.02) 0.975
Pneumonia (x1.02) 0.975
Morbid obesity (x1.02) 0.975
−0.4 −0.2 0.0 0.2 0.4
Expressions
10^5 10^3 10^1
−10 0 10

Signature [384]; 286 To Reach 0.975, (100 Plotted)

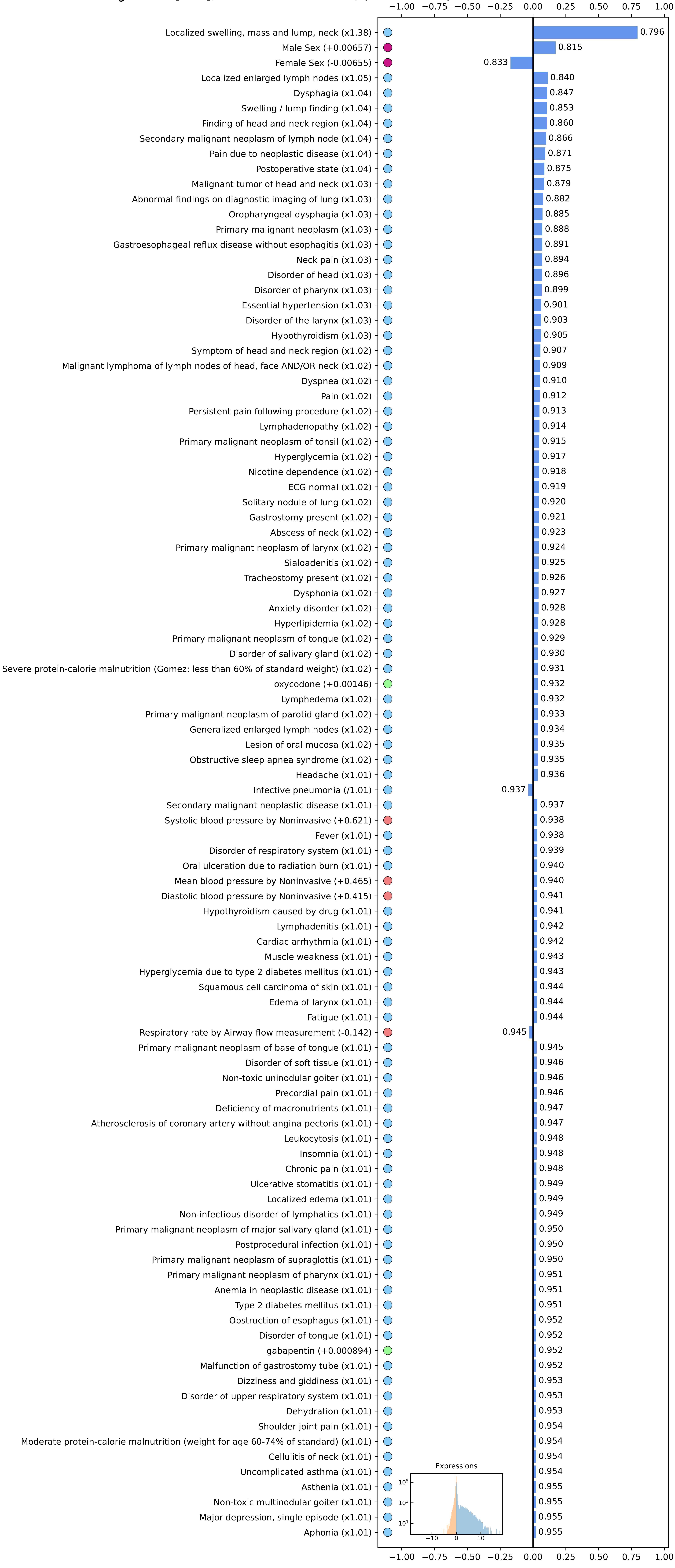

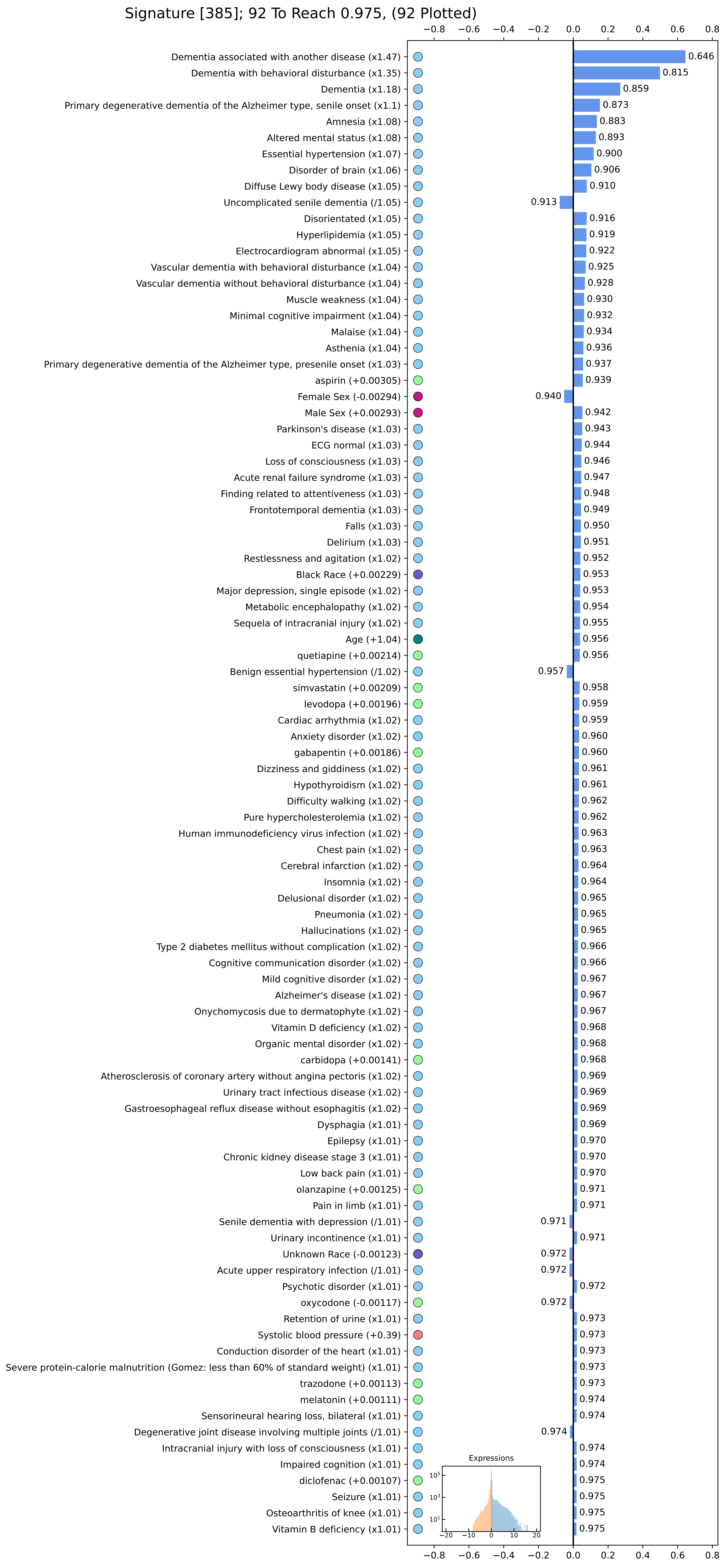
Signature [385]; 92 To Reach 0.975, (92 Plotted)
Dementia associated with another disease (x1.47) 0.646
Dementia with behavioral disturbance (x1.35) 0.815
Dementia (x1.18) 0.859
Primary degenerative dementia of the Alzheimer type, senile onset (x1.1) 0.873
Amnesia (x1.08) 0.883
Altered mental status (x1.08) 0.893
Essential hypertension (x1.07) 0.900
Disorder of brain (x1.06) 0.906
Diffuse Lewy body disease (x1.05) 0.910
Uncomplicated senile dementia (/1.05) 0.913
Disorientated (x1.05) 0.916
Hyperlipidemia (x1.05) 0.919
Electrocardiogram abnormal (x1.05) 0.922
Vascular dementia with behavioral disturbance (x1.04) 0.925
Vascular dementia without behavioral disturbance (x1.04) 0.928
Muscle weakness (x1.04) 0.930
Minimal cognitive impairment (x1.04) 0.932
Malaise (x1.04) 0.934
Asthenia (x1.04) 0.936
Primary degenerative dementia of the Alzheimer type, presenile onset (x1.03) 0.937
aspirin (+0.00305) 0.939
Female Sex (-0.00294) 0.940
Male Sex (+0.00293) 0.942
Parkinson's disease (x1.03) 0.943
ECG normal (x1.03) 0.944
Loss of consciousness (x1.03) 0.946
Acute renal failure syndrome (x1.03) 0.947
Finding related to attentiveness (x1.03) 0.948
Frontotemporal dementia (x1.03) 0.949
Falls (x1.03) 0.950
Delirium (x1.03) 0.951
Restlessness and agitation (x1.02) 0.952
Black Race (+0.00229) 0.953
Major depression, single episode (x1.02) 0.953
Metabolic encephalopathy (x1.02) 0.954
Sequela of intracranial injury (x1.02) 0.955
Age (+1.04) 0.956
quetiapine (+0.00214) 0.956
Benign essential hypertension (/1.02) 0.957
simvastatin (+0.00209) 0.958
levodopa (+0.00196) 0.959
Cardiac arrhythmia (x1.02) 0.959
Anxiety disorder (x1.02) 0.960
gabapentin (+0.00186) 0.960
Dizziness and giddiness (x1.02) 0.961
Hypothyroidism (x1.02) 0.961
Difficulty walking (x1.02) 0.962
Pure hypercholesterolemia (x1.02) 0.962
Human immunodeficiency virus infection (x1.02) 0.963
Chest pain (x1.02) 0.963
Cerebral infarction (x1.02) 0.964
Insomnia (x1.02) 0.964
Delusional disorder (x1.02) 0.965
Pneumonia (x1.02) 0.965
Hallucinations (x1.02) 0.965
Type 2 diabetes mellitus without complication (x1.02) 0.966
Cognitive communication disorder (x1.02) 0.966
Mild cognitive disorder (x1.02) 0.967
Alzheimer's disease (x1.02) 0.967
Onychomycosis due to dermatophyte (x1.02) 0.967
Vitamin D deficiency (x1.02) 0.968
Organic mental disorder (x1.02) 0.968
carbidopa (+0.00141) 0.968
Atherosclerosis of coronary artery without angina pectoris (x1.02) 0.969
Urinary tract infectious disease (x1.02) 0.969
Gastroesophageal reflux disease without esophagitis (x1.02) 0.969
Dysphagia (x1.01) 0.969
Epilepsy (x1.01) 0.970
Chronic kidney disease stage 3 (x1.01) 0.970
Low back pain (x1.01) 0.970
olanzapine (+0.00125) 0.971
Pain in limb (x1.01) 0.971
Senile dementia with depression (/1.01) 0.971
Urinary incontinence (x1.01) 0.971
Unknown Race (-0.00123) 0.972
Acute upper respiratory infection (/1.01) 0.972
Psychotic disorder (x1.01) 0.972
oxycodone (-0.00117) 0.972
Retention of urine (x1.01) 0.973
Systolic blood pressure (+0.39) 0.973
Conduction disorder of the heart (x1.01) 0.973
Severe protein-calorie malnutrition (Gomez: less than 60% of standard weight) (x1.01) 0.973
trazodone (+0.00113) 0.973
melatonin (+0.00111) 0.974
Sensorineural hearing loss, bilateral (x1.01) 0.974
Degenerative joint disease involving multiple joints (/1.01) 0.974
Intracranial injury with loss of consciousness (x1.01) 0.974
Impaired cognition (x1.01) 0.974
diclofenac (+0.00107) 0.975
Seizure (x1.01) 0.975
Osteoarthritis of knee (x1.01) 0.975
Vitamin B deficiency (x1.01) 0.975
−0.8 −0.6 −0.4 −0.2 0.0 0.2 0.4 0.6 0.8
Expressions
−20 −10 0 10 20

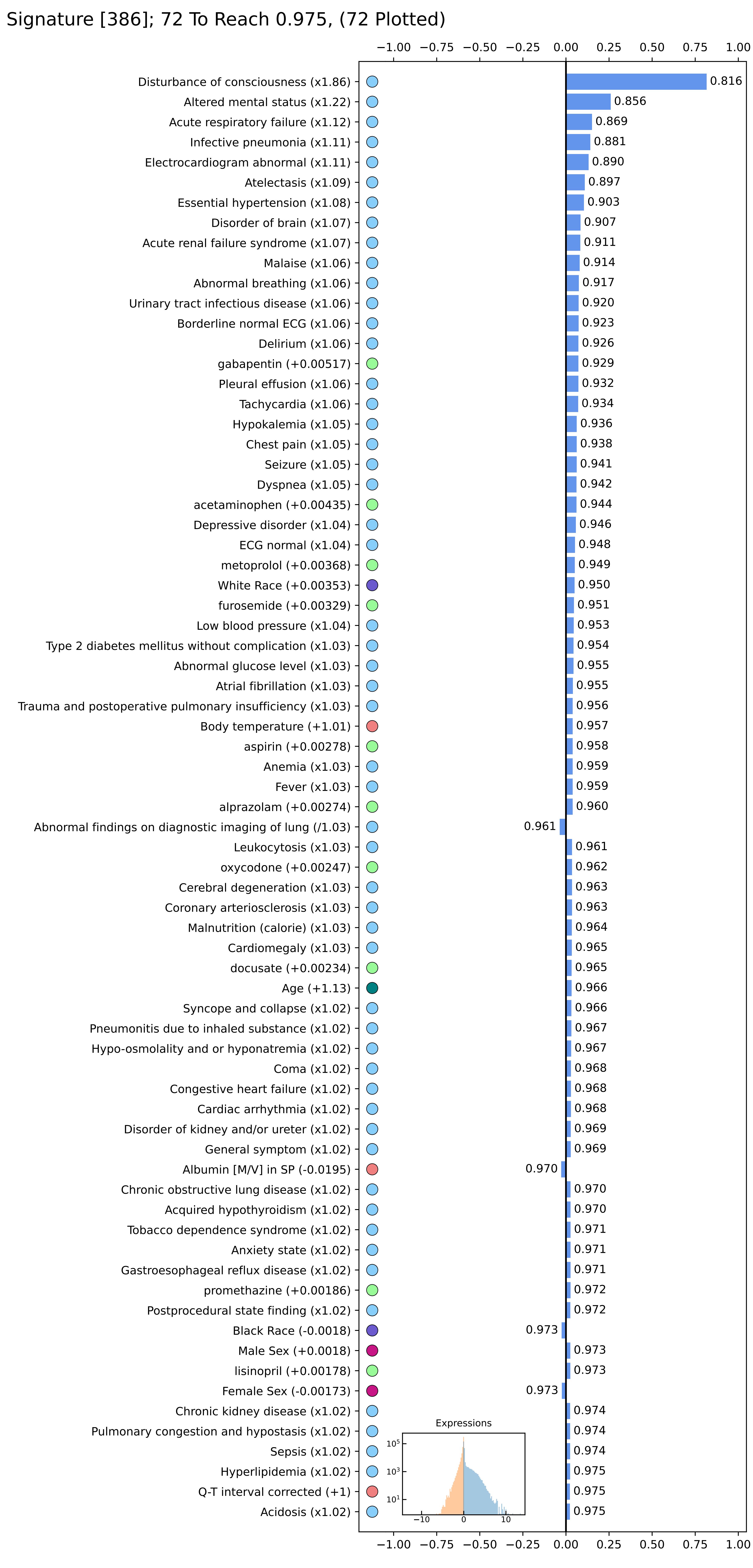
Signature [386]; 72 To Reach 0.975, (72 Plotted)
Disturbance of consciousness (x1.86) 0.816
Altered mental status (x1.22) 0.856
Acute respiratory failure (x1.12) 0.869
Infective pneumonia (x1.11) 0.881
Electrocardiogram abnormal (x1.11) 0.890
Atelectasis (x1.09) 0.897
Essential hypertension (x1.08) 0.903
Disorder of brain (x1.07) 0.907
Acute renal failure syndrome (x1.07) 0.911
Malaise (x1.06) 0.914
Abnormal breathing (x1.06) 0.917
Urinary tract infectious disease (x1.06) 0.920
Borderline normal ECG (x1.06) 0.923
Delirium (x1.06) 0.926
gabapentin (+0.00517) 0.929
Pleural effusion (x1.06) 0.932
Tachycardia (x1.06) 0.934
Hypokalemia (x1.05) 0.936
Chest pain (x1.05) 0.938
Seizure (x1.05) 0.941
Dyspnea (x1.05) 0.942
acetaminophen (+0.00435) 0.944
Depressive disorder (x1.04) 0.946
ECG normal (x1.04) 0.948
metoprolol (+0.00368) 0.949
White Race (+0.00353) 0.950
furosemide (+0.00329) 0.951
Low blood pressure (x1.04) 0.953
Type 2 diabetes mellitus without complication (x1.03) 0.954
Abnormal glucose level (x1.03) 0.955
Atrial fibrillation (x1.03) 0.955
Trauma and postoperative pulmonary insufficiency (x1.03) 0.956
Body temperature (+1.01) 0.957
aspirin (+0.00278) 0.958
Anemia (x1.03) 0.959
Fever (x1.03) 0.959
alprazolam (+0.00274) 0.960
Abnormal findings on diagnostic imaging of lung (/1.03) 0.961
Leukocytosis (x1.03) 0.961
oxycodone (+0.00247) 0.962
Cerebral degeneration (x1.03) 0.963
Coronary arteriosclerosis (x1.03) 0.963
Malnutrition (calorie) (x1.03) 0.964
Cardiomegaly (x1.03) 0.965
docusate (+0.00234) 0.965
Age (+1.13) 0.966
Syncope and collapse (x1.02) 0.966
Pneumonitis due to inhaled substance (x1.02) 0.967
Hypo-osmolality and or hyponatremia (x1.02) 0.967
Coma (x1.02) 0.968
Congestive heart failure (x1.02) 0.968
Cardiac arrhythmia (x1.02) 0.968
Disorder of kidney and/or ureter (x1.02) 0.969
General symptom (x1.02) 0.969
Albumin [M/V] in SP (-0.0195) 0.970
Chronic obstructive lung disease (x1.02) 0.970
Acquired hypothyroidism (x1.02) 0.970
Tobacco dependence syndrome (x1.02) 0.971
Anxiety state (x1.02) 0.971
Gastroesophageal reflux disease (x1.02) 0.971
promethazine (+0.00186) 0.972
Postprocedural state finding (x1.02) 0.972
Black Race (-0.0018) 0.973
Male Sex (+0.0018) 0.973
lisinopril (+0.00178) 0.973
Female Sex (-0.00173) 0.973
Chronic kidney disease (x1.02) 0.974
Pulmonary congestion and hypostasis (x1.02) 0.974
Sepsis (x1.02) 0.974
Hyperlipidemia (x1.02) 0.975
Q-T interval corrected (+1) 0.975
Acidosis (x1.02) 0.975
−1.00 −0.75 −0.50 −0.25 0.00 0.25 0.50 0.75 1.00
Expressions
−10 0 10

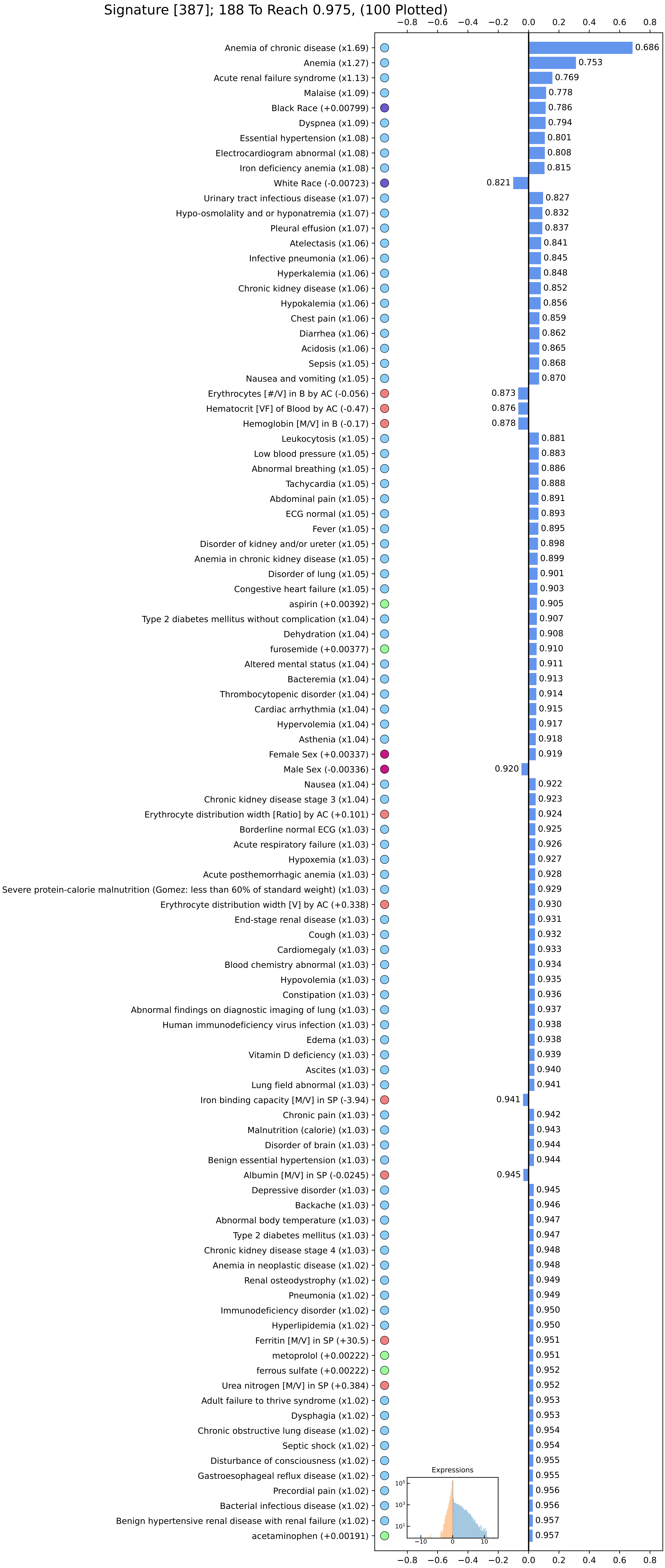

Signature [387]; 188 To Reach 0.975, (100 Plotted)
Anemia of chronic disease (x1.69) 0.686
Anemia (x1.27) 0.753
Acute renal failure syndrome (x1.13) 0.769
Malaise (x1.09) 0.778
Black Race (+0.00799) 0.786
Dyspnea (x1.09) 0.794
Essential hypertension (x1.08) 0.801
Electrocardiogram abnormal (x1.08) 0.808
Iron deficiency anemia (x1.08) 0.815
White Race (-0.00723) 0.821
Urinary tract infectious disease (x1.07) 0.827
Hypo-osmolality and or hyponatremia (x1.07) 0.832
Pleural effusion (x1.07) 0.837
Atelectasis (x1.06) 0.841
Infective pneumonia (x1.06) 0.845
Hyperkalemia (x1.06) 0.848
Chronic kidney disease (x1.06) 0.852
Hypokalemia (x1.06) 0.856
Chest pain (x1.06) 0.859
Diarrhea (x1.06) 0.862
Acidosis (x1.06) 0.865
Sepsis (x1.05) 0.868
Nausea and vomiting (x1.05) 0.870
Erythrocytes [#/V] in B by AC (-0.056) 0.873
Hematocrit [VF] of Blood by AC (-0.47) 0.876
Hemoglobin [M/V] in B (-0.17) 0.878
Leukocytosis (x1.05) 0.881
Low blood pressure (x1.05) 0.883
Abnormal breathing (x1.05) 0.886
Tachycardia (x1.05) 0.888
Abdominal pain (x1.05) 0.891
ECG normal (x1.05) 0.893
Fever (x1.05) 0.895
Disorder of kidney and/or ureter (x1.05) 0.898
Anemia in chronic kidney disease (x1.05) 0.899
Disorder of lung (x1.05) 0.901
Congestive heart failure (x1.05) 0.903
aspirin (+0.00392) 0.905
Type 2 diabetes mellitus without complication (x1.04) 0.907
Dehydration (x1.04) 0.908
furosemide (+0.00377) 0.910
Altered mental status (x1.04) 0.911
Bacteremia (x1.04) 0.913
Thrombocytopenic disorder (x1.04) 0.914
Cardiac arrhythmia (x1.04) 0.915
Hypervolemia (x1.04) 0.917
Asthenia (x1.04) 0.918
Female Sex (+0.00337) 0.919
Male Sex (-0.00336) 0.920
Nausea (x1.04) 0.922
Chronic kidney disease stage 3 (x1.04) 0.923
Erythrocyte distribution width [Ratio] by AC (+0.101) 0.924
Borderline normal ECG (x1.03) 0.925
Acute respiratory failure (x1.03) 0.926
Hypoxemia (x1.03) 0.927
Acute posthemorrhagic anemia (x1.03) 0.928
Severe protein-calorie malnutrition (Gomez: less than 60% of standard weight) (x1.03) 0.929
Erythrocyte distribution width [V] by AC (+0.338) 0.930
End-stage renal disease (x1.03) 0.931
Cough (x1.03) 0.932
Cardiomegaly (x1.03) 0.933
Blood chemistry abnormal (x1.03) 0.934
Hypovolemia (x1.03) 0.935
Constipation (x1.03) 0.936
Abnormal findings on diagnostic imaging of lung (x1.03) 0.937
Human immunodeficiency virus infection (x1.03) 0.938
Edema (x1.03) 0.938
Vitamin D deficiency (x1.03) 0.939
Ascites (x1.03) 0.940
Lung field abnormal (x1.03) 0.941
Iron binding capacity [M/V] in SP (-3.94) 0.941
Chronic pain (x1.03) 0.942
Malnutrition (calorie) (x1.03) 0.943
Disorder of brain (x1.03) 0.944
Benign essential hypertension (x1.03) 0.944
Albumin [M/V] in SP (-0.0245) 0.945
Depressive disorder (x1.03) 0.945
Backache (x1.03) 0.946
Abnormal body temperature (x1.03) 0.947
Type 2 diabetes mellitus (x1.03) 0.947
Chronic kidney disease stage 4 (x1.03) 0.948
Anemia in neoplastic disease (x1.02) 0.948
Renal osteodystrophy (x1.02) 0.949
Pneumonia (x1.02) 0.949
Immunodeficiency disorder (x1.02) 0.950
Hyperlipidemia (x1.02) 0.950
Ferritin [M/V] in SP (+30.5) 0.951
metoprolol (+0.00222) 0.951
ferrous sulfate (+0.00222) 0.952
Urea nitrogen [M/V] in SP (+0.384) 0.952
Adult failure to thrive syndrome (x1.02) 0.953
Dysphagia (x1.02) 0.953
Chronic obstructive lung disease (x1.02) 0.954
Septic shock (x1.02) 0.954
Disturbance of consciousness (x1.02) 0.955
Gastroesophageal reflux disease (x1.02) 0.955
Precordial pain (x1.02) 0.956
Bacterial infectious disease (x1.02) 0.956
Benign hypertensive renal disease with renal failure (x1.02) 0.957
acetaminophen (+0.00191) 0.957
Expressions

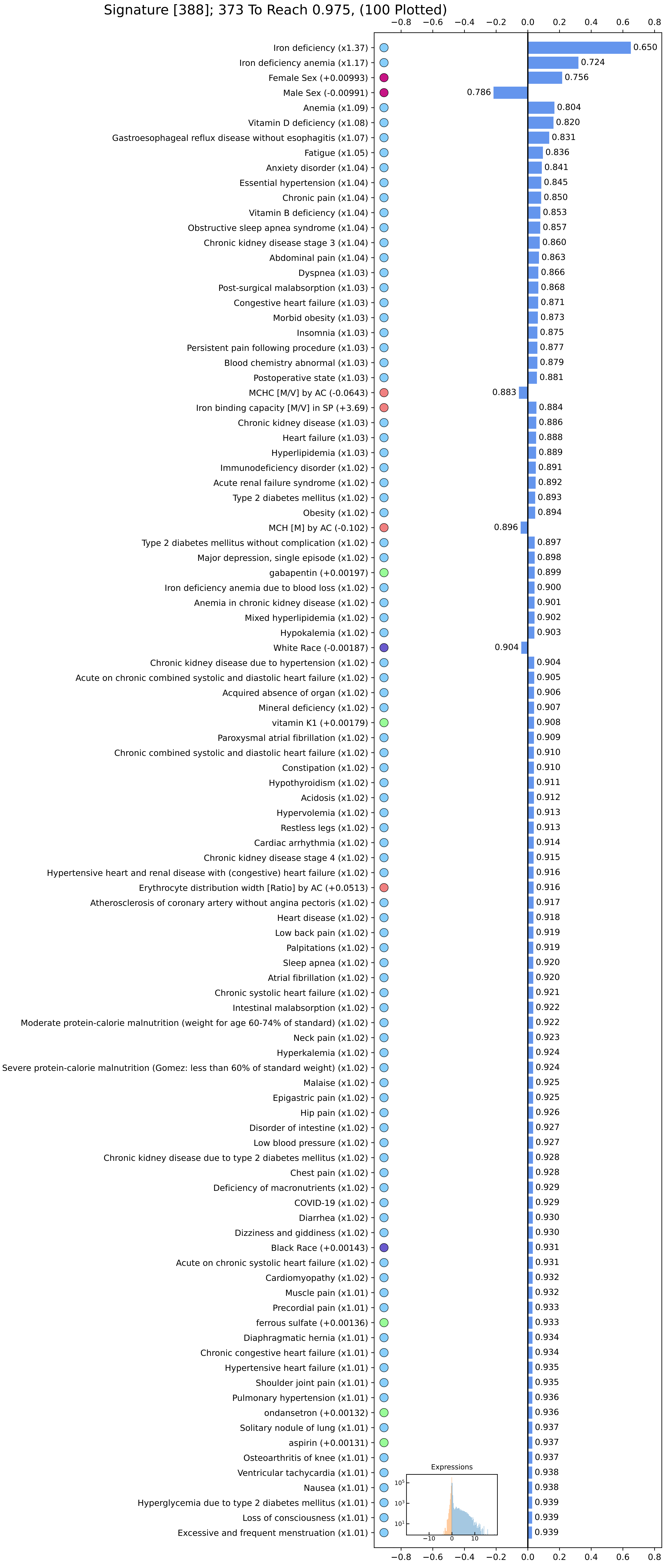

Signature [388]; 373 To Reach 0.975, (100 Plotted)
Iron deficiency (x1.37) 0.650
Iron deficiency anemia (x1.17) 0.724
Female Sex (+0.00993) 0.756
Male Sex (-0.00991) 0.786
Anemia (x1.09) 0.804
Vitamin D deficiency (x1.08) 0.820
Gastroesophageal reflux disease without esophagitis (x1.07) 0.831
Fatigue (x1.05) 0.836
Anxiety disorder (x1.04) 0.841
Essential hypertension (x1.04) 0.845
Chronic pain (x1.04) 0.850
Vitamin B deficiency (x1.04) 0.853
Obstructive sleep apnea syndrome (x1.04) 0.857
Chronic kidney disease stage 3 (x1.04) 0.860
Abdominal pain (x1.04) 0.863
Dyspnea (x1.03) 0.866
Post-surgical malabsorption (x1.03) 0.868
Congestive heart failure (x1.03) 0.871
Morbid obesity (x1.03) 0.873
Insomnia (x1.03) 0.875
Persistent pain following procedure (x1.03) 0.877
Blood chemistry abnormal (x1.03) 0.879
Postoperative state (x1.03) 0.881
MCHC [M/V] by AC (-0.0643) 0.883
Iron binding capacity [M/V] in SP (+3.69) 0.884
Chronic kidney disease (x1.03) 0.886
Heart failure (x1.03) 0.888
Hyperlipidemia (x1.03) 0.889
Immunodeficiency disorder (x1.02) 0.891
Acute renal failure syndrome (x1.02) 0.892
Type 2 diabetes mellitus (x1.02) 0.893
Obesity (x1.02) 0.894
MCH [M] by AC (-0.102) 0.896
Type 2 diabetes mellitus without complication (x1.02) 0.897
Major depression, single episode (x1.02) 0.898
gabapentin (+0.00197) 0.899
Iron deficiency anemia due to blood loss (x1.02) 0.900
Anemia in chronic kidney disease (x1.02) 0.901
Mixed hyperlipidemia (x1.02) 0.902
Hypokalemia (x1.02) 0.903
White Race (-0.00187) 0.904
Chronic kidney disease due to hypertension (x1.02) 0.904
Acute on chronic combined systolic and diastolic heart failure (x1.02) 0.905
Acquired absence of organ (x1.02) 0.906
Mineral deficiency (x1.02) 0.907
vitamin K1 (+0.00179) 0.908
Paroxysmal atrial fibrillation (x1.02) 0.909
Chronic combined systolic and diastolic heart failure (x1.02) 0.910
Constipation (x1.02) 0.910
Hypothyroidism (x1.02) 0.911
Acidosis (x1.02) 0.912
Hypervolemia (x1.02) 0.913
Restless legs (x1.02) 0.913
Cardiac arrhythmia (x1.02) 0.914
Chronic kidney disease stage 4 (x1.02) 0.915
Hypertensive heart and renal disease with (congestive) heart failure (x1.02) 0.916
Erythrocyte distribution width [Ratio] by AC (+0.0513) 0.916
Atherosclerosis of coronary artery without angina pectoris (x1.02) 0.917
Heart disease (x1.02) 0.918
Low back pain (x1.02) 0.919
Palpitations (x1.02) 0.919
Sleep apnea (x1.02) 0.920
Atrial fibrillation (x1.02) 0.920
Chronic systolic heart failure (x1.02) 0.921
Intestinal malabsorption (x1.02) 0.922
Moderate protein-calorie malnutrition (weight for age 60-74% of standard) (x1.02) 0.922
Neck pain (x1.02) 0.923
Hyperkalemia (x1.02) 0.924
Severe protein-calorie malnutrition (Gomez: less than 60% of standard weight) (x1.02) 0.924
Malaise (x1.02) 0.925
Epigastric pain (x1.02) 0.925
Hip pain (x1.02) 0.926
Disorder of intestine (x1.02) 0.927
Low blood pressure (x1.02) 0.927
Chronic kidney disease due to type 2 diabetes mellitus (x1.02) 0.928
Chest pain (x1.02) 0.928
Deficiency of macronutrients (x1.02) 0.929
COVID-19 (x1.02) 0.929
Diarrhea (x1.02) 0.930
Dizziness and giddiness (x1.02) 0.930
Black Race (+0.00143) 0.931
Acute on chronic systolic heart failure (x1.02) 0.931
Cardiomyopathy (x1.02) 0.932
Muscle pain (x1.01) 0.932
Precordial pain (x1.01) 0.933
ferrous sulfate (+0.00136) 0.933
Diaphragmatic hernia (x1.01) 0.934
Chronic congestive heart failure (x1.01) 0.934
Hypertensive heart failure (x1.01) 0.935
Shoulder joint pain (x1.01) 0.935
Pulmonary hypertension (x1.01) 0.936
ondansetron (+0.00132) 0.936
Solitary nodule of lung (x1.01) 0.937
aspirin (+0.00131) 0.937
Osteoarthritis of knee (x1.01) 0.937
Ventricular tachycardia (x1.01) 0.938
Nausea (x1.01) 0.938
Hyperglycemia due to type 2 diabetes mellitus (x1.01) 0.939
Loss of consciousness (x1.01) 0.939
Excessive and frequent menstruation (x1.01) 0.939
−0.8 −0.6 −0.4 −0.2 0.0 0.2 0.4 0.6 0.8
Expressions

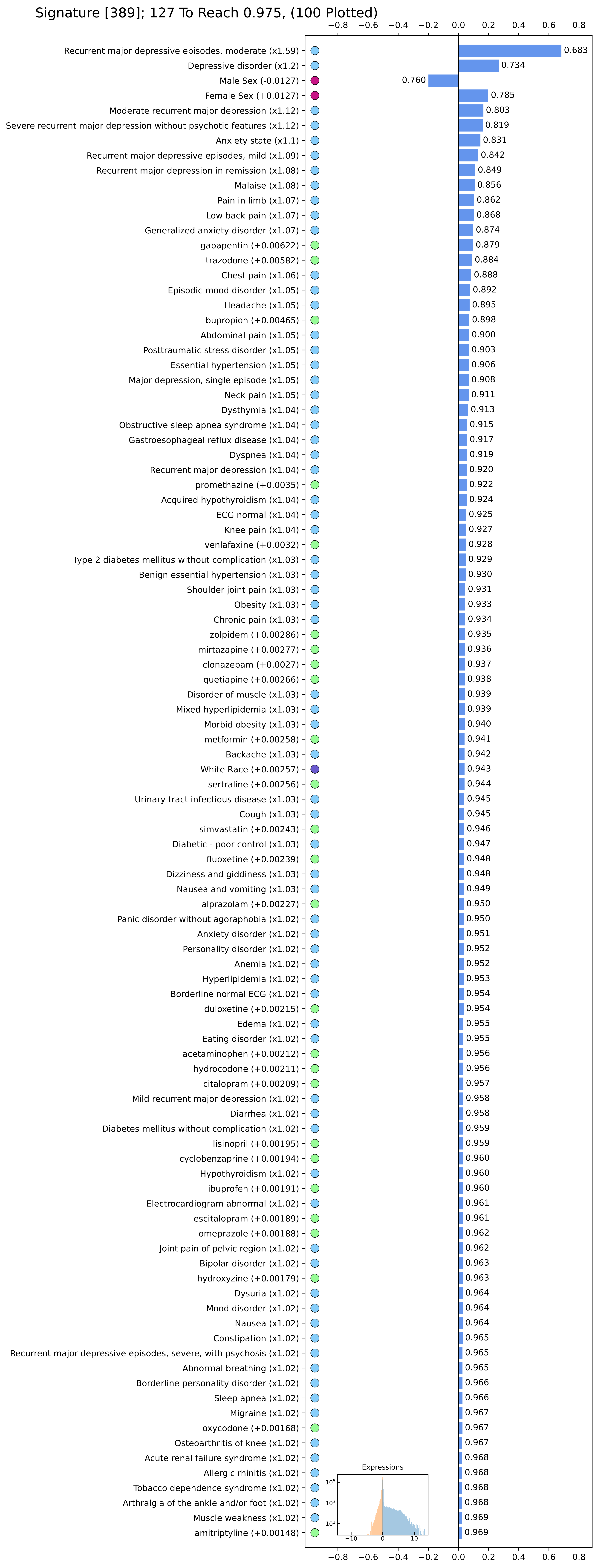

Signature [389]; 127 To Reach 0.975, (100 Plotted)
Recurrent major depressive episodes, moderate (x1.59) 0.683
Depressive disorder (x1.2) 0.734
Male Sex (-0.0127) 0.760
Female Sex (+0.0127) 0.785
Moderate recurrent major depression (x1.12) 0.803
Severe recurrent major depression without psychotic features (x1.12) 0.819
Anxiety state (x1.1) 0.831
Recurrent major depressive episodes, mild (x1.09) 0.842
Recurrent major depression in remission (x1.08) 0.849
Malaise (x1.08) 0.856
Pain in limb (x1.07) 0.862
Low back pain (x1.07) 0.868
Generalized anxiety disorder (x1.07) 0.874
gabapentin (+0.00622) 0.879
trazodone (+0.00582) 0.884
Chest pain (x1.06) 0.888
Episodic mood disorder (x1.05) 0.892
Headache (x1.05) 0.895
bupropion (+0.00465) 0.898
Abdominal pain (x1.05) 0.900
Posttraumatic stress disorder (x1.05) 0.903
Essential hypertension (x1.05) 0.906
Major depression, single episode (x1.05) 0.908
Neck pain (x1.05) 0.911
Dysthymia (x1.04) 0.913
Obstructive sleep apnea syndrome (x1.04) 0.915
Gastroesophageal reflux disease (x1.04) 0.917
Dyspnea (x1.04) 0.919
Recurrent major depression (x1.04) 0.920
promethazine (+0.0035) 0.922
Acquired hypothyroidism (x1.04) 0.924
ECG normal (x1.04) 0.925
Knee pain (x1.04) 0.927
venlafaxine (+0.0032) 0.928
Type 2 diabetes mellitus without complication (x1.03) 0.929
Benign essential hypertension (x1.03) 0.930
Shoulder joint pain (x1.03) 0.931
Obesity (x1.03) 0.933
Chronic pain (x1.03) 0.934
zolpidem (+0.00286) 0.935
mirtazapine (+0.00277) 0.936
clonazepam (+0.0027) 0.937
quetiapine (+0.00266) 0.938
Disorder of muscle (x1.03) 0.939
Mixed hyperlipidemia (x1.03) 0.939
Morbid obesity (x1.03) 0.940
metformin (+0.00258) 0.941
Backache (x1.03) 0.942
White Race (+0.00257) 0.943
sertraline (+0.00256) 0.944
Urinary tract infectious disease (x1.03) 0.945
Cough (x1.03) 0.945
simvastatin (+0.00243) 0.946
Diabetic - poor control (x1.03) 0.947
fluoxetine (+0.00239) 0.948
Dizziness and giddiness (x1.03) 0.948
Nausea and vomiting (x1.03) 0.949
alprazolam (+0.00227) 0.950
Panic disorder without agoraphobia (x1.02) 0.950
Anxiety disorder (x1.02) 0.951
Personality disorder (x1.02) 0.952
Anemia (x1.02) 0.952
Hyperlipidemia (x1.02) 0.953
Borderline normal ECG (x1.02) 0.954
duloxetine (+0.00215) 0.954
Edema (x1.02) 0.955
Eating disorder (x1.02) 0.955
acetaminophen (+0.00212) 0.956
hydrocodone (+0.00211) 0.956
citalopram (+0.00209) 0.957
Mild recurrent major depression (x1.02) 0.958
Diarrhea (x1.02) 0.958
Diabetes mellitus without complication (x1.02) 0.959
lisinopril (+0.00195) 0.959
cyclobenzaprine (+0.00194) 0.960
Hypothyroidism (x1.02) 0.960
ibuprofen (+0.00191) 0.960
Electrocardiogram abnormal (x1.02) 0.961
escitalopram (+0.00189) 0.961
omeprazole (+0.00188) 0.962
Joint pain of pelvic region (x1.02) 0.962
Bipolar disorder (x1.02) 0.963
hydroxyzine (+0.00179) 0.963
Dysuria (x1.02) 0.964
Mood disorder (x1.02) 0.964
Nausea (x1.02) 0.964
Constipation (x1.02) 0.965
Recurrent major depressive episodes, severe, with psychosis (x1.02) 0.965
Abnormal breathing (x1.02) 0.965
Borderline personality disorder (x1.02) 0.966
Sleep apnea (x1.02) 0.966
Migraine (x1.02) 0.967
oxycodone (+0.00168) 0.967
Osteoarthritis of knee (x1.02) 0.967
Acute renal failure syndrome (x1.02) 0.968
Allergic rhinitis (x1.02) 0.968
Tobacco dependence syndrome (x1.02) 0.968
Arthralgia of the ankle and/or foot (x1.02) 0.968
Muscle weakness (x1.02) 0.969
amitriptyline (+0.00148) 0.969
Expressions

Signature [390]; 463 To Reach 0.975, (100 Plotted)

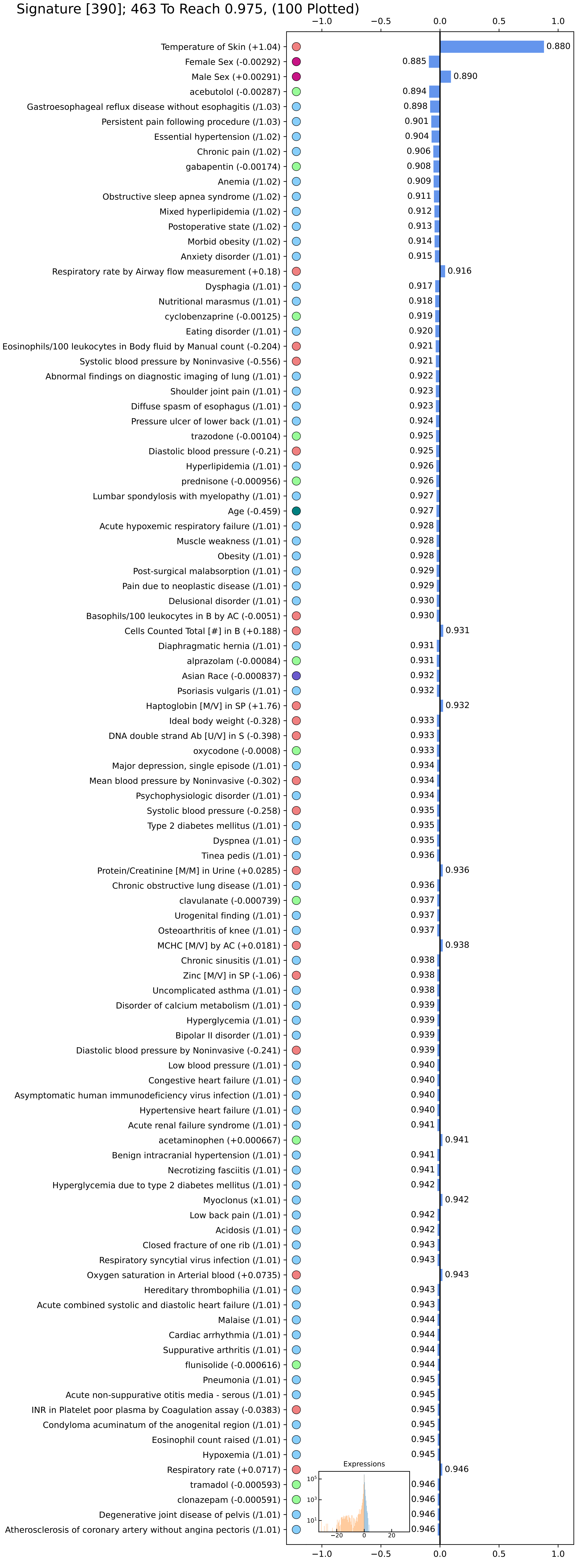

Signature [391]; 2 To Reach 0.975, (10 Plotted)

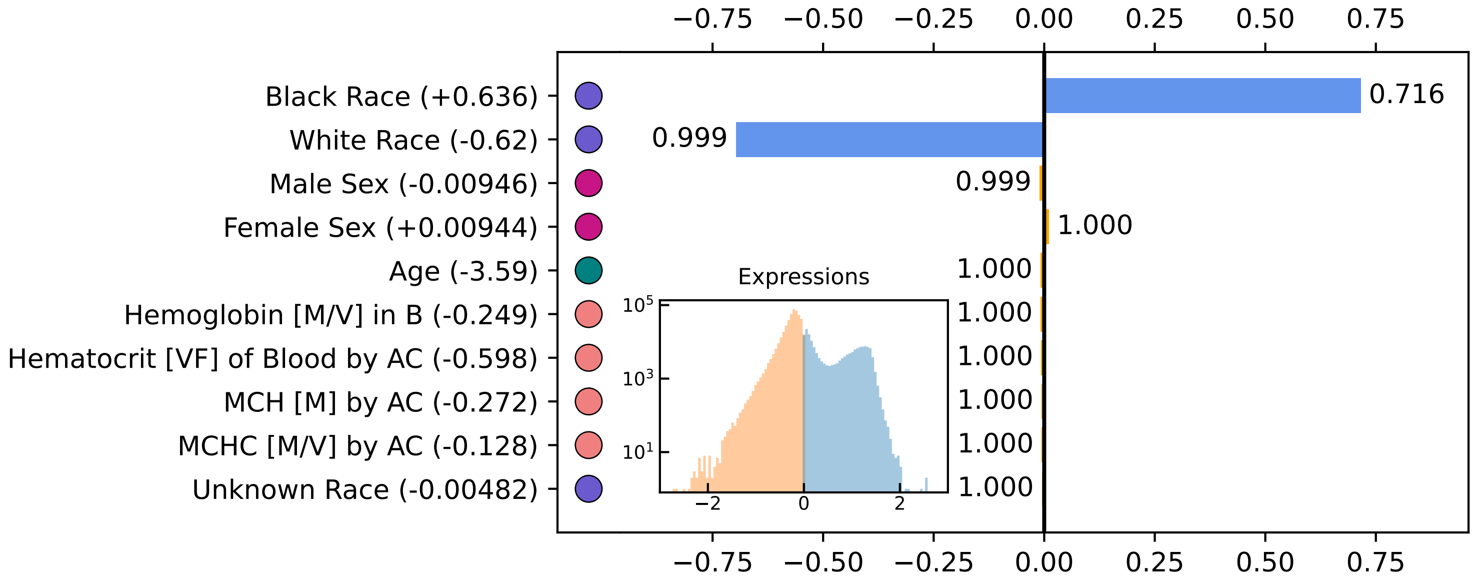

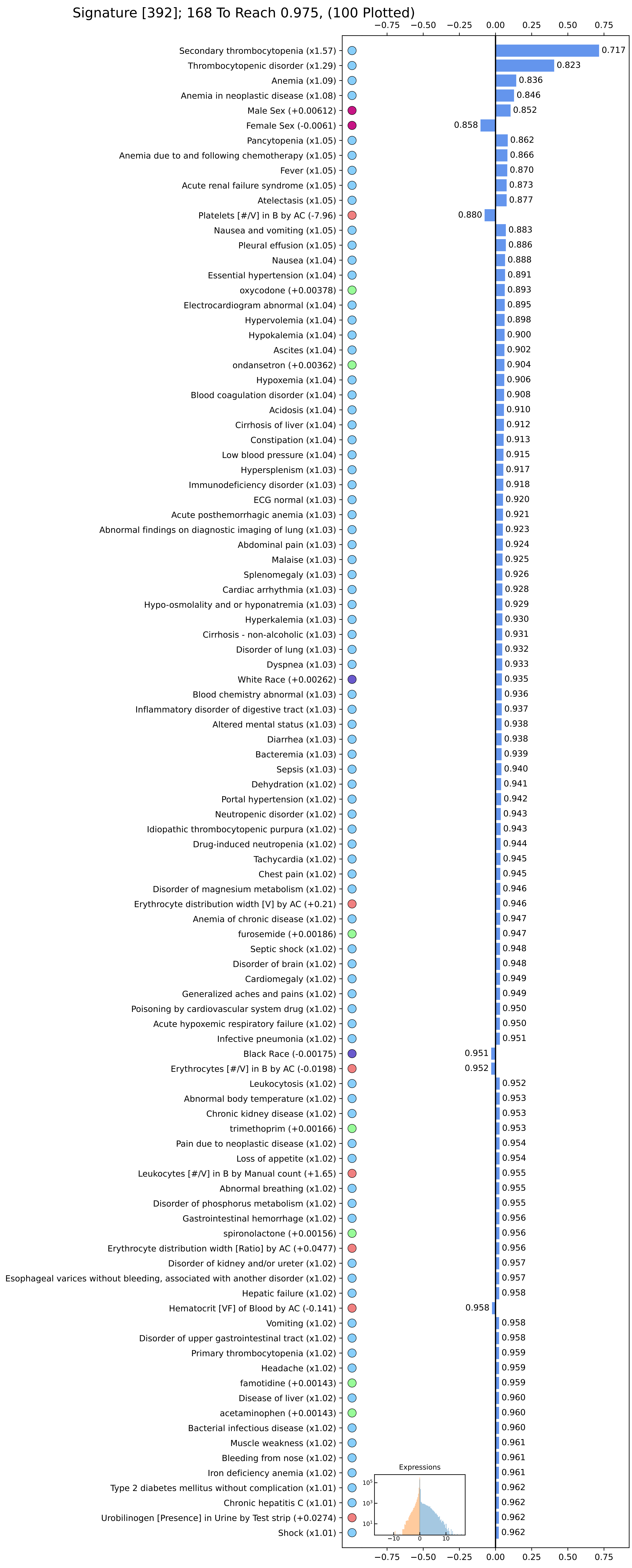
Signature [392]; 168 To Reach 0.975, (100 Plotted)
−0.75 −0.50 −0.25 0.00 0.25 0.50 0.75
Secondary thrombocytopenia (x1.57) 0.717
Thrombocytopenic disorder (x1.29) 0.823
Anemia (x1.09) 0.836
Anemia in neoplastic disease (x1.08) 0.846
Male Sex (+0.00612) 0.852
Female Sex (-0.0061) 0.858
Pancytopenia (x1.05) 0.862
Anemia due to and following chemotherapy (x1.05) 0.866
Fever (x1.05) 0.870
Acute renal failure syndrome (x1.05) 0.873
Atelectasis (x1.05) 0.877
Platelets [#/V] in B by AC (-7.96) 0.880
Nausea and vomiting (x1.05) 0.883
Pleural effusion (x1.05) 0.886
Nausea (x1.04) 0.888
Essential hypertension (x1.04) 0.891
oxycodone (+0.00378) 0.893
Electrocardiogram abnormal (x1.04) 0.895
Hypervolemia (x1.04) 0.898
Hypokalemia (x1.04) 0.900
Ascites (x1.04) 0.902
ondansetron (+0.00362) 0.904
Hypoxemia (x1.04) 0.906
Blood coagulation disorder (x1.04) 0.908
Acidosis (x1.04) 0.910
Cirrhosis of liver (x1.04) 0.912
Constipation (x1.04) 0.913
Low blood pressure (x1.04) 0.915
Hypersplenism (x1.03) 0.917
Immunodeficiency disorder (x1.03) 0.918
ECG normal (x1.03) 0.920
Acute posthemorrhagic anemia (x1.03) 0.921
Abnormal findings on diagnostic imaging of lung (x1.03) 0.923
Abdominal pain (x1.03) 0.924
Malaise (x1.03) 0.925
Splenomegaly (x1.03) 0.926
Cardiac arrhythmia (x1.03) 0.928
Hypo-osmolality and or hyponatremia (x1.03) 0.929
Hyperkalemia (x1.03) 0.930
Cirrhosis - non-alcoholic (x1.03) 0.931
Disorder of lung (x1.03) 0.932
Dyspnea (x1.03) 0.933
White Race (+0.00262) 0.935
Blood chemistry abnormal (x1.03) 0.936
Inflammatory disorder of digestive tract (x1.03) 0.937
Altered mental status (x1.03) 0.938
Diarrhea (x1.03) 0.938
Bacteremia (x1.03) 0.939
Sepsis (x1.03) 0.940
Dehydration (x1.02) 0.941
Portal hypertension (x1.02) 0.942
Neutropenic disorder (x1.02) 0.943
Idiopathic thrombocytopenic purpura (x1.02) 0.943
Drug-induced neutropenia (x1.02) 0.944
Tachycardia (x1.02) 0.945
Chest pain (x1.02) 0.945
Disorder of magnesium metabolism (x1.02) 0.946
Erythrocyte distribution width [V] by AC (+0.21) 0.946
Anemia of chronic disease (x1.02) 0.947
furosemide (+0.00186) 0.947
Septic shock (x1.02) 0.948
Disorder of brain (x1.02) 0.948
Cardiomegaly (x1.02) 0.949
Generalized aches and pains (x1.02) 0.949
Poisoning by cardiovascular system drug (x1.02) 0.950
Acute hypoxemic respiratory failure (x1.02) 0.950
Infective pneumonia (x1.02) 0.951
Black Race (-0.00175) 0.951
Erythrocytes [#/V] in B by AC (-0.0198) 0.952
Leukocytosis (x1.02) 0.952
Abnormal body temperature (x1.02) 0.953
Chronic kidney disease (x1.02) 0.953
trimethoprim (+0.00166) 0.953
Pain due to neoplastic disease (x1.02) 0.954
Loss of appetite (x1.02) 0.954
Leukocytes [#/V] in B by Manual count (+1.65) 0.955
Abnormal breathing (x1.02) 0.955
Disorder of phosphorus metabolism (x1.02) 0.955
Gastrointestinal hemorrhage (x1.02) 0.956
spironolactone (+0.00156) 0.956
Erythrocyte distribution width [Ratio] by AC (+0.0477) 0.956
Disorder of kidney and/or ureter (x1.02) 0.957
Esophageal varices without bleeding, associated with another disorder (x1.02) 0.957
Hepatic failure (x1.02) 0.958
Hematocrit [VF] of Blood by AC (-0.141) 0.958
Vomiting (x1.02) 0.958
Disorder of upper gastrointestinal tract (x1.02) 0.958
Primary thrombocytopenia (x1.02) 0.959
Headache (x1.02) 0.959
famotidine (+0.00143) 0.959
Disease of liver (x1.02) 0.960
acetaminophen (+0.00143) 0.960
Bacterial infectious disease (x1.02) 0.960
Muscle weakness (x1.02) 0.961
Bleeding from nose (x1.02) 0.961
Iron deficiency anemia (x1.02) 0.961
Type 2 diabetes mellitus without complication (x1.01) 0.962
Chronic hepatitis C (x1.01) 0.962
Urobilinogen [Presence] in Urine by Test strip (+0.0274) 0.962
Shock (x1.01) 0.962
Expressions
10^5
10^3
10^1
−10 0 10
−0.75 −0.50 −0.25 0.00 0.25 0.50 0.75

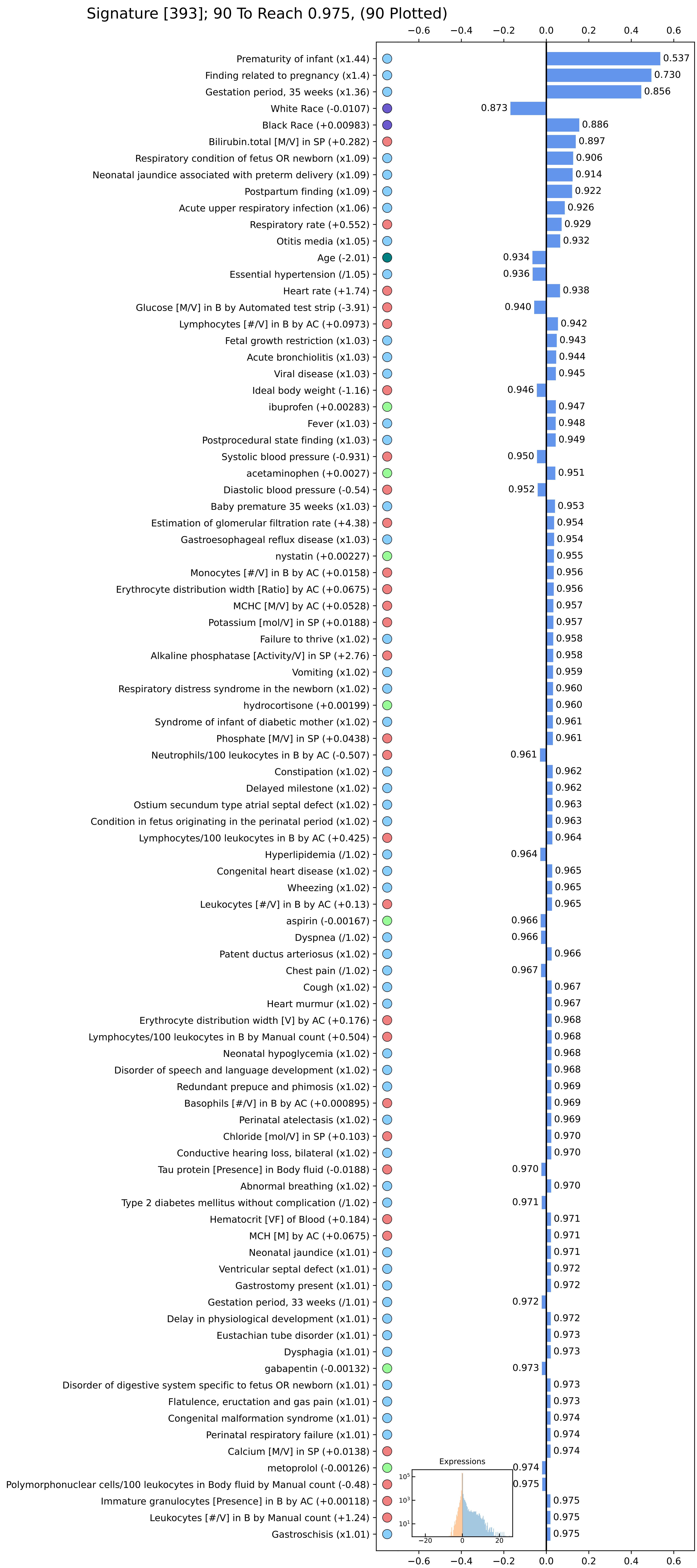

Signature [393]; 90 To Reach 0.975, (90 Plotted)
−0.6
−0.4
−0.2
0.0
0.2
0.4
0.6
Prematurity of infant (x1.44)
0.537
Finding related to pregnancy (x1.4)
0.730
Gestation period, 35 weeks (x1.36)
0.856
White Race (-0.0107)
0.873
Black Race (+0.00983)
0.886
Bilirubin.total [M/V] in SP (+0.282)
0.897
Respiratory condition of fetus OR newborn (x1.09)
0.906
Neonatal jaundice associated with preterm delivery (x1.09)
0.914
Postpartum finding (x1.09)
0.922
Acute upper respiratory infection (x1.06)
0.926
Respiratory rate (+0.552)
0.929
Otitis media (x1.05)
0.932
Age (-2.01)
0.934
Essential hypertension (/1.05)
0.936
Heart rate (+1.74)
0.938
Glucose [M/V] in B by Automated test strip (-3.91)
0.940
Lymphocytes [#/V] in B by AC (+0.0973)
0.942
Fetal growth restriction (x1.03)
0.943
Acute bronchiolitis (x1.03)
0.944
Viral disease (x1.03)
0.945
Ideal body weight (-1.16)
0.946
ibuprofen (+0.00283)
0.947
Fever (x1.03)
0.948
Postprocedural state finding (x1.03)
0.949
Systolic blood pressure (-0.931)
0.950
acetaminophen (+0.0027)
0.951
Diastolic blood pressure (-0.54)
0.952
Baby premature 35 weeks (x1.03)
0.953
Estimation of glomerular filtration rate (+4.38)
0.954
Gastroesophageal reflux disease (x1.03)
0.954
nystatin (+0.00227)
0.955
Monocytes [#/V] in B by AC (+0.0158)
0.956
Erythrocyte distribution width [Ratio] by AC (+0.0675)
0.956
MCHC [M/V] by AC (+0.0528)
0.957
Potassium [mol/V] in SP (+0.0188)
0.957
Failure to thrive (x1.02)
0.958
Alkaline phosphatase [Activity/V] in SP (+2.76)
0.958
Vomiting (x1.02)
0.959
Respiratory distress syndrome in the newborn (x1.02)
0.960
hydrocortisone (+0.00199)
0.960
Syndrome of infant of diabetic mother (x1.02)
0.961
Phosphate [M/V] in SP (+0.0438)
0.961
Neutrophils/100 leukocytes in B by AC (-0.507)
0.961
Constipation (x1.02)
0.962
Delayed milestone (x1.02)
0.962
Ostium secundum type atrial septal defect (x1.02)
0.963
Condition in fetus originating in the perinatal period (x1.02)
0.963
Lymphocytes/100 leukocytes in B by AC (+0.425)
0.964
Hyperlipidemia (/1.02)
0.964
Congenital heart disease (x1.02)
0.965
Wheezing (x1.02)
0.965
Leukocytes [#/V] in B by AC (+0.13)
0.965
aspirin (-0.00167)
0.966
Dyspnea (/1.02)
0.966
Patent ductus arteriosus (x1.02)
0.966
Chest pain (/1.02)
0.967
Cough (x1.02)
0.967
Heart murmur (x1.02)
0.967
Erythrocyte distribution width [V] by AC (+0.176)
0.968
Lymphocytes/100 leukocytes in B by Manual count (+0.504)
0.968
Neonatal hypoglycemia (x1.02)
0.968
Disorder of speech and language development (x1.02)
0.968
Redundant prepuce and phimosis (x1.02)
0.969
Basophils [#/V] in B by AC (+0.000895)
0.969
Perinatal atelectasis (x1.02)
0.969
Chloride [mol/V] in SP (+0.103)
0.970
Conductive hearing loss, bilateral (x1.02)
0.970
Tau protein [Presence] in Body fluid (-0.0188)
0.970
Abnormal breathing (x1.02)
0.970
Type 2 diabetes mellitus without complication (/1.02)
0.971
Hematocrit [VF] of Blood (+0.184)
0.971
MCH [M] by AC (+0.0675)
0.971
Neonatal jaundice (x1.01)
0.971
Ventricular septal defect (x1.01)
0.972
Gastrostomy present (x1.01)
0.972
Gestation period, 33 weeks (/1.01)
0.972
Delay in physiological development (x1.01)
0.972
Eustachian tube disorder (x1.01)
0.973
Dysphagia (x1.01)
0.973
gabapentin (-0.00132)
0.973
Disorder of digestive system specific to fetus OR newborn (x1.01)
0.973
Flatulence, eructation and gas pain (x1.01)
0.973
Congenital malformation syndrome (x1.01)
0.974
Perinatal respiratory failure (x1.01)
0.974
Calcium [M/V] in SP (+0.0138)
0.974
metoprolol (-0.00126)
0.974
Polymorphonuclear cells/100 leukocytes in Body fluid by Manual count (-0.48)
0.975
Immature granulocytes [Presence] in B by AC (+0.00118)
0.975
Leukocytes [#/V] in B by Manual count (+1.24)
0.975
Gastroschisis (x1.01)
0.975
Expressions
10^5
10^3
10^1
−20
0
20

Signature [394]; 118 To Reach 0.975, (100 Plotted)

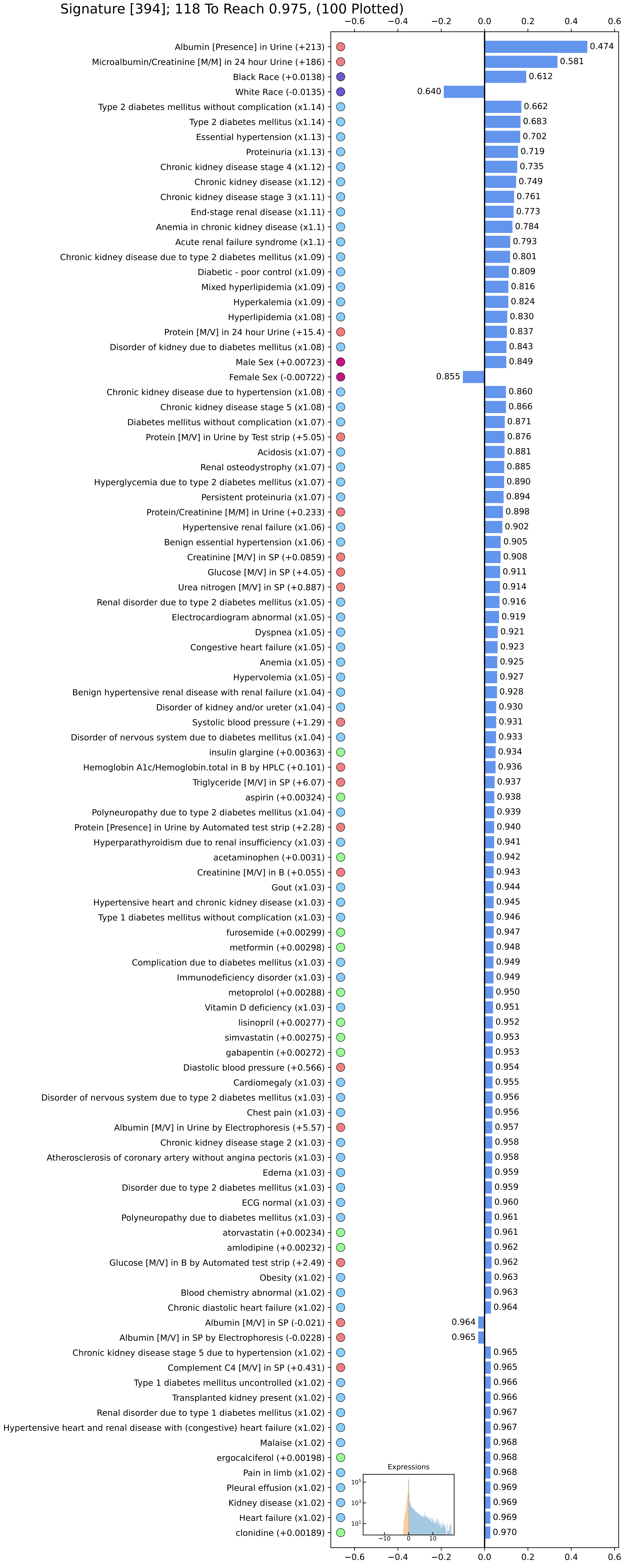

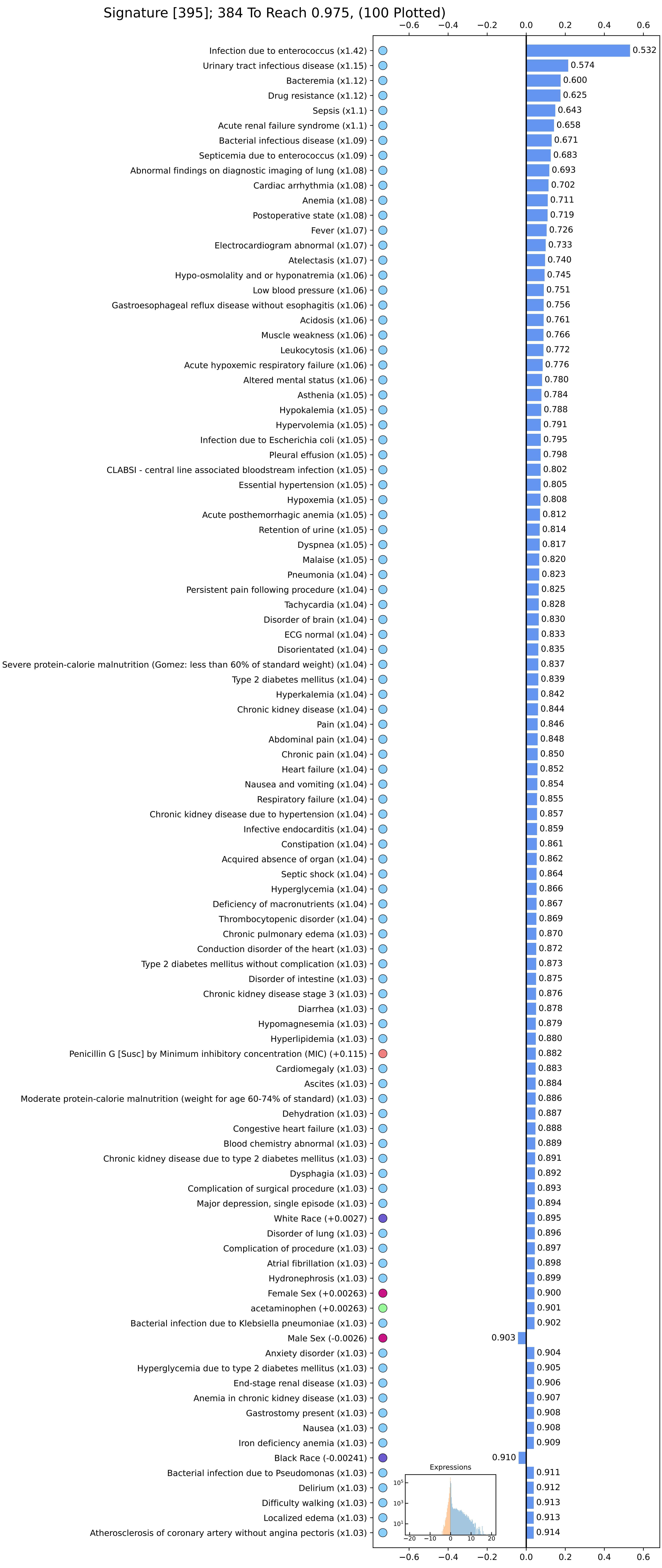

Signature [395]; 384 To Reach 0.975, (100 Plotted)
Infection due to enterococcus (x1.42) 0.532
Urinary tract infectious disease (x1.15) 0.574
Bacteremia (x1.12) 0.600
Drug resistance (x1.12) 0.625
Sepsis (x1.1) 0.643
Acute renal failure syndrome (x1.1) 0.658
Bacterial infectious disease (x1.09) 0.671
Septicemia due to enterococcus (x1.09) 0.683
Abnormal findings on diagnostic imaging of lung (x1.08) 0.693
Cardiac arrhythmia (x1.08) 0.702
Anemia (x1.08) 0.711
Postoperative state (x1.08) 0.719
Fever (x1.07) 0.726
Electrocardiogram abnormal (x1.07) 0.733
Atelectasis (x1.07) 0.740
Hypo-osmolality and or hyponatremia (x1.06) 0.745
Low blood pressure (x1.06) 0.751
Gastroesophageal reflux disease without esophagitis (x1.06) 0.756
Acidosis (x1.06) 0.761
Muscle weakness (x1.06) 0.766
Leukocytosis (x1.06) 0.772
Acute hypoxemic respiratory failure (x1.06) 0.776
Altered mental status (x1.06) 0.780
Asthenia (x1.05) 0.784
Hypokalemia (x1.05) 0.788
Hypervolemia (x1.05) 0.791
Infection due to Escherichia coli (x1.05) 0.795
Pleural effusion (x1.05) 0.798
CLABSI - central line associated bloodstream infection (x1.05) 0.802
Essential hypertension (x1.05) 0.805
Hypoxemia (x1.05) 0.808
Acute posthemorrhagic anemia (x1.05) 0.812
Retention of urine (x1.05) 0.814
Dyspnea (x1.05) 0.817
Malaise (x1.05) 0.820
Pneumonia (x1.04) 0.823
Persistent pain following procedure (x1.04) 0.825
Tachycardia (x1.04) 0.828
Disorder of brain (x1.04) 0.830
ECG normal (x1.04) 0.833
Disorientated (x1.04) 0.835
Severe protein-calorie malnutrition (Gomez: less than 60% of standard weight) (x1.04) 0.837
Type 2 diabetes mellitus (x1.04) 0.839
Hyperkalemia (x1.04) 0.842
Chronic kidney disease (x1.04) 0.844
Pain (x1.04) 0.846
Abdominal pain (x1.04) 0.848
Chronic pain (x1.04) 0.850
Heart failure (x1.04) 0.852
Nausea and vomiting (x1.04) 0.854
Respiratory failure (x1.04) 0.855
Chronic kidney disease due to hypertension (x1.04) 0.857
Infective endocarditis (x1.04) 0.859
Constipation (x1.04) 0.861
Acquired absence of organ (x1.04) 0.862
Septic shock (x1.04) 0.864
Hyperglycemia (x1.04) 0.866
Deficiency of macronutrients (x1.04) 0.867
Thrombocytopenic disorder (x1.04) 0.869
Chronic pulmonary edema (x1.03) 0.870
Conduction disorder of the heart (x1.03) 0.872
Type 2 diabetes mellitus without complication (x1.03) 0.873
Disorder of intestine (x1.03) 0.875
Chronic kidney disease stage 3 (x1.03) 0.876
Diarrhea (x1.03) 0.878
Hypomagnesemia (x1.03) 0.879
Hyperlipidemia (x1.03) 0.880
Penicillin G [Susc] by Minimum inhibitory concentration (MIC) (+0.115) 0.882
Cardiomegaly (x1.03) 0.883
Ascites (x1.03) 0.884
Moderate protein-calorie malnutrition (weight for age 60-74% of standard) (x1.03) 0.886
Dehydration (x1.03) 0.887
Congestive heart failure (x1.03) 0.888
Blood chemistry abnormal (x1.03) 0.889
Chronic kidney disease due to type 2 diabetes mellitus (x1.03) 0.891
Dysphagia (x1.03) 0.892
Complication of surgical procedure (x1.03) 0.893
Major depression, single episode (x1.03) 0.894
White Race (+0.0027) 0.895
Disorder of lung (x1.03) 0.896
Complication of procedure (x1.03) 0.897
Atrial fibrillation (x1.03) 0.898
Hydronephrosis (x1.03) 0.899
Female Sex (+0.00263) 0.900
acetaminophen (+0.00263) 0.901
Bacterial infection due to Klebsiella pneumoniae (x1.03) 0.902
Male Sex (-0.0026) 0.903
Anxiety disorder (x1.03) 0.904
Hyperglycemia due to type 2 diabetes mellitus (x1.03) 0.905
End-stage renal disease (x1.03) 0.906
Anemia in chronic kidney disease (x1.03) 0.907
Gastrostomy present (x1.03) 0.908
Nausea (x1.03) 0.908
Iron deficiency anemia (x1.03) 0.909
Black Race (-0.00241) 0.910
Bacterial infection due to Pseudomonas (x1.03) 0.911
Delirium (x1.03) 0.912
Difficulty walking (x1.03) 0.913
Localized edema (x1.03) 0.913
Atherosclerosis of coronary artery without angina pectoris (x1.03) 0.914
Expressions
−0.6 −0.4 −0.2 0.0 0.2 0.4 0.6

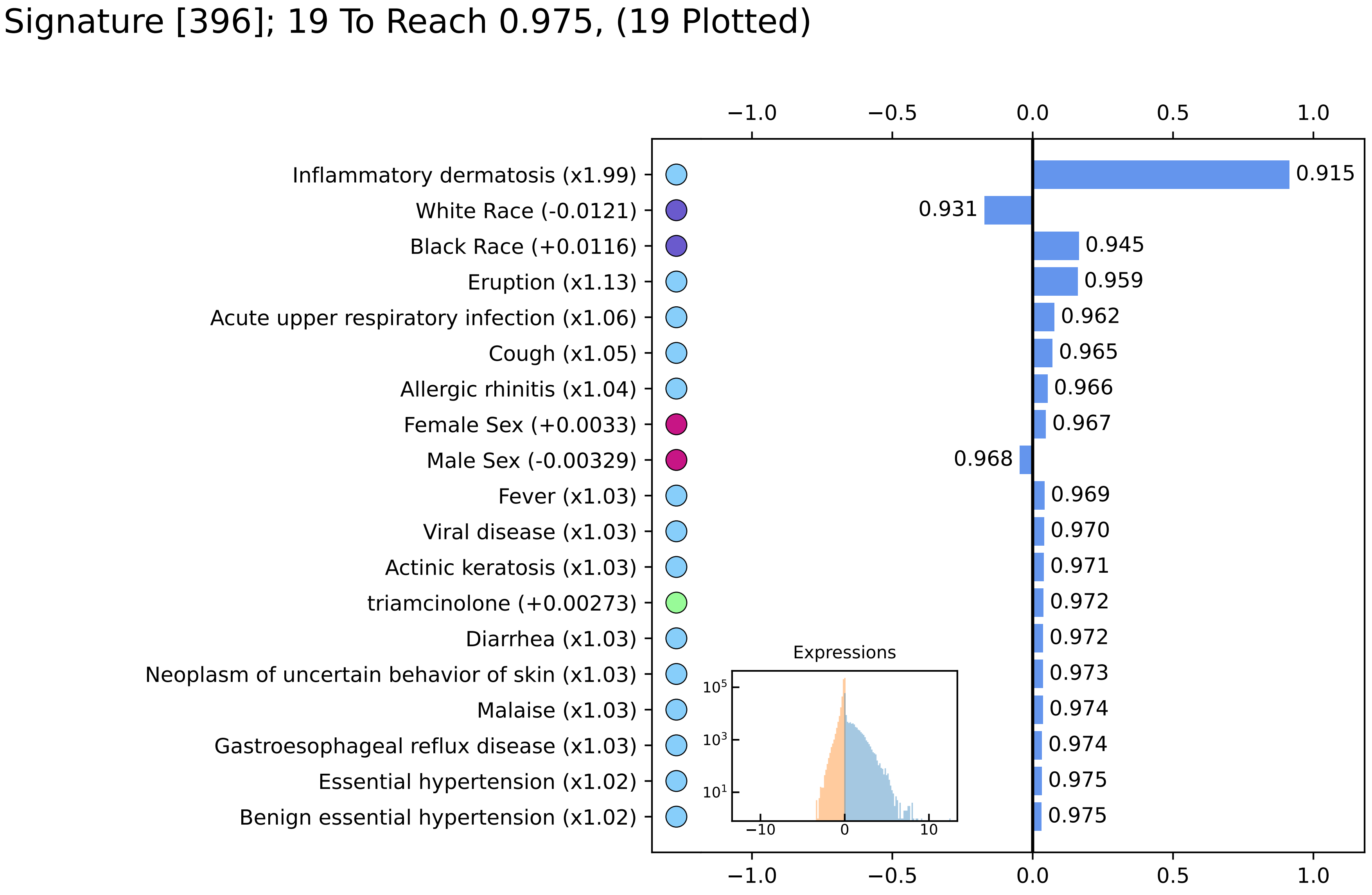

Signature [396]; 19 To Reach 0.975, (19 Plotted)
Inflammatory dermatosis (x1.99)
White Race (-0.0121)
Black Race (+0.0116)
Eruption (x1.13)
Acute upper respiratory infection (x1.06)
Cough (x1.05)
Allergic rhinitis (x1.04)
Female Sex (+0.0033)
Male Sex (-0.00329)
Fever (x1.03)
Viral disease (x1.03)
Actinic keratosis (x1.03)
triamcinolone (+0.00273)
Diarrhea (x1.03)
Neoplasm of uncertain behavior of skin (x1.03)
Malaise (x1.03)
Gastroesophageal reflux disease (x1.03)
Essential hypertension (x1.02)
Benign essential hypertension (x1.02)
0.915
0.931
0.945
0.959
0.962
0.965
0.966
0.967
0.968
0.969
0.970
0.971
0.972
0.972
0.973
0.974
0.974
0.975
0.975
−1.0
−0.5
0.0
0.5
1.0
Expressions

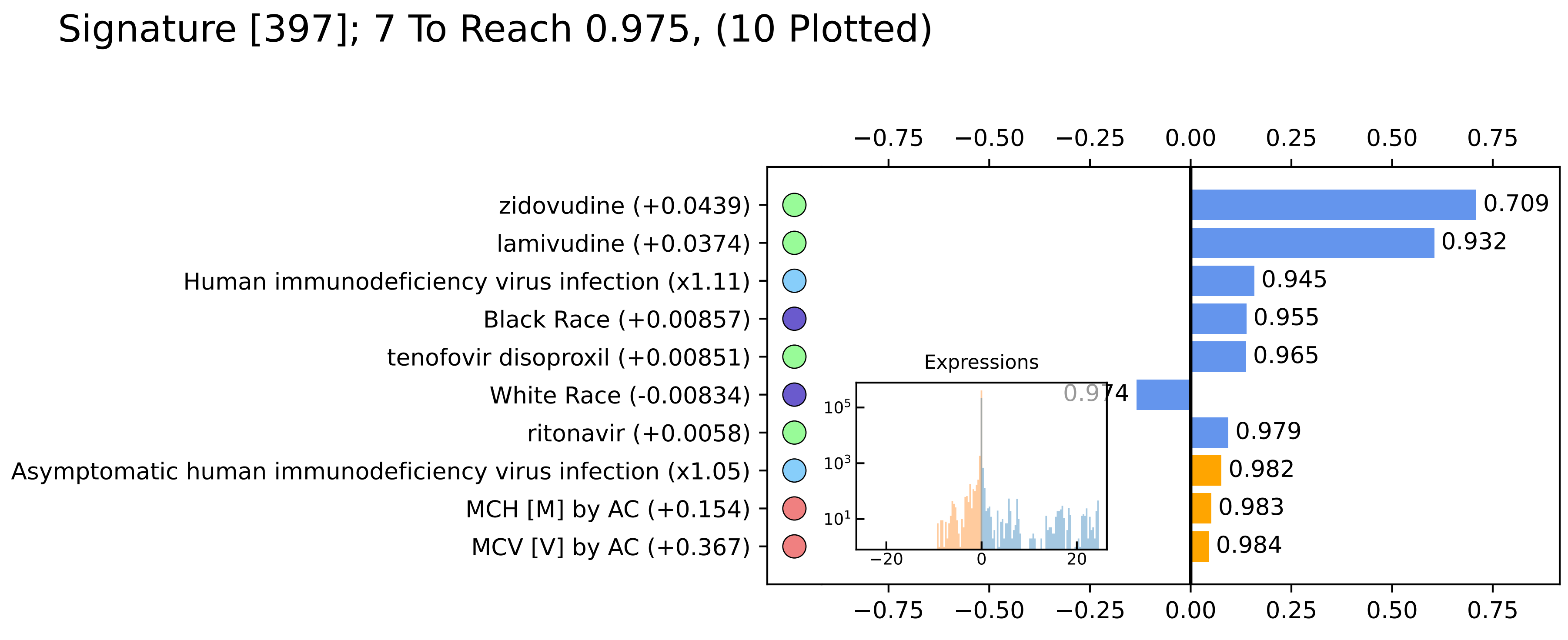

Signature [397]; 7 To Reach 0.975, (10 Plotted)
zidovudine (+0.0439)
lamivudine (+0.0374)
Human immunodeficiency virus infection (x1.11)
Black Race (+0.00857)
tenofovir disoproxil (+0.00851)
White Race (-0.00834)
ritonavir (+0.0058)
Asymptomatic human immunodeficiency virus infection (x1.05)
MCH [M] by AC (+0.154)
MCV [V] by AC (+0.367)
0.709
0.932
0.945
0.955
0.965
0.974
0.979
0.982
0.983
0.984
−0.75
−0.50
−0.25
0.00
0.25
0.50
0.75
Expressions
10^5
10^3
10^1
−20
0
20

Signature [398]; 1 To Reach 0.975, (10 Plotted)

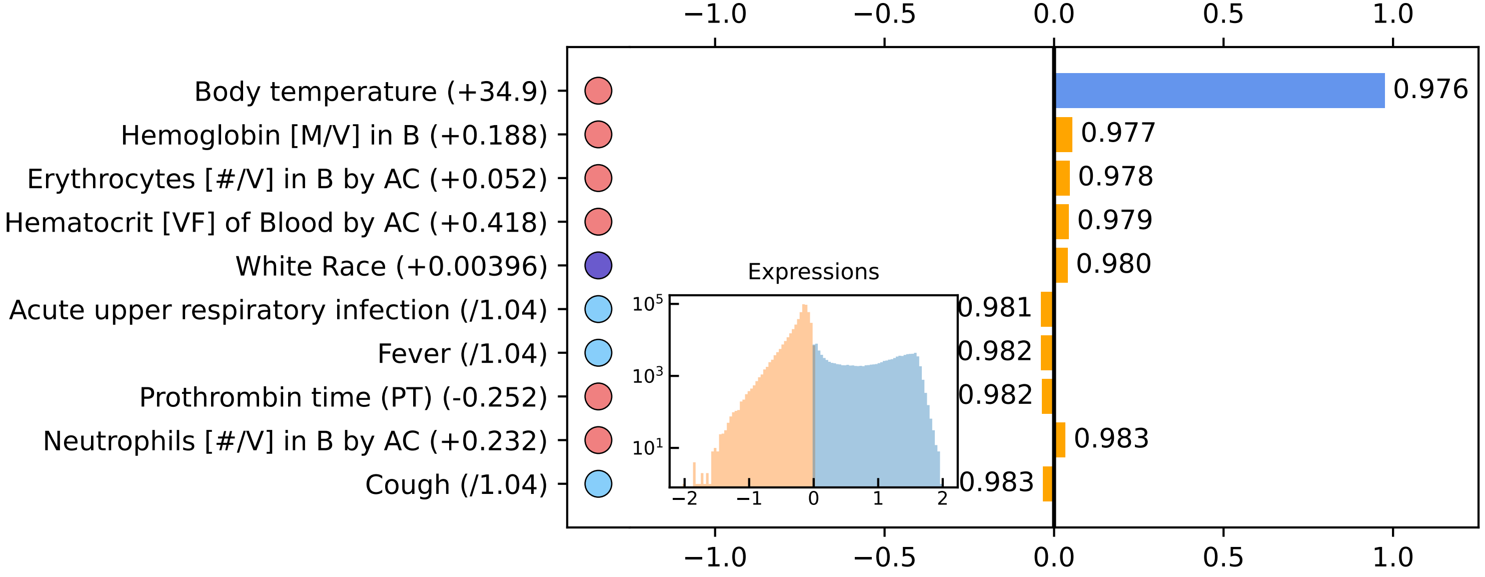

Signature [399]; 58 To Reach 0.975, (58 Plotted)

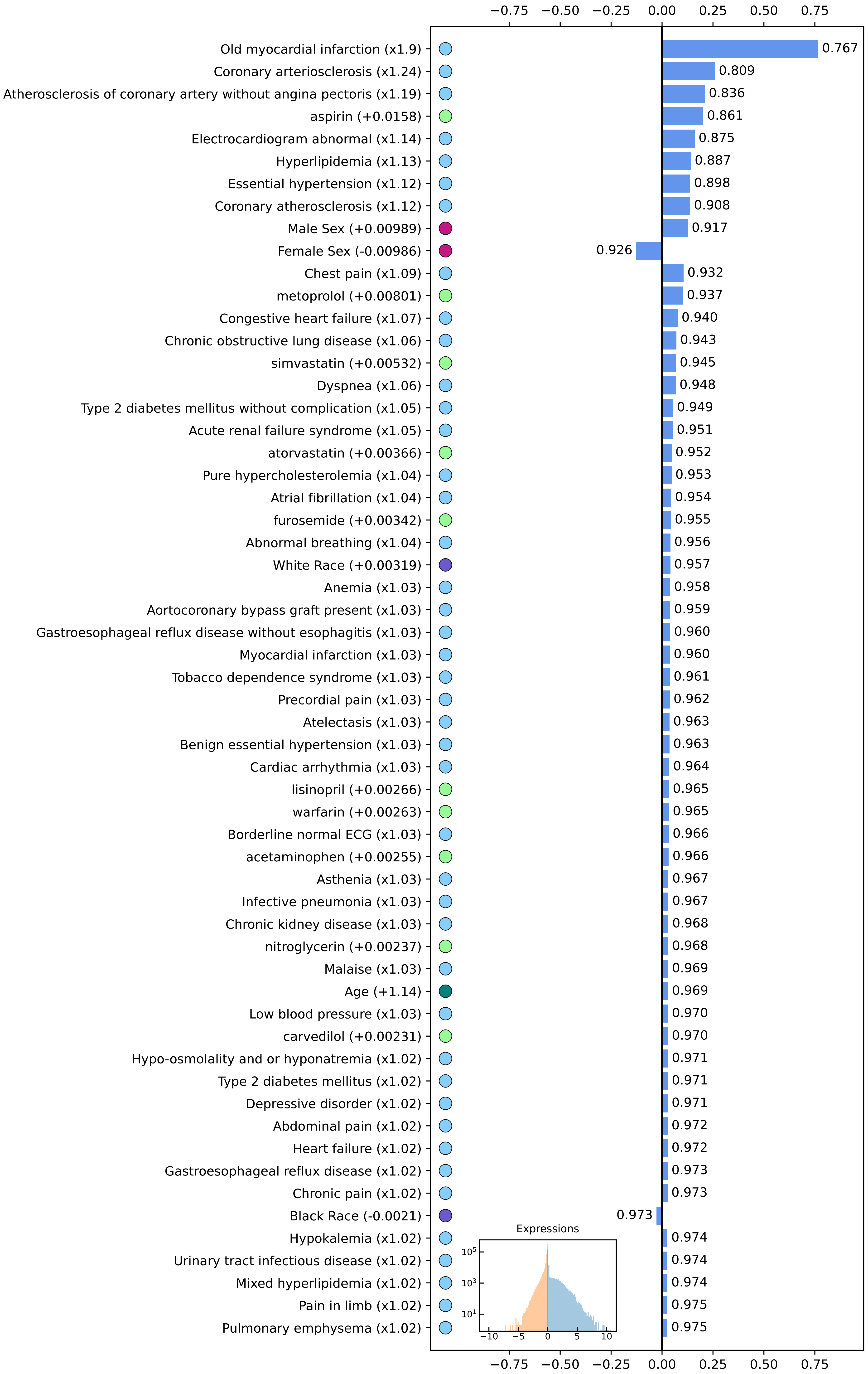

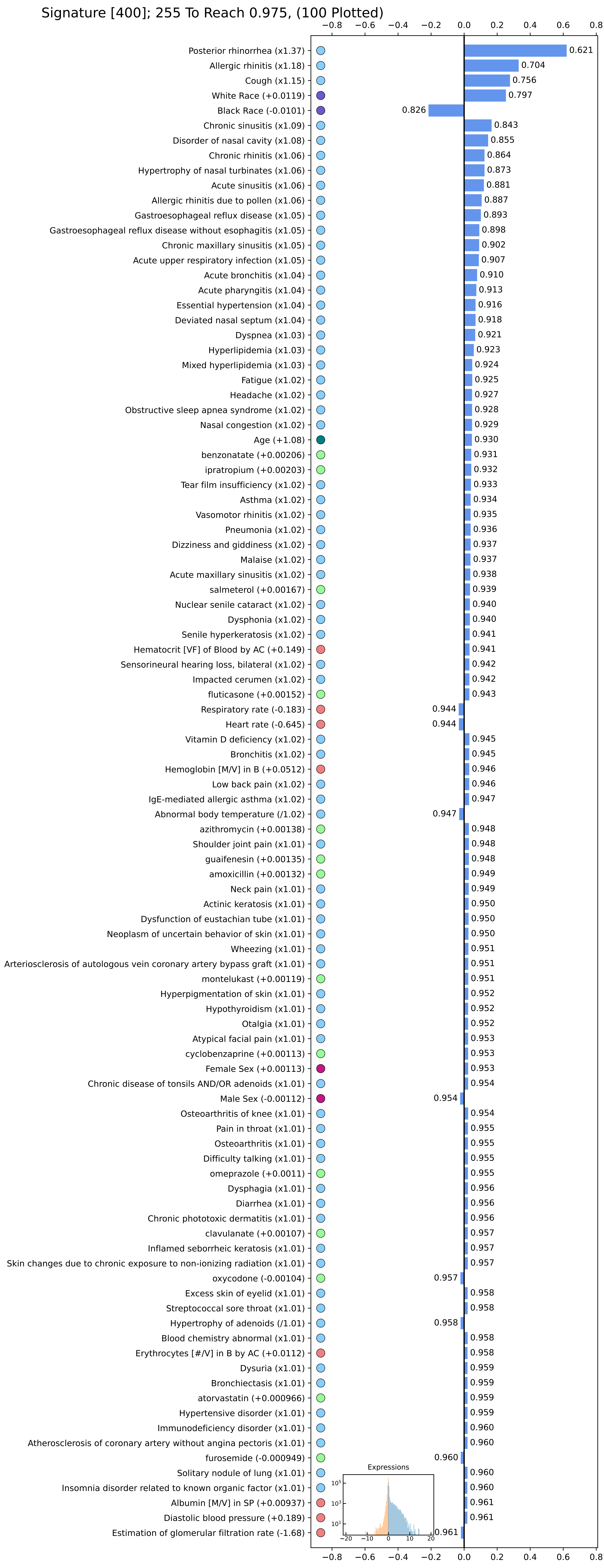

Signature [400]; 255 To Reach 0.975, (100 Plotted)
Posterior rhinorrhea (x1.37) 0.621
Allergic rhinitis (x1.18) 0.704
Cough (x1.15) 0.756
White Race (+0.0119) 0.797
Black Race (-0.0101) 0.826
Chronic sinusitis (x1.09) 0.843
Disorder of nasal cavity (x1.08) 0.855
Chronic rhinitis (x1.06) 0.864
Hypertrophy of nasal turbinates (x1.06) 0.873
Acute sinusitis (x1.06) 0.881
Allergic rhinitis due to pollen (x1.06) 0.887
Gastroesophageal reflux disease (x1.05) 0.893
Gastroesophageal reflux disease without esophagitis (x1.05) 0.898
Chronic maxillary sinusitis (x1.05) 0.902
Acute upper respiratory infection (x1.05) 0.907
Acute bronchitis (x1.04) 0.910
Acute pharyngitis (x1.04) 0.913
Essential hypertension (x1.04) 0.916
Deviated nasal septum (x1.04) 0.918
Dyspnea (x1.03) 0.921
Hyperlipidemia (x1.03) 0.923
Mixed hyperlipidemia (x1.03) 0.924
Fatigue (x1.02) 0.925
Headache (x1.02) 0.927
Obstructive sleep apnea syndrome (x1.02) 0.928
Nasal congestion (x1.02) 0.929
Age (+1.08) 0.930
benzonatate (+0.00206) 0.931
ipratropium (+0.00203) 0.932
Tear film insufficiency (x1.02) 0.933
Asthma (x1.02) 0.934
Vasomotor rhinitis (x1.02) 0.935
Pneumonia (x1.02) 0.936
Dizziness and giddiness (x1.02) 0.937
Malaise (x1.02) 0.937
Acute maxillary sinusitis (x1.02) 0.938
salmeterol (+0.00167) 0.939
Nuclear senile cataract (x1.02) 0.940
Dysphonia (x1.02) 0.940
Senile hyperkeratosis (x1.02) 0.941
Hematocrit [VF] of Blood by AC (+0.149) 0.941
Sensorineural hearing loss, bilateral (x1.02) 0.942
Impacted cerumen (x1.02) 0.942
fluticasone (+0.00152) 0.943
Respiratory rate (-0.183) 0.944
Heart rate (-0.645) 0.944
Vitamin D deficiency (x1.02) 0.945
Bronchitis (x1.02) 0.945
Hemoglobin [M/V] in B (+0.0512) 0.946
Low back pain (x1.02) 0.946
IgE-mediated allergic asthma (x1.02) 0.947
Abnormal body temperature (/1.02) 0.947
azithromycin (+0.00138) 0.948
Shoulder joint pain (x1.01) 0.948
guaifenesin (+0.00135) 0.948
amoxicillin (+0.00132) 0.949
Neck pain (x1.01) 0.949
Actinic keratosis (x1.01) 0.950
Dysfunction of eustachian tube (x1.01) 0.950
Neoplasm of uncertain behavior of skin (x1.01) 0.950
Wheezing (x1.01) 0.951
Arteriosclerosis of autologous vein coronary artery bypass graft (x1.01) 0.951
montelukast (+0.00119) 0.951
Hyperpigmentation of skin (x1.01) 0.952
Hypothyroidism (x1.01) 0.952
Otalgia (x1.01) 0.952
Atypical facial pain (x1.01) 0.953
cyclobenzaprine (+0.00113) 0.953
Female Sex (+0.00113) 0.953
Chronic disease of tonsils AND/OR adenoids (x1.01) 0.954
Male Sex (-0.00112) 0.954
Osteoarthritis of knee (x1.01) 0.954
Pain in throat (x1.01) 0.955
Osteoarthritis (x1.01) 0.955
Difficulty talking (x1.01) 0.955
omeprazole (+0.0011) 0.955
Dysphagia (x1.01) 0.956
Diarrhea (x1.01) 0.956
Chronic phototoxic dermatitis (x1.01) 0.956
clavulanate (+0.00107) 0.957
Inflamed seborrheic keratosis (x1.01) 0.957
Skin changes due to chronic exposure to non-ionizing radiation (x1.01) 0.957
oxycodone (-0.00104) 0.957
Excess skin of eyelid (x1.01) 0.958
Streptococcal sore throat (x1.01) 0.958
Hypertrophy of adenoids (/1.01) 0.958
Blood chemistry abnormal (x1.01) 0.958
Erythrocytes [#/V] in B by AC (+0.0112) 0.958
Dysuria (x1.01) 0.959
Bronchiectasis (x1.01) 0.959
atorvastatin (+0.000966) 0.959
Hypertensive disorder (x1.01) 0.959
Immunodeficiency disorder (x1.01) 0.960
Atherosclerosis of coronary artery without angina pectoris (x1.01) 0.960
furosemide (-0.000949) 0.960
Solitary nodule of lung (x1.01) 0.960
Insomnia disorder related to known organic factor (x1.01) 0.960
Albumin [M/V] in SP (+0.00937) 0.961
Diastolic blood pressure (+0.189) 0.961
Estimation of glomerular filtration rate (-1.68) 0.961
Expressions
−0.8 −0.6 −0.4 −0.2 0.0 0.2 0.4 0.6 0.8

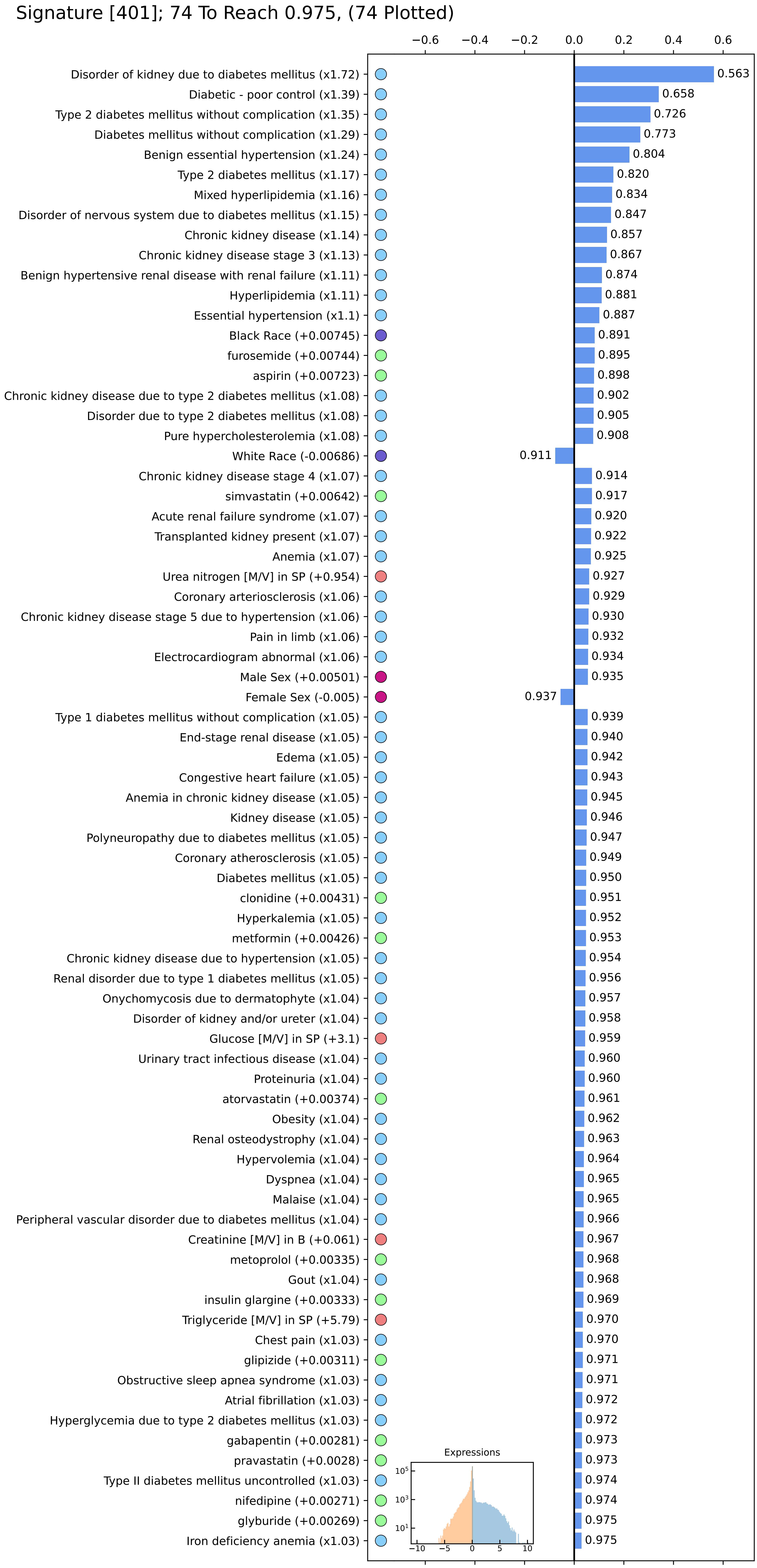
Signature [401]; 74 To Reach 0.975, (74 Plotted)
Disorder of kidney due to diabetes mellitus (x1.72) 0.563
Diabetic - poor control (x1.39) 0.658
Type 2 diabetes mellitus without complication (x1.35) 0.726
Diabetes mellitus without complication (x1.29) 0.773
Benign essential hypertension (x1.24) 0.804
Type 2 diabetes mellitus (x1.17) 0.820
Mixed hyperlipidemia (x1.16) 0.834
Disorder of nervous system due to diabetes mellitus (x1.15) 0.847
Chronic kidney disease (x1.14) 0.857
Chronic kidney disease stage 3 (x1.13) 0.867
Benign hypertensive renal disease with renal failure (x1.11) 0.874
Hyperlipidemia (x1.11) 0.881
Essential hypertension (x1.1) 0.887
Black Race (+0.00745) 0.891
furosemide (+0.00744) 0.895
aspirin (+0.00723) 0.898
Chronic kidney disease due to type 2 diabetes mellitus (x1.08) 0.902
Disorder due to type 2 diabetes mellitus (x1.08) 0.905
Pure hypercholesterolemia (x1.08) 0.908
White Race (-0.00686) 0.911
Chronic kidney disease stage 4 (x1.07) 0.914
simvastatin (+0.00642) 0.917
Acute renal failure syndrome (x1.07) 0.920
Transplanted kidney present (x1.07) 0.922
Anemia (x1.07) 0.925
Urea nitrogen [M/V] in SP (+0.954) 0.927
Coronary arteriosclerosis (x1.06) 0.929
Chronic kidney disease stage 5 due to hypertension (x1.06) 0.930
Pain in limb (x1.06) 0.932
Electrocardiogram abnormal (x1.06) 0.934
Male Sex (+0.00501) 0.935
Female Sex (-0.005) 0.937
Type 1 diabetes mellitus without complication (x1.05) 0.939
End-stage renal disease (x1.05) 0.940
Edema (x1.05) 0.942
Congestive heart failure (x1.05) 0.943
Anemia in chronic kidney disease (x1.05) 0.945
Kidney disease (x1.05) 0.946
Polyneuropathy due to diabetes mellitus (x1.05) 0.947
Coronary atherosclerosis (x1.05) 0.949
Diabetes mellitus (x1.05) 0.950
clonidine (+0.00431) 0.951
Hyperkalemia (x1.05) 0.952
metformin (+0.00426) 0.953
Chronic kidney disease due to hypertension (x1.05) 0.954
Renal disorder due to type 1 diabetes mellitus (x1.05) 0.956
Onychomycosis due to dermatophyte (x1.04) 0.957
Disorder of kidney and/or ureter (x1.04) 0.958
Glucose [M/V] in SP (+3.1) 0.959
Urinary tract infectious disease (x1.04) 0.960
Proteinuria (x1.04) 0.960
atorvastatin (+0.00374) 0.961
Obesity (x1.04) 0.962
Renal osteodystrophy (x1.04) 0.963
Hypervolemia (x1.04) 0.964
Dyspnea (x1.04) 0.965
Malaise (x1.04) 0.965
Peripheral vascular disorder due to diabetes mellitus (x1.04) 0.966
Creatinine [M/V] in B (+0.061) 0.967
metoprolol (+0.00335) 0.968
Gout (x1.04) 0.968
insulin glargine (+0.00333) 0.969
Triglyceride [M/V] in SP (+5.79) 0.970
Chest pain (x1.03) 0.970
glipizide (+0.00311) 0.971
Obstructive sleep apnea syndrome (x1.03) 0.971
Atrial fibrillation (x1.03) 0.972
Hyperglycemia due to type 2 diabetes mellitus (x1.03) 0.972
gabapentin (+0.00281) 0.973
pravastatin (+0.0028) 0.973
Type II diabetes mellitus uncontrolled (x1.03) 0.974
nifedipine (+0.00271) 0.974
glyburide (+0.00269) 0.975
Iron deficiency anemia (x1.03) 0.975
−0.6 −0.4 −0.2 0.0 0.2 0.4 0.6
Expressions
10^5 10^3 10^1
−10 −5 0 5 10

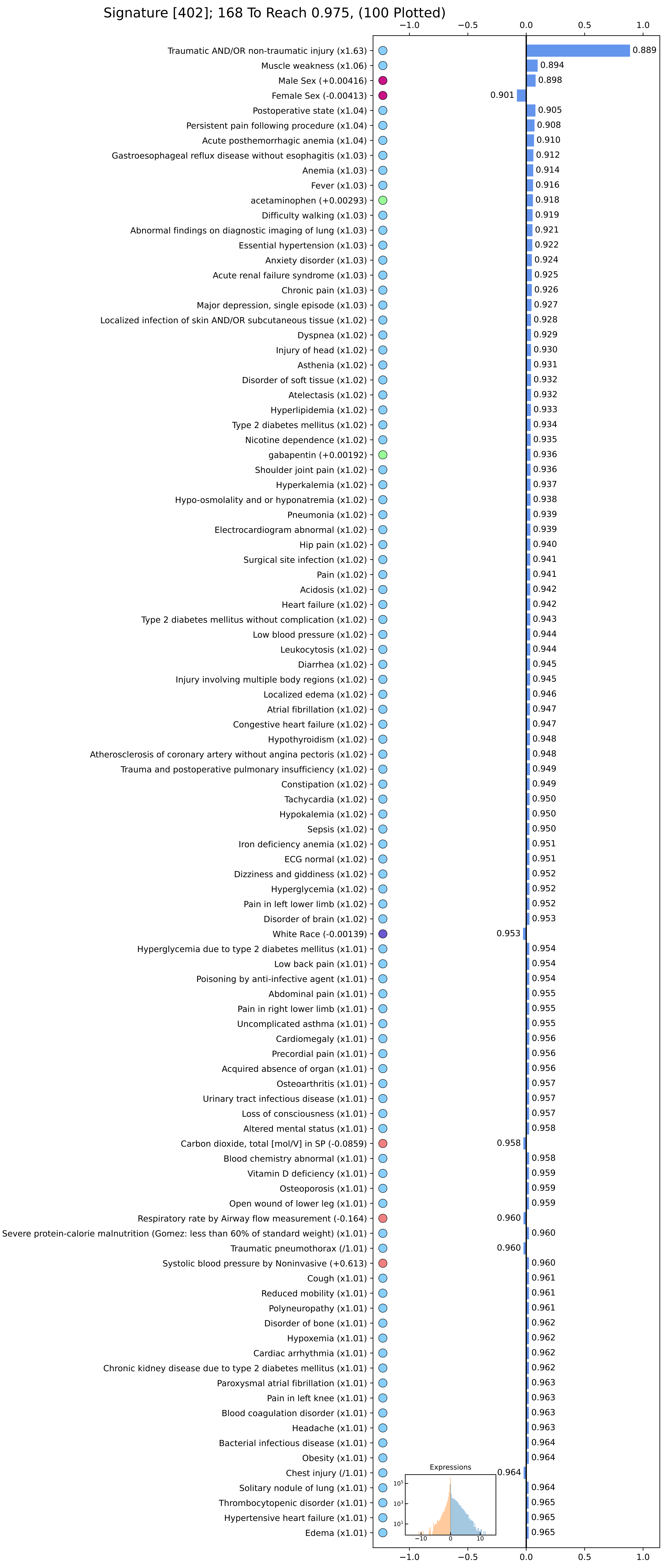

Signature [402]; 168 To Reach 0.975, (100 Plotted)
Traumatic AND/OR non-traumatic injury (x1.63) 0.889
Muscle weakness (x1.06) 0.894
Male Sex (+0.00416) 0.898
Female Sex (-0.00413) 0.901
Postoperative state (x1.04) 0.905
Persistent pain following procedure (x1.04) 0.908
Acute posthemorrhagic anemia (x1.04) 0.910
Gastroesophageal reflux disease without esophagitis (x1.03) 0.912
Anemia (x1.03) 0.914
Fever (x1.03) 0.916
acetaminophen (+0.00293) 0.918
Difficulty walking (x1.03) 0.919
Abnormal findings on diagnostic imaging of lung (x1.03) 0.921
Essential hypertension (x1.03) 0.922
Anxiety disorder (x1.03) 0.924
Acute renal failure syndrome (x1.03) 0.925
Chronic pain (x1.03) 0.926
Major depression, single episode (x1.03) 0.927
Localized infection of skin AND/OR subcutaneous tissue (x1.02) 0.928
Dyspnea (x1.02) 0.929
Injury of head (x1.02) 0.930
Asthenia (x1.02) 0.931
Disorder of soft tissue (x1.02) 0.932
Atelectasis (x1.02) 0.932
Hyperlipidemia (x1.02) 0.933
Type 2 diabetes mellitus (x1.02) 0.934
Nicotine dependence (x1.02) 0.935
gabapentin (+0.00192) 0.936
Shoulder joint pain (x1.02) 0.936
Hyperkalemia (x1.02) 0.937
Hypo-osmolality and or hyponatremia (x1.02) 0.938
Pneumonia (x1.02) 0.939
Electrocardiogram abnormal (x1.02) 0.939
Hip pain (x1.02) 0.940
Surgical site infection (x1.02) 0.941
Pain (x1.02) 0.941
Acidosis (x1.02) 0.942
Heart failure (x1.02) 0.942
Type 2 diabetes mellitus without complication (x1.02) 0.943
Low blood pressure (x1.02) 0.944
Leukocytosis (x1.02) 0.944
Diarrhea (x1.02) 0.945
Injury involving multiple body regions (x1.02) 0.945
Localized edema (x1.02) 0.946
Atrial fibrillation (x1.02) 0.947
Congestive heart failure (x1.02) 0.947
Hypothyroidism (x1.02) 0.948
Atherosclerosis of coronary artery without angina pectoris (x1.02) 0.948
Trauma and postoperative pulmonary insufficiency (x1.02) 0.949
Constipation (x1.02) 0.949
Tachycardia (x1.02) 0.950
Hypokalemia (x1.02) 0.950
Sepsis (x1.02) 0.950
Iron deficiency anemia (x1.02) 0.951
ECG normal (x1.02) 0.951
Dizziness and giddiness (x1.02) 0.952
Hyperglycemia (x1.02) 0.952
Pain in left lower limb (x1.02) 0.952
Disorder of brain (x1.02) 0.953
White Race (-0.00139) 0.953
Hyperglycemia due to type 2 diabetes mellitus (x1.01) 0.954
Low back pain (x1.01) 0.954
Poisoning by anti-infective agent (x1.01) 0.954
Abdominal pain (x1.01) 0.955
Pain in right lower limb (x1.01) 0.955
Uncomplicated asthma (x1.01) 0.955
Cardiomegaly (x1.01) 0.956
Precordial pain (x1.01) 0.956
Acquired absence of organ (x1.01) 0.956
Osteoarthritis (x1.01) 0.957
Urinary tract infectious disease (x1.01) 0.957
Loss of consciousness (x1.01) 0.957
Altered mental status (x1.01) 0.958
Carbon dioxide, total [mol/V] in SP (-0.0859) 0.958
Blood chemistry abnormal (x1.01) 0.958
Vitamin D deficiency (x1.01) 0.959
Osteoporosis (x1.01) 0.959
Open wound of lower leg (x1.01) 0.959
Respiratory rate by Airway flow measurement (-0.164) 0.960
Severe protein-calorie malnutrition (Gomez: less than 60% of standard weight) (x1.01) 0.960
Traumatic pneumothorax (/1.01) 0.960
Systolic blood pressure by Noninvasive (+0.613) 0.960
Cough (x1.01) 0.961
Reduced mobility (x1.01) 0.961
Polyneuropathy (x1.01) 0.961
Disorder of bone (x1.01) 0.962
Hypoxemia (x1.01) 0.962
Cardiac arrhythmia (x1.01) 0.962
Chronic kidney disease due to type 2 diabetes mellitus (x1.01) 0.962
Paroxysmal atrial fibrillation (x1.01) 0.963
Pain in left knee (x1.01) 0.963
Blood coagulation disorder (x1.01) 0.963
Headache (x1.01) 0.963
Bacterial infectious disease (x1.01) 0.964
Obesity (x1.01) 0.964
Chest injury (/1.01) 0.964
Solitary nodule of lung (x1.01) 0.964
Thrombocytopenic disorder (x1.01) 0.965
Hypertensive heart failure (x1.01) 0.965
Edema (x1.01) 0.965
−1.0 −0.5 0.0 0.5 1.0
Expressions

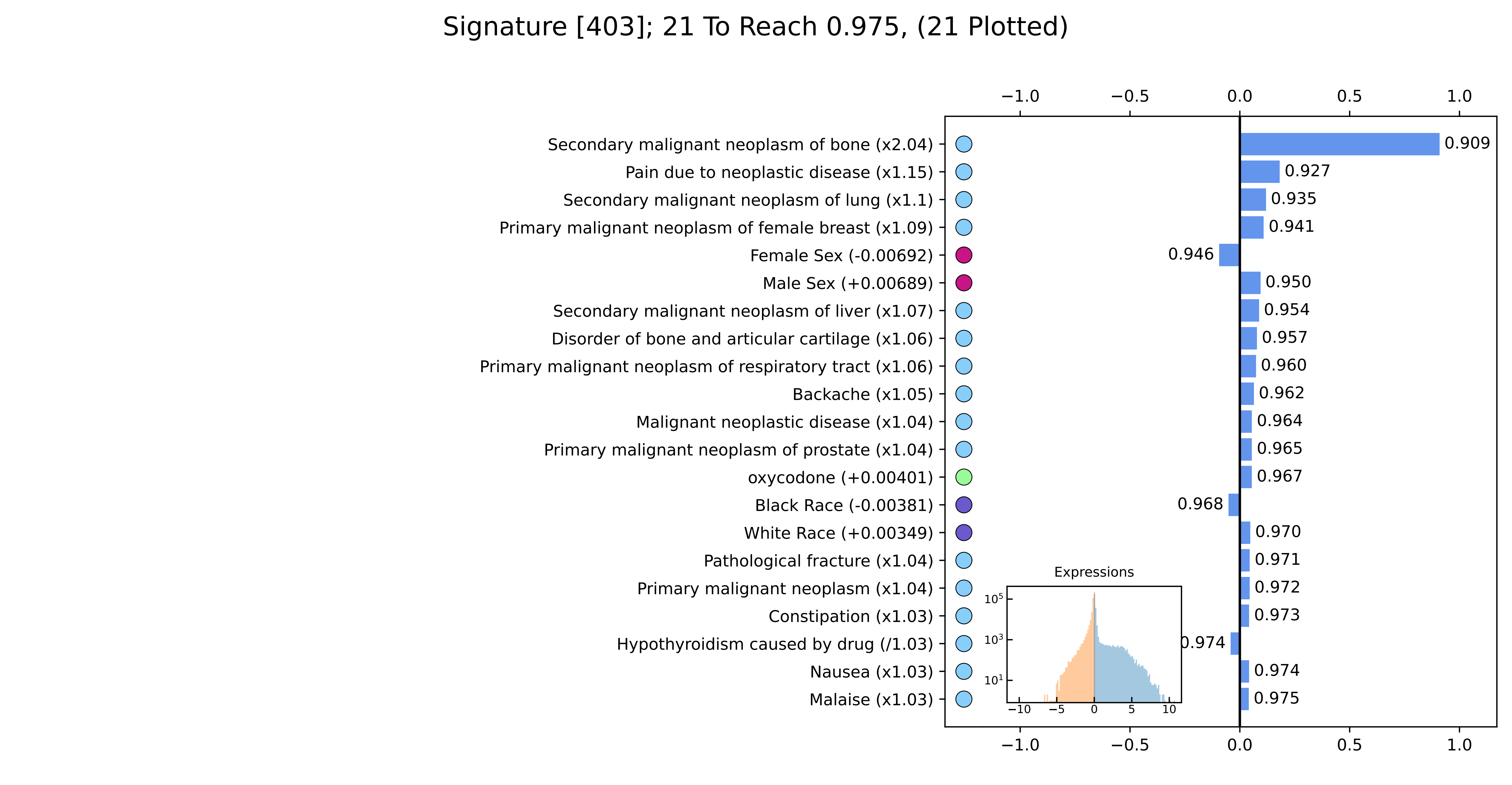

Signature [403]; 21 To Reach 0.975, (21 Plotted)
−1.0
−0.5
0.0
0.5
1.0
Secondary malignant neoplasm of bone (x2.04)
0.909
Pain due to neoplastic disease (x1.15)
0.927
Secondary malignant neoplasm of lung (x1.1)
0.935
Primary malignant neoplasm of female breast (x1.09)
0.941
Female Sex (-0.00692)
0.946
Male Sex (+0.00689)
0.950
Secondary malignant neoplasm of liver (x1.07)
0.954
Disorder of bone and articular cartilage (x1.06)
0.957
Primary malignant neoplasm of respiratory tract (x1.06)
0.960
Backache (x1.05)
0.962
Malignant neoplastic disease (x1.04)
0.964
Primary malignant neoplasm of prostate (x1.04)
0.965
oxycodone (+0.00401)
0.967
Black Race (-0.00381)
0.968
White Race (+0.00349)
0.970
Pathological fracture (x1.04)
0.971
Primary malignant neoplasm (x1.04)
0.972
Constipation (x1.03)
0.973
Hypothyroidism caused by drug (/1.03)
0.974
Nausea (x1.03)
0.974
Malaise (x1.03)
0.975
Expressions

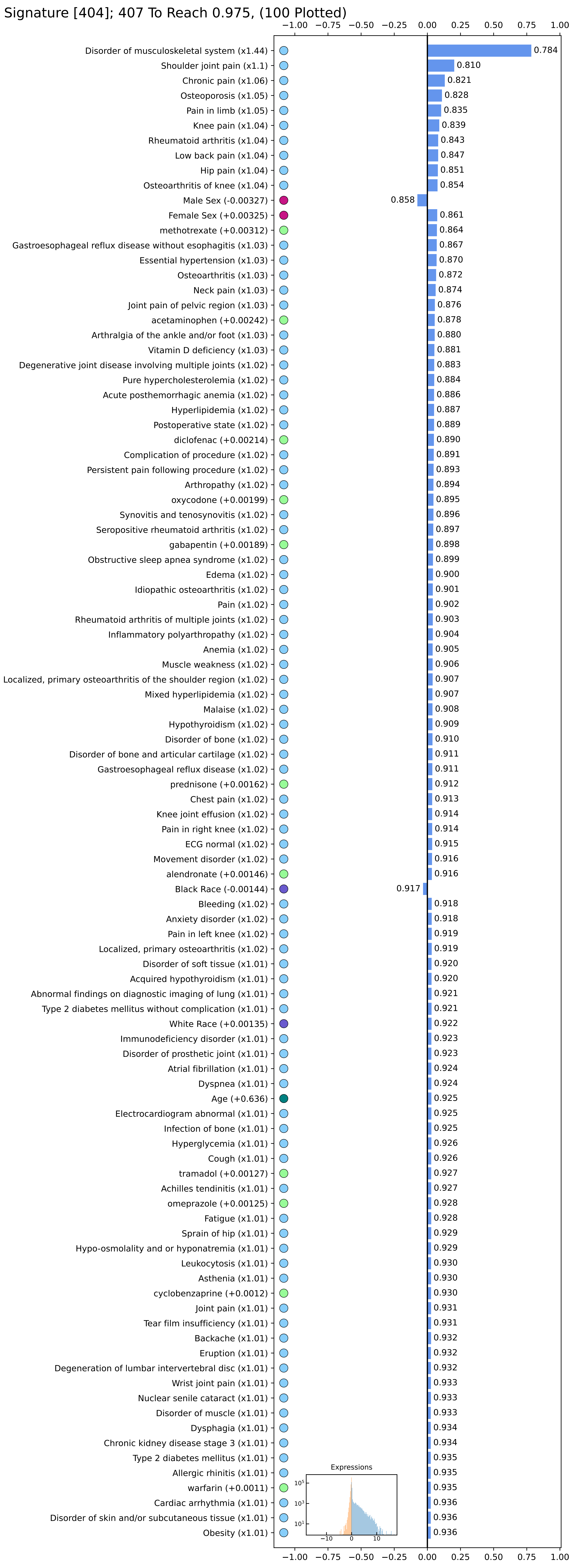

Signature [404]; 407 To Reach 0.975, (100 Plotted)
Disorder of musculoskeletal system (x1.44) 0.784
Shoulder joint pain (x1.1) 0.810
Chronic pain (x1.06) 0.821
Osteoporosis (x1.05) 0.828
Pain in limb (x1.05) 0.835
Knee pain (x1.04) 0.839
Rheumatoid arthritis (x1.04) 0.843
Low back pain (x1.04) 0.847
Hip pain (x1.04) 0.851
Osteoarthritis of knee (x1.04) 0.854
Male Sex (-0.00327) 0.858
Female Sex (+0.00325) 0.861
methotrexate (+0.00312) 0.864
Gastroesophageal reflux disease without esophagitis (x1.03) 0.867
Essential hypertension (x1.03) 0.870
Osteoarthritis (x1.03) 0.872
Neck pain (x1.03) 0.874
Joint pain of pelvic region (x1.03) 0.876
acetaminophen (+0.00242) 0.878
Arthralgia of the ankle and/or foot (x1.03) 0.880
Vitamin D deficiency (x1.03) 0.881
Degenerative joint disease involving multiple joints (x1.02) 0.883
Pure hypercholesterolemia (x1.02) 0.884
Acute posthemorrhagic anemia (x1.02) 0.886
Hyperlipidemia (x1.02) 0.887
Postoperative state (x1.02) 0.889
diclofenac (+0.00214) 0.890
Complication of procedure (x1.02) 0.891
Persistent pain following procedure (x1.02) 0.893
Arthropathy (x1.02) 0.894
oxycodone (+0.00199) 0.895
Synovitis and tenosynovitis (x1.02) 0.896
Seropositive rheumatoid arthritis (x1.02) 0.897
gabapentin (+0.00189) 0.898
Obstructive sleep apnea syndrome (x1.02) 0.899
Edema (x1.02) 0.900
Idiopathic osteoarthritis (x1.02) 0.901
Pain (x1.02) 0.902
Rheumatoid arthritis of multiple joints (x1.02) 0.903
Inflammatory polyarthropathy (x1.02) 0.904
Anemia (x1.02) 0.905
Muscle weakness (x1.02) 0.906
Localized, primary osteoarthritis of the shoulder region (x1.02) 0.907
Mixed hyperlipidemia (x1.02) 0.907
Malaise (x1.02) 0.908
Hypothyroidism (x1.02) 0.909
Disorder of bone (x1.02) 0.910
Disorder of bone and articular cartilage (x1.02) 0.911
Gastroesophageal reflux disease (x1.02) 0.911
prednisone (+0.00162) 0.912
Chest pain (x1.02) 0.913
Knee joint effusion (x1.02) 0.914
Pain in right knee (x1.02) 0.914
ECG normal (x1.02) 0.915
Movement disorder (x1.02) 0.916
alendronate (+0.00146) 0.916
Black Race (-0.00144) 0.917
Bleeding (x1.02) 0.918
Anxiety disorder (x1.02) 0.918
Pain in left knee (x1.02) 0.919
Localized, primary osteoarthritis (x1.02) 0.919
Disorder of soft tissue (x1.01) 0.920
Acquired hypothyroidism (x1.01) 0.920
Abnormal findings on diagnostic imaging of lung (x1.01) 0.921
Type 2 diabetes mellitus without complication (x1.01) 0.921
White Race (+0.00135) 0.922
Immunodeficiency disorder (x1.01) 0.923
Disorder of prosthetic joint (x1.01) 0.923
Atrial fibrillation (x1.01) 0.924
Dyspnea (x1.01) 0.924
Age (+0.636) 0.925
Electrocardiogram abnormal (x1.01) 0.925
Infection of bone (x1.01) 0.925
Hyperglycemia (x1.01) 0.926
Cough (x1.01) 0.926
tramadol (+0.00127) 0.927
Achilles tendinitis (x1.01) 0.927
omeprazole (+0.00125) 0.928
Fatigue (x1.01) 0.928
Sprain of hip (x1.01) 0.929
Hypo-osmolality and or hyponatremia (x1.01) 0.929
Leukocytosis (x1.01) 0.930
Asthenia (x1.01) 0.930
cyclobenzaprine (+0.0012) 0.930
Joint pain (x1.01) 0.931
Tear film insufficiency (x1.01) 0.931
Backache (x1.01) 0.932
Eruption (x1.01) 0.932
Degeneration of lumbar intervertebral disc (x1.01) 0.932
Wrist joint pain (x1.01) 0.933
Nuclear senile cataract (x1.01) 0.933
Disorder of muscle (x1.01) 0.933
Dysphagia (x1.01) 0.934
Chronic kidney disease stage 3 (x1.01) 0.934
Type 2 diabetes mellitus (x1.01) 0.935
Allergic rhinitis (x1.01) 0.935
warfarin (+0.0011) 0.935
Cardiac arrhythmia (x1.01) 0.936
Disorder of skin and/or subcutaneous tissue (x1.01) 0.936
Obesity (x1.01) 0.936
Expressions
−1.00 −0.75 −0.50 −0.25 0.00 0.25 0.50 0.75 1.00

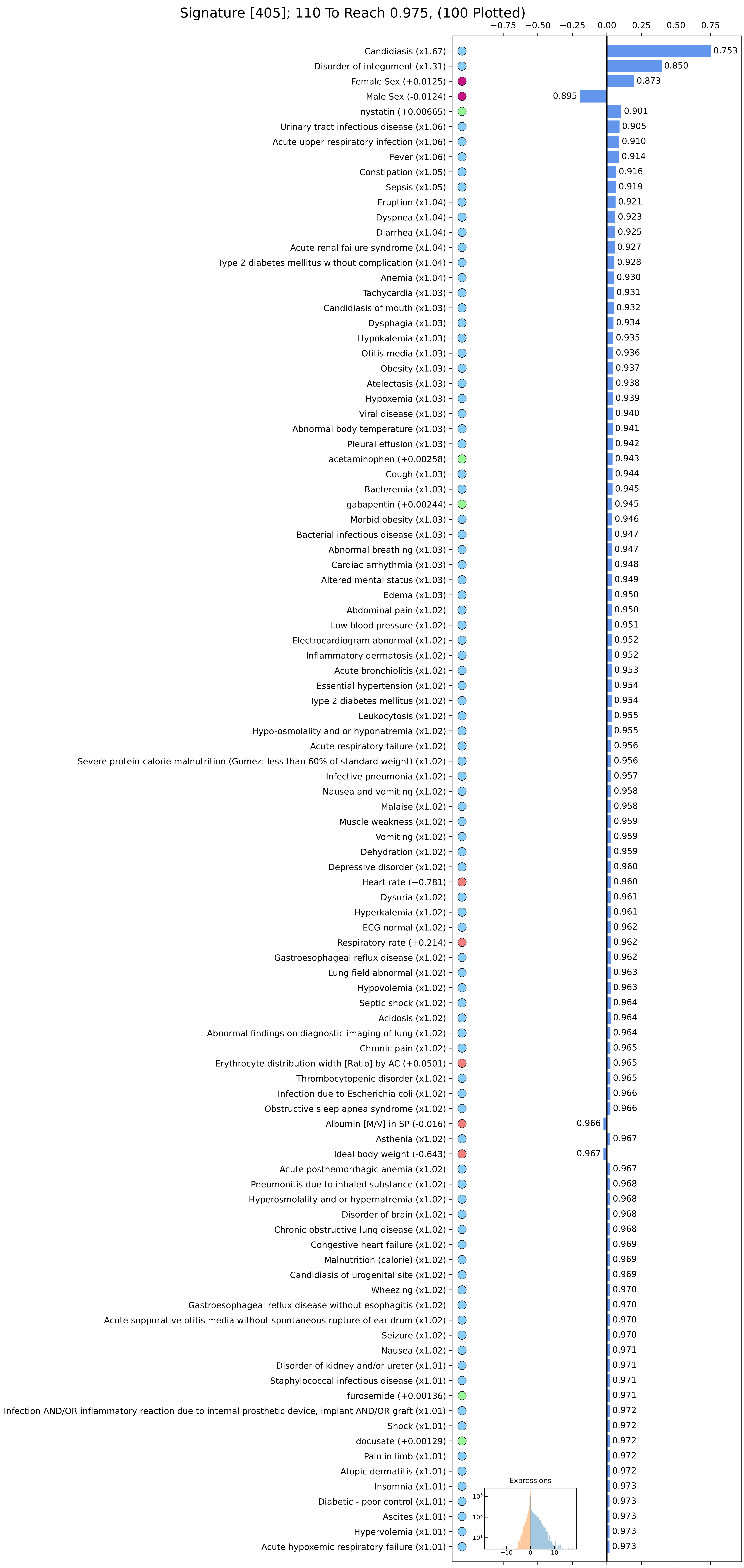

Signature [405]; 110 To Reach 0.975, (100 Plotted)
Candidiasis (x1.67) 0.753
Disorder of integument (x1.31) 0.850
Female Sex (+0.0125) 0.873
Male Sex (-0.0124) 0.895
nystatin (+0.00665) 0.901
Urinary tract infectious disease (x1.06) 0.905
Acute upper respiratory infection (x1.06) 0.910
Fever (x1.06) 0.914
Constipation (x1.05) 0.916
Sepsis (x1.05) 0.919
Eruption (x1.04) 0.921
Dyspnea (x1.04) 0.923
Diarrhea (x1.04) 0.925
Acute renal failure syndrome (x1.04) 0.927
Type 2 diabetes mellitus without complication (x1.04) 0.928
Anemia (x1.04) 0.930
Tachycardia (x1.03) 0.931
Candidiasis of mouth (x1.03) 0.932
Dysphagia (x1.03) 0.934
Hypokalemia (x1.03) 0.935
Otitis media (x1.03) 0.936
Obesity (x1.03) 0.937
Atelectasis (x1.03) 0.938
Hypoxemia (x1.03) 0.939
Viral disease (x1.03) 0.940
Abnormal body temperature (x1.03) 0.941
Pleural effusion (x1.03) 0.942
acetaminophen (+0.00258) 0.943
Cough (x1.03) 0.944
Bacteremia (x1.03) 0.945
gabapentin (+0.00244) 0.945
Morbid obesity (x1.03) 0.946
Bacterial infectious disease (x1.03) 0.947
Abnormal breathing (x1.03) 0.947
Cardiac arrhythmia (x1.03) 0.948
Altered mental status (x1.03) 0.949
Edema (x1.03) 0.950
Abdominal pain (x1.02) 0.950
Low blood pressure (x1.02) 0.951
Electrocardiogram abnormal (x1.02) 0.952
Inflammatory dermatosis (x1.02) 0.952
Acute bronchiolitis (x1.02) 0.953
Essential hypertension (x1.02) 0.954
Type 2 diabetes mellitus (x1.02) 0.954
Leukocytosis (x1.02) 0.955
Hypo-osmolality and or hyponatremia (x1.02) 0.955
Acute respiratory failure (x1.02) 0.956
Severe protein-calorie malnutrition (Gomez: less than 60% of standard weight) (x1.02) 0.956
Infective pneumonia (x1.02) 0.957
Nausea and vomiting (x1.02) 0.958
Malaise (x1.02) 0.958
Muscle weakness (x1.02) 0.959
Vomiting (x1.02) 0.959
Dehydration (x1.02) 0.959
Depressive disorder (x1.02) 0.960
Heart rate (+0.781) 0.960
Dysuria (x1.02) 0.961
Hyperkalemia (x1.02) 0.961
ECG normal (x1.02) 0.962
Respiratory rate (+0.214) 0.962
Gastroesophageal reflux disease (x1.02) 0.962
Lung field abnormal (x1.02) 0.963
Hypovolemia (x1.02) 0.963
Septic shock (x1.02) 0.964
Acidosis (x1.02) 0.964
Abnormal findings on diagnostic imaging of lung (x1.02) 0.964
Chronic pain (x1.02) 0.965
Erythrocyte distribution width [Ratio] by AC (+0.0501) 0.965
Thrombocytopenic disorder (x1.02) 0.965
Infection due to Escherichia coli (x1.02) 0.966
Obstructive sleep apnea syndrome (x1.02) 0.966
Albumin [M/V] in SP (-0.016) 0.966
Asthenia (x1.02) 0.967
Ideal body weight (-0.643) 0.967
Acute posthemorrhagic anemia (x1.02) 0.967
Pneumonitis due to inhaled substance (x1.02) 0.968
Hyperosmolality and or hypernatremia (x1.02) 0.968
Disorder of brain (x1.02) 0.968
Chronic obstructive lung disease (x1.02) 0.968
Congestive heart failure (x1.02) 0.969
Malnutrition (calorie) (x1.02) 0.969
Candidiasis of urogenital site (x1.02) 0.969
Wheezing (x1.02) 0.970
Gastroesophageal reflux disease without esophagitis (x1.02) 0.970
Acute suppurative otitis media without spontaneous rupture of ear drum (x1.02) 0.970
Seizure (x1.02) 0.970
Nausea (x1.02) 0.971
Disorder of kidney and/or ureter (x1.01) 0.971
Staphylococcal infectious disease (x1.01) 0.971
furosemide (+0.00136) 0.971
Infection AND/OR inflammatory reaction due to internal prosthetic device, implant AND/OR graft (x1.01) 0.972
Shock (x1.01) 0.972
docusate (+0.00129) 0.972
Pain in limb (x1.01) 0.972
Atopic dermatitis (x1.01) 0.972
Insomnia (x1.01) 0.973
Diabetic - poor control (x1.01) 0.973
Ascites (x1.01) 0.973
Hypervolemia (x1.01) 0.973
Acute hypoxemic respiratory failure (x1.01) 0.973
−0.75 −0.50 −0.25 0.00 0.25 0.50 0.75
Expressions
−10 0 10

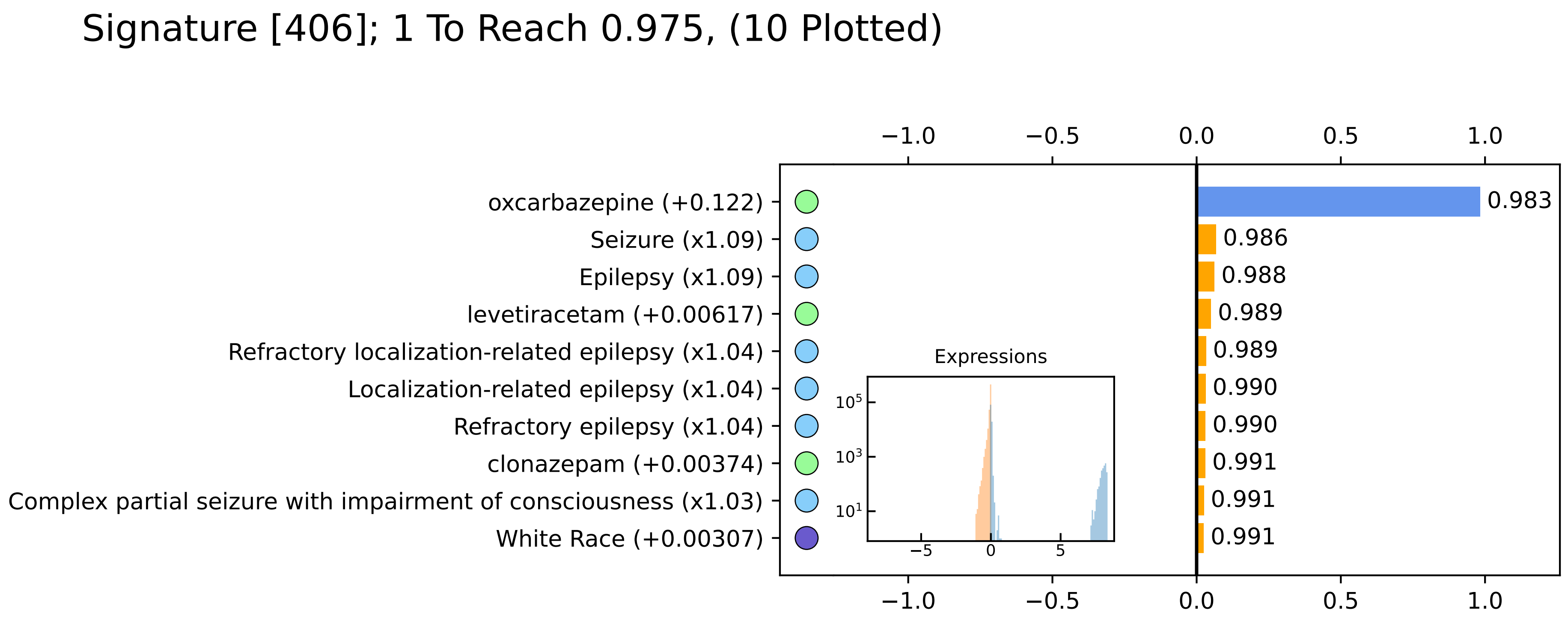

Signature [406]; 1 To Reach 0.975, (10 Plotted)
oxcarbazepine (+0.122)
Seizure (x1.09)
Epilepsy (x1.09)
levetiracetam (+0.00617)
Refractory localization-related epilepsy (x1.04)
Localization-related epilepsy (x1.04)
Refractory epilepsy (x1.04)
clonazepam (+0.00374)
Complex partial seizure with impairment of consciousness (x1.03)
White Race (+0.00307)
0.983
0.986
0.988
0.989
0.989
0.990
0.990
0.991
0.991
0.991
Expressions
−1.0
−0.5
0.0
0.5
1.0

Signature [407]; 2 To Reach 0.975, (10 Plotted)

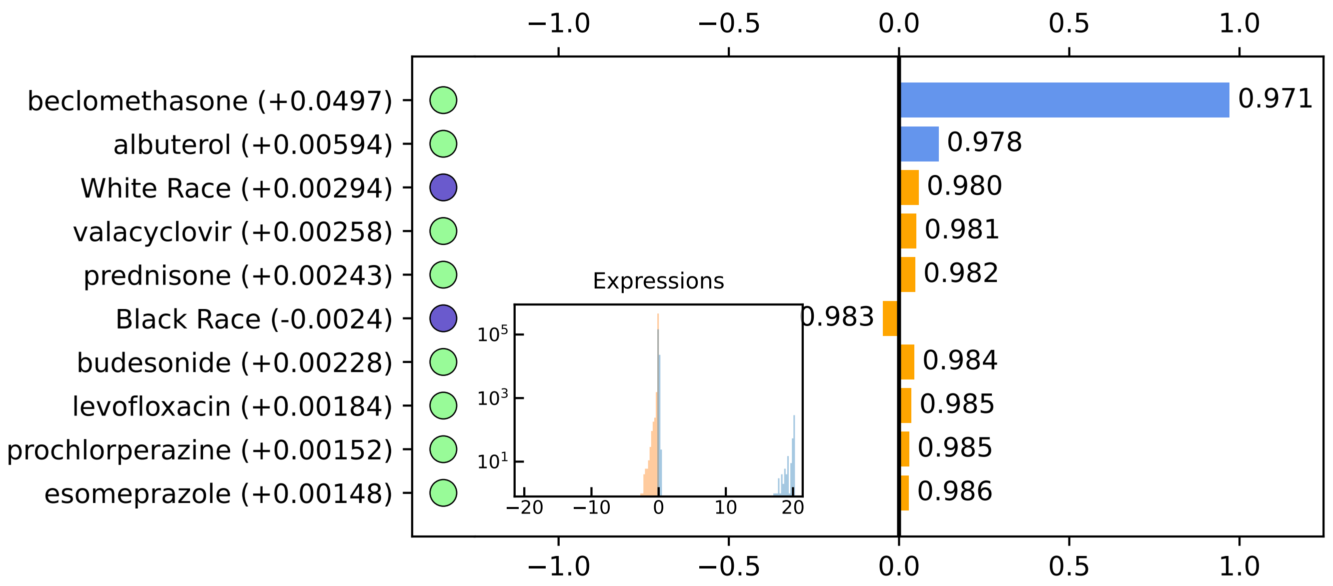

Signature [408]; 2 To Reach 0.975, (10 Plotted)

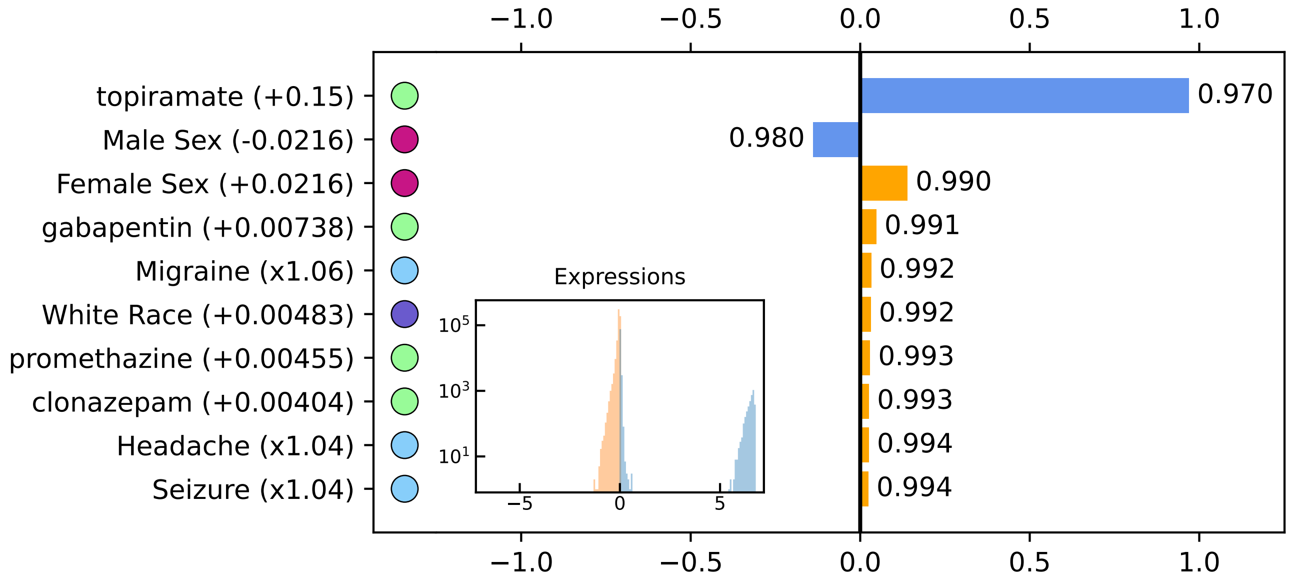

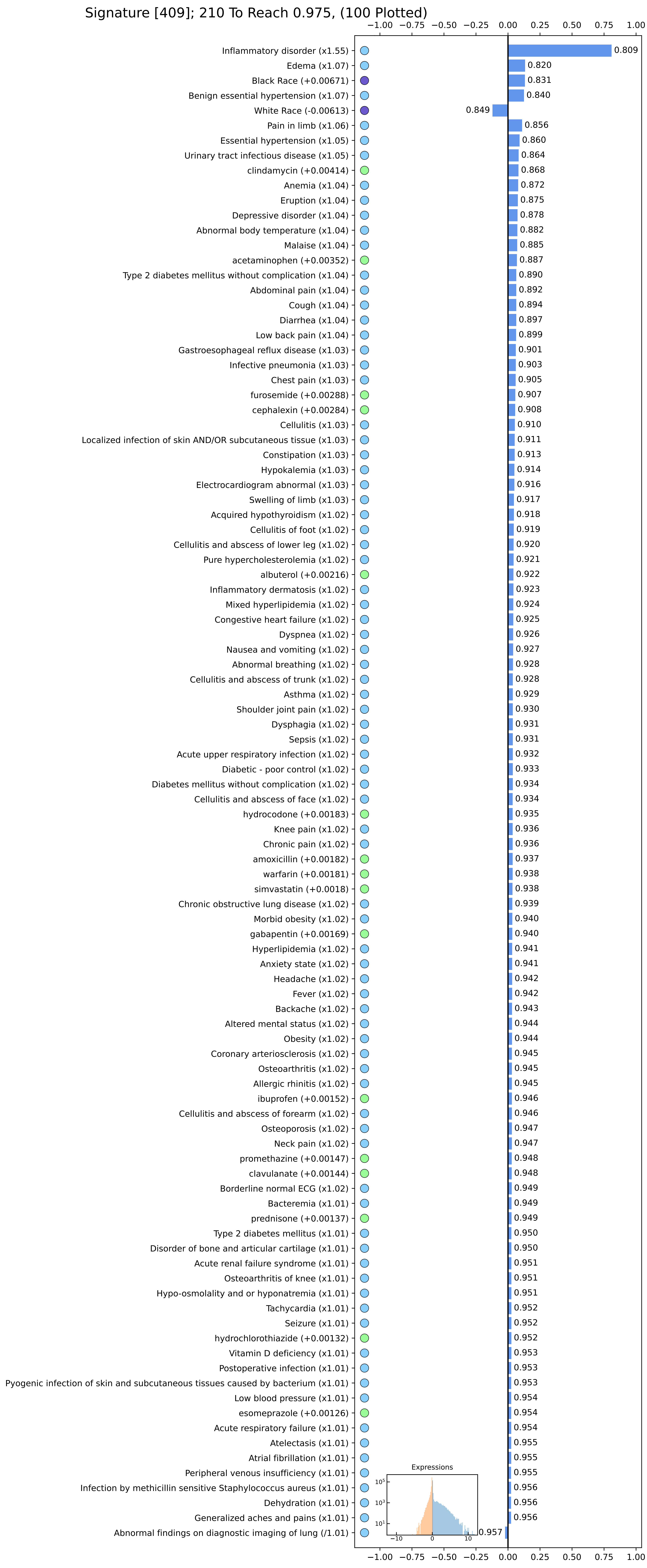
Signature [409]; 210 To Reach 0.975, (100 Plotted)
−1.00 −0.75 −0.50 −0.25 0.00 0.25 0.50 0.75 1.00
Inflammatory disorder (x1.55) 0.809
Edema (x1.07) 0.820
Black Race (+0.00671) 0.831
Benign essential hypertension (x1.07) 0.840
White Race (-0.00613) 0.849
Pain in limb (x1.06) 0.856
Essential hypertension (x1.05) 0.860
Urinary tract infectious disease (x1.05) 0.864
clindamycin (+0.00414) 0.868
Anemia (x1.04) 0.872
Eruption (x1.04) 0.875
Depressive disorder (x1.04) 0.878
Abnormal body temperature (x1.04) 0.882
Malaise (x1.04) 0.885
acetaminophen (+0.00352) 0.887
Type 2 diabetes mellitus without complication (x1.04) 0.890
Abdominal pain (x1.04) 0.892
Cough (x1.04) 0.894
Diarrhea (x1.04) 0.897
Low back pain (x1.04) 0.899
Gastroesophageal reflux disease (x1.03) 0.901
Infective pneumonia (x1.03) 0.903
Chest pain (x1.03) 0.905
furosemide (+0.00288) 0.907
cephalexin (+0.00284) 0.908
Cellulitis (x1.03) 0.910
Localized infection of skin AND/OR subcutaneous tissue (x1.03) 0.911
Constipation (x1.03) 0.913
Hypokalemia (x1.03) 0.914
Electrocardiogram abnormal (x1.03) 0.916
Swelling of limb (x1.03) 0.917
Acquired hypothyroidism (x1.02) 0.918
Cellulitis of foot (x1.02) 0.919
Cellulitis and abscess of lower leg (x1.02) 0.920
Pure hypercholesterolemia (x1.02) 0.921
albuterol (+0.00216) 0.922
Inflammatory dermatosis (x1.02) 0.923
Mixed hyperlipidemia (x1.02) 0.924
Congestive heart failure (x1.02) 0.925
Dyspnea (x1.02) 0.926
Nausea and vomiting (x1.02) 0.927
Abnormal breathing (x1.02) 0.928
Cellulitis and abscess of trunk (x1.02) 0.928
Asthma (x1.02) 0.929
Shoulder joint pain (x1.02) 0.930
Dysphagia (x1.02) 0.931
Sepsis (x1.02) 0.931
Acute upper respiratory infection (x1.02) 0.932
Diabetic - poor control (x1.02) 0.933
Diabetes mellitus without complication (x1.02) 0.934
Cellulitis and abscess of face (x1.02) 0.934
hydrocodone (+0.00183) 0.935
Knee pain (x1.02) 0.936
Chronic pain (x1.02) 0.936
amoxicillin (+0.00182) 0.937
warfarin (+0.00181) 0.938
simvastatin (+0.0018) 0.938
Chronic obstructive lung disease (x1.02) 0.939
Morbid obesity (x1.02) 0.940
gabapentin (+0.00169) 0.940
Hyperlipidemia (x1.02) 0.941
Anxiety state (x1.02) 0.941
Headache (x1.02) 0.942
Fever (x1.02) 0.942
Backache (x1.02) 0.943
Altered mental status (x1.02) 0.944
Obesity (x1.02) 0.944
Coronary arteriosclerosis (x1.02) 0.945
Osteoarthritis (x1.02) 0.945
Allergic rhinitis (x1.02) 0.945
ibuprofen (+0.00152) 0.946
Cellulitis and abscess of forearm (x1.02) 0.946
Osteoporosis (x1.02) 0.947
Neck pain (x1.02) 0.947
promethazine (+0.00147) 0.948
clavulanate (+0.00144) 0.948
Borderline normal ECG (x1.02) 0.949
Bacteremia (x1.01) 0.949
prednisone (+0.00137) 0.949
Type 2 diabetes mellitus (x1.01) 0.950
Disorder of bone and articular cartilage (x1.01) 0.950
Acute renal failure syndrome (x1.01) 0.951
Osteoarthritis of knee (x1.01) 0.951
Hypo-osmolality and or hyponatremia (x1.01) 0.951
Tachycardia (x1.01) 0.952
Seizure (x1.01) 0.952
hydrochlorothiazide (+0.00132) 0.952
Vitamin D deficiency (x1.01) 0.953
Postoperative infection (x1.01) 0.953
Pyogenic infection of skin and subcutaneous tissues caused by bacterium (x1.01) 0.953
Low blood pressure (x1.01) 0.954
esomeprazole (+0.00126) 0.954
Acute respiratory failure (x1.01) 0.954
Atelectasis (x1.01) 0.955
Atrial fibrillation (x1.01) 0.955
Peripheral venous insufficiency (x1.01) 0.955
Infection by methicillin sensitive Staphylococcus aureus (x1.01) 0.956
Dehydration (x1.01) 0.956
Generalized aches and pains (x1.01) 0.956
Abnormal findings on diagnostic imaging of lung (/1.01) 0.957
Expressions
10^5
10^3
10^1
−10 0 10
−1.00 −0.75 −0.50 −0.25 0.00 0.25 0.50 0.75 1.00

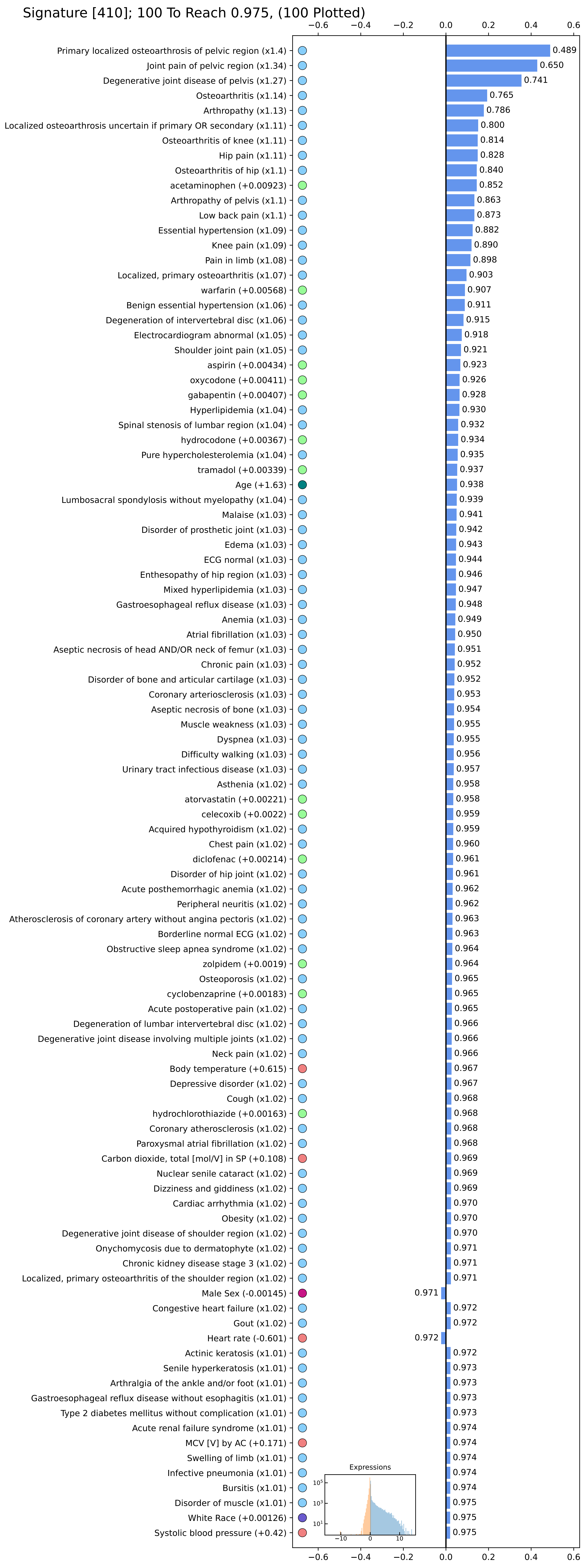
Signature [410]; 100 To Reach 0.975, (100 Plotted)
−0.6 −0.4 −0.2 0.0 0.2 0.4 0.6
Primary localized osteoarthrosis of pelvic region (x1.4) 0.489
Joint pain of pelvic region (x1.34) 0.650
Degenerative joint disease of pelvis (x1.27) 0.741
Osteoarthritis (x1.14) 0.765
Arthropathy (x1.13) 0.786
Localized osteoarthrosis uncertain if primary OR secondary (x1.11) 0.800
Osteoarthritis of knee (x1.11) 0.814
Hip pain (x1.11) 0.828
Osteoarthritis of hip (x1.1) 0.840
acetaminophen (+0.00923) 0.852
Arthropathy of pelvis (x1.1) 0.863
Low back pain (x1.1) 0.873
Essential hypertension (x1.09) 0.882
Knee pain (x1.09) 0.890
Pain in limb (x1.08) 0.898
Localized, primary osteoarthritis (x1.07) 0.903
warfarin (+0.00568) 0.907
Benign essential hypertension (x1.06) 0.911
Degeneration of intervertebral disc (x1.06) 0.915
Electrocardiogram abnormal (x1.05) 0.918
Shoulder joint pain (x1.05) 0.921
aspirin (+0.00434) 0.923
oxycodone (+0.00411) 0.926
gabapentin (+0.00407) 0.928
Hyperlipidemia (x1.04) 0.930
Spinal stenosis of lumbar region (x1.04) 0.932
hydrocodone (+0.00367) 0.934
Pure hypercholesterolemia (x1.04) 0.935
tramadol (+0.00339) 0.937
Age (+1.63) 0.938
Lumbosacral spondylosis without myelopathy (x1.04) 0.939
Malaise (x1.03) 0.941
Disorder of prosthetic joint (x1.03) 0.942
Edema (x1.03) 0.943
ECG normal (x1.03) 0.944
Enthesopathy of hip region (x1.03) 0.946
Mixed hyperlipidemia (x1.03) 0.947
Gastroesophageal reflux disease (x1.03) 0.948
Anemia (x1.03) 0.949
Atrial fibrillation (x1.03) 0.950
Aseptic necrosis of head AND/OR neck of femur (x1.03) 0.951
Chronic pain (x1.03) 0.952
Disorder of bone and articular cartilage (x1.03) 0.952
Coronary arteriosclerosis (x1.03) 0.953
Aseptic necrosis of bone (x1.03) 0.954
Muscle weakness (x1.03) 0.955
Dyspnea (x1.03) 0.955
Difficulty walking (x1.03) 0.956
Urinary tract infectious disease (x1.03) 0.957
Asthenia (x1.02) 0.958
atorvastatin (+0.00221) 0.958
celecoxib (+0.0022) 0.959
Acquired hypothyroidism (x1.02) 0.959
Chest pain (x1.02) 0.960
diclofenac (+0.00214) 0.961
Disorder of hip joint (x1.02) 0.961
Acute posthemorrhagic anemia (x1.02) 0.962
Peripheral neuritis (x1.02) 0.962
Atherosclerosis of coronary artery without angina pectoris (x1.02) 0.963
Borderline normal ECG (x1.02) 0.963
Obstructive sleep apnea syndrome (x1.02) 0.964
zolpidem (+0.0019) 0.964
Osteoporosis (x1.02) 0.965
cyclobenzaprine (+0.00183) 0.965
Acute postoperative pain (x1.02) 0.965
Degeneration of lumbar intervertebral disc (x1.02) 0.966
Degenerative joint disease involving multiple joints (x1.02) 0.966
Neck pain (x1.02) 0.966
Body temperature (+0.615) 0.967
Depressive disorder (x1.02) 0.967
Cough (x1.02) 0.968
hydrochlorothiazide (+0.00163) 0.968
Coronary atherosclerosis (x1.02) 0.968
Paroxysmal atrial fibrillation (x1.02) 0.968
Carbon dioxide, total [mol/V] in SP (+0.108) 0.969
Nuclear senile cataract (x1.02) 0.969
Dizziness and giddiness (x1.02) 0.969
Cardiac arrhythmia (x1.02) 0.970
Obesity (x1.02) 0.970
Degenerative joint disease of shoulder region (x1.02) 0.970
Onychomycosis due to dermatophyte (x1.02) 0.971
Chronic kidney disease stage 3 (x1.02) 0.971
Localized, primary osteoarthritis of the shoulder region (x1.02) 0.971
Male Sex (-0.00145) 0.971
Congestive heart failure (x1.02) 0.972
Gout (x1.02) 0.972
Heart rate (-0.601) 0.972
Actinic keratosis (x1.01) 0.972
Senile hyperkeratosis (x1.01) 0.973
Arthralgia of the ankle and/or foot (x1.01) 0.973
Gastroesophageal reflux disease without esophagitis (x1.01) 0.973
Type 2 diabetes mellitus without complication (x1.01) 0.973
Acute renal failure syndrome (x1.01) 0.974
MCV [V] by AC (+0.171) 0.974
Swelling of limb (x1.01) 0.974
Infective pneumonia (x1.01) 0.974
Bursitis (x1.01) 0.974
Disorder of muscle (x1.01) 0.975
White Race (+0.00126) 0.975
Systolic blood pressure (+0.42) 0.975
Expressions
−10 0 10

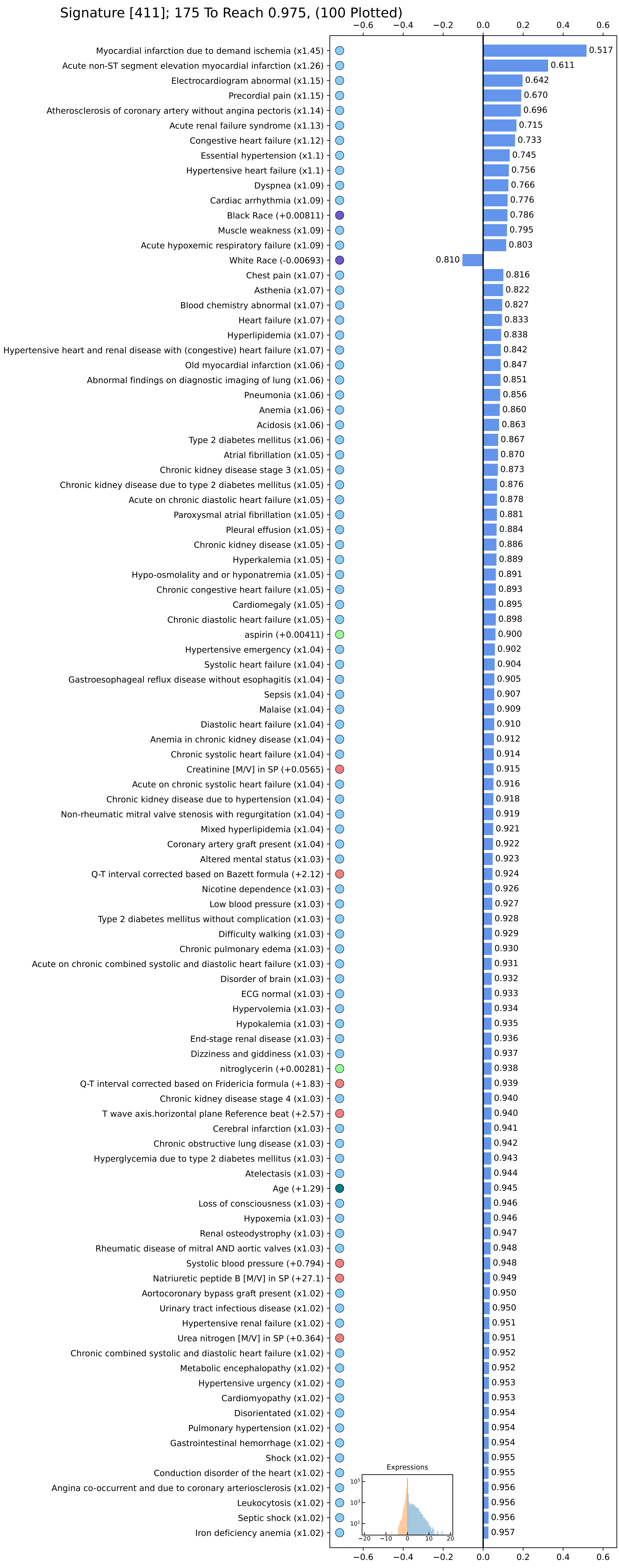

Signature [411]; 175 To Reach 0.975, (100 Plotted)
Myocardial infarction due to demand ischemia (x1.45) 0.517
Acute non-ST segment elevation myocardial infarction (x1.26) 0.611
Electrocardiogram abnormal (x1.15) 0.642
Precordial pain (x1.15) 0.670
Atherosclerosis of coronary artery without angina pectoris (x1.14) 0.696
Acute renal failure syndrome (x1.13) 0.715
Congestive heart failure (x1.12) 0.733
Essential hypertension (x1.1) 0.745
Hypertensive heart failure (x1.1) 0.756
Dyspnea (x1.09) 0.766
Cardiac arrhythmia (x1.09) 0.776
Black Race (+0.00811) 0.786
Muscle weakness (x1.09) 0.795
Acute hypoxemic respiratory failure (x1.09) 0.803
White Race (-0.00693) 0.810
Chest pain (x1.07) 0.816
Asthenia (x1.07) 0.822
Blood chemistry abnormal (x1.07) 0.827
Heart failure (x1.07) 0.833
Hyperlipidemia (x1.07) 0.838
Hypertensive heart and renal disease with (congestive) heart failure (x1.07) 0.842
Old myocardial infarction (x1.06) 0.847
Abnormal findings on diagnostic imaging of lung (x1.06) 0.851
Pneumonia (x1.06) 0.856
Anemia (x1.06) 0.860
Acidosis (x1.06) 0.863
Type 2 diabetes mellitus (x1.06) 0.867
Atrial fibrillation (x1.05) 0.870
Chronic kidney disease stage 3 (x1.05) 0.873
Chronic kidney disease due to type 2 diabetes mellitus (x1.05) 0.876
Acute on chronic diastolic heart failure (x1.05) 0.878
Paroxysmal atrial fibrillation (x1.05) 0.881
Pleural effusion (x1.05) 0.884
Chronic kidney disease (x1.05) 0.886
Hyperkalemia (x1.05) 0.889
Hypo-osmolality and or hyponatremia (x1.05) 0.891
Chronic congestive heart failure (x1.05) 0.893
Cardiomegaly (x1.05) 0.895
Chronic diastolic heart failure (x1.05) 0.898
aspirin (+0.00411) 0.900
Hypertensive emergency (x1.04) 0.902
Systolic heart failure (x1.04) 0.904
Gastroesophageal reflux disease without esophagitis (x1.04) 0.905
Sepsis (x1.04) 0.907
Malaise (x1.04) 0.909
Diastolic heart failure (x1.04) 0.910
Anemia in chronic kidney disease (x1.04) 0.912
Chronic systolic heart failure (x1.04) 0.914
Creatinine [M/V] in SP (+0.0565) 0.915
Acute on chronic systolic heart failure (x1.04) 0.916
Chronic kidney disease due to hypertension (x1.04) 0.918
Non-rheumatic mitral valve stenosis with regurgitation (x1.04) 0.919
Mixed hyperlipidemia (x1.04) 0.921
Coronary artery graft present (x1.04) 0.922
Altered mental status (x1.03) 0.923
Q-T interval corrected based on Bazett formula (+2.12) 0.924
Nicotine dependence (x1.03) 0.926
Low blood pressure (x1.03) 0.927
Type 2 diabetes mellitus without complication (x1.03) 0.928
Difficulty walking (x1.03) 0.929
Chronic pulmonary edema (x1.03) 0.930
Acute on chronic combined systolic and diastolic heart failure (x1.03) 0.931
Disorder of brain (x1.03) 0.932
ECG normal (x1.03) 0.933
Hypervolemia (x1.03) 0.934
Hypokalemia (x1.03) 0.935
End-stage renal disease (x1.03) 0.936
Dizziness and giddiness (x1.03) 0.937
nitroglycerin (+0.00281) 0.938
Q-T interval corrected based on Fridericia formula (+1.83) 0.939
Chronic kidney disease stage 4 (x1.03) 0.940
T wave axis.horizontal plane Reference beat (+2.57) 0.940
Cerebral infarction (x1.03) 0.941
Chronic obstructive lung disease (x1.03) 0.942
Hyperglycemia due to type 2 diabetes mellitus (x1.03) 0.943
Atelectasis (x1.03) 0.944
Age (+1.29) 0.945
Loss of consciousness (x1.03) 0.946
Hypoxemia (x1.03) 0.946
Renal osteodystrophy (x1.03) 0.947
Rheumatic disease of mitral AND aortic valves (x1.03) 0.948
Systolic blood pressure (+0.794) 0.948
Natriuretic peptide B [M/V] in SP (+27.1) 0.949
Aortocoronary bypass graft present (x1.02) 0.950
Urinary tract infectious disease (x1.02) 0.950
Hypertensive renal failure (x1.02) 0.951
Urea nitrogen [M/V] in SP (+0.364) 0.951
Chronic combined systolic and diastolic heart failure (x1.02) 0.952
Metabolic encephalopathy (x1.02) 0.952
Hypertensive urgency (x1.02) 0.953
Cardiomyopathy (x1.02) 0.953
Disorientated (x1.02) 0.954
Pulmonary hypertension (x1.02) 0.954
Gastrointestinal hemorrhage (x1.02) 0.954
Shock (x1.02) 0.955
Conduction disorder of the heart (x1.02) 0.955
Angina co-occurrent and due to coronary arteriosclerosis (x1.02) 0.956
Leukocytosis (x1.02) 0.956
Septic shock (x1.02) 0.956
Iron deficiency anemia (x1.02) 0.957
Expressions
−0.6 −0.4 −0.2 0.0 0.2 0.4 0.6

Signature [412]; 128 To Reach 0.975, (100 Plotted)

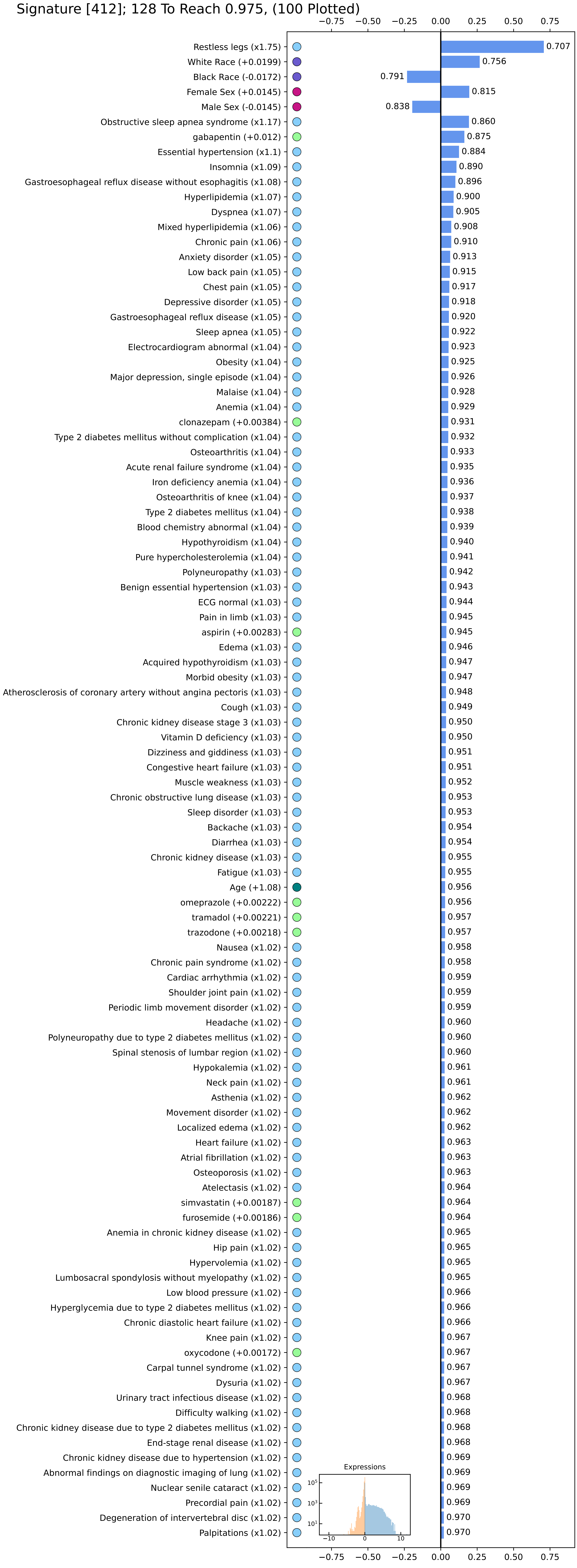

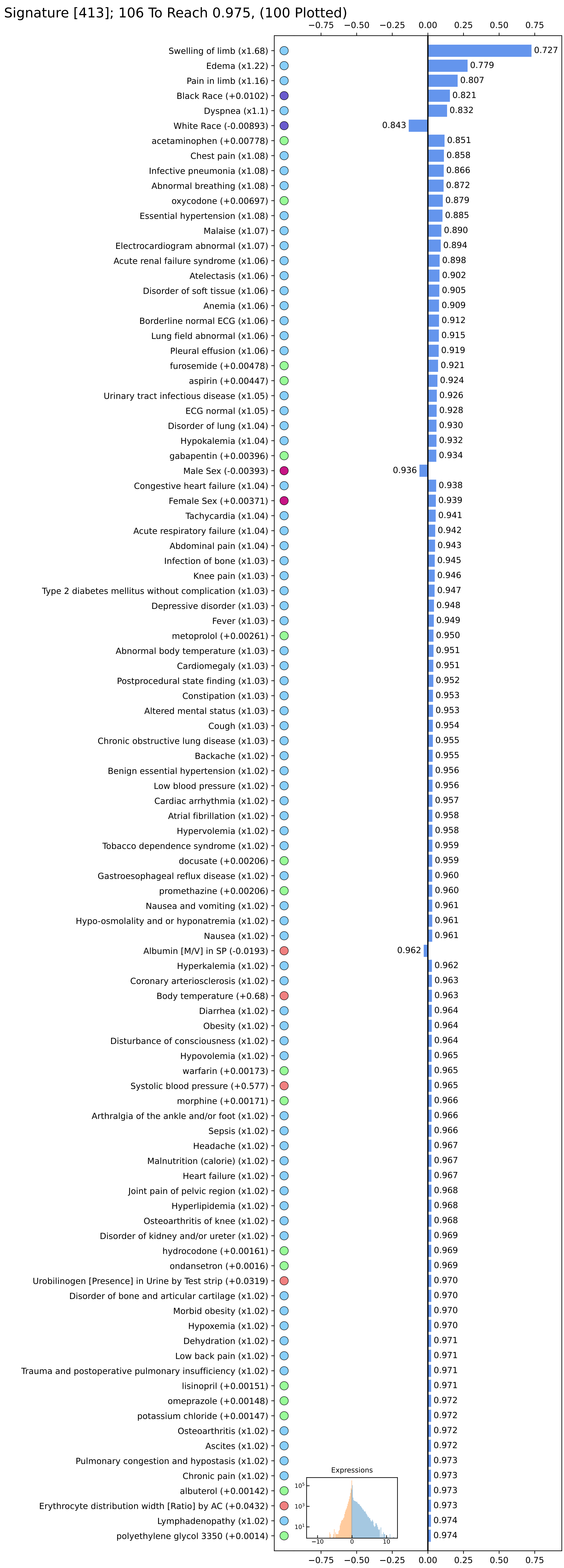

Signature [413]; 106 To Reach 0.975, (100 Plotted)
Swelling of limb (x1.68) 0.727
Edema (x1.22) 0.779
Pain in limb (x1.16) 0.807
Black Race (+0.0102) 0.821
Dyspnea (x1.1) 0.832
White Race (-0.00893) 0.843
acetaminophen (+0.00778) 0.851
Chest pain (x1.08) 0.858
Infective pneumonia (x1.08) 0.866
Abnormal breathing (x1.08) 0.872
oxycodone (+0.00697) 0.879
Essential hypertension (x1.08) 0.885
Malaise (x1.07) 0.890
Electrocardiogram abnormal (x1.07) 0.894
Acute renal failure syndrome (x1.06) 0.898
Atelectasis (x1.06) 0.902
Disorder of soft tissue (x1.06) 0.905
Anemia (x1.06) 0.909
Borderline normal ECG (x1.06) 0.912
Lung field abnormal (x1.06) 0.915
Pleural effusion (x1.06) 0.919
furosemide (+0.00478) 0.921
aspirin (+0.00447) 0.924
Urinary tract infectious disease (x1.05) 0.926
ECG normal (x1.05) 0.928
Disorder of lung (x1.04) 0.930
Hypokalemia (x1.04) 0.932
gabapentin (+0.00396) 0.934
Male Sex (-0.00393) 0.936
Congestive heart failure (x1.04) 0.938
Female Sex (+0.00371) 0.939
Tachycardia (x1.04) 0.941
Acute respiratory failure (x1.04) 0.942
Abdominal pain (x1.04) 0.943
Infection of bone (x1.03) 0.945
Knee pain (x1.03) 0.946
Type 2 diabetes mellitus without complication (x1.03) 0.947
Depressive disorder (x1.03) 0.948
Fever (x1.03) 0.949
metoprolol (+0.00261) 0.950
Abnormal body temperature (x1.03) 0.951
Cardiomegaly (x1.03) 0.951
Postprocedural state finding (x1.03) 0.952
Constipation (x1.03) 0.953
Altered mental status (x1.03) 0.953
Cough (x1.03) 0.954
Chronic obstructive lung disease (x1.03) 0.955
Backache (x1.02) 0.955
Benign essential hypertension (x1.02) 0.956
Low blood pressure (x1.02) 0.956
Cardiac arrhythmia (x1.02) 0.957
Atrial fibrillation (x1.02) 0.958
Hypervolemia (x1.02) 0.958
Tobacco dependence syndrome (x1.02) 0.959
docusate (+0.00206) 0.959
Gastroesophageal reflux disease (x1.02) 0.960
promethazine (+0.00206) 0.960
Nausea and vomiting (x1.02) 0.961
Hypo-osmolality and or hyponatremia (x1.02) 0.961
Nausea (x1.02) 0.961
Albumin [M/V] in SP (-0.0193) 0.962
Hyperkalemia (x1.02) 0.962
Coronary arteriosclerosis (x1.02) 0.963
Body temperature (+0.68) 0.963
Diarrhea (x1.02) 0.964
Obesity (x1.02) 0.964
Disturbance of consciousness (x1.02) 0.964
Hypovolemia (x1.02) 0.965
warfarin (+0.00173) 0.965
Systolic blood pressure (+0.577) 0.965
morphine (+0.00171) 0.966
Arthralgia of the ankle and/or foot (x1.02) 0.966
Sepsis (x1.02) 0.966
Headache (x1.02) 0.967
Malnutrition (calorie) (x1.02) 0.967
Heart failure (x1.02) 0.967
Joint pain of pelvic region (x1.02) 0.968
Hyperlipidemia (x1.02) 0.968
Osteoarthritis of knee (x1.02) 0.968
Disorder of kidney and/or ureter (x1.02) 0.969
hydrocodone (+0.00161) 0.969
ondansetron (+0.0016) 0.969
Urobilinogen [Presence] in Urine by Test strip (+0.0319) 0.970
Disorder of bone and articular cartilage (x1.02) 0.970
Morbid obesity (x1.02) 0.970
Hypoxemia (x1.02) 0.970
Dehydration (x1.02) 0.971
Low back pain (x1.02) 0.971
Trauma and postoperative pulmonary insufficiency (x1.02) 0.971
lisinopril (+0.00151) 0.971
omeprazole (+0.00148) 0.972
potassium chloride (+0.00147) 0.972
Osteoarthritis (x1.02) 0.972
Ascites (x1.02) 0.972
Pulmonary congestion and hypostasis (x1.02) 0.973
Chronic pain (x1.02) 0.973
albuterol (+0.00142) 0.973
Erythrocyte distribution width [Ratio] by AC (+0.0432) 0.973
Lymphadenopathy (x1.02) 0.974
polyethylene glycol 3350 (+0.0014) 0.974
−0.75 −0.50 −0.25 0.00 0.25 0.50 0.75
Expressions
−10 0 10

Signature [414]; 55 To Reach 0.975, (55 Plotted)

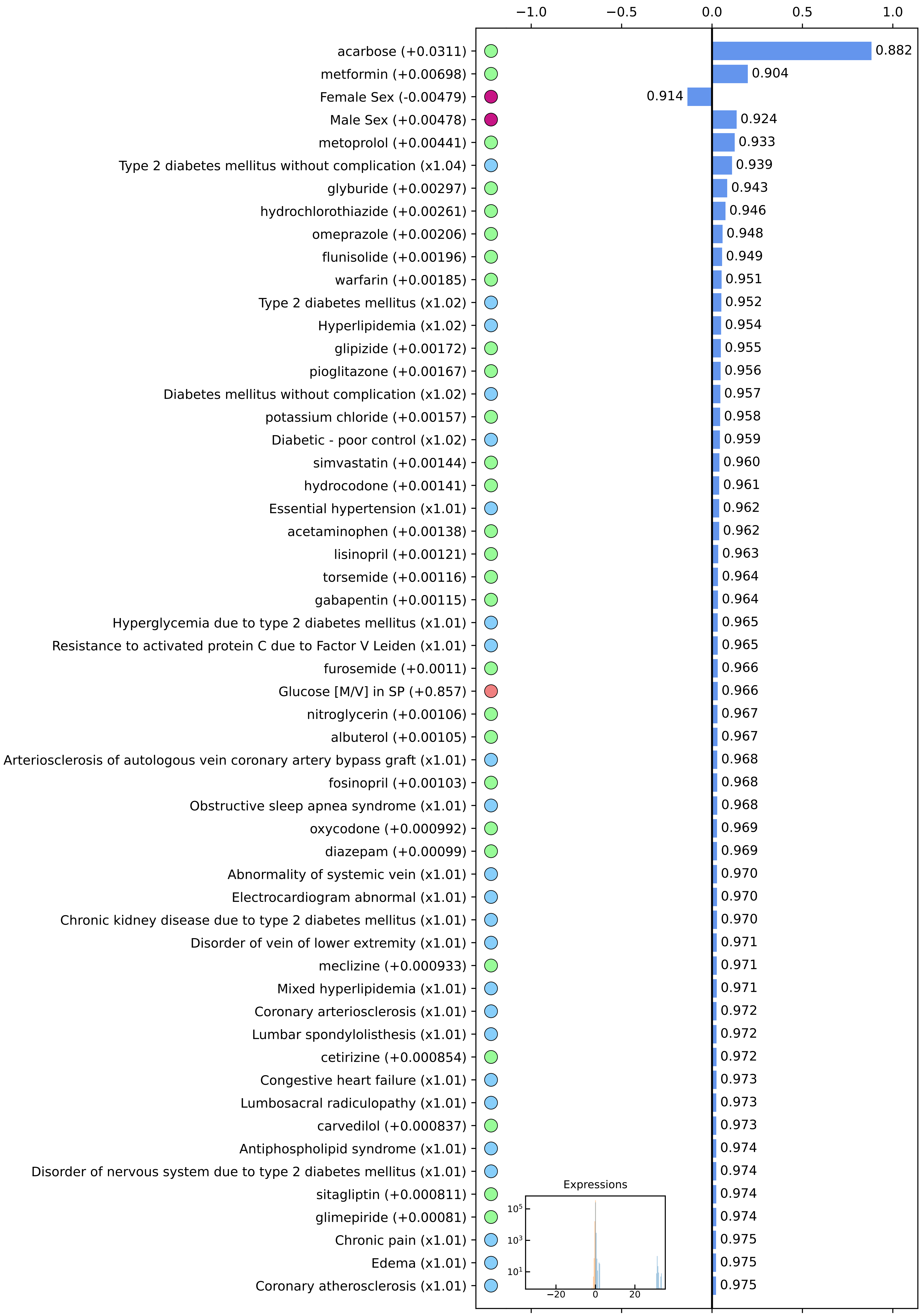

Signature [415]; 22 To Reach 0.975, (22 Plotted)

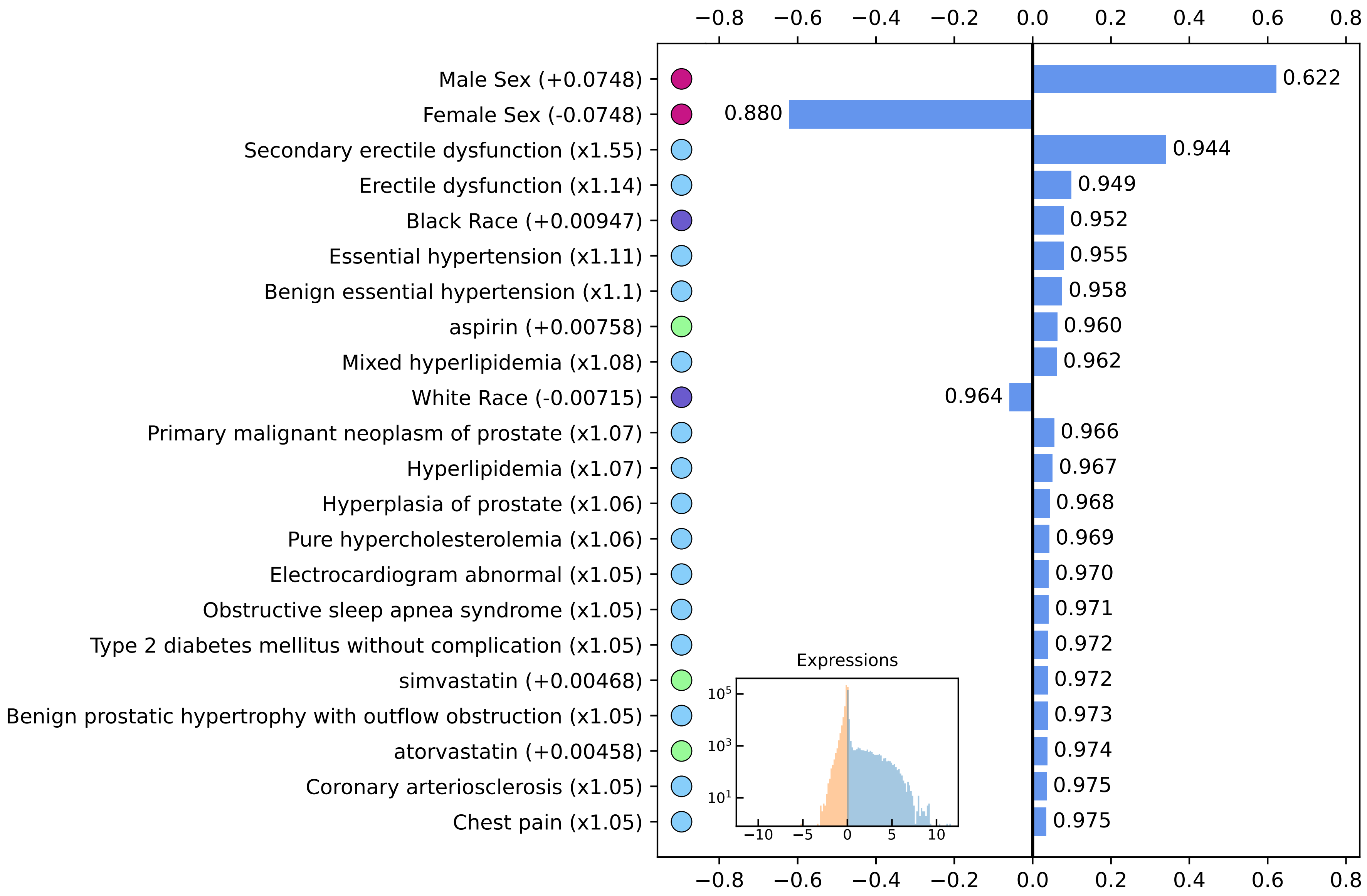

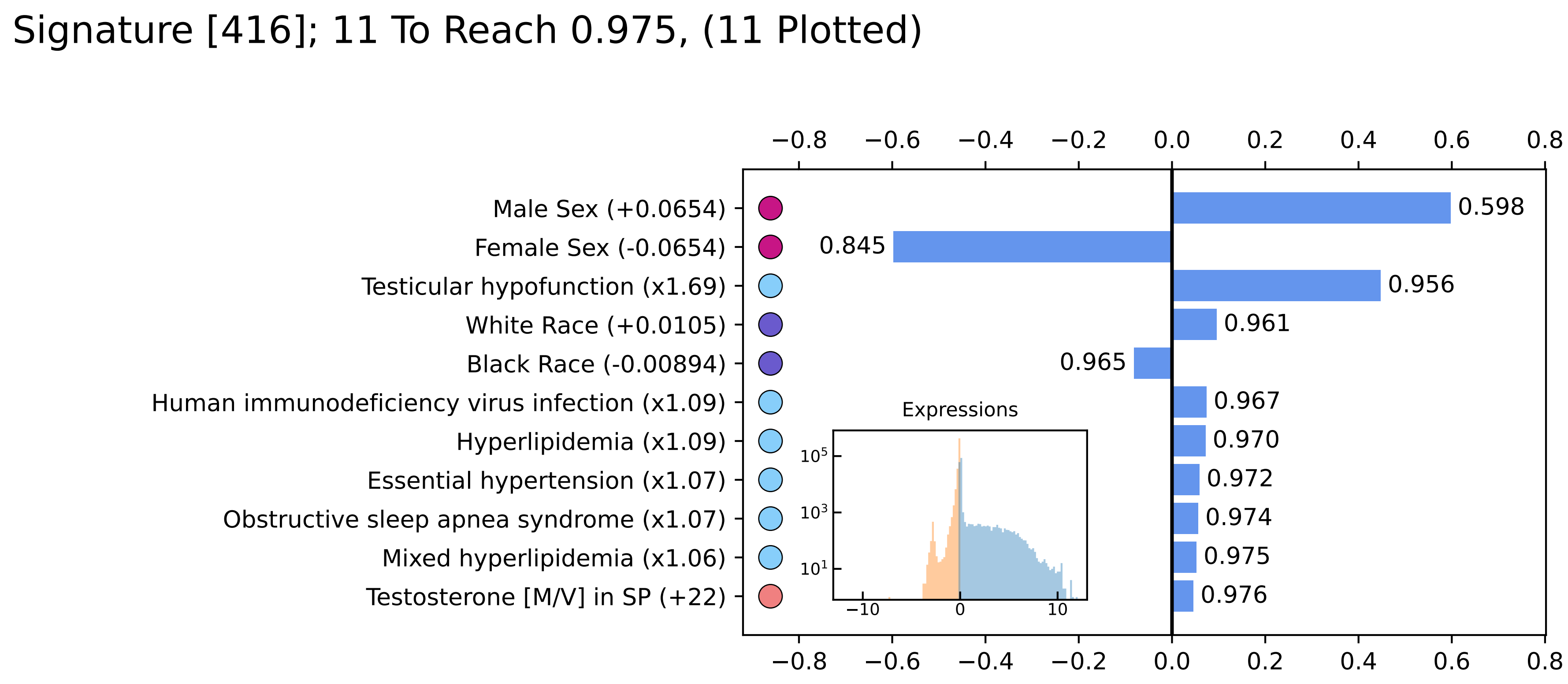

Signature [416]; 11 To Reach 0.975, (11 Plotted)
Male Sex (+0.0654)
Female Sex (-0.0654)
Testicular hypofunction (x1.69)
White Race (+0.0105)
Black Race (-0.00894)
Human immunodeficiency virus infection (x1.09)
Hyperlipidemia (x1.09)
Essential hypertension (x1.07)
Obstructive sleep apnea syndrome (x1.07)
Mixed hyperlipidemia (x1.06)
Testosterone [M/V] in SP (+22)
0.598
0.845
0.956
0.961
0.965
0.967
0.970
0.972
0.974
0.975
0.976
Expressions
−0.8
−0.6
−0.4
−0.2
0.0
0.2
0.4
0.6
0.8

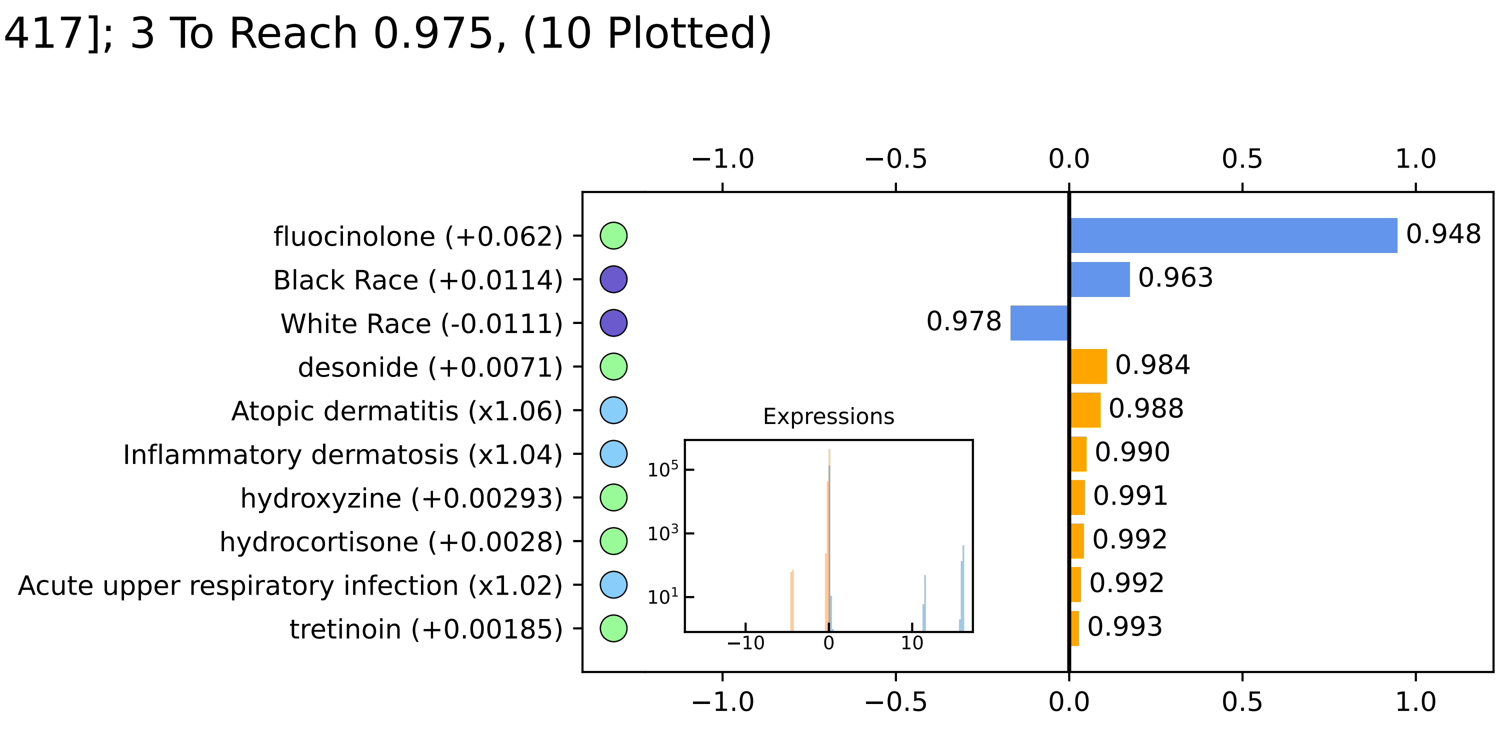
Signature [417]; 3 To Reach 0.975, (10 Plotted)
fluocinolone (+0.062)
Black Race (+0.0114)
White Race (-0.0111)
desonide (+0.0071)
Atopic dermatitis (x1.06)
Inflammatory dermatosis (x1.04)
hydroxyzine (+0.00293)
hydrocortisone (+0.0028)
Acute upper respiratory infection (x1.02)
tretinoin (+0.00185)
0.948
0.963
0.978
0.984
0.988
0.990
0.991
0.992
0.992
0.993
−1.0
−0.5
0.0
0.5
1.0
Expressions
10^5
10^3
10^1
−10
0
10

Signature [418]; 483 To Reach 0.975, (100 Plotted)

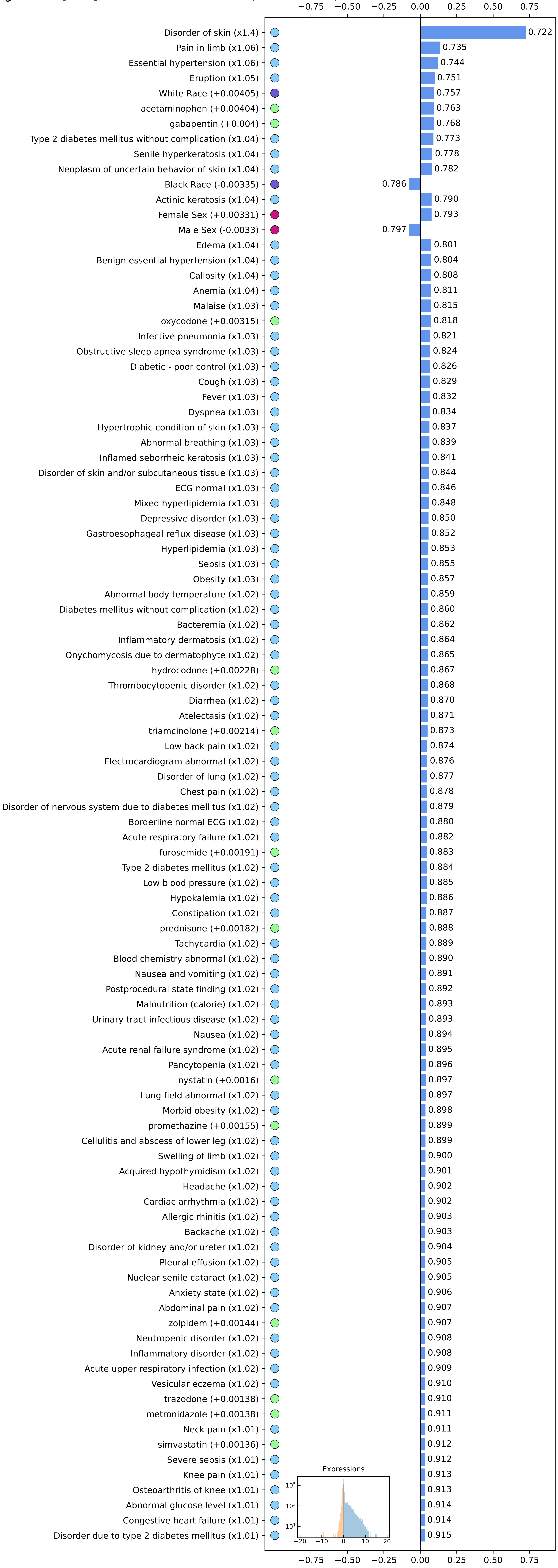

Signature [419]; 4 To Reach 0.975, (10 Plotted)

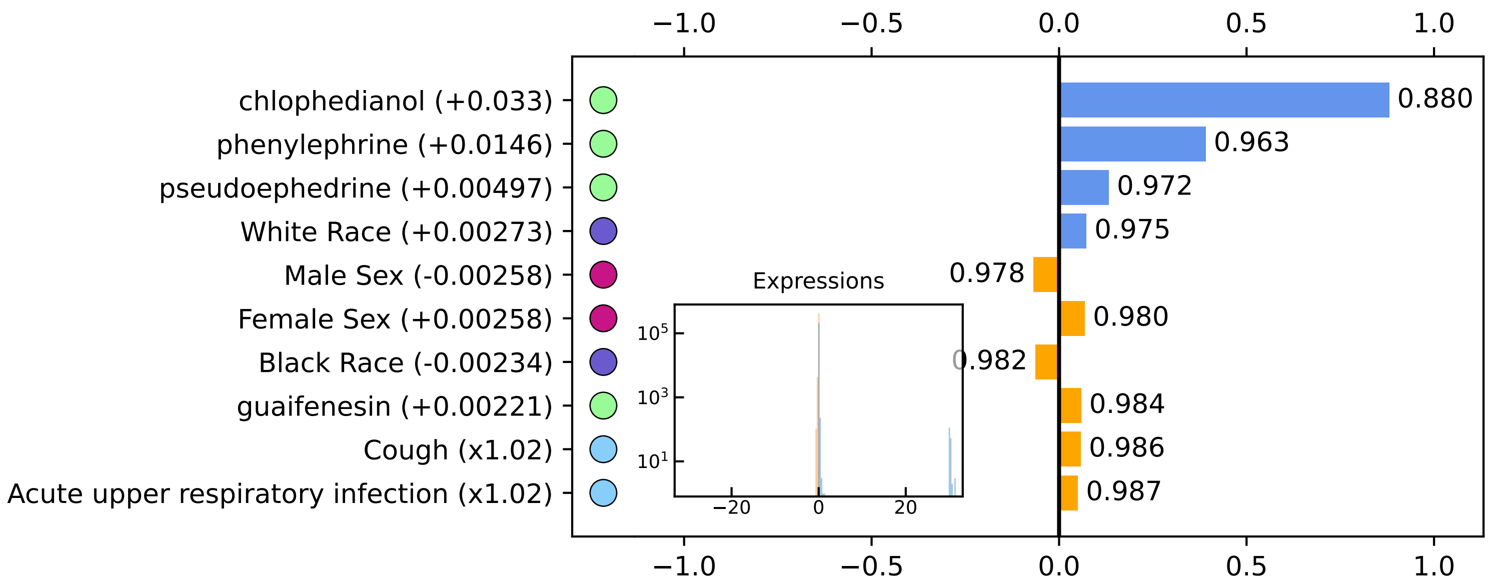

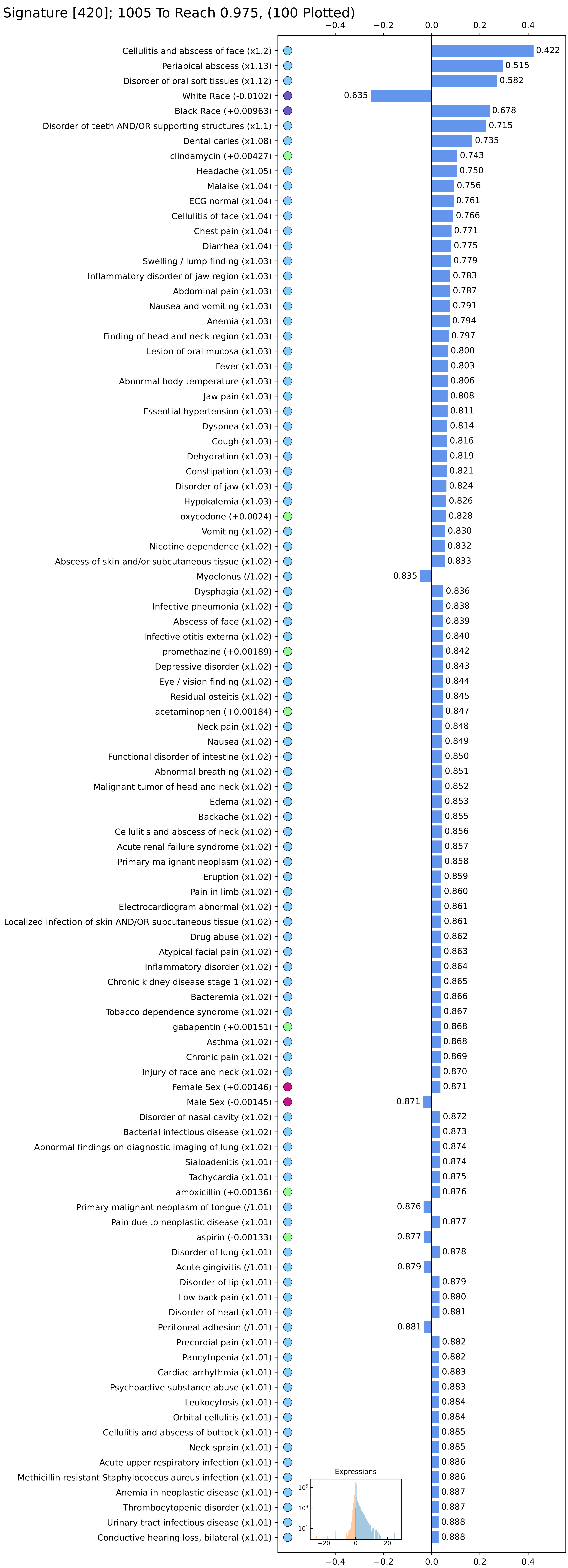
Signature [420]; 1005 To Reach 0.975, (100 Plotted)
−0.4 −0.2 0.0 0.2 0.4
Cellulitis and abscess of face (x1.2) 0.422
Periapical abscess (x1.13) 0.515
Disorder of oral soft tissues (x1.12) 0.582
White Race (-0.0102) 0.635
Black Race (+0.00963) 0.678
Disorder of teeth AND/OR supporting structures (x1.1) 0.715
Dental caries (x1.08) 0.735
clindamycin (+0.00427) 0.743
Headache (x1.05) 0.750
Malaise (x1.04) 0.756
ECG normal (x1.04) 0.761
Cellulitis of face (x1.04) 0.766
Chest pain (x1.04) 0.771
Diarrhea (x1.04) 0.775
Swelling / lump finding (x1.03) 0.779
Inflammatory disorder of jaw region (x1.03) 0.783
Abdominal pain (x1.03) 0.787
Nausea and vomiting (x1.03) 0.791
Anemia (x1.03) 0.794
Finding of head and neck region (x1.03) 0.797
Lesion of oral mucosa (x1.03) 0.800
Fever (x1.03) 0.803
Abnormal body temperature (x1.03) 0.806
Jaw pain (x1.03) 0.808
Essential hypertension (x1.03) 0.811
Dyspnea (x1.03) 0.814
Cough (x1.03) 0.816
Dehydration (x1.03) 0.819
Constipation (x1.03) 0.821
Disorder of jaw (x1.03) 0.824
Hypokalemia (x1.03) 0.826
oxycodone (+0.0024) 0.828
Vomiting (x1.02) 0.830
Nicotine dependence (x1.02) 0.832
Abscess of skin and/or subcutaneous tissue (x1.02) 0.833
Myoclonus (/1.02) 0.835
Dysphagia (x1.02) 0.836
Infective pneumonia (x1.02) 0.838
Abscess of face (x1.02) 0.839
Infective otitis externa (x1.02) 0.840
promethazine (+0.00189) 0.842
Depressive disorder (x1.02) 0.843
Eye / vision finding (x1.02) 0.844
Residual osteitis (x1.02) 0.845
acetaminophen (+0.00184) 0.847
Neck pain (x1.02) 0.848
Nausea (x1.02) 0.849
Functional disorder of intestine (x1.02) 0.850
Abnormal breathing (x1.02) 0.851
Malignant tumor of head and neck (x1.02) 0.852
Edema (x1.02) 0.853
Backache (x1.02) 0.855
Cellulitis and abscess of neck (x1.02) 0.856
Acute renal failure syndrome (x1.02) 0.857
Primary malignant neoplasm (x1.02) 0.858
Eruption (x1.02) 0.859
Pain in limb (x1.02) 0.860
Electrocardiogram abnormal (x1.02) 0.861
Localized infection of skin AND/OR subcutaneous tissue (x1.02) 0.861
Drug abuse (x1.02) 0.862
Atypical facial pain (x1.02) 0.863
Inflammatory disorder (x1.02) 0.864
Chronic kidney disease stage 1 (x1.02) 0.865
Bacteremia (x1.02) 0.866
Tobacco dependence syndrome (x1.02) 0.867
gabapentin (+0.00151) 0.868
Asthma (x1.02) 0.868
Chronic pain (x1.02) 0.869
Injury of face and neck (x1.02) 0.870
Female Sex (+0.00146) 0.871
Male Sex (-0.00145) 0.871
Disorder of nasal cavity (x1.02) 0.872
Bacterial infectious disease (x1.02) 0.873
Abnormal findings on diagnostic imaging of lung (x1.02) 0.874
Sialoadenitis (x1.01) 0.874
Tachycardia (x1.01) 0.875
amoxicillin (+0.00136) 0.876
Primary malignant neoplasm of tongue (/1.01) 0.876
Pain due to neoplastic disease (x1.01) 0.877
aspirin (-0.00133) 0.877
Disorder of lung (x1.01) 0.878
Acute gingivitis (/1.01) 0.879
Disorder of lip (x1.01) 0.879
Low back pain (x1.01) 0.880
Disorder of head (x1.01) 0.881
Peritoneal adhesion (/1.01) 0.881
Precordial pain (x1.01) 0.882
Pancytopenia (x1.01) 0.882
Cardiac arrhythmia (x1.01) 0.883
Psychoactive substance abuse (x1.01) 0.883
Leukocytosis (x1.01) 0.884
Orbital cellulitis (x1.01) 0.884
Cellulitis and abscess of buttock (x1.01) 0.885
Neck sprain (x1.01) 0.885
Acute upper respiratory infection (x1.01) 0.886
Methicillin resistant Staphylococcus aureus infection (x1.01) 0.886
Anemia in neoplastic disease (x1.01) 0.887
Thrombocytopenic disorder (x1.01) 0.887
Urinary tract infectious disease (x1.01) 0.888
Conductive hearing loss, bilateral (x1.01) 0.888
Expressions
−20 0 20
−0.4 −0.2 0.0 0.2 0.4

Signature [421]; 97 To Reach 0.975, (97 Plotted)

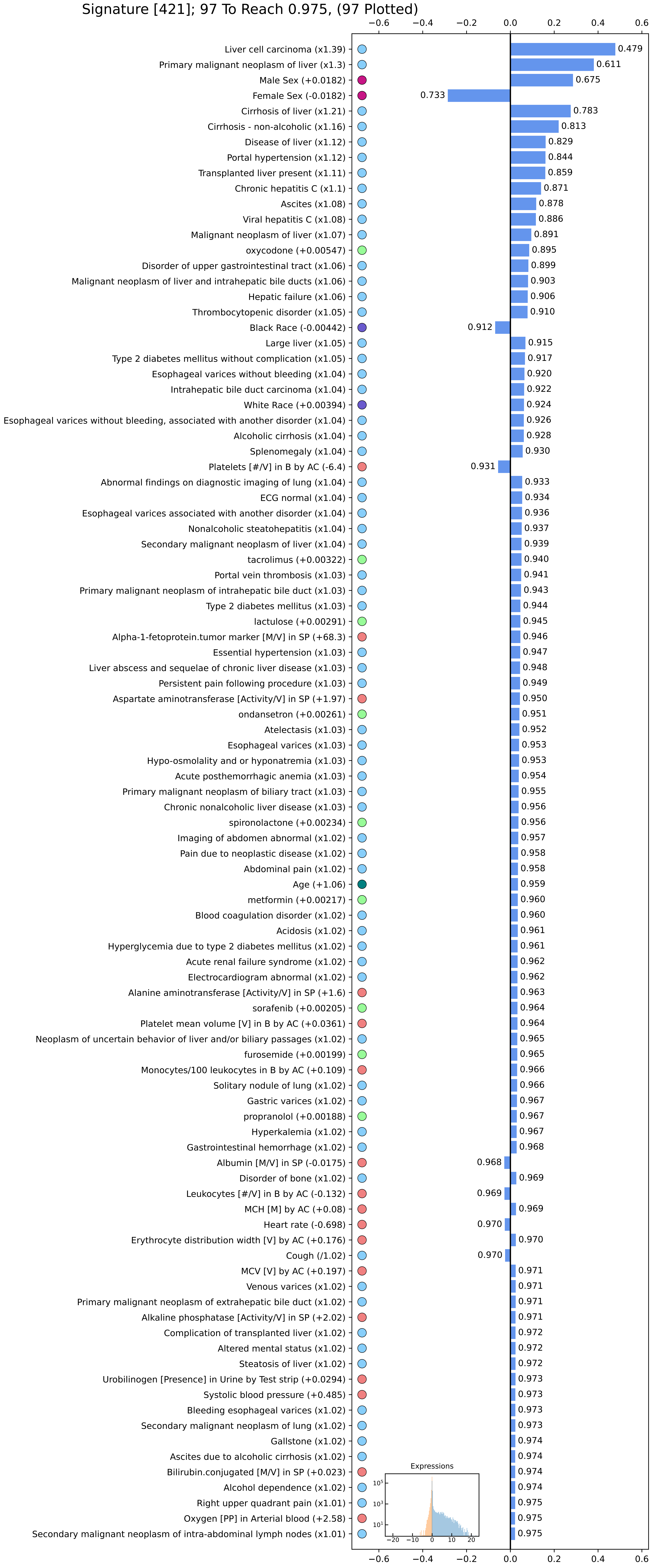

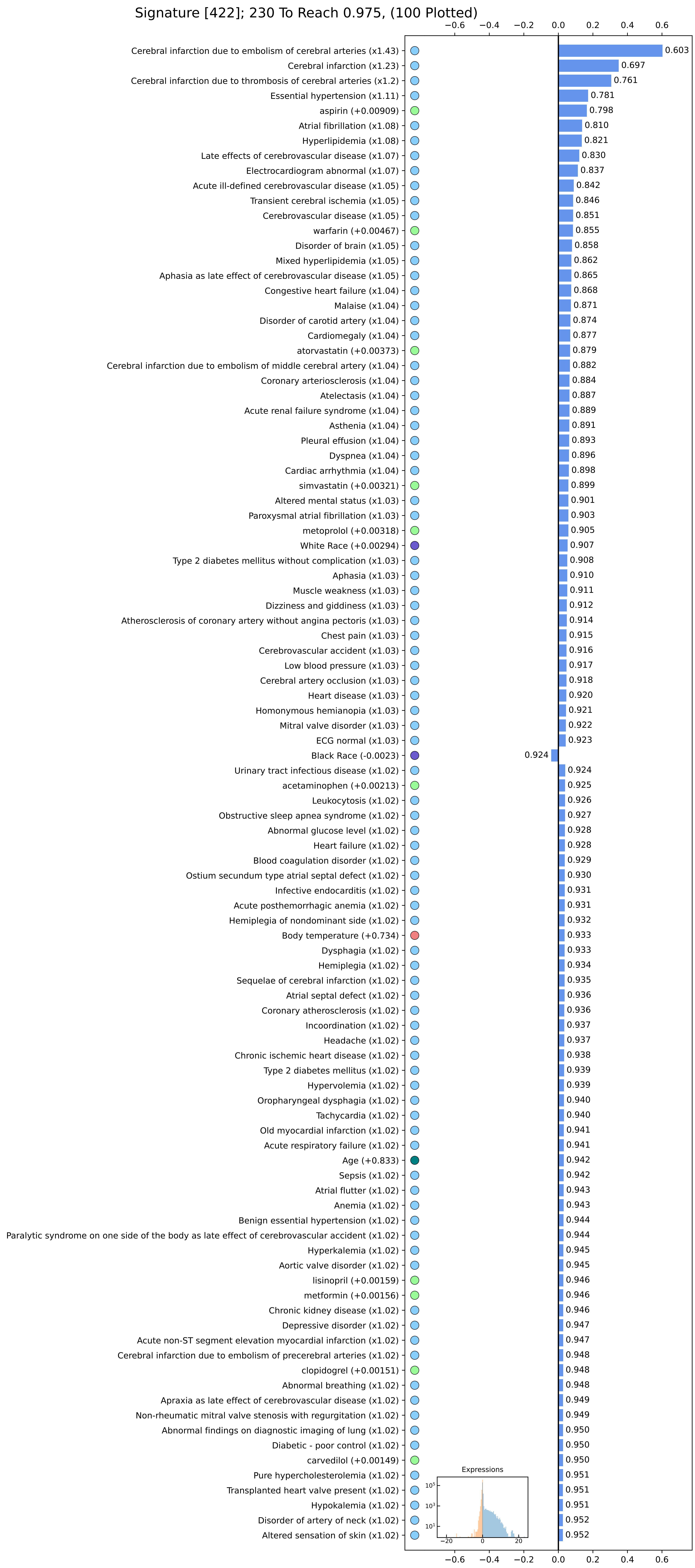
Signature [422]; 230 To Reach 0.975, (100 Plotted)
Cerebral infarction due to embolism of cerebral arteries (x1.43)
0.603
Cerebral infarction (x1.23)
0.697
Cerebral infarction due to thrombosis of cerebral arteries (x1.2)
0.761
Essential hypertension (x1.11)
0.781
aspirin (+0.00909)
0.798
Atrial fibrillation (x1.08)
0.810
Hyperlipidemia (x1.08)
0.821
Late effects of cerebrovascular disease (x1.07)
0.830
Electrocardiogram abnormal (x1.07)
0.837
Acute ill-defined cerebrovascular disease (x1.05)
0.842
Transient cerebral ischemia (x1.05)
0.846
Cerebrovascular disease (x1.05)
0.851
warfarin (+0.00467)
0.855
Disorder of brain (x1.05)
0.858
Mixed hyperlipidemia (x1.05)
0.862
Aphasia as late effect of cerebrovascular disease (x1.05)
0.865
Congestive heart failure (x1.04)
0.868
Malaise (x1.04)
0.871
Disorder of carotid artery (x1.04)
0.874
Cardiomegaly (x1.04)
0.877
atorvastatin (+0.00373)
0.879
Cerebral infarction due to embolism of middle cerebral artery (x1.04)
0.882
Coronary arteriosclerosis (x1.04)
0.884
Atelectasis (x1.04)
0.887
Acute renal failure syndrome (x1.04)
0.889
Asthenia (x1.04)
0.891
Pleural effusion (x1.04)
0.893
Dyspnea (x1.04)
0.896
Cardiac arrhythmia (x1.04)
0.898
simvastatin (+0.00321)
0.899
Altered mental status (x1.03)
0.901
Paroxysmal atrial fibrillation (x1.03)
0.903
metoprolol (+0.00318)
0.905
White Race (+0.00294)
0.907
Type 2 diabetes mellitus without complication (x1.03)
0.908
Aphasia (x1.03)
0.910
Muscle weakness (x1.03)
0.911
Dizziness and giddiness (x1.03)
0.912
Atherosclerosis of coronary artery without angina pectoris (x1.03)
0.914
Chest pain (x1.03)
0.915
Cerebrovascular accident (x1.03)
0.916
Low blood pressure (x1.03)
0.917
Cerebral artery occlusion (x1.03)
0.918
Heart disease (x1.03)
0.920
Homonymous hemianopia (x1.03)
0.921
Mitral valve disorder (x1.03)
0.922
ECG normal (x1.03)
0.923
Black Race (-0.0023)
0.924
Urinary tract infectious disease (x1.02)
0.924
acetaminophen (+0.00213)
0.925
Leukocytosis (x1.02)
0.926
Obstructive sleep apnea syndrome (x1.02)
0.927
Abnormal glucose level (x1.02)
0.928
Heart failure (x1.02)
0.928
Blood coagulation disorder (x1.02)
0.929
Ostium secundum type atrial septal defect (x1.02)
0.930
Infective endocarditis (x1.02)
0.931
Acute posthemorrhagic anemia (x1.02)
0.931
Hemiplegia of nondominant side (x1.02)
0.932
Body temperature (+0.734)
0.933
Dysphagia (x1.02)
0.933
Hemiplegia (x1.02)
0.934
Sequelae of cerebral infarction (x1.02)
0.935
Atrial septal defect (x1.02)
0.936
Coronary atherosclerosis (x1.02)
0.936
Incoordination (x1.02)
0.937
Headache (x1.02)
0.937
Chronic ischemic heart disease (x1.02)
0.938
Type 2 diabetes mellitus (x1.02)
0.939
Hypervolemia (x1.02)
0.939
Oropharyngeal dysphagia (x1.02)
0.940
Tachycardia (x1.02)
0.940
Old myocardial infarction (x1.02)
0.941
Acute respiratory failure (x1.02)
0.941
Age (+0.833)
0.942
Sepsis (x1.02)
0.942
Atrial flutter (x1.02)
0.943
Anemia (x1.02)
0.943
Benign essential hypertension (x1.02)
0.944
Paralytic syndrome on one side of the body as late effect of cerebrovascular accident (x1.02)
0.944
Hyperkalemia (x1.02)
0.945
Aortic valve disorder (x1.02)
0.945
lisinopril (+0.00159)
0.946
metformin (+0.00156)
0.946
Chronic kidney disease (x1.02)
0.946
Depressive disorder (x1.02)
0.947
Acute non-ST segment elevation myocardial infarction (x1.02)
0.947
Cerebral infarction due to embolism of precerebral arteries (x1.02)
0.948
clopidogrel (+0.00151)
0.948
Abnormal breathing (x1.02)
0.948
Apraxia as late effect of cerebrovascular disease (x1.02)
0.949
Non-rheumatic mitral valve stenosis with regurgitation (x1.02)
0.949
Abnormal findings on diagnostic imaging of lung (x1.02)
0.950
Diabetic - poor control (x1.02)
0.950
carvedilol (+0.00149)
0.950
Pure hypercholesterolemia (x1.02)
0.951
Transplanted heart valve present (x1.02)
0.951
Hypokalemia (x1.02)
0.951
Disorder of artery of neck (x1.02)
0.952
Altered sensation of skin (x1.02)
0.952
Expressions
−0.6
−0.4
−0.2
0.0
0.2
0.4
0.6

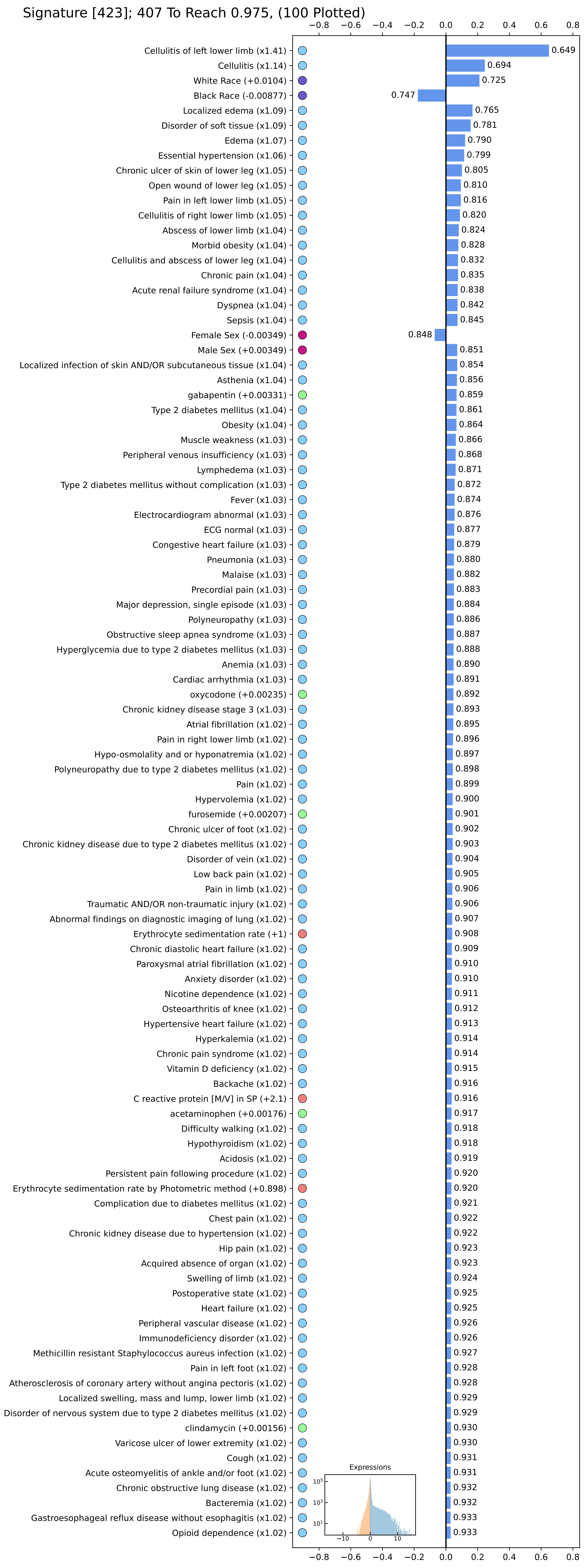
Signature [423]; 407 To Reach 0.975, (100 Plotted)
Cellulitis of left lower limb (x1.41) 0.649
Cellulitis (x1.14) 0.694
White Race (+0.0104) 0.725
Black Race (-0.00877) 0.747
Localized edema (x1.09) 0.765
Disorder of soft tissue (x1.09) 0.781
Edema (x1.07) 0.790
Essential hypertension (x1.06) 0.799
Chronic ulcer of skin of lower leg (x1.05) 0.805
Open wound of lower leg (x1.05) 0.810
Pain in left lower limb (x1.05) 0.816
Cellulitis of right lower limb (x1.05) 0.820
Abscess of lower limb (x1.04) 0.824
Morbid obesity (x1.04) 0.828
Cellulitis and abscess of lower leg (x1.04) 0.832
Chronic pain (x1.04) 0.835
Acute renal failure syndrome (x1.04) 0.838
Dyspnea (x1.04) 0.842
Sepsis (x1.04) 0.845
Female Sex (-0.00349) 0.848
Male Sex (+0.00349) 0.851
Localized infection of skin AND/OR subcutaneous tissue (x1.04) 0.854
Asthenia (x1.04) 0.856
gabapentin (+0.00331) 0.859
Type 2 diabetes mellitus (x1.04) 0.861
Obesity (x1.04) 0.864
Muscle weakness (x1.03) 0.866
Peripheral venous insufficiency (x1.03) 0.868
Lymphedema (x1.03) 0.871
Type 2 diabetes mellitus without complication (x1.03) 0.872
Fever (x1.03) 0.874
Electrocardiogram abnormal (x1.03) 0.876
ECG normal (x1.03) 0.877
Congestive heart failure (x1.03) 0.879
Pneumonia (x1.03) 0.880
Malaise (x1.03) 0.882
Precordial pain (x1.03) 0.883
Major depression, single episode (x1.03) 0.884
Polyneuropathy (x1.03) 0.886
Obstructive sleep apnea syndrome (x1.03) 0.887
Hyperglycemia due to type 2 diabetes mellitus (x1.03) 0.888
Anemia (x1.03) 0.890
Cardiac arrhythmia (x1.03) 0.891
oxycodone (+0.00235) 0.892
Chronic kidney disease stage 3 (x1.03) 0.893
Atrial fibrillation (x1.02) 0.895
Pain in right lower limb (x1.02) 0.896
Hypo-osmolality and or hyponatremia (x1.02) 0.897
Polyneuropathy due to type 2 diabetes mellitus (x1.02) 0.898
Pain (x1.02) 0.899
Hypervolemia (x1.02) 0.900
furosemide (+0.00207) 0.901
Chronic ulcer of foot (x1.02) 0.902
Chronic kidney disease due to type 2 diabetes mellitus (x1.02) 0.903
Disorder of vein (x1.02) 0.904
Low back pain (x1.02) 0.905
Pain in limb (x1.02) 0.906
Traumatic AND/OR non-traumatic injury (x1.02) 0.906
Abnormal findings on diagnostic imaging of lung (x1.02) 0.907
Erythrocyte sedimentation rate (+1) 0.908
Chronic diastolic heart failure (x1.02) 0.909
Paroxysmal atrial fibrillation (x1.02) 0.910
Anxiety disorder (x1.02) 0.910
Nicotine dependence (x1.02) 0.911
Osteoarthritis of knee (x1.02) 0.912
Hypertensive heart failure (x1.02) 0.913
Hyperkalemia (x1.02) 0.914
Chronic pain syndrome (x1.02) 0.914
Vitamin D deficiency (x1.02) 0.915
Backache (x1.02) 0.916
C reactive protein [M/V] in SP (+2.1) 0.916
acetaminophen (+0.00176) 0.917
Difficulty walking (x1.02) 0.918
Hypothyroidism (x1.02) 0.918
Acidosis (x1.02) 0.919
Persistent pain following procedure (x1.02) 0.920
Erythrocyte sedimentation rate by Photometric method (+0.898) 0.920
Complication due to diabetes mellitus (x1.02) 0.921
Chest pain (x1.02) 0.922
Chronic kidney disease due to hypertension (x1.02) 0.922
Hip pain (x1.02) 0.923
Acquired absence of organ (x1.02) 0.923
Swelling of limb (x1.02) 0.924
Postoperative state (x1.02) 0.925
Heart failure (x1.02) 0.925
Peripheral vascular disease (x1.02) 0.926
Immunodeficiency disorder (x1.02) 0.926
Methicillin resistant Staphylococcus aureus infection (x1.02) 0.927
Pain in left foot (x1.02) 0.928
Atherosclerosis of coronary artery without angina pectoris (x1.02) 0.928
Localized swelling, mass and lump, lower limb (x1.02) 0.929
Disorder of nervous system due to type 2 diabetes mellitus (x1.02) 0.929
clindamycin (+0.00156) 0.930
Varicose ulcer of lower extremity (x1.02) 0.930
Cough (x1.02) 0.931
Acute osteomyelitis of ankle and/or foot (x1.02) 0.931
Chronic obstructive lung disease (x1.02) 0.932
Bacteremia (x1.02) 0.932
Gastroesophageal reflux disease without esophagitis (x1.02) 0.933
Opioid dependence (x1.02) 0.933
−0.8 −0.6 −0.4 −0.2 0.0 0.2 0.4 0.6 0.8
Expressions
−10 0 10

Signature [424]; 395 To Reach 0.975, (100 Plotted)

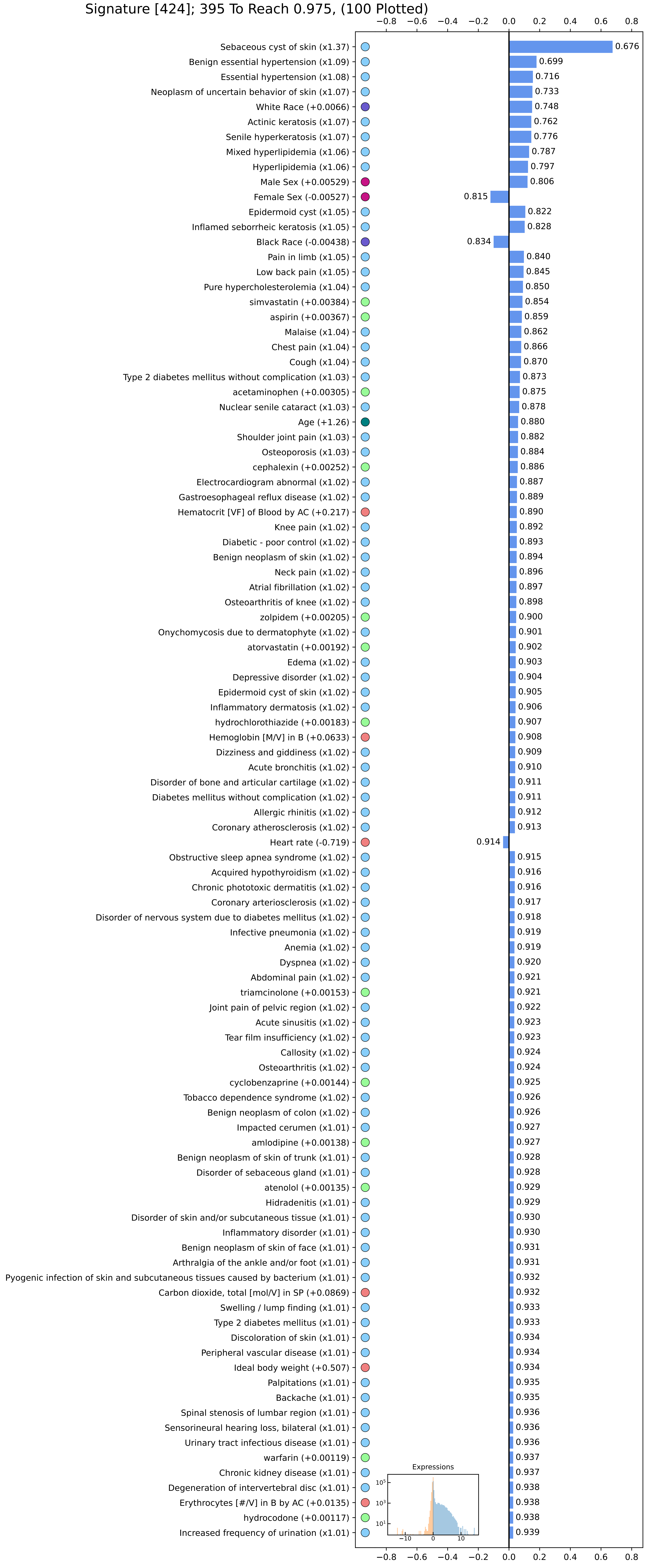

Signature [425]; 1 To Reach 0.975, (10 Plotted)

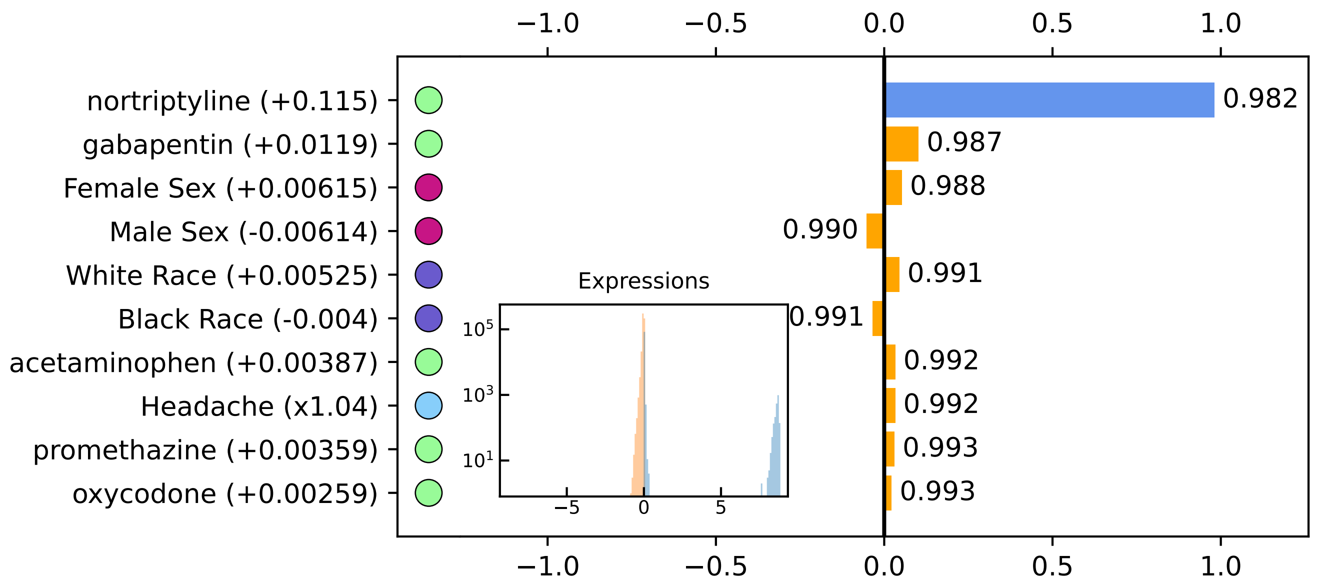

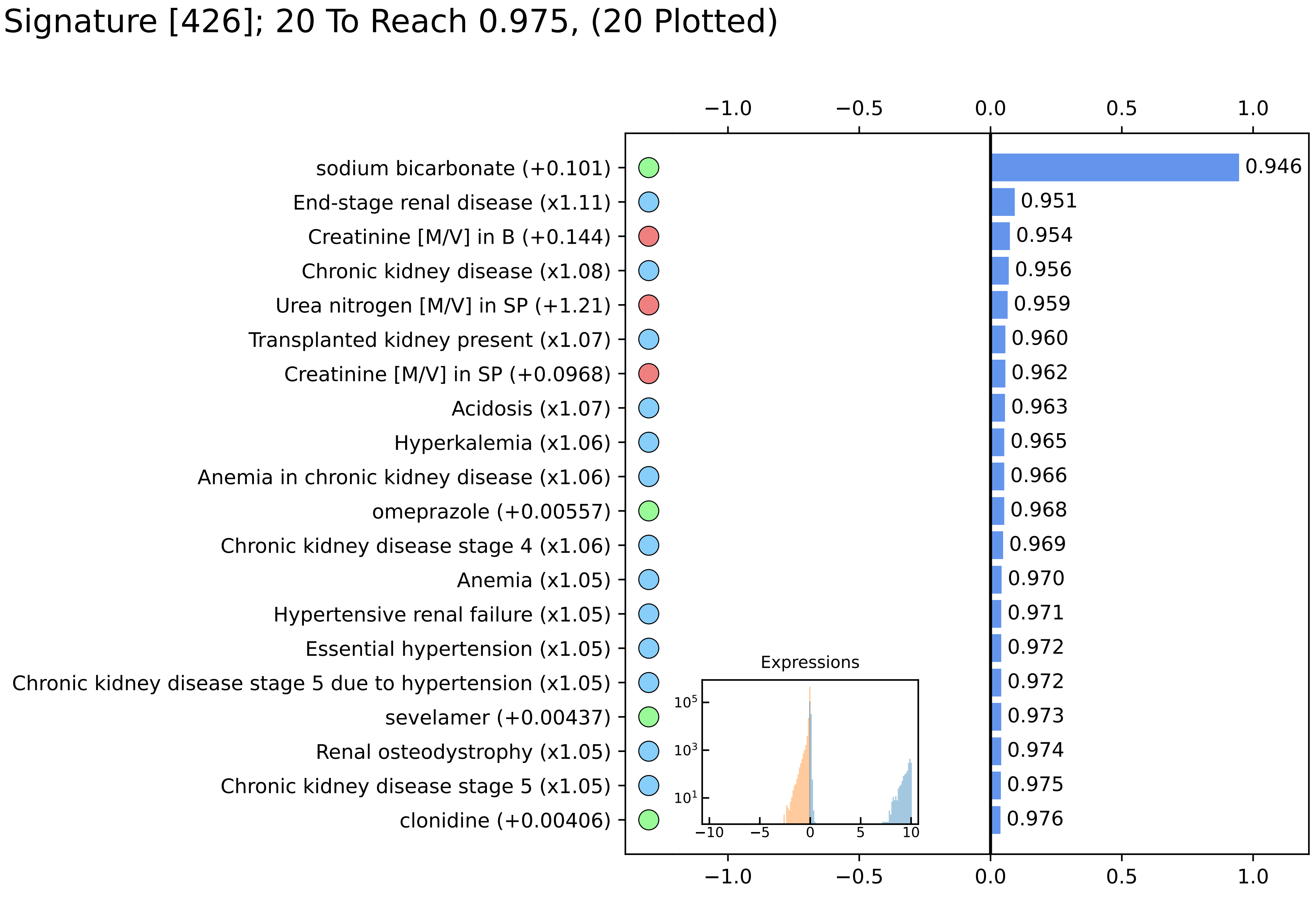

Signature [426]; 20 To Reach 0.975, (20 Plotted)
sodium bicarbonate (+0.101)
End-stage renal disease (x1.11)
Creatinine [M/V] in B (+0.144)
Chronic kidney disease (x1.08)
Urea nitrogen [M/V] in SP (+1.21)
Transplanted kidney present (x1.07)
Creatinine [M/V] in SP (+0.0968)
Acidosis (x1.07)
Hyperkalemia (x1.06)
Anemia in chronic kidney disease (x1.06)
omeprazole (+0.00557)
Chronic kidney disease stage 4 (x1.06)
Anemia (x1.05)
Hypertensive renal failure (x1.05)
Essential hypertension (x1.05)
Chronic kidney disease stage 5 due to hypertension (x1.05)
sevelamer (+0.00437)
Renal osteodystrophy (x1.05)
Chronic kidney disease stage 5 (x1.05)
clonidine (+0.00406)
0.946
0.951
0.954
0.956
0.959
0.960
0.962
0.963
0.965
0.966
0.968
0.969
0.970
0.971
0.972
0.972
0.973
0.974
0.975
0.976
Expressions

Signature [427]; 153 To Reach 0.975, (100 Plotted)

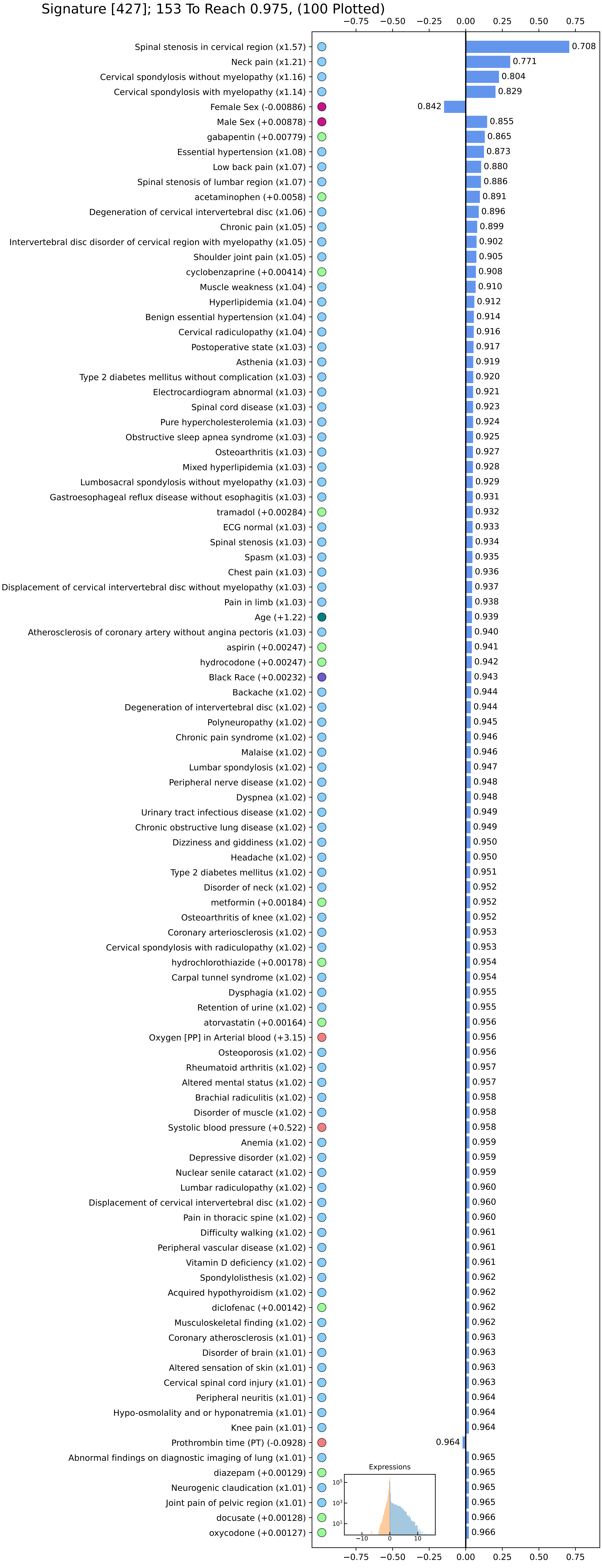

Signature [428]; 208 To Reach 0.975, (100 Plotted)

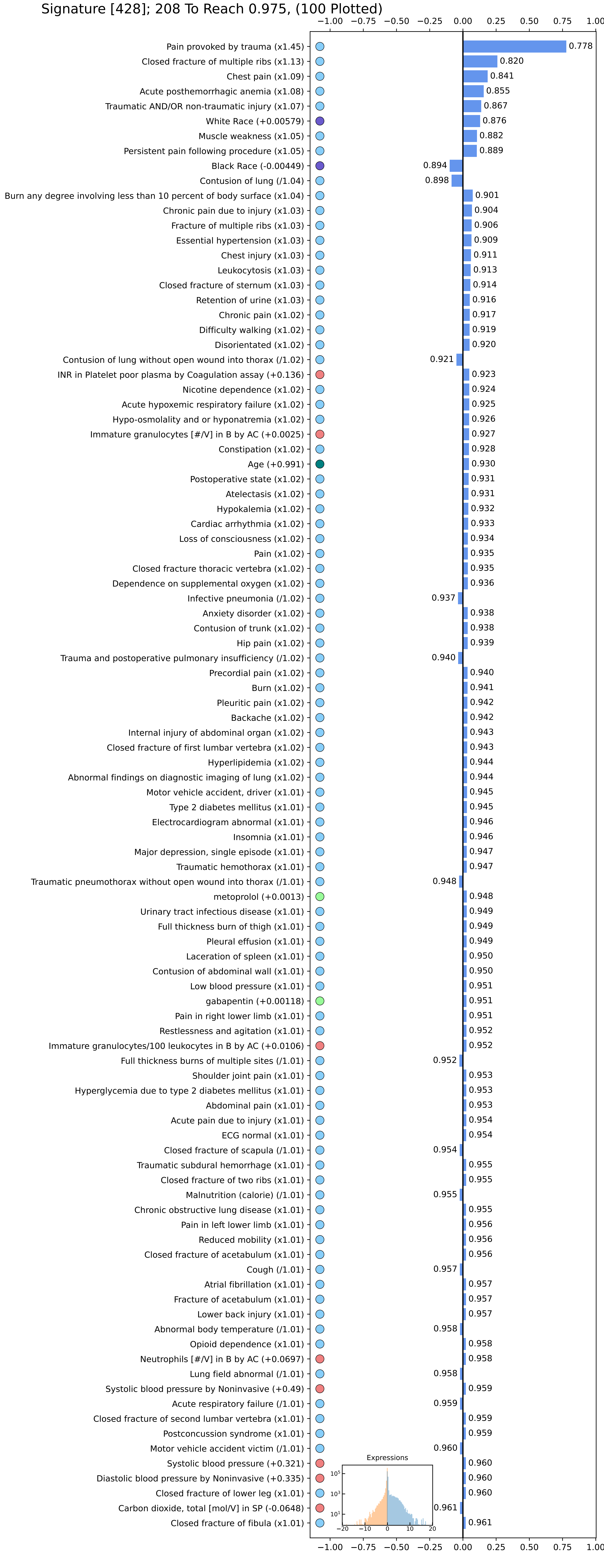

Signature [429]; 1 To Reach 0.975, (10 Plotted)

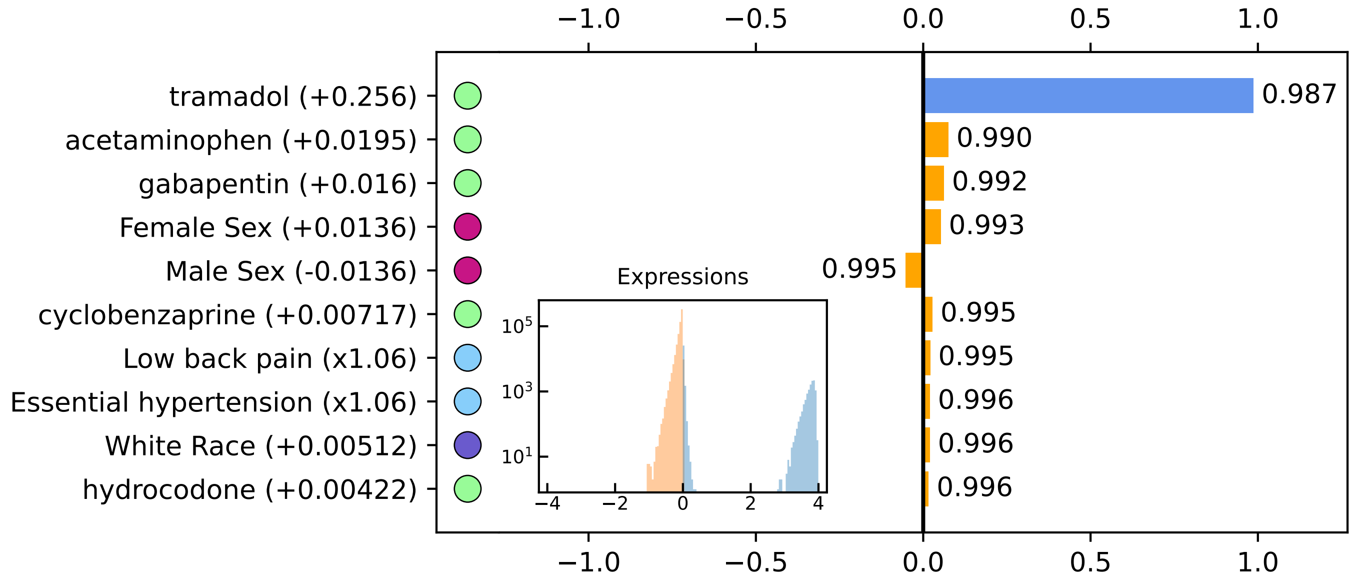

Signature [430]; 7 To Reach 0.975, (10 Plotted)

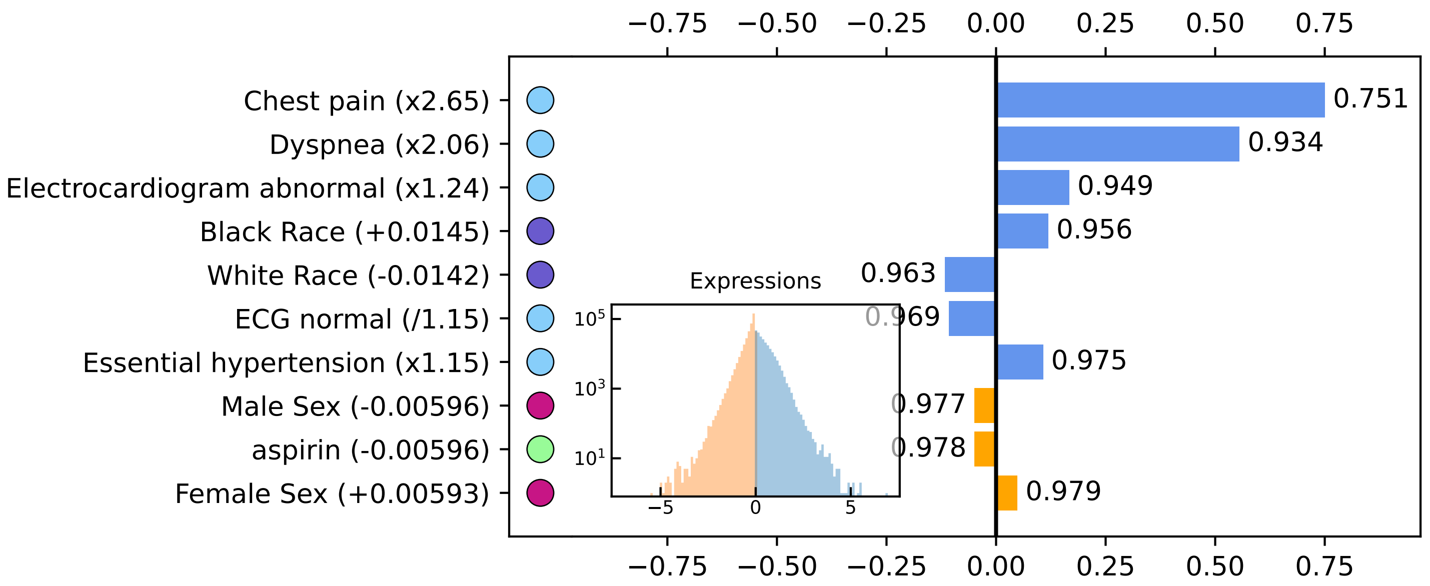

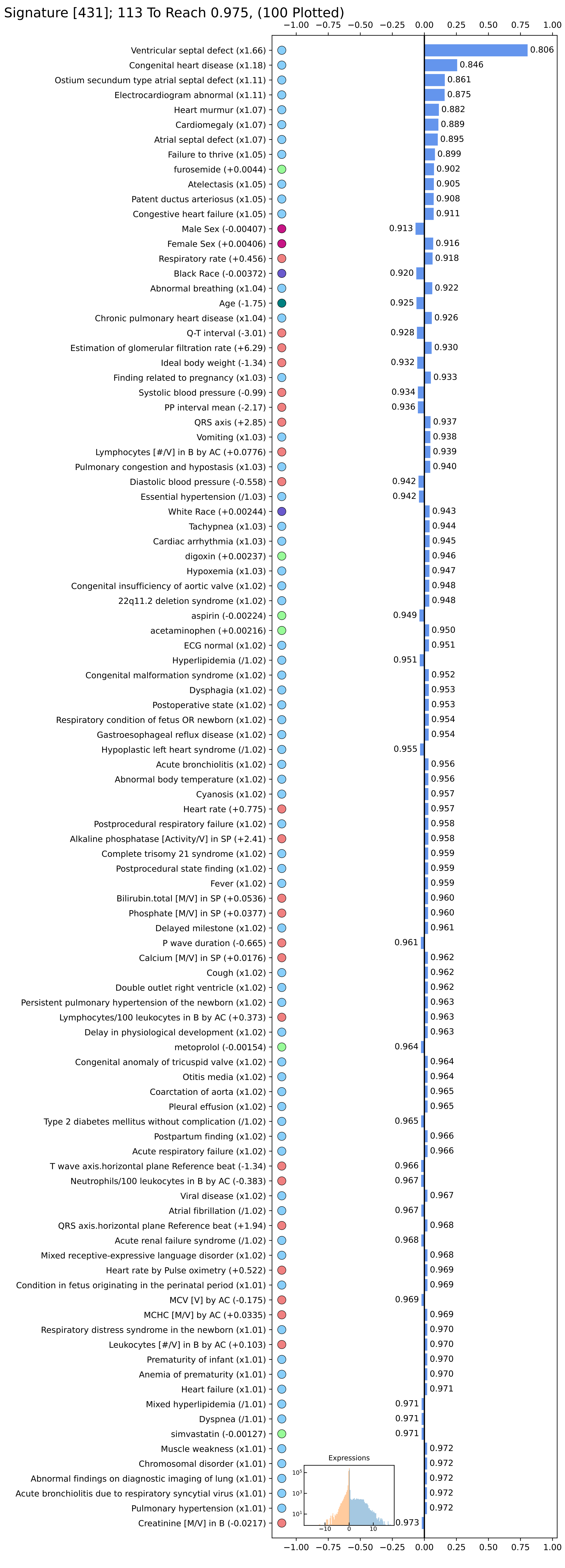

Signature [431]; 113 To Reach 0.975, (100 Plotted)
Ventricular septal defect (x1.66) 0.806
Congenital heart disease (x1.18) 0.846
Ostium secundum type atrial septal defect (x1.11) 0.861
Electrocardiogram abnormal (x1.11) 0.875
Heart murmur (x1.07) 0.882
Cardiomegaly (x1.07) 0.889
Atrial septal defect (x1.07) 0.895
Failure to thrive (x1.05) 0.899
furosemide (+0.0044) 0.902
Atelectasis (x1.05) 0.905
Patent ductus arteriosus (x1.05) 0.908
Congestive heart failure (x1.05) 0.911
Male Sex (-0.00407) 0.913
Female Sex (+0.00406) 0.916
Respiratory rate (+0.456) 0.918
Black Race (-0.00372) 0.920
Abnormal breathing (x1.04) 0.922
Age (-1.75) 0.925
Chronic pulmonary heart disease (x1.04) 0.926
Q-T interval (-3.01) 0.928
Estimation of glomerular filtration rate (+6.29) 0.930
Ideal body weight (-1.34) 0.932
Finding related to pregnancy (x1.03) 0.933
Systolic blood pressure (-0.99) 0.934
PP interval mean (-2.17) 0.936
QRS axis (+2.85) 0.937
Vomiting (x1.03) 0.938
Lymphocytes [#/V] in B by AC (+0.0776) 0.939
Pulmonary congestion and hypostasis (x1.03) 0.940
Diastolic blood pressure (-0.558) 0.942
Essential hypertension (/1.03) 0.942
White Race (+0.00244) 0.943
Tachypnea (x1.03) 0.944
Cardiac arrhythmia (x1.03) 0.945
digoxin (+0.00237) 0.946
Hypoxemia (x1.03) 0.947
Congenital insufficiency of aortic valve (x1.02) 0.948
22q11.2 deletion syndrome (x1.02) 0.948
aspirin (-0.00224) 0.949
acetaminophen (+0.00216) 0.950
ECG normal (x1.02) 0.951
Hyperlipidemia (/1.02) 0.951
Congenital malformation syndrome (x1.02) 0.952
Dysphagia (x1.02) 0.953
Postoperative state (x1.02) 0.953
Respiratory condition of fetus OR newborn (x1.02) 0.954
Gastroesophageal reflux disease (x1.02) 0.954
Hypoplastic left heart syndrome (/1.02) 0.955
Acute bronchiolitis (x1.02) 0.956
Abnormal body temperature (x1.02) 0.956
Cyanosis (x1.02) 0.957
Heart rate (+0.775) 0.957
Postprocedural respiratory failure (x1.02) 0.958
Alkaline phosphatase [Activity/V] in SP (+2.41) 0.958
Complete trisomy 21 syndrome (x1.02) 0.959
Postprocedural state finding (x1.02) 0.959
Fever (x1.02) 0.959
Bilirubin.total [M/V] in SP (+0.0536) 0.960
Phosphate [M/V] in SP (+0.0377) 0.960
Delayed milestone (x1.02) 0.961
P wave duration (-0.665) 0.961
Calcium [M/V] in SP (+0.0176) 0.962
Cough (x1.02) 0.962
Double outlet right ventricle (x1.02) 0.962
Persistent pulmonary hypertension of the newborn (x1.02) 0.963
Lymphocytes/100 leukocytes in B by AC (+0.373) 0.963
Delay in physiological development (x1.02) 0.963
metoprolol (-0.00154) 0.964
Congenital anomaly of tricuspid valve (x1.02) 0.964
Otitis media (x1.02) 0.964
Coarctation of aorta (x1.02) 0.965
Pleural effusion (x1.02) 0.965
Type 2 diabetes mellitus without complication (/1.02) 0.965
Postpartum finding (x1.02) 0.966
Acute respiratory failure (x1.02) 0.966
T wave axis.horizontal plane Reference beat (-1.34) 0.966
Neutrophils/100 leukocytes in B by AC (-0.383) 0.967
Viral disease (x1.02) 0.967
Atrial fibrillation (/1.02) 0.967
QRS axis.horizontal plane Reference beat (+1.94) 0.968
Acute renal failure syndrome (/1.02) 0.968
Mixed receptive-expressive language disorder (x1.02) 0.968
Heart rate by Pulse oximetry (+0.522) 0.969
Condition in fetus originating in the perinatal period (x1.01) 0.969
MCV [V] by AC (-0.175) 0.969
MCHC [M/V] by AC (+0.0335) 0.969
Respiratory distress syndrome in the newborn (x1.01) 0.970
Leukocytes [#/V] in B by AC (+0.103) 0.970
Prematurity of infant (x1.01) 0.970
Anemia of prematurity (x1.01) 0.970
Heart failure (x1.01) 0.971
Mixed hyperlipidemia (/1.01) 0.971
Dyspnea (/1.01) 0.971
simvastatin (-0.00127) 0.971
Muscle weakness (x1.01) 0.972
Chromosomal disorder (x1.01) 0.972
Abnormal findings on diagnostic imaging of lung (x1.01) 0.972
Acute bronchiolitis due to respiratory syncytial virus (x1.01) 0.972
Pulmonary hypertension (x1.01) 0.972
Creatinine [M/V] in B (-0.0217) 0.973
Expressions
−1.00 −0.75 −0.50 −0.25 0.00 0.25 0.50 0.75 1.00

Signature [432]; 2 To Reach 0.975, (10 Plotted)

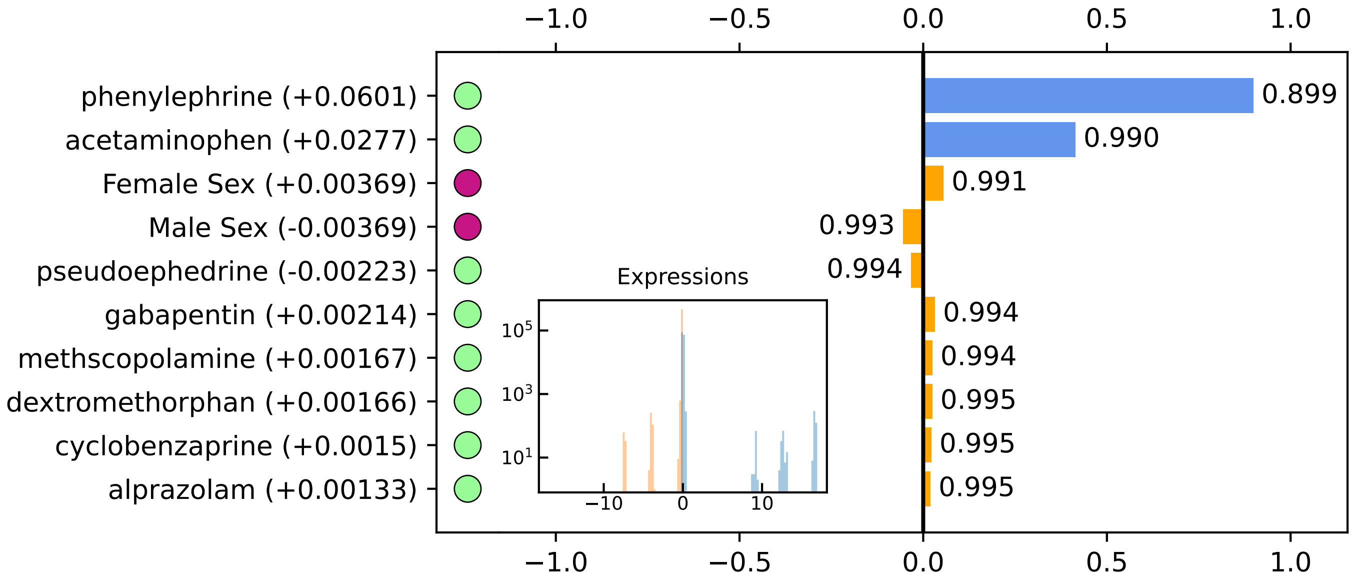

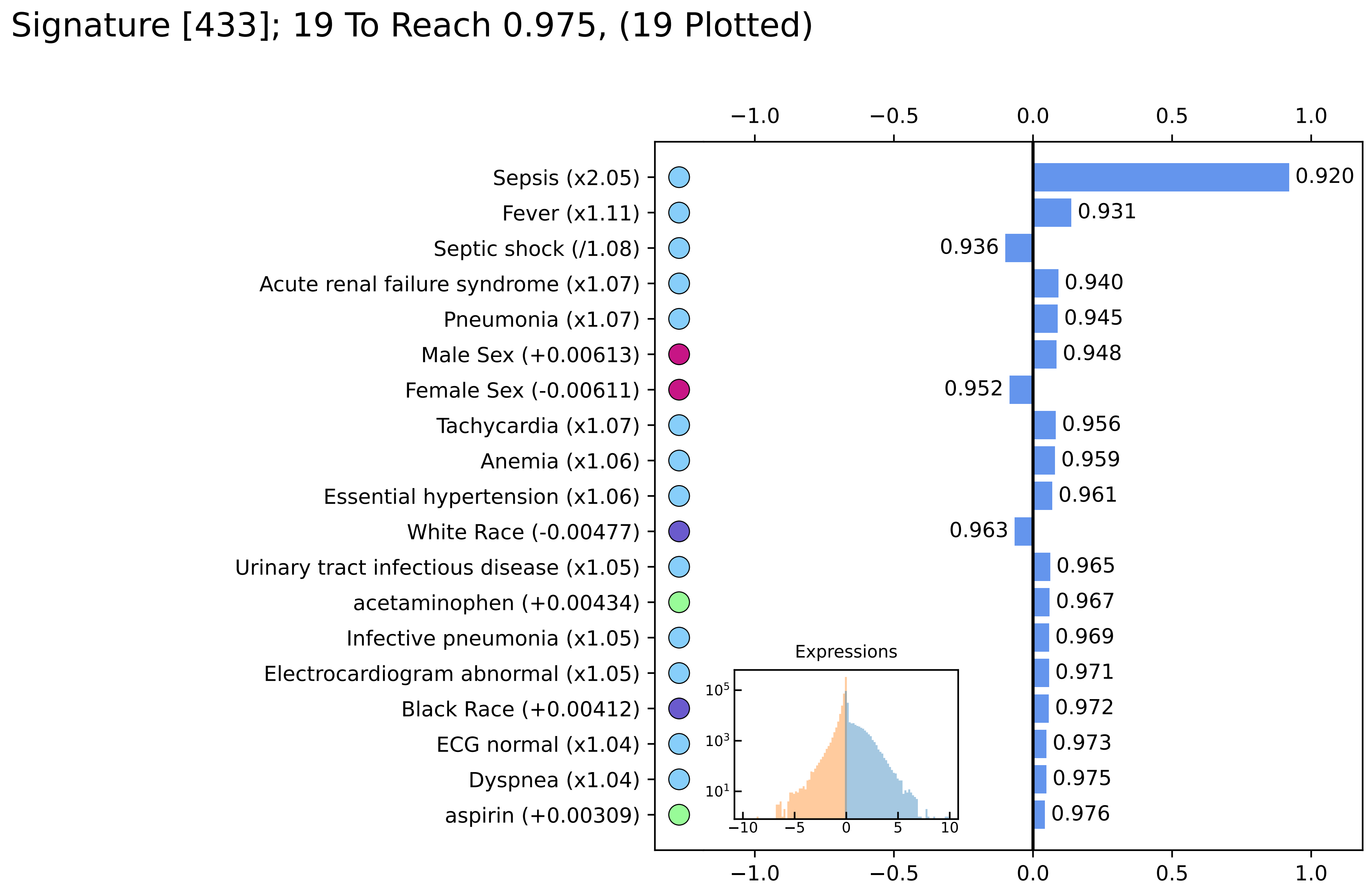

Signature [433]; 19 To Reach 0.975, (19 Plotted)
Sepsis (x2.05)
Fever (x1.11)
Septic shock (/1.08)
Acute renal failure syndrome (x1.07)
Pneumonia (x1.07)
Male Sex (+0.00613)
Female Sex (-0.00611)
Tachycardia (x1.07)
Anemia (x1.06)
Essential hypertension (x1.06)
White Race (-0.00477)
Urinary tract infectious disease (x1.05)
acetaminophen (+0.00434)
Infective pneumonia (x1.05)
Electrocardiogram abnormal (x1.05)
Black Race (+0.00412)
ECG normal (x1.04)
Dyspnea (x1.04)
aspirin (+0.00309)
0.920
0.931
0.936
0.940
0.945
0.948
0.952
0.956
0.959
0.961
0.963
0.965
0.967
0.969
0.971
0.972
0.973
0.975
0.976
−1.0
−0.5
0.0
0.5
1.0
Expressions

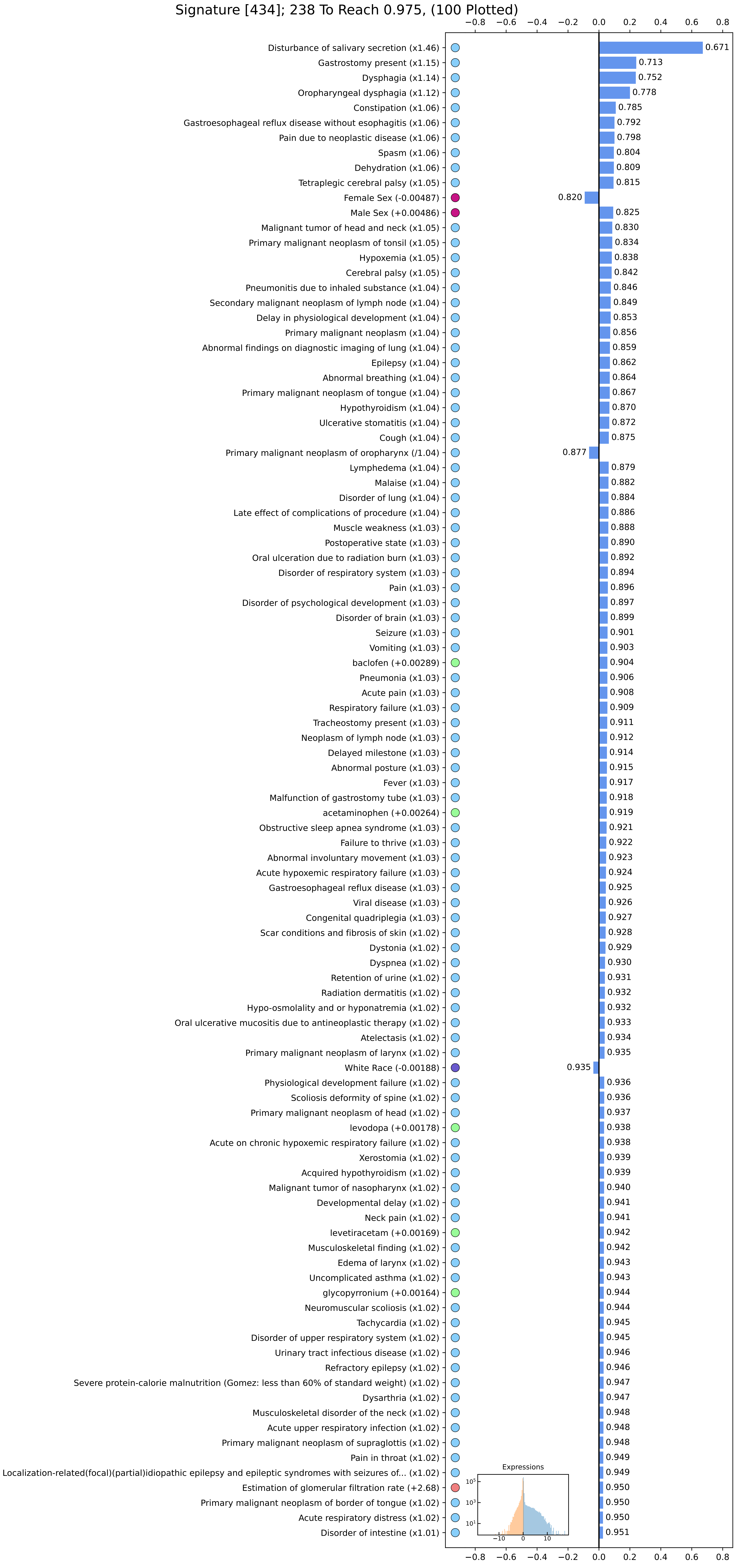
Signature [434]; 238 To Reach 0.975, (100 Plotted)
−0.8 −0.6 −0.4 −0.2 0.0 0.2 0.4 0.6 0.8
Disturbance of salivary secretion (x1.46) 0.671
Gastrostomy present (x1.15) 0.713
Dysphagia (x1.14) 0.752
Oropharyngeal dysphagia (x1.12) 0.778
Constipation (x1.06) 0.785
Gastroesophageal reflux disease without esophagitis (x1.06) 0.792
Pain due to neoplastic disease (x1.06) 0.798
Spasm (x1.06) 0.804
Dehydration (x1.06) 0.809
Tetraplegic cerebral palsy (x1.05) 0.815
Female Sex (-0.00487) 0.820
Male Sex (+0.00486) 0.825
Malignant tumor of head and neck (x1.05) 0.830
Primary malignant neoplasm of tonsil (x1.05) 0.834
Hypoxemia (x1.05) 0.838
Cerebral palsy (x1.05) 0.842
Pneumonitis due to inhaled substance (x1.04) 0.846
Secondary malignant neoplasm of lymph node (x1.04) 0.849
Delay in physiological development (x1.04) 0.853
Primary malignant neoplasm (x1.04) 0.856
Abnormal findings on diagnostic imaging of lung (x1.04) 0.859
Epilepsy (x1.04) 0.862
Abnormal breathing (x1.04) 0.864
Primary malignant neoplasm of tongue (x1.04) 0.867
Hypothyroidism (x1.04) 0.870
Ulcerative stomatitis (x1.04) 0.872
Cough (x1.04) 0.875
Primary malignant neoplasm of oropharynx (/1.04) 0.877
Lymphedema (x1.04) 0.879
Malaise (x1.04) 0.882
Disorder of lung (x1.04) 0.884
Late effect of complications of procedure (x1.04) 0.886
Muscle weakness (x1.03) 0.888
Postoperative state (x1.03) 0.890
Oral ulceration due to radiation burn (x1.03) 0.892
Disorder of respiratory system (x1.03) 0.894
Pain (x1.03) 0.896
Disorder of psychological development (x1.03) 0.897
Disorder of brain (x1.03) 0.899
Seizure (x1.03) 0.901
Vomiting (x1.03) 0.903
baclofen (+0.00289) 0.904
Pneumonia (x1.03) 0.906
Acute pain (x1.03) 0.908
Respiratory failure (x1.03) 0.909
Tracheostomy present (x1.03) 0.911
Neoplasm of lymph node (x1.03) 0.912
Delayed milestone (x1.03) 0.914
Abnormal posture (x1.03) 0.915
Fever (x1.03) 0.917
Malfunction of gastrostomy tube (x1.03) 0.918
acetaminophen (+0.00264) 0.919
Obstructive sleep apnea syndrome (x1.03) 0.921
Failure to thrive (x1.03) 0.922
Abnormal involuntary movement (x1.03) 0.923
Acute hypoxemic respiratory failure (x1.03) 0.924
Gastroesophageal reflux disease (x1.03) 0.925
Viral disease (x1.03) 0.926
Congenital quadriplegia (x1.03) 0.927
Scar conditions and fibrosis of skin (x1.02) 0.928
Dystonia (x1.02) 0.929
Dyspnea (x1.02) 0.930
Retention of urine (x1.02) 0.931
Radiation dermatitis (x1.02) 0.932
Hypo-osmolality and or hyponatremia (x1.02) 0.932
Oral ulcerative mucositis due to antineoplastic therapy (x1.02) 0.933
Atelectasis (x1.02) 0.934
Primary malignant neoplasm of larynx (x1.02) 0.935
White Race (-0.00188) 0.935
Physiological development failure (x1.02) 0.936
Scoliosis deformity of spine (x1.02) 0.936
Primary malignant neoplasm of head (x1.02) 0.937
levodopa (+0.00178) 0.938
Acute on chronic hypoxemic respiratory failure (x1.02) 0.938
Xerostomia (x1.02) 0.939
Acquired hypothyroidism (x1.02) 0.939
Malignant tumor of nasopharynx (x1.02) 0.940
Developmental delay (x1.02) 0.941
Neck pain (x1.02) 0.941
levetiracetam (+0.00169) 0.942
Musculoskeletal finding (x1.02) 0.942
Edema of larynx (x1.02) 0.943
Uncomplicated asthma (x1.02) 0.943
glycopyrronium (+0.00164) 0.944
Neuromuscular scoliosis (x1.02) 0.944
Tachycardia (x1.02) 0.945
Disorder of upper respiratory system (x1.02) 0.945
Urinary tract infectious disease (x1.02) 0.946
Refractory epilepsy (x1.02) 0.946
Severe protein-calorie malnutrition (Gomez: less than 60% of standard weight) (x1.02) 0.947
Dysarthria (x1.02) 0.947
Musculoskeletal disorder of the neck (x1.02) 0.948
Acute upper respiratory infection (x1.02) 0.948
Primary malignant neoplasm of supraglottis (x1.02) 0.948
Pain in throat (x1.02) 0.949
Localization-related(focal)(partial)idiopathic epilepsy and epileptic syndromes with seizures of... (x1.02) 0.949
Estimation of glomerular filtration rate (+2.68) 0.950
Primary malignant neoplasm of border of tongue (x1.02) 0.950
Acute respiratory distress (x1.02) 0.950
Disorder of intestine (x1.01) 0.951
Expressions
10^5
10^3
10^1
−10 0 10
−0.8 −0.6 −0.4 −0.2 0.0 0.2 0.4 0.6 0.8

Signature [435]; 4 To Reach 0.975, (10 Plotted)

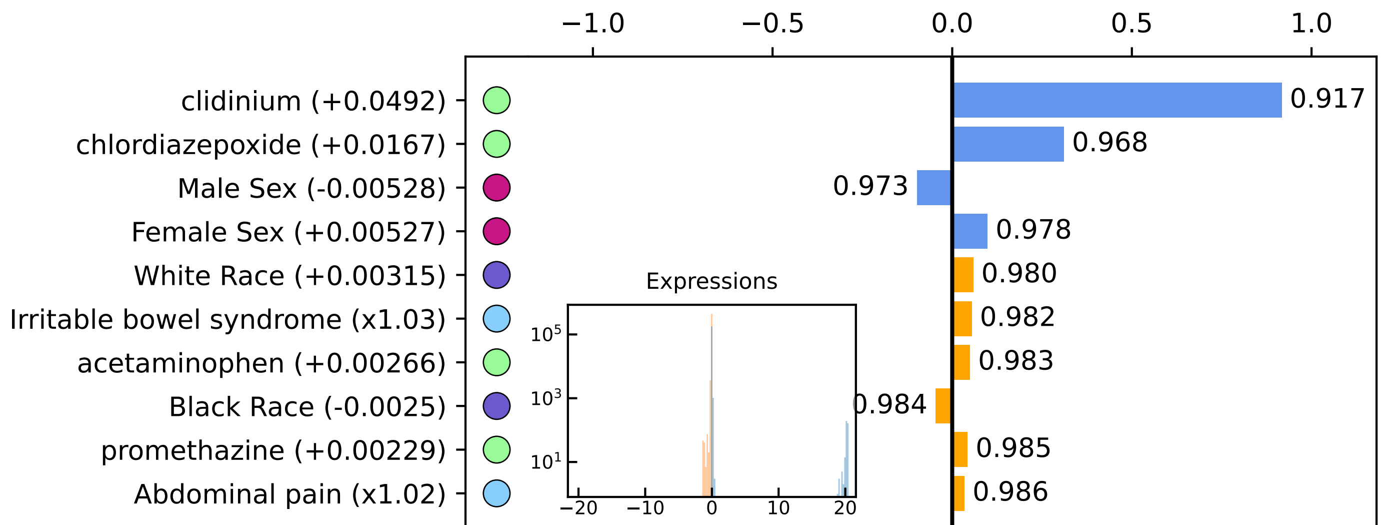

Signature [436]; 5 To Reach 0.975, (10 Plotted)

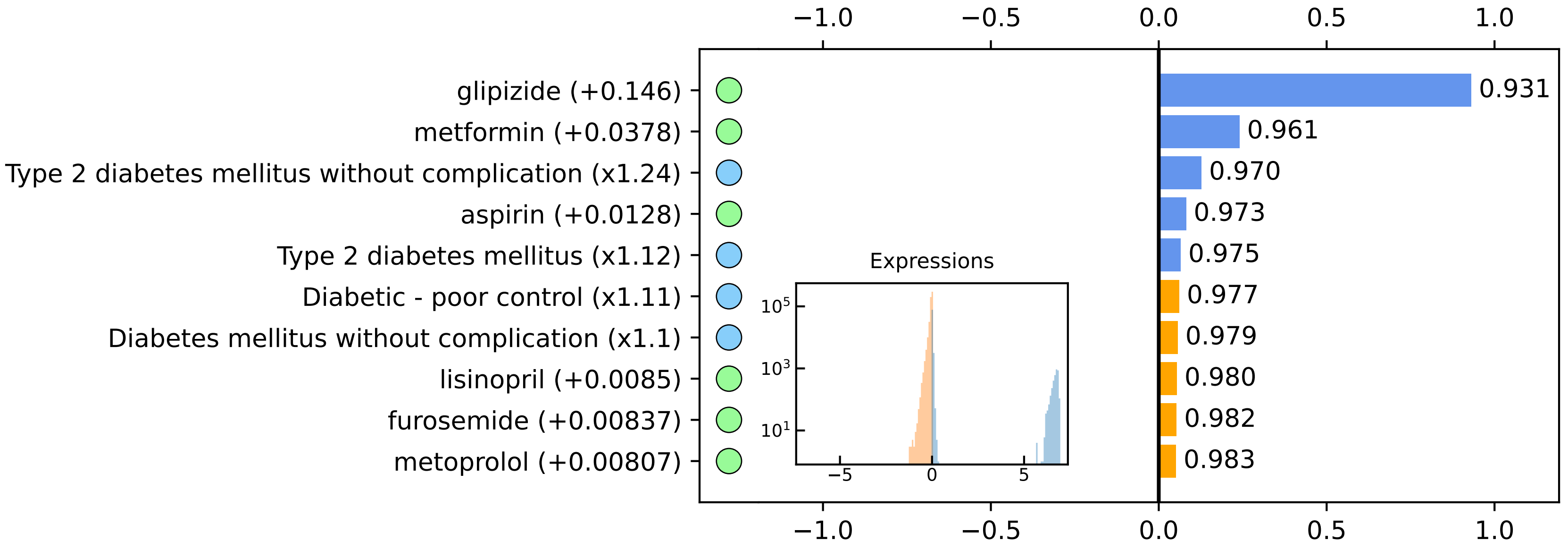

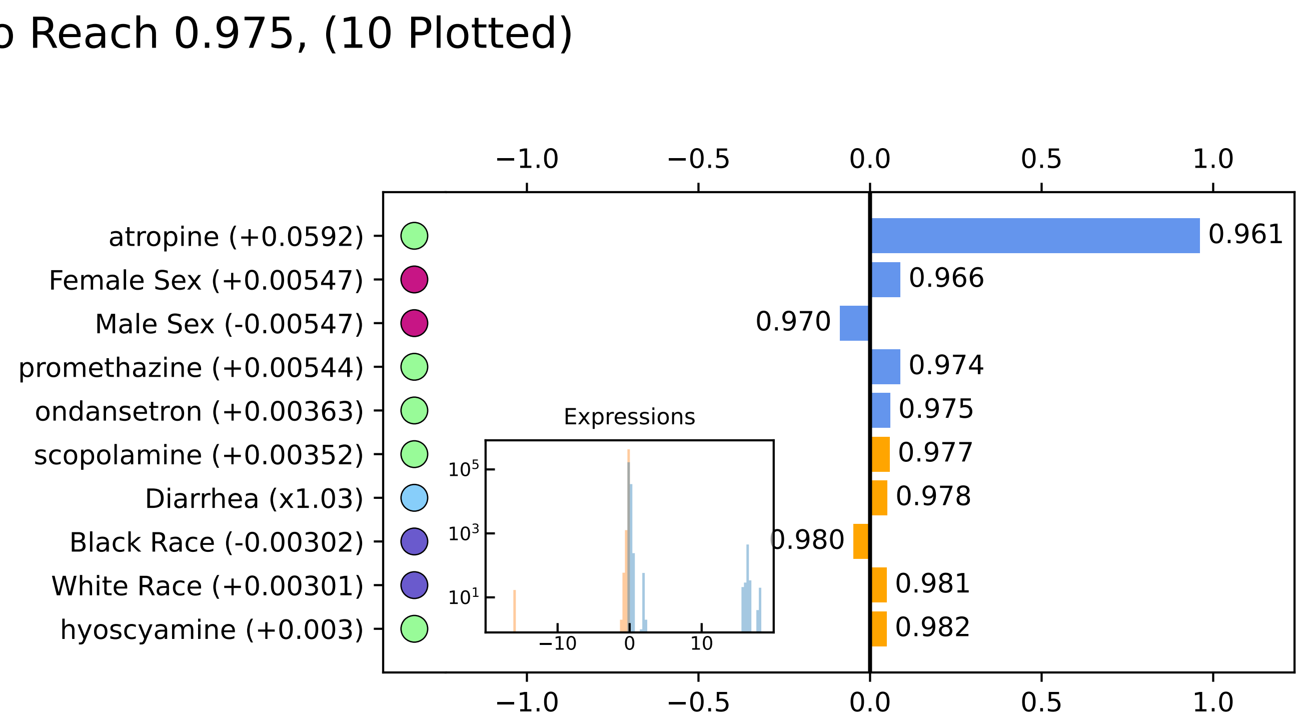
Signature [437]; 5 To Reach 0.975, (10 Plotted)
atropine (+0.0592)
Female Sex (+0.00547)
Male Sex (-0.00547)
promethazine (+0.00544)
ondansetron (+0.00363)
scopolamine (+0.00352)
Diarrhea (x1.03)
Black Race (-0.00302)
White Race (+0.00301)
hyoscyamine (+0.003)
0.961
0.966
0.970
0.974
0.975
0.977
0.978
0.980
0.981
0.982
−1.0
−0.5
0.0
0.5
1.0
Expressions
10^5
10^3
10^1
−10
0
10

Signature [438]; 3 To Reach 0.975, (10 Plotted)

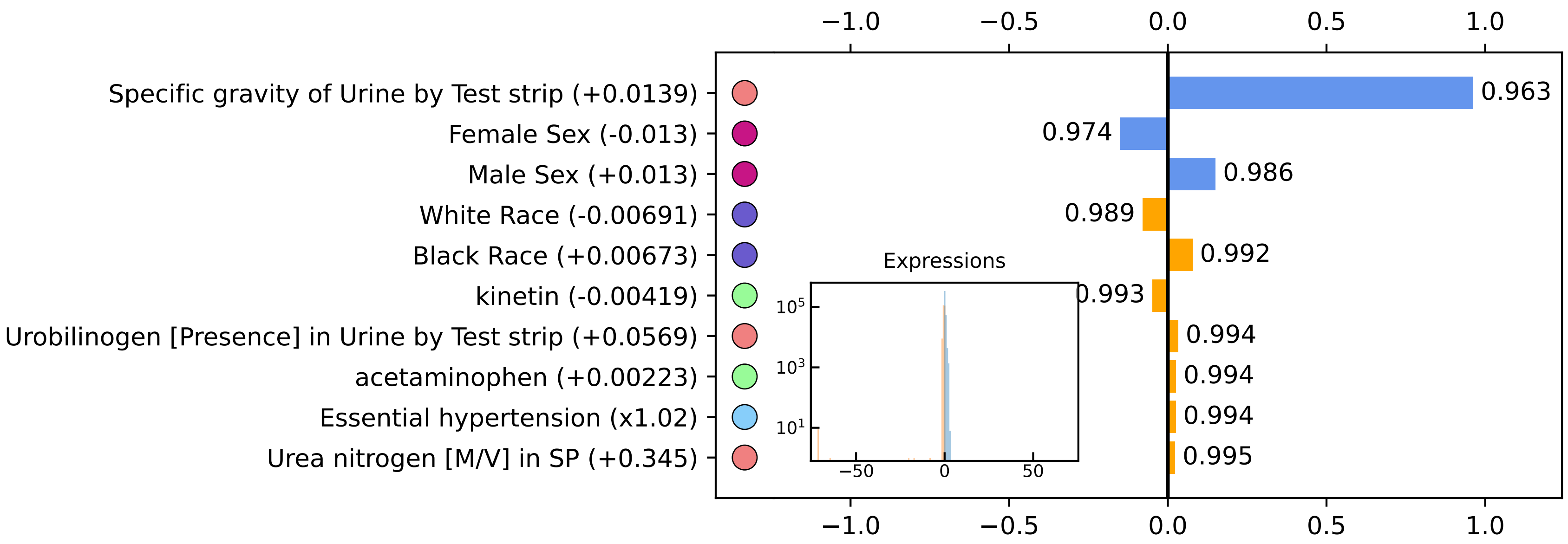

Signature [439]; 13 To Reach 0.975, (13 Plotted)

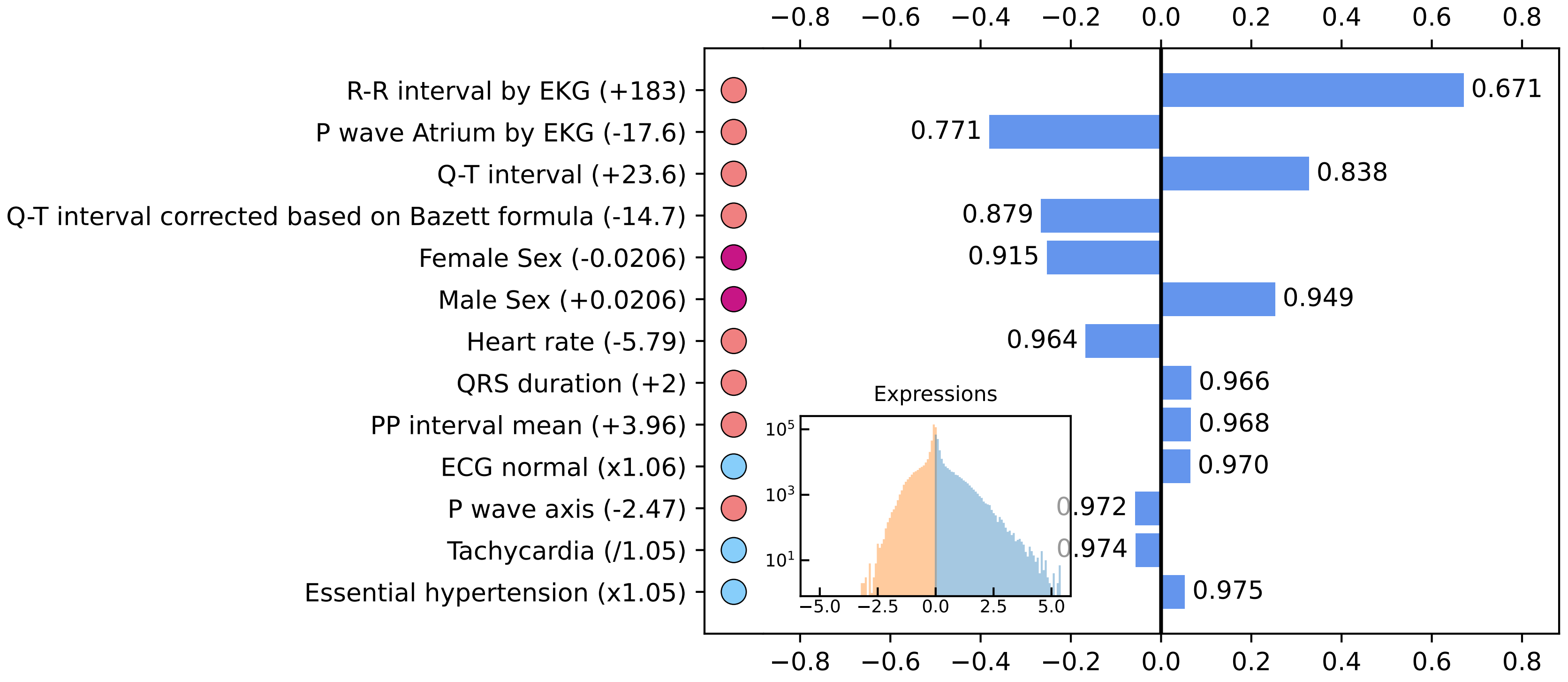

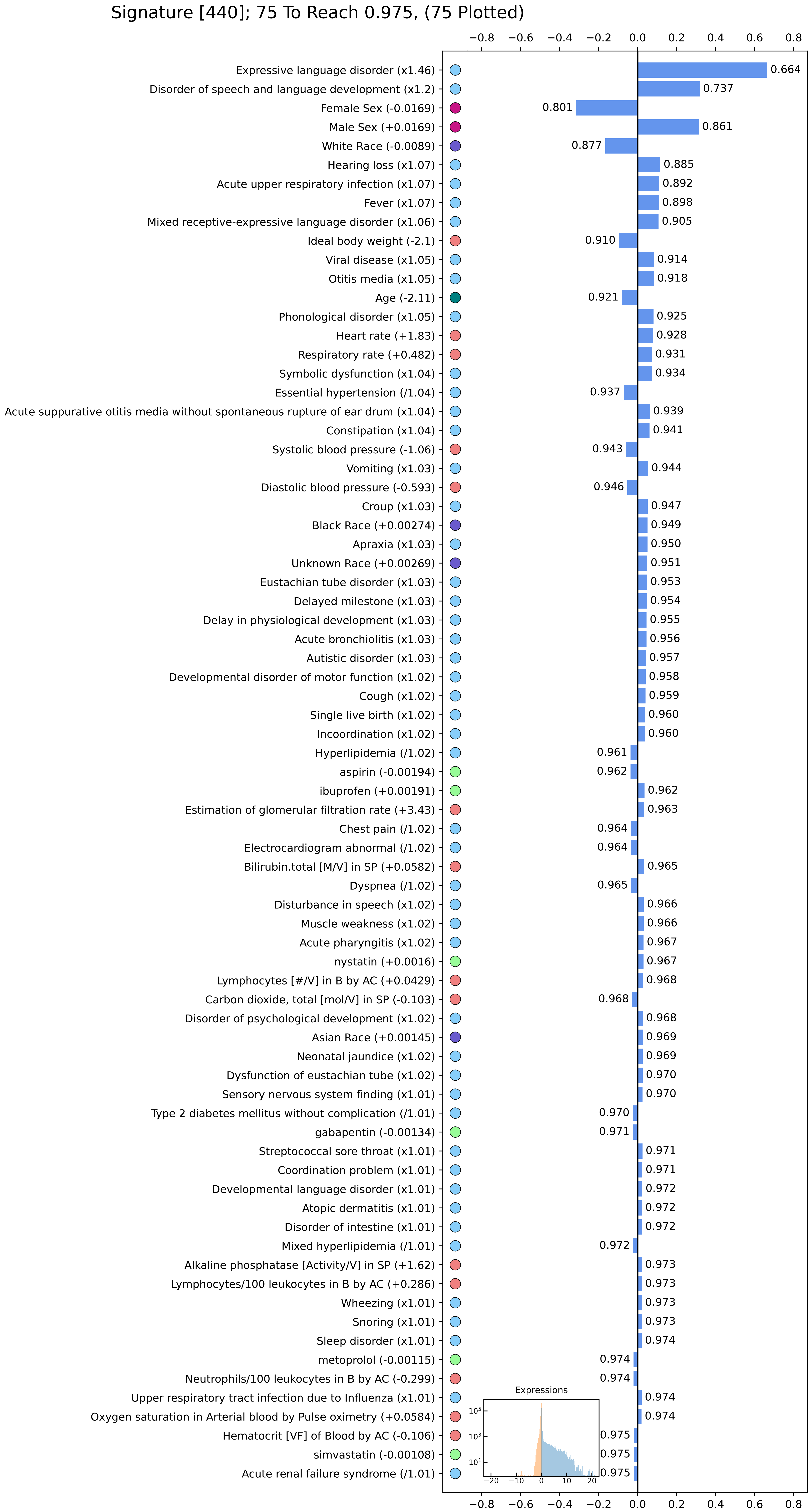
Signature [440]; 75 To Reach 0.975, (75 Plotted)
−0.8 −0.6 −0.4 −0.2 0.0 0.2 0.4 0.6 0.8
Expressive language disorder (x1.46) 0.664
Disorder of speech and language development (x1.2) 0.737
Female Sex (-0.0169) 0.801
Male Sex (+0.0169) 0.861
White Race (-0.0089) 0.877
Hearing loss (x1.07) 0.885
Acute upper respiratory infection (x1.07) 0.892
Fever (x1.07) 0.898
Mixed receptive-expressive language disorder (x1.06) 0.905
Ideal body weight (-2.1) 0.910
Viral disease (x1.05) 0.914
Otitis media (x1.05) 0.918
Age (-2.11) 0.921
Phonological disorder (x1.05) 0.925
Heart rate (+1.83) 0.928
Respiratory rate (+0.482) 0.931
Symbolic dysfunction (x1.04) 0.934
Essential hypertension (/1.04) 0.937
Acute suppurative otitis media without spontaneous rupture of ear drum (x1.04) 0.939
Constipation (x1.04) 0.941
Systolic blood pressure (-1.06) 0.943
Vomiting (x1.03) 0.944
Diastolic blood pressure (-0.593) 0.946
Croup (x1.03) 0.947
Black Race (+0.00274) 0.949
Apraxia (x1.03) 0.950
Unknown Race (+0.00269) 0.951
Eustachian tube disorder (x1.03) 0.953
Delayed milestone (x1.03) 0.954
Delay in physiological development (x1.03) 0.955
Acute bronchiolitis (x1.03) 0.956
Autistic disorder (x1.03) 0.957
Developmental disorder of motor function (x1.02) 0.958
Cough (x1.02) 0.959
Single live birth (x1.02) 0.960
Incoordination (x1.02) 0.960
Hyperlipidemia (/1.02) 0.961
aspirin (-0.00194) 0.962
ibuprofen (+0.00191) 0.962
Estimation of glomerular filtration rate (+3.43) 0.963
Chest pain (/1.02) 0.964
Electrocardiogram abnormal (/1.02) 0.964
Bilirubin.total [M/V] in SP (+0.0582) 0.965
Dyspnea (/1.02) 0.965
Disturbance in speech (x1.02) 0.966
Muscle weakness (x1.02) 0.966
Acute pharyngitis (x1.02) 0.967
nystatin (+0.0016) 0.967
Lymphocytes [#/V] in B by AC (+0.0429) 0.968
Carbon dioxide, total [mol/V] in SP (-0.103) 0.968
Disorder of psychological development (x1.02) 0.968
Asian Race (+0.00145) 0.969
Neonatal jaundice (x1.02) 0.969
Dysfunction of eustachian tube (x1.02) 0.970
Sensory nervous system finding (x1.01) 0.970
Type 2 diabetes mellitus without complication (/1.01) 0.970
gabapentin (-0.00134) 0.971
Streptococcal sore throat (x1.01) 0.971
Coordination problem (x1.01) 0.971
Developmental language disorder (x1.01) 0.972
Atopic dermatitis (x1.01) 0.972
Disorder of intestine (x1.01) 0.972
Mixed hyperlipidemia (/1.01) 0.972
Alkaline phosphatase [Activity/V] in SP (+1.62) 0.973
Lymphocytes/100 leukocytes in B by AC (+0.286) 0.973
Wheezing (x1.01) 0.973
Snoring (x1.01) 0.973
Sleep disorder (x1.01) 0.974
metoprolol (-0.00115) 0.974
Neutrophils/100 leukocytes in B by AC (-0.299) 0.974
Upper respiratory tract infection due to Influenza (x1.01) 0.974
Oxygen saturation in Arterial blood by Pulse oximetry (+0.0584) 0.974
Hematocrit [VF] of Blood by AC (-0.106) 0.975
simvastatin (-0.00108) 0.975
Acute renal failure syndrome (/1.01) 0.975
Expressions
10⁵
10³
10¹
−20 −10 0 10 20
−0.8 −0.6 −0.4 −0.2 0.0 0.2 0.4 0.6 0.8

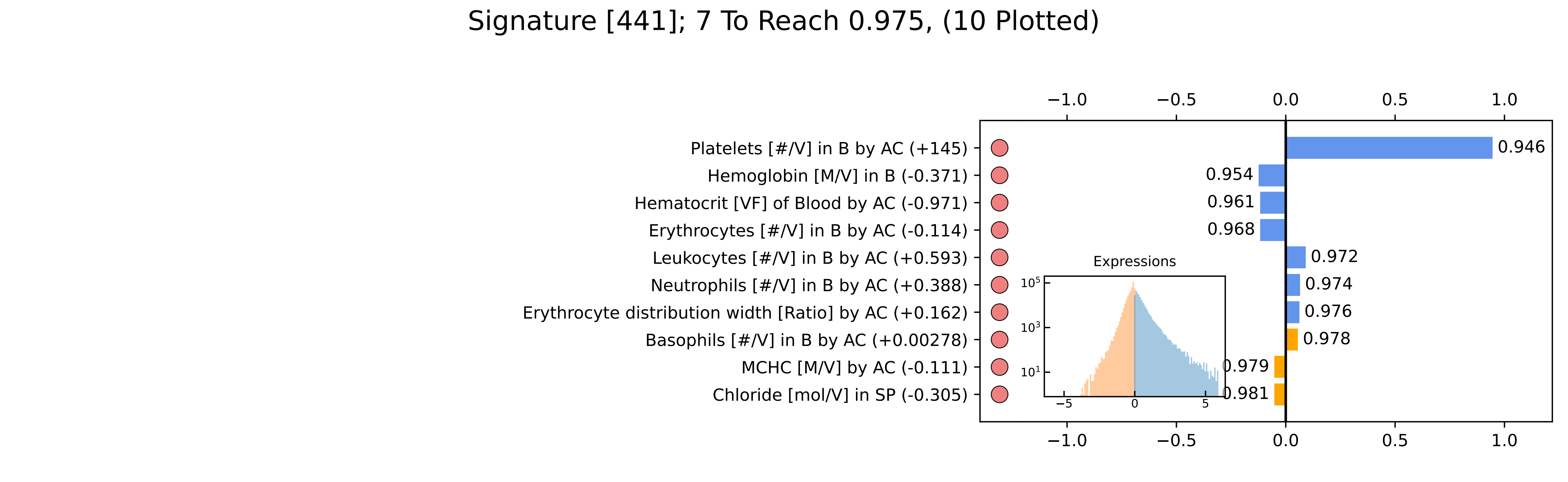
Signature [441]; 7 To Reach 0.975, (10 Plotted)
Platelets [#/V] in B by AC (+145)
Hemoglobin [M/V] in B (-0.371)
Hematocrit [VF] of Blood by AC (-0.971)
Erythrocytes [#/V] in B by AC (-0.114)
Leukocytes [#/V] in B by AC (+0.593)
Neutrophils [#/V] in B by AC (+0.388)
Erythrocyte distribution width [Ratio] by AC (+0.162)
Basophils [#/V] in B by AC (+0.00278)
MCHC [M/V] by AC (-0.111)
Chloride [mol/V] in SP (-0.305)
0.946
0.954
0.961
0.968
0.972
0.974
0.976
0.978
0.979
0.981
Expressions
−1.0
−0.5
0.0
0.5
1.0

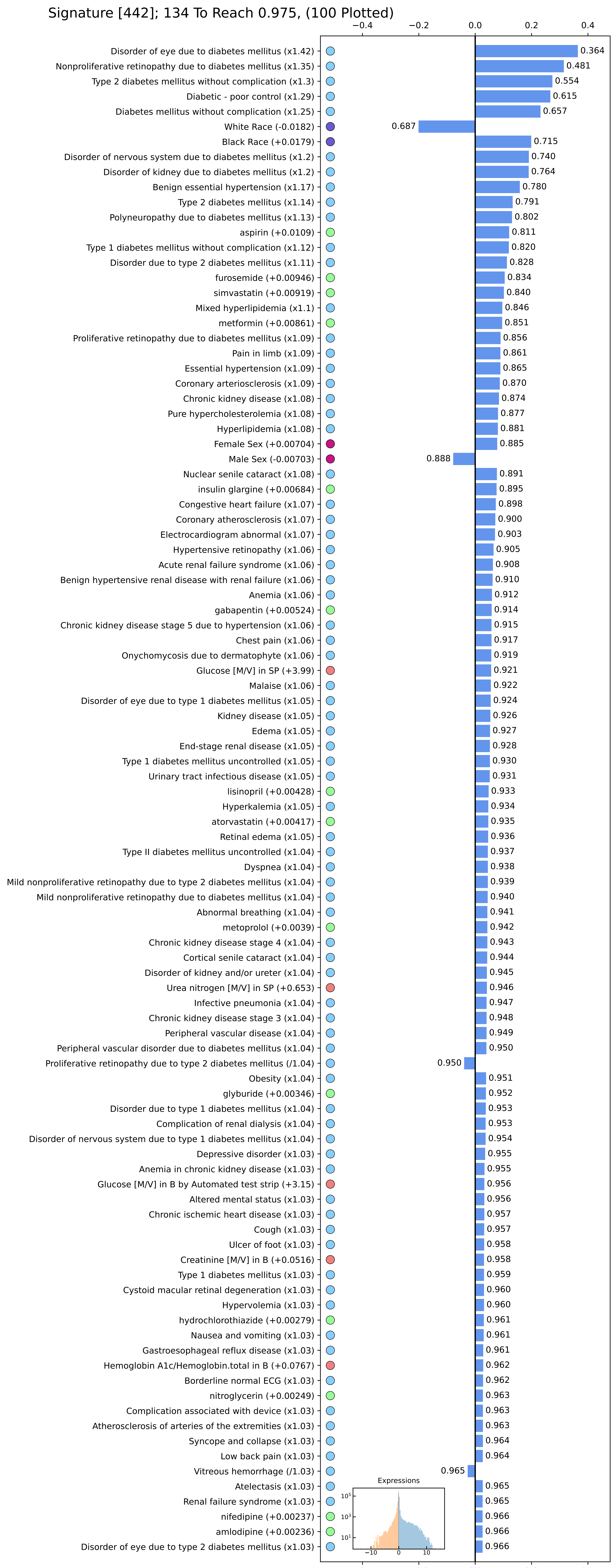

Signature [442]; 134 To Reach 0.975, (100 Plotted)
Disorder of eye due to diabetes mellitus (x1.42) 0.364
Nonproliferative retinopathy due to diabetes mellitus (x1.35) 0.481
Type 2 diabetes mellitus without complication (x1.3) 0.554
Diabetic - poor control (x1.29) 0.615
Diabetes mellitus without complication (x1.25) 0.657
White Race (-0.0182) 0.687
Black Race (+0.0179) 0.715
Disorder of nervous system due to diabetes mellitus (x1.2) 0.740
Disorder of kidney due to diabetes mellitus (x1.2) 0.764
Benign essential hypertension (x1.17) 0.780
Type 2 diabetes mellitus (x1.14) 0.791
Polyneuropathy due to diabetes mellitus (x1.13) 0.802
aspirin (+0.0109) 0.811
Type 1 diabetes mellitus without complication (x1.12) 0.820
Disorder due to type 2 diabetes mellitus (x1.11) 0.828
furosemide (+0.00946) 0.834
simvastatin (+0.00919) 0.840
Mixed hyperlipidemia (x1.1) 0.846
metformin (+0.00861) 0.851
Proliferative retinopathy due to diabetes mellitus (x1.09) 0.856
Pain in limb (x1.09) 0.861
Essential hypertension (x1.09) 0.865
Coronary arteriosclerosis (x1.09) 0.870
Chronic kidney disease (x1.08) 0.874
Pure hypercholesterolemia (x1.08) 0.877
Hyperlipidemia (x1.08) 0.881
Female Sex (+0.00704) 0.885
Male Sex (-0.00703) 0.888
Nuclear senile cataract (x1.08) 0.891
insulin glargine (+0.00684) 0.895
Congestive heart failure (x1.07) 0.898
Coronary atherosclerosis (x1.07) 0.900
Electrocardiogram abnormal (x1.07) 0.903
Hypertensive retinopathy (x1.06) 0.905
Acute renal failure syndrome (x1.06) 0.908
Benign hypertensive renal disease with renal failure (x1.06) 0.910
Anemia (x1.06) 0.912
gabapentin (+0.00524) 0.914
Chronic kidney disease stage 5 due to hypertension (x1.06) 0.915
Chest pain (x1.06) 0.917
Onychomycosis due to dermatophyte (x1.06) 0.919
Glucose [M/V] in SP (+3.99) 0.921
Malaise (x1.06) 0.922
Disorder of eye due to type 1 diabetes mellitus (x1.05) 0.924
Kidney disease (x1.05) 0.926
Edema (x1.05) 0.927
End-stage renal disease (x1.05) 0.928
Type 1 diabetes mellitus uncontrolled (x1.05) 0.930
Urinary tract infectious disease (x1.05) 0.931
lisinopril (+0.00428) 0.933
Hyperkalemia (x1.05) 0.934
atorvastatin (+0.00417) 0.935
Retinal edema (x1.05) 0.936
Type II diabetes mellitus uncontrolled (x1.04) 0.937
Dyspnea (x1.04) 0.938
Mild nonproliferative retinopathy due to type 2 diabetes mellitus (x1.04) 0.939
Mild nonproliferative retinopathy due to diabetes mellitus (x1.04) 0.940
Abnormal breathing (x1.04) 0.941
metoprolol (+0.0039) 0.942
Chronic kidney disease stage 4 (x1.04) 0.943
Cortical senile cataract (x1.04) 0.944
Disorder of kidney and/or ureter (x1.04) 0.945
Urea nitrogen [M/V] in SP (+0.653) 0.946
Infective pneumonia (x1.04) 0.947
Chronic kidney disease stage 3 (x1.04) 0.948
Peripheral vascular disease (x1.04) 0.949
Peripheral vascular disorder due to diabetes mellitus (x1.04) 0.950
Proliferative retinopathy due to type 2 diabetes mellitus (/1.04) 0.950
Obesity (x1.04) 0.951
glyburide (+0.00346) 0.952
Disorder due to type 1 diabetes mellitus (x1.04) 0.953
Complication of renal dialysis (x1.04) 0.953
Disorder of nervous system due to type 1 diabetes mellitus (x1.04) 0.954
Depressive disorder (x1.03) 0.955
Anemia in chronic kidney disease (x1.03) 0.955
Glucose [M/V] in B by Automated test strip (+3.15) 0.956
Altered mental status (x1.03) 0.956
Chronic ischemic heart disease (x1.03) 0.957
Cough (x1.03) 0.957
Ulcer of foot (x1.03) 0.958
Creatinine [M/V] in B (+0.0516) 0.958
Type 1 diabetes mellitus (x1.03) 0.959
Cystoid macular retinal degeneration (x1.03) 0.960
Hypervolemia (x1.03) 0.960
hydrochlorothiazide (+0.00279) 0.961
Nausea and vomiting (x1.03) 0.961
Gastroesophageal reflux disease (x1.03) 0.961
Hemoglobin A1c/Hemoglobin.total in B (+0.0767) 0.962
Borderline normal ECG (x1.03) 0.962
nitroglycerin (+0.00249) 0.963
Complication associated with device (x1.03) 0.963
Atherosclerosis of arteries of the extremities (x1.03) 0.963
Syncope and collapse (x1.03) 0.964
Low back pain (x1.03) 0.964
Vitreous hemorrhage (/1.03) 0.965
Atelectasis (x1.03) 0.965
Renal failure syndrome (x1.03) 0.965
nifedipine (+0.00237) 0.966
amlodipine (+0.00236) 0.966
Disorder of eye due to type 2 diabetes mellitus (x1.03) 0.966
Expressions
−0.4 −0.2 0.0 0.2 0.4

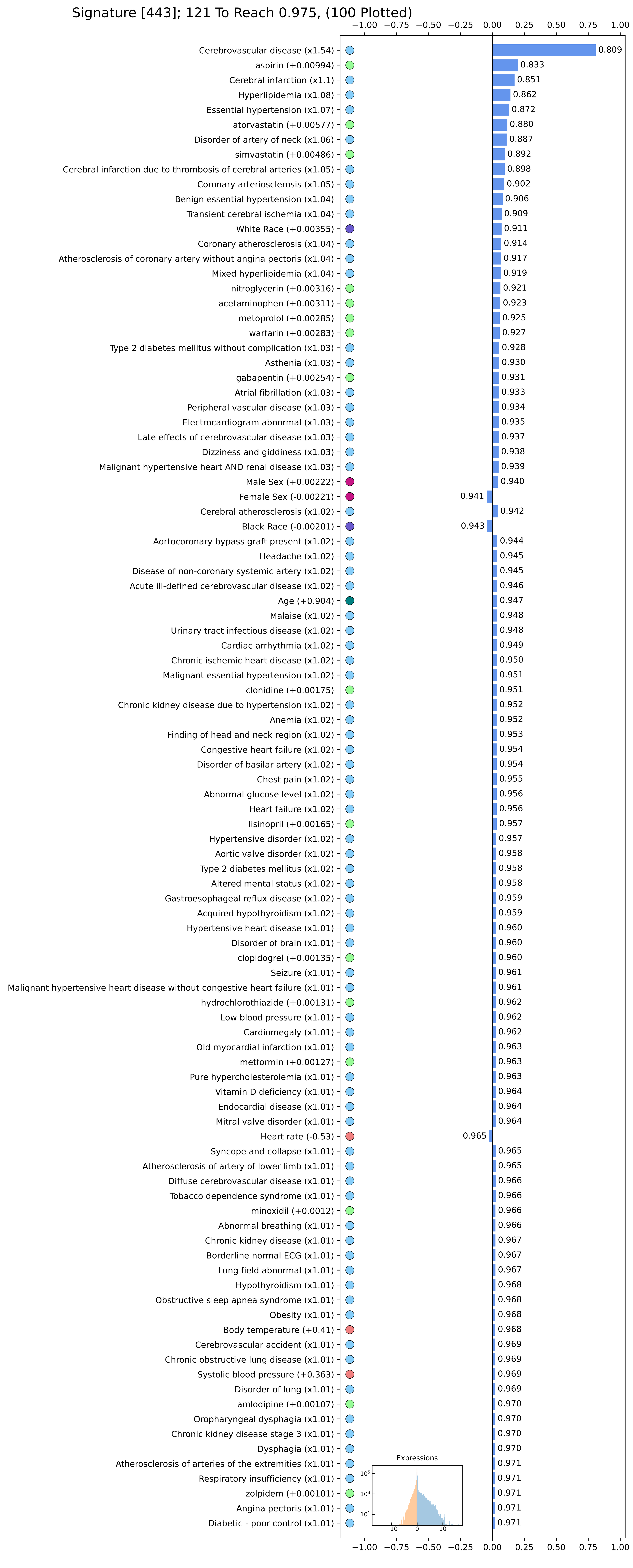

Signature [443]; 121 To Reach 0.975, (100 Plotted)
Cerebrovascular disease (x1.54) 0.809
aspirin (+0.00994) 0.833
Cerebral infarction (x1.1) 0.851
Hyperlipidemia (x1.08) 0.862
Essential hypertension (x1.07) 0.872
atorvastatin (+0.00577) 0.880
Disorder of artery of neck (x1.06) 0.887
simvastatin (+0.00486) 0.892
Cerebral infarction due to thrombosis of cerebral arteries (x1.05) 0.898
Coronary arteriosclerosis (x1.05) 0.902
Benign essential hypertension (x1.04) 0.906
Transient cerebral ischemia (x1.04) 0.909
White Race (+0.00355) 0.911
Coronary atherosclerosis (x1.04) 0.914
Atherosclerosis of coronary artery without angina pectoris (x1.04) 0.917
Mixed hyperlipidemia (x1.04) 0.919
nitroglycerin (+0.00316) 0.921
acetaminophen (+0.00311) 0.923
metoprolol (+0.00285) 0.925
warfarin (+0.00283) 0.927
Type 2 diabetes mellitus without complication (x1.03) 0.928
Asthenia (x1.03) 0.930
gabapentin (+0.00254) 0.931
Atrial fibrillation (x1.03) 0.933
Peripheral vascular disease (x1.03) 0.934
Electrocardiogram abnormal (x1.03) 0.935
Late effects of cerebrovascular disease (x1.03) 0.937
Dizziness and giddiness (x1.03) 0.938
Malignant hypertensive heart AND renal disease (x1.03) 0.939
Male Sex (+0.00222) 0.940
Female Sex (-0.00221) 0.941
Cerebral atherosclerosis (x1.02) 0.942
Black Race (-0.00201) 0.943
Aortocoronary bypass graft present (x1.02) 0.944
Headache (x1.02) 0.945
Disease of non-coronary systemic artery (x1.02) 0.945
Acute ill-defined cerebrovascular disease (x1.02) 0.946
Age (+0.904) 0.947
Malaise (x1.02) 0.948
Urinary tract infectious disease (x1.02) 0.948
Cardiac arrhythmia (x1.02) 0.949
Chronic ischemic heart disease (x1.02) 0.950
Malignant essential hypertension (x1.02) 0.951
clonidine (+0.00175) 0.951
Chronic kidney disease due to hypertension (x1.02) 0.952
Anemia (x1.02) 0.952
Finding of head and neck region (x1.02) 0.953
Congestive heart failure (x1.02) 0.954
Disorder of basilar artery (x1.02) 0.954
Chest pain (x1.02) 0.955
Abnormal glucose level (x1.02) 0.956
Heart failure (x1.02) 0.956
lisinopril (+0.00165) 0.957
Hypertensive disorder (x1.02) 0.957
Aortic valve disorder (x1.02) 0.958
Type 2 diabetes mellitus (x1.02) 0.958
Altered mental status (x1.02) 0.958
Gastroesophageal reflux disease (x1.02) 0.959
Acquired hypothyroidism (x1.02) 0.959
Hypertensive heart disease (x1.01) 0.960
Disorder of brain (x1.01) 0.960
clopidogrel (+0.00135) 0.960
Seizure (x1.01) 0.961
Malignant hypertensive heart disease without congestive heart failure (x1.01) 0.961
hydrochlorothiazide (+0.00131) 0.962
Low blood pressure (x1.01) 0.962
Cardiomegaly (x1.01) 0.962
Old myocardial infarction (x1.01) 0.963
metformin (+0.00127) 0.963
Pure hypercholesterolemia (x1.01) 0.963
Vitamin D deficiency (x1.01) 0.964
Endocardial disease (x1.01) 0.964
Mitral valve disorder (x1.01) 0.964
Heart rate (-0.53) 0.965
Syncope and collapse (x1.01) 0.965
Atherosclerosis of artery of lower limb (x1.01) 0.965
Diffuse cerebrovascular disease (x1.01) 0.966
Tobacco dependence syndrome (x1.01) 0.966
minoxidil (+0.0012) 0.966
Abnormal breathing (x1.01) 0.966
Chronic kidney disease (x1.01) 0.967
Borderline normal ECG (x1.01) 0.967
Lung field abnormal (x1.01) 0.967
Hypothyroidism (x1.01) 0.968
Obstructive sleep apnea syndrome (x1.01) 0.968
Obesity (x1.01) 0.968
Body temperature (+0.41) 0.968
Cerebrovascular accident (x1.01) 0.969
Chronic obstructive lung disease (x1.01) 0.969
Systolic blood pressure (+0.363) 0.969
Disorder of lung (x1.01) 0.969
amlodipine (+0.00107) 0.970
Oropharyngeal dysphagia (x1.01) 0.970
Chronic kidney disease stage 3 (x1.01) 0.970
Dysphagia (x1.01) 0.970
Atherosclerosis of arteries of the extremities (x1.01) 0.971
Respiratory insufficiency (x1.01) 0.971
zolpidem (+0.00101) 0.971
Angina pectoris (x1.01) 0.971
Diabetic - poor control (x1.01) 0.971
−1.00 −0.75 −0.50 −0.25 0.00 0.25 0.50 0.75 1.00
Expressions

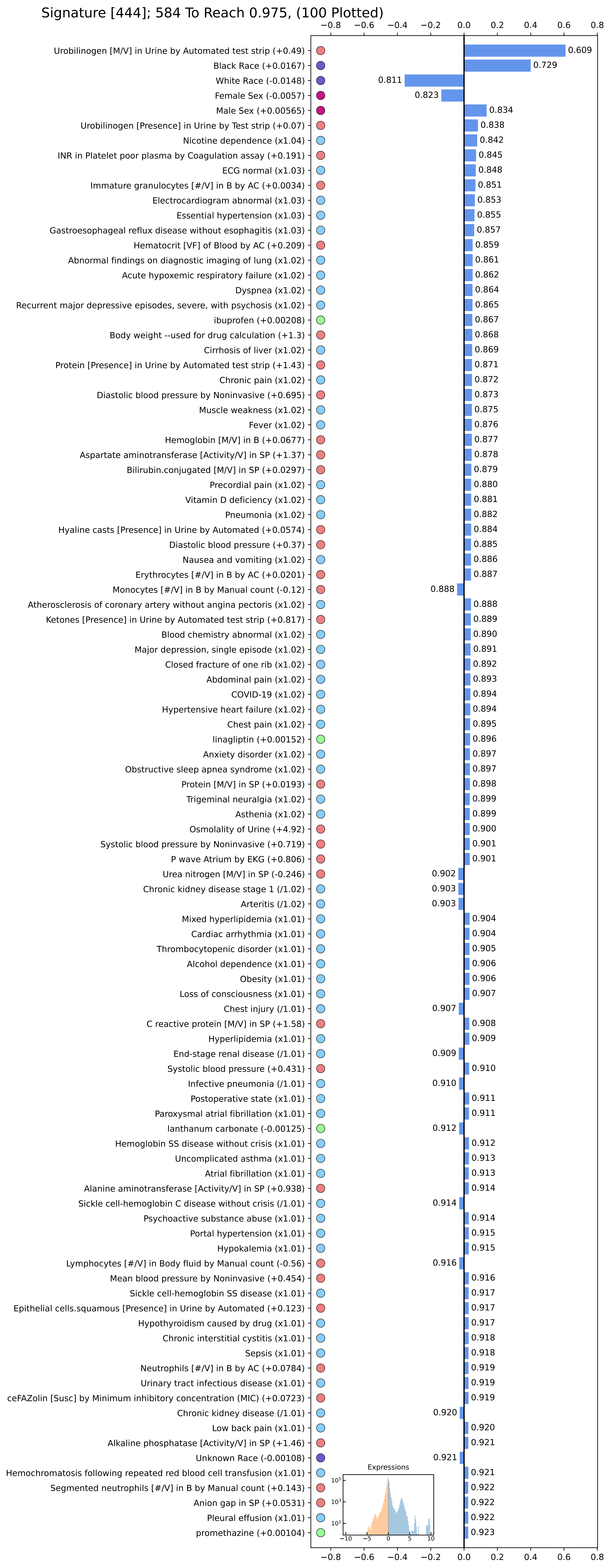

Signature [444]; 584 To Reach 0.975, (100 Plotted)
Urobilinogen [M/V] in Urine by Automated test strip (+0.49) 0.609
Black Race (+0.0167) 0.729
White Race (-0.0148) 0.811
Female Sex (-0.0057) 0.823
Male Sex (+0.00565) 0.834
Urobilinogen [Presence] in Urine by Test strip (+0.07) 0.838
Nicotine dependence (x1.04) 0.842
INR in Platelet poor plasma by Coagulation assay (+0.191) 0.845
ECG normal (x1.03) 0.848
Immature granulocytes [#/V] in B by AC (+0.0034) 0.851
Electrocardiogram abnormal (x1.03) 0.853
Essential hypertension (x1.03) 0.855
Gastroesophageal reflux disease without esophagitis (x1.03) 0.857
Hematocrit [VF] of Blood by AC (+0.209) 0.859
Abnormal findings on diagnostic imaging of lung (x1.02) 0.861
Acute hypoxemic respiratory failure (x1.02) 0.862
Dyspnea (x1.02) 0.864
Recurrent major depressive episodes, severe, with psychosis (x1.02) 0.865
ibuprofen (+0.00208) 0.867
Body weight --used for drug calculation (+1.3) 0.868
Cirrhosis of liver (x1.02) 0.869
Protein [Presence] in Urine by Automated test strip (+1.43) 0.871
Chronic pain (x1.02) 0.872
Diastolic blood pressure by Noninvasive (+0.695) 0.873
Muscle weakness (x1.02) 0.875
Fever (x1.02) 0.876
Hemoglobin [M/V] in B (+0.0677) 0.877
Aspartate aminotransferase [Activity/V] in SP (+1.37) 0.878
Bilirubin.conjugated [M/V] in SP (+0.0297) 0.879
Precordial pain (x1.02) 0.880
Vitamin D deficiency (x1.02) 0.881
Pneumonia (x1.02) 0.882
Hyaline casts [Presence] in Urine by Automated (+0.0574) 0.884
Diastolic blood pressure (+0.37) 0.885
Nausea and vomiting (x1.02) 0.886
Erythrocytes [#/V] in B by AC (+0.0201) 0.887
Monocytes [#/V] in B by Manual count (-0.12) 0.888
Atherosclerosis of coronary artery without angina pectoris (x1.02) 0.888
Ketones [Presence] in Urine by Automated test strip (+0.817) 0.889
Blood chemistry abnormal (x1.02) 0.890
Major depression, single episode (x1.02) 0.891
Closed fracture of one rib (x1.02) 0.892
Abdominal pain (x1.02) 0.893
COVID-19 (x1.02) 0.894
Hypertensive heart failure (x1.02) 0.894
Chest pain (x1.02) 0.895
linagliptin (+0.00152) 0.896
Anxiety disorder (x1.02) 0.897
Obstructive sleep apnea syndrome (x1.02) 0.897
Protein [M/V] in SP (+0.0193) 0.898
Trigeminal neuralgia (x1.02) 0.899
Asthenia (x1.02) 0.899
Osmolality of Urine (+4.92) 0.900
Systolic blood pressure by Noninvasive (+0.719) 0.901
P wave Atrium by EKG (+0.806) 0.901
Urea nitrogen [M/V] in SP (-0.246) 0.902
Chronic kidney disease stage 1 (/1.02) 0.903
Arteritis (/1.02) 0.903
Mixed hyperlipidemia (x1.01) 0.904
Cardiac arrhythmia (x1.01) 0.904
Thrombocytopenic disorder (x1.01) 0.905
Alcohol dependence (x1.01) 0.906
Obesity (x1.01) 0.906
Loss of consciousness (x1.01) 0.907
Chest injury (/1.01) 0.907
C reactive protein [M/V] in SP (+1.58) 0.908
Hyperlipidemia (x1.01) 0.909
End-stage renal disease (/1.01) 0.909
Systolic blood pressure (+0.431) 0.910
Infective pneumonia (/1.01) 0.910
Postoperative state (x1.01) 0.911
Paroxysmal atrial fibrillation (x1.01) 0.911
lanthanum carbonate (-0.00125) 0.912
Hemoglobin SS disease without crisis (x1.01) 0.912
Uncomplicated asthma (x1.01) 0.913
Atrial fibrillation (x1.01) 0.913
Alanine aminotransferase [Activity/V] in SP (+0.938) 0.914
Sickle cell-hemoglobin C disease without crisis (/1.01) 0.914
Psychoactive substance abuse (x1.01) 0.914
Portal hypertension (x1.01) 0.915
Hypokalemia (x1.01) 0.915
Lymphocytes [#/V] in Body fluid by Manual count (-0.56) 0.916
Mean blood pressure by Noninvasive (+0.454) 0.916
Sickle cell-hemoglobin SS disease (x1.01) 0.917
Epithelial cells.squamous [Presence] in Urine by Automated (+0.123) 0.917
Hypothyroidism caused by drug (x1.01) 0.917
Chronic interstitial cystitis (x1.01) 0.918
Sepsis (x1.01) 0.918
Neutrophils [#/V] in B by AC (+0.0784) 0.919
Urinary tract infectious disease (x1.01) 0.919
ceFAZolin [Susc] by Minimum inhibitory concentration (MIC) (+0.0723) 0.919
Chronic kidney disease (/1.01) 0.920
Low back pain (x1.01) 0.920
Alkaline phosphatase [Activity/V] in SP (+1.46) 0.921
Unknown Race (-0.00108) 0.921
Hemochromatosis following repeated red blood cell transfusion (x1.01) 0.921
Segmented neutrophils [#/V] in B by Manual count (+0.143) 0.922
Anion gap in SP (+0.0531) 0.922
Pleural effusion (x1.01) 0.922
promethazine (+0.00104) 0.923
Expressions
−0.8 −0.6 −0.4 −0.2 0.0 0.2 0.4 0.6 0.8

Signature [445]; 1 To Reach 0.975, (10 Plotted)

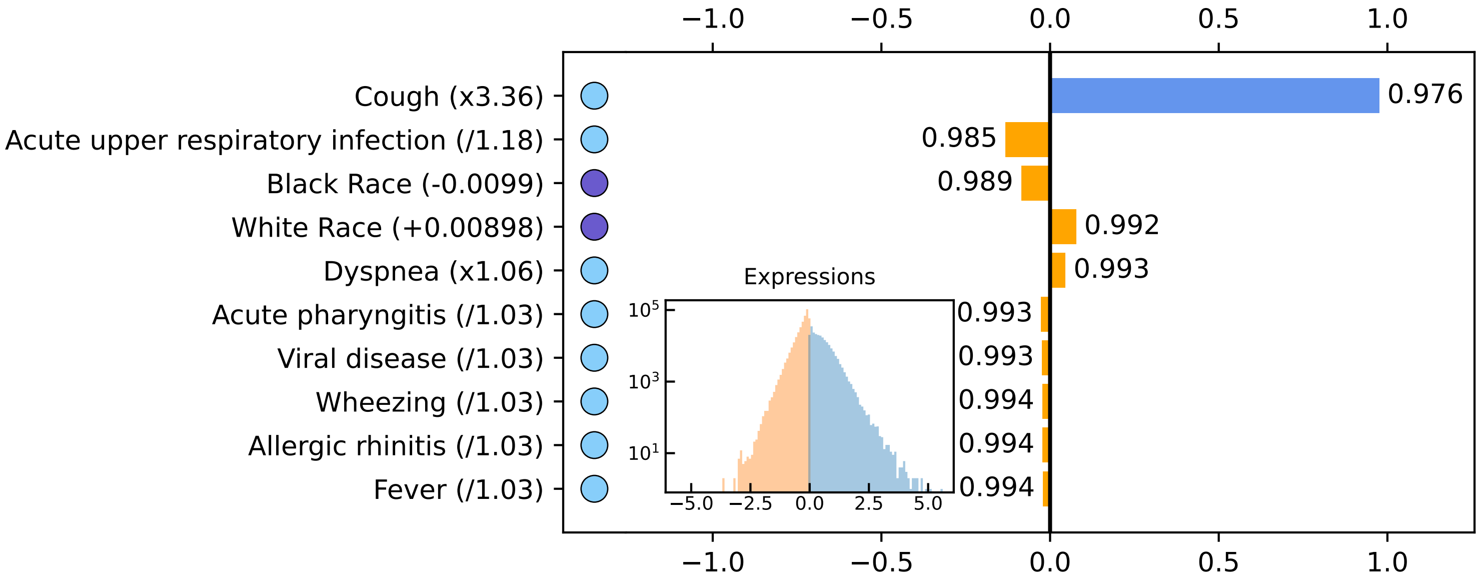

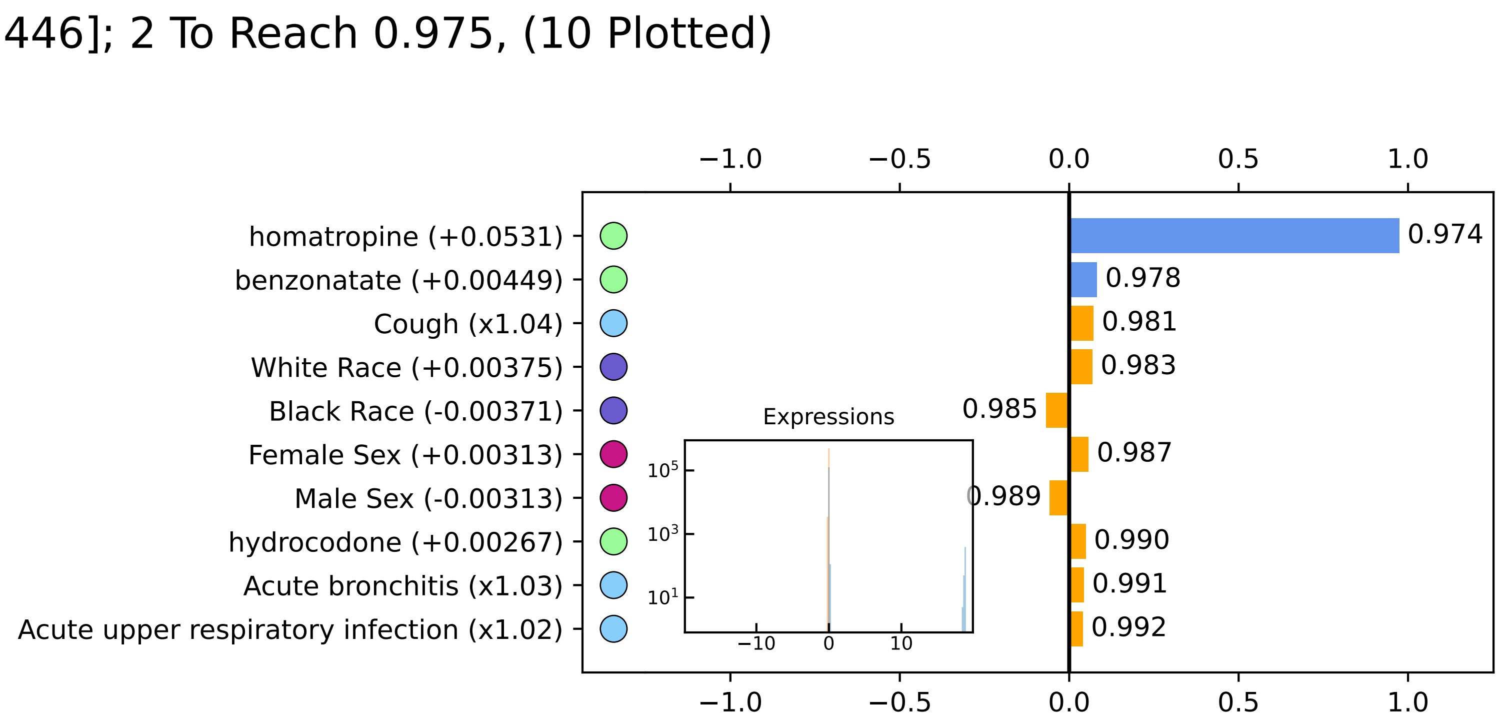

Signature [446]; 2 To Reach 0.975, (10 Plotted)
homatropine (+0.0531)
benzonatate (+0.00449)
Cough (x1.04)
White Race (+0.00375)
Black Race (-0.00371)
Female Sex (+0.00313)
Male Sex (-0.00313)
hydrocodone (+0.00267)
Acute bronchitis (x1.03)
Acute upper respiratory infection (x1.02)
0.974
0.978
0.981
0.983
0.985
0.987
0.989
0.990
0.991
0.992
Expressions
−1.0
−0.5
0.0
0.5
1.0

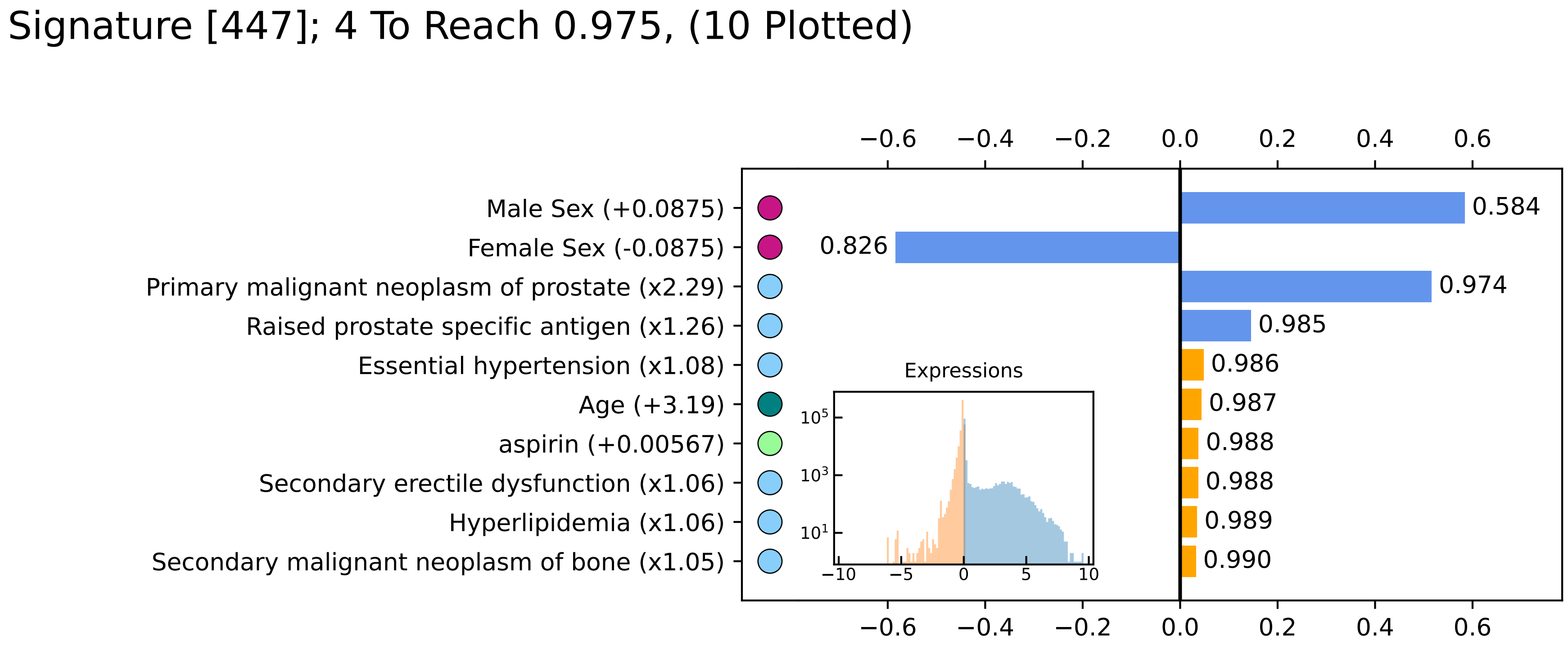

Signature [447]; 4 To Reach 0.975, (10 Plotted)
Male Sex (+0.0875)
Female Sex (-0.0875)
Primary malignant neoplasm of prostate (x2.29)
Raised prostate specific antigen (x1.26)
Essential hypertension (x1.08)
Age (+3.19)
aspirin (+0.00567)
Secondary erectile dysfunction (x1.06)
Hyperlipidemia (x1.06)
Secondary malignant neoplasm of bone (x1.05)
0.584
0.826
0.974
0.985
0.986
0.987
0.988
0.988
0.989
0.990
−0.6
−0.4
−0.2
0.0
0.2
0.4
0.6
Expressions
−10
−5
0
5
10

Signature [448]; 3 To Reach 0.975, (10 Plotted)

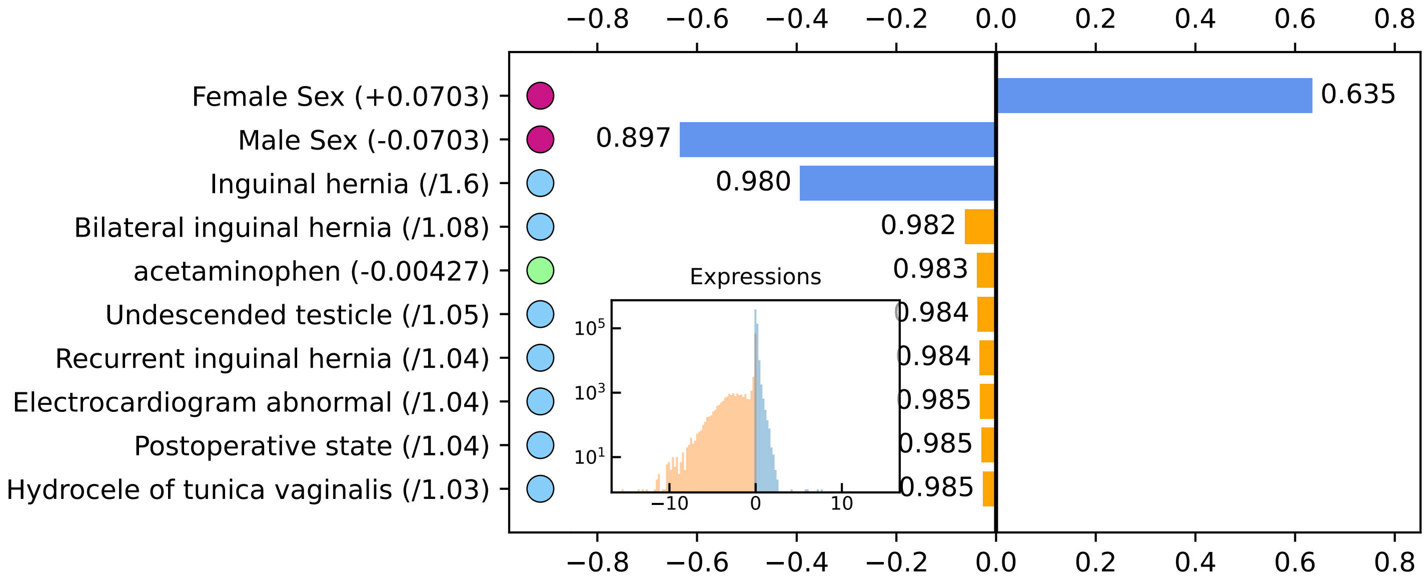

Signature [449]; 2 To Reach 0.975, (10 Plotted)

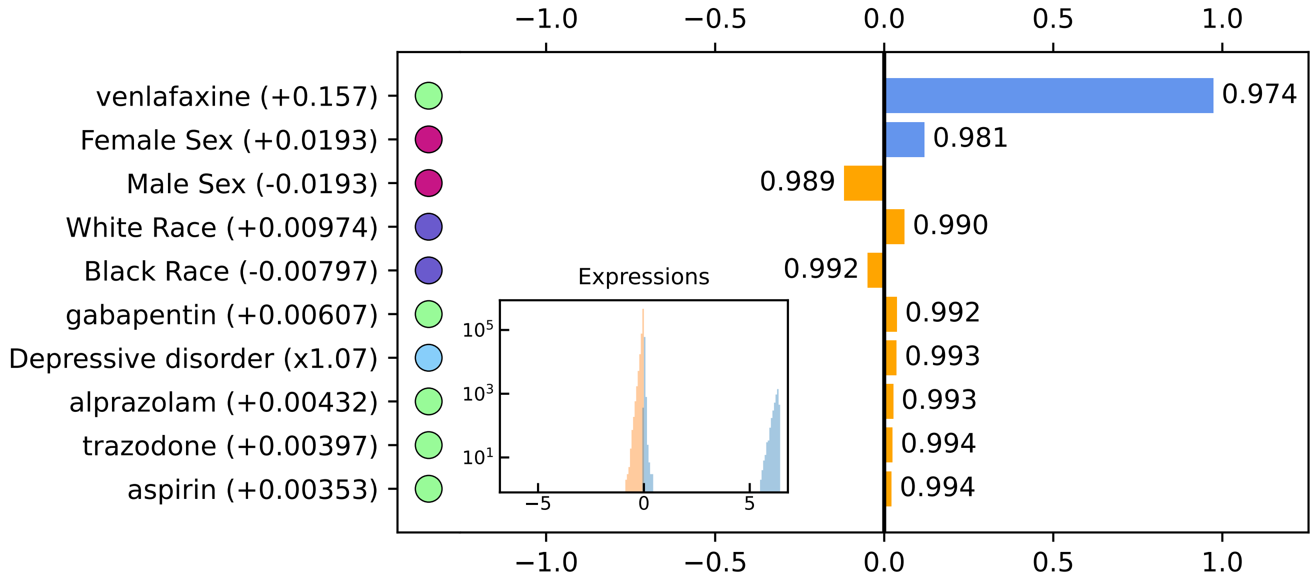

Signature [450]; 3 To Reach 0.975, (10 Plotted)

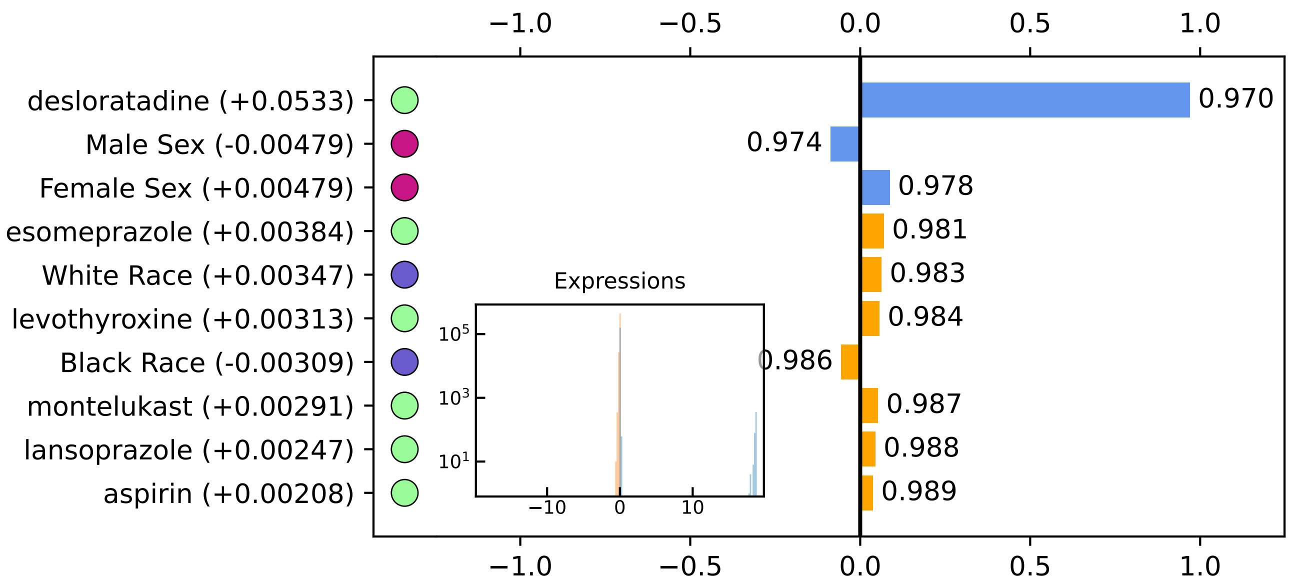

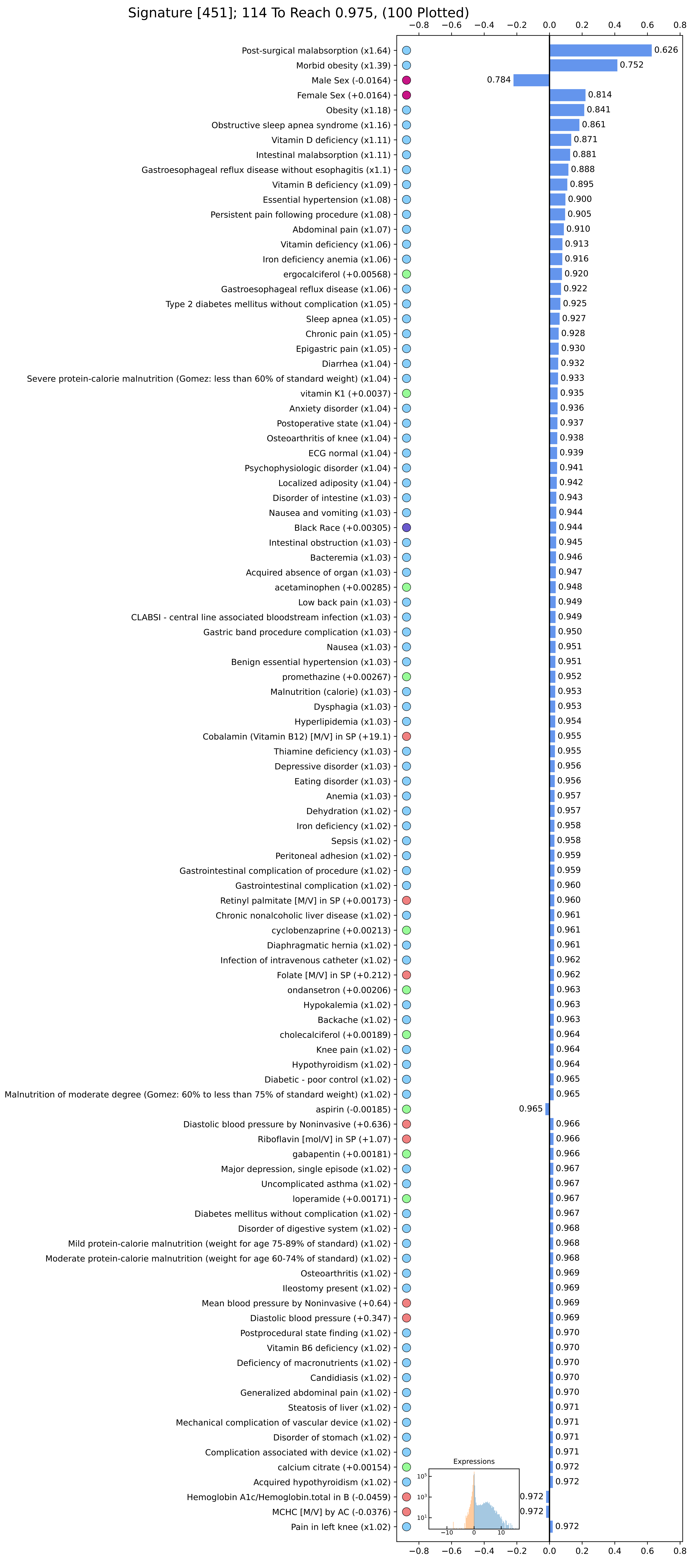

Signature [451]; 114 To Reach 0.975, (100 Plotted)
Post-surgical malabsorption (x1.64) 0.626
Morbid obesity (x1.39) 0.752
Male Sex (-0.0164) 0.784
Female Sex (+0.0164) 0.814
Obesity (x1.18) 0.841
Obstructive sleep apnea syndrome (x1.16) 0.861
Vitamin D deficiency (x1.11) 0.871
Intestinal malabsorption (x1.11) 0.881
Gastroesophageal reflux disease without esophagitis (x1.1) 0.888
Vitamin B deficiency (x1.09) 0.895
Essential hypertension (x1.08) 0.900
Persistent pain following procedure (x1.08) 0.905
Abdominal pain (x1.07) 0.910
Vitamin deficiency (x1.06) 0.913
Iron deficiency anemia (x1.06) 0.916
ergocalciferol (+0.00568) 0.920
Gastroesophageal reflux disease (x1.06) 0.922
Type 2 diabetes mellitus without complication (x1.05) 0.925
Sleep apnea (x1.05) 0.927
Chronic pain (x1.05) 0.928
Epigastric pain (x1.05) 0.930
Diarrhea (x1.04) 0.932
Severe protein-calorie malnutrition (Gomez: less than 60% of standard weight) (x1.04) 0.933
vitamin K1 (+0.0037) 0.935
Anxiety disorder (x1.04) 0.936
Postoperative state (x1.04) 0.937
Osteoarthritis of knee (x1.04) 0.938
ECG normal (x1.04) 0.939
Psychophysiologic disorder (x1.04) 0.941
Localized adiposity (x1.04) 0.942
Disorder of intestine (x1.03) 0.943
Nausea and vomiting (x1.03) 0.944
Black Race (+0.00305) 0.944
Intestinal obstruction (x1.03) 0.945
Bacteremia (x1.03) 0.946
Acquired absence of organ (x1.03) 0.947
acetaminophen (+0.00285) 0.948
Low back pain (x1.03) 0.949
CLABSI - central line associated bloodstream infection (x1.03) 0.949
Gastric band procedure complication (x1.03) 0.950
Nausea (x1.03) 0.951
Benign essential hypertension (x1.03) 0.951
promethazine (+0.00267) 0.952
Malnutrition (calorie) (x1.03) 0.953
Dysphagia (x1.03) 0.953
Hyperlipidemia (x1.03) 0.954
Cobalamin (Vitamin B12) [M/V] in SP (+19.1) 0.955
Thiamine deficiency (x1.03) 0.955
Depressive disorder (x1.03) 0.956
Eating disorder (x1.03) 0.956
Anemia (x1.03) 0.957
Dehydration (x1.02) 0.957
Iron deficiency (x1.02) 0.958
Sepsis (x1.02) 0.958
Peritoneal adhesion (x1.02) 0.959
Gastrointestinal complication of procedure (x1.02) 0.959
Gastrointestinal complication (x1.02) 0.960
Retinyl palmitate [M/V] in SP (+0.00173) 0.960
Chronic nonalcoholic liver disease (x1.02) 0.961
cyclobenzaprine (+0.00213) 0.961
Diaphragmatic hernia (x1.02) 0.961
Infection of intravenous catheter (x1.02) 0.962
Folate [M/V] in SP (+0.212) 0.962
ondansetron (+0.00206) 0.963
Hypokalemia (x1.02) 0.963
Backache (x1.02) 0.963
cholecalciferol (+0.00189) 0.964
Knee pain (x1.02) 0.964
Hypothyroidism (x1.02) 0.964
Diabetic - poor control (x1.02) 0.965
Malnutrition of moderate degree (Gomez: 60% to less than 75% of standard weight) (x1.02) 0.965
aspirin (-0.00185) 0.965
Diastolic blood pressure by Noninvasive (+0.636) 0.966
Riboflavin [mol/V] in SP (+1.07) 0.966
gabapentin (+0.00181) 0.966
Major depression, single episode (x1.02) 0.967
Uncomplicated asthma (x1.02) 0.967
loperamide (+0.00171) 0.967
Diabetes mellitus without complication (x1.02) 0.967
Disorder of digestive system (x1.02) 0.968
Mild protein-calorie malnutrition (weight for age 75-89% of standard) (x1.02) 0.968
Moderate protein-calorie malnutrition (weight for age 60-74% of standard) (x1.02) 0.968
Osteoarthritis (x1.02) 0.969
Ileostomy present (x1.02) 0.969
Mean blood pressure by Noninvasive (+0.64) 0.969
Diastolic blood pressure (+0.347) 0.969
Postprocedural state finding (x1.02) 0.970
Vitamin B6 deficiency (x1.02) 0.970
Deficiency of macronutrients (x1.02) 0.970
Candidiasis (x1.02) 0.970
Generalized abdominal pain (x1.02) 0.970
Steatosis of liver (x1.02) 0.971
Mechanical complication of vascular device (x1.02) 0.971
Disorder of stomach (x1.02) 0.971
Complication associated with device (x1.02) 0.971
calcium citrate (+0.00154) 0.972
Acquired hypothyroidism (x1.02) 0.972
Hemoglobin A1c/Hemoglobin.total in B (-0.0459) 0.972
MCHC [M/V] by AC (-0.0376) 0.972
Pain in left knee (x1.02) 0.972
Expressions
−0.8 −0.6 −0.4 −0.2 0.0 0.2 0.4 0.6 0.8

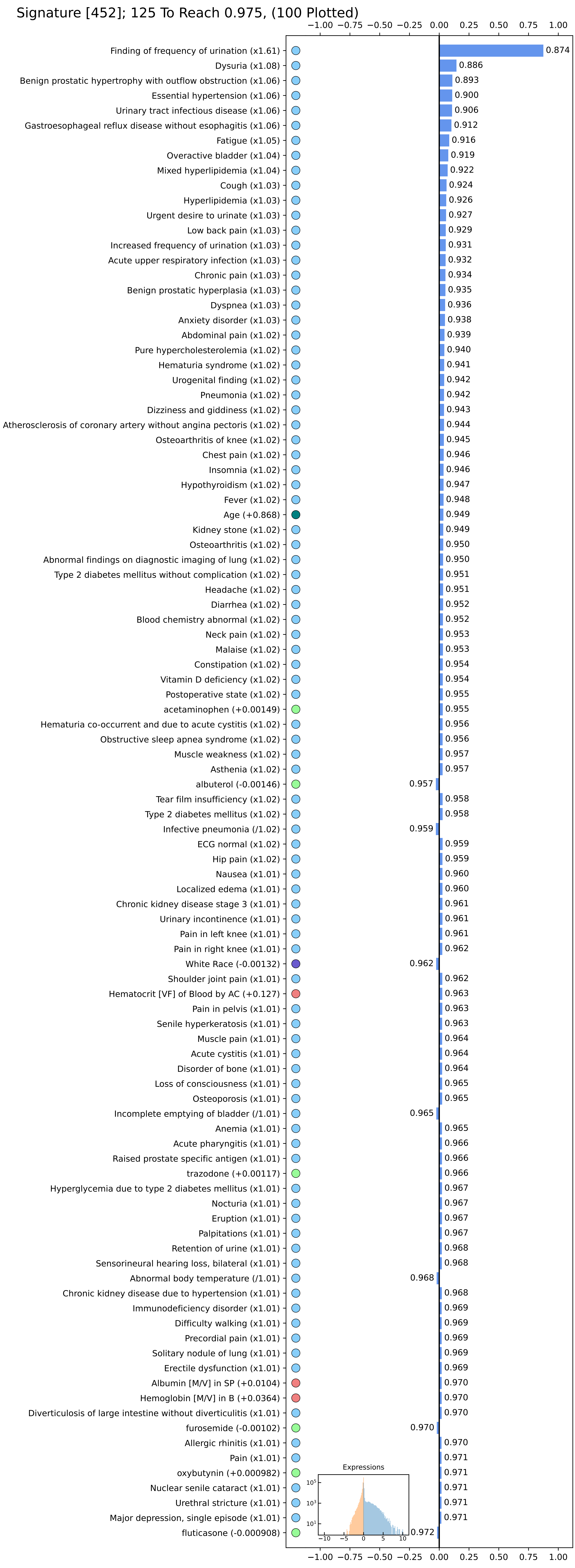

Signature [452]; 125 To Reach 0.975, (100 Plotted)
Finding of frequency of urination (x1.61) 0.874
Dysuria (x1.08) 0.886
Benign prostatic hypertrophy with outflow obstruction (x1.06) 0.893
Essential hypertension (x1.06) 0.900
Urinary tract infectious disease (x1.06) 0.906
Gastroesophageal reflux disease without esophagitis (x1.06) 0.912
Fatigue (x1.05) 0.916
Overactive bladder (x1.04) 0.919
Mixed hyperlipidemia (x1.04) 0.922
Cough (x1.03) 0.924
Hyperlipidemia (x1.03) 0.926
Urgent desire to urinate (x1.03) 0.927
Low back pain (x1.03) 0.929
Increased frequency of urination (x1.03) 0.931
Acute upper respiratory infection (x1.03) 0.932
Chronic pain (x1.03) 0.934
Benign prostatic hyperplasia (x1.03) 0.935
Dyspnea (x1.03) 0.936
Anxiety disorder (x1.03) 0.938
Abdominal pain (x1.02) 0.939
Pure hypercholesterolemia (x1.02) 0.940
Hematuria syndrome (x1.02) 0.941
Urogenital finding (x1.02) 0.942
Pneumonia (x1.02) 0.942
Dizziness and giddiness (x1.02) 0.943
Atherosclerosis of coronary artery without angina pectoris (x1.02) 0.944
Osteoarthritis of knee (x1.02) 0.945
Chest pain (x1.02) 0.946
Insomnia (x1.02) 0.946
Hypothyroidism (x1.02) 0.947
Fever (x1.02) 0.948
Age (+0.868) 0.949
Kidney stone (x1.02) 0.949
Osteoarthritis (x1.02) 0.950
Abnormal findings on diagnostic imaging of lung (x1.02) 0.950
Type 2 diabetes mellitus without complication (x1.02) 0.951
Headache (x1.02) 0.951
Diarrhea (x1.02) 0.952
Blood chemistry abnormal (x1.02) 0.952
Neck pain (x1.02) 0.953
Malaise (x1.02) 0.953
Constipation (x1.02) 0.954
Vitamin D deficiency (x1.02) 0.954
Postoperative state (x1.02) 0.955
acetaminophen (+0.00149) 0.955
Hematuria co-occurrent and due to acute cystitis (x1.02) 0.956
Obstructive sleep apnea syndrome (x1.02) 0.956
Muscle weakness (x1.02) 0.957
Asthenia (x1.02) 0.957
albuterol (-0.00146) 0.957
Tear film insufficiency (x1.02) 0.958
Type 2 diabetes mellitus (x1.02) 0.958
Infective pneumonia (/1.02) 0.959
ECG normal (x1.02) 0.959
Hip pain (x1.02) 0.959
Nausea (x1.01) 0.960
Localized edema (x1.01) 0.960
Chronic kidney disease stage 3 (x1.01) 0.961
Urinary incontinence (x1.01) 0.961
Pain in left knee (x1.01) 0.961
Pain in right knee (x1.01) 0.962
White Race (-0.00132) 0.962
Shoulder joint pain (x1.01) 0.962
Hematocrit [VF] of Blood by AC (+0.127) 0.963
Pain in pelvis (x1.01) 0.963
Senile hyperkeratosis (x1.01) 0.963
Muscle pain (x1.01) 0.964
Acute cystitis (x1.01) 0.964
Disorder of bone (x1.01) 0.964
Loss of consciousness (x1.01) 0.965
Osteoporosis (x1.01) 0.965
Incomplete emptying of bladder (/1.01) 0.965
Anemia (x1.01) 0.965
Acute pharyngitis (x1.01) 0.966
Raised prostate specific antigen (x1.01) 0.966
trazodone (+0.00117) 0.966
Hyperglycemia due to type 2 diabetes mellitus (x1.01) 0.967
Nocturia (x1.01) 0.967
Eruption (x1.01) 0.967
Palpitations (x1.01) 0.967
Retention of urine (x1.01) 0.968
Sensorineural hearing loss, bilateral (x1.01) 0.968
Abnormal body temperature (/1.01) 0.968
Chronic kidney disease due to hypertension (x1.01) 0.968
Immunodeficiency disorder (x1.01) 0.969
Difficulty walking (x1.01) 0.969
Precordial pain (x1.01) 0.969
Solitary nodule of lung (x1.01) 0.969
Erectile dysfunction (x1.01) 0.969
Albumin [M/V] in SP (+0.0104) 0.970
Hemoglobin [M/V] in B (+0.0364) 0.970
Diverticulosis of large intestine without diverticulitis (x1.01) 0.970
furosemide (-0.00102) 0.970
Allergic rhinitis (x1.01) 0.970
Pain (x1.01) 0.971
oxybutynin (+0.000982) 0.971
Nuclear senile cataract (x1.01) 0.971
Urethral stricture (x1.01) 0.971
Major depression, single episode (x1.01) 0.971
fluticasone (-0.000908) 0.972
Expressions
−1.00 −0.75 −0.50 −0.25 0.00 0.25 0.50 0.75 1.00

Signature [453]; 64 To Reach 0.975, (64 Plotted)

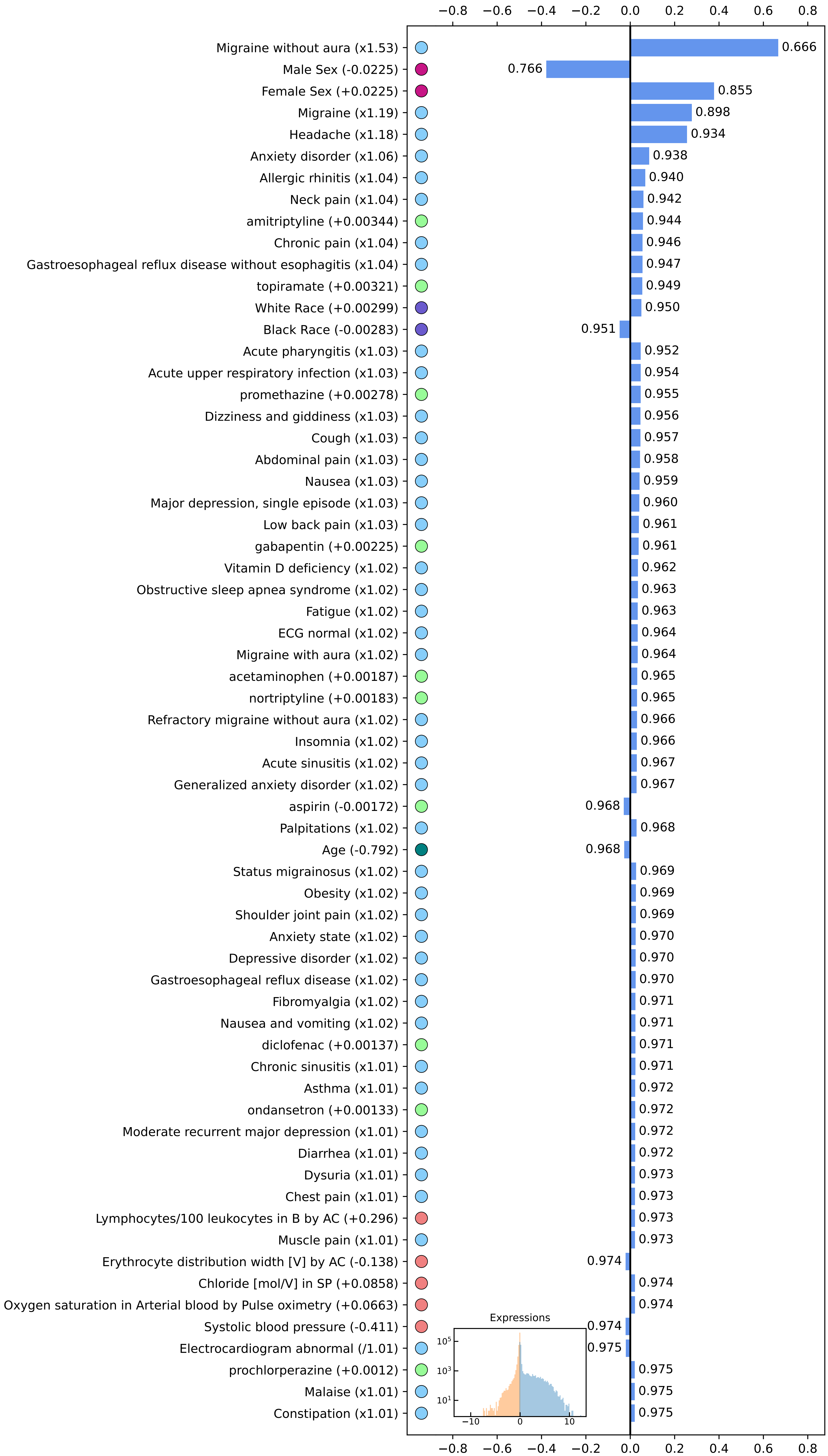

Signature [454]; 8 To Reach 0.975, (10 Plotted)

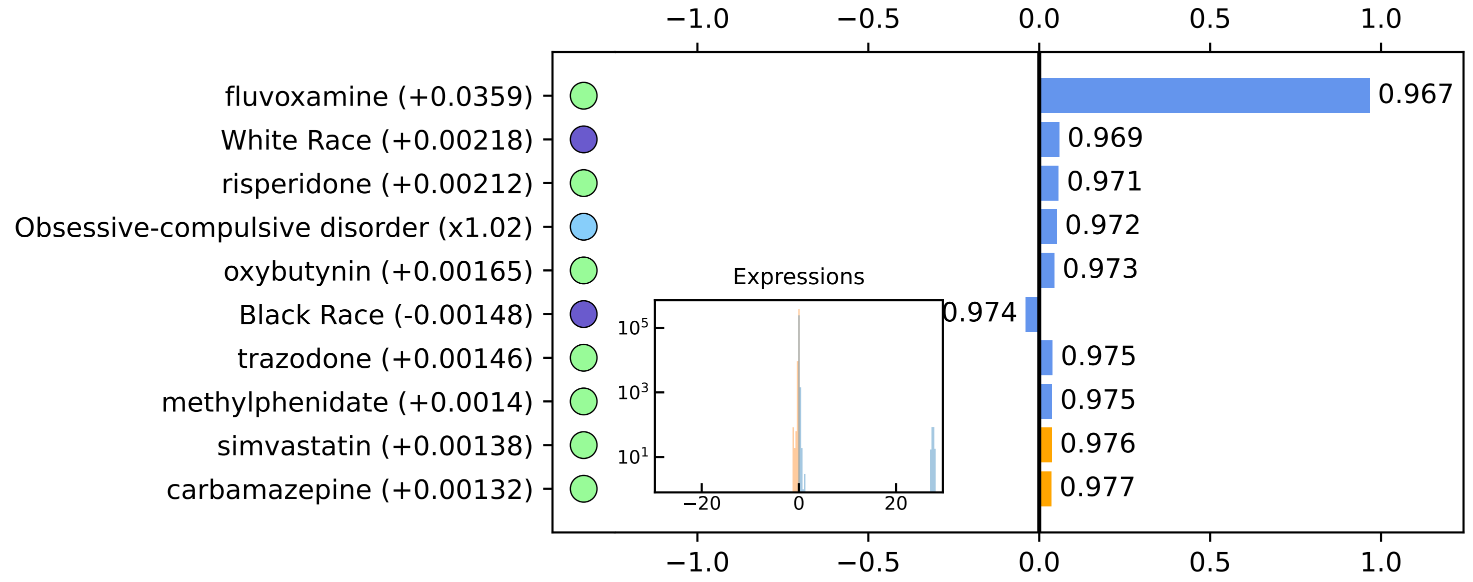

Signature [455]; 1 To Reach 0.975, (10 Plotted)

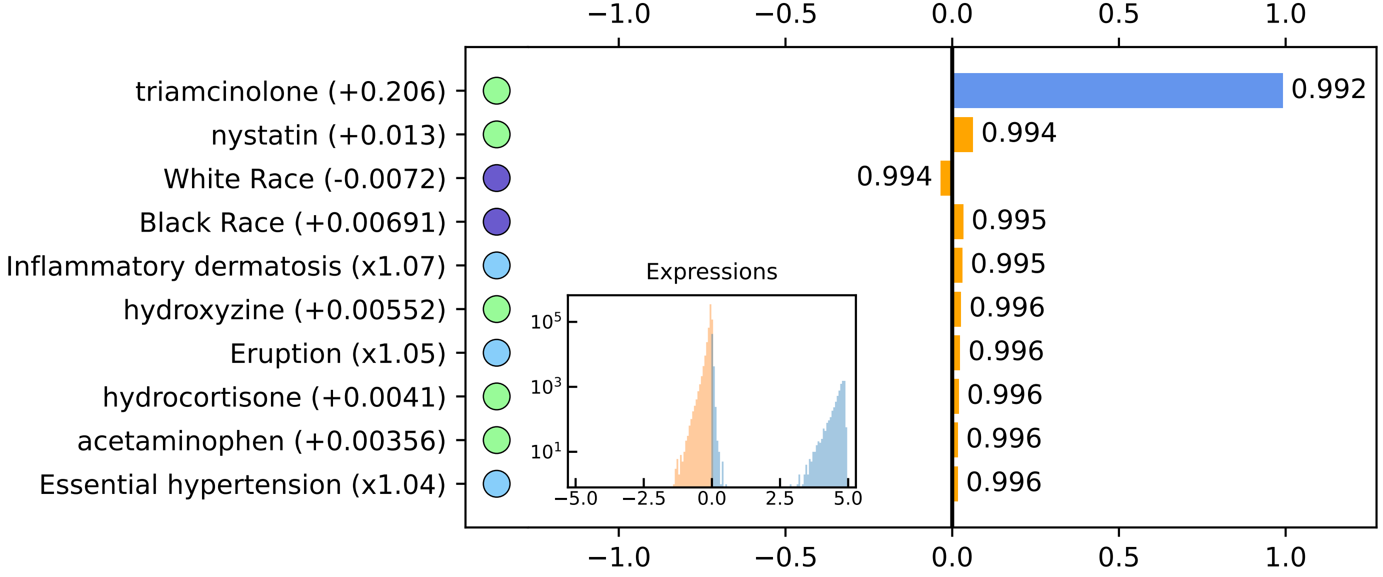

Signature [456]; 86 To Reach 0.975, (86 Plotted)

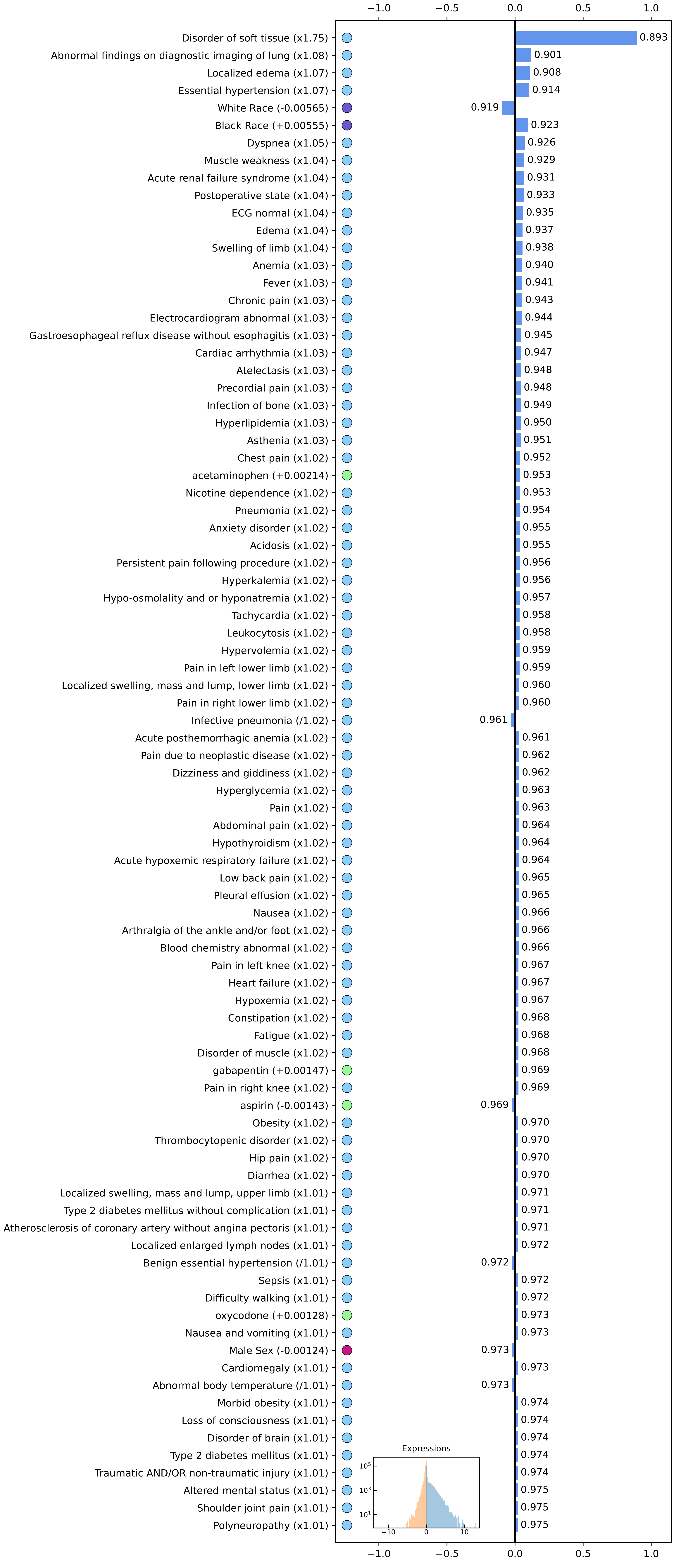

Signature [457]; 158 To Reach 0.975, (100 Plotted)

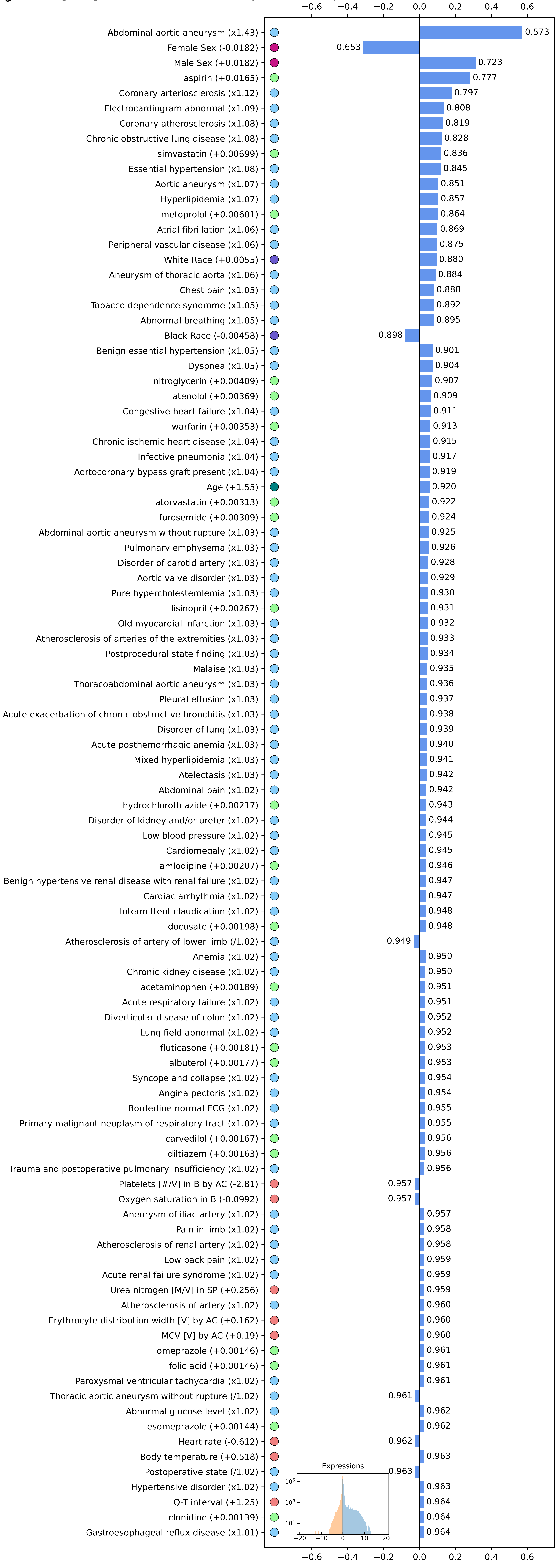

Signature [458]; 144 To Reach 0.975, (100 Plotted)

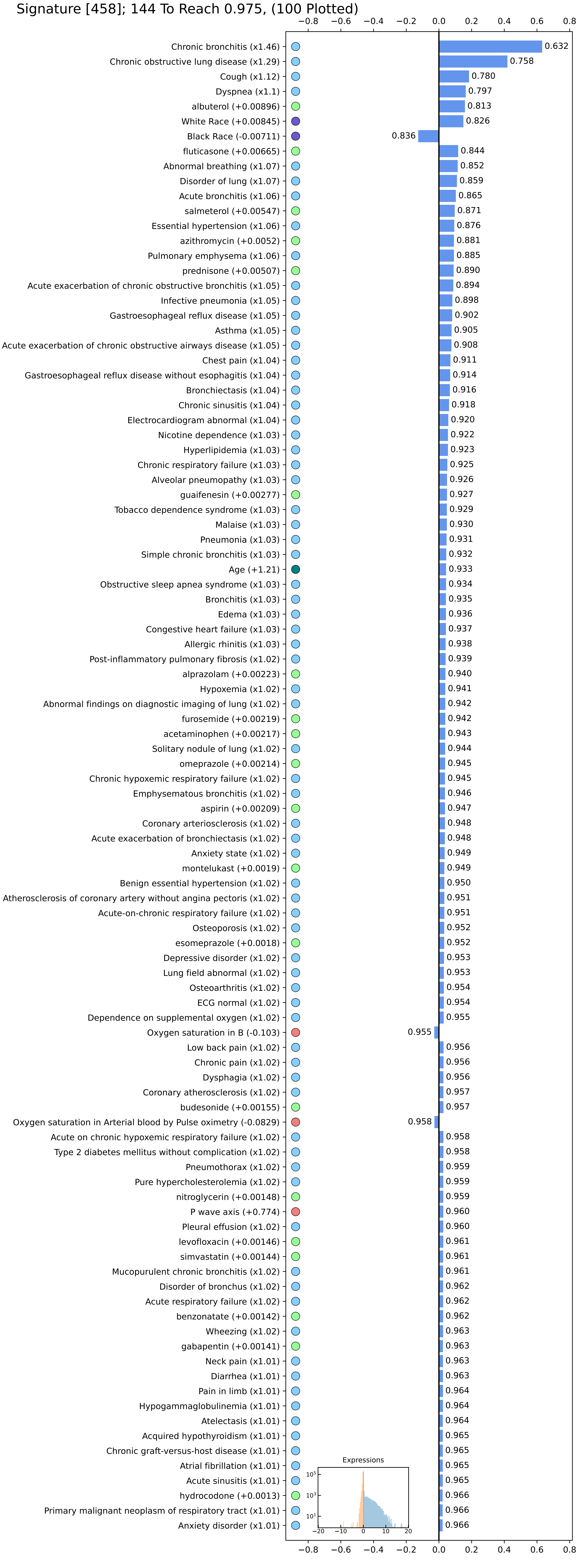

Signature [459]; 8 To Reach 0.975, (10 Plotted)

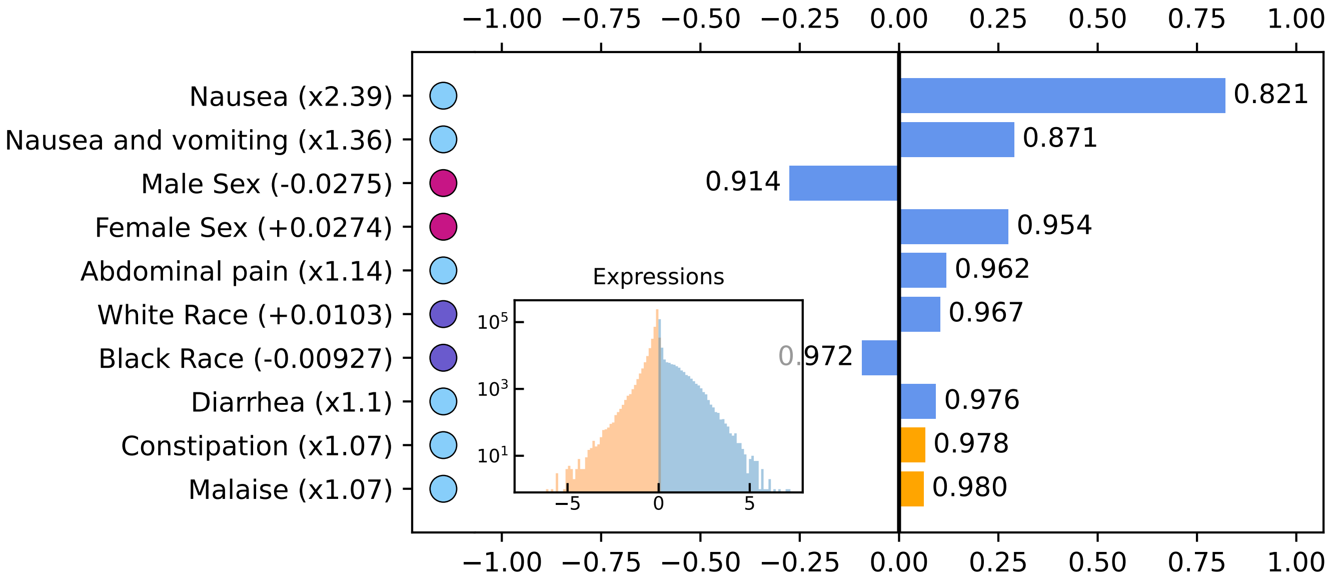

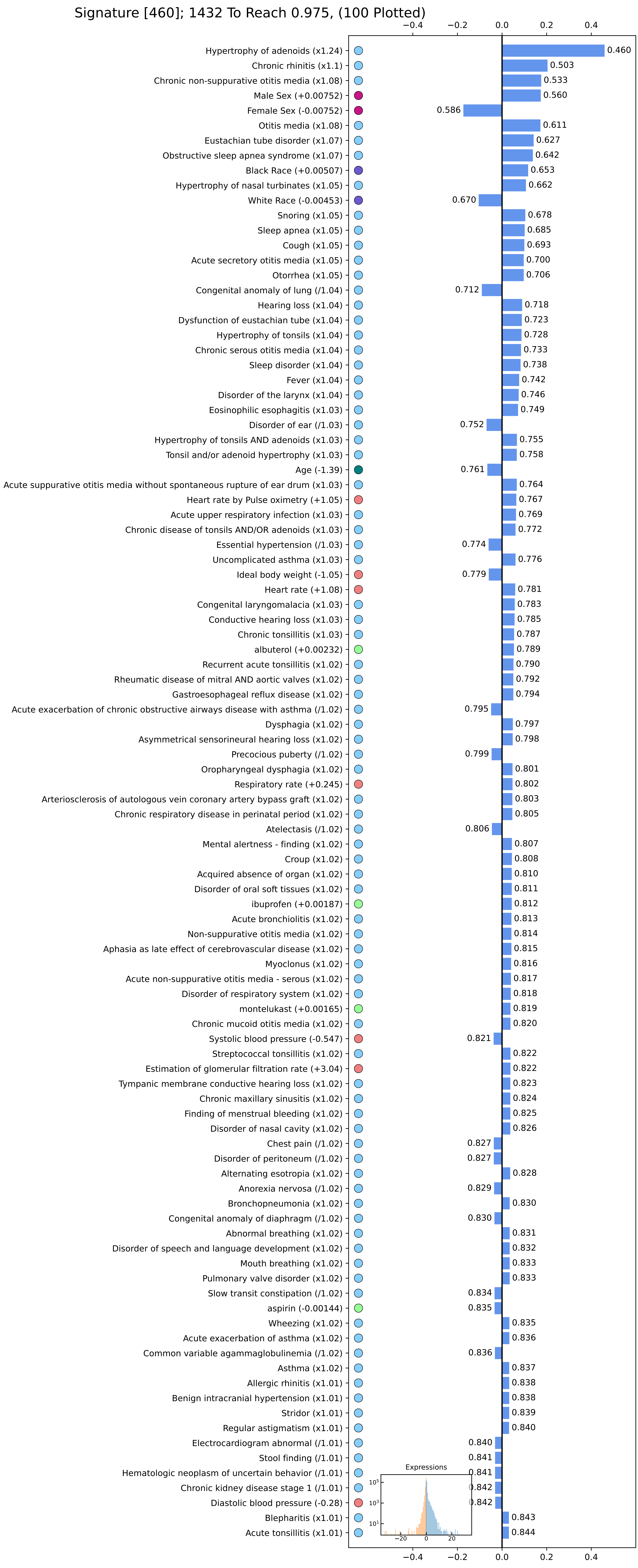

Signature [460]; 1432 To Reach 0.975, (100 Plotted)
Hypertrophy of adenoids (x1.24) 0.460
Chronic rhinitis (x1.1) 0.503
Chronic non-suppurative otitis media (x1.08) 0.533
Male Sex (+0.00752) 0.560
Female Sex (-0.00752) 0.586
Otitis media (x1.08) 0.611
Eustachian tube disorder (x1.07) 0.627
Obstructive sleep apnea syndrome (x1.07) 0.642
Black Race (+0.00507) 0.653
Hypertrophy of nasal turbinates (x1.05) 0.662
White Race (-0.00453) 0.670
Snoring (x1.05) 0.678
Sleep apnea (x1.05) 0.685
Cough (x1.05) 0.693
Acute secretory otitis media (x1.05) 0.700
Otorrhea (x1.05) 0.706
Congenital anomaly of lung (/1.04) 0.712
Hearing loss (x1.04) 0.718
Dysfunction of eustachian tube (x1.04) 0.723
Hypertrophy of tonsils (x1.04) 0.728
Chronic serous otitis media (x1.04) 0.733
Sleep disorder (x1.04) 0.738
Fever (x1.04) 0.742
Disorder of the larynx (x1.04) 0.746
Eosinophilic esophagitis (x1.03) 0.749
Disorder of ear (/1.03) 0.752
Hypertrophy of tonsils AND adenoids (x1.03) 0.755
Tonsil and/or adenoid hypertrophy (x1.03) 0.758
Age (-1.39) 0.761
Acute suppurative otitis media without spontaneous rupture of ear drum (x1.03) 0.764
Heart rate by Pulse oximetry (+1.05) 0.767
Acute upper respiratory infection (x1.03) 0.769
Chronic disease of tonsils AND/OR adenoids (x1.03) 0.772
Essential hypertension (/1.03) 0.774
Uncomplicated asthma (x1.03) 0.776
Ideal body weight (-1.05) 0.779
Heart rate (+1.08) 0.781
Congenital laryngomalacia (x1.03) 0.783
Conductive hearing loss (x1.03) 0.785
Chronic tonsillitis (x1.03) 0.787
albuterol (+0.00232) 0.789
Recurrent acute tonsillitis (x1.02) 0.790
Rheumatic disease of mitral AND aortic valves (x1.02) 0.792
Gastroesophageal reflux disease (x1.02) 0.794
Acute exacerbation of chronic obstructive airways disease with asthma (/1.02) 0.795
Dysphagia (x1.02) 0.797
Asymmetrical sensorineural hearing loss (x1.02) 0.798
Precocious puberty (/1.02) 0.799
Oropharyngeal dysphagia (x1.02) 0.801
Respiratory rate (+0.245) 0.802
Arteriosclerosis of autologous vein coronary artery bypass graft (x1.02) 0.803
Chronic respiratory disease in perinatal period (x1.02) 0.805
Atelectasis (/1.02) 0.806
Mental alertness - finding (x1.02) 0.807
Croup (x1.02) 0.808
Acquired absence of organ (x1.02) 0.810
Disorder of oral soft tissues (x1.02) 0.811
ibuprofen (+0.00187) 0.812
Acute bronchiolitis (x1.02) 0.813
Non-suppurative otitis media (x1.02) 0.814
Aphasia as late effect of cerebrovascular disease (x1.02) 0.815
Myoclonus (x1.02) 0.816
Acute non-suppurative otitis media - serous (x1.02) 0.817
Disorder of respiratory system (x1.02) 0.818
montelukast (+0.00165) 0.819
Chronic mucoid otitis media (x1.02) 0.820
Systolic blood pressure (-0.547) 0.821
Streptococcal tonsillitis (x1.02) 0.822
Estimation of glomerular filtration rate (+3.04) 0.822
Tympanic membrane conductive hearing loss (x1.02) 0.823
Chronic maxillary sinusitis (x1.02) 0.824
Finding of menstrual bleeding (x1.02) 0.825
Disorder of nasal cavity (x1.02) 0.826
Chest pain (/1.02) 0.827
Disorder of peritoneum (/1.02) 0.827
Alternating esotropia (x1.02) 0.828
Anorexia nervosa (/1.02) 0.829
Bronchopneumonia (x1.02) 0.830
Congenital anomaly of diaphragm (/1.02) 0.830
Abnormal breathing (x1.02) 0.831
Disorder of speech and language development (x1.02) 0.832
Mouth breathing (x1.02) 0.833
Pulmonary valve disorder (x1.02) 0.833
Slow transit constipation (/1.02) 0.834
aspirin (-0.00144) 0.835
Wheezing (x1.02) 0.835
Acute exacerbation of asthma (x1.02) 0.836
Common variable agammaglobulinemia (/1.02) 0.836
Asthma (x1.02) 0.837
Allergic rhinitis (x1.01) 0.838
Benign intracranial hypertension (x1.01) 0.838
Stridor (x1.01) 0.839
Regular astigmatism (x1.01) 0.840
Electrocardiogram abnormal (/1.01) 0.840
Stool finding (/1.01) 0.841
Hematologic neoplasm of uncertain behavior (/1.01) 0.841
Chronic kidney disease stage 1 (/1.01) 0.842
Diastolic blood pressure (-0.28) 0.842
Blepharitis (x1.01) 0.843
Acute tonsillitis (x1.01) 0.844
Expressions
−0.4 −0.2 0.0 0.2 0.4

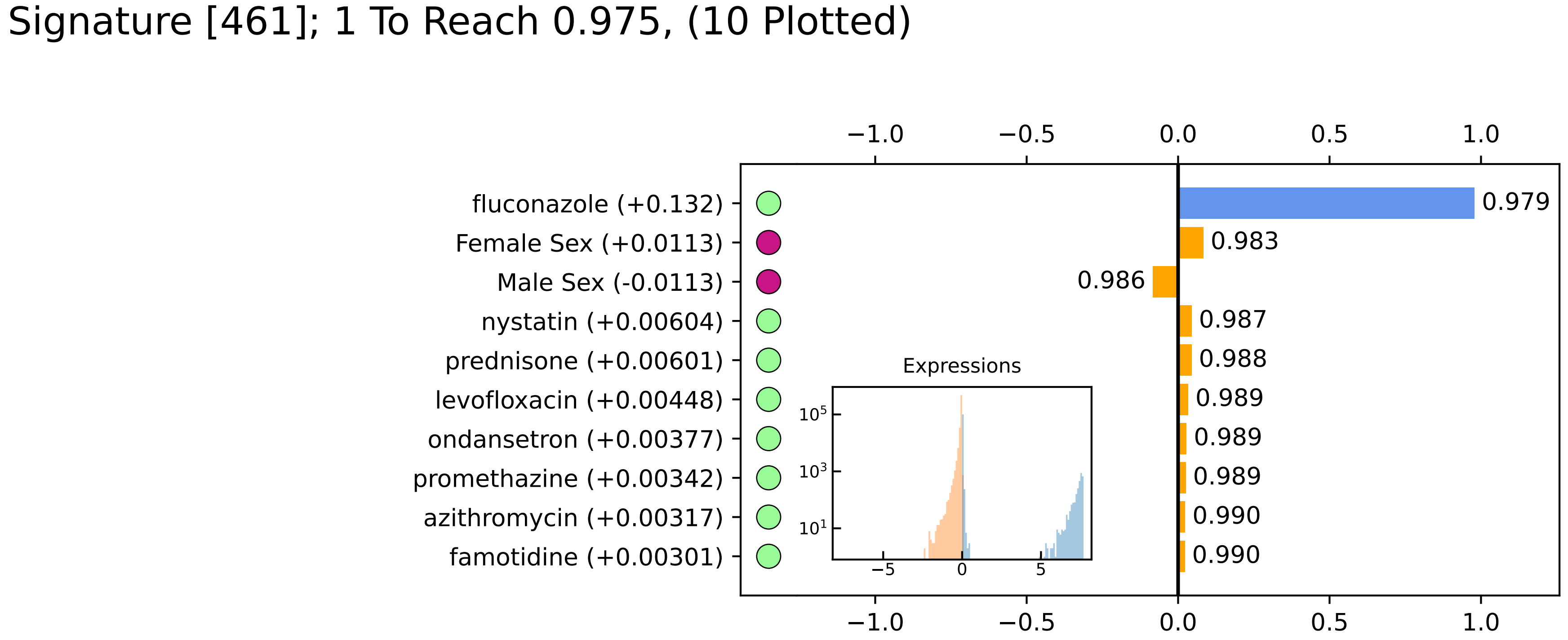
Signature [461]; 1 To Reach 0.975, (10 Plotted)
fluconazole (+0.132)
Female Sex (+0.0113)
Male Sex (-0.0113)
nystatin (+0.00604)
prednisone (+0.00601)
levofloxacin (+0.00448)
ondansetron (+0.00377)
promethazine (+0.00342)
azithromycin (+0.00317)
famotidine (+0.00301)
0.979
0.983
0.986
0.987
0.988
0.989
0.989
0.989
0.990
0.990
Expressions
−1.0
−0.5
0.0
0.5
1.0

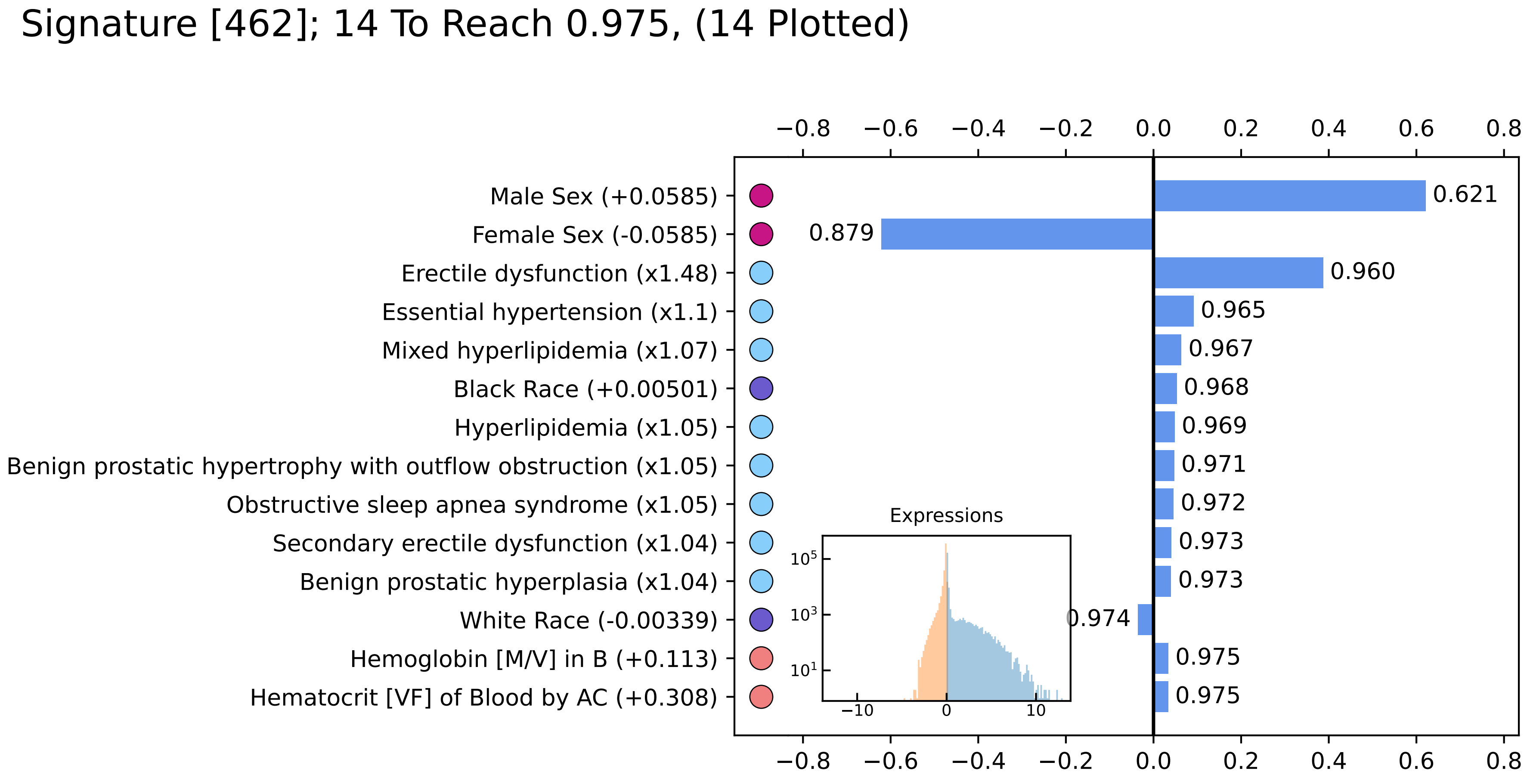

Signature [462]; 14 To Reach 0.975, (14 Plotted)
−0.8
−0.6
−0.4
−0.2
0.0
0.2
0.4
0.6
0.8
Male Sex (+0.0585)
0.621
Female Sex (-0.0585)
0.879
Erectile dysfunction (x1.48)
0.960
Essential hypertension (x1.1)
0.965
Mixed hyperlipidemia (x1.07)
0.967
Black Race (+0.00501)
0.968
Hyperlipidemia (x1.05)
0.969
Benign prostatic hypertrophy with outflow obstruction (x1.05)
0.971
Obstructive sleep apnea syndrome (x1.05)
0.972
Secondary erectile dysfunction (x1.04)
0.973
Benign prostatic hyperplasia (x1.04)
0.973
White Race (-0.00339)
0.974
Hemoglobin [M/V] in B (+0.113)
0.975
Hematocrit [VF] of Blood by AC (+0.308)
0.975
Expressions
10^5
10^3
10^1
−10
0
10

Signature [463]; 1 To Reach 0.975, (10 Plotted)

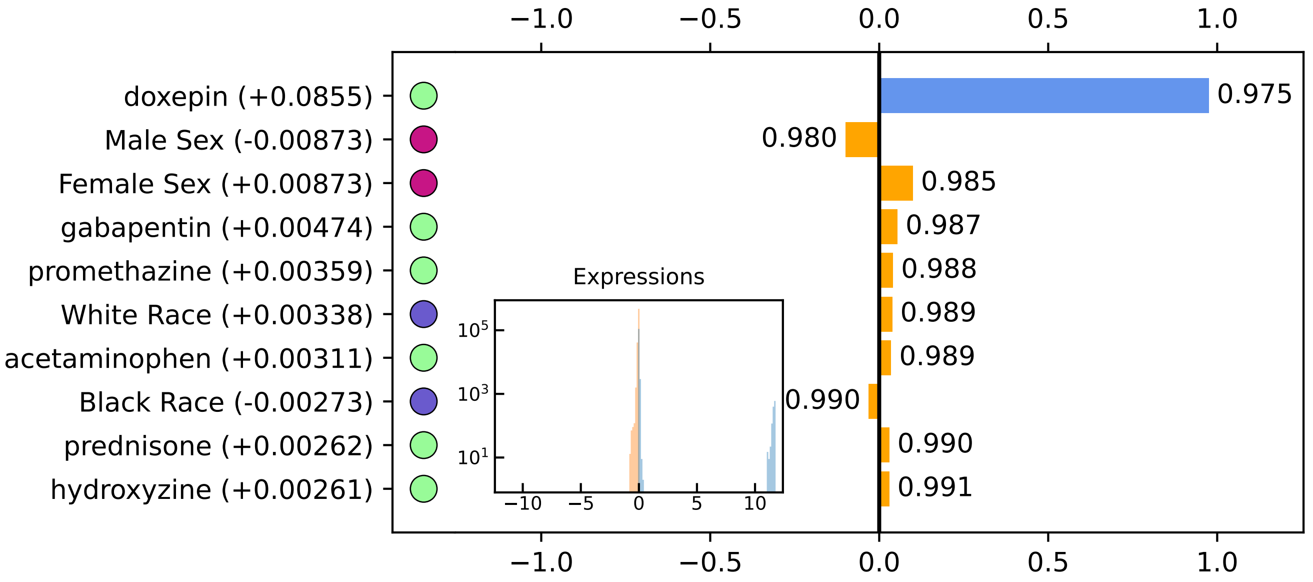

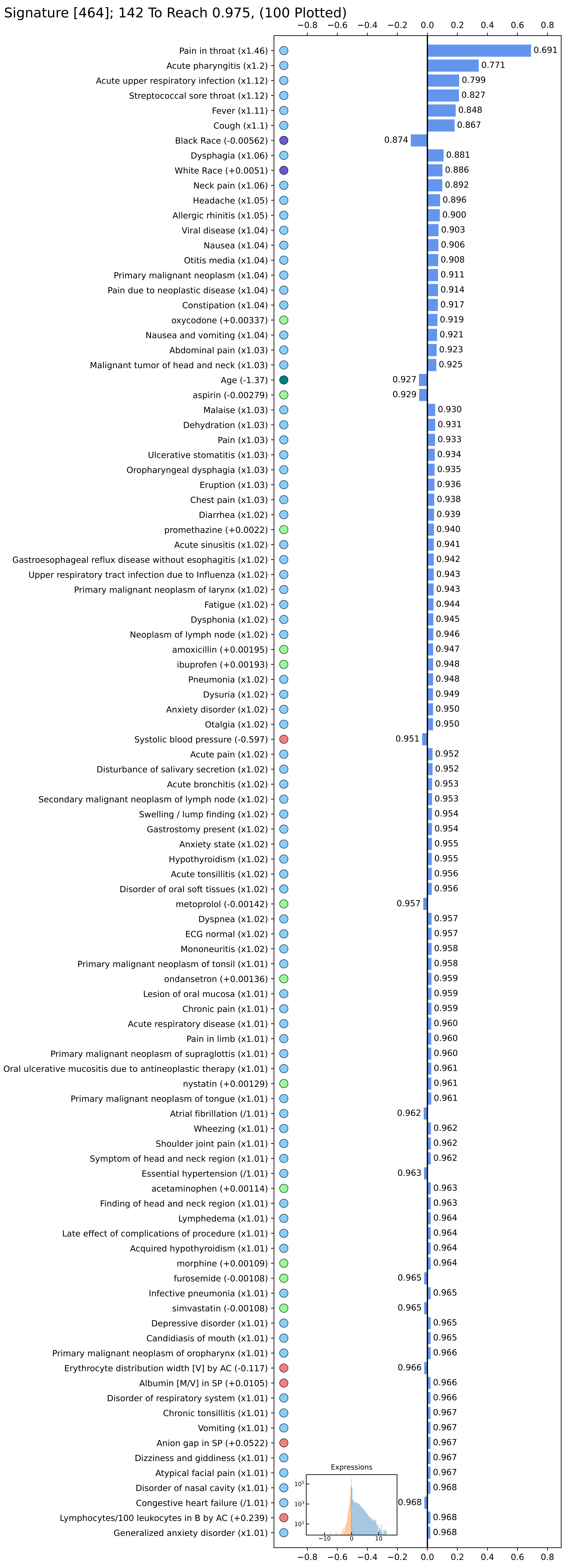

Signature [464]; 142 To Reach 0.975, (100 Plotted)
Pain in throat (x1.46) 0.691
Acute pharyngitis (x1.2) 0.771
Acute upper respiratory infection (x1.12) 0.799
Streptococcal sore throat (x1.12) 0.827
Fever (x1.11) 0.848
Cough (x1.1) 0.867
Black Race (-0.00562) 0.874
Dysphagia (x1.06) 0.881
White Race (+0.0051) 0.886
Neck pain (x1.06) 0.892
Headache (x1.05) 0.896
Allergic rhinitis (x1.05) 0.900
Viral disease (x1.04) 0.903
Nausea (x1.04) 0.906
Otitis media (x1.04) 0.908
Primary malignant neoplasm (x1.04) 0.911
Pain due to neoplastic disease (x1.04) 0.914
Constipation (x1.04) 0.917
oxycodone (+0.00337) 0.919
Nausea and vomiting (x1.04) 0.921
Abdominal pain (x1.03) 0.923
Malignant tumor of head and neck (x1.03) 0.925
Age (-1.37) 0.927
aspirin (-0.00279) 0.929
Malaise (x1.03) 0.930
Dehydration (x1.03) 0.931
Pain (x1.03) 0.933
Ulcerative stomatitis (x1.03) 0.934
Oropharyngeal dysphagia (x1.03) 0.935
Eruption (x1.03) 0.936
Chest pain (x1.03) 0.938
Diarrhea (x1.02) 0.939
promethazine (+0.0022) 0.940
Acute sinusitis (x1.02) 0.941
Gastroesophageal reflux disease without esophagitis (x1.02) 0.942
Upper respiratory tract infection due to Influenza (x1.02) 0.943
Primary malignant neoplasm of larynx (x1.02) 0.943
Fatigue (x1.02) 0.944
Dysphonia (x1.02) 0.945
Neoplasm of lymph node (x1.02) 0.946
amoxicillin (+0.00195) 0.947
ibuprofen (+0.00193) 0.948
Pneumonia (x1.02) 0.948
Dysuria (x1.02) 0.949
Anxiety disorder (x1.02) 0.950
Otalgia (x1.02) 0.950
Systolic blood pressure (-0.597) 0.951
Acute pain (x1.02) 0.952
Disturbance of salivary secretion (x1.02) 0.952
Acute bronchitis (x1.02) 0.953
Secondary malignant neoplasm of lymph node (x1.02) 0.953
Swelling / lump finding (x1.02) 0.954
Gastrostomy present (x1.02) 0.954
Anxiety state (x1.02) 0.955
Hypothyroidism (x1.02) 0.955
Acute tonsillitis (x1.02) 0.956
Disorder of oral soft tissues (x1.02) 0.956
metoprolol (-0.00142) 0.957
Dyspnea (x1.02) 0.957
ECG normal (x1.02) 0.957
Mononeuritis (x1.02) 0.958
Primary malignant neoplasm of tonsil (x1.01) 0.958
ondansetron (+0.00136) 0.959
Lesion of oral mucosa (x1.01) 0.959
Chronic pain (x1.01) 0.959
Acute respiratory disease (x1.01) 0.960
Pain in limb (x1.01) 0.960
Primary malignant neoplasm of supraglottis (x1.01) 0.960
Oral ulcerative mucositis due to antineoplastic therapy (x1.01) 0.961
nystatin (+0.00129) 0.961
Primary malignant neoplasm of tongue (x1.01) 0.961
Atrial fibrillation (/1.01) 0.962
Wheezing (x1.01) 0.962
Shoulder joint pain (x1.01) 0.962
Symptom of head and neck region (x1.01) 0.962
Essential hypertension (/1.01) 0.963
acetaminophen (+0.00114) 0.963
Finding of head and neck region (x1.01) 0.963
Lymphedema (x1.01) 0.964
Late effect of complications of procedure (x1.01) 0.964
Acquired hypothyroidism (x1.01) 0.964
morphine (+0.00109) 0.964
furosemide (-0.00108) 0.965
Infective pneumonia (x1.01) 0.965
simvastatin (-0.00108) 0.965
Depressive disorder (x1.01) 0.965
Candidiasis of mouth (x1.01) 0.965
Primary malignant neoplasm of oropharynx (x1.01) 0.966
Erythrocyte distribution width [V] by AC (-0.117) 0.966
Albumin [M/V] in SP (+0.0105) 0.966
Disorder of respiratory system (x1.01) 0.966
Chronic tonsillitis (x1.01) 0.967
Vomiting (x1.01) 0.967
Anion gap in SP (+0.0522) 0.967
Dizziness and giddiness (x1.01) 0.967
Atypical facial pain (x1.01) 0.967
Disorder of nasal cavity (x1.01) 0.968
Congestive heart failure (/1.01) 0.968
Lymphocytes/100 leukocytes in B by AC (+0.239) 0.968
Generalized anxiety disorder (x1.01) 0.968
−0.8 −0.6 −0.4 −0.2 0.0 0.2 0.4 0.6 0.8
Expressions
10^5 10^3 10^1
−10 0 10

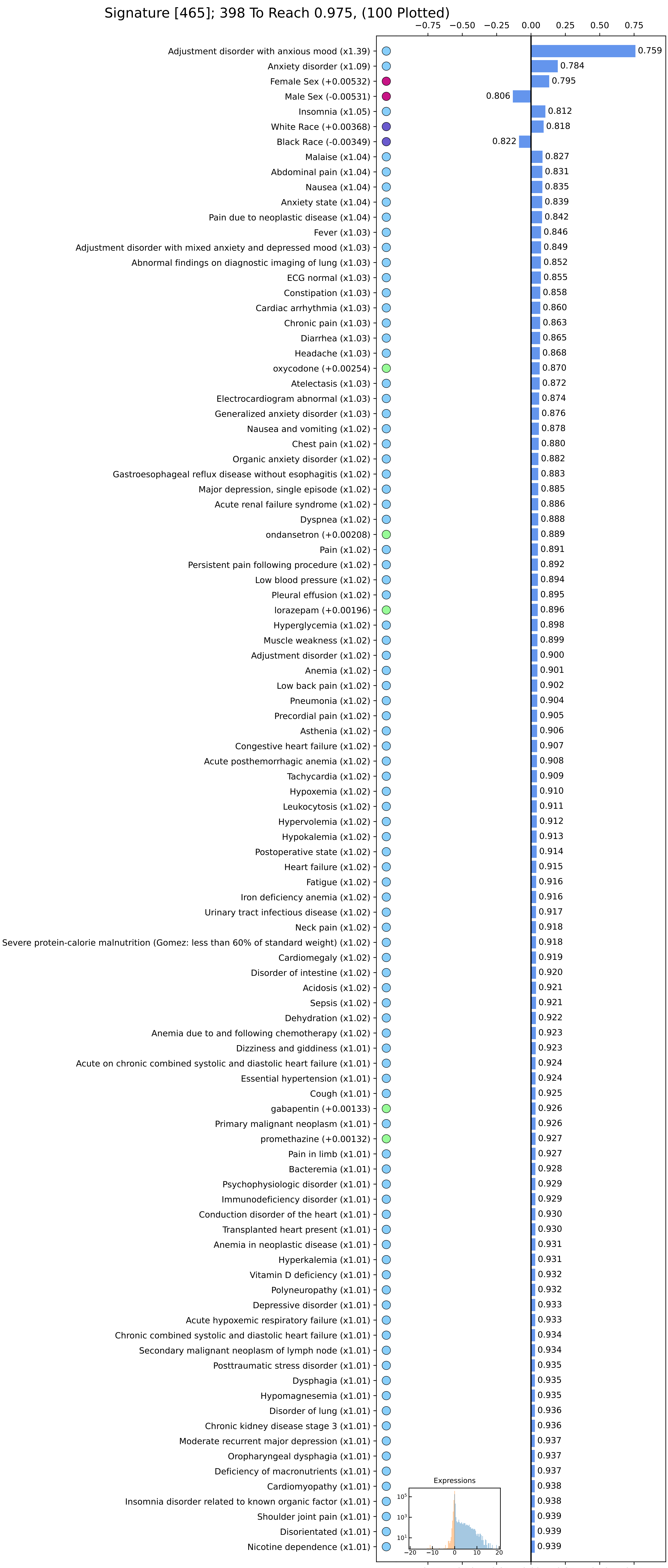

Signature [465]; 398 To Reach 0.975, (100 Plotted)
−0.75 −0.50 −0.25 0.00 0.25 0.50 0.75
Adjustment disorder with anxious mood (x1.39) 0.759
Anxiety disorder (x1.09) 0.784
Female Sex (+0.00532) 0.795
Male Sex (-0.00531) 0.806
Insomnia (x1.05) 0.812
White Race (+0.00368) 0.818
Black Race (-0.00349) 0.822
Malaise (x1.04) 0.827
Abdominal pain (x1.04) 0.831
Nausea (x1.04) 0.835
Anxiety state (x1.04) 0.839
Pain due to neoplastic disease (x1.04) 0.842
Fever (x1.03) 0.846
Adjustment disorder with mixed anxiety and depressed mood (x1.03) 0.849
Abnormal findings on diagnostic imaging of lung (x1.03) 0.852
ECG normal (x1.03) 0.855
Constipation (x1.03) 0.858
Cardiac arrhythmia (x1.03) 0.860
Chronic pain (x1.03) 0.863
Diarrhea (x1.03) 0.865
Headache (x1.03) 0.868
oxycodone (+0.00254) 0.870
Atelectasis (x1.03) 0.872
Electrocardiogram abnormal (x1.03) 0.874
Generalized anxiety disorder (x1.03) 0.876
Nausea and vomiting (x1.02) 0.878
Chest pain (x1.02) 0.880
Organic anxiety disorder (x1.02) 0.882
Gastroesophageal reflux disease without esophagitis (x1.02) 0.883
Major depression, single episode (x1.02) 0.885
Acute renal failure syndrome (x1.02) 0.886
Dyspnea (x1.02) 0.888
ondansetron (+0.00208) 0.889
Pain (x1.02) 0.891
Persistent pain following procedure (x1.02) 0.892
Low blood pressure (x1.02) 0.894
Pleural effusion (x1.02) 0.895
lorazepam (+0.00196) 0.896
Hyperglycemia (x1.02) 0.898
Muscle weakness (x1.02) 0.899
Adjustment disorder (x1.02) 0.900
Anemia (x1.02) 0.901
Low back pain (x1.02) 0.902
Pneumonia (x1.02) 0.904
Precordial pain (x1.02) 0.905
Asthenia (x1.02) 0.906
Congestive heart failure (x1.02) 0.907
Acute posthemorrhagic anemia (x1.02) 0.908
Tachycardia (x1.02) 0.909
Hypoxemia (x1.02) 0.910
Leukocytosis (x1.02) 0.911
Hypervolemia (x1.02) 0.912
Hypokalemia (x1.02) 0.913
Postoperative state (x1.02) 0.914
Heart failure (x1.02) 0.915
Fatigue (x1.02) 0.916
Iron deficiency anemia (x1.02) 0.916
Urinary tract infectious disease (x1.02) 0.917
Neck pain (x1.02) 0.918
Severe protein-calorie malnutrition (Gomez: less than 60% of standard weight) (x1.02) 0.918
Cardiomegaly (x1.02) 0.919
Disorder of intestine (x1.02) 0.920
Acidosis (x1.02) 0.921
Sepsis (x1.02) 0.921
Dehydration (x1.02) 0.922
Anemia due to and following chemotherapy (x1.02) 0.923
Dizziness and giddiness (x1.01) 0.923
Acute on chronic combined systolic and diastolic heart failure (x1.01) 0.924
Essential hypertension (x1.01) 0.924
Cough (x1.01) 0.925
gabapentin (+0.00133) 0.926
Primary malignant neoplasm (x1.01) 0.926
promethazine (+0.00132) 0.927
Pain in limb (x1.01) 0.927
Bacteremia (x1.01) 0.928
Psychophysiologic disorder (x1.01) 0.929
Immunodeficiency disorder (x1.01) 0.929
Conduction disorder of the heart (x1.01) 0.930
Transplanted heart present (x1.01) 0.930
Anemia in neoplastic disease (x1.01) 0.931
Hyperkalemia (x1.01) 0.931
Vitamin D deficiency (x1.01) 0.932
Polyneuropathy (x1.01) 0.932
Depressive disorder (x1.01) 0.933
Acute hypoxemic respiratory failure (x1.01) 0.933
Chronic combined systolic and diastolic heart failure (x1.01) 0.934
Secondary malignant neoplasm of lymph node (x1.01) 0.934
Posttraumatic stress disorder (x1.01) 0.935
Dysphagia (x1.01) 0.935
Hypomagnesemia (x1.01) 0.935
Disorder of lung (x1.01) 0.936
Chronic kidney disease stage 3 (x1.01) 0.936
Moderate recurrent major depression (x1.01) 0.937
Oropharyngeal dysphagia (x1.01) 0.937
Deficiency of macronutrients (x1.01) 0.937
Cardiomyopathy (x1.01) 0.938
Insomnia disorder related to known organic factor (x1.01) 0.938
Shoulder joint pain (x1.01) 0.939
Disorientated (x1.01) 0.939
Nicotine dependence (x1.01) 0.939
Expressions
−20 −10 0 10 20
−0.75 −0.50 −0.25 0.00 0.25 0.50 0.75

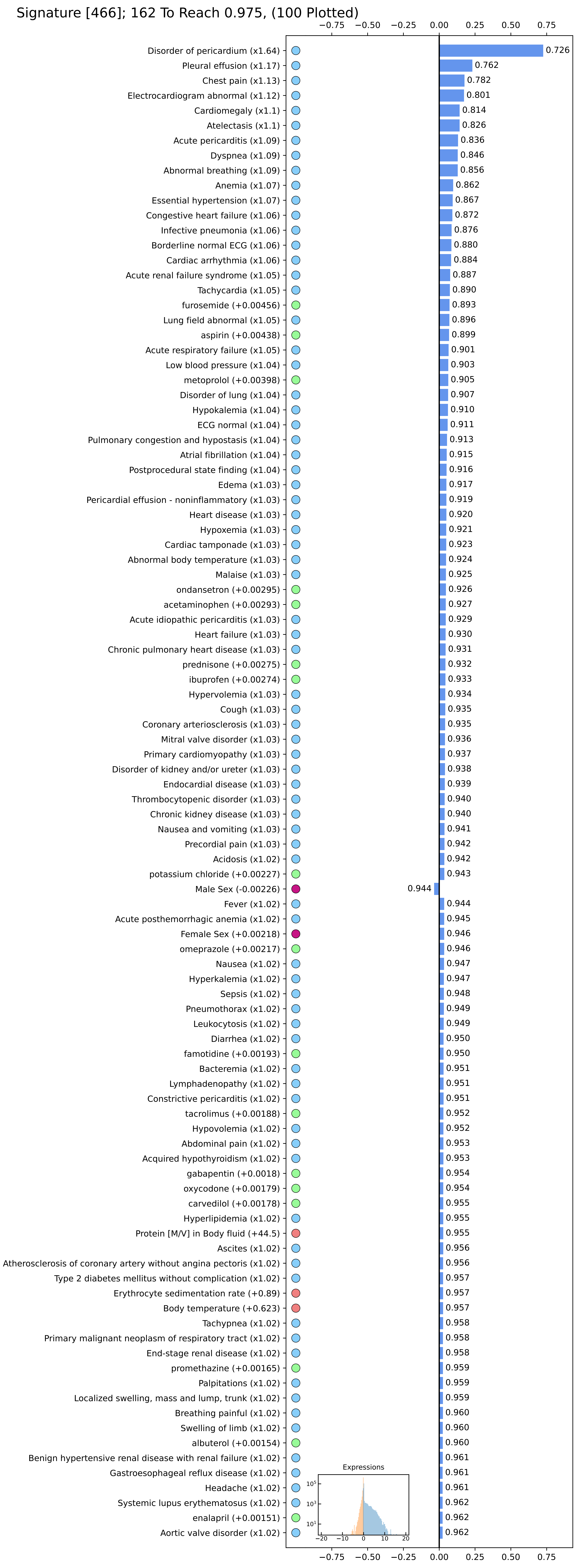
Signature [466]; 162 To Reach 0.975, (100 Plotted)
Disorder of pericardium (x1.64) 0.726
Pleural effusion (x1.17) 0.762
Chest pain (x1.13) 0.782
Electrocardiogram abnormal (x1.12) 0.801
Cardiomegaly (x1.1) 0.814
Atelectasis (x1.1) 0.826
Acute pericarditis (x1.09) 0.836
Dyspnea (x1.09) 0.846
Abnormal breathing (x1.09) 0.856
Anemia (x1.07) 0.862
Essential hypertension (x1.07) 0.867
Congestive heart failure (x1.06) 0.872
Infective pneumonia (x1.06) 0.876
Borderline normal ECG (x1.06) 0.880
Cardiac arrhythmia (x1.06) 0.884
Acute renal failure syndrome (x1.05) 0.887
Tachycardia (x1.05) 0.890
furosemide (+0.00456) 0.893
Lung field abnormal (x1.05) 0.896
aspirin (+0.00438) 0.899
Acute respiratory failure (x1.05) 0.901
Low blood pressure (x1.04) 0.903
metoprolol (+0.00398) 0.905
Disorder of lung (x1.04) 0.907
Hypokalemia (x1.04) 0.910
ECG normal (x1.04) 0.911
Pulmonary congestion and hypostasis (x1.04) 0.913
Atrial fibrillation (x1.04) 0.915
Postprocedural state finding (x1.04) 0.916
Edema (x1.03) 0.917
Pericardial effusion - noninflammatory (x1.03) 0.919
Heart disease (x1.03) 0.920
Hypoxemia (x1.03) 0.921
Cardiac tamponade (x1.03) 0.923
Abnormal body temperature (x1.03) 0.924
Malaise (x1.03) 0.925
ondansetron (+0.00295) 0.926
acetaminophen (+0.00293) 0.927
Acute idiopathic pericarditis (x1.03) 0.929
Heart failure (x1.03) 0.930
Chronic pulmonary heart disease (x1.03) 0.931
prednisone (+0.00275) 0.932
ibuprofen (+0.00274) 0.933
Hypervolemia (x1.03) 0.934
Cough (x1.03) 0.935
Coronary arteriosclerosis (x1.03) 0.935
Mitral valve disorder (x1.03) 0.936
Primary cardiomyopathy (x1.03) 0.937
Disorder of kidney and/or ureter (x1.03) 0.938
Endocardial disease (x1.03) 0.939
Thrombocytopenic disorder (x1.03) 0.940
Chronic kidney disease (x1.03) 0.940
Nausea and vomiting (x1.03) 0.941
Precordial pain (x1.03) 0.942
Acidosis (x1.02) 0.942
potassium chloride (+0.00227) 0.943
Male Sex (-0.00226) 0.944
Fever (x1.02) 0.944
Acute posthemorrhagic anemia (x1.02) 0.945
Female Sex (+0.00218) 0.946
omeprazole (+0.00217) 0.946
Nausea (x1.02) 0.947
Hyperkalemia (x1.02) 0.947
Sepsis (x1.02) 0.948
Pneumothorax (x1.02) 0.949
Leukocytosis (x1.02) 0.949
Diarrhea (x1.02) 0.950
famotidine (+0.00193) 0.950
Bacteremia (x1.02) 0.951
Lymphadenopathy (x1.02) 0.951
Constrictive pericarditis (x1.02) 0.951
tacrolimus (+0.00188) 0.952
Hypovolemia (x1.02) 0.952
Abdominal pain (x1.02) 0.953
Acquired hypothyroidism (x1.02) 0.953
gabapentin (+0.0018) 0.954
oxycodone (+0.00179) 0.954
carvedilol (+0.00178) 0.955
Hyperlipidemia (x1.02) 0.955
Protein [M/V] in Body fluid (+44.5) 0.955
Ascites (x1.02) 0.956
Atherosclerosis of coronary artery without angina pectoris (x1.02) 0.956
Type 2 diabetes mellitus without complication (x1.02) 0.957
Erythrocyte sedimentation rate (+0.89) 0.957
Body temperature (+0.623) 0.957
Tachypnea (x1.02) 0.958
Primary malignant neoplasm of respiratory tract (x1.02) 0.958
End-stage renal disease (x1.02) 0.958
promethazine (+0.00165) 0.959
Palpitations (x1.02) 0.959
Localized swelling, mass and lump, trunk (x1.02) 0.959
Breathing painful (x1.02) 0.960
Swelling of limb (x1.02) 0.960
albuterol (+0.00154) 0.960
Benign hypertensive renal disease with renal failure (x1.02) 0.961
Gastroesophageal reflux disease (x1.02) 0.961
Headache (x1.02) 0.961
Systemic lupus erythematosus (x1.02) 0.962
enalapril (+0.00151) 0.962
Aortic valve disorder (x1.02) 0.962
−0.75 −0.50 −0.25 0.00 0.25 0.50 0.75
Expressions
−20 −10 0 10 20

Signature [467]; 267 To Reach 0.975, (100 Plotted)

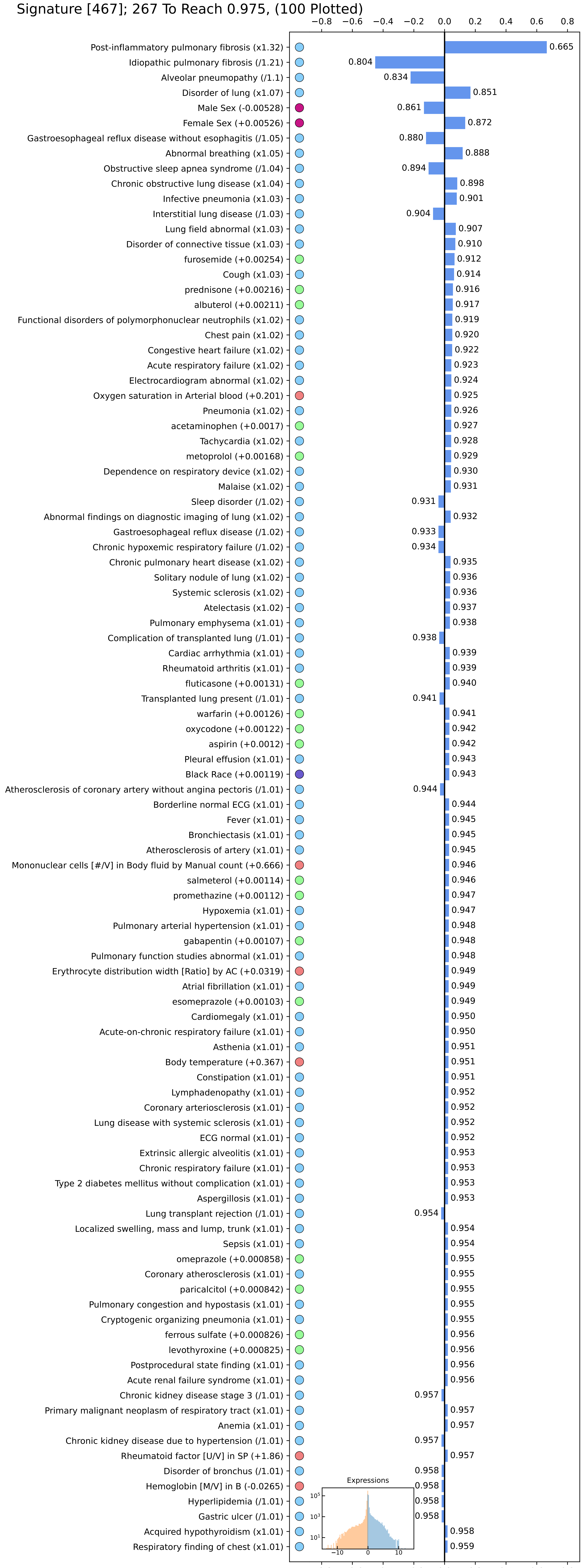

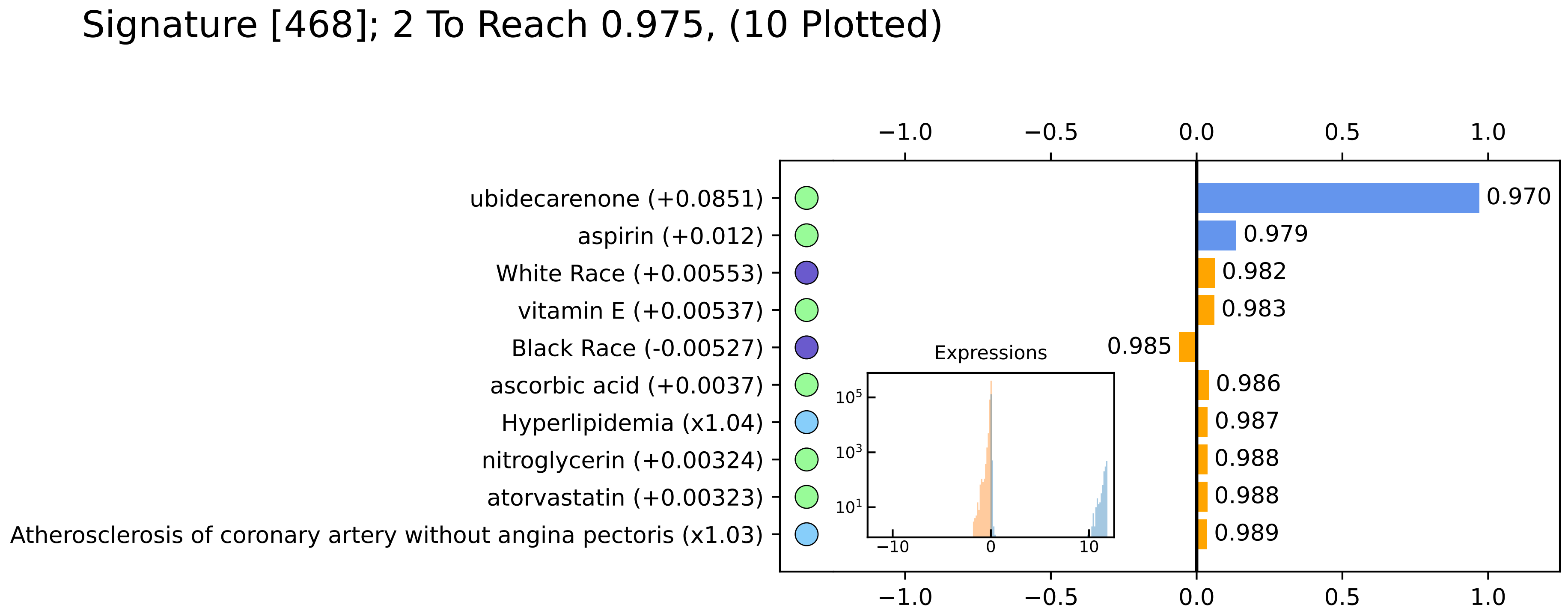

Signature [468]; 2 To Reach 0.975, (10 Plotted)
ubidecarenone (+0.0851)
aspirin (+0.012)
White Race (+0.00553)
vitamin E (+0.00537)
Black Race (-0.00527)
ascorbic acid (+0.0037)
Hyperlipidemia (x1.04)
nitroglycerin (+0.00324)
atorvastatin (+0.00323)
Atherosclerosis of coronary artery without angina pectoris (x1.03)
0.970
0.979
0.982
0.983
0.985
0.986
0.987
0.988
0.988
0.989
Expressions
−1.0
−0.5
0.0
0.5
1.0

Signature [469]; 2 To Reach 0.975, (10 Plotted)

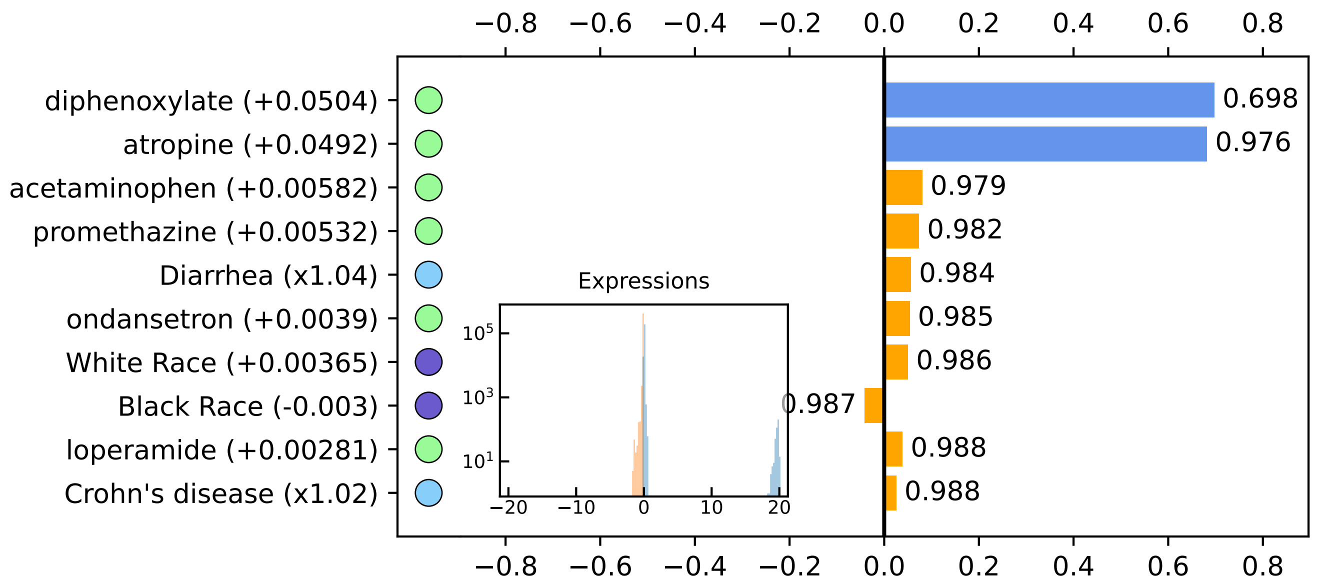

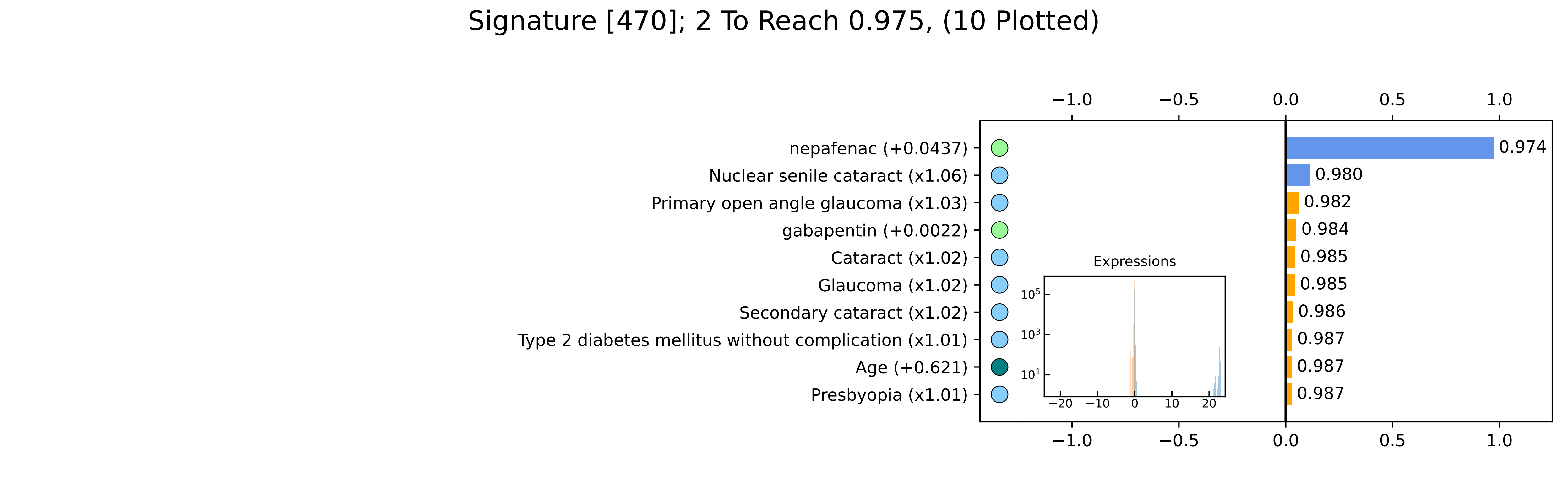
Signature [470]; 2 To Reach 0.975, (10 Plotted)
nepafenac (+0.0437)
Nuclear senile cataract (x1.06)
Primary open angle glaucoma (x1.03)
gabapentin (+0.0022)
Cataract (x1.02)
Glaucoma (x1.02)
Secondary cataract (x1.02)
Type 2 diabetes mellitus without complication (x1.01)
Age (+0.621)
Presbyopia (x1.01)
0.974
0.980
0.982
0.984
0.985
0.985
0.986
0.987
0.987
0.987
Expressions
−1.0
−0.5
0.0
0.5
1.0

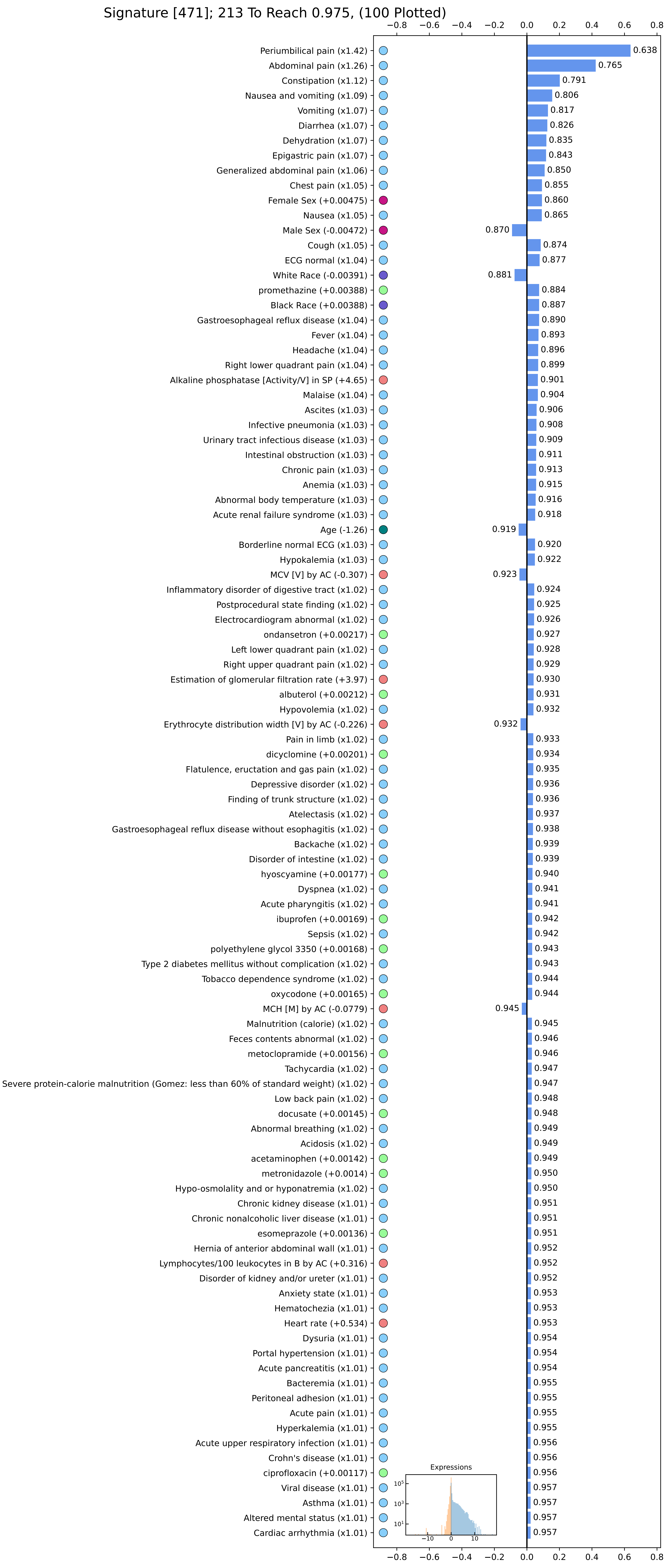

Signature [471]; 213 To Reach 0.975, (100 Plotted)
Periumbilical pain (x1.42) 0.638
Abdominal pain (x1.26) 0.765
Constipation (x1.12) 0.791
Nausea and vomiting (x1.09) 0.806
Vomiting (x1.07) 0.817
Diarrhea (x1.07) 0.826
Dehydration (x1.07) 0.835
Epigastric pain (x1.07) 0.843
Generalized abdominal pain (x1.06) 0.850
Chest pain (x1.05) 0.855
Female Sex (+0.00475) 0.860
Nausea (x1.05) 0.865
Male Sex (-0.00472) 0.870
Cough (x1.05) 0.874
ECG normal (x1.04) 0.877
White Race (-0.00391) 0.881
promethazine (+0.00388) 0.884
Black Race (+0.00388) 0.887
Gastroesophageal reflux disease (x1.04) 0.890
Fever (x1.04) 0.893
Headache (x1.04) 0.896
Right lower quadrant pain (x1.04) 0.899
Alkaline phosphatase [Activity/V] in SP (+4.65) 0.901
Malaise (x1.04) 0.904
Ascites (x1.03) 0.906
Infective pneumonia (x1.03) 0.908
Urinary tract infectious disease (x1.03) 0.909
Intestinal obstruction (x1.03) 0.911
Chronic pain (x1.03) 0.913
Anemia (x1.03) 0.915
Abnormal body temperature (x1.03) 0.916
Acute renal failure syndrome (x1.03) 0.918
Age (-1.26) 0.919
Borderline normal ECG (x1.03) 0.920
Hypokalemia (x1.03) 0.922
MCV [V] by AC (-0.307) 0.923
Inflammatory disorder of digestive tract (x1.02) 0.924
Postprocedural state finding (x1.02) 0.925
Electrocardiogram abnormal (x1.02) 0.926
ondansetron (+0.00217) 0.927
Left lower quadrant pain (x1.02) 0.928
Right upper quadrant pain (x1.02) 0.929
Estimation of glomerular filtration rate (+3.97) 0.930
albuterol (+0.00212) 0.931
Hypovolemia (x1.02) 0.932
Erythrocyte distribution width [V] by AC (-0.226) 0.932
Pain in limb (x1.02) 0.933
dicyclomine (+0.00201) 0.934
Flatulence, eructation and gas pain (x1.02) 0.935
Depressive disorder (x1.02) 0.936
Finding of trunk structure (x1.02) 0.936
Atelectasis (x1.02) 0.937
Gastroesophageal reflux disease without esophagitis (x1.02) 0.938
Backache (x1.02) 0.939
Disorder of intestine (x1.02) 0.939
hyoscyamine (+0.00177) 0.940
Dyspnea (x1.02) 0.941
Acute pharyngitis (x1.02) 0.941
ibuprofen (+0.00169) 0.942
Sepsis (x1.02) 0.942
polyethylene glycol 3350 (+0.00168) 0.943
Type 2 diabetes mellitus without complication (x1.02) 0.943
Tobacco dependence syndrome (x1.02) 0.944
oxycodone (+0.00165) 0.944
MCH [M] by AC (-0.0779) 0.945
Malnutrition (calorie) (x1.02) 0.945
Feces contents abnormal (x1.02) 0.946
metoclopramide (+0.00156) 0.946
Tachycardia (x1.02) 0.947
Severe protein-calorie malnutrition (Gomez: less than 60% of standard weight) (x1.02) 0.947
Low back pain (x1.02) 0.948
docusate (+0.00145) 0.948
Abnormal breathing (x1.02) 0.949
Acidosis (x1.02) 0.949
acetaminophen (+0.00142) 0.949
metronidazole (+0.0014) 0.950
Hypo-osmolality and or hyponatremia (x1.02) 0.950
Chronic kidney disease (x1.01) 0.951
Chronic nonalcoholic liver disease (x1.01) 0.951
esomeprazole (+0.00136) 0.951
Hernia of anterior abdominal wall (x1.01) 0.952
Lymphocytes/100 leukocytes in B by AC (+0.316) 0.952
Disorder of kidney and/or ureter (x1.01) 0.952
Anxiety state (x1.01) 0.953
Hematochezia (x1.01) 0.953
Heart rate (+0.534) 0.953
Dysuria (x1.01) 0.954
Portal hypertension (x1.01) 0.954
Acute pancreatitis (x1.01) 0.954
Bacteremia (x1.01) 0.955
Peritoneal adhesion (x1.01) 0.955
Acute pain (x1.01) 0.955
Hyperkalemia (x1.01) 0.955
Acute upper respiratory infection (x1.01) 0.956
Crohn's disease (x1.01) 0.956
ciprofloxacin (+0.00117) 0.956
Viral disease (x1.01) 0.957
Asthma (x1.01) 0.957
Altered mental status (x1.01) 0.957
Cardiac arrhythmia (x1.01) 0.957
Expressions

Signature [472]; 159 To Reach 0.975, (100 Plotted)

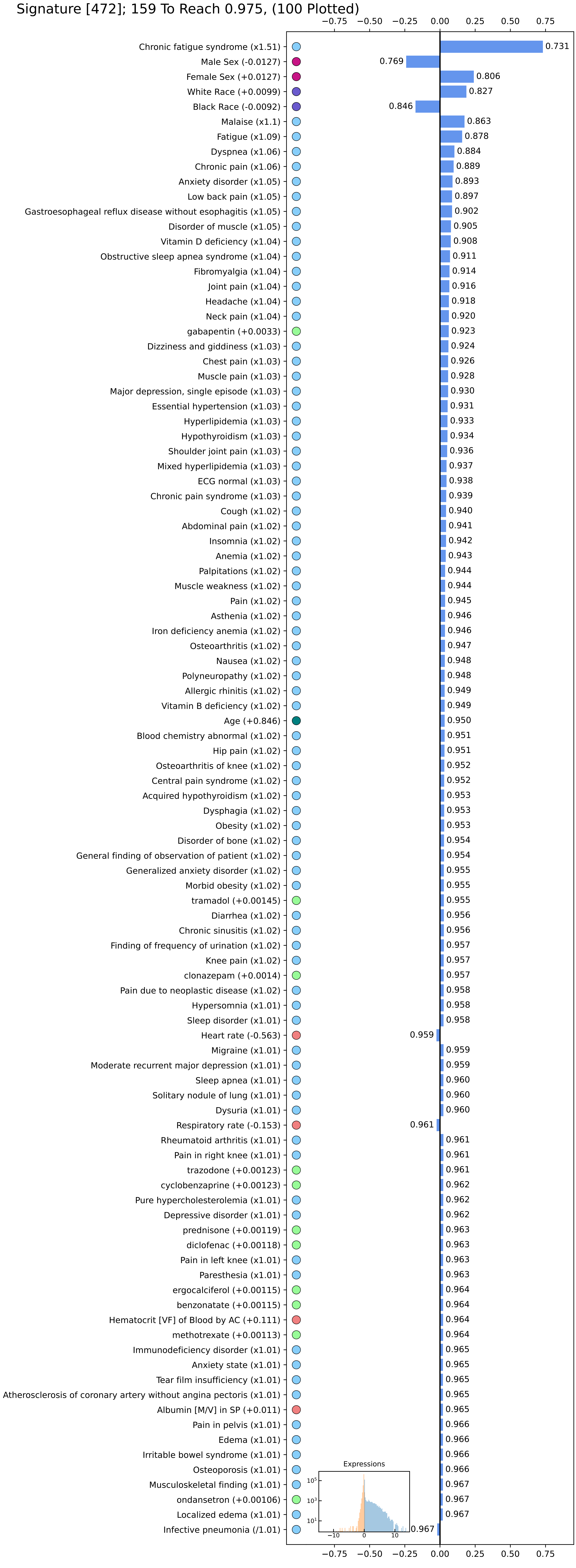

Signature [473]; 2 To Reach 0.975, (10 Plotted)

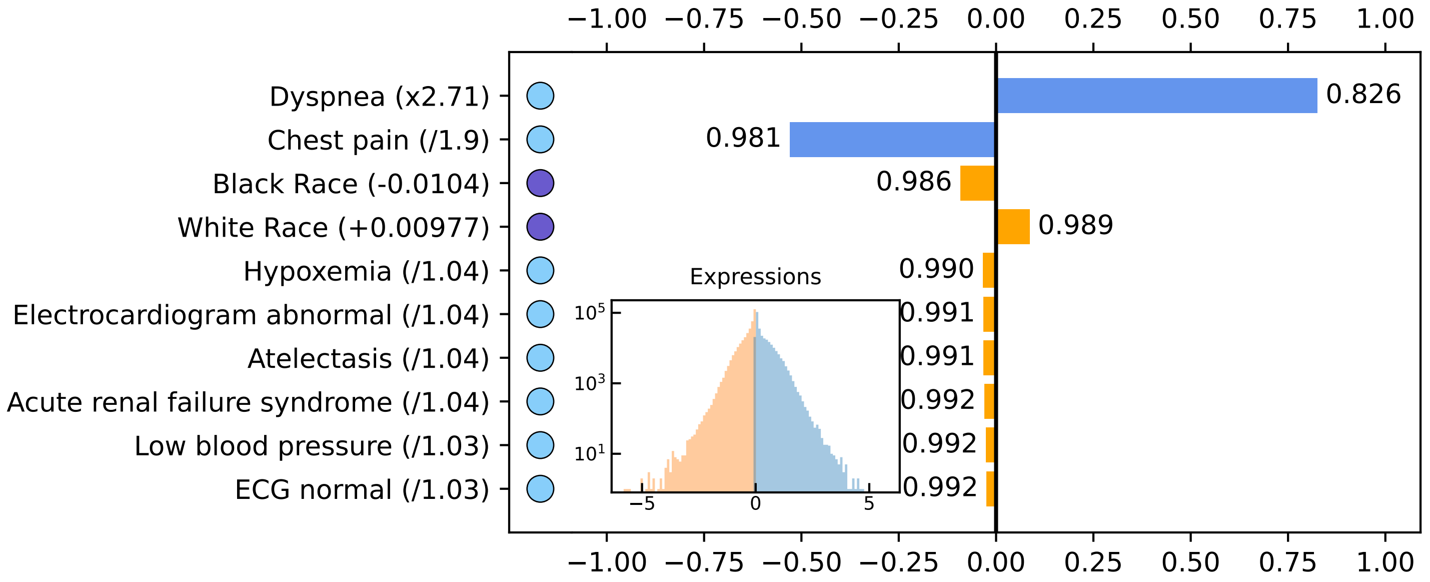

Signature [474]; 2 To Reach 0.975, (10 Plotted)

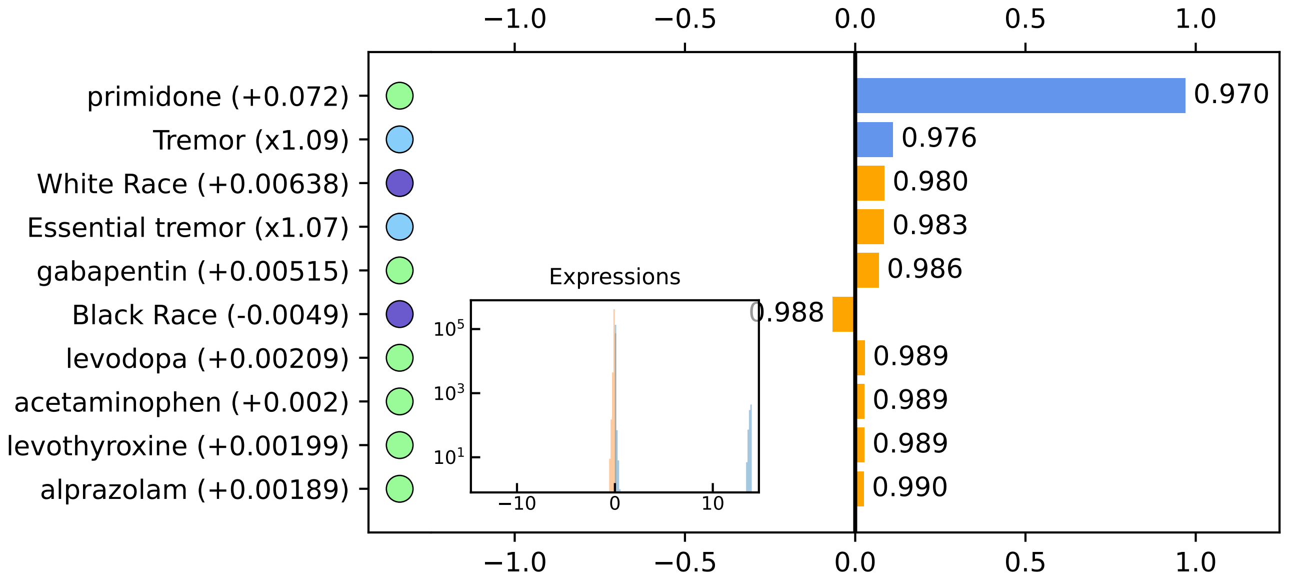

Signature [475]; 3 To Reach 0.975, (10 Plotted)

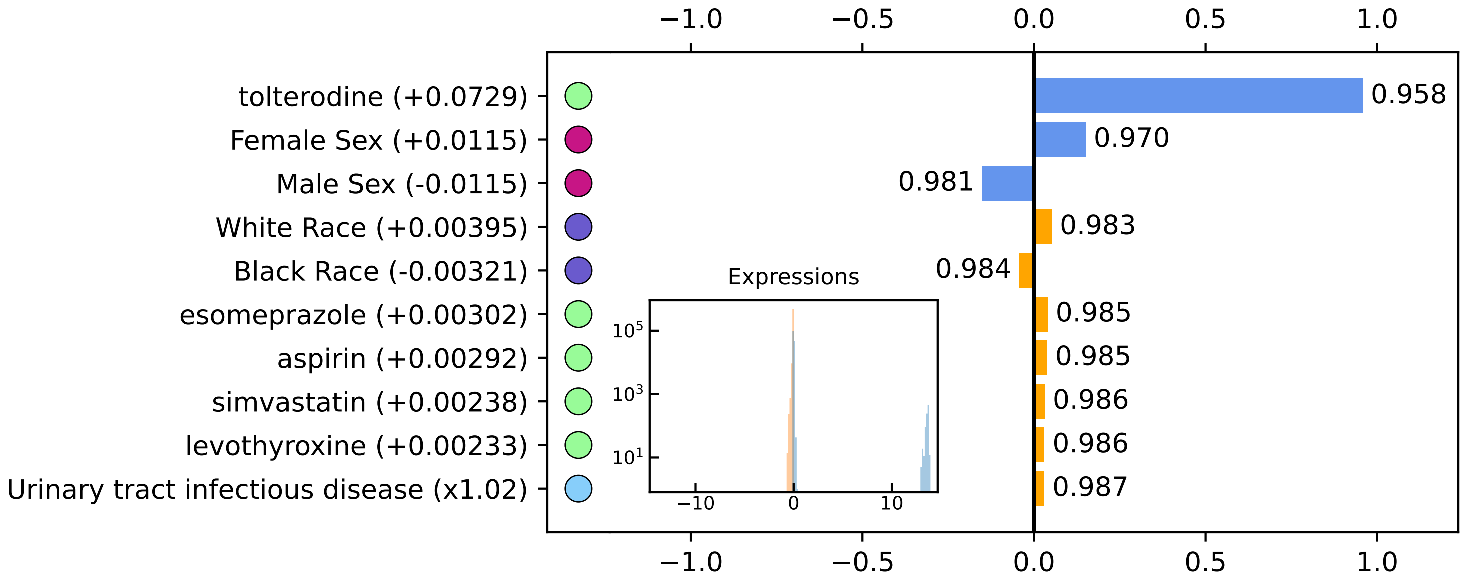

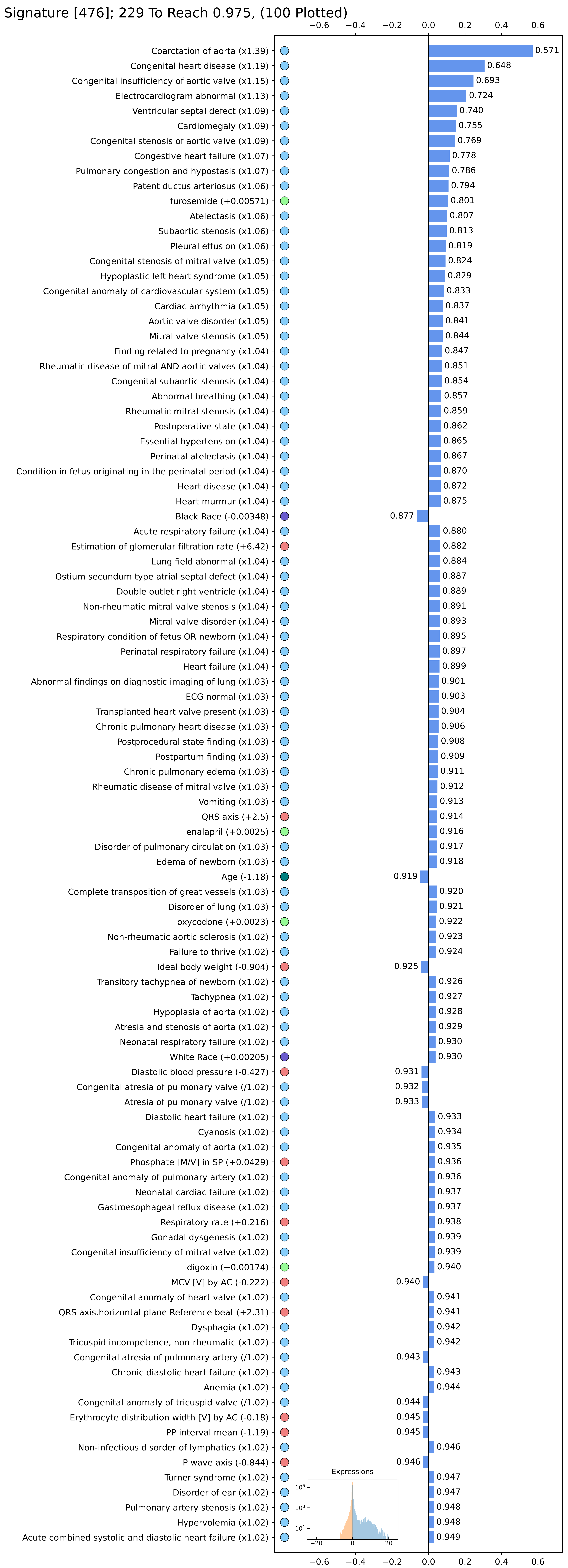

Signature [476]; 229 To Reach 0.975, (100 Plotted)
Coarctation of aorta (x1.39) 0.571
Congenital heart disease (x1.19) 0.648
Congenital insufficiency of aortic valve (x1.15) 0.693
Electrocardiogram abnormal (x1.13) 0.724
Ventricular septal defect (x1.09) 0.740
Cardiomegaly (x1.09) 0.755
Congenital stenosis of aortic valve (x1.09) 0.769
Congestive heart failure (x1.07) 0.778
Pulmonary congestion and hypostasis (x1.07) 0.786
Patent ductus arteriosus (x1.06) 0.794
furosemide (+0.00571) 0.801
Atelectasis (x1.06) 0.807
Subaortic stenosis (x1.06) 0.813
Pleural effusion (x1.06) 0.819
Congenital stenosis of mitral valve (x1.05) 0.824
Hypoplastic left heart syndrome (x1.05) 0.829
Congenital anomaly of cardiovascular system (x1.05) 0.833
Cardiac arrhythmia (x1.05) 0.837
Aortic valve disorder (x1.05) 0.841
Mitral valve stenosis (x1.05) 0.844
Finding related to pregnancy (x1.04) 0.847
Rheumatic disease of mitral AND aortic valves (x1.04) 0.851
Congenital subaortic stenosis (x1.04) 0.854
Abnormal breathing (x1.04) 0.857
Rheumatic mitral stenosis (x1.04) 0.859
Postoperative state (x1.04) 0.862
Essential hypertension (x1.04) 0.865
Perinatal atelectasis (x1.04) 0.867
Condition in fetus originating in the perinatal period (x1.04) 0.870
Heart disease (x1.04) 0.872
Heart murmur (x1.04) 0.875
Black Race (-0.00348) 0.877
Acute respiratory failure (x1.04) 0.880
Estimation of glomerular filtration rate (+6.42) 0.882
Lung field abnormal (x1.04) 0.884
Ostium secundum type atrial septal defect (x1.04) 0.887
Double outlet right ventricle (x1.04) 0.889
Non-rheumatic mitral valve stenosis (x1.04) 0.891
Mitral valve disorder (x1.04) 0.893
Respiratory condition of fetus OR newborn (x1.04) 0.895
Perinatal respiratory failure (x1.04) 0.897
Heart failure (x1.04) 0.899
Abnormal findings on diagnostic imaging of lung (x1.03) 0.901
ECG normal (x1.03) 0.903
Transplanted heart valve present (x1.03) 0.904
Chronic pulmonary heart disease (x1.03) 0.906
Postprocedural state finding (x1.03) 0.908
Postpartum finding (x1.03) 0.909
Chronic pulmonary edema (x1.03) 0.911
Rheumatic disease of mitral valve (x1.03) 0.912
Vomiting (x1.03) 0.913
QRS axis (+2.5) 0.914
enalapril (+0.0025) 0.916
Disorder of pulmonary circulation (x1.03) 0.917
Edema of newborn (x1.03) 0.918
Age (-1.18) 0.919
Complete transposition of great vessels (x1.03) 0.920
Disorder of lung (x1.03) 0.921
oxycodone (+0.0023) 0.922
Non-rheumatic aortic sclerosis (x1.02) 0.923
Failure to thrive (x1.02) 0.924
Ideal body weight (-0.904) 0.925
Transitory tachypnea of newborn (x1.02) 0.926
Tachypnea (x1.02) 0.927
Hypoplasia of aorta (x1.02) 0.928
Atresia and stenosis of aorta (x1.02) 0.929
Neonatal respiratory failure (x1.02) 0.930
White Race (+0.00205) 0.930
Diastolic blood pressure (-0.427) 0.931
Congenital atresia of pulmonary valve (/1.02) 0.932
Atresia of pulmonary valve (/1.02) 0.933
Diastolic heart failure (x1.02) 0.933
Cyanosis (x1.02) 0.934
Congenital anomaly of aorta (x1.02) 0.935
Phosphate [M/V] in SP (+0.0429) 0.936
Congenital anomaly of pulmonary artery (x1.02) 0.936
Neonatal cardiac failure (x1.02) 0.937
Gastroesophageal reflux disease (x1.02) 0.937
Respiratory rate (+0.216) 0.938
Gonadal dysgenesis (x1.02) 0.939
Congenital insufficiency of mitral valve (x1.02) 0.939
digoxin (+0.00174) 0.940
MCV [V] by AC (-0.222) 0.940
Congenital anomaly of heart valve (x1.02) 0.941
QRS axis.horizontal plane Reference beat (+2.31) 0.941
Dysphagia (x1.02) 0.942
Tricuspid incompetence, non-rheumatic (x1.02) 0.942
Congenital atresia of pulmonary artery (/1.02) 0.943
Chronic diastolic heart failure (x1.02) 0.943
Anemia (x1.02) 0.944
Congenital anomaly of tricuspid valve (/1.02) 0.944
Erythrocyte distribution width [V] by AC (-0.18) 0.945
PP interval mean (-1.19) 0.945
Non-infectious disorder of lymphatics (x1.02) 0.946
P wave axis (-0.844) 0.946
Turner syndrome (x1.02) 0.947
Disorder of ear (x1.02) 0.947
Pulmonary artery stenosis (x1.02) 0.948
Hypervolemia (x1.02) 0.948
Acute combined systolic and diastolic heart failure (x1.02) 0.949
−0.6 −0.4 −0.2 0.0 0.2 0.4 0.6
Expressions
10^5 10^3 10^1
−20 0 20

Signature [477]; 2 To Reach 0.975, (10 Plotted)

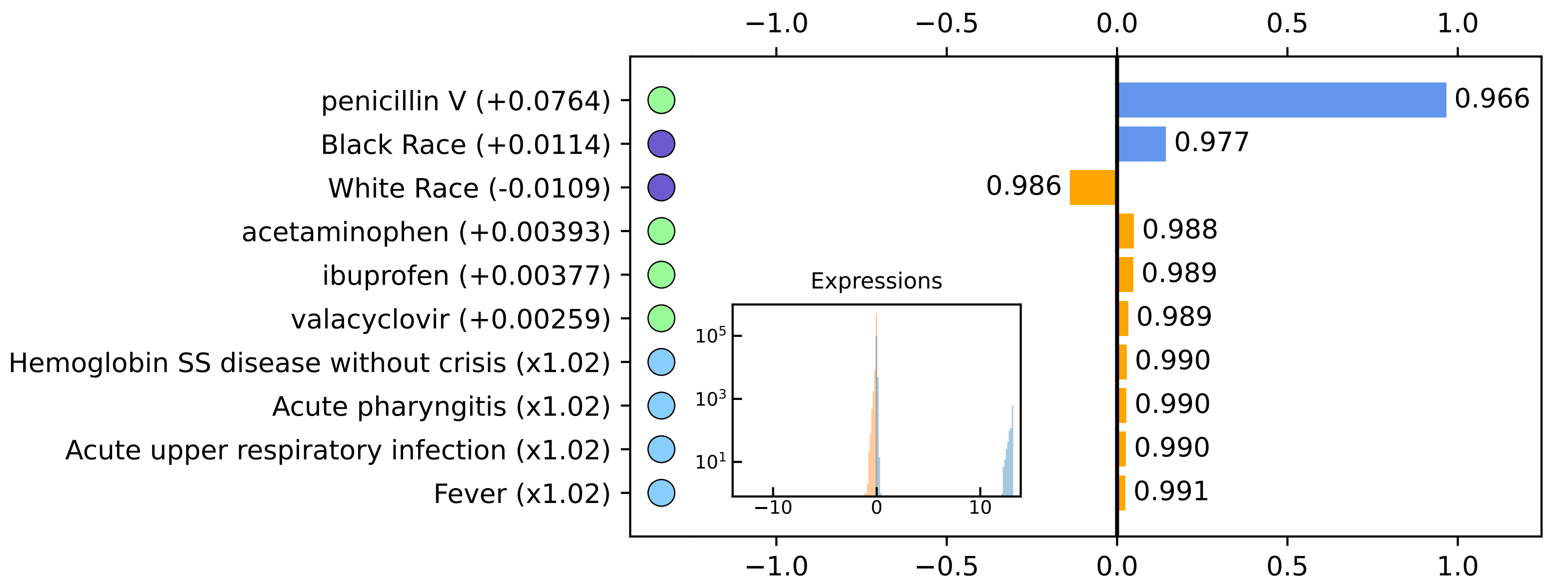

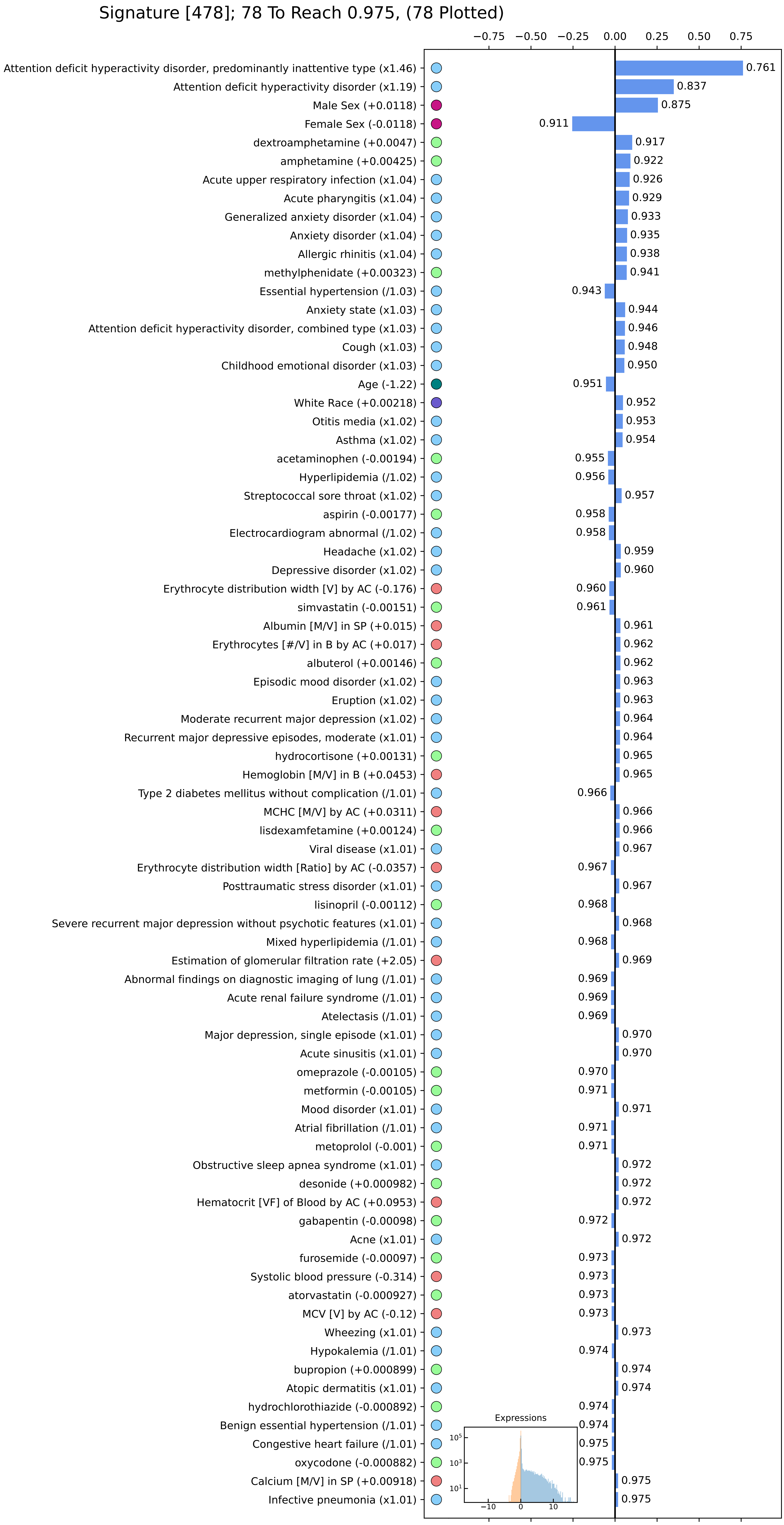

Signature [478]; 78 To Reach 0.975, (78 Plotted)
Attention deficit hyperactivity disorder, predominantly inattentive type (x1.46) 0.761
Attention deficit hyperactivity disorder (x1.19) 0.837
Male Sex (+0.0118) 0.875
Female Sex (-0.0118) 0.911
dextroamphetamine (+0.0047) 0.917
amphetamine (+0.00425) 0.922
Acute upper respiratory infection (x1.04) 0.926
Acute pharyngitis (x1.04) 0.929
Generalized anxiety disorder (x1.04) 0.933
Anxiety disorder (x1.04) 0.935
Allergic rhinitis (x1.04) 0.938
methylphenidate (+0.00323) 0.941
Essential hypertension (/1.03) 0.943
Anxiety state (x1.03) 0.944
Attention deficit hyperactivity disorder, combined type (x1.03) 0.946
Cough (x1.03) 0.948
Childhood emotional disorder (x1.03) 0.950
Age (-1.22) 0.951
White Race (+0.00218) 0.952
Otitis media (x1.02) 0.953
Asthma (x1.02) 0.954
acetaminophen (-0.00194) 0.955
Hyperlipidemia (/1.02) 0.956
Streptococcal sore throat (x1.02) 0.957
aspirin (-0.00177) 0.958
Electrocardiogram abnormal (/1.02) 0.958
Headache (x1.02) 0.959
Depressive disorder (x1.02) 0.960
Erythrocyte distribution width [V] by AC (-0.176) 0.960
simvastatin (-0.00151) 0.961
Albumin [M/V] in SP (+0.015) 0.961
Erythrocytes [#/V] in B by AC (+0.017) 0.962
albuterol (+0.00146) 0.962
Episodic mood disorder (x1.02) 0.963
Eruption (x1.02) 0.963
Moderate recurrent major depression (x1.02) 0.964
Recurrent major depressive episodes, moderate (x1.01) 0.964
hydrocortisone (+0.00131) 0.965
Hemoglobin [M/V] in B (+0.0453) 0.965
Type 2 diabetes mellitus without complication (/1.01) 0.966
MCHC [M/V] by AC (+0.0311) 0.966
lisdexamfetamine (+0.00124) 0.966
Viral disease (x1.01) 0.967
Erythrocyte distribution width [Ratio] by AC (-0.0357) 0.967
Posttraumatic stress disorder (x1.01) 0.967
lisinopril (-0.00112) 0.968
Severe recurrent major depression without psychotic features (x1.01) 0.968
Mixed hyperlipidemia (/1.01) 0.968
Estimation of glomerular filtration rate (+2.05) 0.969
Abnormal findings on diagnostic imaging of lung (/1.01) 0.969
Acute renal failure syndrome (/1.01) 0.969
Atelectasis (/1.01) 0.969
Major depression, single episode (x1.01) 0.970
Acute sinusitis (x1.01) 0.970
omeprazole (-0.00105) 0.970
metformin (-0.00105) 0.971
Mood disorder (x1.01) 0.971
Atrial fibrillation (/1.01) 0.971
metoprolol (-0.001) 0.971
Obstructive sleep apnea syndrome (x1.01) 0.972
desonide (+0.000982) 0.972
Hematocrit [VF] of Blood by AC (+0.0953) 0.972
gabapentin (-0.00098) 0.972
Acne (x1.01) 0.972
furosemide (-0.00097) 0.973
Systolic blood pressure (-0.314) 0.973
atorvastatin (-0.000927) 0.973
MCV [V] by AC (-0.12) 0.973
Wheezing (x1.01) 0.973
Hypokalemia (/1.01) 0.974
bupropion (+0.000899) 0.974
Atopic dermatitis (x1.01) 0.974
hydrochlorothiazide (-0.000892) 0.974
Benign essential hypertension (/1.01) 0.974
Congestive heart failure (/1.01) 0.975
oxycodone (-0.000882) 0.975
Calcium [M/V] in SP (+0.00918) 0.975
Infective pneumonia (x1.01) 0.975
−0.75 −0.50 −0.25 0.00 0.25 0.50 0.75
Expressions
−10 0 10

Signature [479]; 2 To Reach 0.975, (10 Plotted)

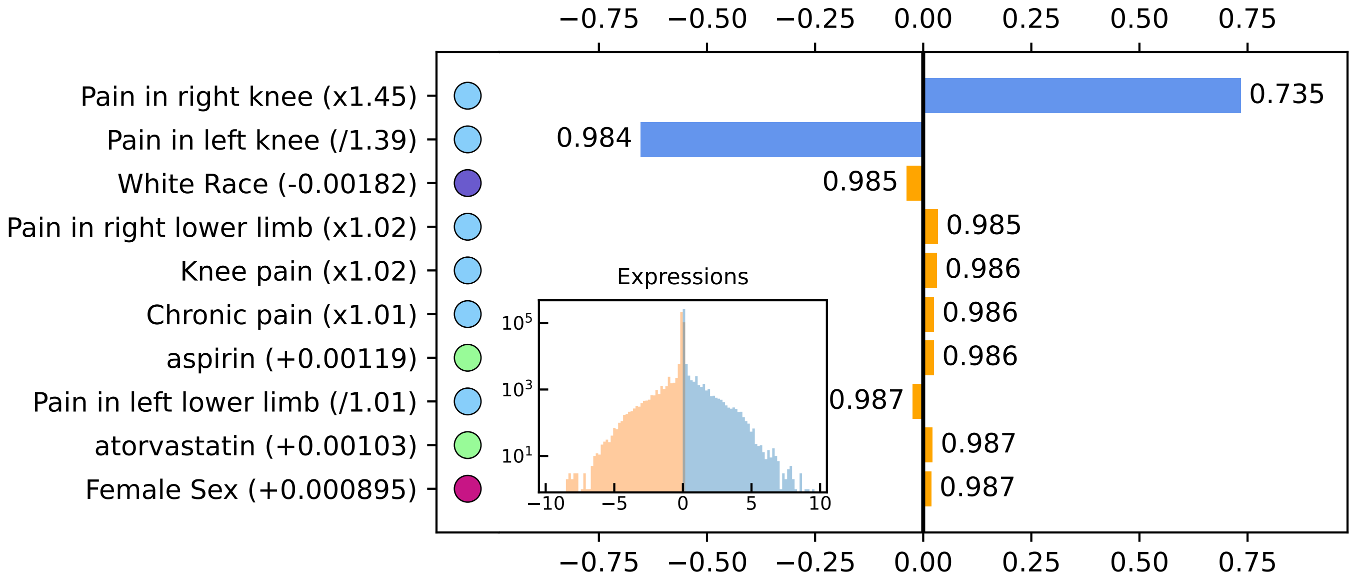

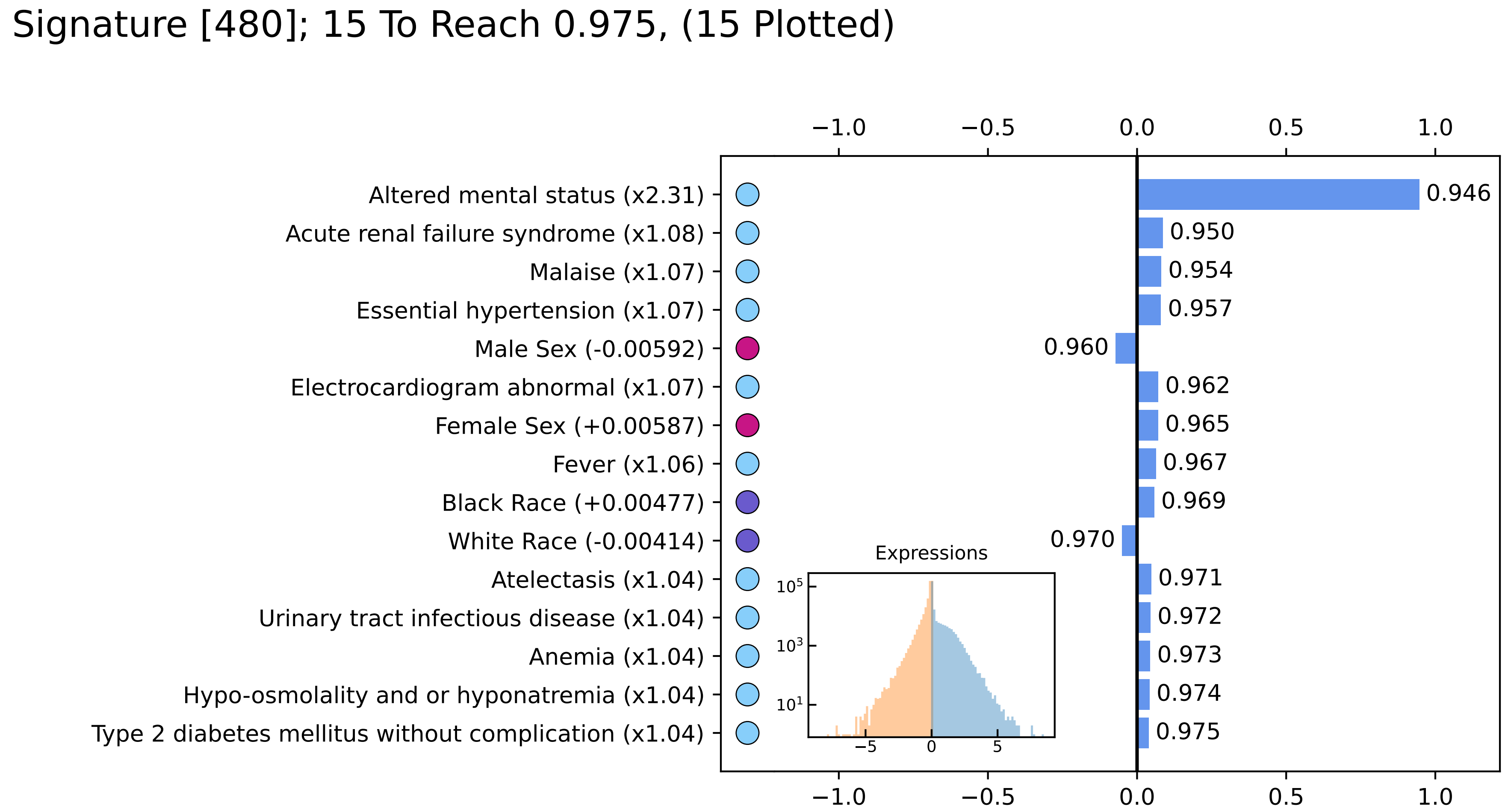

Signature [480]; 15 To Reach 0.975, (15 Plotted)
−1.0
−0.5
0.0
0.5
1.0
Altered mental status (x2.31)
Acute renal failure syndrome (x1.08)
Malaise (x1.07)
Essential hypertension (x1.07)
Male Sex (-0.00592)
Electrocardiogram abnormal (x1.07)
Female Sex (+0.00587)
Fever (x1.06)
Black Race (+0.00477)
White Race (-0.00414)
Atelectasis (x1.04)
Urinary tract infectious disease (x1.04)
Anemia (x1.04)
Hypo-osmolality and or hyponatremia (x1.04)
Type 2 diabetes mellitus without complication (x1.04)
0.946
0.950
0.954
0.957
0.960
0.962
0.965
0.967
0.969
0.970
0.971
0.972
0.973
0.974
0.975
Expressions
10^5
10^3
10^1
−5
0
5

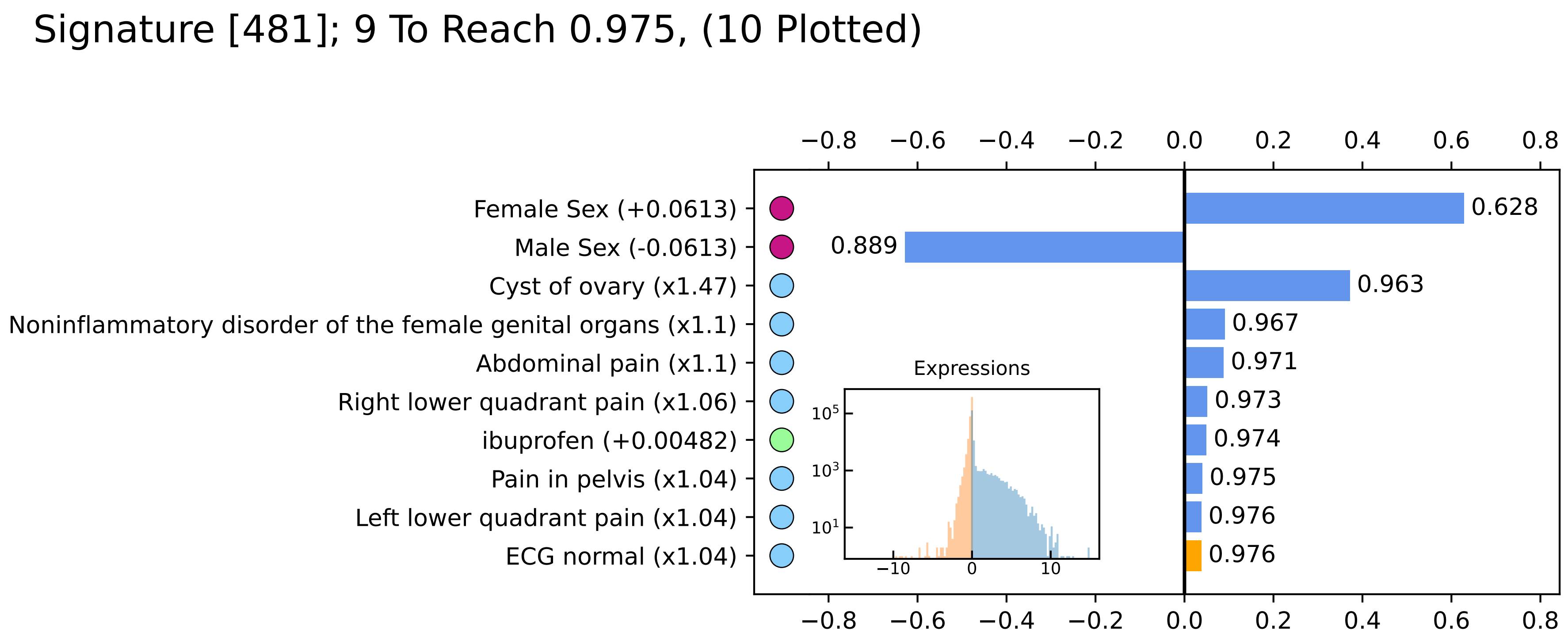

Signature [481]; 9 To Reach 0.975, (10 Plotted)
Female Sex (+0.0613)
Male Sex (-0.0613)
Cyst of ovary (x1.47)
Noninflammatory disorder of the female genital organs (x1.1)
Abdominal pain (x1.1)
Right lower quadrant pain (x1.06)
ibuprofen (+0.00482)
Pain in pelvis (x1.04)
Left lower quadrant pain (x1.04)
ECG normal (x1.04)
0.628
0.889
0.963
0.967
0.971
0.973
0.974
0.975
0.976
0.976
Expressions
−0.8
−0.6
−0.4
−0.2
0.0
0.2
0.4
0.6
0.8

Signature [482]; 9 To Reach 0.975, (10 Plotted)

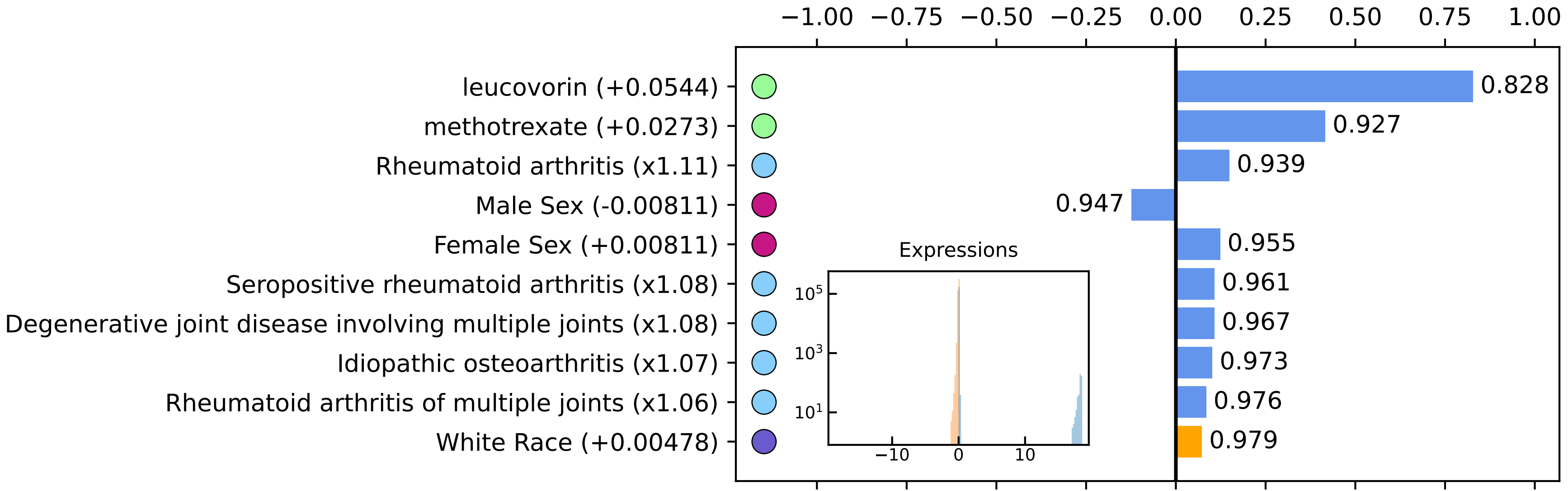

Signature [483]; 1 To Reach 0.975, (10 Plotted)

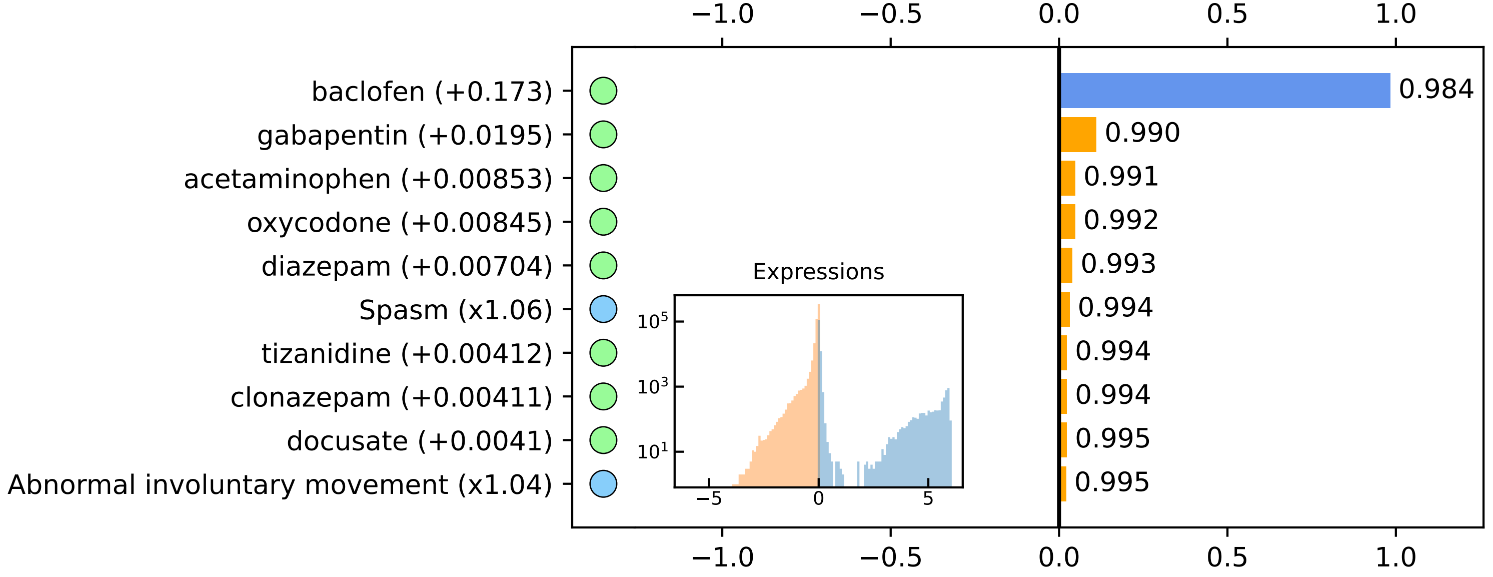

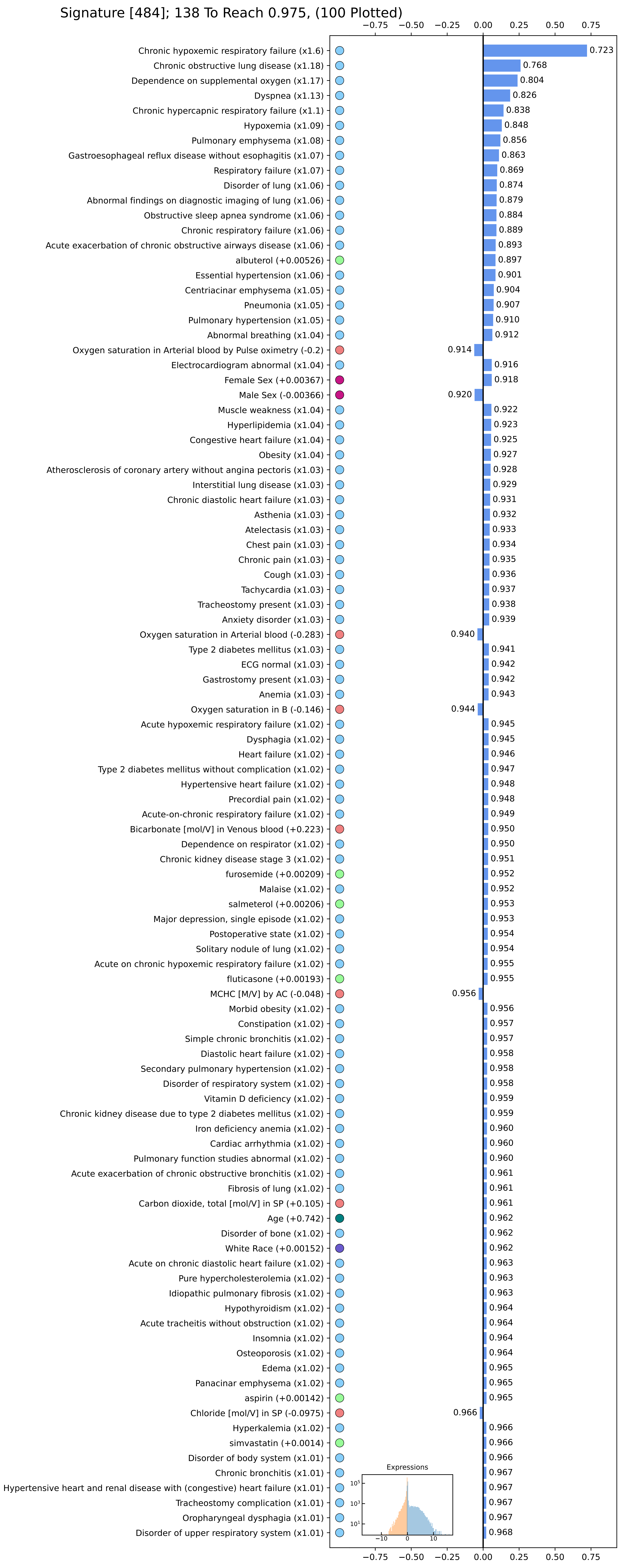

Signature [484]; 138 To Reach 0.975, (100 Plotted)
Chronic hypoxemic respiratory failure (x1.6) 0.723
Chronic obstructive lung disease (x1.18) 0.768
Dependence on supplemental oxygen (x1.17) 0.804
Dyspnea (x1.13) 0.826
Chronic hypercapnic respiratory failure (x1.1) 0.838
Hypoxemia (x1.09) 0.848
Pulmonary emphysema (x1.08) 0.856
Gastroesophageal reflux disease without esophagitis (x1.07) 0.863
Respiratory failure (x1.07) 0.869
Disorder of lung (x1.06) 0.874
Abnormal findings on diagnostic imaging of lung (x1.06) 0.879
Obstructive sleep apnea syndrome (x1.06) 0.884
Chronic respiratory failure (x1.06) 0.889
Acute exacerbation of chronic obstructive airways disease (x1.06) 0.893
albuterol (+0.00526) 0.897
Essential hypertension (x1.06) 0.901
Centriacinar emphysema (x1.05) 0.904
Pneumonia (x1.05) 0.907
Pulmonary hypertension (x1.05) 0.910
Abnormal breathing (x1.04) 0.912
Oxygen saturation in Arterial blood by Pulse oximetry (-0.2) 0.914
Electrocardiogram abnormal (x1.04) 0.916
Female Sex (+0.00367) 0.918
Male Sex (-0.00366) 0.920
Muscle weakness (x1.04) 0.922
Hyperlipidemia (x1.04) 0.923
Congestive heart failure (x1.04) 0.925
Obesity (x1.04) 0.927
Atherosclerosis of coronary artery without angina pectoris (x1.03) 0.928
Interstitial lung disease (x1.03) 0.929
Chronic diastolic heart failure (x1.03) 0.931
Asthenia (x1.03) 0.932
Atelectasis (x1.03) 0.933
Chest pain (x1.03) 0.934
Chronic pain (x1.03) 0.935
Cough (x1.03) 0.936
Tachycardia (x1.03) 0.937
Tracheostomy present (x1.03) 0.938
Anxiety disorder (x1.03) 0.939
Oxygen saturation in Arterial blood (-0.283) 0.940
Type 2 diabetes mellitus (x1.03) 0.941
ECG normal (x1.03) 0.942
Gastrostomy present (x1.03) 0.942
Anemia (x1.03) 0.943
Oxygen saturation in B (-0.146) 0.944
Acute hypoxemic respiratory failure (x1.02) 0.945
Dysphagia (x1.02) 0.945
Heart failure (x1.02) 0.946
Type 2 diabetes mellitus without complication (x1.02) 0.947
Hypertensive heart failure (x1.02) 0.948
Precordial pain (x1.02) 0.948
Acute-on-chronic respiratory failure (x1.02) 0.949
Bicarbonate [mol/V] in Venous blood (+0.223) 0.950
Dependence on respirator (x1.02) 0.950
Chronic kidney disease stage 3 (x1.02) 0.951
furosemide (+0.00209) 0.952
Malaise (x1.02) 0.952
salmeterol (+0.00206) 0.953
Major depression, single episode (x1.02) 0.953
Postoperative state (x1.02) 0.954
Solitary nodule of lung (x1.02) 0.954
Acute on chronic hypoxemic respiratory failure (x1.02) 0.955
fluticasone (+0.00193) 0.955
MCHC [M/V] by AC (-0.048) 0.956
Morbid obesity (x1.02) 0.956
Constipation (x1.02) 0.957
Simple chronic bronchitis (x1.02) 0.957
Diastolic heart failure (x1.02) 0.958
Secondary pulmonary hypertension (x1.02) 0.958
Disorder of respiratory system (x1.02) 0.958
Vitamin D deficiency (x1.02) 0.959
Chronic kidney disease due to type 2 diabetes mellitus (x1.02) 0.959
Iron deficiency anemia (x1.02) 0.960
Cardiac arrhythmia (x1.02) 0.960
Pulmonary function studies abnormal (x1.02) 0.960
Acute exacerbation of chronic obstructive bronchitis (x1.02) 0.961
Fibrosis of lung (x1.02) 0.961
Carbon dioxide, total [mol/V] in SP (+0.105) 0.961
Age (+0.742) 0.962
Disorder of bone (x1.02) 0.962
White Race (+0.00152) 0.962
Acute on chronic diastolic heart failure (x1.02) 0.963
Pure hypercholesterolemia (x1.02) 0.963
Idiopathic pulmonary fibrosis (x1.02) 0.963
Hypothyroidism (x1.02) 0.964
Acute tracheitis without obstruction (x1.02) 0.964
Insomnia (x1.02) 0.964
Osteoporosis (x1.02) 0.964
Edema (x1.02) 0.965
Panacinar emphysema (x1.02) 0.965
aspirin (+0.00142) 0.965
Chloride [mol/V] in SP (-0.0975) 0.966
Hyperkalemia (x1.02) 0.966
simvastatin (+0.0014) 0.966
Disorder of body system (x1.01) 0.966
Chronic bronchitis (x1.01) 0.967
Hypertensive heart and renal disease with (congestive) heart failure (x1.01) 0.967
Tracheostomy complication (x1.01) 0.967
Oropharyngeal dysphagia (x1.01) 0.967
Disorder of upper respiratory system (x1.01) 0.968
−0.75 −0.50 −0.25 0.00 0.25 0.50 0.75
Expressions

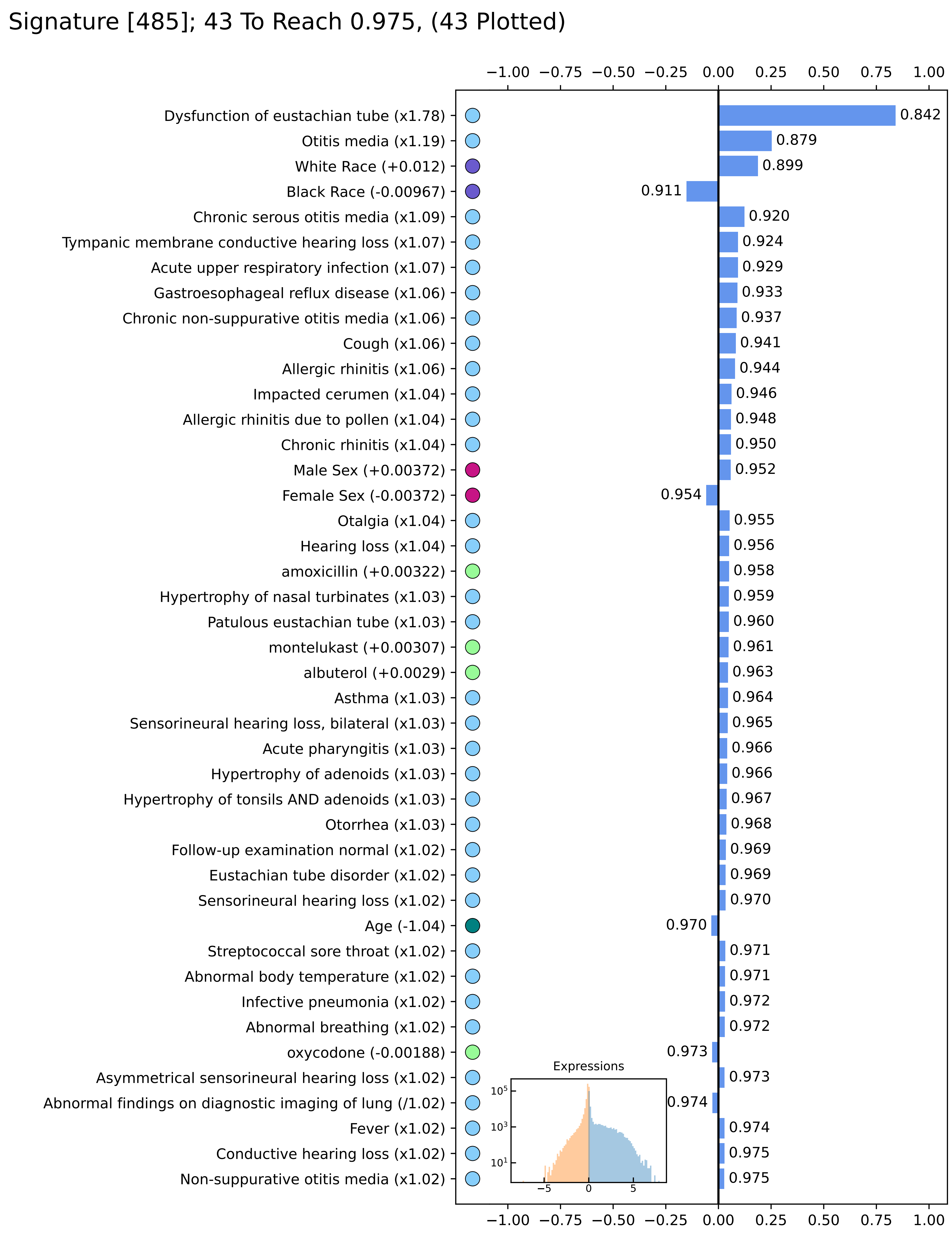
Signature [485]; 43 To Reach 0.975, (43 Plotted)
Dysfunction of eustachian tube (x1.78) 0.842
Otitis media (x1.19) 0.879
White Race (+0.012) 0.899
Black Race (-0.00967) 0.911
Chronic serous otitis media (x1.09) 0.920
Tympanic membrane conductive hearing loss (x1.07) 0.924
Acute upper respiratory infection (x1.07) 0.929
Gastroesophageal reflux disease (x1.06) 0.933
Chronic non-suppurative otitis media (x1.06) 0.937
Cough (x1.06) 0.941
Allergic rhinitis (x1.06) 0.944
Impacted cerumen (x1.04) 0.946
Allergic rhinitis due to pollen (x1.04) 0.948
Chronic rhinitis (x1.04) 0.950
Male Sex (+0.00372) 0.952
Female Sex (-0.00372) 0.954
Otalgia (x1.04) 0.955
Hearing loss (x1.04) 0.956
amoxicillin (+0.00322) 0.958
Hypertrophy of nasal turbinates (x1.03) 0.959
Patulous eustachian tube (x1.03) 0.960
montelukast (+0.00307) 0.961
albuterol (+0.0029) 0.963
Asthma (x1.03) 0.964
Sensorineural hearing loss, bilateral (x1.03) 0.965
Acute pharyngitis (x1.03) 0.966
Hypertrophy of adenoids (x1.03) 0.966
Hypertrophy of tonsils AND adenoids (x1.03) 0.967
Otorrhea (x1.03) 0.968
Follow-up examination normal (x1.02) 0.969
Eustachian tube disorder (x1.02) 0.969
Sensorineural hearing loss (x1.02) 0.970
Age (-1.04) 0.970
Streptococcal sore throat (x1.02) 0.971
Abnormal body temperature (x1.02) 0.971
Infective pneumonia (x1.02) 0.972
Abnormal breathing (x1.02) 0.972
oxycodone (-0.00188) 0.973
Asymmetrical sensorineural hearing loss (x1.02) 0.973
Abnormal findings on diagnostic imaging of lung (/1.02) 0.974
Fever (x1.02) 0.974
Conductive hearing loss (x1.02) 0.975
Non-suppurative otitis media (x1.02) 0.975
−1.00 −0.75 −0.50 −0.25 0.00 0.25 0.50 0.75 1.00
Expressions
10^5
10^3
10^1
−5 0 5

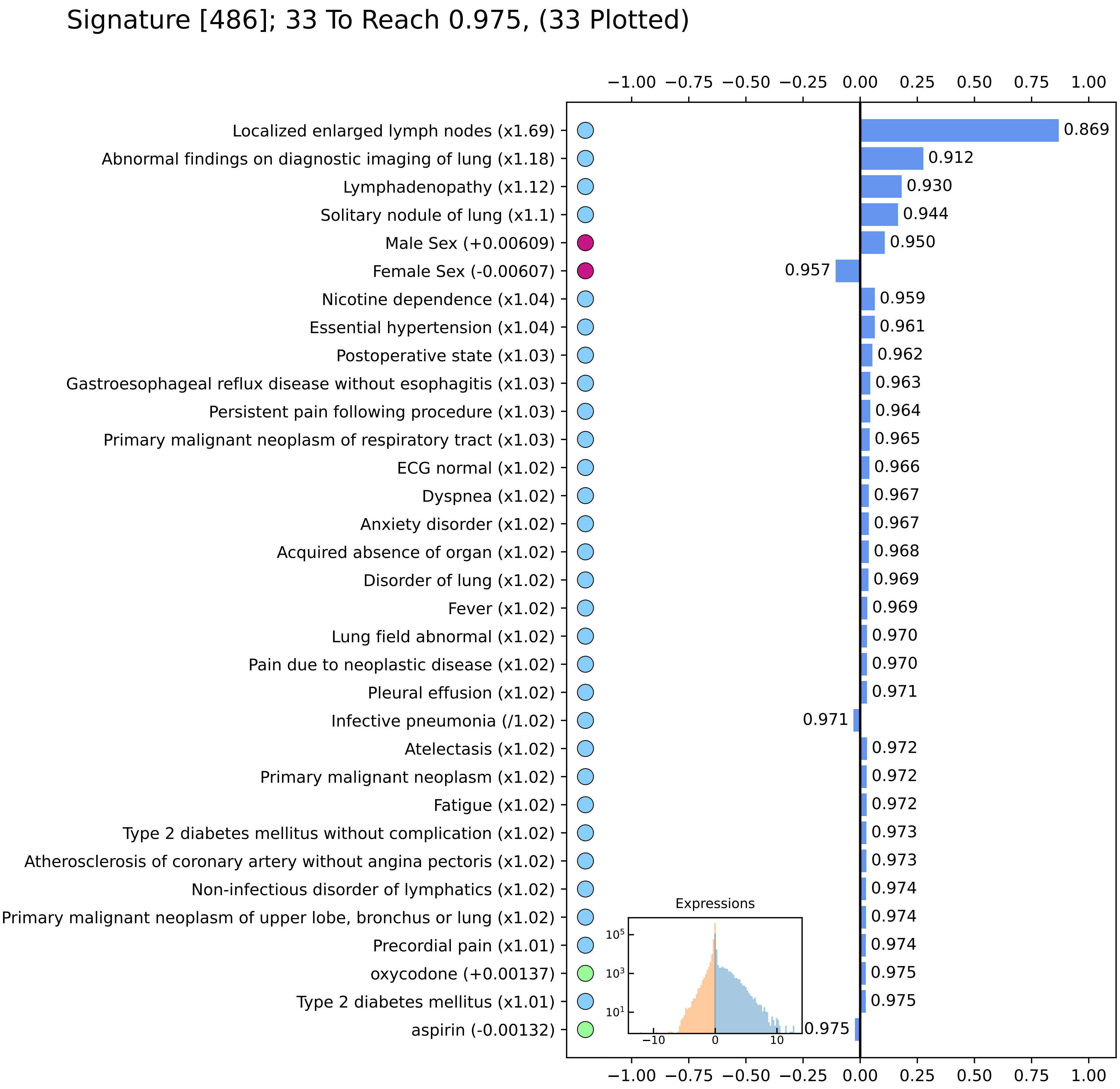

Signature [486]; 33 To Reach 0.975, (33 Plotted)
Localized enlarged lymph nodes (x1.69) 0.869
Abnormal findings on diagnostic imaging of lung (x1.18) 0.912
Lymphadenopathy (x1.12) 0.930
Solitary nodule of lung (x1.1) 0.944
Male Sex (+0.00609) 0.950
Female Sex (-0.00607) 0.957
Nicotine dependence (x1.04) 0.959
Essential hypertension (x1.04) 0.961
Postoperative state (x1.03) 0.962
Gastroesophageal reflux disease without esophagitis (x1.03) 0.963
Persistent pain following procedure (x1.03) 0.964
Primary malignant neoplasm of respiratory tract (x1.03) 0.965
ECG normal (x1.02) 0.966
Dyspnea (x1.02) 0.967
Anxiety disorder (x1.02) 0.967
Acquired absence of organ (x1.02) 0.968
Disorder of lung (x1.02) 0.969
Fever (x1.02) 0.969
Lung field abnormal (x1.02) 0.970
Pain due to neoplastic disease (x1.02) 0.970
Pleural effusion (x1.02) 0.971
Infective pneumonia (/1.02) 0.971
Atelectasis (x1.02) 0.972
Primary malignant neoplasm (x1.02) 0.972
Fatigue (x1.02) 0.972
Type 2 diabetes mellitus without complication (x1.02) 0.973
Atherosclerosis of coronary artery without angina pectoris (x1.02) 0.973
Non-infectious disorder of lymphatics (x1.02) 0.974
Primary malignant neoplasm of upper lobe, bronchus or lung (x1.02) 0.974
Precordial pain (x1.01) 0.974
oxycodone (+0.00137) 0.975
Type 2 diabetes mellitus (x1.01) 0.975
aspirin (-0.00132) 0.975
−1.00 −0.75 −0.50 −0.25 0.00 0.25 0.50 0.75 1.00
Expressions

Signature [487]; 6 To Reach 0.975, (10 Plotted)

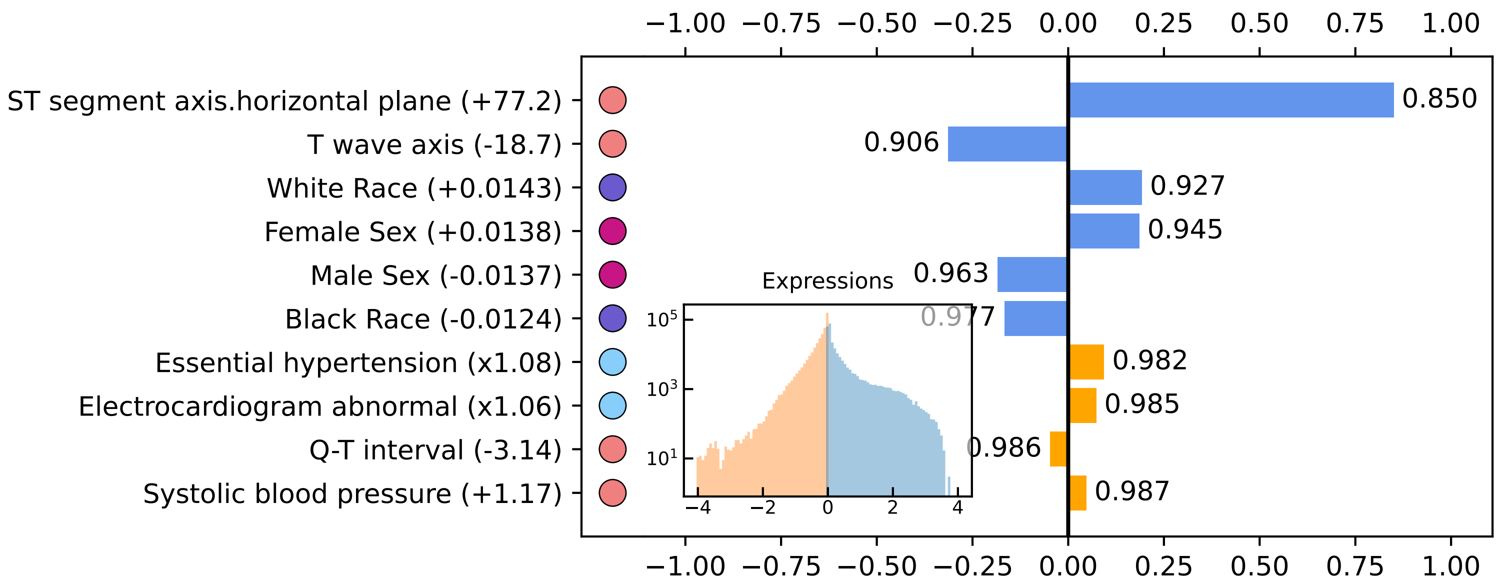

Signature [488]; 2 To Reach 0.975, (10 Plotted)

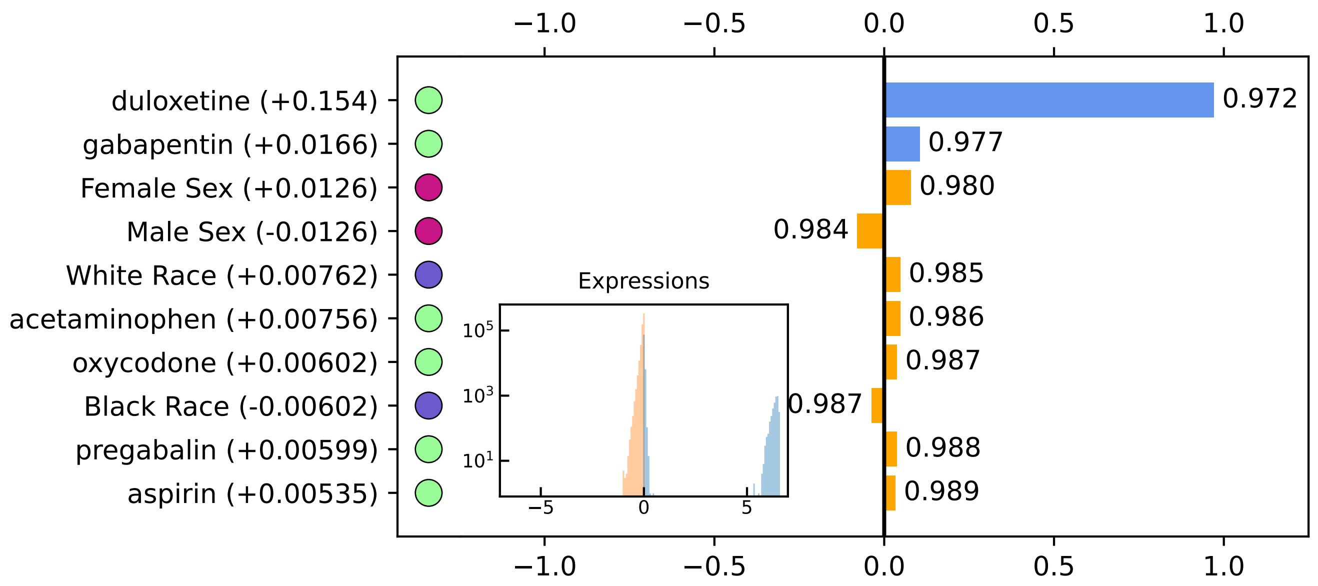

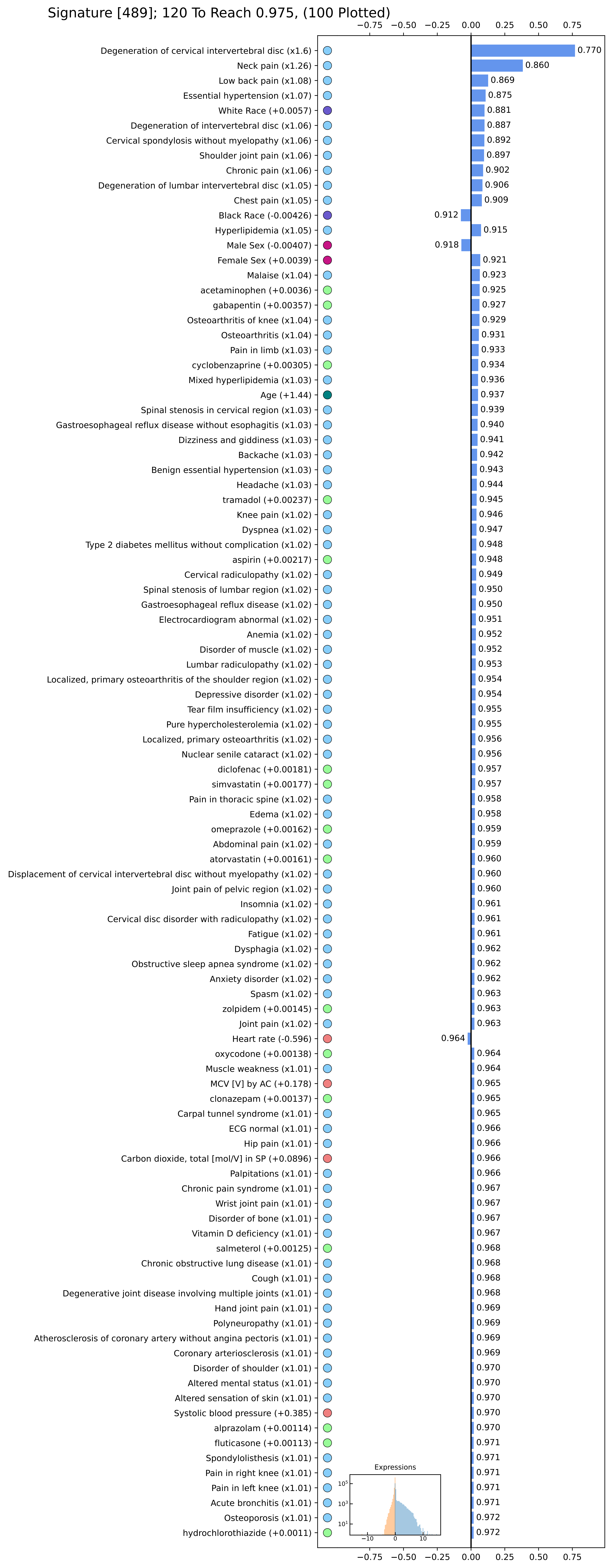

Signature [489]; 120 To Reach 0.975, (100 Plotted)
Degeneration of cervical intervertebral disc (x1.6) 0.770
Neck pain (x1.26) 0.860
Low back pain (x1.08) 0.869
Essential hypertension (x1.07) 0.875
White Race (+0.0057) 0.881
Degeneration of intervertebral disc (x1.06) 0.887
Cervical spondylosis without myelopathy (x1.06) 0.892
Shoulder joint pain (x1.06) 0.897
Chronic pain (x1.06) 0.902
Degeneration of lumbar intervertebral disc (x1.05) 0.906
Chest pain (x1.05) 0.909
Black Race (-0.00426) 0.912
Hyperlipidemia (x1.05) 0.915
Male Sex (-0.00407) 0.918
Female Sex (+0.0039) 0.921
Malaise (x1.04) 0.923
acetaminophen (+0.0036) 0.925
gabapentin (+0.00357) 0.927
Osteoarthritis of knee (x1.04) 0.929
Osteoarthritis (x1.04) 0.931
Pain in limb (x1.03) 0.933
cyclobenzaprine (+0.00305) 0.934
Mixed hyperlipidemia (x1.03) 0.936
Age (+1.44) 0.937
Spinal stenosis in cervical region (x1.03) 0.939
Gastroesophageal reflux disease without esophagitis (x1.03) 0.940
Dizziness and giddiness (x1.03) 0.941
Backache (x1.03) 0.942
Benign essential hypertension (x1.03) 0.943
Headache (x1.03) 0.944
tramadol (+0.00237) 0.945
Knee pain (x1.02) 0.946
Dyspnea (x1.02) 0.947
Type 2 diabetes mellitus without complication (x1.02) 0.948
aspirin (+0.00217) 0.948
Cervical radiculopathy (x1.02) 0.949
Spinal stenosis of lumbar region (x1.02) 0.950
Gastroesophageal reflux disease (x1.02) 0.950
Electrocardiogram abnormal (x1.02) 0.951
Anemia (x1.02) 0.952
Disorder of muscle (x1.02) 0.952
Lumbar radiculopathy (x1.02) 0.953
Localized, primary osteoarthritis of the shoulder region (x1.02) 0.954
Depressive disorder (x1.02) 0.954
Tear film insufficiency (x1.02) 0.955
Pure hypercholesterolemia (x1.02) 0.955
Localized, primary osteoarthritis (x1.02) 0.956
Nuclear senile cataract (x1.02) 0.956
diclofenac (+0.00181) 0.957
simvastatin (+0.00177) 0.957
Pain in thoracic spine (x1.02) 0.958
Edema (x1.02) 0.958
omeprazole (+0.00162) 0.959
Abdominal pain (x1.02) 0.959
atorvastatin (+0.00161) 0.960
Displacement of cervical intervertebral disc without myelopathy (x1.02) 0.960
Joint pain of pelvic region (x1.02) 0.960
Insomnia (x1.02) 0.961
Cervical disc disorder with radiculopathy (x1.02) 0.961
Fatigue (x1.02) 0.961
Dysphagia (x1.02) 0.962
Obstructive sleep apnea syndrome (x1.02) 0.962
Anxiety disorder (x1.02) 0.962
Spasm (x1.02) 0.963
zolpidem (+0.00145) 0.963
Joint pain (x1.02) 0.963
Heart rate (-0.596) 0.964
oxycodone (+0.00138) 0.964
Muscle weakness (x1.01) 0.964
MCV [V] by AC (+0.178) 0.965
clonazepam (+0.00137) 0.965
Carpal tunnel syndrome (x1.01) 0.965
ECG normal (x1.01) 0.966
Hip pain (x1.01) 0.966
Carbon dioxide, total [mol/V] in SP (+0.0896) 0.966
Palpitations (x1.01) 0.966
Chronic pain syndrome (x1.01) 0.967
Wrist joint pain (x1.01) 0.967
Disorder of bone (x1.01) 0.967
Vitamin D deficiency (x1.01) 0.967
salmeterol (+0.00125) 0.968
Chronic obstructive lung disease (x1.01) 0.968
Cough (x1.01) 0.968
Degenerative joint disease involving multiple joints (x1.01) 0.968
Hand joint pain (x1.01) 0.969
Polyneuropathy (x1.01) 0.969
Atherosclerosis of coronary artery without angina pectoris (x1.01) 0.969
Coronary arteriosclerosis (x1.01) 0.969
Disorder of shoulder (x1.01) 0.970
Altered mental status (x1.01) 0.970
Altered sensation of skin (x1.01) 0.970
Systolic blood pressure (+0.385) 0.970
alprazolam (+0.00114) 0.970
fluticasone (+0.00113) 0.971
Spondylolisthesis (x1.01) 0.971
Pain in right knee (x1.01) 0.971
Pain in left knee (x1.01) 0.971
Acute bronchitis (x1.01) 0.971
Osteoporosis (x1.01) 0.972
hydrochlorothiazide (+0.0011) 0.972
−0.75 −0.50 −0.25 0.00 0.25 0.50 0.75
Expressions
10^5
10^3
10^1
−10 0 10

Signature [490]; 1 To Reach 0.975, (10 Plotted)

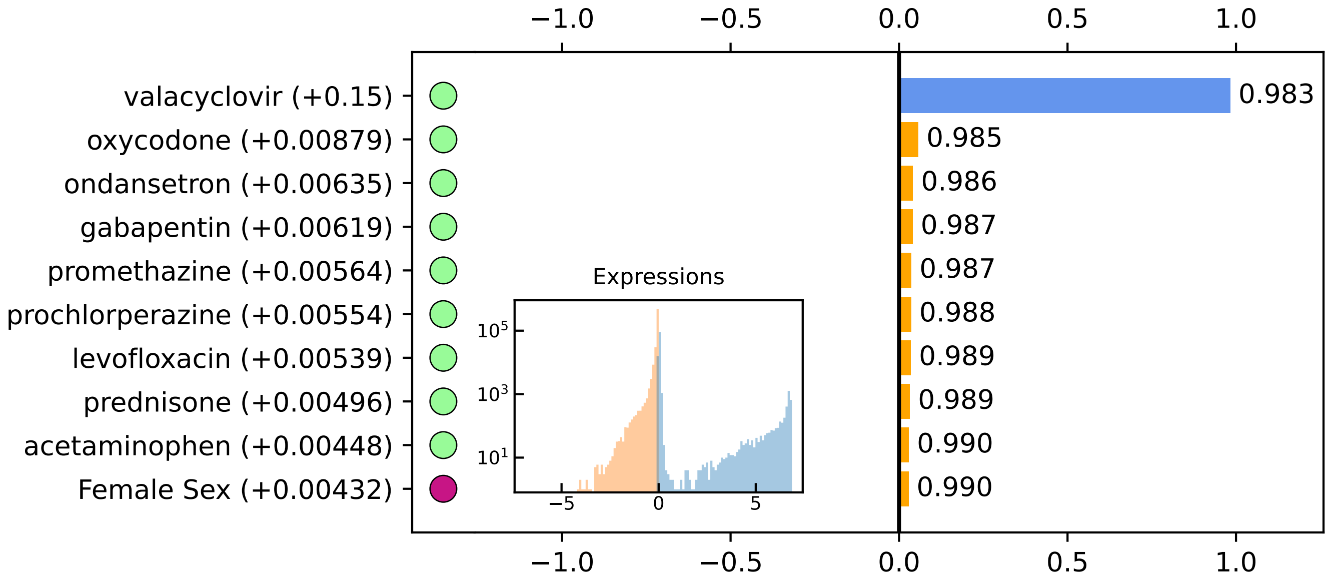

Signature [491]; 2 To Reach 0.975, (10 Plotted)

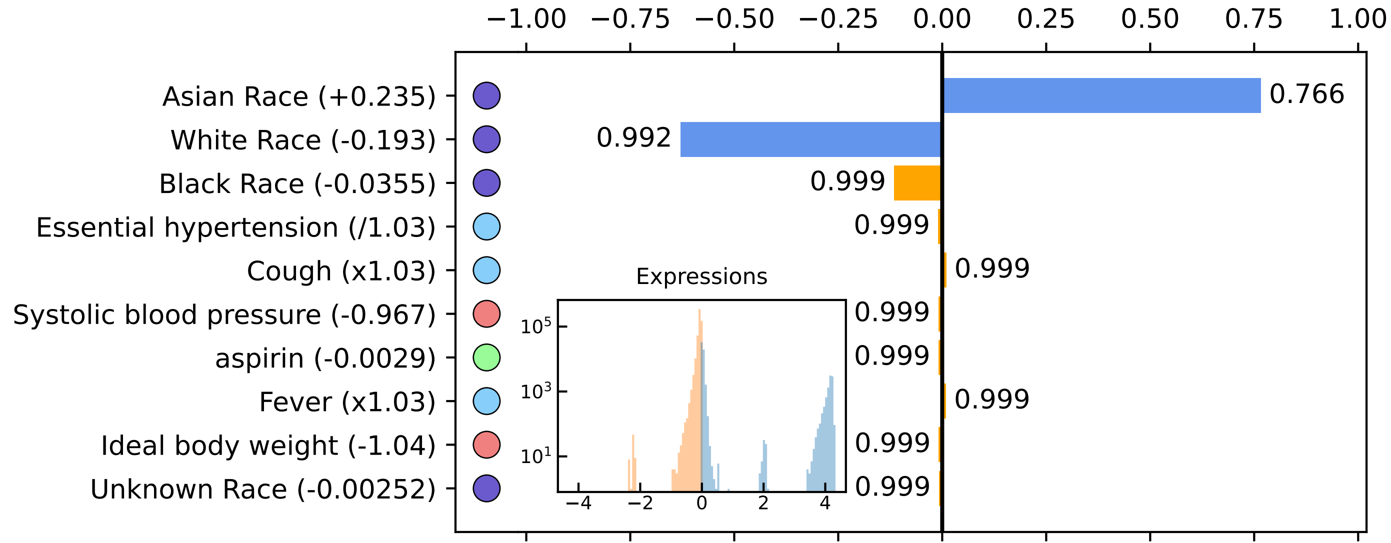

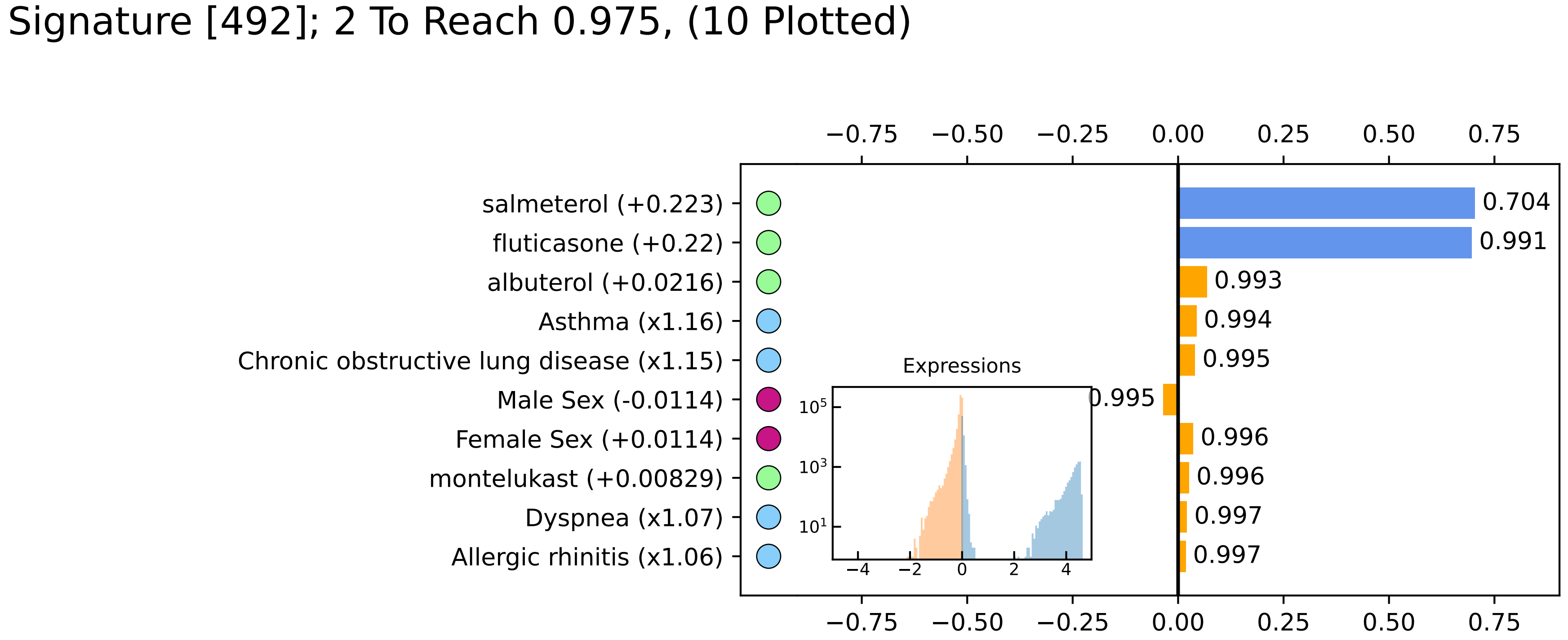

Signature [492]; 2 To Reach 0.975, (10 Plotted)
salmeterol (+0.223)
fluticasone (+0.22)
albuterol (+0.0216)
Asthma (x1.16)
Chronic obstructive lung disease (x1.15)
Male Sex (-0.0114)
Female Sex (+0.0114)
montelukast (+0.00829)
Dyspnea (x1.07)
Allergic rhinitis (x1.06)
0.704
0.991
0.993
0.994
0.995
0.995
0.996
0.996
0.997
0.997
−0.75 −0.50 −0.25 0.00 0.25 0.50 0.75
Expressions

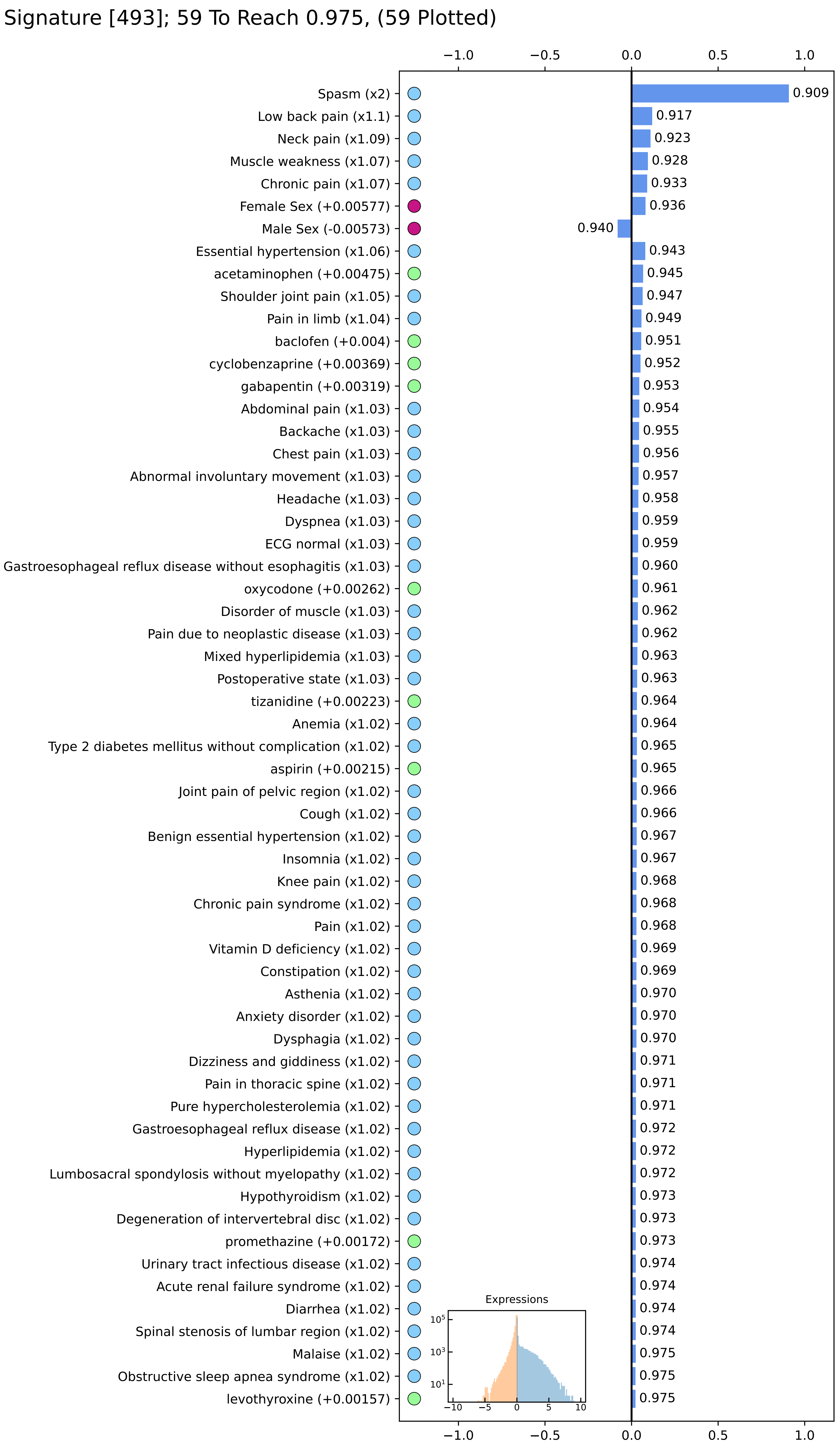

Signature [493]; 59 To Reach 0.975, (59 Plotted)
Spasm (x2) 0.909
Low back pain (x1.1) 0.917
Neck pain (x1.09) 0.923
Muscle weakness (x1.07) 0.928
Chronic pain (x1.07) 0.933
Female Sex (+0.00577) 0.936
Male Sex (-0.00573) 0.940
Essential hypertension (x1.06) 0.943
acetaminophen (+0.00475) 0.945
Shoulder joint pain (x1.05) 0.947
Pain in limb (x1.04) 0.949
baclofen (+0.004) 0.951
cyclobenzaprine (+0.00369) 0.952
gabapentin (+0.00319) 0.953
Abdominal pain (x1.03) 0.954
Backache (x1.03) 0.955
Chest pain (x1.03) 0.956
Abnormal involuntary movement (x1.03) 0.957
Headache (x1.03) 0.958
Dyspnea (x1.03) 0.959
ECG normal (x1.03) 0.959
Gastroesophageal reflux disease without esophagitis (x1.03) 0.960
oxycodone (+0.00262) 0.961
Disorder of muscle (x1.03) 0.962
Pain due to neoplastic disease (x1.03) 0.962
Mixed hyperlipidemia (x1.03) 0.963
Postoperative state (x1.03) 0.963
tizanidine (+0.00223) 0.964
Anemia (x1.02) 0.964
Type 2 diabetes mellitus without complication (x1.02) 0.965
aspirin (+0.00215) 0.965
Joint pain of pelvic region (x1.02) 0.966
Cough (x1.02) 0.966
Benign essential hypertension (x1.02) 0.967
Insomnia (x1.02) 0.967
Knee pain (x1.02) 0.968
Chronic pain syndrome (x1.02) 0.968
Pain (x1.02) 0.968
Vitamin D deficiency (x1.02) 0.969
Constipation (x1.02) 0.969
Asthenia (x1.02) 0.970
Anxiety disorder (x1.02) 0.970
Dysphagia (x1.02) 0.970
Dizziness and giddiness (x1.02) 0.971
Pain in thoracic spine (x1.02) 0.971
Pure hypercholesterolemia (x1.02) 0.971
Gastroesophageal reflux disease (x1.02) 0.972
Hyperlipidemia (x1.02) 0.972
Lumbosacral spondylosis without myelopathy (x1.02) 0.972
Hypothyroidism (x1.02) 0.973
Degeneration of intervertebral disc (x1.02) 0.973
promethazine (+0.00172) 0.973
Urinary tract infectious disease (x1.02) 0.974
Acute renal failure syndrome (x1.02) 0.974
Diarrhea (x1.02) 0.974
Spinal stenosis of lumbar region (x1.02) 0.974
Malaise (x1.02) 0.975
Obstructive sleep apnea syndrome (x1.02) 0.975
levothyroxine (+0.00157) 0.975
−1.0 −0.5 0.0 0.5 1.0
Expressions

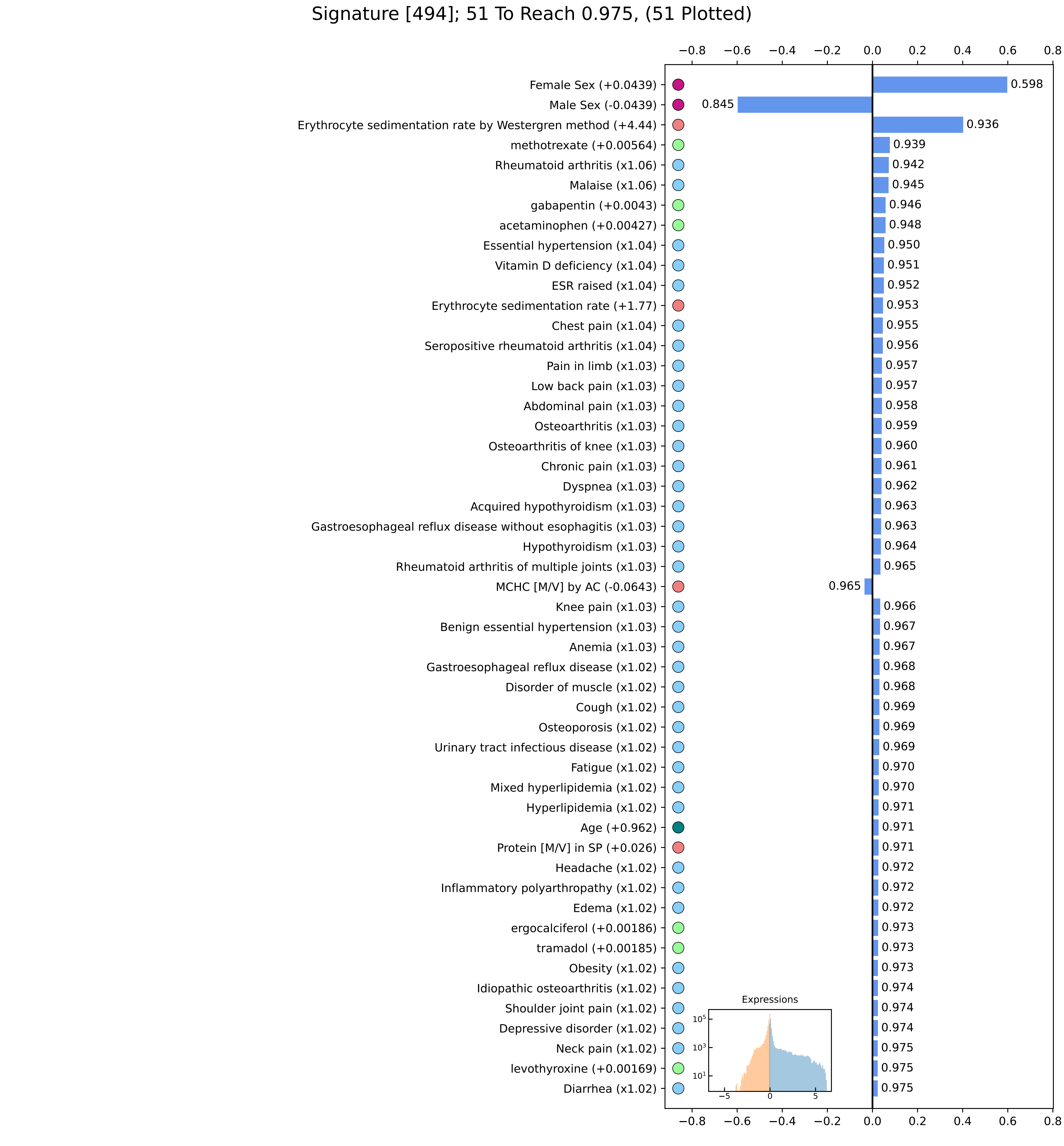

Signature [494]; 51 To Reach 0.975, (51 Plotted)
Female Sex (+0.0439) 0.598
Male Sex (-0.0439) 0.845
Erythrocyte sedimentation rate by Westergren method (+4.44) 0.936
methotrexate (+0.00564) 0.939
Rheumatoid arthritis (x1.06) 0.942
Malaise (x1.06) 0.945
gabapentin (+0.0043) 0.946
acetaminophen (+0.00427) 0.948
Essential hypertension (x1.04) 0.950
Vitamin D deficiency (x1.04) 0.951
ESR raised (x1.04) 0.952
Erythrocyte sedimentation rate (+1.77) 0.953
Chest pain (x1.04) 0.955
Seropositive rheumatoid arthritis (x1.04) 0.956
Pain in limb (x1.03) 0.957
Low back pain (x1.03) 0.957
Abdominal pain (x1.03) 0.958
Osteoarthritis (x1.03) 0.959
Osteoarthritis of knee (x1.03) 0.960
Chronic pain (x1.03) 0.961
Dyspnea (x1.03) 0.962
Acquired hypothyroidism (x1.03) 0.963
Gastroesophageal reflux disease without esophagitis (x1.03) 0.963
Hypothyroidism (x1.03) 0.964
Rheumatoid arthritis of multiple joints (x1.03) 0.965
MCHC [M/V] by AC (-0.0643) 0.965
Knee pain (x1.03) 0.966
Benign essential hypertension (x1.03) 0.967
Anemia (x1.03) 0.967
Gastroesophageal reflux disease (x1.02) 0.968
Disorder of muscle (x1.02) 0.968
Cough (x1.02) 0.969
Osteoporosis (x1.02) 0.969
Urinary tract infectious disease (x1.02) 0.969
Fatigue (x1.02) 0.970
Mixed hyperlipidemia (x1.02) 0.970
Hyperlipidemia (x1.02) 0.971
Age (+0.962) 0.971
Protein [M/V] in SP (+0.026) 0.971
Headache (x1.02) 0.972
Inflammatory polyarthropathy (x1.02) 0.972
Edema (x1.02) 0.972
ergocalciferol (+0.00186) 0.973
tramadol (+0.00185) 0.973
Obesity (x1.02) 0.973
Idiopathic osteoarthritis (x1.02) 0.974
Shoulder joint pain (x1.02) 0.974
Depressive disorder (x1.02) 0.974
Neck pain (x1.02) 0.975
levothyroxine (+0.00169) 0.975
Diarrhea (x1.02) 0.975
−0.8 −0.6 −0.4 −0.2 0.0 0.2 0.4 0.6 0.8
Expressions

Signature [495]; 49 To Reach 0.975, (49 Plotted)

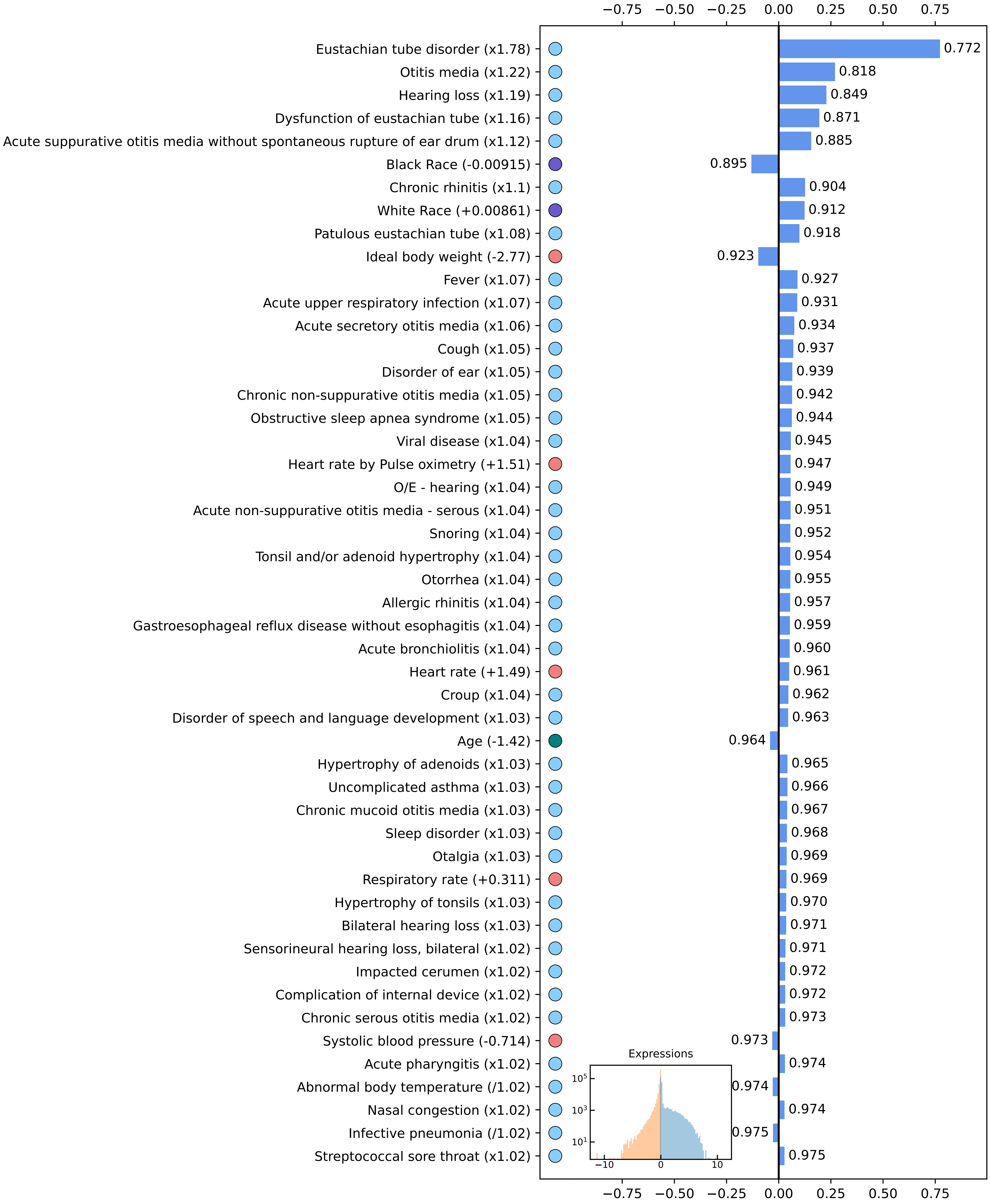

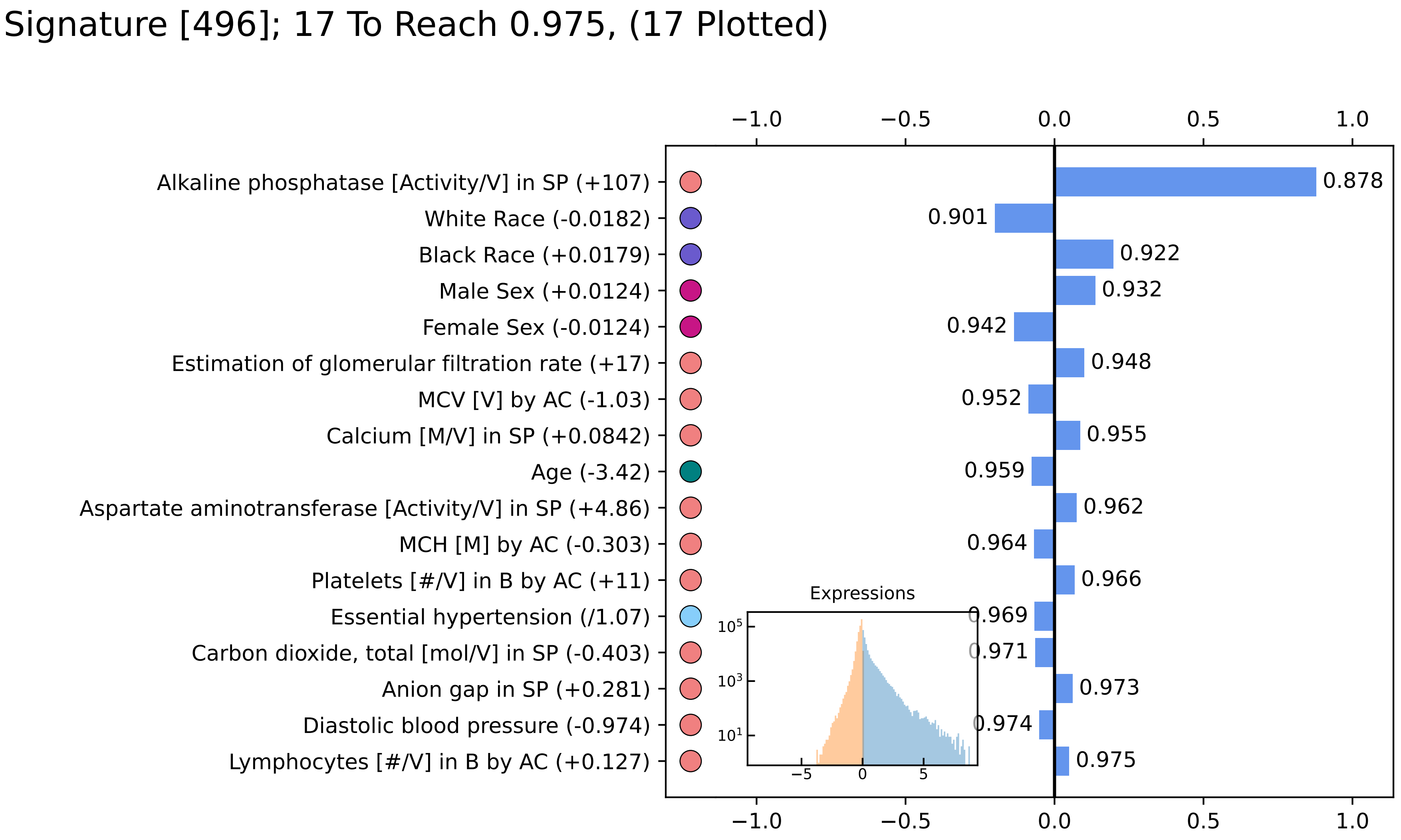
Signature [496]; 17 To Reach 0.975, (17 Plotted)
Alkaline phosphatase [Activity/V] in SP (+107)
White Race (-0.0182)
Black Race (+0.0179)
Male Sex (+0.0124)
Female Sex (-0.0124)
Estimation of glomerular filtration rate (+17)
MCV [V] by AC (-1.03)
Calcium [M/V] in SP (+0.0842)
Age (-3.42)
Aspartate aminotransferase [Activity/V] in SP (+4.86)
MCH [M] by AC (-0.303)
Platelets [#/V] in B by AC (+11)
Essential hypertension (/1.07)
Carbon dioxide, total [mol/V] in SP (-0.403)
Anion gap in SP (+0.281)
Diastolic blood pressure (-0.974)
Lymphocytes [#/V] in B by AC (+0.127)
0.878
0.901
0.922
0.932
0.942
0.948
0.952
0.955
0.959
0.962
0.964
0.966
0.969
0.971
0.973
0.974
0.975
−1.0
−0.5
0.0
0.5
1.0
Expressions
10^5
10^3
10^1
−5
0
5

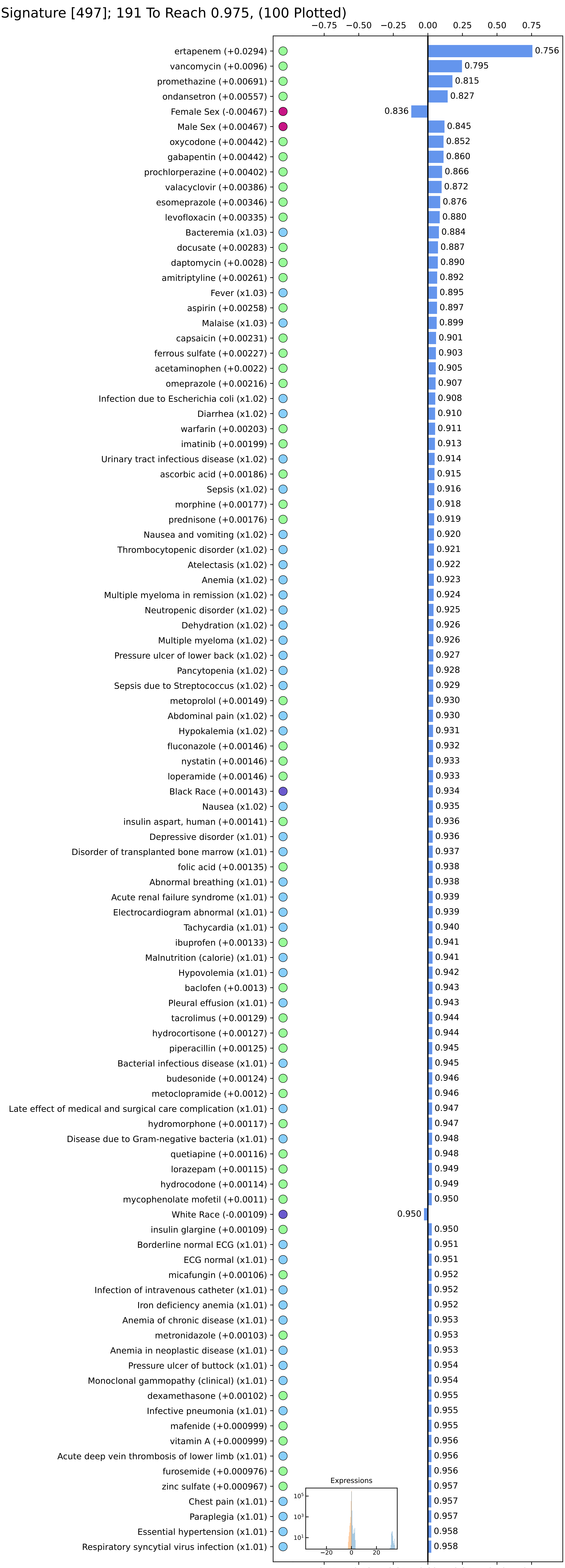

Signature [497]; 191 To Reach 0.975, (100 Plotted)
−0.75 −0.50 −0.25 0.00 0.25 0.50 0.75
ertapenem (+0.0294) 0.756
vancomycin (+0.0096) 0.795
promethazine (+0.00691) 0.815
ondansetron (+0.00557) 0.827
Female Sex (-0.00467) 0.836
Male Sex (+0.00467) 0.845
oxycodone (+0.00442) 0.852
gabapentin (+0.00442) 0.860
prochlorperazine (+0.00402) 0.866
valacyclovir (+0.00386) 0.872
esomeprazole (+0.00346) 0.876
levofloxacin (+0.00335) 0.880
Bacteremia (x1.03) 0.884
docusate (+0.00283) 0.887
daptomycin (+0.0028) 0.890
amitriptyline (+0.00261) 0.892
Fever (x1.03) 0.895
aspirin (+0.00258) 0.897
Malaise (x1.03) 0.899
capsaicin (+0.00231) 0.901
ferrous sulfate (+0.00227) 0.903
acetaminophen (+0.0022) 0.905
omeprazole (+0.00216) 0.907
Infection due to Escherichia coli (x1.02) 0.908
Diarrhea (x1.02) 0.910
warfarin (+0.00203) 0.911
imatinib (+0.00199) 0.913
Urinary tract infectious disease (x1.02) 0.914
ascorbic acid (+0.00186) 0.915
Sepsis (x1.02) 0.916
morphine (+0.00177) 0.918
prednisone (+0.00176) 0.919
Nausea and vomiting (x1.02) 0.920
Thrombocytopenic disorder (x1.02) 0.921
Atelectasis (x1.02) 0.922
Anemia (x1.02) 0.923
Multiple myeloma in remission (x1.02) 0.924
Neutropenic disorder (x1.02) 0.925
Dehydration (x1.02) 0.926
Multiple myeloma (x1.02) 0.926
Pressure ulcer of lower back (x1.02) 0.927
Pancytopenia (x1.02) 0.928
Sepsis due to Streptococcus (x1.02) 0.929
metoprolol (+0.00149) 0.930
Abdominal pain (x1.02) 0.930
Hypokalemia (x1.02) 0.931
fluconazole (+0.00146) 0.932
nystatin (+0.00146) 0.933
loperamide (+0.00146) 0.933
Black Race (+0.00143) 0.934
Nausea (x1.02) 0.935
insulin aspart, human (+0.00141) 0.936
Depressive disorder (x1.01) 0.936
Disorder of transplanted bone marrow (x1.01) 0.937
folic acid (+0.00135) 0.938
Abnormal breathing (x1.01) 0.938
Acute renal failure syndrome (x1.01) 0.939
Electrocardiogram abnormal (x1.01) 0.939
Tachycardia (x1.01) 0.940
ibuprofen (+0.00133) 0.941
Malnutrition (calorie) (x1.01) 0.941
Hypovolemia (x1.01) 0.942
baclofen (+0.0013) 0.943
Pleural effusion (x1.01) 0.943
tacrolimus (+0.00129) 0.944
hydrocortisone (+0.00127) 0.944
piperacillin (+0.00125) 0.945
Bacterial infectious disease (x1.01) 0.945
budesonide (+0.00124) 0.946
metoclopramide (+0.0012) 0.946
Late effect of medical and surgical care complication (x1.01) 0.947
hydromorphone (+0.00117) 0.947
Disease due to Gram-negative bacteria (x1.01) 0.948
quetiapine (+0.00116) 0.948
lorazepam (+0.00115) 0.949
hydrocodone (+0.00114) 0.949
mycophenolate mofetil (+0.0011) 0.950
White Race (-0.00109) 0.950
insulin glargine (+0.00109) 0.950
Borderline normal ECG (x1.01) 0.951
ECG normal (x1.01) 0.951
micafungin (+0.00106) 0.952
Infection of intravenous catheter (x1.01) 0.952
Iron deficiency anemia (x1.01) 0.952
Anemia of chronic disease (x1.01) 0.953
metronidazole (+0.00103) 0.953
Anemia in neoplastic disease (x1.01) 0.953
Pressure ulcer of buttock (x1.01) 0.954
Monoclonal gammopathy (clinical) (x1.01) 0.954
dexamethasone (+0.00102) 0.955
Infective pneumonia (x1.01) 0.955
mafenide (+0.000999) 0.955
vitamin A (+0.000999) 0.956
Acute deep vein thrombosis of lower limb (x1.01) 0.956
furosemide (+0.000976) 0.956
zinc sulfate (+0.000967) 0.957
Chest pain (x1.01) 0.957
Paraplegia (x1.01) 0.957
Essential hypertension (x1.01) 0.958
Respiratory syncytial virus infection (x1.01) 0.958
Expressions
−20 0 20
−0.75 −0.50 −0.25 0.00 0.25 0.50 0.75

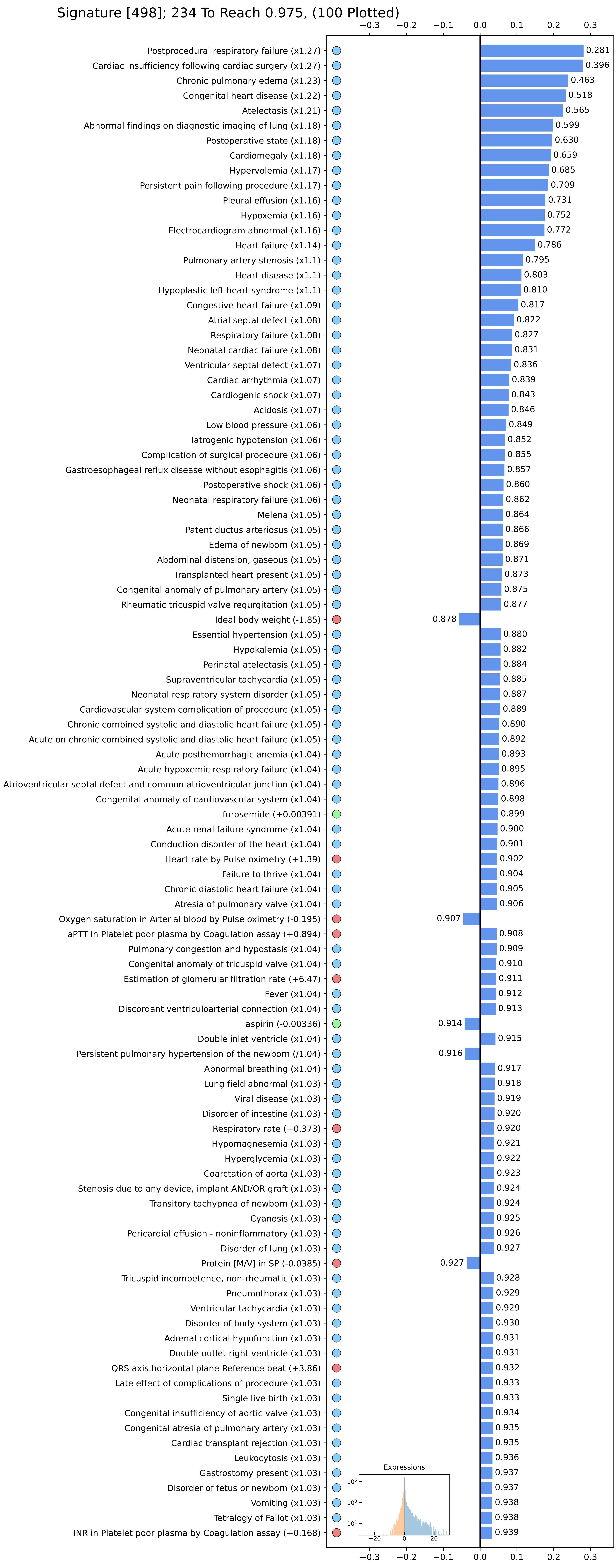

Signature [498]; 234 To Reach 0.975, (100 Plotted)
−0.3
−0.2
−0.1
0.0
0.1
0.2
0.3
Postprocedural respiratory failure (x1.27) 0.281
Cardiac insufficiency following cardiac surgery (x1.27) 0.396
Chronic pulmonary edema (x1.23) 0.463
Congenital heart disease (x1.22) 0.518
Atelectasis (x1.21) 0.565
Abnormal findings on diagnostic imaging of lung (x1.18) 0.599
Postoperative state (x1.18) 0.630
Cardiomegaly (x1.18) 0.659
Hypervolemia (x1.17) 0.685
Persistent pain following procedure (x1.17) 0.709
Pleural effusion (x1.16) 0.731
Hypoxemia (x1.16) 0.752
Electrocardiogram abnormal (x1.16) 0.772
Heart failure (x1.14) 0.786
Pulmonary artery stenosis (x1.1) 0.795
Heart disease (x1.1) 0.803
Hypoplastic left heart syndrome (x1.1) 0.810
Congestive heart failure (x1.09) 0.817
Atrial septal defect (x1.08) 0.822
Respiratory failure (x1.08) 0.827
Neonatal cardiac failure (x1.08) 0.831
Ventricular septal defect (x1.07) 0.836
Cardiac arrhythmia (x1.07) 0.839
Cardiogenic shock (x1.07) 0.843
Acidosis (x1.07) 0.846
Low blood pressure (x1.06) 0.849
Iatrogenic hypotension (x1.06) 0.852
Complication of surgical procedure (x1.06) 0.855
Gastroesophageal reflux disease without esophagitis (x1.06) 0.857
Postoperative shock (x1.06) 0.860
Neonatal respiratory failure (x1.06) 0.862
Melena (x1.05) 0.864
Patent ductus arteriosus (x1.05) 0.866
Edema of newborn (x1.05) 0.869
Abdominal distension, gaseous (x1.05) 0.871
Transplanted heart present (x1.05) 0.873
Congenital anomaly of pulmonary artery (x1.05) 0.875
Rheumatic tricuspid valve regurgitation (x1.05) 0.877
Ideal body weight (-1.85) 0.878
Essential hypertension (x1.05) 0.880
Hypokalemia (x1.05) 0.882
Perinatal atelectasis (x1.05) 0.884
Supraventricular tachycardia (x1.05) 0.885
Neonatal respiratory system disorder (x1.05) 0.887
Cardiovascular system complication of procedure (x1.05) 0.889
Chronic combined systolic and diastolic heart failure (x1.05) 0.890
Acute on chronic combined systolic and diastolic heart failure (x1.05) 0.892
Acute posthemorrhagic anemia (x1.04) 0.893
Acute hypoxemic respiratory failure (x1.04) 0.895
Atrioventricular septal defect and common atrioventricular junction (x1.04) 0.896
Congenital anomaly of cardiovascular system (x1.04) 0.898
furosemide (+0.00391) 0.899
Acute renal failure syndrome (x1.04) 0.900
Conduction disorder of the heart (x1.04) 0.901
Heart rate by Pulse oximetry (+1.39) 0.902
Failure to thrive (x1.04) 0.904
Chronic diastolic heart failure (x1.04) 0.905
Atresia of pulmonary valve (x1.04) 0.906
Oxygen saturation in Arterial blood by Pulse oximetry (-0.195) 0.907
aPTT in Platelet poor plasma by Coagulation assay (+0.894) 0.908
Pulmonary congestion and hypostasis (x1.04) 0.909
Congenital anomaly of tricuspid valve (x1.04) 0.910
Estimation of glomerular filtration rate (+6.47) 0.911
Fever (x1.04) 0.912
Discordant ventriculoarterial connection (x1.04) 0.913
aspirin (-0.00336) 0.914
Double inlet ventricle (x1.04) 0.915
Persistent pulmonary hypertension of the newborn (/1.04) 0.916
Abnormal breathing (x1.04) 0.917
Lung field abnormal (x1.03) 0.918
Viral disease (x1.03) 0.919
Disorder of intestine (x1.03) 0.920
Respiratory rate (+0.373) 0.920
Hypomagnesemia (x1.03) 0.921
Hyperglycemia (x1.03) 0.922
Coarctation of aorta (x1.03) 0.923
Stenosis due to any device, implant AND/OR graft (x1.03) 0.924
Transitory tachypnea of newborn (x1.03) 0.924
Cyanosis (x1.03) 0.925
Pericardial effusion - noninflammatory (x1.03) 0.926
Disorder of lung (x1.03) 0.927
Protein [M/V] in SP (-0.0385) 0.927
Tricuspid incompetence, non-rheumatic (x1.03) 0.928
Pneumothorax (x1.03) 0.929
Ventricular tachycardia (x1.03) 0.929
Disorder of body system (x1.03) 0.930
Adrenal cortical hypofunction (x1.03) 0.931
Double outlet right ventricle (x1.03) 0.931
QRS axis.horizontal plane Reference beat (+3.86) 0.932
Late effect of complications of procedure (x1.03) 0.933
Single live birth (x1.03) 0.933
Congenital insufficiency of aortic valve (x1.03) 0.934
Congenital atresia of pulmonary artery (x1.03) 0.935
Cardiac transplant rejection (x1.03) 0.935
Leukocytosis (x1.03) 0.936
Gastrostomy present (x1.03) 0.937
Disorder of fetus or newborn (x1.03) 0.937
Vomiting (x1.03) 0.938
Tetralogy of Fallot (x1.03) 0.938
INR in Platelet poor plasma by Coagulation assay (+0.168) 0.939
Expressions

Signature [499]; 2 To Reach 0.975, (10 Plotted)

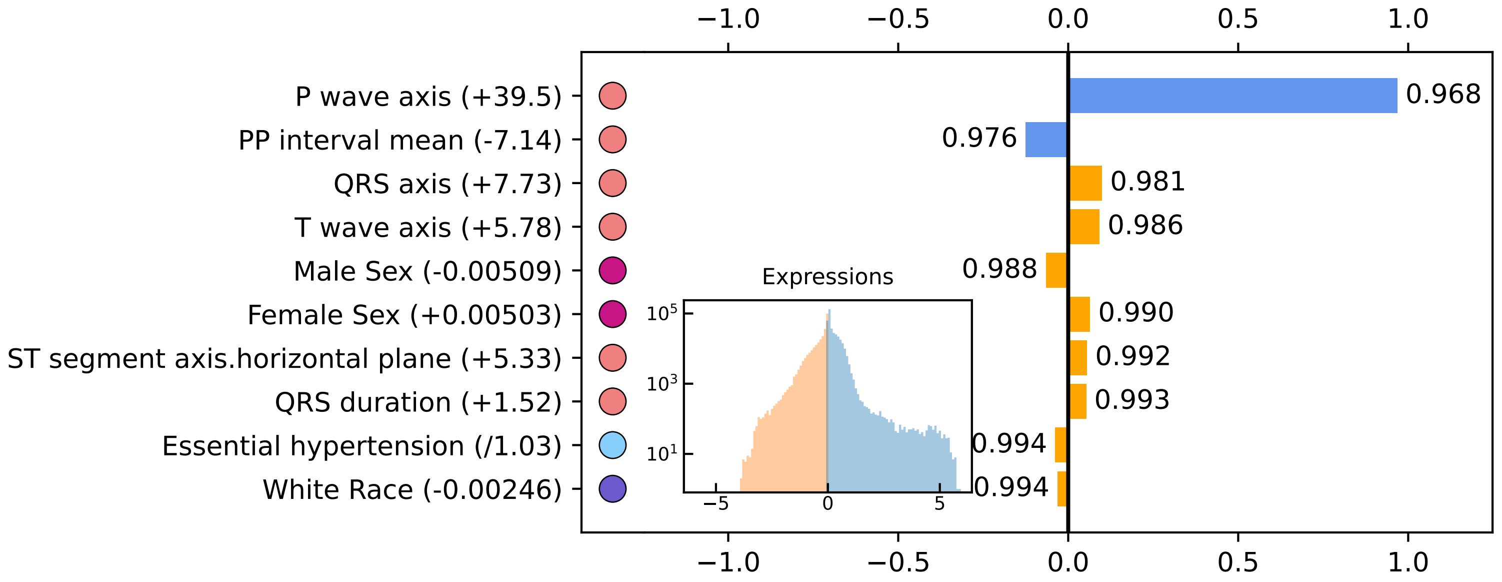

Signature [500]; 1 To Reach 0.975, (10 Plotted)

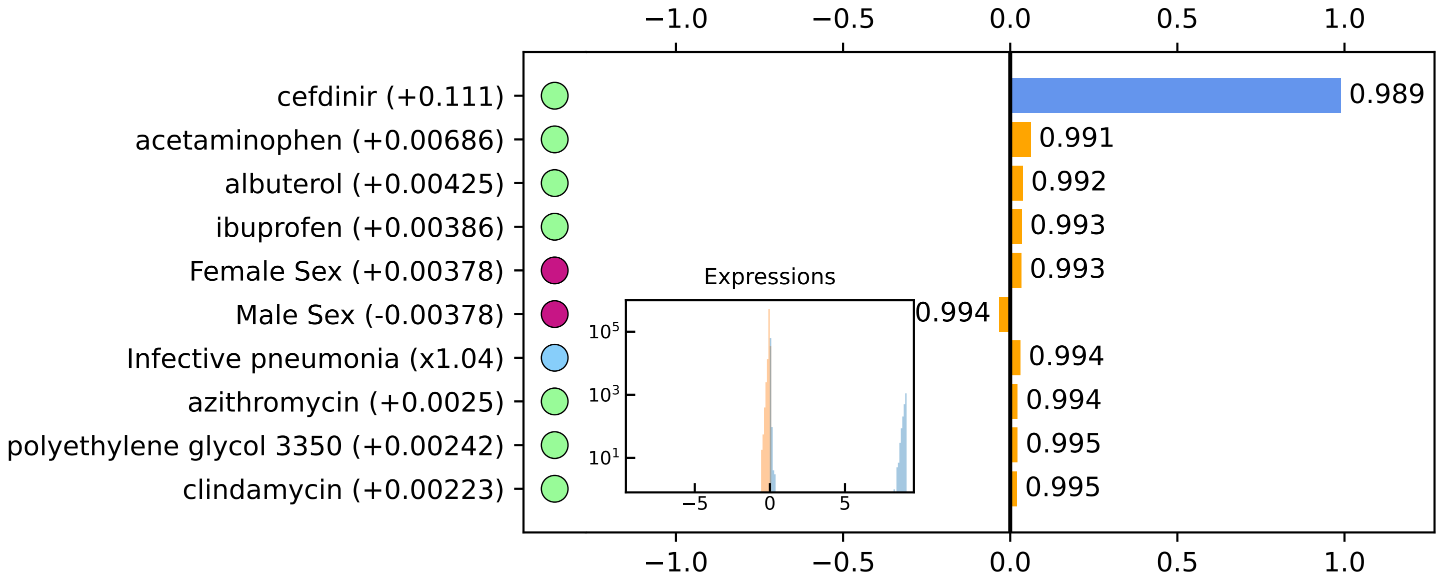

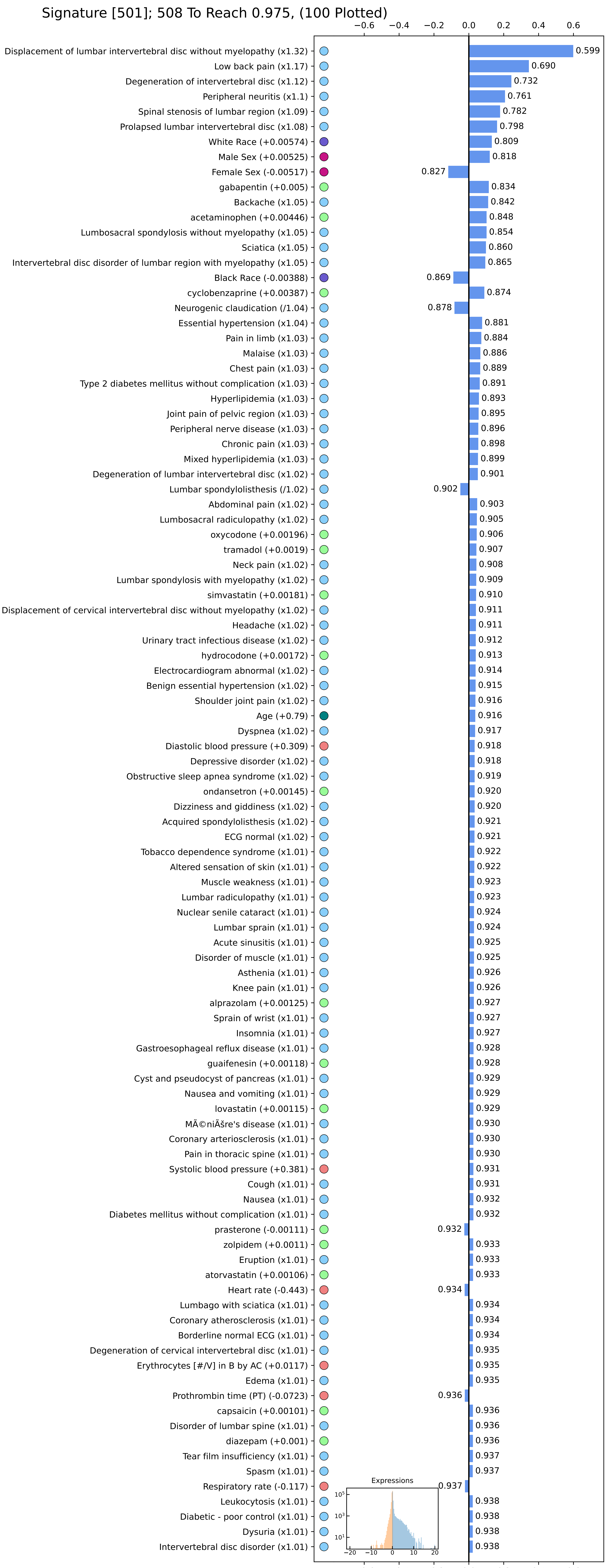

Signature [501]; 508 To Reach 0.975, (100 Plotted)
Displacement of lumbar intervertebral disc without myelopathy (x1.32) 0.599
Low back pain (x1.17) 0.690
Degeneration of intervertebral disc (x1.12) 0.732
Peripheral neuritis (x1.1) 0.761
Spinal stenosis of lumbar region (x1.09) 0.782
Prolapsed lumbar intervertebral disc (x1.08) 0.798
White Race (+0.00574) 0.809
Male Sex (+0.00525) 0.818
Female Sex (-0.00517) 0.827
gabapentin (+0.005) 0.834
Backache (x1.05) 0.842
acetaminophen (+0.00446) 0.848
Lumbosacral spondylosis without myelopathy (x1.05) 0.854
Sciatica (x1.05) 0.860
Intervertebral disc disorder of lumbar region with myelopathy (x1.05) 0.865
Black Race (-0.00388) 0.869
cyclobenzaprine (+0.00387) 0.874
Neurogenic claudication (/1.04) 0.878
Essential hypertension (x1.04) 0.881
Pain in limb (x1.03) 0.884
Malaise (x1.03) 0.886
Chest pain (x1.03) 0.889
Type 2 diabetes mellitus without complication (x1.03) 0.891
Hyperlipidemia (x1.03) 0.893
Joint pain of pelvic region (x1.03) 0.895
Peripheral nerve disease (x1.03) 0.896
Chronic pain (x1.03) 0.898
Mixed hyperlipidemia (x1.03) 0.899
Degeneration of lumbar intervertebral disc (x1.02) 0.901
Lumbar spondylolisthesis (/1.02) 0.902
Abdominal pain (x1.02) 0.903
Lumbosacral radiculopathy (x1.02) 0.905
oxycodone (+0.00196) 0.906
tramadol (+0.0019) 0.907
Neck pain (x1.02) 0.908
Lumbar spondylosis with myelopathy (x1.02) 0.909
simvastatin (+0.00181) 0.910
Displacement of cervical intervertebral disc without myelopathy (x1.02) 0.911
Headache (x1.02) 0.911
Urinary tract infectious disease (x1.02) 0.912
hydrocodone (+0.00172) 0.913
Electrocardiogram abnormal (x1.02) 0.914
Benign essential hypertension (x1.02) 0.915
Shoulder joint pain (x1.02) 0.916
Age (+0.79) 0.916
Dyspnea (x1.02) 0.917
Diastolic blood pressure (+0.309) 0.918
Depressive disorder (x1.02) 0.918
Obstructive sleep apnea syndrome (x1.02) 0.919
ondansetron (+0.00145) 0.920
Dizziness and giddiness (x1.02) 0.920
Acquired spondylolisthesis (x1.02) 0.921
ECG normal (x1.02) 0.921
Tobacco dependence syndrome (x1.01) 0.922
Altered sensation of skin (x1.01) 0.922
Muscle weakness (x1.01) 0.923
Lumbar radiculopathy (x1.01) 0.923
Nuclear senile cataract (x1.01) 0.924
Lumbar sprain (x1.01) 0.924
Acute sinusitis (x1.01) 0.925
Disorder of muscle (x1.01) 0.925
Asthenia (x1.01) 0.926
Knee pain (x1.01) 0.926
alprazolam (+0.00125) 0.927
Sprain of wrist (x1.01) 0.927
Insomnia (x1.01) 0.927
Gastroesophageal reflux disease (x1.01) 0.928
guaifenesin (+0.00118) 0.928
Cyst and pseudocyst of pancreas (x1.01) 0.929
Nausea and vomiting (x1.01) 0.929
lovastatin (+0.00115) 0.929
MÃ©niÃšre's disease (x1.01) 0.930
Coronary arteriosclerosis (x1.01) 0.930
Pain in thoracic spine (x1.01) 0.930
Systolic blood pressure (+0.381) 0.931
Cough (x1.01) 0.931
Nausea (x1.01) 0.932
Diabetes mellitus without complication (x1.01) 0.932
prasterone (-0.00111) 0.932
zolpidem (+0.0011) 0.933
Eruption (x1.01) 0.933
atorvastatin (+0.00106) 0.933
Heart rate (-0.443) 0.934
Lumbago with sciatica (x1.01) 0.934
Coronary atherosclerosis (x1.01) 0.934
Borderline normal ECG (x1.01) 0.934
Degeneration of cervical intervertebral disc (x1.01) 0.935
Erythrocytes [#/V] in B by AC (+0.0117) 0.935
Edema (x1.01) 0.935
Prothrombin time (PT) (-0.0723) 0.936
capsaicin (+0.00101) 0.936
Disorder of lumbar spine (x1.01) 0.936
diazepam (+0.001) 0.936
Tear film insufficiency (x1.01) 0.937
Spasm (x1.01) 0.937
Respiratory rate (-0.117) 0.937
Leukocytosis (x1.01) 0.938
Diabetic - poor control (x1.01) 0.938
Dysuria (x1.01) 0.938
Intervertebral disc disorder (x1.01) 0.938
−0.6 −0.4 −0.2 0.0 0.2 0.4 0.6
Expressions

Signature [502]; 9 To Reach 0.975, (10 Plotted)

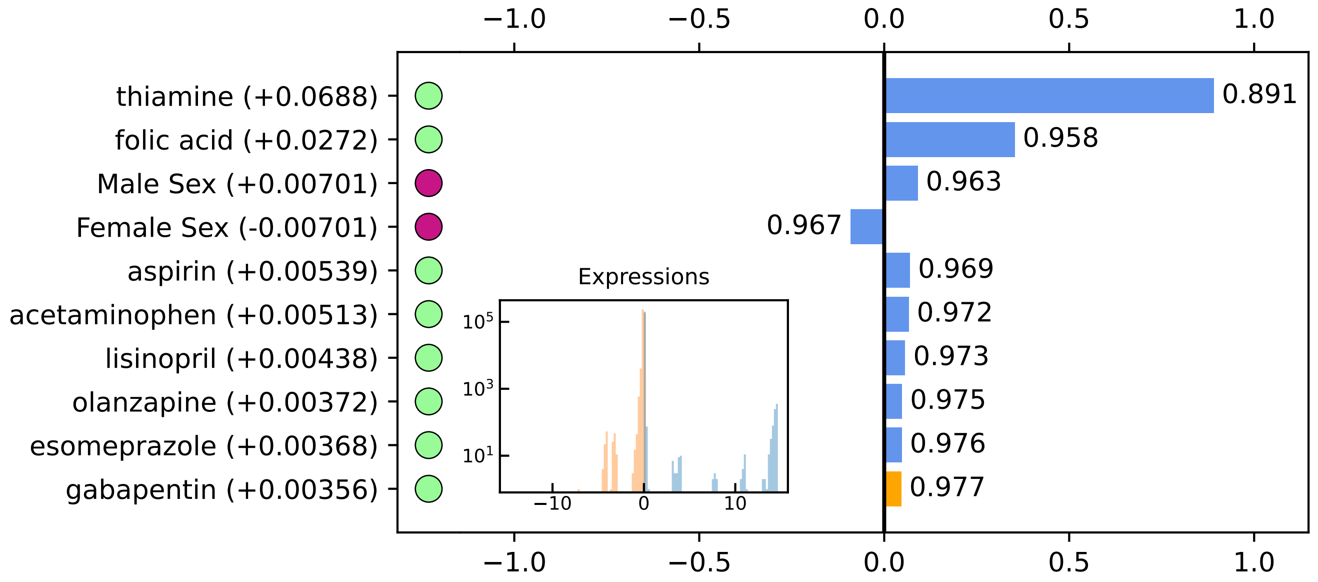

Signature [503]; 938 To Reach 0.975, (100 Plotted)

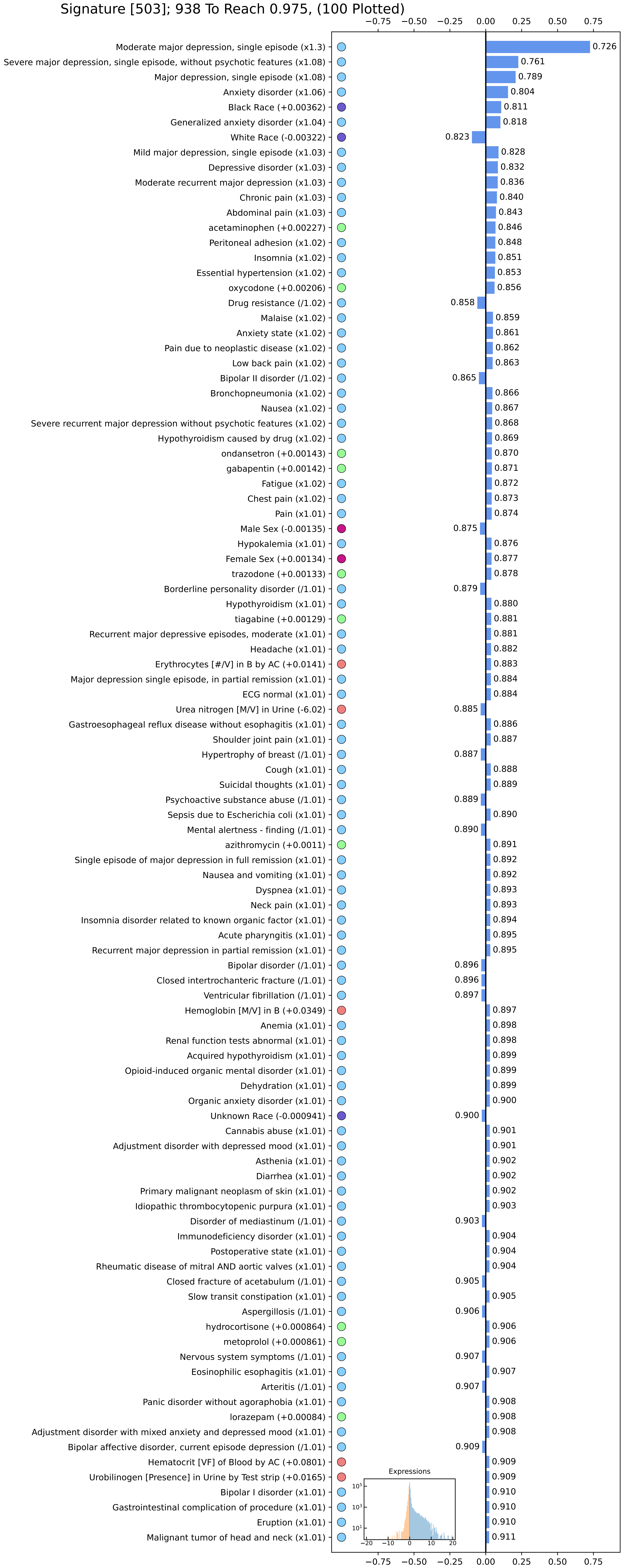

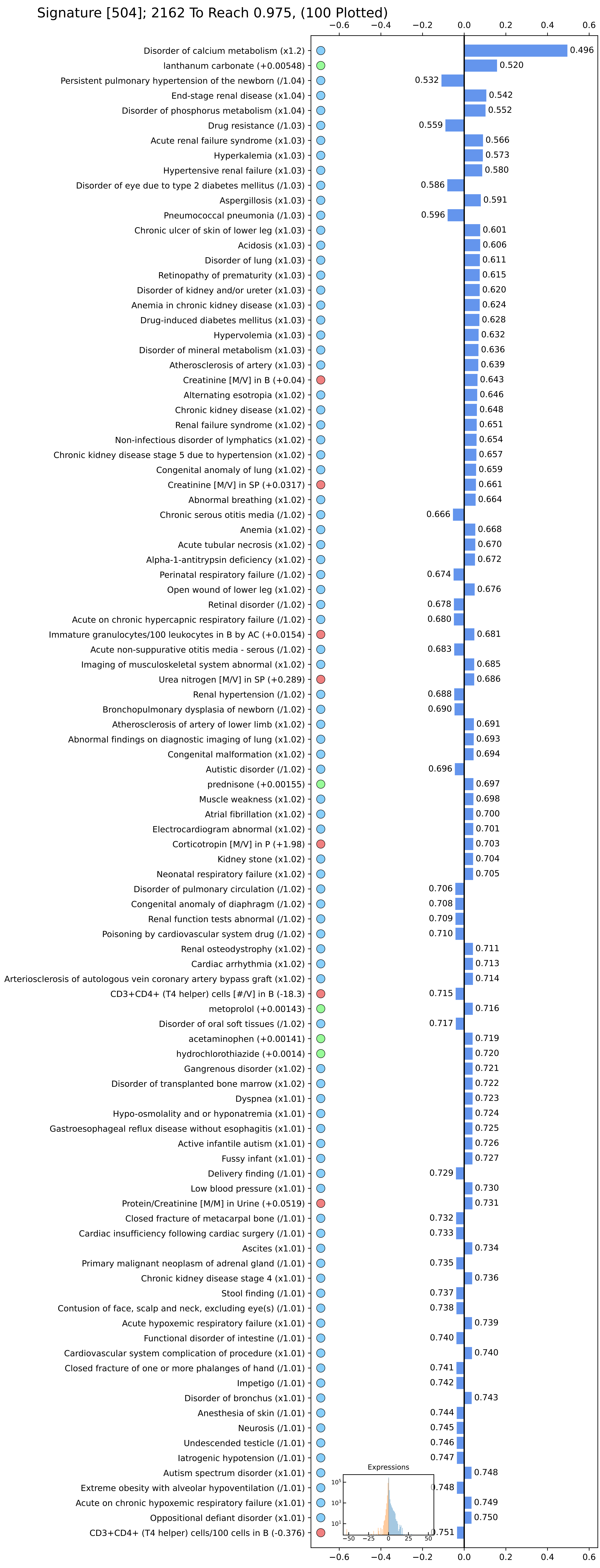

Signature [504]; 2162 To Reach 0.975, (100 Plotted)
Disorder of calcium metabolism (x1.2) 0.496
lanthanum carbonate (+0.00548) 0.520
Persistent pulmonary hypertension of the newborn (/1.04) 0.532
End-stage renal disease (x1.04) 0.542
Disorder of phosphorus metabolism (x1.04) 0.552
Drug resistance (/1.03) 0.559
Acute renal failure syndrome (x1.03) 0.566
Hyperkalemia (x1.03) 0.573
Hypertensive renal failure (x1.03) 0.580
Disorder of eye due to type 2 diabetes mellitus (/1.03) 0.586
Aspergillosis (x1.03) 0.591
Pneumococcal pneumonia (/1.03) 0.596
Chronic ulcer of skin of lower leg (x1.03) 0.601
Acidosis (x1.03) 0.606
Disorder of lung (x1.03) 0.611
Retinopathy of prematurity (x1.03) 0.615
Disorder of kidney and/or ureter (x1.03) 0.620
Anemia in chronic kidney disease (x1.03) 0.624
Drug-induced diabetes mellitus (x1.03) 0.628
Hypervolemia (x1.03) 0.632
Disorder of mineral metabolism (x1.03) 0.636
Atherosclerosis of artery (x1.03) 0.639
Creatinine [M/V] in B (+0.04) 0.643
Alternating esotropia (x1.02) 0.646
Chronic kidney disease (x1.02) 0.648
Renal failure syndrome (x1.02) 0.651
Non-infectious disorder of lymphatics (x1.02) 0.654
Chronic kidney disease stage 5 due to hypertension (x1.02) 0.657
Congenital anomaly of lung (x1.02) 0.659
Creatinine [M/V] in SP (+0.0317) 0.661
Abnormal breathing (x1.02) 0.664
Chronic serous otitis media (/1.02) 0.666
Anemia (x1.02) 0.668
Acute tubular necrosis (x1.02) 0.670
Alpha-1-antitrypsin deficiency (x1.02) 0.672
Perinatal respiratory failure (/1.02) 0.674
Open wound of lower leg (x1.02) 0.676
Retinal disorder (/1.02) 0.678
Acute on chronic hypercapnic respiratory failure (/1.02) 0.680
Immature granulocytes/100 leukocytes in B by AC (+0.0154) 0.681
Acute non-suppurative otitis media - serous (/1.02) 0.683
Imaging of musculoskeletal system abnormal (x1.02) 0.685
Urea nitrogen [M/V] in SP (+0.289) 0.686
Renal hypertension (/1.02) 0.688
Bronchopulmonary dysplasia of newborn (/1.02) 0.690
Atherosclerosis of artery of lower limb (x1.02) 0.691
Abnormal findings on diagnostic imaging of lung (x1.02) 0.693
Congenital malformation (x1.02) 0.694
Autistic disorder (/1.02) 0.696
prednisone (+0.00155) 0.697
Muscle weakness (x1.02) 0.698
Atrial fibrillation (x1.02) 0.700
Electrocardiogram abnormal (x1.02) 0.701
Corticotropin [M/V] in P (+1.98) 0.703
Kidney stone (x1.02) 0.704
Neonatal respiratory failure (x1.02) 0.705
Disorder of pulmonary circulation (/1.02) 0.706
Congenital anomaly of diaphragm (/1.02) 0.708
Renal function tests abnormal (/1.02) 0.709
Poisoning by cardiovascular system drug (/1.02) 0.710
Renal osteodystrophy (x1.02) 0.711
Cardiac arrhythmia (x1.02) 0.713
Arteriosclerosis of autologous vein coronary artery bypass graft (x1.02) 0.714
CD3+CD4+ (T4 helper) cells [#/V] in B (-18.3) 0.715
metoprolol (+0.00143) 0.716
Disorder of oral soft tissues (/1.02) 0.717
acetaminophen (+0.00141) 0.719
hydrochlorothiazide (+0.0014) 0.720
Gangrenous disorder (x1.02) 0.721
Disorder of transplanted bone marrow (x1.02) 0.722
Dyspnea (x1.01) 0.723
Hypo-osmolality and or hyponatremia (x1.01) 0.724
Gastroesophageal reflux disease without esophagitis (x1.01) 0.725
Active infantile autism (x1.01) 0.726
Fussy infant (x1.01) 0.727
Delivery finding (/1.01) 0.729
Low blood pressure (x1.01) 0.730
Protein/Creatinine [M/M] in Urine (+0.0519) 0.731
Closed fracture of metacarpal bone (/1.01) 0.732
Cardiac insufficiency following cardiac surgery (/1.01) 0.733
Ascites (x1.01) 0.734
Primary malignant neoplasm of adrenal gland (/1.01) 0.735
Chronic kidney disease stage 4 (x1.01) 0.736
Stool finding (/1.01) 0.737
Contusion of face, scalp and neck, excluding eye(s) (/1.01) 0.738
Acute hypoxemic respiratory failure (x1.01) 0.739
Functional disorder of intestine (/1.01) 0.740
Cardiovascular system complication of procedure (x1.01) 0.740
Closed fracture of one or more phalanges of hand (/1.01) 0.741
Impetigo (/1.01) 0.742
Disorder of bronchus (x1.01) 0.743
Anesthesia of skin (/1.01) 0.744
Neurosis (/1.01) 0.745
Undescended testicle (/1.01) 0.746
Iatrogenic hypotension (/1.01) 0.747
Autism spectrum disorder (x1.01) 0.748
Extreme obesity with alveolar hypoventilation (/1.01) 0.748
Acute on chronic hypoxemic respiratory failure (x1.01) 0.749
Oppositional defiant disorder (x1.01) 0.750
CD3+CD4+ (T4 helper) cells/100 cells in B (-0.376) 0.751
−0.6 −0.4 −0.2 0.0 0.2 0.4 0.6
Expressions
10^5
10^3
10^1
−50 −25 0 25 50

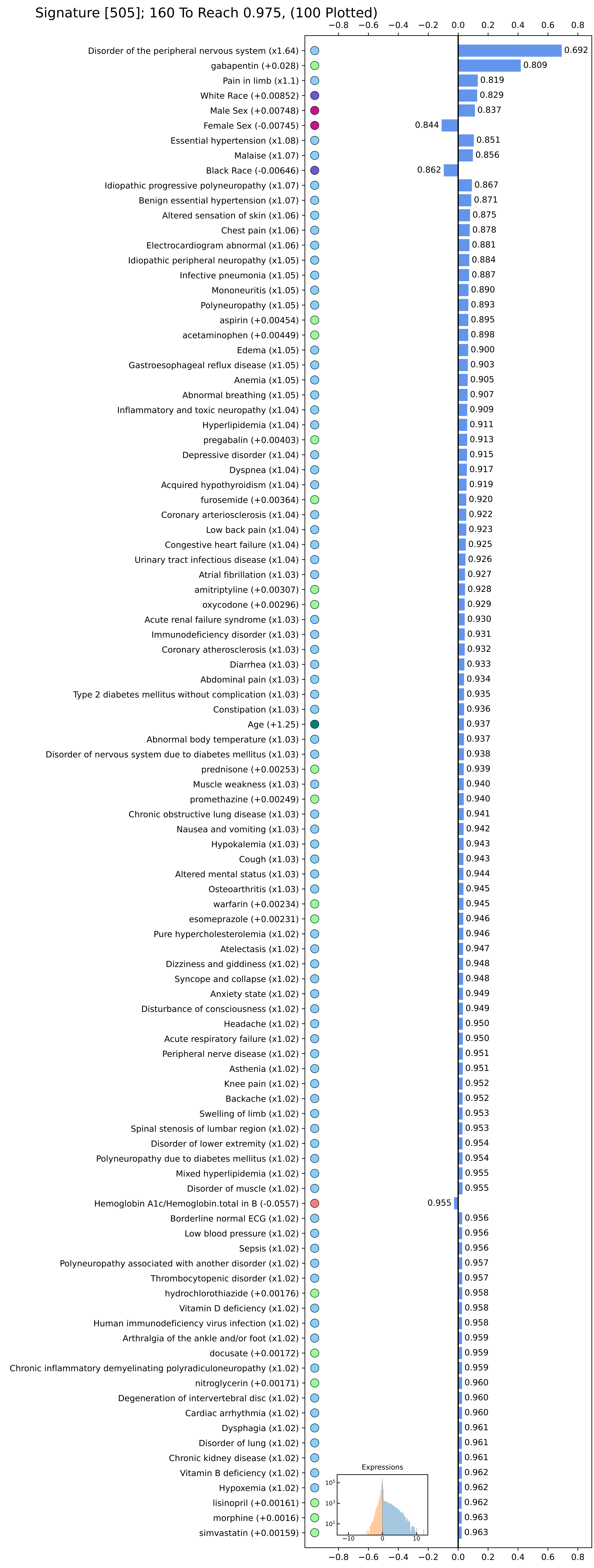
Signature [505]; 160 To Reach 0.975, (100 Plotted)
−0.8 −0.6 −0.4 −0.2 0.0 0.2 0.4 0.6 0.8
Disorder of the peripheral nervous system (x1.64) 0.692
gabapentin (+0.028) 0.809
Pain in limb (x1.1) 0.819
White Race (+0.00852) 0.829
Male Sex (+0.00748) 0.837
Female Sex (-0.00745) 0.844
Essential hypertension (x1.08) 0.851
Malaise (x1.07) 0.856
Black Race (-0.00646) 0.862
Idiopathic progressive polyneuropathy (x1.07) 0.867
Benign essential hypertension (x1.07) 0.871
Altered sensation of skin (x1.06) 0.875
Chest pain (x1.06) 0.878
Electrocardiogram abnormal (x1.06) 0.881
Idiopathic peripheral neuropathy (x1.05) 0.884
Infective pneumonia (x1.05) 0.887
Mononeuritis (x1.05) 0.890
Polyneuropathy (x1.05) 0.893
aspirin (+0.00454) 0.895
acetaminophen (+0.00449) 0.898
Edema (x1.05) 0.900
Gastroesophageal reflux disease (x1.05) 0.903
Anemia (x1.05) 0.905
Abnormal breathing (x1.05) 0.907
Inflammatory and toxic neuropathy (x1.04) 0.909
Hyperlipidemia (x1.04) 0.911
pregabalin (+0.00403) 0.913
Depressive disorder (x1.04) 0.915
Dyspnea (x1.04) 0.917
Acquired hypothyroidism (x1.04) 0.919
furosemide (+0.00364) 0.920
Coronary arteriosclerosis (x1.04) 0.922
Low back pain (x1.04) 0.923
Congestive heart failure (x1.04) 0.925
Urinary tract infectious disease (x1.04) 0.926
Atrial fibrillation (x1.03) 0.927
amitriptyline (+0.00307) 0.928
oxycodone (+0.00296) 0.929
Acute renal failure syndrome (x1.03) 0.930
Immunodeficiency disorder (x1.03) 0.931
Coronary atherosclerosis (x1.03) 0.932
Diarrhea (x1.03) 0.933
Abdominal pain (x1.03) 0.934
Type 2 diabetes mellitus without complication (x1.03) 0.935
Constipation (x1.03) 0.936
Age (+1.25) 0.937
Abnormal body temperature (x1.03) 0.937
Disorder of nervous system due to diabetes mellitus (x1.03) 0.938
prednisone (+0.00253) 0.939
Muscle weakness (x1.03) 0.940
promethazine (+0.00249) 0.940
Chronic obstructive lung disease (x1.03) 0.941
Nausea and vomiting (x1.03) 0.942
Hypokalemia (x1.03) 0.943
Cough (x1.03) 0.943
Altered mental status (x1.03) 0.944
Osteoarthritis (x1.03) 0.945
warfarin (+0.00234) 0.945
esomeprazole (+0.00231) 0.946
Pure hypercholesterolemia (x1.02) 0.946
Atelectasis (x1.02) 0.947
Dizziness and giddiness (x1.02) 0.948
Syncope and collapse (x1.02) 0.948
Anxiety state (x1.02) 0.949
Disturbance of consciousness (x1.02) 0.949
Headache (x1.02) 0.950
Acute respiratory failure (x1.02) 0.950
Peripheral nerve disease (x1.02) 0.951
Asthenia (x1.02) 0.951
Knee pain (x1.02) 0.952
Backache (x1.02) 0.952
Swelling of limb (x1.02) 0.953
Spinal stenosis of lumbar region (x1.02) 0.953
Disorder of lower extremity (x1.02) 0.954
Polyneuropathy due to diabetes mellitus (x1.02) 0.954
Mixed hyperlipidemia (x1.02) 0.955
Disorder of muscle (x1.02) 0.955
Hemoglobin A1c/Hemoglobin.total in B (-0.0557) 0.955
Borderline normal ECG (x1.02) 0.956
Low blood pressure (x1.02) 0.956
Sepsis (x1.02) 0.956
Polyneuropathy associated with another disorder (x1.02) 0.957
Thrombocytopenic disorder (x1.02) 0.957
hydrochlorothiazide (+0.00176) 0.958
Vitamin D deficiency (x1.02) 0.958
Human immunodeficiency virus infection (x1.02) 0.958
Arthralgia of the ankle and/or foot (x1.02) 0.959
docusate (+0.00172) 0.959
Chronic inflammatory demyelinating polyradiculoneuropathy (x1.02) 0.959
nitroglycerin (+0.00171) 0.960
Degeneration of intervertebral disc (x1.02) 0.960
Cardiac arrhythmia (x1.02) 0.960
Dysphagia (x1.02) 0.961
Disorder of lung (x1.02) 0.961
Chronic kidney disease (x1.02) 0.961
Vitamin B deficiency (x1.02) 0.962
Hypoxemia (x1.02) 0.962
lisinopril (+0.00161) 0.962
morphine (+0.0016) 0.963
simvastatin (+0.00159) 0.963
Expressions
−10 0 10
−0.8 −0.6 −0.4 −0.2 0.0 0.2 0.4 0.6 0.8

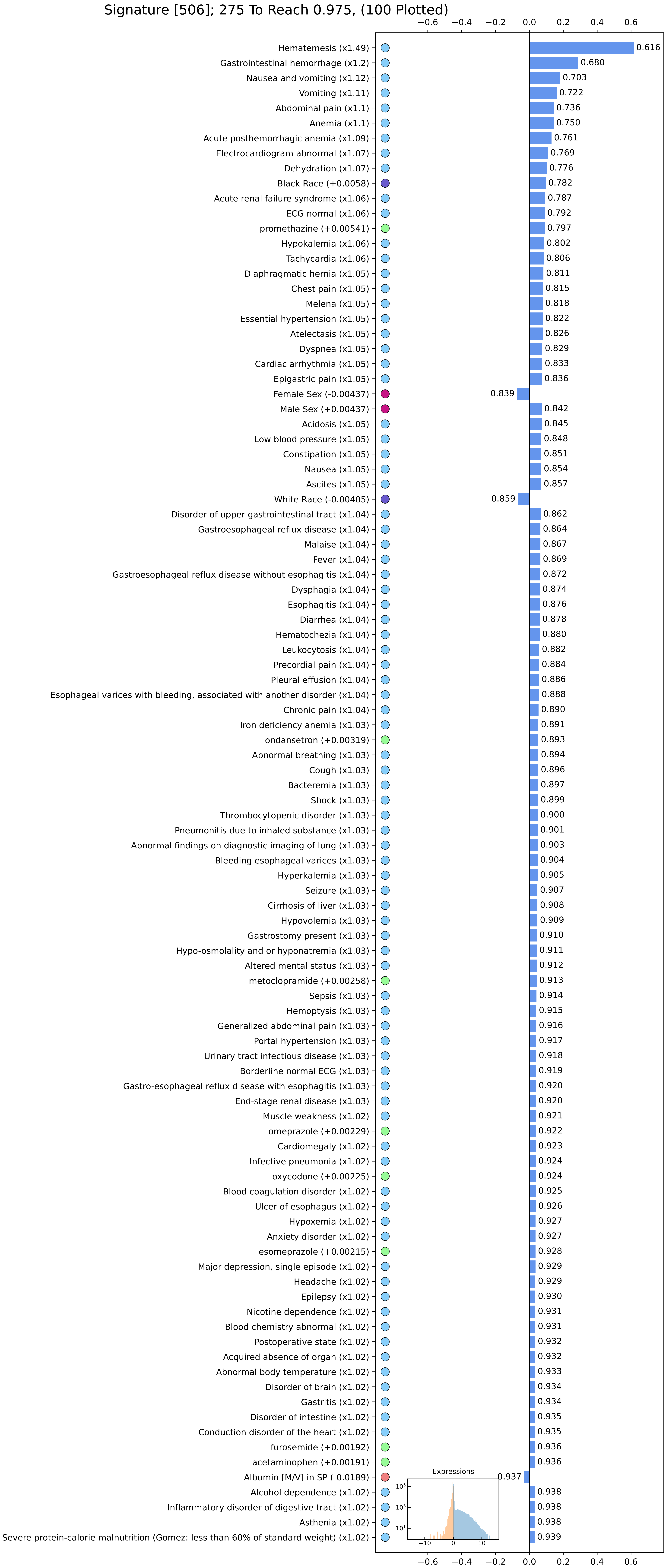

Signature [506]; 275 To Reach 0.975, (100 Plotted)
Hematemesis (x1.49) 0.616
Gastrointestinal hemorrhage (x1.2) 0.680
Nausea and vomiting (x1.12) 0.703
Vomiting (x1.11) 0.722
Abdominal pain (x1.1) 0.736
Anemia (x1.1) 0.750
Acute posthemorrhagic anemia (x1.09) 0.761
Electrocardiogram abnormal (x1.07) 0.769
Dehydration (x1.07) 0.776
Black Race (+0.0058) 0.782
Acute renal failure syndrome (x1.06) 0.787
ECG normal (x1.06) 0.792
promethazine (+0.00541) 0.797
Hypokalemia (x1.06) 0.802
Tachycardia (x1.06) 0.806
Diaphragmatic hernia (x1.05) 0.811
Chest pain (x1.05) 0.815
Melena (x1.05) 0.818
Essential hypertension (x1.05) 0.822
Atelectasis (x1.05) 0.826
Dyspnea (x1.05) 0.829
Cardiac arrhythmia (x1.05) 0.833
Epigastric pain (x1.05) 0.836
Female Sex (-0.00437) 0.839
Male Sex (+0.00437) 0.842
Acidosis (x1.05) 0.845
Low blood pressure (x1.05) 0.848
Constipation (x1.05) 0.851
Nausea (x1.05) 0.854
Ascites (x1.05) 0.857
White Race (-0.00405) 0.859
Disorder of upper gastrointestinal tract (x1.04) 0.862
Gastroesophageal reflux disease (x1.04) 0.864
Malaise (x1.04) 0.867
Fever (x1.04) 0.869
Gastroesophageal reflux disease without esophagitis (x1.04) 0.872
Dysphagia (x1.04) 0.874
Esophagitis (x1.04) 0.876
Diarrhea (x1.04) 0.878
Hematochezia (x1.04) 0.880
Leukocytosis (x1.04) 0.882
Precordial pain (x1.04) 0.884
Pleural effusion (x1.04) 0.886
Esophageal varices with bleeding, associated with another disorder (x1.04) 0.888
Chronic pain (x1.04) 0.890
Iron deficiency anemia (x1.03) 0.891
ondansetron (+0.00319) 0.893
Abnormal breathing (x1.03) 0.894
Cough (x1.03) 0.896
Bacteremia (x1.03) 0.897
Shock (x1.03) 0.899
Thrombocytopenic disorder (x1.03) 0.900
Pneumonitis due to inhaled substance (x1.03) 0.901
Abnormal findings on diagnostic imaging of lung (x1.03) 0.903
Bleeding esophageal varices (x1.03) 0.904
Hyperkalemia (x1.03) 0.905
Seizure (x1.03) 0.907
Cirrhosis of liver (x1.03) 0.908
Hypovolemia (x1.03) 0.909
Gastrostomy present (x1.03) 0.910
Hypo-osmolality and or hyponatremia (x1.03) 0.911
Altered mental status (x1.03) 0.912
metoclopramide (+0.00258) 0.913
Sepsis (x1.03) 0.914
Hemoptysis (x1.03) 0.915
Generalized abdominal pain (x1.03) 0.916
Portal hypertension (x1.03) 0.917
Urinary tract infectious disease (x1.03) 0.918
Borderline normal ECG (x1.03) 0.919
Gastro-esophageal reflux disease with esophagitis (x1.03) 0.920
End-stage renal disease (x1.03) 0.920
Muscle weakness (x1.02) 0.921
omeprazole (+0.00229) 0.922
Cardiomegaly (x1.02) 0.923
Infective pneumonia (x1.02) 0.924
oxycodone (+0.00225) 0.924
Blood coagulation disorder (x1.02) 0.925
Ulcer of esophagus (x1.02) 0.926
Hypoxemia (x1.02) 0.927
Anxiety disorder (x1.02) 0.927
esomeprazole (+0.00215) 0.928
Major depression, single episode (x1.02) 0.929
Headache (x1.02) 0.929
Epilepsy (x1.02) 0.930
Nicotine dependence (x1.02) 0.931
Blood chemistry abnormal (x1.02) 0.931
Postoperative state (x1.02) 0.932
Acquired absence of organ (x1.02) 0.932
Abnormal body temperature (x1.02) 0.933
Disorder of brain (x1.02) 0.934
Gastritis (x1.02) 0.934
Disorder of intestine (x1.02) 0.935
Conduction disorder of the heart (x1.02) 0.935
furosemide (+0.00192) 0.936
acetaminophen (+0.00191) 0.936
Albumin [M/V] in SP (-0.0189) 0.937
Alcohol dependence (x1.02) 0.938
Inflammatory disorder of digestive tract (x1.02) 0.938
Asthenia (x1.02) 0.938
Severe protein-calorie malnutrition (Gomez: less than 60% of standard weight) (x1.02) 0.939
−0.6 −0.4 −0.2 0.0 0.2 0.4 0.6
Expressions
10^5 10^3 10^1
−10 0 10

Signature [507]; 137 To Reach 0.975, (100 Plotted)

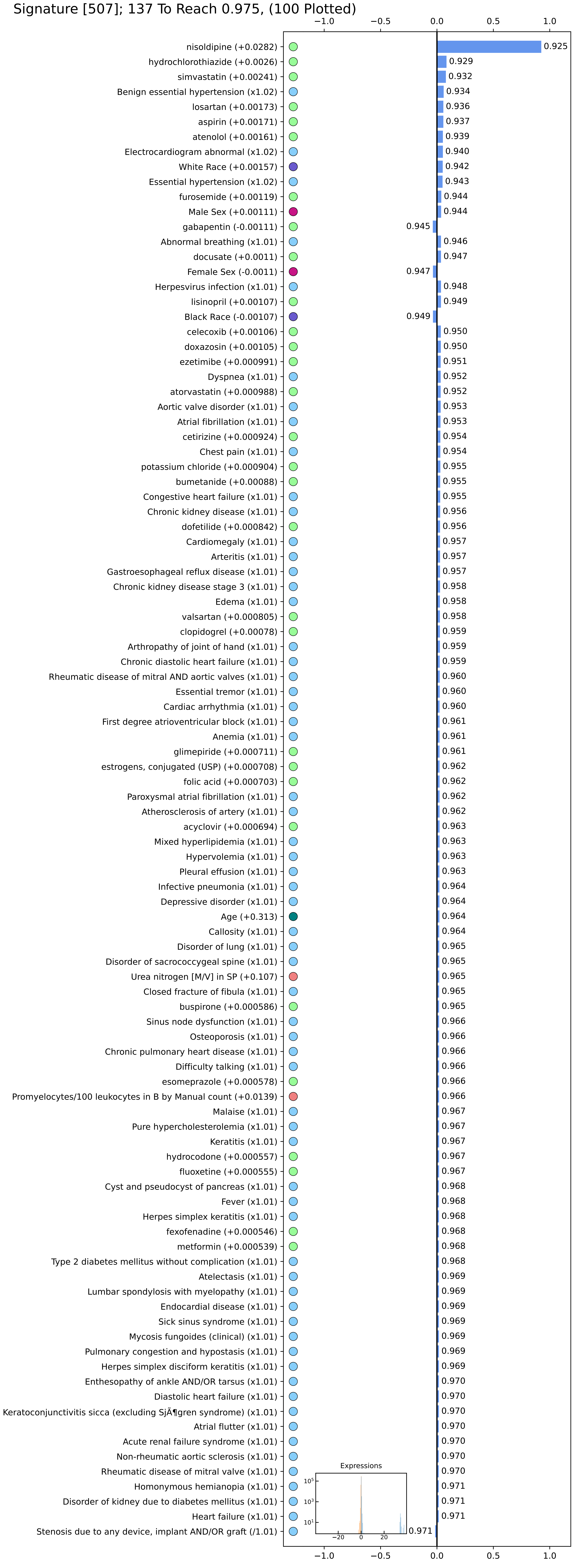

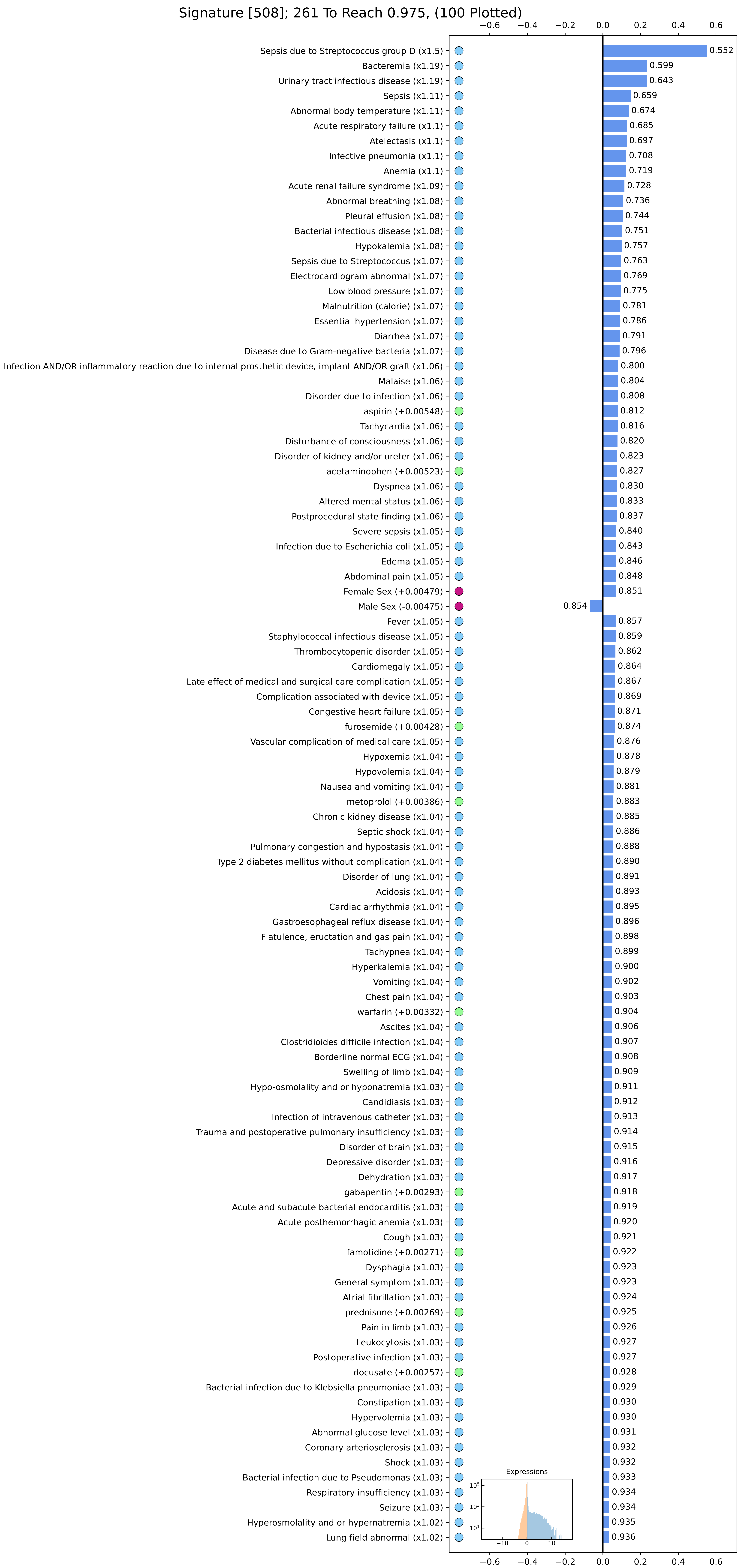

Signature [508]; 261 To Reach 0.975, (100 Plotted)
Sepsis due to Streptococcus group D (x1.5)
Bacteremia (x1.19)
Urinary tract infectious disease (x1.19)
Sepsis (x1.11)
Abnormal body temperature (x1.11)
Acute respiratory failure (x1.1)
Atelectasis (x1.1)
Infective pneumonia (x1.1)
Anemia (x1.1)
Acute renal failure syndrome (x1.09)
Abnormal breathing (x1.08)
Pleural effusion (x1.08)
Bacterial infectious disease (x1.08)
Hypokalemia (x1.08)
Sepsis due to Streptococcus (x1.07)
Electrocardiogram abnormal (x1.07)
Low blood pressure (x1.07)
Malnutrition (calorie) (x1.07)
Essential hypertension (x1.07)
Diarrhea (x1.07)
Disease due to Gram-negative bacteria (x1.07)
Infection AND/OR inflammatory reaction due to internal prosthetic device, implant AND/OR graft (x1.06)
Malaise (x1.06)
Disorder due to infection (x1.06)
aspirin (+0.00548)
Tachycardia (x1.06)
Disturbance of consciousness (x1.06)
Disorder of kidney and/or ureter (x1.06)
acetaminophen (+0.00523)
Dyspnea (x1.06)
Altered mental status (x1.06)
Postprocedural state finding (x1.06)
Severe sepsis (x1.05)
Infection due to Escherichia coli (x1.05)
Edema (x1.05)
Abdominal pain (x1.05)
Female Sex (+0.00479)
Male Sex (-0.00475)
Fever (x1.05)
Staphylococcal infectious disease (x1.05)
Thrombocytopenic disorder (x1.05)
Cardiomegaly (x1.05)
Late effect of medical and surgical care complication (x1.05)
Complication associated with device (x1.05)
Congestive heart failure (x1.05)
furosemide (+0.00428)
Vascular complication of medical care (x1.05)
Hypoxemia (x1.04)
Hypovolemia (x1.04)
Nausea and vomiting (x1.04)
metoprolol (+0.00386)
Chronic kidney disease (x1.04)
Septic shock (x1.04)
Pulmonary congestion and hypostasis (x1.04)
Type 2 diabetes mellitus without complication (x1.04)
Disorder of lung (x1.04)
Acidosis (x1.04)
Cardiac arrhythmia (x1.04)
Gastroesophageal reflux disease (x1.04)
Flatulence, eructation and gas pain (x1.04)
Tachypnea (x1.04)
Hyperkalemia (x1.04)
Vomiting (x1.04)
Chest pain (x1.04)
warfarin (+0.00332)
Ascites (x1.04)
Clostridioides difficile infection (x1.04)
Borderline normal ECG (x1.04)
Swelling of limb (x1.04)
Hypo-osmolality and or hyponatremia (x1.03)
Candidiasis (x1.03)
Infection of intravenous catheter (x1.03)
Trauma and postoperative pulmonary insufficiency (x1.03)
Disorder of brain (x1.03)
Depressive disorder (x1.03)
Dehydration (x1.03)
gabapentin (+0.00293)
Acute and subacute bacterial endocarditis (x1.03)
Acute posthemorrhagic anemia (x1.03)
Cough (x1.03)
famotidine (+0.00271)
Dysphagia (x1.03)
General symptom (x1.03)
Atrial fibrillation (x1.03)
prednisone (+0.00269)
Pain in limb (x1.03)
Leukocytosis (x1.03)
Postoperative infection (x1.03)
docusate (+0.00257)
Bacterial infection due to Klebsiella pneumoniae (x1.03)
Constipation (x1.03)
Hypervolemia (x1.03)
Abnormal glucose level (x1.03)
Coronary arteriosclerosis (x1.03)
Shock (x1.03)
Bacterial infection due to Pseudomonas (x1.03)
Respiratory insufficiency (x1.03)
Seizure (x1.03)
Hyperosmolality and or hypernatremia (x1.02)
Lung field abnormal (x1.02)
0.552
0.599
0.643
0.659
0.674
0.685
0.697
0.708
0.719
0.728
0.736
0.744
0.751
0.757
0.763
0.769
0.775
0.781
0.786
0.791
0.796
0.800
0.804
0.808
0.812
0.816
0.820
0.823
0.827
0.830
0.833
0.837
0.840
0.843
0.846
0.848
0.851
0.854
0.857
0.859
0.862
0.864
0.867
0.869
0.871
0.874
0.876
0.878
0.879
0.881
0.883
0.885
0.886
0.888
0.890
0.891
0.893
0.895
0.896
0.898
0.899
0.900
0.902
0.903
0.904
0.906
0.907
0.908
0.909
0.911
0.912
0.913
0.914
0.915
0.916
0.917
0.918
0.919
0.920
0.921
0.922
0.923
0.923
0.924
0.925
0.926
0.927
0.927
0.928
0.929
0.930
0.930
0.931
0.932
0.932
0.933
0.934
0.934
0.935
0.936
−0.6
−0.4
−0.2
0.0
0.2
0.4
0.6
Expressions

Signature [509]; 2 To Reach 0.975, (10 Plotted)

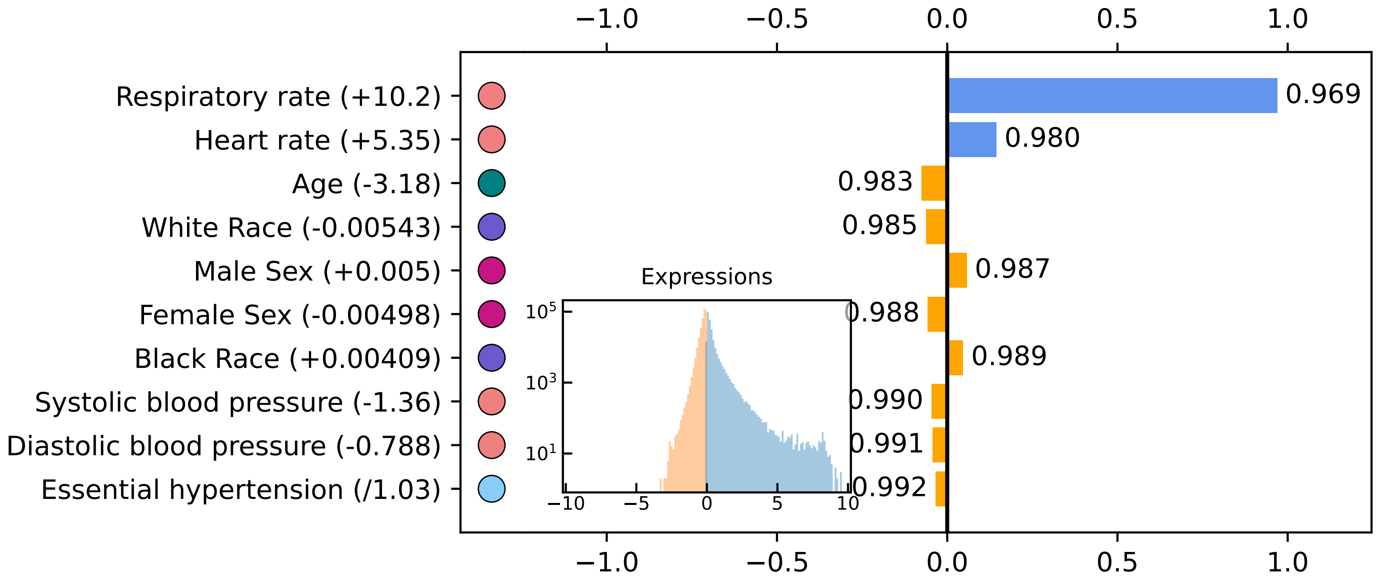

Signature [510]; 15 To Reach 0.975, (15 Plotted)

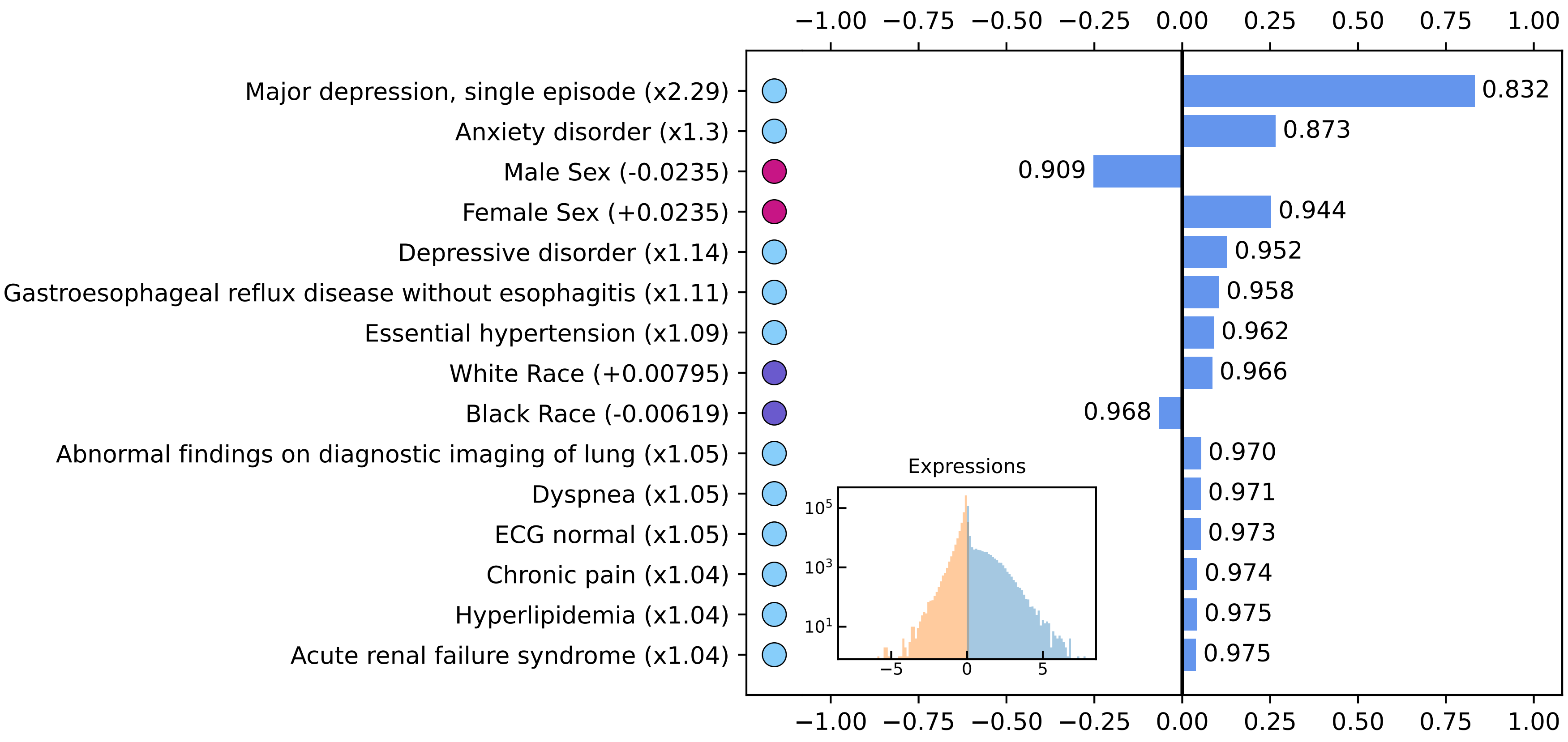

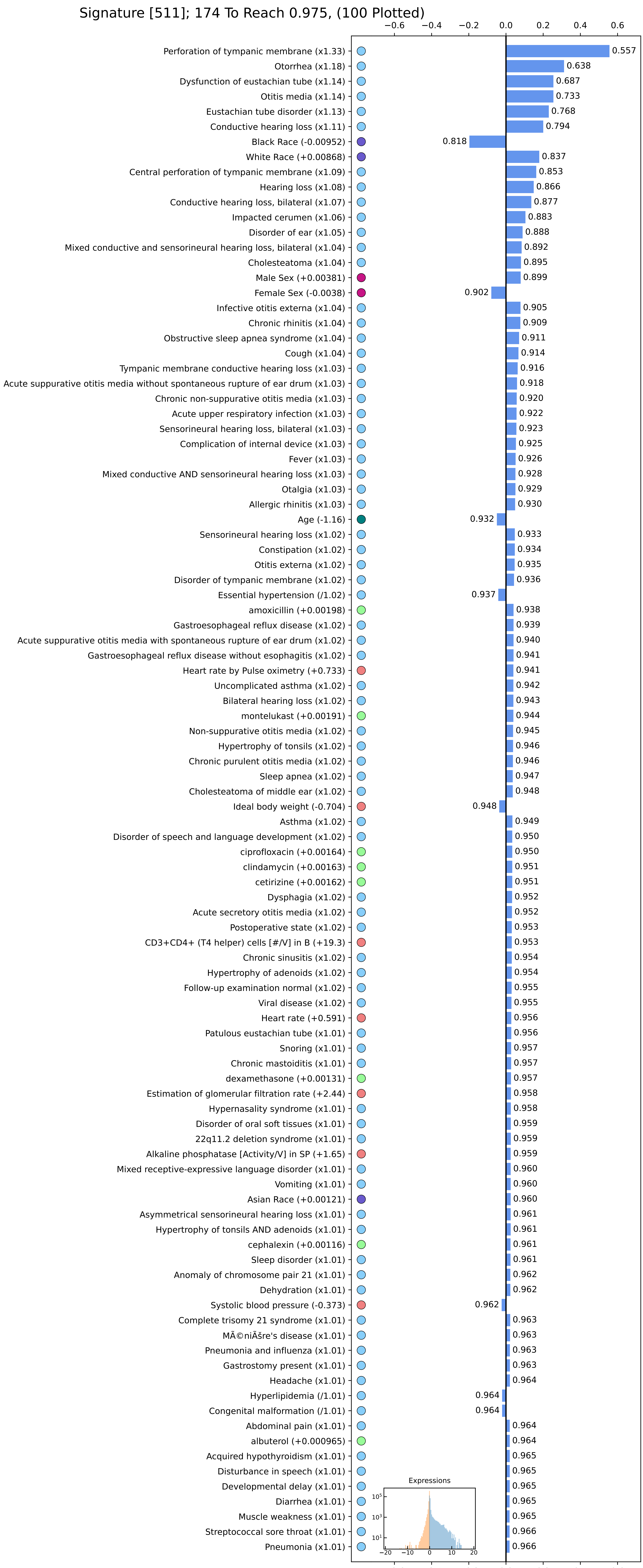

Signature [511]; 174 To Reach 0.975, (100 Plotted)
Perforation of tympanic membrane (x1.33) 0.557
Otorrhea (x1.18) 0.638
Dysfunction of eustachian tube (x1.14) 0.687
Otitis media (x1.14) 0.733
Eustachian tube disorder (x1.13) 0.768
Conductive hearing loss (x1.11) 0.794
Black Race (-0.00952) 0.818
White Race (+0.00868) 0.837
Central perforation of tympanic membrane (x1.09) 0.853
Hearing loss (x1.08) 0.866
Conductive hearing loss, bilateral (x1.07) 0.877
Impacted cerumen (x1.06) 0.883
Disorder of ear (x1.05) 0.888
Mixed conductive and sensorineural hearing loss, bilateral (x1.04) 0.892
Cholesteatoma (x1.04) 0.895
Male Sex (+0.00381) 0.899
Female Sex (-0.0038) 0.902
Infective otitis externa (x1.04) 0.905
Chronic rhinitis (x1.04) 0.909
Obstructive sleep apnea syndrome (x1.04) 0.911
Cough (x1.04) 0.914
Tympanic membrane conductive hearing loss (x1.03) 0.916
Acute suppurative otitis media without spontaneous rupture of ear drum (x1.03) 0.918
Chronic non-suppurative otitis media (x1.03) 0.920
Acute upper respiratory infection (x1.03) 0.922
Sensorineural hearing loss, bilateral (x1.03) 0.923
Complication of internal device (x1.03) 0.925
Fever (x1.03) 0.926
Mixed conductive AND sensorineural hearing loss (x1.03) 0.928
Otalgia (x1.03) 0.929
Allergic rhinitis (x1.03) 0.930
Age (-1.16) 0.932
Sensorineural hearing loss (x1.02) 0.933
Constipation (x1.02) 0.934
Otitis externa (x1.02) 0.935
Disorder of tympanic membrane (x1.02) 0.936
Essential hypertension (/1.02) 0.937
amoxicillin (+0.00198) 0.938
Gastroesophageal reflux disease (x1.02) 0.939
Acute suppurative otitis media with spontaneous rupture of ear drum (x1.02) 0.940
Gastroesophageal reflux disease without esophagitis (x1.02) 0.941
Heart rate by Pulse oximetry (+0.733) 0.941
Uncomplicated asthma (x1.02) 0.942
Bilateral hearing loss (x1.02) 0.943
montelukast (+0.00191) 0.944
Non-suppurative otitis media (x1.02) 0.945
Hypertrophy of tonsils (x1.02) 0.946
Chronic purulent otitis media (x1.02) 0.946
Sleep apnea (x1.02) 0.947
Cholesteatoma of middle ear (x1.02) 0.948
Ideal body weight (-0.704) 0.948
Asthma (x1.02) 0.949
Disorder of speech and language development (x1.02) 0.950
ciprofloxacin (+0.00164) 0.950
clindamycin (+0.00163) 0.951
cetirizine (+0.00162) 0.951
Dysphagia (x1.02) 0.952
Acute secretory otitis media (x1.02) 0.952
Postoperative state (x1.02) 0.953
CD3+CD4+ (T4 helper) cells [#/V] in B (+19.3) 0.953
Chronic sinusitis (x1.02) 0.954
Hypertrophy of adenoids (x1.02) 0.954
Follow-up examination normal (x1.02) 0.955
Viral disease (x1.02) 0.955
Heart rate (+0.591) 0.956
Patulous eustachian tube (x1.01) 0.956
Snoring (x1.01) 0.957
Chronic mastoiditis (x1.01) 0.957
dexamethasone (+0.00131) 0.957
Estimation of glomerular filtration rate (+2.44) 0.958
Hypernasality syndrome (x1.01) 0.958
Disorder of oral soft tissues (x1.01) 0.959
22q11.2 deletion syndrome (x1.01) 0.959
Alkaline phosphatase [Activity/V] in SP (+1.65) 0.959
Mixed receptive-expressive language disorder (x1.01) 0.960
Vomiting (x1.01) 0.960
Asian Race (+0.00121) 0.960
Asymmetrical sensorineural hearing loss (x1.01) 0.961
Hypertrophy of tonsils AND adenoids (x1.01) 0.961
cephalexin (+0.00116) 0.961
Sleep disorder (x1.01) 0.961
Anomaly of chromosome pair 21 (x1.01) 0.962
Dehydration (x1.01) 0.962
Systolic blood pressure (-0.373) 0.962
Complete trisomy 21 syndrome (x1.01) 0.963
MÃ©niÃšre's disease (x1.01) 0.963
Pneumonia and influenza (x1.01) 0.963
Gastrostomy present (x1.01) 0.963
Headache (x1.01) 0.964
Hyperlipidemia (/1.01) 0.964
Congenital malformation (/1.01) 0.964
Abdominal pain (x1.01) 0.964
albuterol (+0.000965) 0.964
Acquired hypothyroidism (x1.01) 0.965
Disturbance in speech (x1.01) 0.965
Developmental delay (x1.01) 0.965
Diarrhea (x1.01) 0.965
Muscle weakness (x1.01) 0.965
Streptococcal sore throat (x1.01) 0.966
Pneumonia (x1.01) 0.966
Expressions
−0.6 −0.4 −0.2 0.0 0.2 0.4 0.6

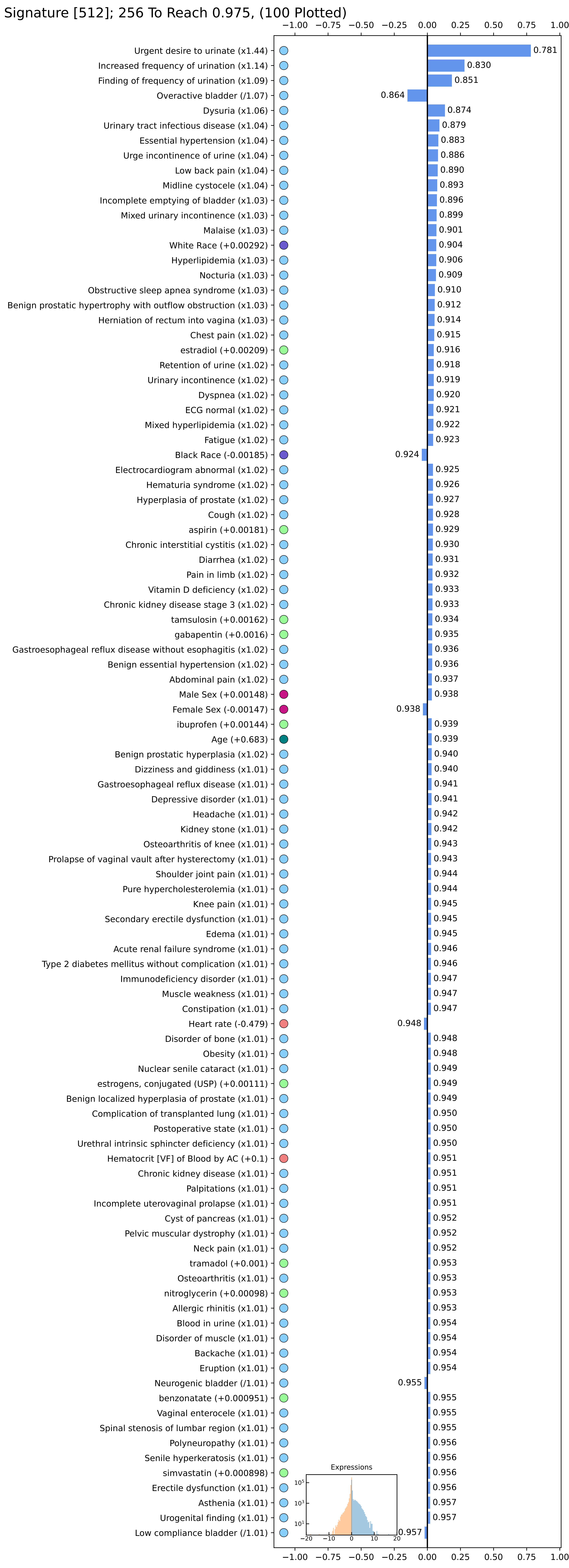
Signature [512]; 256 To Reach 0.975, (100 Plotted)
−1.00 −0.75 −0.50 −0.25 0.00 0.25 0.50 0.75 1.00
Urgent desire to urinate (x1.44) 0.781
Increased frequency of urination (x1.14) 0.830
Finding of frequency of urination (x1.09) 0.851
Overactive bladder (/1.07) 0.864
Dysuria (x1.06) 0.874
Urinary tract infectious disease (x1.04) 0.879
Essential hypertension (x1.04) 0.883
Urge incontinence of urine (x1.04) 0.886
Low back pain (x1.04) 0.890
Midline cystocele (x1.04) 0.893
Incomplete emptying of bladder (x1.03) 0.896
Mixed urinary incontinence (x1.03) 0.899
Malaise (x1.03) 0.901
White Race (+0.00292) 0.904
Hyperlipidemia (x1.03) 0.906
Nocturia (x1.03) 0.909
Obstructive sleep apnea syndrome (x1.03) 0.910
Benign prostatic hypertrophy with outflow obstruction (x1.03) 0.912
Herniation of rectum into vagina (x1.03) 0.914
Chest pain (x1.02) 0.915
estradiol (+0.00209) 0.916
Retention of urine (x1.02) 0.918
Urinary incontinence (x1.02) 0.919
Dyspnea (x1.02) 0.920
ECG normal (x1.02) 0.921
Mixed hyperlipidemia (x1.02) 0.922
Fatigue (x1.02) 0.923
Black Race (-0.00185) 0.924
Electrocardiogram abnormal (x1.02) 0.925
Hematuria syndrome (x1.02) 0.926
Hyperplasia of prostate (x1.02) 0.927
Cough (x1.02) 0.928
aspirin (+0.00181) 0.929
Chronic interstitial cystitis (x1.02) 0.930
Diarrhea (x1.02) 0.931
Pain in limb (x1.02) 0.932
Vitamin D deficiency (x1.02) 0.933
Chronic kidney disease stage 3 (x1.02) 0.933
tamsulosin (+0.00162) 0.934
gabapentin (+0.0016) 0.935
Gastroesophageal reflux disease without esophagitis (x1.02) 0.936
Benign essential hypertension (x1.02) 0.936
Abdominal pain (x1.02) 0.937
Male Sex (+0.00148) 0.938
Female Sex (-0.00147) 0.938
ibuprofen (+0.00144) 0.939
Age (+0.683) 0.939
Benign prostatic hyperplasia (x1.02) 0.940
Dizziness and giddiness (x1.01) 0.940
Gastroesophageal reflux disease (x1.01) 0.941
Depressive disorder (x1.01) 0.941
Headache (x1.01) 0.942
Kidney stone (x1.01) 0.942
Osteoarthritis of knee (x1.01) 0.943
Prolapse of vaginal vault after hysterectomy (x1.01) 0.943
Shoulder joint pain (x1.01) 0.944
Pure hypercholesterolemia (x1.01) 0.944
Knee pain (x1.01) 0.945
Secondary erectile dysfunction (x1.01) 0.945
Edema (x1.01) 0.945
Acute renal failure syndrome (x1.01) 0.946
Type 2 diabetes mellitus without complication (x1.01) 0.946
Immunodeficiency disorder (x1.01) 0.947
Muscle weakness (x1.01) 0.947
Constipation (x1.01) 0.947
Heart rate (-0.479) 0.948
Disorder of bone (x1.01) 0.948
Obesity (x1.01) 0.948
Nuclear senile cataract (x1.01) 0.949
estrogens, conjugated (USP) (+0.00111) 0.949
Benign localized hyperplasia of prostate (x1.01) 0.949
Complication of transplanted lung (x1.01) 0.950
Postoperative state (x1.01) 0.950
Urethral intrinsic sphincter deficiency (x1.01) 0.950
Hematocrit [VF] of Blood by AC (+0.1) 0.951
Chronic kidney disease (x1.01) 0.951
Palpitations (x1.01) 0.951
Incomplete uterovaginal prolapse (x1.01) 0.951
Cyst of pancreas (x1.01) 0.952
Pelvic muscular dystrophy (x1.01) 0.952
Neck pain (x1.01) 0.952
tramadol (+0.001) 0.953
Osteoarthritis (x1.01) 0.953
nitroglycerin (+0.00098) 0.953
Allergic rhinitis (x1.01) 0.953
Blood in urine (x1.01) 0.954
Disorder of muscle (x1.01) 0.954
Backache (x1.01) 0.954
Eruption (x1.01) 0.954
Neurogenic bladder (/1.01) 0.955
benzonatate (+0.000951) 0.955
Vaginal enterocele (x1.01) 0.955
Spinal stenosis of lumbar region (x1.01) 0.955
Polyneuropathy (x1.01) 0.956
Senile hyperkeratosis (x1.01) 0.956
simvastatin (+0.000898) 0.956
Erectile dysfunction (x1.01) 0.956
Asthenia (x1.01) 0.957
Urogenital finding (x1.01) 0.957
Low compliance bladder (/1.01) 0.957
Expressions
10^5 10^3 10^1
−20 −10 0 10 20
−1.00 −0.75 −0.50 −0.25 0.00 0.25 0.50 0.75 1.00

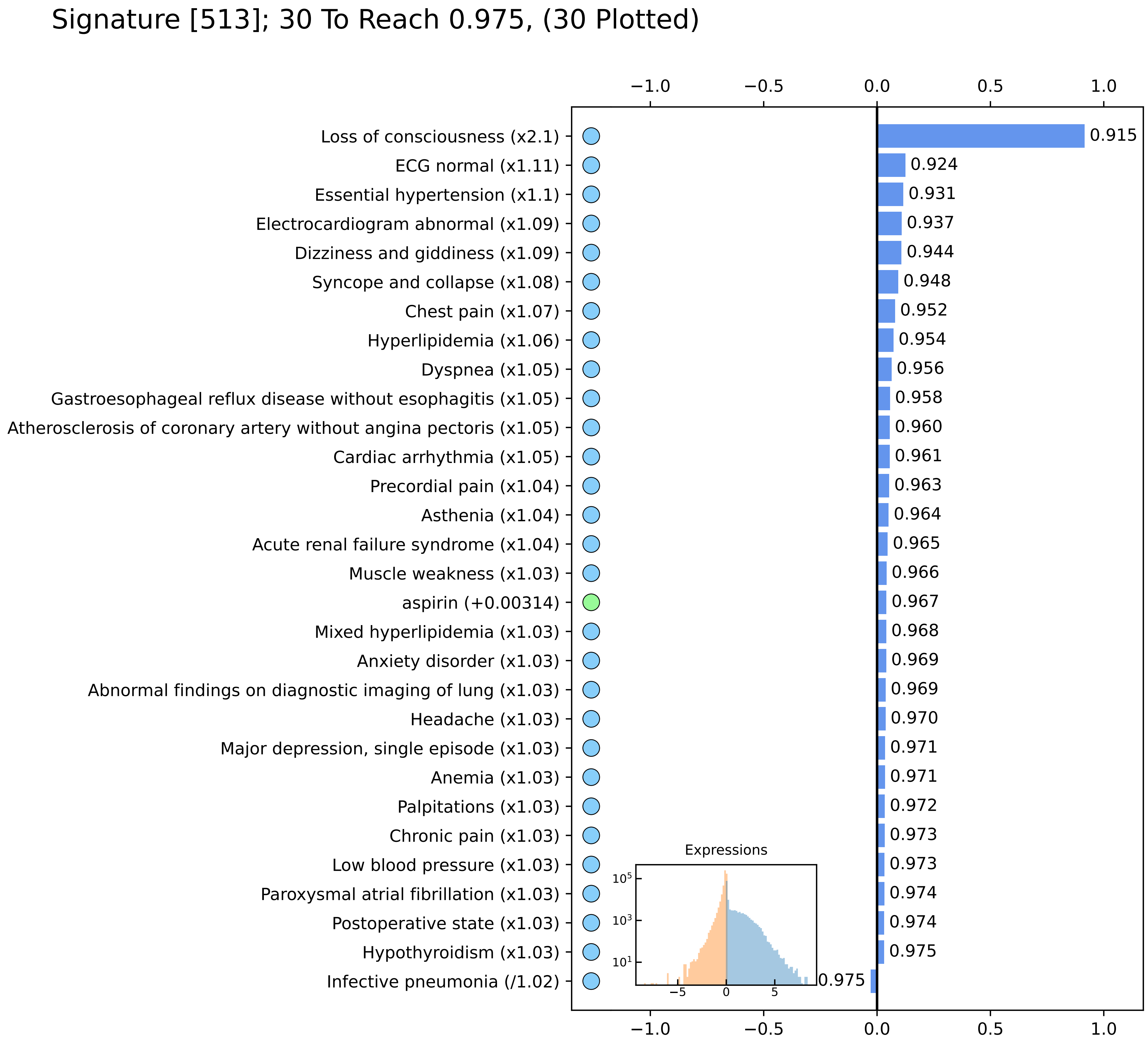

Signature [513]; 30 To Reach 0.975, (30 Plotted)
Loss of consciousness (x2.1)
0.915
ECG normal (x1.11)
0.924
Essential hypertension (x1.1)
0.931
Electrocardiogram abnormal (x1.09)
0.937
Dizziness and giddiness (x1.09)
0.944
Syncope and collapse (x1.08)
0.948
Chest pain (x1.07)
0.952
Hyperlipidemia (x1.06)
0.954
Dyspnea (x1.05)
0.956
Gastroesophageal reflux disease without esophagitis (x1.05)
0.958
Atherosclerosis of coronary artery without angina pectoris (x1.05)
0.960
Cardiac arrhythmia (x1.05)
0.961
Precordial pain (x1.04)
0.963
Asthenia (x1.04)
0.964
Acute renal failure syndrome (x1.04)
0.965
Muscle weakness (x1.03)
0.966
aspirin (+0.00314)
0.967
Mixed hyperlipidemia (x1.03)
0.968
Anxiety disorder (x1.03)
0.969
Abnormal findings on diagnostic imaging of lung (x1.03)
0.969
Headache (x1.03)
0.970
Major depression, single episode (x1.03)
0.971
Anemia (x1.03)
0.971
Palpitations (x1.03)
0.972
Chronic pain (x1.03)
0.973
Low blood pressure (x1.03)
0.973
Paroxysmal atrial fibrillation (x1.03)
0.974
Postoperative state (x1.03)
0.974
Hypothyroidism (x1.03)
0.975
Infective pneumonia (/1.02)
0.975
−1.0
−0.5
0.0
0.5
1.0
Expressions
10^5
10^3
10^1
−5
0
5

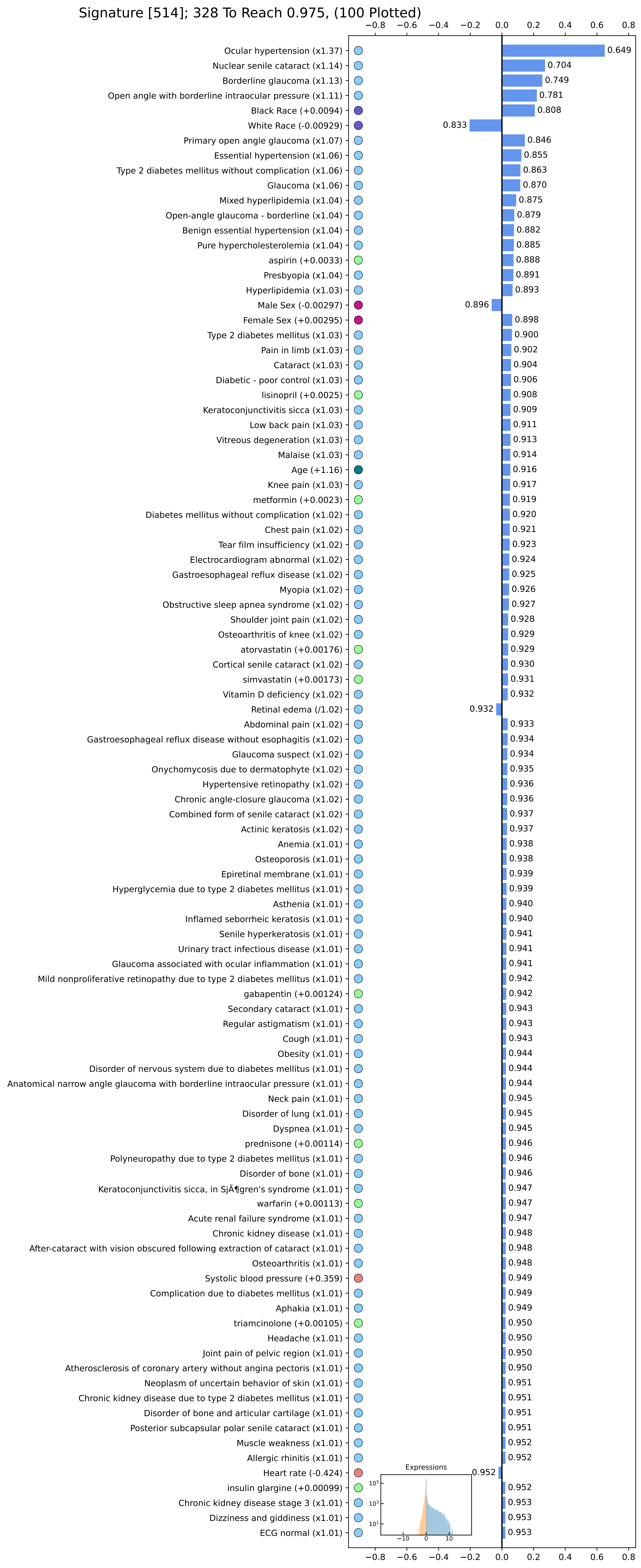

Signature [514]; 328 To Reach 0.975, (100 Plotted)
Ocular hypertension (x1.37) 0.649
Nuclear senile cataract (x1.14) 0.704
Borderline glaucoma (x1.13) 0.749
Open angle with borderline intraocular pressure (x1.11) 0.781
Black Race (+0.0094) 0.808
White Race (-0.00929) 0.833
Primary open angle glaucoma (x1.07) 0.846
Essential hypertension (x1.06) 0.855
Type 2 diabetes mellitus without complication (x1.06) 0.863
Glaucoma (x1.06) 0.870
Mixed hyperlipidemia (x1.04) 0.875
Open-angle glaucoma - borderline (x1.04) 0.879
Benign essential hypertension (x1.04) 0.882
Pure hypercholesterolemia (x1.04) 0.885
aspirin (+0.0033) 0.888
Presbyopia (x1.04) 0.891
Hyperlipidemia (x1.03) 0.893
Male Sex (-0.00297) 0.896
Female Sex (+0.00295) 0.898
Type 2 diabetes mellitus (x1.03) 0.900
Pain in limb (x1.03) 0.902
Cataract (x1.03) 0.904
Diabetic - poor control (x1.03) 0.906
lisinopril (+0.0025) 0.908
Keratoconjunctivitis sicca (x1.03) 0.909
Low back pain (x1.03) 0.911
Vitreous degeneration (x1.03) 0.913
Malaise (x1.03) 0.914
Age (+1.16) 0.916
Knee pain (x1.03) 0.917
metformin (+0.0023) 0.919
Diabetes mellitus without complication (x1.02) 0.920
Chest pain (x1.02) 0.921
Tear film insufficiency (x1.02) 0.923
Electrocardiogram abnormal (x1.02) 0.924
Gastroesophageal reflux disease (x1.02) 0.925
Myopia (x1.02) 0.926
Obstructive sleep apnea syndrome (x1.02) 0.927
Shoulder joint pain (x1.02) 0.928
Osteoarthritis of knee (x1.02) 0.929
atorvastatin (+0.00176) 0.929
Cortical senile cataract (x1.02) 0.930
simvastatin (+0.00173) 0.931
Vitamin D deficiency (x1.02) 0.932
Retinal edema (/1.02) 0.932
Abdominal pain (x1.02) 0.933
Gastroesophageal reflux disease without esophagitis (x1.02) 0.934
Glaucoma suspect (x1.02) 0.934
Onychomycosis due to dermatophyte (x1.02) 0.935
Hypertensive retinopathy (x1.02) 0.936
Chronic angle-closure glaucoma (x1.02) 0.936
Combined form of senile cataract (x1.02) 0.937
Actinic keratosis (x1.02) 0.937
Anemia (x1.01) 0.938
Osteoporosis (x1.01) 0.938
Epiretinal membrane (x1.01) 0.939
Hyperglycemia due to type 2 diabetes mellitus (x1.01) 0.939
Asthenia (x1.01) 0.940
Inflamed seborrheic keratosis (x1.01) 0.940
Senile hyperkeratosis (x1.01) 0.941
Urinary tract infectious disease (x1.01) 0.941
Glaucoma associated with ocular inflammation (x1.01) 0.941
Mild nonproliferative retinopathy due to type 2 diabetes mellitus (x1.01) 0.942
gabapentin (+0.00124) 0.942
Secondary cataract (x1.01) 0.943
Regular astigmatism (x1.01) 0.943
Cough (x1.01) 0.943
Obesity (x1.01) 0.944
Disorder of nervous system due to diabetes mellitus (x1.01) 0.944
Anatomical narrow angle glaucoma with borderline intraocular pressure (x1.01) 0.944
Neck pain (x1.01) 0.945
Disorder of lung (x1.01) 0.945
Dyspnea (x1.01) 0.945
prednisone (+0.00114) 0.946
Polyneuropathy due to type 2 diabetes mellitus (x1.01) 0.946
Disorder of bone (x1.01) 0.946
Keratoconjunctivitis sicca, in SjÃ¶gren's syndrome (x1.01) 0.947
warfarin (+0.00113) 0.947
Acute renal failure syndrome (x1.01) 0.947
Chronic kidney disease (x1.01) 0.948
After-cataract with vision obscured following extraction of cataract (x1.01) 0.948
Osteoarthritis (x1.01) 0.948
Systolic blood pressure (+0.359) 0.949
Complication due to diabetes mellitus (x1.01) 0.949
Aphakia (x1.01) 0.949
triamcinolone (+0.00105) 0.950
Headache (x1.01) 0.950
Joint pain of pelvic region (x1.01) 0.950
Atherosclerosis of coronary artery without angina pectoris (x1.01) 0.950
Neoplasm of uncertain behavior of skin (x1.01) 0.951
Chronic kidney disease due to type 2 diabetes mellitus (x1.01) 0.951
Disorder of bone and articular cartilage (x1.01) 0.951
Posterior subcapsular polar senile cataract (x1.01) 0.951
Muscle weakness (x1.01) 0.952
Allergic rhinitis (x1.01) 0.952
Heart rate (-0.424) 0.952
insulin glargine (+0.00099) 0.952
Chronic kidney disease stage 3 (x1.01) 0.953
Dizziness and giddiness (x1.01) 0.953
ECG normal (x1.01) 0.953
−0.8 −0.6 −0.4 −0.2 0.0 0.2 0.4 0.6 0.8
Expressions
10^5 10^3 10^1
−10 0 10

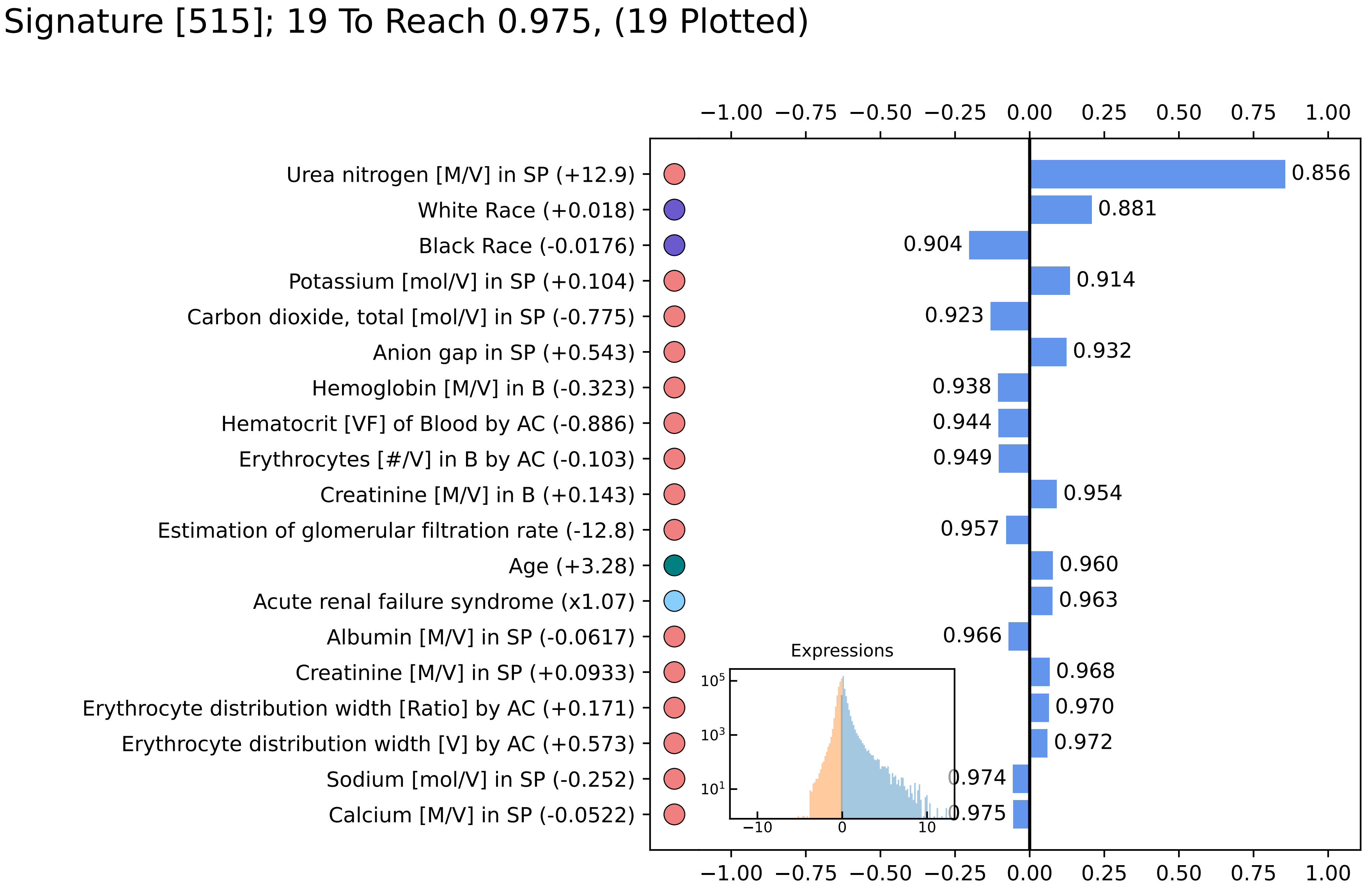

Signature [515]; 19 To Reach 0.975, (19 Plotted)
Urea nitrogen [M/V] in SP (+12.9)
White Race (+0.018)
Black Race (-0.0176)
Potassium [mol/V] in SP (+0.104)
Carbon dioxide, total [mol/V] in SP (-0.775)
Anion gap in SP (+0.543)
Hemoglobin [M/V] in B (-0.323)
Hematocrit [VF] of Blood by AC (-0.886)
Erythrocytes [#/V] in B by AC (-0.103)
Creatinine [M/V] in B (+0.143)
Estimation of glomerular filtration rate (-12.8)
Age (+3.28)
Acute renal failure syndrome (x1.07)
Albumin [M/V] in SP (-0.0617)
Creatinine [M/V] in SP (+0.0933)
Erythrocyte distribution width [Ratio] by AC (+0.171)
Erythrocyte distribution width [V] by AC (+0.573)
Sodium [mol/V] in SP (-0.252)
Calcium [M/V] in SP (-0.0522)
0.856
0.881
0.904
0.914
0.923
0.932
0.938
0.944
0.949
0.954
0.957
0.960
0.963
0.966
0.968
0.970
0.972
0.974
0.975
−1.00 −0.75 −0.50 −0.25 0.00 0.25 0.50 0.75 1.00
Expressions
10^5
10^3
10^1
−10 0 10

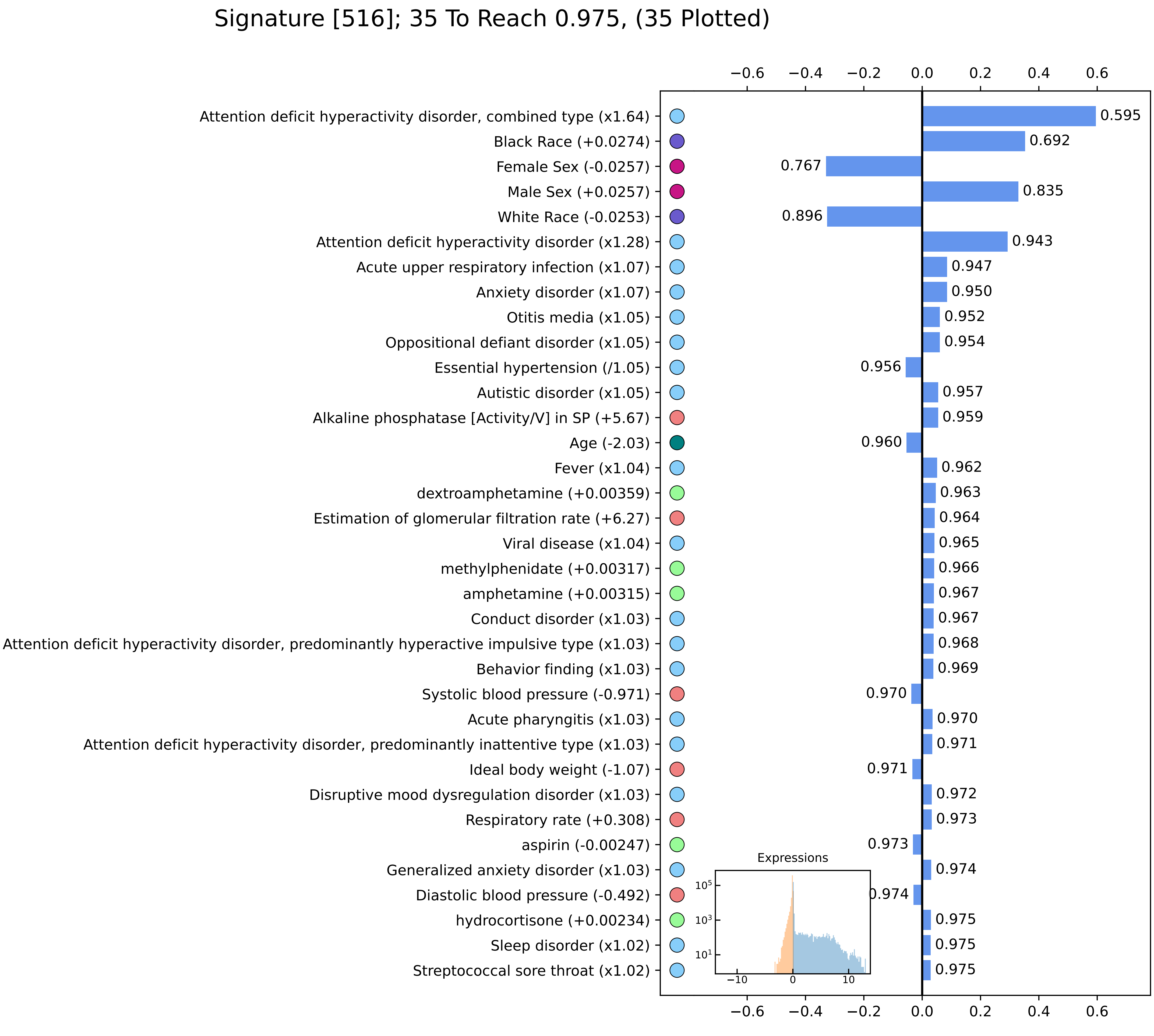

Signature [516]; 35 To Reach 0.975, (35 Plotted)
Attention deficit hyperactivity disorder, combined type (x1.64) 0.595
Black Race (+0.0274) 0.692
Female Sex (-0.0257) 0.767
Male Sex (+0.0257) 0.835
White Race (-0.0253) 0.896
Attention deficit hyperactivity disorder (x1.28) 0.943
Acute upper respiratory infection (x1.07) 0.947
Anxiety disorder (x1.07) 0.950
Otitis media (x1.05) 0.952
Oppositional defiant disorder (x1.05) 0.954
Essential hypertension (/1.05) 0.956
Autistic disorder (x1.05) 0.957
Alkaline phosphatase [Activity/V] in SP (+5.67) 0.959
Age (-2.03) 0.960
Fever (x1.04) 0.962
dextroamphetamine (+0.00359) 0.963
Estimation of glomerular filtration rate (+6.27) 0.964
Viral disease (x1.04) 0.965
methylphenidate (+0.00317) 0.966
amphetamine (+0.00315) 0.967
Conduct disorder (x1.03) 0.967
Attention deficit hyperactivity disorder, predominantly hyperactive impulsive type (x1.03) 0.968
Behavior finding (x1.03) 0.969
Systolic blood pressure (-0.971) 0.970
Acute pharyngitis (x1.03) 0.970
Attention deficit hyperactivity disorder, predominantly inattentive type (x1.03) 0.971
Ideal body weight (-1.07) 0.971
Disruptive mood dysregulation disorder (x1.03) 0.972
Respiratory rate (+0.308) 0.973
aspirin (-0.00247) 0.973
Generalized anxiety disorder (x1.03) 0.974
Diastolic blood pressure (-0.492) 0.974
hydrocortisone (+0.00234) 0.975
Sleep disorder (x1.02) 0.975
Streptococcal sore throat (x1.02) 0.975
−0.6 −0.4 −0.2 0.0 0.2 0.4 0.6
Expressions
10^5 10^3 10^1
−10 0 10

Signature [517]; 4 To Reach 0.975, (10 Plotted)

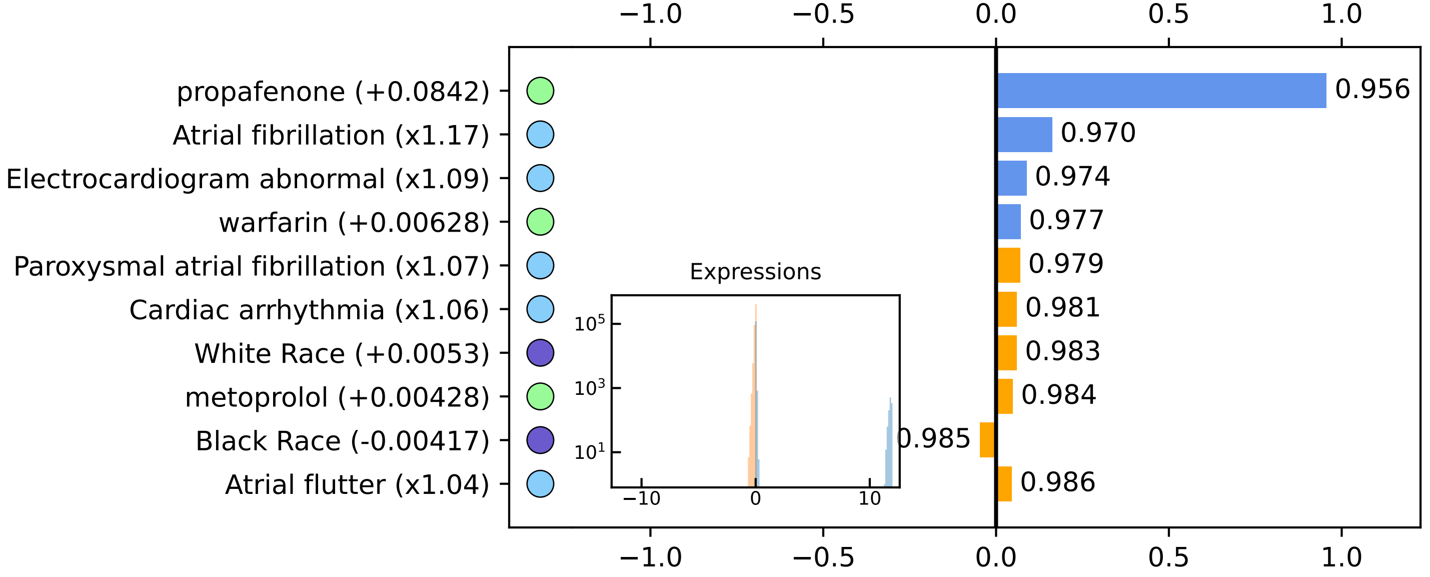

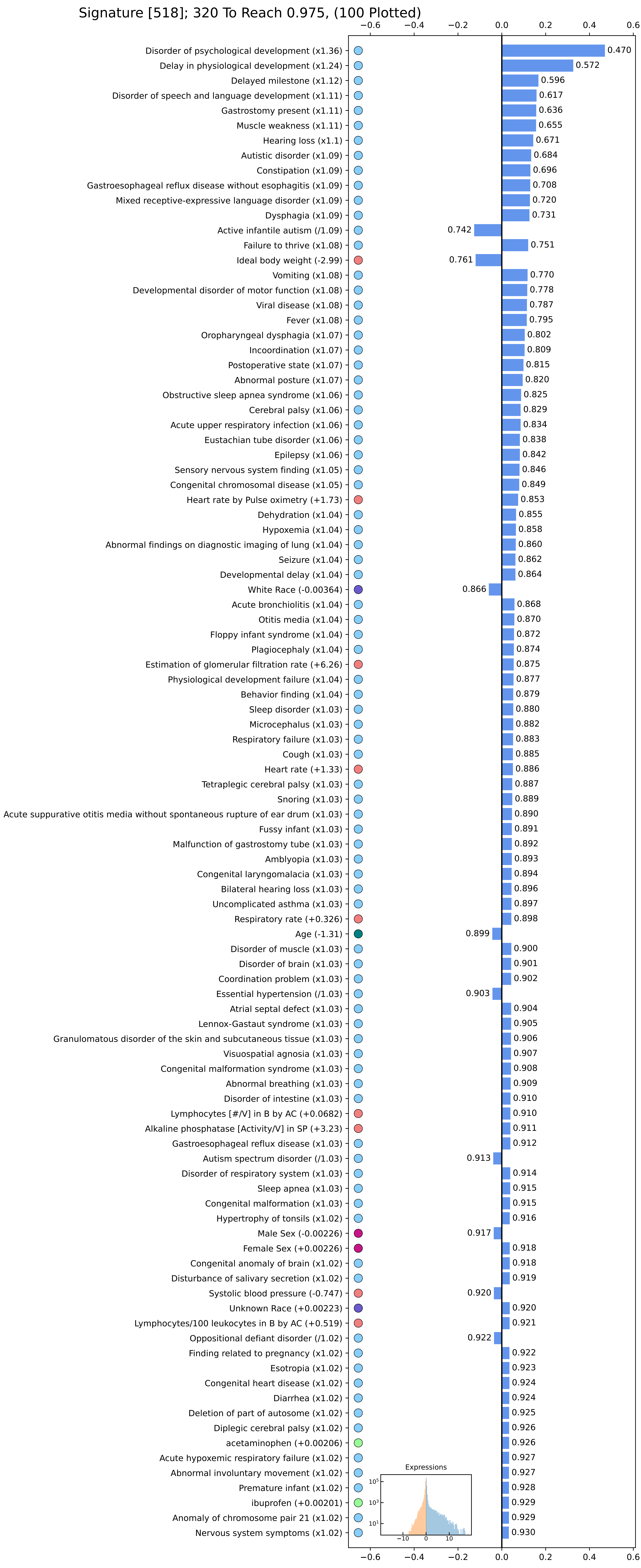
Signature [518]; 320 To Reach 0.975, (100 Plotted)
Disorder of psychological development (x1.36) 0.470
Delay in physiological development (x1.24) 0.572
Delayed milestone (x1.12) 0.596
Disorder of speech and language development (x1.11) 0.617
Gastrostomy present (x1.11) 0.636
Muscle weakness (x1.11) 0.655
Hearing loss (x1.1) 0.671
Autistic disorder (x1.09) 0.684
Constipation (x1.09) 0.696
Gastroesophageal reflux disease without esophagitis (x1.09) 0.708
Mixed receptive-expressive language disorder (x1.09) 0.720
Dysphagia (x1.09) 0.731
Active infantile autism (/1.09) 0.742
Failure to thrive (x1.08) 0.751
Ideal body weight (-2.99) 0.761
Vomiting (x1.08) 0.770
Developmental disorder of motor function (x1.08) 0.778
Viral disease (x1.08) 0.787
Fever (x1.08) 0.795
Oropharyngeal dysphagia (x1.07) 0.802
Incoordination (x1.07) 0.809
Postoperative state (x1.07) 0.815
Abnormal posture (x1.07) 0.820
Obstructive sleep apnea syndrome (x1.06) 0.825
Cerebral palsy (x1.06) 0.829
Acute upper respiratory infection (x1.06) 0.834
Eustachian tube disorder (x1.06) 0.838
Epilepsy (x1.06) 0.842
Sensory nervous system finding (x1.05) 0.846
Congenital chromosomal disease (x1.05) 0.849
Heart rate by Pulse oximetry (+1.73) 0.853
Dehydration (x1.04) 0.855
Hypoxemia (x1.04) 0.858
Abnormal findings on diagnostic imaging of lung (x1.04) 0.860
Seizure (x1.04) 0.862
Developmental delay (x1.04) 0.864
White Race (-0.00364) 0.866
Acute bronchiolitis (x1.04) 0.868
Otitis media (x1.04) 0.870
Floppy infant syndrome (x1.04) 0.872
Plagiocephaly (x1.04) 0.874
Estimation of glomerular filtration rate (+6.26) 0.875
Physiological development failure (x1.04) 0.877
Behavior finding (x1.04) 0.879
Sleep disorder (x1.03) 0.880
Microcephalus (x1.03) 0.882
Respiratory failure (x1.03) 0.883
Cough (x1.03) 0.885
Heart rate (+1.33) 0.886
Tetraplegic cerebral palsy (x1.03) 0.887
Snoring (x1.03) 0.889
Acute suppurative otitis media without spontaneous rupture of ear drum (x1.03) 0.890
Fussy infant (x1.03) 0.891
Malfunction of gastrostomy tube (x1.03) 0.892
Amblyopia (x1.03) 0.893
Congenital laryngomalacia (x1.03) 0.894
Bilateral hearing loss (x1.03) 0.896
Uncomplicated asthma (x1.03) 0.897
Respiratory rate (+0.326) 0.898
Age (-1.31) 0.899
Disorder of muscle (x1.03) 0.900
Disorder of brain (x1.03) 0.901
Coordination problem (x1.03) 0.902
Essential hypertension (/1.03) 0.903
Atrial septal defect (x1.03) 0.904
Lennox-Gastaut syndrome (x1.03) 0.905
Granulomatous disorder of the skin and subcutaneous tissue (x1.03) 0.906
Visuospatial agnosia (x1.03) 0.907
Congenital malformation syndrome (x1.03) 0.908
Abnormal breathing (x1.03) 0.909
Disorder of intestine (x1.03) 0.910
Lymphocytes [#/V] in B by AC (+0.0682) 0.910
Alkaline phosphatase [Activity/V] in SP (+3.23) 0.911
Gastroesophageal reflux disease (x1.03) 0.912
Autism spectrum disorder (/1.03) 0.913
Disorder of respiratory system (x1.03) 0.914
Sleep apnea (x1.03) 0.915
Congenital malformation (x1.03) 0.915
Hypertrophy of tonsils (x1.02) 0.916
Male Sex (-0.00226) 0.917
Female Sex (+0.00226) 0.918
Congenital anomaly of brain (x1.02) 0.918
Disturbance of salivary secretion (x1.02) 0.919
Systolic blood pressure (-0.747) 0.920
Unknown Race (+0.00223) 0.920
Lymphocytes/100 leukocytes in B by AC (+0.519) 0.921
Oppositional defiant disorder (/1.02) 0.922
Finding related to pregnancy (x1.02) 0.922
Esotropia (x1.02) 0.923
Congenital heart disease (x1.02) 0.924
Diarrhea (x1.02) 0.924
Deletion of part of autosome (x1.02) 0.925
Diplegic cerebral palsy (x1.02) 0.926
acetaminophen (+0.00206) 0.926
Acute hypoxemic respiratory failure (x1.02) 0.927
Abnormal involuntary movement (x1.02) 0.927
Premature infant (x1.02) 0.928
ibuprofen (+0.00201) 0.929
Anomaly of chromosome pair 21 (x1.02) 0.929
Nervous system symptoms (x1.02) 0.930
Expressions
−0.6 −0.4 −0.2 0.0 0.2 0.4 0.6

Signature [519]; 519 To Reach 0.975, (100 Plotted)

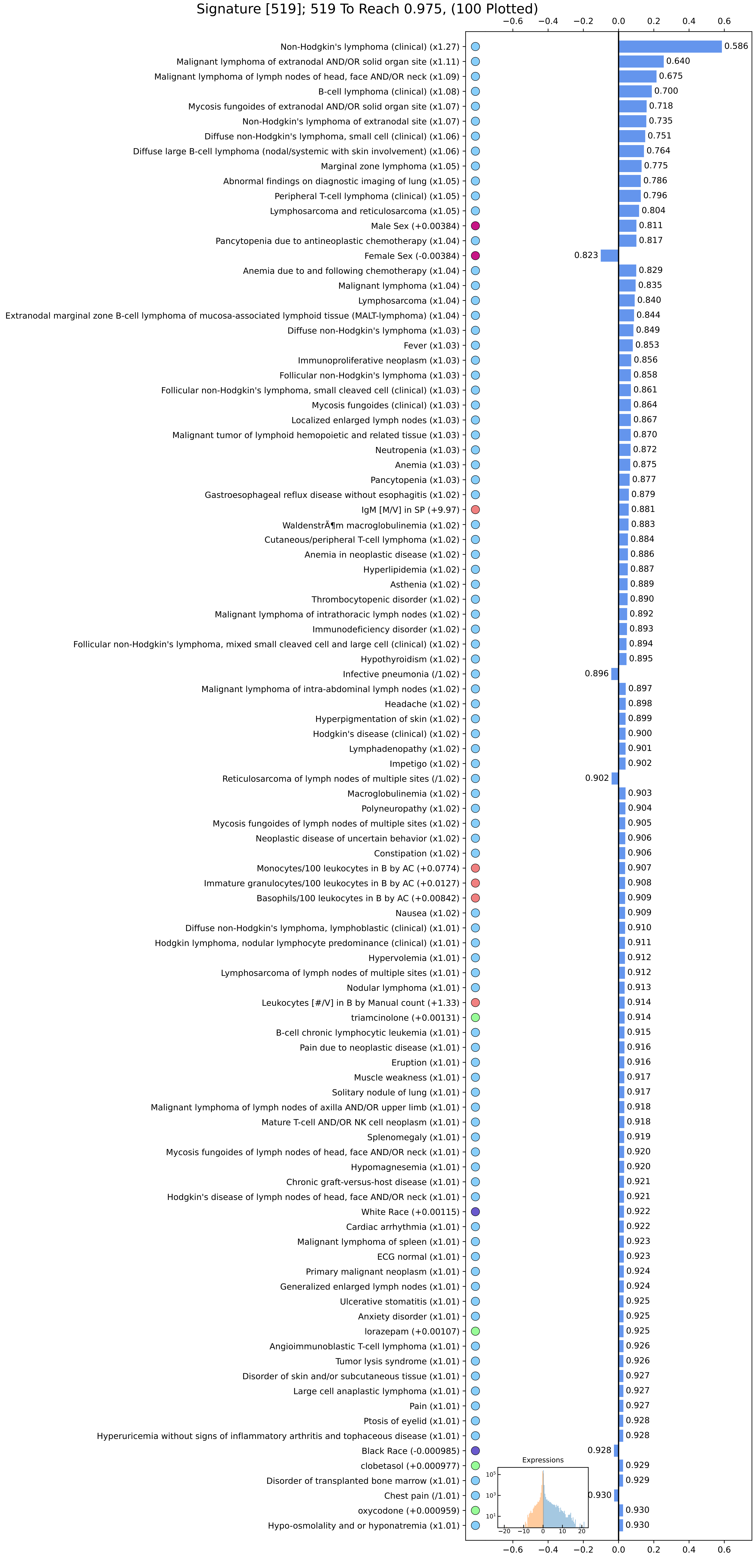

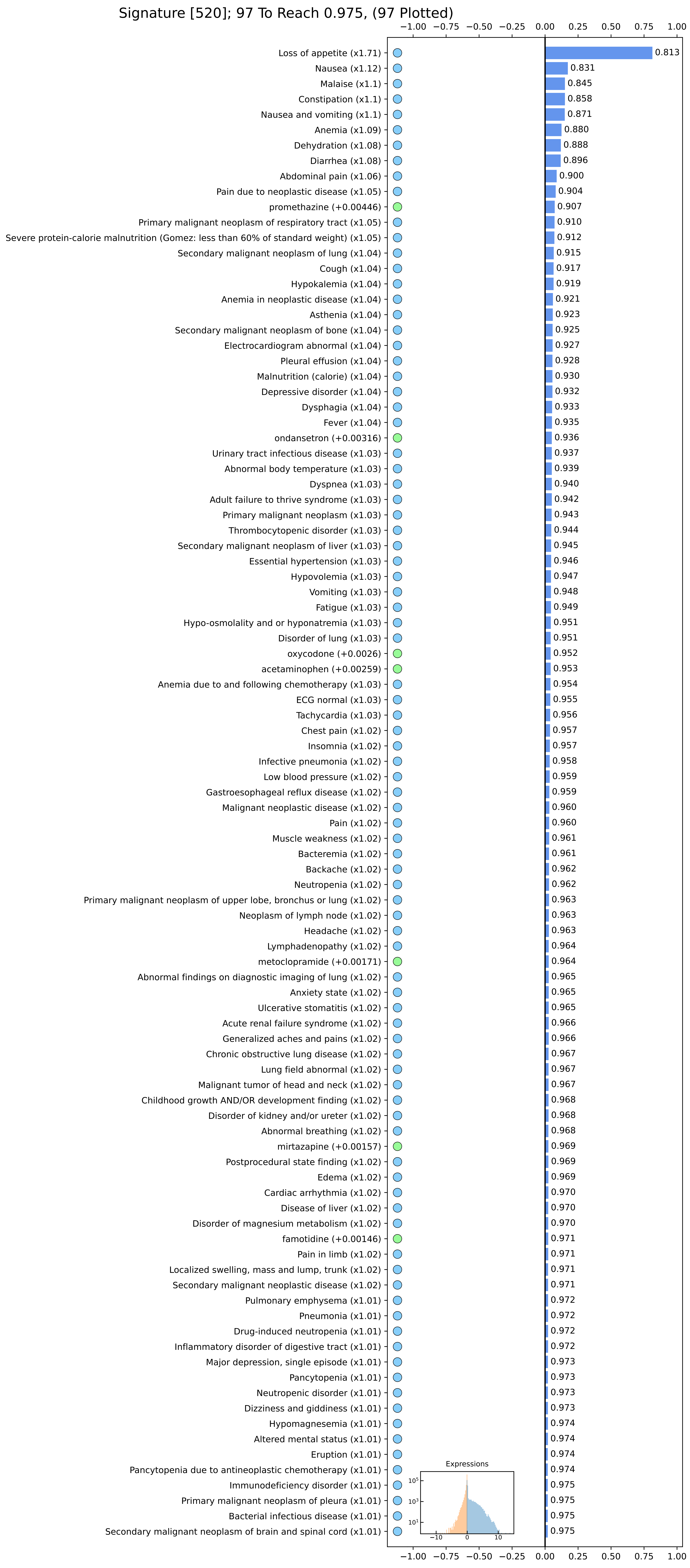
Signature [520]; 97 To Reach 0.975, (97 Plotted)
Loss of appetite (x1.71) 0.813
Nausea (x1.12) 0.831
Malaise (x1.1) 0.845
Constipation (x1.1) 0.858
Nausea and vomiting (x1.1) 0.871
Anemia (x1.09) 0.880
Dehydration (x1.08) 0.888
Diarrhea (x1.08) 0.896
Abdominal pain (x1.06) 0.900
Pain due to neoplastic disease (x1.05) 0.904
promethazine (+0.00446) 0.907
Primary malignant neoplasm of respiratory tract (x1.05) 0.910
Severe protein-calorie malnutrition (Gomez: less than 60% of standard weight) (x1.05) 0.912
Secondary malignant neoplasm of lung (x1.04) 0.915
Cough (x1.04) 0.917
Hypokalemia (x1.04) 0.919
Anemia in neoplastic disease (x1.04) 0.921
Asthenia (x1.04) 0.923
Secondary malignant neoplasm of bone (x1.04) 0.925
Electrocardiogram abnormal (x1.04) 0.927
Pleural effusion (x1.04) 0.928
Malnutrition (calorie) (x1.04) 0.930
Depressive disorder (x1.04) 0.932
Dysphagia (x1.04) 0.933
Fever (x1.04) 0.935
ondansetron (+0.00316) 0.936
Urinary tract infectious disease (x1.03) 0.937
Abnormal body temperature (x1.03) 0.939
Dyspnea (x1.03) 0.940
Adult failure to thrive syndrome (x1.03) 0.942
Primary malignant neoplasm (x1.03) 0.943
Thrombocytopenic disorder (x1.03) 0.944
Secondary malignant neoplasm of liver (x1.03) 0.945
Essential hypertension (x1.03) 0.946
Hypovolemia (x1.03) 0.947
Vomiting (x1.03) 0.948
Fatigue (x1.03) 0.949
Hypo-osmolality and or hyponatremia (x1.03) 0.951
Disorder of lung (x1.03) 0.951
oxycodone (+0.0026) 0.952
acetaminophen (+0.00259) 0.953
Anemia due to and following chemotherapy (x1.03) 0.954
ECG normal (x1.03) 0.955
Tachycardia (x1.03) 0.956
Chest pain (x1.02) 0.957
Insomnia (x1.02) 0.957
Infective pneumonia (x1.02) 0.958
Low blood pressure (x1.02) 0.959
Gastroesophageal reflux disease (x1.02) 0.959
Malignant neoplastic disease (x1.02) 0.960
Pain (x1.02) 0.960
Muscle weakness (x1.02) 0.961
Bacteremia (x1.02) 0.961
Backache (x1.02) 0.962
Neutropenia (x1.02) 0.962
Primary malignant neoplasm of upper lobe, bronchus or lung (x1.02) 0.963
Neoplasm of lymph node (x1.02) 0.963
Headache (x1.02) 0.963
Lymphadenopathy (x1.02) 0.964
metoclopramide (+0.00171) 0.964
Abnormal findings on diagnostic imaging of lung (x1.02) 0.965
Anxiety state (x1.02) 0.965
Ulcerative stomatitis (x1.02) 0.965
Acute renal failure syndrome (x1.02) 0.966
Generalized aches and pains (x1.02) 0.966
Chronic obstructive lung disease (x1.02) 0.967
Lung field abnormal (x1.02) 0.967
Malignant tumor of head and neck (x1.02) 0.967
Childhood growth AND/OR development finding (x1.02) 0.968
Disorder of kidney and/or ureter (x1.02) 0.968
Abnormal breathing (x1.02) 0.968
mirtazapine (+0.00157) 0.969
Postprocedural state finding (x1.02) 0.969
Edema (x1.02) 0.969
Cardiac arrhythmia (x1.02) 0.970
Disease of liver (x1.02) 0.970
Disorder of magnesium metabolism (x1.02) 0.970
famotidine (+0.00146) 0.971
Pain in limb (x1.02) 0.971
Localized swelling, mass and lump, trunk (x1.02) 0.971
Secondary malignant neoplastic disease (x1.02) 0.971
Pulmonary emphysema (x1.01) 0.972
Pneumonia (x1.01) 0.972
Drug-induced neutropenia (x1.01) 0.972
Inflammatory disorder of digestive tract (x1.01) 0.972
Major depression, single episode (x1.01) 0.973
Pancytopenia (x1.01) 0.973
Neutropenic disorder (x1.01) 0.973
Dizziness and giddiness (x1.01) 0.973
Hypomagnesemia (x1.01) 0.974
Altered mental status (x1.01) 0.974
Eruption (x1.01) 0.974
Pancytopenia due to antineoplastic chemotherapy (x1.01) 0.974
Immunodeficiency disorder (x1.01) 0.975
Primary malignant neoplasm of pleura (x1.01) 0.975
Bacterial infectious disease (x1.01) 0.975
Secondary malignant neoplasm of brain and spinal cord (x1.01) 0.975
−1.00 −0.75 −0.50 −0.25 0.00 0.25 0.50 0.75 1.00
Expressions
−10 0 10

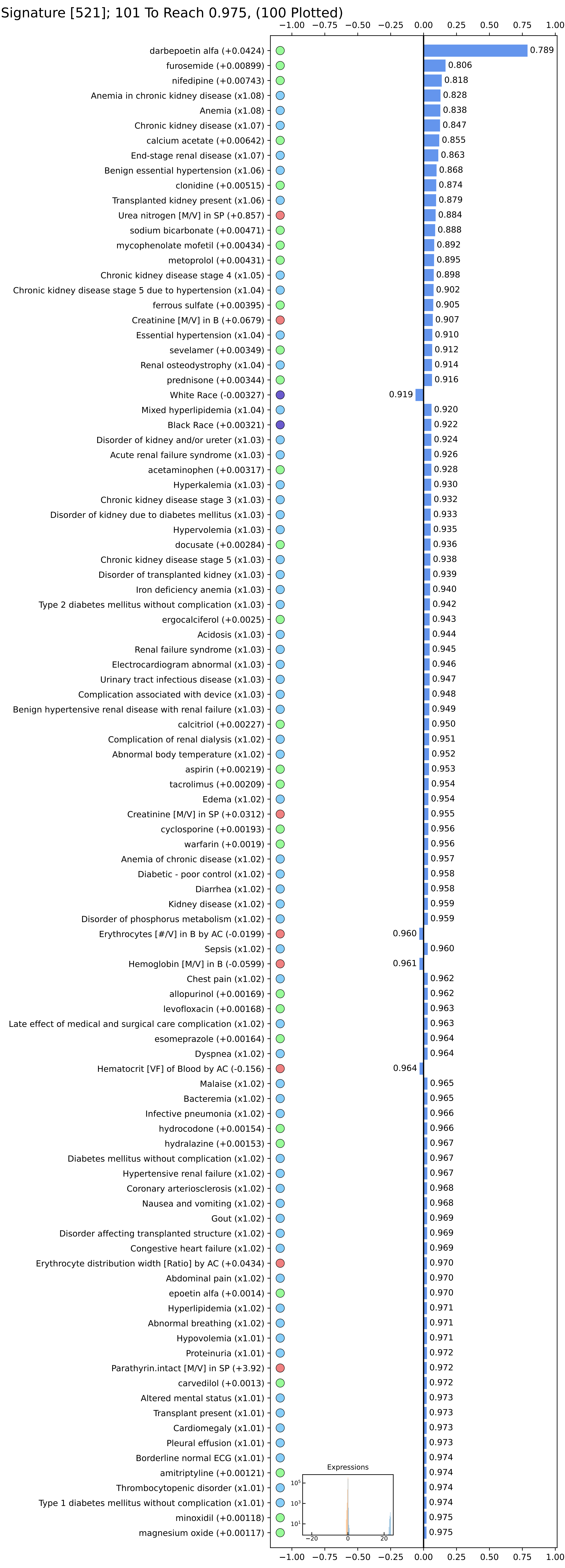
Signature [521]; 101 To Reach 0.975, (100 Plotted)
−1.00 −0.75 −0.50 −0.25 0.00 0.25 0.50 0.75 1.00
darbepoetin alfa (+0.0424) 0.789
furosemide (+0.00899) 0.806
nifedipine (+0.00743) 0.818
Anemia in chronic kidney disease (x1.08) 0.828
Anemia (x1.08) 0.838
Chronic kidney disease (x1.07) 0.847
calcium acetate (+0.00642) 0.855
End-stage renal disease (x1.07) 0.863
Benign essential hypertension (x1.06) 0.868
clonidine (+0.00515) 0.874
Transplanted kidney present (x1.06) 0.879
Urea nitrogen [M/V] in SP (+0.857) 0.884
sodium bicarbonate (+0.00471) 0.888
mycophenolate mofetil (+0.00434) 0.892
metoprolol (+0.00431) 0.895
Chronic kidney disease stage 4 (x1.05) 0.898
Chronic kidney disease stage 5 due to hypertension (x1.04) 0.902
ferrous sulfate (+0.00395) 0.905
Creatinine [M/V] in B (+0.0679) 0.907
Essential hypertension (x1.04) 0.910
sevelamer (+0.00349) 0.912
Renal osteodystrophy (x1.04) 0.914
prednisone (+0.00344) 0.916
White Race (-0.00327) 0.919
Mixed hyperlipidemia (x1.04) 0.920
Black Race (+0.00321) 0.922
Disorder of kidney and/or ureter (x1.03) 0.924
Acute renal failure syndrome (x1.03) 0.926
acetaminophen (+0.00317) 0.928
Hyperkalemia (x1.03) 0.930
Chronic kidney disease stage 3 (x1.03) 0.932
Disorder of kidney due to diabetes mellitus (x1.03) 0.933
Hypervolemia (x1.03) 0.935
docusate (+0.00284) 0.936
Chronic kidney disease stage 5 (x1.03) 0.938
Disorder of transplanted kidney (x1.03) 0.939
Iron deficiency anemia (x1.03) 0.940
Type 2 diabetes mellitus without complication (x1.03) 0.942
ergocalciferol (+0.0025) 0.943
Acidosis (x1.03) 0.944
Renal failure syndrome (x1.03) 0.945
Electrocardiogram abnormal (x1.03) 0.946
Urinary tract infectious disease (x1.03) 0.947
Complication associated with device (x1.03) 0.948
Benign hypertensive renal disease with renal failure (x1.03) 0.949
calcitriol (+0.00227) 0.950
Complication of renal dialysis (x1.02) 0.951
Abnormal body temperature (x1.02) 0.952
aspirin (+0.00219) 0.953
tacrolimus (+0.00209) 0.954
Edema (x1.02) 0.954
Creatinine [M/V] in SP (+0.0312) 0.955
cyclosporine (+0.00193) 0.956
warfarin (+0.0019) 0.956
Anemia of chronic disease (x1.02) 0.957
Diabetic - poor control (x1.02) 0.958
Diarrhea (x1.02) 0.958
Kidney disease (x1.02) 0.959
Disorder of phosphorus metabolism (x1.02) 0.959
Erythrocytes [#/V] in B by AC (-0.0199) 0.960
Sepsis (x1.02) 0.960
Hemoglobin [M/V] in B (-0.0599) 0.961
Chest pain (x1.02) 0.962
allopurinol (+0.00169) 0.962
levofloxacin (+0.00168) 0.963
Late effect of medical and surgical care complication (x1.02) 0.963
esomeprazole (+0.00164) 0.964
Dyspnea (x1.02) 0.964
Hematocrit [VF] of Blood by AC (-0.156) 0.964
Malaise (x1.02) 0.965
Bacteremia (x1.02) 0.965
Infective pneumonia (x1.02) 0.966
hydrocodone (+0.00154) 0.966
hydralazine (+0.00153) 0.967
Diabetes mellitus without complication (x1.02) 0.967
Hypertensive renal failure (x1.02) 0.967
Coronary arteriosclerosis (x1.02) 0.968
Nausea and vomiting (x1.02) 0.968
Gout (x1.02) 0.969
Disorder affecting transplanted structure (x1.02) 0.969
Congestive heart failure (x1.02) 0.969
Erythrocyte distribution width [Ratio] by AC (+0.0434) 0.970
Abdominal pain (x1.02) 0.970
epoetin alfa (+0.0014) 0.970
Hyperlipidemia (x1.02) 0.971
Abnormal breathing (x1.02) 0.971
Hypovolemia (x1.01) 0.971
Proteinuria (x1.01) 0.972
Parathyrin.intact [M/V] in SP (+3.92) 0.972
carvedilol (+0.0013) 0.972
Altered mental status (x1.01) 0.973
Transplant present (x1.01) 0.973
Cardiomegaly (x1.01) 0.973
Pleural effusion (x1.01) 0.973
Borderline normal ECG (x1.01) 0.974
amitriptyline (+0.00121) 0.974
Thrombocytopenic disorder (x1.01) 0.974
Type 1 diabetes mellitus without complication (x1.01) 0.974
minoxidil (+0.00118) 0.975
magnesium oxide (+0.00117) 0.975
Expressions
10^5
10^3
10^1
−20 0 20
−1.00 −0.75 −0.50 −0.25 0.00 0.25 0.50 0.75 1.00

Signature [522]; 1 To Reach 0.975, (10 Plotted)

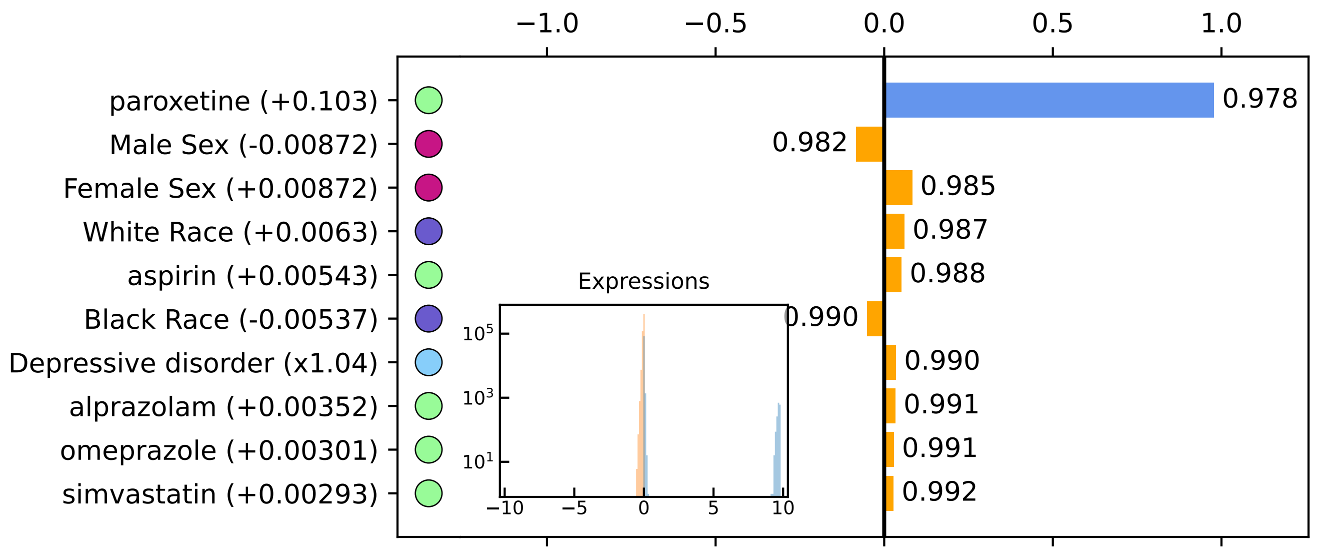

Signature [523]; 1 To Reach 0.975, (10 Plotted)

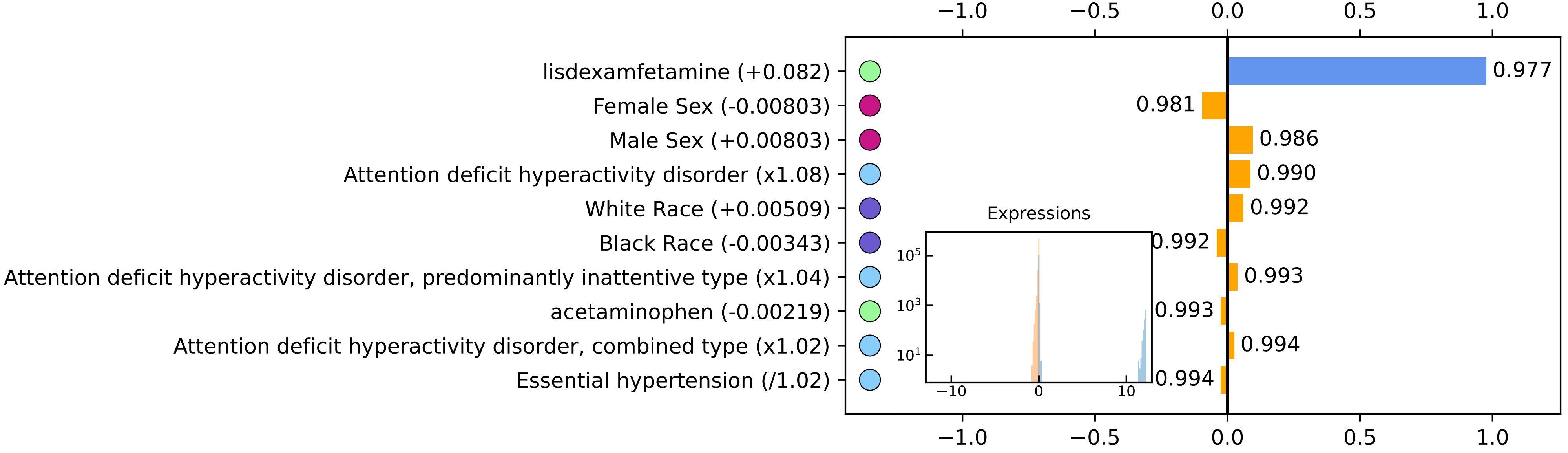

Signature [524]; 110 To Reach 0.975, (100 Plotted)

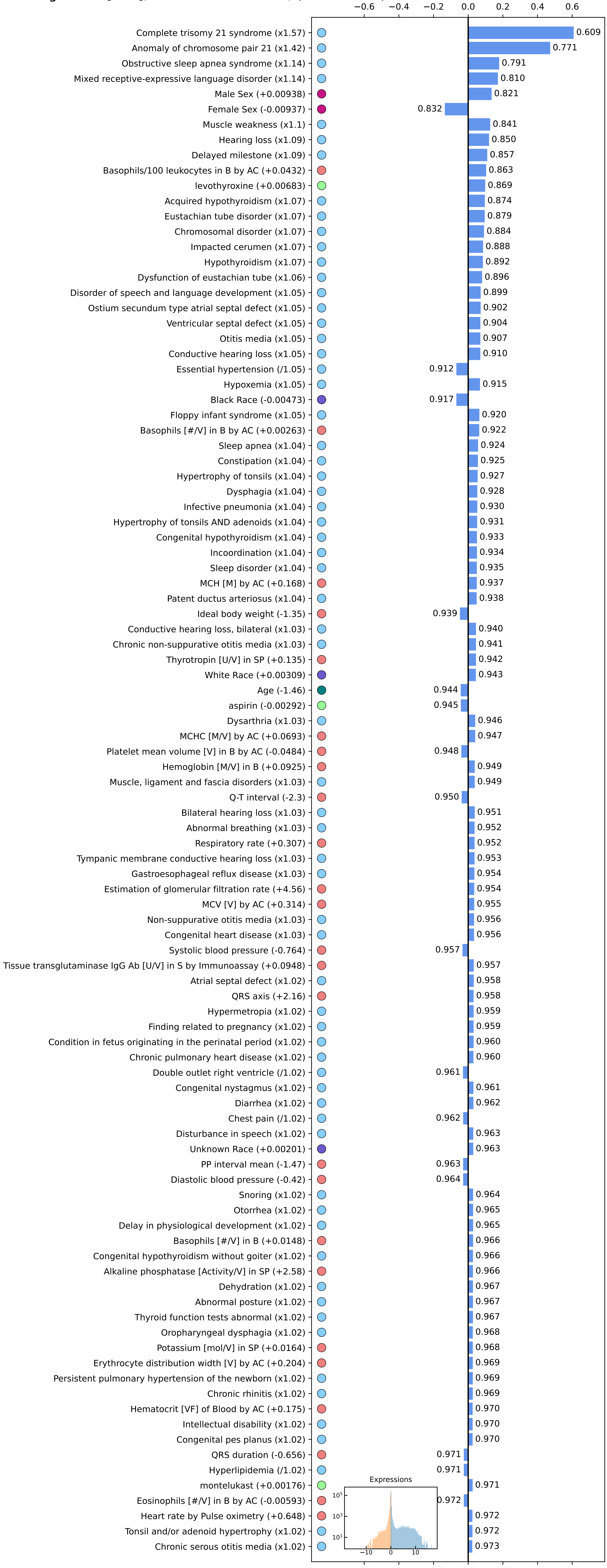

Signature [525]; 142 To Reach 0.975, (100 Plotted)

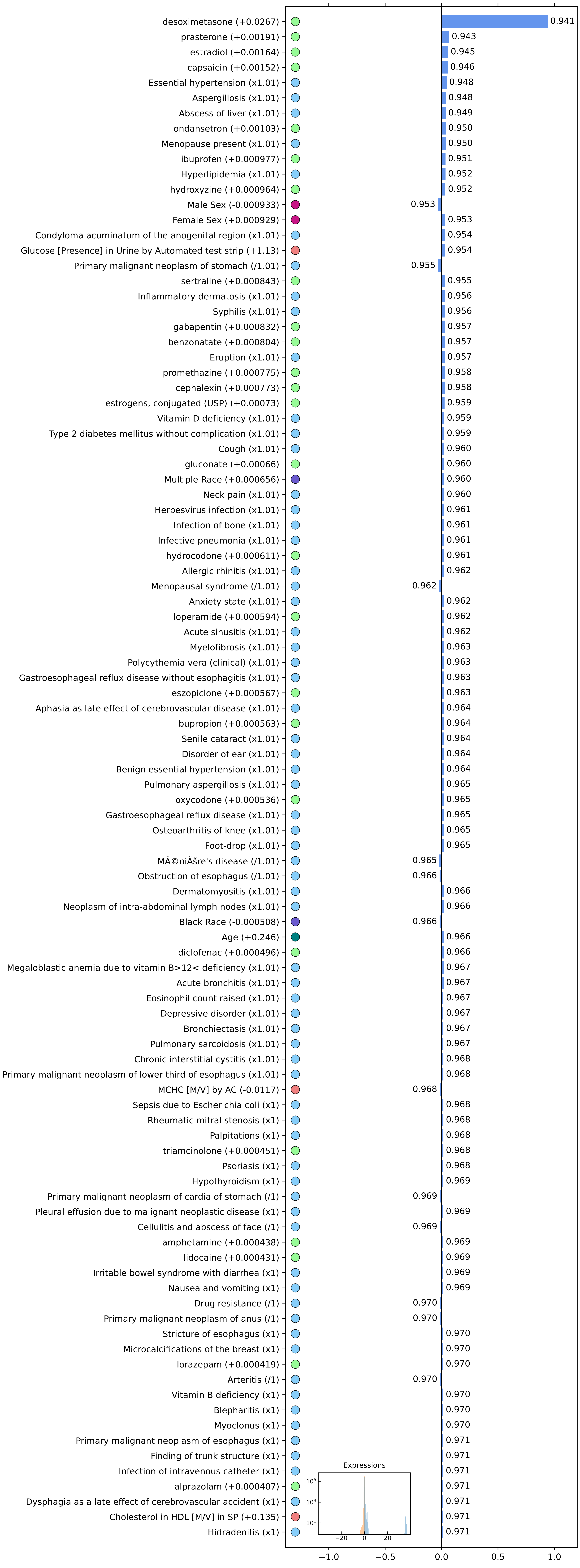

Signature [526]; 3 To Reach 0.975, (10 Plotted)

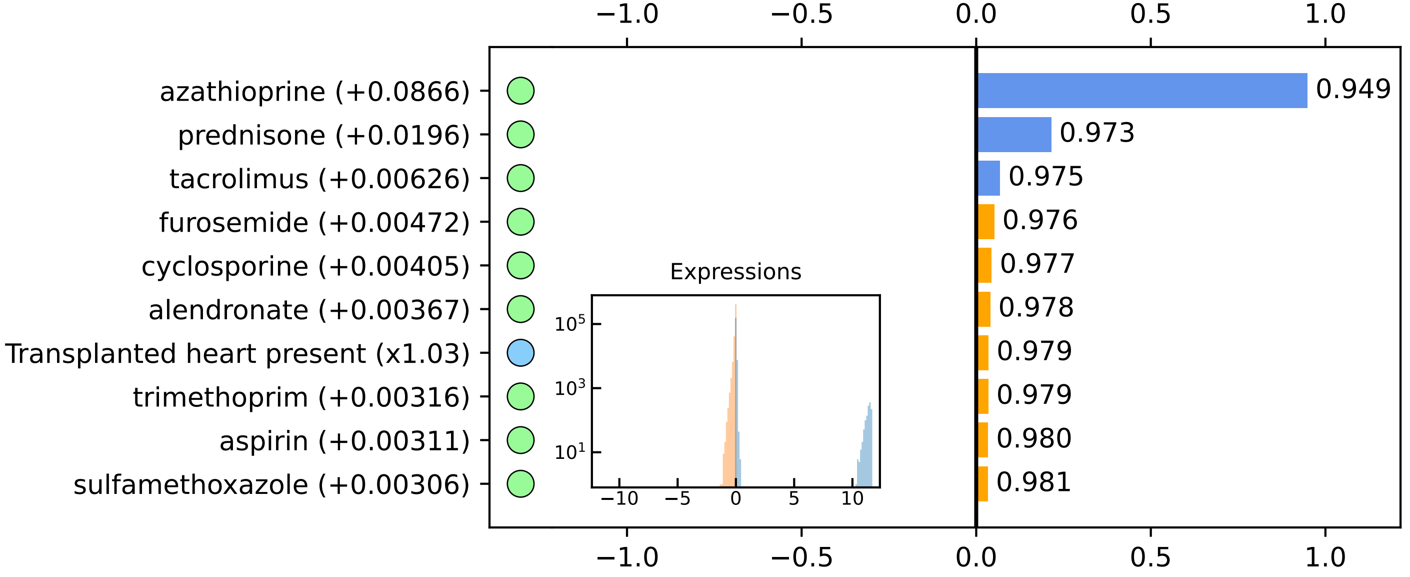

Signature [527]; 42 To Reach 0.975, (42 Plotted)

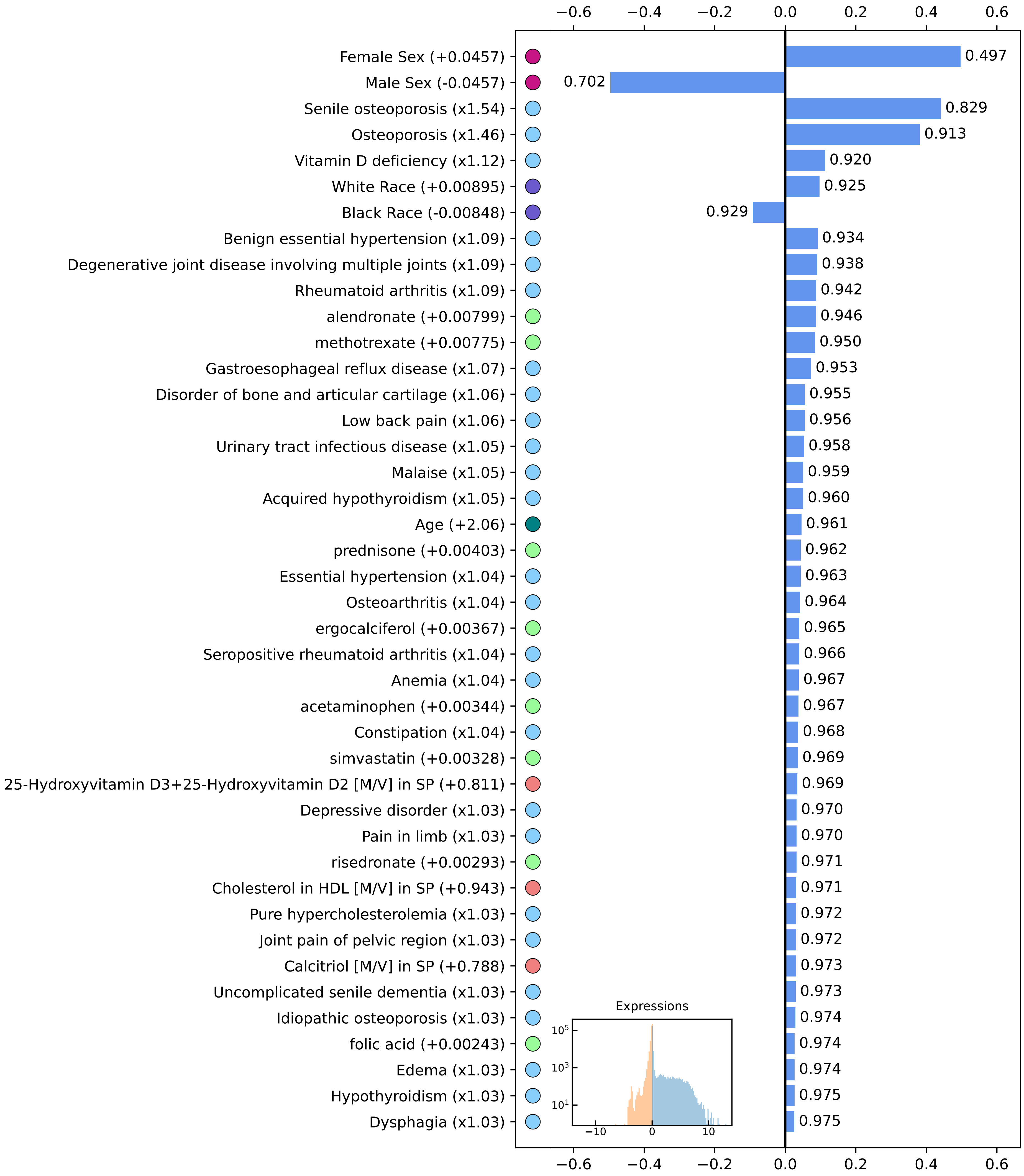

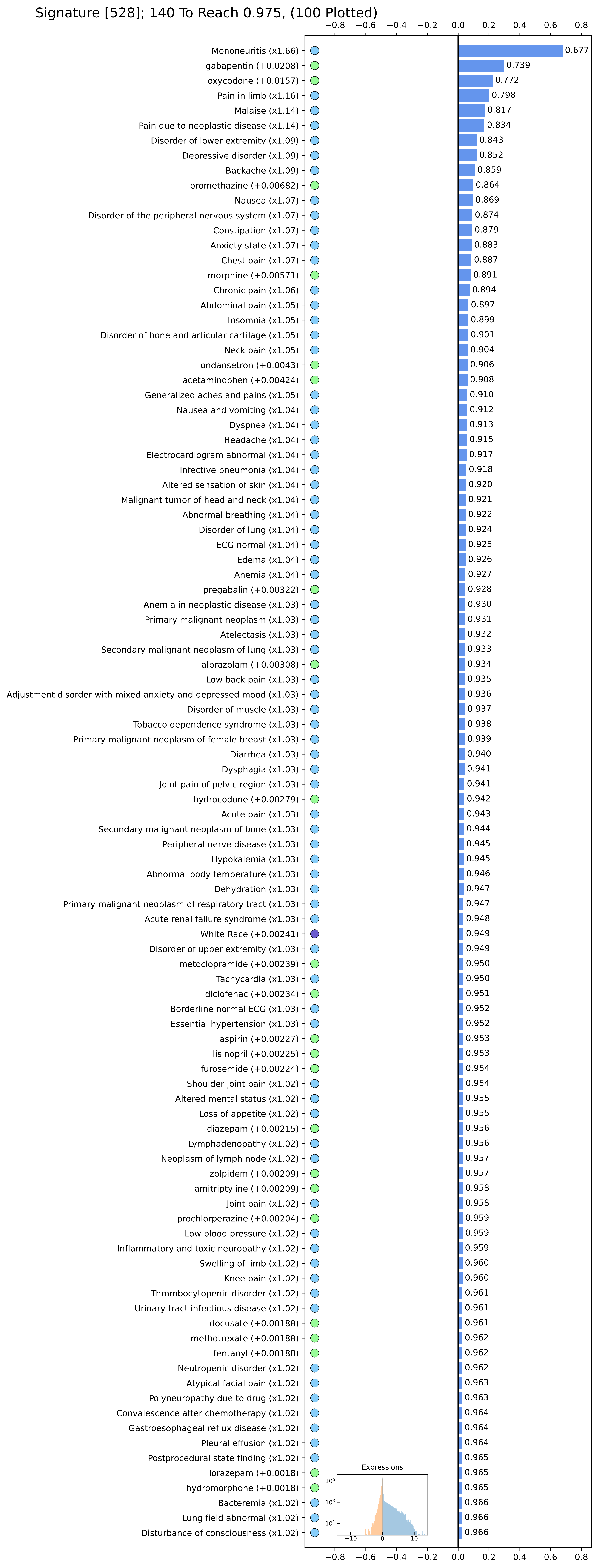
Signature [528]; 140 To Reach 0.975, (100 Plotted)
Mononeuritis (x1.66) 0.677
gabapentin (+0.0208) 0.739
oxycodone (+0.0157) 0.772
Pain in limb (x1.16) 0.798
Malaise (x1.14) 0.817
Pain due to neoplastic disease (x1.14) 0.834
Disorder of lower extremity (x1.09) 0.843
Depressive disorder (x1.09) 0.852
Backache (x1.09) 0.859
promethazine (+0.00682) 0.864
Nausea (x1.07) 0.869
Disorder of the peripheral nervous system (x1.07) 0.874
Constipation (x1.07) 0.879
Anxiety state (x1.07) 0.883
Chest pain (x1.07) 0.887
morphine (+0.00571) 0.891
Chronic pain (x1.06) 0.894
Abdominal pain (x1.05) 0.897
Insomnia (x1.05) 0.899
Disorder of bone and articular cartilage (x1.05) 0.901
Neck pain (x1.05) 0.904
ondansetron (+0.0043) 0.906
acetaminophen (+0.00424) 0.908
Generalized aches and pains (x1.05) 0.910
Nausea and vomiting (x1.04) 0.912
Dyspnea (x1.04) 0.913
Headache (x1.04) 0.915
Electrocardiogram abnormal (x1.04) 0.917
Infective pneumonia (x1.04) 0.918
Altered sensation of skin (x1.04) 0.920
Malignant tumor of head and neck (x1.04) 0.921
Abnormal breathing (x1.04) 0.922
Disorder of lung (x1.04) 0.924
ECG normal (x1.04) 0.925
Edema (x1.04) 0.926
Anemia (x1.04) 0.927
pregabalin (+0.00322) 0.928
Anemia in neoplastic disease (x1.03) 0.930
Primary malignant neoplasm (x1.03) 0.931
Atelectasis (x1.03) 0.932
Secondary malignant neoplasm of lung (x1.03) 0.933
alprazolam (+0.00308) 0.934
Low back pain (x1.03) 0.935
Adjustment disorder with mixed anxiety and depressed mood (x1.03) 0.936
Disorder of muscle (x1.03) 0.937
Tobacco dependence syndrome (x1.03) 0.938
Primary malignant neoplasm of female breast (x1.03) 0.939
Diarrhea (x1.03) 0.940
Dysphagia (x1.03) 0.941
Joint pain of pelvic region (x1.03) 0.941
hydrocodone (+0.00279) 0.942
Acute pain (x1.03) 0.943
Secondary malignant neoplasm of bone (x1.03) 0.944
Peripheral nerve disease (x1.03) 0.945
Hypokalemia (x1.03) 0.945
Abnormal body temperature (x1.03) 0.946
Dehydration (x1.03) 0.947
Primary malignant neoplasm of respiratory tract (x1.03) 0.947
Acute renal failure syndrome (x1.03) 0.948
White Race (+0.00241) 0.949
Disorder of upper extremity (x1.03) 0.949
metoclopramide (+0.00239) 0.950
Tachycardia (x1.03) 0.950
diclofenac (+0.00234) 0.951
Borderline normal ECG (x1.03) 0.952
Essential hypertension (x1.03) 0.952
aspirin (+0.00227) 0.953
lisinopril (+0.00225) 0.953
furosemide (+0.00224) 0.954
Shoulder joint pain (x1.02) 0.954
Altered mental status (x1.02) 0.955
Loss of appetite (x1.02) 0.955
diazepam (+0.00215) 0.956
Lymphadenopathy (x1.02) 0.956
Neoplasm of lymph node (x1.02) 0.957
zolpidem (+0.00209) 0.957
amitriptyline (+0.00209) 0.958
Joint pain (x1.02) 0.958
prochlorperazine (+0.00204) 0.959
Low blood pressure (x1.02) 0.959
Inflammatory and toxic neuropathy (x1.02) 0.959
Swelling of limb (x1.02) 0.960
Knee pain (x1.02) 0.960
Thrombocytopenic disorder (x1.02) 0.961
Urinary tract infectious disease (x1.02) 0.961
docusate (+0.00188) 0.961
methotrexate (+0.00188) 0.962
fentanyl (+0.00188) 0.962
Neutropenic disorder (x1.02) 0.962
Atypical facial pain (x1.02) 0.963
Polyneuropathy due to drug (x1.02) 0.963
Convalescence after chemotherapy (x1.02) 0.964
Gastroesophageal reflux disease (x1.02) 0.964
Pleural effusion (x1.02) 0.964
Postprocedural state finding (x1.02) 0.965
lorazepam (+0.0018) 0.965
hydromorphone (+0.0018) 0.965
Bacteremia (x1.02) 0.966
Lung field abnormal (x1.02) 0.966
Disturbance of consciousness (x1.02) 0.966
−0.8 −0.6 −0.4 −0.2 0.0 0.2 0.4 0.6 0.8
Expressions
−10 0 10

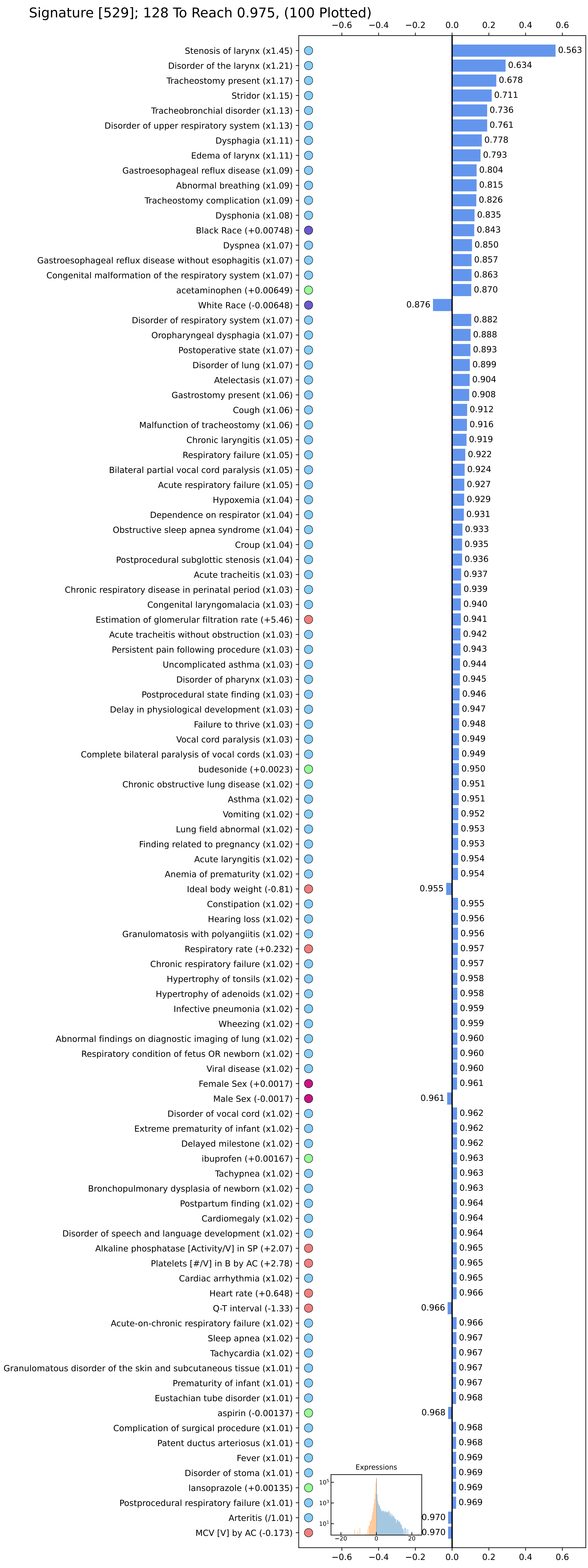

Signature [529]; 128 To Reach 0.975, (100 Plotted)
Stenosis of larynx (x1.45) 0.563
Disorder of the larynx (x1.21) 0.634
Tracheostomy present (x1.17) 0.678
Stridor (x1.15) 0.711
Tracheobronchial disorder (x1.13) 0.736
Disorder of upper respiratory system (x1.13) 0.761
Dysphagia (x1.11) 0.778
Edema of larynx (x1.11) 0.793
Gastroesophageal reflux disease (x1.09) 0.804
Abnormal breathing (x1.09) 0.815
Tracheostomy complication (x1.09) 0.826
Dysphonia (x1.08) 0.835
Black Race (+0.00748) 0.843
Dyspnea (x1.07) 0.850
Gastroesophageal reflux disease without esophagitis (x1.07) 0.857
Congenital malformation of the respiratory system (x1.07) 0.863
acetaminophen (+0.00649) 0.870
White Race (-0.00648) 0.876
Disorder of respiratory system (x1.07) 0.882
Oropharyngeal dysphagia (x1.07) 0.888
Postoperative state (x1.07) 0.893
Disorder of lung (x1.07) 0.899
Atelectasis (x1.07) 0.904
Gastrostomy present (x1.06) 0.908
Cough (x1.06) 0.912
Malfunction of tracheostomy (x1.06) 0.916
Chronic laryngitis (x1.05) 0.919
Respiratory failure (x1.05) 0.922
Bilateral partial vocal cord paralysis (x1.05) 0.924
Acute respiratory failure (x1.05) 0.927
Hypoxemia (x1.04) 0.929
Dependence on respirator (x1.04) 0.931
Obstructive sleep apnea syndrome (x1.04) 0.933
Croup (x1.04) 0.935
Postprocedural subglottic stenosis (x1.04) 0.936
Acute tracheitis (x1.03) 0.937
Chronic respiratory disease in perinatal period (x1.03) 0.939
Congenital laryngomalacia (x1.03) 0.940
Estimation of glomerular filtration rate (+5.46) 0.941
Acute tracheitis without obstruction (x1.03) 0.942
Persistent pain following procedure (x1.03) 0.943
Uncomplicated asthma (x1.03) 0.944
Disorder of pharynx (x1.03) 0.945
Postprocedural state finding (x1.03) 0.946
Delay in physiological development (x1.03) 0.947
Failure to thrive (x1.03) 0.948
Vocal cord paralysis (x1.03) 0.949
Complete bilateral paralysis of vocal cords (x1.03) 0.949
budesonide (+0.0023) 0.950
Chronic obstructive lung disease (x1.02) 0.951
Asthma (x1.02) 0.951
Vomiting (x1.02) 0.952
Lung field abnormal (x1.02) 0.953
Finding related to pregnancy (x1.02) 0.953
Acute laryngitis (x1.02) 0.954
Anemia of prematurity (x1.02) 0.954
Ideal body weight (-0.81) 0.955
Constipation (x1.02) 0.955
Hearing loss (x1.02) 0.956
Granulomatosis with polyangiitis (x1.02) 0.956
Respiratory rate (+0.232) 0.957
Chronic respiratory failure (x1.02) 0.957
Hypertrophy of tonsils (x1.02) 0.958
Hypertrophy of adenoids (x1.02) 0.958
Infective pneumonia (x1.02) 0.959
Wheezing (x1.02) 0.959
Abnormal findings on diagnostic imaging of lung (x1.02) 0.960
Respiratory condition of fetus OR newborn (x1.02) 0.960
Viral disease (x1.02) 0.960
Female Sex (+0.0017) 0.961
Male Sex (-0.0017) 0.961
Disorder of vocal cord (x1.02) 0.962
Extreme prematurity of infant (x1.02) 0.962
Delayed milestone (x1.02) 0.962
ibuprofen (+0.00167) 0.963
Tachypnea (x1.02) 0.963
Bronchopulmonary dysplasia of newborn (x1.02) 0.963
Postpartum finding (x1.02) 0.964
Cardiomegaly (x1.02) 0.964
Disorder of speech and language development (x1.02) 0.964
Alkaline phosphatase [Activity/V] in SP (+2.07) 0.965
Platelets [#/V] in B by AC (+2.78) 0.965
Cardiac arrhythmia (x1.02) 0.965
Heart rate (+0.648) 0.966
Q-T interval (-1.33) 0.966
Acute-on-chronic respiratory failure (x1.02) 0.966
Sleep apnea (x1.02) 0.967
Tachycardia (x1.02) 0.967
Granulomatous disorder of the skin and subcutaneous tissue (x1.01) 0.967
Prematurity of infant (x1.01) 0.967
Eustachian tube disorder (x1.01) 0.968
aspirin (-0.00137) 0.968
Complication of surgical procedure (x1.01) 0.968
Patent ductus arteriosus (x1.01) 0.968
Fever (x1.01) 0.969
Disorder of stoma (x1.01) 0.969
lansoprazole (+0.00135) 0.969
Postprocedural respiratory failure (x1.01) 0.969
Arteritis (/1.01) 0.970
MCV [V] by AC (-0.173) 0.970
Expressions
−0.6 −0.4 −0.2 0.0 0.2 0.4 0.6

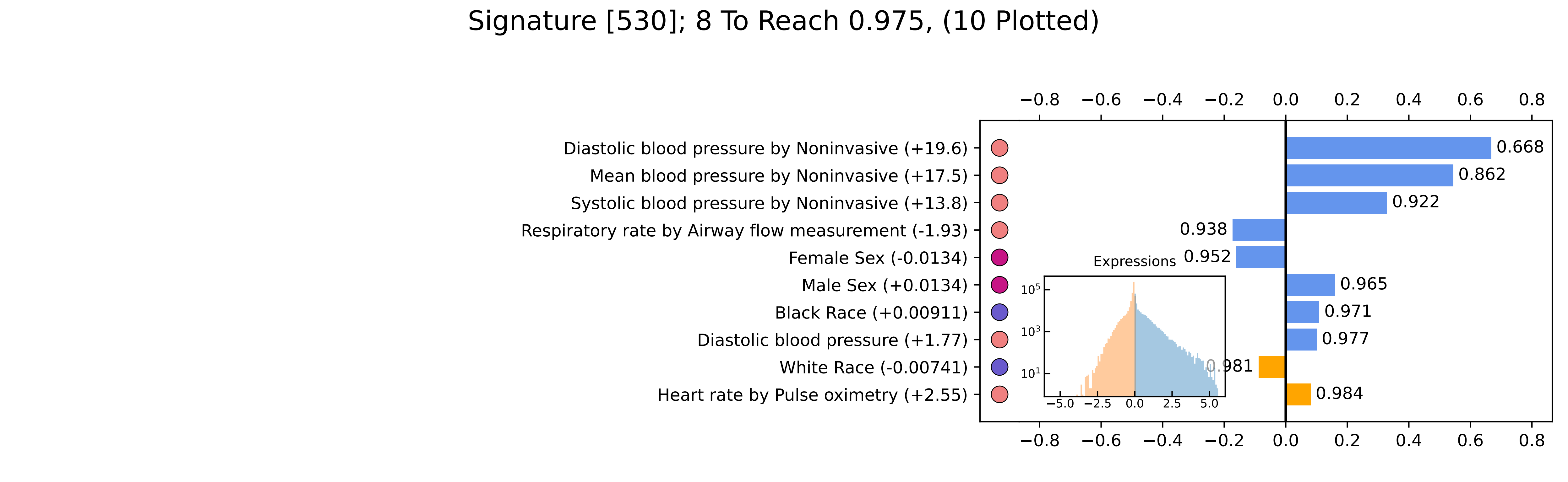

Signature [530]; 8 To Reach 0.975, (10 Plotted)
Diastolic blood pressure by Noninvasive (+19.6)
Mean blood pressure by Noninvasive (+17.5)
Systolic blood pressure by Noninvasive (+13.8)
Respiratory rate by Airway flow measurement (-1.93)
Female Sex (-0.0134)
Male Sex (+0.0134)
Black Race (+0.00911)
Diastolic blood pressure (+1.77)
White Race (-0.00741)
Heart rate by Pulse oximetry (+2.55)
0.668
0.862
0.922
0.938
0.952
0.965
0.971
0.977
0.981
0.984
Expressions
−0.8 −0.6 −0.4 −0.2 0.0 0.2 0.4 0.6 0.8

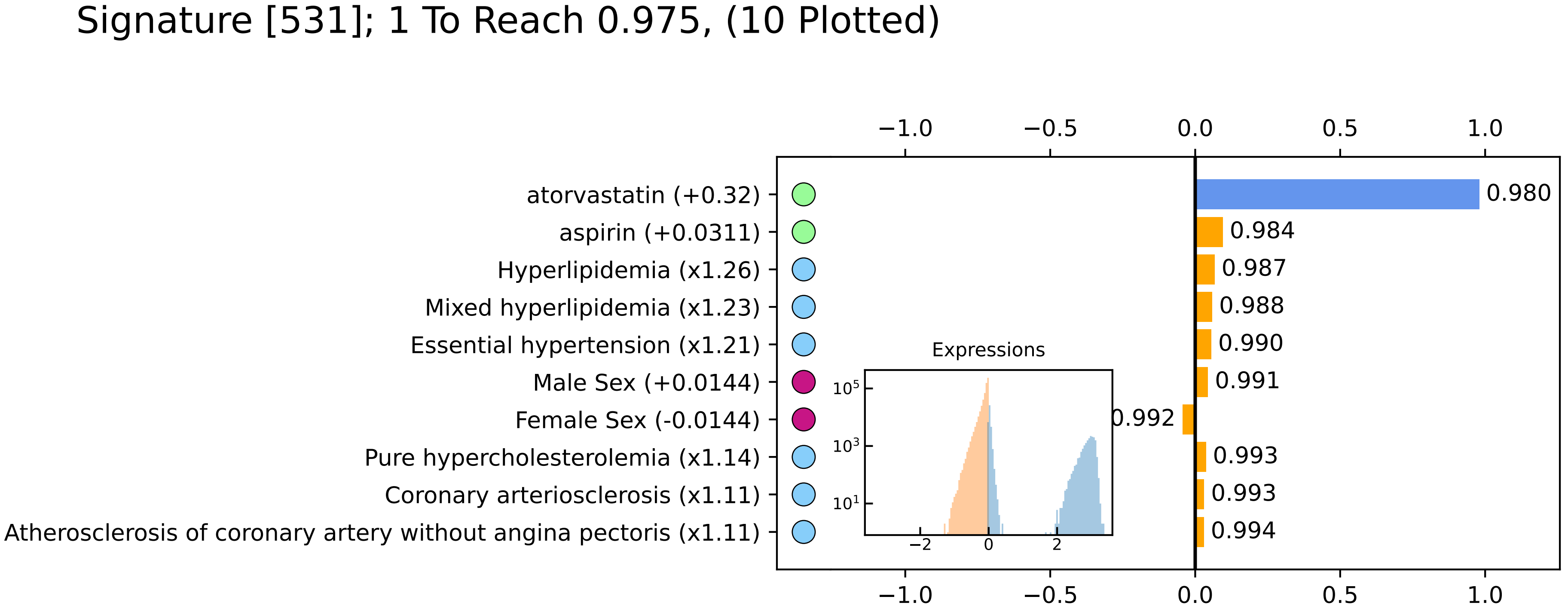

Signature [531]; 1 To Reach 0.975, (10 Plotted)
atorvastatin (+0.32)
aspirin (+0.0311)
Hyperlipidemia (x1.26)
Mixed hyperlipidemia (x1.23)
Essential hypertension (x1.21)
Male Sex (+0.0144)
Female Sex (-0.0144)
Pure hypercholesterolemia (x1.14)
Coronary arteriosclerosis (x1.11)
Atherosclerosis of coronary artery without angina pectoris (x1.11)
0.980
0.984
0.987
0.988
0.990
0.991
0.992
0.993
0.993
0.994
−1.0
−0.5
0.0
0.5
1.0
Expressions

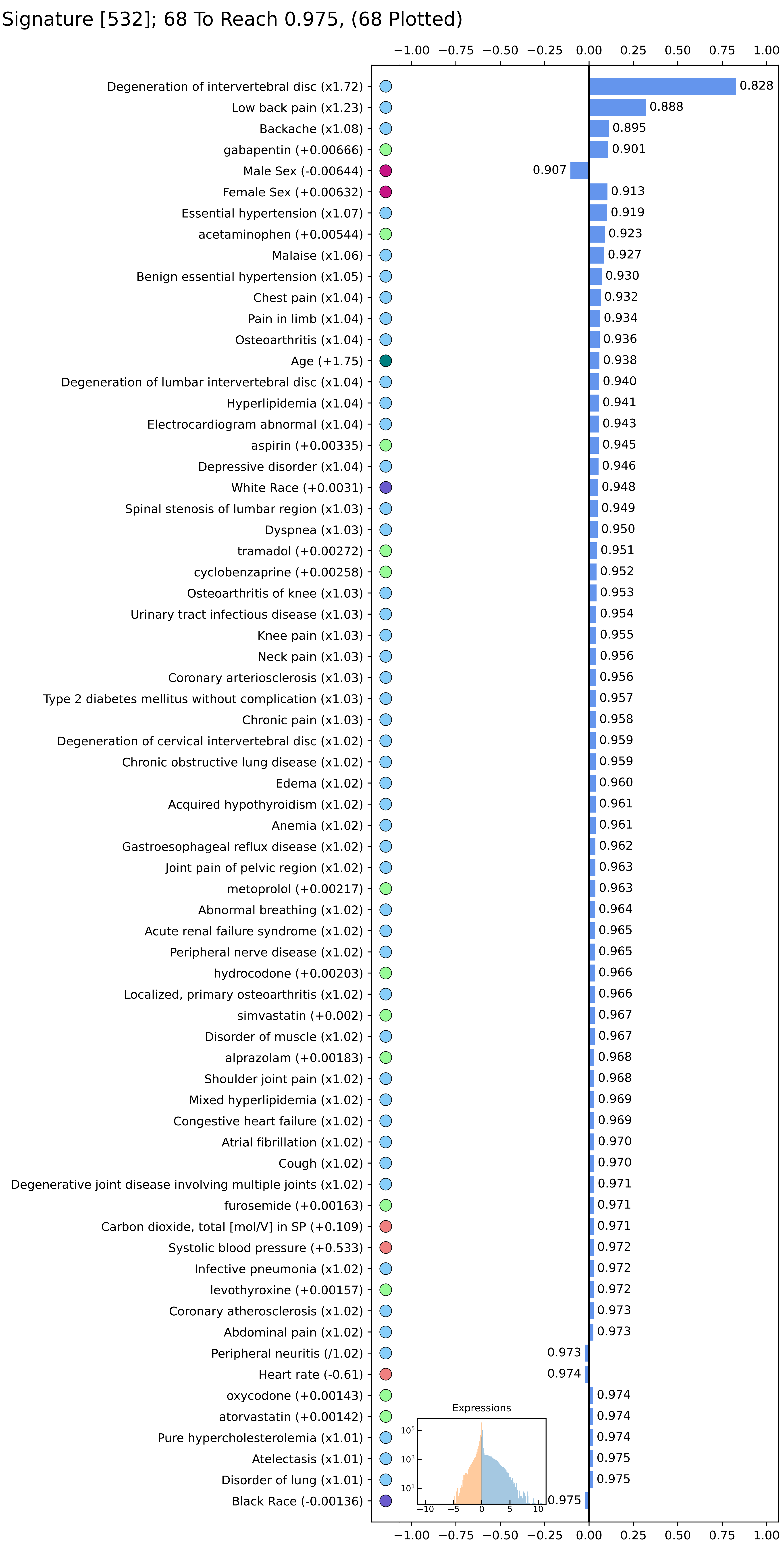

Signature [532]; 68 To Reach 0.975, (68 Plotted)
Degeneration of intervertebral disc (x1.72) 0.828
Low back pain (x1.23) 0.888
Backache (x1.08) 0.895
gabapentin (+0.00666) 0.901
Male Sex (-0.00644) 0.907
Female Sex (+0.00632) 0.913
Essential hypertension (x1.07) 0.919
acetaminophen (+0.00544) 0.923
Malaise (x1.06) 0.927
Benign essential hypertension (x1.05) 0.930
Chest pain (x1.04) 0.932
Pain in limb (x1.04) 0.934
Osteoarthritis (x1.04) 0.936
Age (+1.75) 0.938
Degeneration of lumbar intervertebral disc (x1.04) 0.940
Hyperlipidemia (x1.04) 0.941
Electrocardiogram abnormal (x1.04) 0.943
aspirin (+0.00335) 0.945
Depressive disorder (x1.04) 0.946
White Race (+0.0031) 0.948
Spinal stenosis of lumbar region (x1.03) 0.949
Dyspnea (x1.03) 0.950
tramadol (+0.00272) 0.951
cyclobenzaprine (+0.00258) 0.952
Osteoarthritis of knee (x1.03) 0.953
Urinary tract infectious disease (x1.03) 0.954
Knee pain (x1.03) 0.955
Neck pain (x1.03) 0.956
Coronary arteriosclerosis (x1.03) 0.956
Type 2 diabetes mellitus without complication (x1.03) 0.957
Chronic pain (x1.03) 0.958
Degeneration of cervical intervertebral disc (x1.02) 0.959
Chronic obstructive lung disease (x1.02) 0.959
Edema (x1.02) 0.960
Acquired hypothyroidism (x1.02) 0.961
Anemia (x1.02) 0.961
Gastroesophageal reflux disease (x1.02) 0.962
Joint pain of pelvic region (x1.02) 0.963
metoprolol (+0.00217) 0.963
Abnormal breathing (x1.02) 0.964
Acute renal failure syndrome (x1.02) 0.965
Peripheral nerve disease (x1.02) 0.965
hydrocodone (+0.00203) 0.966
Localized, primary osteoarthritis (x1.02) 0.966
simvastatin (+0.002) 0.967
Disorder of muscle (x1.02) 0.967
alprazolam (+0.00183) 0.968
Shoulder joint pain (x1.02) 0.968
Mixed hyperlipidemia (x1.02) 0.969
Congestive heart failure (x1.02) 0.969
Atrial fibrillation (x1.02) 0.970
Cough (x1.02) 0.970
Degenerative joint disease involving multiple joints (x1.02) 0.971
furosemide (+0.00163) 0.971
Carbon dioxide, total [mol/V] in SP (+0.109) 0.971
Systolic blood pressure (+0.533) 0.972
Infective pneumonia (x1.02) 0.972
levothyroxine (+0.00157) 0.972
Coronary atherosclerosis (x1.02) 0.973
Abdominal pain (x1.02) 0.973
Peripheral neuritis (/1.02) 0.973
Heart rate (-0.61) 0.974
oxycodone (+0.00143) 0.974
atorvastatin (+0.00142) 0.974
Pure hypercholesterolemia (x1.01) 0.974
Atelectasis (x1.01) 0.975
Disorder of lung (x1.01) 0.975
Black Race (-0.00136) 0.975
−1.00 −0.75 −0.50 −0.25 0.00 0.25 0.50 0.75 1.00
Expressions
−10 −5 0 5 10

Signature [533]; 81 To Reach 0.975, (81 Plotted)

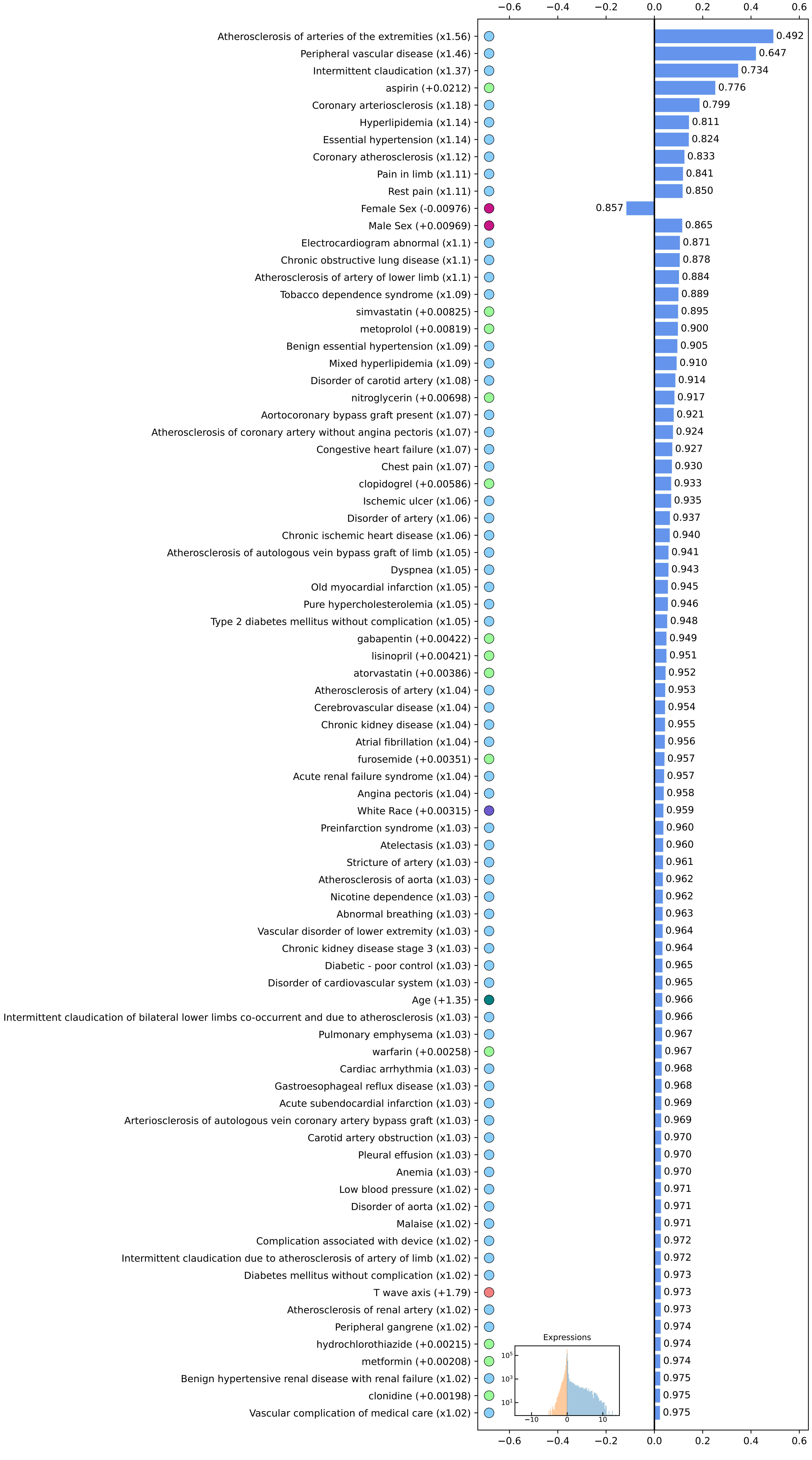

Signature [534]; 8 To Reach 0.975, (10 Plotted)

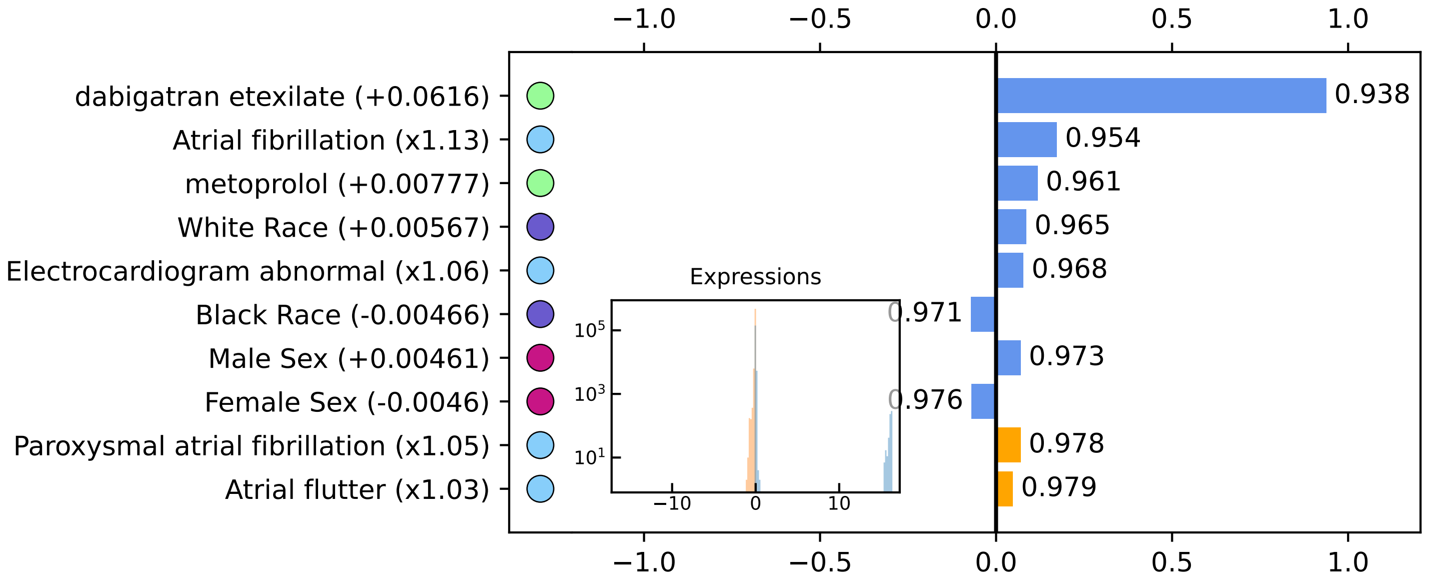

Signature [535]; 12 To Reach 0.975, (12 Plotted)

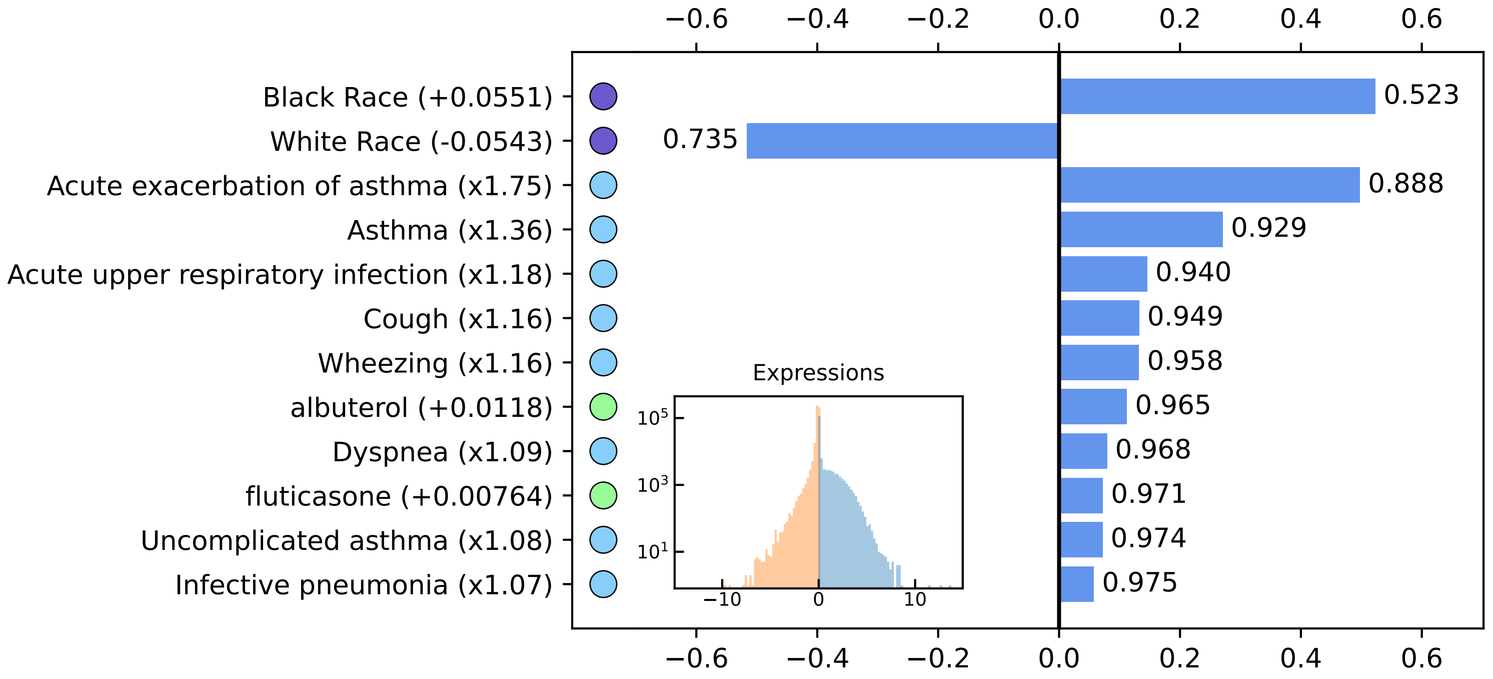

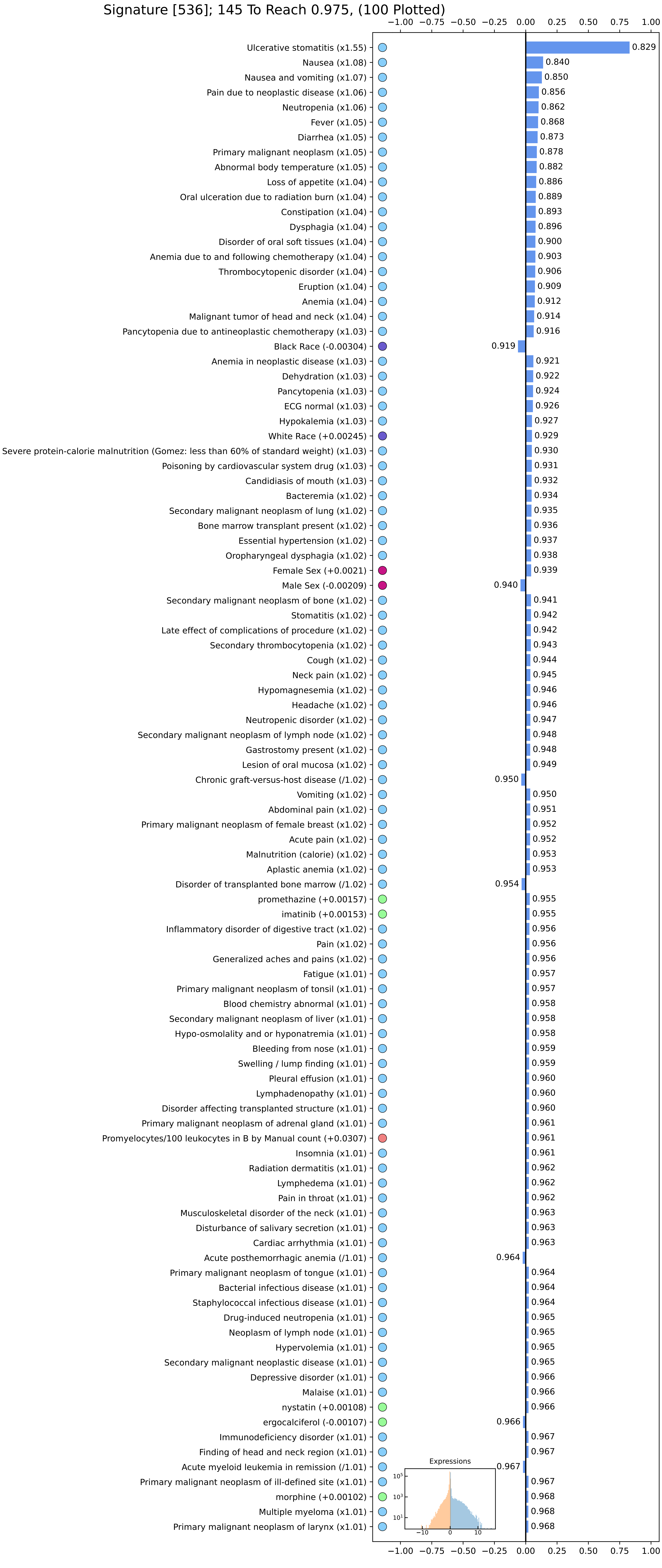

Signature [536]; 145 To Reach 0.975, (100 Plotted)
Ulcerative stomatitis (x1.55) 0.829
Nausea (x1.08) 0.840
Nausea and vomiting (x1.07) 0.850
Pain due to neoplastic disease (x1.06) 0.856
Neutropenia (x1.06) 0.862
Fever (x1.05) 0.868
Diarrhea (x1.05) 0.873
Primary malignant neoplasm (x1.05) 0.878
Abnormal body temperature (x1.05) 0.882
Loss of appetite (x1.04) 0.886
Oral ulceration due to radiation burn (x1.04) 0.889
Constipation (x1.04) 0.893
Dysphagia (x1.04) 0.896
Disorder of oral soft tissues (x1.04) 0.900
Anemia due to and following chemotherapy (x1.04) 0.903
Thrombocytopenic disorder (x1.04) 0.906
Eruption (x1.04) 0.909
Anemia (x1.04) 0.912
Malignant tumor of head and neck (x1.04) 0.914
Pancytopenia due to antineoplastic chemotherapy (x1.03) 0.916
Black Race (-0.00304) 0.919
Anemia in neoplastic disease (x1.03) 0.921
Dehydration (x1.03) 0.922
Pancytopenia (x1.03) 0.924
ECG normal (x1.03) 0.926
Hypokalemia (x1.03) 0.927
White Race (+0.00245) 0.929
Severe protein-calorie malnutrition (Gomez: less than 60% of standard weight) (x1.03) 0.930
Poisoning by cardiovascular system drug (x1.03) 0.931
Candidiasis of mouth (x1.03) 0.932
Bacteremia (x1.02) 0.934
Secondary malignant neoplasm of lung (x1.02) 0.935
Bone marrow transplant present (x1.02) 0.936
Essential hypertension (x1.02) 0.937
Oropharyngeal dysphagia (x1.02) 0.938
Female Sex (+0.0021) 0.939
Male Sex (-0.00209) 0.940
Secondary malignant neoplasm of bone (x1.02) 0.941
Stomatitis (x1.02) 0.942
Late effect of complications of procedure (x1.02) 0.942
Secondary thrombocytopenia (x1.02) 0.943
Cough (x1.02) 0.944
Neck pain (x1.02) 0.945
Hypomagnesemia (x1.02) 0.946
Headache (x1.02) 0.946
Neutropenic disorder (x1.02) 0.947
Secondary malignant neoplasm of lymph node (x1.02) 0.948
Gastrostomy present (x1.02) 0.948
Lesion of oral mucosa (x1.02) 0.949
Chronic graft-versus-host disease (/1.02) 0.950
Vomiting (x1.02) 0.950
Abdominal pain (x1.02) 0.951
Primary malignant neoplasm of female breast (x1.02) 0.952
Acute pain (x1.02) 0.952
Malnutrition (calorie) (x1.02) 0.953
Aplastic anemia (x1.02) 0.953
Disorder of transplanted bone marrow (/1.02) 0.954
promethazine (+0.00157) 0.955
imatinib (+0.00153) 0.955
Inflammatory disorder of digestive tract (x1.02) 0.956
Pain (x1.02) 0.956
Generalized aches and pains (x1.02) 0.956
Fatigue (x1.01) 0.957
Primary malignant neoplasm of tonsil (x1.01) 0.957
Blood chemistry abnormal (x1.01) 0.958
Secondary malignant neoplasm of liver (x1.01) 0.958
Hypo-osmolality and or hyponatremia (x1.01) 0.958
Bleeding from nose (x1.01) 0.959
Swelling / lump finding (x1.01) 0.959
Pleural effusion (x1.01) 0.960
Lymphadenopathy (x1.01) 0.960
Disorder affecting transplanted structure (x1.01) 0.960
Primary malignant neoplasm of adrenal gland (x1.01) 0.961
Promyelocytes/100 leukocytes in B by Manual count (+0.0307) 0.961
Insomnia (x1.01) 0.961
Radiation dermatitis (x1.01) 0.962
Lymphedema (x1.01) 0.962
Pain in throat (x1.01) 0.962
Musculoskeletal disorder of the neck (x1.01) 0.963
Disturbance of salivary secretion (x1.01) 0.963
Cardiac arrhythmia (x1.01) 0.963
Acute posthemorrhagic anemia (/1.01) 0.964
Primary malignant neoplasm of tongue (x1.01) 0.964
Bacterial infectious disease (x1.01) 0.964
Staphylococcal infectious disease (x1.01) 0.964
Drug-induced neutropenia (x1.01) 0.965
Neoplasm of lymph node (x1.01) 0.965
Hypervolemia (x1.01) 0.965
Secondary malignant neoplastic disease (x1.01) 0.965
Depressive disorder (x1.01) 0.966
Malaise (x1.01) 0.966
nystatin (+0.00108) 0.966
ergocalciferol (-0.00107) 0.966
Immunodeficiency disorder (x1.01) 0.967
Finding of head and neck region (x1.01) 0.967
Acute myeloid leukemia in remission (/1.01) 0.967
Primary malignant neoplasm of ill-defined site (x1.01) 0.967
morphine (+0.00102) 0.968
Multiple myeloma (x1.01) 0.968
Primary malignant neoplasm of larynx (x1.01) 0.968
−1.00 −0.75 −0.50 −0.25 0.00 0.25 0.50 0.75 1.00
Expressions

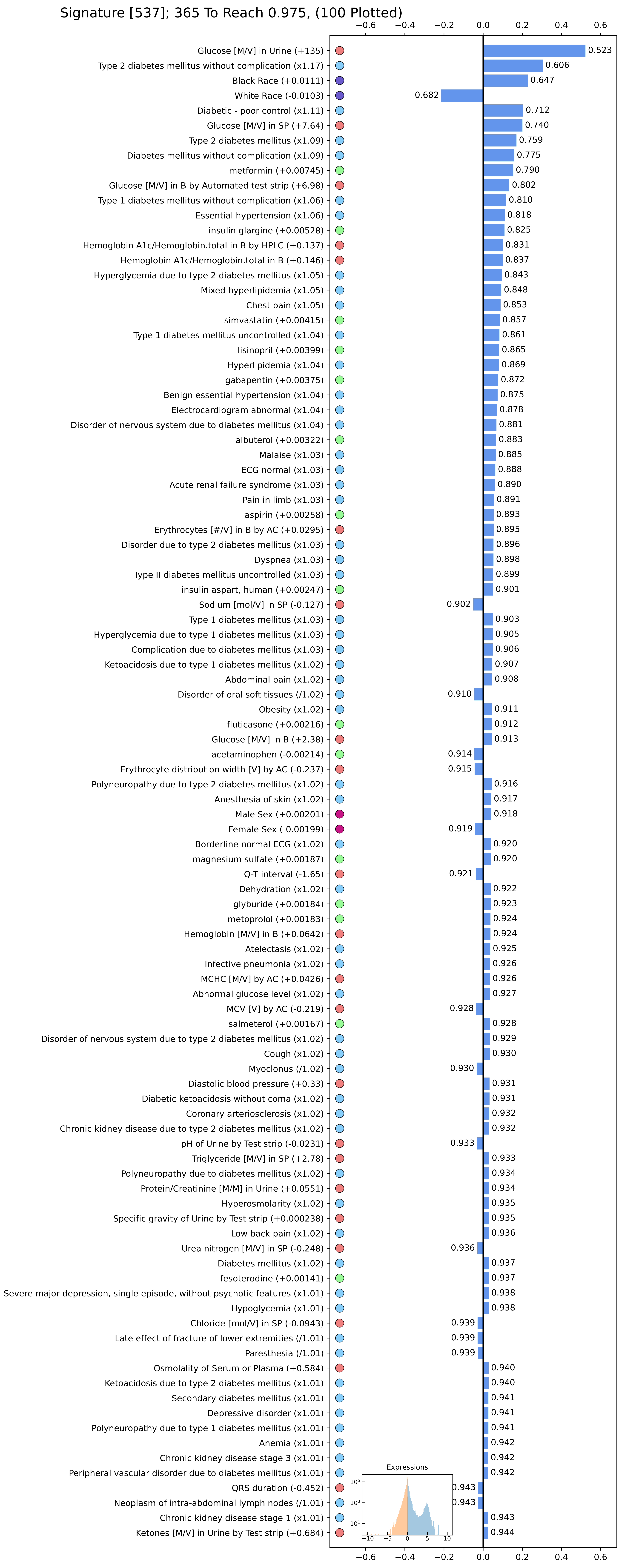
Signature [537]; 365 To Reach 0.975, (100 Plotted)
Glucose [M/V] in Urine (+135)
Type 2 diabetes mellitus without complication (x1.17)
Black Race (+0.0111)
White Race (-0.0103)
Diabetic - poor control (x1.11)
Glucose [M/V] in SP (+7.64)
Type 2 diabetes mellitus (x1.09)
Diabetes mellitus without complication (x1.09)
metformin (+0.00745)
Glucose [M/V] in B by Automated test strip (+6.98)
Type 1 diabetes mellitus without complication (x1.06)
Essential hypertension (x1.06)
insulin glargine (+0.00528)
Hemoglobin A1c/Hemoglobin.total in B by HPLC (+0.137)
Hemoglobin A1c/Hemoglobin.total in B (+0.146)
Hyperglycemia due to type 2 diabetes mellitus (x1.05)
Mixed hyperlipidemia (x1.05)
Chest pain (x1.05)
simvastatin (+0.00415)
Type 1 diabetes mellitus uncontrolled (x1.04)
lisinopril (+0.00399)
Hyperlipidemia (x1.04)
gabapentin (+0.00375)
Benign essential hypertension (x1.04)
Electrocardiogram abnormal (x1.04)
Disorder of nervous system due to diabetes mellitus (x1.04)
albuterol (+0.00322)
Malaise (x1.03)
ECG normal (x1.03)
Acute renal failure syndrome (x1.03)
Pain in limb (x1.03)
aspirin (+0.00258)
Erythrocytes [#/V] in B by AC (+0.0295)
Disorder due to type 2 diabetes mellitus (x1.03)
Dyspnea (x1.03)
Type II diabetes mellitus uncontrolled (x1.03)
insulin aspart, human (+0.00247)
Sodium [mol/V] in SP (-0.127)
Type 1 diabetes mellitus (x1.03)
Hyperglycemia due to type 1 diabetes mellitus (x1.03)
Complication due to diabetes mellitus (x1.03)
Ketoacidosis due to type 1 diabetes mellitus (x1.02)
Abdominal pain (x1.02)
Disorder of oral soft tissues (/1.02)
Obesity (x1.02)
fluticasone (+0.00216)
Glucose [M/V] in B (+2.38)
acetaminophen (-0.00214)
Erythrocyte distribution width [V] by AC (-0.237)
Polyneuropathy due to type 2 diabetes mellitus (x1.02)
Anesthesia of skin (x1.02)
Male Sex (+0.00201)
Female Sex (-0.00199)
Borderline normal ECG (x1.02)
magnesium sulfate (+0.00187)
Q-T interval (-1.65)
Dehydration (x1.02)
glyburide (+0.00184)
metoprolol (+0.00183)
Hemoglobin [M/V] in B (+0.0642)
Atelectasis (x1.02)
Infective pneumonia (x1.02)
MCHC [M/V] by AC (+0.0426)
Abnormal glucose level (x1.02)
MCV [V] by AC (-0.219)
salmeterol (+0.00167)
Disorder of nervous system due to type 2 diabetes mellitus (x1.02)
Cough (x1.02)
Myoclonus (/1.02)
Diastolic blood pressure (+0.33)
Diabetic ketoacidosis without coma (x1.02)
Coronary arteriosclerosis (x1.02)
Chronic kidney disease due to type 2 diabetes mellitus (x1.02)
pH of Urine by Test strip (-0.0231)
Triglyceride [M/V] in SP (+2.78)
Polyneuropathy due to diabetes mellitus (x1.02)
Protein/Creatinine [M/M] in Urine (+0.0551)
Hyperosmolarity (x1.02)
Specific gravity of Urine by Test strip (+0.000238)
Low back pain (x1.02)
Urea nitrogen [M/V] in SP (-0.248)
Diabetes mellitus (x1.02)
fesoterodine (+0.00141)
Severe major depression, single episode, without psychotic features (x1.01)
Hypoglycemia (x1.01)
Chloride [mol/V] in SP (-0.0943)
Late effect of fracture of lower extremities (/1.01)
Paresthesia (/1.01)
Osmolality of Serum or Plasma (+0.584)
Ketoacidosis due to type 2 diabetes mellitus (x1.01)
Secondary diabetes mellitus (x1.01)
Depressive disorder (x1.01)
Polyneuropathy due to type 1 diabetes mellitus (x1.01)
Anemia (x1.01)
Chronic kidney disease stage 3 (x1.01)
Peripheral vascular disorder due to diabetes mellitus (x1.01)
QRS duration (-0.452)
Neoplasm of intra-abdominal lymph nodes (/1.01)
Chronic kidney disease stage 1 (x1.01)
Ketones [M/V] in Urine by Test strip (+0.684)
0.523
0.606
0.647
0.682
0.712
0.740
0.759
0.775
0.790
0.802
0.810
0.818
0.825
0.831
0.837
0.843
0.848
0.853
0.857
0.861
0.865
0.869
0.872
0.875
0.878
0.881
0.883
0.885
0.888
0.890
0.891
0.893
0.895
0.896
0.898
0.899
0.901
0.902
0.903
0.905
0.906
0.907
0.908
0.910
0.911
0.912
0.913
0.914
0.915
0.916
0.917
0.918
0.919
0.920
0.920
0.921
0.922
0.923
0.924
0.924
0.925
0.926
0.926
0.927
0.928
0.928
0.929
0.930
0.930
0.931
0.931
0.932
0.932
0.933
0.933
0.934
0.934
0.935
0.935
0.936
0.936
0.937
0.937
0.938
0.938
0.939
0.939
0.939
0.940
0.940
0.941
0.941
0.941
0.942
0.942
0.942
0.943
0.943
0.943
0.944
Expressions
−0.6
−0.4
−0.2
0.0
0.2
0.4
0.6

Signature [538]; 174 To Reach 0.975, (100 Plotted)

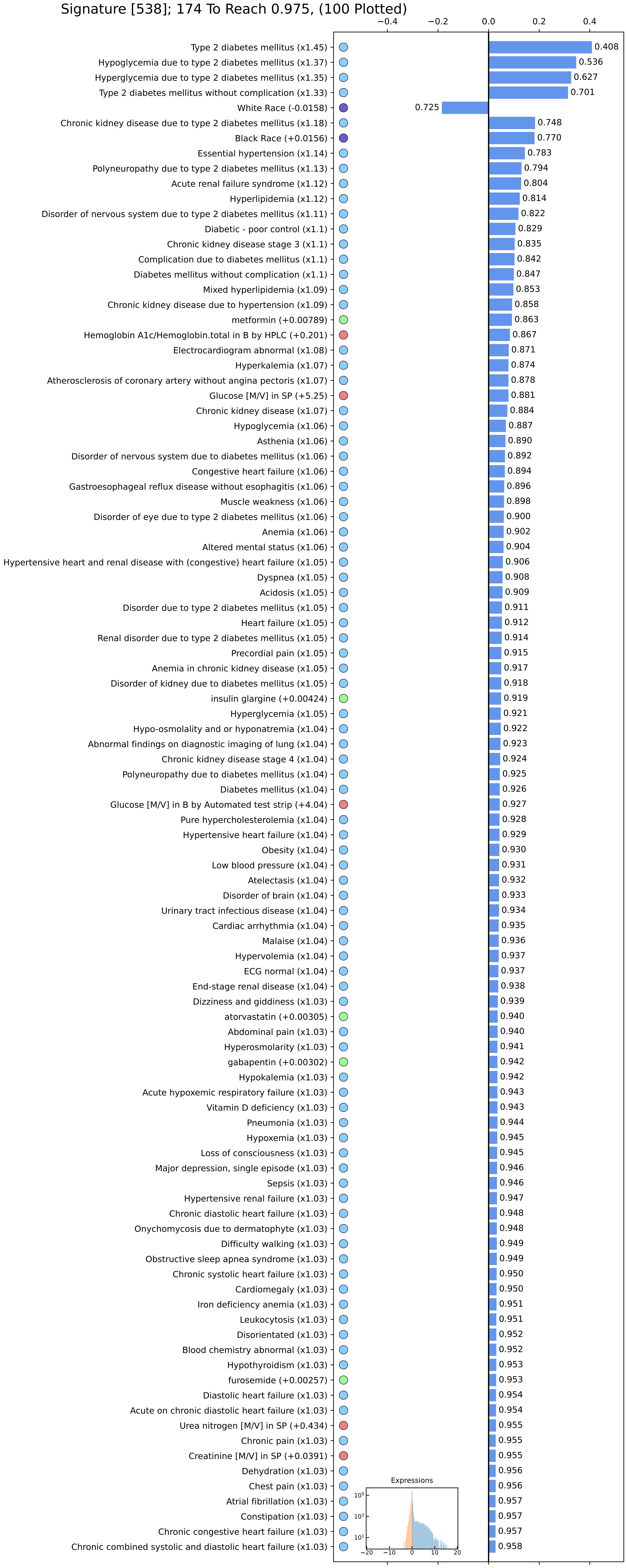

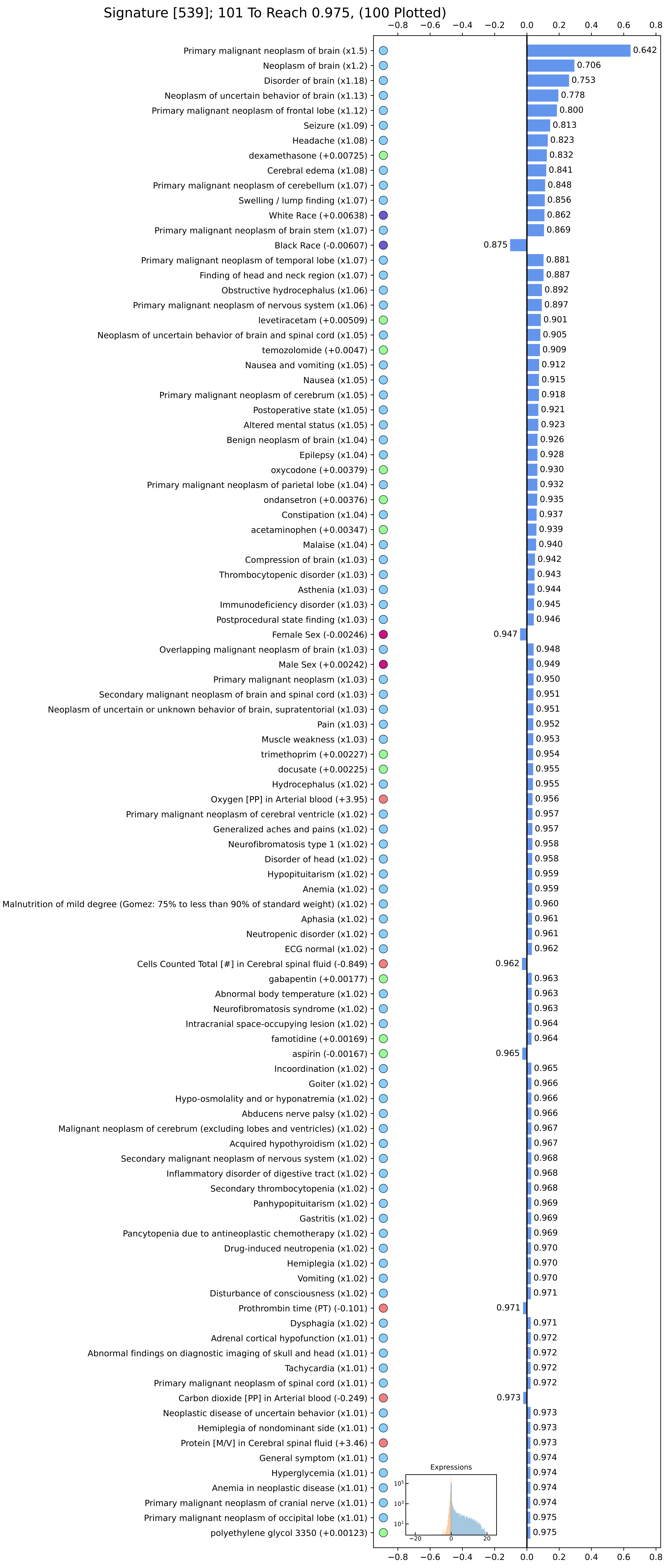

Signature [539]; 101 To Reach 0.975, (100 Plotted)
Primary malignant neoplasm of brain (x1.5) 0.642
Neoplasm of brain (x1.2) 0.706
Disorder of brain (x1.18) 0.753
Neoplasm of uncertain behavior of brain (x1.13) 0.778
Primary malignant neoplasm of frontal lobe (x1.12) 0.800
Seizure (x1.09) 0.813
Headache (x1.08) 0.823
dexamethasone (+0.00725) 0.832
Cerebral edema (x1.08) 0.841
Primary malignant neoplasm of cerebellum (x1.07) 0.848
Swelling / lump finding (x1.07) 0.856
White Race (+0.00638) 0.862
Primary malignant neoplasm of brain stem (x1.07) 0.869
Black Race (-0.00607) 0.875
Primary malignant neoplasm of temporal lobe (x1.07) 0.881
Finding of head and neck region (x1.07) 0.887
Obstructive hydrocephalus (x1.06) 0.892
Primary malignant neoplasm of nervous system (x1.06) 0.897
levetiracetam (+0.00509) 0.901
Neoplasm of uncertain behavior of brain and spinal cord (x1.05) 0.905
temozolomide (+0.0047) 0.909
Nausea and vomiting (x1.05) 0.912
Nausea (x1.05) 0.915
Primary malignant neoplasm of cerebrum (x1.05) 0.918
Postoperative state (x1.05) 0.921
Altered mental status (x1.05) 0.923
Benign neoplasm of brain (x1.04) 0.926
Epilepsy (x1.04) 0.928
oxycodone (+0.00379) 0.930
Primary malignant neoplasm of parietal lobe (x1.04) 0.932
ondansetron (+0.00376) 0.935
Constipation (x1.04) 0.937
acetaminophen (+0.00347) 0.939
Malaise (x1.04) 0.940
Compression of brain (x1.03) 0.942
Thrombocytopenic disorder (x1.03) 0.943
Asthenia (x1.03) 0.944
Immunodeficiency disorder (x1.03) 0.945
Postprocedural state finding (x1.03) 0.946
Female Sex (-0.00246) 0.947
Overlapping malignant neoplasm of brain (x1.03) 0.948
Male Sex (+0.00242) 0.949
Primary malignant neoplasm (x1.03) 0.950
Secondary malignant neoplasm of brain and spinal cord (x1.03) 0.951
Neoplasm of uncertain or unknown behavior of brain, supratentorial (x1.03) 0.951
Pain (x1.03) 0.952
Muscle weakness (x1.03) 0.953
trimethoprim (+0.00227) 0.954
docusate (+0.00225) 0.955
Hydrocephalus (x1.02) 0.955
Oxygen [PP] in Arterial blood (+3.95) 0.956
Primary malignant neoplasm of cerebral ventricle (x1.02) 0.957
Generalized aches and pains (x1.02) 0.957
Neurofibromatosis type 1 (x1.02) 0.958
Disorder of head (x1.02) 0.958
Hypopituitarism (x1.02) 0.959
Anemia (x1.02) 0.959
Malnutrition of mild degree (Gomez: 75% to less than 90% of standard weight) (x1.02) 0.960
Aphasia (x1.02) 0.961
Neutropenic disorder (x1.02) 0.961
ECG normal (x1.02) 0.962
Cells Counted Total [#] in Cerebral spinal fluid (-0.849) 0.962
gabapentin (+0.00177) 0.963
Abnormal body temperature (x1.02) 0.963
Neurofibromatosis syndrome (x1.02) 0.963
Intracranial space-occupying lesion (x1.02) 0.964
famotidine (+0.00169) 0.964
aspirin (-0.00167) 0.965
Incoordination (x1.02) 0.965
Goiter (x1.02) 0.966
Hypo-osmolality and or hyponatremia (x1.02) 0.966
Abducens nerve palsy (x1.02) 0.966
Malignant neoplasm of cerebrum (excluding lobes and ventricles) (x1.02) 0.967
Acquired hypothyroidism (x1.02) 0.967
Secondary malignant neoplasm of nervous system (x1.02) 0.968
Inflammatory disorder of digestive tract (x1.02) 0.968
Secondary thrombocytopenia (x1.02) 0.968
Panhypopituitarism (x1.02) 0.969
Gastritis (x1.02) 0.969
Pancytopenia due to antineoplastic chemotherapy (x1.02) 0.969
Drug-induced neutropenia (x1.02) 0.970
Hemiplegia (x1.02) 0.970
Vomiting (x1.02) 0.970
Disturbance of consciousness (x1.02) 0.971
Prothrombin time (PT) (-0.101) 0.971
Dysphagia (x1.02) 0.971
Adrenal cortical hypofunction (x1.01) 0.972
Abnormal findings on diagnostic imaging of skull and head (x1.01) 0.972
Tachycardia (x1.01) 0.972
Primary malignant neoplasm of spinal cord (x1.01) 0.972
Carbon dioxide [PP] in Arterial blood (-0.249) 0.973
Neoplastic disease of uncertain behavior (x1.01) 0.973
Hemiplegia of nondominant side (x1.01) 0.973
Protein [M/V] in Cerebral spinal fluid (+3.46) 0.973
General symptom (x1.01) 0.974
Hyperglycemia (x1.01) 0.974
Anemia in neoplastic disease (x1.01) 0.974
Primary malignant neoplasm of cranial nerve (x1.01) 0.974
Primary malignant neoplasm of occipital lobe (x1.01) 0.975
polyethylene glycol 3350 (+0.00123) 0.975
Expressions

Signature [540]; 3 To Reach 0.975, (10 Plotted)

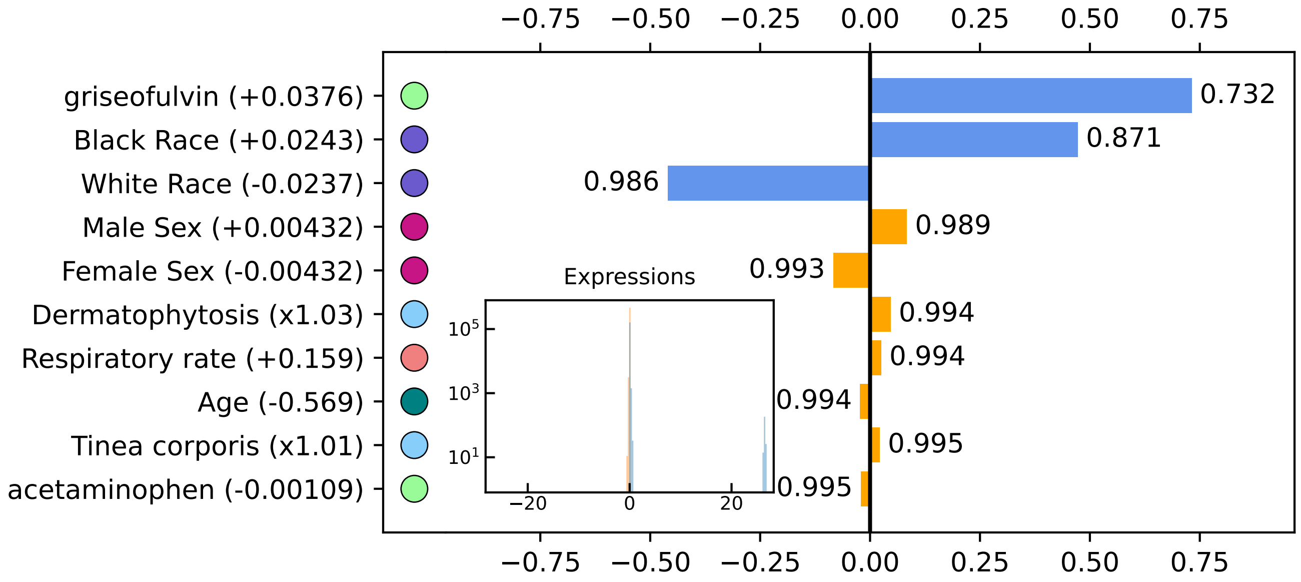

Signature [541]; 35 To Reach 0.975, (35 Plotted)

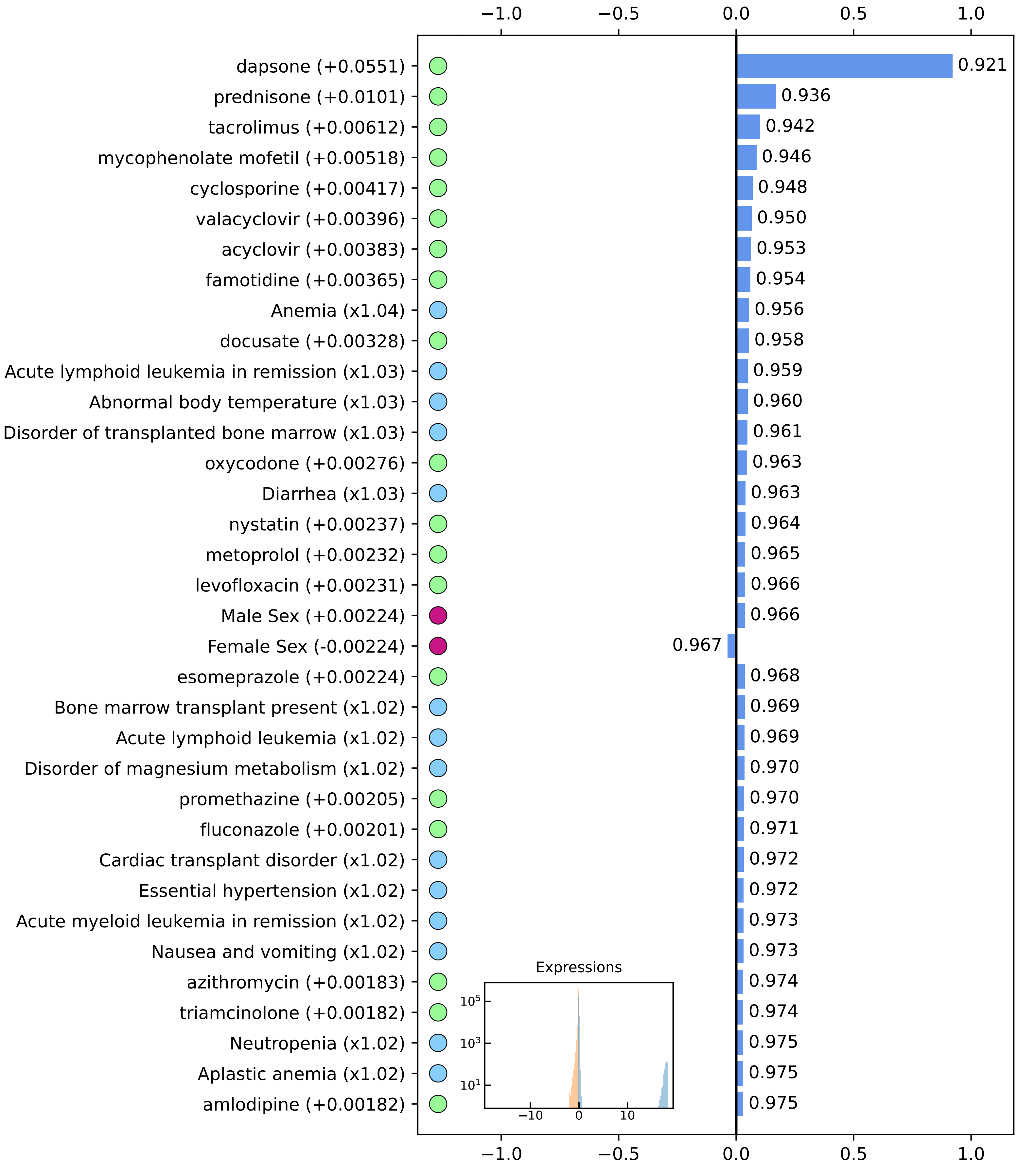

Signature [542]; 55 To Reach 0.975, (55 Plotted)

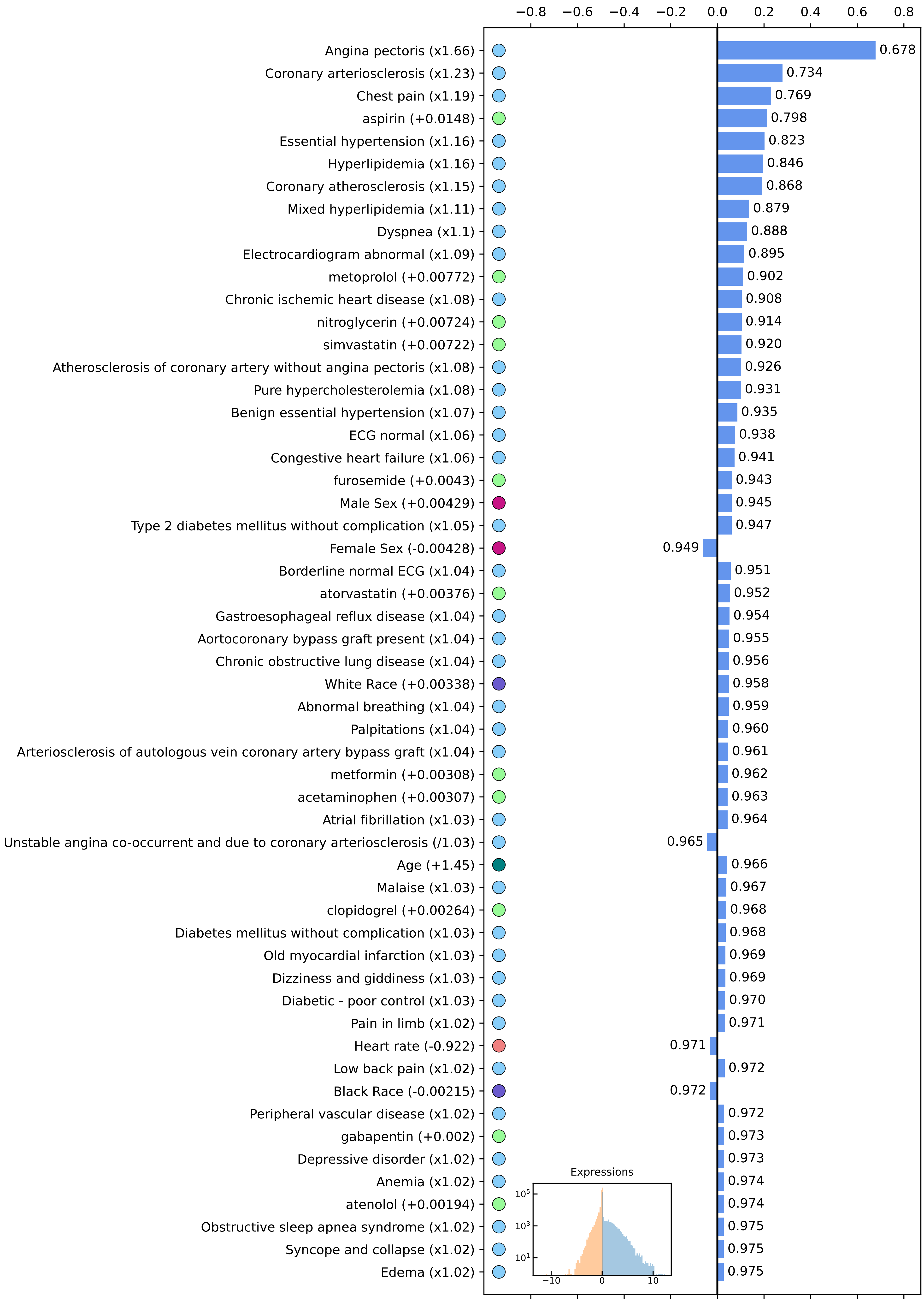

Signature [543]; 3 To Reach 0.975, (10 Plotted)

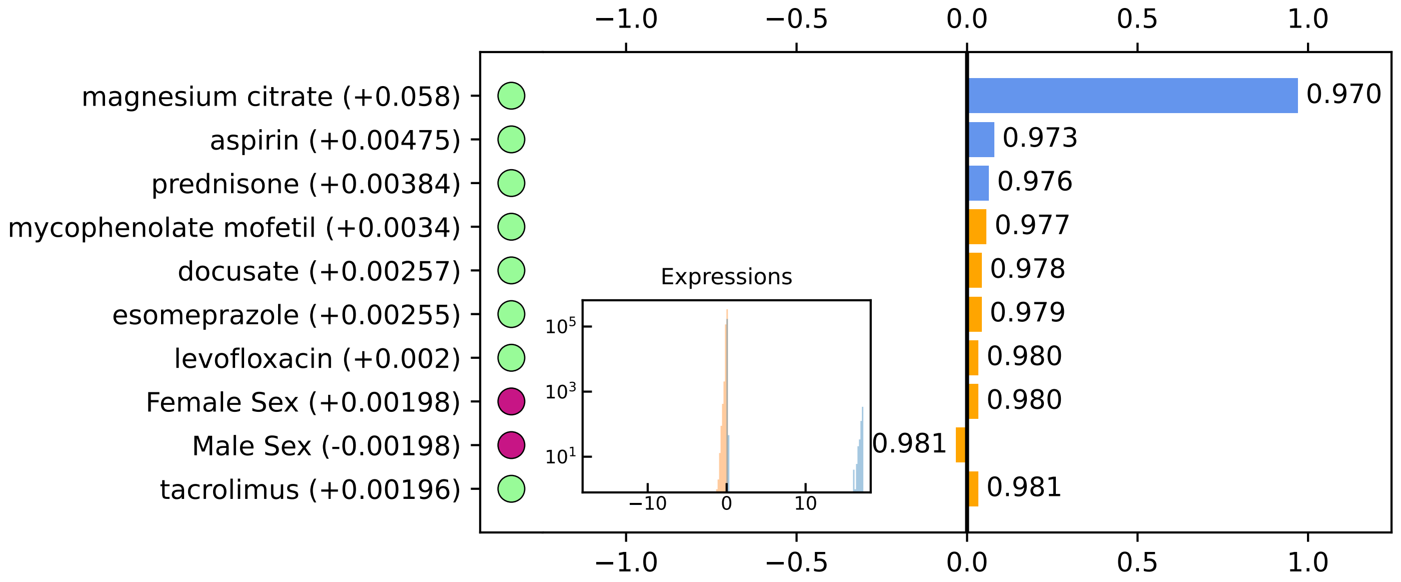

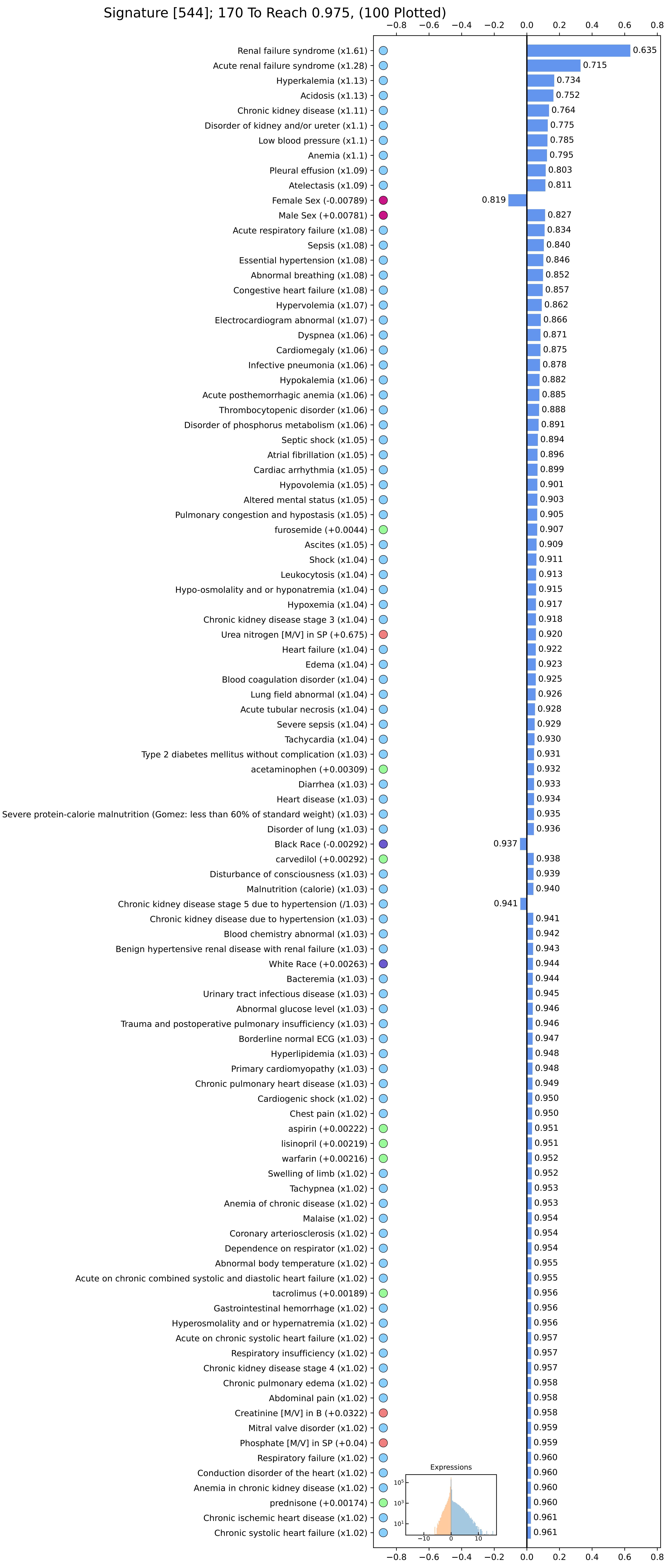

Signature [544]; 170 To Reach 0.975, (100 Plotted)
Renal failure syndrome (x1.61) 0.635
Acute renal failure syndrome (x1.28) 0.715
Hyperkalemia (x1.13) 0.734
Acidosis (x1.13) 0.752
Chronic kidney disease (x1.11) 0.764
Disorder of kidney and/or ureter (x1.1) 0.775
Low blood pressure (x1.1) 0.785
Anemia (x1.1) 0.795
Pleural effusion (x1.09) 0.803
Atelectasis (x1.09) 0.811
Female Sex (-0.00789) 0.819
Male Sex (+0.00781) 0.827
Acute respiratory failure (x1.08) 0.834
Sepsis (x1.08) 0.840
Essential hypertension (x1.08) 0.846
Abnormal breathing (x1.08) 0.852
Congestive heart failure (x1.08) 0.857
Hypervolemia (x1.07) 0.862
Electrocardiogram abnormal (x1.07) 0.866
Dyspnea (x1.06) 0.871
Cardiomegaly (x1.06) 0.875
Infective pneumonia (x1.06) 0.878
Hypokalemia (x1.06) 0.882
Acute posthemorrhagic anemia (x1.06) 0.885
Thrombocytopenic disorder (x1.06) 0.888
Disorder of phosphorus metabolism (x1.06) 0.891
Septic shock (x1.05) 0.894
Atrial fibrillation (x1.05) 0.896
Cardiac arrhythmia (x1.05) 0.899
Hypovolemia (x1.05) 0.901
Altered mental status (x1.05) 0.903
Pulmonary congestion and hypostasis (x1.05) 0.905
furosemide (+0.0044) 0.907
Ascites (x1.05) 0.909
Shock (x1.04) 0.911
Leukocytosis (x1.04) 0.913
Hypo-osmolality and or hyponatremia (x1.04) 0.915
Hypoxemia (x1.04) 0.917
Chronic kidney disease stage 3 (x1.04) 0.918
Urea nitrogen [M/V] in SP (+0.675) 0.920
Heart failure (x1.04) 0.922
Edema (x1.04) 0.923
Blood coagulation disorder (x1.04) 0.925
Lung field abnormal (x1.04) 0.926
Acute tubular necrosis (x1.04) 0.928
Severe sepsis (x1.04) 0.929
Tachycardia (x1.04) 0.930
Type 2 diabetes mellitus without complication (x1.03) 0.931
acetaminophen (+0.00309) 0.932
Diarrhea (x1.03) 0.933
Heart disease (x1.03) 0.934
Severe protein-calorie malnutrition (Gomez: less than 60% of standard weight) (x1.03) 0.935
Disorder of lung (x1.03) 0.936
Black Race (-0.00292) 0.937
carvedilol (+0.00292) 0.938
Disturbance of consciousness (x1.03) 0.939
Malnutrition (calorie) (x1.03) 0.940
Chronic kidney disease stage 5 due to hypertension (/1.03) 0.941
Chronic kidney disease due to hypertension (x1.03) 0.941
Blood chemistry abnormal (x1.03) 0.942
Benign hypertensive renal disease with renal failure (x1.03) 0.943
White Race (+0.00263) 0.944
Bacteremia (x1.03) 0.944
Urinary tract infectious disease (x1.03) 0.945
Abnormal glucose level (x1.03) 0.946
Trauma and postoperative pulmonary insufficiency (x1.03) 0.946
Borderline normal ECG (x1.03) 0.947
Hyperlipidemia (x1.03) 0.948
Primary cardiomyopathy (x1.03) 0.948
Chronic pulmonary heart disease (x1.03) 0.949
Cardiogenic shock (x1.02) 0.950
Chest pain (x1.02) 0.950
aspirin (+0.00222) 0.951
lisinopril (+0.00219) 0.951
warfarin (+0.00216) 0.952
Swelling of limb (x1.02) 0.952
Tachypnea (x1.02) 0.953
Anemia of chronic disease (x1.02) 0.953
Malaise (x1.02) 0.954
Coronary arteriosclerosis (x1.02) 0.954
Dependence on respirator (x1.02) 0.954
Abnormal body temperature (x1.02) 0.955
Acute on chronic combined systolic and diastolic heart failure (x1.02) 0.955
tacrolimus (+0.00189) 0.956
Gastrointestinal hemorrhage (x1.02) 0.956
Hyperosmolality and or hypernatremia (x1.02) 0.956
Acute on chronic systolic heart failure (x1.02) 0.957
Respiratory insufficiency (x1.02) 0.957
Chronic kidney disease stage 4 (x1.02) 0.957
Chronic pulmonary edema (x1.02) 0.958
Abdominal pain (x1.02) 0.958
Creatinine [M/V] in B (+0.0322) 0.958
Mitral valve disorder (x1.02) 0.959
Phosphate [M/V] in SP (+0.04) 0.959
Respiratory failure (x1.02) 0.960
Conduction disorder of the heart (x1.02) 0.960
Anemia in chronic kidney disease (x1.02) 0.960
prednisone (+0.00174) 0.960
Chronic ischemic heart disease (x1.02) 0.961
Chronic systolic heart failure (x1.02) 0.961
−0.8 −0.6 −0.4 −0.2 0.0 0.2 0.4 0.6 0.8
Expressions

Signature [545]; 139 To Reach 0.975, (100 Plotted)

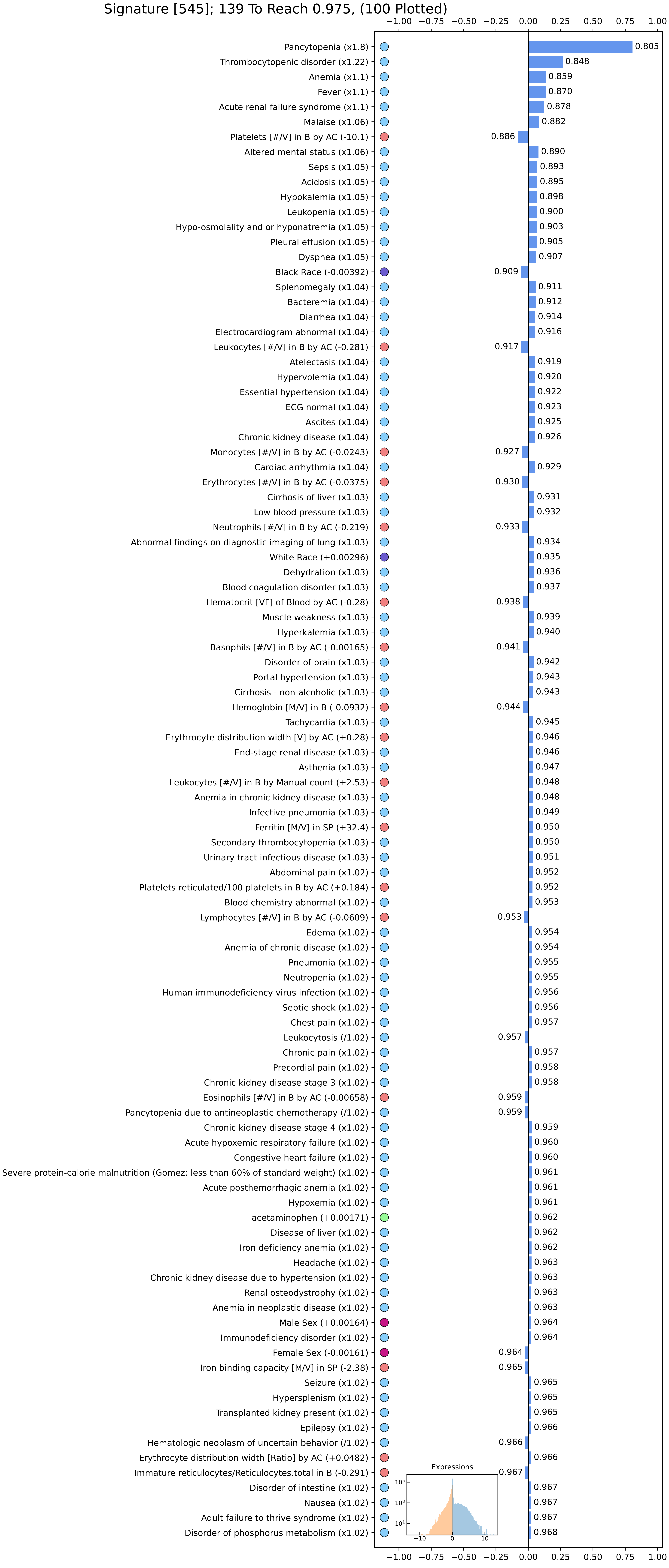

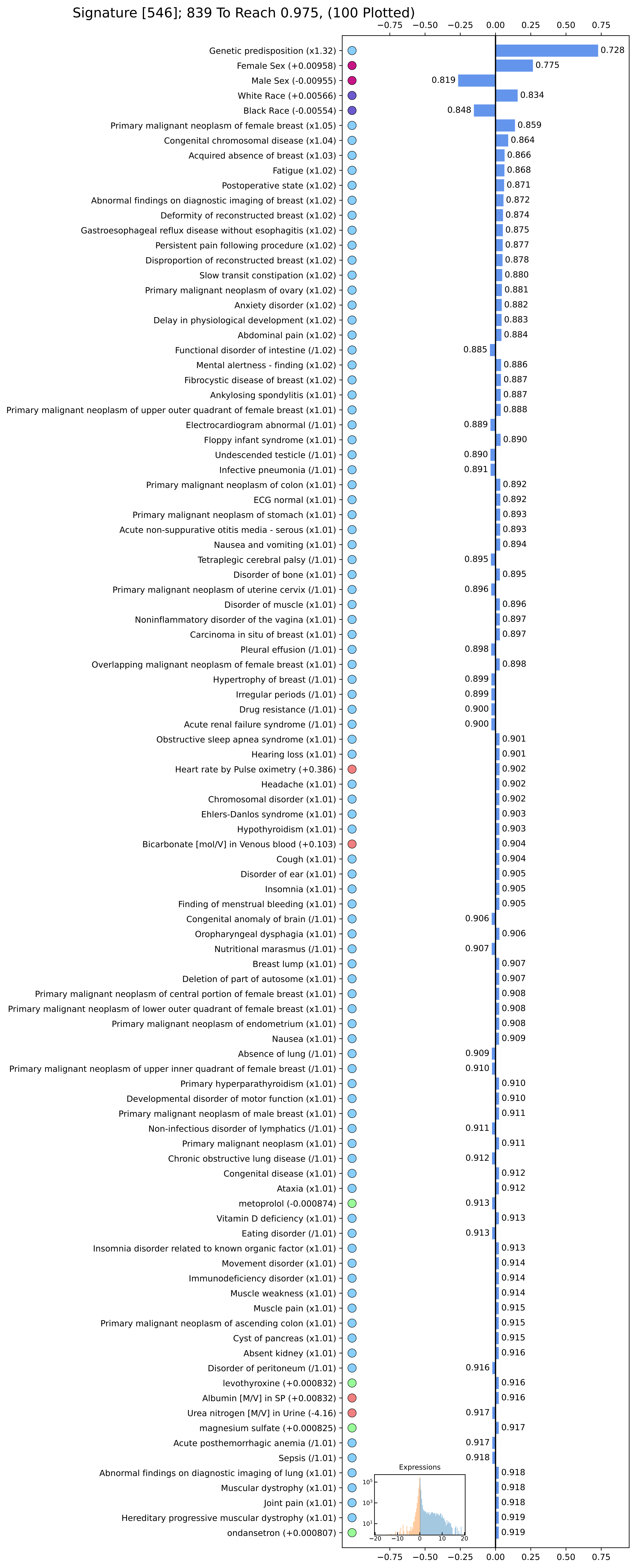

Signature [546]; 839 To Reach 0.975, (100 Plotted)
Genetic predisposition (x1.32) 0.728
Female Sex (+0.00958) 0.775
Male Sex (-0.00955) 0.819
White Race (+0.00566) 0.834
Black Race (-0.00554) 0.848
Primary malignant neoplasm of female breast (x1.05) 0.859
Congenital chromosomal disease (x1.04) 0.864
Acquired absence of breast (x1.03) 0.866
Fatigue (x1.02) 0.868
Postoperative state (x1.02) 0.871
Abnormal findings on diagnostic imaging of breast (x1.02) 0.872
Deformity of reconstructed breast (x1.02) 0.874
Gastroesophageal reflux disease without esophagitis (x1.02) 0.875
Persistent pain following procedure (x1.02) 0.877
Disproportion of reconstructed breast (x1.02) 0.878
Slow transit constipation (x1.02) 0.880
Primary malignant neoplasm of ovary (x1.02) 0.881
Anxiety disorder (x1.02) 0.882
Delay in physiological development (x1.02) 0.883
Abdominal pain (x1.02) 0.884
Functional disorder of intestine (/1.02) 0.885
Mental alertness - finding (x1.02) 0.886
Fibrocystic disease of breast (x1.02) 0.887
Ankylosing spondylitis (x1.01) 0.887
Primary malignant neoplasm of upper outer quadrant of female breast (x1.01) 0.888
Electrocardiogram abnormal (/1.01) 0.889
Floppy infant syndrome (x1.01) 0.890
Undescended testicle (/1.01) 0.890
Infective pneumonia (/1.01) 0.891
Primary malignant neoplasm of colon (x1.01) 0.892
ECG normal (x1.01) 0.892
Primary malignant neoplasm of stomach (x1.01) 0.893
Acute non-suppurative otitis media - serous (x1.01) 0.893
Nausea and vomiting (x1.01) 0.894
Tetraplegic cerebral palsy (/1.01) 0.895
Disorder of bone (x1.01) 0.895
Primary malignant neoplasm of uterine cervix (/1.01) 0.896
Disorder of muscle (x1.01) 0.896
Noninflammatory disorder of the vagina (x1.01) 0.897
Carcinoma in situ of breast (x1.01) 0.897
Pleural effusion (/1.01) 0.898
Overlapping malignant neoplasm of female breast (x1.01) 0.898
Hypertrophy of breast (/1.01) 0.899
Irregular periods (/1.01) 0.899
Drug resistance (/1.01) 0.900
Acute renal failure syndrome (/1.01) 0.900
Obstructive sleep apnea syndrome (x1.01) 0.901
Hearing loss (x1.01) 0.901
Heart rate by Pulse oximetry (+0.386) 0.902
Headache (x1.01) 0.902
Chromosomal disorder (x1.01) 0.902
Ehlers-Danlos syndrome (x1.01) 0.903
Hypothyroidism (x1.01) 0.903
Bicarbonate [mol/V] in Venous blood (+0.103) 0.904
Cough (x1.01) 0.904
Disorder of ear (x1.01) 0.905
Insomnia (x1.01) 0.905
Finding of menstrual bleeding (x1.01) 0.905
Congenital anomaly of brain (/1.01) 0.906
Oropharyngeal dysphagia (x1.01) 0.906
Nutritional marasmus (/1.01) 0.907
Breast lump (x1.01) 0.907
Deletion of part of autosome (x1.01) 0.907
Primary malignant neoplasm of central portion of female breast (x1.01) 0.908
Primary malignant neoplasm of lower outer quadrant of female breast (x1.01) 0.908
Primary malignant neoplasm of endometrium (x1.01) 0.908
Nausea (x1.01) 0.909
Absence of lung (/1.01) 0.909
Primary malignant neoplasm of upper inner quadrant of female breast (/1.01) 0.910
Primary hyperparathyroidism (x1.01) 0.910
Developmental disorder of motor function (x1.01) 0.910
Primary malignant neoplasm of male breast (x1.01) 0.911
Non-infectious disorder of lymphatics (/1.01) 0.911
Primary malignant neoplasm (x1.01) 0.911
Chronic obstructive lung disease (/1.01) 0.912
Congenital disease (x1.01) 0.912
Ataxia (x1.01) 0.912
metoprolol (-0.000874) 0.913
Vitamin D deficiency (x1.01) 0.913
Eating disorder (/1.01) 0.913
Insomnia disorder related to known organic factor (x1.01) 0.913
Movement disorder (x1.01) 0.914
Immunodeficiency disorder (x1.01) 0.914
Muscle weakness (x1.01) 0.914
Muscle pain (x1.01) 0.915
Primary malignant neoplasm of ascending colon (x1.01) 0.915
Cyst of pancreas (x1.01) 0.915
Absent kidney (x1.01) 0.916
Disorder of peritoneum (/1.01) 0.916
levothyroxine (+0.000832) 0.916
Albumin [M/V] in SP (+0.00832) 0.916
Urea nitrogen [M/V] in Urine (-4.16) 0.917
magnesium sulfate (+0.000825) 0.917
Acute posthemorrhagic anemia (/1.01) 0.917
Sepsis (/1.01) 0.918
Abnormal findings on diagnostic imaging of lung (x1.01) 0.918
Muscular dystrophy (x1.01) 0.918
Joint pain (x1.01) 0.918
Hereditary progressive muscular dystrophy (x1.01) 0.919
ondansetron (+0.000807) 0.919
−0.75 −0.50 −0.25 0.00 0.25 0.50 0.75
Expressions
−20 −10 0 10 20

Signature [547]; 1 To Reach 0.975, (10 Plotted)

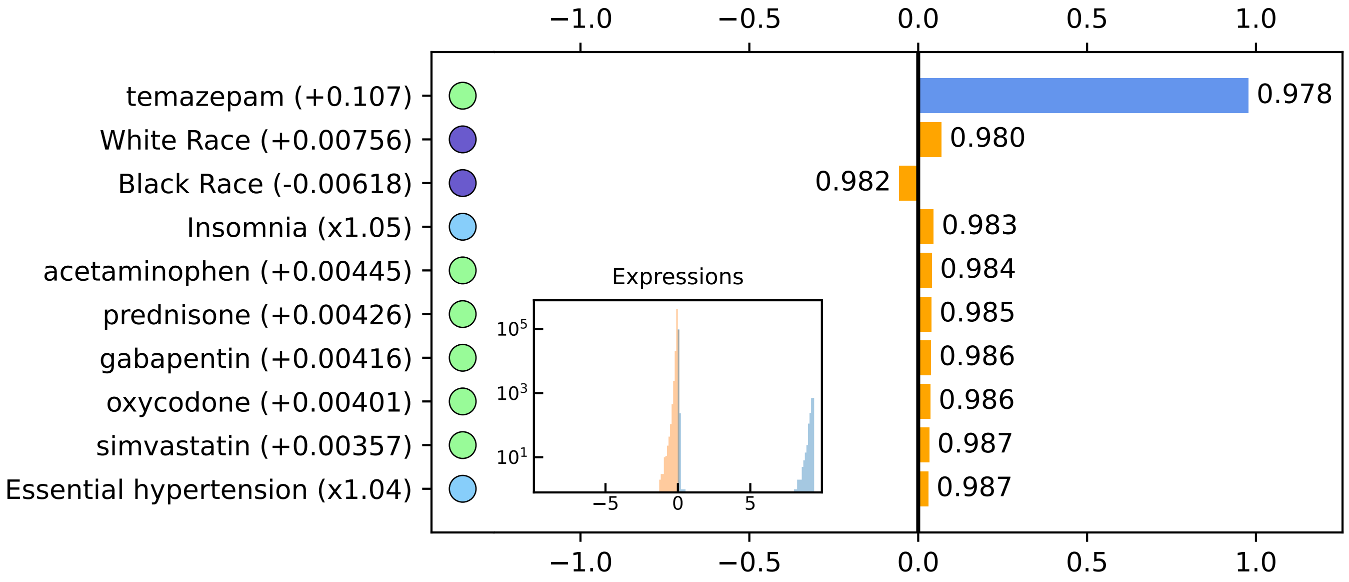

Signature [548]; 3 To Reach 0.975, (10 Plotted)

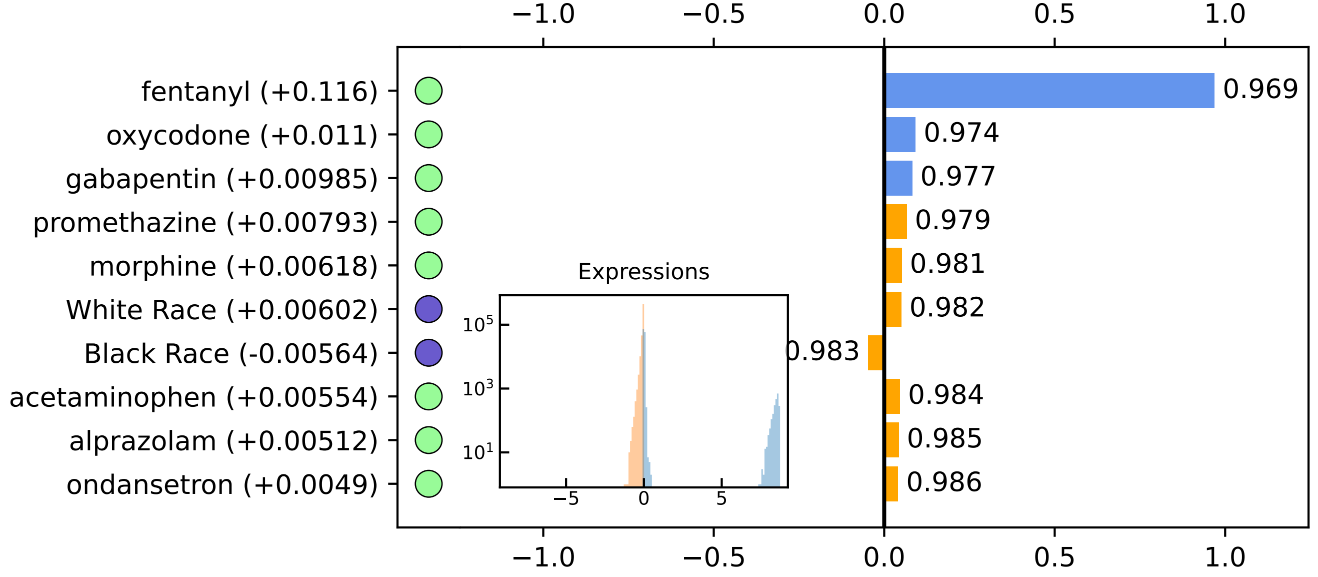

Signature [549]; 138 To Reach 0.975, (100 Plotted)

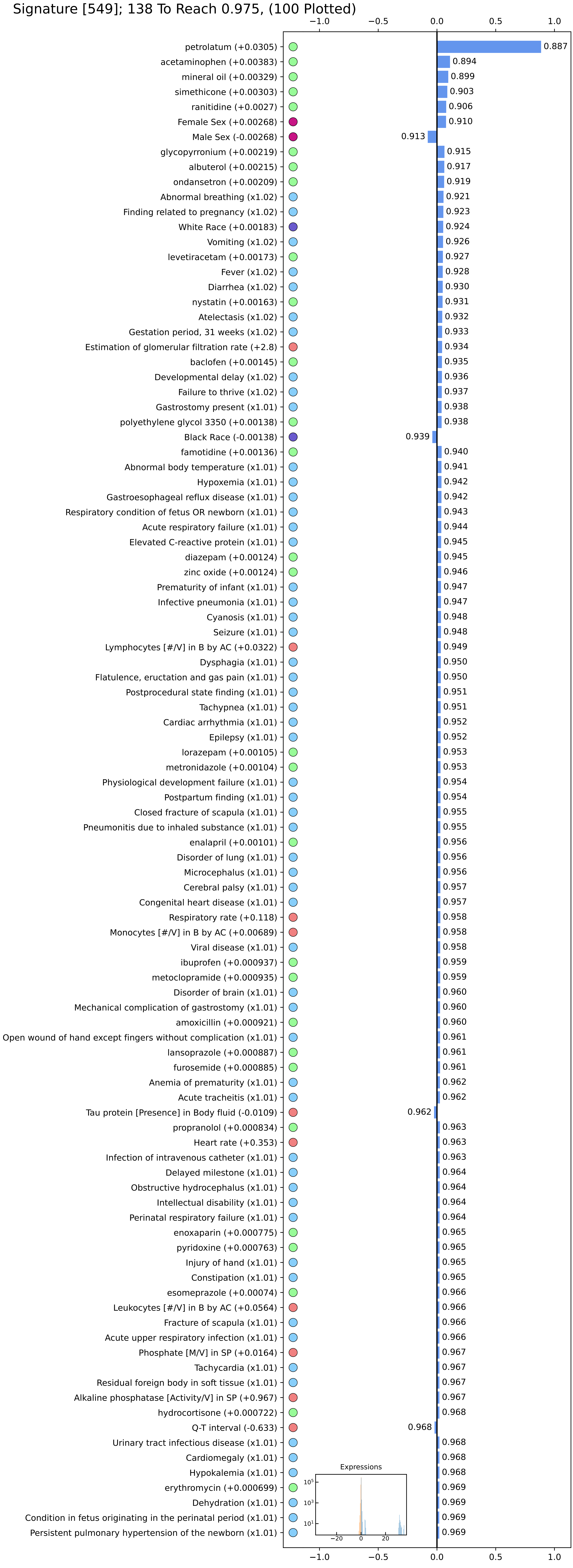

Signature [550]; 6 To Reach 0.975, (10 Plotted)

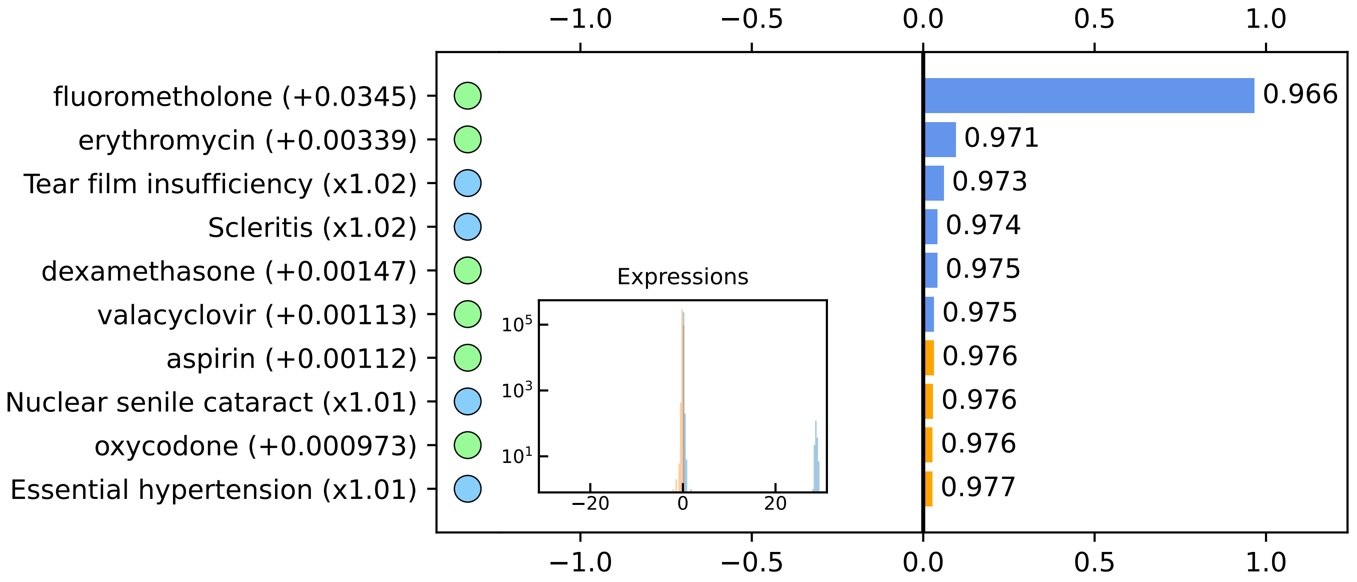

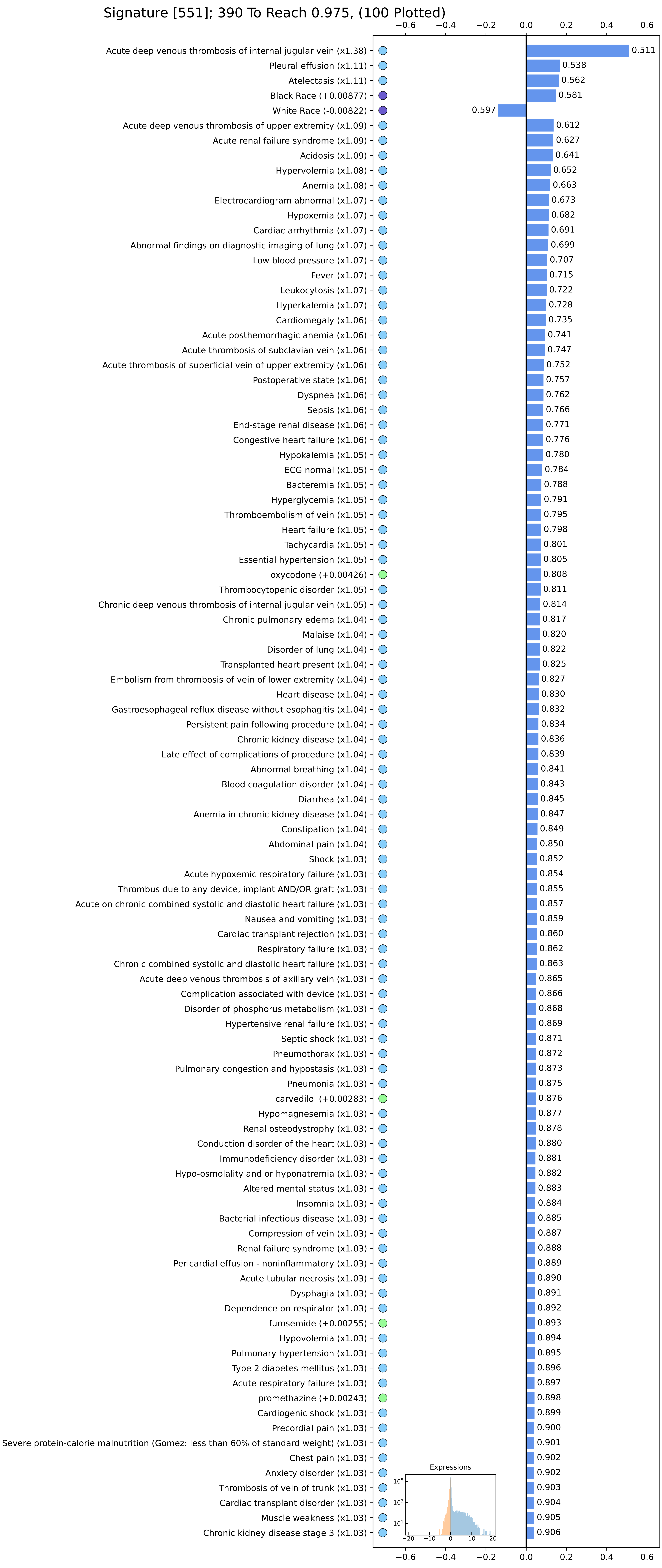

Signature [551]; 390 To Reach 0.975, (100 Plotted)
Acute deep venous thrombosis of internal jugular vein (x1.38) 0.511
Pleural effusion (x1.11) 0.538
Atelectasis (x1.11) 0.562
Black Race (+0.00877) 0.581
White Race (-0.00822) 0.597
Acute deep venous thrombosis of upper extremity (x1.09) 0.612
Acute renal failure syndrome (x1.09) 0.627
Acidosis (x1.09) 0.641
Hypervolemia (x1.08) 0.652
Anemia (x1.08) 0.663
Electrocardiogram abnormal (x1.07) 0.673
Hypoxemia (x1.07) 0.682
Cardiac arrhythmia (x1.07) 0.691
Abnormal findings on diagnostic imaging of lung (x1.07) 0.699
Low blood pressure (x1.07) 0.707
Fever (x1.07) 0.715
Leukocytosis (x1.07) 0.722
Hyperkalemia (x1.07) 0.728
Cardiomegaly (x1.06) 0.735
Acute posthemorrhagic anemia (x1.06) 0.741
Acute thrombosis of subclavian vein (x1.06) 0.747
Acute thrombosis of superficial vein of upper extremity (x1.06) 0.752
Postoperative state (x1.06) 0.757
Dyspnea (x1.06) 0.762
Sepsis (x1.06) 0.766
End-stage renal disease (x1.06) 0.771
Congestive heart failure (x1.06) 0.776
Hypokalemia (x1.05) 0.780
ECG normal (x1.05) 0.784
Bacteremia (x1.05) 0.788
Hyperglycemia (x1.05) 0.791
Thromboembolism of vein (x1.05) 0.795
Heart failure (x1.05) 0.798
Tachycardia (x1.05) 0.801
Essential hypertension (x1.05) 0.805
oxycodone (+0.00426) 0.808
Thrombocytopenic disorder (x1.05) 0.811
Chronic deep venous thrombosis of internal jugular vein (x1.05) 0.814
Chronic pulmonary edema (x1.04) 0.817
Malaise (x1.04) 0.820
Disorder of lung (x1.04) 0.822
Transplanted heart present (x1.04) 0.825
Embolism from thrombosis of vein of lower extremity (x1.04) 0.827
Heart disease (x1.04) 0.830
Gastroesophageal reflux disease without esophagitis (x1.04) 0.832
Persistent pain following procedure (x1.04) 0.834
Chronic kidney disease (x1.04) 0.836
Late effect of complications of procedure (x1.04) 0.839
Abnormal breathing (x1.04) 0.841
Blood coagulation disorder (x1.04) 0.843
Diarrhea (x1.04) 0.845
Anemia in chronic kidney disease (x1.04) 0.847
Constipation (x1.04) 0.849
Abdominal pain (x1.04) 0.850
Shock (x1.03) 0.852
Acute hypoxemic respiratory failure (x1.03) 0.854
Thrombus due to any device, implant AND/OR graft (x1.03) 0.855
Acute on chronic combined systolic and diastolic heart failure (x1.03) 0.857
Nausea and vomiting (x1.03) 0.859
Cardiac transplant rejection (x1.03) 0.860
Respiratory failure (x1.03) 0.862
Chronic combined systolic and diastolic heart failure (x1.03) 0.863
Acute deep venous thrombosis of axillary vein (x1.03) 0.865
Complication associated with device (x1.03) 0.866
Disorder of phosphorus metabolism (x1.03) 0.868
Hypertensive renal failure (x1.03) 0.869
Septic shock (x1.03) 0.871
Pneumothorax (x1.03) 0.872
Pulmonary congestion and hypostasis (x1.03) 0.873
Pneumonia (x1.03) 0.875
carvedilol (+0.00283) 0.876
Hypomagnesemia (x1.03) 0.877
Renal osteodystrophy (x1.03) 0.878
Conduction disorder of the heart (x1.03) 0.880
Immunodeficiency disorder (x1.03) 0.881
Hypo-osmolality and or hyponatremia (x1.03) 0.882
Altered mental status (x1.03) 0.883
Insomnia (x1.03) 0.884
Bacterial infectious disease (x1.03) 0.885
Compression of vein (x1.03) 0.887
Renal failure syndrome (x1.03) 0.888
Pericardial effusion - noninflammatory (x1.03) 0.889
Acute tubular necrosis (x1.03) 0.890
Dysphagia (x1.03) 0.891
Dependence on respirator (x1.03) 0.892
furosemide (+0.00255) 0.893
Hypovolemia (x1.03) 0.894
Pulmonary hypertension (x1.03) 0.895
Type 2 diabetes mellitus (x1.03) 0.896
Acute respiratory failure (x1.03) 0.897
promethazine (+0.00243) 0.898
Cardiogenic shock (x1.03) 0.899
Precordial pain (x1.03) 0.900
Severe protein-calorie malnutrition (Gomez: less than 60% of standard weight) (x1.03) 0.901
Chest pain (x1.03) 0.902
Anxiety disorder (x1.03) 0.902
Thrombosis of vein of trunk (x1.03) 0.903
Cardiac transplant disorder (x1.03) 0.904
Muscle weakness (x1.03) 0.905
Chronic kidney disease stage 3 (x1.03) 0.906
Expressions
−0.6 −0.4 −0.2 0.0 0.2 0.4 0.6

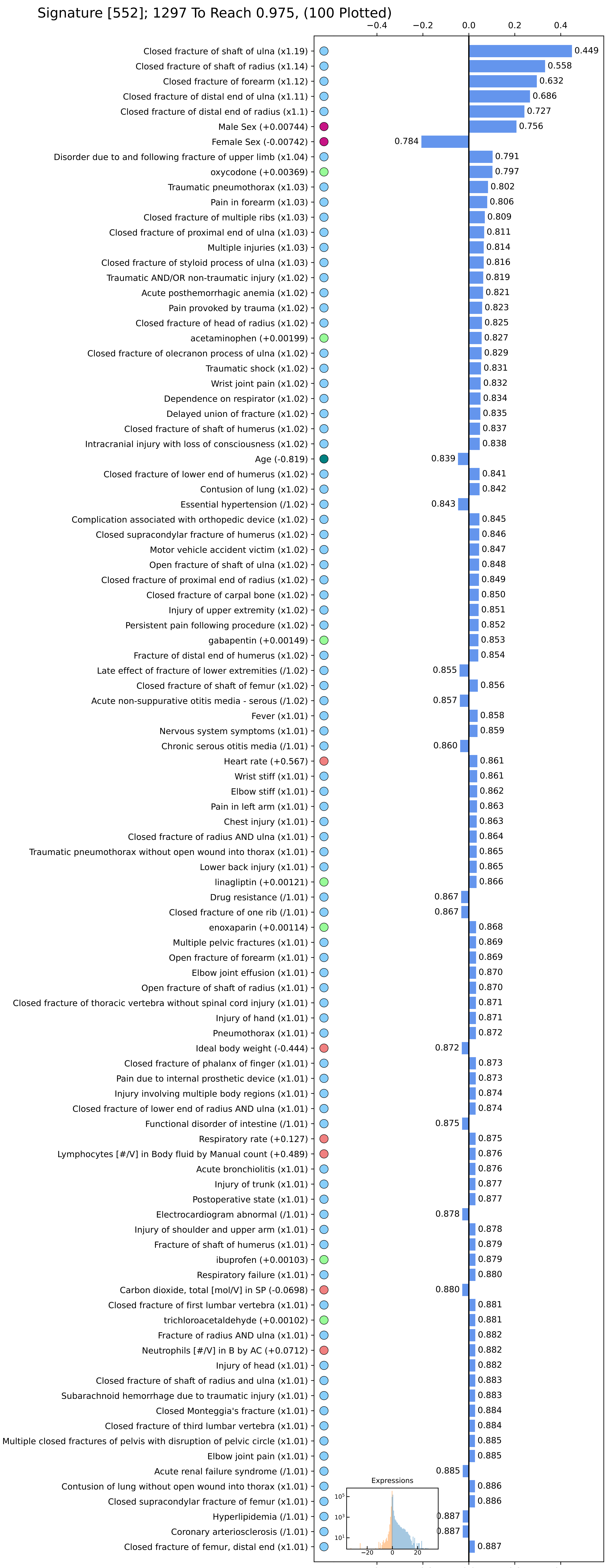

Signature [552]; 1297 To Reach 0.975, (100 Plotted)
Closed fracture of shaft of ulna (x1.19) 0.449
Closed fracture of shaft of radius (x1.14) 0.558
Closed fracture of forearm (x1.12) 0.632
Closed fracture of distal end of ulna (x1.11) 0.686
Closed fracture of distal end of radius (x1.1) 0.727
Male Sex (+0.00744) 0.756
Female Sex (-0.00742) 0.784
Disorder due to and following fracture of upper limb (x1.04) 0.791
oxycodone (+0.00369) 0.797
Traumatic pneumothorax (x1.03) 0.802
Pain in forearm (x1.03) 0.806
Closed fracture of multiple ribs (x1.03) 0.809
Closed fracture of proximal end of ulna (x1.03) 0.811
Multiple injuries (x1.03) 0.814
Closed fracture of styloid process of ulna (x1.03) 0.816
Traumatic AND/OR non-traumatic injury (x1.02) 0.819
Acute posthemorrhagic anemia (x1.02) 0.821
Pain provoked by trauma (x1.02) 0.823
Closed fracture of head of radius (x1.02) 0.825
acetaminophen (+0.00199) 0.827
Closed fracture of olecranon process of ulna (x1.02) 0.829
Traumatic shock (x1.02) 0.831
Wrist joint pain (x1.02) 0.832
Dependence on respirator (x1.02) 0.834
Delayed union of fracture (x1.02) 0.835
Closed fracture of shaft of humerus (x1.02) 0.837
Intracranial injury with loss of consciousness (x1.02) 0.838
Age (-0.819) 0.839
Closed fracture of lower end of humerus (x1.02) 0.841
Contusion of lung (x1.02) 0.842
Essential hypertension (/1.02) 0.843
Complication associated with orthopedic device (x1.02) 0.845
Closed supracondylar fracture of humerus (x1.02) 0.846
Motor vehicle accident victim (x1.02) 0.847
Open fracture of shaft of ulna (x1.02) 0.848
Closed fracture of proximal end of radius (x1.02) 0.849
Closed fracture of carpal bone (x1.02) 0.850
Injury of upper extremity (x1.02) 0.851
Persistent pain following procedure (x1.02) 0.852
gabapentin (+0.00149) 0.853
Fracture of distal end of humerus (x1.02) 0.854
Late effect of fracture of lower extremities (/1.02) 0.855
Closed fracture of shaft of femur (x1.02) 0.856
Acute non-suppurative otitis media - serous (/1.02) 0.857
Fever (x1.01) 0.858
Nervous system symptoms (x1.01) 0.859
Chronic serous otitis media (/1.01) 0.860
Heart rate (+0.567) 0.861
Wrist stiff (x1.01) 0.861
Elbow stiff (x1.01) 0.862
Pain in left arm (x1.01) 0.863
Chest injury (x1.01) 0.863
Closed fracture of radius AND ulna (x1.01) 0.864
Traumatic pneumothorax without open wound into thorax (x1.01) 0.865
Lower back injury (x1.01) 0.865
linagliptin (+0.00121) 0.866
Drug resistance (/1.01) 0.867
Closed fracture of one rib (/1.01) 0.867
enoxaparin (+0.00114) 0.868
Multiple pelvic fractures (x1.01) 0.869
Open fracture of forearm (x1.01) 0.869
Elbow joint effusion (x1.01) 0.870
Open fracture of shaft of radius (x1.01) 0.870
Closed fracture of thoracic vertebra without spinal cord injury (x1.01) 0.871
Injury of hand (x1.01) 0.871
Pneumothorax (x1.01) 0.872
Ideal body weight (-0.444) 0.872
Closed fracture of phalanx of finger (x1.01) 0.873
Pain due to internal prosthetic device (x1.01) 0.873
Injury involving multiple body regions (x1.01) 0.874
Closed fracture of lower end of radius AND ulna (x1.01) 0.874
Functional disorder of intestine (/1.01) 0.875
Respiratory rate (+0.127) 0.875
Lymphocytes [#/V] in Body fluid by Manual count (+0.489) 0.876
Acute bronchiolitis (x1.01) 0.876
Injury of trunk (x1.01) 0.877
Postoperative state (x1.01) 0.877
Electrocardiogram abnormal (/1.01) 0.878
Injury of shoulder and upper arm (x1.01) 0.878
Fracture of shaft of humerus (x1.01) 0.879
ibuprofen (+0.00103) 0.879
Respiratory failure (x1.01) 0.880
Carbon dioxide, total [mol/V] in SP (-0.0698) 0.880
Closed fracture of first lumbar vertebra (x1.01) 0.881
trichloroacetaldehyde (+0.00102) 0.881
Fracture of radius AND ulna (x1.01) 0.882
Neutrophils [#/V] in B by AC (+0.0712) 0.882
Injury of head (x1.01) 0.882
Closed fracture of shaft of radius and ulna (x1.01) 0.883
Subarachnoid hemorrhage due to traumatic injury (x1.01) 0.883
Closed Monteggia's fracture (x1.01) 0.884
Closed fracture of third lumbar vertebra (x1.01) 0.884
Multiple closed fractures of pelvis with disruption of pelvic circle (x1.01) 0.885
Elbow joint pain (x1.01) 0.885
Acute renal failure syndrome (/1.01) 0.885
Contusion of lung without open wound into thorax (x1.01) 0.886
Closed supracondylar fracture of femur (x1.01) 0.886
Hyperlipidemia (/1.01) 0.887
Coronary arteriosclerosis (/1.01) 0.887
Closed fracture of femur, distal end (x1.01) 0.887
−0.4 −0.2 0.0 0.2 0.4
Expressions

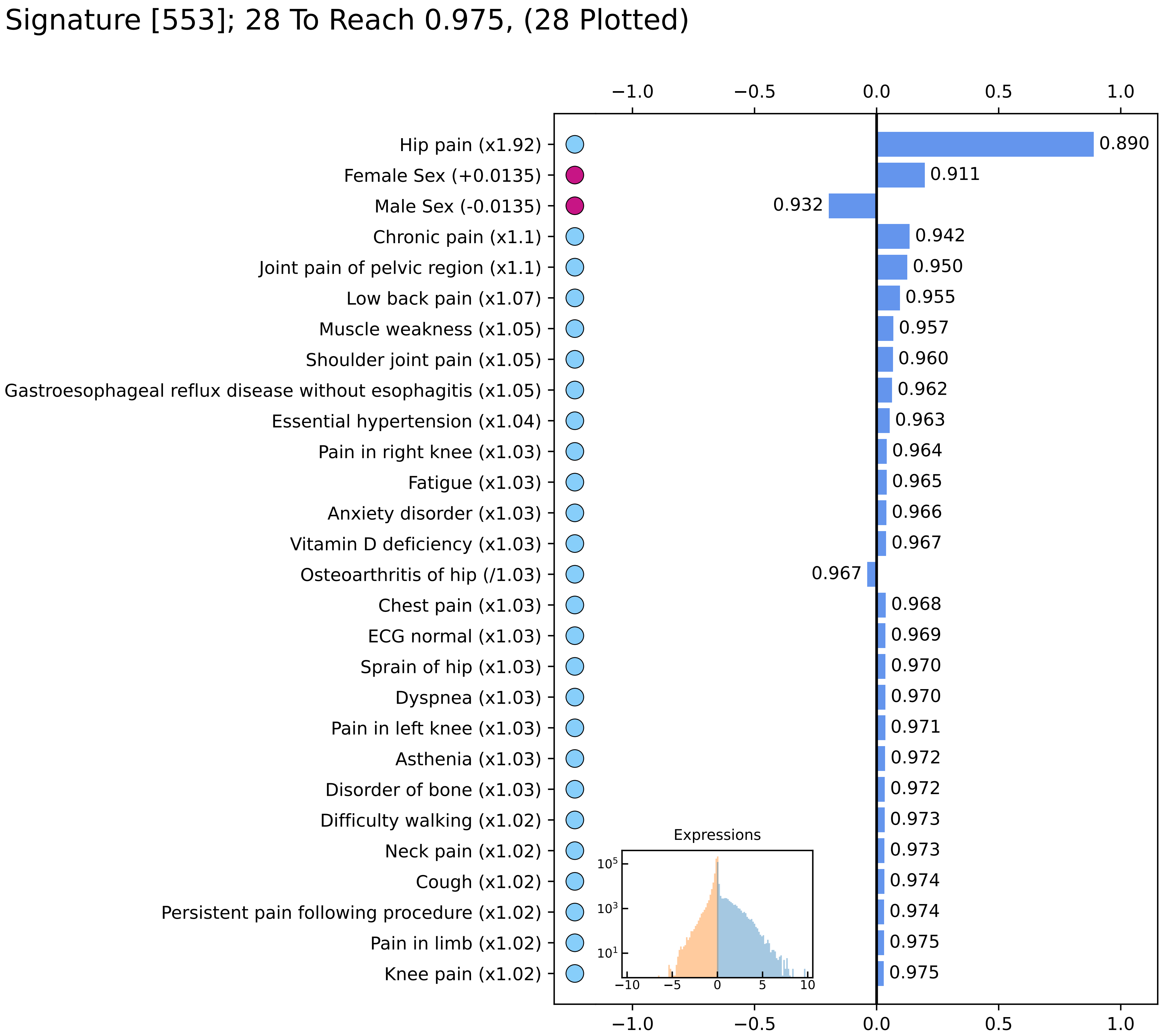
Signature [553]; 28 To Reach 0.975, (28 Plotted)
Hip pain (x1.92) 0.890
Female Sex (+0.0135) 0.911
Male Sex (-0.0135) 0.932
Chronic pain (x1.1) 0.942
Joint pain of pelvic region (x1.1) 0.950
Low back pain (x1.07) 0.955
Muscle weakness (x1.05) 0.957
Shoulder joint pain (x1.05) 0.960
Gastroesophageal reflux disease without esophagitis (x1.05) 0.962
Essential hypertension (x1.04) 0.963
Pain in right knee (x1.03) 0.964
Fatigue (x1.03) 0.965
Anxiety disorder (x1.03) 0.966
Vitamin D deficiency (x1.03) 0.967
Osteoarthritis of hip (/1.03) 0.967
Chest pain (x1.03) 0.968
ECG normal (x1.03) 0.969
Sprain of hip (x1.03) 0.970
Dyspnea (x1.03) 0.970
Pain in left knee (x1.03) 0.971
Asthenia (x1.03) 0.972
Disorder of bone (x1.03) 0.972
Difficulty walking (x1.02) 0.973
Neck pain (x1.02) 0.973
Cough (x1.02) 0.974
Persistent pain following procedure (x1.02) 0.974
Pain in limb (x1.02) 0.975
Knee pain (x1.02) 0.975
−1.0 −0.5 0.0 0.5 1.0
Expressions
10^5 10^3 10^1
−10 −5 0 5 10

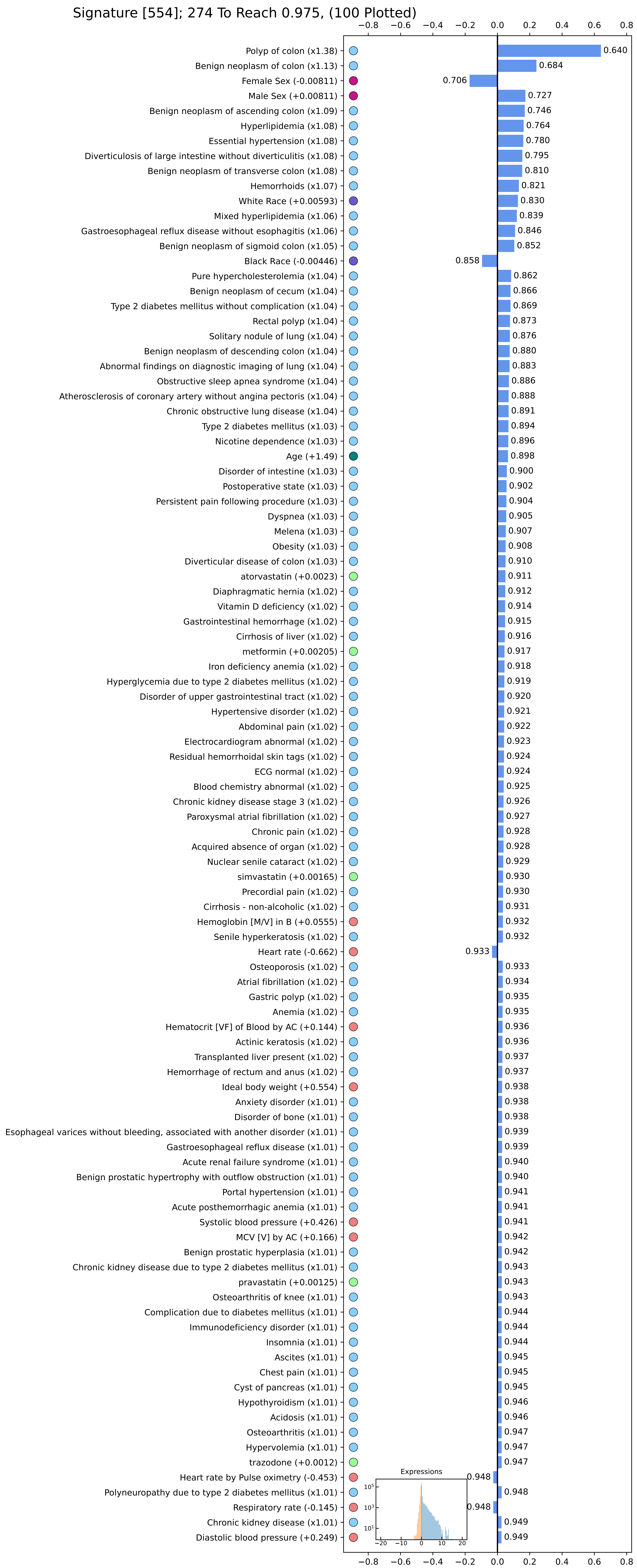

Signature [554]; 274 To Reach 0.975, (100 Plotted)
Polyp of colon (x1.38) 0.640
Benign neoplasm of colon (x1.13) 0.684
Female Sex (-0.00811) 0.706
Male Sex (+0.00811) 0.727
Benign neoplasm of ascending colon (x1.09) 0.746
Hyperlipidemia (x1.08) 0.764
Essential hypertension (x1.08) 0.780
Diverticulosis of large intestine without diverticulitis (x1.08) 0.795
Benign neoplasm of transverse colon (x1.08) 0.810
Hemorrhoids (x1.07) 0.821
White Race (+0.00593) 0.830
Mixed hyperlipidemia (x1.06) 0.839
Gastroesophageal reflux disease without esophagitis (x1.06) 0.846
Benign neoplasm of sigmoid colon (x1.05) 0.852
Black Race (-0.00446) 0.858
Pure hypercholesterolemia (x1.04) 0.862
Benign neoplasm of cecum (x1.04) 0.866
Type 2 diabetes mellitus without complication (x1.04) 0.869
Rectal polyp (x1.04) 0.873
Solitary nodule of lung (x1.04) 0.876
Benign neoplasm of descending colon (x1.04) 0.880
Abnormal findings on diagnostic imaging of lung (x1.04) 0.883
Obstructive sleep apnea syndrome (x1.04) 0.886
Atherosclerosis of coronary artery without angina pectoris (x1.04) 0.888
Chronic obstructive lung disease (x1.04) 0.891
Type 2 diabetes mellitus (x1.03) 0.894
Nicotine dependence (x1.03) 0.896
Age (+1.49) 0.898
Disorder of intestine (x1.03) 0.900
Postoperative state (x1.03) 0.902
Persistent pain following procedure (x1.03) 0.904
Dyspnea (x1.03) 0.905
Melena (x1.03) 0.907
Obesity (x1.03) 0.908
Diverticular disease of colon (x1.03) 0.910
atorvastatin (+0.0023) 0.911
Diaphragmatic hernia (x1.02) 0.912
Vitamin D deficiency (x1.02) 0.914
Gastrointestinal hemorrhage (x1.02) 0.915
Cirrhosis of liver (x1.02) 0.916
metformin (+0.00205) 0.917
Iron deficiency anemia (x1.02) 0.918
Hyperglycemia due to type 2 diabetes mellitus (x1.02) 0.919
Disorder of upper gastrointestinal tract (x1.02) 0.920
Hypertensive disorder (x1.02) 0.921
Abdominal pain (x1.02) 0.922
Electrocardiogram abnormal (x1.02) 0.923
Residual hemorrhoidal skin tags (x1.02) 0.924
ECG normal (x1.02) 0.924
Blood chemistry abnormal (x1.02) 0.925
Chronic kidney disease stage 3 (x1.02) 0.926
Paroxysmal atrial fibrillation (x1.02) 0.927
Chronic pain (x1.02) 0.928
Acquired absence of organ (x1.02) 0.928
Nuclear senile cataract (x1.02) 0.929
simvastatin (+0.00165) 0.930
Precordial pain (x1.02) 0.930
Cirrhosis - non-alcoholic (x1.02) 0.931
Hemoglobin [M/V] in B (+0.0555) 0.932
Senile hyperkeratosis (x1.02) 0.932
Heart rate (-0.662) 0.933
Osteoporosis (x1.02) 0.933
Atrial fibrillation (x1.02) 0.934
Gastric polyp (x1.02) 0.935
Anemia (x1.02) 0.935
Hematocrit [VF] of Blood by AC (+0.144) 0.936
Actinic keratosis (x1.02) 0.936
Transplanted liver present (x1.02) 0.937
Hemorrhage of rectum and anus (x1.02) 0.937
Ideal body weight (+0.554) 0.938
Anxiety disorder (x1.01) 0.938
Disorder of bone (x1.01) 0.938
Esophageal varices without bleeding, associated with another disorder (x1.01) 0.939
Gastroesophageal reflux disease (x1.01) 0.939
Acute renal failure syndrome (x1.01) 0.940
Benign prostatic hypertrophy with outflow obstruction (x1.01) 0.940
Portal hypertension (x1.01) 0.941
Acute posthemorrhagic anemia (x1.01) 0.941
Systolic blood pressure (+0.426) 0.941
MCV [V] by AC (+0.166) 0.942
Benign prostatic hyperplasia (x1.01) 0.942
Chronic kidney disease due to type 2 diabetes mellitus (x1.01) 0.943
pravastatin (+0.00125) 0.943
Osteoarthritis of knee (x1.01) 0.943
Complication due to diabetes mellitus (x1.01) 0.944
Immunodeficiency disorder (x1.01) 0.944
Insomnia (x1.01) 0.944
Ascites (x1.01) 0.945
Chest pain (x1.01) 0.945
Cyst of pancreas (x1.01) 0.945
Hypothyroidism (x1.01) 0.946
Acidosis (x1.01) 0.946
Osteoarthritis (x1.01) 0.947
Hypervolemia (x1.01) 0.947
trazodone (+0.0012) 0.947
Heart rate by Pulse oximetry (-0.453) 0.948
Polyneuropathy due to type 2 diabetes mellitus (x1.01) 0.948
Respiratory rate (-0.145) 0.948
Chronic kidney disease (x1.01) 0.949
Diastolic blood pressure (+0.249) 0.949
−0.8 −0.6 −0.4 −0.2 0.0 0.2 0.4 0.6 0.8
Expressions

Signature [555]; 2 To Reach 0.975, (10 Plotted)

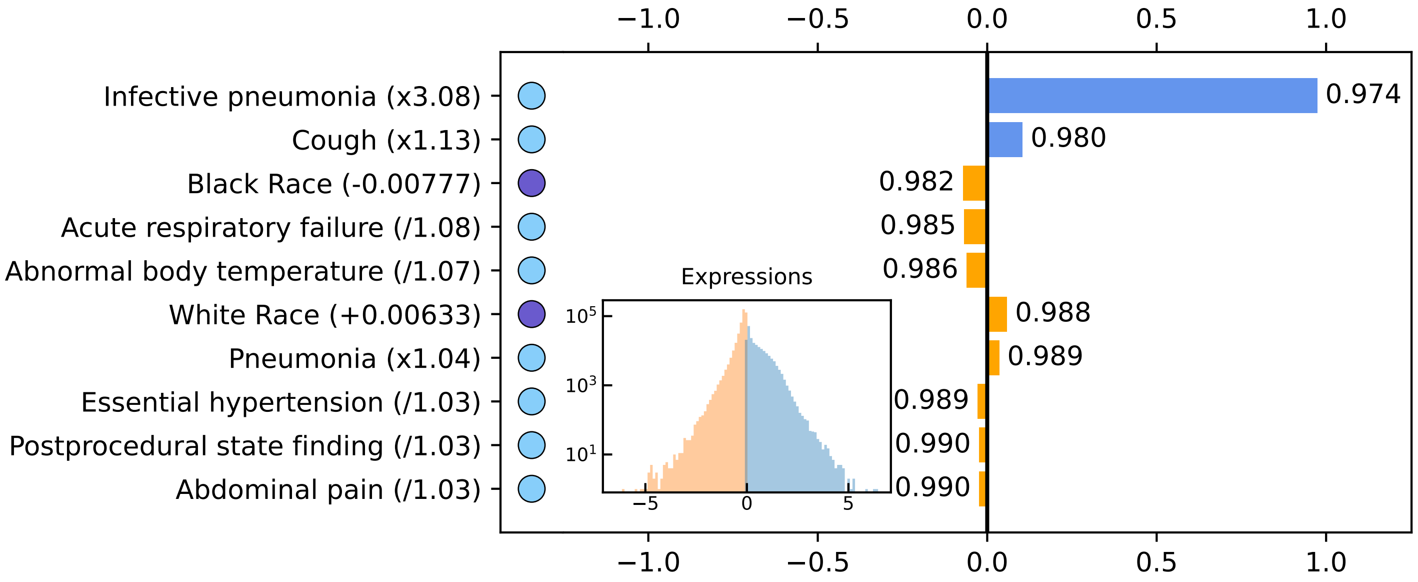

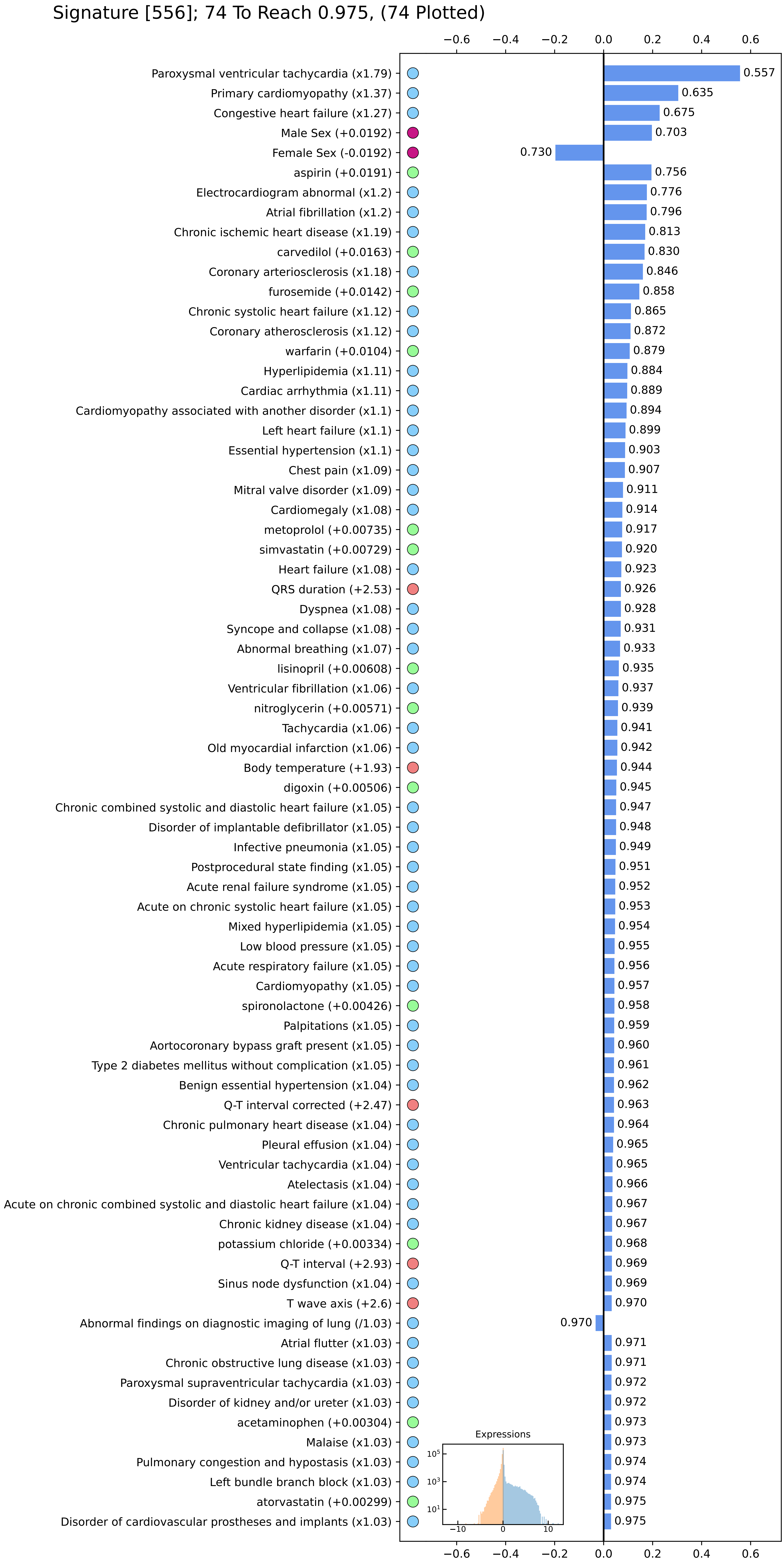

Signature [556]; 74 To Reach 0.975, (74 Plotted)
−0.6 −0.4 −0.2 0.0 0.2 0.4 0.6
Paroxysmal ventricular tachycardia (x1.79) 0.557
Primary cardiomyopathy (x1.37) 0.635
Congestive heart failure (x1.27) 0.675
Male Sex (+0.0192) 0.703
Female Sex (-0.0192) 0.730
aspirin (+0.0191) 0.756
Electrocardiogram abnormal (x1.2) 0.776
Atrial fibrillation (x1.2) 0.796
Chronic ischemic heart disease (x1.19) 0.813
carvedilol (+0.0163) 0.830
Coronary arteriosclerosis (x1.18) 0.846
furosemide (+0.0142) 0.858
Chronic systolic heart failure (x1.12) 0.865
Coronary atherosclerosis (x1.12) 0.872
warfarin (+0.0104) 0.879
Hyperlipidemia (x1.11) 0.884
Cardiac arrhythmia (x1.11) 0.889
Cardiomyopathy associated with another disorder (x1.1) 0.894
Left heart failure (x1.1) 0.899
Essential hypertension (x1.1) 0.903
Chest pain (x1.09) 0.907
Mitral valve disorder (x1.09) 0.911
Cardiomegaly (x1.08) 0.914
metoprolol (+0.00735) 0.917
simvastatin (+0.00729) 0.920
Heart failure (x1.08) 0.923
QRS duration (+2.53) 0.926
Dyspnea (x1.08) 0.928
Syncope and collapse (x1.08) 0.931
Abnormal breathing (x1.07) 0.933
lisinopril (+0.00608) 0.935
Ventricular fibrillation (x1.06) 0.937
nitroglycerin (+0.00571) 0.939
Tachycardia (x1.06) 0.941
Old myocardial infarction (x1.06) 0.942
Body temperature (+1.93) 0.944
digoxin (+0.00506) 0.945
Chronic combined systolic and diastolic heart failure (x1.05) 0.947
Disorder of implantable defibrillator (x1.05) 0.948
Infective pneumonia (x1.05) 0.949
Postprocedural state finding (x1.05) 0.951
Acute renal failure syndrome (x1.05) 0.952
Acute on chronic systolic heart failure (x1.05) 0.953
Mixed hyperlipidemia (x1.05) 0.954
Low blood pressure (x1.05) 0.955
Acute respiratory failure (x1.05) 0.956
Cardiomyopathy (x1.05) 0.957
spironolactone (+0.00426) 0.958
Palpitations (x1.05) 0.959
Aortocoronary bypass graft present (x1.05) 0.960
Type 2 diabetes mellitus without complication (x1.05) 0.961
Benign essential hypertension (x1.04) 0.962
Q-T interval corrected (+2.47) 0.963
Chronic pulmonary heart disease (x1.04) 0.964
Pleural effusion (x1.04) 0.965
Ventricular tachycardia (x1.04) 0.965
Atelectasis (x1.04) 0.966
Acute on chronic combined systolic and diastolic heart failure (x1.04) 0.967
Chronic kidney disease (x1.04) 0.967
potassium chloride (+0.00334) 0.968
Q-T interval (+2.93) 0.969
Sinus node dysfunction (x1.04) 0.969
T wave axis (+2.6) 0.970
Abnormal findings on diagnostic imaging of lung (/1.03) 0.970
Atrial flutter (x1.03) 0.971
Chronic obstructive lung disease (x1.03) 0.971
Paroxysmal supraventricular tachycardia (x1.03) 0.972
Disorder of kidney and/or ureter (x1.03) 0.972
acetaminophen (+0.00304) 0.973
Malaise (x1.03) 0.973
Pulmonary congestion and hypostasis (x1.03) 0.974
Left bundle branch block (x1.03) 0.974
atorvastatin (+0.00299) 0.975
Disorder of cardiovascular prostheses and implants (x1.03) 0.975
Expressions
−10 0 10

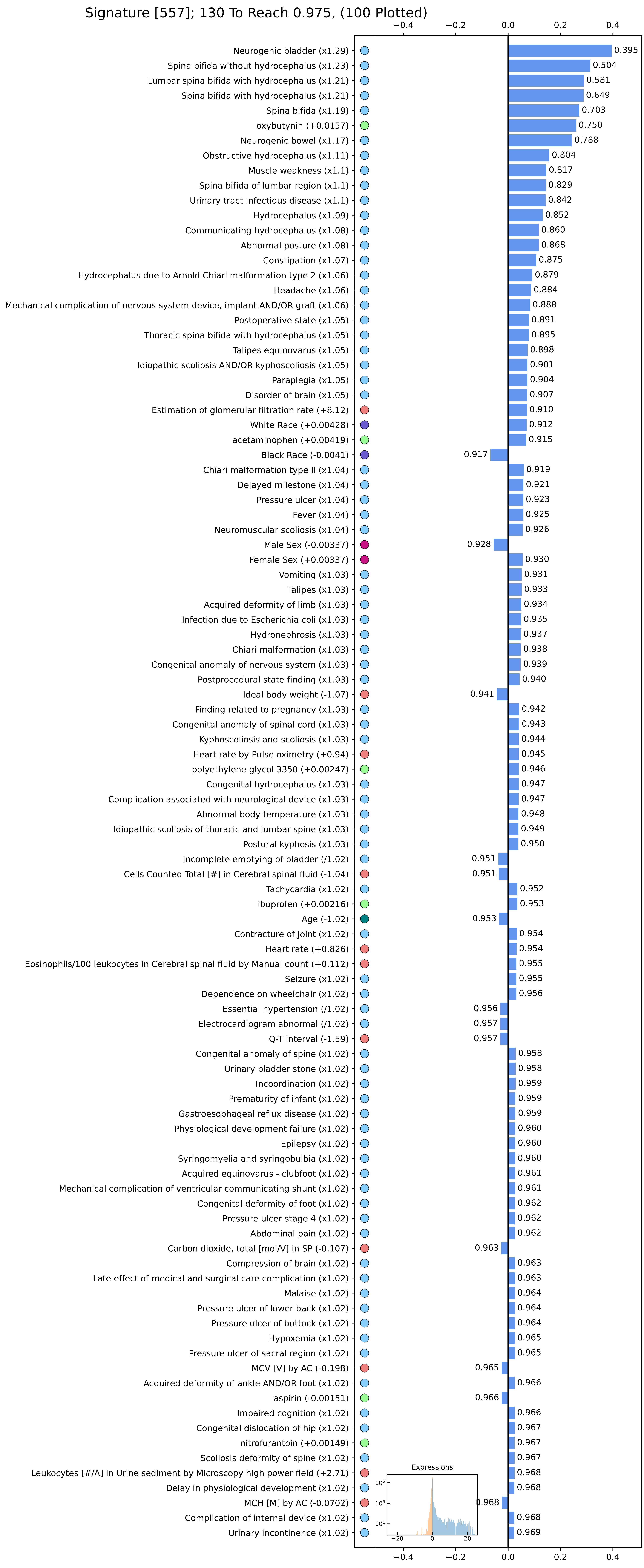
Signature [557]; 130 To Reach 0.975, (100 Plotted)
−0.4
−0.2
0.0
0.2
0.4
Neurogenic bladder (x1.29) 0.395
Spina bifida without hydrocephalus (x1.23) 0.504
Lumbar spina bifida with hydrocephalus (x1.21) 0.581
Spina bifida with hydrocephalus (x1.21) 0.649
Spina bifida (x1.19) 0.703
oxybutynin (+0.0157) 0.750
Neurogenic bowel (x1.17) 0.788
Obstructive hydrocephalus (x1.11) 0.804
Muscle weakness (x1.1) 0.817
Spina bifida of lumbar region (x1.1) 0.829
Urinary tract infectious disease (x1.1) 0.842
Hydrocephalus (x1.09) 0.852
Communicating hydrocephalus (x1.08) 0.860
Abnormal posture (x1.08) 0.868
Constipation (x1.07) 0.875
Hydrocephalus due to Arnold Chiari malformation type 2 (x1.06) 0.879
Headache (x1.06) 0.884
Mechanical complication of nervous system device, implant AND/OR graft (x1.06) 0.888
Postoperative state (x1.05) 0.891
Thoracic spina bifida with hydrocephalus (x1.05) 0.895
Talipes equinovarus (x1.05) 0.898
Idiopathic scoliosis AND/OR kyphoscoliosis (x1.05) 0.901
Paraplegia (x1.05) 0.904
Disorder of brain (x1.05) 0.907
Estimation of glomerular filtration rate (+8.12) 0.910
White Race (+0.00428) 0.912
acetaminophen (+0.00419) 0.915
Black Race (-0.0041) 0.917
Chiari malformation type II (x1.04) 0.919
Delayed milestone (x1.04) 0.921
Pressure ulcer (x1.04) 0.923
Fever (x1.04) 0.925
Neuromuscular scoliosis (x1.04) 0.926
Male Sex (-0.00337) 0.928
Female Sex (+0.00337) 0.930
Vomiting (x1.03) 0.931
Talipes (x1.03) 0.933
Acquired deformity of limb (x1.03) 0.934
Infection due to Escherichia coli (x1.03) 0.935
Hydronephrosis (x1.03) 0.937
Chiari malformation (x1.03) 0.938
Congenital anomaly of nervous system (x1.03) 0.939
Postprocedural state finding (x1.03) 0.940
Ideal body weight (-1.07) 0.941
Finding related to pregnancy (x1.03) 0.942
Congenital anomaly of spinal cord (x1.03) 0.943
Kyphoscoliosis and scoliosis (x1.03) 0.944
Heart rate by Pulse oximetry (+0.94) 0.945
polyethylene glycol 3350 (+0.00247) 0.946
Congenital hydrocephalus (x1.03) 0.947
Complication associated with neurological device (x1.03) 0.947
Abnormal body temperature (x1.03) 0.948
Idiopathic scoliosis of thoracic and lumbar spine (x1.03) 0.949
Postural kyphosis (x1.03) 0.950
Incomplete emptying of bladder (/1.02) 0.951
Cells Counted Total [#] in Cerebral spinal fluid (-1.04) 0.951
Tachycardia (x1.02) 0.952
ibuprofen (+0.00216) 0.953
Age (-1.02) 0.953
Contracture of joint (x1.02) 0.954
Heart rate (+0.826) 0.954
Eosinophils/100 leukocytes in Cerebral spinal fluid by Manual count (+0.112) 0.955
Seizure (x1.02) 0.955
Dependence on wheelchair (x1.02) 0.956
Essential hypertension (/1.02) 0.956
Electrocardiogram abnormal (/1.02) 0.957
Q-T interval (-1.59) 0.957
Congenital anomaly of spine (x1.02) 0.958
Urinary bladder stone (x1.02) 0.958
Incoordination (x1.02) 0.959
Prematurity of infant (x1.02) 0.959
Gastroesophageal reflux disease (x1.02) 0.959
Physiological development failure (x1.02) 0.960
Epilepsy (x1.02) 0.960
Syringomyelia and syringobulbia (x1.02) 0.960
Acquired equinovarus - clubfoot (x1.02) 0.961
Mechanical complication of ventricular communicating shunt (x1.02) 0.961
Congenital deformity of foot (x1.02) 0.962
Pressure ulcer stage 4 (x1.02) 0.962
Abdominal pain (x1.02) 0.962
Carbon dioxide, total [mol/V] in SP (-0.107) 0.963
Compression of brain (x1.02) 0.963
Late effect of medical and surgical care complication (x1.02) 0.963
Malaise (x1.02) 0.964
Pressure ulcer of lower back (x1.02) 0.964
Pressure ulcer of buttock (x1.02) 0.964
Hypoxemia (x1.02) 0.965
Pressure ulcer of sacral region (x1.02) 0.965
MCV [V] by AC (-0.198) 0.965
Acquired deformity of ankle AND/OR foot (x1.02) 0.966
aspirin (-0.00151) 0.966
Impaired cognition (x1.02) 0.966
Congenital dislocation of hip (x1.02) 0.967
nitrofurantoin (+0.00149) 0.967
Scoliosis deformity of spine (x1.02) 0.967
Leukocytes [#/A] in Urine sediment by Microscopy high power field (+2.71) 0.968
Delay in physiological development (x1.02) 0.968
MCH [M] by AC (-0.0702) 0.968
Complication of internal device (x1.02) 0.968
Urinary incontinence (x1.02) 0.969
Expressions
−20
0
20

Signature [558]; 10 To Reach 0.975, (10 Plotted)

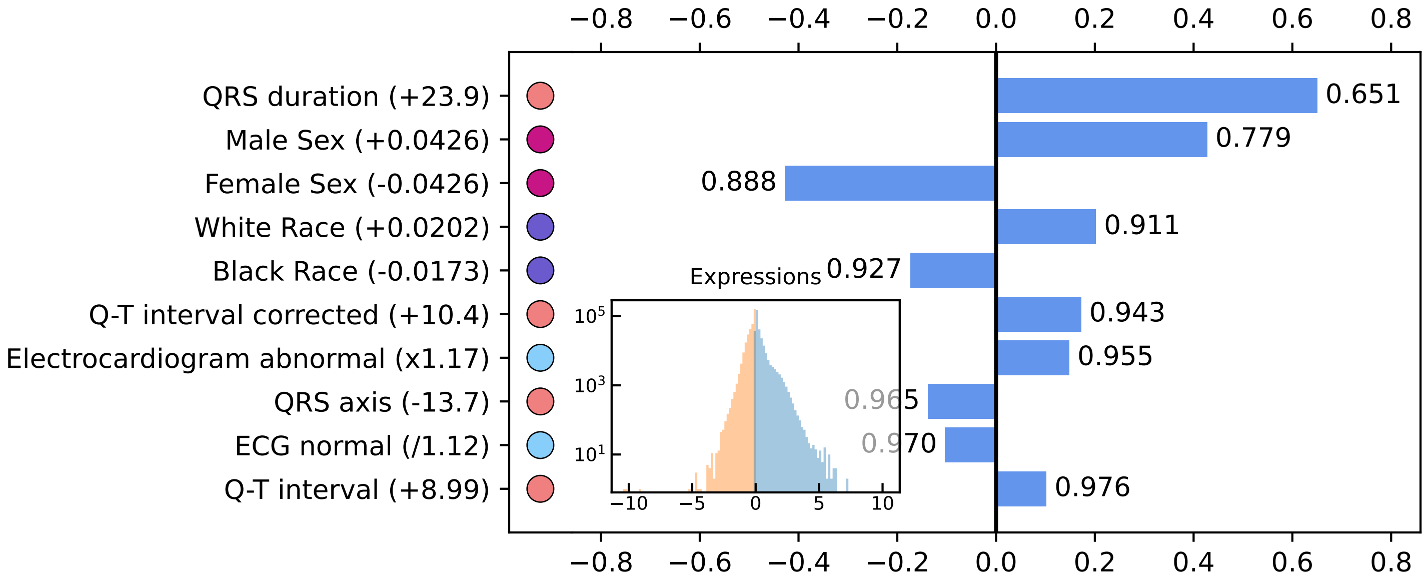

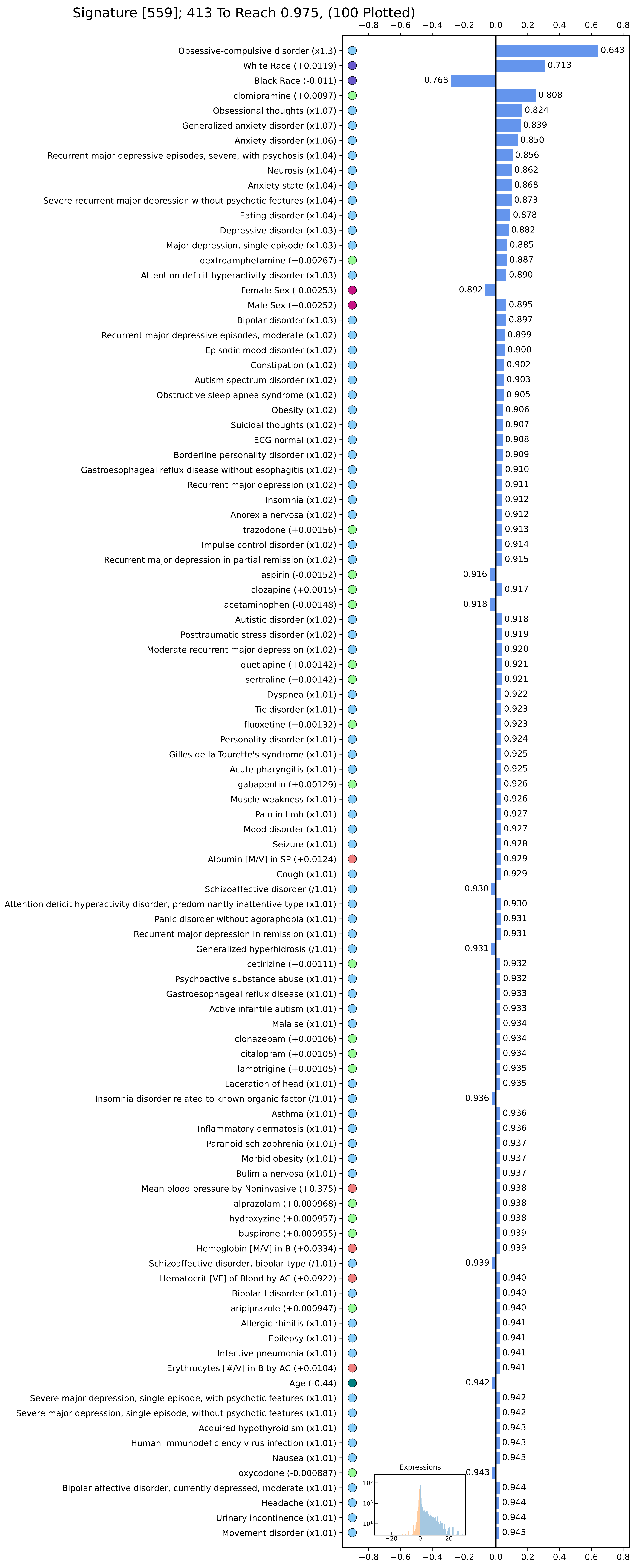
Signature [559]; 413 To Reach 0.975, (100 Plotted)
Obsessive-compulsive disorder (x1.3)
White Race (+0.0119)
Black Race (-0.011)
clomipramine (+0.0097)
Obsessional thoughts (x1.07)
Generalized anxiety disorder (x1.07)
Anxiety disorder (x1.06)
Recurrent major depressive episodes, severe, with psychosis (x1.04)
Neurosis (x1.04)
Anxiety state (x1.04)
Severe recurrent major depression without psychotic features (x1.04)
Eating disorder (x1.04)
Depressive disorder (x1.03)
Major depression, single episode (x1.03)
dextroamphetamine (+0.00267)
Attention deficit hyperactivity disorder (x1.03)
Female Sex (-0.00253)
Male Sex (+0.00252)
Bipolar disorder (x1.03)
Recurrent major depressive episodes, moderate (x1.02)
Episodic mood disorder (x1.02)
Constipation (x1.02)
Autism spectrum disorder (x1.02)
Obstructive sleep apnea syndrome (x1.02)
Obesity (x1.02)
Suicidal thoughts (x1.02)
ECG normal (x1.02)
Borderline personality disorder (x1.02)
Gastroesophageal reflux disease without esophagitis (x1.02)
Recurrent major depression (x1.02)
Insomnia (x1.02)
Anorexia nervosa (x1.02)
trazodone (+0.00156)
Impulse control disorder (x1.02)
Recurrent major depression in partial remission (x1.02)
aspirin (-0.00152)
clozapine (+0.0015)
acetaminophen (-0.00148)
Autistic disorder (x1.02)
Posttraumatic stress disorder (x1.02)
Moderate recurrent major depression (x1.02)
quetiapine (+0.00142)
sertraline (+0.00142)
Dyspnea (x1.01)
Tic disorder (x1.01)
fluoxetine (+0.00132)
Personality disorder (x1.01)
Gilles de la Tourette's syndrome (x1.01)
Acute pharyngitis (x1.01)
gabapentin (+0.00129)
Muscle weakness (x1.01)
Pain in limb (x1.01)
Mood disorder (x1.01)
Seizure (x1.01)
Albumin [M/V] in SP (+0.0124)
Cough (x1.01)
Schizoaffective disorder (/1.01)
Attention deficit hyperactivity disorder, predominantly inattentive type (x1.01)
Panic disorder without agoraphobia (x1.01)
Recurrent major depression in remission (x1.01)
Generalized hyperhidrosis (/1.01)
cetirizine (+0.00111)
Psychoactive substance abuse (x1.01)
Gastroesophageal reflux disease (x1.01)
Active infantile autism (x1.01)
Malaise (x1.01)
clonazepam (+0.00106)
citalopram (+0.00105)
lamotrigine (+0.00105)
Laceration of head (x1.01)
Insomnia disorder related to known organic factor (/1.01)
Asthma (x1.01)
Inflammatory dermatosis (x1.01)
Paranoid schizophrenia (x1.01)
Morbid obesity (x1.01)
Bulimia nervosa (x1.01)
Mean blood pressure by Noninvasive (+0.375)
alprazolam (+0.000968)
hydroxyzine (+0.000957)
buspirone (+0.000955)
Hemoglobin [M/V] in B (+0.0334)
Schizoaffective disorder, bipolar type (/1.01)
Hematocrit [VF] of Blood by AC (+0.0922)
Bipolar I disorder (x1.01)
aripiprazole (+0.000947)
Allergic rhinitis (x1.01)
Epilepsy (x1.01)
Infective pneumonia (x1.01)
Erythrocytes [#/V] in B by AC (+0.0104)
Age (-0.44)
Severe major depression, single episode, with psychotic features (x1.01)
Severe major depression, single episode, without psychotic features (x1.01)
Acquired hypothyroidism (x1.01)
Human immunodeficiency virus infection (x1.01)
Nausea (x1.01)
oxycodone (-0.000887)
Bipolar affective disorder, currently depressed, moderate (x1.01)
Headache (x1.01)
Urinary incontinence (x1.01)
Movement disorder (x1.01)
0.643
0.713
0.768
0.808
0.824
0.839
0.850
0.856
0.862
0.868
0.873
0.878
0.882
0.885
0.887
0.890
0.892
0.895
0.897
0.899
0.900
0.902
0.903
0.905
0.906
0.907
0.908
0.909
0.910
0.911
0.912
0.912
0.913
0.914
0.915
0.916
0.917
0.918
0.918
0.919
0.920
0.921
0.921
0.922
0.923
0.923
0.924
0.925
0.925
0.926
0.926
0.927
0.927
0.928
0.929
0.929
0.930
0.930
0.931
0.931
0.931
0.932
0.932
0.933
0.933
0.934
0.934
0.934
0.935
0.935
0.936
0.936
0.936
0.937
0.937
0.937
0.938
0.938
0.938
0.939
0.939
0.939
0.940
0.940
0.940
0.941
0.941
0.941
0.941
0.942
0.942
0.942
0.943
0.943
0.943
0.943
0.944
0.944
0.944
0.945
−0.8 −0.6 −0.4 −0.2 0.0 0.2 0.4 0.6 0.8
Expressions
−20 0 20

Signature [560]; 240 To Reach 0.975, (100 Plotted)

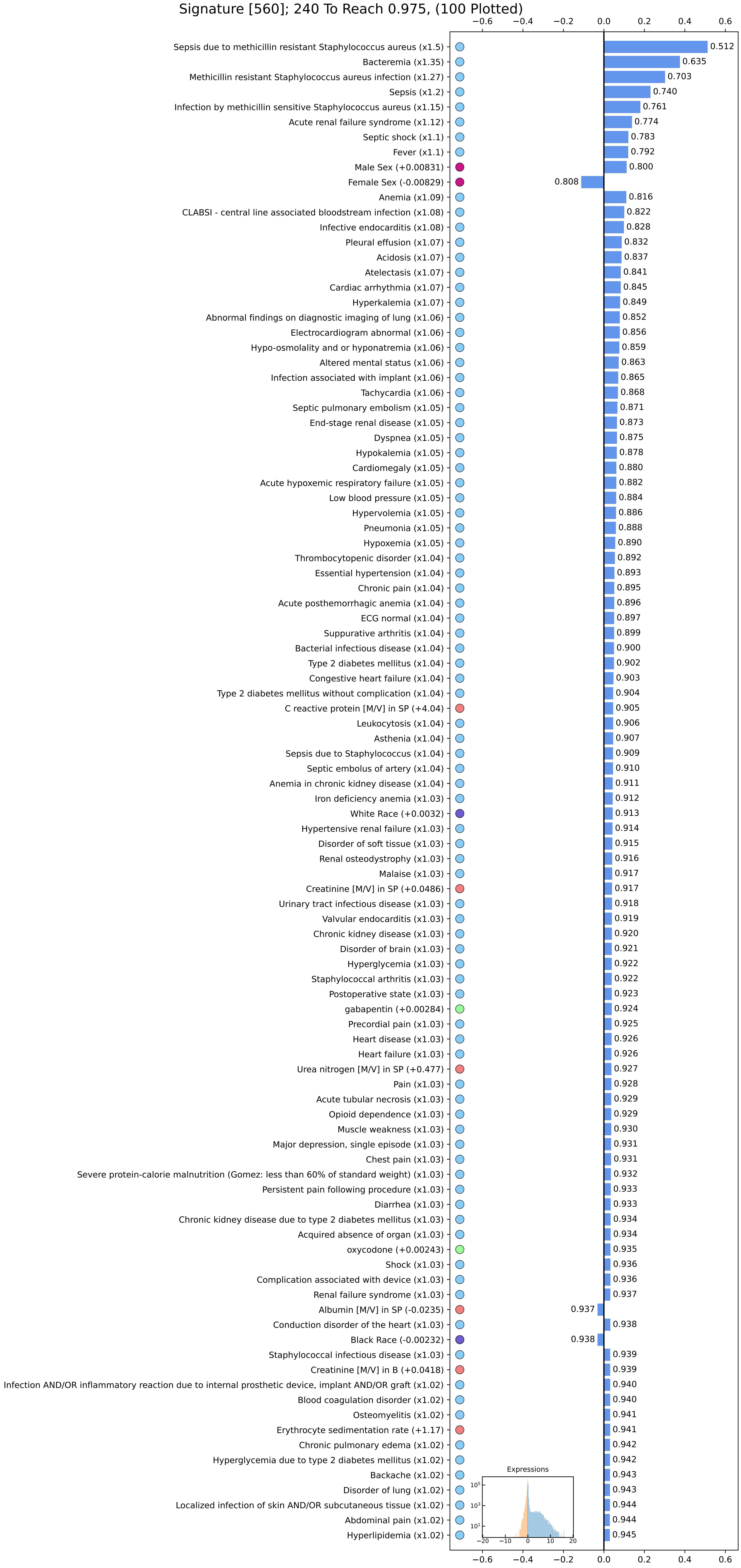

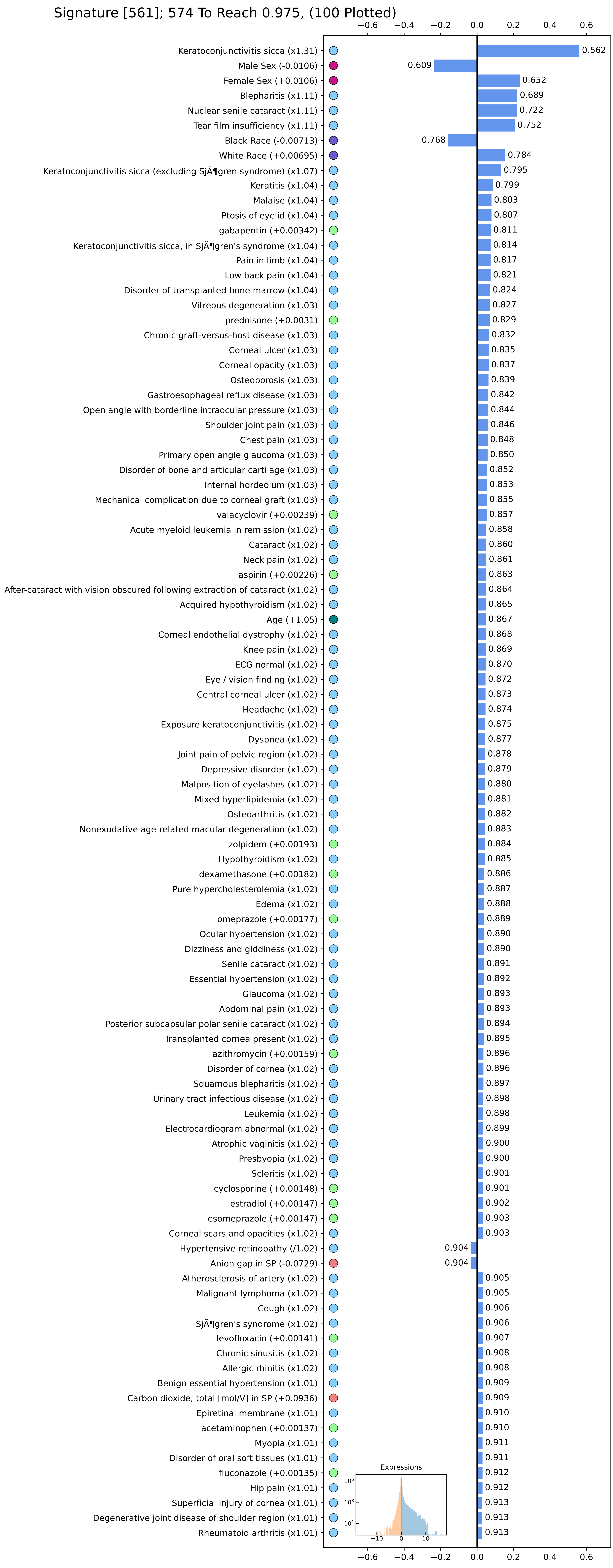
Signature [561]; 574 To Reach 0.975, (100 Plotted)
Keratoconjunctivitis sicca (x1.31) 0.562
Male Sex (-0.0106) 0.609
Female Sex (+0.0106) 0.652
Blepharitis (x1.11) 0.689
Nuclear senile cataract (x1.11) 0.722
Tear film insufficiency (x1.11) 0.752
Black Race (-0.00713) 0.768
White Race (+0.00695) 0.784
Keratoconjunctivitis sicca (excluding SjÃ¶gren syndrome) (x1.07) 0.795
Keratitis (x1.04) 0.799
Malaise (x1.04) 0.803
Ptosis of eyelid (x1.04) 0.807
gabapentin (+0.00342) 0.811
Keratoconjunctivitis sicca, in SjÃ¶gren's syndrome (x1.04) 0.814
Pain in limb (x1.04) 0.817
Low back pain (x1.04) 0.821
Disorder of transplanted bone marrow (x1.04) 0.824
Vitreous degeneration (x1.03) 0.827
prednisone (+0.0031) 0.829
Chronic graft-versus-host disease (x1.03) 0.832
Corneal ulcer (x1.03) 0.835
Corneal opacity (x1.03) 0.837
Osteoporosis (x1.03) 0.839
Gastroesophageal reflux disease (x1.03) 0.842
Open angle with borderline intraocular pressure (x1.03) 0.844
Shoulder joint pain (x1.03) 0.846
Chest pain (x1.03) 0.848
Primary open angle glaucoma (x1.03) 0.850
Disorder of bone and articular cartilage (x1.03) 0.852
Internal hordeolum (x1.03) 0.853
Mechanical complication due to corneal graft (x1.03) 0.855
valacyclovir (+0.00239) 0.857
Acute myeloid leukemia in remission (x1.02) 0.858
Cataract (x1.02) 0.860
Neck pain (x1.02) 0.861
aspirin (+0.00226) 0.863
After-cataract with vision obscured following extraction of cataract (x1.02) 0.864
Acquired hypothyroidism (x1.02) 0.865
Age (+1.05) 0.867
Corneal endothelial dystrophy (x1.02) 0.868
Knee pain (x1.02) 0.869
ECG normal (x1.02) 0.870
Eye / vision finding (x1.02) 0.872
Central corneal ulcer (x1.02) 0.873
Headache (x1.02) 0.874
Exposure keratoconjunctivitis (x1.02) 0.875
Dyspnea (x1.02) 0.877
Joint pain of pelvic region (x1.02) 0.878
Depressive disorder (x1.02) 0.879
Malposition of eyelashes (x1.02) 0.880
Mixed hyperlipidemia (x1.02) 0.881
Osteoarthritis (x1.02) 0.882
Nonexudative age-related macular degeneration (x1.02) 0.883
zolpidem (+0.00193) 0.884
Hypothyroidism (x1.02) 0.885
dexamethasone (+0.00182) 0.886
Pure hypercholesterolemia (x1.02) 0.887
Edema (x1.02) 0.888
omeprazole (+0.00177) 0.889
Ocular hypertension (x1.02) 0.890
Dizziness and giddiness (x1.02) 0.890
Senile cataract (x1.02) 0.891
Essential hypertension (x1.02) 0.892
Glaucoma (x1.02) 0.893
Abdominal pain (x1.02) 0.893
Posterior subcapsular polar senile cataract (x1.02) 0.894
Transplanted cornea present (x1.02) 0.895
azithromycin (+0.00159) 0.896
Disorder of cornea (x1.02) 0.896
Squamous blepharitis (x1.02) 0.897
Urinary tract infectious disease (x1.02) 0.898
Leukemia (x1.02) 0.898
Electrocardiogram abnormal (x1.02) 0.899
Atrophic vaginitis (x1.02) 0.900
Presbyopia (x1.02) 0.900
Scleritis (x1.02) 0.901
cyclosporine (+0.00148) 0.901
estradiol (+0.00147) 0.902
esomeprazole (+0.00147) 0.903
Corneal scars and opacities (x1.02) 0.903
Hypertensive retinopathy (/1.02) 0.904
Anion gap in SP (-0.0729) 0.904
Atherosclerosis of artery (x1.02) 0.905
Malignant lymphoma (x1.02) 0.905
Cough (x1.02) 0.906
SjÃ¶gren's syndrome (x1.02) 0.906
levofloxacin (+0.00141) 0.907
Chronic sinusitis (x1.02) 0.908
Allergic rhinitis (x1.02) 0.908
Benign essential hypertension (x1.01) 0.909
Carbon dioxide, total [mol/V] in SP (+0.0936) 0.909
Epiretinal membrane (x1.01) 0.910
acetaminophen (+0.00137) 0.910
Myopia (x1.01) 0.911
Disorder of oral soft tissues (x1.01) 0.911
fluconazole (+0.00135) 0.912
Hip pain (x1.01) 0.912
Superficial injury of cornea (x1.01) 0.913
Degenerative joint disease of shoulder region (x1.01) 0.913
Rheumatoid arthritis (x1.01) 0.913
−0.6 −0.4 −0.2 0.0 0.2 0.4 0.6
Expressions
−10 0 10

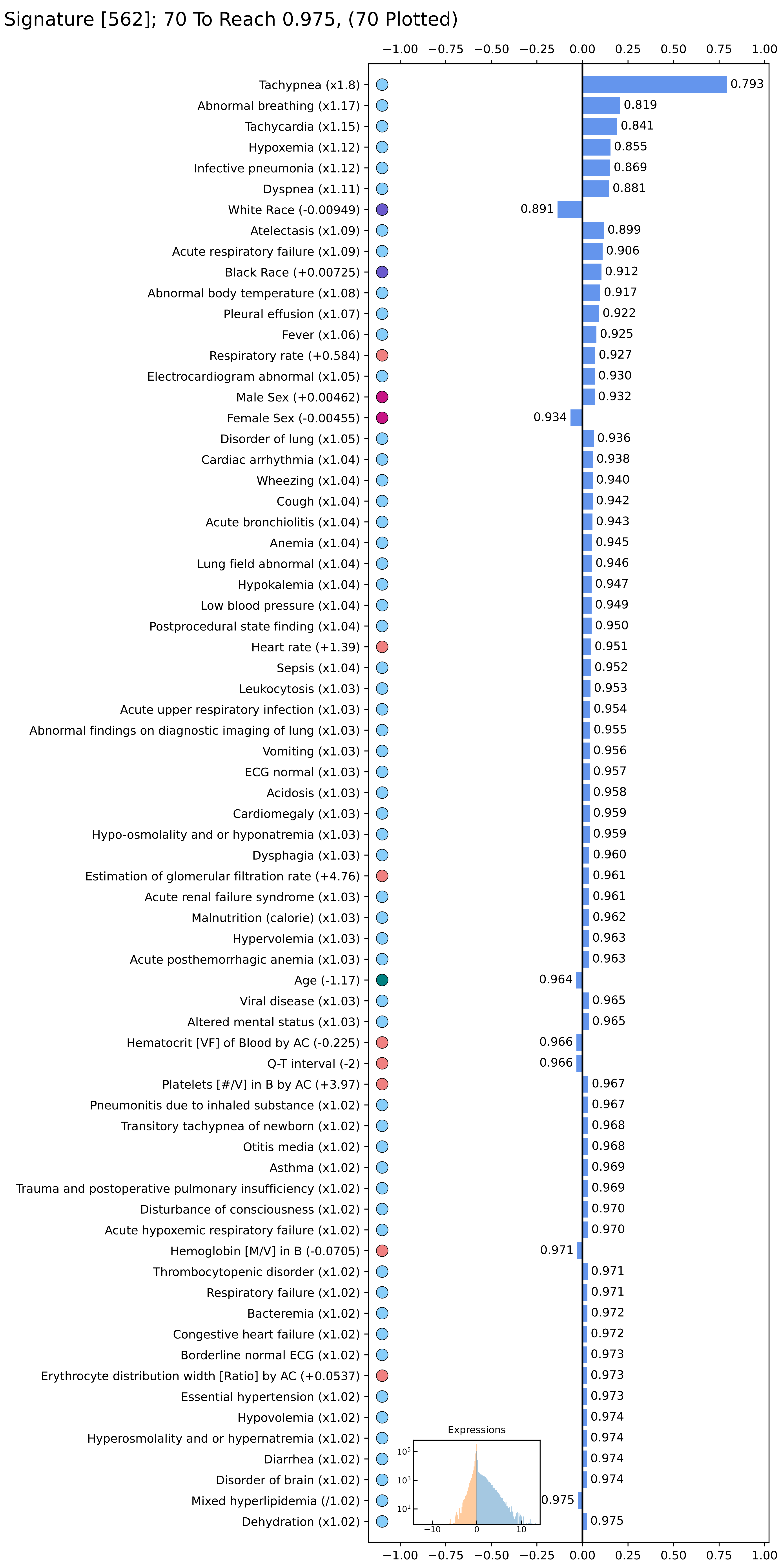

Signature [562]; 70 To Reach 0.975, (70 Plotted)
−1.00 −0.75 −0.50 −0.25 0.00 0.25 0.50 0.75 1.00
Tachypnea (x1.8) 0.793
Abnormal breathing (x1.17) 0.819
Tachycardia (x1.15) 0.841
Hypoxemia (x1.12) 0.855
Infective pneumonia (x1.12) 0.869
Dyspnea (x1.11) 0.881
White Race (-0.00949) 0.891
Atelectasis (x1.09) 0.899
Acute respiratory failure (x1.09) 0.906
Black Race (+0.00725) 0.912
Abnormal body temperature (x1.08) 0.917
Pleural effusion (x1.07) 0.922
Fever (x1.06) 0.925
Respiratory rate (+0.584) 0.927
Electrocardiogram abnormal (x1.05) 0.930
Male Sex (+0.00462) 0.932
Female Sex (-0.00455) 0.934
Disorder of lung (x1.05) 0.936
Cardiac arrhythmia (x1.04) 0.938
Wheezing (x1.04) 0.940
Cough (x1.04) 0.942
Acute bronchiolitis (x1.04) 0.943
Anemia (x1.04) 0.945
Lung field abnormal (x1.04) 0.946
Hypokalemia (x1.04) 0.947
Low blood pressure (x1.04) 0.949
Postprocedural state finding (x1.04) 0.950
Heart rate (+1.39) 0.951
Sepsis (x1.04) 0.952
Leukocytosis (x1.03) 0.953
Acute upper respiratory infection (x1.03) 0.954
Abnormal findings on diagnostic imaging of lung (x1.03) 0.955
Vomiting (x1.03) 0.956
ECG normal (x1.03) 0.957
Acidosis (x1.03) 0.958
Cardiomegaly (x1.03) 0.959
Hypo-osmolality and or hyponatremia (x1.03) 0.959
Dysphagia (x1.03) 0.960
Estimation of glomerular filtration rate (+4.76) 0.961
Acute renal failure syndrome (x1.03) 0.961
Malnutrition (calorie) (x1.03) 0.962
Hypervolemia (x1.03) 0.963
Acute posthemorrhagic anemia (x1.03) 0.963
Age (-1.17) 0.964
Viral disease (x1.03) 0.965
Altered mental status (x1.03) 0.965
Hematocrit [VF] of Blood by AC (-0.225) 0.966
Q-T interval (-2) 0.966
Platelets [#/V] in B by AC (+3.97) 0.967
Pneumonitis due to inhaled substance (x1.02) 0.967
Transitory tachypnea of newborn (x1.02) 0.968
Otitis media (x1.02) 0.968
Asthma (x1.02) 0.969
Trauma and postoperative pulmonary insufficiency (x1.02) 0.969
Disturbance of consciousness (x1.02) 0.970
Acute hypoxemic respiratory failure (x1.02) 0.970
Hemoglobin [M/V] in B (-0.0705) 0.971
Thrombocytopenic disorder (x1.02) 0.971
Respiratory failure (x1.02) 0.971
Bacteremia (x1.02) 0.972
Congestive heart failure (x1.02) 0.972
Borderline normal ECG (x1.02) 0.973
Erythrocyte distribution width [Ratio] by AC (+0.0537) 0.973
Essential hypertension (x1.02) 0.973
Hypovolemia (x1.02) 0.974
Hyperosmolality and or hypernatremia (x1.02) 0.974
Diarrhea (x1.02) 0.974
Disorder of brain (x1.02) 0.974
Mixed hyperlipidemia (/1.02) 0.975
Dehydration (x1.02) 0.975
Expressions

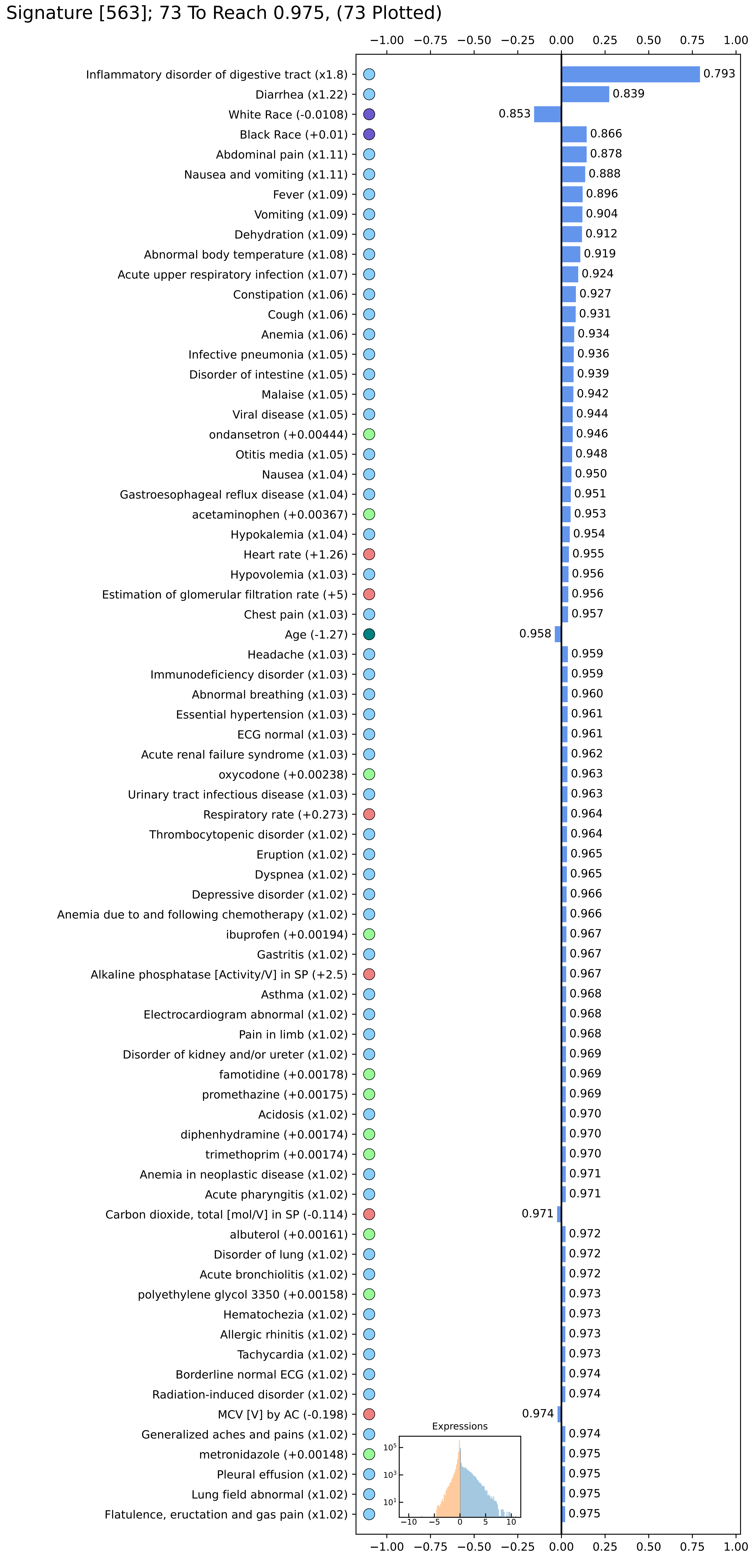

Signature [563]; 73 To Reach 0.975, (73 Plotted)
Inflammatory disorder of digestive tract (x1.8) 0.793
Diarrhea (x1.22) 0.839
White Race (-0.0108) 0.853
Black Race (+0.01) 0.866
Abdominal pain (x1.11) 0.878
Nausea and vomiting (x1.11) 0.888
Fever (x1.09) 0.896
Vomiting (x1.09) 0.904
Dehydration (x1.09) 0.912
Abnormal body temperature (x1.08) 0.919
Acute upper respiratory infection (x1.07) 0.924
Constipation (x1.06) 0.927
Cough (x1.06) 0.931
Anemia (x1.06) 0.934
Infective pneumonia (x1.05) 0.936
Disorder of intestine (x1.05) 0.939
Malaise (x1.05) 0.942
Viral disease (x1.05) 0.944
ondansetron (+0.00444) 0.946
Otitis media (x1.05) 0.948
Nausea (x1.04) 0.950
Gastroesophageal reflux disease (x1.04) 0.951
acetaminophen (+0.00367) 0.953
Hypokalemia (x1.04) 0.954
Heart rate (+1.26) 0.955
Hypovolemia (x1.03) 0.956
Estimation of glomerular filtration rate (+5) 0.956
Chest pain (x1.03) 0.957
Age (-1.27) 0.958
Headache (x1.03) 0.959
Immunodeficiency disorder (x1.03) 0.959
Abnormal breathing (x1.03) 0.960
Essential hypertension (x1.03) 0.961
ECG normal (x1.03) 0.961
Acute renal failure syndrome (x1.03) 0.962
oxycodone (+0.00238) 0.963
Urinary tract infectious disease (x1.03) 0.963
Respiratory rate (+0.273) 0.964
Thrombocytopenic disorder (x1.02) 0.964
Eruption (x1.02) 0.965
Dyspnea (x1.02) 0.965
Depressive disorder (x1.02) 0.966
Anemia due to and following chemotherapy (x1.02) 0.966
ibuprofen (+0.00194) 0.967
Gastritis (x1.02) 0.967
Alkaline phosphatase [Activity/V] in SP (+2.5) 0.967
Asthma (x1.02) 0.968
Electrocardiogram abnormal (x1.02) 0.968
Pain in limb (x1.02) 0.968
Disorder of kidney and/or ureter (x1.02) 0.969
famotidine (+0.00178) 0.969
promethazine (+0.00175) 0.969
Acidosis (x1.02) 0.970
diphenhydramine (+0.00174) 0.970
trimethoprim (+0.00174) 0.970
Anemia in neoplastic disease (x1.02) 0.971
Acute pharyngitis (x1.02) 0.971
Carbon dioxide, total [mol/V] in SP (-0.114) 0.971
albuterol (+0.00161) 0.972
Disorder of lung (x1.02) 0.972
Acute bronchiolitis (x1.02) 0.972
polyethylene glycol 3350 (+0.00158) 0.973
Hematochezia (x1.02) 0.973
Allergic rhinitis (x1.02) 0.973
Tachycardia (x1.02) 0.973
Borderline normal ECG (x1.02) 0.974
Radiation-induced disorder (x1.02) 0.974
MCV [V] by AC (-0.198) 0.974
Generalized aches and pains (x1.02) 0.974
metronidazole (+0.00148) 0.975
Pleural effusion (x1.02) 0.975
Lung field abnormal (x1.02) 0.975
Flatulence, eructation and gas pain (x1.02) 0.975
−1.00 −0.75 −0.50 −0.25 0.00 0.25 0.50 0.75 1.00
Expressions

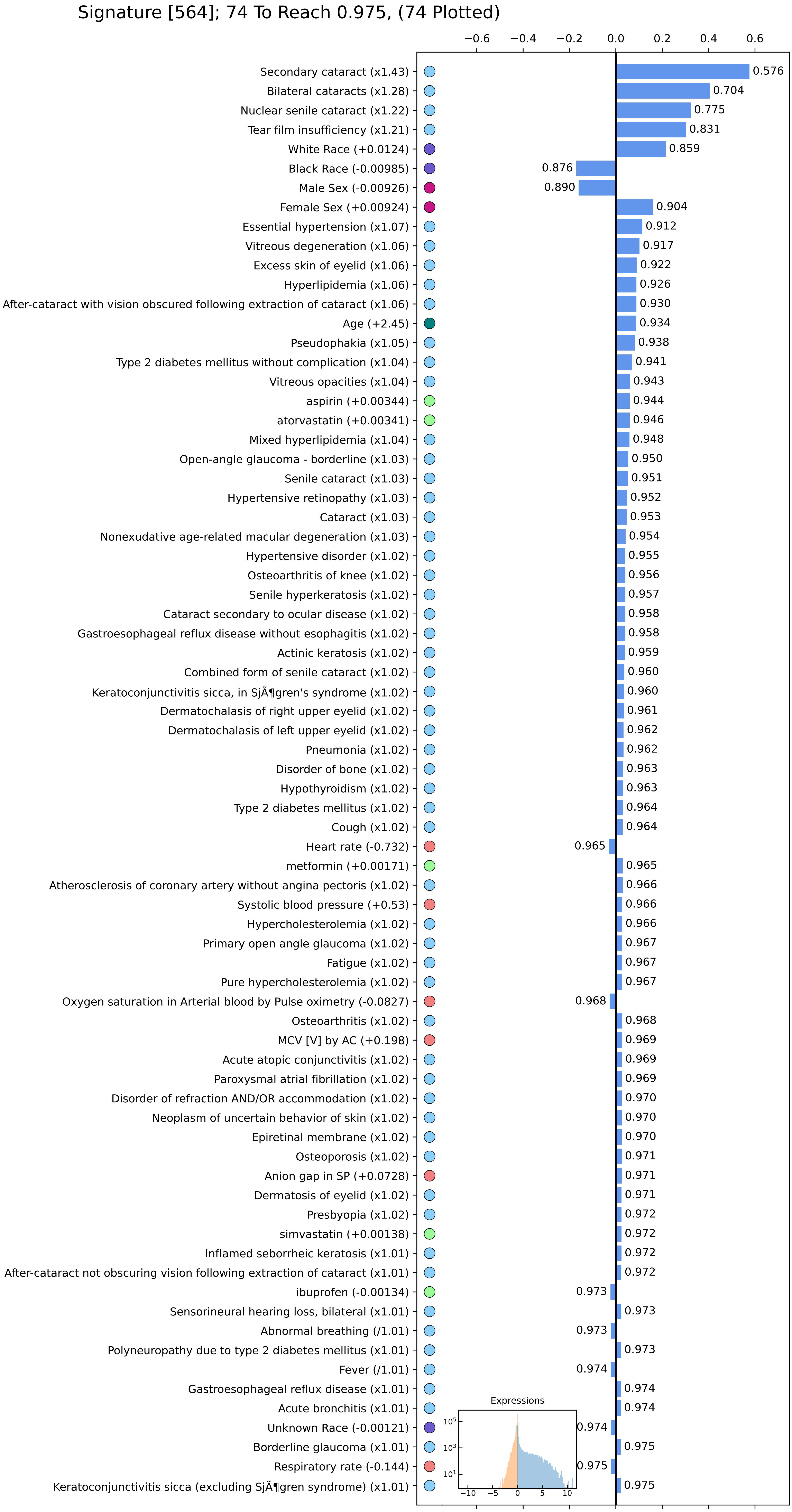

Signature [564]; 74 To Reach 0.975, (74 Plotted)
Secondary cataract (x1.43) 0.576
Bilateral cataracts (x1.28) 0.704
Nuclear senile cataract (x1.22) 0.775
Tear film insufficiency (x1.21) 0.831
White Race (+0.0124) 0.859
Black Race (-0.00985) 0.876
Male Sex (-0.00926) 0.890
Female Sex (+0.00924) 0.904
Essential hypertension (x1.07) 0.912
Vitreous degeneration (x1.06) 0.917
Excess skin of eyelid (x1.06) 0.922
Hyperlipidemia (x1.06) 0.926
After-cataract with vision obscured following extraction of cataract (x1.06) 0.930
Age (+2.45) 0.934
Pseudophakia (x1.05) 0.938
Type 2 diabetes mellitus without complication (x1.04) 0.941
Vitreous opacities (x1.04) 0.943
aspirin (+0.00344) 0.944
atorvastatin (+0.00341) 0.946
Mixed hyperlipidemia (x1.04) 0.948
Open-angle glaucoma - borderline (x1.03) 0.950
Senile cataract (x1.03) 0.951
Hypertensive retinopathy (x1.03) 0.952
Cataract (x1.03) 0.953
Nonexudative age-related macular degeneration (x1.03) 0.954
Hypertensive disorder (x1.02) 0.955
Osteoarthritis of knee (x1.02) 0.956
Senile hyperkeratosis (x1.02) 0.957
Cataract secondary to ocular disease (x1.02) 0.958
Gastroesophageal reflux disease without esophagitis (x1.02) 0.958
Actinic keratosis (x1.02) 0.959
Combined form of senile cataract (x1.02) 0.960
Keratoconjunctivitis sicca, in SjÃ¶gren's syndrome (x1.02) 0.960
Dermatochalasis of right upper eyelid (x1.02) 0.961
Dermatochalasis of left upper eyelid (x1.02) 0.962
Pneumonia (x1.02) 0.962
Disorder of bone (x1.02) 0.963
Hypothyroidism (x1.02) 0.963
Type 2 diabetes mellitus (x1.02) 0.964
Cough (x1.02) 0.964
Heart rate (-0.732) 0.965
metformin (+0.00171) 0.965
Atherosclerosis of coronary artery without angina pectoris (x1.02) 0.966
Systolic blood pressure (+0.53) 0.966
Hypercholesterolemia (x1.02) 0.966
Primary open angle glaucoma (x1.02) 0.967
Fatigue (x1.02) 0.967
Pure hypercholesterolemia (x1.02) 0.967
Oxygen saturation in Arterial blood by Pulse oximetry (-0.0827) 0.968
Osteoarthritis (x1.02) 0.968
MCV [V] by AC (+0.198) 0.969
Acute atopic conjunctivitis (x1.02) 0.969
Paroxysmal atrial fibrillation (x1.02) 0.969
Disorder of refraction AND/OR accommodation (x1.02) 0.970
Neoplasm of uncertain behavior of skin (x1.02) 0.970
Epiretinal membrane (x1.02) 0.970
Osteoporosis (x1.02) 0.971
Anion gap in SP (+0.0728) 0.971
Dermatosis of eyelid (x1.02) 0.971
Presbyopia (x1.02) 0.972
simvastatin (+0.00138) 0.972
Inflamed seborrheic keratosis (x1.01) 0.972
After-cataract not obscuring vision following extraction of cataract (x1.01) 0.972
ibuprofen (-0.00134) 0.973
Sensorineural hearing loss, bilateral (x1.01) 0.973
Abnormal breathing (/1.01) 0.973
Polyneuropathy due to type 2 diabetes mellitus (x1.01) 0.973
Fever (/1.01) 0.974
Gastroesophageal reflux disease (x1.01) 0.974
Acute bronchitis (x1.01) 0.974
Unknown Race (-0.00121) 0.974
Borderline glaucoma (x1.01) 0.975
Respiratory rate (-0.144) 0.975
Keratoconjunctivitis sicca (excluding SjÃ¶gren syndrome) (x1.01) 0.975
−0.6 −0.4 −0.2 0.0 0.2 0.4 0.6
Expressions

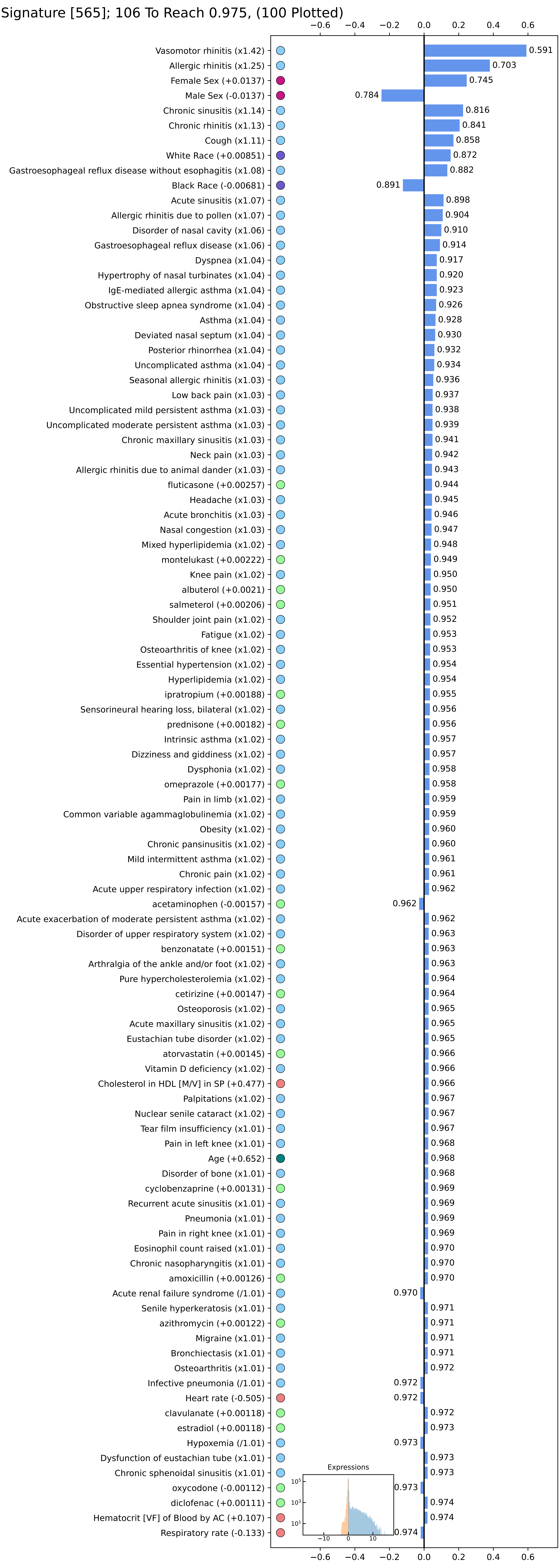

Signature [565]; 106 To Reach 0.975, (100 Plotted)
Vasomotor rhinitis (x1.42) 0.591
Allergic rhinitis (x1.25) 0.703
Female Sex (+0.0137) 0.745
Male Sex (-0.0137) 0.784
Chronic sinusitis (x1.14) 0.816
Chronic rhinitis (x1.13) 0.841
Cough (x1.11) 0.858
White Race (+0.00851) 0.872
Gastroesophageal reflux disease without esophagitis (x1.08) 0.882
Black Race (-0.00681) 0.891
Acute sinusitis (x1.07) 0.898
Allergic rhinitis due to pollen (x1.07) 0.904
Disorder of nasal cavity (x1.06) 0.910
Gastroesophageal reflux disease (x1.06) 0.914
Dyspnea (x1.04) 0.917
Hypertrophy of nasal turbinates (x1.04) 0.920
IgE-mediated allergic asthma (x1.04) 0.923
Obstructive sleep apnea syndrome (x1.04) 0.926
Asthma (x1.04) 0.928
Deviated nasal septum (x1.04) 0.930
Posterior rhinorrhea (x1.04) 0.932
Uncomplicated asthma (x1.04) 0.934
Seasonal allergic rhinitis (x1.03) 0.936
Low back pain (x1.03) 0.937
Uncomplicated mild persistent asthma (x1.03) 0.938
Uncomplicated moderate persistent asthma (x1.03) 0.939
Chronic maxillary sinusitis (x1.03) 0.941
Neck pain (x1.03) 0.942
Allergic rhinitis due to animal dander (x1.03) 0.943
fluticasone (+0.00257) 0.944
Headache (x1.03) 0.945
Acute bronchitis (x1.03) 0.946
Nasal congestion (x1.03) 0.947
Mixed hyperlipidemia (x1.02) 0.948
montelukast (+0.00222) 0.949
Knee pain (x1.02) 0.950
albuterol (+0.0021) 0.950
salmeterol (+0.00206) 0.951
Shoulder joint pain (x1.02) 0.952
Fatigue (x1.02) 0.953
Osteoarthritis of knee (x1.02) 0.953
Essential hypertension (x1.02) 0.954
Hyperlipidemia (x1.02) 0.954
ipratropium (+0.00188) 0.955
Sensorineural hearing loss, bilateral (x1.02) 0.956
prednisone (+0.00182) 0.956
Intrinsic asthma (x1.02) 0.957
Dizziness and giddiness (x1.02) 0.957
Dysphonia (x1.02) 0.958
omeprazole (+0.00177) 0.958
Pain in limb (x1.02) 0.959
Common variable agammaglobulinemia (x1.02) 0.959
Obesity (x1.02) 0.960
Chronic pansinusitis (x1.02) 0.960
Mild intermittent asthma (x1.02) 0.961
Chronic pain (x1.02) 0.961
Acute upper respiratory infection (x1.02) 0.962
acetaminophen (-0.00157) 0.962
Acute exacerbation of moderate persistent asthma (x1.02) 0.962
Disorder of upper respiratory system (x1.02) 0.963
benzonatate (+0.00151) 0.963
Arthralgia of the ankle and/or foot (x1.02) 0.963
Pure hypercholesterolemia (x1.02) 0.964
cetirizine (+0.00147) 0.964
Osteoporosis (x1.02) 0.965
Acute maxillary sinusitis (x1.02) 0.965
Eustachian tube disorder (x1.02) 0.965
atorvastatin (+0.00145) 0.966
Vitamin D deficiency (x1.02) 0.966
Cholesterol in HDL [M/V] in SP (+0.477) 0.966
Palpitations (x1.02) 0.967
Nuclear senile cataract (x1.02) 0.967
Tear film insufficiency (x1.01) 0.967
Pain in left knee (x1.01) 0.968
Age (+0.652) 0.968
Disorder of bone (x1.01) 0.968
cyclobenzaprine (+0.00131) 0.969
Recurrent acute sinusitis (x1.01) 0.969
Pneumonia (x1.01) 0.969
Pain in right knee (x1.01) 0.969
Eosinophil count raised (x1.01) 0.970
Chronic nasopharyngitis (x1.01) 0.970
amoxicillin (+0.00126) 0.970
Acute renal failure syndrome (/1.01) 0.970
Senile hyperkeratosis (x1.01) 0.971
azithromycin (+0.00122) 0.971
Migraine (x1.01) 0.971
Bronchiectasis (x1.01) 0.971
Osteoarthritis (x1.01) 0.972
Infective pneumonia (/1.01) 0.972
Heart rate (-0.505) 0.972
clavulanate (+0.00118) 0.972
estradiol (+0.00118) 0.973
Hypoxemia (/1.01) 0.973
Dysfunction of eustachian tube (x1.01) 0.973
Chronic sphenoidal sinusitis (x1.01) 0.973
oxycodone (-0.00112) 0.973
diclofenac (+0.00111) 0.974
Hematocrit [VF] of Blood by AC (+0.107) 0.974
Respiratory rate (-0.133) 0.974
Expressions
−0.6 −0.4 −0.2 0.0 0.2 0.4 0.6

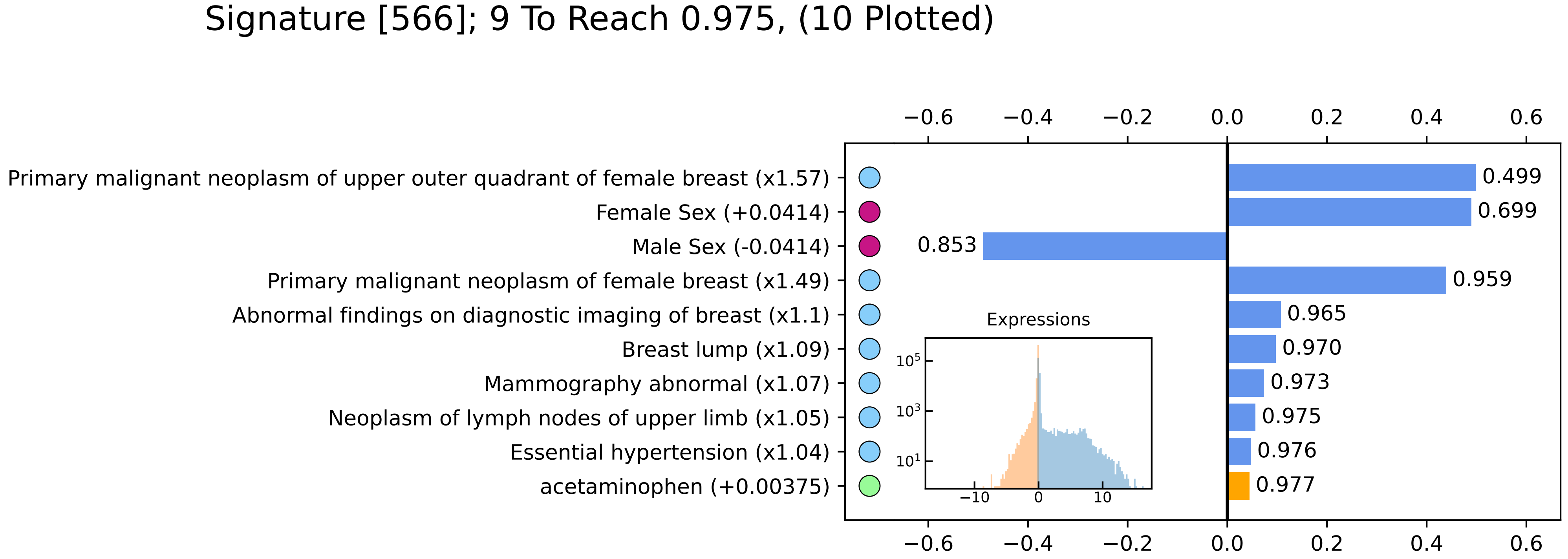

Signature [566]; 9 To Reach 0.975, (10 Plotted)
Primary malignant neoplasm of upper outer quadrant of female breast (x1.57)
Female Sex (+0.0414)
Male Sex (-0.0414)
Primary malignant neoplasm of female breast (x1.49)
Abnormal findings on diagnostic imaging of breast (x1.1)
Breast lump (x1.09)
Mammography abnormal (x1.07)
Neoplasm of lymph nodes of upper limb (x1.05)
Essential hypertension (x1.04)
acetaminophen (+0.00375)
0.499
0.699
0.853
0.959
0.965
0.970
0.973
0.975
0.976
0.977
Expressions
−0.6
−0.4
−0.2
0.0
0.2
0.4
0.6

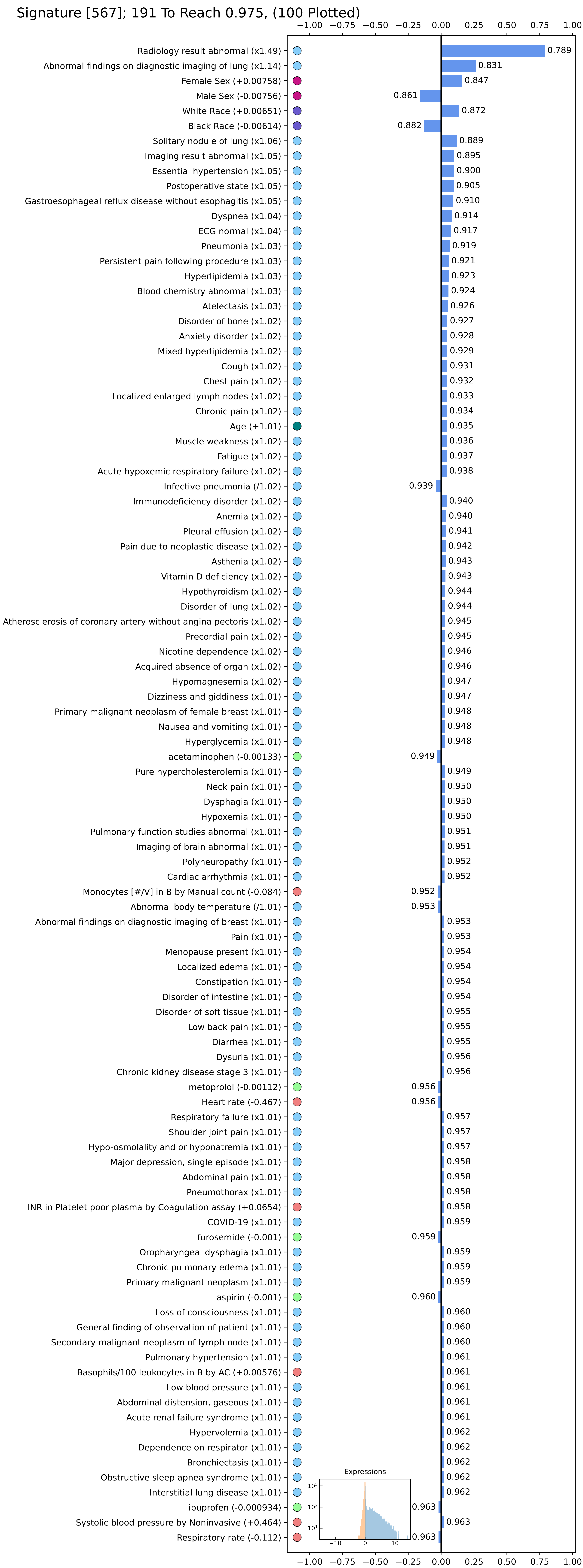
Signature [567]; 191 To Reach 0.975, (100 Plotted)
−1.00 −0.75 −0.50 −0.25 0.00 0.25 0.50 0.75 1.00
Radiology result abnormal (x1.49) 0.789
Abnormal findings on diagnostic imaging of lung (x1.14) 0.831
Female Sex (+0.00758) 0.847
Male Sex (-0.00756) 0.861
White Race (+0.00651) 0.872
Black Race (-0.00614) 0.882
Solitary nodule of lung (x1.06) 0.889
Imaging result abnormal (x1.05) 0.895
Essential hypertension (x1.05) 0.900
Postoperative state (x1.05) 0.905
Gastroesophageal reflux disease without esophagitis (x1.05) 0.910
Dyspnea (x1.04) 0.914
ECG normal (x1.04) 0.917
Pneumonia (x1.03) 0.919
Persistent pain following procedure (x1.03) 0.921
Hyperlipidemia (x1.03) 0.923
Blood chemistry abnormal (x1.03) 0.924
Atelectasis (x1.03) 0.926
Disorder of bone (x1.02) 0.927
Anxiety disorder (x1.02) 0.928
Mixed hyperlipidemia (x1.02) 0.929
Cough (x1.02) 0.931
Chest pain (x1.02) 0.932
Localized enlarged lymph nodes (x1.02) 0.933
Chronic pain (x1.02) 0.934
Age (+1.01) 0.935
Muscle weakness (x1.02) 0.936
Fatigue (x1.02) 0.937
Acute hypoxemic respiratory failure (x1.02) 0.938
Infective pneumonia (/1.02) 0.939
Immunodeficiency disorder (x1.02) 0.940
Anemia (x1.02) 0.940
Pleural effusion (x1.02) 0.941
Pain due to neoplastic disease (x1.02) 0.942
Asthenia (x1.02) 0.943
Vitamin D deficiency (x1.02) 0.943
Hypothyroidism (x1.02) 0.944
Disorder of lung (x1.02) 0.944
Atherosclerosis of coronary artery without angina pectoris (x1.02) 0.945
Precordial pain (x1.02) 0.945
Nicotine dependence (x1.02) 0.946
Acquired absence of organ (x1.02) 0.946
Hypomagnesemia (x1.02) 0.947
Dizziness and giddiness (x1.01) 0.947
Primary malignant neoplasm of female breast (x1.01) 0.948
Nausea and vomiting (x1.01) 0.948
Hyperglycemia (x1.01) 0.948
acetaminophen (-0.00133) 0.949
Pure hypercholesterolemia (x1.01) 0.949
Neck pain (x1.01) 0.950
Dysphagia (x1.01) 0.950
Hypoxemia (x1.01) 0.950
Pulmonary function studies abnormal (x1.01) 0.951
Imaging of brain abnormal (x1.01) 0.951
Polyneuropathy (x1.01) 0.952
Cardiac arrhythmia (x1.01) 0.952
Monocytes [#/V] in B by Manual count (-0.084) 0.952
Abnormal body temperature (/1.01) 0.953
Abnormal findings on diagnostic imaging of breast (x1.01) 0.953
Pain (x1.01) 0.953
Menopause present (x1.01) 0.954
Localized edema (x1.01) 0.954
Constipation (x1.01) 0.954
Disorder of intestine (x1.01) 0.954
Disorder of soft tissue (x1.01) 0.955
Low back pain (x1.01) 0.955
Diarrhea (x1.01) 0.955
Dysuria (x1.01) 0.956
Chronic kidney disease stage 3 (x1.01) 0.956
metoprolol (-0.00112) 0.956
Heart rate (-0.467) 0.956
Respiratory failure (x1.01) 0.957
Shoulder joint pain (x1.01) 0.957
Hypo-osmolality and or hyponatremia (x1.01) 0.957
Major depression, single episode (x1.01) 0.958
Abdominal pain (x1.01) 0.958
Pneumothorax (x1.01) 0.958
INR in Platelet poor plasma by Coagulation assay (+0.0654) 0.958
COVID-19 (x1.01) 0.959
furosemide (-0.001) 0.959
Oropharyngeal dysphagia (x1.01) 0.959
Chronic pulmonary edema (x1.01) 0.959
Primary malignant neoplasm (x1.01) 0.959
aspirin (-0.001) 0.960
Loss of consciousness (x1.01) 0.960
General finding of observation of patient (x1.01) 0.960
Secondary malignant neoplasm of lymph node (x1.01) 0.960
Pulmonary hypertension (x1.01) 0.961
Basophils/100 leukocytes in B by AC (+0.00576) 0.961
Low blood pressure (x1.01) 0.961
Abdominal distension, gaseous (x1.01) 0.961
Acute renal failure syndrome (x1.01) 0.961
Hypervolemia (x1.01) 0.962
Dependence on respirator (x1.01) 0.962
Bronchiectasis (x1.01) 0.962
Obstructive sleep apnea syndrome (x1.01) 0.962
Interstitial lung disease (x1.01) 0.962
ibuprofen (-0.000934) 0.963
Systolic blood pressure by Noninvasive (+0.464) 0.963
Respiratory rate (-0.112) 0.963
Expressions
−10 0 10
−1.00 −0.75 −0.50 −0.25 0.00 0.25 0.50 0.75 1.00

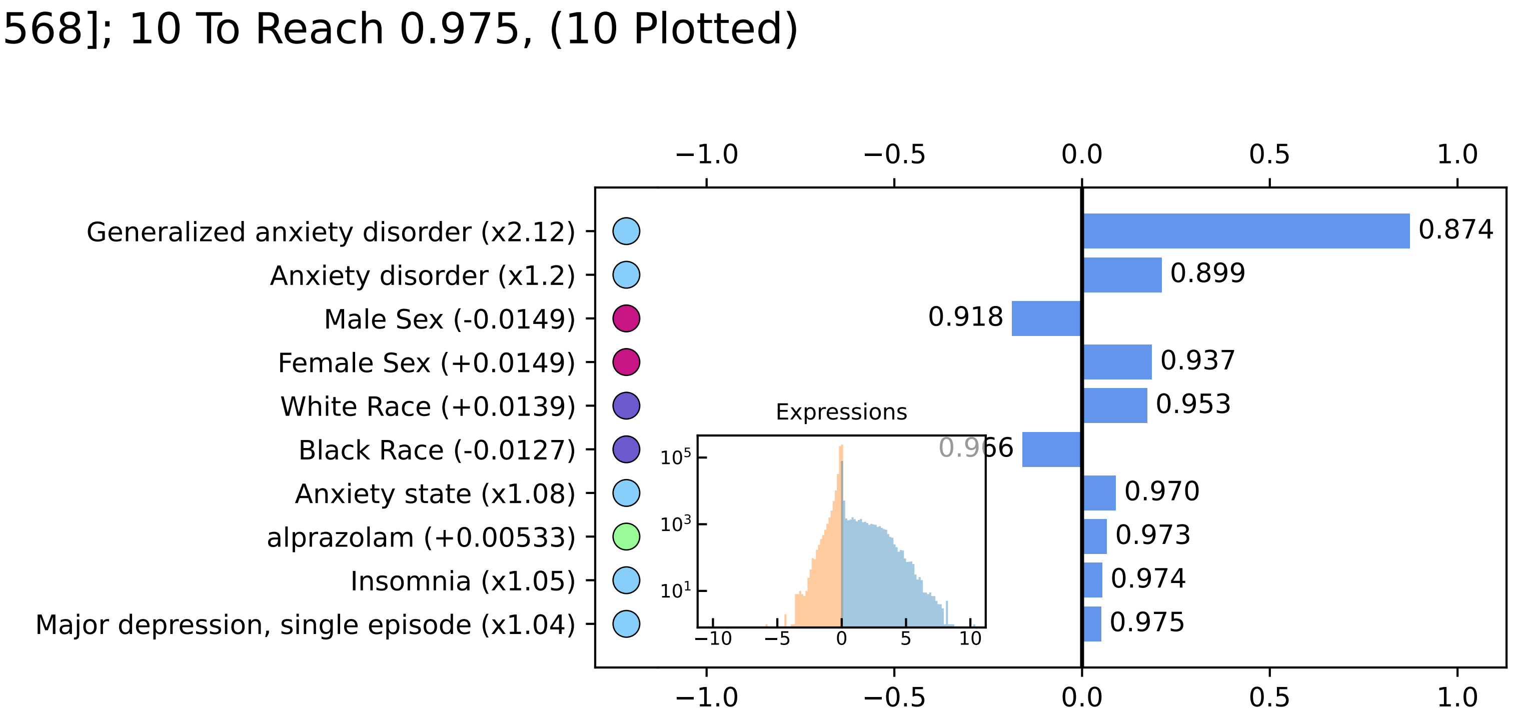
Signature [568]; 10 To Reach 0.975, (10 Plotted)
Generalized anxiety disorder (x2.12)
Anxiety disorder (x1.2)
Male Sex (-0.0149)
Female Sex (+0.0149)
White Race (+0.0139)
Black Race (-0.0127)
Anxiety state (x1.08)
alprazolam (+0.00533)
Insomnia (x1.05)
Major depression, single episode (x1.04)
0.874
0.899
0.918
0.937
0.953
0.966
0.970
0.973
0.974
0.975
−1.0
−0.5
0.0
0.5
1.0
Expressions
10^5
10^3
10^1
−10
−5
0
5
10

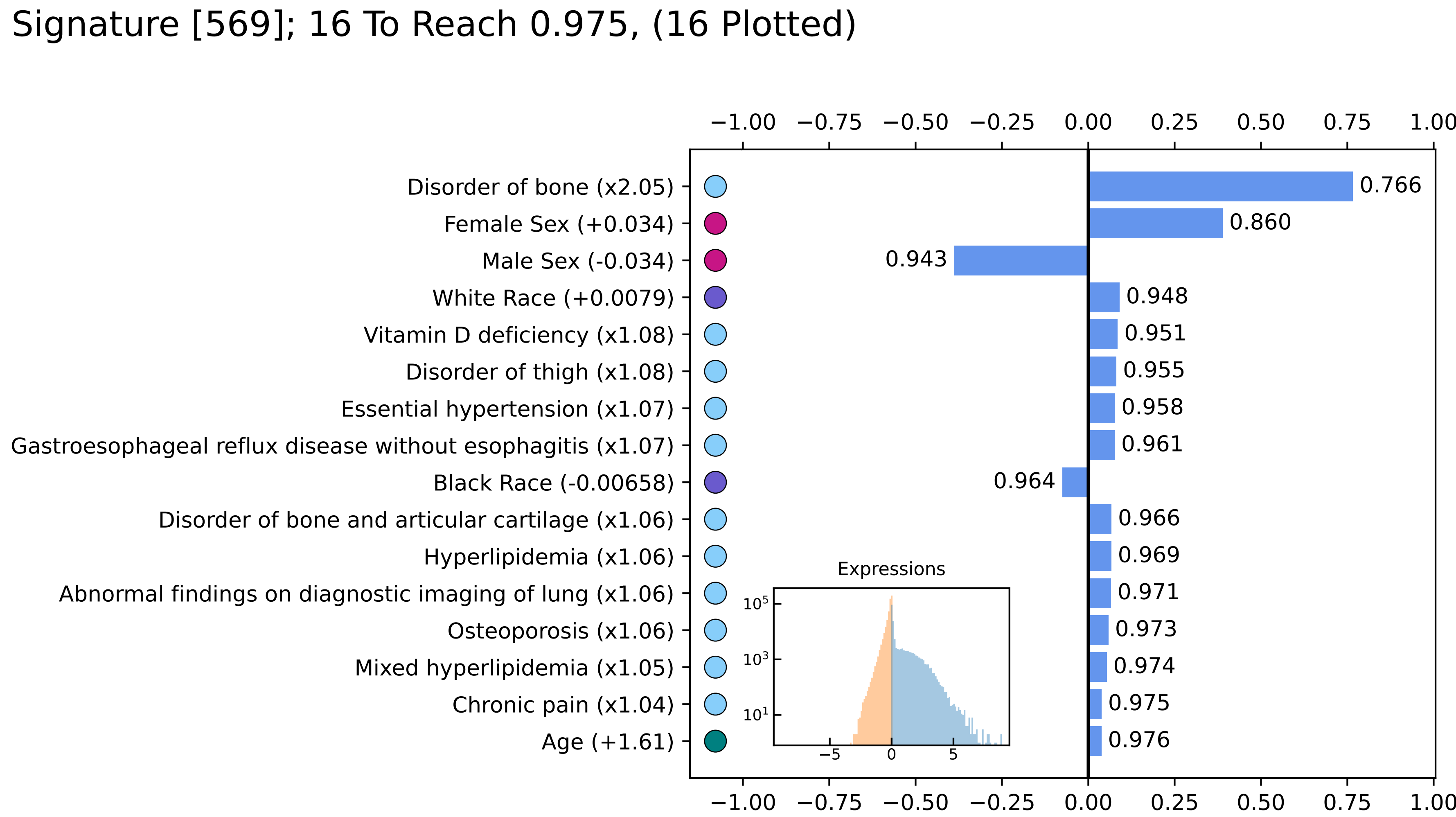

Signature [569]; 16 To Reach 0.975, (16 Plotted)
Disorder of bone (x2.05)
Female Sex (+0.034)
Male Sex (-0.034)
White Race (+0.0079)
Vitamin D deficiency (x1.08)
Disorder of thigh (x1.08)
Essential hypertension (x1.07)
Gastroesophageal reflux disease without esophagitis (x1.07)
Black Race (-0.00658)
Disorder of bone and articular cartilage (x1.06)
Hyperlipidemia (x1.06)
Abnormal findings on diagnostic imaging of lung (x1.06)
Osteoporosis (x1.06)
Mixed hyperlipidemia (x1.05)
Chronic pain (x1.04)
Age (+1.61)
0.766
0.860
0.943
0.948
0.951
0.955
0.958
0.961
0.964
0.966
0.969
0.971
0.973
0.974
0.975
0.976
−1.00 −0.75 −0.50 −0.25 0.00 0.25 0.50 0.75 1.00
Expressions
10^5
10^3
10^1
−5 0 5

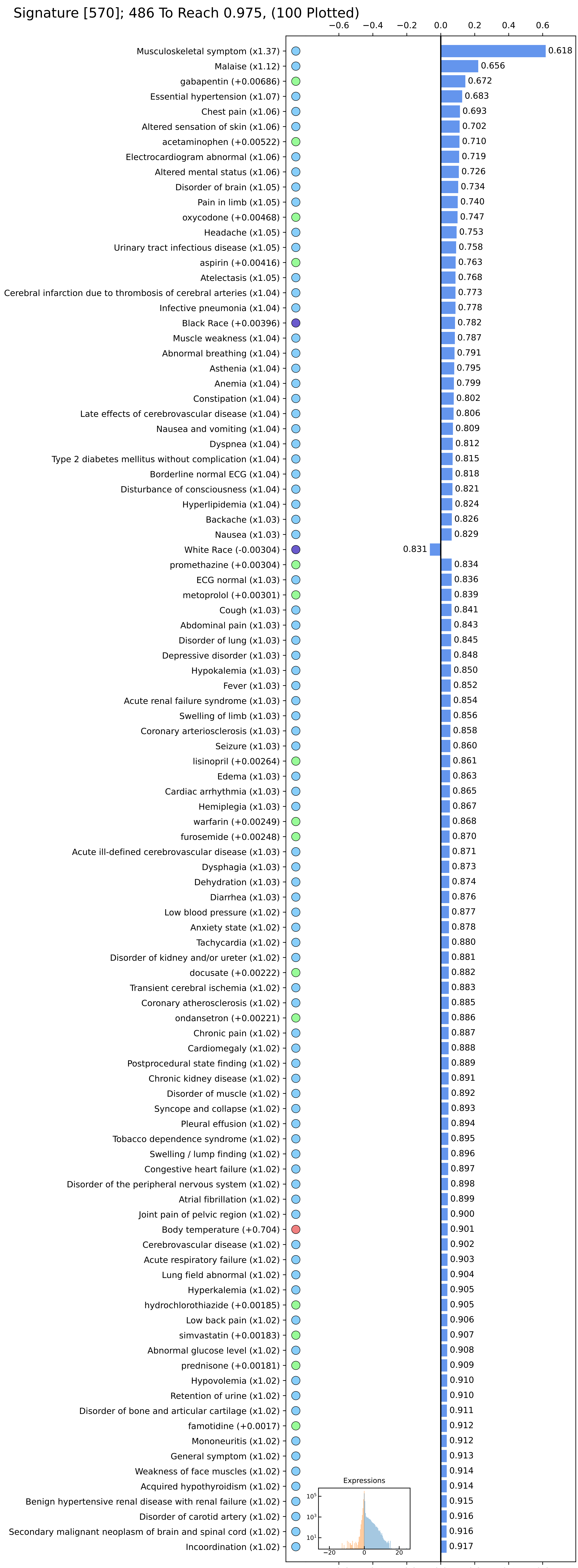

Signature [570]; 486 To Reach 0.975, (100 Plotted)
Musculoskeletal symptom (x1.37) 0.618
Malaise (x1.12) 0.656
gabapentin (+0.00686) 0.672
Essential hypertension (x1.07) 0.683
Chest pain (x1.06) 0.693
Altered sensation of skin (x1.06) 0.702
acetaminophen (+0.00522) 0.710
Electrocardiogram abnormal (x1.06) 0.719
Altered mental status (x1.06) 0.726
Disorder of brain (x1.05) 0.734
Pain in limb (x1.05) 0.740
oxycodone (+0.00468) 0.747
Headache (x1.05) 0.753
Urinary tract infectious disease (x1.05) 0.758
aspirin (+0.00416) 0.763
Atelectasis (x1.05) 0.768
Cerebral infarction due to thrombosis of cerebral arteries (x1.04) 0.773
Infective pneumonia (x1.04) 0.778
Black Race (+0.00396) 0.782
Muscle weakness (x1.04) 0.787
Abnormal breathing (x1.04) 0.791
Asthenia (x1.04) 0.795
Anemia (x1.04) 0.799
Constipation (x1.04) 0.802
Late effects of cerebrovascular disease (x1.04) 0.806
Nausea and vomiting (x1.04) 0.809
Dyspnea (x1.04) 0.812
Type 2 diabetes mellitus without complication (x1.04) 0.815
Borderline normal ECG (x1.04) 0.818
Disturbance of consciousness (x1.04) 0.821
Hyperlipidemia (x1.04) 0.824
Backache (x1.03) 0.826
Nausea (x1.03) 0.829
White Race (-0.00304) 0.831
promethazine (+0.00304) 0.834
ECG normal (x1.03) 0.836
metoprolol (+0.00301) 0.839
Cough (x1.03) 0.841
Abdominal pain (x1.03) 0.843
Disorder of lung (x1.03) 0.845
Depressive disorder (x1.03) 0.848
Hypokalemia (x1.03) 0.850
Fever (x1.03) 0.852
Acute renal failure syndrome (x1.03) 0.854
Swelling of limb (x1.03) 0.856
Coronary arteriosclerosis (x1.03) 0.858
Seizure (x1.03) 0.860
lisinopril (+0.00264) 0.861
Edema (x1.03) 0.863
Cardiac arrhythmia (x1.03) 0.865
Hemiplegia (x1.03) 0.867
warfarin (+0.00249) 0.868
furosemide (+0.00248) 0.870
Acute ill-defined cerebrovascular disease (x1.03) 0.871
Dysphagia (x1.03) 0.873
Dehydration (x1.03) 0.874
Diarrhea (x1.03) 0.876
Low blood pressure (x1.02) 0.877
Anxiety state (x1.02) 0.878
Tachycardia (x1.02) 0.880
Disorder of kidney and/or ureter (x1.02) 0.881
docusate (+0.00222) 0.882
Transient cerebral ischemia (x1.02) 0.883
Coronary atherosclerosis (x1.02) 0.885
ondansetron (+0.00221) 0.886
Chronic pain (x1.02) 0.887
Cardiomegaly (x1.02) 0.888
Postprocedural state finding (x1.02) 0.889
Chronic kidney disease (x1.02) 0.891
Disorder of muscle (x1.02) 0.892
Syncope and collapse (x1.02) 0.893
Pleural effusion (x1.02) 0.894
Tobacco dependence syndrome (x1.02) 0.895
Swelling / lump finding (x1.02) 0.896
Congestive heart failure (x1.02) 0.897
Disorder of the peripheral nervous system (x1.02) 0.898
Atrial fibrillation (x1.02) 0.899
Joint pain of pelvic region (x1.02) 0.900
Body temperature (+0.704) 0.901
Cerebrovascular disease (x1.02) 0.902
Acute respiratory failure (x1.02) 0.903
Lung field abnormal (x1.02) 0.904
Hyperkalemia (x1.02) 0.905
hydrochlorothiazide (+0.00185) 0.905
Low back pain (x1.02) 0.906
simvastatin (+0.00183) 0.907
Abnormal glucose level (x1.02) 0.908
prednisone (+0.00181) 0.909
Hypovolemia (x1.02) 0.910
Retention of urine (x1.02) 0.910
Disorder of bone and articular cartilage (x1.02) 0.911
famotidine (+0.0017) 0.912
Mononeuritis (x1.02) 0.912
General symptom (x1.02) 0.913
Weakness of face muscles (x1.02) 0.914
Acquired hypothyroidism (x1.02) 0.914
Benign hypertensive renal disease with renal failure (x1.02) 0.915
Disorder of carotid artery (x1.02) 0.916
Secondary malignant neoplasm of brain and spinal cord (x1.02) 0.916
Incoordination (x1.02) 0.917
−0.6 −0.4 −0.2 0.0 0.2 0.4 0.6
Expressions

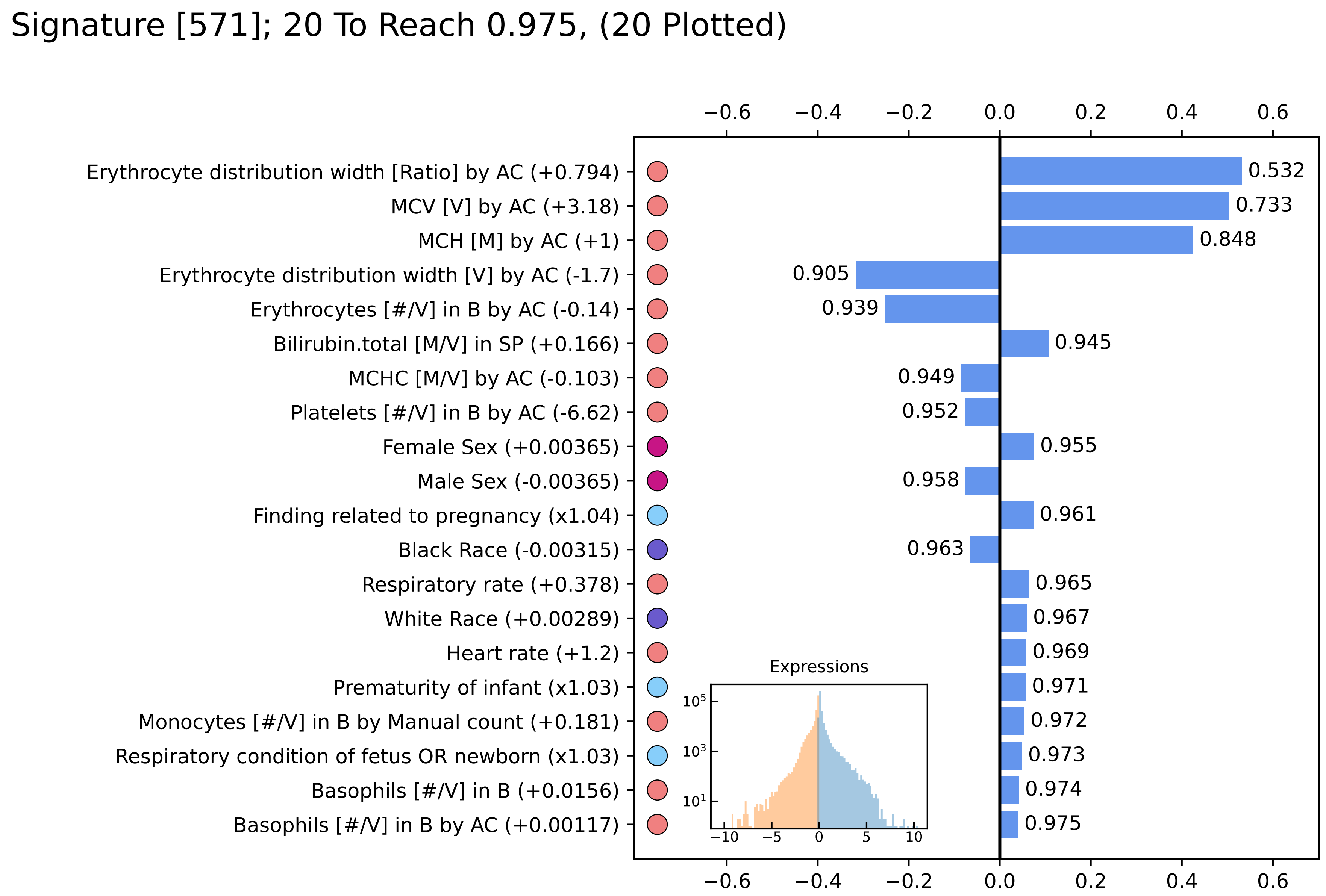

Signature [571]; 20 To Reach 0.975, (20 Plotted)
Erythrocyte distribution width [Ratio] by AC (+0.794)
MCV [V] by AC (+3.18)
MCH [M] by AC (+1)
Erythrocyte distribution width [V] by AC (-1.7)
Erythrocytes [#/V] in B by AC (-0.14)
Bilirubin.total [M/V] in SP (+0.166)
MCHC [M/V] by AC (-0.103)
Platelets [#/V] in B by AC (-6.62)
Female Sex (+0.00365)
Male Sex (-0.00365)
Finding related to pregnancy (x1.04)
Black Race (-0.00315)
Respiratory rate (+0.378)
White Race (+0.00289)
Heart rate (+1.2)
Prematurity of infant (x1.03)
Monocytes [#/V] in B by Manual count (+0.181)
Respiratory condition of fetus OR newborn (x1.03)
Basophils [#/V] in B (+0.0156)
Basophils [#/V] in B by AC (+0.00117)
0.532
0.733
0.848
0.905
0.939
0.945
0.949
0.952
0.955
0.958
0.961
0.963
0.965
0.967
0.969
0.971
0.972
0.973
0.974
0.975
Expressions
−0.6 −0.4 −0.2 0.0 0.2 0.4 0.6

Signature [572]; 1 To Reach 0.975, (10 Plotted)

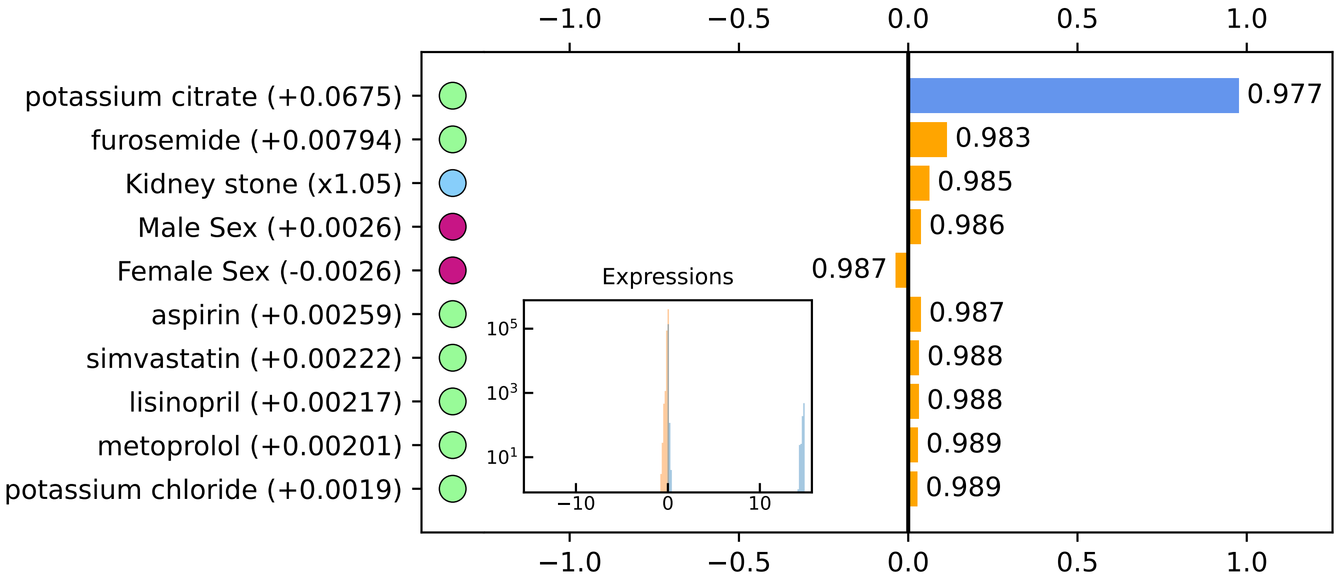

Signature [573]; 145 To Reach 0.975, (100 Plotted)

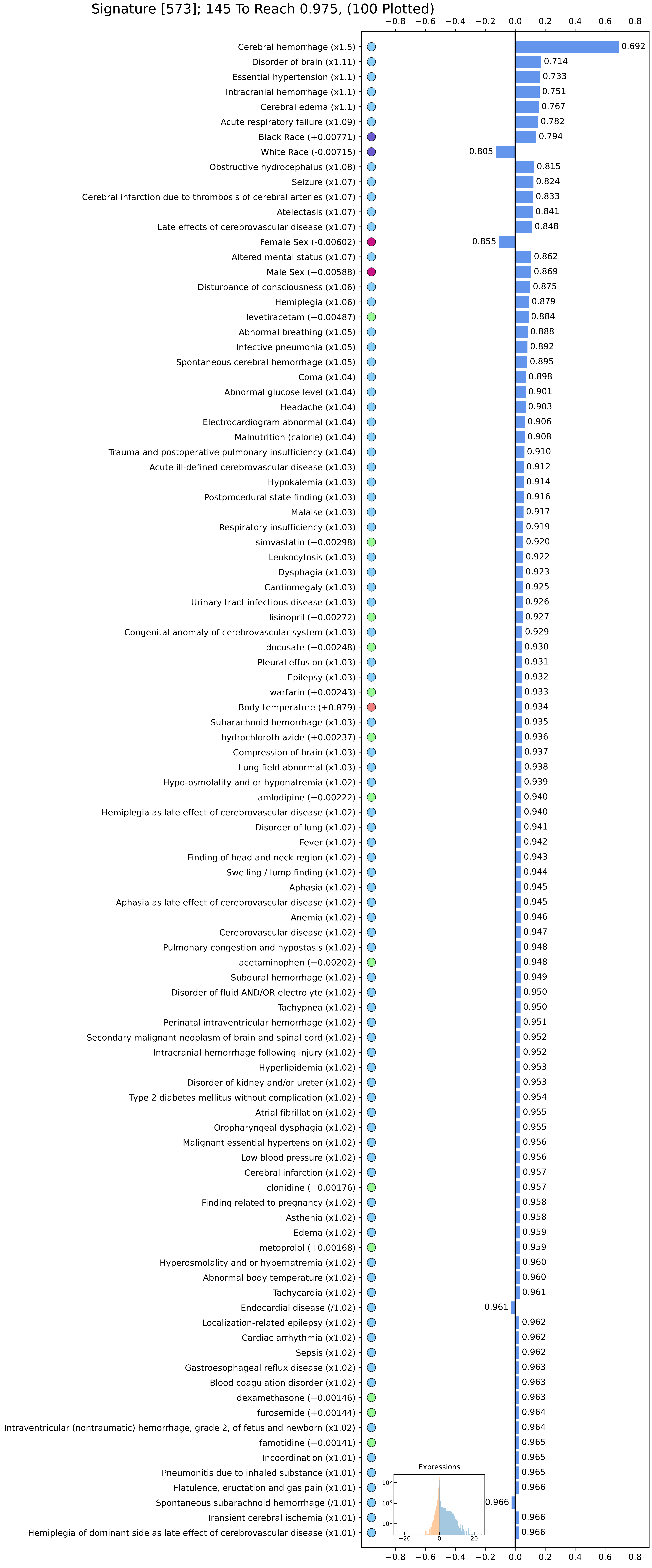

Signature [574]; 5 To Reach 0.975, (10 Plotted)

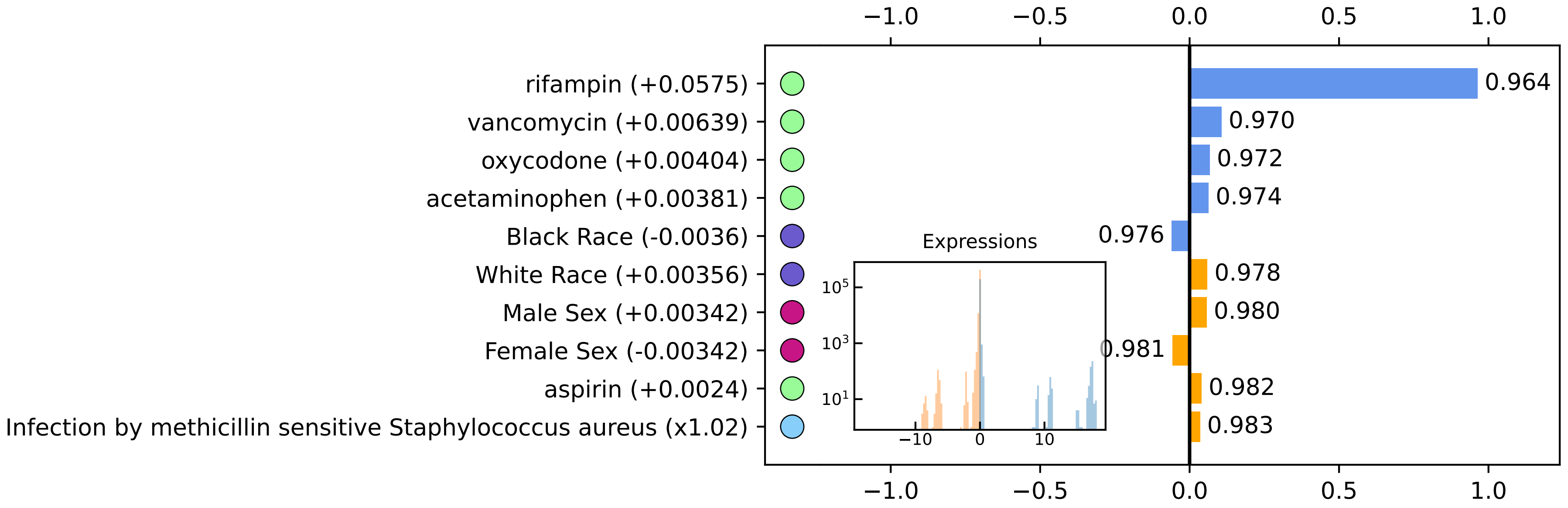

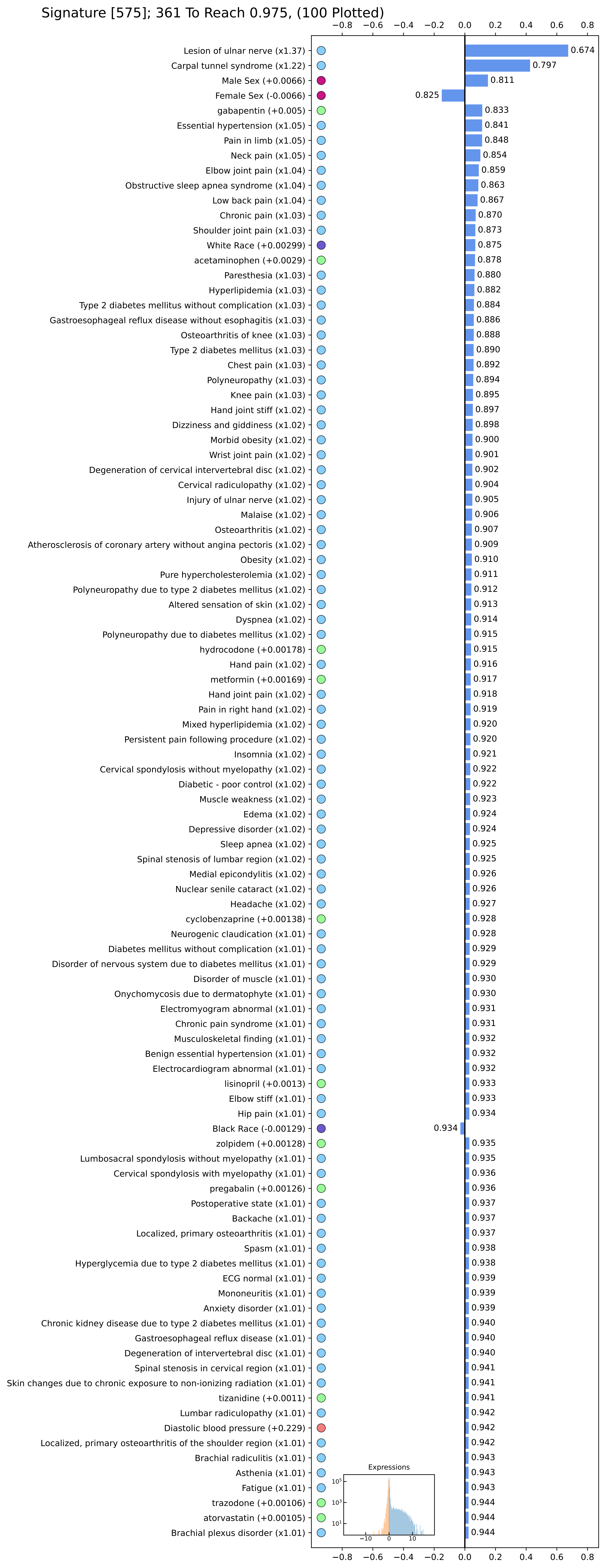

Signature [575]; 361 To Reach 0.975, (100 Plotted)
Lesion of ulnar nerve (x1.37) 0.674
Carpal tunnel syndrome (x1.22) 0.797
Male Sex (+0.0066) 0.811
Female Sex (-0.0066) 0.825
gabapentin (+0.005) 0.833
Essential hypertension (x1.05) 0.841
Pain in limb (x1.05) 0.848
Neck pain (x1.05) 0.854
Elbow joint pain (x1.04) 0.859
Obstructive sleep apnea syndrome (x1.04) 0.863
Low back pain (x1.04) 0.867
Chronic pain (x1.03) 0.870
Shoulder joint pain (x1.03) 0.873
White Race (+0.00299) 0.875
acetaminophen (+0.0029) 0.878
Paresthesia (x1.03) 0.880
Hyperlipidemia (x1.03) 0.882
Type 2 diabetes mellitus without complication (x1.03) 0.884
Gastroesophageal reflux disease without esophagitis (x1.03) 0.886
Osteoarthritis of knee (x1.03) 0.888
Type 2 diabetes mellitus (x1.03) 0.890
Chest pain (x1.03) 0.892
Polyneuropathy (x1.03) 0.894
Knee pain (x1.03) 0.895
Hand joint stiff (x1.02) 0.897
Dizziness and giddiness (x1.02) 0.898
Morbid obesity (x1.02) 0.900
Wrist joint pain (x1.02) 0.901
Degeneration of cervical intervertebral disc (x1.02) 0.902
Cervical radiculopathy (x1.02) 0.904
Injury of ulnar nerve (x1.02) 0.905
Malaise (x1.02) 0.906
Osteoarthritis (x1.02) 0.907
Atherosclerosis of coronary artery without angina pectoris (x1.02) 0.909
Obesity (x1.02) 0.910
Pure hypercholesterolemia (x1.02) 0.911
Polyneuropathy due to type 2 diabetes mellitus (x1.02) 0.912
Altered sensation of skin (x1.02) 0.913
Dyspnea (x1.02) 0.914
Polyneuropathy due to diabetes mellitus (x1.02) 0.915
hydrocodone (+0.00178) 0.915
Hand pain (x1.02) 0.916
metformin (+0.00169) 0.917
Hand joint pain (x1.02) 0.918
Pain in right hand (x1.02) 0.919
Mixed hyperlipidemia (x1.02) 0.920
Persistent pain following procedure (x1.02) 0.920
Insomnia (x1.02) 0.921
Cervical spondylosis without myelopathy (x1.02) 0.922
Diabetic - poor control (x1.02) 0.922
Muscle weakness (x1.02) 0.923
Edema (x1.02) 0.924
Depressive disorder (x1.02) 0.924
Sleep apnea (x1.02) 0.925
Spinal stenosis of lumbar region (x1.02) 0.925
Medial epicondylitis (x1.02) 0.926
Nuclear senile cataract (x1.02) 0.926
Headache (x1.02) 0.927
cyclobenzaprine (+0.00138) 0.928
Neurogenic claudication (x1.01) 0.928
Diabetes mellitus without complication (x1.01) 0.929
Disorder of nervous system due to diabetes mellitus (x1.01) 0.929
Disorder of muscle (x1.01) 0.930
Onychomycosis due to dermatophyte (x1.01) 0.930
Electromyogram abnormal (x1.01) 0.931
Chronic pain syndrome (x1.01) 0.931
Musculoskeletal finding (x1.01) 0.932
Benign essential hypertension (x1.01) 0.932
Electrocardiogram abnormal (x1.01) 0.932
lisinopril (+0.0013) 0.933
Elbow stiff (x1.01) 0.933
Hip pain (x1.01) 0.934
Black Race (-0.00129) 0.934
zolpidem (+0.00128) 0.935
Lumbosacral spondylosis without myelopathy (x1.01) 0.935
Cervical spondylosis with myelopathy (x1.01) 0.936
pregabalin (+0.00126) 0.936
Postoperative state (x1.01) 0.937
Backache (x1.01) 0.937
Localized, primary osteoarthritis (x1.01) 0.937
Spasm (x1.01) 0.938
Hyperglycemia due to type 2 diabetes mellitus (x1.01) 0.938
ECG normal (x1.01) 0.939
Mononeuritis (x1.01) 0.939
Anxiety disorder (x1.01) 0.939
Chronic kidney disease due to type 2 diabetes mellitus (x1.01) 0.940
Gastroesophageal reflux disease (x1.01) 0.940
Degeneration of intervertebral disc (x1.01) 0.940
Spinal stenosis in cervical region (x1.01) 0.941
Skin changes due to chronic exposure to non-ionizing radiation (x1.01) 0.941
tizanidine (+0.0011) 0.941
Lumbar radiculopathy (x1.01) 0.942
Diastolic blood pressure (+0.229) 0.942
Localized, primary osteoarthritis of the shoulder region (x1.01) 0.942
Brachial radiculitis (x1.01) 0.943
Asthenia (x1.01) 0.943
Fatigue (x1.01) 0.943
trazodone (+0.00106) 0.944
atorvastatin (+0.00105) 0.944
Brachial plexus disorder (x1.01) 0.944
−0.8 −0.6 −0.4 −0.2 0.0 0.2 0.4 0.6 0.8
Expressions

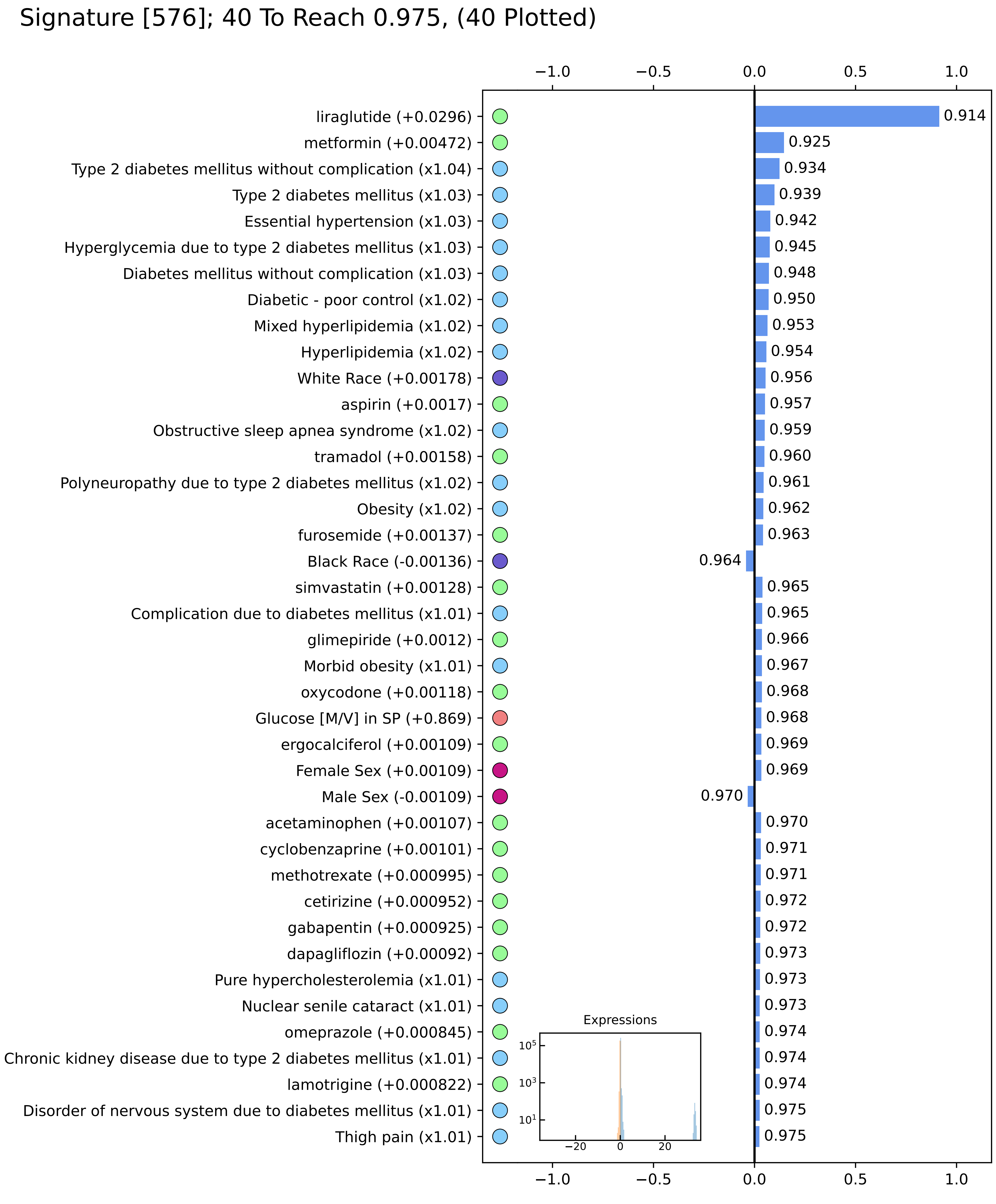
Signature [576]; 40 To Reach 0.975, (40 Plotted)
−1.0
−0.5
0.0
0.5
1.0
liraglutide (+0.0296)
0.914
metformin (+0.00472)
0.925
Type 2 diabetes mellitus without complication (x1.04)
0.934
Type 2 diabetes mellitus (x1.03)
0.939
Essential hypertension (x1.03)
0.942
Hyperglycemia due to type 2 diabetes mellitus (x1.03)
0.945
Diabetes mellitus without complication (x1.03)
0.948
Diabetic - poor control (x1.02)
0.950
Mixed hyperlipidemia (x1.02)
0.953
Hyperlipidemia (x1.02)
0.954
White Race (+0.00178)
0.956
aspirin (+0.0017)
0.957
Obstructive sleep apnea syndrome (x1.02)
0.959
tramadol (+0.00158)
0.960
Polyneuropathy due to type 2 diabetes mellitus (x1.02)
0.961
Obesity (x1.02)
0.962
furosemide (+0.00137)
0.963
Black Race (-0.00136)
0.964
simvastatin (+0.00128)
0.965
Complication due to diabetes mellitus (x1.01)
0.965
glimepiride (+0.0012)
0.966
Morbid obesity (x1.01)
0.967
oxycodone (+0.00118)
0.968
Glucose [M/V] in SP (+0.869)
0.968
ergocalciferol (+0.00109)
0.969
Female Sex (+0.00109)
0.969
Male Sex (-0.00109)
0.970
acetaminophen (+0.00107)
0.970
cyclobenzaprine (+0.00101)
0.971
methotrexate (+0.000995)
0.971
cetirizine (+0.000952)
0.972
gabapentin (+0.000925)
0.972
dapagliflozin (+0.00092)
0.973
Pure hypercholesterolemia (x1.01)
0.973
Nuclear senile cataract (x1.01)
0.973
omeprazole (+0.000845)
0.974
Chronic kidney disease due to type 2 diabetes mellitus (x1.01)
0.974
lamotrigine (+0.000822)
0.974
Disorder of nervous system due to diabetes mellitus (x1.01)
0.975
Thigh pain (x1.01)
0.975
Expressions
10^5
10^3
10^1
−20
0
20

Signature [577]; 7 To Reach 0.975, (10 Plotted)

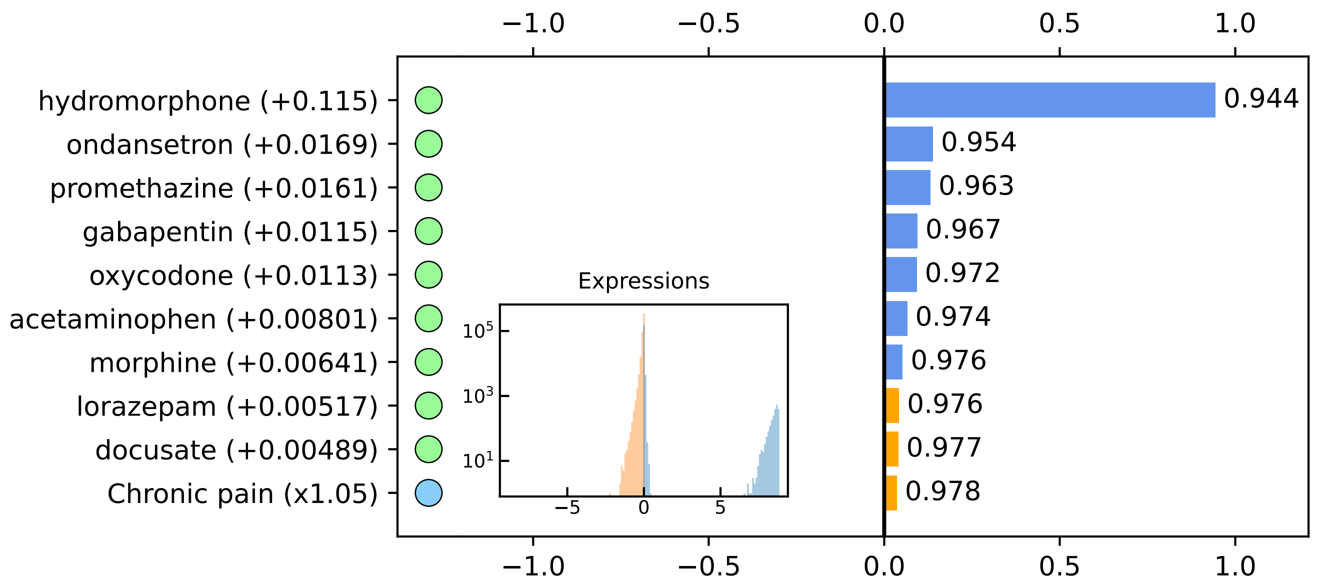

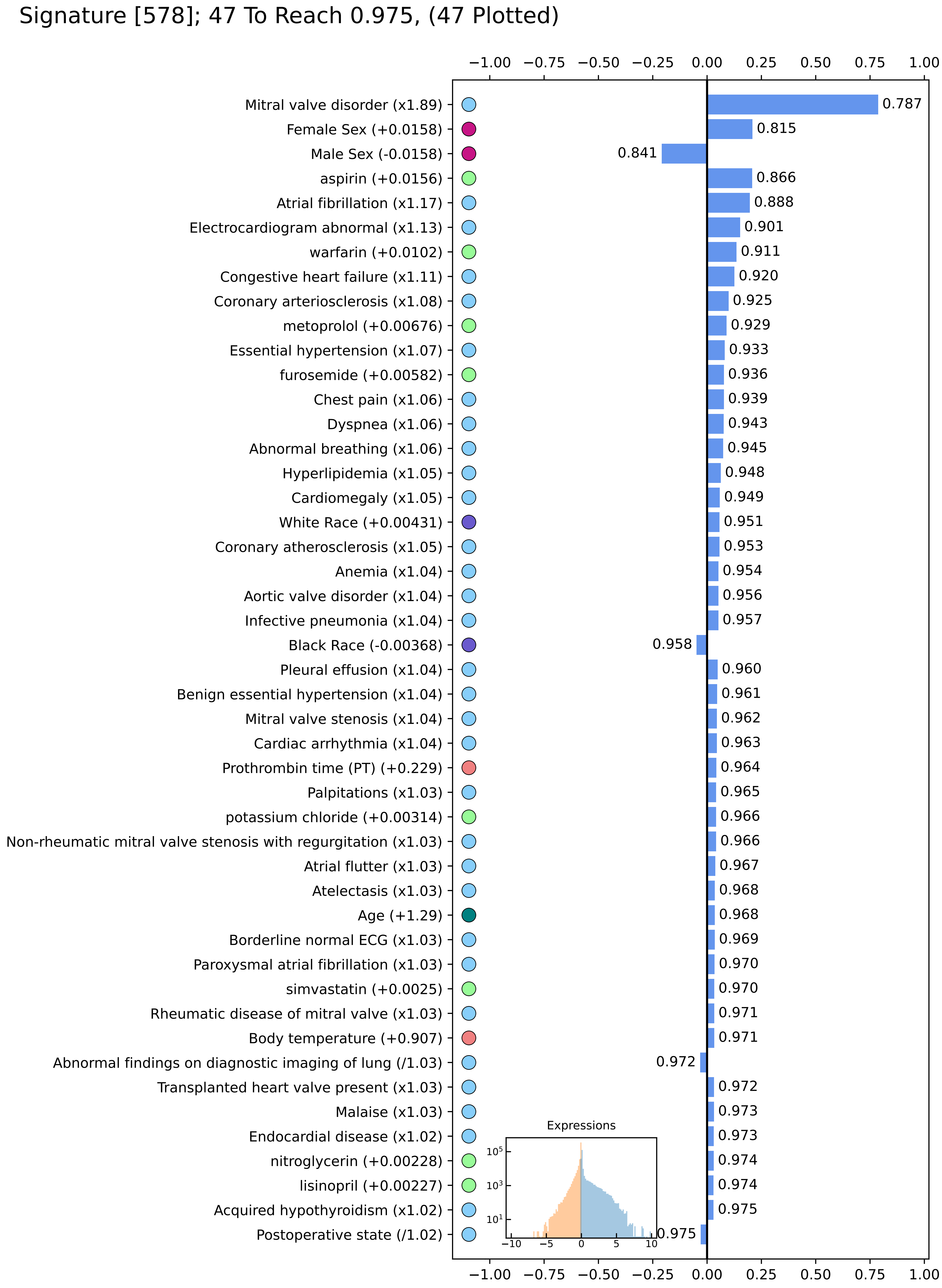

Signature [578]; 47 To Reach 0.975, (47 Plotted)
Mitral valve disorder (x1.89) 0.787
Female Sex (+0.0158) 0.815
Male Sex (-0.0158) 0.841
aspirin (+0.0156) 0.866
Atrial fibrillation (x1.17) 0.888
Electrocardiogram abnormal (x1.13) 0.901
warfarin (+0.0102) 0.911
Congestive heart failure (x1.11) 0.920
Coronary arteriosclerosis (x1.08) 0.925
metoprolol (+0.00676) 0.929
Essential hypertension (x1.07) 0.933
furosemide (+0.00582) 0.936
Chest pain (x1.06) 0.939
Dyspnea (x1.06) 0.943
Abnormal breathing (x1.06) 0.945
Hyperlipidemia (x1.05) 0.948
Cardiomegaly (x1.05) 0.949
White Race (+0.00431) 0.951
Coronary atherosclerosis (x1.05) 0.953
Anemia (x1.04) 0.954
Aortic valve disorder (x1.04) 0.956
Infective pneumonia (x1.04) 0.957
Black Race (-0.00368) 0.958
Pleural effusion (x1.04) 0.960
Benign essential hypertension (x1.04) 0.961
Mitral valve stenosis (x1.04) 0.962
Cardiac arrhythmia (x1.04) 0.963
Prothrombin time (PT) (+0.229) 0.964
Palpitations (x1.03) 0.965
potassium chloride (+0.00314) 0.966
Non-rheumatic mitral valve stenosis with regurgitation (x1.03) 0.966
Atrial flutter (x1.03) 0.967
Atelectasis (x1.03) 0.968
Age (+1.29) 0.968
Borderline normal ECG (x1.03) 0.969
Paroxysmal atrial fibrillation (x1.03) 0.970
simvastatin (+0.0025) 0.970
Rheumatic disease of mitral valve (x1.03) 0.971
Body temperature (+0.907) 0.971
Abnormal findings on diagnostic imaging of lung (/1.03) 0.972
Transplanted heart valve present (x1.03) 0.972
Malaise (x1.03) 0.973
Endocardial disease (x1.02) 0.973
nitroglycerin (+0.00228) 0.974
lisinopril (+0.00227) 0.974
Acquired hypothyroidism (x1.02) 0.975
Postoperative state (/1.02) 0.975
−1.00 −0.75 −0.50 −0.25 0.00 0.25 0.50 0.75 1.00
Expressions
10^5
10^3
10^1
−10 −5 0 5 10

Signature [579]; 82 To Reach 0.975, (82 Plotted)

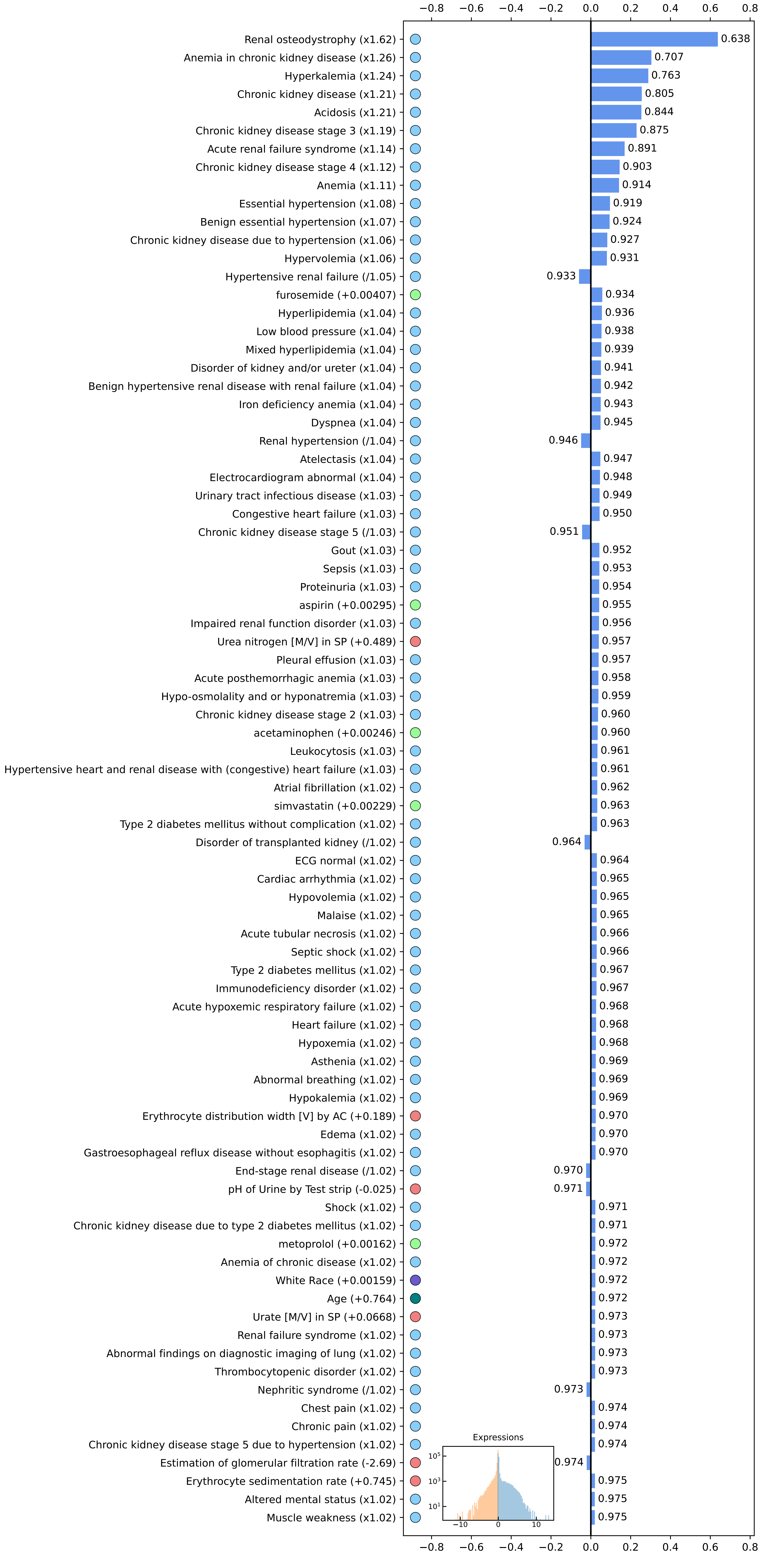

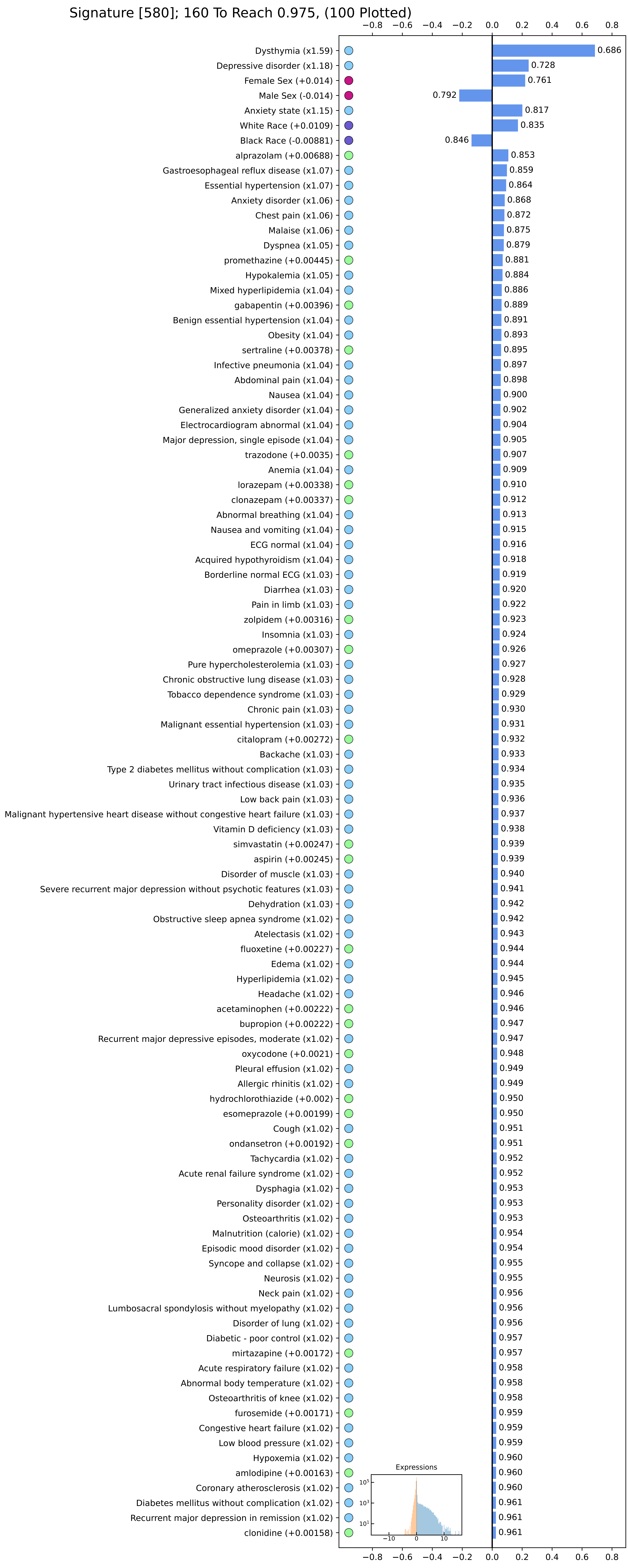

Signature [580]; 160 To Reach 0.975, (100 Plotted)
Dysthymia (x1.59) 0.686
Depressive disorder (x1.18) 0.728
Female Sex (+0.014) 0.761
Male Sex (-0.014) 0.792
Anxiety state (x1.15) 0.817
White Race (+0.0109) 0.835
Black Race (-0.00881) 0.846
alprazolam (+0.00688) 0.853
Gastroesophageal reflux disease (x1.07) 0.859
Essential hypertension (x1.07) 0.864
Anxiety disorder (x1.06) 0.868
Chest pain (x1.06) 0.872
Malaise (x1.06) 0.875
Dyspnea (x1.05) 0.879
promethazine (+0.00445) 0.881
Hypokalemia (x1.05) 0.884
Mixed hyperlipidemia (x1.04) 0.886
gabapentin (+0.00396) 0.889
Benign essential hypertension (x1.04) 0.891
Obesity (x1.04) 0.893
sertraline (+0.00378) 0.895
Infective pneumonia (x1.04) 0.897
Abdominal pain (x1.04) 0.898
Nausea (x1.04) 0.900
Generalized anxiety disorder (x1.04) 0.902
Electrocardiogram abnormal (x1.04) 0.904
Major depression, single episode (x1.04) 0.905
trazodone (+0.0035) 0.907
Anemia (x1.04) 0.909
lorazepam (+0.00338) 0.910
clonazepam (+0.00337) 0.912
Abnormal breathing (x1.04) 0.913
Nausea and vomiting (x1.04) 0.915
ECG normal (x1.04) 0.916
Acquired hypothyroidism (x1.04) 0.918
Borderline normal ECG (x1.03) 0.919
Diarrhea (x1.03) 0.920
Pain in limb (x1.03) 0.922
zolpidem (+0.00316) 0.923
Insomnia (x1.03) 0.924
omeprazole (+0.00307) 0.926
Pure hypercholesterolemia (x1.03) 0.927
Chronic obstructive lung disease (x1.03) 0.928
Tobacco dependence syndrome (x1.03) 0.929
Chronic pain (x1.03) 0.930
Malignant essential hypertension (x1.03) 0.931
citalopram (+0.00272) 0.932
Backache (x1.03) 0.933
Type 2 diabetes mellitus without complication (x1.03) 0.934
Urinary tract infectious disease (x1.03) 0.935
Low back pain (x1.03) 0.936
Malignant hypertensive heart disease without congestive heart failure (x1.03) 0.937
Vitamin D deficiency (x1.03) 0.938
simvastatin (+0.00247) 0.939
aspirin (+0.00245) 0.939
Disorder of muscle (x1.03) 0.940
Severe recurrent major depression without psychotic features (x1.03) 0.941
Dehydration (x1.03) 0.942
Obstructive sleep apnea syndrome (x1.02) 0.942
Atelectasis (x1.02) 0.943
fluoxetine (+0.00227) 0.944
Edema (x1.02) 0.944
Hyperlipidemia (x1.02) 0.945
Headache (x1.02) 0.946
acetaminophen (+0.00222) 0.946
bupropion (+0.00222) 0.947
Recurrent major depressive episodes, moderate (x1.02) 0.947
oxycodone (+0.0021) 0.948
Pleural effusion (x1.02) 0.949
Allergic rhinitis (x1.02) 0.949
hydrochlorothiazide (+0.002) 0.950
esomeprazole (+0.00199) 0.950
Cough (x1.02) 0.951
ondansetron (+0.00192) 0.951
Tachycardia (x1.02) 0.952
Acute renal failure syndrome (x1.02) 0.952
Dysphagia (x1.02) 0.953
Personality disorder (x1.02) 0.953
Osteoarthritis (x1.02) 0.953
Malnutrition (calorie) (x1.02) 0.954
Episodic mood disorder (x1.02) 0.954
Syncope and collapse (x1.02) 0.955
Neurosis (x1.02) 0.955
Neck pain (x1.02) 0.956
Lumbosacral spondylosis without myelopathy (x1.02) 0.956
Disorder of lung (x1.02) 0.956
Diabetic - poor control (x1.02) 0.957
mirtazapine (+0.00172) 0.957
Acute respiratory failure (x1.02) 0.958
Abnormal body temperature (x1.02) 0.958
Osteoarthritis of knee (x1.02) 0.958
furosemide (+0.00171) 0.959
Congestive heart failure (x1.02) 0.959
Low blood pressure (x1.02) 0.959
Hypoxemia (x1.02) 0.960
amlodipine (+0.00163) 0.960
Coronary atherosclerosis (x1.02) 0.960
Diabetes mellitus without complication (x1.02) 0.961
Recurrent major depression in remission (x1.02) 0.961
clonidine (+0.00158) 0.961
−0.8 −0.6 −0.4 −0.2 0.0 0.2 0.4 0.6 0.8
Expressions

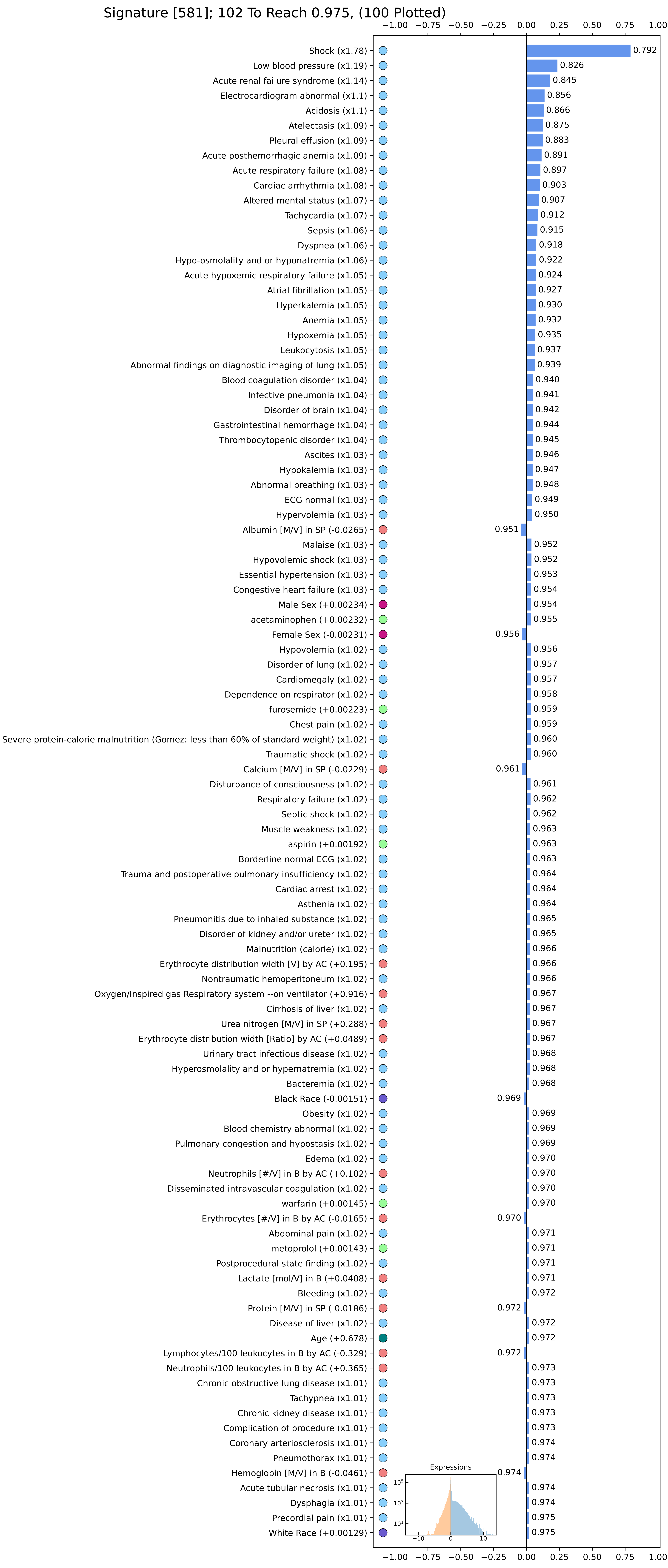

Signature [581]; 102 To Reach 0.975, (100 Plotted)
Shock (x1.78) 0.792
Low blood pressure (x1.19) 0.826
Acute renal failure syndrome (x1.14) 0.845
Electrocardiogram abnormal (x1.1) 0.856
Acidosis (x1.1) 0.866
Atelectasis (x1.09) 0.875
Pleural effusion (x1.09) 0.883
Acute posthemorrhagic anemia (x1.09) 0.891
Acute respiratory failure (x1.08) 0.897
Cardiac arrhythmia (x1.08) 0.903
Altered mental status (x1.07) 0.907
Tachycardia (x1.07) 0.912
Sepsis (x1.06) 0.915
Dyspnea (x1.06) 0.918
Hypo-osmolality and or hyponatremia (x1.06) 0.922
Acute hypoxemic respiratory failure (x1.05) 0.924
Atrial fibrillation (x1.05) 0.927
Hyperkalemia (x1.05) 0.930
Anemia (x1.05) 0.932
Hypoxemia (x1.05) 0.935
Leukocytosis (x1.05) 0.937
Abnormal findings on diagnostic imaging of lung (x1.05) 0.939
Blood coagulation disorder (x1.04) 0.940
Infective pneumonia (x1.04) 0.941
Disorder of brain (x1.04) 0.942
Gastrointestinal hemorrhage (x1.04) 0.944
Thrombocytopenic disorder (x1.04) 0.945
Ascites (x1.03) 0.946
Hypokalemia (x1.03) 0.947
Abnormal breathing (x1.03) 0.948
ECG normal (x1.03) 0.949
Hypervolemia (x1.03) 0.950
Albumin [M/V] in SP (-0.0265) 0.951
Malaise (x1.03) 0.952
Hypovolemic shock (x1.03) 0.952
Essential hypertension (x1.03) 0.953
Congestive heart failure (x1.03) 0.954
Male Sex (+0.00234) 0.954
acetaminophen (+0.00232) 0.955
Female Sex (-0.00231) 0.956
Hypovolemia (x1.02) 0.956
Disorder of lung (x1.02) 0.957
Cardiomegaly (x1.02) 0.957
Dependence on respirator (x1.02) 0.958
furosemide (+0.00223) 0.959
Chest pain (x1.02) 0.959
Severe protein-calorie malnutrition (Gomez: less than 60% of standard weight) (x1.02) 0.960
Traumatic shock (x1.02) 0.960
Calcium [M/V] in SP (-0.0229) 0.961
Disturbance of consciousness (x1.02) 0.961
Respiratory failure (x1.02) 0.962
Septic shock (x1.02) 0.962
Muscle weakness (x1.02) 0.963
aspirin (+0.00192) 0.963
Borderline normal ECG (x1.02) 0.963
Trauma and postoperative pulmonary insufficiency (x1.02) 0.964
Cardiac arrest (x1.02) 0.964
Asthenia (x1.02) 0.964
Pneumonitis due to inhaled substance (x1.02) 0.965
Disorder of kidney and/or ureter (x1.02) 0.965
Malnutrition (calorie) (x1.02) 0.966
Erythrocyte distribution width [V] by AC (+0.195) 0.966
Nontraumatic hemoperitoneum (x1.02) 0.966
Oxygen/Inspired gas Respiratory system --on ventilator (+0.916) 0.967
Cirrhosis of liver (x1.02) 0.967
Urea nitrogen [M/V] in SP (+0.288) 0.967
Erythrocyte distribution width [Ratio] by AC (+0.0489) 0.967
Urinary tract infectious disease (x1.02) 0.968
Hyperosmolality and or hypernatremia (x1.02) 0.968
Bacteremia (x1.02) 0.968
Black Race (-0.00151) 0.969
Obesity (x1.02) 0.969
Blood chemistry abnormal (x1.02) 0.969
Pulmonary congestion and hypostasis (x1.02) 0.969
Edema (x1.02) 0.970
Neutrophils [#/V] in B by AC (+0.102) 0.970
Disseminated intravascular coagulation (x1.02) 0.970
warfarin (+0.00145) 0.970
Erythrocytes [#/V] in B by AC (-0.0165) 0.970
Abdominal pain (x1.02) 0.971
metoprolol (+0.00143) 0.971
Postprocedural state finding (x1.02) 0.971
Lactate [mol/V] in B (+0.0408) 0.971
Bleeding (x1.02) 0.972
Protein [M/V] in SP (-0.0186) 0.972
Disease of liver (x1.02) 0.972
Age (+0.678) 0.972
Lymphocytes/100 leukocytes in B by AC (-0.329) 0.972
Neutrophils/100 leukocytes in B by AC (+0.365) 0.973
Chronic obstructive lung disease (x1.01) 0.973
Tachypnea (x1.01) 0.973
Chronic kidney disease (x1.01) 0.973
Complication of procedure (x1.01) 0.973
Coronary arteriosclerosis (x1.01) 0.974
Pneumothorax (x1.01) 0.974
Hemoglobin [M/V] in B (-0.0461) 0.974
Acute tubular necrosis (x1.01) 0.974
Dysphagia (x1.01) 0.974
Precordial pain (x1.01) 0.975
White Race (+0.00129) 0.975
−1.00 −0.75 −0.50 −0.25 0.00 0.25 0.50 0.75 1.00
Expressions

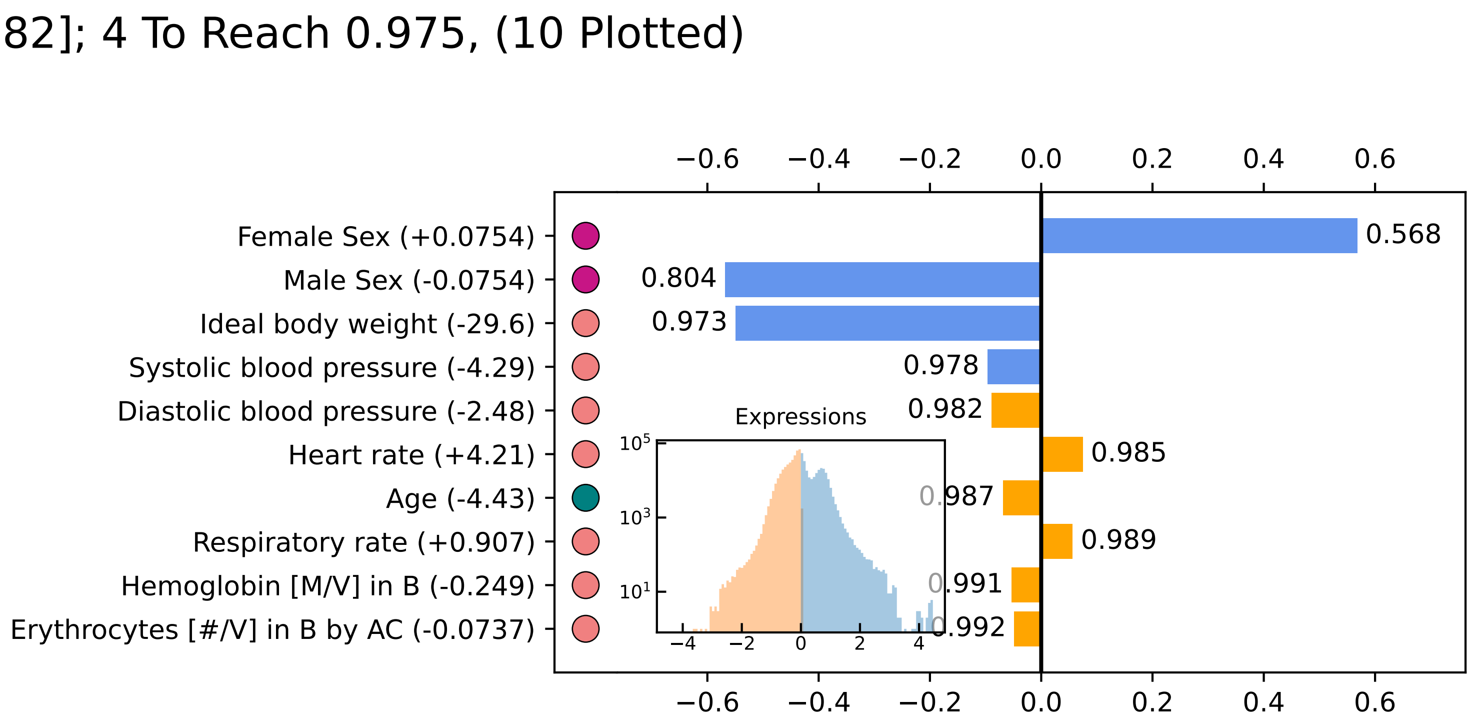

Signature [582]; 4 To Reach 0.975, (10 Plotted)
Female Sex (+0.0754)
Male Sex (-0.0754)
Ideal body weight (-29.6)
Systolic blood pressure (-4.29)
Diastolic blood pressure (-2.48)
Heart rate (+4.21)
Age (-4.43)
Respiratory rate (+0.907)
Hemoglobin [M/V] in B (-0.249)
Erythrocytes [#/V] in B by AC (-0.0737)
0.568
0.804
0.973
0.978
0.982
0.985
0.987
0.989
0.991
0.992
Expressions
−0.6
−0.4
−0.2
0.0
0.2
0.4
0.6

Signature [583]; 8 To Reach 0.975, (10 Plotted)

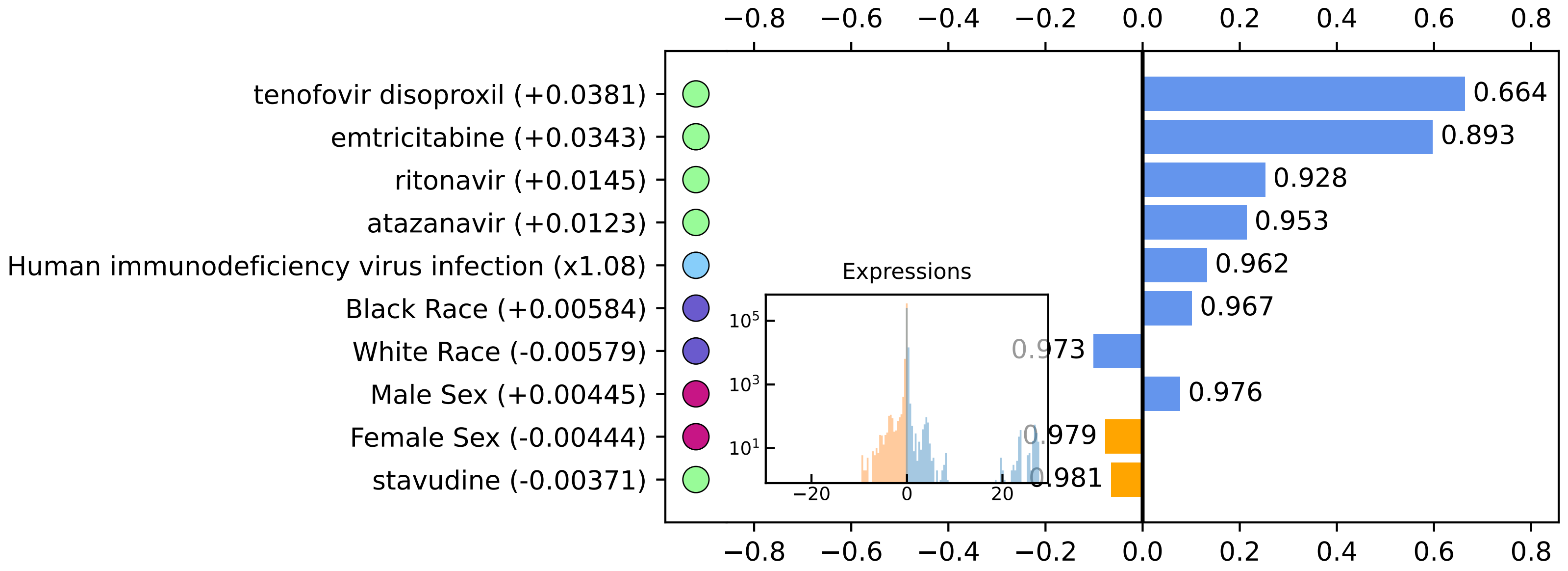

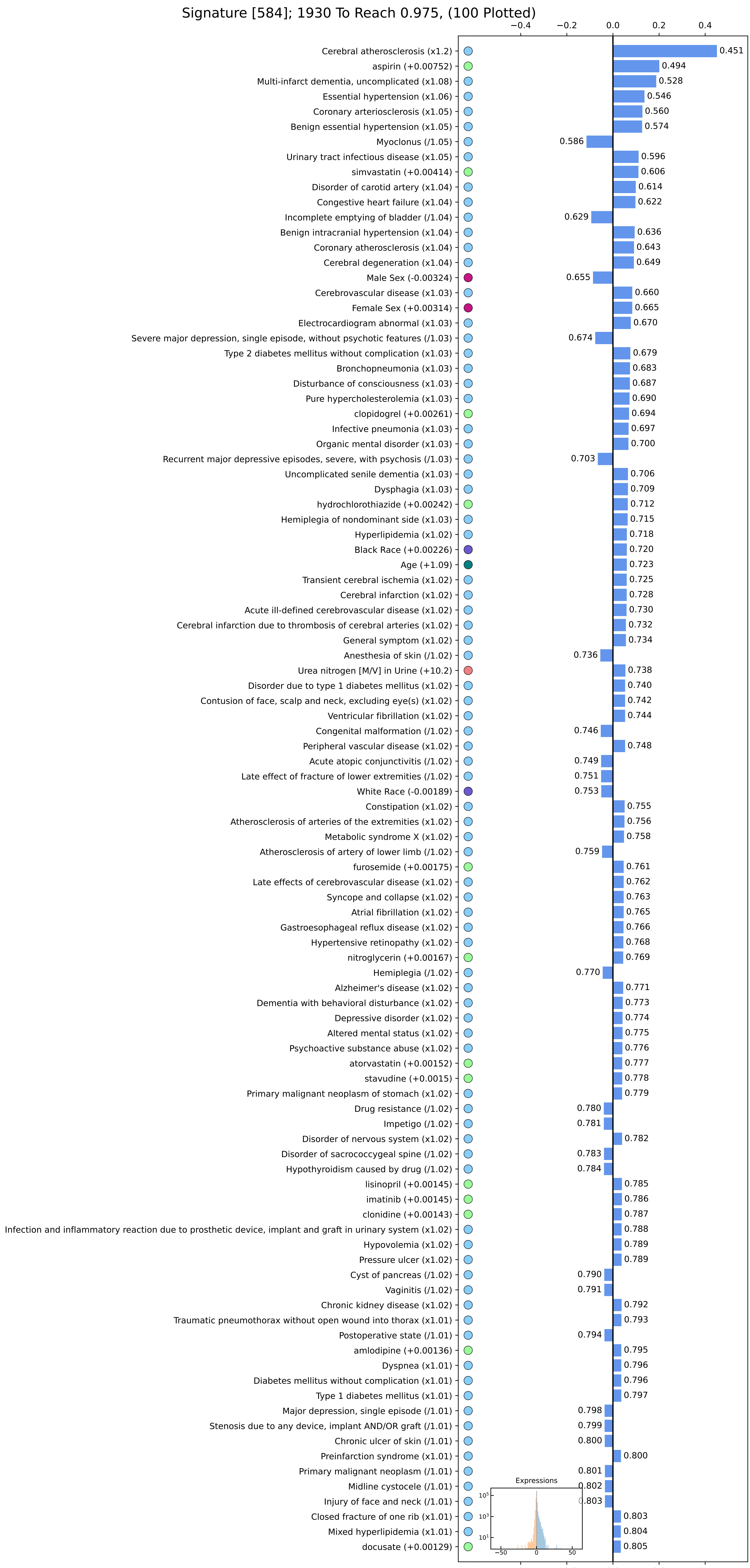

Signature [584]; 1930 To Reach 0.975, (100 Plotted)
Cerebral atherosclerosis (x1.2) 0.451
aspirin (+0.00752) 0.494
Multi-infarct dementia, uncomplicated (x1.08) 0.528
Essential hypertension (x1.06) 0.546
Coronary arteriosclerosis (x1.05) 0.560
Benign essential hypertension (x1.05) 0.574
Myoclonus (/1.05) 0.586
Urinary tract infectious disease (x1.05) 0.596
simvastatin (+0.00414) 0.606
Disorder of carotid artery (x1.04) 0.614
Congestive heart failure (x1.04) 0.622
Incomplete emptying of bladder (/1.04) 0.629
Benign intracranial hypertension (x1.04) 0.636
Coronary atherosclerosis (x1.04) 0.643
Cerebral degeneration (x1.04) 0.649
Male Sex (-0.00324) 0.655
Cerebrovascular disease (x1.03) 0.660
Female Sex (+0.00314) 0.665
Electrocardiogram abnormal (x1.03) 0.670
Severe major depression, single episode, without psychotic features (/1.03) 0.674
Type 2 diabetes mellitus without complication (x1.03) 0.679
Bronchopneumonia (x1.03) 0.683
Disturbance of consciousness (x1.03) 0.687
Pure hypercholesterolemia (x1.03) 0.690
clopidogrel (+0.00261) 0.694
Infective pneumonia (x1.03) 0.697
Organic mental disorder (x1.03) 0.700
Recurrent major depressive episodes, severe, with psychosis (/1.03) 0.703
Uncomplicated senile dementia (x1.03) 0.706
Dysphagia (x1.03) 0.709
hydrochlorothiazide (+0.00242) 0.712
Hemiplegia of nondominant side (x1.03) 0.715
Hyperlipidemia (x1.02) 0.718
Black Race (+0.00226) 0.720
Age (+1.09) 0.723
Transient cerebral ischemia (x1.02) 0.725
Cerebral infarction (x1.02) 0.728
Acute ill-defined cerebrovascular disease (x1.02) 0.730
Cerebral infarction due to thrombosis of cerebral arteries (x1.02) 0.732
General symptom (x1.02) 0.734
Anesthesia of skin (/1.02) 0.736
Urea nitrogen [M/V] in Urine (+10.2) 0.738
Disorder due to type 1 diabetes mellitus (x1.02) 0.740
Contusion of face, scalp and neck, excluding eye(s) (x1.02) 0.742
Ventricular fibrillation (x1.02) 0.744
Congenital malformation (/1.02) 0.746
Peripheral vascular disease (x1.02) 0.748
Acute atopic conjunctivitis (/1.02) 0.749
Late effect of fracture of lower extremities (/1.02) 0.751
White Race (-0.00189) 0.753
Constipation (x1.02) 0.755
Atherosclerosis of arteries of the extremities (x1.02) 0.756
Metabolic syndrome X (x1.02) 0.758
Atherosclerosis of artery of lower limb (/1.02) 0.759
furosemide (+0.00175) 0.761
Late effects of cerebrovascular disease (x1.02) 0.762
Syncope and collapse (x1.02) 0.763
Atrial fibrillation (x1.02) 0.765
Gastroesophageal reflux disease (x1.02) 0.766
Hypertensive retinopathy (x1.02) 0.768
nitroglycerin (+0.00167) 0.769
Hemiplegia (/1.02) 0.770
Alzheimer's disease (x1.02) 0.771
Dementia with behavioral disturbance (x1.02) 0.773
Depressive disorder (x1.02) 0.774
Altered mental status (x1.02) 0.775
Psychoactive substance abuse (x1.02) 0.776
atorvastatin (+0.00152) 0.777
stavudine (+0.0015) 0.778
Primary malignant neoplasm of stomach (x1.02) 0.779
Drug resistance (/1.02) 0.780
Impetigo (/1.02) 0.781
Disorder of nervous system (x1.02) 0.782
Disorder of sacrococcygeal spine (/1.02) 0.783
Hypothyroidism caused by drug (/1.02) 0.784
lisinopril (+0.00145) 0.785
imatinib (+0.00145) 0.786
clonidine (+0.00143) 0.787
Infection and inflammatory reaction due to prosthetic device, implant and graft in urinary system (x1.02) 0.788
Hypovolemia (x1.02) 0.789
Pressure ulcer (x1.02) 0.789
Cyst of pancreas (/1.02) 0.790
Vaginitis (/1.02) 0.791
Chronic kidney disease (x1.02) 0.792
Traumatic pneumothorax without open wound into thorax (x1.01) 0.793
Postoperative state (/1.01) 0.794
amlodipine (+0.00136) 0.795
Dyspnea (x1.01) 0.796
Diabetes mellitus without complication (x1.01) 0.796
Type 1 diabetes mellitus (x1.01) 0.797
Major depression, single episode (/1.01) 0.798
Stenosis due to any device, implant AND/OR graft (/1.01) 0.799
Chronic ulcer of skin (/1.01) 0.800
Preinfarction syndrome (x1.01) 0.800
Primary malignant neoplasm (/1.01) 0.801
Midline cystocele (/1.01) 0.802
Injury of face and neck (/1.01) 0.803
Closed fracture of one rib (x1.01) 0.803
Mixed hyperlipidemia (x1.01) 0.804
docusate (+0.00129) 0.805
Expressions
−0.4 −0.2 0.0 0.2 0.4

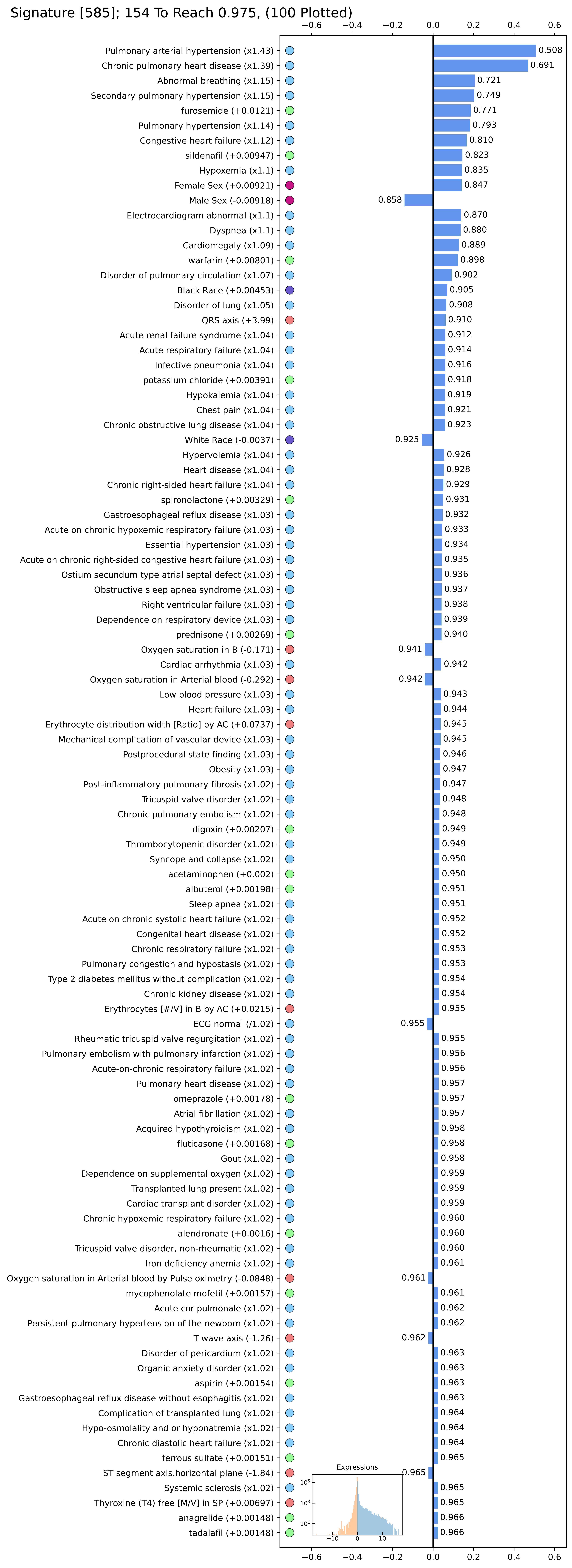

Signature [585]; 154 To Reach 0.975, (100 Plotted)
Pulmonary arterial hypertension (x1.43) 0.508
Chronic pulmonary heart disease (x1.39) 0.691
Abnormal breathing (x1.15) 0.721
Secondary pulmonary hypertension (x1.15) 0.749
furosemide (+0.0121) 0.771
Pulmonary hypertension (x1.14) 0.793
Congestive heart failure (x1.12) 0.810
sildenafil (+0.00947) 0.823
Hypoxemia (x1.1) 0.835
Female Sex (+0.00921) 0.847
Male Sex (-0.00918) 0.858
Electrocardiogram abnormal (x1.1) 0.870
Dyspnea (x1.1) 0.880
Cardiomegaly (x1.09) 0.889
warfarin (+0.00801) 0.898
Disorder of pulmonary circulation (x1.07) 0.902
Black Race (+0.00453) 0.905
Disorder of lung (x1.05) 0.908
QRS axis (+3.99) 0.910
Acute renal failure syndrome (x1.04) 0.912
Acute respiratory failure (x1.04) 0.914
Infective pneumonia (x1.04) 0.916
potassium chloride (+0.00391) 0.918
Hypokalemia (x1.04) 0.919
Chest pain (x1.04) 0.921
Chronic obstructive lung disease (x1.04) 0.923
White Race (-0.0037) 0.925
Hypervolemia (x1.04) 0.926
Heart disease (x1.04) 0.928
Chronic right-sided heart failure (x1.04) 0.929
spironolactone (+0.00329) 0.931
Gastroesophageal reflux disease (x1.03) 0.932
Acute on chronic hypoxemic respiratory failure (x1.03) 0.933
Essential hypertension (x1.03) 0.934
Acute on chronic right-sided congestive heart failure (x1.03) 0.935
Ostium secundum type atrial septal defect (x1.03) 0.936
Obstructive sleep apnea syndrome (x1.03) 0.937
Right ventricular failure (x1.03) 0.938
Dependence on respiratory device (x1.03) 0.939
prednisone (+0.00269) 0.940
Oxygen saturation in B (-0.171) 0.941
Cardiac arrhythmia (x1.03) 0.942
Oxygen saturation in Arterial blood (-0.292) 0.942
Low blood pressure (x1.03) 0.943
Heart failure (x1.03) 0.944
Erythrocyte distribution width [Ratio] by AC (+0.0737) 0.945
Mechanical complication of vascular device (x1.03) 0.945
Postprocedural state finding (x1.03) 0.946
Obesity (x1.03) 0.947
Post-inflammatory pulmonary fibrosis (x1.02) 0.947
Tricuspid valve disorder (x1.02) 0.948
Chronic pulmonary embolism (x1.02) 0.948
digoxin (+0.00207) 0.949
Thrombocytopenic disorder (x1.02) 0.949
Syncope and collapse (x1.02) 0.950
acetaminophen (+0.002) 0.950
albuterol (+0.00198) 0.951
Sleep apnea (x1.02) 0.951
Acute on chronic systolic heart failure (x1.02) 0.952
Congenital heart disease (x1.02) 0.952
Chronic respiratory failure (x1.02) 0.953
Pulmonary congestion and hypostasis (x1.02) 0.953
Type 2 diabetes mellitus without complication (x1.02) 0.954
Chronic kidney disease (x1.02) 0.954
Erythrocytes [#/V] in B by AC (+0.0215) 0.955
ECG normal (/1.02) 0.955
Rheumatic tricuspid valve regurgitation (x1.02) 0.955
Pulmonary embolism with pulmonary infarction (x1.02) 0.956
Acute-on-chronic respiratory failure (x1.02) 0.956
Pulmonary heart disease (x1.02) 0.957
omeprazole (+0.00178) 0.957
Atrial fibrillation (x1.02) 0.957
Acquired hypothyroidism (x1.02) 0.958
fluticasone (+0.00168) 0.958
Gout (x1.02) 0.958
Dependence on supplemental oxygen (x1.02) 0.959
Transplanted lung present (x1.02) 0.959
Cardiac transplant disorder (x1.02) 0.959
Chronic hypoxemic respiratory failure (x1.02) 0.960
alendronate (+0.0016) 0.960
Tricuspid valve disorder, non-rheumatic (x1.02) 0.960
Iron deficiency anemia (x1.02) 0.961
Oxygen saturation in Arterial blood by Pulse oximetry (-0.0848) 0.961
mycophenolate mofetil (+0.00157) 0.961
Acute cor pulmonale (x1.02) 0.962
Persistent pulmonary hypertension of the newborn (x1.02) 0.962
T wave axis (-1.26) 0.962
Disorder of pericardium (x1.02) 0.963
Organic anxiety disorder (x1.02) 0.963
aspirin (+0.00154) 0.963
Gastroesophageal reflux disease without esophagitis (x1.02) 0.963
Complication of transplanted lung (x1.02) 0.964
Hypo-osmolality and or hyponatremia (x1.02) 0.964
Chronic diastolic heart failure (x1.02) 0.964
ferrous sulfate (+0.00151) 0.965
ST segment axis.horizontal plane (-1.84) 0.965
Systemic sclerosis (x1.02) 0.965
Thyroxine (T4) free [M/V] in SP (+0.00697) 0.965
anagrelide (+0.00148) 0.966
tadalafil (+0.00148) 0.966
Expressions
−0.6 −0.4 −0.2 0.0 0.2 0.4 0.6

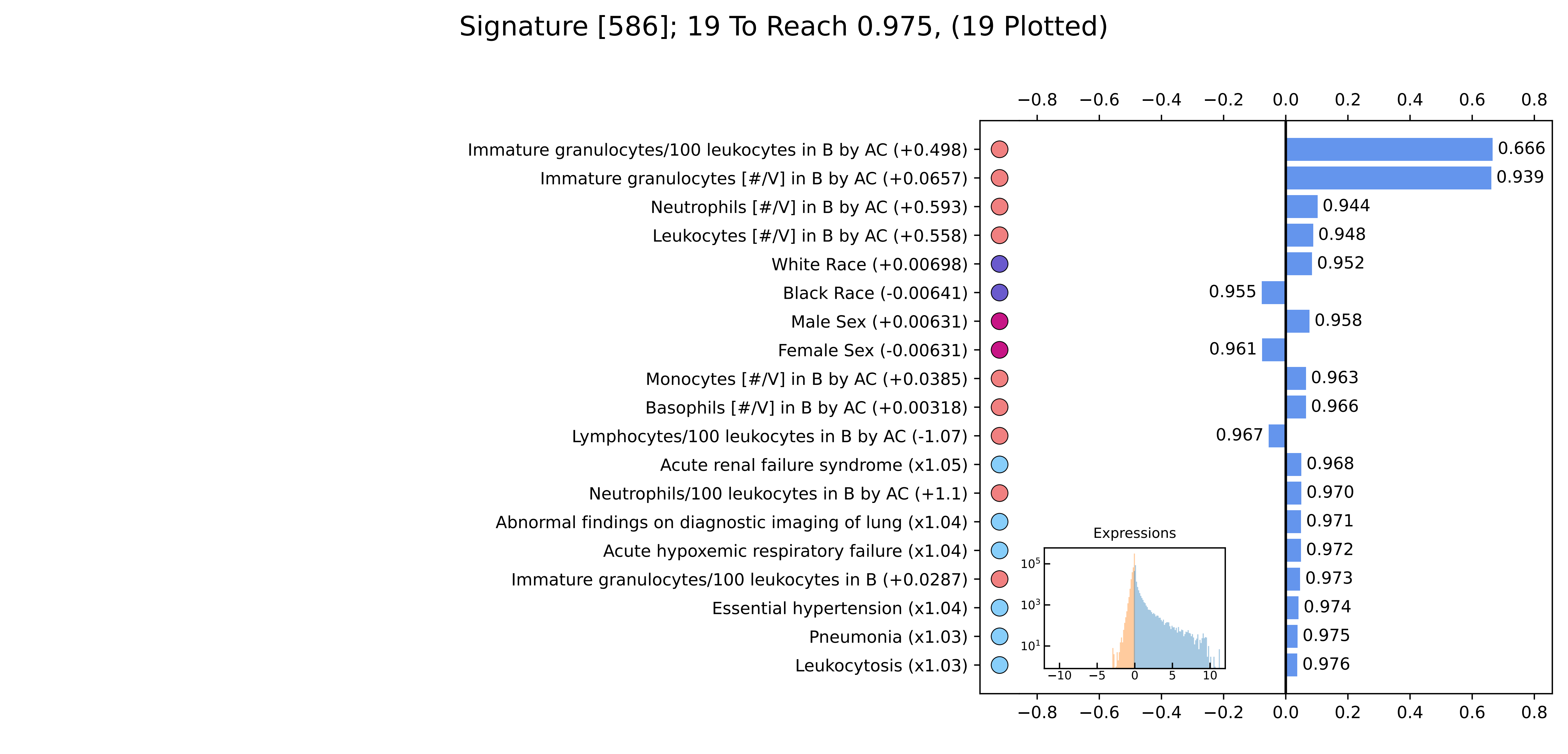
Signature [586]; 19 To Reach 0.975, (19 Plotted)
Immature granulocytes/100 leukocytes in B by AC (+0.498)
Immature granulocytes [#/V] in B by AC (+0.0657)
Neutrophils [#/V] in B by AC (+0.593)
Leukocytes [#/V] in B by AC (+0.558)
White Race (+0.00698)
Black Race (-0.00641)
Male Sex (+0.00631)
Female Sex (-0.00631)
Monocytes [#/V] in B by AC (+0.0385)
Basophils [#/V] in B by AC (+0.00318)
Lymphocytes/100 leukocytes in B by AC (-1.07)
Acute renal failure syndrome (x1.05)
Neutrophils/100 leukocytes in B by AC (+1.1)
Abnormal findings on diagnostic imaging of lung (x1.04)
Acute hypoxemic respiratory failure (x1.04)
Immature granulocytes/100 leukocytes in B (+0.0287)
Essential hypertension (x1.04)
Pneumonia (x1.03)
Leukocytosis (x1.03)
0.666
0.939
0.944
0.948
0.952
0.955
0.958
0.961
0.963
0.966
0.967
0.968
0.970
0.971
0.972
0.973
0.974
0.975
0.976
Expressions

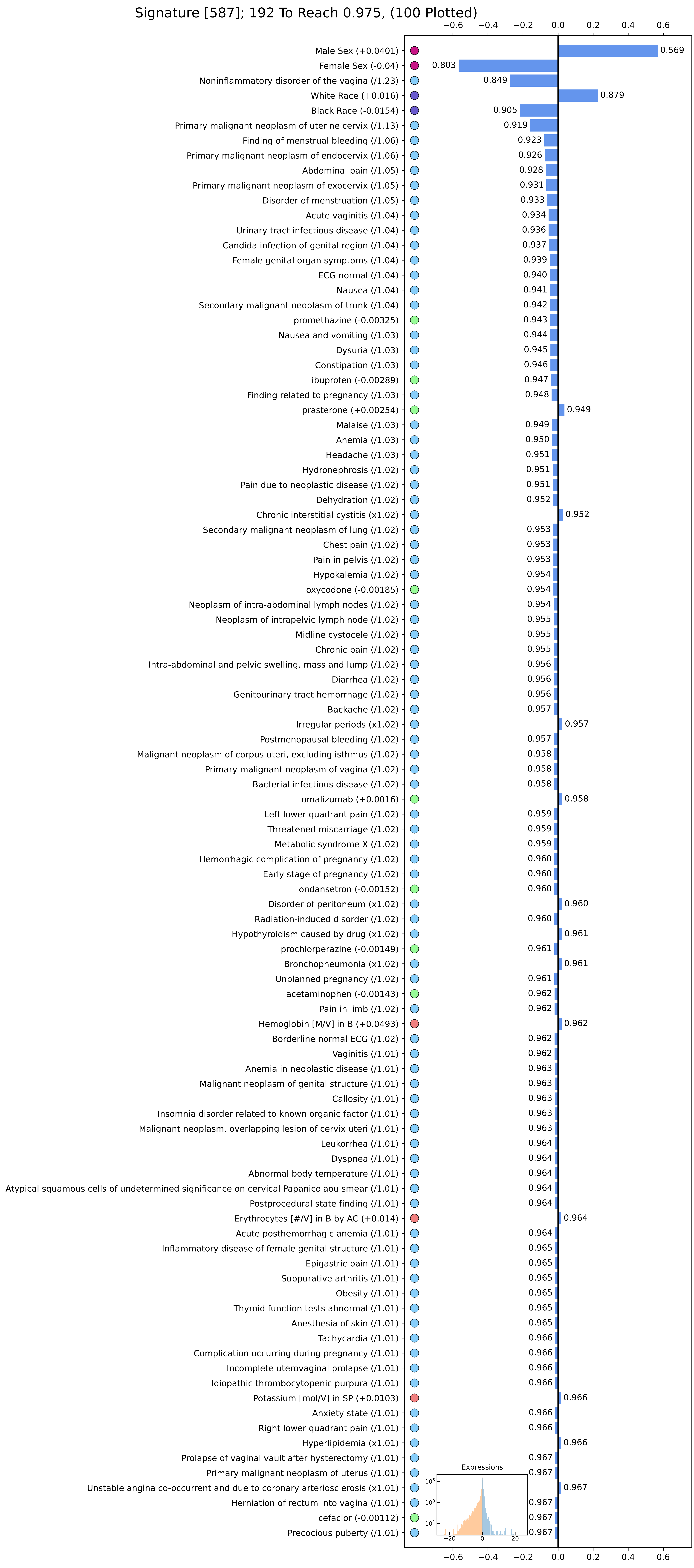

Signature [587]; 192 To Reach 0.975, (100 Plotted)
Male Sex (+0.0401) 0.569
Female Sex (-0.04) 0.803
Noninflammatory disorder of the vagina (/1.23) 0.849
White Race (+0.016) 0.879
Black Race (-0.0154) 0.905
Primary malignant neoplasm of uterine cervix (/1.13) 0.919
Finding of menstrual bleeding (/1.06) 0.923
Primary malignant neoplasm of endocervix (/1.06) 0.926
Abdominal pain (/1.05) 0.928
Primary malignant neoplasm of exocervix (/1.05) 0.931
Disorder of menstruation (/1.05) 0.933
Acute vaginitis (/1.04) 0.934
Urinary tract infectious disease (/1.04) 0.936
Candida infection of genital region (/1.04) 0.937
Female genital organ symptoms (/1.04) 0.939
ECG normal (/1.04) 0.940
Nausea (/1.04) 0.941
Secondary malignant neoplasm of trunk (/1.04) 0.942
promethazine (-0.00325) 0.943
Nausea and vomiting (/1.03) 0.944
Dysuria (/1.03) 0.945
Constipation (/1.03) 0.946
ibuprofen (-0.00289) 0.947
Finding related to pregnancy (/1.03) 0.948
prasterone (+0.00254) 0.949
Malaise (/1.03) 0.949
Anemia (/1.03) 0.950
Headache (/1.03) 0.951
Hydronephrosis (/1.02) 0.951
Pain due to neoplastic disease (/1.02) 0.951
Dehydration (/1.02) 0.952
Chronic interstitial cystitis (x1.02) 0.952
Secondary malignant neoplasm of lung (/1.02) 0.953
Chest pain (/1.02) 0.953
Pain in pelvis (/1.02) 0.953
Hypokalemia (/1.02) 0.954
oxycodone (-0.00185) 0.954
Neoplasm of intra-abdominal lymph nodes (/1.02) 0.954
Neoplasm of intrapelvic lymph node (/1.02) 0.955
Midline cystocele (/1.02) 0.955
Chronic pain (/1.02) 0.955
Intra-abdominal and pelvic swelling, mass and lump (/1.02) 0.956
Diarrhea (/1.02) 0.956
Genitourinary tract hemorrhage (/1.02) 0.956
Backache (/1.02) 0.957
Irregular periods (x1.02) 0.957
Postmenopausal bleeding (/1.02) 0.957
Malignant neoplasm of corpus uteri, excluding isthmus (/1.02) 0.958
Primary malignant neoplasm of vagina (/1.02) 0.958
Bacterial infectious disease (/1.02) 0.958
omalizumab (+0.0016) 0.958
Left lower quadrant pain (/1.02) 0.959
Threatened miscarriage (/1.02) 0.959
Metabolic syndrome X (/1.02) 0.959
Hemorrhagic complication of pregnancy (/1.02) 0.960
Early stage of pregnancy (/1.02) 0.960
ondansetron (-0.00152) 0.960
Disorder of peritoneum (x1.02) 0.960
Radiation-induced disorder (/1.02) 0.960
Hypothyroidism caused by drug (x1.02) 0.961
prochlorperazine (-0.00149) 0.961
Bronchopneumonia (x1.02) 0.961
Unplanned pregnancy (/1.02) 0.961
acetaminophen (-0.00143) 0.962
Pain in limb (/1.02) 0.962
Hemoglobin [M/V] in B (+0.0493) 0.962
Borderline normal ECG (/1.02) 0.962
Vaginitis (/1.01) 0.962
Anemia in neoplastic disease (/1.01) 0.963
Malignant neoplasm of genital structure (/1.01) 0.963
Callosity (/1.01) 0.963
Insomnia disorder related to known organic factor (/1.01) 0.963
Malignant neoplasm, overlapping lesion of cervix uteri (/1.01) 0.963
Leukorrhea (/1.01) 0.964
Dyspnea (/1.01) 0.964
Abnormal body temperature (/1.01) 0.964
Atypical squamous cells of undetermined significance on cervical Papanicolaou smear (/1.01) 0.964
Postprocedural state finding (/1.01) 0.964
Erythrocytes [#/V] in B by AC (+0.014) 0.964
Acute posthemorrhagic anemia (/1.01) 0.964
Inflammatory disease of female genital structure (/1.01) 0.965
Epigastric pain (/1.01) 0.965
Suppurative arthritis (/1.01) 0.965
Obesity (/1.01) 0.965
Thyroid function tests abnormal (/1.01) 0.965
Anesthesia of skin (/1.01) 0.965
Tachycardia (/1.01) 0.966
Complication occurring during pregnancy (/1.01) 0.966
Incomplete uterovaginal prolapse (/1.01) 0.966
Idiopathic thrombocytopenic purpura (/1.01) 0.966
Potassium [mol/V] in SP (+0.0103) 0.966
Anxiety state (/1.01) 0.966
Right lower quadrant pain (/1.01) 0.966
Hyperlipidemia (x1.01) 0.966
Prolapse of vaginal vault after hysterectomy (/1.01) 0.967
Primary malignant neoplasm of uterus (/1.01) 0.967
Unstable angina co-occurrent and due to coronary arteriosclerosis (x1.01) 0.967
Herniation of rectum into vagina (/1.01) 0.967
cefaclor (-0.00112) 0.967
Precocious puberty (/1.01) 0.967
−0.6 −0.4 −0.2 0.0 0.2 0.4 0.6
Expressions

Signature [588]; 6 To Reach 0.975, (10 Plotted)

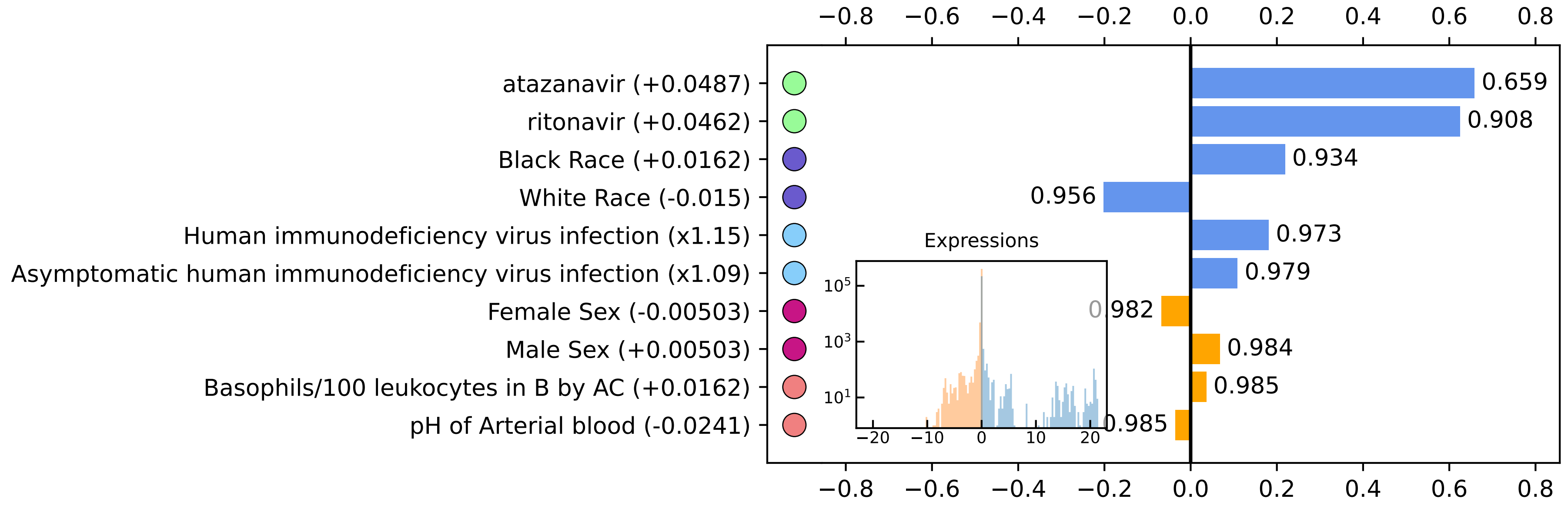

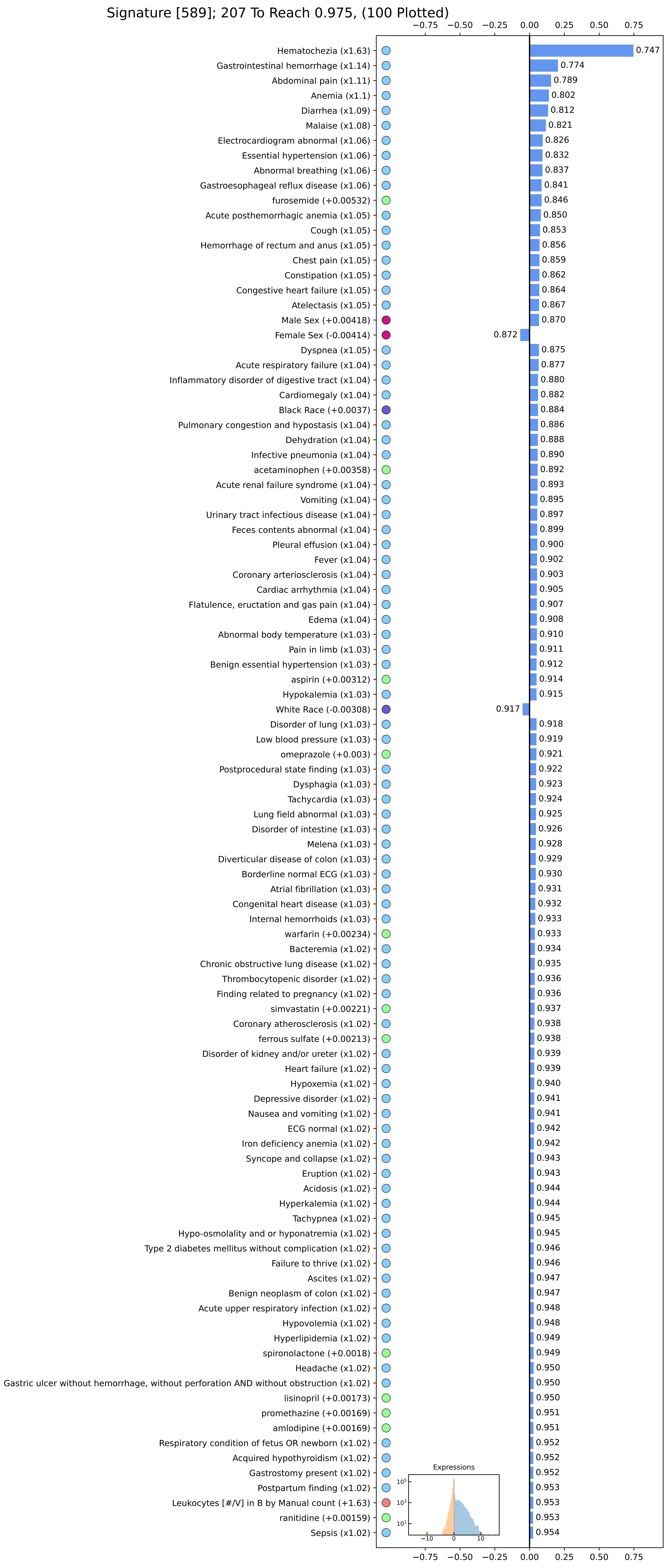
Signature [589]; 207 To Reach 0.975, (100 Plotted)
−0.75 −0.50 −0.25 0.00 0.25 0.50 0.75
Hematochezia (x1.63) 0.747
Gastrointestinal hemorrhage (x1.14) 0.774
Abdominal pain (x1.11) 0.789
Anemia (x1.1) 0.802
Diarrhea (x1.09) 0.812
Malaise (x1.08) 0.821
Electrocardiogram abnormal (x1.06) 0.826
Essential hypertension (x1.06) 0.832
Abnormal breathing (x1.06) 0.837
Gastroesophageal reflux disease (x1.06) 0.841
furosemide (+0.00532) 0.846
Acute posthemorrhagic anemia (x1.05) 0.850
Cough (x1.05) 0.853
Hemorrhage of rectum and anus (x1.05) 0.856
Chest pain (x1.05) 0.859
Constipation (x1.05) 0.862
Congestive heart failure (x1.05) 0.864
Atelectasis (x1.05) 0.867
Male Sex (+0.00418) 0.870
Female Sex (-0.00414) 0.872
Dyspnea (x1.05) 0.875
Acute respiratory failure (x1.04) 0.877
Inflammatory disorder of digestive tract (x1.04) 0.880
Cardiomegaly (x1.04) 0.882
Black Race (+0.0037) 0.884
Pulmonary congestion and hypostasis (x1.04) 0.886
Dehydration (x1.04) 0.888
Infective pneumonia (x1.04) 0.890
acetaminophen (+0.00358) 0.892
Acute renal failure syndrome (x1.04) 0.893
Vomiting (x1.04) 0.895
Urinary tract infectious disease (x1.04) 0.897
Feces contents abnormal (x1.04) 0.899
Pleural effusion (x1.04) 0.900
Fever (x1.04) 0.902
Coronary arteriosclerosis (x1.04) 0.903
Cardiac arrhythmia (x1.04) 0.905
Flatulence, eructation and gas pain (x1.04) 0.907
Edema (x1.04) 0.908
Abnormal body temperature (x1.03) 0.910
Pain in limb (x1.03) 0.911
Benign essential hypertension (x1.03) 0.912
aspirin (+0.00312) 0.914
Hypokalemia (x1.03) 0.915
White Race (-0.00308) 0.917
Disorder of lung (x1.03) 0.918
Low blood pressure (x1.03) 0.919
omeprazole (+0.003) 0.921
Postprocedural state finding (x1.03) 0.922
Dysphagia (x1.03) 0.923
Tachycardia (x1.03) 0.924
Lung field abnormal (x1.03) 0.925
Disorder of intestine (x1.03) 0.926
Melena (x1.03) 0.928
Diverticular disease of colon (x1.03) 0.929
Borderline normal ECG (x1.03) 0.930
Atrial fibrillation (x1.03) 0.931
Congenital heart disease (x1.03) 0.932
Internal hemorrhoids (x1.03) 0.933
warfarin (+0.00234) 0.933
Bacteremia (x1.02) 0.934
Chronic obstructive lung disease (x1.02) 0.935
Thrombocytopenic disorder (x1.02) 0.936
Finding related to pregnancy (x1.02) 0.936
simvastatin (+0.00221) 0.937
Coronary atherosclerosis (x1.02) 0.938
ferrous sulfate (+0.00213) 0.938
Disorder of kidney and/or ureter (x1.02) 0.939
Heart failure (x1.02) 0.939
Hypoxemia (x1.02) 0.940
Depressive disorder (x1.02) 0.941
Nausea and vomiting (x1.02) 0.941
ECG normal (x1.02) 0.942
Iron deficiency anemia (x1.02) 0.942
Syncope and collapse (x1.02) 0.943
Eruption (x1.02) 0.943
Acidosis (x1.02) 0.944
Hyperkalemia (x1.02) 0.944
Tachypnea (x1.02) 0.945
Hypo-osmolality and or hyponatremia (x1.02) 0.945
Type 2 diabetes mellitus without complication (x1.02) 0.946
Failure to thrive (x1.02) 0.946
Ascites (x1.02) 0.947
Benign neoplasm of colon (x1.02) 0.947
Acute upper respiratory infection (x1.02) 0.948
Hypovolemia (x1.02) 0.948
Hyperlipidemia (x1.02) 0.949
spironolactone (+0.0018) 0.949
Headache (x1.02) 0.950
Gastric ulcer without hemorrhage, without perforation AND without obstruction (x1.02) 0.950
lisinopril (+0.00173) 0.950
promethazine (+0.00169) 0.951
amlodipine (+0.00169) 0.951
Respiratory condition of fetus OR newborn (x1.02) 0.952
Acquired hypothyroidism (x1.02) 0.952
Gastrostomy present (x1.02) 0.952
Postpartum finding (x1.02) 0.953
Leukocytes [#/V] in B by Manual count (+1.63) 0.953
ranitidine (+0.00159) 0.953
Sepsis (x1.02) 0.954
Expressions
10^5
10^3
10^1
−10 0 10
−0.75 −0.50 −0.25 0.00 0.25 0.50 0.75

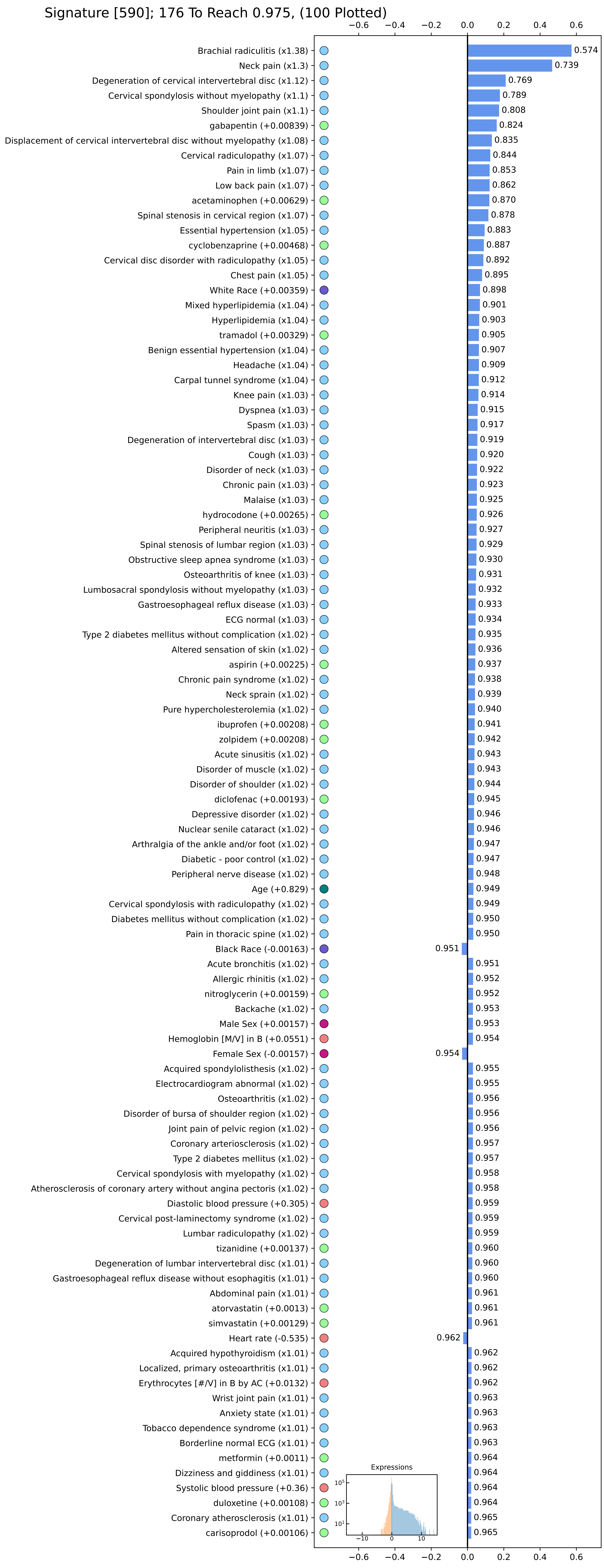

Signature [590]; 176 To Reach 0.975, (100 Plotted)
Brachial radiculitis (x1.38) 0.574
Neck pain (x1.3) 0.739
Degeneration of cervical intervertebral disc (x1.12) 0.769
Cervical spondylosis without myelopathy (x1.1) 0.789
Shoulder joint pain (x1.1) 0.808
gabapentin (+0.00839) 0.824
Displacement of cervical intervertebral disc without myelopathy (x1.08) 0.835
Cervical radiculopathy (x1.07) 0.844
Pain in limb (x1.07) 0.853
Low back pain (x1.07) 0.862
acetaminophen (+0.00629) 0.870
Spinal stenosis in cervical region (x1.07) 0.878
Essential hypertension (x1.05) 0.883
cyclobenzaprine (+0.00468) 0.887
Cervical disc disorder with radiculopathy (x1.05) 0.892
Chest pain (x1.05) 0.895
White Race (+0.00359) 0.898
Mixed hyperlipidemia (x1.04) 0.901
Hyperlipidemia (x1.04) 0.903
tramadol (+0.00329) 0.905
Benign essential hypertension (x1.04) 0.907
Headache (x1.04) 0.909
Carpal tunnel syndrome (x1.04) 0.912
Knee pain (x1.03) 0.914
Dyspnea (x1.03) 0.915
Spasm (x1.03) 0.917
Degeneration of intervertebral disc (x1.03) 0.919
Cough (x1.03) 0.920
Disorder of neck (x1.03) 0.922
Chronic pain (x1.03) 0.923
Malaise (x1.03) 0.925
hydrocodone (+0.00265) 0.926
Peripheral neuritis (x1.03) 0.927
Spinal stenosis of lumbar region (x1.03) 0.929
Obstructive sleep apnea syndrome (x1.03) 0.930
Osteoarthritis of knee (x1.03) 0.931
Lumbosacral spondylosis without myelopathy (x1.03) 0.932
Gastroesophageal reflux disease (x1.03) 0.933
ECG normal (x1.03) 0.934
Type 2 diabetes mellitus without complication (x1.02) 0.935
Altered sensation of skin (x1.02) 0.936
aspirin (+0.00225) 0.937
Chronic pain syndrome (x1.02) 0.938
Neck sprain (x1.02) 0.939
Pure hypercholesterolemia (x1.02) 0.940
ibuprofen (+0.00208) 0.941
zolpidem (+0.00208) 0.942
Acute sinusitis (x1.02) 0.943
Disorder of muscle (x1.02) 0.943
Disorder of shoulder (x1.02) 0.944
diclofenac (+0.00193) 0.945
Depressive disorder (x1.02) 0.946
Nuclear senile cataract (x1.02) 0.946
Arthralgia of the ankle and/or foot (x1.02) 0.947
Diabetic - poor control (x1.02) 0.947
Peripheral nerve disease (x1.02) 0.948
Age (+0.829) 0.949
Cervical spondylosis with radiculopathy (x1.02) 0.949
Diabetes mellitus without complication (x1.02) 0.950
Pain in thoracic spine (x1.02) 0.950
Black Race (-0.00163) 0.951
Acute bronchitis (x1.02) 0.951
Allergic rhinitis (x1.02) 0.952
nitroglycerin (+0.00159) 0.952
Backache (x1.02) 0.953
Male Sex (+0.00157) 0.953
Hemoglobin [M/V] in B (+0.0551) 0.954
Female Sex (-0.00157) 0.954
Acquired spondylolisthesis (x1.02) 0.955
Electrocardiogram abnormal (x1.02) 0.955
Osteoarthritis (x1.02) 0.956
Disorder of bursa of shoulder region (x1.02) 0.956
Joint pain of pelvic region (x1.02) 0.956
Coronary arteriosclerosis (x1.02) 0.957
Type 2 diabetes mellitus (x1.02) 0.957
Cervical spondylosis with myelopathy (x1.02) 0.958
Atherosclerosis of coronary artery without angina pectoris (x1.02) 0.958
Diastolic blood pressure (+0.305) 0.959
Cervical post-laminectomy syndrome (x1.02) 0.959
Lumbar radiculopathy (x1.02) 0.959
tizanidine (+0.00137) 0.960
Degeneration of lumbar intervertebral disc (x1.01) 0.960
Gastroesophageal reflux disease without esophagitis (x1.01) 0.960
Abdominal pain (x1.01) 0.961
atorvastatin (+0.0013) 0.961
simvastatin (+0.00129) 0.961
Heart rate (-0.535) 0.962
Acquired hypothyroidism (x1.01) 0.962
Localized, primary osteoarthritis (x1.01) 0.962
Erythrocytes [#/V] in B by AC (+0.0132) 0.962
Wrist joint pain (x1.01) 0.963
Anxiety state (x1.01) 0.963
Tobacco dependence syndrome (x1.01) 0.963
Borderline normal ECG (x1.01) 0.963
metformin (+0.0011) 0.964
Dizziness and giddiness (x1.01) 0.964
Systolic blood pressure (+0.36) 0.964
duloxetine (+0.00108) 0.964
Coronary atherosclerosis (x1.01) 0.965
carisoprodol (+0.00106) 0.965
−0.6 −0.4 −0.2 0.0 0.2 0.4 0.6
Expressions
10^5 10^3 10^1
−10 0 10

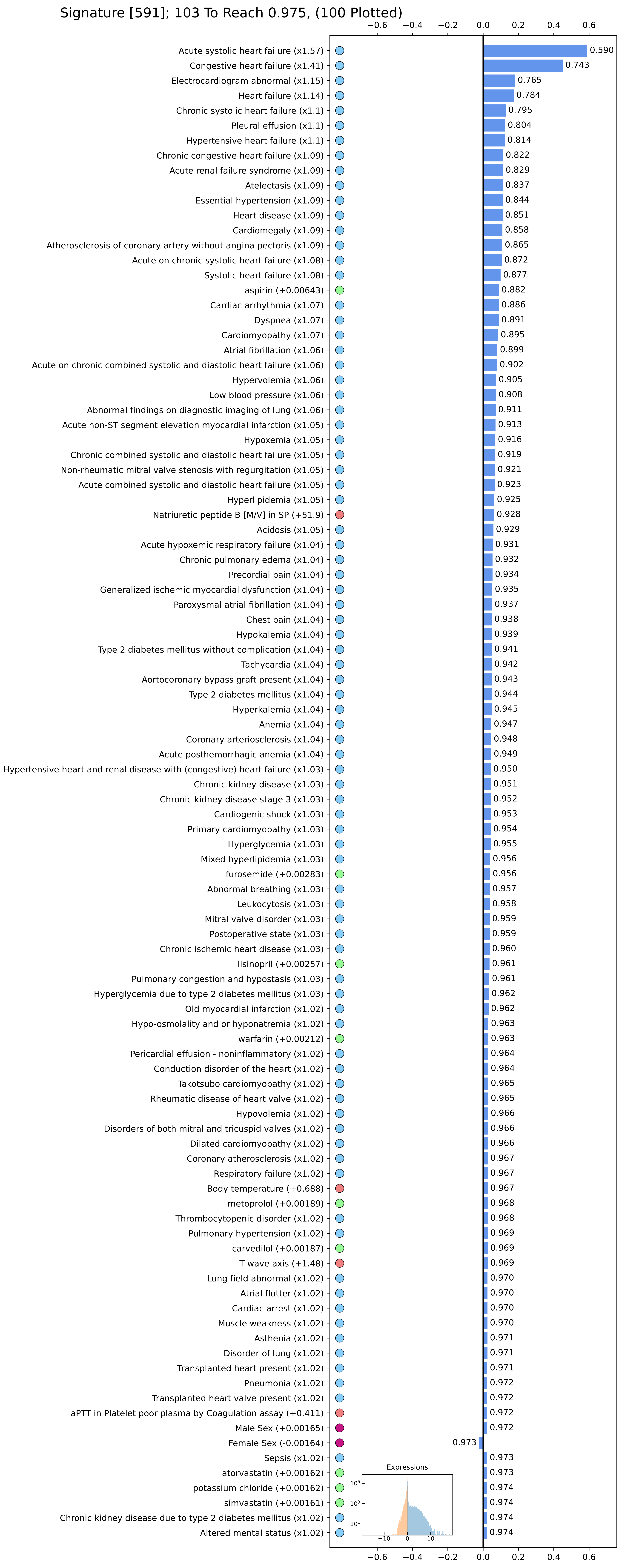

Signature [591]; 103 To Reach 0.975, (100 Plotted)
Acute systolic heart failure (x1.57) 0.590
Congestive heart failure (x1.41) 0.743
Electrocardiogram abnormal (x1.15) 0.765
Heart failure (x1.14) 0.784
Chronic systolic heart failure (x1.1) 0.795
Pleural effusion (x1.1) 0.804
Hypertensive heart failure (x1.1) 0.814
Chronic congestive heart failure (x1.09) 0.822
Acute renal failure syndrome (x1.09) 0.829
Atelectasis (x1.09) 0.837
Essential hypertension (x1.09) 0.844
Heart disease (x1.09) 0.851
Cardiomegaly (x1.09) 0.858
Atherosclerosis of coronary artery without angina pectoris (x1.09) 0.865
Acute on chronic systolic heart failure (x1.08) 0.872
Systolic heart failure (x1.08) 0.877
aspirin (+0.00643) 0.882
Cardiac arrhythmia (x1.07) 0.886
Dyspnea (x1.07) 0.891
Cardiomyopathy (x1.07) 0.895
Atrial fibrillation (x1.06) 0.899
Acute on chronic combined systolic and diastolic heart failure (x1.06) 0.902
Hypervolemia (x1.06) 0.905
Low blood pressure (x1.06) 0.908
Abnormal findings on diagnostic imaging of lung (x1.06) 0.911
Acute non-ST segment elevation myocardial infarction (x1.05) 0.913
Hypoxemia (x1.05) 0.916
Chronic combined systolic and diastolic heart failure (x1.05) 0.919
Non-rheumatic mitral valve stenosis with regurgitation (x1.05) 0.921
Acute combined systolic and diastolic heart failure (x1.05) 0.923
Hyperlipidemia (x1.05) 0.925
Natriuretic peptide B [M/V] in SP (+51.9) 0.928
Acidosis (x1.05) 0.929
Acute hypoxemic respiratory failure (x1.04) 0.931
Chronic pulmonary edema (x1.04) 0.932
Precordial pain (x1.04) 0.934
Generalized ischemic myocardial dysfunction (x1.04) 0.935
Paroxysmal atrial fibrillation (x1.04) 0.937
Chest pain (x1.04) 0.938
Hypokalemia (x1.04) 0.939
Type 2 diabetes mellitus without complication (x1.04) 0.941
Tachycardia (x1.04) 0.942
Aortocoronary bypass graft present (x1.04) 0.943
Type 2 diabetes mellitus (x1.04) 0.944
Hyperkalemia (x1.04) 0.945
Anemia (x1.04) 0.947
Coronary arteriosclerosis (x1.04) 0.948
Acute posthemorrhagic anemia (x1.04) 0.949
Hypertensive heart and renal disease with (congestive) heart failure (x1.03) 0.950
Chronic kidney disease (x1.03) 0.951
Chronic kidney disease stage 3 (x1.03) 0.952
Cardiogenic shock (x1.03) 0.953
Primary cardiomyopathy (x1.03) 0.954
Hyperglycemia (x1.03) 0.955
Mixed hyperlipidemia (x1.03) 0.956
furosemide (+0.00283) 0.956
Abnormal breathing (x1.03) 0.957
Leukocytosis (x1.03) 0.958
Mitral valve disorder (x1.03) 0.959
Postoperative state (x1.03) 0.959
Chronic ischemic heart disease (x1.03) 0.960
lisinopril (+0.00257) 0.961
Pulmonary congestion and hypostasis (x1.03) 0.961
Hyperglycemia due to type 2 diabetes mellitus (x1.03) 0.962
Old myocardial infarction (x1.02) 0.962
Hypo-osmolality and or hyponatremia (x1.02) 0.963
warfarin (+0.00212) 0.963
Pericardial effusion - noninflammatory (x1.02) 0.964
Conduction disorder of the heart (x1.02) 0.964
Takotsubo cardiomyopathy (x1.02) 0.965
Rheumatic disease of heart valve (x1.02) 0.965
Hypovolemia (x1.02) 0.966
Disorders of both mitral and tricuspid valves (x1.02) 0.966
Dilated cardiomyopathy (x1.02) 0.966
Coronary atherosclerosis (x1.02) 0.967
Respiratory failure (x1.02) 0.967
Body temperature (+0.688) 0.967
metoprolol (+0.00189) 0.968
Thrombocytopenic disorder (x1.02) 0.968
Pulmonary hypertension (x1.02) 0.969
carvedilol (+0.00187) 0.969
T wave axis (+1.48) 0.969
Lung field abnormal (x1.02) 0.970
Atrial flutter (x1.02) 0.970
Cardiac arrest (x1.02) 0.970
Muscle weakness (x1.02) 0.970
Asthenia (x1.02) 0.971
Disorder of lung (x1.02) 0.971
Transplanted heart present (x1.02) 0.971
Pneumonia (x1.02) 0.972
Transplanted heart valve present (x1.02) 0.972
aPTT in Platelet poor plasma by Coagulation assay (+0.411) 0.972
Male Sex (+0.00165) 0.972
Female Sex (-0.00164) 0.973
Sepsis (x1.02) 0.973
atorvastatin (+0.00162) 0.973
potassium chloride (+0.00162) 0.974
simvastatin (+0.00161) 0.974
Chronic kidney disease due to type 2 diabetes mellitus (x1.02) 0.974
Altered mental status (x1.02) 0.974
Expressions
−0.6 −0.4 −0.2 0.0 0.2 0.4 0.6

Signature [592]; 18 To Reach 0.975, (18 Plotted)

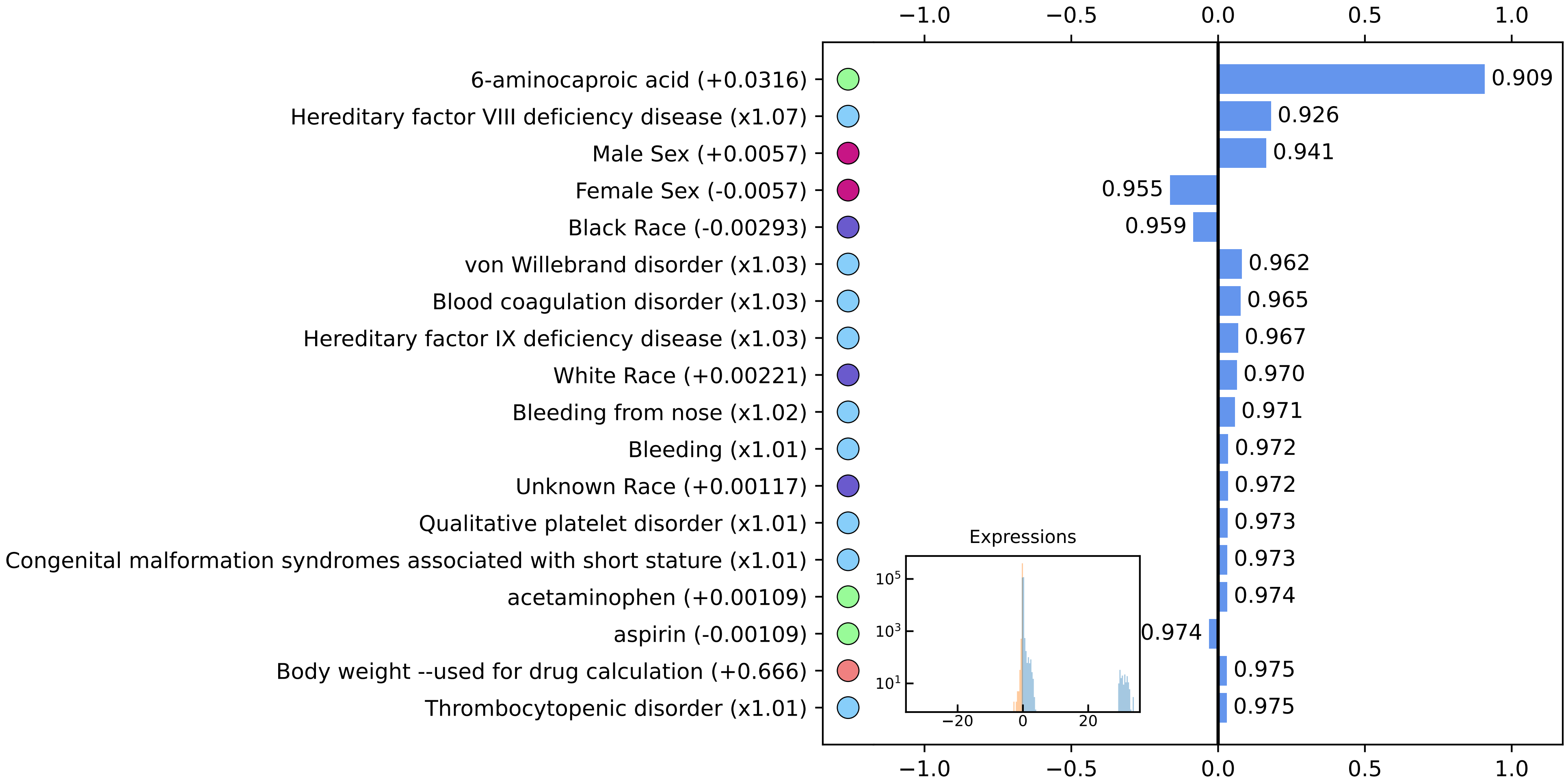

Signature [593]; 2 To Reach 0.975, (10 Plotted)

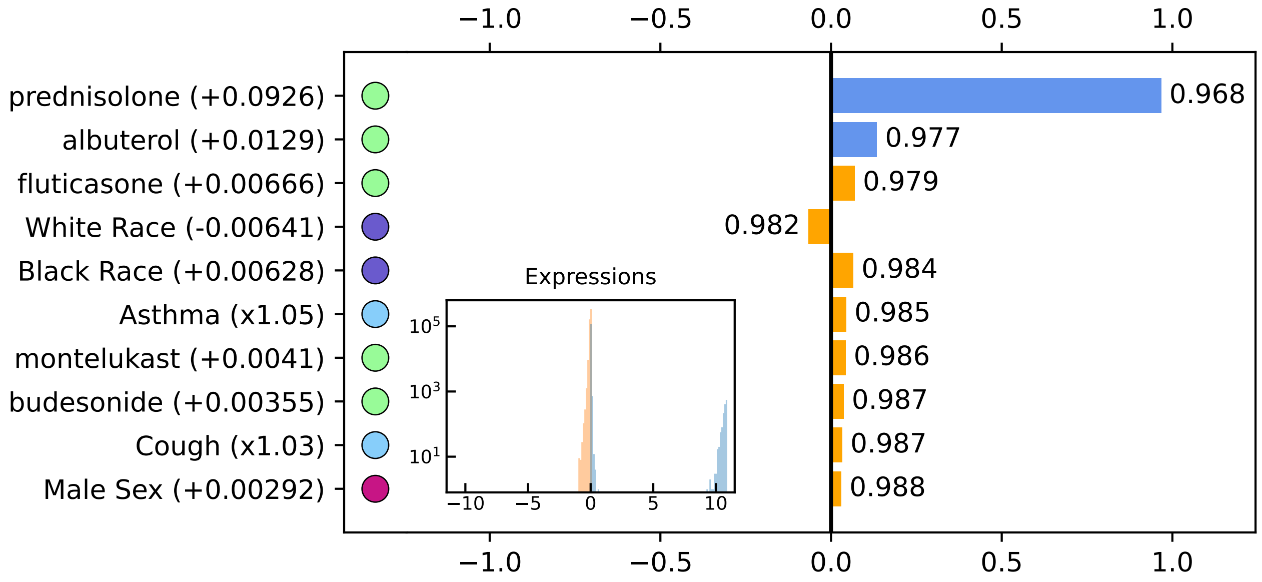

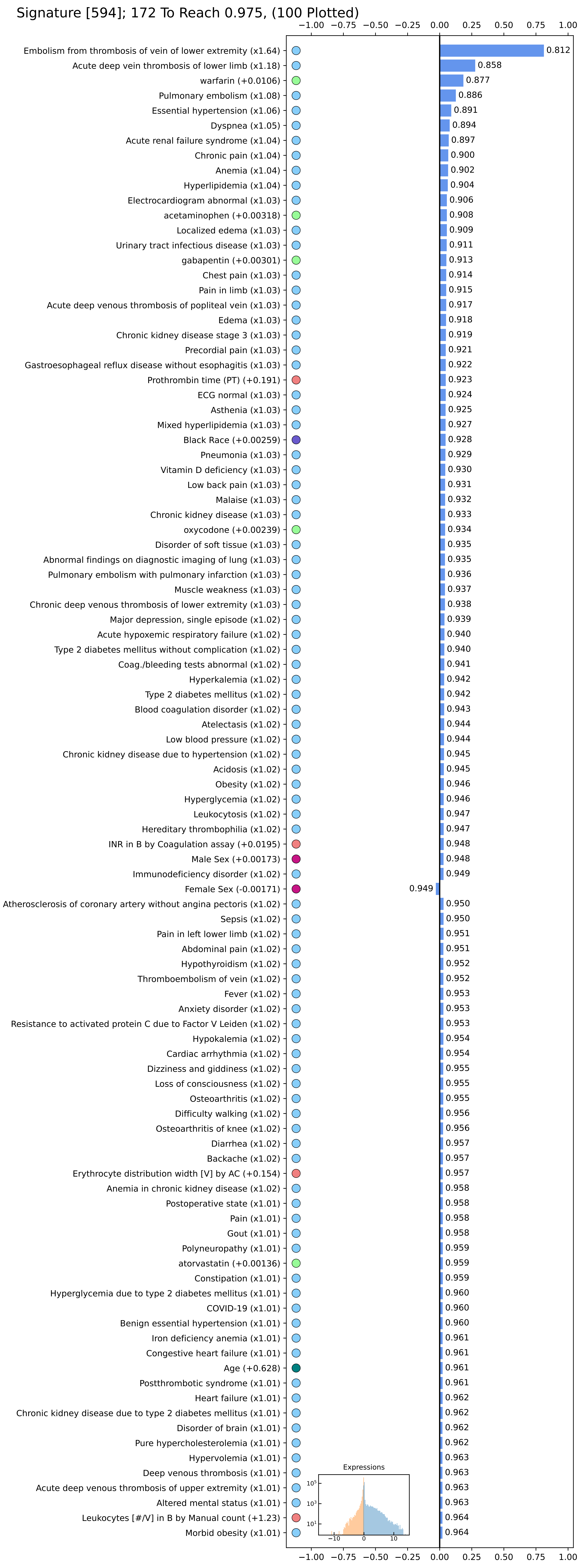
Signature [594]; 172 To Reach 0.975, (100 Plotted)
−1.00 −0.75 −0.50 −0.25 0.00 0.25 0.50 0.75 1.00
Embolism from thrombosis of vein of lower extremity (x1.64) 0.812
Acute deep vein thrombosis of lower limb (x1.18) 0.858
warfarin (+0.0106) 0.877
Pulmonary embolism (x1.08) 0.886
Essential hypertension (x1.06) 0.891
Dyspnea (x1.05) 0.894
Acute renal failure syndrome (x1.04) 0.897
Chronic pain (x1.04) 0.900
Anemia (x1.04) 0.902
Hyperlipidemia (x1.04) 0.904
Electrocardiogram abnormal (x1.03) 0.906
acetaminophen (+0.00318) 0.908
Localized edema (x1.03) 0.909
Urinary tract infectious disease (x1.03) 0.911
gabapentin (+0.00301) 0.913
Chest pain (x1.03) 0.914
Pain in limb (x1.03) 0.915
Acute deep venous thrombosis of popliteal vein (x1.03) 0.917
Edema (x1.03) 0.918
Chronic kidney disease stage 3 (x1.03) 0.919
Precordial pain (x1.03) 0.921
Gastroesophageal reflux disease without esophagitis (x1.03) 0.922
Prothrombin time (PT) (+0.191) 0.923
ECG normal (x1.03) 0.924
Asthenia (x1.03) 0.925
Mixed hyperlipidemia (x1.03) 0.927
Black Race (+0.00259) 0.928
Pneumonia (x1.03) 0.929
Vitamin D deficiency (x1.03) 0.930
Low back pain (x1.03) 0.931
Malaise (x1.03) 0.932
Chronic kidney disease (x1.03) 0.933
oxycodone (+0.00239) 0.934
Disorder of soft tissue (x1.03) 0.935
Abnormal findings on diagnostic imaging of lung (x1.03) 0.935
Pulmonary embolism with pulmonary infarction (x1.03) 0.936
Muscle weakness (x1.03) 0.937
Chronic deep venous thrombosis of lower extremity (x1.03) 0.938
Major depression, single episode (x1.02) 0.939
Acute hypoxemic respiratory failure (x1.02) 0.940
Type 2 diabetes mellitus without complication (x1.02) 0.940
Coag./bleeding tests abnormal (x1.02) 0.941
Hyperkalemia (x1.02) 0.942
Type 2 diabetes mellitus (x1.02) 0.942
Blood coagulation disorder (x1.02) 0.943
Atelectasis (x1.02) 0.944
Low blood pressure (x1.02) 0.944
Chronic kidney disease due to hypertension (x1.02) 0.945
Acidosis (x1.02) 0.945
Obesity (x1.02) 0.946
Hyperglycemia (x1.02) 0.946
Leukocytosis (x1.02) 0.947
Hereditary thrombophilia (x1.02) 0.947
INR in B by Coagulation assay (+0.0195) 0.948
Male Sex (+0.00173) 0.948
Immunodeficiency disorder (x1.02) 0.949
Female Sex (-0.00171) 0.949
Atherosclerosis of coronary artery without angina pectoris (x1.02) 0.950
Sepsis (x1.02) 0.950
Pain in left lower limb (x1.02) 0.951
Abdominal pain (x1.02) 0.951
Hypothyroidism (x1.02) 0.952
Thromboembolism of vein (x1.02) 0.952
Fever (x1.02) 0.953
Anxiety disorder (x1.02) 0.953
Resistance to activated protein C due to Factor V Leiden (x1.02) 0.953
Hypokalemia (x1.02) 0.954
Cardiac arrhythmia (x1.02) 0.954
Dizziness and giddiness (x1.02) 0.955
Loss of consciousness (x1.02) 0.955
Osteoarthritis (x1.02) 0.955
Difficulty walking (x1.02) 0.956
Osteoarthritis of knee (x1.02) 0.956
Diarrhea (x1.02) 0.957
Backache (x1.02) 0.957
Erythrocyte distribution width [V] by AC (+0.154) 0.957
Anemia in chronic kidney disease (x1.02) 0.958
Postoperative state (x1.01) 0.958
Pain (x1.01) 0.958
Gout (x1.01) 0.958
Polyneuropathy (x1.01) 0.959
atorvastatin (+0.00136) 0.959
Constipation (x1.01) 0.959
Hyperglycemia due to type 2 diabetes mellitus (x1.01) 0.960
COVID-19 (x1.01) 0.960
Benign essential hypertension (x1.01) 0.960
Iron deficiency anemia (x1.01) 0.961
Congestive heart failure (x1.01) 0.961
Age (+0.628) 0.961
Postthrombotic syndrome (x1.01) 0.961
Heart failure (x1.01) 0.962
Chronic kidney disease due to type 2 diabetes mellitus (x1.01) 0.962
Disorder of brain (x1.01) 0.962
Pure hypercholesterolemia (x1.01) 0.962
Hypervolemia (x1.01) 0.963
Deep venous thrombosis (x1.01) 0.963
Acute deep venous thrombosis of upper extremity (x1.01) 0.963
Altered mental status (x1.01) 0.963
Leukocytes [#/V] in B by Manual count (+1.23) 0.964
Morbid obesity (x1.01) 0.964
Expressions
−1.00 −0.75 −0.50 −0.25 0.00 0.25 0.50 0.75 1.00

Signature [595]; 136 To Reach 0.975, (100 Plotted)

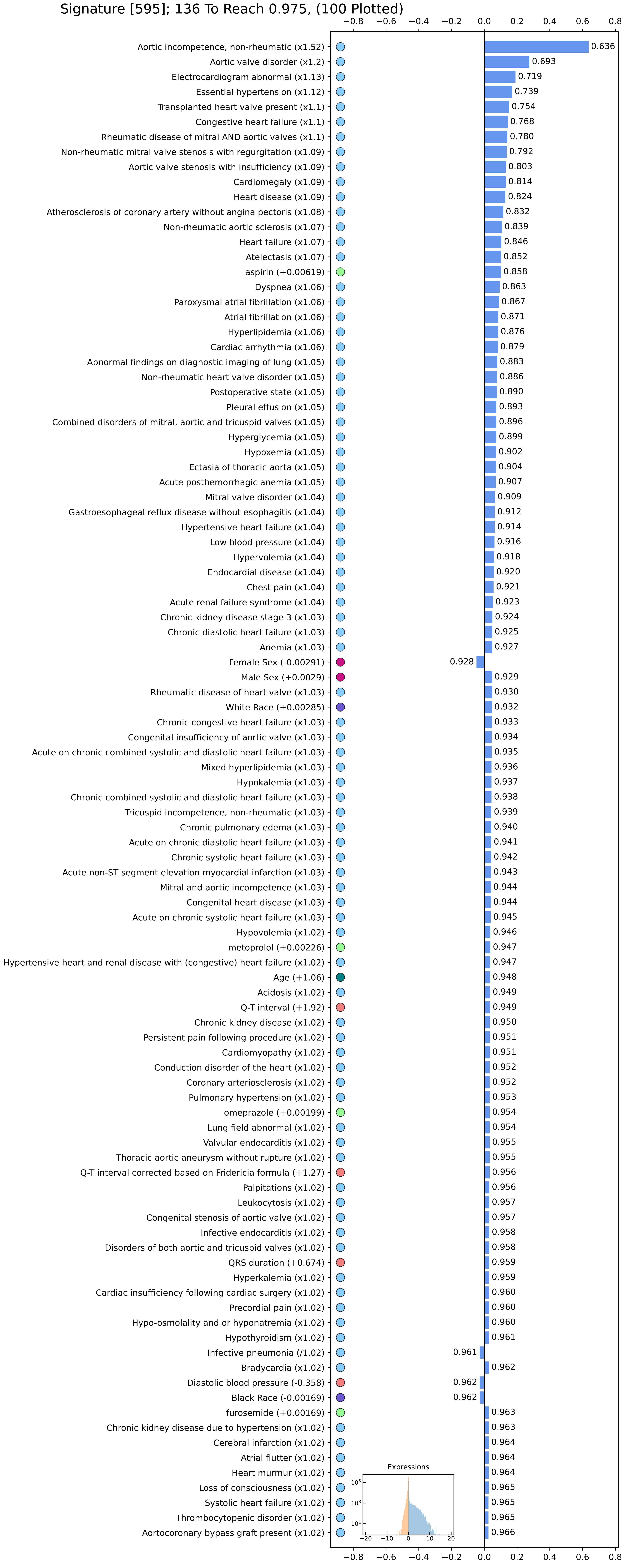

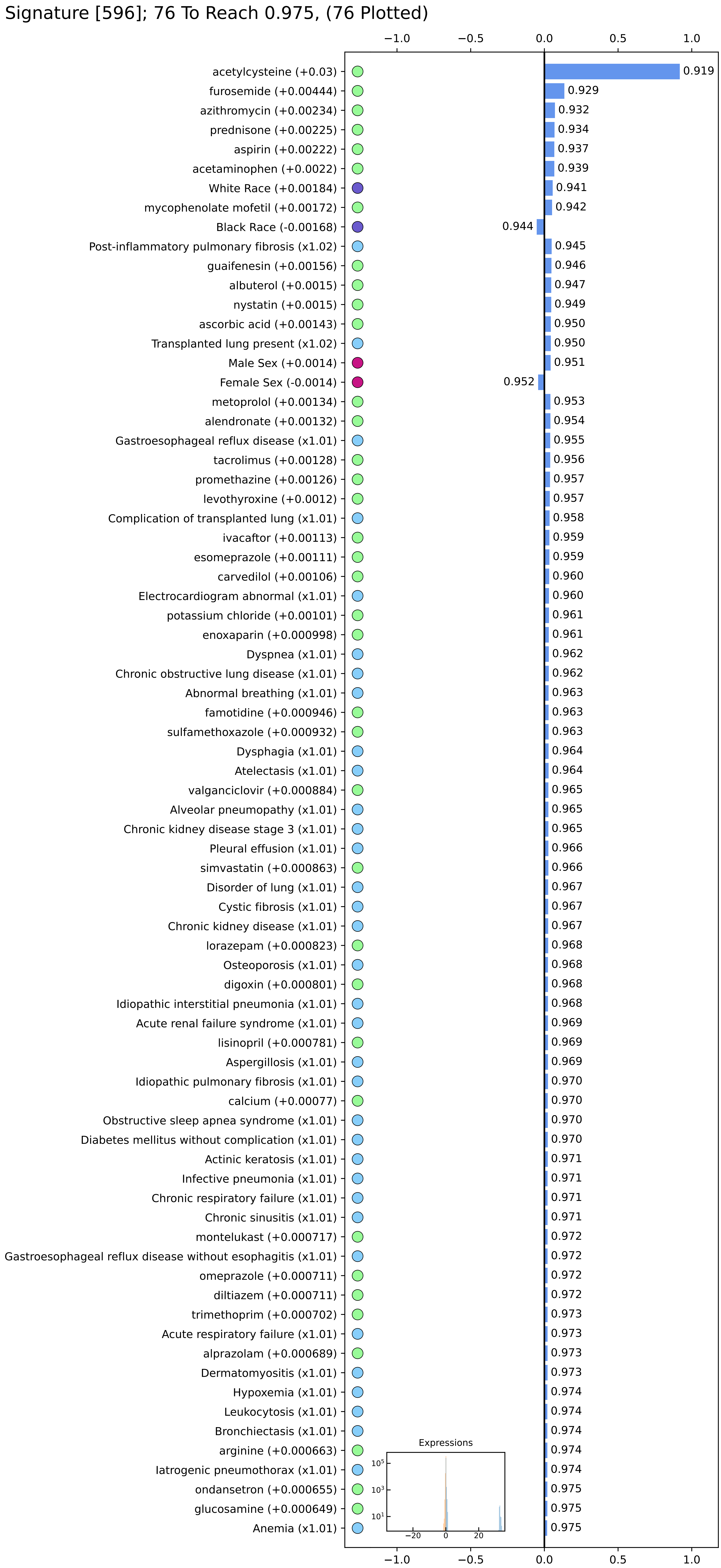

Signature [596]; 76 To Reach 0.975, (76 Plotted)
acetylcysteine (+0.03) 0.919
furosemide (+0.00444) 0.929
azithromycin (+0.00234) 0.932
prednisone (+0.00225) 0.934
aspirin (+0.00222) 0.937
acetaminophen (+0.0022) 0.939
White Race (+0.00184) 0.941
mycophenolate mofetil (+0.00172) 0.942
Black Race (-0.00168) 0.944
Post-inflammatory pulmonary fibrosis (x1.02) 0.945
guaifenesin (+0.00156) 0.946
albuterol (+0.0015) 0.947
nystatin (+0.0015) 0.949
ascorbic acid (+0.00143) 0.950
Transplanted lung present (x1.02) 0.950
Male Sex (+0.0014) 0.951
Female Sex (-0.0014) 0.952
metoprolol (+0.00134) 0.953
alendronate (+0.00132) 0.954
Gastroesophageal reflux disease (x1.01) 0.955
tacrolimus (+0.00128) 0.956
promethazine (+0.00126) 0.957
levothyroxine (+0.0012) 0.957
Complication of transplanted lung (x1.01) 0.958
ivacaftor (+0.00113) 0.959
esomeprazole (+0.00111) 0.959
carvedilol (+0.00106) 0.960
Electrocardiogram abnormal (x1.01) 0.960
potassium chloride (+0.00101) 0.961
enoxaparin (+0.000998) 0.961
Dyspnea (x1.01) 0.962
Chronic obstructive lung disease (x1.01) 0.962
Abnormal breathing (x1.01) 0.963
famotidine (+0.000946) 0.963
sulfamethoxazole (+0.000932) 0.963
Dysphagia (x1.01) 0.964
Atelectasis (x1.01) 0.964
valganciclovir (+0.000884) 0.965
Alveolar pneumopathy (x1.01) 0.965
Chronic kidney disease stage 3 (x1.01) 0.965
Pleural effusion (x1.01) 0.966
simvastatin (+0.000863) 0.966
Disorder of lung (x1.01) 0.967
Cystic fibrosis (x1.01) 0.967
Chronic kidney disease (x1.01) 0.967
lorazepam (+0.000823) 0.968
Osteoporosis (x1.01) 0.968
digoxin (+0.000801) 0.968
Idiopathic interstitial pneumonia (x1.01) 0.968
Acute renal failure syndrome (x1.01) 0.969
lisinopril (+0.000781) 0.969
Aspergillosis (x1.01) 0.969
Idiopathic pulmonary fibrosis (x1.01) 0.970
calcium (+0.00077) 0.970
Obstructive sleep apnea syndrome (x1.01) 0.970
Diabetes mellitus without complication (x1.01) 0.970
Actinic keratosis (x1.01) 0.971
Infective pneumonia (x1.01) 0.971
Chronic respiratory failure (x1.01) 0.971
Chronic sinusitis (x1.01) 0.971
montelukast (+0.000717) 0.972
Gastroesophageal reflux disease without esophagitis (x1.01) 0.972
omeprazole (+0.000711) 0.972
diltiazem (+0.000711) 0.972
trimethoprim (+0.000702) 0.973
Acute respiratory failure (x1.01) 0.973
alprazolam (+0.000689) 0.973
Dermatomyositis (x1.01) 0.973
Hypoxemia (x1.01) 0.974
Leukocytosis (x1.01) 0.974
Bronchiectasis (x1.01) 0.974
arginine (+0.000663) 0.974
Iatrogenic pneumothorax (x1.01) 0.974
ondansetron (+0.000655) 0.975
glucosamine (+0.000649) 0.975
Anemia (x1.01) 0.975
−1.0 −0.5 0.0 0.5 1.0
Expressions

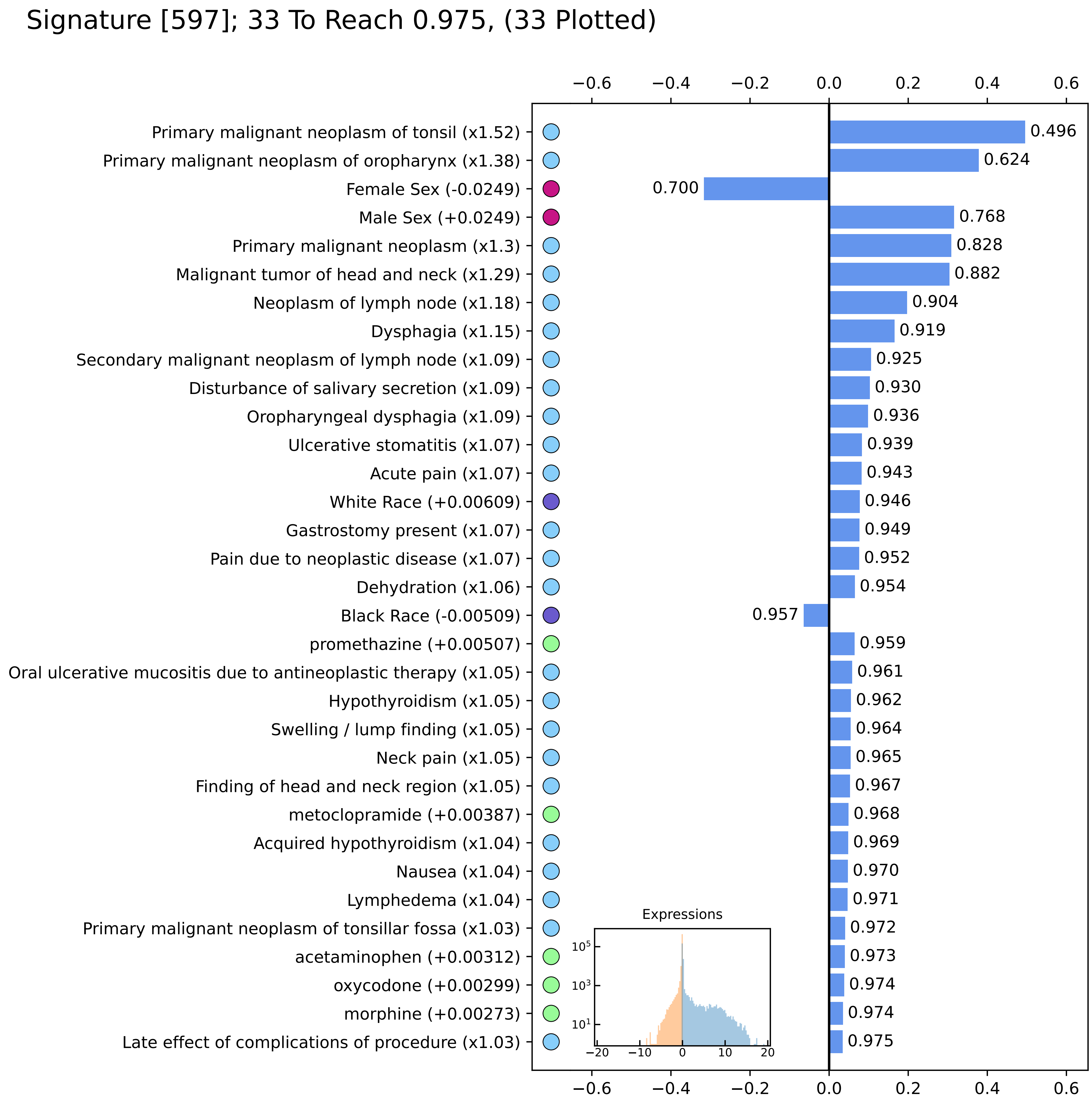

Signature [597]; 33 To Reach 0.975, (33 Plotted)
Primary malignant neoplasm of tonsil (x1.52)
Primary malignant neoplasm of oropharynx (x1.38)
Female Sex (-0.0249)
Male Sex (+0.0249)
Primary malignant neoplasm (x1.3)
Malignant tumor of head and neck (x1.29)
Neoplasm of lymph node (x1.18)
Dysphagia (x1.15)
Secondary malignant neoplasm of lymph node (x1.09)
Disturbance of salivary secretion (x1.09)
Oropharyngeal dysphagia (x1.09)
Ulcerative stomatitis (x1.07)
Acute pain (x1.07)
White Race (+0.00609)
Gastrostomy present (x1.07)
Pain due to neoplastic disease (x1.07)
Dehydration (x1.06)
Black Race (-0.00509)
promethazine (+0.00507)
Oral ulcerative mucositis due to antineoplastic therapy (x1.05)
Hypothyroidism (x1.05)
Swelling / lump finding (x1.05)
Neck pain (x1.05)
Finding of head and neck region (x1.05)
metoclopramide (+0.00387)
Acquired hypothyroidism (x1.04)
Nausea (x1.04)
Lymphedema (x1.04)
Primary malignant neoplasm of tonsillar fossa (x1.03)
acetaminophen (+0.00312)
oxycodone (+0.00299)
morphine (+0.00273)
Late effect of complications of procedure (x1.03)
0.496
0.624
0.700
0.768
0.828
0.882
0.904
0.919
0.925
0.930
0.936
0.939
0.943
0.946
0.949
0.952
0.954
0.957
0.959
0.961
0.962
0.964
0.965
0.967
0.968
0.969
0.970
0.971
0.972
0.973
0.974
0.974
0.975
−0.6 −0.4 −0.2 0.0 0.2 0.4 0.6
Expressions

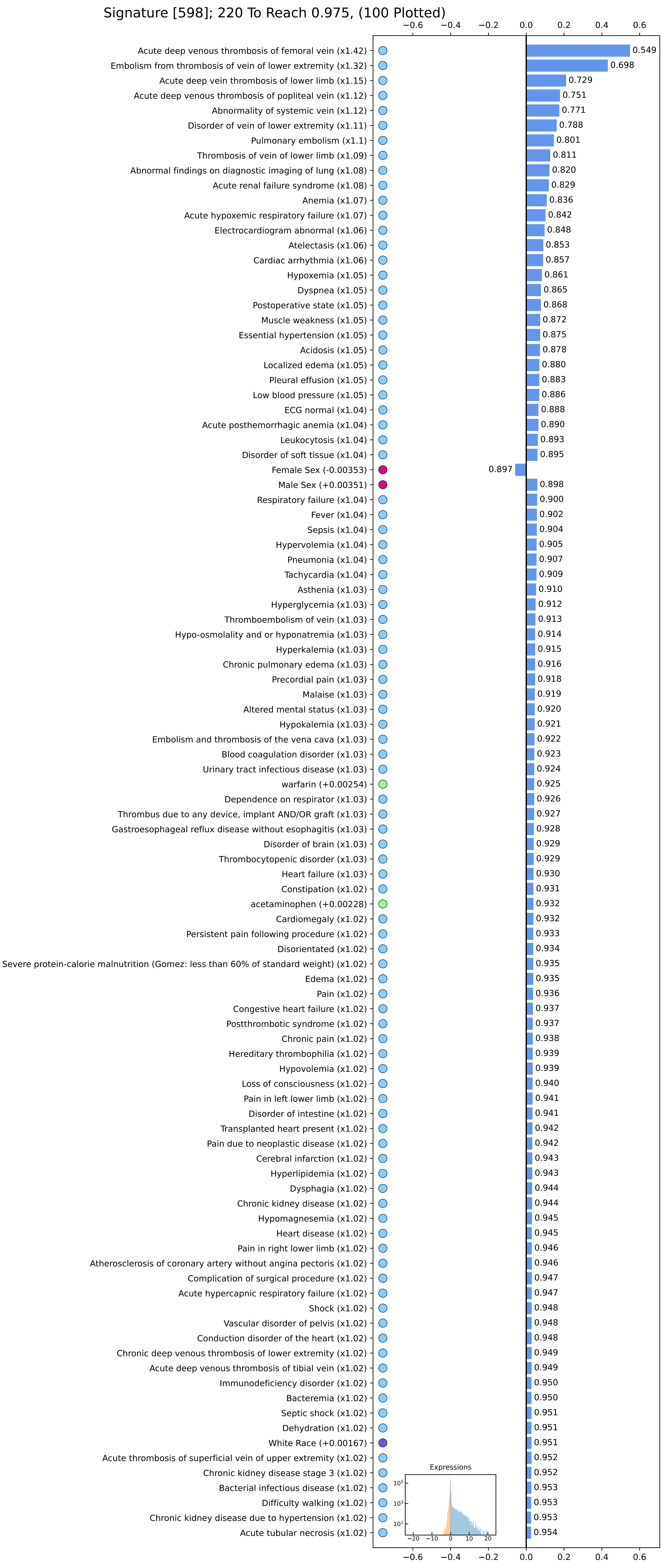

Signature [598]; 220 To Reach 0.975, (100 Plotted)
Acute deep venous thrombosis of femoral vein (x1.42) 0.549
Embolism from thrombosis of vein of lower extremity (x1.32) 0.698
Acute deep vein thrombosis of lower limb (x1.15) 0.729
Acute deep venous thrombosis of popliteal vein (x1.12) 0.751
Abnormality of systemic vein (x1.12) 0.771
Disorder of vein of lower extremity (x1.11) 0.788
Pulmonary embolism (x1.1) 0.801
Thrombosis of vein of lower limb (x1.09) 0.811
Abnormal findings on diagnostic imaging of lung (x1.08) 0.820
Acute renal failure syndrome (x1.08) 0.829
Anemia (x1.07) 0.836
Acute hypoxemic respiratory failure (x1.07) 0.842
Electrocardiogram abnormal (x1.06) 0.848
Atelectasis (x1.06) 0.853
Cardiac arrhythmia (x1.06) 0.857
Hypoxemia (x1.05) 0.861
Dyspnea (x1.05) 0.865
Postoperative state (x1.05) 0.868
Muscle weakness (x1.05) 0.872
Essential hypertension (x1.05) 0.875
Acidosis (x1.05) 0.878
Localized edema (x1.05) 0.880
Pleural effusion (x1.05) 0.883
Low blood pressure (x1.05) 0.886
ECG normal (x1.04) 0.888
Acute posthemorrhagic anemia (x1.04) 0.890
Leukocytosis (x1.04) 0.893
Disorder of soft tissue (x1.04) 0.895
Female Sex (-0.00353) 0.897
Male Sex (+0.00351) 0.898
Respiratory failure (x1.04) 0.900
Fever (x1.04) 0.902
Sepsis (x1.04) 0.904
Hypervolemia (x1.04) 0.905
Pneumonia (x1.04) 0.907
Tachycardia (x1.04) 0.909
Asthenia (x1.03) 0.910
Hyperglycemia (x1.03) 0.912
Thromboembolism of vein (x1.03) 0.913
Hypo-osmolality and or hyponatremia (x1.03) 0.914
Hyperkalemia (x1.03) 0.915
Chronic pulmonary edema (x1.03) 0.916
Precordial pain (x1.03) 0.918
Malaise (x1.03) 0.919
Altered mental status (x1.03) 0.920
Hypokalemia (x1.03) 0.921
Embolism and thrombosis of the vena cava (x1.03) 0.922
Blood coagulation disorder (x1.03) 0.923
Urinary tract infectious disease (x1.03) 0.924
warfarin (+0.00254) 0.925
Dependence on respirator (x1.03) 0.926
Thrombus due to any device, implant AND/OR graft (x1.03) 0.927
Gastroesophageal reflux disease without esophagitis (x1.03) 0.928
Disorder of brain (x1.03) 0.929
Thrombocytopenic disorder (x1.03) 0.929
Heart failure (x1.03) 0.930
Constipation (x1.02) 0.931
acetaminophen (+0.00228) 0.932
Cardiomegaly (x1.02) 0.932
Persistent pain following procedure (x1.02) 0.933
Disorientated (x1.02) 0.934
Severe protein-calorie malnutrition (Gomez: less than 60% of standard weight) (x1.02) 0.935
Edema (x1.02) 0.935
Pain (x1.02) 0.936
Congestive heart failure (x1.02) 0.937
Postthrombotic syndrome (x1.02) 0.937
Chronic pain (x1.02) 0.938
Hereditary thrombophilia (x1.02) 0.939
Hypovolemia (x1.02) 0.939
Loss of consciousness (x1.02) 0.940
Pain in left lower limb (x1.02) 0.941
Disorder of intestine (x1.02) 0.941
Transplanted heart present (x1.02) 0.942
Pain due to neoplastic disease (x1.02) 0.942
Cerebral infarction (x1.02) 0.943
Hyperlipidemia (x1.02) 0.943
Dysphagia (x1.02) 0.944
Chronic kidney disease (x1.02) 0.944
Hypomagnesemia (x1.02) 0.945
Heart disease (x1.02) 0.945
Pain in right lower limb (x1.02) 0.946
Atherosclerosis of coronary artery without angina pectoris (x1.02) 0.946
Complication of surgical procedure (x1.02) 0.947
Acute hypercapnic respiratory failure (x1.02) 0.947
Shock (x1.02) 0.948
Vascular disorder of pelvis (x1.02) 0.948
Conduction disorder of the heart (x1.02) 0.948
Chronic deep venous thrombosis of lower extremity (x1.02) 0.949
Acute deep venous thrombosis of tibial vein (x1.02) 0.949
Immunodeficiency disorder (x1.02) 0.950
Bacteremia (x1.02) 0.950
Septic shock (x1.02) 0.951
Dehydration (x1.02) 0.951
White Race (+0.00167) 0.951
Acute thrombosis of superficial vein of upper extremity (x1.02) 0.952
Chronic kidney disease stage 3 (x1.02) 0.952
Bacterial infectious disease (x1.02) 0.953
Difficulty walking (x1.02) 0.953
Chronic kidney disease due to hypertension (x1.02) 0.953
Acute tubular necrosis (x1.02) 0.954
Expressions
−0.6 −0.4 −0.2 0.0 0.2 0.4 0.6

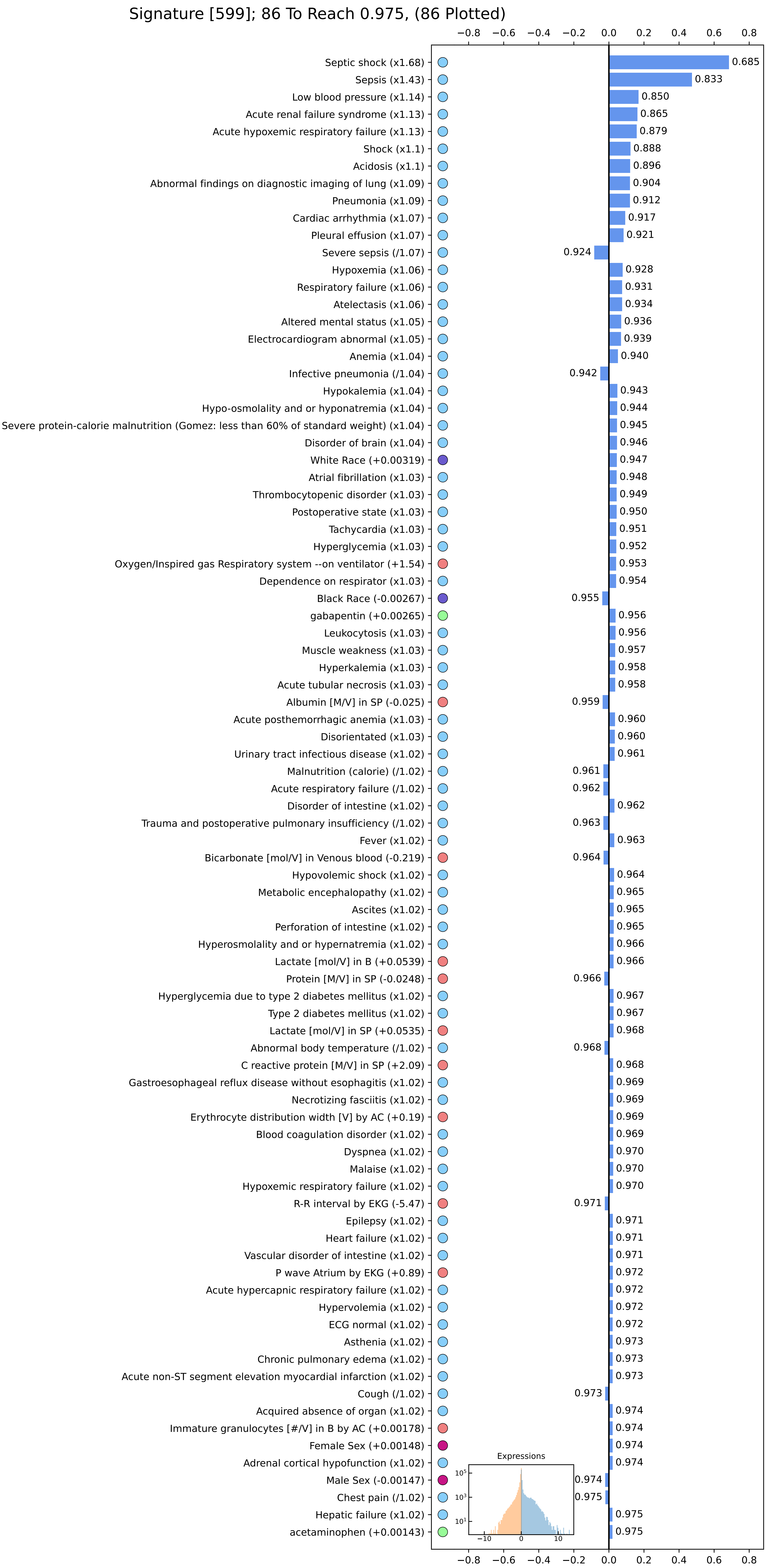
Signature [599]; 86 To Reach 0.975, (86 Plotted)
−0.8 −0.6 −0.4 −0.2 0.0 0.2 0.4 0.6 0.8
Septic shock (x1.68) 0.685
Sepsis (x1.43) 0.833
Low blood pressure (x1.14) 0.850
Acute renal failure syndrome (x1.13) 0.865
Acute hypoxemic respiratory failure (x1.13) 0.879
Shock (x1.1) 0.888
Acidosis (x1.1) 0.896
Abnormal findings on diagnostic imaging of lung (x1.09) 0.904
Pneumonia (x1.09) 0.912
Cardiac arrhythmia (x1.07) 0.917
Pleural effusion (x1.07) 0.921
Severe sepsis (/1.07) 0.924
Hypoxemia (x1.06) 0.928
Respiratory failure (x1.06) 0.931
Atelectasis (x1.06) 0.934
Altered mental status (x1.05) 0.936
Electrocardiogram abnormal (x1.05) 0.939
Anemia (x1.04) 0.940
Infective pneumonia (/1.04) 0.942
Hypokalemia (x1.04) 0.943
Hypo-osmolality and or hyponatremia (x1.04) 0.944
Severe protein-calorie malnutrition (Gomez: less than 60% of standard weight) (x1.04) 0.945
Disorder of brain (x1.04) 0.946
White Race (+0.00319) 0.947
Atrial fibrillation (x1.03) 0.948
Thrombocytopenic disorder (x1.03) 0.949
Postoperative state (x1.03) 0.950
Tachycardia (x1.03) 0.951
Hyperglycemia (x1.03) 0.952
Oxygen/Inspired gas Respiratory system --on ventilator (+1.54) 0.953
Dependence on respirator (x1.03) 0.954
Black Race (-0.00267) 0.955
gabapentin (+0.00265) 0.956
Leukocytosis (x1.03) 0.956
Muscle weakness (x1.03) 0.957
Hyperkalemia (x1.03) 0.958
Acute tubular necrosis (x1.03) 0.958
Albumin [M/V] in SP (-0.025) 0.959
Acute posthemorrhagic anemia (x1.03) 0.960
Disorientated (x1.03) 0.960
Urinary tract infectious disease (x1.02) 0.961
Malnutrition (calorie) (/1.02) 0.961
Acute respiratory failure (/1.02) 0.962
Disorder of intestine (x1.02) 0.962
Trauma and postoperative pulmonary insufficiency (/1.02) 0.963
Fever (x1.02) 0.963
Bicarbonate [mol/V] in Venous blood (-0.219) 0.964
Hypovolemic shock (x1.02) 0.964
Metabolic encephalopathy (x1.02) 0.965
Ascites (x1.02) 0.965
Perforation of intestine (x1.02) 0.965
Hyperosmolality and or hypernatremia (x1.02) 0.966
Lactate [mol/V] in B (+0.0539) 0.966
Protein [M/V] in SP (-0.0248) 0.966
Hyperglycemia due to type 2 diabetes mellitus (x1.02) 0.967
Type 2 diabetes mellitus (x1.02) 0.967
Lactate [mol/V] in SP (+0.0535) 0.968
Abnormal body temperature (/1.02) 0.968
C reactive protein [M/V] in SP (+2.09) 0.968
Gastroesophageal reflux disease without esophagitis (x1.02) 0.969
Necrotizing fasciitis (x1.02) 0.969
Erythrocyte distribution width [V] by AC (+0.19) 0.969
Blood coagulation disorder (x1.02) 0.969
Dyspnea (x1.02) 0.970
Malaise (x1.02) 0.970
Hypoxemic respiratory failure (x1.02) 0.970
R-R interval by EKG (-5.47) 0.971
Epilepsy (x1.02) 0.971
Heart failure (x1.02) 0.971
Vascular disorder of intestine (x1.02) 0.971
P wave Atrium by EKG (+0.89) 0.972
Acute hypercapnic respiratory failure (x1.02) 0.972
Hypervolemia (x1.02) 0.972
ECG normal (x1.02) 0.972
Asthenia (x1.02) 0.973
Chronic pulmonary edema (x1.02) 0.973
Acute non-ST segment elevation myocardial infarction (x1.02) 0.973
Cough (/1.02) 0.973
Acquired absence of organ (x1.02) 0.974
Immature granulocytes [#/V] in B by AC (+0.00178) 0.974
Female Sex (+0.00148) 0.974
Adrenal cortical hypofunction (x1.02) 0.974
Male Sex (-0.00147) 0.974
Chest pain (/1.02) 0.975
Hepatic failure (x1.02) 0.975
acetaminophen (+0.00143) 0.975
Expressions
10⁵
10³
10¹
−10 0 10
−0.8 −0.6 −0.4 −0.2 0.0 0.2 0.4 0.6 0.8

Signature [600]; 152 To Reach 0.975, (100 Plotted)

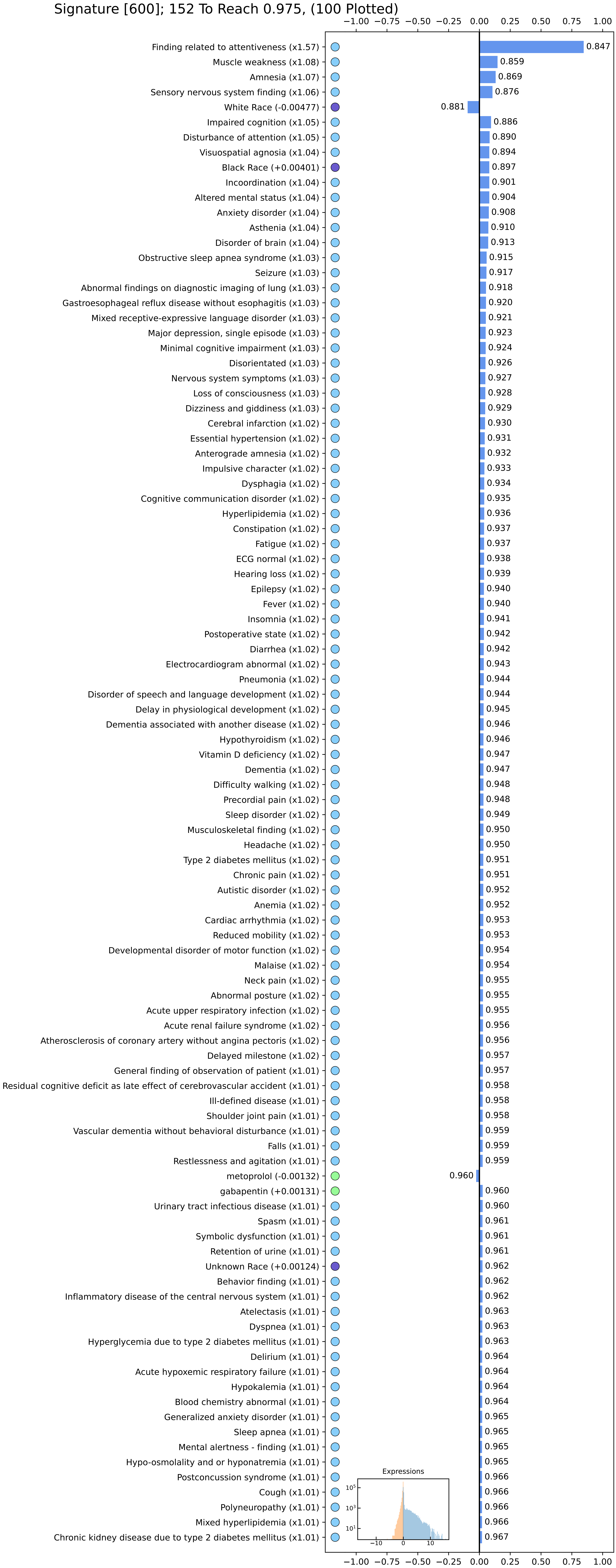

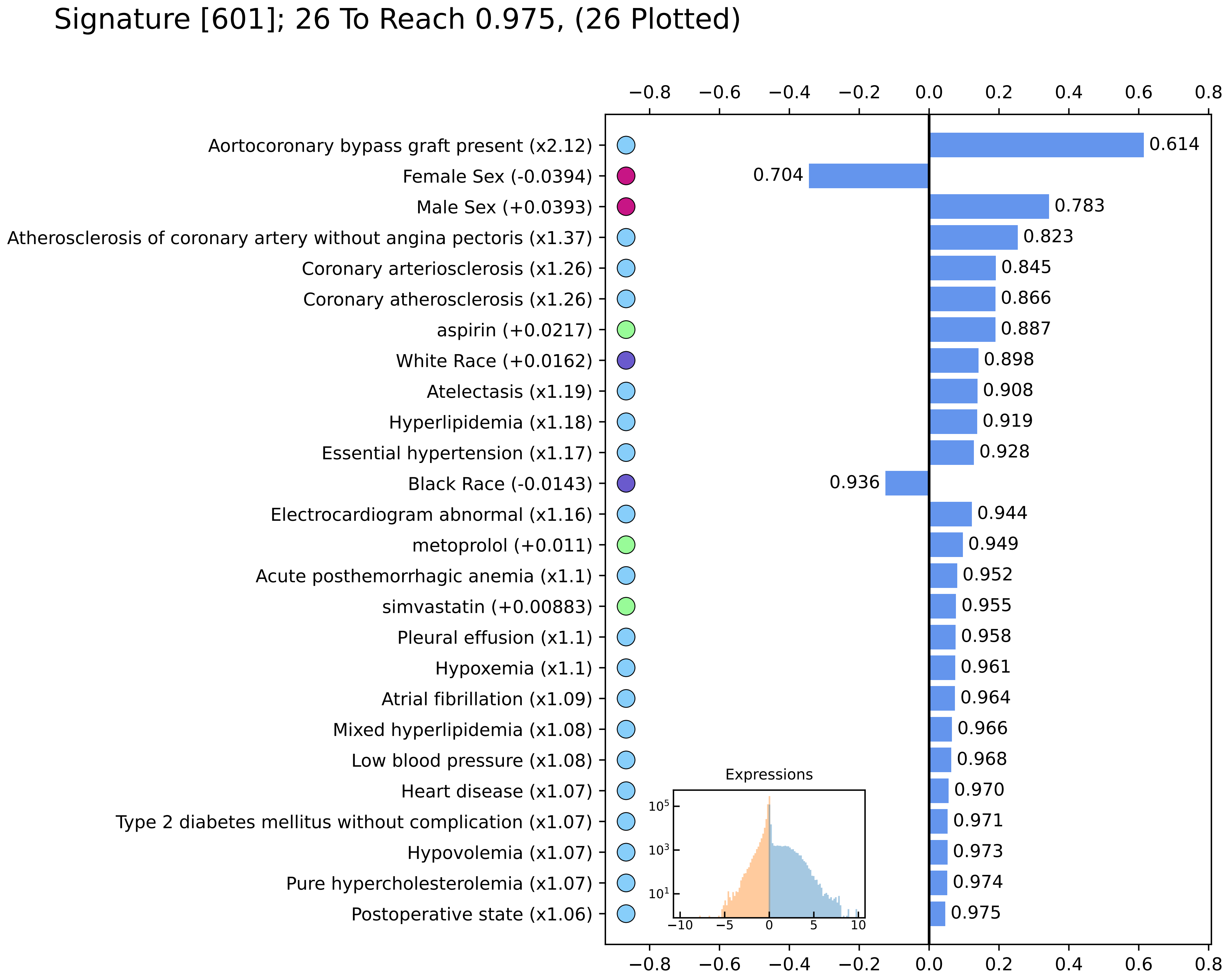
Signature [601]; 26 To Reach 0.975, (26 Plotted)
Aortocoronary bypass graft present (x2.12)
Female Sex (-0.0394)
Male Sex (+0.0393)
Atherosclerosis of coronary artery without angina pectoris (x1.37)
Coronary arteriosclerosis (x1.26)
Coronary atherosclerosis (x1.26)
aspirin (+0.0217)
White Race (+0.0162)
Atelectasis (x1.19)
Hyperlipidemia (x1.18)
Essential hypertension (x1.17)
Black Race (-0.0143)
Electrocardiogram abnormal (x1.16)
metoprolol (+0.011)
Acute posthemorrhagic anemia (x1.1)
simvastatin (+0.00883)
Pleural effusion (x1.1)
Hypoxemia (x1.1)
Atrial fibrillation (x1.09)
Mixed hyperlipidemia (x1.08)
Low blood pressure (x1.08)
Heart disease (x1.07)
Type 2 diabetes mellitus without complication (x1.07)
Hypovolemia (x1.07)
Pure hypercholesterolemia (x1.07)
Postoperative state (x1.06)
0.614
0.704
0.783
0.823
0.845
0.866
0.887
0.898
0.908
0.919
0.928
0.936
0.944
0.949
0.952
0.955
0.958
0.961
0.964
0.966
0.968
0.970
0.971
0.973
0.974
0.975
Expressions
−0.8
−0.6
−0.4
−0.2
0.0
0.2
0.4
0.6
0.8

Signature [602]; 11 To Reach 0.975, (11 Plotted)

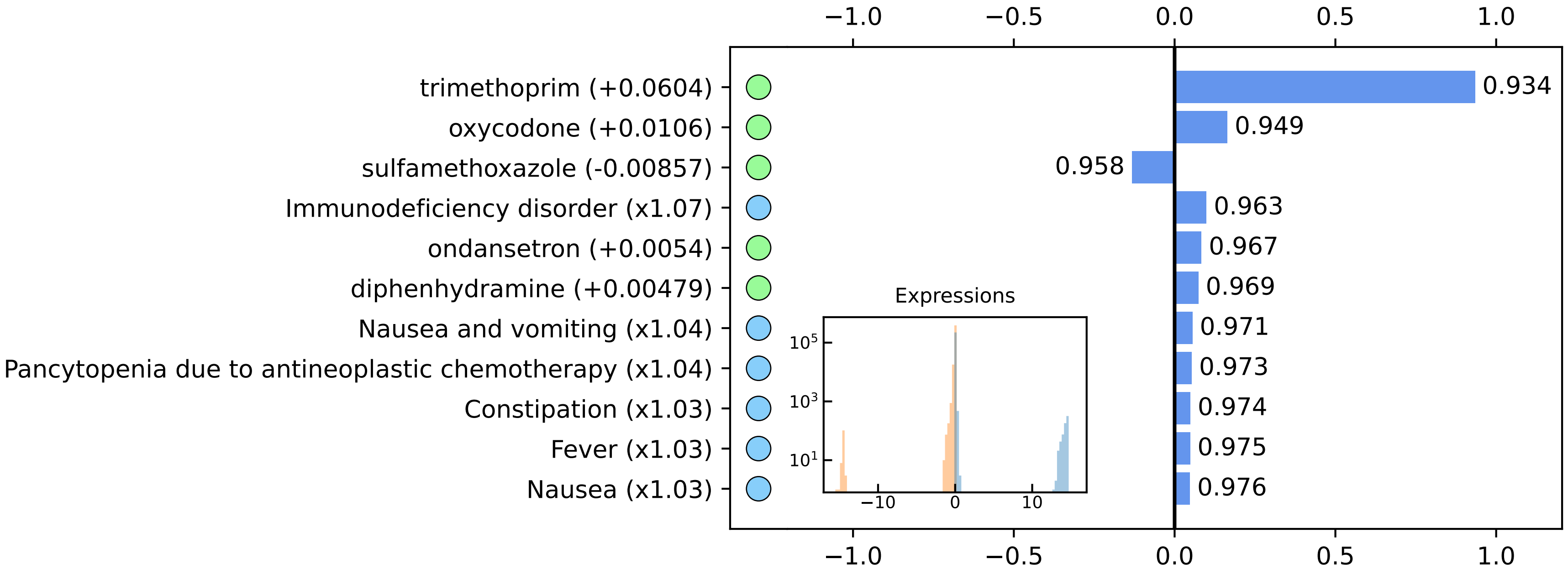

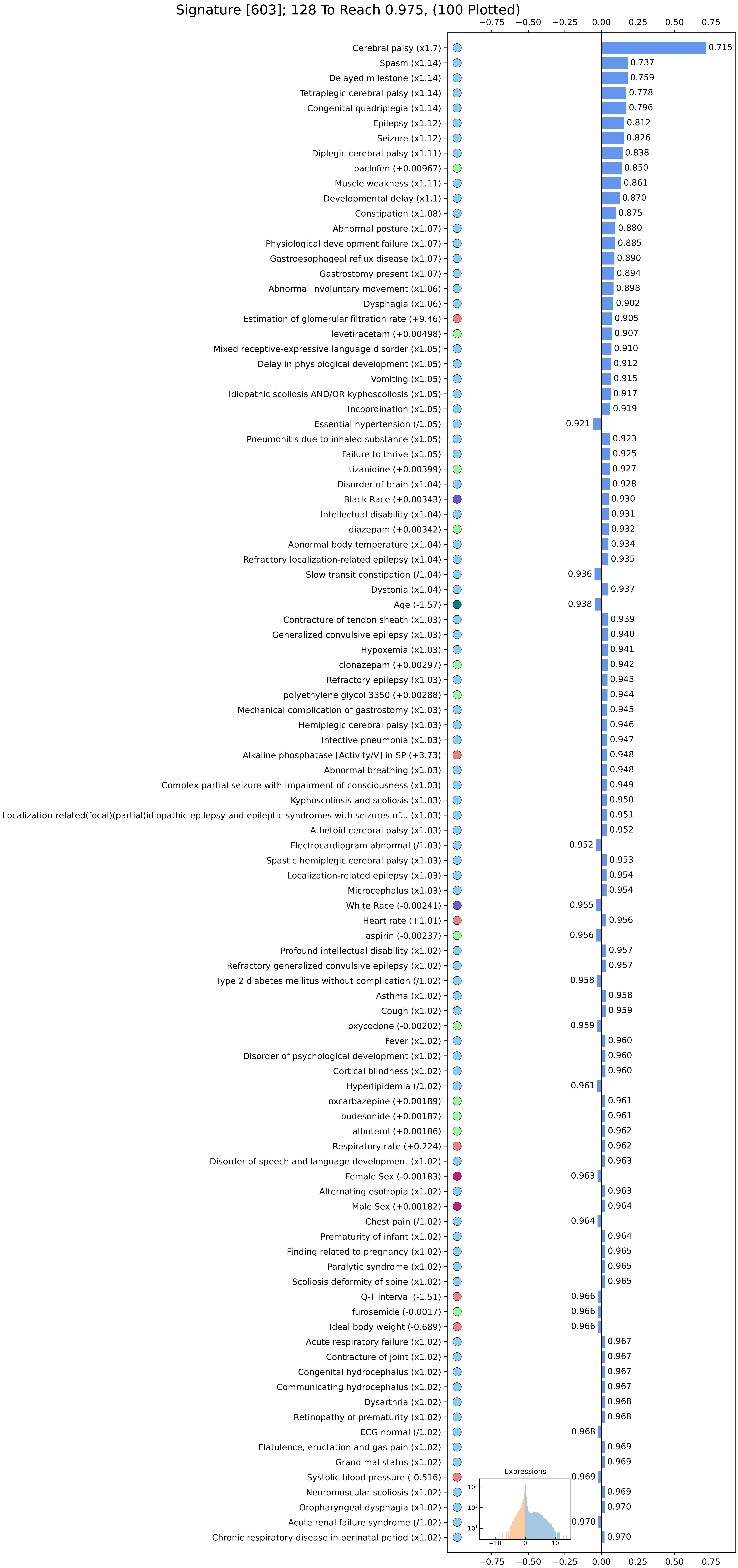

Signature [603]; 128 To Reach 0.975, (100 Plotted)
Cerebral palsy (x1.7) 0.715
Spasm (x1.14) 0.737
Delayed milestone (x1.14) 0.759
Tetraplegic cerebral palsy (x1.14) 0.778
Congenital quadriplegia (x1.14) 0.796
Epilepsy (x1.12) 0.812
Seizure (x1.12) 0.826
Diplegic cerebral palsy (x1.11) 0.838
baclofen (+0.00967) 0.850
Muscle weakness (x1.11) 0.861
Developmental delay (x1.1) 0.870
Constipation (x1.08) 0.875
Abnormal posture (x1.07) 0.880
Physiological development failure (x1.07) 0.885
Gastroesophageal reflux disease (x1.07) 0.890
Gastrostomy present (x1.07) 0.894
Abnormal involuntary movement (x1.06) 0.898
Dysphagia (x1.06) 0.902
Estimation of glomerular filtration rate (+9.46) 0.905
levetiracetam (+0.00498) 0.907
Mixed receptive-expressive language disorder (x1.05) 0.910
Delay in physiological development (x1.05) 0.912
Vomiting (x1.05) 0.915
Idiopathic scoliosis AND/OR kyphoscoliosis (x1.05) 0.917
Incoordination (x1.05) 0.919
Essential hypertension (/1.05) 0.921
Pneumonitis due to inhaled substance (x1.05) 0.923
Failure to thrive (x1.05) 0.925
tizanidine (+0.00399) 0.927
Disorder of brain (x1.04) 0.928
Black Race (+0.00343) 0.930
Intellectual disability (x1.04) 0.931
diazepam (+0.00342) 0.932
Abnormal body temperature (x1.04) 0.934
Refractory localization-related epilepsy (x1.04) 0.935
Slow transit constipation (/1.04) 0.936
Dystonia (x1.04) 0.937
Age (-1.57) 0.938
Contracture of tendon sheath (x1.03) 0.939
Generalized convulsive epilepsy (x1.03) 0.940
Hypoxemia (x1.03) 0.941
clonazepam (+0.00297) 0.942
Refractory epilepsy (x1.03) 0.943
polyethylene glycol 3350 (+0.00288) 0.944
Mechanical complication of gastrostomy (x1.03) 0.945
Hemiplegic cerebral palsy (x1.03) 0.946
Infective pneumonia (x1.03) 0.947
Alkaline phosphatase [Activity/V] in SP (+3.73) 0.948
Abnormal breathing (x1.03) 0.948
Complex partial seizure with impairment of consciousness (x1.03) 0.949
Kyphoscoliosis and scoliosis (x1.03) 0.950
Localization-related(focal)(partial)idiopathic epilepsy and epileptic syndromes with seizures of... (x1.03) 0.951
Athetoid cerebral palsy (x1.03) 0.952
Electrocardiogram abnormal (/1.03) 0.952
Spastic hemiplegic cerebral palsy (x1.03) 0.953
Localization-related epilepsy (x1.03) 0.954
Microcephalus (x1.03) 0.954
White Race (-0.00241) 0.955
Heart rate (+1.01) 0.956
aspirin (-0.00237) 0.956
Profound intellectual disability (x1.02) 0.957
Refractory generalized convulsive epilepsy (x1.02) 0.957
Type 2 diabetes mellitus without complication (/1.02) 0.958
Asthma (x1.02) 0.958
Cough (x1.02) 0.959
oxycodone (-0.00202) 0.959
Fever (x1.02) 0.960
Disorder of psychological development (x1.02) 0.960
Cortical blindness (x1.02) 0.960
Hyperlipidemia (/1.02) 0.961
oxcarbazepine (+0.00189) 0.961
budesonide (+0.00187) 0.961
albuterol (+0.00186) 0.962
Respiratory rate (+0.224) 0.962
Disorder of speech and language development (x1.02) 0.963
Female Sex (-0.00183) 0.963
Alternating esotropia (x1.02) 0.963
Male Sex (+0.00182) 0.964
Chest pain (/1.02) 0.964
Prematurity of infant (x1.02) 0.964
Finding related to pregnancy (x1.02) 0.965
Paralytic syndrome (x1.02) 0.965
Scoliosis deformity of spine (x1.02) 0.965
Q-T interval (-1.51) 0.966
furosemide (-0.0017) 0.966
Ideal body weight (-0.689) 0.966
Acute respiratory failure (x1.02) 0.967
Contracture of joint (x1.02) 0.967
Congenital hydrocephalus (x1.02) 0.967
Communicating hydrocephalus (x1.02) 0.967
Dysarthria (x1.02) 0.968
Retinopathy of prematurity (x1.02) 0.968
ECG normal (/1.02) 0.968
Flatulence, eructation and gas pain (x1.02) 0.969
Grand mal status (x1.02) 0.969
Systolic blood pressure (-0.516) 0.969
Neuromuscular scoliosis (x1.02) 0.969
Oropharyngeal dysphagia (x1.02) 0.970
Acute renal failure syndrome (/1.02) 0.970
Chronic respiratory disease in perinatal period (x1.02) 0.970
−0.75 −0.50 −0.25 0.00 0.25 0.50 0.75
Expressions

Signature [604]; 35 To Reach 0.975, (35 Plotted)

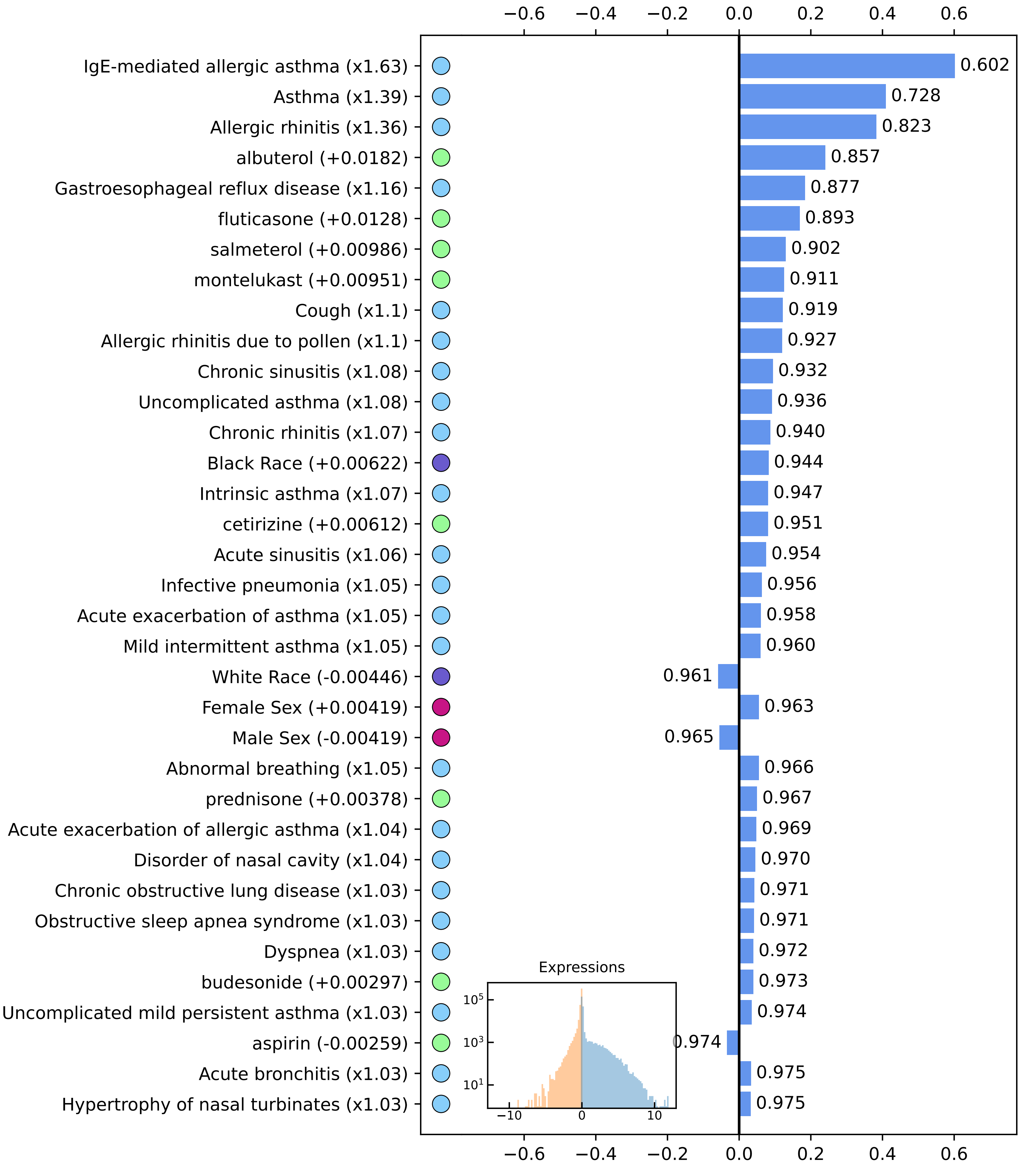

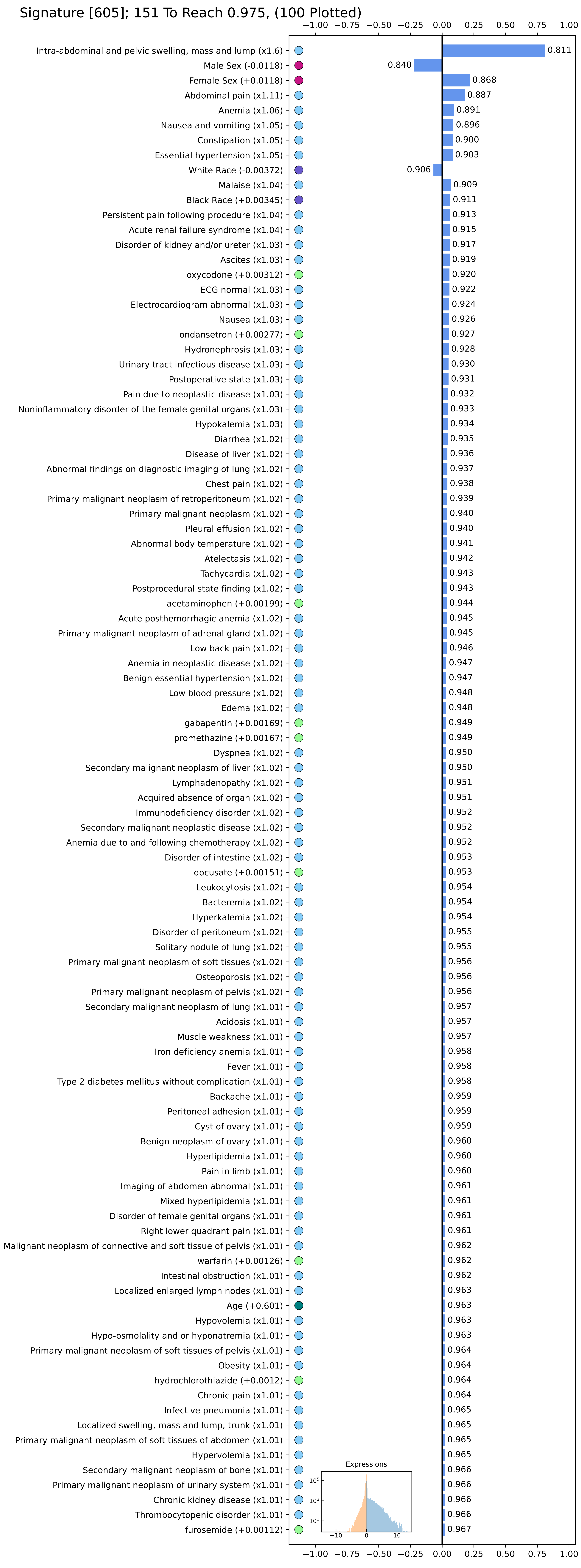

Signature [605]; 151 To Reach 0.975, (100 Plotted)
−1.00 −0.75 −0.50 −0.25 0.00 0.25 0.50 0.75 1.00
Intra-abdominal and pelvic swelling, mass and lump (x1.6) 0.811
Male Sex (-0.0118) 0.840
Female Sex (+0.0118) 0.868
Abdominal pain (x1.11) 0.887
Anemia (x1.06) 0.891
Nausea and vomiting (x1.05) 0.896
Constipation (x1.05) 0.900
Essential hypertension (x1.05) 0.903
White Race (-0.00372) 0.906
Malaise (x1.04) 0.909
Black Race (+0.00345) 0.911
Persistent pain following procedure (x1.04) 0.913
Acute renal failure syndrome (x1.04) 0.915
Disorder of kidney and/or ureter (x1.03) 0.917
Ascites (x1.03) 0.919
oxycodone (+0.00312) 0.920
ECG normal (x1.03) 0.922
Electrocardiogram abnormal (x1.03) 0.924
Nausea (x1.03) 0.926
ondansetron (+0.00277) 0.927
Hydronephrosis (x1.03) 0.928
Urinary tract infectious disease (x1.03) 0.930
Postoperative state (x1.03) 0.931
Pain due to neoplastic disease (x1.03) 0.932
Noninflammatory disorder of the female genital organs (x1.03) 0.933
Hypokalemia (x1.03) 0.934
Diarrhea (x1.02) 0.935
Disease of liver (x1.02) 0.936
Abnormal findings on diagnostic imaging of lung (x1.02) 0.937
Chest pain (x1.02) 0.938
Primary malignant neoplasm of retroperitoneum (x1.02) 0.939
Primary malignant neoplasm (x1.02) 0.940
Pleural effusion (x1.02) 0.940
Abnormal body temperature (x1.02) 0.941
Atelectasis (x1.02) 0.942
Tachycardia (x1.02) 0.943
Postprocedural state finding (x1.02) 0.943
acetaminophen (+0.00199) 0.944
Acute posthemorrhagic anemia (x1.02) 0.945
Primary malignant neoplasm of adrenal gland (x1.02) 0.945
Low back pain (x1.02) 0.946
Anemia in neoplastic disease (x1.02) 0.947
Benign essential hypertension (x1.02) 0.947
Low blood pressure (x1.02) 0.948
Edema (x1.02) 0.948
gabapentin (+0.00169) 0.949
promethazine (+0.00167) 0.949
Dyspnea (x1.02) 0.950
Secondary malignant neoplasm of liver (x1.02) 0.950
Lymphadenopathy (x1.02) 0.951
Acquired absence of organ (x1.02) 0.951
Immunodeficiency disorder (x1.02) 0.952
Secondary malignant neoplastic disease (x1.02) 0.952
Anemia due to and following chemotherapy (x1.02) 0.952
Disorder of intestine (x1.02) 0.953
docusate (+0.00151) 0.953
Leukocytosis (x1.02) 0.954
Bacteremia (x1.02) 0.954
Hyperkalemia (x1.02) 0.954
Disorder of peritoneum (x1.02) 0.955
Solitary nodule of lung (x1.02) 0.955
Primary malignant neoplasm of soft tissues (x1.02) 0.956
Osteoporosis (x1.02) 0.956
Primary malignant neoplasm of pelvis (x1.02) 0.956
Secondary malignant neoplasm of lung (x1.01) 0.957
Acidosis (x1.01) 0.957
Muscle weakness (x1.01) 0.957
Iron deficiency anemia (x1.01) 0.958
Fever (x1.01) 0.958
Type 2 diabetes mellitus without complication (x1.01) 0.958
Backache (x1.01) 0.959
Peritoneal adhesion (x1.01) 0.959
Cyst of ovary (x1.01) 0.959
Benign neoplasm of ovary (x1.01) 0.960
Hyperlipidemia (x1.01) 0.960
Pain in limb (x1.01) 0.960
Imaging of abdomen abnormal (x1.01) 0.961
Mixed hyperlipidemia (x1.01) 0.961
Disorder of female genital organs (x1.01) 0.961
Right lower quadrant pain (x1.01) 0.961
Malignant neoplasm of connective and soft tissue of pelvis (x1.01) 0.962
warfarin (+0.00126) 0.962
Intestinal obstruction (x1.01) 0.962
Localized enlarged lymph nodes (x1.01) 0.963
Age (+0.601) 0.963
Hypovolemia (x1.01) 0.963
Hypo-osmolality and or hyponatremia (x1.01) 0.963
Primary malignant neoplasm of soft tissues of pelvis (x1.01) 0.964
Obesity (x1.01) 0.964
hydrochlorothiazide (+0.0012) 0.964
Chronic pain (x1.01) 0.964
Infective pneumonia (x1.01) 0.965
Localized swelling, mass and lump, trunk (x1.01) 0.965
Primary malignant neoplasm of soft tissues of abdomen (x1.01) 0.965
Hypervolemia (x1.01) 0.965
Secondary malignant neoplasm of bone (x1.01) 0.966
Primary malignant neoplasm of urinary system (x1.01) 0.966
Chronic kidney disease (x1.01) 0.966
Thrombocytopenic disorder (x1.01) 0.966
furosemide (+0.00112) 0.967
Expressions
−10 0 10
−1.00 −0.75 −0.50 −0.25 0.00 0.25 0.50 0.75 1.00

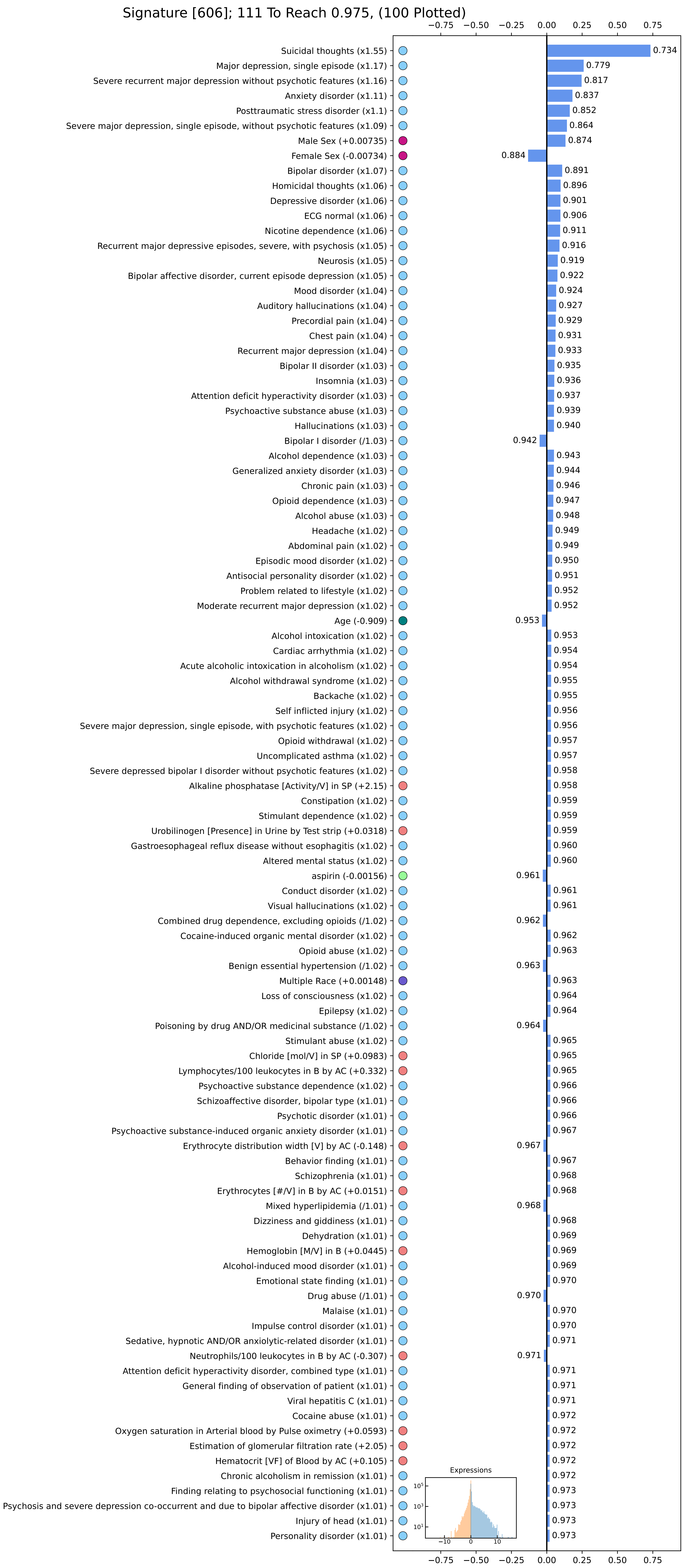

Signature [606]; 111 To Reach 0.975, (100 Plotted)
Suicidal thoughts (x1.55) 0.734
Major depression, single episode (x1.17) 0.779
Severe recurrent major depression without psychotic features (x1.16) 0.817
Anxiety disorder (x1.11) 0.837
Posttraumatic stress disorder (x1.1) 0.852
Severe major depression, single episode, without psychotic features (x1.09) 0.864
Male Sex (+0.00735) 0.874
Female Sex (-0.00734) 0.884
Bipolar disorder (x1.07) 0.891
Homicidal thoughts (x1.06) 0.896
Depressive disorder (x1.06) 0.901
ECG normal (x1.06) 0.906
Nicotine dependence (x1.06) 0.911
Recurrent major depressive episodes, severe, with psychosis (x1.05) 0.916
Neurosis (x1.05) 0.919
Bipolar affective disorder, current episode depression (x1.05) 0.922
Mood disorder (x1.04) 0.924
Auditory hallucinations (x1.04) 0.927
Precordial pain (x1.04) 0.929
Chest pain (x1.04) 0.931
Recurrent major depression (x1.04) 0.933
Bipolar II disorder (x1.03) 0.935
Insomnia (x1.03) 0.936
Attention deficit hyperactivity disorder (x1.03) 0.937
Psychoactive substance abuse (x1.03) 0.939
Hallucinations (x1.03) 0.940
Bipolar I disorder (/1.03) 0.942
Alcohol dependence (x1.03) 0.943
Generalized anxiety disorder (x1.03) 0.944
Chronic pain (x1.03) 0.946
Opioid dependence (x1.03) 0.947
Alcohol abuse (x1.03) 0.948
Headache (x1.02) 0.949
Abdominal pain (x1.02) 0.949
Episodic mood disorder (x1.02) 0.950
Antisocial personality disorder (x1.02) 0.951
Problem related to lifestyle (x1.02) 0.952
Moderate recurrent major depression (x1.02) 0.952
Age (-0.909) 0.953
Alcohol intoxication (x1.02) 0.953
Cardiac arrhythmia (x1.02) 0.954
Acute alcoholic intoxication in alcoholism (x1.02) 0.954
Alcohol withdrawal syndrome (x1.02) 0.955
Backache (x1.02) 0.955
Self inflicted injury (x1.02) 0.956
Severe major depression, single episode, with psychotic features (x1.02) 0.956
Opioid withdrawal (x1.02) 0.957
Uncomplicated asthma (x1.02) 0.957
Severe depressed bipolar I disorder without psychotic features (x1.02) 0.958
Alkaline phosphatase [Activity/V] in SP (+2.15) 0.958
Constipation (x1.02) 0.959
Stimulant dependence (x1.02) 0.959
Urobilinogen [Presence] in Urine by Test strip (+0.0318) 0.959
Gastroesophageal reflux disease without esophagitis (x1.02) 0.960
Altered mental status (x1.02) 0.960
aspirin (-0.00156) 0.961
Conduct disorder (x1.02) 0.961
Visual hallucinations (x1.02) 0.961
Combined drug dependence, excluding opioids (/1.02) 0.962
Cocaine-induced organic mental disorder (x1.02) 0.962
Opioid abuse (x1.02) 0.963
Benign essential hypertension (/1.02) 0.963
Multiple Race (+0.00148) 0.963
Loss of consciousness (x1.02) 0.964
Epilepsy (x1.02) 0.964
Poisoning by drug AND/OR medicinal substance (/1.02) 0.964
Stimulant abuse (x1.02) 0.965
Chloride [mol/V] in SP (+0.0983) 0.965
Lymphocytes/100 leukocytes in B by AC (+0.332) 0.965
Psychoactive substance dependence (x1.02) 0.966
Schizoaffective disorder, bipolar type (x1.01) 0.966
Psychotic disorder (x1.01) 0.966
Psychoactive substance-induced organic anxiety disorder (x1.01) 0.967
Erythrocyte distribution width [V] by AC (-0.148) 0.967
Behavior finding (x1.01) 0.967
Schizophrenia (x1.01) 0.968
Erythrocytes [#/V] in B by AC (+0.0151) 0.968
Mixed hyperlipidemia (/1.01) 0.968
Dizziness and giddiness (x1.01) 0.968
Dehydration (x1.01) 0.969
Hemoglobin [M/V] in B (+0.0445) 0.969
Alcohol-induced mood disorder (x1.01) 0.969
Emotional state finding (x1.01) 0.970
Drug abuse (/1.01) 0.970
Malaise (x1.01) 0.970
Impulse control disorder (x1.01) 0.970
Sedative, hypnotic AND/OR anxiolytic-related disorder (x1.01) 0.971
Neutrophils/100 leukocytes in B by AC (-0.307) 0.971
Attention deficit hyperactivity disorder, combined type (x1.01) 0.971
General finding of observation of patient (x1.01) 0.971
Viral hepatitis C (x1.01) 0.971
Cocaine abuse (x1.01) 0.972
Oxygen saturation in Arterial blood by Pulse oximetry (+0.0593) 0.972
Estimation of glomerular filtration rate (+2.05) 0.972
Hematocrit [VF] of Blood by AC (+0.105) 0.972
Chronic alcoholism in remission (x1.01) 0.972
Finding relating to psychosocial functioning (x1.01) 0.973
Psychosis and severe depression co-occurrent and due to bipolar affective disorder (x1.01) 0.973
Injury of head (x1.01) 0.973
Personality disorder (x1.01) 0.973
−0.75 −0.50 −0.25 0.00 0.25 0.50 0.75
Expressions

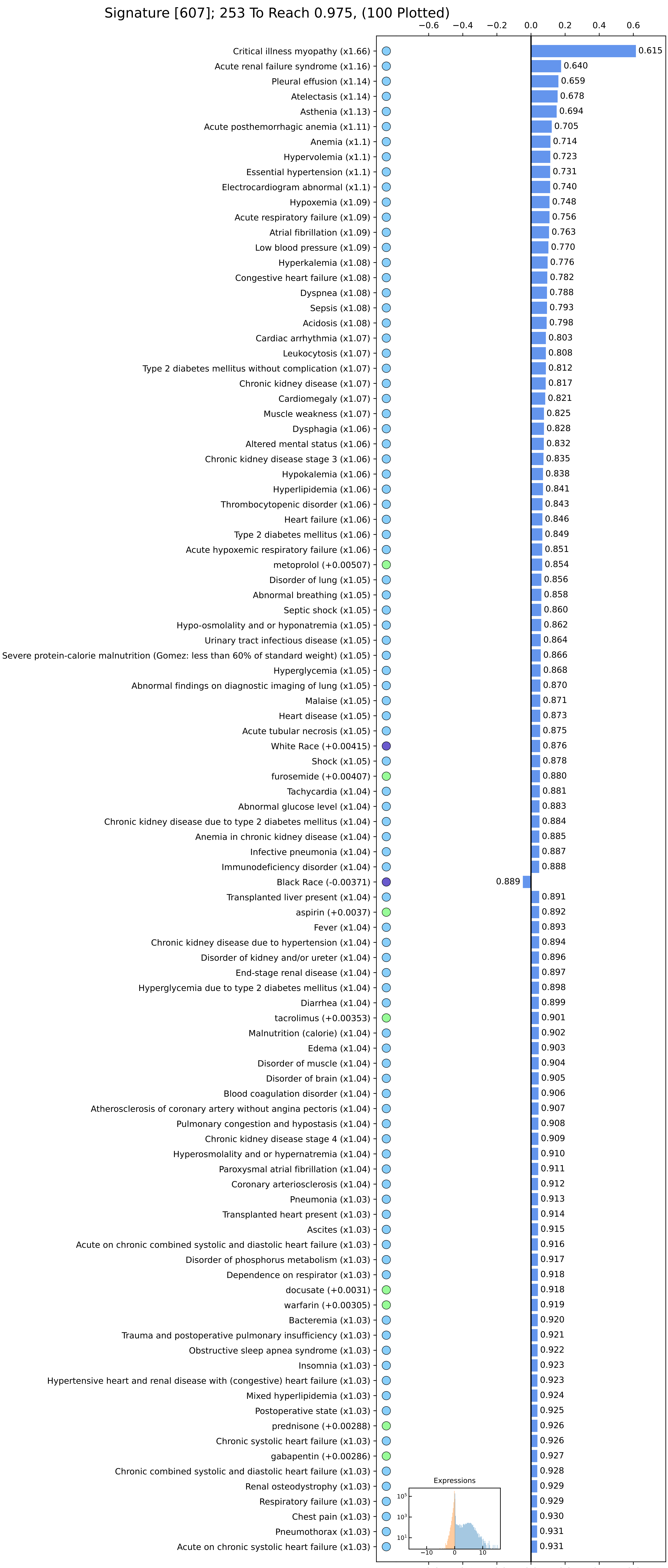
Signature [607]; 253 To Reach 0.975, (100 Plotted)
Critical illness myopathy (x1.66) 0.615
Acute renal failure syndrome (x1.16) 0.640
Pleural effusion (x1.14) 0.659
Atelectasis (x1.14) 0.678
Asthenia (x1.13) 0.694
Acute posthemorrhagic anemia (x1.11) 0.705
Anemia (x1.1) 0.714
Hypervolemia (x1.1) 0.723
Essential hypertension (x1.1) 0.731
Electrocardiogram abnormal (x1.1) 0.740
Hypoxemia (x1.09) 0.748
Acute respiratory failure (x1.09) 0.756
Atrial fibrillation (x1.09) 0.763
Low blood pressure (x1.09) 0.770
Hyperkalemia (x1.08) 0.776
Congestive heart failure (x1.08) 0.782
Dyspnea (x1.08) 0.788
Sepsis (x1.08) 0.793
Acidosis (x1.08) 0.798
Cardiac arrhythmia (x1.07) 0.803
Leukocytosis (x1.07) 0.808
Type 2 diabetes mellitus without complication (x1.07) 0.812
Chronic kidney disease (x1.07) 0.817
Cardiomegaly (x1.07) 0.821
Muscle weakness (x1.07) 0.825
Dysphagia (x1.06) 0.828
Altered mental status (x1.06) 0.832
Chronic kidney disease stage 3 (x1.06) 0.835
Hypokalemia (x1.06) 0.838
Hyperlipidemia (x1.06) 0.841
Thrombocytopenic disorder (x1.06) 0.843
Heart failure (x1.06) 0.846
Type 2 diabetes mellitus (x1.06) 0.849
Acute hypoxemic respiratory failure (x1.06) 0.851
metoprolol (+0.00507) 0.854
Disorder of lung (x1.05) 0.856
Abnormal breathing (x1.05) 0.858
Septic shock (x1.05) 0.860
Hypo-osmolality and or hyponatremia (x1.05) 0.862
Urinary tract infectious disease (x1.05) 0.864
Severe protein-calorie malnutrition (Gomez: less than 60% of standard weight) (x1.05) 0.866
Hyperglycemia (x1.05) 0.868
Abnormal findings on diagnostic imaging of lung (x1.05) 0.870
Malaise (x1.05) 0.871
Heart disease (x1.05) 0.873
Acute tubular necrosis (x1.05) 0.875
White Race (+0.00415) 0.876
Shock (x1.05) 0.878
furosemide (+0.00407) 0.880
Tachycardia (x1.04) 0.881
Abnormal glucose level (x1.04) 0.883
Chronic kidney disease due to type 2 diabetes mellitus (x1.04) 0.884
Anemia in chronic kidney disease (x1.04) 0.885
Infective pneumonia (x1.04) 0.887
Immunodeficiency disorder (x1.04) 0.888
Black Race (-0.00371) 0.889
Transplanted liver present (x1.04) 0.891
aspirin (+0.0037) 0.892
Fever (x1.04) 0.893
Chronic kidney disease due to hypertension (x1.04) 0.894
Disorder of kidney and/or ureter (x1.04) 0.896
End-stage renal disease (x1.04) 0.897
Hyperglycemia due to type 2 diabetes mellitus (x1.04) 0.898
Diarrhea (x1.04) 0.899
tacrolimus (+0.00353) 0.901
Malnutrition (calorie) (x1.04) 0.902
Edema (x1.04) 0.903
Disorder of muscle (x1.04) 0.904
Disorder of brain (x1.04) 0.905
Blood coagulation disorder (x1.04) 0.906
Atherosclerosis of coronary artery without angina pectoris (x1.04) 0.907
Pulmonary congestion and hypostasis (x1.04) 0.908
Chronic kidney disease stage 4 (x1.04) 0.909
Hyperosmolality and or hypernatremia (x1.04) 0.910
Paroxysmal atrial fibrillation (x1.04) 0.911
Coronary arteriosclerosis (x1.04) 0.912
Pneumonia (x1.03) 0.913
Transplanted heart present (x1.03) 0.914
Ascites (x1.03) 0.915
Acute on chronic combined systolic and diastolic heart failure (x1.03) 0.916
Disorder of phosphorus metabolism (x1.03) 0.917
Dependence on respirator (x1.03) 0.918
docusate (+0.0031) 0.918
warfarin (+0.00305) 0.919
Bacteremia (x1.03) 0.920
Trauma and postoperative pulmonary insufficiency (x1.03) 0.921
Obstructive sleep apnea syndrome (x1.03) 0.922
Insomnia (x1.03) 0.923
Hypertensive heart and renal disease with (congestive) heart failure (x1.03) 0.923
Mixed hyperlipidemia (x1.03) 0.924
Postoperative state (x1.03) 0.925
prednisone (+0.00288) 0.926
Chronic systolic heart failure (x1.03) 0.926
gabapentin (+0.00286) 0.927
Chronic combined systolic and diastolic heart failure (x1.03) 0.928
Renal osteodystrophy (x1.03) 0.929
Respiratory failure (x1.03) 0.929
Chest pain (x1.03) 0.930
Pneumothorax (x1.03) 0.931
Acute on chronic systolic heart failure (x1.03) 0.931
−0.6 −0.4 −0.2 0.0 0.2 0.4 0.6
Expressions
10^5
10^3
10^1
−10 0 10

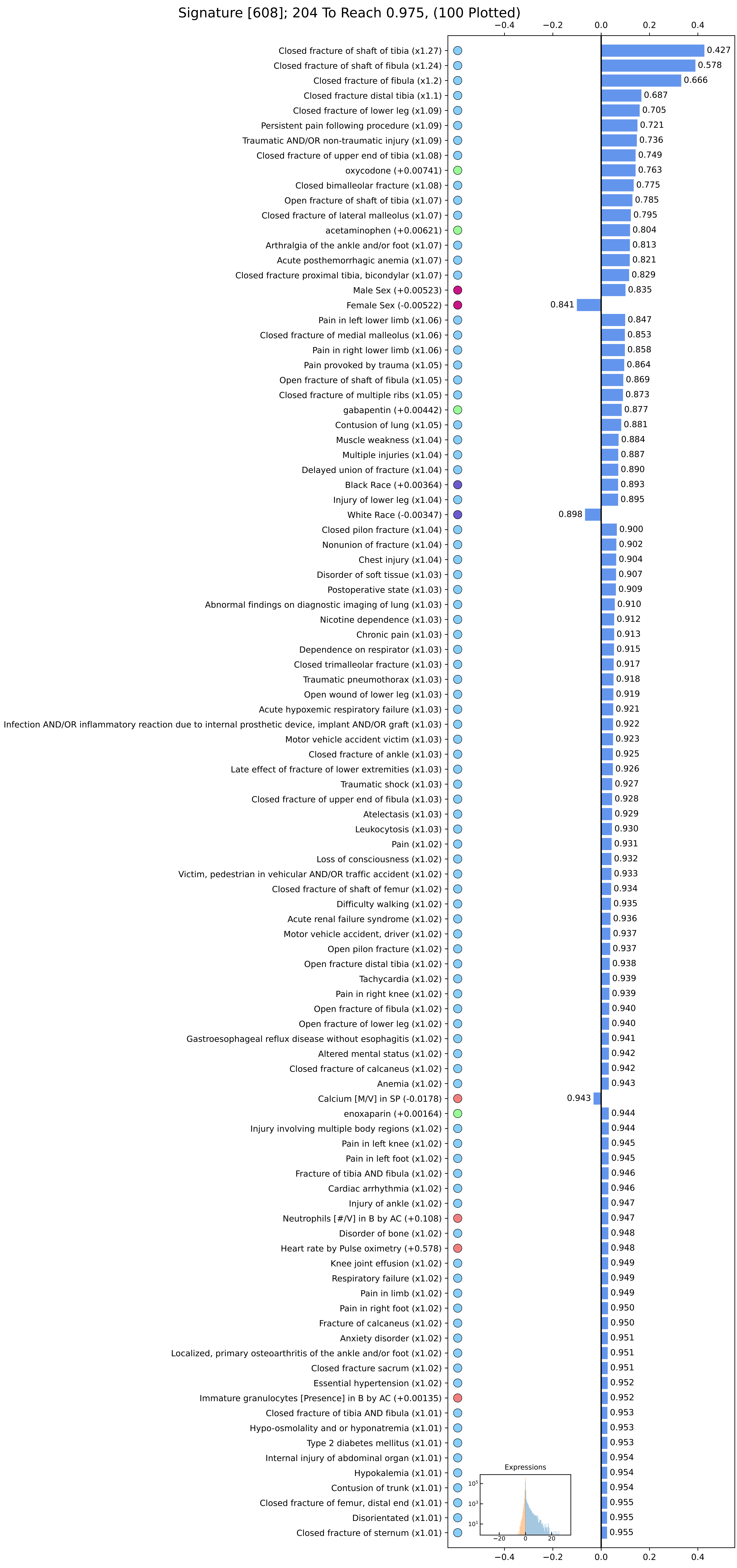
Signature [608]; 204 To Reach 0.975, (100 Plotted)
Closed fracture of shaft of tibia (x1.27) 0.427
Closed fracture of shaft of fibula (x1.24) 0.578
Closed fracture of fibula (x1.2) 0.666
Closed fracture distal tibia (x1.1) 0.687
Closed fracture of lower leg (x1.09) 0.705
Persistent pain following procedure (x1.09) 0.721
Traumatic AND/OR non-traumatic injury (x1.09) 0.736
Closed fracture of upper end of tibia (x1.08) 0.749
oxycodone (+0.00741) 0.763
Closed bimalleolar fracture (x1.08) 0.775
Open fracture of shaft of tibia (x1.07) 0.785
Closed fracture of lateral malleolus (x1.07) 0.795
acetaminophen (+0.00621) 0.804
Arthralgia of the ankle and/or foot (x1.07) 0.813
Acute posthemorrhagic anemia (x1.07) 0.821
Closed fracture proximal tibia, bicondylar (x1.07) 0.829
Male Sex (+0.00523) 0.835
Female Sex (-0.00522) 0.841
Pain in left lower limb (x1.06) 0.847
Closed fracture of medial malleolus (x1.06) 0.853
Pain in right lower limb (x1.06) 0.858
Pain provoked by trauma (x1.05) 0.864
Open fracture of shaft of fibula (x1.05) 0.869
Closed fracture of multiple ribs (x1.05) 0.873
gabapentin (+0.00442) 0.877
Contusion of lung (x1.05) 0.881
Muscle weakness (x1.04) 0.884
Multiple injuries (x1.04) 0.887
Delayed union of fracture (x1.04) 0.890
Black Race (+0.00364) 0.893
Injury of lower leg (x1.04) 0.895
White Race (-0.00347) 0.898
Closed pilon fracture (x1.04) 0.900
Nonunion of fracture (x1.04) 0.902
Chest injury (x1.04) 0.904
Disorder of soft tissue (x1.03) 0.907
Postoperative state (x1.03) 0.909
Abnormal findings on diagnostic imaging of lung (x1.03) 0.910
Nicotine dependence (x1.03) 0.912
Chronic pain (x1.03) 0.913
Dependence on respirator (x1.03) 0.915
Closed trimalleolar fracture (x1.03) 0.917
Traumatic pneumothorax (x1.03) 0.918
Open wound of lower leg (x1.03) 0.919
Acute hypoxemic respiratory failure (x1.03) 0.921
Infection AND/OR inflammatory reaction due to internal prosthetic device, implant AND/OR graft (x1.03) 0.922
Motor vehicle accident victim (x1.03) 0.923
Closed fracture of ankle (x1.03) 0.925
Late effect of fracture of lower extremities (x1.03) 0.926
Traumatic shock (x1.03) 0.927
Closed fracture of upper end of fibula (x1.03) 0.928
Atelectasis (x1.03) 0.929
Leukocytosis (x1.03) 0.930
Pain (x1.02) 0.931
Loss of consciousness (x1.02) 0.932
Victim, pedestrian in vehicular AND/OR traffic accident (x1.02) 0.933
Closed fracture of shaft of femur (x1.02) 0.934
Difficulty walking (x1.02) 0.935
Acute renal failure syndrome (x1.02) 0.936
Motor vehicle accident, driver (x1.02) 0.937
Open pilon fracture (x1.02) 0.937
Open fracture distal tibia (x1.02) 0.938
Tachycardia (x1.02) 0.939
Pain in right knee (x1.02) 0.939
Open fracture of fibula (x1.02) 0.940
Open fracture of lower leg (x1.02) 0.940
Gastroesophageal reflux disease without esophagitis (x1.02) 0.941
Altered mental status (x1.02) 0.942
Closed fracture of calcaneus (x1.02) 0.942
Anemia (x1.02) 0.943
Calcium [M/V] in SP (-0.0178) 0.943
enoxaparin (+0.00164) 0.944
Injury involving multiple body regions (x1.02) 0.944
Pain in left knee (x1.02) 0.945
Pain in left foot (x1.02) 0.945
Fracture of tibia AND fibula (x1.02) 0.946
Cardiac arrhythmia (x1.02) 0.946
Injury of ankle (x1.02) 0.947
Neutrophils [#/V] in B by AC (+0.108) 0.947
Disorder of bone (x1.02) 0.948
Heart rate by Pulse oximetry (+0.578) 0.948
Knee joint effusion (x1.02) 0.949
Respiratory failure (x1.02) 0.949
Pain in limb (x1.02) 0.949
Pain in right foot (x1.02) 0.950
Fracture of calcaneus (x1.02) 0.950
Anxiety disorder (x1.02) 0.951
Localized, primary osteoarthritis of the ankle and/or foot (x1.02) 0.951
Closed fracture sacrum (x1.02) 0.951
Essential hypertension (x1.02) 0.952
Immature granulocytes [Presence] in B by AC (+0.00135) 0.952
Closed fracture of tibia AND fibula (x1.01) 0.953
Hypo-osmolality and or hyponatremia (x1.01) 0.953
Type 2 diabetes mellitus (x1.01) 0.953
Internal injury of abdominal organ (x1.01) 0.954
Hypokalemia (x1.01) 0.954
Contusion of trunk (x1.01) 0.954
Closed fracture of femur, distal end (x1.01) 0.955
Disorientated (x1.01) 0.955
Closed fracture of sternum (x1.01) 0.955
−0.4 −0.2 0.0 0.2 0.4
Expressions
−20 0 20

Signature [609]; 5 To Reach 0.975, (10 Plotted)

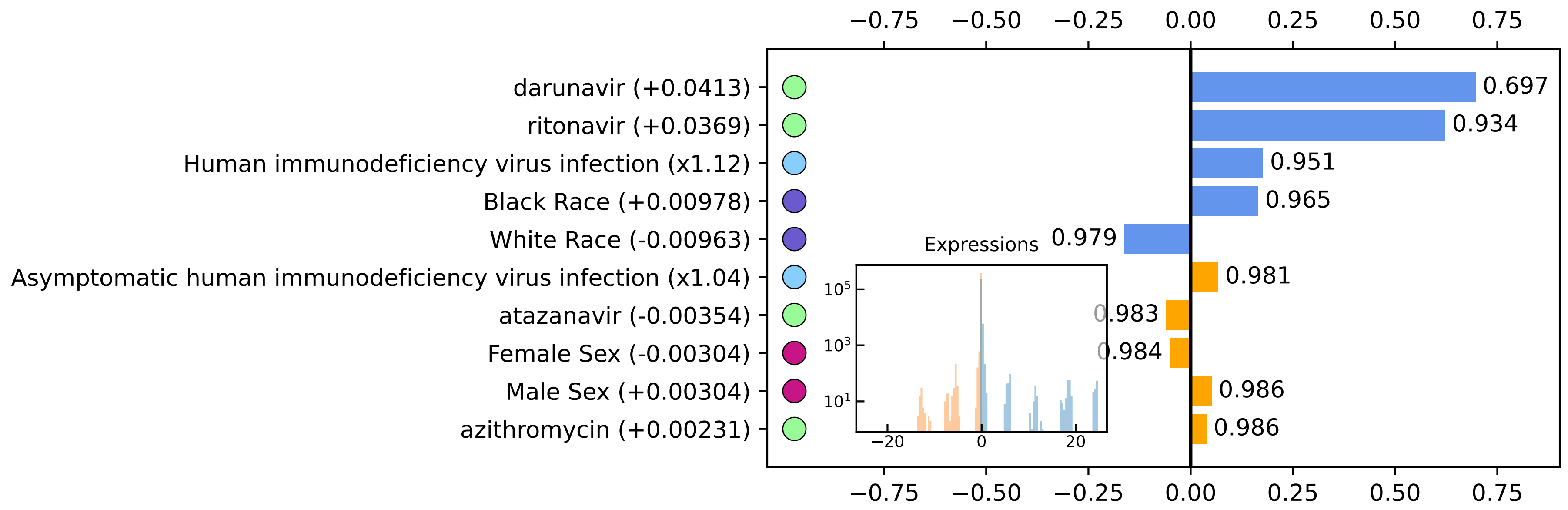

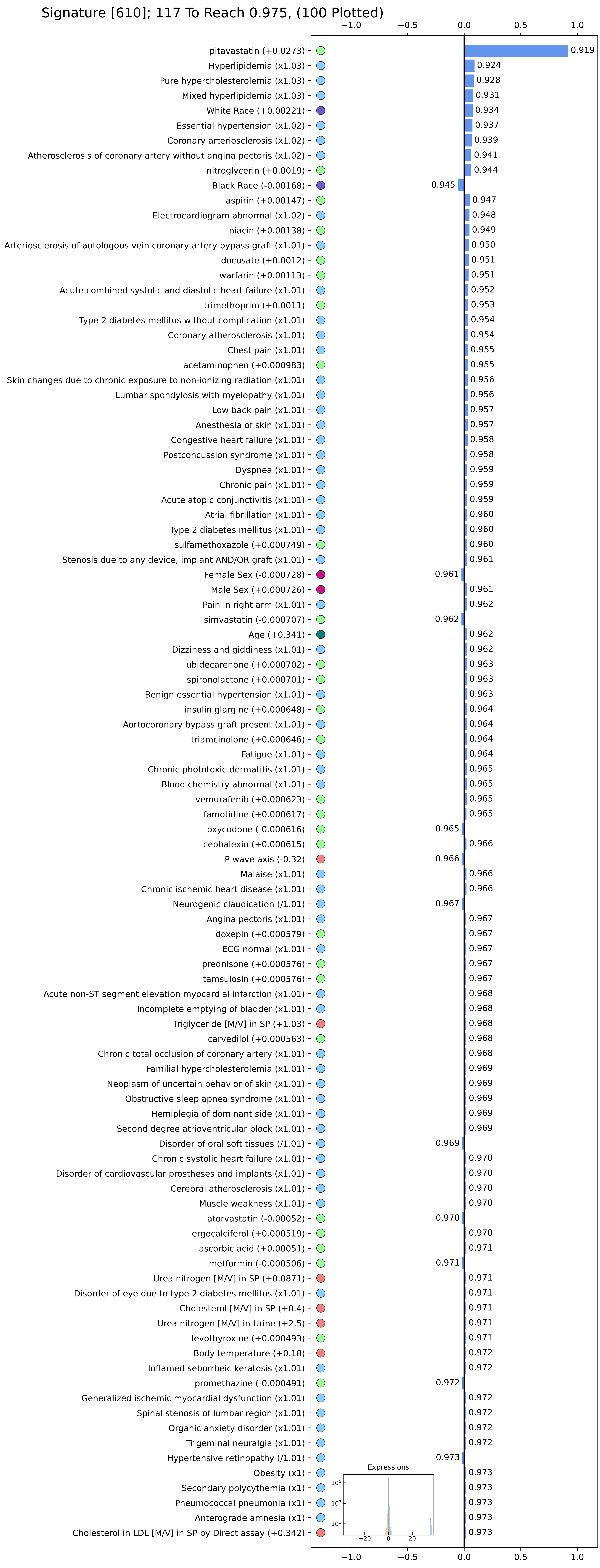
Signature [610]; 117 To Reach 0.975, (100 Plotted)
pitavastatin (+0.0273) 0.919
Hyperlipidemia (x1.03) 0.924
Pure hypercholesterolemia (x1.03) 0.928
Mixed hyperlipidemia (x1.03) 0.931
White Race (+0.00221) 0.934
Essential hypertension (x1.02) 0.937
Coronary arteriosclerosis (x1.02) 0.939
Atherosclerosis of coronary artery without angina pectoris (x1.02) 0.941
nitroglycerin (+0.0019) 0.944
Black Race (-0.00168) 0.945
aspirin (+0.00147) 0.947
Electrocardiogram abnormal (x1.02) 0.948
niacin (+0.00138) 0.949
Arteriosclerosis of autologous vein coronary artery bypass graft (x1.01) 0.950
docusate (+0.0012) 0.951
warfarin (+0.00113) 0.951
Acute combined systolic and diastolic heart failure (x1.01) 0.952
trimethoprim (+0.0011) 0.953
Type 2 diabetes mellitus without complication (x1.01) 0.954
Coronary atherosclerosis (x1.01) 0.954
Chest pain (x1.01) 0.955
acetaminophen (+0.000983) 0.955
Skin changes due to chronic exposure to non-ionizing radiation (x1.01) 0.956
Lumbar spondylosis with myelopathy (x1.01) 0.956
Low back pain (x1.01) 0.957
Anesthesia of skin (x1.01) 0.957
Congestive heart failure (x1.01) 0.958
Postconcussion syndrome (x1.01) 0.958
Dyspnea (x1.01) 0.959
Chronic pain (x1.01) 0.959
Acute atopic conjunctivitis (x1.01) 0.959
Atrial fibrillation (x1.01) 0.960
Type 2 diabetes mellitus (x1.01) 0.960
sulfamethoxazole (+0.000749) 0.960
Stenosis due to any device, implant AND/OR graft (x1.01) 0.961
Female Sex (-0.000728) 0.961
Male Sex (+0.000726) 0.961
Pain in right arm (x1.01) 0.962
simvastatin (-0.000707) 0.962
Age (+0.341) 0.962
Dizziness and giddiness (x1.01) 0.962
ubidecarenone (+0.000702) 0.963
spironolactone (+0.000701) 0.963
Benign essential hypertension (x1.01) 0.963
insulin glargine (+0.000648) 0.964
Aortocoronary bypass graft present (x1.01) 0.964
triamcinolone (+0.000646) 0.964
Fatigue (x1.01) 0.964
Chronic phototoxic dermatitis (x1.01) 0.965
Blood chemistry abnormal (x1.01) 0.965
vemurafenib (+0.000623) 0.965
famotidine (+0.000617) 0.965
oxycodone (-0.000616) 0.965
cephalexin (+0.000615) 0.966
P wave axis (-0.32) 0.966
Malaise (x1.01) 0.966
Chronic ischemic heart disease (x1.01) 0.966
Neurogenic claudication (/1.01) 0.967
Angina pectoris (x1.01) 0.967
doxepin (+0.000579) 0.967
ECG normal (x1.01) 0.967
prednisone (+0.000576) 0.967
tamsulosin (+0.000576) 0.967
Acute non-ST segment elevation myocardial infarction (x1.01) 0.968
Incomplete emptying of bladder (x1.01) 0.968
Triglyceride [M/V] in SP (+1.03) 0.968
carvedilol (+0.000563) 0.968
Chronic total occlusion of coronary artery (x1.01) 0.968
Familial hypercholesterolemia (x1.01) 0.969
Neoplasm of uncertain behavior of skin (x1.01) 0.969
Obstructive sleep apnea syndrome (x1.01) 0.969
Hemiplegia of dominant side (x1.01) 0.969
Second degree atrioventricular block (x1.01) 0.969
Disorder of oral soft tissues (/1.01) 0.969
Chronic systolic heart failure (x1.01) 0.970
Disorder of cardiovascular prostheses and implants (x1.01) 0.970
Cerebral atherosclerosis (x1.01) 0.970
Muscle weakness (x1.01) 0.970
atorvastatin (-0.00052) 0.970
ergocalciferol (+0.000519) 0.970
ascorbic acid (+0.00051) 0.971
metformin (-0.000506) 0.971
Urea nitrogen [M/V] in SP (+0.0871) 0.971
Disorder of eye due to type 2 diabetes mellitus (x1.01) 0.971
Cholesterol [M/V] in SP (+0.4) 0.971
Urea nitrogen [M/V] in Urine (+2.5) 0.971
levothyroxine (+0.000493) 0.971
Body temperature (+0.18) 0.972
Inflamed seborrheic keratosis (x1.01) 0.972
promethazine (-0.000491) 0.972
Generalized ischemic myocardial dysfunction (x1.01) 0.972
Spinal stenosis of lumbar region (x1.01) 0.972
Organic anxiety disorder (x1.01) 0.972
Trigeminal neuralgia (x1.01) 0.972
Hypertensive retinopathy (/1.01) 0.973
Obesity (x1) 0.973
Secondary polycythemia (x1) 0.973
Pneumococcal pneumonia (x1) 0.973
Anterograde amnesia (x1) 0.973
Cholesterol in LDL [M/V] in SP by Direct assay (+0.342) 0.973
−1.0 −0.5 0.0 0.5 1.0
Expressions
10^5 10^3 10^1
−20 0 20

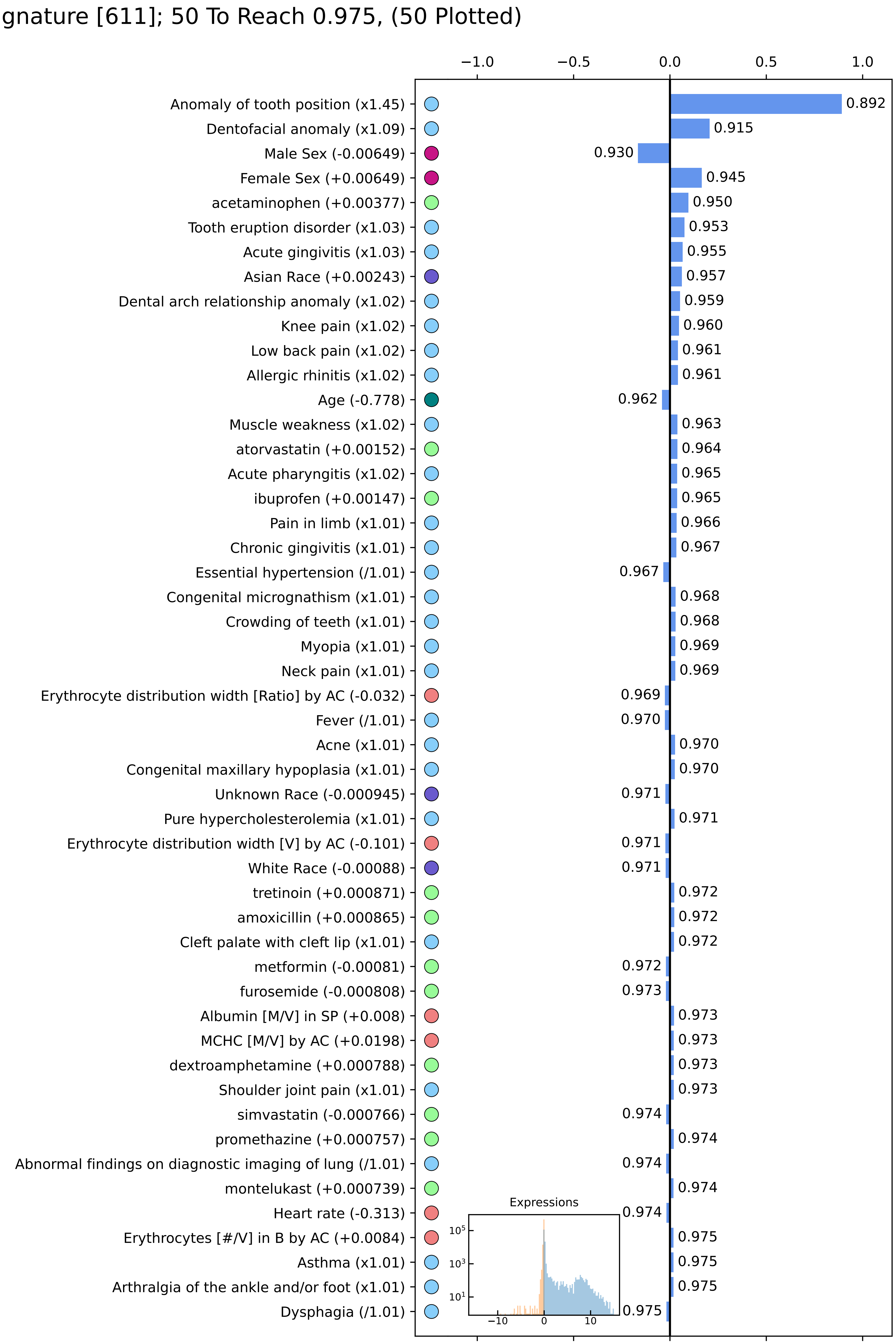

Signature [611]; 50 To Reach 0.975, (50 Plotted)
Anomaly of tooth position (x1.45) 0.892
Dentofacial anomaly (x1.09) 0.915
Male Sex (-0.00649) 0.930
Female Sex (+0.00649) 0.945
acetaminophen (+0.00377) 0.950
Tooth eruption disorder (x1.03) 0.953
Acute gingivitis (x1.03) 0.955
Asian Race (+0.00243) 0.957
Dental arch relationship anomaly (x1.02) 0.959
Knee pain (x1.02) 0.960
Low back pain (x1.02) 0.961
Allergic rhinitis (x1.02) 0.961
Age (-0.778) 0.962
Muscle weakness (x1.02) 0.963
atorvastatin (+0.00152) 0.964
Acute pharyngitis (x1.02) 0.965
ibuprofen (+0.00147) 0.965
Pain in limb (x1.01) 0.966
Chronic gingivitis (x1.01) 0.967
Essential hypertension (/1.01) 0.967
Congenital micrognathism (x1.01) 0.968
Crowding of teeth (x1.01) 0.968
Myopia (x1.01) 0.969
Neck pain (x1.01) 0.969
Erythrocyte distribution width [Ratio] by AC (-0.032) 0.969
Fever (/1.01) 0.970
Acne (x1.01) 0.970
Congenital maxillary hypoplasia (x1.01) 0.970
Unknown Race (-0.000945) 0.971
Pure hypercholesterolemia (x1.01) 0.971
Erythrocyte distribution width [V] by AC (-0.101) 0.971
White Race (-0.00088) 0.971
tretinoin (+0.000871) 0.972
amoxicillin (+0.000865) 0.972
Cleft palate with cleft lip (x1.01) 0.972
metformin (-0.00081) 0.972
furosemide (-0.000808) 0.973
Albumin [M/V] in SP (+0.008) 0.973
MCHC [M/V] by AC (+0.0198) 0.973
dextroamphetamine (+0.000788) 0.973
Shoulder joint pain (x1.01) 0.973
simvastatin (-0.000766) 0.974
promethazine (+0.000757) 0.974
Abnormal findings on diagnostic imaging of lung (/1.01) 0.974
montelukast (+0.000739) 0.974
Heart rate (-0.313) 0.974
Erythrocytes [#/V] in B by AC (+0.0084) 0.975
Asthma (x1.01) 0.975
Arthralgia of the ankle and/or foot (x1.01) 0.975
Dysphagia (/1.01) 0.975
−1.0 −0.5 0.0 0.5 1.0
Expressions
10^5 10^3 10^1
−10 0 10

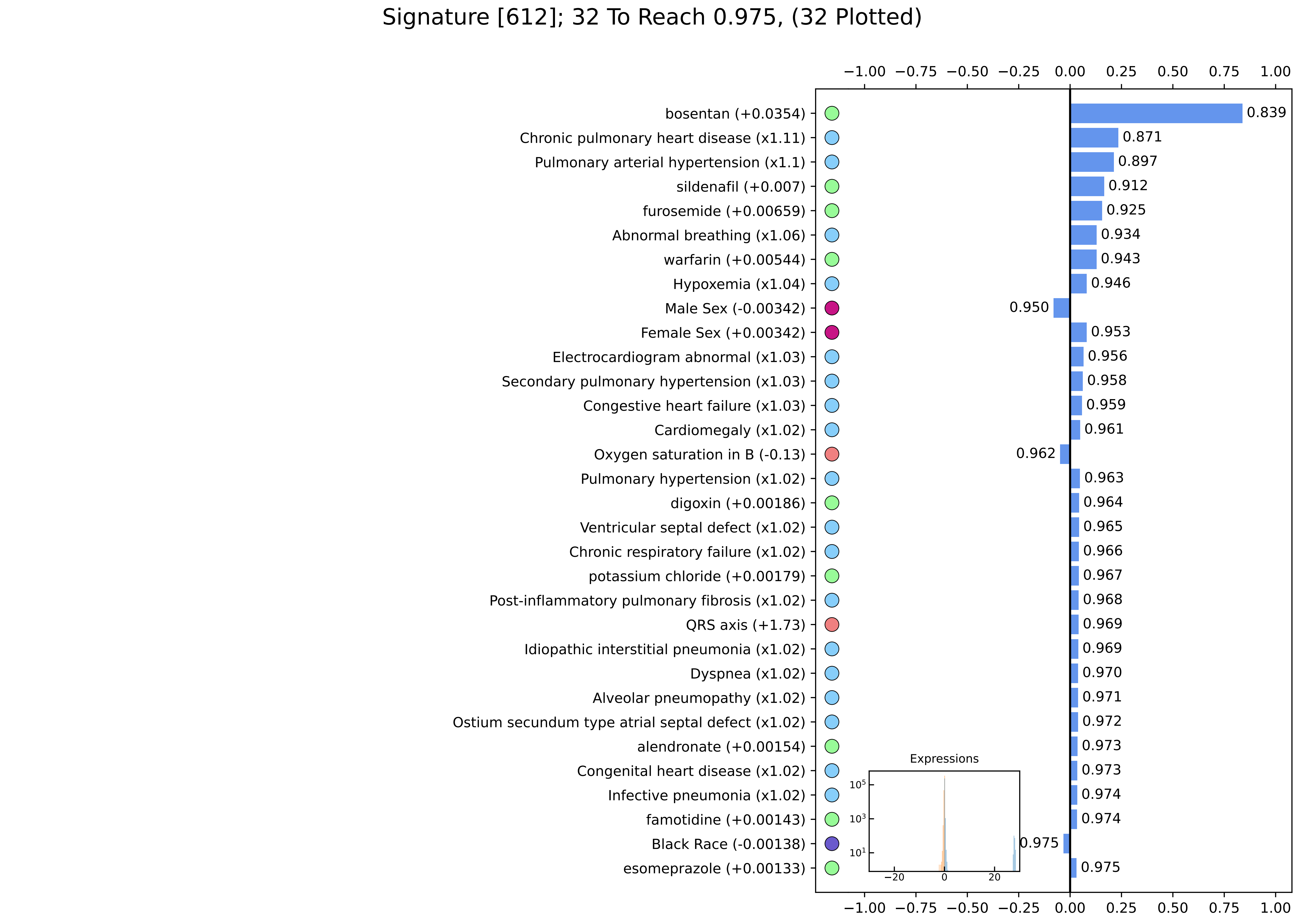

Signature [612]; 32 To Reach 0.975, (32 Plotted)
bosentan (+0.0354) 0.839
Chronic pulmonary heart disease (x1.11) 0.871
Pulmonary arterial hypertension (x1.1) 0.897
sildenafil (+0.007) 0.912
furosemide (+0.00659) 0.925
Abnormal breathing (x1.06) 0.934
warfarin (+0.00544) 0.943
Hypoxemia (x1.04) 0.946
Male Sex (-0.00342) 0.950
Female Sex (+0.00342) 0.953
Electrocardiogram abnormal (x1.03) 0.956
Secondary pulmonary hypertension (x1.03) 0.958
Congestive heart failure (x1.03) 0.959
Cardiomegaly (x1.02) 0.961
Oxygen saturation in B (-0.13) 0.962
Pulmonary hypertension (x1.02) 0.963
digoxin (+0.00186) 0.964
Ventricular septal defect (x1.02) 0.965
Chronic respiratory failure (x1.02) 0.966
potassium chloride (+0.00179) 0.967
Post-inflammatory pulmonary fibrosis (x1.02) 0.968
QRS axis (+1.73) 0.969
Idiopathic interstitial pneumonia (x1.02) 0.969
Dyspnea (x1.02) 0.970
Alveolar pneumopathy (x1.02) 0.971
Ostium secundum type atrial septal defect (x1.02) 0.972
alendronate (+0.00154) 0.973
Congenital heart disease (x1.02) 0.973
Infective pneumonia (x1.02) 0.974
famotidine (+0.00143) 0.974
Black Race (-0.00138) 0.975
esomeprazole (+0.00133) 0.975
−1.00 −0.75 −0.50 −0.25 0.00 0.25 0.50 0.75 1.00
Expressions
10^5
10^3
10^1
−20 0 20

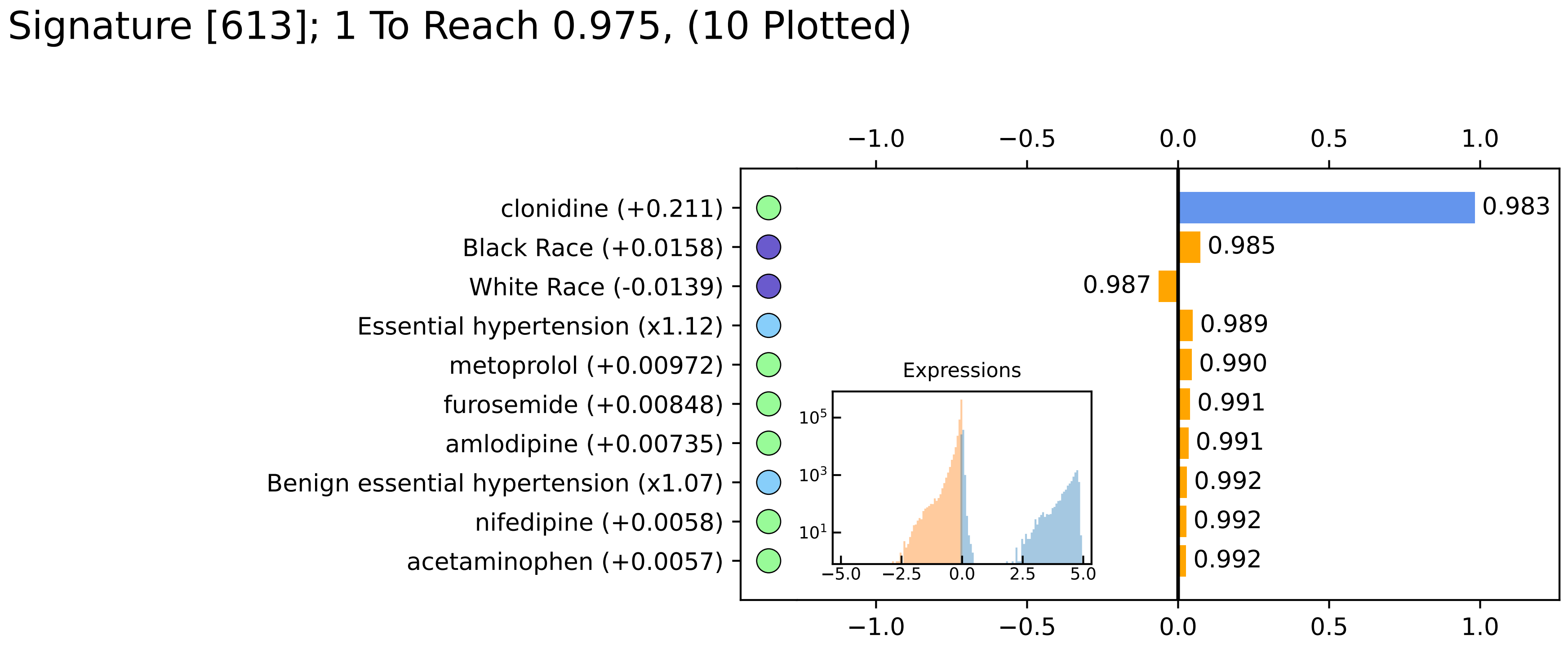
Signature [613]; 1 To Reach 0.975, (10 Plotted)
clonidine (+0.211)
Black Race (+0.0158)
White Race (-0.0139)
Essential hypertension (x1.12)
metoprolol (+0.00972)
furosemide (+0.00848)
amlodipine (+0.00735)
Benign essential hypertension (x1.07)
nifedipine (+0.0058)
acetaminophen (+0.0057)
0.983
0.985
0.987
0.989
0.990
0.991
0.991
0.992
0.992
0.992
Expressions
−1.0
−0.5
0.0
0.5
1.0

Signature [614]; 16 To Reach 0.975, (16 Plotted)

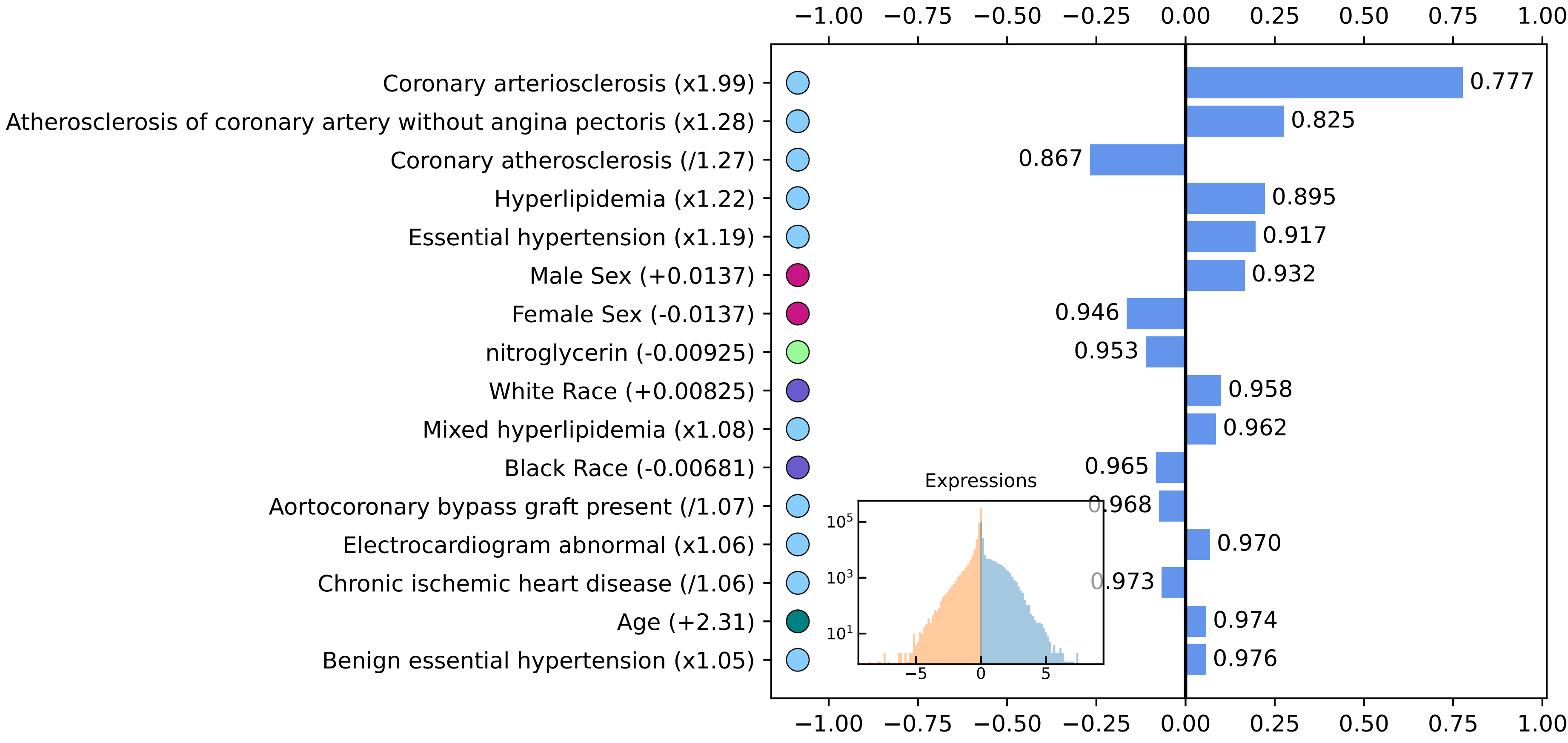

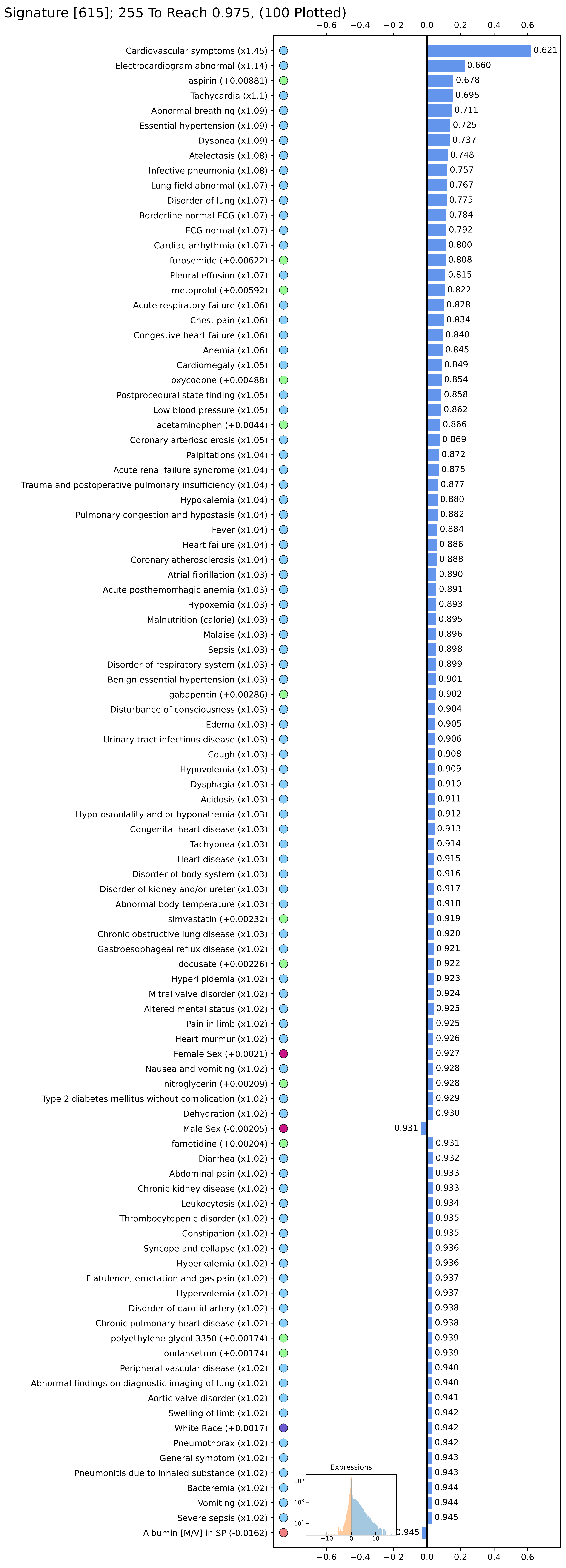

Signature [615]; 255 To Reach 0.975, (100 Plotted)
Cardiovascular symptoms (x1.45) 0.621
Electrocardiogram abnormal (x1.14) 0.660
aspirin (+0.00881) 0.678
Tachycardia (x1.1) 0.695
Abnormal breathing (x1.09) 0.711
Essential hypertension (x1.09) 0.725
Dyspnea (x1.09) 0.737
Atelectasis (x1.08) 0.748
Infective pneumonia (x1.08) 0.757
Lung field abnormal (x1.07) 0.767
Disorder of lung (x1.07) 0.775
Borderline normal ECG (x1.07) 0.784
ECG normal (x1.07) 0.792
Cardiac arrhythmia (x1.07) 0.800
furosemide (+0.00622) 0.808
Pleural effusion (x1.07) 0.815
metoprolol (+0.00592) 0.822
Acute respiratory failure (x1.06) 0.828
Chest pain (x1.06) 0.834
Congestive heart failure (x1.06) 0.840
Anemia (x1.06) 0.845
Cardiomegaly (x1.05) 0.849
oxycodone (+0.00488) 0.854
Postprocedural state finding (x1.05) 0.858
Low blood pressure (x1.05) 0.862
acetaminophen (+0.0044) 0.866
Coronary arteriosclerosis (x1.05) 0.869
Palpitations (x1.04) 0.872
Acute renal failure syndrome (x1.04) 0.875
Trauma and postoperative pulmonary insufficiency (x1.04) 0.877
Hypokalemia (x1.04) 0.880
Pulmonary congestion and hypostasis (x1.04) 0.882
Fever (x1.04) 0.884
Heart failure (x1.04) 0.886
Coronary atherosclerosis (x1.04) 0.888
Atrial fibrillation (x1.03) 0.890
Acute posthemorrhagic anemia (x1.03) 0.891
Hypoxemia (x1.03) 0.893
Malnutrition (calorie) (x1.03) 0.895
Malaise (x1.03) 0.896
Sepsis (x1.03) 0.898
Disorder of respiratory system (x1.03) 0.899
Benign essential hypertension (x1.03) 0.901
gabapentin (+0.00286) 0.902
Disturbance of consciousness (x1.03) 0.904
Edema (x1.03) 0.905
Urinary tract infectious disease (x1.03) 0.906
Cough (x1.03) 0.908
Hypovolemia (x1.03) 0.909
Dysphagia (x1.03) 0.910
Acidosis (x1.03) 0.911
Hypo-osmolality and or hyponatremia (x1.03) 0.912
Congenital heart disease (x1.03) 0.913
Tachypnea (x1.03) 0.914
Heart disease (x1.03) 0.915
Disorder of body system (x1.03) 0.916
Disorder of kidney and/or ureter (x1.03) 0.917
Abnormal body temperature (x1.03) 0.918
simvastatin (+0.00232) 0.919
Chronic obstructive lung disease (x1.03) 0.920
Gastroesophageal reflux disease (x1.02) 0.921
docusate (+0.00226) 0.922
Hyperlipidemia (x1.02) 0.923
Mitral valve disorder (x1.02) 0.924
Altered mental status (x1.02) 0.925
Pain in limb (x1.02) 0.925
Heart murmur (x1.02) 0.926
Female Sex (+0.0021) 0.927
Nausea and vomiting (x1.02) 0.928
nitroglycerin (+0.00209) 0.928
Type 2 diabetes mellitus without complication (x1.02) 0.929
Dehydration (x1.02) 0.930
Male Sex (-0.00205) 0.931
famotidine (+0.00204) 0.931
Diarrhea (x1.02) 0.932
Abdominal pain (x1.02) 0.933
Chronic kidney disease (x1.02) 0.933
Leukocytosis (x1.02) 0.934
Thrombocytopenic disorder (x1.02) 0.935
Constipation (x1.02) 0.935
Syncope and collapse (x1.02) 0.936
Hyperkalemia (x1.02) 0.936
Flatulence, eructation and gas pain (x1.02) 0.937
Hypervolemia (x1.02) 0.937
Disorder of carotid artery (x1.02) 0.938
Chronic pulmonary heart disease (x1.02) 0.938
polyethylene glycol 3350 (+0.00174) 0.939
ondansetron (+0.00174) 0.939
Peripheral vascular disease (x1.02) 0.940
Abnormal findings on diagnostic imaging of lung (x1.02) 0.940
Aortic valve disorder (x1.02) 0.941
Swelling of limb (x1.02) 0.942
White Race (+0.0017) 0.942
Pneumothorax (x1.02) 0.942
General symptom (x1.02) 0.943
Pneumonitis due to inhaled substance (x1.02) 0.943
Bacteremia (x1.02) 0.944
Vomiting (x1.02) 0.944
Severe sepsis (x1.02) 0.945
Albumin [M/V] in SP (-0.0162) 0.945
−0.6 −0.4 −0.2 0.0 0.2 0.4 0.6
Expressions

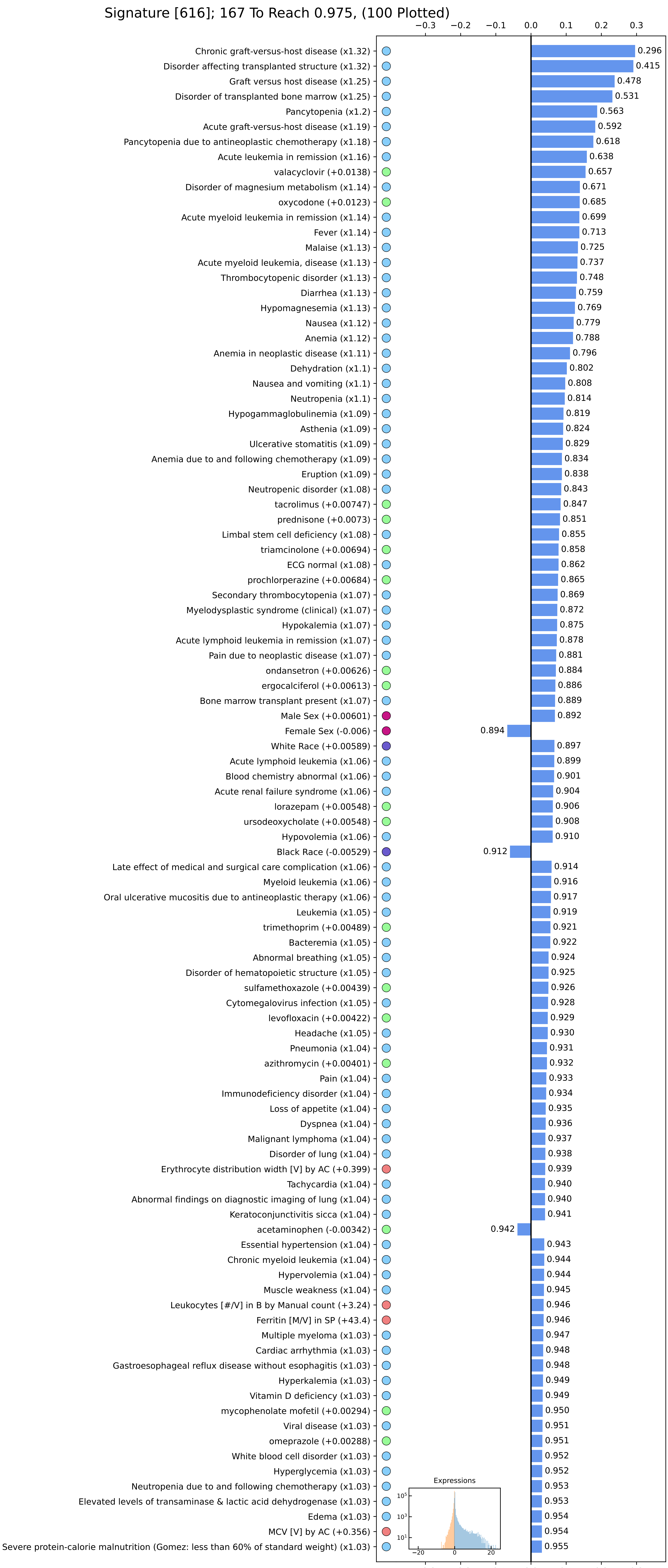

Signature [616]; 167 To Reach 0.975, (100 Plotted)
Chronic graft-versus-host disease (x1.32) 0.296
Disorder affecting transplanted structure (x1.32) 0.415
Graft versus host disease (x1.25) 0.478
Disorder of transplanted bone marrow (x1.25) 0.531
Pancytopenia (x1.2) 0.563
Acute graft-versus-host disease (x1.19) 0.592
Pancytopenia due to antineoplastic chemotherapy (x1.18) 0.618
Acute leukemia in remission (x1.16) 0.638
valacyclovir (+0.0138) 0.657
Disorder of magnesium metabolism (x1.14) 0.671
oxycodone (+0.0123) 0.685
Acute myeloid leukemia in remission (x1.14) 0.699
Fever (x1.14) 0.713
Malaise (x1.13) 0.725
Acute myeloid leukemia, disease (x1.13) 0.737
Thrombocytopenic disorder (x1.13) 0.748
Diarrhea (x1.13) 0.759
Hypomagnesemia (x1.13) 0.769
Nausea (x1.12) 0.779
Anemia (x1.12) 0.788
Anemia in neoplastic disease (x1.11) 0.796
Dehydration (x1.1) 0.802
Nausea and vomiting (x1.1) 0.808
Neutropenia (x1.1) 0.814
Hypogammaglobulinemia (x1.09) 0.819
Asthenia (x1.09) 0.824
Ulcerative stomatitis (x1.09) 0.829
Anemia due to and following chemotherapy (x1.09) 0.834
Eruption (x1.09) 0.838
Neutropenic disorder (x1.08) 0.843
tacrolimus (+0.00747) 0.847
prednisone (+0.0073) 0.851
Limbal stem cell deficiency (x1.08) 0.855
triamcinolone (+0.00694) 0.858
ECG normal (x1.08) 0.862
prochlorperazine (+0.00684) 0.865
Secondary thrombocytopenia (x1.07) 0.869
Myelodysplastic syndrome (clinical) (x1.07) 0.872
Hypokalemia (x1.07) 0.875
Acute lymphoid leukemia in remission (x1.07) 0.878
Pain due to neoplastic disease (x1.07) 0.881
ondansetron (+0.00626) 0.884
ergocalciferol (+0.00613) 0.886
Bone marrow transplant present (x1.07) 0.889
Male Sex (+0.00601) 0.892
Female Sex (-0.006) 0.894
White Race (+0.00589) 0.897
Acute lymphoid leukemia (x1.06) 0.899
Blood chemistry abnormal (x1.06) 0.901
Acute renal failure syndrome (x1.06) 0.904
lorazepam (+0.00548) 0.906
ursodeoxycholate (+0.00548) 0.908
Hypovolemia (x1.06) 0.910
Black Race (-0.00529) 0.912
Late effect of medical and surgical care complication (x1.06) 0.914
Myeloid leukemia (x1.06) 0.916
Oral ulcerative mucositis due to antineoplastic therapy (x1.06) 0.917
Leukemia (x1.05) 0.919
trimethoprim (+0.00489) 0.921
Bacteremia (x1.05) 0.922
Abnormal breathing (x1.05) 0.924
Disorder of hematopoietic structure (x1.05) 0.925
sulfamethoxazole (+0.00439) 0.926
Cytomegalovirus infection (x1.05) 0.928
levofloxacin (+0.00422) 0.929
Headache (x1.05) 0.930
Pneumonia (x1.04) 0.931
azithromycin (+0.00401) 0.932
Pain (x1.04) 0.933
Immunodeficiency disorder (x1.04) 0.934
Loss of appetite (x1.04) 0.935
Dyspnea (x1.04) 0.936
Malignant lymphoma (x1.04) 0.937
Disorder of lung (x1.04) 0.938
Erythrocyte distribution width [V] by AC (+0.399) 0.939
Tachycardia (x1.04) 0.940
Abnormal findings on diagnostic imaging of lung (x1.04) 0.940
Keratoconjunctivitis sicca (x1.04) 0.941
acetaminophen (-0.00342) 0.942
Essential hypertension (x1.04) 0.943
Chronic myeloid leukemia (x1.04) 0.944
Hypervolemia (x1.04) 0.944
Muscle weakness (x1.04) 0.945
Leukocytes [#/V] in B by Manual count (+3.24) 0.946
Ferritin [M/V] in SP (+43.4) 0.946
Multiple myeloma (x1.03) 0.947
Cardiac arrhythmia (x1.03) 0.948
Gastroesophageal reflux disease without esophagitis (x1.03) 0.948
Hyperkalemia (x1.03) 0.949
Vitamin D deficiency (x1.03) 0.949
mycophenolate mofetil (+0.00294) 0.950
Viral disease (x1.03) 0.951
omeprazole (+0.00288) 0.951
White blood cell disorder (x1.03) 0.952
Hyperglycemia (x1.03) 0.952
Neutropenia due to and following chemotherapy (x1.03) 0.953
Elevated levels of transaminase & lactic acid dehydrogenase (x1.03) 0.953
Edema (x1.03) 0.954
MCV [V] by AC (+0.356) 0.954
Severe protein-calorie malnutrition (Gomez: less than 60% of standard weight) (x1.03) 0.955
Expressions
−0.3 −0.2 −0.1 0.0 0.1 0.2 0.3

Signature [617]; 3 To Reach 0.975, (10 Plotted)

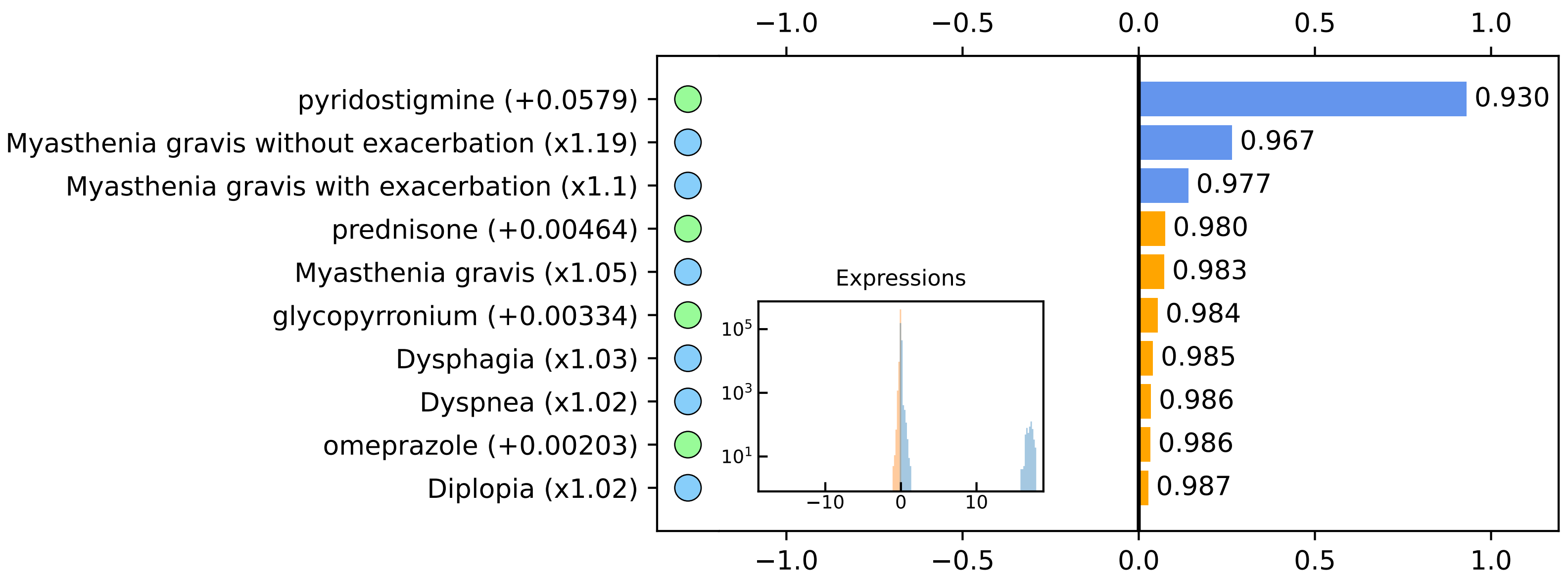

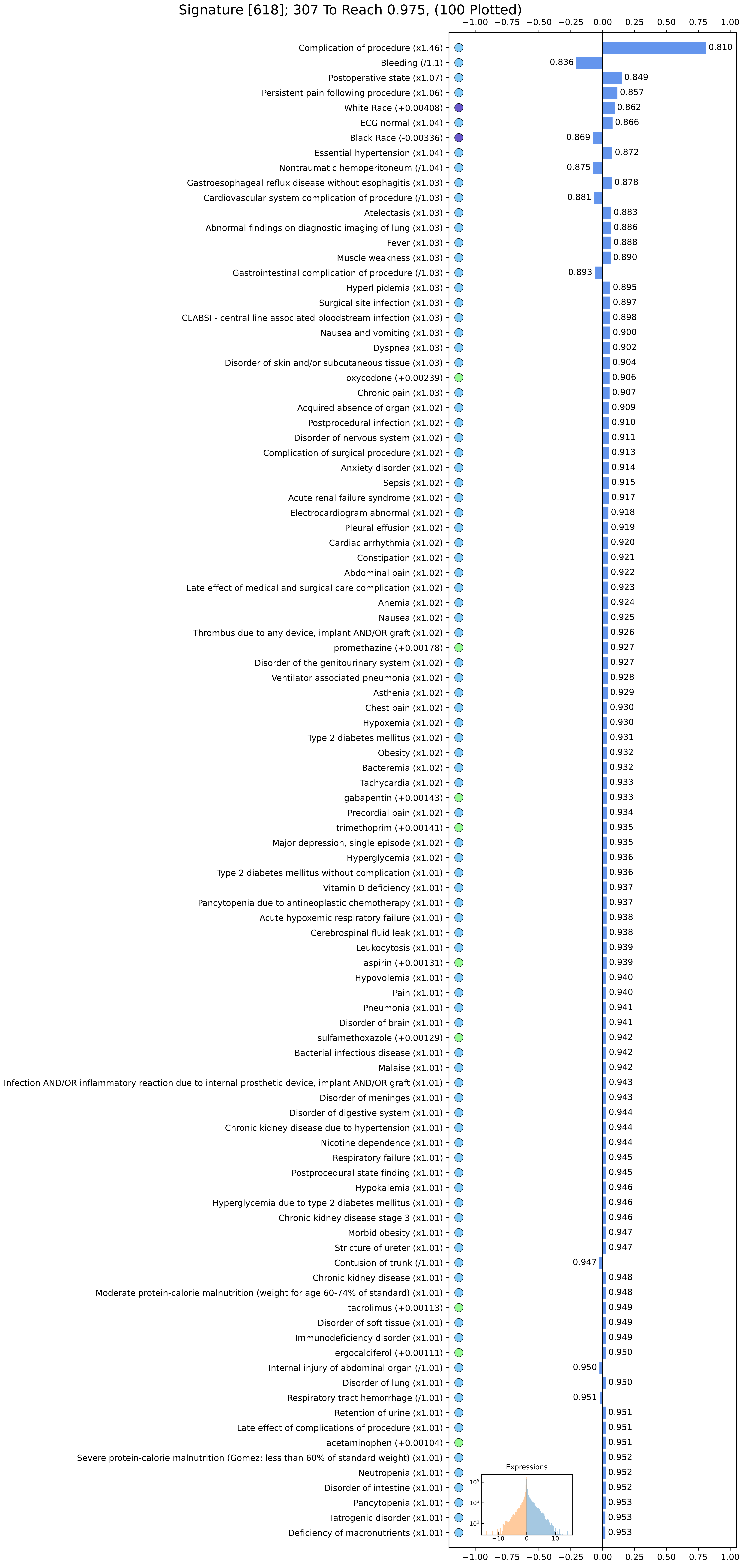

Signature [618]; 307 To Reach 0.975, (100 Plotted)
Complication of procedure (x1.46) 0.810
Bleeding (/1.1) 0.836
Postoperative state (x1.07) 0.849
Persistent pain following procedure (x1.06) 0.857
White Race (+0.00408) 0.862
ECG normal (x1.04) 0.866
Black Race (-0.00336) 0.869
Essential hypertension (x1.04) 0.872
Nontraumatic hemoperitoneum (/1.04) 0.875
Gastroesophageal reflux disease without esophagitis (x1.03) 0.878
Cardiovascular system complication of procedure (/1.03) 0.881
Atelectasis (x1.03) 0.883
Abnormal findings on diagnostic imaging of lung (x1.03) 0.886
Fever (x1.03) 0.888
Muscle weakness (x1.03) 0.890
Gastrointestinal complication of procedure (/1.03) 0.893
Hyperlipidemia (x1.03) 0.895
Surgical site infection (x1.03) 0.897
CLABSI - central line associated bloodstream infection (x1.03) 0.898
Nausea and vomiting (x1.03) 0.900
Dyspnea (x1.03) 0.902
Disorder of skin and/or subcutaneous tissue (x1.03) 0.904
oxycodone (+0.00239) 0.906
Chronic pain (x1.03) 0.907
Acquired absence of organ (x1.02) 0.909
Postprocedural infection (x1.02) 0.910
Disorder of nervous system (x1.02) 0.911
Complication of surgical procedure (x1.02) 0.913
Anxiety disorder (x1.02) 0.914
Sepsis (x1.02) 0.915
Acute renal failure syndrome (x1.02) 0.917
Electrocardiogram abnormal (x1.02) 0.918
Pleural effusion (x1.02) 0.919
Cardiac arrhythmia (x1.02) 0.920
Constipation (x1.02) 0.921
Abdominal pain (x1.02) 0.922
Late effect of medical and surgical care complication (x1.02) 0.923
Anemia (x1.02) 0.924
Nausea (x1.02) 0.925
Thrombus due to any device, implant AND/OR graft (x1.02) 0.926
promethazine (+0.00178) 0.927
Disorder of the genitourinary system (x1.02) 0.927
Ventilator associated pneumonia (x1.02) 0.928
Asthenia (x1.02) 0.929
Chest pain (x1.02) 0.930
Hypoxemia (x1.02) 0.930
Type 2 diabetes mellitus (x1.02) 0.931
Obesity (x1.02) 0.932
Bacteremia (x1.02) 0.932
Tachycardia (x1.02) 0.933
gabapentin (+0.00143) 0.933
Precordial pain (x1.02) 0.934
trimethoprim (+0.00141) 0.935
Major depression, single episode (x1.02) 0.935
Hyperglycemia (x1.02) 0.936
Type 2 diabetes mellitus without complication (x1.01) 0.936
Vitamin D deficiency (x1.01) 0.937
Pancytopenia due to antineoplastic chemotherapy (x1.01) 0.937
Acute hypoxemic respiratory failure (x1.01) 0.938
Cerebrospinal fluid leak (x1.01) 0.938
Leukocytosis (x1.01) 0.939
aspirin (+0.00131) 0.939
Hypovolemia (x1.01) 0.940
Pain (x1.01) 0.940
Pneumonia (x1.01) 0.941
Disorder of brain (x1.01) 0.941
sulfamethoxazole (+0.00129) 0.942
Bacterial infectious disease (x1.01) 0.942
Malaise (x1.01) 0.942
Infection AND/OR inflammatory reaction due to internal prosthetic device, implant AND/OR graft (x1.01) 0.943
Disorder of meninges (x1.01) 0.943
Disorder of digestive system (x1.01) 0.944
Chronic kidney disease due to hypertension (x1.01) 0.944
Nicotine dependence (x1.01) 0.944
Respiratory failure (x1.01) 0.945
Postprocedural state finding (x1.01) 0.945
Hypokalemia (x1.01) 0.946
Hyperglycemia due to type 2 diabetes mellitus (x1.01) 0.946
Chronic kidney disease stage 3 (x1.01) 0.946
Morbid obesity (x1.01) 0.947
Stricture of ureter (x1.01) 0.947
Contusion of trunk (/1.01) 0.947
Chronic kidney disease (x1.01) 0.948
Moderate protein-calorie malnutrition (weight for age 60-74% of standard) (x1.01) 0.948
tacrolimus (+0.00113) 0.949
Disorder of soft tissue (x1.01) 0.949
Immunodeficiency disorder (x1.01) 0.949
ergocalciferol (+0.00111) 0.950
Internal injury of abdominal organ (/1.01) 0.950
Disorder of lung (x1.01) 0.950
Respiratory tract hemorrhage (/1.01) 0.951
Retention of urine (x1.01) 0.951
Late effect of complications of procedure (x1.01) 0.951
acetaminophen (+0.00104) 0.951
Severe protein-calorie malnutrition (Gomez: less than 60% of standard weight) (x1.01) 0.952
Neutropenia (x1.01) 0.952
Disorder of intestine (x1.01) 0.952
Pancytopenia (x1.01) 0.953
Iatrogenic disorder (x1.01) 0.953
Deficiency of macronutrients (x1.01) 0.953
Expressions
−1.00 −0.75 −0.50 −0.25 0.00 0.25 0.50 0.75 1.00

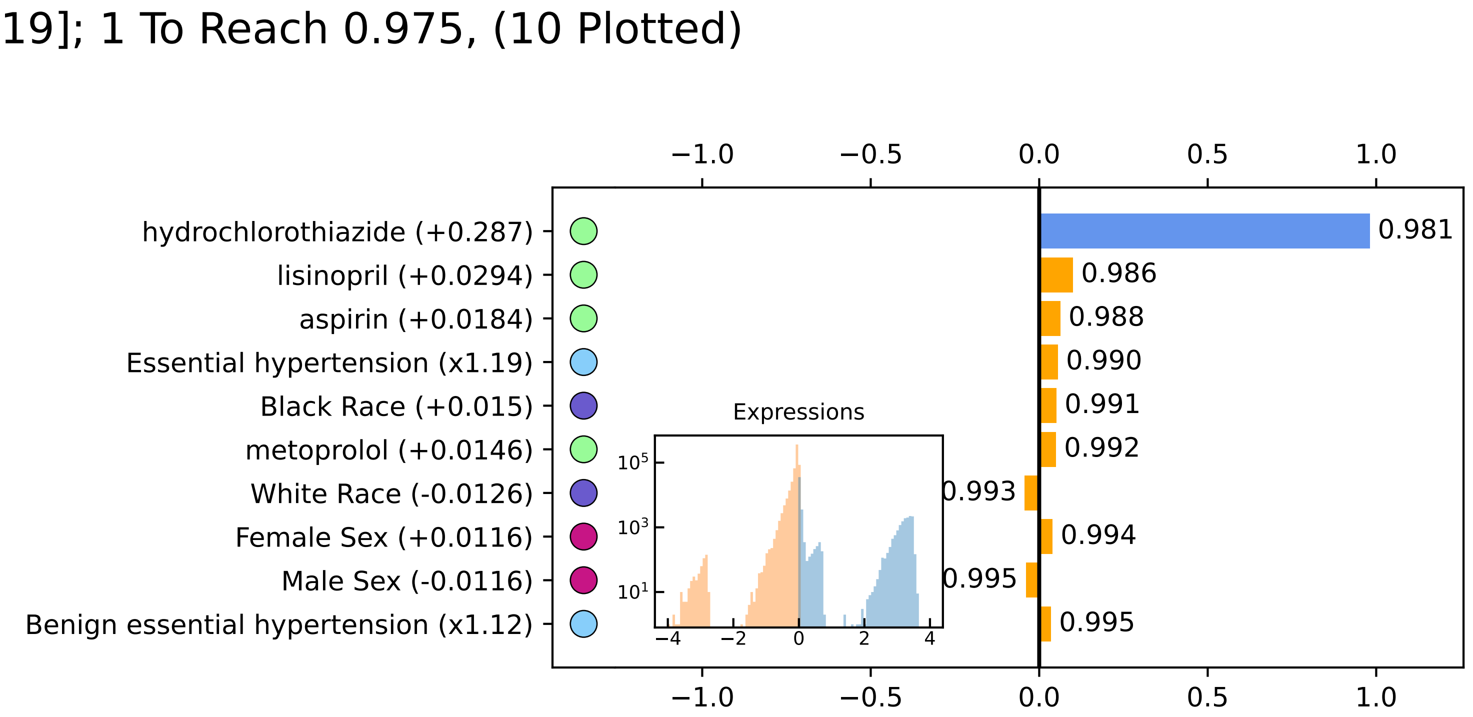

Signature [619]; 1 To Reach 0.975, (10 Plotted)
hydrochlorothiazide (+0.287)
lisinopril (+0.0294)
aspirin (+0.0184)
Essential hypertension (x1.19)
Black Race (+0.015)
metoprolol (+0.0146)
White Race (-0.0126)
Female Sex (+0.0116)
Male Sex (-0.0116)
Benign essential hypertension (x1.12)
0.981
0.986
0.988
0.990
0.991
0.992
0.993
0.994
0.995
0.995
Expressions
−1.0
−0.5
0.0
0.5
1.0

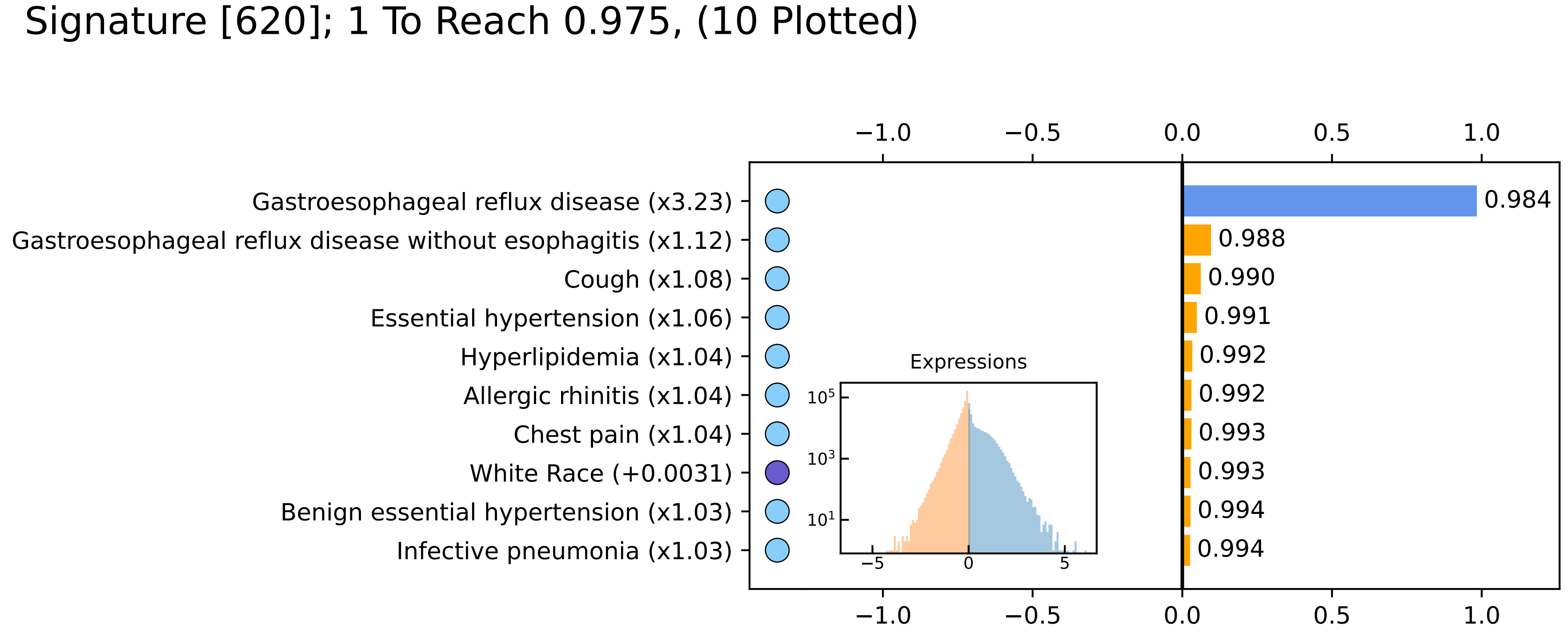
Signature [620]; 1 To Reach 0.975, (10 Plotted)
Gastroesophageal reflux disease (x3.23)
Gastroesophageal reflux disease without esophagitis (x1.12)
Cough (x1.08)
Essential hypertension (x1.06)
Hyperlipidemia (x1.04)
Allergic rhinitis (x1.04)
Chest pain (x1.04)
White Race (+0.0031)
Benign essential hypertension (x1.03)
Infective pneumonia (x1.03)
0.984
0.988
0.990
0.991
0.992
0.992
0.993
0.993
0.994
0.994
Expressions
−1.0
−0.5
0.0
0.5
1.0

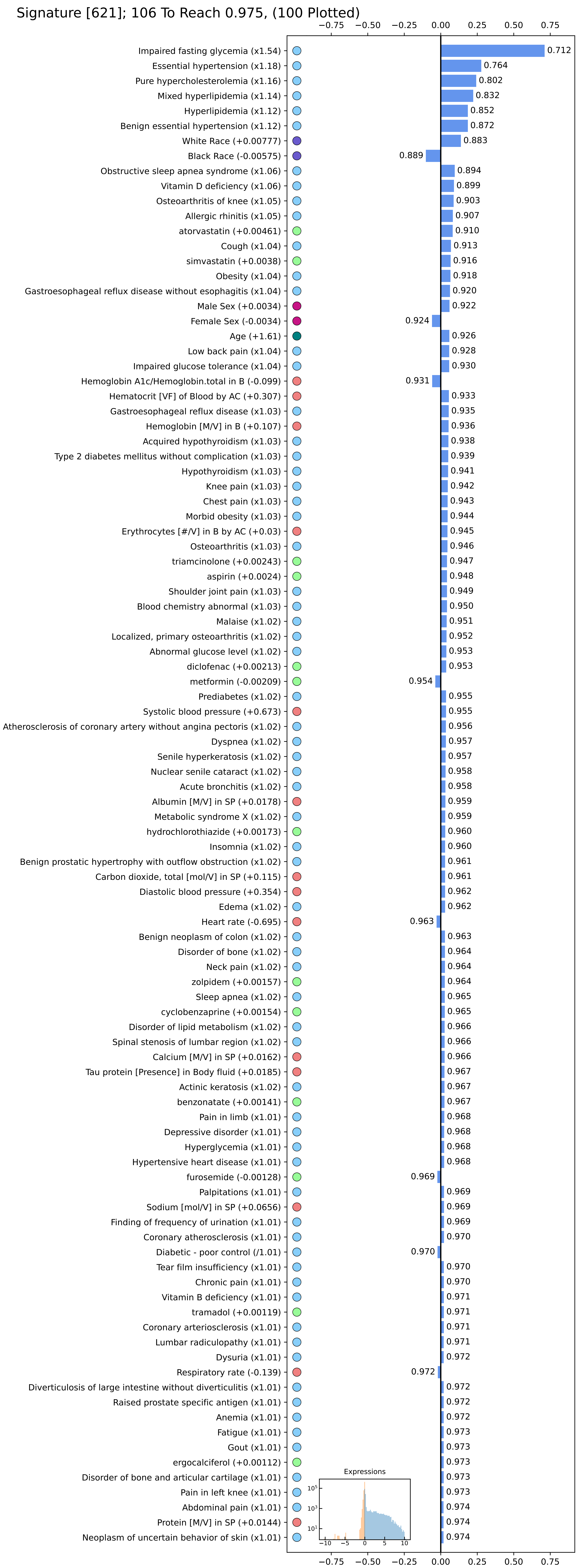

Signature [621]; 106 To Reach 0.975, (100 Plotted)
Impaired fasting glycemia (x1.54) 0.712
Essential hypertension (x1.18) 0.764
Pure hypercholesterolemia (x1.16) 0.802
Mixed hyperlipidemia (x1.14) 0.832
Hyperlipidemia (x1.12) 0.852
Benign essential hypertension (x1.12) 0.872
White Race (+0.00777) 0.883
Black Race (-0.00575) 0.889
Obstructive sleep apnea syndrome (x1.06) 0.894
Vitamin D deficiency (x1.06) 0.899
Osteoarthritis of knee (x1.05) 0.903
Allergic rhinitis (x1.05) 0.907
atorvastatin (+0.00461) 0.910
Cough (x1.04) 0.913
simvastatin (+0.0038) 0.916
Obesity (x1.04) 0.918
Gastroesophageal reflux disease without esophagitis (x1.04) 0.920
Male Sex (+0.0034) 0.922
Female Sex (-0.0034) 0.924
Age (+1.61) 0.926
Low back pain (x1.04) 0.928
Impaired glucose tolerance (x1.04) 0.930
Hemoglobin A1c/Hemoglobin.total in B (-0.099) 0.931
Hematocrit [VF] of Blood by AC (+0.307) 0.933
Gastroesophageal reflux disease (x1.03) 0.935
Hemoglobin [M/V] in B (+0.107) 0.936
Acquired hypothyroidism (x1.03) 0.938
Type 2 diabetes mellitus without complication (x1.03) 0.939
Hypothyroidism (x1.03) 0.941
Knee pain (x1.03) 0.942
Chest pain (x1.03) 0.943
Morbid obesity (x1.03) 0.944
Erythrocytes [#/V] in B by AC (+0.03) 0.945
Osteoarthritis (x1.03) 0.946
triamcinolone (+0.00243) 0.947
aspirin (+0.0024) 0.948
Shoulder joint pain (x1.03) 0.949
Blood chemistry abnormal (x1.03) 0.950
Malaise (x1.02) 0.951
Localized, primary osteoarthritis (x1.02) 0.952
Abnormal glucose level (x1.02) 0.953
diclofenac (+0.00213) 0.953
metformin (-0.00209) 0.954
Prediabetes (x1.02) 0.955
Systolic blood pressure (+0.673) 0.955
Atherosclerosis of coronary artery without angina pectoris (x1.02) 0.956
Dyspnea (x1.02) 0.957
Senile hyperkeratosis (x1.02) 0.957
Nuclear senile cataract (x1.02) 0.958
Acute bronchitis (x1.02) 0.958
Albumin [M/V] in SP (+0.0178) 0.959
Metabolic syndrome X (x1.02) 0.959
hydrochlorothiazide (+0.00173) 0.960
Insomnia (x1.02) 0.960
Benign prostatic hypertrophy with outflow obstruction (x1.02) 0.961
Carbon dioxide, total [mol/V] in SP (+0.115) 0.961
Diastolic blood pressure (+0.354) 0.962
Edema (x1.02) 0.962
Heart rate (-0.695) 0.963
Benign neoplasm of colon (x1.02) 0.963
Disorder of bone (x1.02) 0.964
Neck pain (x1.02) 0.964
zolpidem (+0.00157) 0.964
Sleep apnea (x1.02) 0.965
cyclobenzaprine (+0.00154) 0.965
Disorder of lipid metabolism (x1.02) 0.966
Spinal stenosis of lumbar region (x1.02) 0.966
Calcium [M/V] in SP (+0.0162) 0.966
Tau protein [Presence] in Body fluid (+0.0185) 0.967
Actinic keratosis (x1.02) 0.967
benzonatate (+0.00141) 0.967
Pain in limb (x1.01) 0.968
Depressive disorder (x1.01) 0.968
Hyperglycemia (x1.01) 0.968
Hypertensive heart disease (x1.01) 0.968
furosemide (-0.00128) 0.969
Palpitations (x1.01) 0.969
Sodium [mol/V] in SP (+0.0656) 0.969
Finding of frequency of urination (x1.01) 0.969
Coronary atherosclerosis (x1.01) 0.970
Diabetic - poor control (/1.01) 0.970
Tear film insufficiency (x1.01) 0.970
Chronic pain (x1.01) 0.970
Vitamin B deficiency (x1.01) 0.971
tramadol (+0.00119) 0.971
Coronary arteriosclerosis (x1.01) 0.971
Lumbar radiculopathy (x1.01) 0.971
Dysuria (x1.01) 0.972
Respiratory rate (-0.139) 0.972
Diverticulosis of large intestine without diverticulitis (x1.01) 0.972
Raised prostate specific antigen (x1.01) 0.972
Anemia (x1.01) 0.972
Fatigue (x1.01) 0.973
Gout (x1.01) 0.973
ergocalciferol (+0.00112) 0.973
Disorder of bone and articular cartilage (x1.01) 0.973
Pain in left knee (x1.01) 0.973
Abdominal pain (x1.01) 0.974
Protein [M/V] in SP (+0.0144) 0.974
Neoplasm of uncertain behavior of skin (x1.01) 0.974
−0.75 −0.50 −0.25 0.00 0.25 0.50 0.75
Expressions

Signature [622]; 6 To Reach 0.975, (10 Plotted)

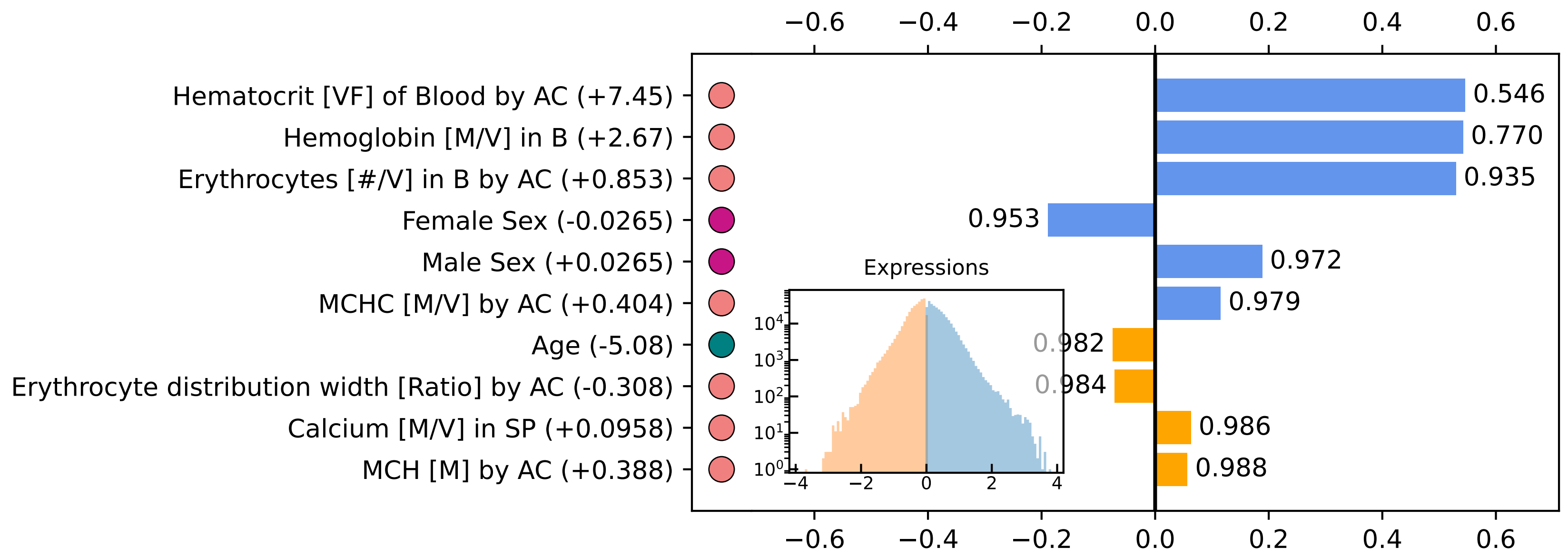

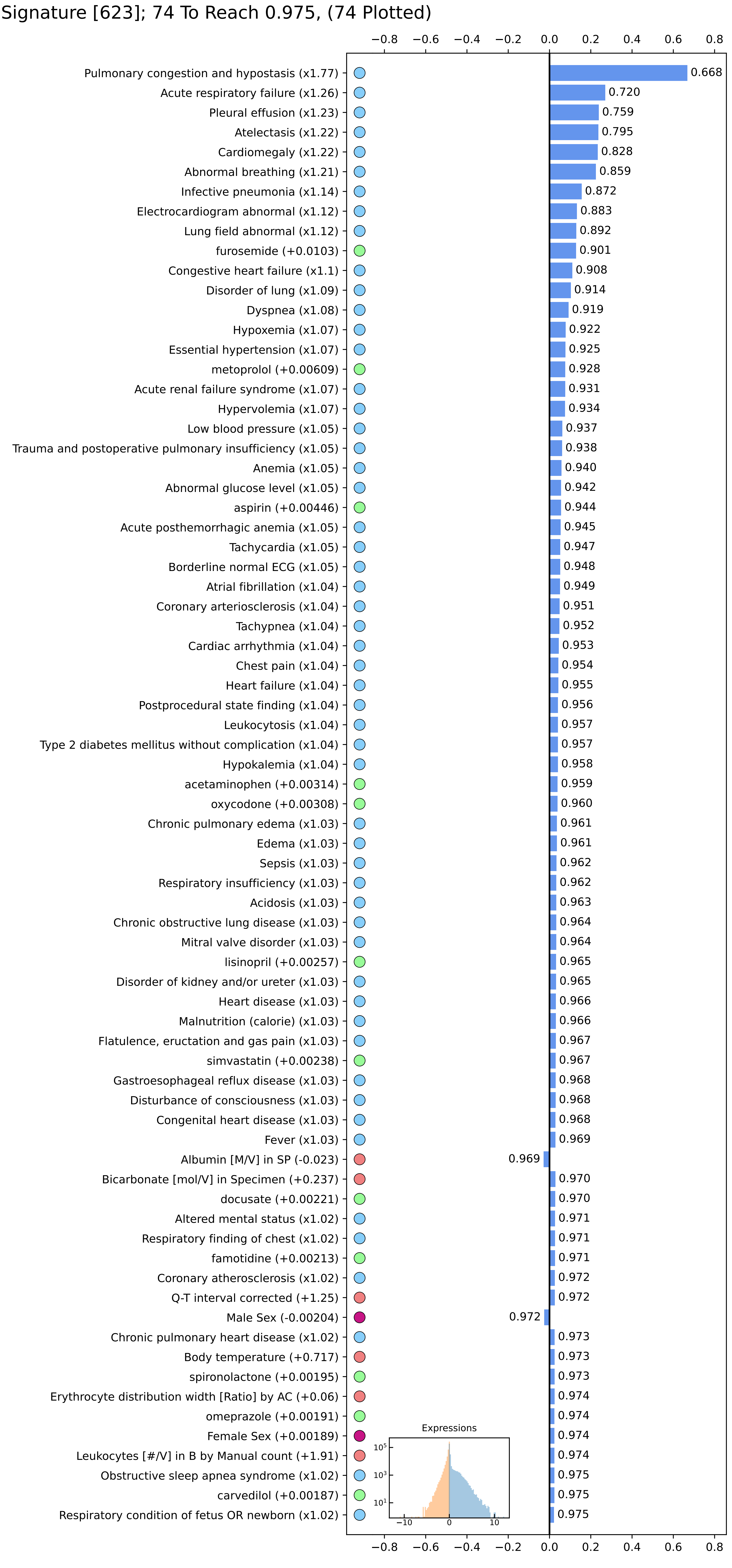

Signature [623]; 74 To Reach 0.975, (74 Plotted)
Pulmonary congestion and hypostasis (x1.77) 0.668
Acute respiratory failure (x1.26) 0.720
Pleural effusion (x1.23) 0.759
Atelectasis (x1.22) 0.795
Cardiomegaly (x1.22) 0.828
Abnormal breathing (x1.21) 0.859
Infective pneumonia (x1.14) 0.872
Electrocardiogram abnormal (x1.12) 0.883
Lung field abnormal (x1.12) 0.892
furosemide (+0.0103) 0.901
Congestive heart failure (x1.1) 0.908
Disorder of lung (x1.09) 0.914
Dyspnea (x1.08) 0.919
Hypoxemia (x1.07) 0.922
Essential hypertension (x1.07) 0.925
metoprolol (+0.00609) 0.928
Acute renal failure syndrome (x1.07) 0.931
Hypervolemia (x1.07) 0.934
Low blood pressure (x1.05) 0.937
Trauma and postoperative pulmonary insufficiency (x1.05) 0.938
Anemia (x1.05) 0.940
Abnormal glucose level (x1.05) 0.942
aspirin (+0.00446) 0.944
Acute posthemorrhagic anemia (x1.05) 0.945
Tachycardia (x1.05) 0.947
Borderline normal ECG (x1.05) 0.948
Atrial fibrillation (x1.04) 0.949
Coronary arteriosclerosis (x1.04) 0.951
Tachypnea (x1.04) 0.952
Cardiac arrhythmia (x1.04) 0.953
Chest pain (x1.04) 0.954
Heart failure (x1.04) 0.955
Postprocedural state finding (x1.04) 0.956
Leukocytosis (x1.04) 0.957
Type 2 diabetes mellitus without complication (x1.04) 0.957
Hypokalemia (x1.04) 0.958
acetaminophen (+0.00314) 0.959
oxycodone (+0.00308) 0.960
Chronic pulmonary edema (x1.03) 0.961
Edema (x1.03) 0.961
Sepsis (x1.03) 0.962
Respiratory insufficiency (x1.03) 0.962
Acidosis (x1.03) 0.963
Chronic obstructive lung disease (x1.03) 0.964
Mitral valve disorder (x1.03) 0.964
lisinopril (+0.00257) 0.965
Disorder of kidney and/or ureter (x1.03) 0.965
Heart disease (x1.03) 0.966
Malnutrition (calorie) (x1.03) 0.966
Flatulence, eructation and gas pain (x1.03) 0.967
simvastatin (+0.00238) 0.967
Gastroesophageal reflux disease (x1.03) 0.968
Disturbance of consciousness (x1.03) 0.968
Congenital heart disease (x1.03) 0.968
Fever (x1.03) 0.969
Albumin [M/V] in SP (-0.023) 0.969
Bicarbonate [mol/V] in Specimen (+0.237) 0.970
docusate (+0.00221) 0.970
Altered mental status (x1.02) 0.971
Respiratory finding of chest (x1.02) 0.971
famotidine (+0.00213) 0.971
Coronary atherosclerosis (x1.02) 0.972
Q-T interval corrected (+1.25) 0.972
Male Sex (-0.00204) 0.972
Chronic pulmonary heart disease (x1.02) 0.973
Body temperature (+0.717) 0.973
spironolactone (+0.00195) 0.973
Erythrocyte distribution width [Ratio] by AC (+0.06) 0.974
omeprazole (+0.00191) 0.974
Female Sex (+0.00189) 0.974
Leukocytes [#/V] in B by Manual count (+1.91) 0.974
Obstructive sleep apnea syndrome (x1.02) 0.975
carvedilol (+0.00187) 0.975
Respiratory condition of fetus OR newborn (x1.02) 0.975
−0.8 −0.6 −0.4 −0.2 0.0 0.2 0.4 0.6 0.8
Expressions
10^5
10^3
10^1
−10 0 10

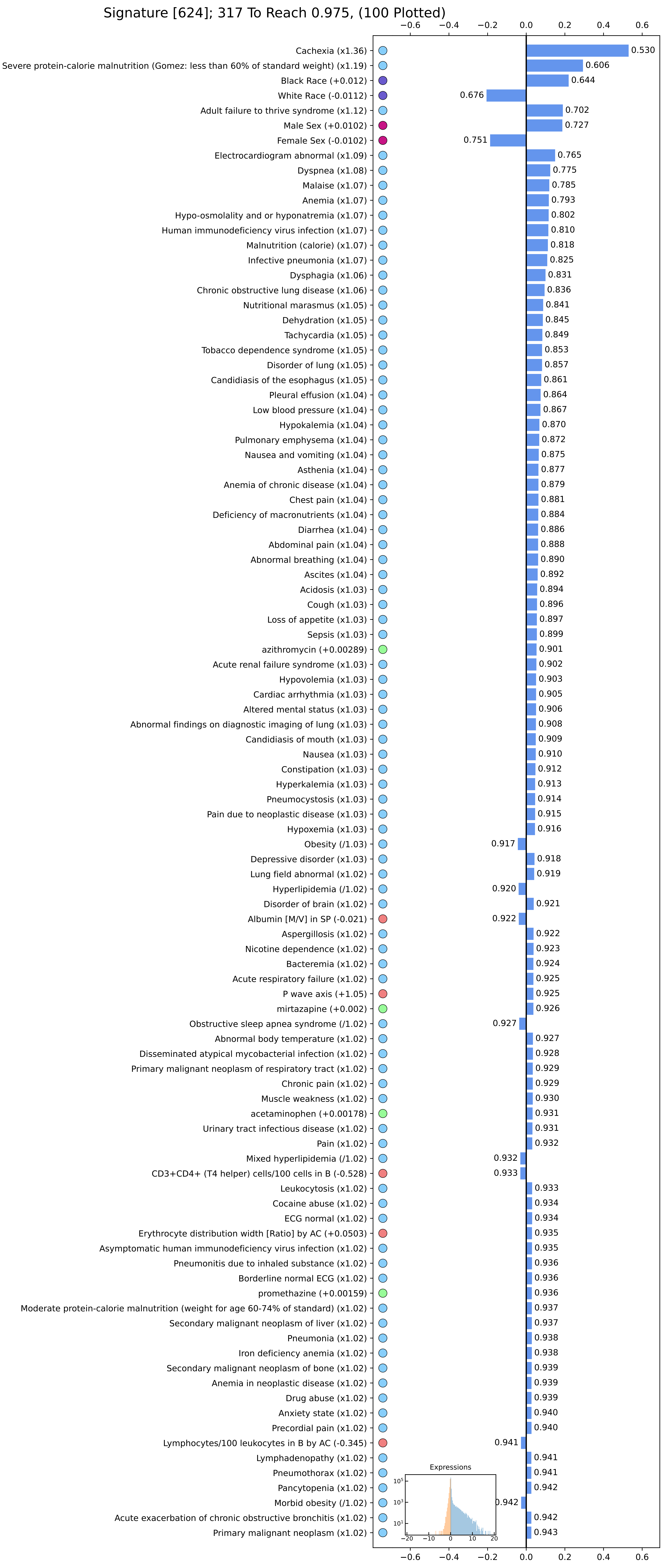

Signature [624]; 317 To Reach 0.975, (100 Plotted)
Cachexia (x1.36) 0.530
Severe protein-calorie malnutrition (Gomez: less than 60% of standard weight) (x1.19) 0.606
Black Race (+0.012) 0.644
White Race (-0.0112) 0.676
Adult failure to thrive syndrome (x1.12) 0.702
Male Sex (+0.0102) 0.727
Female Sex (-0.0102) 0.751
Electrocardiogram abnormal (x1.09) 0.765
Dyspnea (x1.08) 0.775
Malaise (x1.07) 0.785
Anemia (x1.07) 0.793
Hypo-osmolality and or hyponatremia (x1.07) 0.802
Human immunodeficiency virus infection (x1.07) 0.810
Malnutrition (calorie) (x1.07) 0.818
Infective pneumonia (x1.07) 0.825
Dysphagia (x1.06) 0.831
Chronic obstructive lung disease (x1.06) 0.836
Nutritional marasmus (x1.05) 0.841
Dehydration (x1.05) 0.845
Tachycardia (x1.05) 0.849
Tobacco dependence syndrome (x1.05) 0.853
Disorder of lung (x1.05) 0.857
Candidiasis of the esophagus (x1.05) 0.861
Pleural effusion (x1.04) 0.864
Low blood pressure (x1.04) 0.867
Hypokalemia (x1.04) 0.870
Pulmonary emphysema (x1.04) 0.872
Nausea and vomiting (x1.04) 0.875
Asthenia (x1.04) 0.877
Anemia of chronic disease (x1.04) 0.879
Chest pain (x1.04) 0.881
Deficiency of macronutrients (x1.04) 0.884
Diarrhea (x1.04) 0.886
Abdominal pain (x1.04) 0.888
Abnormal breathing (x1.04) 0.890
Ascites (x1.04) 0.892
Acidosis (x1.03) 0.894
Cough (x1.03) 0.896
Loss of appetite (x1.03) 0.897
Sepsis (x1.03) 0.899
azithromycin (+0.00289) 0.901
Acute renal failure syndrome (x1.03) 0.902
Hypovolemia (x1.03) 0.903
Cardiac arrhythmia (x1.03) 0.905
Altered mental status (x1.03) 0.906
Abnormal findings on diagnostic imaging of lung (x1.03) 0.908
Candidiasis of mouth (x1.03) 0.909
Nausea (x1.03) 0.910
Constipation (x1.03) 0.912
Hyperkalemia (x1.03) 0.913
Pneumocystosis (x1.03) 0.914
Pain due to neoplastic disease (x1.03) 0.915
Hypoxemia (x1.03) 0.916
Obesity (/1.03) 0.917
Depressive disorder (x1.03) 0.918
Lung field abnormal (x1.02) 0.919
Hyperlipidemia (/1.02) 0.920
Disorder of brain (x1.02) 0.921
Albumin [M/V] in SP (-0.021) 0.922
Aspergillosis (x1.02) 0.922
Nicotine dependence (x1.02) 0.923
Bacteremia (x1.02) 0.924
Acute respiratory failure (x1.02) 0.925
P wave axis (+1.05) 0.925
mirtazapine (+0.002) 0.926
Obstructive sleep apnea syndrome (/1.02) 0.927
Abnormal body temperature (x1.02) 0.927
Disseminated atypical mycobacterial infection (x1.02) 0.928
Primary malignant neoplasm of respiratory tract (x1.02) 0.929
Chronic pain (x1.02) 0.929
Muscle weakness (x1.02) 0.930
acetaminophen (+0.00178) 0.931
Urinary tract infectious disease (x1.02) 0.931
Pain (x1.02) 0.932
Mixed hyperlipidemia (/1.02) 0.932
CD3+CD4+ (T4 helper) cells/100 cells in B (-0.528) 0.933
Leukocytosis (x1.02) 0.933
Cocaine abuse (x1.02) 0.934
ECG normal (x1.02) 0.934
Erythrocyte distribution width [Ratio] by AC (+0.0503) 0.935
Asymptomatic human immunodeficiency virus infection (x1.02) 0.935
Pneumonitis due to inhaled substance (x1.02) 0.936
Borderline normal ECG (x1.02) 0.936
promethazine (+0.00159) 0.936
Moderate protein-calorie malnutrition (weight for age 60-74% of standard) (x1.02) 0.937
Secondary malignant neoplasm of liver (x1.02) 0.937
Pneumonia (x1.02) 0.938
Iron deficiency anemia (x1.02) 0.938
Secondary malignant neoplasm of bone (x1.02) 0.939
Anemia in neoplastic disease (x1.02) 0.939
Drug abuse (x1.02) 0.939
Anxiety state (x1.02) 0.940
Precordial pain (x1.02) 0.940
Lymphocytes/100 leukocytes in B by AC (-0.345) 0.941
Lymphadenopathy (x1.02) 0.941
Pneumothorax (x1.02) 0.941
Pancytopenia (x1.02) 0.942
Morbid obesity (/1.02) 0.942
Acute exacerbation of chronic obstructive bronchitis (x1.02) 0.942
Primary malignant neoplasm (x1.02) 0.943
−0.6 −0.4 −0.2 0.0 0.2 0.4 0.6
Expressions

Signature [625]; 3 To Reach 0.975, (10 Plotted)

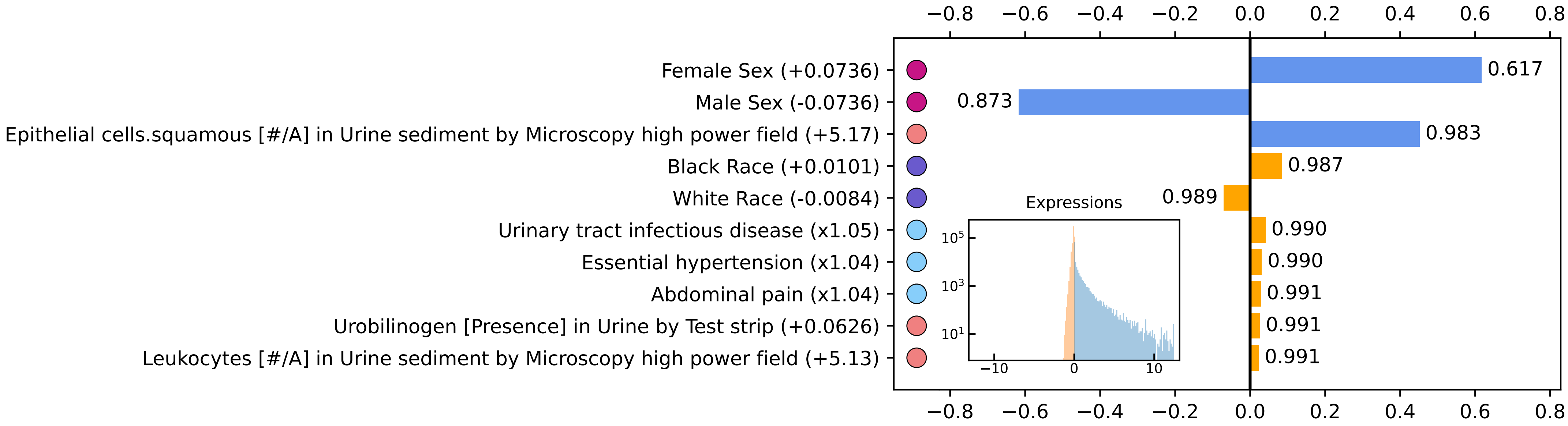

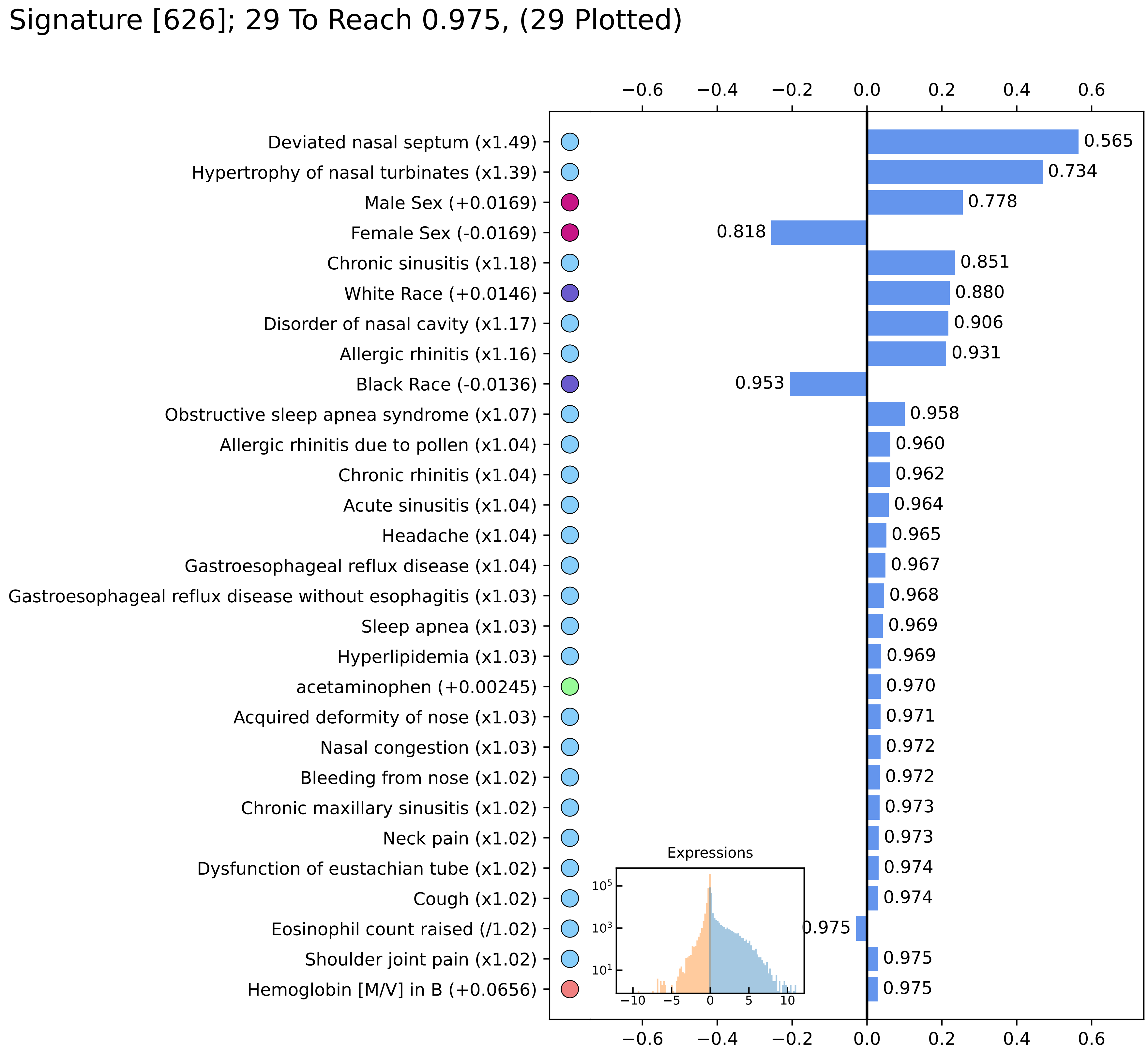

Signature [626]; 29 To Reach 0.975, (29 Plotted)
Deviated nasal septum (x1.49) 0.565
Hypertrophy of nasal turbinates (x1.39) 0.734
Male Sex (+0.0169) 0.778
Female Sex (-0.0169) 0.818
Chronic sinusitis (x1.18) 0.851
White Race (+0.0146) 0.880
Disorder of nasal cavity (x1.17) 0.906
Allergic rhinitis (x1.16) 0.931
Black Race (-0.0136) 0.953
Obstructive sleep apnea syndrome (x1.07) 0.958
Allergic rhinitis due to pollen (x1.04) 0.960
Chronic rhinitis (x1.04) 0.962
Acute sinusitis (x1.04) 0.964
Headache (x1.04) 0.965
Gastroesophageal reflux disease (x1.04) 0.967
Gastroesophageal reflux disease without esophagitis (x1.03) 0.968
Sleep apnea (x1.03) 0.969
Hyperlipidemia (x1.03) 0.969
acetaminophen (+0.00245) 0.970
Acquired deformity of nose (x1.03) 0.971
Nasal congestion (x1.03) 0.972
Bleeding from nose (x1.02) 0.972
Chronic maxillary sinusitis (x1.02) 0.973
Neck pain (x1.02) 0.973
Dysfunction of eustachian tube (x1.02) 0.974
Cough (x1.02) 0.974
Eosinophil count raised (/1.02) 0.975
Shoulder joint pain (x1.02) 0.975
Hemoglobin [M/V] in B (+0.0656) 0.975
−0.6 −0.4 −0.2 0.0 0.2 0.4 0.6
Expressions
10^5 10^3 10^1
−10 −5 0 5 10

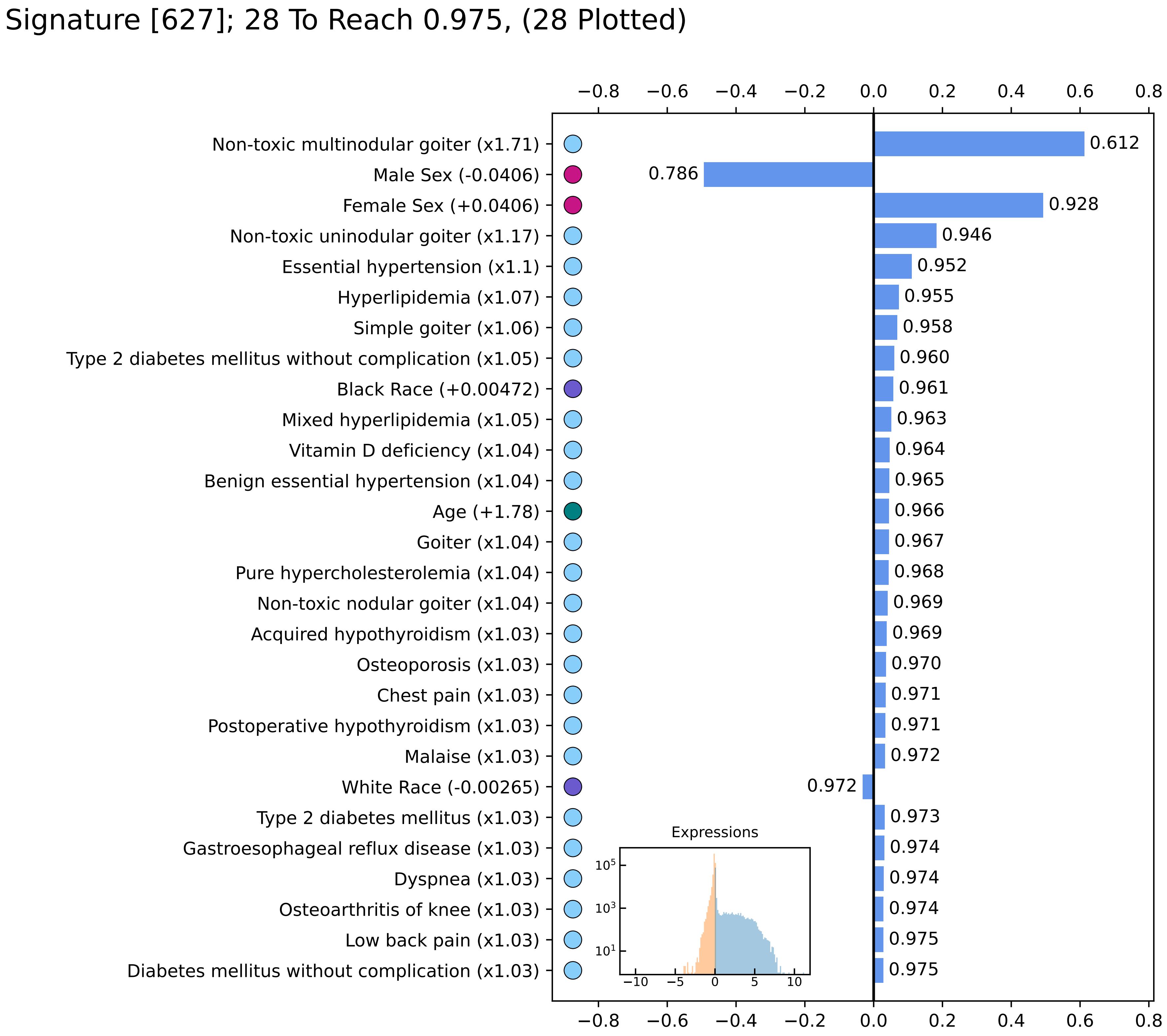

Signature [627]; 28 To Reach 0.975, (28 Plotted)
Non-toxic multinodular goiter (x1.71)
0.612
Male Sex (-0.0406)
0.786
Female Sex (+0.0406)
0.928
Non-toxic uninodular goiter (x1.17)
0.946
Essential hypertension (x1.1)
0.952
Hyperlipidemia (x1.07)
0.955
Simple goiter (x1.06)
0.958
Type 2 diabetes mellitus without complication (x1.05)
0.960
Black Race (+0.00472)
0.961
Mixed hyperlipidemia (x1.05)
0.963
Vitamin D deficiency (x1.04)
0.964
Benign essential hypertension (x1.04)
0.965
Age (+1.78)
0.966
Goiter (x1.04)
0.967
Pure hypercholesterolemia (x1.04)
0.968
Non-toxic nodular goiter (x1.04)
0.969
Acquired hypothyroidism (x1.03)
0.969
Osteoporosis (x1.03)
0.970
Chest pain (x1.03)
0.971
Postoperative hypothyroidism (x1.03)
0.971
Malaise (x1.03)
0.972
White Race (-0.00265)
0.972
Type 2 diabetes mellitus (x1.03)
0.973
Gastroesophageal reflux disease (x1.03)
0.974
Dyspnea (x1.03)
0.974
Osteoarthritis of knee (x1.03)
0.974
Low back pain (x1.03)
0.975
Diabetes mellitus without complication (x1.03)
0.975
Expressions
−0.8 −0.6 −0.4 −0.2 0.0 0.2 0.4 0.6 0.8

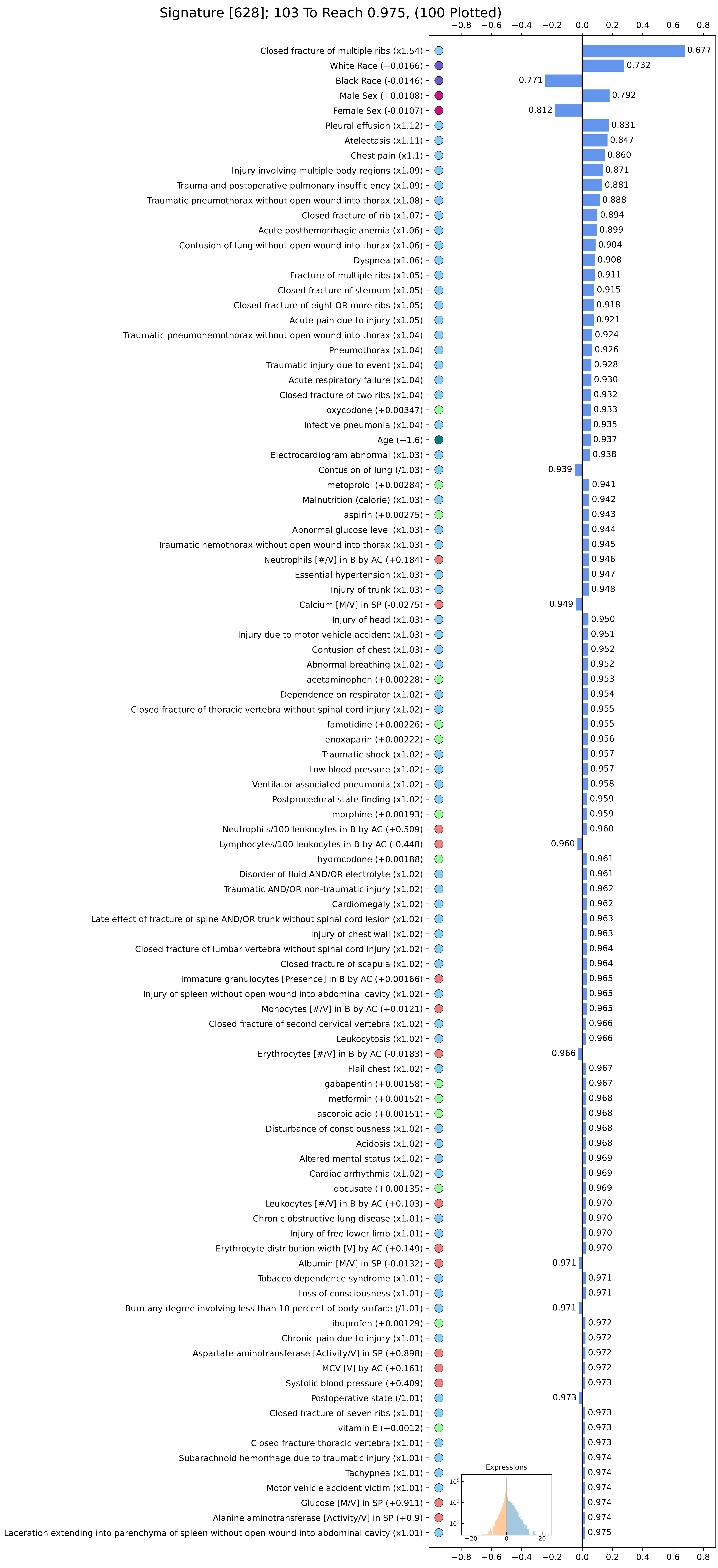

Signature [628]; 103 To Reach 0.975, (100 Plotted)
Closed fracture of multiple ribs (x1.54) 0.677
White Race (+0.0166) 0.732
Black Race (-0.0146) 0.771
Male Sex (+0.0108) 0.792
Female Sex (-0.0107) 0.812
Pleural effusion (x1.12) 0.831
Atelectasis (x1.11) 0.847
Chest pain (x1.1) 0.860
Injury involving multiple body regions (x1.09) 0.871
Trauma and postoperative pulmonary insufficiency (x1.09) 0.881
Traumatic pneumothorax without open wound into thorax (x1.08) 0.888
Closed fracture of rib (x1.07) 0.894
Acute posthemorrhagic anemia (x1.06) 0.899
Contusion of lung without open wound into thorax (x1.06) 0.904
Dyspnea (x1.06) 0.908
Fracture of multiple ribs (x1.05) 0.911
Closed fracture of sternum (x1.05) 0.915
Closed fracture of eight OR more ribs (x1.05) 0.918
Acute pain due to injury (x1.05) 0.921
Traumatic pneumohemothorax without open wound into thorax (x1.04) 0.924
Pneumothorax (x1.04) 0.926
Traumatic injury due to event (x1.04) 0.928
Acute respiratory failure (x1.04) 0.930
Closed fracture of two ribs (x1.04) 0.932
oxycodone (+0.00347) 0.933
Infective pneumonia (x1.04) 0.935
Age (+1.6) 0.937
Electrocardiogram abnormal (x1.03) 0.938
Contusion of lung (/1.03) 0.939
metoprolol (+0.00284) 0.941
Malnutrition (calorie) (x1.03) 0.942
aspirin (+0.00275) 0.943
Abnormal glucose level (x1.03) 0.944
Traumatic hemothorax without open wound into thorax (x1.03) 0.945
Neutrophils [#/V] in B by AC (+0.184) 0.946
Essential hypertension (x1.03) 0.947
Injury of trunk (x1.03) 0.948
Calcium [M/V] in SP (-0.0275) 0.949
Injury of head (x1.03) 0.950
Injury due to motor vehicle accident (x1.03) 0.951
Contusion of chest (x1.03) 0.952
Abnormal breathing (x1.02) 0.952
acetaminophen (+0.00228) 0.953
Dependence on respirator (x1.02) 0.954
Closed fracture of thoracic vertebra without spinal cord injury (x1.02) 0.955
famotidine (+0.00226) 0.955
enoxaparin (+0.00222) 0.956
Traumatic shock (x1.02) 0.957
Low blood pressure (x1.02) 0.957
Ventilator associated pneumonia (x1.02) 0.958
Postprocedural state finding (x1.02) 0.959
morphine (+0.00193) 0.959
Neutrophils/100 leukocytes in B by AC (+0.509) 0.960
Lymphocytes/100 leukocytes in B by AC (-0.448) 0.960
hydrocodone (+0.00188) 0.961
Disorder of fluid AND/OR electrolyte (x1.02) 0.961
Traumatic AND/OR non-traumatic injury (x1.02) 0.962
Cardiomegaly (x1.02) 0.962
Late effect of fracture of spine AND/OR trunk without spinal cord lesion (x1.02) 0.963
Injury of chest wall (x1.02) 0.963
Closed fracture of lumbar vertebra without spinal cord injury (x1.02) 0.964
Closed fracture of scapula (x1.02) 0.964
Immature granulocytes [Presence] in B by AC (+0.00166) 0.965
Injury of spleen without open wound into abdominal cavity (x1.02) 0.965
Monocytes [#/V] in B by AC (+0.0121) 0.965
Closed fracture of second cervical vertebra (x1.02) 0.966
Leukocytosis (x1.02) 0.966
Erythrocytes [#/V] in B by AC (-0.0183) 0.966
Flail chest (x1.02) 0.967
gabapentin (+0.00158) 0.967
metformin (+0.00152) 0.968
ascorbic acid (+0.00151) 0.968
Disturbance of consciousness (x1.02) 0.968
Acidosis (x1.02) 0.968
Altered mental status (x1.02) 0.969
Cardiac arrhythmia (x1.02) 0.969
docusate (+0.00135) 0.969
Leukocytes [#/V] in B by AC (+0.103) 0.970
Chronic obstructive lung disease (x1.01) 0.970
Injury of free lower limb (x1.01) 0.970
Erythrocyte distribution width [V] by AC (+0.149) 0.970
Albumin [M/V] in SP (-0.0132) 0.971
Tobacco dependence syndrome (x1.01) 0.971
Loss of consciousness (x1.01) 0.971
Burn any degree involving less than 10 percent of body surface (/1.01) 0.971
ibuprofen (+0.00129) 0.972
Chronic pain due to injury (x1.01) 0.972
Aspartate aminotransferase [Activity/V] in SP (+0.898) 0.972
MCV [V] by AC (+0.161) 0.972
Systolic blood pressure (+0.409) 0.973
Postoperative state (/1.01) 0.973
Closed fracture of seven ribs (x1.01) 0.973
vitamin E (+0.0012) 0.973
Closed fracture thoracic vertebra (x1.01) 0.973
Subarachnoid hemorrhage due to traumatic injury (x1.01) 0.974
Tachypnea (x1.01) 0.974
Motor vehicle accident victim (x1.01) 0.974
Glucose [M/V] in SP (+0.911) 0.974
Alanine aminotransferase [Activity/V] in SP (+0.9) 0.974
Laceration extending into parenchyma of spleen without open wound into abdominal cavity (x1.01) 0.975
−0.8 −0.6 −0.4 −0.2 0.0 0.2 0.4 0.6 0.8
Expressions

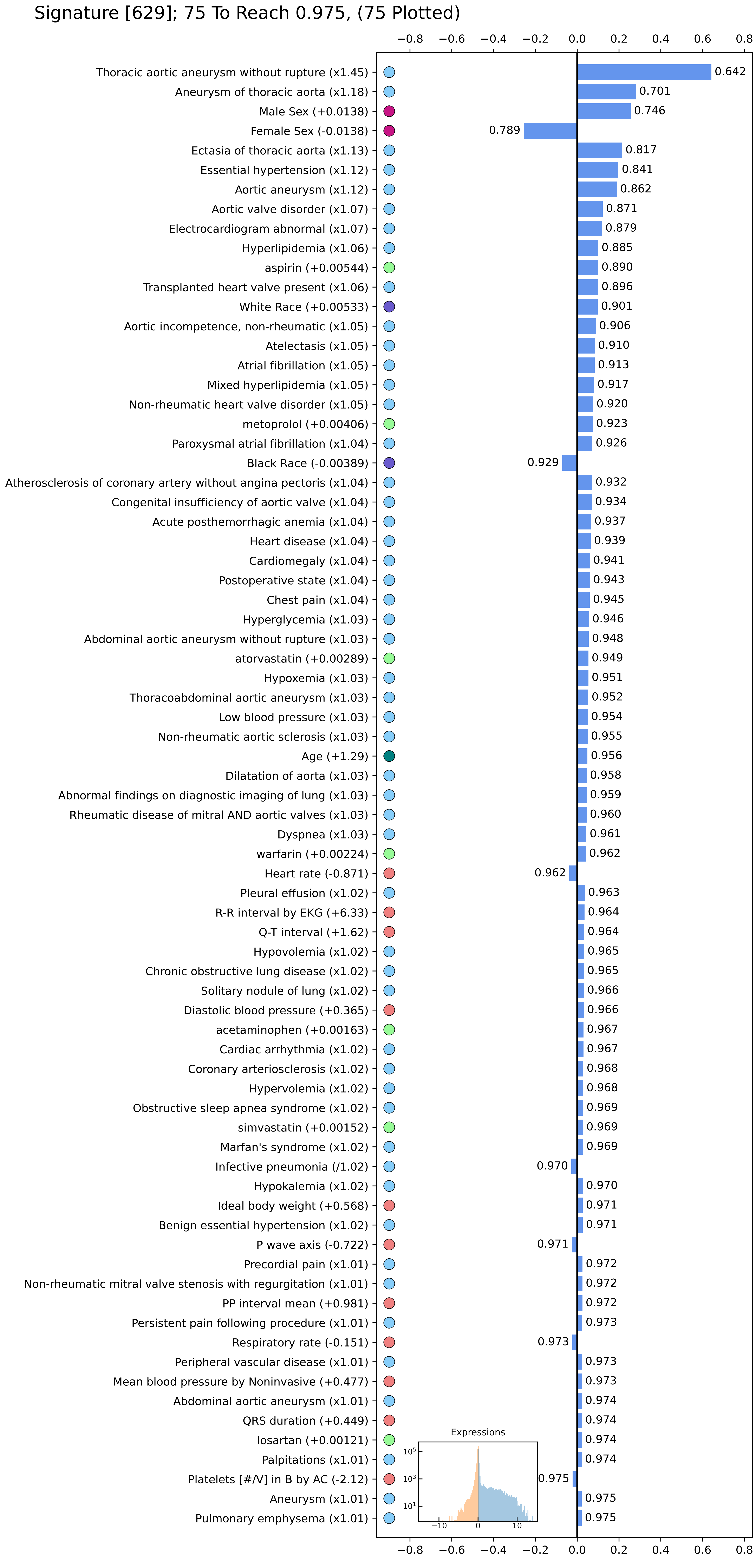

Signature [629]; 75 To Reach 0.975, (75 Plotted)
Thoracic aortic aneurysm without rupture (x1.45) 0.642
Aneurysm of thoracic aorta (x1.18) 0.701
Male Sex (+0.0138) 0.746
Female Sex (-0.0138) 0.789
Ectasia of thoracic aorta (x1.13) 0.817
Essential hypertension (x1.12) 0.841
Aortic aneurysm (x1.12) 0.862
Aortic valve disorder (x1.07) 0.871
Electrocardiogram abnormal (x1.07) 0.879
Hyperlipidemia (x1.06) 0.885
aspirin (+0.00544) 0.890
Transplanted heart valve present (x1.06) 0.896
White Race (+0.00533) 0.901
Aortic incompetence, non-rheumatic (x1.05) 0.906
Atelectasis (x1.05) 0.910
Atrial fibrillation (x1.05) 0.913
Mixed hyperlipidemia (x1.05) 0.917
Non-rheumatic heart valve disorder (x1.05) 0.920
metoprolol (+0.00406) 0.923
Paroxysmal atrial fibrillation (x1.04) 0.926
Black Race (-0.00389) 0.929
Atherosclerosis of coronary artery without angina pectoris (x1.04) 0.932
Congenital insufficiency of aortic valve (x1.04) 0.934
Acute posthemorrhagic anemia (x1.04) 0.937
Heart disease (x1.04) 0.939
Cardiomegaly (x1.04) 0.941
Postoperative state (x1.04) 0.943
Chest pain (x1.04) 0.945
Hyperglycemia (x1.03) 0.946
Abdominal aortic aneurysm without rupture (x1.03) 0.948
atorvastatin (+0.00289) 0.949
Hypoxemia (x1.03) 0.951
Thoracoabdominal aortic aneurysm (x1.03) 0.952
Low blood pressure (x1.03) 0.954
Non-rheumatic aortic sclerosis (x1.03) 0.955
Age (+1.29) 0.956
Dilatation of aorta (x1.03) 0.958
Abnormal findings on diagnostic imaging of lung (x1.03) 0.959
Rheumatic disease of mitral AND aortic valves (x1.03) 0.960
Dyspnea (x1.03) 0.961
warfarin (+0.00224) 0.962
Heart rate (-0.871) 0.962
Pleural effusion (x1.02) 0.963
R-R interval by EKG (+6.33) 0.964
Q-T interval (+1.62) 0.964
Hypovolemia (x1.02) 0.965
Chronic obstructive lung disease (x1.02) 0.965
Solitary nodule of lung (x1.02) 0.966
Diastolic blood pressure (+0.365) 0.966
acetaminophen (+0.00163) 0.967
Cardiac arrhythmia (x1.02) 0.967
Coronary arteriosclerosis (x1.02) 0.968
Hypervolemia (x1.02) 0.968
Obstructive sleep apnea syndrome (x1.02) 0.969
simvastatin (+0.00152) 0.969
Marfan's syndrome (x1.02) 0.969
Infective pneumonia (/1.02) 0.970
Hypokalemia (x1.02) 0.970
Ideal body weight (+0.568) 0.971
Benign essential hypertension (x1.02) 0.971
P wave axis (-0.722) 0.971
Precordial pain (x1.01) 0.972
Non-rheumatic mitral valve stenosis with regurgitation (x1.01) 0.972
PP interval mean (+0.981) 0.972
Persistent pain following procedure (x1.01) 0.973
Respiratory rate (-0.151) 0.973
Peripheral vascular disease (x1.01) 0.973
Mean blood pressure by Noninvasive (+0.477) 0.973
Abdominal aortic aneurysm (x1.01) 0.974
QRS duration (+0.449) 0.974
losartan (+0.00121) 0.974
Palpitations (x1.01) 0.974
Platelets [#/V] in B by AC (-2.12) 0.975
Aneurysm (x1.01) 0.975
Pulmonary emphysema (x1.01) 0.975
Expressions
−0.8 −0.6 −0.4 −0.2 0.0 0.2 0.4 0.6 0.8

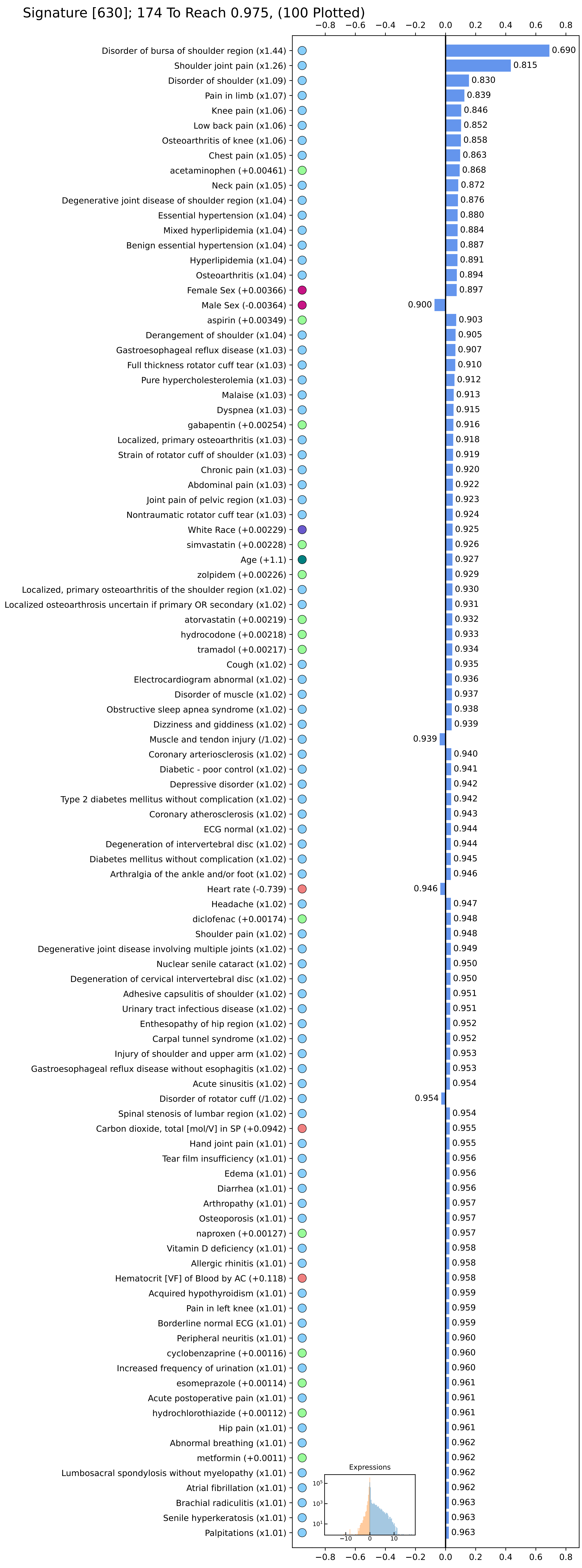
Signature [630]; 174 To Reach 0.975, (100 Plotted)
Disorder of bursa of shoulder region (x1.44) 0.690
Shoulder joint pain (x1.26) 0.815
Disorder of shoulder (x1.09) 0.830
Pain in limb (x1.07) 0.839
Knee pain (x1.06) 0.846
Low back pain (x1.06) 0.852
Osteoarthritis of knee (x1.06) 0.858
Chest pain (x1.05) 0.863
acetaminophen (+0.00461) 0.868
Neck pain (x1.05) 0.872
Degenerative joint disease of shoulder region (x1.04) 0.876
Essential hypertension (x1.04) 0.880
Mixed hyperlipidemia (x1.04) 0.884
Benign essential hypertension (x1.04) 0.887
Hyperlipidemia (x1.04) 0.891
Osteoarthritis (x1.04) 0.894
Female Sex (+0.00366) 0.897
Male Sex (-0.00364) 0.900
aspirin (+0.00349) 0.903
Derangement of shoulder (x1.04) 0.905
Gastroesophageal reflux disease (x1.03) 0.907
Full thickness rotator cuff tear (x1.03) 0.910
Pure hypercholesterolemia (x1.03) 0.912
Malaise (x1.03) 0.913
Dyspnea (x1.03) 0.915
gabapentin (+0.00254) 0.916
Localized, primary osteoarthritis (x1.03) 0.918
Strain of rotator cuff of shoulder (x1.03) 0.919
Chronic pain (x1.03) 0.920
Abdominal pain (x1.03) 0.922
Joint pain of pelvic region (x1.03) 0.923
Nontraumatic rotator cuff tear (x1.03) 0.924
White Race (+0.00229) 0.925
simvastatin (+0.00228) 0.926
Age (+1.1) 0.927
zolpidem (+0.00226) 0.929
Localized, primary osteoarthritis of the shoulder region (x1.02) 0.930
Localized osteoarthrosis uncertain if primary OR secondary (x1.02) 0.931
atorvastatin (+0.00219) 0.932
hydrocodone (+0.00218) 0.933
tramadol (+0.00217) 0.934
Cough (x1.02) 0.935
Electrocardiogram abnormal (x1.02) 0.936
Disorder of muscle (x1.02) 0.937
Obstructive sleep apnea syndrome (x1.02) 0.938
Dizziness and giddiness (x1.02) 0.939
Muscle and tendon injury (/1.02) 0.939
Coronary arteriosclerosis (x1.02) 0.940
Diabetic - poor control (x1.02) 0.941
Depressive disorder (x1.02) 0.942
Type 2 diabetes mellitus without complication (x1.02) 0.942
Coronary atherosclerosis (x1.02) 0.943
ECG normal (x1.02) 0.944
Degeneration of intervertebral disc (x1.02) 0.944
Diabetes mellitus without complication (x1.02) 0.945
Arthralgia of the ankle and/or foot (x1.02) 0.946
Heart rate (-0.739) 0.946
Headache (x1.02) 0.947
diclofenac (+0.00174) 0.948
Shoulder pain (x1.02) 0.948
Degenerative joint disease involving multiple joints (x1.02) 0.949
Nuclear senile cataract (x1.02) 0.950
Degeneration of cervical intervertebral disc (x1.02) 0.950
Adhesive capsulitis of shoulder (x1.02) 0.951
Urinary tract infectious disease (x1.02) 0.951
Enthesopathy of hip region (x1.02) 0.952
Carpal tunnel syndrome (x1.02) 0.952
Injury of shoulder and upper arm (x1.02) 0.953
Gastroesophageal reflux disease without esophagitis (x1.02) 0.953
Acute sinusitis (x1.02) 0.954
Disorder of rotator cuff (/1.02) 0.954
Spinal stenosis of lumbar region (x1.02) 0.954
Carbon dioxide, total [mol/V] in SP (+0.0942) 0.955
Hand joint pain (x1.01) 0.955
Tear film insufficiency (x1.01) 0.956
Edema (x1.01) 0.956
Diarrhea (x1.01) 0.956
Arthropathy (x1.01) 0.957
Osteoporosis (x1.01) 0.957
naproxen (+0.00127) 0.957
Vitamin D deficiency (x1.01) 0.958
Allergic rhinitis (x1.01) 0.958
Hematocrit [VF] of Blood by AC (+0.118) 0.958
Acquired hypothyroidism (x1.01) 0.959
Pain in left knee (x1.01) 0.959
Borderline normal ECG (x1.01) 0.959
Peripheral neuritis (x1.01) 0.960
cyclobenzaprine (+0.00116) 0.960
Increased frequency of urination (x1.01) 0.960
esomeprazole (+0.00114) 0.961
Acute postoperative pain (x1.01) 0.961
hydrochlorothiazide (+0.00112) 0.961
Hip pain (x1.01) 0.961
Abnormal breathing (x1.01) 0.962
metformin (+0.0011) 0.962
Lumbosacral spondylosis without myelopathy (x1.01) 0.962
Atrial fibrillation (x1.01) 0.962
Brachial radiculitis (x1.01) 0.963
Senile hyperkeratosis (x1.01) 0.963
Palpitations (x1.01) 0.963
−0.8 −0.6 −0.4 −0.2 0.0 0.2 0.4 0.6 0.8
Expressions
10⁵
10³
10¹
−10 0 10

Signature [631]; 1 To Reach 0.975, (10 Plotted)

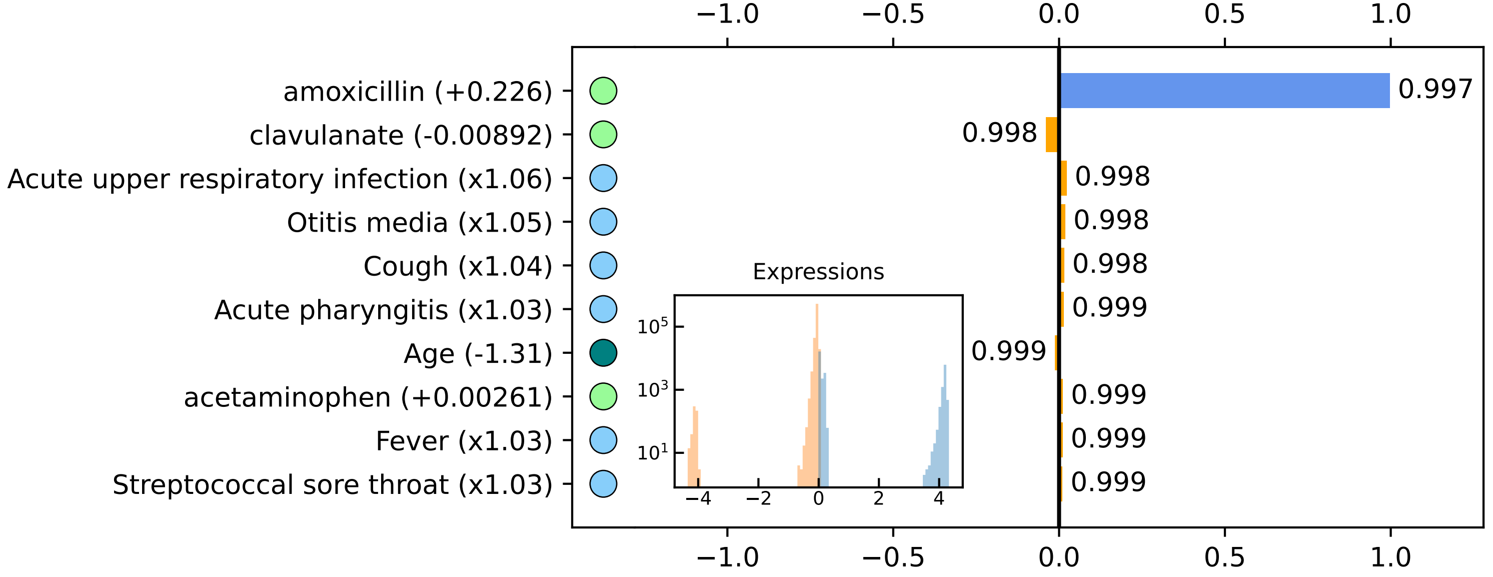

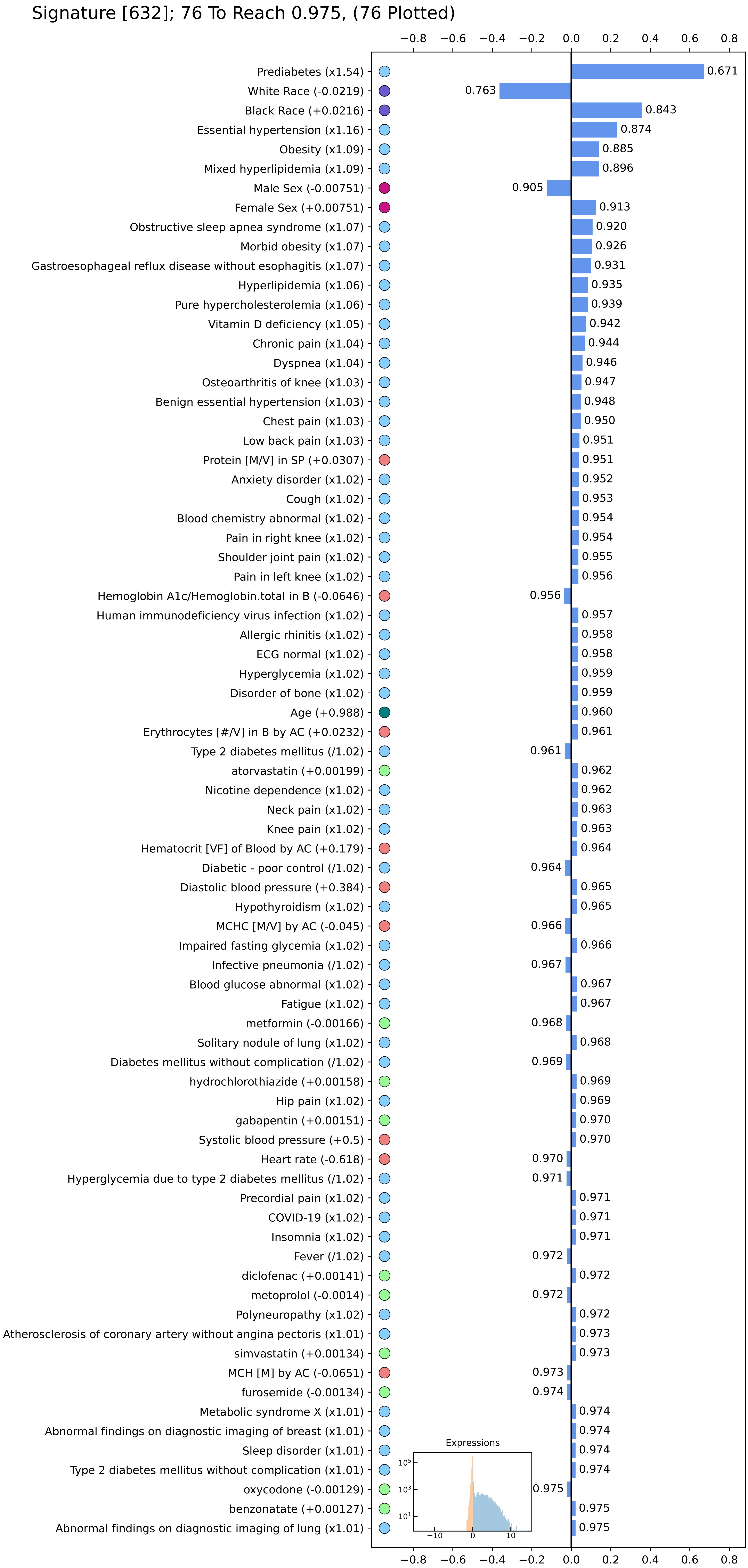

Signature [632]; 76 To Reach 0.975, (76 Plotted)
Prediabetes (x1.54) 0.671
White Race (-0.0219) 0.763
Black Race (+0.0216) 0.843
Essential hypertension (x1.16) 0.874
Obesity (x1.09) 0.885
Mixed hyperlipidemia (x1.09) 0.896
Male Sex (-0.00751) 0.905
Female Sex (+0.00751) 0.913
Obstructive sleep apnea syndrome (x1.07) 0.920
Morbid obesity (x1.07) 0.926
Gastroesophageal reflux disease without esophagitis (x1.07) 0.931
Hyperlipidemia (x1.06) 0.935
Pure hypercholesterolemia (x1.06) 0.939
Vitamin D deficiency (x1.05) 0.942
Chronic pain (x1.04) 0.944
Dyspnea (x1.04) 0.946
Osteoarthritis of knee (x1.03) 0.947
Benign essential hypertension (x1.03) 0.948
Chest pain (x1.03) 0.950
Low back pain (x1.03) 0.951
Protein [M/V] in SP (+0.0307) 0.951
Anxiety disorder (x1.02) 0.952
Cough (x1.02) 0.953
Blood chemistry abnormal (x1.02) 0.954
Pain in right knee (x1.02) 0.954
Shoulder joint pain (x1.02) 0.955
Pain in left knee (x1.02) 0.956
Hemoglobin A1c/Hemoglobin.total in B (-0.0646) 0.956
Human immunodeficiency virus infection (x1.02) 0.957
Allergic rhinitis (x1.02) 0.958
ECG normal (x1.02) 0.958
Hyperglycemia (x1.02) 0.959
Disorder of bone (x1.02) 0.959
Age (+0.988) 0.960
Erythrocytes [#/V] in B by AC (+0.0232) 0.961
Type 2 diabetes mellitus (/1.02) 0.961
atorvastatin (+0.00199) 0.962
Nicotine dependence (x1.02) 0.962
Neck pain (x1.02) 0.963
Knee pain (x1.02) 0.963
Hematocrit [VF] of Blood by AC (+0.179) 0.964
Diabetic - poor control (/1.02) 0.964
Diastolic blood pressure (+0.384) 0.965
Hypothyroidism (x1.02) 0.965
MCHC [M/V] by AC (-0.045) 0.966
Impaired fasting glycemia (x1.02) 0.966
Infective pneumonia (/1.02) 0.967
Blood glucose abnormal (x1.02) 0.967
Fatigue (x1.02) 0.967
metformin (-0.00166) 0.968
Solitary nodule of lung (x1.02) 0.968
Diabetes mellitus without complication (/1.02) 0.969
hydrochlorothiazide (+0.00158) 0.969
Hip pain (x1.02) 0.969
gabapentin (+0.00151) 0.970
Systolic blood pressure (+0.5) 0.970
Heart rate (-0.618) 0.970
Hyperglycemia due to type 2 diabetes mellitus (/1.02) 0.971
Precordial pain (x1.02) 0.971
COVID-19 (x1.02) 0.971
Insomnia (x1.02) 0.971
Fever (/1.02) 0.972
diclofenac (+0.00141) 0.972
metoprolol (-0.0014) 0.972
Polyneuropathy (x1.02) 0.972
Atherosclerosis of coronary artery without angina pectoris (x1.01) 0.973
simvastatin (+0.00134) 0.973
MCH [M] by AC (-0.0651) 0.973
furosemide (-0.00134) 0.974
Metabolic syndrome X (x1.01) 0.974
Abnormal findings on diagnostic imaging of breast (x1.01) 0.974
Sleep disorder (x1.01) 0.974
Type 2 diabetes mellitus without complication (x1.01) 0.974
oxycodone (-0.00129) 0.975
benzonatate (+0.00127) 0.975
Abnormal findings on diagnostic imaging of lung (x1.01) 0.975
−0.8 −0.6 −0.4 −0.2 0.0 0.2 0.4 0.6 0.8
Expressions
−10 0 10

Signature [633]; 24 To Reach 0.975, (24 Plotted)

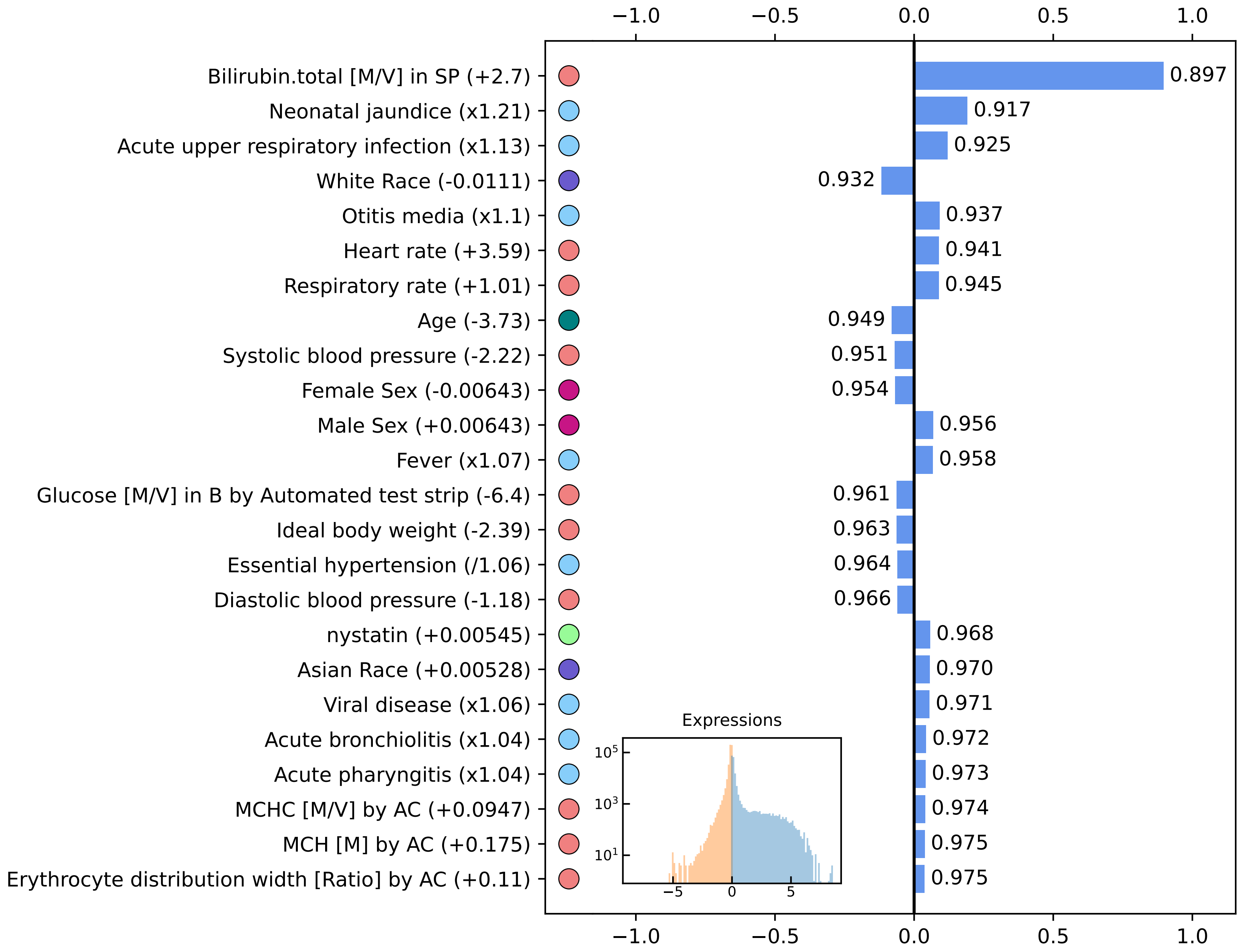

Signature [634]; 164 To Reach 0.975, (100 Plotted)

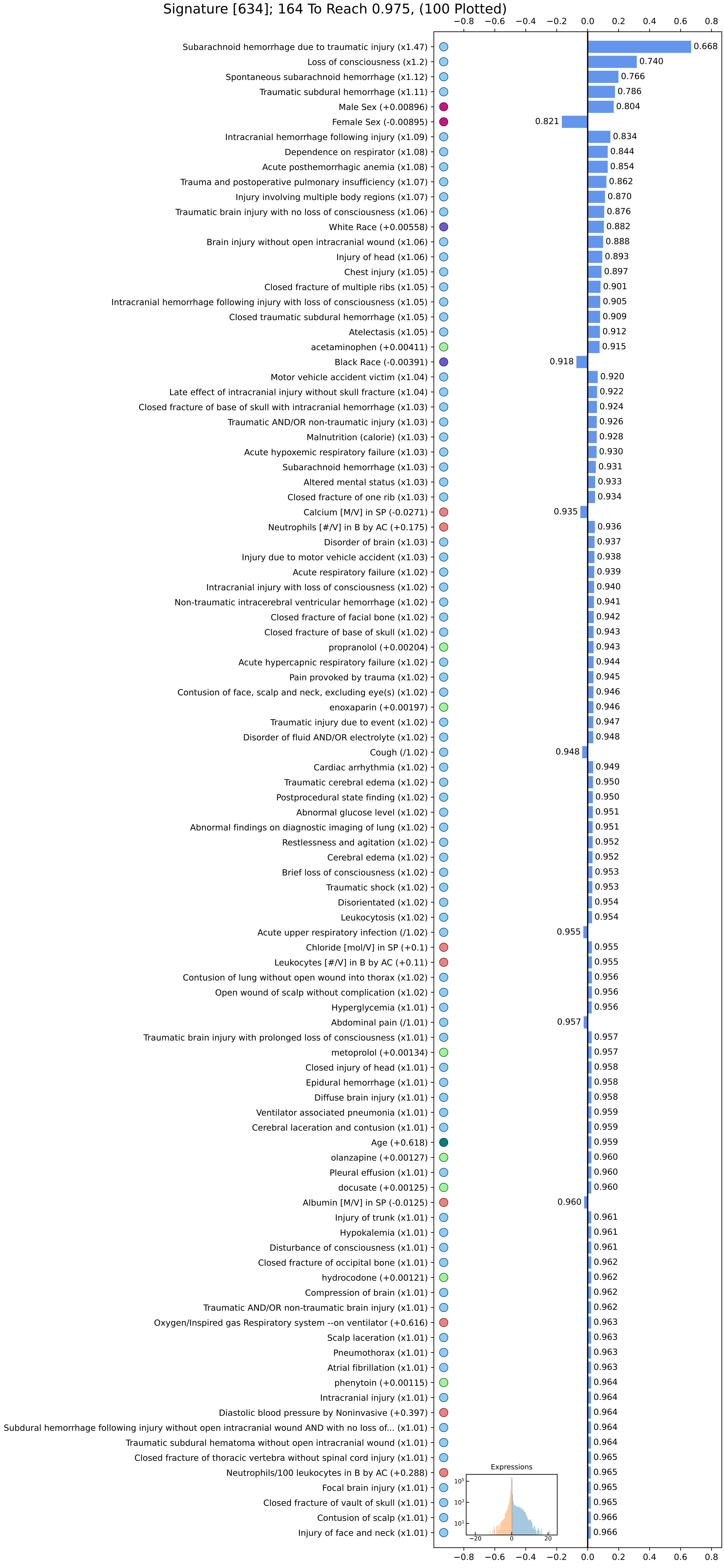

Signature [635]; 1479 To Reach 0.975, (100 Plotted)

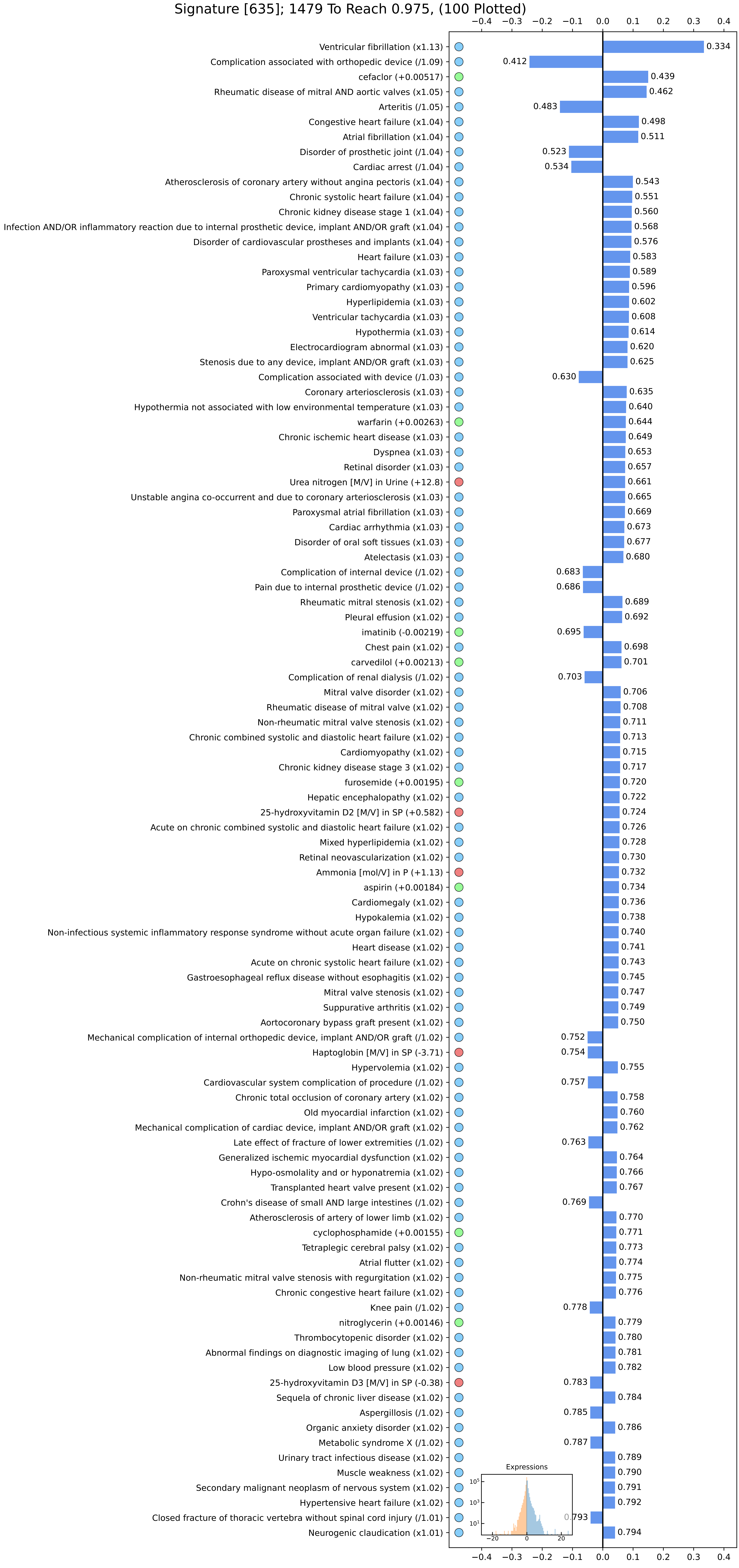

Signature [636]; 3 To Reach 0.975, (10 Plotted)

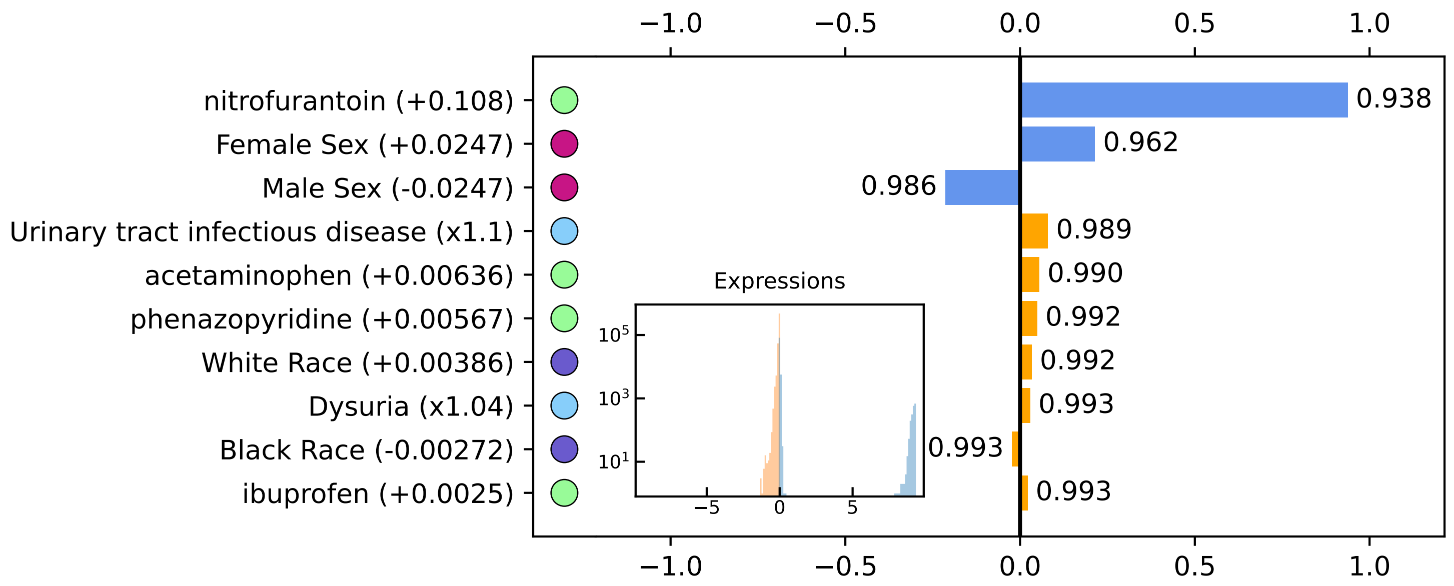

Signature [637]; 49 To Reach 0.975, (49 Plotted)

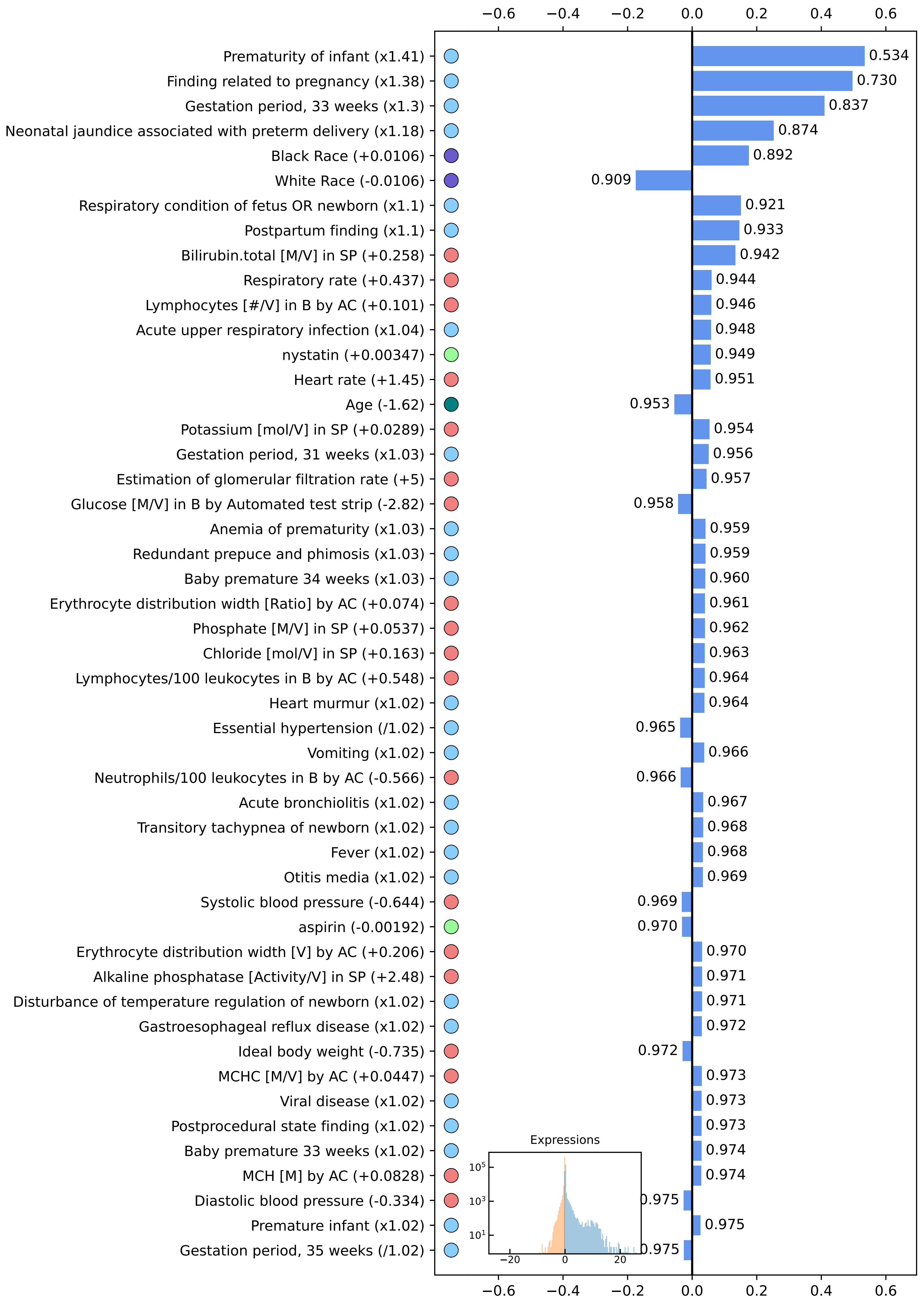

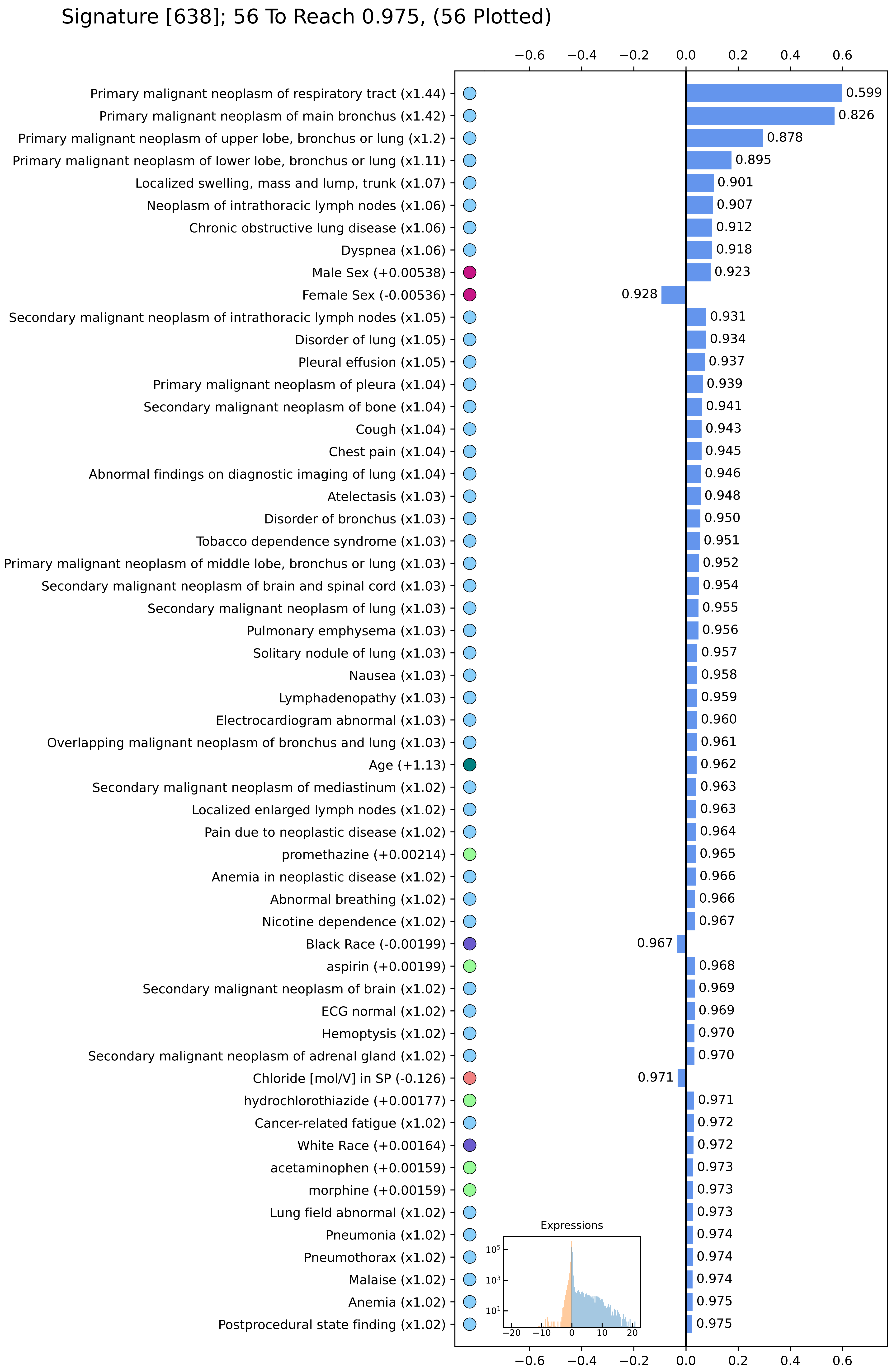
Signature [638]; 56 To Reach 0.975, (56 Plotted)
Primary malignant neoplasm of respiratory tract (x1.44) 0.599
Primary malignant neoplasm of main bronchus (x1.42) 0.826
Primary malignant neoplasm of upper lobe, bronchus or lung (x1.2) 0.878
Primary malignant neoplasm of lower lobe, bronchus or lung (x1.11) 0.895
Localized swelling, mass and lump, trunk (x1.07) 0.901
Neoplasm of intrathoracic lymph nodes (x1.06) 0.907
Chronic obstructive lung disease (x1.06) 0.912
Dyspnea (x1.06) 0.918
Male Sex (+0.00538) 0.923
Female Sex (-0.00536) 0.928
Secondary malignant neoplasm of intrathoracic lymph nodes (x1.05) 0.931
Disorder of lung (x1.05) 0.934
Pleural effusion (x1.05) 0.937
Primary malignant neoplasm of pleura (x1.04) 0.939
Secondary malignant neoplasm of bone (x1.04) 0.941
Cough (x1.04) 0.943
Chest pain (x1.04) 0.945
Abnormal findings on diagnostic imaging of lung (x1.04) 0.946
Atelectasis (x1.03) 0.948
Disorder of bronchus (x1.03) 0.950
Tobacco dependence syndrome (x1.03) 0.951
Primary malignant neoplasm of middle lobe, bronchus or lung (x1.03) 0.952
Secondary malignant neoplasm of brain and spinal cord (x1.03) 0.954
Secondary malignant neoplasm of lung (x1.03) 0.955
Pulmonary emphysema (x1.03) 0.956
Solitary nodule of lung (x1.03) 0.957
Nausea (x1.03) 0.958
Lymphadenopathy (x1.03) 0.959
Electrocardiogram abnormal (x1.03) 0.960
Overlapping malignant neoplasm of bronchus and lung (x1.03) 0.961
Age (+1.13) 0.962
Secondary malignant neoplasm of mediastinum (x1.02) 0.963
Localized enlarged lymph nodes (x1.02) 0.963
Pain due to neoplastic disease (x1.02) 0.964
promethazine (+0.00214) 0.965
Anemia in neoplastic disease (x1.02) 0.966
Abnormal breathing (x1.02) 0.966
Nicotine dependence (x1.02) 0.967
Black Race (-0.00199) 0.967
aspirin (+0.00199) 0.968
Secondary malignant neoplasm of brain (x1.02) 0.969
ECG normal (x1.02) 0.969
Hemoptysis (x1.02) 0.970
Secondary malignant neoplasm of adrenal gland (x1.02) 0.970
Chloride [mol/V] in SP (-0.126) 0.971
hydrochlorothiazide (+0.00177) 0.971
Cancer-related fatigue (x1.02) 0.972
White Race (+0.00164) 0.972
acetaminophen (+0.00159) 0.973
morphine (+0.00159) 0.973
Lung field abnormal (x1.02) 0.973
Pneumonia (x1.02) 0.974
Pneumothorax (x1.02) 0.974
Malaise (x1.02) 0.974
Anemia (x1.02) 0.975
Postprocedural state finding (x1.02) 0.975
−0.6 −0.4 −0.2 0.0 0.2 0.4 0.6
Expressions
10^5
10^3
10^1
−20 −10 0 10 20

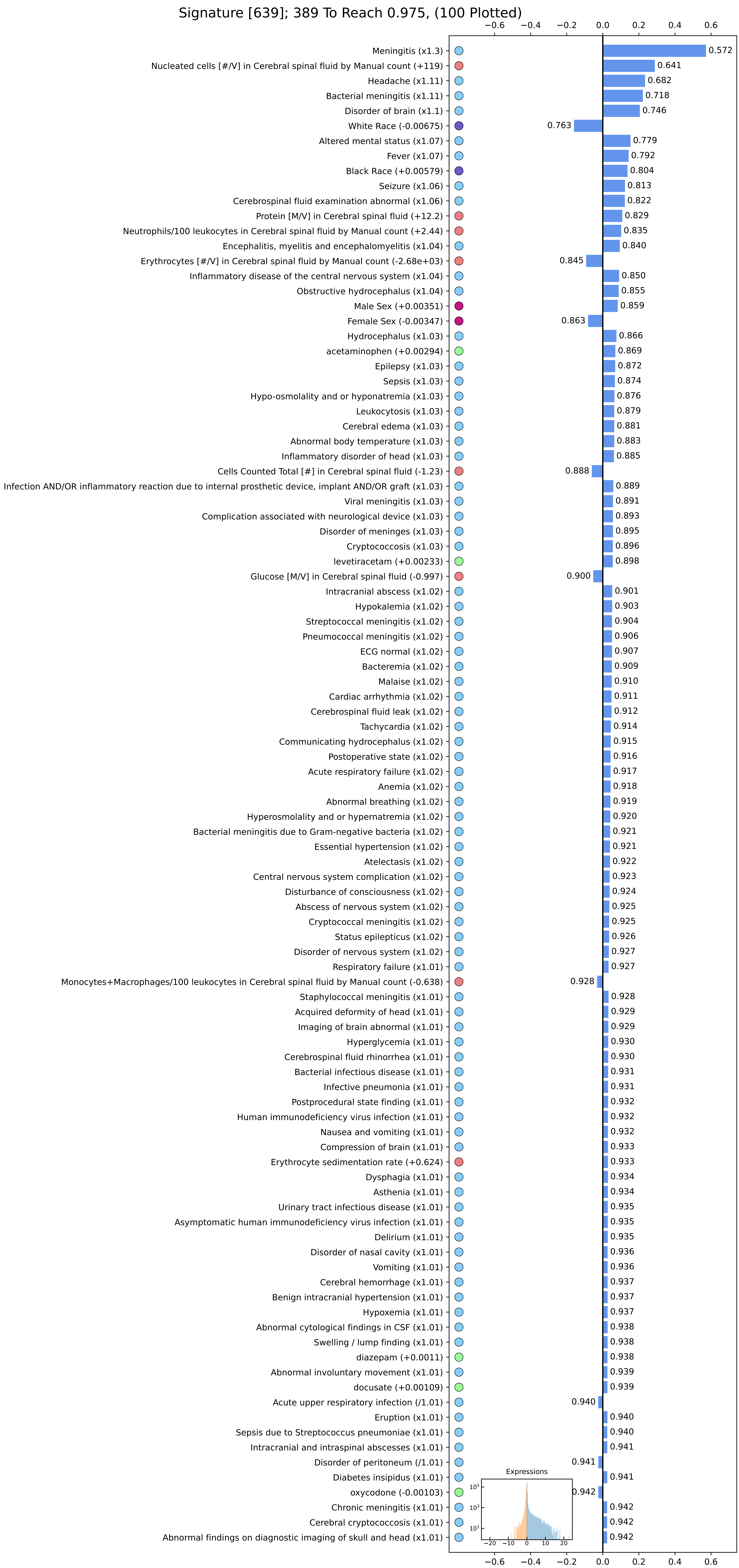
Signature [639]; 389 To Reach 0.975, (100 Plotted)
Meningitis (x1.3)
Nucleated cells [#/V] in Cerebral spinal fluid by Manual count (+119)
Headache (x1.11)
Bacterial meningitis (x1.11)
Disorder of brain (x1.1)
White Race (-0.00675)
Altered mental status (x1.07)
Fever (x1.07)
Black Race (+0.00579)
Seizure (x1.06)
Cerebrospinal fluid examination abnormal (x1.06)
Protein [M/V] in Cerebral spinal fluid (+12.2)
Neutrophils/100 leukocytes in Cerebral spinal fluid by Manual count (+2.44)
Encephalitis, myelitis and encephalomyelitis (x1.04)
Erythrocytes [#/V] in Cerebral spinal fluid by Manual count (-2.68e+03)
Inflammatory disease of the central nervous system (x1.04)
Obstructive hydrocephalus (x1.04)
Male Sex (+0.00351)
Female Sex (-0.00347)
Hydrocephalus (x1.03)
acetaminophen (+0.00294)
Epilepsy (x1.03)
Sepsis (x1.03)
Hypo-osmolality and or hyponatremia (x1.03)
Leukocytosis (x1.03)
Cerebral edema (x1.03)
Abnormal body temperature (x1.03)
Inflammatory disorder of head (x1.03)
Cells Counted Total [#] in Cerebral spinal fluid (-1.23)
Infection AND/OR inflammatory reaction due to internal prosthetic device, implant AND/OR graft (x1.03)
Viral meningitis (x1.03)
Complication associated with neurological device (x1.03)
Disorder of meninges (x1.03)
Cryptococcosis (x1.03)
levetiracetam (+0.00233)
Glucose [M/V] in Cerebral spinal fluid (-0.997)
Intracranial abscess (x1.02)
Hypokalemia (x1.02)
Streptococcal meningitis (x1.02)
Pneumococcal meningitis (x1.02)
ECG normal (x1.02)
Bacteremia (x1.02)
Malaise (x1.02)
Cardiac arrhythmia (x1.02)
Cerebrospinal fluid leak (x1.02)
Tachycardia (x1.02)
Communicating hydrocephalus (x1.02)
Postoperative state (x1.02)
Acute respiratory failure (x1.02)
Anemia (x1.02)
Abnormal breathing (x1.02)
Hyperosmolality and or hypernatremia (x1.02)
Bacterial meningitis due to Gram-negative bacteria (x1.02)
Essential hypertension (x1.02)
Atelectasis (x1.02)
Central nervous system complication (x1.02)
Disturbance of consciousness (x1.02)
Abscess of nervous system (x1.02)
Cryptococcal meningitis (x1.02)
Status epilepticus (x1.02)
Disorder of nervous system (x1.02)
Respiratory failure (x1.01)
Monocytes+Macrophages/100 leukocytes in Cerebral spinal fluid by Manual count (-0.638)
Staphylococcal meningitis (x1.01)
Acquired deformity of head (x1.01)
Imaging of brain abnormal (x1.01)
Hyperglycemia (x1.01)
Cerebrospinal fluid rhinorrhea (x1.01)
Bacterial infectious disease (x1.01)
Infective pneumonia (x1.01)
Postprocedural state finding (x1.01)
Human immunodeficiency virus infection (x1.01)
Nausea and vomiting (x1.01)
Compression of brain (x1.01)
Erythrocyte sedimentation rate (+0.624)
Dysphagia (x1.01)
Asthenia (x1.01)
Urinary tract infectious disease (x1.01)
Asymptomatic human immunodeficiency virus infection (x1.01)
Delirium (x1.01)
Disorder of nasal cavity (x1.01)
Vomiting (x1.01)
Cerebral hemorrhage (x1.01)
Benign intracranial hypertension (x1.01)
Hypoxemia (x1.01)
Abnormal cytological findings in CSF (x1.01)
Swelling / lump finding (x1.01)
diazepam (+0.0011)
Abnormal involuntary movement (x1.01)
docusate (+0.00109)
Acute upper respiratory infection (/1.01)
Eruption (x1.01)
Sepsis due to Streptococcus pneumoniae (x1.01)
Intracranial and intraspinal abscesses (x1.01)
Disorder of peritoneum (/1.01)
Diabetes insipidus (x1.01)
oxycodone (-0.00103)
Chronic meningitis (x1.01)
Cerebral cryptococcosis (x1.01)
Abnormal findings on diagnostic imaging of skull and head (x1.01)
0.572
0.641
0.682
0.718
0.746
0.763
0.779
0.792
0.804
0.813
0.822
0.829
0.835
0.840
0.845
0.850
0.855
0.859
0.863
0.866
0.869
0.872
0.874
0.876
0.879
0.881
0.883
0.885
0.888
0.889
0.891
0.893
0.895
0.896
0.898
0.900
0.901
0.903
0.904
0.906
0.907
0.909
0.910
0.911
0.912
0.914
0.915
0.916
0.917
0.918
0.919
0.920
0.921
0.921
0.922
0.923
0.924
0.925
0.925
0.926
0.927
0.927
0.928
0.928
0.929
0.929
0.930
0.930
0.931
0.931
0.932
0.932
0.932
0.933
0.933
0.934
0.934
0.935
0.935
0.935
0.936
0.936
0.937
0.937
0.937
0.938
0.938
0.938
0.939
0.939
0.940
0.940
0.940
0.941
0.941
0.941
0.942
0.942
0.942
0.942
−0.6
−0.4
−0.2
0.0
0.2
0.4
0.6
Expressions
−20
−10
0
10
20

Signature [640]; 1 To Reach 0.975, (10 Plotted)

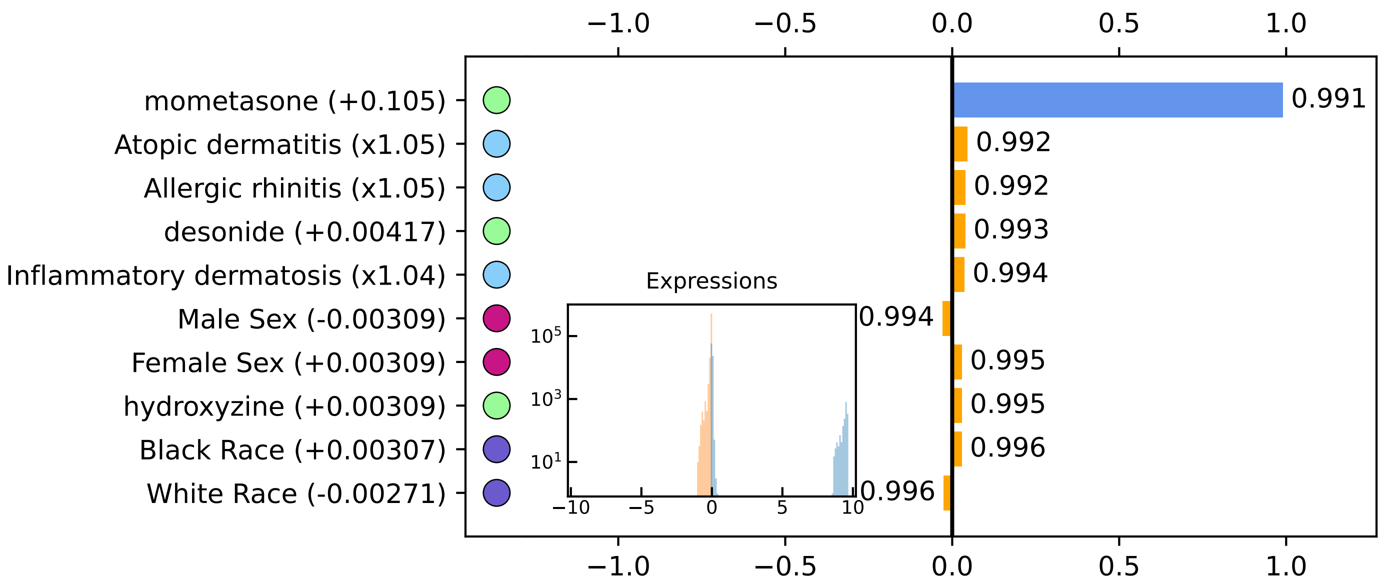

Signature [641]; 214 To Reach 0.975, (100 Plotted)

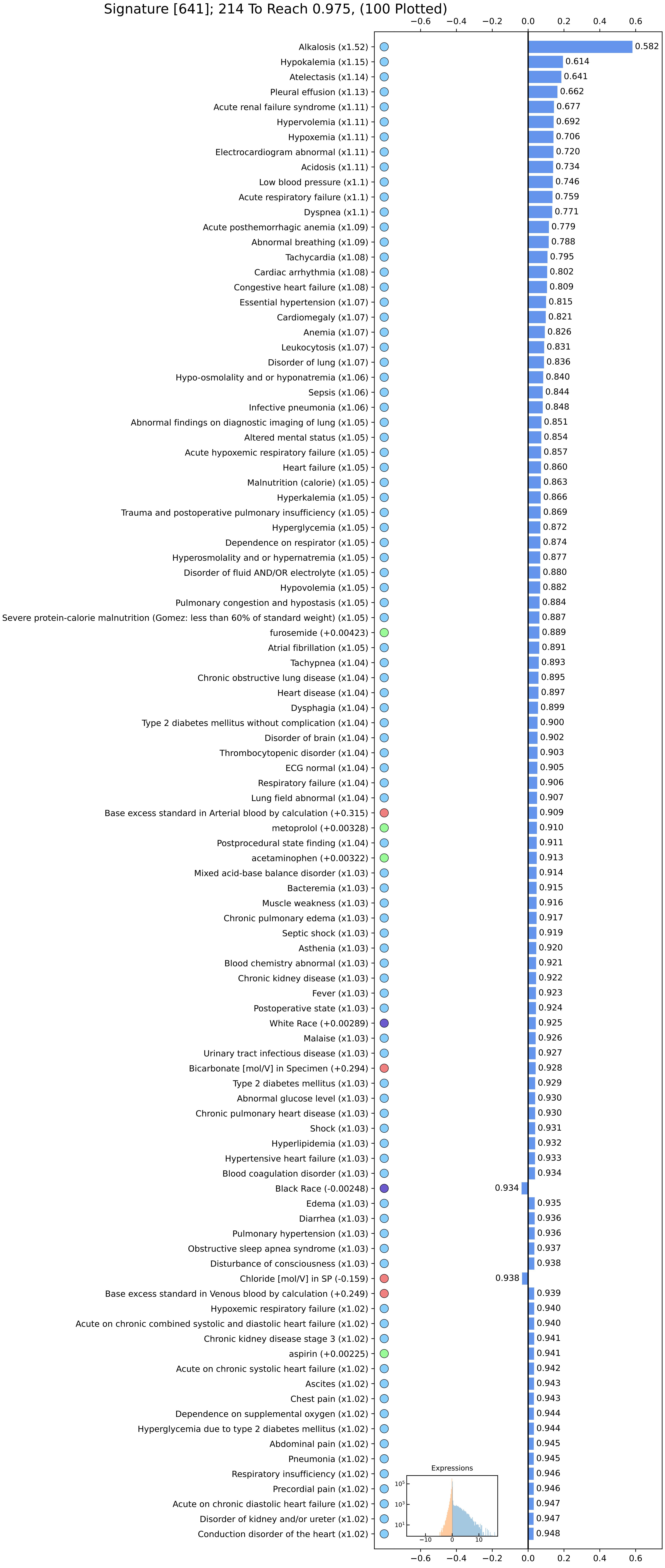

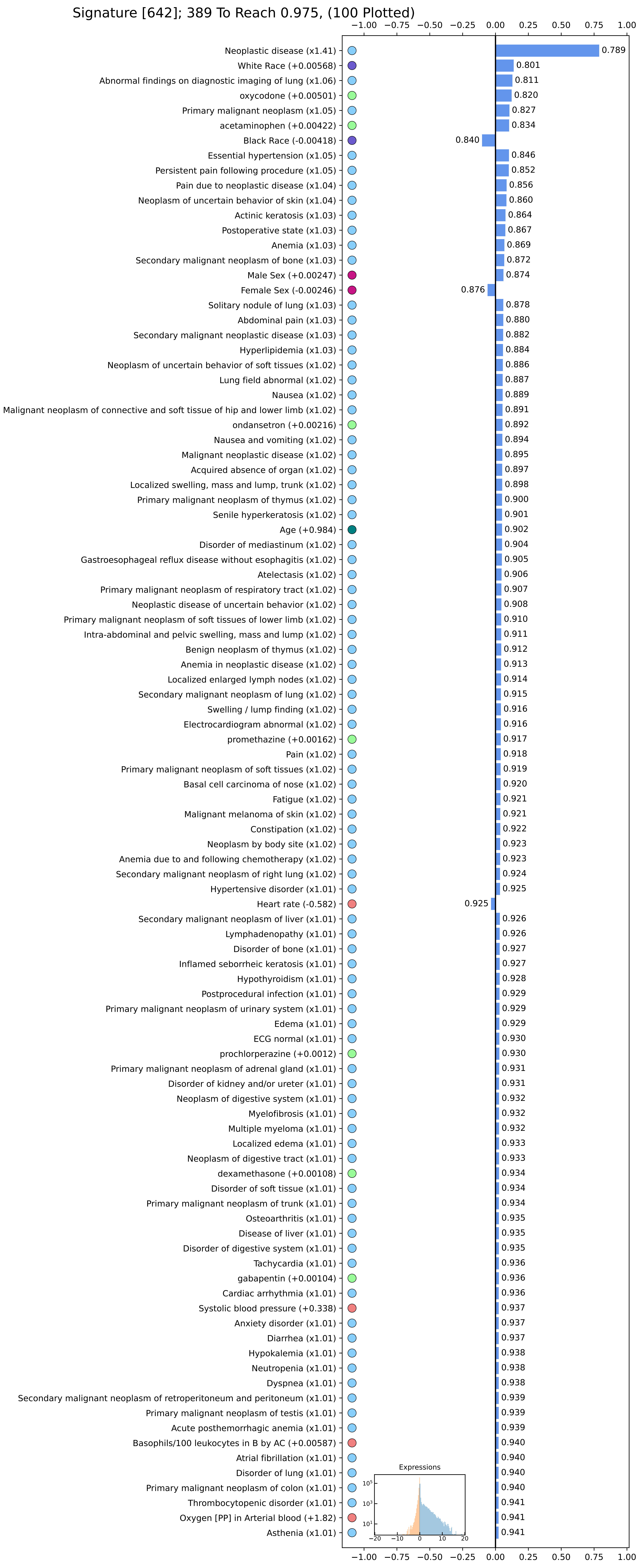

Signature [642]; 389 To Reach 0.975, (100 Plotted)
Neoplastic disease (x1.41) 0.789
White Race (+0.00568) 0.801
Abnormal findings on diagnostic imaging of lung (x1.06) 0.811
oxycodone (+0.00501) 0.820
Primary malignant neoplasm (x1.05) 0.827
acetaminophen (+0.00422) 0.834
Black Race (-0.00418) 0.840
Essential hypertension (x1.05) 0.846
Persistent pain following procedure (x1.05) 0.852
Pain due to neoplastic disease (x1.04) 0.856
Neoplasm of uncertain behavior of skin (x1.04) 0.860
Actinic keratosis (x1.03) 0.864
Postoperative state (x1.03) 0.867
Anemia (x1.03) 0.869
Secondary malignant neoplasm of bone (x1.03) 0.872
Male Sex (+0.00247) 0.874
Female Sex (-0.00246) 0.876
Solitary nodule of lung (x1.03) 0.878
Abdominal pain (x1.03) 0.880
Secondary malignant neoplastic disease (x1.03) 0.882
Hyperlipidemia (x1.03) 0.884
Neoplasm of uncertain behavior of soft tissues (x1.02) 0.886
Lung field abnormal (x1.02) 0.887
Nausea (x1.02) 0.889
Malignant neoplasm of connective and soft tissue of hip and lower limb (x1.02) 0.891
ondansetron (+0.00216) 0.892
Nausea and vomiting (x1.02) 0.894
Malignant neoplastic disease (x1.02) 0.895
Acquired absence of organ (x1.02) 0.897
Localized swelling, mass and lump, trunk (x1.02) 0.898
Primary malignant neoplasm of thymus (x1.02) 0.900
Senile hyperkeratosis (x1.02) 0.901
Age (+0.984) 0.902
Disorder of mediastinum (x1.02) 0.904
Gastroesophageal reflux disease without esophagitis (x1.02) 0.905
Atelectasis (x1.02) 0.906
Primary malignant neoplasm of respiratory tract (x1.02) 0.907
Neoplastic disease of uncertain behavior (x1.02) 0.908
Primary malignant neoplasm of soft tissues of lower limb (x1.02) 0.910
Intra-abdominal and pelvic swelling, mass and lump (x1.02) 0.911
Benign neoplasm of thymus (x1.02) 0.912
Anemia in neoplastic disease (x1.02) 0.913
Localized enlarged lymph nodes (x1.02) 0.914
Secondary malignant neoplasm of lung (x1.02) 0.915
Swelling / lump finding (x1.02) 0.916
Electrocardiogram abnormal (x1.02) 0.916
promethazine (+0.00162) 0.917
Pain (x1.02) 0.918
Primary malignant neoplasm of soft tissues (x1.02) 0.919
Basal cell carcinoma of nose (x1.02) 0.920
Fatigue (x1.02) 0.921
Malignant melanoma of skin (x1.02) 0.921
Constipation (x1.02) 0.922
Neoplasm by body site (x1.02) 0.923
Anemia due to and following chemotherapy (x1.02) 0.923
Secondary malignant neoplasm of right lung (x1.02) 0.924
Hypertensive disorder (x1.01) 0.925
Heart rate (-0.582) 0.925
Secondary malignant neoplasm of liver (x1.01) 0.926
Lymphadenopathy (x1.01) 0.926
Disorder of bone (x1.01) 0.927
Inflamed seborrheic keratosis (x1.01) 0.927
Hypothyroidism (x1.01) 0.928
Postprocedural infection (x1.01) 0.929
Primary malignant neoplasm of urinary system (x1.01) 0.929
Edema (x1.01) 0.929
ECG normal (x1.01) 0.930
prochlorperazine (+0.0012) 0.930
Primary malignant neoplasm of adrenal gland (x1.01) 0.931
Disorder of kidney and/or ureter (x1.01) 0.931
Neoplasm of digestive system (x1.01) 0.932
Myelofibrosis (x1.01) 0.932
Multiple myeloma (x1.01) 0.932
Localized edema (x1.01) 0.933
Neoplasm of digestive tract (x1.01) 0.933
dexamethasone (+0.00108) 0.934
Disorder of soft tissue (x1.01) 0.934
Primary malignant neoplasm of trunk (x1.01) 0.934
Osteoarthritis (x1.01) 0.935
Disease of liver (x1.01) 0.935
Disorder of digestive system (x1.01) 0.935
Tachycardia (x1.01) 0.936
gabapentin (+0.00104) 0.936
Cardiac arrhythmia (x1.01) 0.936
Systolic blood pressure (+0.338) 0.937
Anxiety disorder (x1.01) 0.937
Diarrhea (x1.01) 0.937
Hypokalemia (x1.01) 0.938
Neutropenia (x1.01) 0.938
Dyspnea (x1.01) 0.938
Secondary malignant neoplasm of retroperitoneum and peritoneum (x1.01) 0.939
Primary malignant neoplasm of testis (x1.01) 0.939
Acute posthemorrhagic anemia (x1.01) 0.939
Basophils/100 leukocytes in B by AC (+0.00587) 0.940
Atrial fibrillation (x1.01) 0.940
Disorder of lung (x1.01) 0.940
Primary malignant neoplasm of colon (x1.01) 0.940
Thrombocytopenic disorder (x1.01) 0.941
Oxygen [PP] in Arterial blood (+1.82) 0.941
Asthenia (x1.01) 0.941
−1.00 −0.75 −0.50 −0.25 0.00 0.25 0.50 0.75 1.00
Expressions

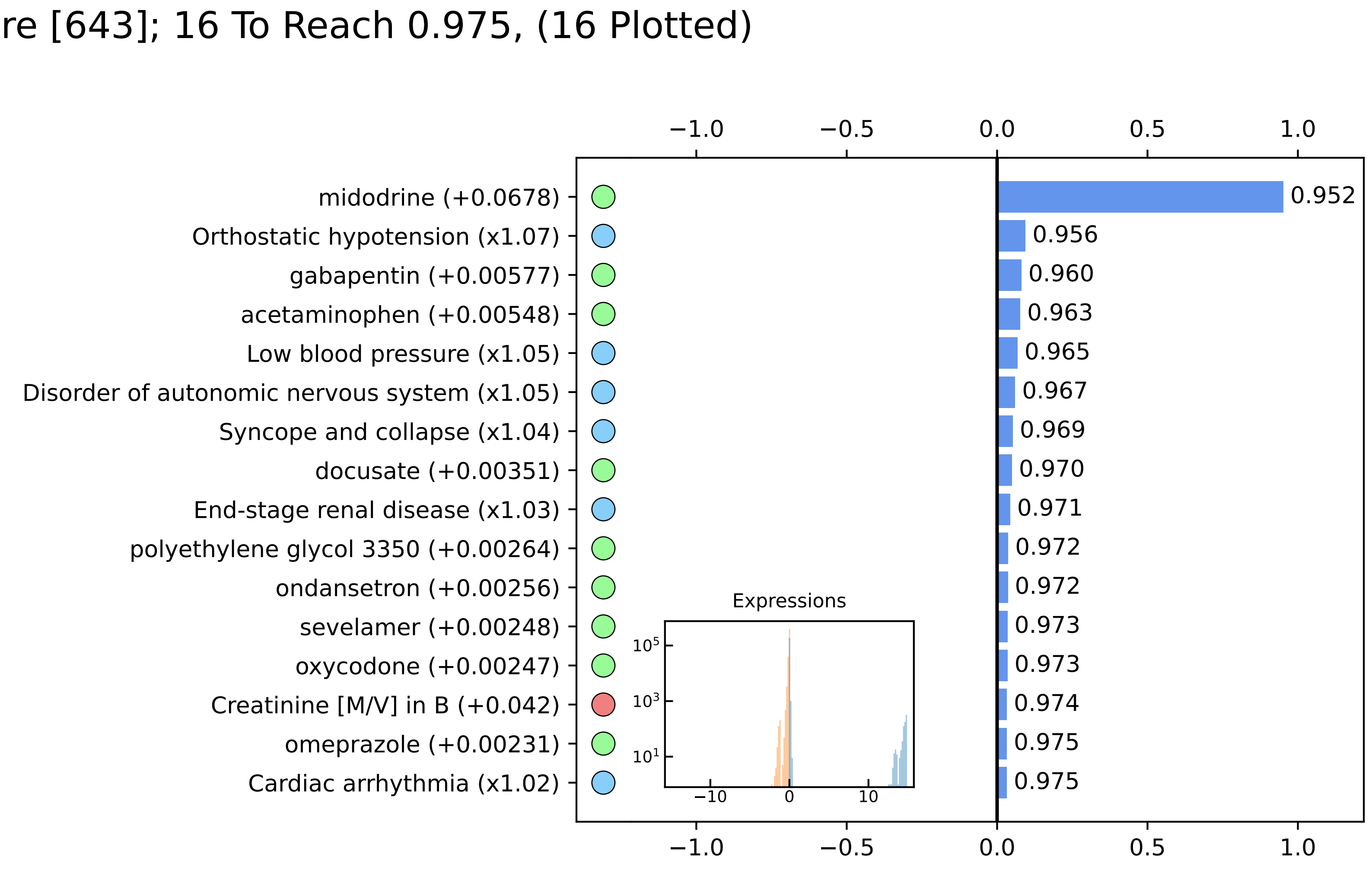

Signature [643]; 16 To Reach 0.975, (16 Plotted)
midodrine (+0.0678)
Orthostatic hypotension (x1.07)
gabapentin (+0.00577)
acetaminophen (+0.00548)
Low blood pressure (x1.05)
Disorder of autonomic nervous system (x1.05)
Syncope and collapse (x1.04)
docusate (+0.00351)
End-stage renal disease (x1.03)
polyethylene glycol 3350 (+0.00264)
ondansetron (+0.00256)
sevelamer (+0.00248)
oxycodone (+0.00247)
Creatinine [M/V] in B (+0.042)
omeprazole (+0.00231)
Cardiac arrhythmia (x1.02)
0.952
0.956
0.960
0.963
0.965
0.967
0.969
0.970
0.971
0.972
0.972
0.973
0.973
0.974
0.975
0.975
Expressions

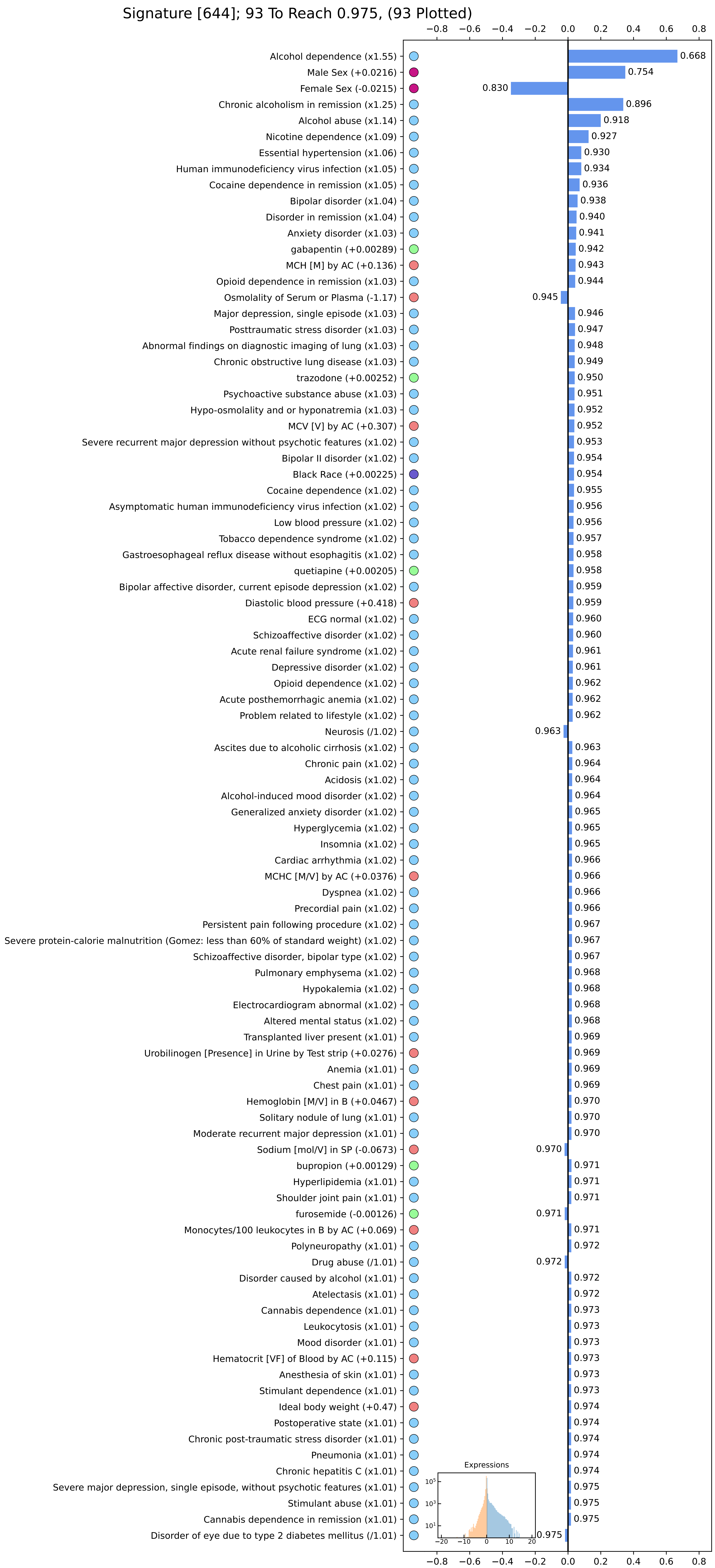

Signature [644]; 93 To Reach 0.975, (93 Plotted)
Alcohol dependence (x1.55) 0.668
Male Sex (+0.0216) 0.754
Female Sex (-0.0215) 0.830
Chronic alcoholism in remission (x1.25) 0.896
Alcohol abuse (x1.14) 0.918
Nicotine dependence (x1.09) 0.927
Essential hypertension (x1.06) 0.930
Human immunodeficiency virus infection (x1.05) 0.934
Cocaine dependence in remission (x1.05) 0.936
Bipolar disorder (x1.04) 0.938
Disorder in remission (x1.04) 0.940
Anxiety disorder (x1.03) 0.941
gabapentin (+0.00289) 0.942
MCH [M] by AC (+0.136) 0.943
Opioid dependence in remission (x1.03) 0.944
Osmolality of Serum or Plasma (-1.17) 0.945
Major depression, single episode (x1.03) 0.946
Posttraumatic stress disorder (x1.03) 0.947
Abnormal findings on diagnostic imaging of lung (x1.03) 0.948
Chronic obstructive lung disease (x1.03) 0.949
trazodone (+0.00252) 0.950
Psychoactive substance abuse (x1.03) 0.951
Hypo-osmolality and or hyponatremia (x1.03) 0.952
MCV [V] by AC (+0.307) 0.952
Severe recurrent major depression without psychotic features (x1.02) 0.953
Bipolar II disorder (x1.02) 0.954
Black Race (+0.00225) 0.954
Cocaine dependence (x1.02) 0.955
Asymptomatic human immunodeficiency virus infection (x1.02) 0.956
Low blood pressure (x1.02) 0.956
Tobacco dependence syndrome (x1.02) 0.957
Gastroesophageal reflux disease without esophagitis (x1.02) 0.958
quetiapine (+0.00205) 0.958
Bipolar affective disorder, current episode depression (x1.02) 0.959
Diastolic blood pressure (+0.418) 0.959
ECG normal (x1.02) 0.960
Schizoaffective disorder (x1.02) 0.960
Acute renal failure syndrome (x1.02) 0.961
Depressive disorder (x1.02) 0.961
Opioid dependence (x1.02) 0.962
Acute posthemorrhagic anemia (x1.02) 0.962
Problem related to lifestyle (x1.02) 0.962
Neurosis (/1.02) 0.963
Ascites due to alcoholic cirrhosis (x1.02) 0.963
Chronic pain (x1.02) 0.964
Acidosis (x1.02) 0.964
Alcohol-induced mood disorder (x1.02) 0.964
Generalized anxiety disorder (x1.02) 0.965
Hyperglycemia (x1.02) 0.965
Insomnia (x1.02) 0.965
Cardiac arrhythmia (x1.02) 0.966
MCHC [M/V] by AC (+0.0376) 0.966
Dyspnea (x1.02) 0.966
Precordial pain (x1.02) 0.966
Persistent pain following procedure (x1.02) 0.967
Severe protein-calorie malnutrition (Gomez: less than 60% of standard weight) (x1.02) 0.967
Schizoaffective disorder, bipolar type (x1.02) 0.967
Pulmonary emphysema (x1.02) 0.968
Hypokalemia (x1.02) 0.968
Electrocardiogram abnormal (x1.02) 0.968
Altered mental status (x1.02) 0.968
Transplanted liver present (x1.01) 0.969
Urobilinogen [Presence] in Urine by Test strip (+0.0276) 0.969
Anemia (x1.01) 0.969
Chest pain (x1.01) 0.969
Hemoglobin [M/V] in B (+0.0467) 0.970
Solitary nodule of lung (x1.01) 0.970
Moderate recurrent major depression (x1.01) 0.970
Sodium [mol/V] in SP (-0.0673) 0.970
bupropion (+0.00129) 0.971
Hyperlipidemia (x1.01) 0.971
Shoulder joint pain (x1.01) 0.971
furosemide (-0.00126) 0.971
Monocytes/100 leukocytes in B by AC (+0.069) 0.971
Polyneuropathy (x1.01) 0.972
Drug abuse (/1.01) 0.972
Disorder caused by alcohol (x1.01) 0.972
Atelectasis (x1.01) 0.972
Cannabis dependence (x1.01) 0.973
Leukocytosis (x1.01) 0.973
Mood disorder (x1.01) 0.973
Hematocrit [VF] of Blood by AC (+0.115) 0.973
Anesthesia of skin (x1.01) 0.973
Stimulant dependence (x1.01) 0.973
Ideal body weight (+0.47) 0.974
Postoperative state (x1.01) 0.974
Chronic post-traumatic stress disorder (x1.01) 0.974
Pneumonia (x1.01) 0.974
Chronic hepatitis C (x1.01) 0.974
Severe major depression, single episode, without psychotic features (x1.01) 0.975
Stimulant abuse (x1.01) 0.975
Cannabis dependence in remission (x1.01) 0.975
Disorder of eye due to type 2 diabetes mellitus (/1.01) 0.975
−0.8 −0.6 −0.4 −0.2 0.0 0.2 0.4 0.6 0.8
Expressions

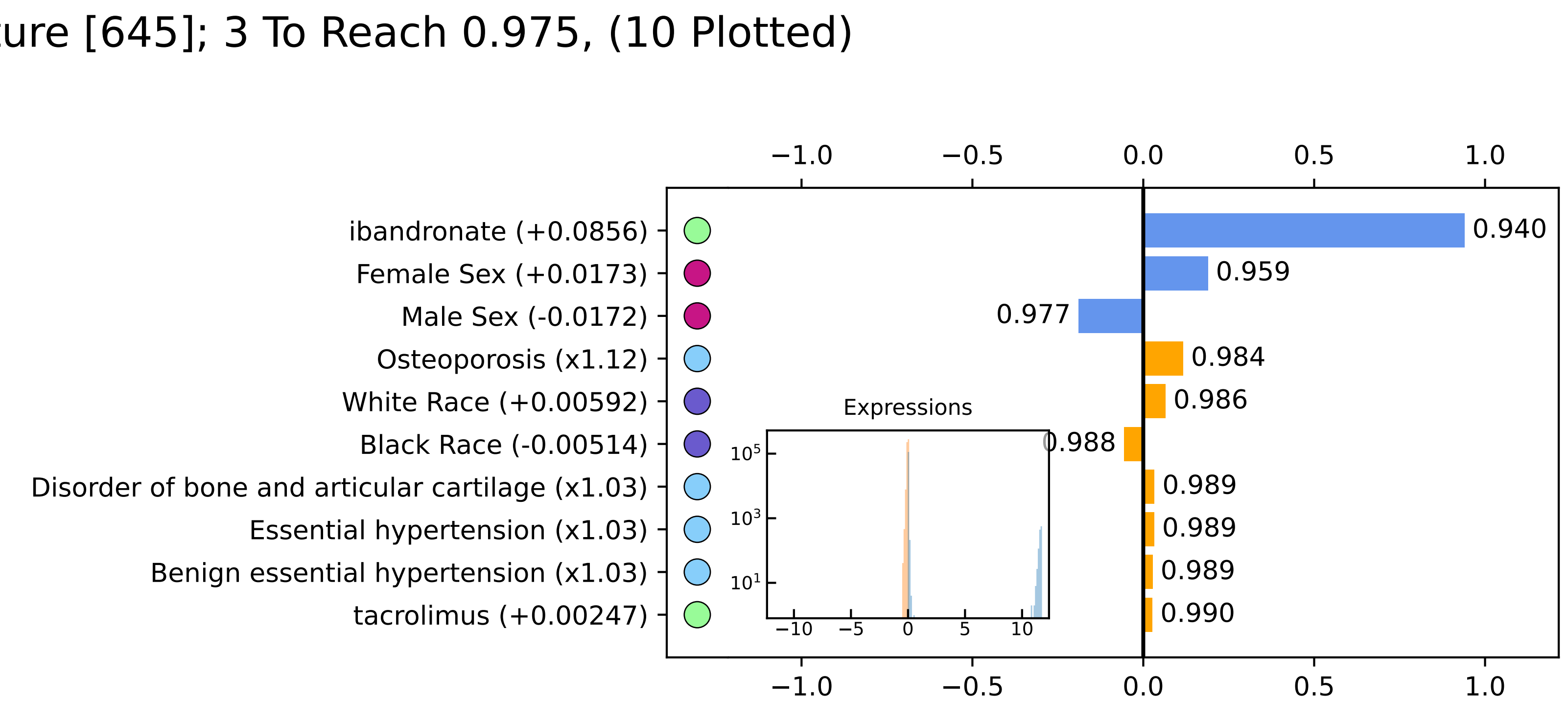

Signature [645]; 3 To Reach 0.975, (10 Plotted)
ibandronate (+0.0856)
Female Sex (+0.0173)
Male Sex (-0.0172)
Osteoporosis (x1.12)
White Race (+0.00592)
Black Race (-0.00514)
Disorder of bone and articular cartilage (x1.03)
Essential hypertension (x1.03)
Benign essential hypertension (x1.03)
tacrolimus (+0.00247)
0.940
0.959
0.977
0.984
0.986
0.988
0.989
0.989
0.989
0.990
−1.0
−0.5
0.0
0.5
1.0
Expressions

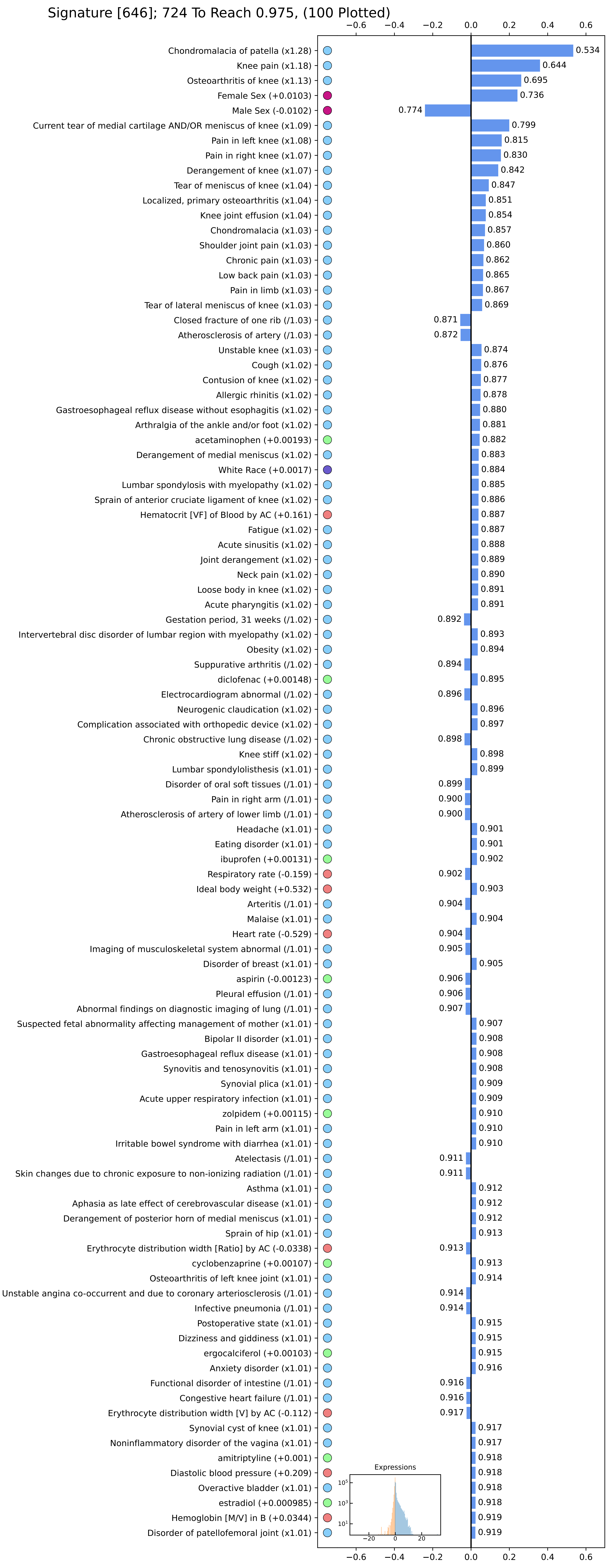

Signature [646]; 724 To Reach 0.975, (100 Plotted)
Chondromalacia of patella (x1.28) 0.534
Knee pain (x1.18) 0.644
Osteoarthritis of knee (x1.13) 0.695
Female Sex (+0.0103) 0.736
Male Sex (-0.0102) 0.774
Current tear of medial cartilage AND/OR meniscus of knee (x1.09) 0.799
Pain in left knee (x1.08) 0.815
Pain in right knee (x1.07) 0.830
Derangement of knee (x1.07) 0.842
Tear of meniscus of knee (x1.04) 0.847
Localized, primary osteoarthritis (x1.04) 0.851
Knee joint effusion (x1.04) 0.854
Chondromalacia (x1.03) 0.857
Shoulder joint pain (x1.03) 0.860
Chronic pain (x1.03) 0.862
Low back pain (x1.03) 0.865
Pain in limb (x1.03) 0.867
Tear of lateral meniscus of knee (x1.03) 0.869
Closed fracture of one rib (/1.03) 0.871
Atherosclerosis of artery (/1.03) 0.872
Unstable knee (x1.03) 0.874
Cough (x1.02) 0.876
Contusion of knee (x1.02) 0.877
Allergic rhinitis (x1.02) 0.878
Gastroesophageal reflux disease without esophagitis (x1.02) 0.880
Arthralgia of the ankle and/or foot (x1.02) 0.881
acetaminophen (+0.00193) 0.882
Derangement of medial meniscus (x1.02) 0.883
White Race (+0.0017) 0.884
Lumbar spondylosis with myelopathy (x1.02) 0.885
Sprain of anterior cruciate ligament of knee (x1.02) 0.886
Hematocrit [VF] of Blood by AC (+0.161) 0.887
Fatigue (x1.02) 0.887
Acute sinusitis (x1.02) 0.888
Joint derangement (x1.02) 0.889
Neck pain (x1.02) 0.890
Loose body in knee (x1.02) 0.891
Acute pharyngitis (x1.02) 0.891
Gestation period, 31 weeks (/1.02) 0.892
Intervertebral disc disorder of lumbar region with myelopathy (x1.02) 0.893
Obesity (x1.02) 0.894
Suppurative arthritis (/1.02) 0.894
diclofenac (+0.00148) 0.895
Electrocardiogram abnormal (/1.02) 0.896
Neurogenic claudication (x1.02) 0.896
Complication associated with orthopedic device (x1.02) 0.897
Chronic obstructive lung disease (/1.02) 0.898
Knee stiff (x1.02) 0.898
Lumbar spondylolisthesis (x1.01) 0.899
Disorder of oral soft tissues (/1.01) 0.899
Pain in right arm (/1.01) 0.900
Atherosclerosis of artery of lower limb (/1.01) 0.900
Headache (x1.01) 0.901
Eating disorder (x1.01) 0.901
ibuprofen (+0.00131) 0.902
Respiratory rate (-0.159) 0.902
Ideal body weight (+0.532) 0.903
Arteritis (/1.01) 0.904
Malaise (x1.01) 0.904
Heart rate (-0.529) 0.904
Imaging of musculoskeletal system abnormal (/1.01) 0.905
Disorder of breast (x1.01) 0.905
aspirin (-0.00123) 0.906
Pleural effusion (/1.01) 0.906
Abnormal findings on diagnostic imaging of lung (/1.01) 0.907
Suspected fetal abnormality affecting management of mother (x1.01) 0.907
Bipolar II disorder (x1.01) 0.908
Gastroesophageal reflux disease (x1.01) 0.908
Synovitis and tenosynovitis (x1.01) 0.908
Synovial plica (x1.01) 0.909
Acute upper respiratory infection (x1.01) 0.909
zolpidem (+0.00115) 0.910
Pain in left arm (x1.01) 0.910
Irritable bowel syndrome with diarrhea (x1.01) 0.910
Atelectasis (/1.01) 0.911
Skin changes due to chronic exposure to non-ionizing radiation (/1.01) 0.911
Asthma (x1.01) 0.912
Aphasia as late effect of cerebrovascular disease (x1.01) 0.912
Derangement of posterior horn of medial meniscus (x1.01) 0.912
Sprain of hip (x1.01) 0.913
Erythrocyte distribution width [Ratio] by AC (-0.0338) 0.913
cyclobenzaprine (+0.00107) 0.913
Osteoarthritis of left knee joint (x1.01) 0.914
Unstable angina co-occurrent and due to coronary arteriosclerosis (/1.01) 0.914
Infective pneumonia (/1.01) 0.914
Postoperative state (x1.01) 0.915
Dizziness and giddiness (x1.01) 0.915
ergocalciferol (+0.00103) 0.915
Anxiety disorder (x1.01) 0.916
Functional disorder of intestine (/1.01) 0.916
Congestive heart failure (/1.01) 0.916
Erythrocyte distribution width [V] by AC (-0.112) 0.917
Synovial cyst of knee (x1.01) 0.917
Noninflammatory disorder of the vagina (x1.01) 0.917
amitriptyline (+0.001) 0.918
Diastolic blood pressure (+0.209) 0.918
Overactive bladder (x1.01) 0.918
estradiol (+0.000985) 0.918
Hemoglobin [M/V] in B (+0.0344) 0.919
Disorder of patellofemoral joint (x1.01) 0.919
Expressions
−0.6 −0.4 −0.2 0.0 0.2 0.4 0.6

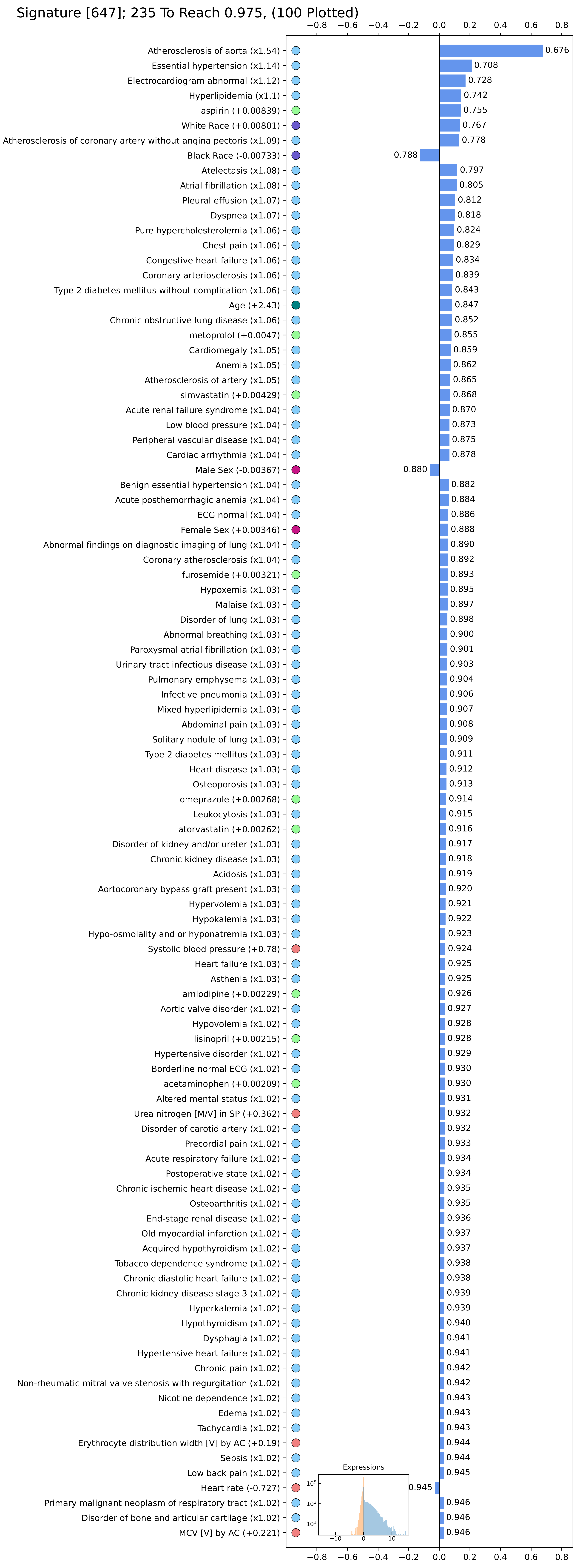
Signature [647]; 235 To Reach 0.975, (100 Plotted)
Atherosclerosis of aorta (x1.54) 0.676
Essential hypertension (x1.14) 0.708
Electrocardiogram abnormal (x1.12) 0.728
Hyperlipidemia (x1.1) 0.742
aspirin (+0.00839) 0.755
White Race (+0.00801) 0.767
Atherosclerosis of coronary artery without angina pectoris (x1.09) 0.778
Black Race (-0.00733) 0.788
Atelectasis (x1.08) 0.797
Atrial fibrillation (x1.08) 0.805
Pleural effusion (x1.07) 0.812
Dyspnea (x1.07) 0.818
Pure hypercholesterolemia (x1.06) 0.824
Chest pain (x1.06) 0.829
Congestive heart failure (x1.06) 0.834
Coronary arteriosclerosis (x1.06) 0.839
Type 2 diabetes mellitus without complication (x1.06) 0.843
Age (+2.43) 0.847
Chronic obstructive lung disease (x1.06) 0.852
metoprolol (+0.0047) 0.855
Cardiomegaly (x1.05) 0.859
Anemia (x1.05) 0.862
Atherosclerosis of artery (x1.05) 0.865
simvastatin (+0.00429) 0.868
Acute renal failure syndrome (x1.04) 0.870
Low blood pressure (x1.04) 0.873
Peripheral vascular disease (x1.04) 0.875
Cardiac arrhythmia (x1.04) 0.878
Male Sex (-0.00367) 0.880
Benign essential hypertension (x1.04) 0.882
Acute posthemorrhagic anemia (x1.04) 0.884
ECG normal (x1.04) 0.886
Female Sex (+0.00346) 0.888
Abnormal findings on diagnostic imaging of lung (x1.04) 0.890
Coronary atherosclerosis (x1.04) 0.892
furosemide (+0.00321) 0.893
Hypoxemia (x1.03) 0.895
Malaise (x1.03) 0.897
Disorder of lung (x1.03) 0.898
Abnormal breathing (x1.03) 0.900
Paroxysmal atrial fibrillation (x1.03) 0.901
Urinary tract infectious disease (x1.03) 0.903
Pulmonary emphysema (x1.03) 0.904
Infective pneumonia (x1.03) 0.906
Mixed hyperlipidemia (x1.03) 0.907
Abdominal pain (x1.03) 0.908
Solitary nodule of lung (x1.03) 0.909
Type 2 diabetes mellitus (x1.03) 0.911
Heart disease (x1.03) 0.912
Osteoporosis (x1.03) 0.913
omeprazole (+0.00268) 0.914
Leukocytosis (x1.03) 0.915
atorvastatin (+0.00262) 0.916
Disorder of kidney and/or ureter (x1.03) 0.917
Chronic kidney disease (x1.03) 0.918
Acidosis (x1.03) 0.919
Aortocoronary bypass graft present (x1.03) 0.920
Hypervolemia (x1.03) 0.921
Hypokalemia (x1.03) 0.922
Hypo-osmolality and or hyponatremia (x1.03) 0.923
Systolic blood pressure (+0.78) 0.924
Heart failure (x1.03) 0.925
Asthenia (x1.03) 0.925
amlodipine (+0.00229) 0.926
Aortic valve disorder (x1.02) 0.927
Hypovolemia (x1.02) 0.928
lisinopril (+0.00215) 0.928
Hypertensive disorder (x1.02) 0.929
Borderline normal ECG (x1.02) 0.930
acetaminophen (+0.00209) 0.930
Altered mental status (x1.02) 0.931
Urea nitrogen [M/V] in SP (+0.362) 0.932
Disorder of carotid artery (x1.02) 0.932
Precordial pain (x1.02) 0.933
Acute respiratory failure (x1.02) 0.934
Postoperative state (x1.02) 0.934
Chronic ischemic heart disease (x1.02) 0.935
Osteoarthritis (x1.02) 0.935
End-stage renal disease (x1.02) 0.936
Old myocardial infarction (x1.02) 0.937
Acquired hypothyroidism (x1.02) 0.937
Tobacco dependence syndrome (x1.02) 0.938
Chronic diastolic heart failure (x1.02) 0.938
Chronic kidney disease stage 3 (x1.02) 0.939
Hyperkalemia (x1.02) 0.939
Hypothyroidism (x1.02) 0.940
Dysphagia (x1.02) 0.941
Hypertensive heart failure (x1.02) 0.941
Chronic pain (x1.02) 0.942
Non-rheumatic mitral valve stenosis with regurgitation (x1.02) 0.942
Nicotine dependence (x1.02) 0.943
Edema (x1.02) 0.943
Tachycardia (x1.02) 0.943
Erythrocyte distribution width [V] by AC (+0.19) 0.944
Sepsis (x1.02) 0.944
Low back pain (x1.02) 0.945
Heart rate (-0.727) 0.945
Primary malignant neoplasm of respiratory tract (x1.02) 0.946
Disorder of bone and articular cartilage (x1.02) 0.946
MCV [V] by AC (+0.221) 0.946
−0.8 −0.6 −0.4 −0.2 0.0 0.2 0.4 0.6 0.8
Expressions
−10 0 10

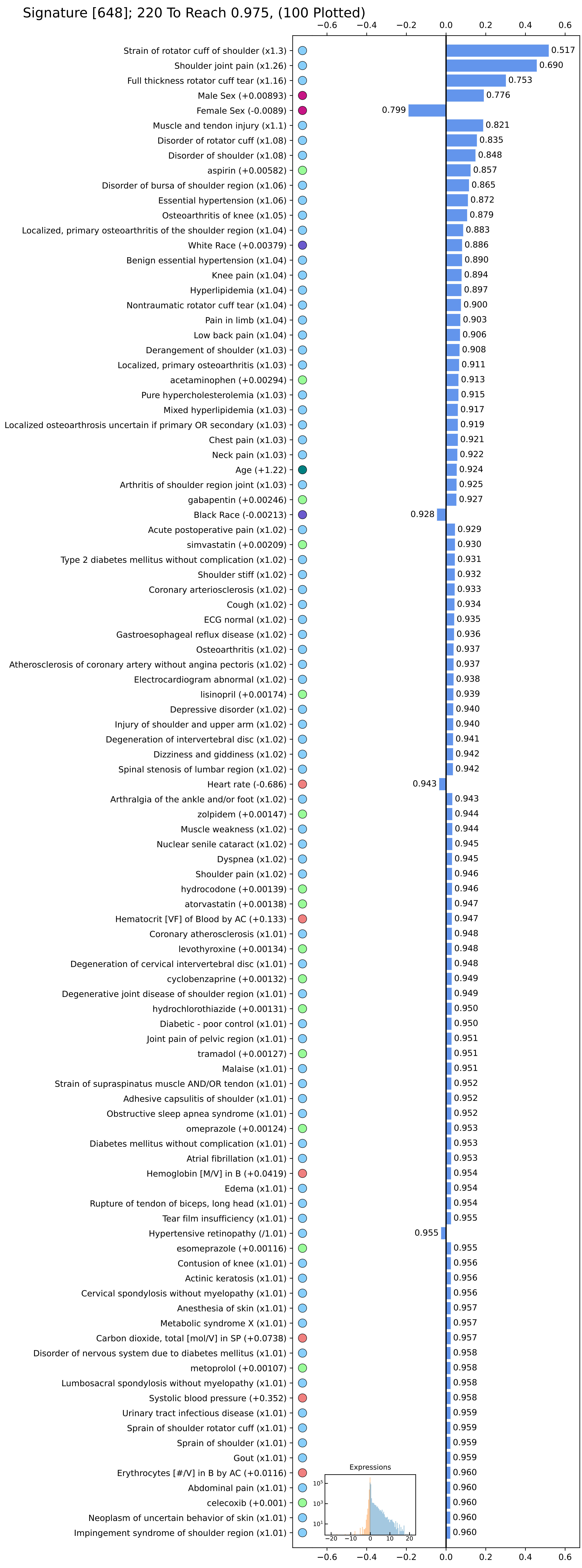

Signature [648]; 220 To Reach 0.975, (100 Plotted)
Strain of rotator cuff of shoulder (x1.3) 0.517
Shoulder joint pain (x1.26) 0.690
Full thickness rotator cuff tear (x1.16) 0.753
Male Sex (+0.00893) 0.776
Female Sex (-0.0089) 0.799
Muscle and tendon injury (x1.1) 0.821
Disorder of rotator cuff (x1.08) 0.835
Disorder of shoulder (x1.08) 0.848
aspirin (+0.00582) 0.857
Disorder of bursa of shoulder region (x1.06) 0.865
Essential hypertension (x1.06) 0.872
Osteoarthritis of knee (x1.05) 0.879
Localized, primary osteoarthritis of the shoulder region (x1.04) 0.883
White Race (+0.00379) 0.886
Benign essential hypertension (x1.04) 0.890
Knee pain (x1.04) 0.894
Hyperlipidemia (x1.04) 0.897
Nontraumatic rotator cuff tear (x1.04) 0.900
Pain in limb (x1.04) 0.903
Low back pain (x1.04) 0.906
Derangement of shoulder (x1.03) 0.908
Localized, primary osteoarthritis (x1.03) 0.911
acetaminophen (+0.00294) 0.913
Pure hypercholesterolemia (x1.03) 0.915
Mixed hyperlipidemia (x1.03) 0.917
Localized osteoarthrosis uncertain if primary OR secondary (x1.03) 0.919
Chest pain (x1.03) 0.921
Neck pain (x1.03) 0.922
Age (+1.22) 0.924
Arthritis of shoulder region joint (x1.03) 0.925
gabapentin (+0.00246) 0.927
Black Race (-0.00213) 0.928
Acute postoperative pain (x1.02) 0.929
simvastatin (+0.00209) 0.930
Type 2 diabetes mellitus without complication (x1.02) 0.931
Shoulder stiff (x1.02) 0.932
Coronary arteriosclerosis (x1.02) 0.933
Cough (x1.02) 0.934
ECG normal (x1.02) 0.935
Gastroesophageal reflux disease (x1.02) 0.936
Osteoarthritis (x1.02) 0.937
Atherosclerosis of coronary artery without angina pectoris (x1.02) 0.937
Electrocardiogram abnormal (x1.02) 0.938
lisinopril (+0.00174) 0.939
Depressive disorder (x1.02) 0.940
Injury of shoulder and upper arm (x1.02) 0.940
Degeneration of intervertebral disc (x1.02) 0.941
Dizziness and giddiness (x1.02) 0.942
Spinal stenosis of lumbar region (x1.02) 0.942
Heart rate (-0.686) 0.943
Arthralgia of the ankle and/or foot (x1.02) 0.943
zolpidem (+0.00147) 0.944
Muscle weakness (x1.02) 0.944
Nuclear senile cataract (x1.02) 0.945
Dyspnea (x1.02) 0.945
Shoulder pain (x1.02) 0.946
hydrocodone (+0.00139) 0.946
atorvastatin (+0.00138) 0.947
Hematocrit [VF] of Blood by AC (+0.133) 0.947
Coronary atherosclerosis (x1.01) 0.948
levothyroxine (+0.00134) 0.948
Degeneration of cervical intervertebral disc (x1.01) 0.948
cyclobenzaprine (+0.00132) 0.949
Degenerative joint disease of shoulder region (x1.01) 0.949
hydrochlorothiazide (+0.00131) 0.950
Diabetic - poor control (x1.01) 0.950
Joint pain of pelvic region (x1.01) 0.951
tramadol (+0.00127) 0.951
Malaise (x1.01) 0.951
Strain of supraspinatus muscle AND/OR tendon (x1.01) 0.952
Adhesive capsulitis of shoulder (x1.01) 0.952
Obstructive sleep apnea syndrome (x1.01) 0.952
omeprazole (+0.00124) 0.953
Diabetes mellitus without complication (x1.01) 0.953
Atrial fibrillation (x1.01) 0.953
Hemoglobin [M/V] in B (+0.0419) 0.954
Edema (x1.01) 0.954
Rupture of tendon of biceps, long head (x1.01) 0.954
Tear film insufficiency (x1.01) 0.955
Hypertensive retinopathy (/1.01) 0.955
esomeprazole (+0.00116) 0.955
Contusion of knee (x1.01) 0.956
Actinic keratosis (x1.01) 0.956
Cervical spondylosis without myelopathy (x1.01) 0.956
Anesthesia of skin (x1.01) 0.957
Metabolic syndrome X (x1.01) 0.957
Carbon dioxide, total [mol/V] in SP (+0.0738) 0.957
Disorder of nervous system due to diabetes mellitus (x1.01) 0.958
metoprolol (+0.00107) 0.958
Lumbosacral spondylosis without myelopathy (x1.01) 0.958
Systolic blood pressure (+0.352) 0.958
Urinary tract infectious disease (x1.01) 0.959
Sprain of shoulder rotator cuff (x1.01) 0.959
Sprain of shoulder (x1.01) 0.959
Gout (x1.01) 0.959
Erythrocytes [#/V] in B by AC (+0.0116) 0.960
Abdominal pain (x1.01) 0.960
celecoxib (+0.001) 0.960
Neoplasm of uncertain behavior of skin (x1.01) 0.960
Impingement syndrome of shoulder region (x1.01) 0.960
Expressions
−0.6 −0.4 −0.2 0.0 0.2 0.4 0.6

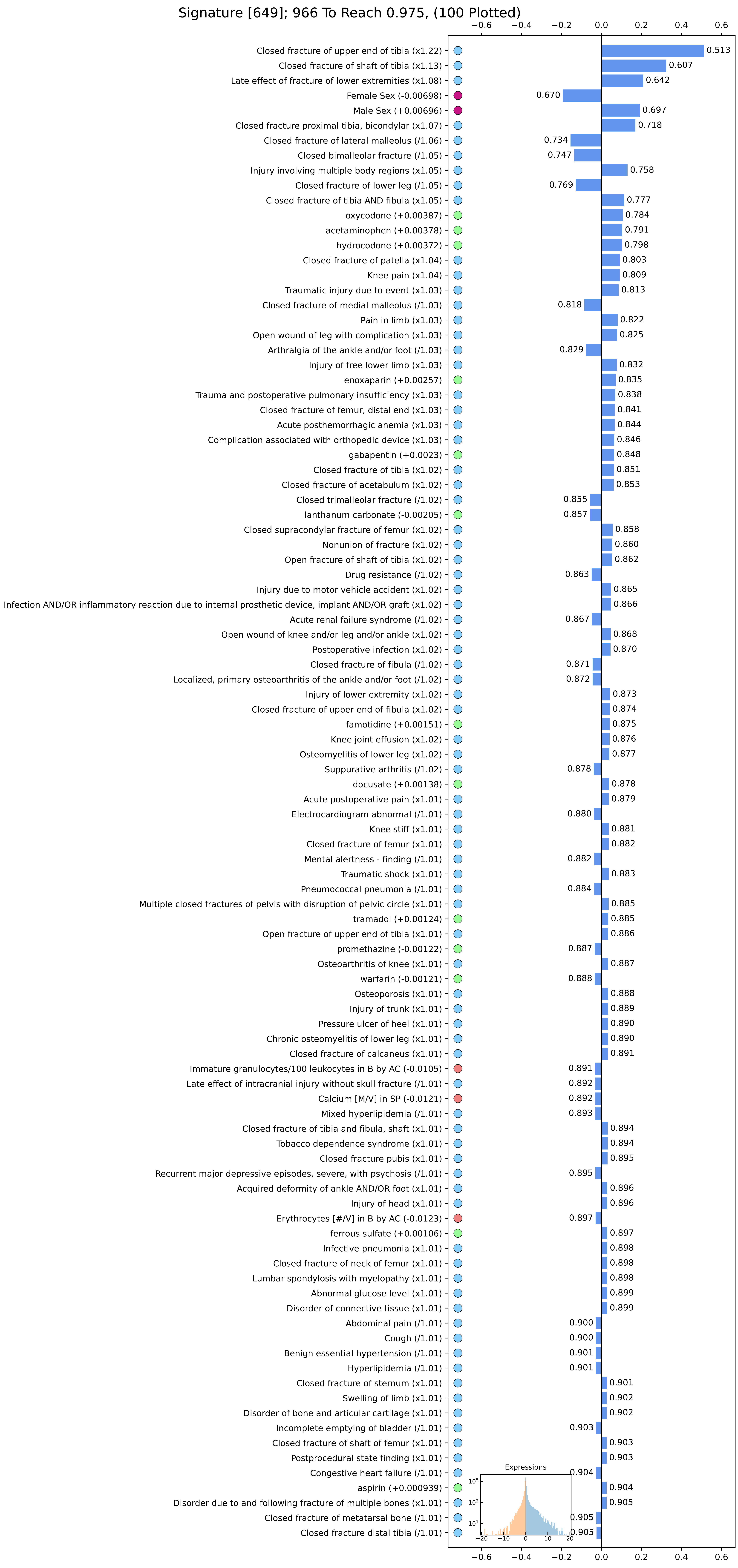

Signature [649]; 966 To Reach 0.975, (100 Plotted)
Closed fracture of upper end of tibia (x1.22) 0.513
Closed fracture of shaft of tibia (x1.13) 0.607
Late effect of fracture of lower extremities (x1.08) 0.642
Female Sex (-0.00698) 0.670
Male Sex (+0.00696) 0.697
Closed fracture proximal tibia, bicondylar (x1.07) 0.718
Closed fracture of lateral malleolus (/1.06) 0.734
Closed bimalleolar fracture (/1.05) 0.747
Injury involving multiple body regions (x1.05) 0.758
Closed fracture of lower leg (/1.05) 0.769
Closed fracture of tibia AND fibula (x1.05) 0.777
oxycodone (+0.00387) 0.784
acetaminophen (+0.00378) 0.791
hydrocodone (+0.00372) 0.798
Closed fracture of patella (x1.04) 0.803
Knee pain (x1.04) 0.809
Traumatic injury due to event (x1.03) 0.813
Closed fracture of medial malleolus (/1.03) 0.818
Pain in limb (x1.03) 0.822
Open wound of leg with complication (x1.03) 0.825
Arthralgia of the ankle and/or foot (/1.03) 0.829
Injury of free lower limb (x1.03) 0.832
enoxaparin (+0.00257) 0.835
Trauma and postoperative pulmonary insufficiency (x1.03) 0.838
Closed fracture of femur, distal end (x1.03) 0.841
Acute posthemorrhagic anemia (x1.03) 0.844
Complication associated with orthopedic device (x1.03) 0.846
gabapentin (+0.0023) 0.848
Closed fracture of tibia (x1.02) 0.851
Closed fracture of acetabulum (x1.02) 0.853
Closed trimalleolar fracture (/1.02) 0.855
lanthanum carbonate (-0.00205) 0.857
Closed supracondylar fracture of femur (x1.02) 0.858
Nonunion of fracture (x1.02) 0.860
Open fracture of shaft of tibia (x1.02) 0.862
Drug resistance (/1.02) 0.863
Injury due to motor vehicle accident (x1.02) 0.865
Infection AND/OR inflammatory reaction due to internal prosthetic device, implant AND/OR graft (x1.02) 0.866
Acute renal failure syndrome (/1.02) 0.867
Open wound of knee and/or leg and/or ankle (x1.02) 0.868
Postoperative infection (x1.02) 0.870
Closed fracture of fibula (/1.02) 0.871
Localized, primary osteoarthritis of the ankle and/or foot (/1.02) 0.872
Injury of lower extremity (x1.02) 0.873
Closed fracture of upper end of fibula (x1.02) 0.874
famotidine (+0.00151) 0.875
Knee joint effusion (x1.02) 0.876
Osteomyelitis of lower leg (x1.02) 0.877
Suppurative arthritis (/1.02) 0.878
docusate (+0.00138) 0.878
Acute postoperative pain (x1.01) 0.879
Electrocardiogram abnormal (/1.01) 0.880
Knee stiff (x1.01) 0.881
Closed fracture of femur (x1.01) 0.882
Mental alertness - finding (/1.01) 0.882
Traumatic shock (x1.01) 0.883
Pneumococcal pneumonia (/1.01) 0.884
Multiple closed fractures of pelvis with disruption of pelvic circle (x1.01) 0.885
tramadol (+0.00124) 0.885
Open fracture of upper end of tibia (x1.01) 0.886
promethazine (-0.00122) 0.887
Osteoarthritis of knee (x1.01) 0.887
warfarin (-0.00121) 0.888
Osteoporosis (x1.01) 0.888
Injury of trunk (x1.01) 0.889
Pressure ulcer of heel (x1.01) 0.890
Chronic osteomyelitis of lower leg (x1.01) 0.890
Closed fracture of calcaneus (x1.01) 0.891
Immature granulocytes/100 leukocytes in B by AC (-0.0105) 0.891
Late effect of intracranial injury without skull fracture (/1.01) 0.892
Calcium [M/V] in SP (-0.0121) 0.892
Mixed hyperlipidemia (/1.01) 0.893
Closed fracture of tibia and fibula, shaft (x1.01) 0.894
Tobacco dependence syndrome (x1.01) 0.894
Closed fracture pubis (x1.01) 0.895
Recurrent major depressive episodes, severe, with psychosis (/1.01) 0.895
Acquired deformity of ankle AND/OR foot (x1.01) 0.896
Injury of head (x1.01) 0.896
Erythrocytes [#/V] in B by AC (-0.0123) 0.897
ferrous sulfate (+0.00106) 0.897
Infective pneumonia (x1.01) 0.898
Closed fracture of neck of femur (x1.01) 0.898
Lumbar spondylosis with myelopathy (x1.01) 0.898
Abnormal glucose level (x1.01) 0.899
Disorder of connective tissue (x1.01) 0.899
Abdominal pain (/1.01) 0.900
Cough (/1.01) 0.900
Benign essential hypertension (/1.01) 0.901
Hyperlipidemia (/1.01) 0.901
Closed fracture of sternum (x1.01) 0.901
Swelling of limb (x1.01) 0.902
Disorder of bone and articular cartilage (x1.01) 0.902
Incomplete emptying of bladder (/1.01) 0.903
Closed fracture of shaft of femur (x1.01) 0.903
Postprocedural state finding (x1.01) 0.903
Congestive heart failure (/1.01) 0.904
aspirin (+0.000939) 0.904
Disorder due to and following fracture of multiple bones (x1.01) 0.905
Closed fracture of metatarsal bone (/1.01) 0.905
Closed fracture distal tibia (/1.01) 0.905
−0.6 −0.4 −0.2 0.0 0.2 0.4 0.6
Expressions
10^5 10^3 10^1
−20 −10 0 10 20

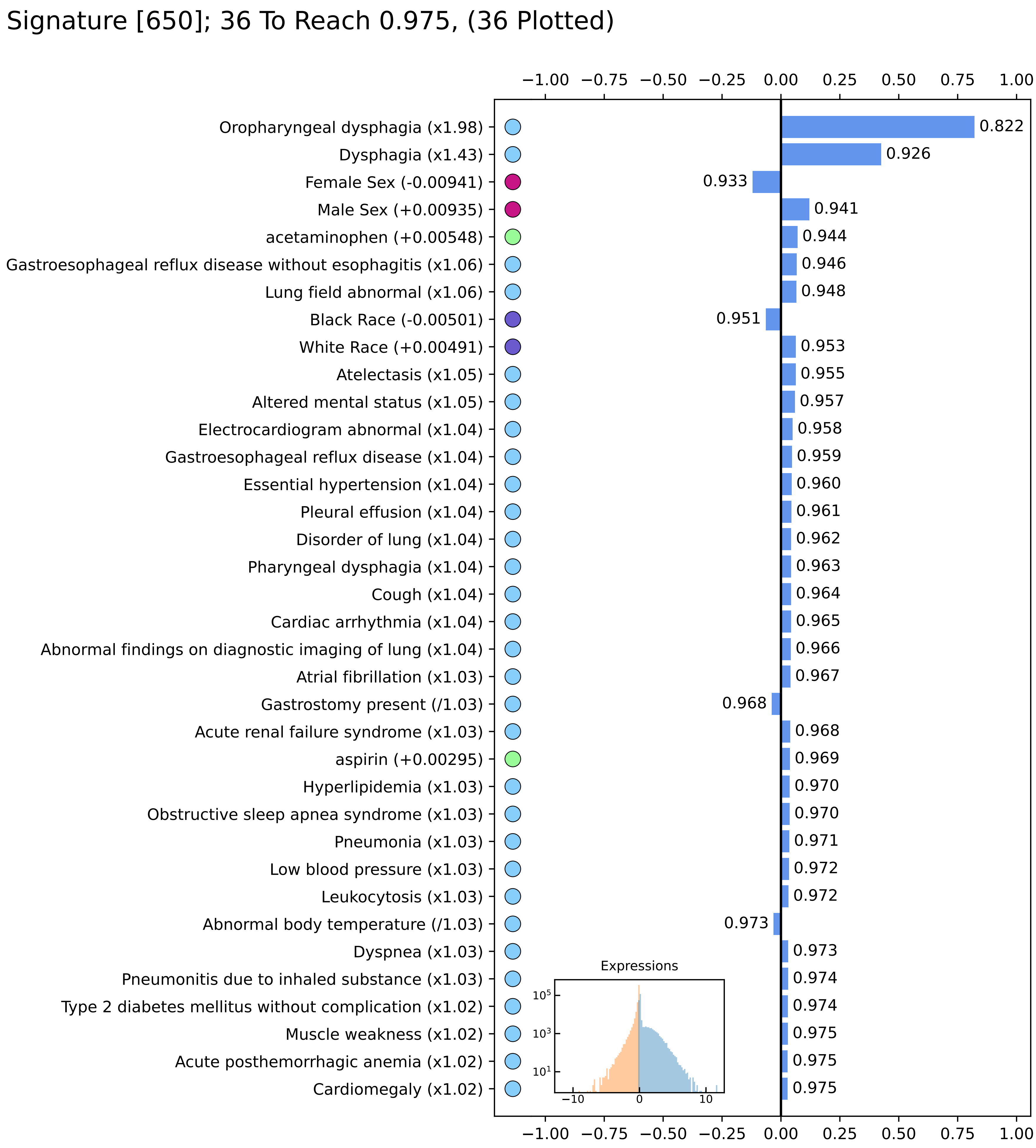

Signature [650]; 36 To Reach 0.975, (36 Plotted)
Oropharyngeal dysphagia (x1.98)
0.822
Dysphagia (x1.43)
0.926
Female Sex (-0.00941)
0.933
Male Sex (+0.00935)
0.941
acetaminophen (+0.00548)
0.944
Gastroesophageal reflux disease without esophagitis (x1.06)
0.946
Lung field abnormal (x1.06)
0.948
Black Race (-0.00501)
0.951
White Race (+0.00491)
0.953
Atelectasis (x1.05)
0.955
Altered mental status (x1.05)
0.957
Electrocardiogram abnormal (x1.04)
0.958
Gastroesophageal reflux disease (x1.04)
0.959
Essential hypertension (x1.04)
0.960
Pleural effusion (x1.04)
0.961
Disorder of lung (x1.04)
0.962
Pharyngeal dysphagia (x1.04)
0.963
Cough (x1.04)
0.964
Cardiac arrhythmia (x1.04)
0.965
Abnormal findings on diagnostic imaging of lung (x1.04)
0.966
Atrial fibrillation (x1.03)
0.967
Gastrostomy present (/1.03)
0.968
Acute renal failure syndrome (x1.03)
0.968
aspirin (+0.00295)
0.969
Hyperlipidemia (x1.03)
0.970
Obstructive sleep apnea syndrome (x1.03)
0.970
Pneumonia (x1.03)
0.971
Low blood pressure (x1.03)
0.972
Leukocytosis (x1.03)
0.972
Abnormal body temperature (/1.03)
0.973
Dyspnea (x1.03)
0.973
Pneumonitis due to inhaled substance (x1.03)
0.974
Type 2 diabetes mellitus without complication (x1.02)
0.974
Muscle weakness (x1.02)
0.975
Acute posthemorrhagic anemia (x1.02)
0.975
Cardiomegaly (x1.02)
0.975
−1.00 −0.75 −0.50 −0.25 0.00 0.25 0.50 0.75 1.00
Expressions
10^5
10^3
10^1
−10 0 10

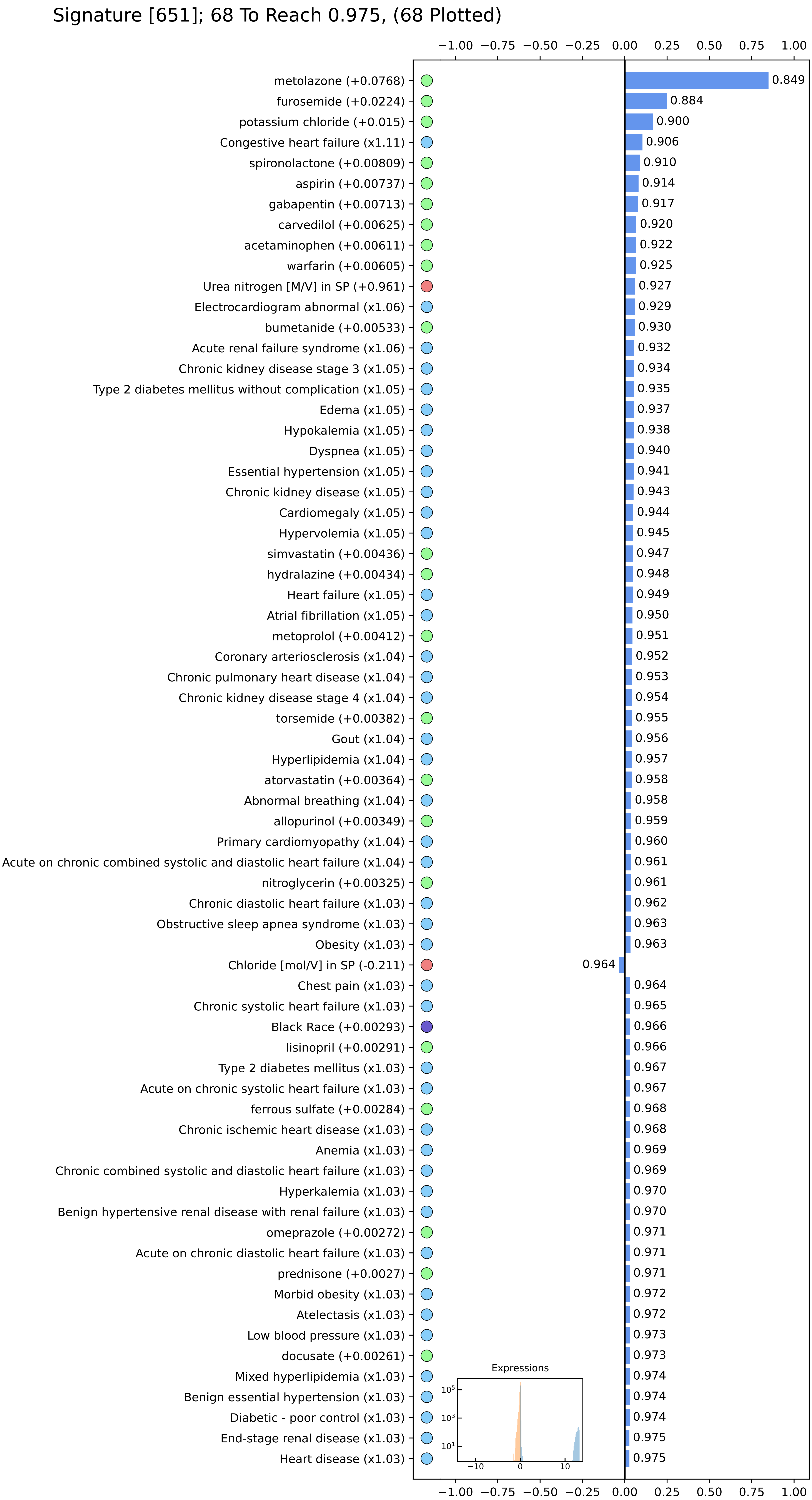

Signature [651]; 68 To Reach 0.975, (68 Plotted)
metolazone (+0.0768) 0.849
furosemide (+0.0224) 0.884
potassium chloride (+0.015) 0.900
Congestive heart failure (x1.11) 0.906
spironolactone (+0.00809) 0.910
aspirin (+0.00737) 0.914
gabapentin (+0.00713) 0.917
carvedilol (+0.00625) 0.920
acetaminophen (+0.00611) 0.922
warfarin (+0.00605) 0.925
Urea nitrogen [M/V] in SP (+0.961) 0.927
Electrocardiogram abnormal (x1.06) 0.929
bumetanide (+0.00533) 0.930
Acute renal failure syndrome (x1.06) 0.932
Chronic kidney disease stage 3 (x1.05) 0.934
Type 2 diabetes mellitus without complication (x1.05) 0.935
Edema (x1.05) 0.937
Hypokalemia (x1.05) 0.938
Dyspnea (x1.05) 0.940
Essential hypertension (x1.05) 0.941
Chronic kidney disease (x1.05) 0.943
Cardiomegaly (x1.05) 0.944
Hypervolemia (x1.05) 0.945
simvastatin (+0.00436) 0.947
hydralazine (+0.00434) 0.948
Heart failure (x1.05) 0.949
Atrial fibrillation (x1.05) 0.950
metoprolol (+0.00412) 0.951
Coronary arteriosclerosis (x1.04) 0.952
Chronic pulmonary heart disease (x1.04) 0.953
Chronic kidney disease stage 4 (x1.04) 0.954
torsemide (+0.00382) 0.955
Gout (x1.04) 0.956
Hyperlipidemia (x1.04) 0.957
atorvastatin (+0.00364) 0.958
Abnormal breathing (x1.04) 0.958
allopurinol (+0.00349) 0.959
Primary cardiomyopathy (x1.04) 0.960
Acute on chronic combined systolic and diastolic heart failure (x1.04) 0.961
nitroglycerin (+0.00325) 0.961
Chronic diastolic heart failure (x1.03) 0.962
Obstructive sleep apnea syndrome (x1.03) 0.963
Obesity (x1.03) 0.963
Chloride [mol/V] in SP (-0.211) 0.964
Chest pain (x1.03) 0.964
Chronic systolic heart failure (x1.03) 0.965
Black Race (+0.00293) 0.966
lisinopril (+0.00291) 0.966
Type 2 diabetes mellitus (x1.03) 0.967
Acute on chronic systolic heart failure (x1.03) 0.967
ferrous sulfate (+0.00284) 0.968
Chronic ischemic heart disease (x1.03) 0.968
Anemia (x1.03) 0.969
Chronic combined systolic and diastolic heart failure (x1.03) 0.969
Hyperkalemia (x1.03) 0.970
Benign hypertensive renal disease with renal failure (x1.03) 0.970
omeprazole (+0.00272) 0.971
Acute on chronic diastolic heart failure (x1.03) 0.971
prednisone (+0.0027) 0.971
Morbid obesity (x1.03) 0.972
Atelectasis (x1.03) 0.972
Low blood pressure (x1.03) 0.973
docusate (+0.00261) 0.973
Mixed hyperlipidemia (x1.03) 0.974
Benign essential hypertension (x1.03) 0.974
Diabetic - poor control (x1.03) 0.974
End-stage renal disease (x1.03) 0.975
Heart disease (x1.03) 0.975
−1.00 −0.75 −0.50 −0.25 0.00 0.25 0.50 0.75 1.00
Expressions

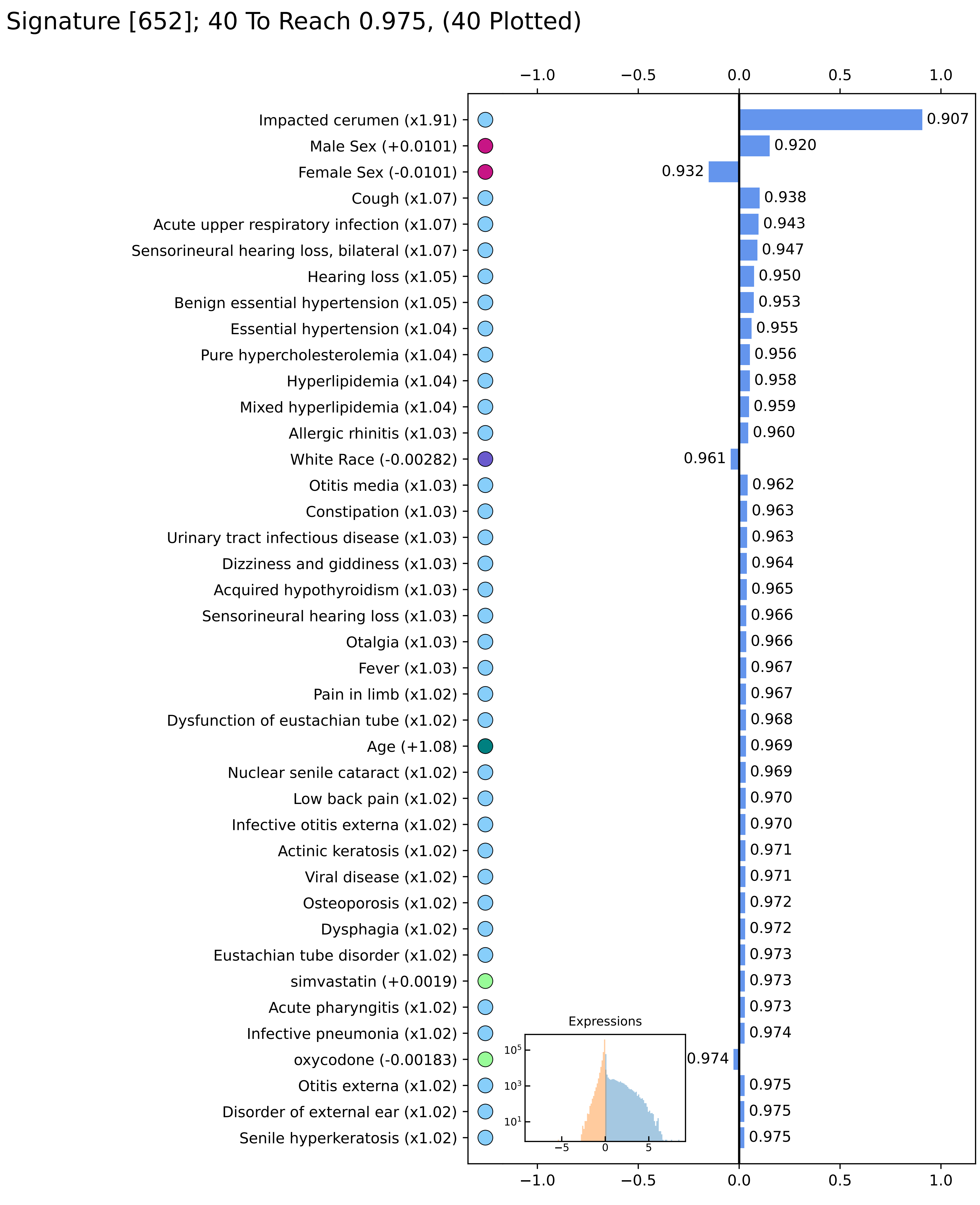

Signature [652]; 40 To Reach 0.975, (40 Plotted)
Impacted cerumen (x1.91)
Male Sex (+0.0101)
Female Sex (-0.0101)
Cough (x1.07)
Acute upper respiratory infection (x1.07)
Sensorineural hearing loss, bilateral (x1.07)
Hearing loss (x1.05)
Benign essential hypertension (x1.05)
Essential hypertension (x1.04)
Pure hypercholesterolemia (x1.04)
Hyperlipidemia (x1.04)
Mixed hyperlipidemia (x1.04)
Allergic rhinitis (x1.03)
White Race (-0.00282)
Otitis media (x1.03)
Constipation (x1.03)
Urinary tract infectious disease (x1.03)
Dizziness and giddiness (x1.03)
Acquired hypothyroidism (x1.03)
Sensorineural hearing loss (x1.03)
Otalgia (x1.03)
Fever (x1.03)
Pain in limb (x1.02)
Dysfunction of eustachian tube (x1.02)
Age (+1.08)
Nuclear senile cataract (x1.02)
Low back pain (x1.02)
Infective otitis externa (x1.02)
Actinic keratosis (x1.02)
Viral disease (x1.02)
Osteoporosis (x1.02)
Dysphagia (x1.02)
Eustachian tube disorder (x1.02)
simvastatin (+0.0019)
Acute pharyngitis (x1.02)
Infective pneumonia (x1.02)
oxycodone (-0.00183)
Otitis externa (x1.02)
Disorder of external ear (x1.02)
Senile hyperkeratosis (x1.02)
0.907
0.920
0.932
0.938
0.943
0.947
0.950
0.953
0.955
0.956
0.958
0.959
0.960
0.961
0.962
0.963
0.963
0.964
0.965
0.966
0.966
0.967
0.967
0.968
0.969
0.969
0.970
0.970
0.971
0.971
0.972
0.972
0.973
0.973
0.973
0.974
0.974
0.975
0.975
0.975
−1.0
−0.5
0.0
0.5
1.0
Expressions

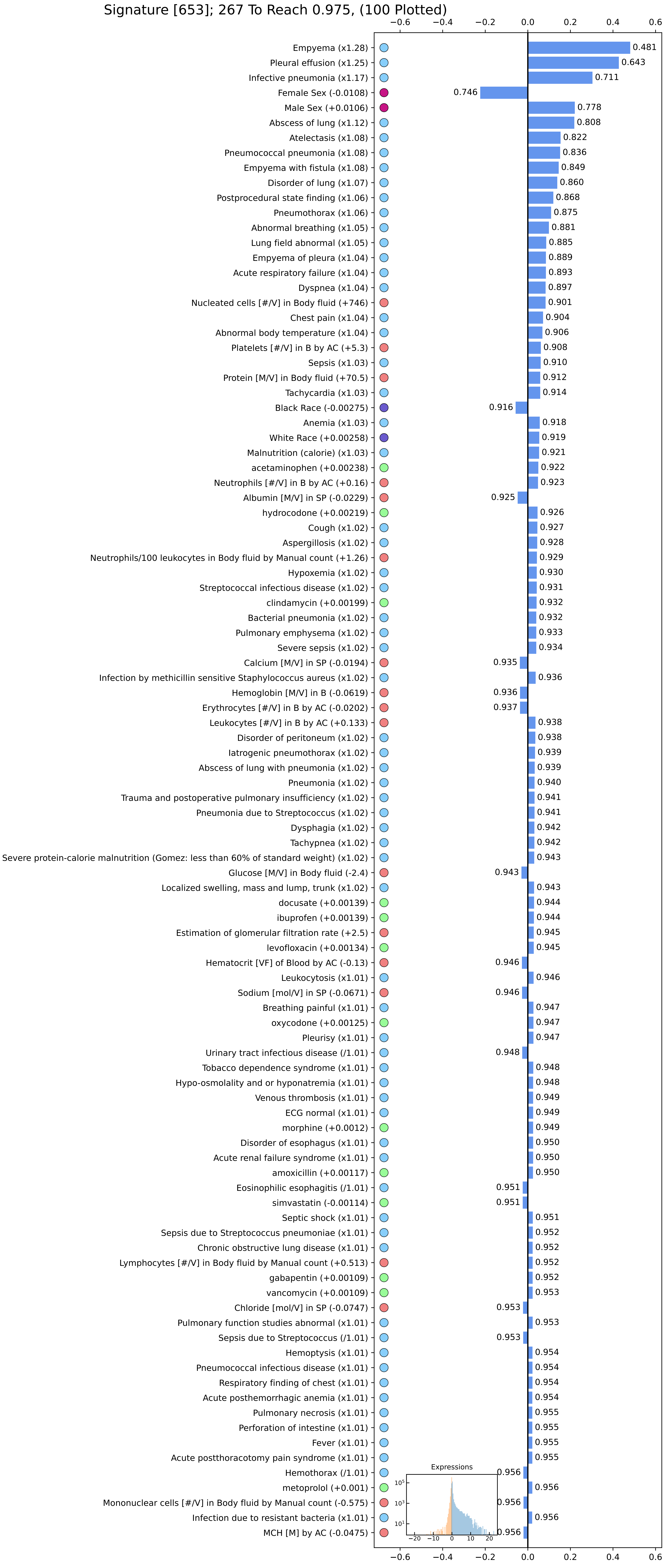

Signature [653]; 267 To Reach 0.975, (100 Plotted)
Empyema (x1.28) 0.481
Pleural effusion (x1.25) 0.643
Infective pneumonia (x1.17) 0.711
Female Sex (-0.0108) 0.746
Male Sex (+0.0106) 0.778
Abscess of lung (x1.12) 0.808
Atelectasis (x1.08) 0.822
Pneumococcal pneumonia (x1.08) 0.836
Empyema with fistula (x1.08) 0.849
Disorder of lung (x1.07) 0.860
Postprocedural state finding (x1.06) 0.868
Pneumothorax (x1.06) 0.875
Abnormal breathing (x1.05) 0.881
Lung field abnormal (x1.05) 0.885
Empyema of pleura (x1.04) 0.889
Acute respiratory failure (x1.04) 0.893
Dyspnea (x1.04) 0.897
Nucleated cells [#/V] in Body fluid (+746) 0.901
Chest pain (x1.04) 0.904
Abnormal body temperature (x1.04) 0.906
Platelets [#/V] in B by AC (+5.3) 0.908
Sepsis (x1.03) 0.910
Protein [M/V] in Body fluid (+70.5) 0.912
Tachycardia (x1.03) 0.914
Black Race (-0.00275) 0.916
Anemia (x1.03) 0.918
White Race (+0.00258) 0.919
Malnutrition (calorie) (x1.03) 0.921
acetaminophen (+0.00238) 0.922
Neutrophils [#/V] in B by AC (+0.16) 0.923
Albumin [M/V] in SP (-0.0229) 0.925
hydrocodone (+0.00219) 0.926
Cough (x1.02) 0.927
Aspergillosis (x1.02) 0.928
Neutrophils/100 leukocytes in Body fluid by Manual count (+1.26) 0.929
Hypoxemia (x1.02) 0.930
Streptococcal infectious disease (x1.02) 0.931
clindamycin (+0.00199) 0.932
Bacterial pneumonia (x1.02) 0.932
Pulmonary emphysema (x1.02) 0.933
Severe sepsis (x1.02) 0.934
Calcium [M/V] in SP (-0.0194) 0.935
Infection by methicillin sensitive Staphylococcus aureus (x1.02) 0.936
Hemoglobin [M/V] in B (-0.0619) 0.936
Erythrocytes [#/V] in B by AC (-0.0202) 0.937
Leukocytes [#/V] in B by AC (+0.133) 0.938
Disorder of peritoneum (x1.02) 0.938
Iatrogenic pneumothorax (x1.02) 0.939
Abscess of lung with pneumonia (x1.02) 0.939
Pneumonia (x1.02) 0.940
Trauma and postoperative pulmonary insufficiency (x1.02) 0.941
Pneumonia due to Streptococcus (x1.02) 0.941
Dysphagia (x1.02) 0.942
Tachypnea (x1.02) 0.942
Severe protein-calorie malnutrition (Gomez: less than 60% of standard weight) (x1.02) 0.943
Glucose [M/V] in Body fluid (-2.4) 0.943
Localized swelling, mass and lump, trunk (x1.02) 0.943
docusate (+0.00139) 0.944
ibuprofen (+0.00139) 0.944
Estimation of glomerular filtration rate (+2.5) 0.945
levofloxacin (+0.00134) 0.945
Hematocrit [VF] of Blood by AC (-0.13) 0.946
Leukocytosis (x1.01) 0.946
Sodium [mol/V] in SP (-0.0671) 0.946
Breathing painful (x1.01) 0.947
oxycodone (+0.00125) 0.947
Pleurisy (x1.01) 0.947
Urinary tract infectious disease (/1.01) 0.948
Tobacco dependence syndrome (x1.01) 0.948
Hypo-osmolality and or hyponatremia (x1.01) 0.948
Venous thrombosis (x1.01) 0.949
ECG normal (x1.01) 0.949
morphine (+0.0012) 0.949
Disorder of esophagus (x1.01) 0.950
Acute renal failure syndrome (x1.01) 0.950
amoxicillin (+0.00117) 0.950
Eosinophilic esophagitis (/1.01) 0.951
simvastatin (-0.00114) 0.951
Septic shock (x1.01) 0.951
Sepsis due to Streptococcus pneumoniae (x1.01) 0.952
Chronic obstructive lung disease (x1.01) 0.952
Lymphocytes [#/V] in Body fluid by Manual count (+0.513) 0.952
gabapentin (+0.00109) 0.952
vancomycin (+0.00109) 0.953
Chloride [mol/V] in SP (-0.0747) 0.953
Pulmonary function studies abnormal (x1.01) 0.953
Sepsis due to Streptococcus (/1.01) 0.953
Hemoptysis (x1.01) 0.954
Pneumococcal infectious disease (x1.01) 0.954
Respiratory finding of chest (x1.01) 0.954
Acute posthemorrhagic anemia (x1.01) 0.954
Pulmonary necrosis (x1.01) 0.955
Perforation of intestine (x1.01) 0.955
Fever (x1.01) 0.955
Acute postthoracotomy pain syndrome (x1.01) 0.955
Hemothorax (/1.01) 0.956
metoprolol (+0.001) 0.956
Mononuclear cells [#/V] in Body fluid by Manual count (-0.575) 0.956
Infection due to resistant bacteria (x1.01) 0.956
MCH [M] by AC (-0.0475) 0.956
−0.6 −0.4 −0.2 0.0 0.2 0.4 0.6
Expressions

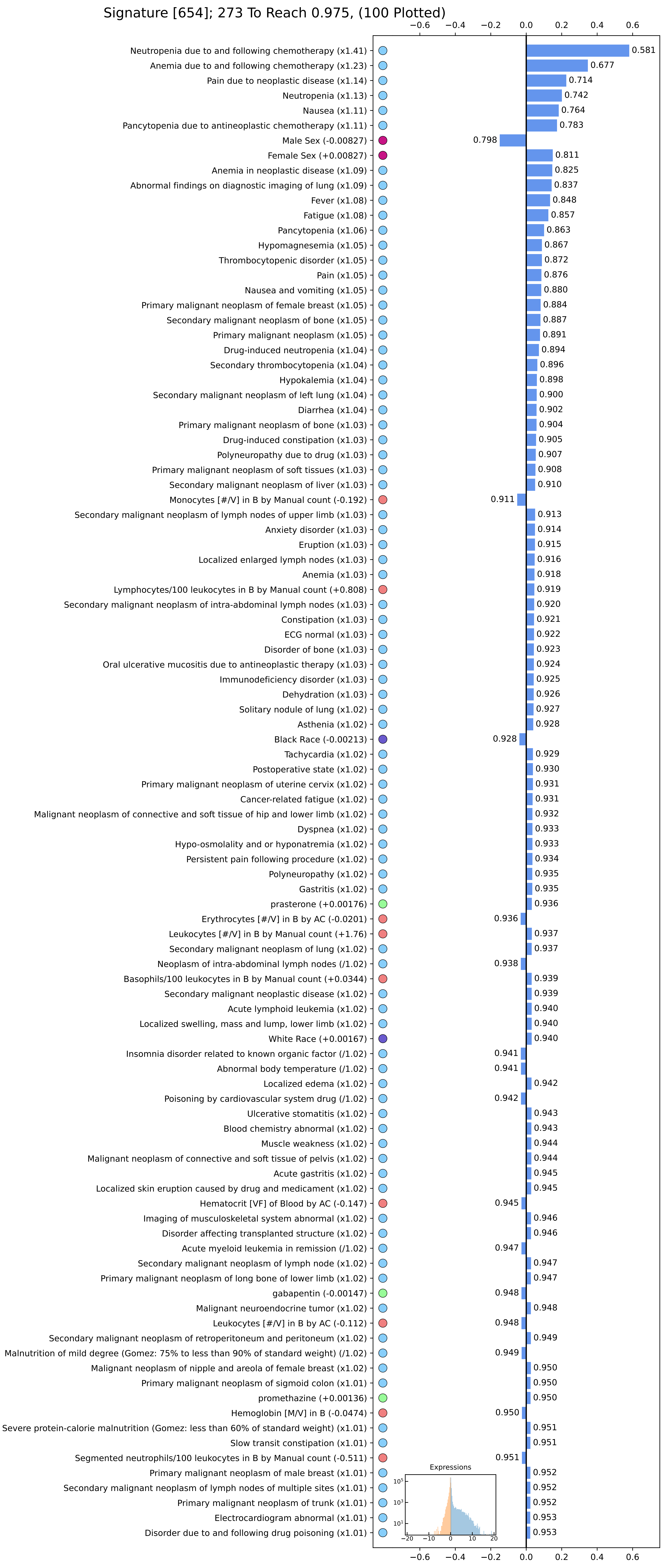

Signature [654]; 273 To Reach 0.975, (100 Plotted)
Neutropenia due to and following chemotherapy (x1.41)
0.581
Anemia due to and following chemotherapy (x1.23)
0.677
Pain due to neoplastic disease (x1.14)
0.714
Neutropenia (x1.13)
0.742
Nausea (x1.11)
0.764
Pancytopenia due to antineoplastic chemotherapy (x1.11)
0.783
Male Sex (-0.00827)
0.798
Female Sex (+0.00827)
0.811
Anemia in neoplastic disease (x1.09)
0.825
Abnormal findings on diagnostic imaging of lung (x1.09)
0.837
Fever (x1.08)
0.848
Fatigue (x1.08)
0.857
Pancytopenia (x1.06)
0.863
Hypomagnesemia (x1.05)
0.867
Thrombocytopenic disorder (x1.05)
0.872
Pain (x1.05)
0.876
Nausea and vomiting (x1.05)
0.880
Primary malignant neoplasm of female breast (x1.05)
0.884
Secondary malignant neoplasm of bone (x1.05)
0.887
Primary malignant neoplasm (x1.05)
0.891
Drug-induced neutropenia (x1.04)
0.894
Secondary thrombocytopenia (x1.04)
0.896
Hypokalemia (x1.04)
0.898
Secondary malignant neoplasm of left lung (x1.04)
0.900
Diarrhea (x1.04)
0.902
Primary malignant neoplasm of bone (x1.03)
0.904
Drug-induced constipation (x1.03)
0.905
Polyneuropathy due to drug (x1.03)
0.907
Primary malignant neoplasm of soft tissues (x1.03)
0.908
Secondary malignant neoplasm of liver (x1.03)
0.910
Monocytes [#/V] in B by Manual count (-0.192)
0.911
Secondary malignant neoplasm of lymph nodes of upper limb (x1.03)
0.913
Anxiety disorder (x1.03)
0.914
Eruption (x1.03)
0.915
Localized enlarged lymph nodes (x1.03)
0.916
Anemia (x1.03)
0.918
Lymphocytes/100 leukocytes in B by Manual count (+0.808)
0.919
Secondary malignant neoplasm of intra-abdominal lymph nodes (x1.03)
0.920
Constipation (x1.03)
0.921
ECG normal (x1.03)
0.922
Disorder of bone (x1.03)
0.923
Oral ulcerative mucositis due to antineoplastic therapy (x1.03)
0.924
Immunodeficiency disorder (x1.03)
0.925
Dehydration (x1.03)
0.926
Solitary nodule of lung (x1.02)
0.927
Asthenia (x1.02)
0.928
Black Race (-0.00213)
0.928
Tachycardia (x1.02)
0.929
Postoperative state (x1.02)
0.930
Primary malignant neoplasm of uterine cervix (x1.02)
0.931
Cancer-related fatigue (x1.02)
0.931
Malignant neoplasm of connective and soft tissue of hip and lower limb (x1.02)
0.932
Dyspnea (x1.02)
0.933
Hypo-osmolality and or hyponatremia (x1.02)
0.933
Persistent pain following procedure (x1.02)
0.934
Polyneuropathy (x1.02)
0.935
Gastritis (x1.02)
0.935
prasterone (+0.00176)
0.936
Erythrocytes [#/V] in B by AC (-0.0201)
0.936
Leukocytes [#/V] in B by Manual count (+1.76)
0.937
Secondary malignant neoplasm of lung (x1.02)
0.937
Neoplasm of intra-abdominal lymph nodes (/1.02)
0.938
Basophils/100 leukocytes in B by Manual count (+0.0344)
0.939
Secondary malignant neoplastic disease (x1.02)
0.939
Acute lymphoid leukemia (x1.02)
0.940
Localized swelling, mass and lump, lower limb (x1.02)
0.940
White Race (+0.00167)
0.940
Insomnia disorder related to known organic factor (/1.02)
0.941
Abnormal body temperature (/1.02)
0.941
Localized edema (x1.02)
0.942
Poisoning by cardiovascular system drug (/1.02)
0.942
Ulcerative stomatitis (x1.02)
0.943
Blood chemistry abnormal (x1.02)
0.943
Muscle weakness (x1.02)
0.944
Malignant neoplasm of connective and soft tissue of pelvis (x1.02)
0.944
Acute gastritis (x1.02)
0.945
Localized skin eruption caused by drug and medicament (x1.02)
0.945
Hematocrit [VF] of Blood by AC (-0.147)
0.945
Imaging of musculoskeletal system abnormal (x1.02)
0.946
Disorder affecting transplanted structure (x1.02)
0.946
Acute myeloid leukemia in remission (/1.02)
0.947
Secondary malignant neoplasm of lymph node (x1.02)
0.947
Primary malignant neoplasm of long bone of lower limb (x1.02)
0.947
gabapentin (-0.00147)
0.948
Malignant neuroendocrine tumor (x1.02)
0.948
Leukocytes [#/V] in B by AC (-0.112)
0.948
Secondary malignant neoplasm of retroperitoneum and peritoneum (x1.02)
0.949
Malnutrition of mild degree (Gomez: 75% to less than 90% of standard weight) (/1.02)
0.949
Malignant neoplasm of nipple and areola of female breast (x1.02)
0.950
Primary malignant neoplasm of sigmoid colon (x1.01)
0.950
promethazine (+0.00136)
0.950
Hemoglobin [M/V] in B (-0.0474)
0.950
Severe protein-calorie malnutrition (Gomez: less than 60% of standard weight) (x1.01)
0.951
Slow transit constipation (x1.01)
0.951
Segmented neutrophils/100 leukocytes in B by Manual count (-0.511)
0.951
Primary malignant neoplasm of male breast (x1.01)
0.952
Secondary malignant neoplasm of lymph nodes of multiple sites (x1.01)
0.952
Primary malignant neoplasm of trunk (x1.01)
0.952
Electrocardiogram abnormal (x1.01)
0.953
Disorder due to and following drug poisoning (x1.01)
0.953
−0.6 −0.4 −0.2 0.0 0.2 0.4 0.6
Expressions
−20 −10 0 10 20

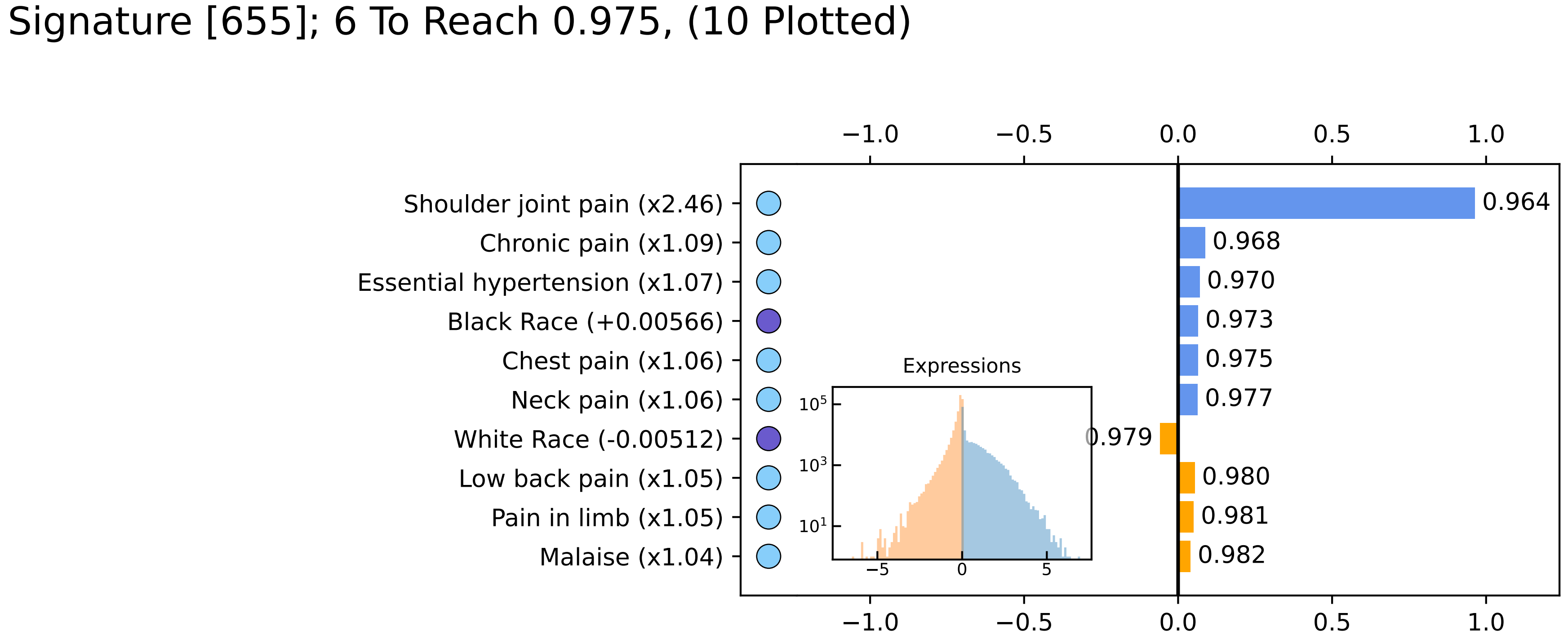

Signature [655]; 6 To Reach 0.975, (10 Plotted)
Shoulder joint pain (x2.46)
Chronic pain (x1.09)
Essential hypertension (x1.07)
Black Race (+0.00566)
Chest pain (x1.06)
Neck pain (x1.06)
White Race (-0.00512)
Low back pain (x1.05)
Pain in limb (x1.05)
Malaise (x1.04)
0.964
0.968
0.970
0.973
0.975
0.977
0.979
0.980
0.981
0.982
Expressions
−1.0
−0.5
0.0
0.5
1.0

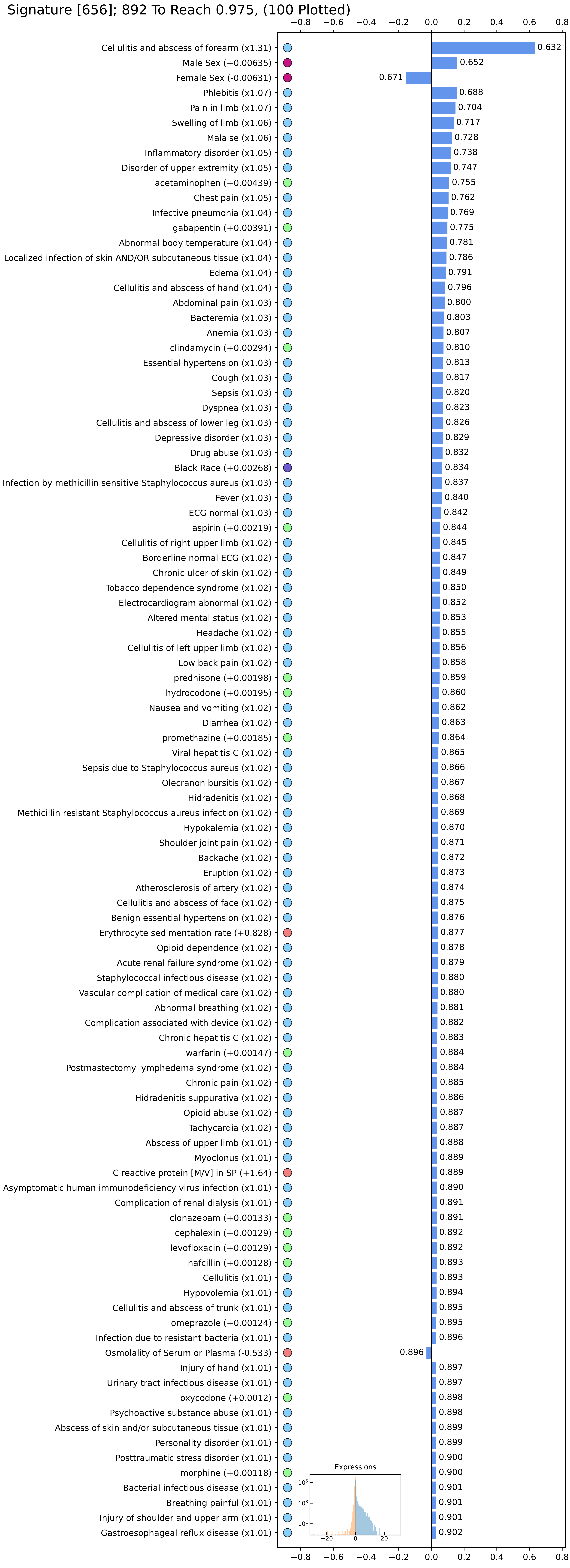
Signature [656]; 892 To Reach 0.975, (100 Plotted)
−0.8 −0.6 −0.4 −0.2 0.0 0.2 0.4 0.6 0.8
Cellulitis and abscess of forearm (x1.31) 0.632
Male Sex (+0.00635) 0.652
Female Sex (-0.00631) 0.671
Phlebitis (x1.07) 0.688
Pain in limb (x1.07) 0.704
Swelling of limb (x1.06) 0.717
Malaise (x1.06) 0.728
Inflammatory disorder (x1.05) 0.738
Disorder of upper extremity (x1.05) 0.747
acetaminophen (+0.00439) 0.755
Chest pain (x1.05) 0.762
Infective pneumonia (x1.04) 0.769
gabapentin (+0.00391) 0.775
Abnormal body temperature (x1.04) 0.781
Localized infection of skin AND/OR subcutaneous tissue (x1.04) 0.786
Edema (x1.04) 0.791
Cellulitis and abscess of hand (x1.04) 0.796
Abdominal pain (x1.03) 0.800
Bacteremia (x1.03) 0.803
Anemia (x1.03) 0.807
clindamycin (+0.00294) 0.810
Essential hypertension (x1.03) 0.813
Cough (x1.03) 0.817
Sepsis (x1.03) 0.820
Dyspnea (x1.03) 0.823
Cellulitis and abscess of lower leg (x1.03) 0.826
Depressive disorder (x1.03) 0.829
Drug abuse (x1.03) 0.832
Black Race (+0.00268) 0.834
Infection by methicillin sensitive Staphylococcus aureus (x1.03) 0.837
Fever (x1.03) 0.840
ECG normal (x1.03) 0.842
aspirin (+0.00219) 0.844
Cellulitis of right upper limb (x1.02) 0.845
Borderline normal ECG (x1.02) 0.847
Chronic ulcer of skin (x1.02) 0.849
Tobacco dependence syndrome (x1.02) 0.850
Electrocardiogram abnormal (x1.02) 0.852
Altered mental status (x1.02) 0.853
Headache (x1.02) 0.855
Cellulitis of left upper limb (x1.02) 0.856
Low back pain (x1.02) 0.858
prednisone (+0.00198) 0.859
hydrocodone (+0.00195) 0.860
Nausea and vomiting (x1.02) 0.862
Diarrhea (x1.02) 0.863
promethazine (+0.00185) 0.864
Viral hepatitis C (x1.02) 0.865
Sepsis due to Staphylococcus aureus (x1.02) 0.866
Olecranon bursitis (x1.02) 0.867
Hidradenitis (x1.02) 0.868
Methicillin resistant Staphylococcus aureus infection (x1.02) 0.869
Hypokalemia (x1.02) 0.870
Shoulder joint pain (x1.02) 0.871
Backache (x1.02) 0.872
Eruption (x1.02) 0.873
Atherosclerosis of artery (x1.02) 0.874
Cellulitis and abscess of face (x1.02) 0.875
Benign essential hypertension (x1.02) 0.876
Erythrocyte sedimentation rate (+0.828) 0.877
Opioid dependence (x1.02) 0.878
Acute renal failure syndrome (x1.02) 0.879
Staphylococcal infectious disease (x1.02) 0.880
Vascular complication of medical care (x1.02) 0.880
Abnormal breathing (x1.02) 0.881
Complication associated with device (x1.02) 0.882
Chronic hepatitis C (x1.02) 0.883
warfarin (+0.00147) 0.884
Postmastectomy lymphedema syndrome (x1.02) 0.884
Chronic pain (x1.02) 0.885
Hidradenitis suppurativa (x1.02) 0.886
Opioid abuse (x1.02) 0.887
Tachycardia (x1.02) 0.887
Abscess of upper limb (x1.01) 0.888
Myoclonus (x1.01) 0.889
C reactive protein [M/V] in SP (+1.64) 0.889
Asymptomatic human immunodeficiency virus infection (x1.01) 0.890
Complication of renal dialysis (x1.01) 0.891
clonazepam (+0.00133) 0.891
cephalexin (+0.00129) 0.892
levofloxacin (+0.00129) 0.892
nafcillin (+0.00128) 0.893
Cellulitis (x1.01) 0.893
Hypovolemia (x1.01) 0.894
Cellulitis and abscess of trunk (x1.01) 0.895
omeprazole (+0.00124) 0.895
Infection due to resistant bacteria (x1.01) 0.896
Osmolality of Serum or Plasma (-0.533) 0.896
Injury of hand (x1.01) 0.897
Urinary tract infectious disease (x1.01) 0.897
oxycodone (+0.0012) 0.898
Psychoactive substance abuse (x1.01) 0.898
Abscess of skin and/or subcutaneous tissue (x1.01) 0.899
Personality disorder (x1.01) 0.899
Posttraumatic stress disorder (x1.01) 0.900
morphine (+0.00118) 0.900
Bacterial infectious disease (x1.01) 0.901
Breathing painful (x1.01) 0.901
Injury of shoulder and upper arm (x1.01) 0.901
Gastroesophageal reflux disease (x1.01) 0.902
Expressions
10^5
10^3
10^1
−20 0 20
−0.8 −0.6 −0.4 −0.2 0.0 0.2 0.4 0.6 0.8

Signature [657]; 67 To Reach 0.975, (67 Plotted)

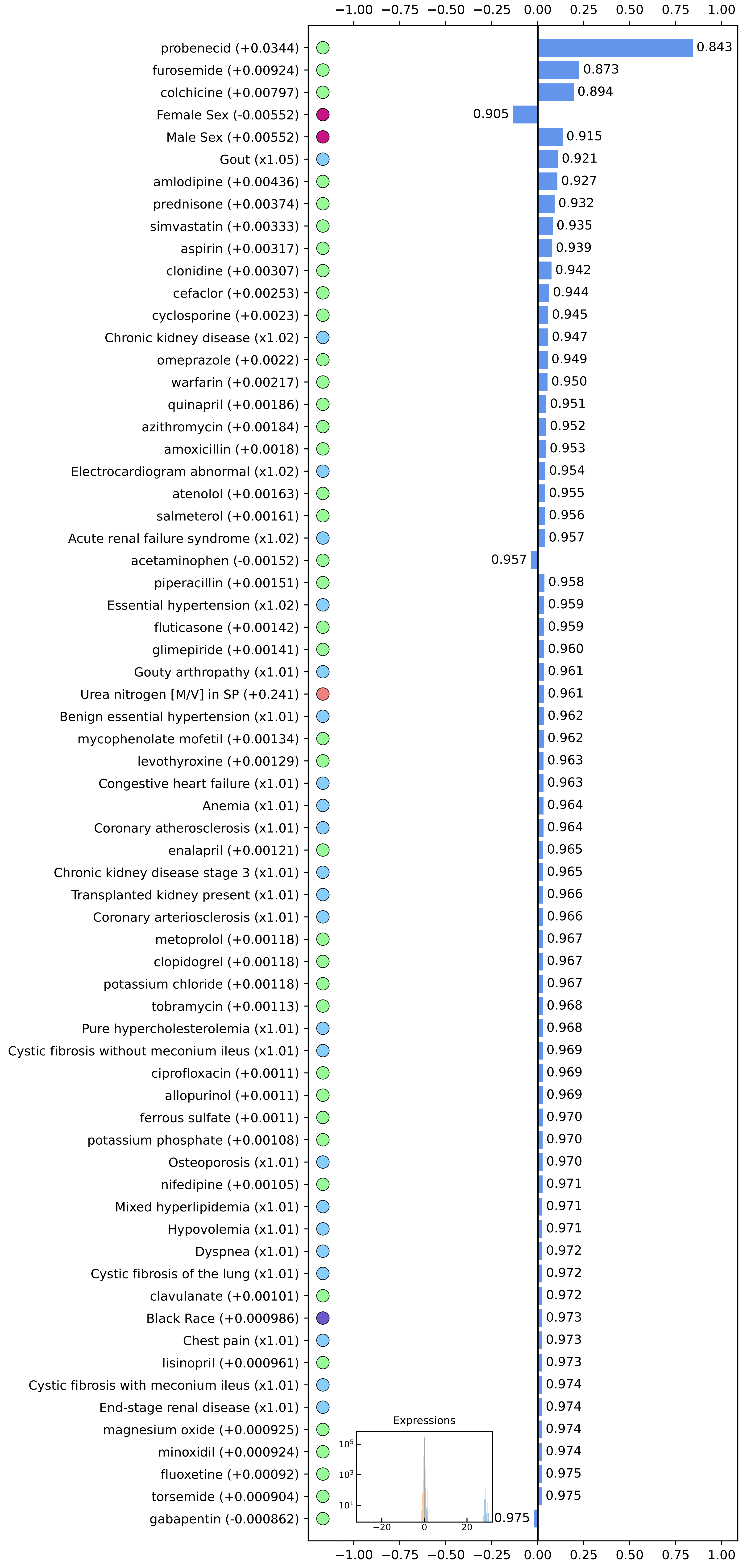

Signature [658]; 72 To Reach 0.975, (72 Plotted)

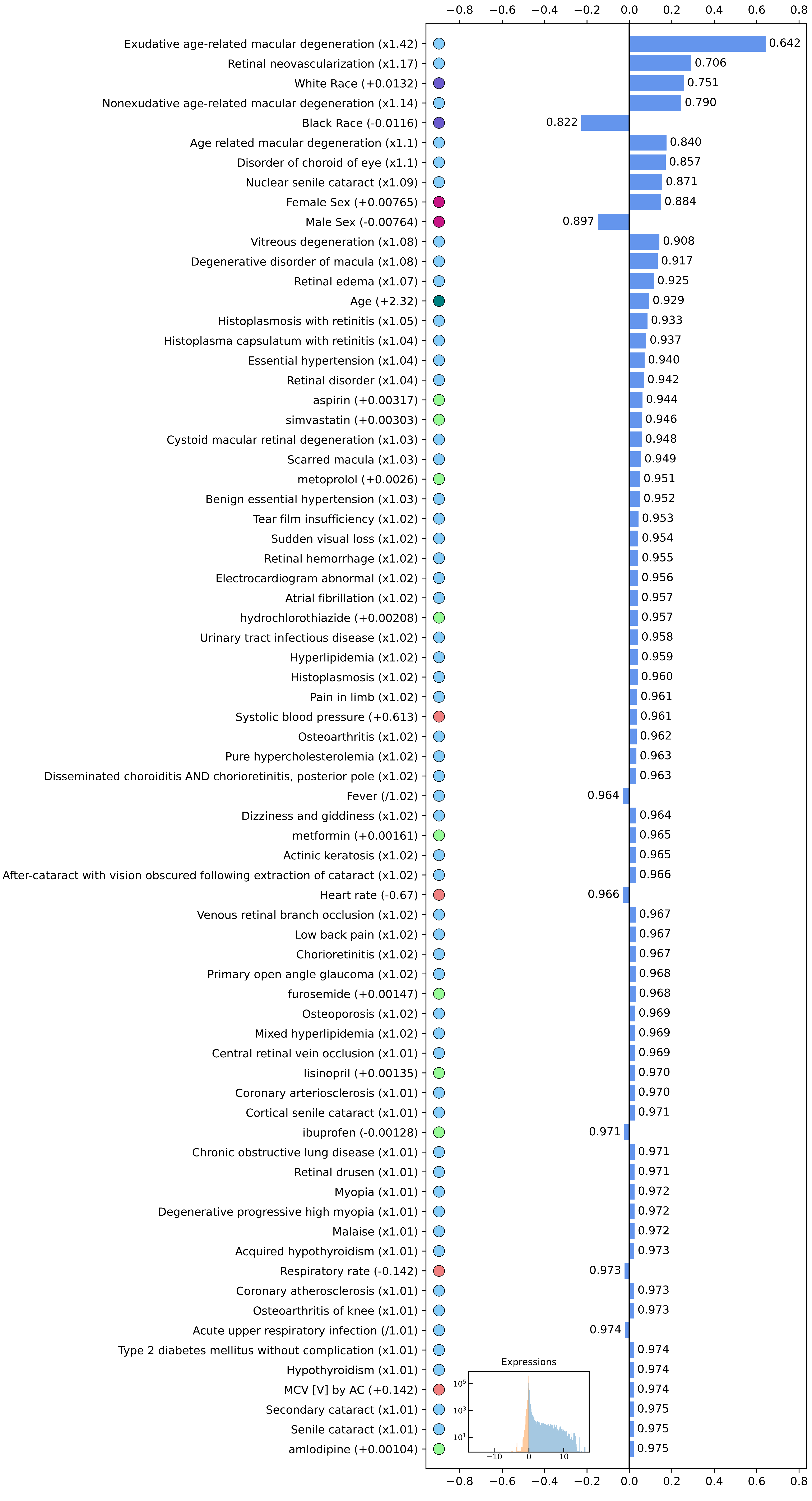

Signature [659]; 1 To Reach 0.975, (10 Plotted)

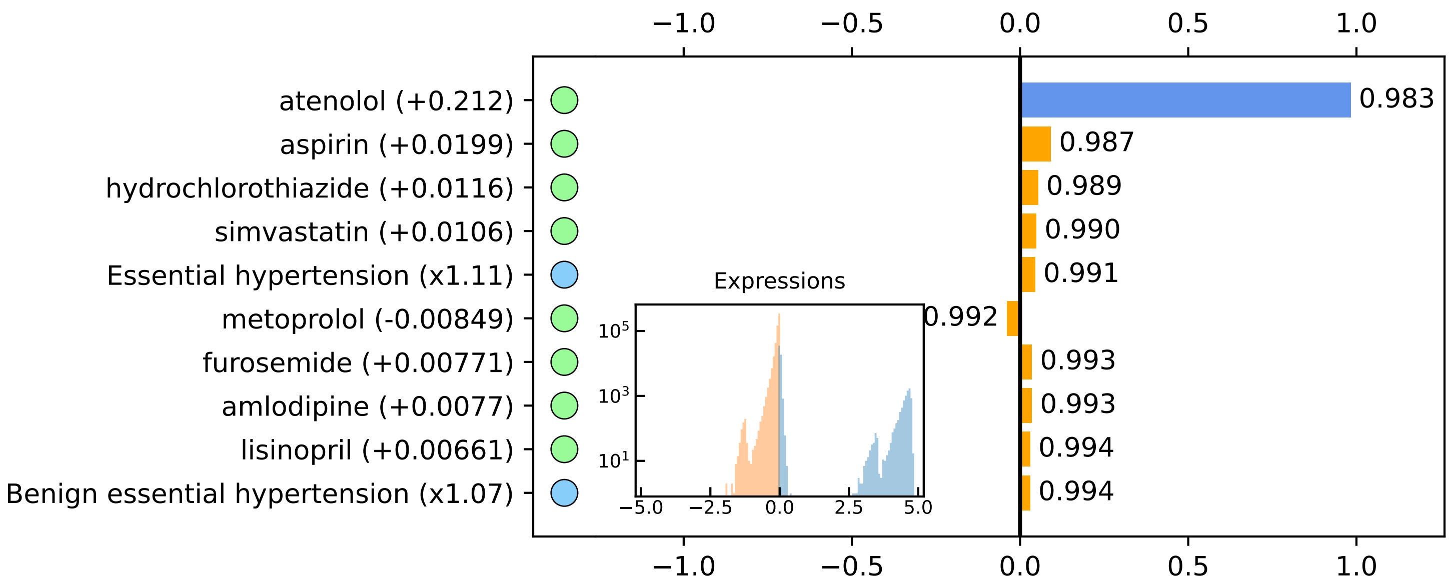

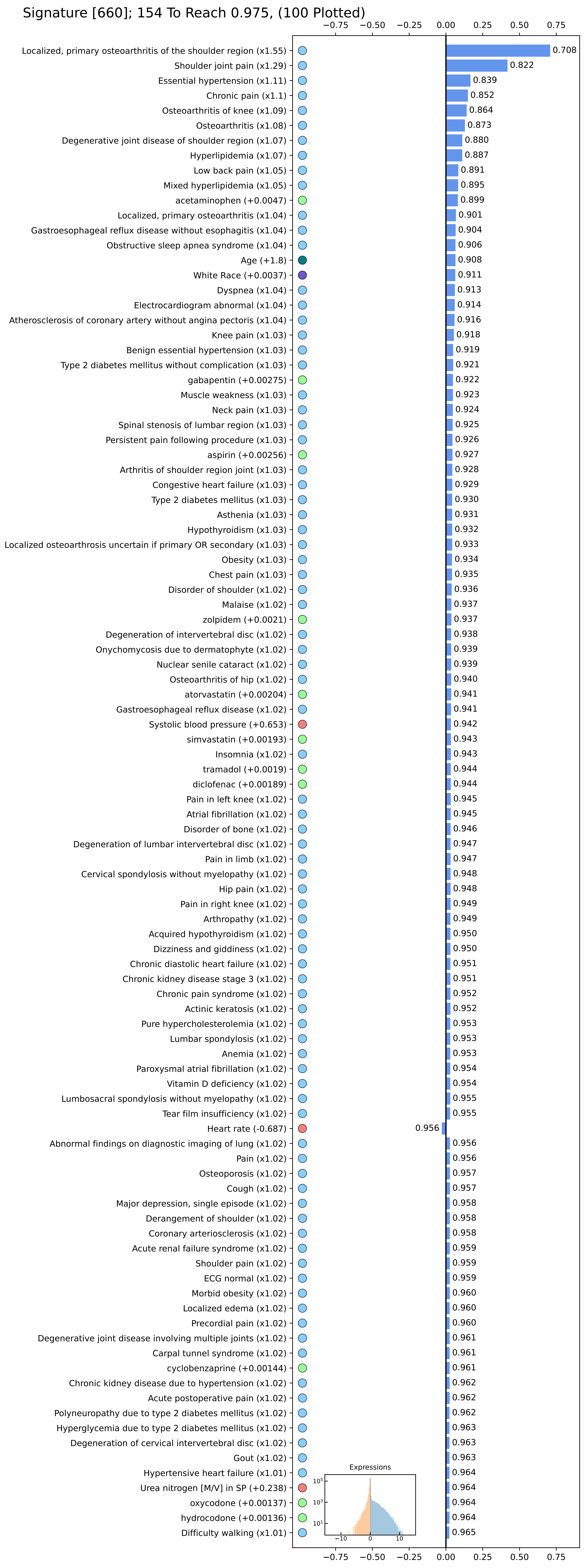

Signature [660]; 154 To Reach 0.975, (100 Plotted)
Localized, primary osteoarthritis of the shoulder region (x1.55) 0.708
Shoulder joint pain (x1.29) 0.822
Essential hypertension (x1.11) 0.839
Chronic pain (x1.1) 0.852
Osteoarthritis of knee (x1.09) 0.864
Osteoarthritis (x1.08) 0.873
Degenerative joint disease of shoulder region (x1.07) 0.880
Hyperlipidemia (x1.07) 0.887
Low back pain (x1.05) 0.891
Mixed hyperlipidemia (x1.05) 0.895
acetaminophen (+0.0047) 0.899
Localized, primary osteoarthritis (x1.04) 0.901
Gastroesophageal reflux disease without esophagitis (x1.04) 0.904
Obstructive sleep apnea syndrome (x1.04) 0.906
Age (+1.8) 0.908
White Race (+0.0037) 0.911
Dyspnea (x1.04) 0.913
Electrocardiogram abnormal (x1.04) 0.914
Atherosclerosis of coronary artery without angina pectoris (x1.04) 0.916
Knee pain (x1.03) 0.918
Benign essential hypertension (x1.03) 0.919
Type 2 diabetes mellitus without complication (x1.03) 0.921
gabapentin (+0.00275) 0.922
Muscle weakness (x1.03) 0.923
Neck pain (x1.03) 0.924
Spinal stenosis of lumbar region (x1.03) 0.925
Persistent pain following procedure (x1.03) 0.926
aspirin (+0.00256) 0.927
Arthritis of shoulder region joint (x1.03) 0.928
Congestive heart failure (x1.03) 0.929
Type 2 diabetes mellitus (x1.03) 0.930
Asthenia (x1.03) 0.931
Hypothyroidism (x1.03) 0.932
Localized osteoarthrosis uncertain if primary OR secondary (x1.03) 0.933
Obesity (x1.03) 0.934
Chest pain (x1.03) 0.935
Disorder of shoulder (x1.02) 0.936
Malaise (x1.02) 0.937
zolpidem (+0.0021) 0.937
Degeneration of intervertebral disc (x1.02) 0.938
Onychomycosis due to dermatophyte (x1.02) 0.939
Nuclear senile cataract (x1.02) 0.939
Osteoarthritis of hip (x1.02) 0.940
atorvastatin (+0.00204) 0.941
Gastroesophageal reflux disease (x1.02) 0.941
Systolic blood pressure (+0.653) 0.942
simvastatin (+0.00193) 0.943
Insomnia (x1.02) 0.943
tramadol (+0.0019) 0.944
diclofenac (+0.00189) 0.944
Pain in left knee (x1.02) 0.945
Atrial fibrillation (x1.02) 0.945
Disorder of bone (x1.02) 0.946
Degeneration of lumbar intervertebral disc (x1.02) 0.947
Pain in limb (x1.02) 0.947
Cervical spondylosis without myelopathy (x1.02) 0.948
Hip pain (x1.02) 0.948
Pain in right knee (x1.02) 0.949
Arthropathy (x1.02) 0.949
Acquired hypothyroidism (x1.02) 0.950
Dizziness and giddiness (x1.02) 0.950
Chronic diastolic heart failure (x1.02) 0.951
Chronic kidney disease stage 3 (x1.02) 0.951
Chronic pain syndrome (x1.02) 0.952
Actinic keratosis (x1.02) 0.952
Pure hypercholesterolemia (x1.02) 0.953
Lumbar spondylosis (x1.02) 0.953
Anemia (x1.02) 0.953
Paroxysmal atrial fibrillation (x1.02) 0.954
Vitamin D deficiency (x1.02) 0.954
Lumbosacral spondylosis without myelopathy (x1.02) 0.955
Tear film insufficiency (x1.02) 0.955
Heart rate (-0.687) 0.956
Abnormal findings on diagnostic imaging of lung (x1.02) 0.956
Pain (x1.02) 0.956
Osteoporosis (x1.02) 0.957
Cough (x1.02) 0.957
Major depression, single episode (x1.02) 0.958
Derangement of shoulder (x1.02) 0.958
Coronary arteriosclerosis (x1.02) 0.958
Acute renal failure syndrome (x1.02) 0.959
Shoulder pain (x1.02) 0.959
ECG normal (x1.02) 0.959
Morbid obesity (x1.02) 0.960
Localized edema (x1.02) 0.960
Precordial pain (x1.02) 0.960
Degenerative joint disease involving multiple joints (x1.02) 0.961
Carpal tunnel syndrome (x1.02) 0.961
cyclobenzaprine (+0.00144) 0.961
Chronic kidney disease due to hypertension (x1.02) 0.962
Acute postoperative pain (x1.02) 0.962
Polyneuropathy due to type 2 diabetes mellitus (x1.02) 0.962
Hyperglycemia due to type 2 diabetes mellitus (x1.02) 0.963
Degeneration of cervical intervertebral disc (x1.02) 0.963
Gout (x1.02) 0.963
Hypertensive heart failure (x1.01) 0.964
Urea nitrogen [M/V] in SP (+0.238) 0.964
oxycodone (+0.00137) 0.964
hydrocodone (+0.00136) 0.964
Difficulty walking (x1.01) 0.965
−0.75 −0.50 −0.25 0.00 0.25 0.50 0.75
Expressions
−10 0 10

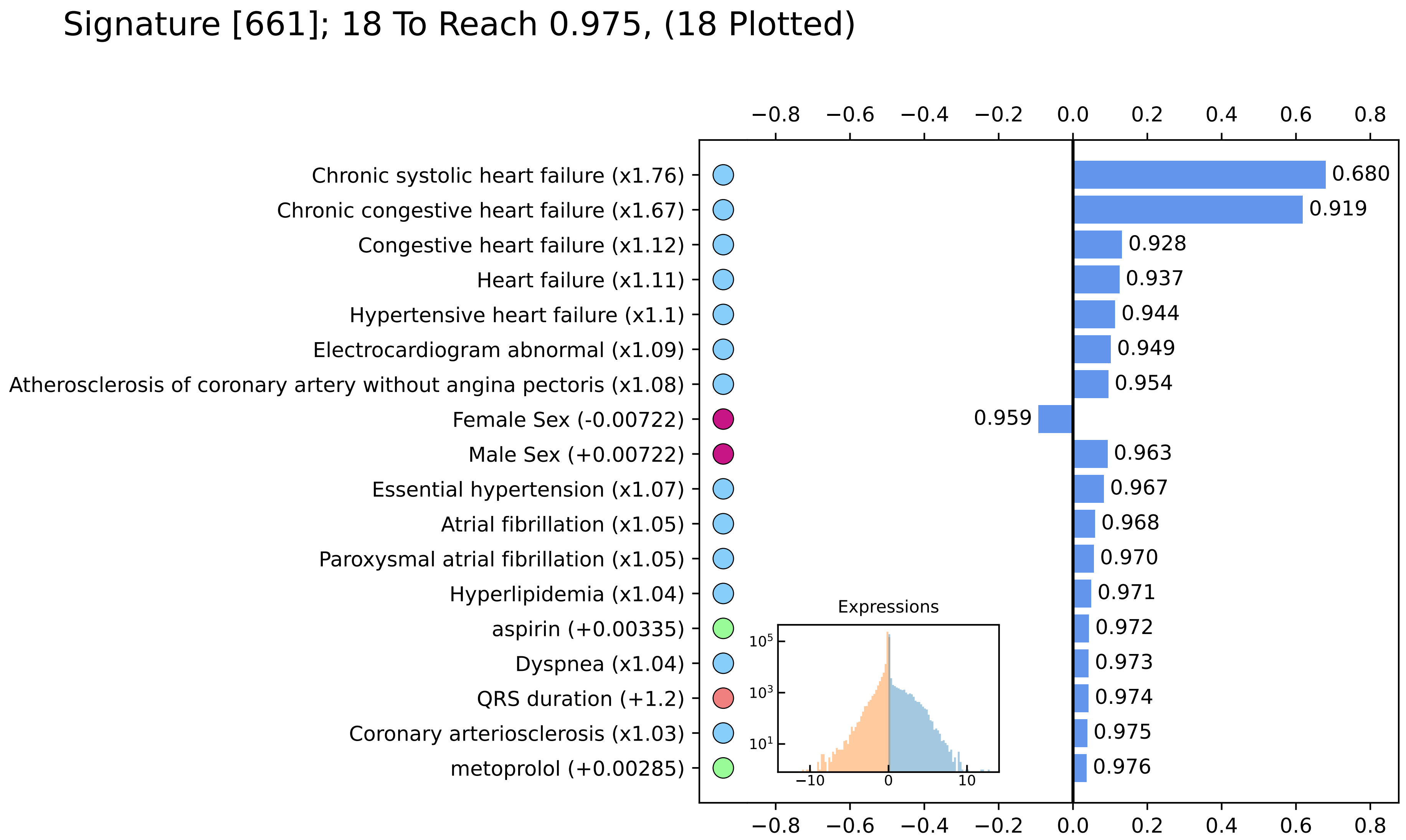

Signature [661]; 18 To Reach 0.975, (18 Plotted)
Chronic systolic heart failure (x1.76)
Chronic congestive heart failure (x1.67)
Congestive heart failure (x1.12)
Heart failure (x1.11)
Hypertensive heart failure (x1.1)
Electrocardiogram abnormal (x1.09)
Atherosclerosis of coronary artery without angina pectoris (x1.08)
Female Sex (-0.00722)
Male Sex (+0.00722)
Essential hypertension (x1.07)
Atrial fibrillation (x1.05)
Paroxysmal atrial fibrillation (x1.05)
Hyperlipidemia (x1.04)
aspirin (+0.00335)
Dyspnea (x1.04)
QRS duration (+1.2)
Coronary arteriosclerosis (x1.03)
metoprolol (+0.00285)
0.680
0.919
0.928
0.937
0.944
0.949
0.954
0.959
0.963
0.967
0.968
0.970
0.971
0.972
0.973
0.974
0.975
0.976
−0.8 −0.6 −0.4 −0.2 0.0 0.2 0.4 0.6 0.8
Expressions

Signature [662]; 33 To Reach 0.975, (33 Plotted)

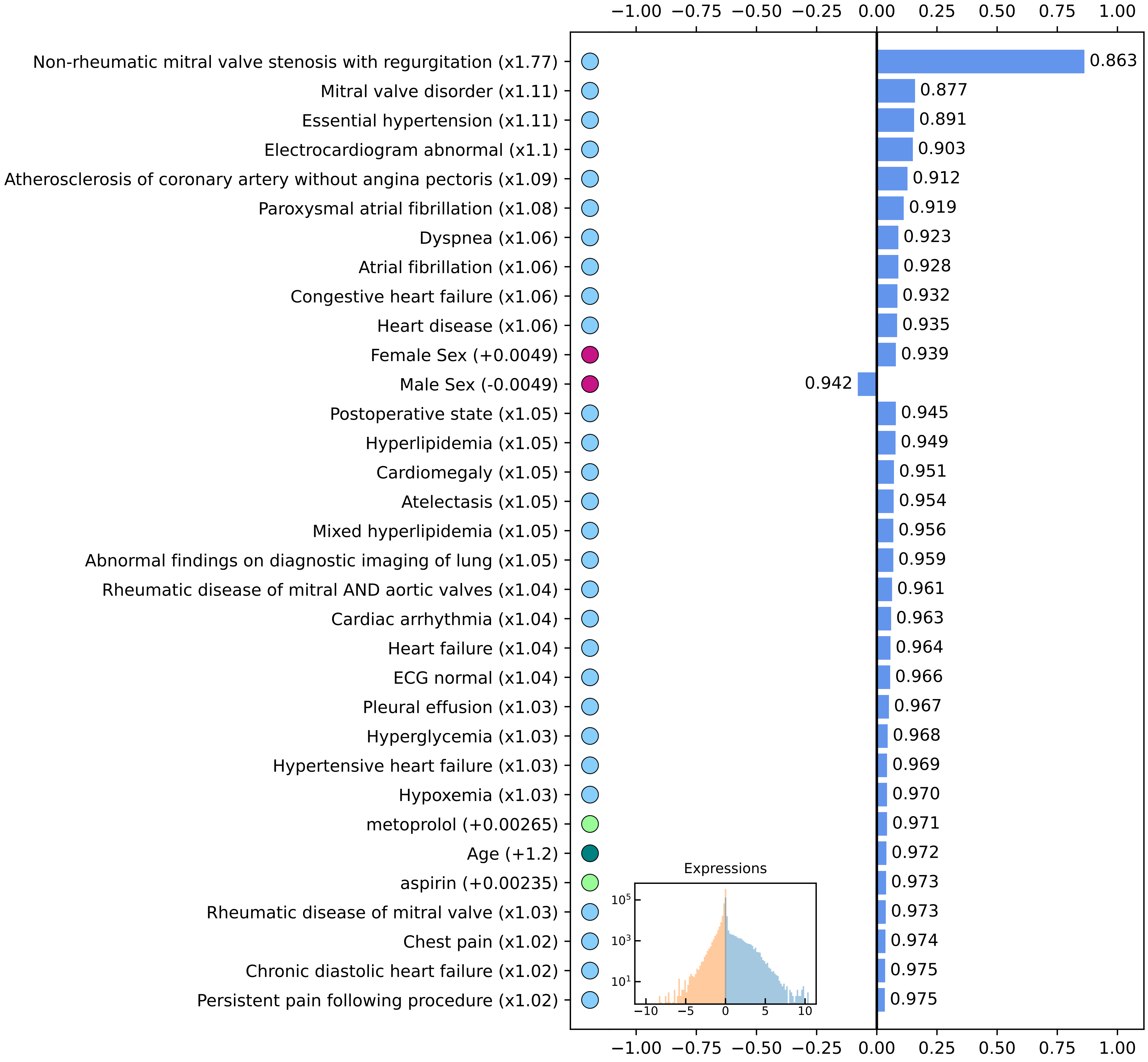

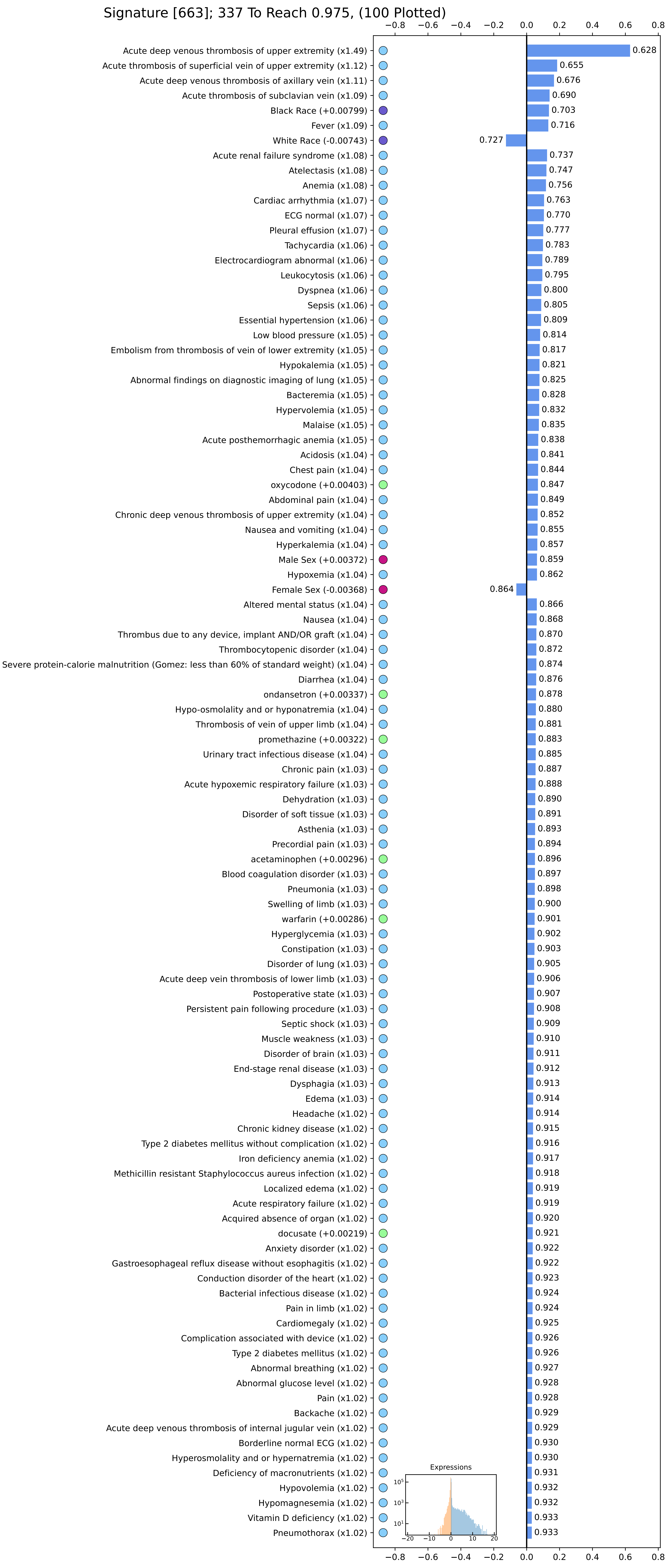

Signature [663]; 337 To Reach 0.975, (100 Plotted)
Acute deep venous thrombosis of upper extremity (x1.49) 0.628
Acute thrombosis of superficial vein of upper extremity (x1.12) 0.655
Acute deep venous thrombosis of axillary vein (x1.11) 0.676
Acute thrombosis of subclavian vein (x1.09) 0.690
Black Race (+0.00799) 0.703
Fever (x1.09) 0.716
White Race (-0.00743) 0.727
Acute renal failure syndrome (x1.08) 0.737
Atelectasis (x1.08) 0.747
Anemia (x1.08) 0.756
Cardiac arrhythmia (x1.07) 0.763
ECG normal (x1.07) 0.770
Pleural effusion (x1.07) 0.777
Tachycardia (x1.06) 0.783
Electrocardiogram abnormal (x1.06) 0.789
Leukocytosis (x1.06) 0.795
Dyspnea (x1.06) 0.800
Sepsis (x1.06) 0.805
Essential hypertension (x1.06) 0.809
Low blood pressure (x1.05) 0.814
Embolism from thrombosis of vein of lower extremity (x1.05) 0.817
Hypokalemia (x1.05) 0.821
Abnormal findings on diagnostic imaging of lung (x1.05) 0.825
Bacteremia (x1.05) 0.828
Hypervolemia (x1.05) 0.832
Malaise (x1.05) 0.835
Acute posthemorrhagic anemia (x1.05) 0.838
Acidosis (x1.04) 0.841
Chest pain (x1.04) 0.844
oxycodone (+0.00403) 0.847
Abdominal pain (x1.04) 0.849
Chronic deep venous thrombosis of upper extremity (x1.04) 0.852
Nausea and vomiting (x1.04) 0.855
Hyperkalemia (x1.04) 0.857
Male Sex (+0.00372) 0.859
Hypoxemia (x1.04) 0.862
Female Sex (-0.00368) 0.864
Altered mental status (x1.04) 0.866
Nausea (x1.04) 0.868
Thrombus due to any device, implant AND/OR graft (x1.04) 0.870
Thrombocytopenic disorder (x1.04) 0.872
Severe protein-calorie malnutrition (Gomez: less than 60% of standard weight) (x1.04) 0.874
Diarrhea (x1.04) 0.876
ondansetron (+0.00337) 0.878
Hypo-osmolality and or hyponatremia (x1.04) 0.880
Thrombosis of vein of upper limb (x1.04) 0.881
promethazine (+0.00322) 0.883
Urinary tract infectious disease (x1.04) 0.885
Chronic pain (x1.03) 0.887
Acute hypoxemic respiratory failure (x1.03) 0.888
Dehydration (x1.03) 0.890
Disorder of soft tissue (x1.03) 0.891
Asthenia (x1.03) 0.893
Precordial pain (x1.03) 0.894
acetaminophen (+0.00296) 0.896
Blood coagulation disorder (x1.03) 0.897
Pneumonia (x1.03) 0.898
Swelling of limb (x1.03) 0.900
warfarin (+0.00286) 0.901
Hyperglycemia (x1.03) 0.902
Constipation (x1.03) 0.903
Disorder of lung (x1.03) 0.905
Acute deep vein thrombosis of lower limb (x1.03) 0.906
Postoperative state (x1.03) 0.907
Persistent pain following procedure (x1.03) 0.908
Septic shock (x1.03) 0.909
Muscle weakness (x1.03) 0.910
Disorder of brain (x1.03) 0.911
End-stage renal disease (x1.03) 0.912
Dysphagia (x1.03) 0.913
Edema (x1.03) 0.914
Headache (x1.02) 0.914
Chronic kidney disease (x1.02) 0.915
Type 2 diabetes mellitus without complication (x1.02) 0.916
Iron deficiency anemia (x1.02) 0.917
Methicillin resistant Staphylococcus aureus infection (x1.02) 0.918
Localized edema (x1.02) 0.919
Acute respiratory failure (x1.02) 0.919
Acquired absence of organ (x1.02) 0.920
docusate (+0.00219) 0.921
Anxiety disorder (x1.02) 0.922
Gastroesophageal reflux disease without esophagitis (x1.02) 0.922
Conduction disorder of the heart (x1.02) 0.923
Bacterial infectious disease (x1.02) 0.924
Pain in limb (x1.02) 0.924
Cardiomegaly (x1.02) 0.925
Complication associated with device (x1.02) 0.926
Type 2 diabetes mellitus (x1.02) 0.926
Abnormal breathing (x1.02) 0.927
Abnormal glucose level (x1.02) 0.928
Pain (x1.02) 0.928
Backache (x1.02) 0.929
Acute deep venous thrombosis of internal jugular vein (x1.02) 0.929
Borderline normal ECG (x1.02) 0.930
Hyperosmolality and or hypernatremia (x1.02) 0.930
Deficiency of macronutrients (x1.02) 0.931
Hypovolemia (x1.02) 0.932
Hypomagnesemia (x1.02) 0.932
Vitamin D deficiency (x1.02) 0.933
Pneumothorax (x1.02) 0.933
−0.8 −0.6 −0.4 −0.2 0.0 0.2 0.4 0.6 0.8
Expressions

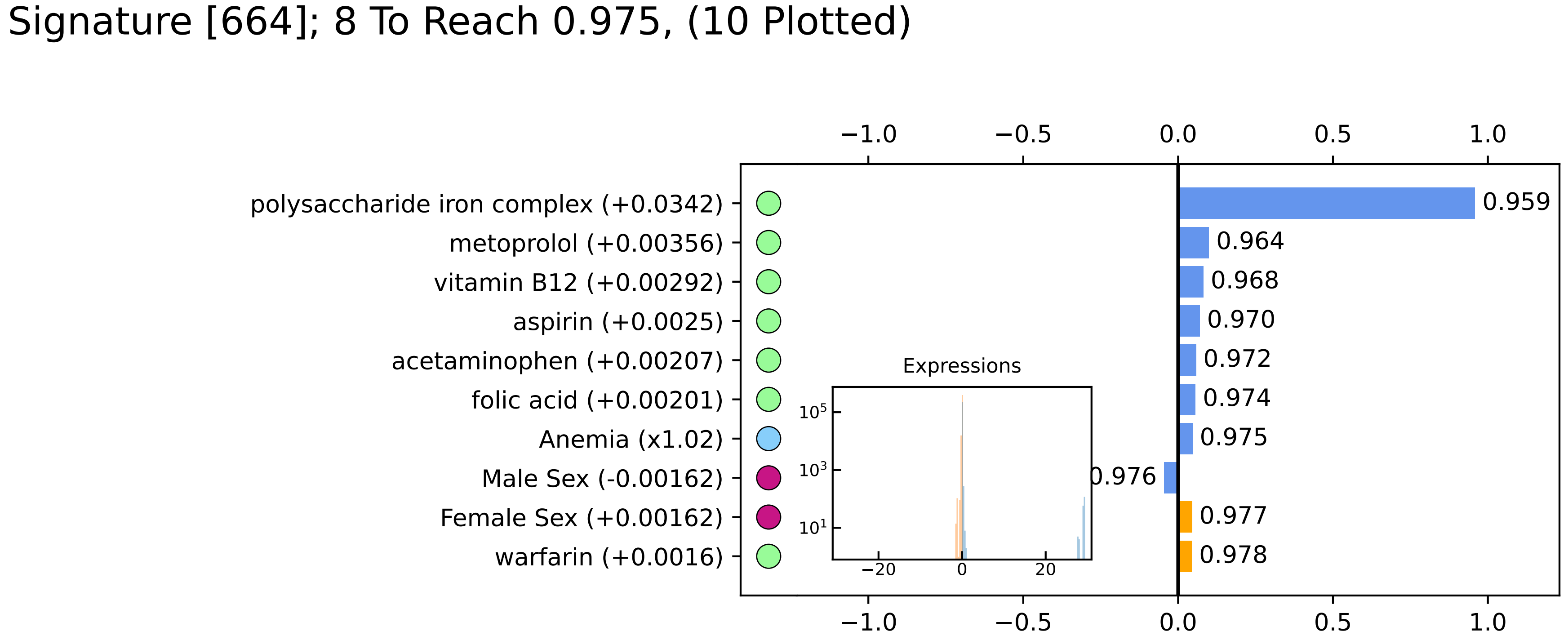

Signature [664]; 8 To Reach 0.975, (10 Plotted)
polysaccharide iron complex (+0.0342)
metoprolol (+0.00356)
vitamin B12 (+0.00292)
aspirin (+0.0025)
acetaminophen (+0.00207)
folic acid (+0.00201)
Anemia (x1.02)
Male Sex (-0.00162)
Female Sex (+0.00162)
warfarin (+0.0016)
0.959
0.964
0.968
0.970
0.972
0.974
0.975
0.976
0.977
0.978
−1.0
−0.5
0.0
0.5
1.0
Expressions
−20
0
20

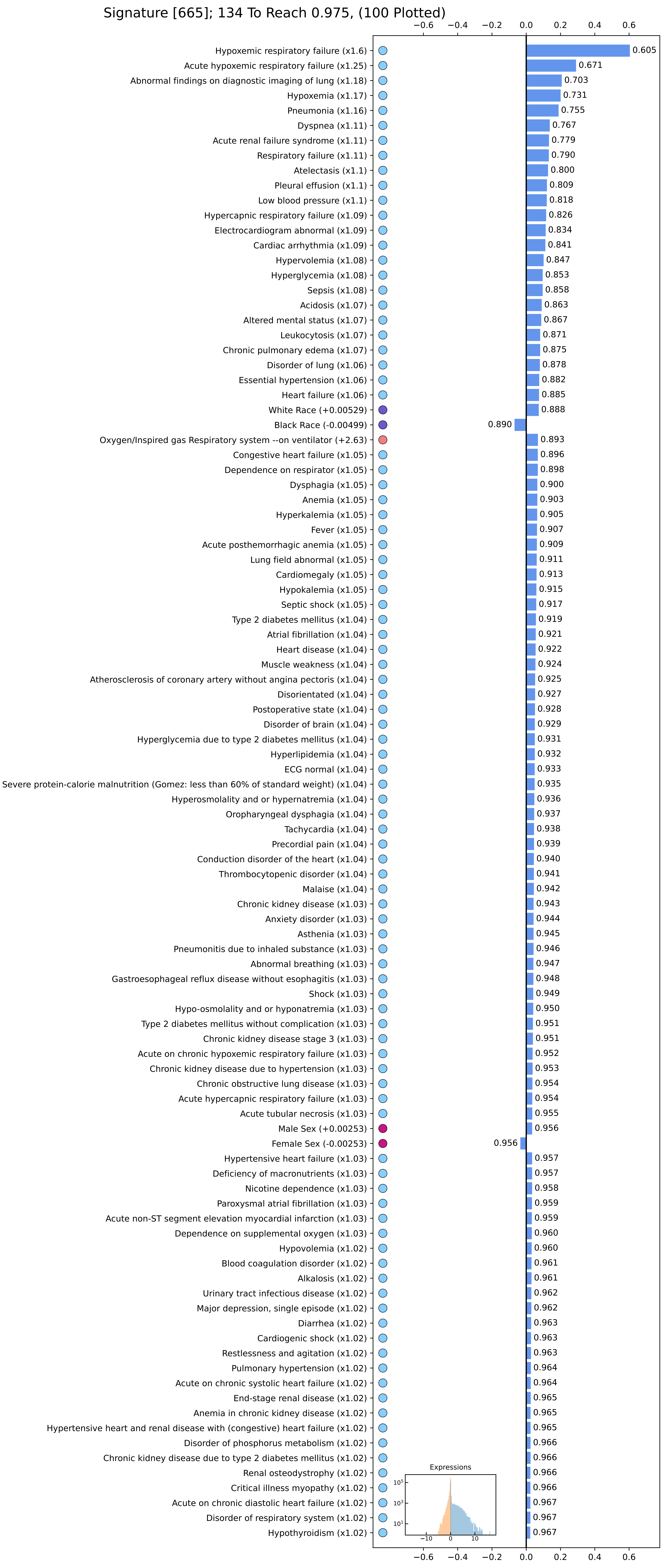

Signature [665]; 134 To Reach 0.975, (100 Plotted)
Hypoxemic respiratory failure (x1.6) 0.605
Acute hypoxemic respiratory failure (x1.25) 0.671
Abnormal findings on diagnostic imaging of lung (x1.18) 0.703
Hypoxemia (x1.17) 0.731
Pneumonia (x1.16) 0.755
Dyspnea (x1.11) 0.767
Acute renal failure syndrome (x1.11) 0.779
Respiratory failure (x1.11) 0.790
Atelectasis (x1.1) 0.800
Pleural effusion (x1.1) 0.809
Low blood pressure (x1.1) 0.818
Hypercapnic respiratory failure (x1.09) 0.826
Electrocardiogram abnormal (x1.09) 0.834
Cardiac arrhythmia (x1.09) 0.841
Hypervolemia (x1.08) 0.847
Hyperglycemia (x1.08) 0.853
Sepsis (x1.08) 0.858
Acidosis (x1.07) 0.863
Altered mental status (x1.07) 0.867
Leukocytosis (x1.07) 0.871
Chronic pulmonary edema (x1.07) 0.875
Disorder of lung (x1.06) 0.878
Essential hypertension (x1.06) 0.882
Heart failure (x1.06) 0.885
White Race (+0.00529) 0.888
Black Race (-0.00499) 0.890
Oxygen/Inspired gas Respiratory system --on ventilator (+2.63) 0.893
Congestive heart failure (x1.05) 0.896
Dependence on respirator (x1.05) 0.898
Dysphagia (x1.05) 0.900
Anemia (x1.05) 0.903
Hyperkalemia (x1.05) 0.905
Fever (x1.05) 0.907
Acute posthemorrhagic anemia (x1.05) 0.909
Lung field abnormal (x1.05) 0.911
Cardiomegaly (x1.05) 0.913
Hypokalemia (x1.05) 0.915
Septic shock (x1.05) 0.917
Type 2 diabetes mellitus (x1.04) 0.919
Atrial fibrillation (x1.04) 0.921
Heart disease (x1.04) 0.922
Muscle weakness (x1.04) 0.924
Atherosclerosis of coronary artery without angina pectoris (x1.04) 0.925
Disorientated (x1.04) 0.927
Postoperative state (x1.04) 0.928
Disorder of brain (x1.04) 0.929
Hyperglycemia due to type 2 diabetes mellitus (x1.04) 0.931
Hyperlipidemia (x1.04) 0.932
ECG normal (x1.04) 0.933
Severe protein-calorie malnutrition (Gomez: less than 60% of standard weight) (x1.04) 0.935
Hyperosmolality and or hypernatremia (x1.04) 0.936
Oropharyngeal dysphagia (x1.04) 0.937
Tachycardia (x1.04) 0.938
Precordial pain (x1.04) 0.939
Conduction disorder of the heart (x1.04) 0.940
Thrombocytopenic disorder (x1.04) 0.941
Malaise (x1.04) 0.942
Chronic kidney disease (x1.03) 0.943
Anxiety disorder (x1.03) 0.944
Asthenia (x1.03) 0.945
Pneumonitis due to inhaled substance (x1.03) 0.946
Abnormal breathing (x1.03) 0.947
Gastroesophageal reflux disease without esophagitis (x1.03) 0.948
Shock (x1.03) 0.949
Hypo-osmolality and or hyponatremia (x1.03) 0.950
Type 2 diabetes mellitus without complication (x1.03) 0.951
Chronic kidney disease stage 3 (x1.03) 0.951
Acute on chronic hypoxemic respiratory failure (x1.03) 0.952
Chronic kidney disease due to hypertension (x1.03) 0.953
Chronic obstructive lung disease (x1.03) 0.954
Acute hypercapnic respiratory failure (x1.03) 0.954
Acute tubular necrosis (x1.03) 0.955
Male Sex (+0.00253) 0.956
Female Sex (-0.00253) 0.956
Hypertensive heart failure (x1.03) 0.957
Deficiency of macronutrients (x1.03) 0.957
Nicotine dependence (x1.03) 0.958
Paroxysmal atrial fibrillation (x1.03) 0.959
Acute non-ST segment elevation myocardial infarction (x1.03) 0.959
Dependence on supplemental oxygen (x1.03) 0.960
Hypovolemia (x1.02) 0.960
Blood coagulation disorder (x1.02) 0.961
Alkalosis (x1.02) 0.961
Urinary tract infectious disease (x1.02) 0.962
Major depression, single episode (x1.02) 0.962
Diarrhea (x1.02) 0.963
Cardiogenic shock (x1.02) 0.963
Restlessness and agitation (x1.02) 0.963
Pulmonary hypertension (x1.02) 0.964
Acute on chronic systolic heart failure (x1.02) 0.964
End-stage renal disease (x1.02) 0.965
Anemia in chronic kidney disease (x1.02) 0.965
Hypertensive heart and renal disease with (congestive) heart failure (x1.02) 0.965
Disorder of phosphorus metabolism (x1.02) 0.966
Chronic kidney disease due to type 2 diabetes mellitus (x1.02) 0.966
Renal osteodystrophy (x1.02) 0.966
Critical illness myopathy (x1.02) 0.966
Acute on chronic diastolic heart failure (x1.02) 0.967
Disorder of respiratory system (x1.02) 0.967
Hypothyroidism (x1.02) 0.967
Expressions
−0.6 −0.4 −0.2 0.0 0.2 0.4 0.6

Signature [666]; 12 To Reach 0.975, (12 Plotted)

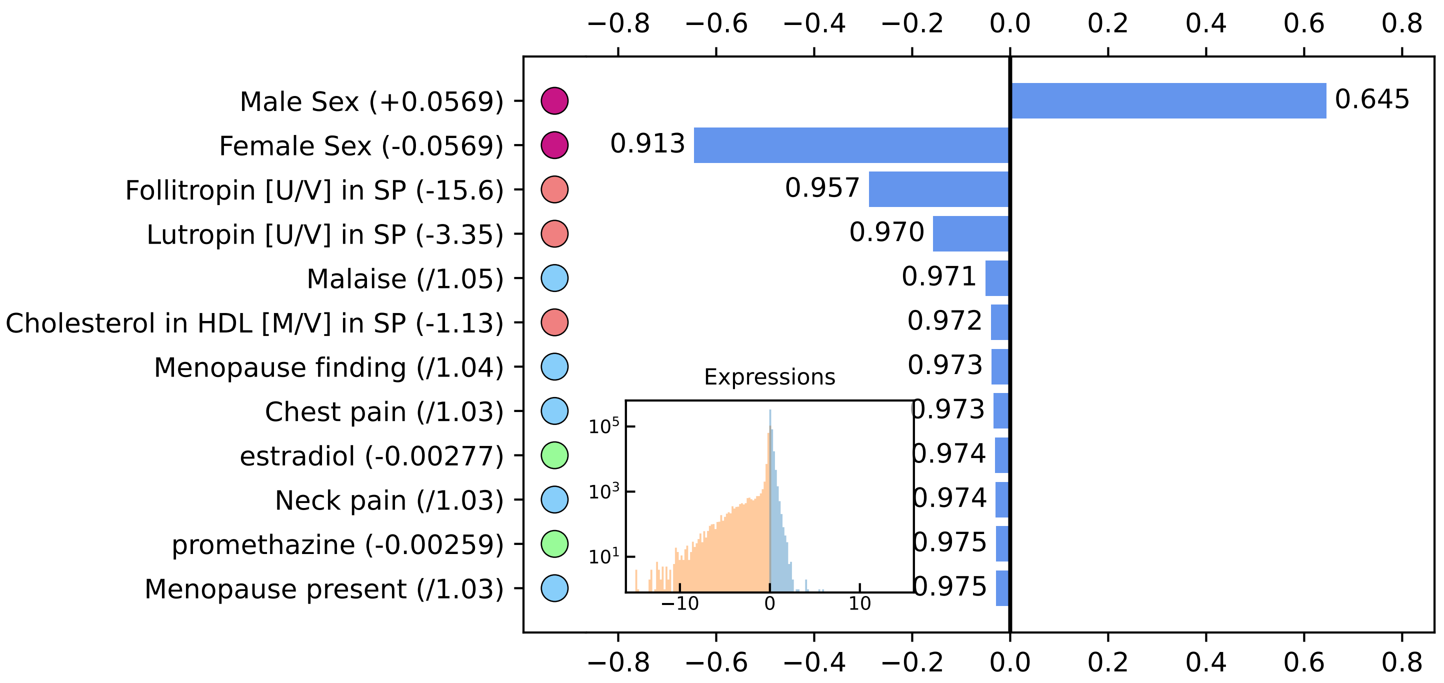

Signature [667]; 75 To Reach 0.975, (75 Plotted)

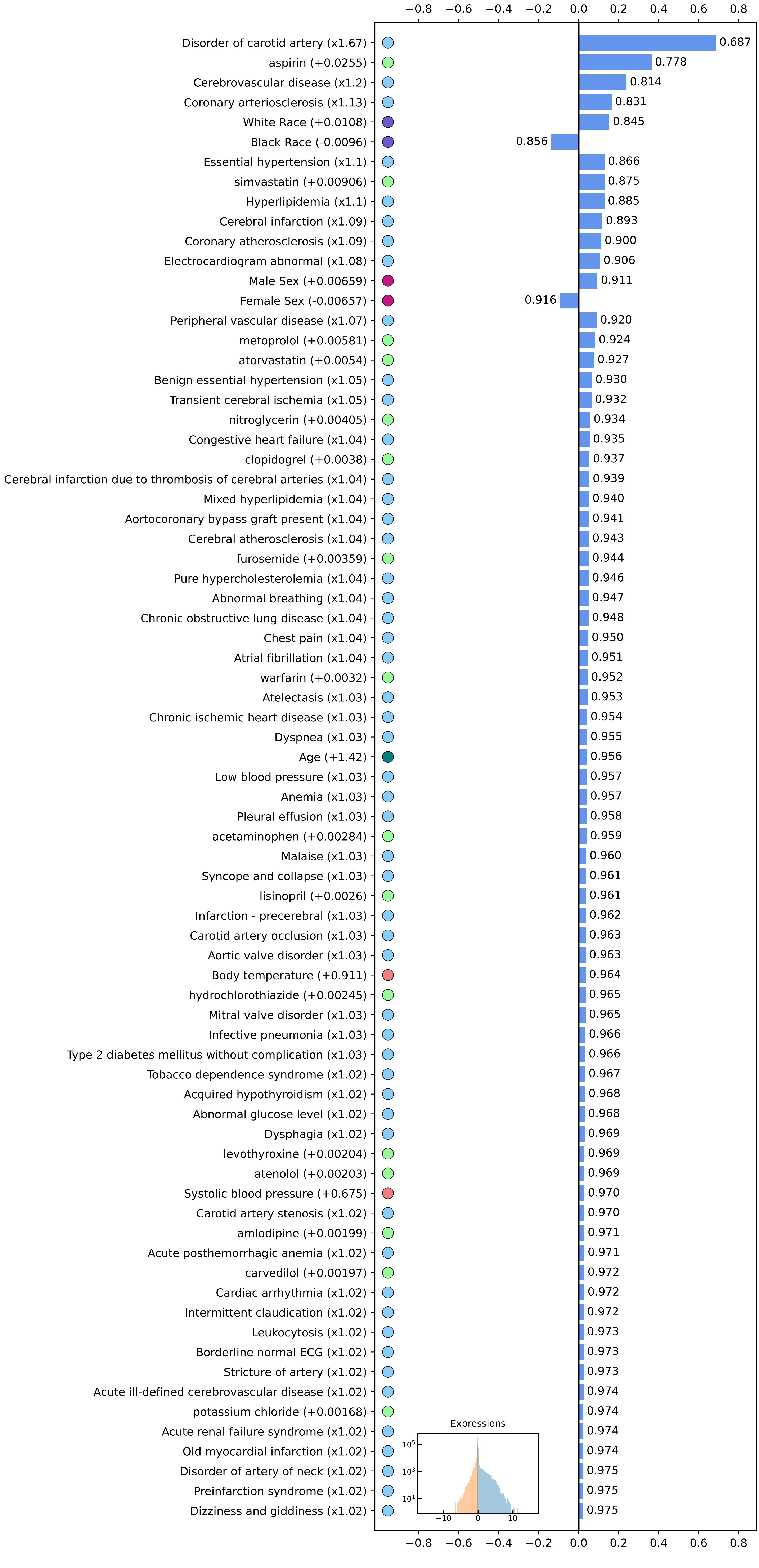

Signature [668]; 5 To Reach 0.975, (10 Plotted)

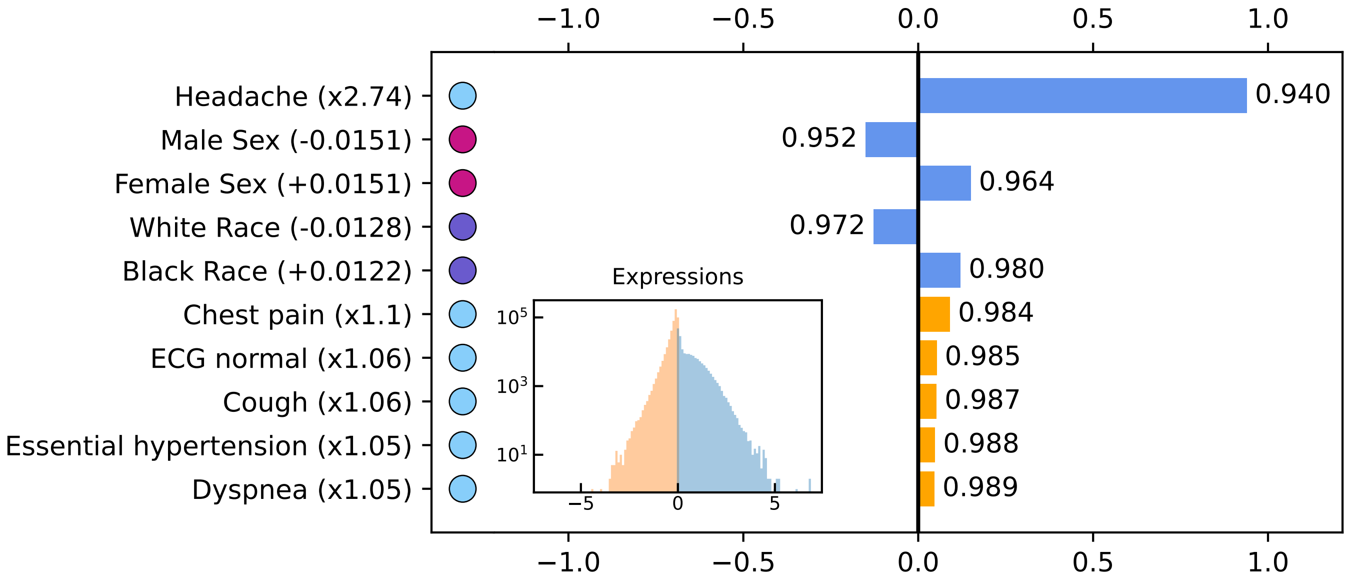

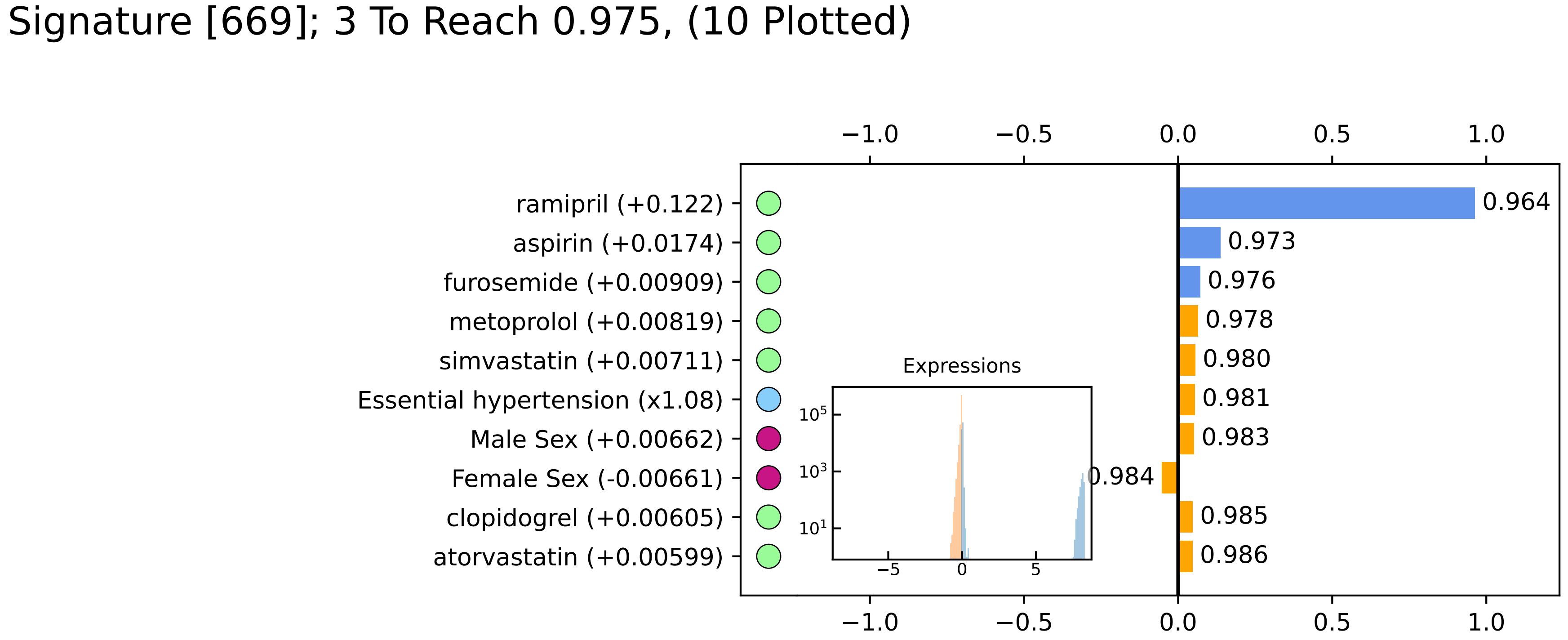

Signature [669]; 3 To Reach 0.975, (10 Plotted)
ramipril (+0.122)
aspirin (+0.0174)
furosemide (+0.00909)
metoprolol (+0.00819)
simvastatin (+0.00711)
Essential hypertension (x1.08)
Male Sex (+0.00662)
Female Sex (-0.00661)
clopidogrel (+0.00605)
atorvastatin (+0.00599)
0.964
0.973
0.976
0.978
0.980
0.981
0.983
0.984
0.985
0.986
−1.0
−0.5
0.0
0.5
1.0
Expressions

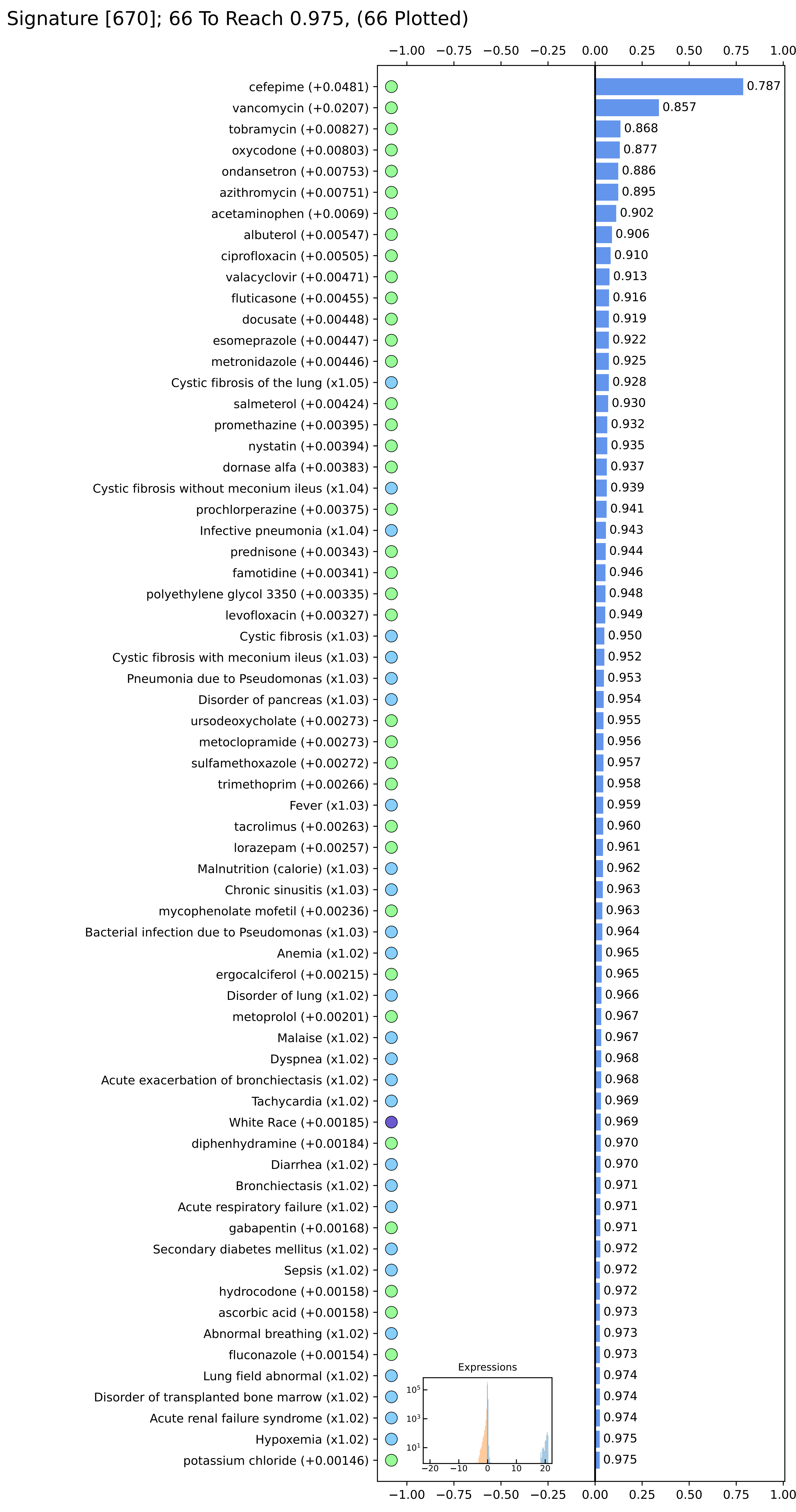

Signature [670]; 66 To Reach 0.975, (66 Plotted)
−1.00 −0.75 −0.50 −0.25 0.00 0.25 0.50 0.75 1.00
cefepime (+0.0481) 0.787
vancomycin (+0.0207) 0.857
tobramycin (+0.00827) 0.868
oxycodone (+0.00803) 0.877
ondansetron (+0.00753) 0.886
azithromycin (+0.00751) 0.895
acetaminophen (+0.0069) 0.902
albuterol (+0.00547) 0.906
ciprofloxacin (+0.00505) 0.910
valacyclovir (+0.00471) 0.913
fluticasone (+0.00455) 0.916
docusate (+0.00448) 0.919
esomeprazole (+0.00447) 0.922
metronidazole (+0.00446) 0.925
Cystic fibrosis of the lung (x1.05) 0.928
salmeterol (+0.00424) 0.930
promethazine (+0.00395) 0.932
nystatin (+0.00394) 0.935
dornase alfa (+0.00383) 0.937
Cystic fibrosis without meconium ileus (x1.04) 0.939
prochlorperazine (+0.00375) 0.941
Infective pneumonia (x1.04) 0.943
prednisone (+0.00343) 0.944
famotidine (+0.00341) 0.946
polyethylene glycol 3350 (+0.00335) 0.948
levofloxacin (+0.00327) 0.949
Cystic fibrosis (x1.03) 0.950
Cystic fibrosis with meconium ileus (x1.03) 0.952
Pneumonia due to Pseudomonas (x1.03) 0.953
Disorder of pancreas (x1.03) 0.954
ursodeoxycholate (+0.00273) 0.955
metoclopramide (+0.00273) 0.956
sulfamethoxazole (+0.00272) 0.957
trimethoprim (+0.00266) 0.958
Fever (x1.03) 0.959
tacrolimus (+0.00263) 0.960
lorazepam (+0.00257) 0.961
Malnutrition (calorie) (x1.03) 0.962
Chronic sinusitis (x1.03) 0.963
mycophenolate mofetil (+0.00236) 0.963
Bacterial infection due to Pseudomonas (x1.03) 0.964
Anemia (x1.02) 0.965
ergocalciferol (+0.00215) 0.965
Disorder of lung (x1.02) 0.966
metoprolol (+0.00201) 0.967
Malaise (x1.02) 0.967
Dyspnea (x1.02) 0.968
Acute exacerbation of bronchiectasis (x1.02) 0.968
Tachycardia (x1.02) 0.969
White Race (+0.00185) 0.969
diphenhydramine (+0.00184) 0.970
Diarrhea (x1.02) 0.970
Bronchiectasis (x1.02) 0.971
Acute respiratory failure (x1.02) 0.971
gabapentin (+0.00168) 0.971
Secondary diabetes mellitus (x1.02) 0.972
Sepsis (x1.02) 0.972
hydrocodone (+0.00158) 0.972
ascorbic acid (+0.00158) 0.973
Abnormal breathing (x1.02) 0.973
fluconazole (+0.00154) 0.973
Lung field abnormal (x1.02) 0.974
Disorder of transplanted bone marrow (x1.02) 0.974
Acute renal failure syndrome (x1.02) 0.974
Hypoxemia (x1.02) 0.975
potassium chloride (+0.00146) 0.975
Expressions
10^5
10^3
10^1
−20 −10 0 10 20
−1.00 −0.75 −0.50 −0.25 0.00 0.25 0.50 0.75 1.00

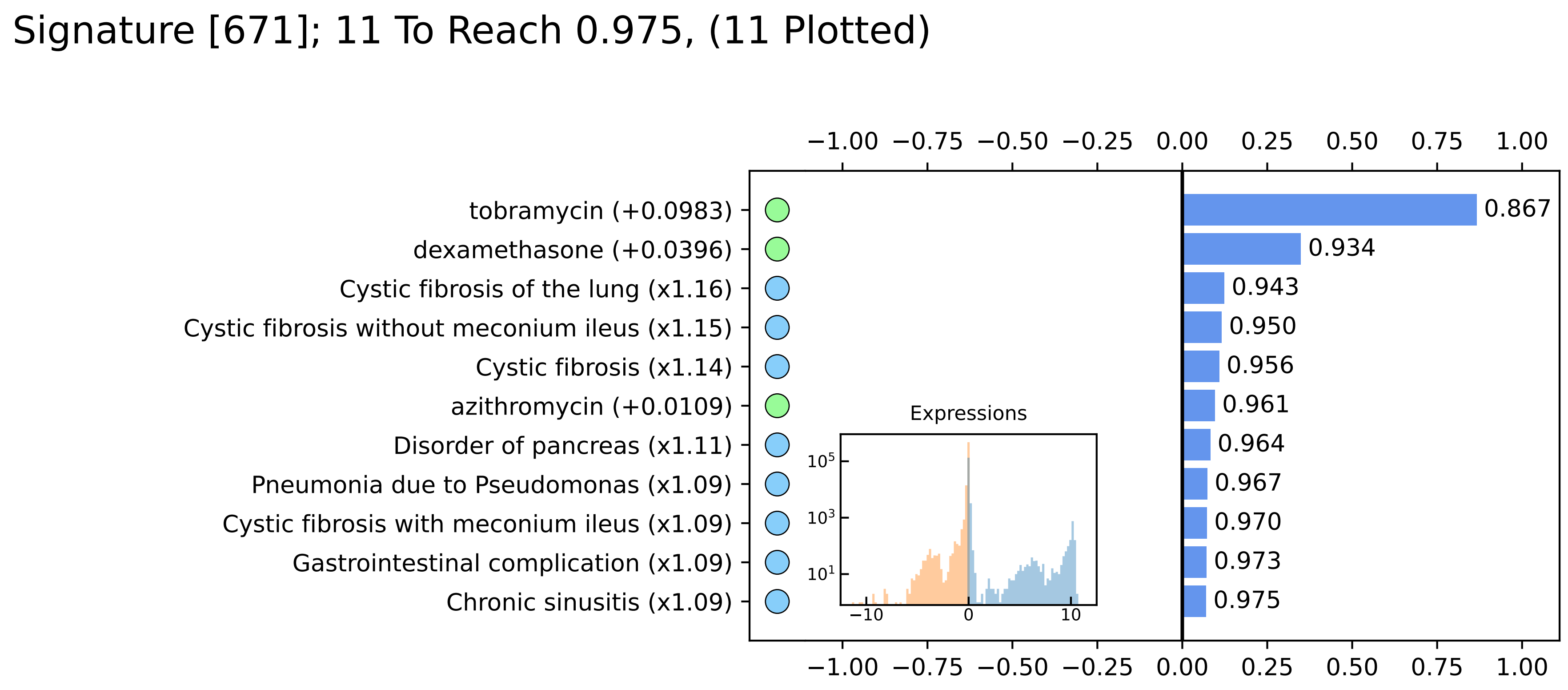

Signature [671]; 11 To Reach 0.975, (11 Plotted)
tobramycin (+0.0983)
dexamethasone (+0.0396)
Cystic fibrosis of the lung (x1.16)
Cystic fibrosis without meconium ileus (x1.15)
Cystic fibrosis (x1.14)
azithromycin (+0.0109)
Disorder of pancreas (x1.11)
Pneumonia due to Pseudomonas (x1.09)
Cystic fibrosis with meconium ileus (x1.09)
Gastrointestinal complication (x1.09)
Chronic sinusitis (x1.09)
0.867
0.934
0.943
0.950
0.956
0.961
0.964
0.967
0.970
0.973
0.975
Expressions
−1.00 −0.75 −0.50 −0.25 0.00 0.25 0.50 0.75 1.00

Signature [672]; 153 To Reach 0.975, (100 Plotted)

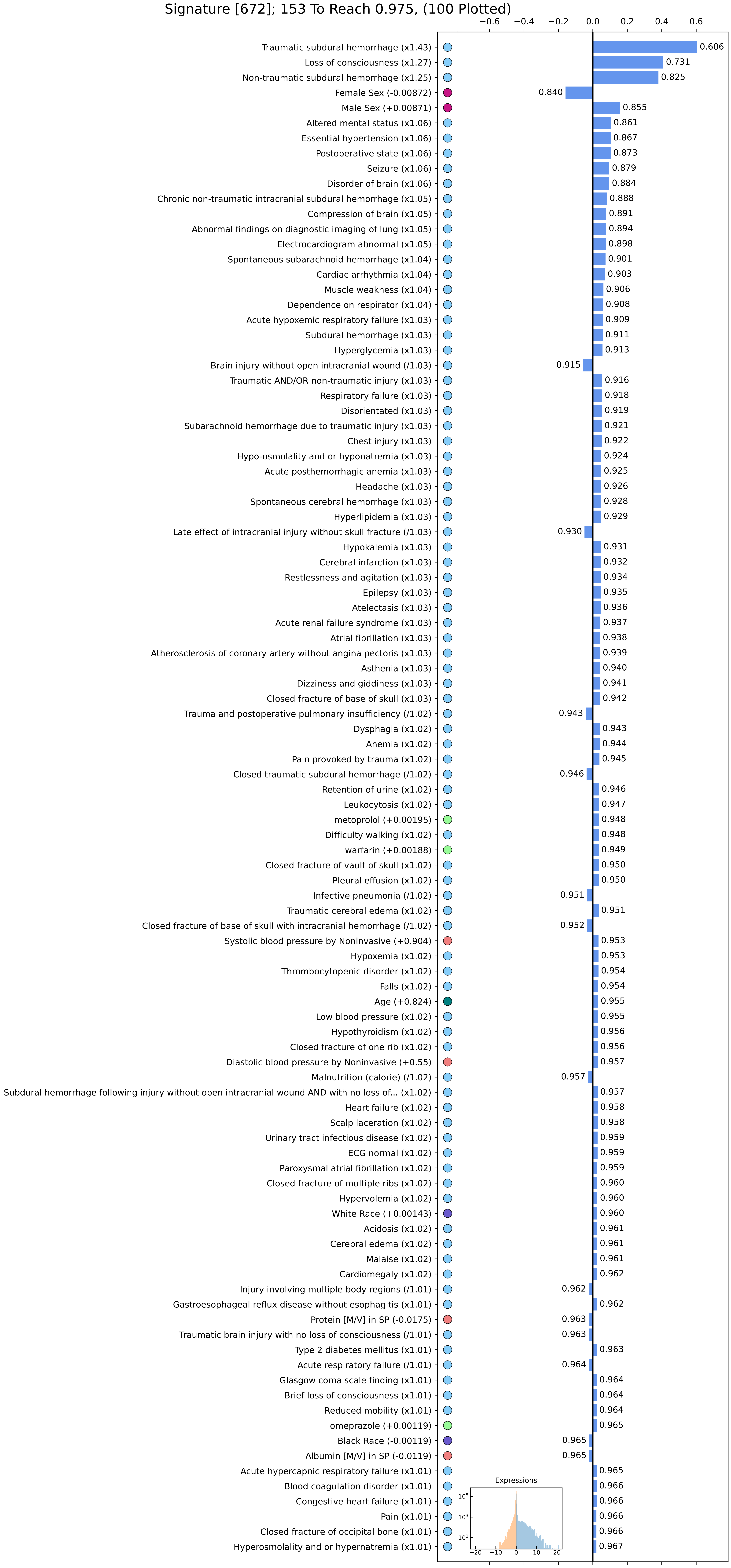

Signature [673]; 6 To Reach 0.975, (10 Plotted)

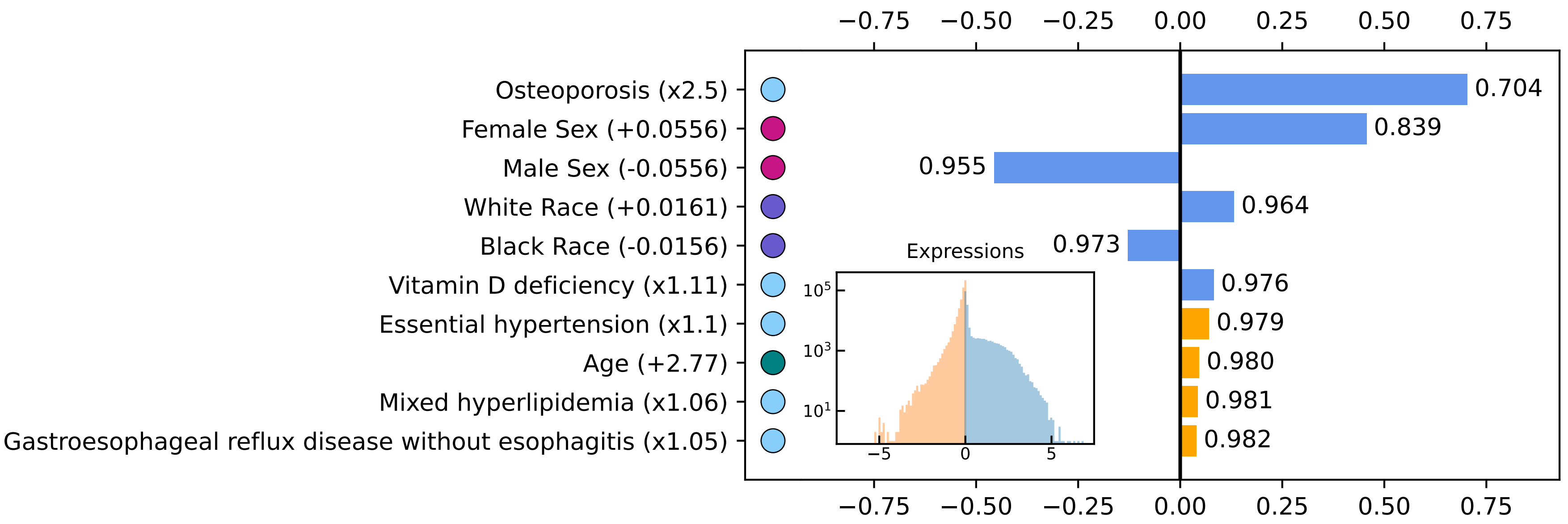

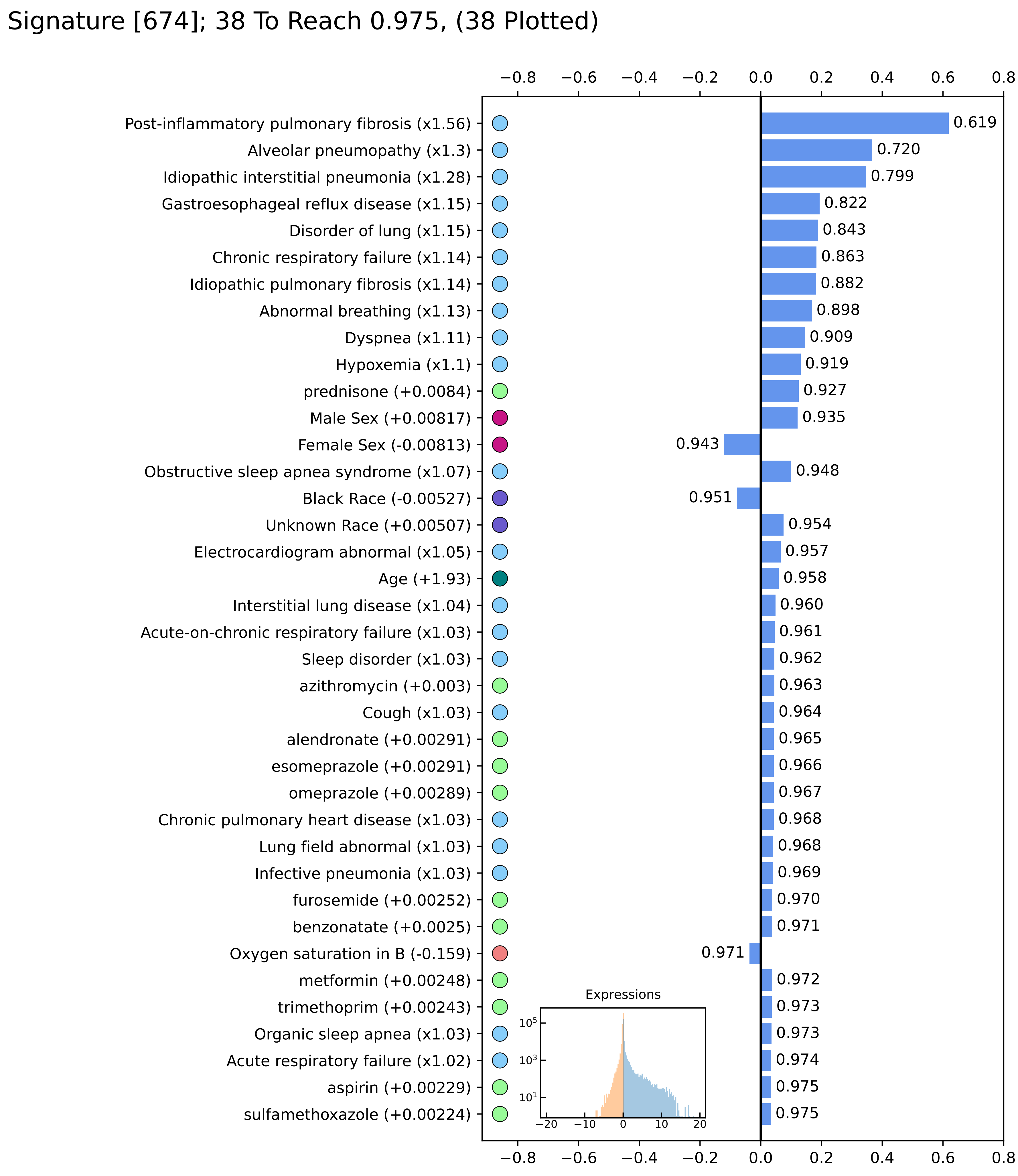

Signature [674]; 38 To Reach 0.975, (38 Plotted)
Post-inflammatory pulmonary fibrosis (x1.56)
Alveolar pneumopathy (x1.3)
Idiopathic interstitial pneumonia (x1.28)
Gastroesophageal reflux disease (x1.15)
Disorder of lung (x1.15)
Chronic respiratory failure (x1.14)
Idiopathic pulmonary fibrosis (x1.14)
Abnormal breathing (x1.13)
Dyspnea (x1.11)
Hypoxemia (x1.1)
prednisone (+0.0084)
Male Sex (+0.00817)
Female Sex (-0.00813)
Obstructive sleep apnea syndrome (x1.07)
Black Race (-0.00527)
Unknown Race (+0.00507)
Electrocardiogram abnormal (x1.05)
Age (+1.93)
Interstitial lung disease (x1.04)
Acute-on-chronic respiratory failure (x1.03)
Sleep disorder (x1.03)
azithromycin (+0.003)
Cough (x1.03)
alendronate (+0.00291)
esomeprazole (+0.00291)
omeprazole (+0.00289)
Chronic pulmonary heart disease (x1.03)
Lung field abnormal (x1.03)
Infective pneumonia (x1.03)
furosemide (+0.00252)
benzonatate (+0.0025)
Oxygen saturation in B (-0.159)
metformin (+0.00248)
trimethoprim (+0.00243)
Organic sleep apnea (x1.03)
Acute respiratory failure (x1.02)
aspirin (+0.00229)
sulfamethoxazole (+0.00224)
0.619
0.720
0.799
0.822
0.843
0.863
0.882
0.898
0.909
0.919
0.927
0.935
0.943
0.948
0.951
0.954
0.957
0.958
0.960
0.961
0.962
0.963
0.964
0.965
0.966
0.967
0.968
0.968
0.969
0.970
0.971
0.971
0.972
0.973
0.973
0.974
0.975
0.975
−0.8
−0.6
−0.4
−0.2
0.0
0.2
0.4
0.6
0.8
Expressions
10^5
10^3
10^1
−20
−10
0
10
20

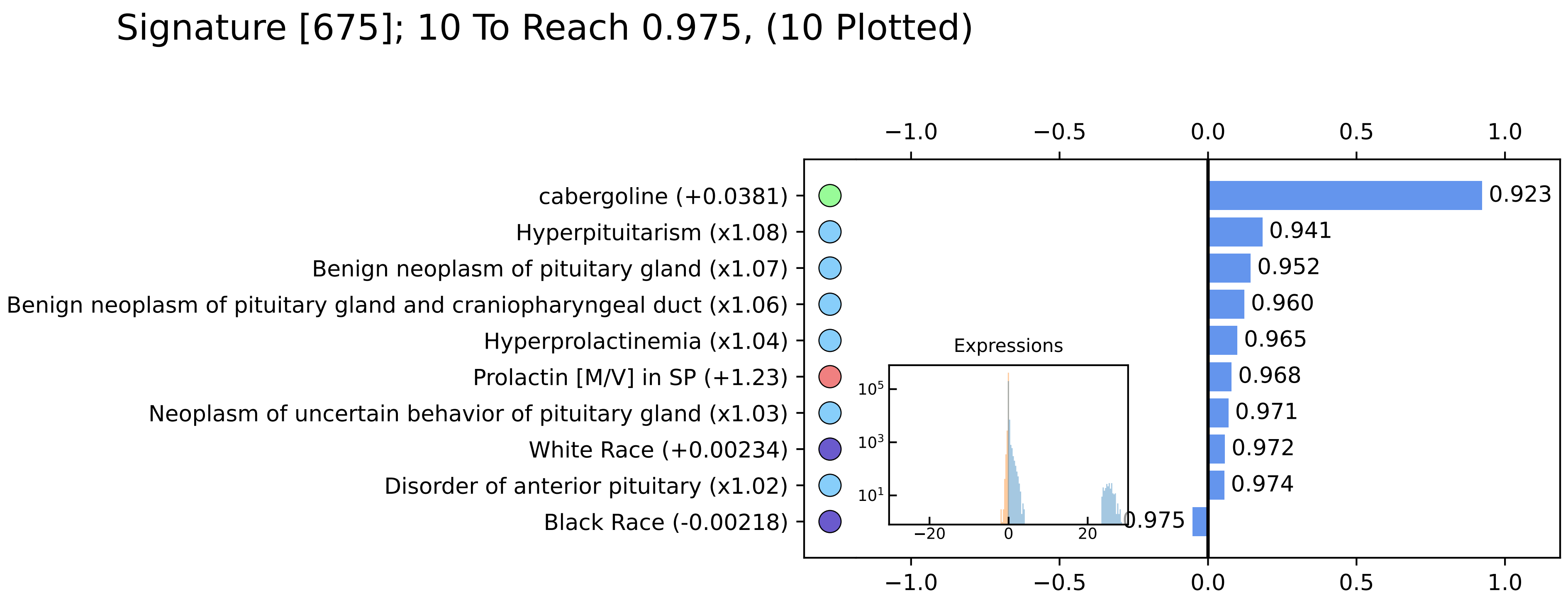
Signature [675]; 10 To Reach 0.975, (10 Plotted)
cabergoline (+0.0381)
Hyperpituitarism (x1.08)
Benign neoplasm of pituitary gland (x1.07)
Benign neoplasm of pituitary gland and craniopharyngeal duct (x1.06)
Hyperprolactinemia (x1.04)
Prolactin [M/V] in SP (+1.23)
Neoplasm of uncertain behavior of pituitary gland (x1.03)
White Race (+0.00234)
Disorder of anterior pituitary (x1.02)
Black Race (-0.00218)
0.923
0.941
0.952
0.960
0.965
0.968
0.971
0.972
0.974
0.975
−1.0
−0.5
0.0
0.5
1.0
Expressions
10^5
10^3
10^1
−20
0
20

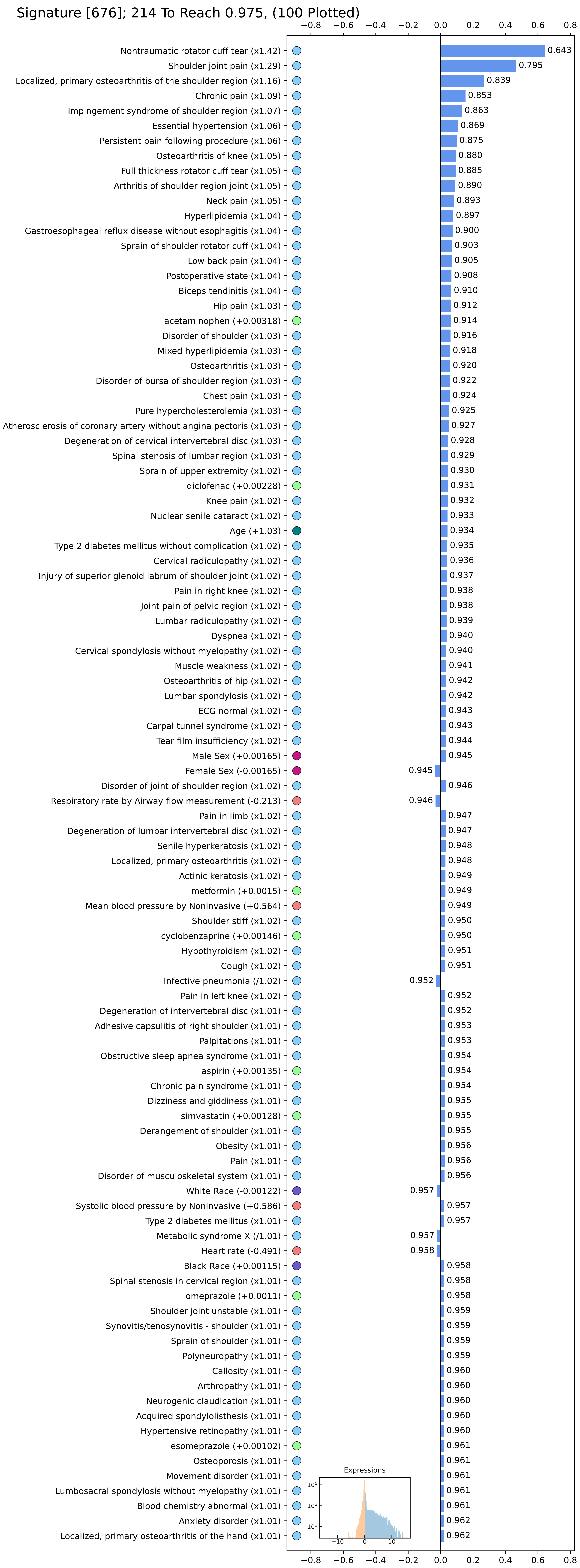

Signature [676]; 214 To Reach 0.975, (100 Plotted)
Nontraumatic rotator cuff tear (x1.42) 0.643
Shoulder joint pain (x1.29) 0.795
Localized, primary osteoarthritis of the shoulder region (x1.16) 0.839
Chronic pain (x1.09) 0.853
Impingement syndrome of shoulder region (x1.07) 0.863
Essential hypertension (x1.06) 0.869
Persistent pain following procedure (x1.06) 0.875
Osteoarthritis of knee (x1.05) 0.880
Full thickness rotator cuff tear (x1.05) 0.885
Arthritis of shoulder region joint (x1.05) 0.890
Neck pain (x1.05) 0.893
Hyperlipidemia (x1.04) 0.897
Gastroesophageal reflux disease without esophagitis (x1.04) 0.900
Sprain of shoulder rotator cuff (x1.04) 0.903
Low back pain (x1.04) 0.905
Postoperative state (x1.04) 0.908
Biceps tendinitis (x1.04) 0.910
Hip pain (x1.03) 0.912
acetaminophen (+0.00318) 0.914
Disorder of shoulder (x1.03) 0.916
Mixed hyperlipidemia (x1.03) 0.918
Osteoarthritis (x1.03) 0.920
Disorder of bursa of shoulder region (x1.03) 0.922
Chest pain (x1.03) 0.924
Pure hypercholesterolemia (x1.03) 0.925
Atherosclerosis of coronary artery without angina pectoris (x1.03) 0.927
Degeneration of cervical intervertebral disc (x1.03) 0.928
Spinal stenosis of lumbar region (x1.03) 0.929
Sprain of upper extremity (x1.02) 0.930
diclofenac (+0.00228) 0.931
Knee pain (x1.02) 0.932
Nuclear senile cataract (x1.02) 0.933
Age (+1.03) 0.934
Type 2 diabetes mellitus without complication (x1.02) 0.935
Cervical radiculopathy (x1.02) 0.936
Injury of superior glenoid labrum of shoulder joint (x1.02) 0.937
Pain in right knee (x1.02) 0.938
Joint pain of pelvic region (x1.02) 0.938
Lumbar radiculopathy (x1.02) 0.939
Dyspnea (x1.02) 0.940
Cervical spondylosis without myelopathy (x1.02) 0.940
Muscle weakness (x1.02) 0.941
Osteoarthritis of hip (x1.02) 0.942
Lumbar spondylosis (x1.02) 0.942
ECG normal (x1.02) 0.943
Carpal tunnel syndrome (x1.02) 0.943
Tear film insufficiency (x1.02) 0.944
Male Sex (+0.00165) 0.945
Female Sex (-0.00165) 0.945
Disorder of joint of shoulder region (x1.02) 0.946
Respiratory rate by Airway flow measurement (-0.213) 0.946
Pain in limb (x1.02) 0.947
Degeneration of lumbar intervertebral disc (x1.02) 0.947
Senile hyperkeratosis (x1.02) 0.948
Localized, primary osteoarthritis (x1.02) 0.948
Actinic keratosis (x1.02) 0.949
metformin (+0.0015) 0.949
Mean blood pressure by Noninvasive (+0.564) 0.949
Shoulder stiff (x1.02) 0.950
cyclobenzaprine (+0.00146) 0.950
Hypothyroidism (x1.02) 0.951
Cough (x1.02) 0.951
Infective pneumonia (/1.02) 0.952
Pain in left knee (x1.02) 0.952
Degeneration of intervertebral disc (x1.01) 0.952
Adhesive capsulitis of right shoulder (x1.01) 0.953
Palpitations (x1.01) 0.953
Obstructive sleep apnea syndrome (x1.01) 0.954
aspirin (+0.00135) 0.954
Chronic pain syndrome (x1.01) 0.954
Dizziness and giddiness (x1.01) 0.955
simvastatin (+0.00128) 0.955
Derangement of shoulder (x1.01) 0.955
Obesity (x1.01) 0.956
Pain (x1.01) 0.956
Disorder of musculoskeletal system (x1.01) 0.956
White Race (-0.00122) 0.957
Systolic blood pressure by Noninvasive (+0.586) 0.957
Type 2 diabetes mellitus (x1.01) 0.957
Metabolic syndrome X (/1.01) 0.957
Heart rate (-0.491) 0.958
Black Race (+0.00115) 0.958
Spinal stenosis in cervical region (x1.01) 0.958
omeprazole (+0.0011) 0.958
Shoulder joint unstable (x1.01) 0.959
Synovitis/tenosynovitis - shoulder (x1.01) 0.959
Sprain of shoulder (x1.01) 0.959
Polyneuropathy (x1.01) 0.959
Callosity (x1.01) 0.960
Arthropathy (x1.01) 0.960
Neurogenic claudication (x1.01) 0.960
Acquired spondylolisthesis (x1.01) 0.960
Hypertensive retinopathy (x1.01) 0.960
esomeprazole (+0.00102) 0.961
Osteoporosis (x1.01) 0.961
Movement disorder (x1.01) 0.961
Lumbosacral spondylosis without myelopathy (x1.01) 0.961
Blood chemistry abnormal (x1.01) 0.961
Anxiety disorder (x1.01) 0.962
Localized, primary osteoarthritis of the hand (x1.01) 0.962
Expressions
−0.8 −0.6 −0.4 −0.2 0.0 0.2 0.4 0.6 0.8

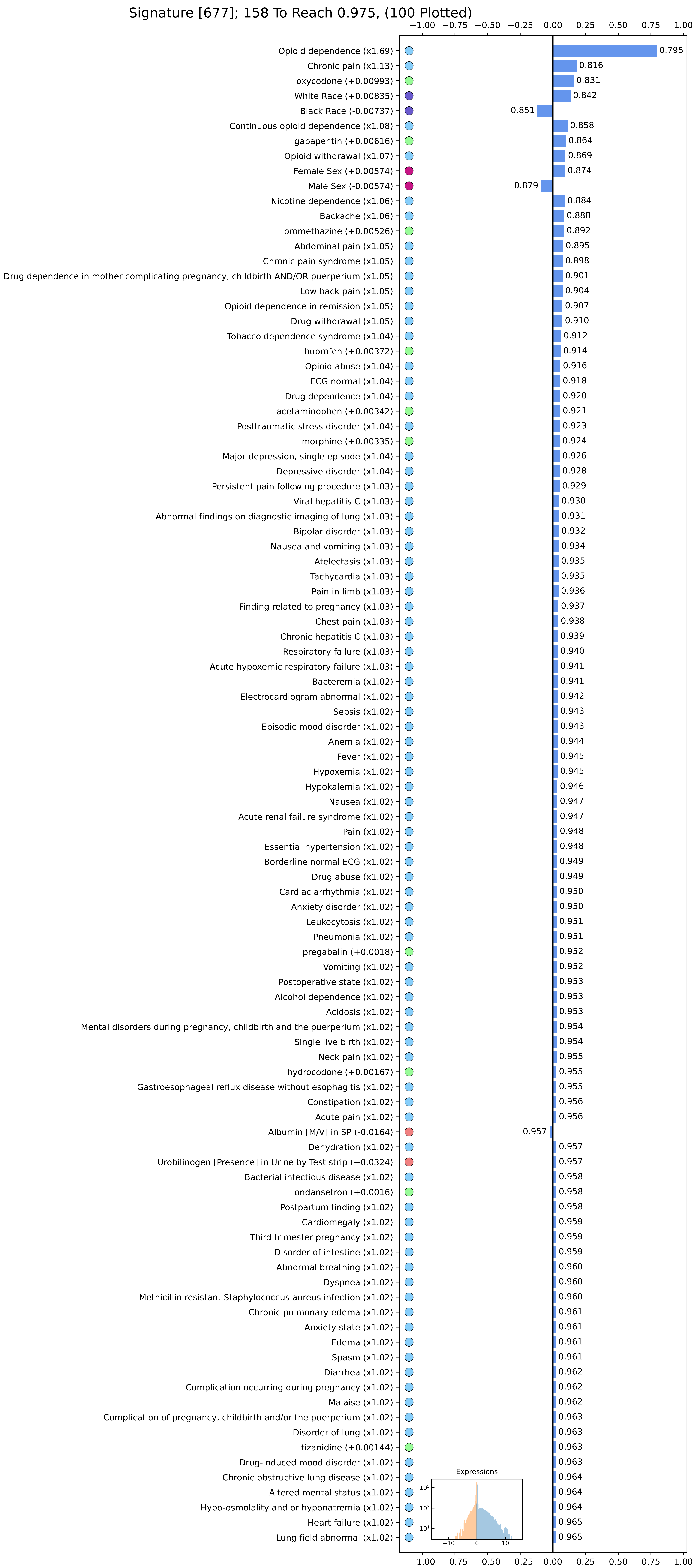

Signature [677]; 158 To Reach 0.975, (100 Plotted)
Opioid dependence (x1.69) 0.795
Chronic pain (x1.13) 0.816
oxycodone (+0.00993) 0.831
White Race (+0.00835) 0.842
Black Race (-0.00737) 0.851
Continuous opioid dependence (x1.08) 0.858
gabapentin (+0.00616) 0.864
Opioid withdrawal (x1.07) 0.869
Female Sex (+0.00574) 0.874
Male Sex (-0.00574) 0.879
Nicotine dependence (x1.06) 0.884
Backache (x1.06) 0.888
promethazine (+0.00526) 0.892
Abdominal pain (x1.05) 0.895
Chronic pain syndrome (x1.05) 0.898
Drug dependence in mother complicating pregnancy, childbirth AND/OR puerperium (x1.05) 0.901
Low back pain (x1.05) 0.904
Opioid dependence in remission (x1.05) 0.907
Drug withdrawal (x1.05) 0.910
Tobacco dependence syndrome (x1.04) 0.912
ibuprofen (+0.00372) 0.914
Opioid abuse (x1.04) 0.916
ECG normal (x1.04) 0.918
Drug dependence (x1.04) 0.920
acetaminophen (+0.00342) 0.921
Posttraumatic stress disorder (x1.04) 0.923
morphine (+0.00335) 0.924
Major depression, single episode (x1.04) 0.926
Depressive disorder (x1.04) 0.928
Persistent pain following procedure (x1.03) 0.929
Viral hepatitis C (x1.03) 0.930
Abnormal findings on diagnostic imaging of lung (x1.03) 0.931
Bipolar disorder (x1.03) 0.932
Nausea and vomiting (x1.03) 0.934
Atelectasis (x1.03) 0.935
Tachycardia (x1.03) 0.935
Pain in limb (x1.03) 0.936
Finding related to pregnancy (x1.03) 0.937
Chest pain (x1.03) 0.938
Chronic hepatitis C (x1.03) 0.939
Respiratory failure (x1.03) 0.940
Acute hypoxemic respiratory failure (x1.03) 0.941
Bacteremia (x1.02) 0.941
Electrocardiogram abnormal (x1.02) 0.942
Sepsis (x1.02) 0.943
Episodic mood disorder (x1.02) 0.943
Anemia (x1.02) 0.944
Fever (x1.02) 0.945
Hypoxemia (x1.02) 0.945
Hypokalemia (x1.02) 0.946
Nausea (x1.02) 0.947
Acute renal failure syndrome (x1.02) 0.947
Pain (x1.02) 0.948
Essential hypertension (x1.02) 0.948
Borderline normal ECG (x1.02) 0.949
Drug abuse (x1.02) 0.949
Cardiac arrhythmia (x1.02) 0.950
Anxiety disorder (x1.02) 0.950
Leukocytosis (x1.02) 0.951
Pneumonia (x1.02) 0.951
pregabalin (+0.0018) 0.952
Vomiting (x1.02) 0.952
Postoperative state (x1.02) 0.953
Alcohol dependence (x1.02) 0.953
Acidosis (x1.02) 0.953
Mental disorders during pregnancy, childbirth and the puerperium (x1.02) 0.954
Single live birth (x1.02) 0.954
Neck pain (x1.02) 0.955
hydrocodone (+0.00167) 0.955
Gastroesophageal reflux disease without esophagitis (x1.02) 0.955
Constipation (x1.02) 0.956
Acute pain (x1.02) 0.956
Albumin [M/V] in SP (-0.0164) 0.957
Dehydration (x1.02) 0.957
Urobilinogen [Presence] in Urine by Test strip (+0.0324) 0.957
Bacterial infectious disease (x1.02) 0.958
ondansetron (+0.0016) 0.958
Postpartum finding (x1.02) 0.958
Cardiomegaly (x1.02) 0.959
Third trimester pregnancy (x1.02) 0.959
Disorder of intestine (x1.02) 0.959
Abnormal breathing (x1.02) 0.960
Dyspnea (x1.02) 0.960
Methicillin resistant Staphylococcus aureus infection (x1.02) 0.960
Chronic pulmonary edema (x1.02) 0.961
Anxiety state (x1.02) 0.961
Edema (x1.02) 0.961
Spasm (x1.02) 0.961
Diarrhea (x1.02) 0.962
Complication occurring during pregnancy (x1.02) 0.962
Malaise (x1.02) 0.962
Complication of pregnancy, childbirth and/or the puerperium (x1.02) 0.963
Disorder of lung (x1.02) 0.963
tizanidine (+0.00144) 0.963
Drug-induced mood disorder (x1.02) 0.963
Chronic obstructive lung disease (x1.02) 0.964
Altered mental status (x1.02) 0.964
Hypo-osmolality and or hyponatremia (x1.02) 0.964
Heart failure (x1.02) 0.965
Lung field abnormal (x1.02) 0.965
Expressions
−1.00 −0.75 −0.50 −0.25 0.00 0.25 0.50 0.75 1.00

Signature [678]; 287 To Reach 0.975, (100 Plotted)

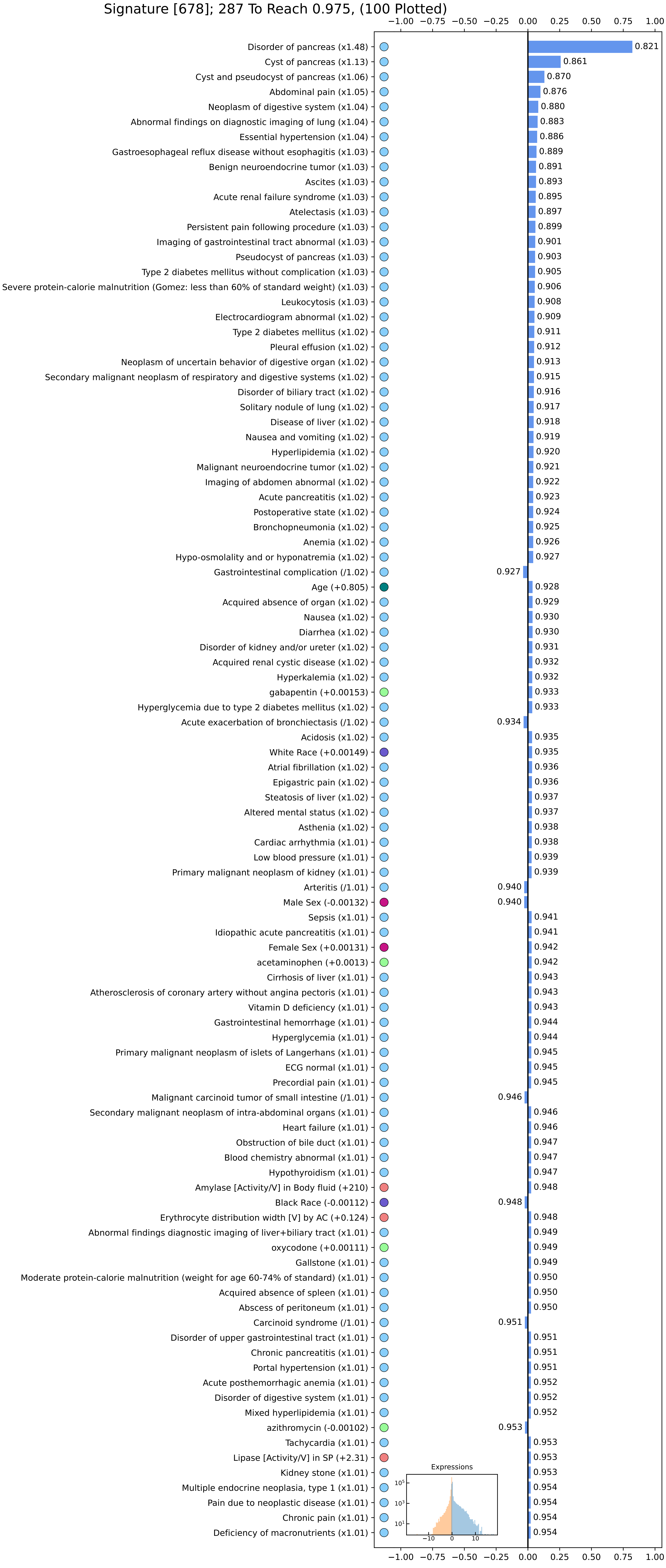

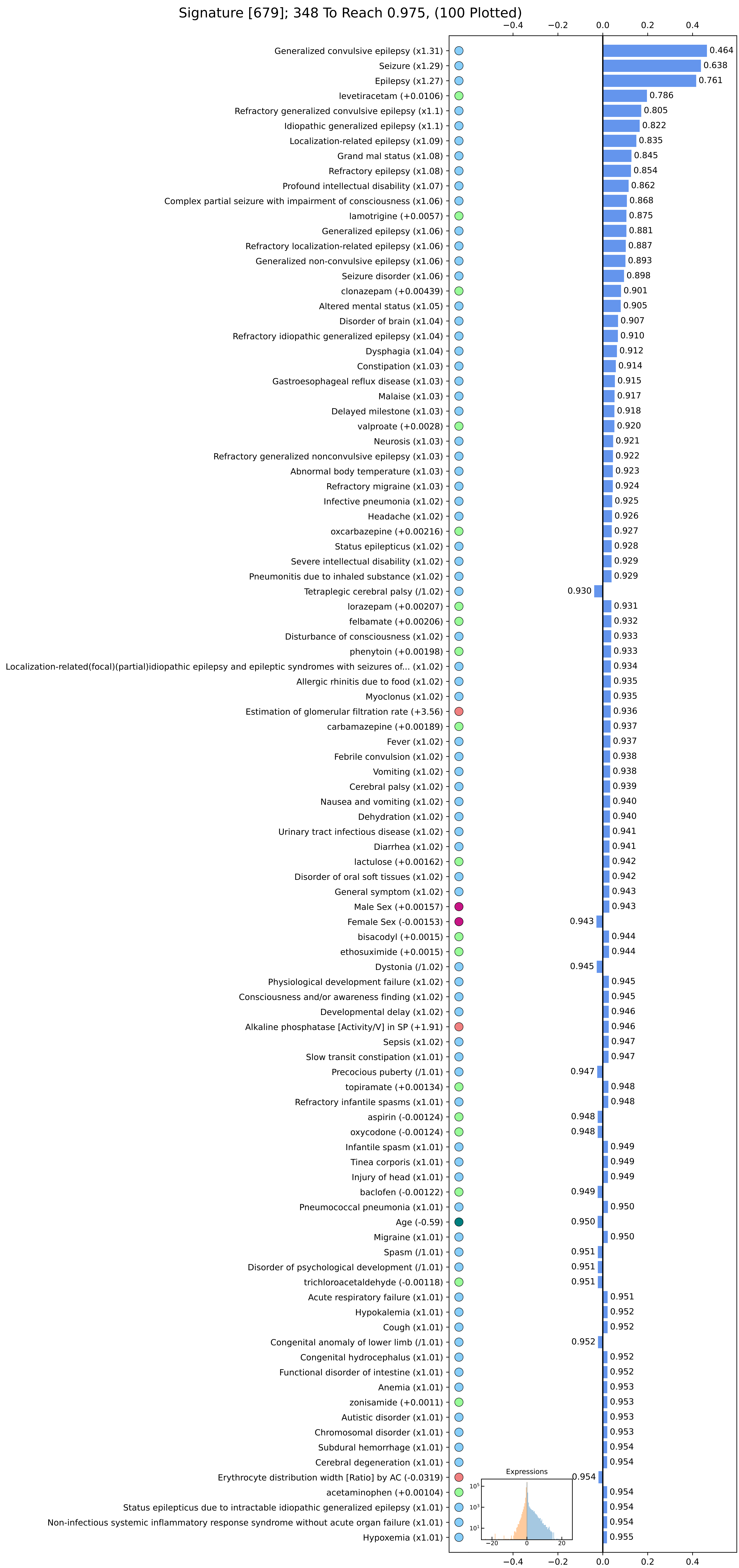

Signature [679]; 348 To Reach 0.975, (100 Plotted)
Generalized convulsive epilepsy (x1.31) 0.464
Seizure (x1.29) 0.638
Epilepsy (x1.27) 0.761
levetiracetam (+0.0106) 0.786
Refractory generalized convulsive epilepsy (x1.1) 0.805
Idiopathic generalized epilepsy (x1.1) 0.822
Localization-related epilepsy (x1.09) 0.835
Grand mal status (x1.08) 0.845
Refractory epilepsy (x1.08) 0.854
Profound intellectual disability (x1.07) 0.862
Complex partial seizure with impairment of consciousness (x1.06) 0.868
lamotrigine (+0.0057) 0.875
Generalized epilepsy (x1.06) 0.881
Refractory localization-related epilepsy (x1.06) 0.887
Generalized non-convulsive epilepsy (x1.06) 0.893
Seizure disorder (x1.06) 0.898
clonazepam (+0.00439) 0.901
Altered mental status (x1.05) 0.905
Disorder of brain (x1.04) 0.907
Refractory idiopathic generalized epilepsy (x1.04) 0.910
Dysphagia (x1.04) 0.912
Constipation (x1.03) 0.914
Gastroesophageal reflux disease (x1.03) 0.915
Malaise (x1.03) 0.917
Delayed milestone (x1.03) 0.918
valproate (+0.0028) 0.920
Neurosis (x1.03) 0.921
Refractory generalized nonconvulsive epilepsy (x1.03) 0.922
Abnormal body temperature (x1.03) 0.923
Refractory migraine (x1.03) 0.924
Infective pneumonia (x1.02) 0.925
Headache (x1.02) 0.926
oxcarbazepine (+0.00216) 0.927
Status epilepticus (x1.02) 0.928
Severe intellectual disability (x1.02) 0.929
Pneumonitis due to inhaled substance (x1.02) 0.929
Tetraplegic cerebral palsy (/1.02) 0.930
lorazepam (+0.00207) 0.931
felbamate (+0.00206) 0.932
Disturbance of consciousness (x1.02) 0.933
phenytoin (+0.00198) 0.933
Localization-related(focal)(partial)idiopathic epilepsy and epileptic syndromes with seizures of... (x1.02) 0.934
Allergic rhinitis due to food (x1.02) 0.935
Myoclonus (x1.02) 0.935
Estimation of glomerular filtration rate (+3.56) 0.936
carbamazepine (+0.00189) 0.937
Fever (x1.02) 0.937
Febrile convulsion (x1.02) 0.938
Vomiting (x1.02) 0.938
Cerebral palsy (x1.02) 0.939
Nausea and vomiting (x1.02) 0.940
Dehydration (x1.02) 0.940
Urinary tract infectious disease (x1.02) 0.941
Diarrhea (x1.02) 0.941
lactulose (+0.00162) 0.942
Disorder of oral soft tissues (x1.02) 0.942
General symptom (x1.02) 0.943
Male Sex (+0.00157) 0.943
Female Sex (-0.00153) 0.943
bisacodyl (+0.0015) 0.944
ethosuximide (+0.0015) 0.944
Dystonia (/1.02) 0.945
Physiological development failure (x1.02) 0.945
Consciousness and/or awareness finding (x1.02) 0.945
Developmental delay (x1.02) 0.946
Alkaline phosphatase [Activity/V] in SP (+1.91) 0.946
Sepsis (x1.02) 0.947
Slow transit constipation (x1.01) 0.947
Precocious puberty (/1.01) 0.947
topiramate (+0.00134) 0.948
Refractory infantile spasms (x1.01) 0.948
aspirin (-0.00124) 0.948
oxycodone (-0.00124) 0.948
Infantile spasm (x1.01) 0.949
Tinea corporis (x1.01) 0.949
Injury of head (x1.01) 0.949
baclofen (-0.00122) 0.949
Pneumococcal pneumonia (x1.01) 0.950
Age (-0.59) 0.950
Migraine (x1.01) 0.950
Spasm (/1.01) 0.951
Disorder of psychological development (/1.01) 0.951
trichloroacetaldehyde (-0.00118) 0.951
Acute respiratory failure (x1.01) 0.951
Hypokalemia (x1.01) 0.952
Cough (x1.01) 0.952
Congenital anomaly of lower limb (/1.01) 0.952
Congenital hydrocephalus (x1.01) 0.952
Functional disorder of intestine (x1.01) 0.952
Anemia (x1.01) 0.953
zonisamide (+0.0011) 0.953
Autistic disorder (x1.01) 0.953
Chromosomal disorder (x1.01) 0.953
Subdural hemorrhage (x1.01) 0.954
Cerebral degeneration (x1.01) 0.954
Erythrocyte distribution width [Ratio] by AC (-0.0319) 0.954
acetaminophen (+0.00104) 0.954
Status epilepticus due to intractable idiopathic generalized epilepsy (x1.01) 0.954
Non-infectious systemic inflammatory response syndrome without acute organ failure (x1.01) 0.954
Hypoxemia (x1.01) 0.955
−0.4 −0.2 0.0 0.2 0.4
Expressions

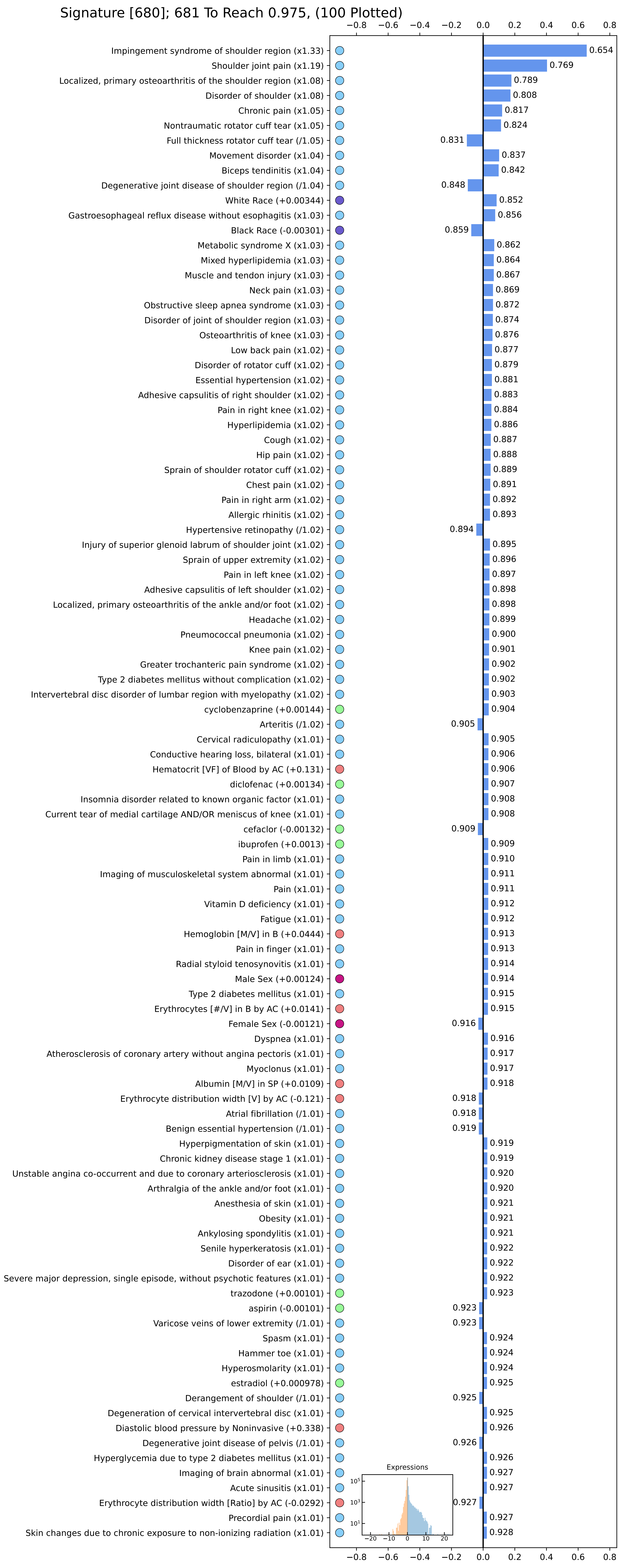

Signature [680]; 681 To Reach 0.975, (100 Plotted)
Impingement syndrome of shoulder region (x1.33) 0.654
Shoulder joint pain (x1.19) 0.769
Localized, primary osteoarthritis of the shoulder region (x1.08) 0.789
Disorder of shoulder (x1.08) 0.808
Chronic pain (x1.05) 0.817
Nontraumatic rotator cuff tear (x1.05) 0.824
Full thickness rotator cuff tear (/1.05) 0.831
Movement disorder (x1.04) 0.837
Biceps tendinitis (x1.04) 0.842
Degenerative joint disease of shoulder region (/1.04) 0.848
White Race (+0.00344) 0.852
Gastroesophageal reflux disease without esophagitis (x1.03) 0.856
Black Race (-0.00301) 0.859
Metabolic syndrome X (x1.03) 0.862
Mixed hyperlipidemia (x1.03) 0.864
Muscle and tendon injury (x1.03) 0.867
Neck pain (x1.03) 0.869
Obstructive sleep apnea syndrome (x1.03) 0.872
Disorder of joint of shoulder region (x1.03) 0.874
Osteoarthritis of knee (x1.03) 0.876
Low back pain (x1.02) 0.877
Disorder of rotator cuff (x1.02) 0.879
Essential hypertension (x1.02) 0.881
Adhesive capsulitis of right shoulder (x1.02) 0.883
Pain in right knee (x1.02) 0.884
Hyperlipidemia (x1.02) 0.886
Cough (x1.02) 0.887
Hip pain (x1.02) 0.888
Sprain of shoulder rotator cuff (x1.02) 0.889
Chest pain (x1.02) 0.891
Pain in right arm (x1.02) 0.892
Allergic rhinitis (x1.02) 0.893
Hypertensive retinopathy (/1.02) 0.894
Injury of superior glenoid labrum of shoulder joint (x1.02) 0.895
Sprain of upper extremity (x1.02) 0.896
Pain in left knee (x1.02) 0.897
Adhesive capsulitis of left shoulder (x1.02) 0.898
Localized, primary osteoarthritis of the ankle and/or foot (x1.02) 0.898
Headache (x1.02) 0.899
Pneumococcal pneumonia (x1.02) 0.900
Knee pain (x1.02) 0.901
Greater trochanteric pain syndrome (x1.02) 0.902
Type 2 diabetes mellitus without complication (x1.02) 0.902
Intervertebral disc disorder of lumbar region with myelopathy (x1.02) 0.903
cyclobenzaprine (+0.00144) 0.904
Arteritis (/1.02) 0.905
Cervical radiculopathy (x1.01) 0.905
Conductive hearing loss, bilateral (x1.01) 0.906
Hematocrit [VF] of Blood by AC (+0.131) 0.906
diclofenac (+0.00134) 0.907
Insomnia disorder related to known organic factor (x1.01) 0.908
Current tear of medial cartilage AND/OR meniscus of knee (x1.01) 0.908
cefaclor (-0.00132) 0.909
ibuprofen (+0.0013) 0.909
Pain in limb (x1.01) 0.910
Imaging of musculoskeletal system abnormal (x1.01) 0.911
Pain (x1.01) 0.911
Vitamin D deficiency (x1.01) 0.912
Fatigue (x1.01) 0.912
Hemoglobin [M/V] in B (+0.0444) 0.913
Pain in finger (x1.01) 0.913
Radial styloid tenosynovitis (x1.01) 0.914
Male Sex (+0.00124) 0.914
Type 2 diabetes mellitus (x1.01) 0.915
Erythrocytes [#/V] in B by AC (+0.0141) 0.915
Female Sex (-0.00121) 0.916
Dyspnea (x1.01) 0.916
Atherosclerosis of coronary artery without angina pectoris (x1.01) 0.917
Myoclonus (x1.01) 0.917
Albumin [M/V] in SP (+0.0109) 0.918
Erythrocyte distribution width [V] by AC (-0.121) 0.918
Atrial fibrillation (/1.01) 0.918
Benign essential hypertension (/1.01) 0.919
Hyperpigmentation of skin (x1.01) 0.919
Chronic kidney disease stage 1 (x1.01) 0.919
Unstable angina co-occurrent and due to coronary arteriosclerosis (x1.01) 0.920
Arthralgia of the ankle and/or foot (x1.01) 0.920
Anesthesia of skin (x1.01) 0.921
Obesity (x1.01) 0.921
Ankylosing spondylitis (x1.01) 0.921
Senile hyperkeratosis (x1.01) 0.922
Disorder of ear (x1.01) 0.922
Severe major depression, single episode, without psychotic features (x1.01) 0.922
trazodone (+0.00101) 0.923
aspirin (-0.00101) 0.923
Varicose veins of lower extremity (/1.01) 0.923
Spasm (x1.01) 0.924
Hammer toe (x1.01) 0.924
Hyperosmolarity (x1.01) 0.924
estradiol (+0.000978) 0.925
Derangement of shoulder (/1.01) 0.925
Degeneration of cervical intervertebral disc (x1.01) 0.925
Diastolic blood pressure by Noninvasive (+0.338) 0.926
Degenerative joint disease of pelvis (/1.01) 0.926
Hyperglycemia due to type 2 diabetes mellitus (x1.01) 0.926
Imaging of brain abnormal (x1.01) 0.927
Acute sinusitis (x1.01) 0.927
Erythrocyte distribution width [Ratio] by AC (-0.0292) 0.927
Precordial pain (x1.01) 0.927
Skin changes due to chronic exposure to non-ionizing radiation (x1.01) 0.928
−0.8 −0.6 −0.4 −0.2 0.0 0.2 0.4 0.6 0.8
Expressions
−20 −10 0 10 20

Signature [681]; 4 To Reach 0.975, (10 Plotted)

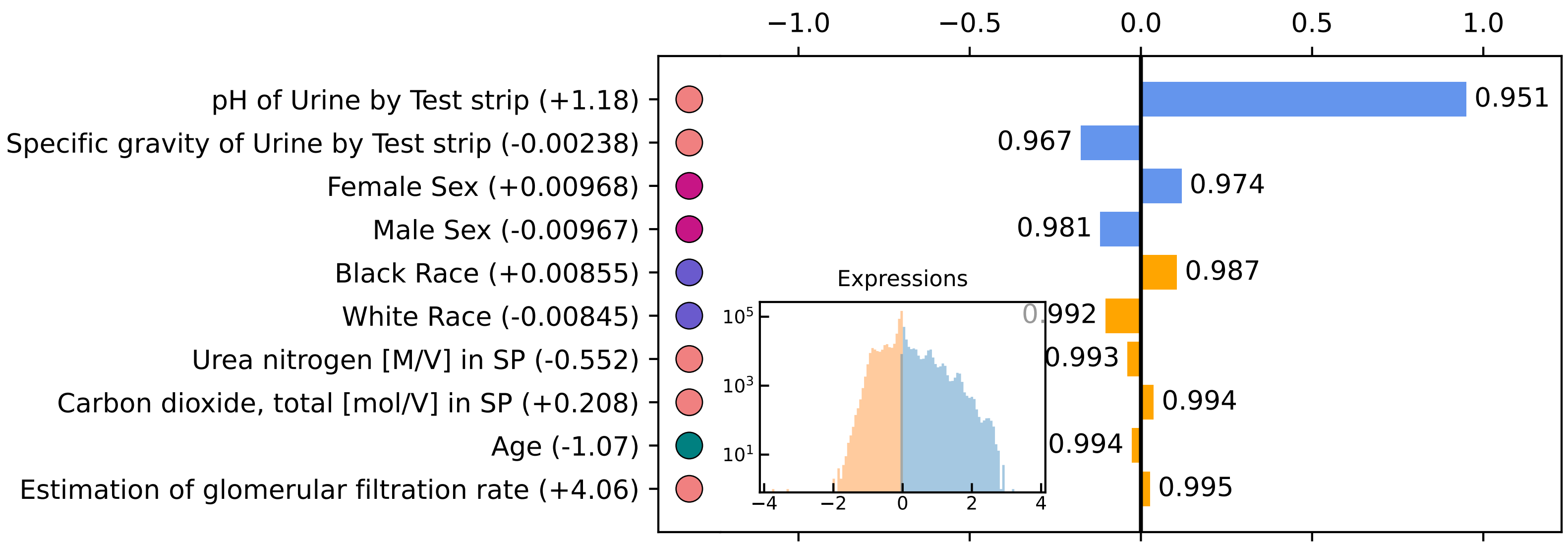

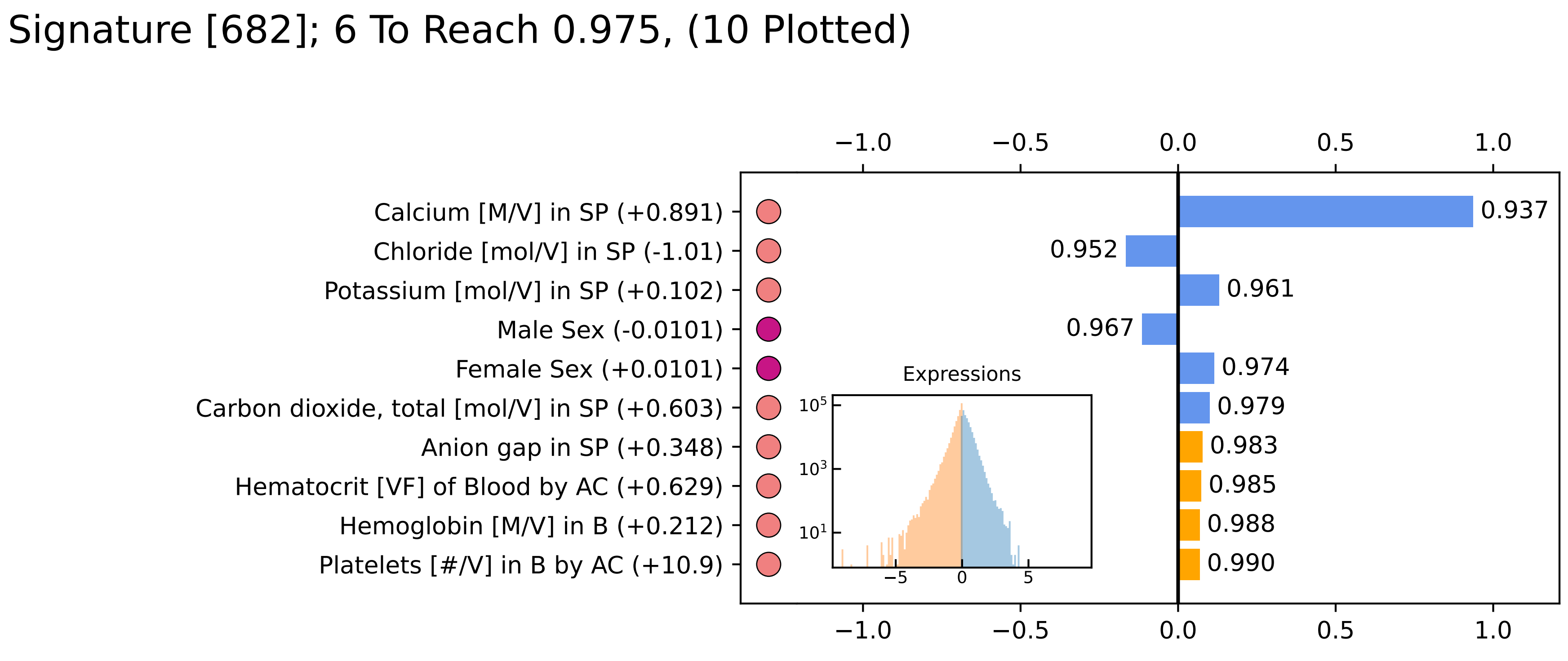

Signature [682]; 6 To Reach 0.975, (10 Plotted)
Calcium [M/V] in SP (+0.891)
Chloride [mol/V] in SP (-1.01)
Potassium [mol/V] in SP (+0.102)
Male Sex (-0.0101)
Female Sex (+0.0101)
Carbon dioxide, total [mol/V] in SP (+0.603)
Anion gap in SP (+0.348)
Hematocrit [VF] of Blood by AC (+0.629)
Hemoglobin [M/V] in B (+0.212)
Platelets [#/V] in B by AC (+10.9)
0.937
0.952
0.961
0.967
0.974
0.979
0.983
0.985
0.988
0.990
−1.0
−0.5
0.0
0.5
1.0
Expressions

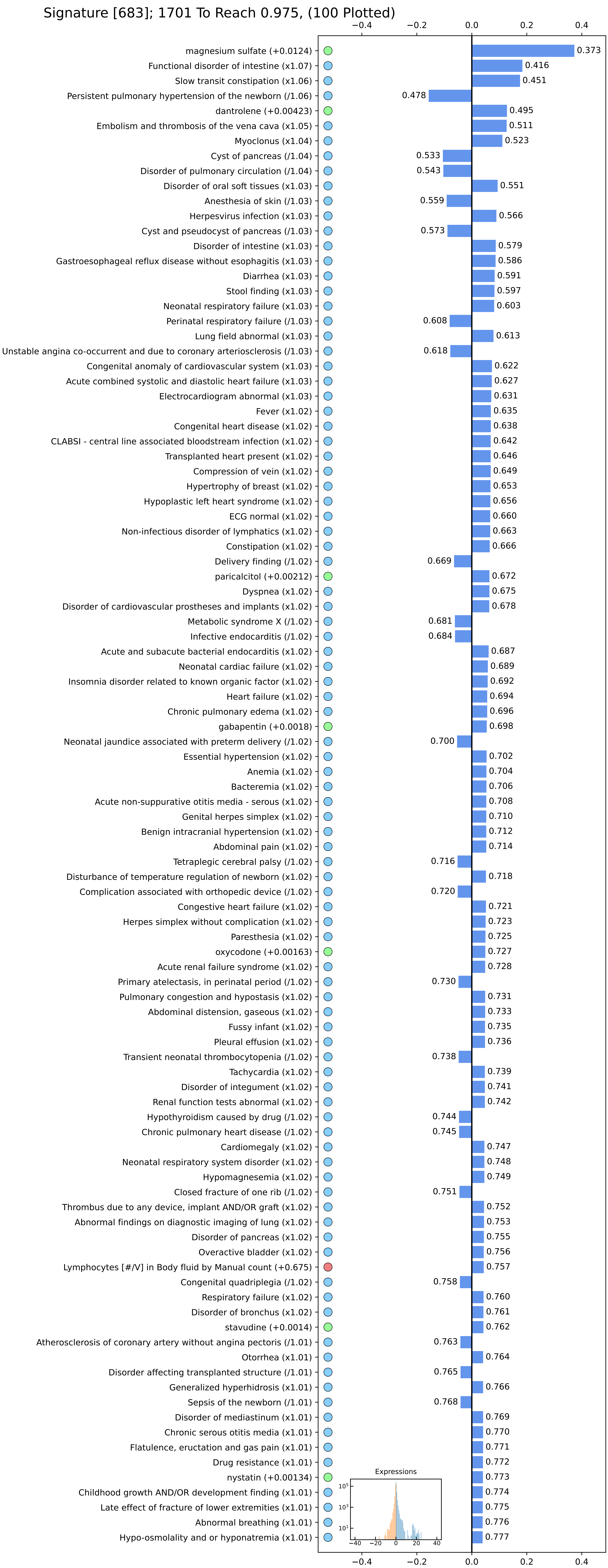

Signature [683]; 1701 To Reach 0.975, (100 Plotted)
magnesium sulfate (+0.0124) 0.373
Functional disorder of intestine (x1.07) 0.416
Slow transit constipation (x1.06) 0.451
Persistent pulmonary hypertension of the newborn (/1.06) 0.478
dantrolene (+0.00423) 0.495
Embolism and thrombosis of the vena cava (x1.05) 0.511
Myoclonus (x1.04) 0.523
Cyst of pancreas (/1.04) 0.533
Disorder of pulmonary circulation (/1.04) 0.543
Disorder of oral soft tissues (x1.03) 0.551
Anesthesia of skin (/1.03) 0.559
Herpesvirus infection (x1.03) 0.566
Cyst and pseudocyst of pancreas (/1.03) 0.573
Disorder of intestine (x1.03) 0.579
Gastroesophageal reflux disease without esophagitis (x1.03) 0.586
Diarrhea (x1.03) 0.591
Stool finding (x1.03) 0.597
Neonatal respiratory failure (x1.03) 0.603
Perinatal respiratory failure (/1.03) 0.608
Lung field abnormal (x1.03) 0.613
Unstable angina co-occurrent and due to coronary arteriosclerosis (/1.03) 0.618
Congenital anomaly of cardiovascular system (x1.03) 0.622
Acute combined systolic and diastolic heart failure (x1.03) 0.627
Electrocardiogram abnormal (x1.03) 0.631
Fever (x1.02) 0.635
Congenital heart disease (x1.02) 0.638
CLABSI - central line associated bloodstream infection (x1.02) 0.642
Transplanted heart present (x1.02) 0.646
Compression of vein (x1.02) 0.649
Hypertrophy of breast (x1.02) 0.653
Hypoplastic left heart syndrome (x1.02) 0.656
ECG normal (x1.02) 0.660
Non-infectious disorder of lymphatics (x1.02) 0.663
Constipation (x1.02) 0.666
Delivery finding (/1.02) 0.669
paricalcitol (+0.00212) 0.672
Dyspnea (x1.02) 0.675
Disorder of cardiovascular prostheses and implants (x1.02) 0.678
Metabolic syndrome X (/1.02) 0.681
Infective endocarditis (/1.02) 0.684
Acute and subacute bacterial endocarditis (x1.02) 0.687
Neonatal cardiac failure (x1.02) 0.689
Insomnia disorder related to known organic factor (x1.02) 0.692
Heart failure (x1.02) 0.694
Chronic pulmonary edema (x1.02) 0.696
gabapentin (+0.0018) 0.698
Neonatal jaundice associated with preterm delivery (/1.02) 0.700
Essential hypertension (x1.02) 0.702
Anemia (x1.02) 0.704
Bacteremia (x1.02) 0.706
Acute non-suppurative otitis media - serous (x1.02) 0.708
Genital herpes simplex (x1.02) 0.710
Benign intracranial hypertension (x1.02) 0.712
Abdominal pain (x1.02) 0.714
Tetraplegic cerebral palsy (/1.02) 0.716
Disturbance of temperature regulation of newborn (x1.02) 0.718
Complication associated with orthopedic device (/1.02) 0.720
Congestive heart failure (x1.02) 0.721
Herpes simplex without complication (x1.02) 0.723
Paresthesia (x1.02) 0.725
oxycodone (+0.00163) 0.727
Acute renal failure syndrome (x1.02) 0.728
Primary atelectasis, in perinatal period (/1.02) 0.730
Pulmonary congestion and hypostasis (x1.02) 0.731
Abdominal distension, gaseous (x1.02) 0.733
Fussy infant (x1.02) 0.735
Pleural effusion (x1.02) 0.736
Transient neonatal thrombocytopenia (/1.02) 0.738
Tachycardia (x1.02) 0.739
Disorder of integument (x1.02) 0.741
Renal function tests abnormal (x1.02) 0.742
Hypothyroidism caused by drug (/1.02) 0.744
Chronic pulmonary heart disease (/1.02) 0.745
Cardiomegaly (x1.02) 0.747
Neonatal respiratory system disorder (x1.02) 0.748
Hypomagnesemia (x1.02) 0.749
Closed fracture of one rib (/1.02) 0.751
Thrombus due to any device, implant AND/OR graft (x1.02) 0.752
Abnormal findings on diagnostic imaging of lung (x1.02) 0.753
Disorder of pancreas (x1.02) 0.755
Overactive bladder (x1.02) 0.756
Lymphocytes [#/V] in Body fluid by Manual count (+0.675) 0.757
Congenital quadriplegia (/1.02) 0.758
Respiratory failure (x1.02) 0.760
Disorder of bronchus (x1.02) 0.761
stavudine (+0.0014) 0.762
Atherosclerosis of coronary artery without angina pectoris (/1.01) 0.763
Otorrhea (x1.01) 0.764
Disorder affecting transplanted structure (/1.01) 0.765
Generalized hyperhidrosis (x1.01) 0.766
Sepsis of the newborn (/1.01) 0.768
Disorder of mediastinum (x1.01) 0.769
Chronic serous otitis media (x1.01) 0.770
Flatulence, eructation and gas pain (x1.01) 0.771
Drug resistance (x1.01) 0.772
nystatin (+0.00134) 0.773
Childhood growth AND/OR development finding (x1.01) 0.774
Late effect of fracture of lower extremities (x1.01) 0.775
Abnormal breathing (x1.01) 0.776
Hypo-osmolality and or hyponatremia (x1.01) 0.777
−0.4 −0.2 0.0 0.2 0.4
Expressions

Signature [684]; 1 To Reach 0.975, (10 Plotted)

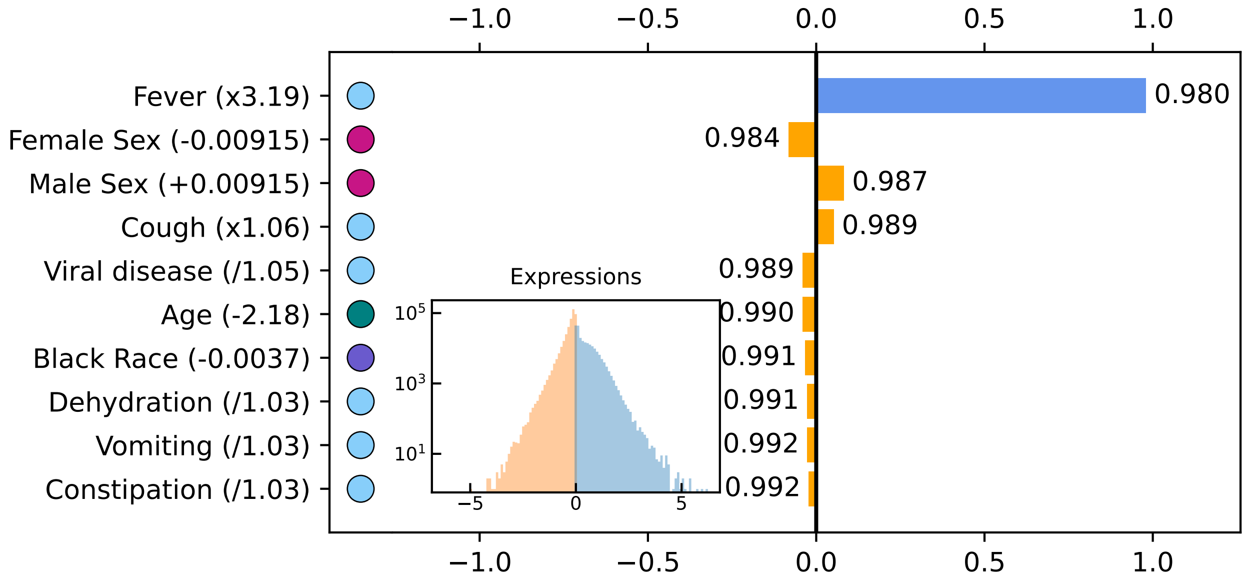

Signature [685]; 1 To Reach 0.975, (10 Plotted)

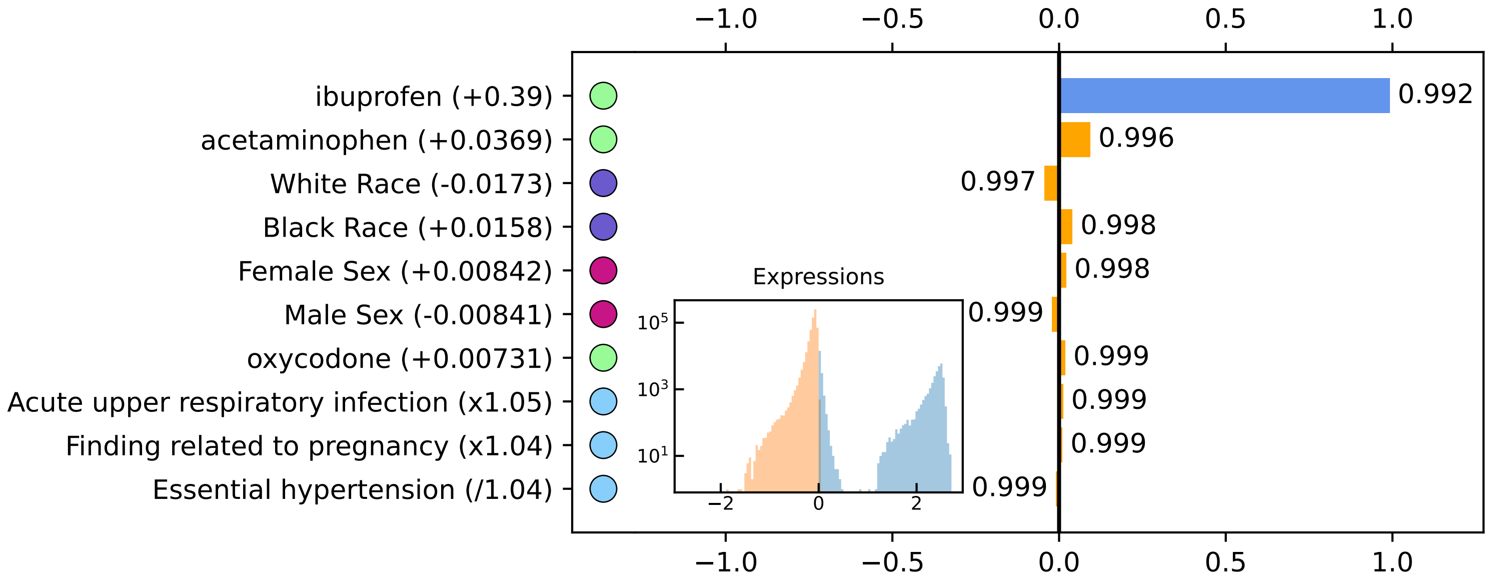

Signature [686]; 119 To Reach 0.975, (100 Plotted)

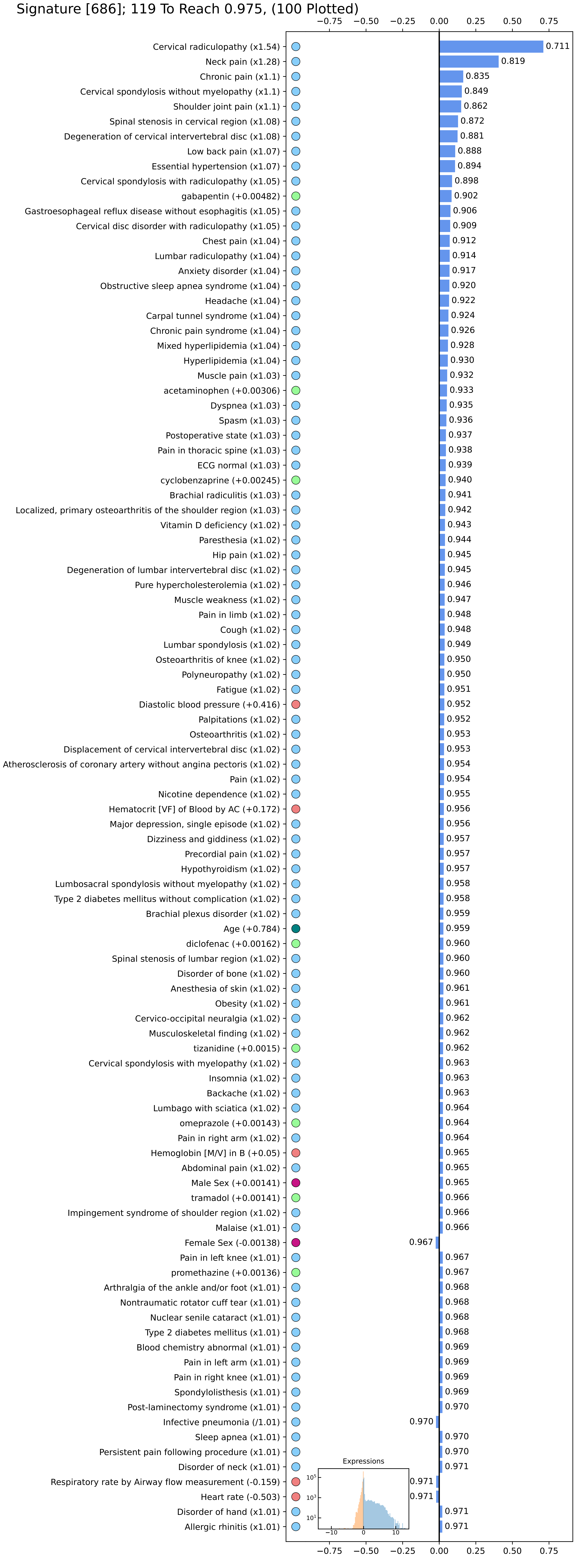

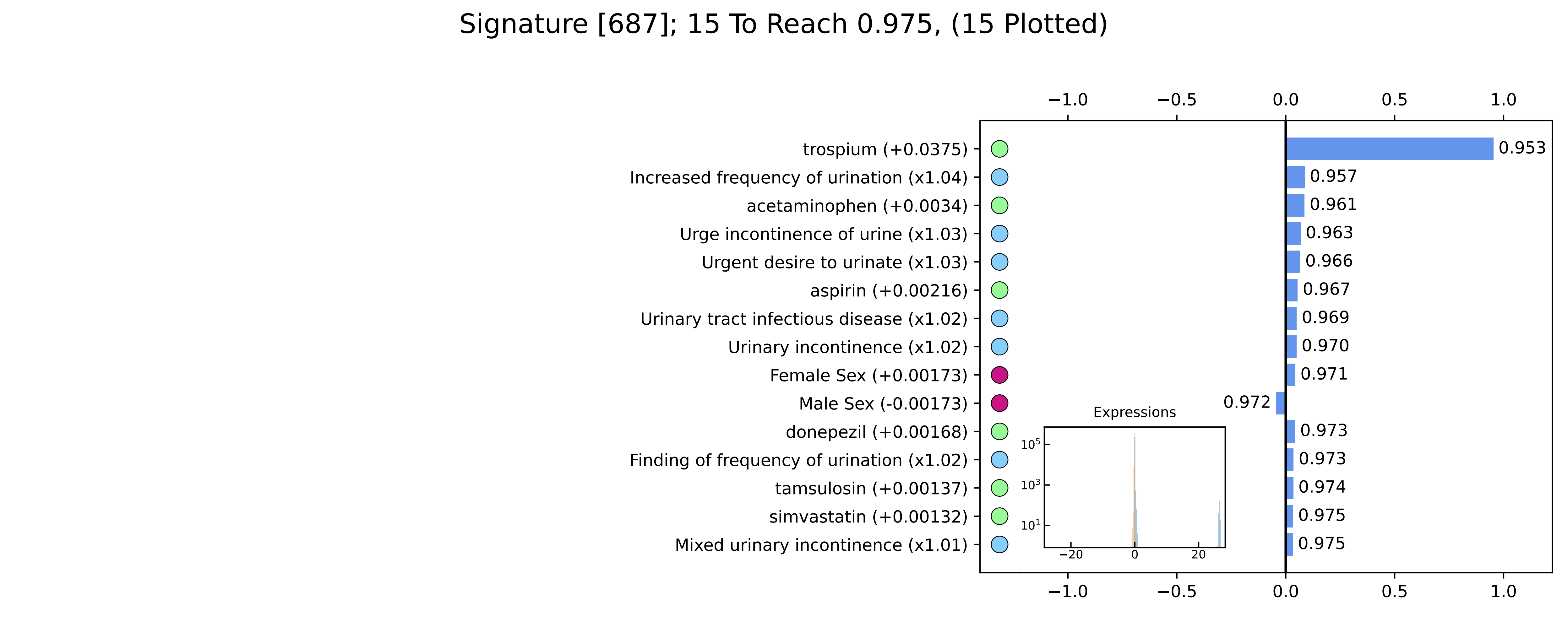

Signature [687]; 15 To Reach 0.975, (15 Plotted)
trospium (+0.0375)
Increased frequency of urination (x1.04)
acetaminophen (+0.0034)
Urge incontinence of urine (x1.03)
Urgent desire to urinate (x1.03)
aspirin (+0.00216)
Urinary tract infectious disease (x1.02)
Urinary incontinence (x1.02)
Female Sex (+0.00173)
Male Sex (-0.00173)
donepezil (+0.00168)
Finding of frequency of urination (x1.02)
tamsulosin (+0.00137)
simvastatin (+0.00132)
Mixed urinary incontinence (x1.01)
0.953
0.957
0.961
0.963
0.966
0.967
0.969
0.970
0.971
0.972
0.973
0.973
0.974
0.975
0.975
Expressions
−1.0
−0.5
0.0
0.5
1.0

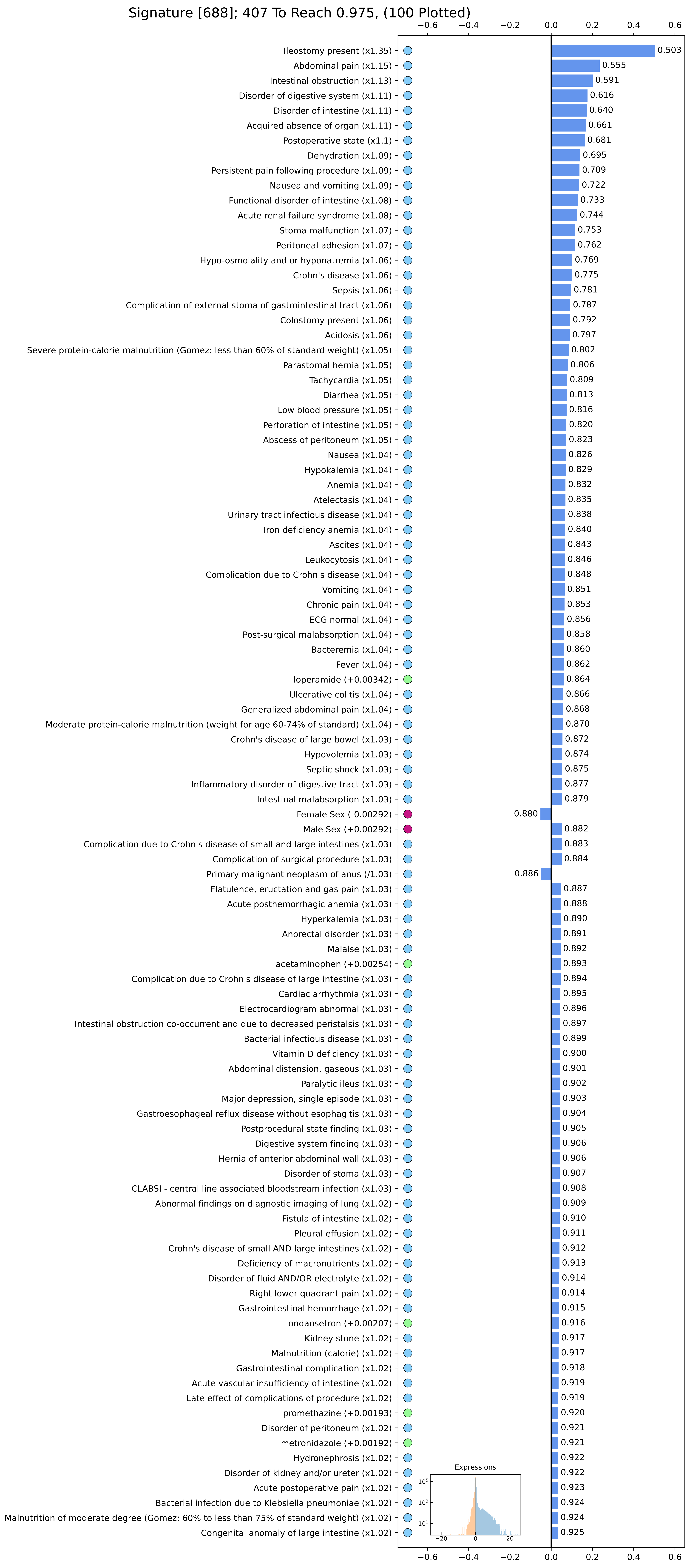

Signature [688]; 407 To Reach 0.975, (100 Plotted)
Ileostomy present (x1.35) 0.503
Abdominal pain (x1.15) 0.555
Intestinal obstruction (x1.13) 0.591
Disorder of digestive system (x1.11) 0.616
Disorder of intestine (x1.11) 0.640
Acquired absence of organ (x1.11) 0.661
Postoperative state (x1.1) 0.681
Dehydration (x1.09) 0.695
Persistent pain following procedure (x1.09) 0.709
Nausea and vomiting (x1.09) 0.722
Functional disorder of intestine (x1.08) 0.733
Acute renal failure syndrome (x1.08) 0.744
Stoma malfunction (x1.07) 0.753
Peritoneal adhesion (x1.07) 0.762
Hypo-osmolality and or hyponatremia (x1.06) 0.769
Crohn's disease (x1.06) 0.775
Sepsis (x1.06) 0.781
Complication of external stoma of gastrointestinal tract (x1.06) 0.787
Colostomy present (x1.06) 0.792
Acidosis (x1.06) 0.797
Severe protein-calorie malnutrition (Gomez: less than 60% of standard weight) (x1.05) 0.802
Parastomal hernia (x1.05) 0.806
Tachycardia (x1.05) 0.809
Diarrhea (x1.05) 0.813
Low blood pressure (x1.05) 0.816
Perforation of intestine (x1.05) 0.820
Abscess of peritoneum (x1.05) 0.823
Nausea (x1.04) 0.826
Hypokalemia (x1.04) 0.829
Anemia (x1.04) 0.832
Atelectasis (x1.04) 0.835
Urinary tract infectious disease (x1.04) 0.838
Iron deficiency anemia (x1.04) 0.840
Ascites (x1.04) 0.843
Leukocytosis (x1.04) 0.846
Complication due to Crohn's disease (x1.04) 0.848
Vomiting (x1.04) 0.851
Chronic pain (x1.04) 0.853
ECG normal (x1.04) 0.856
Post-surgical malabsorption (x1.04) 0.858
Bacteremia (x1.04) 0.860
Fever (x1.04) 0.862
loperamide (+0.00342) 0.864
Ulcerative colitis (x1.04) 0.866
Generalized abdominal pain (x1.04) 0.868
Moderate protein-calorie malnutrition (weight for age 60-74% of standard) (x1.04) 0.870
Crohn's disease of large bowel (x1.03) 0.872
Hypovolemia (x1.03) 0.874
Septic shock (x1.03) 0.875
Inflammatory disorder of digestive tract (x1.03) 0.877
Intestinal malabsorption (x1.03) 0.879
Female Sex (-0.00292) 0.880
Male Sex (+0.00292) 0.882
Complication due to Crohn's disease of small and large intestines (x1.03) 0.883
Complication of surgical procedure (x1.03) 0.884
Primary malignant neoplasm of anus (/1.03) 0.886
Flatulence, eructation and gas pain (x1.03) 0.887
Acute posthemorrhagic anemia (x1.03) 0.888
Hyperkalemia (x1.03) 0.890
Anorectal disorder (x1.03) 0.891
Malaise (x1.03) 0.892
acetaminophen (+0.00254) 0.893
Complication due to Crohn's disease of large intestine (x1.03) 0.894
Cardiac arrhythmia (x1.03) 0.895
Electrocardiogram abnormal (x1.03) 0.896
Intestinal obstruction co-occurrent and due to decreased peristalsis (x1.03) 0.897
Bacterial infectious disease (x1.03) 0.899
Vitamin D deficiency (x1.03) 0.900
Abdominal distension, gaseous (x1.03) 0.901
Paralytic ileus (x1.03) 0.902
Major depression, single episode (x1.03) 0.903
Gastroesophageal reflux disease without esophagitis (x1.03) 0.904
Postprocedural state finding (x1.03) 0.905
Digestive system finding (x1.03) 0.906
Hernia of anterior abdominal wall (x1.03) 0.906
Disorder of stoma (x1.03) 0.907
CLABSI - central line associated bloodstream infection (x1.03) 0.908
Abnormal findings on diagnostic imaging of lung (x1.02) 0.909
Fistula of intestine (x1.02) 0.910
Pleural effusion (x1.02) 0.911
Crohn's disease of small AND large intestines (x1.02) 0.912
Deficiency of macronutrients (x1.02) 0.913
Disorder of fluid AND/OR electrolyte (x1.02) 0.914
Right lower quadrant pain (x1.02) 0.914
Gastrointestinal hemorrhage (x1.02) 0.915
ondansetron (+0.00207) 0.916
Kidney stone (x1.02) 0.917
Malnutrition (calorie) (x1.02) 0.917
Gastrointestinal complication (x1.02) 0.918
Acute vascular insufficiency of intestine (x1.02) 0.919
Late effect of complications of procedure (x1.02) 0.919
promethazine (+0.00193) 0.920
Disorder of peritoneum (x1.02) 0.921
metronidazole (+0.00192) 0.921
Hydronephrosis (x1.02) 0.922
Disorder of kidney and/or ureter (x1.02) 0.922
Acute postoperative pain (x1.02) 0.923
Bacterial infection due to Klebsiella pneumoniae (x1.02) 0.924
Malnutrition of moderate degree (Gomez: 60% to less than 75% of standard weight) (x1.02) 0.924
Congenital anomaly of large intestine (x1.02) 0.925
Expressions
−0.6 −0.4 −0.2 0.0 0.2 0.4 0.6

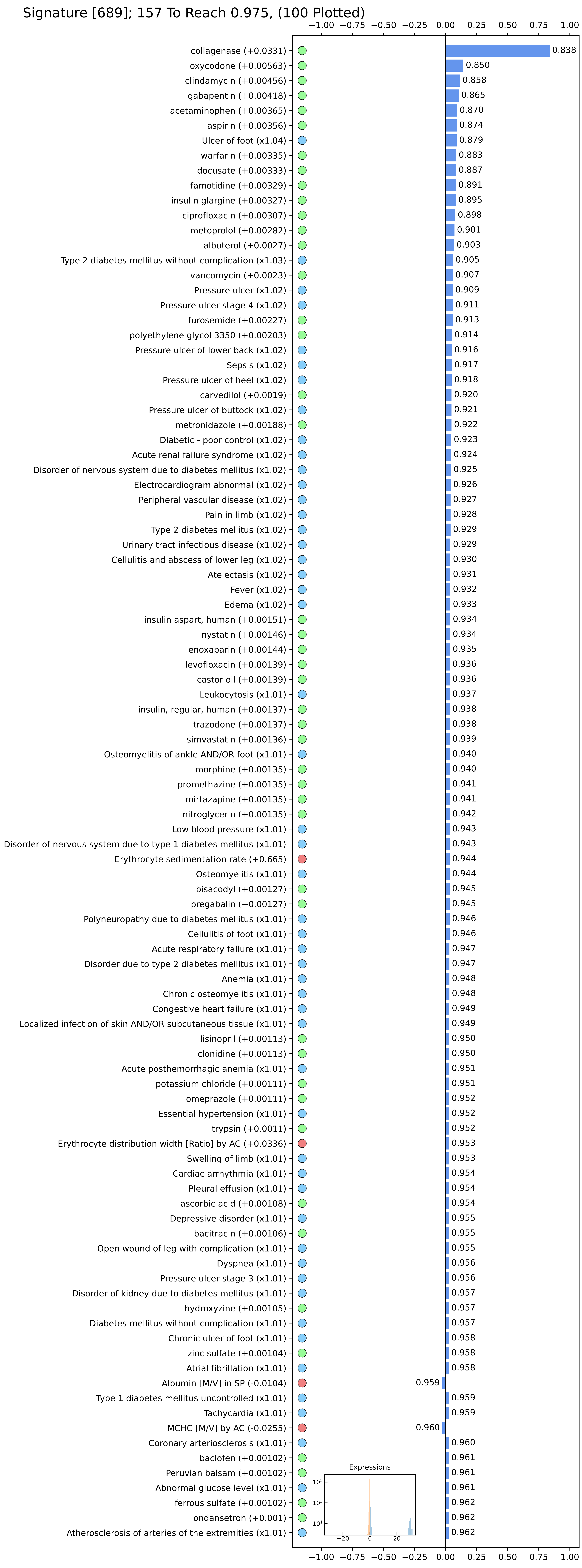

Signature [689]; 157 To Reach 0.975, (100 Plotted)
collagenase (+0.0331) 0.838
oxycodone (+0.00563) 0.850
clindamycin (+0.00456) 0.858
gabapentin (+0.00418) 0.865
acetaminophen (+0.00365) 0.870
aspirin (+0.00356) 0.874
Ulcer of foot (x1.04) 0.879
warfarin (+0.00335) 0.883
docusate (+0.00333) 0.887
famotidine (+0.00329) 0.891
insulin glargine (+0.00327) 0.895
ciprofloxacin (+0.00307) 0.898
metoprolol (+0.00282) 0.901
albuterol (+0.0027) 0.903
Type 2 diabetes mellitus without complication (x1.03) 0.905
vancomycin (+0.0023) 0.907
Pressure ulcer (x1.02) 0.909
Pressure ulcer stage 4 (x1.02) 0.911
furosemide (+0.00227) 0.913
polyethylene glycol 3350 (+0.00203) 0.914
Pressure ulcer of lower back (x1.02) 0.916
Sepsis (x1.02) 0.917
Pressure ulcer of heel (x1.02) 0.918
carvedilol (+0.0019) 0.920
Pressure ulcer of buttock (x1.02) 0.921
metronidazole (+0.00188) 0.922
Diabetic - poor control (x1.02) 0.923
Acute renal failure syndrome (x1.02) 0.924
Disorder of nervous system due to diabetes mellitus (x1.02) 0.925
Electrocardiogram abnormal (x1.02) 0.926
Peripheral vascular disease (x1.02) 0.927
Pain in limb (x1.02) 0.928
Type 2 diabetes mellitus (x1.02) 0.929
Urinary tract infectious disease (x1.02) 0.929
Cellulitis and abscess of lower leg (x1.02) 0.930
Atelectasis (x1.02) 0.931
Fever (x1.02) 0.932
Edema (x1.02) 0.933
insulin aspart, human (+0.00151) 0.934
nystatin (+0.00146) 0.934
enoxaparin (+0.00144) 0.935
levofloxacin (+0.00139) 0.936
castor oil (+0.00139) 0.936
Leukocytosis (x1.01) 0.937
insulin, regular, human (+0.00137) 0.938
trazodone (+0.00137) 0.938
simvastatin (+0.00136) 0.939
Osteomyelitis of ankle AND/OR foot (x1.01) 0.940
morphine (+0.00135) 0.940
promethazine (+0.00135) 0.941
mirtazapine (+0.00135) 0.941
nitroglycerin (+0.00135) 0.942
Low blood pressure (x1.01) 0.943
Disorder of nervous system due to type 1 diabetes mellitus (x1.01) 0.943
Erythrocyte sedimentation rate (+0.665) 0.944
Osteomyelitis (x1.01) 0.944
bisacodyl (+0.00127) 0.945
pregabalin (+0.00127) 0.945
Polyneuropathy due to diabetes mellitus (x1.01) 0.946
Cellulitis of foot (x1.01) 0.946
Acute respiratory failure (x1.01) 0.947
Disorder due to type 2 diabetes mellitus (x1.01) 0.947
Anemia (x1.01) 0.948
Chronic osteomyelitis (x1.01) 0.948
Congestive heart failure (x1.01) 0.949
Localized infection of skin AND/OR subcutaneous tissue (x1.01) 0.949
lisinopril (+0.00113) 0.950
clonidine (+0.00113) 0.950
Acute posthemorrhagic anemia (x1.01) 0.951
potassium chloride (+0.00111) 0.951
omeprazole (+0.00111) 0.952
Essential hypertension (x1.01) 0.952
trypsin (+0.0011) 0.952
Erythrocyte distribution width [Ratio] by AC (+0.0336) 0.953
Swelling of limb (x1.01) 0.953
Cardiac arrhythmia (x1.01) 0.954
Pleural effusion (x1.01) 0.954
ascorbic acid (+0.00108) 0.954
Depressive disorder (x1.01) 0.955
bacitracin (+0.00106) 0.955
Open wound of leg with complication (x1.01) 0.955
Dyspnea (x1.01) 0.956
Pressure ulcer stage 3 (x1.01) 0.956
Disorder of kidney due to diabetes mellitus (x1.01) 0.957
hydroxyzine (+0.00105) 0.957
Diabetes mellitus without complication (x1.01) 0.957
Chronic ulcer of foot (x1.01) 0.958
zinc sulfate (+0.00104) 0.958
Atrial fibrillation (x1.01) 0.958
Albumin [M/V] in SP (-0.0104) 0.959
Type 1 diabetes mellitus uncontrolled (x1.01) 0.959
Tachycardia (x1.01) 0.959
MCHC [M/V] by AC (-0.0255) 0.960
Coronary arteriosclerosis (x1.01) 0.960
baclofen (+0.00102) 0.961
Peruvian balsam (+0.00102) 0.961
Abnormal glucose level (x1.01) 0.961
ferrous sulfate (+0.00102) 0.962
ondansetron (+0.001) 0.962
Atherosclerosis of arteries of the extremities (x1.01) 0.962
−1.00 −0.75 −0.50 −0.25 0.00 0.25 0.50 0.75 1.00
Expressions

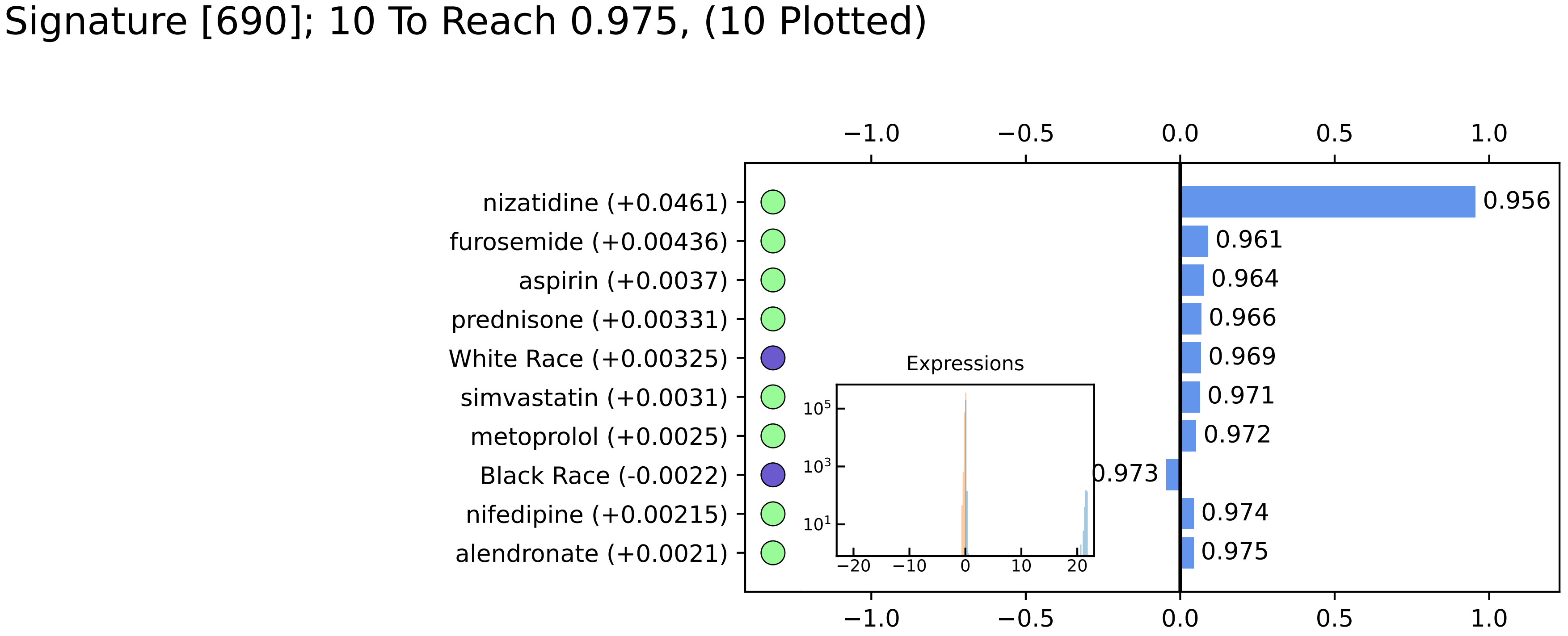
Signature [690]; 10 To Reach 0.975, (10 Plotted)
nizatidine (+0.0461)
furosemide (+0.00436)
aspirin (+0.0037)
prednisone (+0.00331)
White Race (+0.00325)
simvastatin (+0.0031)
metoprolol (+0.0025)
Black Race (-0.0022)
nifedipine (+0.00215)
alendronate (+0.0021)
0.956
0.961
0.964
0.966
0.969
0.971
0.972
0.973
0.974
0.975
−1.0
−0.5
0.0
0.5
1.0
Expressions
−20
−10
0
10
20

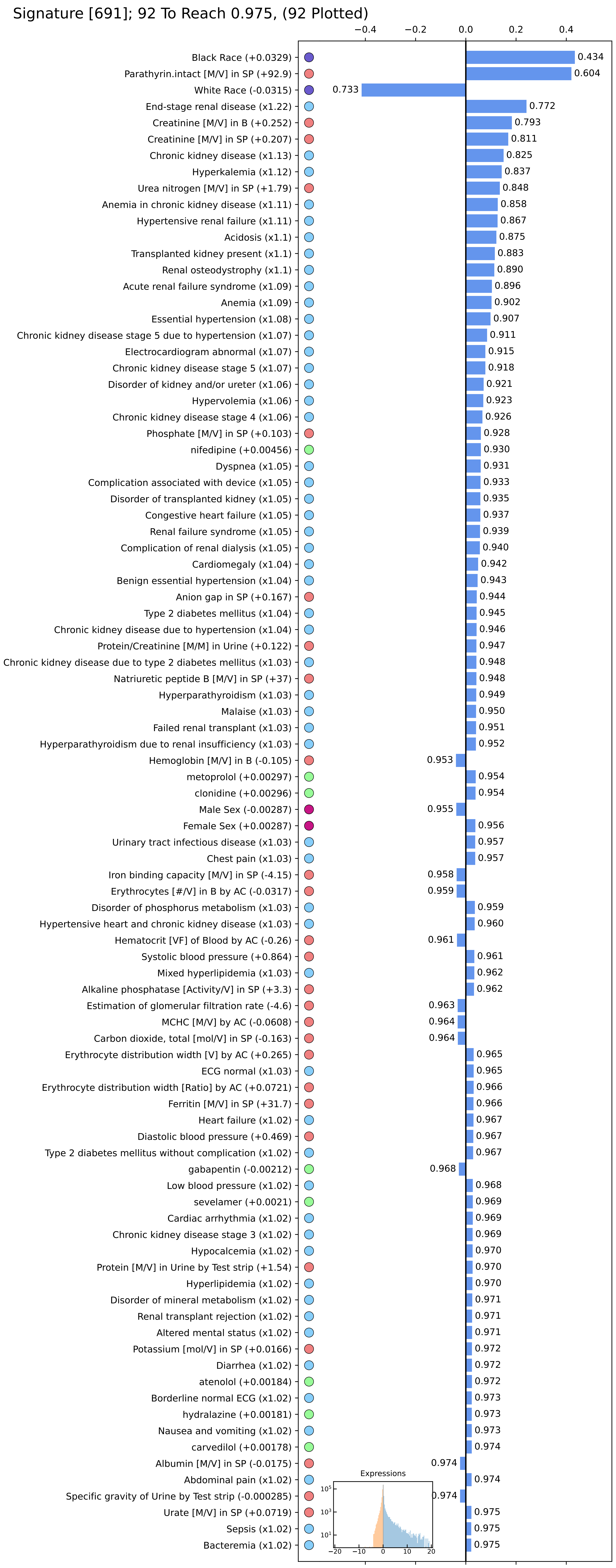

Signature [691]; 92 To Reach 0.975, (92 Plotted)
Black Race (+0.0329) 0.434
Parathyrin.intact [M/V] in SP (+92.9) 0.604
White Race (-0.0315) 0.733
End-stage renal disease (x1.22) 0.772
Creatinine [M/V] in B (+0.252) 0.793
Creatinine [M/V] in SP (+0.207) 0.811
Chronic kidney disease (x1.13) 0.825
Hyperkalemia (x1.12) 0.837
Urea nitrogen [M/V] in SP (+1.79) 0.848
Anemia in chronic kidney disease (x1.11) 0.858
Hypertensive renal failure (x1.11) 0.867
Acidosis (x1.1) 0.875
Transplanted kidney present (x1.1) 0.883
Renal osteodystrophy (x1.1) 0.890
Acute renal failure syndrome (x1.09) 0.896
Anemia (x1.09) 0.902
Essential hypertension (x1.08) 0.907
Chronic kidney disease stage 5 due to hypertension (x1.07) 0.911
Electrocardiogram abnormal (x1.07) 0.915
Chronic kidney disease stage 5 (x1.07) 0.918
Disorder of kidney and/or ureter (x1.06) 0.921
Hypervolemia (x1.06) 0.923
Chronic kidney disease stage 4 (x1.06) 0.926
Phosphate [M/V] in SP (+0.103) 0.928
nifedipine (+0.00456) 0.930
Dyspnea (x1.05) 0.931
Complication associated with device (x1.05) 0.933
Disorder of transplanted kidney (x1.05) 0.935
Congestive heart failure (x1.05) 0.937
Renal failure syndrome (x1.05) 0.939
Complication of renal dialysis (x1.05) 0.940
Cardiomegaly (x1.04) 0.942
Benign essential hypertension (x1.04) 0.943
Anion gap in SP (+0.167) 0.944
Type 2 diabetes mellitus (x1.04) 0.945
Chronic kidney disease due to hypertension (x1.04) 0.946
Protein/Creatinine [M/M] in Urine (+0.122) 0.947
Chronic kidney disease due to type 2 diabetes mellitus (x1.03) 0.948
Natriuretic peptide B [M/V] in SP (+37) 0.948
Hyperparathyroidism (x1.03) 0.949
Malaise (x1.03) 0.950
Failed renal transplant (x1.03) 0.951
Hyperparathyroidism due to renal insufficiency (x1.03) 0.952
Hemoglobin [M/V] in B (-0.105) 0.953
metoprolol (+0.00297) 0.954
clonidine (+0.00296) 0.954
Male Sex (-0.00287) 0.955
Female Sex (+0.00287) 0.956
Urinary tract infectious disease (x1.03) 0.957
Chest pain (x1.03) 0.957
Iron binding capacity [M/V] in SP (-4.15) 0.958
Erythrocytes [#/V] in B by AC (-0.0317) 0.959
Disorder of phosphorus metabolism (x1.03) 0.959
Hypertensive heart and chronic kidney disease (x1.03) 0.960
Hematocrit [VF] of Blood by AC (-0.26) 0.961
Systolic blood pressure (+0.864) 0.961
Mixed hyperlipidemia (x1.03) 0.962
Alkaline phosphatase [Activity/V] in SP (+3.3) 0.962
Estimation of glomerular filtration rate (-4.6) 0.963
MCHC [M/V] by AC (-0.0608) 0.964
Carbon dioxide, total [mol/V] in SP (-0.163) 0.964
Erythrocyte distribution width [V] by AC (+0.265) 0.965
ECG normal (x1.03) 0.965
Erythrocyte distribution width [Ratio] by AC (+0.0721) 0.966
Ferritin [M/V] in SP (+31.7) 0.966
Heart failure (x1.02) 0.967
Diastolic blood pressure (+0.469) 0.967
Type 2 diabetes mellitus without complication (x1.02) 0.967
gabapentin (-0.00212) 0.968
Low blood pressure (x1.02) 0.968
sevelamer (+0.0021) 0.969
Cardiac arrhythmia (x1.02) 0.969
Chronic kidney disease stage 3 (x1.02) 0.969
Hypocalcemia (x1.02) 0.970
Protein [M/V] in Urine by Test strip (+1.54) 0.970
Hyperlipidemia (x1.02) 0.970
Disorder of mineral metabolism (x1.02) 0.971
Renal transplant rejection (x1.02) 0.971
Altered mental status (x1.02) 0.971
Potassium [mol/V] in SP (+0.0166) 0.972
Diarrhea (x1.02) 0.972
atenolol (+0.00184) 0.972
Borderline normal ECG (x1.02) 0.973
hydralazine (+0.00181) 0.973
Nausea and vomiting (x1.02) 0.973
carvedilol (+0.00178) 0.974
Albumin [M/V] in SP (-0.0175) 0.974
Abdominal pain (x1.02) 0.974
Specific gravity of Urine by Test strip (-0.000285) 0.974
Urate [M/V] in SP (+0.0719) 0.975
Sepsis (x1.02) 0.975
Bacteremia (x1.02) 0.975
−0.4 −0.2 0.0 0.2 0.4
Expressions

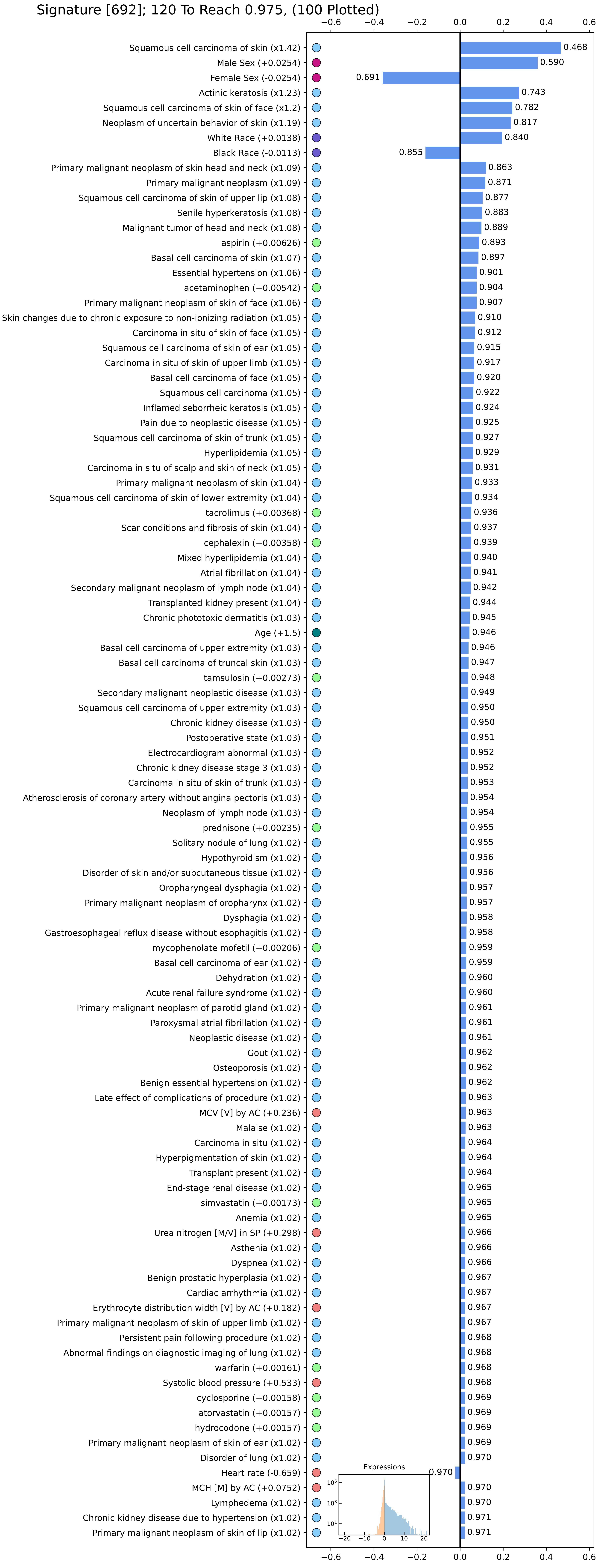

Signature [692]; 120 To Reach 0.975, (100 Plotted)
Squamous cell carcinoma of skin (x1.42) 0.468
Male Sex (+0.0254) 0.590
Female Sex (-0.0254) 0.691
Actinic keratosis (x1.23) 0.743
Squamous cell carcinoma of skin of face (x1.2) 0.782
Neoplasm of uncertain behavior of skin (x1.19) 0.817
White Race (+0.0138) 0.840
Black Race (-0.0113) 0.855
Primary malignant neoplasm of skin head and neck (x1.09) 0.863
Primary malignant neoplasm (x1.09) 0.871
Squamous cell carcinoma of skin of upper lip (x1.08) 0.877
Senile hyperkeratosis (x1.08) 0.883
Malignant tumor of head and neck (x1.08) 0.889
aspirin (+0.00626) 0.893
Basal cell carcinoma of skin (x1.07) 0.897
Essential hypertension (x1.06) 0.901
acetaminophen (+0.00542) 0.904
Primary malignant neoplasm of skin of face (x1.06) 0.907
Skin changes due to chronic exposure to non-ionizing radiation (x1.05) 0.910
Carcinoma in situ of skin of face (x1.05) 0.912
Squamous cell carcinoma of skin of ear (x1.05) 0.915
Carcinoma in situ of skin of upper limb (x1.05) 0.917
Basal cell carcinoma of face (x1.05) 0.920
Squamous cell carcinoma (x1.05) 0.922
Inflamed seborrheic keratosis (x1.05) 0.924
Pain due to neoplastic disease (x1.05) 0.925
Squamous cell carcinoma of skin of trunk (x1.05) 0.927
Hyperlipidemia (x1.05) 0.929
Carcinoma in situ of scalp and skin of neck (x1.05) 0.931
Primary malignant neoplasm of skin (x1.04) 0.933
Squamous cell carcinoma of skin of lower extremity (x1.04) 0.934
tacrolimus (+0.00368) 0.936
Scar conditions and fibrosis of skin (x1.04) 0.937
cephalexin (+0.00358) 0.939
Mixed hyperlipidemia (x1.04) 0.940
Atrial fibrillation (x1.04) 0.941
Secondary malignant neoplasm of lymph node (x1.04) 0.942
Transplanted kidney present (x1.04) 0.944
Chronic phototoxic dermatitis (x1.03) 0.945
Age (+1.5) 0.946
Basal cell carcinoma of upper extremity (x1.03) 0.946
Basal cell carcinoma of truncal skin (x1.03) 0.947
tamsulosin (+0.00273) 0.948
Secondary malignant neoplastic disease (x1.03) 0.949
Squamous cell carcinoma of upper extremity (x1.03) 0.950
Chronic kidney disease (x1.03) 0.950
Postoperative state (x1.03) 0.951
Electrocardiogram abnormal (x1.03) 0.952
Chronic kidney disease stage 3 (x1.03) 0.952
Carcinoma in situ of skin of trunk (x1.03) 0.953
Atherosclerosis of coronary artery without angina pectoris (x1.03) 0.954
Neoplasm of lymph node (x1.03) 0.954
prednisone (+0.00235) 0.955
Solitary nodule of lung (x1.02) 0.955
Hypothyroidism (x1.02) 0.956
Disorder of skin and/or subcutaneous tissue (x1.02) 0.956
Oropharyngeal dysphagia (x1.02) 0.957
Primary malignant neoplasm of oropharynx (x1.02) 0.957
Dysphagia (x1.02) 0.958
Gastroesophageal reflux disease without esophagitis (x1.02) 0.958
mycophenolate mofetil (+0.00206) 0.959
Basal cell carcinoma of ear (x1.02) 0.959
Dehydration (x1.02) 0.960
Acute renal failure syndrome (x1.02) 0.960
Primary malignant neoplasm of parotid gland (x1.02) 0.961
Paroxysmal atrial fibrillation (x1.02) 0.961
Neoplastic disease (x1.02) 0.961
Gout (x1.02) 0.962
Osteoporosis (x1.02) 0.962
Benign essential hypertension (x1.02) 0.962
Late effect of complications of procedure (x1.02) 0.963
MCV [V] by AC (+0.236) 0.963
Malaise (x1.02) 0.963
Carcinoma in situ (x1.02) 0.964
Hyperpigmentation of skin (x1.02) 0.964
Transplant present (x1.02) 0.964
End-stage renal disease (x1.02) 0.965
simvastatin (+0.00173) 0.965
Anemia (x1.02) 0.965
Urea nitrogen [M/V] in SP (+0.298) 0.966
Asthenia (x1.02) 0.966
Dyspnea (x1.02) 0.966
Benign prostatic hyperplasia (x1.02) 0.967
Cardiac arrhythmia (x1.02) 0.967
Erythrocyte distribution width [V] by AC (+0.182) 0.967
Primary malignant neoplasm of skin of upper limb (x1.02) 0.967
Persistent pain following procedure (x1.02) 0.968
Abnormal findings on diagnostic imaging of lung (x1.02) 0.968
warfarin (+0.00161) 0.968
Systolic blood pressure (+0.533) 0.968
cyclosporine (+0.00158) 0.969
atorvastatin (+0.00157) 0.969
hydrocodone (+0.00157) 0.969
Primary malignant neoplasm of skin of ear (x1.02) 0.969
Disorder of lung (x1.02) 0.970
Heart rate (-0.659) 0.970
MCH [M] by AC (+0.0752) 0.970
Lymphedema (x1.02) 0.970
Chronic kidney disease due to hypertension (x1.02) 0.971
Primary malignant neoplasm of skin of lip (x1.02) 0.971
Expressions
−0.6 −0.4 −0.2 0.0 0.2 0.4 0.6

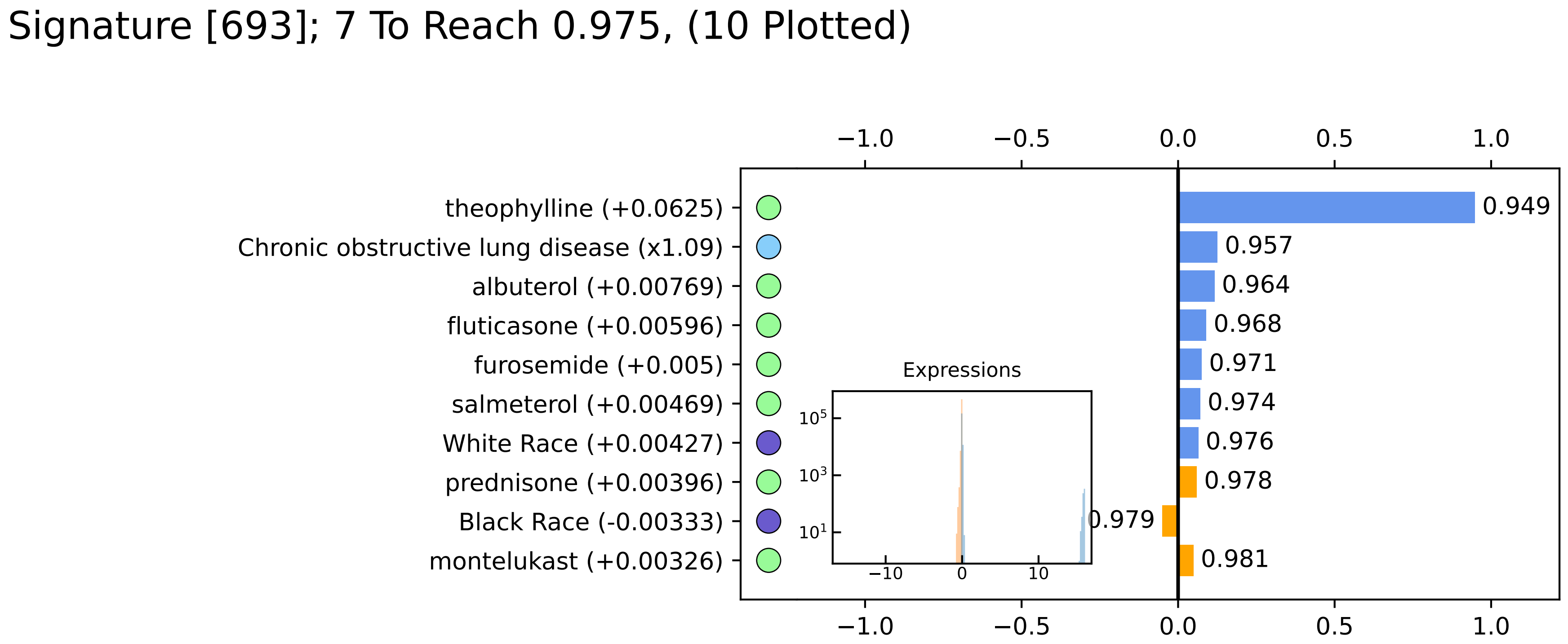

Signature [693]; 7 To Reach 0.975, (10 Plotted)
theophylline (+0.0625)
Chronic obstructive lung disease (x1.09)
albuterol (+0.00769)
fluticasone (+0.00596)
furosemide (+0.005)
salmeterol (+0.00469)
White Race (+0.00427)
prednisone (+0.00396)
Black Race (-0.00333)
montelukast (+0.00326)
0.949
0.957
0.964
0.968
0.971
0.974
0.976
0.978
0.979
0.981
−1.0
−0.5
0.0
0.5
1.0
Expressions
10^5
10^3
10^1
−10
0
10

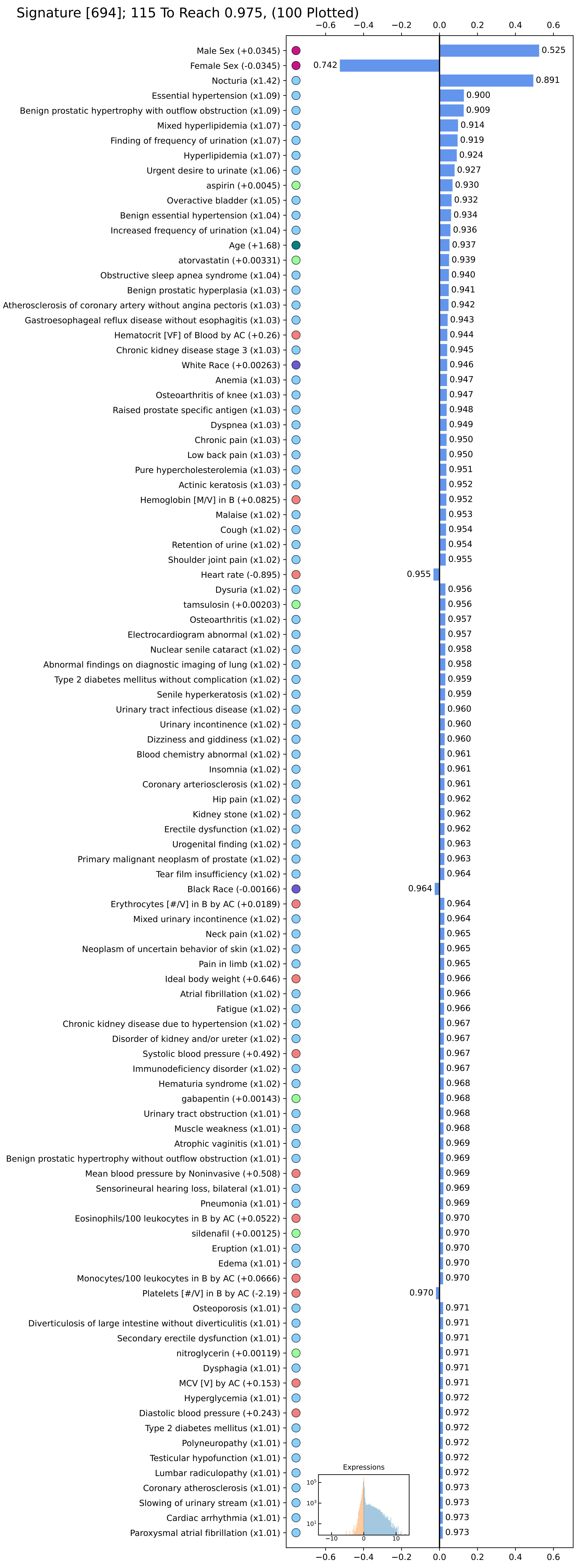
Signature [694]; 115 To Reach 0.975, (100 Plotted)
−0.6 −0.4 −0.2 0.0 0.2 0.4 0.6
Male Sex (+0.0345) 0.525
Female Sex (-0.0345) 0.742
Nocturia (x1.42) 0.891
Essential hypertension (x1.09) 0.900
Benign prostatic hypertrophy with outflow obstruction (x1.09) 0.909
Mixed hyperlipidemia (x1.07) 0.914
Finding of frequency of urination (x1.07) 0.919
Hyperlipidemia (x1.07) 0.924
Urgent desire to urinate (x1.06) 0.927
aspirin (+0.0045) 0.930
Overactive bladder (x1.05) 0.932
Benign essential hypertension (x1.04) 0.934
Increased frequency of urination (x1.04) 0.936
Age (+1.68) 0.937
atorvastatin (+0.00331) 0.939
Obstructive sleep apnea syndrome (x1.04) 0.940
Benign prostatic hyperplasia (x1.03) 0.941
Atherosclerosis of coronary artery without angina pectoris (x1.03) 0.942
Gastroesophageal reflux disease without esophagitis (x1.03) 0.943
Hematocrit [VF] of Blood by AC (+0.26) 0.944
Chronic kidney disease stage 3 (x1.03) 0.945
White Race (+0.00263) 0.946
Anemia (x1.03) 0.947
Osteoarthritis of knee (x1.03) 0.947
Raised prostate specific antigen (x1.03) 0.948
Dyspnea (x1.03) 0.949
Chronic pain (x1.03) 0.950
Low back pain (x1.03) 0.950
Pure hypercholesterolemia (x1.03) 0.951
Actinic keratosis (x1.03) 0.952
Hemoglobin [M/V] in B (+0.0825) 0.952
Malaise (x1.02) 0.953
Cough (x1.02) 0.954
Retention of urine (x1.02) 0.954
Shoulder joint pain (x1.02) 0.955
Heart rate (-0.895) 0.955
Dysuria (x1.02) 0.956
tamsulosin (+0.00203) 0.956
Osteoarthritis (x1.02) 0.957
Electrocardiogram abnormal (x1.02) 0.957
Nuclear senile cataract (x1.02) 0.958
Abnormal findings on diagnostic imaging of lung (x1.02) 0.958
Type 2 diabetes mellitus without complication (x1.02) 0.959
Senile hyperkeratosis (x1.02) 0.959
Urinary tract infectious disease (x1.02) 0.960
Urinary incontinence (x1.02) 0.960
Dizziness and giddiness (x1.02) 0.960
Blood chemistry abnormal (x1.02) 0.961
Insomnia (x1.02) 0.961
Coronary arteriosclerosis (x1.02) 0.961
Hip pain (x1.02) 0.962
Kidney stone (x1.02) 0.962
Erectile dysfunction (x1.02) 0.962
Urogenital finding (x1.02) 0.963
Primary malignant neoplasm of prostate (x1.02) 0.963
Tear film insufficiency (x1.02) 0.964
Black Race (-0.00166) 0.964
Erythrocytes [#/V] in B by AC (+0.0189) 0.964
Mixed urinary incontinence (x1.02) 0.964
Neck pain (x1.02) 0.965
Neoplasm of uncertain behavior of skin (x1.02) 0.965
Pain in limb (x1.02) 0.965
Ideal body weight (+0.646) 0.966
Atrial fibrillation (x1.02) 0.966
Fatigue (x1.02) 0.966
Chronic kidney disease due to hypertension (x1.02) 0.967
Disorder of kidney and/or ureter (x1.02) 0.967
Systolic blood pressure (+0.492) 0.967
Immunodeficiency disorder (x1.02) 0.967
Hematuria syndrome (x1.02) 0.968
gabapentin (+0.00143) 0.968
Urinary tract obstruction (x1.01) 0.968
Muscle weakness (x1.01) 0.968
Atrophic vaginitis (x1.01) 0.969
Benign prostatic hypertrophy without outflow obstruction (x1.01) 0.969
Mean blood pressure by Noninvasive (+0.508) 0.969
Sensorineural hearing loss, bilateral (x1.01) 0.969
Pneumonia (x1.01) 0.969
Eosinophils/100 leukocytes in B by AC (+0.0522) 0.970
sildenafil (+0.00125) 0.970
Eruption (x1.01) 0.970
Edema (x1.01) 0.970
Monocytes/100 leukocytes in B by AC (+0.0666) 0.970
Platelets [#/V] in B by AC (-2.19) 0.970
Osteoporosis (x1.01) 0.971
Diverticulosis of large intestine without diverticulitis (x1.01) 0.971
Secondary erectile dysfunction (x1.01) 0.971
nitroglycerin (+0.00119) 0.971
Dysphagia (x1.01) 0.971
MCV [V] by AC (+0.153) 0.971
Hyperglycemia (x1.01) 0.972
Diastolic blood pressure (+0.243) 0.972
Type 2 diabetes mellitus (x1.01) 0.972
Polyneuropathy (x1.01) 0.972
Testicular hypofunction (x1.01) 0.972
Lumbar radiculopathy (x1.01) 0.972
Coronary atherosclerosis (x1.01) 0.973
Slowing of urinary stream (x1.01) 0.973
Cardiac arrhythmia (x1.01) 0.973
Paroxysmal atrial fibrillation (x1.01) 0.973
Expressions
−10 0 10
−0.6 −0.4 −0.2 0.0 0.2 0.4 0.6

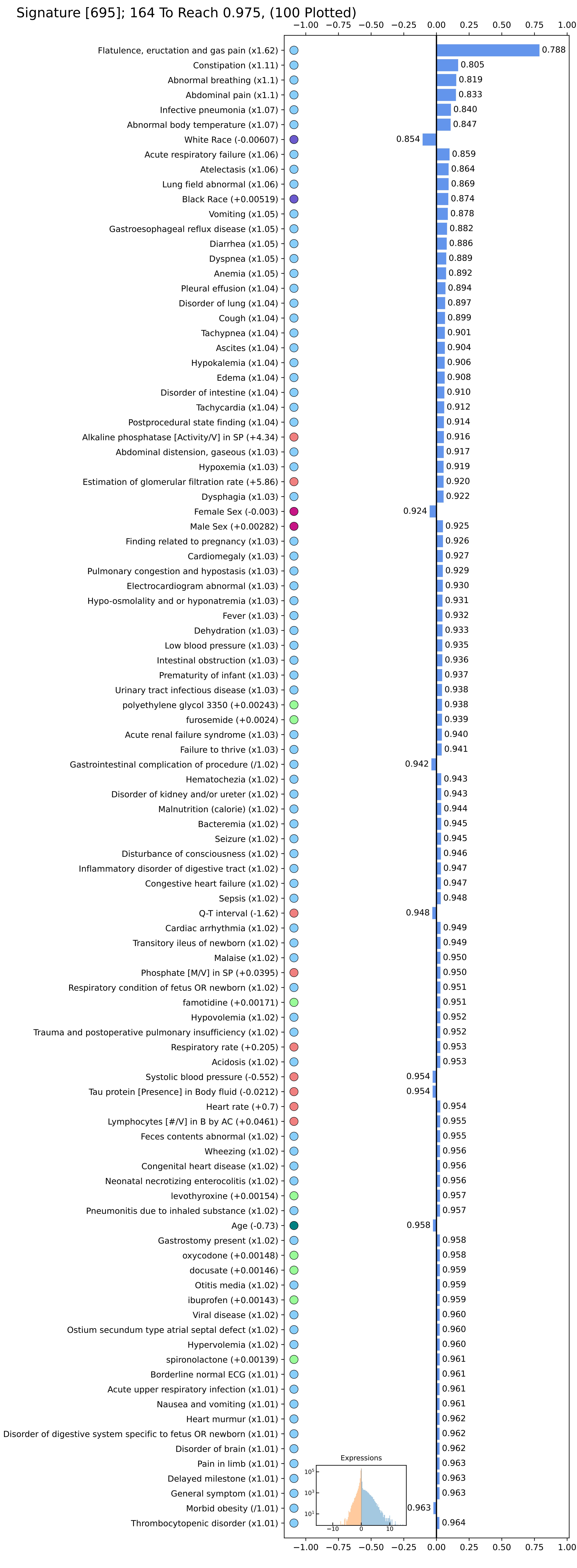

Signature [695]; 164 To Reach 0.975, (100 Plotted)
Flatulence, eructation and gas pain (x1.62) 0.788
Constipation (x1.11) 0.805
Abnormal breathing (x1.1) 0.819
Abdominal pain (x1.1) 0.833
Infective pneumonia (x1.07) 0.840
Abnormal body temperature (x1.07) 0.847
White Race (-0.00607) 0.854
Acute respiratory failure (x1.06) 0.859
Atelectasis (x1.06) 0.864
Lung field abnormal (x1.06) 0.869
Black Race (+0.00519) 0.874
Vomiting (x1.05) 0.878
Gastroesophageal reflux disease (x1.05) 0.882
Diarrhea (x1.05) 0.886
Dyspnea (x1.05) 0.889
Anemia (x1.05) 0.892
Pleural effusion (x1.04) 0.894
Disorder of lung (x1.04) 0.897
Cough (x1.04) 0.899
Tachypnea (x1.04) 0.901
Ascites (x1.04) 0.904
Hypokalemia (x1.04) 0.906
Edema (x1.04) 0.908
Disorder of intestine (x1.04) 0.910
Tachycardia (x1.04) 0.912
Postprocedural state finding (x1.04) 0.914
Alkaline phosphatase [Activity/V] in SP (+4.34) 0.916
Abdominal distension, gaseous (x1.03) 0.917
Hypoxemia (x1.03) 0.919
Estimation of glomerular filtration rate (+5.86) 0.920
Dysphagia (x1.03) 0.922
Female Sex (-0.003) 0.924
Male Sex (+0.00282) 0.925
Finding related to pregnancy (x1.03) 0.926
Cardiomegaly (x1.03) 0.927
Pulmonary congestion and hypostasis (x1.03) 0.929
Electrocardiogram abnormal (x1.03) 0.930
Hypo-osmolality and or hyponatremia (x1.03) 0.931
Fever (x1.03) 0.932
Dehydration (x1.03) 0.933
Low blood pressure (x1.03) 0.935
Intestinal obstruction (x1.03) 0.936
Prematurity of infant (x1.03) 0.937
Urinary tract infectious disease (x1.03) 0.938
polyethylene glycol 3350 (+0.00243) 0.938
furosemide (+0.0024) 0.939
Acute renal failure syndrome (x1.03) 0.940
Failure to thrive (x1.03) 0.941
Gastrointestinal complication of procedure (/1.02) 0.942
Hematochezia (x1.02) 0.943
Disorder of kidney and/or ureter (x1.02) 0.943
Malnutrition (calorie) (x1.02) 0.944
Bacteremia (x1.02) 0.945
Seizure (x1.02) 0.945
Disturbance of consciousness (x1.02) 0.946
Inflammatory disorder of digestive tract (x1.02) 0.947
Congestive heart failure (x1.02) 0.947
Sepsis (x1.02) 0.948
Q-T interval (-1.62) 0.948
Cardiac arrhythmia (x1.02) 0.949
Transitory ileus of newborn (x1.02) 0.949
Malaise (x1.02) 0.950
Phosphate [M/V] in SP (+0.0395) 0.950
Respiratory condition of fetus OR newborn (x1.02) 0.951
famotidine (+0.00171) 0.951
Hypovolemia (x1.02) 0.952
Trauma and postoperative pulmonary insufficiency (x1.02) 0.952
Respiratory rate (+0.205) 0.953
Acidosis (x1.02) 0.953
Systolic blood pressure (-0.552) 0.954
Tau protein [Presence] in Body fluid (-0.0212) 0.954
Heart rate (+0.7) 0.954
Lymphocytes [#/V] in B by AC (+0.0461) 0.955
Feces contents abnormal (x1.02) 0.955
Wheezing (x1.02) 0.956
Congenital heart disease (x1.02) 0.956
Neonatal necrotizing enterocolitis (x1.02) 0.956
levothyroxine (+0.00154) 0.957
Pneumonitis due to inhaled substance (x1.02) 0.957
Age (-0.73) 0.958
Gastrostomy present (x1.02) 0.958
oxycodone (+0.00148) 0.958
docusate (+0.00146) 0.959
Otitis media (x1.02) 0.959
ibuprofen (+0.00143) 0.959
Viral disease (x1.02) 0.960
Ostium secundum type atrial septal defect (x1.02) 0.960
Hypervolemia (x1.02) 0.960
spironolactone (+0.00139) 0.961
Borderline normal ECG (x1.01) 0.961
Acute upper respiratory infection (x1.01) 0.961
Nausea and vomiting (x1.01) 0.961
Heart murmur (x1.01) 0.962
Disorder of digestive system specific to fetus OR newborn (x1.01) 0.962
Disorder of brain (x1.01) 0.962
Pain in limb (x1.01) 0.963
Delayed milestone (x1.01) 0.963
General symptom (x1.01) 0.963
Morbid obesity (/1.01) 0.963
Thrombocytopenic disorder (x1.01) 0.964
−1.00 −0.75 −0.50 −0.25 0.00 0.25 0.50 0.75 1.00
Expressions

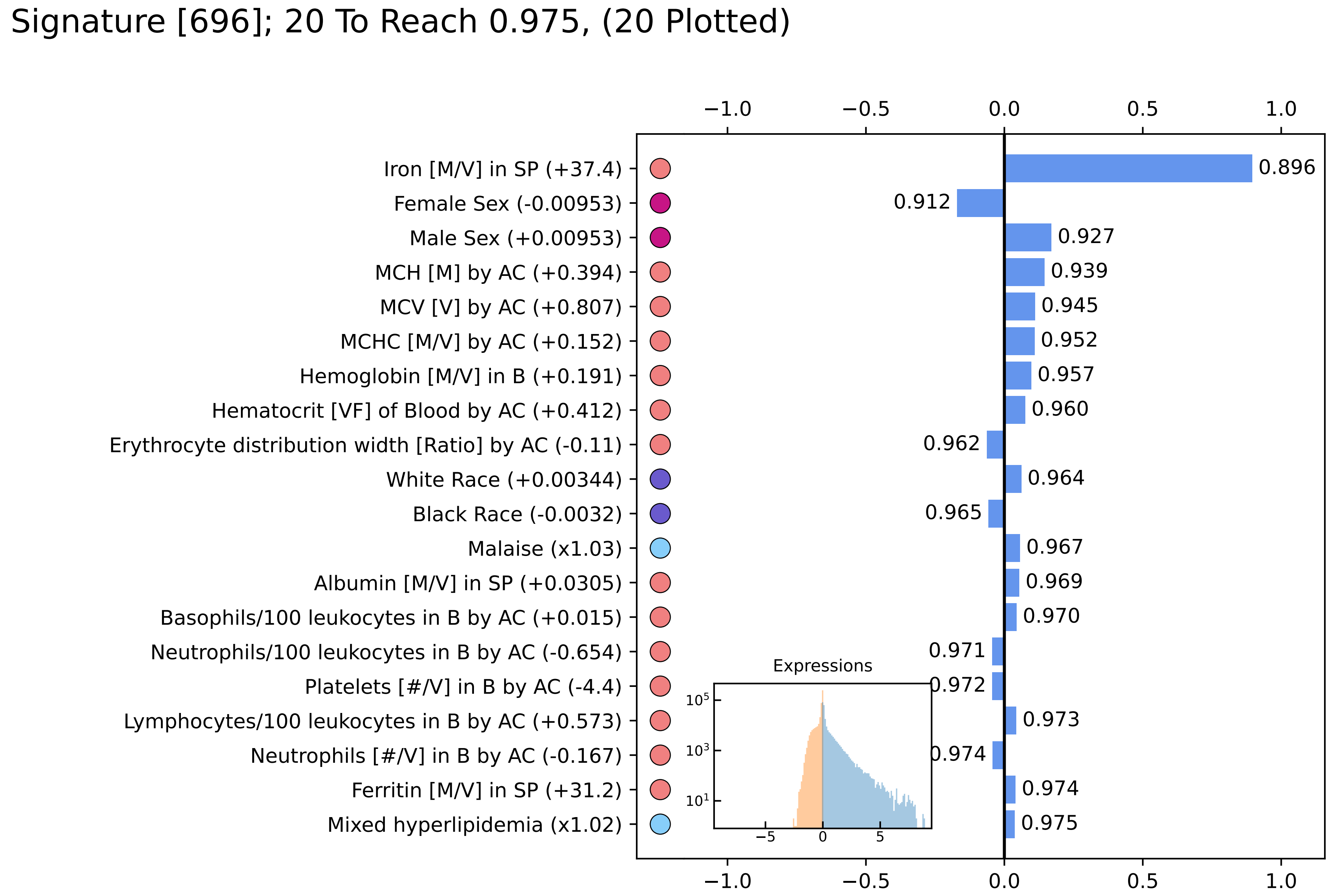

Signature [696]; 20 To Reach 0.975, (20 Plotted)
Iron [M/V] in SP (+37.4)
Female Sex (-0.00953)
Male Sex (+0.00953)
MCH [M] by AC (+0.394)
MCV [V] by AC (+0.807)
MCHC [M/V] by AC (+0.152)
Hemoglobin [M/V] in B (+0.191)
Hematocrit [VF] of Blood by AC (+0.412)
Erythrocyte distribution width [Ratio] by AC (-0.11)
White Race (+0.00344)
Black Race (-0.0032)
Malaise (x1.03)
Albumin [M/V] in SP (+0.0305)
Basophils/100 leukocytes in B by AC (+0.015)
Neutrophils/100 leukocytes in B by AC (-0.654)
Platelets [#/V] in B by AC (-4.4)
Lymphocytes/100 leukocytes in B by AC (+0.573)
Neutrophils [#/V] in B by AC (-0.167)
Ferritin [M/V] in SP (+31.2)
Mixed hyperlipidemia (x1.02)
0.896
0.912
0.927
0.939
0.945
0.952
0.957
0.960
0.962
0.964
0.965
0.967
0.969
0.970
0.971
0.972
0.973
0.974
0.974
0.975
−1.0
−0.5
0.0
0.5
1.0
Expressions
10^5
10^3
10^1
−5
0
5

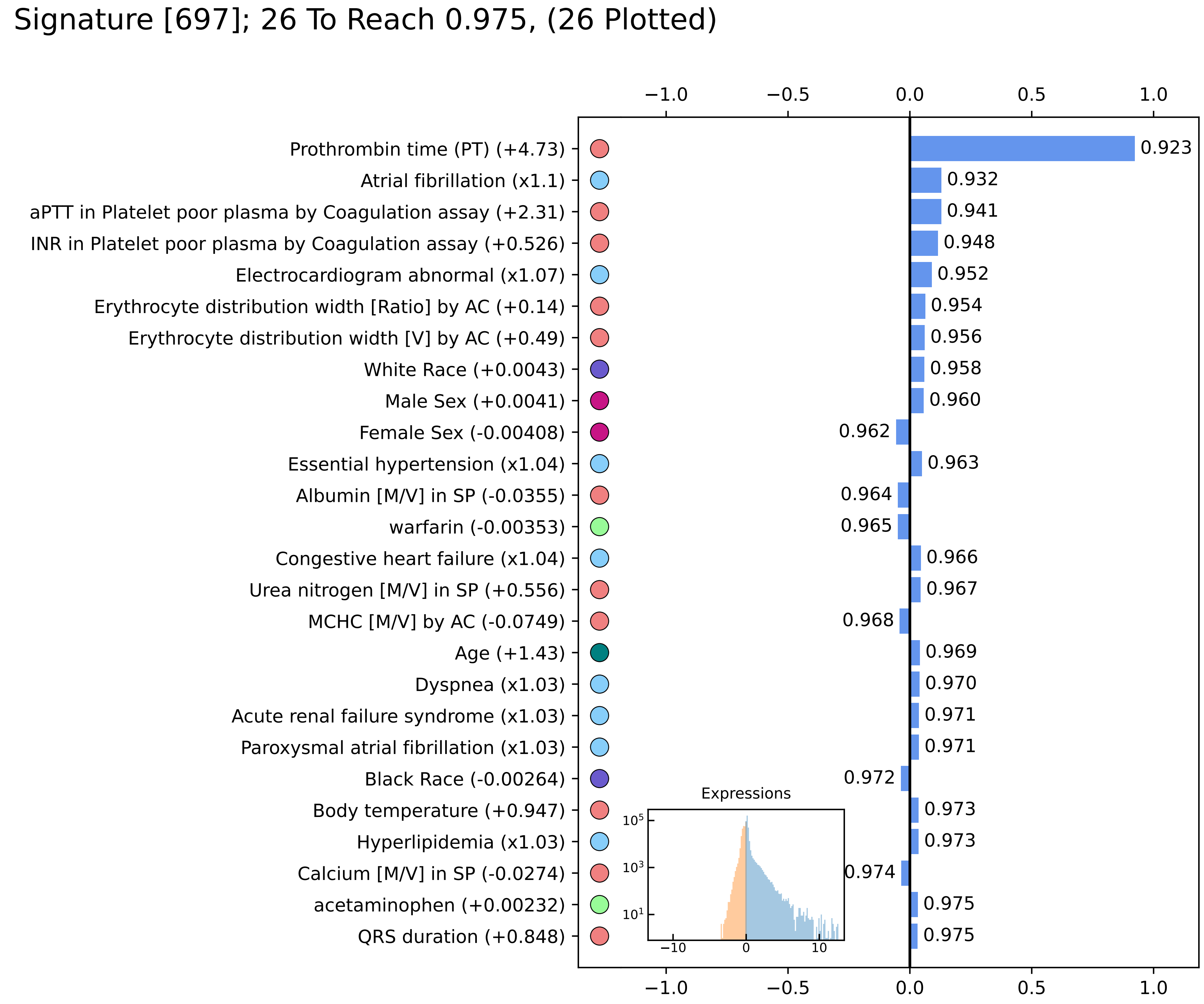
Signature [697]; 26 To Reach 0.975, (26 Plotted)
Prothrombin time (PT) (+4.73)
Atrial fibrillation (x1.1)
aPTT in Platelet poor plasma by Coagulation assay (+2.31)
INR in Platelet poor plasma by Coagulation assay (+0.526)
Electrocardiogram abnormal (x1.07)
Erythrocyte distribution width [Ratio] by AC (+0.14)
Erythrocyte distribution width [V] by AC (+0.49)
White Race (+0.0043)
Male Sex (+0.0041)
Female Sex (-0.00408)
Essential hypertension (x1.04)
Albumin [M/V] in SP (-0.0355)
warfarin (-0.00353)
Congestive heart failure (x1.04)
Urea nitrogen [M/V] in SP (+0.556)
MCHC [M/V] by AC (-0.0749)
Age (+1.43)
Dyspnea (x1.03)
Acute renal failure syndrome (x1.03)
Paroxysmal atrial fibrillation (x1.03)
Black Race (-0.00264)
Body temperature (+0.947)
Hyperlipidemia (x1.03)
Calcium [M/V] in SP (-0.0274)
acetaminophen (+0.00232)
QRS duration (+0.848)
0.923
0.932
0.941
0.948
0.952
0.954
0.956
0.958
0.960
0.962
0.963
0.964
0.965
0.966
0.967
0.968
0.969
0.970
0.971
0.971
0.972
0.973
0.973
0.974
0.975
0.975
−1.0
−0.5
0.0
0.5
1.0
Expressions
10^5
10^3
10^1
−10
0
10

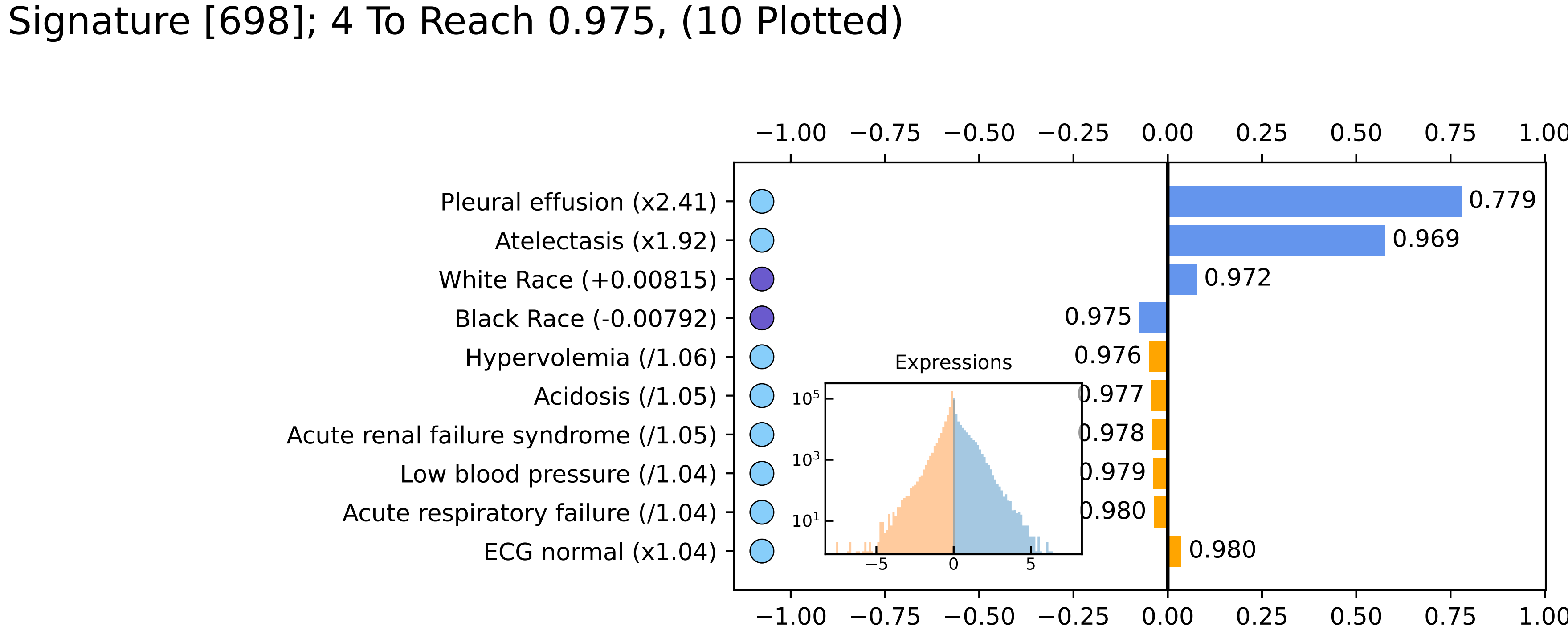

Signature [698]; 4 To Reach 0.975, (10 Plotted)
Pleural effusion (x2.41)
Atelectasis (x1.92)
White Race (+0.00815)
Black Race (-0.00792)
Hypervolemia (/1.06)
Acidosis (/1.05)
Acute renal failure syndrome (/1.05)
Low blood pressure (/1.04)
Acute respiratory failure (/1.04)
ECG normal (x1.04)
0.779
0.969
0.972
0.975
0.976
0.977
0.978
0.979
0.980
0.980
Expressions
−1.00
−0.75
−0.50
−0.25
0.00
0.25
0.50
0.75
1.00

Signature [699]; 4 To Reach 0.975, (10 Plotted)

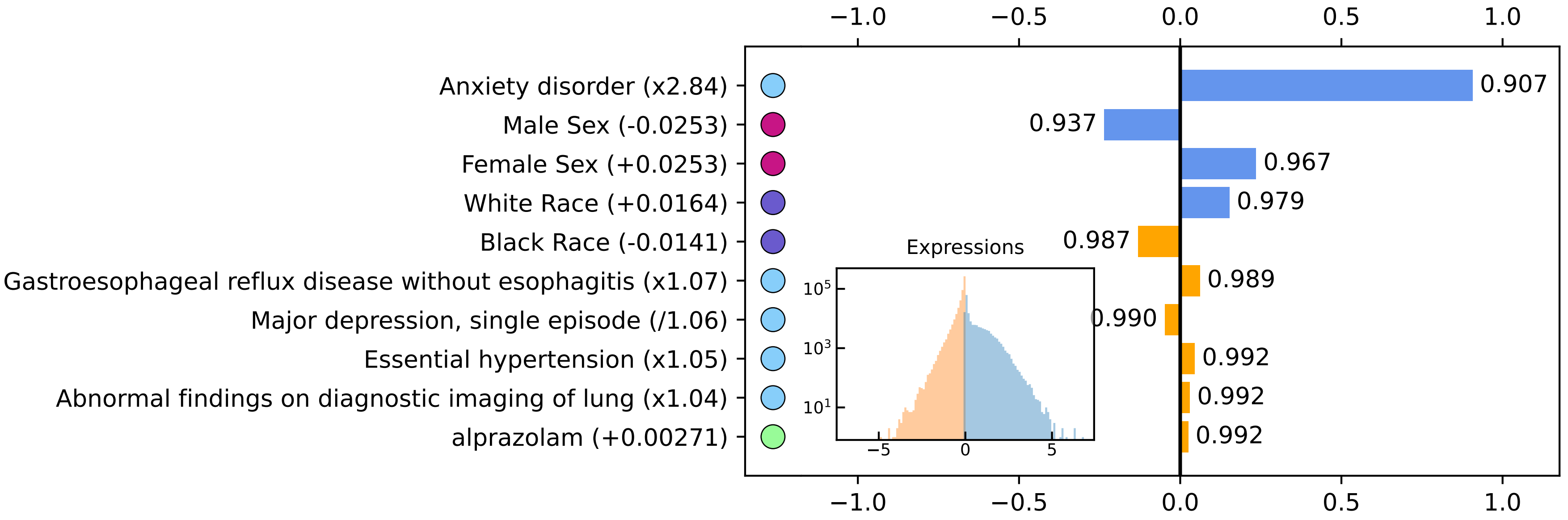

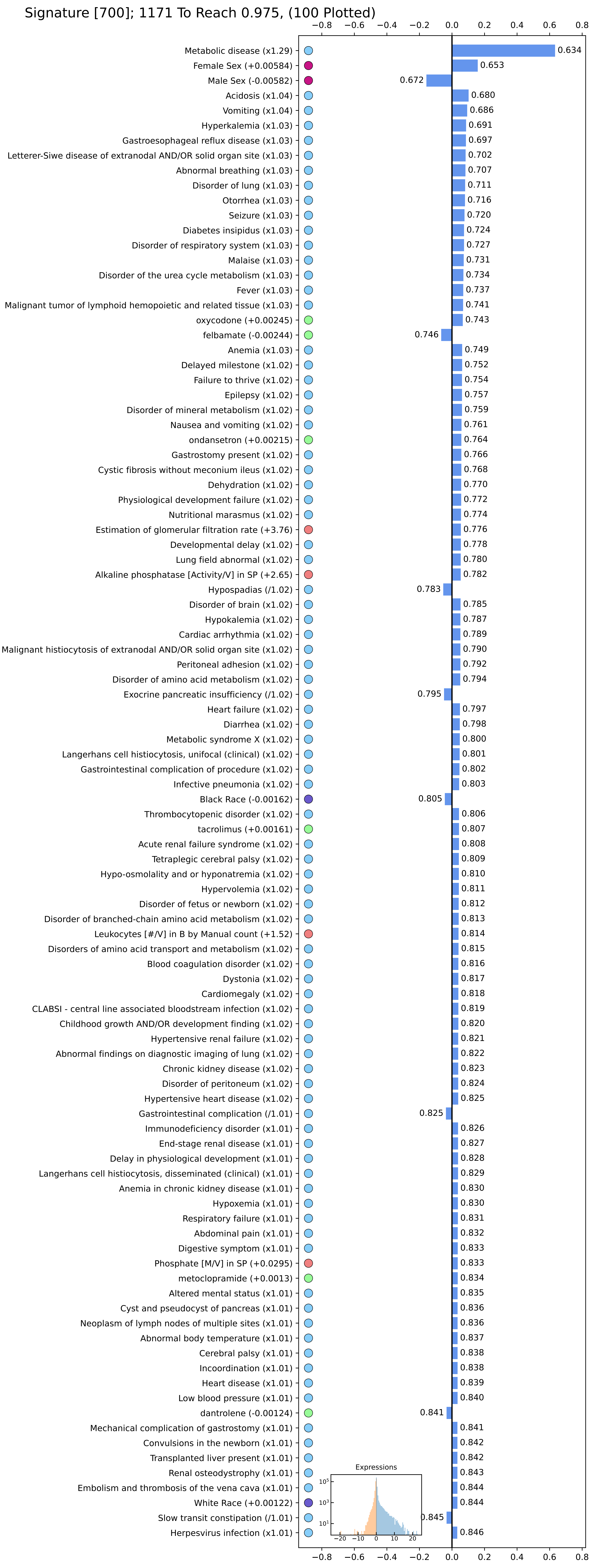

Signature [701]; 23 To Reach 0.975, (23 Plotted)

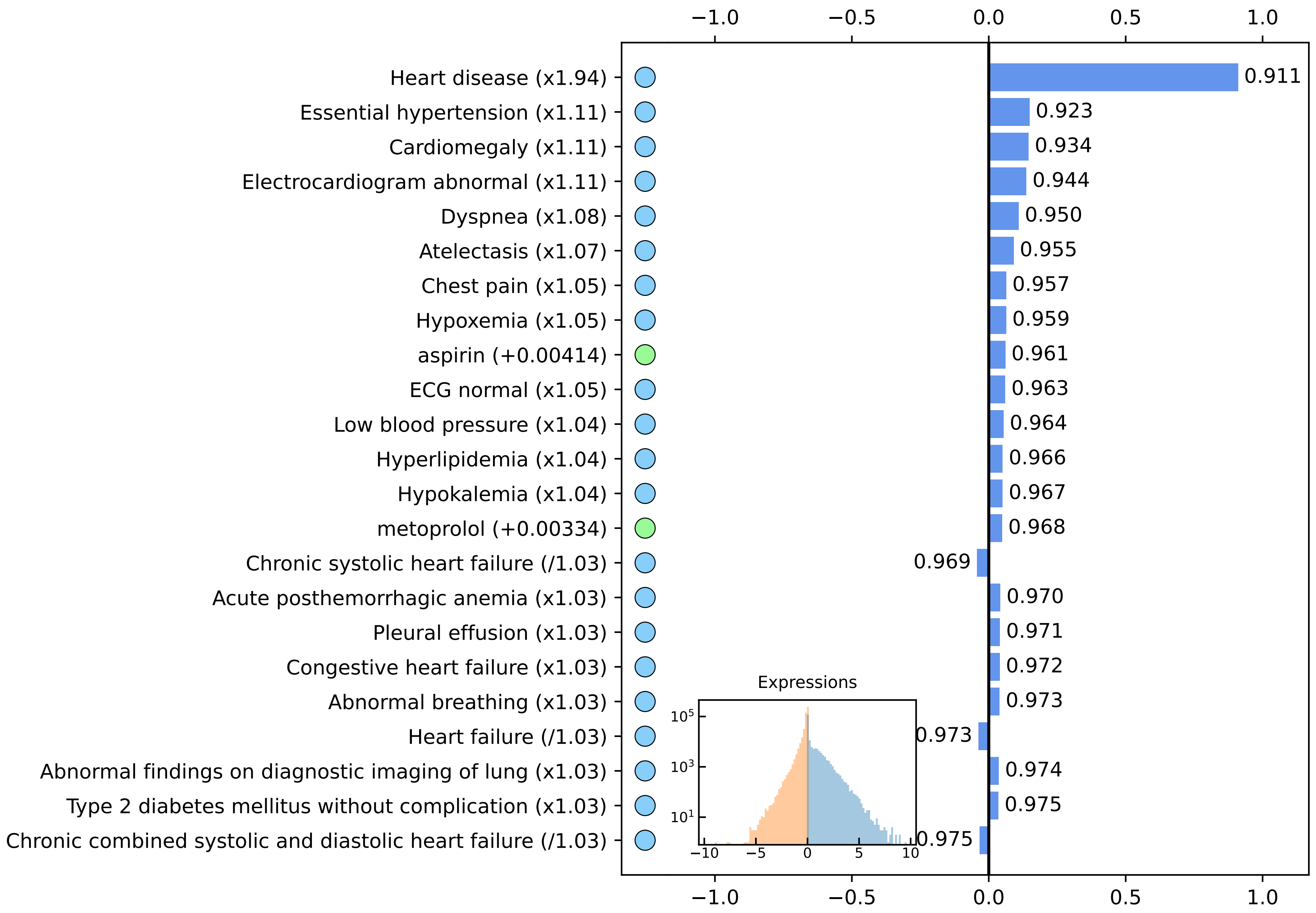

Signature [702]; 1 To Reach 0.975, (10 Plotted)

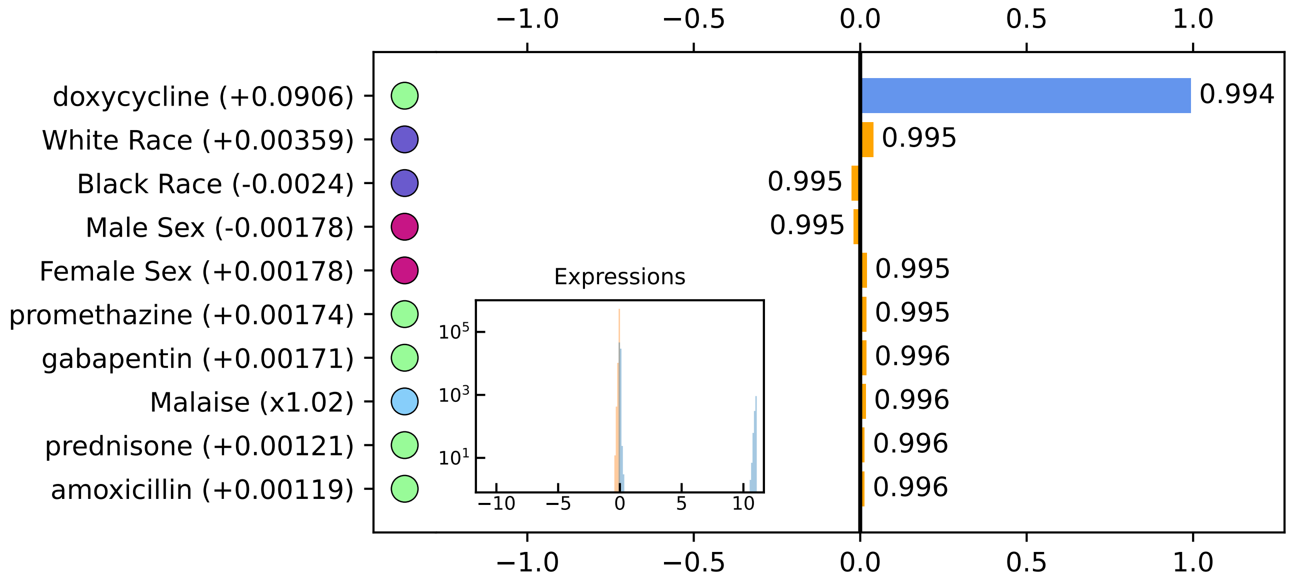

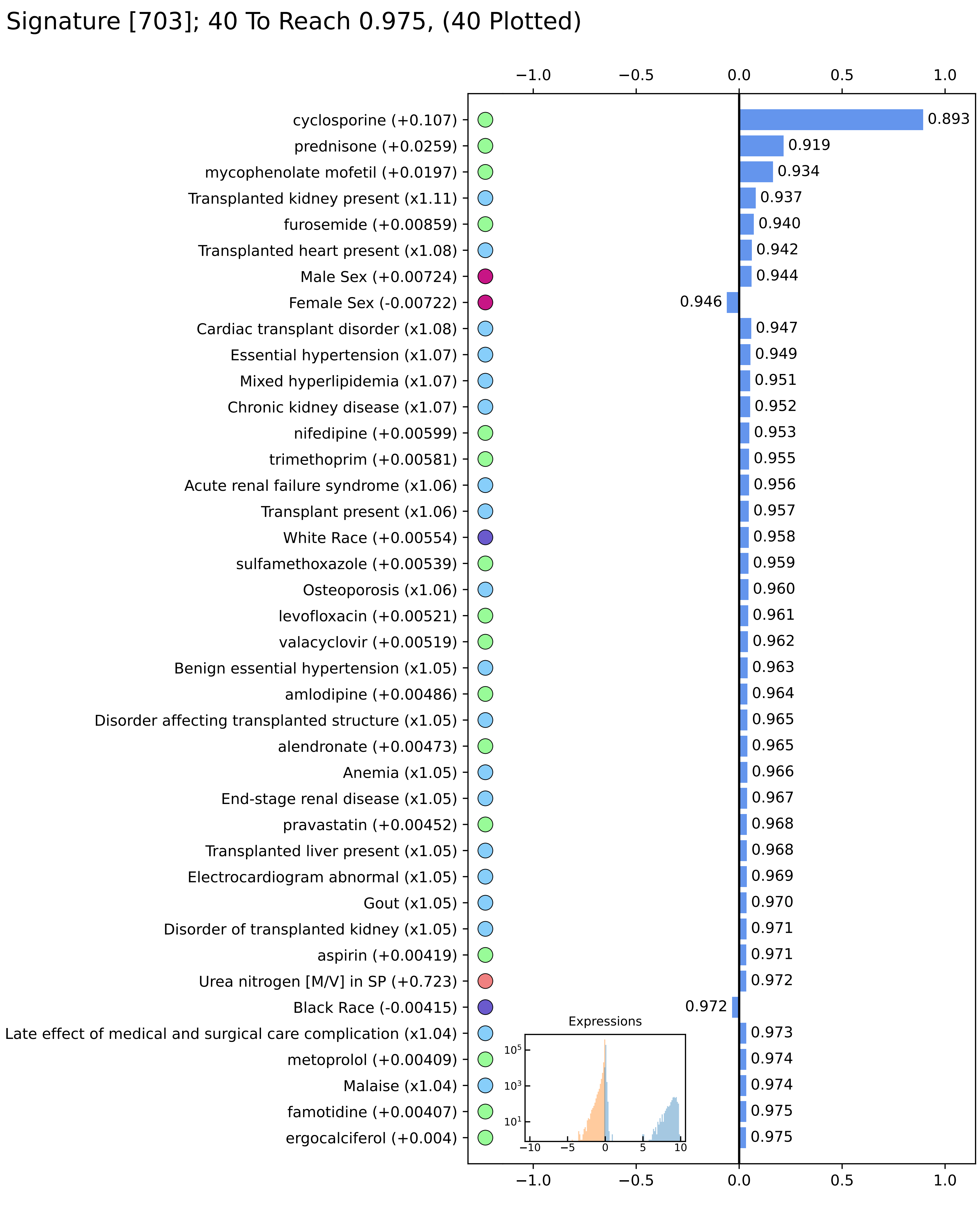

Signature [703]; 40 To Reach 0.975, (40 Plotted)
cyclosporine (+0.107)
0.893
prednisone (+0.0259)
0.919
mycophenolate mofetil (+0.0197)
0.934
Transplanted kidney present (x1.11)
0.937
furosemide (+0.00859)
0.940
Transplanted heart present (x1.08)
0.942
Male Sex (+0.00724)
0.944
Female Sex (-0.00722)
0.946
Cardiac transplant disorder (x1.08)
0.947
Essential hypertension (x1.07)
0.949
Mixed hyperlipidemia (x1.07)
0.951
Chronic kidney disease (x1.07)
0.952
nifedipine (+0.00599)
0.953
trimethoprim (+0.00581)
0.955
Acute renal failure syndrome (x1.06)
0.956
Transplant present (x1.06)
0.957
White Race (+0.00554)
0.958
sulfamethoxazole (+0.00539)
0.959
Osteoporosis (x1.06)
0.960
levofloxacin (+0.00521)
0.961
valacyclovir (+0.00519)
0.962
Benign essential hypertension (x1.05)
0.963
amlodipine (+0.00486)
0.964
Disorder affecting transplanted structure (x1.05)
0.965
alendronate (+0.00473)
0.965
Anemia (x1.05)
0.966
End-stage renal disease (x1.05)
0.967
pravastatin (+0.00452)
0.968
Transplanted liver present (x1.05)
0.968
Electrocardiogram abnormal (x1.05)
0.969
Gout (x1.05)
0.970
Disorder of transplanted kidney (x1.05)
0.971
aspirin (+0.00419)
0.971
Urea nitrogen [M/V] in SP (+0.723)
0.972
Black Race (-0.00415)
0.972
Late effect of medical and surgical care complication (x1.04)
0.973
metoprolol (+0.00409)
0.974
Malaise (x1.04)
0.974
famotidine (+0.00407)
0.975
ergocalciferol (+0.004)
0.975
−1.0
−0.5
0.0
0.5
1.0
Expressions

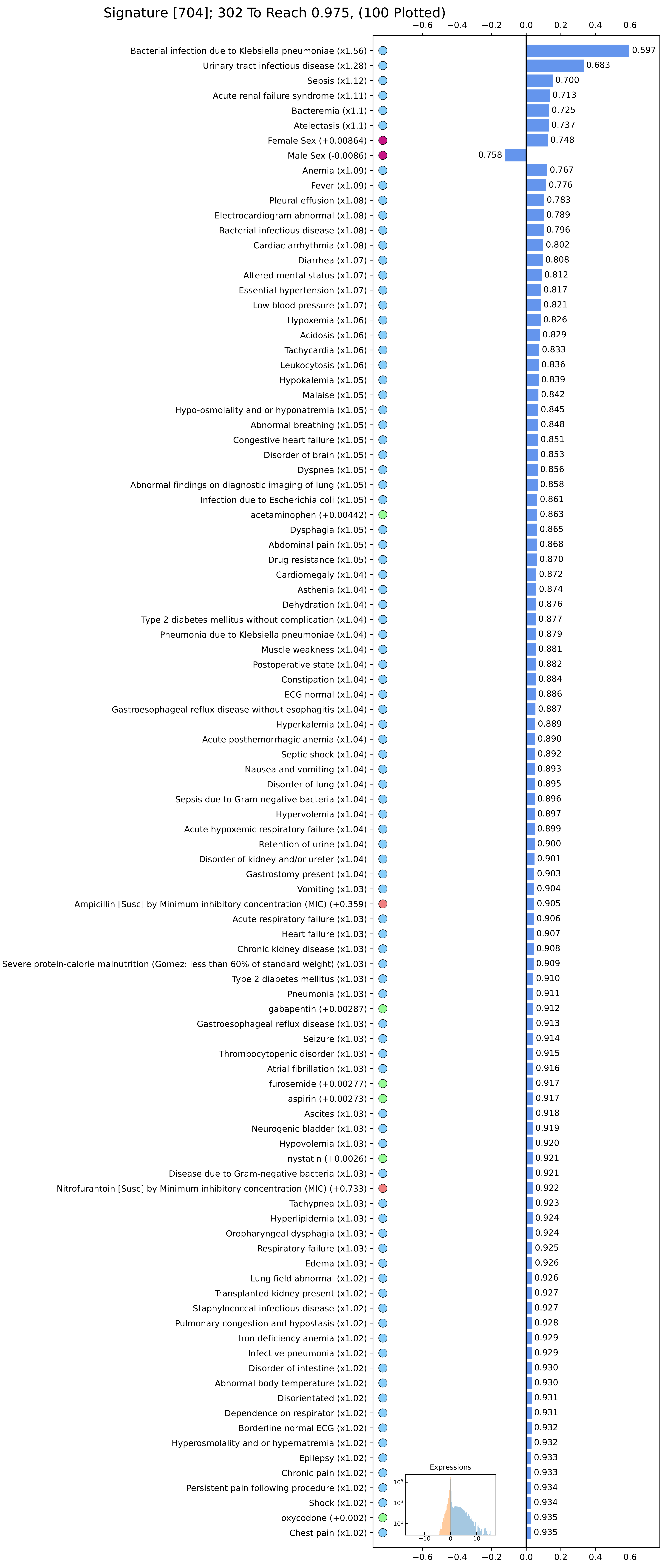

Signature [704]; 302 To Reach 0.975, (100 Plotted)
Bacterial infection due to Klebsiella pneumoniae (x1.56) 0.597
Urinary tract infectious disease (x1.28) 0.683
Sepsis (x1.12) 0.700
Acute renal failure syndrome (x1.11) 0.713
Bacteremia (x1.1) 0.725
Atelectasis (x1.1) 0.737
Female Sex (+0.00864) 0.748
Male Sex (-0.0086) 0.758
Anemia (x1.09) 0.767
Fever (x1.09) 0.776
Pleural effusion (x1.08) 0.783
Electrocardiogram abnormal (x1.08) 0.789
Bacterial infectious disease (x1.08) 0.796
Cardiac arrhythmia (x1.08) 0.802
Diarrhea (x1.07) 0.808
Altered mental status (x1.07) 0.812
Essential hypertension (x1.07) 0.817
Low blood pressure (x1.07) 0.821
Hypoxemia (x1.06) 0.826
Acidosis (x1.06) 0.829
Tachycardia (x1.06) 0.833
Leukocytosis (x1.06) 0.836
Hypokalemia (x1.05) 0.839
Malaise (x1.05) 0.842
Hypo-osmolality and or hyponatremia (x1.05) 0.845
Abnormal breathing (x1.05) 0.848
Congestive heart failure (x1.05) 0.851
Disorder of brain (x1.05) 0.853
Dyspnea (x1.05) 0.856
Abnormal findings on diagnostic imaging of lung (x1.05) 0.858
Infection due to Escherichia coli (x1.05) 0.861
acetaminophen (+0.00442) 0.863
Dysphagia (x1.05) 0.865
Abdominal pain (x1.05) 0.868
Drug resistance (x1.05) 0.870
Cardiomegaly (x1.04) 0.872
Asthenia (x1.04) 0.874
Dehydration (x1.04) 0.876
Type 2 diabetes mellitus without complication (x1.04) 0.877
Pneumonia due to Klebsiella pneumoniae (x1.04) 0.879
Muscle weakness (x1.04) 0.881
Postoperative state (x1.04) 0.882
Constipation (x1.04) 0.884
ECG normal (x1.04) 0.886
Gastroesophageal reflux disease without esophagitis (x1.04) 0.887
Hyperkalemia (x1.04) 0.889
Acute posthemorrhagic anemia (x1.04) 0.890
Septic shock (x1.04) 0.892
Nausea and vomiting (x1.04) 0.893
Disorder of lung (x1.04) 0.895
Sepsis due to Gram negative bacteria (x1.04) 0.896
Hypervolemia (x1.04) 0.897
Acute hypoxemic respiratory failure (x1.04) 0.899
Retention of urine (x1.04) 0.900
Disorder of kidney and/or ureter (x1.04) 0.901
Gastrostomy present (x1.04) 0.903
Vomiting (x1.03) 0.904
Ampicillin [Susc] by Minimum inhibitory concentration (MIC) (+0.359) 0.905
Acute respiratory failure (x1.03) 0.906
Heart failure (x1.03) 0.907
Chronic kidney disease (x1.03) 0.908
Severe protein-calorie malnutrition (Gomez: less than 60% of standard weight) (x1.03) 0.909
Type 2 diabetes mellitus (x1.03) 0.910
Pneumonia (x1.03) 0.911
gabapentin (+0.00287) 0.912
Gastroesophageal reflux disease (x1.03) 0.913
Seizure (x1.03) 0.914
Thrombocytopenic disorder (x1.03) 0.915
Atrial fibrillation (x1.03) 0.916
furosemide (+0.00277) 0.917
aspirin (+0.00273) 0.917
Ascites (x1.03) 0.918
Neurogenic bladder (x1.03) 0.919
Hypovolemia (x1.03) 0.920
nystatin (+0.0026) 0.921
Disease due to Gram-negative bacteria (x1.03) 0.921
Nitrofurantoin [Susc] by Minimum inhibitory concentration (MIC) (+0.733) 0.922
Tachypnea (x1.03) 0.923
Hyperlipidemia (x1.03) 0.924
Oropharyngeal dysphagia (x1.03) 0.924
Respiratory failure (x1.03) 0.925
Edema (x1.03) 0.926
Lung field abnormal (x1.02) 0.926
Transplanted kidney present (x1.02) 0.927
Staphylococcal infectious disease (x1.02) 0.927
Pulmonary congestion and hypostasis (x1.02) 0.928
Iron deficiency anemia (x1.02) 0.929
Infective pneumonia (x1.02) 0.929
Disorder of intestine (x1.02) 0.930
Abnormal body temperature (x1.02) 0.930
Disorientated (x1.02) 0.931
Dependence on respirator (x1.02) 0.931
Borderline normal ECG (x1.02) 0.932
Hyperosmolality and or hypernatremia (x1.02) 0.932
Epilepsy (x1.02) 0.933
Chronic pain (x1.02) 0.933
Persistent pain following procedure (x1.02) 0.934
Shock (x1.02) 0.934
oxycodone (+0.002) 0.935
Chest pain (x1.02) 0.935
Expressions
−0.6 −0.4 −0.2 0.0 0.2 0.4 0.6

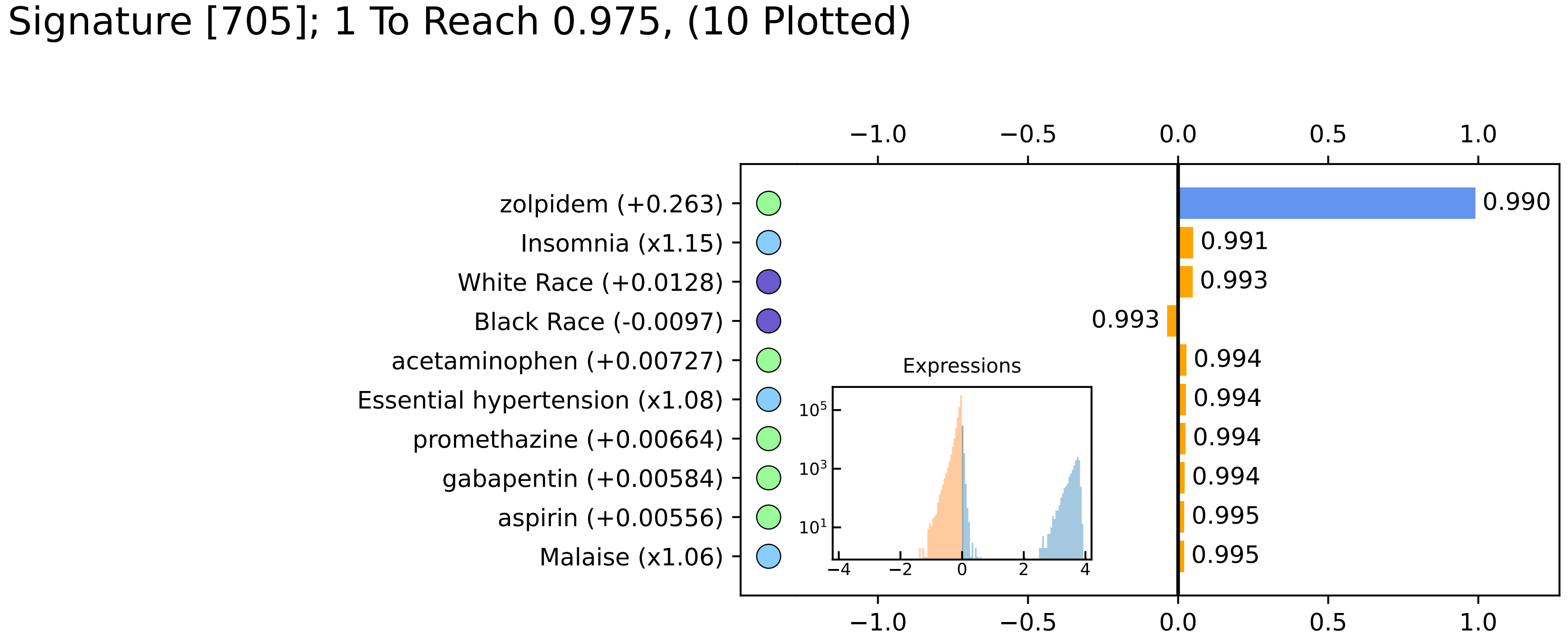

Signature [705]; 1 To Reach 0.975, (10 Plotted)
zolpidem (+0.263)
Insomnia (x1.15)
White Race (+0.0128)
Black Race (-0.0097)
acetaminophen (+0.00727)
Essential hypertension (x1.08)
promethazine (+0.00664)
gabapentin (+0.00584)
aspirin (+0.00556)
Malaise (x1.06)
0.990
0.991
0.993
0.993
0.994
0.994
0.994
0.994
0.995
0.995
Expressions

Signature [706]; 27 To Reach 0.975, (27 Plotted)

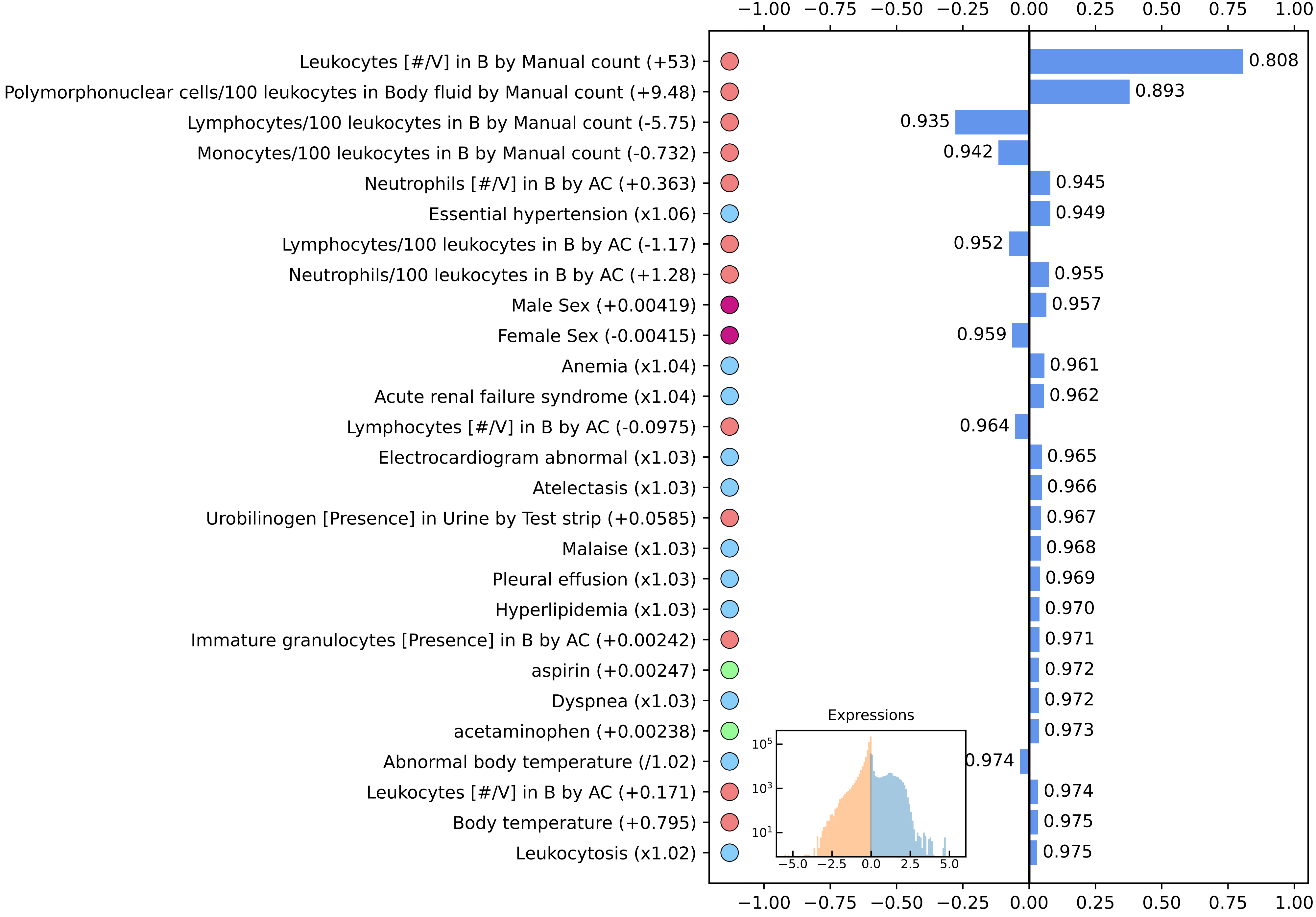

Signature [707]; 21 To Reach 0.975, (21 Plotted)

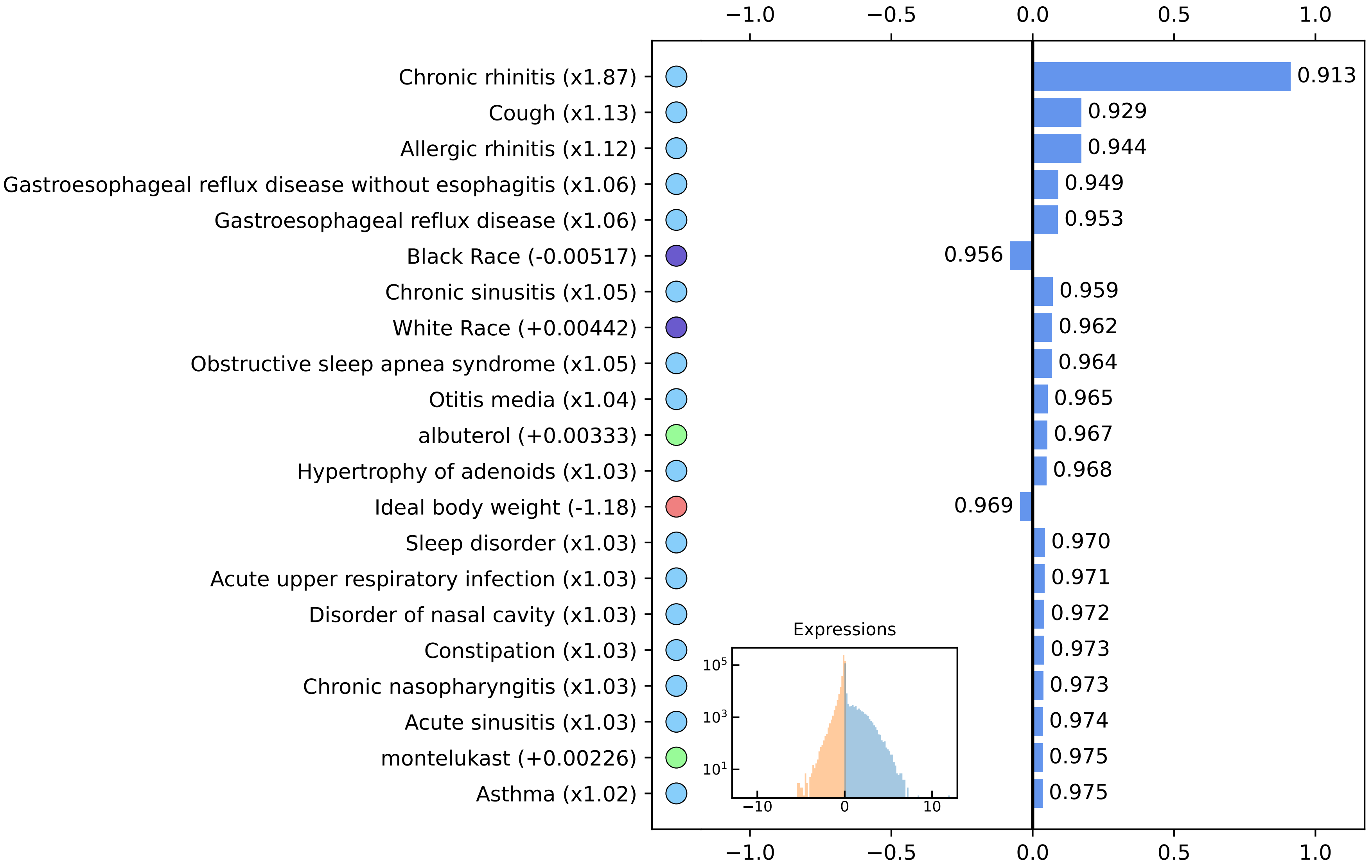

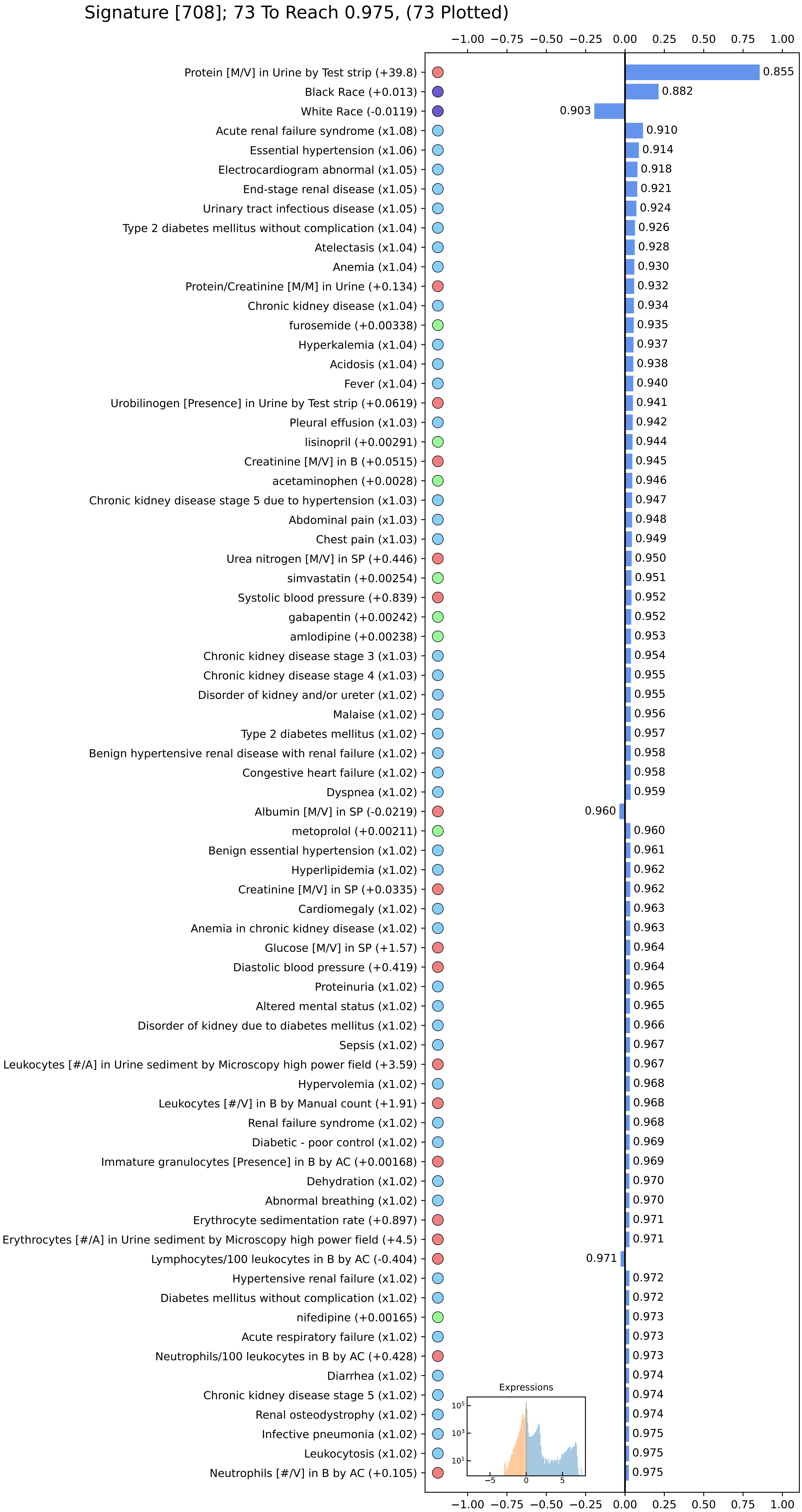

Signature [708]; 73 To Reach 0.975, (73 Plotted)
Protein [M/V] in Urine by Test strip (+39.8) 0.855
Black Race (+0.013) 0.882
White Race (-0.0119) 0.903
Acute renal failure syndrome (x1.08) 0.910
Essential hypertension (x1.06) 0.914
Electrocardiogram abnormal (x1.05) 0.918
End-stage renal disease (x1.05) 0.921
Urinary tract infectious disease (x1.05) 0.924
Type 2 diabetes mellitus without complication (x1.04) 0.926
Atelectasis (x1.04) 0.928
Anemia (x1.04) 0.930
Protein/Creatinine [M/M] in Urine (+0.134) 0.932
Chronic kidney disease (x1.04) 0.934
furosemide (+0.00338) 0.935
Hyperkalemia (x1.04) 0.937
Acidosis (x1.04) 0.938
Fever (x1.04) 0.940
Urobilinogen [Presence] in Urine by Test strip (+0.0619) 0.941
Pleural effusion (x1.03) 0.942
lisinopril (+0.00291) 0.944
Creatinine [M/V] in B (+0.0515) 0.945
acetaminophen (+0.0028) 0.946
Chronic kidney disease stage 5 due to hypertension (x1.03) 0.947
Abdominal pain (x1.03) 0.948
Chest pain (x1.03) 0.949
Urea nitrogen [M/V] in SP (+0.446) 0.950
simvastatin (+0.00254) 0.951
Systolic blood pressure (+0.839) 0.952
gabapentin (+0.00242) 0.952
amlodipine (+0.00238) 0.953
Chronic kidney disease stage 3 (x1.03) 0.954
Chronic kidney disease stage 4 (x1.03) 0.955
Disorder of kidney and/or ureter (x1.02) 0.955
Malaise (x1.02) 0.956
Type 2 diabetes mellitus (x1.02) 0.957
Benign hypertensive renal disease with renal failure (x1.02) 0.958
Congestive heart failure (x1.02) 0.958
Dyspnea (x1.02) 0.959
Albumin [M/V] in SP (-0.0219) 0.960
metoprolol (+0.00211) 0.960
Benign essential hypertension (x1.02) 0.961
Hyperlipidemia (x1.02) 0.962
Creatinine [M/V] in SP (+0.0335) 0.962
Cardiomegaly (x1.02) 0.963
Anemia in chronic kidney disease (x1.02) 0.963
Glucose [M/V] in SP (+1.57) 0.964
Diastolic blood pressure (+0.419) 0.964
Proteinuria (x1.02) 0.965
Altered mental status (x1.02) 0.965
Disorder of kidney due to diabetes mellitus (x1.02) 0.966
Sepsis (x1.02) 0.967
Leukocytes [#/A] in Urine sediment by Microscopy high power field (+3.59) 0.967
Hypervolemia (x1.02) 0.968
Leukocytes [#/V] in B by Manual count (+1.91) 0.968
Renal failure syndrome (x1.02) 0.968
Diabetic - poor control (x1.02) 0.969
Immature granulocytes [Presence] in B by AC (+0.00168) 0.969
Dehydration (x1.02) 0.970
Abnormal breathing (x1.02) 0.970
Erythrocyte sedimentation rate (+0.897) 0.971
Erythrocytes [#/A] in Urine sediment by Microscopy high power field (+4.5) 0.971
Lymphocytes/100 leukocytes in B by AC (-0.404) 0.971
Hypertensive renal failure (x1.02) 0.972
Diabetes mellitus without complication (x1.02) 0.972
nifedipine (+0.00165) 0.973
Acute respiratory failure (x1.02) 0.973
Neutrophils/100 leukocytes in B by AC (+0.428) 0.973
Diarrhea (x1.02) 0.974
Chronic kidney disease stage 5 (x1.02) 0.974
Renal osteodystrophy (x1.02) 0.974
Infective pneumonia (x1.02) 0.975
Leukocytosis (x1.02) 0.975
Neutrophils [#/V] in B by AC (+0.105) 0.975
−1.00 −0.75 −0.50 −0.25 0.00 0.25 0.50 0.75 1.00
Expressions

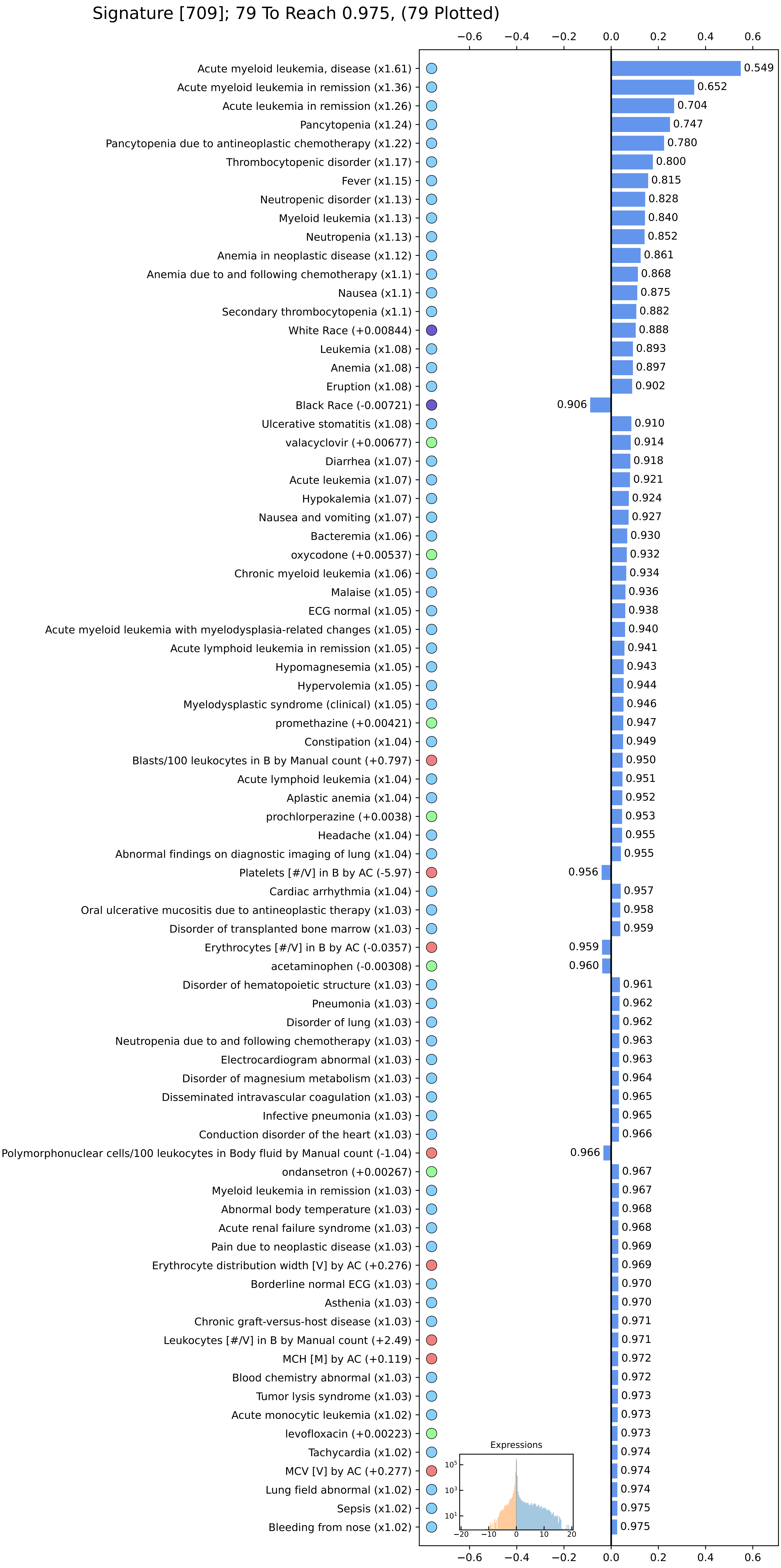

Signature [709]; 79 To Reach 0.975, (79 Plotted)
Acute myeloid leukemia, disease (x1.61) 0.549
Acute myeloid leukemia in remission (x1.36) 0.652
Acute leukemia in remission (x1.26) 0.704
Pancytopenia (x1.24) 0.747
Pancytopenia due to antineoplastic chemotherapy (x1.22) 0.780
Thrombocytopenic disorder (x1.17) 0.800
Fever (x1.15) 0.815
Neutropenic disorder (x1.13) 0.828
Myeloid leukemia (x1.13) 0.840
Neutropenia (x1.13) 0.852
Anemia in neoplastic disease (x1.12) 0.861
Anemia due to and following chemotherapy (x1.1) 0.868
Nausea (x1.1) 0.875
Secondary thrombocytopenia (x1.1) 0.882
White Race (+0.00844) 0.888
Leukemia (x1.08) 0.893
Anemia (x1.08) 0.897
Eruption (x1.08) 0.902
Black Race (-0.00721) 0.906
Ulcerative stomatitis (x1.08) 0.910
valacyclovir (+0.00677) 0.914
Diarrhea (x1.07) 0.918
Acute leukemia (x1.07) 0.921
Hypokalemia (x1.07) 0.924
Nausea and vomiting (x1.07) 0.927
Bacteremia (x1.06) 0.930
oxycodone (+0.00537) 0.932
Chronic myeloid leukemia (x1.06) 0.934
Malaise (x1.05) 0.936
ECG normal (x1.05) 0.938
Acute myeloid leukemia with myelodysplasia-related changes (x1.05) 0.940
Acute lymphoid leukemia in remission (x1.05) 0.941
Hypomagnesemia (x1.05) 0.943
Hypervolemia (x1.05) 0.944
Myelodysplastic syndrome (clinical) (x1.05) 0.946
promethazine (+0.00421) 0.947
Constipation (x1.04) 0.949
Blasts/100 leukocytes in B by Manual count (+0.797) 0.950
Acute lymphoid leukemia (x1.04) 0.951
Aplastic anemia (x1.04) 0.952
prochlorperazine (+0.0038) 0.953
Headache (x1.04) 0.955
Abnormal findings on diagnostic imaging of lung (x1.04) 0.955
Platelets [#/V] in B by AC (-5.97) 0.956
Cardiac arrhythmia (x1.04) 0.957
Oral ulcerative mucositis due to antineoplastic therapy (x1.03) 0.958
Disorder of transplanted bone marrow (x1.03) 0.959
Erythrocytes [#/V] in B by AC (-0.0357) 0.959
acetaminophen (-0.00308) 0.960
Disorder of hematopoietic structure (x1.03) 0.961
Pneumonia (x1.03) 0.962
Disorder of lung (x1.03) 0.962
Neutropenia due to and following chemotherapy (x1.03) 0.963
Electrocardiogram abnormal (x1.03) 0.963
Disorder of magnesium metabolism (x1.03) 0.964
Disseminated intravascular coagulation (x1.03) 0.965
Infective pneumonia (x1.03) 0.965
Conduction disorder of the heart (x1.03) 0.966
Polymorphonuclear cells/100 leukocytes in Body fluid by Manual count (-1.04) 0.966
ondansetron (+0.00267) 0.967
Myeloid leukemia in remission (x1.03) 0.967
Abnormal body temperature (x1.03) 0.968
Acute renal failure syndrome (x1.03) 0.968
Pain due to neoplastic disease (x1.03) 0.969
Erythrocyte distribution width [V] by AC (+0.276) 0.969
Borderline normal ECG (x1.03) 0.970
Asthenia (x1.03) 0.970
Chronic graft-versus-host disease (x1.03) 0.971
Leukocytes [#/V] in B by Manual count (+2.49) 0.971
MCH [M] by AC (+0.119) 0.972
Blood chemistry abnormal (x1.03) 0.972
Tumor lysis syndrome (x1.03) 0.973
Acute monocytic leukemia (x1.02) 0.973
levofloxacin (+0.00223) 0.973
Tachycardia (x1.02) 0.974
MCV [V] by AC (+0.277) 0.974
Lung field abnormal (x1.02) 0.974
Sepsis (x1.02) 0.975
Bleeding from nose (x1.02) 0.975
−0.6 −0.4 −0.2 0.0 0.2 0.4 0.6
Expressions
−20 −10 0 10 20

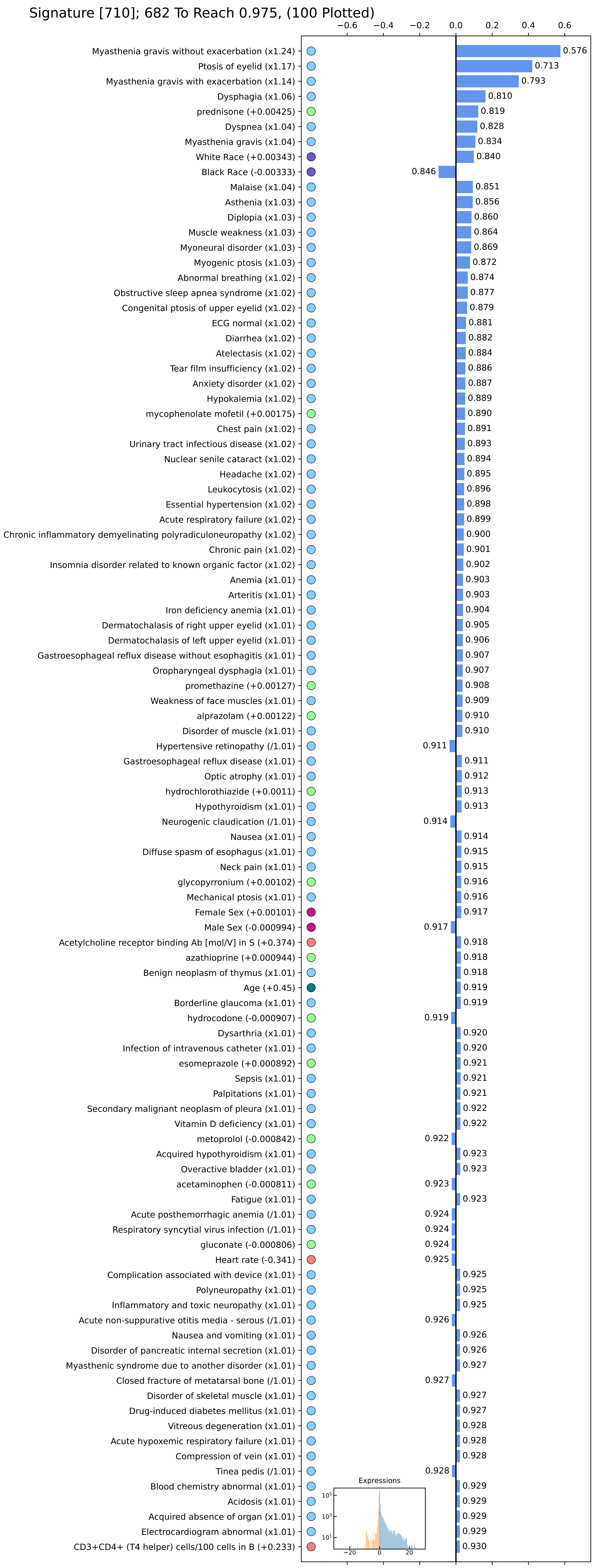

Signature [710]; 682 To Reach 0.975, (100 Plotted)
Myasthenia gravis without exacerbation (x1.24) 0.576
Ptosis of eyelid (x1.17) 0.713
Myasthenia gravis with exacerbation (x1.14) 0.793
Dysphagia (x1.06) 0.810
prednisone (+0.00425) 0.819
Dyspnea (x1.04) 0.828
Myasthenia gravis (x1.04) 0.834
White Race (+0.00343) 0.840
Black Race (-0.00333) 0.846
Malaise (x1.04) 0.851
Asthenia (x1.03) 0.856
Diplopia (x1.03) 0.860
Muscle weakness (x1.03) 0.864
Myoneural disorder (x1.03) 0.869
Myogenic ptosis (x1.03) 0.872
Abnormal breathing (x1.02) 0.874
Obstructive sleep apnea syndrome (x1.02) 0.877
Congenital ptosis of upper eyelid (x1.02) 0.879
ECG normal (x1.02) 0.881
Diarrhea (x1.02) 0.882
Atelectasis (x1.02) 0.884
Tear film insufficiency (x1.02) 0.886
Anxiety disorder (x1.02) 0.887
Hypokalemia (x1.02) 0.889
mycophenolate mofetil (+0.00175) 0.890
Chest pain (x1.02) 0.891
Urinary tract infectious disease (x1.02) 0.893
Nuclear senile cataract (x1.02) 0.894
Headache (x1.02) 0.895
Leukocytosis (x1.02) 0.896
Essential hypertension (x1.02) 0.898
Acute respiratory failure (x1.02) 0.899
Chronic inflammatory demyelinating polyradiculoneuropathy (x1.02) 0.900
Chronic pain (x1.02) 0.901
Insomnia disorder related to known organic factor (x1.02) 0.902
Anemia (x1.01) 0.903
Arteritis (x1.01) 0.903
Iron deficiency anemia (x1.01) 0.904
Dermatochalasis of right upper eyelid (x1.01) 0.905
Dermatochalasis of left upper eyelid (x1.01) 0.906
Gastroesophageal reflux disease without esophagitis (x1.01) 0.907
Oropharyngeal dysphagia (x1.01) 0.907
promethazine (+0.00127) 0.908
Weakness of face muscles (x1.01) 0.909
alprazolam (+0.00122) 0.910
Disorder of muscle (x1.01) 0.910
Hypertensive retinopathy (/1.01) 0.911
Gastroesophageal reflux disease (x1.01) 0.911
Optic atrophy (x1.01) 0.912
hydrochlorothiazide (+0.0011) 0.913
Hypothyroidism (x1.01) 0.913
Neurogenic claudication (/1.01) 0.914
Nausea (x1.01) 0.914
Diffuse spasm of esophagus (x1.01) 0.915
Neck pain (x1.01) 0.915
glycopyrronium (+0.00102) 0.916
Mechanical ptosis (x1.01) 0.916
Female Sex (+0.00101) 0.917
Male Sex (-0.000994) 0.917
Acetylcholine receptor binding Ab [mol/V] in S (+0.374) 0.918
azathioprine (+0.000944) 0.918
Benign neoplasm of thymus (x1.01) 0.918
Age (+0.45) 0.919
Borderline glaucoma (x1.01) 0.919
hydrocodone (-0.000907) 0.919
Dysarthria (x1.01) 0.920
Infection of intravenous catheter (x1.01) 0.920
esomeprazole (+0.000892) 0.921
Sepsis (x1.01) 0.921
Palpitations (x1.01) 0.921
Secondary malignant neoplasm of pleura (x1.01) 0.922
Vitamin D deficiency (x1.01) 0.922
metoprolol (-0.000842) 0.922
Acquired hypothyroidism (x1.01) 0.923
Overactive bladder (x1.01) 0.923
acetaminophen (-0.000811) 0.923
Fatigue (x1.01) 0.923
Acute posthemorrhagic anemia (/1.01) 0.924
Respiratory syncytial virus infection (/1.01) 0.924
gluconate (-0.000806) 0.924
Heart rate (-0.341) 0.925
Complication associated with device (x1.01) 0.925
Polyneuropathy (x1.01) 0.925
Inflammatory and toxic neuropathy (x1.01) 0.925
Acute non-suppurative otitis media - serous (/1.01) 0.926
Nausea and vomiting (x1.01) 0.926
Disorder of pancreatic internal secretion (x1.01) 0.926
Myasthenic syndrome due to another disorder (x1.01) 0.927
Closed fracture of metatarsal bone (/1.01) 0.927
Disorder of skeletal muscle (x1.01) 0.927
Drug-induced diabetes mellitus (x1.01) 0.927
Vitreous degeneration (x1.01) 0.928
Acute hypoxemic respiratory failure (x1.01) 0.928
Compression of vein (x1.01) 0.928
Tinea pedis (/1.01) 0.928
Blood chemistry abnormal (x1.01) 0.929
Acidosis (x1.01) 0.929
Acquired absence of organ (x1.01) 0.929
Electrocardiogram abnormal (x1.01) 0.929
CD3+CD4+ (T4 helper) cells/100 cells in B (+0.233) 0.930
−0.6 −0.4 −0.2 0.0 0.2 0.4 0.6
Expressions

Signature [711]; 56 To Reach 0.975, (56 Plotted)

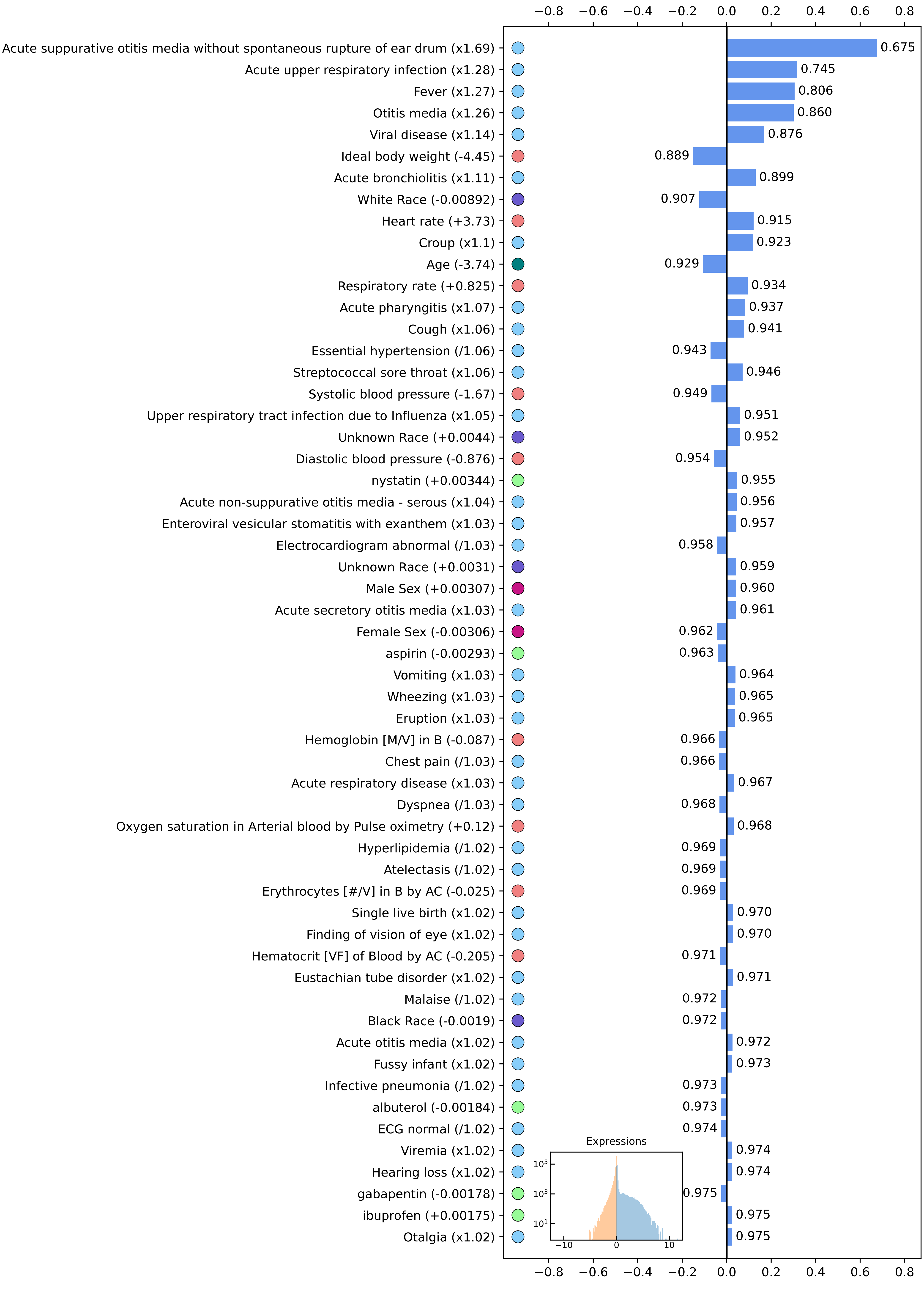

Signature [712]; 6 To Reach 0.975, (10 Plotted)

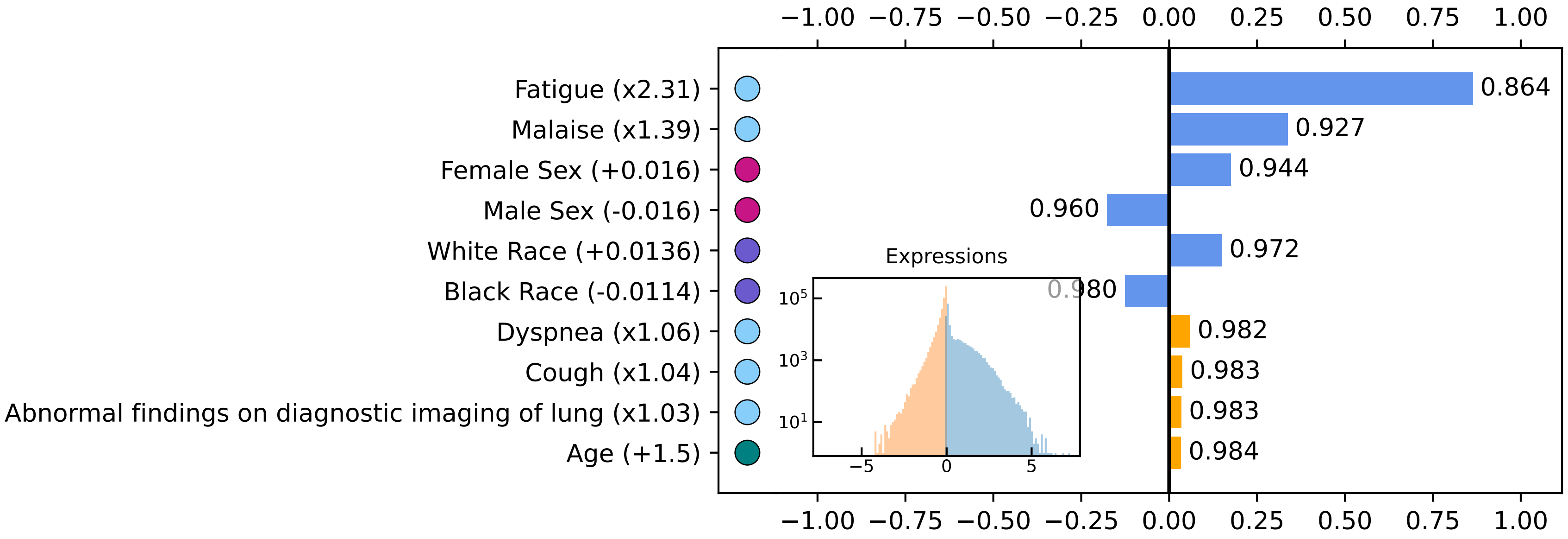

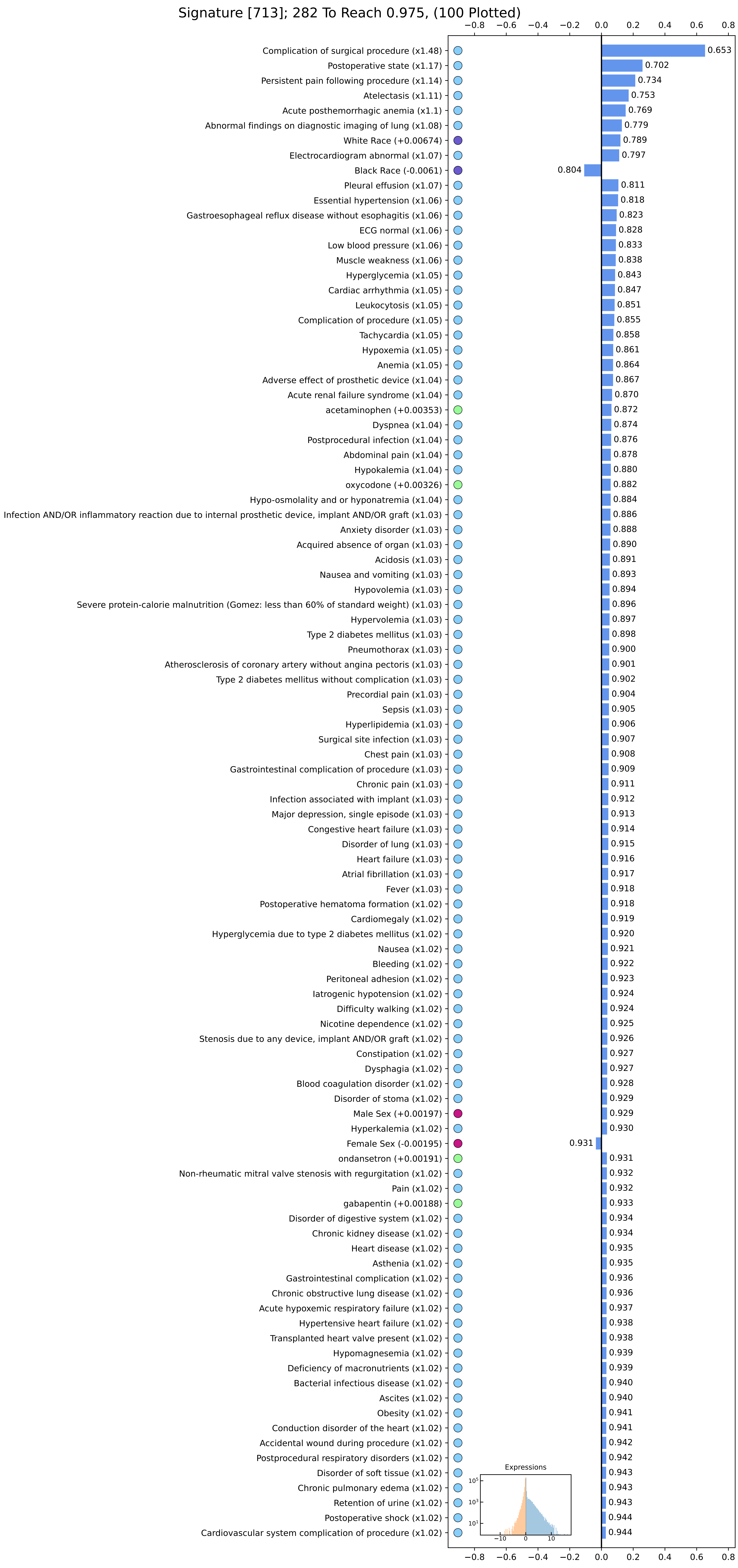

Signature [713]; 282 To Reach 0.975, (100 Plotted)
Complication of surgical procedure (x1.48) 0.653
Postoperative state (x1.17) 0.702
Persistent pain following procedure (x1.14) 0.734
Atelectasis (x1.11) 0.753
Acute posthemorrhagic anemia (x1.1) 0.769
Abnormal findings on diagnostic imaging of lung (x1.08) 0.779
White Race (+0.00674) 0.789
Electrocardiogram abnormal (x1.07) 0.797
Black Race (-0.0061) 0.804
Pleural effusion (x1.07) 0.811
Essential hypertension (x1.06) 0.818
Gastroesophageal reflux disease without esophagitis (x1.06) 0.823
ECG normal (x1.06) 0.828
Low blood pressure (x1.06) 0.833
Muscle weakness (x1.06) 0.838
Hyperglycemia (x1.05) 0.843
Cardiac arrhythmia (x1.05) 0.847
Leukocytosis (x1.05) 0.851
Complication of procedure (x1.05) 0.855
Tachycardia (x1.05) 0.858
Hypoxemia (x1.05) 0.861
Anemia (x1.05) 0.864
Adverse effect of prosthetic device (x1.04) 0.867
Acute renal failure syndrome (x1.04) 0.870
acetaminophen (+0.00353) 0.872
Dyspnea (x1.04) 0.874
Postprocedural infection (x1.04) 0.876
Abdominal pain (x1.04) 0.878
Hypokalemia (x1.04) 0.880
oxycodone (+0.00326) 0.882
Hypo-osmolality and or hyponatremia (x1.04) 0.884
Infection AND/OR inflammatory reaction due to internal prosthetic device, implant AND/OR graft (x1.03) 0.886
Anxiety disorder (x1.03) 0.888
Acquired absence of organ (x1.03) 0.890
Acidosis (x1.03) 0.891
Nausea and vomiting (x1.03) 0.893
Hypovolemia (x1.03) 0.894
Severe protein-calorie malnutrition (Gomez: less than 60% of standard weight) (x1.03) 0.896
Hypervolemia (x1.03) 0.897
Type 2 diabetes mellitus (x1.03) 0.898
Pneumothorax (x1.03) 0.900
Atherosclerosis of coronary artery without angina pectoris (x1.03) 0.901
Type 2 diabetes mellitus without complication (x1.03) 0.902
Precordial pain (x1.03) 0.904
Sepsis (x1.03) 0.905
Hyperlipidemia (x1.03) 0.906
Surgical site infection (x1.03) 0.907
Chest pain (x1.03) 0.908
Gastrointestinal complication of procedure (x1.03) 0.909
Chronic pain (x1.03) 0.911
Infection associated with implant (x1.03) 0.912
Major depression, single episode (x1.03) 0.913
Congestive heart failure (x1.03) 0.914
Disorder of lung (x1.03) 0.915
Heart failure (x1.03) 0.916
Atrial fibrillation (x1.03) 0.917
Fever (x1.03) 0.918
Postoperative hematoma formation (x1.02) 0.918
Cardiomegaly (x1.02) 0.919
Hyperglycemia due to type 2 diabetes mellitus (x1.02) 0.920
Nausea (x1.02) 0.921
Bleeding (x1.02) 0.922
Peritoneal adhesion (x1.02) 0.923
Iatrogenic hypotension (x1.02) 0.924
Difficulty walking (x1.02) 0.924
Nicotine dependence (x1.02) 0.925
Stenosis due to any device, implant AND/OR graft (x1.02) 0.926
Constipation (x1.02) 0.927
Dysphagia (x1.02) 0.927
Blood coagulation disorder (x1.02) 0.928
Disorder of stoma (x1.02) 0.929
Male Sex (+0.00197) 0.929
Hyperkalemia (x1.02) 0.930
Female Sex (-0.00195) 0.931
ondansetron (+0.00191) 0.931
Non-rheumatic mitral valve stenosis with regurgitation (x1.02) 0.932
Pain (x1.02) 0.932
gabapentin (+0.00188) 0.933
Disorder of digestive system (x1.02) 0.934
Chronic kidney disease (x1.02) 0.934
Heart disease (x1.02) 0.935
Asthenia (x1.02) 0.935
Gastrointestinal complication (x1.02) 0.936
Chronic obstructive lung disease (x1.02) 0.936
Acute hypoxemic respiratory failure (x1.02) 0.937
Hypertensive heart failure (x1.02) 0.938
Transplanted heart valve present (x1.02) 0.938
Hypomagnesemia (x1.02) 0.939
Deficiency of macronutrients (x1.02) 0.939
Bacterial infectious disease (x1.02) 0.940
Ascites (x1.02) 0.940
Obesity (x1.02) 0.941
Conduction disorder of the heart (x1.02) 0.941
Accidental wound during procedure (x1.02) 0.942
Postprocedural respiratory disorders (x1.02) 0.942
Disorder of soft tissue (x1.02) 0.943
Chronic pulmonary edema (x1.02) 0.943
Retention of urine (x1.02) 0.943
Postoperative shock (x1.02) 0.944
Cardiovascular system complication of procedure (x1.02) 0.944
−0.8 −0.6 −0.4 −0.2 0.0 0.2 0.4 0.6 0.8
Expressions
10^5 10^3 10^1
−10 0 10

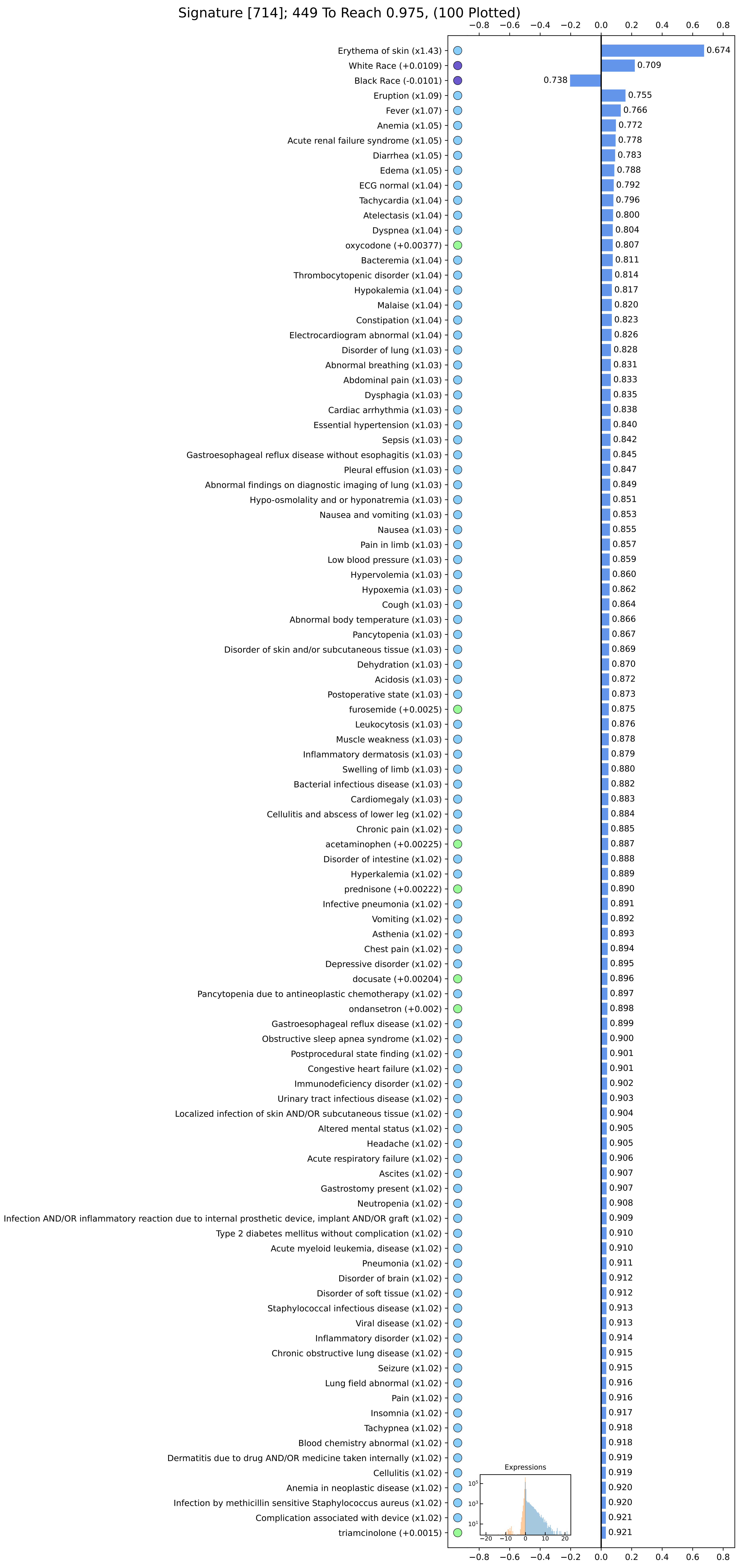

Signature [714]; 449 To Reach 0.975, (100 Plotted)
Erythema of skin (x1.43) 0.674
White Race (+0.0109) 0.709
Black Race (-0.0101) 0.738
Eruption (x1.09) 0.755
Fever (x1.07) 0.766
Anemia (x1.05) 0.772
Acute renal failure syndrome (x1.05) 0.778
Diarrhea (x1.05) 0.783
Edema (x1.05) 0.788
ECG normal (x1.04) 0.792
Tachycardia (x1.04) 0.796
Atelectasis (x1.04) 0.800
Dyspnea (x1.04) 0.804
oxycodone (+0.00377) 0.807
Bacteremia (x1.04) 0.811
Thrombocytopenic disorder (x1.04) 0.814
Hypokalemia (x1.04) 0.817
Malaise (x1.04) 0.820
Constipation (x1.04) 0.823
Electrocardiogram abnormal (x1.04) 0.826
Disorder of lung (x1.03) 0.828
Abnormal breathing (x1.03) 0.831
Abdominal pain (x1.03) 0.833
Dysphagia (x1.03) 0.835
Cardiac arrhythmia (x1.03) 0.838
Essential hypertension (x1.03) 0.840
Sepsis (x1.03) 0.842
Gastroesophageal reflux disease without esophagitis (x1.03) 0.845
Pleural effusion (x1.03) 0.847
Abnormal findings on diagnostic imaging of lung (x1.03) 0.849
Hypo-osmolality and or hyponatremia (x1.03) 0.851
Nausea and vomiting (x1.03) 0.853
Nausea (x1.03) 0.855
Pain in limb (x1.03) 0.857
Low blood pressure (x1.03) 0.859
Hypervolemia (x1.03) 0.860
Hypoxemia (x1.03) 0.862
Cough (x1.03) 0.864
Abnormal body temperature (x1.03) 0.866
Pancytopenia (x1.03) 0.867
Disorder of skin and/or subcutaneous tissue (x1.03) 0.869
Dehydration (x1.03) 0.870
Acidosis (x1.03) 0.872
Postoperative state (x1.03) 0.873
furosemide (+0.0025) 0.875
Leukocytosis (x1.03) 0.876
Muscle weakness (x1.03) 0.878
Inflammatory dermatosis (x1.03) 0.879
Swelling of limb (x1.03) 0.880
Bacterial infectious disease (x1.03) 0.882
Cardiomegaly (x1.03) 0.883
Cellulitis and abscess of lower leg (x1.02) 0.884
Chronic pain (x1.02) 0.885
acetaminophen (+0.00225) 0.887
Disorder of intestine (x1.02) 0.888
Hyperkalemia (x1.02) 0.889
prednisone (+0.00222) 0.890
Infective pneumonia (x1.02) 0.891
Vomiting (x1.02) 0.892
Asthenia (x1.02) 0.893
Chest pain (x1.02) 0.894
Depressive disorder (x1.02) 0.895
docusate (+0.00204) 0.896
Pancytopenia due to antineoplastic chemotherapy (x1.02) 0.897
ondansetron (+0.002) 0.898
Gastroesophageal reflux disease (x1.02) 0.899
Obstructive sleep apnea syndrome (x1.02) 0.900
Postprocedural state finding (x1.02) 0.901
Congestive heart failure (x1.02) 0.901
Immunodeficiency disorder (x1.02) 0.902
Urinary tract infectious disease (x1.02) 0.903
Localized infection of skin AND/OR subcutaneous tissue (x1.02) 0.904
Altered mental status (x1.02) 0.905
Headache (x1.02) 0.905
Acute respiratory failure (x1.02) 0.906
Ascites (x1.02) 0.907
Gastrostomy present (x1.02) 0.907
Neutropenia (x1.02) 0.908
Infection AND/OR inflammatory reaction due to internal prosthetic device, implant AND/OR graft (x1.02) 0.909
Type 2 diabetes mellitus without complication (x1.02) 0.910
Acute myeloid leukemia, disease (x1.02) 0.910
Pneumonia (x1.02) 0.911
Disorder of brain (x1.02) 0.912
Disorder of soft tissue (x1.02) 0.912
Staphylococcal infectious disease (x1.02) 0.913
Viral disease (x1.02) 0.913
Inflammatory disorder (x1.02) 0.914
Chronic obstructive lung disease (x1.02) 0.915
Seizure (x1.02) 0.915
Lung field abnormal (x1.02) 0.916
Pain (x1.02) 0.916
Insomnia (x1.02) 0.917
Tachypnea (x1.02) 0.918
Blood chemistry abnormal (x1.02) 0.918
Dermatitis due to drug AND/OR medicine taken internally (x1.02) 0.919
Cellulitis (x1.02) 0.919
Anemia in neoplastic disease (x1.02) 0.920
Infection by methicillin sensitive Staphylococcus aureus (x1.02) 0.920
Complication associated with device (x1.02) 0.921
triamcinolone (+0.0015) 0.921
Expressions
−0.8 −0.6 −0.4 −0.2 0.0 0.2 0.4 0.6 0.8

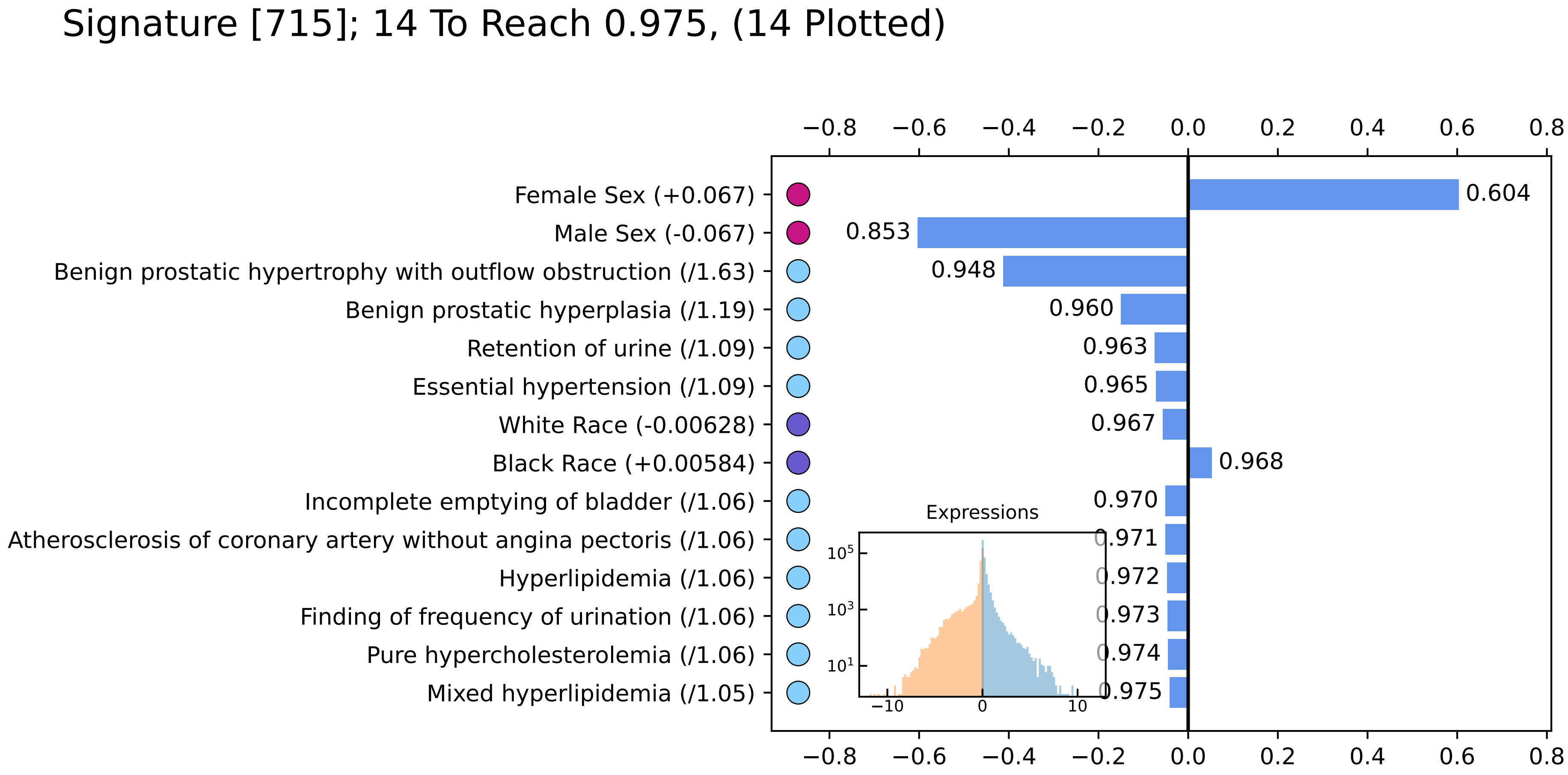
Signature [715]; 14 To Reach 0.975, (14 Plotted)
Female Sex (+0.067)
Male Sex (-0.067)
Benign prostatic hypertrophy with outflow obstruction (/1.63)
Benign prostatic hyperplasia (/1.19)
Retention of urine (/1.09)
Essential hypertension (/1.09)
White Race (-0.00628)
Black Race (+0.00584)
Incomplete emptying of bladder (/1.06)
Atherosclerosis of coronary artery without angina pectoris (/1.06)
Hyperlipidemia (/1.06)
Finding of frequency of urination (/1.06)
Pure hypercholesterolemia (/1.06)
Mixed hyperlipidemia (/1.05)
0.604
0.853
0.948
0.960
0.963
0.965
0.967
0.968
0.970
0.971
0.972
0.973
0.974
0.975
−0.8
−0.6
−0.4
−0.2
0.0
0.2
0.4
0.6
0.8
Expressions
10^5
10^3
10^1
−10
0
10

Signature [716]; 11 To Reach 0.975, (11 Plotted)

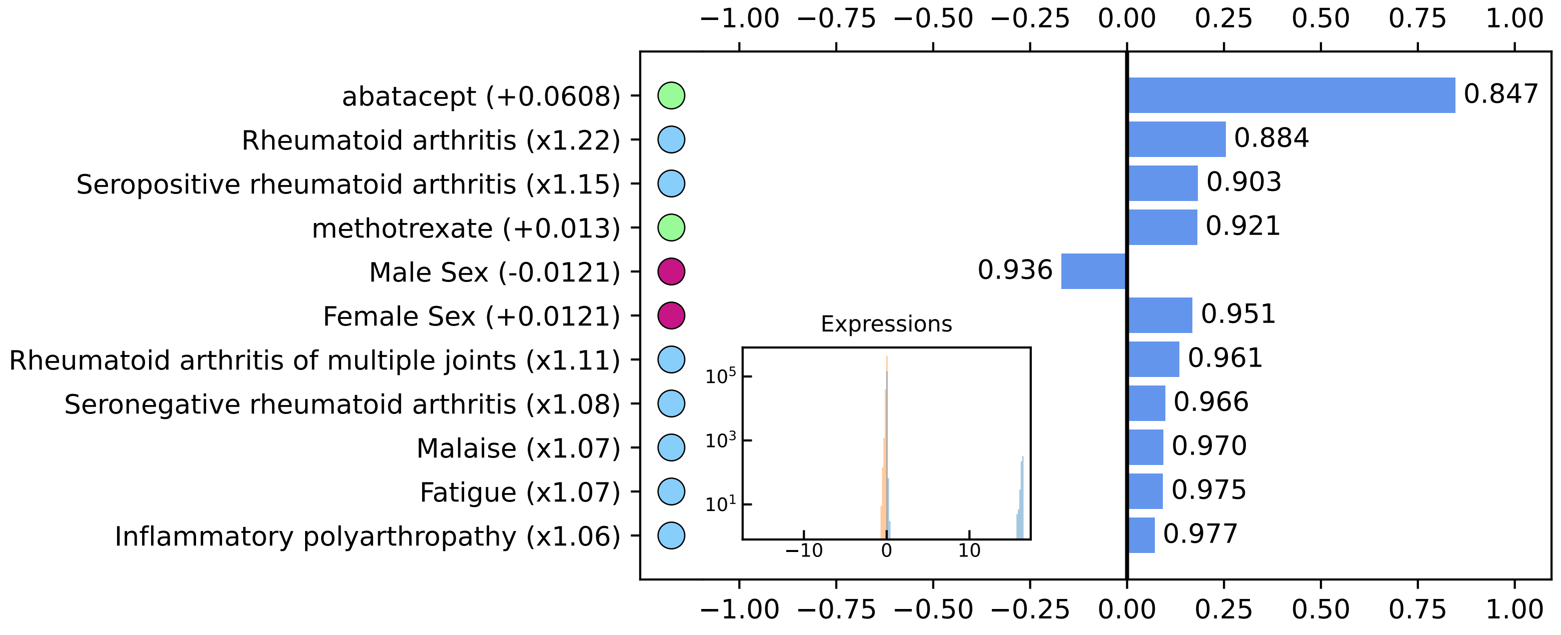

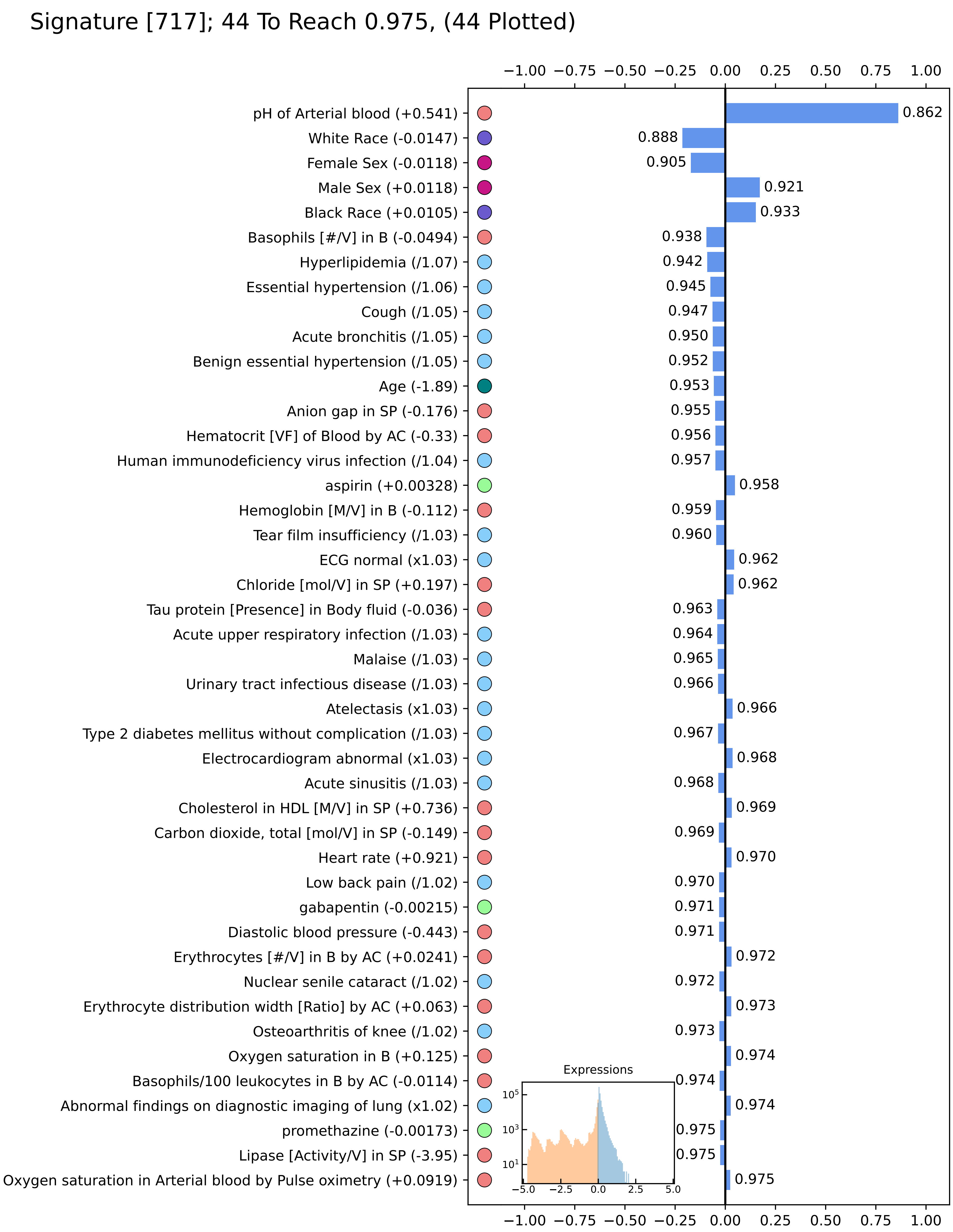

Signature [717]; 44 To Reach 0.975, (44 Plotted)
pH of Arterial blood (+0.541) 0.862
White Race (-0.0147) 0.888
Female Sex (-0.0118) 0.905
Male Sex (+0.0118) 0.921
Black Race (+0.0105) 0.933
Basophils [#/V] in B (-0.0494) 0.938
Hyperlipidemia (/1.07) 0.942
Essential hypertension (/1.06) 0.945
Cough (/1.05) 0.947
Acute bronchitis (/1.05) 0.950
Benign essential hypertension (/1.05) 0.952
Age (-1.89) 0.953
Anion gap in SP (-0.176) 0.955
Hematocrit [VF] of Blood by AC (-0.33) 0.956
Human immunodeficiency virus infection (/1.04) 0.957
aspirin (+0.00328) 0.958
Hemoglobin [M/V] in B (-0.112) 0.959
Tear film insufficiency (/1.03) 0.960
ECG normal (x1.03) 0.962
Chloride [mol/V] in SP (+0.197) 0.962
Tau protein [Presence] in Body fluid (-0.036) 0.963
Acute upper respiratory infection (/1.03) 0.964
Malaise (/1.03) 0.965
Urinary tract infectious disease (/1.03) 0.966
Atelectasis (x1.03) 0.966
Type 2 diabetes mellitus without complication (/1.03) 0.967
Electrocardiogram abnormal (x1.03) 0.968
Acute sinusitis (/1.03) 0.968
Cholesterol in HDL [M/V] in SP (+0.736) 0.969
Carbon dioxide, total [mol/V] in SP (-0.149) 0.969
Heart rate (+0.921) 0.970
Low back pain (/1.02) 0.970
gabapentin (-0.00215) 0.971
Diastolic blood pressure (-0.443) 0.971
Erythrocytes [#/V] in B by AC (+0.0241) 0.972
Nuclear senile cataract (/1.02) 0.972
Erythrocyte distribution width [Ratio] by AC (+0.063) 0.973
Osteoarthritis of knee (/1.02) 0.973
Oxygen saturation in B (+0.125) 0.974
Basophils/100 leukocytes in B by AC (-0.0114) 0.974
Abnormal findings on diagnostic imaging of lung (x1.02) 0.974
promethazine (-0.00173) 0.975
Lipase [Activity/V] in SP (-3.95) 0.975
Oxygen saturation in Arterial blood by Pulse oximetry (+0.0919) 0.975
−1.00 −0.75 −0.50 −0.25 0.00 0.25 0.50 0.75 1.00
Expressions
10^5
10^3
10^1
−5.0 −2.5 0.0 2.5 5.0

Signature [718]; 39 To Reach 0.975, (39 Plotted)

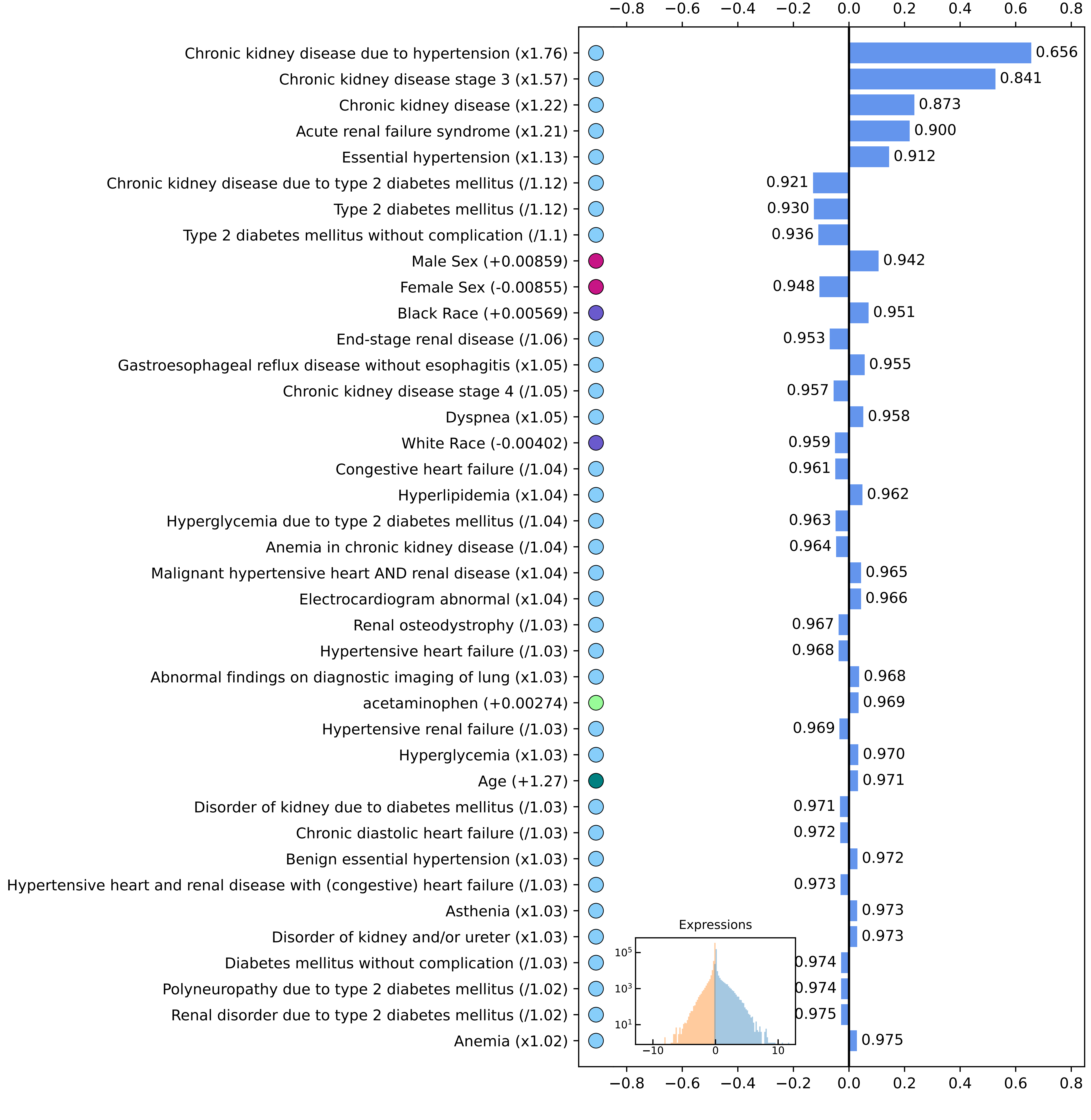

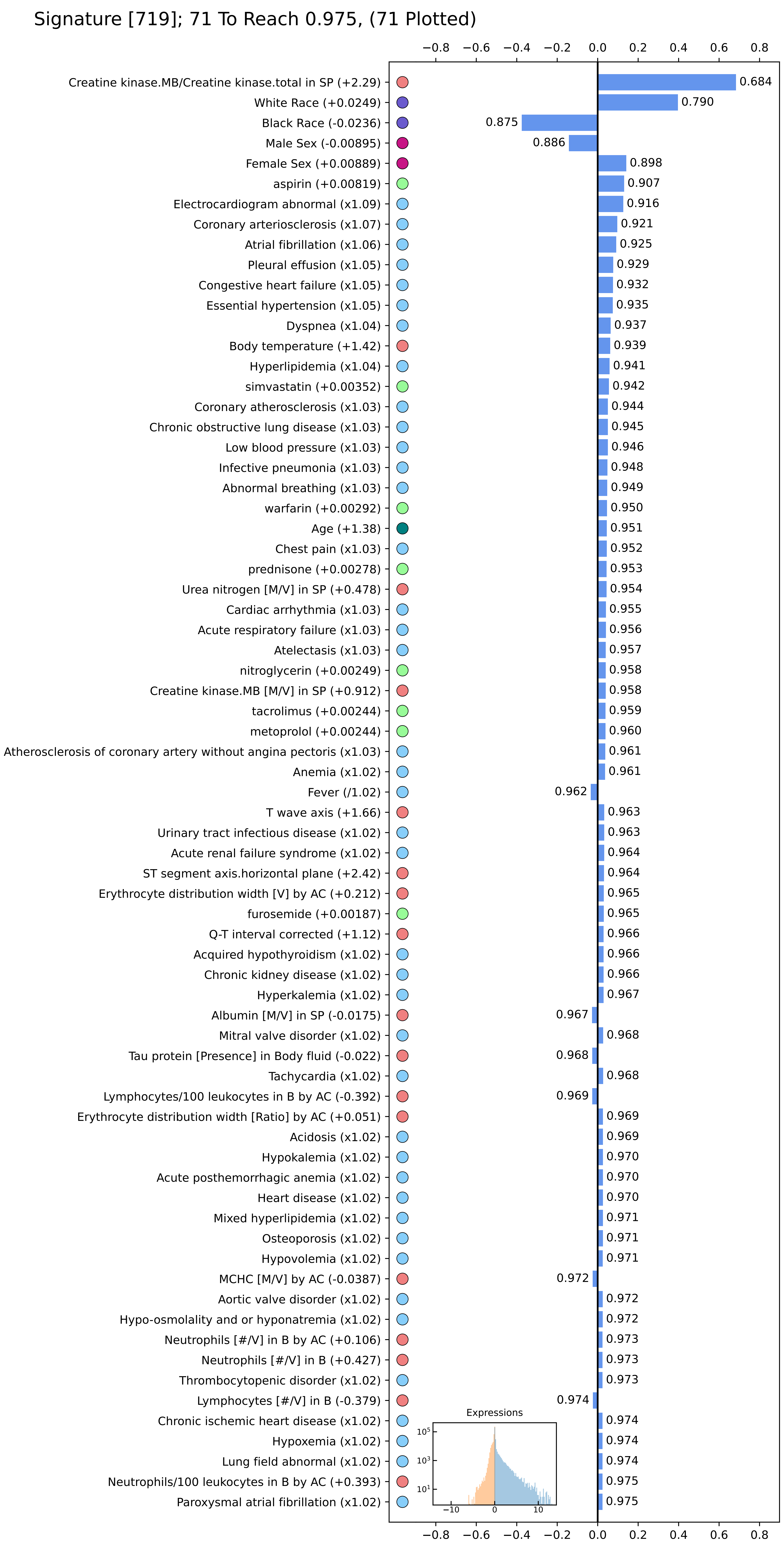

Signature [719]; 71 To Reach 0.975, (71 Plotted)
Creatine kinase.MB/Creatine kinase.total in SP (+2.29) 0.684
White Race (+0.0249) 0.790
Black Race (-0.0236) 0.875
Male Sex (-0.00895) 0.886
Female Sex (+0.00889) 0.898
aspirin (+0.00819) 0.907
Electrocardiogram abnormal (x1.09) 0.916
Coronary arteriosclerosis (x1.07) 0.921
Atrial fibrillation (x1.06) 0.925
Pleural effusion (x1.05) 0.929
Congestive heart failure (x1.05) 0.932
Essential hypertension (x1.05) 0.935
Dyspnea (x1.04) 0.937
Body temperature (+1.42) 0.939
Hyperlipidemia (x1.04) 0.941
simvastatin (+0.00352) 0.942
Coronary atherosclerosis (x1.03) 0.944
Chronic obstructive lung disease (x1.03) 0.945
Low blood pressure (x1.03) 0.946
Infective pneumonia (x1.03) 0.948
Abnormal breathing (x1.03) 0.949
warfarin (+0.00292) 0.950
Age (+1.38) 0.951
Chest pain (x1.03) 0.952
prednisone (+0.00278) 0.953
Urea nitrogen [M/V] in SP (+0.478) 0.954
Cardiac arrhythmia (x1.03) 0.955
Acute respiratory failure (x1.03) 0.956
Atelectasis (x1.03) 0.957
nitroglycerin (+0.00249) 0.958
Creatine kinase.MB [M/V] in SP (+0.912) 0.958
tacrolimus (+0.00244) 0.959
metoprolol (+0.00244) 0.960
Atherosclerosis of coronary artery without angina pectoris (x1.03) 0.961
Anemia (x1.02) 0.961
Fever (/1.02) 0.962
T wave axis (+1.66) 0.963
Urinary tract infectious disease (x1.02) 0.963
Acute renal failure syndrome (x1.02) 0.964
ST segment axis.horizontal plane (+2.42) 0.964
Erythrocyte distribution width [V] by AC (+0.212) 0.965
furosemide (+0.00187) 0.965
Q-T interval corrected (+1.12) 0.966
Acquired hypothyroidism (x1.02) 0.966
Chronic kidney disease (x1.02) 0.966
Hyperkalemia (x1.02) 0.967
Albumin [M/V] in SP (-0.0175) 0.967
Mitral valve disorder (x1.02) 0.968
Tau protein [Presence] in Body fluid (-0.022) 0.968
Tachycardia (x1.02) 0.968
Lymphocytes/100 leukocytes in B by AC (-0.392) 0.969
Erythrocyte distribution width [Ratio] by AC (+0.051) 0.969
Acidosis (x1.02) 0.969
Hypokalemia (x1.02) 0.970
Acute posthemorrhagic anemia (x1.02) 0.970
Heart disease (x1.02) 0.970
Mixed hyperlipidemia (x1.02) 0.971
Osteoporosis (x1.02) 0.971
Hypovolemia (x1.02) 0.971
MCHC [M/V] by AC (-0.0387) 0.972
Aortic valve disorder (x1.02) 0.972
Hypo-osmolality and or hyponatremia (x1.02) 0.972
Neutrophils [#/V] in B by AC (+0.106) 0.973
Neutrophils [#/V] in B (+0.427) 0.973
Thrombocytopenic disorder (x1.02) 0.973
Lymphocytes [#/V] in B (-0.379) 0.974
Chronic ischemic heart disease (x1.02) 0.974
Hypoxemia (x1.02) 0.974
Lung field abnormal (x1.02) 0.974
Neutrophils/100 leukocytes in B by AC (+0.393) 0.975
Paroxysmal atrial fibrillation (x1.02) 0.975
−0.8 −0.6 −0.4 −0.2 0.0 0.2 0.4 0.6 0.8
Expressions
10^5 10^3 10^1
−10 0 10

Signature [720]; 90 To Reach 0.975, (90 Plotted)

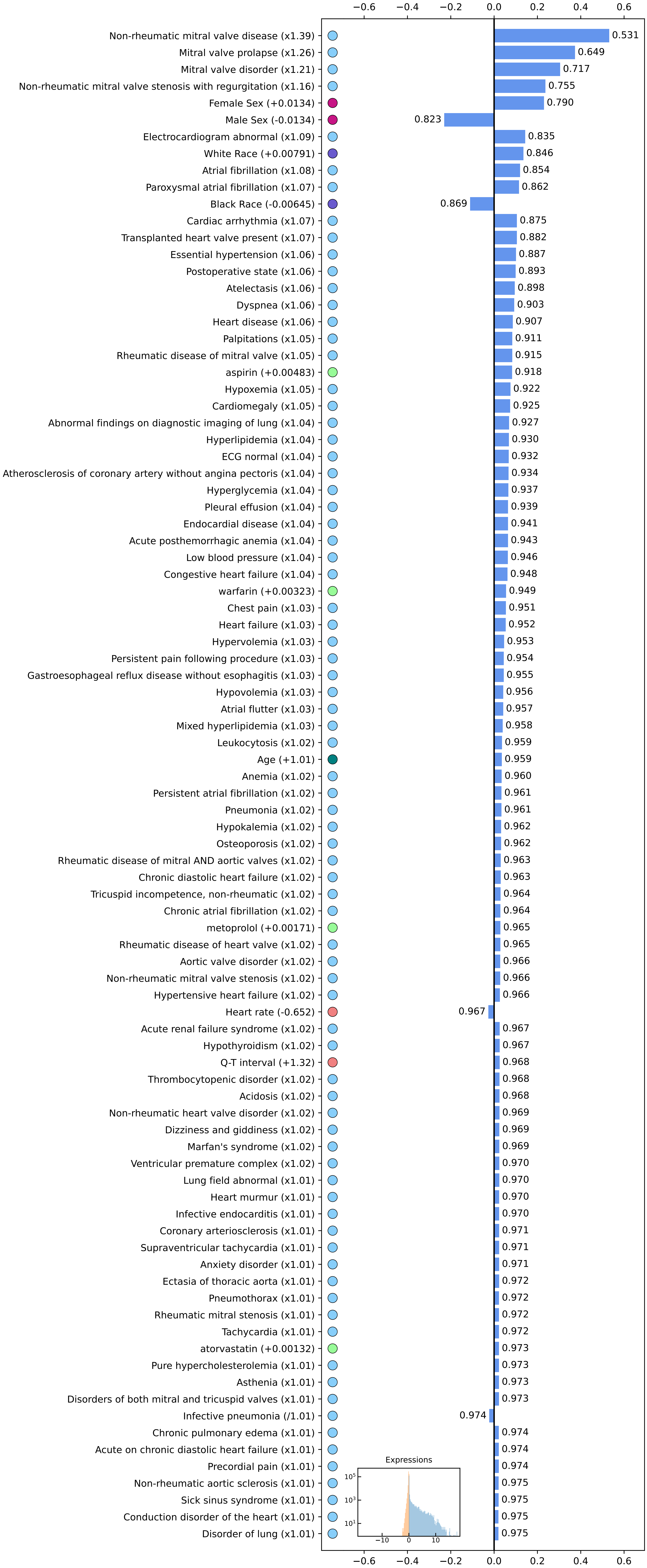

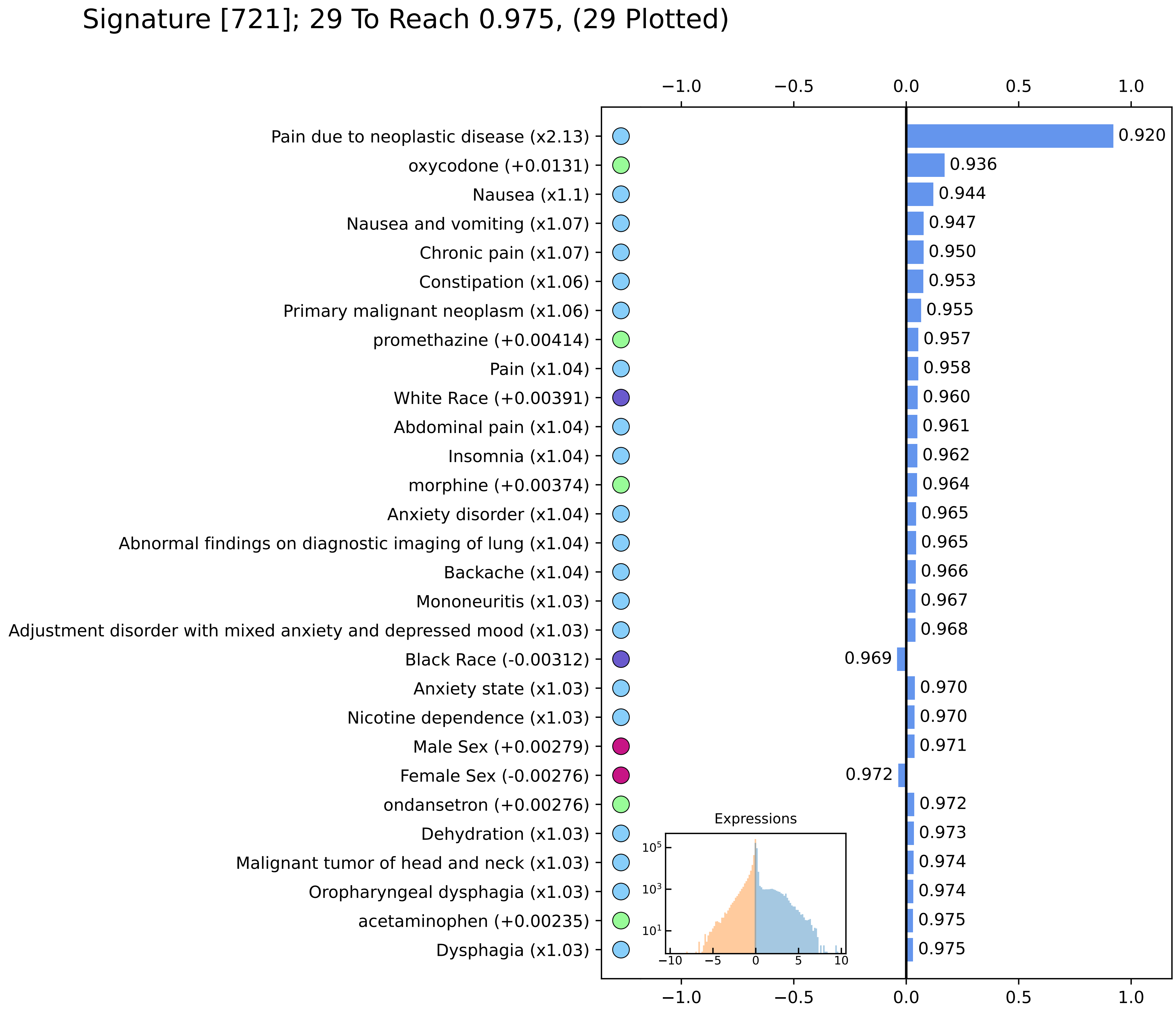

Signature [721]; 29 To Reach 0.975, (29 Plotted)
Pain due to neoplastic disease (x2.13)
oxycodone (+0.0131)
Nausea (x1.1)
Nausea and vomiting (x1.07)
Chronic pain (x1.07)
Constipation (x1.06)
Primary malignant neoplasm (x1.06)
promethazine (+0.00414)
Pain (x1.04)
White Race (+0.00391)
Abdominal pain (x1.04)
Insomnia (x1.04)
morphine (+0.00374)
Anxiety disorder (x1.04)
Abnormal findings on diagnostic imaging of lung (x1.04)
Backache (x1.04)
Mononeuritis (x1.03)
Adjustment disorder with mixed anxiety and depressed mood (x1.03)
Black Race (-0.00312)
Anxiety state (x1.03)
Nicotine dependence (x1.03)
Male Sex (+0.00279)
Female Sex (-0.00276)
ondansetron (+0.00276)
Dehydration (x1.03)
Malignant tumor of head and neck (x1.03)
Oropharyngeal dysphagia (x1.03)
acetaminophen (+0.00235)
Dysphagia (x1.03)
0.920
0.936
0.944
0.947
0.950
0.953
0.955
0.957
0.958
0.960
0.961
0.962
0.964
0.965
0.965
0.966
0.967
0.968
0.969
0.970
0.970
0.971
0.972
0.972
0.973
0.974
0.974
0.975
0.975
−1.0
−0.5
0.0
0.5
1.0
Expressions

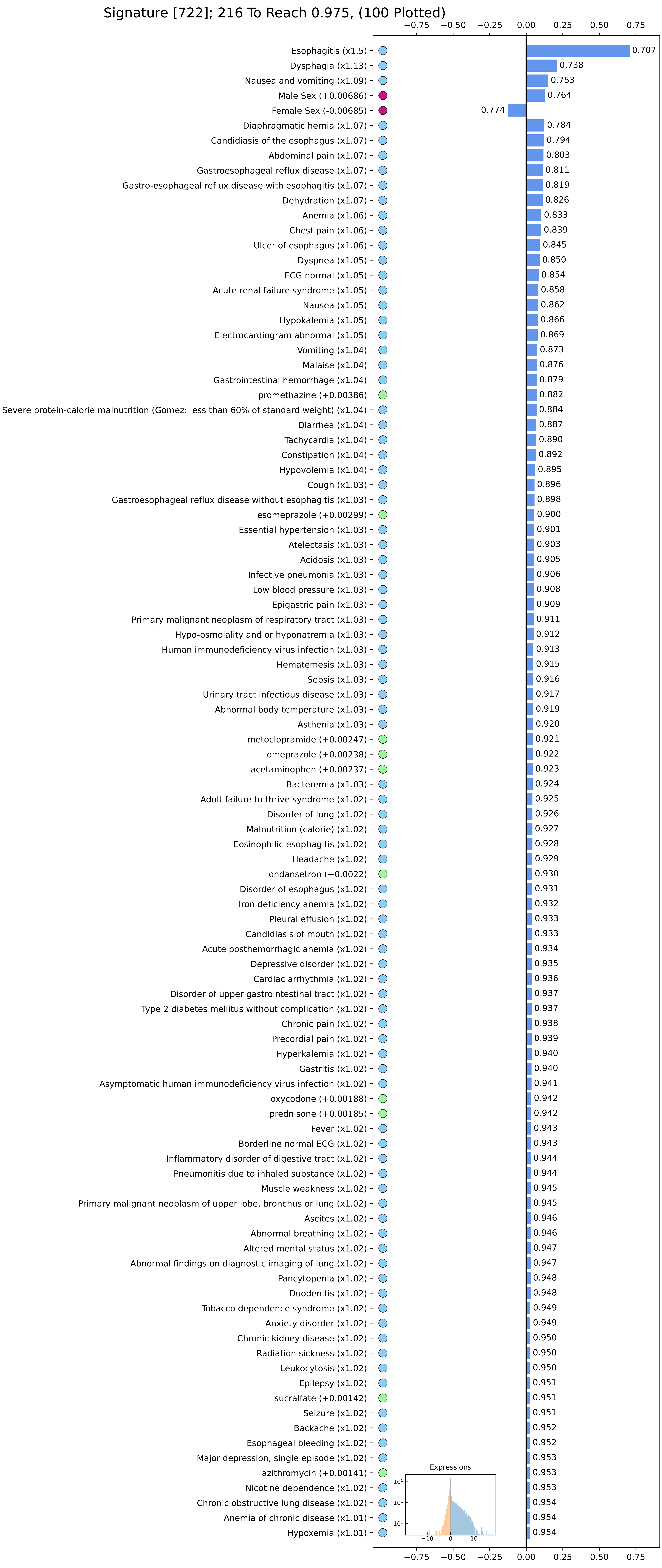

Signature [722]; 216 To Reach 0.975, (100 Plotted)
Esophagitis (x1.5)
Dysphagia (x1.13)
Nausea and vomiting (x1.09)
Male Sex (+0.00686)
Female Sex (-0.00685)
Diaphragmatic hernia (x1.07)
Candidiasis of the esophagus (x1.07)
Abdominal pain (x1.07)
Gastroesophageal reflux disease (x1.07)
Gastro-esophageal reflux disease with esophagitis (x1.07)
Dehydration (x1.07)
Anemia (x1.06)
Chest pain (x1.06)
Ulcer of esophagus (x1.06)
Dyspnea (x1.05)
ECG normal (x1.05)
Acute renal failure syndrome (x1.05)
Nausea (x1.05)
Hypokalemia (x1.05)
Electrocardiogram abnormal (x1.05)
Vomiting (x1.04)
Malaise (x1.04)
Gastrointestinal hemorrhage (x1.04)
promethazine (+0.00386)
Severe protein-calorie malnutrition (Gomez: less than 60% of standard weight) (x1.04)
Diarrhea (x1.04)
Tachycardia (x1.04)
Constipation (x1.04)
Hypovolemia (x1.04)
Cough (x1.03)
Gastroesophageal reflux disease without esophagitis (x1.03)
esomeprazole (+0.00299)
Essential hypertension (x1.03)
Atelectasis (x1.03)
Acidosis (x1.03)
Infective pneumonia (x1.03)
Low blood pressure (x1.03)
Epigastric pain (x1.03)
Primary malignant neoplasm of respiratory tract (x1.03)
Hypo-osmolality and or hyponatremia (x1.03)
Human immunodeficiency virus infection (x1.03)
Hematemesis (x1.03)
Sepsis (x1.03)
Urinary tract infectious disease (x1.03)
Abnormal body temperature (x1.03)
Asthenia (x1.03)
metoclopramide (+0.00247)
omeprazole (+0.00238)
acetaminophen (+0.00237)
Bacteremia (x1.03)
Adult failure to thrive syndrome (x1.02)
Disorder of lung (x1.02)
Malnutrition (calorie) (x1.02)
Eosinophilic esophagitis (x1.02)
Headache (x1.02)
ondansetron (+0.0022)
Disorder of esophagus (x1.02)
Iron deficiency anemia (x1.02)
Pleural effusion (x1.02)
Candidiasis of mouth (x1.02)
Acute posthemorrhagic anemia (x1.02)
Depressive disorder (x1.02)
Cardiac arrhythmia (x1.02)
Disorder of upper gastrointestinal tract (x1.02)
Type 2 diabetes mellitus without complication (x1.02)
Chronic pain (x1.02)
Precordial pain (x1.02)
Hyperkalemia (x1.02)
Gastritis (x1.02)
Asymptomatic human immunodeficiency virus infection (x1.02)
oxycodone (+0.00188)
prednisone (+0.00185)
Fever (x1.02)
Borderline normal ECG (x1.02)
Inflammatory disorder of digestive tract (x1.02)
Pneumonitis due to inhaled substance (x1.02)
Muscle weakness (x1.02)
Primary malignant neoplasm of upper lobe, bronchus or lung (x1.02)
Ascites (x1.02)
Abnormal breathing (x1.02)
Altered mental status (x1.02)
Abnormal findings on diagnostic imaging of lung (x1.02)
Pancytopenia (x1.02)
Duodenitis (x1.02)
Tobacco dependence syndrome (x1.02)
Anxiety disorder (x1.02)
Chronic kidney disease (x1.02)
Radiation sickness (x1.02)
Leukocytosis (x1.02)
Epilepsy (x1.02)
sucralfate (+0.00142)
Seizure (x1.02)
Backache (x1.02)
Esophageal bleeding (x1.02)
Major depression, single episode (x1.02)
azithromycin (+0.00141)
Nicotine dependence (x1.02)
Chronic obstructive lung disease (x1.02)
Anemia of chronic disease (x1.01)
Hypoxemia (x1.01)
0.707
0.738
0.753
0.764
0.774
0.784
0.794
0.803
0.811
0.819
0.826
0.833
0.839
0.845
0.850
0.854
0.858
0.862
0.866
0.869
0.873
0.876
0.879
0.882
0.884
0.887
0.890
0.892
0.895
0.896
0.898
0.900
0.901
0.903
0.905
0.906
0.908
0.909
0.911
0.912
0.913
0.915
0.916
0.917
0.919
0.920
0.921
0.922
0.923
0.924
0.925
0.926
0.927
0.928
0.929
0.930
0.931
0.932
0.933
0.933
0.934
0.935
0.936
0.937
0.937
0.938
0.939
0.940
0.940
0.941
0.942
0.942
0.943
0.943
0.944
0.944
0.945
0.945
0.946
0.946
0.947
0.947
0.948
0.948
0.949
0.949
0.950
0.950
0.950
0.951
0.951
0.951
0.952
0.952
0.953
0.953
0.953
0.954
0.954
0.954
−0.75 −0.50 −0.25 0.00 0.25 0.50 0.75
Expressions

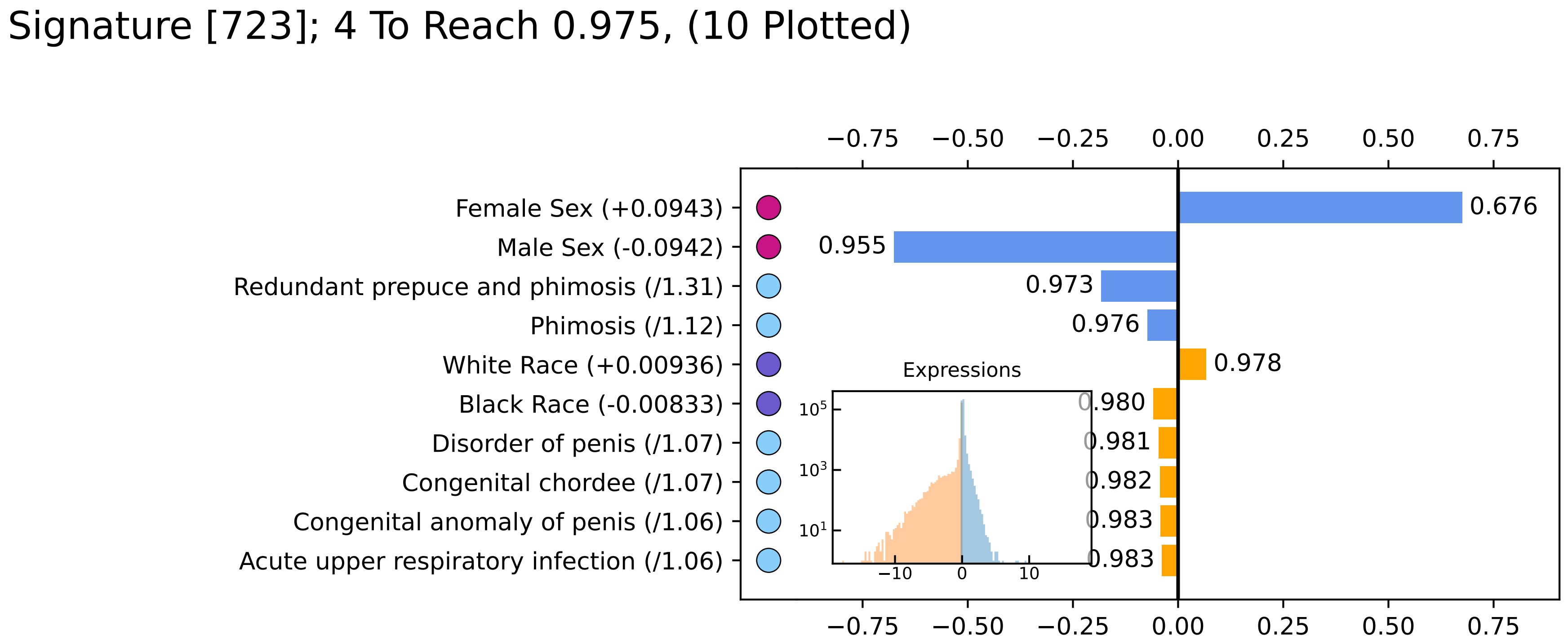

Signature [723]; 4 To Reach 0.975, (10 Plotted)
Female Sex (+0.0943)
Male Sex (-0.0942)
Redundant prepuce and phimosis (/1.31)
Phimosis (/1.12)
White Race (+0.00936)
Black Race (-0.00833)
Disorder of penis (/1.07)
Congenital chordee (/1.07)
Congenital anomaly of penis (/1.06)
Acute upper respiratory infection (/1.06)
0.676
0.955
0.973
0.976
0.978
0.980
0.981
0.982
0.983
0.983
−0.75 −0.50 −0.25 0.00 0.25 0.50 0.75
Expressions
10^5
10^3
10^1
−10 0 10

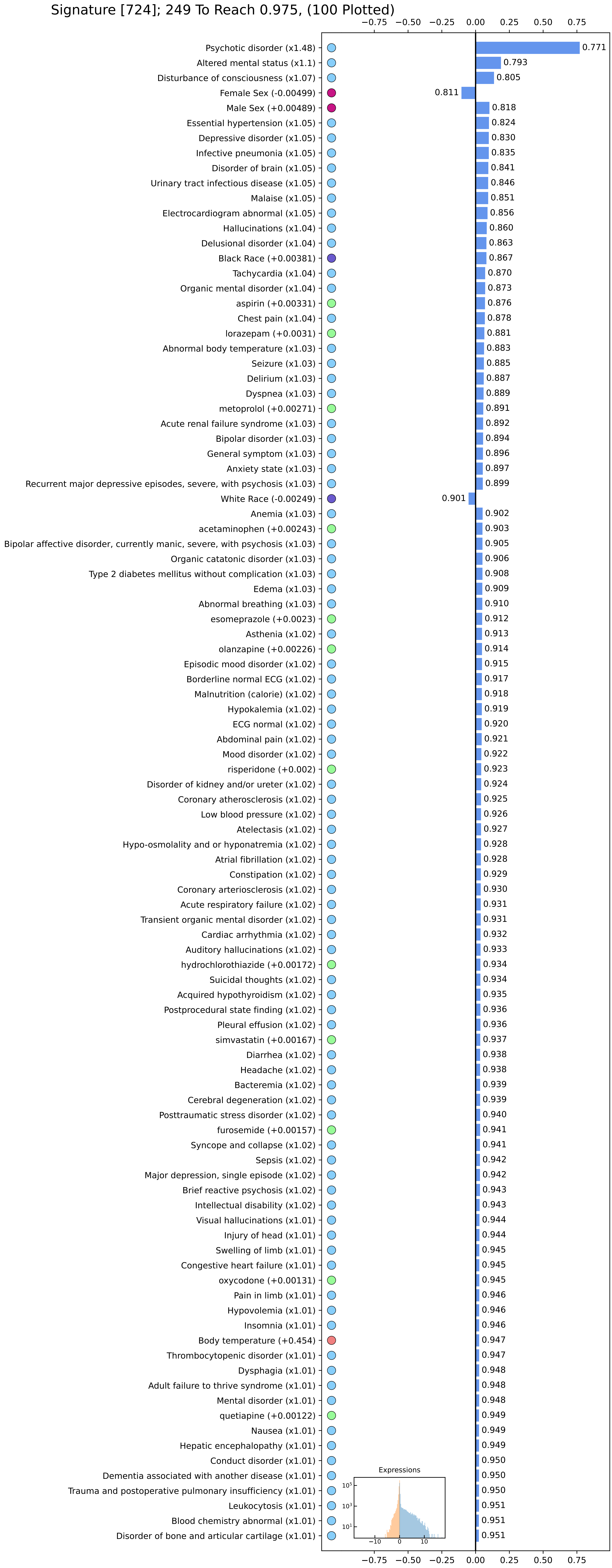

Signature [724]; 249 To Reach 0.975, (100 Plotted)
−0.75 −0.50 −0.25 0.00 0.25 0.50 0.75
Psychotic disorder (x1.48) 0.771
Altered mental status (x1.1) 0.793
Disturbance of consciousness (x1.07) 0.805
Female Sex (-0.00499) 0.811
Male Sex (+0.00489) 0.818
Essential hypertension (x1.05) 0.824
Depressive disorder (x1.05) 0.830
Infective pneumonia (x1.05) 0.835
Disorder of brain (x1.05) 0.841
Urinary tract infectious disease (x1.05) 0.846
Malaise (x1.05) 0.851
Electrocardiogram abnormal (x1.05) 0.856
Hallucinations (x1.04) 0.860
Delusional disorder (x1.04) 0.863
Black Race (+0.00381) 0.867
Tachycardia (x1.04) 0.870
Organic mental disorder (x1.04) 0.873
aspirin (+0.00331) 0.876
Chest pain (x1.04) 0.878
lorazepam (+0.0031) 0.881
Abnormal body temperature (x1.03) 0.883
Seizure (x1.03) 0.885
Delirium (x1.03) 0.887
Dyspnea (x1.03) 0.889
metoprolol (+0.00271) 0.891
Acute renal failure syndrome (x1.03) 0.892
Bipolar disorder (x1.03) 0.894
General symptom (x1.03) 0.896
Anxiety state (x1.03) 0.897
Recurrent major depressive episodes, severe, with psychosis (x1.03) 0.899
White Race (-0.00249) 0.901
Anemia (x1.03) 0.902
acetaminophen (+0.00243) 0.903
Bipolar affective disorder, currently manic, severe, with psychosis (x1.03) 0.905
Organic catatonic disorder (x1.03) 0.906
Type 2 diabetes mellitus without complication (x1.03) 0.908
Edema (x1.03) 0.909
Abnormal breathing (x1.03) 0.910
esomeprazole (+0.0023) 0.912
Asthenia (x1.02) 0.913
olanzapine (+0.00226) 0.914
Episodic mood disorder (x1.02) 0.915
Borderline normal ECG (x1.02) 0.917
Malnutrition (calorie) (x1.02) 0.918
Hypokalemia (x1.02) 0.919
ECG normal (x1.02) 0.920
Abdominal pain (x1.02) 0.921
Mood disorder (x1.02) 0.922
risperidone (+0.002) 0.923
Disorder of kidney and/or ureter (x1.02) 0.924
Coronary atherosclerosis (x1.02) 0.925
Low blood pressure (x1.02) 0.926
Atelectasis (x1.02) 0.927
Hypo-osmolality and or hyponatremia (x1.02) 0.928
Atrial fibrillation (x1.02) 0.928
Constipation (x1.02) 0.929
Coronary arteriosclerosis (x1.02) 0.930
Acute respiratory failure (x1.02) 0.931
Transient organic mental disorder (x1.02) 0.931
Cardiac arrhythmia (x1.02) 0.932
Auditory hallucinations (x1.02) 0.933
hydrochlorothiazide (+0.00172) 0.934
Suicidal thoughts (x1.02) 0.934
Acquired hypothyroidism (x1.02) 0.935
Postprocedural state finding (x1.02) 0.936
Pleural effusion (x1.02) 0.936
simvastatin (+0.00167) 0.937
Diarrhea (x1.02) 0.938
Headache (x1.02) 0.938
Bacteremia (x1.02) 0.939
Cerebral degeneration (x1.02) 0.939
Posttraumatic stress disorder (x1.02) 0.940
furosemide (+0.00157) 0.941
Syncope and collapse (x1.02) 0.941
Sepsis (x1.02) 0.942
Major depression, single episode (x1.02) 0.942
Brief reactive psychosis (x1.02) 0.943
Intellectual disability (x1.02) 0.943
Visual hallucinations (x1.01) 0.944
Injury of head (x1.01) 0.944
Swelling of limb (x1.01) 0.945
Congestive heart failure (x1.01) 0.945
oxycodone (+0.00131) 0.945
Pain in limb (x1.01) 0.946
Hypovolemia (x1.01) 0.946
Insomnia (x1.01) 0.946
Body temperature (+0.454) 0.947
Thrombocytopenic disorder (x1.01) 0.947
Dysphagia (x1.01) 0.948
Adult failure to thrive syndrome (x1.01) 0.948
Mental disorder (x1.01) 0.948
quetiapine (+0.00122) 0.949
Nausea (x1.01) 0.949
Hepatic encephalopathy (x1.01) 0.949
Conduct disorder (x1.01) 0.950
Dementia associated with another disease (x1.01) 0.950
Trauma and postoperative pulmonary insufficiency (x1.01) 0.950
Leukocytosis (x1.01) 0.951
Blood chemistry abnormal (x1.01) 0.951
Disorder of bone and articular cartilage (x1.01) 0.951
Expressions
−10 0 10
−0.75 −0.50 −0.25 0.00 0.25 0.50 0.75

Signature [725]; 217 To Reach 0.975, (100 Plotted)

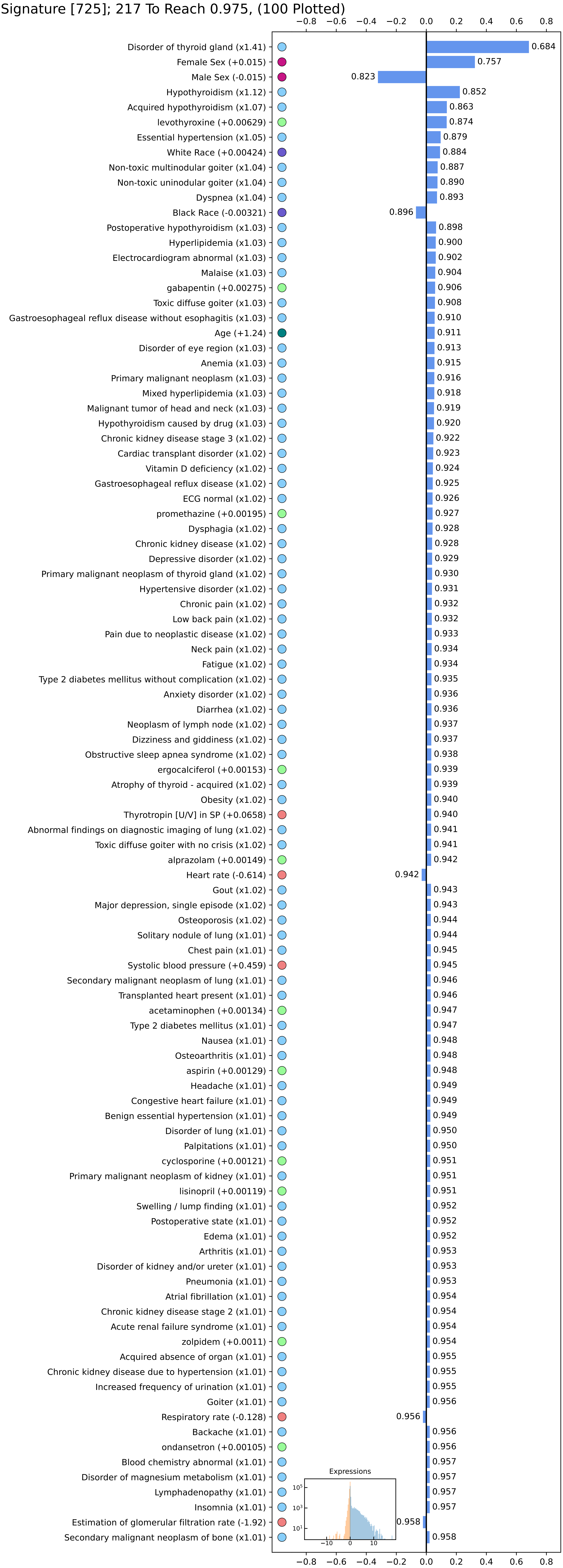

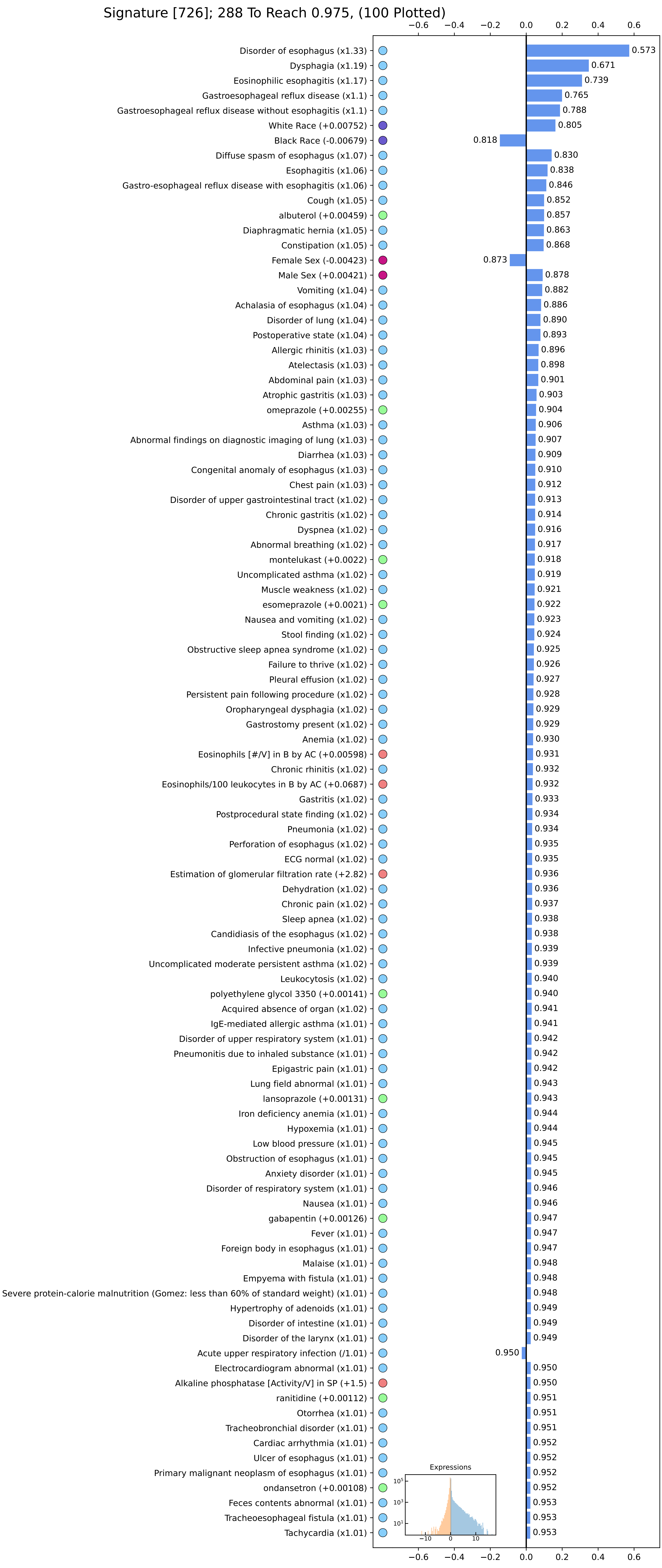
Signature [726]; 288 To Reach 0.975, (100 Plotted)
Disorder of esophagus (x1.33) 0.573
Dysphagia (x1.19) 0.671
Eosinophilic esophagitis (x1.17) 0.739
Gastroesophageal reflux disease (x1.1) 0.765
Gastroesophageal reflux disease without esophagitis (x1.1) 0.788
White Race (+0.00752) 0.805
Black Race (-0.00679) 0.818
Diffuse spasm of esophagus (x1.07) 0.830
Esophagitis (x1.06) 0.838
Gastro-esophageal reflux disease with esophagitis (x1.06) 0.846
Cough (x1.05) 0.852
albuterol (+0.00459) 0.857
Diaphragmatic hernia (x1.05) 0.863
Constipation (x1.05) 0.868
Female Sex (-0.00423) 0.873
Male Sex (+0.00421) 0.878
Vomiting (x1.04) 0.882
Achalasia of esophagus (x1.04) 0.886
Disorder of lung (x1.04) 0.890
Postoperative state (x1.04) 0.893
Allergic rhinitis (x1.03) 0.896
Atelectasis (x1.03) 0.898
Abdominal pain (x1.03) 0.901
Atrophic gastritis (x1.03) 0.903
omeprazole (+0.00255) 0.904
Asthma (x1.03) 0.906
Abnormal findings on diagnostic imaging of lung (x1.03) 0.907
Diarrhea (x1.03) 0.909
Congenital anomaly of esophagus (x1.03) 0.910
Chest pain (x1.03) 0.912
Disorder of upper gastrointestinal tract (x1.02) 0.913
Chronic gastritis (x1.02) 0.914
Dyspnea (x1.02) 0.916
Abnormal breathing (x1.02) 0.917
montelukast (+0.0022) 0.918
Uncomplicated asthma (x1.02) 0.919
Muscle weakness (x1.02) 0.921
esomeprazole (+0.0021) 0.922
Nausea and vomiting (x1.02) 0.923
Stool finding (x1.02) 0.924
Obstructive sleep apnea syndrome (x1.02) 0.925
Failure to thrive (x1.02) 0.926
Pleural effusion (x1.02) 0.927
Persistent pain following procedure (x1.02) 0.928
Oropharyngeal dysphagia (x1.02) 0.929
Gastrostomy present (x1.02) 0.929
Anemia (x1.02) 0.930
Eosinophils [#/V] in B by AC (+0.00598) 0.931
Chronic rhinitis (x1.02) 0.932
Eosinophils/100 leukocytes in B by AC (+0.0687) 0.932
Gastritis (x1.02) 0.933
Postprocedural state finding (x1.02) 0.934
Pneumonia (x1.02) 0.934
Perforation of esophagus (x1.02) 0.935
ECG normal (x1.02) 0.935
Estimation of glomerular filtration rate (+2.82) 0.936
Dehydration (x1.02) 0.936
Chronic pain (x1.02) 0.937
Sleep apnea (x1.02) 0.938
Candidiasis of the esophagus (x1.02) 0.938
Infective pneumonia (x1.02) 0.939
Uncomplicated moderate persistent asthma (x1.02) 0.939
Leukocytosis (x1.02) 0.940
polyethylene glycol 3350 (+0.00141) 0.940
Acquired absence of organ (x1.02) 0.941
IgE-mediated allergic asthma (x1.01) 0.941
Disorder of upper respiratory system (x1.01) 0.942
Pneumonitis due to inhaled substance (x1.01) 0.942
Epigastric pain (x1.01) 0.942
Lung field abnormal (x1.01) 0.943
lansoprazole (+0.00131) 0.943
Iron deficiency anemia (x1.01) 0.944
Hypoxemia (x1.01) 0.944
Low blood pressure (x1.01) 0.945
Obstruction of esophagus (x1.01) 0.945
Anxiety disorder (x1.01) 0.945
Disorder of respiratory system (x1.01) 0.946
Nausea (x1.01) 0.946
gabapentin (+0.00126) 0.947
Fever (x1.01) 0.947
Foreign body in esophagus (x1.01) 0.947
Malaise (x1.01) 0.948
Empyema with fistula (x1.01) 0.948
Severe protein-calorie malnutrition (Gomez: less than 60% of standard weight) (x1.01) 0.948
Hypertrophy of adenoids (x1.01) 0.949
Disorder of intestine (x1.01) 0.949
Disorder of the larynx (x1.01) 0.949
Acute upper respiratory infection (/1.01) 0.950
Electrocardiogram abnormal (x1.01) 0.950
Alkaline phosphatase [Activity/V] in SP (+1.5) 0.950
ranitidine (+0.00112) 0.951
Otorrhea (x1.01) 0.951
Tracheobronchial disorder (x1.01) 0.951
Cardiac arrhythmia (x1.01) 0.952
Ulcer of esophagus (x1.01) 0.952
Primary malignant neoplasm of esophagus (x1.01) 0.952
ondansetron (+0.00108) 0.952
Feces contents abnormal (x1.01) 0.953
Tracheoesophageal fistula (x1.01) 0.953
Tachycardia (x1.01) 0.953
−0.6 −0.4 −0.2 0.0 0.2 0.4 0.6
Expressions
−10 0 10

Signature [727]; 10 To Reach 0.975, (10 Plotted)

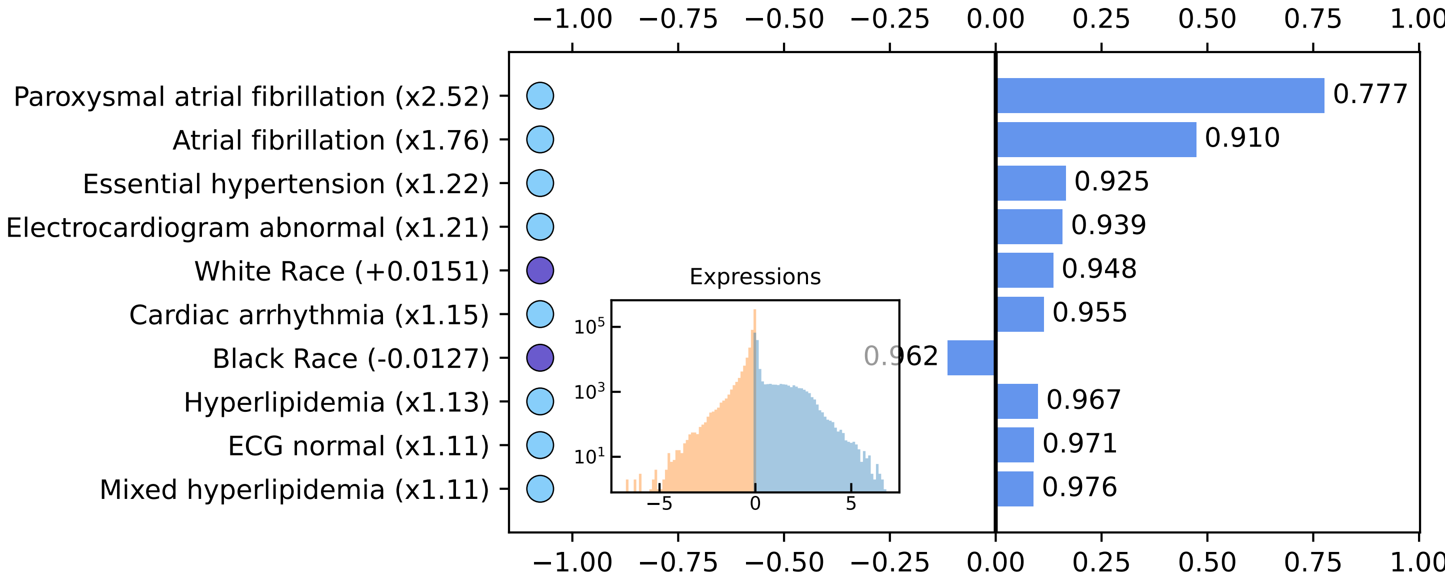

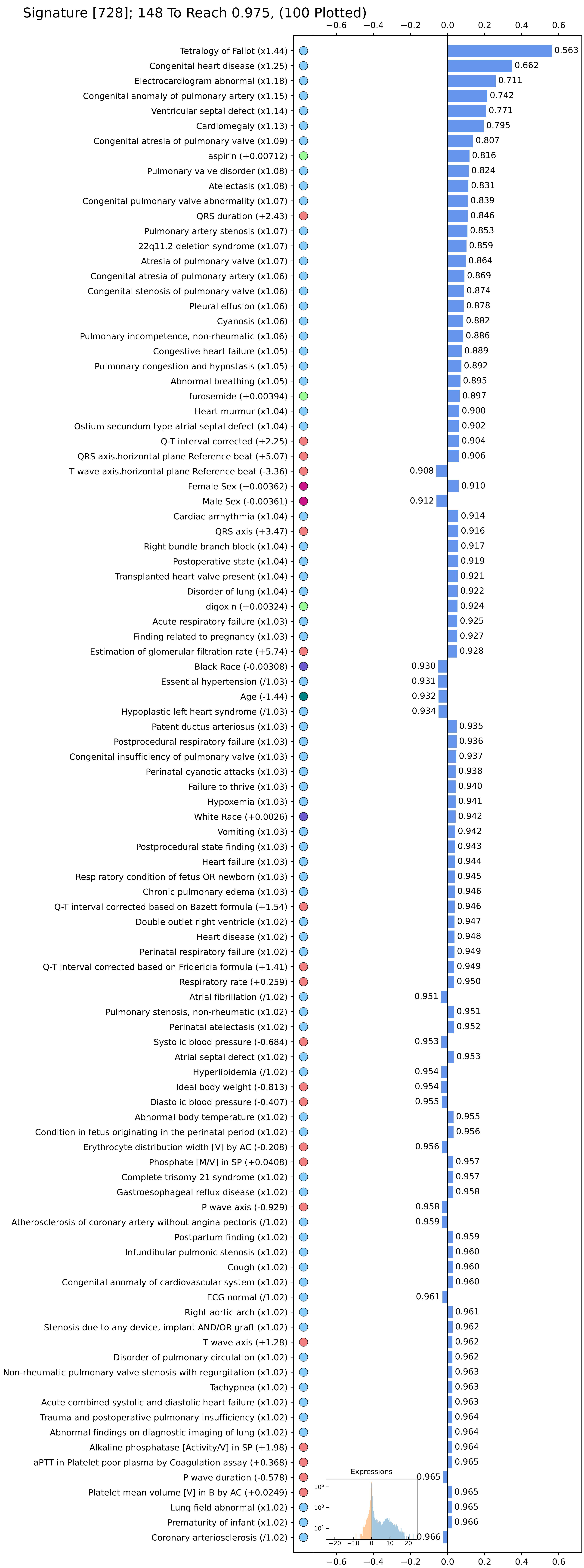

Signature [728]; 148 To Reach 0.975, (100 Plotted)
Tetralogy of Fallot (x1.44) 0.563
Congenital heart disease (x1.25) 0.662
Electrocardiogram abnormal (x1.18) 0.711
Congenital anomaly of pulmonary artery (x1.15) 0.742
Ventricular septal defect (x1.14) 0.771
Cardiomegaly (x1.13) 0.795
Congenital atresia of pulmonary valve (x1.09) 0.807
aspirin (+0.00712) 0.816
Pulmonary valve disorder (x1.08) 0.824
Atelectasis (x1.08) 0.831
Congenital pulmonary valve abnormality (x1.07) 0.839
QRS duration (+2.43) 0.846
Pulmonary artery stenosis (x1.07) 0.853
22q11.2 deletion syndrome (x1.07) 0.859
Atresia of pulmonary valve (x1.07) 0.864
Congenital atresia of pulmonary artery (x1.06) 0.869
Congenital stenosis of pulmonary valve (x1.06) 0.874
Pleural effusion (x1.06) 0.878
Cyanosis (x1.06) 0.882
Pulmonary incompetence, non-rheumatic (x1.06) 0.886
Congestive heart failure (x1.05) 0.889
Pulmonary congestion and hypostasis (x1.05) 0.892
Abnormal breathing (x1.05) 0.895
furosemide (+0.00394) 0.897
Heart murmur (x1.04) 0.900
Ostium secundum type atrial septal defect (x1.04) 0.902
Q-T interval corrected (+2.25) 0.904
QRS axis.horizontal plane Reference beat (+5.07) 0.906
T wave axis.horizontal plane Reference beat (-3.36) 0.908
Female Sex (+0.00362) 0.910
Male Sex (-0.00361) 0.912
Cardiac arrhythmia (x1.04) 0.914
QRS axis (+3.47) 0.916
Right bundle branch block (x1.04) 0.917
Postoperative state (x1.04) 0.919
Transplanted heart valve present (x1.04) 0.921
Disorder of lung (x1.04) 0.922
digoxin (+0.00324) 0.924
Acute respiratory failure (x1.03) 0.925
Finding related to pregnancy (x1.03) 0.927
Estimation of glomerular filtration rate (+5.74) 0.928
Black Race (-0.00308) 0.930
Essential hypertension (/1.03) 0.931
Age (-1.44) 0.932
Hypoplastic left heart syndrome (/1.03) 0.934
Patent ductus arteriosus (x1.03) 0.935
Postprocedural respiratory failure (x1.03) 0.936
Congenital insufficiency of pulmonary valve (x1.03) 0.937
Perinatal cyanotic attacks (x1.03) 0.938
Failure to thrive (x1.03) 0.940
Hypoxemia (x1.03) 0.941
White Race (+0.0026) 0.942
Vomiting (x1.03) 0.942
Postprocedural state finding (x1.03) 0.943
Heart failure (x1.03) 0.944
Respiratory condition of fetus OR newborn (x1.03) 0.945
Chronic pulmonary edema (x1.03) 0.946
Q-T interval corrected based on Bazett formula (+1.54) 0.946
Double outlet right ventricle (x1.02) 0.947
Heart disease (x1.02) 0.948
Perinatal respiratory failure (x1.02) 0.949
Q-T interval corrected based on Fridericia formula (+1.41) 0.949
Respiratory rate (+0.259) 0.950
Atrial fibrillation (/1.02) 0.951
Pulmonary stenosis, non-rheumatic (x1.02) 0.951
Perinatal atelectasis (x1.02) 0.952
Systolic blood pressure (-0.684) 0.953
Atrial septal defect (x1.02) 0.953
Hyperlipidemia (/1.02) 0.954
Ideal body weight (-0.813) 0.954
Diastolic blood pressure (-0.407) 0.955
Abnormal body temperature (x1.02) 0.955
Condition in fetus originating in the perinatal period (x1.02) 0.956
Erythrocyte distribution width [V] by AC (-0.208) 0.956
Phosphate [M/V] in SP (+0.0408) 0.957
Complete trisomy 21 syndrome (x1.02) 0.957
Gastroesophageal reflux disease (x1.02) 0.958
P wave axis (-0.929) 0.958
Atherosclerosis of coronary artery without angina pectoris (/1.02) 0.959
Postpartum finding (x1.02) 0.959
Infundibular pulmonic stenosis (x1.02) 0.960
Cough (x1.02) 0.960
Congenital anomaly of cardiovascular system (x1.02) 0.960
ECG normal (/1.02) 0.961
Right aortic arch (x1.02) 0.961
Stenosis due to any device, implant AND/OR graft (x1.02) 0.962
T wave axis (+1.28) 0.962
Disorder of pulmonary circulation (x1.02) 0.962
Non-rheumatic pulmonary valve stenosis with regurgitation (x1.02) 0.963
Tachypnea (x1.02) 0.963
Acute combined systolic and diastolic heart failure (x1.02) 0.963
Trauma and postoperative pulmonary insufficiency (x1.02) 0.964
Abnormal findings on diagnostic imaging of lung (x1.02) 0.964
Alkaline phosphatase [Activity/V] in SP (+1.98) 0.964
aPTT in Platelet poor plasma by Coagulation assay (+0.368) 0.965
P wave duration (-0.578) 0.965
Platelet mean volume [V] in B by AC (+0.0249) 0.965
Lung field abnormal (x1.02) 0.965
Prematurity of infant (x1.02) 0.966
Coronary arteriosclerosis (/1.02) 0.966
Expressions

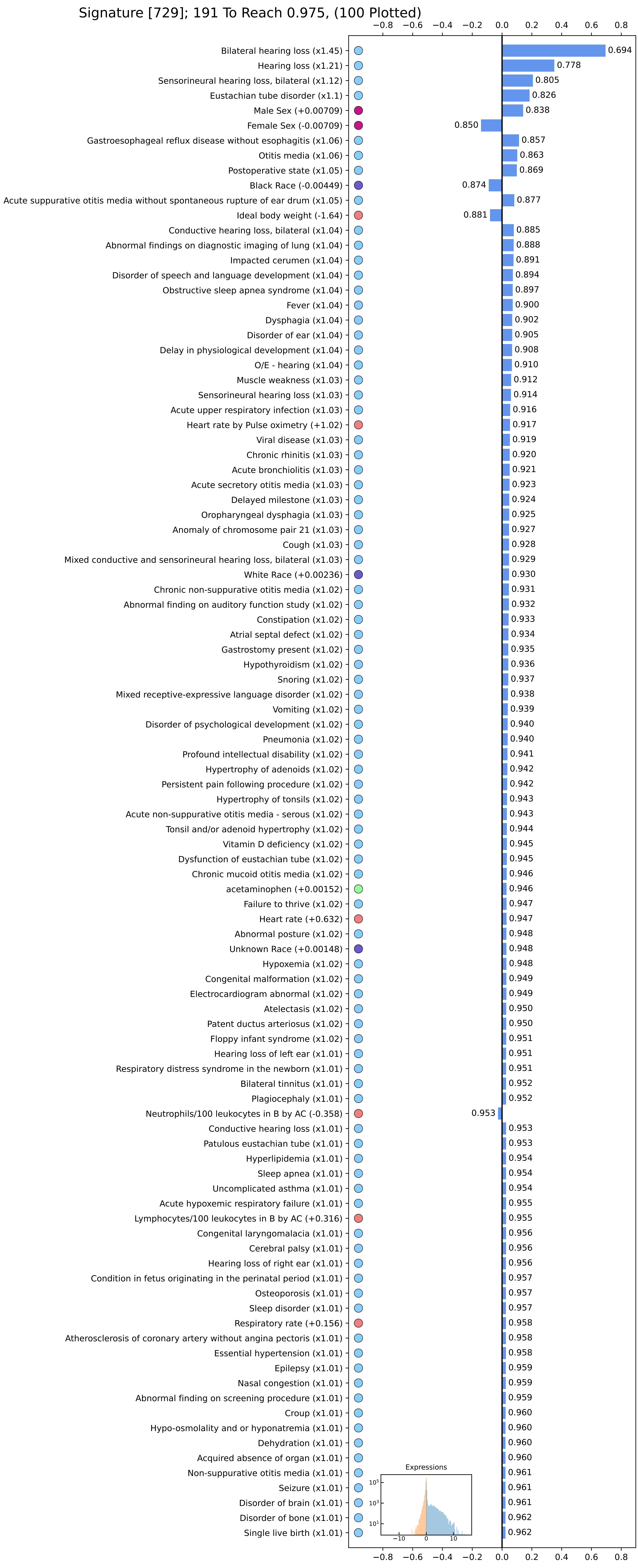

Signature [729]; 191 To Reach 0.975, (100 Plotted)
Bilateral hearing loss (x1.45) 0.694
Hearing loss (x1.21) 0.778
Sensorineural hearing loss, bilateral (x1.12) 0.805
Eustachian tube disorder (x1.1) 0.826
Male Sex (+0.00709) 0.838
Female Sex (-0.00709) 0.850
Gastroesophageal reflux disease without esophagitis (x1.06) 0.857
Otitis media (x1.06) 0.863
Postoperative state (x1.05) 0.869
Black Race (-0.00449) 0.874
Acute suppurative otitis media without spontaneous rupture of ear drum (x1.05) 0.877
Ideal body weight (-1.64) 0.881
Conductive hearing loss, bilateral (x1.04) 0.885
Abnormal findings on diagnostic imaging of lung (x1.04) 0.888
Impacted cerumen (x1.04) 0.891
Disorder of speech and language development (x1.04) 0.894
Obstructive sleep apnea syndrome (x1.04) 0.897
Fever (x1.04) 0.900
Dysphagia (x1.04) 0.902
Disorder of ear (x1.04) 0.905
Delay in physiological development (x1.04) 0.908
O/E - hearing (x1.04) 0.910
Muscle weakness (x1.03) 0.912
Sensorineural hearing loss (x1.03) 0.914
Acute upper respiratory infection (x1.03) 0.916
Heart rate by Pulse oximetry (+1.02) 0.917
Viral disease (x1.03) 0.919
Chronic rhinitis (x1.03) 0.920
Acute bronchiolitis (x1.03) 0.921
Acute secretory otitis media (x1.03) 0.923
Delayed milestone (x1.03) 0.924
Oropharyngeal dysphagia (x1.03) 0.925
Anomaly of chromosome pair 21 (x1.03) 0.927
Cough (x1.03) 0.928
Mixed conductive and sensorineural hearing loss, bilateral (x1.03) 0.929
White Race (+0.00236) 0.930
Chronic non-suppurative otitis media (x1.02) 0.931
Abnormal finding on auditory function study (x1.02) 0.932
Constipation (x1.02) 0.933
Atrial septal defect (x1.02) 0.934
Gastrostomy present (x1.02) 0.935
Hypothyroidism (x1.02) 0.936
Snoring (x1.02) 0.937
Mixed receptive-expressive language disorder (x1.02) 0.938
Vomiting (x1.02) 0.939
Disorder of psychological development (x1.02) 0.940
Pneumonia (x1.02) 0.940
Profound intellectual disability (x1.02) 0.941
Hypertrophy of adenoids (x1.02) 0.942
Persistent pain following procedure (x1.02) 0.942
Hypertrophy of tonsils (x1.02) 0.943
Acute non-suppurative otitis media - serous (x1.02) 0.943
Tonsil and/or adenoid hypertrophy (x1.02) 0.944
Vitamin D deficiency (x1.02) 0.945
Dysfunction of eustachian tube (x1.02) 0.945
Chronic mucoid otitis media (x1.02) 0.946
acetaminophen (+0.00152) 0.946
Failure to thrive (x1.02) 0.947
Heart rate (+0.632) 0.947
Abnormal posture (x1.02) 0.948
Unknown Race (+0.00148) 0.948
Hypoxemia (x1.02) 0.948
Congenital malformation (x1.02) 0.949
Electrocardiogram abnormal (x1.02) 0.949
Atelectasis (x1.02) 0.950
Patent ductus arteriosus (x1.02) 0.950
Floppy infant syndrome (x1.02) 0.951
Hearing loss of left ear (x1.01) 0.951
Respiratory distress syndrome in the newborn (x1.01) 0.951
Bilateral tinnitus (x1.01) 0.952
Plagiocephaly (x1.01) 0.952
Neutrophils/100 leukocytes in B by AC (-0.358) 0.953
Conductive hearing loss (x1.01) 0.953
Patulous eustachian tube (x1.01) 0.953
Hyperlipidemia (x1.01) 0.954
Sleep apnea (x1.01) 0.954
Uncomplicated asthma (x1.01) 0.954
Acute hypoxemic respiratory failure (x1.01) 0.955
Lymphocytes/100 leukocytes in B by AC (+0.316) 0.955
Congenital laryngomalacia (x1.01) 0.956
Cerebral palsy (x1.01) 0.956
Hearing loss of right ear (x1.01) 0.956
Condition in fetus originating in the perinatal period (x1.01) 0.957
Osteoporosis (x1.01) 0.957
Sleep disorder (x1.01) 0.957
Respiratory rate (+0.156) 0.958
Atherosclerosis of coronary artery without angina pectoris (x1.01) 0.958
Essential hypertension (x1.01) 0.958
Epilepsy (x1.01) 0.959
Nasal congestion (x1.01) 0.959
Abnormal finding on screening procedure (x1.01) 0.959
Croup (x1.01) 0.960
Hypo-osmolality and or hyponatremia (x1.01) 0.960
Dehydration (x1.01) 0.960
Acquired absence of organ (x1.01) 0.960
Non-suppurative otitis media (x1.01) 0.961
Seizure (x1.01) 0.961
Disorder of brain (x1.01) 0.961
Disorder of bone (x1.01) 0.962
Single live birth (x1.01) 0.962
Expressions
−0.8 −0.6 −0.4 −0.2 0.0 0.2 0.4 0.6 0.8

Signature [730]; 73 To Reach 0.975, (73 Plotted)

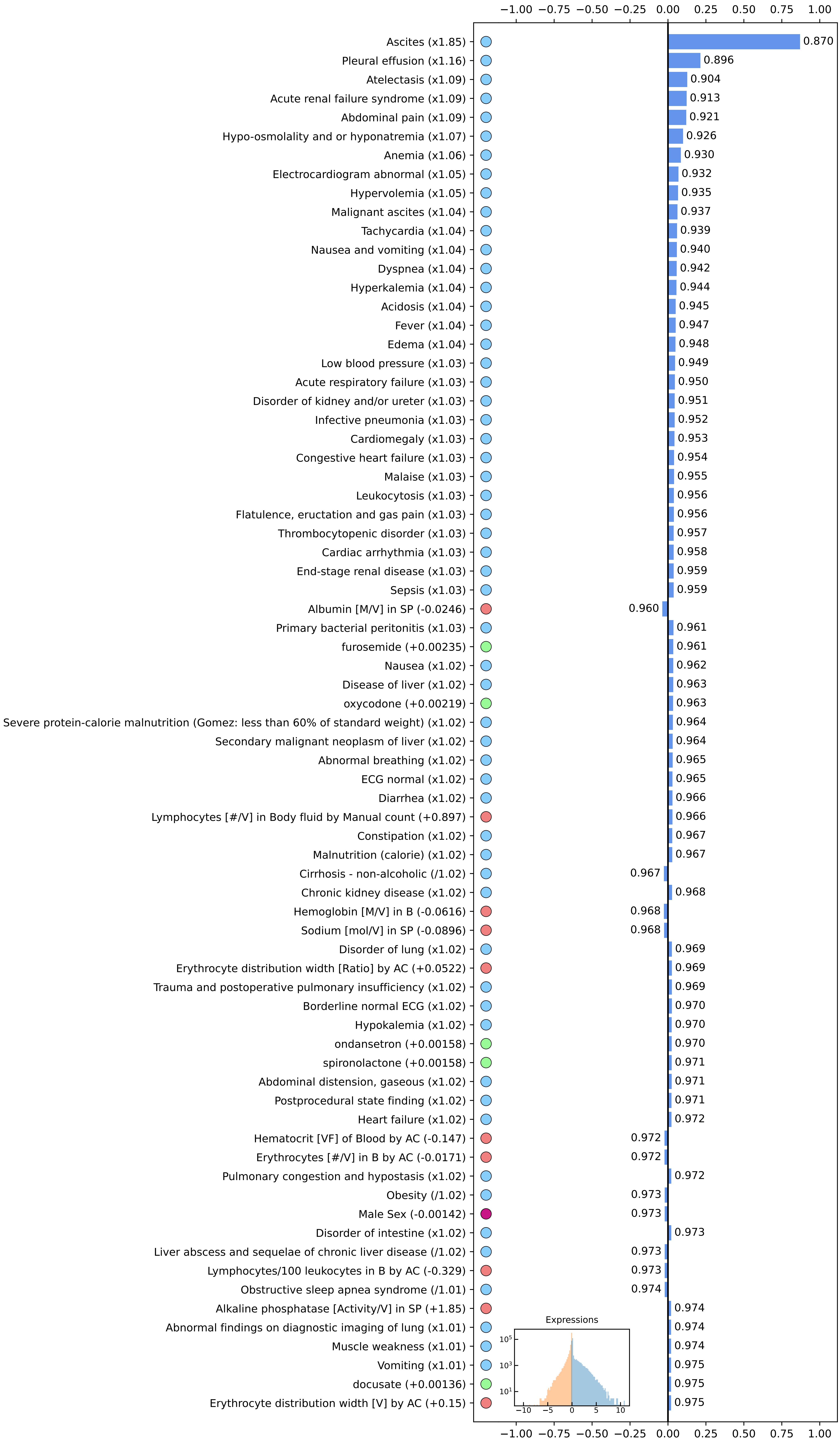

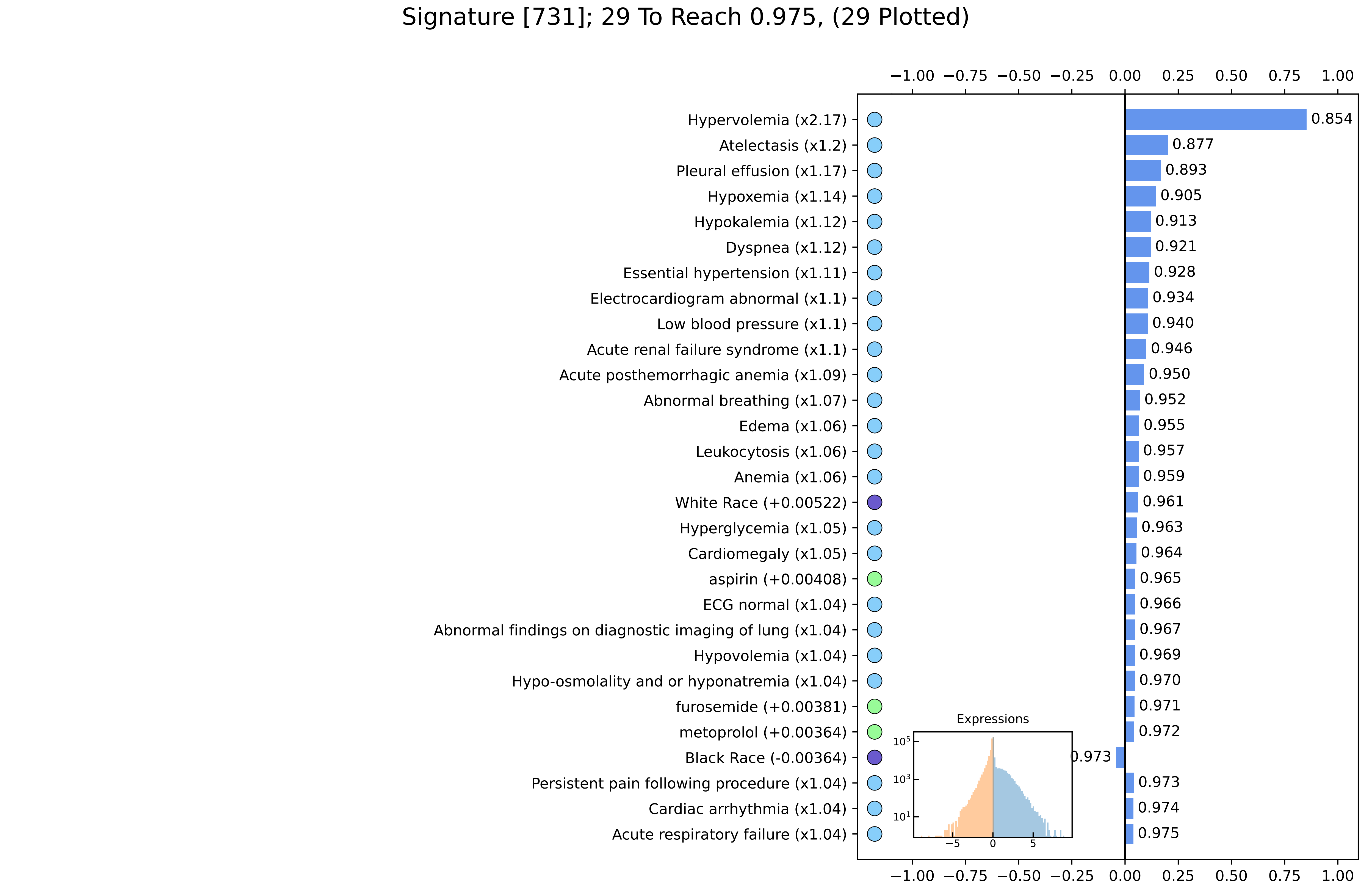

Signature [731]; 29 To Reach 0.975, (29 Plotted)
Hypervolemia (x2.17)
0.854
Atelectasis (x1.2)
0.877
Pleural effusion (x1.17)
0.893
Hypoxemia (x1.14)
0.905
Hypokalemia (x1.12)
0.913
Dyspnea (x1.12)
0.921
Essential hypertension (x1.11)
0.928
Electrocardiogram abnormal (x1.1)
0.934
Low blood pressure (x1.1)
0.940
Acute renal failure syndrome (x1.1)
0.946
Acute posthemorrhagic anemia (x1.09)
0.950
Abnormal breathing (x1.07)
0.952
Edema (x1.06)
0.955
Leukocytosis (x1.06)
0.957
Anemia (x1.06)
0.959
White Race (+0.00522)
0.961
Hyperglycemia (x1.05)
0.963
Cardiomegaly (x1.05)
0.964
aspirin (+0.00408)
0.965
ECG normal (x1.04)
0.966
Abnormal findings on diagnostic imaging of lung (x1.04)
0.967
Hypovolemia (x1.04)
0.969
Hypo-osmolality and or hyponatremia (x1.04)
0.970
furosemide (+0.00381)
0.971
metoprolol (+0.00364)
0.972
Black Race (-0.00364)
0.973
Persistent pain following procedure (x1.04)
0.973
Cardiac arrhythmia (x1.04)
0.974
Acute respiratory failure (x1.04)
0.975
Expressions
−1.00 −0.75 −0.50 −0.25 0.00 0.25 0.50 0.75 1.00

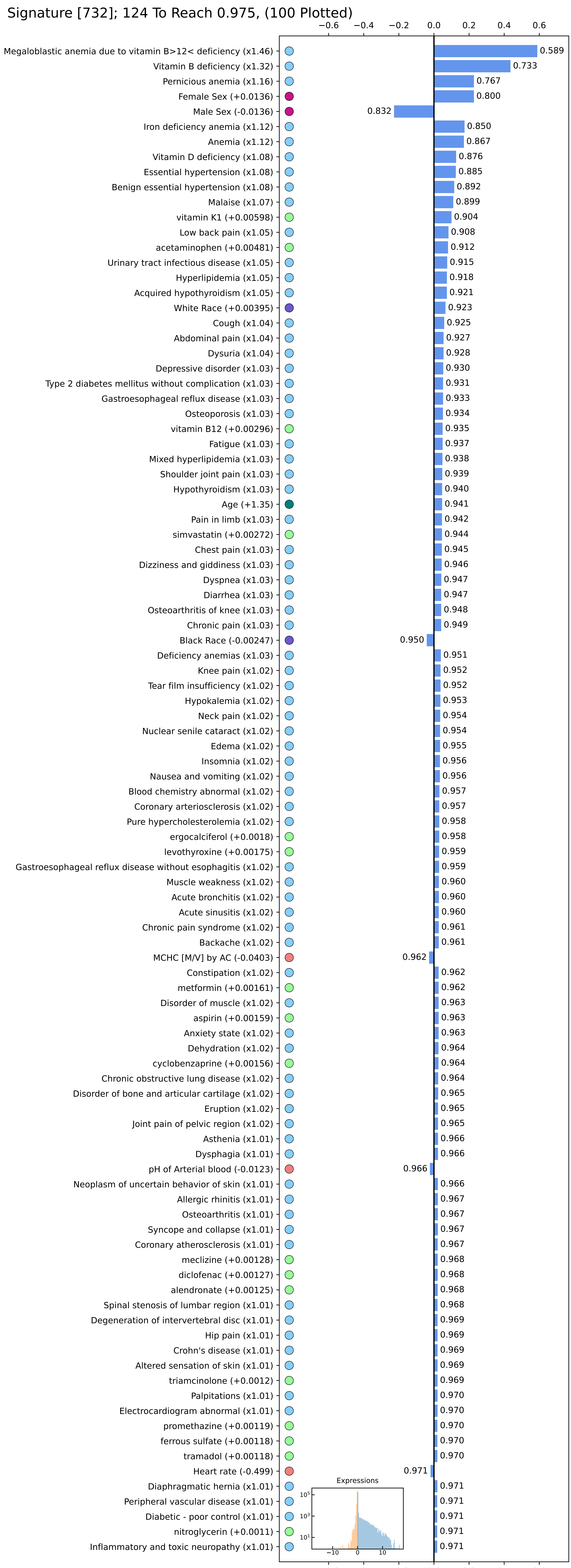

Signature [732]; 124 To Reach 0.975, (100 Plotted)
Megaloblastic anemia due to vitamin B>12< deficiency (x1.46) 0.589
Vitamin B deficiency (x1.32) 0.733
Pernicious anemia (x1.16) 0.767
Female Sex (+0.0136) 0.800
Male Sex (-0.0136) 0.832
Iron deficiency anemia (x1.12) 0.850
Anemia (x1.12) 0.867
Vitamin D deficiency (x1.08) 0.876
Essential hypertension (x1.08) 0.885
Benign essential hypertension (x1.08) 0.892
Malaise (x1.07) 0.899
vitamin K1 (+0.00598) 0.904
Low back pain (x1.05) 0.908
acetaminophen (+0.00481) 0.912
Urinary tract infectious disease (x1.05) 0.915
Hyperlipidemia (x1.05) 0.918
Acquired hypothyroidism (x1.05) 0.921
White Race (+0.00395) 0.923
Cough (x1.04) 0.925
Abdominal pain (x1.04) 0.927
Dysuria (x1.04) 0.928
Depressive disorder (x1.03) 0.930
Type 2 diabetes mellitus without complication (x1.03) 0.931
Gastroesophageal reflux disease (x1.03) 0.933
Osteoporosis (x1.03) 0.934
vitamin B12 (+0.00296) 0.935
Fatigue (x1.03) 0.937
Mixed hyperlipidemia (x1.03) 0.938
Shoulder joint pain (x1.03) 0.939
Hypothyroidism (x1.03) 0.940
Age (+1.35) 0.941
Pain in limb (x1.03) 0.942
simvastatin (+0.00272) 0.944
Chest pain (x1.03) 0.945
Dizziness and giddiness (x1.03) 0.946
Dyspnea (x1.03) 0.947
Diarrhea (x1.03) 0.947
Osteoarthritis of knee (x1.03) 0.948
Chronic pain (x1.03) 0.949
Black Race (-0.00247) 0.950
Deficiency anemias (x1.03) 0.951
Knee pain (x1.02) 0.952
Tear film insufficiency (x1.02) 0.952
Hypokalemia (x1.02) 0.953
Neck pain (x1.02) 0.954
Nuclear senile cataract (x1.02) 0.954
Edema (x1.02) 0.955
Insomnia (x1.02) 0.956
Nausea and vomiting (x1.02) 0.956
Blood chemistry abnormal (x1.02) 0.957
Coronary arteriosclerosis (x1.02) 0.957
Pure hypercholesterolemia (x1.02) 0.958
ergocalciferol (+0.0018) 0.958
levothyroxine (+0.00175) 0.959
Gastroesophageal reflux disease without esophagitis (x1.02) 0.959
Muscle weakness (x1.02) 0.960
Acute bronchitis (x1.02) 0.960
Acute sinusitis (x1.02) 0.960
Chronic pain syndrome (x1.02) 0.961
Backache (x1.02) 0.961
MCHC [M/V] by AC (-0.0403) 0.962
Constipation (x1.02) 0.962
metformin (+0.00161) 0.962
Disorder of muscle (x1.02) 0.963
aspirin (+0.00159) 0.963
Anxiety state (x1.02) 0.963
Dehydration (x1.02) 0.964
cyclobenzaprine (+0.00156) 0.964
Chronic obstructive lung disease (x1.02) 0.964
Disorder of bone and articular cartilage (x1.02) 0.965
Eruption (x1.02) 0.965
Joint pain of pelvic region (x1.02) 0.965
Asthenia (x1.01) 0.966
Dysphagia (x1.01) 0.966
pH of Arterial blood (-0.0123) 0.966
Neoplasm of uncertain behavior of skin (x1.01) 0.966
Allergic rhinitis (x1.01) 0.967
Osteoarthritis (x1.01) 0.967
Syncope and collapse (x1.01) 0.967
Coronary atherosclerosis (x1.01) 0.967
meclizine (+0.00128) 0.968
diclofenac (+0.00127) 0.968
alendronate (+0.00125) 0.968
Spinal stenosis of lumbar region (x1.01) 0.968
Degeneration of intervertebral disc (x1.01) 0.969
Hip pain (x1.01) 0.969
Crohn's disease (x1.01) 0.969
Altered sensation of skin (x1.01) 0.969
triamcinolone (+0.0012) 0.969
Palpitations (x1.01) 0.970
Electrocardiogram abnormal (x1.01) 0.970
promethazine (+0.00119) 0.970
ferrous sulfate (+0.00118) 0.970
tramadol (+0.00118) 0.970
Heart rate (-0.499) 0.971
Diaphragmatic hernia (x1.01) 0.971
Peripheral vascular disease (x1.01) 0.971
Diabetic - poor control (x1.01) 0.971
nitroglycerin (+0.0011) 0.971
Inflammatory and toxic neuropathy (x1.01) 0.971
Expressions
−0.6 −0.4 −0.2 0.0 0.2 0.4 0.6

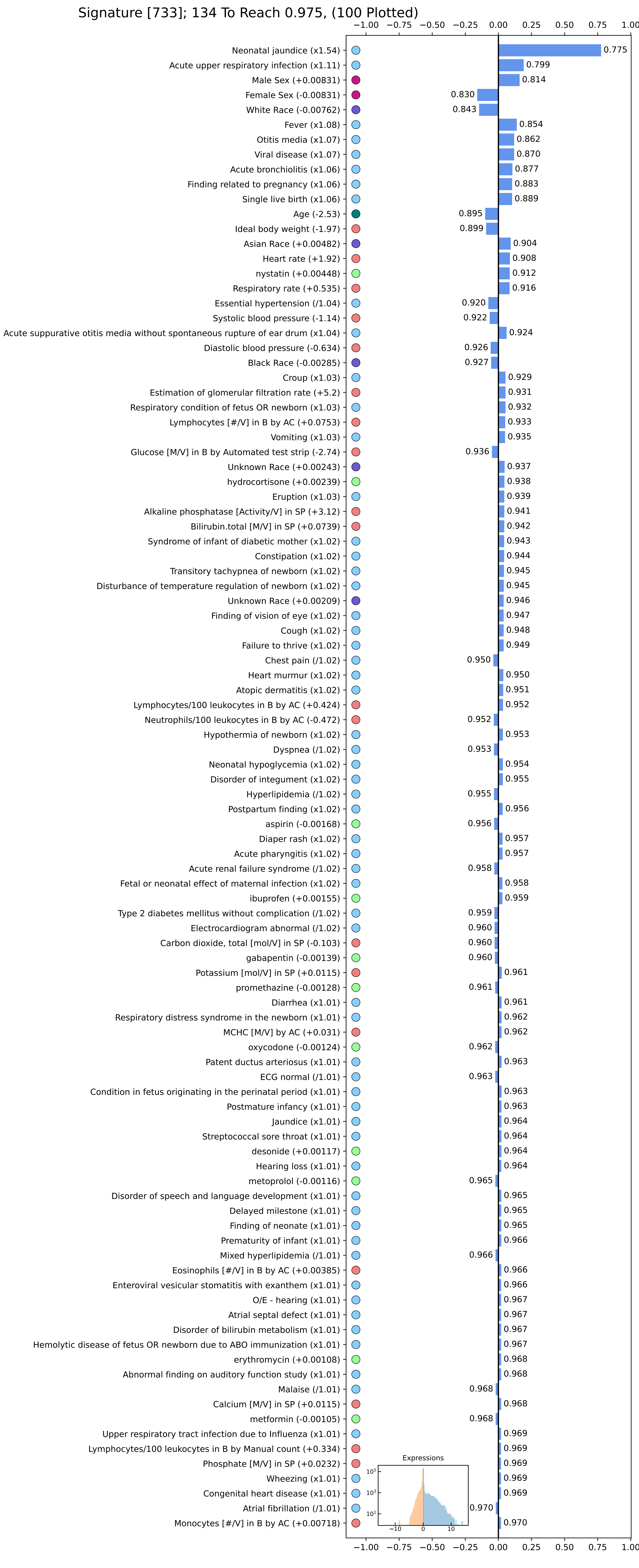

Signature [733]; 134 To Reach 0.975, (100 Plotted)
Neonatal jaundice (x1.54) 0.775
Acute upper respiratory infection (x1.11) 0.799
Male Sex (+0.00831) 0.814
Female Sex (-0.00831) 0.830
White Race (-0.00762) 0.843
Fever (x1.08) 0.854
Otitis media (x1.07) 0.862
Viral disease (x1.07) 0.870
Acute bronchiolitis (x1.06) 0.877
Finding related to pregnancy (x1.06) 0.883
Single live birth (x1.06) 0.889
Age (-2.53) 0.895
Ideal body weight (-1.97) 0.899
Asian Race (+0.00482) 0.904
Heart rate (+1.92) 0.908
nystatin (+0.00448) 0.912
Respiratory rate (+0.535) 0.916
Essential hypertension (/1.04) 0.920
Systolic blood pressure (-1.14) 0.922
Acute suppurative otitis media without spontaneous rupture of ear drum (x1.04) 0.924
Diastolic blood pressure (-0.634) 0.926
Black Race (-0.00285) 0.927
Croup (x1.03) 0.929
Estimation of glomerular filtration rate (+5.2) 0.931
Respiratory condition of fetus OR newborn (x1.03) 0.932
Lymphocytes [#/V] in B by AC (+0.0753) 0.933
Vomiting (x1.03) 0.935
Glucose [M/V] in B by Automated test strip (-2.74) 0.936
Unknown Race (+0.00243) 0.937
hydrocortisone (+0.00239) 0.938
Eruption (x1.03) 0.939
Alkaline phosphatase [Activity/V] in SP (+3.12) 0.941
Bilirubin.total [M/V] in SP (+0.0739) 0.942
Syndrome of infant of diabetic mother (x1.02) 0.943
Constipation (x1.02) 0.944
Transitory tachypnea of newborn (x1.02) 0.945
Disturbance of temperature regulation of newborn (x1.02) 0.945
Unknown Race (+0.00209) 0.946
Finding of vision of eye (x1.02) 0.947
Cough (x1.02) 0.948
Failure to thrive (x1.02) 0.949
Chest pain (/1.02) 0.950
Heart murmur (x1.02) 0.950
Atopic dermatitis (x1.02) 0.951
Lymphocytes/100 leukocytes in B by AC (+0.424) 0.952
Neutrophils/100 leukocytes in B by AC (-0.472) 0.952
Hypothermia of newborn (x1.02) 0.953
Dyspnea (/1.02) 0.953
Neonatal hypoglycemia (x1.02) 0.954
Disorder of integument (x1.02) 0.955
Hyperlipidemia (/1.02) 0.955
Postpartum finding (x1.02) 0.956
aspirin (-0.00168) 0.956
Diaper rash (x1.02) 0.957
Acute pharyngitis (x1.02) 0.957
Acute renal failure syndrome (/1.02) 0.958
Fetal or neonatal effect of maternal infection (x1.02) 0.958
ibuprofen (+0.00155) 0.959
Type 2 diabetes mellitus without complication (/1.02) 0.959
Electrocardiogram abnormal (/1.02) 0.960
Carbon dioxide, total [mol/V] in SP (-0.103) 0.960
gabapentin (-0.00139) 0.960
Potassium [mol/V] in SP (+0.0115) 0.961
promethazine (-0.00128) 0.961
Diarrhea (x1.01) 0.961
Respiratory distress syndrome in the newborn (x1.01) 0.962
MCHC [M/V] by AC (+0.031) 0.962
oxycodone (-0.00124) 0.962
Patent ductus arteriosus (x1.01) 0.963
ECG normal (/1.01) 0.963
Condition in fetus originating in the perinatal period (x1.01) 0.963
Postmature infancy (x1.01) 0.963
Jaundice (x1.01) 0.964
Streptococcal sore throat (x1.01) 0.964
desonide (+0.00117) 0.964
Hearing loss (x1.01) 0.964
metoprolol (-0.00116) 0.965
Disorder of speech and language development (x1.01) 0.965
Delayed milestone (x1.01) 0.965
Finding of neonate (x1.01) 0.965
Prematurity of infant (x1.01) 0.966
Mixed hyperlipidemia (/1.01) 0.966
Eosinophils [#/V] in B by AC (+0.00385) 0.966
Enteroviral vesicular stomatitis with exanthem (x1.01) 0.966
O/E - hearing (x1.01) 0.967
Atrial septal defect (x1.01) 0.967
Disorder of bilirubin metabolism (x1.01) 0.967
Hemolytic disease of fetus OR newborn due to ABO immunization (x1.01) 0.967
erythromycin (+0.00108) 0.968
Abnormal finding on auditory function study (x1.01) 0.968
Malaise (/1.01) 0.968
Calcium [M/V] in SP (+0.0115) 0.968
metformin (-0.00105) 0.968
Upper respiratory tract infection due to Influenza (x1.01) 0.969
Lymphocytes/100 leukocytes in B by Manual count (+0.334) 0.969
Phosphate [M/V] in SP (+0.0232) 0.969
Wheezing (x1.01) 0.969
Congenital heart disease (x1.01) 0.969
Atrial fibrillation (/1.01) 0.970
Monocytes [#/V] in B by AC (+0.00718) 0.970
−1.00 −0.75 −0.50 −0.25 0.00 0.25 0.50 0.75 1.00
Expressions

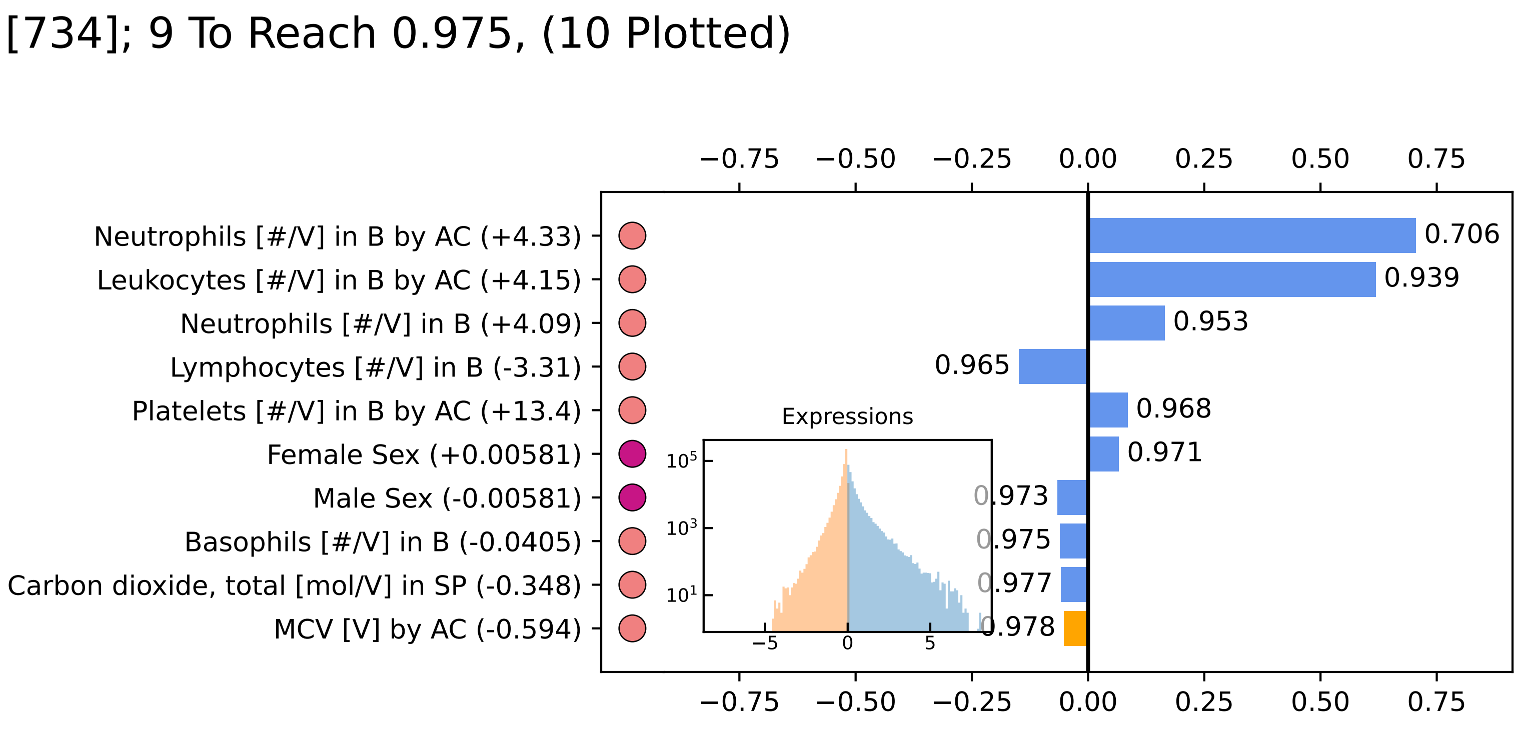

Signature [734]; 9 To Reach 0.975, (10 Plotted)
Neutrophils [#/V] in B by AC (+4.33)
Leukocytes [#/V] in B by AC (+4.15)
Neutrophils [#/V] in B (+4.09)
Lymphocytes [#/V] in B (-3.31)
Platelets [#/V] in B by AC (+13.4)
Female Sex (+0.00581)
Male Sex (-0.00581)
Basophils [#/V] in B (-0.0405)
Carbon dioxide, total [mol/V] in SP (-0.348)
MCV [V] by AC (-0.594)
0.706
0.939
0.953
0.965
0.968
0.971
0.973
0.975
0.977
0.978
−0.75
−0.50
−0.25
0.00
0.25
0.50
0.75
Expressions
10^5
10^3
10^1
−5
0
5

Signature [735]; 1 To Reach 0.975, (10 Plotted)

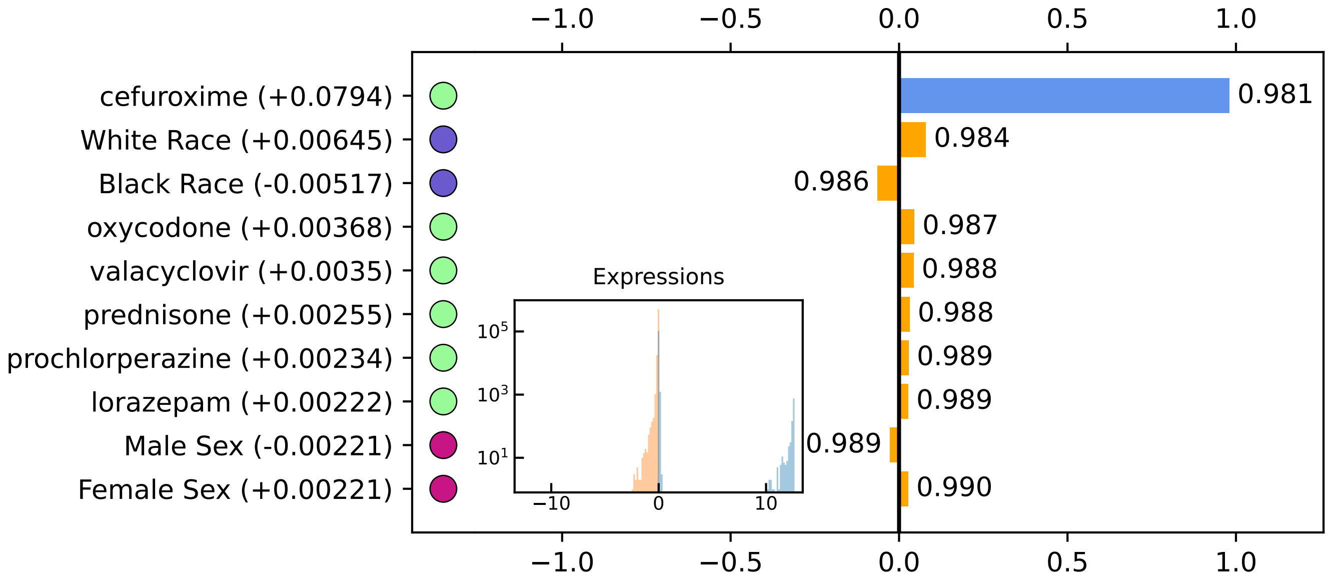

Signature [736]; 11 To Reach 0.975, (11 Plotted)

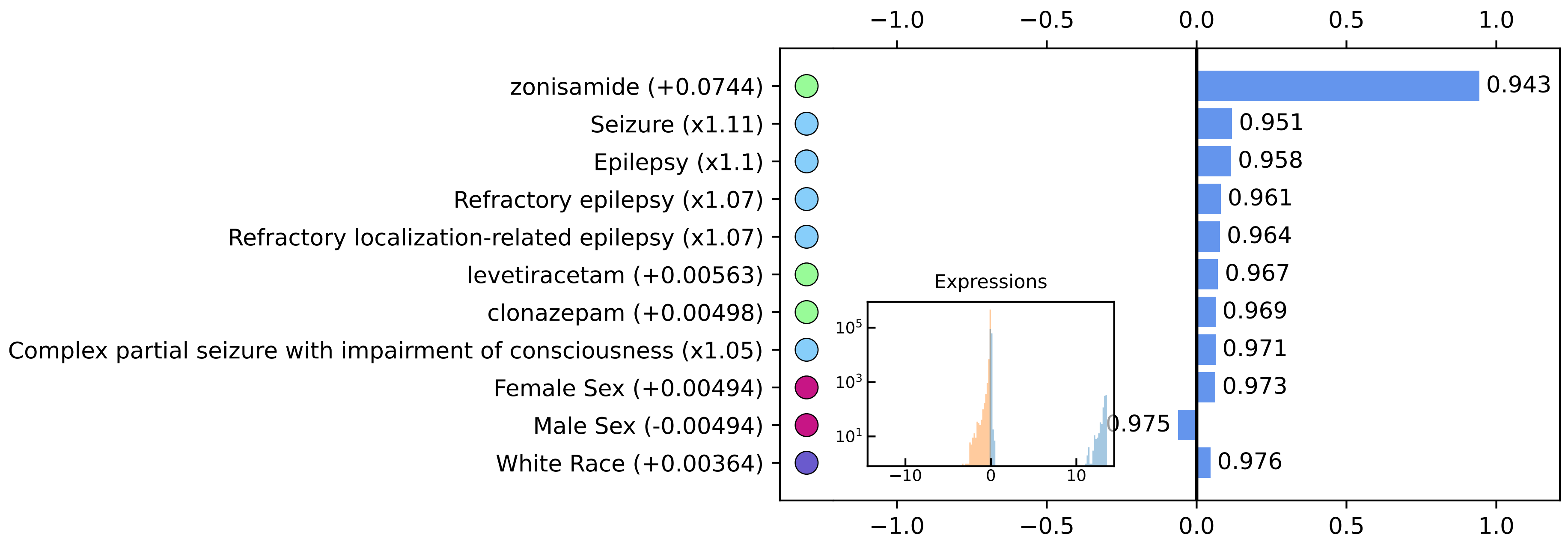

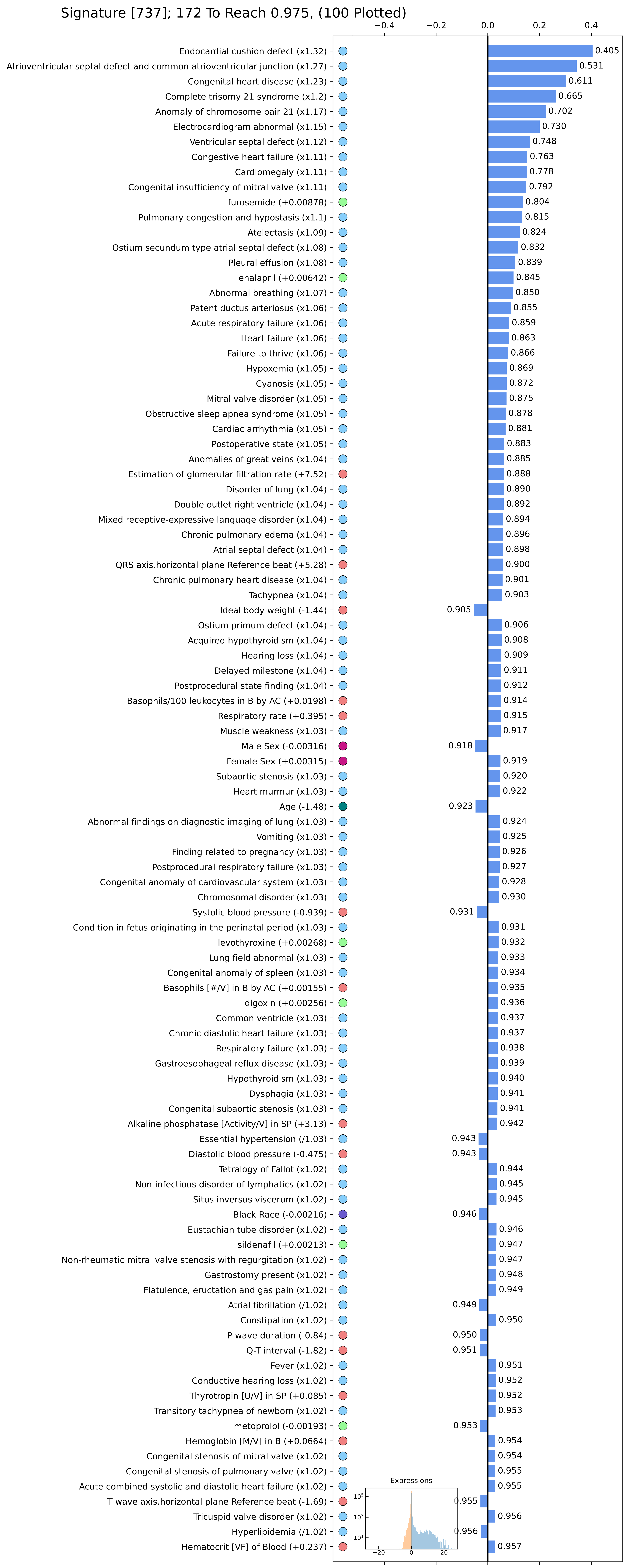

Signature [737]; 172 To Reach 0.975, (100 Plotted)
Endocardial cushion defect (x1.32) 0.405
Atrioventricular septal defect and common atrioventricular junction (x1.27) 0.531
Congenital heart disease (x1.23) 0.611
Complete trisomy 21 syndrome (x1.2) 0.665
Anomaly of chromosome pair 21 (x1.17) 0.702
Electrocardiogram abnormal (x1.15) 0.730
Ventricular septal defect (x1.12) 0.748
Congestive heart failure (x1.11) 0.763
Cardiomegaly (x1.11) 0.778
Congenital insufficiency of mitral valve (x1.11) 0.792
furosemide (+0.00878) 0.804
Pulmonary congestion and hypostasis (x1.1) 0.815
Atelectasis (x1.09) 0.824
Ostium secundum type atrial septal defect (x1.08) 0.832
Pleural effusion (x1.08) 0.839
enalapril (+0.00642) 0.845
Abnormal breathing (x1.07) 0.850
Patent ductus arteriosus (x1.06) 0.855
Acute respiratory failure (x1.06) 0.859
Heart failure (x1.06) 0.863
Failure to thrive (x1.06) 0.866
Hypoxemia (x1.05) 0.869
Cyanosis (x1.05) 0.872
Mitral valve disorder (x1.05) 0.875
Obstructive sleep apnea syndrome (x1.05) 0.878
Cardiac arrhythmia (x1.05) 0.881
Postoperative state (x1.05) 0.883
Anomalies of great veins (x1.04) 0.885
Estimation of glomerular filtration rate (+7.52) 0.888
Disorder of lung (x1.04) 0.890
Double outlet right ventricle (x1.04) 0.892
Mixed receptive-expressive language disorder (x1.04) 0.894
Chronic pulmonary edema (x1.04) 0.896
Atrial septal defect (x1.04) 0.898
QRS axis.horizontal plane Reference beat (+5.28) 0.900
Chronic pulmonary heart disease (x1.04) 0.901
Tachypnea (x1.04) 0.903
Ideal body weight (-1.44) 0.905
Ostium primum defect (x1.04) 0.906
Acquired hypothyroidism (x1.04) 0.908
Hearing loss (x1.04) 0.909
Delayed milestone (x1.04) 0.911
Postprocedural state finding (x1.04) 0.912
Basophils/100 leukocytes in B by AC (+0.0198) 0.914
Respiratory rate (+0.395) 0.915
Muscle weakness (x1.03) 0.917
Male Sex (-0.00316) 0.918
Female Sex (+0.00315) 0.919
Subaortic stenosis (x1.03) 0.920
Heart murmur (x1.03) 0.922
Age (-1.48) 0.923
Abnormal findings on diagnostic imaging of lung (x1.03) 0.924
Vomiting (x1.03) 0.925
Finding related to pregnancy (x1.03) 0.926
Postprocedural respiratory failure (x1.03) 0.927
Congenital anomaly of cardiovascular system (x1.03) 0.928
Chromosomal disorder (x1.03) 0.930
Systolic blood pressure (-0.939) 0.931
Condition in fetus originating in the perinatal period (x1.03) 0.931
levothyroxine (+0.00268) 0.932
Lung field abnormal (x1.03) 0.933
Congenital anomaly of spleen (x1.03) 0.934
Basophils [#/V] in B by AC (+0.00155) 0.935
digoxin (+0.00256) 0.936
Common ventricle (x1.03) 0.937
Chronic diastolic heart failure (x1.03) 0.937
Respiratory failure (x1.03) 0.938
Gastroesophageal reflux disease (x1.03) 0.939
Hypothyroidism (x1.03) 0.940
Dysphagia (x1.03) 0.941
Congenital subaortic stenosis (x1.03) 0.941
Alkaline phosphatase [Activity/V] in SP (+3.13) 0.942
Essential hypertension (/1.03) 0.943
Diastolic blood pressure (-0.475) 0.943
Tetralogy of Fallot (x1.02) 0.944
Non-infectious disorder of lymphatics (x1.02) 0.945
Situs inversus viscerum (x1.02) 0.945
Black Race (-0.00216) 0.946
Eustachian tube disorder (x1.02) 0.946
sildenafil (+0.00213) 0.947
Non-rheumatic mitral valve stenosis with regurgitation (x1.02) 0.947
Gastrostomy present (x1.02) 0.948
Flatulence, eructation and gas pain (x1.02) 0.949
Atrial fibrillation (/1.02) 0.949
Constipation (x1.02) 0.950
P wave duration (-0.84) 0.950
Q-T interval (-1.82) 0.951
Fever (x1.02) 0.951
Conductive hearing loss (x1.02) 0.952
Thyrotropin [U/V] in SP (+0.085) 0.952
Transitory tachypnea of newborn (x1.02) 0.953
metoprolol (-0.00193) 0.953
Hemoglobin [M/V] in B (+0.0664) 0.954
Congenital stenosis of mitral valve (x1.02) 0.954
Congenital stenosis of pulmonary valve (x1.02) 0.955
Acute combined systolic and diastolic heart failure (x1.02) 0.955
T wave axis.horizontal plane Reference beat (-1.69) 0.955
Tricuspid valve disorder (x1.02) 0.956
Hyperlipidemia (/1.02) 0.956
Hematocrit [VF] of Blood (+0.237) 0.957
−0.4 −0.2 0.0 0.2 0.4
Expressions
−20 0 20

Signature [738]; 27 To Reach 0.975, (27 Plotted)

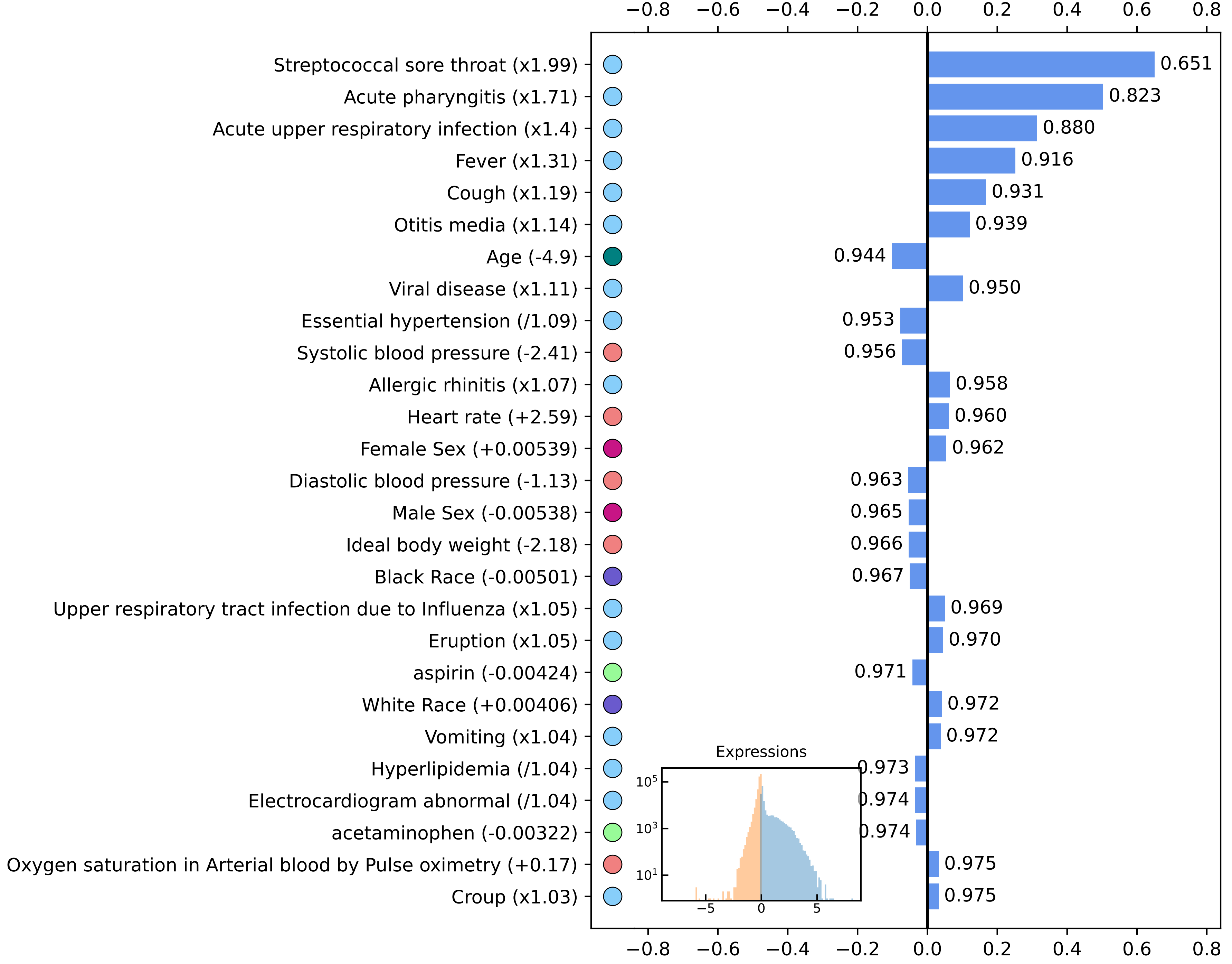

Signature [739]; 6 To Reach 0.975, (10 Plotted)

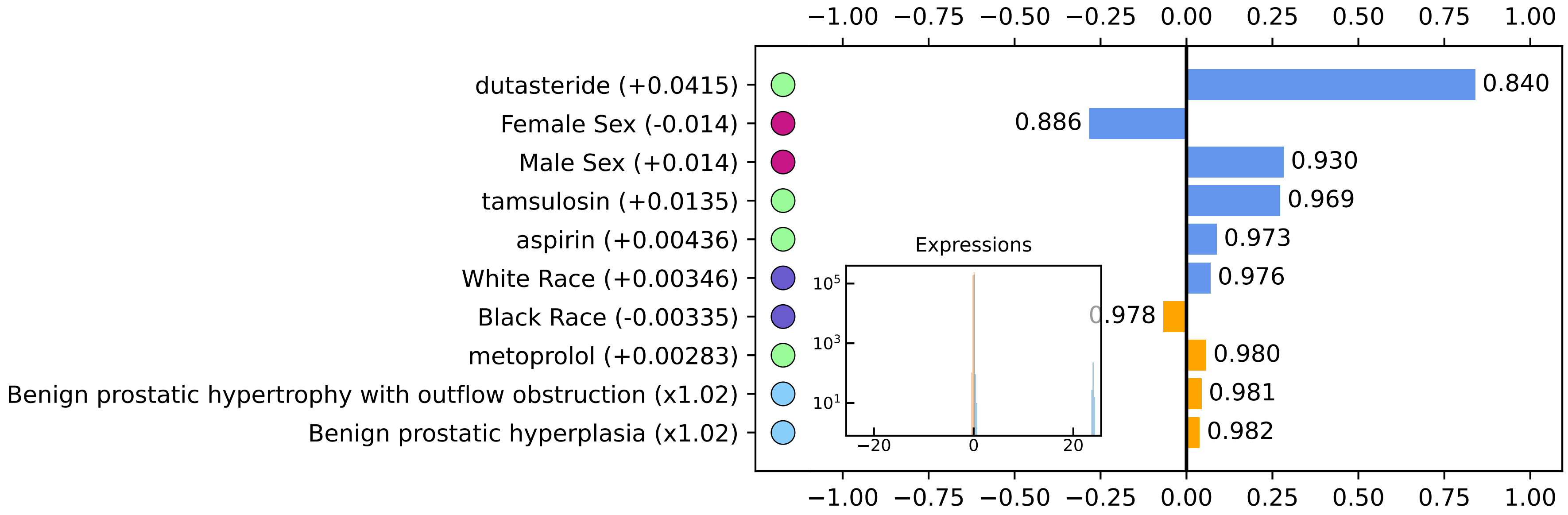

Signature [740]; 76 To Reach 0.975, (76 Plotted)

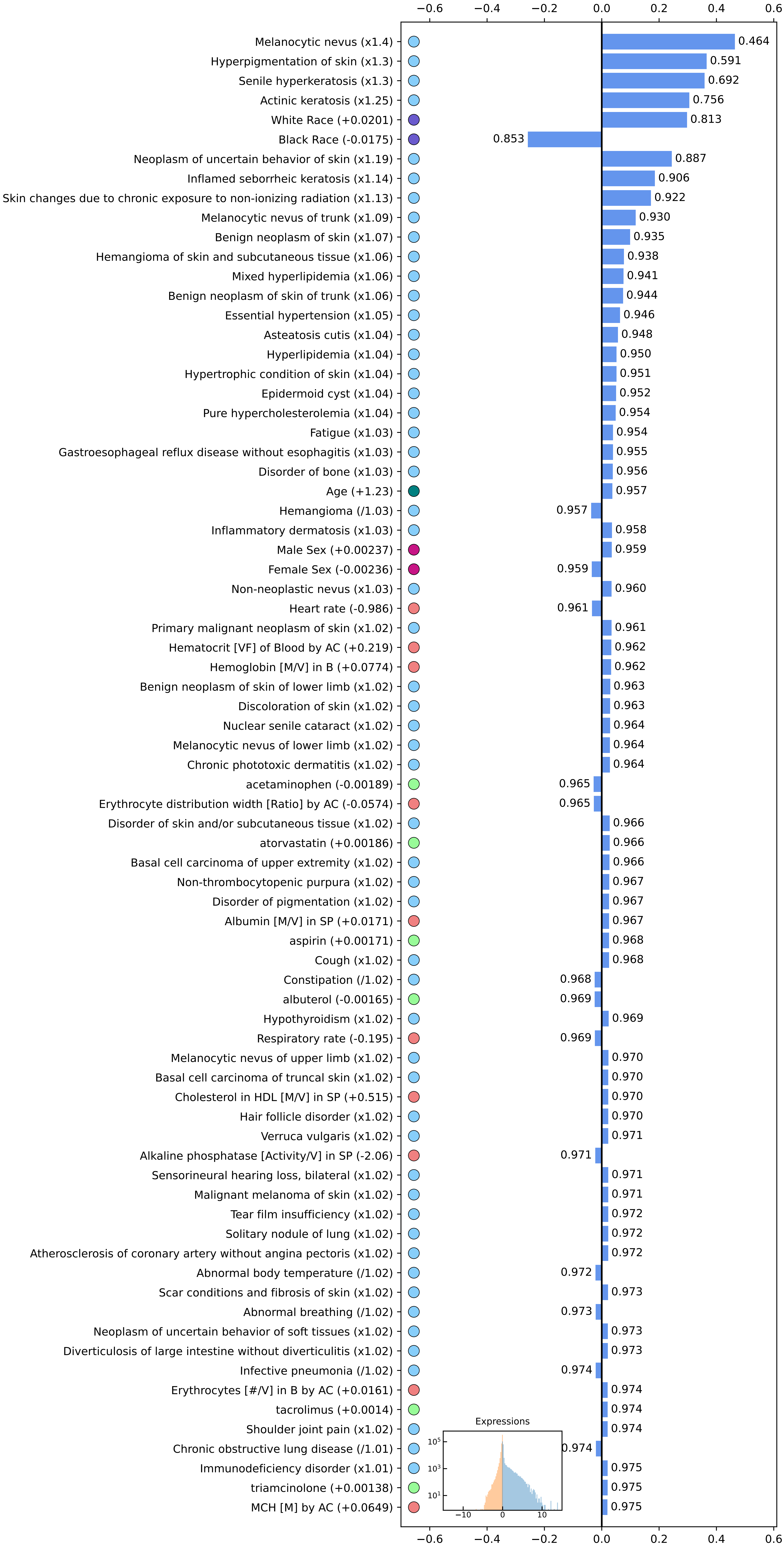

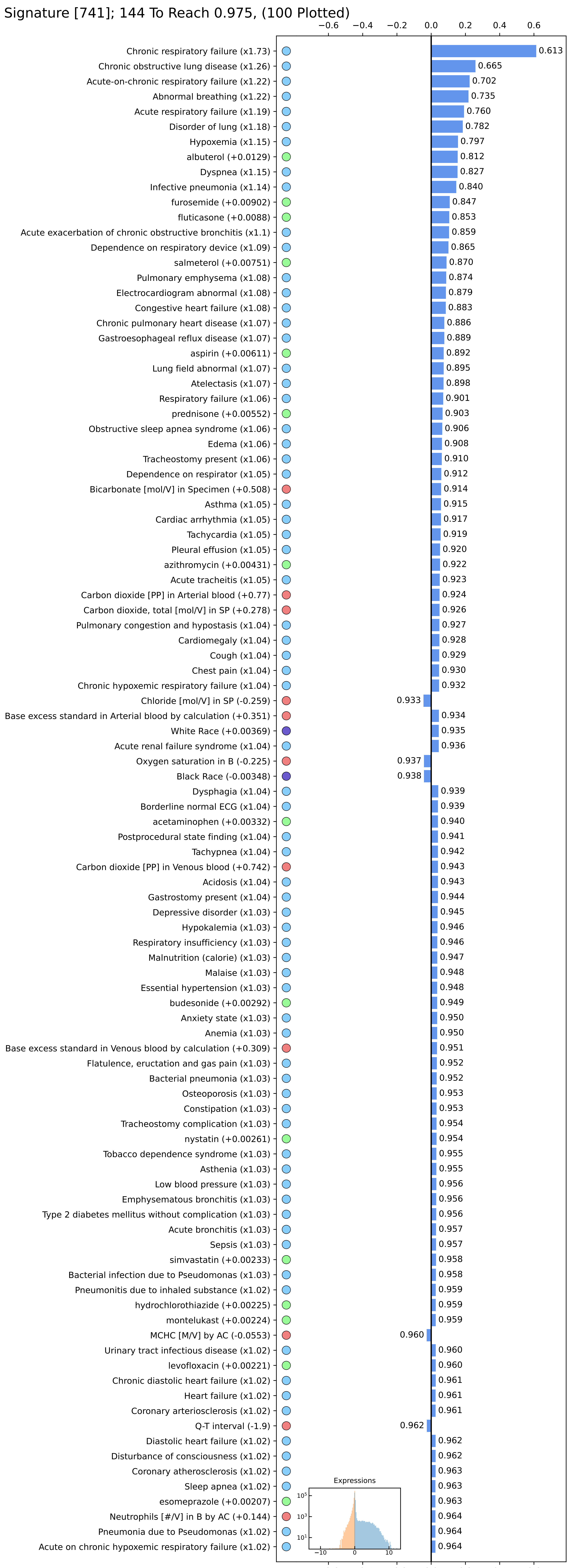

Signature [741]; 144 To Reach 0.975, (100 Plotted)
Chronic respiratory failure (x1.73) 0.613
Chronic obstructive lung disease (x1.26) 0.665
Acute-on-chronic respiratory failure (x1.22) 0.702
Abnormal breathing (x1.22) 0.735
Acute respiratory failure (x1.19) 0.760
Disorder of lung (x1.18) 0.782
Hypoxemia (x1.15) 0.797
albuterol (+0.0129) 0.812
Dyspnea (x1.15) 0.827
Infective pneumonia (x1.14) 0.840
furosemide (+0.00902) 0.847
fluticasone (+0.0088) 0.853
Acute exacerbation of chronic obstructive bronchitis (x1.1) 0.859
Dependence on respiratory device (x1.09) 0.865
salmeterol (+0.00751) 0.870
Pulmonary emphysema (x1.08) 0.874
Electrocardiogram abnormal (x1.08) 0.879
Congestive heart failure (x1.08) 0.883
Chronic pulmonary heart disease (x1.07) 0.886
Gastroesophageal reflux disease (x1.07) 0.889
aspirin (+0.00611) 0.892
Lung field abnormal (x1.07) 0.895
Atelectasis (x1.07) 0.898
Respiratory failure (x1.06) 0.901
prednisone (+0.00552) 0.903
Obstructive sleep apnea syndrome (x1.06) 0.906
Edema (x1.06) 0.908
Tracheostomy present (x1.06) 0.910
Dependence on respirator (x1.05) 0.912
Bicarbonate [mol/V] in Specimen (+0.508) 0.914
Asthma (x1.05) 0.915
Cardiac arrhythmia (x1.05) 0.917
Tachycardia (x1.05) 0.919
Pleural effusion (x1.05) 0.920
azithromycin (+0.00431) 0.922
Acute tracheitis (x1.05) 0.923
Carbon dioxide [PP] in Arterial blood (+0.77) 0.924
Carbon dioxide, total [mol/V] in SP (+0.278) 0.926
Pulmonary congestion and hypostasis (x1.04) 0.927
Cardiomegaly (x1.04) 0.928
Cough (x1.04) 0.929
Chest pain (x1.04) 0.930
Chronic hypoxemic respiratory failure (x1.04) 0.932
Chloride [mol/V] in SP (-0.259) 0.933
Base excess standard in Arterial blood by calculation (+0.351) 0.934
White Race (+0.00369) 0.935
Acute renal failure syndrome (x1.04) 0.936
Oxygen saturation in B (-0.225) 0.937
Black Race (-0.00348) 0.938
Dysphagia (x1.04) 0.939
Borderline normal ECG (x1.04) 0.939
acetaminophen (+0.00332) 0.940
Postprocedural state finding (x1.04) 0.941
Tachypnea (x1.04) 0.942
Carbon dioxide [PP] in Venous blood (+0.742) 0.943
Acidosis (x1.04) 0.943
Gastrostomy present (x1.04) 0.944
Depressive disorder (x1.03) 0.945
Hypokalemia (x1.03) 0.946
Respiratory insufficiency (x1.03) 0.946
Malnutrition (calorie) (x1.03) 0.947
Malaise (x1.03) 0.948
Essential hypertension (x1.03) 0.948
budesonide (+0.00292) 0.949
Anxiety state (x1.03) 0.950
Anemia (x1.03) 0.950
Base excess standard in Venous blood by calculation (+0.309) 0.951
Flatulence, eructation and gas pain (x1.03) 0.952
Bacterial pneumonia (x1.03) 0.952
Osteoporosis (x1.03) 0.953
Constipation (x1.03) 0.953
Tracheostomy complication (x1.03) 0.954
nystatin (+0.00261) 0.954
Tobacco dependence syndrome (x1.03) 0.955
Asthenia (x1.03) 0.955
Low blood pressure (x1.03) 0.956
Emphysematous bronchitis (x1.03) 0.956
Type 2 diabetes mellitus without complication (x1.03) 0.956
Acute bronchitis (x1.03) 0.957
Sepsis (x1.03) 0.957
simvastatin (+0.00233) 0.958
Bacterial infection due to Pseudomonas (x1.03) 0.958
Pneumonitis due to inhaled substance (x1.02) 0.959
hydrochlorothiazide (+0.00225) 0.959
montelukast (+0.00224) 0.959
MCHC [M/V] by AC (-0.0553) 0.960
Urinary tract infectious disease (x1.02) 0.960
levofloxacin (+0.00221) 0.960
Chronic diastolic heart failure (x1.02) 0.961
Heart failure (x1.02) 0.961
Coronary arteriosclerosis (x1.02) 0.961
Q-T interval (-1.9) 0.962
Diastolic heart failure (x1.02) 0.962
Disturbance of consciousness (x1.02) 0.962
Coronary atherosclerosis (x1.02) 0.963
Sleep apnea (x1.02) 0.963
esomeprazole (+0.00207) 0.963
Neutrophils [#/V] in B by AC (+0.144) 0.964
Pneumonia due to Pseudomonas (x1.02) 0.964
Acute on chronic hypoxemic respiratory failure (x1.02) 0.964
−0.6 −0.4 −0.2 0.0 0.2 0.4 0.6
Expressions

Signature [742]; 12 To Reach 0.975, (12 Plotted)

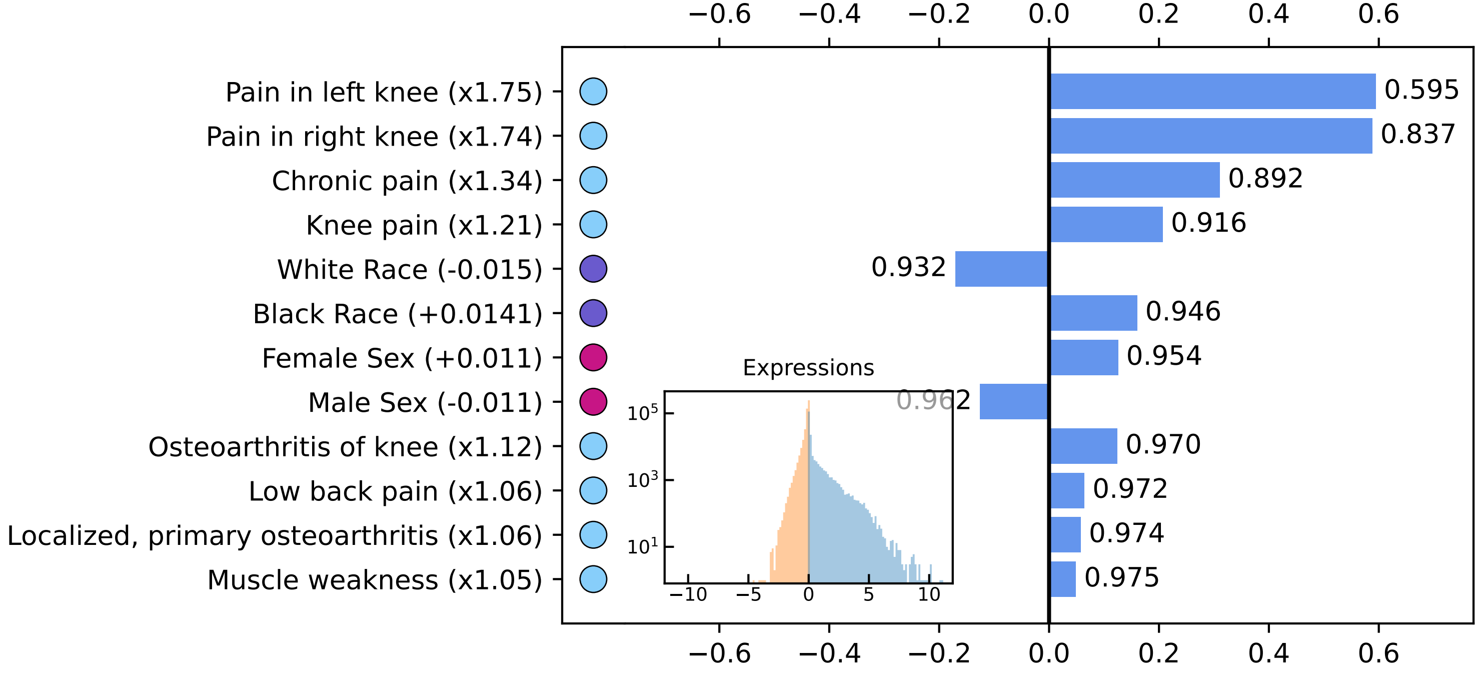

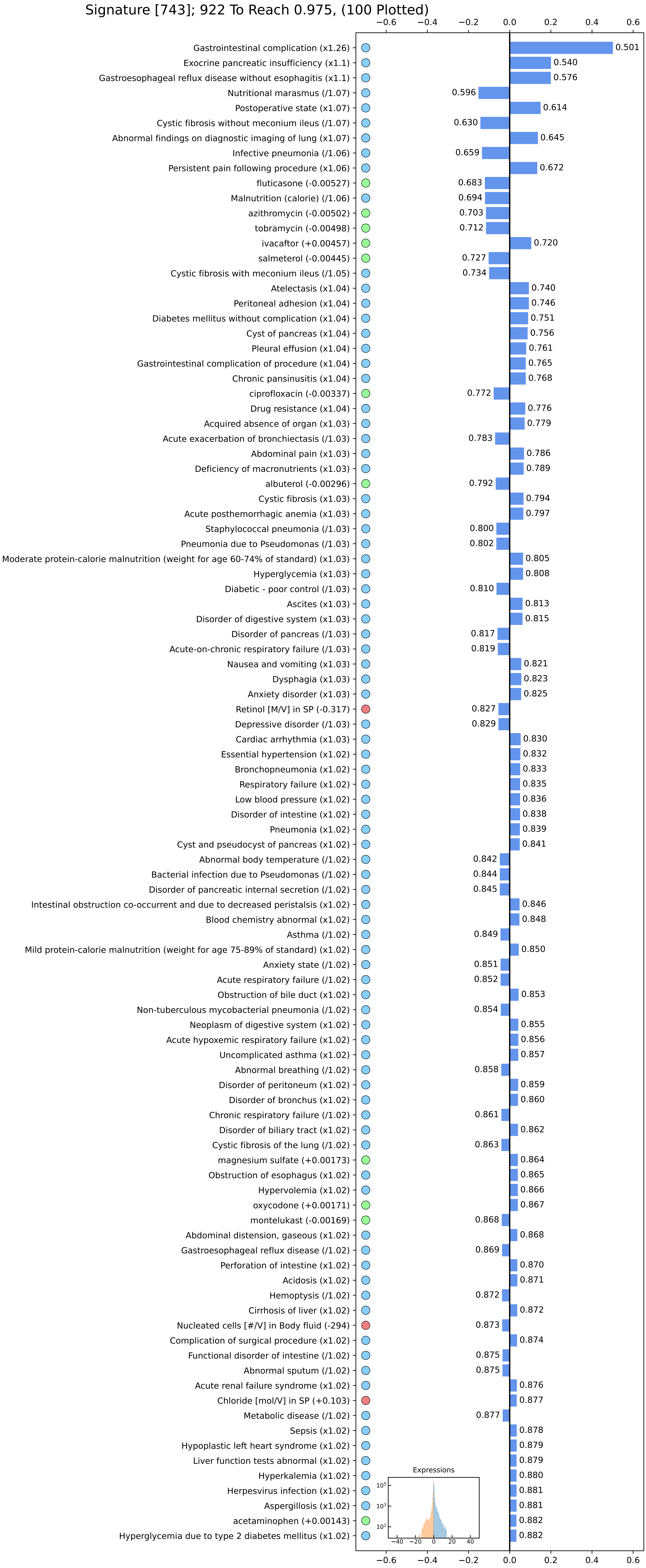

Signature [743]; 922 To Reach 0.975, (100 Plotted)
Gastrointestinal complication (x1.26) 0.501
Exocrine pancreatic insufficiency (x1.1) 0.540
Gastroesophageal reflux disease without esophagitis (x1.1) 0.576
Nutritional marasmus (/1.07) 0.596
Postoperative state (x1.07) 0.614
Cystic fibrosis without meconium ileus (/1.07) 0.630
Abnormal findings on diagnostic imaging of lung (x1.07) 0.645
Infective pneumonia (/1.06) 0.659
Persistent pain following procedure (x1.06) 0.672
fluticasone (-0.00527) 0.683
Malnutrition (calorie) (/1.06) 0.694
azithromycin (-0.00502) 0.703
tobramycin (-0.00498) 0.712
ivacaftor (+0.00457) 0.720
salmeterol (-0.00445) 0.727
Cystic fibrosis with meconium ileus (/1.05) 0.734
Atelectasis (x1.04) 0.740
Peritoneal adhesion (x1.04) 0.746
Diabetes mellitus without complication (x1.04) 0.751
Cyst of pancreas (x1.04) 0.756
Pleural effusion (x1.04) 0.761
Gastrointestinal complication of procedure (x1.04) 0.765
Chronic pansinusitis (x1.04) 0.768
ciprofloxacin (-0.00337) 0.772
Drug resistance (x1.04) 0.776
Acquired absence of organ (x1.03) 0.779
Acute exacerbation of bronchiectasis (/1.03) 0.783
Abdominal pain (x1.03) 0.786
Deficiency of macronutrients (x1.03) 0.789
albuterol (-0.00296) 0.792
Cystic fibrosis (x1.03) 0.794
Acute posthemorrhagic anemia (x1.03) 0.797
Staphylococcal pneumonia (/1.03) 0.800
Pneumonia due to Pseudomonas (/1.03) 0.802
Moderate protein-calorie malnutrition (weight for age 60-74% of standard) (x1.03) 0.805
Hyperglycemia (x1.03) 0.808
Diabetic - poor control (/1.03) 0.810
Ascites (x1.03) 0.813
Disorder of digestive system (x1.03) 0.815
Disorder of pancreas (/1.03) 0.817
Acute-on-chronic respiratory failure (/1.03) 0.819
Nausea and vomiting (x1.03) 0.821
Dysphagia (x1.03) 0.823
Anxiety disorder (x1.03) 0.825
Retinol [M/V] in SP (-0.317) 0.827
Depressive disorder (/1.03) 0.829
Cardiac arrhythmia (x1.03) 0.830
Essential hypertension (x1.02) 0.832
Bronchopneumonia (x1.02) 0.833
Respiratory failure (x1.02) 0.835
Low blood pressure (x1.02) 0.836
Disorder of intestine (x1.02) 0.838
Pneumonia (x1.02) 0.839
Cyst and pseudocyst of pancreas (x1.02) 0.841
Abnormal body temperature (/1.02) 0.842
Bacterial infection due to Pseudomonas (/1.02) 0.844
Disorder of pancreatic internal secretion (/1.02) 0.845
Intestinal obstruction co-occurrent and due to decreased peristalsis (x1.02) 0.846
Blood chemistry abnormal (x1.02) 0.848
Asthma (/1.02) 0.849
Mild protein-calorie malnutrition (weight for age 75-89% of standard) (x1.02) 0.850
Anxiety state (/1.02) 0.851
Acute respiratory failure (/1.02) 0.852
Obstruction of bile duct (x1.02) 0.853
Non-tuberculous mycobacterial pneumonia (/1.02) 0.854
Neoplasm of digestive system (x1.02) 0.855
Acute hypoxemic respiratory failure (x1.02) 0.856
Uncomplicated asthma (x1.02) 0.857
Abnormal breathing (/1.02) 0.858
Disorder of peritoneum (x1.02) 0.859
Disorder of bronchus (x1.02) 0.860
Chronic respiratory failure (/1.02) 0.861
Disorder of biliary tract (x1.02) 0.862
Cystic fibrosis of the lung (/1.02) 0.863
magnesium sulfate (+0.00173) 0.864
Obstruction of esophagus (x1.02) 0.865
Hypervolemia (x1.02) 0.866
oxycodone (+0.00171) 0.867
montelukast (-0.00169) 0.868
Abdominal distension, gaseous (x1.02) 0.868
Gastroesophageal reflux disease (/1.02) 0.869
Perforation of intestine (x1.02) 0.870
Acidosis (x1.02) 0.871
Hemoptysis (/1.02) 0.872
Cirrhosis of liver (x1.02) 0.872
Nucleated cells [#/V] in Body fluid (-294) 0.873
Complication of surgical procedure (x1.02) 0.874
Functional disorder of intestine (/1.02) 0.875
Abnormal sputum (/1.02) 0.875
Acute renal failure syndrome (x1.02) 0.876
Chloride [mol/V] in SP (+0.103) 0.877
Metabolic disease (/1.02) 0.877
Sepsis (x1.02) 0.878
Hypoplastic left heart syndrome (x1.02) 0.879
Liver function tests abnormal (x1.02) 0.879
Hyperkalemia (x1.02) 0.880
Herpesvirus infection (x1.02) 0.881
Aspergillosis (x1.02) 0.881
acetaminophen (+0.00143) 0.882
Hyperglycemia due to type 2 diabetes mellitus (x1.02) 0.882
−0.6 −0.4 −0.2 0.0 0.2 0.4 0.6
Expressions
10^5
10^3
10^1
−40 −20 0 20 40

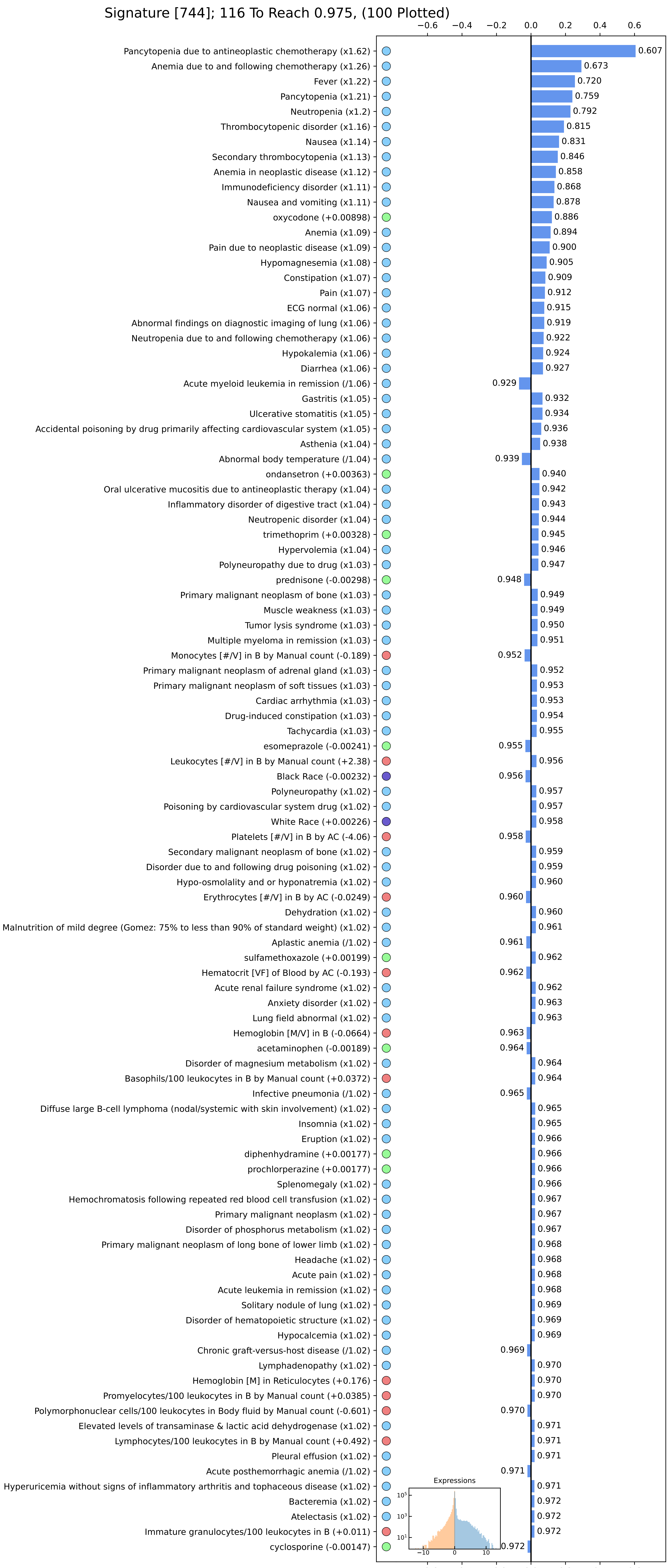
Signature [744]; 116 To Reach 0.975, (100 Plotted)
Pancytopenia due to antineoplastic chemotherapy (x1.62)
Anemia due to and following chemotherapy (x1.26)
Fever (x1.22)
Pancytopenia (x1.21)
Neutropenia (x1.2)
Thrombocytopenic disorder (x1.16)
Nausea (x1.14)
Secondary thrombocytopenia (x1.13)
Anemia in neoplastic disease (x1.12)
Immunodeficiency disorder (x1.11)
Nausea and vomiting (x1.11)
oxycodone (+0.00898)
Anemia (x1.09)
Pain due to neoplastic disease (x1.09)
Hypomagnesemia (x1.08)
Constipation (x1.07)
Pain (x1.07)
ECG normal (x1.06)
Abnormal findings on diagnostic imaging of lung (x1.06)
Neutropenia due to and following chemotherapy (x1.06)
Hypokalemia (x1.06)
Diarrhea (x1.06)
Acute myeloid leukemia in remission (/1.06)
Gastritis (x1.05)
Ulcerative stomatitis (x1.05)
Accidental poisoning by drug primarily affecting cardiovascular system (x1.05)
Asthenia (x1.04)
Abnormal body temperature (/1.04)
ondansetron (+0.00363)
Oral ulcerative mucositis due to antineoplastic therapy (x1.04)
Inflammatory disorder of digestive tract (x1.04)
Neutropenic disorder (x1.04)
trimethoprim (+0.00328)
Hypervolemia (x1.04)
Polyneuropathy due to drug (x1.03)
prednisone (-0.00298)
Primary malignant neoplasm of bone (x1.03)
Muscle weakness (x1.03)
Tumor lysis syndrome (x1.03)
Multiple myeloma in remission (x1.03)
Monocytes [#/V] in B by Manual count (-0.189)
Primary malignant neoplasm of adrenal gland (x1.03)
Primary malignant neoplasm of soft tissues (x1.03)
Cardiac arrhythmia (x1.03)
Drug-induced constipation (x1.03)
Tachycardia (x1.03)
esomeprazole (-0.00241)
Leukocytes [#/V] in B by Manual count (+2.38)
Black Race (-0.00232)
Polyneuropathy (x1.02)
Poisoning by cardiovascular system drug (x1.02)
White Race (+0.00226)
Platelets [#/V] in B by AC (-4.06)
Secondary malignant neoplasm of bone (x1.02)
Disorder due to and following drug poisoning (x1.02)
Hypo-osmolality and or hyponatremia (x1.02)
Erythrocytes [#/V] in B by AC (-0.0249)
Dehydration (x1.02)
Malnutrition of mild degree (Gomez: 75% to less than 90% of standard weight) (x1.02)
Aplastic anemia (/1.02)
sulfamethoxazole (+0.00199)
Hematocrit [VF] of Blood by AC (-0.193)
Acute renal failure syndrome (x1.02)
Anxiety disorder (x1.02)
Lung field abnormal (x1.02)
Hemoglobin [M/V] in B (-0.0664)
acetaminophen (-0.00189)
Disorder of magnesium metabolism (x1.02)
Basophils/100 leukocytes in B by Manual count (+0.0372)
Infective pneumonia (/1.02)
Diffuse large B-cell lymphoma (nodal/systemic with skin involvement) (x1.02)
Insomnia (x1.02)
Eruption (x1.02)
diphenhydramine (+0.00177)
prochlorperazine (+0.00177)
Splenomegaly (x1.02)
Hemochromatosis following repeated red blood cell transfusion (x1.02)
Primary malignant neoplasm (x1.02)
Disorder of phosphorus metabolism (x1.02)
Primary malignant neoplasm of long bone of lower limb (x1.02)
Headache (x1.02)
Acute pain (x1.02)
Acute leukemia in remission (x1.02)
Solitary nodule of lung (x1.02)
Disorder of hematopoietic structure (x1.02)
Hypocalcemia (x1.02)
Chronic graft-versus-host disease (/1.02)
Lymphadenopathy (x1.02)
Hemoglobin [M] in Reticulocytes (+0.176)
Promyelocytes/100 leukocytes in B by Manual count (+0.0385)
Polymorphonuclear cells/100 leukocytes in Body fluid by Manual count (-0.601)
Elevated levels of transaminase & lactic acid dehydrogenase (x1.02)
Lymphocytes/100 leukocytes in B by Manual count (+0.492)
Pleural effusion (x1.02)
Acute posthemorrhagic anemia (/1.02)
Hyperuricemia without signs of inflammatory arthritis and tophaceous disease (x1.02)
Bacteremia (x1.02)
Atelectasis (x1.02)
Immature granulocytes/100 leukocytes in B (+0.011)
cyclosporine (-0.00147)
0.607
0.673
0.720
0.759
0.792
0.815
0.831
0.846
0.858
0.868
0.878
0.886
0.894
0.900
0.905
0.909
0.912
0.915
0.919
0.922
0.924
0.927
0.929
0.932
0.934
0.936
0.938
0.939
0.940
0.942
0.943
0.944
0.945
0.946
0.947
0.948
0.949
0.949
0.950
0.951
0.952
0.952
0.953
0.953
0.954
0.955
0.955
0.956
0.956
0.957
0.957
0.958
0.958
0.959
0.959
0.960
0.960
0.960
0.961
0.961
0.962
0.962
0.962
0.963
0.963
0.963
0.964
0.964
0.964
0.965
0.965
0.965
0.966
0.966
0.966
0.966
0.967
0.967
0.967
0.968
0.968
0.968
0.968
0.969
0.969
0.969
0.969
0.970
0.970
0.970
0.970
0.971
0.971
0.971
0.971
0.971
0.972
0.972
0.972
0.972
−0.6
−0.4
−0.2
0.0
0.2
0.4
0.6
Expressions
−10
0
10

Signature [745]; 14 To Reach 0.975, (14 Plotted)

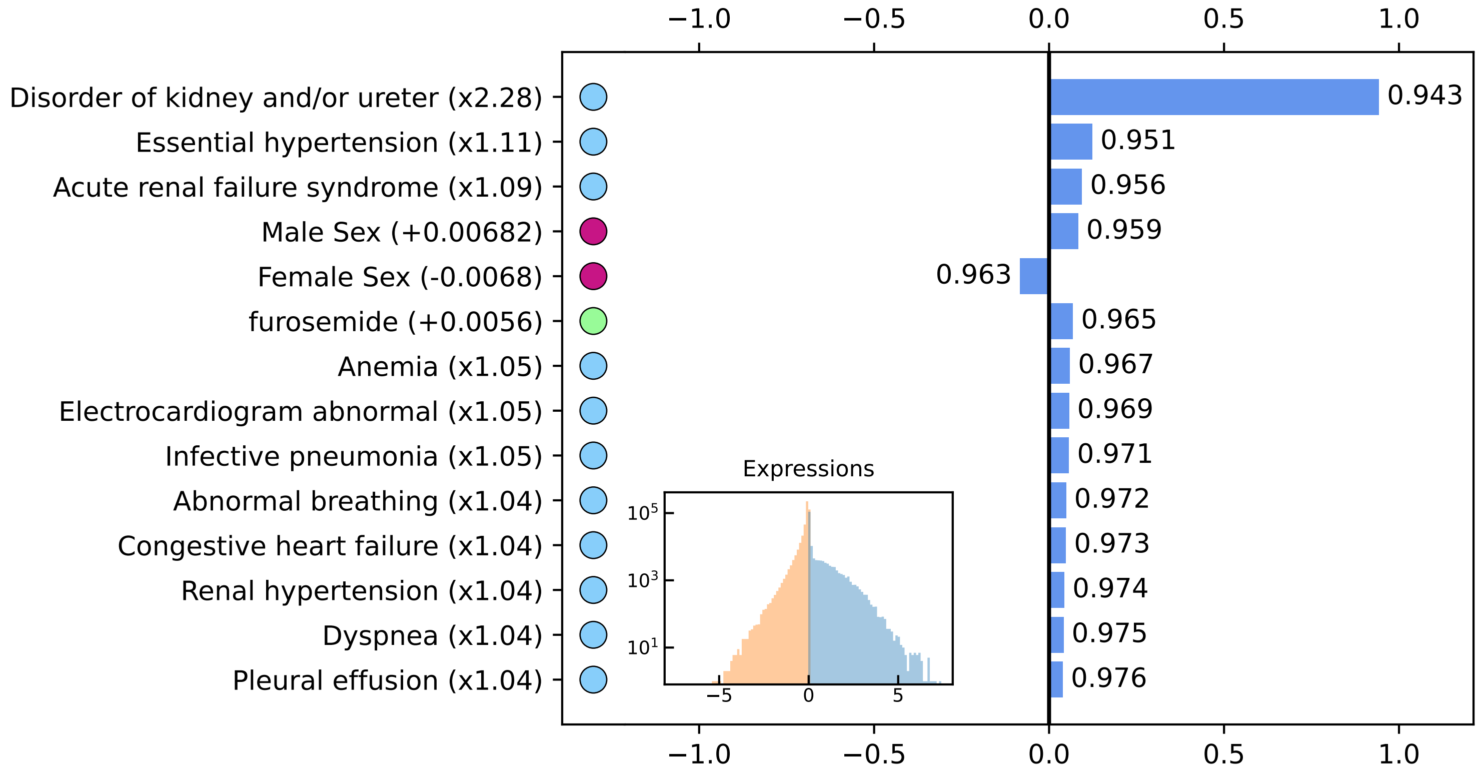

Signature [746]; 2 To Reach 0.975, (10 Plotted)

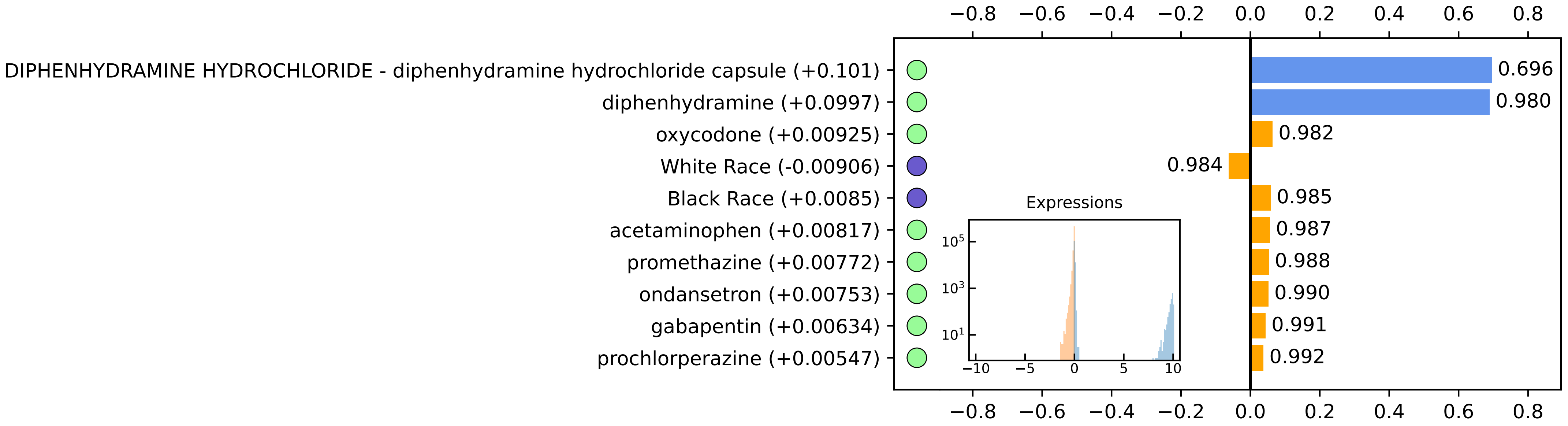

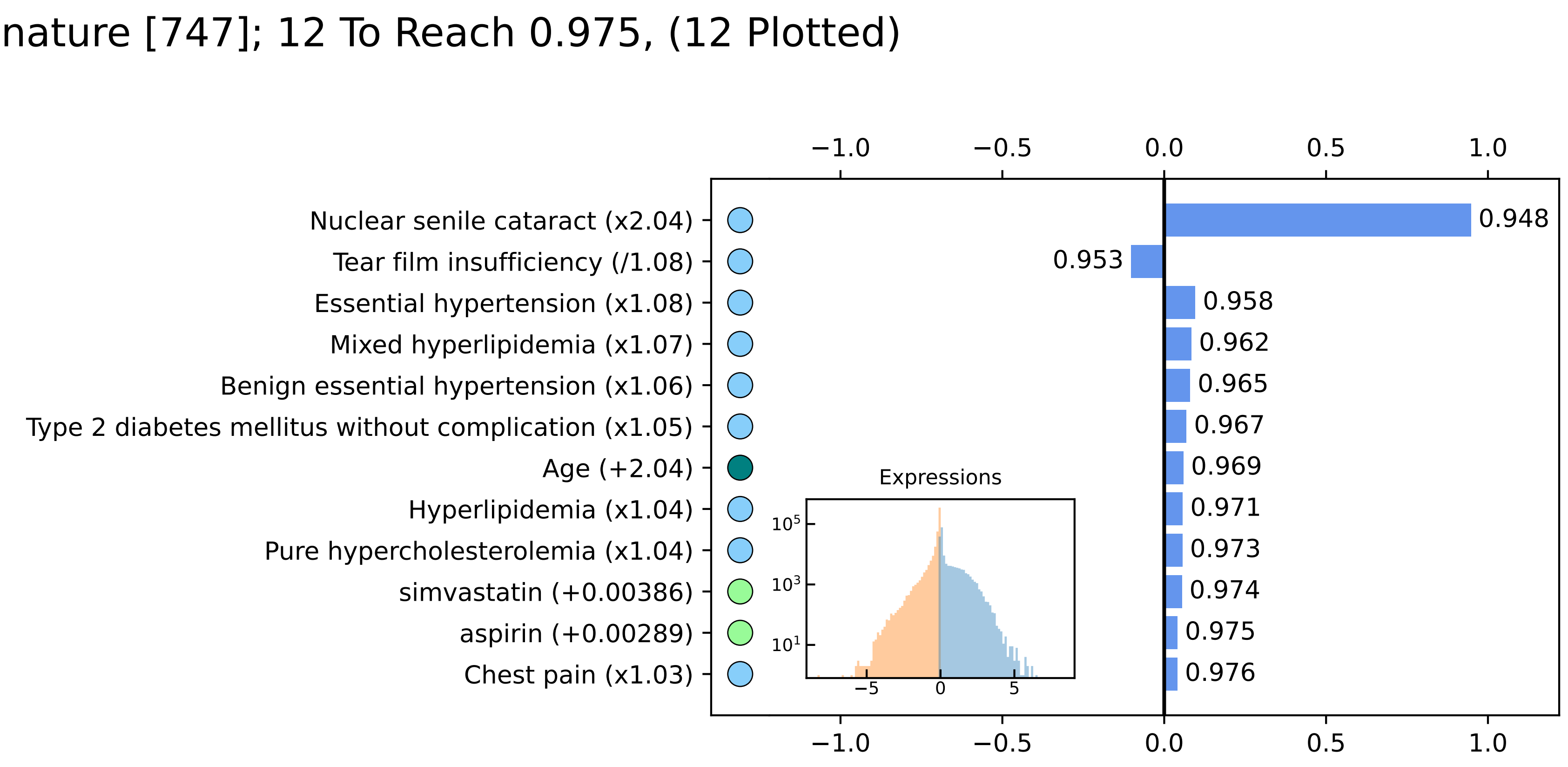

Signature [747]; 12 To Reach 0.975, (12 Plotted)
Nuclear senile cataract (x2.04)
Tear film insufficiency (/1.08)
Essential hypertension (x1.08)
Mixed hyperlipidemia (x1.07)
Benign essential hypertension (x1.06)
Type 2 diabetes mellitus without complication (x1.05)
Age (+2.04)
Hyperlipidemia (x1.04)
Pure hypercholesterolemia (x1.04)
simvastatin (+0.00386)
aspirin (+0.00289)
Chest pain (x1.03)
0.948
0.953
0.958
0.962
0.965
0.967
0.969
0.971
0.973
0.974
0.975
0.976
−1.0
−0.5
0.0
0.5
1.0
Expressions

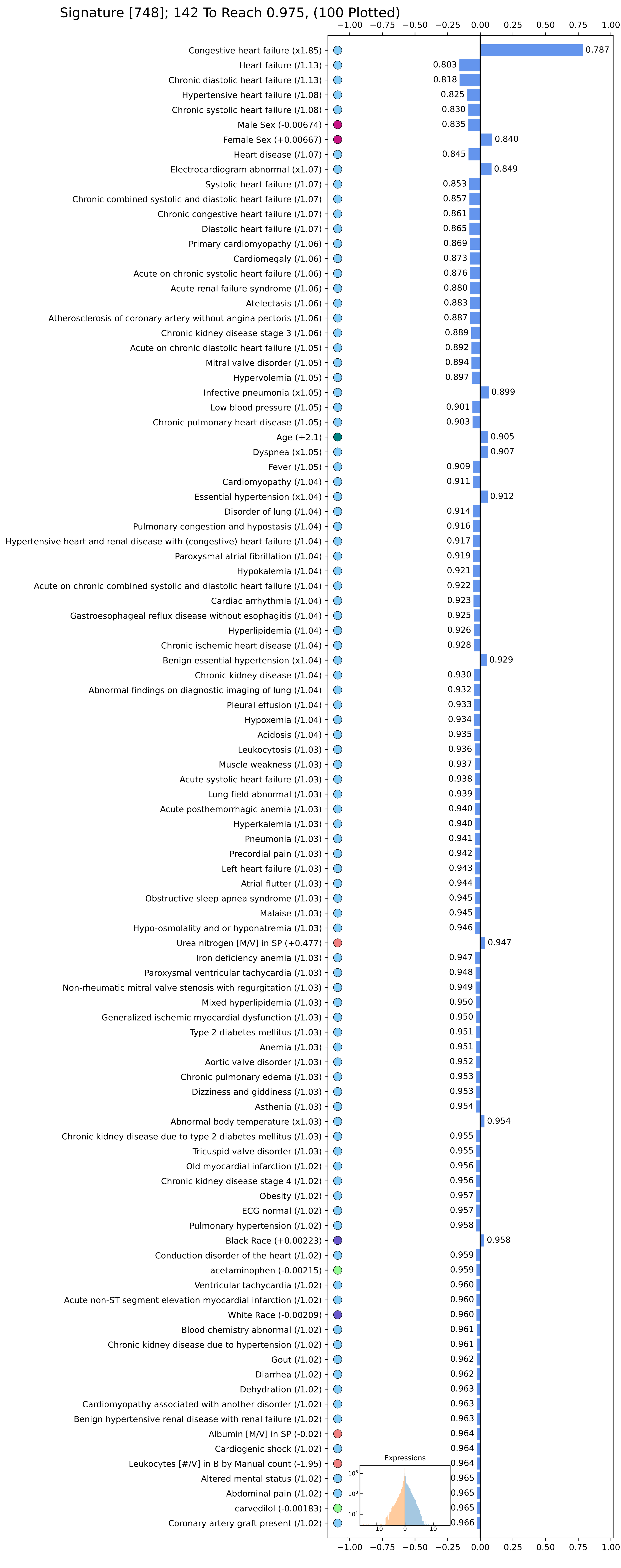

Signature [748]; 142 To Reach 0.975, (100 Plotted)
Congestive heart failure (x1.85) 0.787
Heart failure (/1.13) 0.803
Chronic diastolic heart failure (/1.13) 0.818
Hypertensive heart failure (/1.08) 0.825
Chronic systolic heart failure (/1.08) 0.830
Male Sex (-0.00674) 0.835
Female Sex (+0.00667) 0.840
Heart disease (/1.07) 0.845
Electrocardiogram abnormal (x1.07) 0.849
Systolic heart failure (/1.07) 0.853
Chronic combined systolic and diastolic heart failure (/1.07) 0.857
Chronic congestive heart failure (/1.07) 0.861
Diastolic heart failure (/1.07) 0.865
Primary cardiomyopathy (/1.06) 0.869
Cardiomegaly (/1.06) 0.873
Acute on chronic systolic heart failure (/1.06) 0.876
Acute renal failure syndrome (/1.06) 0.880
Atelectasis (/1.06) 0.883
Atherosclerosis of coronary artery without angina pectoris (/1.06) 0.887
Chronic kidney disease stage 3 (/1.06) 0.889
Acute on chronic diastolic heart failure (/1.05) 0.892
Mitral valve disorder (/1.05) 0.894
Hypervolemia (/1.05) 0.897
Infective pneumonia (x1.05) 0.899
Low blood pressure (/1.05) 0.901
Chronic pulmonary heart disease (/1.05) 0.903
Age (+2.1) 0.905
Dyspnea (x1.05) 0.907
Fever (/1.05) 0.909
Cardiomyopathy (/1.04) 0.911
Essential hypertension (x1.04) 0.912
Disorder of lung (/1.04) 0.914
Pulmonary congestion and hypostasis (/1.04) 0.916
Hypertensive heart and renal disease with (congestive) heart failure (/1.04) 0.917
Paroxysmal atrial fibrillation (/1.04) 0.919
Hypokalemia (/1.04) 0.921
Acute on chronic combined systolic and diastolic heart failure (/1.04) 0.922
Cardiac arrhythmia (/1.04) 0.923
Gastroesophageal reflux disease without esophagitis (/1.04) 0.925
Hyperlipidemia (/1.04) 0.926
Chronic ischemic heart disease (/1.04) 0.928
Benign essential hypertension (x1.04) 0.929
Chronic kidney disease (/1.04) 0.930
Abnormal findings on diagnostic imaging of lung (/1.04) 0.932
Pleural effusion (/1.04) 0.933
Hypoxemia (/1.04) 0.934
Acidosis (/1.04) 0.935
Leukocytosis (/1.03) 0.936
Muscle weakness (/1.03) 0.937
Acute systolic heart failure (/1.03) 0.938
Lung field abnormal (/1.03) 0.939
Acute posthemorrhagic anemia (/1.03) 0.940
Hyperkalemia (/1.03) 0.940
Pneumonia (/1.03) 0.941
Precordial pain (/1.03) 0.942
Left heart failure (/1.03) 0.943
Atrial flutter (/1.03) 0.944
Obstructive sleep apnea syndrome (/1.03) 0.945
Malaise (/1.03) 0.945
Hypo-osmolality and or hyponatremia (/1.03) 0.946
Urea nitrogen [M/V] in SP (+0.477) 0.947
Iron deficiency anemia (/1.03) 0.947
Paroxysmal ventricular tachycardia (/1.03) 0.948
Non-rheumatic mitral valve stenosis with regurgitation (/1.03) 0.949
Mixed hyperlipidemia (/1.03) 0.950
Generalized ischemic myocardial dysfunction (/1.03) 0.950
Type 2 diabetes mellitus (/1.03) 0.951
Anemia (/1.03) 0.951
Aortic valve disorder (/1.03) 0.952
Chronic pulmonary edema (/1.03) 0.953
Dizziness and giddiness (/1.03) 0.953
Asthenia (/1.03) 0.954
Abnormal body temperature (x1.03) 0.954
Chronic kidney disease due to type 2 diabetes mellitus (/1.03) 0.955
Tricuspid valve disorder (/1.03) 0.955
Old myocardial infarction (/1.02) 0.956
Chronic kidney disease stage 4 (/1.02) 0.956
Obesity (/1.02) 0.957
ECG normal (/1.02) 0.957
Pulmonary hypertension (/1.02) 0.958
Black Race (+0.00223) 0.958
Conduction disorder of the heart (/1.02) 0.959
acetaminophen (-0.00215) 0.959
Ventricular tachycardia (/1.02) 0.960
Acute non-ST segment elevation myocardial infarction (/1.02) 0.960
White Race (-0.00209) 0.960
Blood chemistry abnormal (/1.02) 0.961
Chronic kidney disease due to hypertension (/1.02) 0.961
Gout (/1.02) 0.962
Diarrhea (/1.02) 0.962
Dehydration (/1.02) 0.963
Cardiomyopathy associated with another disorder (/1.02) 0.963
Benign hypertensive renal disease with renal failure (/1.02) 0.963
Albumin [M/V] in SP (-0.02) 0.964
Cardiogenic shock (/1.02) 0.964
Leukocytes [#/V] in B by Manual count (-1.95) 0.964
Altered mental status (/1.02) 0.965
Abdominal pain (/1.02) 0.965
carvedilol (-0.00183) 0.965
Coronary artery graft present (/1.02) 0.966
Expressions
−1.00 −0.75 −0.50 −0.25 0.00 0.25 0.50 0.75 1.00

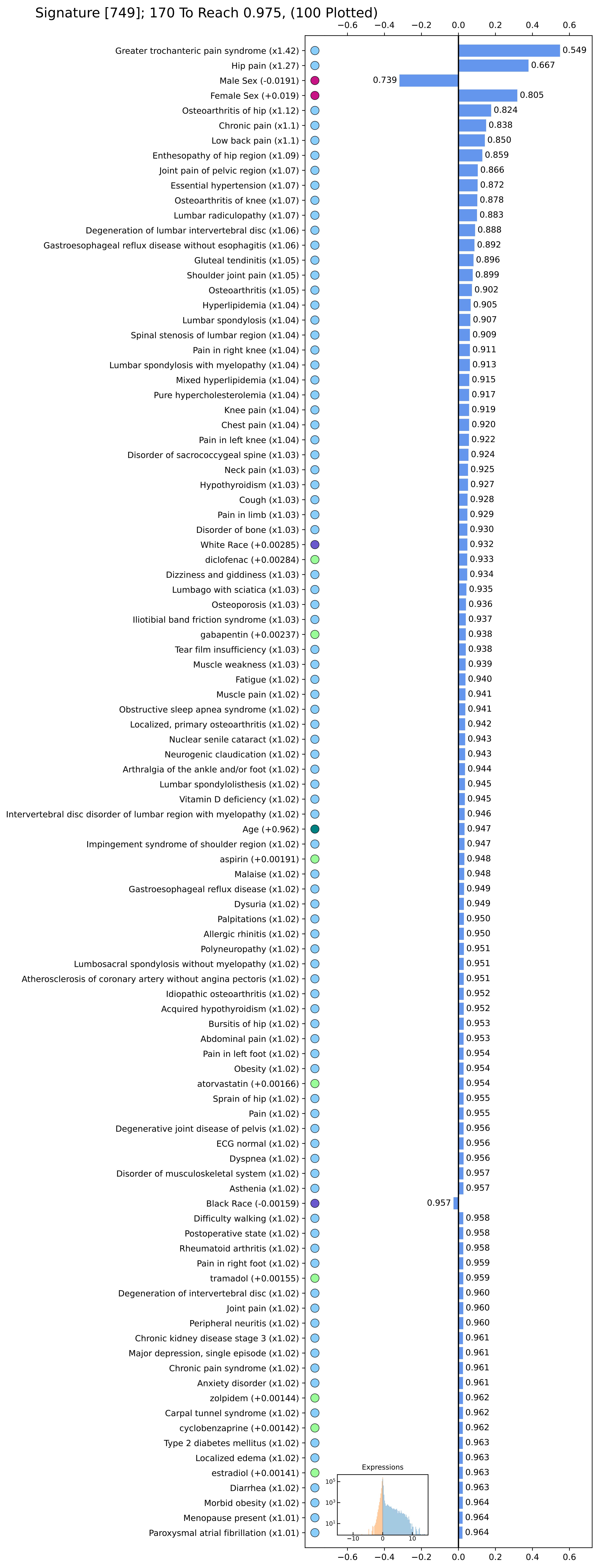
Signature [749]; 170 To Reach 0.975, (100 Plotted)
−0.6 −0.4 −0.2 0.0 0.2 0.4 0.6
Greater trochanteric pain syndrome (x1.42) 0.549
Hip pain (x1.27) 0.667
Male Sex (-0.0191) 0.739
Female Sex (+0.019) 0.805
Osteoarthritis of hip (x1.12) 0.824
Chronic pain (x1.1) 0.838
Low back pain (x1.1) 0.850
Enthesopathy of hip region (x1.09) 0.859
Joint pain of pelvic region (x1.07) 0.866
Essential hypertension (x1.07) 0.872
Osteoarthritis of knee (x1.07) 0.878
Lumbar radiculopathy (x1.07) 0.883
Degeneration of lumbar intervertebral disc (x1.06) 0.888
Gastroesophageal reflux disease without esophagitis (x1.06) 0.892
Gluteal tendinitis (x1.05) 0.896
Shoulder joint pain (x1.05) 0.899
Osteoarthritis (x1.05) 0.902
Hyperlipidemia (x1.04) 0.905
Lumbar spondylosis (x1.04) 0.907
Spinal stenosis of lumbar region (x1.04) 0.909
Pain in right knee (x1.04) 0.911
Lumbar spondylosis with myelopathy (x1.04) 0.913
Mixed hyperlipidemia (x1.04) 0.915
Pure hypercholesterolemia (x1.04) 0.917
Knee pain (x1.04) 0.919
Chest pain (x1.04) 0.920
Pain in left knee (x1.04) 0.922
Disorder of sacrococcygeal spine (x1.03) 0.924
Neck pain (x1.03) 0.925
Hypothyroidism (x1.03) 0.927
Cough (x1.03) 0.928
Pain in limb (x1.03) 0.929
Disorder of bone (x1.03) 0.930
White Race (+0.00285) 0.932
diclofenac (+0.00284) 0.933
Dizziness and giddiness (x1.03) 0.934
Lumbago with sciatica (x1.03) 0.935
Osteoporosis (x1.03) 0.936
Iliotibial band friction syndrome (x1.03) 0.937
gabapentin (+0.00237) 0.938
Tear film insufficiency (x1.03) 0.938
Muscle weakness (x1.03) 0.939
Fatigue (x1.02) 0.940
Muscle pain (x1.02) 0.941
Obstructive sleep apnea syndrome (x1.02) 0.941
Localized, primary osteoarthritis (x1.02) 0.942
Nuclear senile cataract (x1.02) 0.943
Neurogenic claudication (x1.02) 0.943
Arthralgia of the ankle and/or foot (x1.02) 0.944
Lumbar spondylolisthesis (x1.02) 0.945
Vitamin D deficiency (x1.02) 0.945
Intervertebral disc disorder of lumbar region with myelopathy (x1.02) 0.946
Age (+0.962) 0.947
Impingement syndrome of shoulder region (x1.02) 0.947
aspirin (+0.00191) 0.948
Malaise (x1.02) 0.948
Gastroesophageal reflux disease (x1.02) 0.949
Dysuria (x1.02) 0.949
Palpitations (x1.02) 0.950
Allergic rhinitis (x1.02) 0.950
Polyneuropathy (x1.02) 0.951
Lumbosacral spondylosis without myelopathy (x1.02) 0.951
Atherosclerosis of coronary artery without angina pectoris (x1.02) 0.951
Idiopathic osteoarthritis (x1.02) 0.952
Acquired hypothyroidism (x1.02) 0.952
Bursitis of hip (x1.02) 0.953
Abdominal pain (x1.02) 0.953
Pain in left foot (x1.02) 0.954
Obesity (x1.02) 0.954
atorvastatin (+0.00166) 0.954
Sprain of hip (x1.02) 0.955
Pain (x1.02) 0.955
Degenerative joint disease of pelvis (x1.02) 0.956
ECG normal (x1.02) 0.956
Dyspnea (x1.02) 0.956
Disorder of musculoskeletal system (x1.02) 0.957
Asthenia (x1.02) 0.957
Black Race (-0.00159) 0.957
Difficulty walking (x1.02) 0.958
Postoperative state (x1.02) 0.958
Rheumatoid arthritis (x1.02) 0.958
Pain in right foot (x1.02) 0.959
tramadol (+0.00155) 0.959
Degeneration of intervertebral disc (x1.02) 0.960
Joint pain (x1.02) 0.960
Peripheral neuritis (x1.02) 0.960
Chronic kidney disease stage 3 (x1.02) 0.961
Major depression, single episode (x1.02) 0.961
Chronic pain syndrome (x1.02) 0.961
Anxiety disorder (x1.02) 0.961
zolpidem (+0.00144) 0.962
Carpal tunnel syndrome (x1.02) 0.962
cyclobenzaprine (+0.00142) 0.962
Type 2 diabetes mellitus (x1.02) 0.963
Localized edema (x1.02) 0.963
estradiol (+0.00141) 0.963
Diarrhea (x1.02) 0.963
Morbid obesity (x1.02) 0.964
Menopause present (x1.01) 0.964
Paroxysmal atrial fibrillation (x1.01) 0.964
Expressions
10^5
10^3
10^1
−10 0 10
−0.6 −0.4 −0.2 0.0 0.2 0.4 0.6

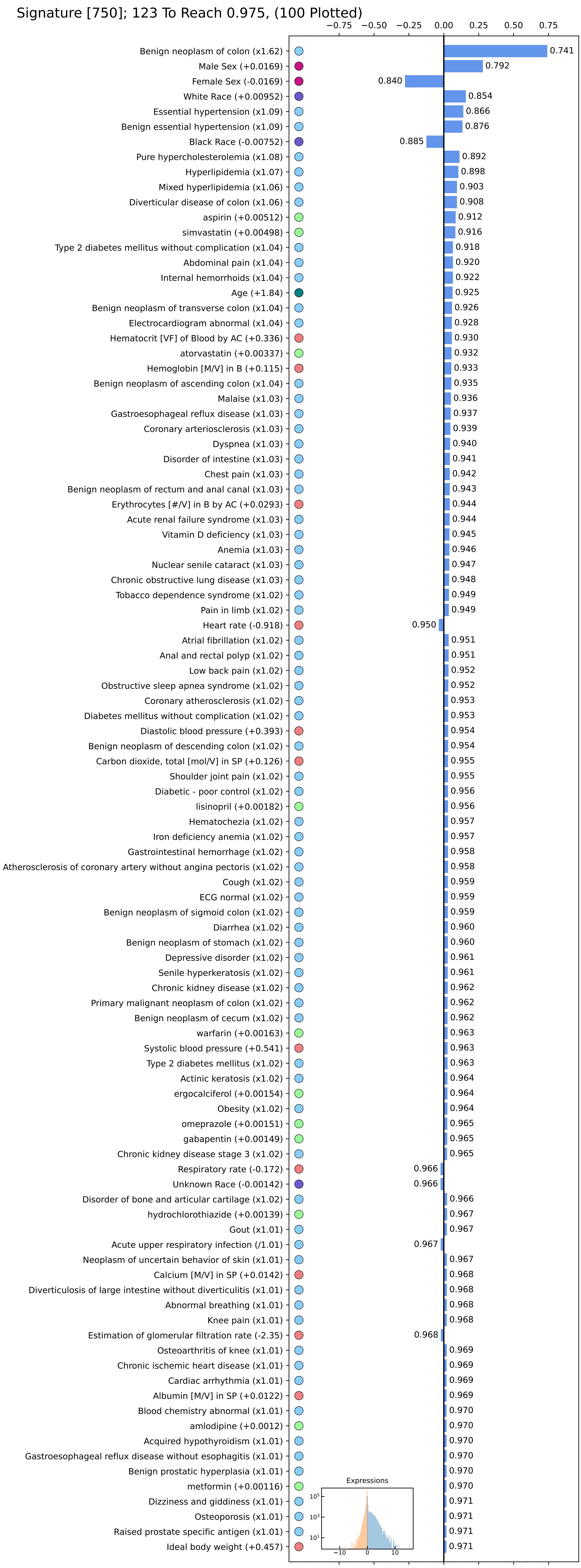

Signature [750]; 123 To Reach 0.975, (100 Plotted)
Benign neoplasm of colon (x1.62) 0.741
Male Sex (+0.0169) 0.792
Female Sex (-0.0169) 0.840
White Race (+0.00952) 0.854
Essential hypertension (x1.09) 0.866
Benign essential hypertension (x1.09) 0.876
Black Race (-0.00752) 0.885
Pure hypercholesterolemia (x1.08) 0.892
Hyperlipidemia (x1.07) 0.898
Mixed hyperlipidemia (x1.06) 0.903
Diverticular disease of colon (x1.06) 0.908
aspirin (+0.00512) 0.912
simvastatin (+0.00498) 0.916
Type 2 diabetes mellitus without complication (x1.04) 0.918
Abdominal pain (x1.04) 0.920
Internal hemorrhoids (x1.04) 0.922
Age (+1.84) 0.925
Benign neoplasm of transverse colon (x1.04) 0.926
Electrocardiogram abnormal (x1.04) 0.928
Hematocrit [VF] of Blood by AC (+0.336) 0.930
atorvastatin (+0.00337) 0.932
Hemoglobin [M/V] in B (+0.115) 0.933
Benign neoplasm of ascending colon (x1.04) 0.935
Malaise (x1.03) 0.936
Gastroesophageal reflux disease (x1.03) 0.937
Coronary arteriosclerosis (x1.03) 0.939
Dyspnea (x1.03) 0.940
Disorder of intestine (x1.03) 0.941
Chest pain (x1.03) 0.942
Benign neoplasm of rectum and anal canal (x1.03) 0.943
Erythrocytes [#/V] in B by AC (+0.0293) 0.944
Acute renal failure syndrome (x1.03) 0.944
Vitamin D deficiency (x1.03) 0.945
Anemia (x1.03) 0.946
Nuclear senile cataract (x1.03) 0.947
Chronic obstructive lung disease (x1.03) 0.948
Tobacco dependence syndrome (x1.02) 0.949
Pain in limb (x1.02) 0.949
Heart rate (-0.918) 0.950
Atrial fibrillation (x1.02) 0.951
Anal and rectal polyp (x1.02) 0.951
Low back pain (x1.02) 0.952
Obstructive sleep apnea syndrome (x1.02) 0.952
Coronary atherosclerosis (x1.02) 0.953
Diabetes mellitus without complication (x1.02) 0.953
Diastolic blood pressure (+0.393) 0.954
Benign neoplasm of descending colon (x1.02) 0.954
Carbon dioxide, total [mol/V] in SP (+0.126) 0.955
Shoulder joint pain (x1.02) 0.955
Diabetic - poor control (x1.02) 0.956
lisinopril (+0.00182) 0.956
Hematochezia (x1.02) 0.957
Iron deficiency anemia (x1.02) 0.957
Gastrointestinal hemorrhage (x1.02) 0.958
Atherosclerosis of coronary artery without angina pectoris (x1.02) 0.958
Cough (x1.02) 0.959
ECG normal (x1.02) 0.959
Benign neoplasm of sigmoid colon (x1.02) 0.959
Diarrhea (x1.02) 0.960
Benign neoplasm of stomach (x1.02) 0.960
Depressive disorder (x1.02) 0.961
Senile hyperkeratosis (x1.02) 0.961
Chronic kidney disease (x1.02) 0.962
Primary malignant neoplasm of colon (x1.02) 0.962
Benign neoplasm of cecum (x1.02) 0.962
warfarin (+0.00163) 0.963
Systolic blood pressure (+0.541) 0.963
Type 2 diabetes mellitus (x1.02) 0.963
Actinic keratosis (x1.02) 0.964
ergocalciferol (+0.00154) 0.964
Obesity (x1.02) 0.964
omeprazole (+0.00151) 0.965
gabapentin (+0.00149) 0.965
Chronic kidney disease stage 3 (x1.02) 0.965
Respiratory rate (-0.172) 0.966
Unknown Race (-0.00142) 0.966
Disorder of bone and articular cartilage (x1.02) 0.966
hydrochlorothiazide (+0.00139) 0.967
Gout (x1.01) 0.967
Acute upper respiratory infection (/1.01) 0.967
Neoplasm of uncertain behavior of skin (x1.01) 0.967
Calcium [M/V] in SP (+0.0142) 0.968
Diverticulosis of large intestine without diverticulitis (x1.01) 0.968
Abnormal breathing (x1.01) 0.968
Knee pain (x1.01) 0.968
Estimation of glomerular filtration rate (-2.35) 0.968
Osteoarthritis of knee (x1.01) 0.969
Chronic ischemic heart disease (x1.01) 0.969
Cardiac arrhythmia (x1.01) 0.969
Albumin [M/V] in SP (+0.0122) 0.969
Blood chemistry abnormal (x1.01) 0.970
amlodipine (+0.0012) 0.970
Acquired hypothyroidism (x1.01) 0.970
Gastroesophageal reflux disease without esophagitis (x1.01) 0.970
Benign prostatic hyperplasia (x1.01) 0.970
metformin (+0.00116) 0.970
Dizziness and giddiness (x1.01) 0.971
Osteoporosis (x1.01) 0.971
Raised prostate specific antigen (x1.01) 0.971
Ideal body weight (+0.457) 0.971
−0.75 −0.50 −0.25 0.00 0.25 0.50 0.75
Expressions

Signature [751]; 27 To Reach 0.975, (27 Plotted)

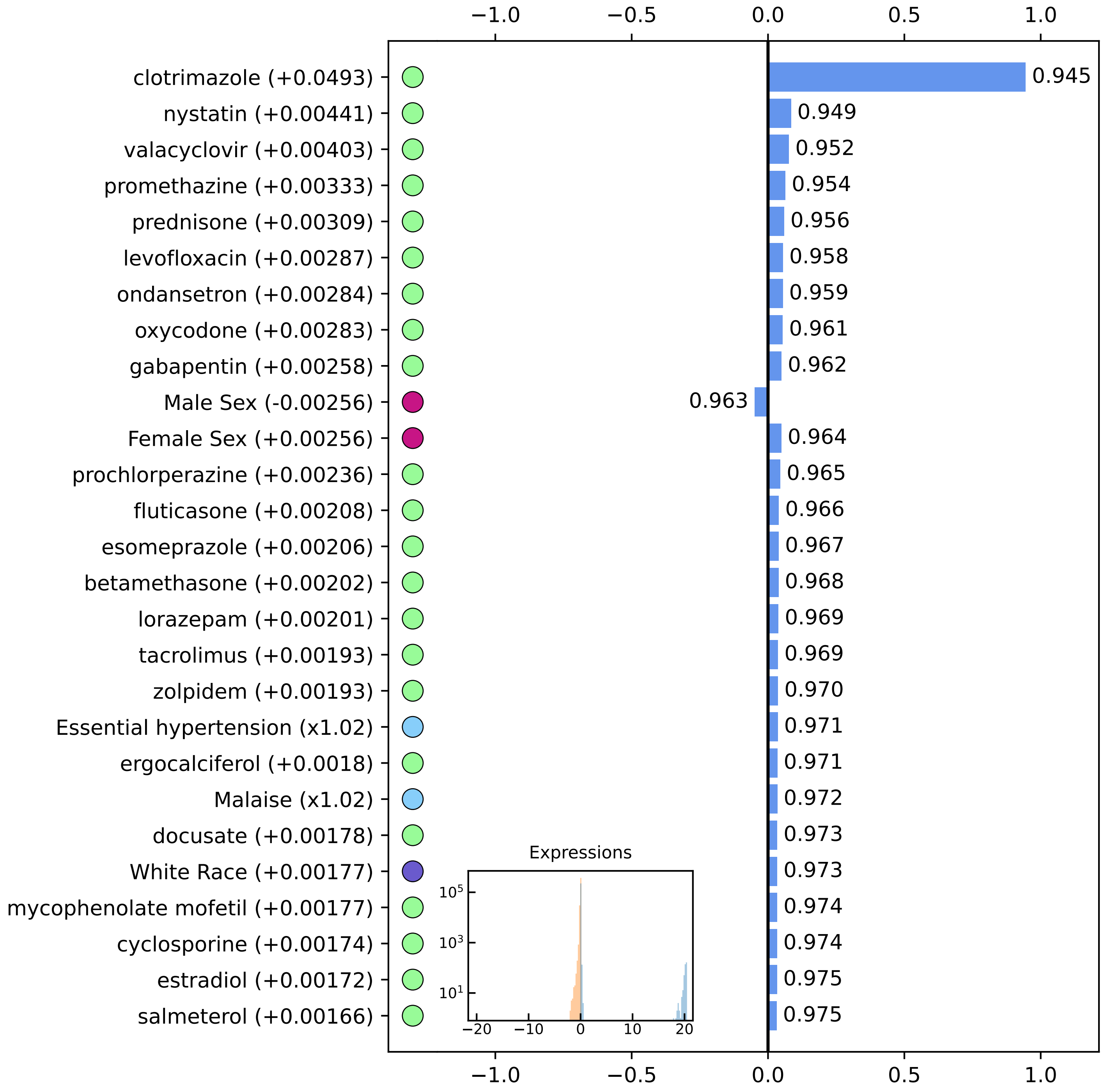

Signature [752]; 1 To Reach 0.975, (10 Plotted)

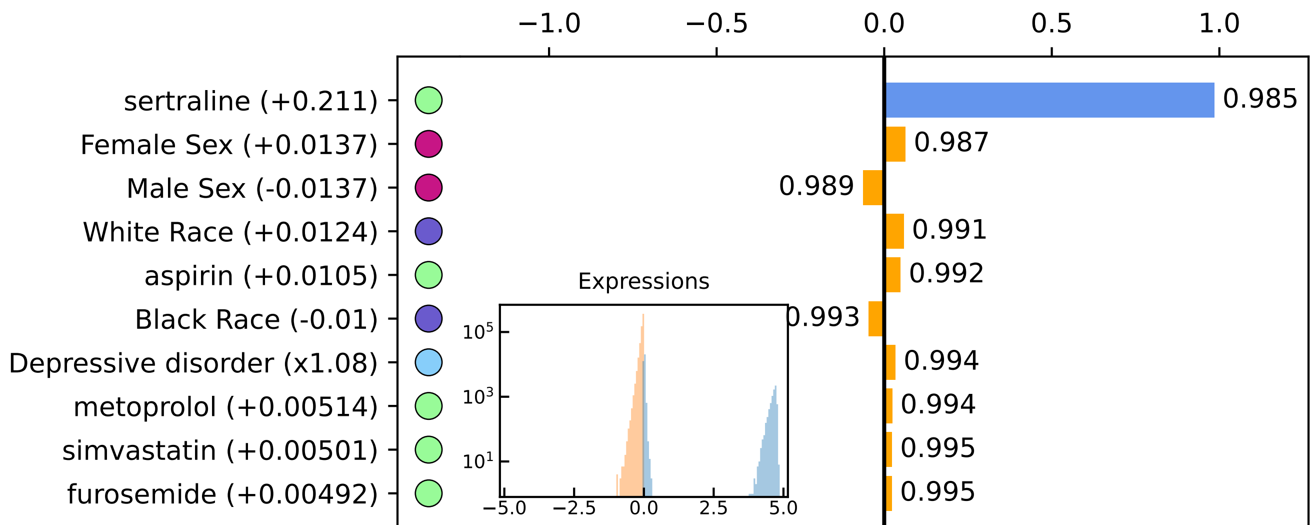

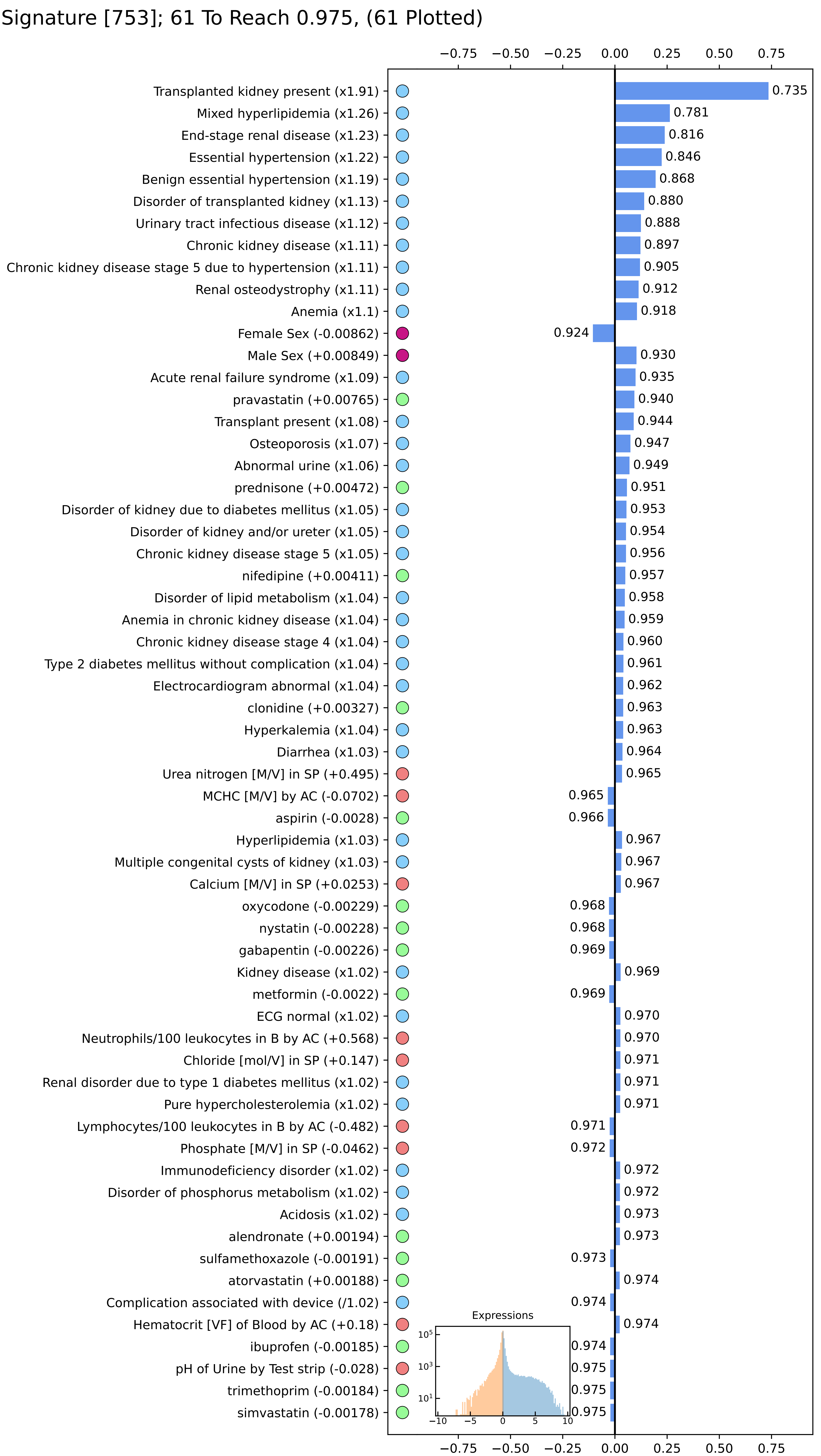
Signature [753]; 61 To Reach 0.975, (61 Plotted)
Transplanted kidney present (x1.91) 0.735
Mixed hyperlipidemia (x1.26) 0.781
End-stage renal disease (x1.23) 0.816
Essential hypertension (x1.22) 0.846
Benign essential hypertension (x1.19) 0.868
Disorder of transplanted kidney (x1.13) 0.880
Urinary tract infectious disease (x1.12) 0.888
Chronic kidney disease (x1.11) 0.897
Chronic kidney disease stage 5 due to hypertension (x1.11) 0.905
Renal osteodystrophy (x1.11) 0.912
Anemia (x1.1) 0.918
Female Sex (-0.00862) 0.924
Male Sex (+0.00849) 0.930
Acute renal failure syndrome (x1.09) 0.935
pravastatin (+0.00765) 0.940
Transplant present (x1.08) 0.944
Osteoporosis (x1.07) 0.947
Abnormal urine (x1.06) 0.949
prednisone (+0.00472) 0.951
Disorder of kidney due to diabetes mellitus (x1.05) 0.953
Disorder of kidney and/or ureter (x1.05) 0.954
Chronic kidney disease stage 5 (x1.05) 0.956
nifedipine (+0.00411) 0.957
Disorder of lipid metabolism (x1.04) 0.958
Anemia in chronic kidney disease (x1.04) 0.959
Chronic kidney disease stage 4 (x1.04) 0.960
Type 2 diabetes mellitus without complication (x1.04) 0.961
Electrocardiogram abnormal (x1.04) 0.962
clonidine (+0.00327) 0.963
Hyperkalemia (x1.04) 0.963
Diarrhea (x1.03) 0.964
Urea nitrogen [M/V] in SP (+0.495) 0.965
MCHC [M/V] by AC (-0.0702) 0.965
aspirin (-0.0028) 0.966
Hyperlipidemia (x1.03) 0.967
Multiple congenital cysts of kidney (x1.03) 0.967
Calcium [M/V] in SP (+0.0253) 0.967
oxycodone (-0.00229) 0.968
nystatin (-0.00228) 0.968
gabapentin (-0.00226) 0.969
Kidney disease (x1.02) 0.969
metformin (-0.0022) 0.969
ECG normal (x1.02) 0.970
Neutrophils/100 leukocytes in B by AC (+0.568) 0.970
Chloride [mol/V] in SP (+0.147) 0.971
Renal disorder due to type 1 diabetes mellitus (x1.02) 0.971
Pure hypercholesterolemia (x1.02) 0.971
Lymphocytes/100 leukocytes in B by AC (-0.482) 0.971
Phosphate [M/V] in SP (-0.0462) 0.972
Immunodeficiency disorder (x1.02) 0.972
Disorder of phosphorus metabolism (x1.02) 0.972
Acidosis (x1.02) 0.973
alendronate (+0.00194) 0.973
sulfamethoxazole (-0.00191) 0.973
atorvastatin (+0.00188) 0.974
Complication associated with device (/1.02) 0.974
Hematocrit [VF] of Blood by AC (+0.18) 0.974
ibuprofen (-0.00185) 0.974
pH of Urine by Test strip (-0.028) 0.975
trimethoprim (-0.00184) 0.975
simvastatin (-0.00178) 0.975
−0.75 −0.50 −0.25 0.00 0.25 0.50 0.75
Expressions
−10 −5 0 5 10

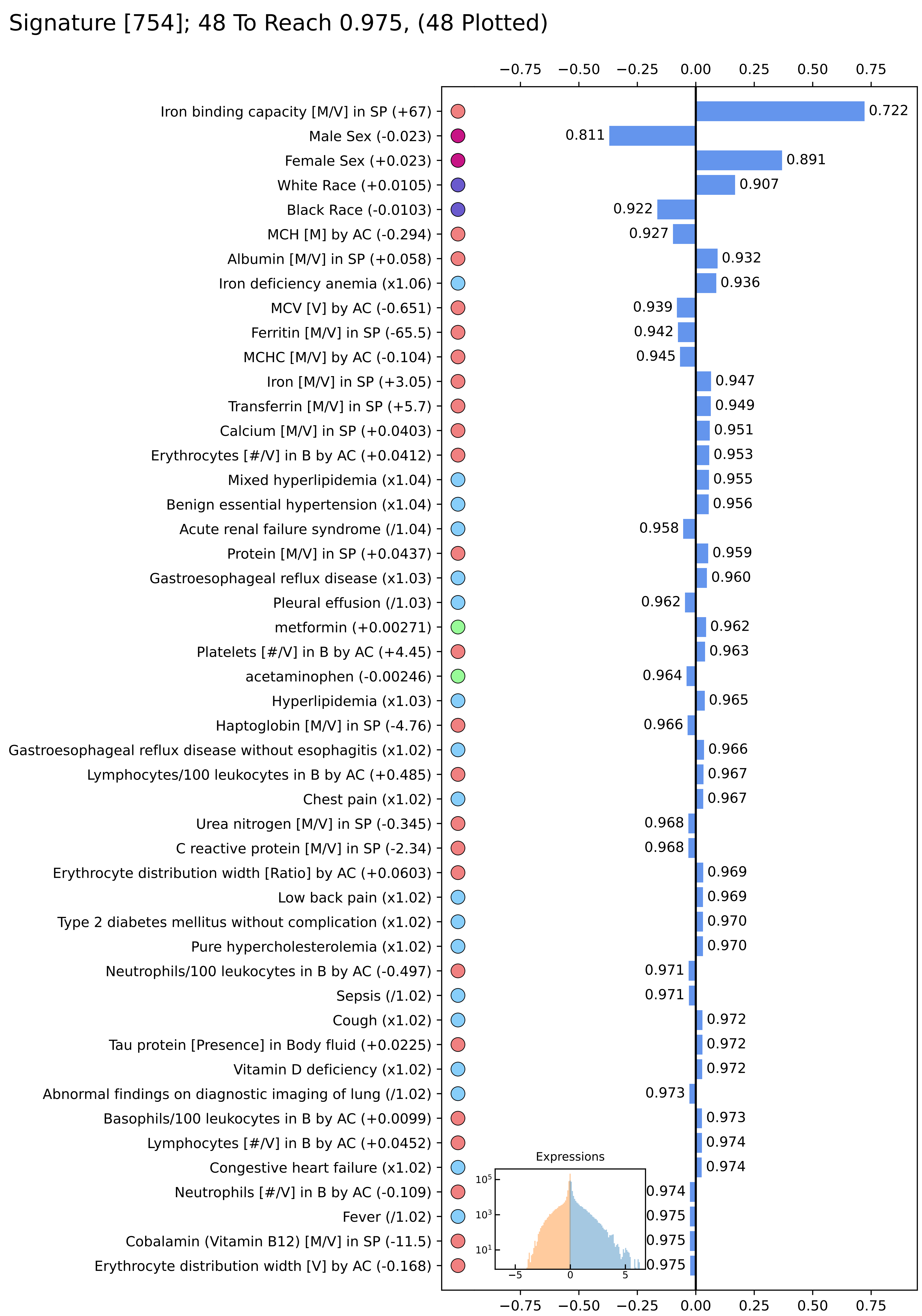

Signature [754]; 48 To Reach 0.975, (48 Plotted)
Iron binding capacity [M/V] in SP (+67) 0.722
Male Sex (-0.023) 0.811
Female Sex (+0.023) 0.891
White Race (+0.0105) 0.907
Black Race (-0.0103) 0.922
MCH [M] by AC (-0.294) 0.927
Albumin [M/V] in SP (+0.058) 0.932
Iron deficiency anemia (x1.06) 0.936
MCV [V] by AC (-0.651) 0.939
Ferritin [M/V] in SP (-65.5) 0.942
MCHC [M/V] by AC (-0.104) 0.945
Iron [M/V] in SP (+3.05) 0.947
Transferrin [M/V] in SP (+5.7) 0.949
Calcium [M/V] in SP (+0.0403) 0.951
Erythrocytes [#/V] in B by AC (+0.0412) 0.953
Mixed hyperlipidemia (x1.04) 0.955
Benign essential hypertension (x1.04) 0.956
Acute renal failure syndrome (/1.04) 0.958
Protein [M/V] in SP (+0.0437) 0.959
Gastroesophageal reflux disease (x1.03) 0.960
Pleural effusion (/1.03) 0.962
metformin (+0.00271) 0.962
Platelets [#/V] in B by AC (+4.45) 0.963
acetaminophen (-0.00246) 0.964
Hyperlipidemia (x1.03) 0.965
Haptoglobin [M/V] in SP (-4.76) 0.966
Gastroesophageal reflux disease without esophagitis (x1.02) 0.966
Lymphocytes/100 leukocytes in B by AC (+0.485) 0.967
Chest pain (x1.02) 0.967
Urea nitrogen [M/V] in SP (-0.345) 0.968
C reactive protein [M/V] in SP (-2.34) 0.968
Erythrocyte distribution width [Ratio] by AC (+0.0603) 0.969
Low back pain (x1.02) 0.969
Type 2 diabetes mellitus without complication (x1.02) 0.970
Pure hypercholesterolemia (x1.02) 0.970
Neutrophils/100 leukocytes in B by AC (-0.497) 0.971
Sepsis (/1.02) 0.971
Cough (x1.02) 0.972
Tau protein [Presence] in Body fluid (+0.0225) 0.972
Vitamin D deficiency (x1.02) 0.972
Abnormal findings on diagnostic imaging of lung (/1.02) 0.973
Basophils/100 leukocytes in B by AC (+0.0099) 0.973
Lymphocytes [#/V] in B by AC (+0.0452) 0.974
Congestive heart failure (x1.02) 0.974
Neutrophils [#/V] in B by AC (-0.109) 0.974
Fever (/1.02) 0.975
Cobalamin (Vitamin B12) [M/V] in SP (-11.5) 0.975
Erythrocyte distribution width [V] by AC (-0.168) 0.975
−0.75 −0.50 −0.25 0.00 0.25 0.50 0.75
Expressions

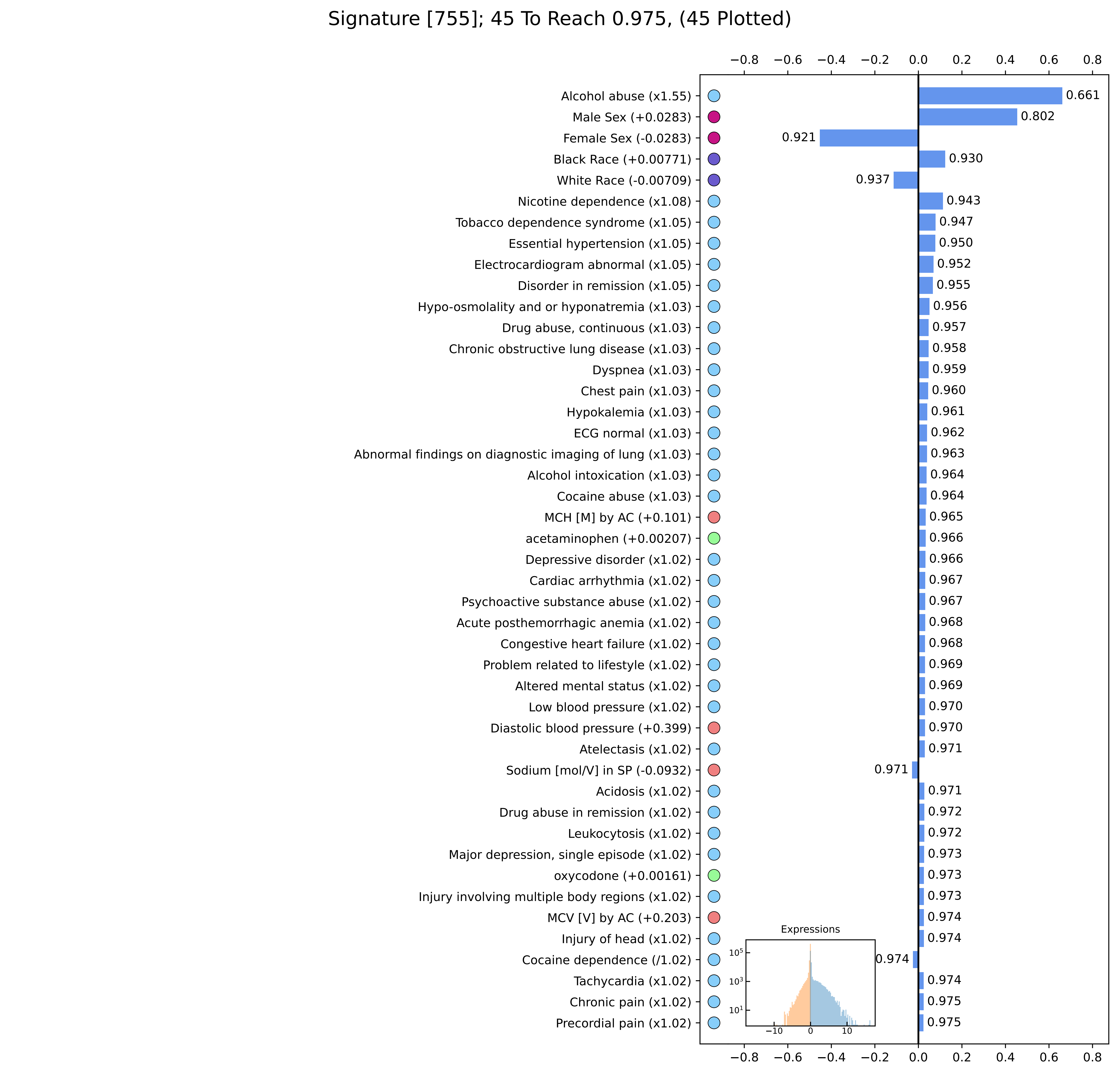

Signature [755]; 45 To Reach 0.975, (45 Plotted)
Alcohol abuse (x1.55) 0.661
Male Sex (+0.0283) 0.802
Female Sex (-0.0283) 0.921
Black Race (+0.00771) 0.930
White Race (-0.00709) 0.937
Nicotine dependence (x1.08) 0.943
Tobacco dependence syndrome (x1.05) 0.947
Essential hypertension (x1.05) 0.950
Electrocardiogram abnormal (x1.05) 0.952
Disorder in remission (x1.05) 0.955
Hypo-osmolality and or hyponatremia (x1.03) 0.956
Drug abuse, continuous (x1.03) 0.957
Chronic obstructive lung disease (x1.03) 0.958
Dyspnea (x1.03) 0.959
Chest pain (x1.03) 0.960
Hypokalemia (x1.03) 0.961
ECG normal (x1.03) 0.962
Abnormal findings on diagnostic imaging of lung (x1.03) 0.963
Alcohol intoxication (x1.03) 0.964
Cocaine abuse (x1.03) 0.964
MCH [M] by AC (+0.101) 0.965
acetaminophen (+0.00207) 0.966
Depressive disorder (x1.02) 0.966
Cardiac arrhythmia (x1.02) 0.967
Psychoactive substance abuse (x1.02) 0.967
Acute posthemorrhagic anemia (x1.02) 0.968
Congestive heart failure (x1.02) 0.968
Problem related to lifestyle (x1.02) 0.969
Altered mental status (x1.02) 0.969
Low blood pressure (x1.02) 0.970
Diastolic blood pressure (+0.399) 0.970
Atelectasis (x1.02) 0.971
Sodium [mol/V] in SP (-0.0932) 0.971
Acidosis (x1.02) 0.971
Drug abuse in remission (x1.02) 0.972
Leukocytosis (x1.02) 0.972
Major depression, single episode (x1.02) 0.973
oxycodone (+0.00161) 0.973
Injury involving multiple body regions (x1.02) 0.973
MCV [V] by AC (+0.203) 0.974
Injury of head (x1.02) 0.974
Cocaine dependence (/1.02) 0.974
Tachycardia (x1.02) 0.974
Chronic pain (x1.02) 0.975
Precordial pain (x1.02) 0.975
−0.8 −0.6 −0.4 −0.2 0.0 0.2 0.4 0.6 0.8
Expressions
10^5
10^3
10^1
−10 0 10

Signature [756]; 36 To Reach 0.975, (36 Plotted)

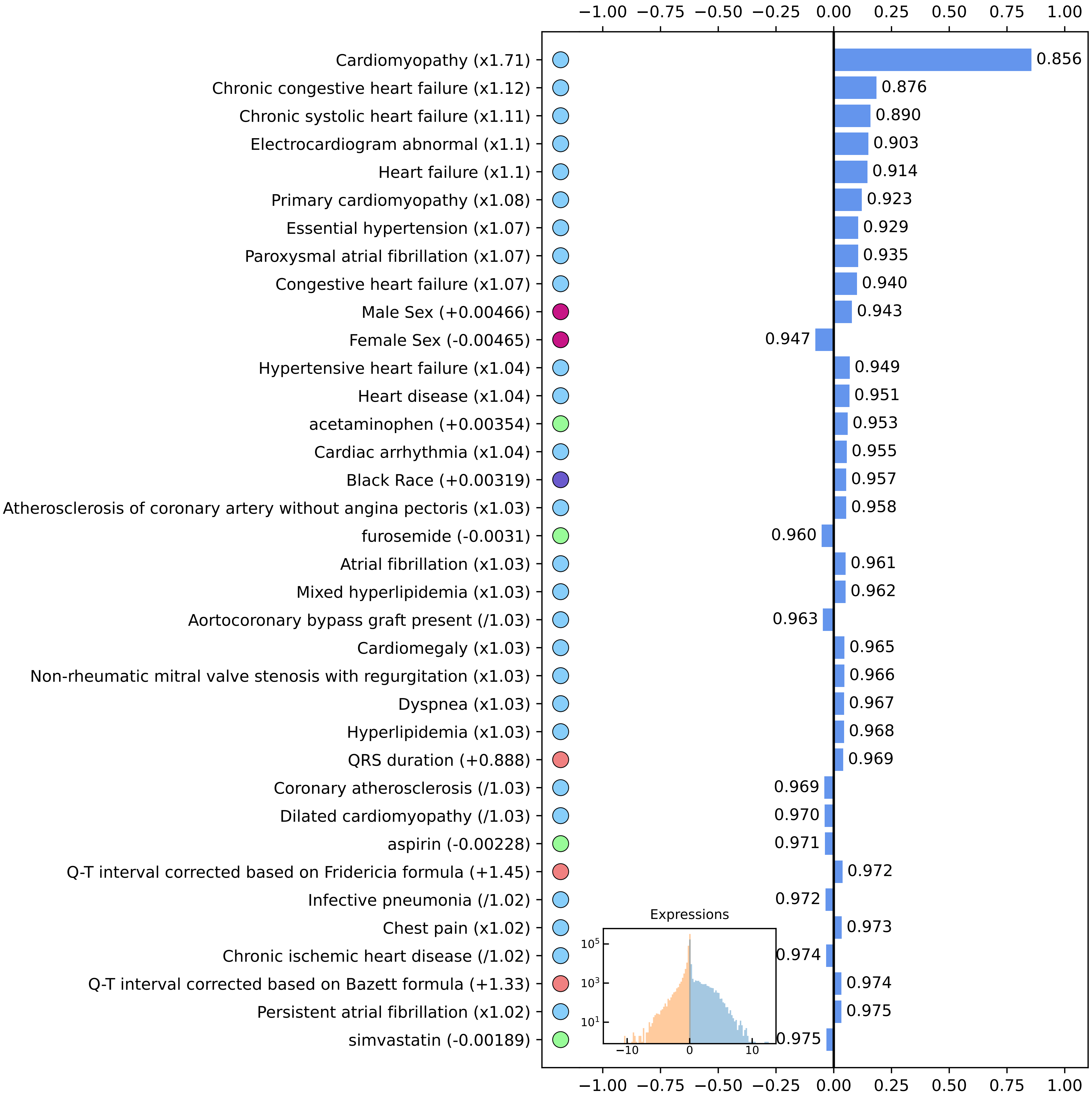

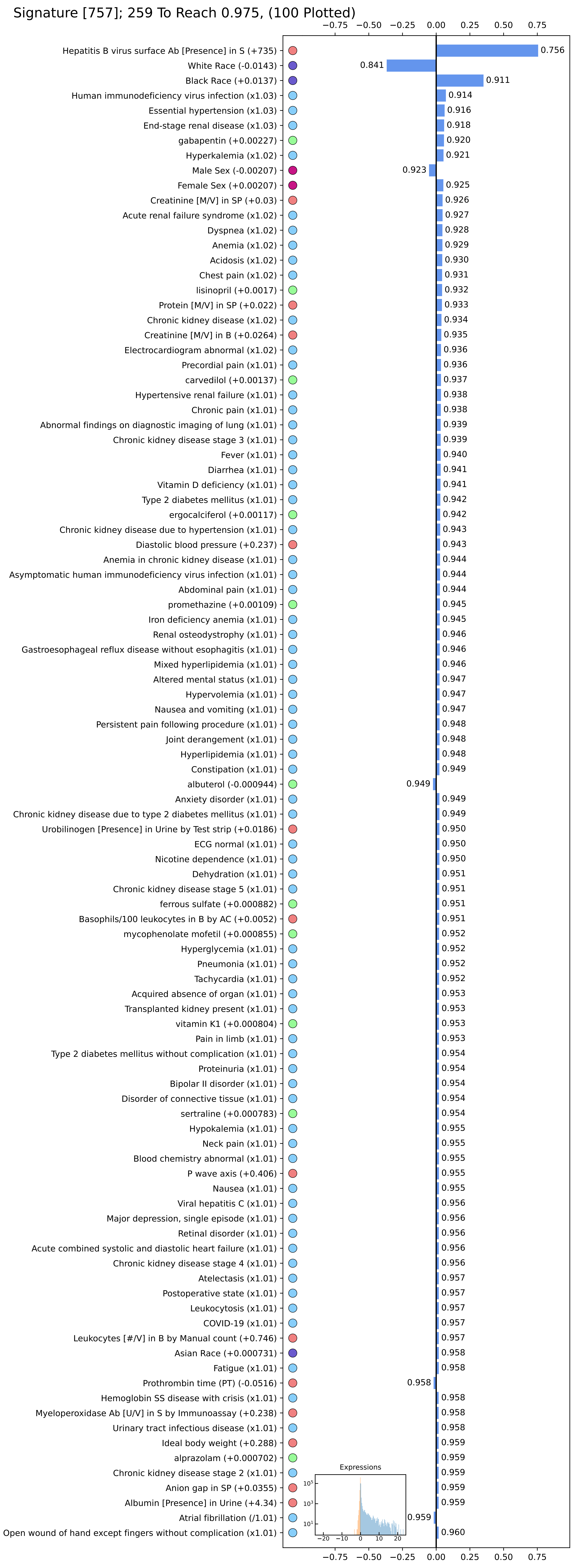

Signature [757]; 259 To Reach 0.975, (100 Plotted)
Hepatitis B virus surface Ab [Presence] in S (+735)
White Race (-0.0143)
Black Race (+0.0137)
Human immunodeficiency virus infection (x1.03)
Essential hypertension (x1.03)
End-stage renal disease (x1.03)
gabapentin (+0.00227)
Hyperkalemia (x1.02)
Male Sex (-0.00207)
Female Sex (+0.00207)
Creatinine [M/V] in SP (+0.03)
Acute renal failure syndrome (x1.02)
Dyspnea (x1.02)
Anemia (x1.02)
Acidosis (x1.02)
Chest pain (x1.02)
lisinopril (+0.0017)
Protein [M/V] in SP (+0.022)
Chronic kidney disease (x1.02)
Creatinine [M/V] in B (+0.0264)
Electrocardiogram abnormal (x1.02)
Precordial pain (x1.01)
carvedilol (+0.00137)
Hypertensive renal failure (x1.01)
Chronic pain (x1.01)
Abnormal findings on diagnostic imaging of lung (x1.01)
Chronic kidney disease stage 3 (x1.01)
Fever (x1.01)
Diarrhea (x1.01)
Vitamin D deficiency (x1.01)
Type 2 diabetes mellitus (x1.01)
ergocalciferol (+0.00117)
Chronic kidney disease due to hypertension (x1.01)
Diastolic blood pressure (+0.237)
Anemia in chronic kidney disease (x1.01)
Asymptomatic human immunodeficiency virus infection (x1.01)
Abdominal pain (x1.01)
promethazine (+0.00109)
Iron deficiency anemia (x1.01)
Renal osteodystrophy (x1.01)
Gastroesophageal reflux disease without esophagitis (x1.01)
Mixed hyperlipidemia (x1.01)
Altered mental status (x1.01)
Hypervolemia (x1.01)
Nausea and vomiting (x1.01)
Persistent pain following procedure (x1.01)
Joint derangement (x1.01)
Hyperlipidemia (x1.01)
Constipation (x1.01)
albuterol (-0.000944)
Anxiety disorder (x1.01)
Chronic kidney disease due to type 2 diabetes mellitus (x1.01)
Urobilinogen [Presence] in Urine by Test strip (+0.0186)
ECG normal (x1.01)
Nicotine dependence (x1.01)
Dehydration (x1.01)
Chronic kidney disease stage 5 (x1.01)
ferrous sulfate (+0.000882)
Basophils/100 leukocytes in B by AC (+0.0052)
mycophenolate mofetil (+0.000855)
Hyperglycemia (x1.01)
Pneumonia (x1.01)
Tachycardia (x1.01)
Acquired absence of organ (x1.01)
Transplanted kidney present (x1.01)
vitamin K1 (+0.000804)
Pain in limb (x1.01)
Type 2 diabetes mellitus without complication (x1.01)
Proteinuria (x1.01)
Bipolar II disorder (x1.01)
Disorder of connective tissue (x1.01)
sertraline (+0.000783)
Hypokalemia (x1.01)
Neck pain (x1.01)
Blood chemistry abnormal (x1.01)
P wave axis (+0.406)
Nausea (x1.01)
Viral hepatitis C (x1.01)
Major depression, single episode (x1.01)
Retinal disorder (x1.01)
Acute combined systolic and diastolic heart failure (x1.01)
Chronic kidney disease stage 4 (x1.01)
Atelectasis (x1.01)
Postoperative state (x1.01)
Leukocytosis (x1.01)
COVID-19 (x1.01)
Leukocytes [#/V] in B by Manual count (+0.746)
Asian Race (+0.000731)
Fatigue (x1.01)
Prothrombin time (PT) (-0.0516)
Hemoglobin SS disease with crisis (x1.01)
Myeloperoxidase Ab [U/V] in S by Immunoassay (+0.238)
Urinary tract infectious disease (x1.01)
Ideal body weight (+0.288)
alprazolam (+0.000702)
Chronic kidney disease stage 2 (x1.01)
Anion gap in SP (+0.0355)
Albumin [Presence] in Urine (+4.34)
Atrial fibrillation (/1.01)
Open wound of hand except fingers without complication (x1.01)
0.756
0.841
0.911
0.914
0.916
0.918
0.920
0.921
0.923
0.925
0.926
0.927
0.928
0.929
0.930
0.931
0.932
0.933
0.934
0.935
0.936
0.936
0.937
0.938
0.938
0.939
0.939
0.940
0.941
0.941
0.942
0.942
0.943
0.943
0.944
0.944
0.944
0.945
0.945
0.946
0.946
0.946
0.947
0.947
0.947
0.948
0.948
0.948
0.949
0.949
0.949
0.949
0.950
0.950
0.950
0.951
0.951
0.951
0.951
0.952
0.952
0.952
0.952
0.953
0.953
0.953
0.953
0.954
0.954
0.954
0.954
0.954
0.955
0.955
0.955
0.955
0.955
0.956
0.956
0.956
0.956
0.956
0.957
0.957
0.957
0.957
0.957
0.958
0.958
0.958
0.958
0.958
0.958
0.959
0.959
0.959
0.959
0.959
0.959
0.960
−0.75 −0.50 −0.25 0.00 0.25 0.50 0.75
Expressions

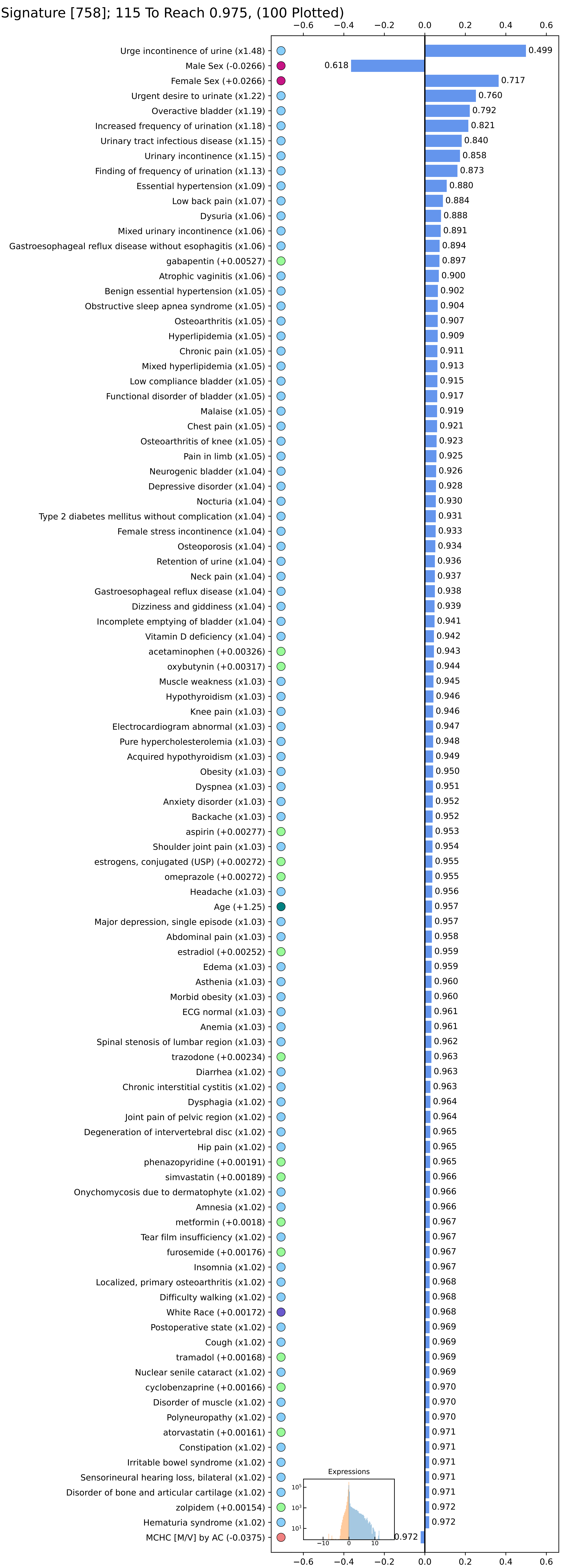

Signature [758]; 115 To Reach 0.975, (100 Plotted)
Urge incontinence of urine (x1.48) 0.499
Male Sex (-0.0266) 0.618
Female Sex (+0.0266) 0.717
Urgent desire to urinate (x1.22) 0.760
Overactive bladder (x1.19) 0.792
Increased frequency of urination (x1.18) 0.821
Urinary tract infectious disease (x1.15) 0.840
Urinary incontinence (x1.15) 0.858
Finding of frequency of urination (x1.13) 0.873
Essential hypertension (x1.09) 0.880
Low back pain (x1.07) 0.884
Dysuria (x1.06) 0.888
Mixed urinary incontinence (x1.06) 0.891
Gastroesophageal reflux disease without esophagitis (x1.06) 0.894
gabapentin (+0.00527) 0.897
Atrophic vaginitis (x1.06) 0.900
Benign essential hypertension (x1.05) 0.902
Obstructive sleep apnea syndrome (x1.05) 0.904
Osteoarthritis (x1.05) 0.907
Hyperlipidemia (x1.05) 0.909
Chronic pain (x1.05) 0.911
Mixed hyperlipidemia (x1.05) 0.913
Low compliance bladder (x1.05) 0.915
Functional disorder of bladder (x1.05) 0.917
Malaise (x1.05) 0.919
Chest pain (x1.05) 0.921
Osteoarthritis of knee (x1.05) 0.923
Pain in limb (x1.05) 0.925
Neurogenic bladder (x1.04) 0.926
Depressive disorder (x1.04) 0.928
Nocturia (x1.04) 0.930
Type 2 diabetes mellitus without complication (x1.04) 0.931
Female stress incontinence (x1.04) 0.933
Osteoporosis (x1.04) 0.934
Retention of urine (x1.04) 0.936
Neck pain (x1.04) 0.937
Gastroesophageal reflux disease (x1.04) 0.938
Dizziness and giddiness (x1.04) 0.939
Incomplete emptying of bladder (x1.04) 0.941
Vitamin D deficiency (x1.04) 0.942
acetaminophen (+0.00326) 0.943
oxybutynin (+0.00317) 0.944
Muscle weakness (x1.03) 0.945
Hypothyroidism (x1.03) 0.946
Knee pain (x1.03) 0.946
Electrocardiogram abnormal (x1.03) 0.947
Pure hypercholesterolemia (x1.03) 0.948
Acquired hypothyroidism (x1.03) 0.949
Obesity (x1.03) 0.950
Dyspnea (x1.03) 0.951
Anxiety disorder (x1.03) 0.952
Backache (x1.03) 0.952
aspirin (+0.00277) 0.953
Shoulder joint pain (x1.03) 0.954
estrogens, conjugated (USP) (+0.00272) 0.955
omeprazole (+0.00272) 0.955
Headache (x1.03) 0.956
Age (+1.25) 0.957
Major depression, single episode (x1.03) 0.957
Abdominal pain (x1.03) 0.958
estradiol (+0.00252) 0.959
Edema (x1.03) 0.959
Asthenia (x1.03) 0.960
Morbid obesity (x1.03) 0.960
ECG normal (x1.03) 0.961
Anemia (x1.03) 0.961
Spinal stenosis of lumbar region (x1.03) 0.962
trazodone (+0.00234) 0.963
Diarrhea (x1.02) 0.963
Chronic interstitial cystitis (x1.02) 0.963
Dysphagia (x1.02) 0.964
Joint pain of pelvic region (x1.02) 0.964
Degeneration of intervertebral disc (x1.02) 0.965
Hip pain (x1.02) 0.965
phenazopyridine (+0.00191) 0.965
simvastatin (+0.00189) 0.966
Onychomycosis due to dermatophyte (x1.02) 0.966
Amnesia (x1.02) 0.966
metformin (+0.0018) 0.967
Tear film insufficiency (x1.02) 0.967
furosemide (+0.00176) 0.967
Insomnia (x1.02) 0.967
Localized, primary osteoarthritis (x1.02) 0.968
Difficulty walking (x1.02) 0.968
White Race (+0.00172) 0.968
Postoperative state (x1.02) 0.969
Cough (x1.02) 0.969
tramadol (+0.00168) 0.969
Nuclear senile cataract (x1.02) 0.969
cyclobenzaprine (+0.00166) 0.970
Disorder of muscle (x1.02) 0.970
Polyneuropathy (x1.02) 0.970
atorvastatin (+0.00161) 0.971
Constipation (x1.02) 0.971
Irritable bowel syndrome (x1.02) 0.971
Sensorineural hearing loss, bilateral (x1.02) 0.971
Disorder of bone and articular cartilage (x1.02) 0.971
zolpidem (+0.00154) 0.972
Hematuria syndrome (x1.02) 0.972
MCHC [M/V] by AC (-0.0375) 0.972
−0.6 −0.4 −0.2 0.0 0.2 0.4 0.6
Expressions

Signature [759]; 27 To Reach 0.975, (27 Plotted)

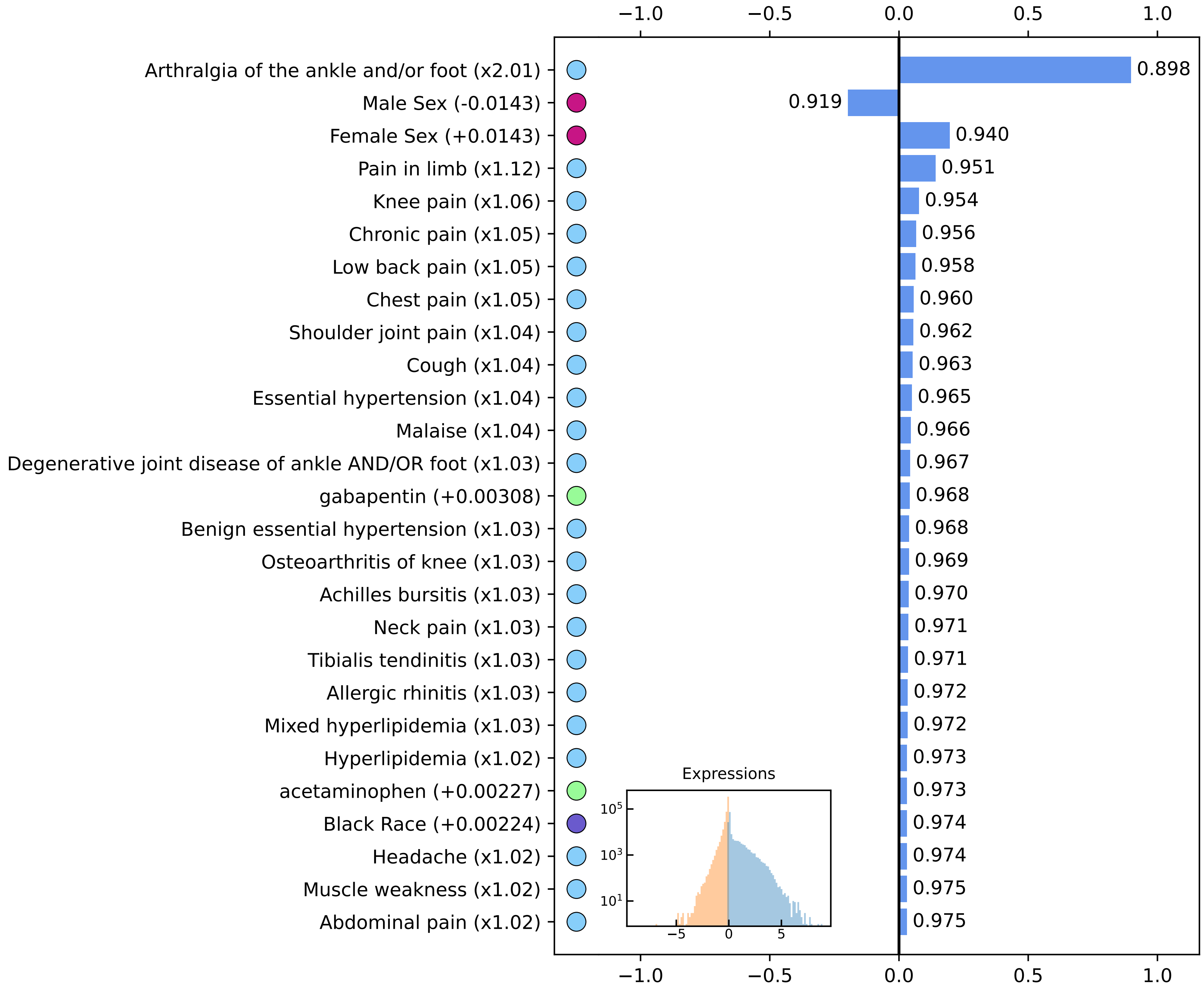

Signature [760]; 94 To Reach 0.975, (94 Plotted)

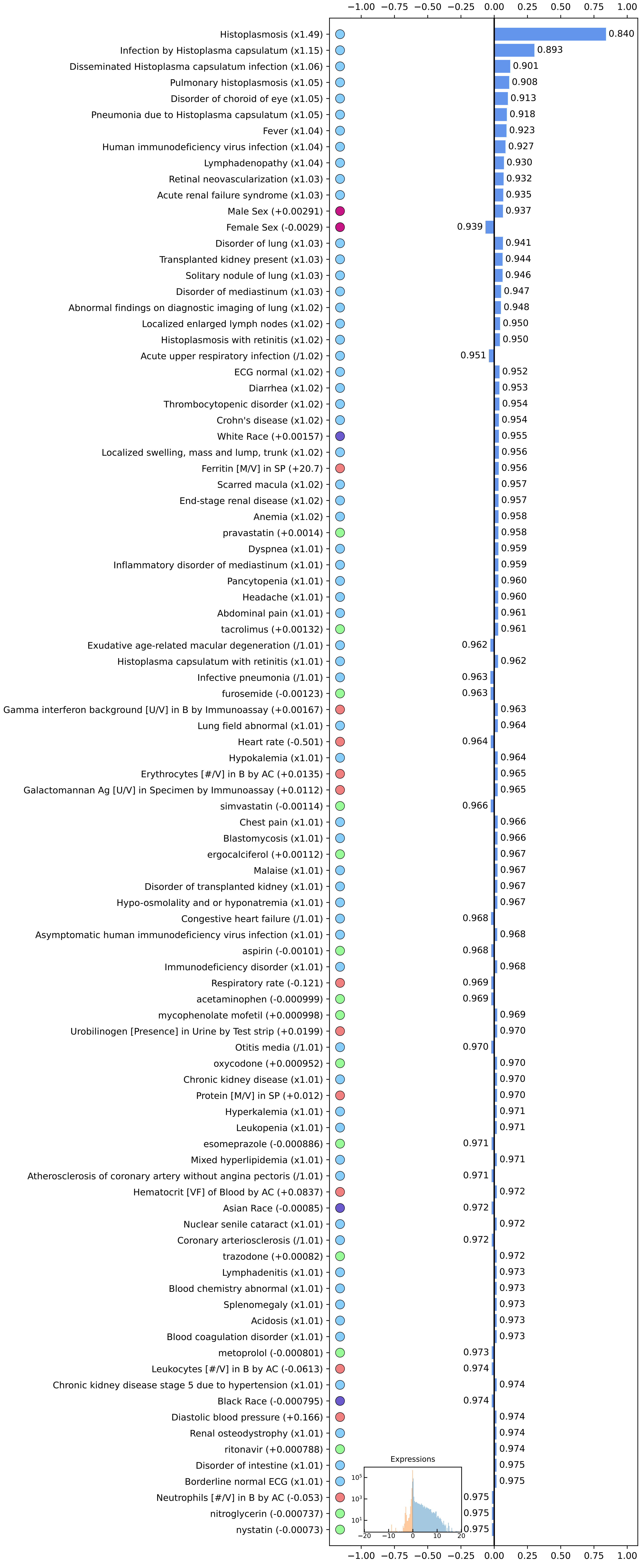

Signature [761]; 5 To Reach 0.975, (10 Plotted)

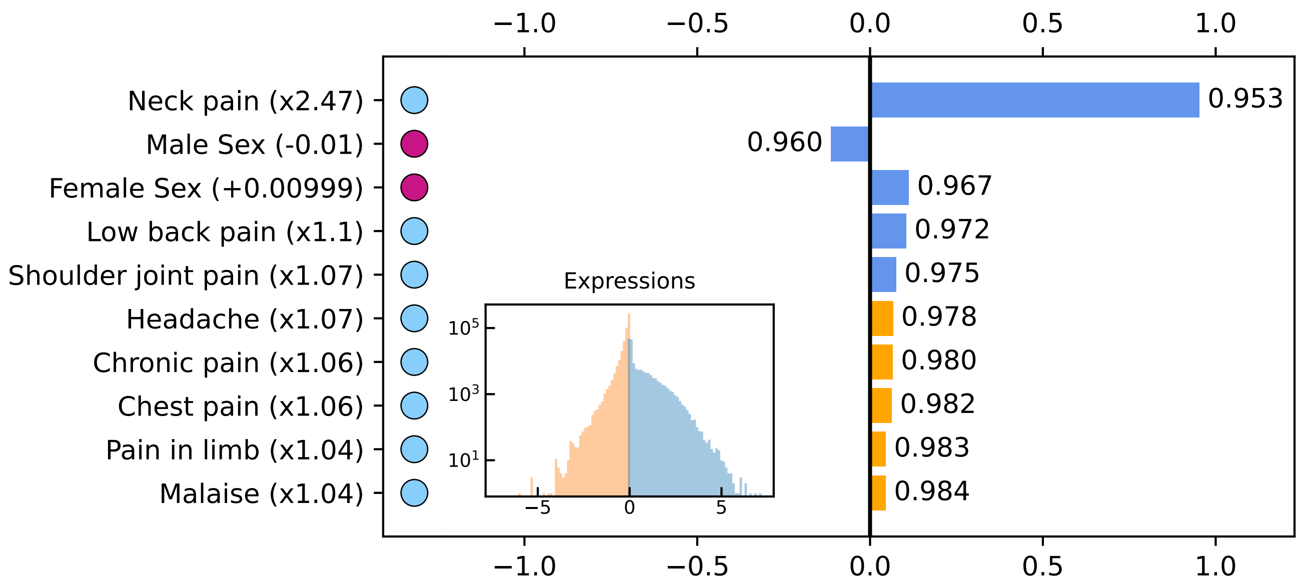

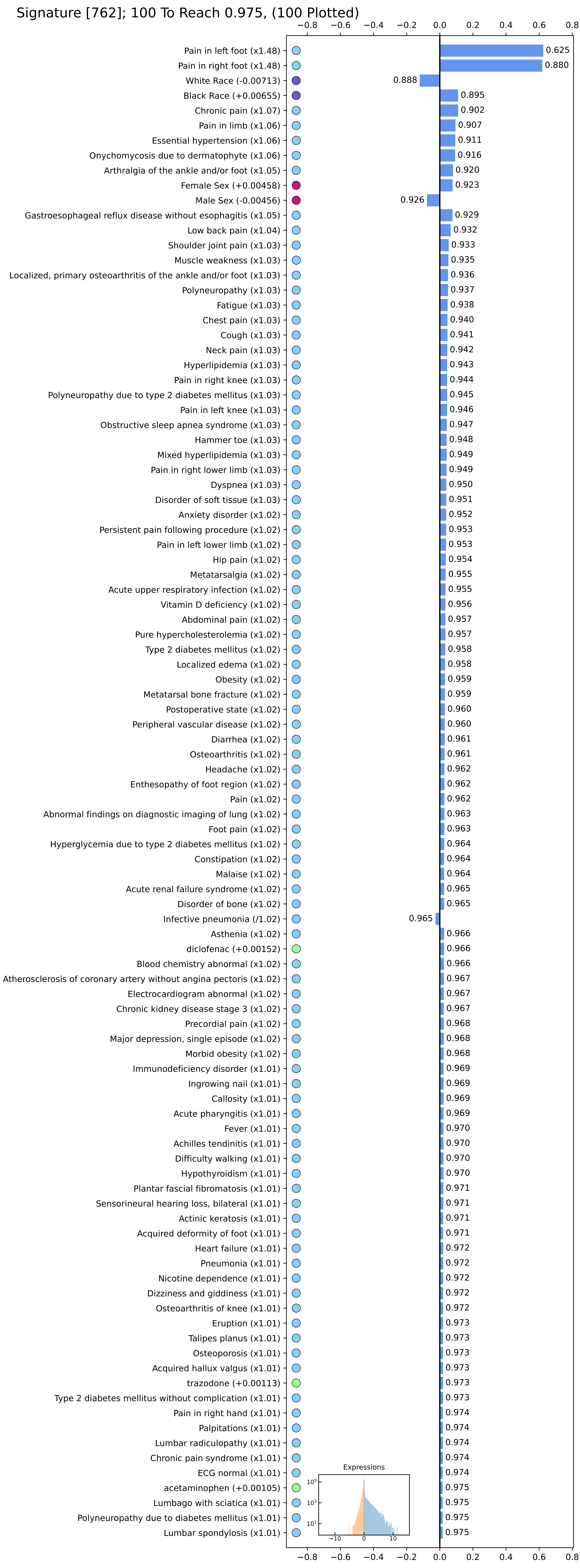

Signature [762]; 100 To Reach 0.975, (100 Plotted)
Pain in left foot (x1.48) 0.625
Pain in right foot (x1.48) 0.880
White Race (-0.00713) 0.888
Black Race (+0.00655) 0.895
Chronic pain (x1.07) 0.902
Pain in limb (x1.06) 0.907
Essential hypertension (x1.06) 0.911
Onychomycosis due to dermatophyte (x1.06) 0.916
Arthralgia of the ankle and/or foot (x1.05) 0.920
Female Sex (+0.00458) 0.923
Male Sex (-0.00456) 0.926
Gastroesophageal reflux disease without esophagitis (x1.05) 0.929
Low back pain (x1.04) 0.932
Shoulder joint pain (x1.03) 0.933
Muscle weakness (x1.03) 0.935
Localized, primary osteoarthritis of the ankle and/or foot (x1.03) 0.936
Polyneuropathy (x1.03) 0.937
Fatigue (x1.03) 0.938
Chest pain (x1.03) 0.940
Cough (x1.03) 0.941
Neck pain (x1.03) 0.942
Hyperlipidemia (x1.03) 0.943
Pain in right knee (x1.03) 0.944
Polyneuropathy due to type 2 diabetes mellitus (x1.03) 0.945
Pain in left knee (x1.03) 0.946
Obstructive sleep apnea syndrome (x1.03) 0.947
Hammer toe (x1.03) 0.948
Mixed hyperlipidemia (x1.03) 0.949
Pain in right lower limb (x1.03) 0.949
Dyspnea (x1.03) 0.950
Disorder of soft tissue (x1.03) 0.951
Anxiety disorder (x1.02) 0.952
Persistent pain following procedure (x1.02) 0.953
Pain in left lower limb (x1.02) 0.953
Hip pain (x1.02) 0.954
Metatarsalgia (x1.02) 0.955
Acute upper respiratory infection (x1.02) 0.955
Vitamin D deficiency (x1.02) 0.956
Abdominal pain (x1.02) 0.957
Pure hypercholesterolemia (x1.02) 0.957
Type 2 diabetes mellitus (x1.02) 0.958
Localized edema (x1.02) 0.958
Obesity (x1.02) 0.959
Metatarsal bone fracture (x1.02) 0.959
Postoperative state (x1.02) 0.960
Peripheral vascular disease (x1.02) 0.960
Diarrhea (x1.02) 0.961
Osteoarthritis (x1.02) 0.961
Headache (x1.02) 0.962
Enthesopathy of foot region (x1.02) 0.962
Pain (x1.02) 0.962
Abnormal findings on diagnostic imaging of lung (x1.02) 0.963
Foot pain (x1.02) 0.963
Hyperglycemia due to type 2 diabetes mellitus (x1.02) 0.964
Constipation (x1.02) 0.964
Malaise (x1.02) 0.964
Acute renal failure syndrome (x1.02) 0.965
Disorder of bone (x1.02) 0.965
Infective pneumonia (/1.02) 0.965
Asthenia (x1.02) 0.966
diclofenac (+0.00152) 0.966
Blood chemistry abnormal (x1.02) 0.966
Atherosclerosis of coronary artery without angina pectoris (x1.02) 0.967
Electrocardiogram abnormal (x1.02) 0.967
Chronic kidney disease stage 3 (x1.02) 0.967
Precordial pain (x1.02) 0.968
Major depression, single episode (x1.02) 0.968
Morbid obesity (x1.02) 0.968
Immunodeficiency disorder (x1.01) 0.969
Ingrowing nail (x1.01) 0.969
Callosity (x1.01) 0.969
Acute pharyngitis (x1.01) 0.969
Fever (x1.01) 0.970
Achilles tendinitis (x1.01) 0.970
Difficulty walking (x1.01) 0.970
Hypothyroidism (x1.01) 0.970
Plantar fascial fibromatosis (x1.01) 0.971
Sensorineural hearing loss, bilateral (x1.01) 0.971
Actinic keratosis (x1.01) 0.971
Acquired deformity of foot (x1.01) 0.971
Heart failure (x1.01) 0.972
Pneumonia (x1.01) 0.972
Nicotine dependence (x1.01) 0.972
Dizziness and giddiness (x1.01) 0.972
Osteoarthritis of knee (x1.01) 0.972
Eruption (x1.01) 0.973
Talipes planus (x1.01) 0.973
Osteoporosis (x1.01) 0.973
Acquired hallux valgus (x1.01) 0.973
trazodone (+0.00113) 0.973
Type 2 diabetes mellitus without complication (x1.01) 0.973
Pain in right hand (x1.01) 0.974
Palpitations (x1.01) 0.974
Lumbar radiculopathy (x1.01) 0.974
Chronic pain syndrome (x1.01) 0.974
ECG normal (x1.01) 0.974
acetaminophen (+0.00105) 0.975
Lumbago with sciatica (x1.01) 0.975
Polyneuropathy due to diabetes mellitus (x1.01) 0.975
Lumbar spondylosis (x1.01) 0.975
−0.8 −0.6 −0.4 −0.2 0.0 0.2 0.4 0.6 0.8
Expressions

Signature [763]; 2 To Reach 0.975, (10 Plotted)

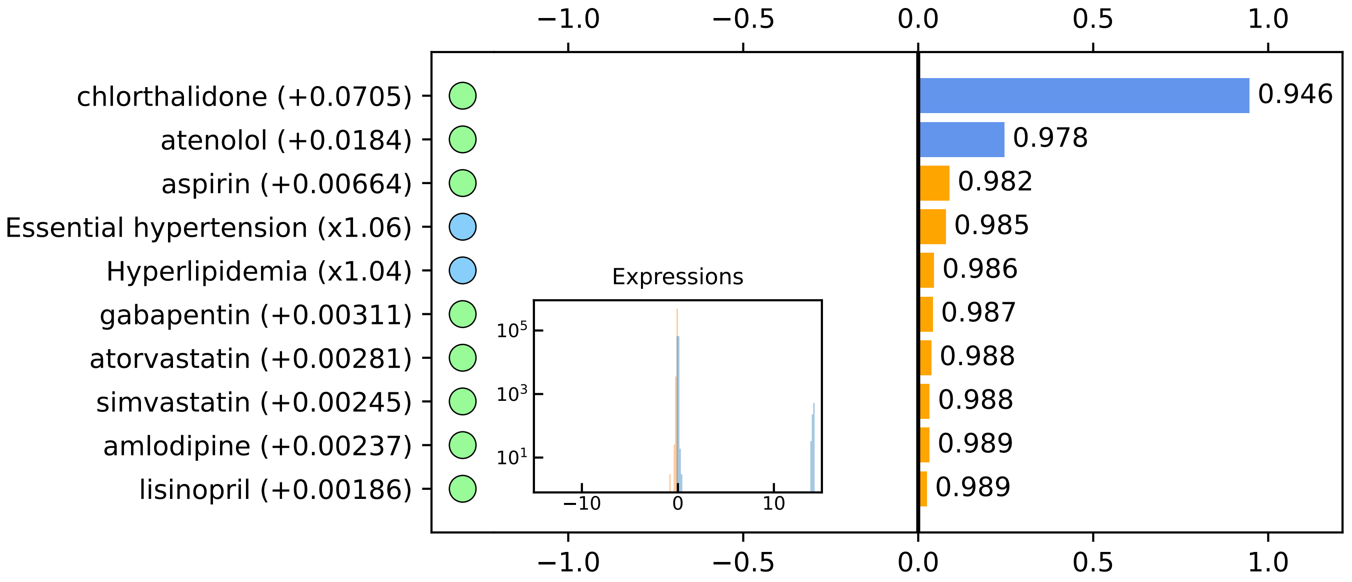

Signature [764]; 76 To Reach 0.975, (76 Plotted)

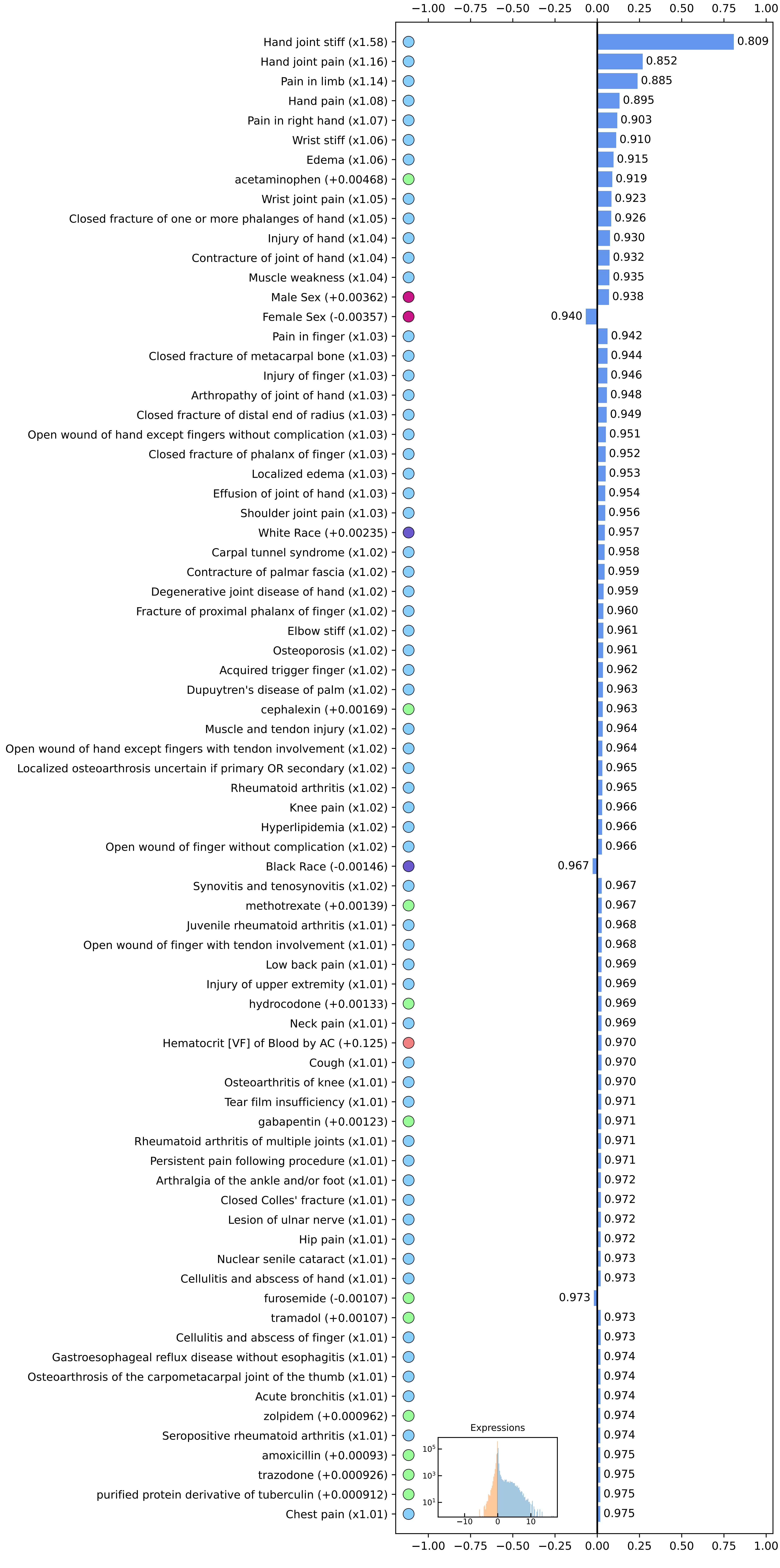

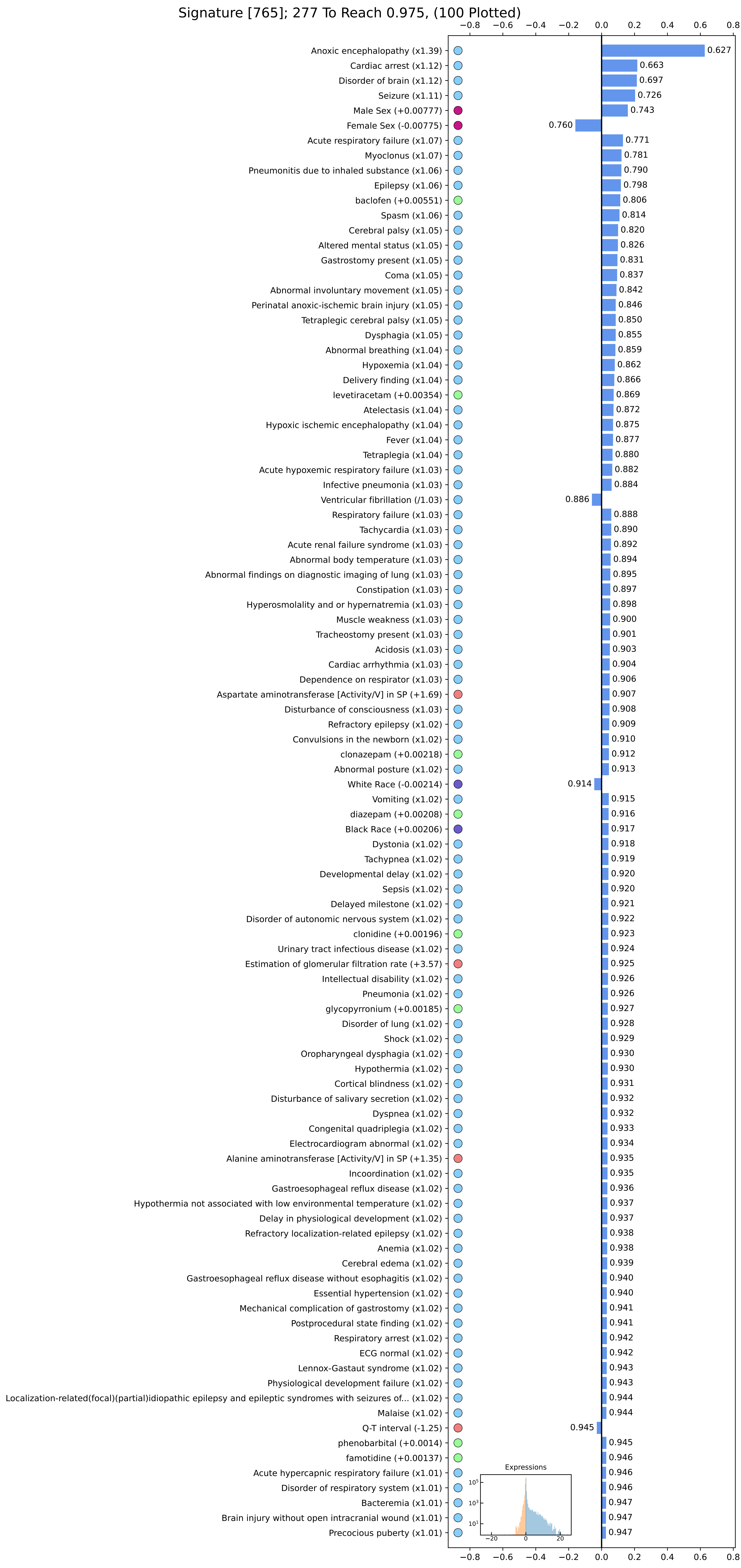

Signature [765]; 277 To Reach 0.975, (100 Plotted)
−0.8 −0.6 −0.4 −0.2 0.0 0.2 0.4 0.6 0.8
Anoxic encephalopathy (x1.39) 0.627
Cardiac arrest (x1.12) 0.663
Disorder of brain (x1.12) 0.697
Seizure (x1.11) 0.726
Male Sex (+0.00777) 0.743
Female Sex (-0.00775) 0.760
Acute respiratory failure (x1.07) 0.771
Myoclonus (x1.07) 0.781
Pneumonitis due to inhaled substance (x1.06) 0.790
Epilepsy (x1.06) 0.798
baclofen (+0.00551) 0.806
Spasm (x1.06) 0.814
Cerebral palsy (x1.05) 0.820
Altered mental status (x1.05) 0.826
Gastrostomy present (x1.05) 0.831
Coma (x1.05) 0.837
Abnormal involuntary movement (x1.05) 0.842
Perinatal anoxic-ischemic brain injury (x1.05) 0.846
Tetraplegic cerebral palsy (x1.05) 0.850
Dysphagia (x1.05) 0.855
Abnormal breathing (x1.04) 0.859
Hypoxemia (x1.04) 0.862
Delivery finding (x1.04) 0.866
levetiracetam (+0.00354) 0.869
Atelectasis (x1.04) 0.872
Hypoxic ischemic encephalopathy (x1.04) 0.875
Fever (x1.04) 0.877
Tetraplegia (x1.04) 0.880
Acute hypoxemic respiratory failure (x1.03) 0.882
Infective pneumonia (x1.03) 0.884
Ventricular fibrillation (/1.03) 0.886
Respiratory failure (x1.03) 0.888
Tachycardia (x1.03) 0.890
Acute renal failure syndrome (x1.03) 0.892
Abnormal body temperature (x1.03) 0.894
Abnormal findings on diagnostic imaging of lung (x1.03) 0.895
Constipation (x1.03) 0.897
Hyperosmolality and or hypernatremia (x1.03) 0.898
Muscle weakness (x1.03) 0.900
Tracheostomy present (x1.03) 0.901
Acidosis (x1.03) 0.903
Cardiac arrhythmia (x1.03) 0.904
Dependence on respirator (x1.03) 0.906
Aspartate aminotransferase [Activity/V] in SP (+1.69) 0.907
Disturbance of consciousness (x1.03) 0.908
Refractory epilepsy (x1.02) 0.909
Convulsions in the newborn (x1.02) 0.910
clonazepam (+0.00218) 0.912
Abnormal posture (x1.02) 0.913
White Race (-0.00214) 0.914
Vomiting (x1.02) 0.915
diazepam (+0.00208) 0.916
Black Race (+0.00206) 0.917
Dystonia (x1.02) 0.918
Tachypnea (x1.02) 0.919
Developmental delay (x1.02) 0.920
Sepsis (x1.02) 0.920
Delayed milestone (x1.02) 0.921
Disorder of autonomic nervous system (x1.02) 0.922
clonidine (+0.00196) 0.923
Urinary tract infectious disease (x1.02) 0.924
Estimation of glomerular filtration rate (+3.57) 0.925
Intellectual disability (x1.02) 0.926
Pneumonia (x1.02) 0.926
glycopyrronium (+0.00185) 0.927
Disorder of lung (x1.02) 0.928
Shock (x1.02) 0.929
Oropharyngeal dysphagia (x1.02) 0.930
Hypothermia (x1.02) 0.930
Cortical blindness (x1.02) 0.931
Disturbance of salivary secretion (x1.02) 0.932
Dyspnea (x1.02) 0.932
Congenital quadriplegia (x1.02) 0.933
Electrocardiogram abnormal (x1.02) 0.934
Alanine aminotransferase [Activity/V] in SP (+1.35) 0.935
Incoordination (x1.02) 0.935
Gastroesophageal reflux disease (x1.02) 0.936
Hypothermia not associated with low environmental temperature (x1.02) 0.937
Delay in physiological development (x1.02) 0.937
Refractory localization-related epilepsy (x1.02) 0.938
Anemia (x1.02) 0.938
Cerebral edema (x1.02) 0.939
Gastroesophageal reflux disease without esophagitis (x1.02) 0.940
Essential hypertension (x1.02) 0.940
Mechanical complication of gastrostomy (x1.02) 0.941
Postprocedural state finding (x1.02) 0.941
Respiratory arrest (x1.02) 0.942
ECG normal (x1.02) 0.942
Lennox-Gastaut syndrome (x1.02) 0.943
Physiological development failure (x1.02) 0.943
Localization-related(focal)(partial)idiopathic epilepsy and epileptic syndromes with seizures of... (x1.02) 0.944
Malaise (x1.02) 0.944
Q-T interval (-1.25) 0.945
phenobarbital (+0.0014) 0.945
famotidine (+0.00137) 0.946
Acute hypercapnic respiratory failure (x1.01) 0.946
Disorder of respiratory system (x1.01) 0.946
Bacteremia (x1.01) 0.947
Brain injury without open intracranial wound (x1.01) 0.947
Precocious puberty (x1.01) 0.947
Expressions
−20 0 20
−0.8 −0.6 −0.4 −0.2 0.0 0.2 0.4 0.6 0.8

Signature [766]; 2 To Reach 0.975, (10 Plotted)

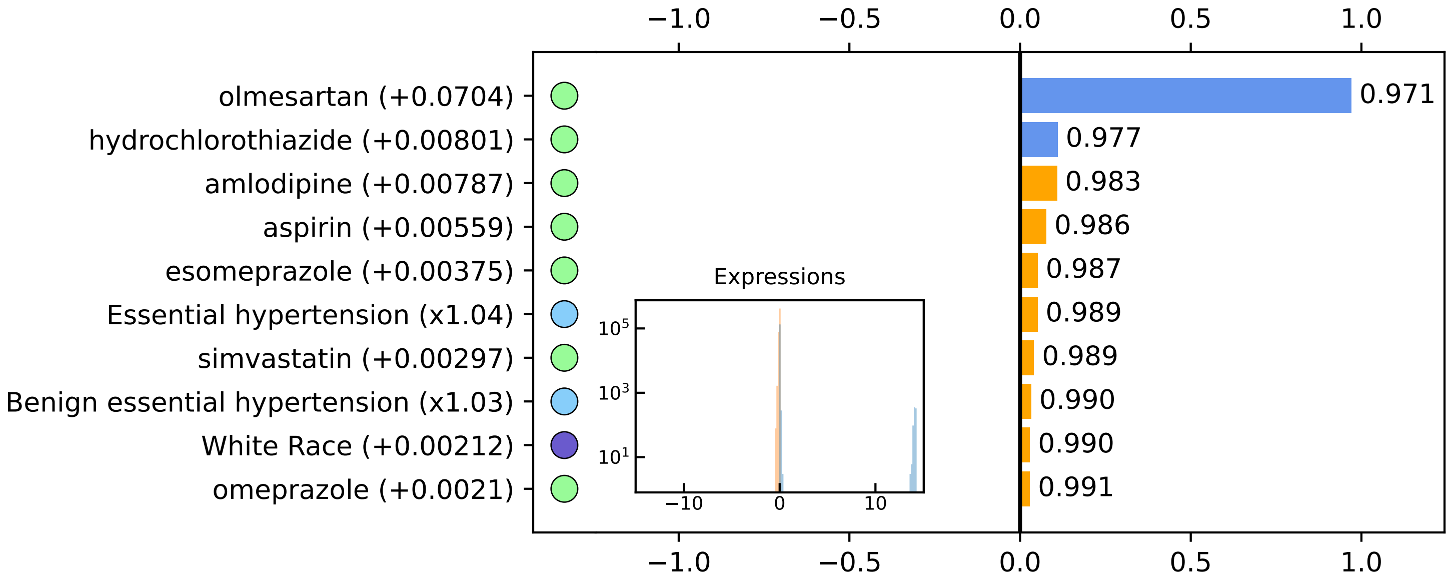

Signature [767]; 504 To Reach 0.975, (100 Plotted)

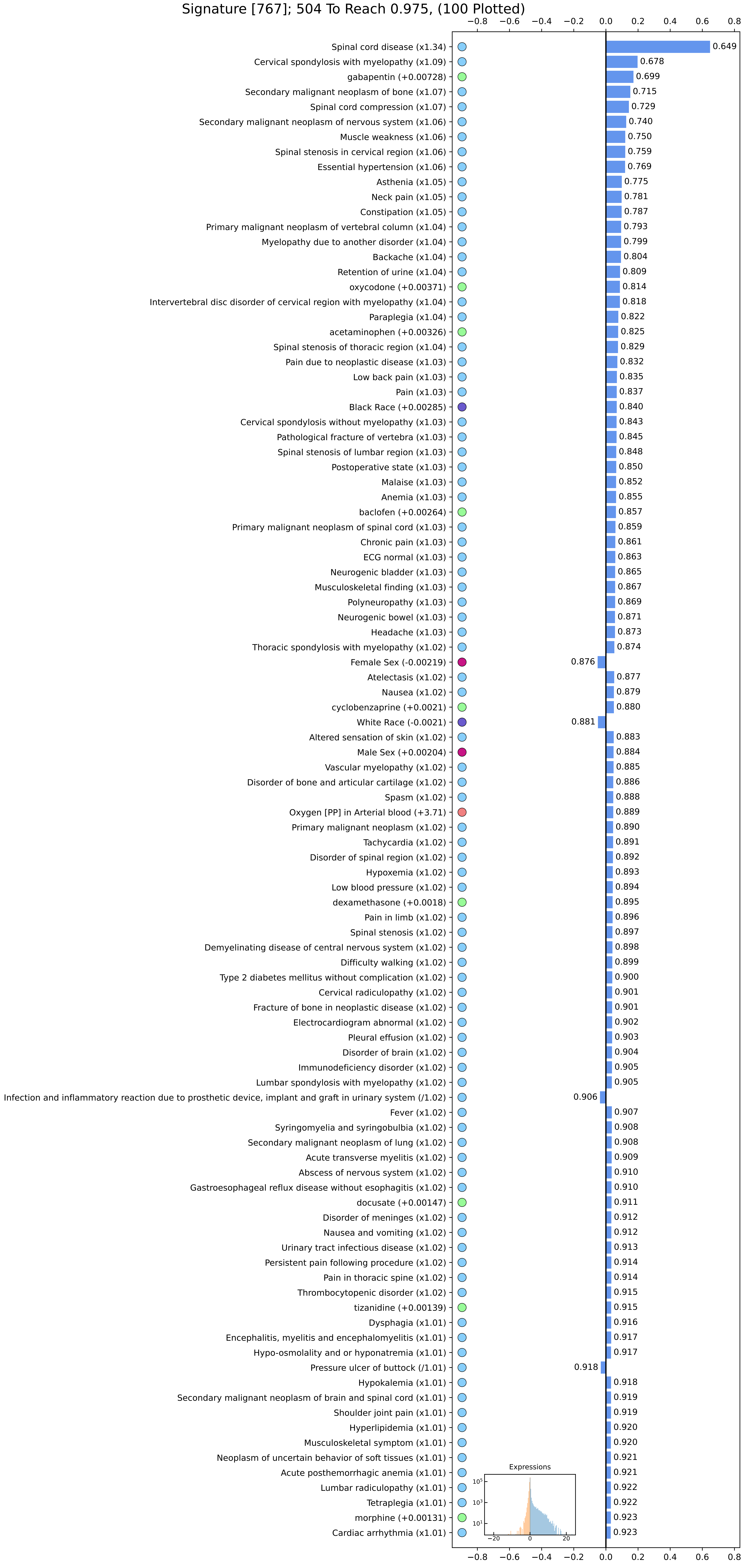

Signature [768]; 44 To Reach 0.975, (44 Plotted)

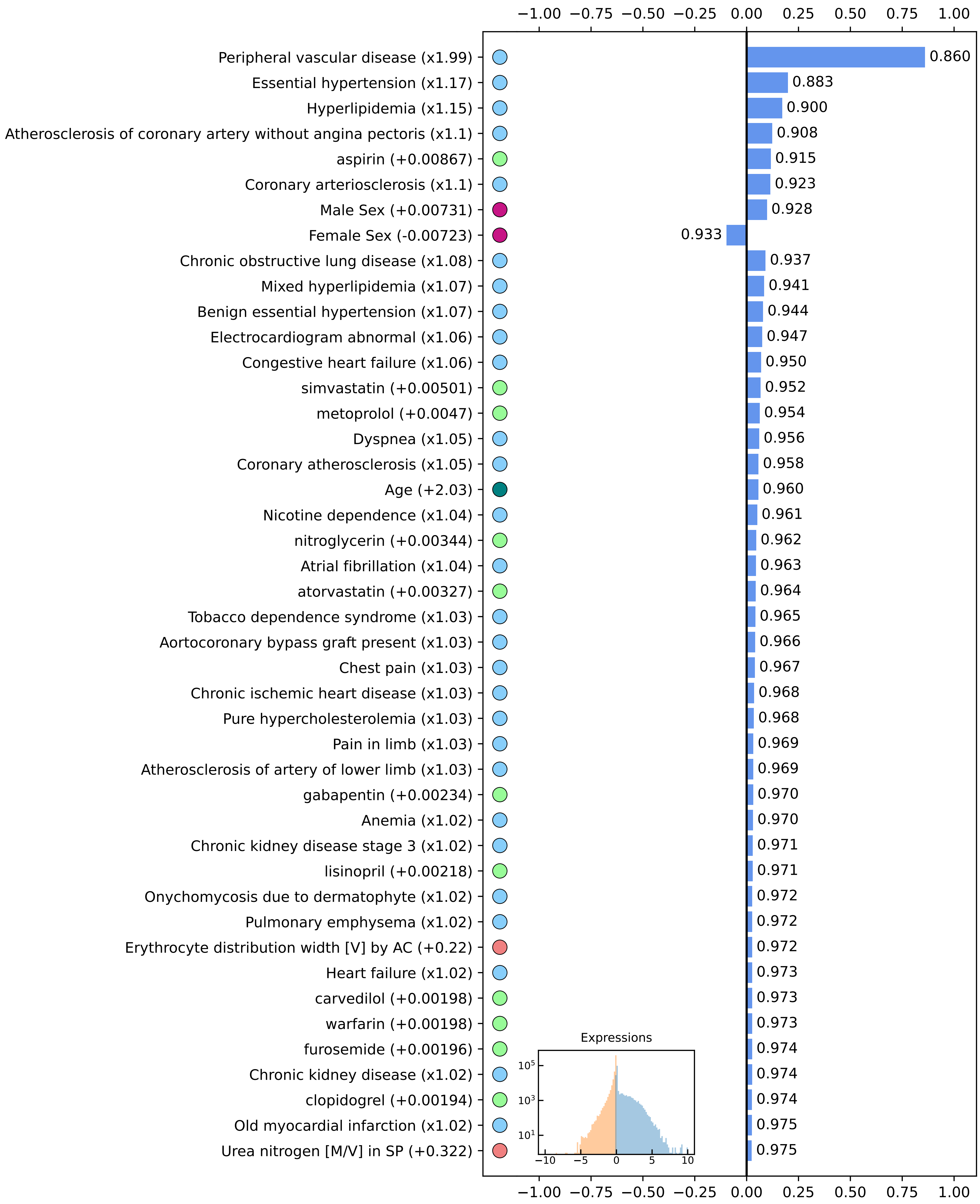

Signature [769]; 326 To Reach 0.975, (100 Plotted)

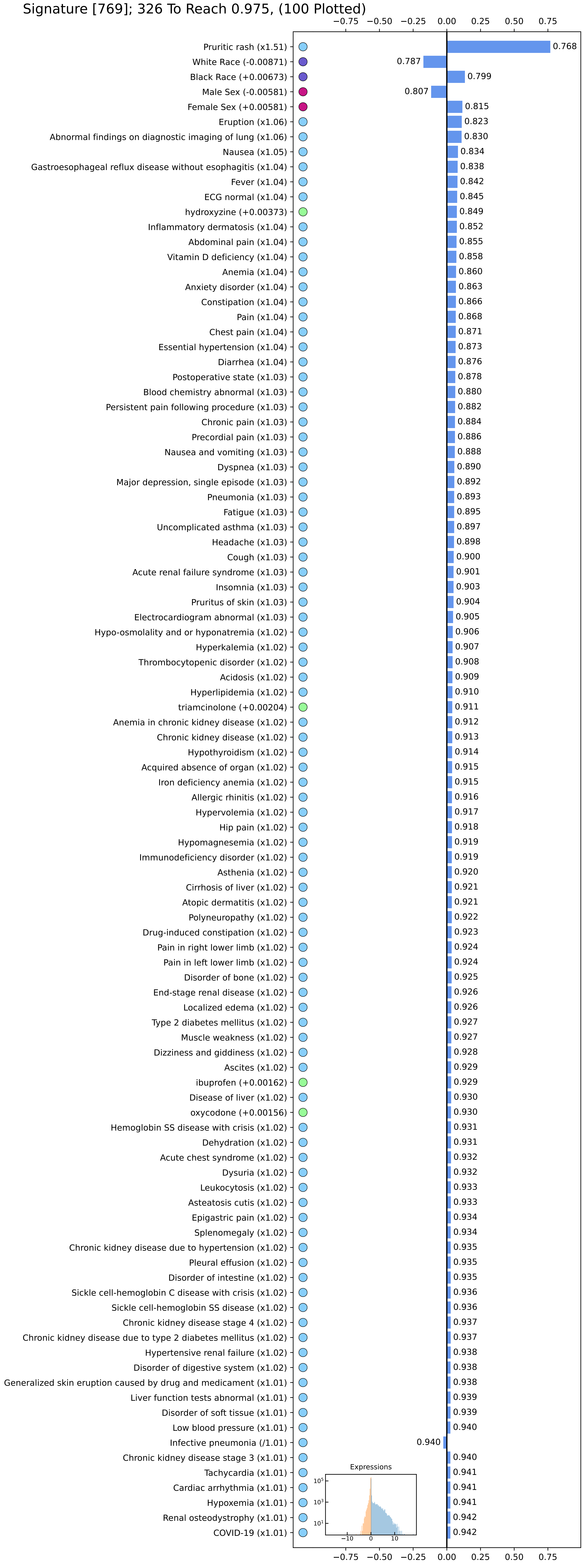

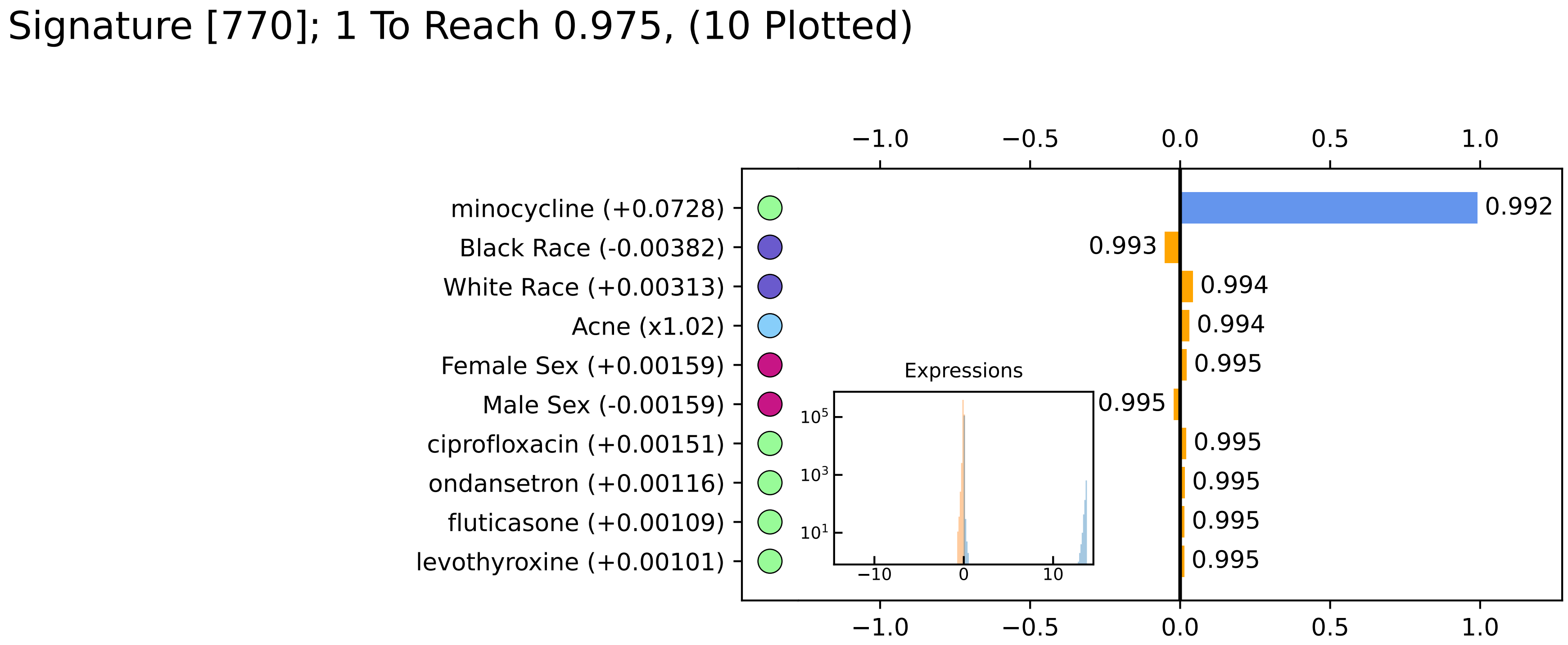

Signature [770]; 1 To Reach 0.975, (10 Plotted)
minocycline (+0.0728)
Black Race (-0.00382)
White Race (+0.00313)
Acne (x1.02)
Female Sex (+0.00159)
Male Sex (-0.00159)
ciprofloxacin (+0.00151)
ondansetron (+0.00116)
fluticasone (+0.00109)
levothyroxine (+0.00101)
0.992
0.993
0.994
0.994
0.995
0.995
0.995
0.995
0.995
0.995
−1.0
−0.5
0.0
0.5
1.0
Expressions

Signature [771]; 1 To Reach 0.975, (10 Plotted)

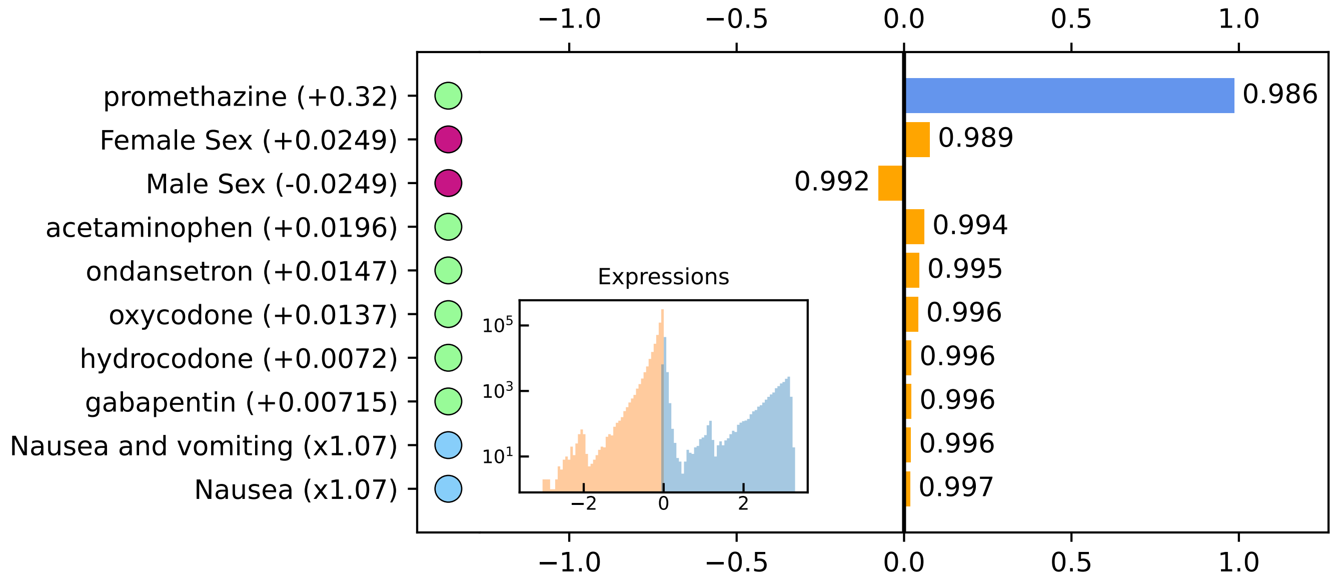

Signature [772]; 178 To Reach 0.975, (100 Plotted)

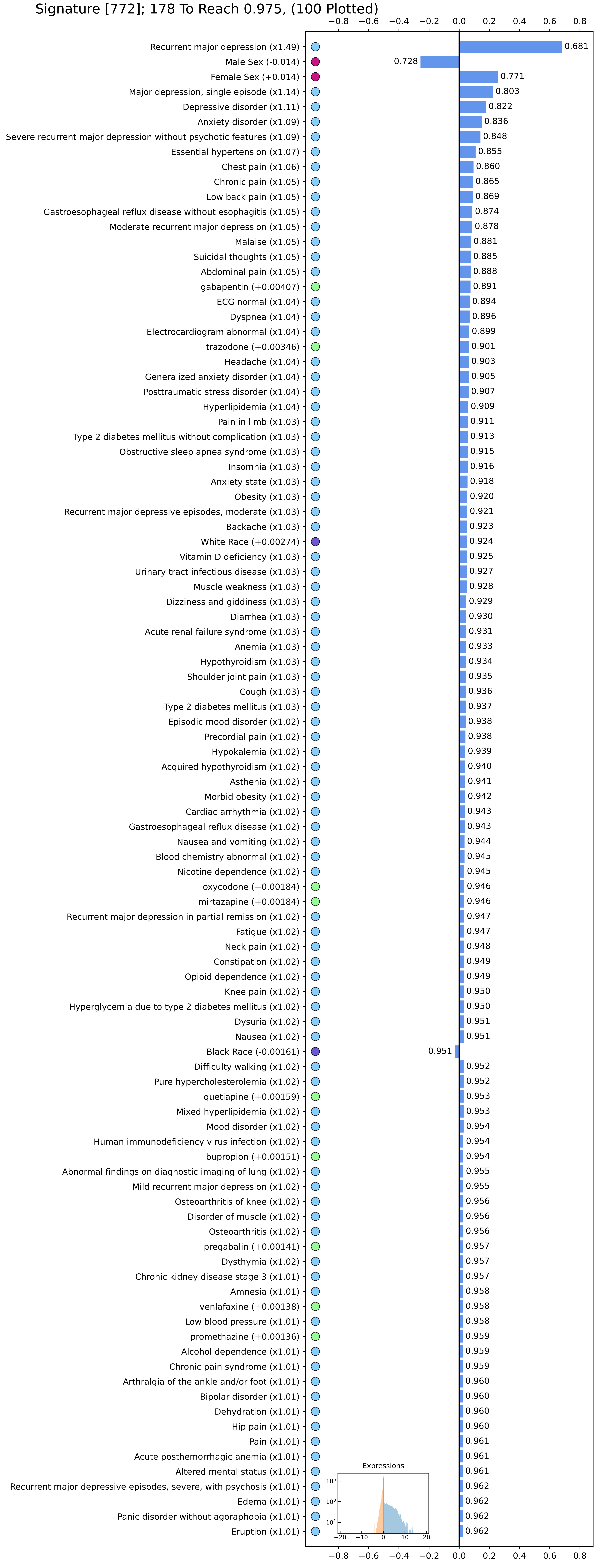

Signature [773]; 135 To Reach 0.975, (100 Plotted)

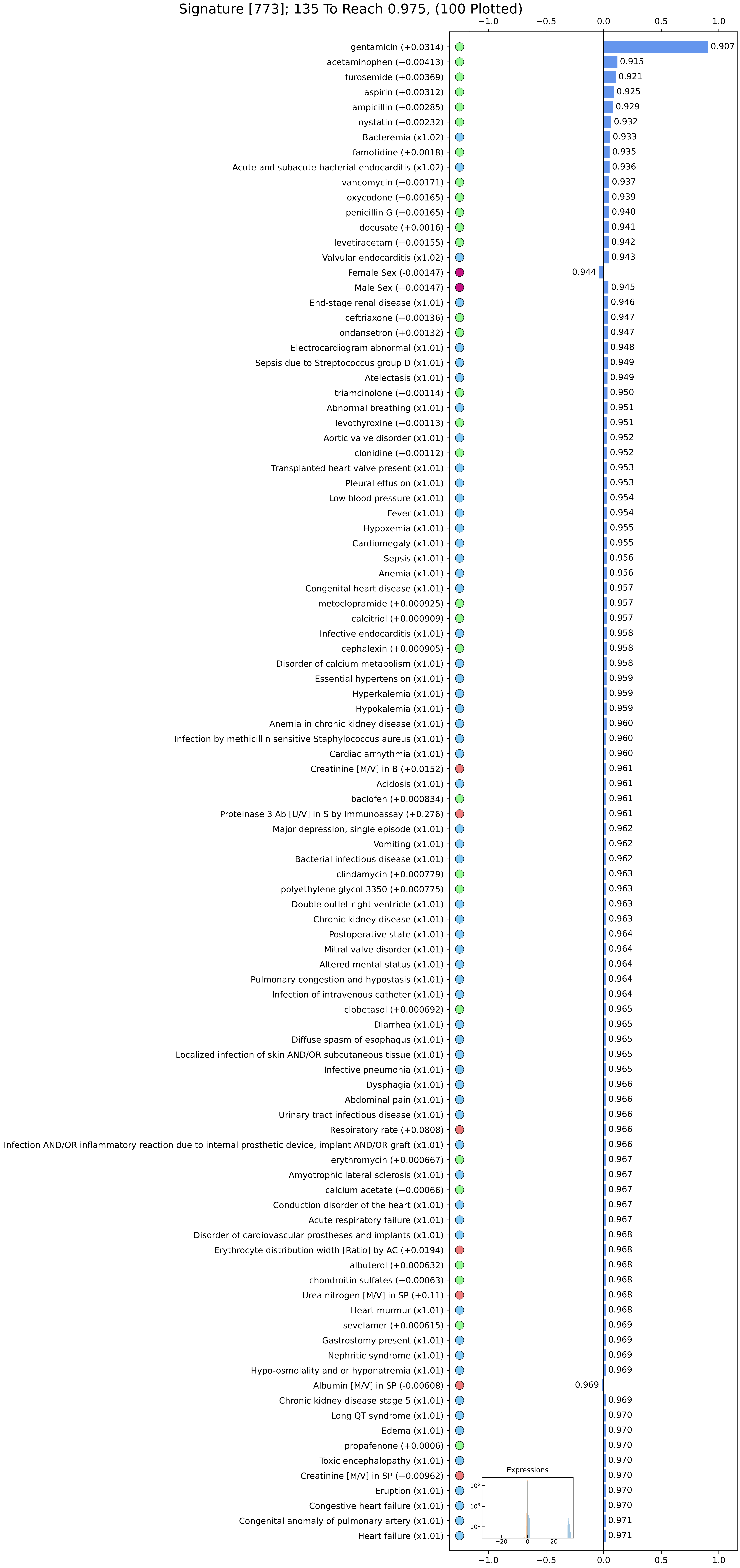

Signature [774]; 1 To Reach 0.975, (10 Plotted)

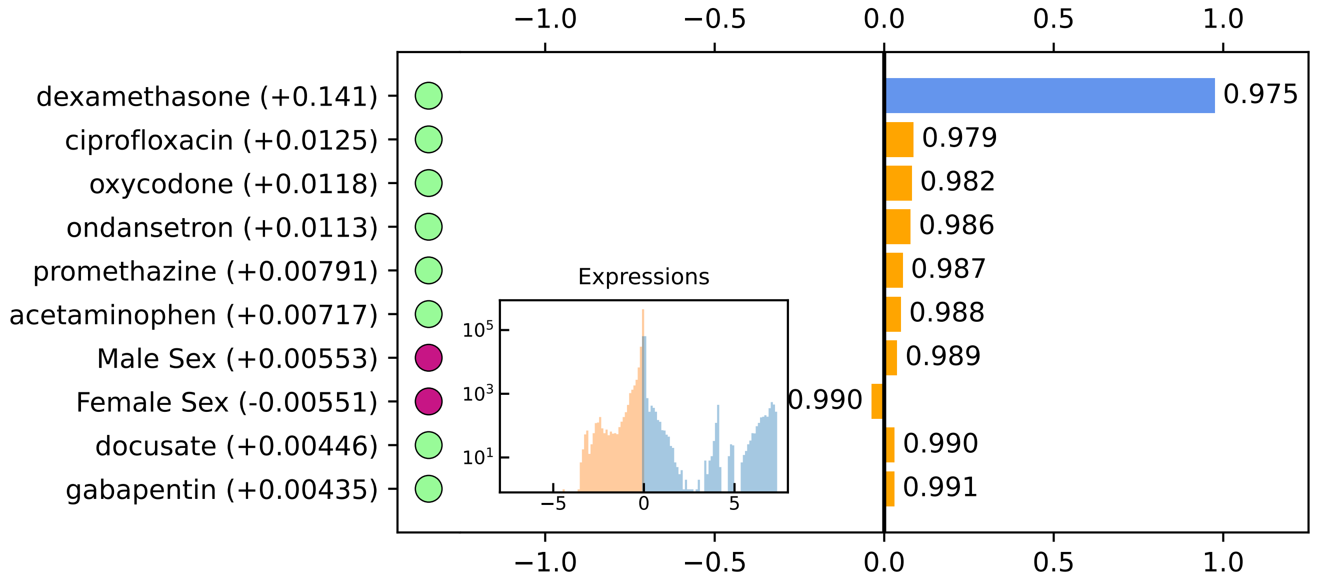

Signature [775]; 71 To Reach 0.975, (71 Plotted)

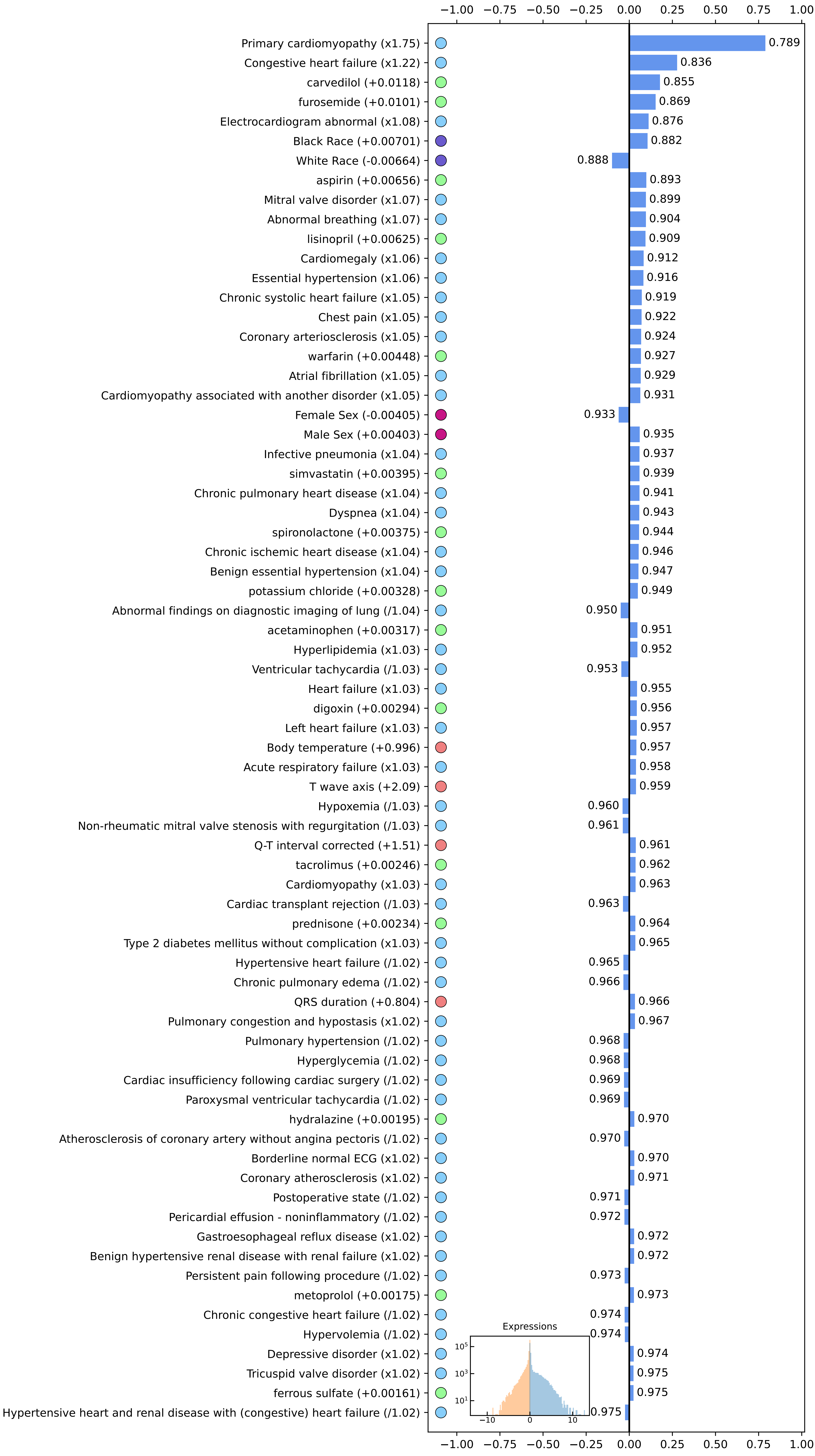

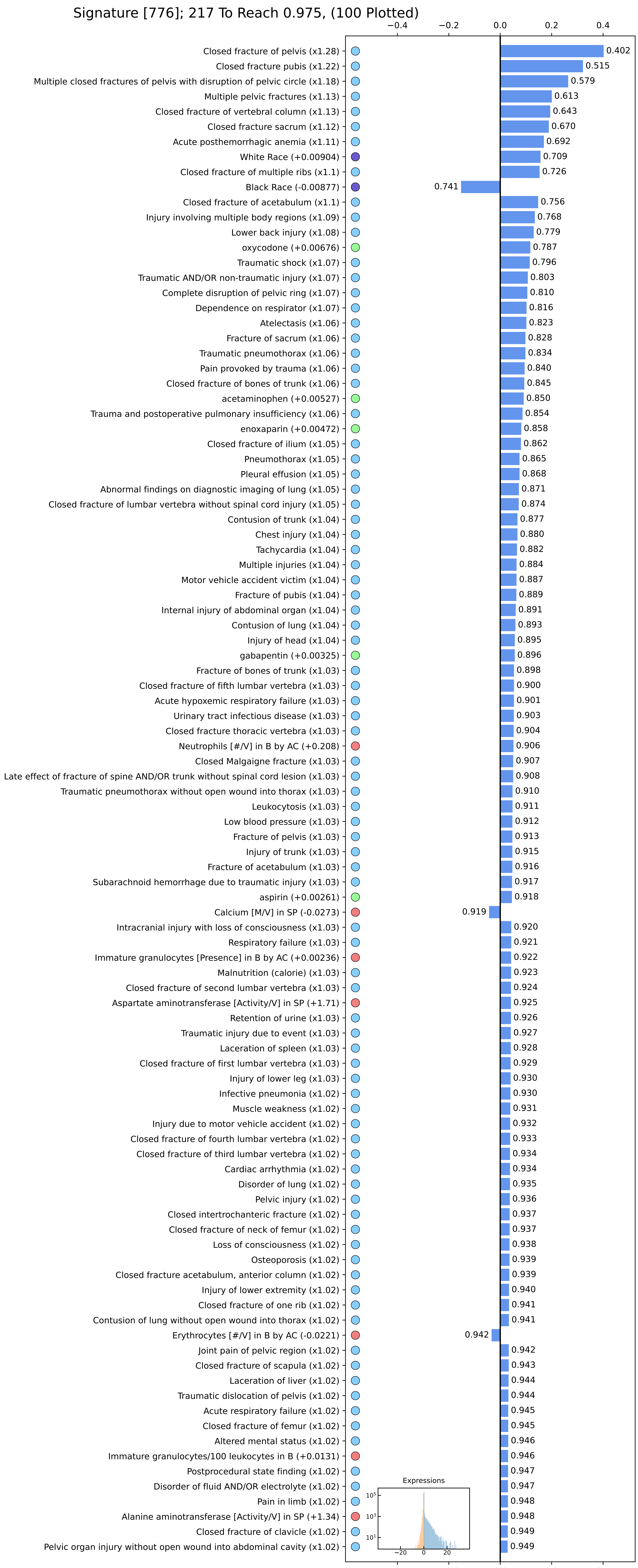
Signature [776]; 217 To Reach 0.975, (100 Plotted)
Closed fracture of pelvis (x1.28) 0.402
Closed fracture pubis (x1.22) 0.515
Multiple closed fractures of pelvis with disruption of pelvic circle (x1.18) 0.579
Multiple pelvic fractures (x1.13) 0.613
Closed fracture of vertebral column (x1.13) 0.643
Closed fracture sacrum (x1.12) 0.670
Acute posthemorrhagic anemia (x1.11) 0.692
White Race (+0.00904) 0.709
Closed fracture of multiple ribs (x1.1) 0.726
Black Race (-0.00877) 0.741
Closed fracture of acetabulum (x1.1) 0.756
Injury involving multiple body regions (x1.09) 0.768
Lower back injury (x1.08) 0.779
oxycodone (+0.00676) 0.787
Traumatic shock (x1.07) 0.796
Traumatic AND/OR non-traumatic injury (x1.07) 0.803
Complete disruption of pelvic ring (x1.07) 0.810
Dependence on respirator (x1.07) 0.816
Atelectasis (x1.06) 0.823
Fracture of sacrum (x1.06) 0.828
Traumatic pneumothorax (x1.06) 0.834
Pain provoked by trauma (x1.06) 0.840
Closed fracture of bones of trunk (x1.06) 0.845
acetaminophen (+0.00527) 0.850
Trauma and postoperative pulmonary insufficiency (x1.06) 0.854
enoxaparin (+0.00472) 0.858
Closed fracture of ilium (x1.05) 0.862
Pneumothorax (x1.05) 0.865
Pleural effusion (x1.05) 0.868
Abnormal findings on diagnostic imaging of lung (x1.05) 0.871
Closed fracture of lumbar vertebra without spinal cord injury (x1.05) 0.874
Contusion of trunk (x1.04) 0.877
Chest injury (x1.04) 0.880
Tachycardia (x1.04) 0.882
Multiple injuries (x1.04) 0.884
Motor vehicle accident victim (x1.04) 0.887
Fracture of pubis (x1.04) 0.889
Internal injury of abdominal organ (x1.04) 0.891
Contusion of lung (x1.04) 0.893
Injury of head (x1.04) 0.895
gabapentin (+0.00325) 0.896
Fracture of bones of trunk (x1.03) 0.898
Closed fracture of fifth lumbar vertebra (x1.03) 0.900
Acute hypoxemic respiratory failure (x1.03) 0.901
Urinary tract infectious disease (x1.03) 0.903
Closed fracture thoracic vertebra (x1.03) 0.904
Neutrophils [#/V] in B by AC (+0.208) 0.906
Closed Malgaigne fracture (x1.03) 0.907
Late effect of fracture of spine AND/OR trunk without spinal cord lesion (x1.03) 0.908
Traumatic pneumothorax without open wound into thorax (x1.03) 0.910
Leukocytosis (x1.03) 0.911
Low blood pressure (x1.03) 0.912
Fracture of pelvis (x1.03) 0.913
Injury of trunk (x1.03) 0.915
Fracture of acetabulum (x1.03) 0.916
Subarachnoid hemorrhage due to traumatic injury (x1.03) 0.917
aspirin (+0.00261) 0.918
Calcium [M/V] in SP (-0.0273) 0.919
Intracranial injury with loss of consciousness (x1.03) 0.920
Respiratory failure (x1.03) 0.921
Immature granulocytes [Presence] in B by AC (+0.00236) 0.922
Malnutrition (calorie) (x1.03) 0.923
Closed fracture of second lumbar vertebra (x1.03) 0.924
Aspartate aminotransferase [Activity/V] in SP (+1.71) 0.925
Retention of urine (x1.03) 0.926
Traumatic injury due to event (x1.03) 0.927
Laceration of spleen (x1.03) 0.928
Closed fracture of first lumbar vertebra (x1.03) 0.929
Injury of lower leg (x1.03) 0.930
Infective pneumonia (x1.02) 0.930
Muscle weakness (x1.02) 0.931
Injury due to motor vehicle accident (x1.02) 0.932
Closed fracture of fourth lumbar vertebra (x1.02) 0.933
Closed fracture of third lumbar vertebra (x1.02) 0.934
Cardiac arrhythmia (x1.02) 0.934
Disorder of lung (x1.02) 0.935
Pelvic injury (x1.02) 0.936
Closed intertrochanteric fracture (x1.02) 0.937
Closed fracture of neck of femur (x1.02) 0.937
Loss of consciousness (x1.02) 0.938
Osteoporosis (x1.02) 0.939
Closed fracture acetabulum, anterior column (x1.02) 0.939
Injury of lower extremity (x1.02) 0.940
Closed fracture of one rib (x1.02) 0.941
Contusion of lung without open wound into thorax (x1.02) 0.941
Erythrocytes [#/V] in B by AC (-0.0221) 0.942
Joint pain of pelvic region (x1.02) 0.942
Closed fracture of scapula (x1.02) 0.943
Laceration of liver (x1.02) 0.944
Traumatic dislocation of pelvis (x1.02) 0.944
Acute respiratory failure (x1.02) 0.945
Closed fracture of femur (x1.02) 0.945
Altered mental status (x1.02) 0.946
Immature granulocytes/100 leukocytes in B (+0.0131) 0.946
Postprocedural state finding (x1.02) 0.947
Disorder of fluid AND/OR electrolyte (x1.02) 0.947
Pain in limb (x1.02) 0.948
Alanine aminotransferase [Activity/V] in SP (+1.34) 0.948
Closed fracture of clavicle (x1.02) 0.949
Pelvic organ injury without open wound into abdominal cavity (x1.02) 0.949
Expressions
−0.4 −0.2 0.0 0.2 0.4

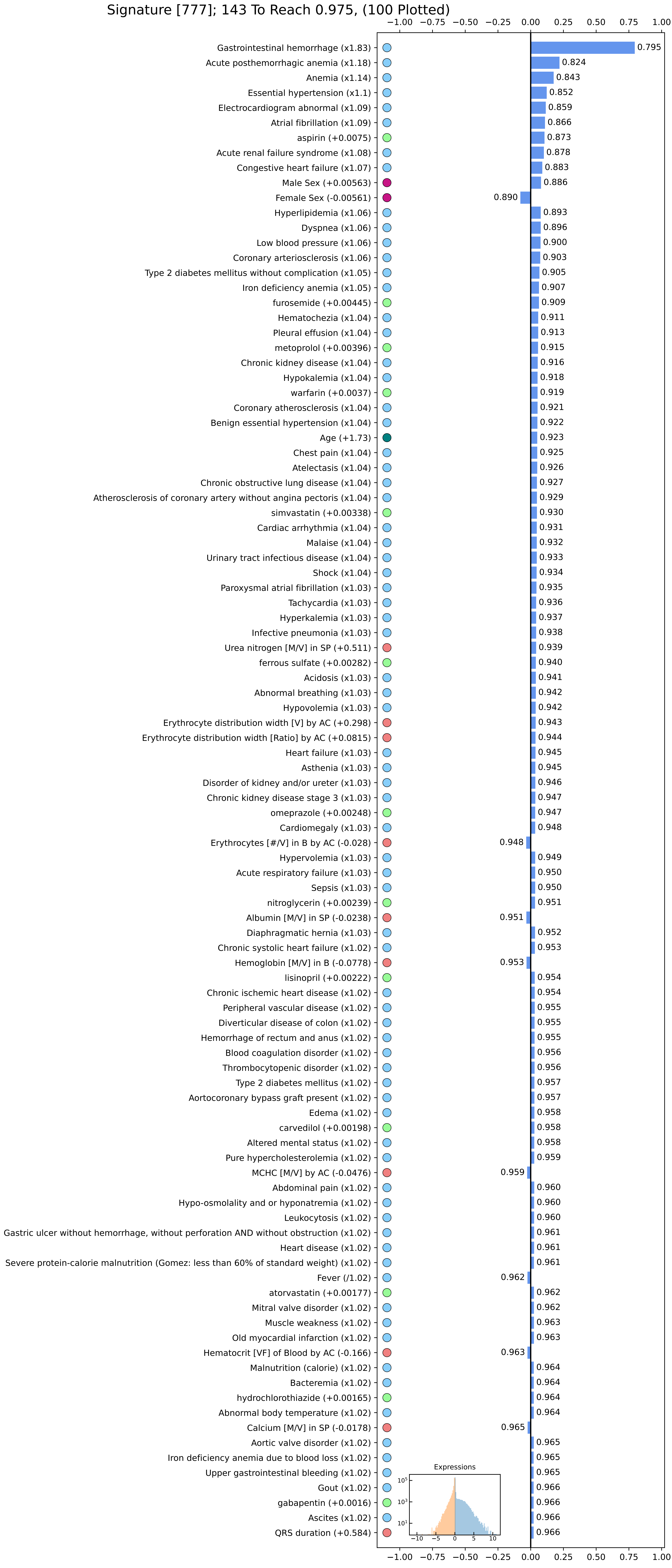
Signature [777]; 143 To Reach 0.975, (100 Plotted)
Gastrointestinal hemorrhage (x1.83)
Acute posthemorrhagic anemia (x1.18)
Anemia (x1.14)
Essential hypertension (x1.1)
Electrocardiogram abnormal (x1.09)
Atrial fibrillation (x1.09)
aspirin (+0.0075)
Acute renal failure syndrome (x1.08)
Congestive heart failure (x1.07)
Male Sex (+0.00563)
Female Sex (-0.00561)
Hyperlipidemia (x1.06)
Dyspnea (x1.06)
Low blood pressure (x1.06)
Coronary arteriosclerosis (x1.06)
Type 2 diabetes mellitus without complication (x1.05)
Iron deficiency anemia (x1.05)
furosemide (+0.00445)
Hematochezia (x1.04)
Pleural effusion (x1.04)
metoprolol (+0.00396)
Chronic kidney disease (x1.04)
Hypokalemia (x1.04)
warfarin (+0.0037)
Coronary atherosclerosis (x1.04)
Benign essential hypertension (x1.04)
Age (+1.73)
Chest pain (x1.04)
Atelectasis (x1.04)
Chronic obstructive lung disease (x1.04)
Atherosclerosis of coronary artery without angina pectoris (x1.04)
simvastatin (+0.00338)
Cardiac arrhythmia (x1.04)
Malaise (x1.04)
Urinary tract infectious disease (x1.04)
Shock (x1.04)
Paroxysmal atrial fibrillation (x1.03)
Tachycardia (x1.03)
Hyperkalemia (x1.03)
Infective pneumonia (x1.03)
Urea nitrogen [M/V] in SP (+0.511)
ferrous sulfate (+0.00282)
Acidosis (x1.03)
Abnormal breathing (x1.03)
Hypovolemia (x1.03)
Erythrocyte distribution width [V] by AC (+0.298)
Erythrocyte distribution width [Ratio] by AC (+0.0815)
Heart failure (x1.03)
Asthenia (x1.03)
Disorder of kidney and/or ureter (x1.03)
Chronic kidney disease stage 3 (x1.03)
omeprazole (+0.00248)
Cardiomegaly (x1.03)
Erythrocytes [#/V] in B by AC (-0.028)
Hypervolemia (x1.03)
Acute respiratory failure (x1.03)
Sepsis (x1.03)
nitroglycerin (+0.00239)
Albumin [M/V] in SP (-0.0238)
Diaphragmatic hernia (x1.03)
Chronic systolic heart failure (x1.02)
Hemoglobin [M/V] in B (-0.0778)
lisinopril (+0.00222)
Chronic ischemic heart disease (x1.02)
Peripheral vascular disease (x1.02)
Diverticular disease of colon (x1.02)
Hemorrhage of rectum and anus (x1.02)
Blood coagulation disorder (x1.02)
Thrombocytopenic disorder (x1.02)
Type 2 diabetes mellitus (x1.02)
Aortocoronary bypass graft present (x1.02)
Edema (x1.02)
carvedilol (+0.00198)
Altered mental status (x1.02)
Pure hypercholesterolemia (x1.02)
MCHC [M/V] by AC (-0.0476)
Abdominal pain (x1.02)
Hypo-osmolality and or hyponatremia (x1.02)
Leukocytosis (x1.02)
Gastric ulcer without hemorrhage, without perforation AND without obstruction (x1.02)
Heart disease (x1.02)
Severe protein-calorie malnutrition (Gomez: less than 60% of standard weight) (x1.02)
Fever (/1.02)
atorvastatin (+0.00177)
Mitral valve disorder (x1.02)
Muscle weakness (x1.02)
Old myocardial infarction (x1.02)
Hematocrit [VF] of Blood by AC (-0.166)
Malnutrition (calorie) (x1.02)
Bacteremia (x1.02)
hydrochlorothiazide (+0.00165)
Abnormal body temperature (x1.02)
Calcium [M/V] in SP (-0.0178)
Aortic valve disorder (x1.02)
Iron deficiency anemia due to blood loss (x1.02)
Upper gastrointestinal bleeding (x1.02)
Gout (x1.02)
gabapentin (+0.0016)
Ascites (x1.02)
QRS duration (+0.584)
0.795
0.824
0.843
0.852
0.859
0.866
0.873
0.878
0.883
0.886
0.890
0.893
0.896
0.900
0.903
0.905
0.907
0.909
0.911
0.913
0.915
0.916
0.918
0.919
0.921
0.922
0.923
0.925
0.926
0.927
0.929
0.930
0.931
0.932
0.933
0.934
0.935
0.936
0.937
0.938
0.939
0.940
0.941
0.942
0.942
0.943
0.944
0.945
0.945
0.946
0.947
0.947
0.948
0.948
0.949
0.950
0.950
0.951
0.951
0.952
0.953
0.953
0.954
0.954
0.955
0.955
0.955
0.956
0.956
0.957
0.957
0.958
0.958
0.958
0.959
0.959
0.960
0.960
0.960
0.961
0.961
0.961
0.962
0.962
0.962
0.963
0.963
0.963
0.964
0.964
0.964
0.964
0.965
0.965
0.965
0.965
0.966
0.966
0.966
0.966
Expressions
−1.00 −0.75 −0.50 −0.25 0.00 0.25 0.50 0.75 1.00

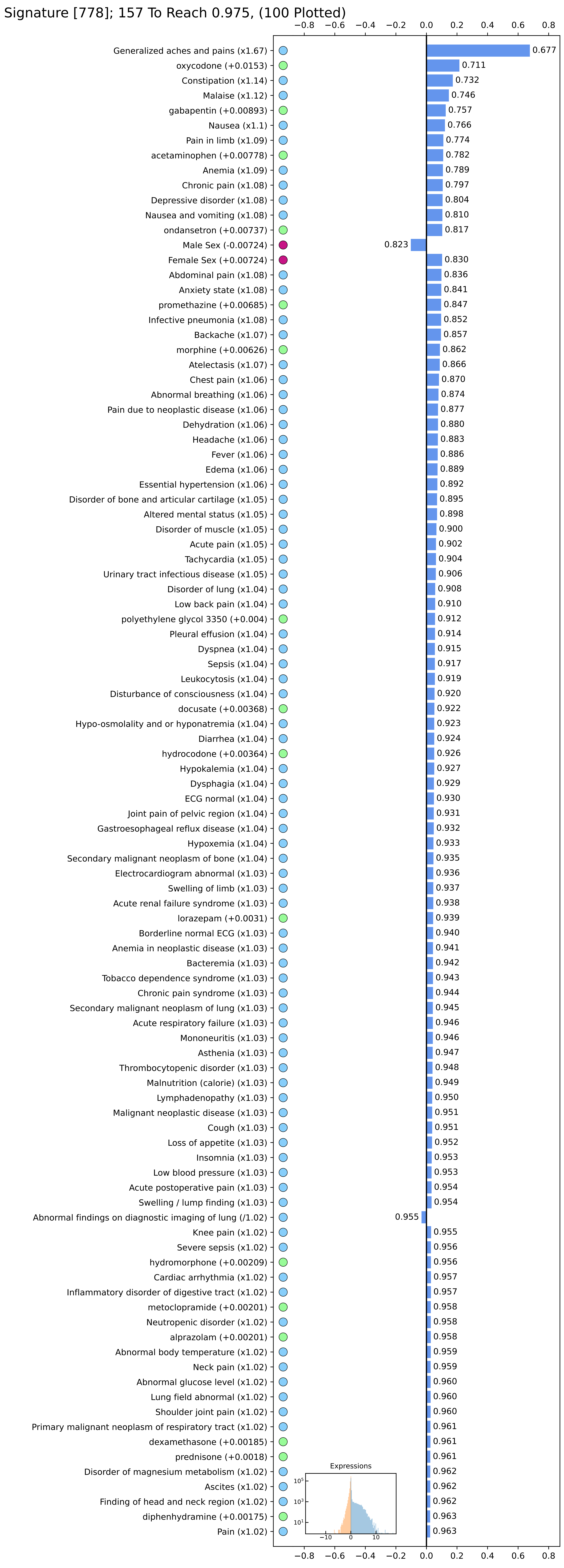
Signature [778]; 157 To Reach 0.975, (100 Plotted)
Generalized aches and pains (x1.67) 0.677
oxycodone (+0.0153) 0.711
Constipation (x1.14) 0.732
Malaise (x1.12) 0.746
gabapentin (+0.00893) 0.757
Nausea (x1.1) 0.766
Pain in limb (x1.09) 0.774
acetaminophen (+0.00778) 0.782
Anemia (x1.09) 0.789
Chronic pain (x1.08) 0.797
Depressive disorder (x1.08) 0.804
Nausea and vomiting (x1.08) 0.810
ondansetron (+0.00737) 0.817
Male Sex (-0.00724) 0.823
Female Sex (+0.00724) 0.830
Abdominal pain (x1.08) 0.836
Anxiety state (x1.08) 0.841
promethazine (+0.00685) 0.847
Infective pneumonia (x1.08) 0.852
Backache (x1.07) 0.857
morphine (+0.00626) 0.862
Atelectasis (x1.07) 0.866
Chest pain (x1.06) 0.870
Abnormal breathing (x1.06) 0.874
Pain due to neoplastic disease (x1.06) 0.877
Dehydration (x1.06) 0.880
Headache (x1.06) 0.883
Fever (x1.06) 0.886
Edema (x1.06) 0.889
Essential hypertension (x1.06) 0.892
Disorder of bone and articular cartilage (x1.05) 0.895
Altered mental status (x1.05) 0.898
Disorder of muscle (x1.05) 0.900
Acute pain (x1.05) 0.902
Tachycardia (x1.05) 0.904
Urinary tract infectious disease (x1.05) 0.906
Disorder of lung (x1.04) 0.908
Low back pain (x1.04) 0.910
polyethylene glycol 3350 (+0.004) 0.912
Pleural effusion (x1.04) 0.914
Dyspnea (x1.04) 0.915
Sepsis (x1.04) 0.917
Leukocytosis (x1.04) 0.919
Disturbance of consciousness (x1.04) 0.920
docusate (+0.00368) 0.922
Hypo-osmolality and or hyponatremia (x1.04) 0.923
Diarrhea (x1.04) 0.924
hydrocodone (+0.00364) 0.926
Hypokalemia (x1.04) 0.927
Dysphagia (x1.04) 0.929
ECG normal (x1.04) 0.930
Joint pain of pelvic region (x1.04) 0.931
Gastroesophageal reflux disease (x1.04) 0.932
Hypoxemia (x1.04) 0.933
Secondary malignant neoplasm of bone (x1.04) 0.935
Electrocardiogram abnormal (x1.03) 0.936
Swelling of limb (x1.03) 0.937
Acute renal failure syndrome (x1.03) 0.938
lorazepam (+0.0031) 0.939
Borderline normal ECG (x1.03) 0.940
Anemia in neoplastic disease (x1.03) 0.941
Bacteremia (x1.03) 0.942
Tobacco dependence syndrome (x1.03) 0.943
Chronic pain syndrome (x1.03) 0.944
Secondary malignant neoplasm of lung (x1.03) 0.945
Acute respiratory failure (x1.03) 0.946
Mononeuritis (x1.03) 0.946
Asthenia (x1.03) 0.947
Thrombocytopenic disorder (x1.03) 0.948
Malnutrition (calorie) (x1.03) 0.949
Lymphadenopathy (x1.03) 0.950
Malignant neoplastic disease (x1.03) 0.951
Cough (x1.03) 0.951
Loss of appetite (x1.03) 0.952
Insomnia (x1.03) 0.953
Low blood pressure (x1.03) 0.953
Acute postoperative pain (x1.03) 0.954
Swelling / lump finding (x1.03) 0.954
Abnormal findings on diagnostic imaging of lung (/1.02) 0.955
Knee pain (x1.02) 0.955
Severe sepsis (x1.02) 0.956
hydromorphone (+0.00209) 0.956
Cardiac arrhythmia (x1.02) 0.957
Inflammatory disorder of digestive tract (x1.02) 0.957
metoclopramide (+0.00201) 0.958
Neutropenic disorder (x1.02) 0.958
alprazolam (+0.00201) 0.958
Abnormal body temperature (x1.02) 0.959
Neck pain (x1.02) 0.959
Abnormal glucose level (x1.02) 0.960
Lung field abnormal (x1.02) 0.960
Shoulder joint pain (x1.02) 0.960
Primary malignant neoplasm of respiratory tract (x1.02) 0.961
dexamethasone (+0.00185) 0.961
prednisone (+0.0018) 0.961
Disorder of magnesium metabolism (x1.02) 0.962
Ascites (x1.02) 0.962
Finding of head and neck region (x1.02) 0.962
diphenhydramine (+0.00175) 0.963
Pain (x1.02) 0.963
Expressions
−0.8 −0.6 −0.4 −0.2 0.0 0.2 0.4 0.6 0.8

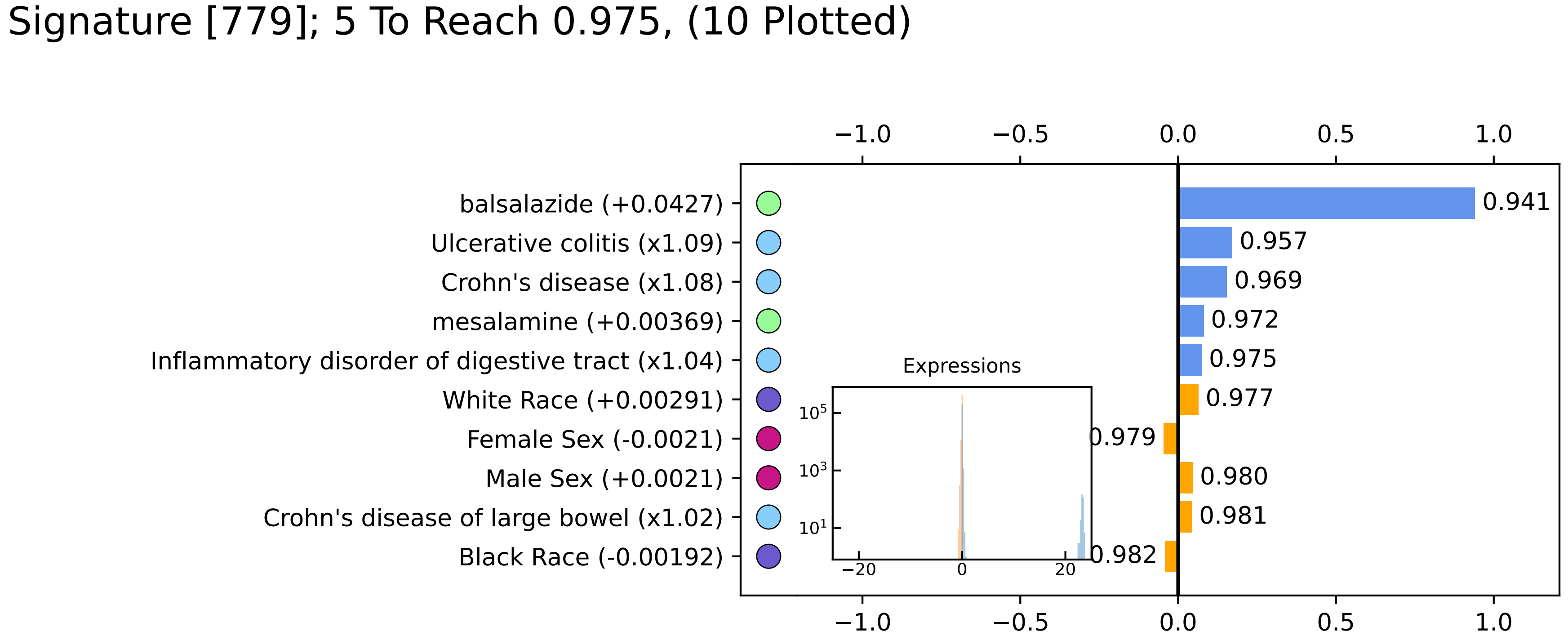
Signature [779]; 5 To Reach 0.975, (10 Plotted)
balsalazide (+0.0427)
Ulcerative colitis (x1.09)
Crohn's disease (x1.08)
mesalamine (+0.00369)
Inflammatory disorder of digestive tract (x1.04)
White Race (+0.00291)
Female Sex (-0.0021)
Male Sex (+0.0021)
Crohn's disease of large bowel (x1.02)
Black Race (-0.00192)
0.941
0.957
0.969
0.972
0.975
0.977
0.979
0.980
0.981
0.982
−1.0
−0.5
0.0
0.5
1.0
Expressions
10^5
10^3
10^1
−20
0
20

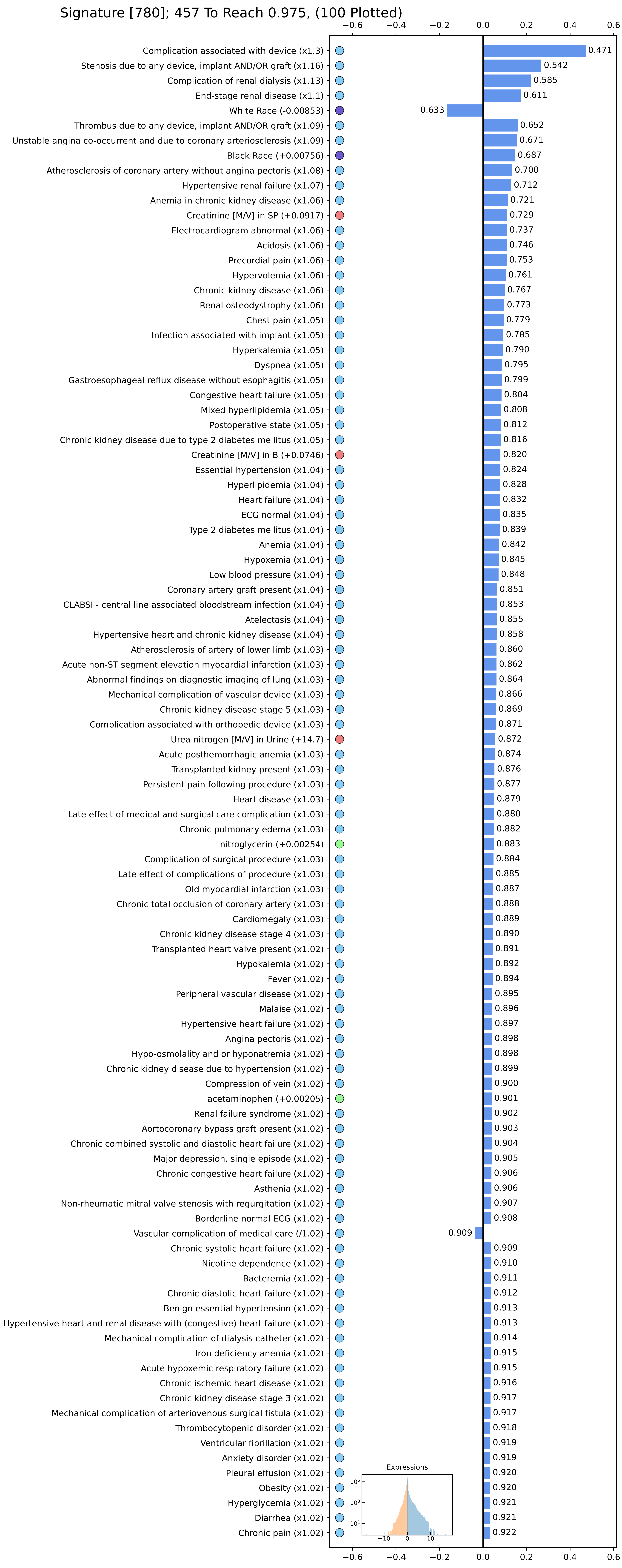

Signature [780]; 457 To Reach 0.975, (100 Plotted)
Complication associated with device (x1.3) 0.471
Stenosis due to any device, implant AND/OR graft (x1.16) 0.542
Complication of renal dialysis (x1.13) 0.585
End-stage renal disease (x1.1) 0.611
White Race (-0.00853) 0.633
Thrombus due to any device, implant AND/OR graft (x1.09) 0.652
Unstable angina co-occurrent and due to coronary arteriosclerosis (x1.09) 0.671
Black Race (+0.00756) 0.687
Atherosclerosis of coronary artery without angina pectoris (x1.08) 0.700
Hypertensive renal failure (x1.07) 0.712
Anemia in chronic kidney disease (x1.06) 0.721
Creatinine [M/V] in SP (+0.0917) 0.729
Electrocardiogram abnormal (x1.06) 0.737
Acidosis (x1.06) 0.746
Precordial pain (x1.06) 0.753
Hypervolemia (x1.06) 0.761
Chronic kidney disease (x1.06) 0.767
Renal osteodystrophy (x1.06) 0.773
Chest pain (x1.05) 0.779
Infection associated with implant (x1.05) 0.785
Hyperkalemia (x1.05) 0.790
Dyspnea (x1.05) 0.795
Gastroesophageal reflux disease without esophagitis (x1.05) 0.799
Congestive heart failure (x1.05) 0.804
Mixed hyperlipidemia (x1.05) 0.808
Postoperative state (x1.05) 0.812
Chronic kidney disease due to type 2 diabetes mellitus (x1.05) 0.816
Creatinine [M/V] in B (+0.0746) 0.820
Essential hypertension (x1.04) 0.824
Hyperlipidemia (x1.04) 0.828
Heart failure (x1.04) 0.832
ECG normal (x1.04) 0.835
Type 2 diabetes mellitus (x1.04) 0.839
Anemia (x1.04) 0.842
Hypoxemia (x1.04) 0.845
Low blood pressure (x1.04) 0.848
Coronary artery graft present (x1.04) 0.851
CLABSI - central line associated bloodstream infection (x1.04) 0.853
Atelectasis (x1.04) 0.855
Hypertensive heart and chronic kidney disease (x1.04) 0.858
Atherosclerosis of artery of lower limb (x1.03) 0.860
Acute non-ST segment elevation myocardial infarction (x1.03) 0.862
Abnormal findings on diagnostic imaging of lung (x1.03) 0.864
Mechanical complication of vascular device (x1.03) 0.866
Chronic kidney disease stage 5 (x1.03) 0.869
Complication associated with orthopedic device (x1.03) 0.871
Urea nitrogen [M/V] in Urine (+14.7) 0.872
Acute posthemorrhagic anemia (x1.03) 0.874
Transplanted kidney present (x1.03) 0.876
Persistent pain following procedure (x1.03) 0.877
Heart disease (x1.03) 0.879
Late effect of medical and surgical care complication (x1.03) 0.880
Chronic pulmonary edema (x1.03) 0.882
nitroglycerin (+0.00254) 0.883
Complication of surgical procedure (x1.03) 0.884
Late effect of complications of procedure (x1.03) 0.885
Old myocardial infarction (x1.03) 0.887
Chronic total occlusion of coronary artery (x1.03) 0.888
Cardiomegaly (x1.03) 0.889
Chronic kidney disease stage 4 (x1.03) 0.890
Transplanted heart valve present (x1.02) 0.891
Hypokalemia (x1.02) 0.892
Fever (x1.02) 0.894
Peripheral vascular disease (x1.02) 0.895
Malaise (x1.02) 0.896
Hypertensive heart failure (x1.02) 0.897
Angina pectoris (x1.02) 0.898
Hypo-osmolality and or hyponatremia (x1.02) 0.898
Chronic kidney disease due to hypertension (x1.02) 0.899
Compression of vein (x1.02) 0.900
acetaminophen (+0.00205) 0.901
Renal failure syndrome (x1.02) 0.902
Aortocoronary bypass graft present (x1.02) 0.903
Chronic combined systolic and diastolic heart failure (x1.02) 0.904
Major depression, single episode (x1.02) 0.905
Chronic congestive heart failure (x1.02) 0.906
Asthenia (x1.02) 0.906
Non-rheumatic mitral valve stenosis with regurgitation (x1.02) 0.907
Borderline normal ECG (x1.02) 0.908
Vascular complication of medical care (/1.02) 0.909
Chronic systolic heart failure (x1.02) 0.909
Nicotine dependence (x1.02) 0.910
Bacteremia (x1.02) 0.911
Chronic diastolic heart failure (x1.02) 0.912
Benign essential hypertension (x1.02) 0.913
Hypertensive heart and renal disease with (congestive) heart failure (x1.02) 0.913
Mechanical complication of dialysis catheter (x1.02) 0.914
Iron deficiency anemia (x1.02) 0.915
Acute hypoxemic respiratory failure (x1.02) 0.915
Chronic ischemic heart disease (x1.02) 0.916
Chronic kidney disease stage 3 (x1.02) 0.917
Mechanical complication of arteriovenous surgical fistula (x1.02) 0.917
Thrombocytopenic disorder (x1.02) 0.918
Ventricular fibrillation (x1.02) 0.919
Anxiety disorder (x1.02) 0.919
Pleural effusion (x1.02) 0.920
Obesity (x1.02) 0.920
Hyperglycemia (x1.02) 0.921
Diarrhea (x1.02) 0.921
Chronic pain (x1.02) 0.922
−0.6 −0.4 −0.2 0.0 0.2 0.4 0.6
Expressions

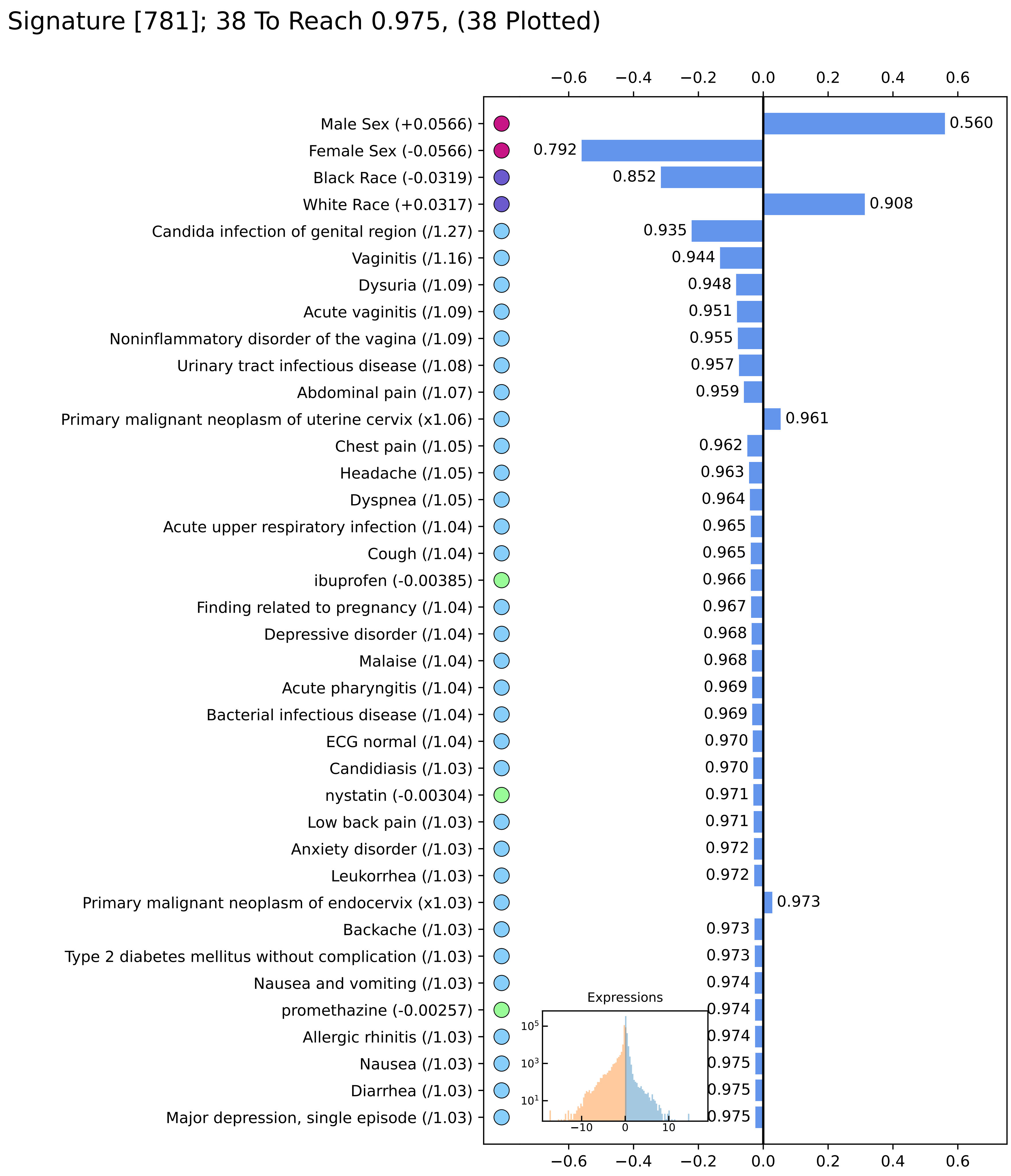
Signature [781]; 38 To Reach 0.975, (38 Plotted)
Male Sex (+0.0566) 0.560
Female Sex (-0.0566) 0.792
Black Race (-0.0319) 0.852
White Race (+0.0317) 0.908
Candida infection of genital region (/1.27) 0.935
Vaginitis (/1.16) 0.944
Dysuria (/1.09) 0.948
Acute vaginitis (/1.09) 0.951
Noninflammatory disorder of the vagina (/1.09) 0.955
Urinary tract infectious disease (/1.08) 0.957
Abdominal pain (/1.07) 0.959
Primary malignant neoplasm of uterine cervix (x1.06) 0.961
Chest pain (/1.05) 0.962
Headache (/1.05) 0.963
Dyspnea (/1.05) 0.964
Acute upper respiratory infection (/1.04) 0.965
Cough (/1.04) 0.965
ibuprofen (-0.00385) 0.966
Finding related to pregnancy (/1.04) 0.967
Depressive disorder (/1.04) 0.968
Malaise (/1.04) 0.968
Acute pharyngitis (/1.04) 0.969
Bacterial infectious disease (/1.04) 0.969
ECG normal (/1.04) 0.970
Candidiasis (/1.03) 0.970
nystatin (-0.00304) 0.971
Low back pain (/1.03) 0.971
Anxiety disorder (/1.03) 0.972
Leukorrhea (/1.03) 0.972
Primary malignant neoplasm of endocervix (x1.03) 0.973
Backache (/1.03) 0.973
Type 2 diabetes mellitus without complication (/1.03) 0.973
Nausea and vomiting (/1.03) 0.974
promethazine (-0.00257) 0.974
Allergic rhinitis (/1.03) 0.974
Nausea (/1.03) 0.975
Diarrhea (/1.03) 0.975
Major depression, single episode (/1.03) 0.975
Expressions
−0.6 −0.4 −0.2 0.0 0.2 0.4 0.6

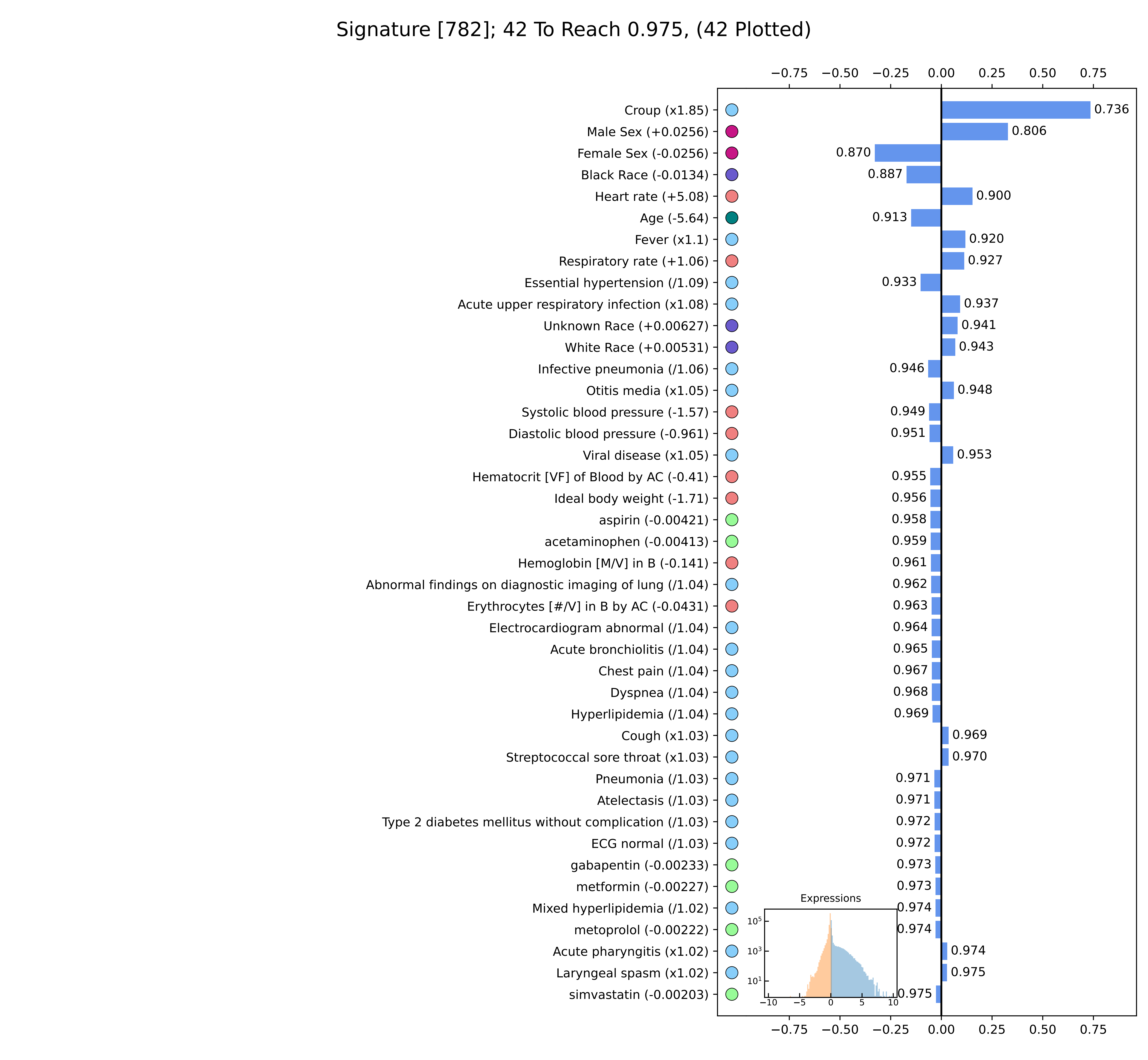

Signature [782]; 42 To Reach 0.975, (42 Plotted)
Croup (x1.85) 0.736
Male Sex (+0.0256) 0.806
Female Sex (-0.0256) 0.870
Black Race (-0.0134) 0.887
Heart rate (+5.08) 0.900
Age (-5.64) 0.913
Fever (x1.1) 0.920
Respiratory rate (+1.06) 0.927
Essential hypertension (/1.09) 0.933
Acute upper respiratory infection (x1.08) 0.937
Unknown Race (+0.00627) 0.941
White Race (+0.00531) 0.943
Infective pneumonia (/1.06) 0.946
Otitis media (x1.05) 0.948
Systolic blood pressure (-1.57) 0.949
Diastolic blood pressure (-0.961) 0.951
Viral disease (x1.05) 0.953
Hematocrit [VF] of Blood by AC (-0.41) 0.955
Ideal body weight (-1.71) 0.956
aspirin (-0.00421) 0.958
acetaminophen (-0.00413) 0.959
Hemoglobin [M/V] in B (-0.141) 0.961
Abnormal findings on diagnostic imaging of lung (/1.04) 0.962
Erythrocytes [#/V] in B by AC (-0.0431) 0.963
Electrocardiogram abnormal (/1.04) 0.964
Acute bronchiolitis (/1.04) 0.965
Chest pain (/1.04) 0.967
Dyspnea (/1.04) 0.968
Hyperlipidemia (/1.04) 0.969
Cough (x1.03) 0.969
Streptococcal sore throat (x1.03) 0.970
Pneumonia (/1.03) 0.971
Atelectasis (/1.03) 0.971
Type 2 diabetes mellitus without complication (/1.03) 0.972
ECG normal (/1.03) 0.972
gabapentin (-0.00233) 0.973
metformin (-0.00227) 0.973
Mixed hyperlipidemia (/1.02) 0.974
metoprolol (-0.00222) 0.974
Acute pharyngitis (x1.02) 0.974
Laryngeal spasm (x1.02) 0.975
simvastatin (-0.00203) 0.975
−0.75 −0.50 −0.25 0.00 0.25 0.50 0.75
Expressions

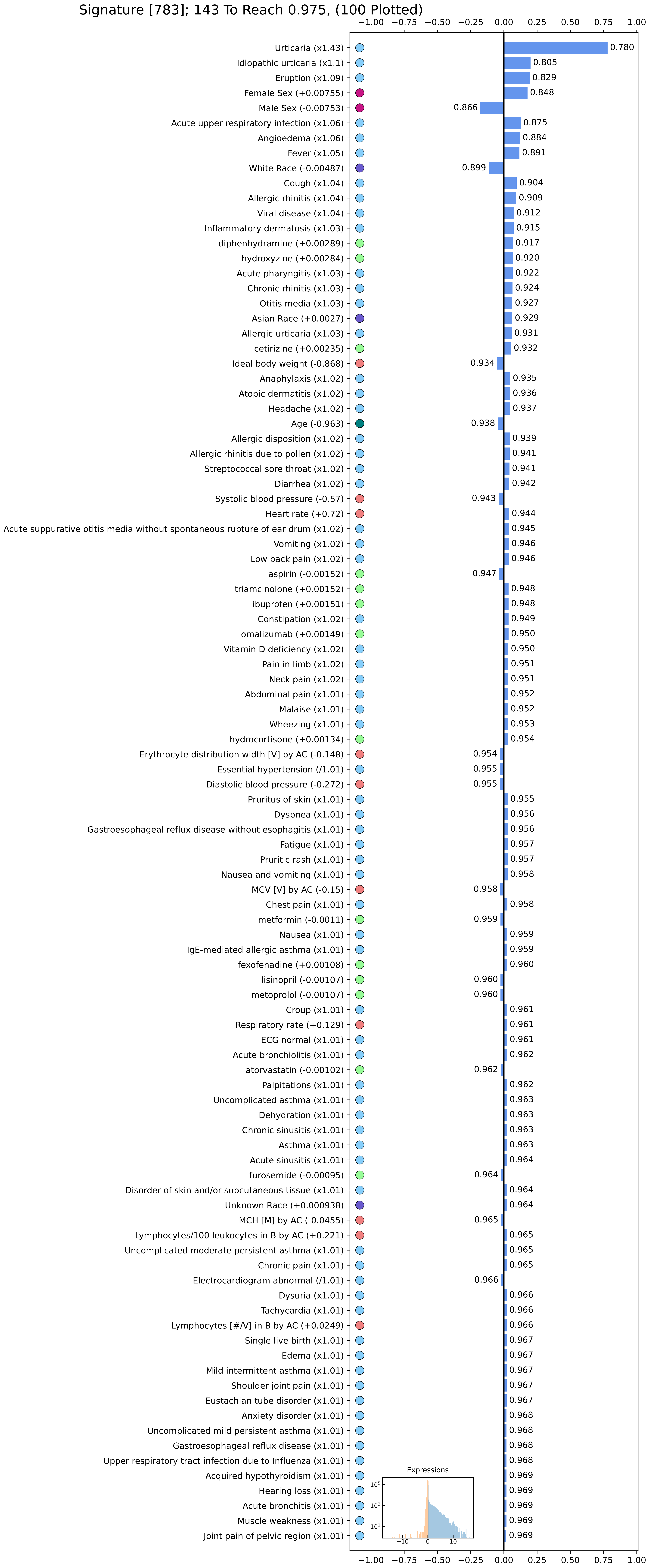

Signature [783]; 143 To Reach 0.975, (100 Plotted)
−1.00 −0.75 −0.50 −0.25 0.00 0.25 0.50 0.75 1.00
Urticaria (x1.43) 0.780
Idiopathic urticaria (x1.1) 0.805
Eruption (x1.09) 0.829
Female Sex (+0.00755) 0.848
Male Sex (-0.00753) 0.866
Acute upper respiratory infection (x1.06) 0.875
Angioedema (x1.06) 0.884
Fever (x1.05) 0.891
White Race (-0.00487) 0.899
Cough (x1.04) 0.904
Allergic rhinitis (x1.04) 0.909
Viral disease (x1.04) 0.912
Inflammatory dermatosis (x1.03) 0.915
diphenhydramine (+0.00289) 0.917
hydroxyzine (+0.00284) 0.920
Acute pharyngitis (x1.03) 0.922
Chronic rhinitis (x1.03) 0.924
Otitis media (x1.03) 0.927
Asian Race (+0.0027) 0.929
Allergic urticaria (x1.03) 0.931
cetirizine (+0.00235) 0.932
Ideal body weight (-0.868) 0.934
Anaphylaxis (x1.02) 0.935
Atopic dermatitis (x1.02) 0.936
Headache (x1.02) 0.937
Age (-0.963) 0.938
Allergic disposition (x1.02) 0.939
Allergic rhinitis due to pollen (x1.02) 0.941
Streptococcal sore throat (x1.02) 0.941
Diarrhea (x1.02) 0.942
Systolic blood pressure (-0.57) 0.943
Heart rate (+0.72) 0.944
Acute suppurative otitis media without spontaneous rupture of ear drum (x1.02) 0.945
Vomiting (x1.02) 0.946
Low back pain (x1.02) 0.946
aspirin (-0.00152) 0.947
triamcinolone (+0.00152) 0.948
ibuprofen (+0.00151) 0.948
Constipation (x1.02) 0.949
omalizumab (+0.00149) 0.950
Vitamin D deficiency (x1.02) 0.950
Pain in limb (x1.02) 0.951
Neck pain (x1.02) 0.951
Abdominal pain (x1.01) 0.952
Malaise (x1.01) 0.952
Wheezing (x1.01) 0.953
hydrocortisone (+0.00134) 0.954
Erythrocyte distribution width [V] by AC (-0.148) 0.954
Essential hypertension (/1.01) 0.955
Diastolic blood pressure (-0.272) 0.955
Pruritus of skin (x1.01) 0.955
Dyspnea (x1.01) 0.956
Gastroesophageal reflux disease without esophagitis (x1.01) 0.956
Fatigue (x1.01) 0.957
Pruritic rash (x1.01) 0.957
Nausea and vomiting (x1.01) 0.958
MCV [V] by AC (-0.15) 0.958
Chest pain (x1.01) 0.958
metformin (-0.0011) 0.959
Nausea (x1.01) 0.959
IgE-mediated allergic asthma (x1.01) 0.959
fexofenadine (+0.00108) 0.960
lisinopril (-0.00107) 0.960
metoprolol (-0.00107) 0.960
Croup (x1.01) 0.961
Respiratory rate (+0.129) 0.961
ECG normal (x1.01) 0.961
Acute bronchiolitis (x1.01) 0.962
atorvastatin (-0.00102) 0.962
Palpitations (x1.01) 0.962
Uncomplicated asthma (x1.01) 0.963
Dehydration (x1.01) 0.963
Chronic sinusitis (x1.01) 0.963
Asthma (x1.01) 0.963
Acute sinusitis (x1.01) 0.964
furosemide (-0.00095) 0.964
Disorder of skin and/or subcutaneous tissue (x1.01) 0.964
Unknown Race (+0.000938) 0.964
MCH [M] by AC (-0.0455) 0.965
Lymphocytes/100 leukocytes in B by AC (+0.221) 0.965
Uncomplicated moderate persistent asthma (x1.01) 0.965
Chronic pain (x1.01) 0.965
Electrocardiogram abnormal (/1.01) 0.966
Dysuria (x1.01) 0.966
Tachycardia (x1.01) 0.966
Lymphocytes [#/V] in B by AC (+0.0249) 0.966
Single live birth (x1.01) 0.967
Edema (x1.01) 0.967
Mild intermittent asthma (x1.01) 0.967
Shoulder joint pain (x1.01) 0.967
Eustachian tube disorder (x1.01) 0.967
Anxiety disorder (x1.01) 0.968
Uncomplicated mild persistent asthma (x1.01) 0.968
Gastroesophageal reflux disease (x1.01) 0.968
Upper respiratory tract infection due to Influenza (x1.01) 0.968
Acquired hypothyroidism (x1.01) 0.969
Hearing loss (x1.01) 0.969
Acute bronchitis (x1.01) 0.969
Muscle weakness (x1.01) 0.969
Joint pain of pelvic region (x1.01) 0.969
Expressions
10^5
10^3
10^1
−10 0 10
−1.00 −0.75 −0.50 −0.25 0.00 0.25 0.50 0.75 1.00

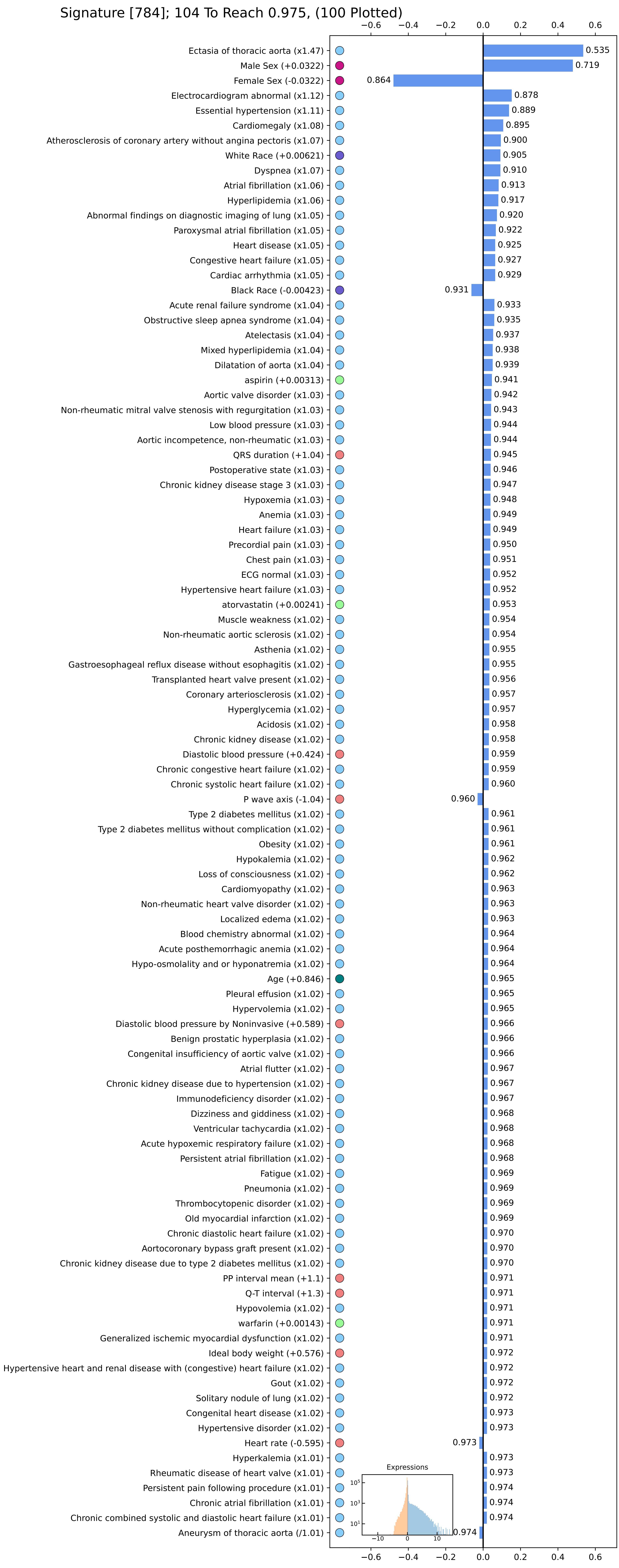

Signature [784]; 104 To Reach 0.975, (100 Plotted)
Ectasia of thoracic aorta (x1.47)
Male Sex (+0.0322)
Female Sex (-0.0322)
Electrocardiogram abnormal (x1.12)
Essential hypertension (x1.11)
Cardiomegaly (x1.08)
Atherosclerosis of coronary artery without angina pectoris (x1.07)
White Race (+0.00621)
Dyspnea (x1.07)
Atrial fibrillation (x1.06)
Hyperlipidemia (x1.06)
Abnormal findings on diagnostic imaging of lung (x1.05)
Paroxysmal atrial fibrillation (x1.05)
Heart disease (x1.05)
Congestive heart failure (x1.05)
Cardiac arrhythmia (x1.05)
Black Race (-0.00423)
Acute renal failure syndrome (x1.04)
Obstructive sleep apnea syndrome (x1.04)
Atelectasis (x1.04)
Mixed hyperlipidemia (x1.04)
Dilatation of aorta (x1.04)
aspirin (+0.00313)
Aortic valve disorder (x1.03)
Non-rheumatic mitral valve stenosis with regurgitation (x1.03)
Low blood pressure (x1.03)
Aortic incompetence, non-rheumatic (x1.03)
QRS duration (+1.04)
Postoperative state (x1.03)
Chronic kidney disease stage 3 (x1.03)
Hypoxemia (x1.03)
Anemia (x1.03)
Heart failure (x1.03)
Precordial pain (x1.03)
Chest pain (x1.03)
ECG normal (x1.03)
Hypertensive heart failure (x1.03)
atorvastatin (+0.00241)
Muscle weakness (x1.02)
Non-rheumatic aortic sclerosis (x1.02)
Asthenia (x1.02)
Gastroesophageal reflux disease without esophagitis (x1.02)
Transplanted heart valve present (x1.02)
Coronary arteriosclerosis (x1.02)
Hyperglycemia (x1.02)
Acidosis (x1.02)
Chronic kidney disease (x1.02)
Diastolic blood pressure (+0.424)
Chronic congestive heart failure (x1.02)
Chronic systolic heart failure (x1.02)
P wave axis (-1.04)
Type 2 diabetes mellitus (x1.02)
Type 2 diabetes mellitus without complication (x1.02)
Obesity (x1.02)
Hypokalemia (x1.02)
Loss of consciousness (x1.02)
Cardiomyopathy (x1.02)
Non-rheumatic heart valve disorder (x1.02)
Localized edema (x1.02)
Blood chemistry abnormal (x1.02)
Acute posthemorrhagic anemia (x1.02)
Hypo-osmolality and or hyponatremia (x1.02)
Age (+0.846)
Pleural effusion (x1.02)
Hypervolemia (x1.02)
Diastolic blood pressure by Noninvasive (+0.589)
Benign prostatic hyperplasia (x1.02)
Congenital insufficiency of aortic valve (x1.02)
Atrial flutter (x1.02)
Chronic kidney disease due to hypertension (x1.02)
Immunodeficiency disorder (x1.02)
Dizziness and giddiness (x1.02)
Ventricular tachycardia (x1.02)
Acute hypoxemic respiratory failure (x1.02)
Persistent atrial fibrillation (x1.02)
Fatigue (x1.02)
Pneumonia (x1.02)
Thrombocytopenic disorder (x1.02)
Old myocardial infarction (x1.02)
Chronic diastolic heart failure (x1.02)
Aortocoronary bypass graft present (x1.02)
Chronic kidney disease due to type 2 diabetes mellitus (x1.02)
PP interval mean (+1.1)
Q-T interval (+1.3)
Hypovolemia (x1.02)
warfarin (+0.00143)
Generalized ischemic myocardial dysfunction (x1.02)
Ideal body weight (+0.576)
Hypertensive heart and renal disease with (congestive) heart failure (x1.02)
Gout (x1.02)
Solitary nodule of lung (x1.02)
Congenital heart disease (x1.02)
Hypertensive disorder (x1.02)
Heart rate (-0.595)
Hyperkalemia (x1.01)
Rheumatic disease of heart valve (x1.01)
Persistent pain following procedure (x1.01)
Chronic atrial fibrillation (x1.01)
Chronic combined systolic and diastolic heart failure (x1.01)
Aneurysm of thoracic aorta (/1.01)
0.535
0.719
0.864
0.878
0.889
0.895
0.900
0.905
0.910
0.913
0.917
0.920
0.922
0.925
0.927
0.929
0.931
0.933
0.935
0.937
0.938
0.939
0.941
0.942
0.943
0.944
0.944
0.945
0.946
0.947
0.948
0.949
0.949
0.950
0.951
0.952
0.952
0.953
0.954
0.954
0.955
0.955
0.956
0.957
0.957
0.958
0.958
0.959
0.959
0.960
0.960
0.961
0.961
0.961
0.962
0.962
0.963
0.963
0.963
0.964
0.964
0.964
0.965
0.965
0.965
0.966
0.966
0.966
0.967
0.967
0.967
0.968
0.968
0.968
0.968
0.969
0.969
0.969
0.969
0.970
0.970
0.970
0.971
0.971
0.971
0.971
0.971
0.972
0.972
0.972
0.972
0.973
0.973
0.973
0.973
0.973
0.974
0.974
0.974
0.974
−0.6
−0.4
−0.2
0.0
0.2
0.4
0.6
Expressions
10^5
10^3
10^1
−10
0
10

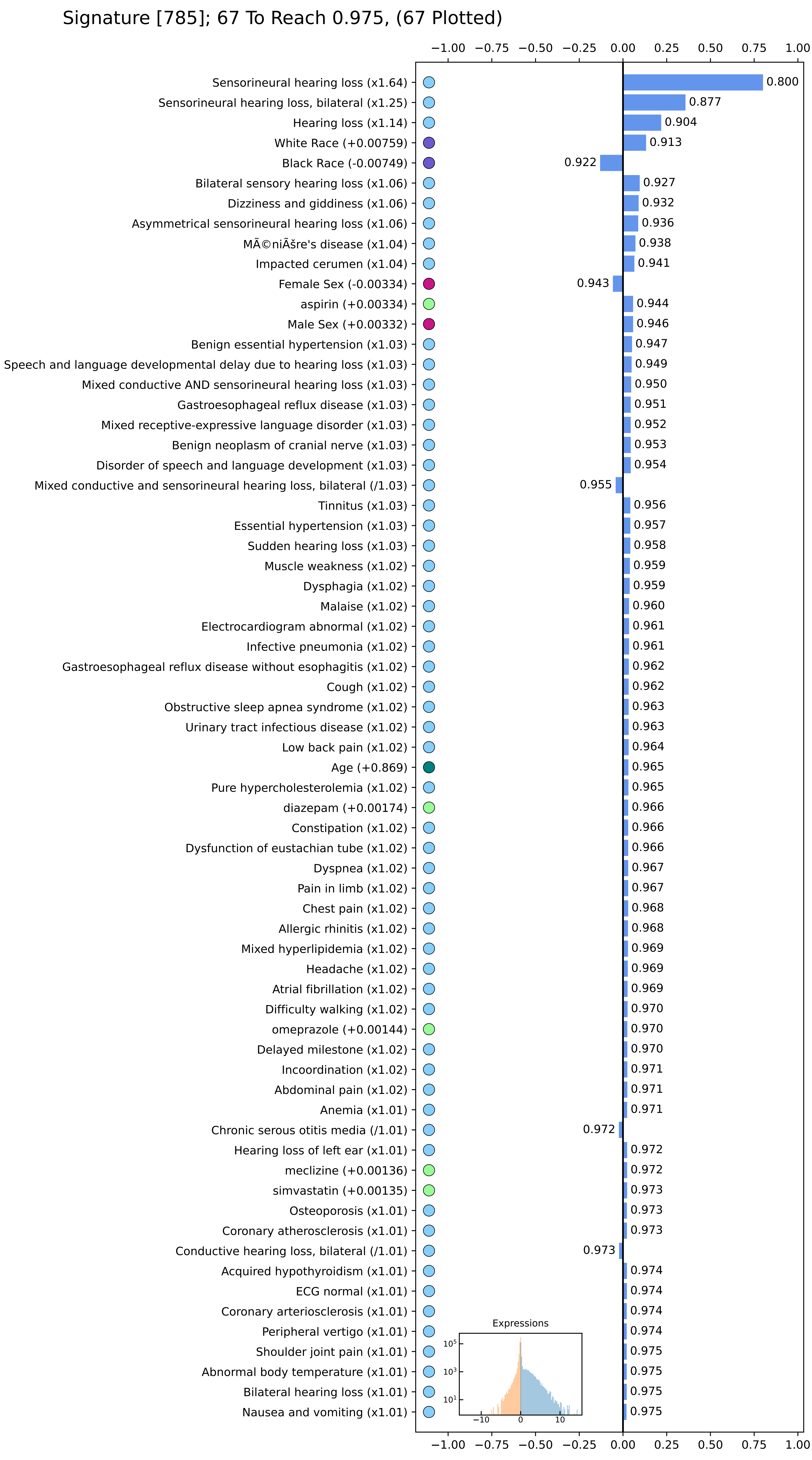

Signature [785]; 67 To Reach 0.975, (67 Plotted)
−1.00 −0.75 −0.50 −0.25 0.00 0.25 0.50 0.75 1.00
Sensorineural hearing loss (x1.64) 0.800
Sensorineural hearing loss, bilateral (x1.25) 0.877
Hearing loss (x1.14) 0.904
White Race (+0.00759) 0.913
Black Race (-0.00749) 0.922
Bilateral sensory hearing loss (x1.06) 0.927
Dizziness and giddiness (x1.06) 0.932
Asymmetrical sensorineural hearing loss (x1.06) 0.936
MÃ©niÃšre's disease (x1.04) 0.938
Impacted cerumen (x1.04) 0.941
Female Sex (-0.00334) 0.943
aspirin (+0.00334) 0.944
Male Sex (+0.00332) 0.946
Benign essential hypertension (x1.03) 0.947
Speech and language developmental delay due to hearing loss (x1.03) 0.949
Mixed conductive AND sensorineural hearing loss (x1.03) 0.950
Gastroesophageal reflux disease (x1.03) 0.951
Mixed receptive-expressive language disorder (x1.03) 0.952
Benign neoplasm of cranial nerve (x1.03) 0.953
Disorder of speech and language development (x1.03) 0.954
Mixed conductive and sensorineural hearing loss, bilateral (/1.03) 0.955
Tinnitus (x1.03) 0.956
Essential hypertension (x1.03) 0.957
Sudden hearing loss (x1.03) 0.958
Muscle weakness (x1.02) 0.959
Dysphagia (x1.02) 0.959
Malaise (x1.02) 0.960
Electrocardiogram abnormal (x1.02) 0.961
Infective pneumonia (x1.02) 0.961
Gastroesophageal reflux disease without esophagitis (x1.02) 0.962
Cough (x1.02) 0.962
Obstructive sleep apnea syndrome (x1.02) 0.963
Urinary tract infectious disease (x1.02) 0.963
Low back pain (x1.02) 0.964
Age (+0.869) 0.965
Pure hypercholesterolemia (x1.02) 0.965
diazepam (+0.00174) 0.966
Constipation (x1.02) 0.966
Dysfunction of eustachian tube (x1.02) 0.966
Dyspnea (x1.02) 0.967
Pain in limb (x1.02) 0.967
Chest pain (x1.02) 0.968
Allergic rhinitis (x1.02) 0.968
Mixed hyperlipidemia (x1.02) 0.969
Headache (x1.02) 0.969
Atrial fibrillation (x1.02) 0.969
Difficulty walking (x1.02) 0.970
omeprazole (+0.00144) 0.970
Delayed milestone (x1.02) 0.970
Incoordination (x1.02) 0.971
Abdominal pain (x1.02) 0.971
Anemia (x1.01) 0.971
Chronic serous otitis media (/1.01) 0.972
Hearing loss of left ear (x1.01) 0.972
meclizine (+0.00136) 0.972
simvastatin (+0.00135) 0.973
Osteoporosis (x1.01) 0.973
Coronary atherosclerosis (x1.01) 0.973
Conductive hearing loss, bilateral (/1.01) 0.973
Acquired hypothyroidism (x1.01) 0.974
ECG normal (x1.01) 0.974
Coronary arteriosclerosis (x1.01) 0.974
Peripheral vertigo (x1.01) 0.974
Shoulder joint pain (x1.01) 0.975
Abnormal body temperature (x1.01) 0.975
Bilateral hearing loss (x1.01) 0.975
Nausea and vomiting (x1.01) 0.975
Expressions
10^5
10^3
10^1
−10
0
10

Signature [786]; 6 To Reach 0.975, (10 Plotted)

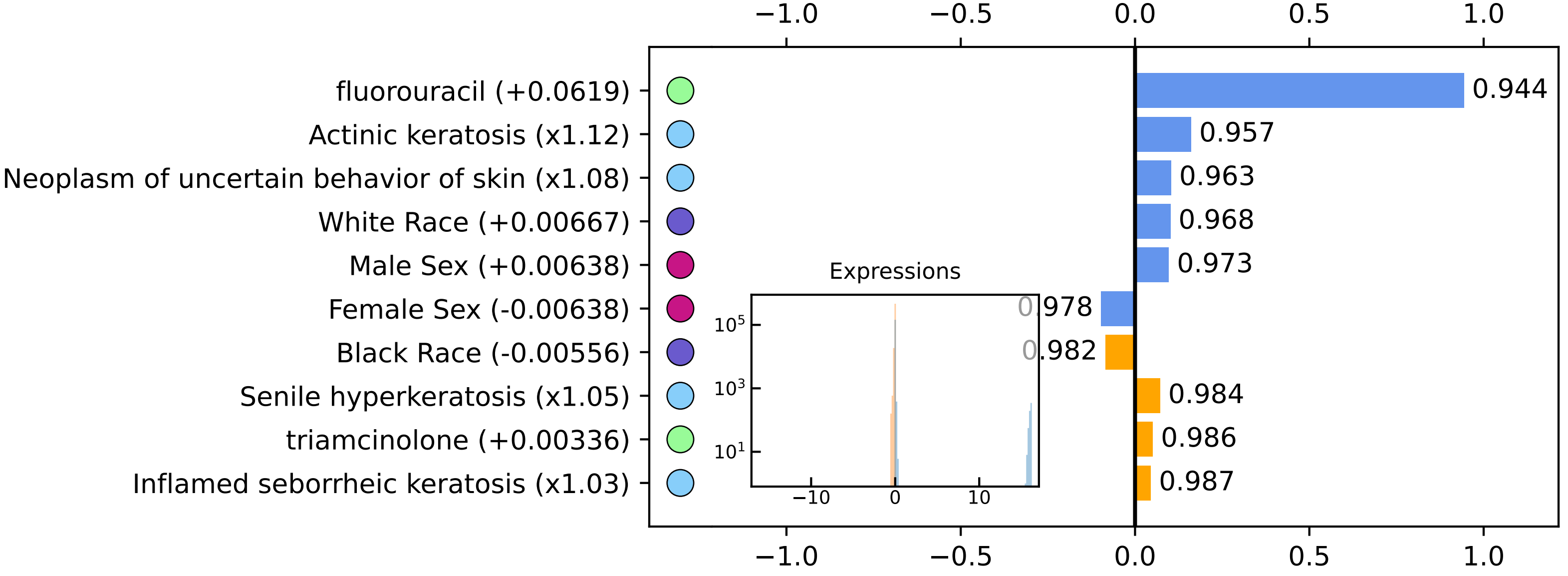

Signature [787]; 1 To Reach 0.975, (10 Plotted)

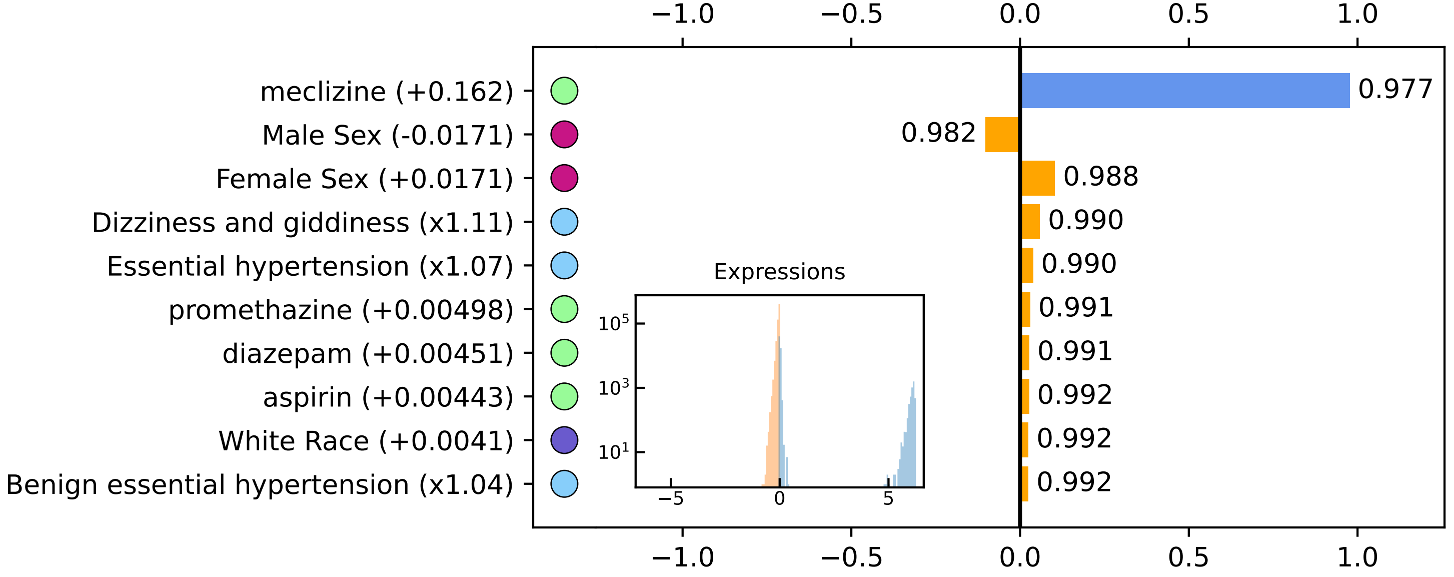

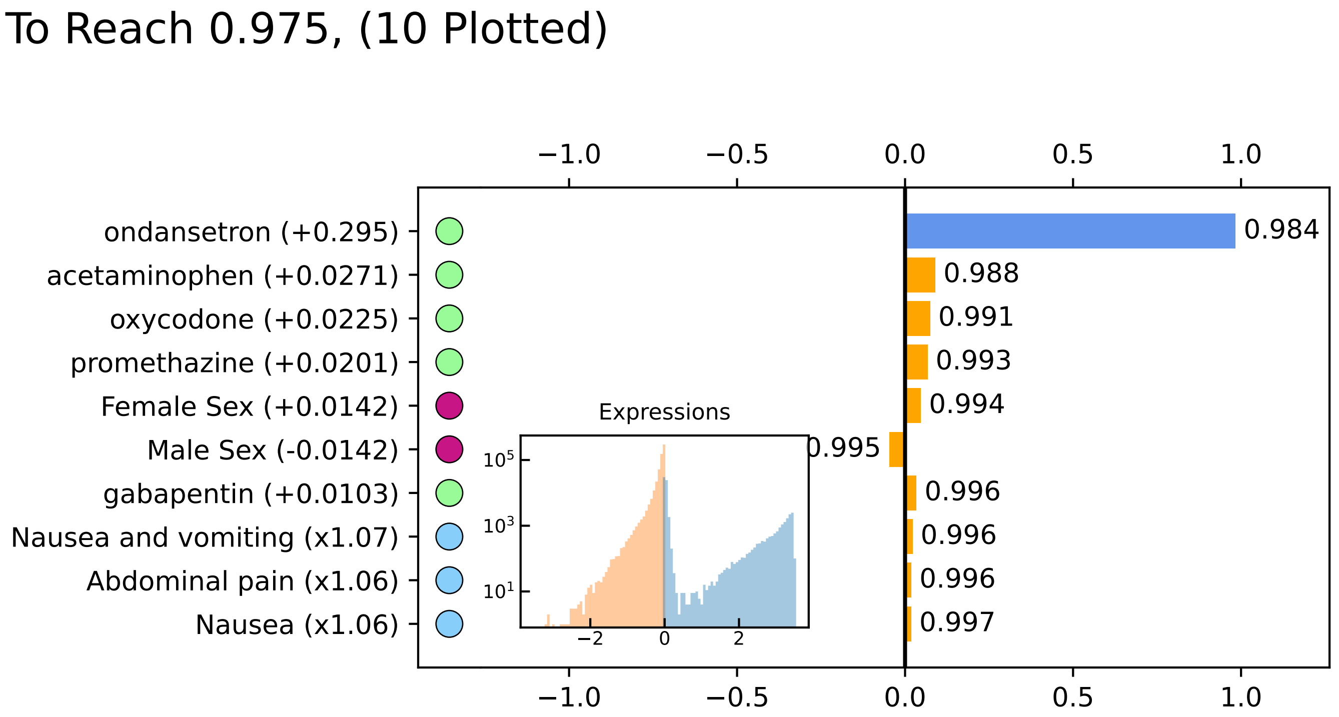

Signature [788]; 1 To Reach 0.975, (10 Plotted)
ondansetron (+0.295)
acetaminophen (+0.0271)
oxycodone (+0.0225)
promethazine (+0.0201)
Female Sex (+0.0142)
Male Sex (-0.0142)
gabapentin (+0.0103)
Nausea and vomiting (x1.07)
Abdominal pain (x1.06)
Nausea (x1.06)
0.984
0.988
0.991
0.993
0.994
0.995
0.996
0.996
0.996
0.997
Expressions
−1.0
−0.5
0.0
0.5
1.0

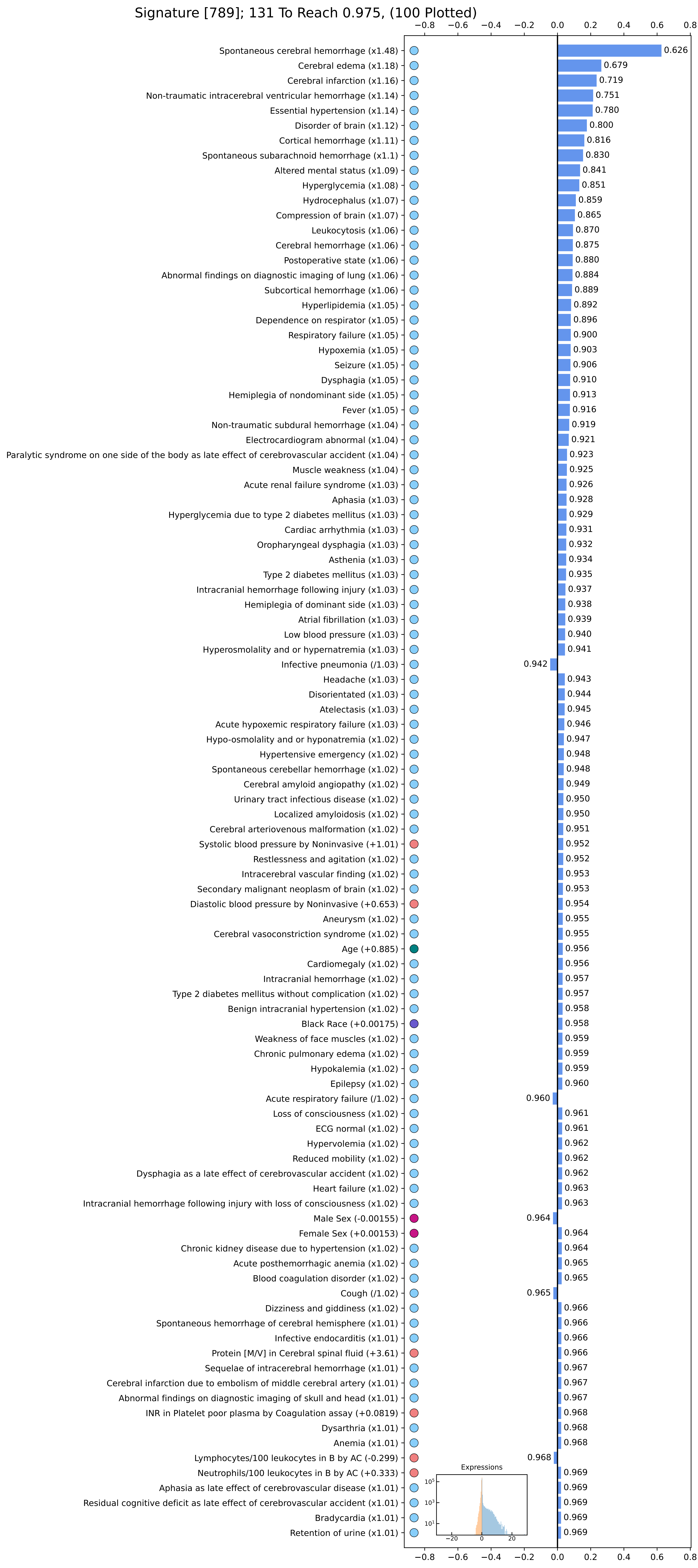
Signature [789]; 131 To Reach 0.975, (100 Plotted)
Spontaneous cerebral hemorrhage (x1.48) 0.626
Cerebral edema (x1.18) 0.679
Cerebral infarction (x1.16) 0.719
Non-traumatic intracerebral ventricular hemorrhage (x1.14) 0.751
Essential hypertension (x1.14) 0.780
Disorder of brain (x1.12) 0.800
Cortical hemorrhage (x1.11) 0.816
Spontaneous subarachnoid hemorrhage (x1.1) 0.830
Altered mental status (x1.09) 0.841
Hyperglycemia (x1.08) 0.851
Hydrocephalus (x1.07) 0.859
Compression of brain (x1.07) 0.865
Leukocytosis (x1.06) 0.870
Cerebral hemorrhage (x1.06) 0.875
Postoperative state (x1.06) 0.880
Abnormal findings on diagnostic imaging of lung (x1.06) 0.884
Subcortical hemorrhage (x1.06) 0.889
Hyperlipidemia (x1.05) 0.892
Dependence on respirator (x1.05) 0.896
Respiratory failure (x1.05) 0.900
Hypoxemia (x1.05) 0.903
Seizure (x1.05) 0.906
Dysphagia (x1.05) 0.910
Hemiplegia of nondominant side (x1.05) 0.913
Fever (x1.05) 0.916
Non-traumatic subdural hemorrhage (x1.04) 0.919
Electrocardiogram abnormal (x1.04) 0.921
Paralytic syndrome on one side of the body as late effect of cerebrovascular accident (x1.04) 0.923
Muscle weakness (x1.04) 0.925
Acute renal failure syndrome (x1.03) 0.926
Aphasia (x1.03) 0.928
Hyperglycemia due to type 2 diabetes mellitus (x1.03) 0.929
Cardiac arrhythmia (x1.03) 0.931
Oropharyngeal dysphagia (x1.03) 0.932
Asthenia (x1.03) 0.934
Type 2 diabetes mellitus (x1.03) 0.935
Intracranial hemorrhage following injury (x1.03) 0.937
Hemiplegia of dominant side (x1.03) 0.938
Atrial fibrillation (x1.03) 0.939
Low blood pressure (x1.03) 0.940
Hyperosmolality and or hypernatremia (x1.03) 0.941
Infective pneumonia (/1.03) 0.942
Headache (x1.03) 0.943
Disorientated (x1.03) 0.944
Atelectasis (x1.03) 0.945
Acute hypoxemic respiratory failure (x1.03) 0.946
Hypo-osmolality and or hyponatremia (x1.02) 0.947
Hypertensive emergency (x1.02) 0.948
Spontaneous cerebellar hemorrhage (x1.02) 0.948
Cerebral amyloid angiopathy (x1.02) 0.949
Urinary tract infectious disease (x1.02) 0.950
Localized amyloidosis (x1.02) 0.950
Cerebral arteriovenous malformation (x1.02) 0.951
Systolic blood pressure by Noninvasive (+1.01) 0.952
Restlessness and agitation (x1.02) 0.952
Intracerebral vascular finding (x1.02) 0.953
Secondary malignant neoplasm of brain (x1.02) 0.953
Diastolic blood pressure by Noninvasive (+0.653) 0.954
Aneurysm (x1.02) 0.955
Cerebral vasoconstriction syndrome (x1.02) 0.955
Age (+0.885) 0.956
Cardiomegaly (x1.02) 0.956
Intracranial hemorrhage (x1.02) 0.957
Type 2 diabetes mellitus without complication (x1.02) 0.957
Benign intracranial hypertension (x1.02) 0.958
Black Race (+0.00175) 0.958
Weakness of face muscles (x1.02) 0.959
Chronic pulmonary edema (x1.02) 0.959
Hypokalemia (x1.02) 0.959
Epilepsy (x1.02) 0.960
Acute respiratory failure (/1.02) 0.960
Loss of consciousness (x1.02) 0.961
ECG normal (x1.02) 0.961
Hypervolemia (x1.02) 0.962
Reduced mobility (x1.02) 0.962
Dysphagia as a late effect of cerebrovascular accident (x1.02) 0.962
Heart failure (x1.02) 0.963
Intracranial hemorrhage following injury with loss of consciousness (x1.02) 0.963
Male Sex (-0.00155) 0.964
Female Sex (+0.00153) 0.964
Chronic kidney disease due to hypertension (x1.02) 0.964
Acute posthemorrhagic anemia (x1.02) 0.965
Blood coagulation disorder (x1.02) 0.965
Cough (/1.02) 0.965
Dizziness and giddiness (x1.02) 0.966
Spontaneous hemorrhage of cerebral hemisphere (x1.01) 0.966
Infective endocarditis (x1.01) 0.966
Protein [M/V] in Cerebral spinal fluid (+3.61) 0.966
Sequelae of intracerebral hemorrhage (x1.01) 0.967
Cerebral infarction due to embolism of middle cerebral artery (x1.01) 0.967
Abnormal findings on diagnostic imaging of skull and head (x1.01) 0.967
INR in Platelet poor plasma by Coagulation assay (+0.0819) 0.968
Dysarthria (x1.01) 0.968
Anemia (x1.01) 0.968
Lymphocytes/100 leukocytes in B by AC (-0.299) 0.968
Neutrophils/100 leukocytes in B by AC (+0.333) 0.969
Aphasia as late effect of cerebrovascular disease (x1.01) 0.969
Residual cognitive deficit as late effect of cerebrovascular accident (x1.01) 0.969
Bradycardia (x1.01) 0.969
Retention of urine (x1.01) 0.969
Expressions
−0.8 −0.6 −0.4 −0.2 0.0 0.2 0.4 0.6 0.8

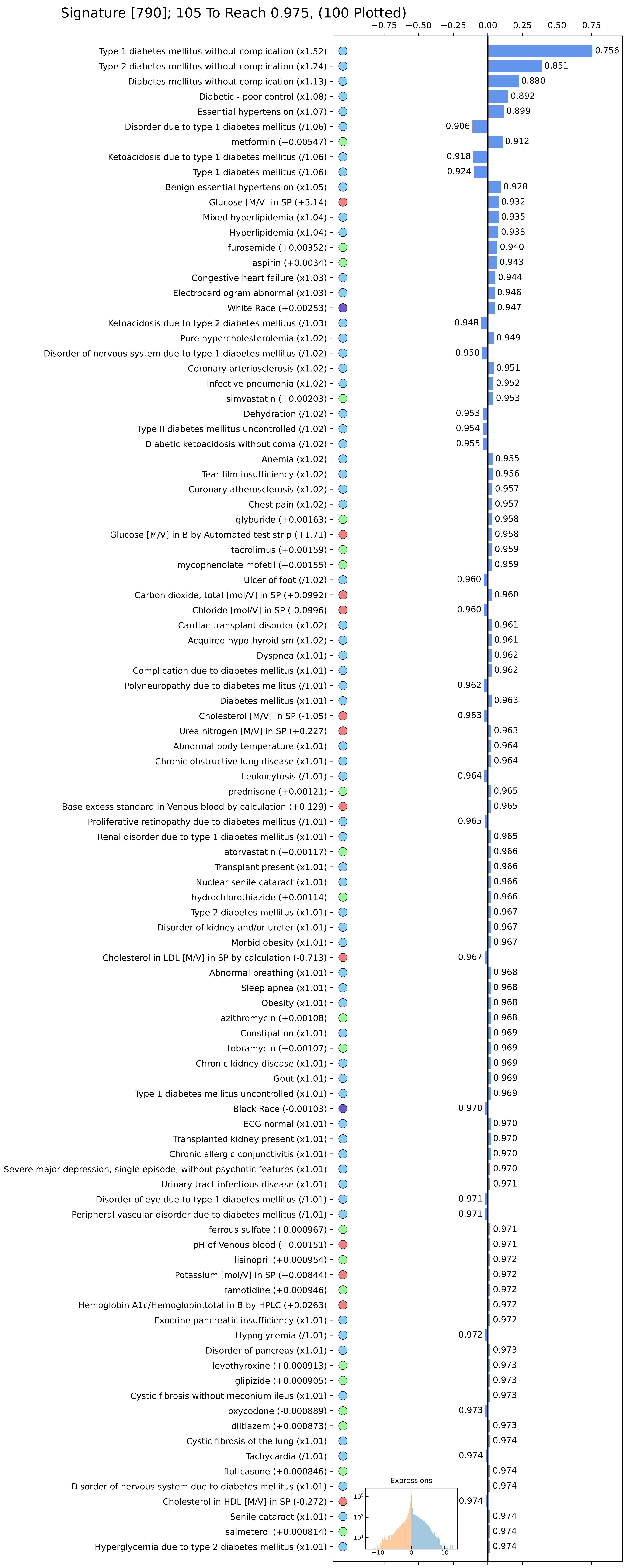

Signature [790]; 105 To Reach 0.975, (100 Plotted)
Type 1 diabetes mellitus without complication (x1.52) 0.756
Type 2 diabetes mellitus without complication (x1.24) 0.851
Diabetes mellitus without complication (x1.13) 0.880
Diabetic - poor control (x1.08) 0.892
Essential hypertension (x1.07) 0.899
Disorder due to type 1 diabetes mellitus (/1.06) 0.906
metformin (+0.00547) 0.912
Ketoacidosis due to type 1 diabetes mellitus (/1.06) 0.918
Type 1 diabetes mellitus (/1.06) 0.924
Benign essential hypertension (x1.05) 0.928
Glucose [M/V] in SP (+3.14) 0.932
Mixed hyperlipidemia (x1.04) 0.935
Hyperlipidemia (x1.04) 0.938
furosemide (+0.00352) 0.940
aspirin (+0.0034) 0.943
Congestive heart failure (x1.03) 0.944
Electrocardiogram abnormal (x1.03) 0.946
White Race (+0.00253) 0.947
Ketoacidosis due to type 2 diabetes mellitus (/1.03) 0.948
Pure hypercholesterolemia (x1.02) 0.949
Disorder of nervous system due to type 1 diabetes mellitus (/1.02) 0.950
Coronary arteriosclerosis (x1.02) 0.951
Infective pneumonia (x1.02) 0.952
simvastatin (+0.00203) 0.953
Dehydration (/1.02) 0.953
Type II diabetes mellitus uncontrolled (/1.02) 0.954
Diabetic ketoacidosis without coma (/1.02) 0.955
Anemia (x1.02) 0.955
Tear film insufficiency (x1.02) 0.956
Coronary atherosclerosis (x1.02) 0.957
Chest pain (x1.02) 0.957
glyburide (+0.00163) 0.958
Glucose [M/V] in B by Automated test strip (+1.71) 0.958
tacrolimus (+0.00159) 0.959
mycophenolate mofetil (+0.00155) 0.959
Ulcer of foot (/1.02) 0.960
Carbon dioxide, total [mol/V] in SP (+0.0992) 0.960
Chloride [mol/V] in SP (-0.0996) 0.960
Cardiac transplant disorder (x1.02) 0.961
Acquired hypothyroidism (x1.02) 0.961
Dyspnea (x1.01) 0.962
Complication due to diabetes mellitus (x1.01) 0.962
Polyneuropathy due to diabetes mellitus (/1.01) 0.962
Diabetes mellitus (x1.01) 0.963
Cholesterol [M/V] in SP (-1.05) 0.963
Urea nitrogen [M/V] in SP (+0.227) 0.963
Abnormal body temperature (x1.01) 0.964
Chronic obstructive lung disease (x1.01) 0.964
Leukocytosis (/1.01) 0.964
prednisone (+0.00121) 0.965
Base excess standard in Venous blood by calculation (+0.129) 0.965
Proliferative retinopathy due to diabetes mellitus (/1.01) 0.965
Renal disorder due to type 1 diabetes mellitus (x1.01) 0.965
atorvastatin (+0.00117) 0.966
Transplant present (x1.01) 0.966
Nuclear senile cataract (x1.01) 0.966
hydrochlorothiazide (+0.00114) 0.966
Type 2 diabetes mellitus (x1.01) 0.967
Disorder of kidney and/or ureter (x1.01) 0.967
Morbid obesity (x1.01) 0.967
Cholesterol in LDL [M/V] in SP by calculation (-0.713) 0.967
Abnormal breathing (x1.01) 0.968
Sleep apnea (x1.01) 0.968
Obesity (x1.01) 0.968
azithromycin (+0.00108) 0.968
Constipation (x1.01) 0.969
tobramycin (+0.00107) 0.969
Chronic kidney disease (x1.01) 0.969
Gout (x1.01) 0.969
Type 1 diabetes mellitus uncontrolled (x1.01) 0.969
Black Race (-0.00103) 0.970
ECG normal (x1.01) 0.970
Transplanted kidney present (x1.01) 0.970
Chronic allergic conjunctivitis (x1.01) 0.970
Severe major depression, single episode, without psychotic features (x1.01) 0.970
Urinary tract infectious disease (x1.01) 0.971
Disorder of eye due to type 1 diabetes mellitus (/1.01) 0.971
Peripheral vascular disorder due to diabetes mellitus (/1.01) 0.971
ferrous sulfate (+0.000967) 0.971
pH of Venous blood (+0.00151) 0.971
lisinopril (+0.000954) 0.972
Potassium [mol/V] in SP (+0.00844) 0.972
famotidine (+0.000946) 0.972
Hemoglobin A1c/Hemoglobin.total in B by HPLC (+0.0263) 0.972
Exocrine pancreatic insufficiency (x1.01) 0.972
Hypoglycemia (/1.01) 0.972
Disorder of pancreas (x1.01) 0.973
levothyroxine (+0.000913) 0.973
glipizide (+0.000905) 0.973
Cystic fibrosis without meconium ileus (x1.01) 0.973
oxycodone (-0.000889) 0.973
diltiazem (+0.000873) 0.973
Cystic fibrosis of the lung (x1.01) 0.974
Tachycardia (/1.01) 0.974
fluticasone (+0.000846) 0.974
Disorder of nervous system due to diabetes mellitus (x1.01) 0.974
Cholesterol in HDL [M/V] in SP (-0.272) 0.974
Senile cataract (x1.01) 0.974
salmeterol (+0.000814) 0.974
Hyperglycemia due to type 2 diabetes mellitus (x1.01) 0.974
−0.75 −0.50 −0.25 0.00 0.25 0.50 0.75
Expressions

Signature [791]; 74 To Reach 0.975, (74 Plotted)

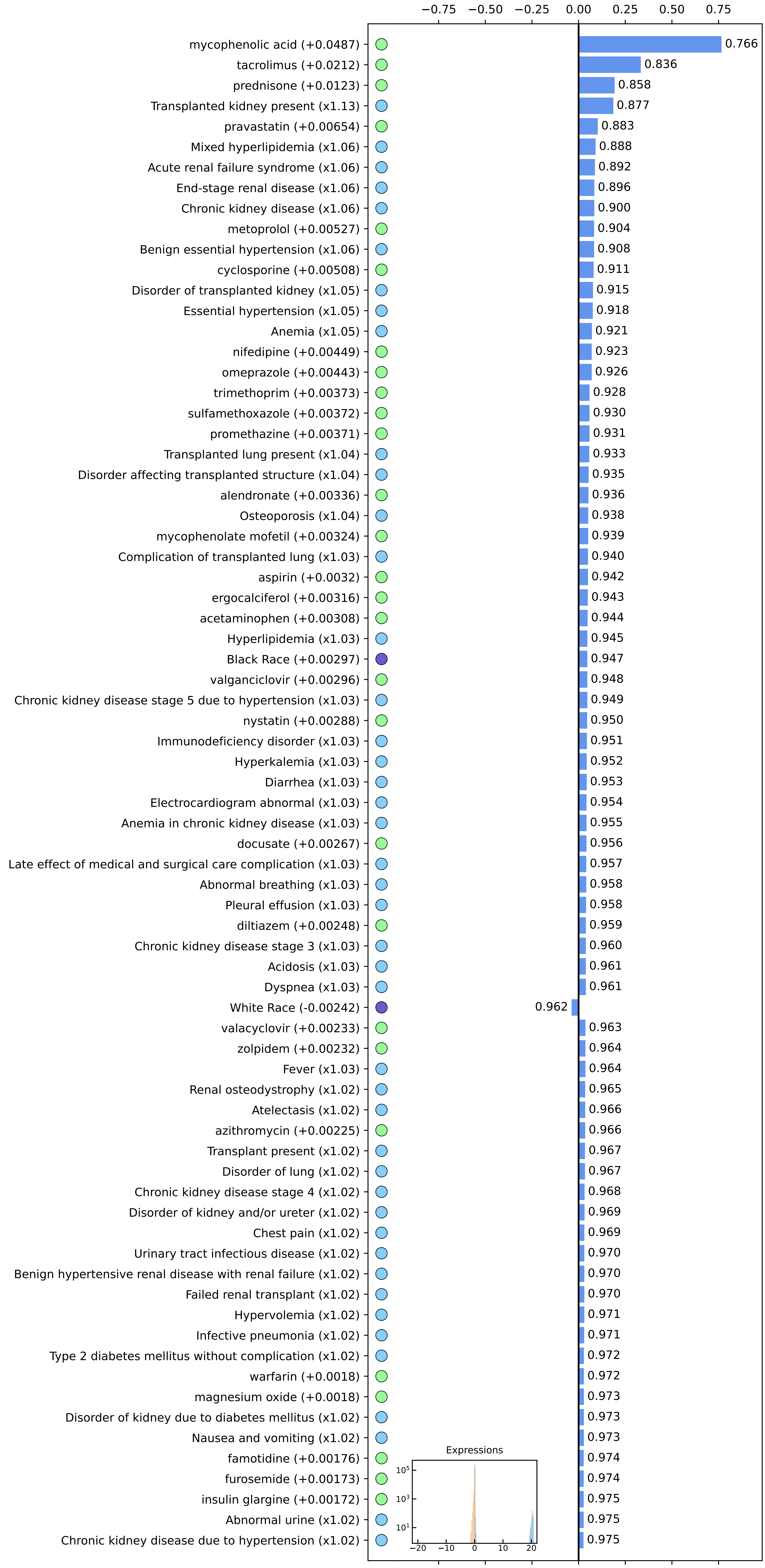

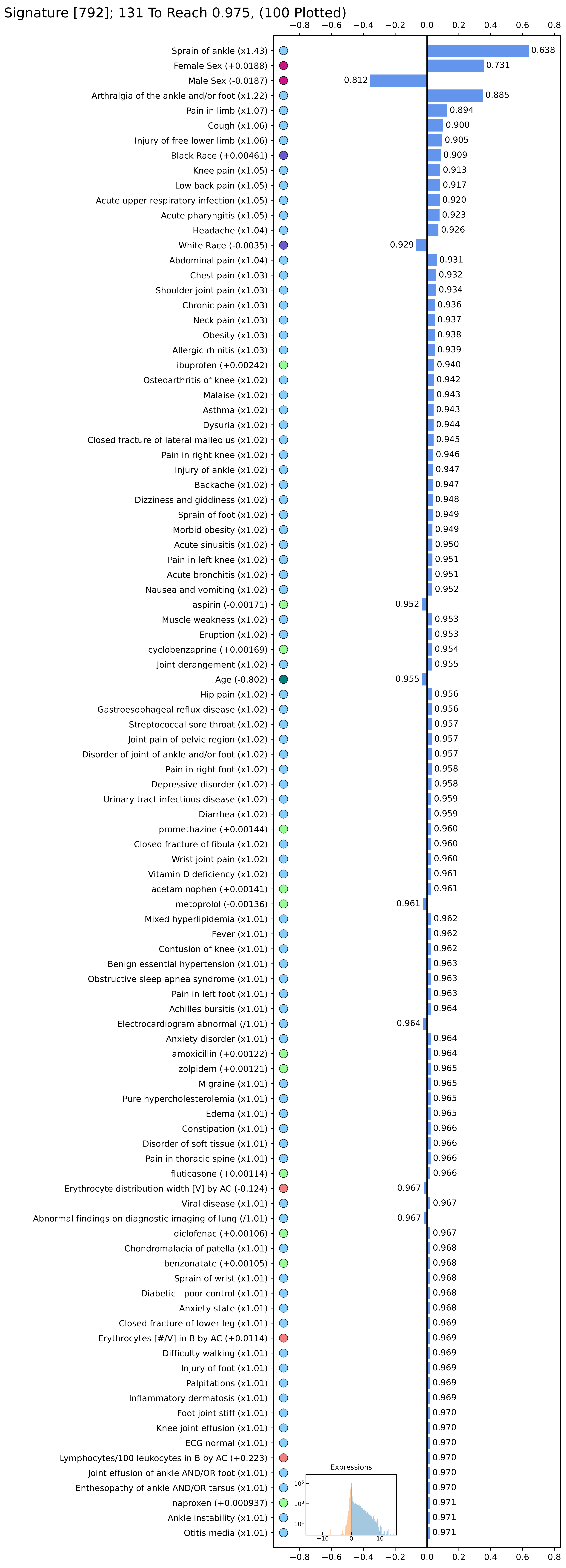

Signature [792]; 131 To Reach 0.975, (100 Plotted)
Sprain of ankle (x1.43) 0.638
Female Sex (+0.0188) 0.731
Male Sex (-0.0187) 0.812
Arthralgia of the ankle and/or foot (x1.22) 0.885
Pain in limb (x1.07) 0.894
Cough (x1.06) 0.900
Injury of free lower limb (x1.06) 0.905
Black Race (+0.00461) 0.909
Knee pain (x1.05) 0.913
Low back pain (x1.05) 0.917
Acute upper respiratory infection (x1.05) 0.920
Acute pharyngitis (x1.05) 0.923
Headache (x1.04) 0.926
White Race (-0.0035) 0.929
Abdominal pain (x1.04) 0.931
Chest pain (x1.03) 0.932
Shoulder joint pain (x1.03) 0.934
Chronic pain (x1.03) 0.936
Neck pain (x1.03) 0.937
Obesity (x1.03) 0.938
Allergic rhinitis (x1.03) 0.939
ibuprofen (+0.00242) 0.940
Osteoarthritis of knee (x1.02) 0.942
Malaise (x1.02) 0.943
Asthma (x1.02) 0.943
Dysuria (x1.02) 0.944
Closed fracture of lateral malleolus (x1.02) 0.945
Pain in right knee (x1.02) 0.946
Injury of ankle (x1.02) 0.947
Backache (x1.02) 0.947
Dizziness and giddiness (x1.02) 0.948
Sprain of foot (x1.02) 0.949
Morbid obesity (x1.02) 0.949
Acute sinusitis (x1.02) 0.950
Pain in left knee (x1.02) 0.951
Acute bronchitis (x1.02) 0.951
Nausea and vomiting (x1.02) 0.952
aspirin (-0.00171) 0.952
Muscle weakness (x1.02) 0.953
Eruption (x1.02) 0.953
cyclobenzaprine (+0.00169) 0.954
Joint derangement (x1.02) 0.955
Age (-0.802) 0.955
Hip pain (x1.02) 0.956
Gastroesophageal reflux disease (x1.02) 0.956
Streptococcal sore throat (x1.02) 0.957
Joint pain of pelvic region (x1.02) 0.957
Disorder of joint of ankle and/or foot (x1.02) 0.957
Pain in right foot (x1.02) 0.958
Depressive disorder (x1.02) 0.958
Urinary tract infectious disease (x1.02) 0.959
Diarrhea (x1.02) 0.959
promethazine (+0.00144) 0.960
Closed fracture of fibula (x1.02) 0.960
Wrist joint pain (x1.02) 0.960
Vitamin D deficiency (x1.02) 0.961
acetaminophen (+0.00141) 0.961
metoprolol (-0.00136) 0.961
Mixed hyperlipidemia (x1.01) 0.962
Fever (x1.01) 0.962
Contusion of knee (x1.01) 0.962
Benign essential hypertension (x1.01) 0.963
Obstructive sleep apnea syndrome (x1.01) 0.963
Pain in left foot (x1.01) 0.963
Achilles bursitis (x1.01) 0.964
Electrocardiogram abnormal (/1.01) 0.964
Anxiety disorder (x1.01) 0.964
amoxicillin (+0.00122) 0.964
zolpidem (+0.00121) 0.965
Migraine (x1.01) 0.965
Pure hypercholesterolemia (x1.01) 0.965
Edema (x1.01) 0.965
Constipation (x1.01) 0.966
Disorder of soft tissue (x1.01) 0.966
Pain in thoracic spine (x1.01) 0.966
fluticasone (+0.00114) 0.966
Erythrocyte distribution width [V] by AC (-0.124) 0.967
Viral disease (x1.01) 0.967
Abnormal findings on diagnostic imaging of lung (/1.01) 0.967
diclofenac (+0.00106) 0.967
Chondromalacia of patella (x1.01) 0.968
benzonatate (+0.00105) 0.968
Sprain of wrist (x1.01) 0.968
Diabetic - poor control (x1.01) 0.968
Anxiety state (x1.01) 0.968
Closed fracture of lower leg (x1.01) 0.969
Erythrocytes [#/V] in B by AC (+0.0114) 0.969
Difficulty walking (x1.01) 0.969
Injury of foot (x1.01) 0.969
Palpitations (x1.01) 0.969
Inflammatory dermatosis (x1.01) 0.969
Foot joint stiff (x1.01) 0.970
Knee joint effusion (x1.01) 0.970
ECG normal (x1.01) 0.970
Lymphocytes/100 leukocytes in B by AC (+0.223) 0.970
Joint effusion of ankle AND/OR foot (x1.01) 0.970
Enthesopathy of ankle AND/OR tarsus (x1.01) 0.970
naproxen (+0.000937) 0.971
Ankle instability (x1.01) 0.971
Otitis media (x1.01) 0.971
Expressions
−0.8 −0.6 −0.4 −0.2 0.0 0.2 0.4 0.6 0.8

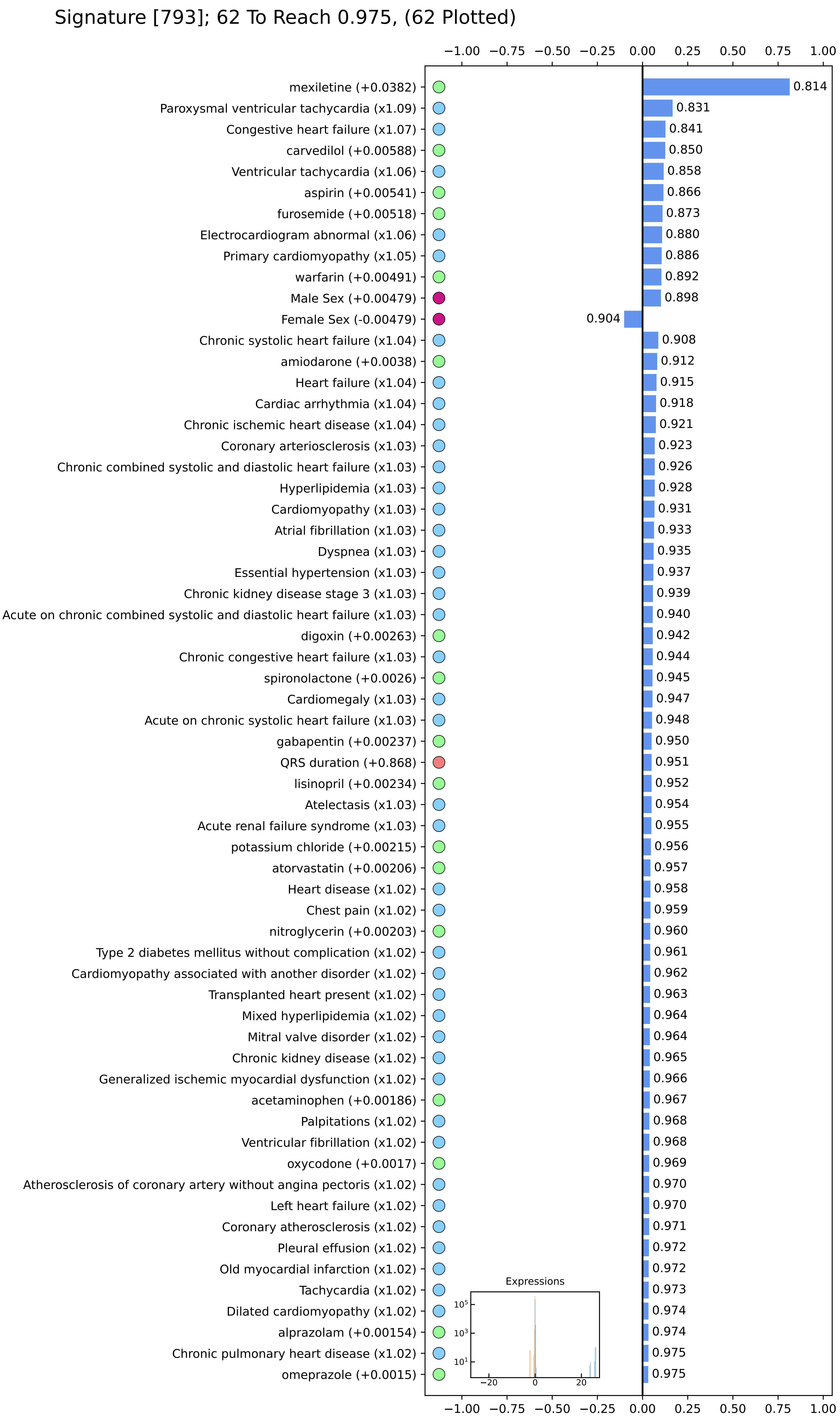

Signature [793]; 62 To Reach 0.975, (62 Plotted)
−1.00 −0.75 −0.50 −0.25 0.00 0.25 0.50 0.75 1.00
mexiletine (+0.0382) 0.814
Paroxysmal ventricular tachycardia (x1.09) 0.831
Congestive heart failure (x1.07) 0.841
carvedilol (+0.00588) 0.850
Ventricular tachycardia (x1.06) 0.858
aspirin (+0.00541) 0.866
furosemide (+0.00518) 0.873
Electrocardiogram abnormal (x1.06) 0.880
Primary cardiomyopathy (x1.05) 0.886
warfarin (+0.00491) 0.892
Male Sex (+0.00479) 0.898
Female Sex (-0.00479) 0.904
Chronic systolic heart failure (x1.04) 0.908
amiodarone (+0.0038) 0.912
Heart failure (x1.04) 0.915
Cardiac arrhythmia (x1.04) 0.918
Chronic ischemic heart disease (x1.04) 0.921
Coronary arteriosclerosis (x1.03) 0.923
Chronic combined systolic and diastolic heart failure (x1.03) 0.926
Hyperlipidemia (x1.03) 0.928
Cardiomyopathy (x1.03) 0.931
Atrial fibrillation (x1.03) 0.933
Dyspnea (x1.03) 0.935
Essential hypertension (x1.03) 0.937
Chronic kidney disease stage 3 (x1.03) 0.939
Acute on chronic combined systolic and diastolic heart failure (x1.03) 0.940
digoxin (+0.00263) 0.942
Chronic congestive heart failure (x1.03) 0.944
spironolactone (+0.0026) 0.945
Cardiomegaly (x1.03) 0.947
Acute on chronic systolic heart failure (x1.03) 0.948
gabapentin (+0.00237) 0.950
QRS duration (+0.868) 0.951
lisinopril (+0.00234) 0.952
Atelectasis (x1.03) 0.954
Acute renal failure syndrome (x1.03) 0.955
potassium chloride (+0.00215) 0.956
atorvastatin (+0.00206) 0.957
Heart disease (x1.02) 0.958
Chest pain (x1.02) 0.959
nitroglycerin (+0.00203) 0.960
Type 2 diabetes mellitus without complication (x1.02) 0.961
Cardiomyopathy associated with another disorder (x1.02) 0.962
Transplanted heart present (x1.02) 0.963
Mixed hyperlipidemia (x1.02) 0.964
Mitral valve disorder (x1.02) 0.964
Chronic kidney disease (x1.02) 0.965
Generalized ischemic myocardial dysfunction (x1.02) 0.966
acetaminophen (+0.00186) 0.967
Palpitations (x1.02) 0.968
Ventricular fibrillation (x1.02) 0.968
oxycodone (+0.0017) 0.969
Atherosclerosis of coronary artery without angina pectoris (x1.02) 0.970
Left heart failure (x1.02) 0.970
Coronary atherosclerosis (x1.02) 0.971
Pleural effusion (x1.02) 0.972
Old myocardial infarction (x1.02) 0.972
Tachycardia (x1.02) 0.973
Dilated cardiomyopathy (x1.02) 0.974
alprazolam (+0.00154) 0.974
Chronic pulmonary heart disease (x1.02) 0.975
omeprazole (+0.0015) 0.975
Expressions
10^5
10^3
10^1
−20 0 20

Signature [794]; 1 To Reach 0.975, (10 Plotted)

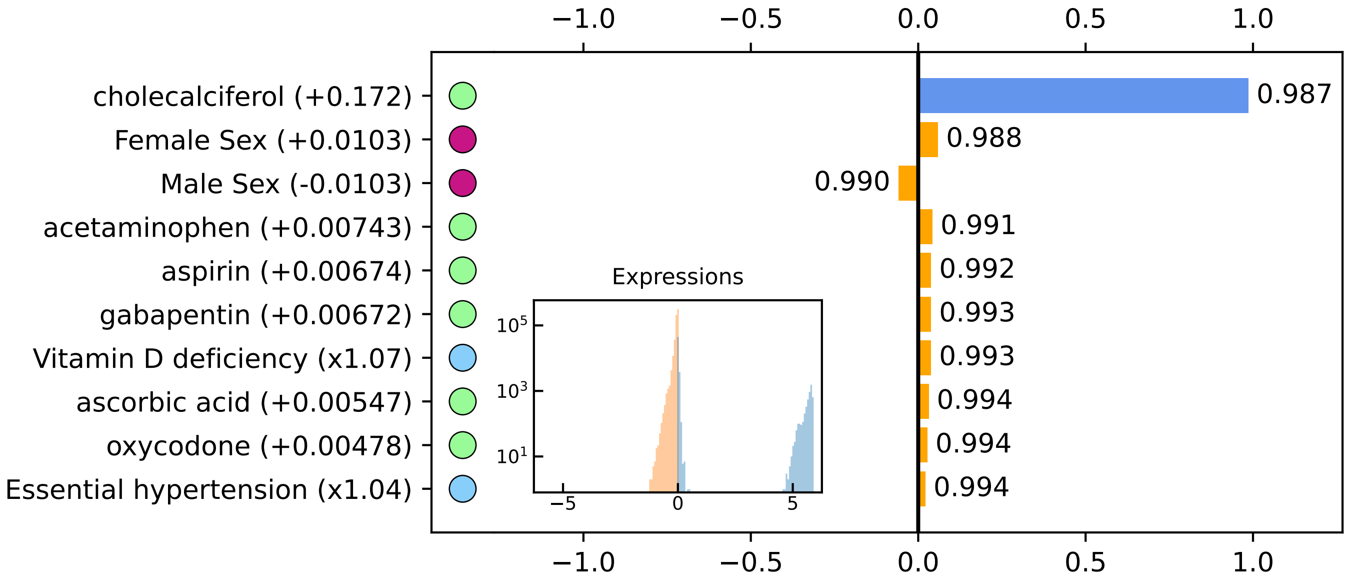

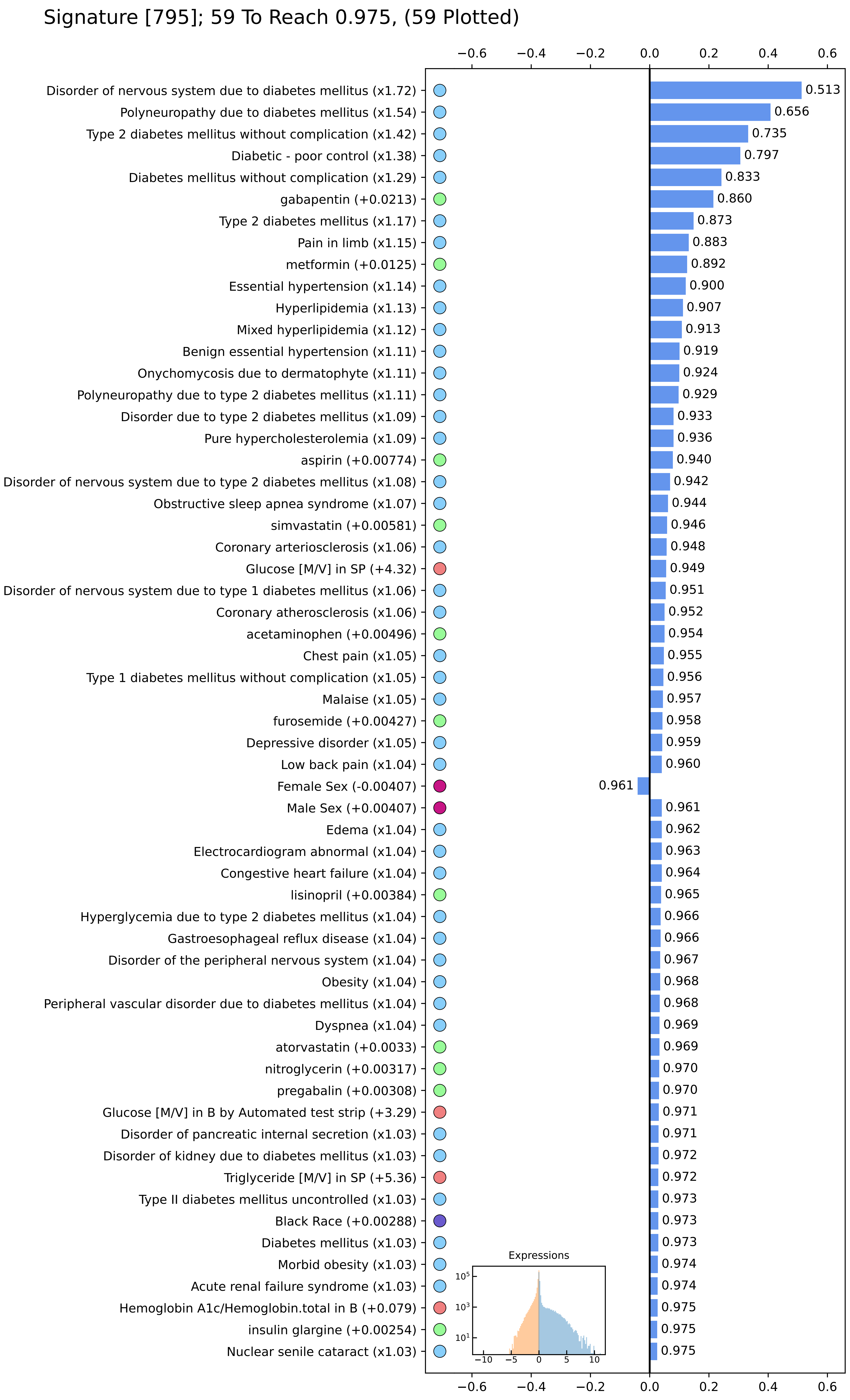

Signature [795]; 59 To Reach 0.975, (59 Plotted)
Disorder of nervous system due to diabetes mellitus (x1.72) 0.513
Polyneuropathy due to diabetes mellitus (x1.54) 0.656
Type 2 diabetes mellitus without complication (x1.42) 0.735
Diabetic - poor control (x1.38) 0.797
Diabetes mellitus without complication (x1.29) 0.833
gabapentin (+0.0213) 0.860
Type 2 diabetes mellitus (x1.17) 0.873
Pain in limb (x1.15) 0.883
metformin (+0.0125) 0.892
Essential hypertension (x1.14) 0.900
Hyperlipidemia (x1.13) 0.907
Mixed hyperlipidemia (x1.12) 0.913
Benign essential hypertension (x1.11) 0.919
Onychomycosis due to dermatophyte (x1.11) 0.924
Polyneuropathy due to type 2 diabetes mellitus (x1.11) 0.929
Disorder due to type 2 diabetes mellitus (x1.09) 0.933
Pure hypercholesterolemia (x1.09) 0.936
aspirin (+0.00774) 0.940
Disorder of nervous system due to type 2 diabetes mellitus (x1.08) 0.942
Obstructive sleep apnea syndrome (x1.07) 0.944
simvastatin (+0.00581) 0.946
Coronary arteriosclerosis (x1.06) 0.948
Glucose [M/V] in SP (+4.32) 0.949
Disorder of nervous system due to type 1 diabetes mellitus (x1.06) 0.951
Coronary atherosclerosis (x1.06) 0.952
acetaminophen (+0.00496) 0.954
Chest pain (x1.05) 0.955
Type 1 diabetes mellitus without complication (x1.05) 0.956
Malaise (x1.05) 0.957
furosemide (+0.00427) 0.958
Depressive disorder (x1.05) 0.959
Low back pain (x1.04) 0.960
Female Sex (-0.00407) 0.961
Male Sex (+0.00407) 0.961
Edema (x1.04) 0.962
Electrocardiogram abnormal (x1.04) 0.963
Congestive heart failure (x1.04) 0.964
lisinopril (+0.00384) 0.965
Hyperglycemia due to type 2 diabetes mellitus (x1.04) 0.966
Gastroesophageal reflux disease (x1.04) 0.966
Disorder of the peripheral nervous system (x1.04) 0.967
Obesity (x1.04) 0.968
Peripheral vascular disorder due to diabetes mellitus (x1.04) 0.968
Dyspnea (x1.04) 0.969
atorvastatin (+0.0033) 0.969
nitroglycerin (+0.00317) 0.970
pregabalin (+0.00308) 0.970
Glucose [M/V] in B by Automated test strip (+3.29) 0.971
Disorder of pancreatic internal secretion (x1.03) 0.971
Disorder of kidney due to diabetes mellitus (x1.03) 0.972
Triglyceride [M/V] in SP (+5.36) 0.972
Type II diabetes mellitus uncontrolled (x1.03) 0.973
Black Race (+0.00288) 0.973
Diabetes mellitus (x1.03) 0.973
Morbid obesity (x1.03) 0.974
Acute renal failure syndrome (x1.03) 0.974
Hemoglobin A1c/Hemoglobin.total in B (+0.079) 0.975
insulin glargine (+0.00254) 0.975
Nuclear senile cataract (x1.03) 0.975
−0.6 −0.4 −0.2 0.0 0.2 0.4 0.6
Expressions
10^5 10^3 10^1
−10 −5 0 5 10

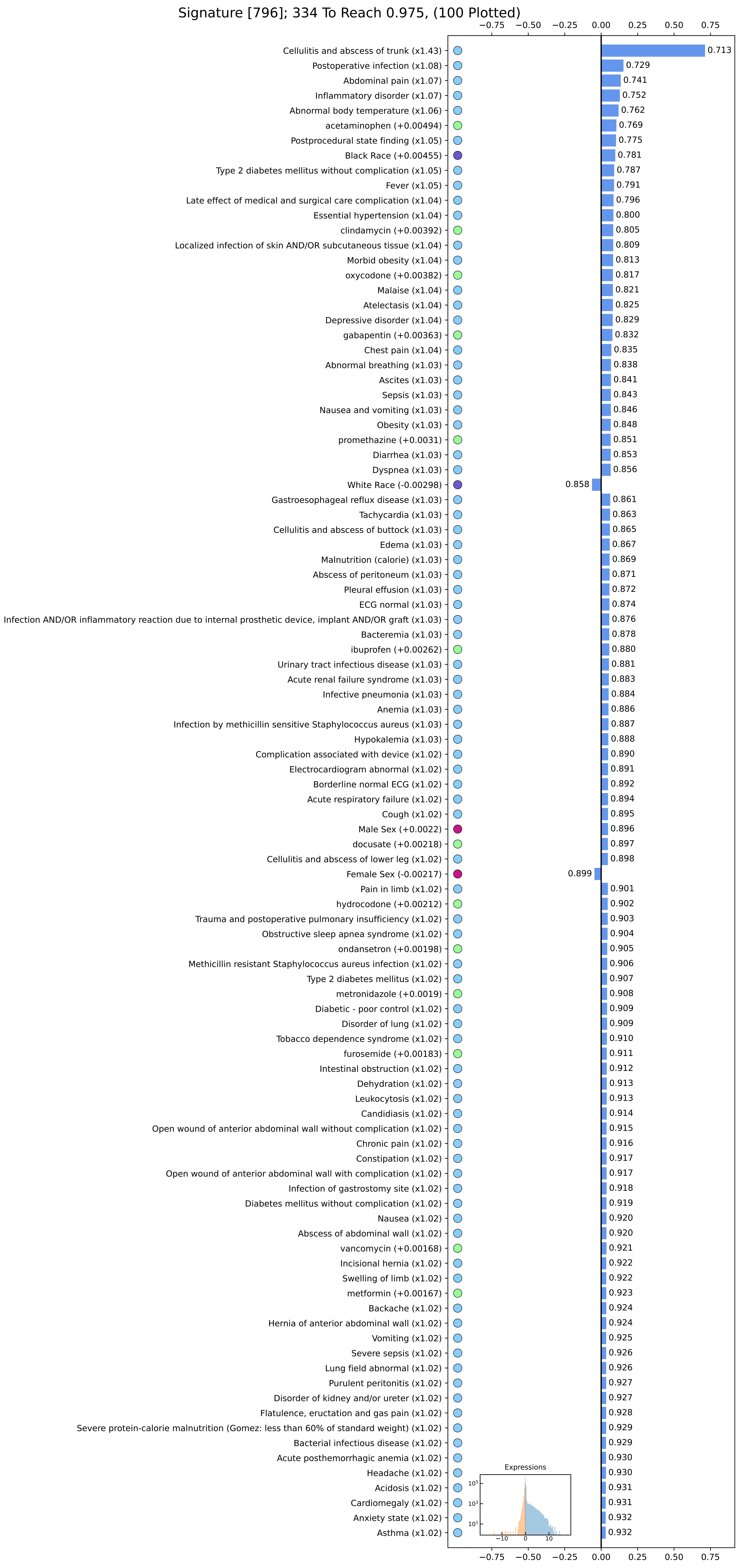

Signature [796]; 334 To Reach 0.975, (100 Plotted)
Cellulitis and abscess of trunk (x1.43) 0.713
Postoperative infection (x1.08) 0.729
Abdominal pain (x1.07) 0.741
Inflammatory disorder (x1.07) 0.752
Abnormal body temperature (x1.06) 0.762
acetaminophen (+0.00494) 0.769
Postprocedural state finding (x1.05) 0.775
Black Race (+0.00455) 0.781
Type 2 diabetes mellitus without complication (x1.05) 0.787
Fever (x1.05) 0.791
Late effect of medical and surgical care complication (x1.04) 0.796
Essential hypertension (x1.04) 0.800
clindamycin (+0.00392) 0.805
Localized infection of skin AND/OR subcutaneous tissue (x1.04) 0.809
Morbid obesity (x1.04) 0.813
oxycodone (+0.00382) 0.817
Malaise (x1.04) 0.821
Atelectasis (x1.04) 0.825
Depressive disorder (x1.04) 0.829
gabapentin (+0.00363) 0.832
Chest pain (x1.04) 0.835
Abnormal breathing (x1.03) 0.838
Ascites (x1.03) 0.841
Sepsis (x1.03) 0.843
Nausea and vomiting (x1.03) 0.846
Obesity (x1.03) 0.848
promethazine (+0.0031) 0.851
Diarrhea (x1.03) 0.853
Dyspnea (x1.03) 0.856
White Race (-0.00298) 0.858
Gastroesophageal reflux disease (x1.03) 0.861
Tachycardia (x1.03) 0.863
Cellulitis and abscess of buttock (x1.03) 0.865
Edema (x1.03) 0.867
Malnutrition (calorie) (x1.03) 0.869
Abscess of peritoneum (x1.03) 0.871
Pleural effusion (x1.03) 0.872
ECG normal (x1.03) 0.874
Infection AND/OR inflammatory reaction due to internal prosthetic device, implant AND/OR graft (x1.03) 0.876
Bacteremia (x1.03) 0.878
ibuprofen (+0.00262) 0.880
Urinary tract infectious disease (x1.03) 0.881
Acute renal failure syndrome (x1.03) 0.883
Infective pneumonia (x1.03) 0.884
Anemia (x1.03) 0.886
Infection by methicillin sensitive Staphylococcus aureus (x1.03) 0.887
Hypokalemia (x1.03) 0.888
Complication associated with device (x1.02) 0.890
Electrocardiogram abnormal (x1.02) 0.891
Borderline normal ECG (x1.02) 0.892
Acute respiratory failure (x1.02) 0.894
Cough (x1.02) 0.895
Male Sex (+0.0022) 0.896
docusate (+0.00218) 0.897
Cellulitis and abscess of lower leg (x1.02) 0.898
Female Sex (-0.00217) 0.899
Pain in limb (x1.02) 0.901
hydrocodone (+0.00212) 0.902
Trauma and postoperative pulmonary insufficiency (x1.02) 0.903
Obstructive sleep apnea syndrome (x1.02) 0.904
ondansetron (+0.00198) 0.905
Methicillin resistant Staphylococcus aureus infection (x1.02) 0.906
Type 2 diabetes mellitus (x1.02) 0.907
metronidazole (+0.0019) 0.908
Diabetic - poor control (x1.02) 0.909
Disorder of lung (x1.02) 0.909
Tobacco dependence syndrome (x1.02) 0.910
furosemide (+0.00183) 0.911
Intestinal obstruction (x1.02) 0.912
Dehydration (x1.02) 0.913
Leukocytosis (x1.02) 0.913
Candidiasis (x1.02) 0.914
Open wound of anterior abdominal wall without complication (x1.02) 0.915
Chronic pain (x1.02) 0.916
Constipation (x1.02) 0.917
Open wound of anterior abdominal wall with complication (x1.02) 0.917
Infection of gastrostomy site (x1.02) 0.918
Diabetes mellitus without complication (x1.02) 0.919
Nausea (x1.02) 0.920
Abscess of abdominal wall (x1.02) 0.920
vancomycin (+0.00168) 0.921
Incisional hernia (x1.02) 0.922
Swelling of limb (x1.02) 0.922
metformin (+0.00167) 0.923
Backache (x1.02) 0.924
Hernia of anterior abdominal wall (x1.02) 0.924
Vomiting (x1.02) 0.925
Severe sepsis (x1.02) 0.926
Lung field abnormal (x1.02) 0.926
Purulent peritonitis (x1.02) 0.927
Disorder of kidney and/or ureter (x1.02) 0.927
Flatulence, eructation and gas pain (x1.02) 0.928
Severe protein-calorie malnutrition (Gomez: less than 60% of standard weight) (x1.02) 0.929
Bacterial infectious disease (x1.02) 0.929
Acute posthemorrhagic anemia (x1.02) 0.930
Headache (x1.02) 0.930
Acidosis (x1.02) 0.931
Cardiomegaly (x1.02) 0.931
Anxiety state (x1.02) 0.932
Asthma (x1.02) 0.932
Expressions
−0.75 −0.50 −0.25 0.00 0.25 0.50 0.75

Signature [797]; 56 To Reach 0.975, (56 Plotted)

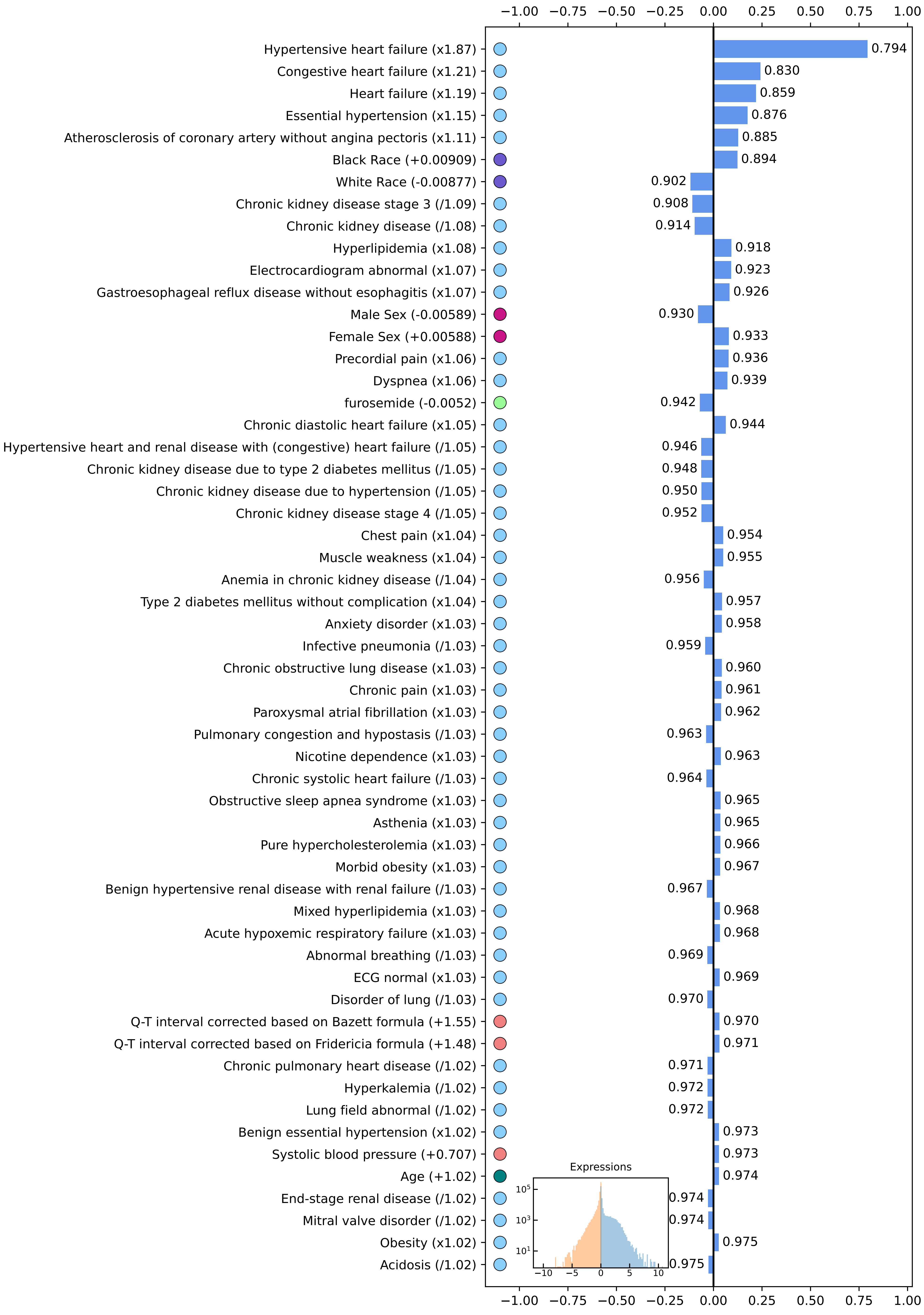

Signature [798]; 121 To Reach 0.975, (100 Plotted)

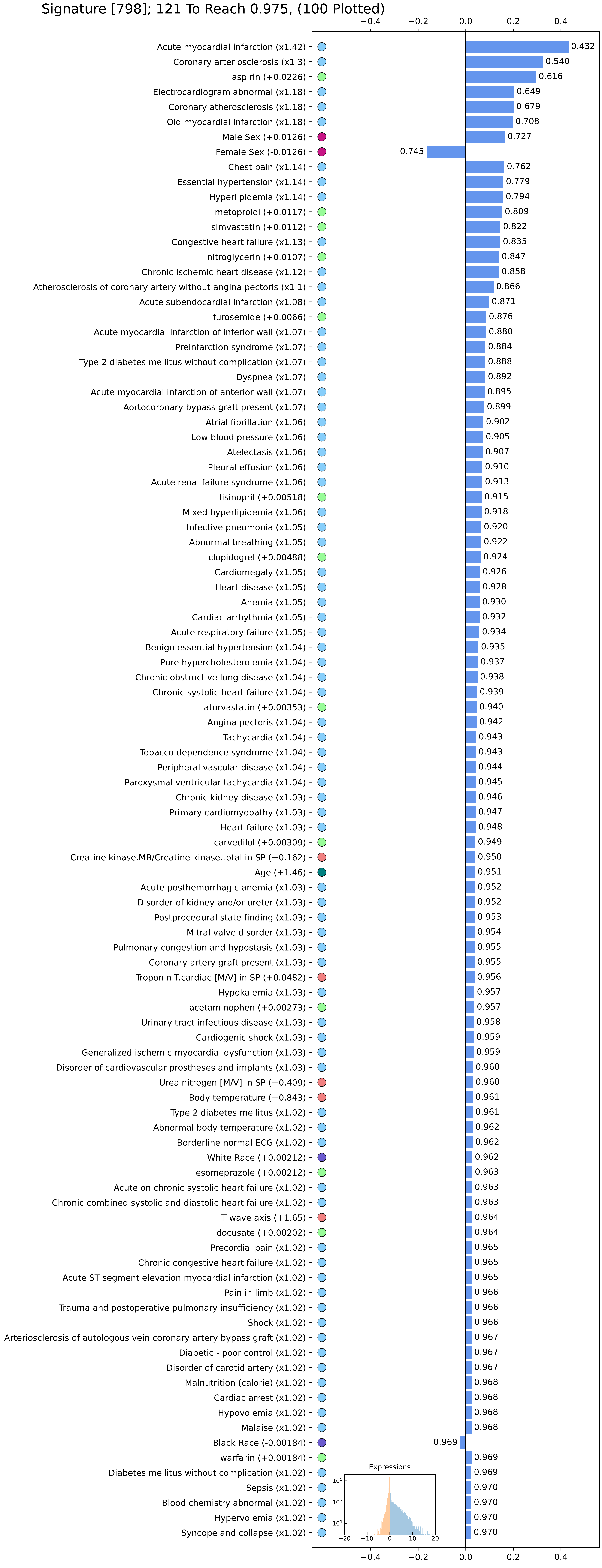

Signature [799]; 801 To Reach 0.975, (100 Plotted)

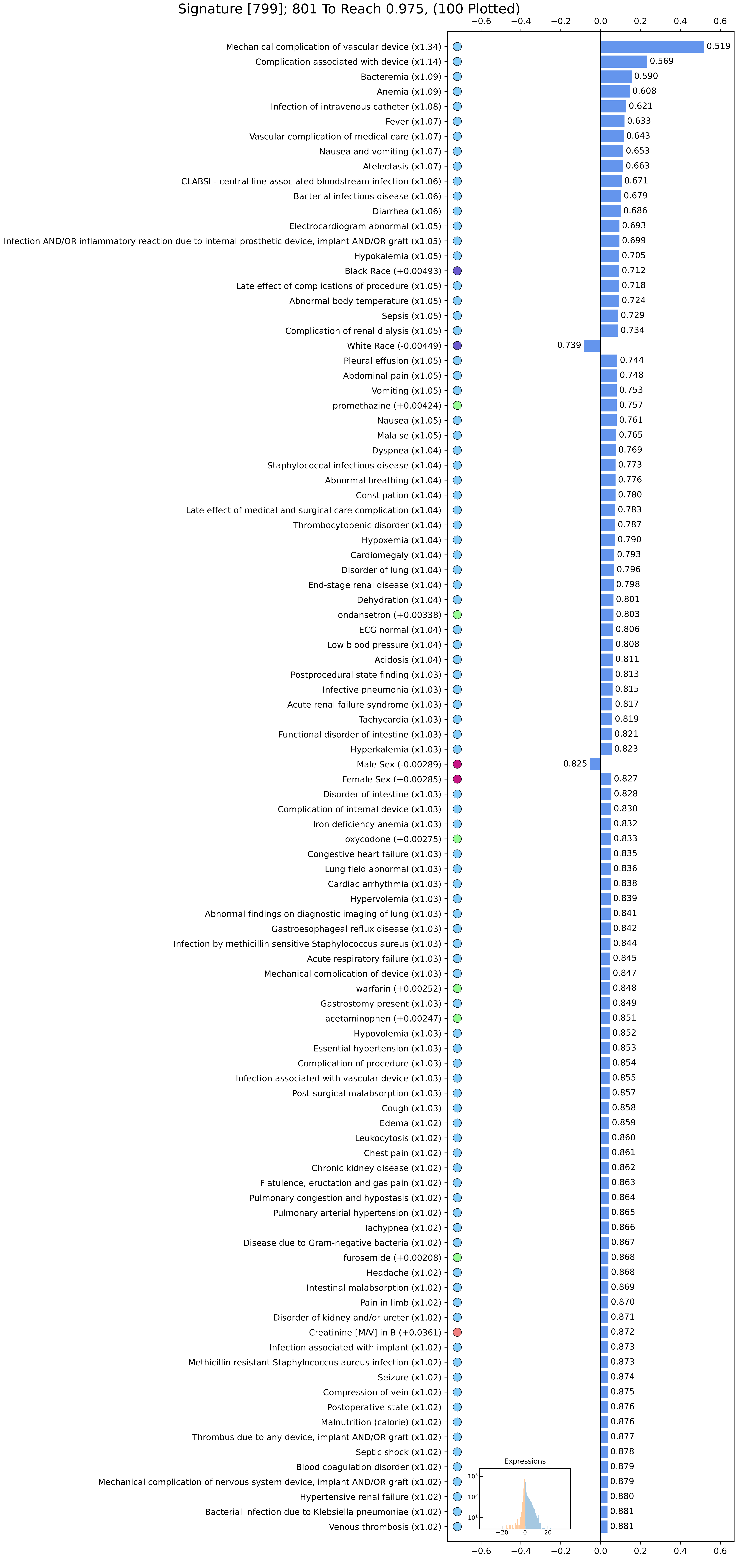

Signature [800]; 17 To Reach 0.975, (17 Plotted)

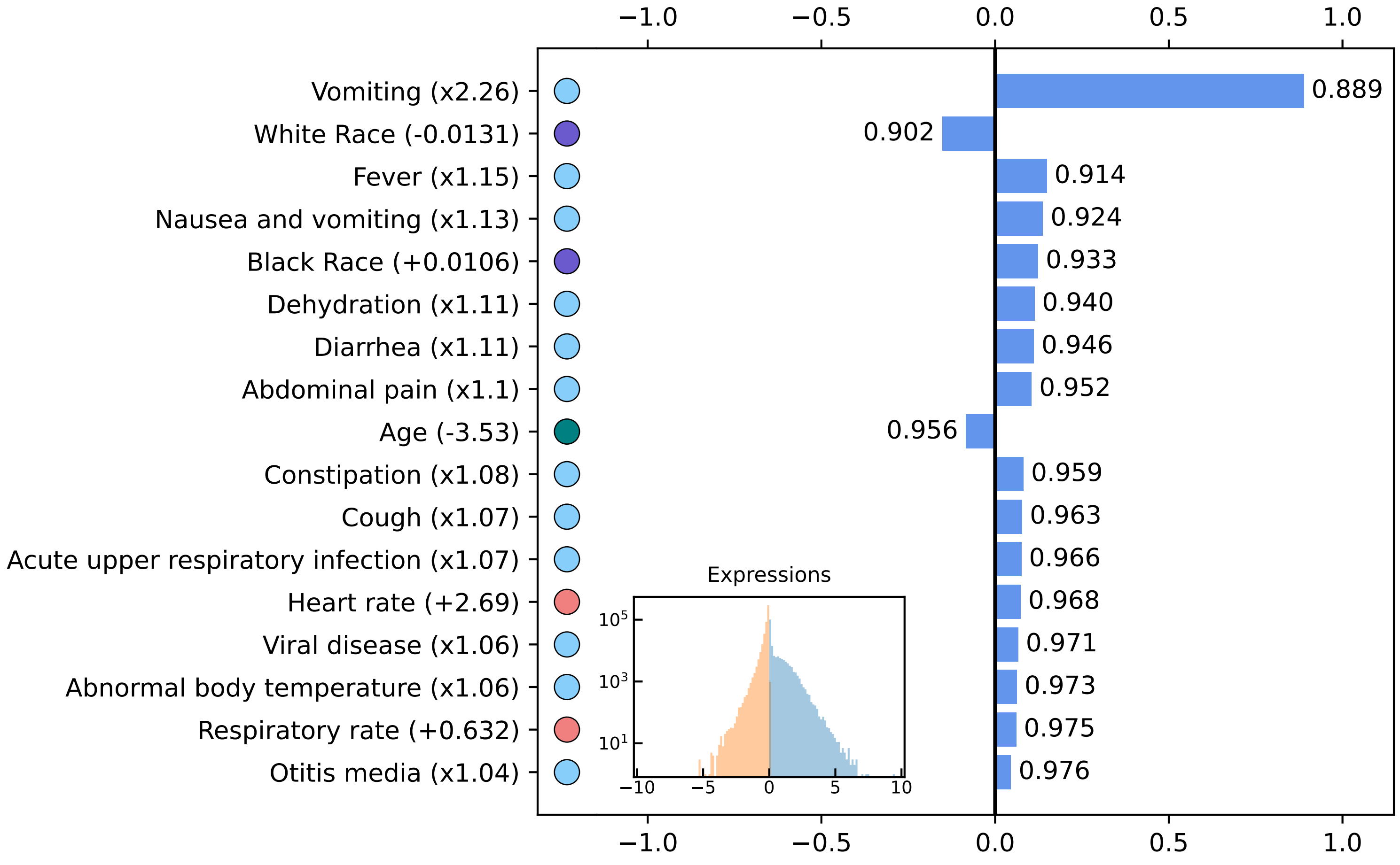

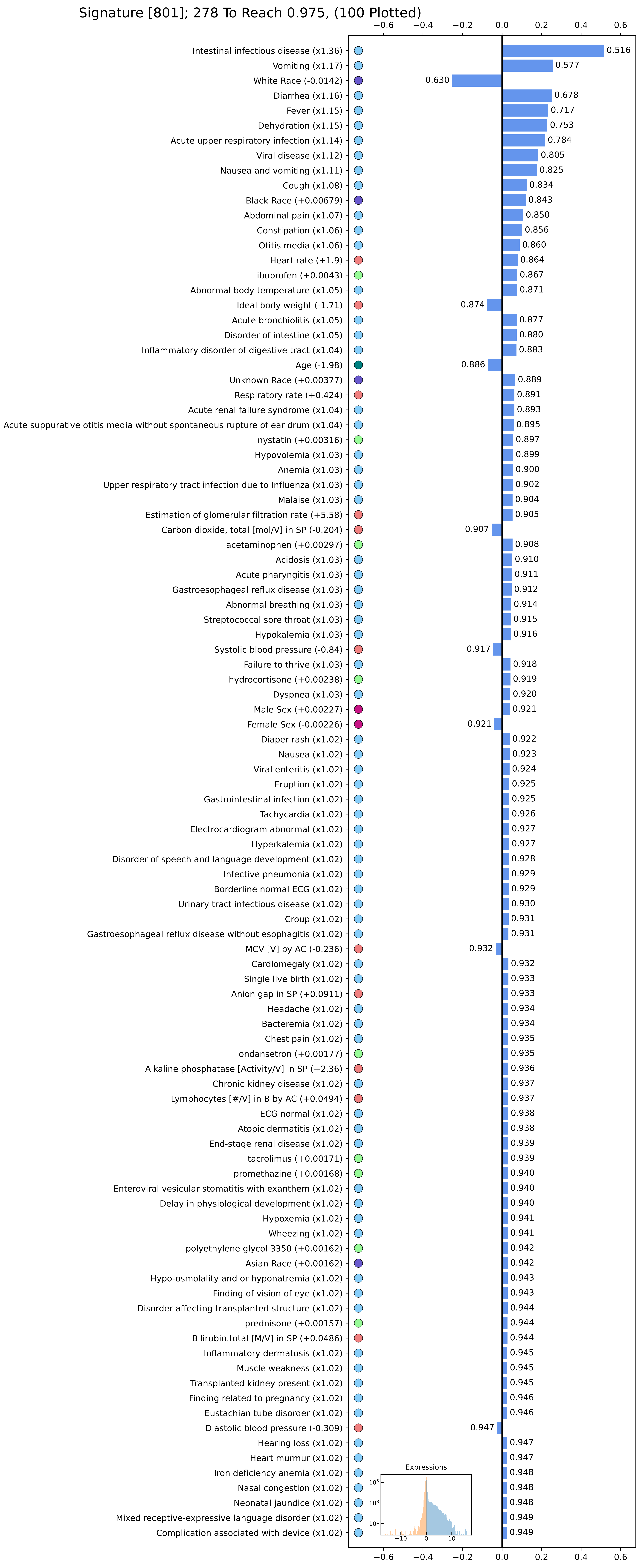

Signature [801]; 278 To Reach 0.975, (100 Plotted)
Intestinal infectious disease (x1.36) 0.516
Vomiting (x1.17) 0.577
White Race (-0.0142) 0.630
Diarrhea (x1.16) 0.678
Fever (x1.15) 0.717
Dehydration (x1.15) 0.753
Acute upper respiratory infection (x1.14) 0.784
Viral disease (x1.12) 0.805
Nausea and vomiting (x1.11) 0.825
Cough (x1.08) 0.834
Black Race (+0.00679) 0.843
Abdominal pain (x1.07) 0.850
Constipation (x1.06) 0.856
Otitis media (x1.06) 0.860
Heart rate (+1.9) 0.864
ibuprofen (+0.0043) 0.867
Abnormal body temperature (x1.05) 0.871
Ideal body weight (-1.71) 0.874
Acute bronchiolitis (x1.05) 0.877
Disorder of intestine (x1.05) 0.880
Inflammatory disorder of digestive tract (x1.04) 0.883
Age (-1.98) 0.886
Unknown Race (+0.00377) 0.889
Respiratory rate (+0.424) 0.891
Acute renal failure syndrome (x1.04) 0.893
Acute suppurative otitis media without spontaneous rupture of ear drum (x1.04) 0.895
nystatin (+0.00316) 0.897
Hypovolemia (x1.03) 0.899
Anemia (x1.03) 0.900
Upper respiratory tract infection due to Influenza (x1.03) 0.902
Malaise (x1.03) 0.904
Estimation of glomerular filtration rate (+5.58) 0.905
Carbon dioxide, total [mol/V] in SP (-0.204) 0.907
acetaminophen (+0.00297) 0.908
Acidosis (x1.03) 0.910
Acute pharyngitis (x1.03) 0.911
Gastroesophageal reflux disease (x1.03) 0.912
Abnormal breathing (x1.03) 0.914
Streptococcal sore throat (x1.03) 0.915
Hypokalemia (x1.03) 0.916
Systolic blood pressure (-0.84) 0.917
Failure to thrive (x1.03) 0.918
hydrocortisone (+0.00238) 0.919
Dyspnea (x1.03) 0.920
Male Sex (+0.00227) 0.921
Female Sex (-0.00226) 0.921
Diaper rash (x1.02) 0.922
Nausea (x1.02) 0.923
Viral enteritis (x1.02) 0.924
Eruption (x1.02) 0.925
Gastrointestinal infection (x1.02) 0.925
Tachycardia (x1.02) 0.926
Electrocardiogram abnormal (x1.02) 0.927
Hyperkalemia (x1.02) 0.927
Disorder of speech and language development (x1.02) 0.928
Infective pneumonia (x1.02) 0.929
Borderline normal ECG (x1.02) 0.929
Urinary tract infectious disease (x1.02) 0.930
Croup (x1.02) 0.931
Gastroesophageal reflux disease without esophagitis (x1.02) 0.931
MCV [V] by AC (-0.236) 0.932
Cardiomegaly (x1.02) 0.932
Single live birth (x1.02) 0.933
Anion gap in SP (+0.0911) 0.933
Headache (x1.02) 0.934
Bacteremia (x1.02) 0.934
Chest pain (x1.02) 0.935
ondansetron (+0.00177) 0.935
Alkaline phosphatase [Activity/V] in SP (+2.36) 0.936
Chronic kidney disease (x1.02) 0.937
Lymphocytes [#/V] in B by AC (+0.0494) 0.937
ECG normal (x1.02) 0.938
Atopic dermatitis (x1.02) 0.938
End-stage renal disease (x1.02) 0.939
tacrolimus (+0.00171) 0.939
promethazine (+0.00168) 0.940
Enteroviral vesicular stomatitis with exanthem (x1.02) 0.940
Delay in physiological development (x1.02) 0.940
Hypoxemia (x1.02) 0.941
Wheezing (x1.02) 0.941
polyethylene glycol 3350 (+0.00162) 0.942
Asian Race (+0.00162) 0.942
Hypo-osmolality and or hyponatremia (x1.02) 0.943
Finding of vision of eye (x1.02) 0.943
Disorder affecting transplanted structure (x1.02) 0.944
prednisone (+0.00157) 0.944
Bilirubin.total [M/V] in SP (+0.0486) 0.944
Inflammatory dermatosis (x1.02) 0.945
Muscle weakness (x1.02) 0.945
Transplanted kidney present (x1.02) 0.945
Finding related to pregnancy (x1.02) 0.946
Eustachian tube disorder (x1.02) 0.946
Diastolic blood pressure (-0.309) 0.947
Hearing loss (x1.02) 0.947
Heart murmur (x1.02) 0.947
Iron deficiency anemia (x1.02) 0.948
Nasal congestion (x1.02) 0.948
Neonatal jaundice (x1.02) 0.948
Mixed receptive-expressive language disorder (x1.02) 0.949
Complication associated with device (x1.02) 0.949
−0.6 −0.4 −0.2 0.0 0.2 0.4 0.6
Expressions
−10 0 10

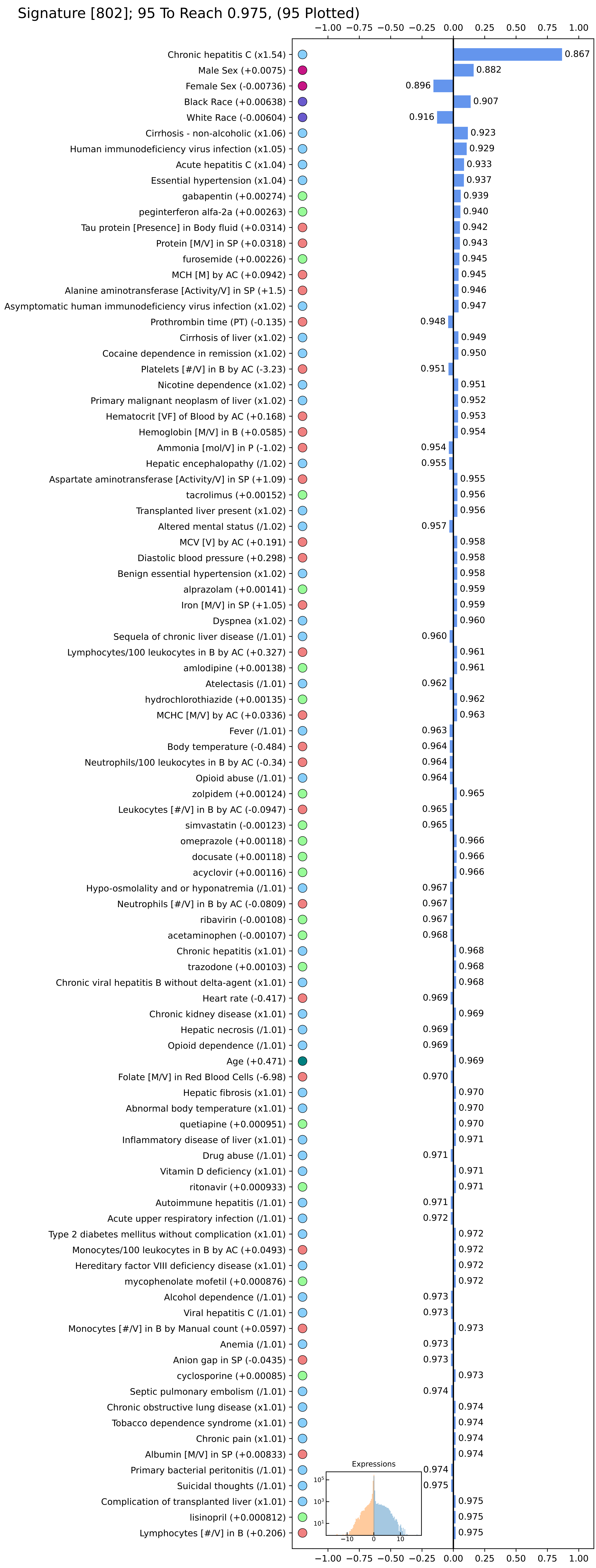
Signature [802]; 95 To Reach 0.975, (95 Plotted)
−1.00 −0.75 −0.50 −0.25 0.00 0.25 0.50 0.75 1.00
Chronic hepatitis C (x1.54) 0.867
Male Sex (+0.0075) 0.882
Female Sex (-0.00736) 0.896
Black Race (+0.00638) 0.907
White Race (-0.00604) 0.916
Cirrhosis - non-alcoholic (x1.06) 0.923
Human immunodeficiency virus infection (x1.05) 0.929
Acute hepatitis C (x1.04) 0.933
Essential hypertension (x1.04) 0.937
gabapentin (+0.00274) 0.939
peginterferon alfa-2a (+0.00263) 0.940
Tau protein [Presence] in Body fluid (+0.0314) 0.942
Protein [M/V] in SP (+0.0318) 0.943
furosemide (+0.00226) 0.945
MCH [M] by AC (+0.0942) 0.945
Alanine aminotransferase [Activity/V] in SP (+1.5) 0.946
Asymptomatic human immunodeficiency virus infection (x1.02) 0.947
Prothrombin time (PT) (-0.135) 0.948
Cirrhosis of liver (x1.02) 0.949
Cocaine dependence in remission (x1.02) 0.950
Platelets [#/V] in B by AC (-3.23) 0.951
Nicotine dependence (x1.02) 0.951
Primary malignant neoplasm of liver (x1.02) 0.952
Hematocrit [VF] of Blood by AC (+0.168) 0.953
Hemoglobin [M/V] in B (+0.0585) 0.954
Ammonia [mol/V] in P (-1.02) 0.954
Hepatic encephalopathy (/1.02) 0.955
Aspartate aminotransferase [Activity/V] in SP (+1.09) 0.955
tacrolimus (+0.00152) 0.956
Transplanted liver present (x1.02) 0.956
Altered mental status (/1.02) 0.957
MCV [V] by AC (+0.191) 0.958
Diastolic blood pressure (+0.298) 0.958
Benign essential hypertension (x1.02) 0.958
alprazolam (+0.00141) 0.959
Iron [M/V] in SP (+1.05) 0.959
Dyspnea (x1.02) 0.960
Sequela of chronic liver disease (/1.01) 0.960
Lymphocytes/100 leukocytes in B by AC (+0.327) 0.961
amlodipine (+0.00138) 0.961
Atelectasis (/1.01) 0.962
hydrochlorothiazide (+0.00135) 0.962
MCHC [M/V] by AC (+0.0336) 0.963
Fever (/1.01) 0.963
Body temperature (-0.484) 0.964
Neutrophils/100 leukocytes in B by AC (-0.34) 0.964
Opioid abuse (/1.01) 0.964
zolpidem (+0.00124) 0.965
Leukocytes [#/V] in B by AC (-0.0947) 0.965
simvastatin (-0.00123) 0.965
omeprazole (+0.00118) 0.966
docusate (+0.00118) 0.966
acyclovir (+0.00116) 0.966
Hypo-osmolality and or hyponatremia (/1.01) 0.967
Neutrophils [#/V] in B by AC (-0.0809) 0.967
ribavirin (-0.00108) 0.967
acetaminophen (-0.00107) 0.968
Chronic hepatitis (x1.01) 0.968
trazodone (+0.00103) 0.968
Chronic viral hepatitis B without delta-agent (x1.01) 0.968
Heart rate (-0.417) 0.969
Chronic kidney disease (x1.01) 0.969
Hepatic necrosis (/1.01) 0.969
Opioid dependence (/1.01) 0.969
Age (+0.471) 0.969
Folate [M/V] in Red Blood Cells (-6.98) 0.970
Hepatic fibrosis (x1.01) 0.970
Abnormal body temperature (x1.01) 0.970
quetiapine (+0.000951) 0.970
Inflammatory disease of liver (x1.01) 0.971
Drug abuse (/1.01) 0.971
Vitamin D deficiency (x1.01) 0.971
ritonavir (+0.000933) 0.971
Autoimmune hepatitis (/1.01) 0.971
Acute upper respiratory infection (/1.01) 0.972
Type 2 diabetes mellitus without complication (x1.01) 0.972
Monocytes/100 leukocytes in B by AC (+0.0493) 0.972
Hereditary factor VIII deficiency disease (x1.01) 0.972
mycophenolate mofetil (+0.000876) 0.972
Alcohol dependence (/1.01) 0.973
Viral hepatitis C (/1.01) 0.973
Monocytes [#/V] in B by Manual count (+0.0597) 0.973
Anemia (/1.01) 0.973
Anion gap in SP (-0.0435) 0.973
cyclosporine (+0.00085) 0.973
Septic pulmonary embolism (/1.01) 0.974
Chronic obstructive lung disease (x1.01) 0.974
Tobacco dependence syndrome (x1.01) 0.974
Chronic pain (x1.01) 0.974
Albumin [M/V] in SP (+0.00833) 0.974
Primary bacterial peritonitis (/1.01) 0.974
Suicidal thoughts (/1.01) 0.975
Complication of transplanted liver (x1.01) 0.975
lisinopril (+0.000812) 0.975
Lymphocytes [#/V] in B (+0.206) 0.975
Expressions
10^5
10^3
10^1
−10 0 10
−1.00 −0.75 −0.50 −0.25 0.00 0.25 0.50 0.75 1.00

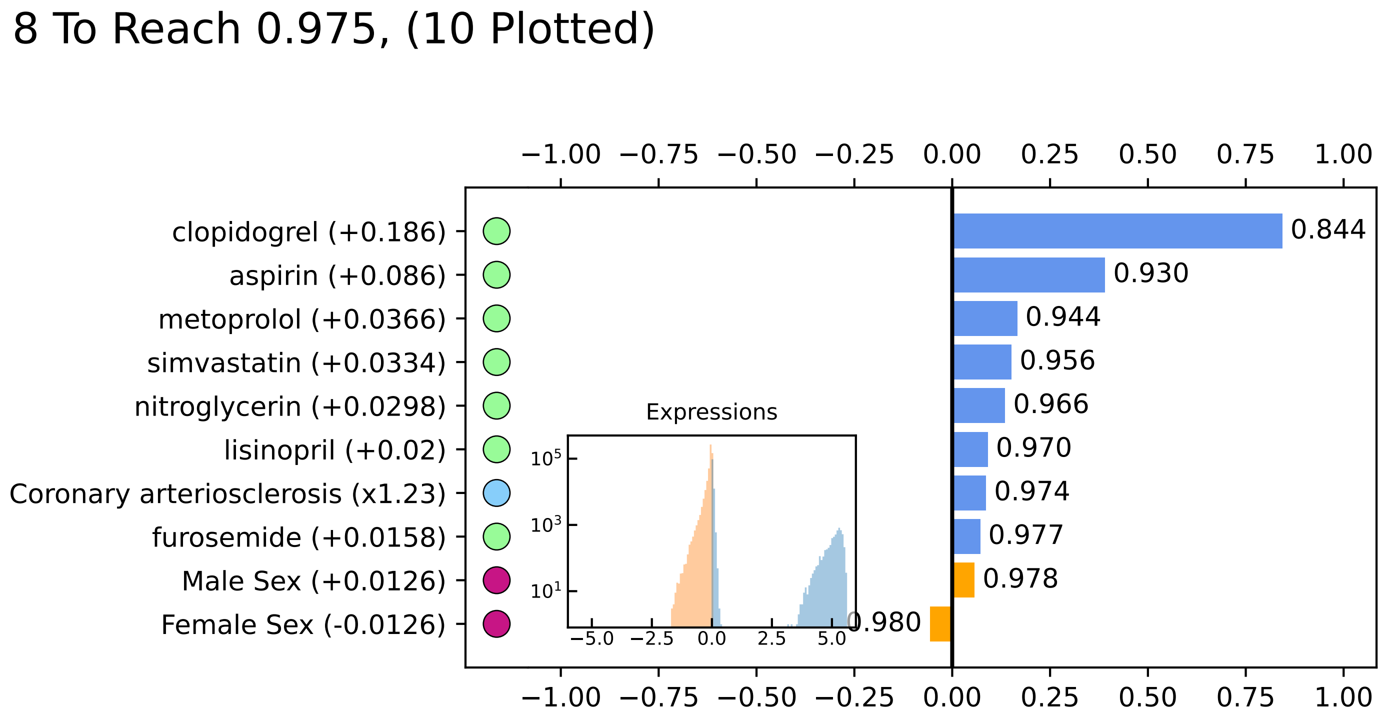
Signature [803]; 8 To Reach 0.975, (10 Plotted)
clopidogrel (+0.186)
aspirin (+0.086)
metoprolol (+0.0366)
simvastatin (+0.0334)
nitroglycerin (+0.0298)
lisinopril (+0.02)
Coronary arteriosclerosis (x1.23)
furosemide (+0.0158)
Male Sex (+0.0126)
Female Sex (-0.0126)
0.844
0.930
0.944
0.956
0.966
0.970
0.974
0.977
0.978
0.980
Expressions
−1.00 −0.75 −0.50 −0.25 0.00 0.25 0.50 0.75 1.00
−5.0 −2.5 0.0 2.5 5.0

Signature [804]; 5 To Reach 0.975, (10 Plotted)

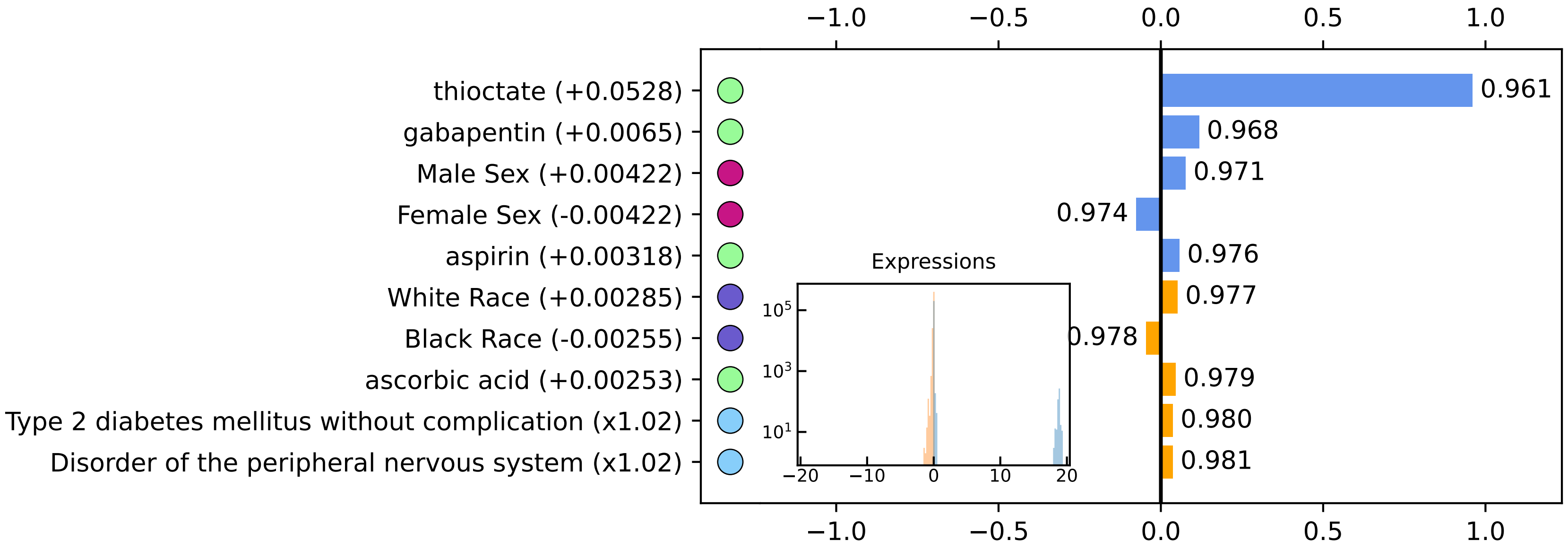

Signature [805]; 2 To Reach 0.975, (10 Plotted)

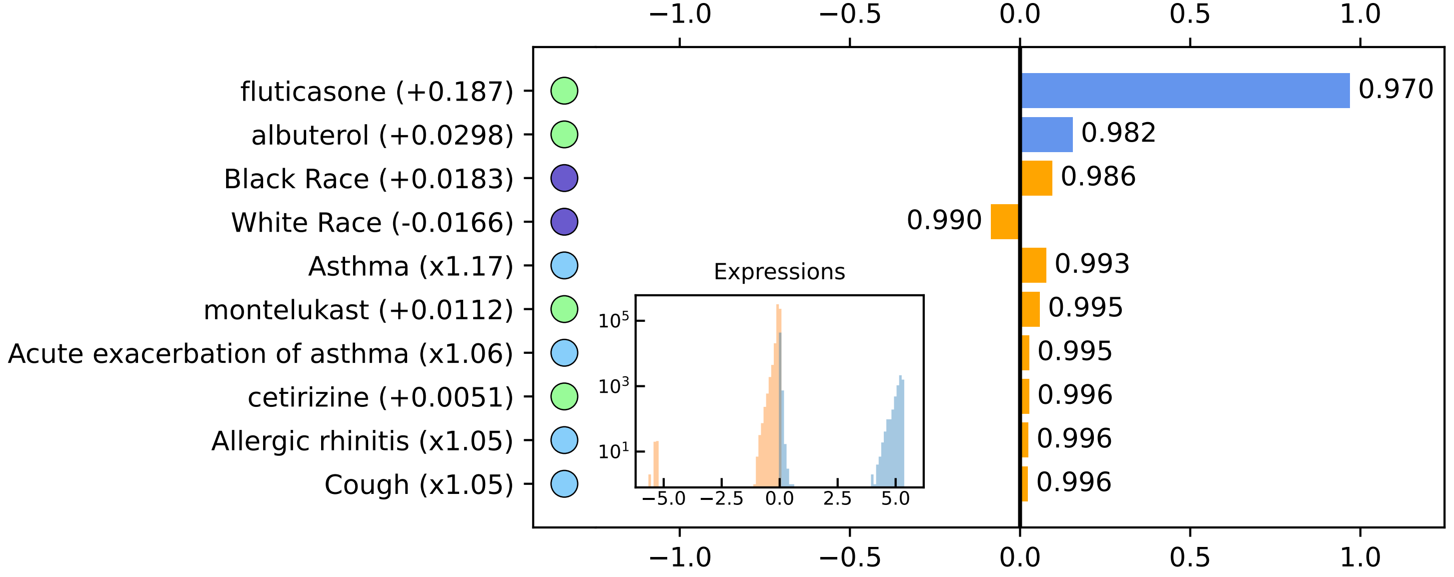

Signature [806]; 129 To Reach 0.975, (100 Plotted)

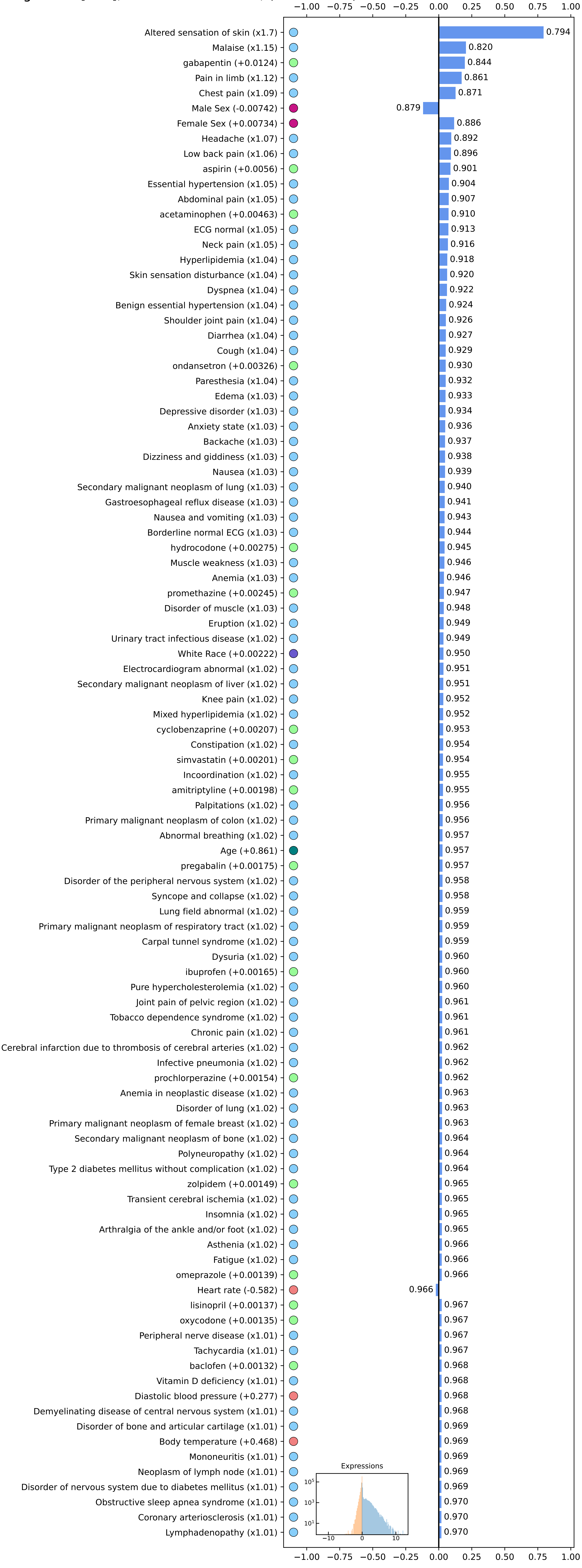

Signature [807]; 1 To Reach 0.975, (10 Plotted)

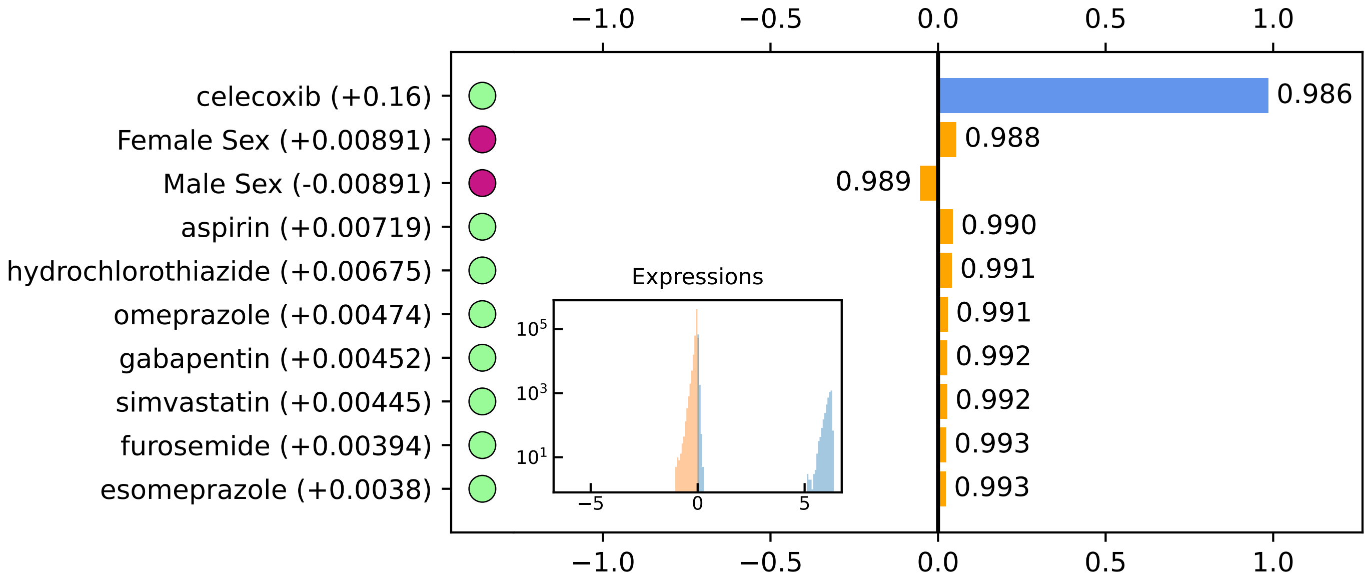

Signature [808]; 87 To Reach 0.975, (87 Plotted)

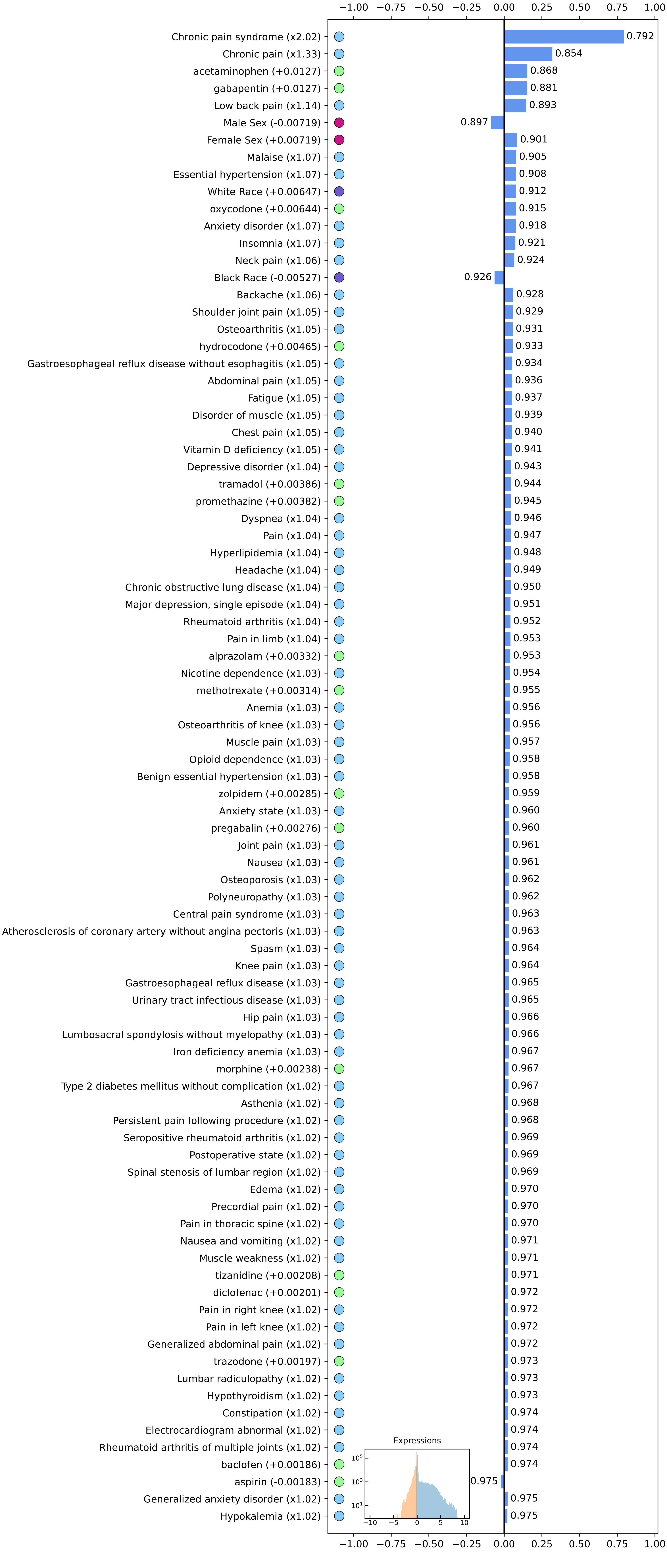

Signature [809]; 96 To Reach 0.975, (96 Plotted)

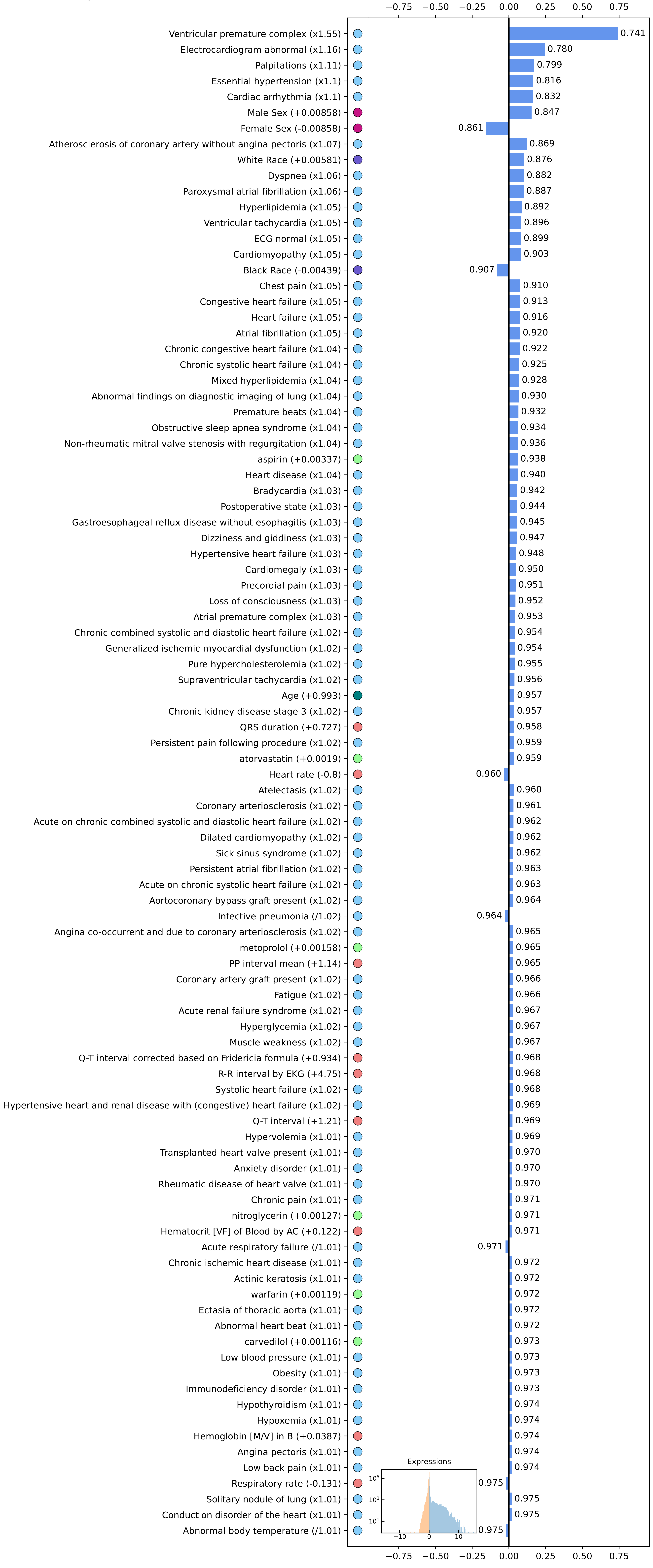

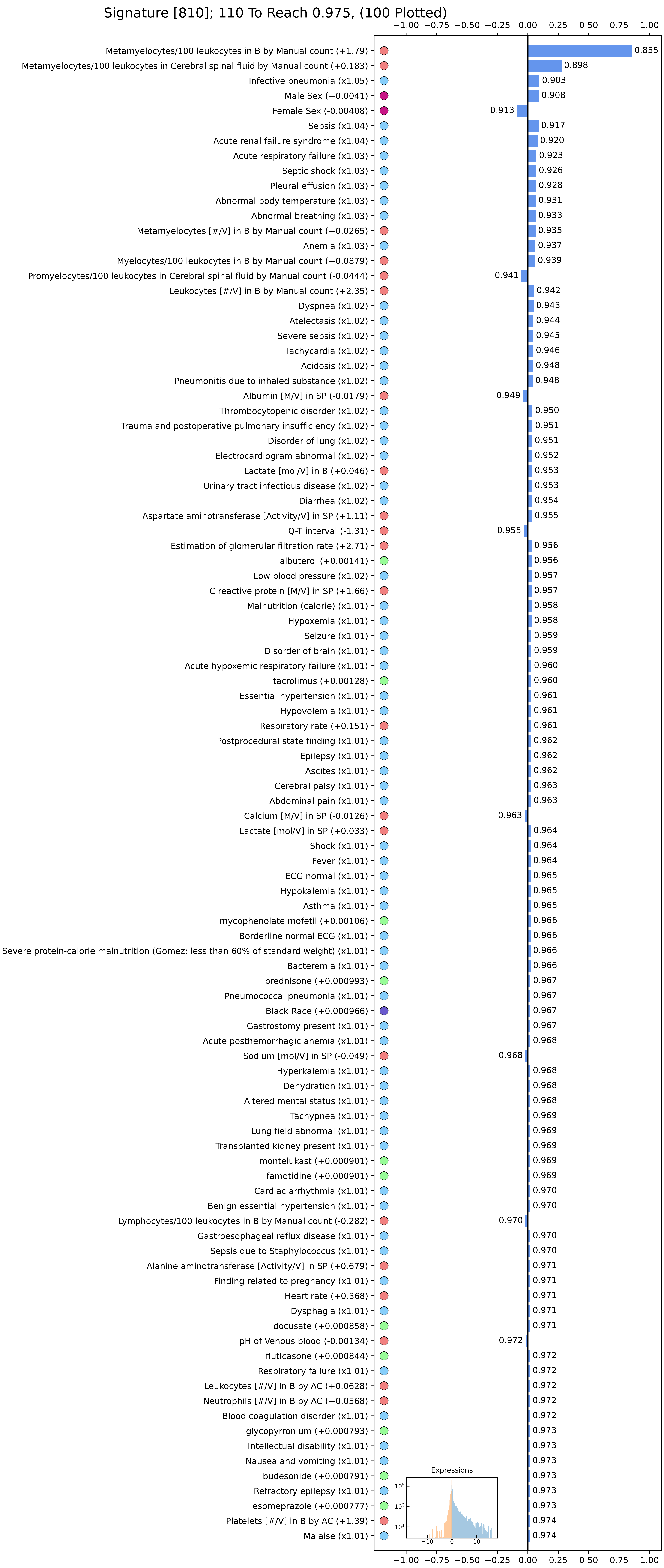

Signature [810]; 110 To Reach 0.975, (100 Plotted)
Metamyelocytes/100 leukocytes in B by Manual count (+1.79) 0.855
Metamyelocytes/100 leukocytes in Cerebral spinal fluid by Manual count (+0.183) 0.898
Infective pneumonia (x1.05) 0.903
Male Sex (+0.0041) 0.908
Female Sex (-0.00408) 0.913
Sepsis (x1.04) 0.917
Acute renal failure syndrome (x1.04) 0.920
Acute respiratory failure (x1.03) 0.923
Septic shock (x1.03) 0.926
Pleural effusion (x1.03) 0.928
Abnormal body temperature (x1.03) 0.931
Abnormal breathing (x1.03) 0.933
Metamyelocytes [#/V] in B by Manual count (+0.0265) 0.935
Anemia (x1.03) 0.937
Myelocytes/100 leukocytes in B by Manual count (+0.0879) 0.939
Promyelocytes/100 leukocytes in Cerebral spinal fluid by Manual count (-0.0444) 0.941
Leukocytes [#/V] in B by Manual count (+2.35) 0.942
Dyspnea (x1.02) 0.943
Atelectasis (x1.02) 0.944
Severe sepsis (x1.02) 0.945
Tachycardia (x1.02) 0.946
Acidosis (x1.02) 0.948
Pneumonitis due to inhaled substance (x1.02) 0.948
Albumin [M/V] in SP (-0.0179) 0.949
Thrombocytopenic disorder (x1.02) 0.950
Trauma and postoperative pulmonary insufficiency (x1.02) 0.951
Disorder of lung (x1.02) 0.951
Electrocardiogram abnormal (x1.02) 0.952
Lactate [mol/V] in B (+0.046) 0.953
Urinary tract infectious disease (x1.02) 0.953
Diarrhea (x1.02) 0.954
Aspartate aminotransferase [Activity/V] in SP (+1.11) 0.955
Q-T interval (-1.31) 0.955
Estimation of glomerular filtration rate (+2.71) 0.956
albuterol (+0.00141) 0.956
Low blood pressure (x1.02) 0.957
C reactive protein [M/V] in SP (+1.66) 0.957
Malnutrition (calorie) (x1.01) 0.958
Hypoxemia (x1.01) 0.958
Seizure (x1.01) 0.959
Disorder of brain (x1.01) 0.959
Acute hypoxemic respiratory failure (x1.01) 0.960
tacrolimus (+0.00128) 0.960
Essential hypertension (x1.01) 0.961
Hypovolemia (x1.01) 0.961
Respiratory rate (+0.151) 0.961
Postprocedural state finding (x1.01) 0.962
Epilepsy (x1.01) 0.962
Ascites (x1.01) 0.962
Cerebral palsy (x1.01) 0.963
Abdominal pain (x1.01) 0.963
Calcium [M/V] in SP (-0.0126) 0.963
Lactate [mol/V] in SP (+0.033) 0.964
Shock (x1.01) 0.964
Fever (x1.01) 0.964
ECG normal (x1.01) 0.965
Hypokalemia (x1.01) 0.965
Asthma (x1.01) 0.965
mycophenolate mofetil (+0.00106) 0.966
Borderline normal ECG (x1.01) 0.966
Severe protein-calorie malnutrition (Gomez: less than 60% of standard weight) (x1.01) 0.966
Bacteremia (x1.01) 0.966
prednisone (+0.000993) 0.967
Pneumococcal pneumonia (x1.01) 0.967
Black Race (+0.000966) 0.967
Gastrostomy present (x1.01) 0.967
Acute posthemorrhagic anemia (x1.01) 0.968
Sodium [mol/V] in SP (-0.049) 0.968
Hyperkalemia (x1.01) 0.968
Dehydration (x1.01) 0.968
Altered mental status (x1.01) 0.968
Tachypnea (x1.01) 0.969
Lung field abnormal (x1.01) 0.969
Transplanted kidney present (x1.01) 0.969
montelukast (+0.000901) 0.969
famotidine (+0.000901) 0.969
Cardiac arrhythmia (x1.01) 0.970
Benign essential hypertension (x1.01) 0.970
Lymphocytes/100 leukocytes in B by Manual count (-0.282) 0.970
Gastroesophageal reflux disease (x1.01) 0.970
Sepsis due to Staphylococcus (x1.01) 0.970
Alanine aminotransferase [Activity/V] in SP (+0.679) 0.971
Finding related to pregnancy (x1.01) 0.971
Heart rate (+0.368) 0.971
Dysphagia (x1.01) 0.971
docusate (+0.000858) 0.971
pH of Venous blood (-0.00134) 0.972
fluticasone (+0.000844) 0.972
Respiratory failure (x1.01) 0.972
Leukocytes [#/V] in B by AC (+0.0628) 0.972
Neutrophils [#/V] in B by AC (+0.0568) 0.972
Blood coagulation disorder (x1.01) 0.972
glycopyrronium (+0.000793) 0.973
Intellectual disability (x1.01) 0.973
Nausea and vomiting (x1.01) 0.973
budesonide (+0.000791) 0.973
Refractory epilepsy (x1.01) 0.973
esomeprazole (+0.000777) 0.973
Platelets [#/V] in B by AC (+1.39) 0.974
Malaise (x1.01) 0.974
−1.00 −0.75 −0.50 −0.25 0.00 0.25 0.50 0.75 1.00
Expressions

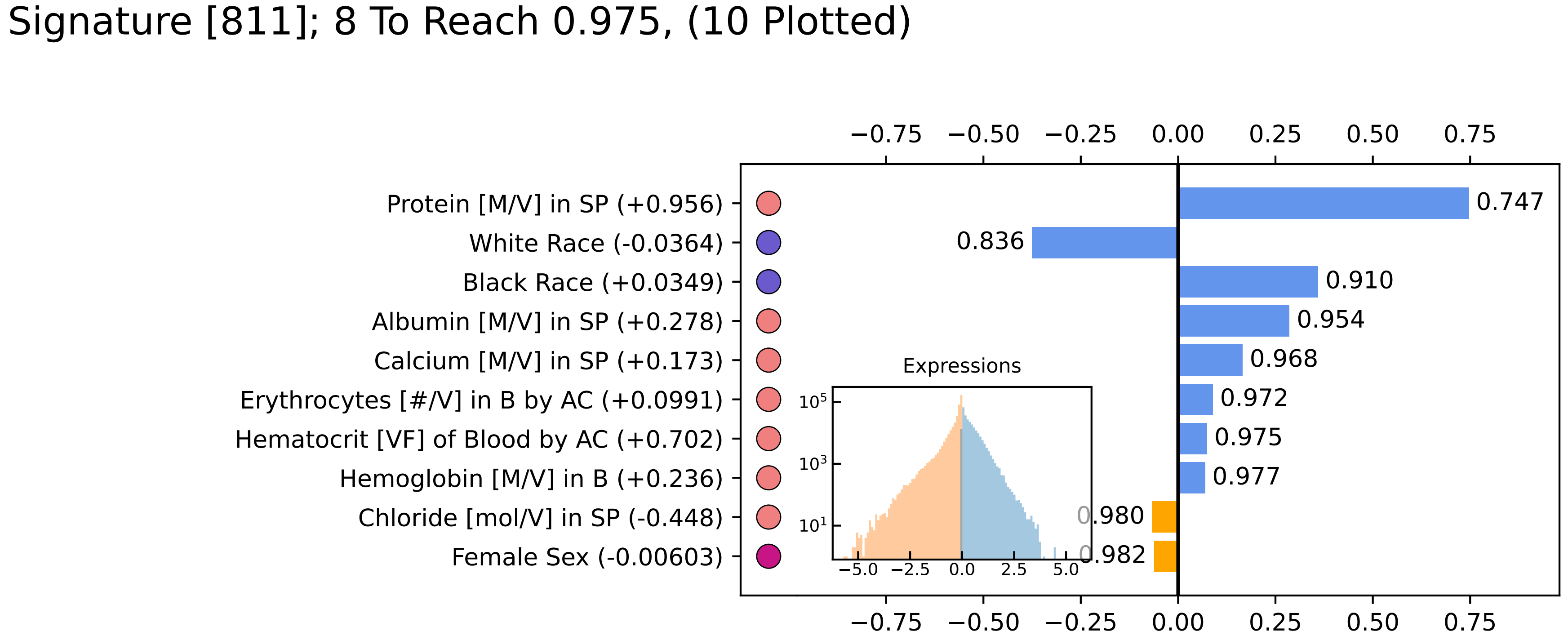

Signature [811]; 8 To Reach 0.975, (10 Plotted)
Protein [M/V] in SP (+0.956)
White Race (-0.0364)
Black Race (+0.0349)
Albumin [M/V] in SP (+0.278)
Calcium [M/V] in SP (+0.173)
Erythrocytes [#/V] in B by AC (+0.0991)
Hematocrit [VF] of Blood by AC (+0.702)
Hemoglobin [M/V] in B (+0.236)
Chloride [mol/V] in SP (-0.448)
Female Sex (-0.00603)
0.747
0.836
0.910
0.954
0.968
0.972
0.975
0.977
0.980
0.982
Expressions
−0.75
−0.50
−0.25
0.00
0.25
0.50
0.75

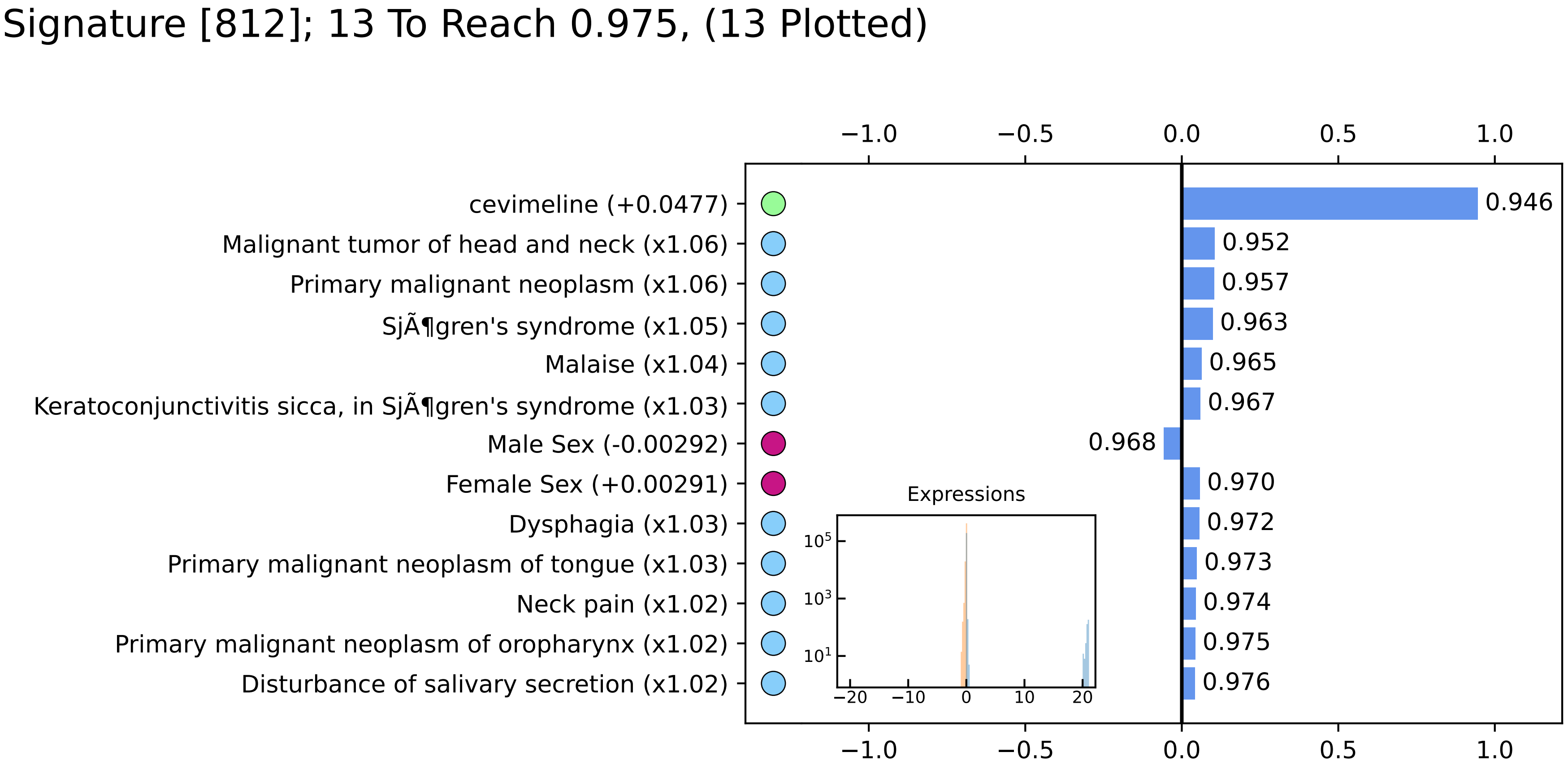

Signature [812]; 13 To Reach 0.975, (13 Plotted)
cevimeline (+0.0477)
Malignant tumor of head and neck (x1.06)
Primary malignant neoplasm (x1.06)
SjÃ¶gren's syndrome (x1.05)
Malaise (x1.04)
Keratoconjunctivitis sicca, in SjÃ¶gren's syndrome (x1.03)
Male Sex (-0.00292)
Female Sex (+0.00291)
Dysphagia (x1.03)
Primary malignant neoplasm of tongue (x1.03)
Neck pain (x1.02)
Primary malignant neoplasm of oropharynx (x1.02)
Disturbance of salivary secretion (x1.02)
0.946
0.952
0.957
0.963
0.965
0.967
0.968
0.970
0.972
0.973
0.974
0.975
0.976
Expressions
−1.0
−0.5
0.0
0.5
1.0

Signature [813]; 22 To Reach 0.975, (22 Plotted)

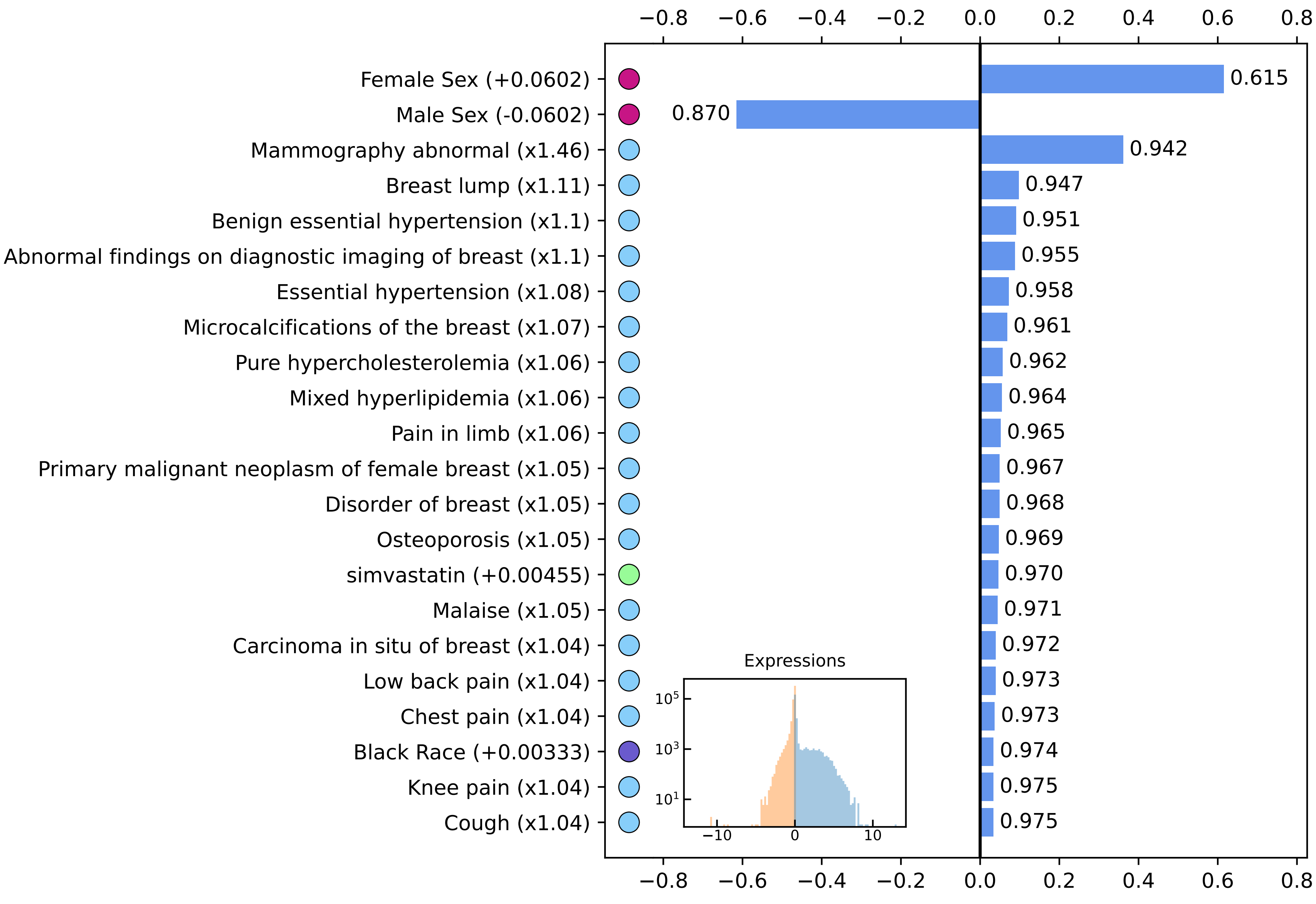

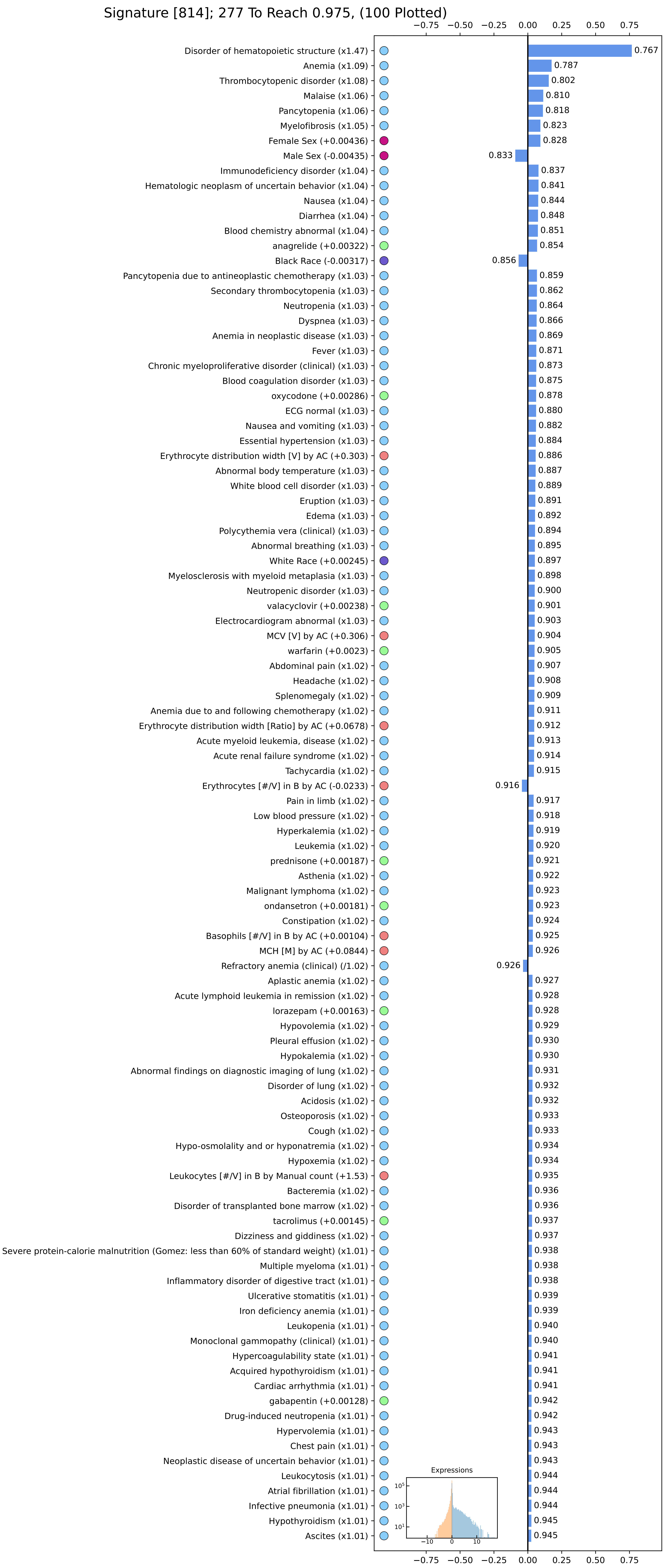

Signature [814]; 277 To Reach 0.975, (100 Plotted)
Disorder of hematopoietic structure (x1.47) 0.767
Anemia (x1.09) 0.787
Thrombocytopenic disorder (x1.08) 0.802
Malaise (x1.06) 0.810
Pancytopenia (x1.06) 0.818
Myelofibrosis (x1.05) 0.823
Female Sex (+0.00436) 0.828
Male Sex (-0.00435) 0.833
Immunodeficiency disorder (x1.04) 0.837
Hematologic neoplasm of uncertain behavior (x1.04) 0.841
Nausea (x1.04) 0.844
Diarrhea (x1.04) 0.848
Blood chemistry abnormal (x1.04) 0.851
anagrelide (+0.00322) 0.854
Black Race (-0.00317) 0.856
Pancytopenia due to antineoplastic chemotherapy (x1.03) 0.859
Secondary thrombocytopenia (x1.03) 0.862
Neutropenia (x1.03) 0.864
Dyspnea (x1.03) 0.866
Anemia in neoplastic disease (x1.03) 0.869
Fever (x1.03) 0.871
Chronic myeloproliferative disorder (clinical) (x1.03) 0.873
Blood coagulation disorder (x1.03) 0.875
oxycodone (+0.00286) 0.878
ECG normal (x1.03) 0.880
Nausea and vomiting (x1.03) 0.882
Essential hypertension (x1.03) 0.884
Erythrocyte distribution width [V] by AC (+0.303) 0.886
Abnormal body temperature (x1.03) 0.887
White blood cell disorder (x1.03) 0.889
Eruption (x1.03) 0.891
Edema (x1.03) 0.892
Polycythemia vera (clinical) (x1.03) 0.894
Abnormal breathing (x1.03) 0.895
White Race (+0.00245) 0.897
Myelosclerosis with myeloid metaplasia (x1.03) 0.898
Neutropenic disorder (x1.03) 0.900
valacyclovir (+0.00238) 0.901
Electrocardiogram abnormal (x1.03) 0.903
MCV [V] by AC (+0.306) 0.904
warfarin (+0.0023) 0.905
Abdominal pain (x1.02) 0.907
Headache (x1.02) 0.908
Splenomegaly (x1.02) 0.909
Anemia due to and following chemotherapy (x1.02) 0.911
Erythrocyte distribution width [Ratio] by AC (+0.0678) 0.912
Acute myeloid leukemia, disease (x1.02) 0.913
Acute renal failure syndrome (x1.02) 0.914
Tachycardia (x1.02) 0.915
Erythrocytes [#/V] in B by AC (-0.0233) 0.916
Pain in limb (x1.02) 0.917
Low blood pressure (x1.02) 0.918
Hyperkalemia (x1.02) 0.919
Leukemia (x1.02) 0.920
prednisone (+0.00187) 0.921
Asthenia (x1.02) 0.922
Malignant lymphoma (x1.02) 0.923
ondansetron (+0.00181) 0.923
Constipation (x1.02) 0.924
Basophils [#/V] in B by AC (+0.00104) 0.925
MCH [M] by AC (+0.0844) 0.926
Refractory anemia (clinical) (/1.02) 0.926
Aplastic anemia (x1.02) 0.927
Acute lymphoid leukemia in remission (x1.02) 0.928
lorazepam (+0.00163) 0.928
Hypovolemia (x1.02) 0.929
Pleural effusion (x1.02) 0.930
Hypokalemia (x1.02) 0.930
Abnormal findings on diagnostic imaging of lung (x1.02) 0.931
Disorder of lung (x1.02) 0.932
Acidosis (x1.02) 0.932
Osteoporosis (x1.02) 0.933
Cough (x1.02) 0.933
Hypo-osmolality and or hyponatremia (x1.02) 0.934
Hypoxemia (x1.02) 0.934
Leukocytes [#/V] in B by Manual count (+1.53) 0.935
Bacteremia (x1.02) 0.936
Disorder of transplanted bone marrow (x1.02) 0.936
tacrolimus (+0.00145) 0.937
Dizziness and giddiness (x1.02) 0.937
Severe protein-calorie malnutrition (Gomez: less than 60% of standard weight) (x1.01) 0.938
Multiple myeloma (x1.01) 0.938
Inflammatory disorder of digestive tract (x1.01) 0.938
Ulcerative stomatitis (x1.01) 0.939
Iron deficiency anemia (x1.01) 0.939
Leukopenia (x1.01) 0.940
Monoclonal gammopathy (clinical) (x1.01) 0.940
Hypercoagulability state (x1.01) 0.941
Acquired hypothyroidism (x1.01) 0.941
Cardiac arrhythmia (x1.01) 0.941
gabapentin (+0.00128) 0.942
Drug-induced neutropenia (x1.01) 0.942
Hypervolemia (x1.01) 0.943
Chest pain (x1.01) 0.943
Neoplastic disease of uncertain behavior (x1.01) 0.943
Leukocytosis (x1.01) 0.944
Atrial fibrillation (x1.01) 0.944
Infective pneumonia (x1.01) 0.944
Hypothyroidism (x1.01) 0.945
Ascites (x1.01) 0.945
−0.75 −0.50 −0.25 0.00 0.25 0.50 0.75
Expressions
−10 0 10

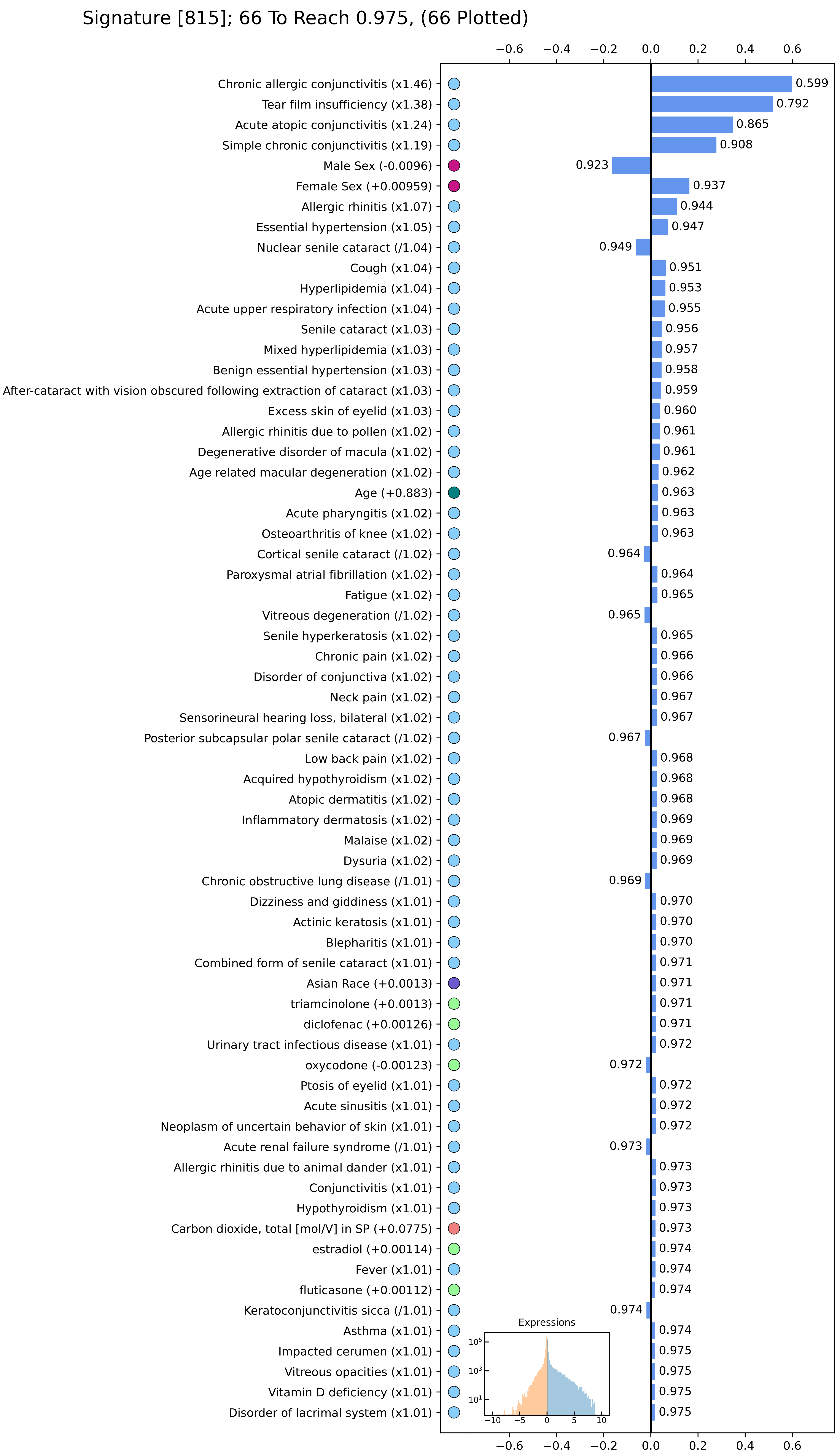

Signature [815]; 66 To Reach 0.975, (66 Plotted)
Chronic allergic conjunctivitis (x1.46) 0.599
Tear film insufficiency (x1.38) 0.792
Acute atopic conjunctivitis (x1.24) 0.865
Simple chronic conjunctivitis (x1.19) 0.908
Male Sex (-0.0096) 0.923
Female Sex (+0.00959) 0.937
Allergic rhinitis (x1.07) 0.944
Essential hypertension (x1.05) 0.947
Nuclear senile cataract (/1.04) 0.949
Cough (x1.04) 0.951
Hyperlipidemia (x1.04) 0.953
Acute upper respiratory infection (x1.04) 0.955
Senile cataract (x1.03) 0.956
Mixed hyperlipidemia (x1.03) 0.957
Benign essential hypertension (x1.03) 0.958
After-cataract with vision obscured following extraction of cataract (x1.03) 0.959
Excess skin of eyelid (x1.03) 0.960
Allergic rhinitis due to pollen (x1.02) 0.961
Degenerative disorder of macula (x1.02) 0.961
Age related macular degeneration (x1.02) 0.962
Age (+0.883) 0.963
Acute pharyngitis (x1.02) 0.963
Osteoarthritis of knee (x1.02) 0.963
Cortical senile cataract (/1.02) 0.964
Paroxysmal atrial fibrillation (x1.02) 0.964
Fatigue (x1.02) 0.965
Vitreous degeneration (/1.02) 0.965
Senile hyperkeratosis (x1.02) 0.965
Chronic pain (x1.02) 0.966
Disorder of conjunctiva (x1.02) 0.966
Neck pain (x1.02) 0.967
Sensorineural hearing loss, bilateral (x1.02) 0.967
Posterior subcapsular polar senile cataract (/1.02) 0.967
Low back pain (x1.02) 0.968
Acquired hypothyroidism (x1.02) 0.968
Atopic dermatitis (x1.02) 0.968
Inflammatory dermatosis (x1.02) 0.969
Malaise (x1.02) 0.969
Dysuria (x1.02) 0.969
Chronic obstructive lung disease (/1.01) 0.969
Dizziness and giddiness (x1.01) 0.970
Actinic keratosis (x1.01) 0.970
Blepharitis (x1.01) 0.970
Combined form of senile cataract (x1.01) 0.971
Asian Race (+0.0013) 0.971
triamcinolone (+0.0013) 0.971
diclofenac (+0.00126) 0.971
Urinary tract infectious disease (x1.01) 0.972
oxycodone (-0.00123) 0.972
Ptosis of eyelid (x1.01) 0.972
Acute sinusitis (x1.01) 0.972
Neoplasm of uncertain behavior of skin (x1.01) 0.972
Acute renal failure syndrome (/1.01) 0.973
Allergic rhinitis due to animal dander (x1.01) 0.973
Conjunctivitis (x1.01) 0.973
Hypothyroidism (x1.01) 0.973
Carbon dioxide, total [mol/V] in SP (+0.0775) 0.973
estradiol (+0.00114) 0.974
Fever (x1.01) 0.974
fluticasone (+0.00112) 0.974
Keratoconjunctivitis sicca (/1.01) 0.974
Asthma (x1.01) 0.974
Impacted cerumen (x1.01) 0.975
Vitreous opacities (x1.01) 0.975
Vitamin D deficiency (x1.01) 0.975
Disorder of lacrimal system (x1.01) 0.975
−0.6 −0.4 −0.2 0.0 0.2 0.4 0.6
Expressions
−10 −5 0 5 10

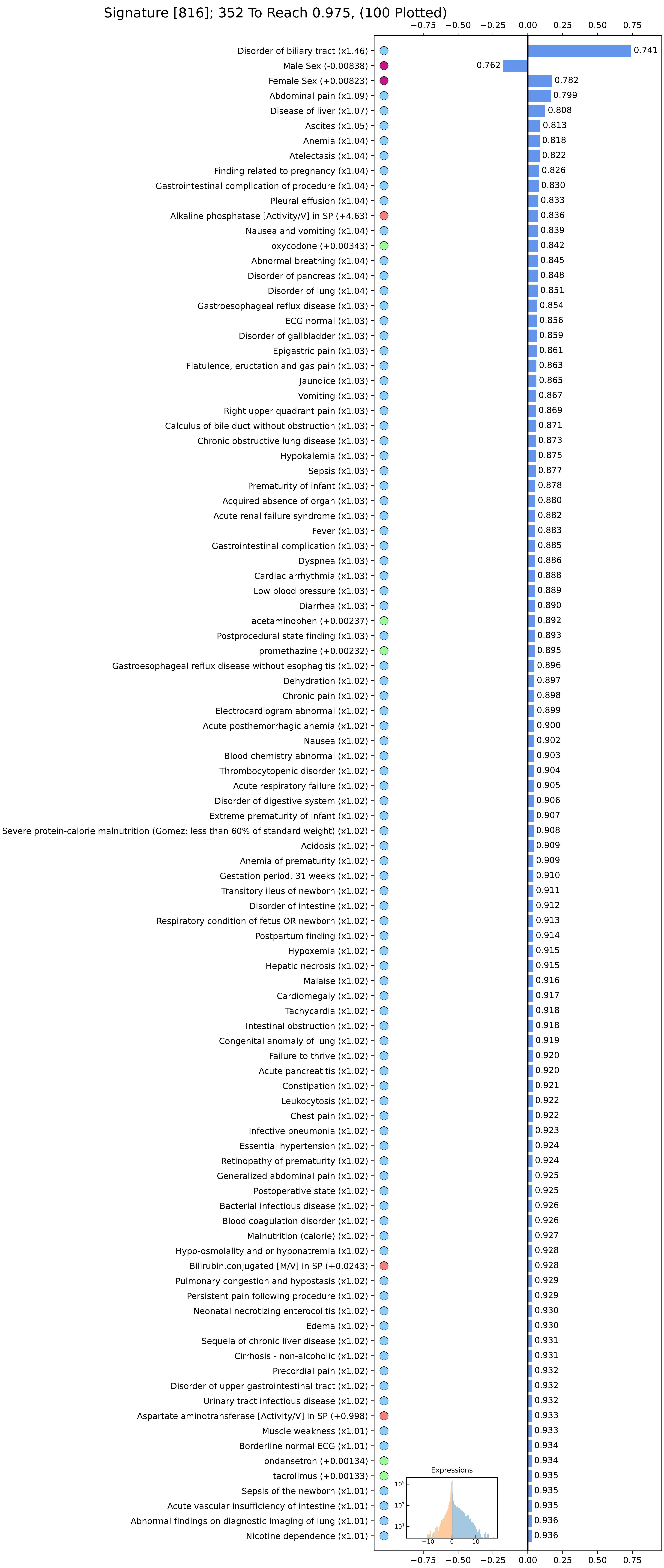

Signature [816]; 352 To Reach 0.975, (100 Plotted)
Disorder of biliary tract (x1.46) 0.741
Male Sex (-0.00838) 0.762
Female Sex (+0.00823) 0.782
Abdominal pain (x1.09) 0.799
Disease of liver (x1.07) 0.808
Ascites (x1.05) 0.813
Anemia (x1.04) 0.818
Atelectasis (x1.04) 0.822
Finding related to pregnancy (x1.04) 0.826
Gastrointestinal complication of procedure (x1.04) 0.830
Pleural effusion (x1.04) 0.833
Alkaline phosphatase [Activity/V] in SP (+4.63) 0.836
Nausea and vomiting (x1.04) 0.839
oxycodone (+0.00343) 0.842
Abnormal breathing (x1.04) 0.845
Disorder of pancreas (x1.04) 0.848
Disorder of lung (x1.04) 0.851
Gastroesophageal reflux disease (x1.03) 0.854
ECG normal (x1.03) 0.856
Disorder of gallbladder (x1.03) 0.859
Epigastric pain (x1.03) 0.861
Flatulence, eructation and gas pain (x1.03) 0.863
Jaundice (x1.03) 0.865
Vomiting (x1.03) 0.867
Right upper quadrant pain (x1.03) 0.869
Calculus of bile duct without obstruction (x1.03) 0.871
Chronic obstructive lung disease (x1.03) 0.873
Hypokalemia (x1.03) 0.875
Sepsis (x1.03) 0.877
Prematurity of infant (x1.03) 0.878
Acquired absence of organ (x1.03) 0.880
Acute renal failure syndrome (x1.03) 0.882
Fever (x1.03) 0.883
Gastrointestinal complication (x1.03) 0.885
Dyspnea (x1.03) 0.886
Cardiac arrhythmia (x1.03) 0.888
Low blood pressure (x1.03) 0.889
Diarrhea (x1.03) 0.890
acetaminophen (+0.00237) 0.892
Postprocedural state finding (x1.03) 0.893
promethazine (+0.00232) 0.895
Gastroesophageal reflux disease without esophagitis (x1.02) 0.896
Dehydration (x1.02) 0.897
Chronic pain (x1.02) 0.898
Electrocardiogram abnormal (x1.02) 0.899
Acute posthemorrhagic anemia (x1.02) 0.900
Nausea (x1.02) 0.902
Blood chemistry abnormal (x1.02) 0.903
Thrombocytopenic disorder (x1.02) 0.904
Acute respiratory failure (x1.02) 0.905
Disorder of digestive system (x1.02) 0.906
Extreme prematurity of infant (x1.02) 0.907
Severe protein-calorie malnutrition (Gomez: less than 60% of standard weight) (x1.02) 0.908
Acidosis (x1.02) 0.909
Anemia of prematurity (x1.02) 0.909
Gestation period, 31 weeks (x1.02) 0.910
Transitory ileus of newborn (x1.02) 0.911
Disorder of intestine (x1.02) 0.912
Respiratory condition of fetus OR newborn (x1.02) 0.913
Postpartum finding (x1.02) 0.914
Hypoxemia (x1.02) 0.915
Hepatic necrosis (x1.02) 0.915
Malaise (x1.02) 0.916
Cardiomegaly (x1.02) 0.917
Tachycardia (x1.02) 0.918
Intestinal obstruction (x1.02) 0.918
Congenital anomaly of lung (x1.02) 0.919
Failure to thrive (x1.02) 0.920
Acute pancreatitis (x1.02) 0.920
Constipation (x1.02) 0.921
Leukocytosis (x1.02) 0.922
Chest pain (x1.02) 0.922
Infective pneumonia (x1.02) 0.923
Essential hypertension (x1.02) 0.924
Retinopathy of prematurity (x1.02) 0.924
Generalized abdominal pain (x1.02) 0.925
Postoperative state (x1.02) 0.925
Bacterial infectious disease (x1.02) 0.926
Blood coagulation disorder (x1.02) 0.926
Malnutrition (calorie) (x1.02) 0.927
Hypo-osmolality and or hyponatremia (x1.02) 0.928
Bilirubin.conjugated [M/V] in SP (+0.0243) 0.928
Pulmonary congestion and hypostasis (x1.02) 0.929
Persistent pain following procedure (x1.02) 0.929
Neonatal necrotizing enterocolitis (x1.02) 0.930
Edema (x1.02) 0.930
Sequela of chronic liver disease (x1.02) 0.931
Cirrhosis - non-alcoholic (x1.02) 0.931
Precordial pain (x1.02) 0.932
Disorder of upper gastrointestinal tract (x1.02) 0.932
Urinary tract infectious disease (x1.02) 0.932
Aspartate aminotransferase [Activity/V] in SP (+0.998) 0.933
Muscle weakness (x1.01) 0.933
Borderline normal ECG (x1.01) 0.934
ondansetron (+0.00134) 0.934
tacrolimus (+0.00133) 0.935
Sepsis of the newborn (x1.01) 0.935
Acute vascular insufficiency of intestine (x1.01) 0.935
Abnormal findings on diagnostic imaging of lung (x1.01) 0.936
Nicotine dependence (x1.01) 0.936
Expressions
−0.75 −0.50 −0.25 0.00 0.25 0.50 0.75

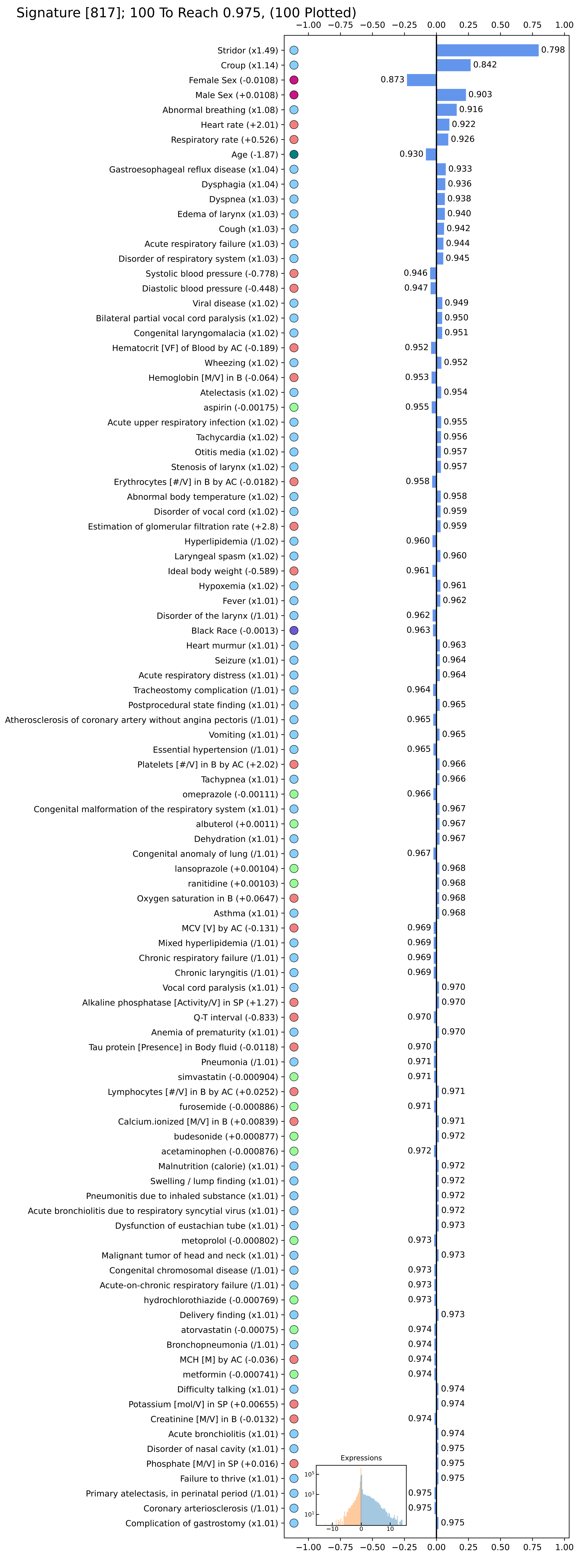

Signature [817]; 100 To Reach 0.975, (100 Plotted)
Stridor (x1.49) 0.798
Croup (x1.14) 0.842
Female Sex (-0.0108) 0.873
Male Sex (+0.0108) 0.903
Abnormal breathing (x1.08) 0.916
Heart rate (+2.01) 0.922
Respiratory rate (+0.526) 0.926
Age (-1.87) 0.930
Gastroesophageal reflux disease (x1.04) 0.933
Dysphagia (x1.04) 0.936
Dyspnea (x1.03) 0.938
Edema of larynx (x1.03) 0.940
Cough (x1.03) 0.942
Acute respiratory failure (x1.03) 0.944
Disorder of respiratory system (x1.03) 0.945
Systolic blood pressure (-0.778) 0.946
Diastolic blood pressure (-0.448) 0.947
Viral disease (x1.02) 0.949
Bilateral partial vocal cord paralysis (x1.02) 0.950
Congenital laryngomalacia (x1.02) 0.951
Hematocrit [VF] of Blood by AC (-0.189) 0.952
Wheezing (x1.02) 0.952
Hemoglobin [M/V] in B (-0.064) 0.953
Atelectasis (x1.02) 0.954
aspirin (-0.00175) 0.955
Acute upper respiratory infection (x1.02) 0.955
Tachycardia (x1.02) 0.956
Otitis media (x1.02) 0.957
Stenosis of larynx (x1.02) 0.957
Erythrocytes [#/V] in B by AC (-0.0182) 0.958
Abnormal body temperature (x1.02) 0.958
Disorder of vocal cord (x1.02) 0.959
Estimation of glomerular filtration rate (+2.8) 0.959
Hyperlipidemia (/1.02) 0.960
Laryngeal spasm (x1.02) 0.960
Ideal body weight (-0.589) 0.961
Hypoxemia (x1.02) 0.961
Fever (x1.01) 0.962
Disorder of the larynx (/1.01) 0.962
Black Race (-0.0013) 0.963
Heart murmur (x1.01) 0.963
Seizure (x1.01) 0.964
Acute respiratory distress (x1.01) 0.964
Tracheostomy complication (/1.01) 0.964
Postprocedural state finding (x1.01) 0.965
Atherosclerosis of coronary artery without angina pectoris (/1.01) 0.965
Vomiting (x1.01) 0.965
Essential hypertension (/1.01) 0.965
Platelets [#/V] in B by AC (+2.02) 0.966
Tachypnea (x1.01) 0.966
omeprazole (-0.00111) 0.966
Congenital malformation of the respiratory system (x1.01) 0.967
albuterol (+0.0011) 0.967
Dehydration (x1.01) 0.967
Congenital anomaly of lung (/1.01) 0.967
lansoprazole (+0.00104) 0.968
ranitidine (+0.00103) 0.968
Oxygen saturation in B (+0.0647) 0.968
Asthma (x1.01) 0.968
MCV [V] by AC (-0.131) 0.969
Mixed hyperlipidemia (/1.01) 0.969
Chronic respiratory failure (/1.01) 0.969
Chronic laryngitis (/1.01) 0.969
Vocal cord paralysis (x1.01) 0.970
Alkaline phosphatase [Activity/V] in SP (+1.27) 0.970
Q-T interval (-0.833) 0.970
Anemia of prematurity (x1.01) 0.970
Tau protein [Presence] in Body fluid (-0.0118) 0.970
Pneumonia (/1.01) 0.971
simvastatin (-0.000904) 0.971
Lymphocytes [#/V] in B by AC (+0.0252) 0.971
furosemide (-0.000886) 0.971
Calcium.ionized [M/V] in B (+0.00839) 0.971
budesonide (+0.000877) 0.972
acetaminophen (-0.000876) 0.972
Malnutrition (calorie) (x1.01) 0.972
Swelling / lump finding (x1.01) 0.972
Pneumonitis due to inhaled substance (x1.01) 0.972
Acute bronchiolitis due to respiratory syncytial virus (x1.01) 0.972
Dysfunction of eustachian tube (x1.01) 0.973
metoprolol (-0.000802) 0.973
Malignant tumor of head and neck (x1.01) 0.973
Congenital chromosomal disease (/1.01) 0.973
Acute-on-chronic respiratory failure (/1.01) 0.973
hydrochlorothiazide (-0.000769) 0.973
Delivery finding (x1.01) 0.973
atorvastatin (-0.00075) 0.974
Bronchopneumonia (/1.01) 0.974
MCH [M] by AC (-0.036) 0.974
metformin (-0.000741) 0.974
Difficulty talking (x1.01) 0.974
Potassium [mol/V] in SP (+0.00655) 0.974
Creatinine [M/V] in B (-0.0132) 0.974
Acute bronchiolitis (x1.01) 0.974
Disorder of nasal cavity (x1.01) 0.975
Phosphate [M/V] in SP (+0.016) 0.975
Failure to thrive (x1.01) 0.975
Primary atelectasis, in perinatal period (/1.01) 0.975
Coronary arteriosclerosis (/1.01) 0.975
Complication of gastrostomy (x1.01) 0.975
−1.00 −0.75 −0.50 −0.25 0.00 0.25 0.50 0.75 1.00
Expressions
10^5 10^3 10^1
−10 0 10

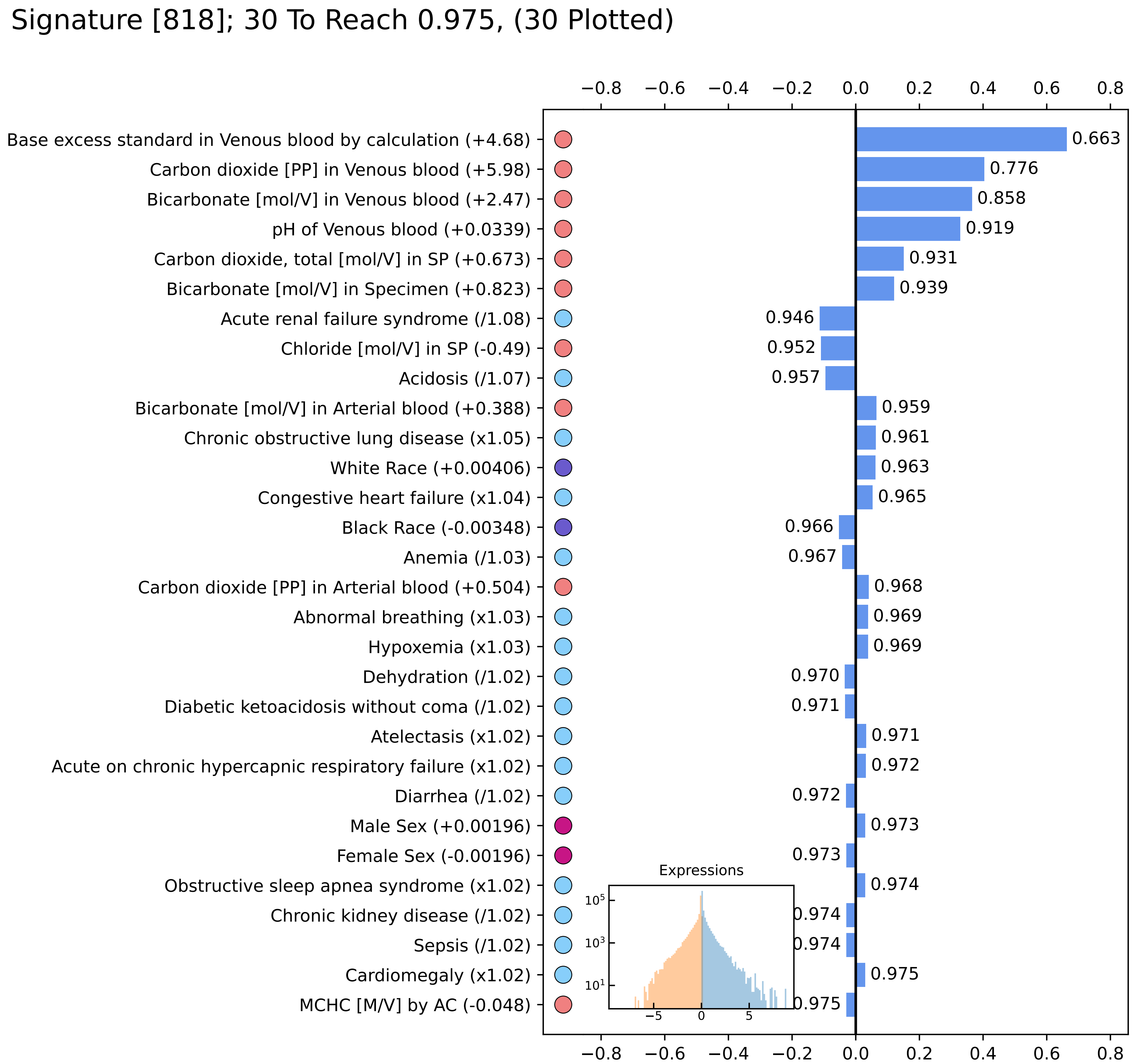

Signature [818]; 30 To Reach 0.975, (30 Plotted)
Base excess standard in Venous blood by calculation (+4.68) 0.663
Carbon dioxide [PP] in Venous blood (+5.98) 0.776
Bicarbonate [mol/V] in Venous blood (+2.47) 0.858
pH of Venous blood (+0.0339) 0.919
Carbon dioxide, total [mol/V] in SP (+0.673) 0.931
Bicarbonate [mol/V] in Specimen (+0.823) 0.939
Acute renal failure syndrome (/1.08) 0.946
Chloride [mol/V] in SP (-0.49) 0.952
Acidosis (/1.07) 0.957
Bicarbonate [mol/V] in Arterial blood (+0.388) 0.959
Chronic obstructive lung disease (x1.05) 0.961
White Race (+0.00406) 0.963
Congestive heart failure (x1.04) 0.965
Black Race (-0.00348) 0.966
Anemia (/1.03) 0.967
Carbon dioxide [PP] in Arterial blood (+0.504) 0.968
Abnormal breathing (x1.03) 0.969
Hypoxemia (x1.03) 0.969
Dehydration (/1.02) 0.970
Diabetic ketoacidosis without coma (/1.02) 0.971
Atelectasis (x1.02) 0.971
Acute on chronic hypercapnic respiratory failure (x1.02) 0.972
Diarrhea (/1.02) 0.972
Male Sex (+0.00196) 0.973
Female Sex (-0.00196) 0.973
Obstructive sleep apnea syndrome (x1.02) 0.974
Chronic kidney disease (/1.02) 0.974
Sepsis (/1.02) 0.974
Cardiomegaly (x1.02) 0.975
MCHC [M/V] by AC (-0.048) 0.975
−0.8 −0.6 −0.4 −0.2 0.0 0.2 0.4 0.6 0.8
Expressions
10^5
10^3
10^1
−5 0 5

Signature [819]; 361 To Reach 0.975, (100 Plotted)

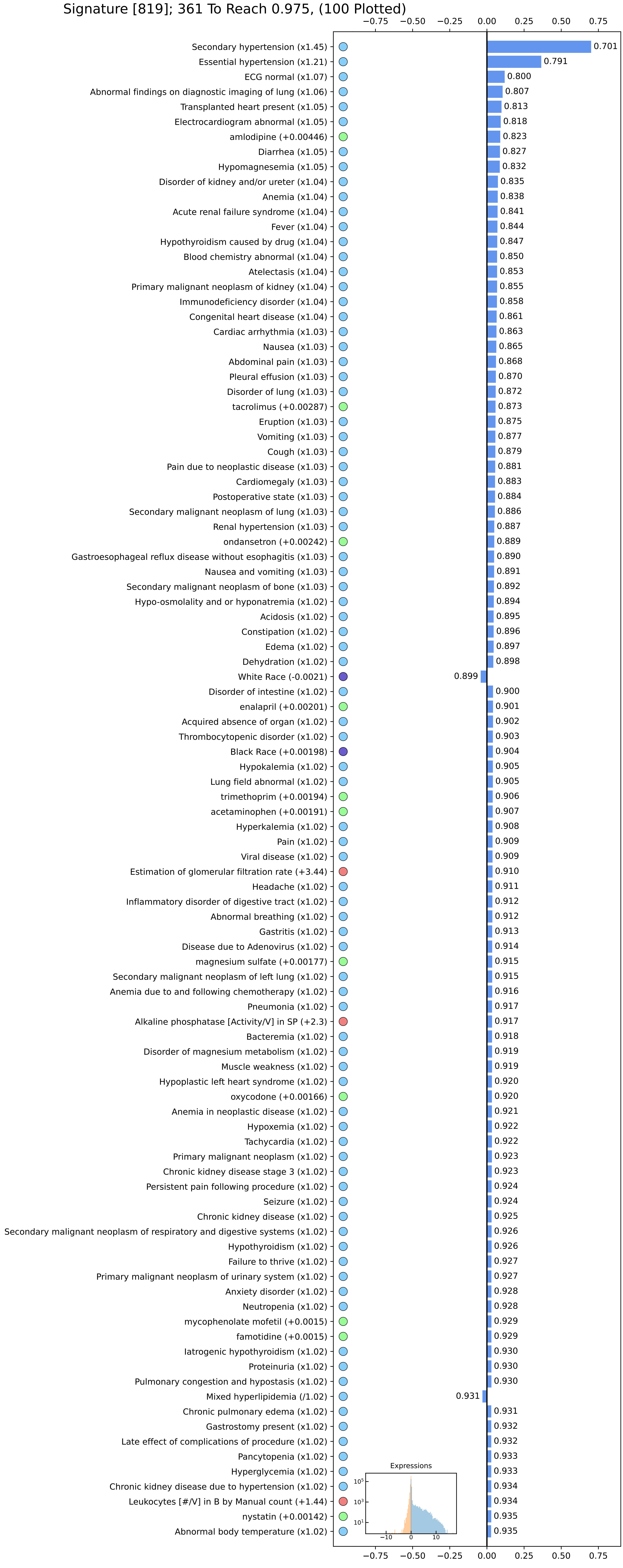

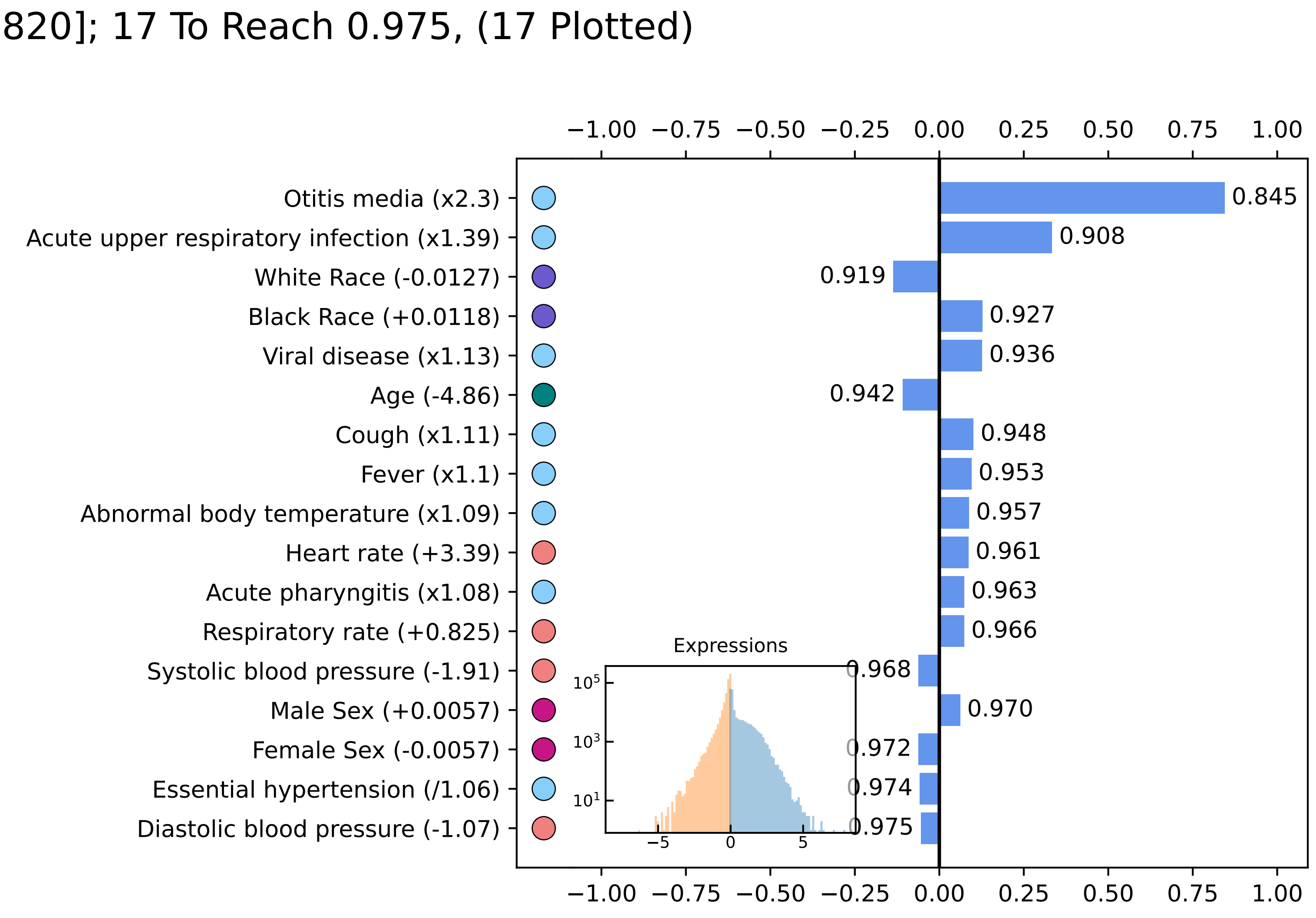

Signature [820]; 17 To Reach 0.975, (17 Plotted)
Otitis media (x2.3)
Acute upper respiratory infection (x1.39)
White Race (-0.0127)
Black Race (+0.0118)
Viral disease (x1.13)
Age (-4.86)
Cough (x1.11)
Fever (x1.1)
Abnormal body temperature (x1.09)
Heart rate (+3.39)
Acute pharyngitis (x1.08)
Respiratory rate (+0.825)
Systolic blood pressure (-1.91)
Male Sex (+0.0057)
Female Sex (-0.0057)
Essential hypertension (/1.06)
Diastolic blood pressure (-1.07)
0.845
0.908
0.919
0.927
0.936
0.942
0.948
0.953
0.957
0.961
0.963
0.966
0.968
0.970
0.972
0.974
0.975
−1.00 −0.75 −0.50 −0.25 0.00 0.25 0.50 0.75 1.00
Expressions

Signature [821]; 7 To Reach 0.975, (10 Plotted)

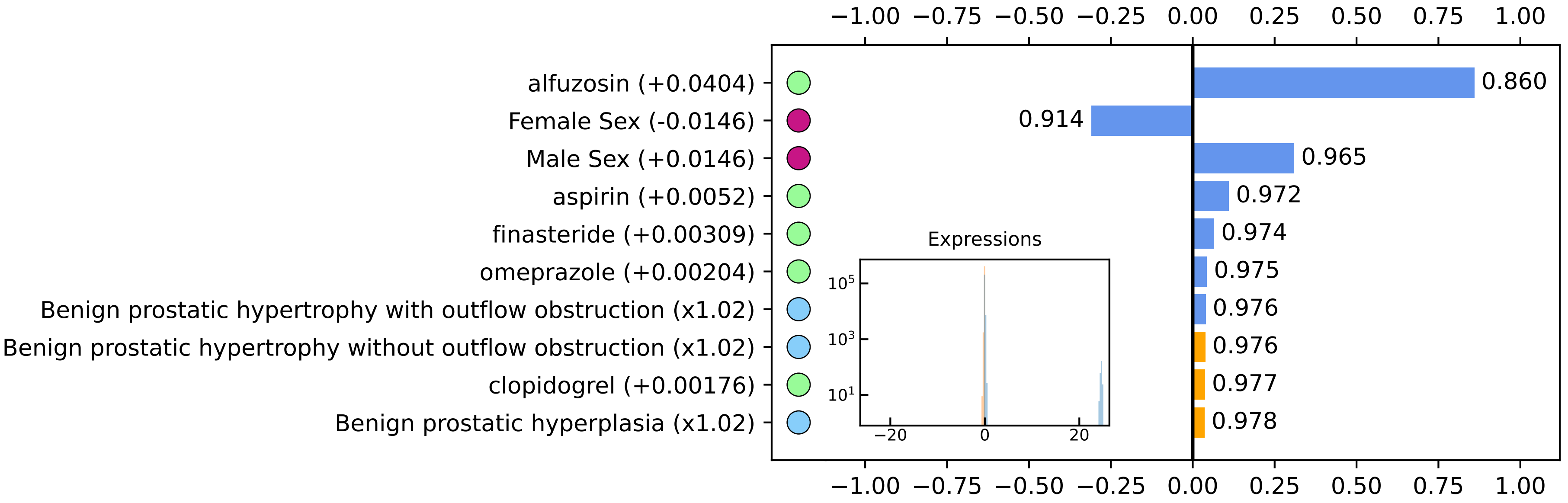

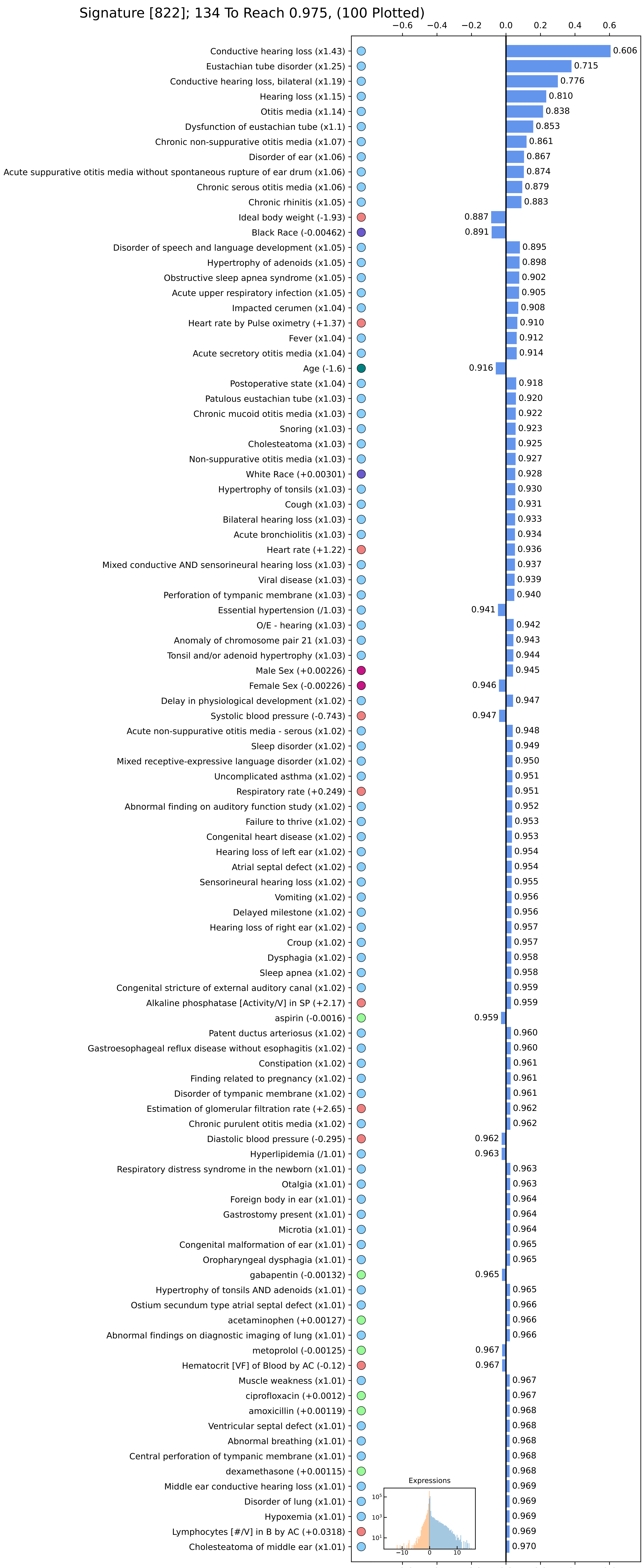

Signature [822]; 134 To Reach 0.975, (100 Plotted)
Conductive hearing loss (x1.43) 0.606
Eustachian tube disorder (x1.25) 0.715
Conductive hearing loss, bilateral (x1.19) 0.776
Hearing loss (x1.15) 0.810
Otitis media (x1.14) 0.838
Dysfunction of eustachian tube (x1.1) 0.853
Chronic non-suppurative otitis media (x1.07) 0.861
Disorder of ear (x1.06) 0.867
Acute suppurative otitis media without spontaneous rupture of ear drum (x1.06) 0.874
Chronic serous otitis media (x1.06) 0.879
Chronic rhinitis (x1.05) 0.883
Ideal body weight (-1.93) 0.887
Black Race (-0.00462) 0.891
Disorder of speech and language development (x1.05) 0.895
Hypertrophy of adenoids (x1.05) 0.898
Obstructive sleep apnea syndrome (x1.05) 0.902
Acute upper respiratory infection (x1.05) 0.905
Impacted cerumen (x1.04) 0.908
Heart rate by Pulse oximetry (+1.37) 0.910
Fever (x1.04) 0.912
Acute secretory otitis media (x1.04) 0.914
Age (-1.6) 0.916
Postoperative state (x1.04) 0.918
Patulous eustachian tube (x1.03) 0.920
Chronic mucoid otitis media (x1.03) 0.922
Snoring (x1.03) 0.923
Cholesteatoma (x1.03) 0.925
Non-suppurative otitis media (x1.03) 0.927
White Race (+0.00301) 0.928
Hypertrophy of tonsils (x1.03) 0.930
Cough (x1.03) 0.931
Bilateral hearing loss (x1.03) 0.933
Acute bronchiolitis (x1.03) 0.934
Heart rate (+1.22) 0.936
Mixed conductive AND sensorineural hearing loss (x1.03) 0.937
Viral disease (x1.03) 0.939
Perforation of tympanic membrane (x1.03) 0.940
Essential hypertension (/1.03) 0.941
O/E - hearing (x1.03) 0.942
Anomaly of chromosome pair 21 (x1.03) 0.943
Tonsil and/or adenoid hypertrophy (x1.03) 0.944
Male Sex (+0.00226) 0.945
Female Sex (-0.00226) 0.946
Delay in physiological development (x1.02) 0.947
Systolic blood pressure (-0.743) 0.947
Acute non-suppurative otitis media - serous (x1.02) 0.948
Sleep disorder (x1.02) 0.949
Mixed receptive-expressive language disorder (x1.02) 0.950
Uncomplicated asthma (x1.02) 0.951
Respiratory rate (+0.249) 0.951
Abnormal finding on auditory function study (x1.02) 0.952
Failure to thrive (x1.02) 0.953
Congenital heart disease (x1.02) 0.953
Hearing loss of left ear (x1.02) 0.954
Atrial septal defect (x1.02) 0.954
Sensorineural hearing loss (x1.02) 0.955
Vomiting (x1.02) 0.956
Delayed milestone (x1.02) 0.956
Hearing loss of right ear (x1.02) 0.957
Croup (x1.02) 0.957
Dysphagia (x1.02) 0.958
Sleep apnea (x1.02) 0.958
Congenital stricture of external auditory canal (x1.02) 0.959
Alkaline phosphatase [Activity/V] in SP (+2.17) 0.959
aspirin (-0.0016) 0.959
Patent ductus arteriosus (x1.02) 0.960
Gastroesophageal reflux disease without esophagitis (x1.02) 0.960
Constipation (x1.02) 0.961
Finding related to pregnancy (x1.02) 0.961
Disorder of tympanic membrane (x1.02) 0.961
Estimation of glomerular filtration rate (+2.65) 0.962
Chronic purulent otitis media (x1.02) 0.962
Diastolic blood pressure (-0.295) 0.962
Hyperlipidemia (/1.01) 0.963
Respiratory distress syndrome in the newborn (x1.01) 0.963
Otalgia (x1.01) 0.963
Foreign body in ear (x1.01) 0.964
Gastrostomy present (x1.01) 0.964
Microtia (x1.01) 0.964
Congenital malformation of ear (x1.01) 0.965
Oropharyngeal dysphagia (x1.01) 0.965
gabapentin (-0.00132) 0.965
Hypertrophy of tonsils AND adenoids (x1.01) 0.965
Ostium secundum type atrial septal defect (x1.01) 0.966
acetaminophen (+0.00127) 0.966
Abnormal findings on diagnostic imaging of lung (x1.01) 0.966
metoprolol (-0.00125) 0.967
Hematocrit [VF] of Blood by AC (-0.12) 0.967
Muscle weakness (x1.01) 0.967
ciprofloxacin (+0.0012) 0.967
amoxicillin (+0.00119) 0.968
Ventricular septal defect (x1.01) 0.968
Abnormal breathing (x1.01) 0.968
Central perforation of tympanic membrane (x1.01) 0.968
dexamethasone (+0.00115) 0.968
Middle ear conductive hearing loss (x1.01) 0.969
Disorder of lung (x1.01) 0.969
Hypoxemia (x1.01) 0.969
Lymphocytes [#/V] in B by AC (+0.0318) 0.969
Cholesteatoma of middle ear (x1.01) 0.970
Expressions
−0.6 −0.4 −0.2 0.0 0.2 0.4 0.6

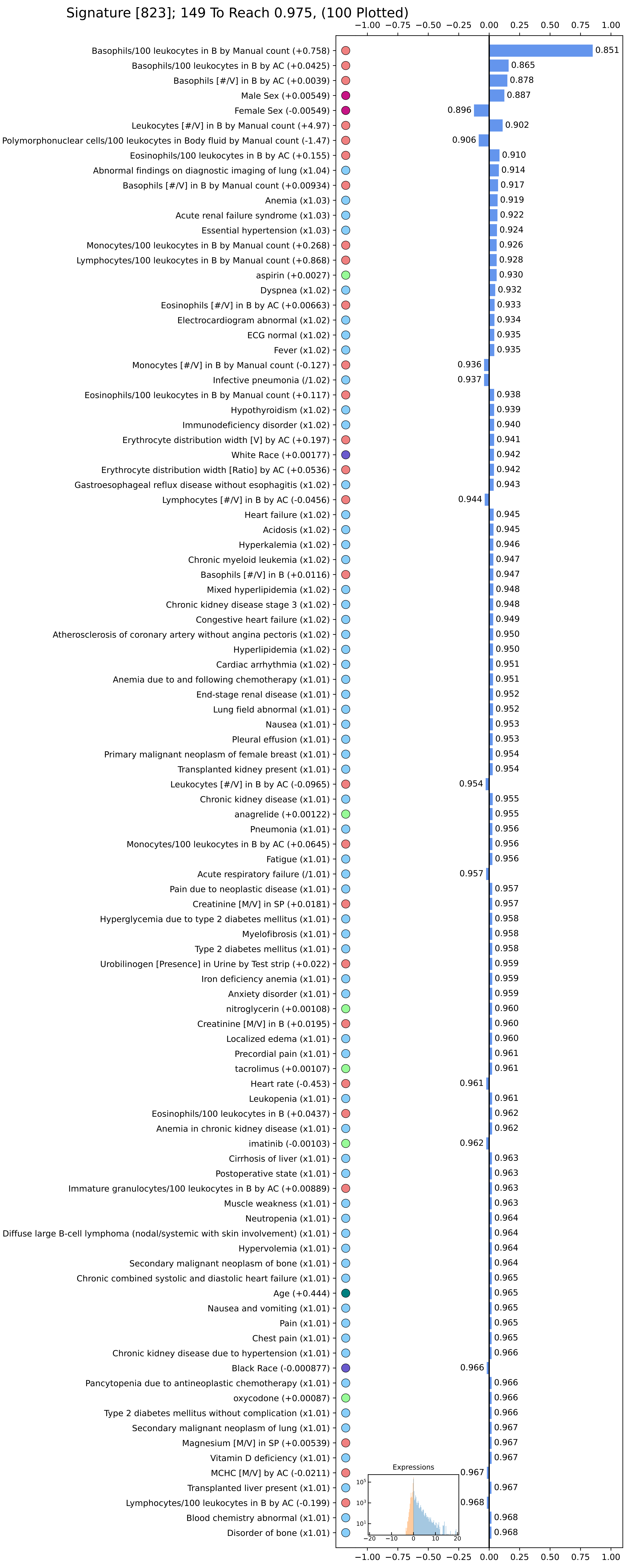

Signature [823]; 149 To Reach 0.975, (100 Plotted)
Basophils/100 leukocytes in B by Manual count (+0.758)
Basophils/100 leukocytes in B by AC (+0.0425)
Basophils [#/V] in B by AC (+0.0039)
Male Sex (+0.00549)
Female Sex (-0.00549)
Leukocytes [#/V] in B by Manual count (+4.97)
Polymorphonuclear cells/100 leukocytes in Body fluid by Manual count (-1.47)
Eosinophils/100 leukocytes in B by AC (+0.155)
Abnormal findings on diagnostic imaging of lung (x1.04)
Basophils [#/V] in B by Manual count (+0.00934)
Anemia (x1.03)
Acute renal failure syndrome (x1.03)
Essential hypertension (x1.03)
Monocytes/100 leukocytes in B by Manual count (+0.268)
Lymphocytes/100 leukocytes in B by Manual count (+0.868)
aspirin (+0.0027)
Dyspnea (x1.02)
Eosinophils [#/V] in B by AC (+0.00663)
Electrocardiogram abnormal (x1.02)
ECG normal (x1.02)
Fever (x1.02)
Monocytes [#/V] in B by Manual count (-0.127)
Infective pneumonia (/1.02)
Eosinophils/100 leukocytes in B by Manual count (+0.117)
Hypothyroidism (x1.02)
Immunodeficiency disorder (x1.02)
Erythrocyte distribution width [V] by AC (+0.197)
White Race (+0.00177)
Erythrocyte distribution width [Ratio] by AC (+0.0536)
Gastroesophageal reflux disease without esophagitis (x1.02)
Lymphocytes [#/V] in B by AC (-0.0456)
Heart failure (x1.02)
Acidosis (x1.02)
Hyperkalemia (x1.02)
Chronic myeloid leukemia (x1.02)
Basophils [#/V] in B (+0.0116)
Mixed hyperlipidemia (x1.02)
Chronic kidney disease stage 3 (x1.02)
Congestive heart failure (x1.02)
Atherosclerosis of coronary artery without angina pectoris (x1.02)
Hyperlipidemia (x1.02)
Cardiac arrhythmia (x1.02)
Anemia due to and following chemotherapy (x1.01)
End-stage renal disease (x1.01)
Lung field abnormal (x1.01)
Nausea (x1.01)
Pleural effusion (x1.01)
Primary malignant neoplasm of female breast (x1.01)
Transplanted kidney present (x1.01)
Leukocytes [#/V] in B by AC (-0.0965)
Chronic kidney disease (x1.01)
anagrelide (+0.00122)
Pneumonia (x1.01)
Monocytes/100 leukocytes in B by AC (+0.0645)
Fatigue (x1.01)
Acute respiratory failure (/1.01)
Pain due to neoplastic disease (x1.01)
Creatinine [M/V] in SP (+0.0181)
Hyperglycemia due to type 2 diabetes mellitus (x1.01)
Myelofibrosis (x1.01)
Type 2 diabetes mellitus (x1.01)
Urobilinogen [Presence] in Urine by Test strip (+0.022)
Iron deficiency anemia (x1.01)
Anxiety disorder (x1.01)
nitroglycerin (+0.00108)
Creatinine [M/V] in B (+0.0195)
Localized edema (x1.01)
Precordial pain (x1.01)
tacrolimus (+0.00107)
Heart rate (-0.453)
Leukopenia (x1.01)
Eosinophils/100 leukocytes in B (+0.0437)
Anemia in chronic kidney disease (x1.01)
imatinib (-0.00103)
Cirrhosis of liver (x1.01)
Postoperative state (x1.01)
Immature granulocytes/100 leukocytes in B by AC (+0.00889)
Muscle weakness (x1.01)
Neutropenia (x1.01)
Diffuse large B-cell lymphoma (nodal/systemic with skin involvement) (x1.01)
Hypervolemia (x1.01)
Secondary malignant neoplasm of bone (x1.01)
Chronic combined systolic and diastolic heart failure (x1.01)
Age (+0.444)
Nausea and vomiting (x1.01)
Pain (x1.01)
Chest pain (x1.01)
Chronic kidney disease due to hypertension (x1.01)
Black Race (-0.000877)
Pancytopenia due to antineoplastic chemotherapy (x1.01)
oxycodone (+0.00087)
Type 2 diabetes mellitus without complication (x1.01)
Secondary malignant neoplasm of lung (x1.01)
Magnesium [M/V] in SP (+0.00539)
Vitamin D deficiency (x1.01)
MCHC [M/V] by AC (-0.0211)
Transplanted liver present (x1.01)
Lymphocytes/100 leukocytes in B by AC (-0.199)
Blood chemistry abnormal (x1.01)
Disorder of bone (x1.01)
0.851
0.865
0.878
0.887
0.896
0.902
0.906
0.910
0.914
0.917
0.919
0.922
0.924
0.926
0.928
0.930
0.932
0.933
0.934
0.935
0.935
0.936
0.937
0.938
0.939
0.940
0.941
0.942
0.942
0.943
0.944
0.945
0.945
0.946
0.947
0.947
0.948
0.948
0.949
0.950
0.950
0.951
0.951
0.952
0.952
0.953
0.953
0.954
0.954
0.954
0.955
0.955
0.956
0.956
0.956
0.957
0.957
0.957
0.958
0.958
0.958
0.959
0.959
0.959
0.960
0.960
0.960
0.961
0.961
0.961
0.961
0.962
0.962
0.962
0.963
0.963
0.963
0.963
0.964
0.964
0.964
0.964
0.965
0.965
0.965
0.965
0.965
0.966
0.966
0.966
0.966
0.966
0.967
0.967
0.967
0.967
0.967
0.968
0.968
0.968
−1.00 −0.75 −0.50 −0.25 0.00 0.25 0.50 0.75 1.00
Expressions

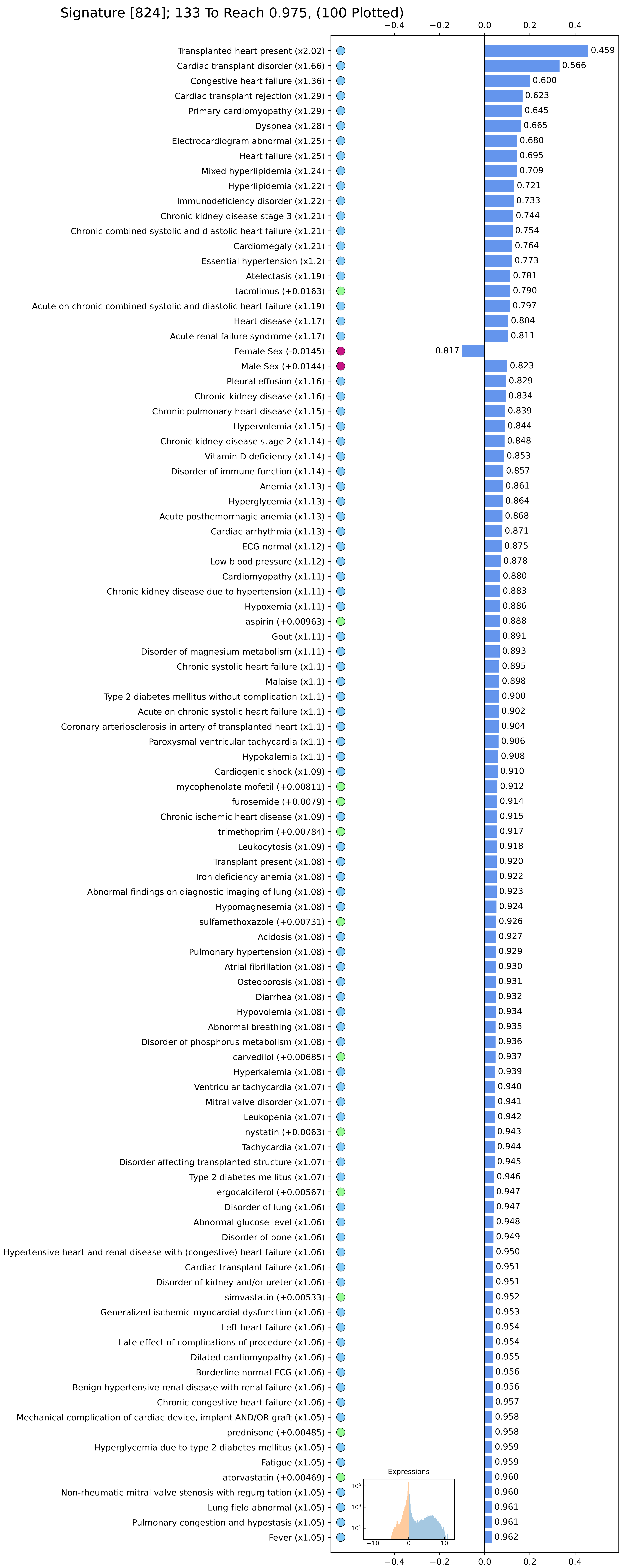

Signature [824]; 133 To Reach 0.975, (100 Plotted)
Transplanted heart present (x2.02) 0.459
Cardiac transplant disorder (x1.66) 0.566
Congestive heart failure (x1.36) 0.600
Cardiac transplant rejection (x1.29) 0.623
Primary cardiomyopathy (x1.29) 0.645
Dyspnea (x1.28) 0.665
Electrocardiogram abnormal (x1.25) 0.680
Heart failure (x1.25) 0.695
Mixed hyperlipidemia (x1.24) 0.709
Hyperlipidemia (x1.22) 0.721
Immunodeficiency disorder (x1.22) 0.733
Chronic kidney disease stage 3 (x1.21) 0.744
Chronic combined systolic and diastolic heart failure (x1.21) 0.754
Cardiomegaly (x1.21) 0.764
Essential hypertension (x1.2) 0.773
Atelectasis (x1.19) 0.781
tacrolimus (+0.0163) 0.790
Acute on chronic combined systolic and diastolic heart failure (x1.19) 0.797
Heart disease (x1.17) 0.804
Acute renal failure syndrome (x1.17) 0.811
Female Sex (-0.0145) 0.817
Male Sex (+0.0144) 0.823
Pleural effusion (x1.16) 0.829
Chronic kidney disease (x1.16) 0.834
Chronic pulmonary heart disease (x1.15) 0.839
Hypervolemia (x1.15) 0.844
Chronic kidney disease stage 2 (x1.14) 0.848
Vitamin D deficiency (x1.14) 0.853
Disorder of immune function (x1.14) 0.857
Anemia (x1.13) 0.861
Hyperglycemia (x1.13) 0.864
Acute posthemorrhagic anemia (x1.13) 0.868
Cardiac arrhythmia (x1.13) 0.871
ECG normal (x1.12) 0.875
Low blood pressure (x1.12) 0.878
Cardiomyopathy (x1.11) 0.880
Chronic kidney disease due to hypertension (x1.11) 0.883
Hypoxemia (x1.11) 0.886
aspirin (+0.00963) 0.888
Gout (x1.11) 0.891
Disorder of magnesium metabolism (x1.11) 0.893
Chronic systolic heart failure (x1.1) 0.895
Malaise (x1.1) 0.898
Type 2 diabetes mellitus without complication (x1.1) 0.900
Acute on chronic systolic heart failure (x1.1) 0.902
Coronary arteriosclerosis in artery of transplanted heart (x1.1) 0.904
Paroxysmal ventricular tachycardia (x1.1) 0.906
Hypokalemia (x1.1) 0.908
Cardiogenic shock (x1.09) 0.910
mycophenolate mofetil (+0.00811) 0.912
furosemide (+0.0079) 0.914
Chronic ischemic heart disease (x1.09) 0.915
trimethoprim (+0.00784) 0.917
Leukocytosis (x1.09) 0.918
Transplant present (x1.08) 0.920
Iron deficiency anemia (x1.08) 0.922
Abnormal findings on diagnostic imaging of lung (x1.08) 0.923
Hypomagnesemia (x1.08) 0.924
sulfamethoxazole (+0.00731) 0.926
Acidosis (x1.08) 0.927
Pulmonary hypertension (x1.08) 0.929
Atrial fibrillation (x1.08) 0.930
Osteoporosis (x1.08) 0.931
Diarrhea (x1.08) 0.932
Hypovolemia (x1.08) 0.934
Abnormal breathing (x1.08) 0.935
Disorder of phosphorus metabolism (x1.08) 0.936
carvedilol (+0.00685) 0.937
Hyperkalemia (x1.08) 0.939
Ventricular tachycardia (x1.07) 0.940
Mitral valve disorder (x1.07) 0.941
Leukopenia (x1.07) 0.942
nystatin (+0.0063) 0.943
Tachycardia (x1.07) 0.944
Disorder affecting transplanted structure (x1.07) 0.945
Type 2 diabetes mellitus (x1.07) 0.946
ergocalciferol (+0.00567) 0.947
Disorder of lung (x1.06) 0.947
Abnormal glucose level (x1.06) 0.948
Disorder of bone (x1.06) 0.949
Hypertensive heart and renal disease with (congestive) heart failure (x1.06) 0.950
Cardiac transplant failure (x1.06) 0.951
Disorder of kidney and/or ureter (x1.06) 0.951
simvastatin (+0.00533) 0.952
Generalized ischemic myocardial dysfunction (x1.06) 0.953
Left heart failure (x1.06) 0.954
Late effect of complications of procedure (x1.06) 0.954
Dilated cardiomyopathy (x1.06) 0.955
Borderline normal ECG (x1.06) 0.956
Benign hypertensive renal disease with renal failure (x1.06) 0.956
Chronic congestive heart failure (x1.06) 0.957
Mechanical complication of cardiac device, implant AND/OR graft (x1.05) 0.958
prednisone (+0.00485) 0.958
Hyperglycemia due to type 2 diabetes mellitus (x1.05) 0.959
Fatigue (x1.05) 0.959
atorvastatin (+0.00469) 0.960
Non-rheumatic mitral valve stenosis with regurgitation (x1.05) 0.960
Lung field abnormal (x1.05) 0.961
Pulmonary congestion and hypostasis (x1.05) 0.961
Fever (x1.05) 0.962
−0.4 −0.2 0.0 0.2 0.4
Expressions

Signature [825]; 10 To Reach 0.975, (10 Plotted)

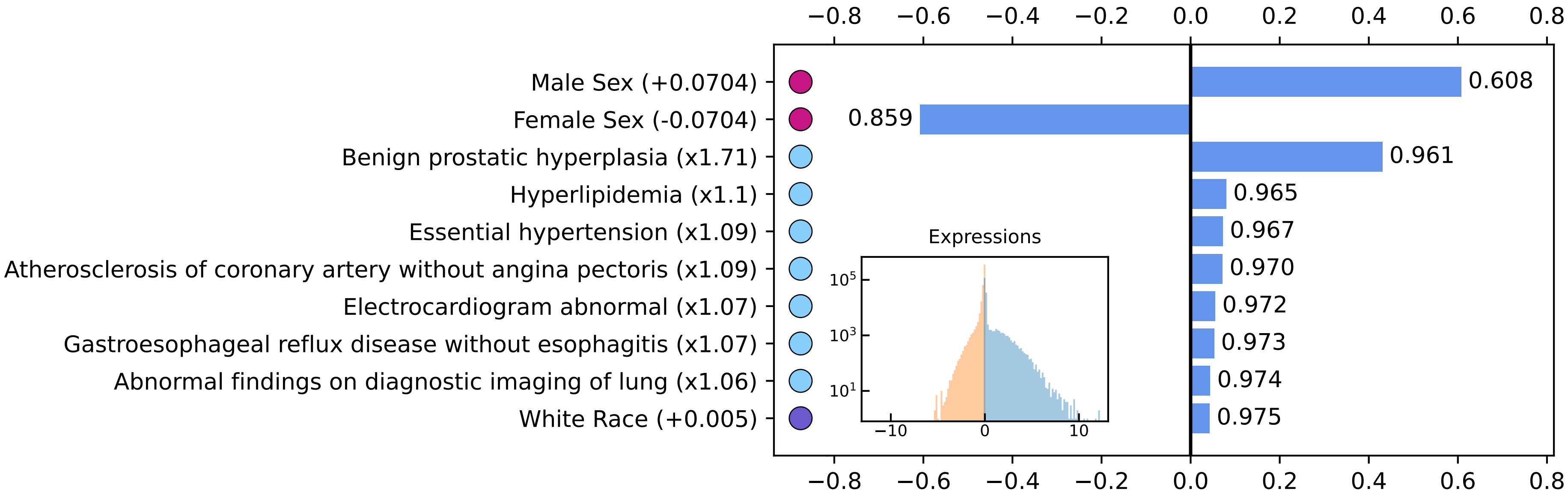

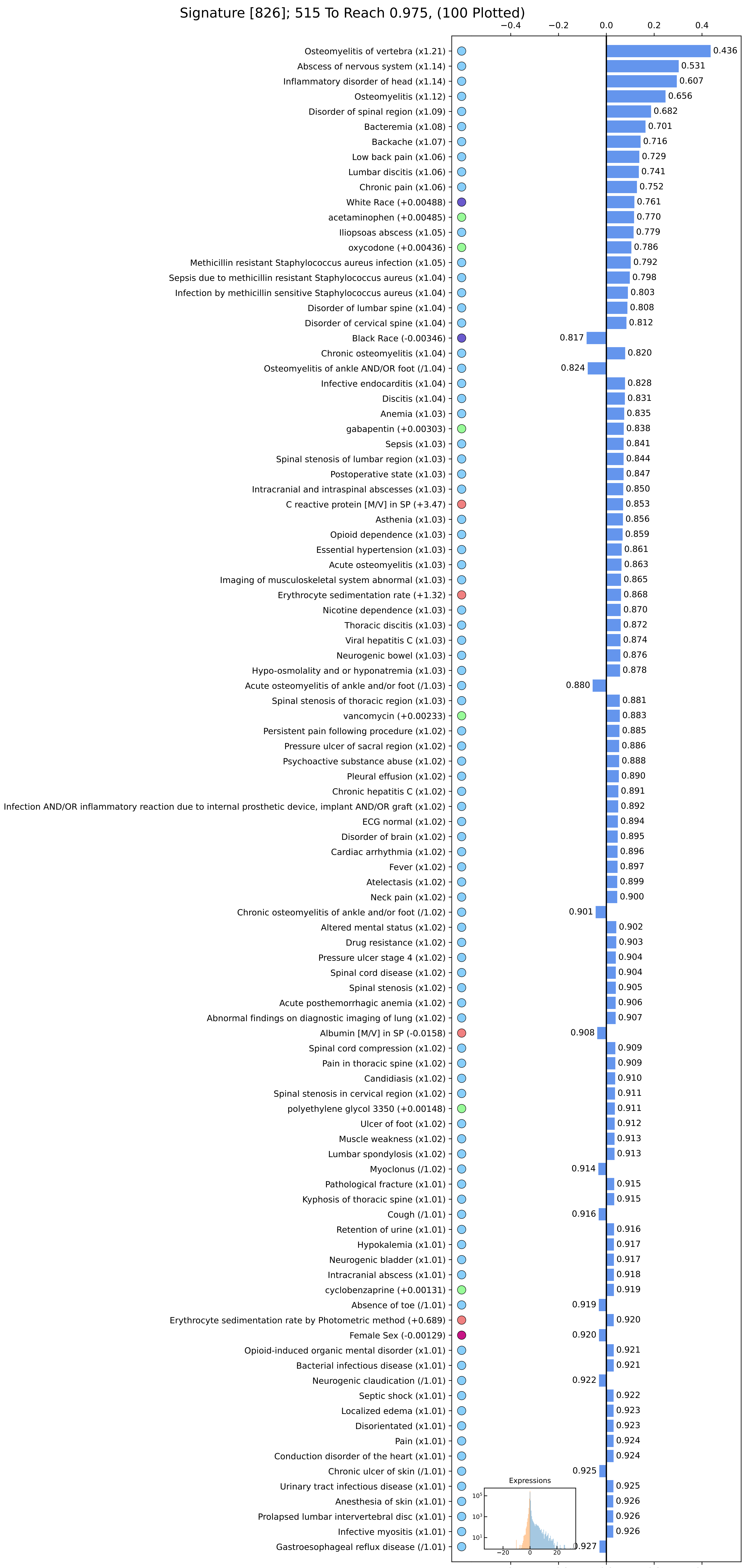

Signature [826]; 515 To Reach 0.975, (100 Plotted)
Osteomyelitis of vertebra (x1.21) 0.436
Abscess of nervous system (x1.14) 0.531
Inflammatory disorder of head (x1.14) 0.607
Osteomyelitis (x1.12) 0.656
Disorder of spinal region (x1.09) 0.682
Bacteremia (x1.08) 0.701
Backache (x1.07) 0.716
Low back pain (x1.06) 0.729
Lumbar discitis (x1.06) 0.741
Chronic pain (x1.06) 0.752
White Race (+0.00488) 0.761
acetaminophen (+0.00485) 0.770
Iliopsoas abscess (x1.05) 0.779
oxycodone (+0.00436) 0.786
Methicillin resistant Staphylococcus aureus infection (x1.05) 0.792
Sepsis due to methicillin resistant Staphylococcus aureus (x1.04) 0.798
Infection by methicillin sensitive Staphylococcus aureus (x1.04) 0.803
Disorder of lumbar spine (x1.04) 0.808
Disorder of cervical spine (x1.04) 0.812
Black Race (-0.00346) 0.817
Chronic osteomyelitis (x1.04) 0.820
Osteomyelitis of ankle AND/OR foot (/1.04) 0.824
Infective endocarditis (x1.04) 0.828
Discitis (x1.04) 0.831
Anemia (x1.03) 0.835
gabapentin (+0.00303) 0.838
Sepsis (x1.03) 0.841
Spinal stenosis of lumbar region (x1.03) 0.844
Postoperative state (x1.03) 0.847
Intracranial and intraspinal abscesses (x1.03) 0.850
C reactive protein [M/V] in SP (+3.47) 0.853
Asthenia (x1.03) 0.856
Opioid dependence (x1.03) 0.859
Essential hypertension (x1.03) 0.861
Acute osteomyelitis (x1.03) 0.863
Imaging of musculoskeletal system abnormal (x1.03) 0.865
Erythrocyte sedimentation rate (+1.32) 0.868
Nicotine dependence (x1.03) 0.870
Thoracic discitis (x1.03) 0.872
Viral hepatitis C (x1.03) 0.874
Neurogenic bowel (x1.03) 0.876
Hypo-osmolality and or hyponatremia (x1.03) 0.878
Acute osteomyelitis of ankle and/or foot (/1.03) 0.880
Spinal stenosis of thoracic region (x1.03) 0.881
vancomycin (+0.00233) 0.883
Persistent pain following procedure (x1.02) 0.885
Pressure ulcer of sacral region (x1.02) 0.886
Psychoactive substance abuse (x1.02) 0.888
Pleural effusion (x1.02) 0.890
Chronic hepatitis C (x1.02) 0.891
Infection AND/OR inflammatory reaction due to internal prosthetic device, implant AND/OR graft (x1.02) 0.892
ECG normal (x1.02) 0.894
Disorder of brain (x1.02) 0.895
Cardiac arrhythmia (x1.02) 0.896
Fever (x1.02) 0.897
Atelectasis (x1.02) 0.899
Neck pain (x1.02) 0.900
Chronic osteomyelitis of ankle and/or foot (/1.02) 0.901
Altered mental status (x1.02) 0.902
Drug resistance (x1.02) 0.903
Pressure ulcer stage 4 (x1.02) 0.904
Spinal cord disease (x1.02) 0.904
Spinal stenosis (x1.02) 0.905
Acute posthemorrhagic anemia (x1.02) 0.906
Abnormal findings on diagnostic imaging of lung (x1.02) 0.907
Albumin [M/V] in SP (-0.0158) 0.908
Spinal cord compression (x1.02) 0.909
Pain in thoracic spine (x1.02) 0.909
Candidiasis (x1.02) 0.910
Spinal stenosis in cervical region (x1.02) 0.911
polyethylene glycol 3350 (+0.00148) 0.911
Ulcer of foot (x1.02) 0.912
Muscle weakness (x1.02) 0.913
Lumbar spondylosis (x1.02) 0.913
Myoclonus (/1.02) 0.914
Pathological fracture (x1.01) 0.915
Kyphosis of thoracic spine (x1.01) 0.915
Cough (/1.01) 0.916
Retention of urine (x1.01) 0.916
Hypokalemia (x1.01) 0.917
Neurogenic bladder (x1.01) 0.917
Intracranial abscess (x1.01) 0.918
cyclobenzaprine (+0.00131) 0.919
Absence of toe (/1.01) 0.919
Erythrocyte sedimentation rate by Photometric method (+0.689) 0.920
Female Sex (-0.00129) 0.920
Opioid-induced organic mental disorder (x1.01) 0.921
Bacterial infectious disease (x1.01) 0.921
Neurogenic claudication (/1.01) 0.922
Septic shock (x1.01) 0.922
Localized edema (x1.01) 0.923
Disorientated (x1.01) 0.923
Pain (x1.01) 0.924
Conduction disorder of the heart (x1.01) 0.924
Chronic ulcer of skin (/1.01) 0.925
Urinary tract infectious disease (x1.01) 0.925
Anesthesia of skin (x1.01) 0.926
Prolapsed lumbar intervertebral disc (x1.01) 0.926
Infective myositis (x1.01) 0.926
Gastroesophageal reflux disease (/1.01) 0.927
Expressions
−0.4 −0.2 0.0 0.2 0.4

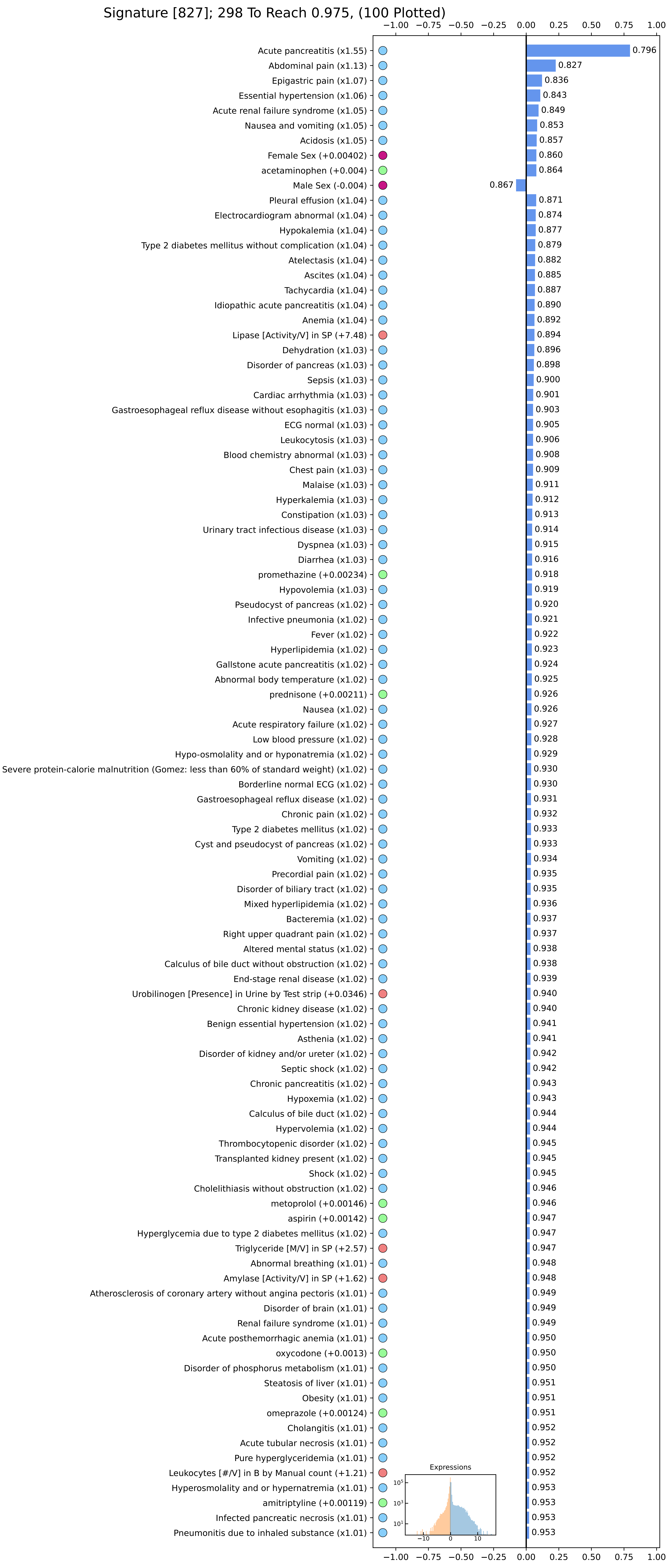

Signature [827]; 298 To Reach 0.975, (100 Plotted)
Acute pancreatitis (x1.55) 0.796
Abdominal pain (x1.13) 0.827
Epigastric pain (x1.07) 0.836
Essential hypertension (x1.06) 0.843
Acute renal failure syndrome (x1.05) 0.849
Nausea and vomiting (x1.05) 0.853
Acidosis (x1.05) 0.857
Female Sex (+0.00402) 0.860
acetaminophen (+0.004) 0.864
Male Sex (-0.004) 0.867
Pleural effusion (x1.04) 0.871
Electrocardiogram abnormal (x1.04) 0.874
Hypokalemia (x1.04) 0.877
Type 2 diabetes mellitus without complication (x1.04) 0.879
Atelectasis (x1.04) 0.882
Ascites (x1.04) 0.885
Tachycardia (x1.04) 0.887
Idiopathic acute pancreatitis (x1.04) 0.890
Anemia (x1.04) 0.892
Lipase [Activity/V] in SP (+7.48) 0.894
Dehydration (x1.03) 0.896
Disorder of pancreas (x1.03) 0.898
Sepsis (x1.03) 0.900
Cardiac arrhythmia (x1.03) 0.901
Gastroesophageal reflux disease without esophagitis (x1.03) 0.903
ECG normal (x1.03) 0.905
Leukocytosis (x1.03) 0.906
Blood chemistry abnormal (x1.03) 0.908
Chest pain (x1.03) 0.909
Malaise (x1.03) 0.911
Hyperkalemia (x1.03) 0.912
Constipation (x1.03) 0.913
Urinary tract infectious disease (x1.03) 0.914
Dyspnea (x1.03) 0.915
Diarrhea (x1.03) 0.916
promethazine (+0.00234) 0.918
Hypovolemia (x1.03) 0.919
Pseudocyst of pancreas (x1.02) 0.920
Infective pneumonia (x1.02) 0.921
Fever (x1.02) 0.922
Hyperlipidemia (x1.02) 0.923
Gallstone acute pancreatitis (x1.02) 0.924
Abnormal body temperature (x1.02) 0.925
prednisone (+0.00211) 0.926
Nausea (x1.02) 0.926
Acute respiratory failure (x1.02) 0.927
Low blood pressure (x1.02) 0.928
Hypo-osmolality and or hyponatremia (x1.02) 0.929
Severe protein-calorie malnutrition (Gomez: less than 60% of standard weight) (x1.02) 0.930
Borderline normal ECG (x1.02) 0.930
Gastroesophageal reflux disease (x1.02) 0.931
Chronic pain (x1.02) 0.932
Type 2 diabetes mellitus (x1.02) 0.933
Cyst and pseudocyst of pancreas (x1.02) 0.933
Vomiting (x1.02) 0.934
Precordial pain (x1.02) 0.935
Disorder of biliary tract (x1.02) 0.935
Mixed hyperlipidemia (x1.02) 0.936
Bacteremia (x1.02) 0.937
Right upper quadrant pain (x1.02) 0.937
Altered mental status (x1.02) 0.938
Calculus of bile duct without obstruction (x1.02) 0.938
End-stage renal disease (x1.02) 0.939
Urobilinogen [Presence] in Urine by Test strip (+0.0346) 0.940
Chronic kidney disease (x1.02) 0.940
Benign essential hypertension (x1.02) 0.941
Asthenia (x1.02) 0.941
Disorder of kidney and/or ureter (x1.02) 0.942
Septic shock (x1.02) 0.942
Chronic pancreatitis (x1.02) 0.943
Hypoxemia (x1.02) 0.943
Calculus of bile duct (x1.02) 0.944
Hypervolemia (x1.02) 0.944
Thrombocytopenic disorder (x1.02) 0.945
Transplanted kidney present (x1.02) 0.945
Shock (x1.02) 0.945
Cholelithiasis without obstruction (x1.02) 0.946
metoprolol (+0.00146) 0.946
aspirin (+0.00142) 0.947
Hyperglycemia due to type 2 diabetes mellitus (x1.02) 0.947
Triglyceride [M/V] in SP (+2.57) 0.947
Abnormal breathing (x1.01) 0.948
Amylase [Activity/V] in SP (+1.62) 0.948
Atherosclerosis of coronary artery without angina pectoris (x1.01) 0.949
Disorder of brain (x1.01) 0.949
Renal failure syndrome (x1.01) 0.949
Acute posthemorrhagic anemia (x1.01) 0.950
oxycodone (+0.0013) 0.950
Disorder of phosphorus metabolism (x1.01) 0.950
Steatosis of liver (x1.01) 0.951
Obesity (x1.01) 0.951
omeprazole (+0.00124) 0.951
Cholangitis (x1.01) 0.952
Acute tubular necrosis (x1.01) 0.952
Pure hyperglyceridemia (x1.01) 0.952
Leukocytes [#/V] in B by Manual count (+1.21) 0.952
Hyperosmolality and or hypernatremia (x1.01) 0.953
amitriptyline (+0.00119) 0.953
Infected pancreatic necrosis (x1.01) 0.953
Pneumonitis due to inhaled substance (x1.01) 0.953
Expressions
−1.00 −0.75 −0.50 −0.25 0.00 0.25 0.50 0.75 1.00

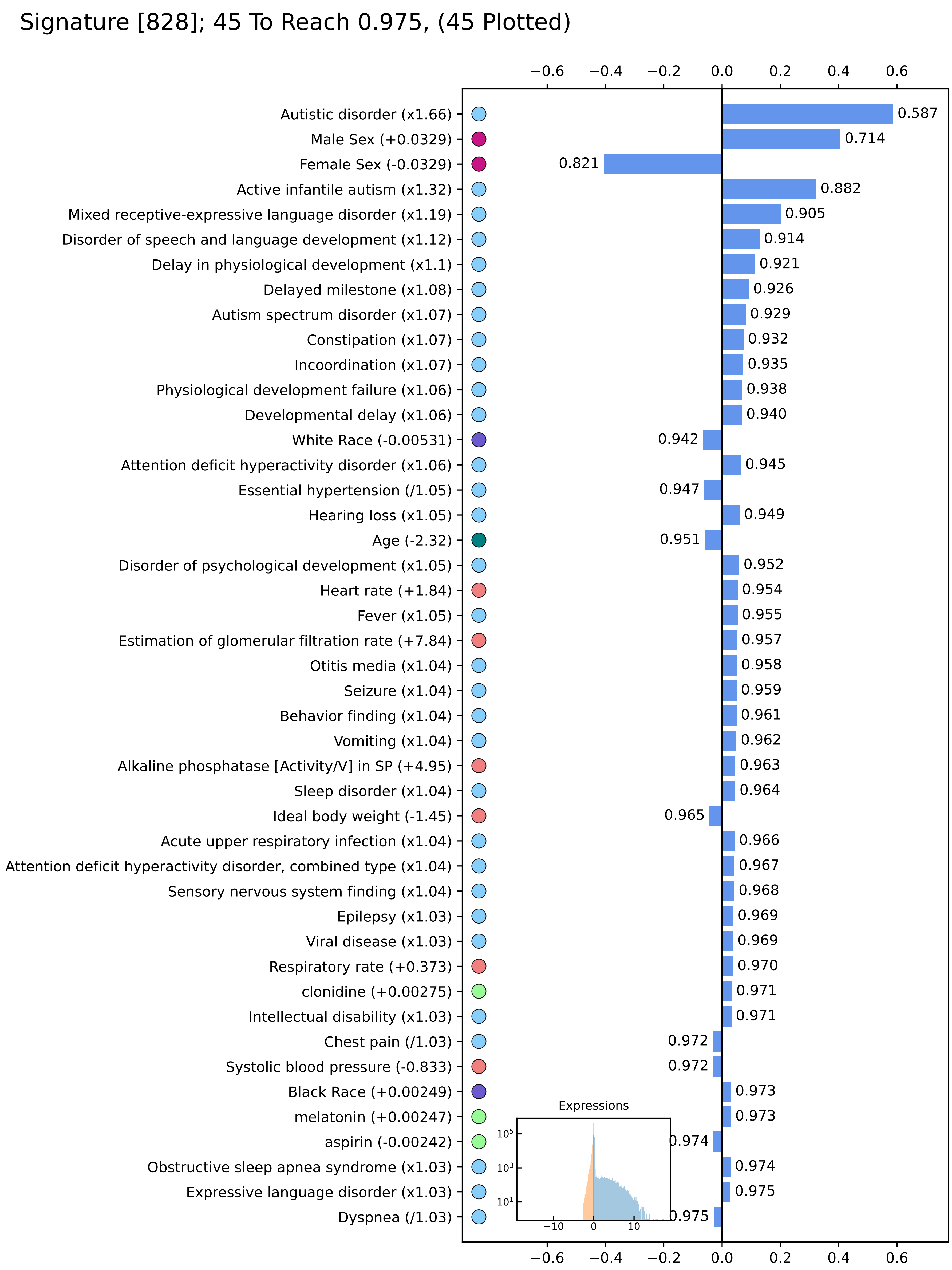

Signature [828]; 45 To Reach 0.975, (45 Plotted)
Autistic disorder (x1.66) 0.587
Male Sex (+0.0329) 0.714
Female Sex (-0.0329) 0.821
Active infantile autism (x1.32) 0.882
Mixed receptive-expressive language disorder (x1.19) 0.905
Disorder of speech and language development (x1.12) 0.914
Delay in physiological development (x1.1) 0.921
Delayed milestone (x1.08) 0.926
Autism spectrum disorder (x1.07) 0.929
Constipation (x1.07) 0.932
Incoordination (x1.07) 0.935
Physiological development failure (x1.06) 0.938
Developmental delay (x1.06) 0.940
White Race (-0.00531) 0.942
Attention deficit hyperactivity disorder (x1.06) 0.945
Essential hypertension (/1.05) 0.947
Hearing loss (x1.05) 0.949
Age (-2.32) 0.951
Disorder of psychological development (x1.05) 0.952
Heart rate (+1.84) 0.954
Fever (x1.05) 0.955
Estimation of glomerular filtration rate (+7.84) 0.957
Otitis media (x1.04) 0.958
Seizure (x1.04) 0.959
Behavior finding (x1.04) 0.961
Vomiting (x1.04) 0.962
Alkaline phosphatase [Activity/V] in SP (+4.95) 0.963
Sleep disorder (x1.04) 0.964
Ideal body weight (-1.45) 0.965
Acute upper respiratory infection (x1.04) 0.966
Attention deficit hyperactivity disorder, combined type (x1.04) 0.967
Sensory nervous system finding (x1.04) 0.968
Epilepsy (x1.03) 0.969
Viral disease (x1.03) 0.969
Respiratory rate (+0.373) 0.970
clonidine (+0.00275) 0.971
Intellectual disability (x1.03) 0.971
Chest pain (/1.03) 0.972
Systolic blood pressure (-0.833) 0.972
Black Race (+0.00249) 0.973
melatonin (+0.00247) 0.973
aspirin (-0.00242) 0.974
Obstructive sleep apnea syndrome (x1.03) 0.974
Expressive language disorder (x1.03) 0.975
Dyspnea (/1.03) 0.975
−0.6 −0.4 −0.2 0.0 0.2 0.4 0.6
Expressions

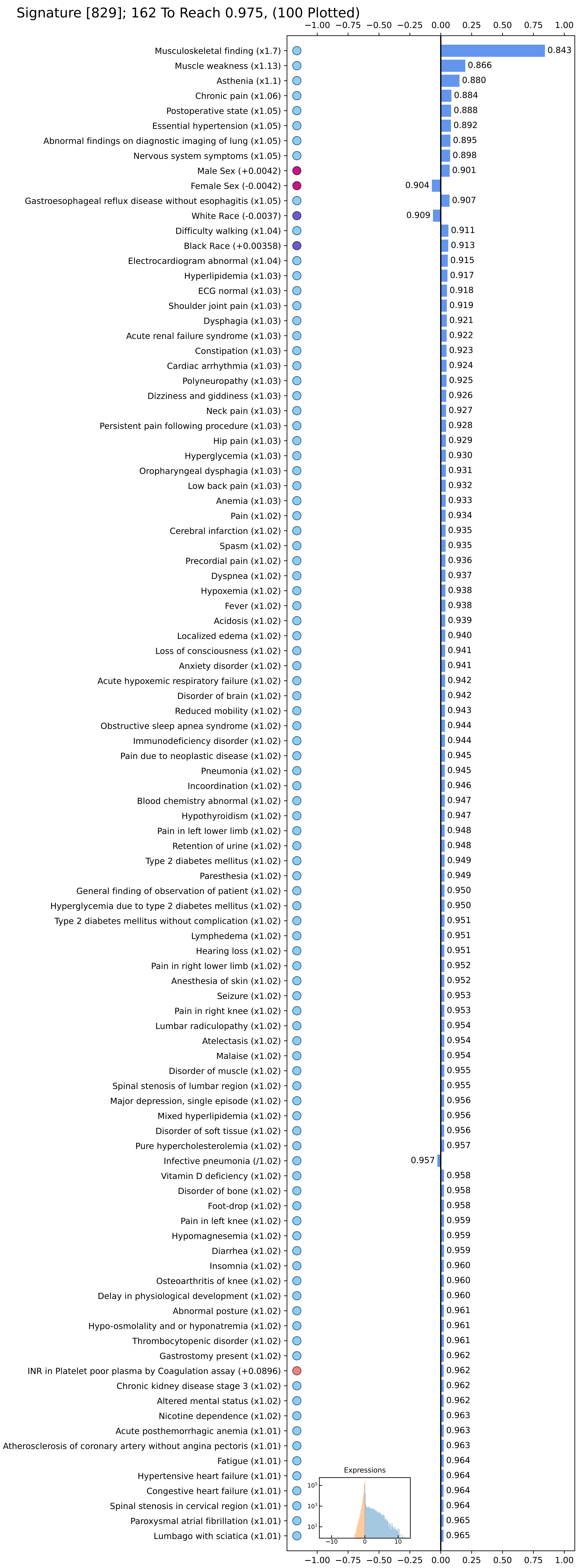

Signature [829]; 162 To Reach 0.975, (100 Plotted)
Musculoskeletal finding (x1.7) 0.843
Muscle weakness (x1.13) 0.866
Asthenia (x1.1) 0.880
Chronic pain (x1.06) 0.884
Postoperative state (x1.05) 0.888
Essential hypertension (x1.05) 0.892
Abnormal findings on diagnostic imaging of lung (x1.05) 0.895
Nervous system symptoms (x1.05) 0.898
Male Sex (+0.0042) 0.901
Female Sex (-0.0042) 0.904
Gastroesophageal reflux disease without esophagitis (x1.05) 0.907
White Race (-0.0037) 0.909
Difficulty walking (x1.04) 0.911
Black Race (+0.00358) 0.913
Electrocardiogram abnormal (x1.04) 0.915
Hyperlipidemia (x1.03) 0.917
ECG normal (x1.03) 0.918
Shoulder joint pain (x1.03) 0.919
Dysphagia (x1.03) 0.921
Acute renal failure syndrome (x1.03) 0.922
Constipation (x1.03) 0.923
Cardiac arrhythmia (x1.03) 0.924
Polyneuropathy (x1.03) 0.925
Dizziness and giddiness (x1.03) 0.926
Neck pain (x1.03) 0.927
Persistent pain following procedure (x1.03) 0.928
Hip pain (x1.03) 0.929
Hyperglycemia (x1.03) 0.930
Oropharyngeal dysphagia (x1.03) 0.931
Low back pain (x1.03) 0.932
Anemia (x1.03) 0.933
Pain (x1.02) 0.934
Cerebral infarction (x1.02) 0.935
Spasm (x1.02) 0.935
Precordial pain (x1.02) 0.936
Dyspnea (x1.02) 0.937
Hypoxemia (x1.02) 0.938
Fever (x1.02) 0.938
Acidosis (x1.02) 0.939
Localized edema (x1.02) 0.940
Loss of consciousness (x1.02) 0.941
Anxiety disorder (x1.02) 0.941
Acute hypoxemic respiratory failure (x1.02) 0.942
Disorder of brain (x1.02) 0.942
Reduced mobility (x1.02) 0.943
Obstructive sleep apnea syndrome (x1.02) 0.944
Immunodeficiency disorder (x1.02) 0.944
Pain due to neoplastic disease (x1.02) 0.945
Pneumonia (x1.02) 0.945
Incoordination (x1.02) 0.946
Blood chemistry abnormal (x1.02) 0.947
Hypothyroidism (x1.02) 0.947
Pain in left lower limb (x1.02) 0.948
Retention of urine (x1.02) 0.948
Type 2 diabetes mellitus (x1.02) 0.949
Paresthesia (x1.02) 0.949
General finding of observation of patient (x1.02) 0.950
Hyperglycemia due to type 2 diabetes mellitus (x1.02) 0.950
Type 2 diabetes mellitus without complication (x1.02) 0.951
Lymphedema (x1.02) 0.951
Hearing loss (x1.02) 0.951
Pain in right lower limb (x1.02) 0.952
Anesthesia of skin (x1.02) 0.952
Seizure (x1.02) 0.953
Pain in right knee (x1.02) 0.953
Lumbar radiculopathy (x1.02) 0.954
Atelectasis (x1.02) 0.954
Malaise (x1.02) 0.954
Disorder of muscle (x1.02) 0.955
Spinal stenosis of lumbar region (x1.02) 0.955
Major depression, single episode (x1.02) 0.956
Mixed hyperlipidemia (x1.02) 0.956
Disorder of soft tissue (x1.02) 0.956
Pure hypercholesterolemia (x1.02) 0.957
Infective pneumonia (/1.02) 0.957
Vitamin D deficiency (x1.02) 0.958
Disorder of bone (x1.02) 0.958
Foot-drop (x1.02) 0.958
Pain in left knee (x1.02) 0.959
Hypomagnesemia (x1.02) 0.959
Diarrhea (x1.02) 0.959
Insomnia (x1.02) 0.960
Osteoarthritis of knee (x1.02) 0.960
Delay in physiological development (x1.02) 0.960
Abnormal posture (x1.02) 0.961
Hypo-osmolality and or hyponatremia (x1.02) 0.961
Thrombocytopenic disorder (x1.02) 0.961
Gastrostomy present (x1.02) 0.962
INR in Platelet poor plasma by Coagulation assay (+0.0896) 0.962
Chronic kidney disease stage 3 (x1.02) 0.962
Altered mental status (x1.02) 0.962
Nicotine dependence (x1.02) 0.963
Acute posthemorrhagic anemia (x1.01) 0.963
Atherosclerosis of coronary artery without angina pectoris (x1.01) 0.963
Fatigue (x1.01) 0.964
Hypertensive heart failure (x1.01) 0.964
Congestive heart failure (x1.01) 0.964
Spinal stenosis in cervical region (x1.01) 0.964
Paroxysmal atrial fibrillation (x1.01) 0.965
Lumbago with sciatica (x1.01) 0.965
−1.00 −0.75 −0.50 −0.25 0.00 0.25 0.50 0.75 1.00
Expressions

Signature [830]; 62 To Reach 0.975, (62 Plotted)

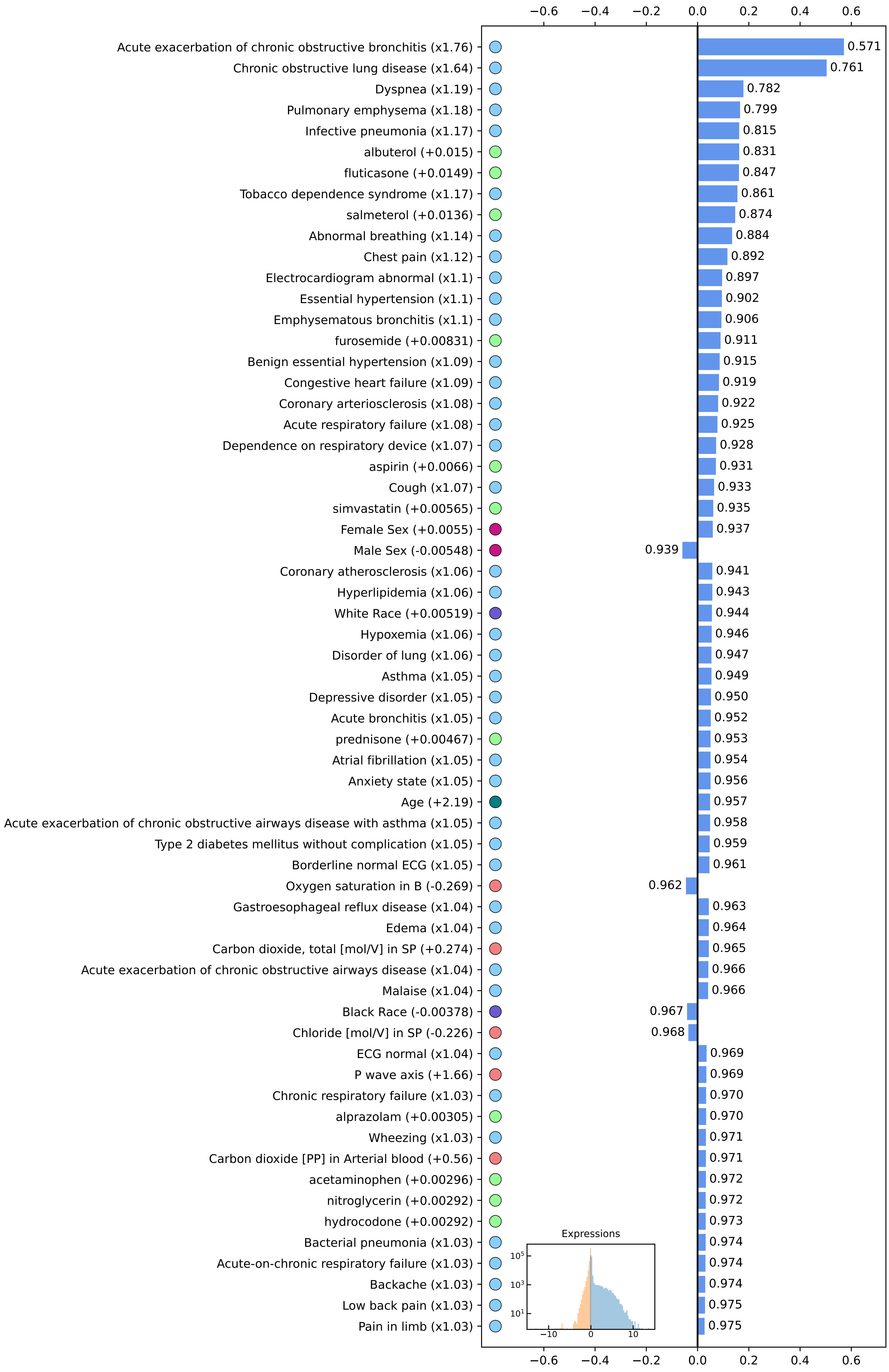

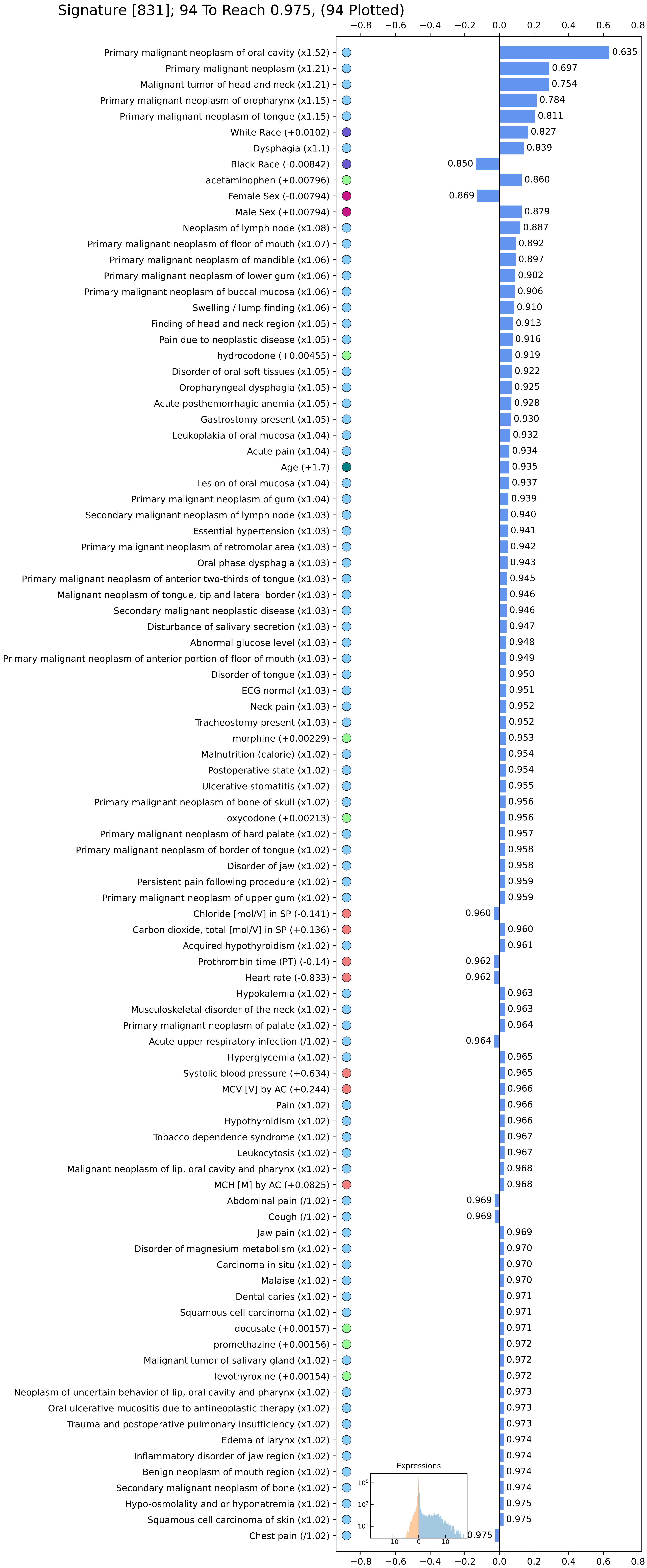

Signature [831]; 94 To Reach 0.975, (94 Plotted)
Primary malignant neoplasm of oral cavity (x1.52) 0.635
Primary malignant neoplasm (x1.21) 0.697
Malignant tumor of head and neck (x1.21) 0.754
Primary malignant neoplasm of oropharynx (x1.15) 0.784
Primary malignant neoplasm of tongue (x1.15) 0.811
White Race (+0.0102) 0.827
Dysphagia (x1.1) 0.839
Black Race (-0.00842) 0.850
acetaminophen (+0.00796) 0.860
Female Sex (-0.00794) 0.869
Male Sex (+0.00794) 0.879
Neoplasm of lymph node (x1.08) 0.887
Primary malignant neoplasm of floor of mouth (x1.07) 0.892
Primary malignant neoplasm of mandible (x1.06) 0.897
Primary malignant neoplasm of lower gum (x1.06) 0.902
Primary malignant neoplasm of buccal mucosa (x1.06) 0.906
Swelling / lump finding (x1.06) 0.910
Finding of head and neck region (x1.05) 0.913
Pain due to neoplastic disease (x1.05) 0.916
hydrocodone (+0.00455) 0.919
Disorder of oral soft tissues (x1.05) 0.922
Oropharyngeal dysphagia (x1.05) 0.925
Acute posthemorrhagic anemia (x1.05) 0.928
Gastrostomy present (x1.05) 0.930
Leukoplakia of oral mucosa (x1.04) 0.932
Acute pain (x1.04) 0.934
Age (+1.7) 0.935
Lesion of oral mucosa (x1.04) 0.937
Primary malignant neoplasm of gum (x1.04) 0.939
Secondary malignant neoplasm of lymph node (x1.03) 0.940
Essential hypertension (x1.03) 0.941
Primary malignant neoplasm of retromolar area (x1.03) 0.942
Oral phase dysphagia (x1.03) 0.943
Primary malignant neoplasm of anterior two-thirds of tongue (x1.03) 0.945
Malignant neoplasm of tongue, tip and lateral border (x1.03) 0.946
Secondary malignant neoplastic disease (x1.03) 0.946
Disturbance of salivary secretion (x1.03) 0.947
Abnormal glucose level (x1.03) 0.948
Primary malignant neoplasm of anterior portion of floor of mouth (x1.03) 0.949
Disorder of tongue (x1.03) 0.950
ECG normal (x1.03) 0.951
Neck pain (x1.03) 0.952
Tracheostomy present (x1.03) 0.952
morphine (+0.00229) 0.953
Malnutrition (calorie) (x1.02) 0.954
Postoperative state (x1.02) 0.954
Ulcerative stomatitis (x1.02) 0.955
Primary malignant neoplasm of bone of skull (x1.02) 0.956
oxycodone (+0.00213) 0.956
Primary malignant neoplasm of hard palate (x1.02) 0.957
Primary malignant neoplasm of border of tongue (x1.02) 0.958
Disorder of jaw (x1.02) 0.958
Persistent pain following procedure (x1.02) 0.959
Primary malignant neoplasm of upper gum (x1.02) 0.959
Chloride [mol/V] in SP (-0.141) 0.960
Carbon dioxide, total [mol/V] in SP (+0.136) 0.960
Acquired hypothyroidism (x1.02) 0.961
Prothrombin time (PT) (-0.14) 0.962
Heart rate (-0.833) 0.962
Hypokalemia (x1.02) 0.963
Musculoskeletal disorder of the neck (x1.02) 0.963
Primary malignant neoplasm of palate (x1.02) 0.964
Acute upper respiratory infection (/1.02) 0.964
Hyperglycemia (x1.02) 0.965
Systolic blood pressure (+0.634) 0.965
MCV [V] by AC (+0.244) 0.966
Pain (x1.02) 0.966
Hypothyroidism (x1.02) 0.966
Tobacco dependence syndrome (x1.02) 0.967
Leukocytosis (x1.02) 0.967
Malignant neoplasm of lip, oral cavity and pharynx (x1.02) 0.968
MCH [M] by AC (+0.0825) 0.968
Abdominal pain (/1.02) 0.969
Cough (/1.02) 0.969
Jaw pain (x1.02) 0.969
Disorder of magnesium metabolism (x1.02) 0.970
Carcinoma in situ (x1.02) 0.970
Malaise (x1.02) 0.970
Dental caries (x1.02) 0.971
Squamous cell carcinoma (x1.02) 0.971
docusate (+0.00157) 0.971
promethazine (+0.00156) 0.972
Malignant tumor of salivary gland (x1.02) 0.972
levothyroxine (+0.00154) 0.972
Neoplasm of uncertain behavior of lip, oral cavity and pharynx (x1.02) 0.973
Oral ulcerative mucositis due to antineoplastic therapy (x1.02) 0.973
Trauma and postoperative pulmonary insufficiency (x1.02) 0.973
Edema of larynx (x1.02) 0.974
Inflammatory disorder of jaw region (x1.02) 0.974
Benign neoplasm of mouth region (x1.02) 0.974
Secondary malignant neoplasm of bone (x1.02) 0.974
Hypo-osmolality and or hyponatremia (x1.02) 0.975
Squamous cell carcinoma of skin (x1.02) 0.975
Chest pain (/1.02) 0.975
−0.8 −0.6 −0.4 −0.2 0.0 0.2 0.4 0.6 0.8
Expressions

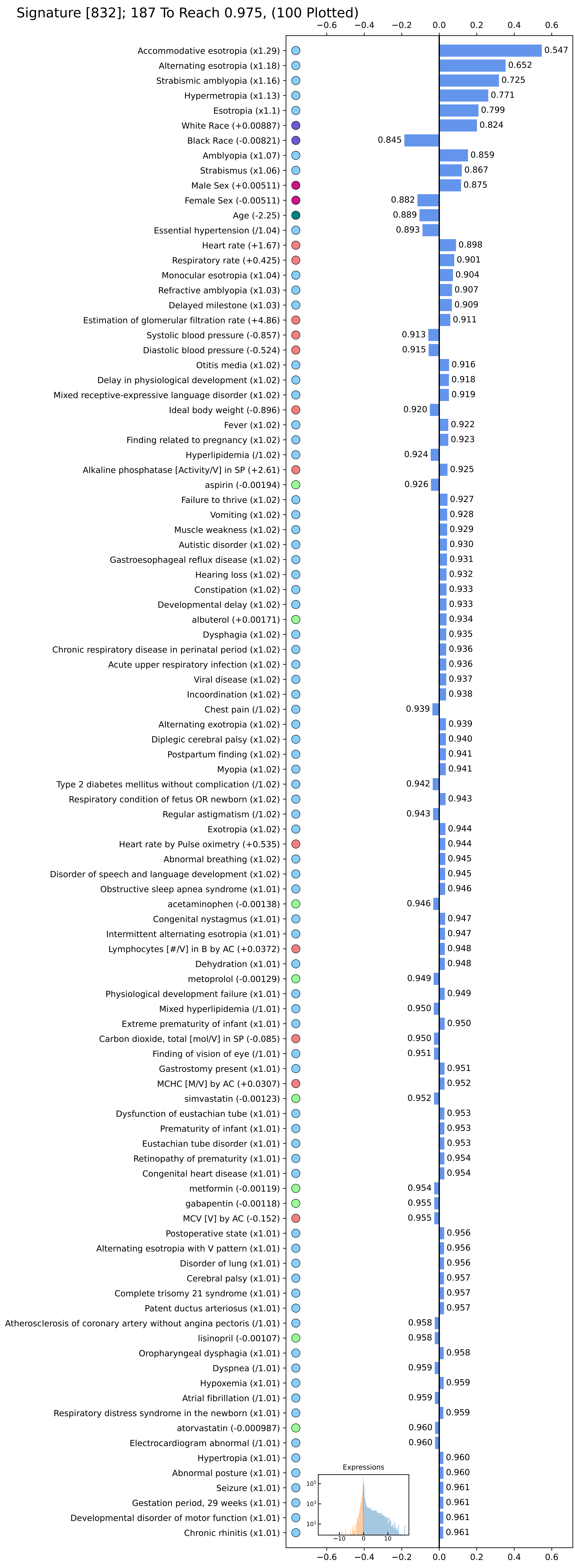

Signature [832]; 187 To Reach 0.975, (100 Plotted)
Accommodative esotropia (x1.29) 0.547
Alternating esotropia (x1.18) 0.652
Strabismic amblyopia (x1.16) 0.725
Hypermetropia (x1.13) 0.771
Esotropia (x1.1) 0.799
White Race (+0.00887) 0.824
Black Race (-0.00821) 0.845
Amblyopia (x1.07) 0.859
Strabismus (x1.06) 0.867
Male Sex (+0.00511) 0.875
Female Sex (-0.00511) 0.882
Age (-2.25) 0.889
Essential hypertension (/1.04) 0.893
Heart rate (+1.67) 0.898
Respiratory rate (+0.425) 0.901
Monocular esotropia (x1.04) 0.904
Refractive amblyopia (x1.03) 0.907
Delayed milestone (x1.03) 0.909
Estimation of glomerular filtration rate (+4.86) 0.911
Systolic blood pressure (-0.857) 0.913
Diastolic blood pressure (-0.524) 0.915
Otitis media (x1.02) 0.916
Delay in physiological development (x1.02) 0.918
Mixed receptive-expressive language disorder (x1.02) 0.919
Ideal body weight (-0.896) 0.920
Fever (x1.02) 0.922
Finding related to pregnancy (x1.02) 0.923
Hyperlipidemia (/1.02) 0.924
Alkaline phosphatase [Activity/V] in SP (+2.61) 0.925
aspirin (-0.00194) 0.926
Failure to thrive (x1.02) 0.927
Vomiting (x1.02) 0.928
Muscle weakness (x1.02) 0.929
Autistic disorder (x1.02) 0.930
Gastroesophageal reflux disease (x1.02) 0.931
Hearing loss (x1.02) 0.932
Constipation (x1.02) 0.933
Developmental delay (x1.02) 0.933
albuterol (+0.00171) 0.934
Dysphagia (x1.02) 0.935
Chronic respiratory disease in perinatal period (x1.02) 0.936
Acute upper respiratory infection (x1.02) 0.936
Viral disease (x1.02) 0.937
Incoordination (x1.02) 0.938
Chest pain (/1.02) 0.939
Alternating exotropia (x1.02) 0.939
Diplegic cerebral palsy (x1.02) 0.940
Postpartum finding (x1.02) 0.941
Myopia (x1.02) 0.941
Type 2 diabetes mellitus without complication (/1.02) 0.942
Respiratory condition of fetus OR newborn (x1.02) 0.943
Regular astigmatism (/1.02) 0.943
Exotropia (x1.02) 0.944
Heart rate by Pulse oximetry (+0.535) 0.944
Abnormal breathing (x1.02) 0.945
Disorder of speech and language development (x1.02) 0.945
Obstructive sleep apnea syndrome (x1.01) 0.946
acetaminophen (-0.00138) 0.946
Congenital nystagmus (x1.01) 0.947
Intermittent alternating esotropia (x1.01) 0.947
Lymphocytes [#/V] in B by AC (+0.0372) 0.948
Dehydration (x1.01) 0.948
metoprolol (-0.00129) 0.949
Physiological development failure (x1.01) 0.949
Mixed hyperlipidemia (/1.01) 0.950
Extreme prematurity of infant (x1.01) 0.950
Carbon dioxide, total [mol/V] in SP (-0.085) 0.950
Finding of vision of eye (/1.01) 0.951
Gastrostomy present (x1.01) 0.951
MCHC [M/V] by AC (+0.0307) 0.952
simvastatin (-0.00123) 0.952
Dysfunction of eustachian tube (x1.01) 0.953
Prematurity of infant (x1.01) 0.953
Eustachian tube disorder (x1.01) 0.953
Retinopathy of prematurity (x1.01) 0.954
Congenital heart disease (x1.01) 0.954
metformin (-0.00119) 0.954
gabapentin (-0.00118) 0.955
MCV [V] by AC (-0.152) 0.955
Postoperative state (x1.01) 0.956
Alternating esotropia with V pattern (x1.01) 0.956
Disorder of lung (x1.01) 0.956
Cerebral palsy (x1.01) 0.957
Complete trisomy 21 syndrome (x1.01) 0.957
Patent ductus arteriosus (x1.01) 0.957
Atherosclerosis of coronary artery without angina pectoris (/1.01) 0.958
lisinopril (-0.00107) 0.958
Oropharyngeal dysphagia (x1.01) 0.958
Dyspnea (/1.01) 0.959
Hypoxemia (x1.01) 0.959
Atrial fibrillation (/1.01) 0.959
Respiratory distress syndrome in the newborn (x1.01) 0.959
atorvastatin (-0.000987) 0.960
Electrocardiogram abnormal (/1.01) 0.960
Hypertropia (x1.01) 0.960
Abnormal posture (x1.01) 0.960
Seizure (x1.01) 0.961
Gestation period, 29 weeks (x1.01) 0.961
Developmental disorder of motor function (x1.01) 0.961
Chronic rhinitis (x1.01) 0.961
−0.6 −0.4 −0.2 0.0 0.2 0.4 0.6
Expressions

Signature [833]; 507 To Reach 0.975, (100 Plotted)

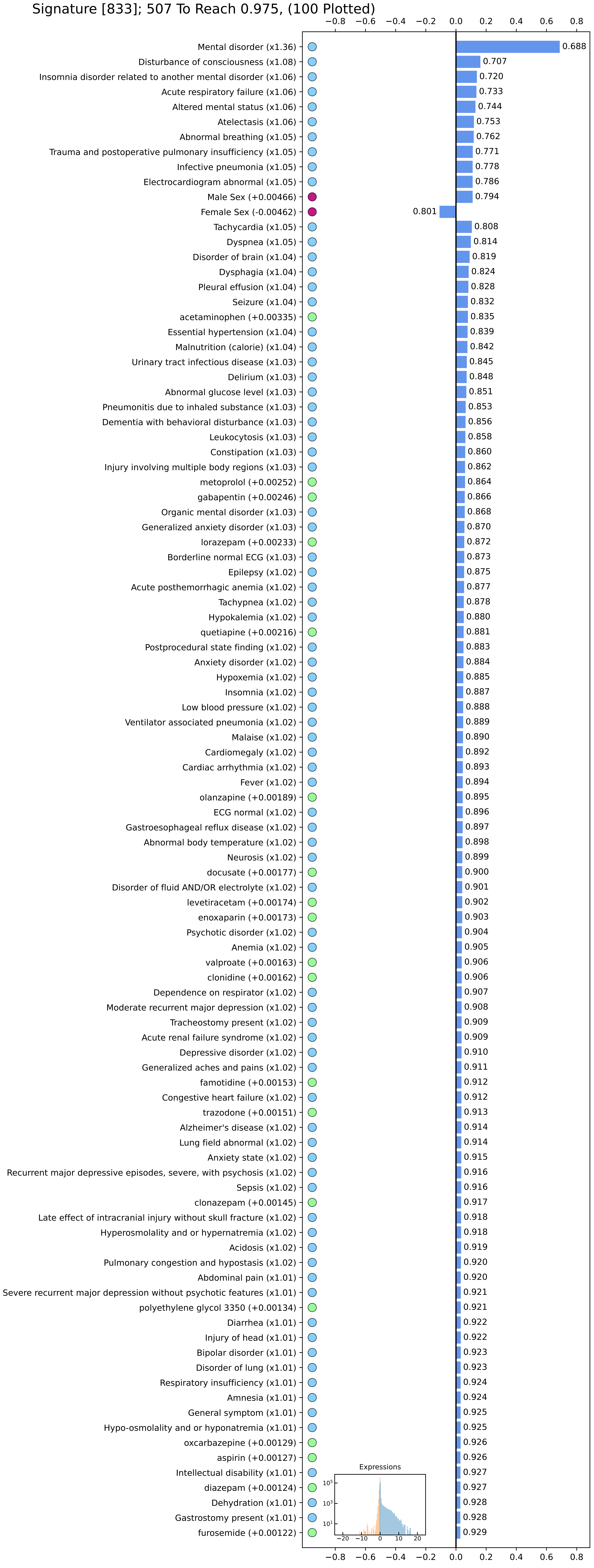

Signature [834]; 58 To Reach 0.975, (58 Plotted)

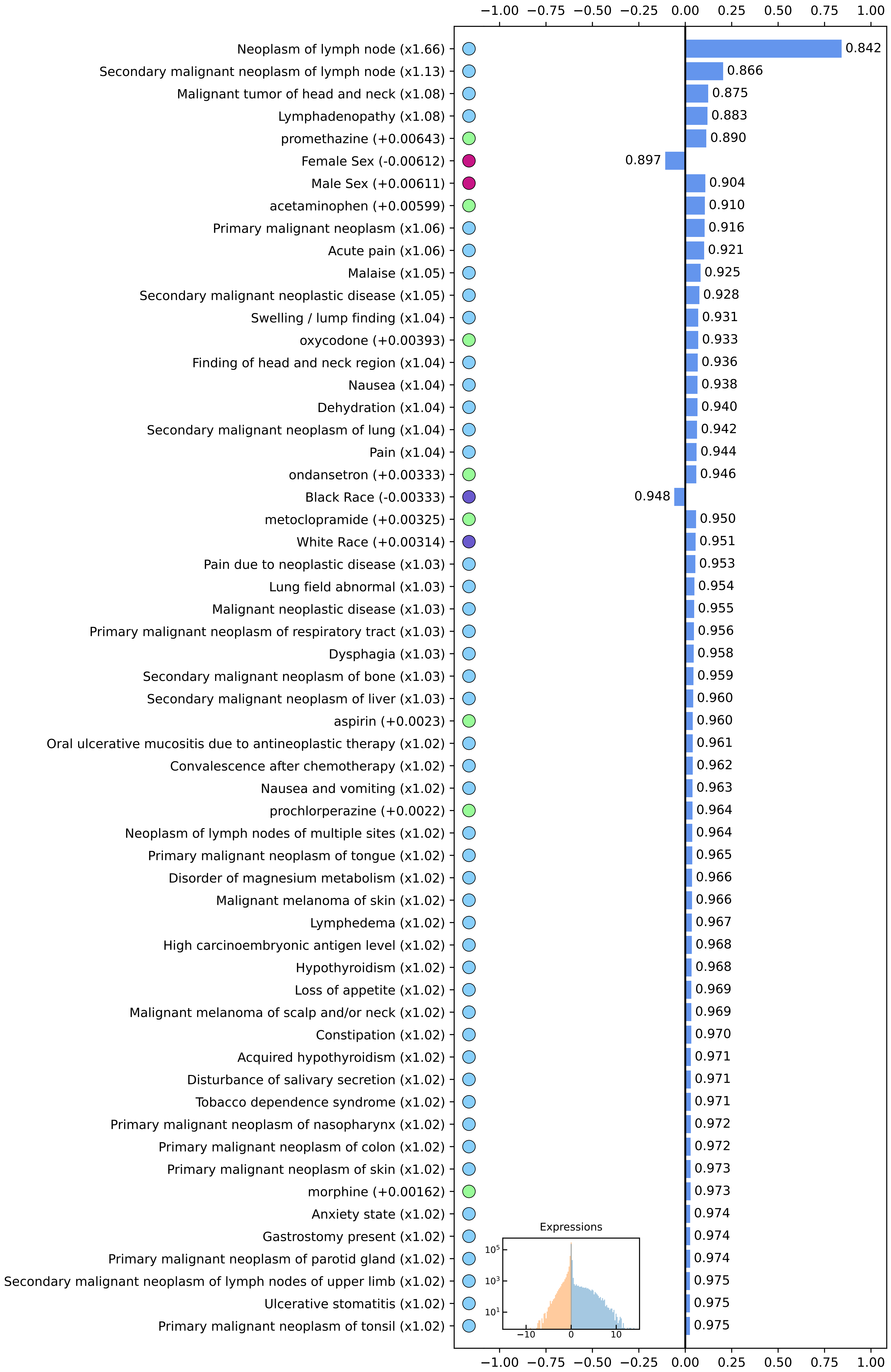

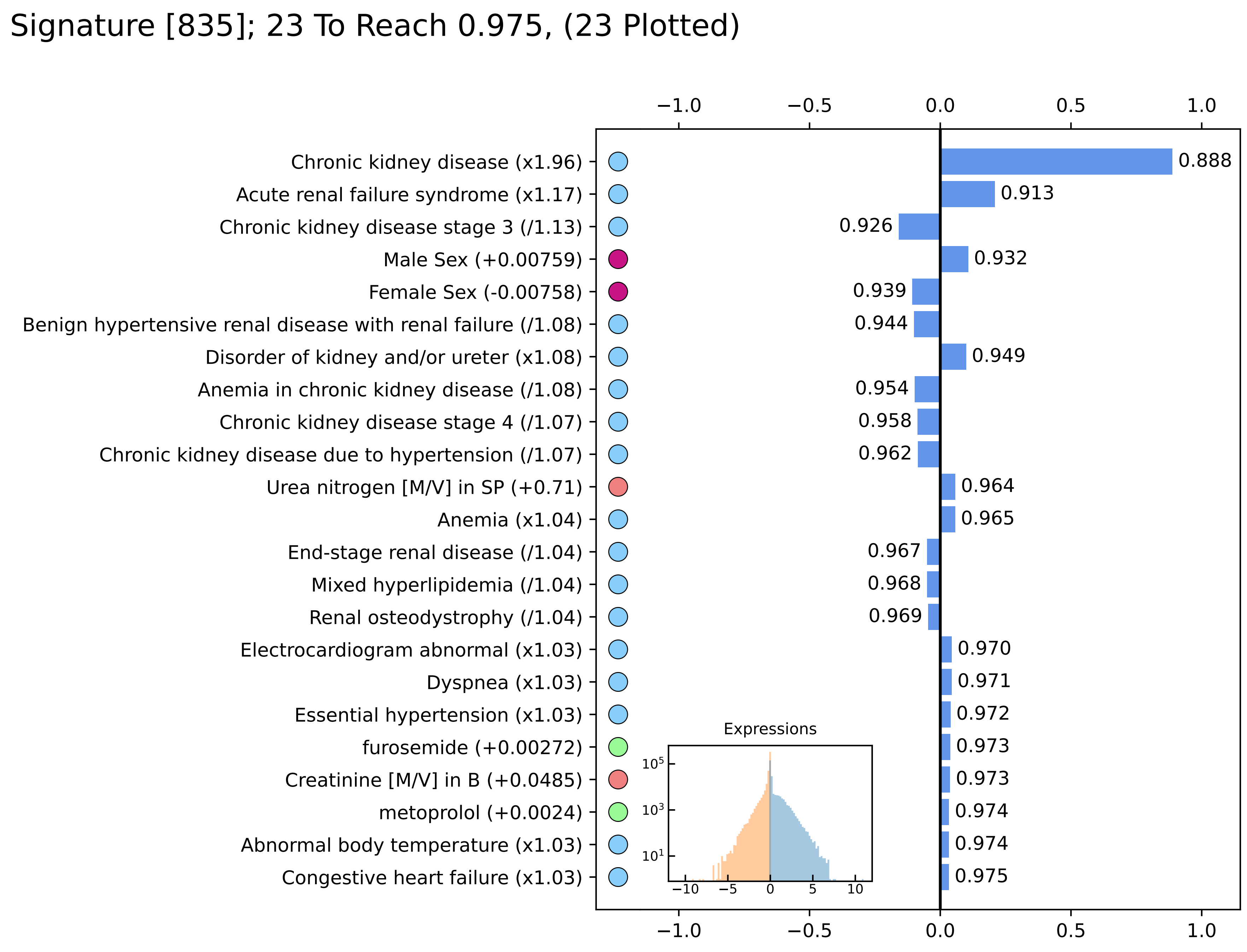
Signature [835]; 23 To Reach 0.975, (23 Plotted)
Chronic kidney disease (x1.96)
Acute renal failure syndrome (x1.17)
Chronic kidney disease stage 3 (/1.13)
Male Sex (+0.00759)
Female Sex (-0.00758)
Benign hypertensive renal disease with renal failure (/1.08)
Disorder of kidney and/or ureter (x1.08)
Anemia in chronic kidney disease (/1.08)
Chronic kidney disease stage 4 (/1.07)
Chronic kidney disease due to hypertension (/1.07)
Urea nitrogen [M/V] in SP (+0.71)
Anemia (x1.04)
End-stage renal disease (/1.04)
Mixed hyperlipidemia (/1.04)
Renal osteodystrophy (/1.04)
Electrocardiogram abnormal (x1.03)
Dyspnea (x1.03)
Essential hypertension (x1.03)
furosemide (+0.00272)
Creatinine [M/V] in B (+0.0485)
metoprolol (+0.0024)
Abnormal body temperature (x1.03)
Congestive heart failure (x1.03)
0.888
0.913
0.926
0.932
0.939
0.944
0.949
0.954
0.958
0.962
0.964
0.965
0.967
0.968
0.969
0.970
0.971
0.972
0.973
0.973
0.974
0.974
0.975
−1.0
−0.5
0.0
0.5
1.0
Expressions
10^5
10^3
10^1
−10
−5
0
5
10

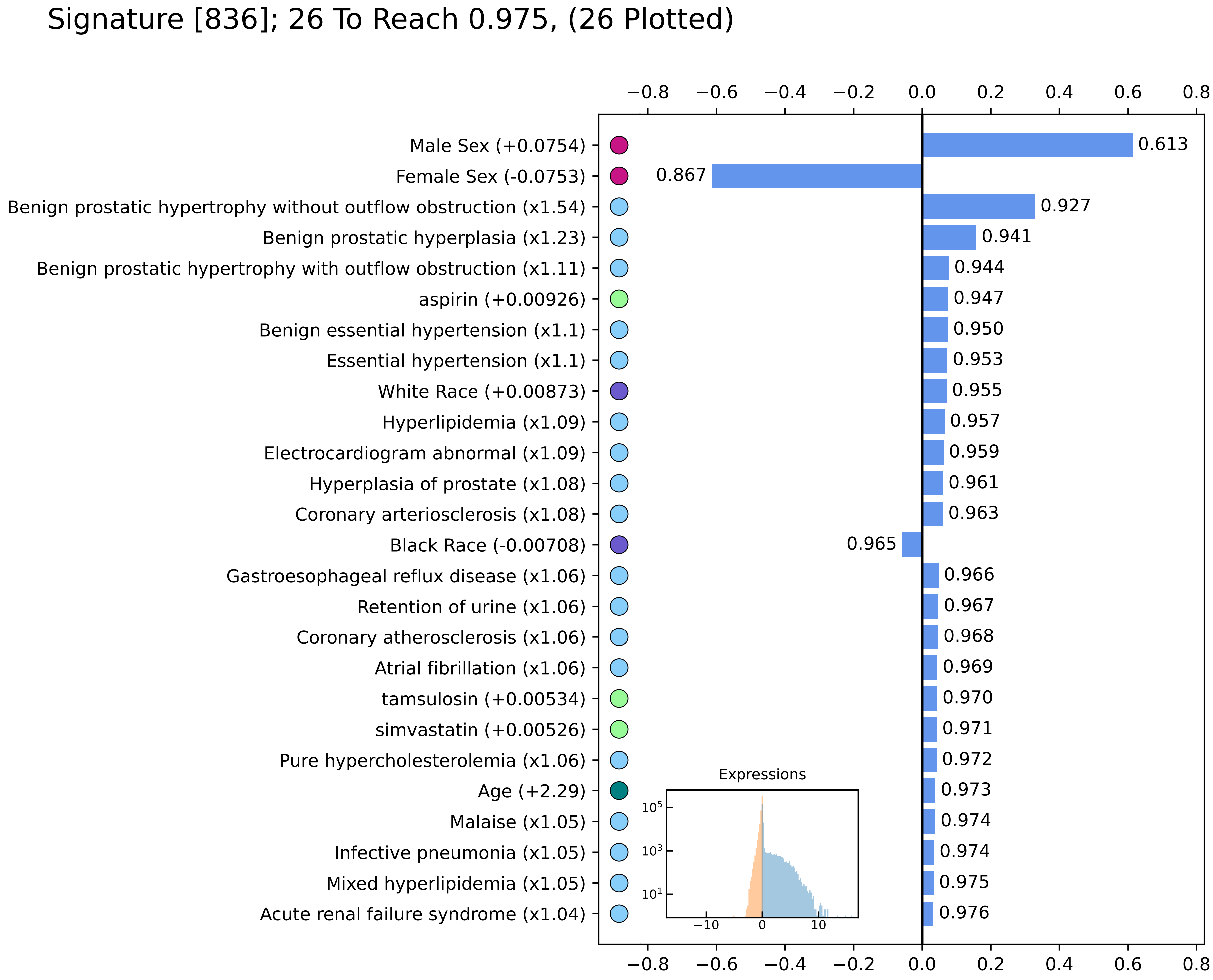
Signature [836]; 26 To Reach 0.975, (26 Plotted)
Male Sex (+0.0754)
Female Sex (-0.0753)
Benign prostatic hypertrophy without outflow obstruction (x1.54)
Benign prostatic hyperplasia (x1.23)
Benign prostatic hypertrophy with outflow obstruction (x1.11)
aspirin (+0.00926)
Benign essential hypertension (x1.1)
Essential hypertension (x1.1)
White Race (+0.00873)
Hyperlipidemia (x1.09)
Electrocardiogram abnormal (x1.09)
Hyperplasia of prostate (x1.08)
Coronary arteriosclerosis (x1.08)
Black Race (-0.00708)
Gastroesophageal reflux disease (x1.06)
Retention of urine (x1.06)
Coronary atherosclerosis (x1.06)
Atrial fibrillation (x1.06)
tamsulosin (+0.00534)
simvastatin (+0.00526)
Pure hypercholesterolemia (x1.06)
Age (+2.29)
Malaise (x1.05)
Infective pneumonia (x1.05)
Mixed hyperlipidemia (x1.05)
Acute renal failure syndrome (x1.04)
0.613
0.867
0.927
0.941
0.944
0.947
0.950
0.953
0.955
0.957
0.959
0.961
0.963
0.965
0.966
0.967
0.968
0.969
0.970
0.971
0.972
0.973
0.974
0.974
0.975
0.976
−0.8 −0.6 −0.4 −0.2 0.0 0.2 0.4 0.6 0.8
Expressions
10^5
10^3
10^1
−10 0 10

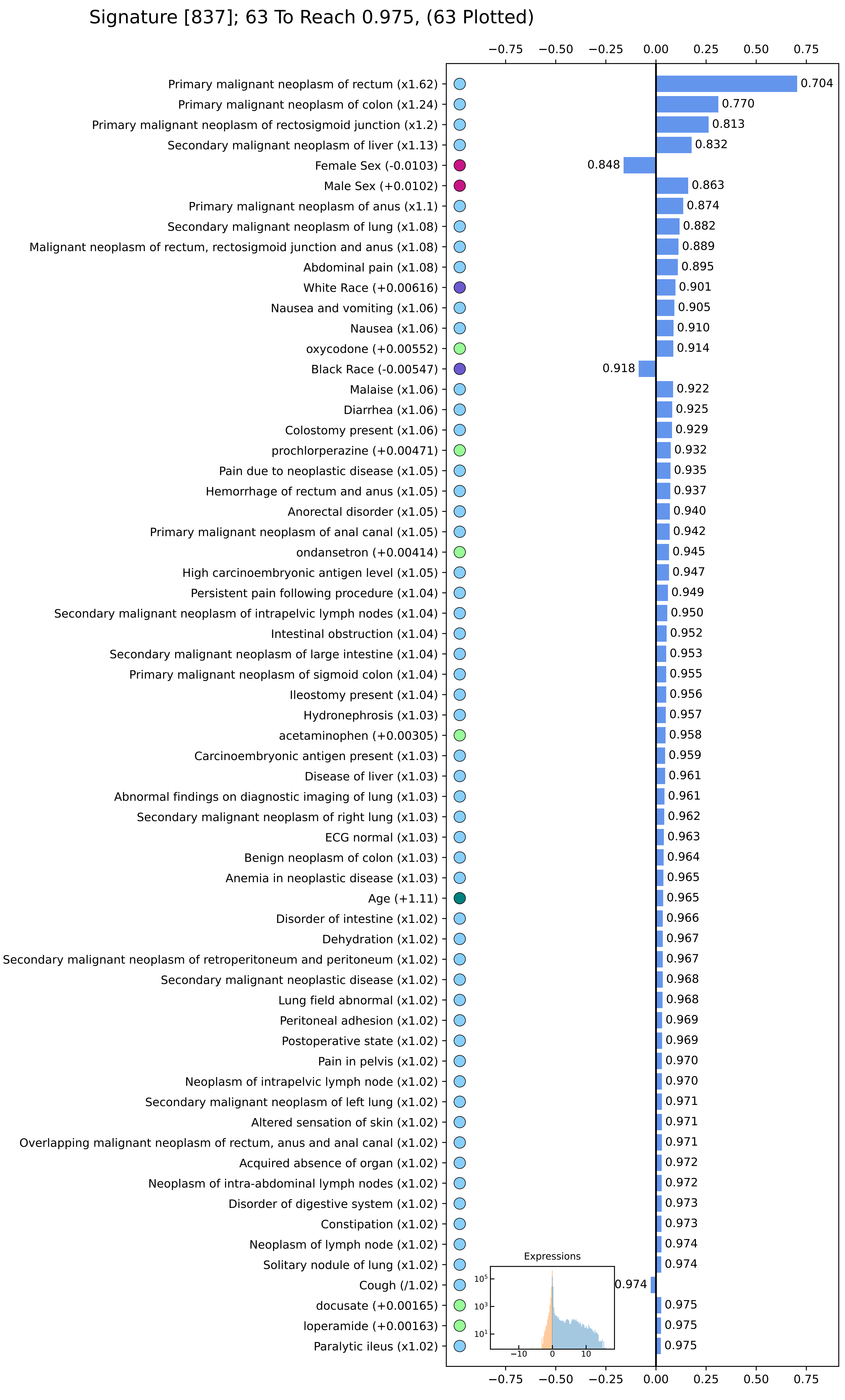

Signature [837]; 63 To Reach 0.975, (63 Plotted)
Primary malignant neoplasm of rectum (x1.62) 0.704
Primary malignant neoplasm of colon (x1.24) 0.770
Primary malignant neoplasm of rectosigmoid junction (x1.2) 0.813
Secondary malignant neoplasm of liver (x1.13) 0.832
Female Sex (-0.0103) 0.848
Male Sex (+0.0102) 0.863
Primary malignant neoplasm of anus (x1.1) 0.874
Secondary malignant neoplasm of lung (x1.08) 0.882
Malignant neoplasm of rectum, rectosigmoid junction and anus (x1.08) 0.889
Abdominal pain (x1.08) 0.895
White Race (+0.00616) 0.901
Nausea and vomiting (x1.06) 0.905
Nausea (x1.06) 0.910
oxycodone (+0.00552) 0.914
Black Race (-0.00547) 0.918
Malaise (x1.06) 0.922
Diarrhea (x1.06) 0.925
Colostomy present (x1.06) 0.929
prochlorperazine (+0.00471) 0.932
Pain due to neoplastic disease (x1.05) 0.935
Hemorrhage of rectum and anus (x1.05) 0.937
Anorectal disorder (x1.05) 0.940
Primary malignant neoplasm of anal canal (x1.05) 0.942
ondansetron (+0.00414) 0.945
High carcinoembryonic antigen level (x1.05) 0.947
Persistent pain following procedure (x1.04) 0.949
Secondary malignant neoplasm of intrapelvic lymph nodes (x1.04) 0.950
Intestinal obstruction (x1.04) 0.952
Secondary malignant neoplasm of large intestine (x1.04) 0.953
Primary malignant neoplasm of sigmoid colon (x1.04) 0.955
Ileostomy present (x1.04) 0.956
Hydronephrosis (x1.03) 0.957
acetaminophen (+0.00305) 0.958
Carcinoembryonic antigen present (x1.03) 0.959
Disease of liver (x1.03) 0.961
Abnormal findings on diagnostic imaging of lung (x1.03) 0.961
Secondary malignant neoplasm of right lung (x1.03) 0.962
ECG normal (x1.03) 0.963
Benign neoplasm of colon (x1.03) 0.964
Anemia in neoplastic disease (x1.03) 0.965
Age (+1.11) 0.965
Disorder of intestine (x1.02) 0.966
Dehydration (x1.02) 0.967
Secondary malignant neoplasm of retroperitoneum and peritoneum (x1.02) 0.967
Secondary malignant neoplastic disease (x1.02) 0.968
Lung field abnormal (x1.02) 0.968
Peritoneal adhesion (x1.02) 0.969
Postoperative state (x1.02) 0.969
Pain in pelvis (x1.02) 0.970
Neoplasm of intrapelvic lymph node (x1.02) 0.970
Secondary malignant neoplasm of left lung (x1.02) 0.971
Altered sensation of skin (x1.02) 0.971
Overlapping malignant neoplasm of rectum, anus and anal canal (x1.02) 0.971
Acquired absence of organ (x1.02) 0.972
Neoplasm of intra-abdominal lymph nodes (x1.02) 0.972
Disorder of digestive system (x1.02) 0.973
Constipation (x1.02) 0.973
Neoplasm of lymph node (x1.02) 0.974
Solitary nodule of lung (x1.02) 0.974
Cough (/1.02) 0.974
docusate (+0.00165) 0.975
loperamide (+0.00163) 0.975
Paralytic ileus (x1.02) 0.975
−0.75 −0.50 −0.25 0.00 0.25 0.50 0.75
Expressions
10^5 10^3 10^1
−10 0 10

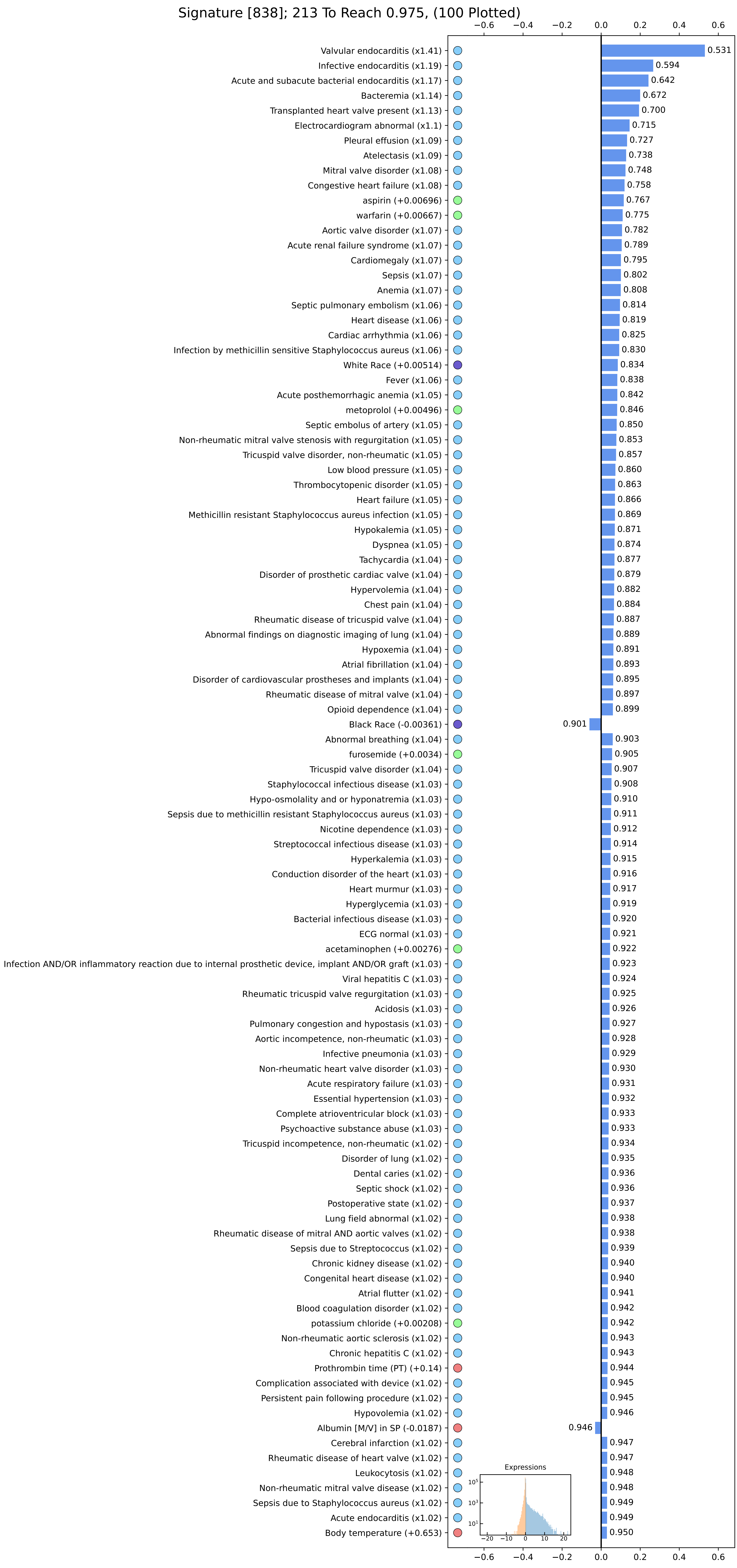

Signature [838]; 213 To Reach 0.975, (100 Plotted)
Valvular endocarditis (x1.41) 0.531
Infective endocarditis (x1.19) 0.594
Acute and subacute bacterial endocarditis (x1.17) 0.642
Bacteremia (x1.14) 0.672
Transplanted heart valve present (x1.13) 0.700
Electrocardiogram abnormal (x1.1) 0.715
Pleural effusion (x1.09) 0.727
Atelectasis (x1.09) 0.738
Mitral valve disorder (x1.08) 0.748
Congestive heart failure (x1.08) 0.758
aspirin (+0.00696) 0.767
warfarin (+0.00667) 0.775
Aortic valve disorder (x1.07) 0.782
Acute renal failure syndrome (x1.07) 0.789
Cardiomegaly (x1.07) 0.795
Sepsis (x1.07) 0.802
Anemia (x1.07) 0.808
Septic pulmonary embolism (x1.06) 0.814
Heart disease (x1.06) 0.819
Cardiac arrhythmia (x1.06) 0.825
Infection by methicillin sensitive Staphylococcus aureus (x1.06) 0.830
White Race (+0.00514) 0.834
Fever (x1.06) 0.838
Acute posthemorrhagic anemia (x1.05) 0.842
metoprolol (+0.00496) 0.846
Septic embolus of artery (x1.05) 0.850
Non-rheumatic mitral valve stenosis with regurgitation (x1.05) 0.853
Tricuspid valve disorder, non-rheumatic (x1.05) 0.857
Low blood pressure (x1.05) 0.860
Thrombocytopenic disorder (x1.05) 0.863
Heart failure (x1.05) 0.866
Methicillin resistant Staphylococcus aureus infection (x1.05) 0.869
Hypokalemia (x1.05) 0.871
Dyspnea (x1.05) 0.874
Tachycardia (x1.04) 0.877
Disorder of prosthetic cardiac valve (x1.04) 0.879
Hypervolemia (x1.04) 0.882
Chest pain (x1.04) 0.884
Rheumatic disease of tricuspid valve (x1.04) 0.887
Abnormal findings on diagnostic imaging of lung (x1.04) 0.889
Hypoxemia (x1.04) 0.891
Atrial fibrillation (x1.04) 0.893
Disorder of cardiovascular prostheses and implants (x1.04) 0.895
Rheumatic disease of mitral valve (x1.04) 0.897
Opioid dependence (x1.04) 0.899
Black Race (-0.00361) 0.901
Abnormal breathing (x1.04) 0.903
furosemide (+0.0034) 0.905
Tricuspid valve disorder (x1.04) 0.907
Staphylococcal infectious disease (x1.03) 0.908
Hypo-osmolality and or hyponatremia (x1.03) 0.910
Sepsis due to methicillin resistant Staphylococcus aureus (x1.03) 0.911
Nicotine dependence (x1.03) 0.912
Streptococcal infectious disease (x1.03) 0.914
Hyperkalemia (x1.03) 0.915
Conduction disorder of the heart (x1.03) 0.916
Heart murmur (x1.03) 0.917
Hyperglycemia (x1.03) 0.919
Bacterial infectious disease (x1.03) 0.920
ECG normal (x1.03) 0.921
acetaminophen (+0.00276) 0.922
Infection AND/OR inflammatory reaction due to internal prosthetic device, implant AND/OR graft (x1.03) 0.923
Viral hepatitis C (x1.03) 0.924
Rheumatic tricuspid valve regurgitation (x1.03) 0.925
Acidosis (x1.03) 0.926
Pulmonary congestion and hypostasis (x1.03) 0.927
Aortic incompetence, non-rheumatic (x1.03) 0.928
Infective pneumonia (x1.03) 0.929
Non-rheumatic heart valve disorder (x1.03) 0.930
Acute respiratory failure (x1.03) 0.931
Essential hypertension (x1.03) 0.932
Complete atrioventricular block (x1.03) 0.933
Psychoactive substance abuse (x1.03) 0.933
Tricuspid incompetence, non-rheumatic (x1.02) 0.934
Disorder of lung (x1.02) 0.935
Dental caries (x1.02) 0.936
Septic shock (x1.02) 0.936
Postoperative state (x1.02) 0.937
Lung field abnormal (x1.02) 0.938
Rheumatic disease of mitral AND aortic valves (x1.02) 0.938
Sepsis due to Streptococcus (x1.02) 0.939
Chronic kidney disease (x1.02) 0.940
Congenital heart disease (x1.02) 0.940
Atrial flutter (x1.02) 0.941
Blood coagulation disorder (x1.02) 0.942
potassium chloride (+0.00208) 0.942
Non-rheumatic aortic sclerosis (x1.02) 0.943
Chronic hepatitis C (x1.02) 0.943
Prothrombin time (PT) (+0.14) 0.944
Complication associated with device (x1.02) 0.945
Persistent pain following procedure (x1.02) 0.945
Hypovolemia (x1.02) 0.946
Albumin [M/V] in SP (-0.0187) 0.946
Cerebral infarction (x1.02) 0.947
Rheumatic disease of heart valve (x1.02) 0.947
Leukocytosis (x1.02) 0.948
Non-rheumatic mitral valve disease (x1.02) 0.948
Sepsis due to Staphylococcus aureus (x1.02) 0.949
Acute endocarditis (x1.02) 0.949
Body temperature (+0.653) 0.950
−0.6 −0.4 −0.2 0.0 0.2 0.4 0.6
Expressions

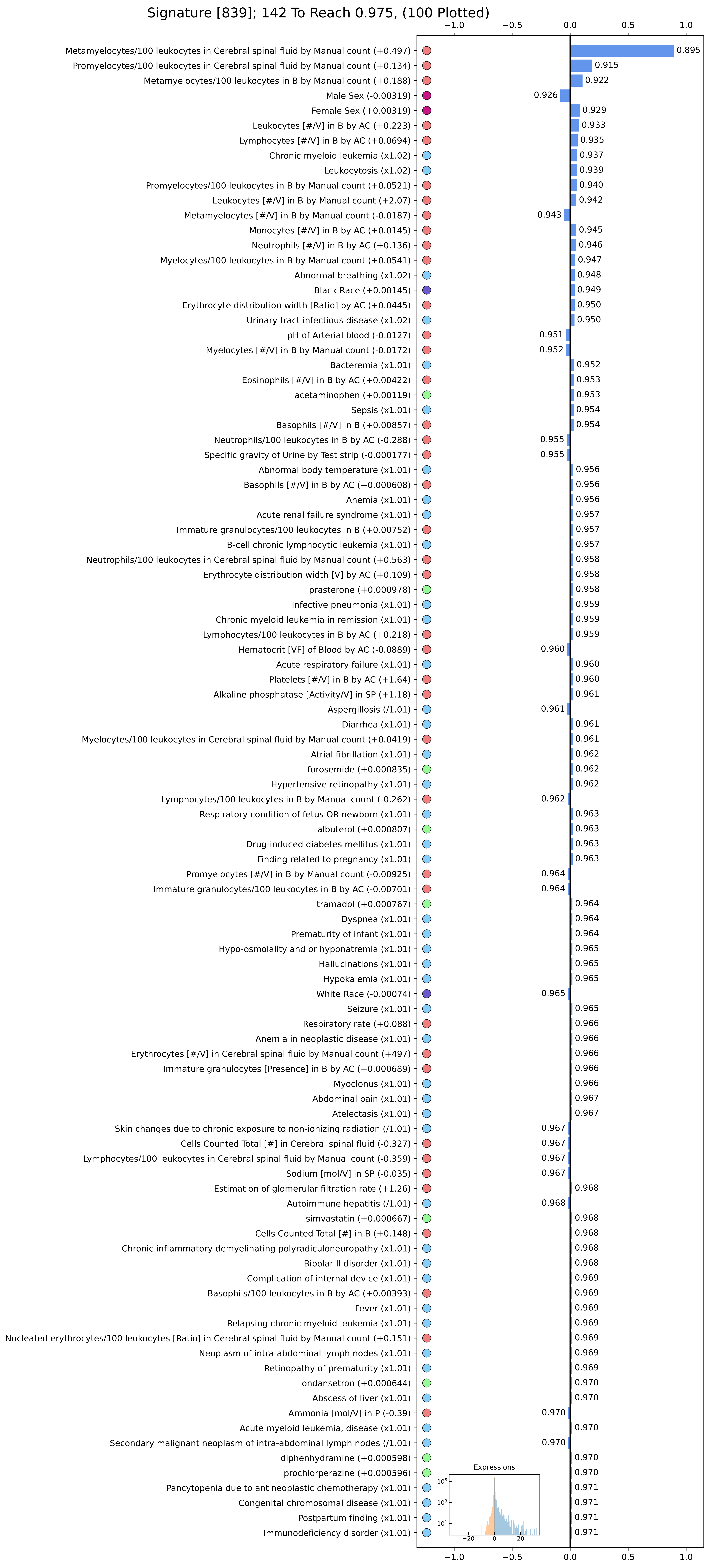

Signature [839]; 142 To Reach 0.975, (100 Plotted)
Metamyelocytes/100 leukocytes in Cerebral spinal fluid by Manual count (+0.497) 0.895
Promyelocytes/100 leukocytes in Cerebral spinal fluid by Manual count (+0.134) 0.915
Metamyelocytes/100 leukocytes in B by Manual count (+0.188) 0.922
Male Sex (-0.00319) 0.926
Female Sex (+0.00319) 0.929
Leukocytes [#/V] in B by AC (+0.223) 0.933
Lymphocytes [#/V] in B by AC (+0.0694) 0.935
Chronic myeloid leukemia (x1.02) 0.937
Leukocytosis (x1.02) 0.939
Promyelocytes/100 leukocytes in B by Manual count (+0.0521) 0.940
Leukocytes [#/V] in B by Manual count (+2.07) 0.942
Metamyelocytes [#/V] in B by Manual count (-0.0187) 0.943
Monocytes [#/V] in B by AC (+0.0145) 0.945
Neutrophils [#/V] in B by AC (+0.136) 0.946
Myelocytes/100 leukocytes in B by Manual count (+0.0541) 0.947
Abnormal breathing (x1.02) 0.948
Black Race (+0.00145) 0.949
Erythrocyte distribution width [Ratio] by AC (+0.0445) 0.950
Urinary tract infectious disease (x1.02) 0.950
pH of Arterial blood (-0.0127) 0.951
Myelocytes [#/V] in B by Manual count (-0.0172) 0.952
Bacteremia (x1.01) 0.952
Eosinophils [#/V] in B by AC (+0.00422) 0.953
acetaminophen (+0.00119) 0.953
Sepsis (x1.01) 0.954
Basophils [#/V] in B (+0.00857) 0.954
Neutrophils/100 leukocytes in B by AC (-0.288) 0.955
Specific gravity of Urine by Test strip (-0.000177) 0.955
Abnormal body temperature (x1.01) 0.956
Basophils [#/V] in B by AC (+0.000608) 0.956
Anemia (x1.01) 0.956
Acute renal failure syndrome (x1.01) 0.957
Immature granulocytes/100 leukocytes in B (+0.00752) 0.957
B-cell chronic lymphocytic leukemia (x1.01) 0.957
Neutrophils/100 leukocytes in Cerebral spinal fluid by Manual count (+0.563) 0.958
Erythrocyte distribution width [V] by AC (+0.109) 0.958
prasterone (+0.000978) 0.958
Infective pneumonia (x1.01) 0.959
Chronic myeloid leukemia in remission (x1.01) 0.959
Lymphocytes/100 leukocytes in B by AC (+0.218) 0.959
Hematocrit [VF] of Blood by AC (-0.0889) 0.960
Acute respiratory failure (x1.01) 0.960
Platelets [#/V] in B by AC (+1.64) 0.960
Alkaline phosphatase [Activity/V] in SP (+1.18) 0.961
Aspergillosis (/1.01) 0.961
Diarrhea (x1.01) 0.961
Myelocytes/100 leukocytes in Cerebral spinal fluid by Manual count (+0.0419) 0.961
Atrial fibrillation (x1.01) 0.962
furosemide (+0.000835) 0.962
Hypertensive retinopathy (x1.01) 0.962
Lymphocytes/100 leukocytes in B by Manual count (-0.262) 0.962
Respiratory condition of fetus OR newborn (x1.01) 0.963
albuterol (+0.000807) 0.963
Drug-induced diabetes mellitus (x1.01) 0.963
Finding related to pregnancy (x1.01) 0.963
Promyelocytes [#/V] in B by Manual count (-0.00925) 0.964
Immature granulocytes/100 leukocytes in B by AC (-0.00701) 0.964
tramadol (+0.000767) 0.964
Dyspnea (x1.01) 0.964
Prematurity of infant (x1.01) 0.964
Hypo-osmolality and or hyponatremia (x1.01) 0.965
Hallucinations (x1.01) 0.965
Hypokalemia (x1.01) 0.965
White Race (-0.00074) 0.965
Seizure (x1.01) 0.965
Respiratory rate (+0.088) 0.966
Anemia in neoplastic disease (x1.01) 0.966
Erythrocytes [#/V] in Cerebral spinal fluid by Manual count (+497) 0.966
Immature granulocytes [Presence] in B by AC (+0.000689) 0.966
Myoclonus (x1.01) 0.966
Abdominal pain (x1.01) 0.967
Atelectasis (x1.01) 0.967
Skin changes due to chronic exposure to non-ionizing radiation (/1.01) 0.967
Cells Counted Total [#] in Cerebral spinal fluid (-0.327) 0.967
Lymphocytes/100 leukocytes in Cerebral spinal fluid by Manual count (-0.359) 0.967
Sodium [mol/V] in SP (-0.035) 0.967
Estimation of glomerular filtration rate (+1.26) 0.968
Autoimmune hepatitis (/1.01) 0.968
simvastatin (+0.000667) 0.968
Cells Counted Total [#] in B (+0.148) 0.968
Chronic inflammatory demyelinating polyradiculoneuropathy (x1.01) 0.968
Bipolar II disorder (x1.01) 0.968
Complication of internal device (x1.01) 0.969
Basophils/100 leukocytes in B by AC (+0.00393) 0.969
Fever (x1.01) 0.969
Relapsing chronic myeloid leukemia (x1.01) 0.969
Nucleated erythrocytes/100 leukocytes [Ratio] in Cerebral spinal fluid by Manual count (+0.151) 0.969
Neoplasm of intra-abdominal lymph nodes (x1.01) 0.969
Retinopathy of prematurity (x1.01) 0.969
ondansetron (+0.000644) 0.970
Abscess of liver (x1.01) 0.970
Ammonia [mol/V] in P (-0.39) 0.970
Acute myeloid leukemia, disease (x1.01) 0.970
Secondary malignant neoplasm of intra-abdominal lymph nodes (/1.01) 0.970
diphenhydramine (+0.000598) 0.970
prochlorperazine (+0.000596) 0.970
Pancytopenia due to antineoplastic chemotherapy (x1.01) 0.971
Congenital chromosomal disease (x1.01) 0.971
Postpartum finding (x1.01) 0.971
Immunodeficiency disorder (x1.01) 0.971
−1.0 −0.5 0.0 0.5 1.0
Expressions

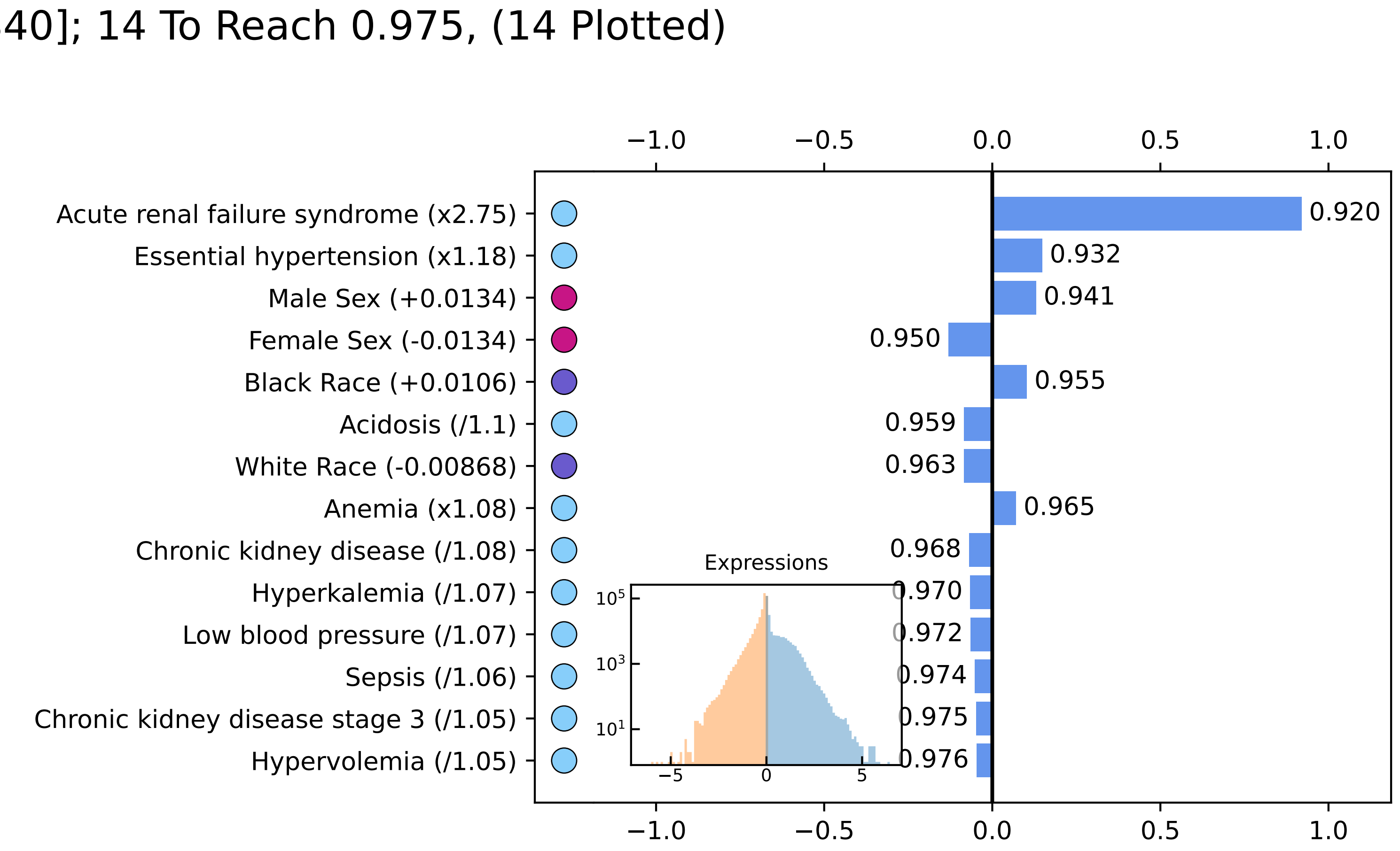

Signature [840]; 14 To Reach 0.975, (14 Plotted)
Acute renal failure syndrome (x2.75)
Essential hypertension (x1.18)
Male Sex (+0.0134)
Female Sex (-0.0134)
Black Race (+0.0106)
Acidosis (/1.1)
White Race (-0.00868)
Anemia (x1.08)
Chronic kidney disease (/1.08)
Hyperkalemia (/1.07)
Low blood pressure (/1.07)
Sepsis (/1.06)
Chronic kidney disease stage 3 (/1.05)
Hypervolemia (/1.05)
0.920
0.932
0.941
0.950
0.955
0.959
0.963
0.965
0.968
0.970
0.972
0.974
0.975
0.976
−1.0
−0.5
0.0
0.5
1.0
Expressions

Signature [841]; 1 To Reach 0.975, (10 Plotted)

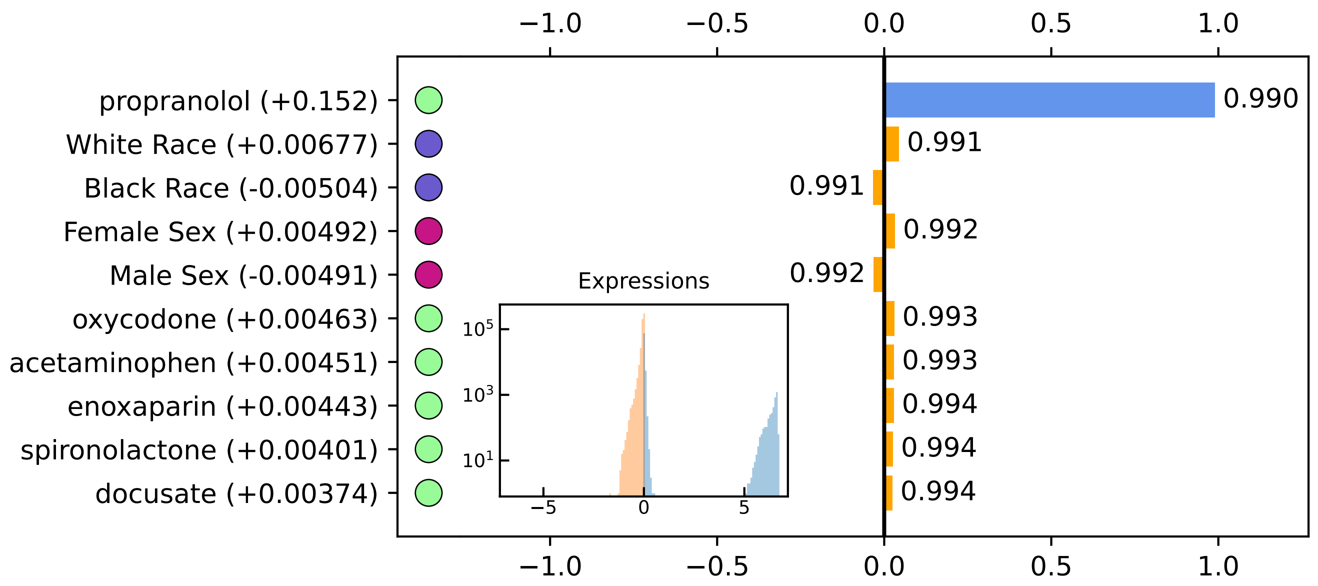

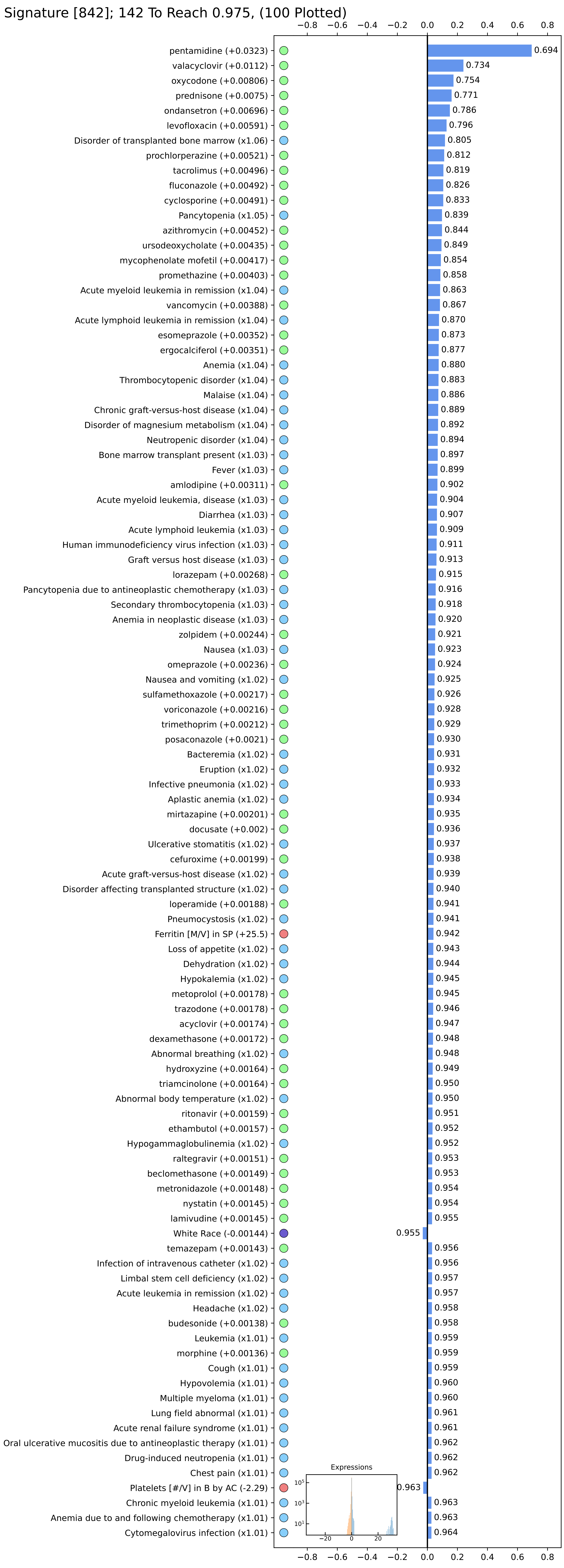

Signature [842]; 142 To Reach 0.975, (100 Plotted)
pentamidine (+0.0323) 0.694
valacyclovir (+0.0112) 0.734
oxycodone (+0.00806) 0.754
prednisone (+0.0075) 0.771
ondansetron (+0.00696) 0.786
levofloxacin (+0.00591) 0.796
Disorder of transplanted bone marrow (x1.06) 0.805
prochlorperazine (+0.00521) 0.812
tacrolimus (+0.00496) 0.819
fluconazole (+0.00492) 0.826
cyclosporine (+0.00491) 0.833
Pancytopenia (x1.05) 0.839
azithromycin (+0.00452) 0.844
ursodeoxycholate (+0.00435) 0.849
mycophenolate mofetil (+0.00417) 0.854
promethazine (+0.00403) 0.858
Acute myeloid leukemia in remission (x1.04) 0.863
vancomycin (+0.00388) 0.867
Acute lymphoid leukemia in remission (x1.04) 0.870
esomeprazole (+0.00352) 0.873
ergocalciferol (+0.00351) 0.877
Anemia (x1.04) 0.880
Thrombocytopenic disorder (x1.04) 0.883
Malaise (x1.04) 0.886
Chronic graft-versus-host disease (x1.04) 0.889
Disorder of magnesium metabolism (x1.04) 0.892
Neutropenic disorder (x1.04) 0.894
Bone marrow transplant present (x1.03) 0.897
Fever (x1.03) 0.899
amlodipine (+0.00311) 0.902
Acute myeloid leukemia, disease (x1.03) 0.904
Diarrhea (x1.03) 0.907
Acute lymphoid leukemia (x1.03) 0.909
Human immunodeficiency virus infection (x1.03) 0.911
Graft versus host disease (x1.03) 0.913
lorazepam (+0.00268) 0.915
Pancytopenia due to antineoplastic chemotherapy (x1.03) 0.916
Secondary thrombocytopenia (x1.03) 0.918
Anemia in neoplastic disease (x1.03) 0.920
zolpidem (+0.00244) 0.921
Nausea (x1.03) 0.923
omeprazole (+0.00236) 0.924
Nausea and vomiting (x1.02) 0.925
sulfamethoxazole (+0.00217) 0.926
voriconazole (+0.00216) 0.928
trimethoprim (+0.00212) 0.929
posaconazole (+0.0021) 0.930
Bacteremia (x1.02) 0.931
Eruption (x1.02) 0.932
Infective pneumonia (x1.02) 0.933
Aplastic anemia (x1.02) 0.934
mirtazapine (+0.00201) 0.935
docusate (+0.002) 0.936
Ulcerative stomatitis (x1.02) 0.937
cefuroxime (+0.00199) 0.938
Acute graft-versus-host disease (x1.02) 0.939
Disorder affecting transplanted structure (x1.02) 0.940
loperamide (+0.00188) 0.941
Pneumocystosis (x1.02) 0.941
Ferritin [M/V] in SP (+25.5) 0.942
Loss of appetite (x1.02) 0.943
Dehydration (x1.02) 0.944
Hypokalemia (x1.02) 0.945
metoprolol (+0.00178) 0.945
trazodone (+0.00178) 0.946
acyclovir (+0.00174) 0.947
dexamethasone (+0.00172) 0.948
Abnormal breathing (x1.02) 0.948
hydroxyzine (+0.00164) 0.949
triamcinolone (+0.00164) 0.950
Abnormal body temperature (x1.02) 0.950
ritonavir (+0.00159) 0.951
ethambutol (+0.00157) 0.952
Hypogammaglobulinemia (x1.02) 0.952
raltegravir (+0.00151) 0.953
beclomethasone (+0.00149) 0.953
metronidazole (+0.00148) 0.954
nystatin (+0.00145) 0.954
lamivudine (+0.00145) 0.955
White Race (-0.00144) 0.955
temazepam (+0.00143) 0.956
Infection of intravenous catheter (x1.02) 0.956
Limbal stem cell deficiency (x1.02) 0.957
Acute leukemia in remission (x1.02) 0.957
Headache (x1.02) 0.958
budesonide (+0.00138) 0.958
Leukemia (x1.01) 0.959
morphine (+0.00136) 0.959
Cough (x1.01) 0.959
Hypovolemia (x1.01) 0.960
Multiple myeloma (x1.01) 0.960
Lung field abnormal (x1.01) 0.961
Acute renal failure syndrome (x1.01) 0.961
Oral ulcerative mucositis due to antineoplastic therapy (x1.01) 0.962
Drug-induced neutropenia (x1.01) 0.962
Chest pain (x1.01) 0.962
Platelets [#/V] in B by AC (-2.29) 0.963
Chronic myeloid leukemia (x1.01) 0.963
Anemia due to and following chemotherapy (x1.01) 0.963
Cytomegalovirus infection (x1.01) 0.964
−0.8 −0.6 −0.4 −0.2 0.0 0.2 0.4 0.6 0.8
Expressions

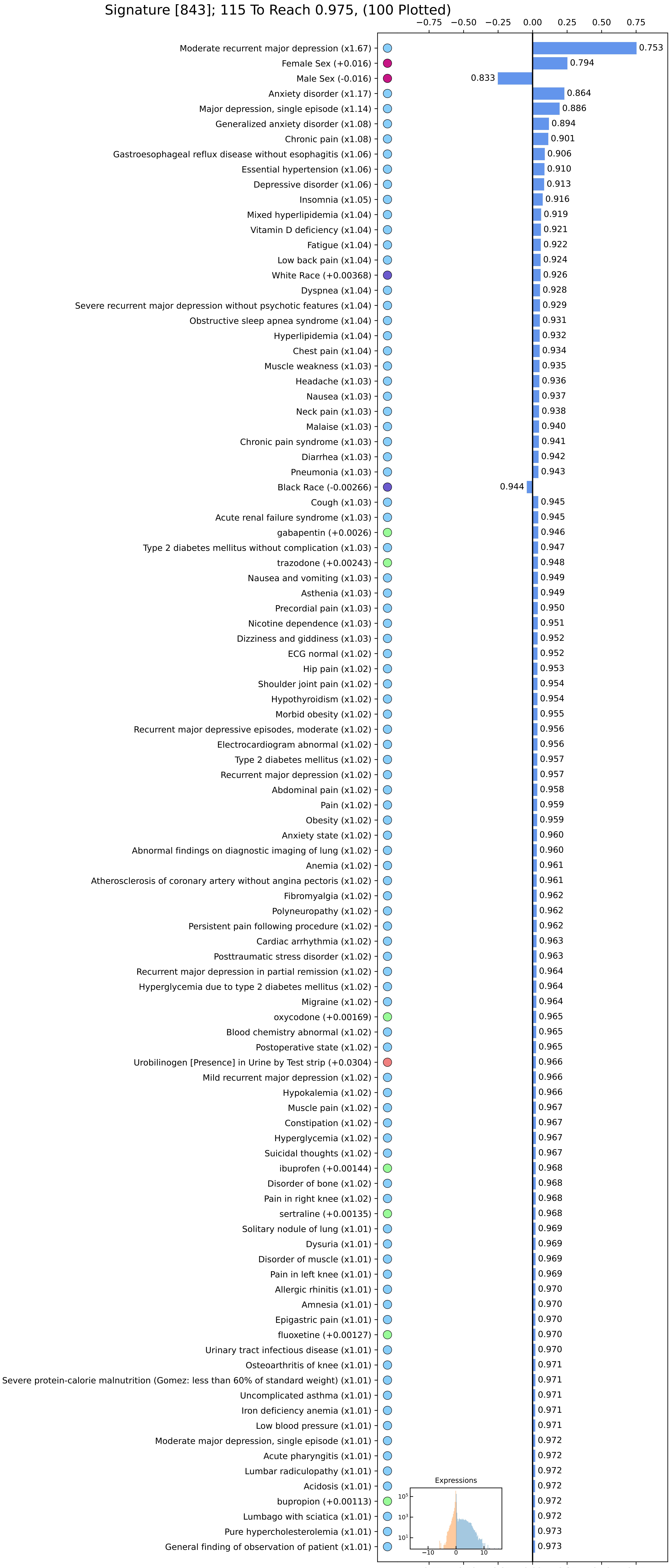
Signature [843]; 115 To Reach 0.975, (100 Plotted)
Moderate recurrent major depression (x1.67) 0.753
Female Sex (+0.016) 0.794
Male Sex (-0.016) 0.833
Anxiety disorder (x1.17) 0.864
Major depression, single episode (x1.14) 0.886
Generalized anxiety disorder (x1.08) 0.894
Chronic pain (x1.08) 0.901
Gastroesophageal reflux disease without esophagitis (x1.06) 0.906
Essential hypertension (x1.06) 0.910
Depressive disorder (x1.06) 0.913
Insomnia (x1.05) 0.916
Mixed hyperlipidemia (x1.04) 0.919
Vitamin D deficiency (x1.04) 0.921
Fatigue (x1.04) 0.922
Low back pain (x1.04) 0.924
White Race (+0.00368) 0.926
Dyspnea (x1.04) 0.928
Severe recurrent major depression without psychotic features (x1.04) 0.929
Obstructive sleep apnea syndrome (x1.04) 0.931
Hyperlipidemia (x1.04) 0.932
Chest pain (x1.04) 0.934
Muscle weakness (x1.03) 0.935
Headache (x1.03) 0.936
Nausea (x1.03) 0.937
Neck pain (x1.03) 0.938
Malaise (x1.03) 0.940
Chronic pain syndrome (x1.03) 0.941
Diarrhea (x1.03) 0.942
Pneumonia (x1.03) 0.943
Black Race (-0.00266) 0.944
Cough (x1.03) 0.945
Acute renal failure syndrome (x1.03) 0.945
gabapentin (+0.0026) 0.946
Type 2 diabetes mellitus without complication (x1.03) 0.947
trazodone (+0.00243) 0.948
Nausea and vomiting (x1.03) 0.949
Asthenia (x1.03) 0.949
Precordial pain (x1.03) 0.950
Nicotine dependence (x1.03) 0.951
Dizziness and giddiness (x1.03) 0.952
ECG normal (x1.02) 0.952
Hip pain (x1.02) 0.953
Shoulder joint pain (x1.02) 0.954
Hypothyroidism (x1.02) 0.954
Morbid obesity (x1.02) 0.955
Recurrent major depressive episodes, moderate (x1.02) 0.956
Electrocardiogram abnormal (x1.02) 0.956
Type 2 diabetes mellitus (x1.02) 0.957
Recurrent major depression (x1.02) 0.957
Abdominal pain (x1.02) 0.958
Pain (x1.02) 0.959
Obesity (x1.02) 0.959
Anxiety state (x1.02) 0.960
Abnormal findings on diagnostic imaging of lung (x1.02) 0.960
Anemia (x1.02) 0.961
Atherosclerosis of coronary artery without angina pectoris (x1.02) 0.961
Fibromyalgia (x1.02) 0.962
Polyneuropathy (x1.02) 0.962
Persistent pain following procedure (x1.02) 0.962
Cardiac arrhythmia (x1.02) 0.963
Posttraumatic stress disorder (x1.02) 0.963
Recurrent major depression in partial remission (x1.02) 0.964
Hyperglycemia due to type 2 diabetes mellitus (x1.02) 0.964
Migraine (x1.02) 0.964
oxycodone (+0.00169) 0.965
Blood chemistry abnormal (x1.02) 0.965
Postoperative state (x1.02) 0.965
Urobilinogen [Presence] in Urine by Test strip (+0.0304) 0.966
Mild recurrent major depression (x1.02) 0.966
Hypokalemia (x1.02) 0.966
Muscle pain (x1.02) 0.967
Constipation (x1.02) 0.967
Hyperglycemia (x1.02) 0.967
Suicidal thoughts (x1.02) 0.967
ibuprofen (+0.00144) 0.968
Disorder of bone (x1.02) 0.968
Pain in right knee (x1.02) 0.968
sertraline (+0.00135) 0.968
Solitary nodule of lung (x1.01) 0.969
Dysuria (x1.01) 0.969
Disorder of muscle (x1.01) 0.969
Pain in left knee (x1.01) 0.969
Allergic rhinitis (x1.01) 0.970
Amnesia (x1.01) 0.970
Epigastric pain (x1.01) 0.970
fluoxetine (+0.00127) 0.970
Urinary tract infectious disease (x1.01) 0.970
Osteoarthritis of knee (x1.01) 0.971
Severe protein-calorie malnutrition (Gomez: less than 60% of standard weight) (x1.01) 0.971
Uncomplicated asthma (x1.01) 0.971
Iron deficiency anemia (x1.01) 0.971
Low blood pressure (x1.01) 0.971
Moderate major depression, single episode (x1.01) 0.972
Acute pharyngitis (x1.01) 0.972
Lumbar radiculopathy (x1.01) 0.972
Acidosis (x1.01) 0.972
bupropion (+0.00113) 0.972
Lumbago with sciatica (x1.01) 0.972
Pure hypercholesterolemia (x1.01) 0.973
General finding of observation of patient (x1.01) 0.973
−0.75 −0.50 −0.25 0.00 0.25 0.50 0.75
Expressions
10^5 10^3 10^1
−10 0 10

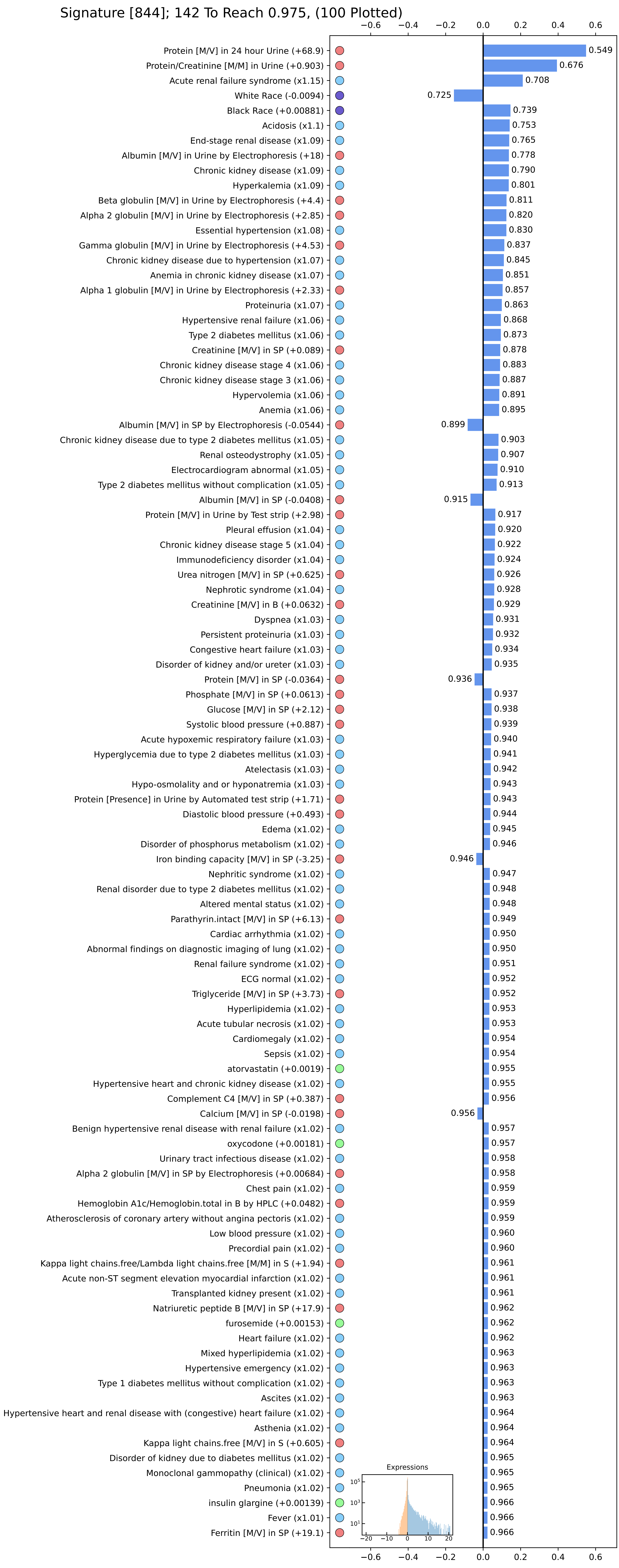

Signature [844]; 142 To Reach 0.975, (100 Plotted)
Protein [M/V] in 24 hour Urine (+68.9)
Protein/Creatinine [M/M] in Urine (+0.903)
Acute renal failure syndrome (x1.15)
White Race (-0.0094)
Black Race (+0.00881)
Acidosis (x1.1)
End-stage renal disease (x1.09)
Albumin [M/V] in Urine by Electrophoresis (+18)
Chronic kidney disease (x1.09)
Hyperkalemia (x1.09)
Beta globulin [M/V] in Urine by Electrophoresis (+4.4)
Alpha 2 globulin [M/V] in Urine by Electrophoresis (+2.85)
Essential hypertension (x1.08)
Gamma globulin [M/V] in Urine by Electrophoresis (+4.53)
Chronic kidney disease due to hypertension (x1.07)
Anemia in chronic kidney disease (x1.07)
Alpha 1 globulin [M/V] in Urine by Electrophoresis (+2.33)
Proteinuria (x1.07)
Hypertensive renal failure (x1.06)
Type 2 diabetes mellitus (x1.06)
Creatinine [M/V] in SP (+0.089)
Chronic kidney disease stage 4 (x1.06)
Chronic kidney disease stage 3 (x1.06)
Hypervolemia (x1.06)
Anemia (x1.06)
Albumin [M/V] in SP by Electrophoresis (-0.0544)
Chronic kidney disease due to type 2 diabetes mellitus (x1.05)
Renal osteodystrophy (x1.05)
Electrocardiogram abnormal (x1.05)
Type 2 diabetes mellitus without complication (x1.05)
Albumin [M/V] in SP (-0.0408)
Protein [M/V] in Urine by Test strip (+2.98)
Pleural effusion (x1.04)
Chronic kidney disease stage 5 (x1.04)
Immunodeficiency disorder (x1.04)
Urea nitrogen [M/V] in SP (+0.625)
Nephrotic syndrome (x1.04)
Creatinine [M/V] in B (+0.0632)
Dyspnea (x1.03)
Persistent proteinuria (x1.03)
Congestive heart failure (x1.03)
Disorder of kidney and/or ureter (x1.03)
Protein [M/V] in SP (-0.0364)
Phosphate [M/V] in SP (+0.0613)
Glucose [M/V] in SP (+2.12)
Systolic blood pressure (+0.887)
Acute hypoxemic respiratory failure (x1.03)
Hyperglycemia due to type 2 diabetes mellitus (x1.03)
Atelectasis (x1.03)
Hypo-osmolality and or hyponatremia (x1.03)
Protein [Presence] in Urine by Automated test strip (+1.71)
Diastolic blood pressure (+0.493)
Edema (x1.02)
Disorder of phosphorus metabolism (x1.02)
Iron binding capacity [M/V] in SP (-3.25)
Nephritic syndrome (x1.02)
Renal disorder due to type 2 diabetes mellitus (x1.02)
Altered mental status (x1.02)
Parathyrin.intact [M/V] in SP (+6.13)
Cardiac arrhythmia (x1.02)
Abnormal findings on diagnostic imaging of lung (x1.02)
Renal failure syndrome (x1.02)
ECG normal (x1.02)
Triglyceride [M/V] in SP (+3.73)
Hyperlipidemia (x1.02)
Acute tubular necrosis (x1.02)
Cardiomegaly (x1.02)
Sepsis (x1.02)
atorvastatin (+0.0019)
Hypertensive heart and chronic kidney disease (x1.02)
Complement C4 [M/V] in SP (+0.387)
Calcium [M/V] in SP (-0.0198)
Benign hypertensive renal disease with renal failure (x1.02)
oxycodone (+0.00181)
Urinary tract infectious disease (x1.02)
Alpha 2 globulin [M/V] in SP by Electrophoresis (+0.00684)
Chest pain (x1.02)
Hemoglobin A1c/Hemoglobin.total in B by HPLC (+0.0482)
Atherosclerosis of coronary artery without angina pectoris (x1.02)
Low blood pressure (x1.02)
Precordial pain (x1.02)
Kappa light chains.free/Lambda light chains.free [M/M] in S (+1.94)
Acute non-ST segment elevation myocardial infarction (x1.02)
Transplanted kidney present (x1.02)
Natriuretic peptide B [M/V] in SP (+17.9)
furosemide (+0.00153)
Heart failure (x1.02)
Mixed hyperlipidemia (x1.02)
Hypertensive emergency (x1.02)
Type 1 diabetes mellitus without complication (x1.02)
Ascites (x1.02)
Hypertensive heart and renal disease with (congestive) heart failure (x1.02)
Asthenia (x1.02)
Kappa light chains.free [M/V] in S (+0.605)
Disorder of kidney due to diabetes mellitus (x1.02)
Monoclonal gammopathy (clinical) (x1.02)
Pneumonia (x1.02)
insulin glargine (+0.00139)
Fever (x1.01)
Ferritin [M/V] in SP (+19.1)
0.549
0.676
0.708
0.725
0.739
0.753
0.765
0.778
0.790
0.801
0.811
0.820
0.830
0.837
0.845
0.851
0.857
0.863
0.868
0.873
0.878
0.883
0.887
0.891
0.895
0.899
0.903
0.907
0.910
0.913
0.915
0.917
0.920
0.922
0.924
0.926
0.928
0.929
0.931
0.932
0.934
0.935
0.936
0.937
0.938
0.939
0.940
0.941
0.942
0.943
0.943
0.944
0.945
0.946
0.946
0.947
0.948
0.948
0.949
0.950
0.950
0.951
0.952
0.952
0.953
0.953
0.954
0.954
0.955
0.955
0.956
0.956
0.957
0.957
0.958
0.958
0.959
0.959
0.959
0.960
0.960
0.961
0.961
0.961
0.962
0.962
0.962
0.963
0.963
0.963
0.963
0.964
0.964
0.964
0.965
0.965
0.965
0.966
0.966
0.966
−0.6 −0.4 −0.2 0.0 0.2 0.4 0.6
Expressions

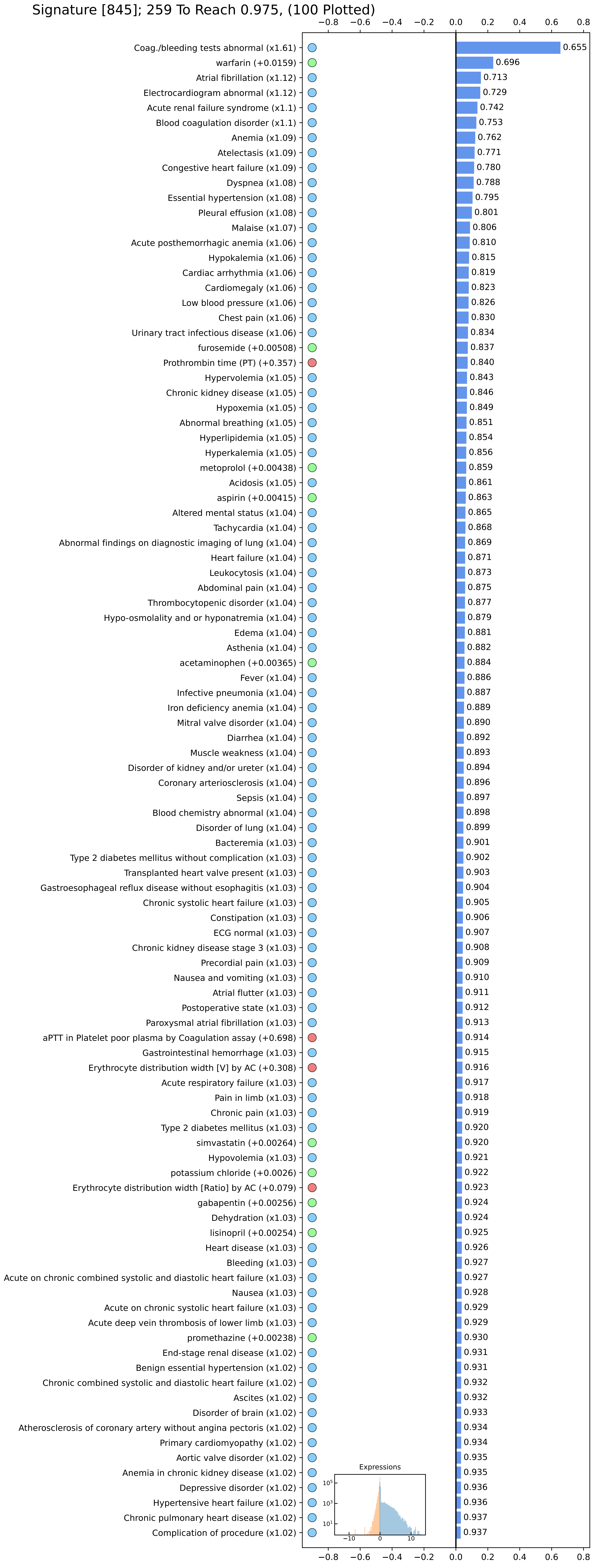

Signature [845]; 259 To Reach 0.975, (100 Plotted)
Coag./bleeding tests abnormal (x1.61) 0.655
warfarin (+0.0159) 0.696
Atrial fibrillation (x1.12) 0.713
Electrocardiogram abnormal (x1.12) 0.729
Acute renal failure syndrome (x1.1) 0.742
Blood coagulation disorder (x1.1) 0.753
Anemia (x1.09) 0.762
Atelectasis (x1.09) 0.771
Congestive heart failure (x1.09) 0.780
Dyspnea (x1.08) 0.788
Essential hypertension (x1.08) 0.795
Pleural effusion (x1.08) 0.801
Malaise (x1.07) 0.806
Acute posthemorrhagic anemia (x1.06) 0.810
Hypokalemia (x1.06) 0.815
Cardiac arrhythmia (x1.06) 0.819
Cardiomegaly (x1.06) 0.823
Low blood pressure (x1.06) 0.826
Chest pain (x1.06) 0.830
Urinary tract infectious disease (x1.06) 0.834
furosemide (+0.00508) 0.837
Prothrombin time (PT) (+0.357) 0.840
Hypervolemia (x1.05) 0.843
Chronic kidney disease (x1.05) 0.846
Hypoxemia (x1.05) 0.849
Abnormal breathing (x1.05) 0.851
Hyperlipidemia (x1.05) 0.854
Hyperkalemia (x1.05) 0.856
metoprolol (+0.00438) 0.859
Acidosis (x1.05) 0.861
aspirin (+0.00415) 0.863
Altered mental status (x1.04) 0.865
Tachycardia (x1.04) 0.868
Abnormal findings on diagnostic imaging of lung (x1.04) 0.869
Heart failure (x1.04) 0.871
Leukocytosis (x1.04) 0.873
Abdominal pain (x1.04) 0.875
Thrombocytopenic disorder (x1.04) 0.877
Hypo-osmolality and or hyponatremia (x1.04) 0.879
Edema (x1.04) 0.881
Asthenia (x1.04) 0.882
acetaminophen (+0.00365) 0.884
Fever (x1.04) 0.886
Infective pneumonia (x1.04) 0.887
Iron deficiency anemia (x1.04) 0.889
Mitral valve disorder (x1.04) 0.890
Diarrhea (x1.04) 0.892
Muscle weakness (x1.04) 0.893
Disorder of kidney and/or ureter (x1.04) 0.894
Coronary arteriosclerosis (x1.04) 0.896
Sepsis (x1.04) 0.897
Blood chemistry abnormal (x1.04) 0.898
Disorder of lung (x1.04) 0.899
Bacteremia (x1.03) 0.901
Type 2 diabetes mellitus without complication (x1.03) 0.902
Transplanted heart valve present (x1.03) 0.903
Gastroesophageal reflux disease without esophagitis (x1.03) 0.904
Chronic systolic heart failure (x1.03) 0.905
Constipation (x1.03) 0.906
ECG normal (x1.03) 0.907
Chronic kidney disease stage 3 (x1.03) 0.908
Precordial pain (x1.03) 0.909
Nausea and vomiting (x1.03) 0.910
Atrial flutter (x1.03) 0.911
Postoperative state (x1.03) 0.912
Paroxysmal atrial fibrillation (x1.03) 0.913
aPTT in Platelet poor plasma by Coagulation assay (+0.698) 0.914
Gastrointestinal hemorrhage (x1.03) 0.915
Erythrocyte distribution width [V] by AC (+0.308) 0.916
Acute respiratory failure (x1.03) 0.917
Pain in limb (x1.03) 0.918
Chronic pain (x1.03) 0.919
Type 2 diabetes mellitus (x1.03) 0.920
simvastatin (+0.00264) 0.920
Hypovolemia (x1.03) 0.921
potassium chloride (+0.0026) 0.922
Erythrocyte distribution width [Ratio] by AC (+0.079) 0.923
gabapentin (+0.00256) 0.924
Dehydration (x1.03) 0.924
lisinopril (+0.00254) 0.925
Heart disease (x1.03) 0.926
Bleeding (x1.03) 0.927
Acute on chronic combined systolic and diastolic heart failure (x1.03) 0.927
Nausea (x1.03) 0.928
Acute on chronic systolic heart failure (x1.03) 0.929
Acute deep vein thrombosis of lower limb (x1.03) 0.929
promethazine (+0.00238) 0.930
End-stage renal disease (x1.02) 0.931
Benign essential hypertension (x1.02) 0.931
Chronic combined systolic and diastolic heart failure (x1.02) 0.932
Ascites (x1.02) 0.932
Disorder of brain (x1.02) 0.933
Atherosclerosis of coronary artery without angina pectoris (x1.02) 0.934
Primary cardiomyopathy (x1.02) 0.934
Aortic valve disorder (x1.02) 0.935
Anemia in chronic kidney disease (x1.02) 0.935
Depressive disorder (x1.02) 0.936
Hypertensive heart failure (x1.02) 0.936
Chronic pulmonary heart disease (x1.02) 0.937
Complication of procedure (x1.02) 0.937
−0.8 −0.6 −0.4 −0.2 0.0 0.2 0.4 0.6 0.8
Expressions

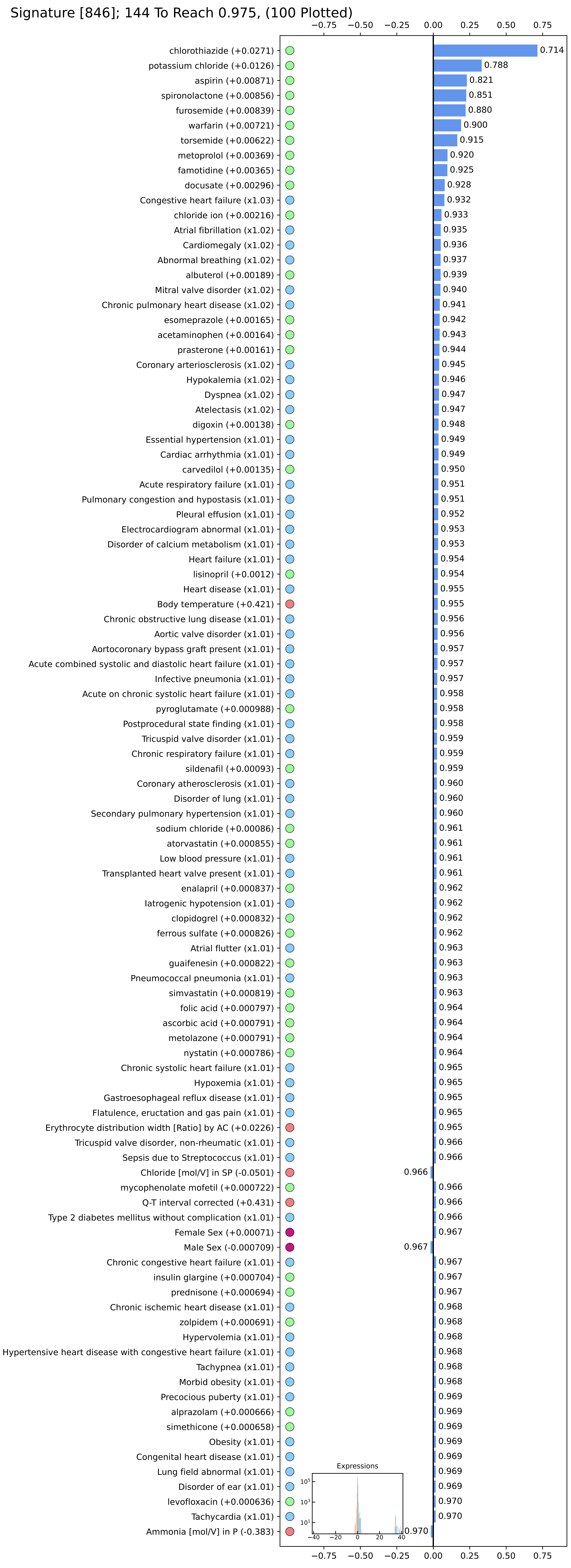

Signature [846]; 144 To Reach 0.975, (100 Plotted)
−0.75 −0.50 −0.25 0.00 0.25 0.50 0.75
chlorothiazide (+0.0271) 0.714
potassium chloride (+0.0126) 0.788
aspirin (+0.00871) 0.821
spironolactone (+0.00856) 0.851
furosemide (+0.00839) 0.880
warfarin (+0.00721) 0.900
torsemide (+0.00622) 0.915
metoprolol (+0.00369) 0.920
famotidine (+0.00365) 0.925
docusate (+0.00296) 0.928
Congestive heart failure (x1.03) 0.932
chloride ion (+0.00216) 0.933
Atrial fibrillation (x1.02) 0.935
Cardiomegaly (x1.02) 0.936
Abnormal breathing (x1.02) 0.937
albuterol (+0.00189) 0.939
Mitral valve disorder (x1.02) 0.940
Chronic pulmonary heart disease (x1.02) 0.941
esomeprazole (+0.00165) 0.942
acetaminophen (+0.00164) 0.943
prasterone (+0.00161) 0.944
Coronary arteriosclerosis (x1.02) 0.945
Hypokalemia (x1.02) 0.946
Dyspnea (x1.02) 0.947
Atelectasis (x1.02) 0.947
digoxin (+0.00138) 0.948
Essential hypertension (x1.01) 0.949
Cardiac arrhythmia (x1.01) 0.949
carvedilol (+0.00135) 0.950
Acute respiratory failure (x1.01) 0.951
Pulmonary congestion and hypostasis (x1.01) 0.951
Pleural effusion (x1.01) 0.952
Electrocardiogram abnormal (x1.01) 0.953
Disorder of calcium metabolism (x1.01) 0.953
Heart failure (x1.01) 0.954
lisinopril (+0.0012) 0.954
Heart disease (x1.01) 0.955
Body temperature (+0.421) 0.955
Chronic obstructive lung disease (x1.01) 0.956
Aortic valve disorder (x1.01) 0.956
Aortocoronary bypass graft present (x1.01) 0.957
Acute combined systolic and diastolic heart failure (x1.01) 0.957
Infective pneumonia (x1.01) 0.957
Acute on chronic systolic heart failure (x1.01) 0.958
pyroglutamate (+0.000988) 0.958
Postprocedural state finding (x1.01) 0.958
Tricuspid valve disorder (x1.01) 0.959
Chronic respiratory failure (x1.01) 0.959
sildenafil (+0.00093) 0.959
Coronary atherosclerosis (x1.01) 0.960
Disorder of lung (x1.01) 0.960
Secondary pulmonary hypertension (x1.01) 0.960
sodium chloride (+0.00086) 0.961
atorvastatin (+0.000855) 0.961
Low blood pressure (x1.01) 0.961
Transplanted heart valve present (x1.01) 0.961
enalapril (+0.000837) 0.962
Iatrogenic hypotension (x1.01) 0.962
clopidogrel (+0.000832) 0.962
ferrous sulfate (+0.000826) 0.962
Atrial flutter (x1.01) 0.963
guaifenesin (+0.000822) 0.963
Pneumococcal pneumonia (x1.01) 0.963
simvastatin (+0.000819) 0.963
folic acid (+0.000797) 0.964
ascorbic acid (+0.000791) 0.964
metolazone (+0.000791) 0.964
nystatin (+0.000786) 0.964
Chronic systolic heart failure (x1.01) 0.965
Hypoxemia (x1.01) 0.965
Gastroesophageal reflux disease (x1.01) 0.965
Flatulence, eructation and gas pain (x1.01) 0.965
Erythrocyte distribution width [Ratio] by AC (+0.0226) 0.965
Tricuspid valve disorder, non-rheumatic (x1.01) 0.966
Sepsis due to Streptococcus (x1.01) 0.966
Chloride [mol/V] in SP (-0.0501) 0.966
mycophenolate mofetil (+0.000722) 0.966
Q-T interval corrected (+0.431) 0.966
Type 2 diabetes mellitus without complication (x1.01) 0.966
Female Sex (+0.00071) 0.967
Male Sex (-0.000709) 0.967
Chronic congestive heart failure (x1.01) 0.967
insulin glargine (+0.000704) 0.967
prednisone (+0.000694) 0.967
Chronic ischemic heart disease (x1.01) 0.968
zolpidem (+0.000691) 0.968
Hypervolemia (x1.01) 0.968
Hypertensive heart disease with congestive heart failure (x1.01) 0.968
Tachypnea (x1.01) 0.968
Morbid obesity (x1.01) 0.968
Precocious puberty (x1.01) 0.969
alprazolam (+0.000666) 0.969
simethicone (+0.000658) 0.969
Obesity (x1.01) 0.969
Congenital heart disease (x1.01) 0.969
Lung field abnormal (x1.01) 0.969
Disorder of ear (x1.01) 0.969
levofloxacin (+0.000636) 0.970
Tachycardia (x1.01) 0.970
Ammonia [mol/V] in P (-0.383) 0.970
Expressions
−40 −20 0 20 40
−0.75 −0.50 −0.25 0.00 0.25 0.50 0.75

Signature [847]; 1 To Reach 0.975, (10 Plotted)

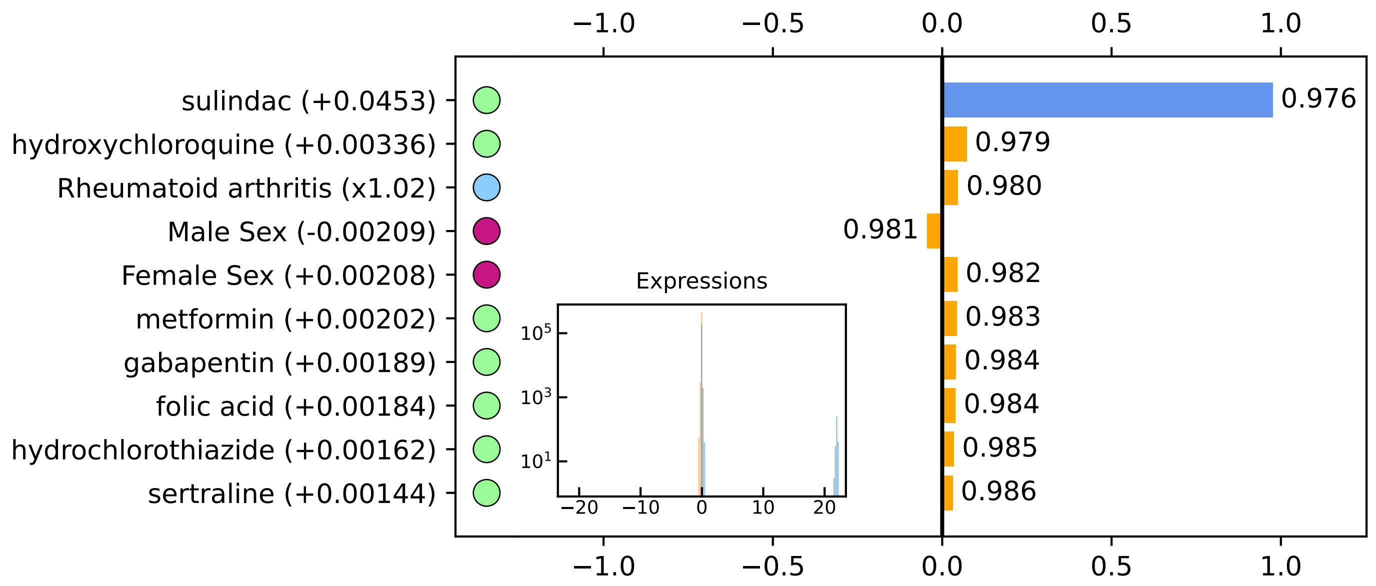

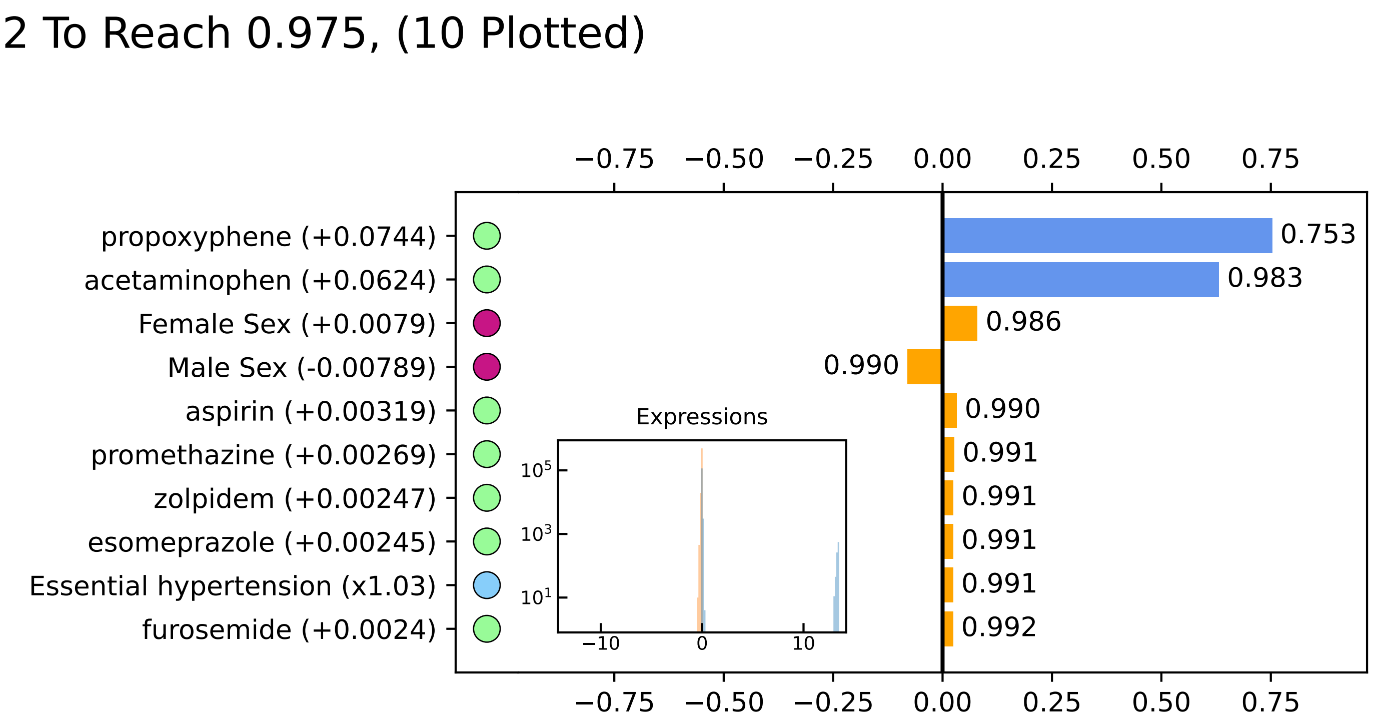
Signature [848]; 2 To Reach 0.975, (10 Plotted)
propoxyphene (+0.0744)
acetaminophen (+0.0624)
Female Sex (+0.0079)
Male Sex (-0.00789)
aspirin (+0.00319)
promethazine (+0.00269)
zolpidem (+0.00247)
esomeprazole (+0.00245)
Essential hypertension (x1.03)
furosemide (+0.0024)
0.753
0.983
0.986
0.990
0.990
0.991
0.991
0.991
0.991
0.992
−0.75
−0.50
−0.25
0.00
0.25
0.50
0.75
Expressions
10^5
10^3
10^1
−10
0
10

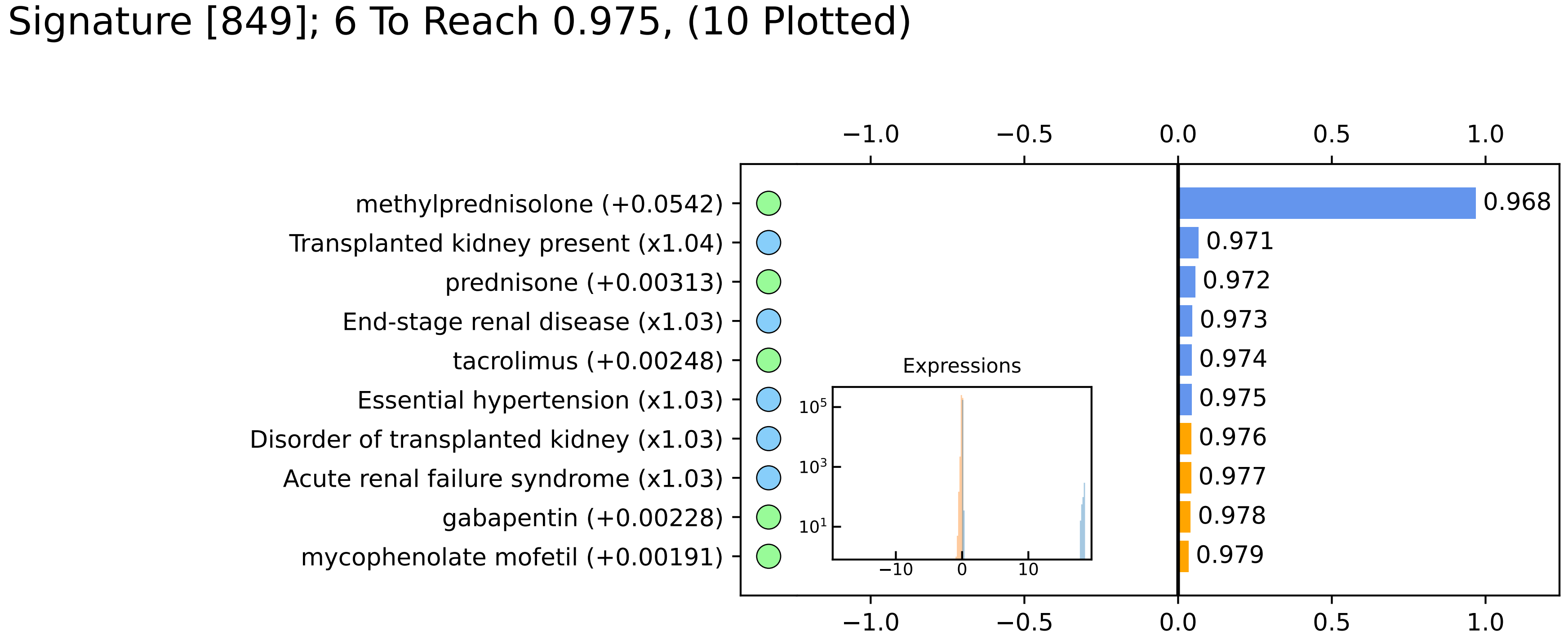
Signature [849]; 6 To Reach 0.975, (10 Plotted)
methylprednisolone (+0.0542)
Transplanted kidney present (x1.04)
prednisone (+0.00313)
End-stage renal disease (x1.03)
tacrolimus (+0.00248)
Essential hypertension (x1.03)
Disorder of transplanted kidney (x1.03)
Acute renal failure syndrome (x1.03)
gabapentin (+0.00228)
mycophenolate mofetil (+0.00191)
0.968
0.971
0.972
0.973
0.974
0.975
0.976
0.977
0.978
0.979
Expressions
−1.0
−0.5
0.0
0.5
1.0

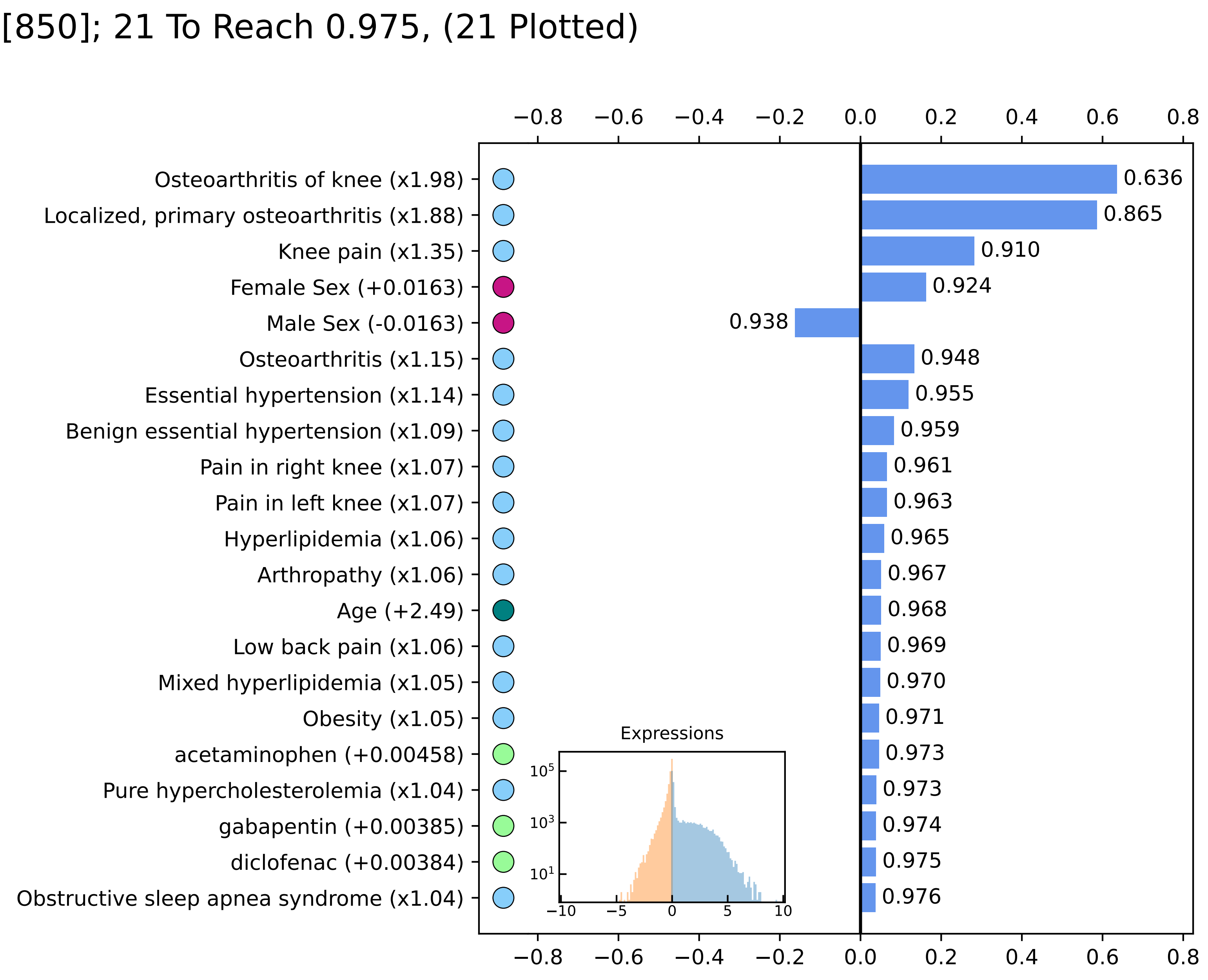
Signature [850]; 21 To Reach 0.975, (21 Plotted)
Osteoarthritis of knee (x1.98)
Localized, primary osteoarthritis (x1.88)
Knee pain (x1.35)
Female Sex (+0.0163)
Male Sex (-0.0163)
Osteoarthritis (x1.15)
Essential hypertension (x1.14)
Benign essential hypertension (x1.09)
Pain in right knee (x1.07)
Pain in left knee (x1.07)
Hyperlipidemia (x1.06)
Arthropathy (x1.06)
Age (+2.49)
Low back pain (x1.06)
Mixed hyperlipidemia (x1.05)
Obesity (x1.05)
acetaminophen (+0.00458)
Pure hypercholesterolemia (x1.04)
gabapentin (+0.00385)
diclofenac (+0.00384)
Obstructive sleep apnea syndrome (x1.04)
0.636
0.865
0.910
0.924
0.938
0.948
0.955
0.959
0.961
0.963
0.965
0.967
0.968
0.969
0.970
0.971
0.973
0.973
0.974
0.975
0.976
−0.8
−0.6
−0.4
−0.2
0.0
0.2
0.4
0.6
0.8
Expressions
−10
−5
0
5
10

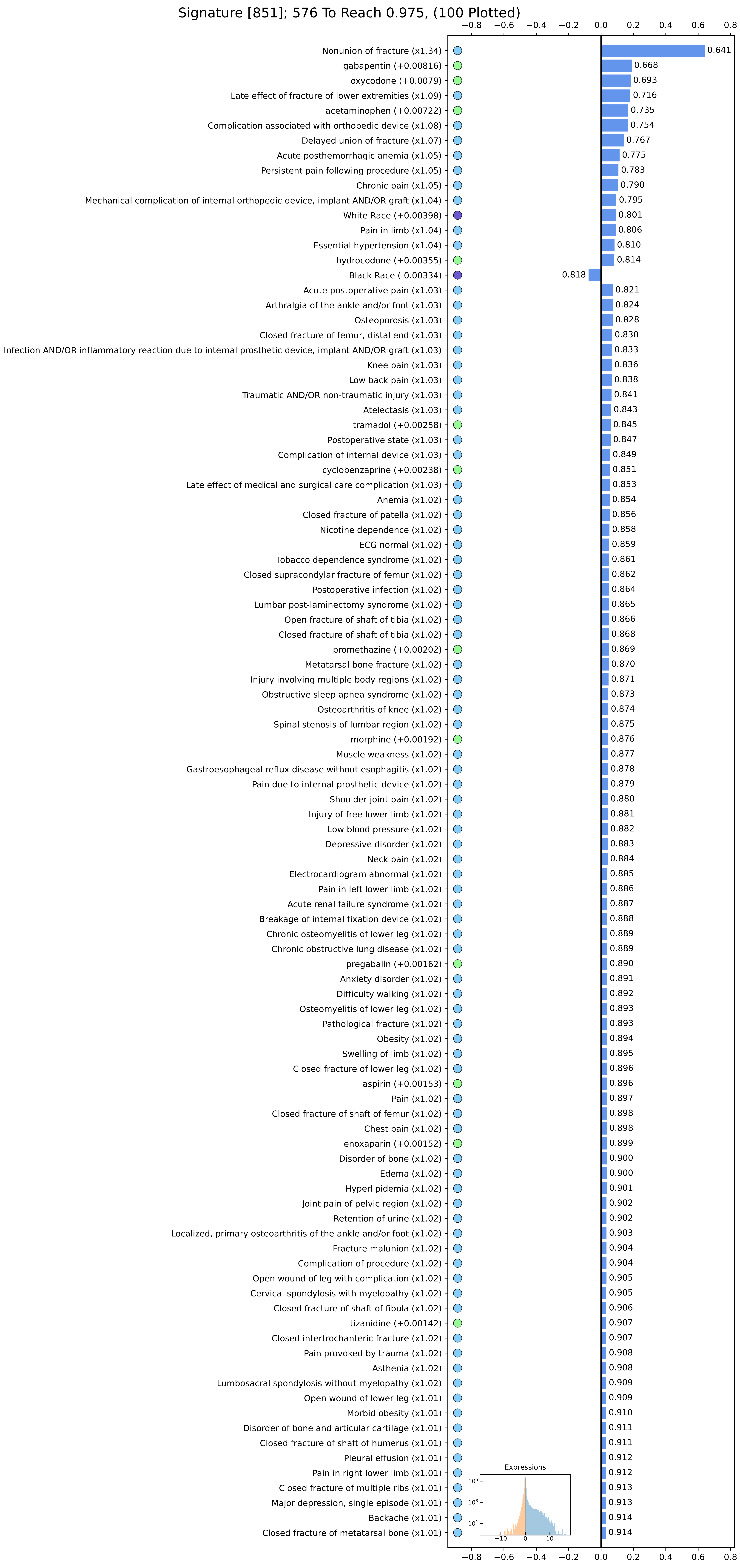

Signature [851]; 576 To Reach 0.975, (100 Plotted)
Nonunion of fracture (x1.34) 0.641
gabapentin (+0.00816) 0.668
oxycodone (+0.0079) 0.693
Late effect of fracture of lower extremities (x1.09) 0.716
acetaminophen (+0.00722) 0.735
Complication associated with orthopedic device (x1.08) 0.754
Delayed union of fracture (x1.07) 0.767
Acute posthemorrhagic anemia (x1.05) 0.775
Persistent pain following procedure (x1.05) 0.783
Chronic pain (x1.05) 0.790
Mechanical complication of internal orthopedic device, implant AND/OR graft (x1.04) 0.795
White Race (+0.00398) 0.801
Pain in limb (x1.04) 0.806
Essential hypertension (x1.04) 0.810
hydrocodone (+0.00355) 0.814
Black Race (-0.00334) 0.818
Acute postoperative pain (x1.03) 0.821
Arthralgia of the ankle and/or foot (x1.03) 0.824
Osteoporosis (x1.03) 0.828
Closed fracture of femur, distal end (x1.03) 0.830
Infection AND/OR inflammatory reaction due to internal prosthetic device, implant AND/OR graft (x1.03) 0.833
Knee pain (x1.03) 0.836
Low back pain (x1.03) 0.838
Traumatic AND/OR non-traumatic injury (x1.03) 0.841
Atelectasis (x1.03) 0.843
tramadol (+0.00258) 0.845
Postoperative state (x1.03) 0.847
Complication of internal device (x1.03) 0.849
cyclobenzaprine (+0.00238) 0.851
Late effect of medical and surgical care complication (x1.03) 0.853
Anemia (x1.02) 0.854
Closed fracture of patella (x1.02) 0.856
Nicotine dependence (x1.02) 0.858
ECG normal (x1.02) 0.859
Tobacco dependence syndrome (x1.02) 0.861
Closed supracondylar fracture of femur (x1.02) 0.862
Postoperative infection (x1.02) 0.864
Lumbar post-laminectomy syndrome (x1.02) 0.865
Open fracture of shaft of tibia (x1.02) 0.866
Closed fracture of shaft of tibia (x1.02) 0.868
promethazine (+0.00202) 0.869
Metatarsal bone fracture (x1.02) 0.870
Injury involving multiple body regions (x1.02) 0.871
Obstructive sleep apnea syndrome (x1.02) 0.873
Osteoarthritis of knee (x1.02) 0.874
Spinal stenosis of lumbar region (x1.02) 0.875
morphine (+0.00192) 0.876
Muscle weakness (x1.02) 0.877
Gastroesophageal reflux disease without esophagitis (x1.02) 0.878
Pain due to internal prosthetic device (x1.02) 0.879
Shoulder joint pain (x1.02) 0.880
Injury of free lower limb (x1.02) 0.881
Low blood pressure (x1.02) 0.882
Depressive disorder (x1.02) 0.883
Neck pain (x1.02) 0.884
Electrocardiogram abnormal (x1.02) 0.885
Pain in left lower limb (x1.02) 0.886
Acute renal failure syndrome (x1.02) 0.887
Breakage of internal fixation device (x1.02) 0.888
Chronic osteomyelitis of lower leg (x1.02) 0.889
Chronic obstructive lung disease (x1.02) 0.889
pregabalin (+0.00162) 0.890
Anxiety disorder (x1.02) 0.891
Difficulty walking (x1.02) 0.892
Osteomyelitis of lower leg (x1.02) 0.893
Pathological fracture (x1.02) 0.893
Obesity (x1.02) 0.894
Swelling of limb (x1.02) 0.895
Closed fracture of lower leg (x1.02) 0.896
aspirin (+0.00153) 0.896
Pain (x1.02) 0.897
Closed fracture of shaft of femur (x1.02) 0.898
Chest pain (x1.02) 0.898
enoxaparin (+0.00152) 0.899
Disorder of bone (x1.02) 0.900
Edema (x1.02) 0.900
Hyperlipidemia (x1.02) 0.901
Joint pain of pelvic region (x1.02) 0.902
Retention of urine (x1.02) 0.902
Localized, primary osteoarthritis of the ankle and/or foot (x1.02) 0.903
Fracture malunion (x1.02) 0.904
Complication of procedure (x1.02) 0.904
Open wound of leg with complication (x1.02) 0.905
Cervical spondylosis with myelopathy (x1.02) 0.905
Closed fracture of shaft of fibula (x1.02) 0.906
tizanidine (+0.00142) 0.907
Closed intertrochanteric fracture (x1.02) 0.907
Pain provoked by trauma (x1.02) 0.908
Asthenia (x1.02) 0.908
Lumbosacral spondylosis without myelopathy (x1.02) 0.909
Open wound of lower leg (x1.01) 0.909
Morbid obesity (x1.01) 0.910
Disorder of bone and articular cartilage (x1.01) 0.911
Closed fracture of shaft of humerus (x1.01) 0.911
Pleural effusion (x1.01) 0.912
Pain in right lower limb (x1.01) 0.912
Closed fracture of multiple ribs (x1.01) 0.913
Major depression, single episode (x1.01) 0.913
Backache (x1.01) 0.914
Closed fracture of metatarsal bone (x1.01) 0.914
−0.8 −0.6 −0.4 −0.2 0.0 0.2 0.4 0.6 0.8
Expressions
10^5
10^3
10^1
−10 0 10

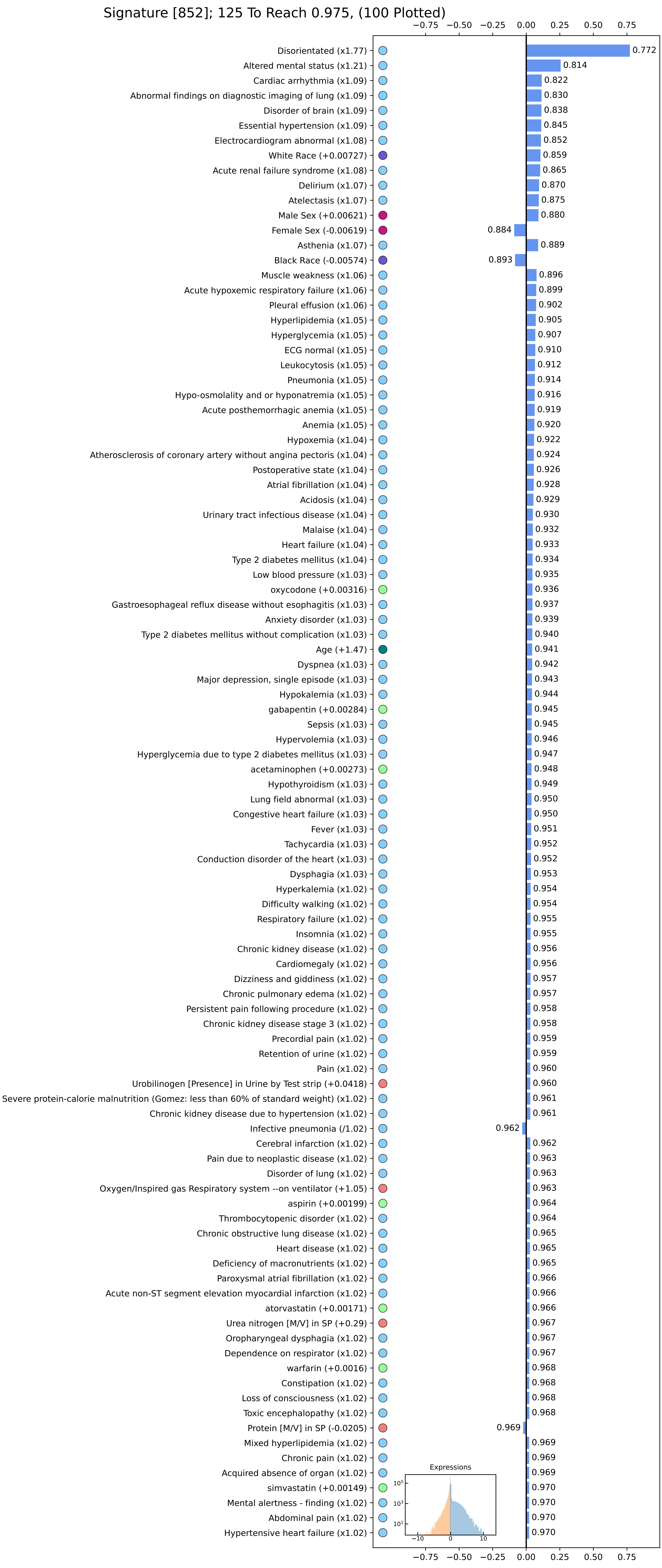

Signature [852]; 125 To Reach 0.975, (100 Plotted)
Disorientated (x1.77) 0.772
Altered mental status (x1.21) 0.814
Cardiac arrhythmia (x1.09) 0.822
Abnormal findings on diagnostic imaging of lung (x1.09) 0.830
Disorder of brain (x1.09) 0.838
Essential hypertension (x1.09) 0.845
Electrocardiogram abnormal (x1.08) 0.852
White Race (+0.00727) 0.859
Acute renal failure syndrome (x1.08) 0.865
Delirium (x1.07) 0.870
Atelectasis (x1.07) 0.875
Male Sex (+0.00621) 0.880
Female Sex (-0.00619) 0.884
Asthenia (x1.07) 0.889
Black Race (-0.00574) 0.893
Muscle weakness (x1.06) 0.896
Acute hypoxemic respiratory failure (x1.06) 0.899
Pleural effusion (x1.06) 0.902
Hyperlipidemia (x1.05) 0.905
Hyperglycemia (x1.05) 0.907
ECG normal (x1.05) 0.910
Leukocytosis (x1.05) 0.912
Pneumonia (x1.05) 0.914
Hypo-osmolality and or hyponatremia (x1.05) 0.916
Acute posthemorrhagic anemia (x1.05) 0.919
Anemia (x1.05) 0.920
Hypoxemia (x1.04) 0.922
Atherosclerosis of coronary artery without angina pectoris (x1.04) 0.924
Postoperative state (x1.04) 0.926
Atrial fibrillation (x1.04) 0.928
Acidosis (x1.04) 0.929
Urinary tract infectious disease (x1.04) 0.930
Malaise (x1.04) 0.932
Heart failure (x1.04) 0.933
Type 2 diabetes mellitus (x1.04) 0.934
Low blood pressure (x1.03) 0.935
oxycodone (+0.00316) 0.936
Gastroesophageal reflux disease without esophagitis (x1.03) 0.937
Anxiety disorder (x1.03) 0.939
Type 2 diabetes mellitus without complication (x1.03) 0.940
Age (+1.47) 0.941
Dyspnea (x1.03) 0.942
Major depression, single episode (x1.03) 0.943
Hypokalemia (x1.03) 0.944
gabapentin (+0.00284) 0.945
Sepsis (x1.03) 0.945
Hypervolemia (x1.03) 0.946
Hyperglycemia due to type 2 diabetes mellitus (x1.03) 0.947
acetaminophen (+0.00273) 0.948
Hypothyroidism (x1.03) 0.949
Lung field abnormal (x1.03) 0.950
Congestive heart failure (x1.03) 0.950
Fever (x1.03) 0.951
Tachycardia (x1.03) 0.952
Conduction disorder of the heart (x1.03) 0.952
Dysphagia (x1.03) 0.953
Hyperkalemia (x1.02) 0.954
Difficulty walking (x1.02) 0.954
Respiratory failure (x1.02) 0.955
Insomnia (x1.02) 0.955
Chronic kidney disease (x1.02) 0.956
Cardiomegaly (x1.02) 0.956
Dizziness and giddiness (x1.02) 0.957
Chronic pulmonary edema (x1.02) 0.957
Persistent pain following procedure (x1.02) 0.958
Chronic kidney disease stage 3 (x1.02) 0.958
Precordial pain (x1.02) 0.959
Retention of urine (x1.02) 0.959
Pain (x1.02) 0.960
Urobilinogen [Presence] in Urine by Test strip (+0.0418) 0.960
Severe protein-calorie malnutrition (Gomez: less than 60% of standard weight) (x1.02) 0.961
Chronic kidney disease due to hypertension (x1.02) 0.961
Infective pneumonia (/1.02) 0.962
Cerebral infarction (x1.02) 0.962
Pain due to neoplastic disease (x1.02) 0.963
Disorder of lung (x1.02) 0.963
Oxygen/Inspired gas Respiratory system --on ventilator (+1.05) 0.963
aspirin (+0.00199) 0.964
Thrombocytopenic disorder (x1.02) 0.964
Chronic obstructive lung disease (x1.02) 0.965
Heart disease (x1.02) 0.965
Deficiency of macronutrients (x1.02) 0.965
Paroxysmal atrial fibrillation (x1.02) 0.966
Acute non-ST segment elevation myocardial infarction (x1.02) 0.966
atorvastatin (+0.00171) 0.966
Urea nitrogen [M/V] in SP (+0.29) 0.967
Oropharyngeal dysphagia (x1.02) 0.967
Dependence on respirator (x1.02) 0.967
warfarin (+0.0016) 0.968
Constipation (x1.02) 0.968
Loss of consciousness (x1.02) 0.968
Toxic encephalopathy (x1.02) 0.968
Protein [M/V] in SP (-0.0205) 0.969
Mixed hyperlipidemia (x1.02) 0.969
Chronic pain (x1.02) 0.969
Acquired absence of organ (x1.02) 0.969
simvastatin (+0.00149) 0.970
Mental alertness - finding (x1.02) 0.970
Abdominal pain (x1.02) 0.970
Hypertensive heart failure (x1.02) 0.970
−0.75 −0.50 −0.25 0.00 0.25 0.50 0.75
Expressions

Signature [853]; 1 To Reach 0.975, (10 Plotted)

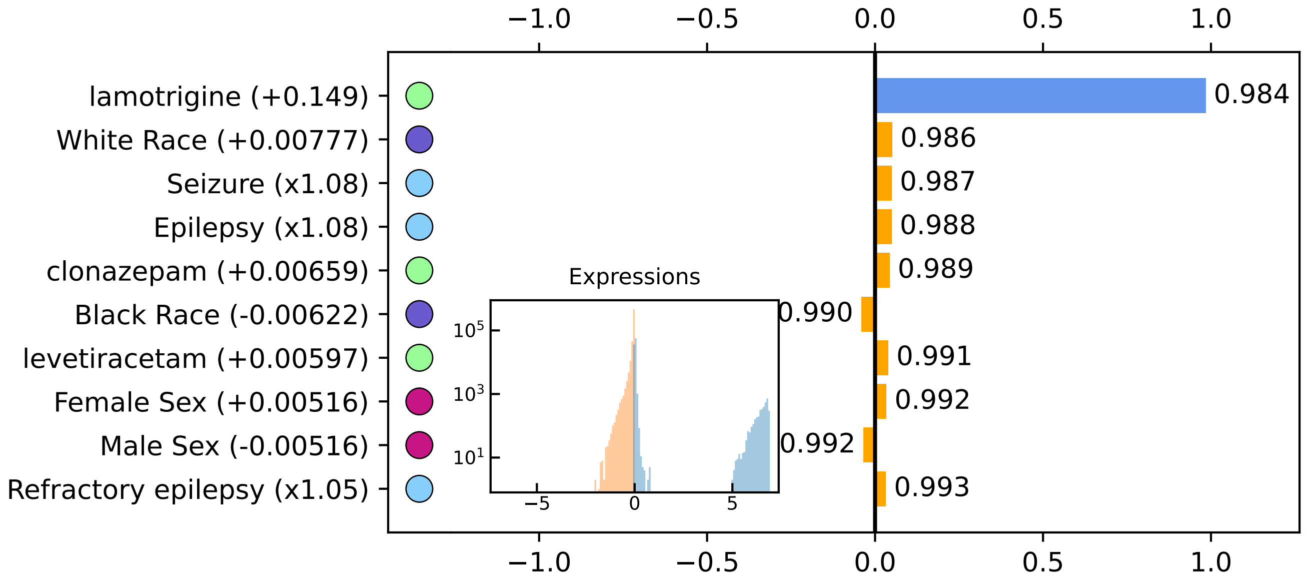

Signature [854]; 129 To Reach 0.975, (100 Plotted)

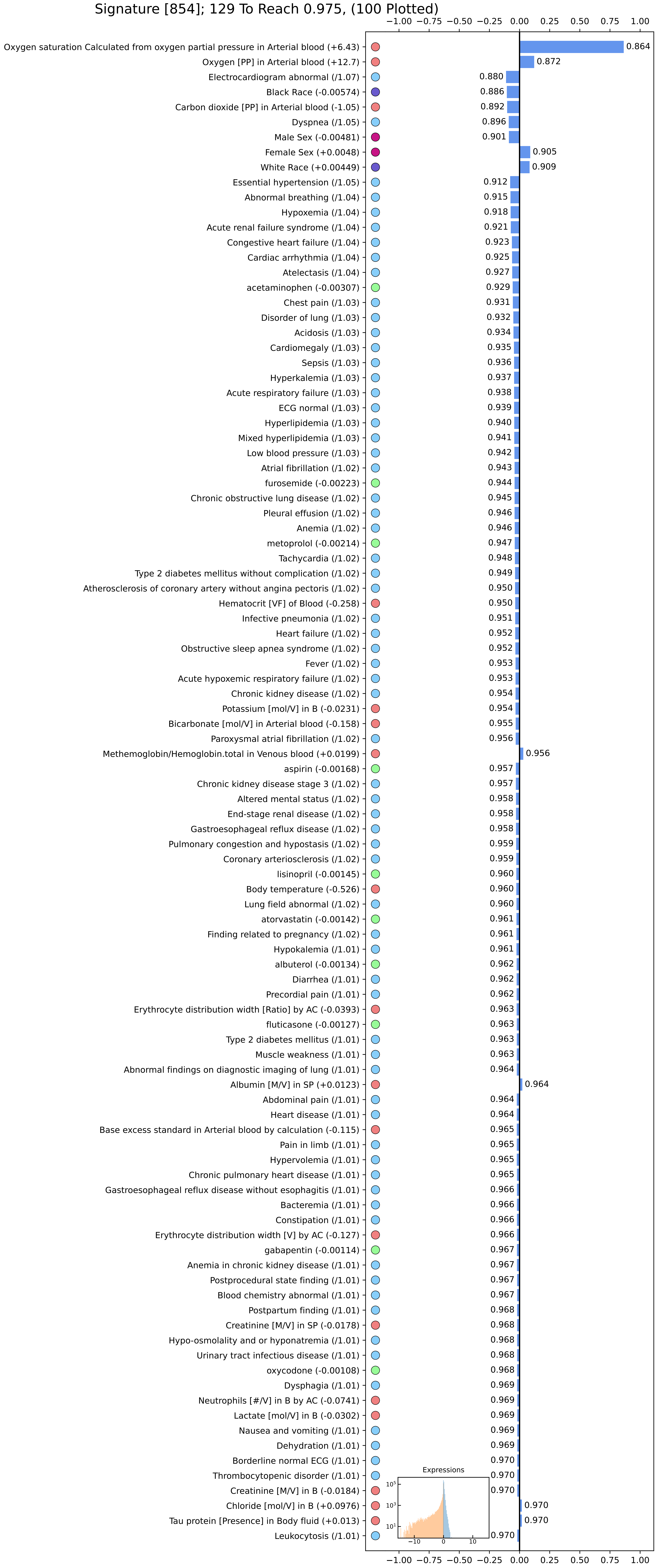

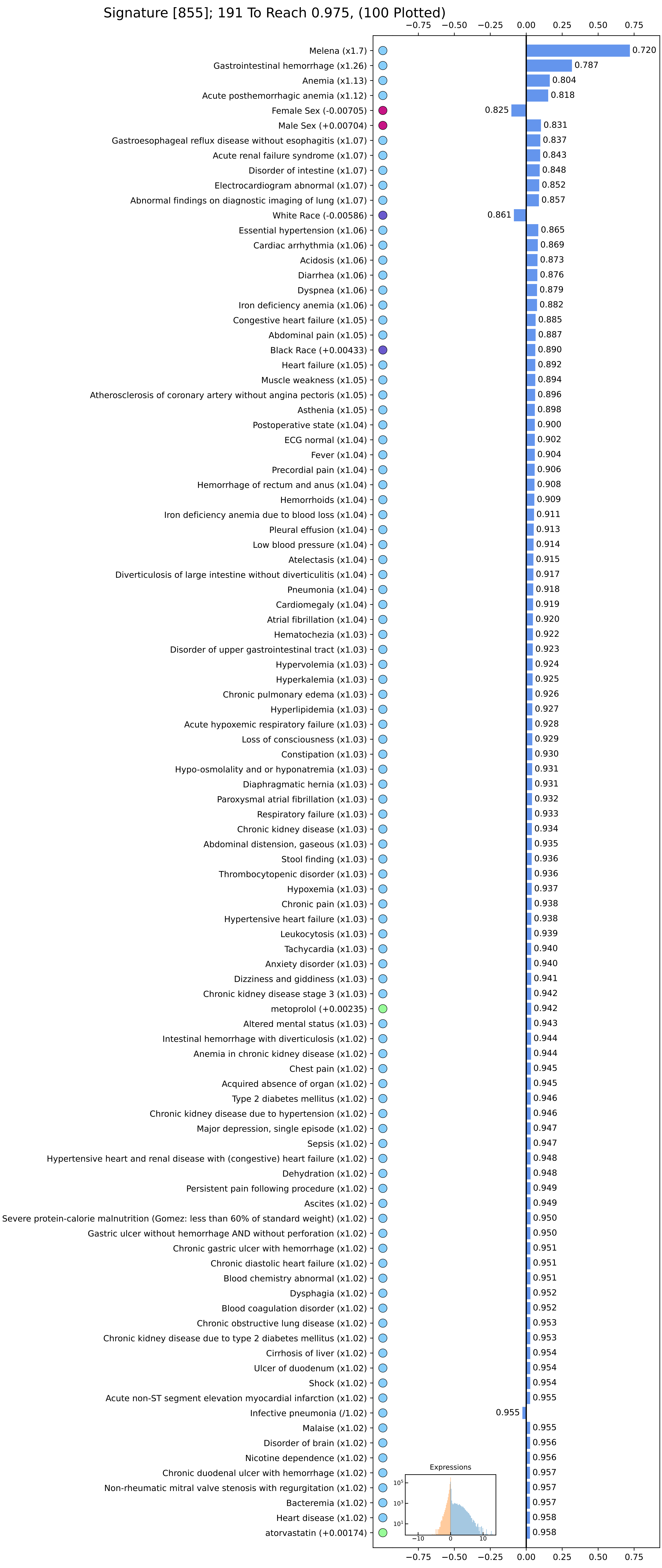

Signature [855]; 191 To Reach 0.975, (100 Plotted)
Melena (x1.7) 0.720
Gastrointestinal hemorrhage (x1.26) 0.787
Anemia (x1.13) 0.804
Acute posthemorrhagic anemia (x1.12) 0.818
Female Sex (-0.00705) 0.825
Male Sex (+0.00704) 0.831
Gastroesophageal reflux disease without esophagitis (x1.07) 0.837
Acute renal failure syndrome (x1.07) 0.843
Disorder of intestine (x1.07) 0.848
Electrocardiogram abnormal (x1.07) 0.852
Abnormal findings on diagnostic imaging of lung (x1.07) 0.857
White Race (-0.00586) 0.861
Essential hypertension (x1.06) 0.865
Cardiac arrhythmia (x1.06) 0.869
Acidosis (x1.06) 0.873
Diarrhea (x1.06) 0.876
Dyspnea (x1.06) 0.879
Iron deficiency anemia (x1.06) 0.882
Congestive heart failure (x1.05) 0.885
Abdominal pain (x1.05) 0.887
Black Race (+0.00433) 0.890
Heart failure (x1.05) 0.892
Muscle weakness (x1.05) 0.894
Atherosclerosis of coronary artery without angina pectoris (x1.05) 0.896
Asthenia (x1.05) 0.898
Postoperative state (x1.04) 0.900
ECG normal (x1.04) 0.902
Fever (x1.04) 0.904
Precordial pain (x1.04) 0.906
Hemorrhage of rectum and anus (x1.04) 0.908
Hemorrhoids (x1.04) 0.909
Iron deficiency anemia due to blood loss (x1.04) 0.911
Pleural effusion (x1.04) 0.913
Low blood pressure (x1.04) 0.914
Atelectasis (x1.04) 0.915
Diverticulosis of large intestine without diverticulitis (x1.04) 0.917
Pneumonia (x1.04) 0.918
Cardiomegaly (x1.04) 0.919
Atrial fibrillation (x1.04) 0.920
Hematochezia (x1.03) 0.922
Disorder of upper gastrointestinal tract (x1.03) 0.923
Hypervolemia (x1.03) 0.924
Hyperkalemia (x1.03) 0.925
Chronic pulmonary edema (x1.03) 0.926
Hyperlipidemia (x1.03) 0.927
Acute hypoxemic respiratory failure (x1.03) 0.928
Loss of consciousness (x1.03) 0.929
Constipation (x1.03) 0.930
Hypo-osmolality and or hyponatremia (x1.03) 0.931
Diaphragmatic hernia (x1.03) 0.931
Paroxysmal atrial fibrillation (x1.03) 0.932
Respiratory failure (x1.03) 0.933
Chronic kidney disease (x1.03) 0.934
Abdominal distension, gaseous (x1.03) 0.935
Stool finding (x1.03) 0.936
Thrombocytopenic disorder (x1.03) 0.936
Hypoxemia (x1.03) 0.937
Chronic pain (x1.03) 0.938
Hypertensive heart failure (x1.03) 0.938
Leukocytosis (x1.03) 0.939
Tachycardia (x1.03) 0.940
Anxiety disorder (x1.03) 0.940
Dizziness and giddiness (x1.03) 0.941
Chronic kidney disease stage 3 (x1.03) 0.942
metoprolol (+0.00235) 0.942
Altered mental status (x1.03) 0.943
Intestinal hemorrhage with diverticulosis (x1.02) 0.944
Anemia in chronic kidney disease (x1.02) 0.944
Chest pain (x1.02) 0.945
Acquired absence of organ (x1.02) 0.945
Type 2 diabetes mellitus (x1.02) 0.946
Chronic kidney disease due to hypertension (x1.02) 0.946
Major depression, single episode (x1.02) 0.947
Sepsis (x1.02) 0.947
Hypertensive heart and renal disease with (congestive) heart failure (x1.02) 0.948
Dehydration (x1.02) 0.948
Persistent pain following procedure (x1.02) 0.949
Ascites (x1.02) 0.949
Severe protein-calorie malnutrition (Gomez: less than 60% of standard weight) (x1.02) 0.950
Gastric ulcer without hemorrhage AND without perforation (x1.02) 0.950
Chronic gastric ulcer with hemorrhage (x1.02) 0.951
Chronic diastolic heart failure (x1.02) 0.951
Blood chemistry abnormal (x1.02) 0.951
Dysphagia (x1.02) 0.952
Blood coagulation disorder (x1.02) 0.952
Chronic obstructive lung disease (x1.02) 0.953
Chronic kidney disease due to type 2 diabetes mellitus (x1.02) 0.953
Cirrhosis of liver (x1.02) 0.954
Ulcer of duodenum (x1.02) 0.954
Shock (x1.02) 0.954
Acute non-ST segment elevation myocardial infarction (x1.02) 0.955
Infective pneumonia (/1.02) 0.955
Malaise (x1.02) 0.955
Disorder of brain (x1.02) 0.956
Nicotine dependence (x1.02) 0.956
Chronic duodenal ulcer with hemorrhage (x1.02) 0.957
Non-rheumatic mitral valve stenosis with regurgitation (x1.02) 0.957
Bacteremia (x1.02) 0.957
Heart disease (x1.02) 0.958
atorvastatin (+0.00174) 0.958
−0.75 −0.50 −0.25 0.00 0.25 0.50 0.75
Expressions

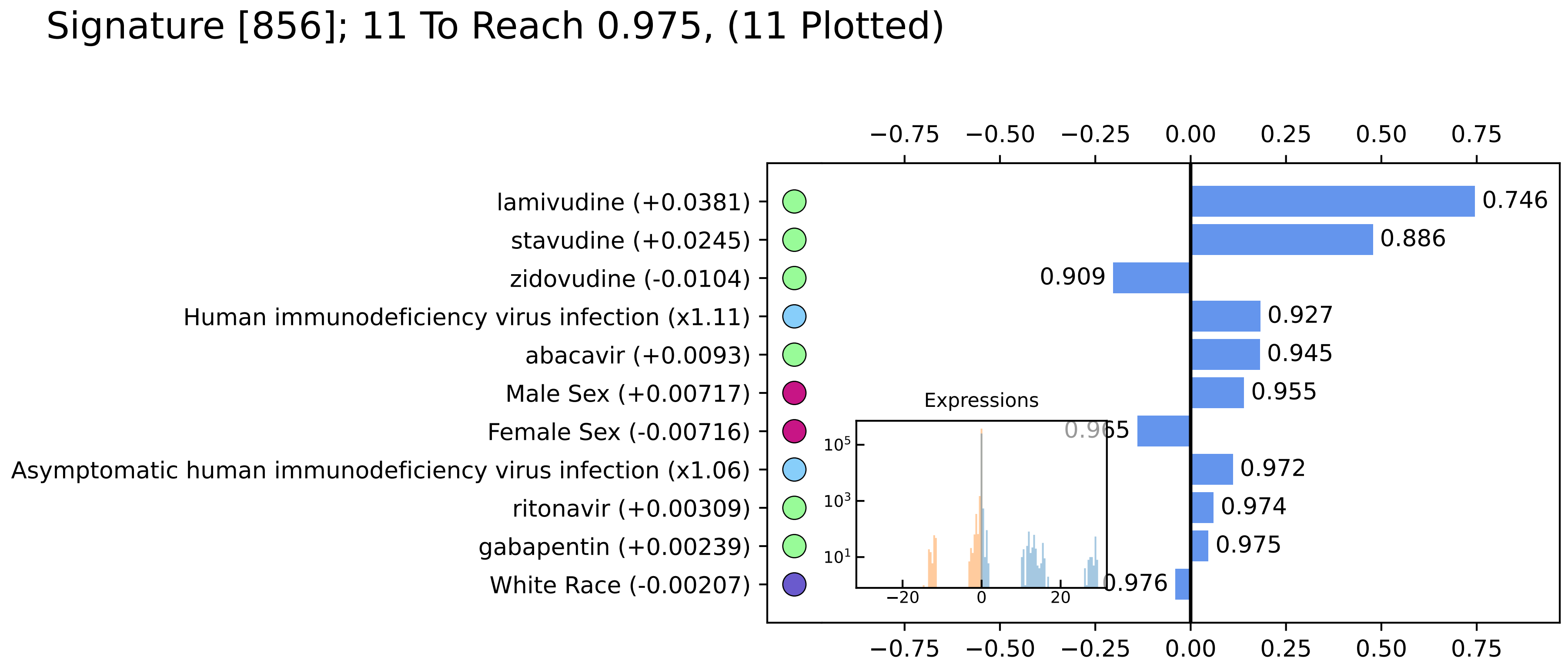
Signature [856]; 11 To Reach 0.975, (11 Plotted)
lamivudine (+0.0381)
stavudine (+0.0245)
zidovudine (-0.0104)
Human immunodeficiency virus infection (x1.11)
abacavir (+0.0093)
Male Sex (+0.00717)
Female Sex (-0.00716)
Asymptomatic human immunodeficiency virus infection (x1.06)
ritonavir (+0.00309)
gabapentin (+0.00239)
White Race (-0.00207)
0.746
0.886
0.909
0.927
0.945
0.955
0.965
0.972
0.974
0.975
0.976
−0.75
−0.50
−0.25
0.00
0.25
0.50
0.75
Expressions
−20
0
20

Signature [857]; 168 To Reach 0.975, (100 Plotted)

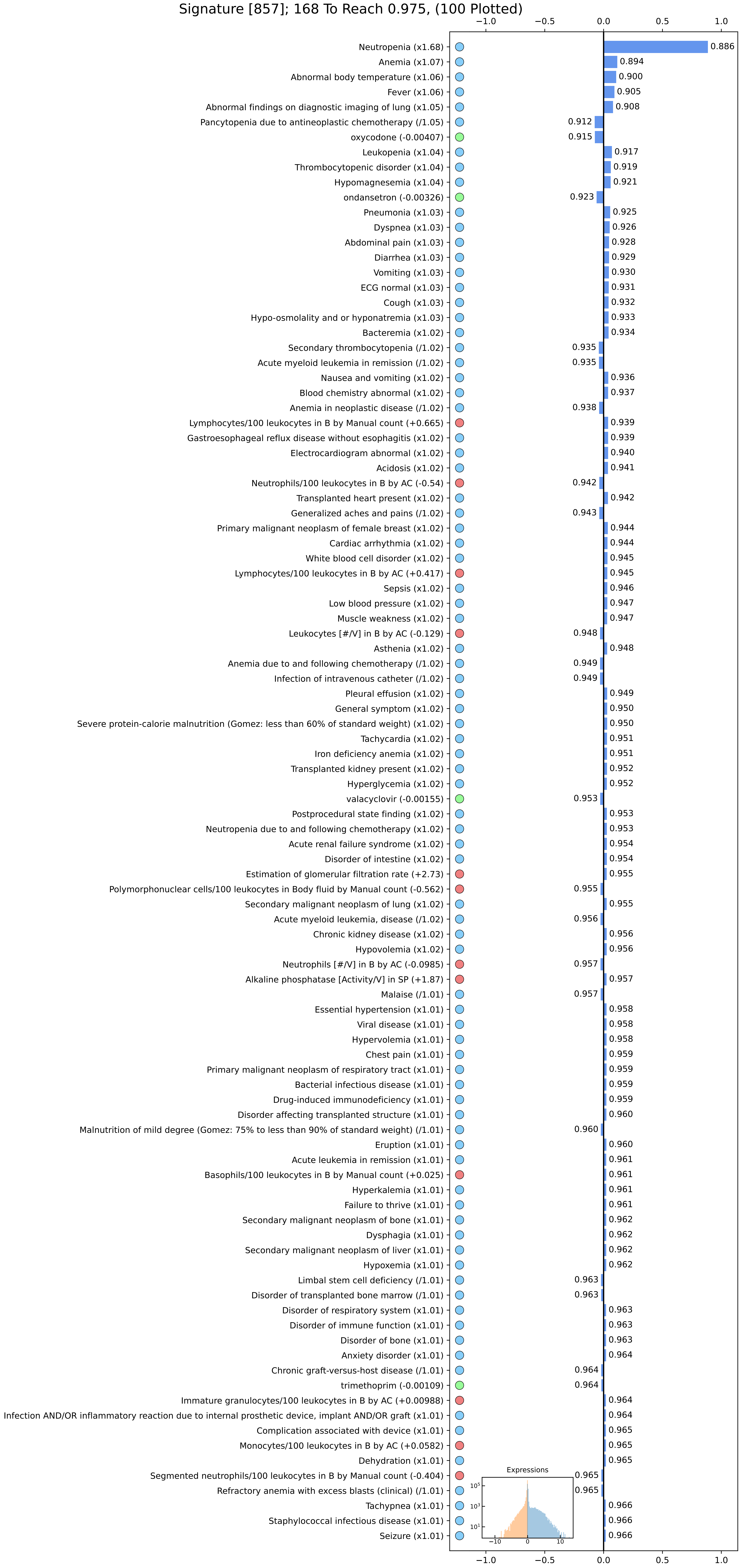

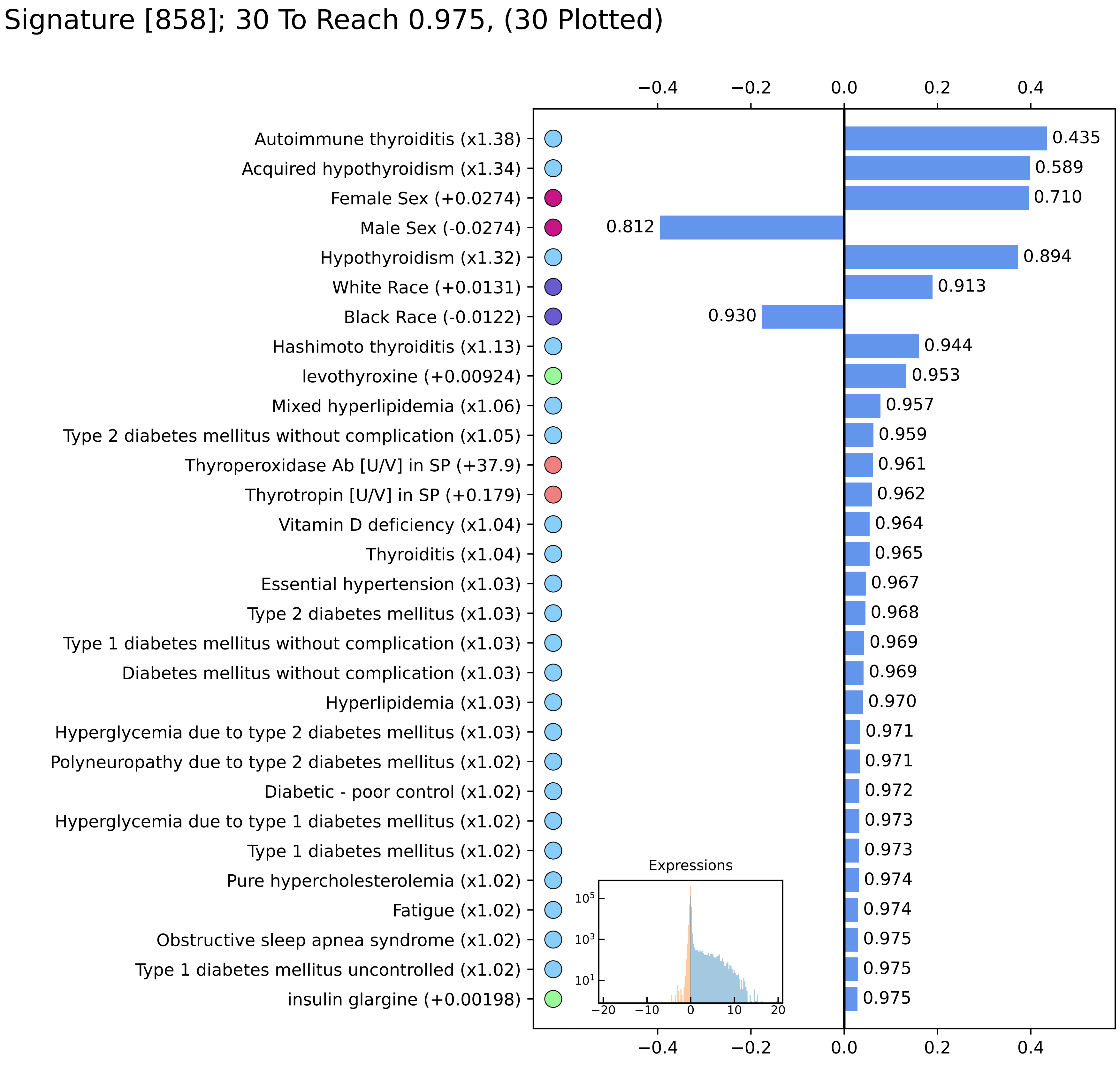

Signature [858]; 30 To Reach 0.975, (30 Plotted)
Autoimmune thyroiditis (x1.38)
Acquired hypothyroidism (x1.34)
Female Sex (+0.0274)
Male Sex (-0.0274)
Hypothyroidism (x1.32)
White Race (+0.0131)
Black Race (-0.0122)
Hashimoto thyroiditis (x1.13)
levothyroxine (+0.00924)
Mixed hyperlipidemia (x1.06)
Type 2 diabetes mellitus without complication (x1.05)
Thyroperoxidase Ab [U/V] in SP (+37.9)
Thyrotropin [U/V] in SP (+0.179)
Vitamin D deficiency (x1.04)
Thyroiditis (x1.04)
Essential hypertension (x1.03)
Type 2 diabetes mellitus (x1.03)
Type 1 diabetes mellitus without complication (x1.03)
Diabetes mellitus without complication (x1.03)
Hyperlipidemia (x1.03)
Hyperglycemia due to type 2 diabetes mellitus (x1.03)
Polyneuropathy due to type 2 diabetes mellitus (x1.02)
Diabetic - poor control (x1.02)
Hyperglycemia due to type 1 diabetes mellitus (x1.02)
Type 1 diabetes mellitus (x1.02)
Pure hypercholesterolemia (x1.02)
Fatigue (x1.02)
Obstructive sleep apnea syndrome (x1.02)
Type 1 diabetes mellitus uncontrolled (x1.02)
insulin glargine (+0.00198)
0.435
0.589
0.710
0.812
0.894
0.913
0.930
0.944
0.953
0.957
0.959
0.961
0.962
0.964
0.965
0.967
0.968
0.969
0.969
0.970
0.971
0.971
0.972
0.973
0.973
0.974
0.974
0.975
0.975
0.975
−0.4
−0.2
0.0
0.2
0.4
Expressions
10^5
10^3
10^1
−20
−10
0
10
20

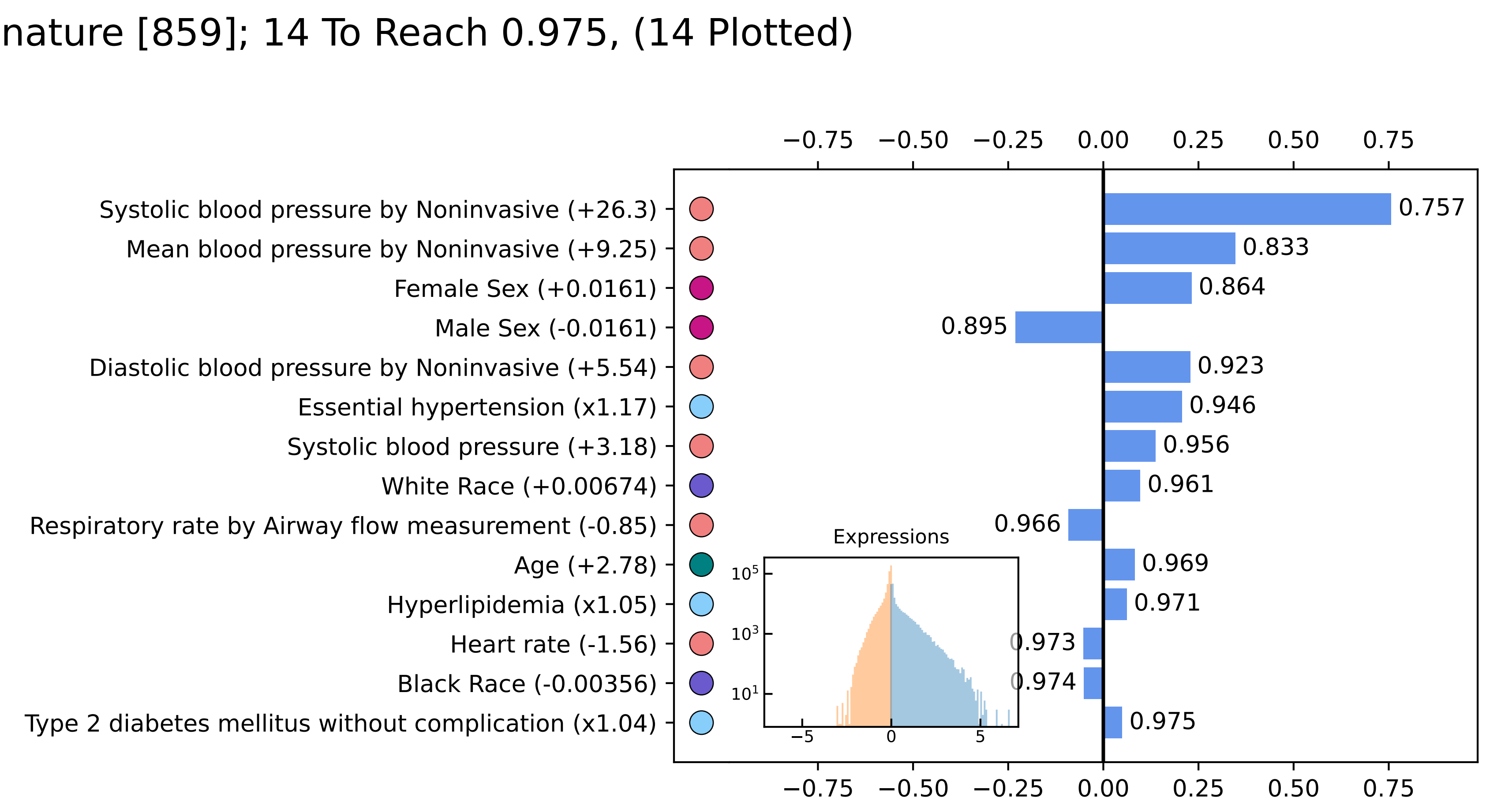

Signature [859]; 14 To Reach 0.975, (14 Plotted)
Systolic blood pressure by Noninvasive (+26.3)
Mean blood pressure by Noninvasive (+9.25)
Female Sex (+0.0161)
Male Sex (-0.0161)
Diastolic blood pressure by Noninvasive (+5.54)
Essential hypertension (x1.17)
Systolic blood pressure (+3.18)
White Race (+0.00674)
Respiratory rate by Airway flow measurement (-0.85)
Age (+2.78)
Hyperlipidemia (x1.05)
Heart rate (-1.56)
Black Race (-0.00356)
Type 2 diabetes mellitus without complication (x1.04)
0.757
0.833
0.864
0.895
0.923
0.946
0.956
0.961
0.966
0.969
0.971
0.973
0.974
0.975
Expressions
−0.75
−0.50
−0.25
0.00
0.25
0.50
0.75

Signature [860]; 3 To Reach 0.975, (10 Plotted)

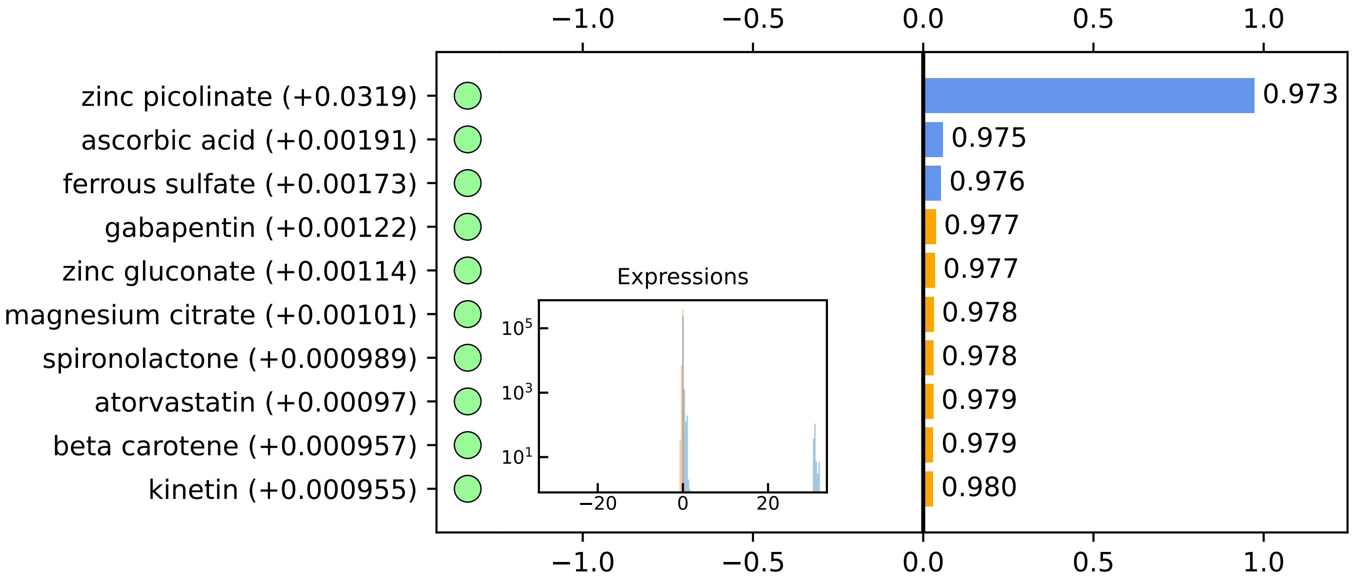

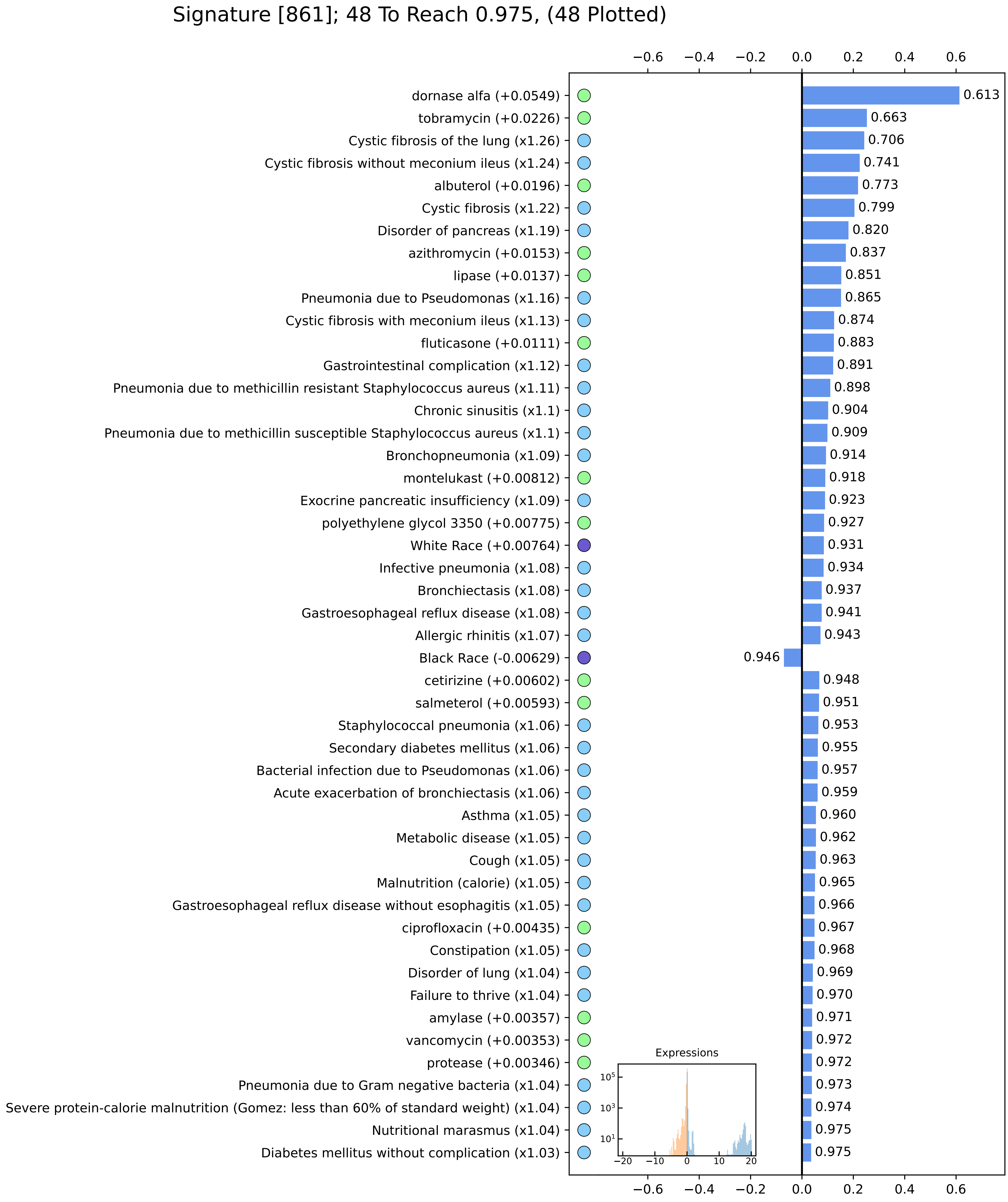

Signature [861]; 48 To Reach 0.975, (48 Plotted)
dornase alfa (+0.0549) 0.613
tobramycin (+0.0226) 0.663
Cystic fibrosis of the lung (x1.26) 0.706
Cystic fibrosis without meconium ileus (x1.24) 0.741
albuterol (+0.0196) 0.773
Cystic fibrosis (x1.22) 0.799
Disorder of pancreas (x1.19) 0.820
azithromycin (+0.0153) 0.837
lipase (+0.0137) 0.851
Pneumonia due to Pseudomonas (x1.16) 0.865
Cystic fibrosis with meconium ileus (x1.13) 0.874
fluticasone (+0.0111) 0.883
Gastrointestinal complication (x1.12) 0.891
Pneumonia due to methicillin resistant Staphylococcus aureus (x1.11) 0.898
Chronic sinusitis (x1.1) 0.904
Pneumonia due to methicillin susceptible Staphylococcus aureus (x1.1) 0.909
Bronchopneumonia (x1.09) 0.914
montelukast (+0.00812) 0.918
Exocrine pancreatic insufficiency (x1.09) 0.923
polyethylene glycol 3350 (+0.00775) 0.927
White Race (+0.00764) 0.931
Infective pneumonia (x1.08) 0.934
Bronchiectasis (x1.08) 0.937
Gastroesophageal reflux disease (x1.08) 0.941
Allergic rhinitis (x1.07) 0.943
Black Race (-0.00629) 0.946
cetirizine (+0.00602) 0.948
salmeterol (+0.00593) 0.951
Staphylococcal pneumonia (x1.06) 0.953
Secondary diabetes mellitus (x1.06) 0.955
Bacterial infection due to Pseudomonas (x1.06) 0.957
Acute exacerbation of bronchiectasis (x1.06) 0.959
Asthma (x1.05) 0.960
Metabolic disease (x1.05) 0.962
Cough (x1.05) 0.963
Malnutrition (calorie) (x1.05) 0.965
Gastroesophageal reflux disease without esophagitis (x1.05) 0.966
ciprofloxacin (+0.00435) 0.967
Constipation (x1.05) 0.968
Disorder of lung (x1.04) 0.969
Failure to thrive (x1.04) 0.970
amylase (+0.00357) 0.971
vancomycin (+0.00353) 0.972
protease (+0.00346) 0.972
Pneumonia due to Gram negative bacteria (x1.04) 0.973
Severe protein-calorie malnutrition (Gomez: less than 60% of standard weight) (x1.04) 0.974
Nutritional marasmus (x1.04) 0.975
Diabetes mellitus without complication (x1.03) 0.975
−0.6 −0.4 −0.2 0.0 0.2 0.4 0.6
Expressions
10^5
10^3
10^1
−20 −10 0 10 20

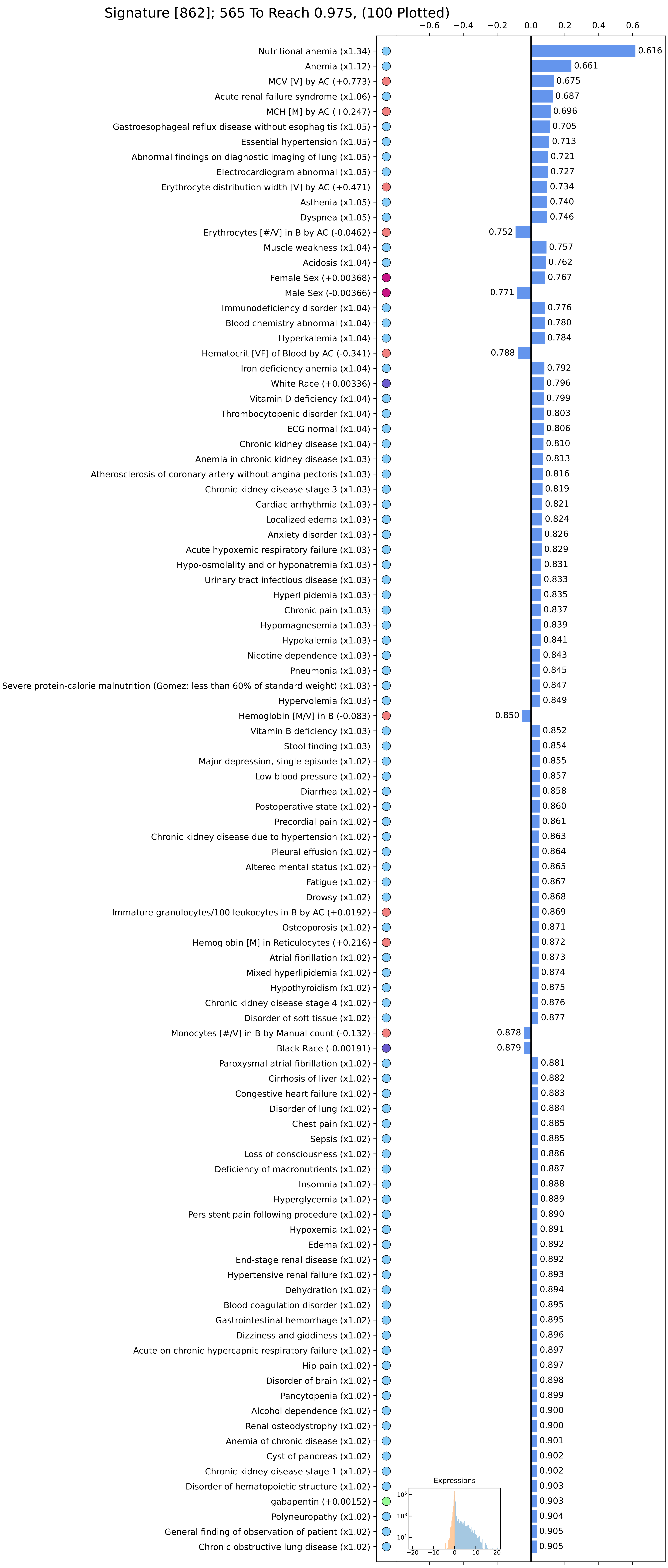

Signature [862]; 565 To Reach 0.975, (100 Plotted)
Nutritional anemia (x1.34) 0.616
Anemia (x1.12) 0.661
MCV [V] by AC (+0.773) 0.675
Acute renal failure syndrome (x1.06) 0.687
MCH [M] by AC (+0.247) 0.696
Gastroesophageal reflux disease without esophagitis (x1.05) 0.705
Essential hypertension (x1.05) 0.713
Abnormal findings on diagnostic imaging of lung (x1.05) 0.721
Electrocardiogram abnormal (x1.05) 0.727
Erythrocyte distribution width [V] by AC (+0.471) 0.734
Asthenia (x1.05) 0.740
Dyspnea (x1.05) 0.746
Erythrocytes [#/V] in B by AC (-0.0462) 0.752
Muscle weakness (x1.04) 0.757
Acidosis (x1.04) 0.762
Female Sex (+0.00368) 0.767
Male Sex (-0.00366) 0.771
Immunodeficiency disorder (x1.04) 0.776
Blood chemistry abnormal (x1.04) 0.780
Hyperkalemia (x1.04) 0.784
Hematocrit [VF] of Blood by AC (-0.341) 0.788
Iron deficiency anemia (x1.04) 0.792
White Race (+0.00336) 0.796
Vitamin D deficiency (x1.04) 0.799
Thrombocytopenic disorder (x1.04) 0.803
ECG normal (x1.04) 0.806
Chronic kidney disease (x1.04) 0.810
Anemia in chronic kidney disease (x1.03) 0.813
Atherosclerosis of coronary artery without angina pectoris (x1.03) 0.816
Chronic kidney disease stage 3 (x1.03) 0.819
Cardiac arrhythmia (x1.03) 0.821
Localized edema (x1.03) 0.824
Anxiety disorder (x1.03) 0.826
Acute hypoxemic respiratory failure (x1.03) 0.829
Hypo-osmolality and or hyponatremia (x1.03) 0.831
Urinary tract infectious disease (x1.03) 0.833
Hyperlipidemia (x1.03) 0.835
Chronic pain (x1.03) 0.837
Hypomagnesemia (x1.03) 0.839
Hypokalemia (x1.03) 0.841
Nicotine dependence (x1.03) 0.843
Pneumonia (x1.03) 0.845
Severe protein-calorie malnutrition (Gomez: less than 60% of standard weight) (x1.03) 0.847
Hypervolemia (x1.03) 0.849
Hemoglobin [M/V] in B (-0.083) 0.850
Vitamin B deficiency (x1.03) 0.852
Stool finding (x1.03) 0.854
Major depression, single episode (x1.02) 0.855
Low blood pressure (x1.02) 0.857
Diarrhea (x1.02) 0.858
Postoperative state (x1.02) 0.860
Precordial pain (x1.02) 0.861
Chronic kidney disease due to hypertension (x1.02) 0.863
Pleural effusion (x1.02) 0.864
Altered mental status (x1.02) 0.865
Fatigue (x1.02) 0.867
Drowsy (x1.02) 0.868
Immature granulocytes/100 leukocytes in B by AC (+0.0192) 0.869
Osteoporosis (x1.02) 0.871
Hemoglobin [M] in Reticulocytes (+0.216) 0.872
Atrial fibrillation (x1.02) 0.873
Mixed hyperlipidemia (x1.02) 0.874
Hypothyroidism (x1.02) 0.875
Chronic kidney disease stage 4 (x1.02) 0.876
Disorder of soft tissue (x1.02) 0.877
Monocytes [#/V] in B by Manual count (-0.132) 0.878
Black Race (-0.00191) 0.879
Paroxysmal atrial fibrillation (x1.02) 0.881
Cirrhosis of liver (x1.02) 0.882
Congestive heart failure (x1.02) 0.883
Disorder of lung (x1.02) 0.884
Chest pain (x1.02) 0.885
Sepsis (x1.02) 0.885
Loss of consciousness (x1.02) 0.886
Deficiency of macronutrients (x1.02) 0.887
Insomnia (x1.02) 0.888
Hyperglycemia (x1.02) 0.889
Persistent pain following procedure (x1.02) 0.890
Hypoxemia (x1.02) 0.891
Edema (x1.02) 0.892
End-stage renal disease (x1.02) 0.892
Hypertensive renal failure (x1.02) 0.893
Dehydration (x1.02) 0.894
Blood coagulation disorder (x1.02) 0.895
Gastrointestinal hemorrhage (x1.02) 0.895
Dizziness and giddiness (x1.02) 0.896
Acute on chronic hypercapnic respiratory failure (x1.02) 0.897
Hip pain (x1.02) 0.897
Disorder of brain (x1.02) 0.898
Pancytopenia (x1.02) 0.899
Alcohol dependence (x1.02) 0.900
Renal osteodystrophy (x1.02) 0.900
Anemia of chronic disease (x1.02) 0.901
Cyst of pancreas (x1.02) 0.902
Chronic kidney disease stage 1 (x1.02) 0.902
Disorder of hematopoietic structure (x1.02) 0.903
gabapentin (+0.00152) 0.903
Polyneuropathy (x1.02) 0.904
General finding of observation of patient (x1.02) 0.905
Chronic obstructive lung disease (x1.02) 0.905
Expressions
−0.6 −0.4 −0.2 0.0 0.2 0.4 0.6

Signature [863]; 90 To Reach 0.975, (90 Plotted)

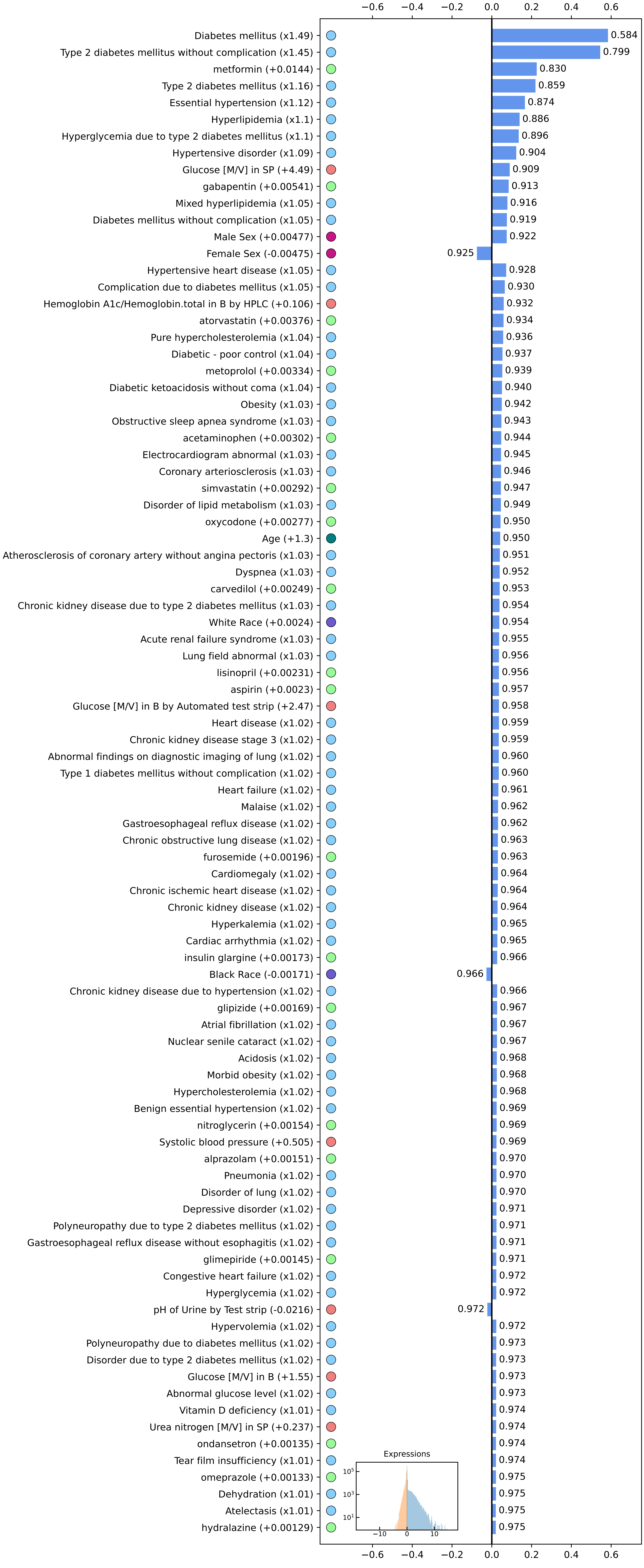

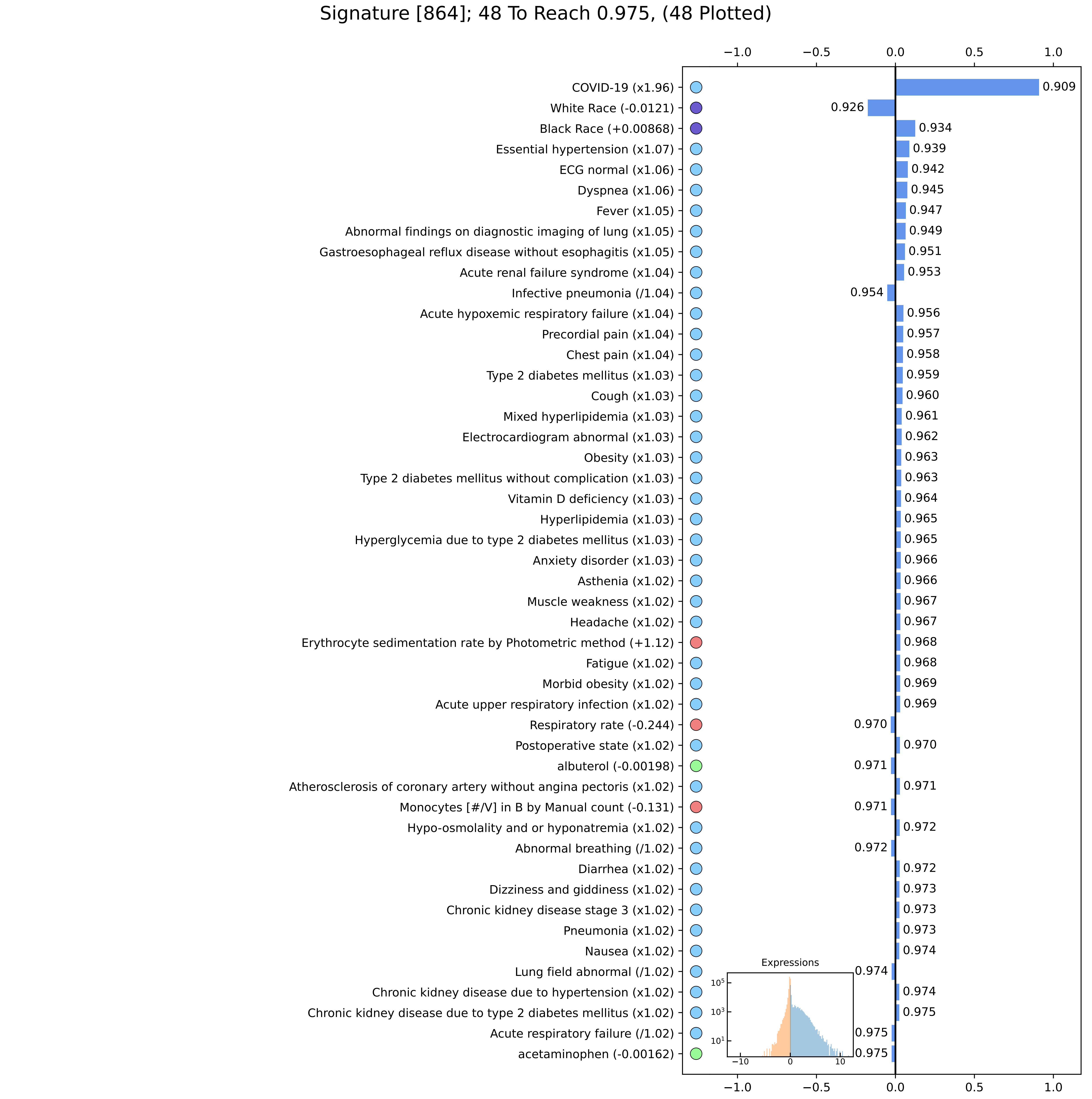
Signature [864]; 48 To Reach 0.975, (48 Plotted)
COVID-19 (x1.96)
0.909
White Race (-0.0121)
0.926
Black Race (+0.00868)
0.934
Essential hypertension (x1.07)
0.939
ECG normal (x1.06)
0.942
Dyspnea (x1.06)
0.945
Fever (x1.05)
0.947
Abnormal findings on diagnostic imaging of lung (x1.05)
0.949
Gastroesophageal reflux disease without esophagitis (x1.05)
0.951
Acute renal failure syndrome (x1.04)
0.953
Infective pneumonia (/1.04)
0.954
Acute hypoxemic respiratory failure (x1.04)
0.956
Precordial pain (x1.04)
0.957
Chest pain (x1.04)
0.958
Type 2 diabetes mellitus (x1.03)
0.959
Cough (x1.03)
0.960
Mixed hyperlipidemia (x1.03)
0.961
Electrocardiogram abnormal (x1.03)
0.962
Obesity (x1.03)
0.963
Type 2 diabetes mellitus without complication (x1.03)
0.963
Vitamin D deficiency (x1.03)
0.964
Hyperlipidemia (x1.03)
0.965
Hyperglycemia due to type 2 diabetes mellitus (x1.03)
0.965
Anxiety disorder (x1.03)
0.966
Asthenia (x1.02)
0.966
Muscle weakness (x1.02)
0.967
Headache (x1.02)
0.967
Erythrocyte sedimentation rate by Photometric method (+1.12)
0.968
Fatigue (x1.02)
0.968
Morbid obesity (x1.02)
0.969
Acute upper respiratory infection (x1.02)
0.969
Respiratory rate (-0.244)
0.970
Postoperative state (x1.02)
0.970
albuterol (-0.00198)
0.971
Atherosclerosis of coronary artery without angina pectoris (x1.02)
0.971
Monocytes [#/V] in B by Manual count (-0.131)
0.971
Hypo-osmolality and or hyponatremia (x1.02)
0.972
Abnormal breathing (/1.02)
0.972
Diarrhea (x1.02)
0.972
Dizziness and giddiness (x1.02)
0.973
Chronic kidney disease stage 3 (x1.02)
0.973
Pneumonia (x1.02)
0.973
Nausea (x1.02)
0.974
Lung field abnormal (/1.02)
0.974
Chronic kidney disease due to hypertension (x1.02)
0.974
Chronic kidney disease due to type 2 diabetes mellitus (x1.02)
0.975
Acute respiratory failure (/1.02)
0.975
acetaminophen (-0.00162)
0.975
−1.0
−0.5
0.0
0.5
1.0
Expressions
10^5
10^3
10^1
−10
0
10

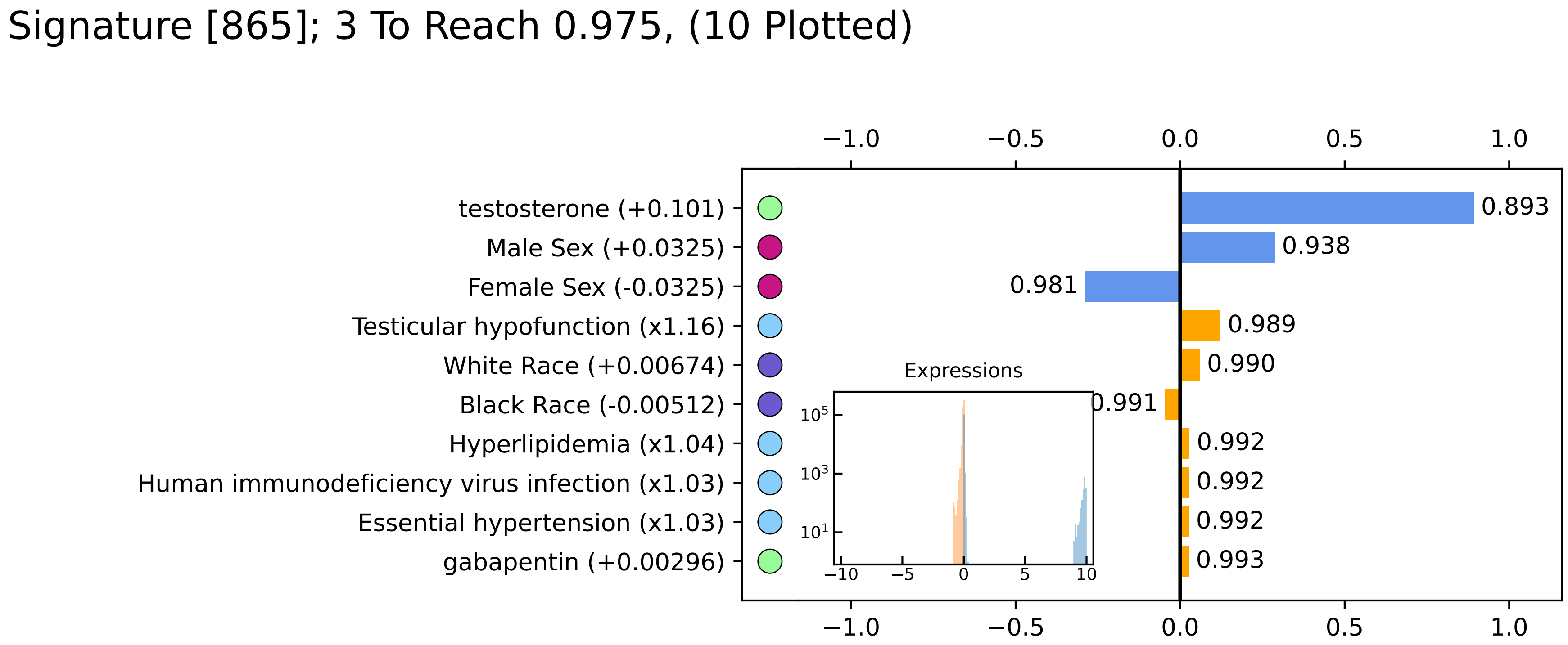
Signature [865]; 3 To Reach 0.975, (10 Plotted)
testosterone (+0.101)
Male Sex (+0.0325)
Female Sex (-0.0325)
Testicular hypofunction (x1.16)
White Race (+0.00674)
Black Race (-0.00512)
Hyperlipidemia (x1.04)
Human immunodeficiency virus infection (x1.03)
Essential hypertension (x1.03)
gabapentin (+0.00296)
0.893
0.938
0.981
0.989
0.990
0.991
0.992
0.992
0.992
0.993
−1.0
−0.5
0.0
0.5
1.0
Expressions

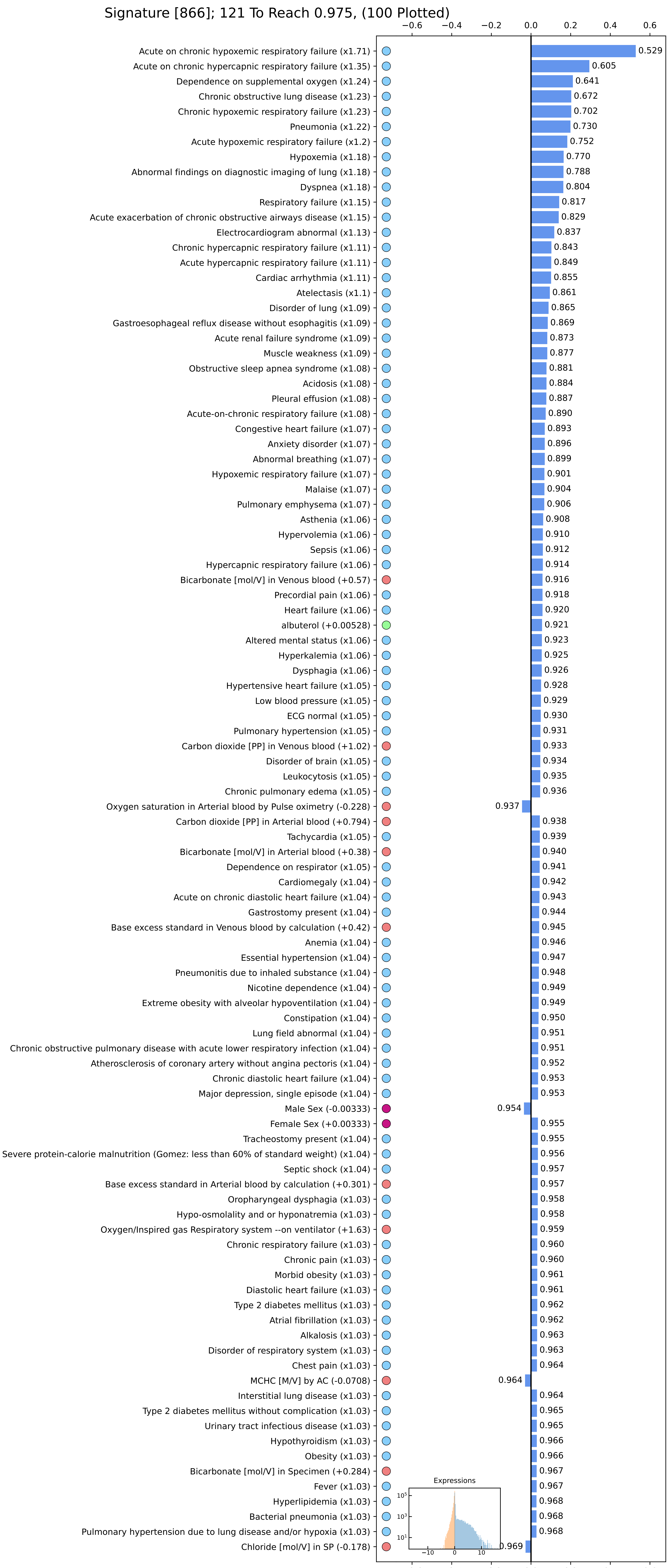

Signature [866]; 121 To Reach 0.975, (100 Plotted)
Acute on chronic hypoxemic respiratory failure (x1.71) 0.529
Acute on chronic hypercapnic respiratory failure (x1.35) 0.605
Dependence on supplemental oxygen (x1.24) 0.641
Chronic obstructive lung disease (x1.23) 0.672
Chronic hypoxemic respiratory failure (x1.23) 0.702
Pneumonia (x1.22) 0.730
Acute hypoxemic respiratory failure (x1.2) 0.752
Hypoxemia (x1.18) 0.770
Abnormal findings on diagnostic imaging of lung (x1.18) 0.788
Dyspnea (x1.18) 0.804
Respiratory failure (x1.15) 0.817
Acute exacerbation of chronic obstructive airways disease (x1.15) 0.829
Electrocardiogram abnormal (x1.13) 0.837
Chronic hypercapnic respiratory failure (x1.11) 0.843
Acute hypercapnic respiratory failure (x1.11) 0.849
Cardiac arrhythmia (x1.11) 0.855
Atelectasis (x1.1) 0.861
Disorder of lung (x1.09) 0.865
Gastroesophageal reflux disease without esophagitis (x1.09) 0.869
Acute renal failure syndrome (x1.09) 0.873
Muscle weakness (x1.09) 0.877
Obstructive sleep apnea syndrome (x1.08) 0.881
Acidosis (x1.08) 0.884
Pleural effusion (x1.08) 0.887
Acute-on-chronic respiratory failure (x1.08) 0.890
Congestive heart failure (x1.07) 0.893
Anxiety disorder (x1.07) 0.896
Abnormal breathing (x1.07) 0.899
Hypoxemic respiratory failure (x1.07) 0.901
Malaise (x1.07) 0.904
Pulmonary emphysema (x1.07) 0.906
Asthenia (x1.06) 0.908
Hypervolemia (x1.06) 0.910
Sepsis (x1.06) 0.912
Hypercapnic respiratory failure (x1.06) 0.914
Bicarbonate [mol/V] in Venous blood (+0.57) 0.916
Precordial pain (x1.06) 0.918
Heart failure (x1.06) 0.920
albuterol (+0.00528) 0.921
Altered mental status (x1.06) 0.923
Hyperkalemia (x1.06) 0.925
Dysphagia (x1.06) 0.926
Hypertensive heart failure (x1.05) 0.928
Low blood pressure (x1.05) 0.929
ECG normal (x1.05) 0.930
Pulmonary hypertension (x1.05) 0.931
Carbon dioxide [PP] in Venous blood (+1.02) 0.933
Disorder of brain (x1.05) 0.934
Leukocytosis (x1.05) 0.935
Chronic pulmonary edema (x1.05) 0.936
Oxygen saturation in Arterial blood by Pulse oximetry (-0.228) 0.937
Carbon dioxide [PP] in Arterial blood (+0.794) 0.938
Tachycardia (x1.05) 0.939
Bicarbonate [mol/V] in Arterial blood (+0.38) 0.940
Dependence on respirator (x1.05) 0.941
Cardiomegaly (x1.04) 0.942
Acute on chronic diastolic heart failure (x1.04) 0.943
Gastrostomy present (x1.04) 0.944
Base excess standard in Venous blood by calculation (+0.42) 0.945
Anemia (x1.04) 0.946
Essential hypertension (x1.04) 0.947
Pneumonitis due to inhaled substance (x1.04) 0.948
Nicotine dependence (x1.04) 0.949
Extreme obesity with alveolar hypoventilation (x1.04) 0.949
Constipation (x1.04) 0.950
Lung field abnormal (x1.04) 0.951
Chronic obstructive pulmonary disease with acute lower respiratory infection (x1.04) 0.951
Atherosclerosis of coronary artery without angina pectoris (x1.04) 0.952
Chronic diastolic heart failure (x1.04) 0.953
Major depression, single episode (x1.04) 0.953
Male Sex (-0.00333) 0.954
Female Sex (+0.00333) 0.955
Tracheostomy present (x1.04) 0.955
Severe protein-calorie malnutrition (Gomez: less than 60% of standard weight) (x1.04) 0.956
Septic shock (x1.04) 0.957
Base excess standard in Arterial blood by calculation (+0.301) 0.957
Oropharyngeal dysphagia (x1.03) 0.958
Hypo-osmolality and or hyponatremia (x1.03) 0.958
Oxygen/Inspired gas Respiratory system --on ventilator (+1.63) 0.959
Chronic respiratory failure (x1.03) 0.960
Chronic pain (x1.03) 0.960
Morbid obesity (x1.03) 0.961
Diastolic heart failure (x1.03) 0.961
Type 2 diabetes mellitus (x1.03) 0.962
Atrial fibrillation (x1.03) 0.962
Alkalosis (x1.03) 0.963
Disorder of respiratory system (x1.03) 0.963
Chest pain (x1.03) 0.964
MCHC [M/V] by AC (-0.0708) 0.964
Interstitial lung disease (x1.03) 0.964
Type 2 diabetes mellitus without complication (x1.03) 0.965
Urinary tract infectious disease (x1.03) 0.965
Hypothyroidism (x1.03) 0.966
Obesity (x1.03) 0.966
Bicarbonate [mol/V] in Specimen (+0.284) 0.967
Fever (x1.03) 0.967
Hyperlipidemia (x1.03) 0.968
Bacterial pneumonia (x1.03) 0.968
Pulmonary hypertension due to lung disease and/or hypoxia (x1.03) 0.968
Chloride [mol/V] in SP (-0.178) 0.969
−0.6 −0.4 −0.2 0.0 0.2 0.4 0.6
Expressions

Signature [867]; 100 To Reach 0.975, (100 Plotted)

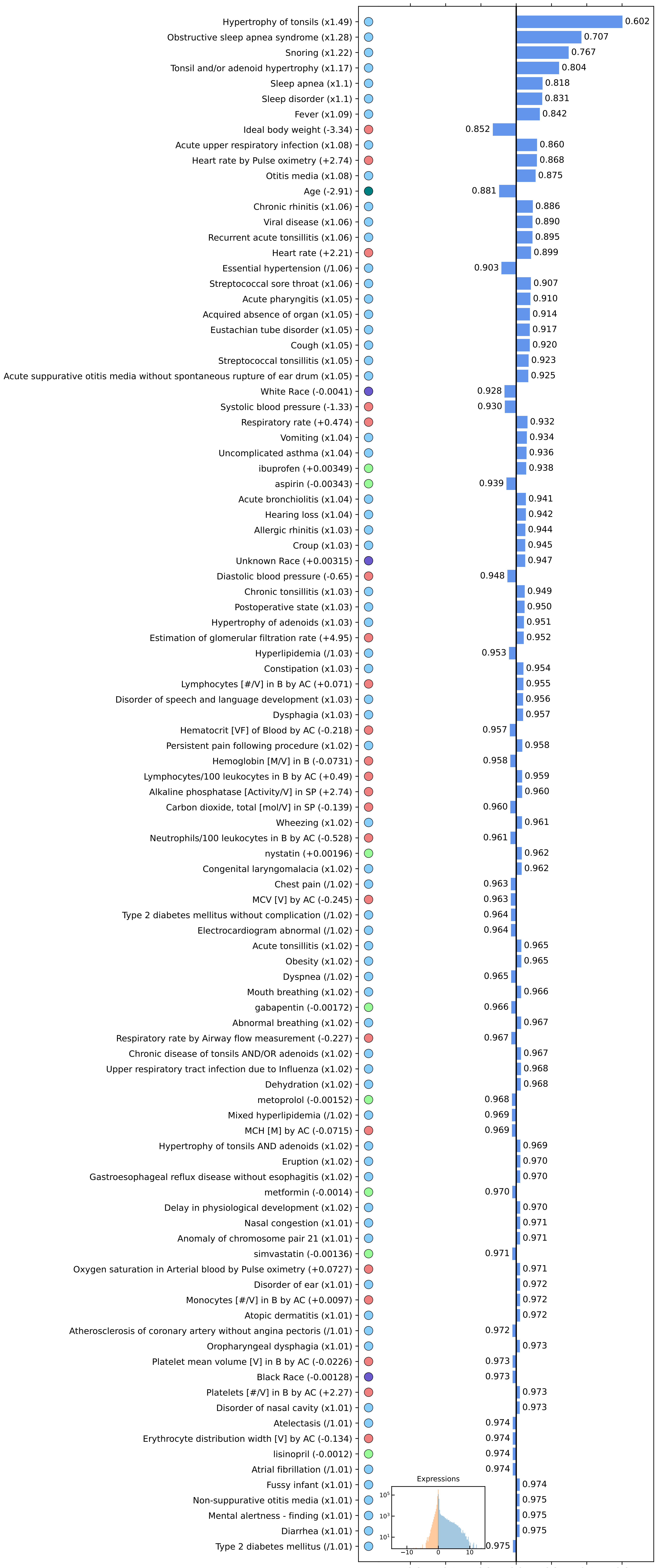

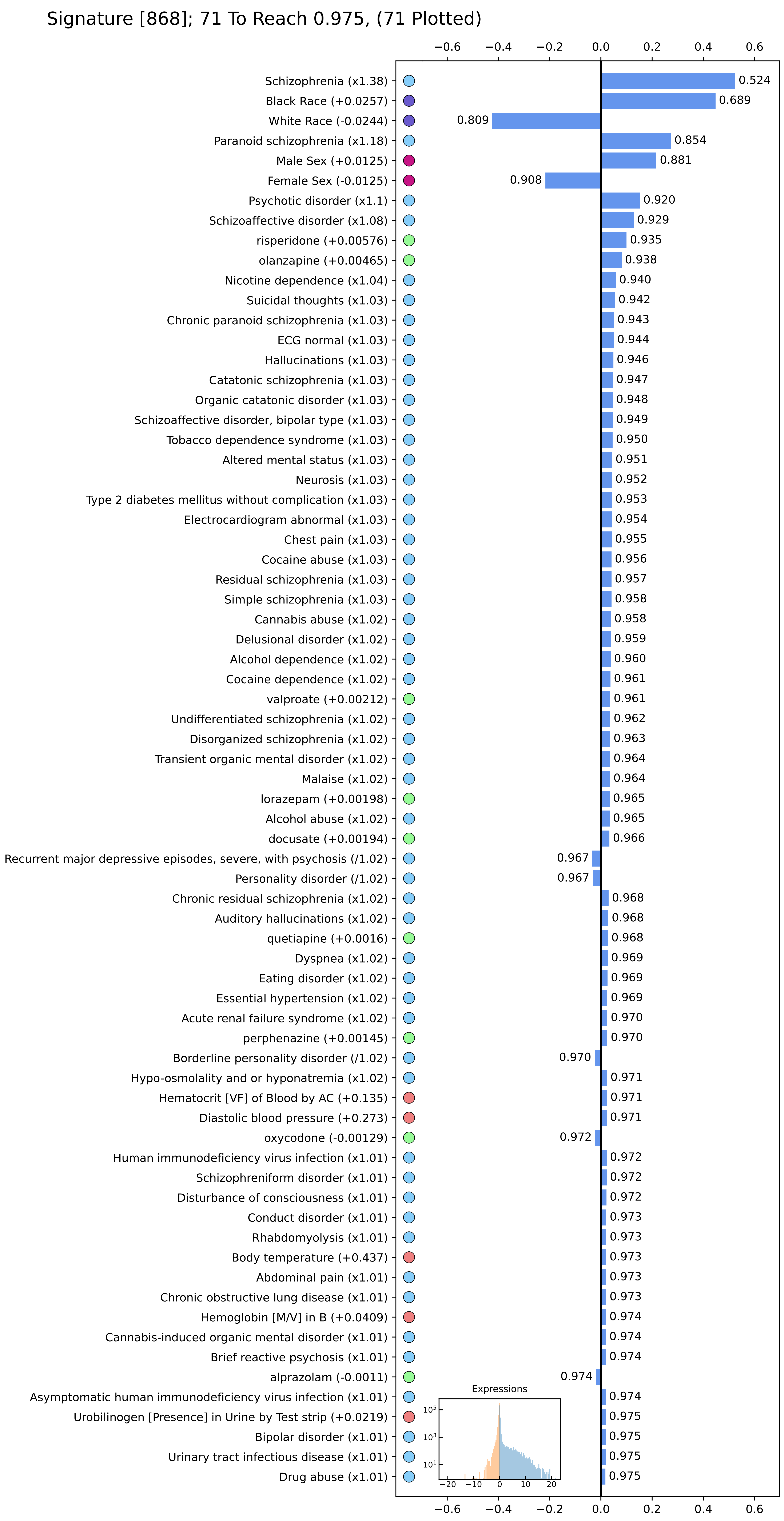

Signature [868]; 71 To Reach 0.975, (71 Plotted)
Schizophrenia (x1.38) 0.524
Black Race (+0.0257) 0.689
White Race (-0.0244) 0.809
Paranoid schizophrenia (x1.18) 0.854
Male Sex (+0.0125) 0.881
Female Sex (-0.0125) 0.908
Psychotic disorder (x1.1) 0.920
Schizoaffective disorder (x1.08) 0.929
risperidone (+0.00576) 0.935
olanzapine (+0.00465) 0.938
Nicotine dependence (x1.04) 0.940
Suicidal thoughts (x1.03) 0.942
Chronic paranoid schizophrenia (x1.03) 0.943
ECG normal (x1.03) 0.944
Hallucinations (x1.03) 0.946
Catatonic schizophrenia (x1.03) 0.947
Organic catatonic disorder (x1.03) 0.948
Schizoaffective disorder, bipolar type (x1.03) 0.949
Tobacco dependence syndrome (x1.03) 0.950
Altered mental status (x1.03) 0.951
Neurosis (x1.03) 0.952
Type 2 diabetes mellitus without complication (x1.03) 0.953
Electrocardiogram abnormal (x1.03) 0.954
Chest pain (x1.03) 0.955
Cocaine abuse (x1.03) 0.956
Residual schizophrenia (x1.03) 0.957
Simple schizophrenia (x1.03) 0.958
Cannabis abuse (x1.02) 0.958
Delusional disorder (x1.02) 0.959
Alcohol dependence (x1.02) 0.960
Cocaine dependence (x1.02) 0.961
valproate (+0.00212) 0.961
Undifferentiated schizophrenia (x1.02) 0.962
Disorganized schizophrenia (x1.02) 0.963
Transient organic mental disorder (x1.02) 0.964
Malaise (x1.02) 0.964
lorazepam (+0.00198) 0.965
Alcohol abuse (x1.02) 0.965
docusate (+0.00194) 0.966
Recurrent major depressive episodes, severe, with psychosis (/1.02) 0.967
Personality disorder (/1.02) 0.967
Chronic residual schizophrenia (x1.02) 0.968
Auditory hallucinations (x1.02) 0.968
quetiapine (+0.0016) 0.968
Dyspnea (x1.02) 0.969
Eating disorder (x1.02) 0.969
Essential hypertension (x1.02) 0.969
Acute renal failure syndrome (x1.02) 0.970
perphenazine (+0.00145) 0.970
Borderline personality disorder (/1.02) 0.970
Hypo-osmolality and or hyponatremia (x1.02) 0.971
Hematocrit [VF] of Blood by AC (+0.135) 0.971
Diastolic blood pressure (+0.273) 0.971
oxycodone (-0.00129) 0.972
Human immunodeficiency virus infection (x1.01) 0.972
Schizophreniform disorder (x1.01) 0.972
Disturbance of consciousness (x1.01) 0.972
Conduct disorder (x1.01) 0.973
Rhabdomyolysis (x1.01) 0.973
Body temperature (+0.437) 0.973
Abdominal pain (x1.01) 0.973
Chronic obstructive lung disease (x1.01) 0.973
Hemoglobin [M/V] in B (+0.0409) 0.974
Cannabis-induced organic mental disorder (x1.01) 0.974
Brief reactive psychosis (x1.01) 0.974
alprazolam (-0.0011) 0.974
Asymptomatic human immunodeficiency virus infection (x1.01) 0.974
Urobilinogen [Presence] in Urine by Test strip (+0.0219) 0.975
Bipolar disorder (x1.01) 0.975
Urinary tract infectious disease (x1.01) 0.975
Drug abuse (x1.01) 0.975
−0.6 −0.4 −0.2 0.0 0.2 0.4 0.6
Expressions
−20 −10 0 10 20

Signature [869]; 77 To Reach 0.975, (77 Plotted)

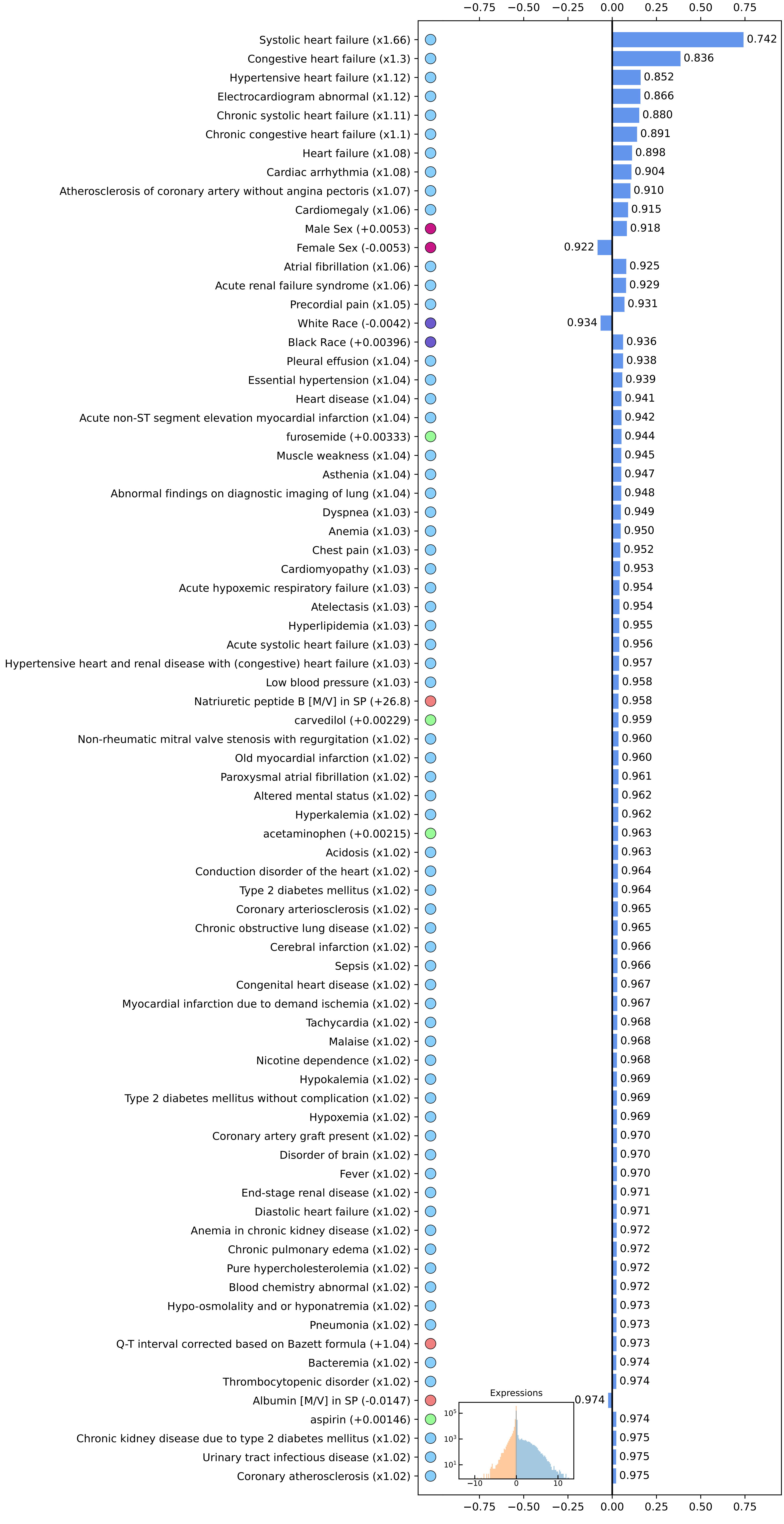

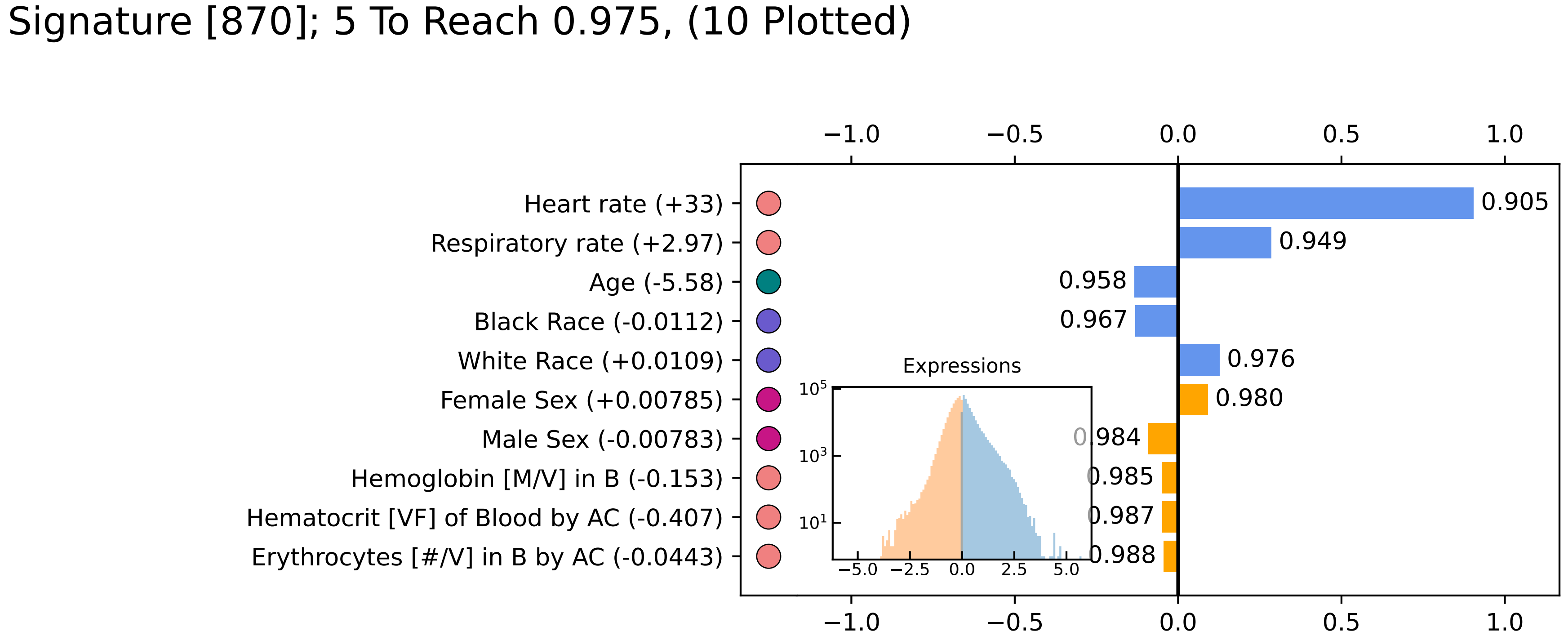

Signature [870]; 5 To Reach 0.975, (10 Plotted)
Heart rate (+33)
Respiratory rate (+2.97)
Age (-5.58)
Black Race (-0.0112)
White Race (+0.0109)
Female Sex (+0.00785)
Male Sex (-0.00783)
Hemoglobin [M/V] in B (-0.153)
Hematocrit [VF] of Blood by AC (-0.407)
Erythrocytes [#/V] in B by AC (-0.0443)
0.905
0.949
0.958
0.967
0.976
0.980
0.984
0.985
0.987
0.988
Expressions
−1.0
−0.5
0.0
0.5
1.0

Signature [871]; 2 To Reach 0.975, (10 Plotted)

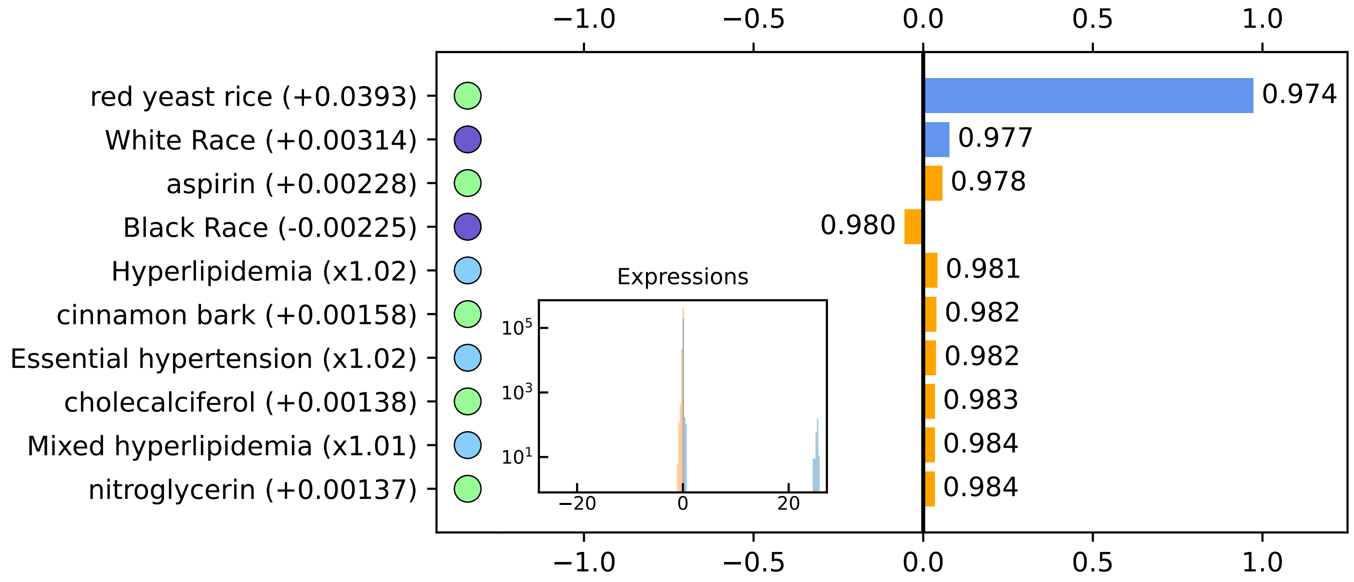

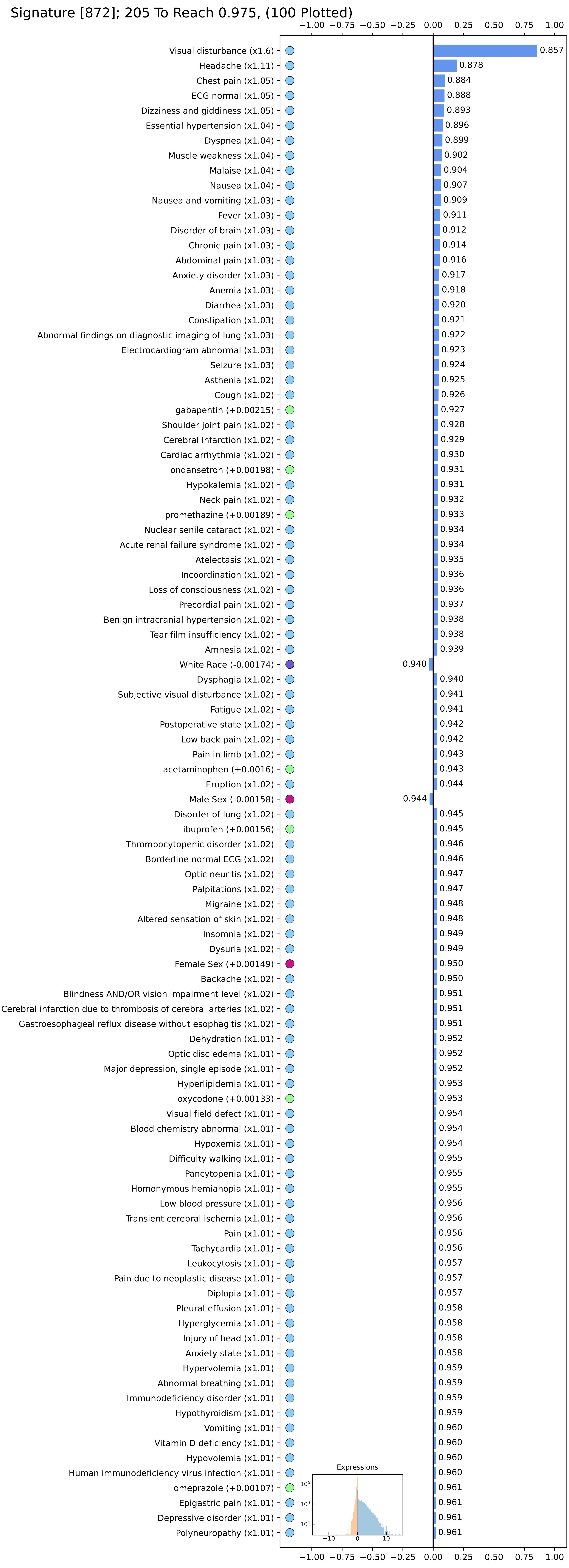

Signature [872]; 205 To Reach 0.975, (100 Plotted)
Visual disturbance (x1.6) 0.857
Headache (x1.11) 0.878
Chest pain (x1.05) 0.884
ECG normal (x1.05) 0.888
Dizziness and giddiness (x1.05) 0.893
Essential hypertension (x1.04) 0.896
Dyspnea (x1.04) 0.899
Muscle weakness (x1.04) 0.902
Malaise (x1.04) 0.904
Nausea (x1.04) 0.907
Nausea and vomiting (x1.03) 0.909
Fever (x1.03) 0.911
Disorder of brain (x1.03) 0.912
Chronic pain (x1.03) 0.914
Abdominal pain (x1.03) 0.916
Anxiety disorder (x1.03) 0.917
Anemia (x1.03) 0.918
Diarrhea (x1.03) 0.920
Constipation (x1.03) 0.921
Abnormal findings on diagnostic imaging of lung (x1.03) 0.922
Electrocardiogram abnormal (x1.03) 0.923
Seizure (x1.03) 0.924
Asthenia (x1.02) 0.925
Cough (x1.02) 0.926
gabapentin (+0.00215) 0.927
Shoulder joint pain (x1.02) 0.928
Cerebral infarction (x1.02) 0.929
Cardiac arrhythmia (x1.02) 0.930
ondansetron (+0.00198) 0.931
Hypokalemia (x1.02) 0.931
Neck pain (x1.02) 0.932
promethazine (+0.00189) 0.933
Nuclear senile cataract (x1.02) 0.934
Acute renal failure syndrome (x1.02) 0.934
Atelectasis (x1.02) 0.935
Incoordination (x1.02) 0.936
Loss of consciousness (x1.02) 0.936
Precordial pain (x1.02) 0.937
Benign intracranial hypertension (x1.02) 0.938
Tear film insufficiency (x1.02) 0.938
Amnesia (x1.02) 0.939
White Race (-0.00174) 0.940
Dysphagia (x1.02) 0.940
Subjective visual disturbance (x1.02) 0.941
Fatigue (x1.02) 0.941
Postoperative state (x1.02) 0.942
Low back pain (x1.02) 0.942
Pain in limb (x1.02) 0.943
acetaminophen (+0.0016) 0.943
Eruption (x1.02) 0.944
Male Sex (-0.00158) 0.944
Disorder of lung (x1.02) 0.945
ibuprofen (+0.00156) 0.945
Thrombocytopenic disorder (x1.02) 0.946
Borderline normal ECG (x1.02) 0.946
Optic neuritis (x1.02) 0.947
Palpitations (x1.02) 0.947
Migraine (x1.02) 0.948
Altered sensation of skin (x1.02) 0.948
Insomnia (x1.02) 0.949
Dysuria (x1.02) 0.949
Female Sex (+0.00149) 0.950
Backache (x1.02) 0.950
Blindness AND/OR vision impairment level (x1.02) 0.951
Cerebral infarction due to thrombosis of cerebral arteries (x1.02) 0.951
Gastroesophageal reflux disease without esophagitis (x1.02) 0.951
Dehydration (x1.01) 0.952
Optic disc edema (x1.01) 0.952
Major depression, single episode (x1.01) 0.952
Hyperlipidemia (x1.01) 0.953
oxycodone (+0.00133) 0.953
Visual field defect (x1.01) 0.954
Blood chemistry abnormal (x1.01) 0.954
Hypoxemia (x1.01) 0.954
Difficulty walking (x1.01) 0.955
Pancytopenia (x1.01) 0.955
Homonymous hemianopia (x1.01) 0.955
Low blood pressure (x1.01) 0.956
Transient cerebral ischemia (x1.01) 0.956
Pain (x1.01) 0.956
Tachycardia (x1.01) 0.956
Leukocytosis (x1.01) 0.957
Pain due to neoplastic disease (x1.01) 0.957
Diplopia (x1.01) 0.957
Pleural effusion (x1.01) 0.958
Hyperglycemia (x1.01) 0.958
Injury of head (x1.01) 0.958
Anxiety state (x1.01) 0.958
Hypervolemia (x1.01) 0.959
Abnormal breathing (x1.01) 0.959
Immunodeficiency disorder (x1.01) 0.959
Hypothyroidism (x1.01) 0.959
Vomiting (x1.01) 0.960
Vitamin D deficiency (x1.01) 0.960
Hypovolemia (x1.01) 0.960
Human immunodeficiency virus infection (x1.01) 0.960
omeprazole (+0.00107) 0.961
Epigastric pain (x1.01) 0.961
Depressive disorder (x1.01) 0.961
Polyneuropathy (x1.01) 0.961
Expressions
−1.00 −0.75 −0.50 −0.25 0.00 0.25 0.50 0.75 1.00

Signature [873]; 5 To Reach 0.975, (10 Plotted)

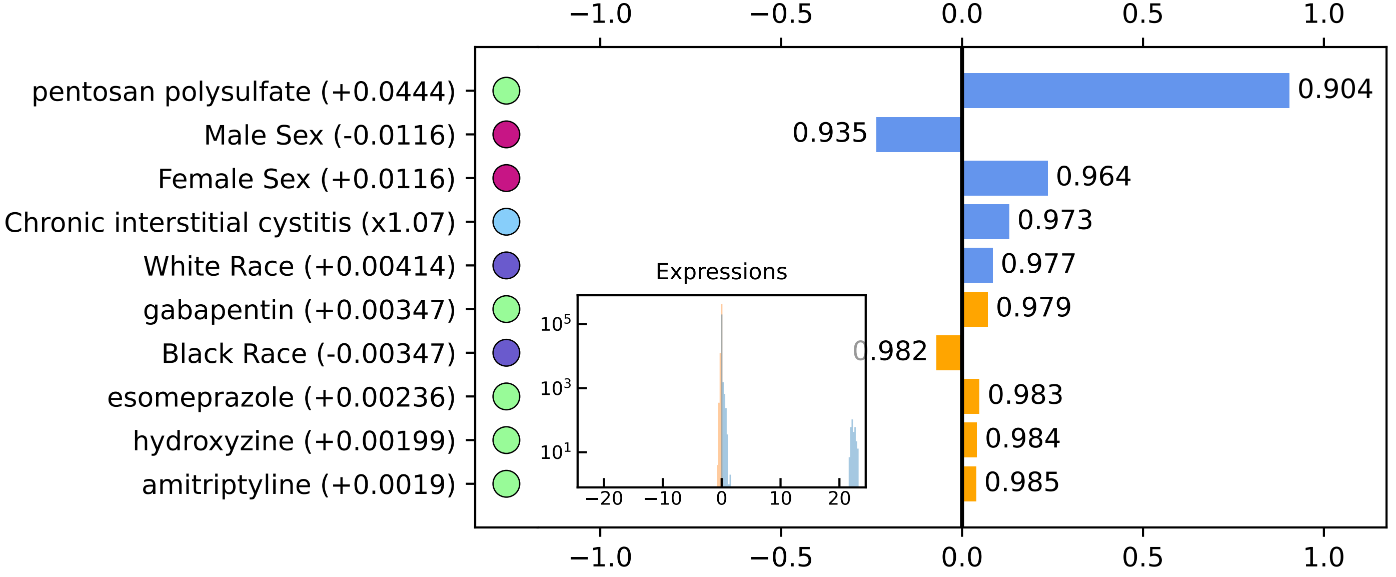

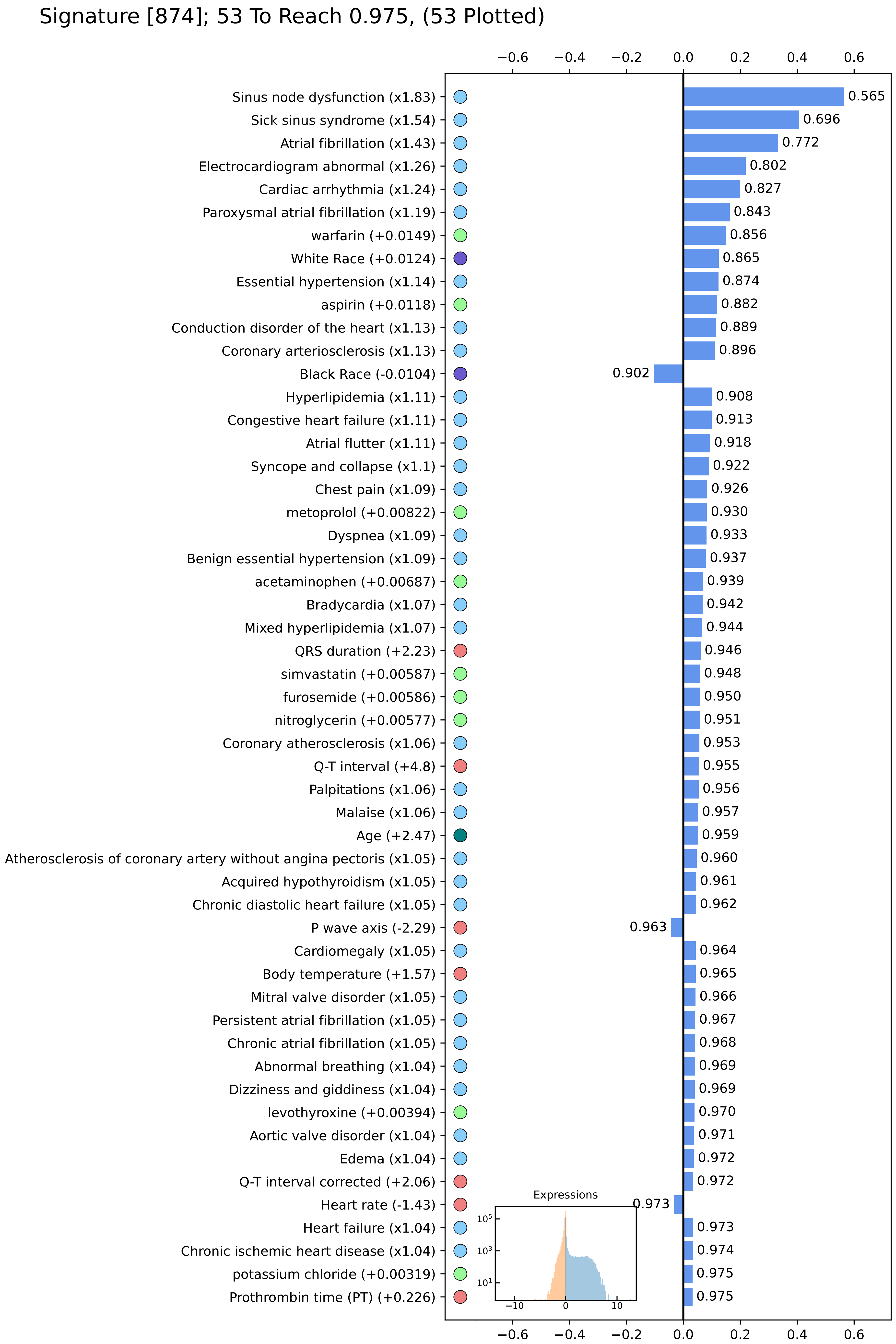
Signature [874]; 53 To Reach 0.975, (53 Plotted)
Sinus node dysfunction (x1.83) 0.565
Sick sinus syndrome (x1.54) 0.696
Atrial fibrillation (x1.43) 0.772
Electrocardiogram abnormal (x1.26) 0.802
Cardiac arrhythmia (x1.24) 0.827
Paroxysmal atrial fibrillation (x1.19) 0.843
warfarin (+0.0149) 0.856
White Race (+0.0124) 0.865
Essential hypertension (x1.14) 0.874
aspirin (+0.0118) 0.882
Conduction disorder of the heart (x1.13) 0.889
Coronary arteriosclerosis (x1.13) 0.896
Black Race (-0.0104) 0.902
Hyperlipidemia (x1.11) 0.908
Congestive heart failure (x1.11) 0.913
Atrial flutter (x1.11) 0.918
Syncope and collapse (x1.1) 0.922
Chest pain (x1.09) 0.926
metoprolol (+0.00822) 0.930
Dyspnea (x1.09) 0.933
Benign essential hypertension (x1.09) 0.937
acetaminophen (+0.00687) 0.939
Bradycardia (x1.07) 0.942
Mixed hyperlipidemia (x1.07) 0.944
QRS duration (+2.23) 0.946
simvastatin (+0.00587) 0.948
furosemide (+0.00586) 0.950
nitroglycerin (+0.00577) 0.951
Coronary atherosclerosis (x1.06) 0.953
Q-T interval (+4.8) 0.955
Palpitations (x1.06) 0.956
Malaise (x1.06) 0.957
Age (+2.47) 0.959
Atherosclerosis of coronary artery without angina pectoris (x1.05) 0.960
Acquired hypothyroidism (x1.05) 0.961
Chronic diastolic heart failure (x1.05) 0.962
P wave axis (-2.29) 0.963
Cardiomegaly (x1.05) 0.964
Body temperature (+1.57) 0.965
Mitral valve disorder (x1.05) 0.966
Persistent atrial fibrillation (x1.05) 0.967
Chronic atrial fibrillation (x1.05) 0.968
Abnormal breathing (x1.04) 0.969
Dizziness and giddiness (x1.04) 0.969
levothyroxine (+0.00394) 0.970
Aortic valve disorder (x1.04) 0.971
Edema (x1.04) 0.972
Q-T interval corrected (+2.06) 0.972
Heart rate (-1.43) 0.973
Heart failure (x1.04) 0.973
Chronic ischemic heart disease (x1.04) 0.974
potassium chloride (+0.00319) 0.975
Prothrombin time (PT) (+0.226) 0.975
−0.6 −0.4 −0.2 0.0 0.2 0.4 0.6
Expressions
10^5
10^3
10^1
−10 0 10

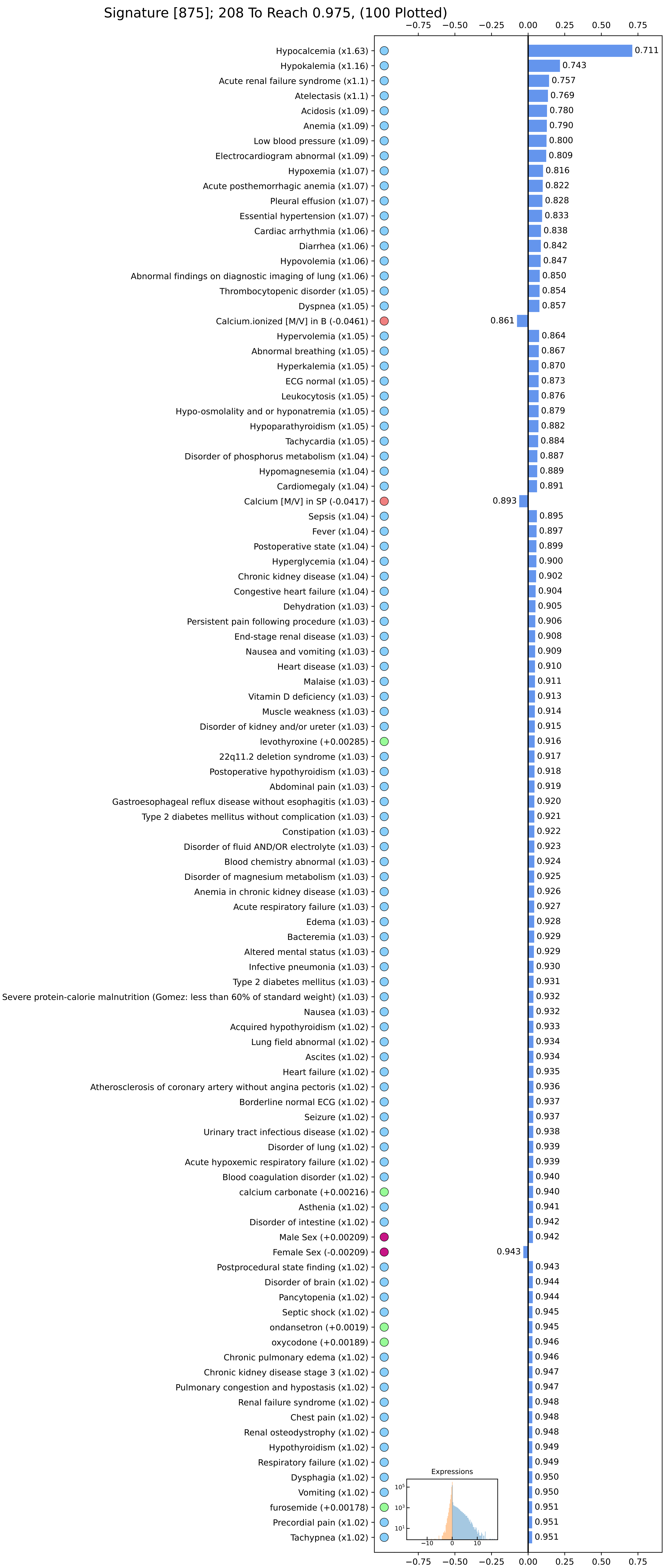
Signature [875]; 208 To Reach 0.975, (100 Plotted)
−0.75 −0.50 −0.25 0.00 0.25 0.50 0.75
Hypocalcemia (x1.63) 0.711
Hypokalemia (x1.16) 0.743
Acute renal failure syndrome (x1.1) 0.757
Atelectasis (x1.1) 0.769
Acidosis (x1.09) 0.780
Anemia (x1.09) 0.790
Low blood pressure (x1.09) 0.800
Electrocardiogram abnormal (x1.09) 0.809
Hypoxemia (x1.07) 0.816
Acute posthemorrhagic anemia (x1.07) 0.822
Pleural effusion (x1.07) 0.828
Essential hypertension (x1.07) 0.833
Cardiac arrhythmia (x1.06) 0.838
Diarrhea (x1.06) 0.842
Hypovolemia (x1.06) 0.847
Abnormal findings on diagnostic imaging of lung (x1.06) 0.850
Thrombocytopenic disorder (x1.05) 0.854
Dyspnea (x1.05) 0.857
Calcium.ionized [M/V] in B (-0.0461) 0.861
Hypervolemia (x1.05) 0.864
Abnormal breathing (x1.05) 0.867
Hyperkalemia (x1.05) 0.870
ECG normal (x1.05) 0.873
Leukocytosis (x1.05) 0.876
Hypo-osmolality and or hyponatremia (x1.05) 0.879
Hypoparathyroidism (x1.05) 0.882
Tachycardia (x1.05) 0.884
Disorder of phosphorus metabolism (x1.04) 0.887
Hypomagnesemia (x1.04) 0.889
Cardiomegaly (x1.04) 0.891
Calcium [M/V] in SP (-0.0417) 0.893
Sepsis (x1.04) 0.895
Fever (x1.04) 0.897
Postoperative state (x1.04) 0.899
Hyperglycemia (x1.04) 0.900
Chronic kidney disease (x1.04) 0.902
Congestive heart failure (x1.04) 0.904
Dehydration (x1.03) 0.905
Persistent pain following procedure (x1.03) 0.906
End-stage renal disease (x1.03) 0.908
Nausea and vomiting (x1.03) 0.909
Heart disease (x1.03) 0.910
Malaise (x1.03) 0.911
Vitamin D deficiency (x1.03) 0.913
Muscle weakness (x1.03) 0.914
Disorder of kidney and/or ureter (x1.03) 0.915
levothyroxine (+0.00285) 0.916
22q11.2 deletion syndrome (x1.03) 0.917
Postoperative hypothyroidism (x1.03) 0.918
Abdominal pain (x1.03) 0.919
Gastroesophageal reflux disease without esophagitis (x1.03) 0.920
Type 2 diabetes mellitus without complication (x1.03) 0.921
Constipation (x1.03) 0.922
Disorder of fluid AND/OR electrolyte (x1.03) 0.923
Blood chemistry abnormal (x1.03) 0.924
Disorder of magnesium metabolism (x1.03) 0.925
Anemia in chronic kidney disease (x1.03) 0.926
Acute respiratory failure (x1.03) 0.927
Edema (x1.03) 0.928
Bacteremia (x1.03) 0.929
Altered mental status (x1.03) 0.929
Infective pneumonia (x1.03) 0.930
Type 2 diabetes mellitus (x1.03) 0.931
Severe protein-calorie malnutrition (Gomez: less than 60% of standard weight) (x1.03) 0.932
Nausea (x1.03) 0.932
Acquired hypothyroidism (x1.02) 0.933
Lung field abnormal (x1.02) 0.934
Ascites (x1.02) 0.934
Heart failure (x1.02) 0.935
Atherosclerosis of coronary artery without angina pectoris (x1.02) 0.936
Borderline normal ECG (x1.02) 0.937
Seizure (x1.02) 0.937
Urinary tract infectious disease (x1.02) 0.938
Disorder of lung (x1.02) 0.939
Acute hypoxemic respiratory failure (x1.02) 0.939
Blood coagulation disorder (x1.02) 0.940
calcium carbonate (+0.00216) 0.940
Asthenia (x1.02) 0.941
Disorder of intestine (x1.02) 0.942
Male Sex (+0.00209) 0.942
Female Sex (-0.00209) 0.943
Postprocedural state finding (x1.02) 0.943
Disorder of brain (x1.02) 0.944
Pancytopenia (x1.02) 0.944
Septic shock (x1.02) 0.945
ondansetron (+0.0019) 0.945
oxycodone (+0.00189) 0.946
Chronic pulmonary edema (x1.02) 0.946
Chronic kidney disease stage 3 (x1.02) 0.947
Pulmonary congestion and hypostasis (x1.02) 0.947
Renal failure syndrome (x1.02) 0.948
Chest pain (x1.02) 0.948
Renal osteodystrophy (x1.02) 0.948
Hypothyroidism (x1.02) 0.949
Respiratory failure (x1.02) 0.949
Dysphagia (x1.02) 0.950
Vomiting (x1.02) 0.950
furosemide (+0.00178) 0.951
Precordial pain (x1.02) 0.951
Tachypnea (x1.02) 0.951
Expressions
10^5
10^3
10^1
−10 0 10
−0.75 −0.50 −0.25 0.00 0.25 0.50 0.75

Signature [876]; 2 To Reach 0.975, (10 Plotted)

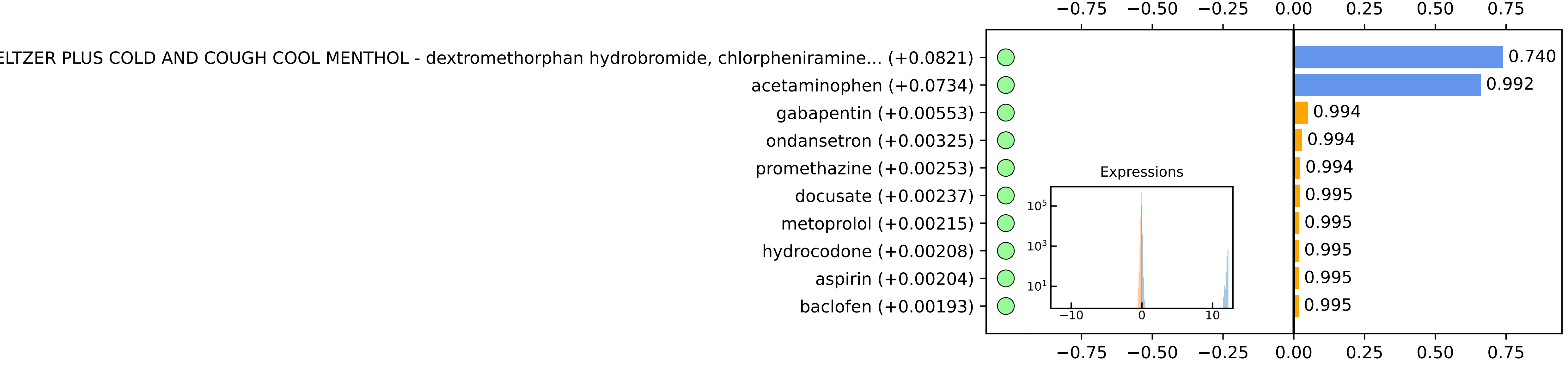

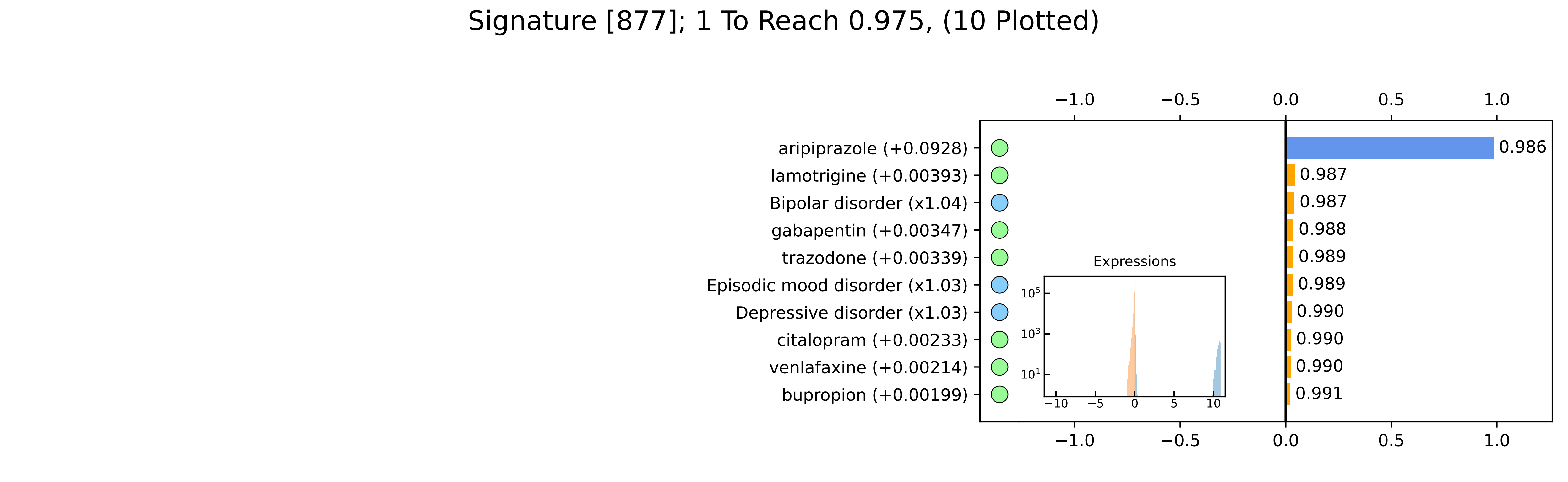

Signature [877]; 1 To Reach 0.975, (10 Plotted)
aripiprazole (+0.0928)
lamotrigine (+0.00393)
Bipolar disorder (x1.04)
gabapentin (+0.00347)
trazodone (+0.00339)
Episodic mood disorder (x1.03)
Depressive disorder (x1.03)
citalopram (+0.00233)
venlafaxine (+0.00214)
bupropion (+0.00199)
0.986
0.987
0.987
0.988
0.989
0.989
0.990
0.990
0.990
0.991
−1.0
−0.5
0.0
0.5
1.0
Expressions
10^5
10^3
10^1
−10
−5
0
5
10

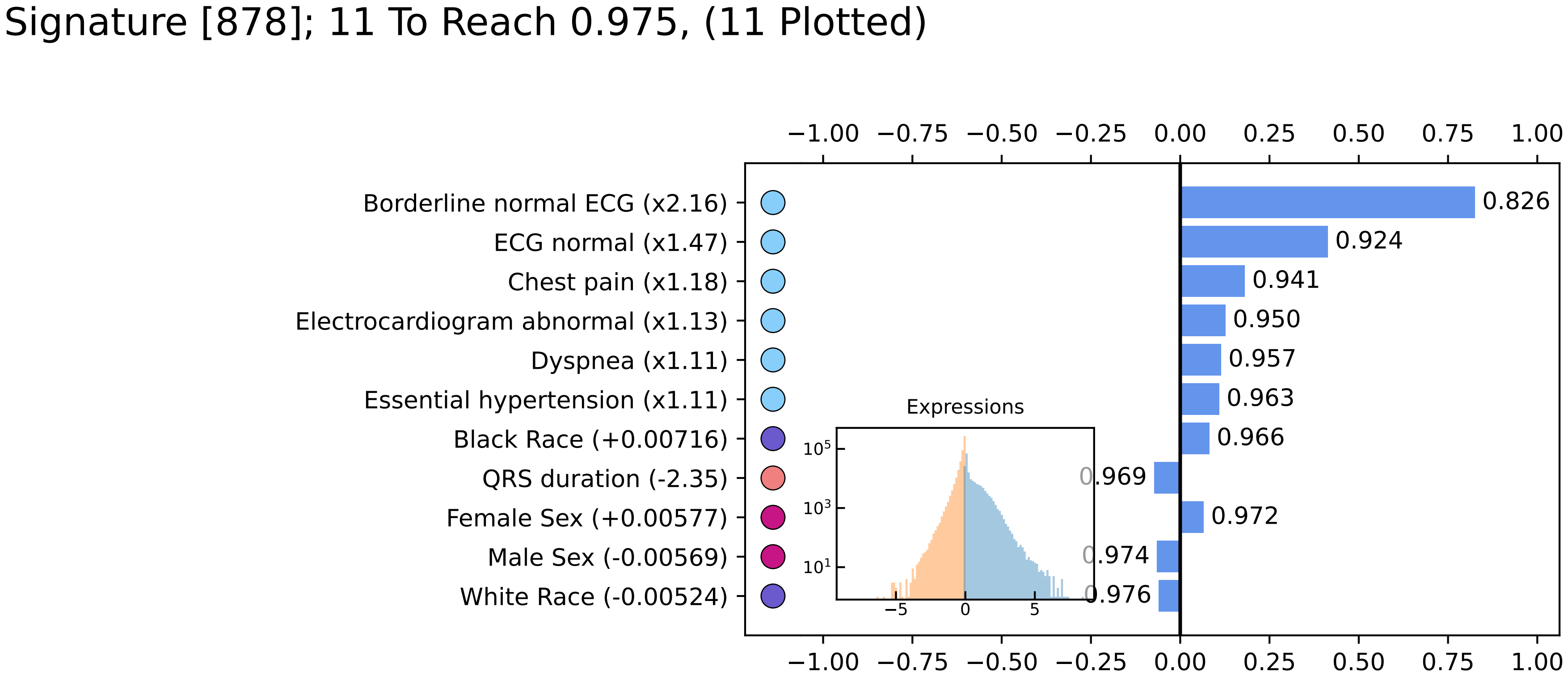

Signature [878]; 11 To Reach 0.975, (11 Plotted)
Borderline normal ECG (x2.16)
ECG normal (x1.47)
Chest pain (x1.18)
Electrocardiogram abnormal (x1.13)
Dyspnea (x1.11)
Essential hypertension (x1.11)
Black Race (+0.00716)
QRS duration (-2.35)
Female Sex (+0.00577)
Male Sex (-0.00569)
White Race (-0.00524)
0.826
0.924
0.941
0.950
0.957
0.963
0.966
0.969
0.972
0.974
0.976
Expressions
−1.00 −0.75 −0.50 −0.25 0.00 0.25 0.50 0.75 1.00

Signature [879]; 2 To Reach 0.975, (10 Plotted)

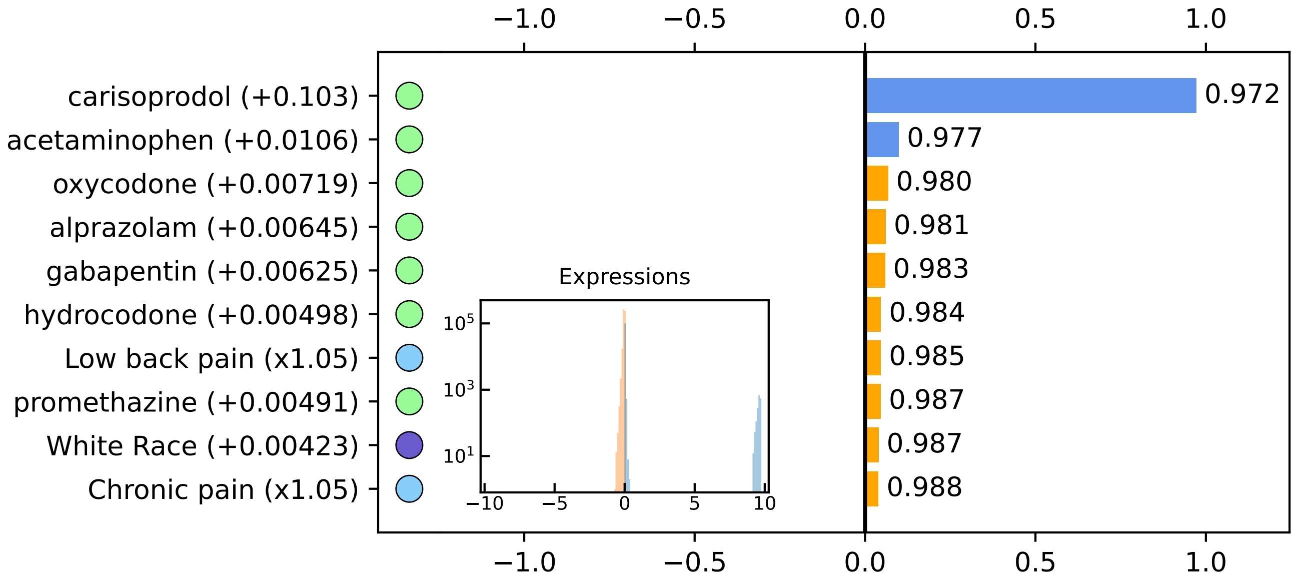

Signature [880]; 5 To Reach 0.975, (10 Plotted)

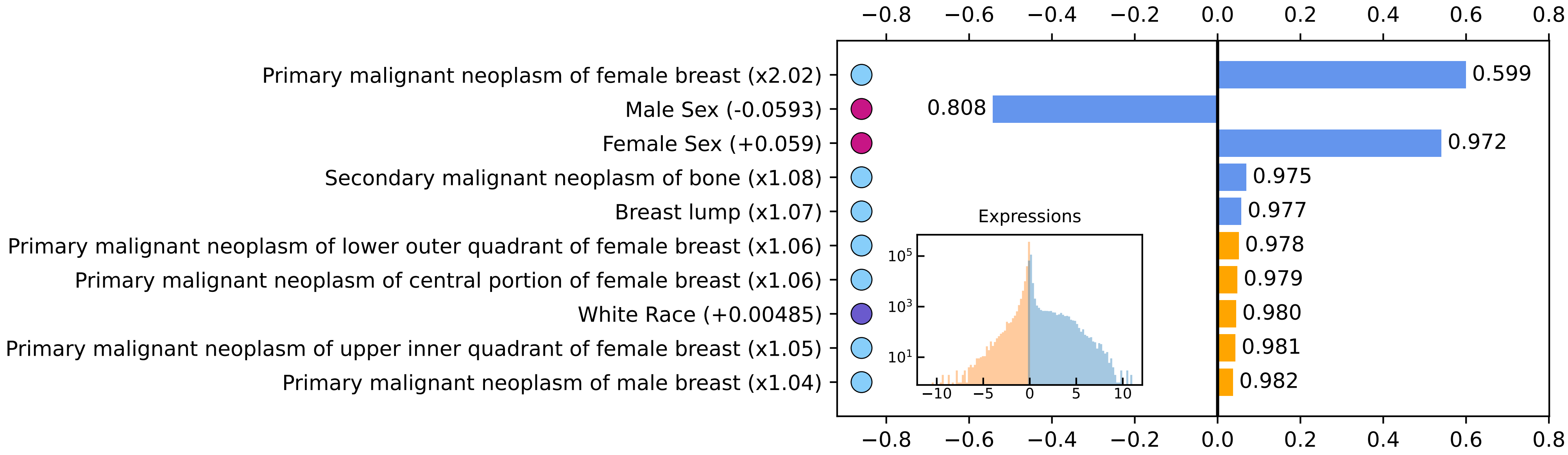

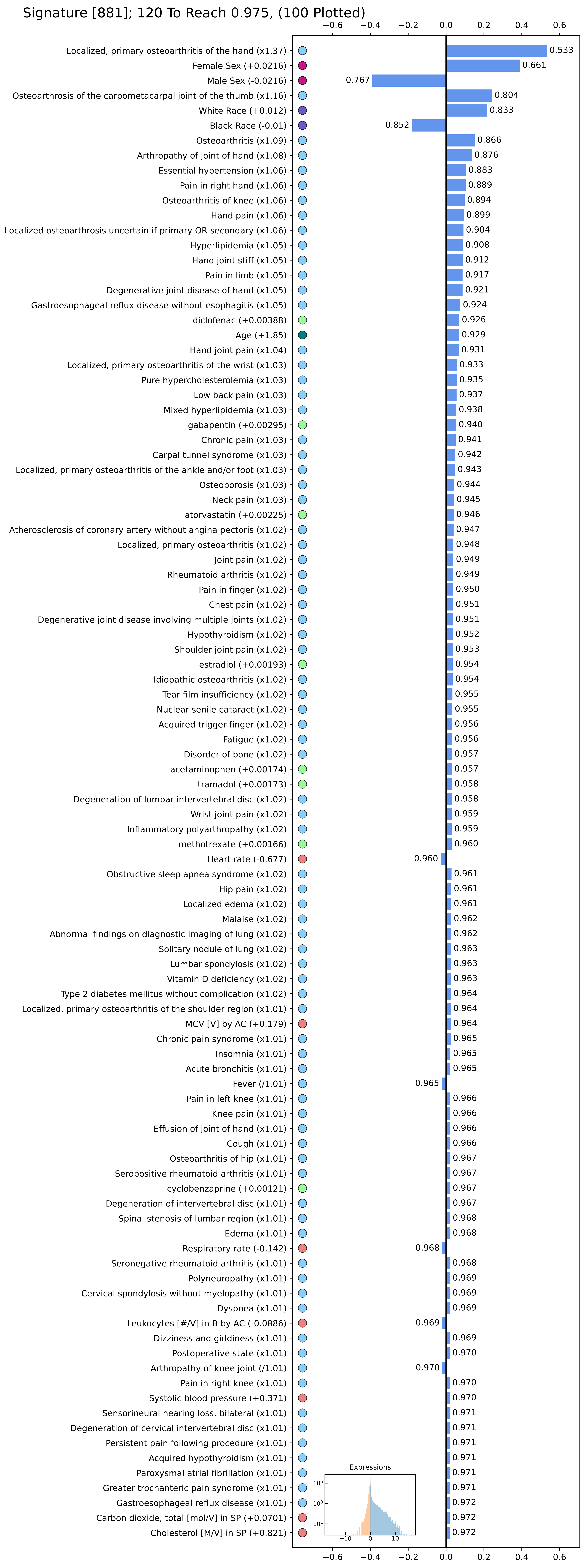

Signature [881]; 120 To Reach 0.975, (100 Plotted)
Localized, primary osteoarthritis of the hand (x1.37) 0.533
Female Sex (+0.0216) 0.661
Male Sex (-0.0216) 0.767
Osteoarthrosis of the carpometacarpal joint of the thumb (x1.16) 0.804
White Race (+0.012) 0.833
Black Race (-0.01) 0.852
Osteoarthritis (x1.09) 0.866
Arthropathy of joint of hand (x1.08) 0.876
Essential hypertension (x1.06) 0.883
Pain in right hand (x1.06) 0.889
Osteoarthritis of knee (x1.06) 0.894
Hand pain (x1.06) 0.899
Localized osteoarthrosis uncertain if primary OR secondary (x1.06) 0.904
Hyperlipidemia (x1.05) 0.908
Hand joint stiff (x1.05) 0.912
Pain in limb (x1.05) 0.917
Degenerative joint disease of hand (x1.05) 0.921
Gastroesophageal reflux disease without esophagitis (x1.05) 0.924
diclofenac (+0.00388) 0.926
Age (+1.85) 0.929
Hand joint pain (x1.04) 0.931
Localized, primary osteoarthritis of the wrist (x1.03) 0.933
Pure hypercholesterolemia (x1.03) 0.935
Low back pain (x1.03) 0.937
Mixed hyperlipidemia (x1.03) 0.938
gabapentin (+0.00295) 0.940
Chronic pain (x1.03) 0.941
Carpal tunnel syndrome (x1.03) 0.942
Localized, primary osteoarthritis of the ankle and/or foot (x1.03) 0.943
Osteoporosis (x1.03) 0.944
Neck pain (x1.03) 0.945
atorvastatin (+0.00225) 0.946
Atherosclerosis of coronary artery without angina pectoris (x1.02) 0.947
Localized, primary osteoarthritis (x1.02) 0.948
Joint pain (x1.02) 0.949
Rheumatoid arthritis (x1.02) 0.949
Pain in finger (x1.02) 0.950
Chest pain (x1.02) 0.951
Degenerative joint disease involving multiple joints (x1.02) 0.951
Hypothyroidism (x1.02) 0.952
Shoulder joint pain (x1.02) 0.953
estradiol (+0.00193) 0.954
Idiopathic osteoarthritis (x1.02) 0.954
Tear film insufficiency (x1.02) 0.955
Nuclear senile cataract (x1.02) 0.955
Acquired trigger finger (x1.02) 0.956
Fatigue (x1.02) 0.956
Disorder of bone (x1.02) 0.957
acetaminophen (+0.00174) 0.957
tramadol (+0.00173) 0.958
Degeneration of lumbar intervertebral disc (x1.02) 0.958
Wrist joint pain (x1.02) 0.959
Inflammatory polyarthropathy (x1.02) 0.959
methotrexate (+0.00166) 0.960
Heart rate (-0.677) 0.960
Obstructive sleep apnea syndrome (x1.02) 0.961
Hip pain (x1.02) 0.961
Localized edema (x1.02) 0.961
Malaise (x1.02) 0.962
Abnormal findings on diagnostic imaging of lung (x1.02) 0.962
Solitary nodule of lung (x1.02) 0.963
Lumbar spondylosis (x1.02) 0.963
Vitamin D deficiency (x1.02) 0.963
Type 2 diabetes mellitus without complication (x1.02) 0.964
Localized, primary osteoarthritis of the shoulder region (x1.01) 0.964
MCV [V] by AC (+0.179) 0.964
Chronic pain syndrome (x1.01) 0.965
Insomnia (x1.01) 0.965
Acute bronchitis (x1.01) 0.965
Fever (/1.01) 0.965
Pain in left knee (x1.01) 0.966
Knee pain (x1.01) 0.966
Effusion of joint of hand (x1.01) 0.966
Cough (x1.01) 0.966
Osteoarthritis of hip (x1.01) 0.967
Seropositive rheumatoid arthritis (x1.01) 0.967
cyclobenzaprine (+0.00121) 0.967
Degeneration of intervertebral disc (x1.01) 0.967
Spinal stenosis of lumbar region (x1.01) 0.968
Edema (x1.01) 0.968
Respiratory rate (-0.142) 0.968
Seronegative rheumatoid arthritis (x1.01) 0.968
Polyneuropathy (x1.01) 0.969
Cervical spondylosis without myelopathy (x1.01) 0.969
Dyspnea (x1.01) 0.969
Leukocytes [#/V] in B by AC (-0.0886) 0.969
Dizziness and giddiness (x1.01) 0.969
Postoperative state (x1.01) 0.970
Arthropathy of knee joint (/1.01) 0.970
Pain in right knee (x1.01) 0.970
Systolic blood pressure (+0.371) 0.970
Sensorineural hearing loss, bilateral (x1.01) 0.971
Degeneration of cervical intervertebral disc (x1.01) 0.971
Persistent pain following procedure (x1.01) 0.971
Acquired hypothyroidism (x1.01) 0.971
Paroxysmal atrial fibrillation (x1.01) 0.971
Greater trochanteric pain syndrome (x1.01) 0.971
Gastroesophageal reflux disease (x1.01) 0.972
Carbon dioxide, total [mol/V] in SP (+0.0701) 0.972
Cholesterol [M/V] in SP (+0.821) 0.972
Expressions
−0.6 −0.4 −0.2 0.0 0.2 0.4 0.6

Signature [882]; 3 To Reach 0.975, (10 Plotted)

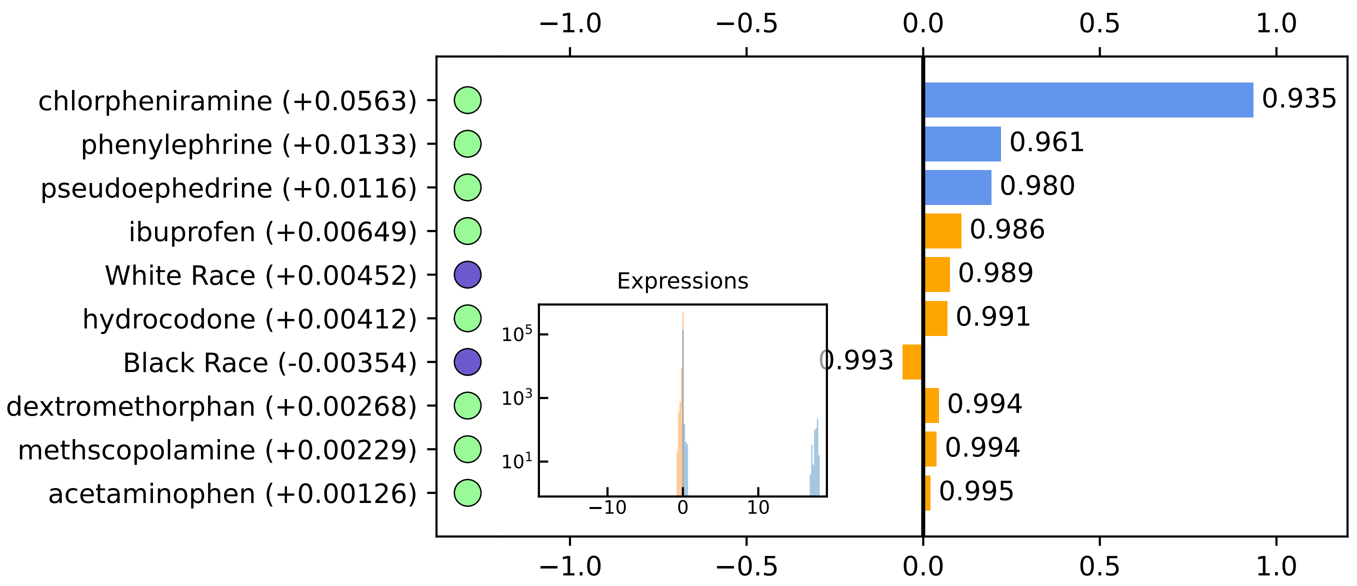

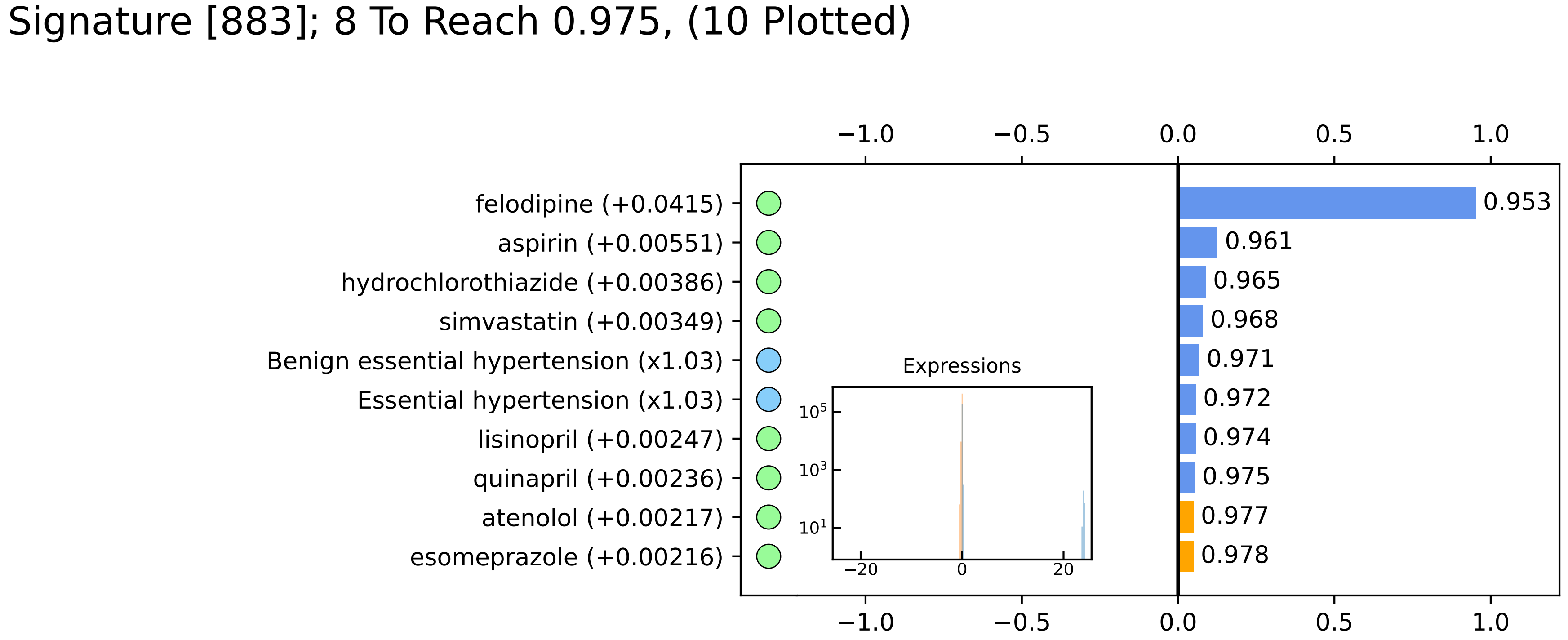

Signature [883]; 8 To Reach 0.975, (10 Plotted)
felodipine (+0.0415)
aspirin (+0.00551)
hydrochlorothiazide (+0.00386)
simvastatin (+0.00349)
Benign essential hypertension (x1.03)
Essential hypertension (x1.03)
lisinopril (+0.00247)
quinapril (+0.00236)
atenolol (+0.00217)
esomeprazole (+0.00216)
0.953
0.961
0.965
0.968
0.971
0.972
0.974
0.975
0.977
0.978
Expressions
−1.0
−0.5
0.0
0.5
1.0
−20
0
20

Signature [884]; 42 To Reach 0.975, (42 Plotted)

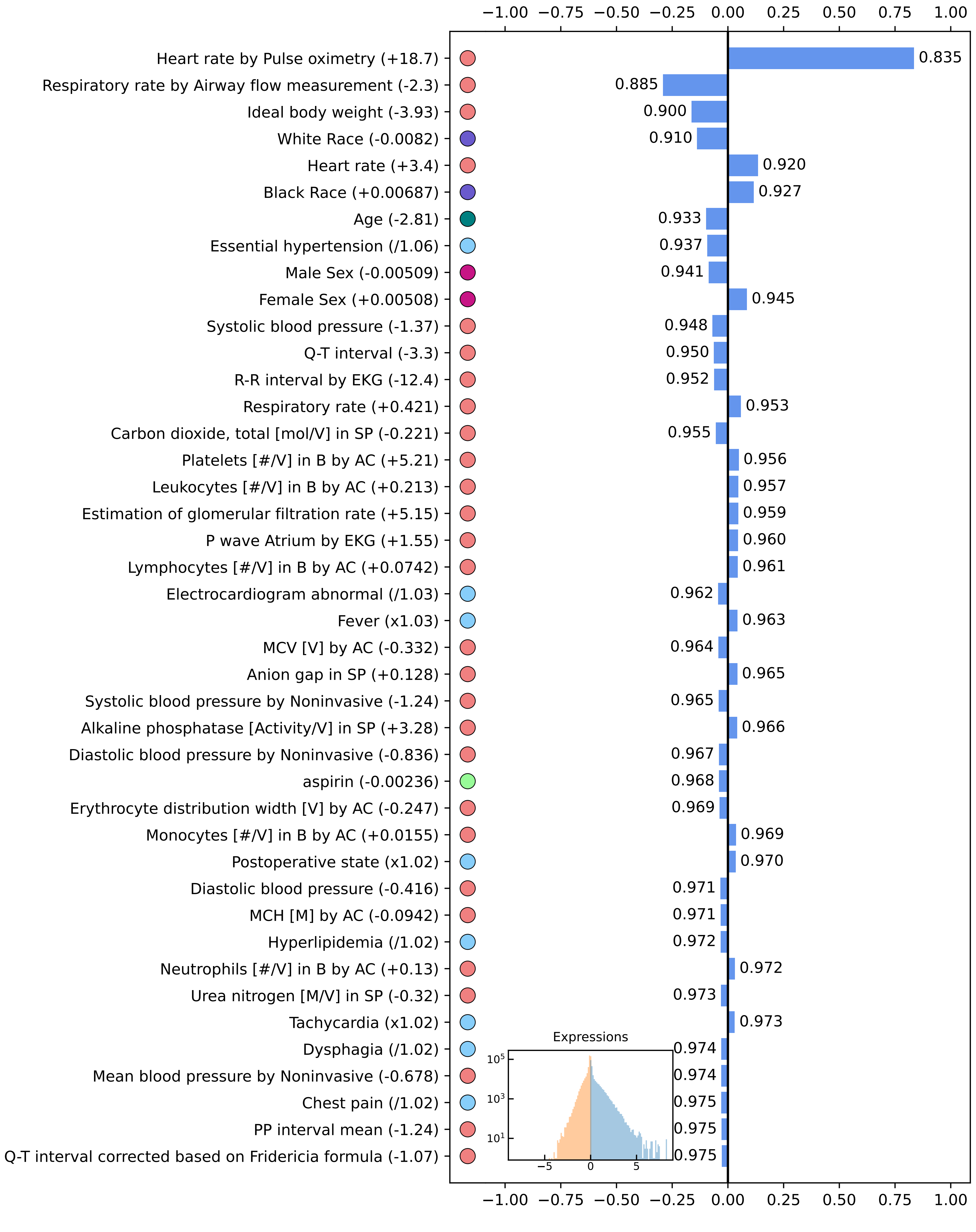

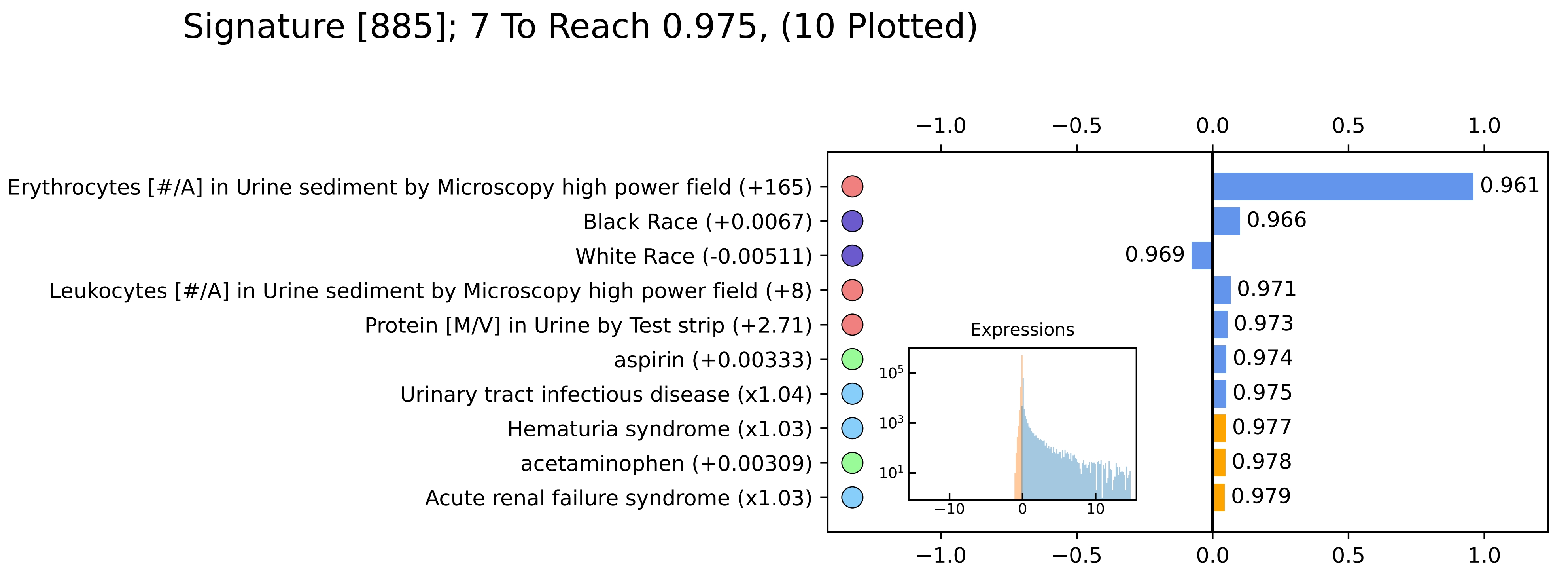
Signature [885]; 7 To Reach 0.975, (10 Plotted)
Erythrocytes [#/A] in Urine sediment by Microscopy high power field (+165)
Black Race (+0.0067)
White Race (-0.00511)
Leukocytes [#/A] in Urine sediment by Microscopy high power field (+8)
Protein [M/V] in Urine by Test strip (+2.71)
aspirin (+0.00333)
Urinary tract infectious disease (x1.04)
Hematuria syndrome (x1.03)
acetaminophen (+0.00309)
Acute renal failure syndrome (x1.03)
0.961
0.966
0.969
0.971
0.973
0.974
0.975
0.977
0.978
0.979
Expressions
−1.0
−0.5
0.0
0.5
1.0

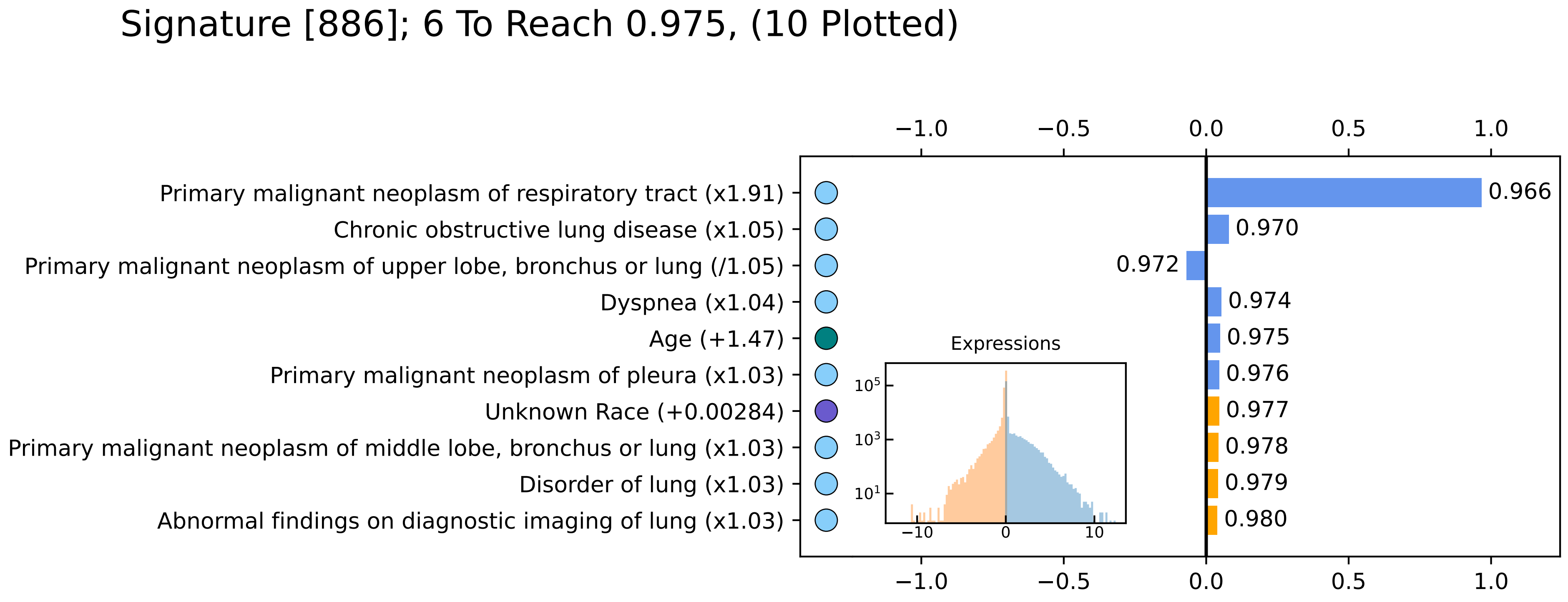

Signature [886]; 6 To Reach 0.975, (10 Plotted)
Primary malignant neoplasm of respiratory tract (x1.91)
Chronic obstructive lung disease (x1.05)
Primary malignant neoplasm of upper lobe, bronchus or lung (/1.05)
Dyspnea (x1.04)
Age (+1.47)
Primary malignant neoplasm of pleura (x1.03)
Unknown Race (+0.00284)
Primary malignant neoplasm of middle lobe, bronchus or lung (x1.03)
Disorder of lung (x1.03)
Abnormal findings on diagnostic imaging of lung (x1.03)
0.966
0.970
0.972
0.974
0.975
0.976
0.977
0.978
0.979
0.980
−1.0
−0.5
0.0
0.5
1.0
Expressions
10^5
10^3
10^1
−10
0
10

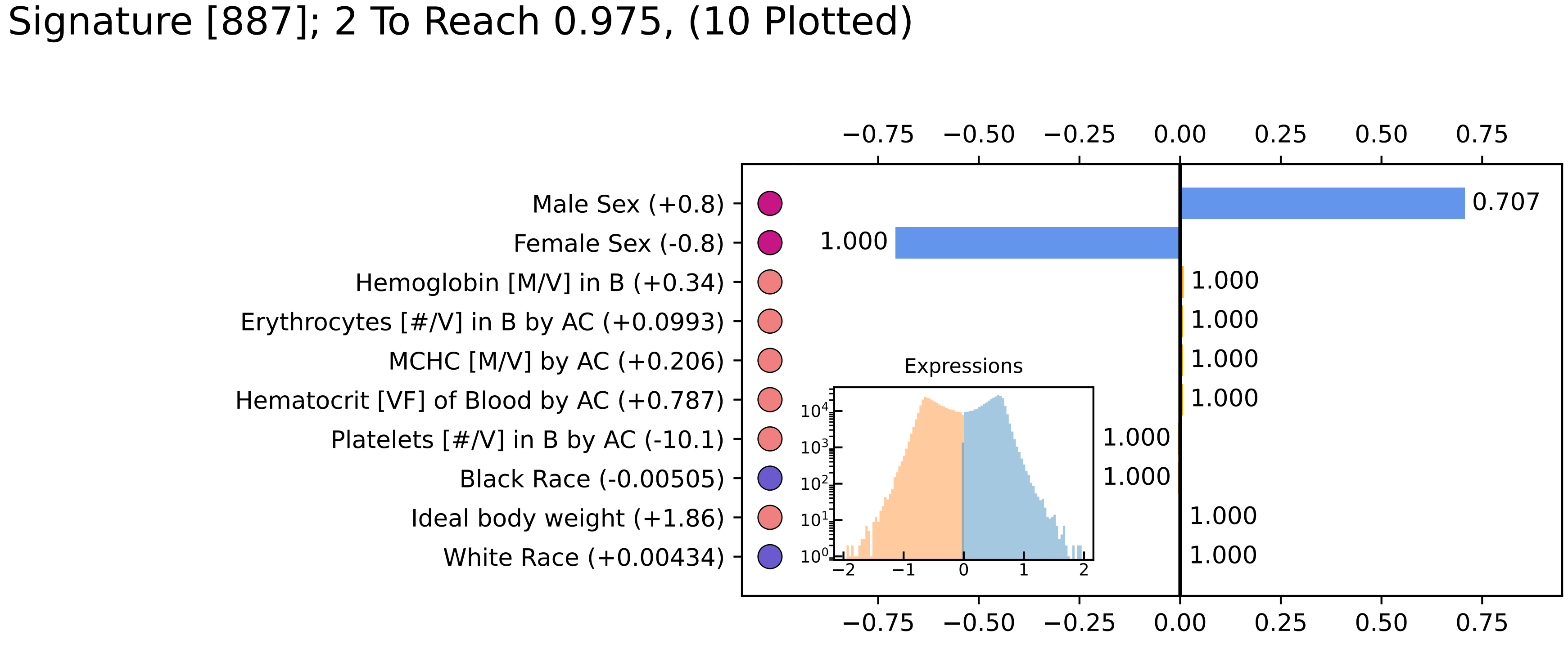

Signature [887]; 2 To Reach 0.975, (10 Plotted)
Male Sex (+0.8)
Female Sex (-0.8)
Hemoglobin [M/V] in B (+0.34)
Erythrocytes [#/V] in B by AC (+0.0993)
MCHC [M/V] by AC (+0.206)
Hematocrit [VF] of Blood by AC (+0.787)
Platelets [#/V] in B by AC (-10.1)
Black Race (-0.00505)
Ideal body weight (+1.86)
White Race (+0.00434)
0.707
1.000
1.000
1.000
1.000
1.000
1.000
1.000
1.000
1.000
Expressions
−0.75
−0.50
−0.25
0.00
0.25
0.50
0.75

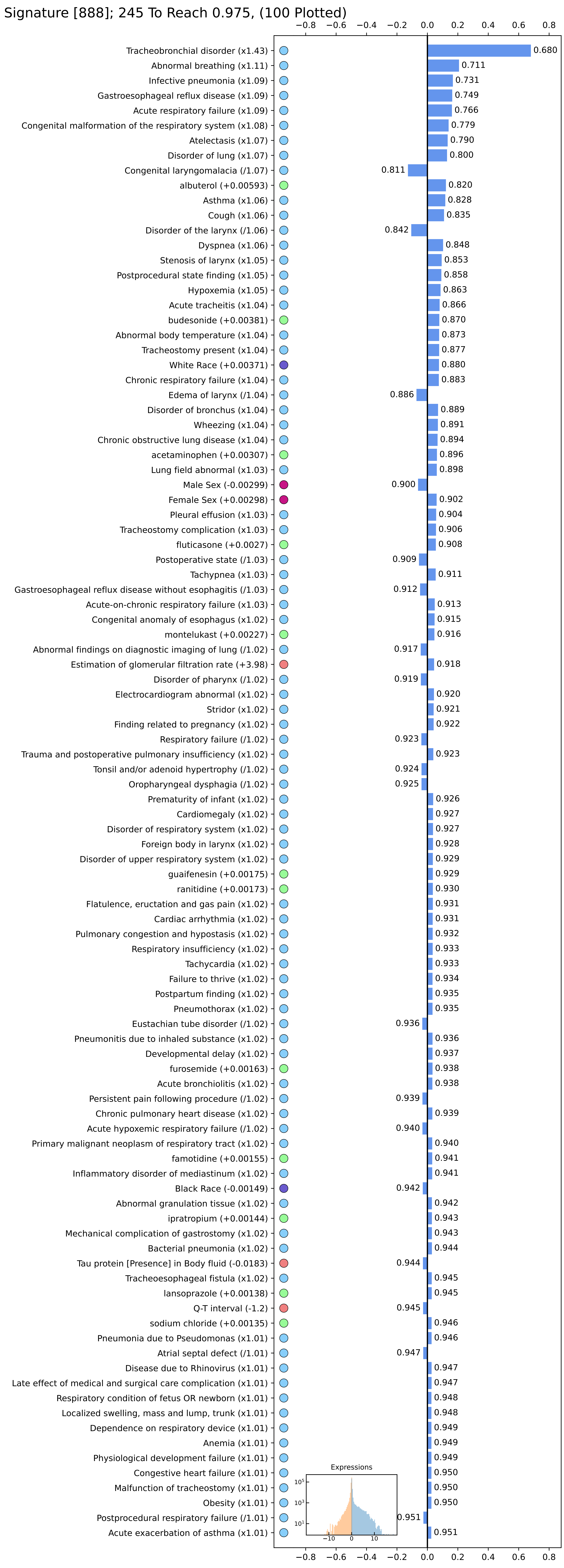

Signature [888]; 245 To Reach 0.975, (100 Plotted)
Tracheobronchial disorder (x1.43) 0.680
Abnormal breathing (x1.11) 0.711
Infective pneumonia (x1.09) 0.731
Gastroesophageal reflux disease (x1.09) 0.749
Acute respiratory failure (x1.09) 0.766
Congenital malformation of the respiratory system (x1.08) 0.779
Atelectasis (x1.07) 0.790
Disorder of lung (x1.07) 0.800
Congenital laryngomalacia (/1.07) 0.811
albuterol (+0.00593) 0.820
Asthma (x1.06) 0.828
Cough (x1.06) 0.835
Disorder of the larynx (/1.06) 0.842
Dyspnea (x1.06) 0.848
Stenosis of larynx (x1.05) 0.853
Postprocedural state finding (x1.05) 0.858
Hypoxemia (x1.05) 0.863
Acute tracheitis (x1.04) 0.866
budesonide (+0.00381) 0.870
Abnormal body temperature (x1.04) 0.873
Tracheostomy present (x1.04) 0.877
White Race (+0.00371) 0.880
Chronic respiratory failure (x1.04) 0.883
Edema of larynx (/1.04) 0.886
Disorder of bronchus (x1.04) 0.889
Wheezing (x1.04) 0.891
Chronic obstructive lung disease (x1.04) 0.894
acetaminophen (+0.00307) 0.896
Lung field abnormal (x1.03) 0.898
Male Sex (-0.00299) 0.900
Female Sex (+0.00298) 0.902
Pleural effusion (x1.03) 0.904
Tracheostomy complication (x1.03) 0.906
fluticasone (+0.0027) 0.908
Postoperative state (/1.03) 0.909
Tachypnea (x1.03) 0.911
Gastroesophageal reflux disease without esophagitis (/1.03) 0.912
Acute-on-chronic respiratory failure (x1.03) 0.913
Congenital anomaly of esophagus (x1.02) 0.915
montelukast (+0.00227) 0.916
Abnormal findings on diagnostic imaging of lung (/1.02) 0.917
Estimation of glomerular filtration rate (+3.98) 0.918
Disorder of pharynx (/1.02) 0.919
Electrocardiogram abnormal (x1.02) 0.920
Stridor (x1.02) 0.921
Finding related to pregnancy (x1.02) 0.922
Respiratory failure (/1.02) 0.923
Trauma and postoperative pulmonary insufficiency (x1.02) 0.923
Tonsil and/or adenoid hypertrophy (/1.02) 0.924
Oropharyngeal dysphagia (/1.02) 0.925
Prematurity of infant (x1.02) 0.926
Cardiomegaly (x1.02) 0.927
Disorder of respiratory system (x1.02) 0.927
Foreign body in larynx (x1.02) 0.928
Disorder of upper respiratory system (x1.02) 0.929
guaifenesin (+0.00175) 0.929
ranitidine (+0.00173) 0.930
Flatulence, eructation and gas pain (x1.02) 0.931
Cardiac arrhythmia (x1.02) 0.931
Pulmonary congestion and hypostasis (x1.02) 0.932
Respiratory insufficiency (x1.02) 0.933
Tachycardia (x1.02) 0.933
Failure to thrive (x1.02) 0.934
Postpartum finding (x1.02) 0.935
Pneumothorax (x1.02) 0.935
Eustachian tube disorder (/1.02) 0.936
Pneumonitis due to inhaled substance (x1.02) 0.936
Developmental delay (x1.02) 0.937
furosemide (+0.00163) 0.938
Acute bronchiolitis (x1.02) 0.938
Persistent pain following procedure (/1.02) 0.939
Chronic pulmonary heart disease (x1.02) 0.939
Acute hypoxemic respiratory failure (/1.02) 0.940
Primary malignant neoplasm of respiratory tract (x1.02) 0.940
famotidine (+0.00155) 0.941
Inflammatory disorder of mediastinum (x1.02) 0.941
Black Race (-0.00149) 0.942
Abnormal granulation tissue (x1.02) 0.942
ipratropium (+0.00144) 0.943
Mechanical complication of gastrostomy (x1.02) 0.943
Bacterial pneumonia (x1.02) 0.944
Tau protein [Presence] in Body fluid (-0.0183) 0.944
Tracheoesophageal fistula (x1.02) 0.945
lansoprazole (+0.00138) 0.945
Q-T interval (-1.2) 0.945
sodium chloride (+0.00135) 0.946
Pneumonia due to Pseudomonas (x1.01) 0.946
Atrial septal defect (/1.01) 0.947
Disease due to Rhinovirus (x1.01) 0.947
Late effect of medical and surgical care complication (x1.01) 0.947
Respiratory condition of fetus OR newborn (x1.01) 0.948
Localized swelling, mass and lump, trunk (x1.01) 0.948
Dependence on respiratory device (x1.01) 0.949
Anemia (x1.01) 0.949
Physiological development failure (x1.01) 0.949
Congestive heart failure (x1.01) 0.950
Malfunction of tracheostomy (x1.01) 0.950
Obesity (x1.01) 0.950
Postprocedural respiratory failure (/1.01) 0.951
Acute exacerbation of asthma (x1.01) 0.951
−0.8 −0.6 −0.4 −0.2 0.0 0.2 0.4 0.6 0.8
Expressions
−10 0 10

Signature [889]; 1 To Reach 0.975, (10 Plotted)

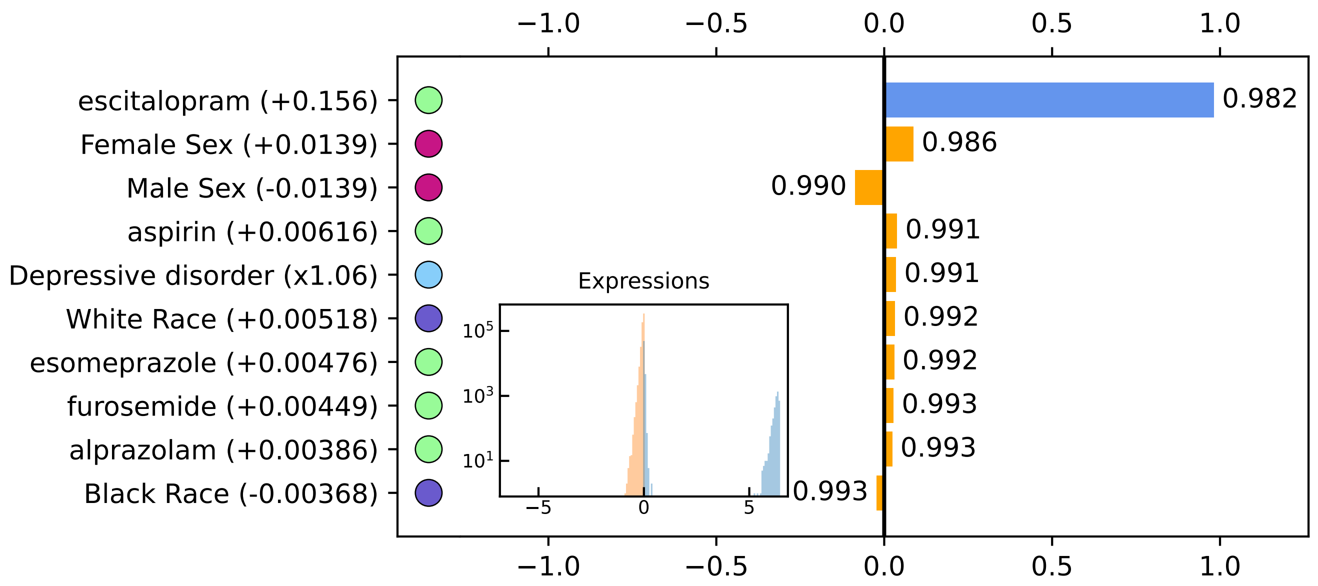

Signature [890]; 331 To Reach 0.975, (100 Plotted)

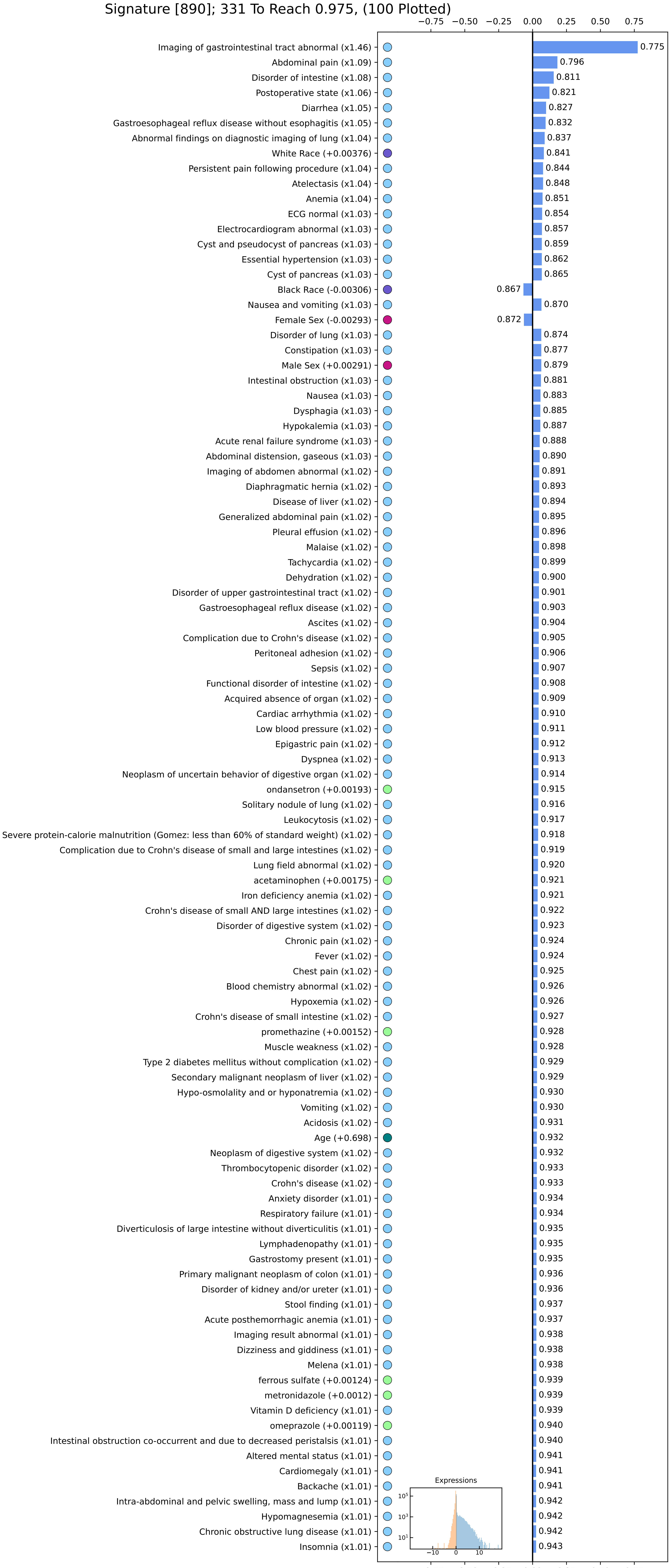

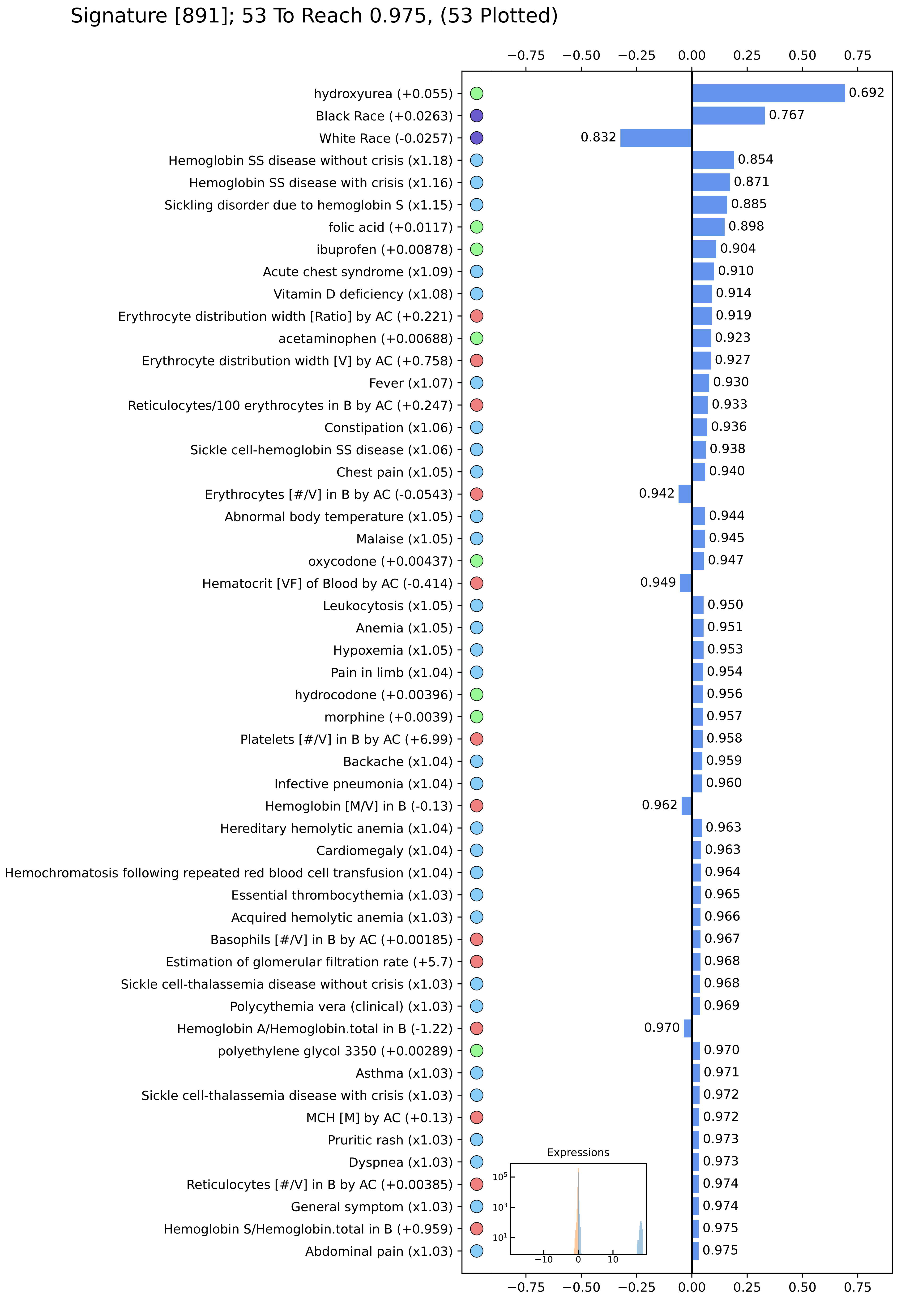

Signature [891]; 53 To Reach 0.975, (53 Plotted)
hydroxyurea (+0.055) 0.692
Black Race (+0.0263) 0.767
White Race (-0.0257) 0.832
Hemoglobin SS disease without crisis (x1.18) 0.854
Hemoglobin SS disease with crisis (x1.16) 0.871
Sickling disorder due to hemoglobin S (x1.15) 0.885
folic acid (+0.0117) 0.898
ibuprofen (+0.00878) 0.904
Acute chest syndrome (x1.09) 0.910
Vitamin D deficiency (x1.08) 0.914
Erythrocyte distribution width [Ratio] by AC (+0.221) 0.919
acetaminophen (+0.00688) 0.923
Erythrocyte distribution width [V] by AC (+0.758) 0.927
Fever (x1.07) 0.930
Reticulocytes/100 erythrocytes in B by AC (+0.247) 0.933
Constipation (x1.06) 0.936
Sickle cell-hemoglobin SS disease (x1.06) 0.938
Chest pain (x1.05) 0.940
Erythrocytes [#/V] in B by AC (-0.0543) 0.942
Abnormal body temperature (x1.05) 0.944
Malaise (x1.05) 0.945
oxycodone (+0.00437) 0.947
Hematocrit [VF] of Blood by AC (-0.414) 0.949
Leukocytosis (x1.05) 0.950
Anemia (x1.05) 0.951
Hypoxemia (x1.05) 0.953
Pain in limb (x1.04) 0.954
hydrocodone (+0.00396) 0.956
morphine (+0.0039) 0.957
Platelets [#/V] in B by AC (+6.99) 0.958
Backache (x1.04) 0.959
Infective pneumonia (x1.04) 0.960
Hemoglobin [M/V] in B (-0.13) 0.962
Hereditary hemolytic anemia (x1.04) 0.963
Cardiomegaly (x1.04) 0.963
Hemochromatosis following repeated red blood cell transfusion (x1.04) 0.964
Essential thrombocythemia (x1.03) 0.965
Acquired hemolytic anemia (x1.03) 0.966
Basophils [#/V] in B by AC (+0.00185) 0.967
Estimation of glomerular filtration rate (+5.7) 0.968
Sickle cell-thalassemia disease without crisis (x1.03) 0.968
Polycythemia vera (clinical) (x1.03) 0.969
Hemoglobin A/Hemoglobin.total in B (-1.22) 0.970
polyethylene glycol 3350 (+0.00289) 0.970
Asthma (x1.03) 0.971
Sickle cell-thalassemia disease with crisis (x1.03) 0.972
MCH [M] by AC (+0.13) 0.972
Pruritic rash (x1.03) 0.973
Dyspnea (x1.03) 0.973
Reticulocytes [#/V] in B by AC (+0.00385) 0.974
General symptom (x1.03) 0.974
Hemoglobin S/Hemoglobin.total in B (+0.959) 0.975
Abdominal pain (x1.03) 0.975
−0.75 −0.50 −0.25 0.00 0.25 0.50 0.75
Expressions
10^5 10^3 10^1
−10 0 10

Signature [892]; 72 To Reach 0.975, (72 Plotted)

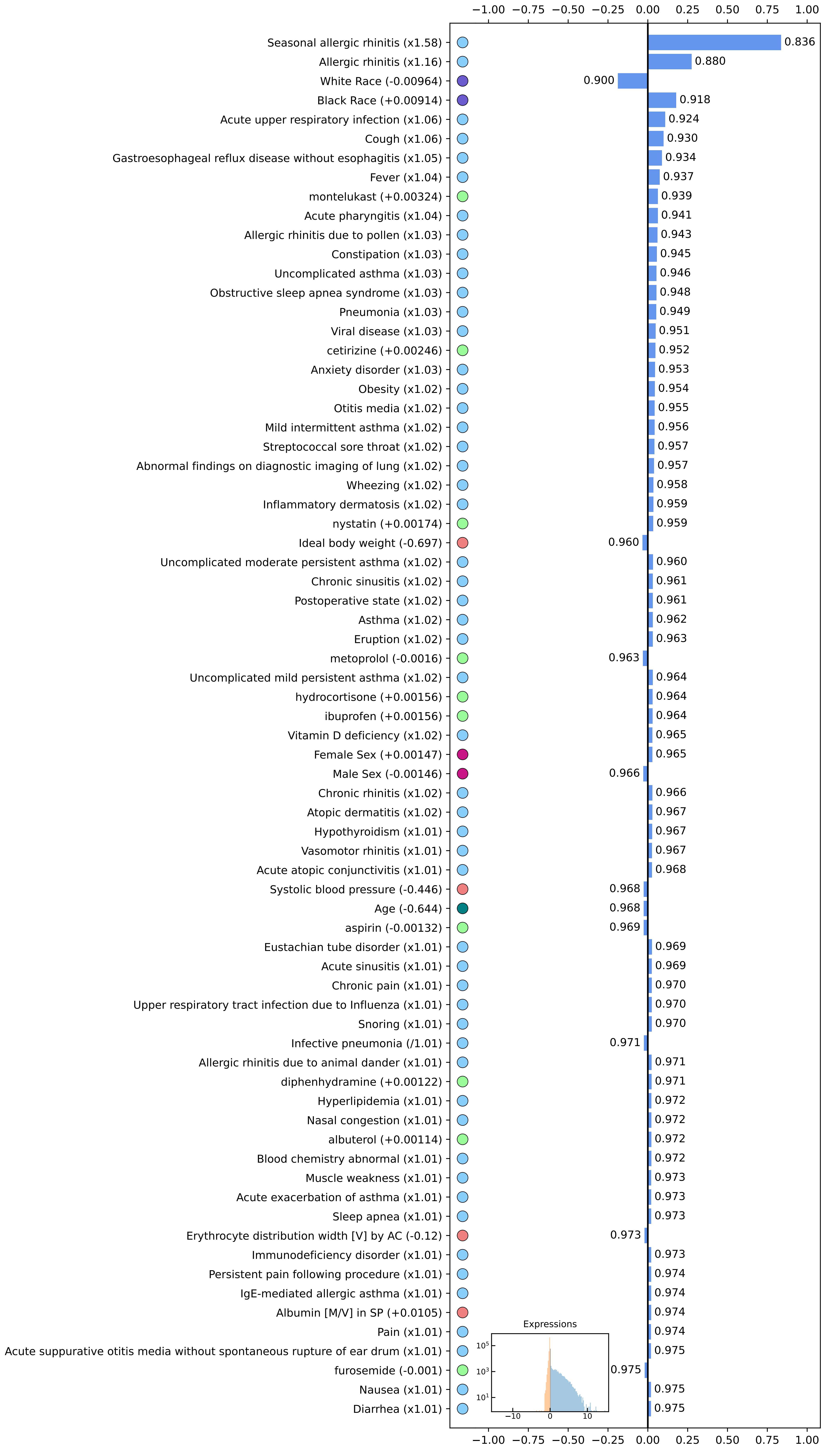

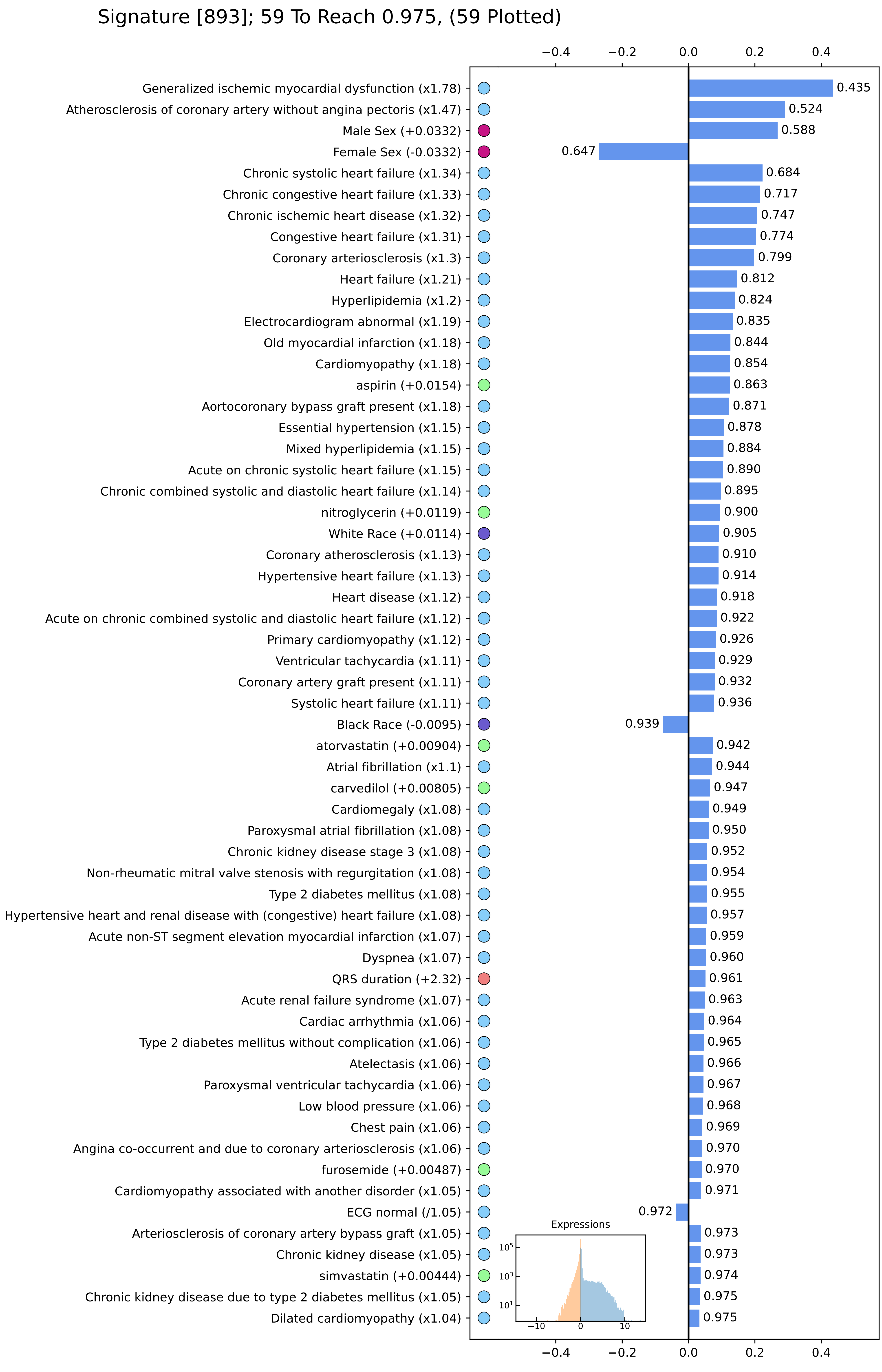

Signature [893]; 59 To Reach 0.975, (59 Plotted)
Generalized ischemic myocardial dysfunction (x1.78)
Atherosclerosis of coronary artery without angina pectoris (x1.47)
Male Sex (+0.0332)
Female Sex (-0.0332)
Chronic systolic heart failure (x1.34)
Chronic congestive heart failure (x1.33)
Chronic ischemic heart disease (x1.32)
Congestive heart failure (x1.31)
Coronary arteriosclerosis (x1.3)
Heart failure (x1.21)
Hyperlipidemia (x1.2)
Electrocardiogram abnormal (x1.19)
Old myocardial infarction (x1.18)
Cardiomyopathy (x1.18)
aspirin (+0.0154)
Aortocoronary bypass graft present (x1.18)
Essential hypertension (x1.15)
Mixed hyperlipidemia (x1.15)
Acute on chronic systolic heart failure (x1.15)
Chronic combined systolic and diastolic heart failure (x1.14)
nitroglycerin (+0.0119)
White Race (+0.0114)
Coronary atherosclerosis (x1.13)
Hypertensive heart failure (x1.13)
Heart disease (x1.12)
Acute on chronic combined systolic and diastolic heart failure (x1.12)
Primary cardiomyopathy (x1.12)
Ventricular tachycardia (x1.11)
Coronary artery graft present (x1.11)
Systolic heart failure (x1.11)
Black Race (-0.0095)
atorvastatin (+0.00904)
Atrial fibrillation (x1.1)
carvedilol (+0.00805)
Cardiomegaly (x1.08)
Paroxysmal atrial fibrillation (x1.08)
Chronic kidney disease stage 3 (x1.08)
Non-rheumatic mitral valve stenosis with regurgitation (x1.08)
Type 2 diabetes mellitus (x1.08)
Hypertensive heart and renal disease with (congestive) heart failure (x1.08)
Acute non-ST segment elevation myocardial infarction (x1.07)
Dyspnea (x1.07)
QRS duration (+2.32)
Acute renal failure syndrome (x1.07)
Cardiac arrhythmia (x1.06)
Type 2 diabetes mellitus without complication (x1.06)
Atelectasis (x1.06)
Paroxysmal ventricular tachycardia (x1.06)
Low blood pressure (x1.06)
Chest pain (x1.06)
Angina co-occurrent and due to coronary arteriosclerosis (x1.06)
furosemide (+0.00487)
Cardiomyopathy associated with another disorder (x1.05)
ECG normal (/1.05)
Arteriosclerosis of coronary artery bypass graft (x1.05)
Chronic kidney disease (x1.05)
simvastatin (+0.00444)
Chronic kidney disease due to type 2 diabetes mellitus (x1.05)
Dilated cardiomyopathy (x1.04)
0.435
0.524
0.588
0.647
0.684
0.717
0.747
0.774
0.799
0.812
0.824
0.835
0.844
0.854
0.863
0.871
0.878
0.884
0.890
0.895
0.900
0.905
0.910
0.914
0.918
0.922
0.926
0.929
0.932
0.936
0.939
0.942
0.944
0.947
0.949
0.950
0.952
0.954
0.955
0.957
0.959
0.960
0.961
0.963
0.964
0.965
0.966
0.967
0.968
0.969
0.970
0.970
0.971
0.972
0.973
0.973
0.974
0.975
0.975
−0.4
−0.2
0.0
0.2
0.4
Expressions

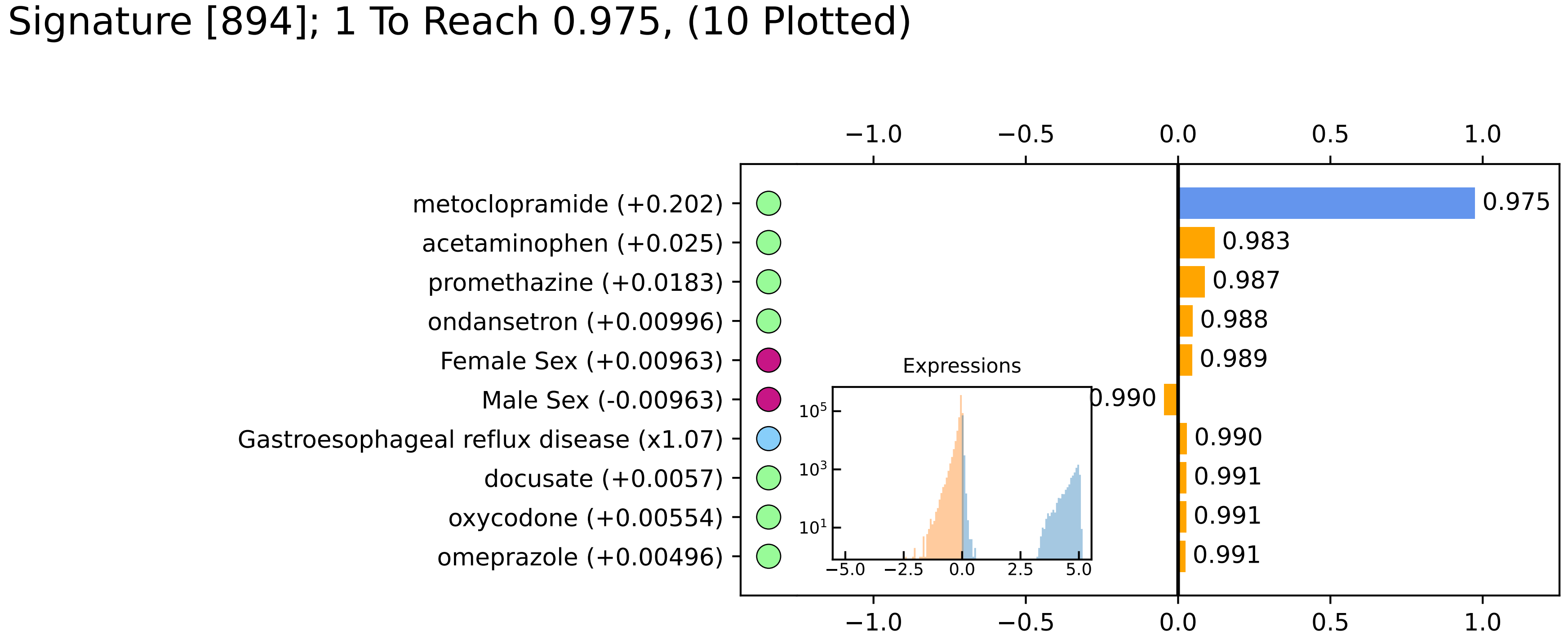
Signature [894]; 1 To Reach 0.975, (10 Plotted)
metoclopramide (+0.202)
acetaminophen (+0.025)
promethazine (+0.0183)
ondansetron (+0.00996)
Female Sex (+0.00963)
Male Sex (-0.00963)
Gastroesophageal reflux disease (x1.07)
docusate (+0.0057)
oxycodone (+0.00554)
omeprazole (+0.00496)
0.975
0.983
0.987
0.988
0.989
0.990
0.990
0.991
0.991
0.991
Expressions
−1.0
−0.5
0.0
0.5
1.0

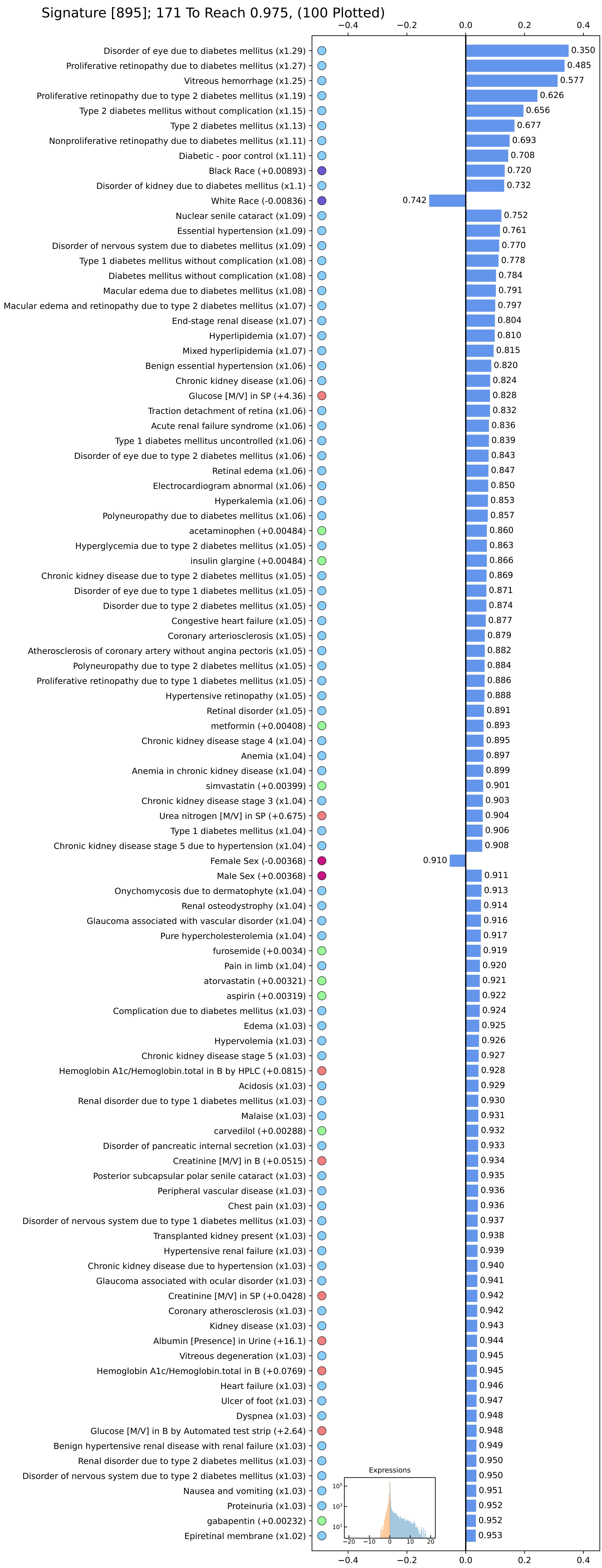

Signature [895]; 171 To Reach 0.975, (100 Plotted)
−0.4 −0.2 0.0 0.2 0.4
Disorder of eye due to diabetes mellitus (x1.29) 0.350
Proliferative retinopathy due to diabetes mellitus (x1.27) 0.485
Vitreous hemorrhage (x1.25) 0.577
Proliferative retinopathy due to type 2 diabetes mellitus (x1.19) 0.626
Type 2 diabetes mellitus without complication (x1.15) 0.656
Type 2 diabetes mellitus (x1.13) 0.677
Nonproliferative retinopathy due to diabetes mellitus (x1.11) 0.693
Diabetic - poor control (x1.11) 0.708
Black Race (+0.00893) 0.720
Disorder of kidney due to diabetes mellitus (x1.1) 0.732
White Race (-0.00836) 0.742
Nuclear senile cataract (x1.09) 0.752
Essential hypertension (x1.09) 0.761
Disorder of nervous system due to diabetes mellitus (x1.09) 0.770
Type 1 diabetes mellitus without complication (x1.08) 0.778
Diabetes mellitus without complication (x1.08) 0.784
Macular edema due to diabetes mellitus (x1.08) 0.791
Macular edema and retinopathy due to type 2 diabetes mellitus (x1.07) 0.797
End-stage renal disease (x1.07) 0.804
Hyperlipidemia (x1.07) 0.810
Mixed hyperlipidemia (x1.07) 0.815
Benign essential hypertension (x1.06) 0.820
Chronic kidney disease (x1.06) 0.824
Glucose [M/V] in SP (+4.36) 0.828
Traction detachment of retina (x1.06) 0.832
Acute renal failure syndrome (x1.06) 0.836
Type 1 diabetes mellitus uncontrolled (x1.06) 0.839
Disorder of eye due to type 2 diabetes mellitus (x1.06) 0.843
Retinal edema (x1.06) 0.847
Electrocardiogram abnormal (x1.06) 0.850
Hyperkalemia (x1.06) 0.853
Polyneuropathy due to diabetes mellitus (x1.06) 0.857
acetaminophen (+0.00484) 0.860
Hyperglycemia due to type 2 diabetes mellitus (x1.05) 0.863
insulin glargine (+0.00484) 0.866
Chronic kidney disease due to type 2 diabetes mellitus (x1.05) 0.869
Disorder of eye due to type 1 diabetes mellitus (x1.05) 0.871
Disorder due to type 2 diabetes mellitus (x1.05) 0.874
Congestive heart failure (x1.05) 0.877
Coronary arteriosclerosis (x1.05) 0.879
Atherosclerosis of coronary artery without angina pectoris (x1.05) 0.882
Polyneuropathy due to type 2 diabetes mellitus (x1.05) 0.884
Proliferative retinopathy due to type 1 diabetes mellitus (x1.05) 0.886
Hypertensive retinopathy (x1.05) 0.888
Retinal disorder (x1.05) 0.891
metformin (+0.00408) 0.893
Chronic kidney disease stage 4 (x1.04) 0.895
Anemia (x1.04) 0.897
Anemia in chronic kidney disease (x1.04) 0.899
simvastatin (+0.00399) 0.901
Chronic kidney disease stage 3 (x1.04) 0.903
Urea nitrogen [M/V] in SP (+0.675) 0.904
Type 1 diabetes mellitus (x1.04) 0.906
Chronic kidney disease stage 5 due to hypertension (x1.04) 0.908
Female Sex (-0.00368) 0.910
Male Sex (+0.00368) 0.911
Onychomycosis due to dermatophyte (x1.04) 0.913
Renal osteodystrophy (x1.04) 0.914
Glaucoma associated with vascular disorder (x1.04) 0.916
Pure hypercholesterolemia (x1.04) 0.917
furosemide (+0.0034) 0.919
Pain in limb (x1.04) 0.920
atorvastatin (+0.00321) 0.921
aspirin (+0.00319) 0.922
Complication due to diabetes mellitus (x1.03) 0.924
Edema (x1.03) 0.925
Hypervolemia (x1.03) 0.926
Chronic kidney disease stage 5 (x1.03) 0.927
Hemoglobin A1c/Hemoglobin.total in B by HPLC (+0.0815) 0.928
Acidosis (x1.03) 0.929
Renal disorder due to type 1 diabetes mellitus (x1.03) 0.930
Malaise (x1.03) 0.931
carvedilol (+0.00288) 0.932
Disorder of pancreatic internal secretion (x1.03) 0.933
Creatinine [M/V] in B (+0.0515) 0.934
Posterior subcapsular polar senile cataract (x1.03) 0.935
Peripheral vascular disease (x1.03) 0.936
Chest pain (x1.03) 0.936
Disorder of nervous system due to type 1 diabetes mellitus (x1.03) 0.937
Transplanted kidney present (x1.03) 0.938
Hypertensive renal failure (x1.03) 0.939
Chronic kidney disease due to hypertension (x1.03) 0.940
Glaucoma associated with ocular disorder (x1.03) 0.941
Creatinine [M/V] in SP (+0.0428) 0.942
Coronary atherosclerosis (x1.03) 0.942
Kidney disease (x1.03) 0.943
Albumin [Presence] in Urine (+16.1) 0.944
Vitreous degeneration (x1.03) 0.945
Hemoglobin A1c/Hemoglobin.total in B (+0.0769) 0.945
Heart failure (x1.03) 0.946
Ulcer of foot (x1.03) 0.947
Dyspnea (x1.03) 0.948
Glucose [M/V] in B by Automated test strip (+2.64) 0.948
Benign hypertensive renal disease with renal failure (x1.03) 0.949
Renal disorder due to type 2 diabetes mellitus (x1.03) 0.950
Disorder of nervous system due to type 2 diabetes mellitus (x1.03) 0.950
Nausea and vomiting (x1.03) 0.951
Proteinuria (x1.03) 0.952
gabapentin (+0.00232) 0.952
Epiretinal membrane (x1.02) 0.953
Expressions
10^5 10^3 10^1
−20 −10 0 10 20
−0.4 −0.2 0.0 0.2 0.4

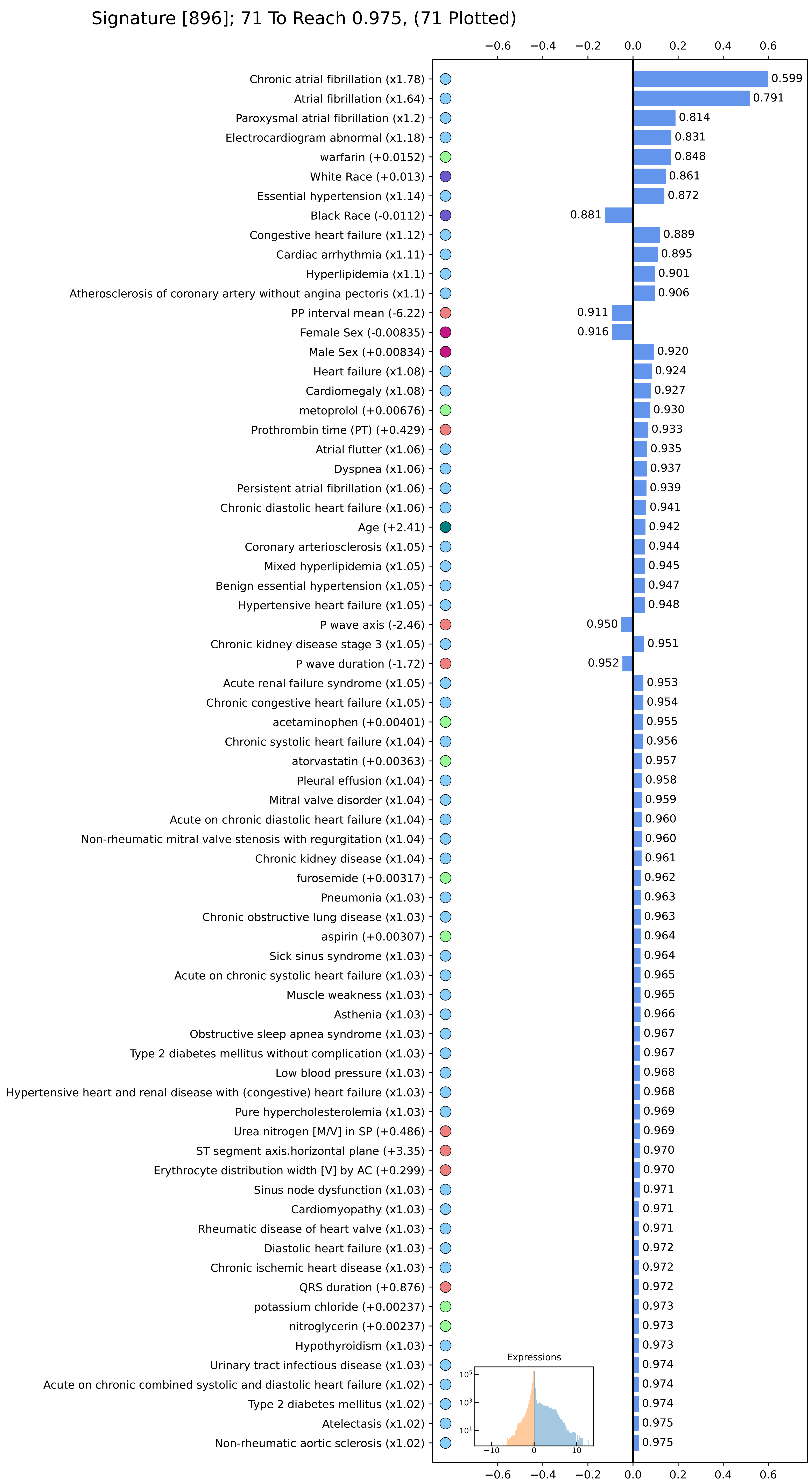
Signature [896]; 71 To Reach 0.975, (71 Plotted)
−0.6 −0.4 −0.2 0.0 0.2 0.4 0.6
Chronic atrial fibrillation (x1.78) 0.599
Atrial fibrillation (x1.64) 0.791
Paroxysmal atrial fibrillation (x1.2) 0.814
Electrocardiogram abnormal (x1.18) 0.831
warfarin (+0.0152) 0.848
White Race (+0.013) 0.861
Essential hypertension (x1.14) 0.872
Black Race (-0.0112) 0.881
Congestive heart failure (x1.12) 0.889
Cardiac arrhythmia (x1.11) 0.895
Hyperlipidemia (x1.1) 0.901
Atherosclerosis of coronary artery without angina pectoris (x1.1) 0.906
PP interval mean (-6.22) 0.911
Female Sex (-0.00835) 0.916
Male Sex (+0.00834) 0.920
Heart failure (x1.08) 0.924
Cardiomegaly (x1.08) 0.927
metoprolol (+0.00676) 0.930
Prothrombin time (PT) (+0.429) 0.933
Atrial flutter (x1.06) 0.935
Dyspnea (x1.06) 0.937
Persistent atrial fibrillation (x1.06) 0.939
Chronic diastolic heart failure (x1.06) 0.941
Age (+2.41) 0.942
Coronary arteriosclerosis (x1.05) 0.944
Mixed hyperlipidemia (x1.05) 0.945
Benign essential hypertension (x1.05) 0.947
Hypertensive heart failure (x1.05) 0.948
P wave axis (-2.46) 0.950
Chronic kidney disease stage 3 (x1.05) 0.951
P wave duration (-1.72) 0.952
Acute renal failure syndrome (x1.05) 0.953
Chronic congestive heart failure (x1.05) 0.954
acetaminophen (+0.00401) 0.955
Chronic systolic heart failure (x1.04) 0.956
atorvastatin (+0.00363) 0.957
Pleural effusion (x1.04) 0.958
Mitral valve disorder (x1.04) 0.959
Acute on chronic diastolic heart failure (x1.04) 0.960
Non-rheumatic mitral valve stenosis with regurgitation (x1.04) 0.960
Chronic kidney disease (x1.04) 0.961
furosemide (+0.00317) 0.962
Pneumonia (x1.03) 0.963
Chronic obstructive lung disease (x1.03) 0.963
aspirin (+0.00307) 0.964
Sick sinus syndrome (x1.03) 0.964
Acute on chronic systolic heart failure (x1.03) 0.965
Muscle weakness (x1.03) 0.965
Asthenia (x1.03) 0.966
Obstructive sleep apnea syndrome (x1.03) 0.967
Type 2 diabetes mellitus without complication (x1.03) 0.967
Low blood pressure (x1.03) 0.968
Hypertensive heart and renal disease with (congestive) heart failure (x1.03) 0.968
Pure hypercholesterolemia (x1.03) 0.969
Urea nitrogen [M/V] in SP (+0.486) 0.969
ST segment axis.horizontal plane (+3.35) 0.970
Erythrocyte distribution width [V] by AC (+0.299) 0.970
Sinus node dysfunction (x1.03) 0.971
Cardiomyopathy (x1.03) 0.971
Rheumatic disease of heart valve (x1.03) 0.971
Diastolic heart failure (x1.03) 0.972
Chronic ischemic heart disease (x1.03) 0.972
QRS duration (+0.876) 0.972
potassium chloride (+0.00237) 0.973
nitroglycerin (+0.00237) 0.973
Hypothyroidism (x1.03) 0.973
Urinary tract infectious disease (x1.03) 0.974
Acute on chronic combined systolic and diastolic heart failure (x1.02) 0.974
Type 2 diabetes mellitus (x1.02) 0.974
Atelectasis (x1.02) 0.975
Non-rheumatic aortic sclerosis (x1.02) 0.975
Expressions
10^5
10^3
10^1
−10 0 10

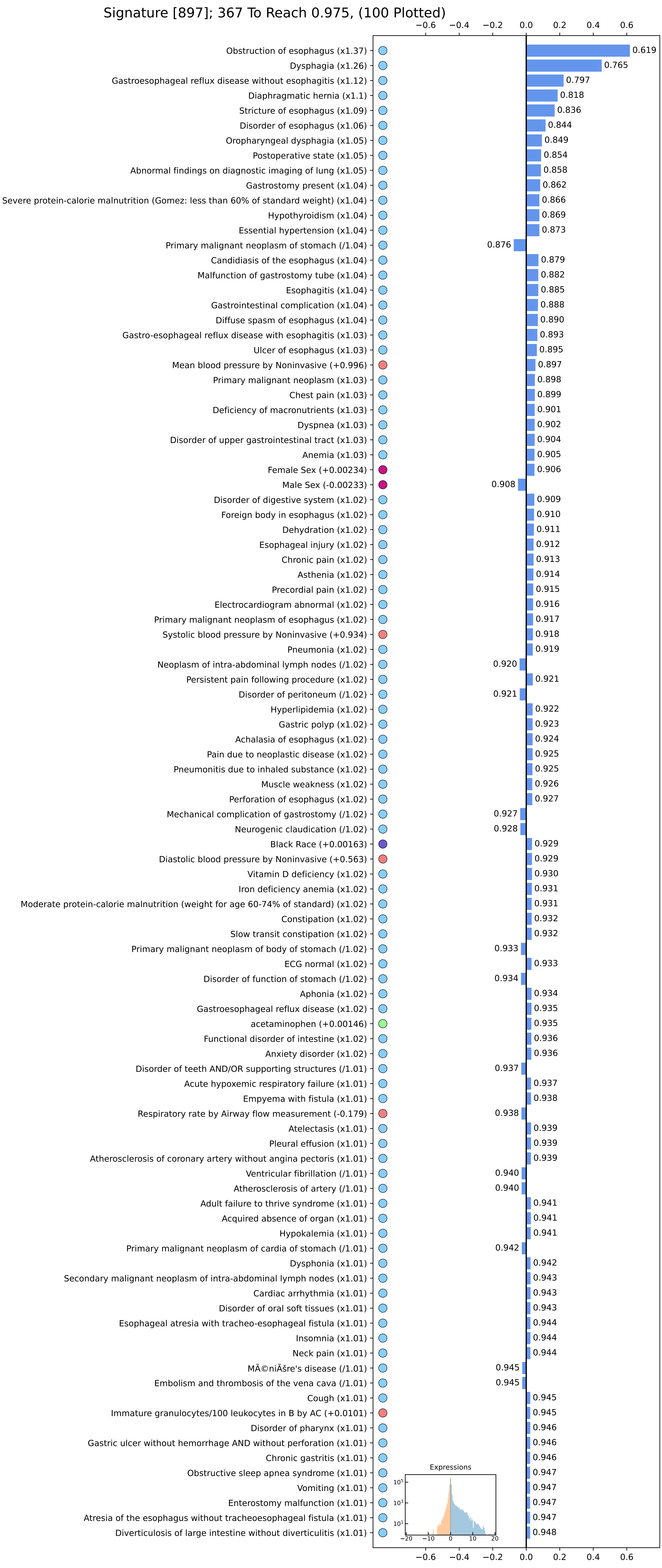

Signature [897]; 367 To Reach 0.975, (100 Plotted)
Obstruction of esophagus (x1.37) 0.619
Dysphagia (x1.26) 0.765
Gastroesophageal reflux disease without esophagitis (x1.12) 0.797
Diaphragmatic hernia (x1.1) 0.818
Stricture of esophagus (x1.09) 0.836
Disorder of esophagus (x1.06) 0.844
Oropharyngeal dysphagia (x1.05) 0.849
Postoperative state (x1.05) 0.854
Abnormal findings on diagnostic imaging of lung (x1.05) 0.858
Gastrostomy present (x1.04) 0.862
Severe protein-calorie malnutrition (Gomez: less than 60% of standard weight) (x1.04) 0.866
Hypothyroidism (x1.04) 0.869
Essential hypertension (x1.04) 0.873
Primary malignant neoplasm of stomach (/1.04) 0.876
Candidiasis of the esophagus (x1.04) 0.879
Malfunction of gastrostomy tube (x1.04) 0.882
Esophagitis (x1.04) 0.885
Gastrointestinal complication (x1.04) 0.888
Diffuse spasm of esophagus (x1.04) 0.890
Gastro-esophageal reflux disease with esophagitis (x1.03) 0.893
Ulcer of esophagus (x1.03) 0.895
Mean blood pressure by Noninvasive (+0.996) 0.897
Primary malignant neoplasm (x1.03) 0.898
Chest pain (x1.03) 0.899
Deficiency of macronutrients (x1.03) 0.901
Dyspnea (x1.03) 0.902
Disorder of upper gastrointestinal tract (x1.03) 0.904
Anemia (x1.03) 0.905
Female Sex (+0.00234) 0.906
Male Sex (-0.00233) 0.908
Disorder of digestive system (x1.02) 0.909
Foreign body in esophagus (x1.02) 0.910
Dehydration (x1.02) 0.911
Esophageal injury (x1.02) 0.912
Chronic pain (x1.02) 0.913
Asthenia (x1.02) 0.914
Precordial pain (x1.02) 0.915
Electrocardiogram abnormal (x1.02) 0.916
Primary malignant neoplasm of esophagus (x1.02) 0.917
Systolic blood pressure by Noninvasive (+0.934) 0.918
Pneumonia (x1.02) 0.919
Neoplasm of intra-abdominal lymph nodes (/1.02) 0.920
Persistent pain following procedure (x1.02) 0.921
Disorder of peritoneum (/1.02) 0.921
Hyperlipidemia (x1.02) 0.922
Gastric polyp (x1.02) 0.923
Achalasia of esophagus (x1.02) 0.924
Pain due to neoplastic disease (x1.02) 0.925
Pneumonitis due to inhaled substance (x1.02) 0.925
Muscle weakness (x1.02) 0.926
Perforation of esophagus (x1.02) 0.927
Mechanical complication of gastrostomy (/1.02) 0.927
Neurogenic claudication (/1.02) 0.928
Black Race (+0.00163) 0.929
Diastolic blood pressure by Noninvasive (+0.563) 0.929
Vitamin D deficiency (x1.02) 0.930
Iron deficiency anemia (x1.02) 0.931
Moderate protein-calorie malnutrition (weight for age 60-74% of standard) (x1.02) 0.931
Constipation (x1.02) 0.932
Slow transit constipation (x1.02) 0.932
Primary malignant neoplasm of body of stomach (/1.02) 0.933
ECG normal (x1.02) 0.933
Disorder of function of stomach (/1.02) 0.934
Aphonia (x1.02) 0.934
Gastroesophageal reflux disease (x1.02) 0.935
acetaminophen (+0.00146) 0.935
Functional disorder of intestine (x1.02) 0.936
Anxiety disorder (x1.02) 0.936
Disorder of teeth AND/OR supporting structures (/1.01) 0.937
Acute hypoxemic respiratory failure (x1.01) 0.937
Empyema with fistula (x1.01) 0.938
Respiratory rate by Airway flow measurement (-0.179) 0.938
Atelectasis (x1.01) 0.939
Pleural effusion (x1.01) 0.939
Atherosclerosis of coronary artery without angina pectoris (x1.01) 0.939
Ventricular fibrillation (/1.01) 0.940
Atherosclerosis of artery (/1.01) 0.940
Adult failure to thrive syndrome (x1.01) 0.941
Acquired absence of organ (x1.01) 0.941
Hypokalemia (x1.01) 0.941
Primary malignant neoplasm of cardia of stomach (/1.01) 0.942
Dysphonia (x1.01) 0.942
Secondary malignant neoplasm of intra-abdominal lymph nodes (x1.01) 0.943
Cardiac arrhythmia (x1.01) 0.943
Disorder of oral soft tissues (x1.01) 0.943
Esophageal atresia with tracheo-esophageal fistula (x1.01) 0.944
Insomnia (x1.01) 0.944
Neck pain (x1.01) 0.944
MÃ©niÃšre's disease (/1.01) 0.945
Embolism and thrombosis of the vena cava (/1.01) 0.945
Cough (x1.01) 0.945
Immature granulocytes/100 leukocytes in B by AC (+0.0101) 0.945
Disorder of pharynx (x1.01) 0.946
Gastric ulcer without hemorrhage AND without perforation (x1.01) 0.946
Chronic gastritis (x1.01) 0.946
Obstructive sleep apnea syndrome (x1.01) 0.947
Vomiting (x1.01) 0.947
Enterostomy malfunction (x1.01) 0.947
Atresia of the esophagus without tracheoesophageal fistula (x1.01) 0.947
Diverticulosis of large intestine without diverticulitis (x1.01) 0.948
−0.6 −0.4 −0.2 0.0 0.2 0.4 0.6
Expressions

Signature [898]; 6 To Reach 0.975, (10 Plotted)

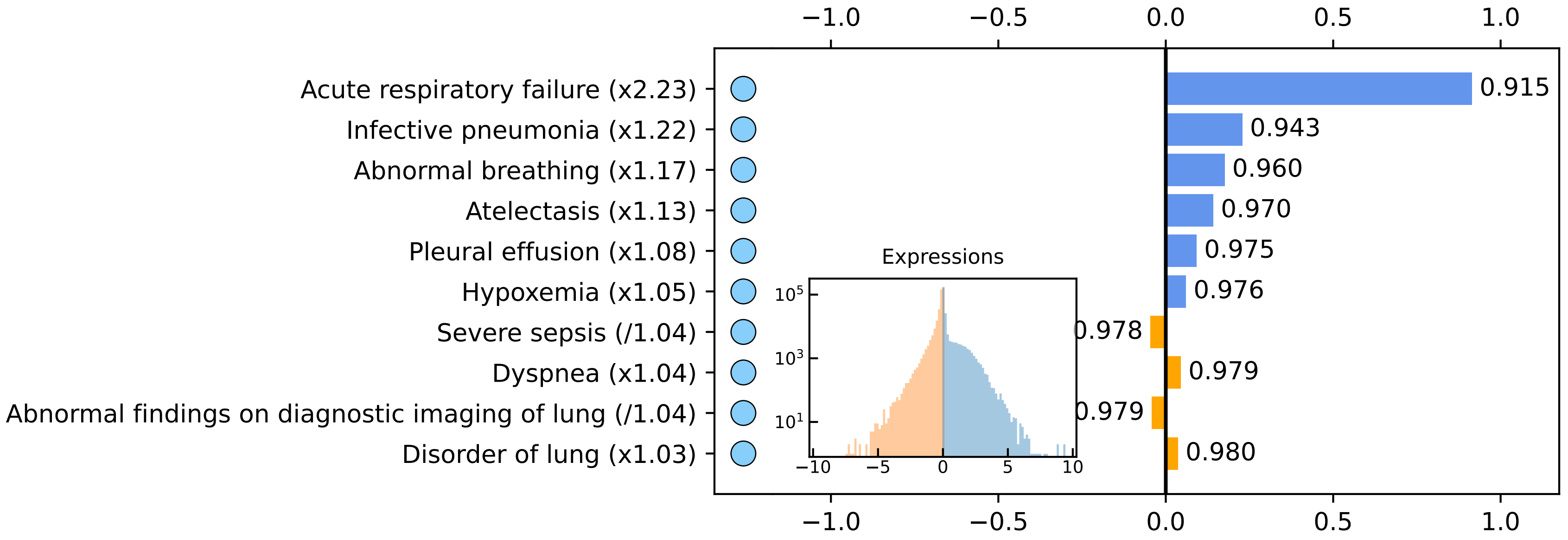

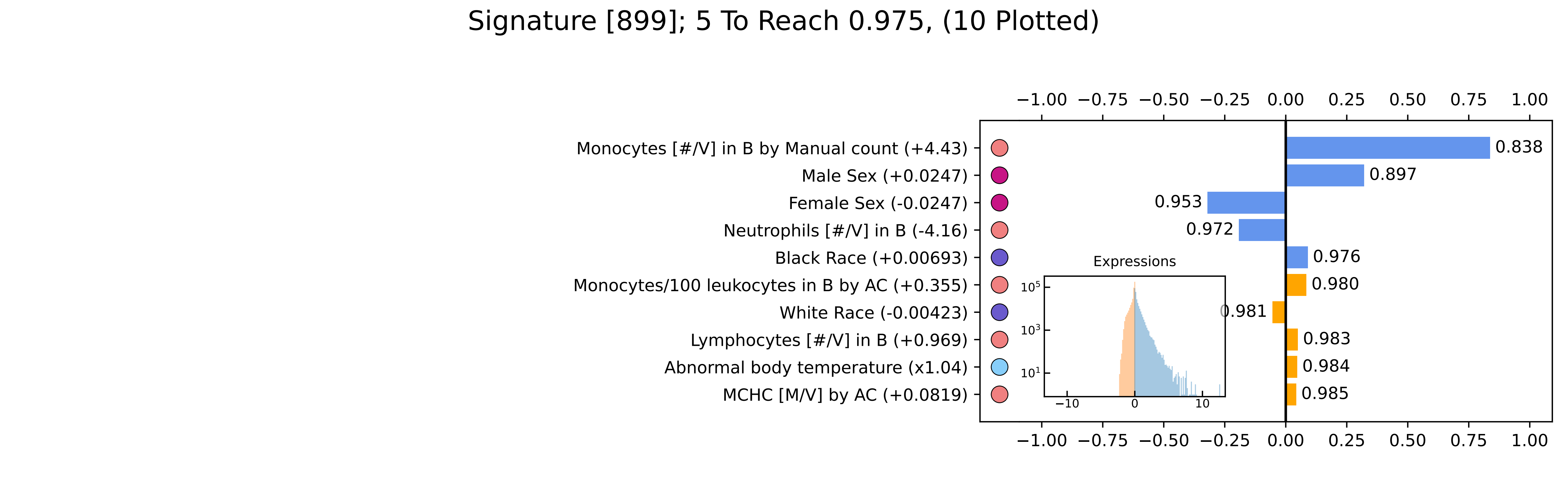

Signature [899]; 5 To Reach 0.975, (10 Plotted)
Monocytes [#/V] in B by Manual count (+4.43)
Male Sex (+0.0247)
Female Sex (-0.0247)
Neutrophils [#/V] in B (-4.16)
Black Race (+0.00693)
Monocytes/100 leukocytes in B by AC (+0.355)
White Race (-0.00423)
Lymphocytes [#/V] in B (+0.969)
Abnormal body temperature (x1.04)
MCHC [M/V] by AC (+0.0819)
0.838
0.897
0.953
0.972
0.976
0.980
0.981
0.983
0.984
0.985
Expressions
−1.00 −0.75 −0.50 −0.25 0.00 0.25 0.50 0.75 1.00

Signature [900]; 3 To Reach 0.975, (10 Plotted)

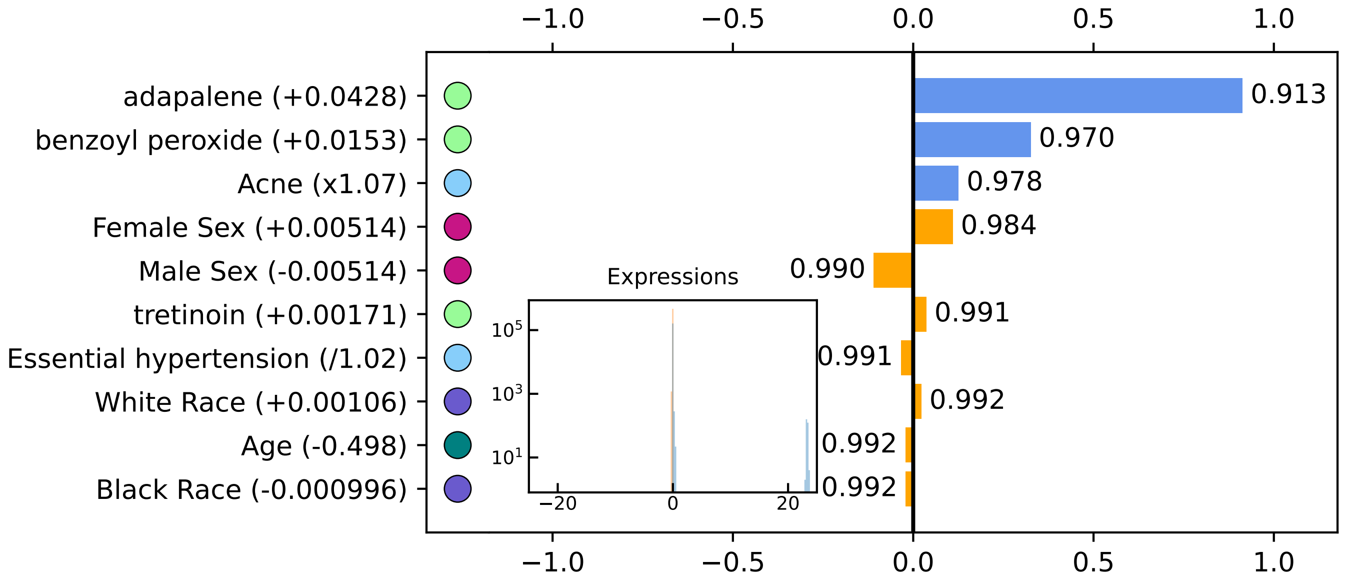

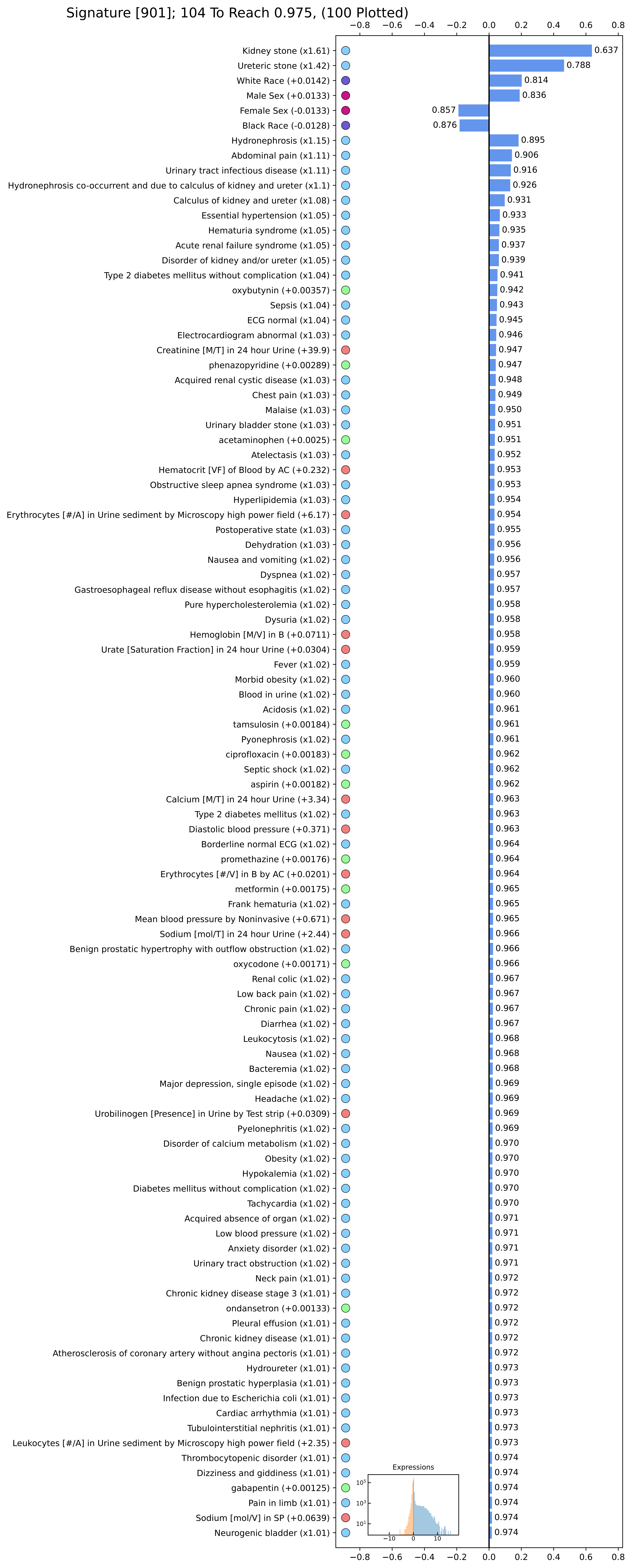
Signature [901]; 104 To Reach 0.975, (100 Plotted)
−0.8 −0.6 −0.4 −0.2 0.0 0.2 0.4 0.6 0.8
Kidney stone (x1.61)
Ureteric stone (x1.42)
White Race (+0.0142)
Male Sex (+0.0133)
Female Sex (-0.0133)
Black Race (-0.0128)
Hydronephrosis (x1.15)
Abdominal pain (x1.11)
Urinary tract infectious disease (x1.11)
Hydronephrosis co-occurrent and due to calculus of kidney and ureter (x1.1)
Calculus of kidney and ureter (x1.08)
Essential hypertension (x1.05)
Hematuria syndrome (x1.05)
Acute renal failure syndrome (x1.05)
Disorder of kidney and/or ureter (x1.05)
Type 2 diabetes mellitus without complication (x1.04)
oxybutynin (+0.00357)
Sepsis (x1.04)
ECG normal (x1.04)
Electrocardiogram abnormal (x1.03)
Creatinine [M/T] in 24 hour Urine (+39.9)
phenazopyridine (+0.00289)
Acquired renal cystic disease (x1.03)
Chest pain (x1.03)
Malaise (x1.03)
Urinary bladder stone (x1.03)
acetaminophen (+0.0025)
Atelectasis (x1.03)
Hematocrit [VF] of Blood by AC (+0.232)
Obstructive sleep apnea syndrome (x1.03)
Hyperlipidemia (x1.03)
Erythrocytes [#/A] in Urine sediment by Microscopy high power field (+6.17)
Postoperative state (x1.03)
Dehydration (x1.03)
Nausea and vomiting (x1.02)
Dyspnea (x1.02)
Gastroesophageal reflux disease without esophagitis (x1.02)
Pure hypercholesterolemia (x1.02)
Dysuria (x1.02)
Hemoglobin [M/V] in B (+0.0711)
Urate [Saturation Fraction] in 24 hour Urine (+0.0304)
Fever (x1.02)
Morbid obesity (x1.02)
Blood in urine (x1.02)
Acidosis (x1.02)
tamsulosin (+0.00184)
Pyonephrosis (x1.02)
ciprofloxacin (+0.00183)
Septic shock (x1.02)
aspirin (+0.00182)
Calcium [M/T] in 24 hour Urine (+3.34)
Type 2 diabetes mellitus (x1.02)
Diastolic blood pressure (+0.371)
Borderline normal ECG (x1.02)
promethazine (+0.00176)
Erythrocytes [#/V] in B by AC (+0.0201)
metformin (+0.00175)
Frank hematuria (x1.02)
Mean blood pressure by Noninvasive (+0.671)
Sodium [mol/T] in 24 hour Urine (+2.44)
Benign prostatic hypertrophy with outflow obstruction (x1.02)
oxycodone (+0.00171)
Renal colic (x1.02)
Low back pain (x1.02)
Chronic pain (x1.02)
Diarrhea (x1.02)
Leukocytosis (x1.02)
Nausea (x1.02)
Bacteremia (x1.02)
Major depression, single episode (x1.02)
Headache (x1.02)
Urobilinogen [Presence] in Urine by Test strip (+0.0309)
Pyelonephritis (x1.02)
Disorder of calcium metabolism (x1.02)
Obesity (x1.02)
Hypokalemia (x1.02)
Diabetes mellitus without complication (x1.02)
Tachycardia (x1.02)
Acquired absence of organ (x1.02)
Low blood pressure (x1.02)
Anxiety disorder (x1.02)
Urinary tract obstruction (x1.02)
Neck pain (x1.01)
Chronic kidney disease stage 3 (x1.01)
ondansetron (+0.00133)
Pleural effusion (x1.01)
Chronic kidney disease (x1.01)
Atherosclerosis of coronary artery without angina pectoris (x1.01)
Hydroureter (x1.01)
Benign prostatic hyperplasia (x1.01)
Infection due to Escherichia coli (x1.01)
Cardiac arrhythmia (x1.01)
Tubulointerstitial nephritis (x1.01)
Leukocytes [#/A] in Urine sediment by Microscopy high power field (+2.35)
Thrombocytopenic disorder (x1.01)
Dizziness and giddiness (x1.01)
gabapentin (+0.00125)
Pain in limb (x1.01)
Sodium [mol/V] in SP (+0.0639)
Neurogenic bladder (x1.01)
0.637
0.788
0.814
0.836
0.857
0.876
0.895
0.906
0.916
0.926
0.931
0.933
0.935
0.937
0.939
0.941
0.942
0.943
0.945
0.946
0.947
0.947
0.948
0.949
0.950
0.951
0.951
0.952
0.953
0.953
0.954
0.954
0.955
0.956
0.956
0.957
0.957
0.958
0.958
0.958
0.959
0.959
0.960
0.960
0.961
0.961
0.961
0.962
0.962
0.962
0.963
0.963
0.963
0.964
0.964
0.964
0.965
0.965
0.965
0.966
0.966
0.966
0.967
0.967
0.967
0.967
0.968
0.968
0.968
0.969
0.969
0.969
0.969
0.970
0.970
0.970
0.970
0.970
0.971
0.971
0.971
0.971
0.972
0.972
0.972
0.972
0.972
0.972
0.973
0.973
0.973
0.973
0.973
0.973
0.974
0.974
0.974
0.974
0.974
0.974
Expressions
−0.8 −0.6 −0.4 −0.2 0.0 0.2 0.4 0.6 0.8

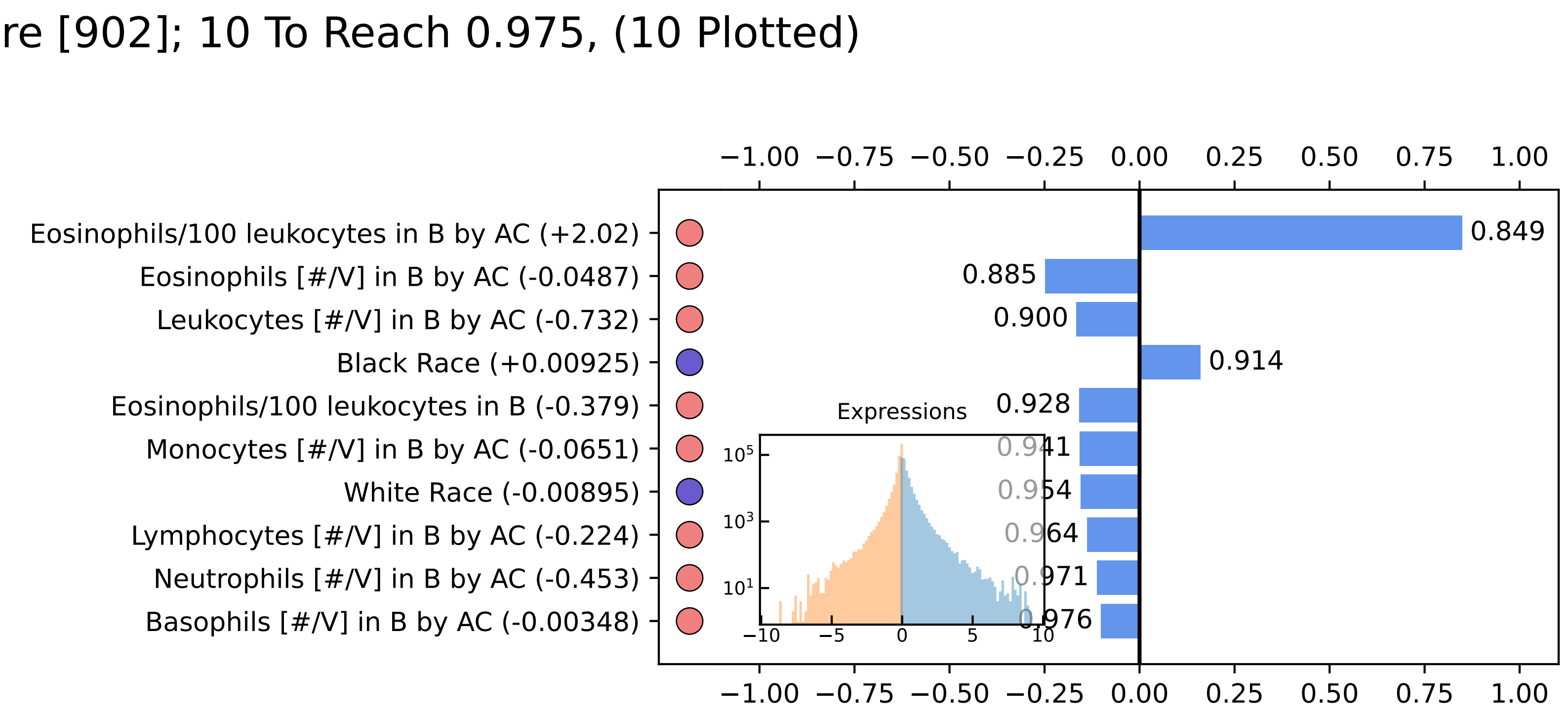

Signature [902]; 10 To Reach 0.975, (10 Plotted)
Eosinophils/100 leukocytes in B by AC (+2.02)
Eosinophils [#/V] in B by AC (-0.0487)
Leukocytes [#/V] in B by AC (-0.732)
Black Race (+0.00925)
Eosinophils/100 leukocytes in B (-0.379)
Monocytes [#/V] in B by AC (-0.0651)
White Race (-0.00895)
Lymphocytes [#/V] in B by AC (-0.224)
Neutrophils [#/V] in B by AC (-0.453)
Basophils [#/V] in B by AC (-0.00348)
0.849
0.885
0.900
0.914
0.928
0.941
0.954
0.964
0.971
0.976
Expressions
−1.00
−0.75
−0.50
−0.25
0.00
0.25
0.50
0.75
1.00

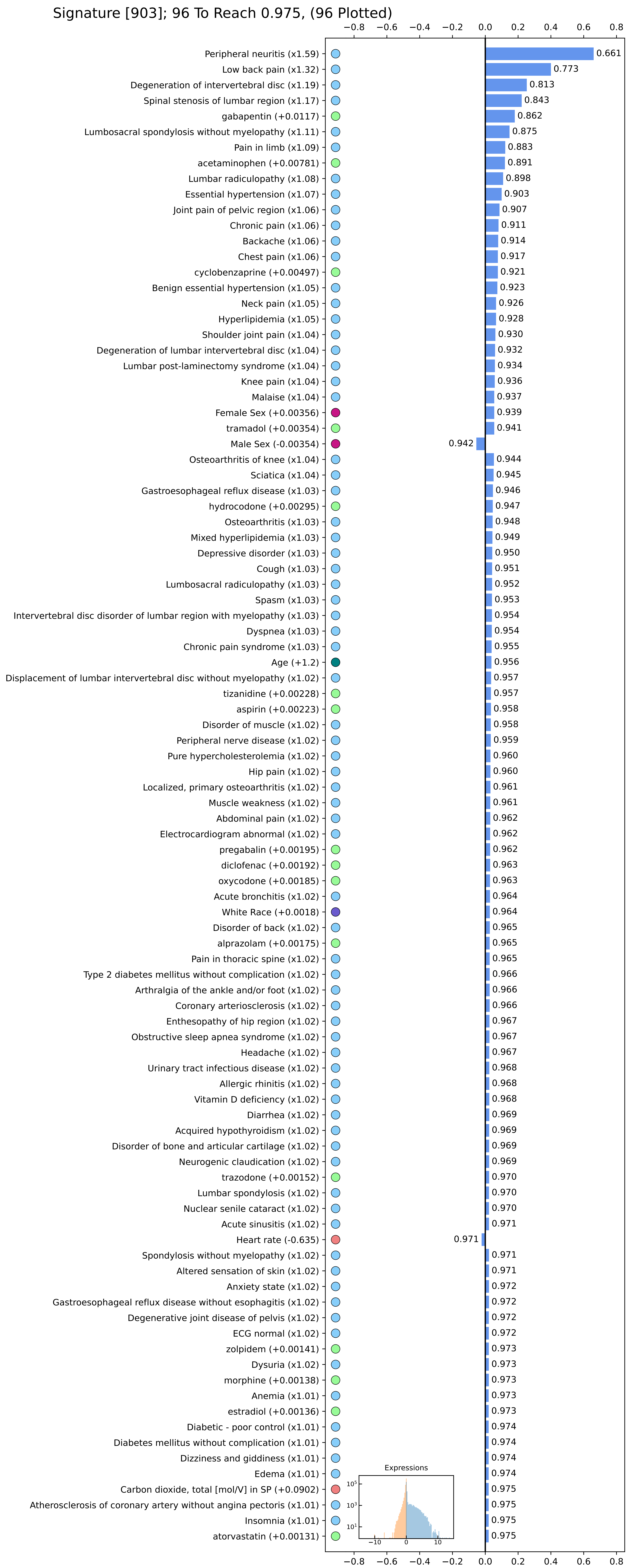

Signature [903]; 96 To Reach 0.975, (96 Plotted)
Peripheral neuritis (x1.59) 0.661
Low back pain (x1.32) 0.773
Degeneration of intervertebral disc (x1.19) 0.813
Spinal stenosis of lumbar region (x1.17) 0.843
gabapentin (+0.0117) 0.862
Lumbosacral spondylosis without myelopathy (x1.11) 0.875
Pain in limb (x1.09) 0.883
acetaminophen (+0.00781) 0.891
Lumbar radiculopathy (x1.08) 0.898
Essential hypertension (x1.07) 0.903
Joint pain of pelvic region (x1.06) 0.907
Chronic pain (x1.06) 0.911
Backache (x1.06) 0.914
Chest pain (x1.06) 0.917
cyclobenzaprine (+0.00497) 0.921
Benign essential hypertension (x1.05) 0.923
Neck pain (x1.05) 0.926
Hyperlipidemia (x1.05) 0.928
Shoulder joint pain (x1.04) 0.930
Degeneration of lumbar intervertebral disc (x1.04) 0.932
Lumbar post-laminectomy syndrome (x1.04) 0.934
Knee pain (x1.04) 0.936
Malaise (x1.04) 0.937
Female Sex (+0.00356) 0.939
tramadol (+0.00354) 0.941
Male Sex (-0.00354) 0.942
Osteoarthritis of knee (x1.04) 0.944
Sciatica (x1.04) 0.945
Gastroesophageal reflux disease (x1.03) 0.946
hydrocodone (+0.00295) 0.947
Osteoarthritis (x1.03) 0.948
Mixed hyperlipidemia (x1.03) 0.949
Depressive disorder (x1.03) 0.950
Cough (x1.03) 0.951
Lumbosacral radiculopathy (x1.03) 0.952
Spasm (x1.03) 0.953
Intervertebral disc disorder of lumbar region with myelopathy (x1.03) 0.954
Dyspnea (x1.03) 0.954
Chronic pain syndrome (x1.03) 0.955
Age (+1.2) 0.956
Displacement of lumbar intervertebral disc without myelopathy (x1.02) 0.957
tizanidine (+0.00228) 0.957
aspirin (+0.00223) 0.958
Disorder of muscle (x1.02) 0.958
Peripheral nerve disease (x1.02) 0.959
Pure hypercholesterolemia (x1.02) 0.960
Hip pain (x1.02) 0.960
Localized, primary osteoarthritis (x1.02) 0.961
Muscle weakness (x1.02) 0.961
Abdominal pain (x1.02) 0.962
Electrocardiogram abnormal (x1.02) 0.962
pregabalin (+0.00195) 0.962
diclofenac (+0.00192) 0.963
oxycodone (+0.00185) 0.963
Acute bronchitis (x1.02) 0.964
White Race (+0.0018) 0.964
Disorder of back (x1.02) 0.965
alprazolam (+0.00175) 0.965
Pain in thoracic spine (x1.02) 0.965
Type 2 diabetes mellitus without complication (x1.02) 0.966
Arthralgia of the ankle and/or foot (x1.02) 0.966
Coronary arteriosclerosis (x1.02) 0.966
Enthesopathy of hip region (x1.02) 0.967
Obstructive sleep apnea syndrome (x1.02) 0.967
Headache (x1.02) 0.967
Urinary tract infectious disease (x1.02) 0.968
Allergic rhinitis (x1.02) 0.968
Vitamin D deficiency (x1.02) 0.968
Diarrhea (x1.02) 0.969
Acquired hypothyroidism (x1.02) 0.969
Disorder of bone and articular cartilage (x1.02) 0.969
Neurogenic claudication (x1.02) 0.969
trazodone (+0.00152) 0.970
Lumbar spondylosis (x1.02) 0.970
Nuclear senile cataract (x1.02) 0.970
Acute sinusitis (x1.02) 0.971
Heart rate (-0.635) 0.971
Spondylosis without myelopathy (x1.02) 0.971
Altered sensation of skin (x1.02) 0.971
Anxiety state (x1.02) 0.972
Gastroesophageal reflux disease without esophagitis (x1.02) 0.972
Degenerative joint disease of pelvis (x1.02) 0.972
ECG normal (x1.02) 0.972
zolpidem (+0.00141) 0.973
Dysuria (x1.02) 0.973
morphine (+0.00138) 0.973
Anemia (x1.01) 0.973
estradiol (+0.00136) 0.973
Diabetic - poor control (x1.01) 0.974
Diabetes mellitus without complication (x1.01) 0.974
Dizziness and giddiness (x1.01) 0.974
Edema (x1.01) 0.974
Carbon dioxide, total [mol/V] in SP (+0.0902) 0.975
Atherosclerosis of coronary artery without angina pectoris (x1.01) 0.975
Insomnia (x1.01) 0.975
atorvastatin (+0.00131) 0.975
−0.8 −0.6 −0.4 −0.2 0.0 0.2 0.4 0.6 0.8
Expressions
10^5
10^3
10^1
−10 0 10

Signature [904]; 5 To Reach 0.975, (10 Plotted)

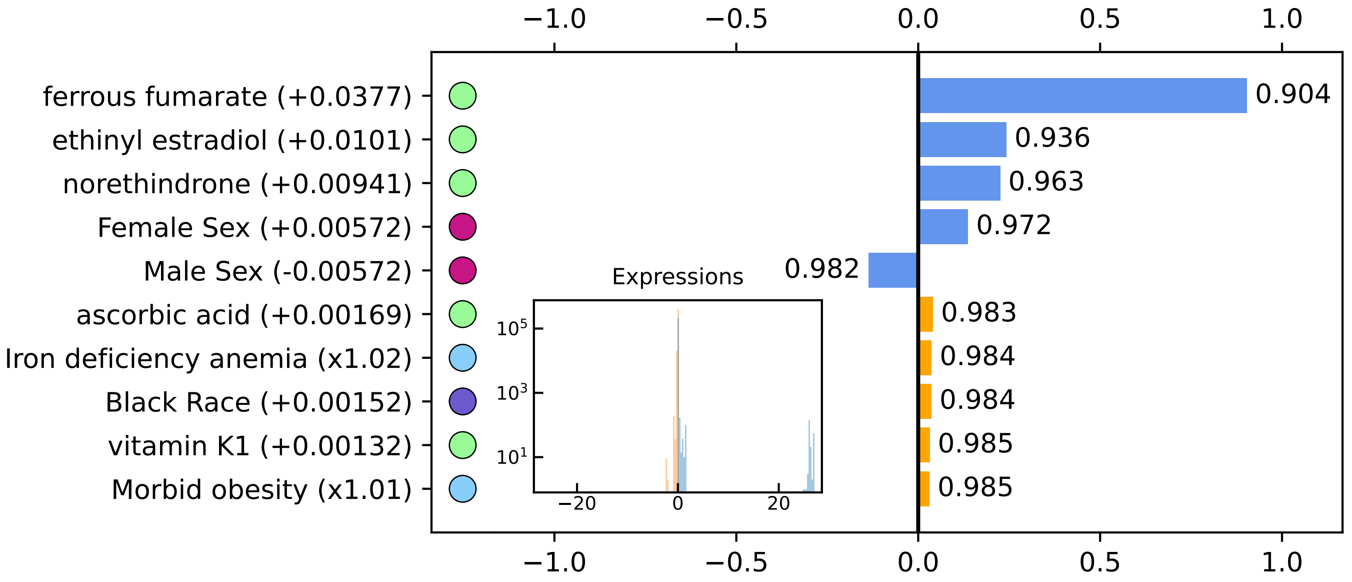

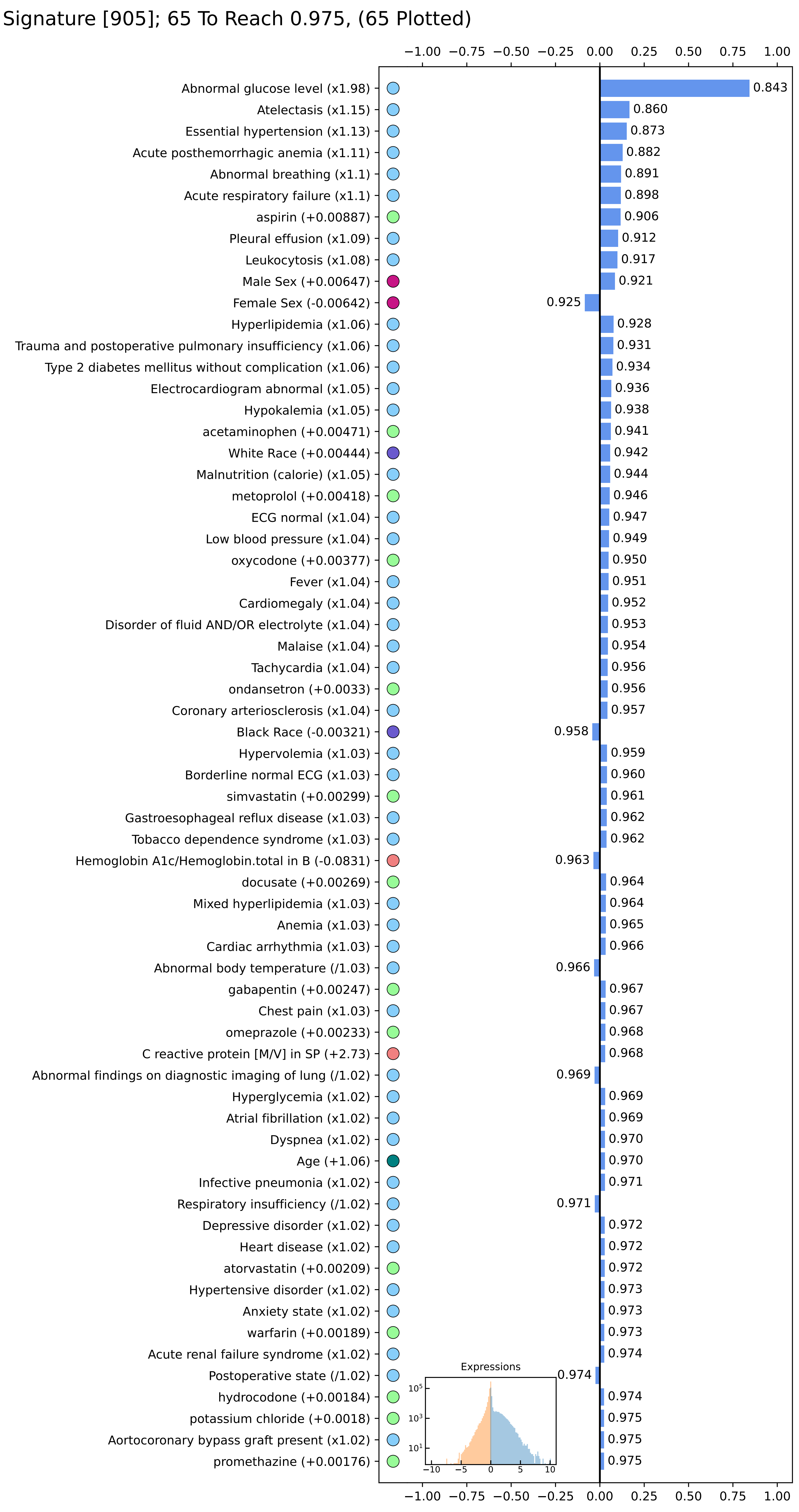
Signature [905]; 65 To Reach 0.975, (65 Plotted)
Abnormal glucose level (x1.98) 0.843
Atelectasis (x1.15) 0.860
Essential hypertension (x1.13) 0.873
Acute posthemorrhagic anemia (x1.11) 0.882
Abnormal breathing (x1.1) 0.891
Acute respiratory failure (x1.1) 0.898
aspirin (+0.00887) 0.906
Pleural effusion (x1.09) 0.912
Leukocytosis (x1.08) 0.917
Male Sex (+0.00647) 0.921
Female Sex (-0.00642) 0.925
Hyperlipidemia (x1.06) 0.928
Trauma and postoperative pulmonary insufficiency (x1.06) 0.931
Type 2 diabetes mellitus without complication (x1.06) 0.934
Electrocardiogram abnormal (x1.05) 0.936
Hypokalemia (x1.05) 0.938
acetaminophen (+0.00471) 0.941
White Race (+0.00444) 0.942
Malnutrition (calorie) (x1.05) 0.944
metoprolol (+0.00418) 0.946
ECG normal (x1.04) 0.947
Low blood pressure (x1.04) 0.949
oxycodone (+0.00377) 0.950
Fever (x1.04) 0.951
Cardiomegaly (x1.04) 0.952
Disorder of fluid AND/OR electrolyte (x1.04) 0.953
Malaise (x1.04) 0.954
Tachycardia (x1.04) 0.956
ondansetron (+0.0033) 0.956
Coronary arteriosclerosis (x1.04) 0.957
Black Race (-0.00321) 0.958
Hypervolemia (x1.03) 0.959
Borderline normal ECG (x1.03) 0.960
simvastatin (+0.00299) 0.961
Gastroesophageal reflux disease (x1.03) 0.962
Tobacco dependence syndrome (x1.03) 0.962
Hemoglobin A1c/Hemoglobin.total in B (-0.0831) 0.963
docusate (+0.00269) 0.964
Mixed hyperlipidemia (x1.03) 0.964
Anemia (x1.03) 0.965
Cardiac arrhythmia (x1.03) 0.966
Abnormal body temperature (/1.03) 0.966
gabapentin (+0.00247) 0.967
Chest pain (x1.03) 0.967
omeprazole (+0.00233) 0.968
C reactive protein [M/V] in SP (+2.73) 0.968
Abnormal findings on diagnostic imaging of lung (/1.02) 0.969
Hyperglycemia (x1.02) 0.969
Atrial fibrillation (x1.02) 0.969
Dyspnea (x1.02) 0.970
Age (+1.06) 0.970
Infective pneumonia (x1.02) 0.971
Respiratory insufficiency (/1.02) 0.971
Depressive disorder (x1.02) 0.972
Heart disease (x1.02) 0.972
atorvastatin (+0.00209) 0.972
Hypertensive disorder (x1.02) 0.973
Anxiety state (x1.02) 0.973
warfarin (+0.00189) 0.973
Acute renal failure syndrome (x1.02) 0.974
Postoperative state (/1.02) 0.974
hydrocodone (+0.00184) 0.974
potassium chloride (+0.0018) 0.975
Aortocoronary bypass graft present (x1.02) 0.975
promethazine (+0.00176) 0.975
−1.00 −0.75 −0.50 −0.25 0.00 0.25 0.50 0.75 1.00
Expressions
10^5
10^3
10^1
−10 −5 0 5 10

Signature [906]; 51 To Reach 0.975, (51 Plotted)

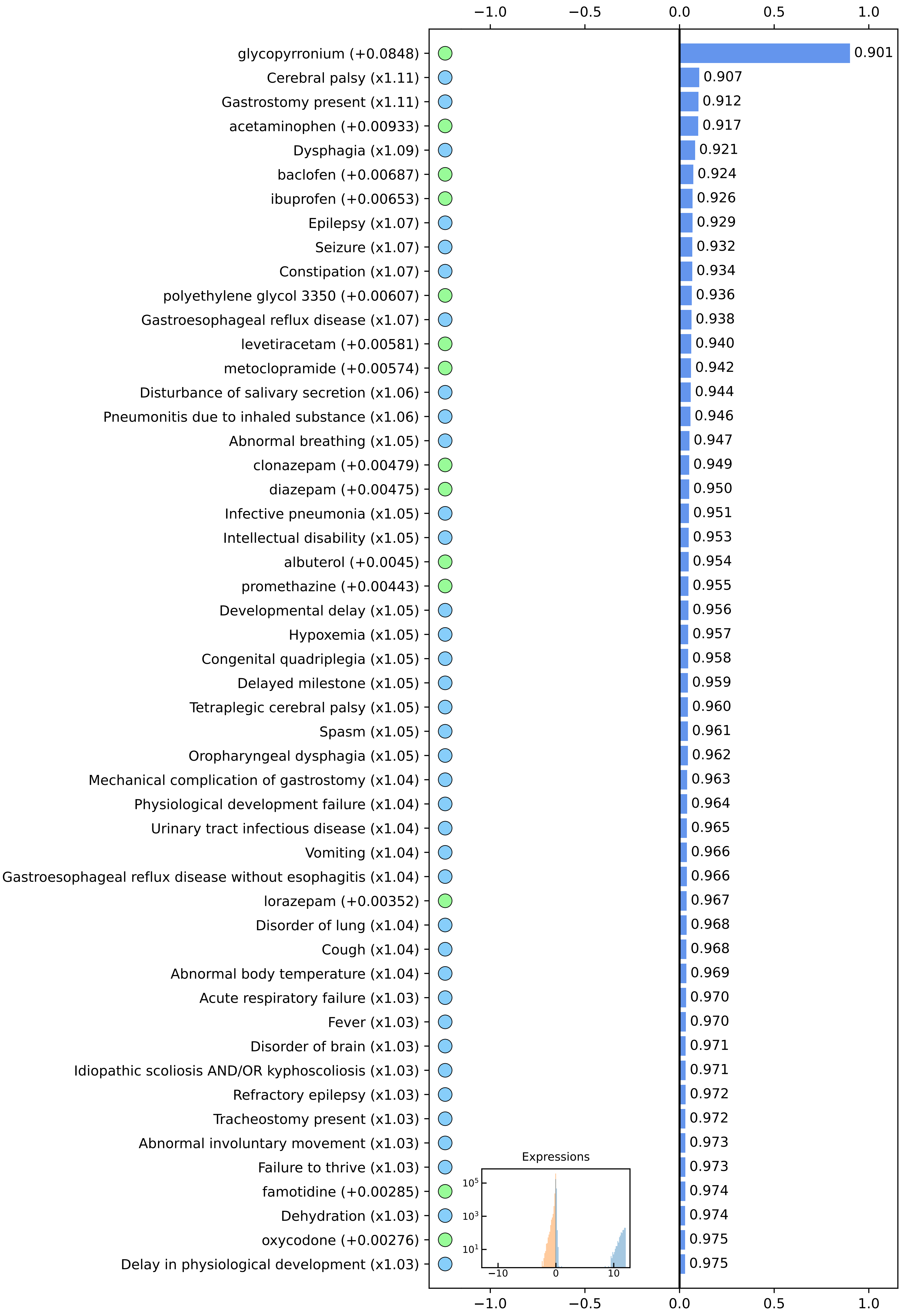

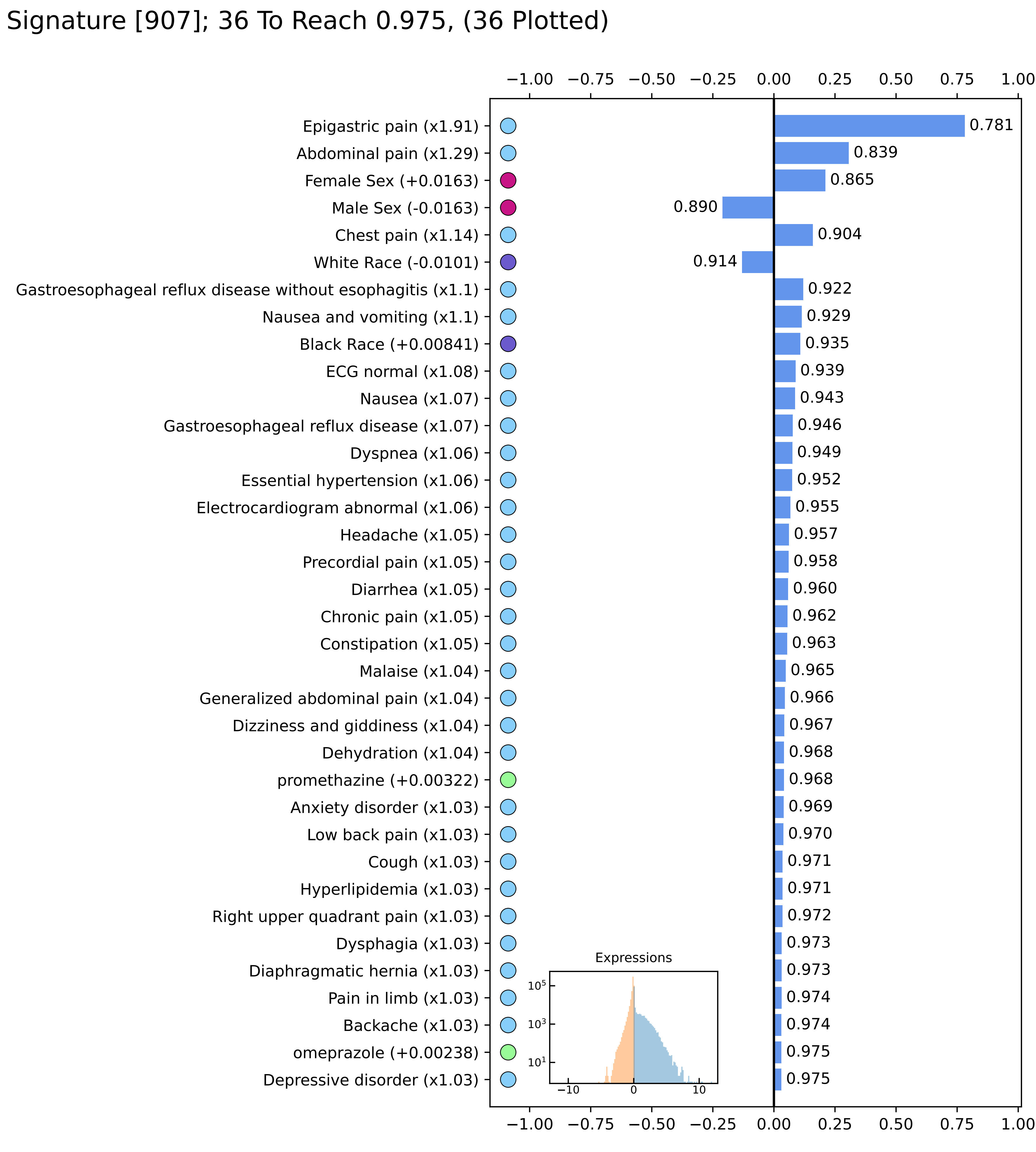
Signature [907]; 36 To Reach 0.975, (36 Plotted)
Epigastric pain (x1.91)
0.781
Abdominal pain (x1.29)
0.839
Female Sex (+0.0163)
0.865
Male Sex (-0.0163)
0.890
Chest pain (x1.14)
0.904
White Race (-0.0101)
0.914
Gastroesophageal reflux disease without esophagitis (x1.1)
0.922
Nausea and vomiting (x1.1)
0.929
Black Race (+0.00841)
0.935
ECG normal (x1.08)
0.939
Nausea (x1.07)
0.943
Gastroesophageal reflux disease (x1.07)
0.946
Dyspnea (x1.06)
0.949
Essential hypertension (x1.06)
0.952
Electrocardiogram abnormal (x1.06)
0.955
Headache (x1.05)
0.957
Precordial pain (x1.05)
0.958
Diarrhea (x1.05)
0.960
Chronic pain (x1.05)
0.962
Constipation (x1.05)
0.963
Malaise (x1.04)
0.965
Generalized abdominal pain (x1.04)
0.966
Dizziness and giddiness (x1.04)
0.967
Dehydration (x1.04)
0.968
promethazine (+0.00322)
0.968
Anxiety disorder (x1.03)
0.969
Low back pain (x1.03)
0.970
Cough (x1.03)
0.971
Hyperlipidemia (x1.03)
0.971
Right upper quadrant pain (x1.03)
0.972
Dysphagia (x1.03)
0.973
Diaphragmatic hernia (x1.03)
0.973
Pain in limb (x1.03)
0.974
Backache (x1.03)
0.974
omeprazole (+0.00238)
0.975
Depressive disorder (x1.03)
0.975
−1.00 −0.75 −0.50 −0.25 0.00 0.25 0.50 0.75 1.00
Expressions
10^5
10^3
10^1
−10 0 10

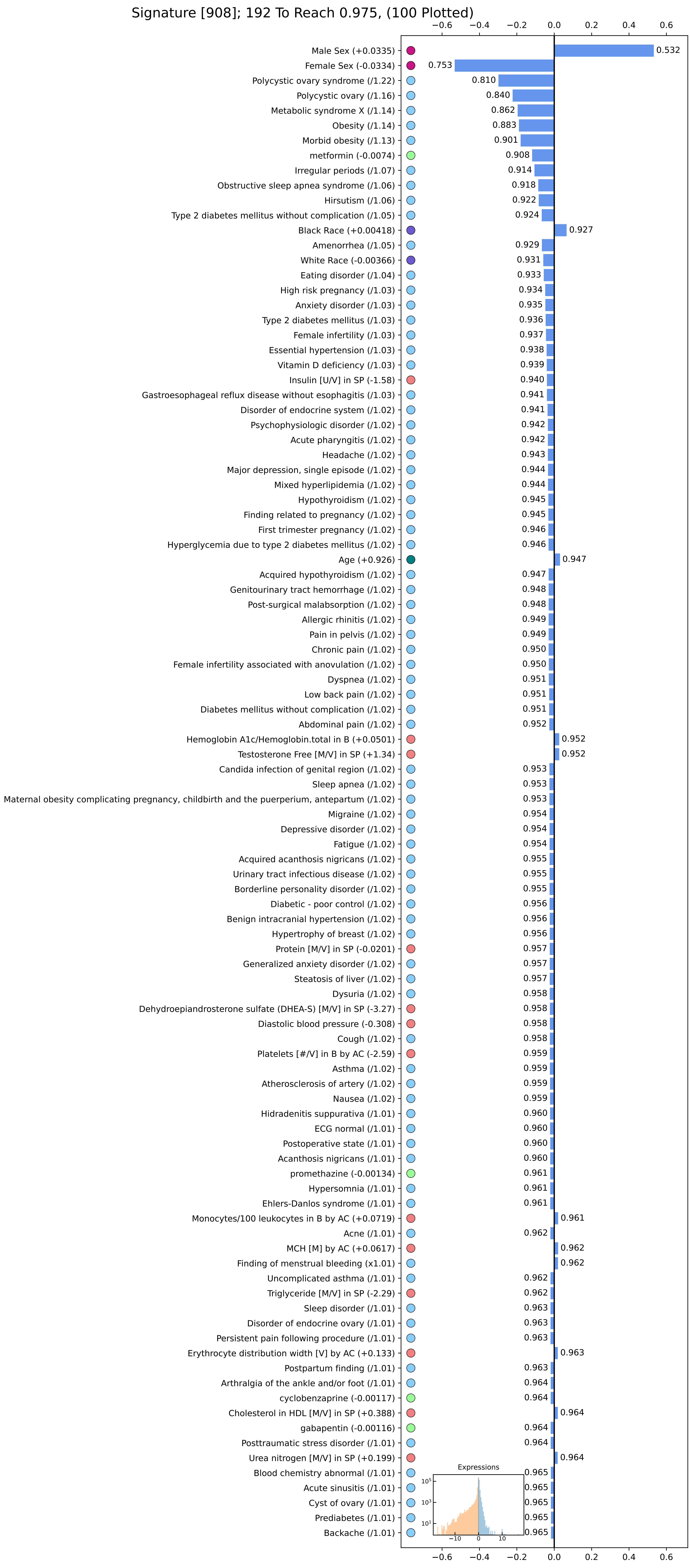

Signature [908]; 192 To Reach 0.975, (100 Plotted)
Male Sex (+0.0335) 0.532
Female Sex (-0.0334) 0.753
Polycystic ovary syndrome (/1.22) 0.810
Polycystic ovary (/1.16) 0.840
Metabolic syndrome X (/1.14) 0.862
Obesity (/1.14) 0.883
Morbid obesity (/1.13) 0.901
metformin (-0.0074) 0.908
Irregular periods (/1.07) 0.914
Obstructive sleep apnea syndrome (/1.06) 0.918
Hirsutism (/1.06) 0.922
Type 2 diabetes mellitus without complication (/1.05) 0.924
Black Race (+0.00418) 0.927
Amenorrhea (/1.05) 0.929
White Race (-0.00366) 0.931
Eating disorder (/1.04) 0.933
High risk pregnancy (/1.03) 0.934
Anxiety disorder (/1.03) 0.935
Type 2 diabetes mellitus (/1.03) 0.936
Female infertility (/1.03) 0.937
Essential hypertension (/1.03) 0.938
Vitamin D deficiency (/1.03) 0.939
Insulin [U/V] in SP (-1.58) 0.940
Gastroesophageal reflux disease without esophagitis (/1.03) 0.941
Disorder of endocrine system (/1.02) 0.941
Psychophysiologic disorder (/1.02) 0.942
Acute pharyngitis (/1.02) 0.942
Headache (/1.02) 0.943
Major depression, single episode (/1.02) 0.944
Mixed hyperlipidemia (/1.02) 0.944
Hypothyroidism (/1.02) 0.945
Finding related to pregnancy (/1.02) 0.945
First trimester pregnancy (/1.02) 0.946
Hyperglycemia due to type 2 diabetes mellitus (/1.02) 0.946
Age (+0.926) 0.947
Acquired hypothyroidism (/1.02) 0.947
Genitourinary tract hemorrhage (/1.02) 0.948
Post-surgical malabsorption (/1.02) 0.948
Allergic rhinitis (/1.02) 0.949
Pain in pelvis (/1.02) 0.949
Chronic pain (/1.02) 0.950
Female infertility associated with anovulation (/1.02) 0.950
Dyspnea (/1.02) 0.951
Low back pain (/1.02) 0.951
Diabetes mellitus without complication (/1.02) 0.951
Abdominal pain (/1.02) 0.952
Hemoglobin A1c/Hemoglobin.total in B (+0.0501) 0.952
Testosterone Free [M/V] in SP (+1.34) 0.952
Candida infection of genital region (/1.02) 0.953
Sleep apnea (/1.02) 0.953
Maternal obesity complicating pregnancy, childbirth and the puerperium, antepartum (/1.02) 0.953
Migraine (/1.02) 0.954
Depressive disorder (/1.02) 0.954
Fatigue (/1.02) 0.954
Acquired acanthosis nigricans (/1.02) 0.955
Urinary tract infectious disease (/1.02) 0.955
Borderline personality disorder (/1.02) 0.955
Diabetic - poor control (/1.02) 0.956
Benign intracranial hypertension (/1.02) 0.956
Hypertrophy of breast (/1.02) 0.956
Protein [M/V] in SP (-0.0201) 0.957
Generalized anxiety disorder (/1.02) 0.957
Steatosis of liver (/1.02) 0.957
Dysuria (/1.02) 0.958
Dehydroepiandrosterone sulfate (DHEA-S) [M/V] in SP (-3.27) 0.958
Diastolic blood pressure (-0.308) 0.958
Cough (/1.02) 0.958
Platelets [#/V] in B by AC (-2.59) 0.959
Asthma (/1.02) 0.959
Atherosclerosis of artery (/1.02) 0.959
Nausea (/1.02) 0.959
Hidradenitis suppurativa (/1.01) 0.960
ECG normal (/1.01) 0.960
Postoperative state (/1.01) 0.960
Acanthosis nigricans (/1.01) 0.960
promethazine (-0.00134) 0.961
Hypersomnia (/1.01) 0.961
Ehlers-Danlos syndrome (/1.01) 0.961
Monocytes/100 leukocytes in B by AC (+0.0719) 0.961
Acne (/1.01) 0.962
MCH [M] by AC (+0.0617) 0.962
Finding of menstrual bleeding (x1.01) 0.962
Uncomplicated asthma (/1.01) 0.962
Triglyceride [M/V] in SP (-2.29) 0.962
Sleep disorder (/1.01) 0.963
Disorder of endocrine ovary (/1.01) 0.963
Persistent pain following procedure (/1.01) 0.963
Erythrocyte distribution width [V] by AC (+0.133) 0.963
Postpartum finding (/1.01) 0.963
Arthralgia of the ankle and/or foot (/1.01) 0.964
cyclobenzaprine (-0.00117) 0.964
Cholesterol in HDL [M/V] in SP (+0.388) 0.964
gabapentin (-0.00116) 0.964
Posttraumatic stress disorder (/1.01) 0.964
Urea nitrogen [M/V] in SP (+0.199) 0.964
Blood chemistry abnormal (/1.01) 0.965
Acute sinusitis (/1.01) 0.965
Cyst of ovary (/1.01) 0.965
Prediabetes (/1.01) 0.965
Backache (/1.01) 0.965
−0.6 −0.4 −0.2 0.0 0.2 0.4 0.6
Expressions

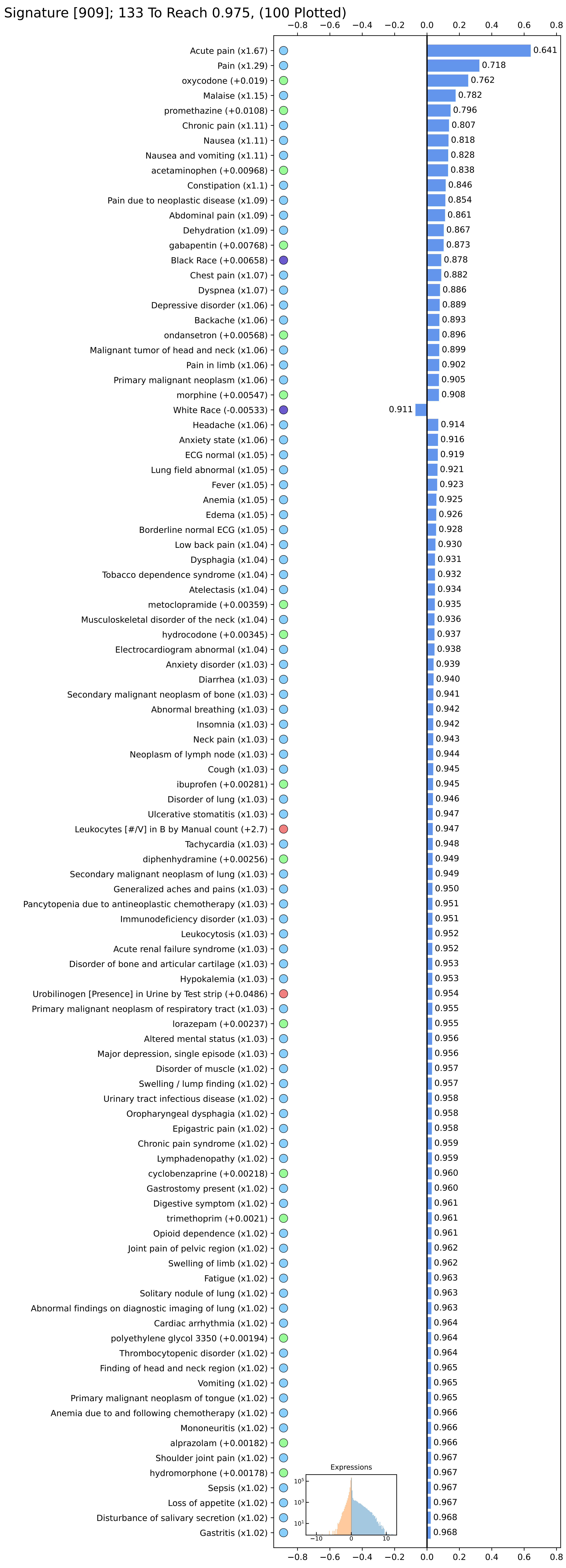

Signature [909]; 133 To Reach 0.975, (100 Plotted)
Acute pain (x1.67) 0.641
Pain (x1.29) 0.718
oxycodone (+0.019) 0.762
Malaise (x1.15) 0.782
promethazine (+0.0108) 0.796
Chronic pain (x1.11) 0.807
Nausea (x1.11) 0.818
Nausea and vomiting (x1.11) 0.828
acetaminophen (+0.00968) 0.838
Constipation (x1.1) 0.846
Pain due to neoplastic disease (x1.09) 0.854
Abdominal pain (x1.09) 0.861
Dehydration (x1.09) 0.867
gabapentin (+0.00768) 0.873
Black Race (+0.00658) 0.878
Chest pain (x1.07) 0.882
Dyspnea (x1.07) 0.886
Depressive disorder (x1.06) 0.889
Backache (x1.06) 0.893
ondansetron (+0.00568) 0.896
Malignant tumor of head and neck (x1.06) 0.899
Pain in limb (x1.06) 0.902
Primary malignant neoplasm (x1.06) 0.905
morphine (+0.00547) 0.908
White Race (-0.00533) 0.911
Headache (x1.06) 0.914
Anxiety state (x1.06) 0.916
ECG normal (x1.05) 0.919
Lung field abnormal (x1.05) 0.921
Fever (x1.05) 0.923
Anemia (x1.05) 0.925
Edema (x1.05) 0.926
Borderline normal ECG (x1.05) 0.928
Low back pain (x1.04) 0.930
Dysphagia (x1.04) 0.931
Tobacco dependence syndrome (x1.04) 0.932
Atelectasis (x1.04) 0.934
metoclopramide (+0.00359) 0.935
Musculoskeletal disorder of the neck (x1.04) 0.936
hydrocodone (+0.00345) 0.937
Electrocardiogram abnormal (x1.04) 0.938
Anxiety disorder (x1.03) 0.939
Diarrhea (x1.03) 0.940
Secondary malignant neoplasm of bone (x1.03) 0.941
Abnormal breathing (x1.03) 0.942
Insomnia (x1.03) 0.942
Neck pain (x1.03) 0.943
Neoplasm of lymph node (x1.03) 0.944
Cough (x1.03) 0.945
ibuprofen (+0.00281) 0.945
Disorder of lung (x1.03) 0.946
Ulcerative stomatitis (x1.03) 0.947
Leukocytes [#/V] in B by Manual count (+2.7) 0.947
Tachycardia (x1.03) 0.948
diphenhydramine (+0.00256) 0.949
Secondary malignant neoplasm of lung (x1.03) 0.949
Generalized aches and pains (x1.03) 0.950
Pancytopenia due to antineoplastic chemotherapy (x1.03) 0.951
Immunodeficiency disorder (x1.03) 0.951
Leukocytosis (x1.03) 0.952
Acute renal failure syndrome (x1.03) 0.952
Disorder of bone and articular cartilage (x1.03) 0.953
Hypokalemia (x1.03) 0.953
Urobilinogen [Presence] in Urine by Test strip (+0.0486) 0.954
Primary malignant neoplasm of respiratory tract (x1.03) 0.955
lorazepam (+0.00237) 0.955
Altered mental status (x1.03) 0.956
Major depression, single episode (x1.03) 0.956
Disorder of muscle (x1.02) 0.957
Swelling / lump finding (x1.02) 0.957
Urinary tract infectious disease (x1.02) 0.958
Oropharyngeal dysphagia (x1.02) 0.958
Epigastric pain (x1.02) 0.958
Chronic pain syndrome (x1.02) 0.959
Lymphadenopathy (x1.02) 0.959
cyclobenzaprine (+0.00218) 0.960
Gastrostomy present (x1.02) 0.960
Digestive symptom (x1.02) 0.961
trimethoprim (+0.0021) 0.961
Opioid dependence (x1.02) 0.961
Joint pain of pelvic region (x1.02) 0.962
Swelling of limb (x1.02) 0.962
Fatigue (x1.02) 0.963
Solitary nodule of lung (x1.02) 0.963
Abnormal findings on diagnostic imaging of lung (x1.02) 0.963
Cardiac arrhythmia (x1.02) 0.964
polyethylene glycol 3350 (+0.00194) 0.964
Thrombocytopenic disorder (x1.02) 0.964
Finding of head and neck region (x1.02) 0.965
Vomiting (x1.02) 0.965
Primary malignant neoplasm of tongue (x1.02) 0.965
Anemia due to and following chemotherapy (x1.02) 0.966
Mononeuritis (x1.02) 0.966
alprazolam (+0.00182) 0.966
Shoulder joint pain (x1.02) 0.967
hydromorphone (+0.00178) 0.967
Sepsis (x1.02) 0.967
Loss of appetite (x1.02) 0.967
Disturbance of salivary secretion (x1.02) 0.968
Gastritis (x1.02) 0.968
Expressions
−0.8 −0.6 −0.4 −0.2 0.0 0.2 0.4 0.6 0.8

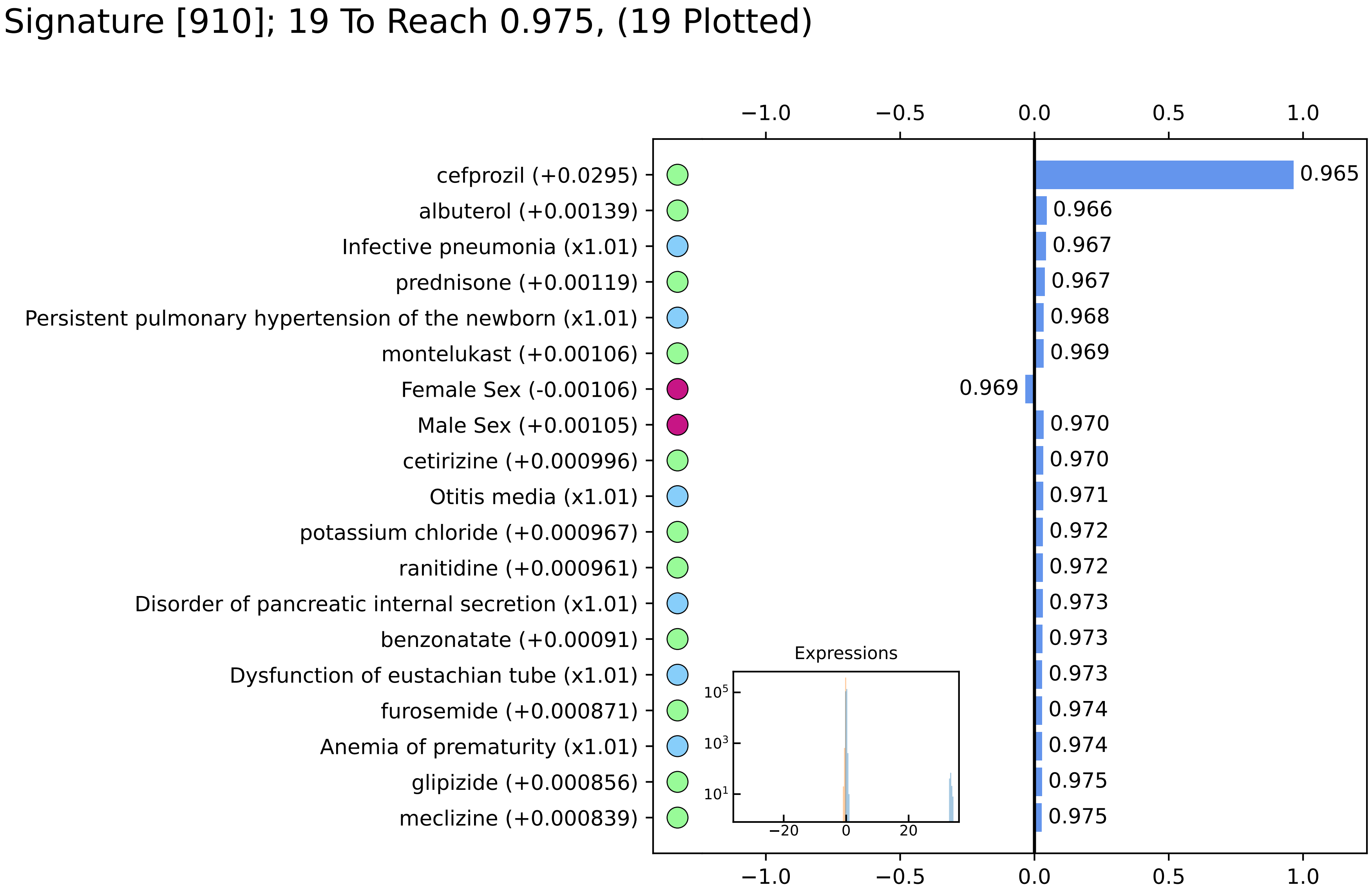

Signature [910]; 19 To Reach 0.975, (19 Plotted)
cefprozil (+0.0295)
albuterol (+0.00139)
Infective pneumonia (x1.01)
prednisone (+0.00119)
Persistent pulmonary hypertension of the newborn (x1.01)
montelukast (+0.00106)
Female Sex (-0.00106)
Male Sex (+0.00105)
cetirizine (+0.000996)
Otitis media (x1.01)
potassium chloride (+0.000967)
ranitidine (+0.000961)
Disorder of pancreatic internal secretion (x1.01)
benzonatate (+0.00091)
Dysfunction of eustachian tube (x1.01)
furosemide (+0.000871)
Anemia of prematurity (x1.01)
glipizide (+0.000856)
meclizine (+0.000839)
0.965
0.966
0.967
0.967
0.968
0.969
0.969
0.970
0.970
0.971
0.972
0.972
0.973
0.973
0.973
0.974
0.974
0.975
0.975
Expressions
−1.0
−0.5
0.0
0.5
1.0

Signature [911]; 52 To Reach 0.975, (52 Plotted)

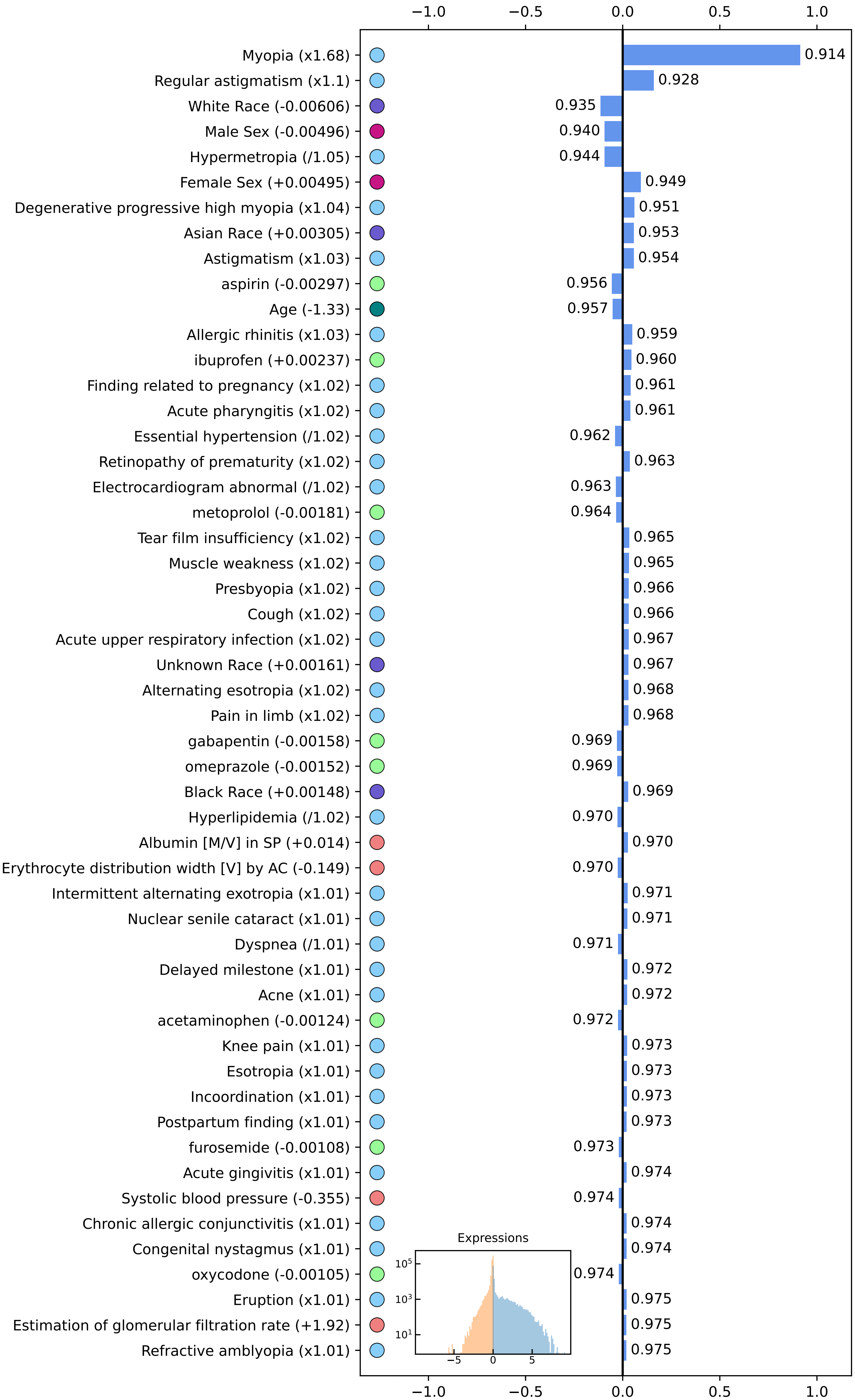

Signature [912]; 2 To Reach 0.975, (10 Plotted)

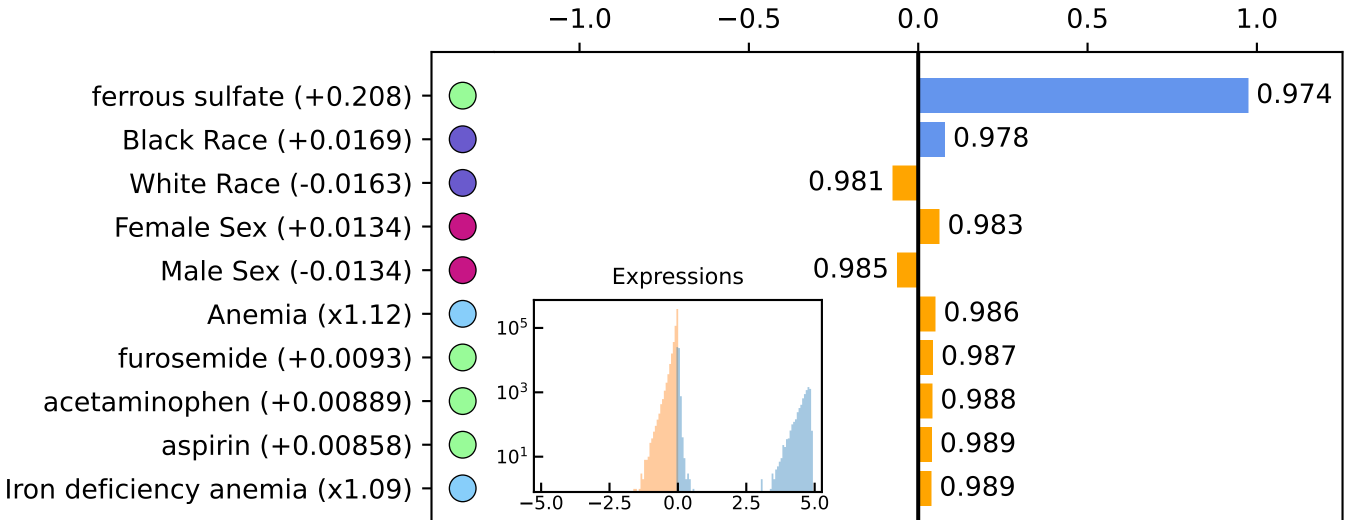

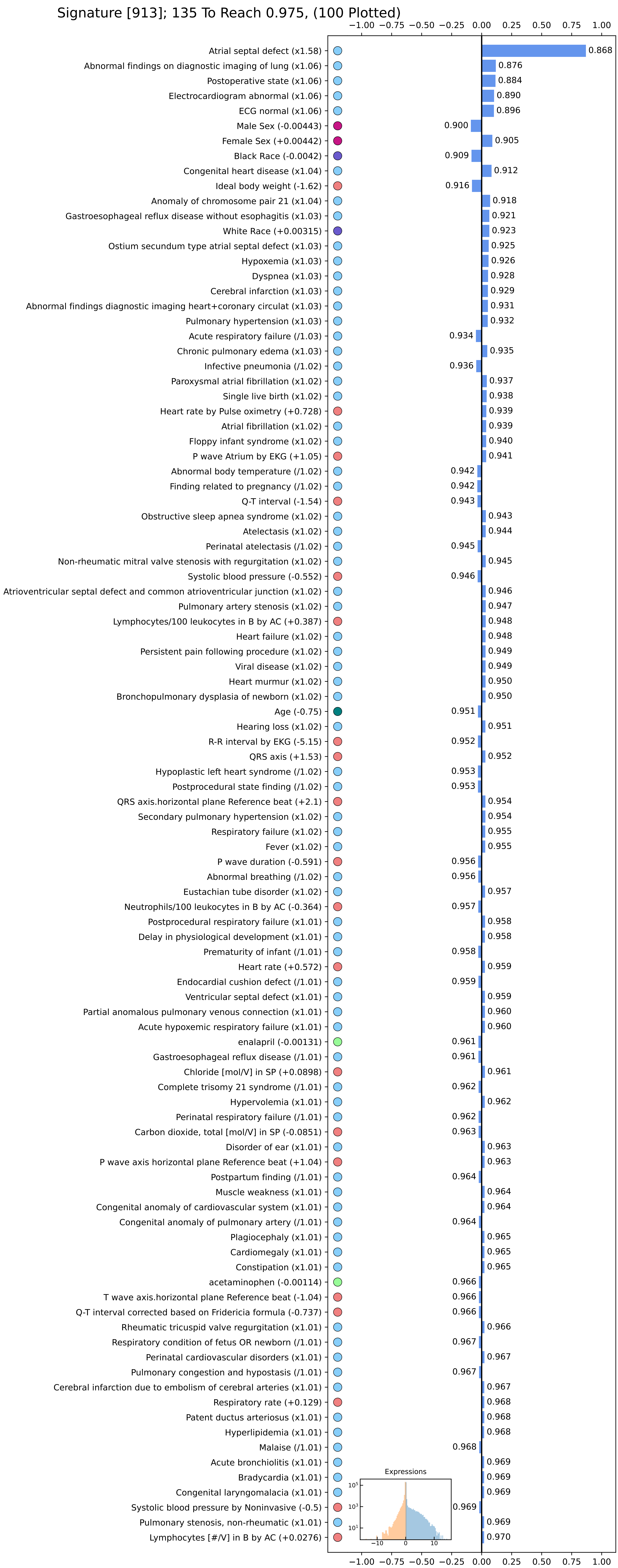

Signature [913]; 135 To Reach 0.975, (100 Plotted)
Atrial septal defect (x1.58) 0.868
Abnormal findings on diagnostic imaging of lung (x1.06) 0.876
Postoperative state (x1.06) 0.884
Electrocardiogram abnormal (x1.06) 0.890
ECG normal (x1.06) 0.896
Male Sex (-0.00443) 0.900
Female Sex (+0.00442) 0.905
Black Race (-0.0042) 0.909
Congenital heart disease (x1.04) 0.912
Ideal body weight (-1.62) 0.916
Anomaly of chromosome pair 21 (x1.04) 0.918
Gastroesophageal reflux disease without esophagitis (x1.03) 0.921
White Race (+0.00315) 0.923
Ostium secundum type atrial septal defect (x1.03) 0.925
Hypoxemia (x1.03) 0.926
Dyspnea (x1.03) 0.928
Cerebral infarction (x1.03) 0.929
Abnormal findings diagnostic imaging heart+coronary circulat (x1.03) 0.931
Pulmonary hypertension (x1.03) 0.932
Acute respiratory failure (/1.03) 0.934
Chronic pulmonary edema (x1.03) 0.935
Infective pneumonia (/1.02) 0.936
Paroxysmal atrial fibrillation (x1.02) 0.937
Single live birth (x1.02) 0.938
Heart rate by Pulse oximetry (+0.728) 0.939
Atrial fibrillation (x1.02) 0.939
Floppy infant syndrome (x1.02) 0.940
P wave Atrium by EKG (+1.05) 0.941
Abnormal body temperature (/1.02) 0.942
Finding related to pregnancy (/1.02) 0.942
Q-T interval (-1.54) 0.943
Obstructive sleep apnea syndrome (x1.02) 0.943
Atelectasis (x1.02) 0.944
Perinatal atelectasis (/1.02) 0.945
Non-rheumatic mitral valve stenosis with regurgitation (x1.02) 0.945
Systolic blood pressure (-0.552) 0.946
Atrioventricular septal defect and common atrioventricular junction (x1.02) 0.946
Pulmonary artery stenosis (x1.02) 0.947
Lymphocytes/100 leukocytes in B by AC (+0.387) 0.948
Heart failure (x1.02) 0.948
Persistent pain following procedure (x1.02) 0.949
Viral disease (x1.02) 0.949
Heart murmur (x1.02) 0.950
Bronchopulmonary dysplasia of newborn (x1.02) 0.950
Age (-0.75) 0.951
Hearing loss (x1.02) 0.951
R-R interval by EKG (-5.15) 0.952
QRS axis (+1.53) 0.952
Hypoplastic left heart syndrome (/1.02) 0.953
Postprocedural state finding (/1.02) 0.953
QRS axis.horizontal plane Reference beat (+2.1) 0.954
Secondary pulmonary hypertension (x1.02) 0.954
Respiratory failure (x1.02) 0.955
Fever (x1.02) 0.955
P wave duration (-0.591) 0.956
Abnormal breathing (/1.02) 0.956
Eustachian tube disorder (x1.02) 0.957
Neutrophils/100 leukocytes in B by AC (-0.364) 0.957
Postprocedural respiratory failure (x1.01) 0.958
Delay in physiological development (x1.01) 0.958
Prematurity of infant (/1.01) 0.958
Heart rate (+0.572) 0.959
Endocardial cushion defect (/1.01) 0.959
Ventricular septal defect (x1.01) 0.959
Partial anomalous pulmonary venous connection (x1.01) 0.960
Acute hypoxemic respiratory failure (x1.01) 0.960
enalapril (-0.00131) 0.961
Gastroesophageal reflux disease (/1.01) 0.961
Chloride [mol/V] in SP (+0.0898) 0.961
Complete trisomy 21 syndrome (/1.01) 0.962
Hypervolemia (x1.01) 0.962
Perinatal respiratory failure (/1.01) 0.962
Carbon dioxide, total [mol/V] in SP (-0.0851) 0.963
Disorder of ear (x1.01) 0.963
P wave axis horizontal plane Reference beat (+1.04) 0.963
Postpartum finding (/1.01) 0.964
Muscle weakness (x1.01) 0.964
Congenital anomaly of cardiovascular system (x1.01) 0.964
Congenital anomaly of pulmonary artery (/1.01) 0.964
Plagiocephaly (x1.01) 0.965
Cardiomegaly (x1.01) 0.965
Constipation (x1.01) 0.965
acetaminophen (-0.00114) 0.966
T wave axis.horizontal plane Reference beat (-1.04) 0.966
Q-T interval corrected based on Fridericia formula (-0.737) 0.966
Rheumatic tricuspid valve regurgitation (x1.01) 0.966
Respiratory condition of fetus OR newborn (/1.01) 0.967
Perinatal cardiovascular disorders (x1.01) 0.967
Pulmonary congestion and hypostasis (/1.01) 0.967
Cerebral infarction due to embolism of cerebral arteries (x1.01) 0.967
Respiratory rate (+0.129) 0.968
Patent ductus arteriosus (x1.01) 0.968
Hyperlipidemia (x1.01) 0.968
Malaise (/1.01) 0.968
Acute bronchiolitis (x1.01) 0.969
Bradycardia (x1.01) 0.969
Congenital laryngomalacia (x1.01) 0.969
Systolic blood pressure by Noninvasive (-0.5) 0.969
Pulmonary stenosis, non-rheumatic (x1.01) 0.969
Lymphocytes [#/V] in B by AC (+0.0276) 0.970
−1.00 −0.75 −0.50 −0.25 0.00 0.25 0.50 0.75 1.00
Expressions

Signature [914]; 161 To Reach 0.975, (100 Plotted)

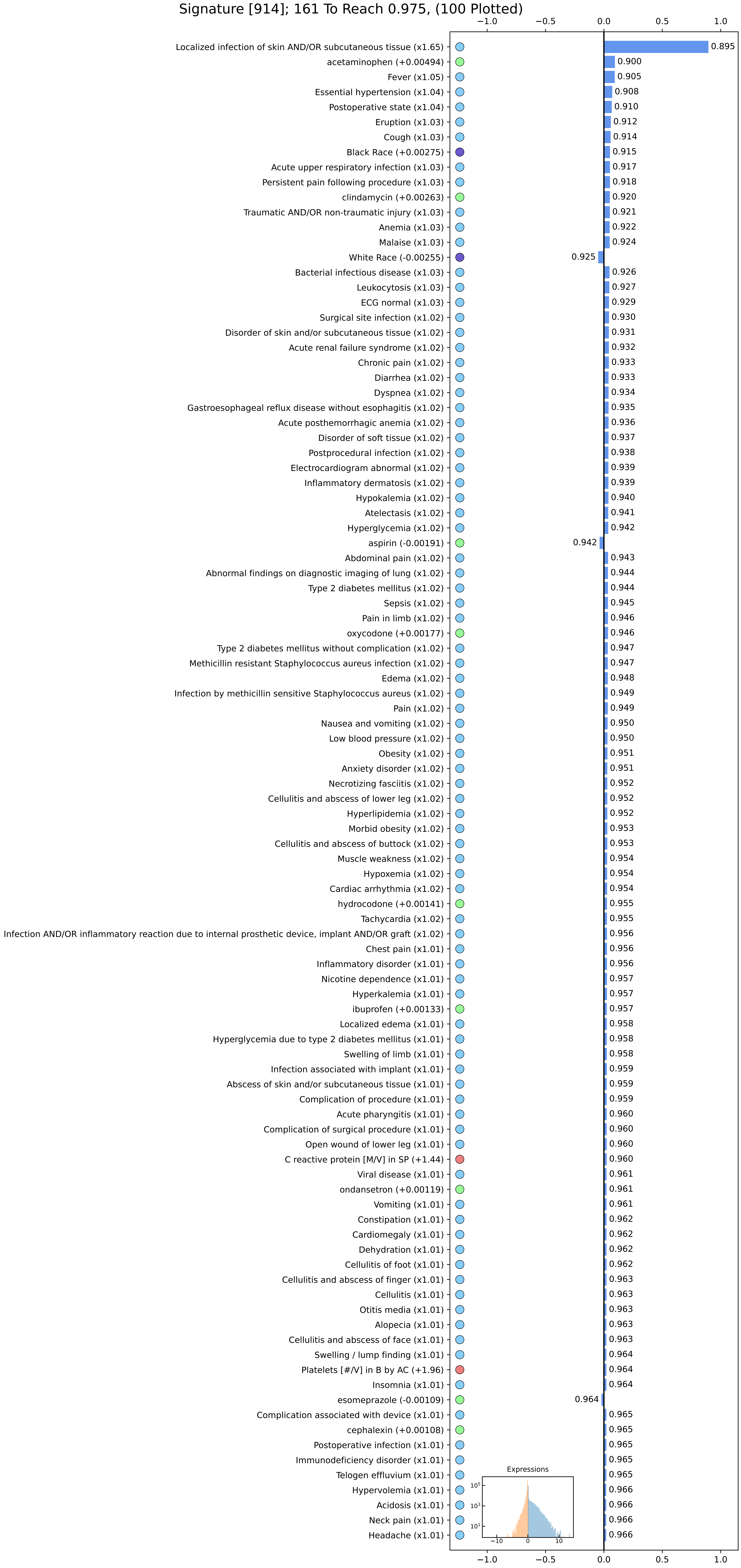

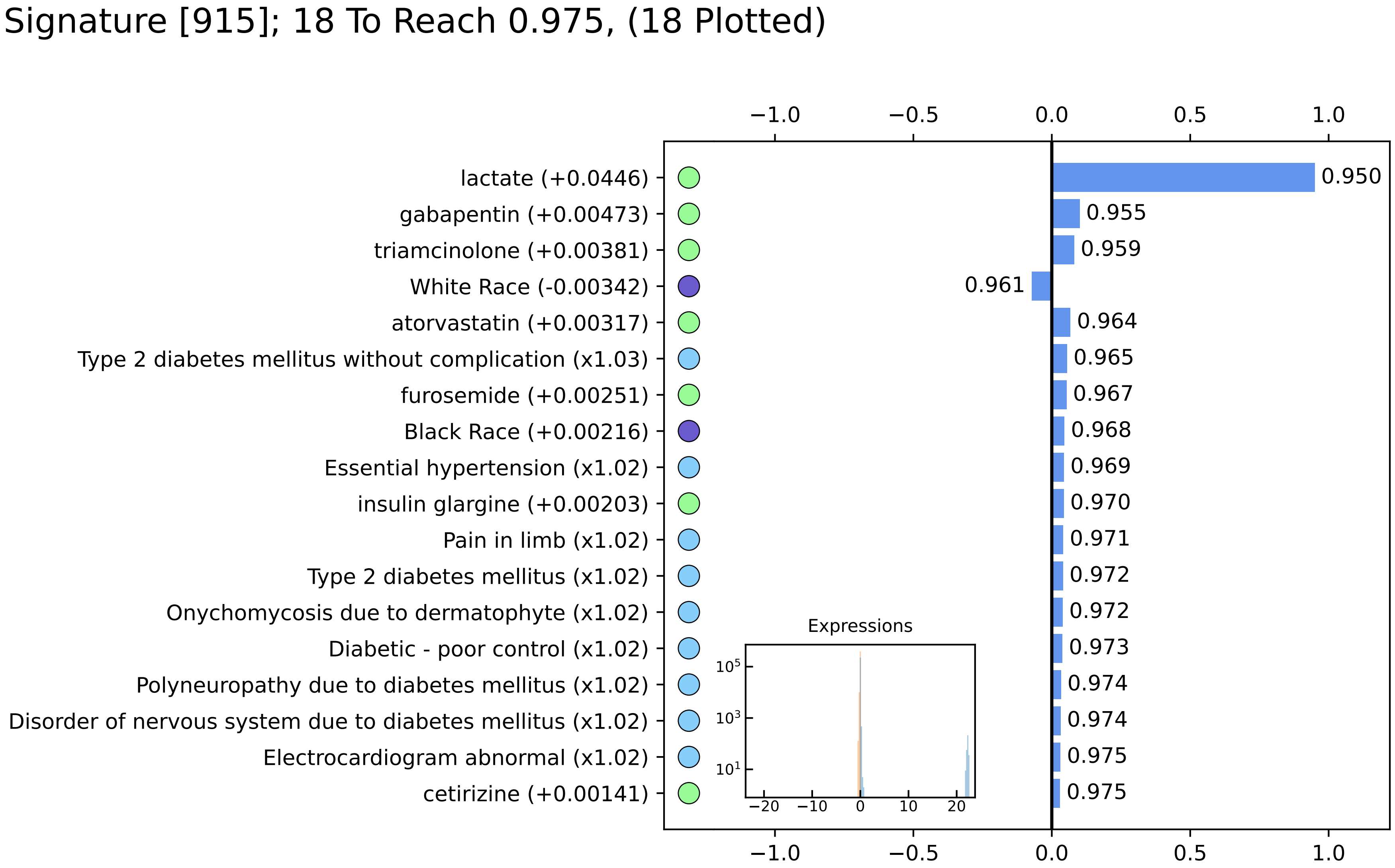
Signature [915]; 18 To Reach 0.975, (18 Plotted)
lactate (+0.0446)
gabapentin (+0.00473)
triamcinolone (+0.00381)
White Race (-0.00342)
atorvastatin (+0.00317)
Type 2 diabetes mellitus without complication (x1.03)
furosemide (+0.00251)
Black Race (+0.00216)
Essential hypertension (x1.02)
insulin glargine (+0.00203)
Pain in limb (x1.02)
Type 2 diabetes mellitus (x1.02)
Onychomycosis due to dermatophyte (x1.02)
Diabetic - poor control (x1.02)
Polyneuropathy due to diabetes mellitus (x1.02)
Disorder of nervous system due to diabetes mellitus (x1.02)
Electrocardiogram abnormal (x1.02)
cetirizine (+0.00141)
0.950
0.955
0.959
0.961
0.964
0.965
0.967
0.968
0.969
0.970
0.971
0.972
0.972
0.973
0.974
0.974
0.975
0.975
−1.0
−0.5
0.0
0.5
1.0
Expressions
−20
−10
0
10
20

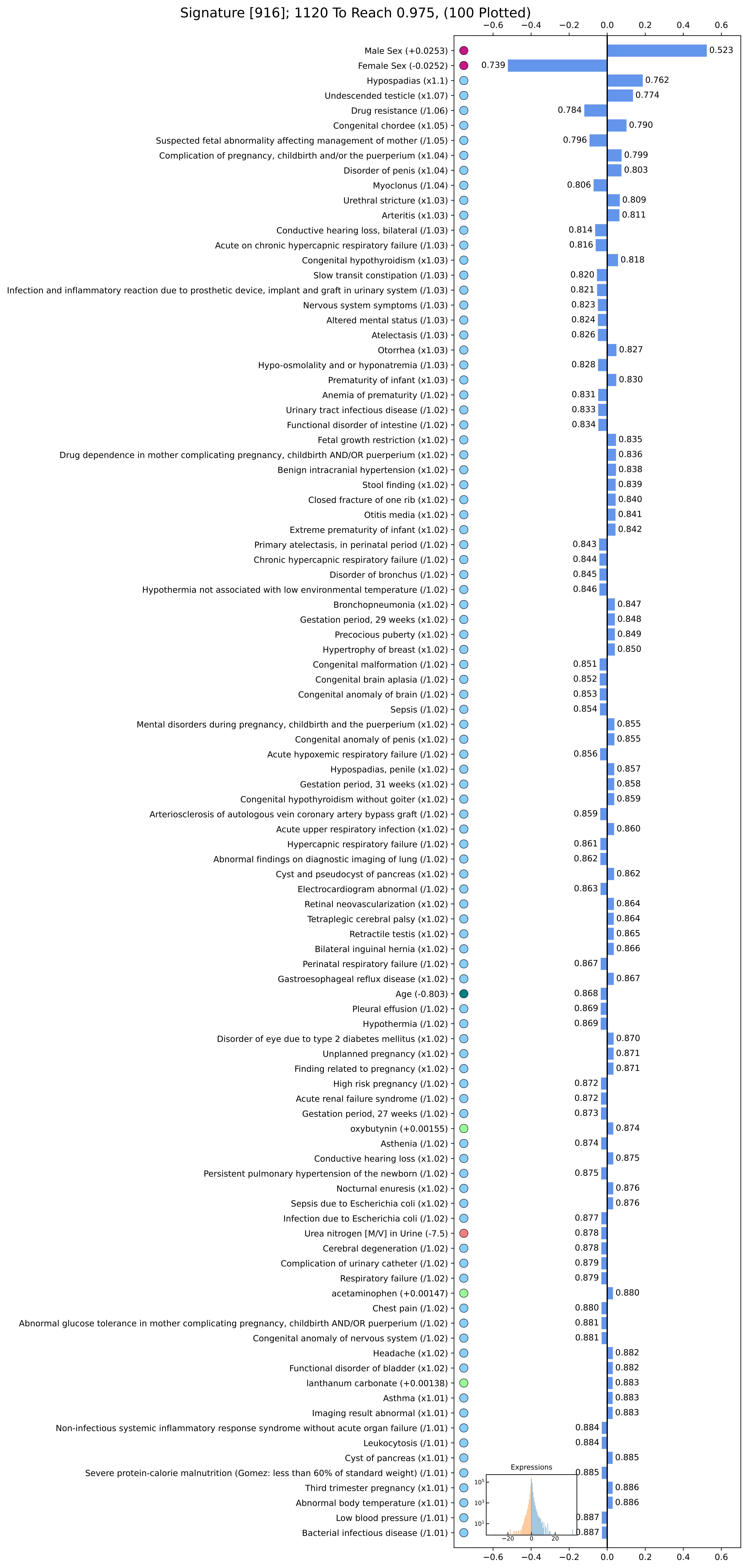

Signature [916]; 1120 To Reach 0.975, (100 Plotted)
Male Sex (+0.0253)
Female Sex (-0.0252)
Hypospadias (x1.1)
Undescended testicle (x1.07)
Drug resistance (/1.06)
Congenital chordee (x1.05)
Suspected fetal abnormality affecting management of mother (/1.05)
Complication of pregnancy, childbirth and/or the puerperium (x1.04)
Disorder of penis (x1.04)
Myoclonus (/1.04)
Urethral stricture (x1.03)
Arteritis (x1.03)
Conductive hearing loss, bilateral (/1.03)
Acute on chronic hypercapnic respiratory failure (/1.03)
Congenital hypothyroidism (x1.03)
Slow transit constipation (/1.03)
Infection and inflammatory reaction due to prosthetic device, implant and graft in urinary system (/1.03)
Nervous system symptoms (/1.03)
Altered mental status (/1.03)
Atelectasis (/1.03)
Otorrhea (x1.03)
Hypo-osmolality and or hyponatremia (/1.03)
Prematurity of infant (x1.03)
Anemia of prematurity (/1.02)
Urinary tract infectious disease (/1.02)
Functional disorder of intestine (/1.02)
Fetal growth restriction (x1.02)
Drug dependence in mother complicating pregnancy, childbirth AND/OR puerperium (x1.02)
Benign intracranial hypertension (x1.02)
Stool finding (x1.02)
Closed fracture of one rib (x1.02)
Otitis media (x1.02)
Extreme prematurity of infant (x1.02)
Primary atelectasis, in perinatal period (/1.02)
Chronic hypercapnic respiratory failure (/1.02)
Disorder of bronchus (/1.02)
Hypothermia not associated with low environmental temperature (/1.02)
Bronchopneumonia (x1.02)
Gestation period, 29 weeks (x1.02)
Precocious puberty (x1.02)
Hypertrophy of breast (x1.02)
Congenital malformation (/1.02)
Congenital brain aplasia (/1.02)
Congenital anomaly of brain (/1.02)
Sepsis (/1.02)
Mental disorders during pregnancy, childbirth and the puerperium (x1.02)
Congenital anomaly of penis (x1.02)
Acute hypoxemic respiratory failure (/1.02)
Hypospadias, penile (x1.02)
Gestation period, 31 weeks (x1.02)
Congenital hypothyroidism without goiter (x1.02)
Arteriosclerosis of autologous vein coronary artery bypass graft (/1.02)
Acute upper respiratory infection (x1.02)
Hypercapnic respiratory failure (/1.02)
Abnormal findings on diagnostic imaging of lung (/1.02)
Cyst and pseudocyst of pancreas (x1.02)
Electrocardiogram abnormal (/1.02)
Retinal neovascularization (x1.02)
Tetraplegic cerebral palsy (x1.02)
Retractile testis (x1.02)
Bilateral inguinal hernia (x1.02)
Perinatal respiratory failure (/1.02)
Gastroesophageal reflux disease (x1.02)
Age (-0.803)
Pleural effusion (/1.02)
Hypothermia (/1.02)
Disorder of eye due to type 2 diabetes mellitus (x1.02)
Unplanned pregnancy (x1.02)
Finding related to pregnancy (x1.02)
High risk pregnancy (/1.02)
Acute renal failure syndrome (/1.02)
Gestation period, 27 weeks (/1.02)
oxybutynin (+0.00155)
Asthenia (/1.02)
Conductive hearing loss (x1.02)
Persistent pulmonary hypertension of the newborn (/1.02)
Nocturnal enuresis (x1.02)
Sepsis due to Escherichia coli (x1.02)
Infection due to Escherichia coli (/1.02)
Urea nitrogen [M/V] in Urine (-7.5)
Cerebral degeneration (/1.02)
Complication of urinary catheter (/1.02)
Respiratory failure (/1.02)
acetaminophen (+0.00147)
Chest pain (/1.02)
Abnormal glucose tolerance in mother complicating pregnancy, childbirth AND/OR puerperium (/1.02)
Congenital anomaly of nervous system (/1.02)
Headache (x1.02)
Functional disorder of bladder (x1.02)
lanthanum carbonate (+0.00138)
Asthma (x1.01)
Imaging result abnormal (x1.01)
Non-infectious systemic inflammatory response syndrome without acute organ failure (/1.01)
Leukocytosis (/1.01)
Cyst of pancreas (x1.01)
Severe protein-calorie malnutrition (Gomez: less than 60% of standard weight) (/1.01)
Third trimester pregnancy (x1.01)
Abnormal body temperature (x1.01)
Low blood pressure (/1.01)
Bacterial infectious disease (/1.01)
0.523
0.739
0.762
0.774
0.784
0.790
0.796
0.799
0.803
0.806
0.809
0.811
0.814
0.816
0.818
0.820
0.821
0.823
0.824
0.826
0.827
0.828
0.830
0.831
0.833
0.834
0.835
0.836
0.838
0.839
0.840
0.841
0.842
0.843
0.844
0.845
0.846
0.847
0.848
0.849
0.850
0.851
0.852
0.853
0.854
0.855
0.855
0.856
0.857
0.858
0.859
0.859
0.860
0.861
0.862
0.862
0.863
0.864
0.864
0.865
0.866
0.867
0.867
0.868
0.869
0.869
0.870
0.871
0.871
0.872
0.872
0.873
0.874
0.874
0.875
0.875
0.876
0.876
0.877
0.878
0.878
0.879
0.879
0.880
0.880
0.881
0.881
0.882
0.882
0.883
0.883
0.883
0.884
0.884
0.885
0.885
0.886
0.886
0.887
0.887
−0.6
−0.4
−0.2
0.0
0.2
0.4
0.6
Expressions
10^5
10^3
10^1
−20
0
20

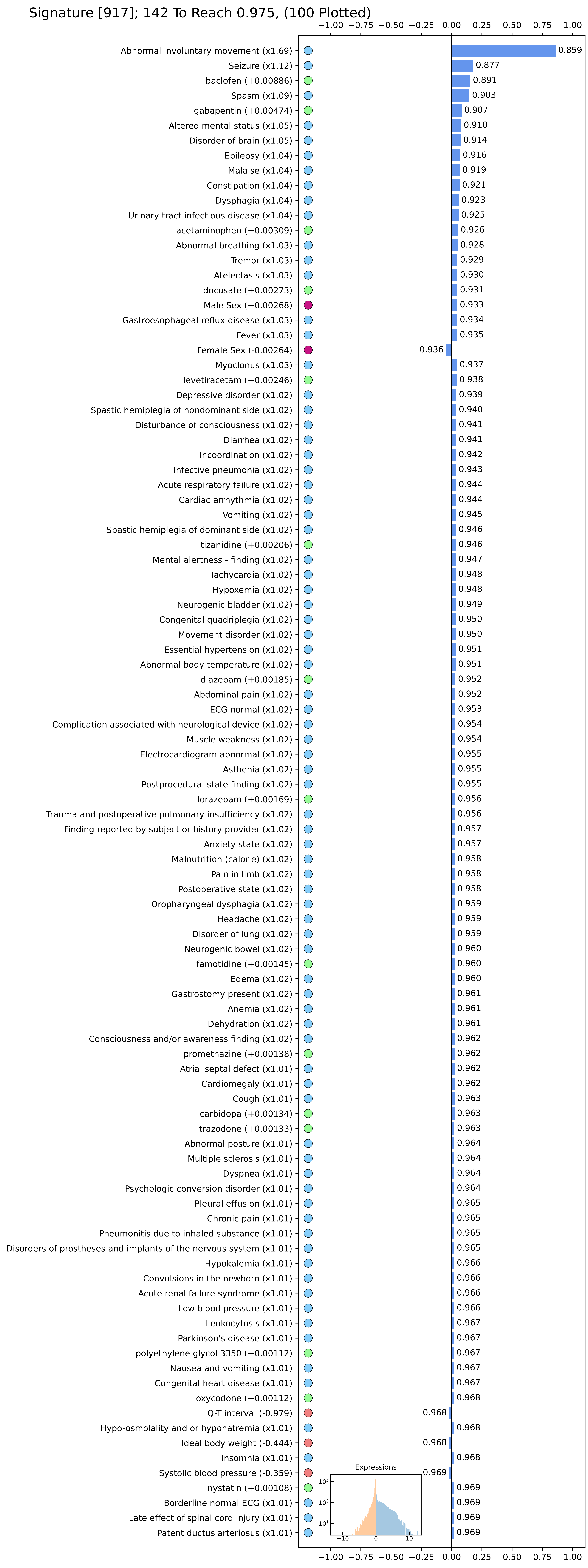

Signature [917]; 142 To Reach 0.975, (100 Plotted)
Abnormal involuntary movement (x1.69) 0.859
Seizure (x1.12) 0.877
baclofen (+0.00886) 0.891
Spasm (x1.09) 0.903
gabapentin (+0.00474) 0.907
Altered mental status (x1.05) 0.910
Disorder of brain (x1.05) 0.914
Epilepsy (x1.04) 0.916
Malaise (x1.04) 0.919
Constipation (x1.04) 0.921
Dysphagia (x1.04) 0.923
Urinary tract infectious disease (x1.04) 0.925
acetaminophen (+0.00309) 0.926
Abnormal breathing (x1.03) 0.928
Tremor (x1.03) 0.929
Atelectasis (x1.03) 0.930
docusate (+0.00273) 0.931
Male Sex (+0.00268) 0.933
Gastroesophageal reflux disease (x1.03) 0.934
Fever (x1.03) 0.935
Female Sex (-0.00264) 0.936
Myoclonus (x1.03) 0.937
levetiracetam (+0.00246) 0.938
Depressive disorder (x1.02) 0.939
Spastic hemiplegia of nondominant side (x1.02) 0.940
Disturbance of consciousness (x1.02) 0.941
Diarrhea (x1.02) 0.941
Incoordination (x1.02) 0.942
Infective pneumonia (x1.02) 0.943
Acute respiratory failure (x1.02) 0.944
Cardiac arrhythmia (x1.02) 0.944
Vomiting (x1.02) 0.945
Spastic hemiplegia of dominant side (x1.02) 0.946
tizanidine (+0.00206) 0.946
Mental alertness - finding (x1.02) 0.947
Tachycardia (x1.02) 0.948
Hypoxemia (x1.02) 0.948
Neurogenic bladder (x1.02) 0.949
Congenital quadriplegia (x1.02) 0.950
Movement disorder (x1.02) 0.950
Essential hypertension (x1.02) 0.951
Abnormal body temperature (x1.02) 0.951
diazepam (+0.00185) 0.952
Abdominal pain (x1.02) 0.952
ECG normal (x1.02) 0.953
Complication associated with neurological device (x1.02) 0.954
Muscle weakness (x1.02) 0.954
Electrocardiogram abnormal (x1.02) 0.955
Asthenia (x1.02) 0.955
Postprocedural state finding (x1.02) 0.955
lorazepam (+0.00169) 0.956
Trauma and postoperative pulmonary insufficiency (x1.02) 0.956
Finding reported by subject or history provider (x1.02) 0.957
Anxiety state (x1.02) 0.957
Malnutrition (calorie) (x1.02) 0.958
Pain in limb (x1.02) 0.958
Postoperative state (x1.02) 0.958
Oropharyngeal dysphagia (x1.02) 0.959
Headache (x1.02) 0.959
Disorder of lung (x1.02) 0.959
Neurogenic bowel (x1.02) 0.960
famotidine (+0.00145) 0.960
Edema (x1.02) 0.960
Gastrostomy present (x1.02) 0.961
Anemia (x1.02) 0.961
Dehydration (x1.02) 0.961
Consciousness and/or awareness finding (x1.02) 0.962
promethazine (+0.00138) 0.962
Atrial septal defect (x1.01) 0.962
Cardiomegaly (x1.01) 0.962
Cough (x1.01) 0.963
carbidopa (+0.00134) 0.963
trazodone (+0.00133) 0.963
Abnormal posture (x1.01) 0.964
Multiple sclerosis (x1.01) 0.964
Dyspnea (x1.01) 0.964
Psychologic conversion disorder (x1.01) 0.964
Pleural effusion (x1.01) 0.965
Chronic pain (x1.01) 0.965
Pneumonitis due to inhaled substance (x1.01) 0.965
Disorders of prostheses and implants of the nervous system (x1.01) 0.965
Hypokalemia (x1.01) 0.966
Convulsions in the newborn (x1.01) 0.966
Acute renal failure syndrome (x1.01) 0.966
Low blood pressure (x1.01) 0.966
Leukocytosis (x1.01) 0.967
Parkinson's disease (x1.01) 0.967
polyethylene glycol 3350 (+0.00112) 0.967
Nausea and vomiting (x1.01) 0.967
Congenital heart disease (x1.01) 0.967
oxycodone (+0.00112) 0.968
Q-T interval (-0.979) 0.968
Hypo-osmolality and or hyponatremia (x1.01) 0.968
Ideal body weight (-0.444) 0.968
Insomnia (x1.01) 0.968
Systolic blood pressure (-0.359) 0.969
nystatin (+0.00108) 0.969
Borderline normal ECG (x1.01) 0.969
Late effect of spinal cord injury (x1.01) 0.969
Patent ductus arteriosus (x1.01) 0.969
−1.00 −0.75 −0.50 −0.25 0.00 0.25 0.50 0.75 1.00
Expressions

Signature [918]; 4 To Reach 0.975, (10 Plotted)

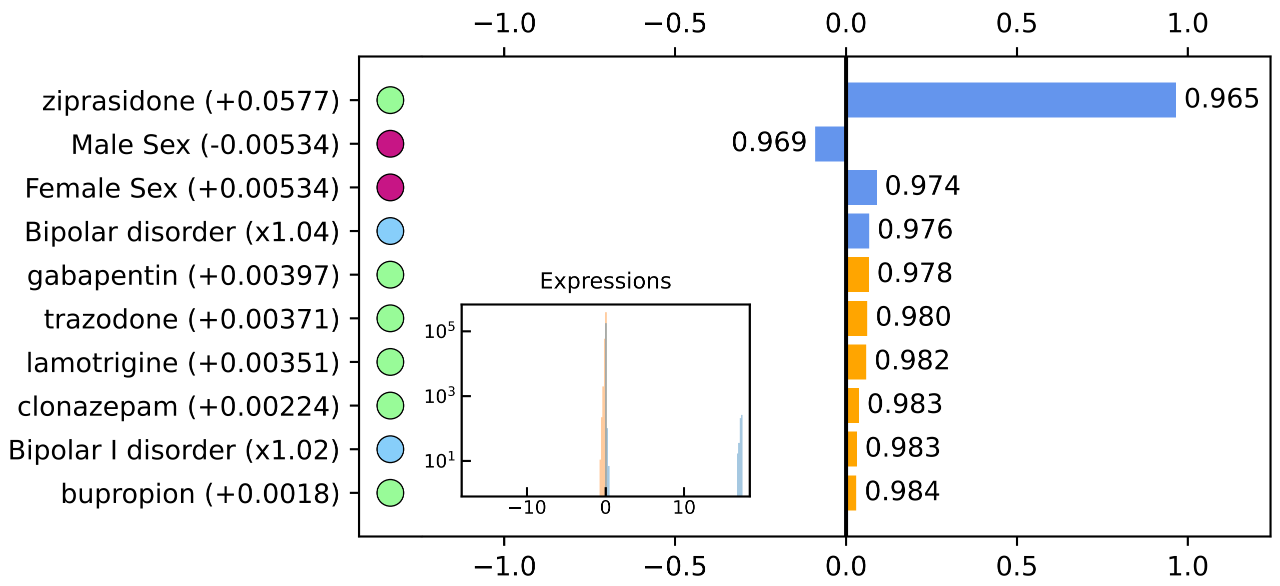

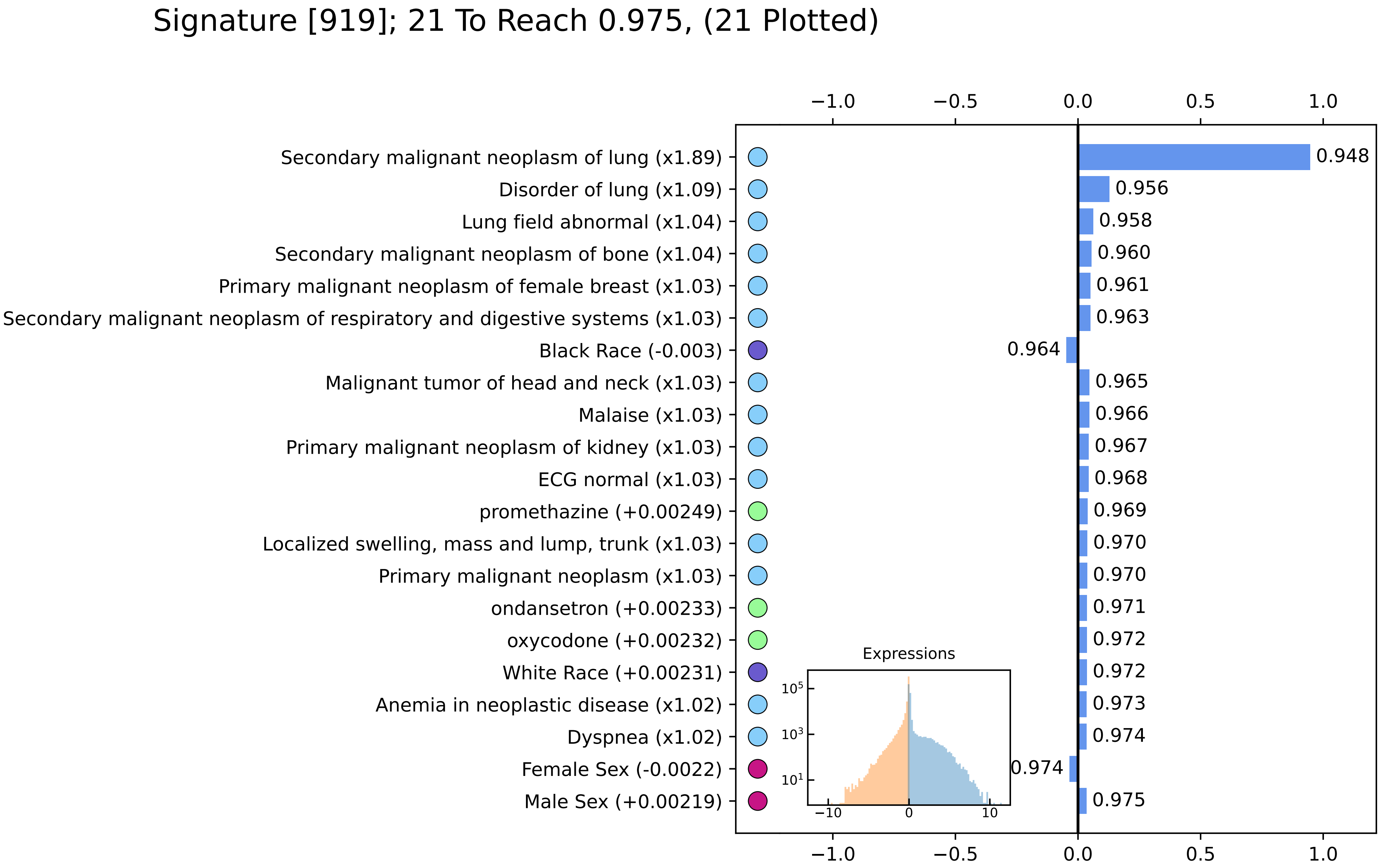

Signature [919]; 21 To Reach 0.975, (21 Plotted)
Secondary malignant neoplasm of lung (x1.89)
0.948
Disorder of lung (x1.09)
0.956
Lung field abnormal (x1.04)
0.958
Secondary malignant neoplasm of bone (x1.04)
0.960
Primary malignant neoplasm of female breast (x1.03)
0.961
Secondary malignant neoplasm of respiratory and digestive systems (x1.03)
0.963
Black Race (-0.003)
0.964
Malignant tumor of head and neck (x1.03)
0.965
Malaise (x1.03)
0.966
Primary malignant neoplasm of kidney (x1.03)
0.967
ECG normal (x1.03)
0.968
promethazine (+0.00249)
0.969
Localized swelling, mass and lump, trunk (x1.03)
0.970
Primary malignant neoplasm (x1.03)
0.970
ondansetron (+0.00233)
0.971
oxycodone (+0.00232)
0.972
White Race (+0.00231)
0.972
Anemia in neoplastic disease (x1.02)
0.973
Dyspnea (x1.02)
0.974
Female Sex (-0.0022)
0.974
Male Sex (+0.00219)
0.975
−1.0
−0.5
0.0
0.5
1.0
Expressions
10^5
10^3
10^1
−10
0
10

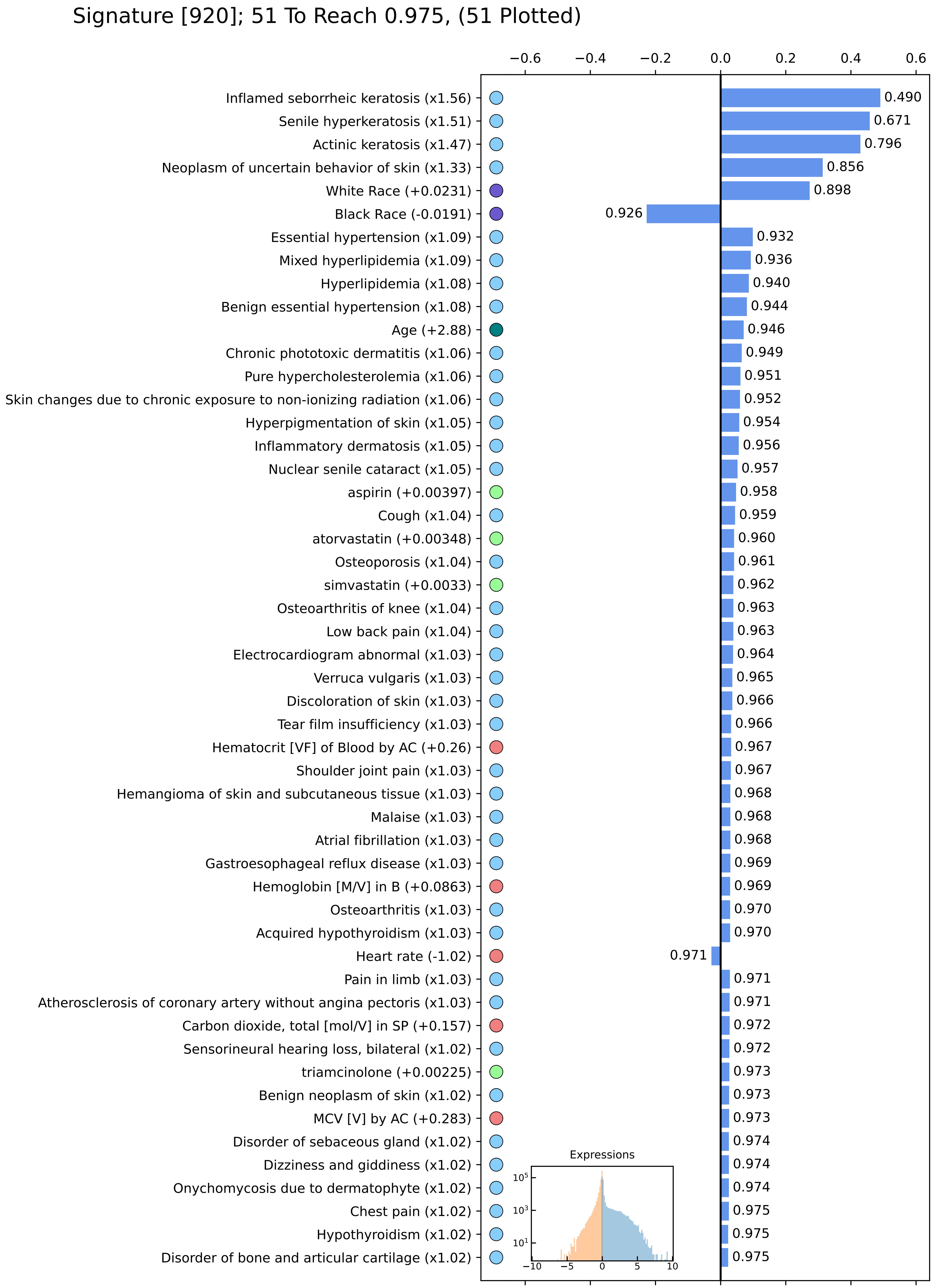
Signature [920]; 51 To Reach 0.975, (51 Plotted)
Inflamed seborrheic keratosis (x1.56) 0.490
Senile hyperkeratosis (x1.51) 0.671
Actinic keratosis (x1.47) 0.796
Neoplasm of uncertain behavior of skin (x1.33) 0.856
White Race (+0.0231) 0.898
Black Race (-0.0191) 0.926
Essential hypertension (x1.09) 0.932
Mixed hyperlipidemia (x1.09) 0.936
Hyperlipidemia (x1.08) 0.940
Benign essential hypertension (x1.08) 0.944
Age (+2.88) 0.946
Chronic phototoxic dermatitis (x1.06) 0.949
Pure hypercholesterolemia (x1.06) 0.951
Skin changes due to chronic exposure to non-ionizing radiation (x1.06) 0.952
Hyperpigmentation of skin (x1.05) 0.954
Inflammatory dermatosis (x1.05) 0.956
Nuclear senile cataract (x1.05) 0.957
aspirin (+0.00397) 0.958
Cough (x1.04) 0.959
atorvastatin (+0.00348) 0.960
Osteoporosis (x1.04) 0.961
simvastatin (+0.0033) 0.962
Osteoarthritis of knee (x1.04) 0.963
Low back pain (x1.04) 0.963
Electrocardiogram abnormal (x1.03) 0.964
Verruca vulgaris (x1.03) 0.965
Discoloration of skin (x1.03) 0.966
Tear film insufficiency (x1.03) 0.966
Hematocrit [VF] of Blood by AC (+0.26) 0.967
Shoulder joint pain (x1.03) 0.967
Hemangioma of skin and subcutaneous tissue (x1.03) 0.968
Malaise (x1.03) 0.968
Atrial fibrillation (x1.03) 0.968
Gastroesophageal reflux disease (x1.03) 0.969
Hemoglobin [M/V] in B (+0.0863) 0.969
Osteoarthritis (x1.03) 0.970
Acquired hypothyroidism (x1.03) 0.970
Heart rate (-1.02) 0.971
Pain in limb (x1.03) 0.971
Atherosclerosis of coronary artery without angina pectoris (x1.03) 0.971
Carbon dioxide, total [mol/V] in SP (+0.157) 0.972
Sensorineural hearing loss, bilateral (x1.02) 0.972
triamcinolone (+0.00225) 0.973
Benign neoplasm of skin (x1.02) 0.973
MCV [V] by AC (+0.283) 0.973
Disorder of sebaceous gland (x1.02) 0.974
Dizziness and giddiness (x1.02) 0.974
Onychomycosis due to dermatophyte (x1.02) 0.974
Chest pain (x1.02) 0.975
Hypothyroidism (x1.02) 0.975
Disorder of bone and articular cartilage (x1.02) 0.975
−0.6 −0.4 −0.2 0.0 0.2 0.4 0.6
Expressions
−10 −5 0 5 10

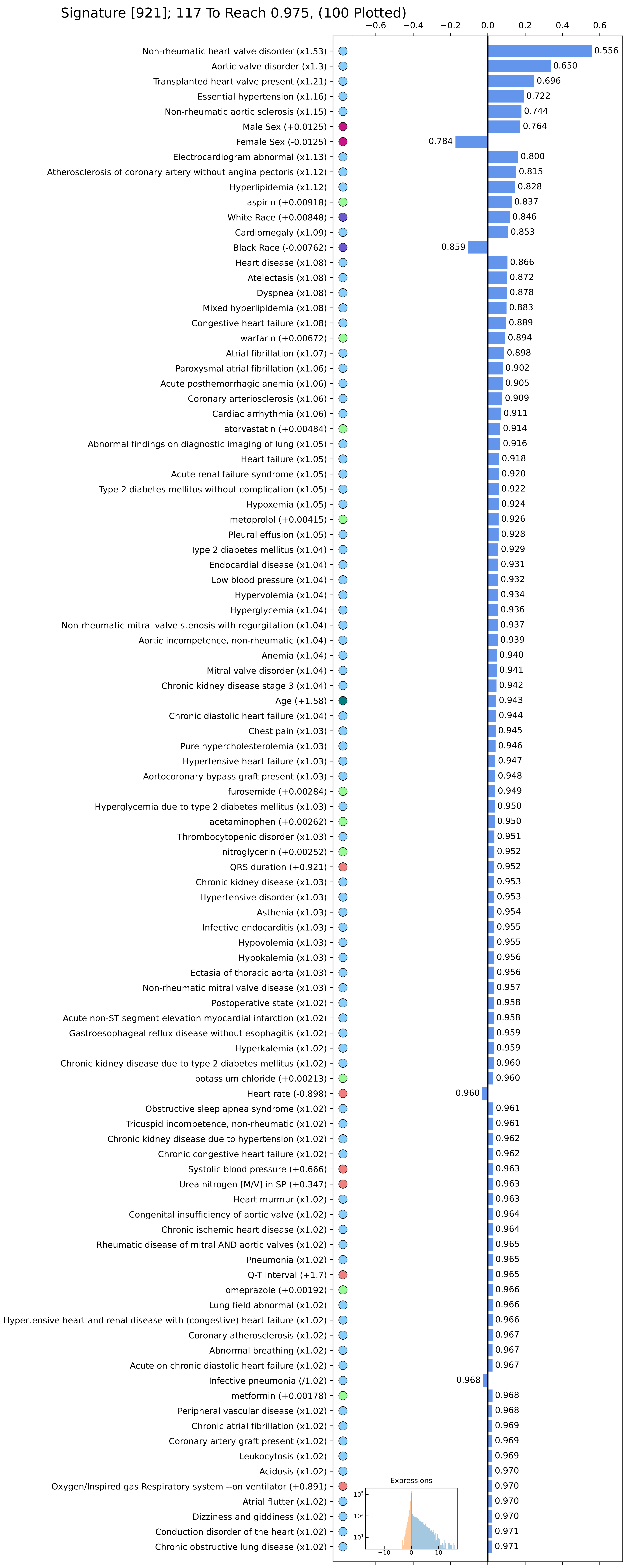

Signature [921]; 117 To Reach 0.975, (100 Plotted)
Non-rheumatic heart valve disorder (x1.53) 0.556
Aortic valve disorder (x1.3) 0.650
Transplanted heart valve present (x1.21) 0.696
Essential hypertension (x1.16) 0.722
Non-rheumatic aortic sclerosis (x1.15) 0.744
Male Sex (+0.0125) 0.764
Female Sex (-0.0125) 0.784
Electrocardiogram abnormal (x1.13) 0.800
Atherosclerosis of coronary artery without angina pectoris (x1.12) 0.815
Hyperlipidemia (x1.12) 0.828
aspirin (+0.00918) 0.837
White Race (+0.00848) 0.846
Cardiomegaly (x1.09) 0.853
Black Race (-0.00762) 0.859
Heart disease (x1.08) 0.866
Atelectasis (x1.08) 0.872
Dyspnea (x1.08) 0.878
Mixed hyperlipidemia (x1.08) 0.883
Congestive heart failure (x1.08) 0.889
warfarin (+0.00672) 0.894
Atrial fibrillation (x1.07) 0.898
Paroxysmal atrial fibrillation (x1.06) 0.902
Acute posthemorrhagic anemia (x1.06) 0.905
Coronary arteriosclerosis (x1.06) 0.909
Cardiac arrhythmia (x1.06) 0.911
atorvastatin (+0.00484) 0.914
Abnormal findings on diagnostic imaging of lung (x1.05) 0.916
Heart failure (x1.05) 0.918
Acute renal failure syndrome (x1.05) 0.920
Type 2 diabetes mellitus without complication (x1.05) 0.922
Hypoxemia (x1.05) 0.924
metoprolol (+0.00415) 0.926
Pleural effusion (x1.05) 0.928
Type 2 diabetes mellitus (x1.04) 0.929
Endocardial disease (x1.04) 0.931
Low blood pressure (x1.04) 0.932
Hypervolemia (x1.04) 0.934
Hyperglycemia (x1.04) 0.936
Non-rheumatic mitral valve stenosis with regurgitation (x1.04) 0.937
Aortic incompetence, non-rheumatic (x1.04) 0.939
Anemia (x1.04) 0.940
Mitral valve disorder (x1.04) 0.941
Chronic kidney disease stage 3 (x1.04) 0.942
Age (+1.58) 0.943
Chronic diastolic heart failure (x1.04) 0.944
Chest pain (x1.03) 0.945
Pure hypercholesterolemia (x1.03) 0.946
Hypertensive heart failure (x1.03) 0.947
Aortocoronary bypass graft present (x1.03) 0.948
furosemide (+0.00284) 0.949
Hyperglycemia due to type 2 diabetes mellitus (x1.03) 0.950
acetaminophen (+0.00262) 0.950
Thrombocytopenic disorder (x1.03) 0.951
nitroglycerin (+0.00252) 0.952
QRS duration (+0.921) 0.952
Chronic kidney disease (x1.03) 0.953
Hypertensive disorder (x1.03) 0.953
Asthenia (x1.03) 0.954
Infective endocarditis (x1.03) 0.955
Hypovolemia (x1.03) 0.955
Hypokalemia (x1.03) 0.956
Ectasia of thoracic aorta (x1.03) 0.956
Non-rheumatic mitral valve disease (x1.03) 0.957
Postoperative state (x1.02) 0.958
Acute non-ST segment elevation myocardial infarction (x1.02) 0.958
Gastroesophageal reflux disease without esophagitis (x1.02) 0.959
Hyperkalemia (x1.02) 0.959
Chronic kidney disease due to type 2 diabetes mellitus (x1.02) 0.960
potassium chloride (+0.00213) 0.960
Heart rate (-0.898) 0.960
Obstructive sleep apnea syndrome (x1.02) 0.961
Tricuspid incompetence, non-rheumatic (x1.02) 0.961
Chronic kidney disease due to hypertension (x1.02) 0.962
Chronic congestive heart failure (x1.02) 0.962
Systolic blood pressure (+0.666) 0.963
Urea nitrogen [M/V] in SP (+0.347) 0.963
Heart murmur (x1.02) 0.963
Congenital insufficiency of aortic valve (x1.02) 0.964
Chronic ischemic heart disease (x1.02) 0.964
Rheumatic disease of mitral AND aortic valves (x1.02) 0.965
Pneumonia (x1.02) 0.965
Q-T interval (+1.7) 0.965
omeprazole (+0.00192) 0.966
Lung field abnormal (x1.02) 0.966
Hypertensive heart and renal disease with (congestive) heart failure (x1.02) 0.966
Coronary atherosclerosis (x1.02) 0.967
Abnormal breathing (x1.02) 0.967
Acute on chronic diastolic heart failure (x1.02) 0.967
Infective pneumonia (/1.02) 0.968
metformin (+0.00178) 0.968
Peripheral vascular disease (x1.02) 0.968
Chronic atrial fibrillation (x1.02) 0.969
Coronary artery graft present (x1.02) 0.969
Leukocytosis (x1.02) 0.969
Acidosis (x1.02) 0.970
Oxygen/Inspired gas Respiratory system --on ventilator (+0.891) 0.970
Atrial flutter (x1.02) 0.970
Dizziness and giddiness (x1.02) 0.970
Conduction disorder of the heart (x1.02) 0.971
Chronic obstructive lung disease (x1.02) 0.971
−0.6 −0.4 −0.2 0.0 0.2 0.4 0.6
Expressions
10^5 10^3 10^1
−10 0 10

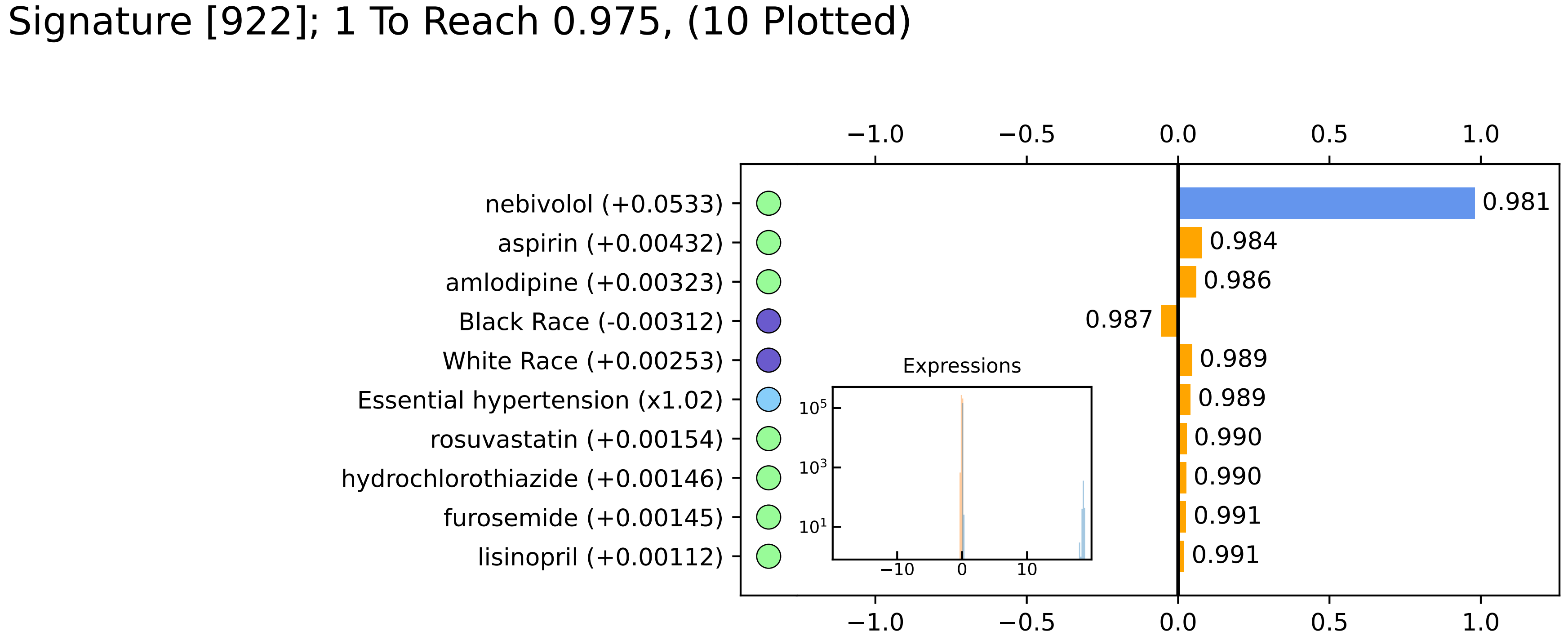
Signature [922]; 1 To Reach 0.975, (10 Plotted)
nebivolol (+0.0533)
aspirin (+0.00432)
amlodipine (+0.00323)
Black Race (-0.00312)
White Race (+0.00253)
Essential hypertension (x1.02)
rosuvastatin (+0.00154)
hydrochlorothiazide (+0.00146)
furosemide (+0.00145)
lisinopril (+0.00112)
0.981
0.984
0.986
0.987
0.989
0.989
0.990
0.990
0.991
0.991
Expressions
−1.0
−0.5
0.0
0.5
1.0

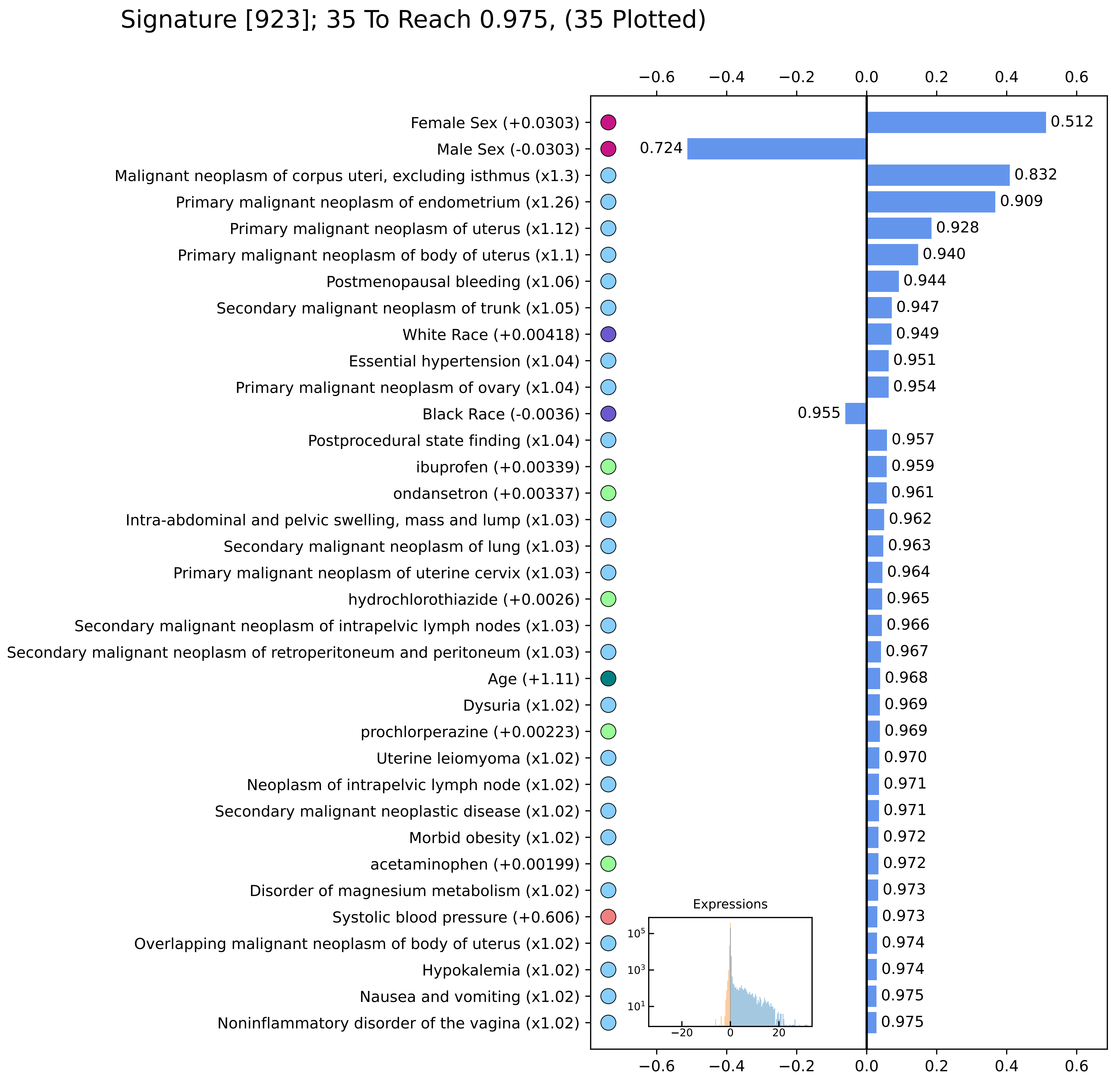

Signature [923]; 35 To Reach 0.975, (35 Plotted)
Female Sex (+0.0303)
Male Sex (-0.0303)
Malignant neoplasm of corpus uteri, excluding isthmus (x1.3)
Primary malignant neoplasm of endometrium (x1.26)
Primary malignant neoplasm of uterus (x1.12)
Primary malignant neoplasm of body of uterus (x1.1)
Postmenopausal bleeding (x1.06)
Secondary malignant neoplasm of trunk (x1.05)
White Race (+0.00418)
Essential hypertension (x1.04)
Primary malignant neoplasm of ovary (x1.04)
Black Race (-0.0036)
Postprocedural state finding (x1.04)
ibuprofen (+0.00339)
ondansetron (+0.00337)
Intra-abdominal and pelvic swelling, mass and lump (x1.03)
Secondary malignant neoplasm of lung (x1.03)
Primary malignant neoplasm of uterine cervix (x1.03)
hydrochlorothiazide (+0.0026)
Secondary malignant neoplasm of intrapelvic lymph nodes (x1.03)
Secondary malignant neoplasm of retroperitoneum and peritoneum (x1.03)
Age (+1.11)
Dysuria (x1.02)
prochlorperazine (+0.00223)
Uterine leiomyoma (x1.02)
Neoplasm of intrapelvic lymph node (x1.02)
Secondary malignant neoplastic disease (x1.02)
Morbid obesity (x1.02)
acetaminophen (+0.00199)
Disorder of magnesium metabolism (x1.02)
Systolic blood pressure (+0.606)
Overlapping malignant neoplasm of body of uterus (x1.02)
Hypokalemia (x1.02)
Nausea and vomiting (x1.02)
Noninflammatory disorder of the vagina (x1.02)
0.512
0.724
0.832
0.909
0.928
0.940
0.944
0.947
0.949
0.951
0.954
0.955
0.957
0.959
0.961
0.962
0.963
0.964
0.965
0.966
0.967
0.968
0.969
0.969
0.970
0.971
0.971
0.972
0.972
0.973
0.973
0.974
0.974
0.975
0.975
−0.6
−0.4
−0.2
0.0
0.2
0.4
0.6
Expressions

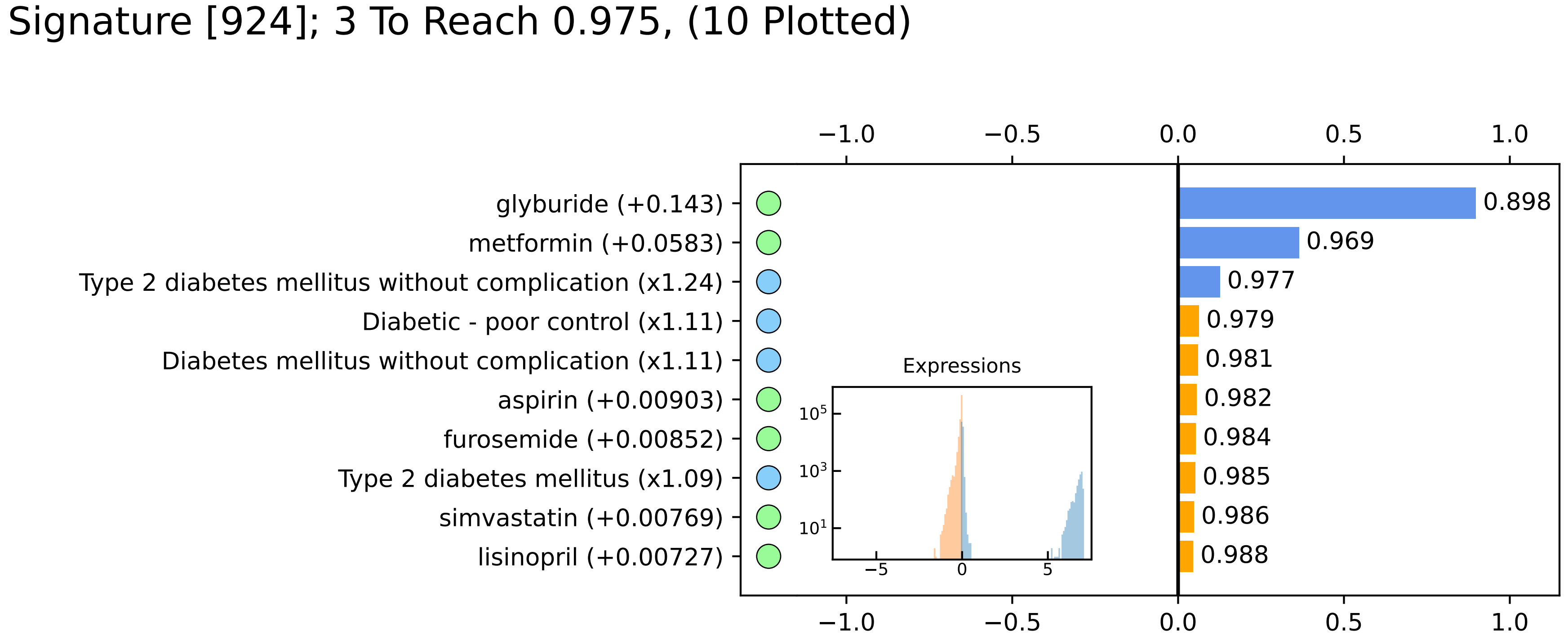

Signature [924]; 3 To Reach 0.975, (10 Plotted)
glyburide (+0.143)
metformin (+0.0583)
Type 2 diabetes mellitus without complication (x1.24)
Diabetic - poor control (x1.11)
Diabetes mellitus without complication (x1.11)
aspirin (+0.00903)
furosemide (+0.00852)
Type 2 diabetes mellitus (x1.09)
simvastatin (+0.00769)
lisinopril (+0.00727)
0.898
0.969
0.977
0.979
0.981
0.982
0.984
0.985
0.986
0.988
−1.0
−0.5
0.0
0.5
1.0
Expressions

Signature [925]; 5 To Reach 0.975, (10 Plotted)

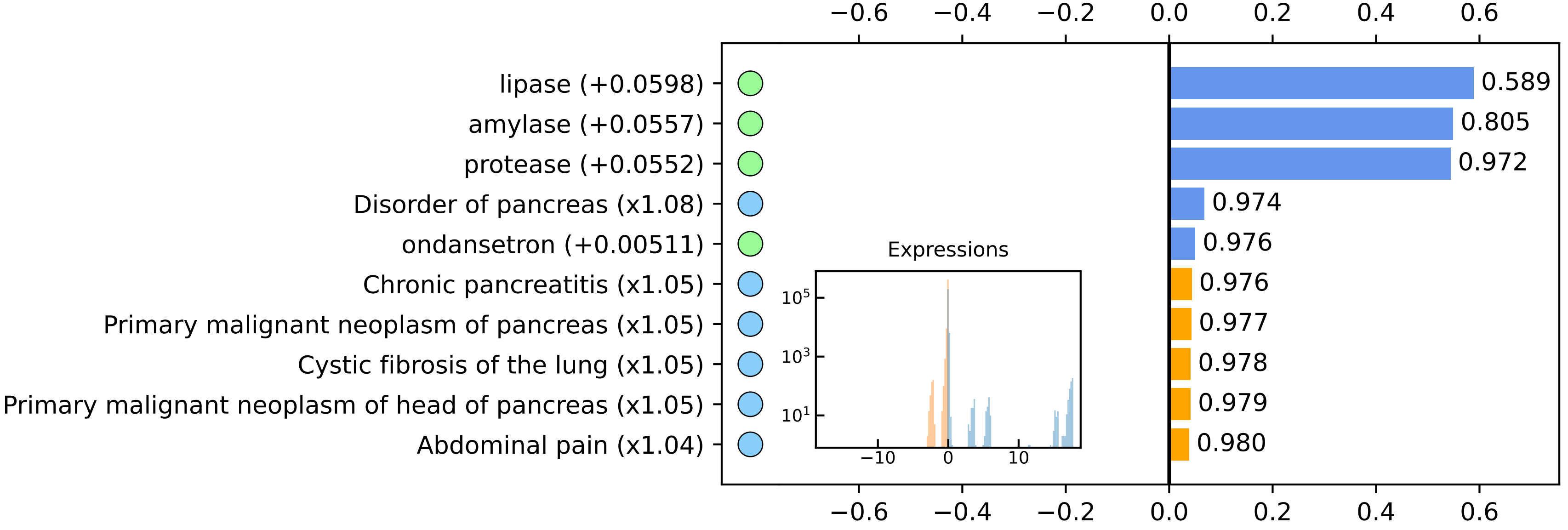

Signature [926]; 3 To Reach 0.975, (10 Plotted)

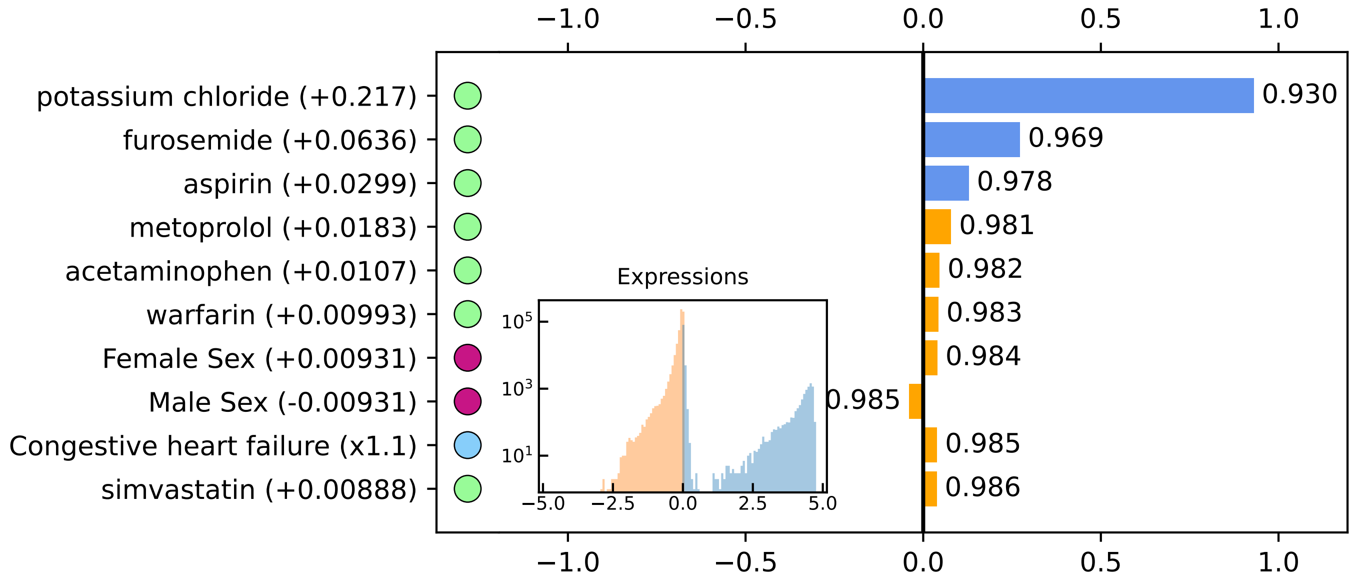

Signature [927]; 198 To Reach 0.975, (100 Plotted)

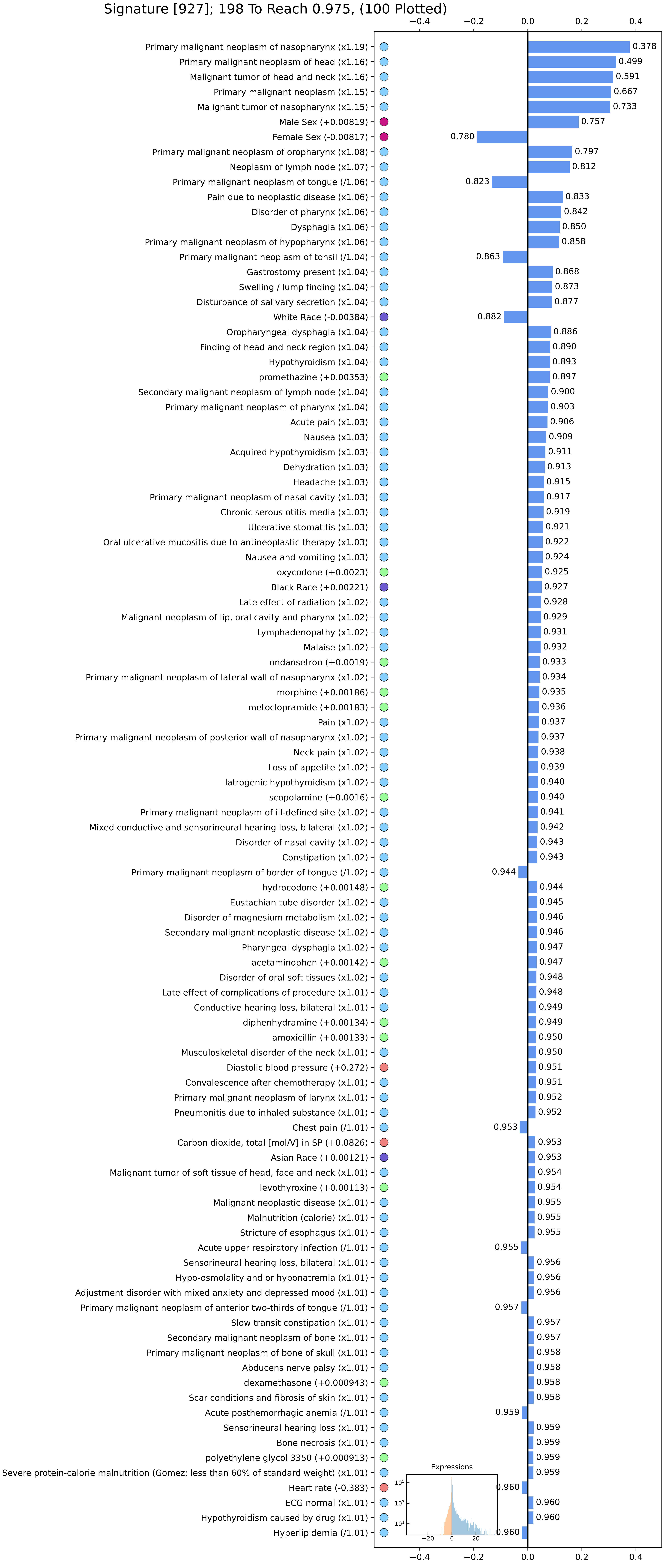

Signature [928]; 119 To Reach 0.975, (100 Plotted)

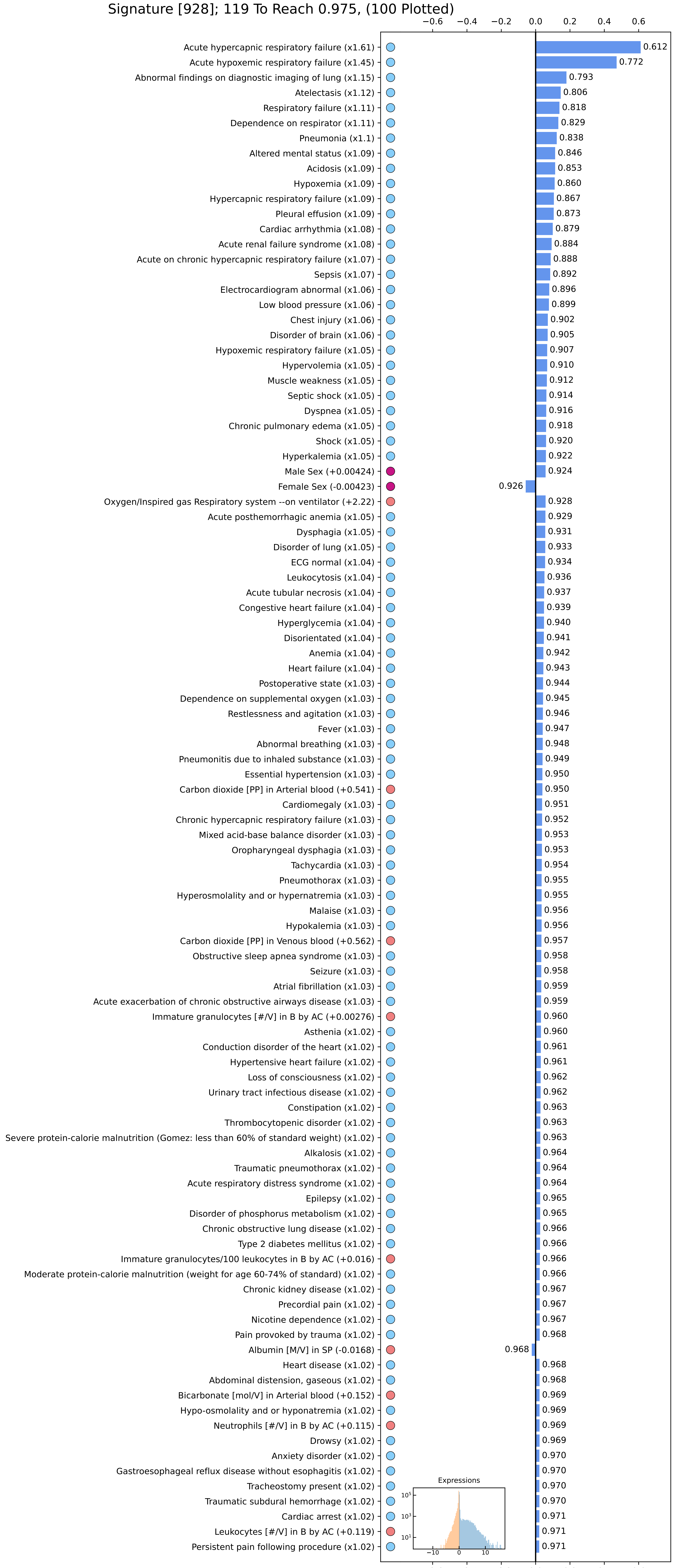

Signature [929]; 4 To Reach 0.975, (10 Plotted)

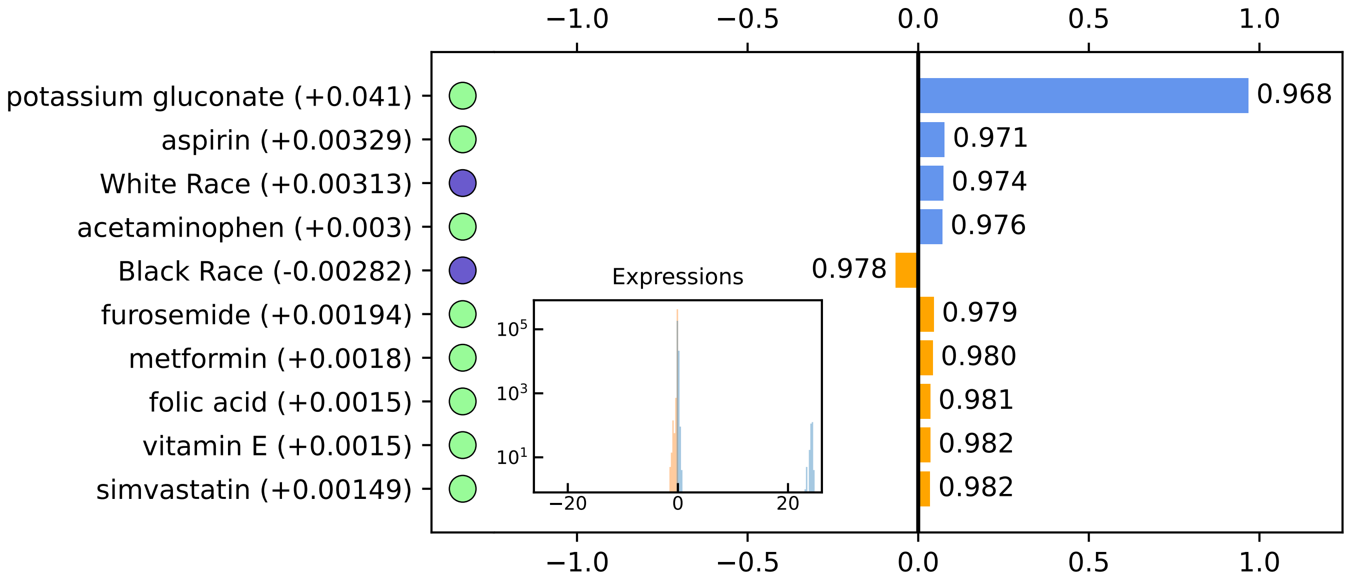

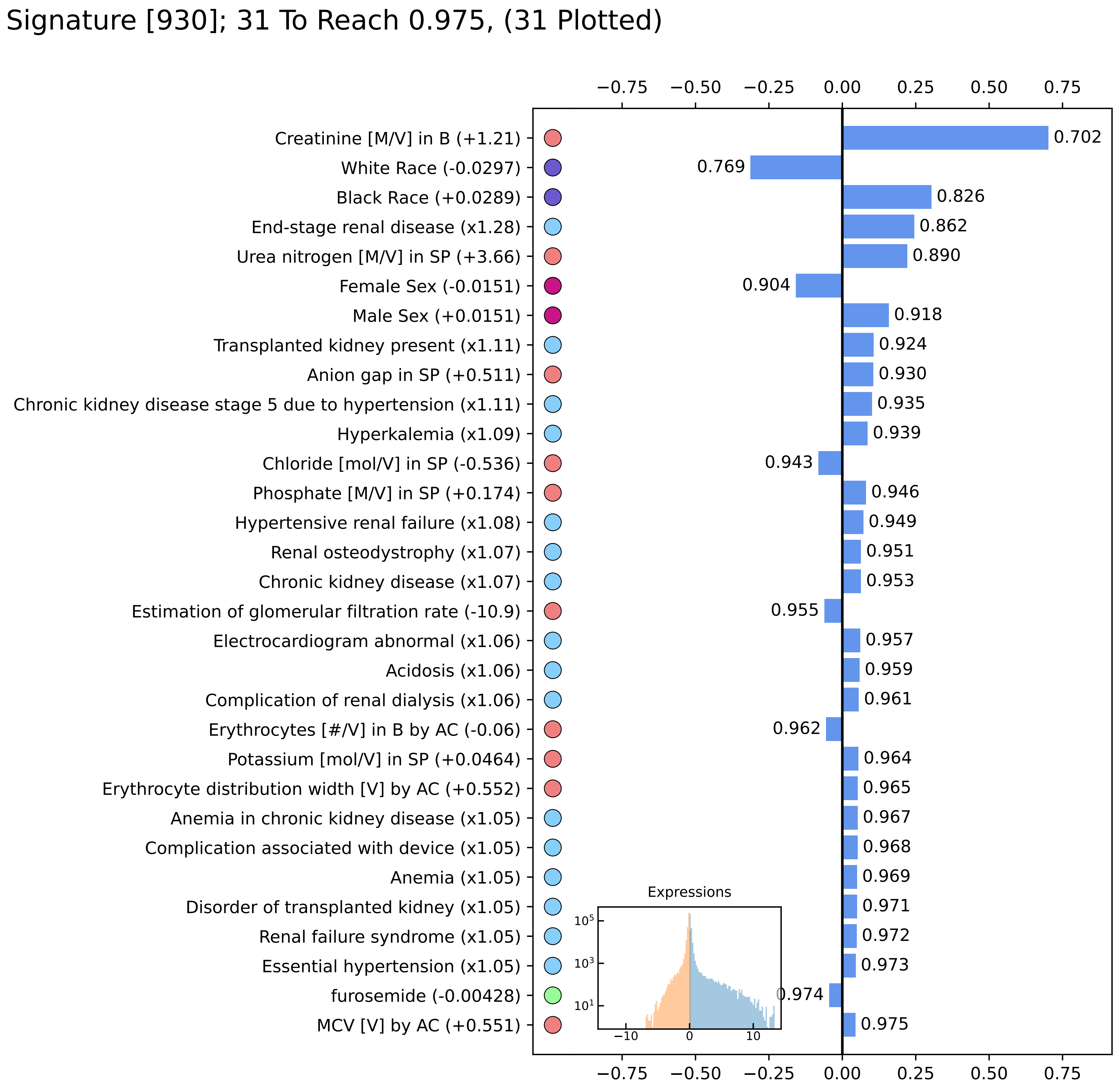

Signature [930]; 31 To Reach 0.975, (31 Plotted)
Creatinine [M/V] in B (+1.21)
White Race (-0.0297)
Black Race (+0.0289)
End-stage renal disease (x1.28)
Urea nitrogen [M/V] in SP (+3.66)
Female Sex (-0.0151)
Male Sex (+0.0151)
Transplanted kidney present (x1.11)
Anion gap in SP (+0.511)
Chronic kidney disease stage 5 due to hypertension (x1.11)
Hyperkalemia (x1.09)
Chloride [mol/V] in SP (-0.536)
Phosphate [M/V] in SP (+0.174)
Hypertensive renal failure (x1.08)
Renal osteodystrophy (x1.07)
Chronic kidney disease (x1.07)
Estimation of glomerular filtration rate (-10.9)
Electrocardiogram abnormal (x1.06)
Acidosis (x1.06)
Complication of renal dialysis (x1.06)
Erythrocytes [#/V] in B by AC (-0.06)
Potassium [mol/V] in SP (+0.0464)
Erythrocyte distribution width [V] by AC (+0.552)
Anemia in chronic kidney disease (x1.05)
Complication associated with device (x1.05)
Anemia (x1.05)
Disorder of transplanted kidney (x1.05)
Renal failure syndrome (x1.05)
Essential hypertension (x1.05)
furosemide (-0.00428)
MCV [V] by AC (+0.551)
0.702
0.769
0.826
0.862
0.890
0.904
0.918
0.924
0.930
0.935
0.939
0.943
0.946
0.949
0.951
0.953
0.955
0.957
0.959
0.961
0.962
0.964
0.965
0.967
0.968
0.969
0.971
0.972
0.973
0.974
0.975
−0.75
−0.50
−0.25
0.00
0.25
0.50
0.75
Expressions
−10
0
10

Signature [931]; 1 To Reach 0.975, (10 Plotted)

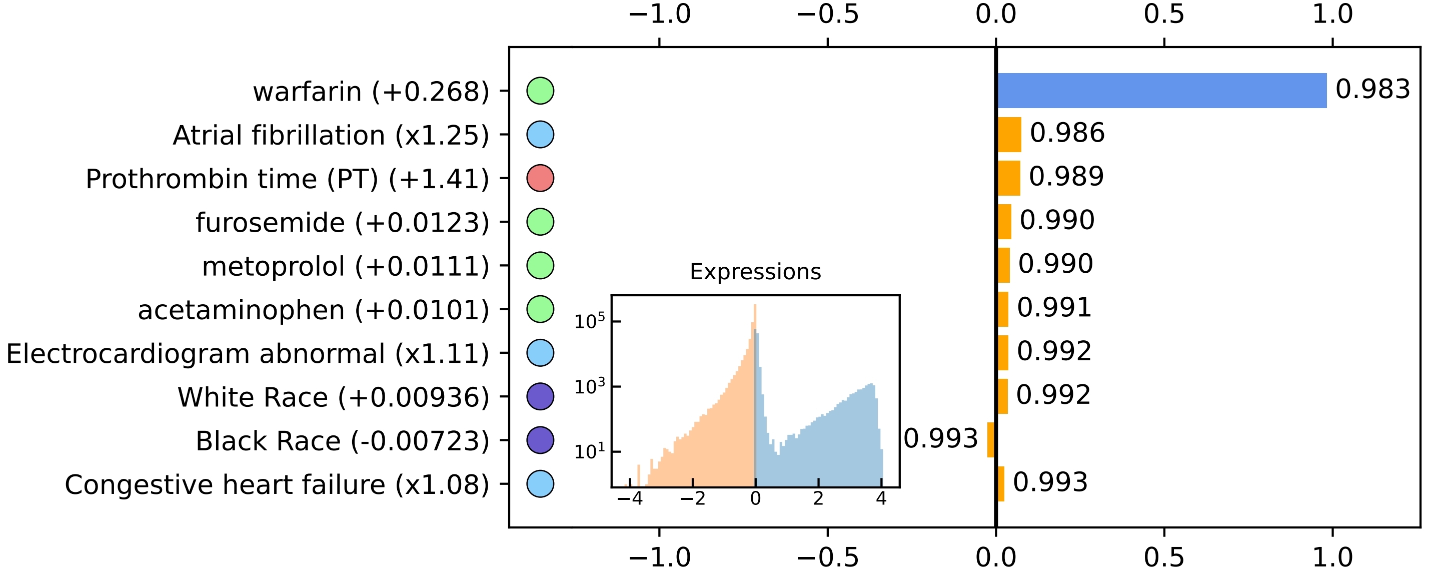

Signature [932]; 8 To Reach 0.975, (10 Plotted)

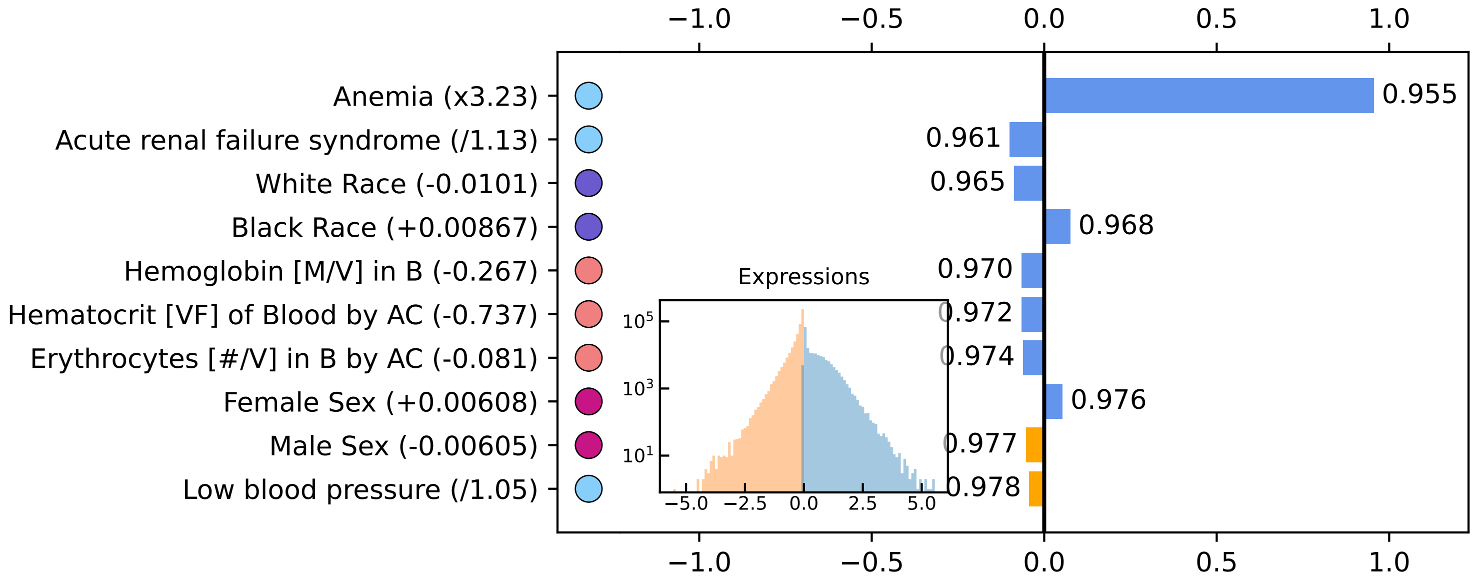

Signature [933]; 78 To Reach 0.975, (78 Plotted)

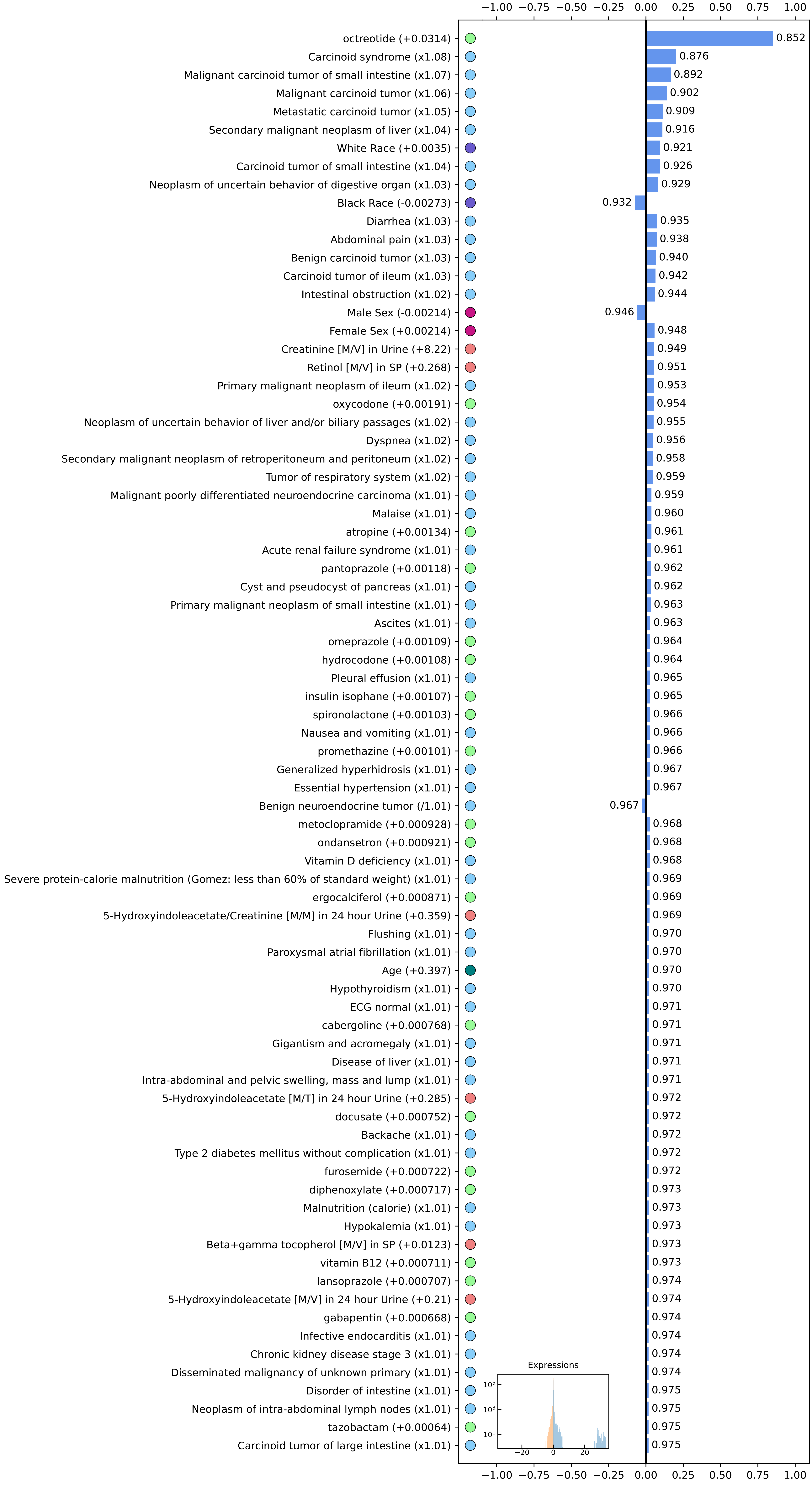

Signature [934]; 22 To Reach 0.975, (22 Plotted)

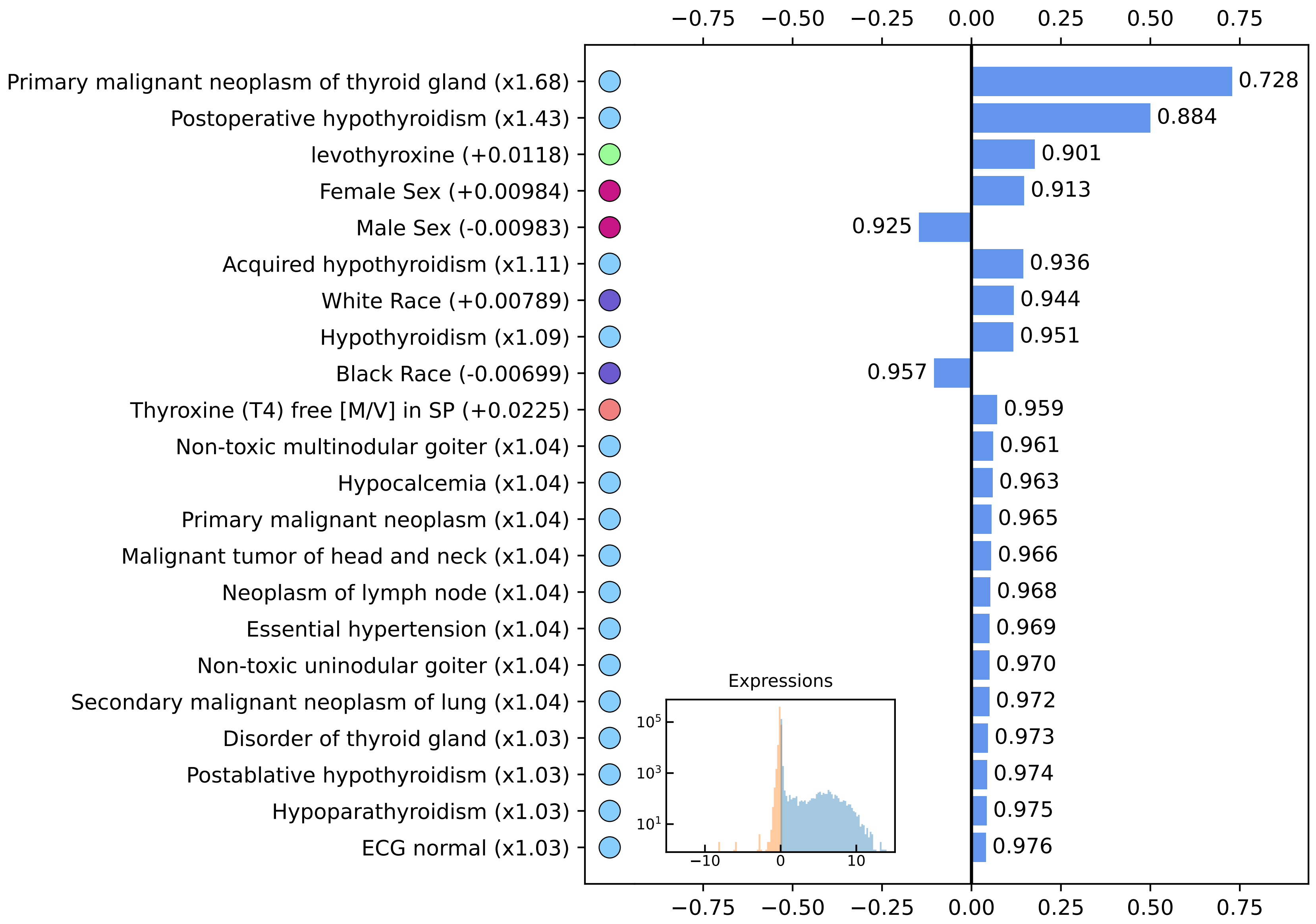

Signature [935]; 2 To Reach 0.975, (10 Plotted)

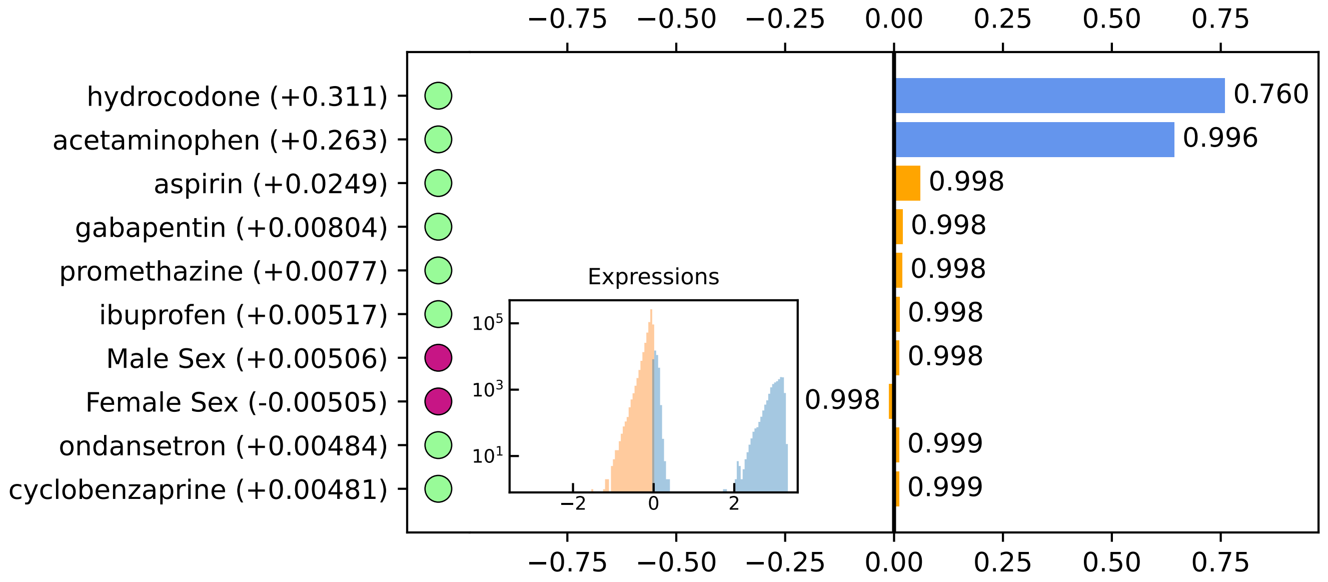

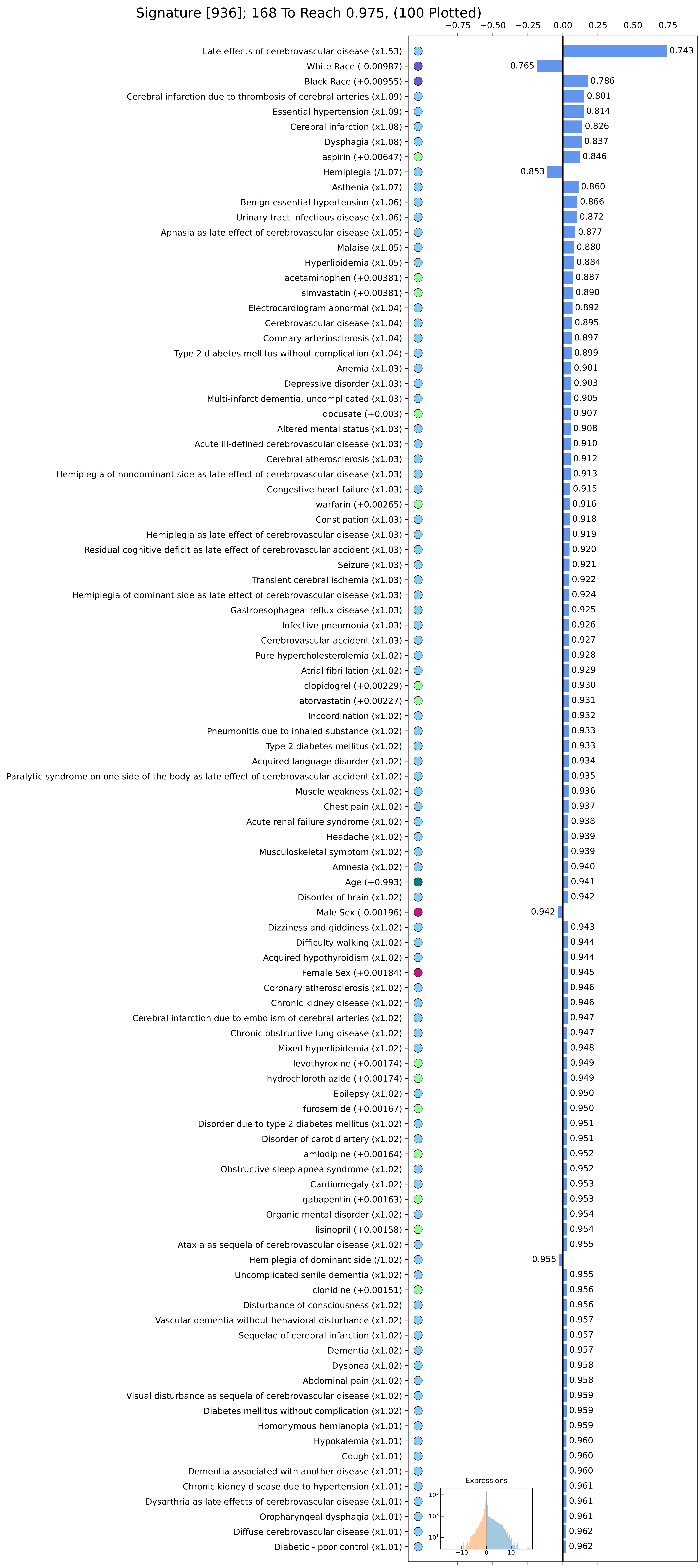

Signature [936]; 168 To Reach 0.975, (100 Plotted)
Late effects of cerebrovascular disease (x1.53) 0.743
White Race (-0.00987) 0.765
Black Race (+0.00955) 0.786
Cerebral infarction due to thrombosis of cerebral arteries (x1.09) 0.801
Essential hypertension (x1.09) 0.814
Cerebral infarction (x1.08) 0.826
Dysphagia (x1.08) 0.837
aspirin (+0.00647) 0.846
Hemiplegia (/1.07) 0.853
Asthenia (x1.07) 0.860
Benign essential hypertension (x1.06) 0.866
Urinary tract infectious disease (x1.06) 0.872
Aphasia as late effect of cerebrovascular disease (x1.05) 0.877
Malaise (x1.05) 0.880
Hyperlipidemia (x1.05) 0.884
acetaminophen (+0.00381) 0.887
simvastatin (+0.00381) 0.890
Electrocardiogram abnormal (x1.04) 0.892
Cerebrovascular disease (x1.04) 0.895
Coronary arteriosclerosis (x1.04) 0.897
Type 2 diabetes mellitus without complication (x1.04) 0.899
Anemia (x1.03) 0.901
Depressive disorder (x1.03) 0.903
Multi-infarct dementia, uncomplicated (x1.03) 0.905
docusate (+0.003) 0.907
Altered mental status (x1.03) 0.908
Acute ill-defined cerebrovascular disease (x1.03) 0.910
Cerebral atherosclerosis (x1.03) 0.912
Hemiplegia of nondominant side as late effect of cerebrovascular disease (x1.03) 0.913
Congestive heart failure (x1.03) 0.915
warfarin (+0.00265) 0.916
Constipation (x1.03) 0.918
Hemiplegia as late effect of cerebrovascular disease (x1.03) 0.919
Residual cognitive deficit as late effect of cerebrovascular accident (x1.03) 0.920
Seizure (x1.03) 0.921
Transient cerebral ischemia (x1.03) 0.922
Hemiplegia of dominant side as late effect of cerebrovascular disease (x1.03) 0.924
Gastroesophageal reflux disease (x1.03) 0.925
Infective pneumonia (x1.03) 0.926
Cerebrovascular accident (x1.03) 0.927
Pure hypercholesterolemia (x1.02) 0.928
Atrial fibrillation (x1.02) 0.929
clopidogrel (+0.00229) 0.930
atorvastatin (+0.00227) 0.931
Incoordination (x1.02) 0.932
Pneumonitis due to inhaled substance (x1.02) 0.933
Type 2 diabetes mellitus (x1.02) 0.933
Acquired language disorder (x1.02) 0.934
Paralytic syndrome on one side of the body as late effect of cerebrovascular accident (x1.02) 0.935
Muscle weakness (x1.02) 0.936
Chest pain (x1.02) 0.937
Acute renal failure syndrome (x1.02) 0.938
Headache (x1.02) 0.939
Musculoskeletal symptom (x1.02) 0.939
Amnesia (x1.02) 0.940
Age (+0.993) 0.941
Disorder of brain (x1.02) 0.942
Male Sex (-0.00196) 0.942
Dizziness and giddiness (x1.02) 0.943
Difficulty walking (x1.02) 0.944
Acquired hypothyroidism (x1.02) 0.944
Female Sex (+0.00184) 0.945
Coronary atherosclerosis (x1.02) 0.946
Chronic kidney disease (x1.02) 0.946
Cerebral infarction due to embolism of cerebral arteries (x1.02) 0.947
Chronic obstructive lung disease (x1.02) 0.947
Mixed hyperlipidemia (x1.02) 0.948
levothyroxine (+0.00174) 0.949
hydrochlorothiazide (+0.00174) 0.949
Epilepsy (x1.02) 0.950
furosemide (+0.00167) 0.950
Disorder due to type 2 diabetes mellitus (x1.02) 0.951
Disorder of carotid artery (x1.02) 0.951
amlodipine (+0.00164) 0.952
Obstructive sleep apnea syndrome (x1.02) 0.952
Cardiomegaly (x1.02) 0.953
gabapentin (+0.00163) 0.953
Organic mental disorder (x1.02) 0.954
lisinopril (+0.00158) 0.954
Ataxia as sequela of cerebrovascular disease (x1.02) 0.955
Hemiplegia of dominant side (/1.02) 0.955
Uncomplicated senile dementia (x1.02) 0.955
clonidine (+0.00151) 0.956
Disturbance of consciousness (x1.02) 0.956
Vascular dementia without behavioral disturbance (x1.02) 0.957
Sequelae of cerebral infarction (x1.02) 0.957
Dementia (x1.02) 0.957
Dyspnea (x1.02) 0.958
Abdominal pain (x1.02) 0.958
Visual disturbance as sequela of cerebrovascular disease (x1.02) 0.959
Diabetes mellitus without complication (x1.02) 0.959
Homonymous hemianopia (x1.01) 0.959
Hypokalemia (x1.01) 0.960
Cough (x1.01) 0.960
Dementia associated with another disease (x1.01) 0.960
Chronic kidney disease due to hypertension (x1.01) 0.961
Dysarthria as late effects of cerebrovascular disease (x1.01) 0.961
Oropharyngeal dysphagia (x1.01) 0.961
Diffuse cerebrovascular disease (x1.01) 0.962
Diabetic - poor control (x1.01) 0.962
−0.75 −0.50 −0.25 0.00 0.25 0.50 0.75
Expressions

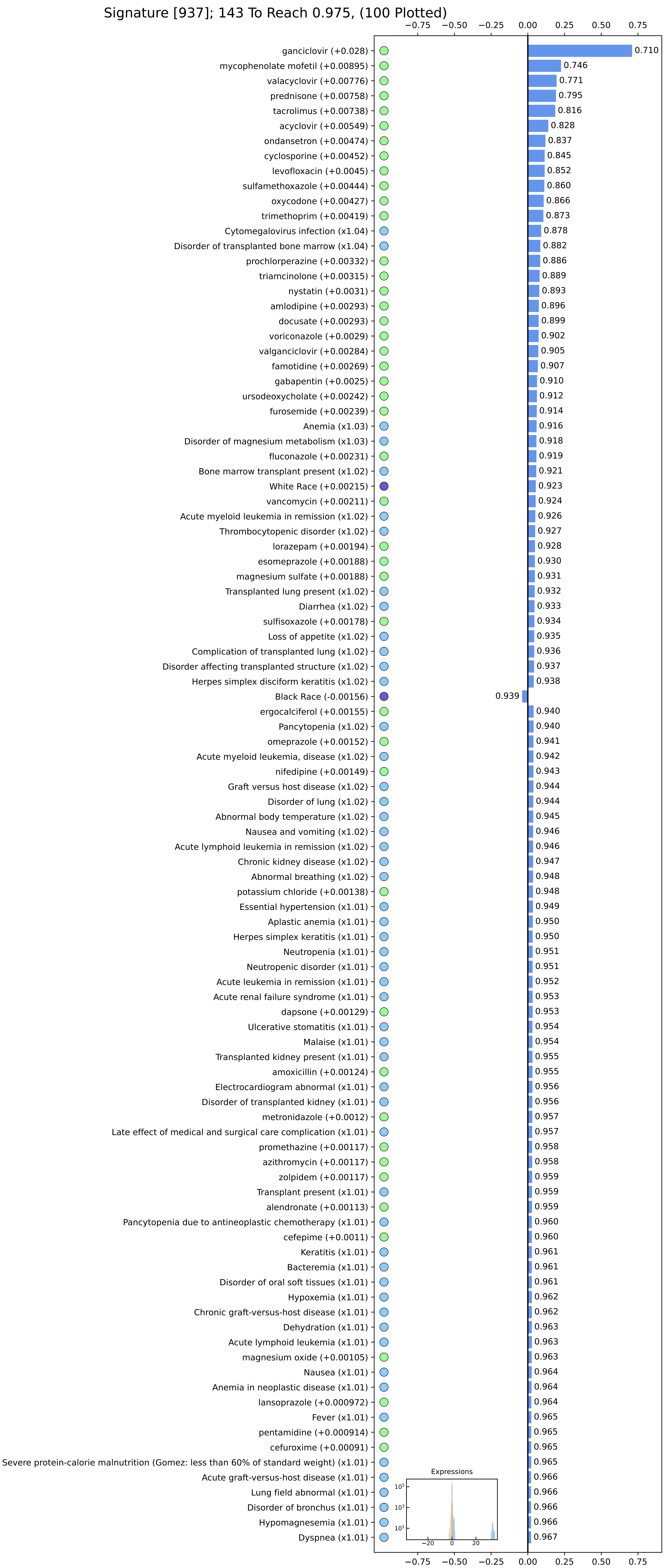

Signature [937]; 143 To Reach 0.975, (100 Plotted)
ganciclovir (+0.028) 0.710
mycophenolate mofetil (+0.00895) 0.746
valacyclovir (+0.00776) 0.771
prednisone (+0.00758) 0.795
tacrolimus (+0.00738) 0.816
acyclovir (+0.00549) 0.828
ondansetron (+0.00474) 0.837
cyclosporine (+0.00452) 0.845
levofloxacin (+0.0045) 0.852
sulfamethoxazole (+0.00444) 0.860
oxycodone (+0.00427) 0.866
trimethoprim (+0.00419) 0.873
Cytomegalovirus infection (x1.04) 0.878
Disorder of transplanted bone marrow (x1.04) 0.882
prochlorperazine (+0.00332) 0.886
triamcinolone (+0.00315) 0.889
nystatin (+0.0031) 0.893
amlodipine (+0.00293) 0.896
docusate (+0.00293) 0.899
voriconazole (+0.0029) 0.902
valganciclovir (+0.00284) 0.905
famotidine (+0.00269) 0.907
gabapentin (+0.0025) 0.910
ursodeoxycholate (+0.00242) 0.912
furosemide (+0.00239) 0.914
Anemia (x1.03) 0.916
Disorder of magnesium metabolism (x1.03) 0.918
fluconazole (+0.00231) 0.919
Bone marrow transplant present (x1.02) 0.921
White Race (+0.00215) 0.923
vancomycin (+0.00211) 0.924
Acute myeloid leukemia in remission (x1.02) 0.926
Thrombocytopenic disorder (x1.02) 0.927
lorazepam (+0.00194) 0.928
esomeprazole (+0.00188) 0.930
magnesium sulfate (+0.00188) 0.931
Transplanted lung present (x1.02) 0.932
Diarrhea (x1.02) 0.933
sulfisoxazole (+0.00178) 0.934
Loss of appetite (x1.02) 0.935
Complication of transplanted lung (x1.02) 0.936
Disorder affecting transplanted structure (x1.02) 0.937
Herpes simplex disciform keratitis (x1.02) 0.938
Black Race (-0.00156) 0.939
ergocalciferol (+0.00155) 0.940
Pancytopenia (x1.02) 0.940
omeprazole (+0.00152) 0.941
Acute myeloid leukemia, disease (x1.02) 0.942
nifedipine (+0.00149) 0.943
Graft versus host disease (x1.02) 0.944
Disorder of lung (x1.02) 0.944
Abnormal body temperature (x1.02) 0.945
Nausea and vomiting (x1.02) 0.946
Acute lymphoid leukemia in remission (x1.02) 0.946
Chronic kidney disease (x1.02) 0.947
Abnormal breathing (x1.02) 0.948
potassium chloride (+0.00138) 0.948
Essential hypertension (x1.01) 0.949
Aplastic anemia (x1.01) 0.950
Herpes simplex keratitis (x1.01) 0.950
Neutropenia (x1.01) 0.951
Neutropenic disorder (x1.01) 0.951
Acute leukemia in remission (x1.01) 0.952
Acute renal failure syndrome (x1.01) 0.953
dapsone (+0.00129) 0.953
Ulcerative stomatitis (x1.01) 0.954
Malaise (x1.01) 0.954
Transplanted kidney present (x1.01) 0.955
amoxicillin (+0.00124) 0.955
Electrocardiogram abnormal (x1.01) 0.956
Disorder of transplanted kidney (x1.01) 0.956
metronidazole (+0.0012) 0.957
Late effect of medical and surgical care complication (x1.01) 0.957
promethazine (+0.00117) 0.958
azithromycin (+0.00117) 0.958
zolpidem (+0.00117) 0.959
Transplant present (x1.01) 0.959
alendronate (+0.00113) 0.959
Pancytopenia due to antineoplastic chemotherapy (x1.01) 0.960
cefepime (+0.0011) 0.960
Keratitis (x1.01) 0.961
Bacteremia (x1.01) 0.961
Disorder of oral soft tissues (x1.01) 0.961
Hypoxemia (x1.01) 0.962
Chronic graft-versus-host disease (x1.01) 0.962
Dehydration (x1.01) 0.963
Acute lymphoid leukemia (x1.01) 0.963
magnesium oxide (+0.00105) 0.963
Nausea (x1.01) 0.964
Anemia in neoplastic disease (x1.01) 0.964
lansoprazole (+0.000972) 0.964
Fever (x1.01) 0.965
pentamidine (+0.000914) 0.965
cefuroxime (+0.00091) 0.965
Severe protein-calorie malnutrition (Gomez: less than 60% of standard weight) (x1.01) 0.965
Acute graft-versus-host disease (x1.01) 0.966
Lung field abnormal (x1.01) 0.966
Disorder of bronchus (x1.01) 0.966
Hypomagnesemia (x1.01) 0.966
Dyspnea (x1.01) 0.967
Expressions
−0.75 −0.50 −0.25 0.00 0.25 0.50 0.75

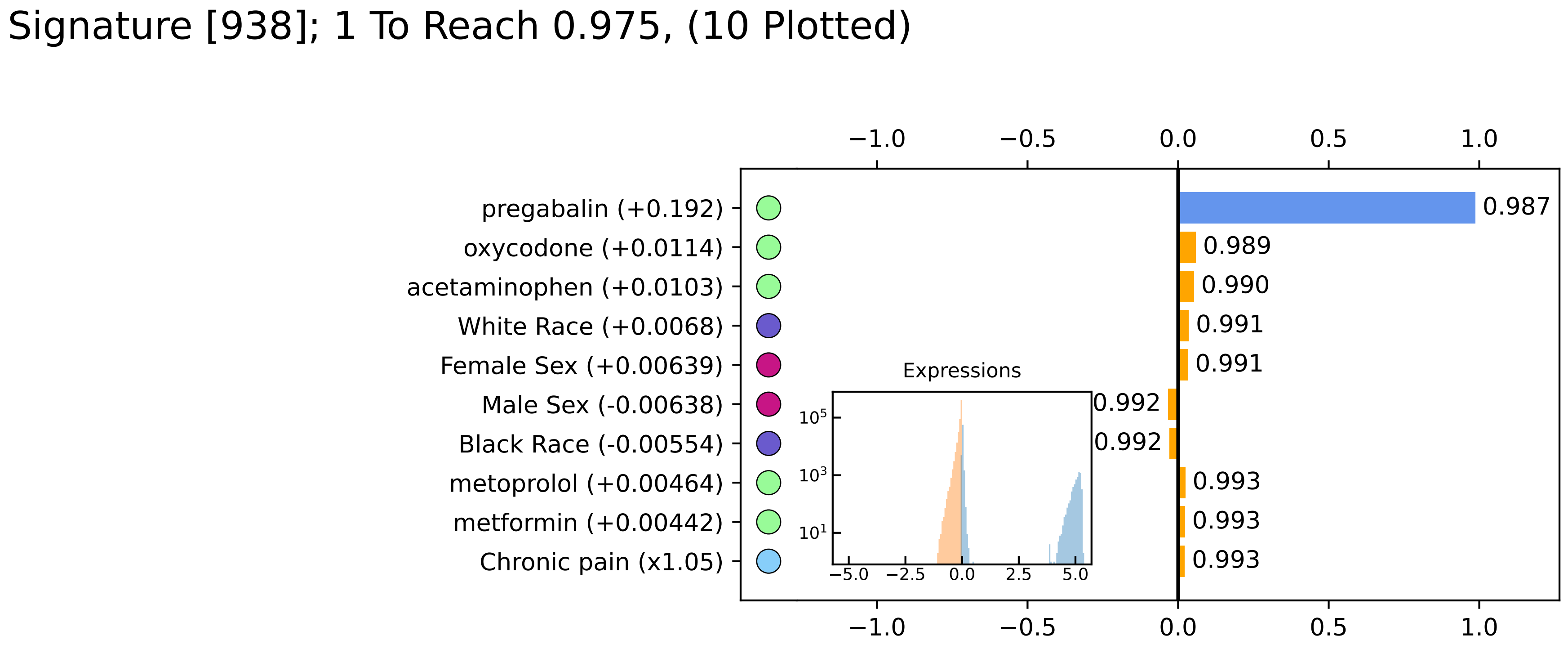

Signature [938]; 1 To Reach 0.975, (10 Plotted)
pregabalin (+0.192)
oxycodone (+0.0114)
acetaminophen (+0.0103)
White Race (+0.0068)
Female Sex (+0.00639)
Male Sex (-0.00638)
Black Race (-0.00554)
metoprolol (+0.00464)
metformin (+0.00442)
Chronic pain (x1.05)
0.987
0.989
0.990
0.991
0.991
0.992
0.992
0.993
0.993
0.993
Expressions
−1.0
−0.5
0.0
0.5
1.0

Signature [939]; 4 To Reach 0.975, (10 Plotted)

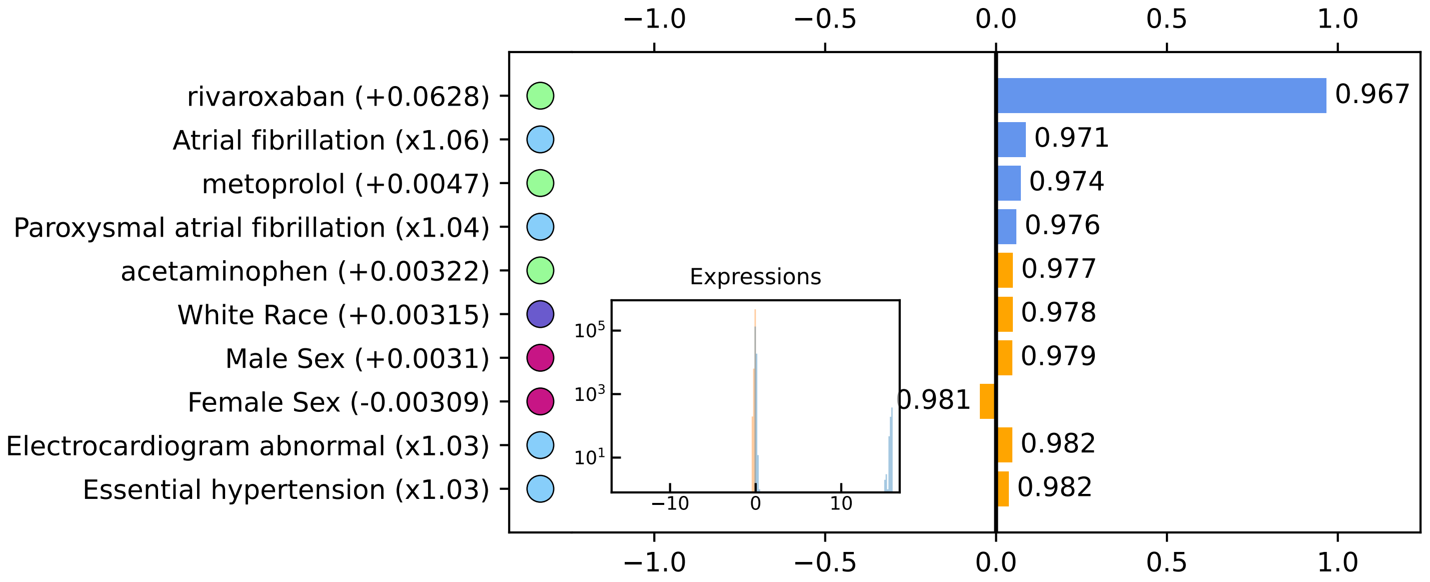

Signature [940]; 7 To Reach 0.975, (10 Plotted)

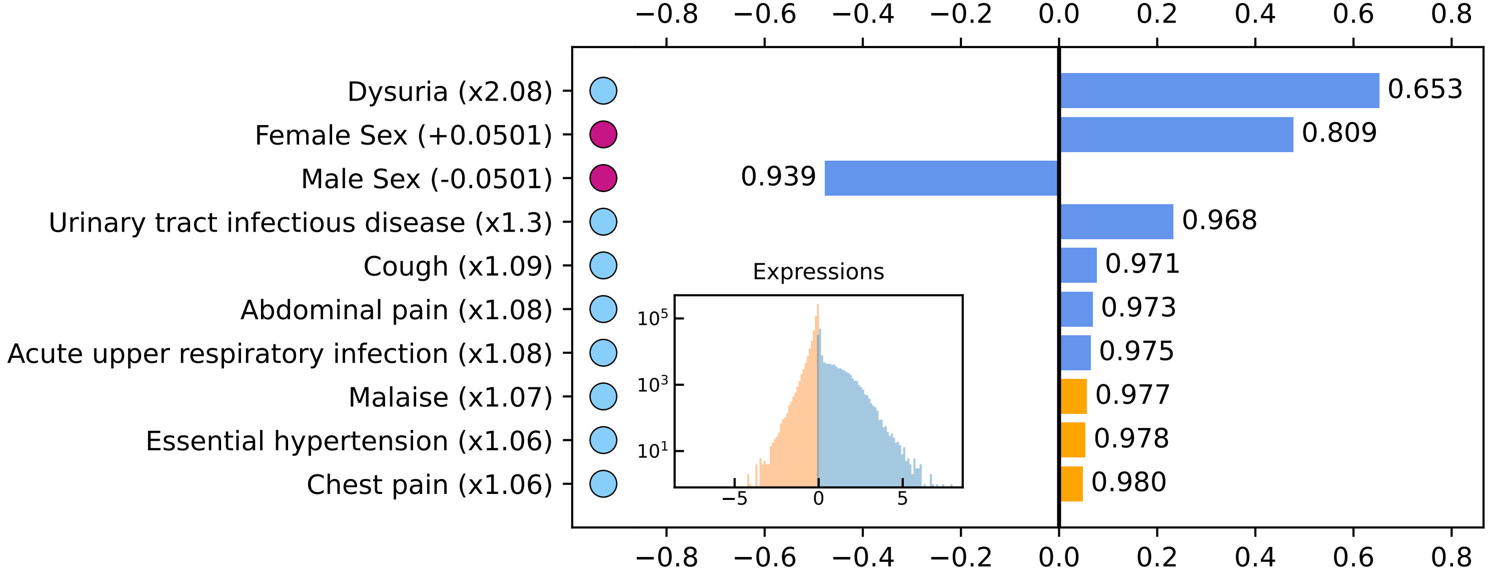

Signature [941]; 42 To Reach 0.975, (42 Plotted)

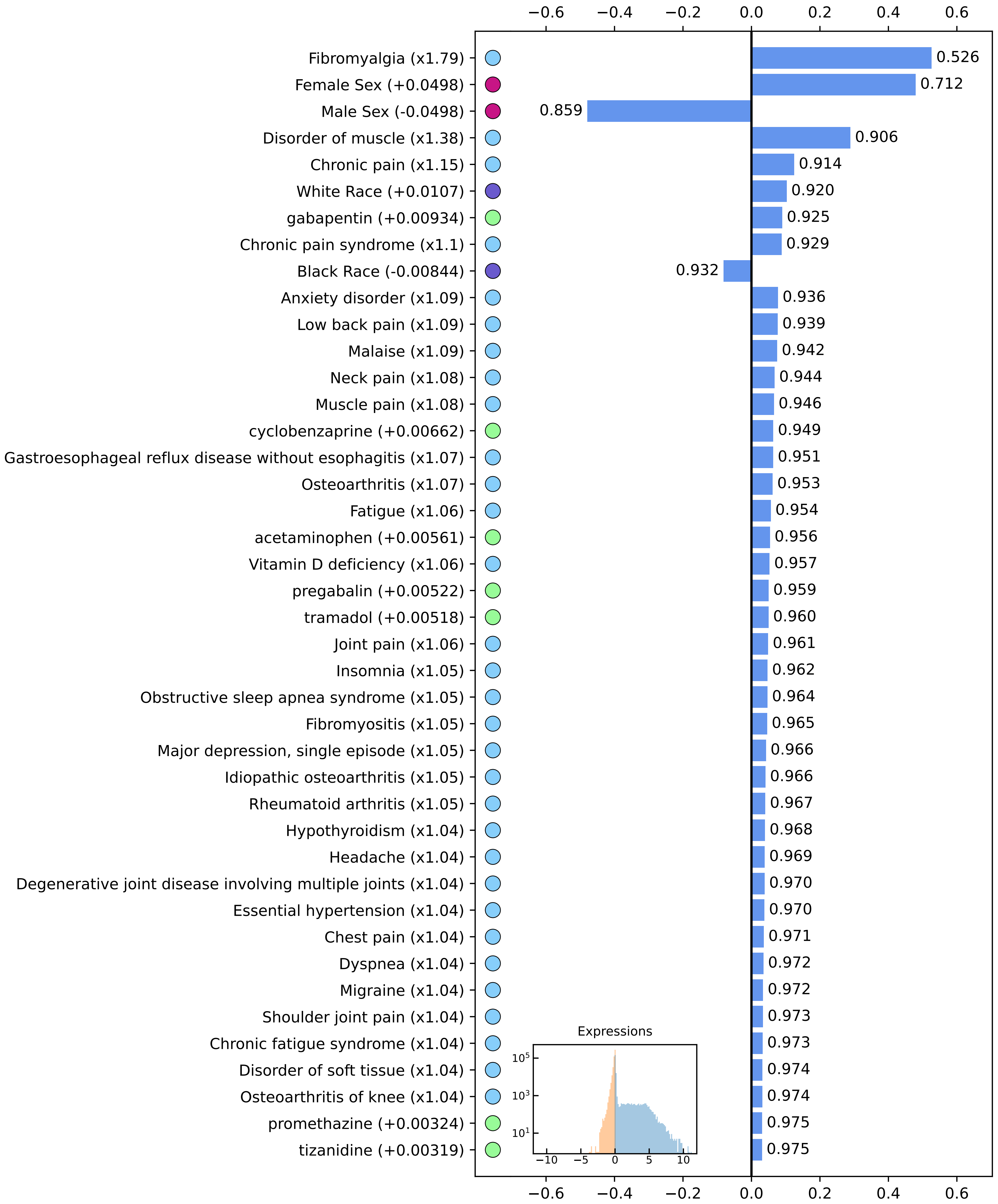

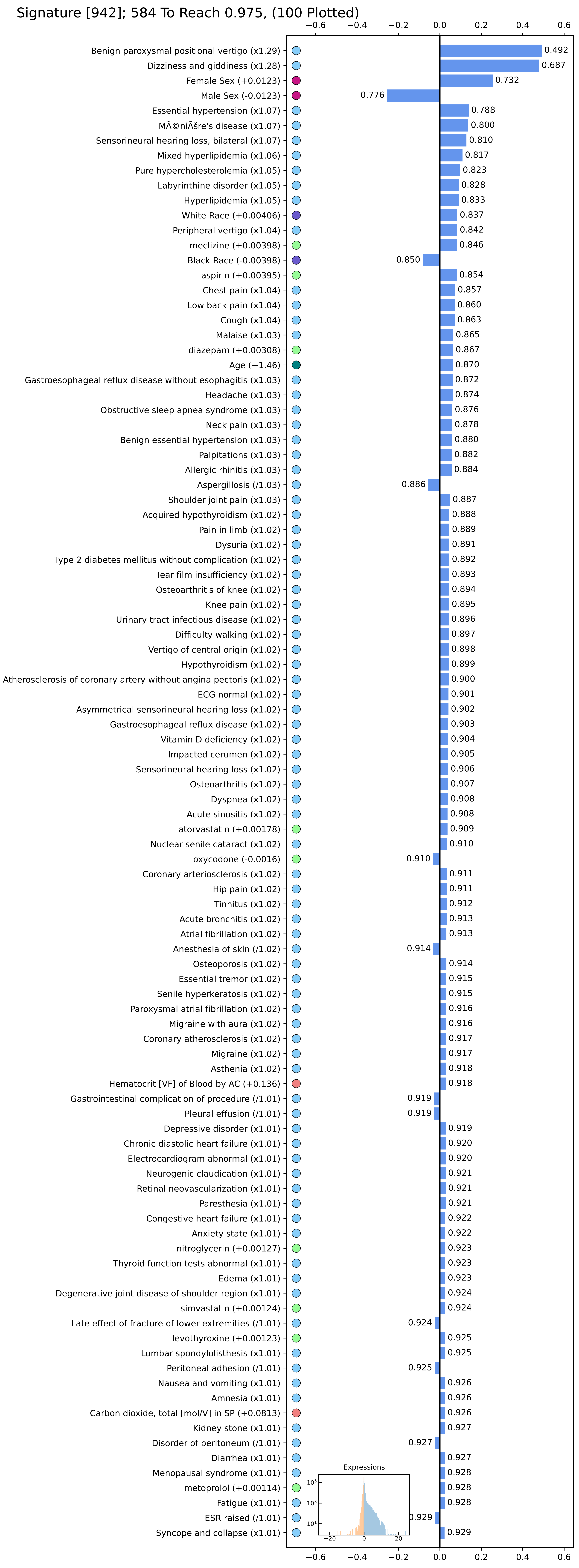

Signature [942]; 584 To Reach 0.975, (100 Plotted)
Benign paroxysmal positional vertigo (x1.29)
Dizziness and giddiness (x1.28)
Female Sex (+0.0123)
Male Sex (-0.0123)
Essential hypertension (x1.07)
MÃ©niÃšre's disease (x1.07)
Sensorineural hearing loss, bilateral (x1.07)
Mixed hyperlipidemia (x1.06)
Pure hypercholesterolemia (x1.05)
Labyrinthine disorder (x1.05)
Hyperlipidemia (x1.05)
White Race (+0.00406)
Peripheral vertigo (x1.04)
meclizine (+0.00398)
Black Race (-0.00398)
aspirin (+0.00395)
Chest pain (x1.04)
Low back pain (x1.04)
Cough (x1.04)
Malaise (x1.03)
diazepam (+0.00308)
Age (+1.46)
Gastroesophageal reflux disease without esophagitis (x1.03)
Headache (x1.03)
Obstructive sleep apnea syndrome (x1.03)
Neck pain (x1.03)
Benign essential hypertension (x1.03)
Palpitations (x1.03)
Allergic rhinitis (x1.03)
Aspergillosis (/1.03)
Shoulder joint pain (x1.03)
Acquired hypothyroidism (x1.02)
Pain in limb (x1.02)
Dysuria (x1.02)
Type 2 diabetes mellitus without complication (x1.02)
Tear film insufficiency (x1.02)
Osteoarthritis of knee (x1.02)
Knee pain (x1.02)
Urinary tract infectious disease (x1.02)
Difficulty walking (x1.02)
Vertigo of central origin (x1.02)
Hypothyroidism (x1.02)
Atherosclerosis of coronary artery without angina pectoris (x1.02)
ECG normal (x1.02)
Asymmetrical sensorineural hearing loss (x1.02)
Gastroesophageal reflux disease (x1.02)
Vitamin D deficiency (x1.02)
Impacted cerumen (x1.02)
Sensorineural hearing loss (x1.02)
Osteoarthritis (x1.02)
Dyspnea (x1.02)
Acute sinusitis (x1.02)
atorvastatin (+0.00178)
Nuclear senile cataract (x1.02)
oxycodone (-0.0016)
Coronary arteriosclerosis (x1.02)
Hip pain (x1.02)
Tinnitus (x1.02)
Acute bronchitis (x1.02)
Atrial fibrillation (x1.02)
Anesthesia of skin (/1.02)
Osteoporosis (x1.02)
Essential tremor (x1.02)
Senile hyperkeratosis (x1.02)
Paroxysmal atrial fibrillation (x1.02)
Migraine with aura (x1.02)
Coronary atherosclerosis (x1.02)
Migraine (x1.02)
Asthenia (x1.02)
Hematocrit [VF] of Blood by AC (+0.136)
Gastrointestinal complication of procedure (/1.01)
Pleural effusion (/1.01)
Depressive disorder (x1.01)
Chronic diastolic heart failure (x1.01)
Electrocardiogram abnormal (x1.01)
Neurogenic claudication (x1.01)
Retinal neovascularization (x1.01)
Paresthesia (x1.01)
Congestive heart failure (x1.01)
Anxiety state (x1.01)
nitroglycerin (+0.00127)
Thyroid function tests abnormal (x1.01)
Edema (x1.01)
Degenerative joint disease of shoulder region (x1.01)
simvastatin (+0.00124)
Late effect of fracture of lower extremities (/1.01)
levothyroxine (+0.00123)
Lumbar spondylolisthesis (x1.01)
Peritoneal adhesion (/1.01)
Nausea and vomiting (x1.01)
Amnesia (x1.01)
Carbon dioxide, total [mol/V] in SP (+0.0813)
Kidney stone (x1.01)
Disorder of peritoneum (/1.01)
Diarrhea (x1.01)
Menopausal syndrome (x1.01)
metoprolol (+0.00114)
Fatigue (x1.01)
ESR raised (/1.01)
Syncope and collapse (x1.01)
0.492
0.687
0.732
0.776
0.788
0.800
0.810
0.817
0.823
0.828
0.833
0.837
0.842
0.846
0.850
0.854
0.857
0.860
0.863
0.865
0.867
0.870
0.872
0.874
0.876
0.878
0.880
0.882
0.884
0.886
0.887
0.888
0.889
0.891
0.892
0.893
0.894
0.895
0.896
0.897
0.898
0.899
0.900
0.901
0.902
0.903
0.904
0.905
0.906
0.907
0.908
0.908
0.909
0.910
0.910
0.911
0.911
0.912
0.913
0.913
0.914
0.914
0.915
0.915
0.916
0.916
0.917
0.917
0.918
0.918
0.919
0.919
0.919
0.920
0.920
0.921
0.921
0.921
0.922
0.922
0.923
0.923
0.923
0.924
0.924
0.924
0.925
0.925
0.925
0.926
0.926
0.926
0.927
0.927
0.927
0.928
0.928
0.928
0.929
0.929
−0.6
−0.4
−0.2
0.0
0.2
0.4
0.6
Expressions
−20
0
20

Signature [943]; 1 To Reach 0.975, (10 Plotted)

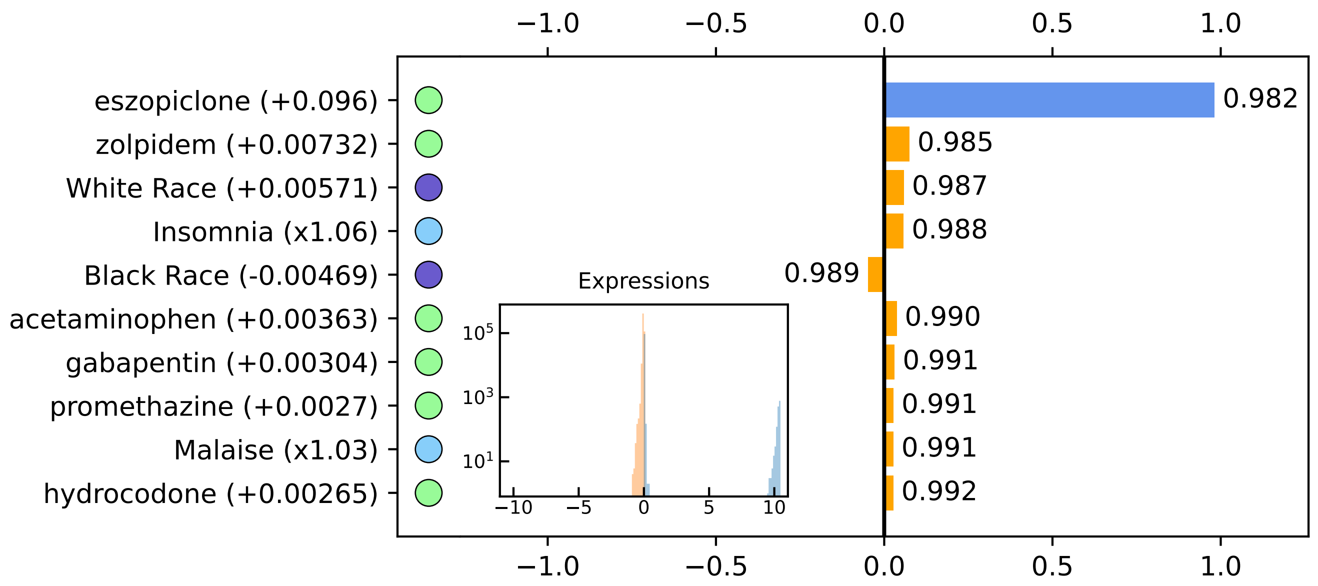

Signature [944]; 96 To Reach 0.975, (96 Plotted)

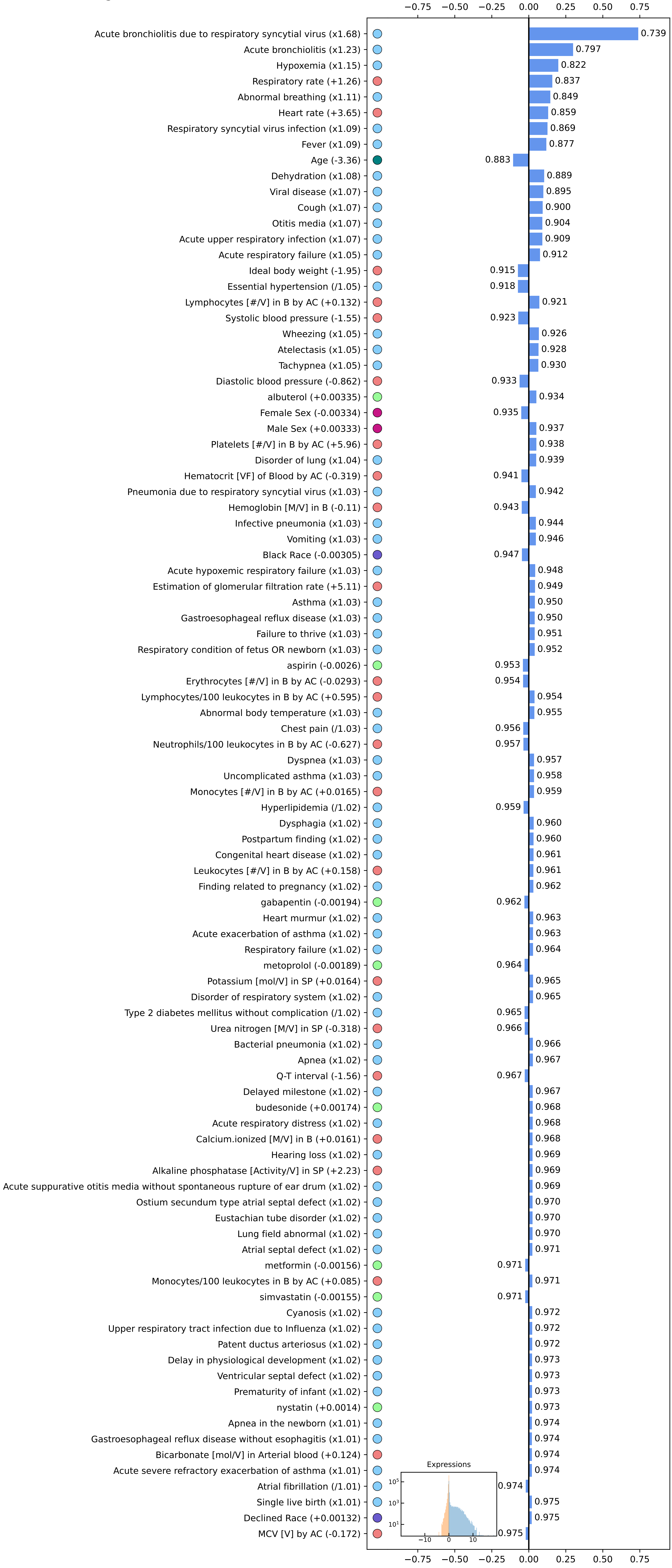

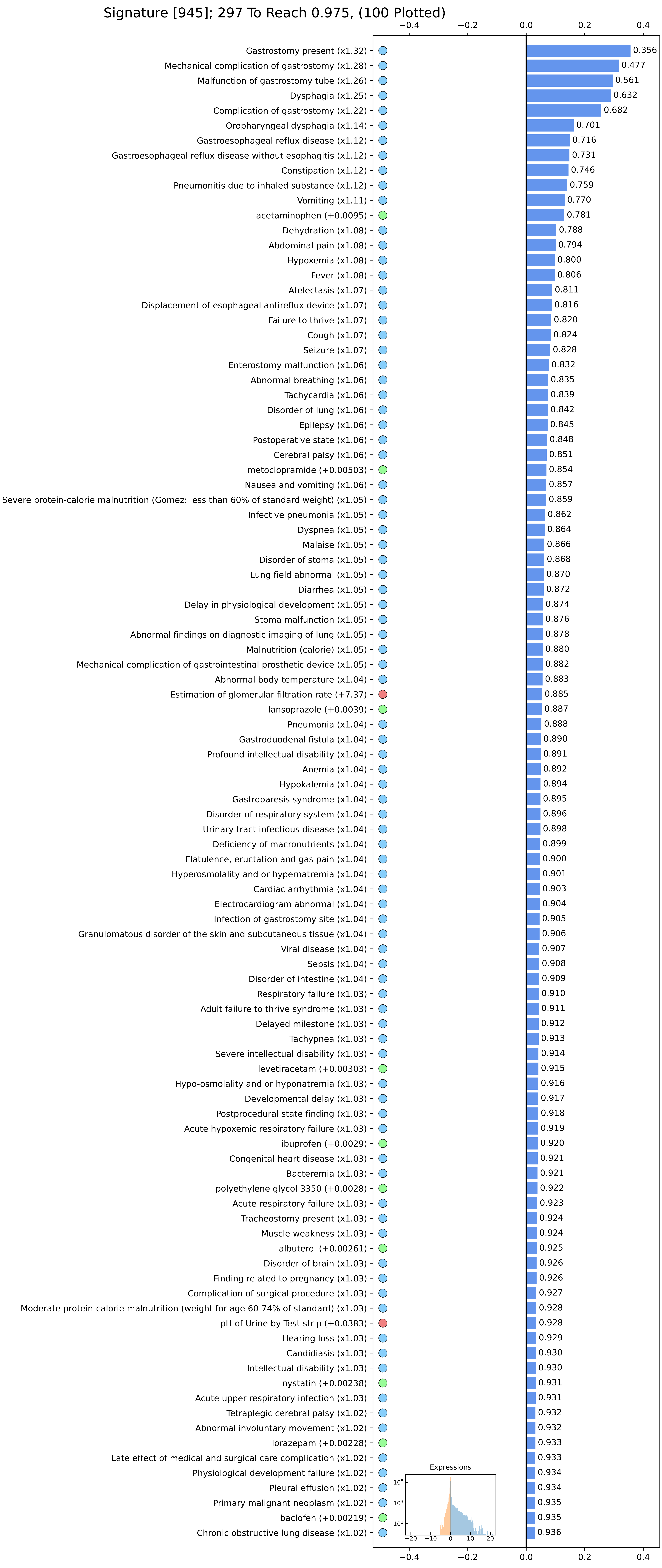

Signature [945]; 297 To Reach 0.975, (100 Plotted)
Gastrostomy present (x1.32) 0.356
Mechanical complication of gastrostomy (x1.28) 0.477
Malfunction of gastrostomy tube (x1.26) 0.561
Dysphagia (x1.25) 0.632
Complication of gastrostomy (x1.22) 0.682
Oropharyngeal dysphagia (x1.14) 0.701
Gastroesophageal reflux disease (x1.12) 0.716
Gastroesophageal reflux disease without esophagitis (x1.12) 0.731
Constipation (x1.12) 0.746
Pneumonitis due to inhaled substance (x1.12) 0.759
Vomiting (x1.11) 0.770
acetaminophen (+0.0095) 0.781
Dehydration (x1.08) 0.788
Abdominal pain (x1.08) 0.794
Hypoxemia (x1.08) 0.800
Fever (x1.08) 0.806
Atelectasis (x1.07) 0.811
Displacement of esophageal antireflux device (x1.07) 0.816
Failure to thrive (x1.07) 0.820
Cough (x1.07) 0.824
Seizure (x1.07) 0.828
Enterostomy malfunction (x1.06) 0.832
Abnormal breathing (x1.06) 0.835
Tachycardia (x1.06) 0.839
Disorder of lung (x1.06) 0.842
Epilepsy (x1.06) 0.845
Postoperative state (x1.06) 0.848
Cerebral palsy (x1.06) 0.851
metoclopramide (+0.00503) 0.854
Nausea and vomiting (x1.06) 0.857
Severe protein-calorie malnutrition (Gomez: less than 60% of standard weight) (x1.05) 0.859
Infective pneumonia (x1.05) 0.862
Dyspnea (x1.05) 0.864
Malaise (x1.05) 0.866
Disorder of stoma (x1.05) 0.868
Lung field abnormal (x1.05) 0.870
Diarrhea (x1.05) 0.872
Delay in physiological development (x1.05) 0.874
Stoma malfunction (x1.05) 0.876
Abnormal findings on diagnostic imaging of lung (x1.05) 0.878
Malnutrition (calorie) (x1.05) 0.880
Mechanical complication of gastrointestinal prosthetic device (x1.05) 0.882
Abnormal body temperature (x1.04) 0.883
Estimation of glomerular filtration rate (+7.37) 0.885
lansoprazole (+0.0039) 0.887
Pneumonia (x1.04) 0.888
Gastroduodenal fistula (x1.04) 0.890
Profound intellectual disability (x1.04) 0.891
Anemia (x1.04) 0.892
Hypokalemia (x1.04) 0.894
Gastroparesis syndrome (x1.04) 0.895
Disorder of respiratory system (x1.04) 0.896
Urinary tract infectious disease (x1.04) 0.898
Deficiency of macronutrients (x1.04) 0.899
Flatulence, eructation and gas pain (x1.04) 0.900
Hyperosmolality and or hypernatremia (x1.04) 0.901
Cardiac arrhythmia (x1.04) 0.903
Electrocardiogram abnormal (x1.04) 0.904
Infection of gastrostomy site (x1.04) 0.905
Granulomatous disorder of the skin and subcutaneous tissue (x1.04) 0.906
Viral disease (x1.04) 0.907
Sepsis (x1.04) 0.908
Disorder of intestine (x1.04) 0.909
Respiratory failure (x1.03) 0.910
Adult failure to thrive syndrome (x1.03) 0.911
Delayed milestone (x1.03) 0.912
Tachypnea (x1.03) 0.913
Severe intellectual disability (x1.03) 0.914
levetiracetam (+0.00303) 0.915
Hypo-osmolality and or hyponatremia (x1.03) 0.916
Developmental delay (x1.03) 0.917
Postprocedural state finding (x1.03) 0.918
Acute hypoxemic respiratory failure (x1.03) 0.919
ibuprofen (+0.0029) 0.920
Congenital heart disease (x1.03) 0.921
Bacteremia (x1.03) 0.921
polyethylene glycol 3350 (+0.0028) 0.922
Acute respiratory failure (x1.03) 0.923
Tracheostomy present (x1.03) 0.924
Muscle weakness (x1.03) 0.924
albuterol (+0.00261) 0.925
Disorder of brain (x1.03) 0.926
Finding related to pregnancy (x1.03) 0.926
Complication of surgical procedure (x1.03) 0.927
Moderate protein-calorie malnutrition (weight for age 60-74% of standard) (x1.03) 0.928
pH of Urine by Test strip (+0.0383) 0.928
Hearing loss (x1.03) 0.929
Candidiasis (x1.03) 0.930
Intellectual disability (x1.03) 0.930
nystatin (+0.00238) 0.931
Acute upper respiratory infection (x1.03) 0.931
Tetraplegic cerebral palsy (x1.02) 0.932
Abnormal involuntary movement (x1.02) 0.932
lorazepam (+0.00228) 0.933
Late effect of medical and surgical care complication (x1.02) 0.933
Physiological development failure (x1.02) 0.934
Pleural effusion (x1.02) 0.934
Primary malignant neoplasm (x1.02) 0.935
baclofen (+0.00219) 0.935
Chronic obstructive lung disease (x1.02) 0.936
Expressions
−0.4 −0.2 0.0 0.2 0.4

Signature [946]; 180 To Reach 0.975, (100 Plotted)

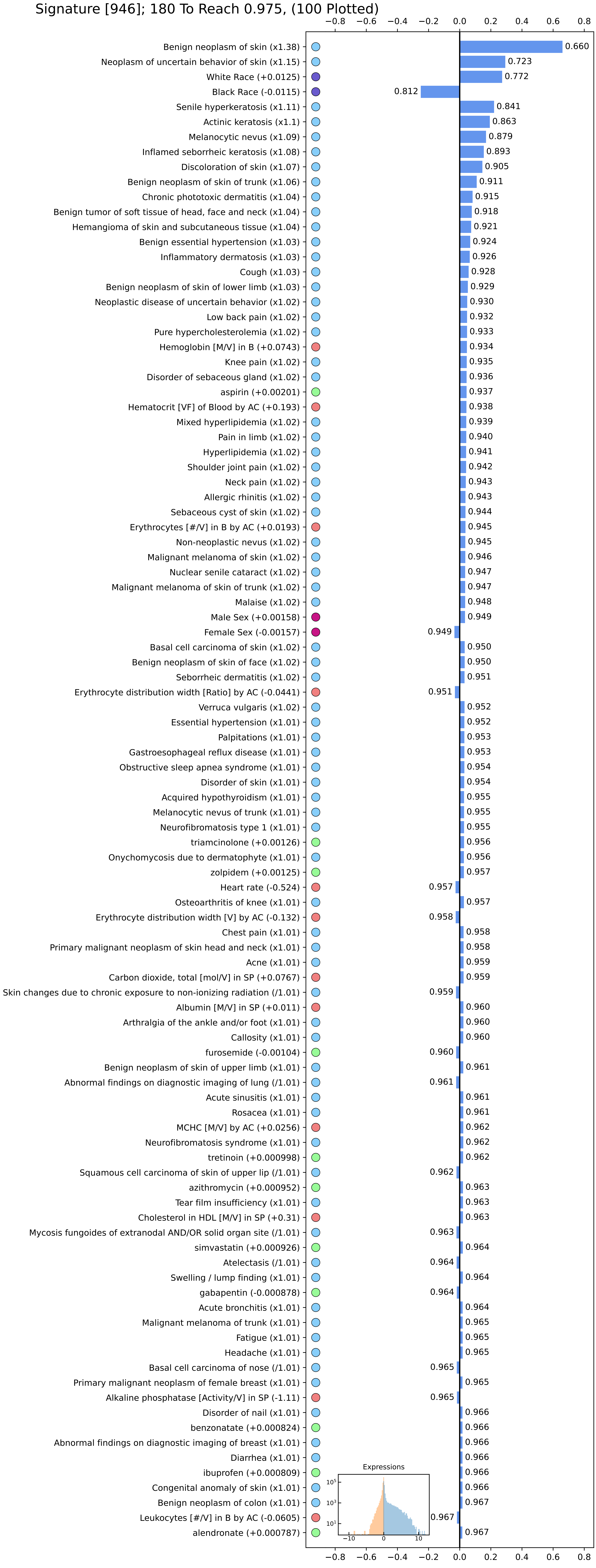

Signature [947]; 4 To Reach 0.975, (10 Plotted)

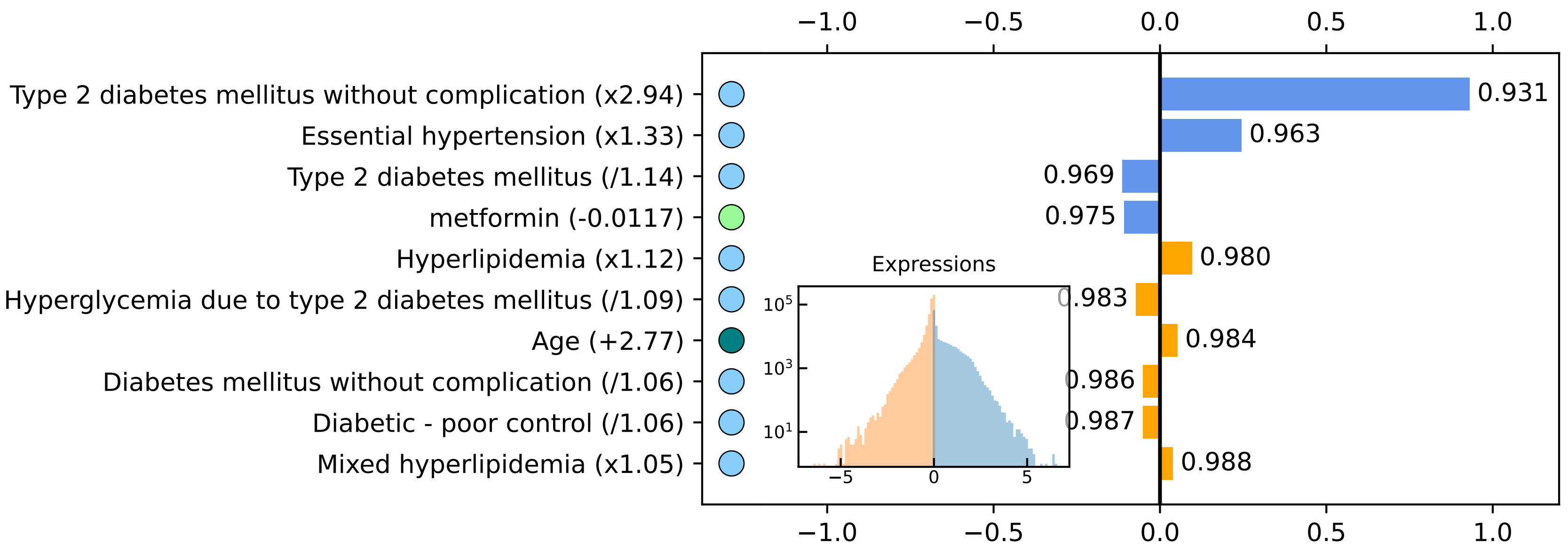

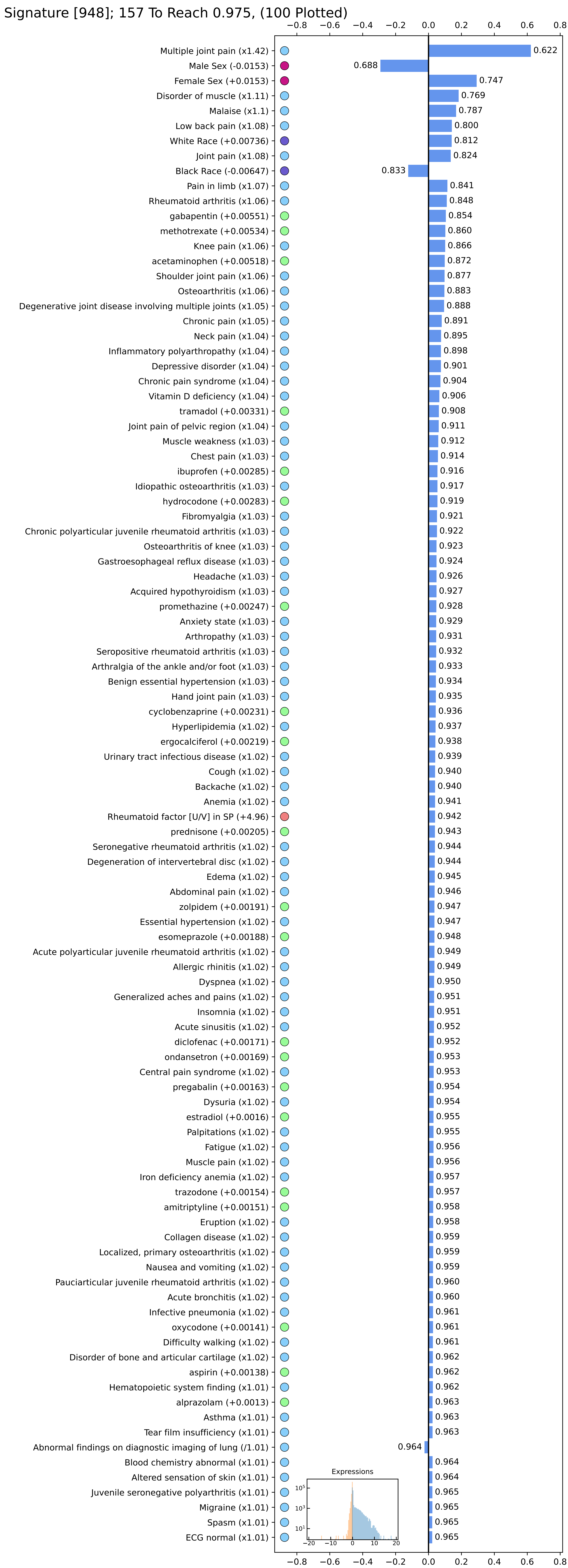

Signature [948]; 157 To Reach 0.975, (100 Plotted)
Multiple joint pain (x1.42) 0.622
Male Sex (-0.0153) 0.688
Female Sex (+0.0153) 0.747
Disorder of muscle (x1.11) 0.769
Malaise (x1.1) 0.787
Low back pain (x1.08) 0.800
White Race (+0.00736) 0.812
Joint pain (x1.08) 0.824
Black Race (-0.00647) 0.833
Pain in limb (x1.07) 0.841
Rheumatoid arthritis (x1.06) 0.848
gabapentin (+0.00551) 0.854
methotrexate (+0.00534) 0.860
Knee pain (x1.06) 0.866
acetaminophen (+0.00518) 0.872
Shoulder joint pain (x1.06) 0.877
Osteoarthritis (x1.06) 0.883
Degenerative joint disease involving multiple joints (x1.05) 0.888
Chronic pain (x1.05) 0.891
Neck pain (x1.04) 0.895
Inflammatory polyarthropathy (x1.04) 0.898
Depressive disorder (x1.04) 0.901
Chronic pain syndrome (x1.04) 0.904
Vitamin D deficiency (x1.04) 0.906
tramadol (+0.00331) 0.908
Joint pain of pelvic region (x1.04) 0.911
Muscle weakness (x1.03) 0.912
Chest pain (x1.03) 0.914
ibuprofen (+0.00285) 0.916
Idiopathic osteoarthritis (x1.03) 0.917
hydrocodone (+0.00283) 0.919
Fibromyalgia (x1.03) 0.921
Chronic polyarticular juvenile rheumatoid arthritis (x1.03) 0.922
Osteoarthritis of knee (x1.03) 0.923
Gastroesophageal reflux disease (x1.03) 0.924
Headache (x1.03) 0.926
Acquired hypothyroidism (x1.03) 0.927
promethazine (+0.00247) 0.928
Anxiety state (x1.03) 0.929
Arthropathy (x1.03) 0.931
Seropositive rheumatoid arthritis (x1.03) 0.932
Arthralgia of the ankle and/or foot (x1.03) 0.933
Benign essential hypertension (x1.03) 0.934
Hand joint pain (x1.03) 0.935
cyclobenzaprine (+0.00231) 0.936
Hyperlipidemia (x1.02) 0.937
ergocalciferol (+0.00219) 0.938
Urinary tract infectious disease (x1.02) 0.939
Cough (x1.02) 0.940
Backache (x1.02) 0.940
Anemia (x1.02) 0.941
Rheumatoid factor [U/V] in SP (+4.96) 0.942
prednisone (+0.00205) 0.943
Seronegative rheumatoid arthritis (x1.02) 0.944
Degeneration of intervertebral disc (x1.02) 0.944
Edema (x1.02) 0.945
Abdominal pain (x1.02) 0.946
zolpidem (+0.00191) 0.947
Essential hypertension (x1.02) 0.947
esomeprazole (+0.00188) 0.948
Acute polyarticular juvenile rheumatoid arthritis (x1.02) 0.949
Allergic rhinitis (x1.02) 0.949
Dyspnea (x1.02) 0.950
Generalized aches and pains (x1.02) 0.951
Insomnia (x1.02) 0.951
Acute sinusitis (x1.02) 0.952
diclofenac (+0.00171) 0.952
ondansetron (+0.00169) 0.953
Central pain syndrome (x1.02) 0.953
pregabalin (+0.00163) 0.954
Dysuria (x1.02) 0.954
estradiol (+0.0016) 0.955
Palpitations (x1.02) 0.955
Fatigue (x1.02) 0.956
Muscle pain (x1.02) 0.956
Iron deficiency anemia (x1.02) 0.957
trazodone (+0.00154) 0.957
amitriptyline (+0.00151) 0.958
Eruption (x1.02) 0.958
Collagen disease (x1.02) 0.959
Localized, primary osteoarthritis (x1.02) 0.959
Nausea and vomiting (x1.02) 0.959
Pauciarticular juvenile rheumatoid arthritis (x1.02) 0.960
Acute bronchitis (x1.02) 0.960
Infective pneumonia (x1.02) 0.961
oxycodone (+0.00141) 0.961
Difficulty walking (x1.02) 0.961
Disorder of bone and articular cartilage (x1.02) 0.962
aspirin (+0.00138) 0.962
Hematopoietic system finding (x1.01) 0.962
alprazolam (+0.0013) 0.963
Asthma (x1.01) 0.963
Tear film insufficiency (x1.01) 0.963
Abnormal findings on diagnostic imaging of lung (/1.01) 0.964
Blood chemistry abnormal (x1.01) 0.964
Altered sensation of skin (x1.01) 0.964
Juvenile seronegative polyarthritis (x1.01) 0.965
Migraine (x1.01) 0.965
Spasm (x1.01) 0.965
ECG normal (x1.01) 0.965
Expressions
−0.8 −0.6 −0.4 −0.2 0.0 0.2 0.4 0.6 0.8

Signature [949]; 11 To Reach 0.975, (11 Plotted)

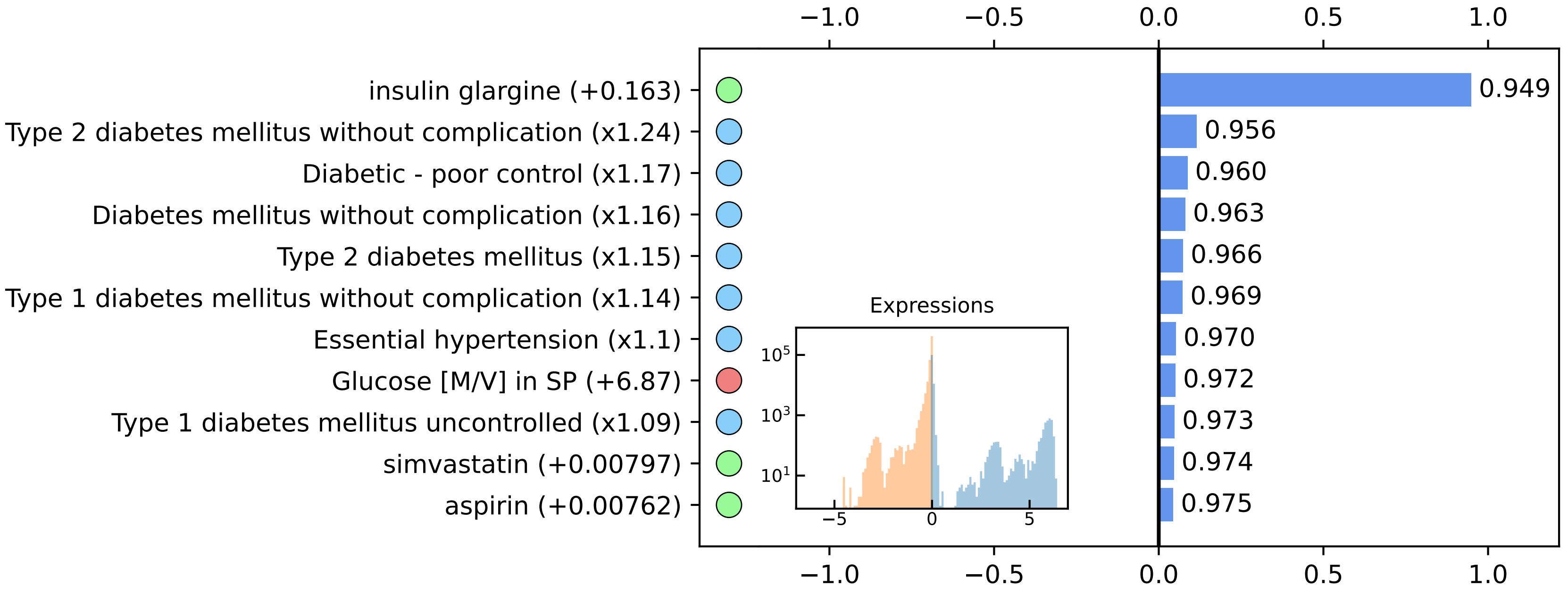

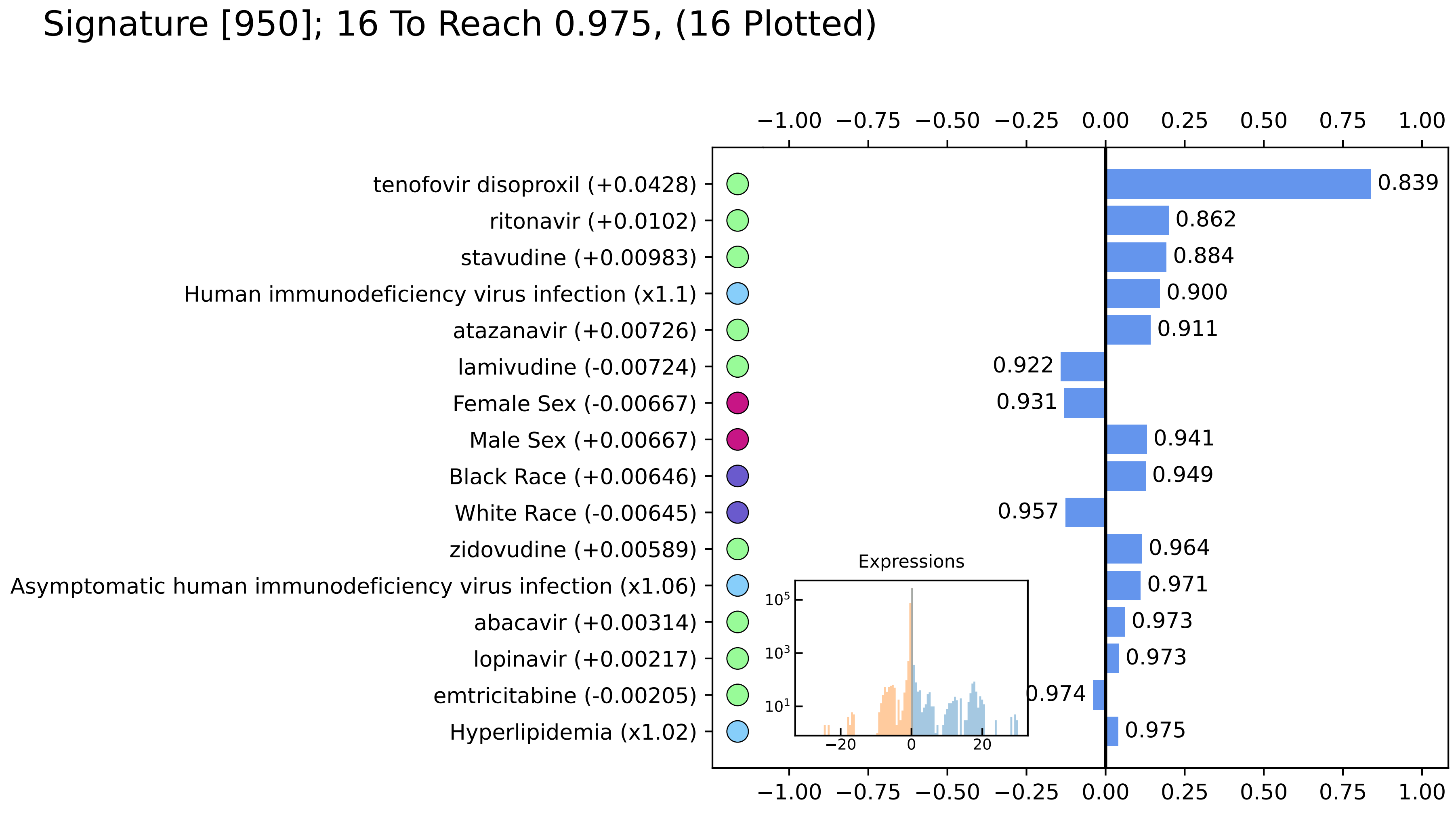

Signature [950]; 16 To Reach 0.975, (16 Plotted)
tenofovir disoproxil (+0.0428) 0.839
ritonavir (+0.0102) 0.862
stavudine (+0.00983) 0.884
Human immunodeficiency virus infection (x1.1) 0.900
atazanavir (+0.00726) 0.911
lamivudine (-0.00724) 0.922
Female Sex (-0.00667) 0.931
Male Sex (+0.00667) 0.941
Black Race (+0.00646) 0.949
White Race (-0.00645) 0.957
zidovudine (+0.00589) 0.964
Asymptomatic human immunodeficiency virus infection (x1.06) 0.971
abacavir (+0.00314) 0.973
lopinavir (+0.00217) 0.973
emtricitabine (-0.00205) 0.974
Hyperlipidemia (x1.02) 0.975
−1.00 −0.75 −0.50 −0.25 0.00 0.25 0.50 0.75 1.00
Expressions
10^5
10^3
10^1
−20
0
20

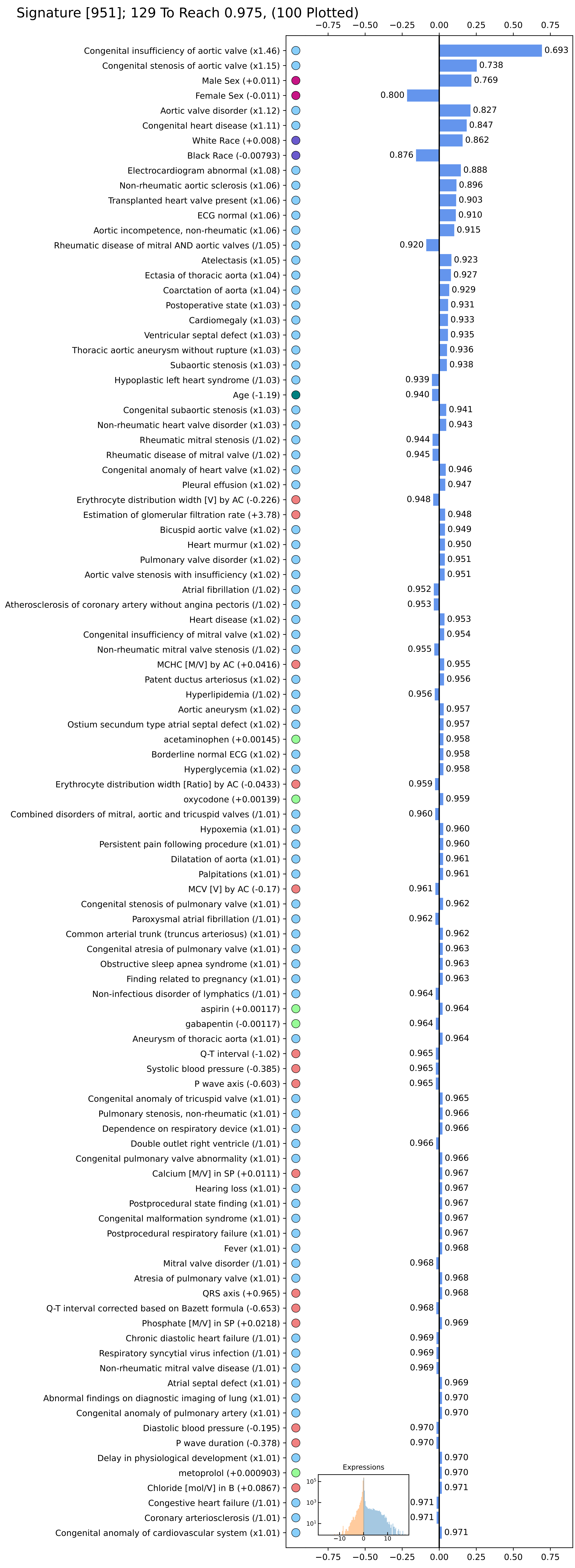

Signature [951]; 129 To Reach 0.975, (100 Plotted)
Congenital insufficiency of aortic valve (x1.46)
Congenital stenosis of aortic valve (x1.15)
Male Sex (+0.011)
Female Sex (-0.011)
Aortic valve disorder (x1.12)
Congenital heart disease (x1.11)
White Race (+0.008)
Black Race (-0.00793)
Electrocardiogram abnormal (x1.08)
Non-rheumatic aortic sclerosis (x1.06)
Transplanted heart valve present (x1.06)
ECG normal (x1.06)
Aortic incompetence, non-rheumatic (x1.06)
Rheumatic disease of mitral AND aortic valves (/1.05)
Atelectasis (x1.05)
Ectasia of thoracic aorta (x1.04)
Coarctation of aorta (x1.04)
Postoperative state (x1.03)
Cardiomegaly (x1.03)
Ventricular septal defect (x1.03)
Thoracic aortic aneurysm without rupture (x1.03)
Subaortic stenosis (x1.03)
Hypoplastic left heart syndrome (/1.03)
Age (-1.19)
Congenital subaortic stenosis (x1.03)
Non-rheumatic heart valve disorder (x1.03)
Rheumatic mitral stenosis (/1.02)
Rheumatic disease of mitral valve (/1.02)
Congenital anomaly of heart valve (x1.02)
Pleural effusion (x1.02)
Erythrocyte distribution width [V] by AC (-0.226)
Estimation of glomerular filtration rate (+3.78)
Bicuspid aortic valve (x1.02)
Heart murmur (x1.02)
Pulmonary valve disorder (x1.02)
Aortic valve stenosis with insufficiency (x1.02)
Atrial fibrillation (/1.02)
Atherosclerosis of coronary artery without angina pectoris (/1.02)
Heart disease (x1.02)
Congenital insufficiency of mitral valve (x1.02)
Non-rheumatic mitral valve stenosis (/1.02)
MCHC [M/V] by AC (+0.0416)
Patent ductus arteriosus (x1.02)
Hyperlipidemia (/1.02)
Aortic aneurysm (x1.02)
Ostium secundum type atrial septal defect (x1.02)
acetaminophen (+0.00145)
Borderline normal ECG (x1.02)
Hyperglycemia (x1.02)
Erythrocyte distribution width [Ratio] by AC (-0.0433)
oxycodone (+0.00139)
Combined disorders of mitral, aortic and tricuspid valves (/1.01)
Hypoxemia (x1.01)
Persistent pain following procedure (x1.01)
Dilatation of aorta (x1.01)
Palpitations (x1.01)
MCV [V] by AC (-0.17)
Congenital stenosis of pulmonary valve (x1.01)
Paroxysmal atrial fibrillation (/1.01)
Common arterial trunk (truncus arteriosus) (x1.01)
Congenital atresia of pulmonary valve (x1.01)
Obstructive sleep apnea syndrome (x1.01)
Finding related to pregnancy (x1.01)
Non-infectious disorder of lymphatics (/1.01)
aspirin (+0.00117)
gabapentin (-0.00117)
Aneurysm of thoracic aorta (x1.01)
Q-T interval (-1.02)
Systolic blood pressure (-0.385)
P wave axis (-0.603)
Congenital anomaly of tricuspid valve (x1.01)
Pulmonary stenosis, non-rheumatic (x1.01)
Dependence on respiratory device (x1.01)
Double outlet right ventricle (/1.01)
Congenital pulmonary valve abnormality (x1.01)
Calcium [M/V] in SP (+0.0111)
Hearing loss (x1.01)
Postprocedural state finding (x1.01)
Congenital malformation syndrome (x1.01)
Postprocedural respiratory failure (x1.01)
Fever (x1.01)
Mitral valve disorder (/1.01)
Atresia of pulmonary valve (x1.01)
QRS axis (+0.965)
Q-T interval corrected based on Bazett formula (-0.653)
Phosphate [M/V] in SP (+0.0218)
Chronic diastolic heart failure (/1.01)
Respiratory syncytial virus infection (/1.01)
Non-rheumatic mitral valve disease (/1.01)
Atrial septal defect (x1.01)
Abnormal findings on diagnostic imaging of lung (x1.01)
Congenital anomaly of pulmonary artery (x1.01)
Diastolic blood pressure (-0.195)
P wave duration (-0.378)
Delay in physiological development (x1.01)
metoprolol (+0.000903)
Chloride [mol/V] in B (+0.0867)
Congestive heart failure (/1.01)
Coronary arteriosclerosis (/1.01)
Congenital anomaly of cardiovascular system (x1.01)
0.693
0.738
0.769
0.800
0.827
0.847
0.862
0.876
0.888
0.896
0.903
0.910
0.915
0.920
0.923
0.927
0.929
0.931
0.933
0.935
0.936
0.938
0.939
0.940
0.941
0.943
0.944
0.945
0.946
0.947
0.948
0.948
0.949
0.950
0.951
0.951
0.952
0.953
0.953
0.954
0.955
0.955
0.956
0.956
0.957
0.957
0.958
0.958
0.958
0.959
0.959
0.960
0.960
0.960
0.961
0.961
0.961
0.962
0.962
0.962
0.963
0.963
0.963
0.964
0.964
0.964
0.964
0.965
0.965
0.965
0.965
0.966
0.966
0.966
0.966
0.967
0.967
0.967
0.967
0.967
0.968
0.968
0.968
0.968
0.968
0.969
0.969
0.969
0.969
0.969
0.970
0.970
0.970
0.970
0.970
0.970
0.971
0.971
0.971
0.971
−0.75 −0.50 −0.25 0.00 0.25 0.50 0.75
Expressions
10^5
10^3
10^1
−10 0 10

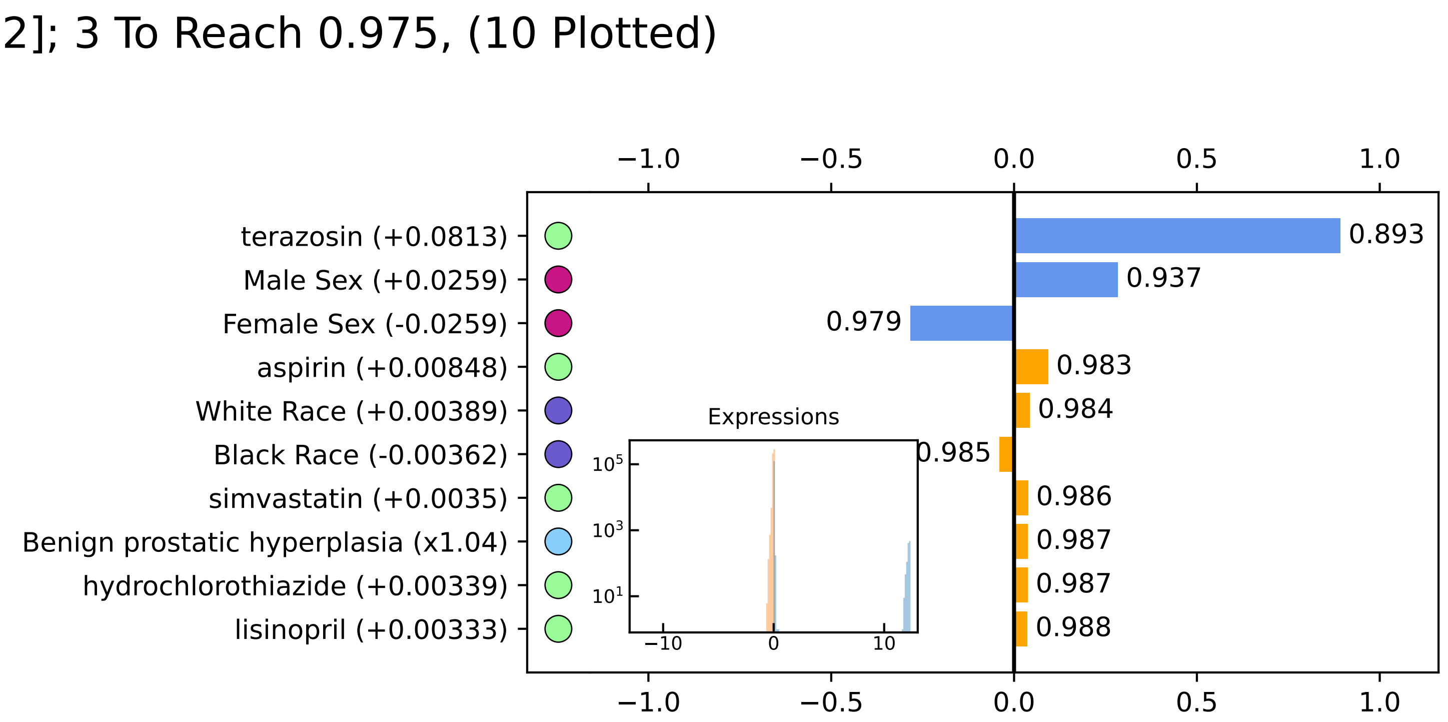
Signature [952]; 3 To Reach 0.975, (10 Plotted)
terazosin (+0.0813)
Male Sex (+0.0259)
Female Sex (-0.0259)
aspirin (+0.00848)
White Race (+0.00389)
Black Race (-0.00362)
simvastatin (+0.0035)
Benign prostatic hyperplasia (x1.04)
hydrochlorothiazide (+0.00339)
lisinopril (+0.00333)
0.893
0.937
0.979
0.983
0.984
0.985
0.986
0.987
0.987
0.988
−1.0
−0.5
0.0
0.5
1.0
Expressions
10^5
10^3
10^1
−10
0
10

Signature [953]; 2 To Reach 0.975, (10 Plotted)

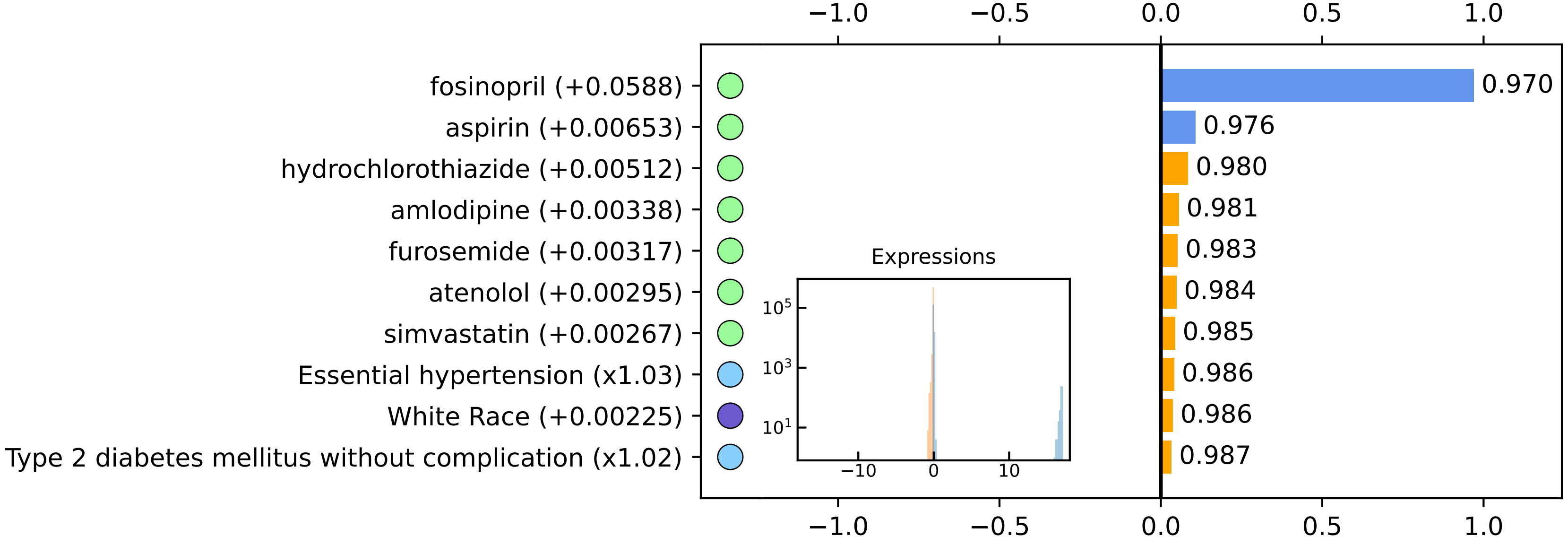

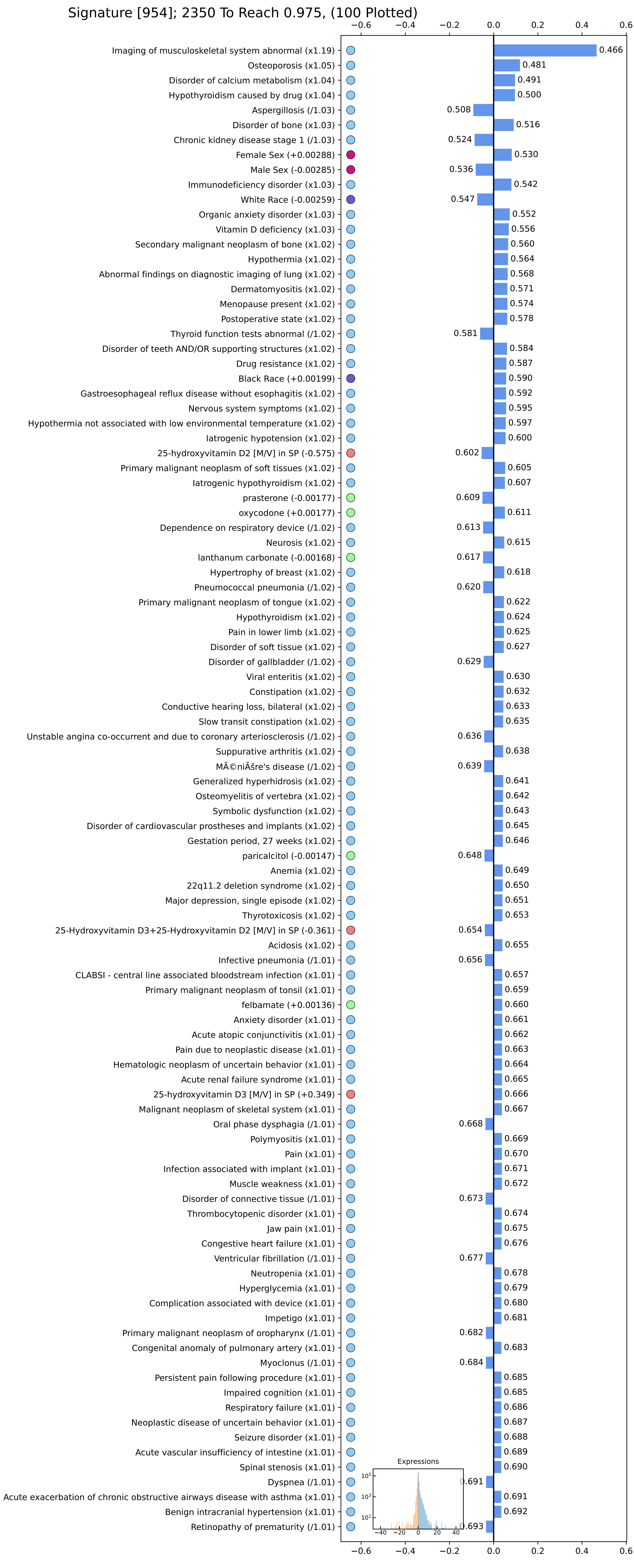

Signature [954]; 2350 To Reach 0.975, (100 Plotted)
Imaging of musculoskeletal system abnormal (x1.19) 0.466
Osteoporosis (x1.05) 0.481
Disorder of calcium metabolism (x1.04) 0.491
Hypothyroidism caused by drug (x1.04) 0.500
Aspergillosis (/1.03) 0.508
Disorder of bone (x1.03) 0.516
Chronic kidney disease stage 1 (/1.03) 0.524
Female Sex (+0.00288) 0.530
Male Sex (-0.00285) 0.536
Immunodeficiency disorder (x1.03) 0.542
White Race (-0.00259) 0.547
Organic anxiety disorder (x1.03) 0.552
Vitamin D deficiency (x1.03) 0.556
Secondary malignant neoplasm of bone (x1.02) 0.560
Hypothermia (x1.02) 0.564
Abnormal findings on diagnostic imaging of lung (x1.02) 0.568
Dermatomyositis (x1.02) 0.571
Menopause present (x1.02) 0.574
Postoperative state (x1.02) 0.578
Thyroid function tests abnormal (/1.02) 0.581
Disorder of teeth AND/OR supporting structures (x1.02) 0.584
Drug resistance (x1.02) 0.587
Black Race (+0.00199) 0.590
Gastroesophageal reflux disease without esophagitis (x1.02) 0.592
Nervous system symptoms (x1.02) 0.595
Hypothermia not associated with low environmental temperature (x1.02) 0.597
Iatrogenic hypotension (x1.02) 0.600
25-hydroxyvitamin D2 [M/V] in SP (-0.575) 0.602
Primary malignant neoplasm of soft tissues (x1.02) 0.605
Iatrogenic hypothyroidism (x1.02) 0.607
prasterone (-0.00177) 0.609
oxycodone (+0.00177) 0.611
Dependence on respiratory device (/1.02) 0.613
Neurosis (x1.02) 0.615
lanthanum carbonate (-0.00168) 0.617
Hypertrophy of breast (x1.02) 0.618
Pneumococcal pneumonia (/1.02) 0.620
Primary malignant neoplasm of tongue (x1.02) 0.622
Hypothyroidism (x1.02) 0.624
Pain in lower limb (x1.02) 0.625
Disorder of soft tissue (x1.02) 0.627
Disorder of gallbladder (/1.02) 0.629
Viral enteritis (x1.02) 0.630
Constipation (x1.02) 0.632
Conductive hearing loss, bilateral (x1.02) 0.633
Slow transit constipation (x1.02) 0.635
Unstable angina co-occurrent and due to coronary arteriosclerosis (/1.02) 0.636
Suppurative arthritis (x1.02) 0.638
MÃ©niÃšre's disease (/1.02) 0.639
Generalized hyperhidrosis (x1.02) 0.641
Osteomyelitis of vertebra (x1.02) 0.642
Symbolic dysfunction (x1.02) 0.643
Disorder of cardiovascular prostheses and implants (x1.02) 0.645
Gestation period, 27 weeks (x1.02) 0.646
paricalcitol (-0.00147) 0.648
Anemia (x1.02) 0.649
22q11.2 deletion syndrome (x1.02) 0.650
Major depression, single episode (x1.02) 0.651
Thyrotoxicosis (x1.02) 0.653
25-Hydroxyvitamin D3+25-Hydroxyvitamin D2 [M/V] in SP (-0.361) 0.654
Acidosis (x1.02) 0.655
Infective pneumonia (/1.01) 0.656
CLABSI - central line associated bloodstream infection (x1.01) 0.657
Primary malignant neoplasm of tonsil (x1.01) 0.659
felbamate (+0.00136) 0.660
Anxiety disorder (x1.01) 0.661
Acute atopic conjunctivitis (x1.01) 0.662
Pain due to neoplastic disease (x1.01) 0.663
Hematologic neoplasm of uncertain behavior (x1.01) 0.664
Acute renal failure syndrome (x1.01) 0.665
25-hydroxyvitamin D3 [M/V] in SP (+0.349) 0.666
Malignant neoplasm of skeletal system (x1.01) 0.667
Oral phase dysphagia (/1.01) 0.668
Polymyositis (x1.01) 0.669
Pain (x1.01) 0.670
Infection associated with implant (x1.01) 0.671
Muscle weakness (x1.01) 0.672
Disorder of connective tissue (/1.01) 0.673
Thrombocytopenic disorder (x1.01) 0.674
Jaw pain (x1.01) 0.675
Congestive heart failure (x1.01) 0.676
Ventricular fibrillation (/1.01) 0.677
Neutropenia (x1.01) 0.678
Hyperglycemia (x1.01) 0.679
Complication associated with device (x1.01) 0.680
Impetigo (x1.01) 0.681
Primary malignant neoplasm of oropharynx (/1.01) 0.682
Congenital anomaly of pulmonary artery (x1.01) 0.683
Myoclonus (/1.01) 0.684
Persistent pain following procedure (x1.01) 0.685
Impaired cognition (x1.01) 0.685
Respiratory failure (x1.01) 0.686
Neoplastic disease of uncertain behavior (x1.01) 0.687
Seizure disorder (x1.01) 0.688
Acute vascular insufficiency of intestine (x1.01) 0.689
Spinal stenosis (x1.01) 0.690
Dyspnea (/1.01) 0.691
Acute exacerbation of chronic obstructive airways disease with asthma (x1.01) 0.691
Benign intracranial hypertension (x1.01) 0.692
Retinopathy of prematurity (/1.01) 0.693
−0.6 −0.4 −0.2 0.0 0.2 0.4 0.6
Expressions

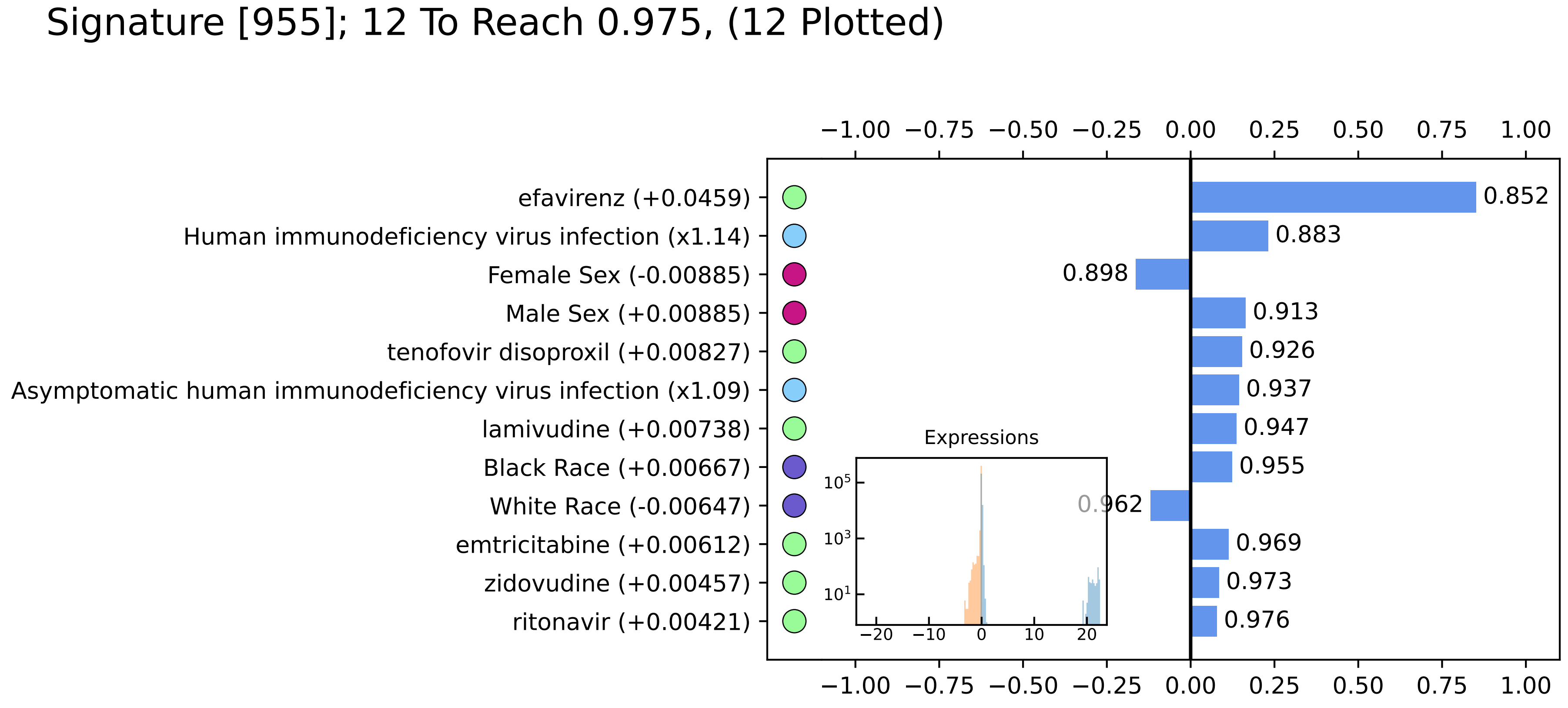
Signature [955]; 12 To Reach 0.975, (12 Plotted)
efavirenz (+0.0459)
Human immunodeficiency virus infection (x1.14)
Female Sex (-0.00885)
Male Sex (+0.00885)
tenofovir disoproxil (+0.00827)
Asymptomatic human immunodeficiency virus infection (x1.09)
lamivudine (+0.00738)
Black Race (+0.00667)
White Race (-0.00647)
emtricitabine (+0.00612)
zidovudine (+0.00457)
ritonavir (+0.00421)
0.852
0.883
0.898
0.913
0.926
0.937
0.947
0.955
0.962
0.969
0.973
0.976
−1.00
−0.75
−0.50
−0.25
0.00
0.25
0.50
0.75
1.00
Expressions
10^5
10^3
10^1
−20
−10
0
10
20

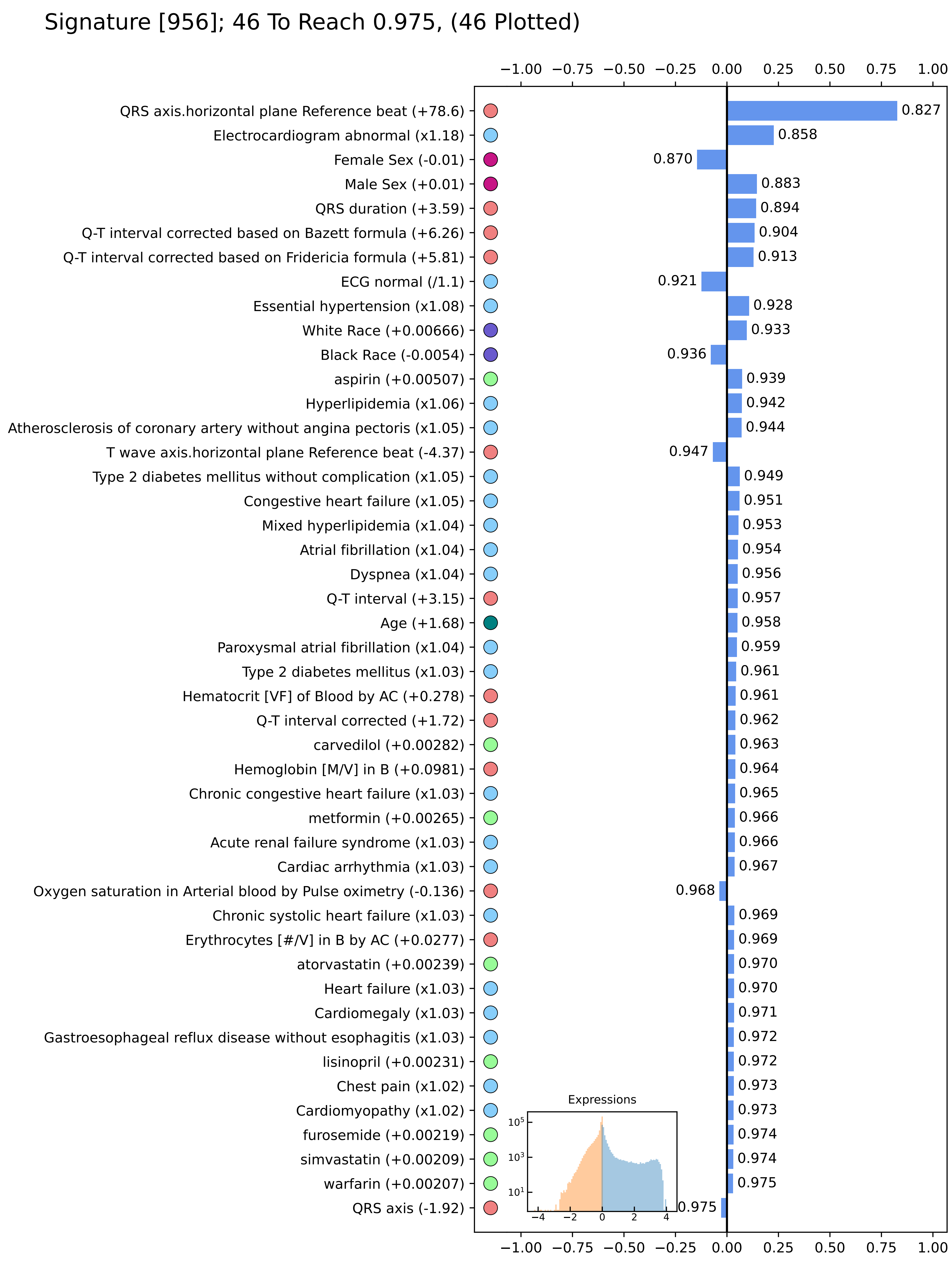

Signature [956]; 46 To Reach 0.975, (46 Plotted)
QRS axis.horizontal plane Reference beat (+78.6)
Electrocardiogram abnormal (x1.18)
Female Sex (-0.01)
Male Sex (+0.01)
QRS duration (+3.59)
Q-T interval corrected based on Bazett formula (+6.26)
Q-T interval corrected based on Fridericia formula (+5.81)
ECG normal (/1.1)
Essential hypertension (x1.08)
White Race (+0.00666)
Black Race (-0.0054)
aspirin (+0.00507)
Hyperlipidemia (x1.06)
Atherosclerosis of coronary artery without angina pectoris (x1.05)
T wave axis.horizontal plane Reference beat (-4.37)
Type 2 diabetes mellitus without complication (x1.05)
Congestive heart failure (x1.05)
Mixed hyperlipidemia (x1.04)
Atrial fibrillation (x1.04)
Dyspnea (x1.04)
Q-T interval (+3.15)
Age (+1.68)
Paroxysmal atrial fibrillation (x1.04)
Type 2 diabetes mellitus (x1.03)
Hematocrit [VF] of Blood by AC (+0.278)
Q-T interval corrected (+1.72)
carvedilol (+0.00282)
Hemoglobin [M/V] in B (+0.0981)
Chronic congestive heart failure (x1.03)
metformin (+0.00265)
Acute renal failure syndrome (x1.03)
Cardiac arrhythmia (x1.03)
Oxygen saturation in Arterial blood by Pulse oximetry (-0.136)
Chronic systolic heart failure (x1.03)
Erythrocytes [#/V] in B by AC (+0.0277)
atorvastatin (+0.00239)
Heart failure (x1.03)
Cardiomegaly (x1.03)
Gastroesophageal reflux disease without esophagitis (x1.03)
lisinopril (+0.00231)
Chest pain (x1.02)
Cardiomyopathy (x1.02)
furosemide (+0.00219)
simvastatin (+0.00209)
warfarin (+0.00207)
QRS axis (-1.92)
0.827
0.858
0.870
0.883
0.894
0.904
0.913
0.921
0.928
0.933
0.936
0.939
0.942
0.944
0.947
0.949
0.951
0.953
0.954
0.956
0.957
0.958
0.959
0.961
0.961
0.962
0.963
0.964
0.965
0.966
0.966
0.967
0.968
0.969
0.969
0.970
0.970
0.971
0.972
0.972
0.973
0.973
0.974
0.974
0.975
0.975
−1.00 −0.75 −0.50 −0.25 0.00 0.25 0.50 0.75 1.00
Expressions

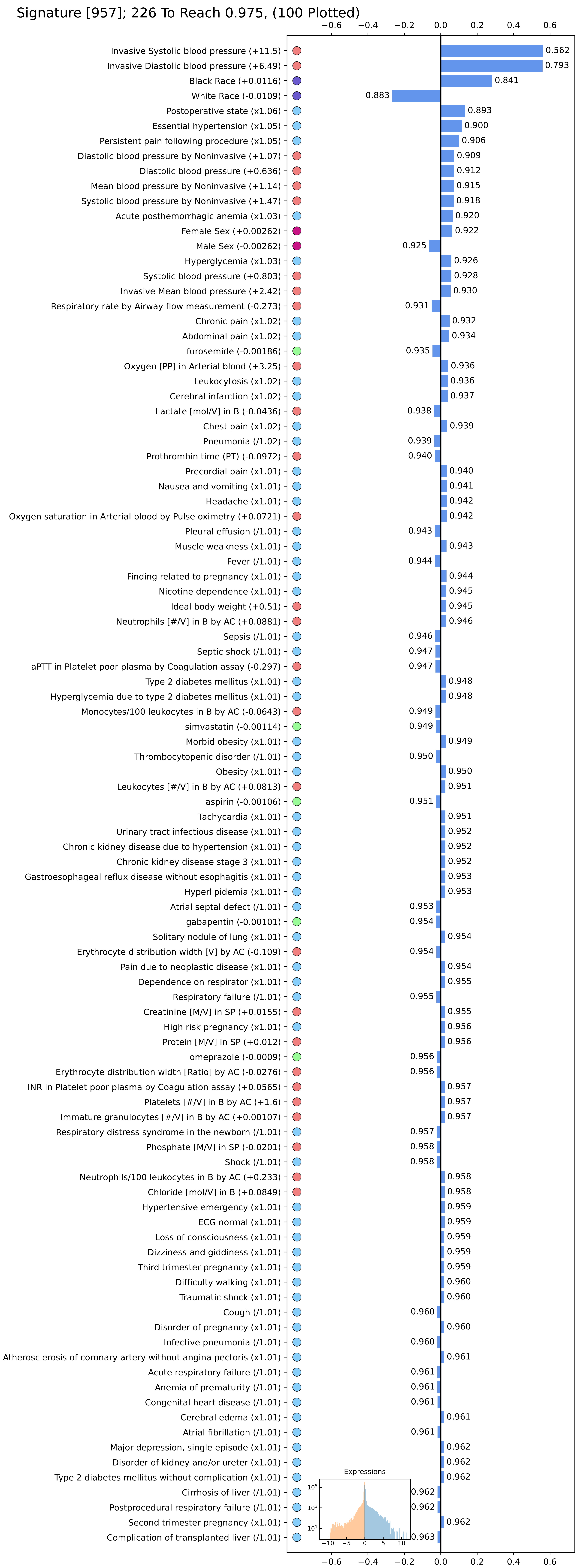

Signature [957]; 226 To Reach 0.975, (100 Plotted)
Invasive Systolic blood pressure (+11.5)
Invasive Diastolic blood pressure (+6.49)
Black Race (+0.0116)
White Race (-0.0109)
Postoperative state (x1.06)
Essential hypertension (x1.05)
Persistent pain following procedure (x1.05)
Diastolic blood pressure by Noninvasive (+1.07)
Diastolic blood pressure (+0.636)
Mean blood pressure by Noninvasive (+1.14)
Systolic blood pressure by Noninvasive (+1.47)
Acute posthemorrhagic anemia (x1.03)
Female Sex (+0.00262)
Male Sex (-0.00262)
Hyperglycemia (x1.03)
Systolic blood pressure (+0.803)
Invasive Mean blood pressure (+2.42)
Respiratory rate by Airway flow measurement (-0.273)
Chronic pain (x1.02)
Abdominal pain (x1.02)
furosemide (-0.00186)
Oxygen [PP] in Arterial blood (+3.25)
Leukocytosis (x1.02)
Cerebral infarction (x1.02)
Lactate [mol/V] in B (-0.0436)
Chest pain (x1.02)
Pneumonia (/1.02)
Prothrombin time (PT) (-0.0972)
Precordial pain (x1.01)
Nausea and vomiting (x1.01)
Headache (x1.01)
Oxygen saturation in Arterial blood by Pulse oximetry (+0.0721)
Pleural effusion (/1.01)
Muscle weakness (x1.01)
Fever (/1.01)
Finding related to pregnancy (x1.01)
Nicotine dependence (x1.01)
Ideal body weight (+0.51)
Neutrophils [#/V] in B by AC (+0.0881)
Sepsis (/1.01)
Septic shock (/1.01)
aPTT in Platelet poor plasma by Coagulation assay (-0.297)
Type 2 diabetes mellitus (x1.01)
Hyperglycemia due to type 2 diabetes mellitus (x1.01)
Monocytes/100 leukocytes in B by AC (-0.0643)
simvastatin (-0.00114)
Morbid obesity (x1.01)
Thrombocytopenic disorder (/1.01)
Obesity (x1.01)
Leukocytes [#/V] in B by AC (+0.0813)
aspirin (-0.00106)
Tachycardia (x1.01)
Urinary tract infectious disease (x1.01)
Chronic kidney disease due to hypertension (x1.01)
Chronic kidney disease stage 3 (x1.01)
Gastroesophageal reflux disease without esophagitis (x1.01)
Hyperlipidemia (x1.01)
Atrial septal defect (/1.01)
gabapentin (-0.00101)
Solitary nodule of lung (x1.01)
Erythrocyte distribution width [V] by AC (-0.109)
Pain due to neoplastic disease (x1.01)
Dependence on respirator (x1.01)
Respiratory failure (/1.01)
Creatinine [M/V] in SP (+0.0155)
High risk pregnancy (x1.01)
Protein [M/V] in SP (+0.012)
omeprazole (-0.0009)
Erythrocyte distribution width [Ratio] by AC (-0.0276)
INR in Platelet poor plasma by Coagulation assay (+0.0565)
Platelets [#/V] in B by AC (+1.6)
Immature granulocytes [#/V] in B by AC (+0.00107)
Respiratory distress syndrome in the newborn (/1.01)
Phosphate [M/V] in SP (-0.0201)
Shock (/1.01)
Neutrophils/100 leukocytes in B by AC (+0.233)
Chloride [mol/V] in B (+0.0849)
Hypertensive emergency (x1.01)
ECG normal (x1.01)
Loss of consciousness (x1.01)
Dizziness and giddiness (x1.01)
Third trimester pregnancy (x1.01)
Difficulty walking (x1.01)
Traumatic shock (x1.01)
Cough (/1.01)
Disorder of pregnancy (x1.01)
Infective pneumonia (/1.01)
Atherosclerosis of coronary artery without angina pectoris (x1.01)
Acute respiratory failure (/1.01)
Anemia of prematurity (/1.01)
Congenital heart disease (/1.01)
Cerebral edema (x1.01)
Atrial fibrillation (/1.01)
Major depression, single episode (x1.01)
Disorder of kidney and/or ureter (x1.01)
Type 2 diabetes mellitus without complication (x1.01)
Cirrhosis of liver (/1.01)
Postprocedural respiratory failure (/1.01)
Second trimester pregnancy (x1.01)
Complication of transplanted liver (/1.01)
0.562
0.793
0.841
0.883
0.893
0.900
0.906
0.909
0.912
0.915
0.918
0.920
0.922
0.925
0.926
0.928
0.930
0.931
0.932
0.934
0.935
0.936
0.936
0.937
0.938
0.939
0.939
0.940
0.940
0.941
0.942
0.942
0.943
0.943
0.944
0.944
0.945
0.945
0.946
0.946
0.947
0.947
0.948
0.948
0.949
0.949
0.949
0.950
0.950
0.951
0.951
0.951
0.952
0.952
0.952
0.953
0.953
0.953
0.954
0.954
0.954
0.954
0.955
0.955
0.955
0.956
0.956
0.956
0.956
0.957
0.957
0.957
0.957
0.958
0.958
0.958
0.958
0.959
0.959
0.959
0.959
0.959
0.960
0.960
0.960
0.960
0.960
0.961
0.961
0.961
0.961
0.961
0.961
0.962
0.962
0.962
0.962
0.962
0.962
0.963
Expressions
−0.6
−0.4
−0.2
0.0
0.2
0.4
0.6

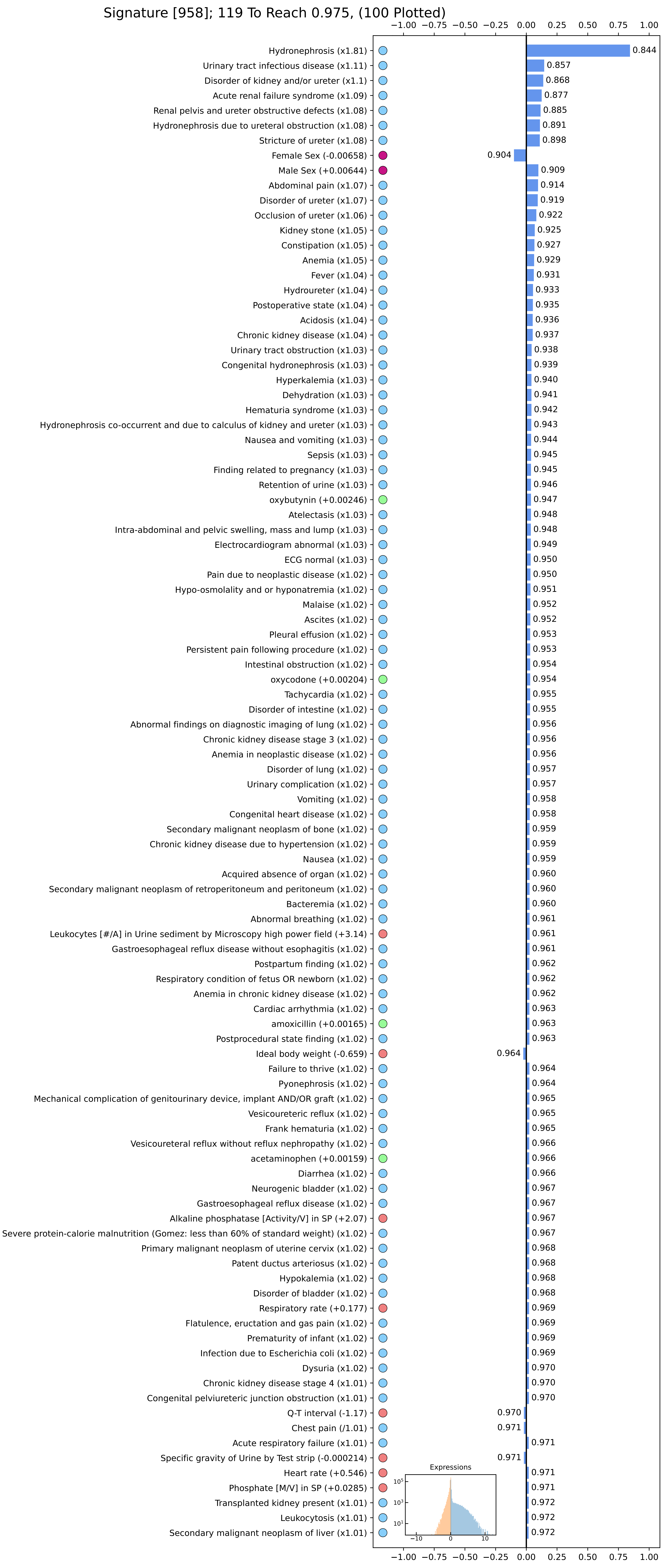

Signature [958]; 119 To Reach 0.975, (100 Plotted)
Hydronephrosis (x1.81) 0.844
Urinary tract infectious disease (x1.11) 0.857
Disorder of kidney and/or ureter (x1.1) 0.868
Acute renal failure syndrome (x1.09) 0.877
Renal pelvis and ureter obstructive defects (x1.08) 0.885
Hydronephrosis due to ureteral obstruction (x1.08) 0.891
Stricture of ureter (x1.08) 0.898
Female Sex (-0.00658) 0.904
Male Sex (+0.00644) 0.909
Abdominal pain (x1.07) 0.914
Disorder of ureter (x1.07) 0.919
Occlusion of ureter (x1.06) 0.922
Kidney stone (x1.05) 0.925
Constipation (x1.05) 0.927
Anemia (x1.05) 0.929
Fever (x1.04) 0.931
Hydroureter (x1.04) 0.933
Postoperative state (x1.04) 0.935
Acidosis (x1.04) 0.936
Chronic kidney disease (x1.04) 0.937
Urinary tract obstruction (x1.03) 0.938
Congenital hydronephrosis (x1.03) 0.939
Hyperkalemia (x1.03) 0.940
Dehydration (x1.03) 0.941
Hematuria syndrome (x1.03) 0.942
Hydronephrosis co-occurrent and due to calculus of kidney and ureter (x1.03) 0.943
Nausea and vomiting (x1.03) 0.944
Sepsis (x1.03) 0.945
Finding related to pregnancy (x1.03) 0.945
Retention of urine (x1.03) 0.946
oxybutynin (+0.00246) 0.947
Atelectasis (x1.03) 0.948
Intra-abdominal and pelvic swelling, mass and lump (x1.03) 0.948
Electrocardiogram abnormal (x1.03) 0.949
ECG normal (x1.03) 0.950
Pain due to neoplastic disease (x1.02) 0.950
Hypo-osmolality and or hyponatremia (x1.02) 0.951
Malaise (x1.02) 0.952
Ascites (x1.02) 0.952
Pleural effusion (x1.02) 0.953
Persistent pain following procedure (x1.02) 0.953
Intestinal obstruction (x1.02) 0.954
oxycodone (+0.00204) 0.954
Tachycardia (x1.02) 0.955
Disorder of intestine (x1.02) 0.955
Abnormal findings on diagnostic imaging of lung (x1.02) 0.956
Chronic kidney disease stage 3 (x1.02) 0.956
Anemia in neoplastic disease (x1.02) 0.956
Disorder of lung (x1.02) 0.957
Urinary complication (x1.02) 0.957
Vomiting (x1.02) 0.958
Congenital heart disease (x1.02) 0.958
Secondary malignant neoplasm of bone (x1.02) 0.959
Chronic kidney disease due to hypertension (x1.02) 0.959
Nausea (x1.02) 0.959
Acquired absence of organ (x1.02) 0.960
Secondary malignant neoplasm of retroperitoneum and peritoneum (x1.02) 0.960
Bacteremia (x1.02) 0.960
Abnormal breathing (x1.02) 0.961
Leukocytes [#/A] in Urine sediment by Microscopy high power field (+3.14) 0.961
Gastroesophageal reflux disease without esophagitis (x1.02) 0.961
Postpartum finding (x1.02) 0.962
Respiratory condition of fetus OR newborn (x1.02) 0.962
Anemia in chronic kidney disease (x1.02) 0.962
Cardiac arrhythmia (x1.02) 0.963
amoxicillin (+0.00165) 0.963
Postprocedural state finding (x1.02) 0.963
Ideal body weight (-0.659) 0.964
Failure to thrive (x1.02) 0.964
Pyonephrosis (x1.02) 0.964
Mechanical complication of genitourinary device, implant AND/OR graft (x1.02) 0.965
Vesicoureteric reflux (x1.02) 0.965
Frank hematuria (x1.02) 0.965
Vesicoureteral reflux without reflux nephropathy (x1.02) 0.966
acetaminophen (+0.00159) 0.966
Diarrhea (x1.02) 0.966
Neurogenic bladder (x1.02) 0.967
Gastroesophageal reflux disease (x1.02) 0.967
Alkaline phosphatase [Activity/V] in SP (+2.07) 0.967
Severe protein-calorie malnutrition (Gomez: less than 60% of standard weight) (x1.02) 0.967
Primary malignant neoplasm of uterine cervix (x1.02) 0.968
Patent ductus arteriosus (x1.02) 0.968
Hypokalemia (x1.02) 0.968
Disorder of bladder (x1.02) 0.968
Respiratory rate (+0.177) 0.969
Flatulence, eructation and gas pain (x1.02) 0.969
Prematurity of infant (x1.02) 0.969
Infection due to Escherichia coli (x1.02) 0.969
Dysuria (x1.02) 0.970
Chronic kidney disease stage 4 (x1.01) 0.970
Congenital pelviureteric junction obstruction (x1.01) 0.970
Q-T interval (-1.17) 0.970
Chest pain (/1.01) 0.971
Acute respiratory failure (x1.01) 0.971
Specific gravity of Urine by Test strip (-0.000214) 0.971
Heart rate (+0.546) 0.971
Phosphate [M/V] in SP (+0.0285) 0.971
Transplanted kidney present (x1.01) 0.972
Leukocytosis (x1.01) 0.972
Secondary malignant neoplasm of liver (x1.01) 0.972
−1.00 −0.75 −0.50 −0.25 0.00 0.25 0.50 0.75 1.00
Expressions
10^5
10^3
10^1
−10 0 10

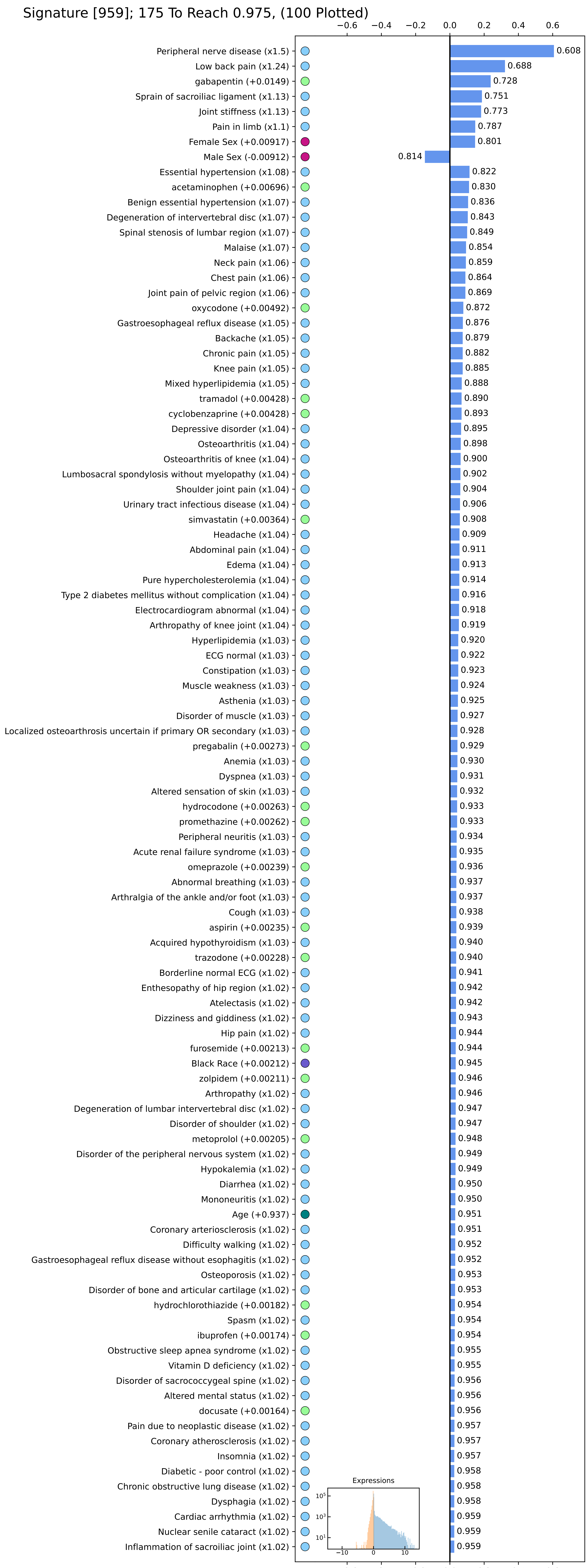
Signature [959]; 175 To Reach 0.975, (100 Plotted)
Peripheral nerve disease (x1.5) 0.608
Low back pain (x1.24) 0.688
gabapentin (+0.0149) 0.728
Sprain of sacroiliac ligament (x1.13) 0.751
Joint stiffness (x1.13) 0.773
Pain in limb (x1.1) 0.787
Female Sex (+0.00917) 0.801
Male Sex (-0.00912) 0.814
Essential hypertension (x1.08) 0.822
acetaminophen (+0.00696) 0.830
Benign essential hypertension (x1.07) 0.836
Degeneration of intervertebral disc (x1.07) 0.843
Spinal stenosis of lumbar region (x1.07) 0.849
Malaise (x1.07) 0.854
Neck pain (x1.06) 0.859
Chest pain (x1.06) 0.864
Joint pain of pelvic region (x1.06) 0.869
oxycodone (+0.00492) 0.872
Gastroesophageal reflux disease (x1.05) 0.876
Backache (x1.05) 0.879
Chronic pain (x1.05) 0.882
Knee pain (x1.05) 0.885
Mixed hyperlipidemia (x1.05) 0.888
tramadol (+0.00428) 0.890
cyclobenzaprine (+0.00428) 0.893
Depressive disorder (x1.04) 0.895
Osteoarthritis (x1.04) 0.898
Osteoarthritis of knee (x1.04) 0.900
Lumbosacral spondylosis without myelopathy (x1.04) 0.902
Shoulder joint pain (x1.04) 0.904
Urinary tract infectious disease (x1.04) 0.906
simvastatin (+0.00364) 0.908
Headache (x1.04) 0.909
Abdominal pain (x1.04) 0.911
Edema (x1.04) 0.913
Pure hypercholesterolemia (x1.04) 0.914
Type 2 diabetes mellitus without complication (x1.04) 0.916
Electrocardiogram abnormal (x1.04) 0.918
Arthropathy of knee joint (x1.04) 0.919
Hyperlipidemia (x1.03) 0.920
ECG normal (x1.03) 0.922
Constipation (x1.03) 0.923
Muscle weakness (x1.03) 0.924
Asthenia (x1.03) 0.925
Disorder of muscle (x1.03) 0.927
Localized osteoarthrosis uncertain if primary OR secondary (x1.03) 0.928
pregabalin (+0.00273) 0.929
Anemia (x1.03) 0.930
Dyspnea (x1.03) 0.931
Altered sensation of skin (x1.03) 0.932
hydrocodone (+0.00263) 0.933
promethazine (+0.00262) 0.933
Peripheral neuritis (x1.03) 0.934
Acute renal failure syndrome (x1.03) 0.935
omeprazole (+0.00239) 0.936
Abnormal breathing (x1.03) 0.937
Arthralgia of the ankle and/or foot (x1.03) 0.937
Cough (x1.03) 0.938
aspirin (+0.00235) 0.939
Acquired hypothyroidism (x1.03) 0.940
trazodone (+0.00228) 0.940
Borderline normal ECG (x1.02) 0.941
Enthesopathy of hip region (x1.02) 0.942
Atelectasis (x1.02) 0.942
Dizziness and giddiness (x1.02) 0.943
Hip pain (x1.02) 0.944
furosemide (+0.00213) 0.944
Black Race (+0.00212) 0.945
zolpidem (+0.00211) 0.946
Arthropathy (x1.02) 0.946
Degeneration of lumbar intervertebral disc (x1.02) 0.947
Disorder of shoulder (x1.02) 0.947
metoprolol (+0.00205) 0.948
Disorder of the peripheral nervous system (x1.02) 0.949
Hypokalemia (x1.02) 0.949
Diarrhea (x1.02) 0.950
Mononeuritis (x1.02) 0.950
Age (+0.937) 0.951
Coronary arteriosclerosis (x1.02) 0.951
Difficulty walking (x1.02) 0.952
Gastroesophageal reflux disease without esophagitis (x1.02) 0.952
Osteoporosis (x1.02) 0.953
Disorder of bone and articular cartilage (x1.02) 0.953
hydrochlorothiazide (+0.00182) 0.954
Spasm (x1.02) 0.954
ibuprofen (+0.00174) 0.954
Obstructive sleep apnea syndrome (x1.02) 0.955
Vitamin D deficiency (x1.02) 0.955
Disorder of sacrococcygeal spine (x1.02) 0.956
Altered mental status (x1.02) 0.956
docusate (+0.00164) 0.956
Pain due to neoplastic disease (x1.02) 0.957
Coronary atherosclerosis (x1.02) 0.957
Insomnia (x1.02) 0.957
Diabetic - poor control (x1.02) 0.958
Chronic obstructive lung disease (x1.02) 0.958
Dysphagia (x1.02) 0.958
Cardiac arrhythmia (x1.02) 0.959
Nuclear senile cataract (x1.02) 0.959
Inflammation of sacroiliac joint (x1.02) 0.959
−0.6 −0.4 −0.2 0.0 0.2 0.4 0.6
Expressions
10^5
10^3
10^1
−10 0 10

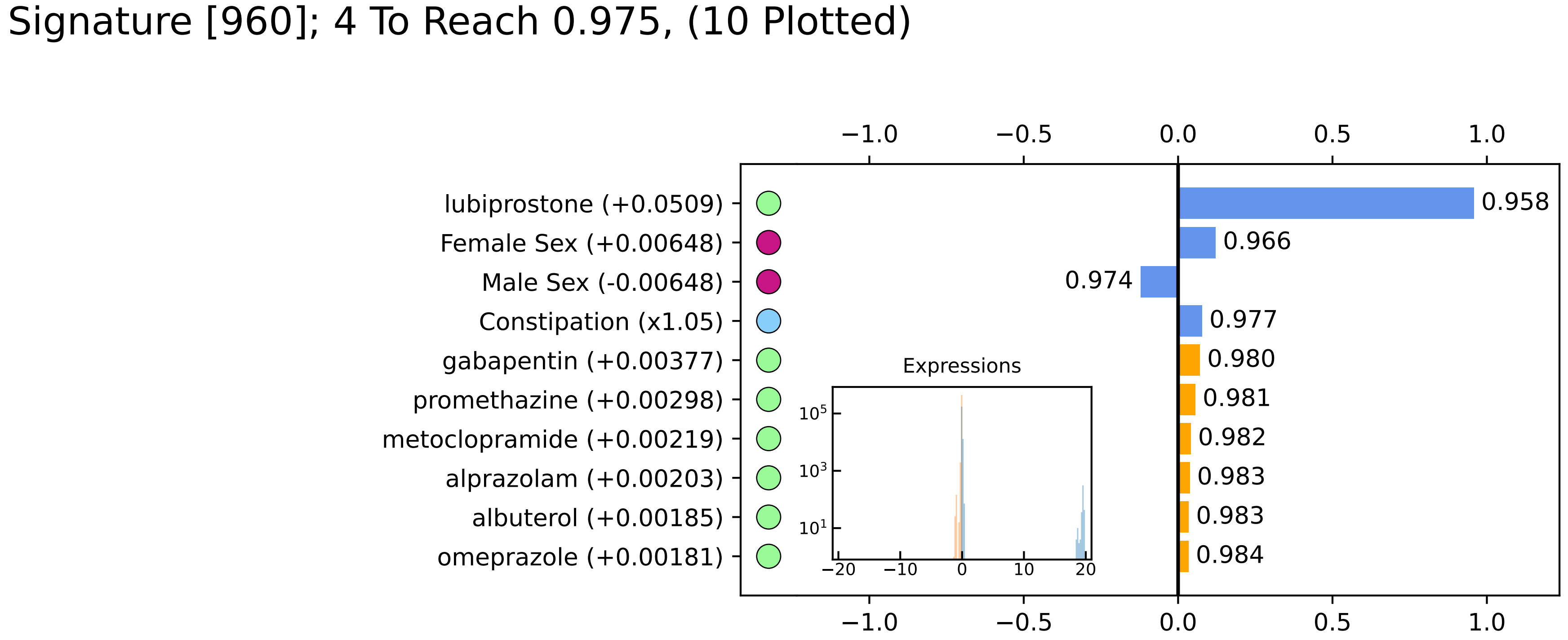

Signature [960]; 4 To Reach 0.975, (10 Plotted)
lubiprostone (+0.0509)
Female Sex (+0.00648)
Male Sex (-0.00648)
Constipation (x1.05)
gabapentin (+0.00377)
promethazine (+0.00298)
metoclopramide (+0.00219)
alprazolam (+0.00203)
albuterol (+0.00185)
omeprazole (+0.00181)
0.958
0.966
0.974
0.977
0.980
0.981
0.982
0.983
0.983
0.984
−1.0
−0.5
0.0
0.5
1.0
Expressions
10^5
10^3
10^1
−20
−10
0
10
20

Signature [961]; 38 To Reach 0.975, (38 Plotted)

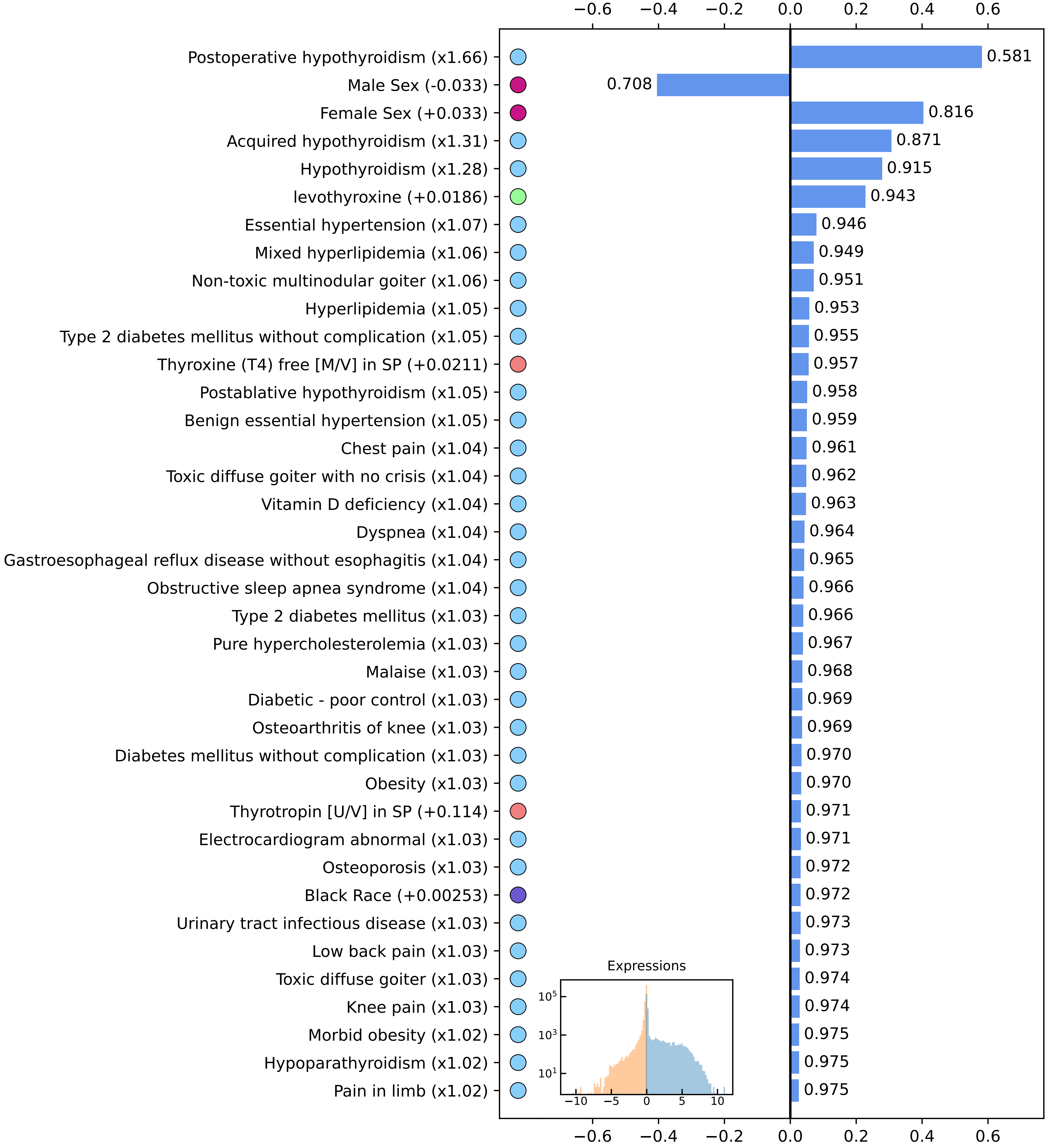

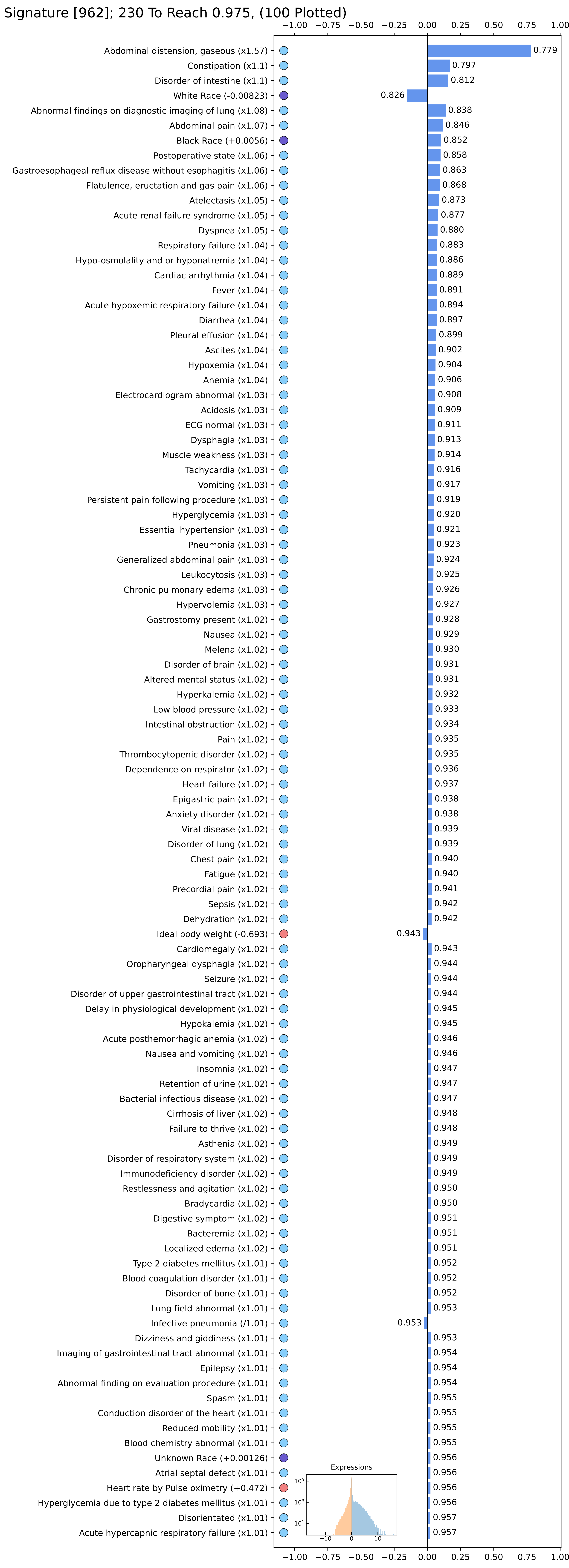

Signature [962]; 230 To Reach 0.975, (100 Plotted)
Abdominal distension, gaseous (x1.57) 0.779
Constipation (x1.1) 0.797
Disorder of intestine (x1.1) 0.812
White Race (-0.00823) 0.826
Abnormal findings on diagnostic imaging of lung (x1.08) 0.838
Abdominal pain (x1.07) 0.846
Black Race (+0.0056) 0.852
Postoperative state (x1.06) 0.858
Gastroesophageal reflux disease without esophagitis (x1.06) 0.863
Flatulence, eructation and gas pain (x1.06) 0.868
Atelectasis (x1.05) 0.873
Acute renal failure syndrome (x1.05) 0.877
Dyspnea (x1.05) 0.880
Respiratory failure (x1.04) 0.883
Hypo-osmolality and or hyponatremia (x1.04) 0.886
Cardiac arrhythmia (x1.04) 0.889
Fever (x1.04) 0.891
Acute hypoxemic respiratory failure (x1.04) 0.894
Diarrhea (x1.04) 0.897
Pleural effusion (x1.04) 0.899
Ascites (x1.04) 0.902
Hypoxemia (x1.04) 0.904
Anemia (x1.04) 0.906
Electrocardiogram abnormal (x1.03) 0.908
Acidosis (x1.03) 0.909
ECG normal (x1.03) 0.911
Dysphagia (x1.03) 0.913
Muscle weakness (x1.03) 0.914
Tachycardia (x1.03) 0.916
Vomiting (x1.03) 0.917
Persistent pain following procedure (x1.03) 0.919
Hyperglycemia (x1.03) 0.920
Essential hypertension (x1.03) 0.921
Pneumonia (x1.03) 0.923
Generalized abdominal pain (x1.03) 0.924
Leukocytosis (x1.03) 0.925
Chronic pulmonary edema (x1.03) 0.926
Hypervolemia (x1.03) 0.927
Gastrostomy present (x1.02) 0.928
Nausea (x1.02) 0.929
Melena (x1.02) 0.930
Disorder of brain (x1.02) 0.931
Altered mental status (x1.02) 0.931
Hyperkalemia (x1.02) 0.932
Low blood pressure (x1.02) 0.933
Intestinal obstruction (x1.02) 0.934
Pain (x1.02) 0.935
Thrombocytopenic disorder (x1.02) 0.935
Dependence on respirator (x1.02) 0.936
Heart failure (x1.02) 0.937
Epigastric pain (x1.02) 0.938
Anxiety disorder (x1.02) 0.938
Viral disease (x1.02) 0.939
Disorder of lung (x1.02) 0.939
Chest pain (x1.02) 0.940
Fatigue (x1.02) 0.940
Precordial pain (x1.02) 0.941
Sepsis (x1.02) 0.942
Dehydration (x1.02) 0.942
Ideal body weight (-0.693) 0.943
Cardiomegaly (x1.02) 0.943
Oropharyngeal dysphagia (x1.02) 0.944
Seizure (x1.02) 0.944
Disorder of upper gastrointestinal tract (x1.02) 0.944
Delay in physiological development (x1.02) 0.945
Hypokalemia (x1.02) 0.945
Acute posthemorrhagic anemia (x1.02) 0.946
Nausea and vomiting (x1.02) 0.946
Insomnia (x1.02) 0.947
Retention of urine (x1.02) 0.947
Bacterial infectious disease (x1.02) 0.947
Cirrhosis of liver (x1.02) 0.948
Failure to thrive (x1.02) 0.948
Asthenia (x1.02) 0.949
Disorder of respiratory system (x1.02) 0.949
Immunodeficiency disorder (x1.02) 0.949
Restlessness and agitation (x1.02) 0.950
Bradycardia (x1.02) 0.950
Digestive symptom (x1.02) 0.951
Bacteremia (x1.02) 0.951
Localized edema (x1.02) 0.951
Type 2 diabetes mellitus (x1.01) 0.952
Blood coagulation disorder (x1.01) 0.952
Disorder of bone (x1.01) 0.952
Lung field abnormal (x1.01) 0.953
Infective pneumonia (/1.01) 0.953
Dizziness and giddiness (x1.01) 0.953
Imaging of gastrointestinal tract abnormal (x1.01) 0.954
Epilepsy (x1.01) 0.954
Abnormal finding on evaluation procedure (x1.01) 0.954
Spasm (x1.01) 0.955
Conduction disorder of the heart (x1.01) 0.955
Reduced mobility (x1.01) 0.955
Blood chemistry abnormal (x1.01) 0.955
Unknown Race (+0.00126) 0.956
Atrial septal defect (x1.01) 0.956
Heart rate by Pulse oximetry (+0.472) 0.956
Hyperglycemia due to type 2 diabetes mellitus (x1.01) 0.956
Disorientated (x1.01) 0.957
Acute hypercapnic respiratory failure (x1.01) 0.957
Expressions

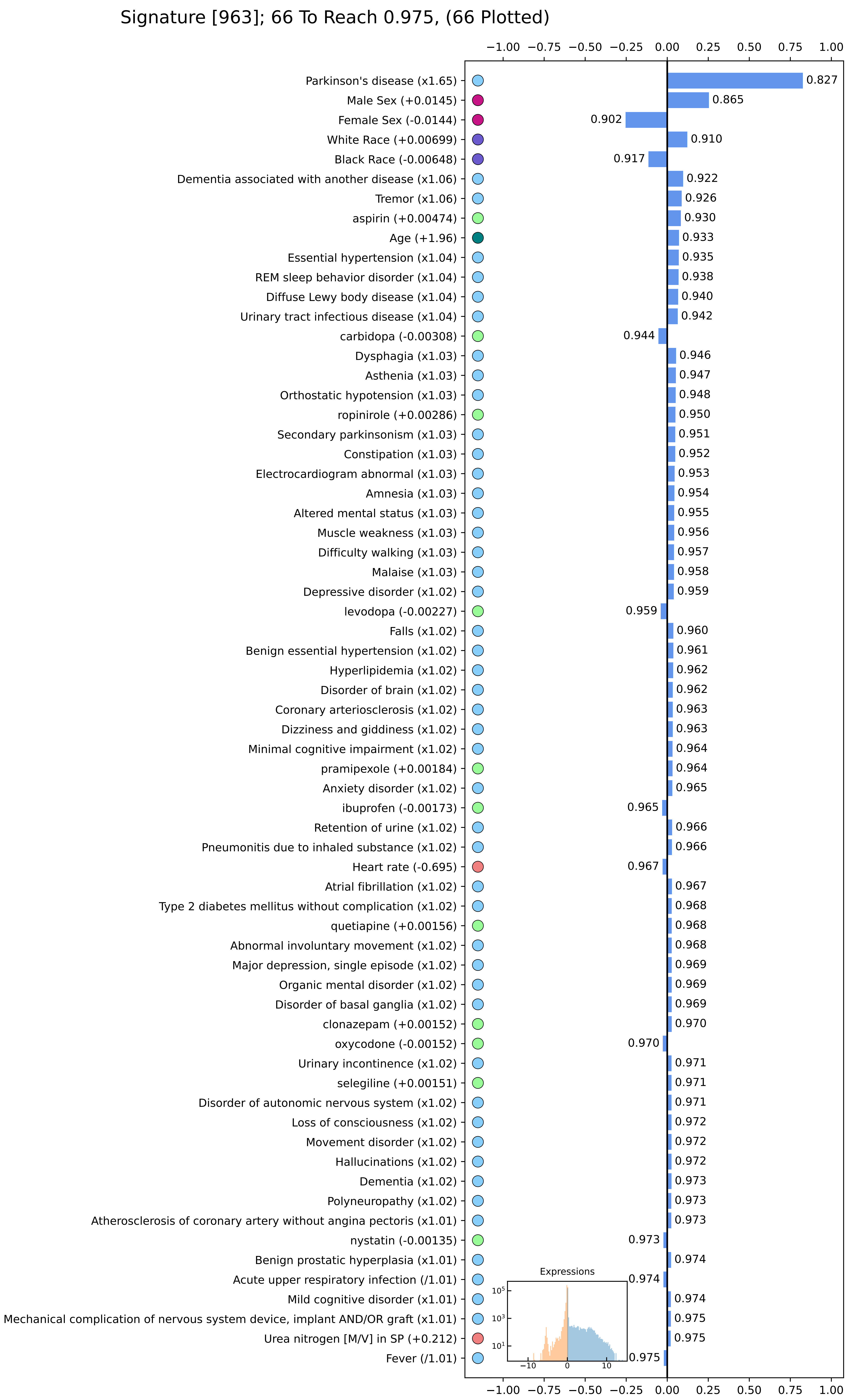

Signature [963]; 66 To Reach 0.975, (66 Plotted)
Parkinson's disease (x1.65) 0.827
Male Sex (+0.0145) 0.865
Female Sex (-0.0144) 0.902
White Race (+0.00699) 0.910
Black Race (-0.00648) 0.917
Dementia associated with another disease (x1.06) 0.922
Tremor (x1.06) 0.926
aspirin (+0.00474) 0.930
Age (+1.96) 0.933
Essential hypertension (x1.04) 0.935
REM sleep behavior disorder (x1.04) 0.938
Diffuse Lewy body disease (x1.04) 0.940
Urinary tract infectious disease (x1.04) 0.942
carbidopa (-0.00308) 0.944
Dysphagia (x1.03) 0.946
Asthenia (x1.03) 0.947
Orthostatic hypotension (x1.03) 0.948
ropinirole (+0.00286) 0.950
Secondary parkinsonism (x1.03) 0.951
Constipation (x1.03) 0.952
Electrocardiogram abnormal (x1.03) 0.953
Amnesia (x1.03) 0.954
Altered mental status (x1.03) 0.955
Muscle weakness (x1.03) 0.956
Difficulty walking (x1.03) 0.957
Malaise (x1.03) 0.958
Depressive disorder (x1.02) 0.959
levodopa (-0.00227) 0.959
Falls (x1.02) 0.960
Benign essential hypertension (x1.02) 0.961
Hyperlipidemia (x1.02) 0.962
Disorder of brain (x1.02) 0.962
Coronary arteriosclerosis (x1.02) 0.963
Dizziness and giddiness (x1.02) 0.963
Minimal cognitive impairment (x1.02) 0.964
pramipexole (+0.00184) 0.964
Anxiety disorder (x1.02) 0.965
ibuprofen (-0.00173) 0.965
Retention of urine (x1.02) 0.966
Pneumonitis due to inhaled substance (x1.02) 0.966
Heart rate (-0.695) 0.967
Atrial fibrillation (x1.02) 0.967
Type 2 diabetes mellitus without complication (x1.02) 0.968
quetiapine (+0.00156) 0.968
Abnormal involuntary movement (x1.02) 0.968
Major depression, single episode (x1.02) 0.969
Organic mental disorder (x1.02) 0.969
Disorder of basal ganglia (x1.02) 0.969
clonazepam (+0.00152) 0.970
oxycodone (-0.00152) 0.970
Urinary incontinence (x1.02) 0.971
selegiline (+0.00151) 0.971
Disorder of autonomic nervous system (x1.02) 0.971
Loss of consciousness (x1.02) 0.972
Movement disorder (x1.02) 0.972
Hallucinations (x1.02) 0.972
Dementia (x1.02) 0.973
Polyneuropathy (x1.02) 0.973
Atherosclerosis of coronary artery without angina pectoris (x1.01) 0.973
nystatin (-0.00135) 0.973
Benign prostatic hyperplasia (x1.01) 0.974
Acute upper respiratory infection (/1.01) 0.974
Mild cognitive disorder (x1.01) 0.974
Mechanical complication of nervous system device, implant AND/OR graft (x1.01) 0.975
Urea nitrogen [M/V] in SP (+0.212) 0.975
Fever (/1.01) 0.975
−1.00 −0.75 −0.50 −0.25 0.00 0.25 0.50 0.75 1.00
Expressions

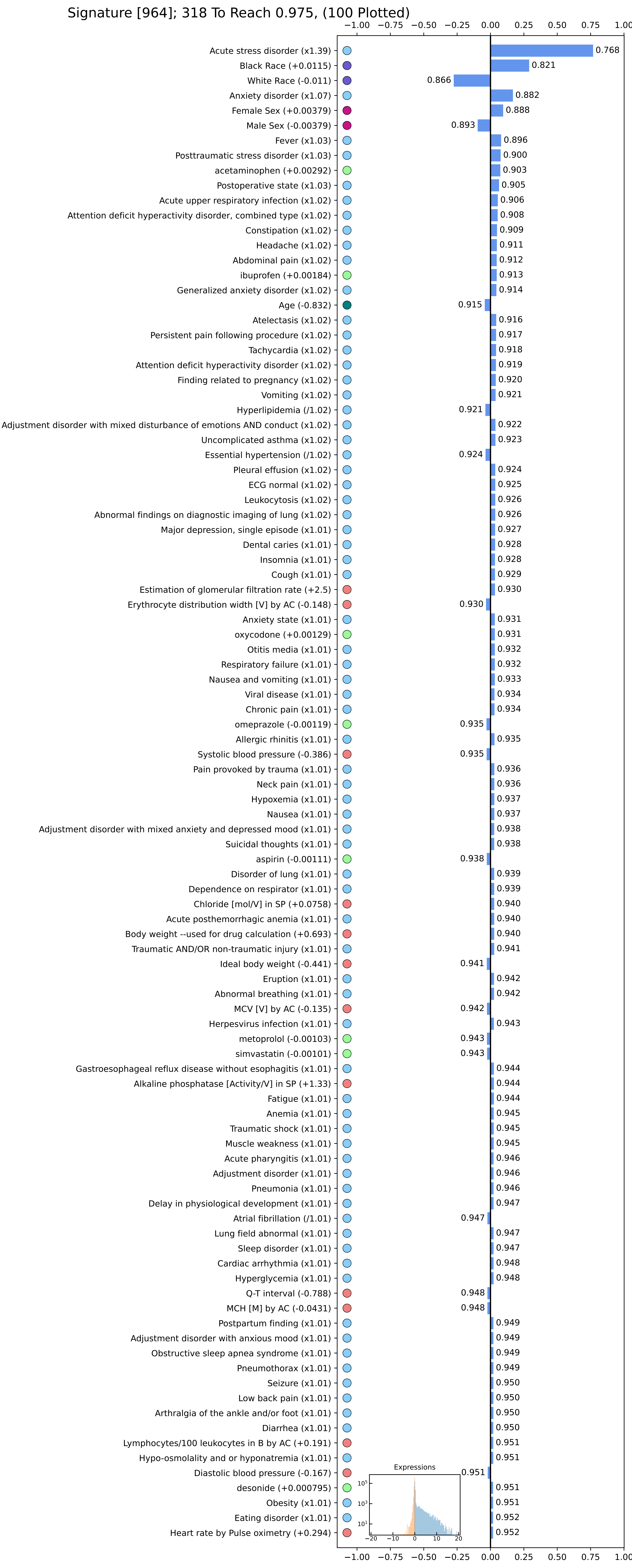

Signature [964]; 318 To Reach 0.975, (100 Plotted)
Acute stress disorder (x1.39) 0.768
Black Race (+0.0115) 0.821
White Race (-0.011) 0.866
Anxiety disorder (x1.07) 0.882
Female Sex (+0.00379) 0.888
Male Sex (-0.00379) 0.893
Fever (x1.03) 0.896
Posttraumatic stress disorder (x1.03) 0.900
acetaminophen (+0.00292) 0.903
Postoperative state (x1.03) 0.905
Acute upper respiratory infection (x1.02) 0.906
Attention deficit hyperactivity disorder, combined type (x1.02) 0.908
Constipation (x1.02) 0.909
Headache (x1.02) 0.911
Abdominal pain (x1.02) 0.912
ibuprofen (+0.00184) 0.913
Generalized anxiety disorder (x1.02) 0.914
Age (-0.832) 0.915
Atelectasis (x1.02) 0.916
Persistent pain following procedure (x1.02) 0.917
Tachycardia (x1.02) 0.918
Attention deficit hyperactivity disorder (x1.02) 0.919
Finding related to pregnancy (x1.02) 0.920
Vomiting (x1.02) 0.921
Hyperlipidemia (/1.02) 0.921
Adjustment disorder with mixed disturbance of emotions AND conduct (x1.02) 0.922
Uncomplicated asthma (x1.02) 0.923
Essential hypertension (/1.02) 0.924
Pleural effusion (x1.02) 0.924
ECG normal (x1.02) 0.925
Leukocytosis (x1.02) 0.926
Abnormal findings on diagnostic imaging of lung (x1.02) 0.926
Major depression, single episode (x1.01) 0.927
Dental caries (x1.01) 0.928
Insomnia (x1.01) 0.928
Cough (x1.01) 0.929
Estimation of glomerular filtration rate (+2.5) 0.930
Erythrocyte distribution width [V] by AC (-0.148) 0.930
Anxiety state (x1.01) 0.931
oxycodone (+0.00129) 0.931
Otitis media (x1.01) 0.932
Respiratory failure (x1.01) 0.932
Nausea and vomiting (x1.01) 0.933
Viral disease (x1.01) 0.934
Chronic pain (x1.01) 0.934
omeprazole (-0.00119) 0.935
Allergic rhinitis (x1.01) 0.935
Systolic blood pressure (-0.386) 0.935
Pain provoked by trauma (x1.01) 0.936
Neck pain (x1.01) 0.936
Hypoxemia (x1.01) 0.937
Nausea (x1.01) 0.937
Adjustment disorder with mixed anxiety and depressed mood (x1.01) 0.938
Suicidal thoughts (x1.01) 0.938
aspirin (-0.00111) 0.938
Disorder of lung (x1.01) 0.939
Dependence on respirator (x1.01) 0.939
Chloride [mol/V] in SP (+0.0758) 0.940
Acute posthemorrhagic anemia (x1.01) 0.940
Body weight --used for drug calculation (+0.693) 0.940
Traumatic AND/OR non-traumatic injury (x1.01) 0.941
Ideal body weight (-0.441) 0.941
Eruption (x1.01) 0.942
Abnormal breathing (x1.01) 0.942
MCV [V] by AC (-0.135) 0.942
Herpesvirus infection (x1.01) 0.943
metoprolol (-0.00103) 0.943
simvastatin (-0.00101) 0.943
Gastroesophageal reflux disease without esophagitis (x1.01) 0.944
Alkaline phosphatase [Activity/V] in SP (+1.33) 0.944
Fatigue (x1.01) 0.944
Anemia (x1.01) 0.945
Traumatic shock (x1.01) 0.945
Muscle weakness (x1.01) 0.945
Acute pharyngitis (x1.01) 0.946
Adjustment disorder (x1.01) 0.946
Pneumonia (x1.01) 0.946
Delay in physiological development (x1.01) 0.947
Atrial fibrillation (/1.01) 0.947
Lung field abnormal (x1.01) 0.947
Sleep disorder (x1.01) 0.947
Cardiac arrhythmia (x1.01) 0.948
Hyperglycemia (x1.01) 0.948
Q-T interval (-0.788) 0.948
MCH [M] by AC (-0.0431) 0.948
Postpartum finding (x1.01) 0.949
Adjustment disorder with anxious mood (x1.01) 0.949
Obstructive sleep apnea syndrome (x1.01) 0.949
Pneumothorax (x1.01) 0.949
Seizure (x1.01) 0.950
Low back pain (x1.01) 0.950
Arthralgia of the ankle and/or foot (x1.01) 0.950
Diarrhea (x1.01) 0.950
Lymphocytes/100 leukocytes in B by AC (+0.191) 0.951
Hypo-osmolality and or hyponatremia (x1.01) 0.951
Diastolic blood pressure (-0.167) 0.951
desonide (+0.000795) 0.951
Obesity (x1.01) 0.951
Eating disorder (x1.01) 0.952
Heart rate by Pulse oximetry (+0.294) 0.952
Expressions
−1.00 −0.75 −0.50 −0.25 0.00 0.25 0.50 0.75 1.00

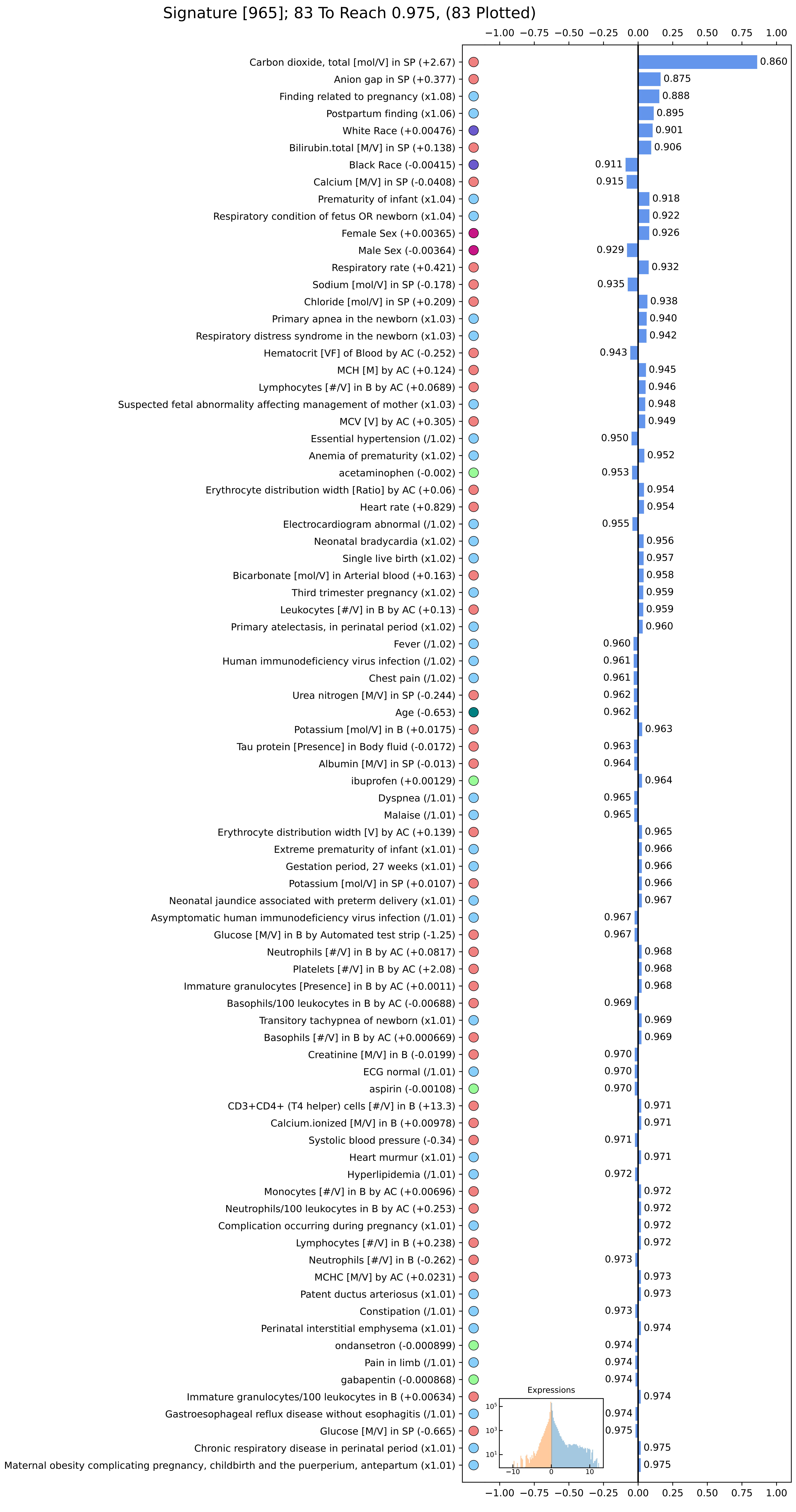
Signature [965]; 83 To Reach 0.975, (83 Plotted)
−1.00 −0.75 −0.50 −0.25 0.00 0.25 0.50 0.75 1.00
Carbon dioxide, total [mol/V] in SP (+2.67) 0.860
Anion gap in SP (+0.377) 0.875
Finding related to pregnancy (x1.08) 0.888
Postpartum finding (x1.06) 0.895
White Race (+0.00476) 0.901
Bilirubin.total [M/V] in SP (+0.138) 0.906
Black Race (-0.00415) 0.911
Calcium [M/V] in SP (-0.0408) 0.915
Prematurity of infant (x1.04) 0.918
Respiratory condition of fetus OR newborn (x1.04) 0.922
Female Sex (+0.00365) 0.926
Male Sex (-0.00364) 0.929
Respiratory rate (+0.421) 0.932
Sodium [mol/V] in SP (-0.178) 0.935
Chloride [mol/V] in SP (+0.209) 0.938
Primary apnea in the newborn (x1.03) 0.940
Respiratory distress syndrome in the newborn (x1.03) 0.942
Hematocrit [VF] of Blood by AC (-0.252) 0.943
MCH [M] by AC (+0.124) 0.945
Lymphocytes [#/V] in B by AC (+0.0689) 0.946
Suspected fetal abnormality affecting management of mother (x1.03) 0.948
MCV [V] by AC (+0.305) 0.949
Essential hypertension (/1.02) 0.950
Anemia of prematurity (x1.02) 0.952
acetaminophen (-0.002) 0.953
Erythrocyte distribution width [Ratio] by AC (+0.06) 0.954
Heart rate (+0.829) 0.954
Electrocardiogram abnormal (/1.02) 0.955
Neonatal bradycardia (x1.02) 0.956
Single live birth (x1.02) 0.957
Bicarbonate [mol/V] in Arterial blood (+0.163) 0.958
Third trimester pregnancy (x1.02) 0.959
Leukocytes [#/V] in B by AC (+0.13) 0.959
Primary atelectasis, in perinatal period (x1.02) 0.960
Fever (/1.02) 0.960
Human immunodeficiency virus infection (/1.02) 0.961
Chest pain (/1.02) 0.961
Urea nitrogen [M/V] in SP (-0.244) 0.962
Age (-0.653) 0.962
Potassium [mol/V] in B (+0.0175) 0.963
Tau protein [Presence] in Body fluid (-0.0172) 0.963
Albumin [M/V] in SP (-0.013) 0.964
ibuprofen (+0.00129) 0.964
Dyspnea (/1.01) 0.965
Malaise (/1.01) 0.965
Erythrocyte distribution width [V] by AC (+0.139) 0.965
Extreme prematurity of infant (x1.01) 0.966
Gestation period, 27 weeks (x1.01) 0.966
Potassium [mol/V] in SP (+0.0107) 0.966
Neonatal jaundice associated with preterm delivery (x1.01) 0.967
Asymptomatic human immunodeficiency virus infection (/1.01) 0.967
Glucose [M/V] in B by Automated test strip (-1.25) 0.967
Neutrophils [#/V] in B by AC (+0.0817) 0.968
Platelets [#/V] in B by AC (+2.08) 0.968
Immature granulocytes [Presence] in B by AC (+0.0011) 0.968
Basophils/100 leukocytes in B by AC (-0.00688) 0.969
Transitory tachypnea of newborn (x1.01) 0.969
Basophils [#/V] in B by AC (+0.000669) 0.969
Creatinine [M/V] in B (-0.0199) 0.970
ECG normal (/1.01) 0.970
aspirin (-0.00108) 0.970
CD3+CD4+ (T4 helper) cells [#/V] in B (+13.3) 0.971
Calcium.ionized [M/V] in B (+0.00978) 0.971
Systolic blood pressure (-0.34) 0.971
Heart murmur (x1.01) 0.971
Hyperlipidemia (/1.01) 0.972
Monocytes [#/V] in B by AC (+0.00696) 0.972
Neutrophils/100 leukocytes in B by AC (+0.253) 0.972
Complication occurring during pregnancy (x1.01) 0.972
Lymphocytes [#/V] in B (+0.238) 0.972
Neutrophils [#/V] in B (-0.262) 0.973
MCHC [M/V] by AC (+0.0231) 0.973
Patent ductus arteriosus (x1.01) 0.973
Constipation (/1.01) 0.973
Perinatal interstitial emphysema (x1.01) 0.974
ondansetron (-0.000899) 0.974
Pain in limb (/1.01) 0.974
gabapentin (-0.000868) 0.974
Immature granulocytes/100 leukocytes in B (+0.00634) 0.974
Gastroesophageal reflux disease without esophagitis (/1.01) 0.974
Glucose [M/V] in SP (-0.665) 0.975
Chronic respiratory disease in perinatal period (x1.01) 0.975
Maternal obesity complicating pregnancy, childbirth and the puerperium, antepartum (x1.01) 0.975
Expressions
10⁵
10³
10¹
−10 0 10
−1.00 −0.75 −0.50 −0.25 0.00 0.25 0.50 0.75 1.00

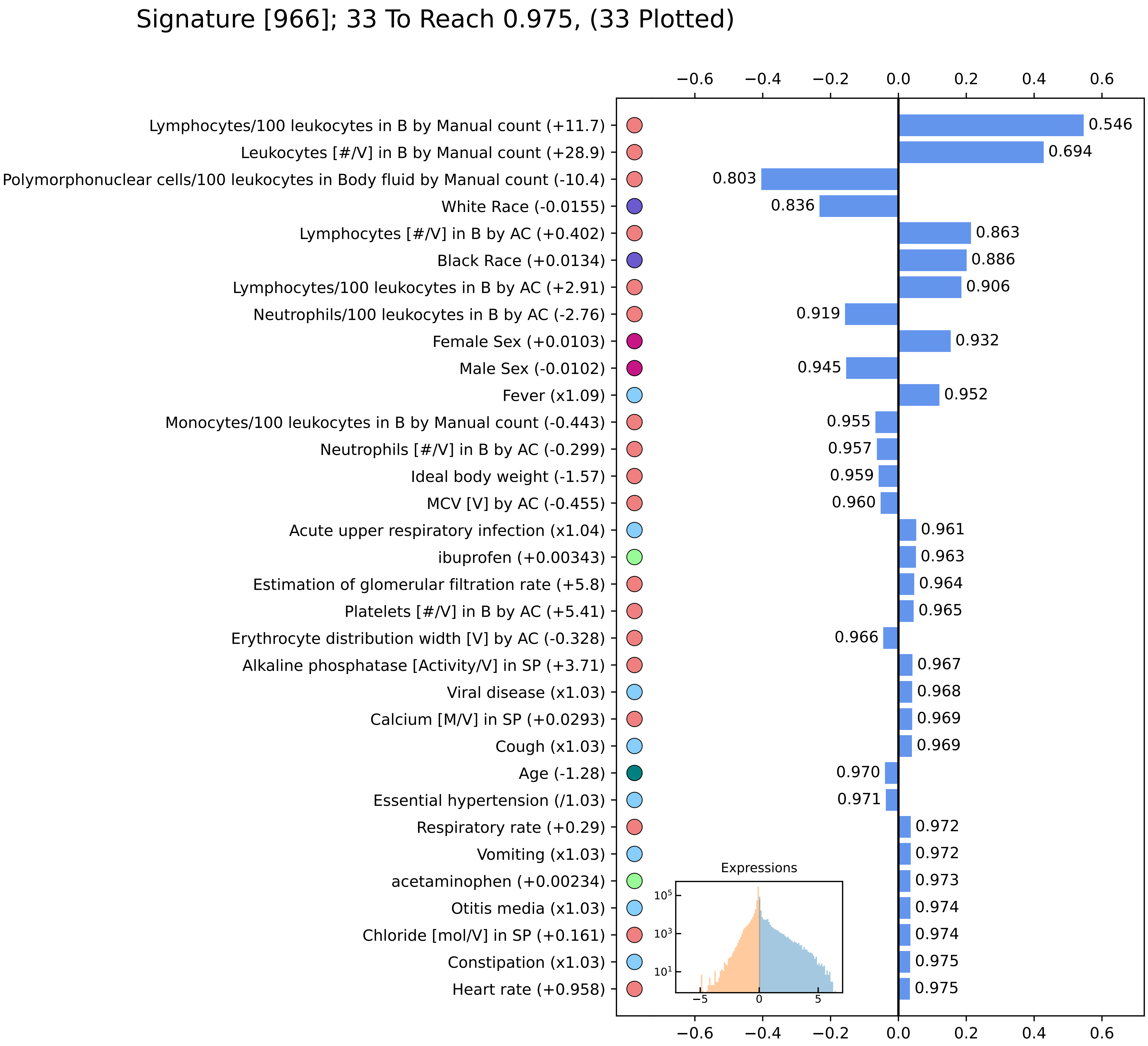

Signature [966]; 33 To Reach 0.975, (33 Plotted)
−0.6
−0.4
−0.2
0.0
0.2
0.4
0.6
Lymphocytes/100 leukocytes in B by Manual count (+11.7)
0.546
Leukocytes [#/V] in B by Manual count (+28.9)
0.694
Polymorphonuclear cells/100 leukocytes in Body fluid by Manual count (-10.4)
0.803
White Race (-0.0155)
0.836
Lymphocytes [#/V] in B by AC (+0.402)
0.863
Black Race (+0.0134)
0.886
Lymphocytes/100 leukocytes in B by AC (+2.91)
0.906
Neutrophils/100 leukocytes in B by AC (-2.76)
0.919
Female Sex (+0.0103)
0.932
Male Sex (-0.0102)
0.945
Fever (x1.09)
0.952
Monocytes/100 leukocytes in B by Manual count (-0.443)
0.955
Neutrophils [#/V] in B by AC (-0.299)
0.957
Ideal body weight (-1.57)
0.959
MCV [V] by AC (-0.455)
0.960
Acute upper respiratory infection (x1.04)
0.961
ibuprofen (+0.00343)
0.963
Estimation of glomerular filtration rate (+5.8)
0.964
Platelets [#/V] in B by AC (+5.41)
0.965
Erythrocyte distribution width [V] by AC (-0.328)
0.966
Alkaline phosphatase [Activity/V] in SP (+3.71)
0.967
Viral disease (x1.03)
0.968
Calcium [M/V] in SP (+0.0293)
0.969
Cough (x1.03)
0.969
Age (-1.28)
0.970
Essential hypertension (/1.03)
0.971
Respiratory rate (+0.29)
0.972
Vomiting (x1.03)
0.972
acetaminophen (+0.00234)
0.973
Otitis media (x1.03)
0.974
Chloride [mol/V] in SP (+0.161)
0.974
Constipation (x1.03)
0.975
Heart rate (+0.958)
0.975
Expressions
10^5
10^3
10^1
−5
0
5

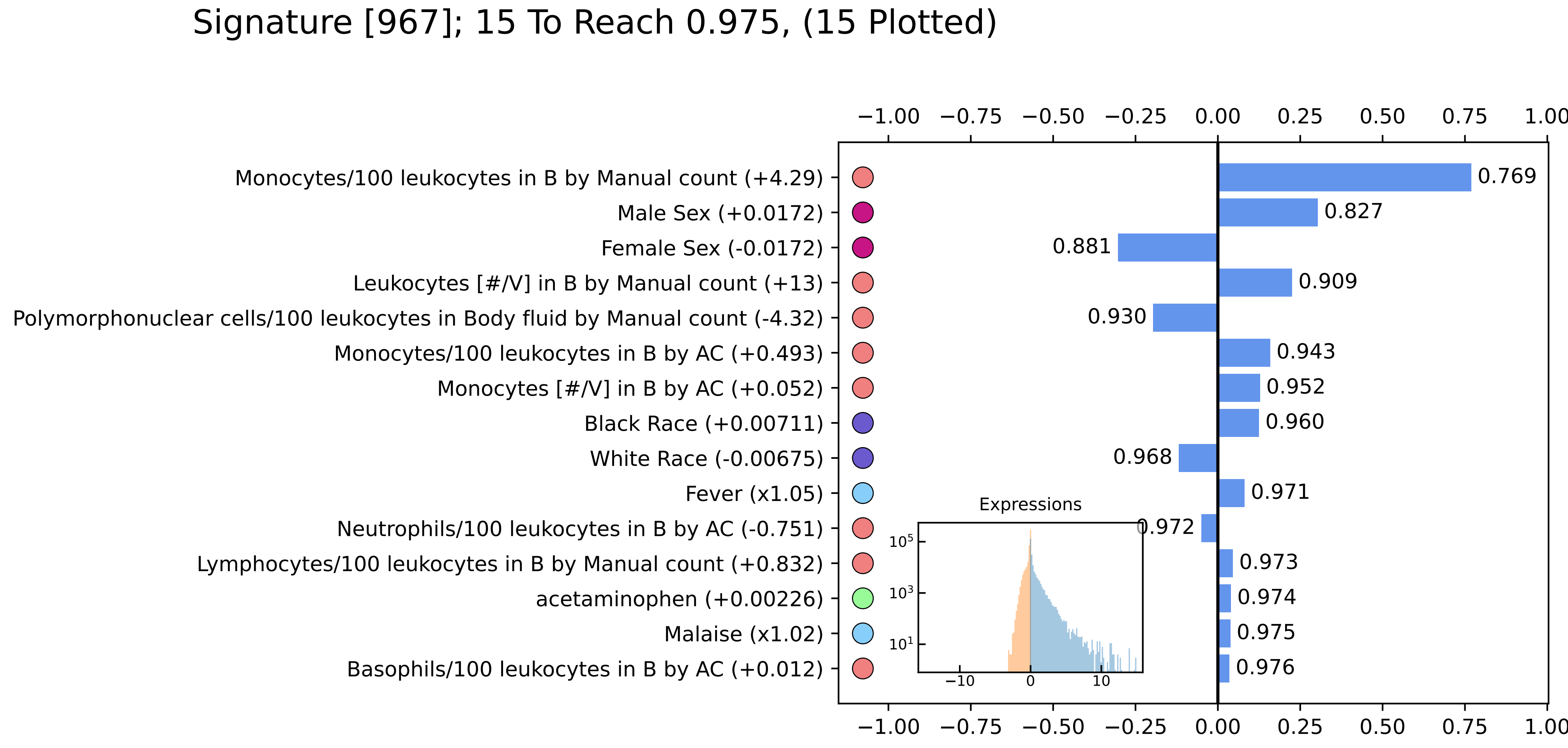

Signature [967]; 15 To Reach 0.975, (15 Plotted)
Monocytes/100 leukocytes in B by Manual count (+4.29)
Male Sex (+0.0172)
Female Sex (-0.0172)
Leukocytes [#/V] in B by Manual count (+13)
Polymorphonuclear cells/100 leukocytes in Body fluid by Manual count (-4.32)
Monocytes/100 leukocytes in B by AC (+0.493)
Monocytes [#/V] in B by AC (+0.052)
Black Race (+0.00711)
White Race (-0.00675)
Fever (x1.05)
Neutrophils/100 leukocytes in B by AC (-0.751)
Lymphocytes/100 leukocytes in B by Manual count (+0.832)
acetaminophen (+0.00226)
Malaise (x1.02)
Basophils/100 leukocytes in B by AC (+0.012)
0.769
0.827
0.881
0.909
0.930
0.943
0.952
0.960
0.968
0.971
0.972
0.973
0.974
0.975
0.976
−1.00 −0.75 −0.50 −0.25 0.00 0.25 0.50 0.75 1.00
Expressions
10^5
10^3
10^1
−10 0 10

Signature [968]; 670 To Reach 0.975, (100 Plotted)

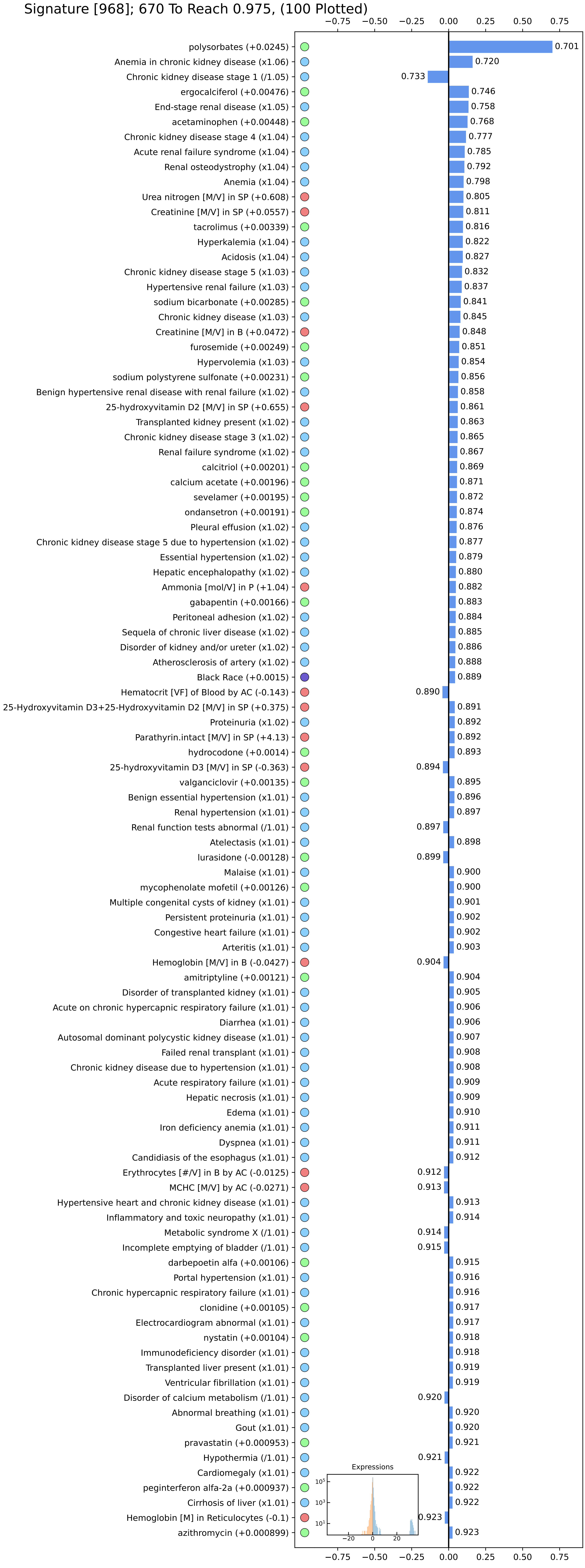

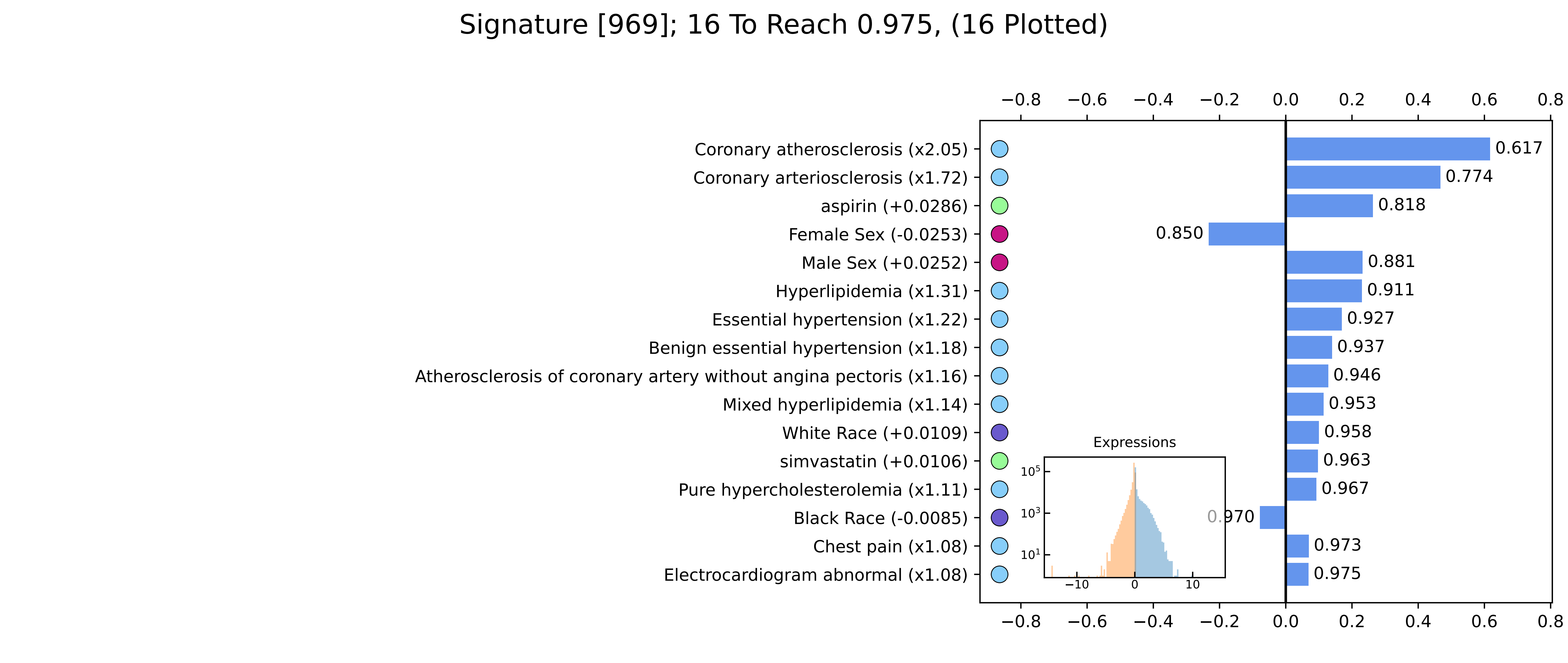
Signature [969]; 16 To Reach 0.975, (16 Plotted)
Coronary atherosclerosis (x2.05)
Coronary arteriosclerosis (x1.72)
aspirin (+0.0286)
Female Sex (-0.0253)
Male Sex (+0.0252)
Hyperlipidemia (x1.31)
Essential hypertension (x1.22)
Benign essential hypertension (x1.18)
Atherosclerosis of coronary artery without angina pectoris (x1.16)
Mixed hyperlipidemia (x1.14)
White Race (+0.0109)
simvastatin (+0.0106)
Pure hypercholesterolemia (x1.11)
Black Race (-0.0085)
Chest pain (x1.08)
Electrocardiogram abnormal (x1.08)
0.617
0.774
0.818
0.850
0.881
0.911
0.927
0.937
0.946
0.953
0.958
0.963
0.967
0.970
0.973
0.975
Expressions
−0.8
−0.6
−0.4
−0.2
0.0
0.2
0.4
0.6
0.8

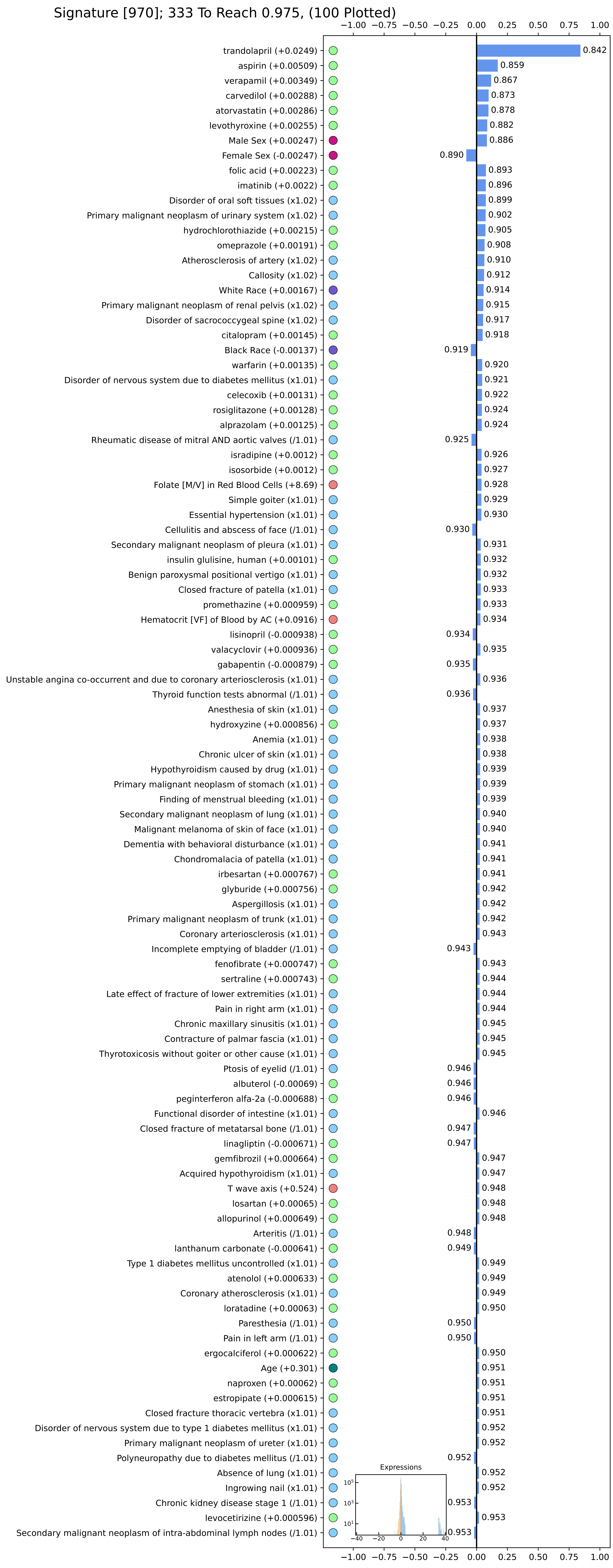

Signature [970]; 333 To Reach 0.975, (100 Plotted)
trandolapril (+0.0249) 0.842
aspirin (+0.00509) 0.859
verapamil (+0.00349) 0.867
carvedilol (+0.00288) 0.873
atorvastatin (+0.00286) 0.878
levothyroxine (+0.00255) 0.882
Male Sex (+0.00247) 0.886
Female Sex (-0.00247) 0.890
folic acid (+0.00223) 0.893
imatinib (+0.0022) 0.896
Disorder of oral soft tissues (x1.02) 0.899
Primary malignant neoplasm of urinary system (x1.02) 0.902
hydrochlorothiazide (+0.00215) 0.905
omeprazole (+0.00191) 0.908
Atherosclerosis of artery (x1.02) 0.910
Callosity (x1.02) 0.912
White Race (+0.00167) 0.914
Primary malignant neoplasm of renal pelvis (x1.02) 0.915
Disorder of sacrococcygeal spine (x1.02) 0.917
citalopram (+0.00145) 0.918
Black Race (-0.00137) 0.919
warfarin (+0.00135) 0.920
Disorder of nervous system due to diabetes mellitus (x1.01) 0.921
celecoxib (+0.00131) 0.922
rosiglitazone (+0.00128) 0.924
alprazolam (+0.00125) 0.924
Rheumatic disease of mitral AND aortic valves (/1.01) 0.925
isradipine (+0.0012) 0.926
isosorbide (+0.0012) 0.927
Folate [M/V] in Red Blood Cells (+8.69) 0.928
Simple goiter (x1.01) 0.929
Essential hypertension (x1.01) 0.930
Cellulitis and abscess of face (/1.01) 0.930
Secondary malignant neoplasm of pleura (x1.01) 0.931
insulin glulisine, human (+0.00101) 0.932
Benign paroxysmal positional vertigo (x1.01) 0.932
Closed fracture of patella (x1.01) 0.933
promethazine (+0.000959) 0.933
Hematocrit [VF] of Blood by AC (+0.0916) 0.934
lisinopril (-0.000938) 0.934
valacyclovir (+0.000936) 0.935
gabapentin (-0.000879) 0.935
Unstable angina co-occurrent and due to coronary arteriosclerosis (x1.01) 0.936
Thyroid function tests abnormal (/1.01) 0.936
Anesthesia of skin (x1.01) 0.937
hydroxyzine (+0.000856) 0.937
Anemia (x1.01) 0.938
Chronic ulcer of skin (x1.01) 0.938
Hypothyroidism caused by drug (x1.01) 0.939
Primary malignant neoplasm of stomach (x1.01) 0.939
Finding of menstrual bleeding (x1.01) 0.939
Secondary malignant neoplasm of lung (x1.01) 0.940
Malignant melanoma of skin of face (x1.01) 0.940
Dementia with behavioral disturbance (x1.01) 0.941
Chondromalacia of patella (x1.01) 0.941
irbesartan (+0.000767) 0.941
glyburide (+0.000756) 0.942
Aspergillosis (x1.01) 0.942
Primary malignant neoplasm of trunk (x1.01) 0.942
Coronary arteriosclerosis (x1.01) 0.943
Incomplete emptying of bladder (/1.01) 0.943
fenofibrate (+0.000747) 0.943
sertraline (+0.000743) 0.944
Late effect of fracture of lower extremities (x1.01) 0.944
Pain in right arm (x1.01) 0.944
Chronic maxillary sinusitis (x1.01) 0.945
Contracture of palmar fascia (x1.01) 0.945
Thyrotoxicosis without goiter or other cause (x1.01) 0.945
Ptosis of eyelid (/1.01) 0.946
albuterol (-0.00069) 0.946
peginterferon alfa-2a (-0.000688) 0.946
Functional disorder of intestine (x1.01) 0.946
Closed fracture of metatarsal bone (/1.01) 0.947
linagliptin (-0.000671) 0.947
gemfibrozil (+0.000664) 0.947
Acquired hypothyroidism (x1.01) 0.947
T wave axis (+0.524) 0.948
losartan (+0.00065) 0.948
allopurinol (+0.000649) 0.948
Arteritis (/1.01) 0.948
lanthanum carbonate (-0.000641) 0.949
Type 1 diabetes mellitus uncontrolled (x1.01) 0.949
atenolol (+0.000633) 0.949
Coronary atherosclerosis (x1.01) 0.949
loratadine (+0.00063) 0.950
Paresthesia (/1.01) 0.950
Pain in left arm (/1.01) 0.950
ergocalciferol (+0.000622) 0.950
Age (+0.301) 0.951
naproxen (+0.00062) 0.951
estropipate (+0.000615) 0.951
Closed fracture thoracic vertebra (x1.01) 0.951
Disorder of nervous system due to type 1 diabetes mellitus (x1.01) 0.952
Primary malignant neoplasm of ureter (x1.01) 0.952
Polyneuropathy due to diabetes mellitus (/1.01) 0.952
Absence of lung (x1.01) 0.952
Ingrowing nail (x1.01) 0.952
Chronic kidney disease stage 1 (/1.01) 0.953
levocetirizine (+0.000596) 0.953
Secondary malignant neoplasm of intra-abdominal lymph nodes (/1.01) 0.953
−1.00 −0.75 −0.50 −0.25 0.00 0.25 0.50 0.75 1.00
Expressions

Signature [971]; 65 To Reach 0.975, (65 Plotted)

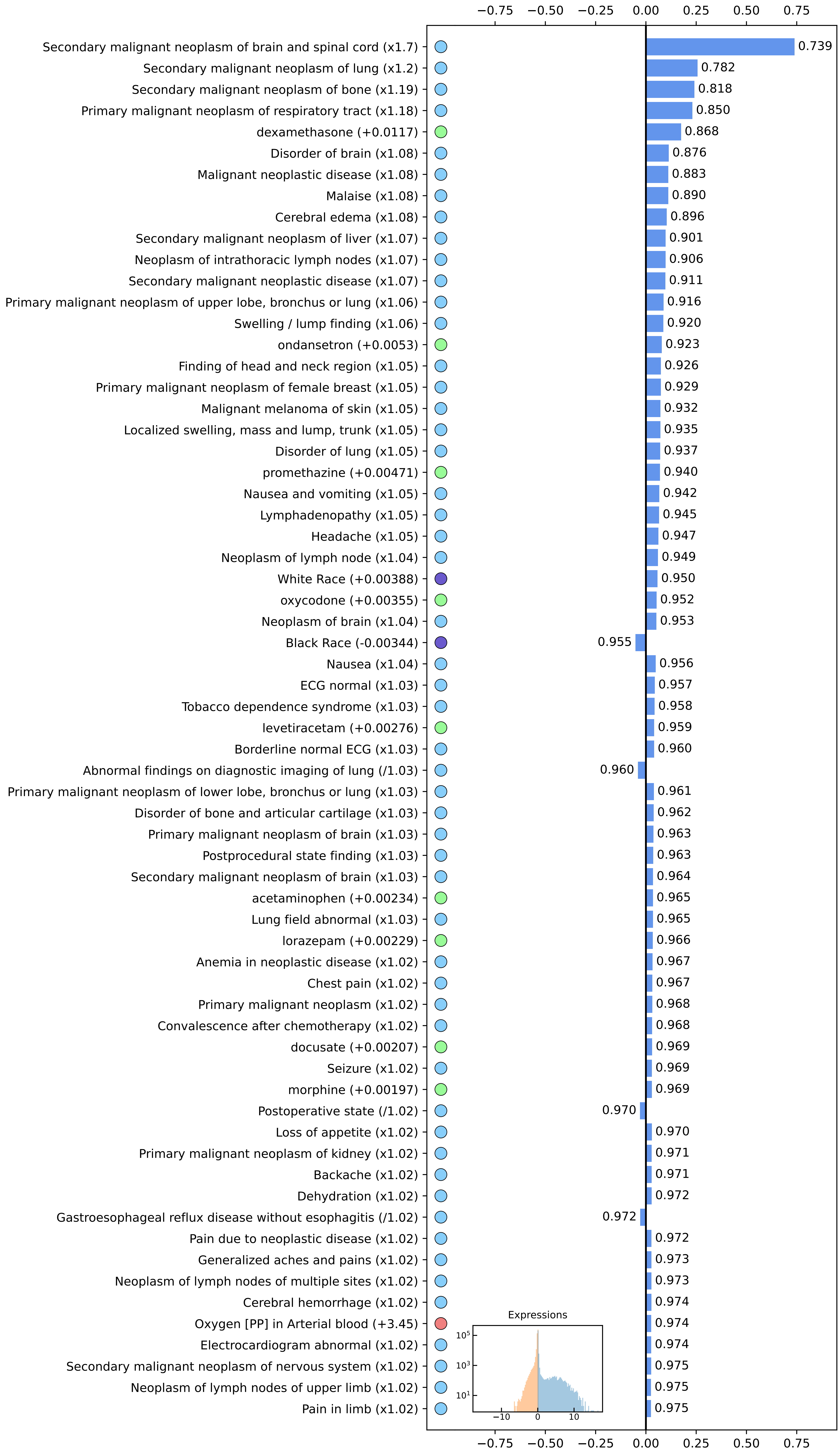

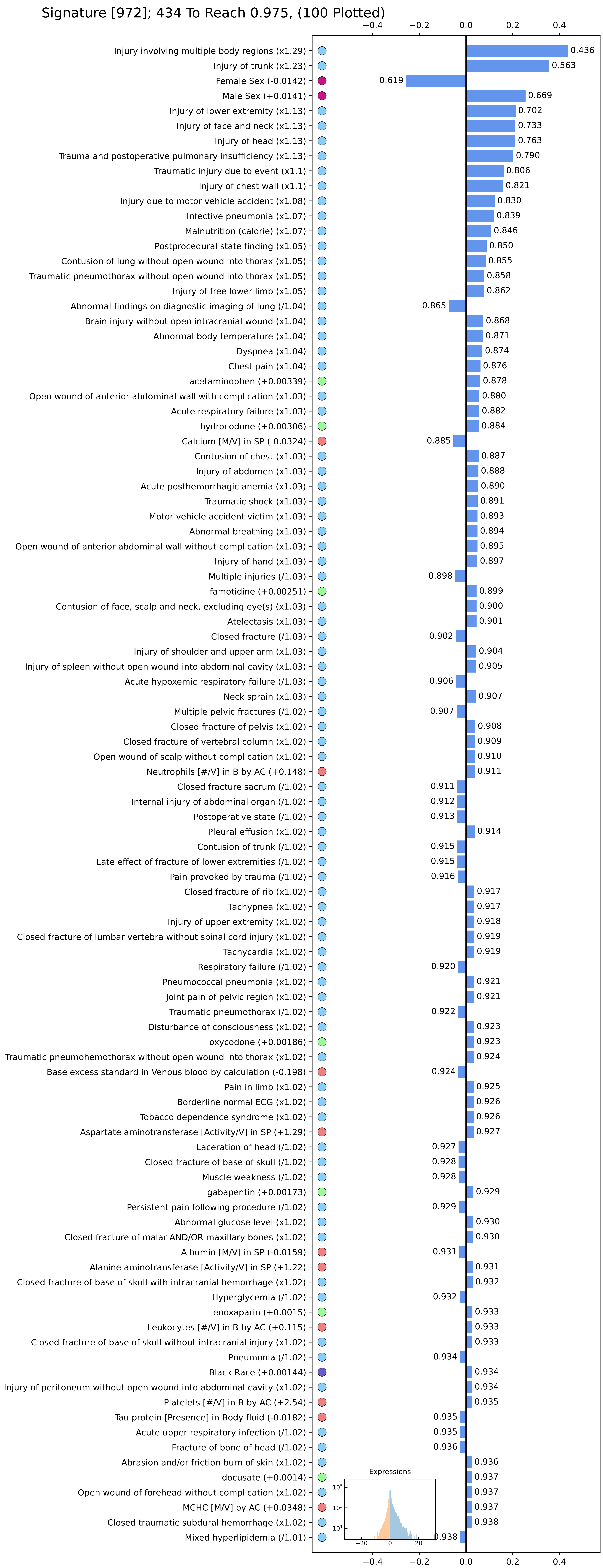

Signature [972]; 434 To Reach 0.975, (100 Plotted)
Injury involving multiple body regions (x1.29) 0.436
Injury of trunk (x1.23) 0.563
Female Sex (-0.0142) 0.619
Male Sex (+0.0141) 0.669
Injury of lower extremity (x1.13) 0.702
Injury of face and neck (x1.13) 0.733
Injury of head (x1.13) 0.763
Trauma and postoperative pulmonary insufficiency (x1.13) 0.790
Traumatic injury due to event (x1.1) 0.806
Injury of chest wall (x1.1) 0.821
Injury due to motor vehicle accident (x1.08) 0.830
Infective pneumonia (x1.07) 0.839
Malnutrition (calorie) (x1.07) 0.846
Postprocedural state finding (x1.05) 0.850
Contusion of lung without open wound into thorax (x1.05) 0.855
Traumatic pneumothorax without open wound into thorax (x1.05) 0.858
Injury of free lower limb (x1.05) 0.862
Abnormal findings on diagnostic imaging of lung (/1.04) 0.865
Brain injury without open intracranial wound (x1.04) 0.868
Abnormal body temperature (x1.04) 0.871
Dyspnea (x1.04) 0.874
Chest pain (x1.04) 0.876
acetaminophen (+0.00339) 0.878
Open wound of anterior abdominal wall with complication (x1.03) 0.880
Acute respiratory failure (x1.03) 0.882
hydrocodone (+0.00306) 0.884
Calcium [M/V] in SP (-0.0324) 0.885
Contusion of chest (x1.03) 0.887
Injury of abdomen (x1.03) 0.888
Acute posthemorrhagic anemia (x1.03) 0.890
Traumatic shock (x1.03) 0.891
Motor vehicle accident victim (x1.03) 0.893
Abnormal breathing (x1.03) 0.894
Open wound of anterior abdominal wall without complication (x1.03) 0.895
Injury of hand (x1.03) 0.897
Multiple injuries (/1.03) 0.898
famotidine (+0.00251) 0.899
Contusion of face, scalp and neck, excluding eye(s) (x1.03) 0.900
Atelectasis (x1.03) 0.901
Closed fracture (/1.03) 0.902
Injury of shoulder and upper arm (x1.03) 0.904
Injury of spleen without open wound into abdominal cavity (x1.03) 0.905
Acute hypoxemic respiratory failure (/1.03) 0.906
Neck sprain (x1.03) 0.907
Multiple pelvic fractures (/1.02) 0.907
Closed fracture of pelvis (x1.02) 0.908
Closed fracture of vertebral column (x1.02) 0.909
Open wound of scalp without complication (x1.02) 0.910
Neutrophils [#/V] in B by AC (+0.148) 0.911
Closed fracture sacrum (/1.02) 0.911
Internal injury of abdominal organ (/1.02) 0.912
Postoperative state (/1.02) 0.913
Pleural effusion (x1.02) 0.914
Contusion of trunk (/1.02) 0.915
Late effect of fracture of lower extremities (/1.02) 0.915
Pain provoked by trauma (/1.02) 0.916
Closed fracture of rib (x1.02) 0.917
Tachypnea (x1.02) 0.917
Injury of upper extremity (x1.02) 0.918
Closed fracture of lumbar vertebra without spinal cord injury (x1.02) 0.919
Tachycardia (x1.02) 0.919
Respiratory failure (/1.02) 0.920
Pneumococcal pneumonia (x1.02) 0.921
Joint pain of pelvic region (x1.02) 0.921
Traumatic pneumothorax (/1.02) 0.922
Disturbance of consciousness (x1.02) 0.923
oxycodone (+0.00186) 0.923
Traumatic pneumohemothorax without open wound into thorax (x1.02) 0.924
Base excess standard in Venous blood by calculation (-0.198) 0.924
Pain in limb (x1.02) 0.925
Borderline normal ECG (x1.02) 0.926
Tobacco dependence syndrome (x1.02) 0.926
Aspartate aminotransferase [Activity/V] in SP (+1.29) 0.927
Laceration of head (/1.02) 0.927
Closed fracture of base of skull (/1.02) 0.928
Muscle weakness (/1.02) 0.928
gabapentin (+0.00173) 0.929
Persistent pain following procedure (/1.02) 0.929
Abnormal glucose level (x1.02) 0.930
Closed fracture of malar AND/OR maxillary bones (x1.02) 0.930
Albumin [M/V] in SP (-0.0159) 0.931
Alanine aminotransferase [Activity/V] in SP (+1.22) 0.931
Closed fracture of base of skull with intracranial hemorrhage (x1.02) 0.932
Hyperglycemia (/1.02) 0.932
enoxaparin (+0.0015) 0.933
Leukocytes [#/V] in B by AC (+0.115) 0.933
Closed fracture of base of skull without intracranial injury (x1.02) 0.933
Pneumonia (/1.02) 0.934
Black Race (+0.00144) 0.934
Injury of peritoneum without open wound into abdominal cavity (x1.02) 0.934
Platelets [#/V] in B by AC (+2.54) 0.935
Tau protein [Presence] in Body fluid (-0.0182) 0.935
Acute upper respiratory infection (/1.02) 0.935
Fracture of bone of head (/1.02) 0.936
Abrasion and/or friction burn of skin (x1.02) 0.936
docusate (+0.0014) 0.937
Open wound of forehead without complication (x1.02) 0.937
MCHC [M/V] by AC (+0.0348) 0.937
Closed traumatic subdural hemorrhage (x1.02) 0.938
Mixed hyperlipidemia (/1.01) 0.938
−0.4 −0.2 0.0 0.2 0.4
Expressions
−20 0 20

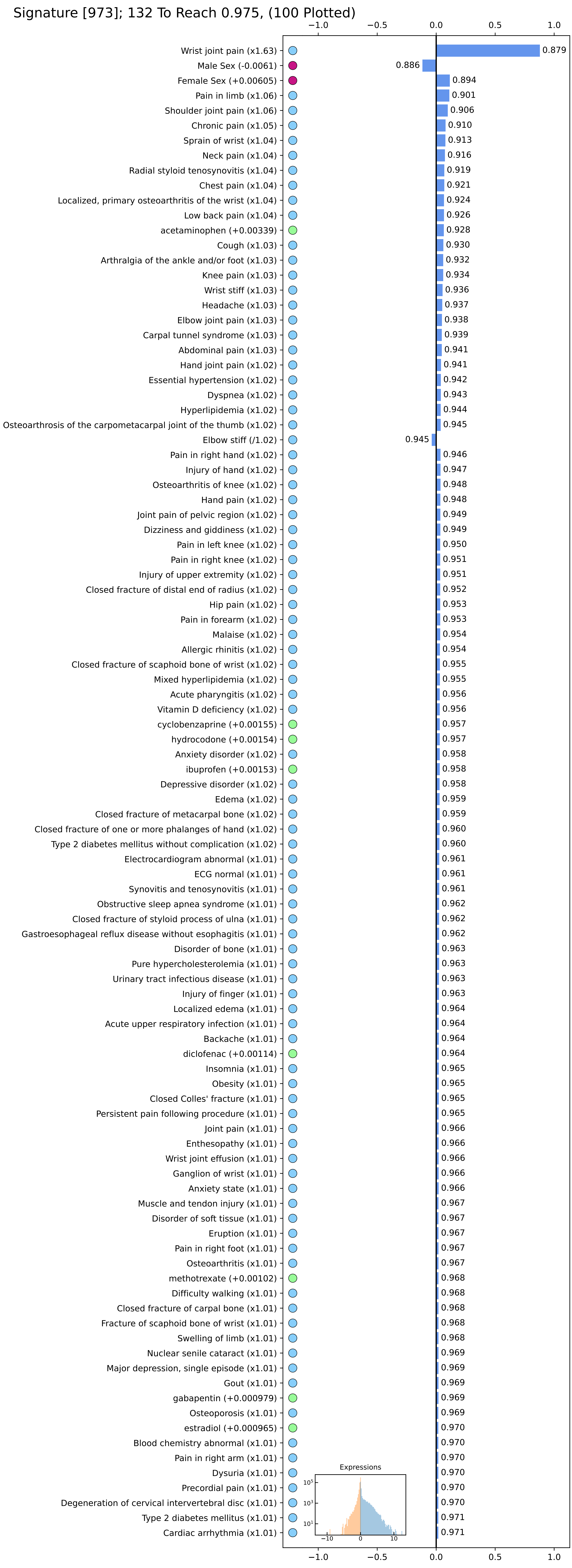
Signature [973]; 132 To Reach 0.975, (100 Plotted)
−1.0 −0.5 0.0 0.5 1.0
Wrist joint pain (x1.63) 0.879
Male Sex (-0.0061) 0.886
Female Sex (+0.00605) 0.894
Pain in limb (x1.06) 0.901
Shoulder joint pain (x1.06) 0.906
Chronic pain (x1.05) 0.910
Sprain of wrist (x1.04) 0.913
Neck pain (x1.04) 0.916
Radial styloid tenosynovitis (x1.04) 0.919
Chest pain (x1.04) 0.921
Localized, primary osteoarthritis of the wrist (x1.04) 0.924
Low back pain (x1.04) 0.926
acetaminophen (+0.00339) 0.928
Cough (x1.03) 0.930
Arthralgia of the ankle and/or foot (x1.03) 0.932
Knee pain (x1.03) 0.934
Wrist stiff (x1.03) 0.936
Headache (x1.03) 0.937
Elbow joint pain (x1.03) 0.938
Carpal tunnel syndrome (x1.03) 0.939
Abdominal pain (x1.03) 0.941
Hand joint pain (x1.02) 0.941
Essential hypertension (x1.02) 0.942
Dyspnea (x1.02) 0.943
Hyperlipidemia (x1.02) 0.944
Osteoarthrosis of the carpometacarpal joint of the thumb (x1.02) 0.945
Elbow stiff (/1.02) 0.945
Pain in right hand (x1.02) 0.946
Injury of hand (x1.02) 0.947
Osteoarthritis of knee (x1.02) 0.948
Hand pain (x1.02) 0.948
Joint pain of pelvic region (x1.02) 0.949
Dizziness and giddiness (x1.02) 0.949
Pain in left knee (x1.02) 0.950
Pain in right knee (x1.02) 0.951
Injury of upper extremity (x1.02) 0.951
Closed fracture of distal end of radius (x1.02) 0.952
Hip pain (x1.02) 0.953
Pain in forearm (x1.02) 0.953
Malaise (x1.02) 0.954
Allergic rhinitis (x1.02) 0.954
Closed fracture of scaphoid bone of wrist (x1.02) 0.955
Mixed hyperlipidemia (x1.02) 0.955
Acute pharyngitis (x1.02) 0.956
Vitamin D deficiency (x1.02) 0.956
cyclobenzaprine (+0.00155) 0.957
hydrocodone (+0.00154) 0.957
Anxiety disorder (x1.02) 0.958
ibuprofen (+0.00153) 0.958
Depressive disorder (x1.02) 0.958
Edema (x1.02) 0.959
Closed fracture of metacarpal bone (x1.02) 0.959
Closed fracture of one or more phalanges of hand (x1.02) 0.960
Type 2 diabetes mellitus without complication (x1.02) 0.960
Electrocardiogram abnormal (x1.01) 0.961
ECG normal (x1.01) 0.961
Synovitis and tenosynovitis (x1.01) 0.961
Obstructive sleep apnea syndrome (x1.01) 0.962
Closed fracture of styloid process of ulna (x1.01) 0.962
Gastroesophageal reflux disease without esophagitis (x1.01) 0.962
Disorder of bone (x1.01) 0.963
Pure hypercholesterolemia (x1.01) 0.963
Urinary tract infectious disease (x1.01) 0.963
Injury of finger (x1.01) 0.963
Localized edema (x1.01) 0.964
Acute upper respiratory infection (x1.01) 0.964
Backache (x1.01) 0.964
diclofenac (+0.00114) 0.964
Insomnia (x1.01) 0.965
Obesity (x1.01) 0.965
Closed Colles' fracture (x1.01) 0.965
Persistent pain following procedure (x1.01) 0.965
Joint pain (x1.01) 0.966
Enthesopathy (x1.01) 0.966
Wrist joint effusion (x1.01) 0.966
Ganglion of wrist (x1.01) 0.966
Anxiety state (x1.01) 0.966
Muscle and tendon injury (x1.01) 0.967
Disorder of soft tissue (x1.01) 0.967
Eruption (x1.01) 0.967
Pain in right foot (x1.01) 0.967
Osteoarthritis (x1.01) 0.967
methotrexate (+0.00102) 0.968
Difficulty walking (x1.01) 0.968
Closed fracture of carpal bone (x1.01) 0.968
Fracture of scaphoid bone of wrist (x1.01) 0.968
Swelling of limb (x1.01) 0.968
Nuclear senile cataract (x1.01) 0.969
Major depression, single episode (x1.01) 0.969
Gout (x1.01) 0.969
gabapentin (+0.000979) 0.969
Osteoporosis (x1.01) 0.969
estradiol (+0.000965) 0.970
Blood chemistry abnormal (x1.01) 0.970
Pain in right arm (x1.01) 0.970
Dysuria (x1.01) 0.970
Precordial pain (x1.01) 0.970
Degeneration of cervical intervertebral disc (x1.01) 0.970
Type 2 diabetes mellitus (x1.01) 0.971
Cardiac arrhythmia (x1.01) 0.971
Expressions
10^5 10^3 10^1
−10 0 10
−1.0 −0.5 0.0 0.5 1.0

Signature [974]; 87 To Reach 0.975, (87 Plotted)

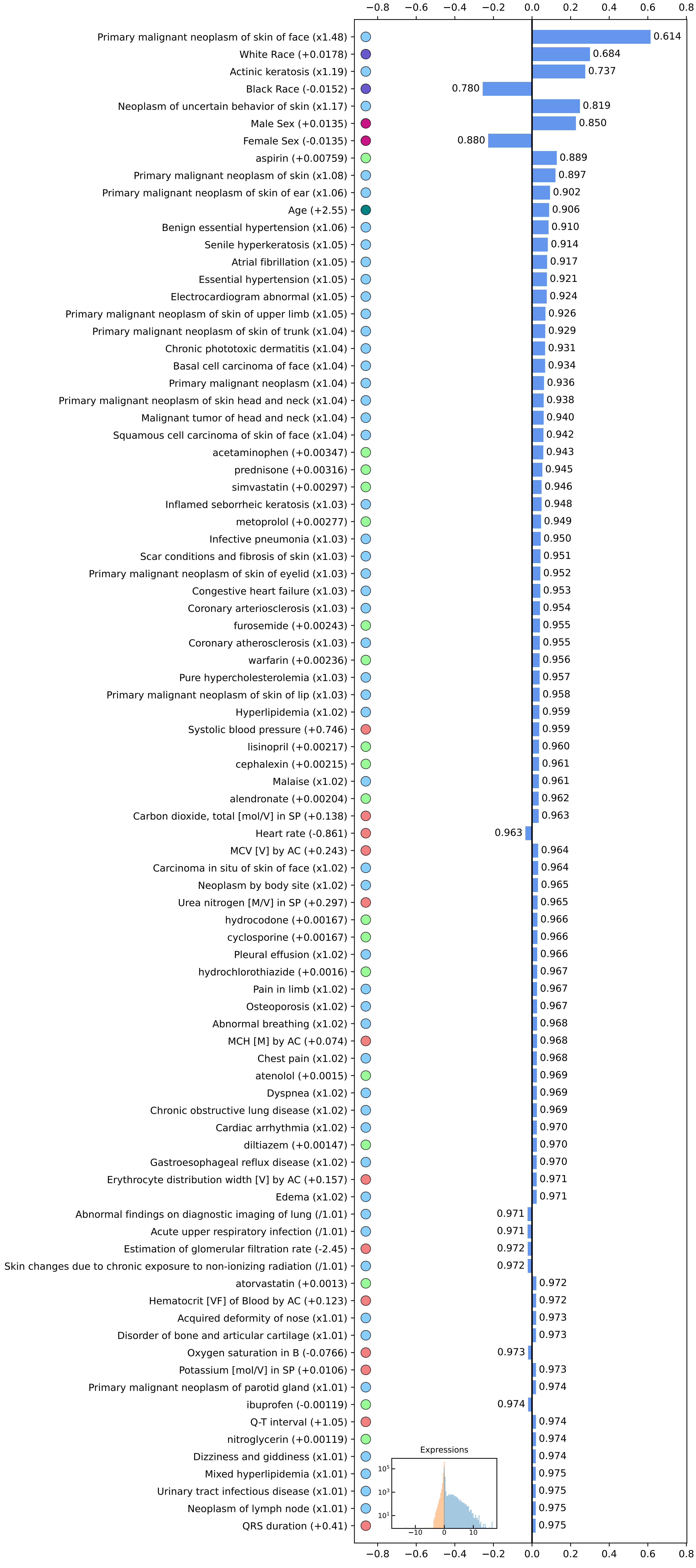

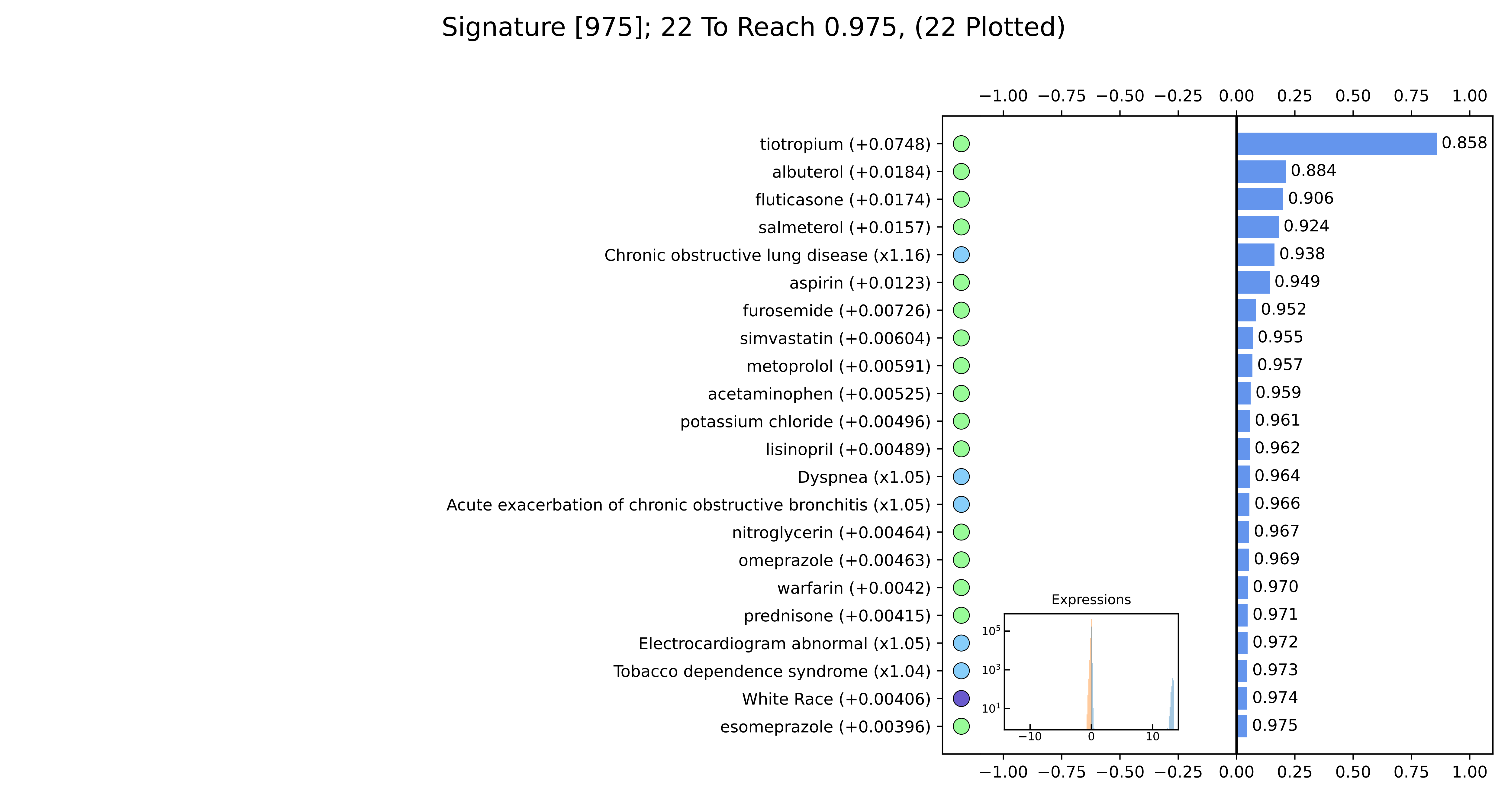
Signature [975]; 22 To Reach 0.975, (22 Plotted)
tiotropium (+0.0748)
albuterol (+0.0184)
fluticasone (+0.0174)
salmeterol (+0.0157)
Chronic obstructive lung disease (x1.16)
aspirin (+0.0123)
furosemide (+0.00726)
simvastatin (+0.00604)
metoprolol (+0.00591)
acetaminophen (+0.00525)
potassium chloride (+0.00496)
lisinopril (+0.00489)
Dyspnea (x1.05)
Acute exacerbation of chronic obstructive bronchitis (x1.05)
nitroglycerin (+0.00464)
omeprazole (+0.00463)
warfarin (+0.0042)
prednisone (+0.00415)
Electrocardiogram abnormal (x1.05)
Tobacco dependence syndrome (x1.04)
White Race (+0.00406)
esomeprazole (+0.00396)
0.858
0.884
0.906
0.924
0.938
0.949
0.952
0.955
0.957
0.959
0.961
0.962
0.964
0.966
0.967
0.969
0.970
0.971
0.972
0.973
0.974
0.975
−1.00 −0.75 −0.50 −0.25 0.00 0.25 0.50 0.75 1.00
Expressions
10^5
10^3
10^1
−10 0 10

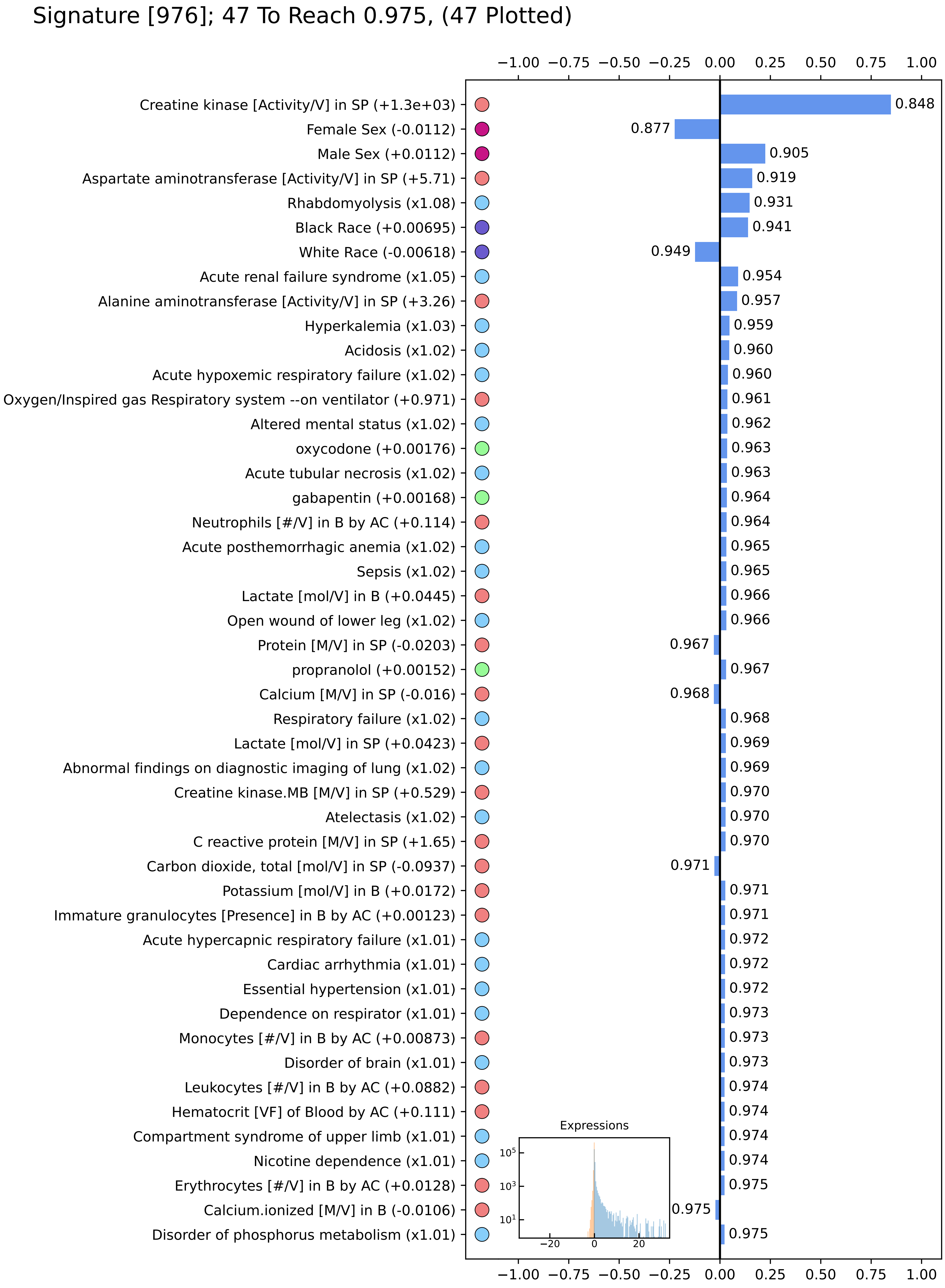
Signature [976]; 47 To Reach 0.975, (47 Plotted)
Creatine kinase [Activity/V] in SP (+1.3e+03) 0.848
Female Sex (-0.0112) 0.877
Male Sex (+0.0112) 0.905
Aspartate aminotransferase [Activity/V] in SP (+5.71) 0.919
Rhabdomyolysis (x1.08) 0.931
Black Race (+0.00695) 0.941
White Race (-0.00618) 0.949
Acute renal failure syndrome (x1.05) 0.954
Alanine aminotransferase [Activity/V] in SP (+3.26) 0.957
Hyperkalemia (x1.03) 0.959
Acidosis (x1.02) 0.960
Acute hypoxemic respiratory failure (x1.02) 0.960
Oxygen/Inspired gas Respiratory system --on ventilator (+0.971) 0.961
Altered mental status (x1.02) 0.962
oxycodone (+0.00176) 0.963
Acute tubular necrosis (x1.02) 0.963
gabapentin (+0.00168) 0.964
Neutrophils [#/V] in B by AC (+0.114) 0.964
Acute posthemorrhagic anemia (x1.02) 0.965
Sepsis (x1.02) 0.965
Lactate [mol/V] in B (+0.0445) 0.966
Open wound of lower leg (x1.02) 0.966
Protein [M/V] in SP (-0.0203) 0.967
propranolol (+0.00152) 0.967
Calcium [M/V] in SP (-0.016) 0.968
Respiratory failure (x1.02) 0.968
Lactate [mol/V] in SP (+0.0423) 0.969
Abnormal findings on diagnostic imaging of lung (x1.02) 0.969
Creatine kinase.MB [M/V] in SP (+0.529) 0.970
Atelectasis (x1.02) 0.970
C reactive protein [M/V] in SP (+1.65) 0.970
Carbon dioxide, total [mol/V] in SP (-0.0937) 0.971
Potassium [mol/V] in B (+0.0172) 0.971
Immature granulocytes [Presence] in B by AC (+0.00123) 0.971
Acute hypercapnic respiratory failure (x1.01) 0.972
Cardiac arrhythmia (x1.01) 0.972
Essential hypertension (x1.01) 0.972
Dependence on respirator (x1.01) 0.973
Monocytes [#/V] in B by AC (+0.00873) 0.973
Disorder of brain (x1.01) 0.973
Leukocytes [#/V] in B by AC (+0.0882) 0.974
Hematocrit [VF] of Blood by AC (+0.111) 0.974
Compartment syndrome of upper limb (x1.01) 0.974
Nicotine dependence (x1.01) 0.974
Erythrocytes [#/V] in B by AC (+0.0128) 0.975
Calcium.ionized [M/V] in B (-0.0106) 0.975
Disorder of phosphorus metabolism (x1.01) 0.975
−1.00 −0.75 −0.50 −0.25 0.00 0.25 0.50 0.75 1.00
Expressions
10^5
10^3
10^1
−20 0 20

Signature [977]; 17 To Reach 0.975, (17 Plotted)

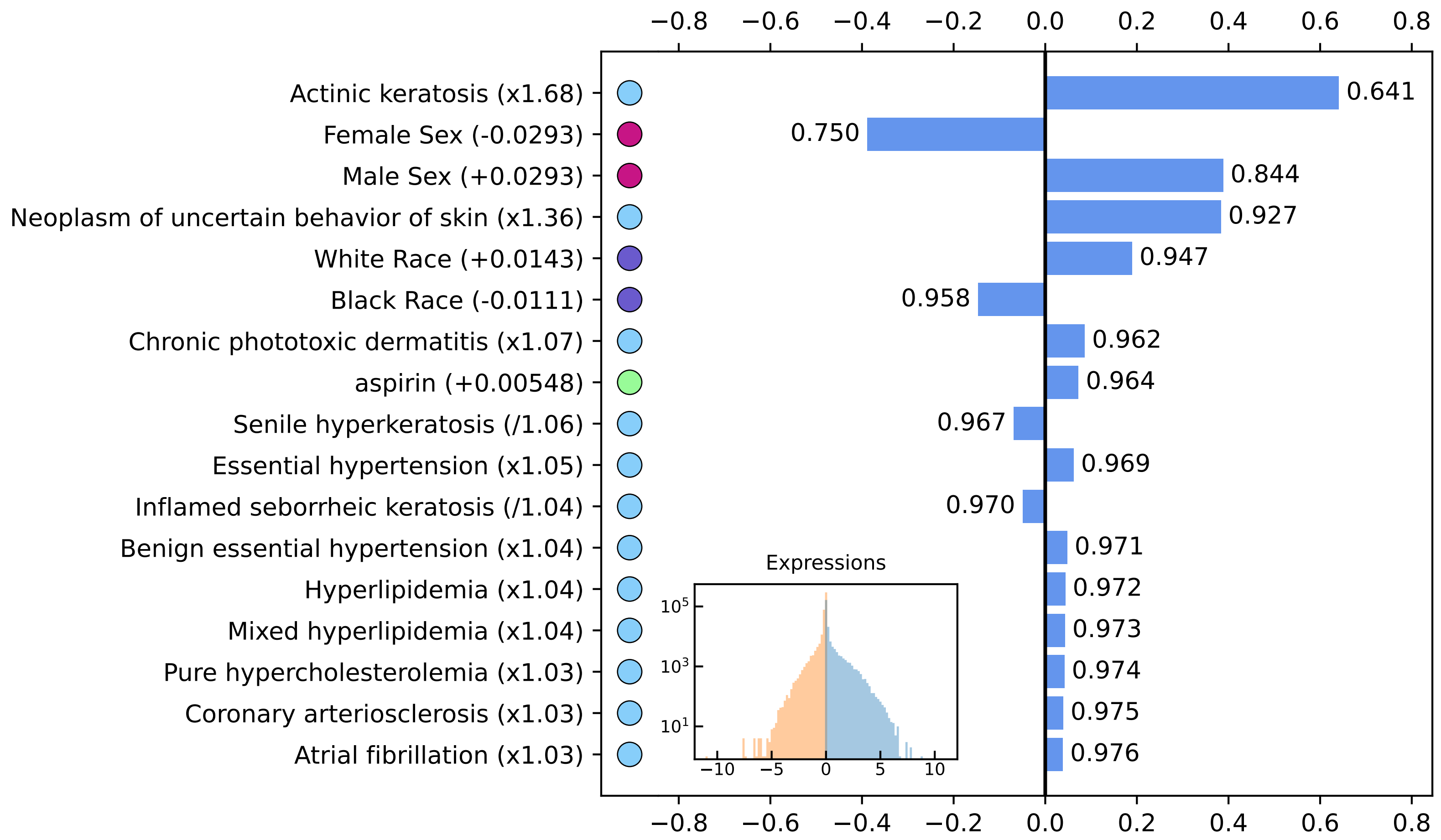

Signature [978]; 4 To Reach 0.975, (10 Plotted)

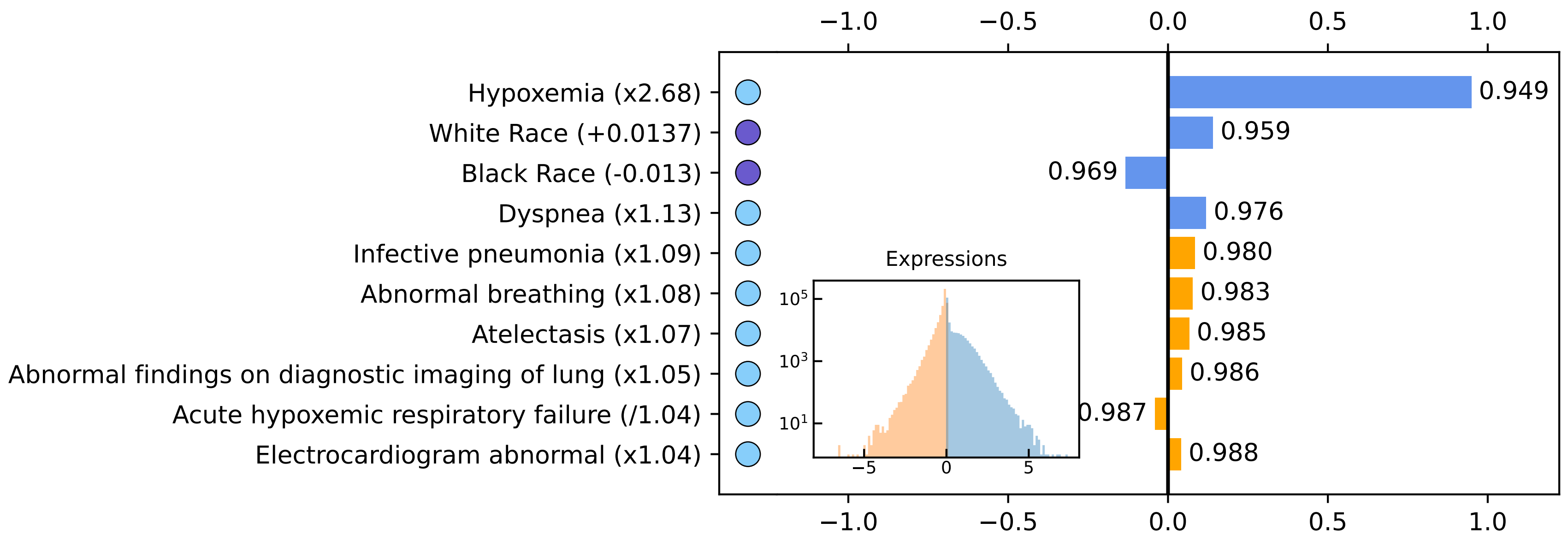

Signature [979]; 1 To Reach 0.975, (10 Plotted)

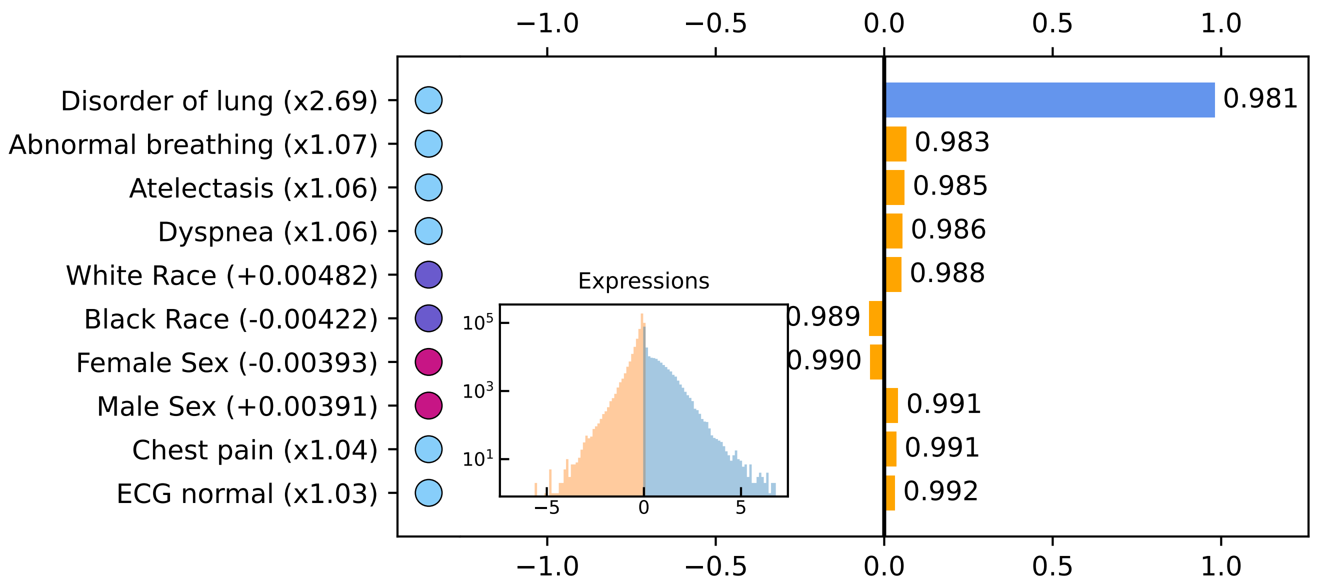

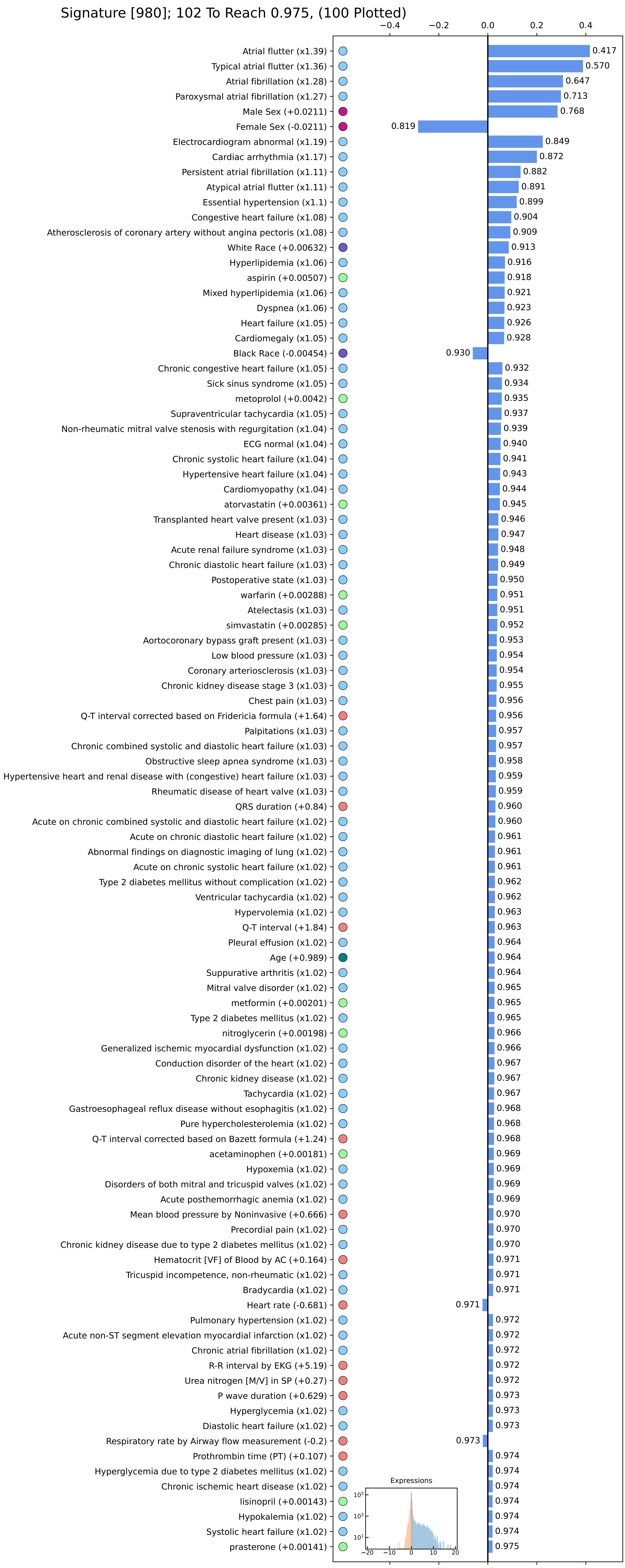

Signature [980]; 102 To Reach 0.975, (100 Plotted)
Atrial flutter (x1.39) 0.417
Typical atrial flutter (x1.36) 0.570
Atrial fibrillation (x1.28) 0.647
Paroxysmal atrial fibrillation (x1.27) 0.713
Male Sex (+0.0211) 0.768
Female Sex (-0.0211) 0.819
Electrocardiogram abnormal (x1.19) 0.849
Cardiac arrhythmia (x1.17) 0.872
Persistent atrial fibrillation (x1.11) 0.882
Atypical atrial flutter (x1.11) 0.891
Essential hypertension (x1.1) 0.899
Congestive heart failure (x1.08) 0.904
Atherosclerosis of coronary artery without angina pectoris (x1.08) 0.909
White Race (+0.00632) 0.913
Hyperlipidemia (x1.06) 0.916
aspirin (+0.00507) 0.918
Mixed hyperlipidemia (x1.06) 0.921
Dyspnea (x1.06) 0.923
Heart failure (x1.05) 0.926
Cardiomegaly (x1.05) 0.928
Black Race (-0.00454) 0.930
Chronic congestive heart failure (x1.05) 0.932
Sick sinus syndrome (x1.05) 0.934
metoprolol (+0.0042) 0.935
Supraventricular tachycardia (x1.05) 0.937
Non-rheumatic mitral valve stenosis with regurgitation (x1.04) 0.939
ECG normal (x1.04) 0.940
Chronic systolic heart failure (x1.04) 0.941
Hypertensive heart failure (x1.04) 0.943
Cardiomyopathy (x1.04) 0.944
atorvastatin (+0.00361) 0.945
Transplanted heart valve present (x1.03) 0.946
Heart disease (x1.03) 0.947
Acute renal failure syndrome (x1.03) 0.948
Chronic diastolic heart failure (x1.03) 0.949
Postoperative state (x1.03) 0.950
warfarin (+0.00288) 0.951
Atelectasis (x1.03) 0.951
simvastatin (+0.00285) 0.952
Aortocoronary bypass graft present (x1.03) 0.953
Low blood pressure (x1.03) 0.954
Coronary arteriosclerosis (x1.03) 0.954
Chronic kidney disease stage 3 (x1.03) 0.955
Chest pain (x1.03) 0.956
Q-T interval corrected based on Fridericia formula (+1.64) 0.956
Palpitations (x1.03) 0.957
Chronic combined systolic and diastolic heart failure (x1.03) 0.957
Obstructive sleep apnea syndrome (x1.03) 0.958
Hypertensive heart and renal disease with (congestive) heart failure (x1.03) 0.959
Rheumatic disease of heart valve (x1.03) 0.959
QRS duration (+0.84) 0.960
Acute on chronic combined systolic and diastolic heart failure (x1.02) 0.960
Acute on chronic diastolic heart failure (x1.02) 0.961
Abnormal findings on diagnostic imaging of lung (x1.02) 0.961
Acute on chronic systolic heart failure (x1.02) 0.961
Type 2 diabetes mellitus without complication (x1.02) 0.962
Ventricular tachycardia (x1.02) 0.962
Hypervolemia (x1.02) 0.963
Q-T interval (+1.84) 0.963
Pleural effusion (x1.02) 0.964
Age (+0.989) 0.964
Suppurative arthritis (x1.02) 0.964
Mitral valve disorder (x1.02) 0.965
metformin (+0.00201) 0.965
Type 2 diabetes mellitus (x1.02) 0.965
nitroglycerin (+0.00198) 0.966
Generalized ischemic myocardial dysfunction (x1.02) 0.966
Conduction disorder of the heart (x1.02) 0.967
Chronic kidney disease (x1.02) 0.967
Tachycardia (x1.02) 0.967
Gastroesophageal reflux disease without esophagitis (x1.02) 0.968
Pure hypercholesterolemia (x1.02) 0.968
Q-T interval corrected based on Bazett formula (+1.24) 0.968
acetaminophen (+0.00181) 0.969
Hypoxemia (x1.02) 0.969
Disorders of both mitral and tricuspid valves (x1.02) 0.969
Acute posthemorrhagic anemia (x1.02) 0.969
Mean blood pressure by Noninvasive (+0.666) 0.970
Precordial pain (x1.02) 0.970
Chronic kidney disease due to type 2 diabetes mellitus (x1.02) 0.970
Hematocrit [VF] of Blood by AC (+0.164) 0.971
Tricuspid incompetence, non-rheumatic (x1.02) 0.971
Bradycardia (x1.02) 0.971
Heart rate (-0.681) 0.971
Pulmonary hypertension (x1.02) 0.972
Acute non-ST segment elevation myocardial infarction (x1.02) 0.972
Chronic atrial fibrillation (x1.02) 0.972
R-R interval by EKG (+5.19) 0.972
Urea nitrogen [M/V] in SP (+0.27) 0.972
P wave duration (+0.629) 0.973
Hyperglycemia (x1.02) 0.973
Diastolic heart failure (x1.02) 0.973
Respiratory rate by Airway flow measurement (-0.2) 0.973
Prothrombin time (PT) (+0.107) 0.974
Hyperglycemia due to type 2 diabetes mellitus (x1.02) 0.974
Chronic ischemic heart disease (x1.02) 0.974
lisinopril (+0.00143) 0.974
Hypokalemia (x1.02) 0.974
Systolic heart failure (x1.02) 0.974
prasterone (+0.00141) 0.975
Expressions
−0.4 −0.2 0.0 0.2 0.4

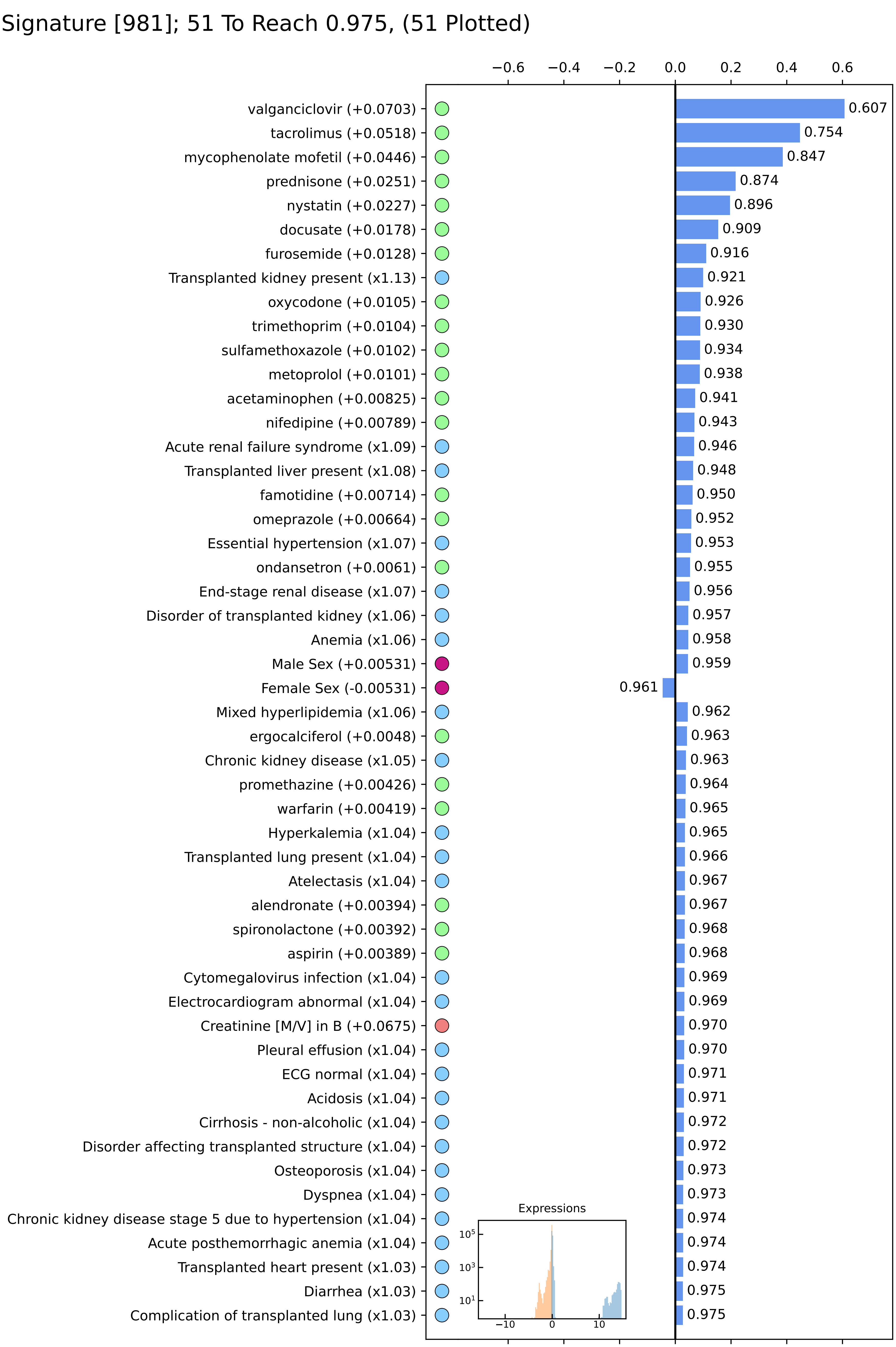
Signature [981]; 51 To Reach 0.975, (51 Plotted)
valganciclovir (+0.0703) 0.607
tacrolimus (+0.0518) 0.754
mycophenolate mofetil (+0.0446) 0.847
prednisone (+0.0251) 0.874
nystatin (+0.0227) 0.896
docusate (+0.0178) 0.909
furosemide (+0.0128) 0.916
Transplanted kidney present (x1.13) 0.921
oxycodone (+0.0105) 0.926
trimethoprim (+0.0104) 0.930
sulfamethoxazole (+0.0102) 0.934
metoprolol (+0.0101) 0.938
acetaminophen (+0.00825) 0.941
nifedipine (+0.00789) 0.943
Acute renal failure syndrome (x1.09) 0.946
Transplanted liver present (x1.08) 0.948
famotidine (+0.00714) 0.950
omeprazole (+0.00664) 0.952
Essential hypertension (x1.07) 0.953
ondansetron (+0.0061) 0.955
End-stage renal disease (x1.07) 0.956
Disorder of transplanted kidney (x1.06) 0.957
Anemia (x1.06) 0.958
Male Sex (+0.00531) 0.959
Female Sex (-0.00531) 0.961
Mixed hyperlipidemia (x1.06) 0.962
ergocalciferol (+0.0048) 0.963
Chronic kidney disease (x1.05) 0.963
promethazine (+0.00426) 0.964
warfarin (+0.00419) 0.965
Hyperkalemia (x1.04) 0.965
Transplanted lung present (x1.04) 0.966
Atelectasis (x1.04) 0.967
alendronate (+0.00394) 0.967
spironolactone (+0.00392) 0.968
aspirin (+0.00389) 0.968
Cytomegalovirus infection (x1.04) 0.969
Electrocardiogram abnormal (x1.04) 0.969
Creatinine [M/V] in B (+0.0675) 0.970
Pleural effusion (x1.04) 0.970
ECG normal (x1.04) 0.971
Acidosis (x1.04) 0.971
Cirrhosis - non-alcoholic (x1.04) 0.972
Disorder affecting transplanted structure (x1.04) 0.972
Osteoporosis (x1.04) 0.973
Dyspnea (x1.04) 0.973
Chronic kidney disease stage 5 due to hypertension (x1.04) 0.974
Acute posthemorrhagic anemia (x1.04) 0.974
Transplanted heart present (x1.03) 0.974
Diarrhea (x1.03) 0.975
Complication of transplanted lung (x1.03) 0.975
−0.6 −0.4 −0.2 0.0 0.2 0.4 0.6
Expressions
10^5 10^3 10^1
−10 0 10

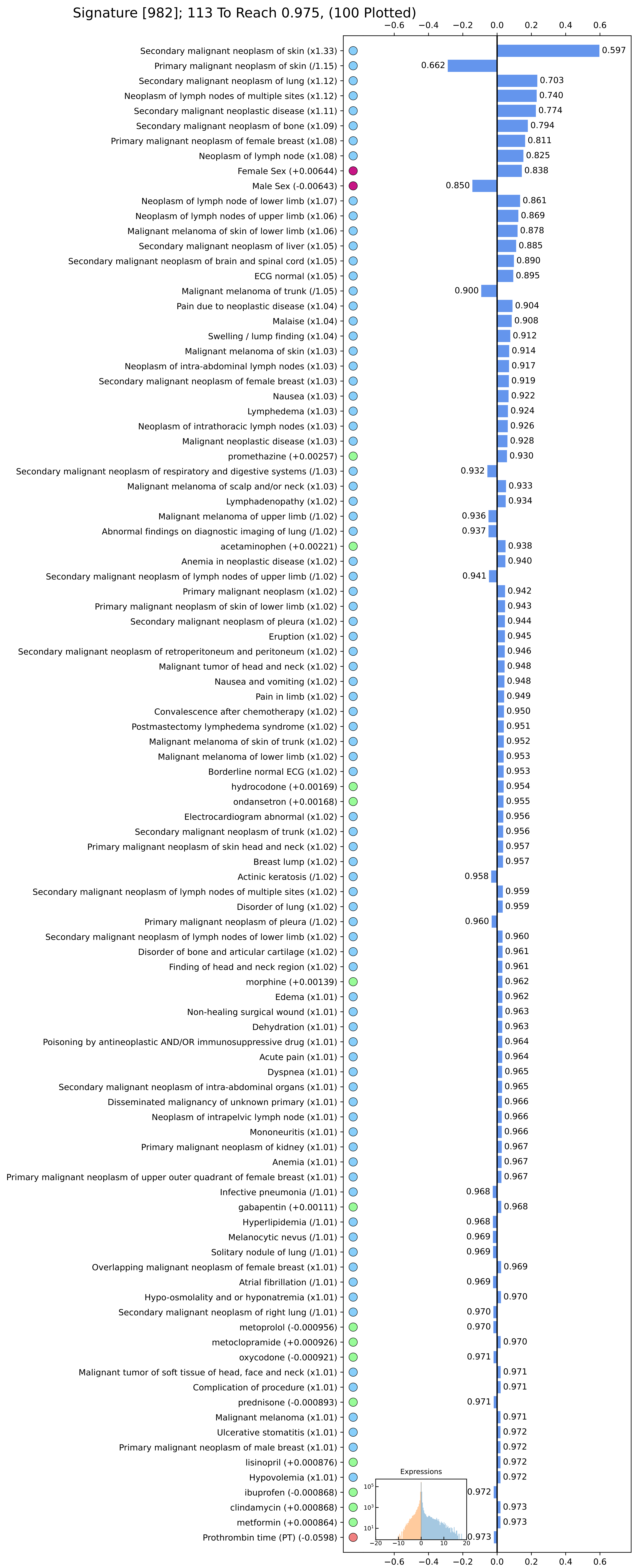

Signature [982]; 113 To Reach 0.975, (100 Plotted)
−0.6
−0.4
−0.2
0.0
0.2
0.4
0.6
Secondary malignant neoplasm of skin (x1.33)
0.597
Primary malignant neoplasm of skin (/1.15)
0.662
Secondary malignant neoplasm of lung (x1.12)
0.703
Neoplasm of lymph nodes of multiple sites (x1.12)
0.740
Secondary malignant neoplastic disease (x1.11)
0.774
Secondary malignant neoplasm of bone (x1.09)
0.794
Primary malignant neoplasm of female breast (x1.08)
0.811
Neoplasm of lymph node (x1.08)
0.825
Female Sex (+0.00644)
0.838
Male Sex (-0.00643)
0.850
Neoplasm of lymph node of lower limb (x1.07)
0.861
Neoplasm of lymph nodes of upper limb (x1.06)
0.869
Malignant melanoma of skin of lower limb (x1.06)
0.878
Secondary malignant neoplasm of liver (x1.05)
0.885
Secondary malignant neoplasm of brain and spinal cord (x1.05)
0.890
ECG normal (x1.05)
0.895
Malignant melanoma of trunk (/1.05)
0.900
Pain due to neoplastic disease (x1.04)
0.904
Malaise (x1.04)
0.908
Swelling / lump finding (x1.04)
0.912
Malignant melanoma of skin (x1.03)
0.914
Neoplasm of intra-abdominal lymph nodes (x1.03)
0.917
Secondary malignant neoplasm of female breast (x1.03)
0.919
Nausea (x1.03)
0.922
Lymphedema (x1.03)
0.924
Neoplasm of intrathoracic lymph nodes (x1.03)
0.926
Malignant neoplastic disease (x1.03)
0.928
promethazine (+0.00257)
0.930
Secondary malignant neoplasm of respiratory and digestive systems (/1.03)
0.932
Malignant melanoma of scalp and/or neck (x1.03)
0.933
Lymphadenopathy (x1.02)
0.934
Malignant melanoma of upper limb (/1.02)
0.936
Abnormal findings on diagnostic imaging of lung (/1.02)
0.937
acetaminophen (+0.00221)
0.938
Anemia in neoplastic disease (x1.02)
0.940
Secondary malignant neoplasm of lymph nodes of upper limb (/1.02)
0.941
Primary malignant neoplasm (x1.02)
0.942
Primary malignant neoplasm of skin of lower limb (x1.02)
0.943
Secondary malignant neoplasm of pleura (x1.02)
0.944
Eruption (x1.02)
0.945
Secondary malignant neoplasm of retroperitoneum and peritoneum (x1.02)
0.946
Malignant tumor of head and neck (x1.02)
0.948
Nausea and vomiting (x1.02)
0.948
Pain in limb (x1.02)
0.949
Convalescence after chemotherapy (x1.02)
0.950
Postmastectomy lymphedema syndrome (x1.02)
0.951
Malignant melanoma of skin of trunk (x1.02)
0.952
Malignant melanoma of lower limb (x1.02)
0.953
Borderline normal ECG (x1.02)
0.953
hydrocodone (+0.00169)
0.954
ondansetron (+0.00168)
0.955
Electrocardiogram abnormal (x1.02)
0.956
Secondary malignant neoplasm of trunk (x1.02)
0.956
Primary malignant neoplasm of skin head and neck (x1.02)
0.957
Breast lump (x1.02)
0.957
Actinic keratosis (/1.02)
0.958
Secondary malignant neoplasm of lymph nodes of multiple sites (x1.02)
0.959
Disorder of lung (x1.02)
0.959
Primary malignant neoplasm of pleura (/1.02)
0.960
Secondary malignant neoplasm of lymph nodes of lower limb (x1.02)
0.960
Disorder of bone and articular cartilage (x1.02)
0.961
Finding of head and neck region (x1.02)
0.961
morphine (+0.00139)
0.962
Edema (x1.01)
0.962
Non-healing surgical wound (x1.01)
0.963
Dehydration (x1.01)
0.963
Poisoning by antineoplastic AND/OR immunosuppressive drug (x1.01)
0.964
Acute pain (x1.01)
0.964
Dyspnea (x1.01)
0.965
Secondary malignant neoplasm of intra-abdominal organs (x1.01)
0.965
Disseminated malignancy of unknown primary (x1.01)
0.966
Neoplasm of intrapelvic lymph node (x1.01)
0.966
Mononeuritis (x1.01)
0.966
Primary malignant neoplasm of kidney (x1.01)
0.967
Anemia (x1.01)
0.967
Primary malignant neoplasm of upper outer quadrant of female breast (x1.01)
0.967
Infective pneumonia (/1.01)
0.968
gabapentin (+0.00111)
0.968
Hyperlipidemia (/1.01)
0.968
Melanocytic nevus (/1.01)
0.969
Solitary nodule of lung (/1.01)
0.969
Overlapping malignant neoplasm of female breast (x1.01)
0.969
Atrial fibrillation (/1.01)
0.969
Hypo-osmolality and or hyponatremia (x1.01)
0.970
Secondary malignant neoplasm of right lung (/1.01)
0.970
metoprolol (-0.000956)
0.970
metoclopramide (+0.000926)
0.970
oxycodone (-0.000921)
0.971
Malignant tumor of soft tissue of head, face and neck (x1.01)
0.971
Complication of procedure (x1.01)
0.971
prednisone (-0.000893)
0.971
Malignant melanoma (x1.01)
0.971
Ulcerative stomatitis (x1.01)
0.972
Primary malignant neoplasm of male breast (x1.01)
0.972
lisinopril (+0.000876)
0.972
Hypovolemia (x1.01)
0.972
ibuprofen (-0.000868)
0.972
clindamycin (+0.000868)
0.973
metformin (+0.000864)
0.973
Prothrombin time (PT) (-0.0598)
0.973
Expressions
10^5
10^3
10^1
−20
−10
0
10
20

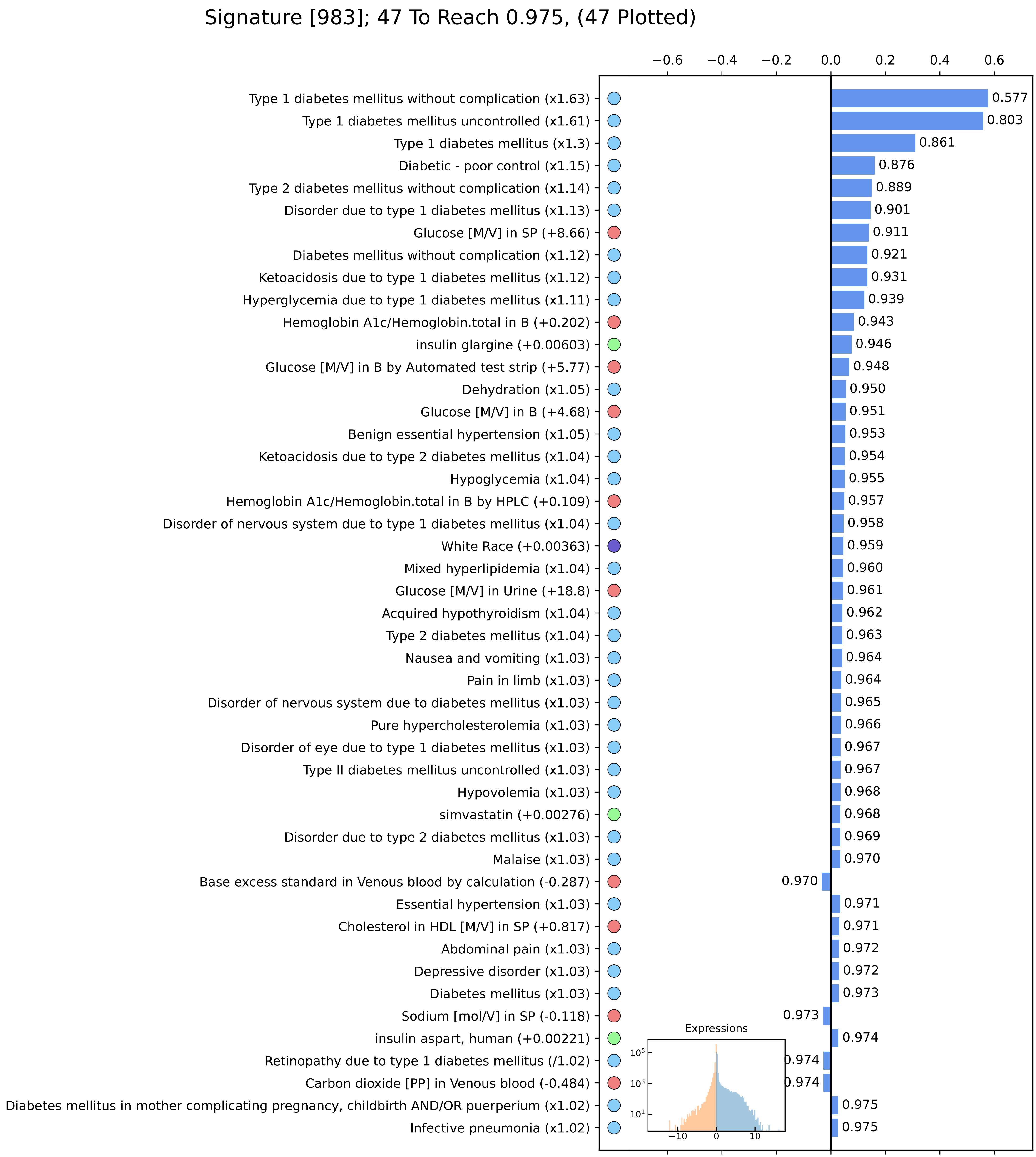

Signature [983]; 47 To Reach 0.975, (47 Plotted)
Type 1 diabetes mellitus without complication (x1.63) 0.577
Type 1 diabetes mellitus uncontrolled (x1.61) 0.803
Type 1 diabetes mellitus (x1.3) 0.861
Diabetic - poor control (x1.15) 0.876
Type 2 diabetes mellitus without complication (x1.14) 0.889
Disorder due to type 1 diabetes mellitus (x1.13) 0.901
Glucose [M/V] in SP (+8.66) 0.911
Diabetes mellitus without complication (x1.12) 0.921
Ketoacidosis due to type 1 diabetes mellitus (x1.12) 0.931
Hyperglycemia due to type 1 diabetes mellitus (x1.11) 0.939
Hemoglobin A1c/Hemoglobin.total in B (+0.202) 0.943
insulin glargine (+0.00603) 0.946
Glucose [M/V] in B by Automated test strip (+5.77) 0.948
Dehydration (x1.05) 0.950
Glucose [M/V] in B (+4.68) 0.951
Benign essential hypertension (x1.05) 0.953
Ketoacidosis due to type 2 diabetes mellitus (x1.04) 0.954
Hypoglycemia (x1.04) 0.955
Hemoglobin A1c/Hemoglobin.total in B by HPLC (+0.109) 0.957
Disorder of nervous system due to type 1 diabetes mellitus (x1.04) 0.958
White Race (+0.00363) 0.959
Mixed hyperlipidemia (x1.04) 0.960
Glucose [M/V] in Urine (+18.8) 0.961
Acquired hypothyroidism (x1.04) 0.962
Type 2 diabetes mellitus (x1.04) 0.963
Nausea and vomiting (x1.03) 0.964
Pain in limb (x1.03) 0.964
Disorder of nervous system due to diabetes mellitus (x1.03) 0.965
Pure hypercholesterolemia (x1.03) 0.966
Disorder of eye due to type 1 diabetes mellitus (x1.03) 0.967
Type II diabetes mellitus uncontrolled (x1.03) 0.967
Hypovolemia (x1.03) 0.968
simvastatin (+0.00276) 0.968
Disorder due to type 2 diabetes mellitus (x1.03) 0.969
Malaise (x1.03) 0.970
Base excess standard in Venous blood by calculation (-0.287) 0.970
Essential hypertension (x1.03) 0.971
Cholesterol in HDL [M/V] in SP (+0.817) 0.971
Abdominal pain (x1.03) 0.972
Depressive disorder (x1.03) 0.972
Diabetes mellitus (x1.03) 0.973
Sodium [mol/V] in SP (-0.118) 0.973
insulin aspart, human (+0.00221) 0.974
Retinopathy due to type 1 diabetes mellitus (/1.02) 0.974
Carbon dioxide [PP] in Venous blood (-0.484) 0.974
Diabetes mellitus in mother complicating pregnancy, childbirth AND/OR puerperium (x1.02) 0.975
Infective pneumonia (x1.02) 0.975
−0.6 −0.4 −0.2 0.0 0.2 0.4 0.6
Expressions
10^5
10^3
10^1
−10 0 10

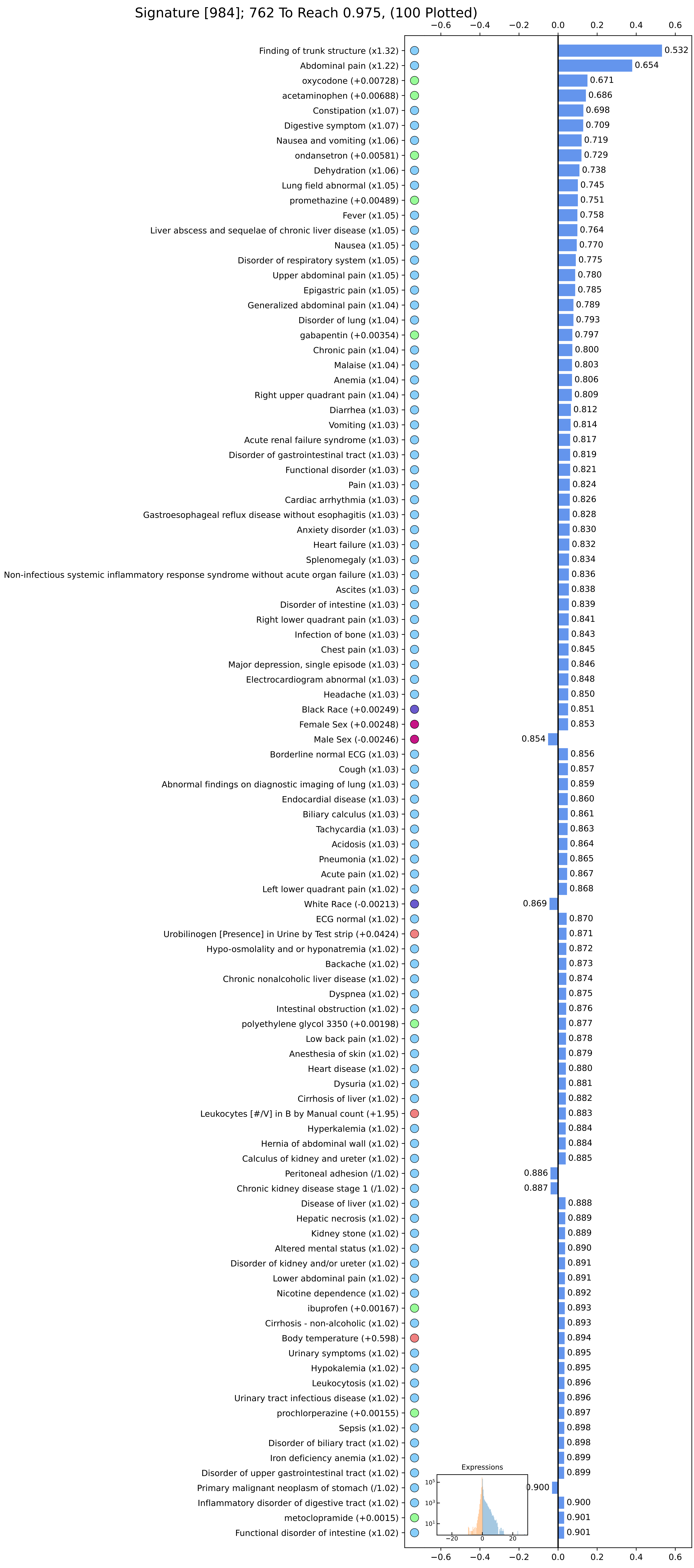
Signature [984]; 762 To Reach 0.975, (100 Plotted)
Finding of trunk structure (x1.32) 0.532
Abdominal pain (x1.22) 0.654
oxycodone (+0.00728) 0.671
acetaminophen (+0.00688) 0.686
Constipation (x1.07) 0.698
Digestive symptom (x1.07) 0.709
Nausea and vomiting (x1.06) 0.719
ondansetron (+0.00581) 0.729
Dehydration (x1.06) 0.738
Lung field abnormal (x1.05) 0.745
promethazine (+0.00489) 0.751
Fever (x1.05) 0.758
Liver abscess and sequelae of chronic liver disease (x1.05) 0.764
Nausea (x1.05) 0.770
Disorder of respiratory system (x1.05) 0.775
Upper abdominal pain (x1.05) 0.780
Epigastric pain (x1.05) 0.785
Generalized abdominal pain (x1.04) 0.789
Disorder of lung (x1.04) 0.793
gabapentin (+0.00354) 0.797
Chronic pain (x1.04) 0.800
Malaise (x1.04) 0.803
Anemia (x1.04) 0.806
Right upper quadrant pain (x1.04) 0.809
Diarrhea (x1.03) 0.812
Vomiting (x1.03) 0.814
Acute renal failure syndrome (x1.03) 0.817
Disorder of gastrointestinal tract (x1.03) 0.819
Functional disorder (x1.03) 0.821
Pain (x1.03) 0.824
Cardiac arrhythmia (x1.03) 0.826
Gastroesophageal reflux disease without esophagitis (x1.03) 0.828
Anxiety disorder (x1.03) 0.830
Heart failure (x1.03) 0.832
Splenomegaly (x1.03) 0.834
Non-infectious systemic inflammatory response syndrome without acute organ failure (x1.03) 0.836
Ascites (x1.03) 0.838
Disorder of intestine (x1.03) 0.839
Right lower quadrant pain (x1.03) 0.841
Infection of bone (x1.03) 0.843
Chest pain (x1.03) 0.845
Major depression, single episode (x1.03) 0.846
Electrocardiogram abnormal (x1.03) 0.848
Headache (x1.03) 0.850
Black Race (+0.00249) 0.851
Female Sex (+0.00248) 0.853
Male Sex (-0.00246) 0.854
Borderline normal ECG (x1.03) 0.856
Cough (x1.03) 0.857
Abnormal findings on diagnostic imaging of lung (x1.03) 0.859
Endocardial disease (x1.03) 0.860
Biliary calculus (x1.03) 0.861
Tachycardia (x1.03) 0.863
Acidosis (x1.03) 0.864
Pneumonia (x1.02) 0.865
Acute pain (x1.02) 0.867
Left lower quadrant pain (x1.02) 0.868
White Race (-0.00213) 0.869
ECG normal (x1.02) 0.870
Urobilinogen [Presence] in Urine by Test strip (+0.0424) 0.871
Hypo-osmolality and or hyponatremia (x1.02) 0.872
Backache (x1.02) 0.873
Chronic nonalcoholic liver disease (x1.02) 0.874
Dyspnea (x1.02) 0.875
Intestinal obstruction (x1.02) 0.876
polyethylene glycol 3350 (+0.00198) 0.877
Low back pain (x1.02) 0.878
Anesthesia of skin (x1.02) 0.879
Heart disease (x1.02) 0.880
Dysuria (x1.02) 0.881
Cirrhosis of liver (x1.02) 0.882
Leukocytes [#/V] in B by Manual count (+1.95) 0.883
Hyperkalemia (x1.02) 0.884
Hernia of abdominal wall (x1.02) 0.884
Calculus of kidney and ureter (x1.02) 0.885
Peritoneal adhesion (/1.02) 0.886
Chronic kidney disease stage 1 (/1.02) 0.887
Disease of liver (x1.02) 0.888
Hepatic necrosis (x1.02) 0.889
Kidney stone (x1.02) 0.889
Altered mental status (x1.02) 0.890
Disorder of kidney and/or ureter (x1.02) 0.891
Lower abdominal pain (x1.02) 0.891
Nicotine dependence (x1.02) 0.892
ibuprofen (+0.00167) 0.893
Cirrhosis - non-alcoholic (x1.02) 0.893
Body temperature (+0.598) 0.894
Urinary symptoms (x1.02) 0.895
Hypokalemia (x1.02) 0.895
Leukocytosis (x1.02) 0.896
Urinary tract infectious disease (x1.02) 0.896
prochlorperazine (+0.00155) 0.897
Sepsis (x1.02) 0.898
Disorder of biliary tract (x1.02) 0.898
Iron deficiency anemia (x1.02) 0.899
Disorder of upper gastrointestinal tract (x1.02) 0.899
Primary malignant neoplasm of stomach (/1.02) 0.900
Inflammatory disorder of digestive tract (x1.02) 0.900
metoclopramide (+0.0015) 0.901
Functional disorder of intestine (x1.02) 0.901
Expressions
−0.6 −0.4 −0.2 0.0 0.2 0.4 0.6

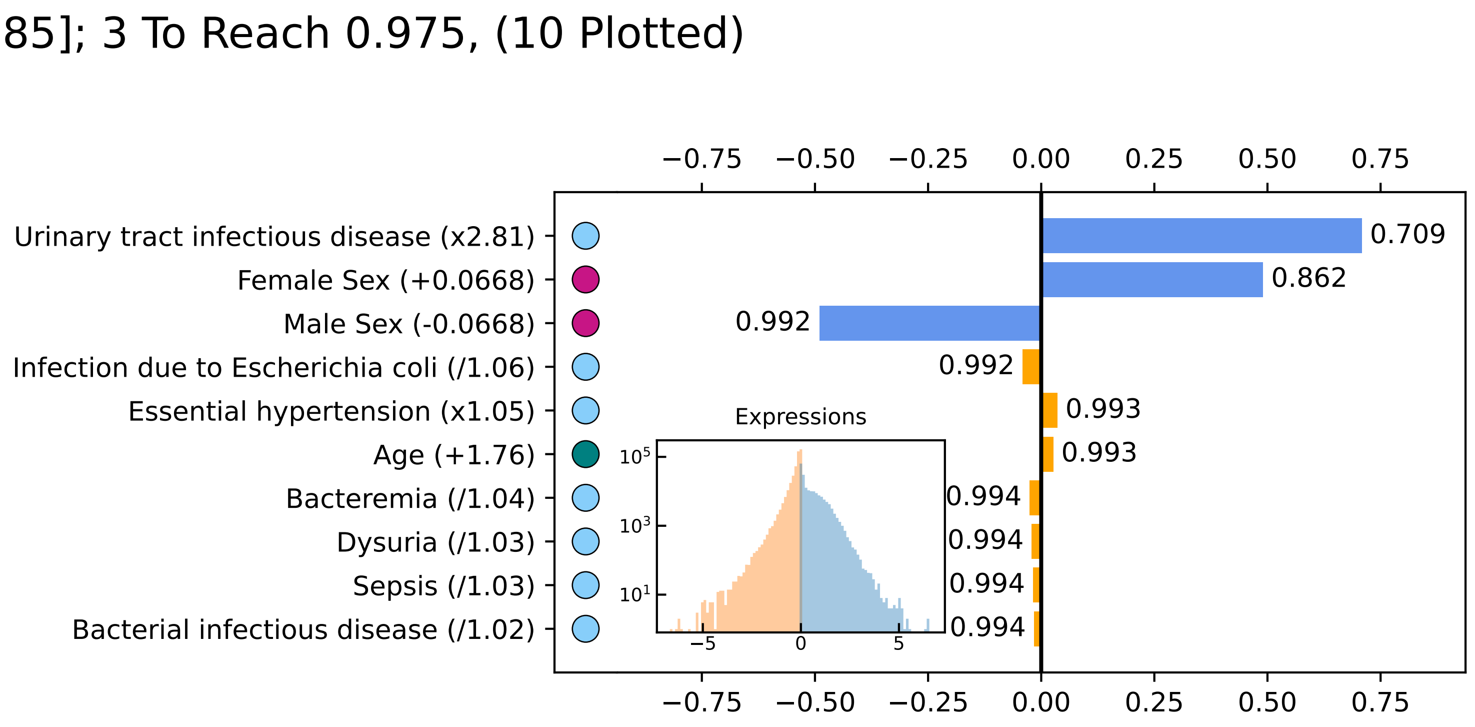
Signature [985]; 3 To Reach 0.975, (10 Plotted)
Urinary tract infectious disease (x2.81)
Female Sex (+0.0668)
Male Sex (-0.0668)
Infection due to Escherichia coli (/1.06)
Essential hypertension (x1.05)
Age (+1.76)
Bacteremia (/1.04)
Dysuria (/1.03)
Sepsis (/1.03)
Bacterial infectious disease (/1.02)
0.709
0.862
0.992
0.992
0.993
0.993
0.994
0.994
0.994
0.994
−0.75
−0.50
−0.25
0.00
0.25
0.50
0.75
Expressions
10^5
10^3
10^1
−5
0
5

Signature [986]; 107 To Reach 0.975, (100 Plotted)

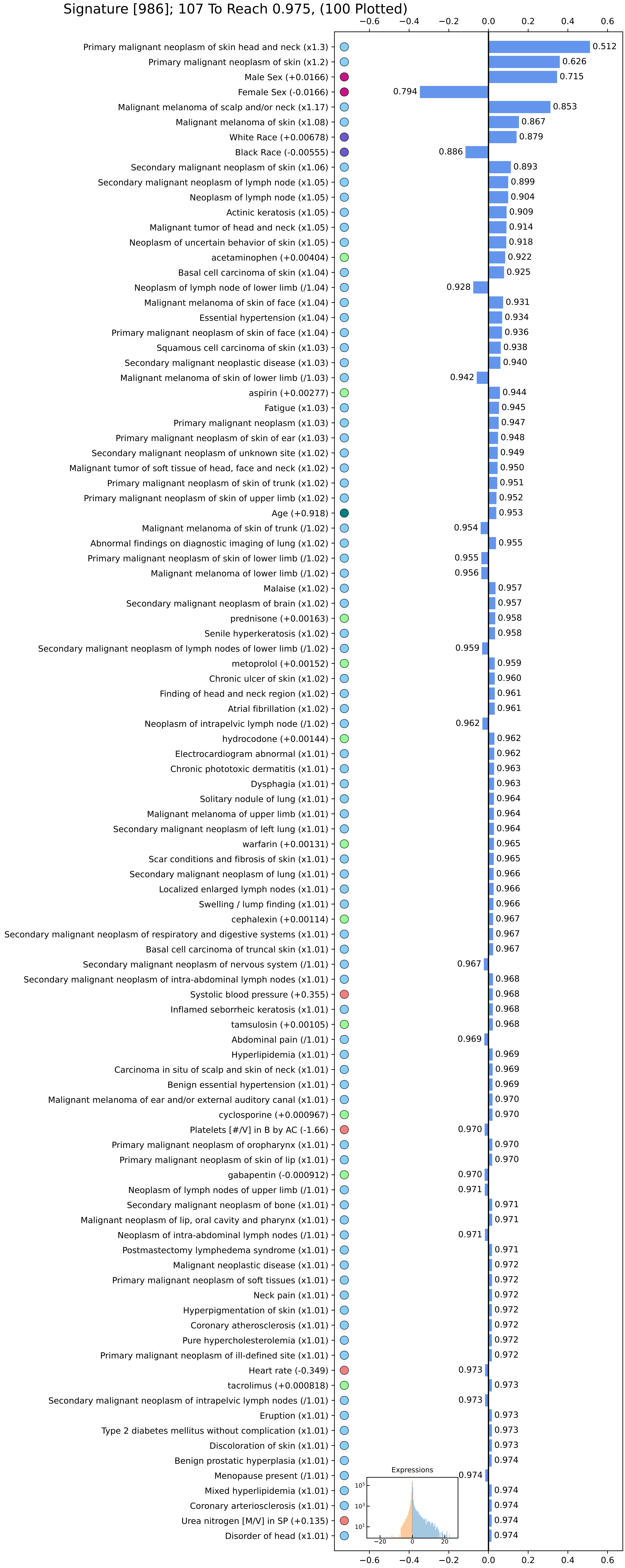

Signature [987]; 250 To Reach 0.975, (100 Plotted)

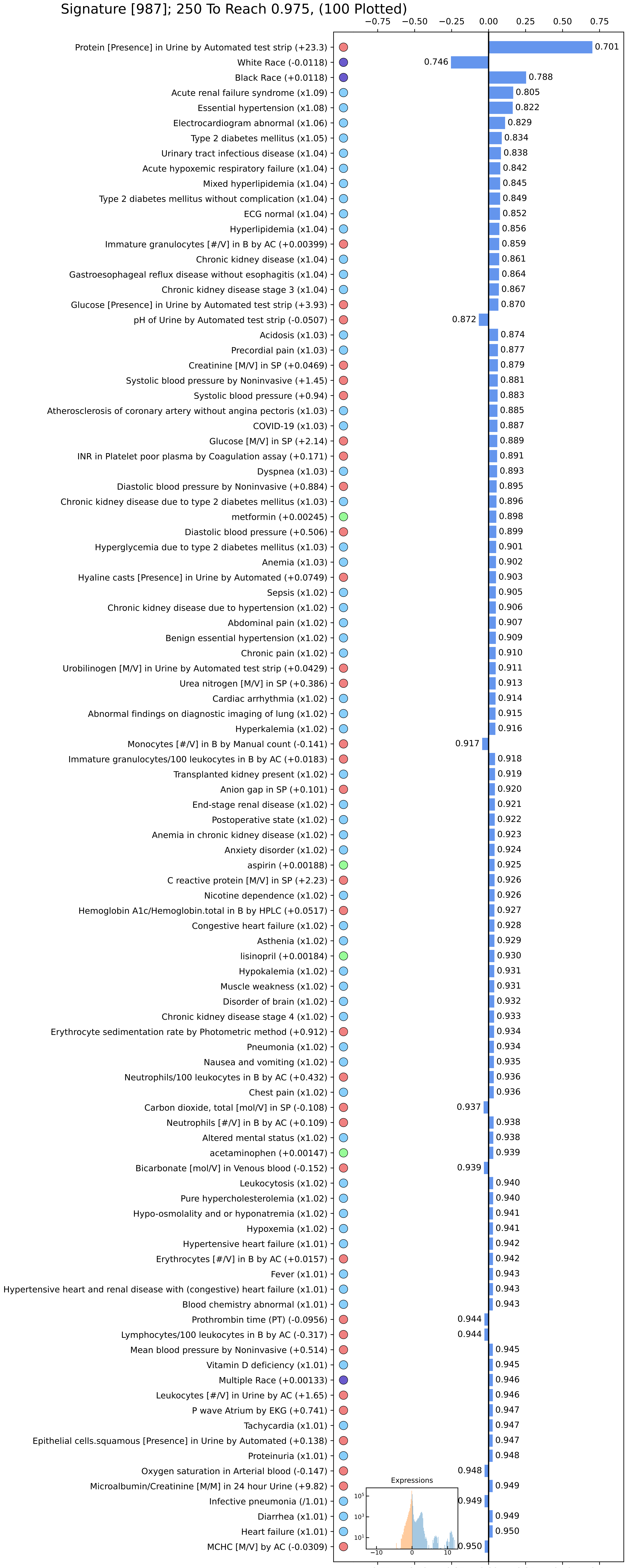

Signature [988]; 8 To Reach 0.975, (10 Plotted)

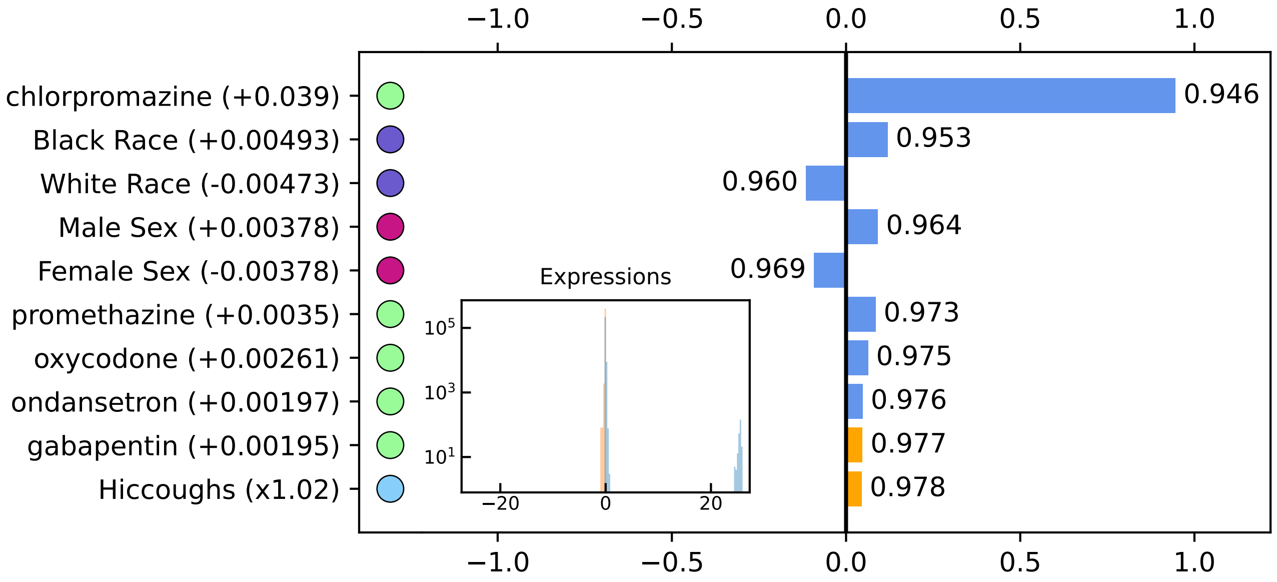

Signature [989]; 11 To Reach 0.975, (11 Plotted)

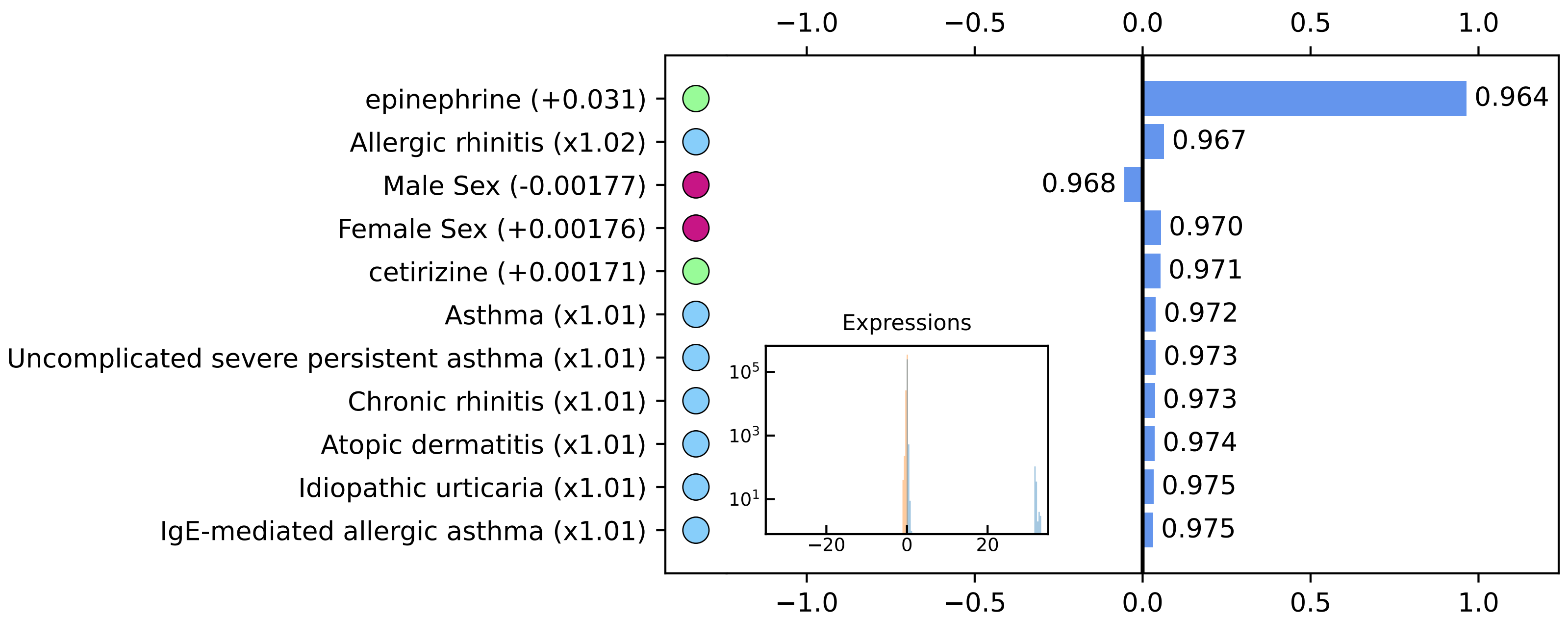

Signature [990]; 323 To Reach 0.975, (100 Plotted)

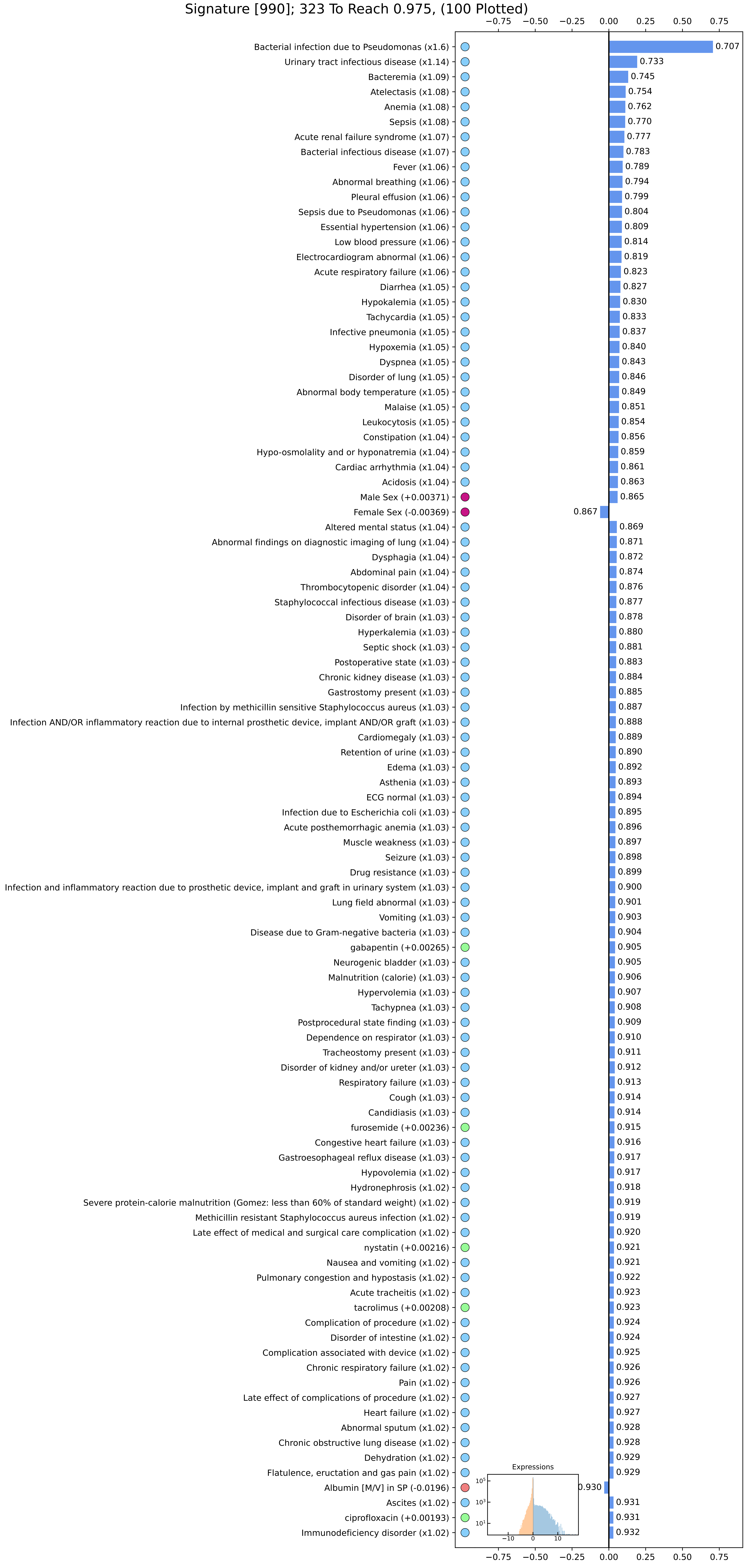

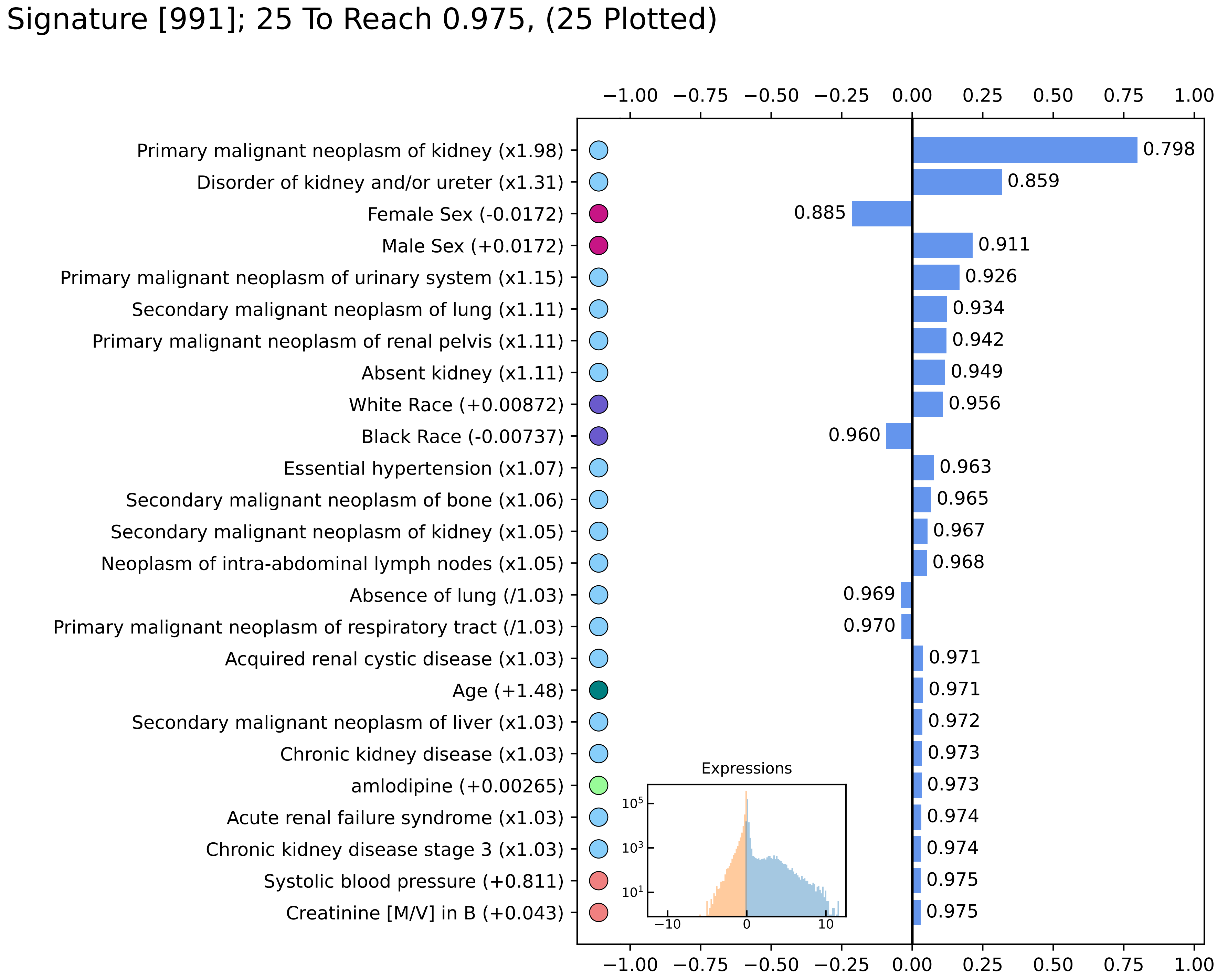

Signature [991]; 25 To Reach 0.975, (25 Plotted)
Primary malignant neoplasm of kidney (x1.98)
0.798
Disorder of kidney and/or ureter (x1.31)
0.859
Female Sex (-0.0172)
0.885
Male Sex (+0.0172)
0.911
Primary malignant neoplasm of urinary system (x1.15)
0.926
Secondary malignant neoplasm of lung (x1.11)
0.934
Primary malignant neoplasm of renal pelvis (x1.11)
0.942
Absent kidney (x1.11)
0.949
White Race (+0.00872)
0.956
Black Race (-0.00737)
0.960
Essential hypertension (x1.07)
0.963
Secondary malignant neoplasm of bone (x1.06)
0.965
Secondary malignant neoplasm of kidney (x1.05)
0.967
Neoplasm of intra-abdominal lymph nodes (x1.05)
0.968
Absence of lung (/1.03)
0.969
Primary malignant neoplasm of respiratory tract (/1.03)
0.970
Acquired renal cystic disease (x1.03)
0.971
Age (+1.48)
0.971
Secondary malignant neoplasm of liver (x1.03)
0.972
Chronic kidney disease (x1.03)
0.973
amlodipine (+0.00265)
0.973
Acute renal failure syndrome (x1.03)
0.974
Chronic kidney disease stage 3 (x1.03)
0.974
Systolic blood pressure (+0.811)
0.975
Creatinine [M/V] in B (+0.043)
0.975
−1.00 −0.75 −0.50 −0.25 0.00 0.25 0.50 0.75 1.00
Expressions

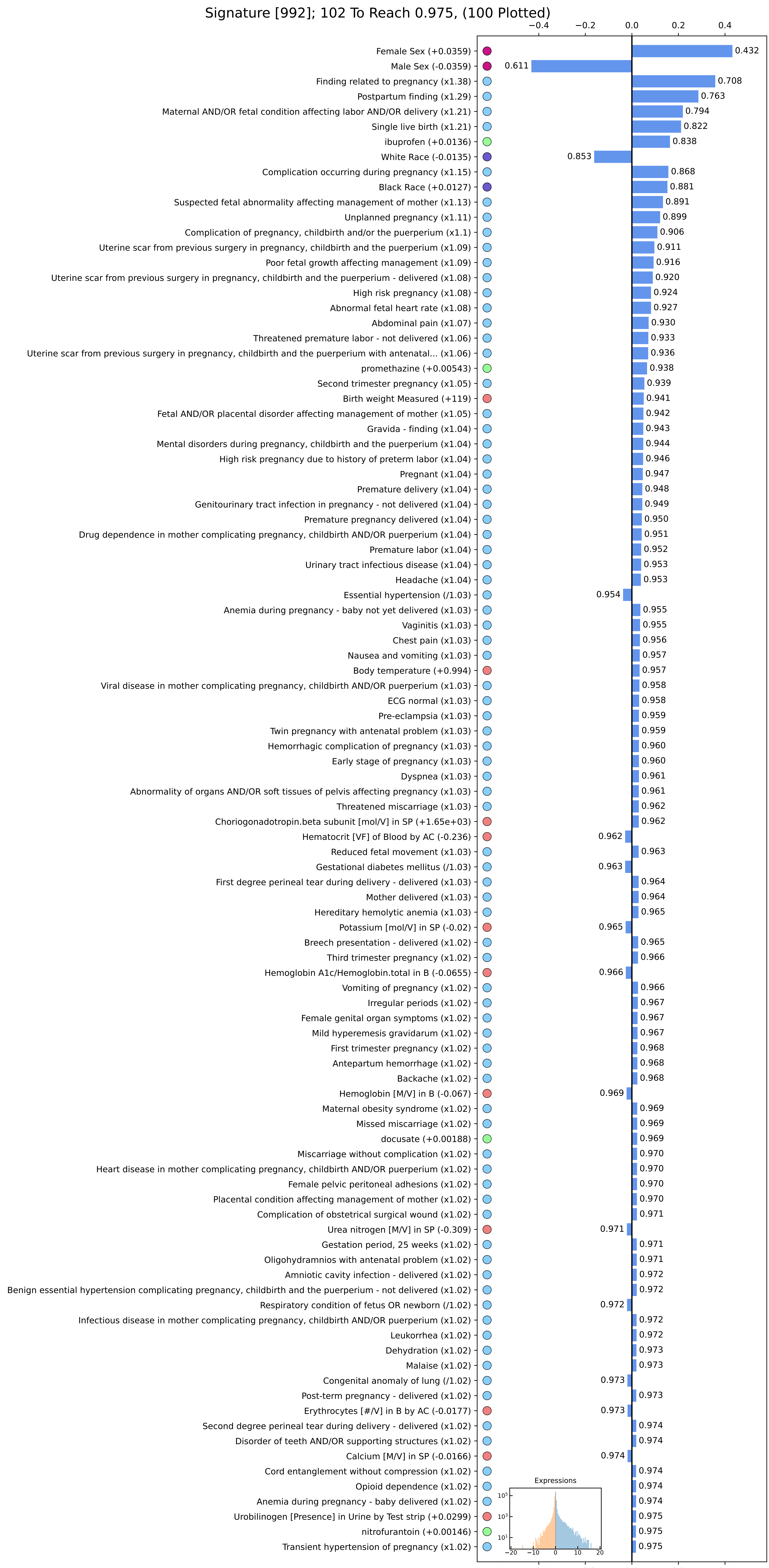

Signature [992]; 102 To Reach 0.975, (100 Plotted)
Female Sex (+0.0359)
Male Sex (-0.0359)
Finding related to pregnancy (x1.38)
Postpartum finding (x1.29)
Maternal AND/OR fetal condition affecting labor AND/OR delivery (x1.21)
Single live birth (x1.21)
ibuprofen (+0.0136)
White Race (-0.0135)
Complication occurring during pregnancy (x1.15)
Black Race (+0.0127)
Suspected fetal abnormality affecting management of mother (x1.13)
Unplanned pregnancy (x1.11)
Complication of pregnancy, childbirth and/or the puerperium (x1.1)
Uterine scar from previous surgery in pregnancy, childbirth and the puerperium (x1.09)
Poor fetal growth affecting management (x1.09)
Uterine scar from previous surgery in pregnancy, childbirth and the puerperium - delivered (x1.08)
High risk pregnancy (x1.08)
Abnormal fetal heart rate (x1.08)
Abdominal pain (x1.07)
Threatened premature labor - not delivered (x1.06)
Uterine scar from previous surgery in pregnancy, childbirth and the puerperium with antenatal... (x1.06)
promethazine (+0.00543)
Second trimester pregnancy (x1.05)
Birth weight Measured (+119)
Fetal AND/OR placental disorder affecting management of mother (x1.05)
Gravida - finding (x1.04)
Mental disorders during pregnancy, childbirth and the puerperium (x1.04)
High risk pregnancy due to history of preterm labor (x1.04)
Pregnant (x1.04)
Premature delivery (x1.04)
Genitourinary tract infection in pregnancy - not delivered (x1.04)
Premature pregnancy delivered (x1.04)
Drug dependence in mother complicating pregnancy, childbirth AND/OR puerperium (x1.04)
Premature labor (x1.04)
Urinary tract infectious disease (x1.04)
Headache (x1.04)
Essential hypertension (/1.03)
Anemia during pregnancy - baby not yet delivered (x1.03)
Vaginitis (x1.03)
Chest pain (x1.03)
Nausea and vomiting (x1.03)
Body temperature (+0.994)
Viral disease in mother complicating pregnancy, childbirth AND/OR puerperium (x1.03)
ECG normal (x1.03)
Pre-eclampsia (x1.03)
Twin pregnancy with antenatal problem (x1.03)
Hemorrhagic complication of pregnancy (x1.03)
Early stage of pregnancy (x1.03)
Dyspnea (x1.03)
Abnormality of organs AND/OR soft tissues of pelvis affecting pregnancy (x1.03)
Threatened miscarriage (x1.03)
Choriogonadotropin.beta subunit [mol/V] in SP (+1.65e+03)
Hematocrit [VF] of Blood by AC (-0.236)
Reduced fetal movement (x1.03)
Gestational diabetes mellitus (/1.03)
First degree perineal tear during delivery - delivered (x1.03)
Mother delivered (x1.03)
Hereditary hemolytic anemia (x1.03)
Potassium [mol/V] in SP (-0.02)
Breech presentation - delivered (x1.02)
Third trimester pregnancy (x1.02)
Hemoglobin A1c/Hemoglobin.total in B (-0.0655)
Vomiting of pregnancy (x1.02)
Irregular periods (x1.02)
Female genital organ symptoms (x1.02)
Mild hyperemesis gravidarum (x1.02)
First trimester pregnancy (x1.02)
Antepartum hemorrhage (x1.02)
Backache (x1.02)
Hemoglobin [M/V] in B (-0.067)
Maternal obesity syndrome (x1.02)
Missed miscarriage (x1.02)
docusate (+0.00188)
Miscarriage without complication (x1.02)
Heart disease in mother complicating pregnancy, childbirth AND/OR puerperium (x1.02)
Female pelvic peritoneal adhesions (x1.02)
Placental condition affecting management of mother (x1.02)
Complication of obstetrical surgical wound (x1.02)
Urea nitrogen [M/V] in SP (-0.309)
Gestation period, 25 weeks (x1.02)
Oligohydramnios with antenatal problem (x1.02)
Amniotic cavity infection - delivered (x1.02)
Benign essential hypertension complicating pregnancy, childbirth and the puerperium - not delivered (x1.02)
Respiratory condition of fetus OR newborn (/1.02)
Infectious disease in mother complicating pregnancy, childbirth AND/OR puerperium (x1.02)
Leukorrhea (x1.02)
Dehydration (x1.02)
Malaise (x1.02)
Congenital anomaly of lung (/1.02)
Post-term pregnancy - delivered (x1.02)
Erythrocytes [#/V] in B by AC (-0.0177)
Second degree perineal tear during delivery - delivered (x1.02)
Disorder of teeth AND/OR supporting structures (x1.02)
Calcium [M/V] in SP (-0.0166)
Cord entanglement without compression (x1.02)
Opioid dependence (x1.02)
Anemia during pregnancy - baby delivered (x1.02)
Urobilinogen [Presence] in Urine by Test strip (+0.0299)
nitrofurantoin (+0.00146)
Transient hypertension of pregnancy (x1.02)
0.432
0.611
0.708
0.763
0.794
0.822
0.838
0.853
0.868
0.881
0.891
0.899
0.906
0.911
0.916
0.920
0.924
0.927
0.930
0.933
0.936
0.938
0.939
0.941
0.942
0.943
0.944
0.946
0.947
0.948
0.949
0.950
0.951
0.952
0.953
0.953
0.954
0.955
0.955
0.956
0.957
0.957
0.958
0.958
0.959
0.959
0.960
0.960
0.961
0.961
0.962
0.962
0.962
0.963
0.963
0.964
0.964
0.965
0.965
0.965
0.966
0.966
0.966
0.967
0.967
0.967
0.968
0.968
0.968
0.969
0.969
0.969
0.969
0.970
0.970
0.970
0.970
0.971
0.971
0.971
0.971
0.972
0.972
0.972
0.972
0.972
0.973
0.973
0.973
0.973
0.973
0.974
0.974
0.974
0.974
0.974
0.974
0.975
0.975
0.975
−0.4
−0.2
0.0
0.2
0.4
Expressions

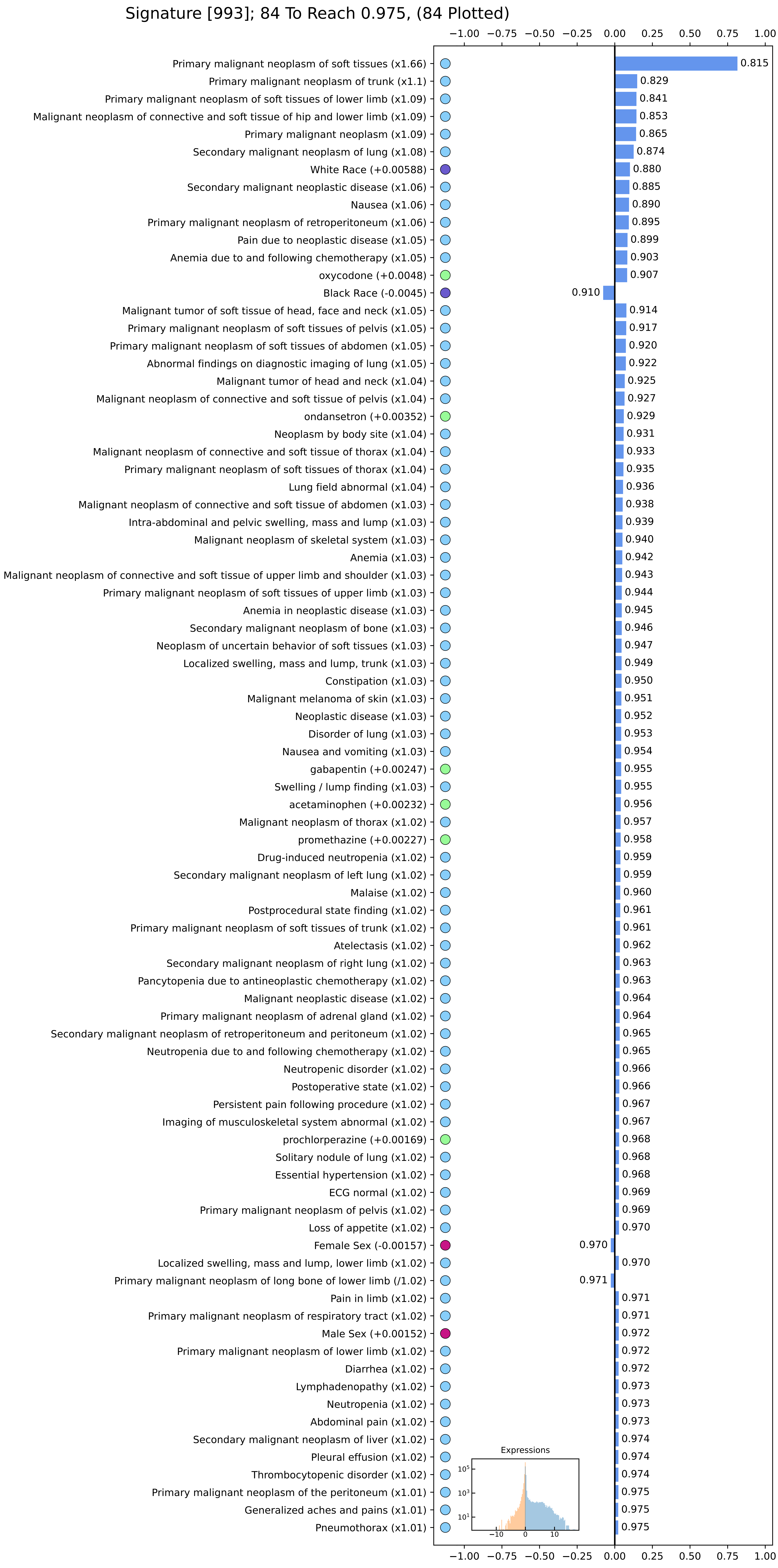

Signature [993]; 84 To Reach 0.975, (84 Plotted)
Primary malignant neoplasm of soft tissues (x1.66) 0.815
Primary malignant neoplasm of trunk (x1.1) 0.829
Primary malignant neoplasm of soft tissues of lower limb (x1.09) 0.841
Malignant neoplasm of connective and soft tissue of hip and lower limb (x1.09) 0.853
Primary malignant neoplasm (x1.09) 0.865
Secondary malignant neoplasm of lung (x1.08) 0.874
White Race (+0.00588) 0.880
Secondary malignant neoplastic disease (x1.06) 0.885
Nausea (x1.06) 0.890
Primary malignant neoplasm of retroperitoneum (x1.06) 0.895
Pain due to neoplastic disease (x1.05) 0.899
Anemia due to and following chemotherapy (x1.05) 0.903
oxycodone (+0.0048) 0.907
Black Race (-0.0045) 0.910
Malignant tumor of soft tissue of head, face and neck (x1.05) 0.914
Primary malignant neoplasm of soft tissues of pelvis (x1.05) 0.917
Primary malignant neoplasm of soft tissues of abdomen (x1.05) 0.920
Abnormal findings on diagnostic imaging of lung (x1.05) 0.922
Malignant tumor of head and neck (x1.04) 0.925
Malignant neoplasm of connective and soft tissue of pelvis (x1.04) 0.927
ondansetron (+0.00352) 0.929
Neoplasm by body site (x1.04) 0.931
Malignant neoplasm of connective and soft tissue of thorax (x1.04) 0.933
Primary malignant neoplasm of soft tissues of thorax (x1.04) 0.935
Lung field abnormal (x1.04) 0.936
Malignant neoplasm of connective and soft tissue of abdomen (x1.03) 0.938
Intra-abdominal and pelvic swelling, mass and lump (x1.03) 0.939
Malignant neoplasm of skeletal system (x1.03) 0.940
Anemia (x1.03) 0.942
Malignant neoplasm of connective and soft tissue of upper limb and shoulder (x1.03) 0.943
Primary malignant neoplasm of soft tissues of upper limb (x1.03) 0.944
Anemia in neoplastic disease (x1.03) 0.945
Secondary malignant neoplasm of bone (x1.03) 0.946
Neoplasm of uncertain behavior of soft tissues (x1.03) 0.947
Localized swelling, mass and lump, trunk (x1.03) 0.949
Constipation (x1.03) 0.950
Malignant melanoma of skin (x1.03) 0.951
Neoplastic disease (x1.03) 0.952
Disorder of lung (x1.03) 0.953
Nausea and vomiting (x1.03) 0.954
gabapentin (+0.00247) 0.955
Swelling / lump finding (x1.03) 0.955
acetaminophen (+0.00232) 0.956
Malignant neoplasm of thorax (x1.02) 0.957
promethazine (+0.00227) 0.958
Drug-induced neutropenia (x1.02) 0.959
Secondary malignant neoplasm of left lung (x1.02) 0.959
Malaise (x1.02) 0.960
Postprocedural state finding (x1.02) 0.961
Primary malignant neoplasm of soft tissues of trunk (x1.02) 0.961
Atelectasis (x1.02) 0.962
Secondary malignant neoplasm of right lung (x1.02) 0.963
Pancytopenia due to antineoplastic chemotherapy (x1.02) 0.963
Malignant neoplastic disease (x1.02) 0.964
Primary malignant neoplasm of adrenal gland (x1.02) 0.964
Secondary malignant neoplasm of retroperitoneum and peritoneum (x1.02) 0.965
Neutropenia due to and following chemotherapy (x1.02) 0.965
Neutropenic disorder (x1.02) 0.966
Postoperative state (x1.02) 0.966
Persistent pain following procedure (x1.02) 0.967
Imaging of musculoskeletal system abnormal (x1.02) 0.967
prochlorperazine (+0.00169) 0.968
Solitary nodule of lung (x1.02) 0.968
Essential hypertension (x1.02) 0.968
ECG normal (x1.02) 0.969
Primary malignant neoplasm of pelvis (x1.02) 0.969
Loss of appetite (x1.02) 0.970
Female Sex (-0.00157) 0.970
Localized swelling, mass and lump, lower limb (x1.02) 0.970
Primary malignant neoplasm of long bone of lower limb (/1.02) 0.971
Pain in limb (x1.02) 0.971
Primary malignant neoplasm of respiratory tract (x1.02) 0.971
Male Sex (+0.00152) 0.972
Primary malignant neoplasm of lower limb (x1.02) 0.972
Diarrhea (x1.02) 0.972
Lymphadenopathy (x1.02) 0.973
Neutropenia (x1.02) 0.973
Abdominal pain (x1.02) 0.973
Secondary malignant neoplasm of liver (x1.02) 0.974
Pleural effusion (x1.02) 0.974
Thrombocytopenic disorder (x1.02) 0.974
Primary malignant neoplasm of the peritoneum (x1.01) 0.975
Generalized aches and pains (x1.01) 0.975
Pneumothorax (x1.01) 0.975
−1.00 −0.75 −0.50 −0.25 0.00 0.25 0.50 0.75 1.00
Expressions
10^5 10^3 10^1
−10 0 10

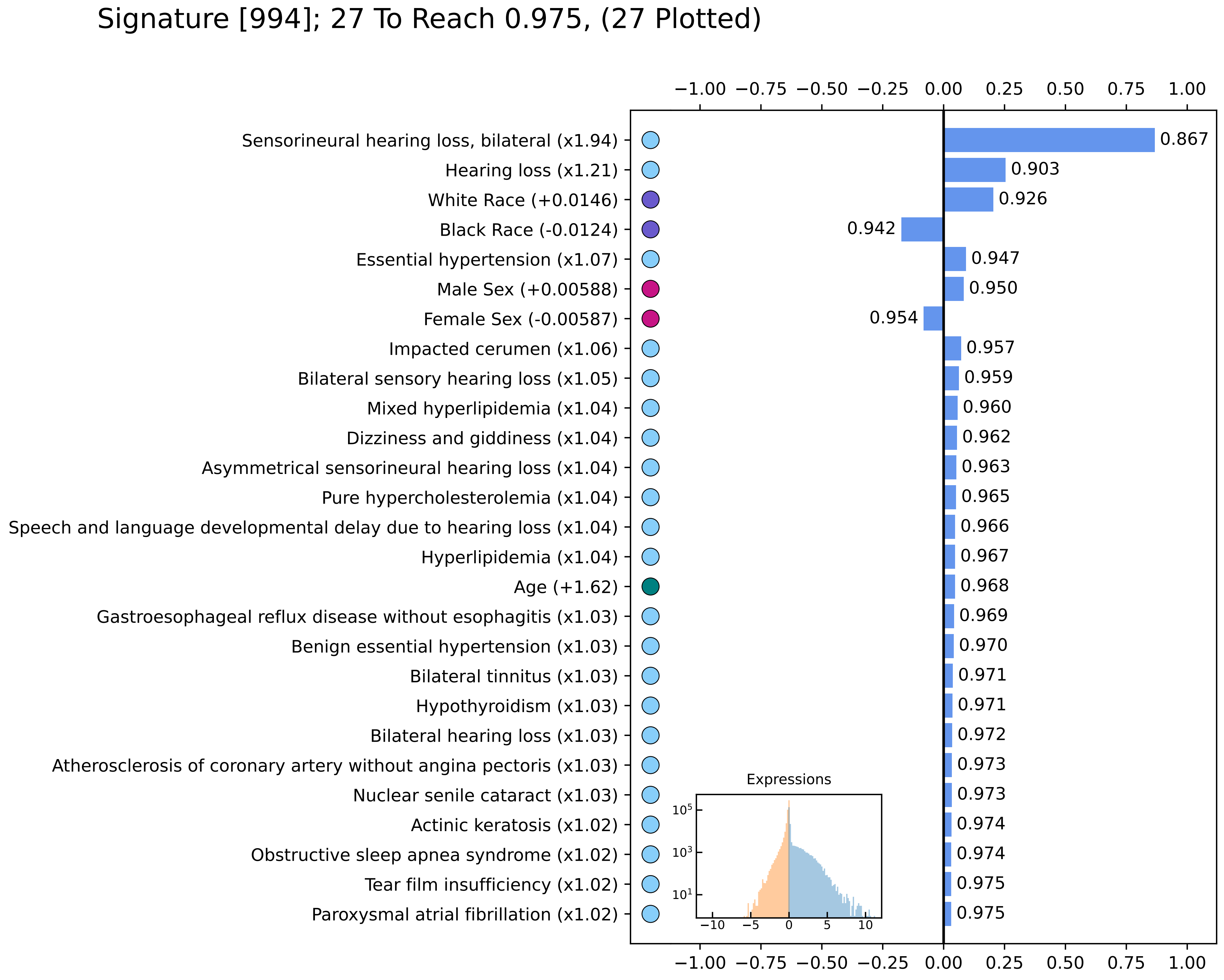

Signature [994]; 27 To Reach 0.975, (27 Plotted)
Sensorineural hearing loss, bilateral (x1.94)
Hearing loss (x1.21)
White Race (+0.0146)
Black Race (-0.0124)
Essential hypertension (x1.07)
Male Sex (+0.00588)
Female Sex (-0.00587)
Impacted cerumen (x1.06)
Bilateral sensory hearing loss (x1.05)
Mixed hyperlipidemia (x1.04)
Dizziness and giddiness (x1.04)
Asymmetrical sensorineural hearing loss (x1.04)
Pure hypercholesterolemia (x1.04)
Speech and language developmental delay due to hearing loss (x1.04)
Hyperlipidemia (x1.04)
Age (+1.62)
Gastroesophageal reflux disease without esophagitis (x1.03)
Benign essential hypertension (x1.03)
Bilateral tinnitus (x1.03)
Hypothyroidism (x1.03)
Bilateral hearing loss (x1.03)
Atherosclerosis of coronary artery without angina pectoris (x1.03)
Nuclear senile cataract (x1.03)
Actinic keratosis (x1.02)
Obstructive sleep apnea syndrome (x1.02)
Tear film insufficiency (x1.02)
Paroxysmal atrial fibrillation (x1.02)
0.867
0.903
0.926
0.942
0.947
0.950
0.954
0.957
0.959
0.960
0.962
0.963
0.965
0.966
0.967
0.968
0.969
0.970
0.971
0.971
0.972
0.973
0.973
0.974
0.974
0.975
0.975
−1.00 −0.75 −0.50 −0.25 0.00 0.25 0.50 0.75 1.00
Expressions
10^5
10^3
10^1
−10 −5 0 5 10

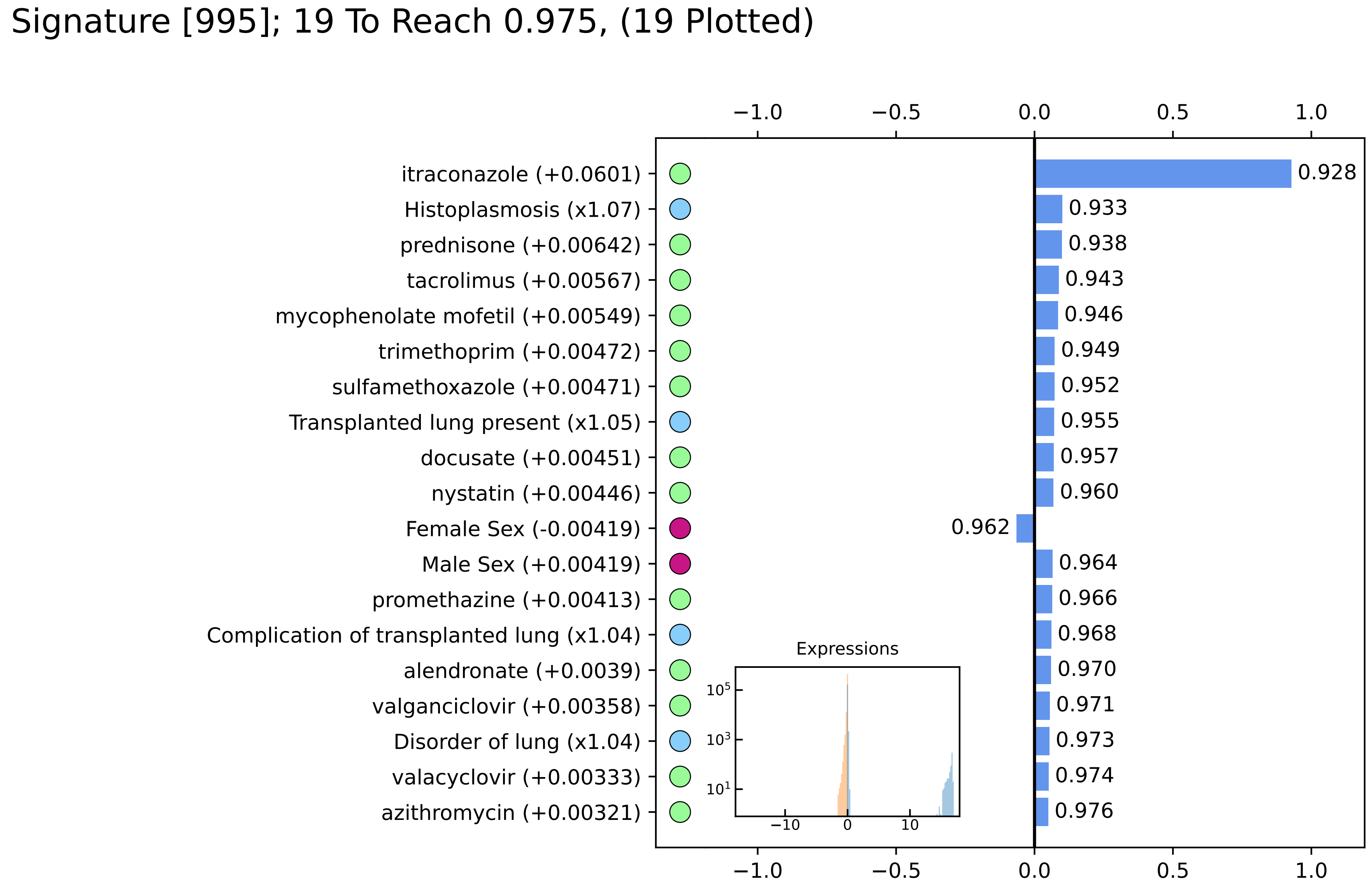

Signature [995]; 19 To Reach 0.975, (19 Plotted)
itraconazole (+0.0601)
Histoplasmosis (x1.07)
prednisone (+0.00642)
tacrolimus (+0.00567)
mycophenolate mofetil (+0.00549)
trimethoprim (+0.00472)
sulfamethoxazole (+0.00471)
Transplanted lung present (x1.05)
docusate (+0.00451)
nystatin (+0.00446)
Female Sex (-0.00419)
Male Sex (+0.00419)
promethazine (+0.00413)
Complication of transplanted lung (x1.04)
alendronate (+0.0039)
valganciclovir (+0.00358)
Disorder of lung (x1.04)
valacyclovir (+0.00333)
azithromycin (+0.00321)
0.928
0.933
0.938
0.943
0.946
0.949
0.952
0.955
0.957
0.960
0.962
0.964
0.966
0.968
0.970
0.971
0.973
0.974
0.976
−1.0
−0.5
0.0
0.5
1.0
Expressions
10^5
10^3
10^1
−10
0
10

Signature [996]; 1 To Reach 0.975, (10 Plotted)

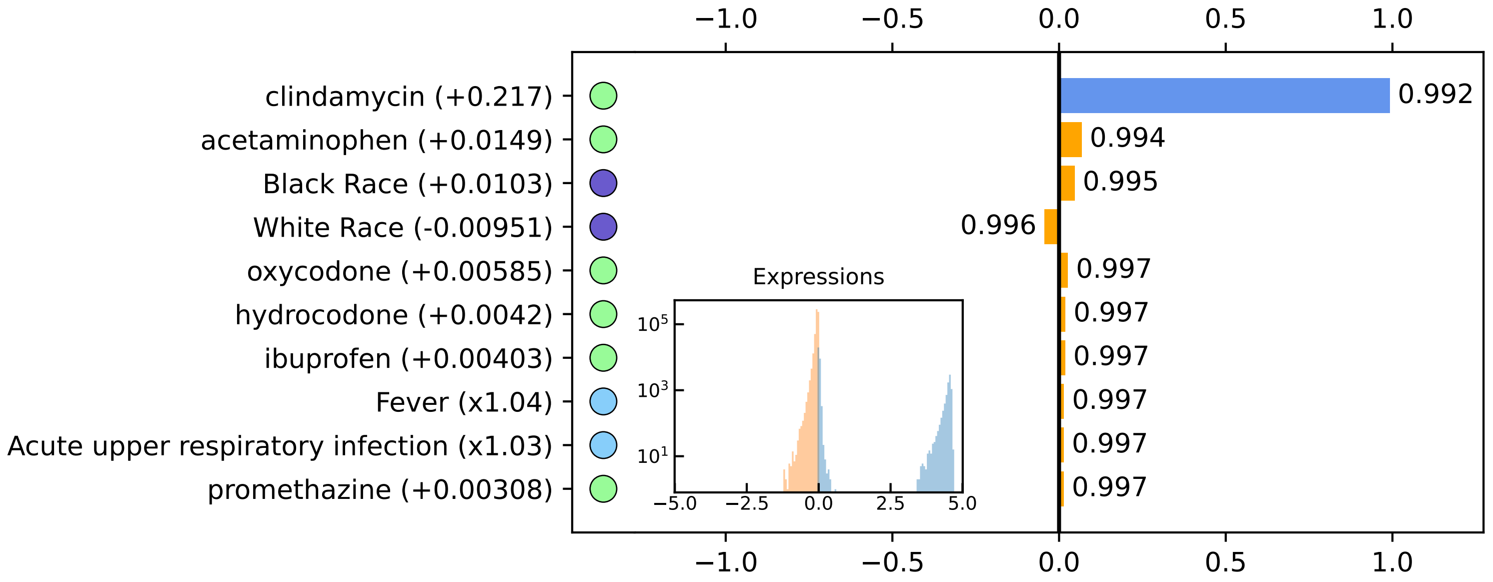

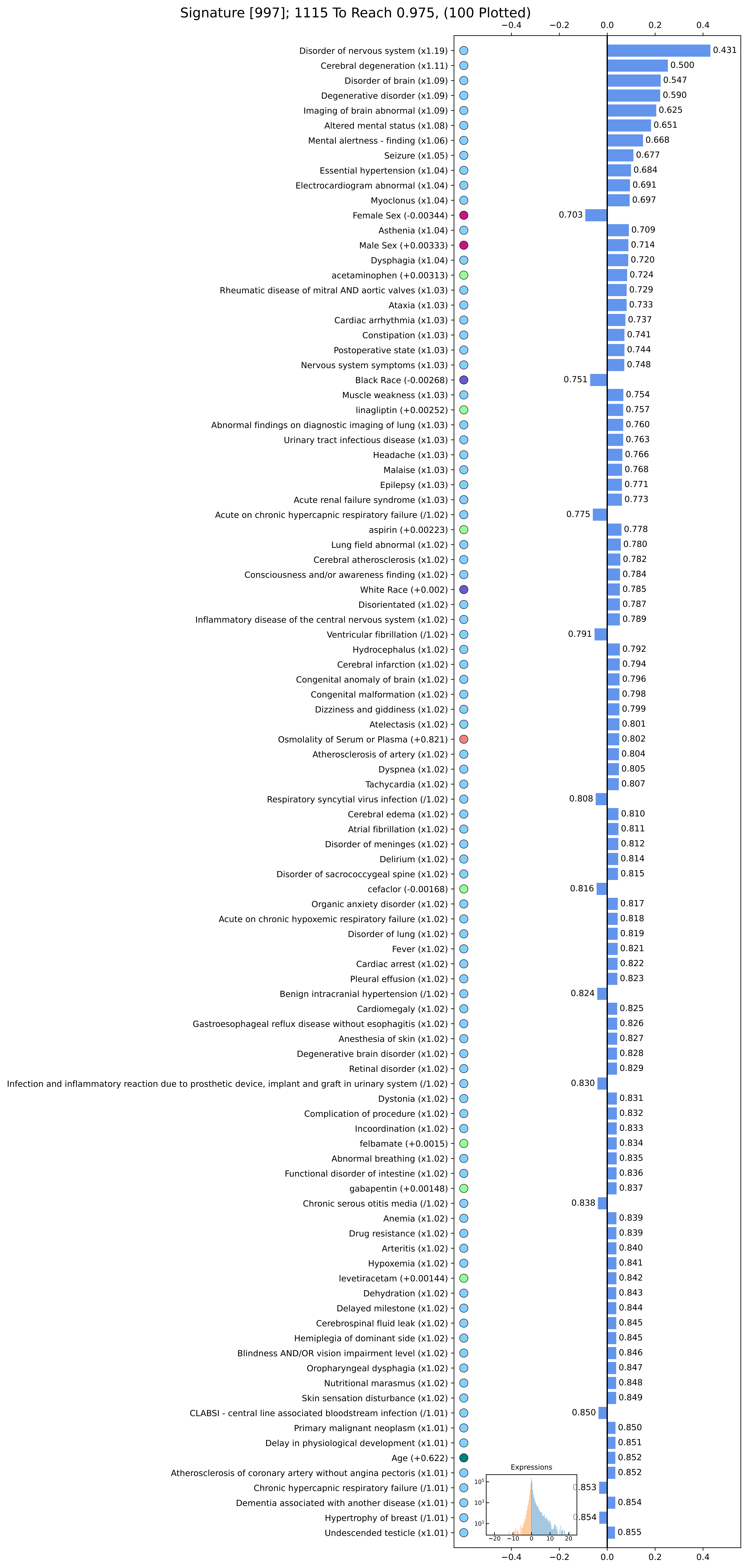

Signature [997]; 1115 To Reach 0.975, (100 Plotted)
Disorder of nervous system (x1.19) 0.431
Cerebral degeneration (x1.11) 0.500
Disorder of brain (x1.09) 0.547
Degenerative disorder (x1.09) 0.590
Imaging of brain abnormal (x1.09) 0.625
Altered mental status (x1.08) 0.651
Mental alertness - finding (x1.06) 0.668
Seizure (x1.05) 0.677
Essential hypertension (x1.04) 0.684
Electrocardiogram abnormal (x1.04) 0.691
Myoclonus (x1.04) 0.697
Female Sex (-0.00344) 0.703
Asthenia (x1.04) 0.709
Male Sex (+0.00333) 0.714
Dysphagia (x1.04) 0.720
acetaminophen (+0.00313) 0.724
Rheumatic disease of mitral AND aortic valves (x1.03) 0.729
Ataxia (x1.03) 0.733
Cardiac arrhythmia (x1.03) 0.737
Constipation (x1.03) 0.741
Postoperative state (x1.03) 0.744
Nervous system symptoms (x1.03) 0.748
Black Race (-0.00268) 0.751
Muscle weakness (x1.03) 0.754
linagliptin (+0.00252) 0.757
Abnormal findings on diagnostic imaging of lung (x1.03) 0.760
Urinary tract infectious disease (x1.03) 0.763
Headache (x1.03) 0.766
Malaise (x1.03) 0.768
Epilepsy (x1.03) 0.771
Acute renal failure syndrome (x1.03) 0.773
Acute on chronic hypercapnic respiratory failure (/1.02) 0.775
aspirin (+0.00223) 0.778
Lung field abnormal (x1.02) 0.780
Cerebral atherosclerosis (x1.02) 0.782
Consciousness and/or awareness finding (x1.02) 0.784
White Race (+0.002) 0.785
Disorientated (x1.02) 0.787
Inflammatory disease of the central nervous system (x1.02) 0.789
Ventricular fibrillation (/1.02) 0.791
Hydrocephalus (x1.02) 0.792
Cerebral infarction (x1.02) 0.794
Congenital anomaly of brain (x1.02) 0.796
Congenital malformation (x1.02) 0.798
Dizziness and giddiness (x1.02) 0.799
Atelectasis (x1.02) 0.801
Osmolality of Serum or Plasma (+0.821) 0.802
Atherosclerosis of artery (x1.02) 0.804
Dyspnea (x1.02) 0.805
Tachycardia (x1.02) 0.807
Respiratory syncytial virus infection (/1.02) 0.808
Cerebral edema (x1.02) 0.810
Atrial fibrillation (x1.02) 0.811
Disorder of meninges (x1.02) 0.812
Delirium (x1.02) 0.814
Disorder of sacrococcygeal spine (x1.02) 0.815
cefaclor (-0.00168) 0.816
Organic anxiety disorder (x1.02) 0.817
Acute on chronic hypoxemic respiratory failure (x1.02) 0.818
Disorder of lung (x1.02) 0.819
Fever (x1.02) 0.821
Cardiac arrest (x1.02) 0.822
Pleural effusion (x1.02) 0.823
Benign intracranial hypertension (/1.02) 0.824
Cardiomegaly (x1.02) 0.825
Gastroesophageal reflux disease without esophagitis (x1.02) 0.826
Anesthesia of skin (x1.02) 0.827
Degenerative brain disorder (x1.02) 0.828
Retinal disorder (x1.02) 0.829
Infection and inflammatory reaction due to prosthetic device, implant and graft in urinary system (/1.02) 0.830
Dystonia (x1.02) 0.831
Complication of procedure (x1.02) 0.832
Incoordination (x1.02) 0.833
felbamate (+0.0015) 0.834
Abnormal breathing (x1.02) 0.835
Functional disorder of intestine (x1.02) 0.836
gabapentin (+0.00148) 0.837
Chronic serous otitis media (/1.02) 0.838
Anemia (x1.02) 0.839
Drug resistance (x1.02) 0.839
Arteritis (x1.02) 0.840
Hypoxemia (x1.02) 0.841
levetiracetam (+0.00144) 0.842
Dehydration (x1.02) 0.843
Delayed milestone (x1.02) 0.844
Cerebrospinal fluid leak (x1.02) 0.845
Hemiplegia of dominant side (x1.02) 0.845
Blindness AND/OR vision impairment level (x1.02) 0.846
Oropharyngeal dysphagia (x1.02) 0.847
Nutritional marasmus (x1.02) 0.848
Skin sensation disturbance (x1.02) 0.849
CLABSI - central line associated bloodstream infection (/1.01) 0.850
Primary malignant neoplasm (x1.01) 0.850
Delay in physiological development (x1.01) 0.851
Age (+0.622) 0.852
Atherosclerosis of coronary artery without angina pectoris (x1.01) 0.852
Chronic hypercapnic respiratory failure (/1.01) 0.853
Dementia associated with another disease (x1.01) 0.854
Hypertrophy of breast (/1.01) 0.854
Undescended testicle (x1.01) 0.855
Expressions
−0.4 −0.2 0.0 0.2 0.4

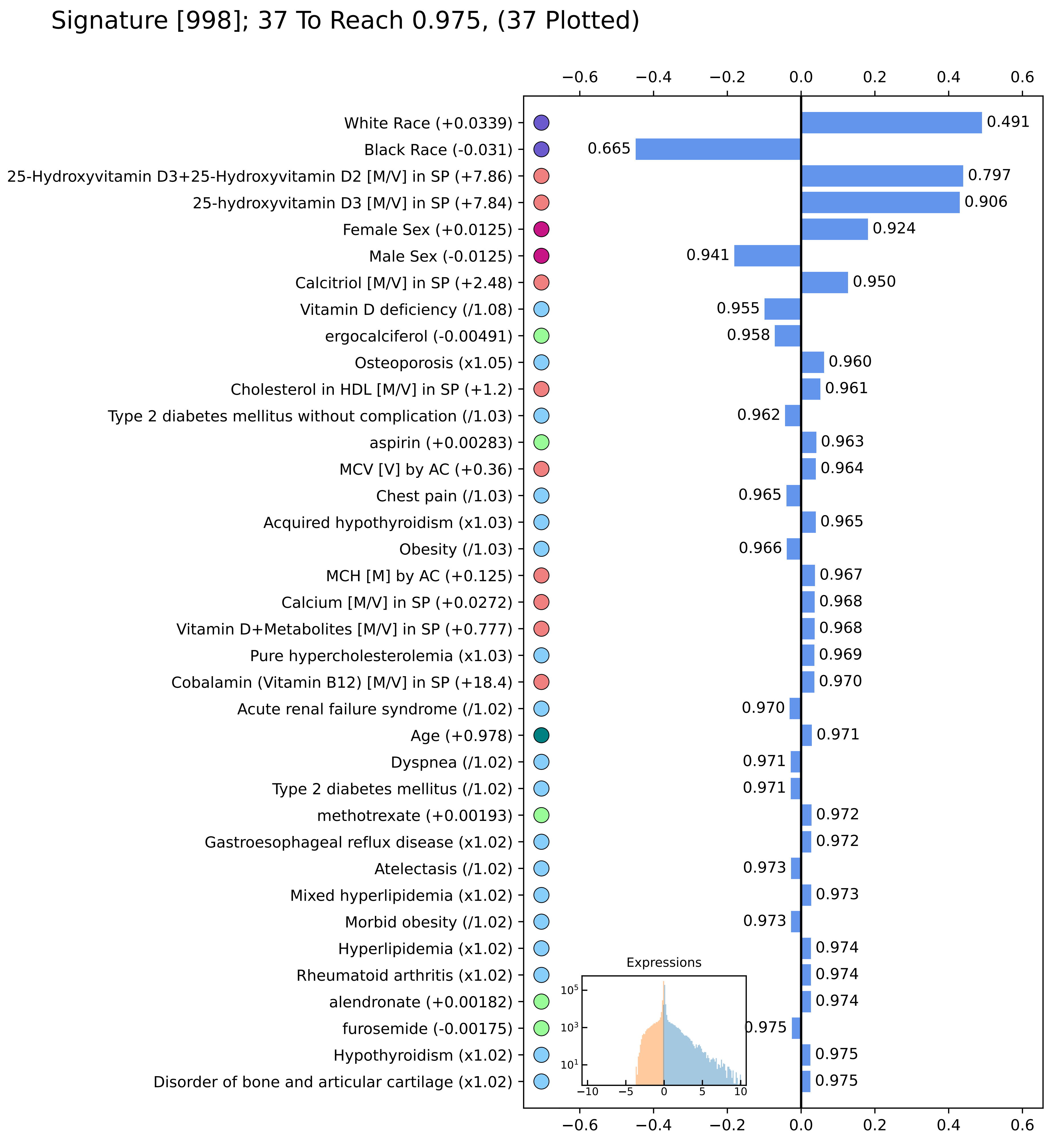

Signature [998]; 37 To Reach 0.975, (37 Plotted)
White Race (+0.0339)
Black Race (-0.031)
25-Hydroxyvitamin D3+25-Hydroxyvitamin D2 [M/V] in SP (+7.86)
25-hydroxyvitamin D3 [M/V] in SP (+7.84)
Female Sex (+0.0125)
Male Sex (-0.0125)
Calcitriol [M/V] in SP (+2.48)
Vitamin D deficiency (/1.08)
ergocalciferol (-0.00491)
Osteoporosis (x1.05)
Cholesterol in HDL [M/V] in SP (+1.2)
Type 2 diabetes mellitus without complication (/1.03)
aspirin (+0.00283)
MCV [V] by AC (+0.36)
Chest pain (/1.03)
Acquired hypothyroidism (x1.03)
Obesity (/1.03)
MCH [M] by AC (+0.125)
Calcium [M/V] in SP (+0.0272)
Vitamin D+Metabolites [M/V] in SP (+0.777)
Pure hypercholesterolemia (x1.03)
Cobalamin (Vitamin B12) [M/V] in SP (+18.4)
Acute renal failure syndrome (/1.02)
Age (+0.978)
Dyspnea (/1.02)
Type 2 diabetes mellitus (/1.02)
methotrexate (+0.00193)
Gastroesophageal reflux disease (x1.02)
Atelectasis (/1.02)
Mixed hyperlipidemia (x1.02)
Morbid obesity (/1.02)
Hyperlipidemia (x1.02)
Rheumatoid arthritis (x1.02)
alendronate (+0.00182)
furosemide (-0.00175)
Hypothyroidism (x1.02)
Disorder of bone and articular cartilage (x1.02)
0.491
0.665
0.797
0.906
0.924
0.941
0.950
0.955
0.958
0.960
0.961
0.962
0.963
0.964
0.965
0.965
0.966
0.967
0.968
0.968
0.969
0.970
0.970
0.971
0.971
0.971
0.972
0.972
0.973
0.973
0.973
0.974
0.974
0.974
0.975
0.975
0.975
−0.6 −0.4 −0.2 0.0 0.2 0.4 0.6
Expressions
10^5
10^3
10^1
−10 −5 0 5 10

Signature [999]; 4 To Reach 0.975, (10 Plotted)

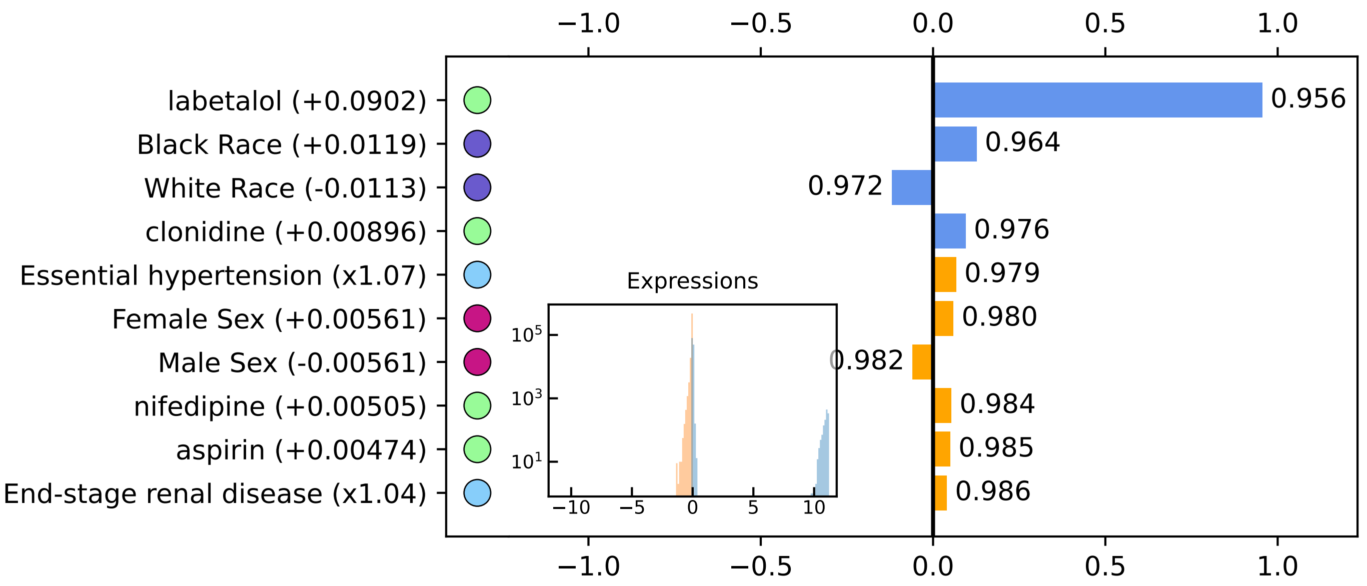

Signature [1000]; 206 To Reach 0.975, (100 Plotted)

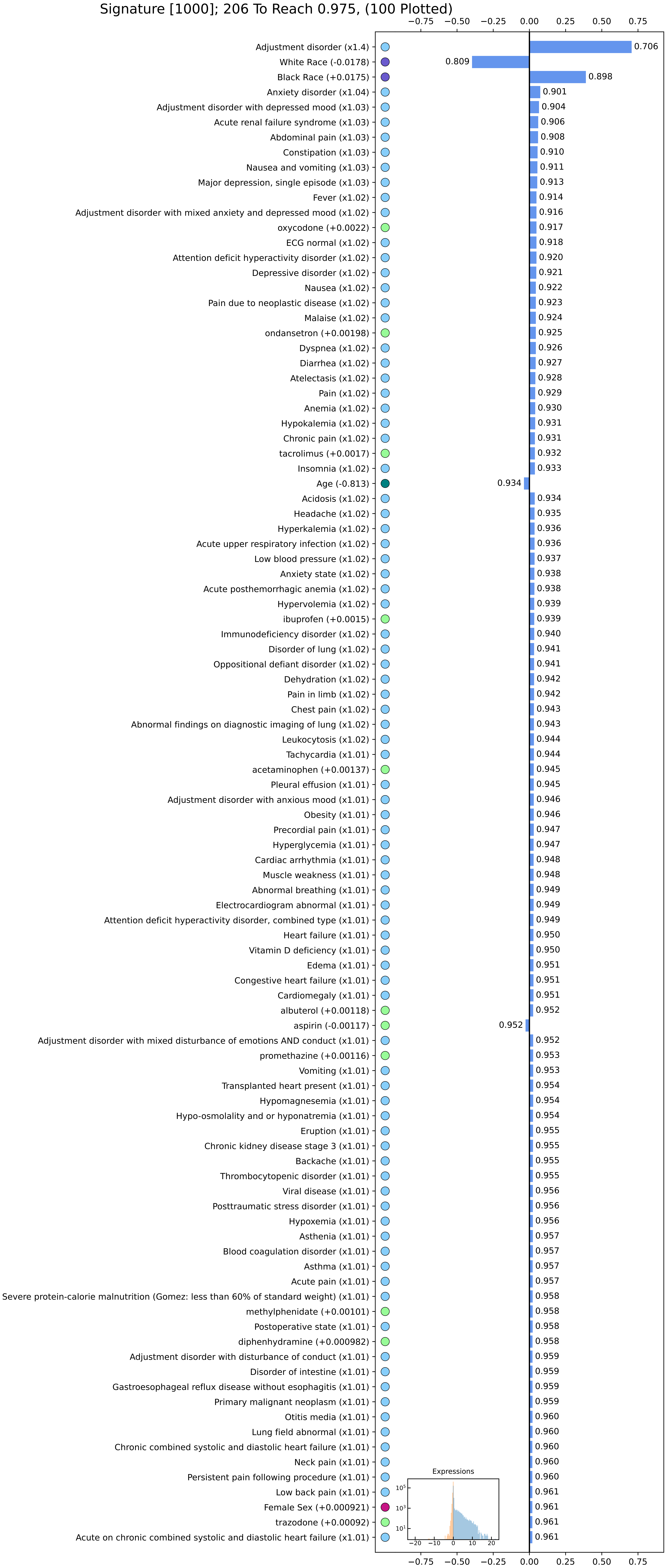

Signature [1001]; 253 To Reach 0.975, (100 Plotted)

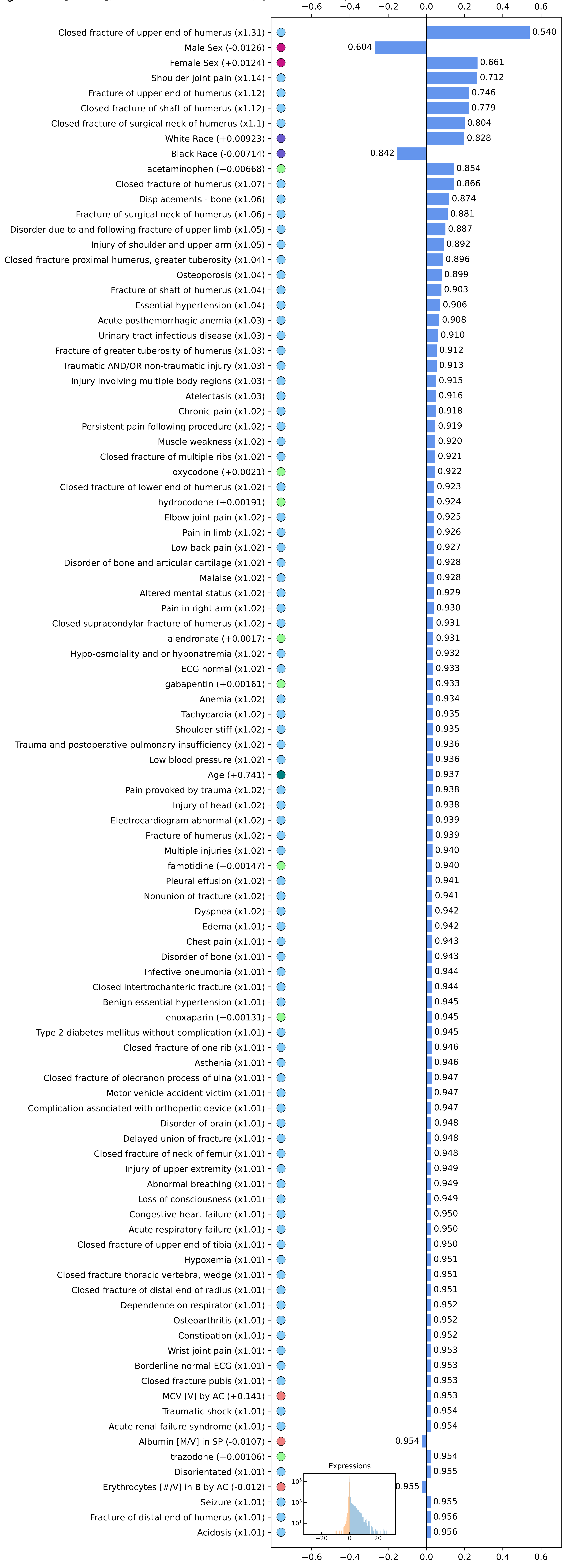

Signature [1002]; 477 To Reach 0.975, (100 Plotted)

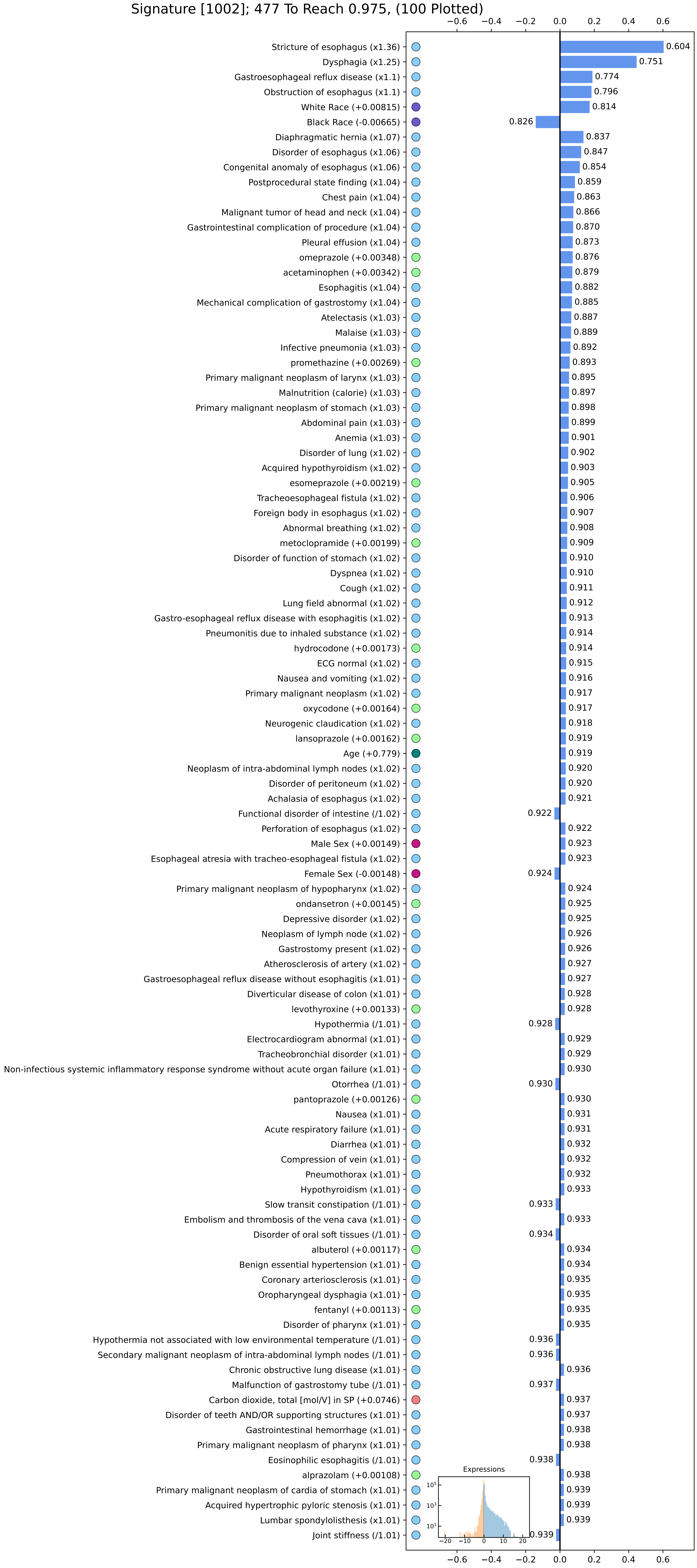

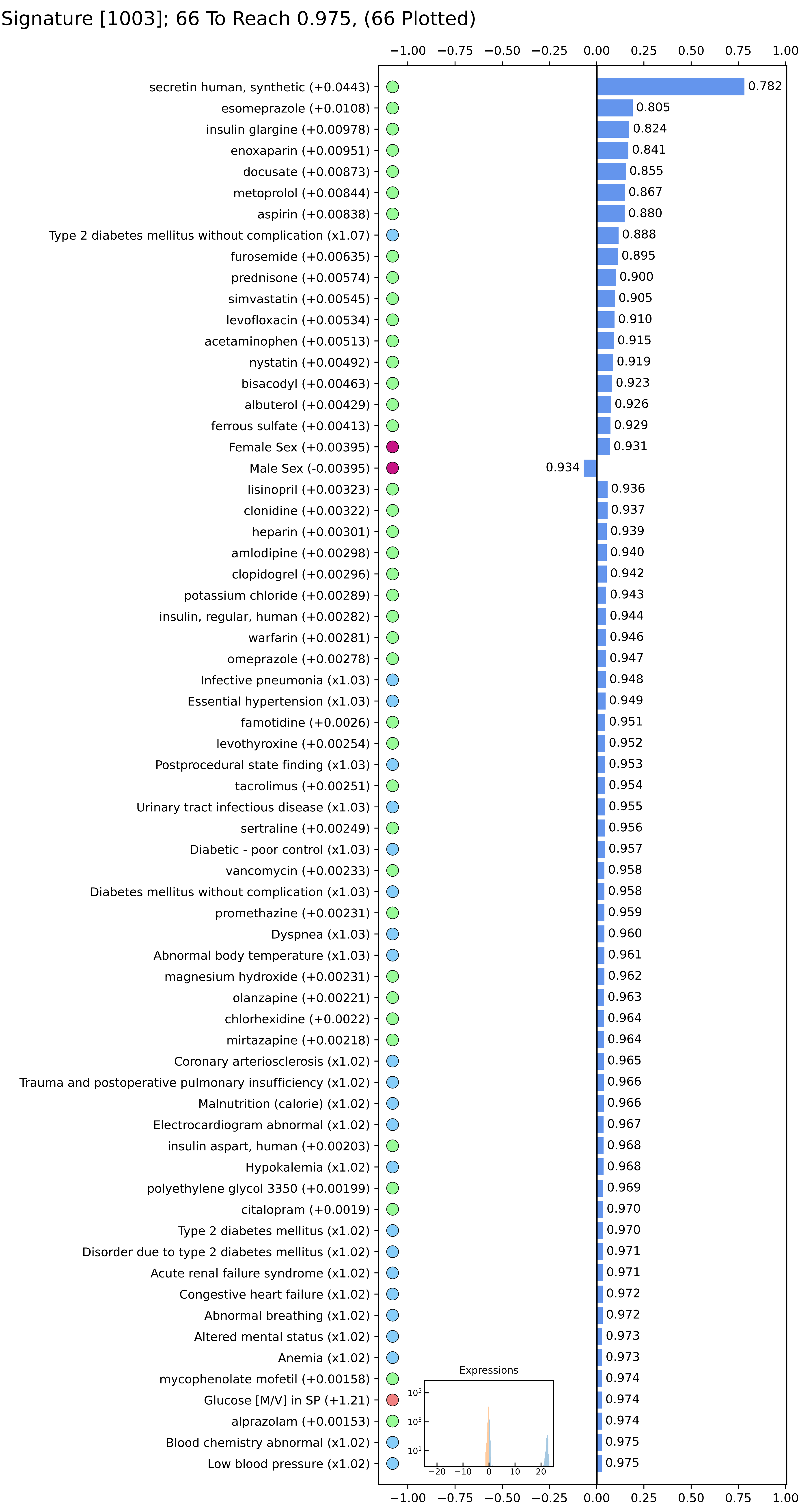
Signature [1003]; 66 To Reach 0.975, (66 Plotted)
−1.00 −0.75 −0.50 −0.25 0.00 0.25 0.50 0.75 1.00
secretin human, synthetic (+0.0443) 0.782
esomeprazole (+0.0108) 0.805
insulin glargine (+0.00978) 0.824
enoxaparin (+0.00951) 0.841
docusate (+0.00873) 0.855
metoprolol (+0.00844) 0.867
aspirin (+0.00838) 0.880
Type 2 diabetes mellitus without complication (x1.07) 0.888
furosemide (+0.00635) 0.895
prednisone (+0.00574) 0.900
simvastatin (+0.00545) 0.905
levofloxacin (+0.00534) 0.910
acetaminophen (+0.00513) 0.915
nystatin (+0.00492) 0.919
bisacodyl (+0.00463) 0.923
albuterol (+0.00429) 0.926
ferrous sulfate (+0.00413) 0.929
Female Sex (+0.00395) 0.931
Male Sex (-0.00395) 0.934
lisinopril (+0.00323) 0.936
clonidine (+0.00322) 0.937
heparin (+0.00301) 0.939
amlodipine (+0.00298) 0.940
clopidogrel (+0.00296) 0.942
potassium chloride (+0.00289) 0.943
insulin, regular, human (+0.00282) 0.944
warfarin (+0.00281) 0.946
omeprazole (+0.00278) 0.947
Infective pneumonia (x1.03) 0.948
Essential hypertension (x1.03) 0.949
famotidine (+0.0026) 0.951
levothyroxine (+0.00254) 0.952
Postprocedural state finding (x1.03) 0.953
tacrolimus (+0.00251) 0.954
Urinary tract infectious disease (x1.03) 0.955
sertraline (+0.00249) 0.956
Diabetic - poor control (x1.03) 0.957
vancomycin (+0.00233) 0.958
Diabetes mellitus without complication (x1.03) 0.958
promethazine (+0.00231) 0.959
Dyspnea (x1.03) 0.960
Abnormal body temperature (x1.03) 0.961
magnesium hydroxide (+0.00231) 0.962
olanzapine (+0.00221) 0.963
chlorhexidine (+0.0022) 0.964
mirtazapine (+0.00218) 0.964
Coronary arteriosclerosis (x1.02) 0.965
Trauma and postoperative pulmonary insufficiency (x1.02) 0.966
Malnutrition (calorie) (x1.02) 0.966
Electrocardiogram abnormal (x1.02) 0.967
insulin aspart, human (+0.00203) 0.968
Hypokalemia (x1.02) 0.968
polyethylene glycol 3350 (+0.00199) 0.969
citalopram (+0.0019) 0.970
Type 2 diabetes mellitus (x1.02) 0.970
Disorder due to type 2 diabetes mellitus (x1.02) 0.971
Acute renal failure syndrome (x1.02) 0.971
Congestive heart failure (x1.02) 0.972
Abnormal breathing (x1.02) 0.972
Altered mental status (x1.02) 0.973
Anemia (x1.02) 0.973
mycophenolate mofetil (+0.00158) 0.974
Glucose [M/V] in SP (+1.21) 0.974
alprazolam (+0.00153) 0.974
Blood chemistry abnormal (x1.02) 0.975
Low blood pressure (x1.02) 0.975
Expressions
10^5
10^3
10^1
−20 −10 0 10 20
−1.00 −0.75 −0.50 −0.25 0.00 0.25 0.50 0.75 1.00

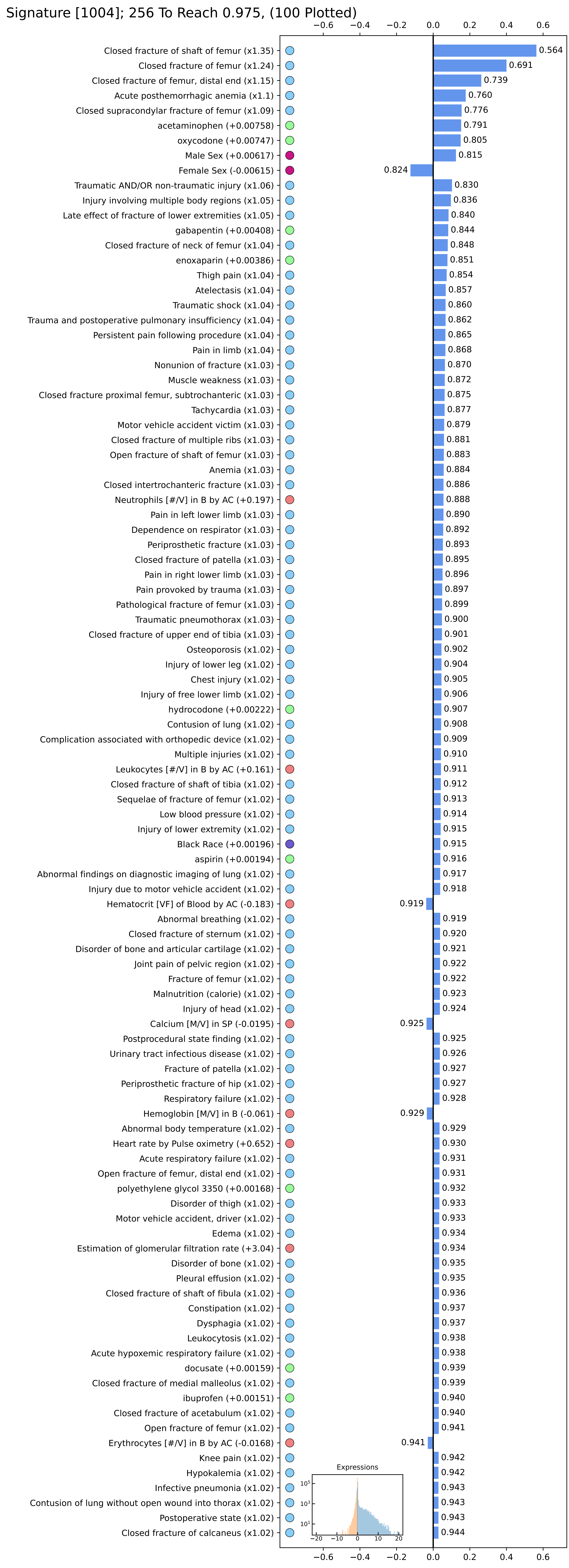

Signature [1004]; 256 To Reach 0.975, (100 Plotted)
Closed fracture of shaft of femur (x1.35) 0.564
Closed fracture of femur (x1.24) 0.691
Closed fracture of femur, distal end (x1.15) 0.739
Acute posthemorrhagic anemia (x1.1) 0.760
Closed supracondylar fracture of femur (x1.09) 0.776
acetaminophen (+0.00758) 0.791
oxycodone (+0.00747) 0.805
Male Sex (+0.00617) 0.815
Female Sex (-0.00615) 0.824
Traumatic AND/OR non-traumatic injury (x1.06) 0.830
Injury involving multiple body regions (x1.05) 0.836
Late effect of fracture of lower extremities (x1.05) 0.840
gabapentin (+0.00408) 0.844
Closed fracture of neck of femur (x1.04) 0.848
enoxaparin (+0.00386) 0.851
Thigh pain (x1.04) 0.854
Atelectasis (x1.04) 0.857
Traumatic shock (x1.04) 0.860
Trauma and postoperative pulmonary insufficiency (x1.04) 0.862
Persistent pain following procedure (x1.04) 0.865
Pain in limb (x1.04) 0.868
Nonunion of fracture (x1.03) 0.870
Muscle weakness (x1.03) 0.872
Closed fracture proximal femur, subtrochanteric (x1.03) 0.875
Tachycardia (x1.03) 0.877
Motor vehicle accident victim (x1.03) 0.879
Closed fracture of multiple ribs (x1.03) 0.881
Open fracture of shaft of femur (x1.03) 0.883
Anemia (x1.03) 0.884
Closed intertrochanteric fracture (x1.03) 0.886
Neutrophils [#/V] in B by AC (+0.197) 0.888
Pain in left lower limb (x1.03) 0.890
Dependence on respirator (x1.03) 0.892
Periprosthetic fracture (x1.03) 0.893
Closed fracture of patella (x1.03) 0.895
Pain in right lower limb (x1.03) 0.896
Pain provoked by trauma (x1.03) 0.897
Pathological fracture of femur (x1.03) 0.899
Traumatic pneumothorax (x1.03) 0.900
Closed fracture of upper end of tibia (x1.03) 0.901
Osteoporosis (x1.02) 0.902
Injury of lower leg (x1.02) 0.904
Chest injury (x1.02) 0.905
Injury of free lower limb (x1.02) 0.906
hydrocodone (+0.00222) 0.907
Contusion of lung (x1.02) 0.908
Complication associated with orthopedic device (x1.02) 0.909
Multiple injuries (x1.02) 0.910
Leukocytes [#/V] in B by AC (+0.161) 0.911
Closed fracture of shaft of tibia (x1.02) 0.912
Sequelae of fracture of femur (x1.02) 0.913
Low blood pressure (x1.02) 0.914
Injury of lower extremity (x1.02) 0.915
Black Race (+0.00196) 0.915
aspirin (+0.00194) 0.916
Abnormal findings on diagnostic imaging of lung (x1.02) 0.917
Injury due to motor vehicle accident (x1.02) 0.918
Hematocrit [VF] of Blood by AC (-0.183) 0.919
Abnormal breathing (x1.02) 0.919
Closed fracture of sternum (x1.02) 0.920
Disorder of bone and articular cartilage (x1.02) 0.921
Joint pain of pelvic region (x1.02) 0.922
Fracture of femur (x1.02) 0.922
Malnutrition (calorie) (x1.02) 0.923
Injury of head (x1.02) 0.924
Calcium [M/V] in SP (-0.0195) 0.925
Postprocedural state finding (x1.02) 0.925
Urinary tract infectious disease (x1.02) 0.926
Fracture of patella (x1.02) 0.927
Periprosthetic fracture of hip (x1.02) 0.927
Respiratory failure (x1.02) 0.928
Hemoglobin [M/V] in B (-0.061) 0.929
Abnormal body temperature (x1.02) 0.929
Heart rate by Pulse oximetry (+0.652) 0.930
Acute respiratory failure (x1.02) 0.931
Open fracture of femur, distal end (x1.02) 0.931
polyethylene glycol 3350 (+0.00168) 0.932
Disorder of thigh (x1.02) 0.933
Motor vehicle accident, driver (x1.02) 0.933
Edema (x1.02) 0.934
Estimation of glomerular filtration rate (+3.04) 0.934
Disorder of bone (x1.02) 0.935
Pleural effusion (x1.02) 0.935
Closed fracture of shaft of fibula (x1.02) 0.936
Constipation (x1.02) 0.937
Dysphagia (x1.02) 0.937
Leukocytosis (x1.02) 0.938
Acute hypoxemic respiratory failure (x1.02) 0.938
docusate (+0.00159) 0.939
Closed fracture of medial malleolus (x1.02) 0.939
ibuprofen (+0.00151) 0.940
Closed fracture of acetabulum (x1.02) 0.940
Open fracture of femur (x1.02) 0.941
Erythrocytes [#/V] in B by AC (-0.0168) 0.941
Knee pain (x1.02) 0.942
Hypokalemia (x1.02) 0.942
Infective pneumonia (x1.02) 0.943
Contusion of lung without open wound into thorax (x1.02) 0.943
Postoperative state (x1.02) 0.943
Closed fracture of calcaneus (x1.02) 0.944
−0.6 −0.4 −0.2 0.0 0.2 0.4 0.6
Expressions

Signature [1005]; 6 To Reach 0.975, (10 Plotted)

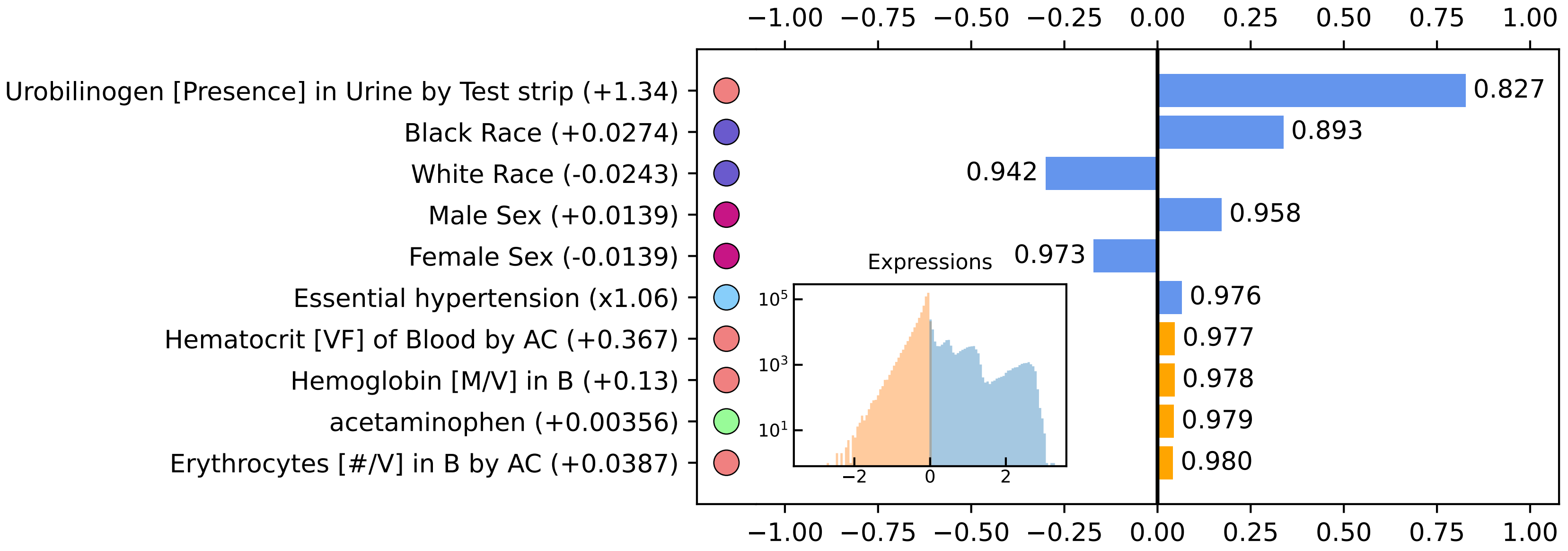

Signature [1006]; 90 To Reach 0.975, (90 Plotted)

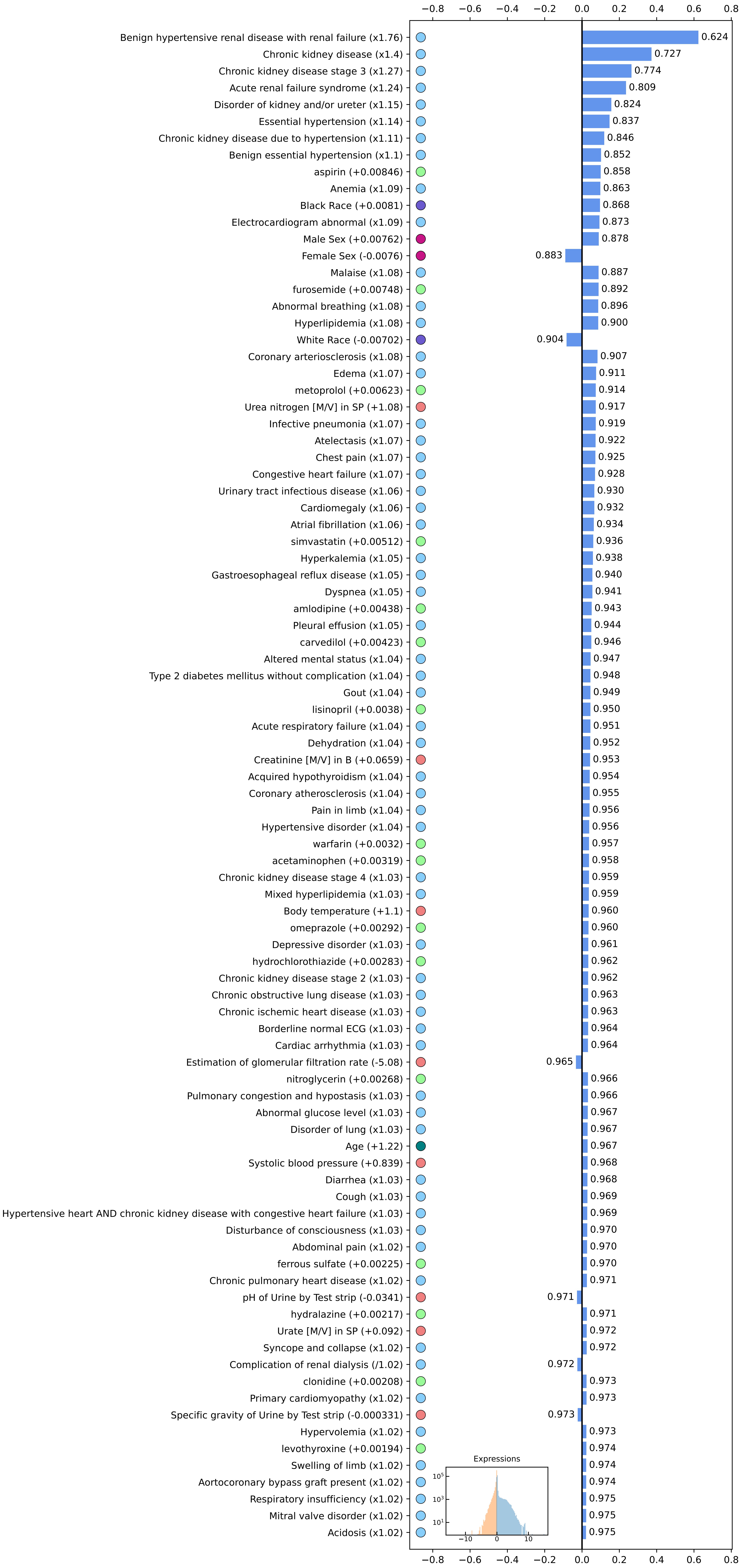

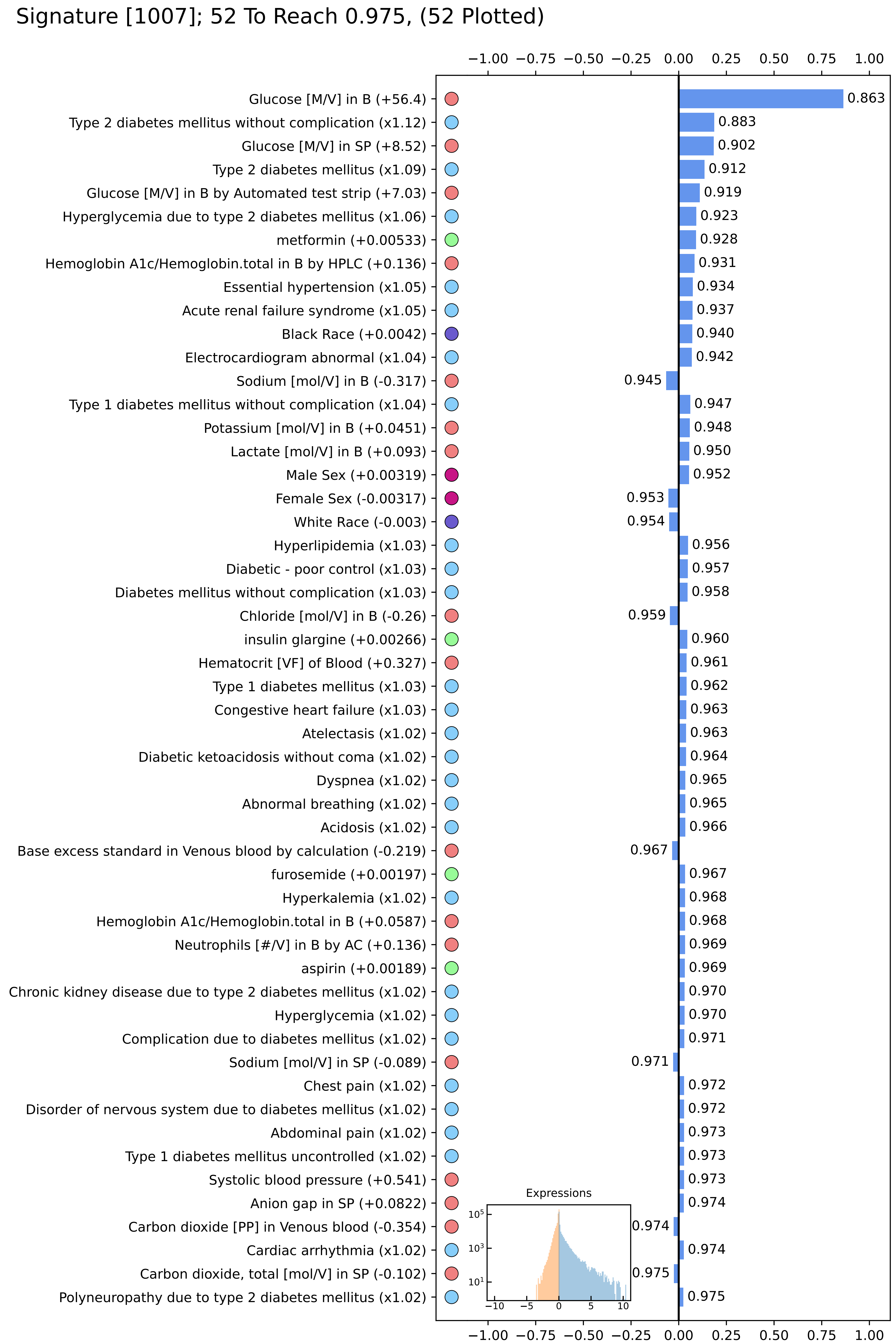

Signature [1007]; 52 To Reach 0.975, (52 Plotted)
Glucose [M/V] in B (+56.4) 0.863
Type 2 diabetes mellitus without complication (x1.12) 0.883
Glucose [M/V] in SP (+8.52) 0.902
Type 2 diabetes mellitus (x1.09) 0.912
Glucose [M/V] in B by Automated test strip (+7.03) 0.919
Hyperglycemia due to type 2 diabetes mellitus (x1.06) 0.923
metformin (+0.00533) 0.928
Hemoglobin A1c/Hemoglobin.total in B by HPLC (+0.136) 0.931
Essential hypertension (x1.05) 0.934
Acute renal failure syndrome (x1.05) 0.937
Black Race (+0.0042) 0.940
Electrocardiogram abnormal (x1.04) 0.942
Sodium [mol/V] in B (-0.317) 0.945
Type 1 diabetes mellitus without complication (x1.04) 0.947
Potassium [mol/V] in B (+0.0451) 0.948
Lactate [mol/V] in B (+0.093) 0.950
Male Sex (+0.00319) 0.952
Female Sex (-0.00317) 0.953
White Race (-0.003) 0.954
Hyperlipidemia (x1.03) 0.956
Diabetic - poor control (x1.03) 0.957
Diabetes mellitus without complication (x1.03) 0.958
Chloride [mol/V] in B (-0.26) 0.959
insulin glargine (+0.00266) 0.960
Hematocrit [VF] of Blood (+0.327) 0.961
Type 1 diabetes mellitus (x1.03) 0.962
Congestive heart failure (x1.03) 0.963
Atelectasis (x1.02) 0.963
Diabetic ketoacidosis without coma (x1.02) 0.964
Dyspnea (x1.02) 0.965
Abnormal breathing (x1.02) 0.965
Acidosis (x1.02) 0.966
Base excess standard in Venous blood by calculation (-0.219) 0.967
furosemide (+0.00197) 0.967
Hyperkalemia (x1.02) 0.968
Hemoglobin A1c/Hemoglobin.total in B (+0.0587) 0.968
Neutrophils [#/V] in B by AC (+0.136) 0.969
aspirin (+0.00189) 0.969
Chronic kidney disease due to type 2 diabetes mellitus (x1.02) 0.970
Hyperglycemia (x1.02) 0.970
Complication due to diabetes mellitus (x1.02) 0.971
Sodium [mol/V] in SP (-0.089) 0.971
Chest pain (x1.02) 0.972
Disorder of nervous system due to diabetes mellitus (x1.02) 0.972
Abdominal pain (x1.02) 0.973
Type 1 diabetes mellitus uncontrolled (x1.02) 0.973
Systolic blood pressure (+0.541) 0.973
Anion gap in SP (+0.0822) 0.974
Carbon dioxide [PP] in Venous blood (-0.354) 0.974
Cardiac arrhythmia (x1.02) 0.974
Carbon dioxide, total [mol/V] in SP (-0.102) 0.975
Polyneuropathy due to type 2 diabetes mellitus (x1.02) 0.975
−1.00 −0.75 −0.50 −0.25 0.00 0.25 0.50 0.75 1.00
Expressions

Signature [1008]; 5 To Reach 0.975, (10 Plotted)

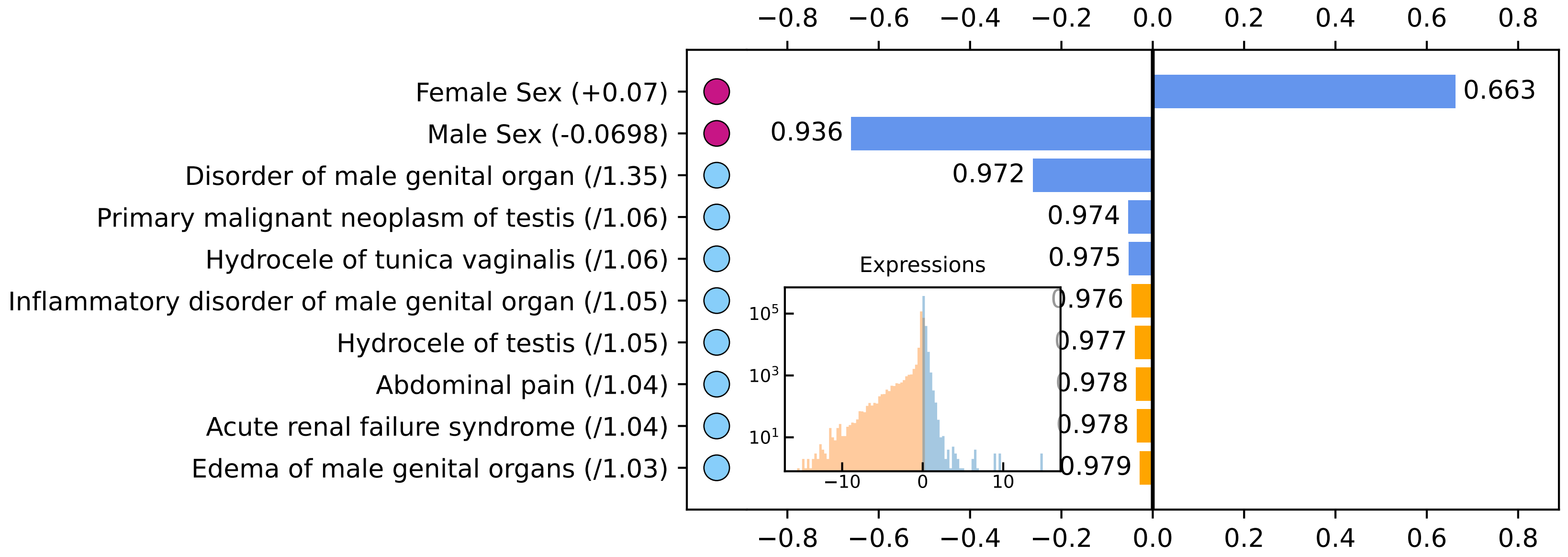

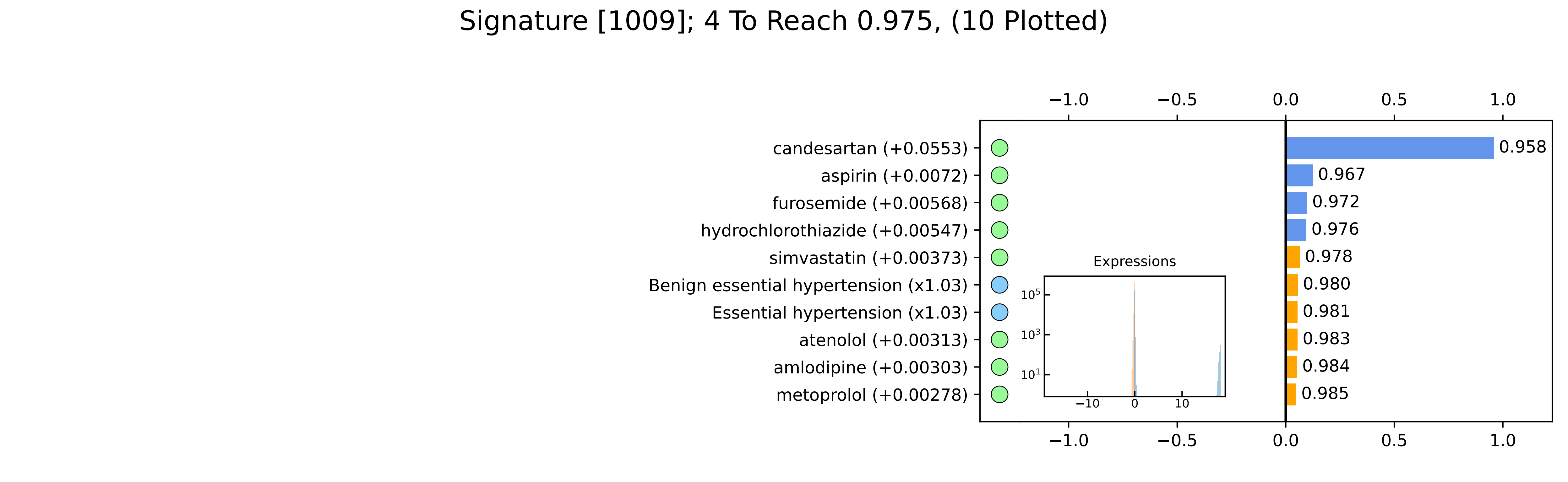

Signature [1009]; 4 To Reach 0.975, (10 Plotted)
candesartan (+0.0553)
aspirin (+0.0072)
furosemide (+0.00568)
hydrochlorothiazide (+0.00547)
simvastatin (+0.00373)
Benign essential hypertension (x1.03)
Essential hypertension (x1.03)
atenolol (+0.00313)
amlodipine (+0.00303)
metoprolol (+0.00278)
0.958
0.967
0.972
0.976
0.978
0.980
0.981
0.983
0.984
0.985
−1.0
−0.5
0.0
0.5
1.0
Expressions
−10
0
10

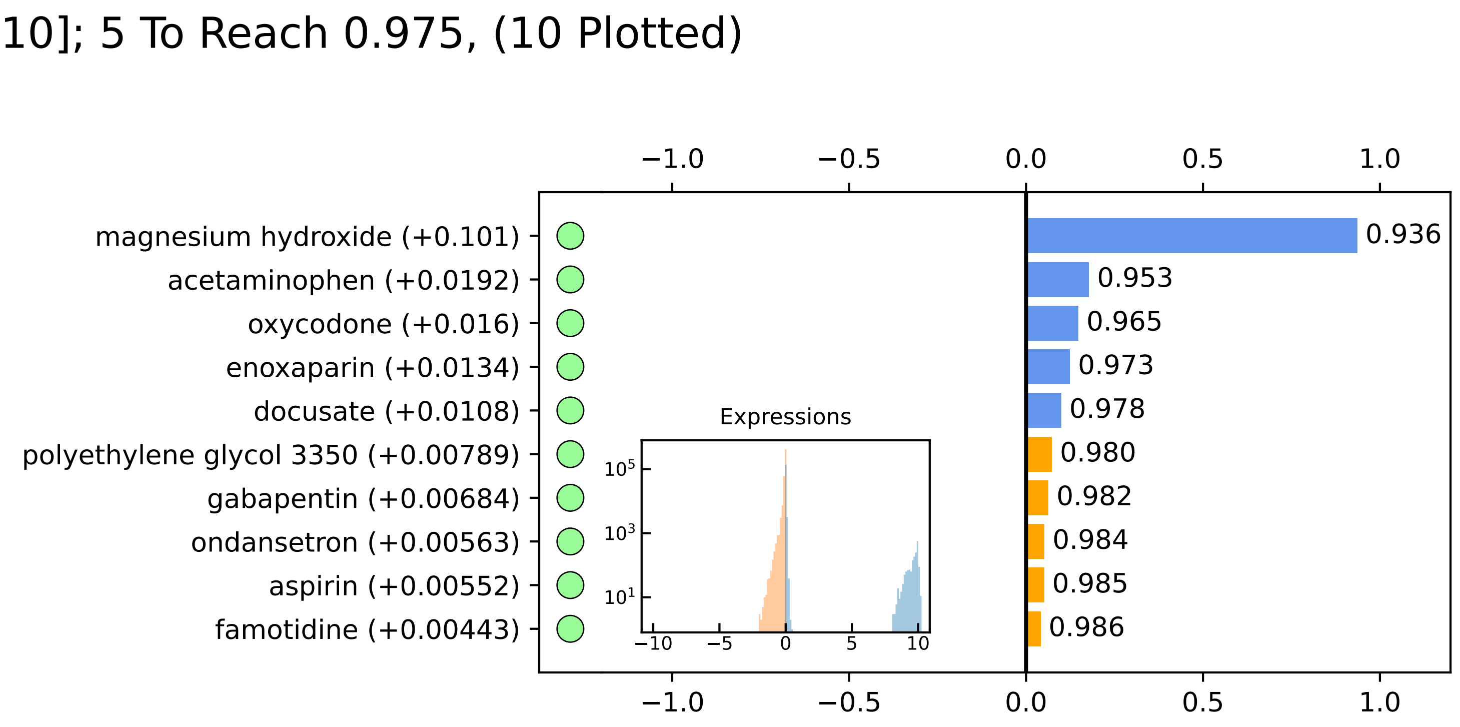

Signature [1010]; 5 To Reach 0.975, (10 Plotted)
magnesium hydroxide (+0.101)
acetaminophen (+0.0192)
oxycodone (+0.016)
enoxaparin (+0.0134)
docusate (+0.0108)
polyethylene glycol 3350 (+0.00789)
gabapentin (+0.00684)
ondansetron (+0.00563)
aspirin (+0.00552)
famotidine (+0.00443)
0.936
0.953
0.965
0.973
0.978
0.980
0.982
0.984
0.985
0.986
−1.0
−0.5
0.0
0.5
1.0
Expressions
10^5
10^3
10^1
−10
−5
0
5
10

Signature [1011]; 11 To Reach 0.975, (11 Plotted)

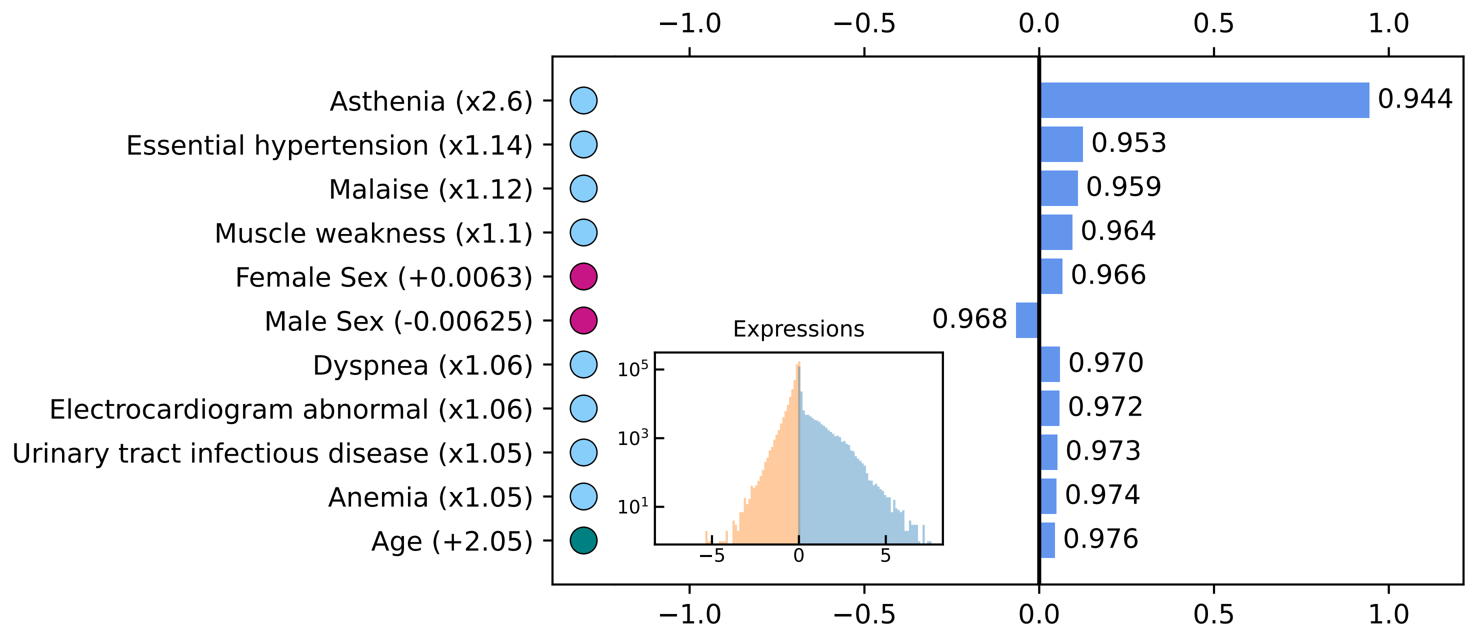

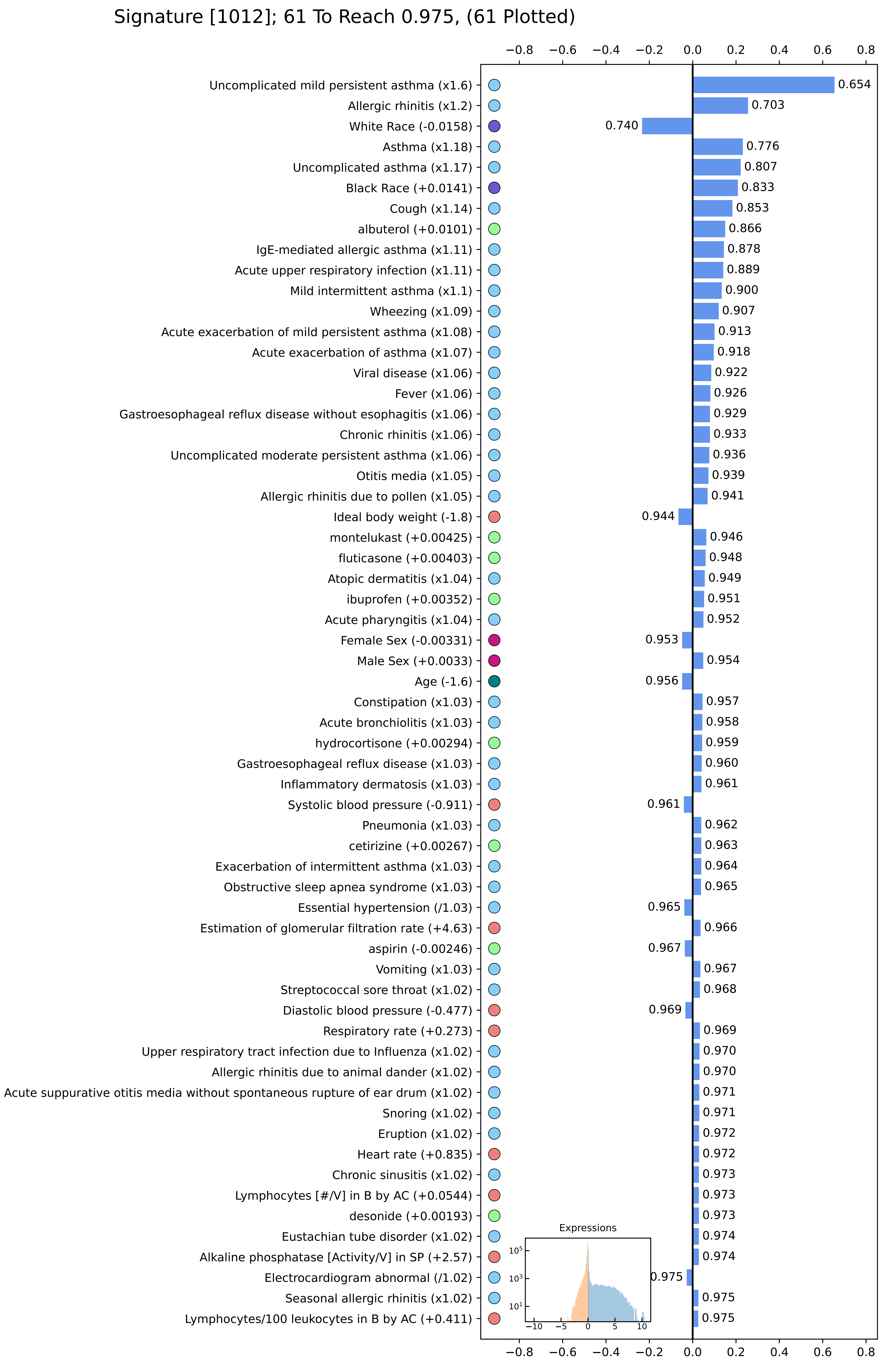
Signature [1012]; 61 To Reach 0.975, (61 Plotted)
Uncomplicated mild persistent asthma (x1.6) 0.654
Allergic rhinitis (x1.2) 0.703
White Race (-0.0158) 0.740
Asthma (x1.18) 0.776
Uncomplicated asthma (x1.17) 0.807
Black Race (+0.0141) 0.833
Cough (x1.14) 0.853
albuterol (+0.0101) 0.866
IgE-mediated allergic asthma (x1.11) 0.878
Acute upper respiratory infection (x1.11) 0.889
Mild intermittent asthma (x1.1) 0.900
Wheezing (x1.09) 0.907
Acute exacerbation of mild persistent asthma (x1.08) 0.913
Acute exacerbation of asthma (x1.07) 0.918
Viral disease (x1.06) 0.922
Fever (x1.06) 0.926
Gastroesophageal reflux disease without esophagitis (x1.06) 0.929
Chronic rhinitis (x1.06) 0.933
Uncomplicated moderate persistent asthma (x1.06) 0.936
Otitis media (x1.05) 0.939
Allergic rhinitis due to pollen (x1.05) 0.941
Ideal body weight (-1.8) 0.944
montelukast (+0.00425) 0.946
fluticasone (+0.00403) 0.948
Atopic dermatitis (x1.04) 0.949
ibuprofen (+0.00352) 0.951
Acute pharyngitis (x1.04) 0.952
Female Sex (-0.00331) 0.953
Male Sex (+0.0033) 0.954
Age (-1.6) 0.956
Constipation (x1.03) 0.957
Acute bronchiolitis (x1.03) 0.958
hydrocortisone (+0.00294) 0.959
Gastroesophageal reflux disease (x1.03) 0.960
Inflammatory dermatosis (x1.03) 0.961
Systolic blood pressure (-0.911) 0.961
Pneumonia (x1.03) 0.962
cetirizine (+0.00267) 0.963
Exacerbation of intermittent asthma (x1.03) 0.964
Obstructive sleep apnea syndrome (x1.03) 0.965
Essential hypertension (/1.03) 0.965
Estimation of glomerular filtration rate (+4.63) 0.966
aspirin (-0.00246) 0.967
Vomiting (x1.03) 0.967
Streptococcal sore throat (x1.02) 0.968
Diastolic blood pressure (-0.477) 0.969
Respiratory rate (+0.273) 0.969
Upper respiratory tract infection due to Influenza (x1.02) 0.970
Allergic rhinitis due to animal dander (x1.02) 0.970
Acute suppurative otitis media without spontaneous rupture of ear drum (x1.02) 0.971
Snoring (x1.02) 0.971
Eruption (x1.02) 0.972
Heart rate (+0.835) 0.972
Chronic sinusitis (x1.02) 0.973
Lymphocytes [#/V] in B by AC (+0.0544) 0.973
desonide (+0.00193) 0.973
Eustachian tube disorder (x1.02) 0.974
Alkaline phosphatase [Activity/V] in SP (+2.57) 0.974
Electrocardiogram abnormal (/1.02) 0.975
Seasonal allergic rhinitis (x1.02) 0.975
Lymphocytes/100 leukocytes in B by AC (+0.411) 0.975
−0.8 −0.6 −0.4 −0.2 0.0 0.2 0.4 0.6 0.8
Expressions
−10 −5 0 5 10

Signature [1013]; 7 To Reach 0.975, (10 Plotted)

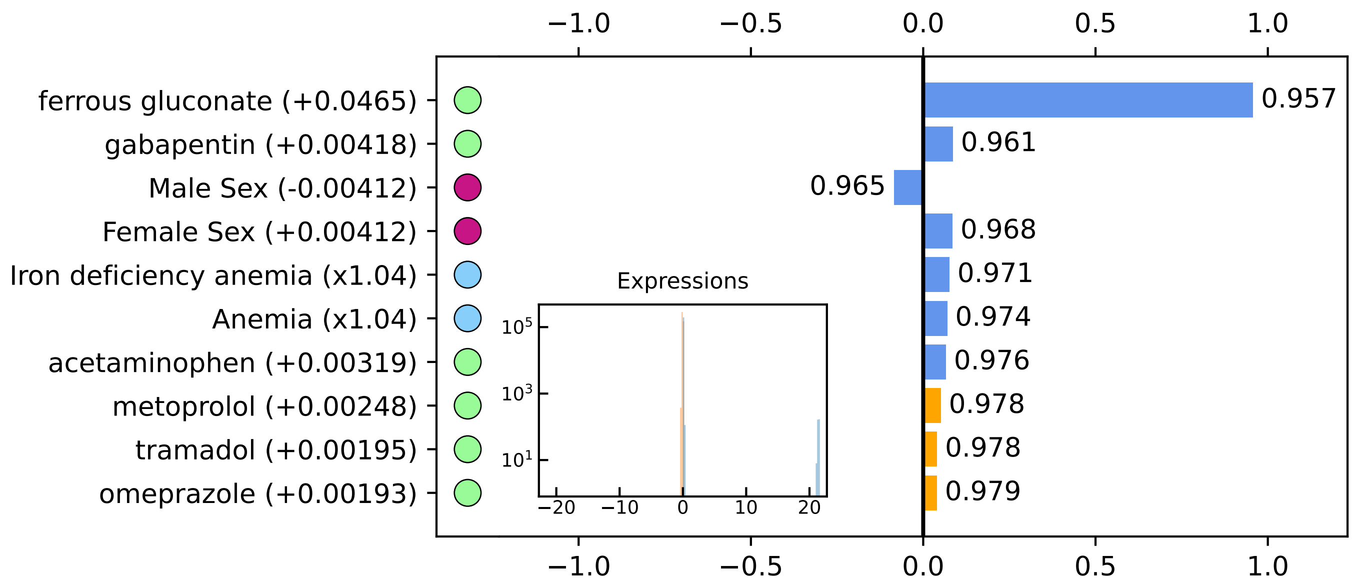

Signature [1014]; 3130 To Reach 0.975, (100 Plotted)

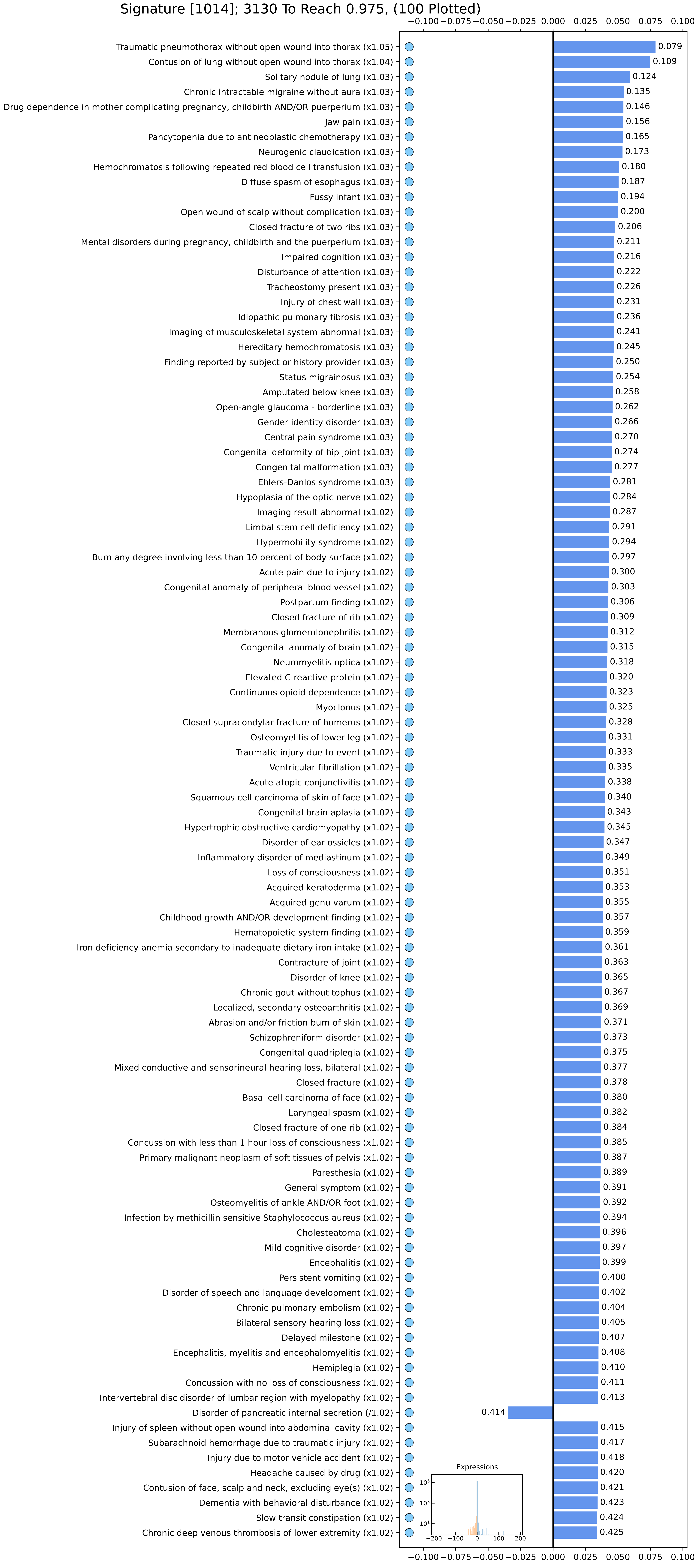

Signature [1015]; 1104 To Reach 0.975, (100 Plotted)

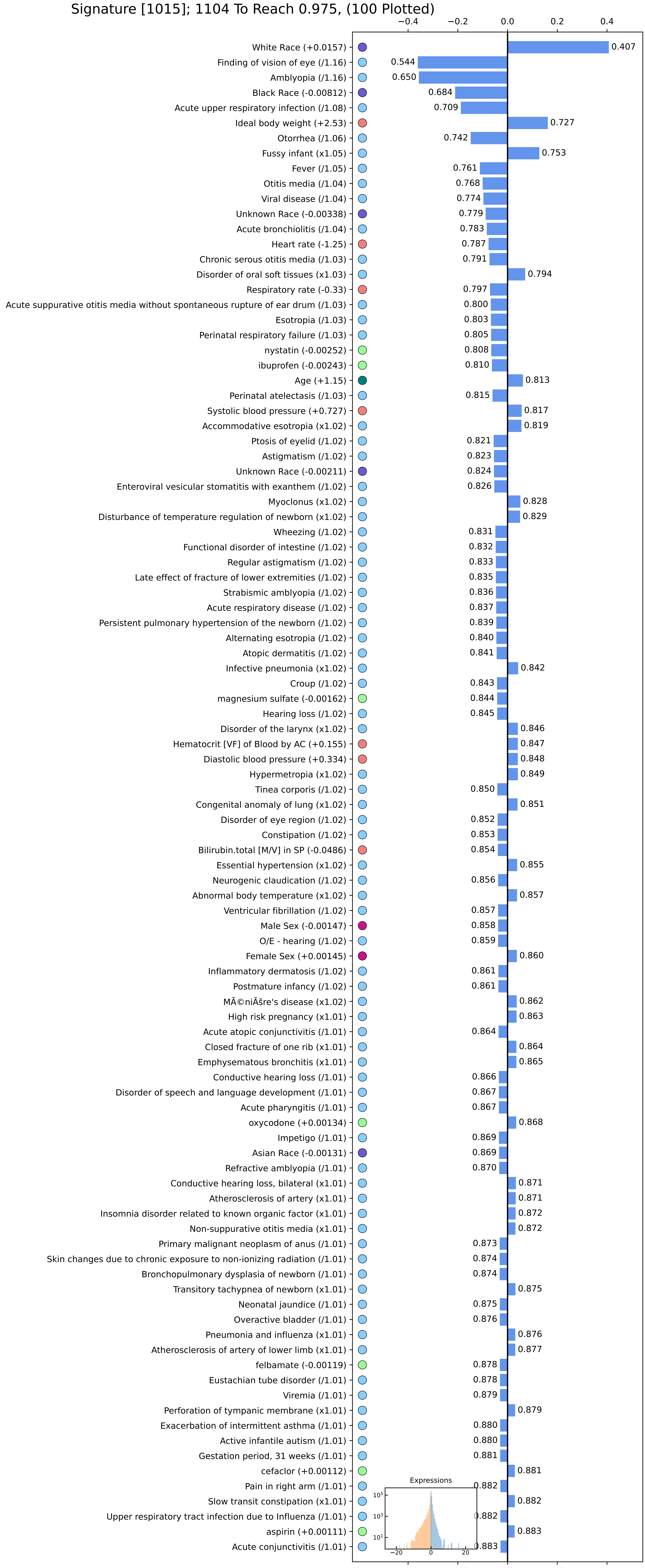

Signature [1016]; 263 To Reach 0.975, (100 Plotted)

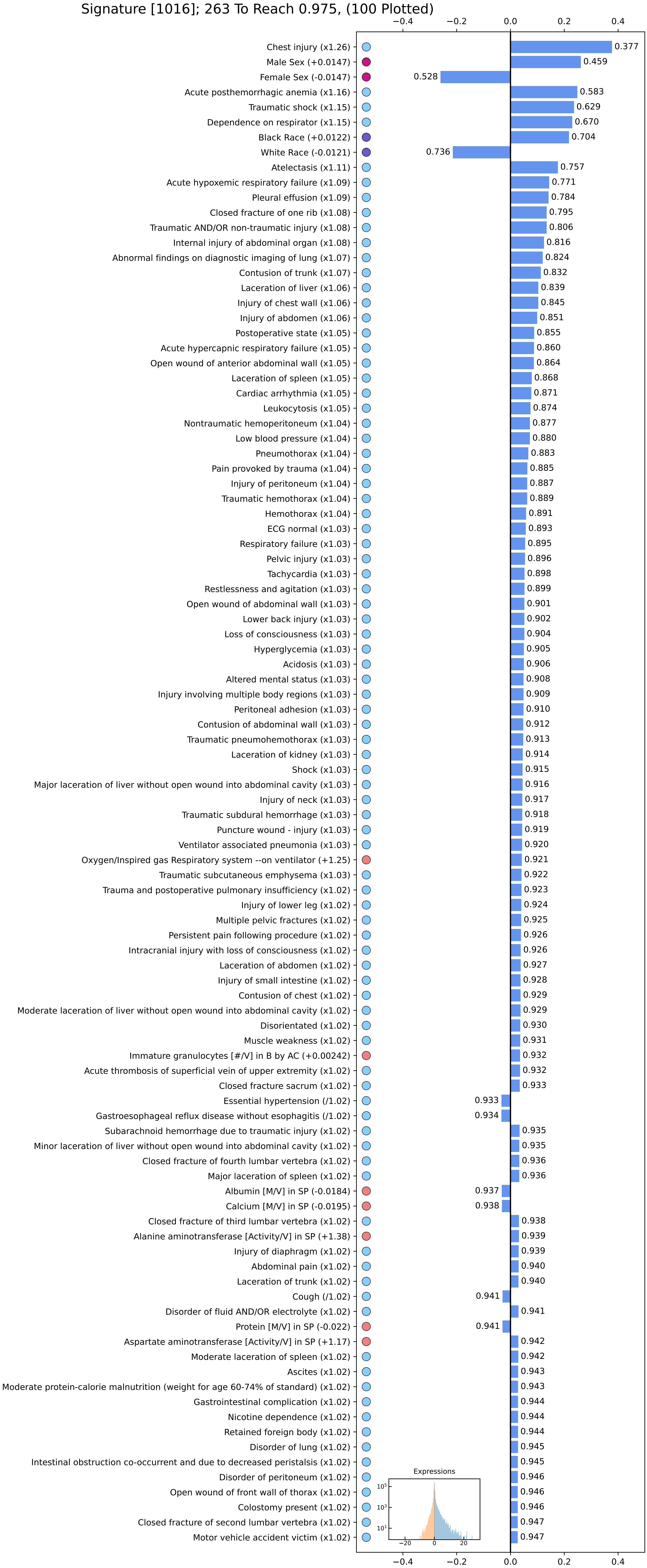

Signature [1017]; 1 To Reach 0.975, (10 Plotted)

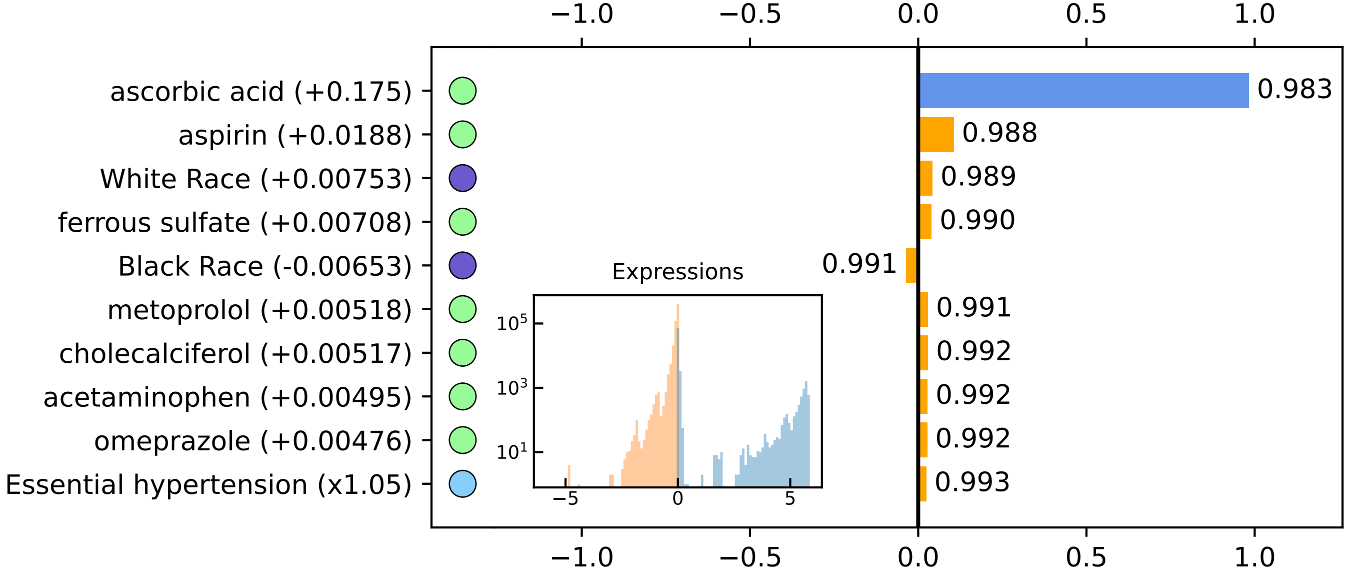

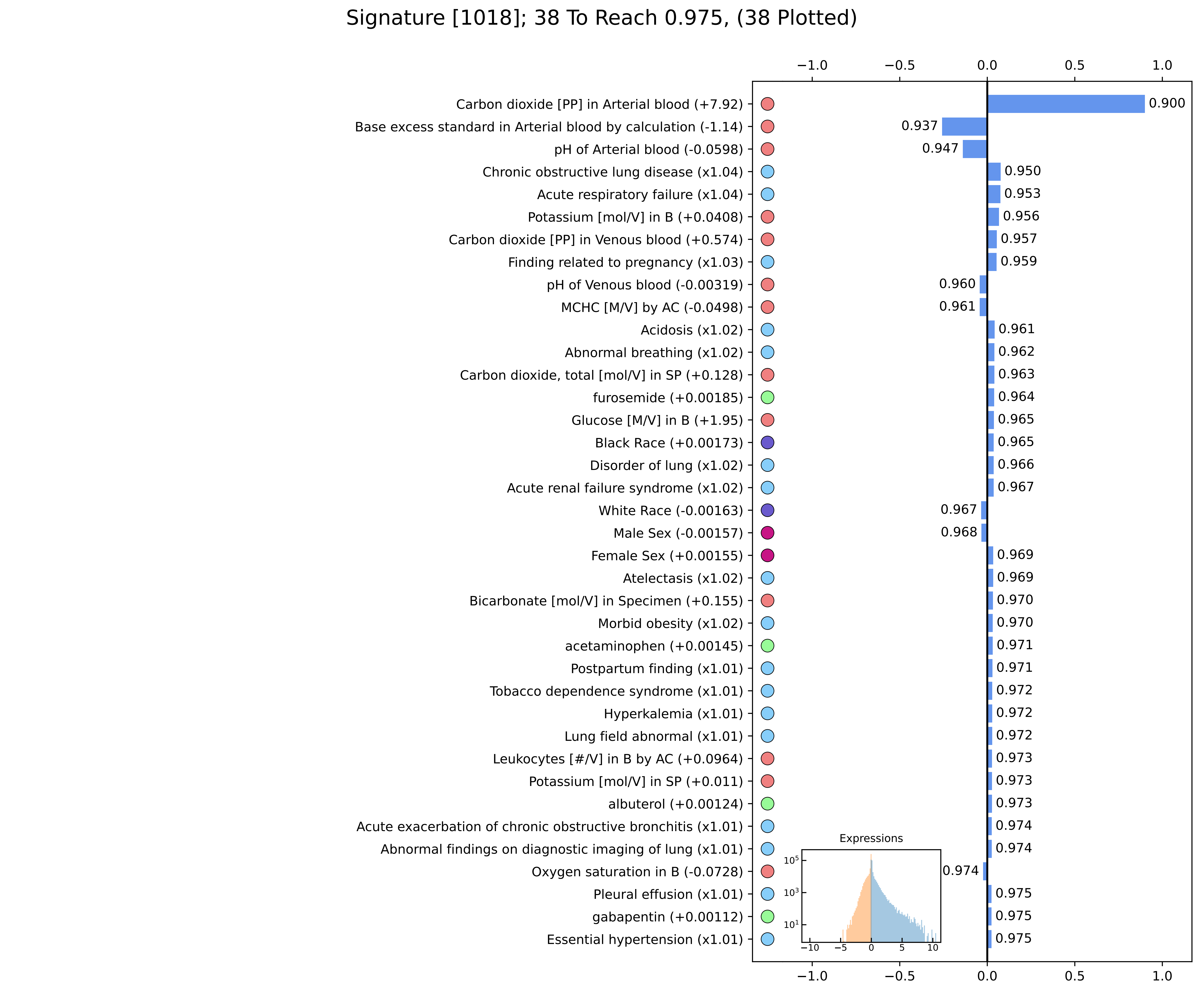
Signature [1018]; 38 To Reach 0.975, (38 Plotted)
Carbon dioxide [PP] in Arterial blood (+7.92) 0.900
Base excess standard in Arterial blood by calculation (-1.14) 0.937
pH of Arterial blood (-0.0598) 0.947
Chronic obstructive lung disease (x1.04) 0.950
Acute respiratory failure (x1.04) 0.953
Potassium [mol/V] in B (+0.0408) 0.956
Carbon dioxide [PP] in Venous blood (+0.574) 0.957
Finding related to pregnancy (x1.03) 0.959
pH of Venous blood (-0.00319) 0.960
MCHC [M/V] by AC (-0.0498) 0.961
Acidosis (x1.02) 0.961
Abnormal breathing (x1.02) 0.962
Carbon dioxide, total [mol/V] in SP (+0.128) 0.963
furosemide (+0.00185) 0.964
Glucose [M/V] in B (+1.95) 0.965
Black Race (+0.00173) 0.965
Disorder of lung (x1.02) 0.966
Acute renal failure syndrome (x1.02) 0.967
White Race (-0.00163) 0.967
Male Sex (-0.00157) 0.968
Female Sex (+0.00155) 0.969
Atelectasis (x1.02) 0.969
Bicarbonate [mol/V] in Specimen (+0.155) 0.970
Morbid obesity (x1.02) 0.970
acetaminophen (+0.00145) 0.971
Postpartum finding (x1.01) 0.971
Tobacco dependence syndrome (x1.01) 0.972
Hyperkalemia (x1.01) 0.972
Lung field abnormal (x1.01) 0.972
Leukocytes [#/V] in B by AC (+0.0964) 0.973
Potassium [mol/V] in SP (+0.011) 0.973
albuterol (+0.00124) 0.973
Acute exacerbation of chronic obstructive bronchitis (x1.01) 0.974
Abnormal findings on diagnostic imaging of lung (x1.01) 0.974
Oxygen saturation in B (-0.0728) 0.974
Pleural effusion (x1.01) 0.975
gabapentin (+0.00112) 0.975
Essential hypertension (x1.01) 0.975
−1.0 −0.5 0.0 0.5 1.0
Expressions

Signature [1019]; 2067 To Reach 0.975, (100 Plotted)

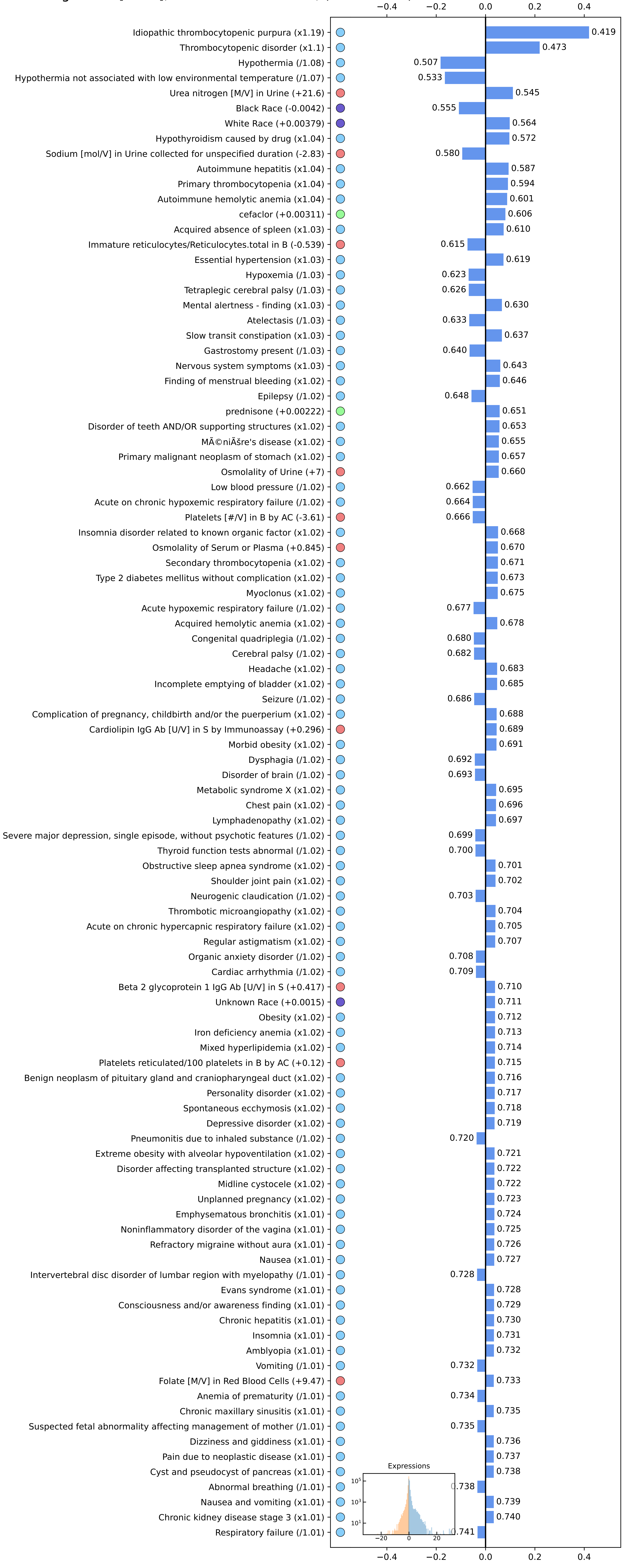

Signature [1020]; 9 To Reach 0.975, (10 Plotted)

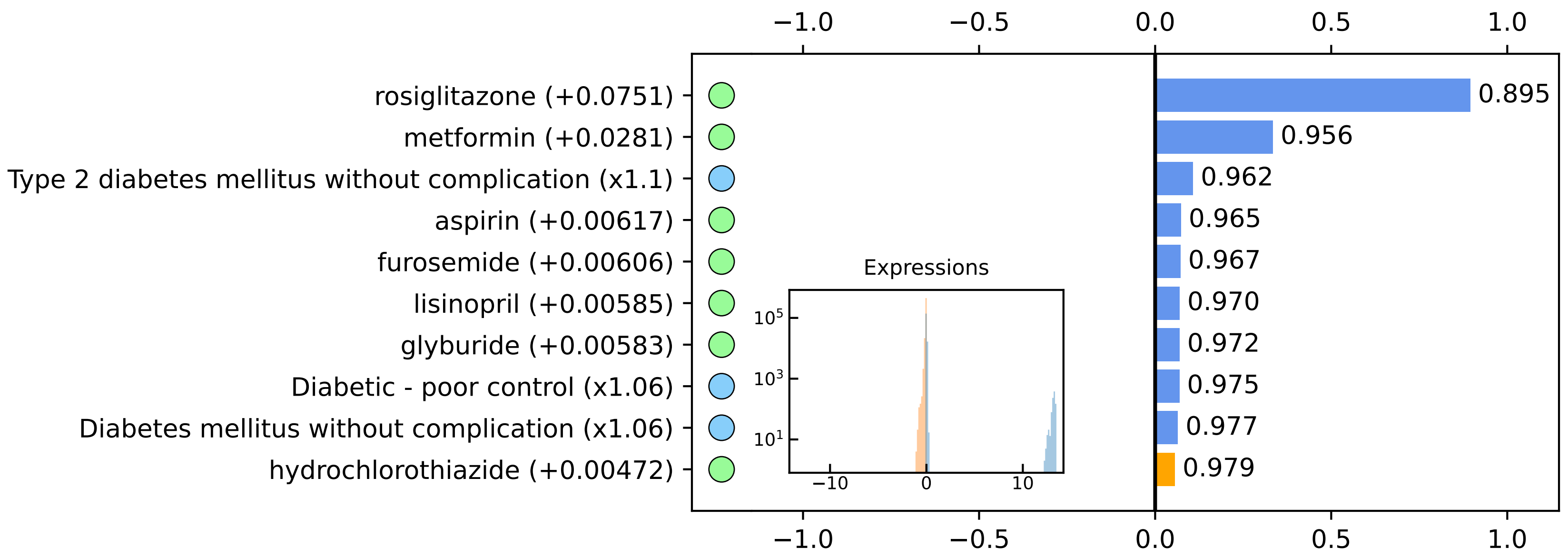

Signature [1021]; 261 To Reach 0.975, (100 Plotted)

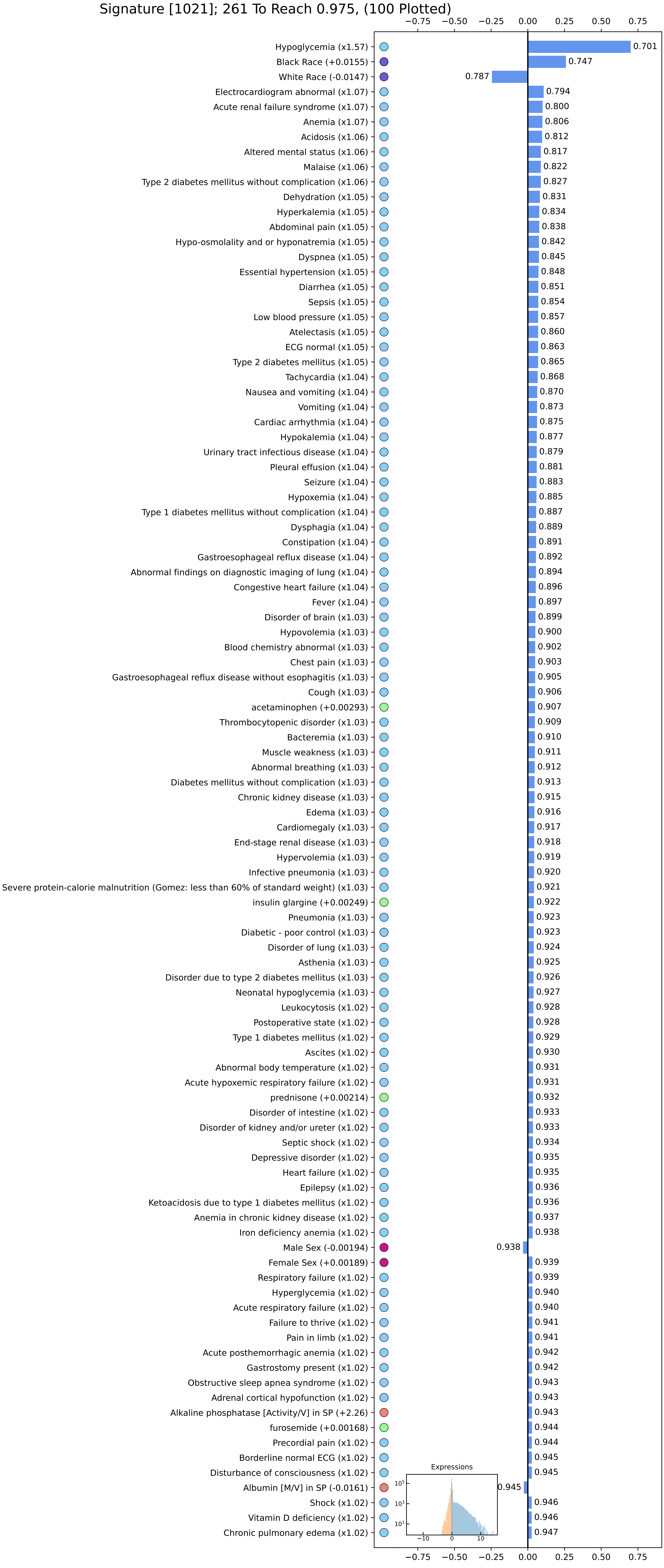

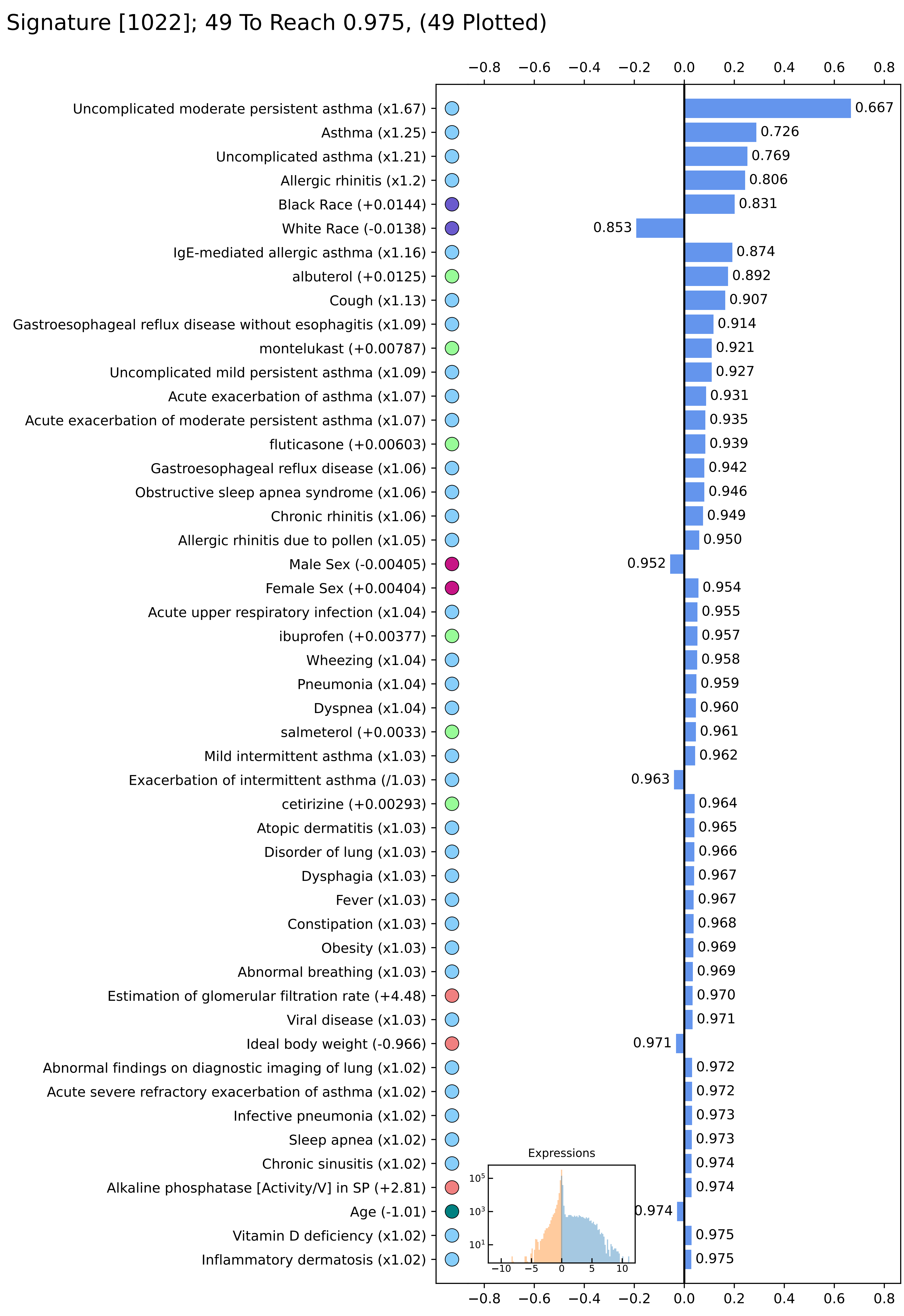

Signature [1022]; 49 To Reach 0.975, (49 Plotted)
Uncomplicated moderate persistent asthma (x1.67) 0.667
Asthma (x1.25) 0.726
Uncomplicated asthma (x1.21) 0.769
Allergic rhinitis (x1.2) 0.806
Black Race (+0.0144) 0.831
White Race (-0.0138) 0.853
IgE-mediated allergic asthma (x1.16) 0.874
albuterol (+0.0125) 0.892
Cough (x1.13) 0.907
Gastroesophageal reflux disease without esophagitis (x1.09) 0.914
montelukast (+0.00787) 0.921
Uncomplicated mild persistent asthma (x1.09) 0.927
Acute exacerbation of asthma (x1.07) 0.931
Acute exacerbation of moderate persistent asthma (x1.07) 0.935
fluticasone (+0.00603) 0.939
Gastroesophageal reflux disease (x1.06) 0.942
Obstructive sleep apnea syndrome (x1.06) 0.946
Chronic rhinitis (x1.06) 0.949
Allergic rhinitis due to pollen (x1.05) 0.950
Male Sex (-0.00405) 0.952
Female Sex (+0.00404) 0.954
Acute upper respiratory infection (x1.04) 0.955
ibuprofen (+0.00377) 0.957
Wheezing (x1.04) 0.958
Pneumonia (x1.04) 0.959
Dyspnea (x1.04) 0.960
salmeterol (+0.0033) 0.961
Mild intermittent asthma (x1.03) 0.962
Exacerbation of intermittent asthma (/1.03) 0.963
cetirizine (+0.00293) 0.964
Atopic dermatitis (x1.03) 0.965
Disorder of lung (x1.03) 0.966
Dysphagia (x1.03) 0.967
Fever (x1.03) 0.967
Constipation (x1.03) 0.968
Obesity (x1.03) 0.969
Abnormal breathing (x1.03) 0.969
Estimation of glomerular filtration rate (+4.48) 0.970
Viral disease (x1.03) 0.971
Ideal body weight (-0.966) 0.971
Abnormal findings on diagnostic imaging of lung (x1.02) 0.972
Acute severe refractory exacerbation of asthma (x1.02) 0.972
Infective pneumonia (x1.02) 0.973
Sleep apnea (x1.02) 0.973
Chronic sinusitis (x1.02) 0.974
Alkaline phosphatase [Activity/V] in SP (+2.81) 0.974
Age (-1.01) 0.974
Vitamin D deficiency (x1.02) 0.975
Inflammatory dermatosis (x1.02) 0.975
−0.8 −0.6 −0.4 −0.2 0.0 0.2 0.4 0.6 0.8
Expressions
10^5
10^3
10^1
−10 −5 0 5 10

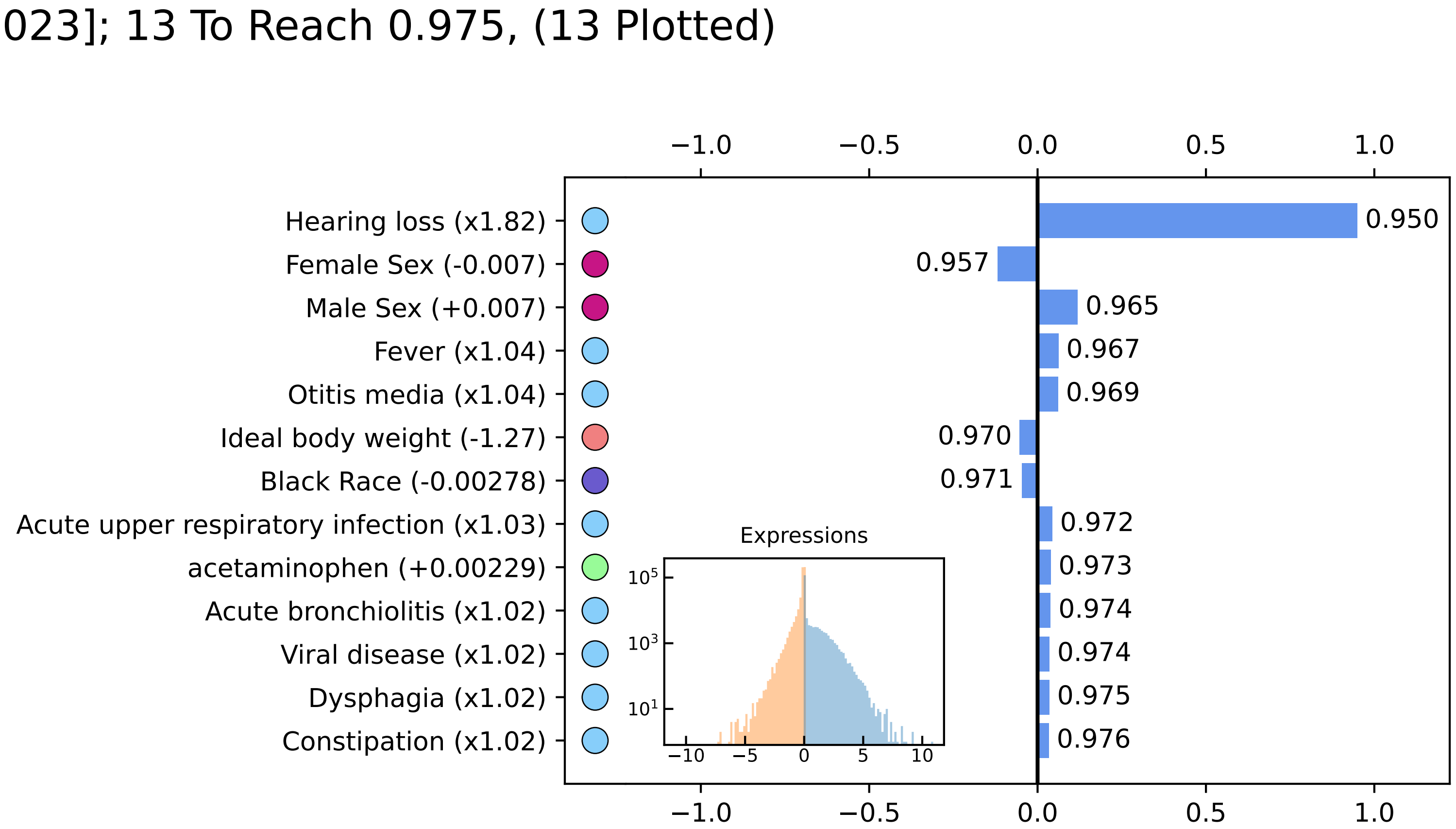
Signature [1023]; 13 To Reach 0.975, (13 Plotted)
Hearing loss (x1.82)
Female Sex (-0.007)
Male Sex (+0.007)
Fever (x1.04)
Otitis media (x1.04)
Ideal body weight (-1.27)
Black Race (-0.00278)
Acute upper respiratory infection (x1.03)
acetaminophen (+0.00229)
Acute bronchiolitis (x1.02)
Viral disease (x1.02)
Dysphagia (x1.02)
Constipation (x1.02)
0.950
0.957
0.965
0.967
0.969
0.970
0.971
0.972
0.973
0.974
0.974
0.975
0.976
−1.0
−0.5
0.0
0.5
1.0
Expressions

Signature [1024]; 13 To Reach 0.975, (13 Plotted)

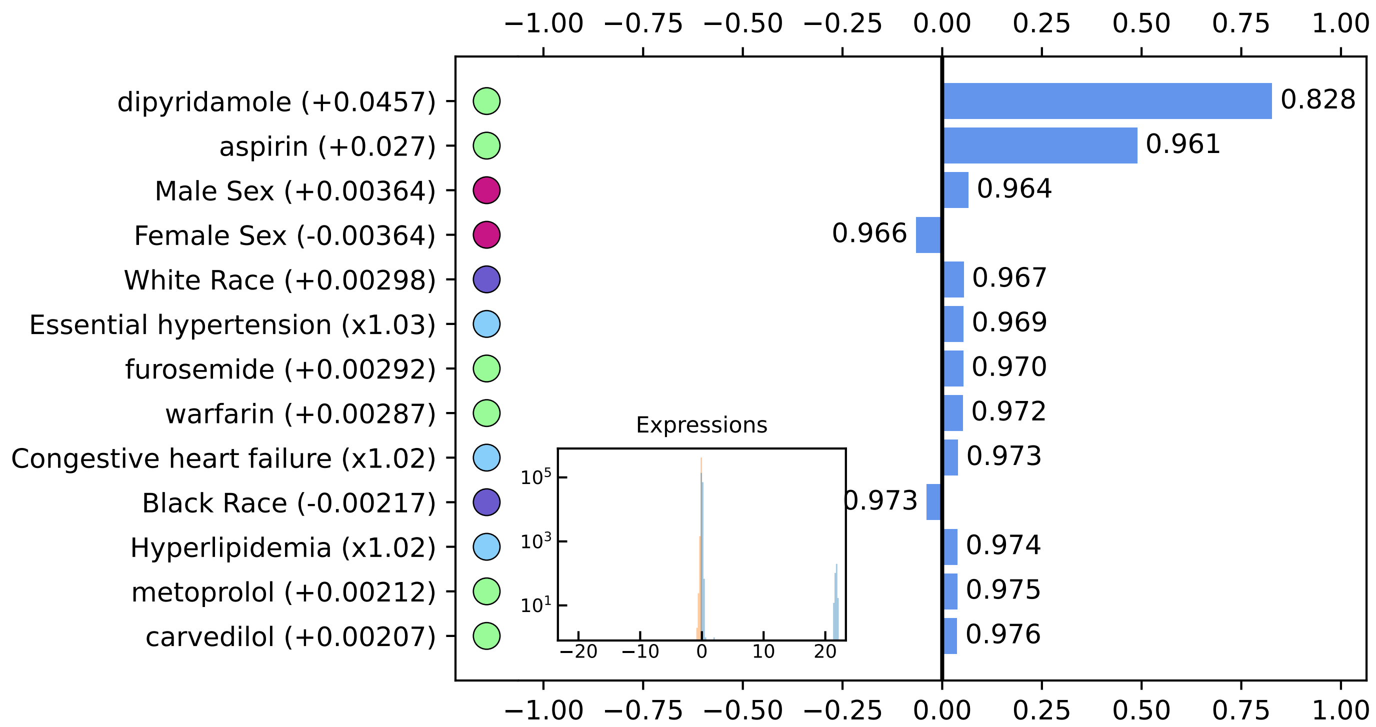

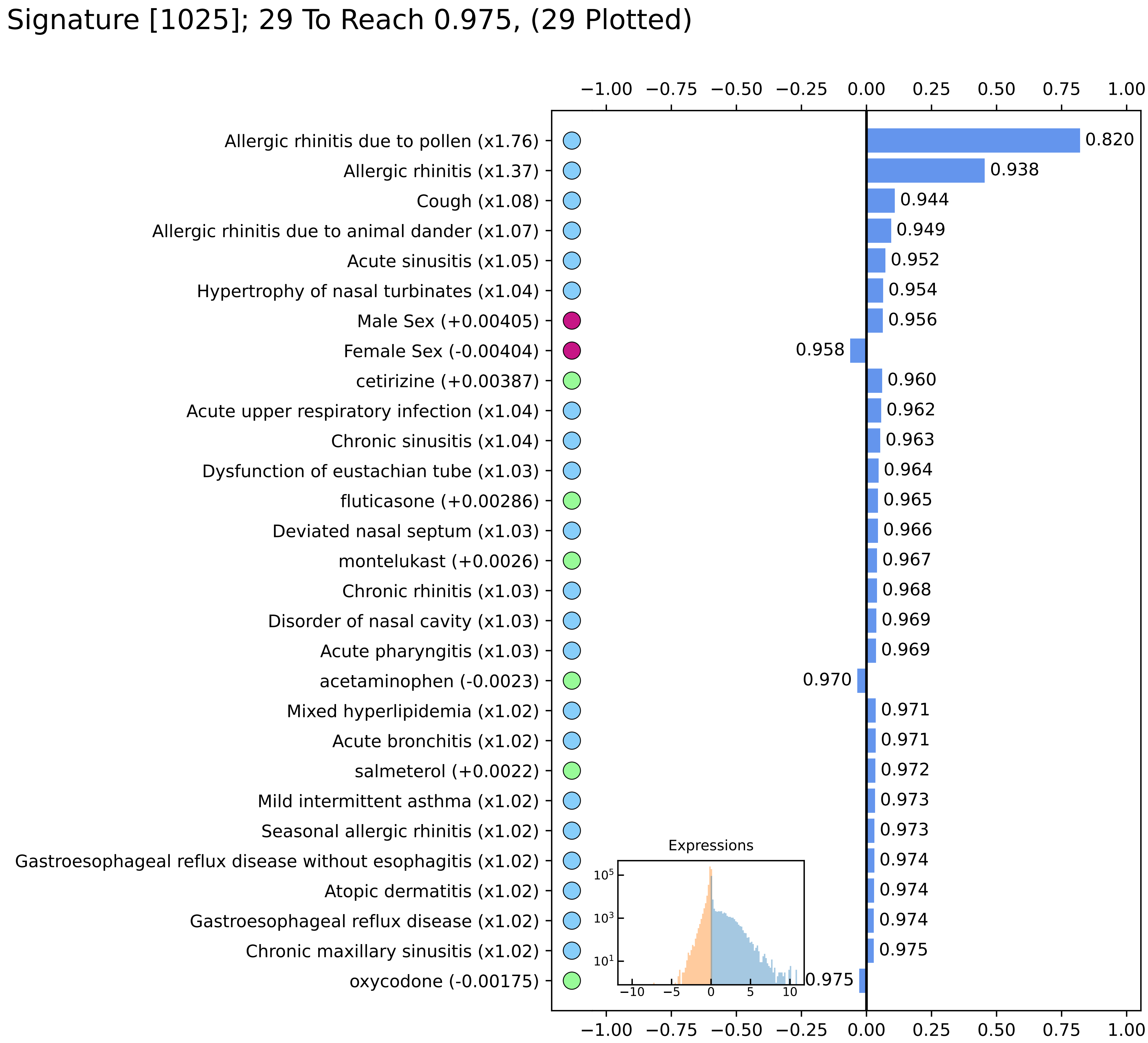

Signature [1025]; 29 To Reach 0.975, (29 Plotted)
Allergic rhinitis due to pollen (x1.76)
Allergic rhinitis (x1.37)
Cough (x1.08)
Allergic rhinitis due to animal dander (x1.07)
Acute sinusitis (x1.05)
Hypertrophy of nasal turbinates (x1.04)
Male Sex (+0.00405)
Female Sex (-0.00404)
cetirizine (+0.00387)
Acute upper respiratory infection (x1.04)
Chronic sinusitis (x1.04)
Dysfunction of eustachian tube (x1.03)
fluticasone (+0.00286)
Deviated nasal septum (x1.03)
montelukast (+0.0026)
Chronic rhinitis (x1.03)
Disorder of nasal cavity (x1.03)
Acute pharyngitis (x1.03)
acetaminophen (-0.0023)
Mixed hyperlipidemia (x1.02)
Acute bronchitis (x1.02)
salmeterol (+0.0022)
Mild intermittent asthma (x1.02)
Seasonal allergic rhinitis (x1.02)
Gastroesophageal reflux disease without esophagitis (x1.02)
Atopic dermatitis (x1.02)
Gastroesophageal reflux disease (x1.02)
Chronic maxillary sinusitis (x1.02)
oxycodone (-0.00175)
0.820
0.938
0.944
0.949
0.952
0.954
0.956
0.958
0.960
0.962
0.963
0.964
0.965
0.966
0.967
0.968
0.969
0.969
0.970
0.971
0.971
0.972
0.973
0.973
0.974
0.974
0.974
0.975
0.975
−1.00 −0.75 −0.50 −0.25 0.00 0.25 0.50 0.75 1.00
Expressions

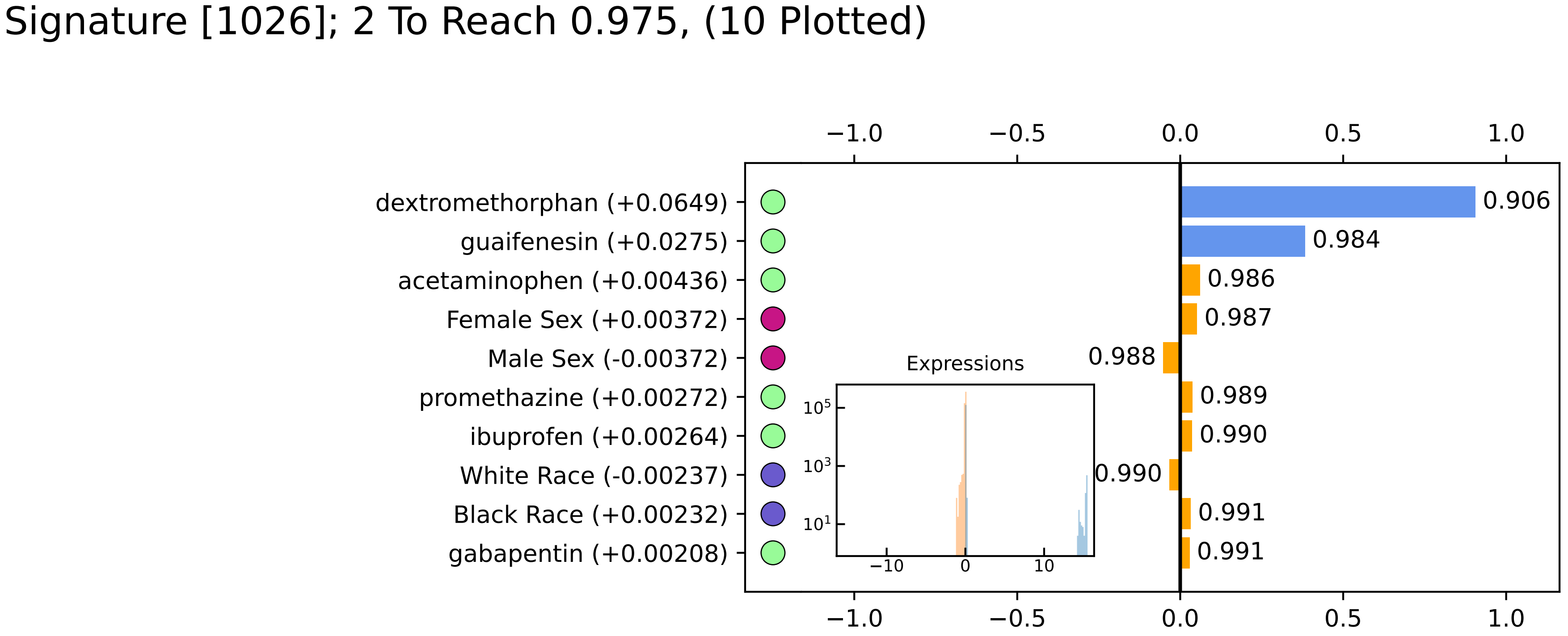

Signature [1026]; 2 To Reach 0.975, (10 Plotted)
dextromethorphan (+0.0649)
guaifenesin (+0.0275)
acetaminophen (+0.00436)
Female Sex (+0.00372)
Male Sex (-0.00372)
promethazine (+0.00272)
ibuprofen (+0.00264)
White Race (-0.00237)
Black Race (+0.00232)
gabapentin (+0.00208)
0.906
0.984
0.986
0.987
0.988
0.989
0.990
0.990
0.991
0.991
Expressions
−1.0
−0.5
0.0
0.5
1.0

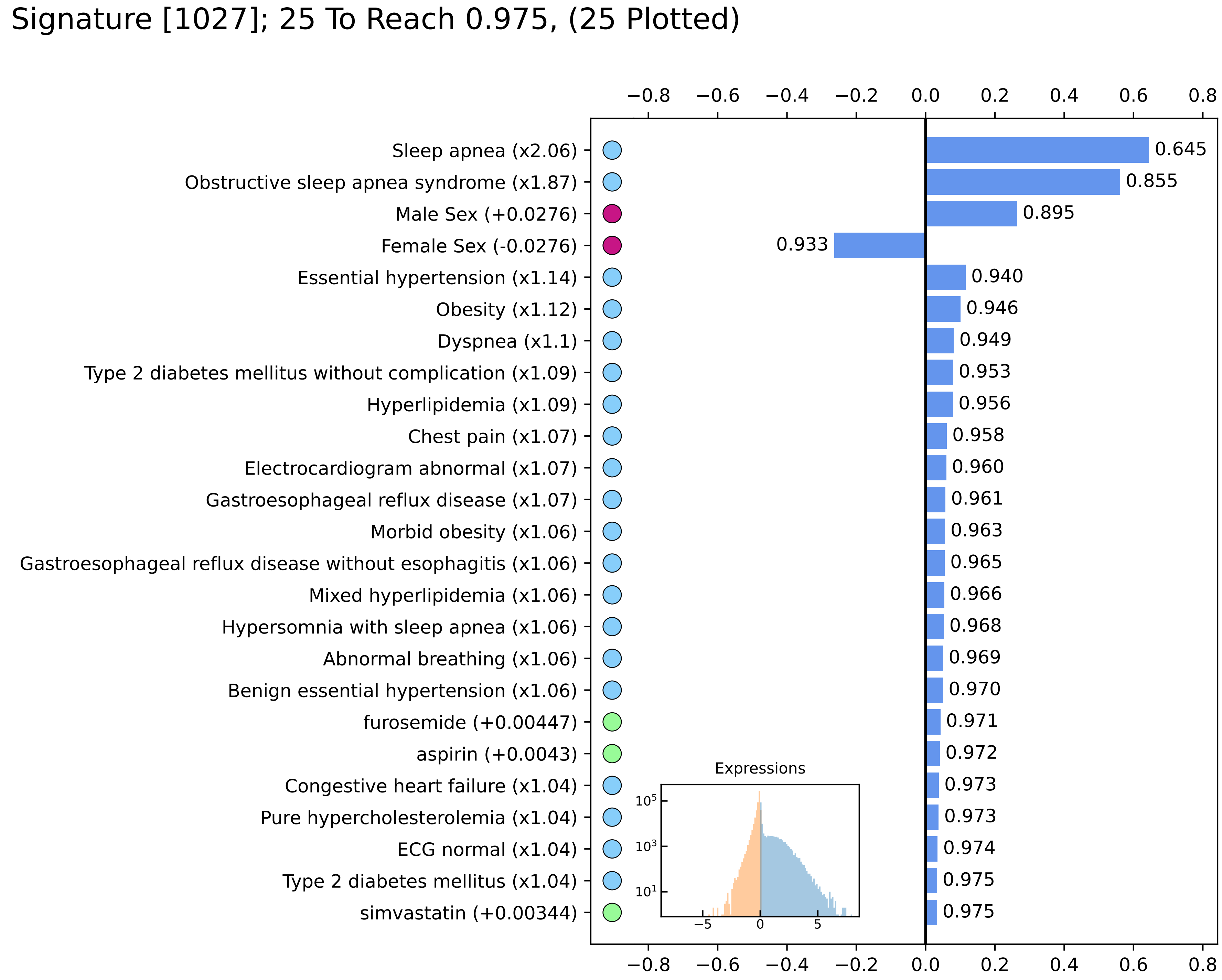

Signature [1027]; 25 To Reach 0.975, (25 Plotted)
Sleep apnea (x2.06)
Obstructive sleep apnea syndrome (x1.87)
Male Sex (+0.0276)
Female Sex (-0.0276)
Essential hypertension (x1.14)
Obesity (x1.12)
Dyspnea (x1.1)
Type 2 diabetes mellitus without complication (x1.09)
Hyperlipidemia (x1.09)
Chest pain (x1.07)
Electrocardiogram abnormal (x1.07)
Gastroesophageal reflux disease (x1.07)
Morbid obesity (x1.06)
Gastroesophageal reflux disease without esophagitis (x1.06)
Mixed hyperlipidemia (x1.06)
Hypersomnia with sleep apnea (x1.06)
Abnormal breathing (x1.06)
Benign essential hypertension (x1.06)
furosemide (+0.00447)
aspirin (+0.0043)
Congestive heart failure (x1.04)
Pure hypercholesterolemia (x1.04)
ECG normal (x1.04)
Type 2 diabetes mellitus (x1.04)
simvastatin (+0.00344)
0.645
0.855
0.895
0.933
0.940
0.946
0.949
0.953
0.956
0.958
0.960
0.961
0.963
0.965
0.966
0.968
0.969
0.970
0.971
0.972
0.973
0.973
0.974
0.975
0.975
Expressions
−0.8 −0.6 −0.4 −0.2 0.0 0.2 0.4 0.6 0.8

Signature [1028]; 51 To Reach 0.975, (51 Plotted)

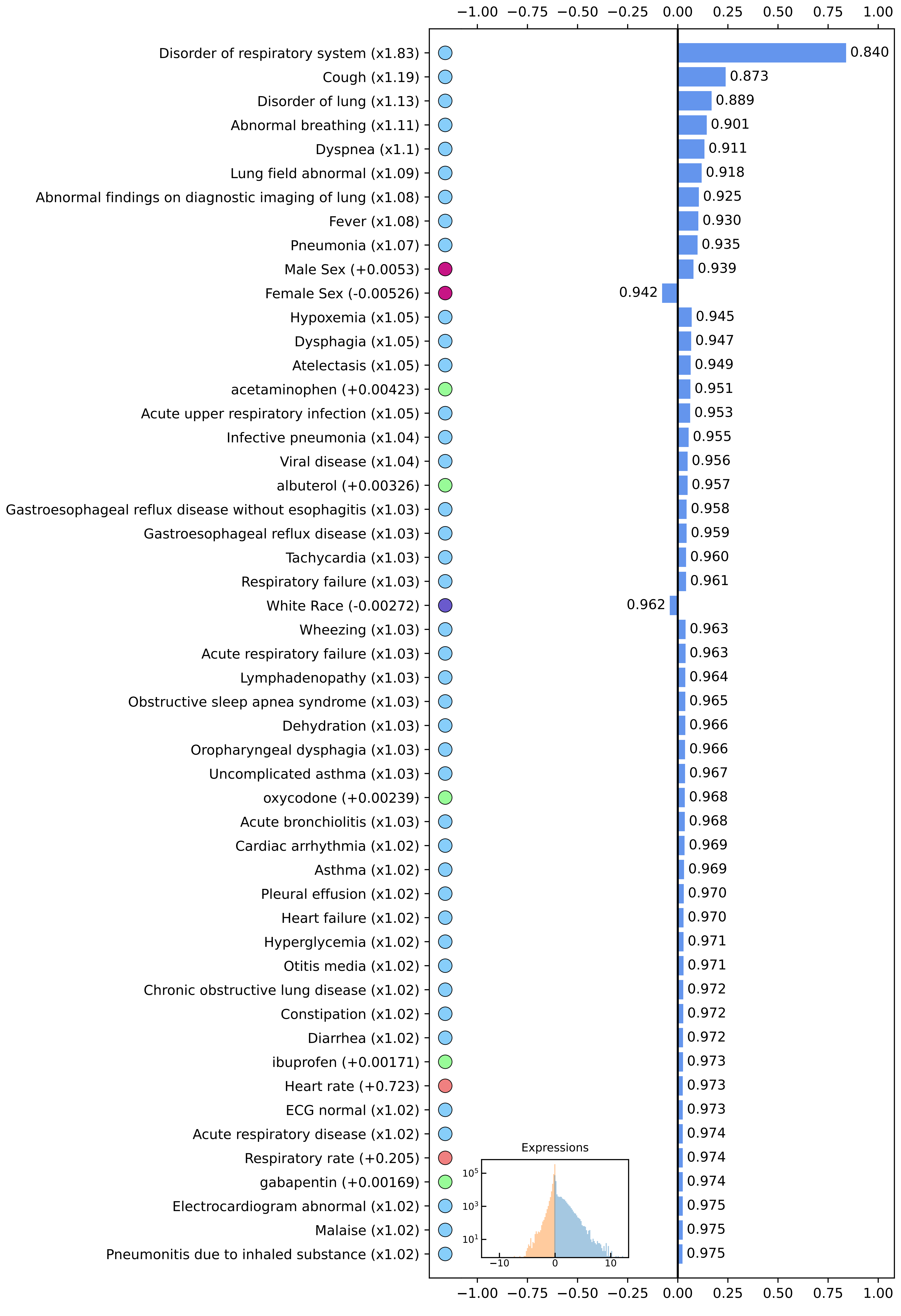

Signature [1029]; 6 To Reach 0.975, (10 Plotted)

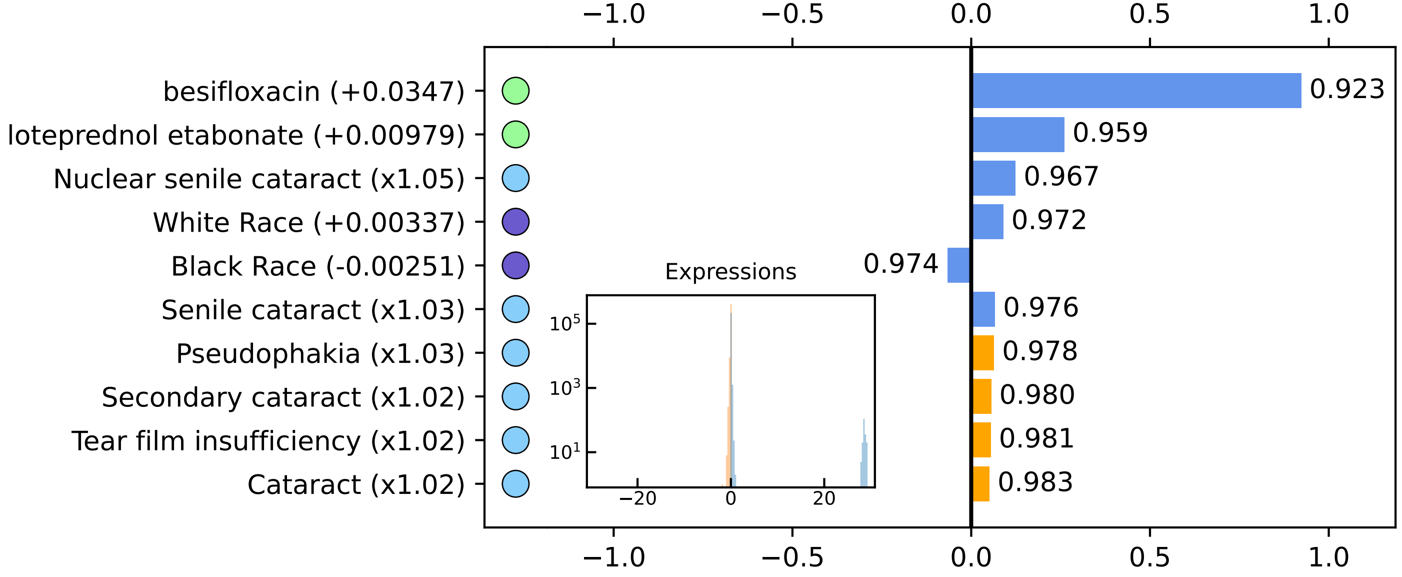

Signature [1030]; 1290 To Reach 0.975, (100 Plotted)

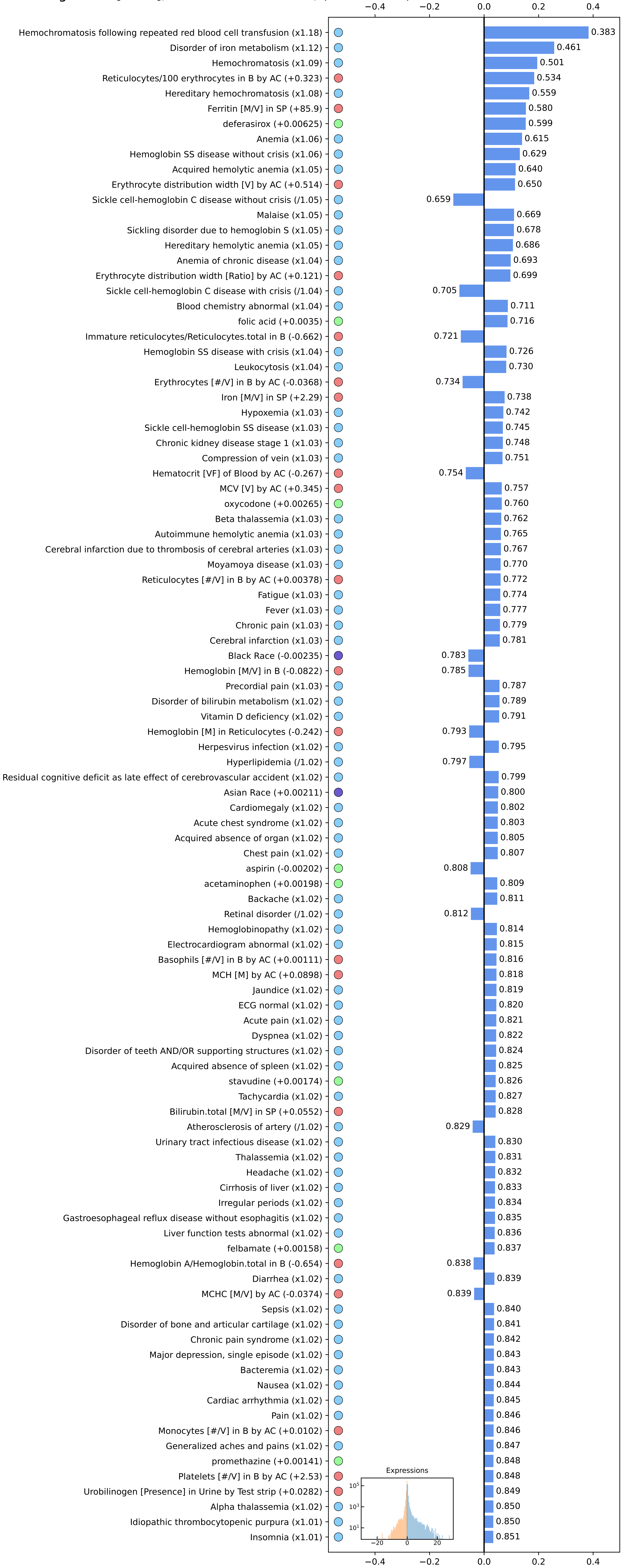

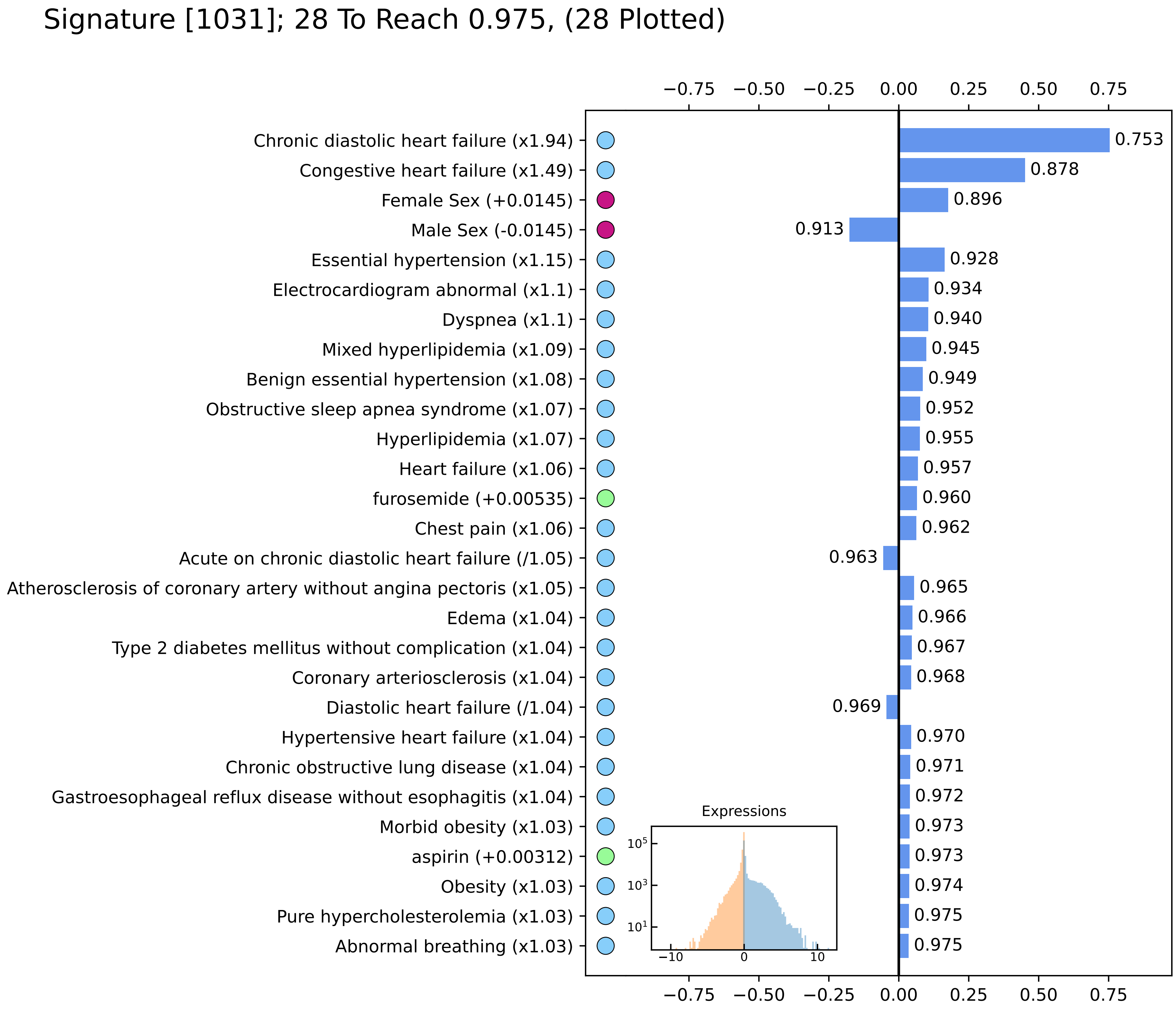

Signature [1031]; 28 To Reach 0.975, (28 Plotted)
Chronic diastolic heart failure (x1.94)
0.753
Congestive heart failure (x1.49)
0.878
Female Sex (+0.0145)
0.896
Male Sex (-0.0145)
0.913
Essential hypertension (x1.15)
0.928
Electrocardiogram abnormal (x1.1)
0.934
Dyspnea (x1.1)
0.940
Mixed hyperlipidemia (x1.09)
0.945
Benign essential hypertension (x1.08)
0.949
Obstructive sleep apnea syndrome (x1.07)
0.952
Hyperlipidemia (x1.07)
0.955
Heart failure (x1.06)
0.957
furosemide (+0.00535)
0.960
Chest pain (x1.06)
0.962
Acute on chronic diastolic heart failure (/1.05)
0.963
Atherosclerosis of coronary artery without angina pectoris (x1.05)
0.965
Edema (x1.04)
0.966
Type 2 diabetes mellitus without complication (x1.04)
0.967
Coronary arteriosclerosis (x1.04)
0.968
Diastolic heart failure (/1.04)
0.969
Hypertensive heart failure (x1.04)
0.970
Chronic obstructive lung disease (x1.04)
0.971
Gastroesophageal reflux disease without esophagitis (x1.04)
0.972
Morbid obesity (x1.03)
0.973
aspirin (+0.00312)
0.973
Obesity (x1.03)
0.974
Pure hypercholesterolemia (x1.03)
0.975
Abnormal breathing (x1.03)
0.975
−0.75 −0.50 −0.25 0.00 0.25 0.50 0.75
Expressions
10^5
10^3
10^1
−10 0 10

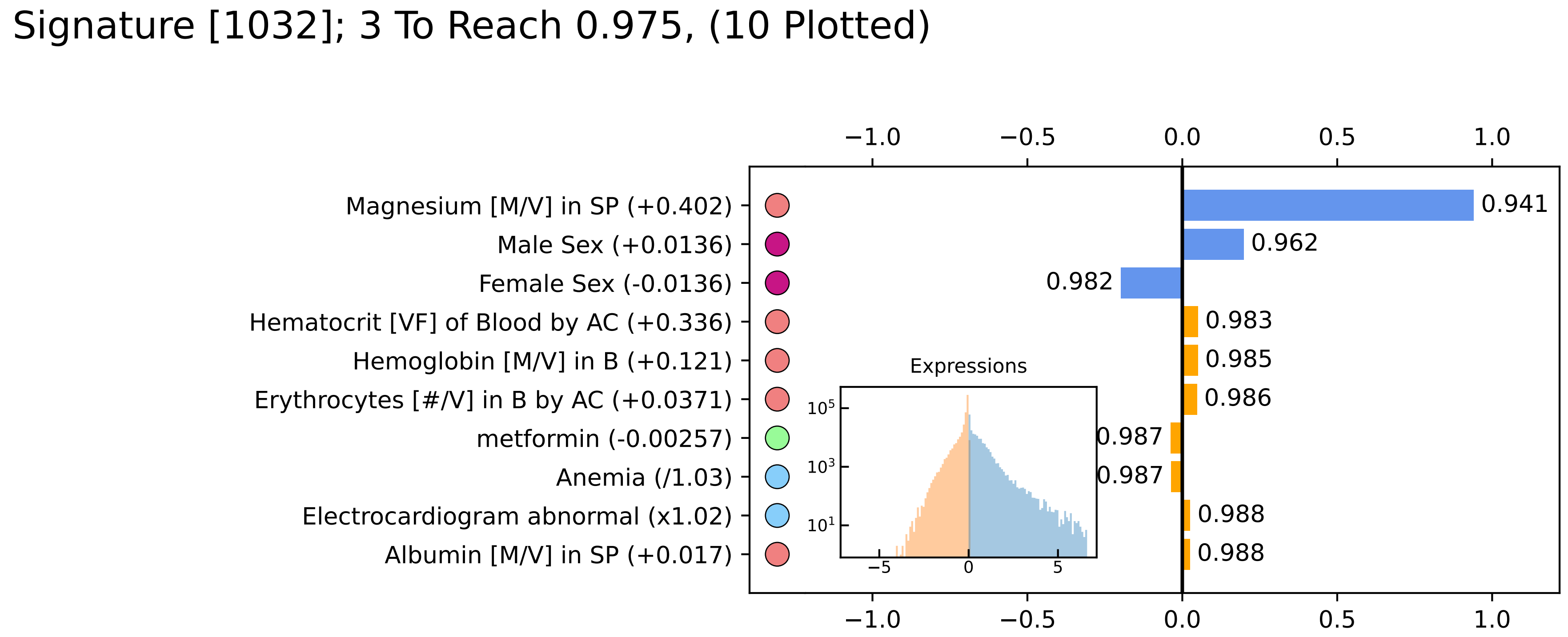

Signature [1032]; 3 To Reach 0.975, (10 Plotted)
Magnesium [M/V] in SP (+0.402)
Male Sex (+0.0136)
Female Sex (-0.0136)
Hematocrit [VF] of Blood by AC (+0.336)
Hemoglobin [M/V] in B (+0.121)
Erythrocytes [#/V] in B by AC (+0.0371)
metformin (-0.00257)
Anemia (/1.03)
Electrocardiogram abnormal (x1.02)
Albumin [M/V] in SP (+0.017)
0.941
0.962
0.982
0.983
0.985
0.986
0.987
0.987
0.988
0.988
Expressions
−1.0
−0.5
0.0
0.5
1.0

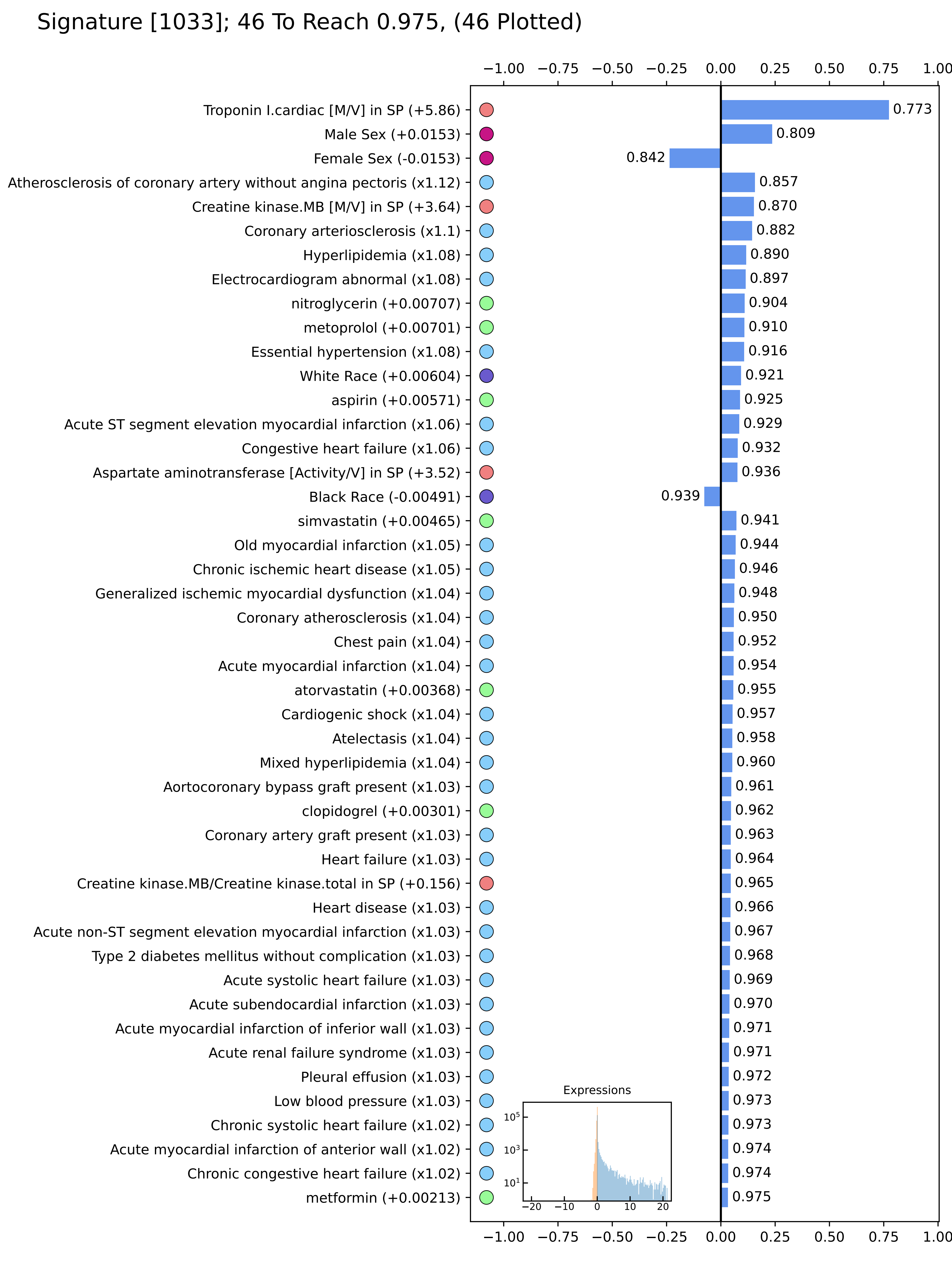
Signature [1033]; 46 To Reach 0.975, (46 Plotted)
Troponin I.cardiac [M/V] in SP (+5.86) 0.773
Male Sex (+0.0153) 0.809
Female Sex (-0.0153) 0.842
Atherosclerosis of coronary artery without angina pectoris (x1.12) 0.857
Creatine kinase.MB [M/V] in SP (+3.64) 0.870
Coronary arteriosclerosis (x1.1) 0.882
Hyperlipidemia (x1.08) 0.890
Electrocardiogram abnormal (x1.08) 0.897
nitroglycerin (+0.00707) 0.904
metoprolol (+0.00701) 0.910
Essential hypertension (x1.08) 0.916
White Race (+0.00604) 0.921
aspirin (+0.00571) 0.925
Acute ST segment elevation myocardial infarction (x1.06) 0.929
Congestive heart failure (x1.06) 0.932
Aspartate aminotransferase [Activity/V] in SP (+3.52) 0.936
Black Race (-0.00491) 0.939
simvastatin (+0.00465) 0.941
Old myocardial infarction (x1.05) 0.944
Chronic ischemic heart disease (x1.05) 0.946
Generalized ischemic myocardial dysfunction (x1.04) 0.948
Coronary atherosclerosis (x1.04) 0.950
Chest pain (x1.04) 0.952
Acute myocardial infarction (x1.04) 0.954
atorvastatin (+0.00368) 0.955
Cardiogenic shock (x1.04) 0.957
Atelectasis (x1.04) 0.958
Mixed hyperlipidemia (x1.04) 0.960
Aortocoronary bypass graft present (x1.03) 0.961
clopidogrel (+0.00301) 0.962
Coronary artery graft present (x1.03) 0.963
Heart failure (x1.03) 0.964
Creatine kinase.MB/Creatine kinase.total in SP (+0.156) 0.965
Heart disease (x1.03) 0.966
Acute non-ST segment elevation myocardial infarction (x1.03) 0.967
Type 2 diabetes mellitus without complication (x1.03) 0.968
Acute systolic heart failure (x1.03) 0.969
Acute subendocardial infarction (x1.03) 0.970
Acute myocardial infarction of inferior wall (x1.03) 0.971
Acute renal failure syndrome (x1.03) 0.971
Pleural effusion (x1.03) 0.972
Low blood pressure (x1.03) 0.973
Chronic systolic heart failure (x1.02) 0.973
Acute myocardial infarction of anterior wall (x1.02) 0.974
Chronic congestive heart failure (x1.02) 0.974
metformin (+0.00213) 0.975
−1.00 −0.75 −0.50 −0.25 0.00 0.25 0.50 0.75 1.00
Expressions
10^5
10^3
10^1
−20 −10 0 10 20

Signature [1034]; 103 To Reach 0.975, (100 Plotted)

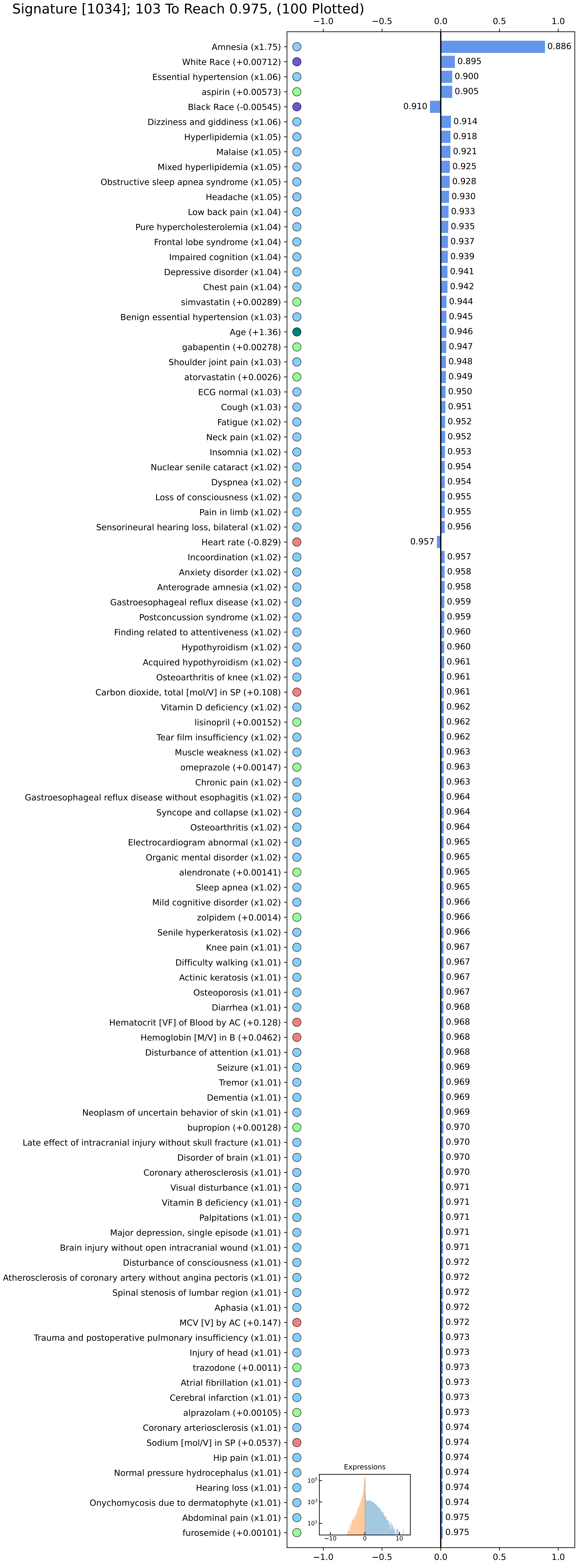

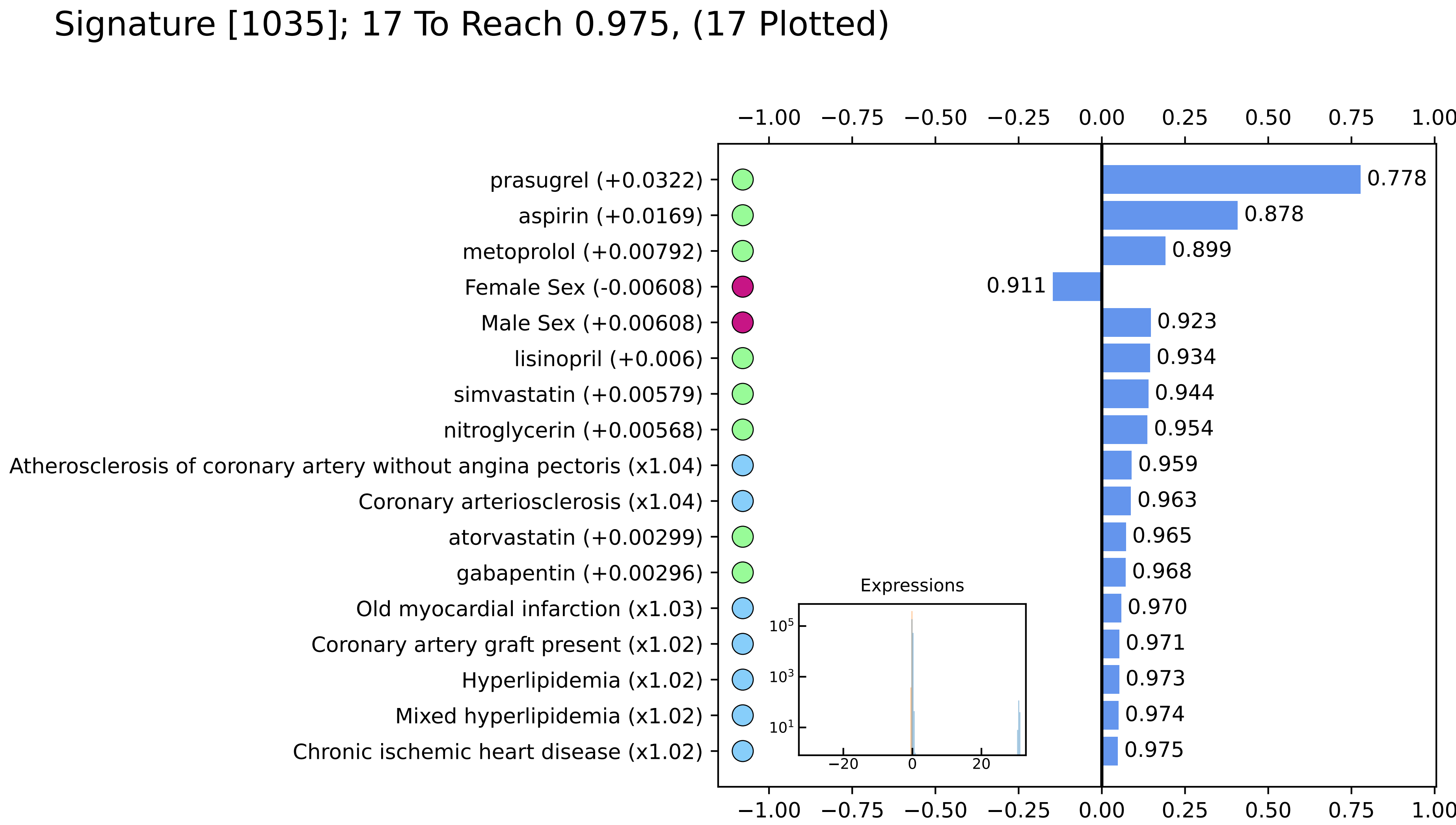

Signature [1035]; 17 To Reach 0.975, (17 Plotted)
−1.00
−0.75
−0.50
−0.25
0.00
0.25
0.50
0.75
1.00
prasugrel (+0.0322)
aspirin (+0.0169)
metoprolol (+0.00792)
Female Sex (-0.00608)
Male Sex (+0.00608)
lisinopril (+0.006)
simvastatin (+0.00579)
nitroglycerin (+0.00568)
Atherosclerosis of coronary artery without angina pectoris (x1.04)
Coronary arteriosclerosis (x1.04)
atorvastatin (+0.00299)
gabapentin (+0.00296)
Old myocardial infarction (x1.03)
Coronary artery graft present (x1.02)
Hyperlipidemia (x1.02)
Mixed hyperlipidemia (x1.02)
Chronic ischemic heart disease (x1.02)
0.778
0.878
0.899
0.911
0.923
0.934
0.944
0.954
0.959
0.963
0.965
0.968
0.970
0.971
0.973
0.974
0.975
Expressions
10^5
10^3
10^1
−20
0
20

Signature [1036]; 6 To Reach 0.975, (10 Plotted)

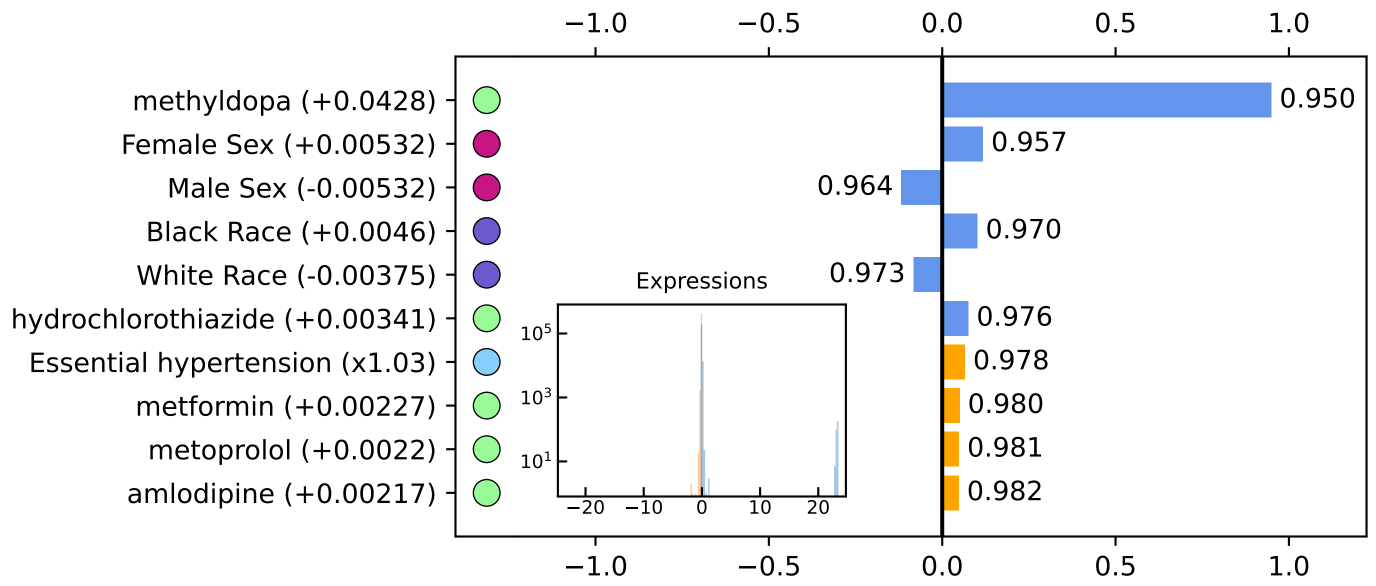

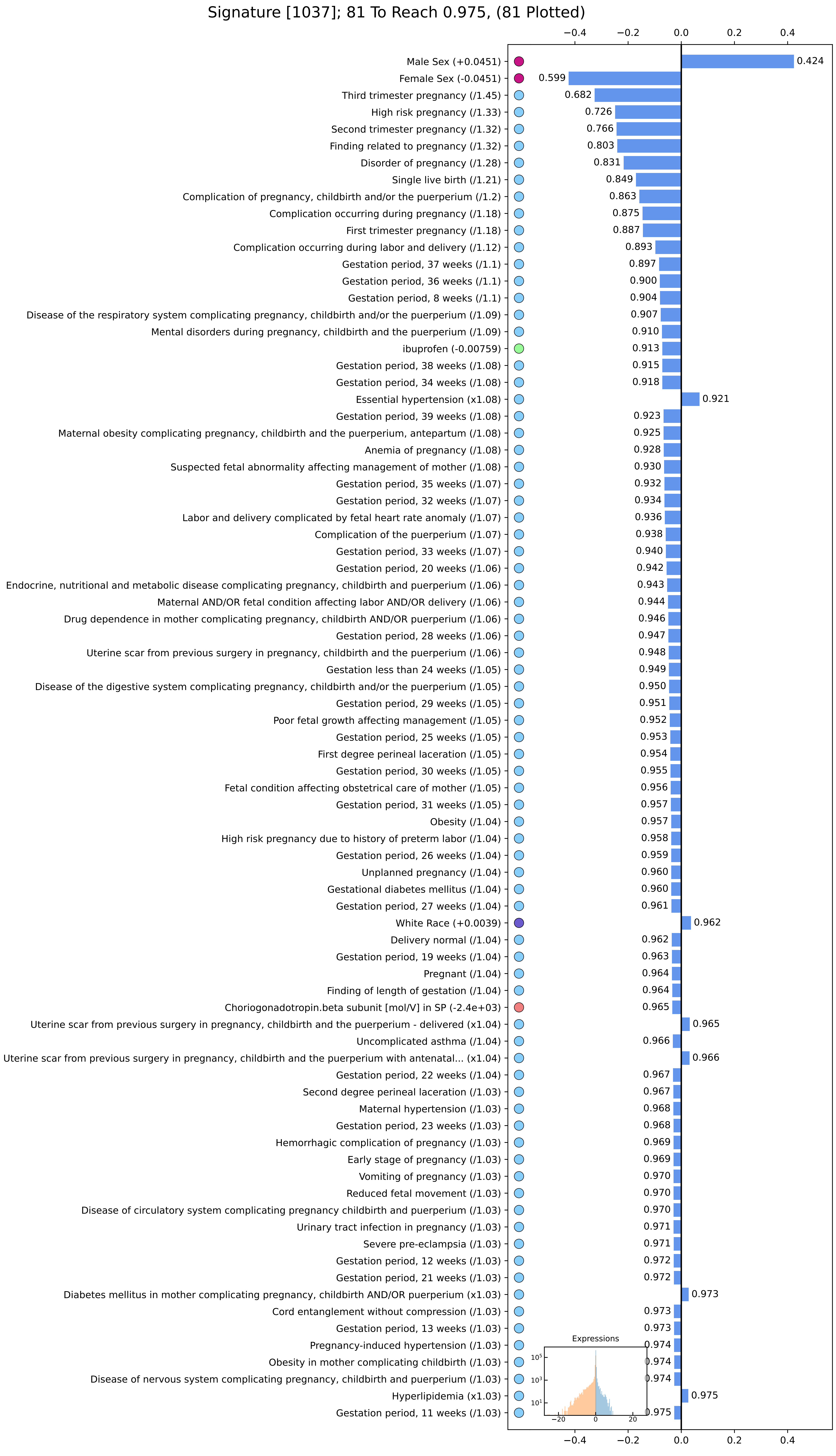
Signature [1037]; 81 To Reach 0.975, (81 Plotted)
Male Sex (+0.0451) 0.424
Female Sex (-0.0451) 0.599
Third trimester pregnancy (/1.45) 0.682
High risk pregnancy (/1.33) 0.726
Second trimester pregnancy (/1.32) 0.766
Finding related to pregnancy (/1.32) 0.803
Disorder of pregnancy (/1.28) 0.831
Single live birth (/1.21) 0.849
Complication of pregnancy, childbirth and/or the puerperium (/1.2) 0.863
Complication occurring during pregnancy (/1.18) 0.875
First trimester pregnancy (/1.18) 0.887
Complication occurring during labor and delivery (/1.12) 0.893
Gestation period, 37 weeks (/1.1) 0.897
Gestation period, 36 weeks (/1.1) 0.900
Gestation period, 8 weeks (/1.1) 0.904
Disease of the respiratory system complicating pregnancy, childbirth and/or the puerperium (/1.09) 0.907
Mental disorders during pregnancy, childbirth and the puerperium (/1.09) 0.910
ibuprofen (-0.00759) 0.913
Gestation period, 38 weeks (/1.08) 0.915
Gestation period, 34 weeks (/1.08) 0.918
Essential hypertension (x1.08) 0.921
Gestation period, 39 weeks (/1.08) 0.923
Maternal obesity complicating pregnancy, childbirth and the puerperium, antepartum (/1.08) 0.925
Anemia of pregnancy (/1.08) 0.928
Suspected fetal abnormality affecting management of mother (/1.08) 0.930
Gestation period, 35 weeks (/1.07) 0.932
Gestation period, 32 weeks (/1.07) 0.934
Labor and delivery complicated by fetal heart rate anomaly (/1.07) 0.936
Complication of the puerperium (/1.07) 0.938
Gestation period, 33 weeks (/1.07) 0.940
Gestation period, 20 weeks (/1.06) 0.942
Endocrine, nutritional and metabolic disease complicating pregnancy, childbirth and puerperium (/1.06) 0.943
Maternal AND/OR fetal condition affecting labor AND/OR delivery (/1.06) 0.944
Drug dependence in mother complicating pregnancy, childbirth AND/OR puerperium (/1.06) 0.946
Gestation period, 28 weeks (/1.06) 0.947
Uterine scar from previous surgery in pregnancy, childbirth and the puerperium (/1.06) 0.948
Gestation less than 24 weeks (/1.05) 0.949
Disease of the digestive system complicating pregnancy, childbirth and/or the puerperium (/1.05) 0.950
Gestation period, 29 weeks (/1.05) 0.951
Poor fetal growth affecting management (/1.05) 0.952
Gestation period, 25 weeks (/1.05) 0.953
First degree perineal laceration (/1.05) 0.954
Gestation period, 30 weeks (/1.05) 0.955
Fetal condition affecting obstetrical care of mother (/1.05) 0.956
Gestation period, 31 weeks (/1.05) 0.957
Obesity (/1.04) 0.957
High risk pregnancy due to history of preterm labor (/1.04) 0.958
Gestation period, 26 weeks (/1.04) 0.959
Unplanned pregnancy (/1.04) 0.960
Gestational diabetes mellitus (/1.04) 0.960
Gestation period, 27 weeks (/1.04) 0.961
White Race (+0.0039) 0.962
Delivery normal (/1.04) 0.962
Gestation period, 19 weeks (/1.04) 0.963
Pregnant (/1.04) 0.964
Finding of length of gestation (/1.04) 0.964
Choriogonadotropin.beta subunit [mol/V] in SP (-2.4e+03) 0.965
Uterine scar from previous surgery in pregnancy, childbirth and the puerperium - delivered (x1.04) 0.965
Uncomplicated asthma (/1.04) 0.966
Uterine scar from previous surgery in pregnancy, childbirth and the puerperium with antenatal... (x1.04) 0.966
Gestation period, 22 weeks (/1.04) 0.967
Second degree perineal laceration (/1.03) 0.967
Maternal hypertension (/1.03) 0.968
Gestation period, 23 weeks (/1.03) 0.968
Hemorrhagic complication of pregnancy (/1.03) 0.969
Early stage of pregnancy (/1.03) 0.969
Vomiting of pregnancy (/1.03) 0.970
Reduced fetal movement (/1.03) 0.970
Disease of circulatory system complicating pregnancy childbirth and puerperium (/1.03) 0.970
Urinary tract infection in pregnancy (/1.03) 0.971
Severe pre-eclampsia (/1.03) 0.971
Gestation period, 12 weeks (/1.03) 0.972
Gestation period, 21 weeks (/1.03) 0.972
Diabetes mellitus in mother complicating pregnancy, childbirth AND/OR puerperium (x1.03) 0.973
Cord entanglement without compression (/1.03) 0.973
Gestation period, 13 weeks (/1.03) 0.973
Pregnancy-induced hypertension (/1.03) 0.974
Obesity in mother complicating childbirth (/1.03) 0.974
Disease of nervous system complicating pregnancy, childbirth and puerperium (/1.03) 0.974
Hyperlipidemia (x1.03) 0.975
Gestation period, 11 weeks (/1.03) 0.975
−0.4 −0.2 0.0 0.2 0.4
Expressions
−20 0 20

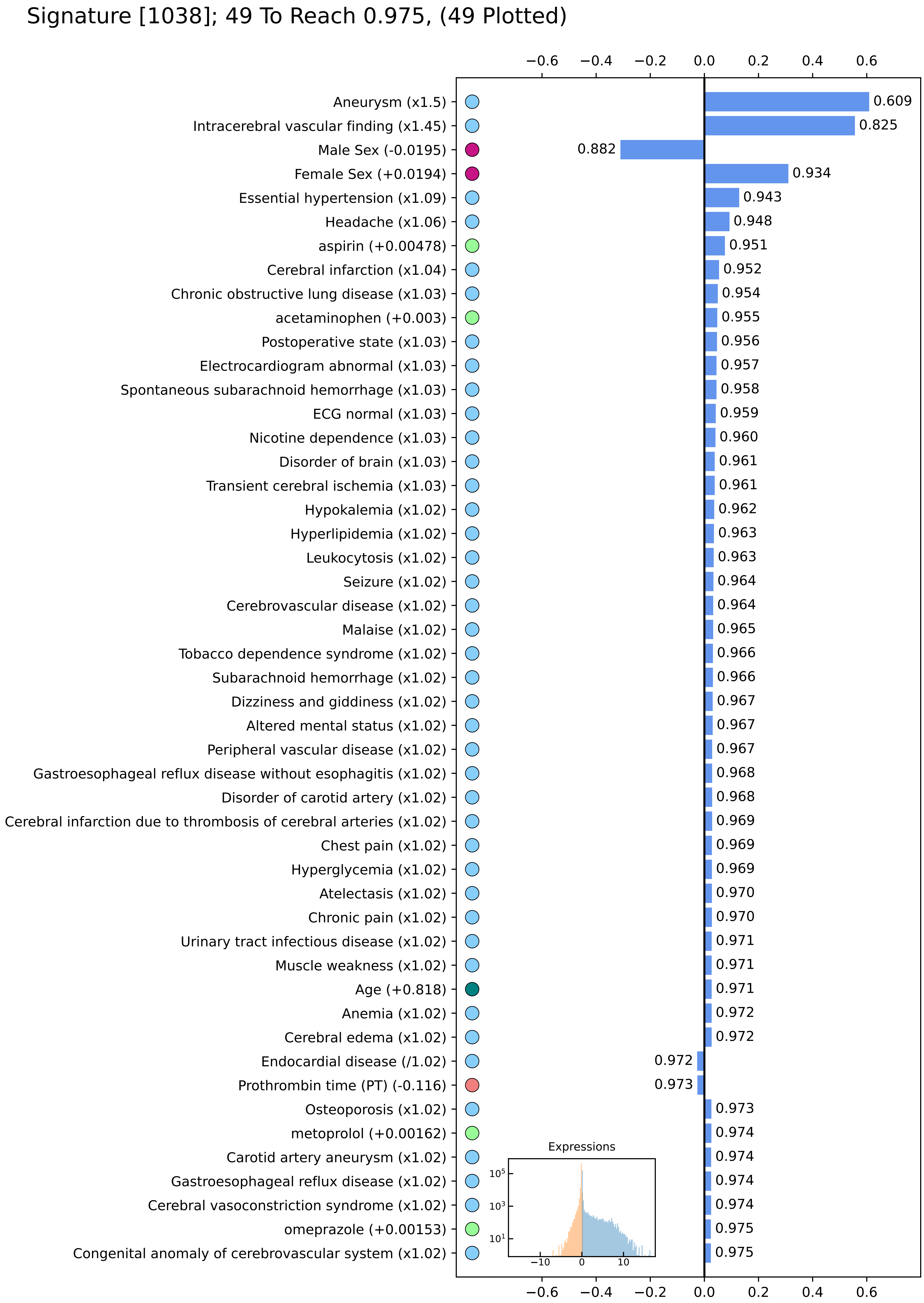

Signature [1038]; 49 To Reach 0.975, (49 Plotted)
Aneurysm (x1.5) 0.609
Intracerebral vascular finding (x1.45) 0.825
Male Sex (-0.0195) 0.882
Female Sex (+0.0194) 0.934
Essential hypertension (x1.09) 0.943
Headache (x1.06) 0.948
aspirin (+0.00478) 0.951
Cerebral infarction (x1.04) 0.952
Chronic obstructive lung disease (x1.03) 0.954
acetaminophen (+0.003) 0.955
Postoperative state (x1.03) 0.956
Electrocardiogram abnormal (x1.03) 0.957
Spontaneous subarachnoid hemorrhage (x1.03) 0.958
ECG normal (x1.03) 0.959
Nicotine dependence (x1.03) 0.960
Disorder of brain (x1.03) 0.961
Transient cerebral ischemia (x1.03) 0.961
Hypokalemia (x1.02) 0.962
Hyperlipidemia (x1.02) 0.963
Leukocytosis (x1.02) 0.963
Seizure (x1.02) 0.964
Cerebrovascular disease (x1.02) 0.964
Malaise (x1.02) 0.965
Tobacco dependence syndrome (x1.02) 0.966
Subarachnoid hemorrhage (x1.02) 0.966
Dizziness and giddiness (x1.02) 0.967
Altered mental status (x1.02) 0.967
Peripheral vascular disease (x1.02) 0.967
Gastroesophageal reflux disease without esophagitis (x1.02) 0.968
Disorder of carotid artery (x1.02) 0.968
Cerebral infarction due to thrombosis of cerebral arteries (x1.02) 0.969
Chest pain (x1.02) 0.969
Hyperglycemia (x1.02) 0.969
Atelectasis (x1.02) 0.970
Chronic pain (x1.02) 0.970
Urinary tract infectious disease (x1.02) 0.971
Muscle weakness (x1.02) 0.971
Age (+0.818) 0.971
Anemia (x1.02) 0.972
Cerebral edema (x1.02) 0.972
Endocardial disease (/1.02) 0.972
Prothrombin time (PT) (-0.116) 0.973
Osteoporosis (x1.02) 0.973
metoprolol (+0.00162) 0.974
Carotid artery aneurysm (x1.02) 0.974
Gastroesophageal reflux disease (x1.02) 0.974
Cerebral vasoconstriction syndrome (x1.02) 0.974
omeprazole (+0.00153) 0.975
Congenital anomaly of cerebrovascular system (x1.02) 0.975
−0.6 −0.4 −0.2 0.0 0.2 0.4 0.6
Expressions

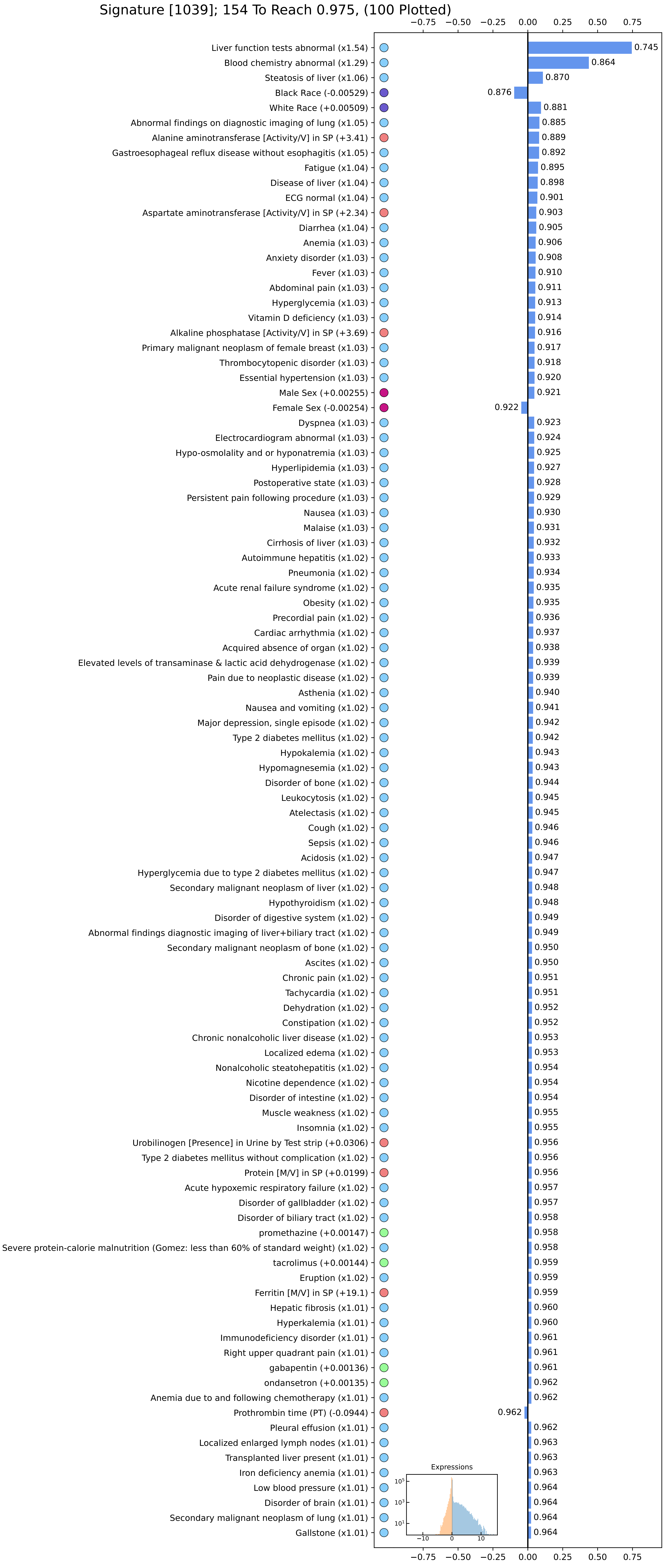

Signature [1039]; 154 To Reach 0.975, (100 Plotted)
Liver function tests abnormal (x1.54) 0.745
Blood chemistry abnormal (x1.29) 0.864
Steatosis of liver (x1.06) 0.870
Black Race (-0.00529) 0.876
White Race (+0.00509) 0.881
Abnormal findings on diagnostic imaging of lung (x1.05) 0.885
Alanine aminotransferase [Activity/V] in SP (+3.41) 0.889
Gastroesophageal reflux disease without esophagitis (x1.05) 0.892
Fatigue (x1.04) 0.895
Disease of liver (x1.04) 0.898
ECG normal (x1.04) 0.901
Aspartate aminotransferase [Activity/V] in SP (+2.34) 0.903
Diarrhea (x1.04) 0.905
Anemia (x1.03) 0.906
Anxiety disorder (x1.03) 0.908
Fever (x1.03) 0.910
Abdominal pain (x1.03) 0.911
Hyperglycemia (x1.03) 0.913
Vitamin D deficiency (x1.03) 0.914
Alkaline phosphatase [Activity/V] in SP (+3.69) 0.916
Primary malignant neoplasm of female breast (x1.03) 0.917
Thrombocytopenic disorder (x1.03) 0.918
Essential hypertension (x1.03) 0.920
Male Sex (+0.00255) 0.921
Female Sex (-0.00254) 0.922
Dyspnea (x1.03) 0.923
Electrocardiogram abnormal (x1.03) 0.924
Hypo-osmolality and or hyponatremia (x1.03) 0.925
Hyperlipidemia (x1.03) 0.927
Postoperative state (x1.03) 0.928
Persistent pain following procedure (x1.03) 0.929
Nausea (x1.03) 0.930
Malaise (x1.03) 0.931
Cirrhosis of liver (x1.03) 0.932
Autoimmune hepatitis (x1.02) 0.933
Pneumonia (x1.02) 0.934
Acute renal failure syndrome (x1.02) 0.935
Obesity (x1.02) 0.935
Precordial pain (x1.02) 0.936
Cardiac arrhythmia (x1.02) 0.937
Acquired absence of organ (x1.02) 0.938
Elevated levels of transaminase & lactic acid dehydrogenase (x1.02) 0.939
Pain due to neoplastic disease (x1.02) 0.939
Asthenia (x1.02) 0.940
Nausea and vomiting (x1.02) 0.941
Major depression, single episode (x1.02) 0.942
Type 2 diabetes mellitus (x1.02) 0.942
Hypokalemia (x1.02) 0.943
Hypomagnesemia (x1.02) 0.943
Disorder of bone (x1.02) 0.944
Leukocytosis (x1.02) 0.945
Atelectasis (x1.02) 0.945
Cough (x1.02) 0.946
Sepsis (x1.02) 0.946
Acidosis (x1.02) 0.947
Hyperglycemia due to type 2 diabetes mellitus (x1.02) 0.947
Secondary malignant neoplasm of liver (x1.02) 0.948
Hypothyroidism (x1.02) 0.948
Disorder of digestive system (x1.02) 0.949
Abnormal findings diagnostic imaging of liver+biliary tract (x1.02) 0.949
Secondary malignant neoplasm of bone (x1.02) 0.950
Ascites (x1.02) 0.950
Chronic pain (x1.02) 0.951
Tachycardia (x1.02) 0.951
Dehydration (x1.02) 0.952
Constipation (x1.02) 0.952
Chronic nonalcoholic liver disease (x1.02) 0.953
Localized edema (x1.02) 0.953
Nonalcoholic steatohepatitis (x1.02) 0.954
Nicotine dependence (x1.02) 0.954
Disorder of intestine (x1.02) 0.954
Muscle weakness (x1.02) 0.955
Insomnia (x1.02) 0.955
Urobilinogen [Presence] in Urine by Test strip (+0.0306) 0.956
Type 2 diabetes mellitus without complication (x1.02) 0.956
Protein [M/V] in SP (+0.0199) 0.956
Acute hypoxemic respiratory failure (x1.02) 0.957
Disorder of gallbladder (x1.02) 0.957
Disorder of biliary tract (x1.02) 0.958
promethazine (+0.00147) 0.958
Severe protein-calorie malnutrition (Gomez: less than 60% of standard weight) (x1.02) 0.958
tacrolimus (+0.00144) 0.959
Eruption (x1.02) 0.959
Ferritin [M/V] in SP (+19.1) 0.959
Hepatic fibrosis (x1.01) 0.960
Hyperkalemia (x1.01) 0.960
Immunodeficiency disorder (x1.01) 0.961
Right upper quadrant pain (x1.01) 0.961
gabapentin (+0.00136) 0.961
ondansetron (+0.00135) 0.962
Anemia due to and following chemotherapy (x1.01) 0.962
Prothrombin time (PT) (-0.0944) 0.962
Pleural effusion (x1.01) 0.962
Localized enlarged lymph nodes (x1.01) 0.963
Transplanted liver present (x1.01) 0.963
Iron deficiency anemia (x1.01) 0.963
Low blood pressure (x1.01) 0.964
Disorder of brain (x1.01) 0.964
Secondary malignant neoplasm of lung (x1.01) 0.964
Gallstone (x1.01) 0.964
−0.75 −0.50 −0.25 0.00 0.25 0.50 0.75
Expressions

Signature [1040]; 8 To Reach 0.975, (10 Plotted)

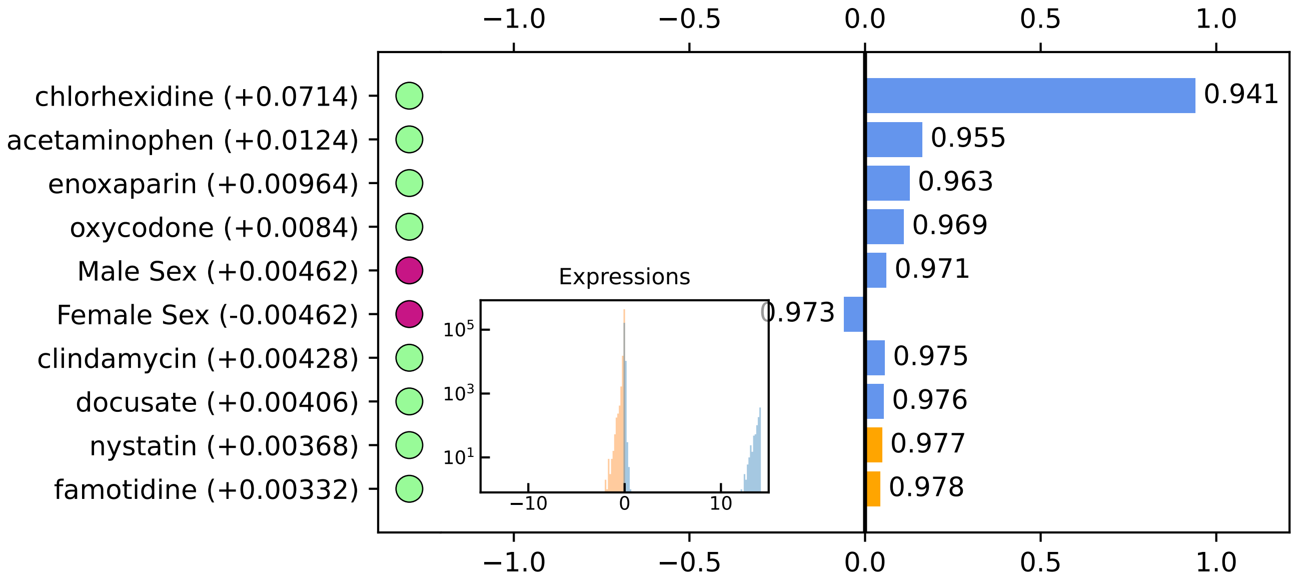

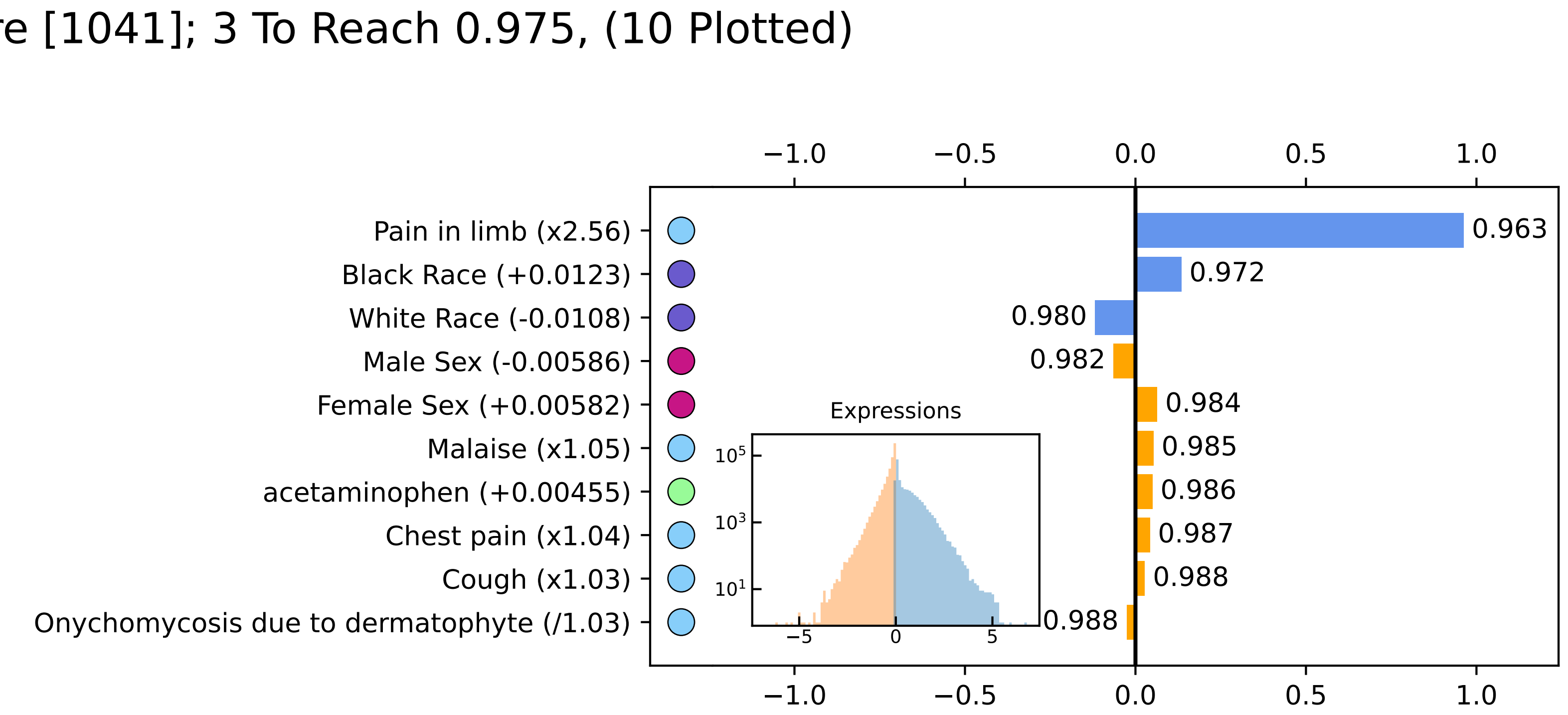

Signature [1041]; 3 To Reach 0.975, (10 Plotted)
Pain in limb (x2.56)
Black Race (+0.0123)
White Race (-0.0108)
Male Sex (-0.00586)
Female Sex (+0.00582)
Malaise (x1.05)
acetaminophen (+0.00455)
Chest pain (x1.04)
Cough (x1.03)
Onychomycosis due to dermatophyte (/1.03)
0.963
0.972
0.980
0.982
0.984
0.985
0.986
0.987
0.988
0.988
−1.0
−0.5
0.0
0.5
1.0
Expressions
10^5
10^3
10^1
−5
0
5

Signature [1042]; 171 To Reach 0.975, (100 Plotted)

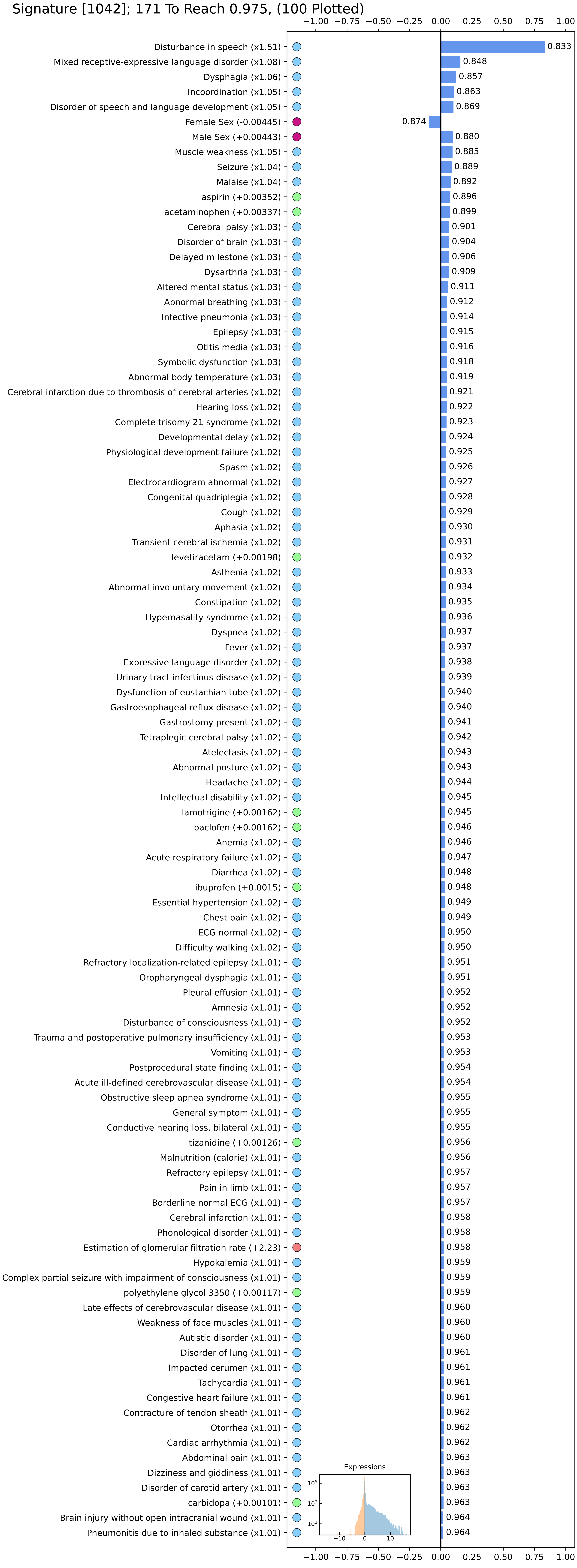

Signature [1043]; 393 To Reach 0.975, (100 Plotted)

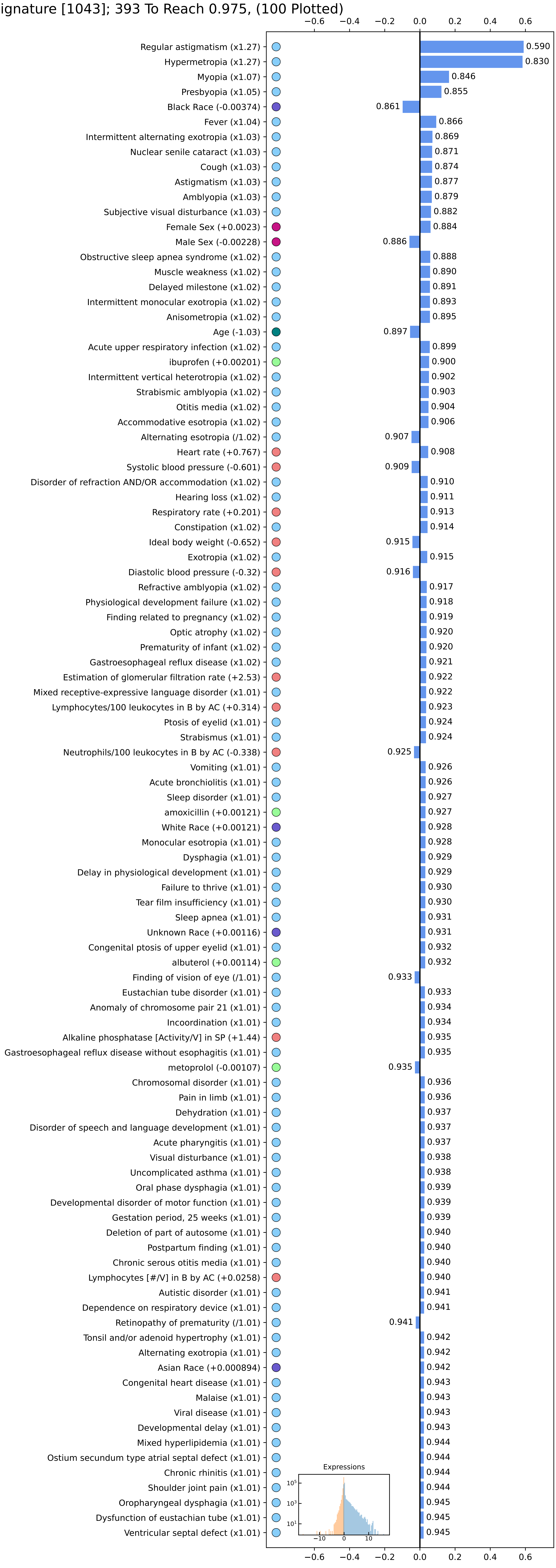

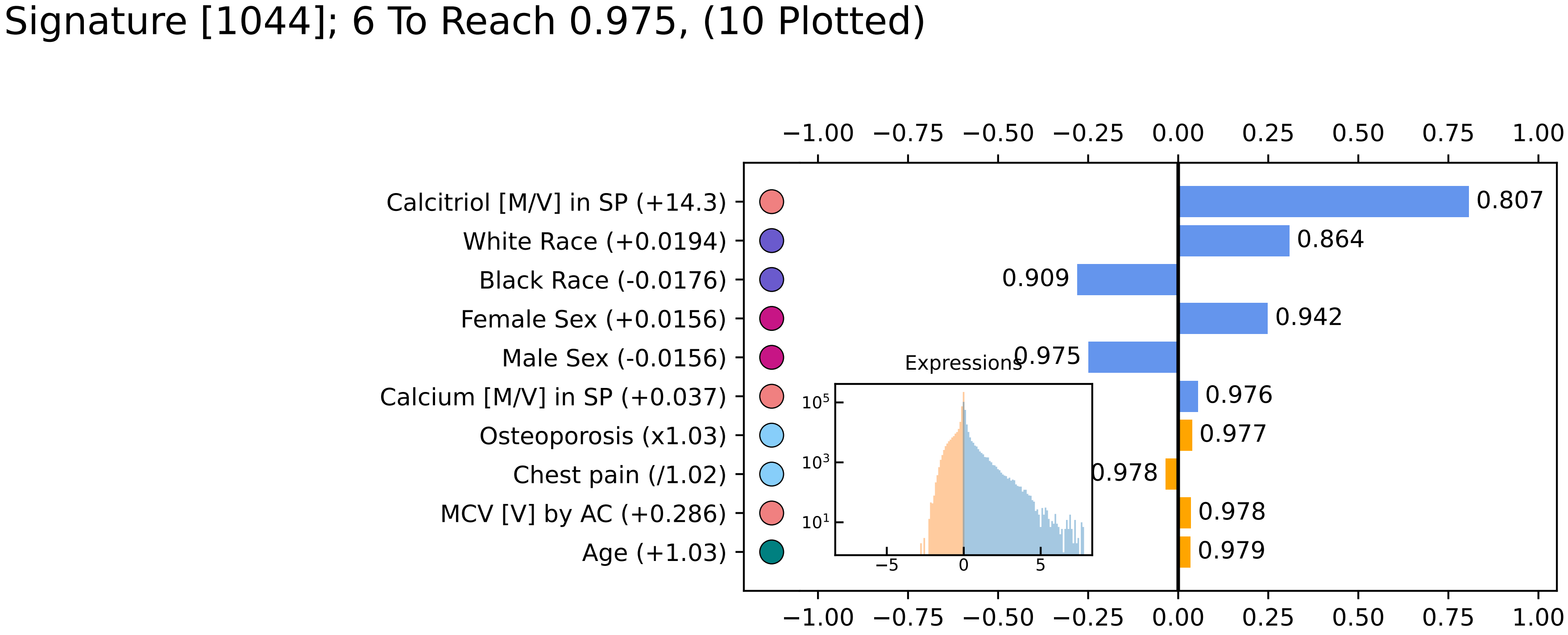

Signature [1044]; 6 To Reach 0.975, (10 Plotted)
Calcitriol [M/V] in SP (+14.3)
White Race (+0.0194)
Black Race (-0.0176)
Female Sex (+0.0156)
Male Sex (-0.0156)
Calcium [M/V] in SP (+0.037)
Osteoporosis (x1.03)
Chest pain (/1.02)
MCV [V] by AC (+0.286)
Age (+1.03)
0.807
0.864
0.909
0.942
0.975
0.976
0.977
0.978
0.978
0.979
Expressions
−1.00 −0.75 −0.50 −0.25 0.00 0.25 0.50 0.75 1.00

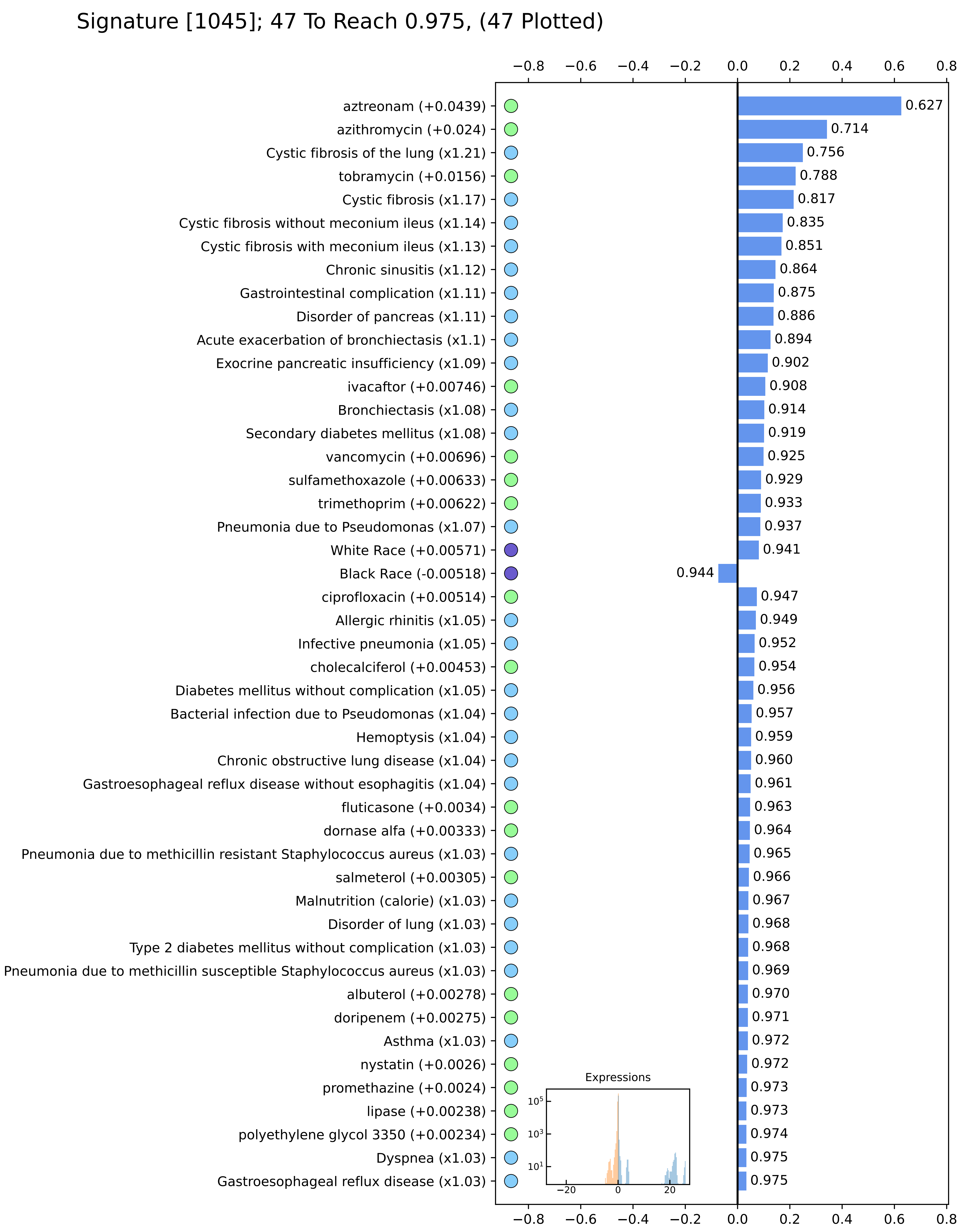

Signature [1045]; 47 To Reach 0.975, (47 Plotted)
aztreonam (+0.0439)
azithromycin (+0.024)
Cystic fibrosis of the lung (x1.21)
tobramycin (+0.0156)
Cystic fibrosis (x1.17)
Cystic fibrosis without meconium ileus (x1.14)
Cystic fibrosis with meconium ileus (x1.13)
Chronic sinusitis (x1.12)
Gastrointestinal complication (x1.11)
Disorder of pancreas (x1.11)
Acute exacerbation of bronchiectasis (x1.1)
Exocrine pancreatic insufficiency (x1.09)
ivacaftor (+0.00746)
Bronchiectasis (x1.08)
Secondary diabetes mellitus (x1.08)
vancomycin (+0.00696)
sulfamethoxazole (+0.00633)
trimethoprim (+0.00622)
Pneumonia due to Pseudomonas (x1.07)
White Race (+0.00571)
Black Race (-0.00518)
ciprofloxacin (+0.00514)
Allergic rhinitis (x1.05)
Infective pneumonia (x1.05)
cholecalciferol (+0.00453)
Diabetes mellitus without complication (x1.05)
Bacterial infection due to Pseudomonas (x1.04)
Hemoptysis (x1.04)
Chronic obstructive lung disease (x1.04)
Gastroesophageal reflux disease without esophagitis (x1.04)
fluticasone (+0.0034)
dornase alfa (+0.00333)
Pneumonia due to methicillin resistant Staphylococcus aureus (x1.03)
salmeterol (+0.00305)
Malnutrition (calorie) (x1.03)
Disorder of lung (x1.03)
Type 2 diabetes mellitus without complication (x1.03)
Pneumonia due to methicillin susceptible Staphylococcus aureus (x1.03)
albuterol (+0.00278)
doripenem (+0.00275)
Asthma (x1.03)
nystatin (+0.0026)
promethazine (+0.0024)
lipase (+0.00238)
polyethylene glycol 3350 (+0.00234)
Dyspnea (x1.03)
Gastroesophageal reflux disease (x1.03)
0.627
0.714
0.756
0.788
0.817
0.835
0.851
0.864
0.875
0.886
0.894
0.902
0.908
0.914
0.919
0.925
0.929
0.933
0.937
0.941
0.944
0.947
0.949
0.952
0.954
0.956
0.957
0.959
0.960
0.961
0.963
0.964
0.965
0.966
0.967
0.968
0.968
0.969
0.970
0.971
0.972
0.972
0.973
0.973
0.974
0.975
0.975
−0.8
−0.6
−0.4
−0.2
0.0
0.2
0.4
0.6
0.8
Expressions
10^5
10^3
10^1
−20
0
20

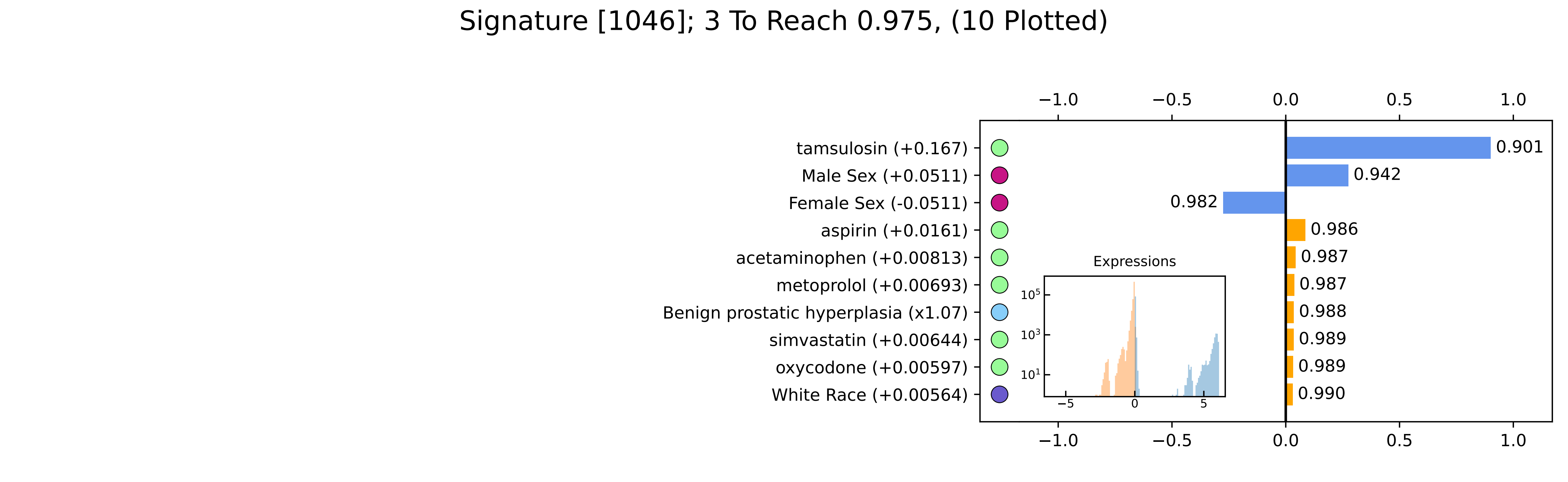

Signature [1046]; 3 To Reach 0.975, (10 Plotted)
tamsulosin (+0.167)
Male Sex (+0.0511)
Female Sex (-0.0511)
aspirin (+0.0161)
acetaminophen (+0.00813)
metoprolol (+0.00693)
Benign prostatic hyperplasia (x1.07)
simvastatin (+0.00644)
oxycodone (+0.00597)
White Race (+0.00564)
0.901
0.942
0.982
0.986
0.987
0.987
0.988
0.989
0.989
0.990
Expressions
−1.0
−0.5
0.0
0.5
1.0

Signature [1047]; 187 To Reach 0.975, (100 Plotted)

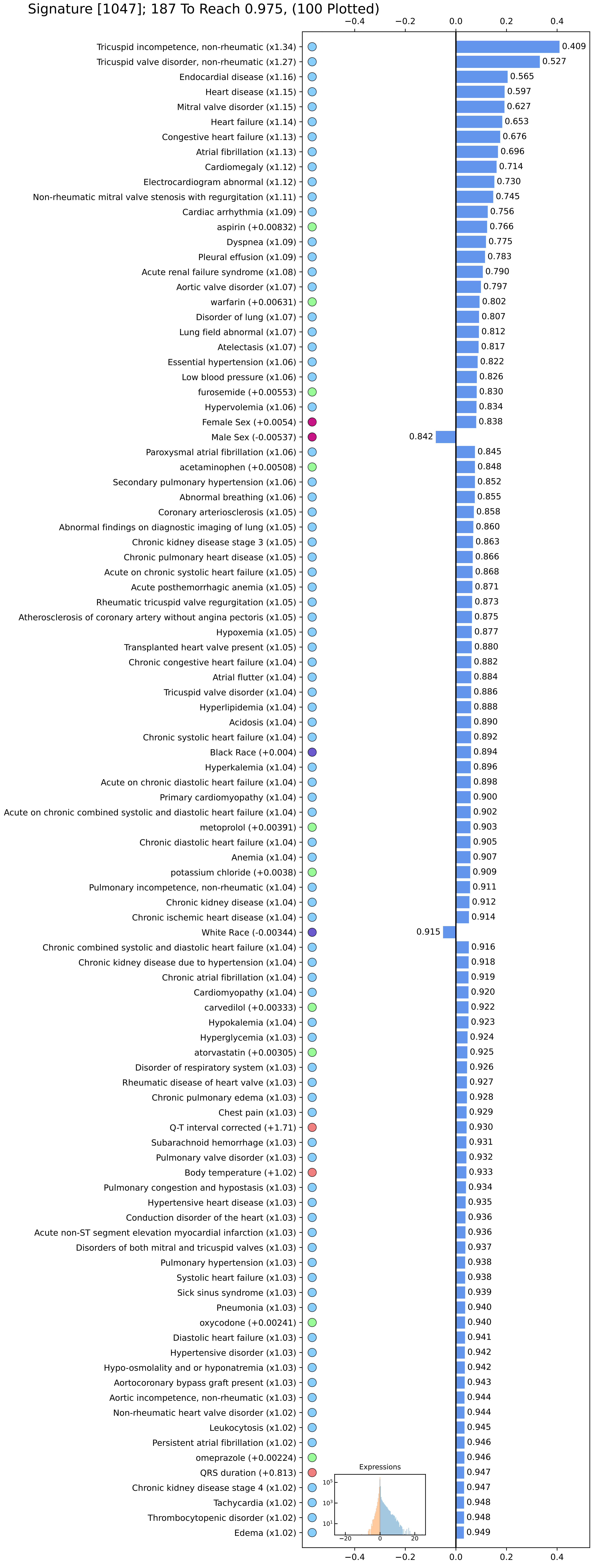

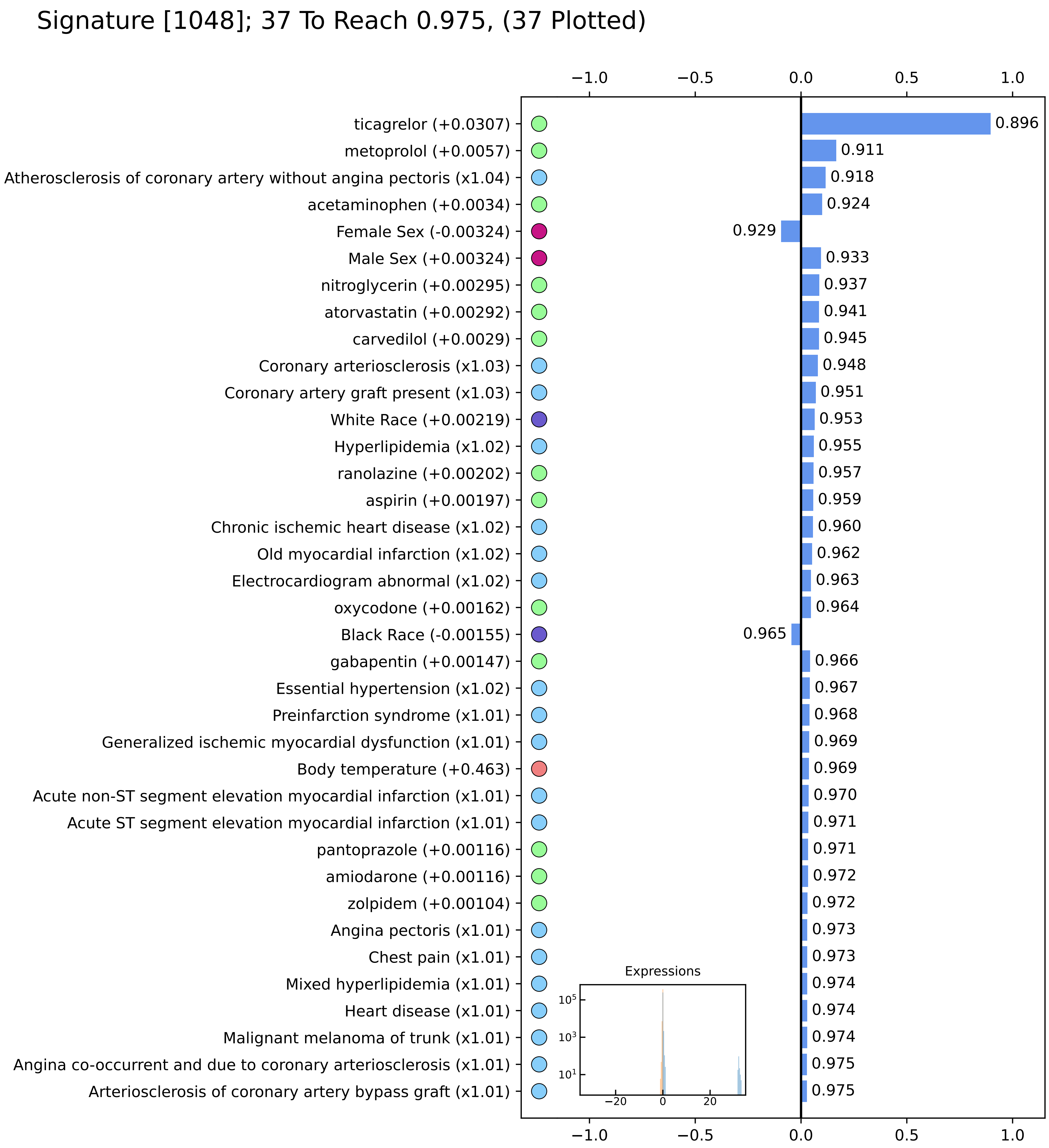

Signature [1048]; 37 To Reach 0.975, (37 Plotted)
ticagrelor (+0.0307) 0.896
metoprolol (+0.0057) 0.911
Atherosclerosis of coronary artery without angina pectoris (x1.04) 0.918
acetaminophen (+0.0034) 0.924
Female Sex (-0.00324) 0.929
Male Sex (+0.00324) 0.933
nitroglycerin (+0.00295) 0.937
atorvastatin (+0.00292) 0.941
carvedilol (+0.0029) 0.945
Coronary arteriosclerosis (x1.03) 0.948
Coronary artery graft present (x1.03) 0.951
White Race (+0.00219) 0.953
Hyperlipidemia (x1.02) 0.955
ranolazine (+0.00202) 0.957
aspirin (+0.00197) 0.959
Chronic ischemic heart disease (x1.02) 0.960
Old myocardial infarction (x1.02) 0.962
Electrocardiogram abnormal (x1.02) 0.963
oxycodone (+0.00162) 0.964
Black Race (-0.00155) 0.965
gabapentin (+0.00147) 0.966
Essential hypertension (x1.02) 0.967
Preinfarction syndrome (x1.01) 0.968
Generalized ischemic myocardial dysfunction (x1.01) 0.969
Body temperature (+0.463) 0.969
Acute non-ST segment elevation myocardial infarction (x1.01) 0.970
Acute ST segment elevation myocardial infarction (x1.01) 0.971
pantoprazole (+0.00116) 0.971
amiodarone (+0.00116) 0.972
zolpidem (+0.00104) 0.972
Angina pectoris (x1.01) 0.973
Chest pain (x1.01) 0.973
Mixed hyperlipidemia (x1.01) 0.974
Heart disease (x1.01) 0.974
Malignant melanoma of trunk (x1.01) 0.974
Angina co-occurrent and due to coronary arteriosclerosis (x1.01) 0.975
Arteriosclerosis of coronary artery bypass graft (x1.01) 0.975
−1.0 −0.5 0.0 0.5 1.0
Expressions

Signature [1049]; 7 To Reach 0.975, (10 Plotted)

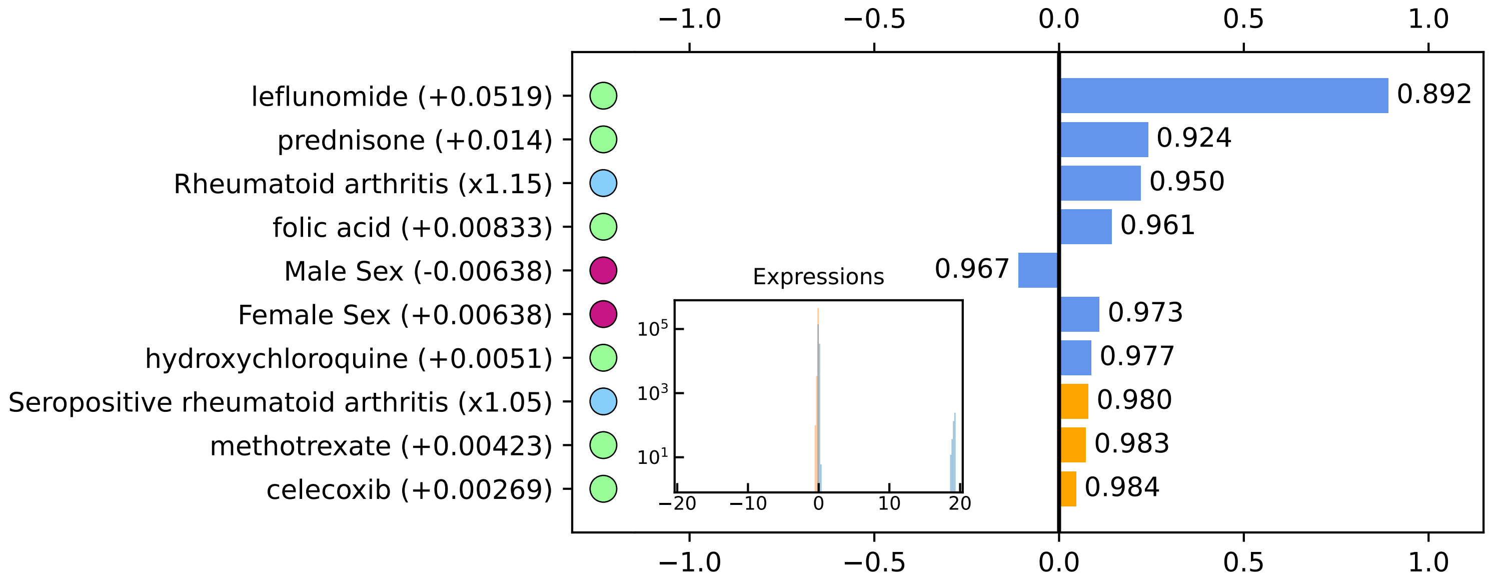

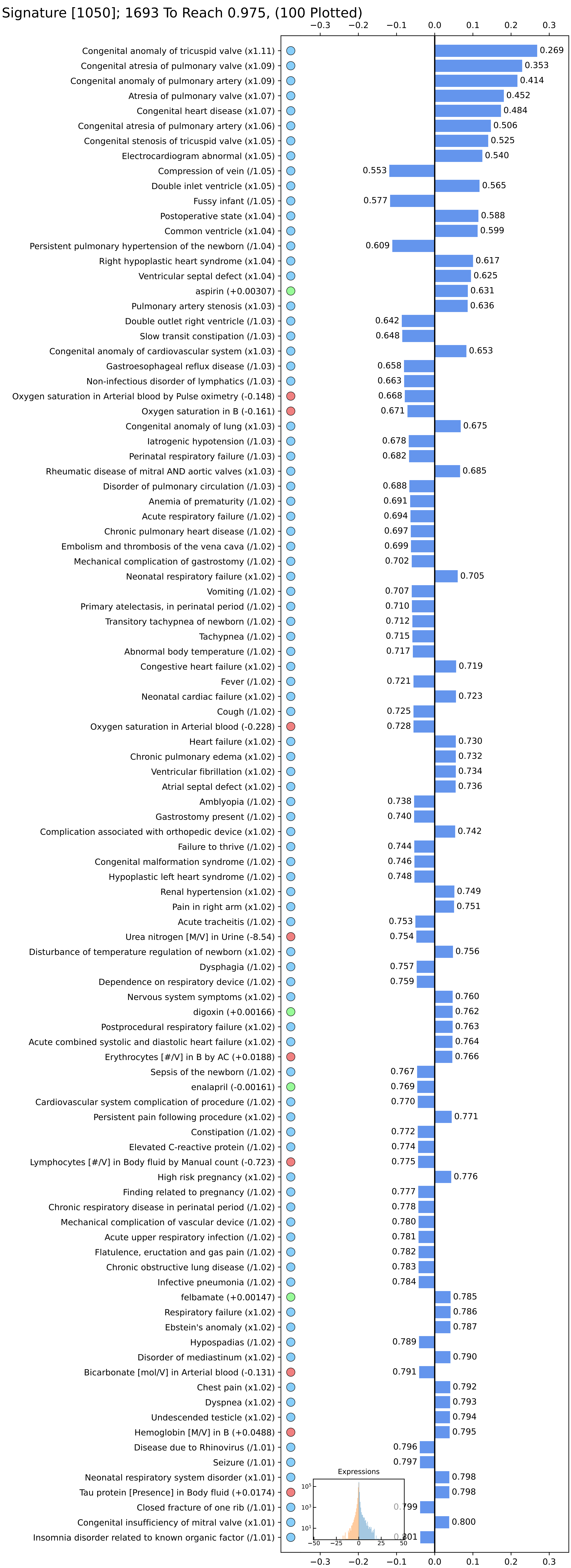
Signature [1050]; 1693 To Reach 0.975, (100 Plotted)
Congenital anomaly of tricuspid valve (x1.11) 0.269
Congenital atresia of pulmonary valve (x1.09) 0.353
Congenital anomaly of pulmonary artery (x1.09) 0.414
Atresia of pulmonary valve (x1.07) 0.452
Congenital heart disease (x1.07) 0.484
Congenital atresia of pulmonary artery (x1.06) 0.506
Congenital stenosis of tricuspid valve (x1.05) 0.525
Electrocardiogram abnormal (x1.05) 0.540
Compression of vein (/1.05) 0.553
Double inlet ventricle (x1.05) 0.565
Fussy infant (/1.05) 0.577
Postoperative state (x1.04) 0.588
Common ventricle (x1.04) 0.599
Persistent pulmonary hypertension of the newborn (/1.04) 0.609
Right hypoplastic heart syndrome (x1.04) 0.617
Ventricular septal defect (x1.04) 0.625
aspirin (+0.00307) 0.631
Pulmonary artery stenosis (x1.03) 0.636
Double outlet right ventricle (/1.03) 0.642
Slow transit constipation (/1.03) 0.648
Congenital anomaly of cardiovascular system (x1.03) 0.653
Gastroesophageal reflux disease (/1.03) 0.658
Non-infectious disorder of lymphatics (/1.03) 0.663
Oxygen saturation in Arterial blood by Pulse oximetry (-0.148) 0.668
Oxygen saturation in B (-0.161) 0.671
Congenital anomaly of lung (x1.03) 0.675
Iatrogenic hypotension (/1.03) 0.678
Perinatal respiratory failure (/1.03) 0.682
Rheumatic disease of mitral AND aortic valves (x1.03) 0.685
Disorder of pulmonary circulation (/1.03) 0.688
Anemia of prematurity (/1.02) 0.691
Acute respiratory failure (/1.02) 0.694
Chronic pulmonary heart disease (/1.02) 0.697
Embolism and thrombosis of the vena cava (/1.02) 0.699
Mechanical complication of gastrostomy (/1.02) 0.702
Neonatal respiratory failure (x1.02) 0.705
Vomiting (/1.02) 0.707
Primary atelectasis, in perinatal period (/1.02) 0.710
Transitory tachypnea of newborn (/1.02) 0.712
Tachypnea (/1.02) 0.715
Abnormal body temperature (/1.02) 0.717
Congestive heart failure (x1.02) 0.719
Fever (/1.02) 0.721
Neonatal cardiac failure (x1.02) 0.723
Cough (/1.02) 0.725
Oxygen saturation in Arterial blood (-0.228) 0.728
Heart failure (x1.02) 0.730
Chronic pulmonary edema (x1.02) 0.732
Ventricular fibrillation (x1.02) 0.734
Atrial septal defect (x1.02) 0.736
Amblyopia (/1.02) 0.738
Gastrostomy present (/1.02) 0.740
Complication associated with orthopedic device (x1.02) 0.742
Failure to thrive (/1.02) 0.744
Congenital malformation syndrome (/1.02) 0.746
Hypoplastic left heart syndrome (/1.02) 0.748
Renal hypertension (x1.02) 0.749
Pain in right arm (x1.02) 0.751
Acute tracheitis (/1.02) 0.753
Urea nitrogen [M/V] in Urine (-8.54) 0.754
Disturbance of temperature regulation of newborn (x1.02) 0.756
Dysphagia (/1.02) 0.757
Dependence on respiratory device (/1.02) 0.759
Nervous system symptoms (x1.02) 0.760
digoxin (+0.00166) 0.762
Postprocedural respiratory failure (x1.02) 0.763
Acute combined systolic and diastolic heart failure (x1.02) 0.764
Erythrocytes [#/V] in B by AC (+0.0188) 0.766
Sepsis of the newborn (/1.02) 0.767
enalapril (-0.00161) 0.769
Cardiovascular system complication of procedure (/1.02) 0.770
Persistent pain following procedure (x1.02) 0.771
Constipation (/1.02) 0.772
Elevated C-reactive protein (/1.02) 0.774
Lymphocytes [#/V] in Body fluid by Manual count (-0.723) 0.775
High risk pregnancy (x1.02) 0.776
Finding related to pregnancy (/1.02) 0.777
Chronic respiratory disease in perinatal period (/1.02) 0.778
Mechanical complication of vascular device (/1.02) 0.780
Acute upper respiratory infection (/1.02) 0.781
Flatulence, eructation and gas pain (/1.02) 0.782
Chronic obstructive lung disease (/1.02) 0.783
Infective pneumonia (/1.02) 0.784
felbamate (+0.00147) 0.785
Respiratory failure (x1.02) 0.786
Ebstein's anomaly (x1.02) 0.787
Hypospadias (/1.02) 0.789
Disorder of mediastinum (x1.02) 0.790
Bicarbonate [mol/V] in Arterial blood (-0.131) 0.791
Chest pain (x1.02) 0.792
Dyspnea (x1.02) 0.793
Undescended testicle (x1.02) 0.794
Hemoglobin [M/V] in B (+0.0488) 0.795
Disease due to Rhinovirus (/1.01) 0.796
Seizure (/1.01) 0.797
Neonatal respiratory system disorder (x1.01) 0.798
Tau protein [Presence] in Body fluid (+0.0174) 0.798
Closed fracture of one rib (/1.01) 0.799
Congenital insufficiency of mitral valve (x1.01) 0.800
Insomnia disorder related to known organic factor (/1.01) 0.801
−0.3 −0.2 −0.1 0.0 0.1 0.2 0.3
Expressions
−50 −25 0 25 50
10^5 10^3 10^1

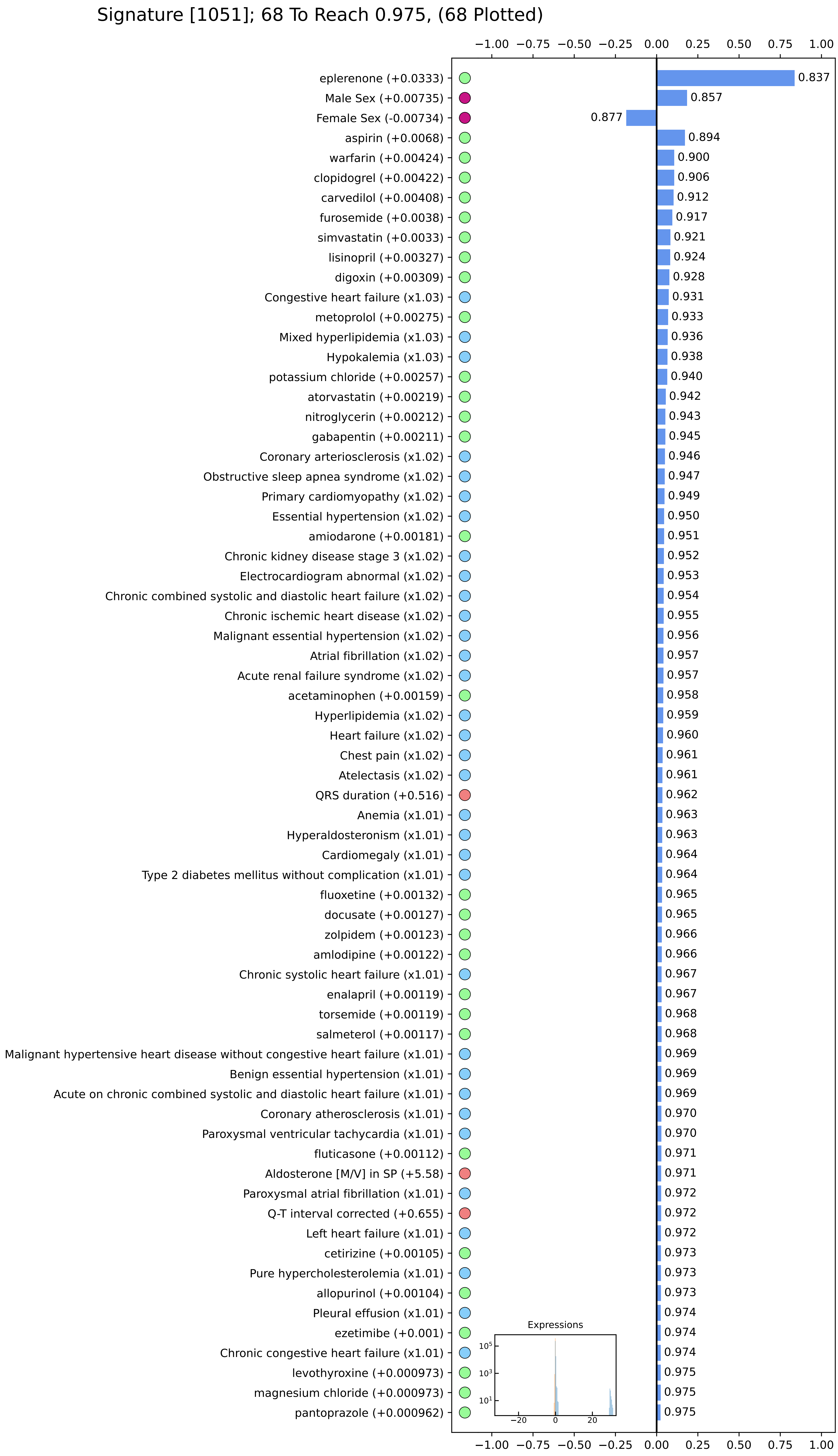

Signature [1051]; 68 To Reach 0.975, (68 Plotted)
−1.00 −0.75 −0.50 −0.25 0.00 0.25 0.50 0.75 1.00
eplerenone (+0.0333) 0.837
Male Sex (+0.00735) 0.857
Female Sex (-0.00734) 0.877
aspirin (+0.0068) 0.894
warfarin (+0.00424) 0.900
clopidogrel (+0.00422) 0.906
carvedilol (+0.00408) 0.912
furosemide (+0.0038) 0.917
simvastatin (+0.0033) 0.921
lisinopril (+0.00327) 0.924
digoxin (+0.00309) 0.928
Congestive heart failure (x1.03) 0.931
metoprolol (+0.00275) 0.933
Mixed hyperlipidemia (x1.03) 0.936
Hypokalemia (x1.03) 0.938
potassium chloride (+0.00257) 0.940
atorvastatin (+0.00219) 0.942
nitroglycerin (+0.00212) 0.943
gabapentin (+0.00211) 0.945
Coronary arteriosclerosis (x1.02) 0.946
Obstructive sleep apnea syndrome (x1.02) 0.947
Primary cardiomyopathy (x1.02) 0.949
Essential hypertension (x1.02) 0.950
amiodarone (+0.00181) 0.951
Chronic kidney disease stage 3 (x1.02) 0.952
Electrocardiogram abnormal (x1.02) 0.953
Chronic combined systolic and diastolic heart failure (x1.02) 0.954
Chronic ischemic heart disease (x1.02) 0.955
Malignant essential hypertension (x1.02) 0.956
Atrial fibrillation (x1.02) 0.957
Acute renal failure syndrome (x1.02) 0.957
acetaminophen (+0.00159) 0.958
Hyperlipidemia (x1.02) 0.959
Heart failure (x1.02) 0.960
Chest pain (x1.02) 0.961
Atelectasis (x1.02) 0.961
QRS duration (+0.516) 0.962
Anemia (x1.01) 0.963
Hyperaldosteronism (x1.01) 0.963
Cardiomegaly (x1.01) 0.964
Type 2 diabetes mellitus without complication (x1.01) 0.964
fluoxetine (+0.00132) 0.965
docusate (+0.00127) 0.965
zolpidem (+0.00123) 0.966
amlodipine (+0.00122) 0.966
Chronic systolic heart failure (x1.01) 0.967
enalapril (+0.00119) 0.967
torsemide (+0.00119) 0.968
salmeterol (+0.00117) 0.968
Malignant hypertensive heart disease without congestive heart failure (x1.01) 0.969
Benign essential hypertension (x1.01) 0.969
Acute on chronic combined systolic and diastolic heart failure (x1.01) 0.969
Coronary atherosclerosis (x1.01) 0.970
Paroxysmal ventricular tachycardia (x1.01) 0.970
fluticasone (+0.00112) 0.971
Aldosterone [M/V] in SP (+5.58) 0.971
Paroxysmal atrial fibrillation (x1.01) 0.972
Q-T interval corrected (+0.655) 0.972
Left heart failure (x1.01) 0.972
cetirizine (+0.00105) 0.973
Pure hypercholesterolemia (x1.01) 0.973
allopurinol (+0.00104) 0.973
Pleural effusion (x1.01) 0.974
ezetimibe (+0.001) 0.974
Chronic congestive heart failure (x1.01) 0.974
levothyroxine (+0.000973) 0.975
magnesium chloride (+0.000973) 0.975
pantoprazole (+0.000962) 0.975
Expressions
10^5
10^3
10^1
−20 0 20
−1.00 −0.75 −0.50 −0.25 0.00 0.25 0.50 0.75 1.00

Signature [1052]; 6 To Reach 0.975, (10 Plotted)

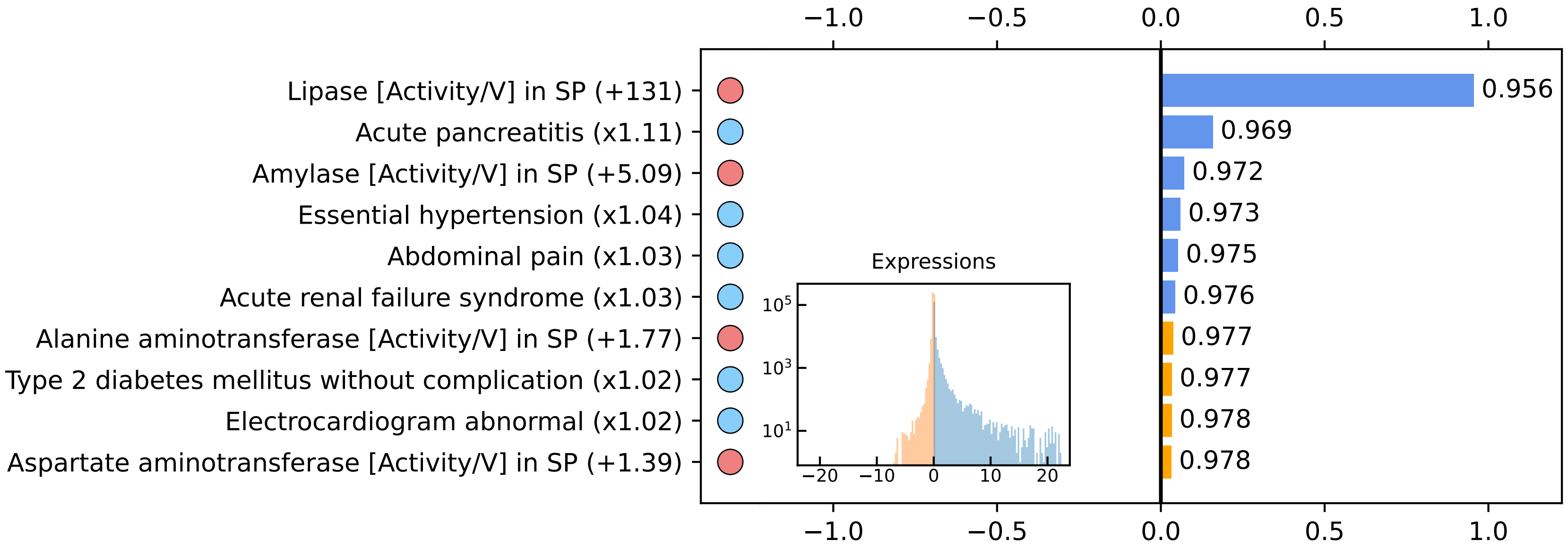

Signature [1053]; 8 To Reach 0.975, (10 Plotted)

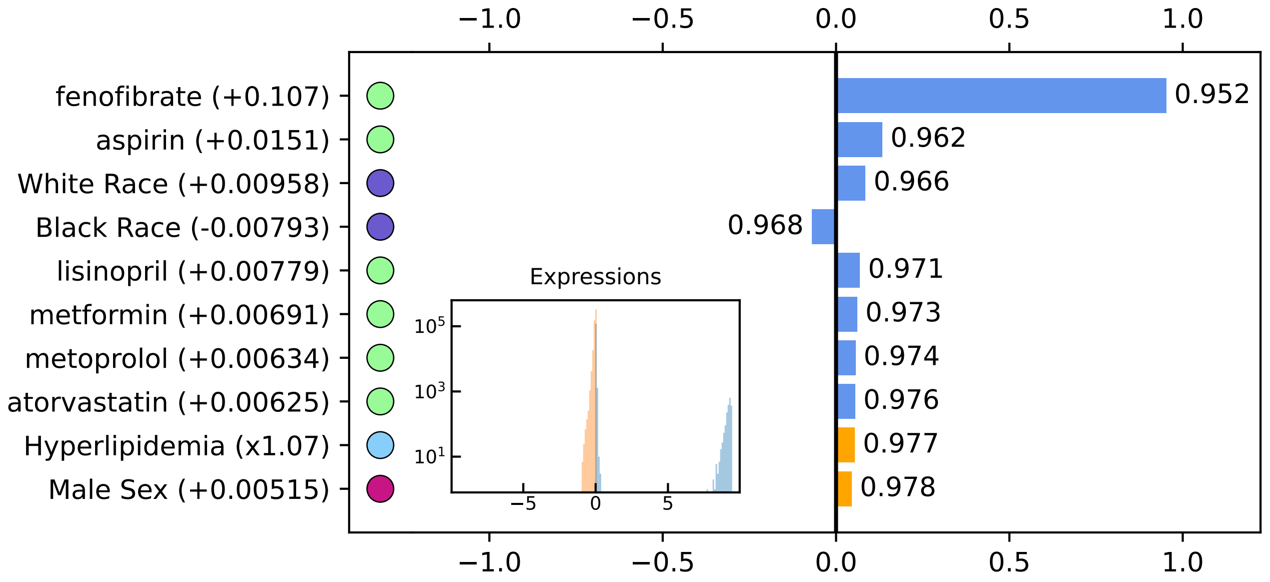

Signature [1054]; 5 To Reach 0.975, (10 Plotted)

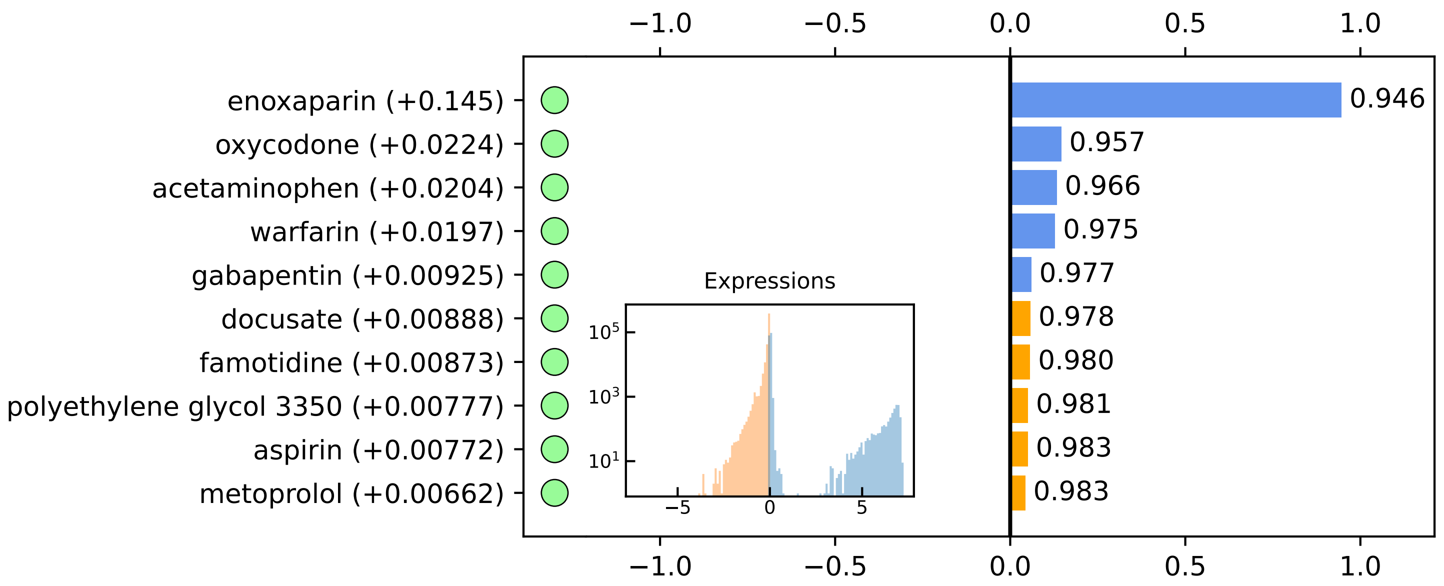

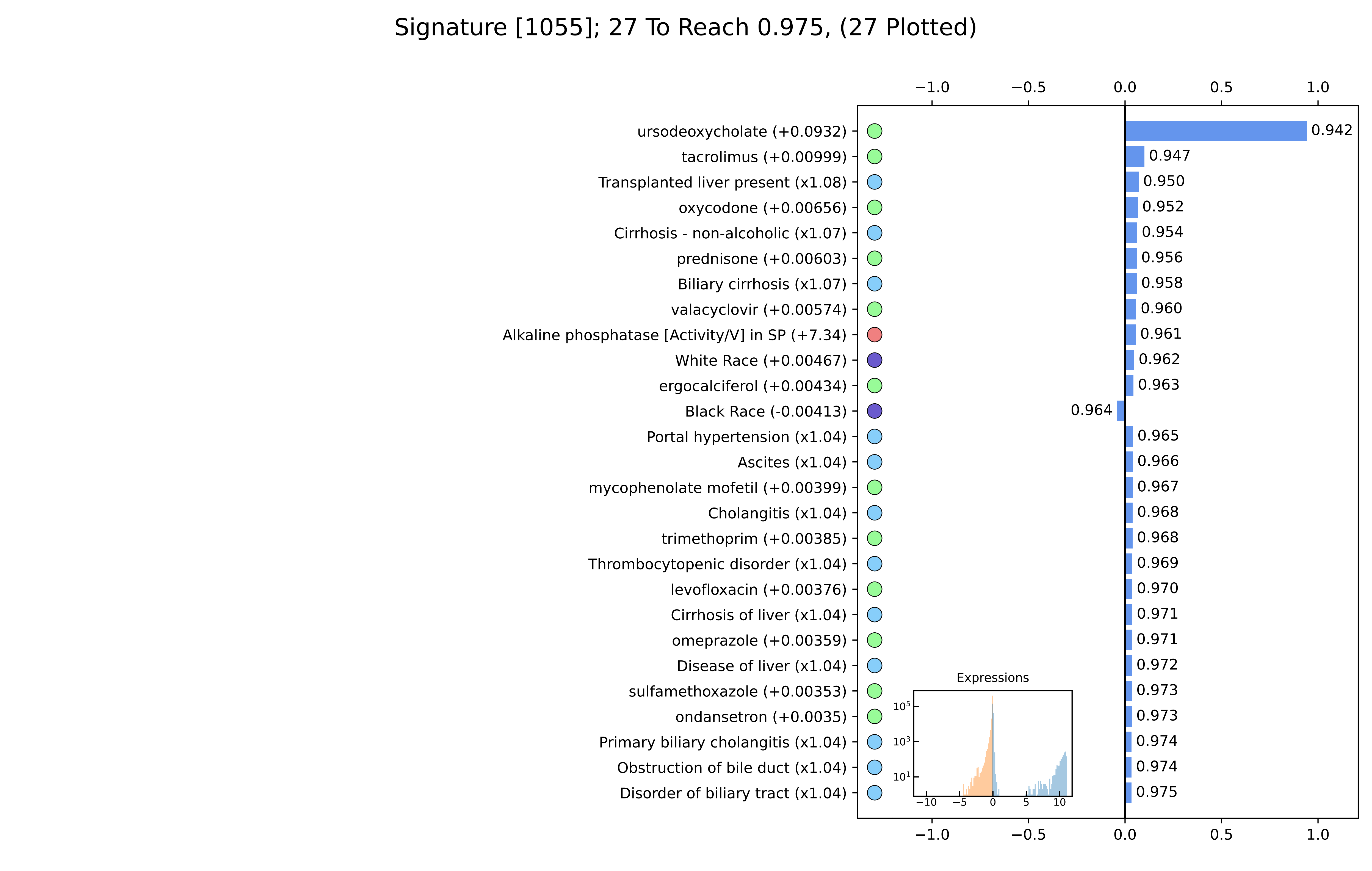

Signature [1055]; 27 To Reach 0.975, (27 Plotted)
ursodeoxycholate (+0.0932)
tacrolimus (+0.00999)
Transplanted liver present (x1.08)
oxycodone (+0.00656)
Cirrhosis - non-alcoholic (x1.07)
prednisone (+0.00603)
Biliary cirrhosis (x1.07)
valacyclovir (+0.00574)
Alkaline phosphatase [Activity/V] in SP (+7.34)
White Race (+0.00467)
ergocalciferol (+0.00434)
Black Race (-0.00413)
Portal hypertension (x1.04)
Ascites (x1.04)
mycophenolate mofetil (+0.00399)
Cholangitis (x1.04)
trimethoprim (+0.00385)
Thrombocytopenic disorder (x1.04)
levofloxacin (+0.00376)
Cirrhosis of liver (x1.04)
omeprazole (+0.00359)
Disease of liver (x1.04)
sulfamethoxazole (+0.00353)
ondansetron (+0.0035)
Primary biliary cholangitis (x1.04)
Obstruction of bile duct (x1.04)
Disorder of biliary tract (x1.04)
0.942
0.947
0.950
0.952
0.954
0.956
0.958
0.960
0.961
0.962
0.963
0.964
0.965
0.966
0.967
0.968
0.968
0.969
0.970
0.971
0.971
0.972
0.973
0.973
0.974
0.974
0.975
−1.0
−0.5
0.0
0.5
1.0
Expressions

Signature [1056]; 151 To Reach 0.975, (100 Plotted)

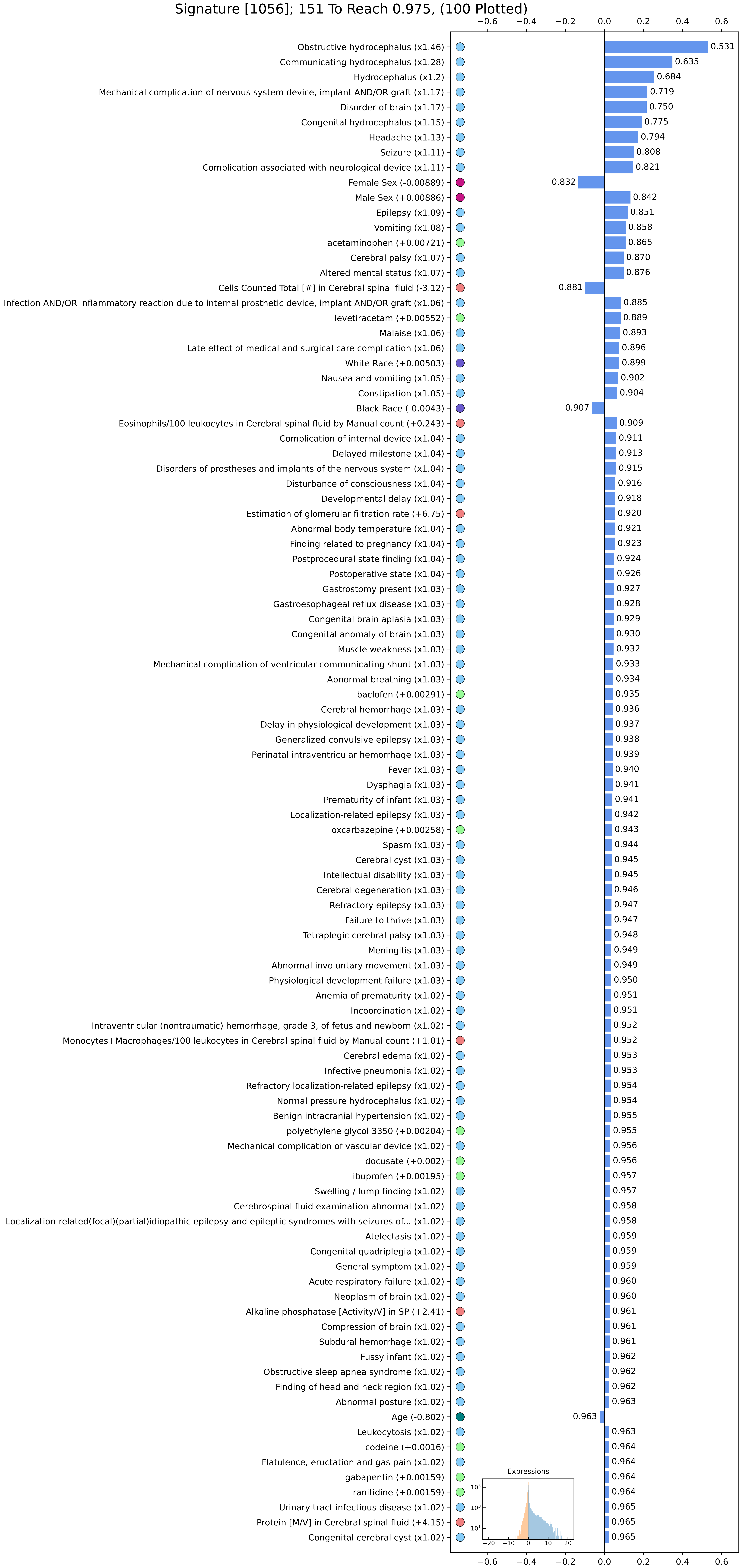

Signature [1057]; 315 To Reach 0.975, (100 Plotted)

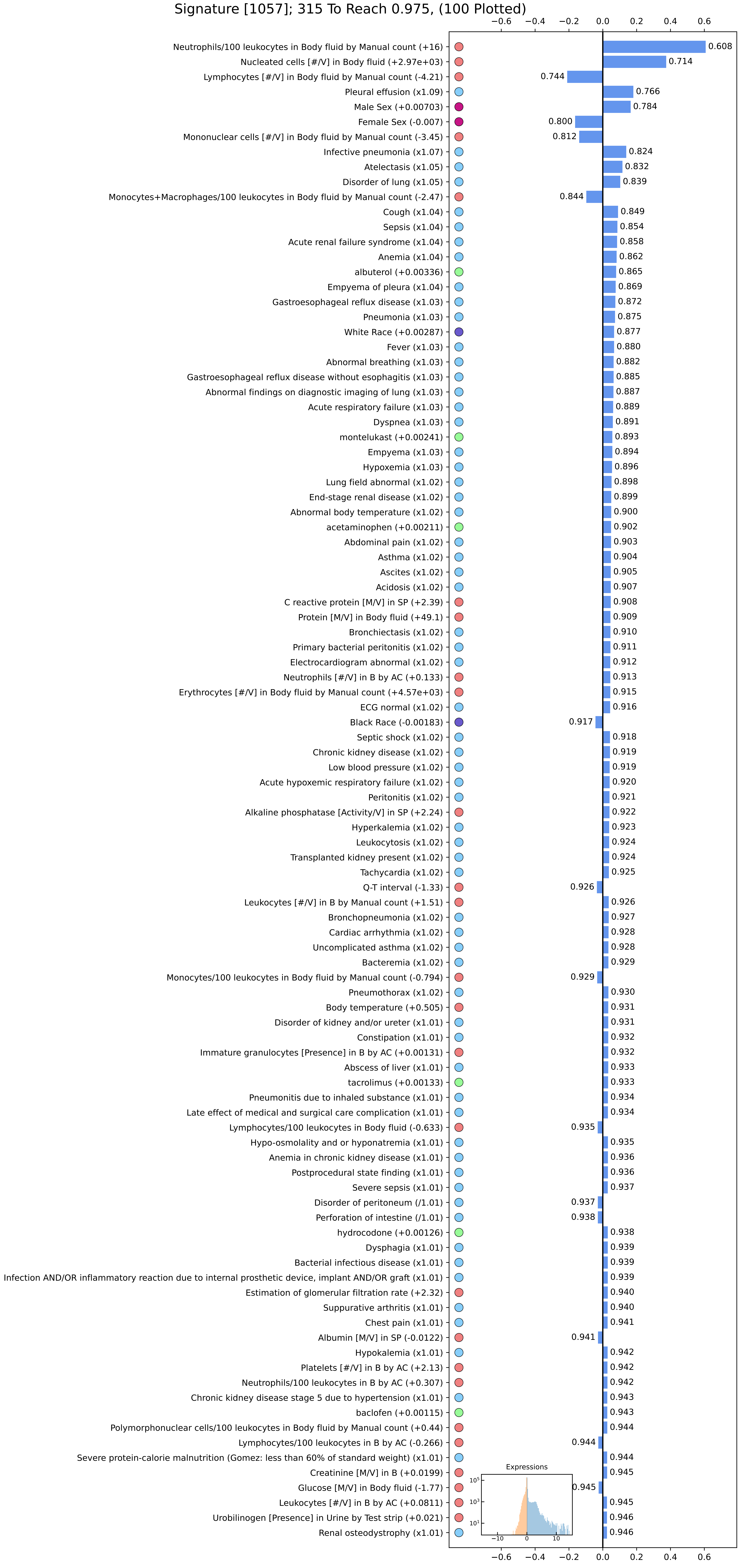

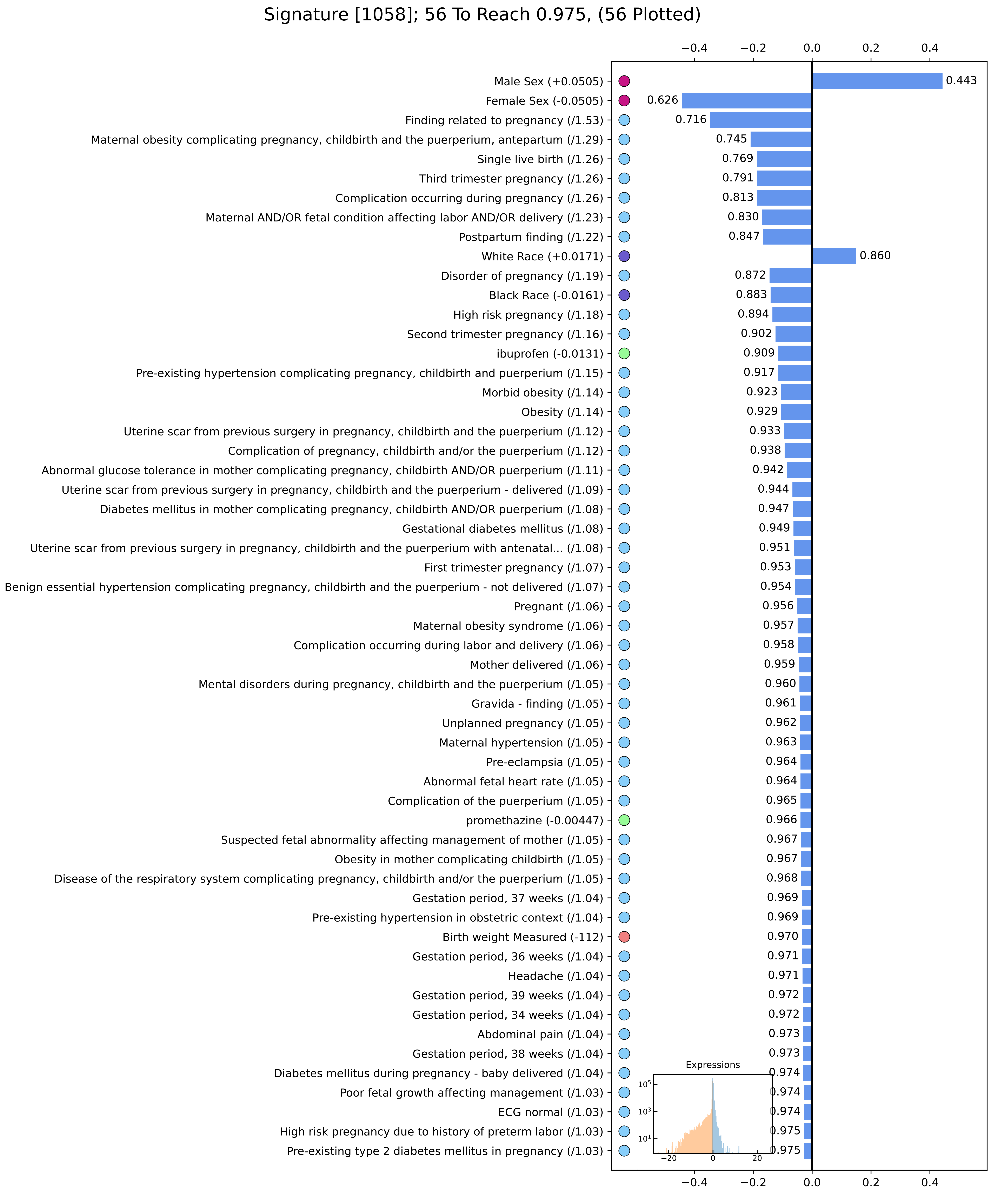

Signature [1058]; 56 To Reach 0.975, (56 Plotted)
Male Sex (+0.0505) 0.443
Female Sex (-0.0505) 0.626
Finding related to pregnancy (/1.53) 0.716
Maternal obesity complicating pregnancy, childbirth and the puerperium, antepartum (/1.29) 0.745
Single live birth (/1.26) 0.769
Third trimester pregnancy (/1.26) 0.791
Complication occurring during pregnancy (/1.26) 0.813
Maternal AND/OR fetal condition affecting labor AND/OR delivery (/1.23) 0.830
Postpartum finding (/1.22) 0.847
White Race (+0.0171) 0.860
Disorder of pregnancy (/1.19) 0.872
Black Race (-0.0161) 0.883
High risk pregnancy (/1.18) 0.894
Second trimester pregnancy (/1.16) 0.902
ibuprofen (-0.0131) 0.909
Pre-existing hypertension complicating pregnancy, childbirth and puerperium (/1.15) 0.917
Morbid obesity (/1.14) 0.923
Obesity (/1.14) 0.929
Uterine scar from previous surgery in pregnancy, childbirth and the puerperium (/1.12) 0.933
Complication of pregnancy, childbirth and/or the puerperium (/1.12) 0.938
Abnormal glucose tolerance in mother complicating pregnancy, childbirth AND/OR puerperium (/1.11) 0.942
Uterine scar from previous surgery in pregnancy, childbirth and the puerperium - delivered (/1.09) 0.944
Diabetes mellitus in mother complicating pregnancy, childbirth AND/OR puerperium (/1.08) 0.947
Gestational diabetes mellitus (/1.08) 0.949
Uterine scar from previous surgery in pregnancy, childbirth and the puerperium with antenatal... (/1.08) 0.951
First trimester pregnancy (/1.07) 0.953
Benign essential hypertension complicating pregnancy, childbirth and the puerperium - not delivered (/1.07) 0.954
Pregnant (/1.06) 0.956
Maternal obesity syndrome (/1.06) 0.957
Complication occurring during labor and delivery (/1.06) 0.958
Mother delivered (/1.06) 0.959
Mental disorders during pregnancy, childbirth and the puerperium (/1.05) 0.960
Gravida - finding (/1.05) 0.961
Unplanned pregnancy (/1.05) 0.962
Maternal hypertension (/1.05) 0.963
Pre-eclampsia (/1.05) 0.964
Abnormal fetal heart rate (/1.05) 0.964
Complication of the puerperium (/1.05) 0.965
promethazine (-0.00447) 0.966
Suspected fetal abnormality affecting management of mother (/1.05) 0.967
Obesity in mother complicating childbirth (/1.05) 0.967
Disease of the respiratory system complicating pregnancy, childbirth and/or the puerperium (/1.05) 0.968
Gestation period, 37 weeks (/1.04) 0.969
Pre-existing hypertension in obstetric context (/1.04) 0.969
Birth weight Measured (-112) 0.970
Gestation period, 36 weeks (/1.04) 0.971
Headache (/1.04) 0.971
Gestation period, 39 weeks (/1.04) 0.972
Gestation period, 34 weeks (/1.04) 0.972
Abdominal pain (/1.04) 0.973
Gestation period, 38 weeks (/1.04) 0.973
Diabetes mellitus during pregnancy - baby delivered (/1.04) 0.974
Poor fetal growth affecting management (/1.03) 0.974
ECG normal (/1.03) 0.974
High risk pregnancy due to history of preterm labor (/1.03) 0.975
Pre-existing type 2 diabetes mellitus in pregnancy (/1.03) 0.975
−0.4 −0.2 0.0 0.2 0.4
Expressions
10^5 10^3 10^1
−20 0 20

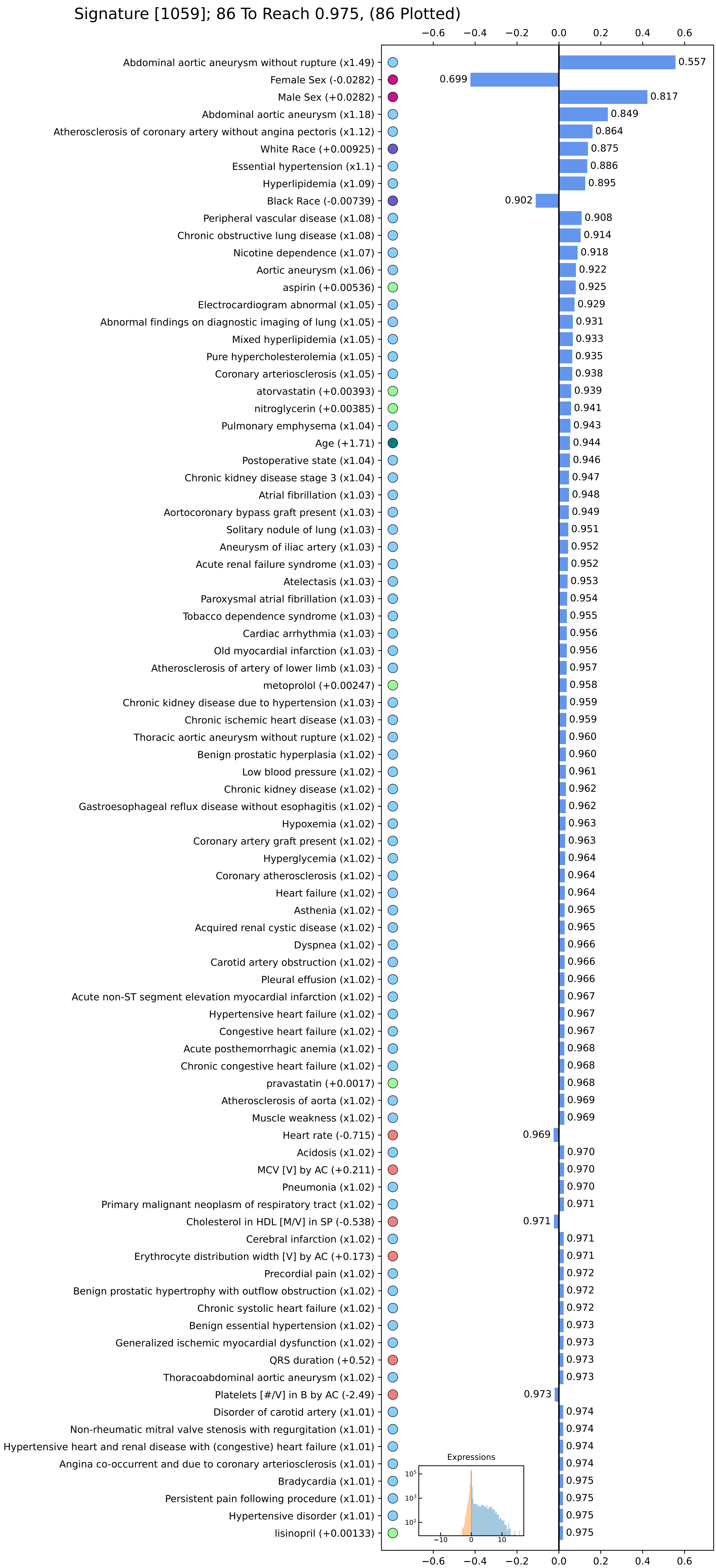

Signature [1059]; 86 To Reach 0.975, (86 Plotted)
Abdominal aortic aneurysm without rupture (x1.49) 0.557
Female Sex (-0.0282) 0.699
Male Sex (+0.0282) 0.817
Abdominal aortic aneurysm (x1.18) 0.849
Atherosclerosis of coronary artery without angina pectoris (x1.12) 0.864
White Race (+0.00925) 0.875
Essential hypertension (x1.1) 0.886
Hyperlipidemia (x1.09) 0.895
Black Race (-0.00739) 0.902
Peripheral vascular disease (x1.08) 0.908
Chronic obstructive lung disease (x1.08) 0.914
Nicotine dependence (x1.07) 0.918
Aortic aneurysm (x1.06) 0.922
aspirin (+0.00536) 0.925
Electrocardiogram abnormal (x1.05) 0.929
Abnormal findings on diagnostic imaging of lung (x1.05) 0.931
Mixed hyperlipidemia (x1.05) 0.933
Pure hypercholesterolemia (x1.05) 0.935
Coronary arteriosclerosis (x1.05) 0.938
atorvastatin (+0.00393) 0.939
nitroglycerin (+0.00385) 0.941
Pulmonary emphysema (x1.04) 0.943
Age (+1.71) 0.944
Postoperative state (x1.04) 0.946
Chronic kidney disease stage 3 (x1.04) 0.947
Atrial fibrillation (x1.03) 0.948
Aortocoronary bypass graft present (x1.03) 0.949
Solitary nodule of lung (x1.03) 0.951
Aneurysm of iliac artery (x1.03) 0.952
Acute renal failure syndrome (x1.03) 0.952
Atelectasis (x1.03) 0.953
Paroxysmal atrial fibrillation (x1.03) 0.954
Tobacco dependence syndrome (x1.03) 0.955
Cardiac arrhythmia (x1.03) 0.956
Old myocardial infarction (x1.03) 0.956
Atherosclerosis of artery of lower limb (x1.03) 0.957
metoprolol (+0.00247) 0.958
Chronic kidney disease due to hypertension (x1.03) 0.959
Chronic ischemic heart disease (x1.03) 0.959
Thoracic aortic aneurysm without rupture (x1.02) 0.960
Benign prostatic hyperplasia (x1.02) 0.960
Low blood pressure (x1.02) 0.961
Chronic kidney disease (x1.02) 0.962
Gastroesophageal reflux disease without esophagitis (x1.02) 0.962
Hypoxemia (x1.02) 0.963
Coronary artery graft present (x1.02) 0.963
Hyperglycemia (x1.02) 0.964
Coronary atherosclerosis (x1.02) 0.964
Heart failure (x1.02) 0.964
Asthenia (x1.02) 0.965
Acquired renal cystic disease (x1.02) 0.965
Dyspnea (x1.02) 0.966
Carotid artery obstruction (x1.02) 0.966
Pleural effusion (x1.02) 0.966
Acute non-ST segment elevation myocardial infarction (x1.02) 0.967
Hypertensive heart failure (x1.02) 0.967
Congestive heart failure (x1.02) 0.967
Acute posthemorrhagic anemia (x1.02) 0.968
Chronic congestive heart failure (x1.02) 0.968
pravastatin (+0.0017) 0.968
Atherosclerosis of aorta (x1.02) 0.969
Muscle weakness (x1.02) 0.969
Heart rate (-0.715) 0.969
Acidosis (x1.02) 0.970
MCV [V] by AC (+0.211) 0.970
Pneumonia (x1.02) 0.970
Primary malignant neoplasm of respiratory tract (x1.02) 0.971
Cholesterol in HDL [M/V] in SP (-0.538) 0.971
Cerebral infarction (x1.02) 0.971
Erythrocyte distribution width [V] by AC (+0.173) 0.971
Precordial pain (x1.02) 0.972
Benign prostatic hypertrophy with outflow obstruction (x1.02) 0.972
Chronic systolic heart failure (x1.02) 0.972
Benign essential hypertension (x1.02) 0.973
Generalized ischemic myocardial dysfunction (x1.02) 0.973
QRS duration (+0.52) 0.973
Thoracoabdominal aortic aneurysm (x1.02) 0.973
Platelets [#/V] in B by AC (-2.49) 0.973
Disorder of carotid artery (x1.01) 0.974
Non-rheumatic mitral valve stenosis with regurgitation (x1.01) 0.974
Hypertensive heart and renal disease with (congestive) heart failure (x1.01) 0.974
Angina co-occurrent and due to coronary arteriosclerosis (x1.01) 0.974
Bradycardia (x1.01) 0.975
Persistent pain following procedure (x1.01) 0.975
Hypertensive disorder (x1.01) 0.975
lisinopril (+0.00133) 0.975
−0.6 −0.4 −0.2 0.0 0.2 0.4 0.6
Expressions

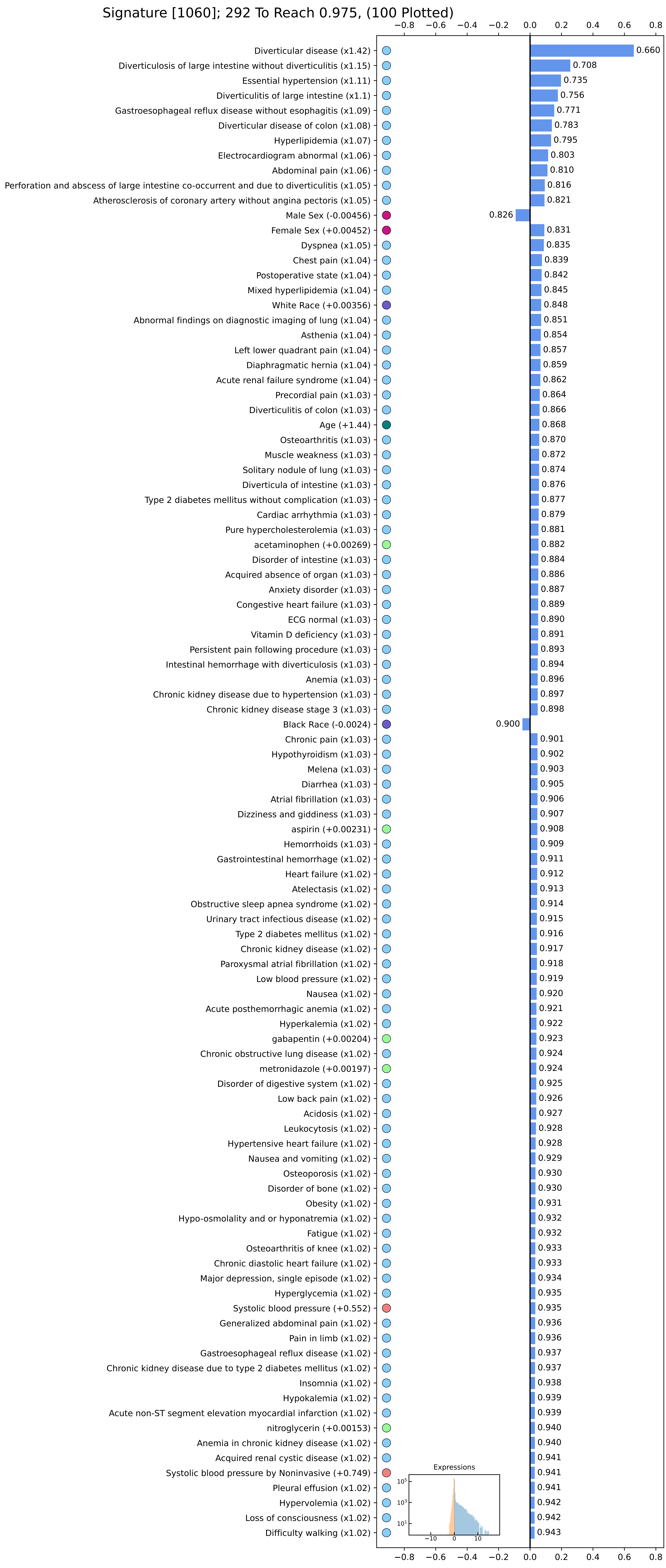

Signature [1060]; 292 To Reach 0.975, (100 Plotted)
Diverticular disease (x1.42) 0.660
Diverticulosis of large intestine without diverticulitis (x1.15) 0.708
Essential hypertension (x1.11) 0.735
Diverticulitis of large intestine (x1.1) 0.756
Gastroesophageal reflux disease without esophagitis (x1.09) 0.771
Diverticular disease of colon (x1.08) 0.783
Hyperlipidemia (x1.07) 0.795
Electrocardiogram abnormal (x1.06) 0.803
Abdominal pain (x1.06) 0.810
Perforation and abscess of large intestine co-occurrent and due to diverticulitis (x1.05) 0.816
Atherosclerosis of coronary artery without angina pectoris (x1.05) 0.821
Male Sex (-0.00456) 0.826
Female Sex (+0.00452) 0.831
Dyspnea (x1.05) 0.835
Chest pain (x1.04) 0.839
Postoperative state (x1.04) 0.842
Mixed hyperlipidemia (x1.04) 0.845
White Race (+0.00356) 0.848
Abnormal findings on diagnostic imaging of lung (x1.04) 0.851
Asthenia (x1.04) 0.854
Left lower quadrant pain (x1.04) 0.857
Diaphragmatic hernia (x1.04) 0.859
Acute renal failure syndrome (x1.04) 0.862
Precordial pain (x1.03) 0.864
Diverticulitis of colon (x1.03) 0.866
Age (+1.44) 0.868
Osteoarthritis (x1.03) 0.870
Muscle weakness (x1.03) 0.872
Solitary nodule of lung (x1.03) 0.874
Diverticula of intestine (x1.03) 0.876
Type 2 diabetes mellitus without complication (x1.03) 0.877
Cardiac arrhythmia (x1.03) 0.879
Pure hypercholesterolemia (x1.03) 0.881
acetaminophen (+0.00269) 0.882
Disorder of intestine (x1.03) 0.884
Acquired absence of organ (x1.03) 0.886
Anxiety disorder (x1.03) 0.887
Congestive heart failure (x1.03) 0.889
ECG normal (x1.03) 0.890
Vitamin D deficiency (x1.03) 0.891
Persistent pain following procedure (x1.03) 0.893
Intestinal hemorrhage with diverticulosis (x1.03) 0.894
Anemia (x1.03) 0.896
Chronic kidney disease due to hypertension (x1.03) 0.897
Chronic kidney disease stage 3 (x1.03) 0.898
Black Race (-0.0024) 0.900
Chronic pain (x1.03) 0.901
Hypothyroidism (x1.03) 0.902
Melena (x1.03) 0.903
Diarrhea (x1.03) 0.905
Atrial fibrillation (x1.03) 0.906
Dizziness and giddiness (x1.03) 0.907
aspirin (+0.00231) 0.908
Hemorrhoids (x1.03) 0.909
Gastrointestinal hemorrhage (x1.02) 0.911
Heart failure (x1.02) 0.912
Atelectasis (x1.02) 0.913
Obstructive sleep apnea syndrome (x1.02) 0.914
Urinary tract infectious disease (x1.02) 0.915
Type 2 diabetes mellitus (x1.02) 0.916
Chronic kidney disease (x1.02) 0.917
Paroxysmal atrial fibrillation (x1.02) 0.918
Low blood pressure (x1.02) 0.919
Nausea (x1.02) 0.920
Acute posthemorrhagic anemia (x1.02) 0.921
Hyperkalemia (x1.02) 0.922
gabapentin (+0.00204) 0.923
Chronic obstructive lung disease (x1.02) 0.924
metronidazole (+0.00197) 0.924
Disorder of digestive system (x1.02) 0.925
Low back pain (x1.02) 0.926
Acidosis (x1.02) 0.927
Leukocytosis (x1.02) 0.928
Hypertensive heart failure (x1.02) 0.928
Nausea and vomiting (x1.02) 0.929
Osteoporosis (x1.02) 0.930
Disorder of bone (x1.02) 0.930
Obesity (x1.02) 0.931
Hypo-osmolality and or hyponatremia (x1.02) 0.932
Fatigue (x1.02) 0.932
Osteoarthritis of knee (x1.02) 0.933
Chronic diastolic heart failure (x1.02) 0.933
Major depression, single episode (x1.02) 0.934
Hyperglycemia (x1.02) 0.935
Systolic blood pressure (+0.552) 0.935
Generalized abdominal pain (x1.02) 0.936
Pain in limb (x1.02) 0.936
Gastroesophageal reflux disease (x1.02) 0.937
Chronic kidney disease due to type 2 diabetes mellitus (x1.02) 0.937
Insomnia (x1.02) 0.938
Hypokalemia (x1.02) 0.939
Acute non-ST segment elevation myocardial infarction (x1.02) 0.939
nitroglycerin (+0.00153) 0.940
Anemia in chronic kidney disease (x1.02) 0.940
Acquired renal cystic disease (x1.02) 0.941
Systolic blood pressure by Noninvasive (+0.749) 0.941
Pleural effusion (x1.02) 0.941
Hypervolemia (x1.02) 0.942
Loss of consciousness (x1.02) 0.942
Difficulty walking (x1.02) 0.943
−0.8 −0.6 −0.4 −0.2 0.0 0.2 0.4 0.6 0.8
Expressions

Signature [1061]; 91 To Reach 0.975, (91 Plotted)

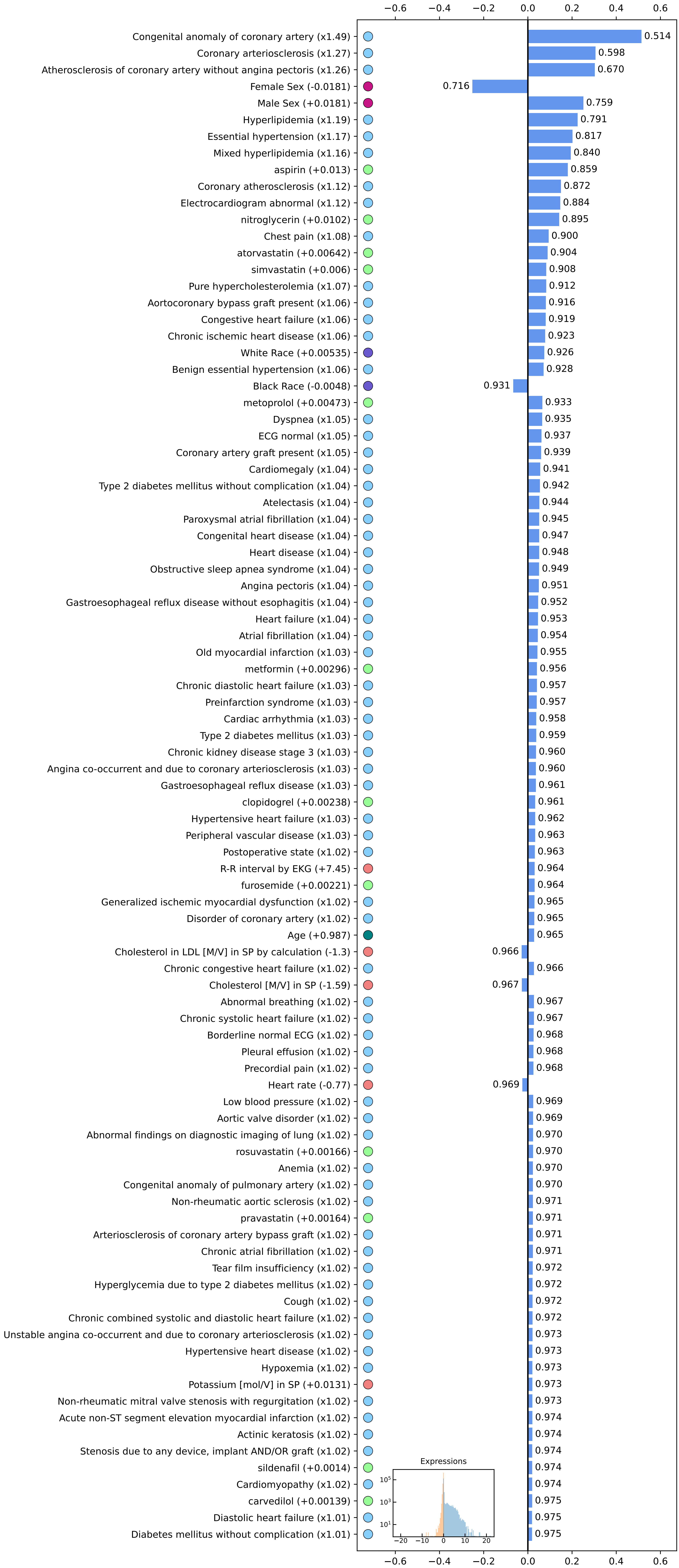

Signature [1062]; 1216 To Reach 0.975, (100 Plotted)

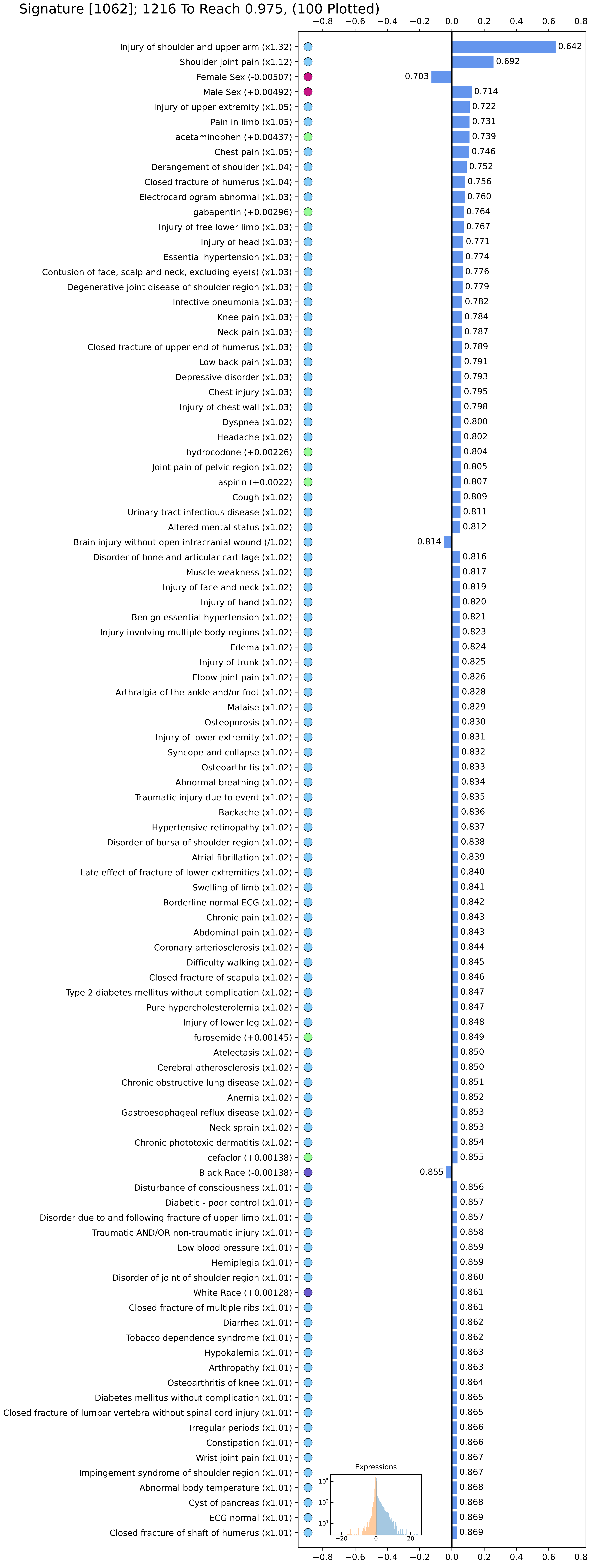

Signature [1063]; 4 To Reach 0.975, (10 Plotted)

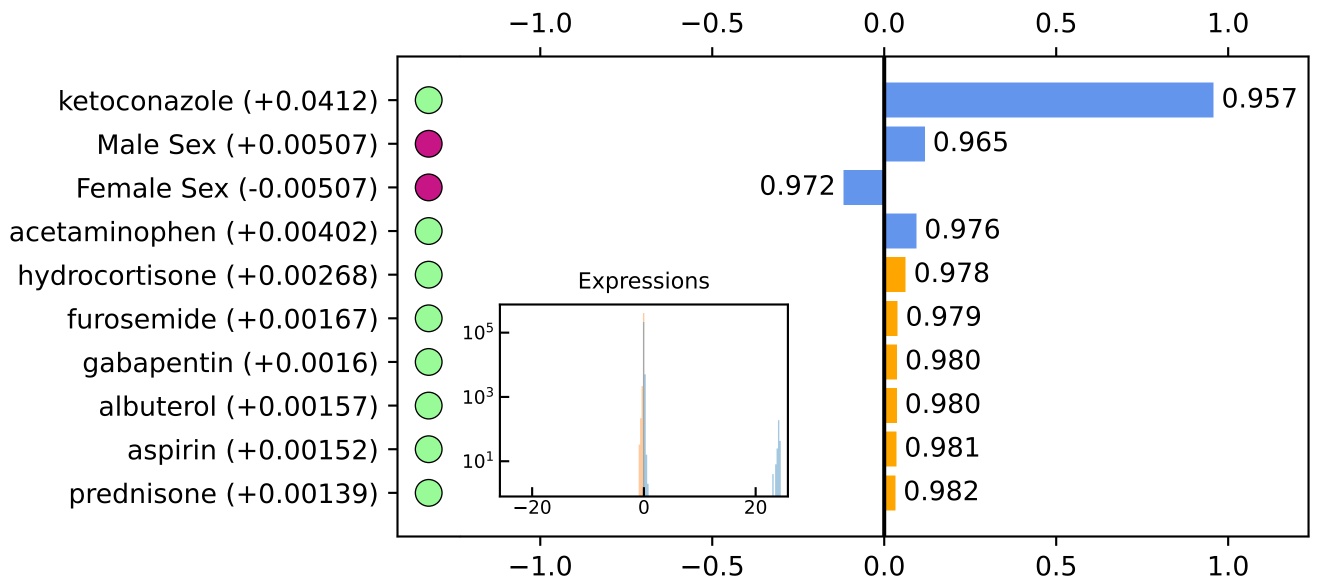

Signature [1064]; 1053 To Reach 0.975, (100 Plotted)

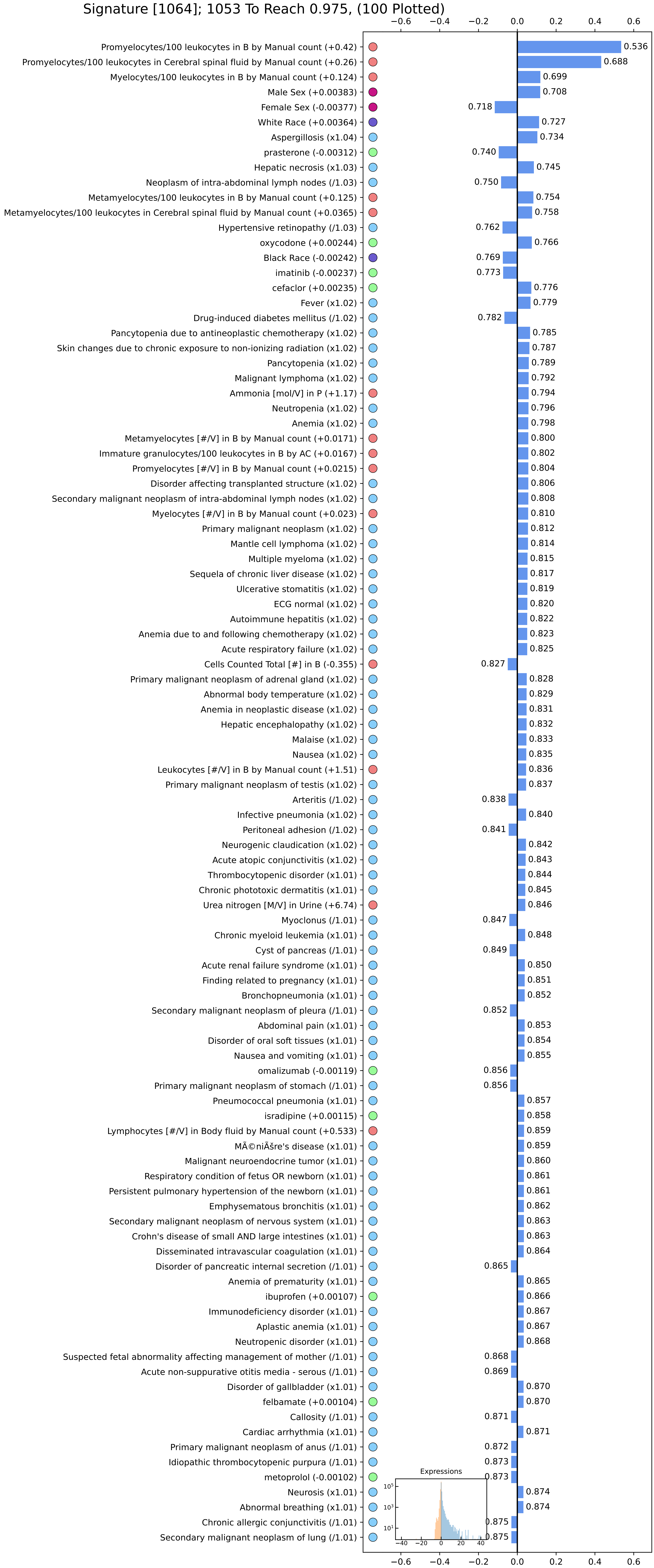

Signature [1065]; 129 To Reach 0.975, (100 Plotted)

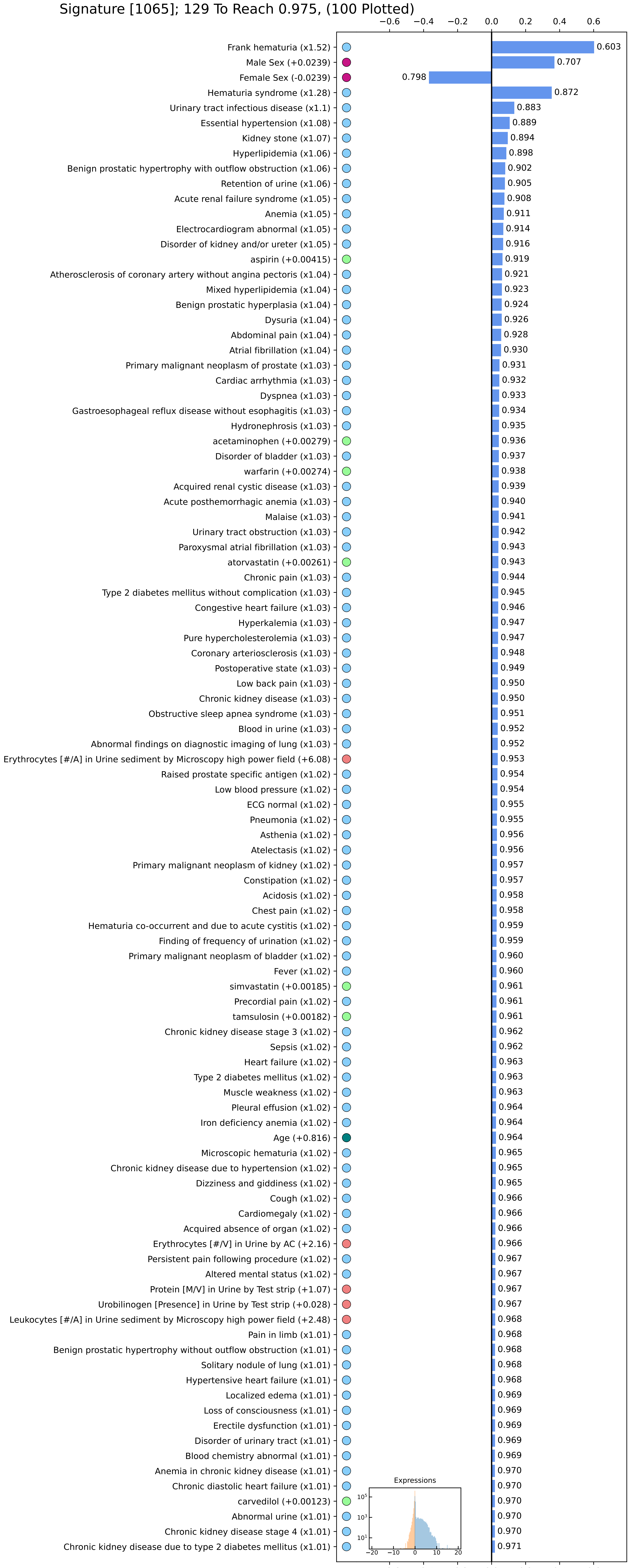

Signature [1066]; 25 To Reach 0.975, (25 Plotted)

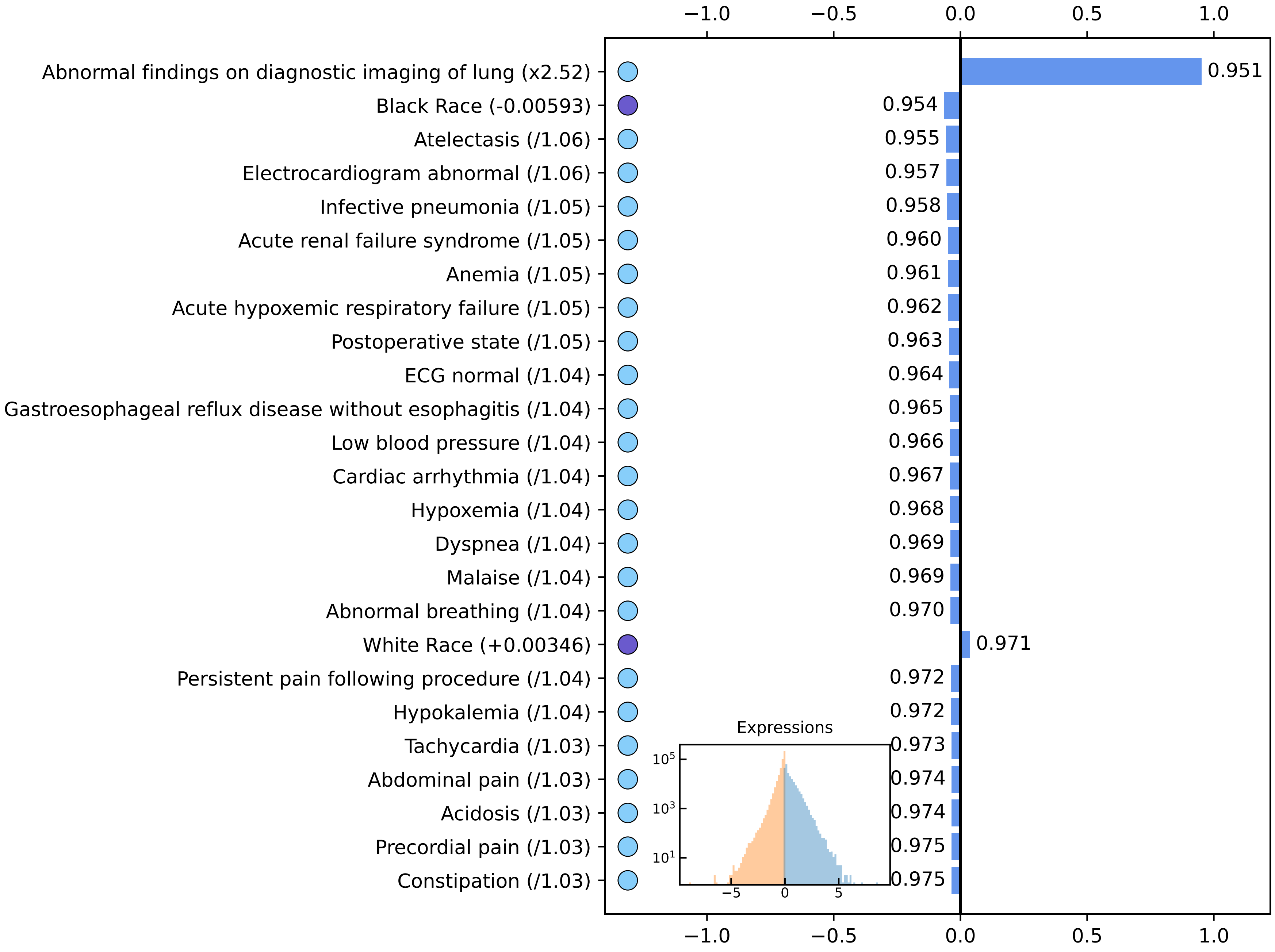

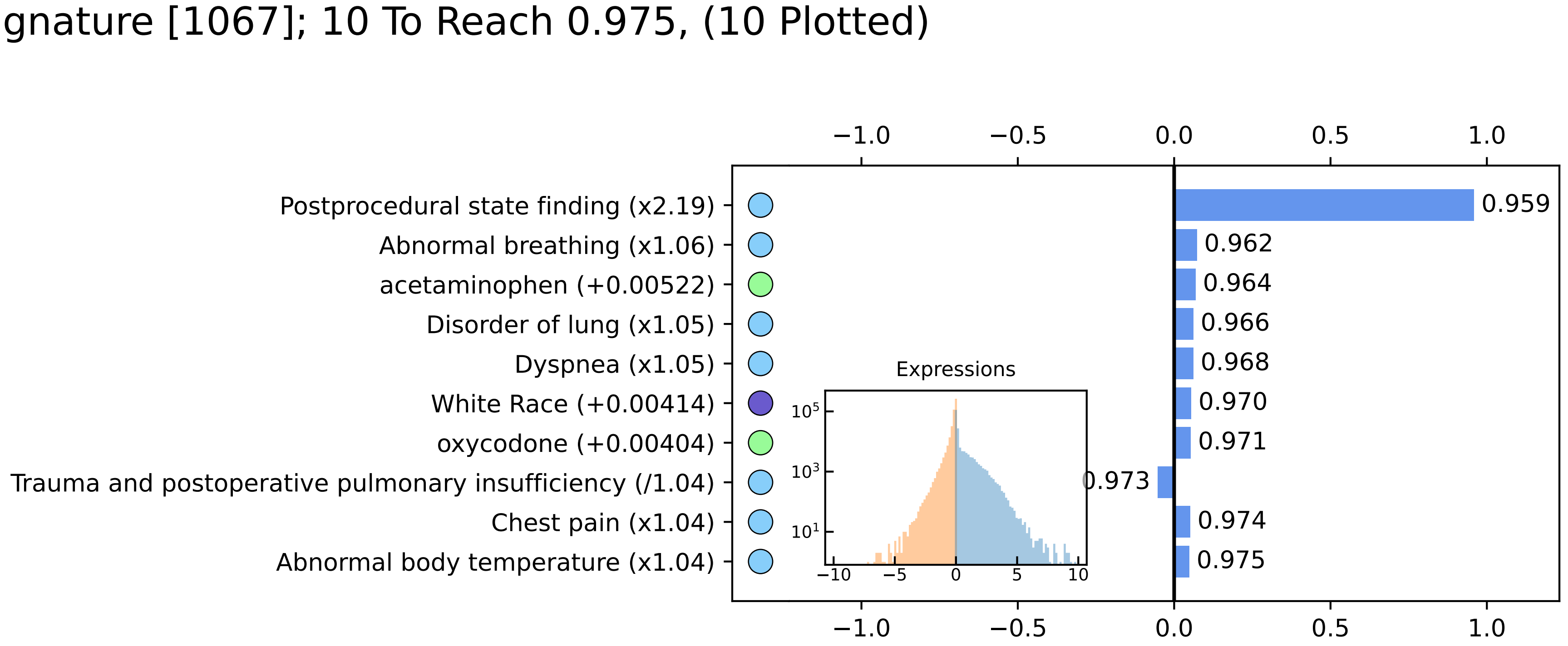
Signature [1067]; 10 To Reach 0.975, (10 Plotted)
Postprocedural state finding (x2.19)
Abnormal breathing (x1.06)
acetaminophen (+0.00522)
Disorder of lung (x1.05)
Dyspnea (x1.05)
White Race (+0.00414)
oxycodone (+0.00404)
Trauma and postoperative pulmonary insufficiency (/1.04)
Chest pain (x1.04)
Abnormal body temperature (x1.04)
0.959
0.962
0.964
0.966
0.968
0.970
0.971
0.973
0.974
0.975
−1.0
−0.5
0.0
0.5
1.0
Expressions
−10
−5
0
5
10

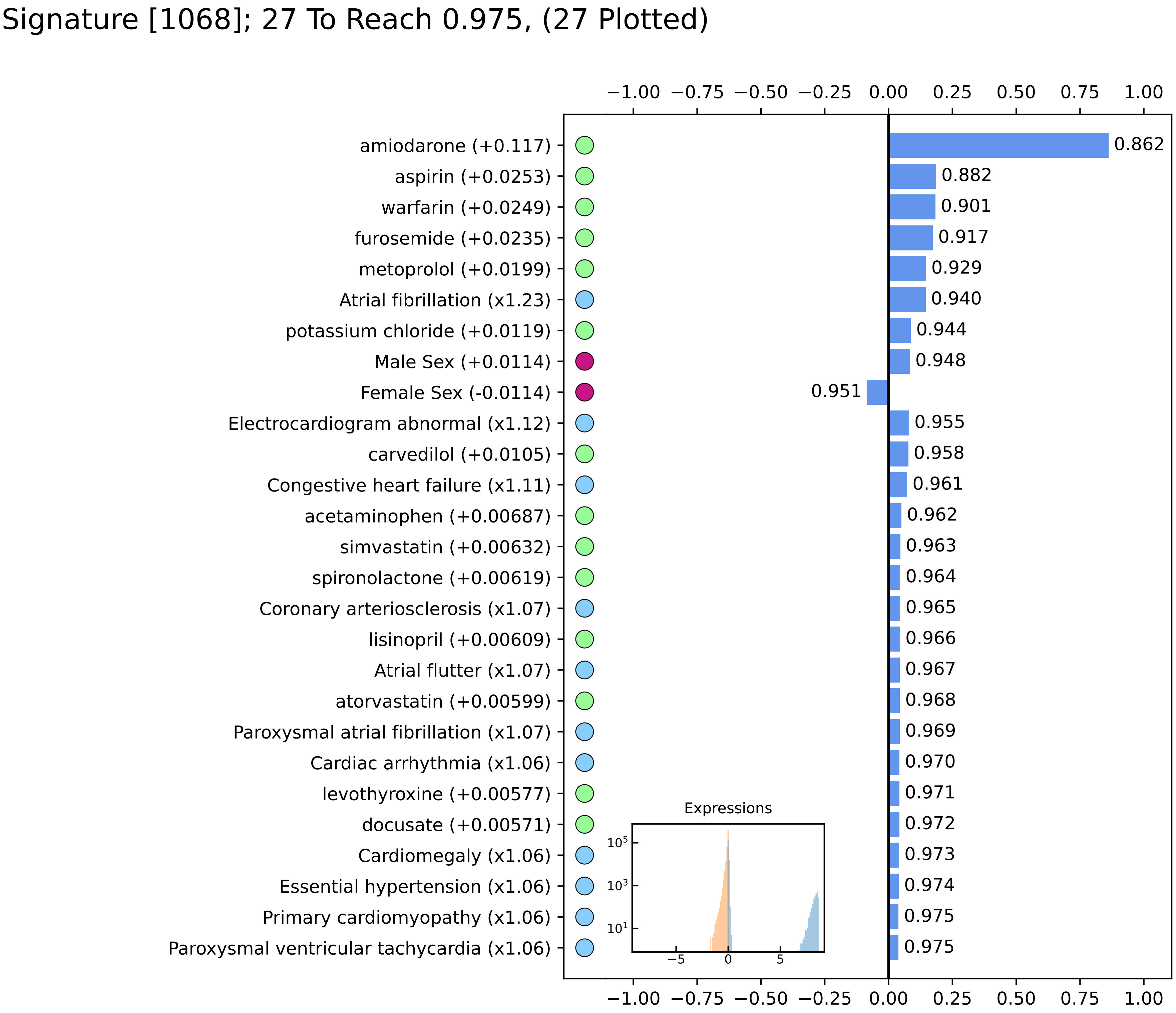
Signature [1068]; 27 To Reach 0.975, (27 Plotted)
amiodarone (+0.117)
aspirin (+0.0253)
warfarin (+0.0249)
furosemide (+0.0235)
metoprolol (+0.0199)
Atrial fibrillation (x1.23)
potassium chloride (+0.0119)
Male Sex (+0.0114)
Female Sex (-0.0114)
Electrocardiogram abnormal (x1.12)
carvedilol (+0.0105)
Congestive heart failure (x1.11)
acetaminophen (+0.00687)
simvastatin (+0.00632)
spironolactone (+0.00619)
Coronary arteriosclerosis (x1.07)
lisinopril (+0.00609)
Atrial flutter (x1.07)
atorvastatin (+0.00599)
Paroxysmal atrial fibrillation (x1.07)
Cardiac arrhythmia (x1.06)
levothyroxine (+0.00577)
docusate (+0.00571)
Cardiomegaly (x1.06)
Essential hypertension (x1.06)
Primary cardiomyopathy (x1.06)
Paroxysmal ventricular tachycardia (x1.06)
0.862
0.882
0.901
0.917
0.929
0.940
0.944
0.948
0.951
0.955
0.958
0.961
0.962
0.963
0.964
0.965
0.966
0.967
0.968
0.969
0.970
0.971
0.972
0.973
0.974
0.975
0.975
−1.00 −0.75 −0.50 −0.25 0.00 0.25 0.50 0.75 1.00
Expressions
10^5
10^3
10^1
−5
0
5

Signature [1069]; 259 To Reach 0.975, (100 Plotted)

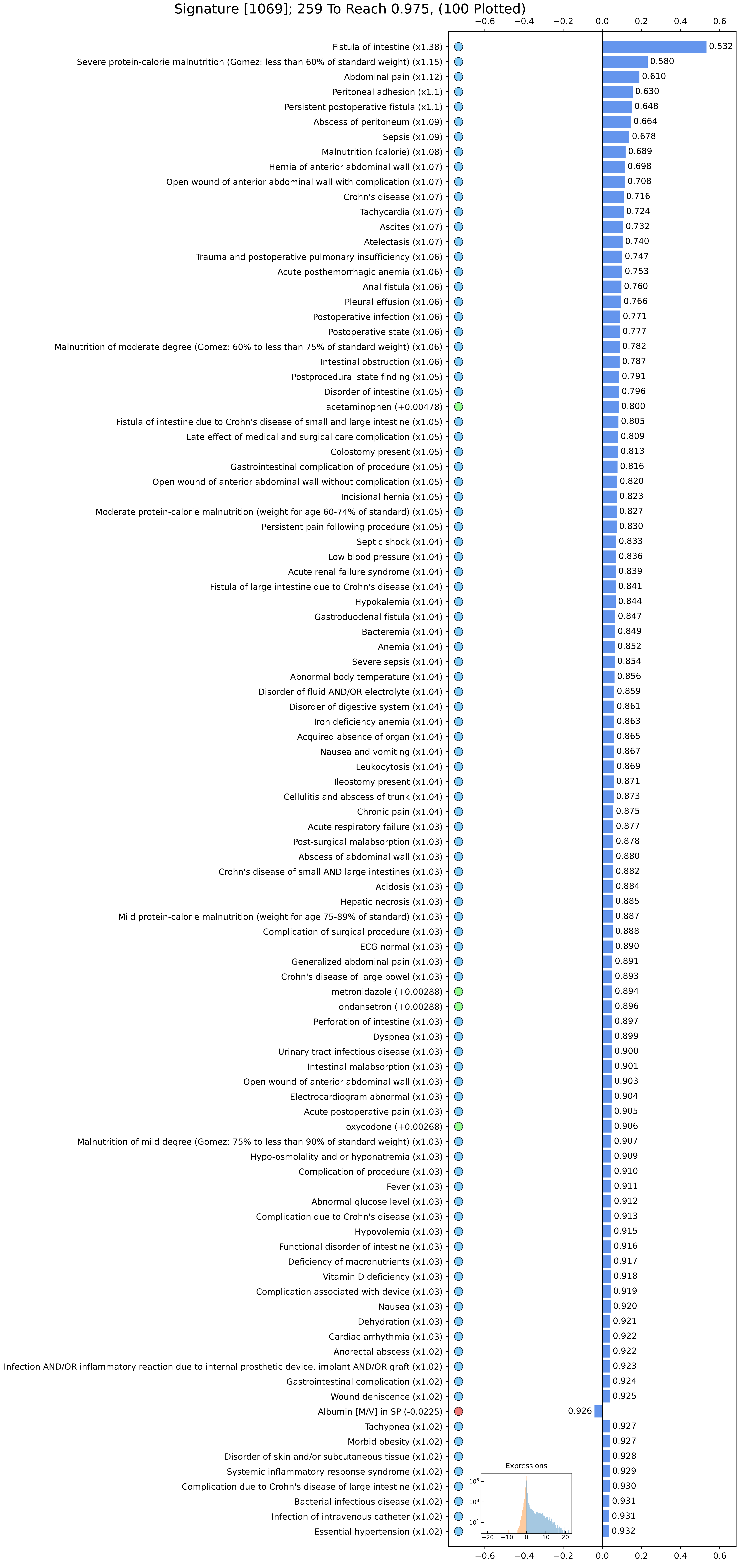

Signature [1070]; 1 To Reach 0.975, (10 Plotted)

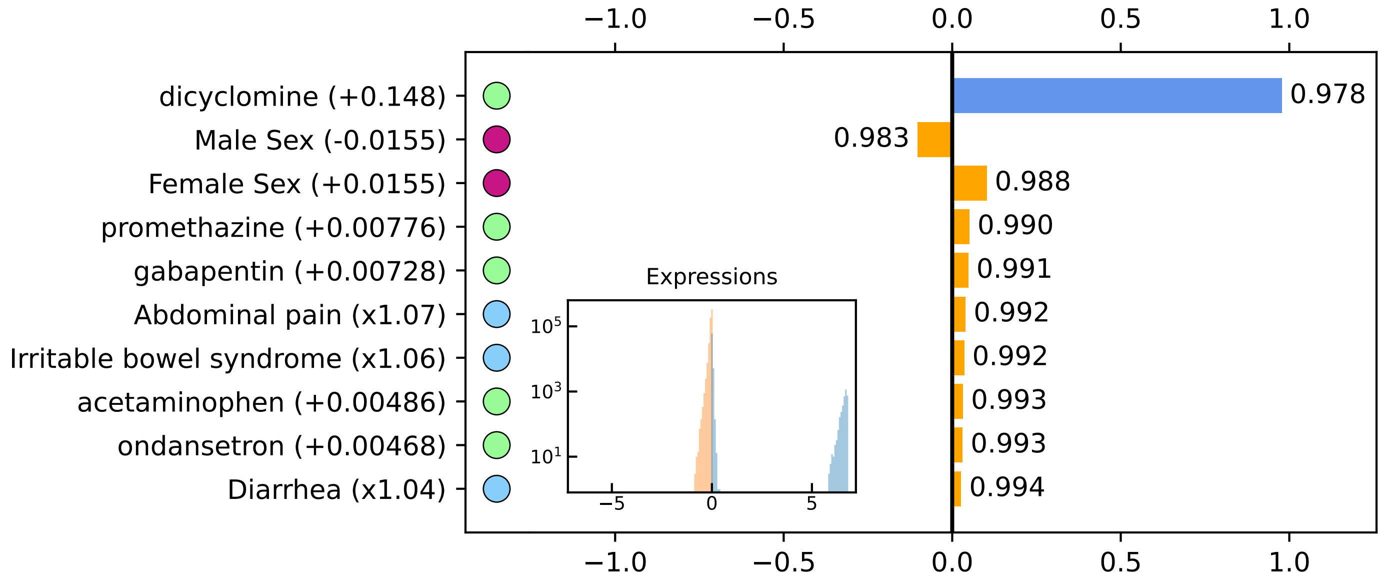

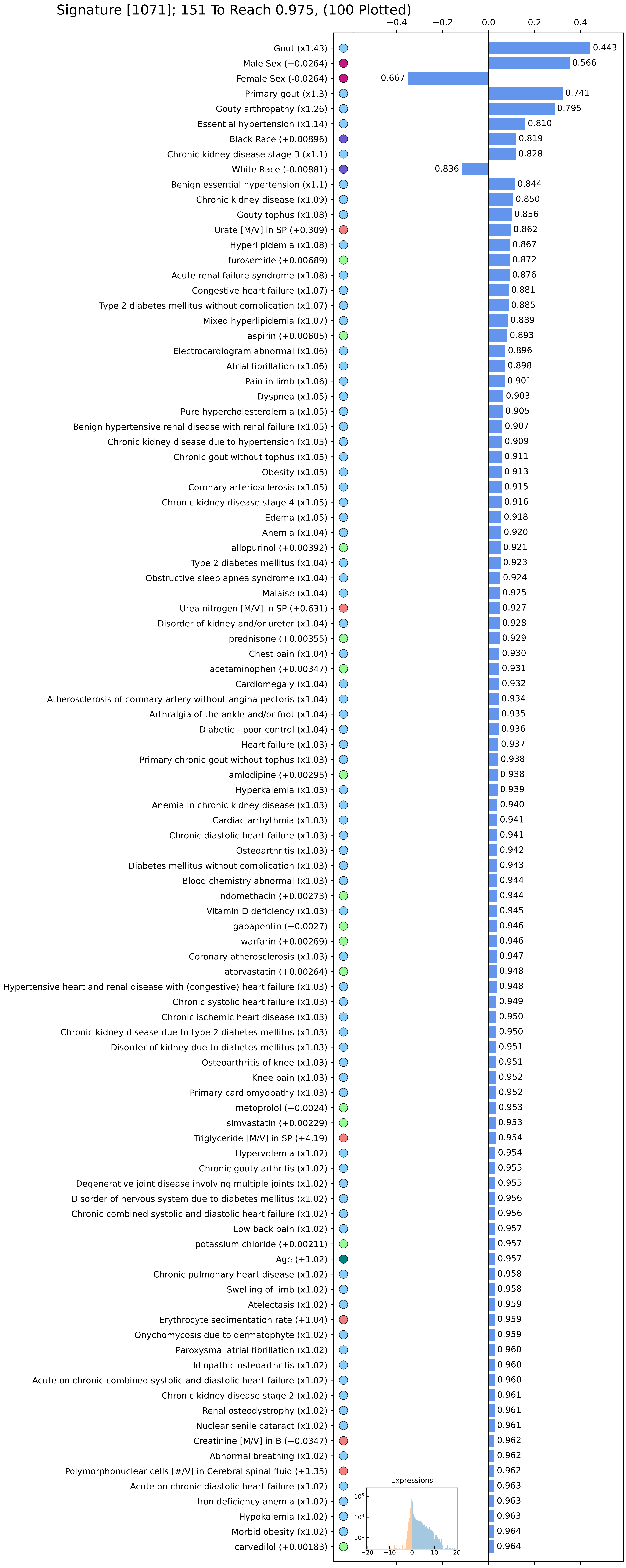

Signature [1071]; 151 To Reach 0.975, (100 Plotted)
Gout (x1.43) 0.443
Male Sex (+0.0264) 0.566
Female Sex (-0.0264) 0.667
Primary gout (x1.3) 0.741
Gouty arthropathy (x1.26) 0.795
Essential hypertension (x1.14) 0.810
Black Race (+0.00896) 0.819
Chronic kidney disease stage 3 (x1.1) 0.828
White Race (-0.00881) 0.836
Benign essential hypertension (x1.1) 0.844
Chronic kidney disease (x1.09) 0.850
Gouty tophus (x1.08) 0.856
Urate [M/V] in SP (+0.309) 0.862
Hyperlipidemia (x1.08) 0.867
furosemide (+0.00689) 0.872
Acute renal failure syndrome (x1.08) 0.876
Congestive heart failure (x1.07) 0.881
Type 2 diabetes mellitus without complication (x1.07) 0.885
Mixed hyperlipidemia (x1.07) 0.889
aspirin (+0.00605) 0.893
Electrocardiogram abnormal (x1.06) 0.896
Atrial fibrillation (x1.06) 0.898
Pain in limb (x1.06) 0.901
Dyspnea (x1.05) 0.903
Pure hypercholesterolemia (x1.05) 0.905
Benign hypertensive renal disease with renal failure (x1.05) 0.907
Chronic kidney disease due to hypertension (x1.05) 0.909
Chronic gout without tophus (x1.05) 0.911
Obesity (x1.05) 0.913
Coronary arteriosclerosis (x1.05) 0.915
Chronic kidney disease stage 4 (x1.05) 0.916
Edema (x1.05) 0.918
Anemia (x1.04) 0.920
allopurinol (+0.00392) 0.921
Type 2 diabetes mellitus (x1.04) 0.923
Obstructive sleep apnea syndrome (x1.04) 0.924
Malaise (x1.04) 0.925
Urea nitrogen [M/V] in SP (+0.631) 0.927
Disorder of kidney and/or ureter (x1.04) 0.928
prednisone (+0.00355) 0.929
Chest pain (x1.04) 0.930
acetaminophen (+0.00347) 0.931
Cardiomegaly (x1.04) 0.932
Atherosclerosis of coronary artery without angina pectoris (x1.04) 0.934
Arthralgia of the ankle and/or foot (x1.04) 0.935
Diabetic - poor control (x1.04) 0.936
Heart failure (x1.03) 0.937
Primary chronic gout without tophus (x1.03) 0.938
amlodipine (+0.00295) 0.938
Hyperkalemia (x1.03) 0.939
Anemia in chronic kidney disease (x1.03) 0.940
Cardiac arrhythmia (x1.03) 0.941
Chronic diastolic heart failure (x1.03) 0.941
Osteoarthritis (x1.03) 0.942
Diabetes mellitus without complication (x1.03) 0.943
Blood chemistry abnormal (x1.03) 0.944
indomethacin (+0.00273) 0.944
Vitamin D deficiency (x1.03) 0.945
gabapentin (+0.0027) 0.946
warfarin (+0.00269) 0.946
Coronary atherosclerosis (x1.03) 0.947
atorvastatin (+0.00264) 0.948
Hypertensive heart and renal disease with (congestive) heart failure (x1.03) 0.948
Chronic systolic heart failure (x1.03) 0.949
Chronic ischemic heart disease (x1.03) 0.950
Chronic kidney disease due to type 2 diabetes mellitus (x1.03) 0.950
Disorder of kidney due to diabetes mellitus (x1.03) 0.951
Osteoarthritis of knee (x1.03) 0.951
Knee pain (x1.03) 0.952
Primary cardiomyopathy (x1.03) 0.952
metoprolol (+0.0024) 0.953
simvastatin (+0.00229) 0.953
Triglyceride [M/V] in SP (+4.19) 0.954
Hypervolemia (x1.02) 0.954
Chronic gouty arthritis (x1.02) 0.955
Degenerative joint disease involving multiple joints (x1.02) 0.955
Disorder of nervous system due to diabetes mellitus (x1.02) 0.956
Chronic combined systolic and diastolic heart failure (x1.02) 0.956
Low back pain (x1.02) 0.957
potassium chloride (+0.00211) 0.957
Age (+1.02) 0.957
Chronic pulmonary heart disease (x1.02) 0.958
Swelling of limb (x1.02) 0.958
Atelectasis (x1.02) 0.959
Erythrocyte sedimentation rate (+1.04) 0.959
Onychomycosis due to dermatophyte (x1.02) 0.959
Paroxysmal atrial fibrillation (x1.02) 0.960
Idiopathic osteoarthritis (x1.02) 0.960
Acute on chronic combined systolic and diastolic heart failure (x1.02) 0.960
Chronic kidney disease stage 2 (x1.02) 0.961
Renal osteodystrophy (x1.02) 0.961
Nuclear senile cataract (x1.02) 0.961
Creatinine [M/V] in B (+0.0347) 0.962
Abnormal breathing (x1.02) 0.962
Polymorphonuclear cells [#/V] in Cerebral spinal fluid (+1.35) 0.962
Acute on chronic diastolic heart failure (x1.02) 0.963
Iron deficiency anemia (x1.02) 0.963
Hypokalemia (x1.02) 0.963
Morbid obesity (x1.02) 0.964
carvedilol (+0.00183) 0.964
−0.4 −0.2 0.0 0.2 0.4
Expressions

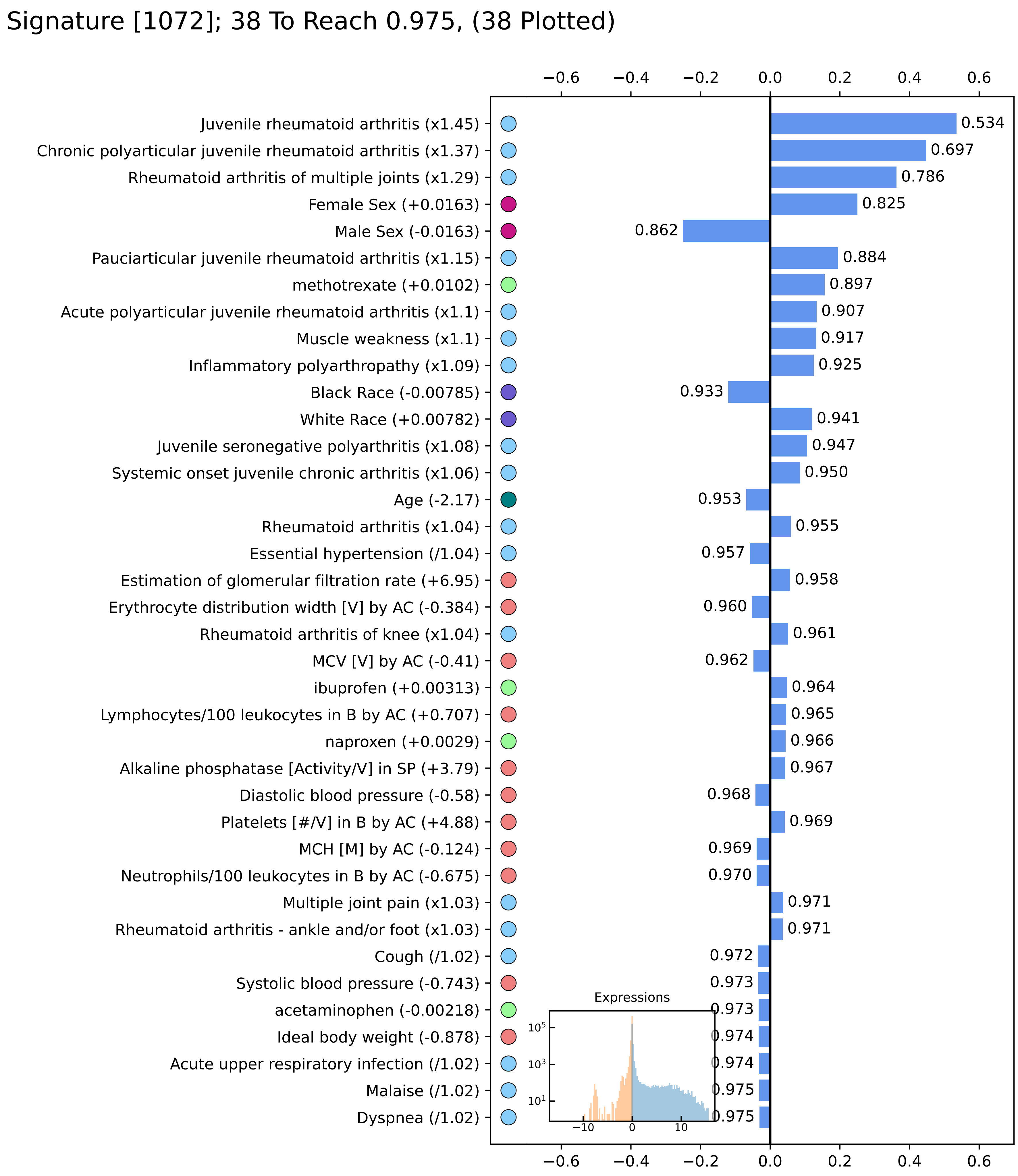

Signature [1072]; 38 To Reach 0.975, (38 Plotted)
Juvenile rheumatoid arthritis (x1.45)
0.534
Chronic polyarticular juvenile rheumatoid arthritis (x1.37)
0.697
Rheumatoid arthritis of multiple joints (x1.29)
0.786
Female Sex (+0.0163)
0.825
Male Sex (-0.0163)
0.862
Pauciarticular juvenile rheumatoid arthritis (x1.15)
0.884
methotrexate (+0.0102)
0.897
Acute polyarticular juvenile rheumatoid arthritis (x1.1)
0.907
Muscle weakness (x1.1)
0.917
Inflammatory polyarthropathy (x1.09)
0.925
Black Race (-0.00785)
0.933
White Race (+0.00782)
0.941
Juvenile seronegative polyarthritis (x1.08)
0.947
Systemic onset juvenile chronic arthritis (x1.06)
0.950
Age (-2.17)
0.953
Rheumatoid arthritis (x1.04)
0.955
Essential hypertension (/1.04)
0.957
Estimation of glomerular filtration rate (+6.95)
0.958
Erythrocyte distribution width [V] by AC (-0.384)
0.960
Rheumatoid arthritis of knee (x1.04)
0.961
MCV [V] by AC (-0.41)
0.962
ibuprofen (+0.00313)
0.964
Lymphocytes/100 leukocytes in B by AC (+0.707)
0.965
naproxen (+0.0029)
0.966
Alkaline phosphatase [Activity/V] in SP (+3.79)
0.967
Diastolic blood pressure (-0.58)
0.968
Platelets [#/V] in B by AC (+4.88)
0.969
MCH [M] by AC (-0.124)
0.969
Neutrophils/100 leukocytes in B by AC (-0.675)
0.970
Multiple joint pain (x1.03)
0.971
Rheumatoid arthritis - ankle and/or foot (x1.03)
0.971
Cough (/1.02)
0.972
Systolic blood pressure (-0.743)
0.973
acetaminophen (-0.00218)
0.973
Ideal body weight (-0.878)
0.974
Acute upper respiratory infection (/1.02)
0.974
Malaise (/1.02)
0.975
Dyspnea (/1.02)
0.975
−0.6
−0.4
−0.2
0.0
0.2
0.4
0.6
Expressions
10^5
10^3
10^1
−10
0
10

Signature [1073]; 86 To Reach 0.975, (86 Plotted)

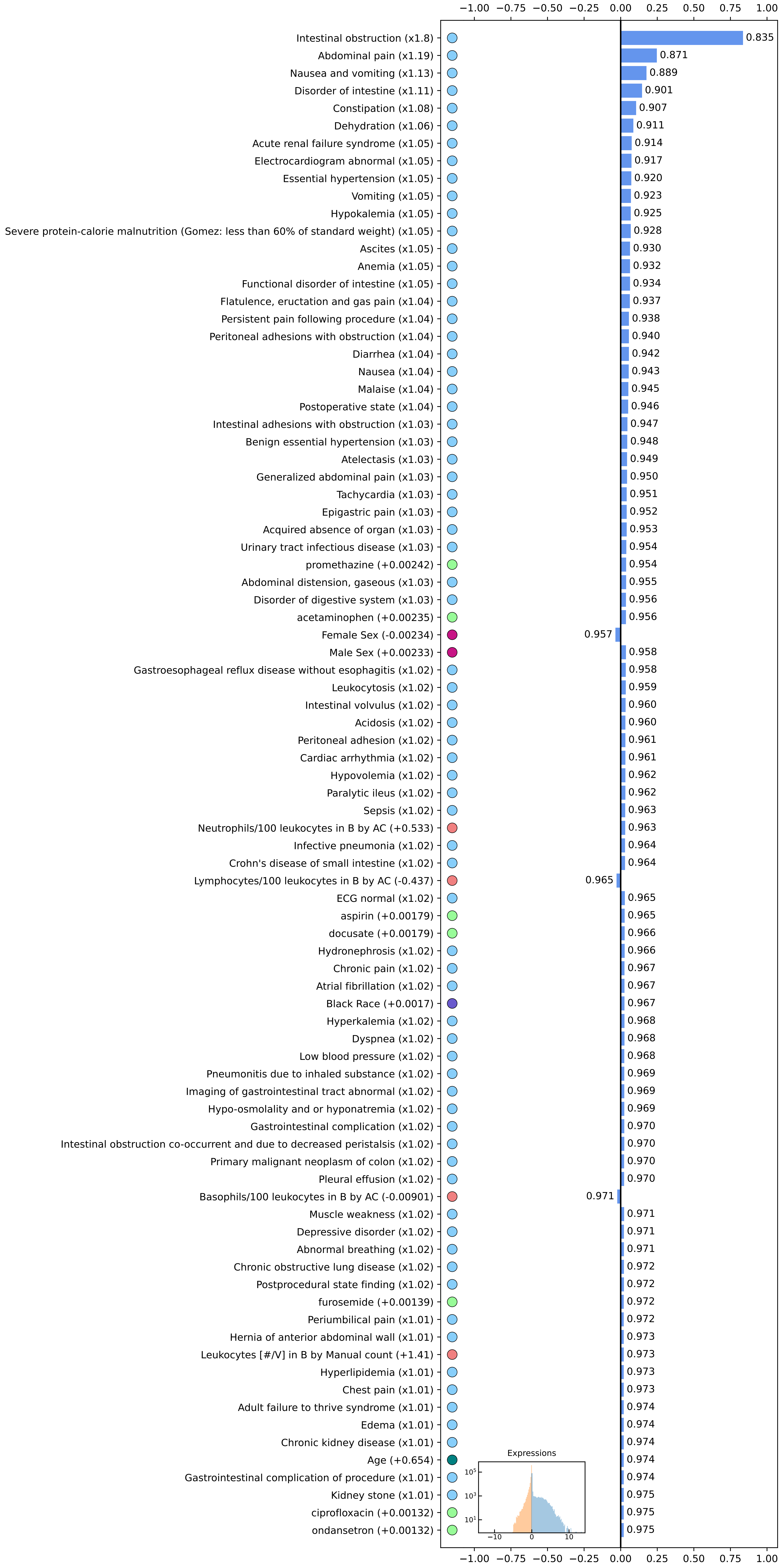

Signature [1074]; 339 To Reach 0.975, (100 Plotted)

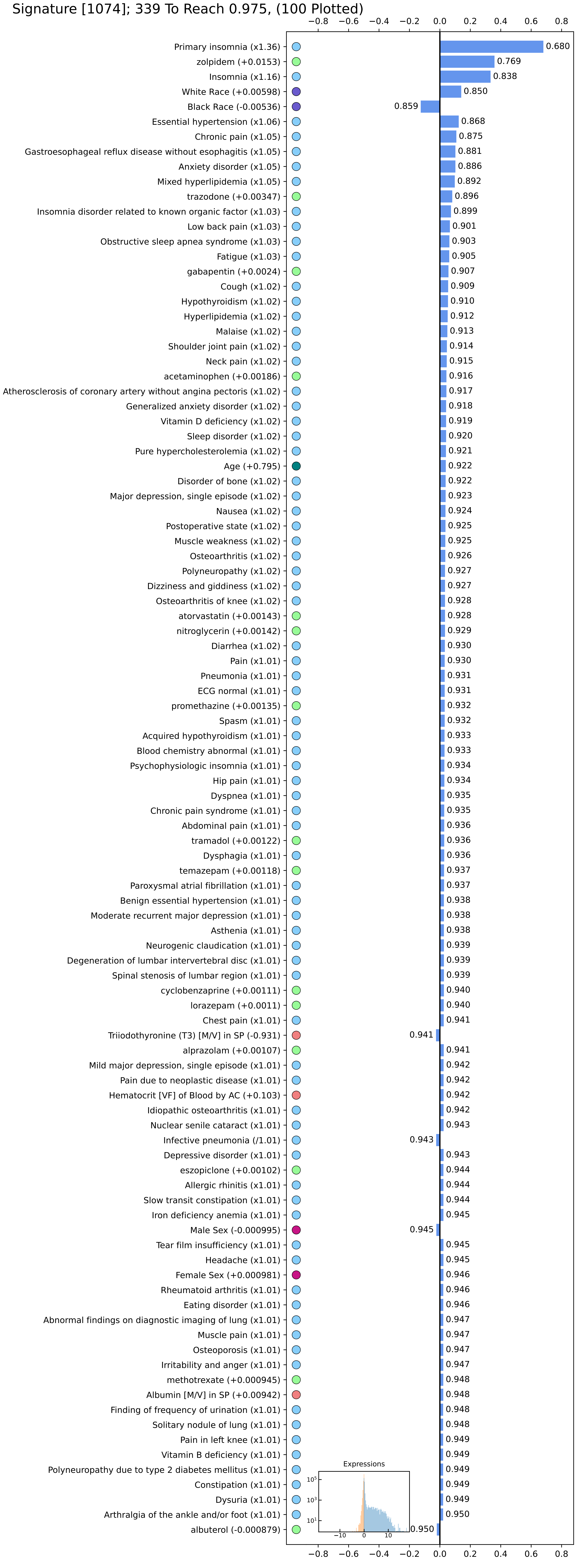

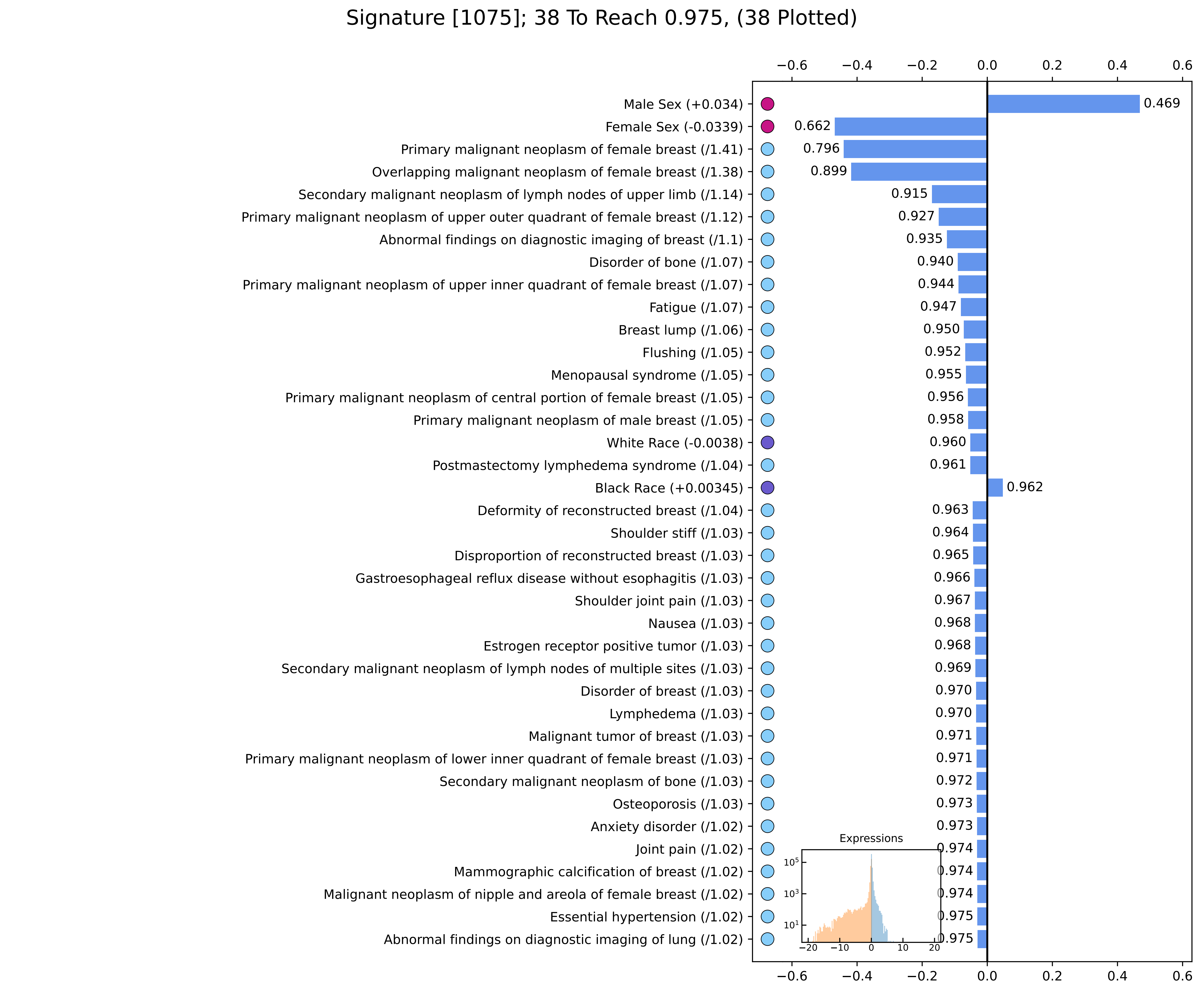

Signature [1075]; 38 To Reach 0.975, (38 Plotted)
Male Sex (+0.034)
Female Sex (-0.0339)
Primary malignant neoplasm of female breast (/1.41)
Overlapping malignant neoplasm of female breast (/1.38)
Secondary malignant neoplasm of lymph nodes of upper limb (/1.14)
Primary malignant neoplasm of upper outer quadrant of female breast (/1.12)
Abnormal findings on diagnostic imaging of breast (/1.1)
Disorder of bone (/1.07)
Primary malignant neoplasm of upper inner quadrant of female breast (/1.07)
Fatigue (/1.07)
Breast lump (/1.06)
Flushing (/1.05)
Menopausal syndrome (/1.05)
Primary malignant neoplasm of central portion of female breast (/1.05)
Primary malignant neoplasm of male breast (/1.05)
White Race (-0.0038)
Postmastectomy lymphedema syndrome (/1.04)
Black Race (+0.00345)
Deformity of reconstructed breast (/1.04)
Shoulder stiff (/1.03)
Disproportion of reconstructed breast (/1.03)
Gastroesophageal reflux disease without esophagitis (/1.03)
Shoulder joint pain (/1.03)
Nausea (/1.03)
Estrogen receptor positive tumor (/1.03)
Secondary malignant neoplasm of lymph nodes of multiple sites (/1.03)
Disorder of breast (/1.03)
Lymphedema (/1.03)
Malignant tumor of breast (/1.03)
Primary malignant neoplasm of lower inner quadrant of female breast (/1.03)
Secondary malignant neoplasm of bone (/1.03)
Osteoporosis (/1.03)
Anxiety disorder (/1.02)
Joint pain (/1.02)
Mammographic calcification of breast (/1.02)
Malignant neoplasm of nipple and areola of female breast (/1.02)
Essential hypertension (/1.02)
Abnormal findings on diagnostic imaging of lung (/1.02)
0.469
0.662
0.796
0.899
0.915
0.927
0.935
0.940
0.944
0.947
0.950
0.952
0.955
0.956
0.958
0.960
0.961
0.962
0.963
0.964
0.965
0.966
0.967
0.968
0.968
0.969
0.970
0.970
0.971
0.971
0.972
0.973
0.973
0.974
0.974
0.974
0.975
0.975
−0.6 −0.4 −0.2 0.0 0.2 0.4 0.6
Expressions
10^5 10^3 10^1
−20 −10 0 10 20

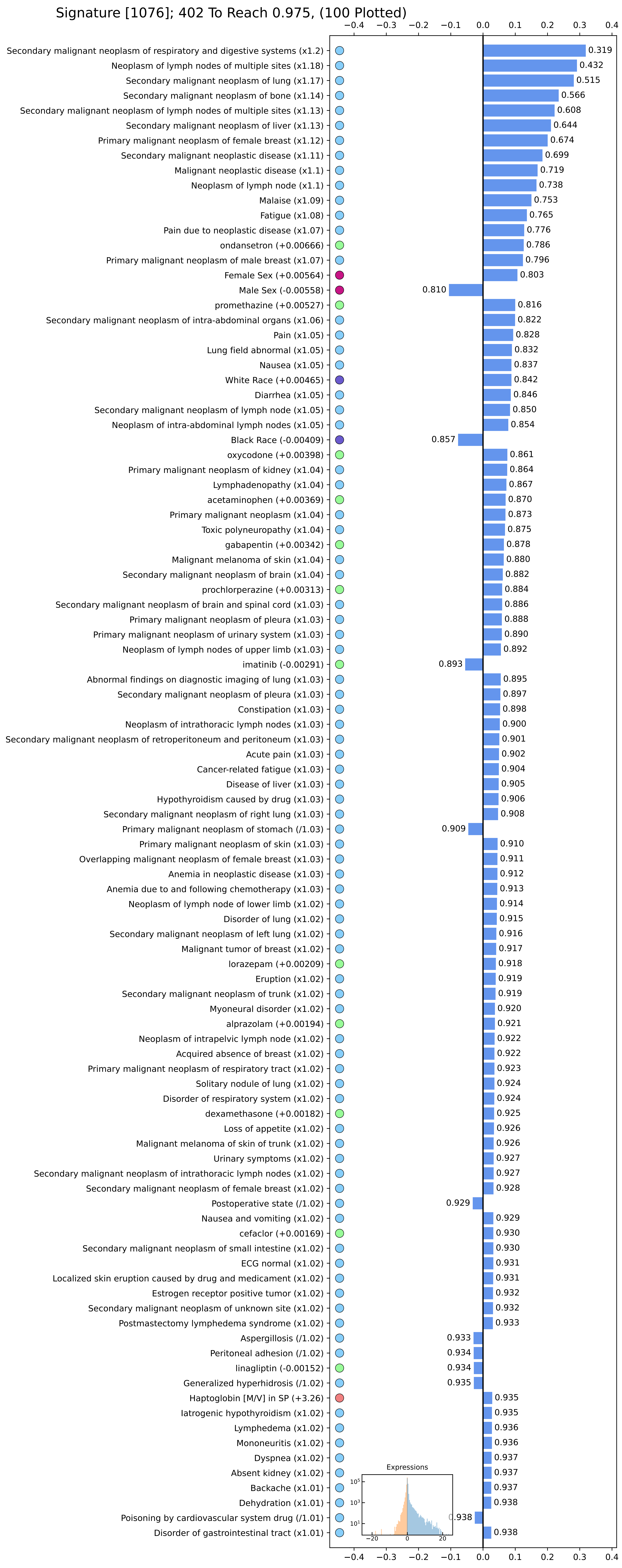

Signature [1076]; 402 To Reach 0.975, (100 Plotted)
Secondary malignant neoplasm of respiratory and digestive systems (x1.2) 0.319
Neoplasm of lymph nodes of multiple sites (x1.18) 0.432
Secondary malignant neoplasm of lung (x1.17) 0.515
Secondary malignant neoplasm of bone (x1.14) 0.566
Secondary malignant neoplasm of lymph nodes of multiple sites (x1.13) 0.608
Secondary malignant neoplasm of liver (x1.13) 0.644
Primary malignant neoplasm of female breast (x1.12) 0.674
Secondary malignant neoplastic disease (x1.11) 0.699
Malignant neoplastic disease (x1.1) 0.719
Neoplasm of lymph node (x1.1) 0.738
Malaise (x1.09) 0.753
Fatigue (x1.08) 0.765
Pain due to neoplastic disease (x1.07) 0.776
ondansetron (+0.00666) 0.786
Primary malignant neoplasm of male breast (x1.07) 0.796
Female Sex (+0.00564) 0.803
Male Sex (-0.00558) 0.810
promethazine (+0.00527) 0.816
Secondary malignant neoplasm of intra-abdominal organs (x1.06) 0.822
Pain (x1.05) 0.828
Lung field abnormal (x1.05) 0.832
Nausea (x1.05) 0.837
White Race (+0.00465) 0.842
Diarrhea (x1.05) 0.846
Secondary malignant neoplasm of lymph node (x1.05) 0.850
Neoplasm of intra-abdominal lymph nodes (x1.05) 0.854
Black Race (-0.00409) 0.857
oxycodone (+0.00398) 0.861
Primary malignant neoplasm of kidney (x1.04) 0.864
Lymphadenopathy (x1.04) 0.867
acetaminophen (+0.00369) 0.870
Primary malignant neoplasm (x1.04) 0.873
Toxic polyneuropathy (x1.04) 0.875
gabapentin (+0.00342) 0.878
Malignant melanoma of skin (x1.04) 0.880
Secondary malignant neoplasm of brain (x1.04) 0.882
prochlorperazine (+0.00313) 0.884
Secondary malignant neoplasm of brain and spinal cord (x1.03) 0.886
Primary malignant neoplasm of pleura (x1.03) 0.888
Primary malignant neoplasm of urinary system (x1.03) 0.890
Neoplasm of lymph nodes of upper limb (x1.03) 0.892
imatinib (-0.00291) 0.893
Abnormal findings on diagnostic imaging of lung (x1.03) 0.895
Secondary malignant neoplasm of pleura (x1.03) 0.897
Constipation (x1.03) 0.898
Neoplasm of intrathoracic lymph nodes (x1.03) 0.900
Secondary malignant neoplasm of retroperitoneum and peritoneum (x1.03) 0.901
Acute pain (x1.03) 0.902
Cancer-related fatigue (x1.03) 0.904
Disease of liver (x1.03) 0.905
Hypothyroidism caused by drug (x1.03) 0.906
Secondary malignant neoplasm of right lung (x1.03) 0.908
Primary malignant neoplasm of stomach (/1.03) 0.909
Primary malignant neoplasm of skin (x1.03) 0.910
Overlapping malignant neoplasm of female breast (x1.03) 0.911
Anemia in neoplastic disease (x1.03) 0.912
Anemia due to and following chemotherapy (x1.03) 0.913
Neoplasm of lymph node of lower limb (x1.02) 0.914
Disorder of lung (x1.02) 0.915
Secondary malignant neoplasm of left lung (x1.02) 0.916
Malignant tumor of breast (x1.02) 0.917
lorazepam (+0.00209) 0.918
Eruption (x1.02) 0.919
Secondary malignant neoplasm of trunk (x1.02) 0.919
Myoneural disorder (x1.02) 0.920
alprazolam (+0.00194) 0.921
Neoplasm of intrapelvic lymph node (x1.02) 0.922
Acquired absence of breast (x1.02) 0.922
Primary malignant neoplasm of respiratory tract (x1.02) 0.923
Solitary nodule of lung (x1.02) 0.924
Disorder of respiratory system (x1.02) 0.924
dexamethasone (+0.00182) 0.925
Loss of appetite (x1.02) 0.926
Malignant melanoma of skin of trunk (x1.02) 0.926
Urinary symptoms (x1.02) 0.927
Secondary malignant neoplasm of intrathoracic lymph nodes (x1.02) 0.927
Secondary malignant neoplasm of female breast (x1.02) 0.928
Postoperative state (/1.02) 0.929
Nausea and vomiting (x1.02) 0.929
cefaclor (+0.00169) 0.930
Secondary malignant neoplasm of small intestine (x1.02) 0.930
ECG normal (x1.02) 0.931
Localized skin eruption caused by drug and medicament (x1.02) 0.931
Estrogen receptor positive tumor (x1.02) 0.932
Secondary malignant neoplasm of unknown site (x1.02) 0.932
Postmastectomy lymphedema syndrome (x1.02) 0.933
Aspergillosis (/1.02) 0.933
Peritoneal adhesion (/1.02) 0.934
linagliptin (-0.00152) 0.934
Generalized hyperhidrosis (/1.02) 0.935
Haptoglobin [M/V] in SP (+3.26) 0.935
Iatrogenic hypothyroidism (x1.02) 0.935
Lymphedema (x1.02) 0.936
Mononeuritis (x1.02) 0.936
Dyspnea (x1.02) 0.937
Absent kidney (x1.02) 0.937
Backache (x1.01) 0.937
Dehydration (x1.01) 0.938
Poisoning by cardiovascular system drug (/1.01) 0.938
Disorder of gastrointestinal tract (x1.01) 0.938
−0.4 −0.3 −0.2 −0.1 0.0 0.1 0.2 0.3 0.4
Expressions

Signature [1077]; 22 To Reach 0.975, (22 Plotted)

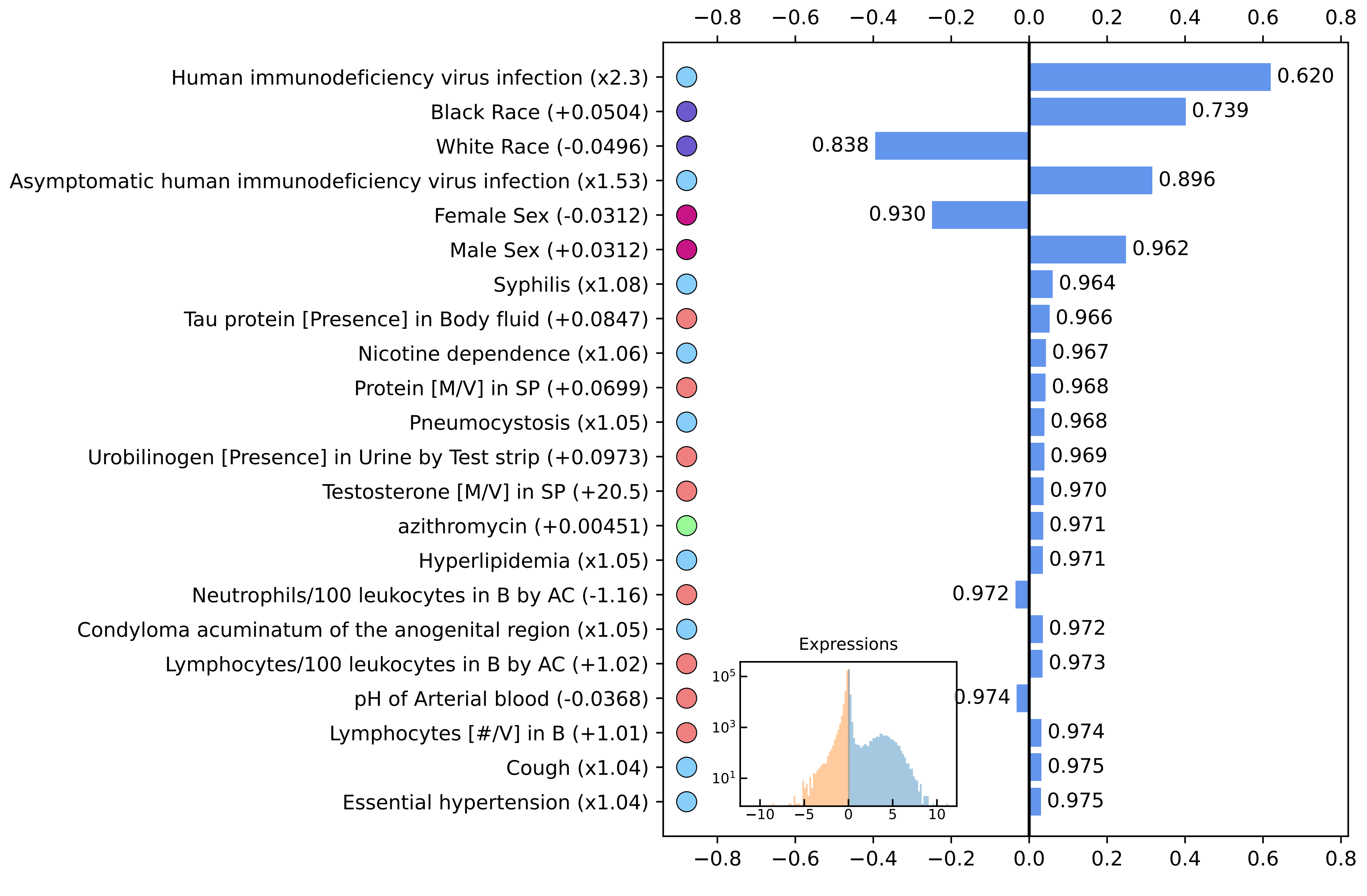

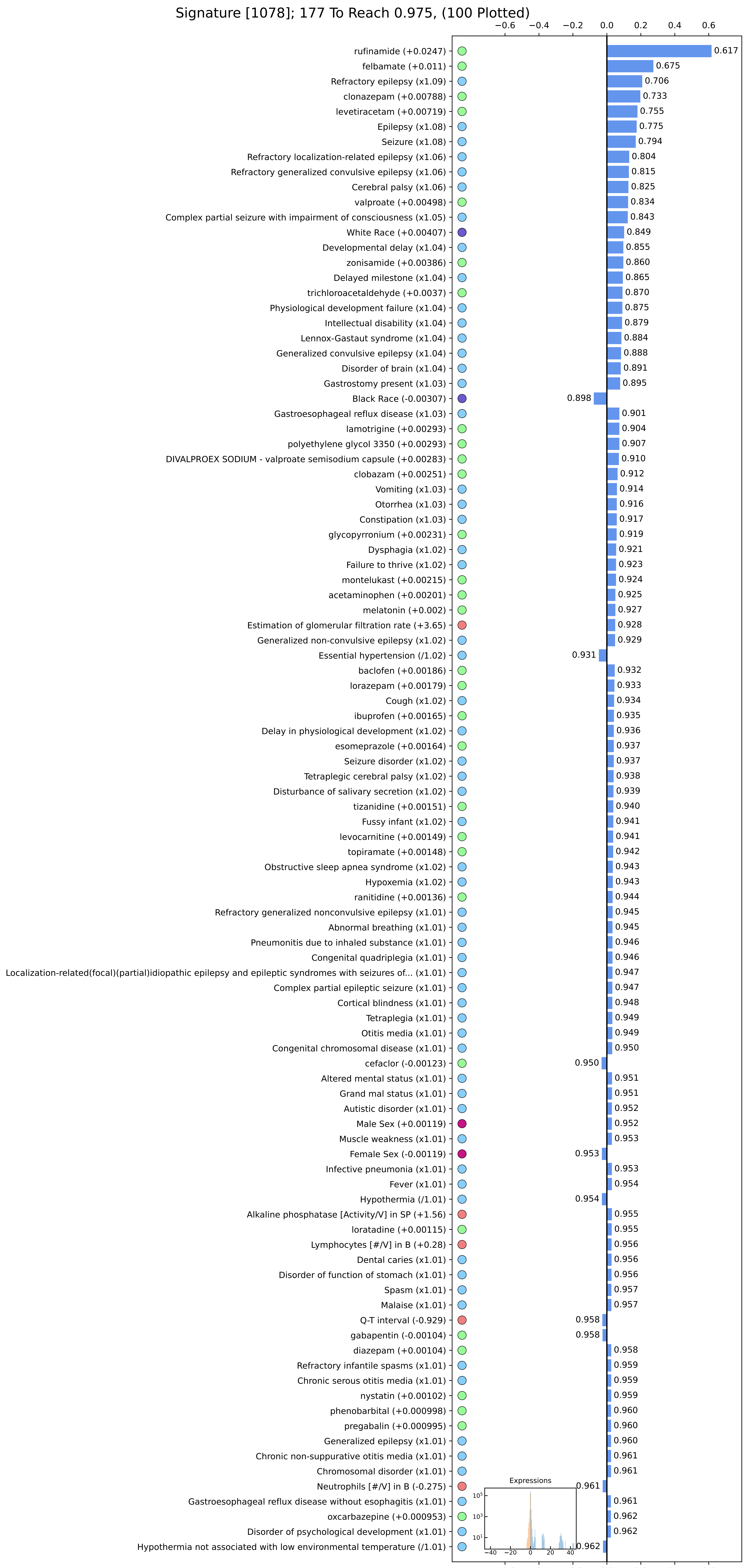

Signature [1078]; 177 To Reach 0.975, (100 Plotted)
−0.6 −0.4 −0.2 0.0 0.2 0.4 0.6
rufinamide (+0.0247) 0.617
felbamate (+0.011) 0.675
Refractory epilepsy (x1.09) 0.706
clonazepam (+0.00788) 0.733
levetiracetam (+0.00719) 0.755
Epilepsy (x1.08) 0.775
Seizure (x1.08) 0.794
Refractory localization-related epilepsy (x1.06) 0.804
Refractory generalized convulsive epilepsy (x1.06) 0.815
Cerebral palsy (x1.06) 0.825
valproate (+0.00498) 0.834
Complex partial seizure with impairment of consciousness (x1.05) 0.843
White Race (+0.00407) 0.849
Developmental delay (x1.04) 0.855
zonisamide (+0.00386) 0.860
Delayed milestone (x1.04) 0.865
trichloroacetaldehyde (+0.0037) 0.870
Physiological development failure (x1.04) 0.875
Intellectual disability (x1.04) 0.879
Lennox-Gastaut syndrome (x1.04) 0.884
Generalized convulsive epilepsy (x1.04) 0.888
Disorder of brain (x1.04) 0.891
Gastrostomy present (x1.03) 0.895
Black Race (-0.00307) 0.898
Gastroesophageal reflux disease (x1.03) 0.901
lamotrigine (+0.00293) 0.904
polyethylene glycol 3350 (+0.00293) 0.907
DIVALPROEX SODIUM - valproate semisodium capsule (+0.00283) 0.910
clobazam (+0.00251) 0.912
Vomiting (x1.03) 0.914
Otorrhea (x1.03) 0.916
Constipation (x1.03) 0.917
glycopyrronium (+0.00231) 0.919
Dysphagia (x1.02) 0.921
Failure to thrive (x1.02) 0.923
montelukast (+0.00215) 0.924
acetaminophen (+0.00201) 0.925
melatonin (+0.002) 0.927
Estimation of glomerular filtration rate (+3.65) 0.928
Generalized non-convulsive epilepsy (x1.02) 0.929
Essential hypertension (/1.02) 0.931
baclofen (+0.00186) 0.932
lorazepam (+0.00179) 0.933
Cough (x1.02) 0.934
ibuprofen (+0.00165) 0.935
Delay in physiological development (x1.02) 0.936
esomeprazole (+0.00164) 0.937
Seizure disorder (x1.02) 0.937
Tetraplegic cerebral palsy (x1.02) 0.938
Disturbance of salivary secretion (x1.02) 0.939
tizanidine (+0.00151) 0.940
Fussy infant (x1.02) 0.941
levocarnitine (+0.00149) 0.941
topiramate (+0.00148) 0.942
Obstructive sleep apnea syndrome (x1.02) 0.943
Hypoxemia (x1.02) 0.943
ranitidine (+0.00136) 0.944
Refractory generalized nonconvulsive epilepsy (x1.01) 0.945
Abnormal breathing (x1.01) 0.945
Pneumonitis due to inhaled substance (x1.01) 0.946
Congenital quadriplegia (x1.01) 0.946
Localization-related(focal)(partial)idiopathic epilepsy and epileptic syndromes with seizures of... (x1.01) 0.947
Complex partial epileptic seizure (x1.01) 0.947
Cortical blindness (x1.01) 0.948
Tetraplegia (x1.01) 0.949
Otitis media (x1.01) 0.949
Congenital chromosomal disease (x1.01) 0.950
cefaclor (-0.00123) 0.950
Altered mental status (x1.01) 0.951
Grand mal status (x1.01) 0.951
Autistic disorder (x1.01) 0.952
Male Sex (+0.00119) 0.952
Muscle weakness (x1.01) 0.953
Female Sex (-0.00119) 0.953
Infective pneumonia (x1.01) 0.953
Fever (x1.01) 0.954
Hypothermia (/1.01) 0.954
Alkaline phosphatase [Activity/V] in SP (+1.56) 0.955
loratadine (+0.00115) 0.955
Lymphocytes [#/V] in B (+0.28) 0.956
Dental caries (x1.01) 0.956
Disorder of function of stomach (x1.01) 0.956
Spasm (x1.01) 0.957
Malaise (x1.01) 0.957
Q-T interval (-0.929) 0.958
gabapentin (-0.00104) 0.958
diazepam (+0.00104) 0.958
Refractory infantile spasms (x1.01) 0.959
Chronic serous otitis media (x1.01) 0.959
nystatin (+0.00102) 0.959
phenobarbital (+0.000998) 0.960
pregabalin (+0.000995) 0.960
Generalized epilepsy (x1.01) 0.960
Chronic non-suppurative otitis media (x1.01) 0.961
Chromosomal disorder (x1.01) 0.961
Neutrophils [#/V] in B (-0.275) 0.961
Gastroesophageal reflux disease without esophagitis (x1.01) 0.961
oxcarbazepine (+0.000953) 0.962
Disorder of psychological development (x1.01) 0.962
Hypothermia not associated with low environmental temperature (/1.01) 0.962
Expressions
−40 −20 0 20 40

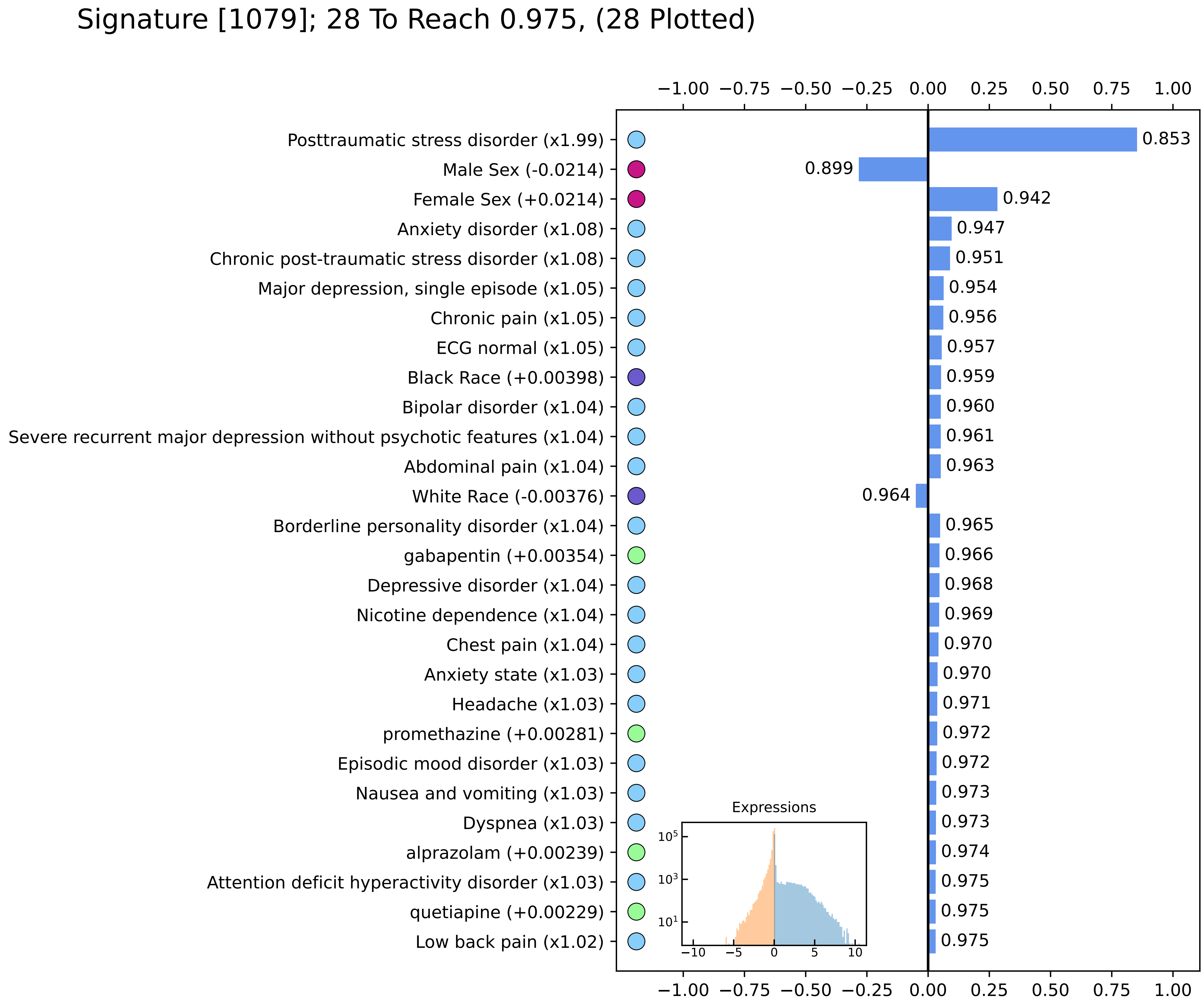

Signature [1079]; 28 To Reach 0.975, (28 Plotted)
−1.00 −0.75 −0.50 −0.25 0.00 0.25 0.50 0.75 1.00
Posttraumatic stress disorder (x1.99) 0.853
Male Sex (-0.0214) 0.899
Female Sex (+0.0214) 0.942
Anxiety disorder (x1.08) 0.947
Chronic post-traumatic stress disorder (x1.08) 0.951
Major depression, single episode (x1.05) 0.954
Chronic pain (x1.05) 0.956
ECG normal (x1.05) 0.957
Black Race (+0.00398) 0.959
Bipolar disorder (x1.04) 0.960
Severe recurrent major depression without psychotic features (x1.04) 0.961
Abdominal pain (x1.04) 0.963
White Race (-0.00376) 0.964
Borderline personality disorder (x1.04) 0.965
gabapentin (+0.00354) 0.966
Depressive disorder (x1.04) 0.968
Nicotine dependence (x1.04) 0.969
Chest pain (x1.04) 0.970
Anxiety state (x1.03) 0.970
Headache (x1.03) 0.971
promethazine (+0.00281) 0.972
Episodic mood disorder (x1.03) 0.972
Nausea and vomiting (x1.03) 0.973
Dyspnea (x1.03) 0.973
alprazolam (+0.00239) 0.974
Attention deficit hyperactivity disorder (x1.03) 0.975
quetiapine (+0.00229) 0.975
Low back pain (x1.02) 0.975
Expressions
10⁵
10³
10¹
−10 −5 0 5 10

Signature [1080]; 18 To Reach 0.975, (18 Plotted)

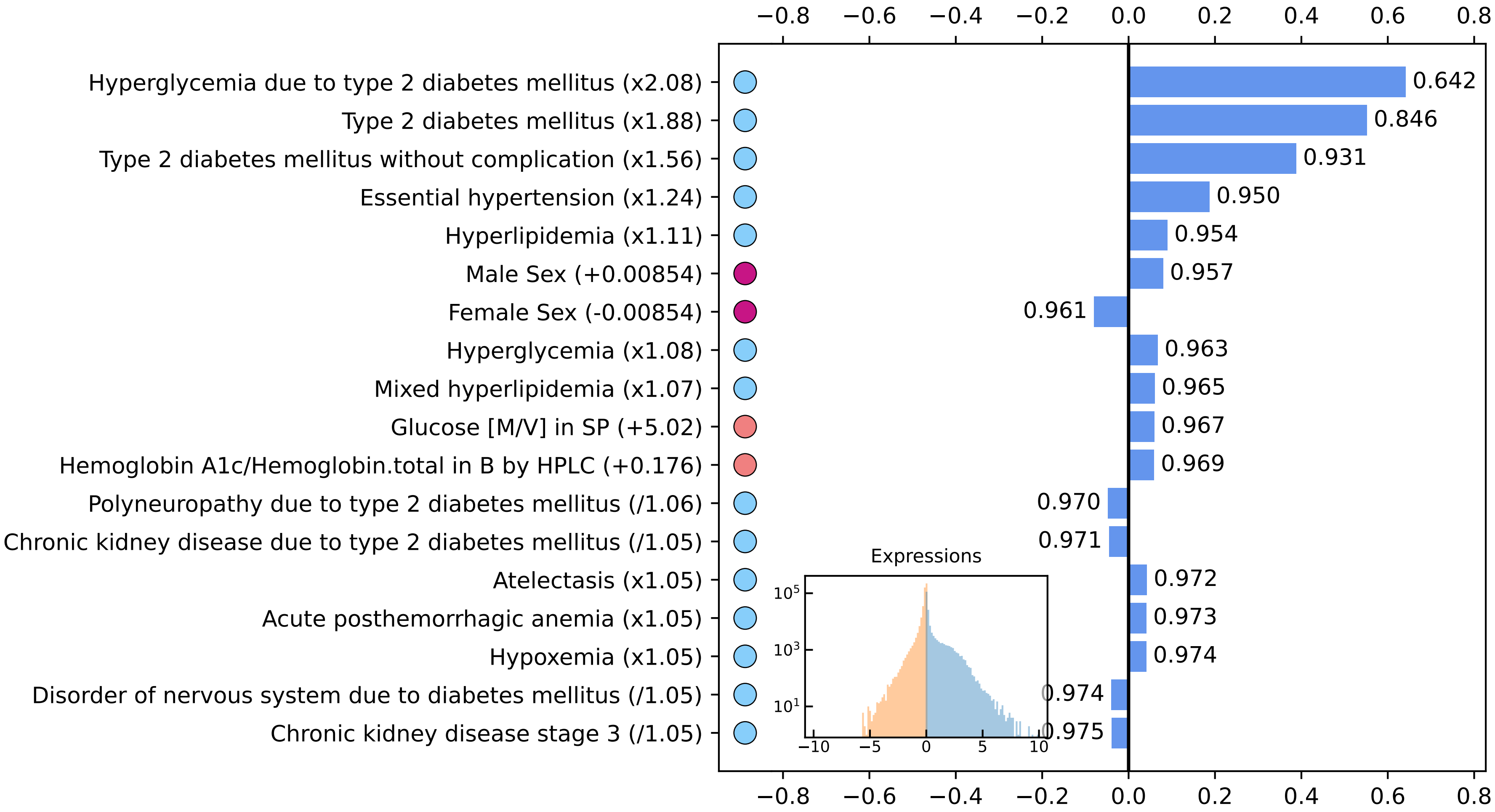

Signature [1081]; 13 To Reach 0.975, (13 Plotted)

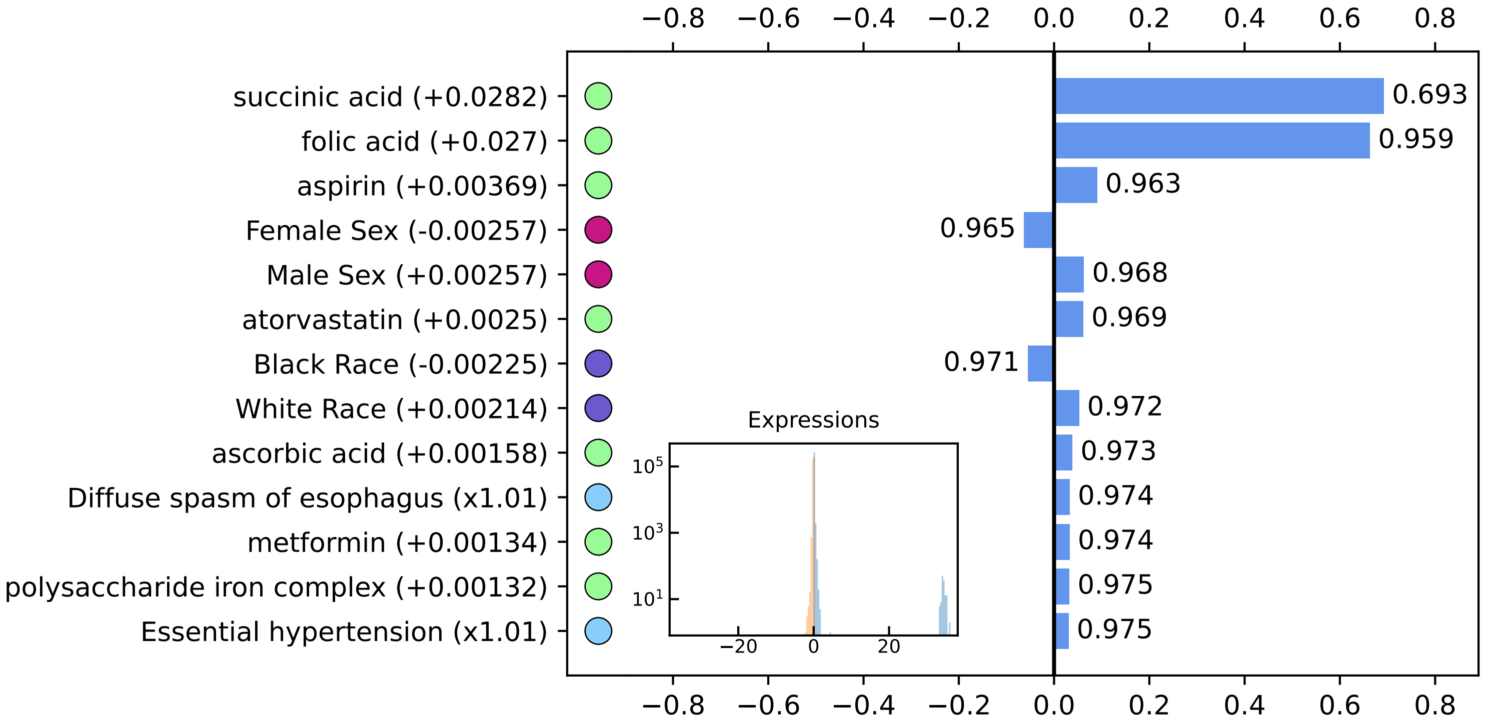

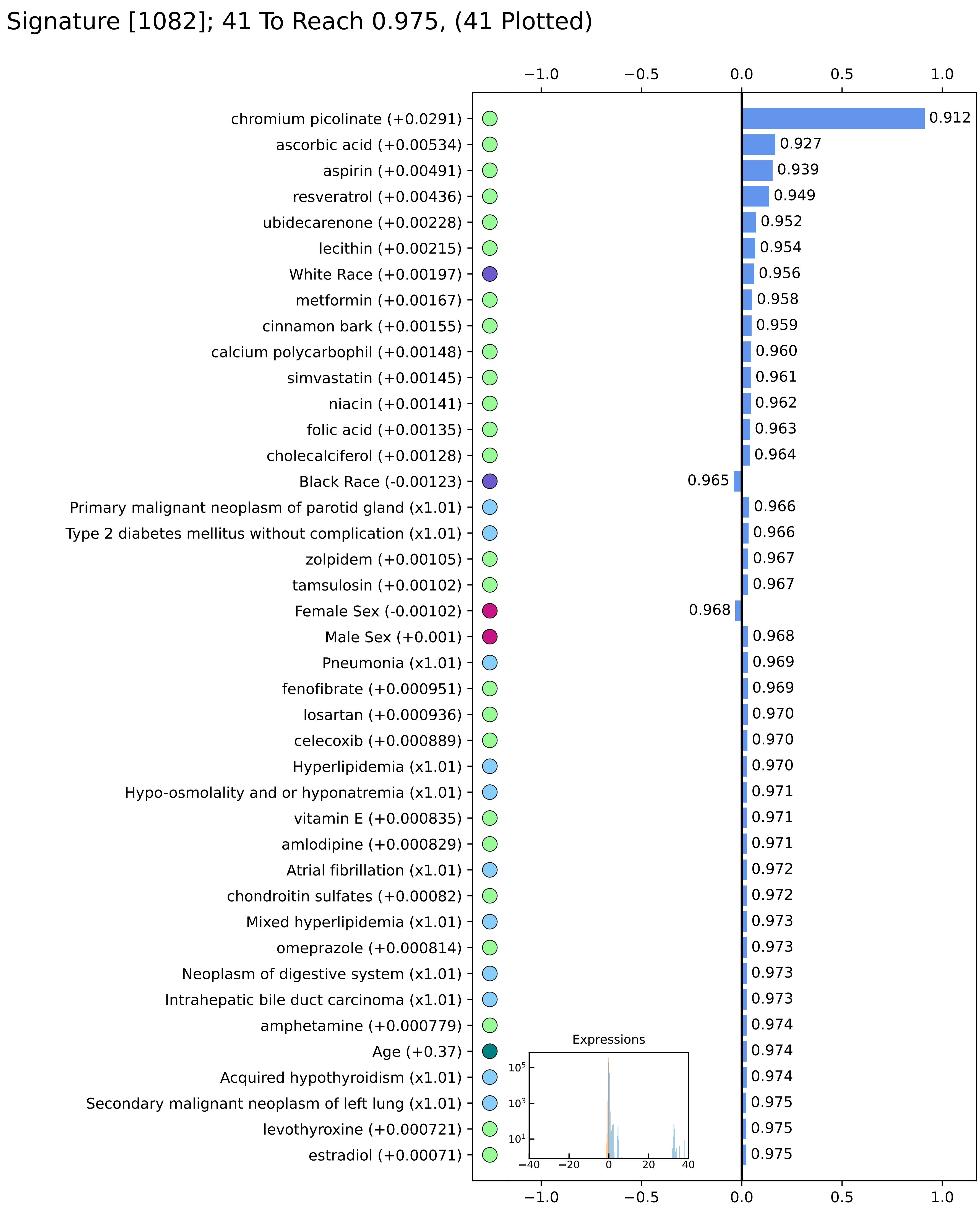

Signature [1082]; 41 To Reach 0.975, (41 Plotted)
chromium picolinate (+0.0291)
ascorbic acid (+0.00534)
aspirin (+0.00491)
resveratrol (+0.00436)
ubidecarenone (+0.00228)
lecithin (+0.00215)
White Race (+0.00197)
metformin (+0.00167)
cinnamon bark (+0.00155)
calcium polycarbophil (+0.00148)
simvastatin (+0.00145)
niacin (+0.00141)
folic acid (+0.00135)
cholecalciferol (+0.00128)
Black Race (-0.00123)
Primary malignant neoplasm of parotid gland (x1.01)
Type 2 diabetes mellitus without complication (x1.01)
zolpidem (+0.00105)
tamsulosin (+0.00102)
Female Sex (-0.00102)
Male Sex (+0.001)
Pneumonia (x1.01)
fenofibrate (+0.000951)
losartan (+0.000936)
celecoxib (+0.000889)
Hyperlipidemia (x1.01)
Hypo-osmolality and or hyponatremia (x1.01)
vitamin E (+0.000835)
amlodipine (+0.000829)
Atrial fibrillation (x1.01)
chondroitin sulfates (+0.00082)
Mixed hyperlipidemia (x1.01)
omeprazole (+0.000814)
Neoplasm of digestive system (x1.01)
Intrahepatic bile duct carcinoma (x1.01)
amphetamine (+0.000779)
Age (+0.37)
Acquired hypothyroidism (x1.01)
Secondary malignant neoplasm of left lung (x1.01)
levothyroxine (+0.000721)
estradiol (+0.00071)
0.912
0.927
0.939
0.949
0.952
0.954
0.956
0.958
0.959
0.960
0.961
0.962
0.963
0.964
0.965
0.966
0.966
0.967
0.967
0.968
0.968
0.969
0.969
0.970
0.970
0.970
0.971
0.971
0.971
0.972
0.972
0.973
0.973
0.973
0.973
0.974
0.974
0.974
0.975
0.975
0.975
−1.0
−0.5
0.0
0.5
1.0
Expressions
−40
−20
0
20
40

Signature [1083]; 177 To Reach 0.975, (100 Plotted)

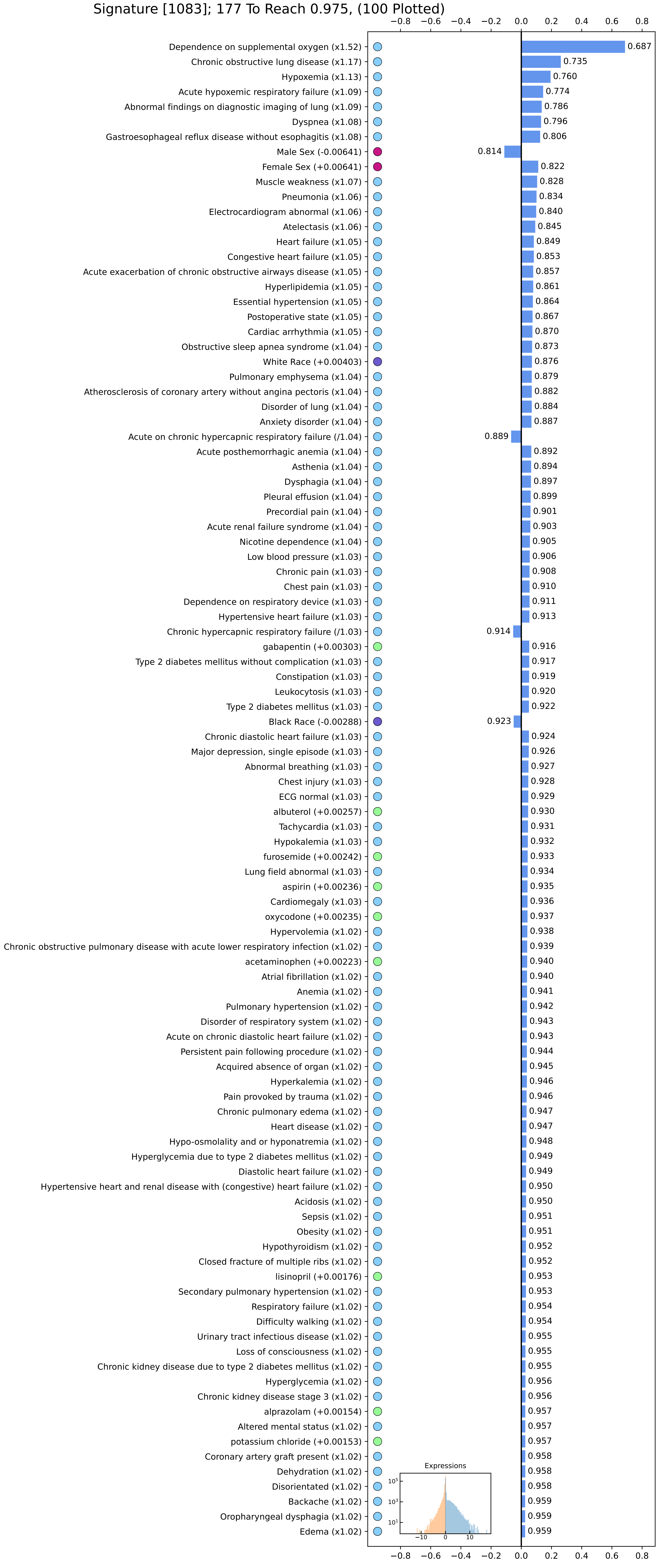

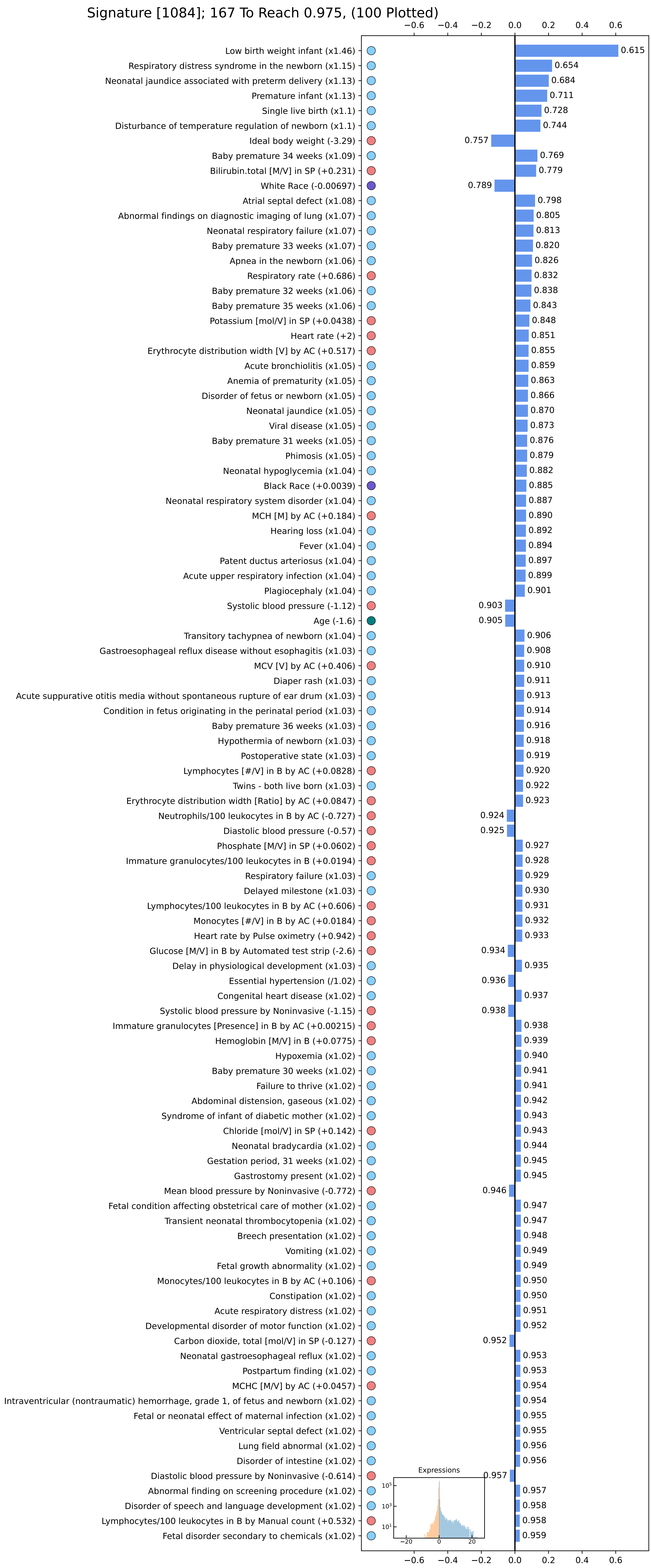

Signature [1084]; 167 To Reach 0.975, (100 Plotted)
Low birth weight infant (x1.46) 0.615
Respiratory distress syndrome in the newborn (x1.15) 0.654
Neonatal jaundice associated with preterm delivery (x1.13) 0.684
Premature infant (x1.13) 0.711
Single live birth (x1.1) 0.728
Disturbance of temperature regulation of newborn (x1.1) 0.744
Ideal body weight (-3.29) 0.757
Baby premature 34 weeks (x1.09) 0.769
Bilirubin.total [M/V] in SP (+0.231) 0.779
White Race (-0.00697) 0.789
Atrial septal defect (x1.08) 0.798
Abnormal findings on diagnostic imaging of lung (x1.07) 0.805
Neonatal respiratory failure (x1.07) 0.813
Baby premature 33 weeks (x1.07) 0.820
Apnea in the newborn (x1.06) 0.826
Respiratory rate (+0.686) 0.832
Baby premature 32 weeks (x1.06) 0.838
Baby premature 35 weeks (x1.06) 0.843
Potassium [mol/V] in SP (+0.0438) 0.848
Heart rate (+2) 0.851
Erythrocyte distribution width [V] by AC (+0.517) 0.855
Acute bronchiolitis (x1.05) 0.859
Anemia of prematurity (x1.05) 0.863
Disorder of fetus or newborn (x1.05) 0.866
Neonatal jaundice (x1.05) 0.870
Viral disease (x1.05) 0.873
Baby premature 31 weeks (x1.05) 0.876
Phimosis (x1.05) 0.879
Neonatal hypoglycemia (x1.04) 0.882
Black Race (+0.0039) 0.885
Neonatal respiratory system disorder (x1.04) 0.887
MCH [M] by AC (+0.184) 0.890
Hearing loss (x1.04) 0.892
Fever (x1.04) 0.894
Patent ductus arteriosus (x1.04) 0.897
Acute upper respiratory infection (x1.04) 0.899
Plagiocephaly (x1.04) 0.901
Systolic blood pressure (-1.12) 0.903
Age (-1.6) 0.905
Transitory tachypnea of newborn (x1.04) 0.906
Gastroesophageal reflux disease without esophagitis (x1.03) 0.908
MCV [V] by AC (+0.406) 0.910
Diaper rash (x1.03) 0.911
Acute suppurative otitis media without spontaneous rupture of ear drum (x1.03) 0.913
Condition in fetus originating in the perinatal period (x1.03) 0.914
Baby premature 36 weeks (x1.03) 0.916
Hypothermia of newborn (x1.03) 0.918
Postoperative state (x1.03) 0.919
Lymphocytes [#/V] in B by AC (+0.0828) 0.920
Twins - both live born (x1.03) 0.922
Erythrocyte distribution width [Ratio] by AC (+0.0847) 0.923
Neutrophils/100 leukocytes in B by AC (-0.727) 0.924
Diastolic blood pressure (-0.57) 0.925
Phosphate [M/V] in SP (+0.0602) 0.927
Immature granulocytes/100 leukocytes in B (+0.0194) 0.928
Respiratory failure (x1.03) 0.929
Delayed milestone (x1.03) 0.930
Lymphocytes/100 leukocytes in B by AC (+0.606) 0.931
Monocytes [#/V] in B by AC (+0.0184) 0.932
Heart rate by Pulse oximetry (+0.942) 0.933
Glucose [M/V] in B by Automated test strip (-2.6) 0.934
Delay in physiological development (x1.03) 0.935
Essential hypertension (/1.02) 0.936
Congenital heart disease (x1.02) 0.937
Systolic blood pressure by Noninvasive (-1.15) 0.938
Immature granulocytes [Presence] in B by AC (+0.00215) 0.938
Hemoglobin [M/V] in B (+0.0775) 0.939
Hypoxemia (x1.02) 0.940
Baby premature 30 weeks (x1.02) 0.941
Failure to thrive (x1.02) 0.941
Abdominal distension, gaseous (x1.02) 0.942
Syndrome of infant of diabetic mother (x1.02) 0.943
Chloride [mol/V] in SP (+0.142) 0.943
Neonatal bradycardia (x1.02) 0.944
Gestation period, 31 weeks (x1.02) 0.945
Gastrostomy present (x1.02) 0.945
Mean blood pressure by Noninvasive (-0.772) 0.946
Fetal condition affecting obstetrical care of mother (x1.02) 0.947
Transient neonatal thrombocytopenia (x1.02) 0.947
Breech presentation (x1.02) 0.948
Vomiting (x1.02) 0.949
Fetal growth abnormality (x1.02) 0.949
Monocytes/100 leukocytes in B by AC (+0.106) 0.950
Constipation (x1.02) 0.950
Acute respiratory distress (x1.02) 0.951
Developmental disorder of motor function (x1.02) 0.952
Carbon dioxide, total [mol/V] in SP (-0.127) 0.952
Neonatal gastroesophageal reflux (x1.02) 0.953
Postpartum finding (x1.02) 0.953
MCHC [M/V] by AC (+0.0457) 0.954
Intraventricular (nontraumatic) hemorrhage, grade 1, of fetus and newborn (x1.02) 0.954
Fetal or neonatal effect of maternal infection (x1.02) 0.955
Ventricular septal defect (x1.02) 0.955
Lung field abnormal (x1.02) 0.956
Disorder of intestine (x1.02) 0.956
Diastolic blood pressure by Noninvasive (-0.614) 0.957
Abnormal finding on screening procedure (x1.02) 0.957
Disorder of speech and language development (x1.02) 0.958
Lymphocytes/100 leukocytes in B by Manual count (+0.532) 0.958
Fetal disorder secondary to chemicals (x1.02) 0.959
−0.6 −0.4 −0.2 0.0 0.2 0.4 0.6
Expressions
10^5
10^3
10^1
−20 0 20

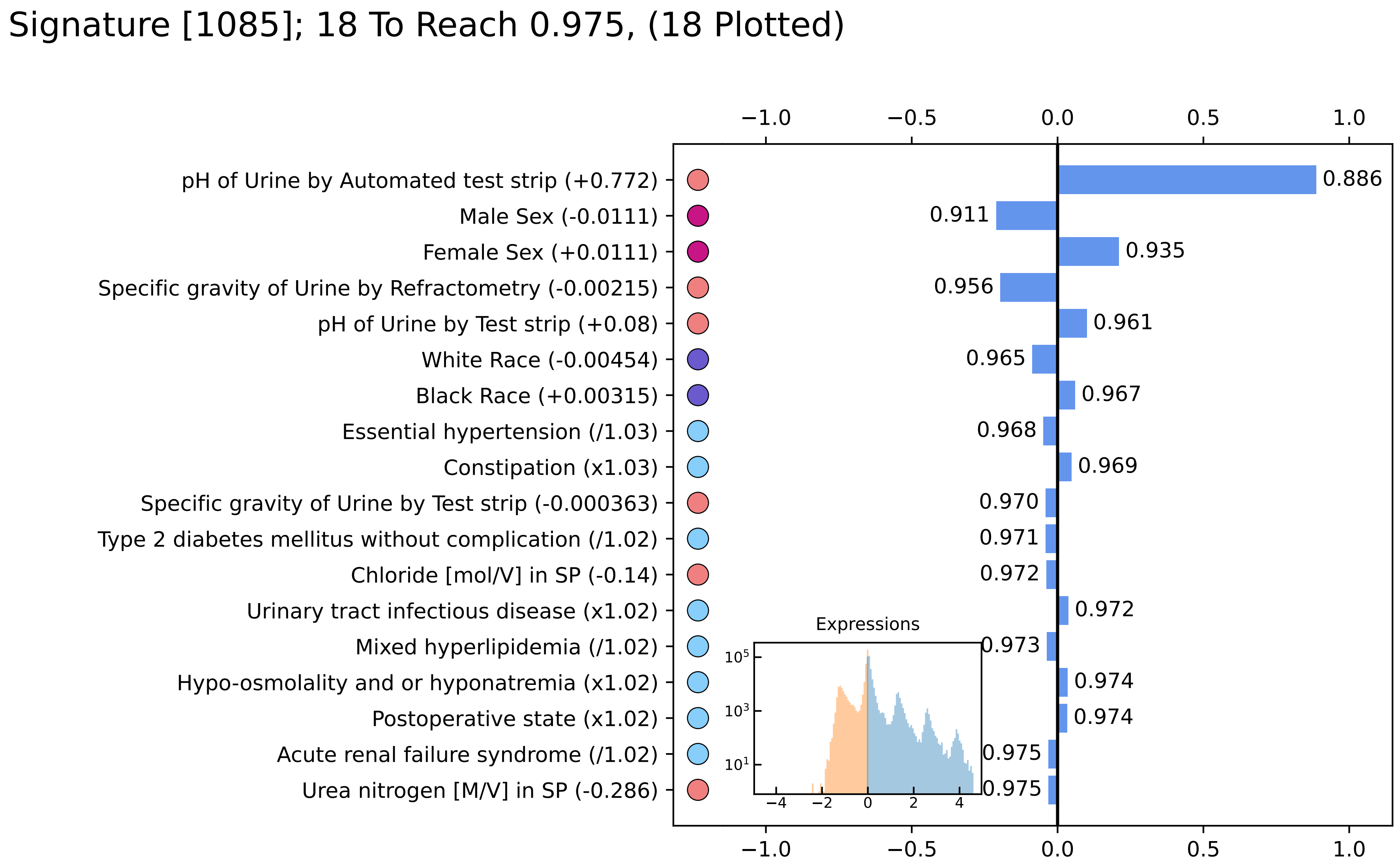

Signature [1085]; 18 To Reach 0.975, (18 Plotted)
pH of Urine by Automated test strip (+0.772)
Male Sex (-0.0111)
Female Sex (+0.0111)
Specific gravity of Urine by Refractometry (-0.00215)
pH of Urine by Test strip (+0.08)
White Race (-0.00454)
Black Race (+0.00315)
Essential hypertension (/1.03)
Constipation (x1.03)
Specific gravity of Urine by Test strip (-0.000363)
Type 2 diabetes mellitus without complication (/1.02)
Chloride [mol/V] in SP (-0.14)
Urinary tract infectious disease (x1.02)
Mixed hyperlipidemia (/1.02)
Hypo-osmolality and or hyponatremia (x1.02)
Postoperative state (x1.02)
Acute renal failure syndrome (/1.02)
Urea nitrogen [M/V] in SP (-0.286)
0.886
0.911
0.935
0.956
0.961
0.965
0.967
0.968
0.969
0.970
0.971
0.972
0.972
0.973
0.974
0.974
0.975
0.975
−1.0
−0.5
0.0
0.5
1.0
Expressions

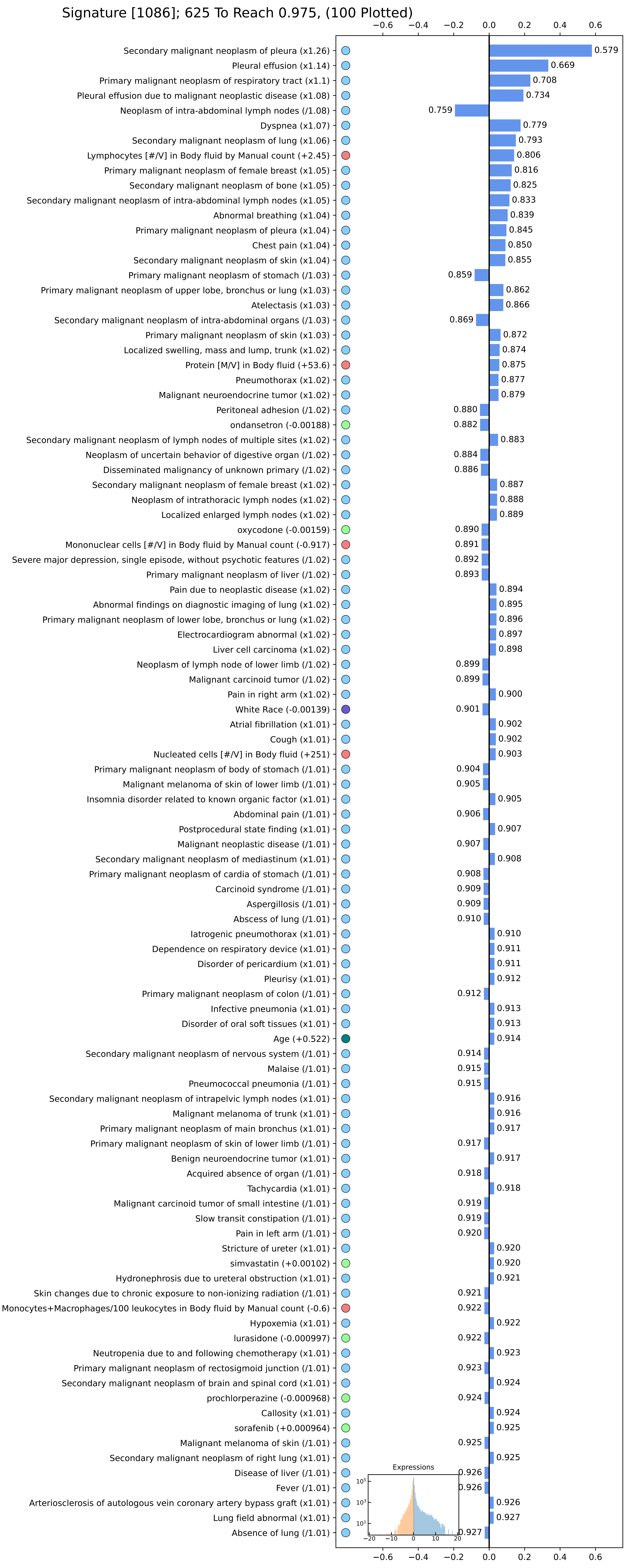

Signature [1086]; 625 To Reach 0.975, (100 Plotted)
Secondary malignant neoplasm of pleura (x1.26) 0.579
Pleural effusion (x1.14) 0.669
Primary malignant neoplasm of respiratory tract (x1.1) 0.708
Pleural effusion due to malignant neoplastic disease (x1.08) 0.734
Neoplasm of intra-abdominal lymph nodes (/1.08) 0.759
Dyspnea (x1.07) 0.779
Secondary malignant neoplasm of lung (x1.06) 0.793
Lymphocytes [#/V] in Body fluid by Manual count (+2.45) 0.806
Primary malignant neoplasm of female breast (x1.05) 0.816
Secondary malignant neoplasm of bone (x1.05) 0.825
Secondary malignant neoplasm of intra-abdominal lymph nodes (x1.05) 0.833
Abnormal breathing (x1.04) 0.839
Primary malignant neoplasm of pleura (x1.04) 0.845
Chest pain (x1.04) 0.850
Secondary malignant neoplasm of skin (x1.04) 0.855
Primary malignant neoplasm of stomach (/1.03) 0.859
Primary malignant neoplasm of upper lobe, bronchus or lung (x1.03) 0.862
Atelectasis (x1.03) 0.866
Secondary malignant neoplasm of intra-abdominal organs (/1.03) 0.869
Primary malignant neoplasm of skin (x1.03) 0.872
Localized swelling, mass and lump, trunk (x1.02) 0.874
Protein [M/V] in Body fluid (+53.6) 0.875
Pneumothorax (x1.02) 0.877
Malignant neuroendocrine tumor (x1.02) 0.879
Peritoneal adhesion (/1.02) 0.880
ondansetron (-0.00188) 0.882
Secondary malignant neoplasm of lymph nodes of multiple sites (x1.02) 0.883
Neoplasm of uncertain behavior of digestive organ (/1.02) 0.884
Disseminated malignancy of unknown primary (/1.02) 0.886
Secondary malignant neoplasm of female breast (x1.02) 0.887
Neoplasm of intrathoracic lymph nodes (x1.02) 0.888
Localized enlarged lymph nodes (x1.02) 0.889
oxycodone (-0.00159) 0.890
Mononuclear cells [#/V] in Body fluid by Manual count (-0.917) 0.891
Severe major depression, single episode, without psychotic features (/1.02) 0.892
Primary malignant neoplasm of liver (/1.02) 0.893
Pain due to neoplastic disease (x1.02) 0.894
Abnormal findings on diagnostic imaging of lung (x1.02) 0.895
Primary malignant neoplasm of lower lobe, bronchus or lung (x1.02) 0.896
Electrocardiogram abnormal (x1.02) 0.897
Liver cell carcinoma (x1.02) 0.898
Neoplasm of lymph node of lower limb (/1.02) 0.899
Malignant carcinoid tumor (/1.02) 0.899
Pain in right arm (x1.02) 0.900
White Race (-0.00139) 0.901
Atrial fibrillation (x1.01) 0.902
Cough (x1.01) 0.902
Nucleated cells [#/V] in Body fluid (+251) 0.903
Primary malignant neoplasm of body of stomach (/1.01) 0.904
Malignant melanoma of skin of lower limb (/1.01) 0.905
Insomnia disorder related to known organic factor (x1.01) 0.905
Abdominal pain (/1.01) 0.906
Postprocedural state finding (x1.01) 0.907
Malignant neoplastic disease (/1.01) 0.907
Secondary malignant neoplasm of mediastinum (x1.01) 0.908
Primary malignant neoplasm of cardia of stomach (/1.01) 0.908
Carcinoid syndrome (/1.01) 0.909
Aspergillosis (/1.01) 0.909
Abscess of lung (/1.01) 0.910
Iatrogenic pneumothorax (x1.01) 0.910
Dependence on respiratory device (x1.01) 0.911
Disorder of pericardium (x1.01) 0.911
Pleurisy (x1.01) 0.912
Primary malignant neoplasm of colon (/1.01) 0.912
Infective pneumonia (x1.01) 0.913
Disorder of oral soft tissues (x1.01) 0.913
Age (+0.522) 0.914
Secondary malignant neoplasm of nervous system (/1.01) 0.914
Malaise (/1.01) 0.915
Pneumococcal pneumonia (/1.01) 0.915
Secondary malignant neoplasm of intrapelvic lymph nodes (x1.01) 0.916
Malignant melanoma of trunk (x1.01) 0.916
Primary malignant neoplasm of main bronchus (x1.01) 0.917
Primary malignant neoplasm of skin of lower limb (/1.01) 0.917
Benign neuroendocrine tumor (x1.01) 0.917
Acquired absence of organ (/1.01) 0.918
Tachycardia (x1.01) 0.918
Malignant carcinoid tumor of small intestine (/1.01) 0.919
Slow transit constipation (/1.01) 0.919
Pain in left arm (/1.01) 0.920
Stricture of ureter (x1.01) 0.920
simvastatin (+0.00102) 0.920
Hydronephrosis due to ureteral obstruction (x1.01) 0.921
Skin changes due to chronic exposure to non-ionizing radiation (/1.01) 0.921
Monocytes+Macrophages/100 leukocytes in Body fluid by Manual count (-0.6) 0.922
Hypoxemia (x1.01) 0.922
lurasidone (-0.000997) 0.922
Neutropenia due to and following chemotherapy (x1.01) 0.923
Primary malignant neoplasm of rectosigmoid junction (/1.01) 0.923
Secondary malignant neoplasm of brain and spinal cord (x1.01) 0.924
prochlorperazine (-0.000968) 0.924
Callosity (x1.01) 0.924
sorafenib (+0.000964) 0.925
Malignant melanoma of skin (/1.01) 0.925
Secondary malignant neoplasm of right lung (x1.01) 0.925
Disease of liver (/1.01) 0.926
Fever (/1.01) 0.926
Arteriosclerosis of autologous vein coronary artery bypass graft (x1.01) 0.926
Lung field abnormal (x1.01) 0.927
Absence of lung (/1.01) 0.927
Expressions
−0.6 −0.4 −0.2 0.0 0.2 0.4 0.6

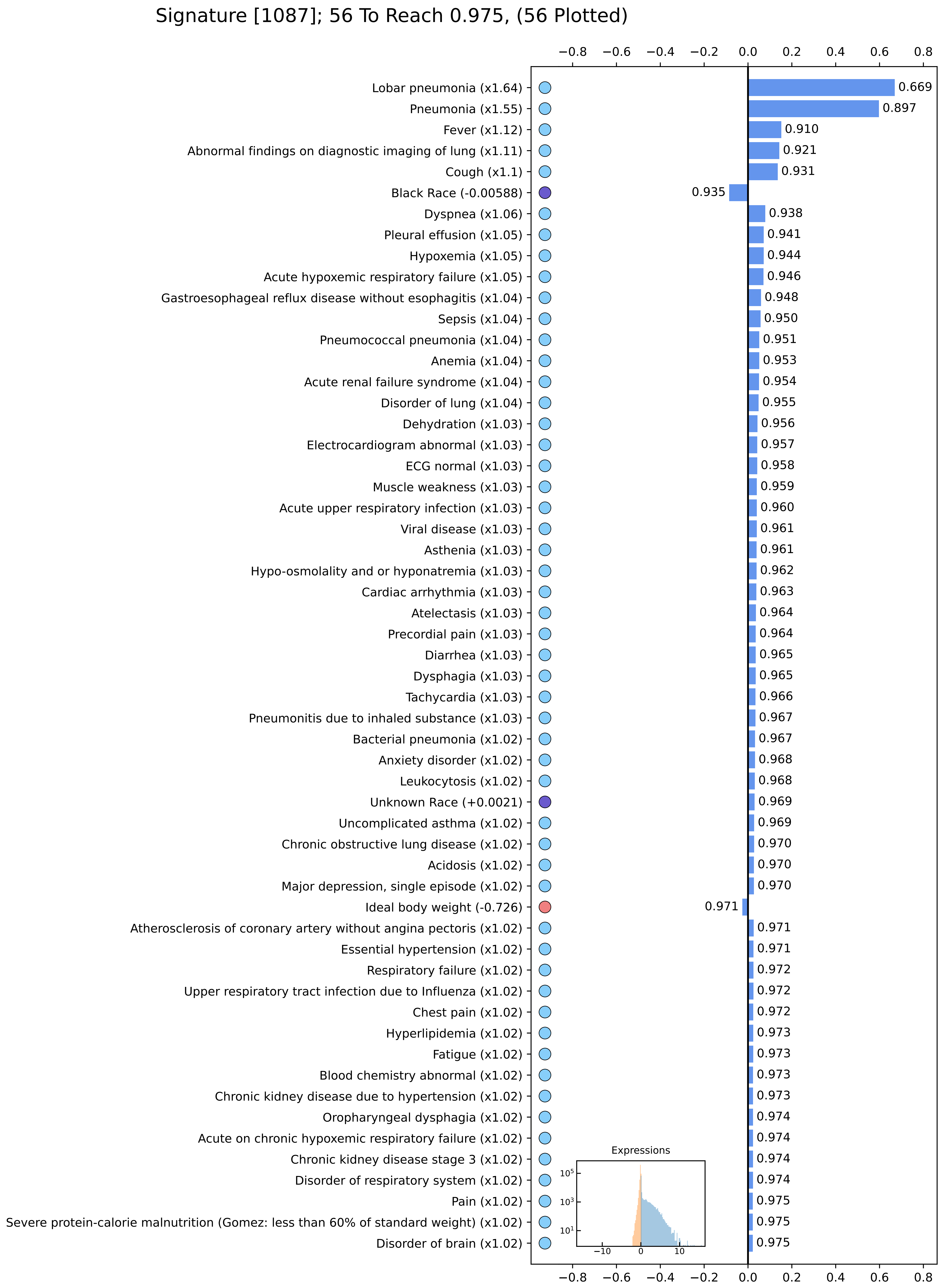
Signature [1087]; 56 To Reach 0.975, (56 Plotted)
Lobar pneumonia (x1.64) 0.669
Pneumonia (x1.55) 0.897
Fever (x1.12) 0.910
Abnormal findings on diagnostic imaging of lung (x1.11) 0.921
Cough (x1.1) 0.931
Black Race (-0.00588) 0.935
Dyspnea (x1.06) 0.938
Pleural effusion (x1.05) 0.941
Hypoxemia (x1.05) 0.944
Acute hypoxemic respiratory failure (x1.05) 0.946
Gastroesophageal reflux disease without esophagitis (x1.04) 0.948
Sepsis (x1.04) 0.950
Pneumococcal pneumonia (x1.04) 0.951
Anemia (x1.04) 0.953
Acute renal failure syndrome (x1.04) 0.954
Disorder of lung (x1.04) 0.955
Dehydration (x1.03) 0.956
Electrocardiogram abnormal (x1.03) 0.957
ECG normal (x1.03) 0.958
Muscle weakness (x1.03) 0.959
Acute upper respiratory infection (x1.03) 0.960
Viral disease (x1.03) 0.961
Asthenia (x1.03) 0.961
Hypo-osmolality and or hyponatremia (x1.03) 0.962
Cardiac arrhythmia (x1.03) 0.963
Atelectasis (x1.03) 0.964
Precordial pain (x1.03) 0.964
Diarrhea (x1.03) 0.965
Dysphagia (x1.03) 0.965
Tachycardia (x1.03) 0.966
Pneumonitis due to inhaled substance (x1.03) 0.967
Bacterial pneumonia (x1.02) 0.967
Anxiety disorder (x1.02) 0.968
Leukocytosis (x1.02) 0.968
Unknown Race (+0.0021) 0.969
Uncomplicated asthma (x1.02) 0.969
Chronic obstructive lung disease (x1.02) 0.970
Acidosis (x1.02) 0.970
Major depression, single episode (x1.02) 0.970
Ideal body weight (-0.726) 0.971
Atherosclerosis of coronary artery without angina pectoris (x1.02) 0.971
Essential hypertension (x1.02) 0.971
Respiratory failure (x1.02) 0.972
Upper respiratory tract infection due to Influenza (x1.02) 0.972
Chest pain (x1.02) 0.972
Hyperlipidemia (x1.02) 0.973
Fatigue (x1.02) 0.973
Blood chemistry abnormal (x1.02) 0.973
Chronic kidney disease due to hypertension (x1.02) 0.973
Oropharyngeal dysphagia (x1.02) 0.974
Acute on chronic hypoxemic respiratory failure (x1.02) 0.974
Chronic kidney disease stage 3 (x1.02) 0.974
Disorder of respiratory system (x1.02) 0.974
Pain (x1.02) 0.975
Severe protein-calorie malnutrition (Gomez: less than 60% of standard weight) (x1.02) 0.975
Disorder of brain (x1.02) 0.975
−0.8 −0.6 −0.4 −0.2 0.0 0.2 0.4 0.6 0.8
Expressions
10^5
10^3
10^1
−10 0 10

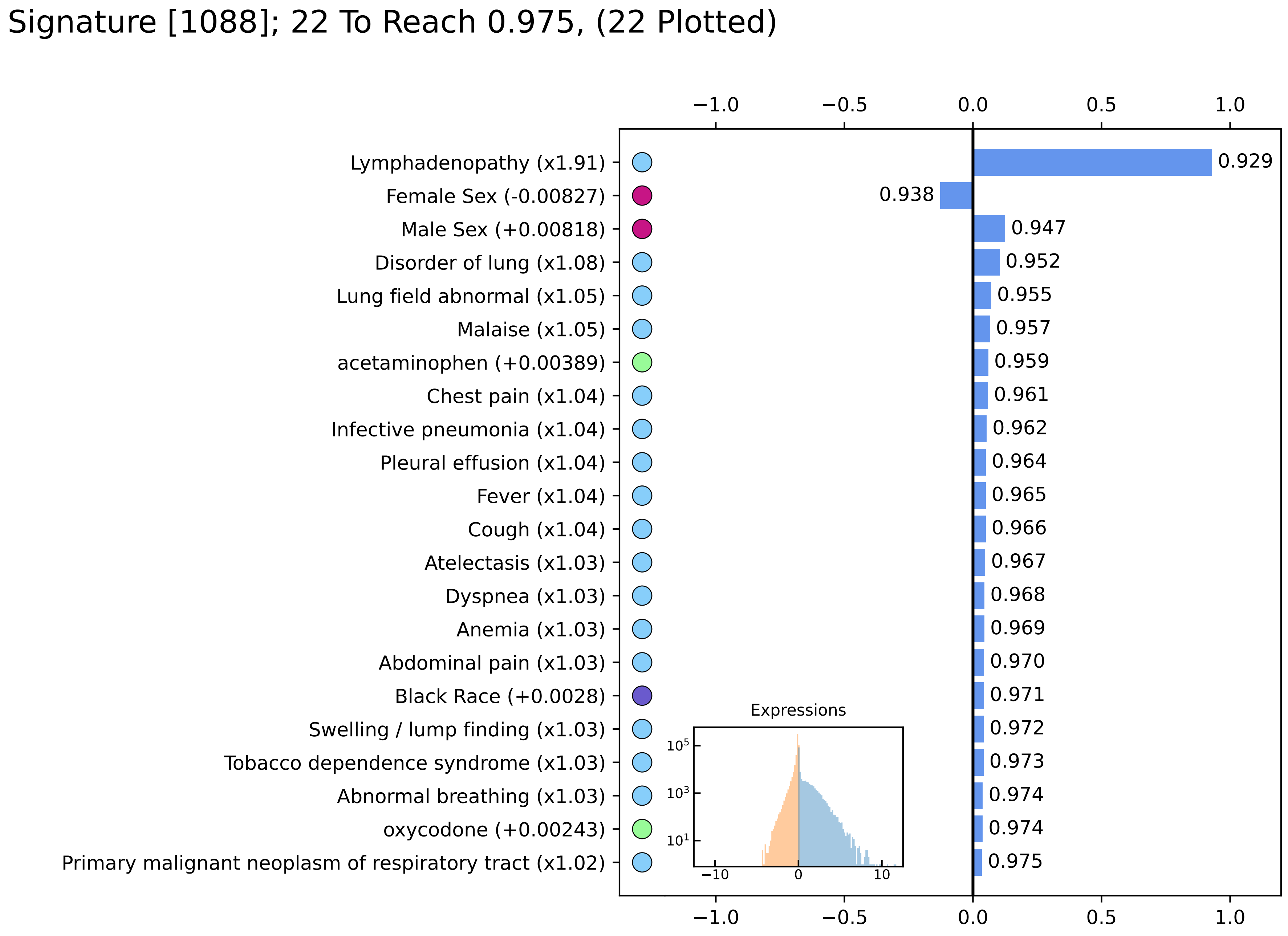

Signature [1088]; 22 To Reach 0.975, (22 Plotted)
Lymphadenopathy (x1.91)
Female Sex (-0.00827)
Male Sex (+0.00818)
Disorder of lung (x1.08)
Lung field abnormal (x1.05)
Malaise (x1.05)
acetaminophen (+0.00389)
Chest pain (x1.04)
Infective pneumonia (x1.04)
Pleural effusion (x1.04)
Fever (x1.04)
Cough (x1.04)
Atelectasis (x1.03)
Dyspnea (x1.03)
Anemia (x1.03)
Abdominal pain (x1.03)
Black Race (+0.0028)
Swelling / lump finding (x1.03)
Tobacco dependence syndrome (x1.03)
Abnormal breathing (x1.03)
oxycodone (+0.00243)
Primary malignant neoplasm of respiratory tract (x1.02)
0.929
0.938
0.947
0.952
0.955
0.957
0.959
0.961
0.962
0.964
0.965
0.966
0.967
0.968
0.969
0.970
0.971
0.972
0.973
0.974
0.974
0.975
−1.0
−0.5
0.0
0.5
1.0
Expressions
10^5
10^3
10^1
−10
0
10

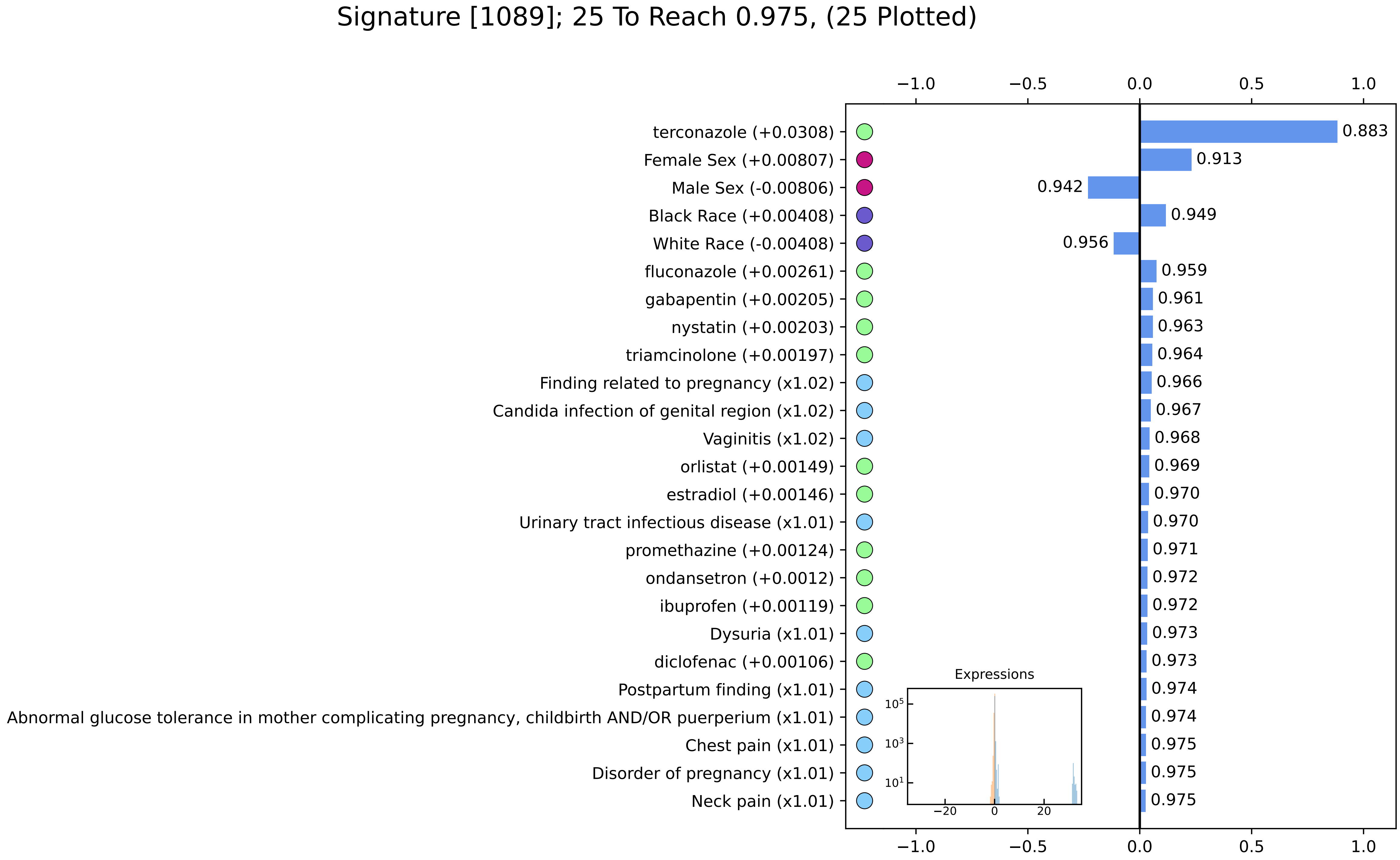

Signature [1089]; 25 To Reach 0.975, (25 Plotted)
terconazole (+0.0308)
Female Sex (+0.00807)
Male Sex (-0.00806)
Black Race (+0.00408)
White Race (-0.00408)
fluconazole (+0.00261)
gabapentin (+0.00205)
nystatin (+0.00203)
triamcinolone (+0.00197)
Finding related to pregnancy (x1.02)
Candida infection of genital region (x1.02)
Vaginitis (x1.02)
orlistat (+0.00149)
estradiol (+0.00146)
Urinary tract infectious disease (x1.01)
promethazine (+0.00124)
ondansetron (+0.0012)
ibuprofen (+0.00119)
Dysuria (x1.01)
diclofenac (+0.00106)
Postpartum finding (x1.01)
Abnormal glucose tolerance in mother complicating pregnancy, childbirth AND/OR puerperium (x1.01)
Chest pain (x1.01)
Disorder of pregnancy (x1.01)
Neck pain (x1.01)
0.883
0.913
0.942
0.949
0.956
0.959
0.961
0.963
0.964
0.966
0.967
0.968
0.969
0.970
0.970
0.971
0.972
0.972
0.973
0.973
0.974
0.974
0.975
0.975
0.975
−1.0
−0.5
0.0
0.5
1.0
Expressions
10^5
10^3
10^1
−20
0
20

Signature [1090]; 78 To Reach 0.975, (78 Plotted)

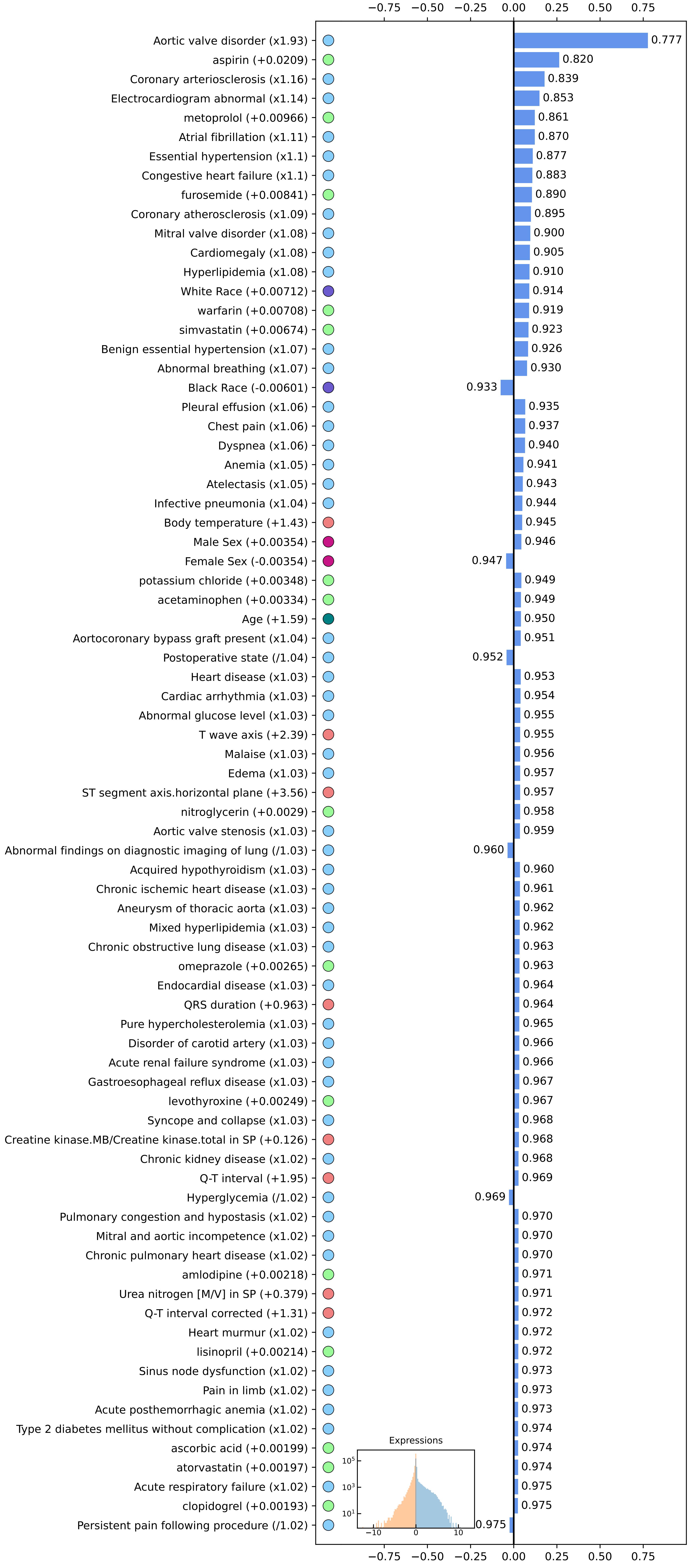

Signature [1091]; 8 To Reach 0.975, (10 Plotted)

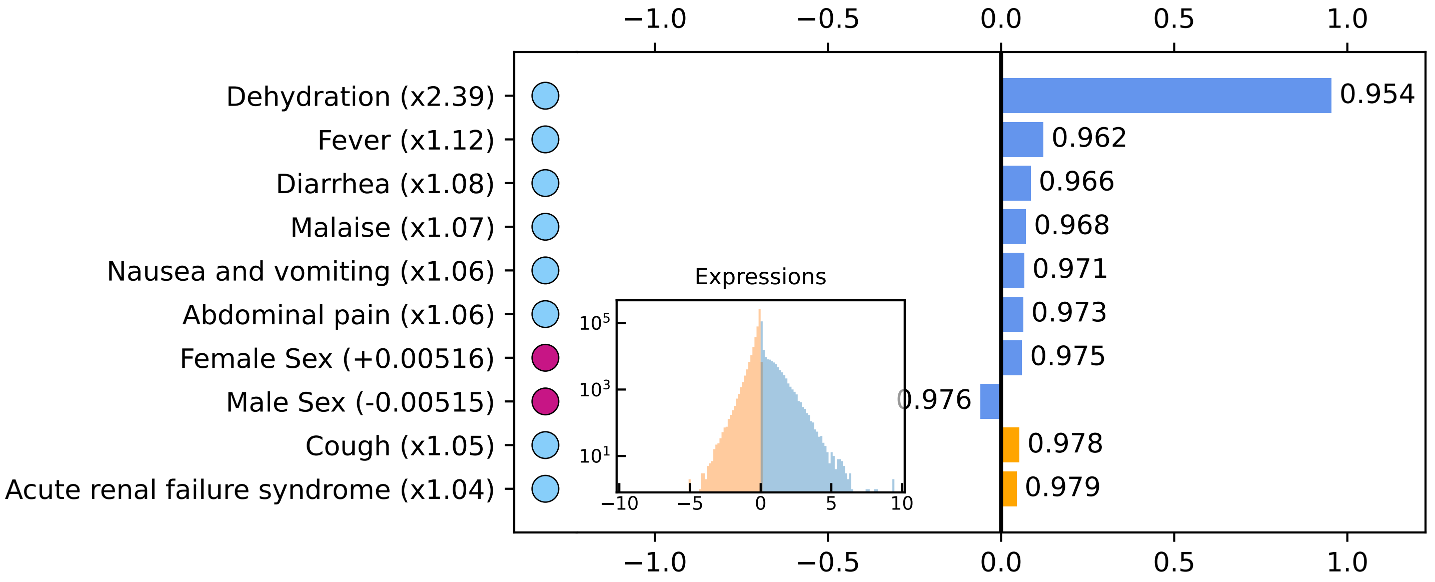

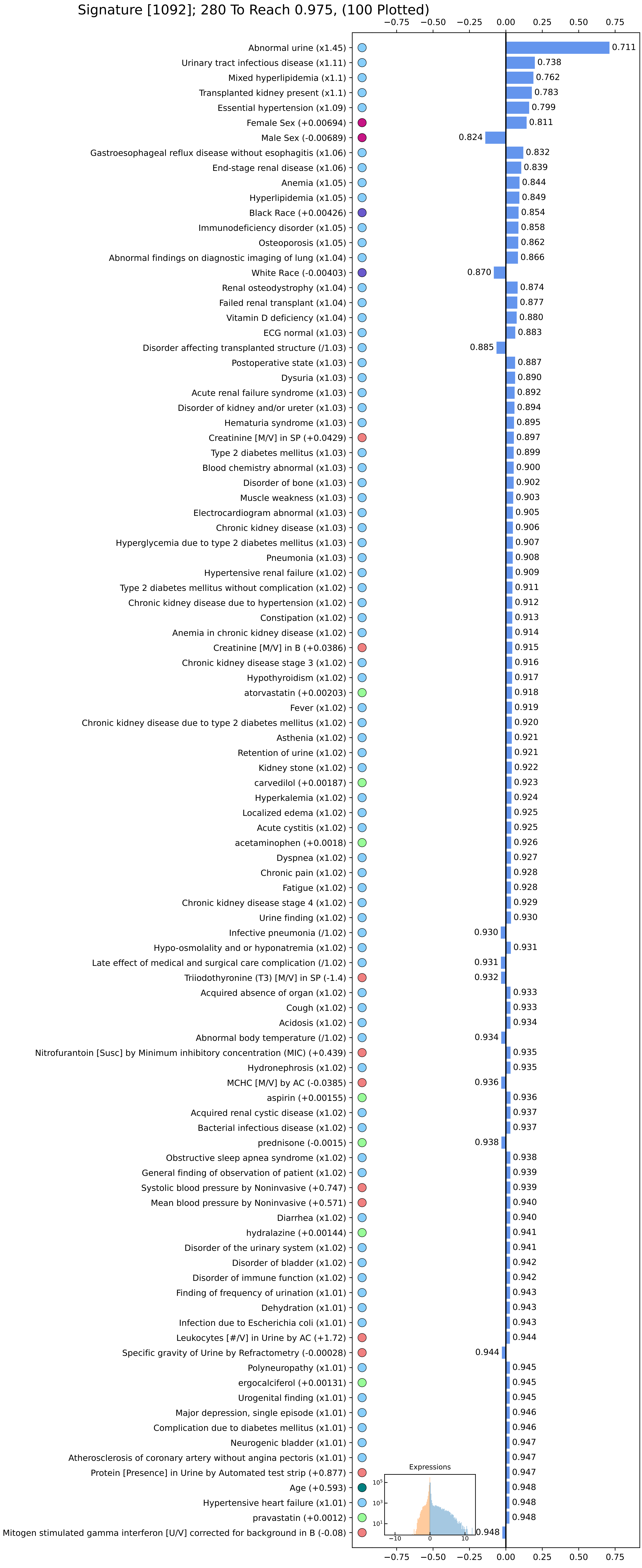
Signature [1092]; 280 To Reach 0.975, (100 Plotted)
−0.75 −0.50 −0.25 0.00 0.25 0.50 0.75
Abnormal urine (x1.45) 0.711
Urinary tract infectious disease (x1.11) 0.738
Mixed hyperlipidemia (x1.1) 0.762
Transplanted kidney present (x1.1) 0.783
Essential hypertension (x1.09) 0.799
Female Sex (+0.00694) 0.811
Male Sex (-0.00689) 0.824
Gastroesophageal reflux disease without esophagitis (x1.06) 0.832
End-stage renal disease (x1.06) 0.839
Anemia (x1.05) 0.844
Hyperlipidemia (x1.05) 0.849
Black Race (+0.00426) 0.854
Immunodeficiency disorder (x1.05) 0.858
Osteoporosis (x1.05) 0.862
Abnormal findings on diagnostic imaging of lung (x1.04) 0.866
White Race (-0.00403) 0.870
Renal osteodystrophy (x1.04) 0.874
Failed renal transplant (x1.04) 0.877
Vitamin D deficiency (x1.04) 0.880
ECG normal (x1.03) 0.883
Disorder affecting transplanted structure (/1.03) 0.885
Postoperative state (x1.03) 0.887
Dysuria (x1.03) 0.890
Acute renal failure syndrome (x1.03) 0.892
Disorder of kidney and/or ureter (x1.03) 0.894
Hematuria syndrome (x1.03) 0.895
Creatinine [M/V] in SP (+0.0429) 0.897
Type 2 diabetes mellitus (x1.03) 0.899
Blood chemistry abnormal (x1.03) 0.900
Disorder of bone (x1.03) 0.902
Muscle weakness (x1.03) 0.903
Electrocardiogram abnormal (x1.03) 0.905
Chronic kidney disease (x1.03) 0.906
Hyperglycemia due to type 2 diabetes mellitus (x1.03) 0.907
Pneumonia (x1.03) 0.908
Hypertensive renal failure (x1.02) 0.909
Type 2 diabetes mellitus without complication (x1.02) 0.911
Chronic kidney disease due to hypertension (x1.02) 0.912
Constipation (x1.02) 0.913
Anemia in chronic kidney disease (x1.02) 0.914
Creatinine [M/V] in B (+0.0386) 0.915
Chronic kidney disease stage 3 (x1.02) 0.916
Hypothyroidism (x1.02) 0.917
atorvastatin (+0.00203) 0.918
Fever (x1.02) 0.919
Chronic kidney disease due to type 2 diabetes mellitus (x1.02) 0.920
Asthenia (x1.02) 0.921
Retention of urine (x1.02) 0.921
Kidney stone (x1.02) 0.922
carvedilol (+0.00187) 0.923
Hyperkalemia (x1.02) 0.924
Localized edema (x1.02) 0.925
Acute cystitis (x1.02) 0.925
acetaminophen (+0.0018) 0.926
Dyspnea (x1.02) 0.927
Chronic pain (x1.02) 0.928
Fatigue (x1.02) 0.928
Chronic kidney disease stage 4 (x1.02) 0.929
Urine finding (x1.02) 0.930
Infective pneumonia (/1.02) 0.930
Hypo-osmolality and or hyponatremia (x1.02) 0.931
Late effect of medical and surgical care complication (/1.02) 0.931
Triiodothyronine (T3) [M/V] in SP (-1.4) 0.932
Acquired absence of organ (x1.02) 0.933
Cough (x1.02) 0.933
Acidosis (x1.02) 0.934
Abnormal body temperature (/1.02) 0.934
Nitrofurantoin [Susc] by Minimum inhibitory concentration (MIC) (+0.439) 0.935
Hydronephrosis (x1.02) 0.935
MCHC [M/V] by AC (-0.0385) 0.936
aspirin (+0.00155) 0.936
Acquired renal cystic disease (x1.02) 0.937
Bacterial infectious disease (x1.02) 0.937
prednisone (-0.0015) 0.938
Obstructive sleep apnea syndrome (x1.02) 0.938
General finding of observation of patient (x1.02) 0.939
Systolic blood pressure by Noninvasive (+0.747) 0.939
Mean blood pressure by Noninvasive (+0.571) 0.940
Diarrhea (x1.02) 0.940
hydralazine (+0.00144) 0.941
Disorder of the urinary system (x1.02) 0.941
Disorder of bladder (x1.02) 0.942
Disorder of immune function (x1.02) 0.942
Finding of frequency of urination (x1.01) 0.943
Dehydration (x1.01) 0.943
Infection due to Escherichia coli (x1.01) 0.943
Leukocytes [#/V] in Urine by AC (+1.72) 0.944
Specific gravity of Urine by Refractometry (-0.00028) 0.944
Polyneuropathy (x1.01) 0.945
ergocalciferol (+0.00131) 0.945
Urogenital finding (x1.01) 0.945
Major depression, single episode (x1.01) 0.946
Complication due to diabetes mellitus (x1.01) 0.946
Neurogenic bladder (x1.01) 0.947
Atherosclerosis of coronary artery without angina pectoris (x1.01) 0.947
Protein [Presence] in Urine by Automated test strip (+0.877) 0.947
Age (+0.593) 0.948
Hypertensive heart failure (x1.01) 0.948
pravastatin (+0.0012) 0.948
Mitogen stimulated gamma interferon [U/V] corrected for background in B (-0.08) 0.948
Expressions
10^5 10^3 10^1
−10 0 10
−0.75 −0.50 −0.25 0.00 0.25 0.50 0.75

Signature [1093]; 1 To Reach 0.975, (10 Plotted)

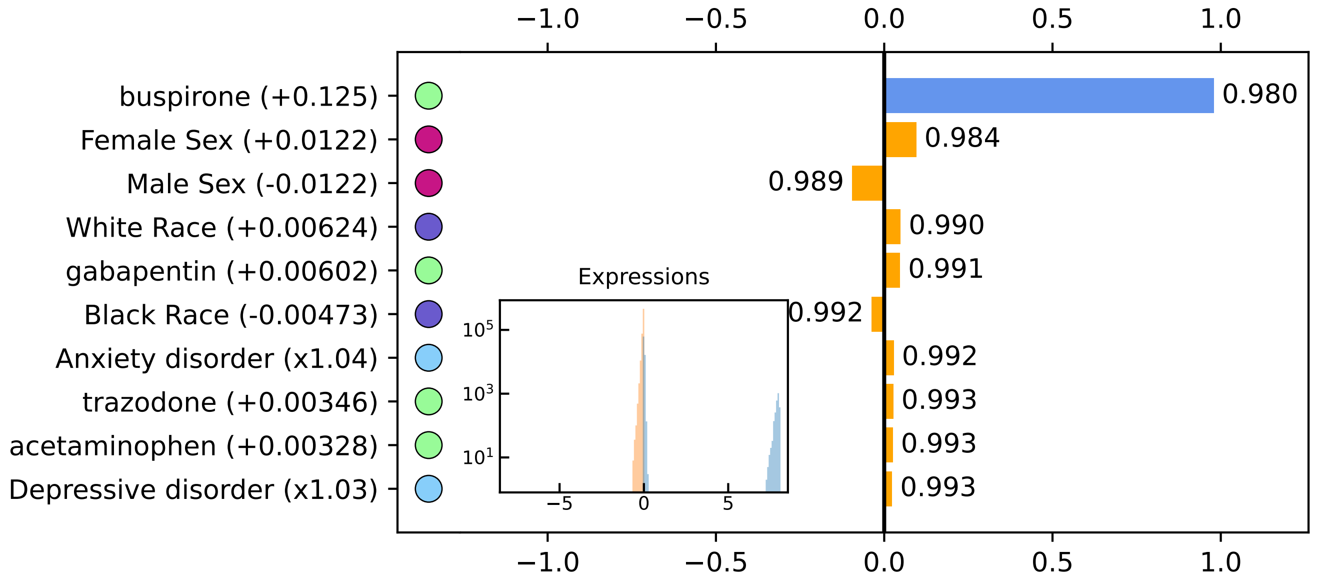

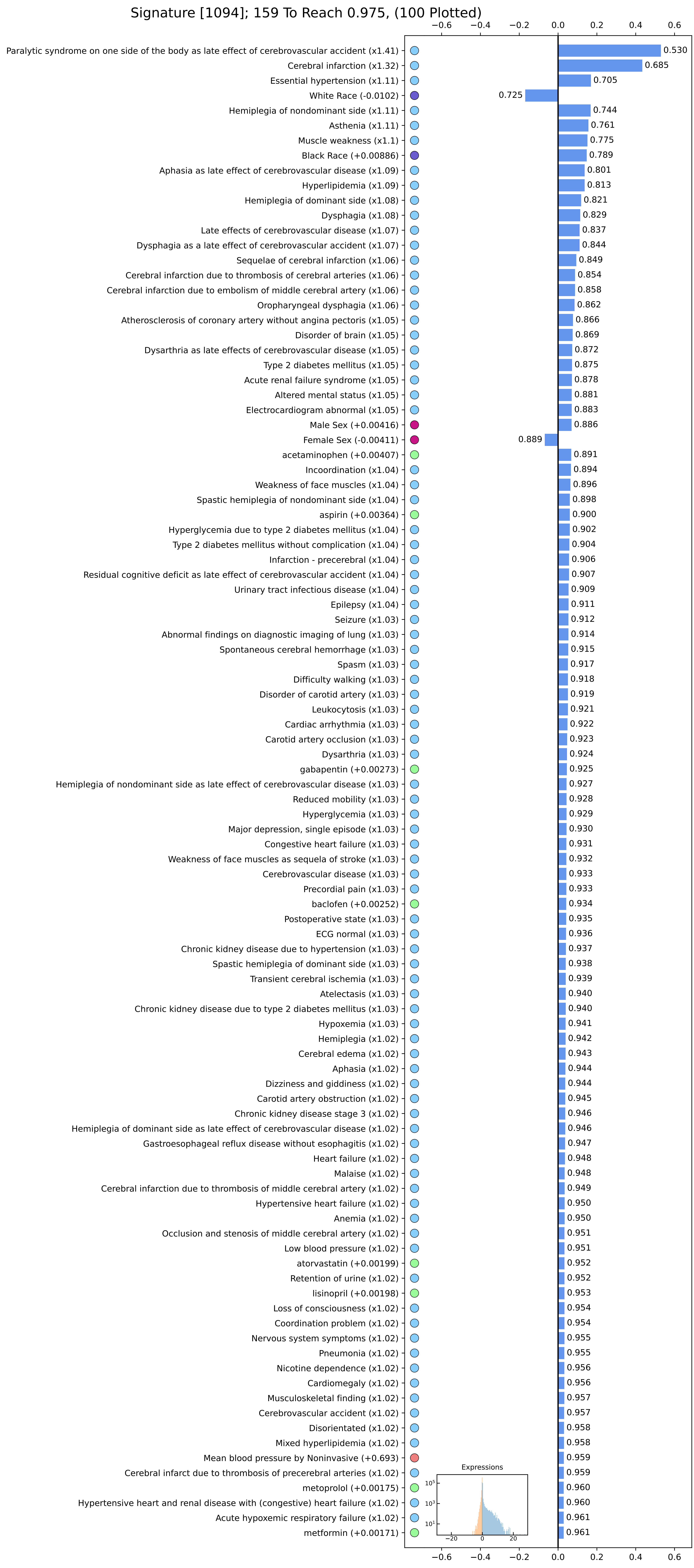

Signature [1094]; 159 To Reach 0.975, (100 Plotted)
−0.6 −0.4 −0.2 0.0 0.2 0.4 0.6
Paralytic syndrome on one side of the body as late effect of cerebrovascular accident (x1.41) 0.530
Cerebral infarction (x1.32) 0.685
Essential hypertension (x1.11) 0.705
White Race (-0.0102) 0.725
Hemiplegia of nondominant side (x1.11) 0.744
Asthenia (x1.11) 0.761
Muscle weakness (x1.1) 0.775
Black Race (+0.00886) 0.789
Aphasia as late effect of cerebrovascular disease (x1.09) 0.801
Hyperlipidemia (x1.09) 0.813
Hemiplegia of dominant side (x1.08) 0.821
Dysphagia (x1.08) 0.829
Late effects of cerebrovascular disease (x1.07) 0.837
Dysphagia as a late effect of cerebrovascular accident (x1.07) 0.844
Sequelae of cerebral infarction (x1.06) 0.849
Cerebral infarction due to thrombosis of cerebral arteries (x1.06) 0.854
Cerebral infarction due to embolism of middle cerebral artery (x1.06) 0.858
Oropharyngeal dysphagia (x1.06) 0.862
Atherosclerosis of coronary artery without angina pectoris (x1.05) 0.866
Disorder of brain (x1.05) 0.869
Dysarthria as late effects of cerebrovascular disease (x1.05) 0.872
Type 2 diabetes mellitus (x1.05) 0.875
Acute renal failure syndrome (x1.05) 0.878
Altered mental status (x1.05) 0.881
Electrocardiogram abnormal (x1.05) 0.883
Male Sex (+0.00416) 0.886
Female Sex (-0.00411) 0.889
acetaminophen (+0.00407) 0.891
Incoordination (x1.04) 0.894
Weakness of face muscles (x1.04) 0.896
Spastic hemiplegia of nondominant side (x1.04) 0.898
aspirin (+0.00364) 0.900
Hyperglycemia due to type 2 diabetes mellitus (x1.04) 0.902
Type 2 diabetes mellitus without complication (x1.04) 0.904
Infarction - precerebral (x1.04) 0.906
Residual cognitive deficit as late effect of cerebrovascular accident (x1.04) 0.907
Urinary tract infectious disease (x1.04) 0.909
Epilepsy (x1.04) 0.911
Seizure (x1.03) 0.912
Abnormal findings on diagnostic imaging of lung (x1.03) 0.914
Spontaneous cerebral hemorrhage (x1.03) 0.915
Spasm (x1.03) 0.917
Difficulty walking (x1.03) 0.918
Disorder of carotid artery (x1.03) 0.919
Leukocytosis (x1.03) 0.921
Cardiac arrhythmia (x1.03) 0.922
Carotid artery occlusion (x1.03) 0.923
Dysarthria (x1.03) 0.924
gabapentin (+0.00273) 0.925
Hemiplegia of nondominant side as late effect of cerebrovascular disease (x1.03) 0.927
Reduced mobility (x1.03) 0.928
Hyperglycemia (x1.03) 0.929
Major depression, single episode (x1.03) 0.930
Congestive heart failure (x1.03) 0.931
Weakness of face muscles as sequela of stroke (x1.03) 0.932
Cerebrovascular disease (x1.03) 0.933
Precordial pain (x1.03) 0.933
baclofen (+0.00252) 0.934
Postoperative state (x1.03) 0.935
ECG normal (x1.03) 0.936
Chronic kidney disease due to hypertension (x1.03) 0.937
Spastic hemiplegia of dominant side (x1.03) 0.938
Transient cerebral ischemia (x1.03) 0.939
Atelectasis (x1.03) 0.940
Chronic kidney disease due to type 2 diabetes mellitus (x1.03) 0.940
Hypoxemia (x1.03) 0.941
Hemiplegia (x1.02) 0.942
Cerebral edema (x1.02) 0.943
Aphasia (x1.02) 0.944
Dizziness and giddiness (x1.02) 0.944
Carotid artery obstruction (x1.02) 0.945
Chronic kidney disease stage 3 (x1.02) 0.946
Hemiplegia of dominant side as late effect of cerebrovascular disease (x1.02) 0.946
Gastroesophageal reflux disease without esophagitis (x1.02) 0.947
Heart failure (x1.02) 0.948
Malaise (x1.02) 0.948
Cerebral infarction due to thrombosis of middle cerebral artery (x1.02) 0.949
Hypertensive heart failure (x1.02) 0.950
Anemia (x1.02) 0.950
Occlusion and stenosis of middle cerebral artery (x1.02) 0.951
Low blood pressure (x1.02) 0.951
atorvastatin (+0.00199) 0.952
Retention of urine (x1.02) 0.952
lisinopril (+0.00198) 0.953
Loss of consciousness (x1.02) 0.954
Coordination problem (x1.02) 0.954
Nervous system symptoms (x1.02) 0.955
Pneumonia (x1.02) 0.955
Nicotine dependence (x1.02) 0.956
Cardiomegaly (x1.02) 0.956
Musculoskeletal finding (x1.02) 0.957
Cerebrovascular accident (x1.02) 0.957
Disorientated (x1.02) 0.958
Mixed hyperlipidemia (x1.02) 0.958
Mean blood pressure by Noninvasive (+0.693) 0.959
Cerebral infarct due to thrombosis of precerebral arteries (x1.02) 0.959
metoprolol (+0.00175) 0.960
Hypertensive heart and renal disease with (congestive) heart failure (x1.02) 0.960
Acute hypoxemic respiratory failure (x1.02) 0.961
metformin (+0.00171) 0.961
Expressions
−20 0 20

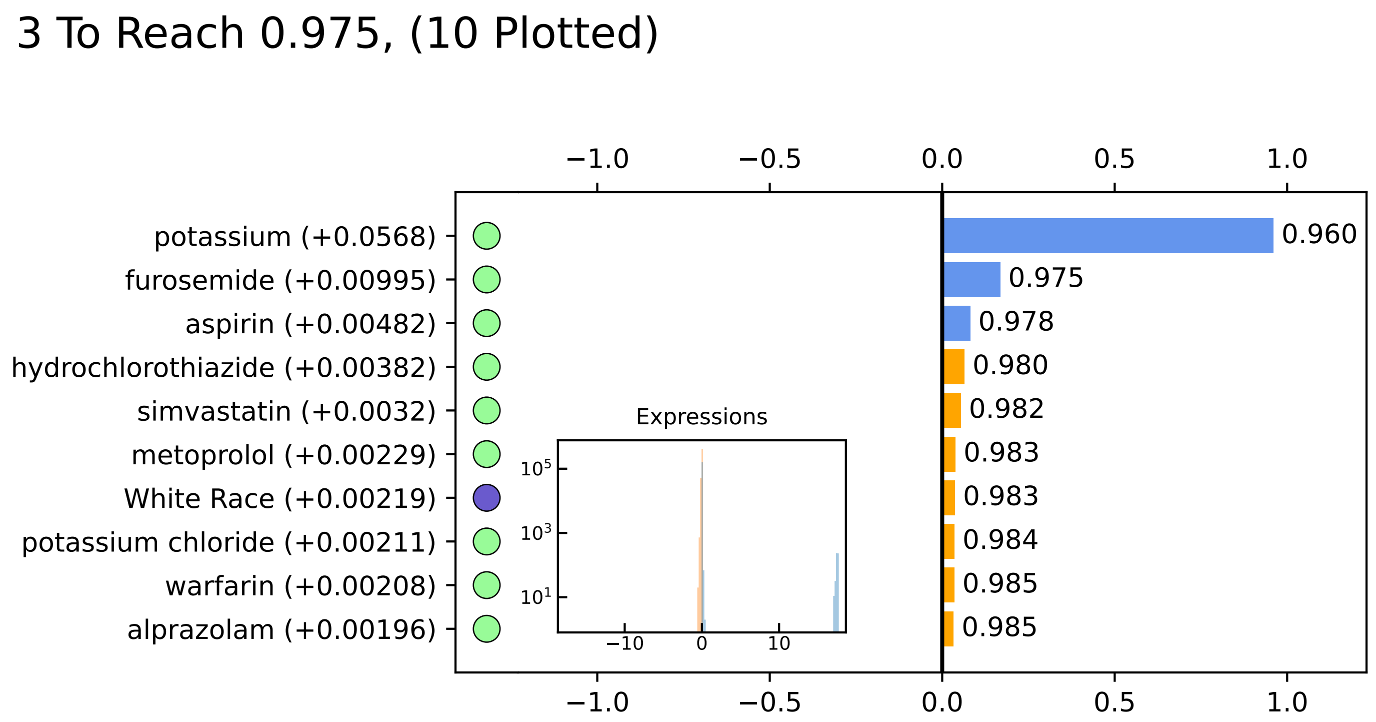
Signature [1095]; 3 To Reach 0.975, (10 Plotted)
potassium (+0.0568)
furosemide (+0.00995)
aspirin (+0.00482)
hydrochlorothiazide (+0.00382)
simvastatin (+0.0032)
metoprolol (+0.00229)
White Race (+0.00219)
potassium chloride (+0.00211)
warfarin (+0.00208)
alprazolam (+0.00196)
0.960
0.975
0.978
0.980
0.982
0.983
0.983
0.984
0.985
0.985
Expressions
−1.0
−0.5
0.0
0.5
1.0

Signature [1096]; 8 To Reach 0.975, (10 Plotted)

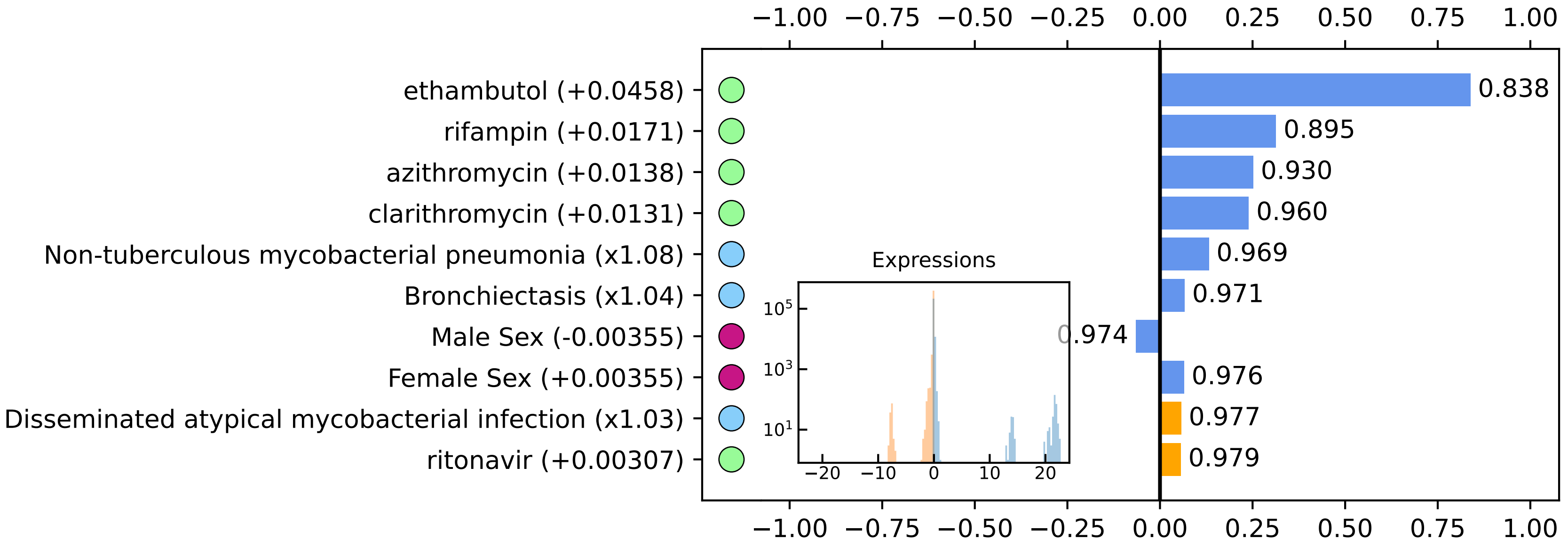

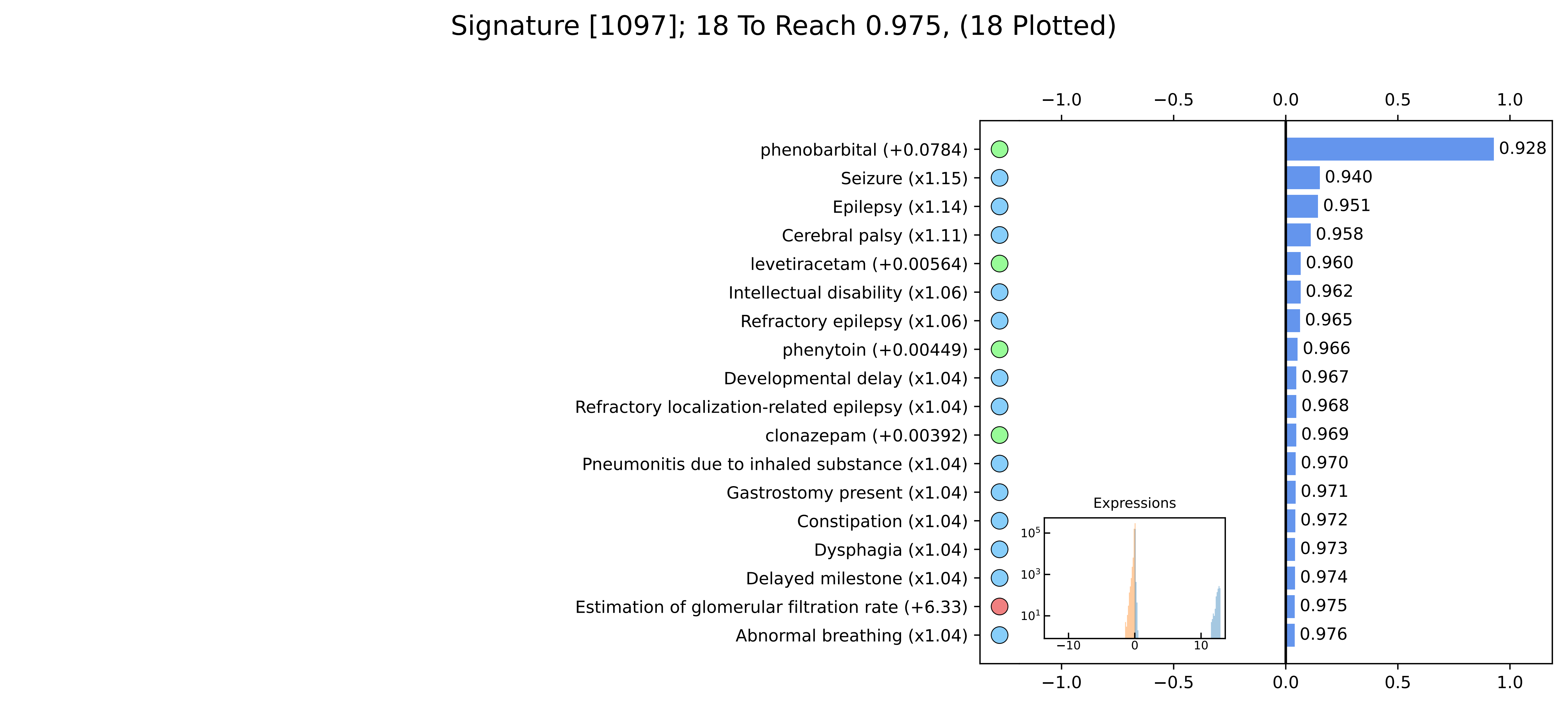
Signature [1097]; 18 To Reach 0.975, (18 Plotted)
phenobarbital (+0.0784)
Seizure (x1.15)
Epilepsy (x1.14)
Cerebral palsy (x1.11)
levetiracetam (+0.00564)
Intellectual disability (x1.06)
Refractory epilepsy (x1.06)
phenytoin (+0.00449)
Developmental delay (x1.04)
Refractory localization-related epilepsy (x1.04)
clonazepam (+0.00392)
Pneumonitis due to inhaled substance (x1.04)
Gastrostomy present (x1.04)
Constipation (x1.04)
Dysphagia (x1.04)
Delayed milestone (x1.04)
Estimation of glomerular filtration rate (+6.33)
Abnormal breathing (x1.04)
0.928
0.940
0.951
0.958
0.960
0.962
0.965
0.966
0.967
0.968
0.969
0.970
0.971
0.972
0.973
0.974
0.975
0.976
−1.0
−0.5
0.0
0.5
1.0
Expressions
−10
0
10

Signature [1098]; 3 To Reach 0.975, (10 Plotted)

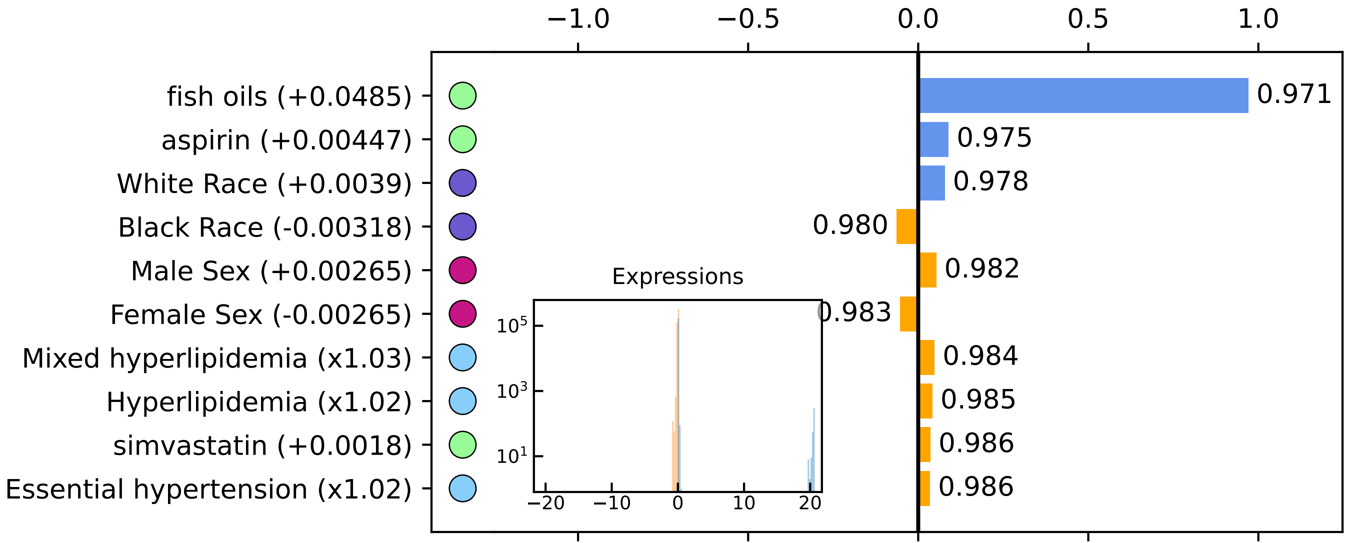

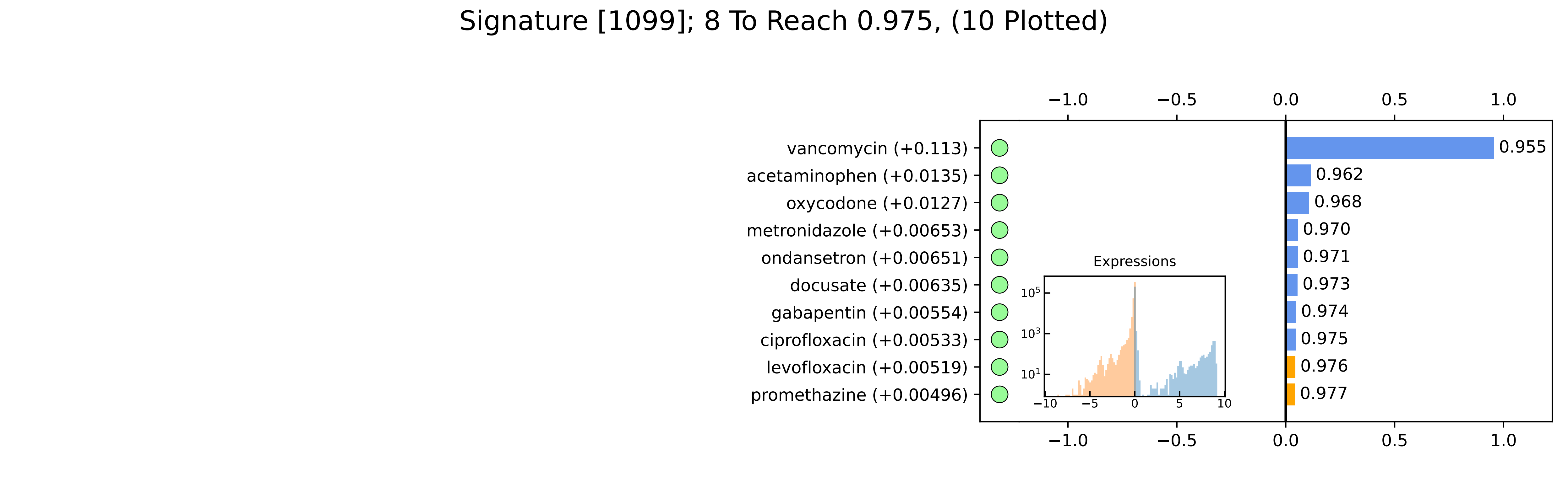

Signature [1099]; 8 To Reach 0.975, (10 Plotted)
vancomycin (+0.113)
acetaminophen (+0.0135)
oxycodone (+0.0127)
metronidazole (+0.00653)
ondansetron (+0.00651)
docusate (+0.00635)
gabapentin (+0.00554)
ciprofloxacin (+0.00533)
levofloxacin (+0.00519)
promethazine (+0.00496)
0.955
0.962
0.968
0.970
0.971
0.973
0.974
0.975
0.976
0.977
Expressions
−1.0
−0.5
0.0
0.5
1.0

Signature [1100]; 1 To Reach 0.975, (10 Plotted)

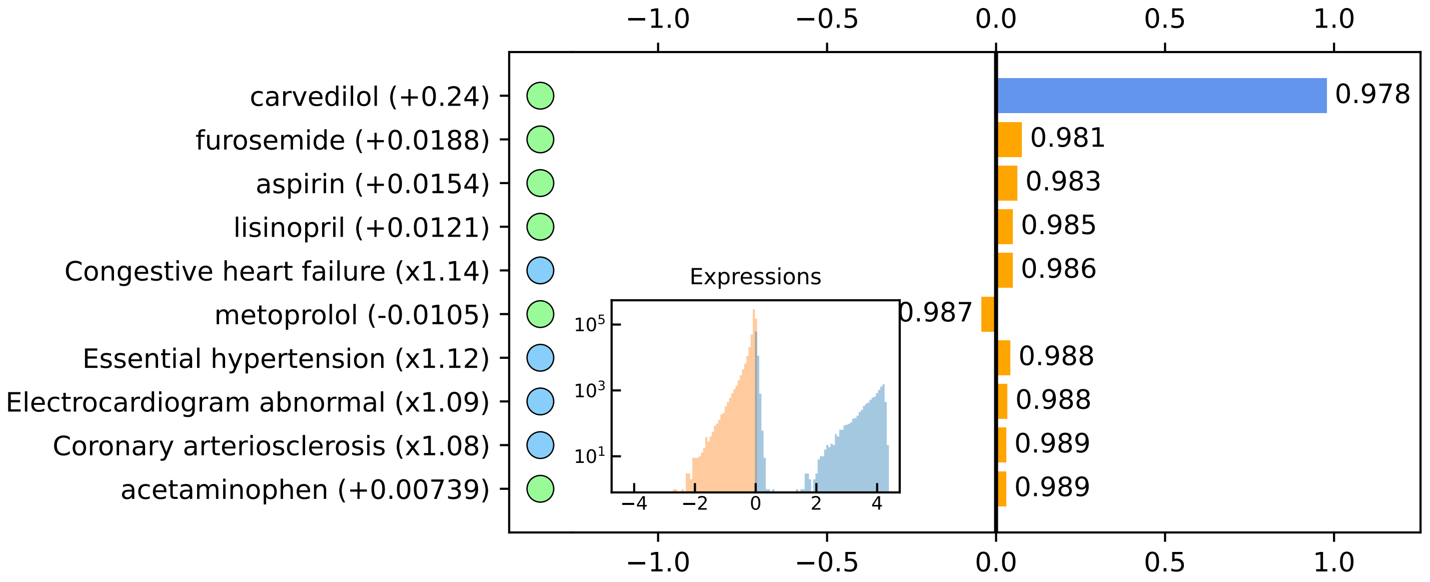

Signature [1101]; 3 To Reach 0.975, (10 Plotted)

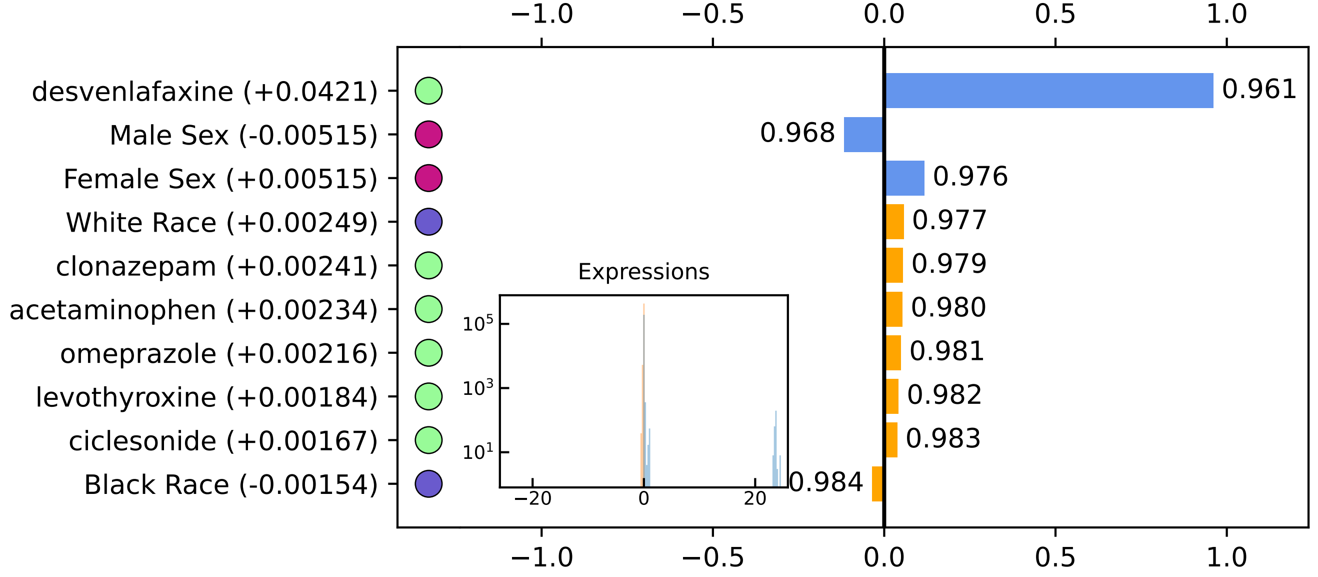

Signature [1102]; 223 To Reach 0.975, (100 Plotted)

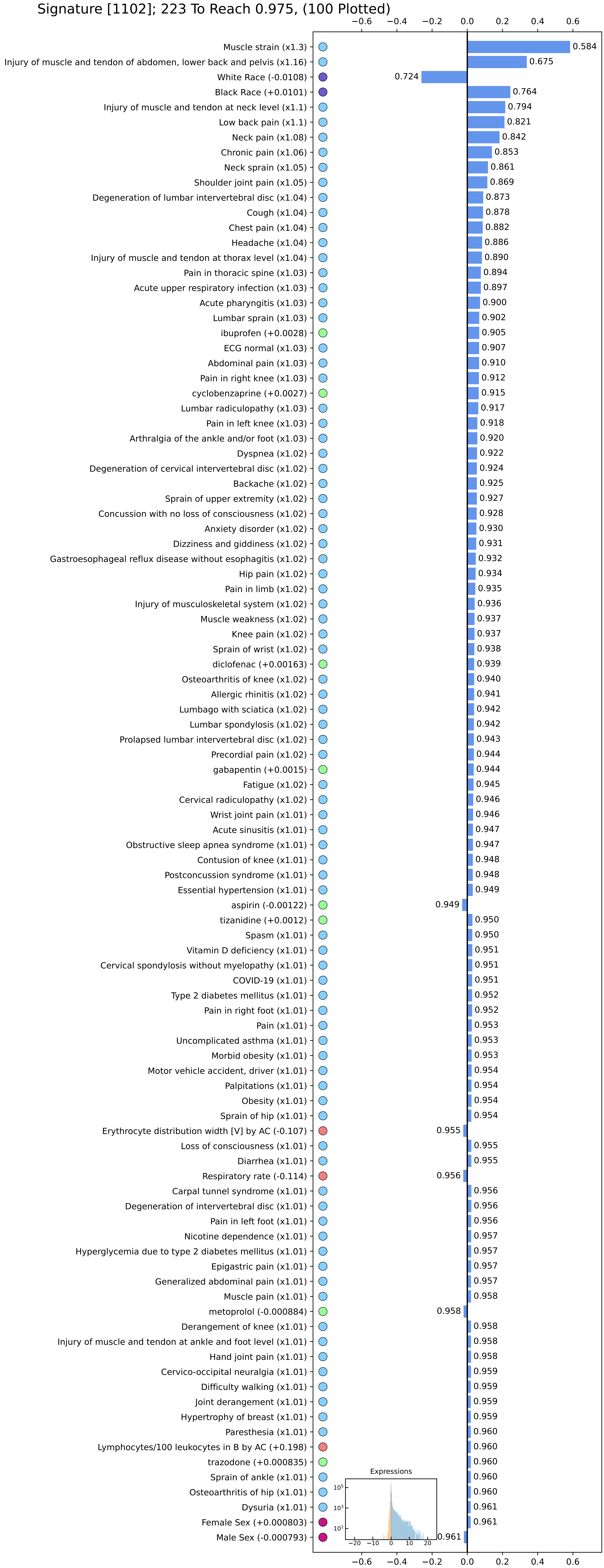

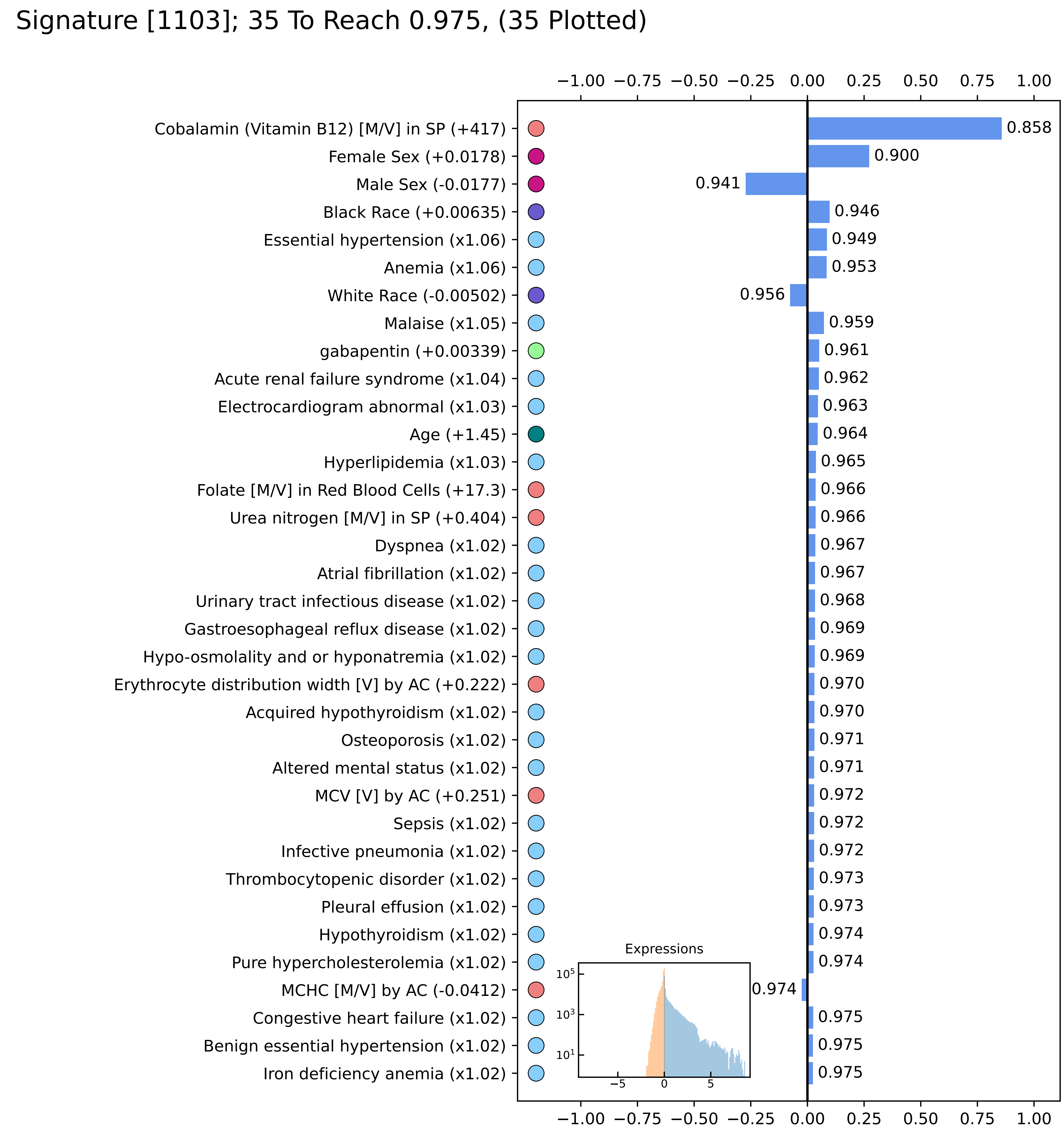
Signature [1103]; 35 To Reach 0.975, (35 Plotted)
Cobalamin (Vitamin B12) [M/V] in SP (+417)
Female Sex (+0.0178)
Male Sex (-0.0177)
Black Race (+0.00635)
Essential hypertension (x1.06)
Anemia (x1.06)
White Race (-0.00502)
Malaise (x1.05)
gabapentin (+0.00339)
Acute renal failure syndrome (x1.04)
Electrocardiogram abnormal (x1.03)
Age (+1.45)
Hyperlipidemia (x1.03)
Folate [M/V] in Red Blood Cells (+17.3)
Urea nitrogen [M/V] in SP (+0.404)
Dyspnea (x1.02)
Atrial fibrillation (x1.02)
Urinary tract infectious disease (x1.02)
Gastroesophageal reflux disease (x1.02)
Hypo-osmolality and or hyponatremia (x1.02)
Erythrocyte distribution width [V] by AC (+0.222)
Acquired hypothyroidism (x1.02)
Osteoporosis (x1.02)
Altered mental status (x1.02)
MCV [V] by AC (+0.251)
Sepsis (x1.02)
Infective pneumonia (x1.02)
Thrombocytopenic disorder (x1.02)
Pleural effusion (x1.02)
Hypothyroidism (x1.02)
Pure hypercholesterolemia (x1.02)
MCHC [M/V] by AC (-0.0412)
Congestive heart failure (x1.02)
Benign essential hypertension (x1.02)
Iron deficiency anemia (x1.02)
0.858
0.900
0.941
0.946
0.949
0.953
0.956
0.959
0.961
0.962
0.963
0.964
0.965
0.966
0.966
0.967
0.967
0.968
0.969
0.969
0.970
0.970
0.971
0.971
0.972
0.972
0.972
0.973
0.973
0.974
0.974
0.974
0.975
0.975
0.975
−1.00 −0.75 −0.50 −0.25 0.00 0.25 0.50 0.75 1.00
Expressions
10^5
10^3
10^1
−5 0 5

Signature [1104]; 43 To Reach 0.975, (43 Plotted)

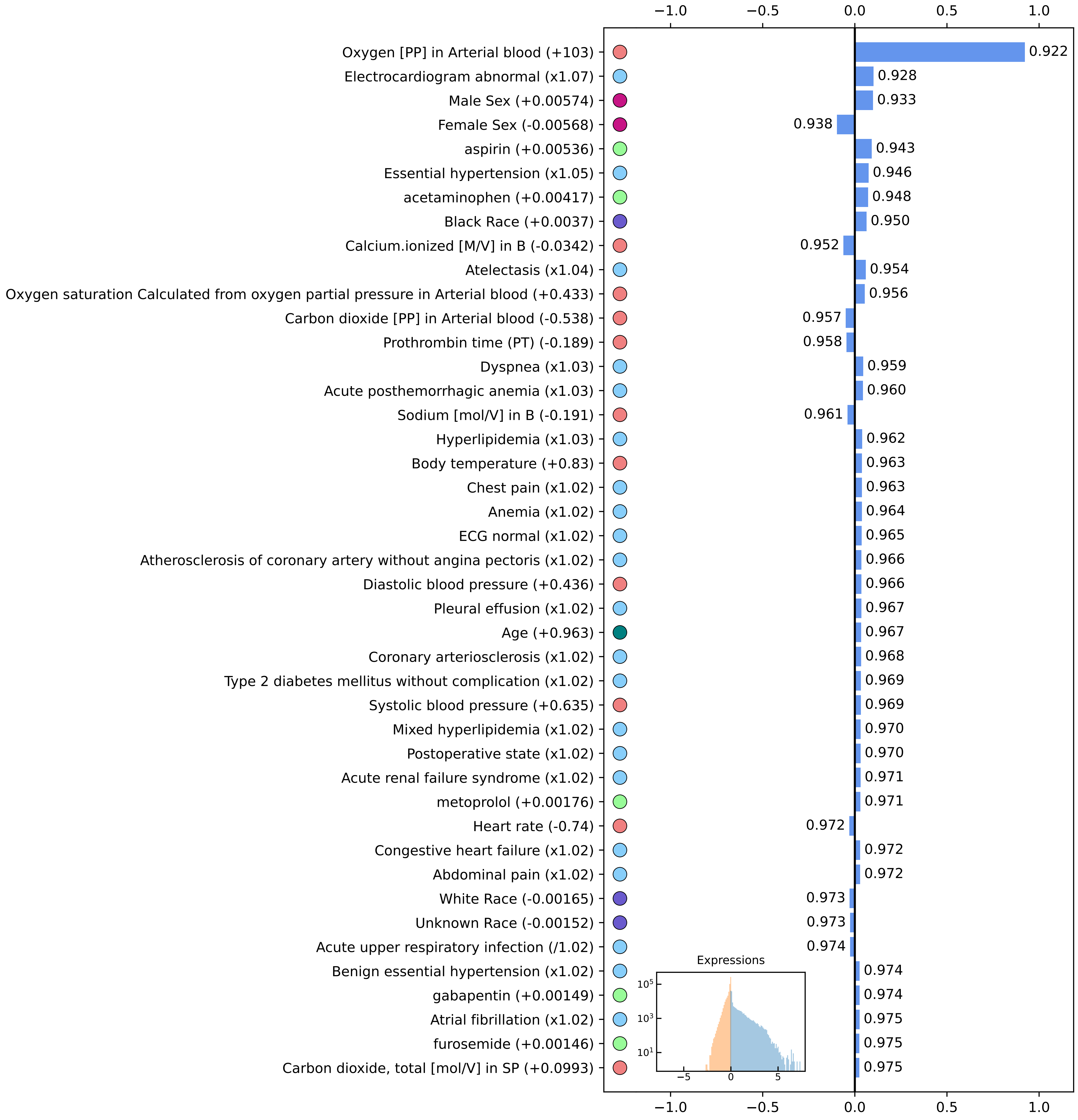

Signature [1105]; 75 To Reach 0.975, (75 Plotted)

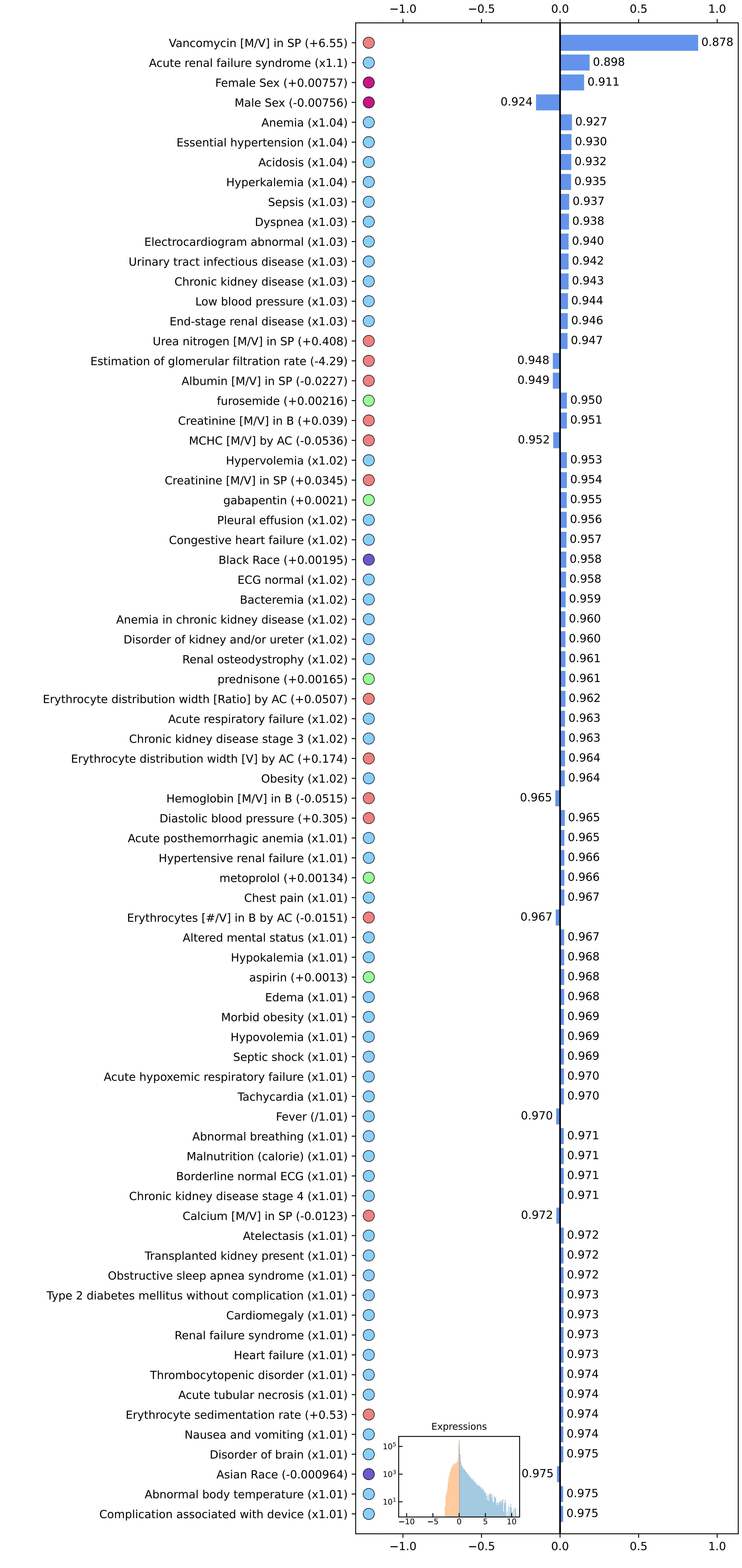

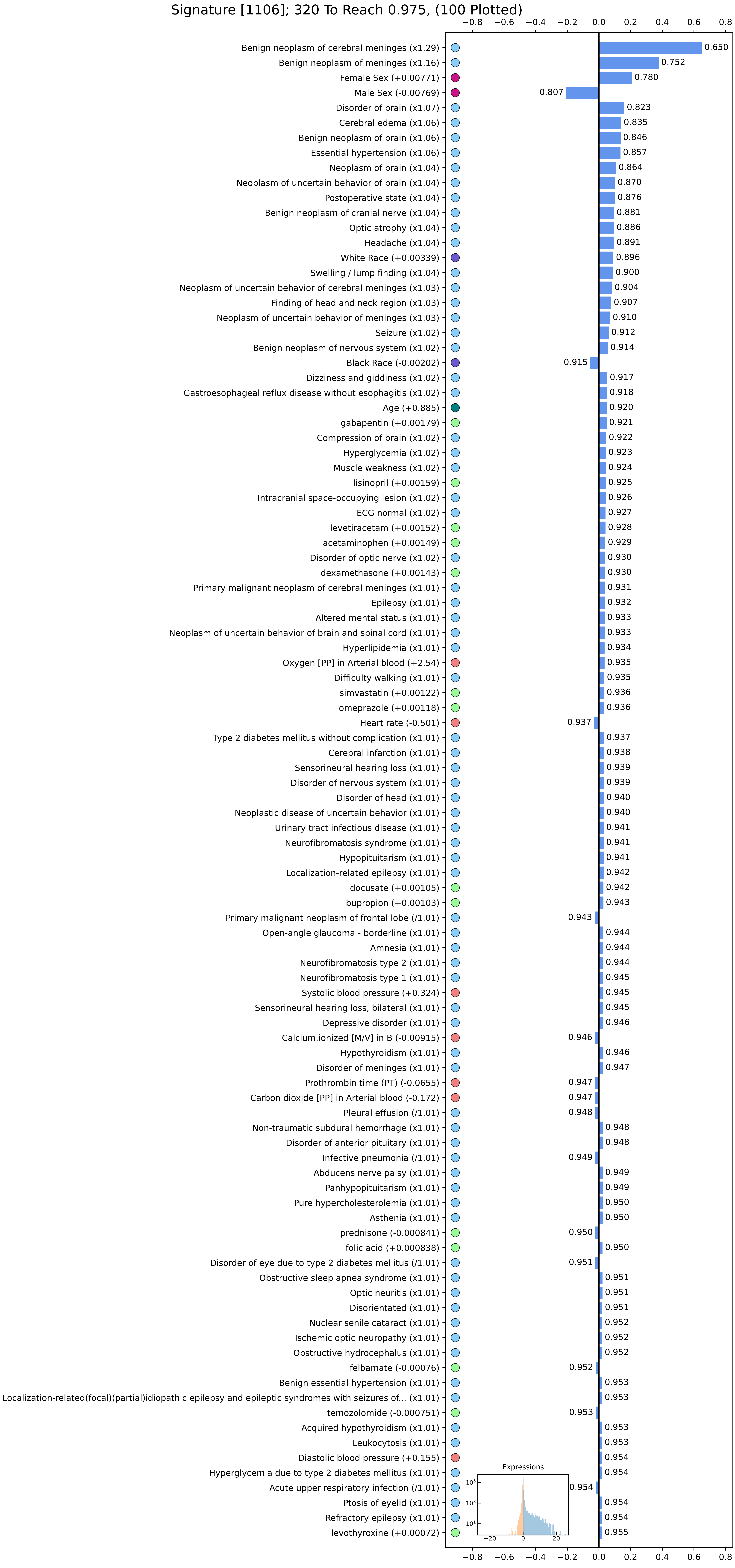

Signature [1106]; 320 To Reach 0.975, (100 Plotted)
−0.8 −0.6 −0.4 −0.2 0.0 0.2 0.4 0.6 0.8
Benign neoplasm of cerebral meninges (x1.29) 0.650
Benign neoplasm of meninges (x1.16) 0.752
Female Sex (+0.00771) 0.780
Male Sex (-0.00769) 0.807
Disorder of brain (x1.07) 0.823
Cerebral edema (x1.06) 0.835
Benign neoplasm of brain (x1.06) 0.846
Essential hypertension (x1.06) 0.857
Neoplasm of brain (x1.04) 0.864
Neoplasm of uncertain behavior of brain (x1.04) 0.870
Postoperative state (x1.04) 0.876
Benign neoplasm of cranial nerve (x1.04) 0.881
Optic atrophy (x1.04) 0.886
Headache (x1.04) 0.891
White Race (+0.00339) 0.896
Swelling / lump finding (x1.04) 0.900
Neoplasm of uncertain behavior of cerebral meninges (x1.03) 0.904
Finding of head and neck region (x1.03) 0.907
Neoplasm of uncertain behavior of meninges (x1.03) 0.910
Seizure (x1.02) 0.912
Benign neoplasm of nervous system (x1.02) 0.914
Black Race (-0.00202) 0.915
Dizziness and giddiness (x1.02) 0.917
Gastroesophageal reflux disease without esophagitis (x1.02) 0.918
Age (+0.885) 0.920
gabapentin (+0.00179) 0.921
Compression of brain (x1.02) 0.922
Hyperglycemia (x1.02) 0.923
Muscle weakness (x1.02) 0.924
lisinopril (+0.00159) 0.925
Intracranial space-occupying lesion (x1.02) 0.926
ECG normal (x1.02) 0.927
levetiracetam (+0.00152) 0.928
acetaminophen (+0.00149) 0.929
Disorder of optic nerve (x1.02) 0.930
dexamethasone (+0.00143) 0.930
Primary malignant neoplasm of cerebral meninges (x1.01) 0.931
Epilepsy (x1.01) 0.932
Altered mental status (x1.01) 0.933
Neoplasm of uncertain behavior of brain and spinal cord (x1.01) 0.933
Hyperlipidemia (x1.01) 0.934
Oxygen [PP] in Arterial blood (+2.54) 0.935
Difficulty walking (x1.01) 0.935
simvastatin (+0.00122) 0.936
omeprazole (+0.00118) 0.936
Heart rate (-0.501) 0.937
Type 2 diabetes mellitus without complication (x1.01) 0.937
Cerebral infarction (x1.01) 0.938
Sensorineural hearing loss (x1.01) 0.939
Disorder of nervous system (x1.01) 0.939
Disorder of head (x1.01) 0.940
Neoplastic disease of uncertain behavior (x1.01) 0.940
Urinary tract infectious disease (x1.01) 0.941
Neurofibromatosis syndrome (x1.01) 0.941
Hypopituitarism (x1.01) 0.941
Localization-related epilepsy (x1.01) 0.942
docusate (+0.00105) 0.942
bupropion (+0.00103) 0.943
Primary malignant neoplasm of frontal lobe (/1.01) 0.943
Open-angle glaucoma - borderline (x1.01) 0.944
Amnesia (x1.01) 0.944
Neurofibromatosis type 2 (x1.01) 0.944
Neurofibromatosis type 1 (x1.01) 0.945
Systolic blood pressure (+0.324) 0.945
Sensorineural hearing loss, bilateral (x1.01) 0.945
Depressive disorder (x1.01) 0.946
Calcium.ionized [M/V] in B (-0.00915) 0.946
Hypothyroidism (x1.01) 0.946
Disorder of meninges (x1.01) 0.947
Prothrombin time (PT) (-0.0655) 0.947
Carbon dioxide [PP] in Arterial blood (-0.172) 0.947
Pleural effusion (/1.01) 0.948
Non-traumatic subdural hemorrhage (x1.01) 0.948
Disorder of anterior pituitary (x1.01) 0.948
Infective pneumonia (/1.01) 0.949
Abducens nerve palsy (x1.01) 0.949
Panhypopituitarism (x1.01) 0.949
Pure hypercholesterolemia (x1.01) 0.950
Asthenia (x1.01) 0.950
prednisone (-0.000841) 0.950
folic acid (+0.000838) 0.950
Disorder of eye due to type 2 diabetes mellitus (/1.01) 0.951
Obstructive sleep apnea syndrome (x1.01) 0.951
Optic neuritis (x1.01) 0.951
Disorientated (x1.01) 0.951
Nuclear senile cataract (x1.01) 0.952
Ischemic optic neuropathy (x1.01) 0.952
Obstructive hydrocephalus (x1.01) 0.952
felbamate (-0.00076) 0.952
Benign essential hypertension (x1.01) 0.953
Localization-related(focal)(partial)idiopathic epilepsy and epileptic syndromes with seizures of... (x1.01) 0.953
temozolomide (-0.000751) 0.953
Acquired hypothyroidism (x1.01) 0.953
Leukocytosis (x1.01) 0.953
Diastolic blood pressure (+0.155) 0.954
Hyperglycemia due to type 2 diabetes mellitus (x1.01) 0.954
Acute upper respiratory infection (/1.01) 0.954
Ptosis of eyelid (x1.01) 0.954
Refractory epilepsy (x1.01) 0.954
levothyroxine (+0.00072) 0.955
Expressions
10^5
10^3
10^1
−20 0 20

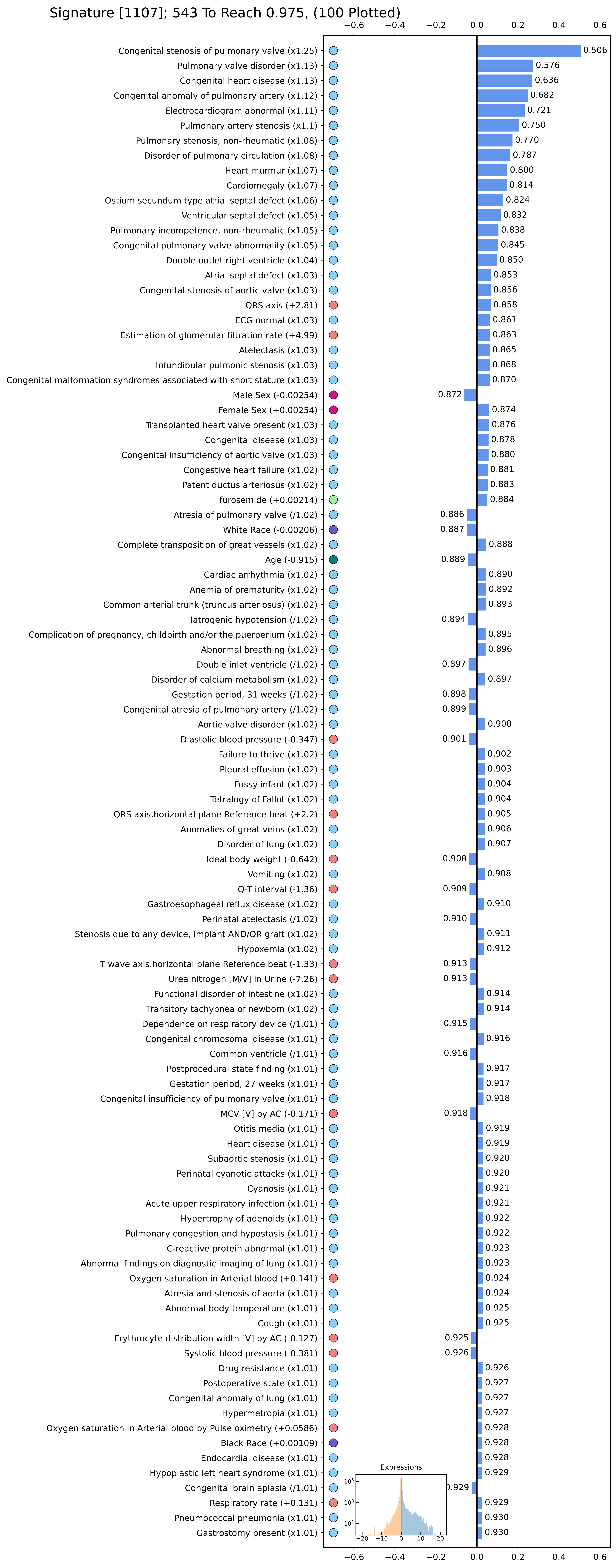

Signature [1107]; 543 To Reach 0.975, (100 Plotted)
Congenital stenosis of pulmonary valve (x1.25) 0.506
Pulmonary valve disorder (x1.13) 0.576
Congenital heart disease (x1.13) 0.636
Congenital anomaly of pulmonary artery (x1.12) 0.682
Electrocardiogram abnormal (x1.11) 0.721
Pulmonary artery stenosis (x1.1) 0.750
Pulmonary stenosis, non-rheumatic (x1.08) 0.770
Disorder of pulmonary circulation (x1.08) 0.787
Heart murmur (x1.07) 0.800
Cardiomegaly (x1.07) 0.814
Ostium secundum type atrial septal defect (x1.06) 0.824
Ventricular septal defect (x1.05) 0.832
Pulmonary incompetence, non-rheumatic (x1.05) 0.838
Congenital pulmonary valve abnormality (x1.05) 0.845
Double outlet right ventricle (x1.04) 0.850
Atrial septal defect (x1.03) 0.853
Congenital stenosis of aortic valve (x1.03) 0.856
QRS axis (+2.81) 0.858
ECG normal (x1.03) 0.861
Estimation of glomerular filtration rate (+4.99) 0.863
Atelectasis (x1.03) 0.865
Infundibular pulmonic stenosis (x1.03) 0.868
Congenital malformation syndromes associated with short stature (x1.03) 0.870
Male Sex (-0.00254) 0.872
Female Sex (+0.00254) 0.874
Transplanted heart valve present (x1.03) 0.876
Congenital disease (x1.03) 0.878
Congenital insufficiency of aortic valve (x1.03) 0.880
Congestive heart failure (x1.02) 0.881
Patent ductus arteriosus (x1.02) 0.883
furosemide (+0.00214) 0.884
Atresia of pulmonary valve (/1.02) 0.886
White Race (-0.00206) 0.887
Complete transposition of great vessels (x1.02) 0.888
Age (-0.915) 0.889
Cardiac arrhythmia (x1.02) 0.890
Anemia of prematurity (x1.02) 0.892
Common arterial trunk (truncus arteriosus) (x1.02) 0.893
Iatrogenic hypotension (/1.02) 0.894
Complication of pregnancy, childbirth and/or the puerperium (x1.02) 0.895
Abnormal breathing (x1.02) 0.896
Double inlet ventricle (/1.02) 0.897
Disorder of calcium metabolism (x1.02) 0.897
Gestation period, 31 weeks (/1.02) 0.898
Congenital atresia of pulmonary artery (/1.02) 0.899
Aortic valve disorder (x1.02) 0.900
Diastolic blood pressure (-0.347) 0.901
Failure to thrive (x1.02) 0.902
Pleural effusion (x1.02) 0.903
Fussy infant (x1.02) 0.904
Tetralogy of Fallot (x1.02) 0.904
QRS axis.horizontal plane Reference beat (+2.2) 0.905
Anomalies of great veins (x1.02) 0.906
Disorder of lung (x1.02) 0.907
Ideal body weight (-0.642) 0.908
Vomiting (x1.02) 0.908
Q-T interval (-1.36) 0.909
Gastroesophageal reflux disease (x1.02) 0.910
Perinatal atelectasis (/1.02) 0.910
Stenosis due to any device, implant AND/OR graft (x1.02) 0.911
Hypoxemia (x1.02) 0.912
T wave axis.horizontal plane Reference beat (-1.33) 0.913
Urea nitrogen [M/V] in Urine (-7.26) 0.913
Functional disorder of intestine (x1.02) 0.914
Transitory tachypnea of newborn (x1.02) 0.914
Dependence on respiratory device (/1.01) 0.915
Congenital chromosomal disease (x1.01) 0.916
Common ventricle (/1.01) 0.916
Postprocedural state finding (x1.01) 0.917
Gestation period, 27 weeks (x1.01) 0.917
Congenital insufficiency of pulmonary valve (x1.01) 0.918
MCV [V] by AC (-0.171) 0.918
Otitis media (x1.01) 0.919
Heart disease (x1.01) 0.919
Subaortic stenosis (x1.01) 0.920
Perinatal cyanotic attacks (x1.01) 0.920
Cyanosis (x1.01) 0.921
Acute upper respiratory infection (x1.01) 0.921
Hypertrophy of adenoids (x1.01) 0.922
Pulmonary congestion and hypostasis (x1.01) 0.922
C-reactive protein abnormal (x1.01) 0.923
Abnormal findings on diagnostic imaging of lung (x1.01) 0.923
Oxygen saturation in Arterial blood (+0.141) 0.924
Atresia and stenosis of aorta (x1.01) 0.924
Abnormal body temperature (x1.01) 0.925
Cough (x1.01) 0.925
Erythrocyte distribution width [V] by AC (-0.127) 0.925
Systolic blood pressure (-0.381) 0.926
Drug resistance (x1.01) 0.926
Postoperative state (x1.01) 0.927
Congenital anomaly of lung (x1.01) 0.927
Hypermetropia (x1.01) 0.927
Oxygen saturation in Arterial blood by Pulse oximetry (+0.0586) 0.928
Black Race (+0.00109) 0.928
Endocardial disease (x1.01) 0.928
Hypoplastic left heart syndrome (x1.01) 0.929
Congenital brain aplasia (/1.01) 0.929
Respiratory rate (+0.131) 0.929
Pneumococcal pneumonia (x1.01) 0.930
Gastrostomy present (x1.01) 0.930
Expressions

Signature [1108]; 186 To Reach 0.975, (100 Plotted)

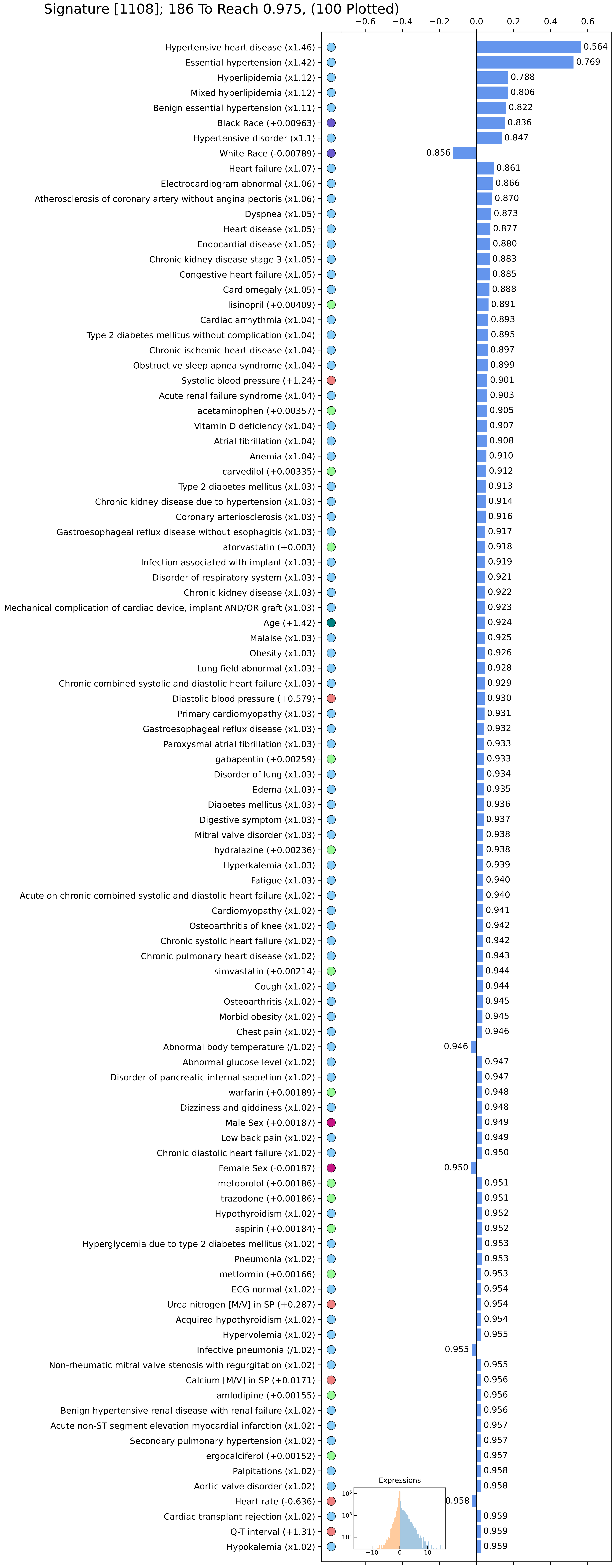

Signature [1109]; 6 To Reach 0.975, (10 Plotted)

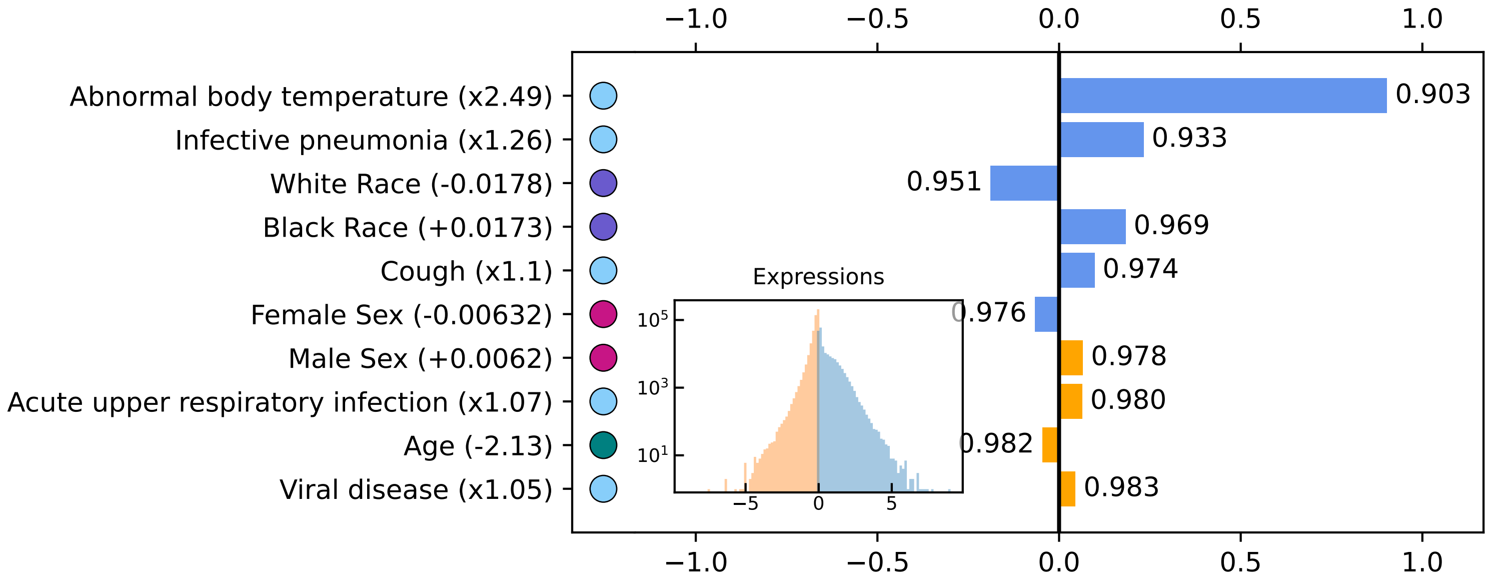

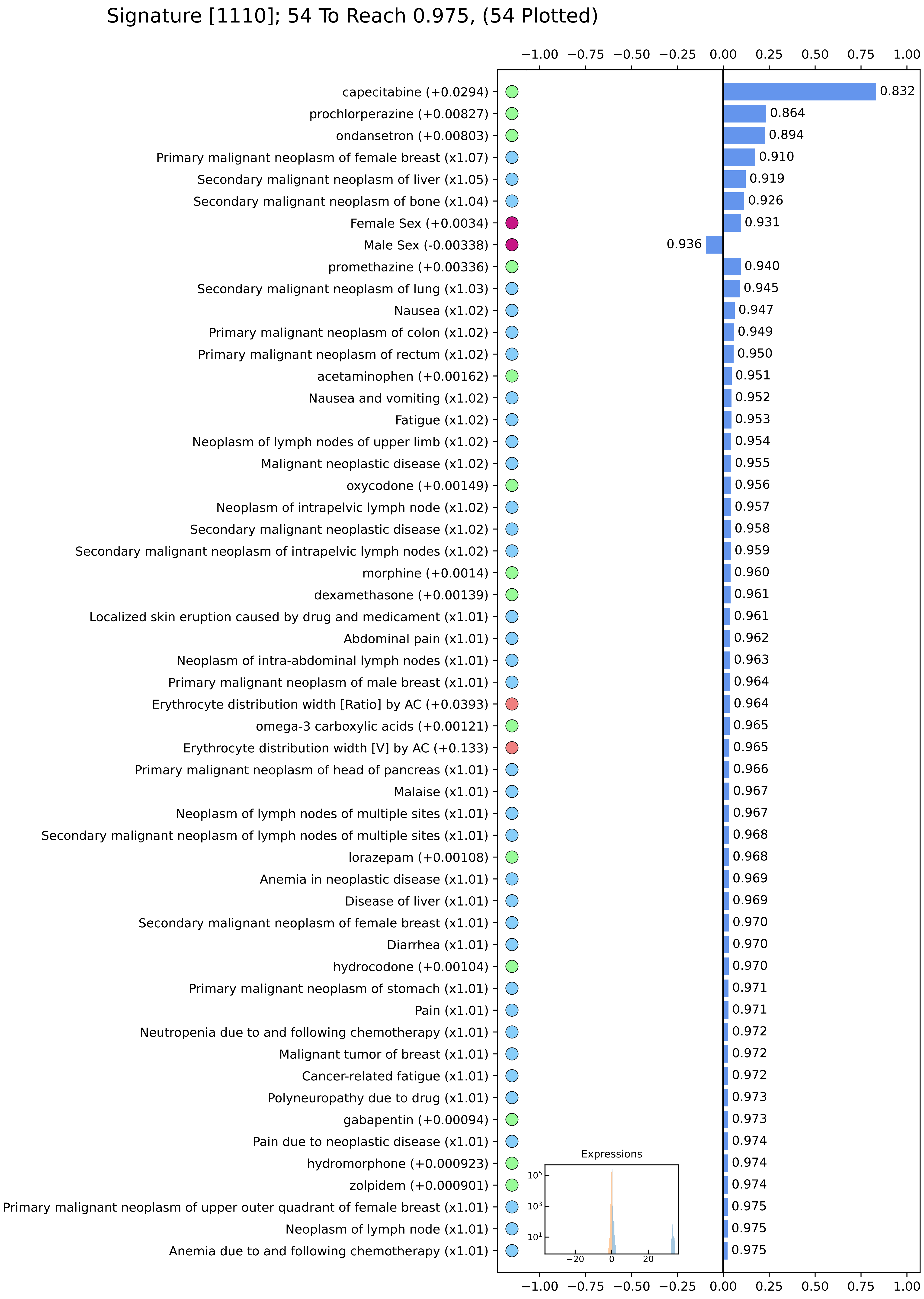

Signature [1110]; 54 To Reach 0.975, (54 Plotted)
capecitabine (+0.0294) 0.832
prochlorperazine (+0.00827) 0.864
ondansetron (+0.00803) 0.894
Primary malignant neoplasm of female breast (x1.07) 0.910
Secondary malignant neoplasm of liver (x1.05) 0.919
Secondary malignant neoplasm of bone (x1.04) 0.926
Female Sex (+0.0034) 0.931
Male Sex (-0.00338) 0.936
promethazine (+0.00336) 0.940
Secondary malignant neoplasm of lung (x1.03) 0.945
Nausea (x1.02) 0.947
Primary malignant neoplasm of colon (x1.02) 0.949
Primary malignant neoplasm of rectum (x1.02) 0.950
acetaminophen (+0.00162) 0.951
Nausea and vomiting (x1.02) 0.952
Fatigue (x1.02) 0.953
Neoplasm of lymph nodes of upper limb (x1.02) 0.954
Malignant neoplastic disease (x1.02) 0.955
oxycodone (+0.00149) 0.956
Neoplasm of intrapelvic lymph node (x1.02) 0.957
Secondary malignant neoplastic disease (x1.02) 0.958
Secondary malignant neoplasm of intrapelvic lymph nodes (x1.02) 0.959
morphine (+0.0014) 0.960
dexamethasone (+0.00139) 0.961
Localized skin eruption caused by drug and medicament (x1.01) 0.961
Abdominal pain (x1.01) 0.962
Neoplasm of intra-abdominal lymph nodes (x1.01) 0.963
Primary malignant neoplasm of male breast (x1.01) 0.964
Erythrocyte distribution width [Ratio] by AC (+0.0393) 0.964
omega-3 carboxylic acids (+0.00121) 0.965
Erythrocyte distribution width [V] by AC (+0.133) 0.965
Primary malignant neoplasm of head of pancreas (x1.01) 0.966
Malaise (x1.01) 0.967
Neoplasm of lymph nodes of multiple sites (x1.01) 0.967
Secondary malignant neoplasm of lymph nodes of multiple sites (x1.01) 0.968
lorazepam (+0.00108) 0.968
Anemia in neoplastic disease (x1.01) 0.969
Disease of liver (x1.01) 0.969
Secondary malignant neoplasm of female breast (x1.01) 0.970
Diarrhea (x1.01) 0.970
hydrocodone (+0.00104) 0.970
Primary malignant neoplasm of stomach (x1.01) 0.971
Pain (x1.01) 0.971
Neutropenia due to and following chemotherapy (x1.01) 0.972
Malignant tumor of breast (x1.01) 0.972
Cancer-related fatigue (x1.01) 0.972
Polyneuropathy due to drug (x1.01) 0.973
gabapentin (+0.00094) 0.973
Pain due to neoplastic disease (x1.01) 0.974
hydromorphone (+0.000923) 0.974
zolpidem (+0.000901) 0.974
Primary malignant neoplasm of upper outer quadrant of female breast (x1.01) 0.975
Neoplasm of lymph node (x1.01) 0.975
Anemia due to and following chemotherapy (x1.01) 0.975
−1.00 −0.75 −0.50 −0.25 0.00 0.25 0.50 0.75 1.00
Expressions
10^5
10^3
10^1
−20 0 20

Signature [1111]; 232 To Reach 0.975, (100 Plotted)

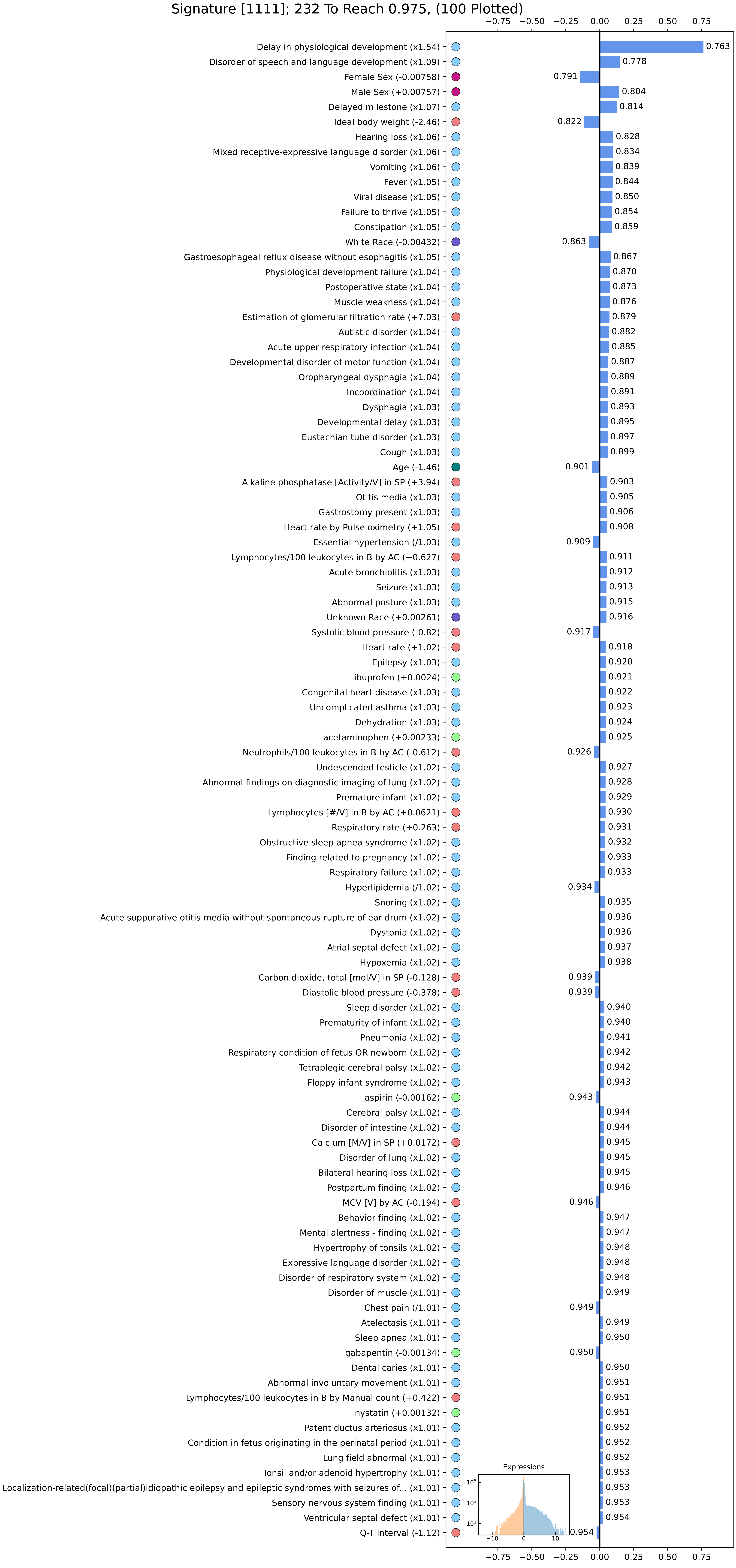

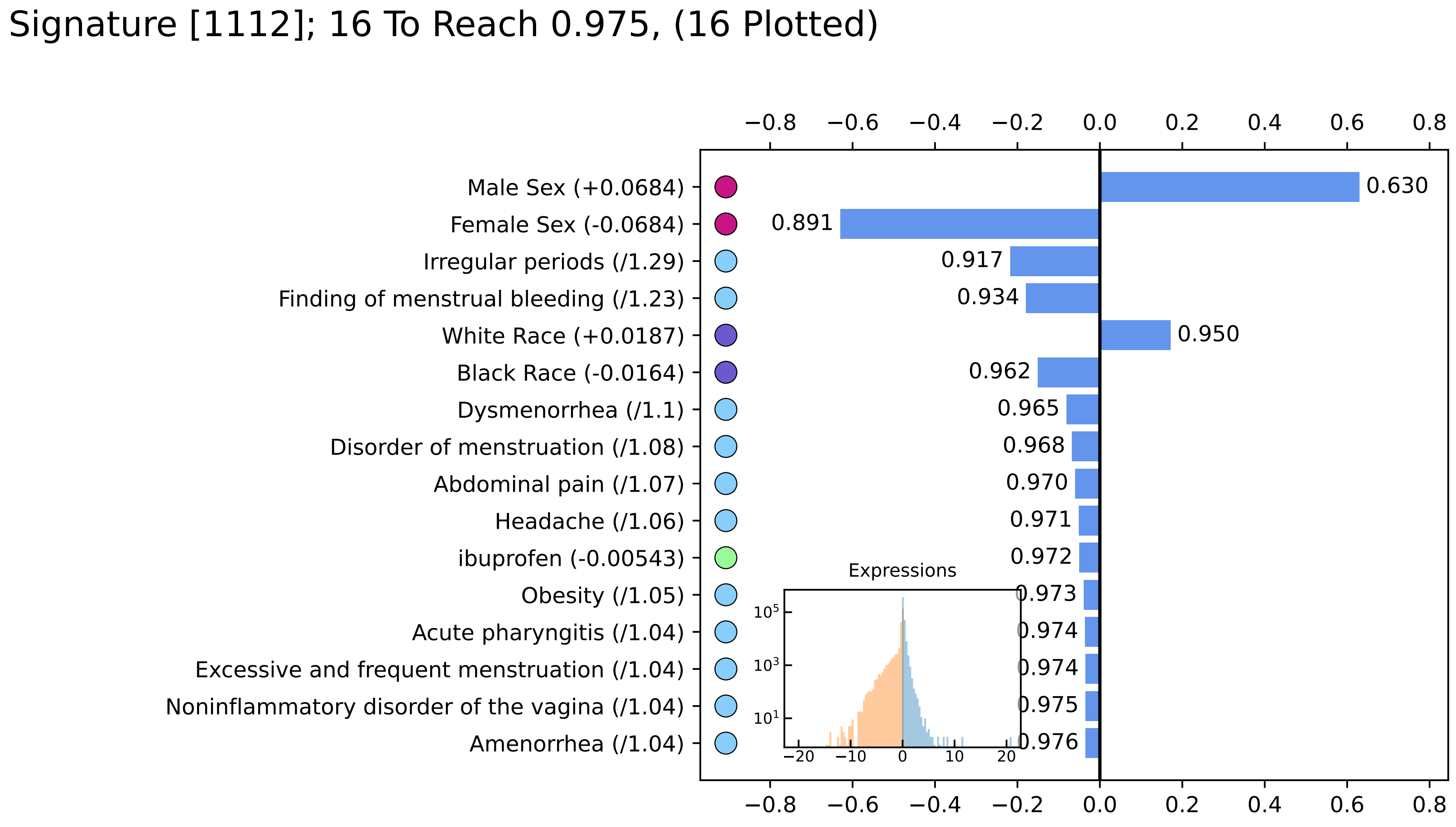
Signature [1112]; 16 To Reach 0.975, (16 Plotted)
Male Sex (+0.0684)
Female Sex (-0.0684)
Irregular periods (/1.29)
Finding of menstrual bleeding (/1.23)
White Race (+0.0187)
Black Race (-0.0164)
Dysmenorrhea (/1.1)
Disorder of menstruation (/1.08)
Abdominal pain (/1.07)
Headache (/1.06)
ibuprofen (-0.00543)
Obesity (/1.05)
Acute pharyngitis (/1.04)
Excessive and frequent menstruation (/1.04)
Noninflammatory disorder of the vagina (/1.04)
Amenorrhea (/1.04)
0.630
0.891
0.917
0.934
0.950
0.962
0.965
0.968
0.970
0.971
0.972
0.973
0.974
0.974
0.975
0.976
Expressions
−0.8 −0.6 −0.4 −0.2 0.0 0.2 0.4 0.6 0.8

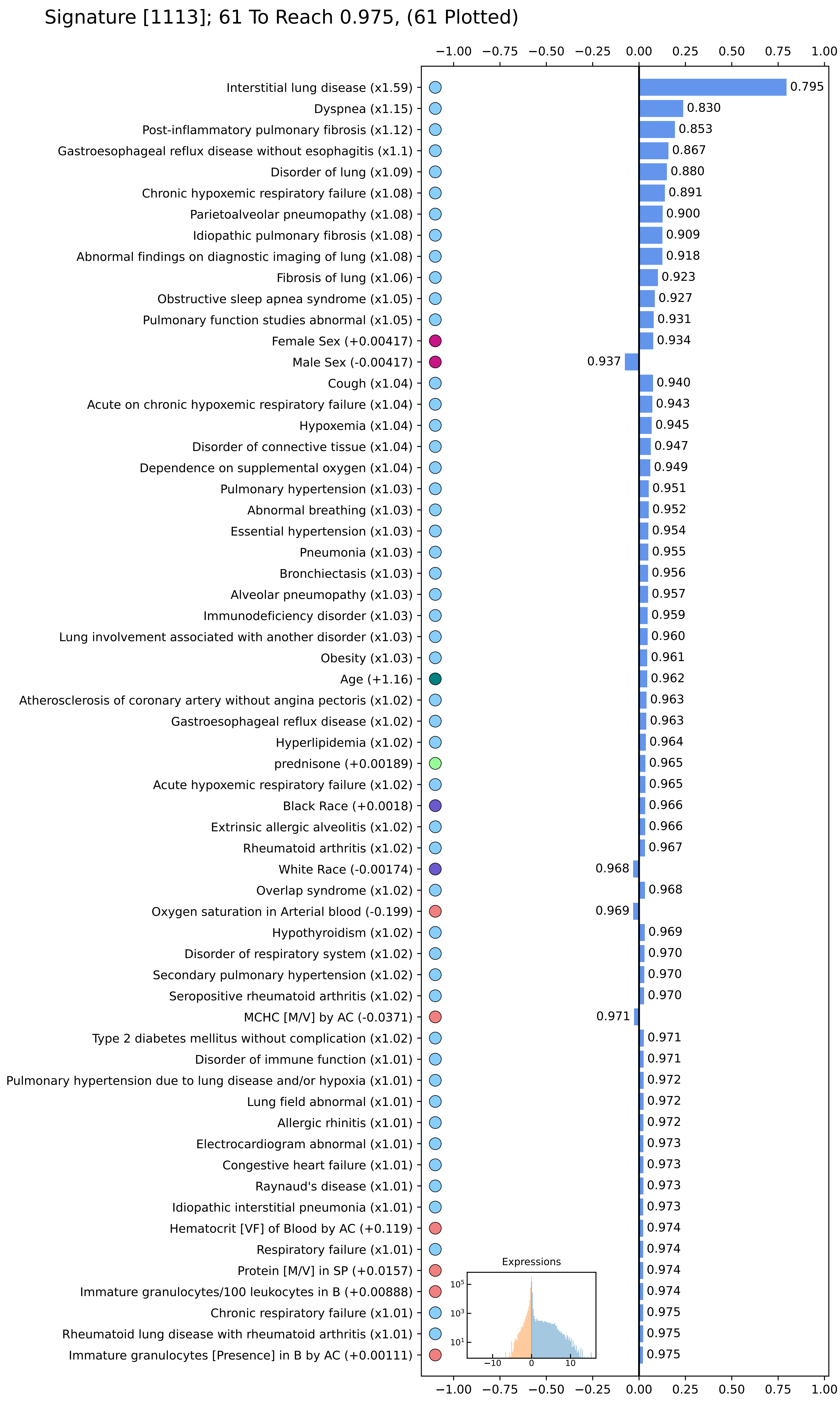

Signature [1113]; 61 To Reach 0.975, (61 Plotted)
Interstitial lung disease (x1.59) 0.795
Dyspnea (x1.15) 0.830
Post-inflammatory pulmonary fibrosis (x1.12) 0.853
Gastroesophageal reflux disease without esophagitis (x1.1) 0.867
Disorder of lung (x1.09) 0.880
Chronic hypoxemic respiratory failure (x1.08) 0.891
Parietoalveolar pneumopathy (x1.08) 0.900
Idiopathic pulmonary fibrosis (x1.08) 0.909
Abnormal findings on diagnostic imaging of lung (x1.08) 0.918
Fibrosis of lung (x1.06) 0.923
Obstructive sleep apnea syndrome (x1.05) 0.927
Pulmonary function studies abnormal (x1.05) 0.931
Female Sex (+0.00417) 0.934
Male Sex (-0.00417) 0.937
Cough (x1.04) 0.940
Acute on chronic hypoxemic respiratory failure (x1.04) 0.943
Hypoxemia (x1.04) 0.945
Disorder of connective tissue (x1.04) 0.947
Dependence on supplemental oxygen (x1.04) 0.949
Pulmonary hypertension (x1.03) 0.951
Abnormal breathing (x1.03) 0.952
Essential hypertension (x1.03) 0.954
Pneumonia (x1.03) 0.955
Bronchiectasis (x1.03) 0.956
Alveolar pneumopathy (x1.03) 0.957
Immunodeficiency disorder (x1.03) 0.959
Lung involvement associated with another disorder (x1.03) 0.960
Obesity (x1.03) 0.961
Age (+1.16) 0.962
Atherosclerosis of coronary artery without angina pectoris (x1.02) 0.963
Gastroesophageal reflux disease (x1.02) 0.963
Hyperlipidemia (x1.02) 0.964
prednisone (+0.00189) 0.965
Acute hypoxemic respiratory failure (x1.02) 0.965
Black Race (+0.0018) 0.966
Extrinsic allergic alveolitis (x1.02) 0.966
Rheumatoid arthritis (x1.02) 0.967
White Race (-0.00174) 0.968
Overlap syndrome (x1.02) 0.968
Oxygen saturation in Arterial blood (-0.199) 0.969
Hypothyroidism (x1.02) 0.969
Disorder of respiratory system (x1.02) 0.970
Secondary pulmonary hypertension (x1.02) 0.970
Seropositive rheumatoid arthritis (x1.02) 0.970
MCHC [M/V] by AC (-0.0371) 0.971
Type 2 diabetes mellitus without complication (x1.02) 0.971
Disorder of immune function (x1.01) 0.971
Pulmonary hypertension due to lung disease and/or hypoxia (x1.01) 0.972
Lung field abnormal (x1.01) 0.972
Allergic rhinitis (x1.01) 0.972
Electrocardiogram abnormal (x1.01) 0.973
Congestive heart failure (x1.01) 0.973
Raynaud's disease (x1.01) 0.973
Idiopathic interstitial pneumonia (x1.01) 0.973
Hematocrit [VF] of Blood by AC (+0.119) 0.974
Respiratory failure (x1.01) 0.974
Protein [M/V] in SP (+0.0157) 0.974
Immature granulocytes/100 leukocytes in B (+0.00888) 0.974
Chronic respiratory failure (x1.01) 0.975
Rheumatoid lung disease with rheumatoid arthritis (x1.01) 0.975
Immature granulocytes [Presence] in B by AC (+0.00111) 0.975
−1.00 −0.75 −0.50 −0.25 0.00 0.25 0.50 0.75 1.00
Expressions

Signature [1114]; 3 To Reach 0.975, (10 Plotted)

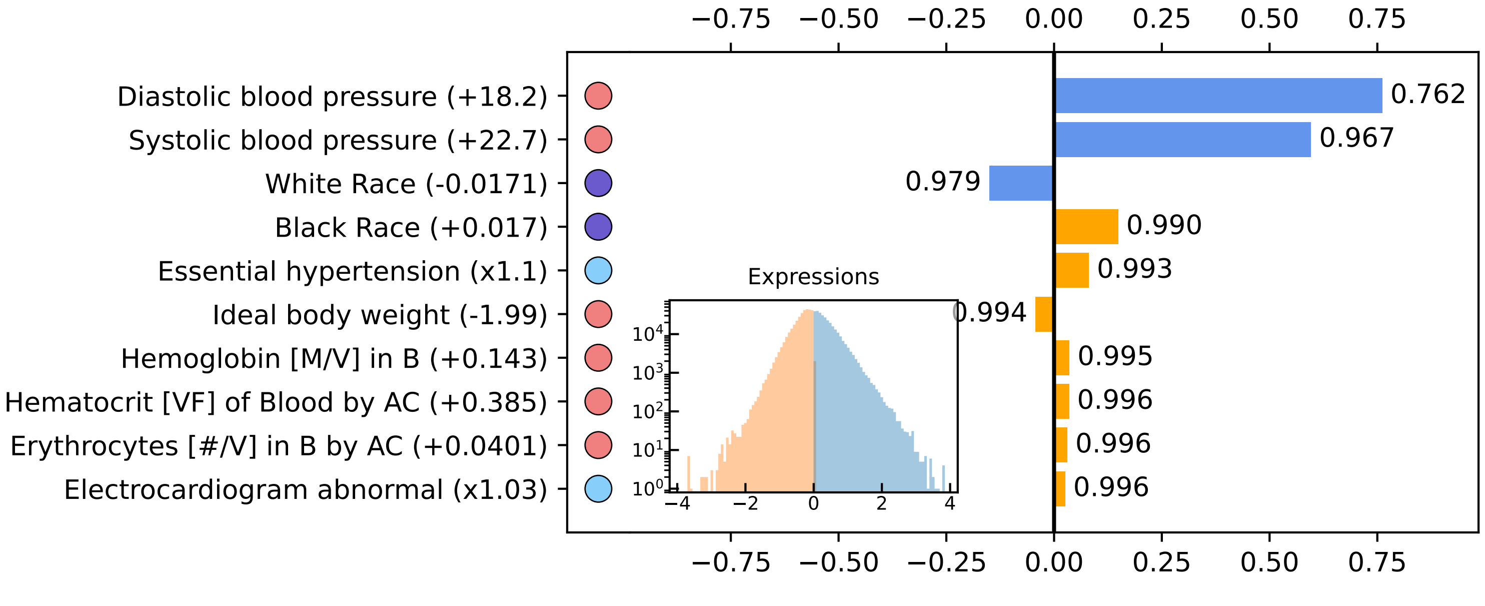

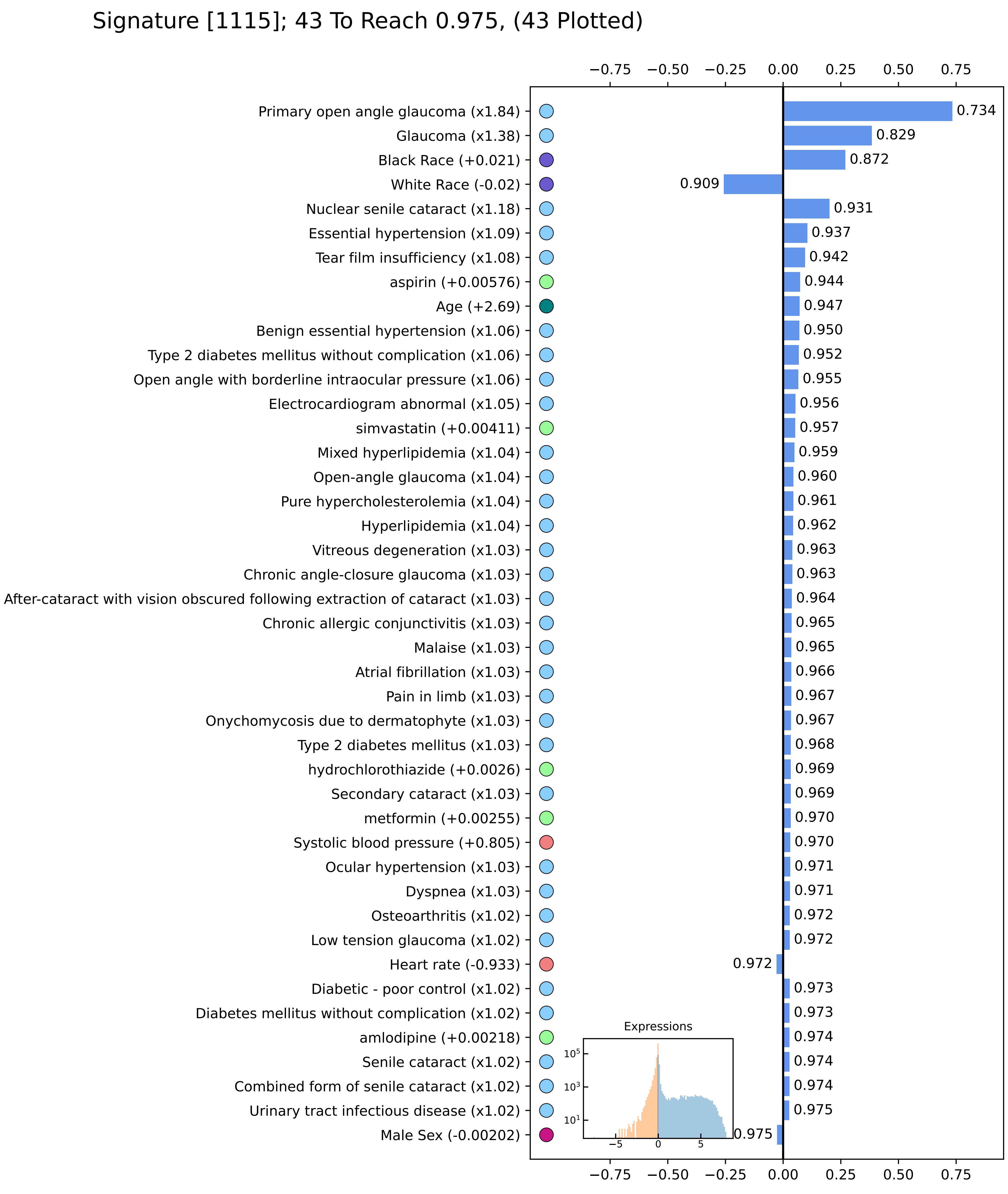

Signature [1115]; 43 To Reach 0.975, (43 Plotted)
Primary open angle glaucoma (x1.84)
Glaucoma (x1.38)
Black Race (+0.021)
White Race (-0.02)
Nuclear senile cataract (x1.18)
Essential hypertension (x1.09)
Tear film insufficiency (x1.08)
aspirin (+0.00576)
Age (+2.69)
Benign essential hypertension (x1.06)
Type 2 diabetes mellitus without complication (x1.06)
Open angle with borderline intraocular pressure (x1.06)
Electrocardiogram abnormal (x1.05)
simvastatin (+0.00411)
Mixed hyperlipidemia (x1.04)
Open-angle glaucoma (x1.04)
Pure hypercholesterolemia (x1.04)
Hyperlipidemia (x1.04)
Vitreous degeneration (x1.03)
Chronic angle-closure glaucoma (x1.03)
After-cataract with vision obscured following extraction of cataract (x1.03)
Chronic allergic conjunctivitis (x1.03)
Malaise (x1.03)
Atrial fibrillation (x1.03)
Pain in limb (x1.03)
Onychomycosis due to dermatophyte (x1.03)
Type 2 diabetes mellitus (x1.03)
hydrochlorothiazide (+0.0026)
Secondary cataract (x1.03)
metformin (+0.00255)
Systolic blood pressure (+0.805)
Ocular hypertension (x1.03)
Dyspnea (x1.03)
Osteoarthritis (x1.02)
Low tension glaucoma (x1.02)
Heart rate (-0.933)
Diabetic - poor control (x1.02)
Diabetes mellitus without complication (x1.02)
amlodipine (+0.00218)
Senile cataract (x1.02)
Combined form of senile cataract (x1.02)
Urinary tract infectious disease (x1.02)
Male Sex (-0.00202)
0.734
0.829
0.872
0.909
0.931
0.937
0.942
0.944
0.947
0.950
0.952
0.955
0.956
0.957
0.959
0.960
0.961
0.962
0.963
0.963
0.964
0.965
0.965
0.966
0.967
0.967
0.968
0.969
0.969
0.970
0.970
0.971
0.971
0.972
0.972
0.972
0.973
0.973
0.974
0.974
0.974
0.975
0.975
−0.75 −0.50 −0.25 0.00 0.25 0.50 0.75
Expressions
10^5
10^3
10^1
−5 0 5

Signature [1116]; 446 To Reach 0.975, (100 Plotted)

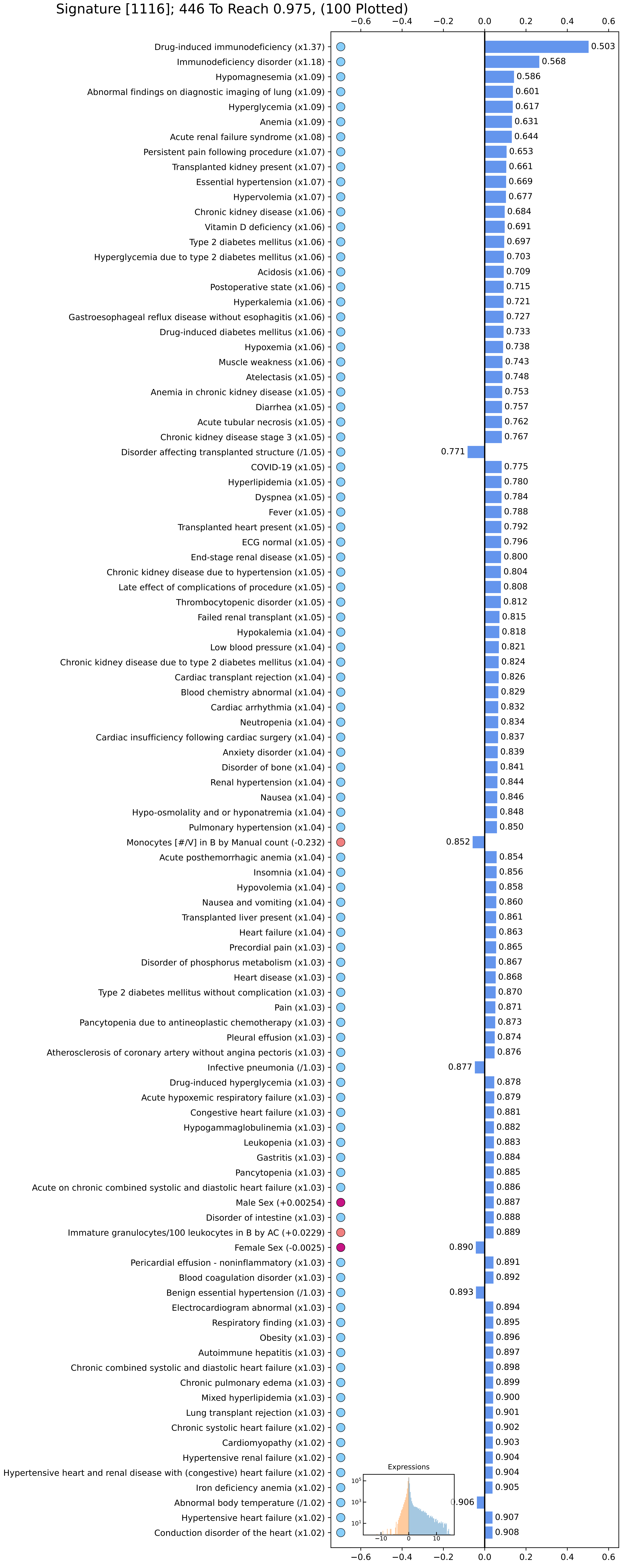

Signature [1117]; 235 To Reach 0.975, (100 Plotted)

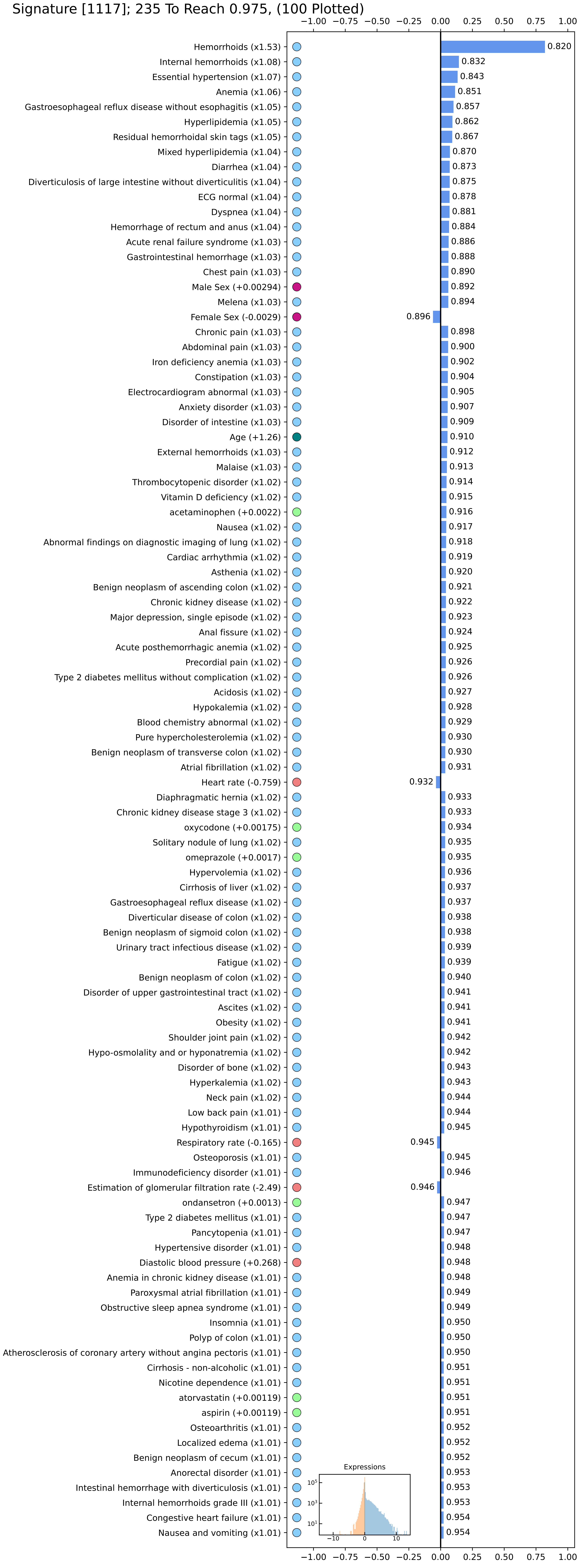

Signature [1118]; 1 To Reach 0.975, (10 Plotted)

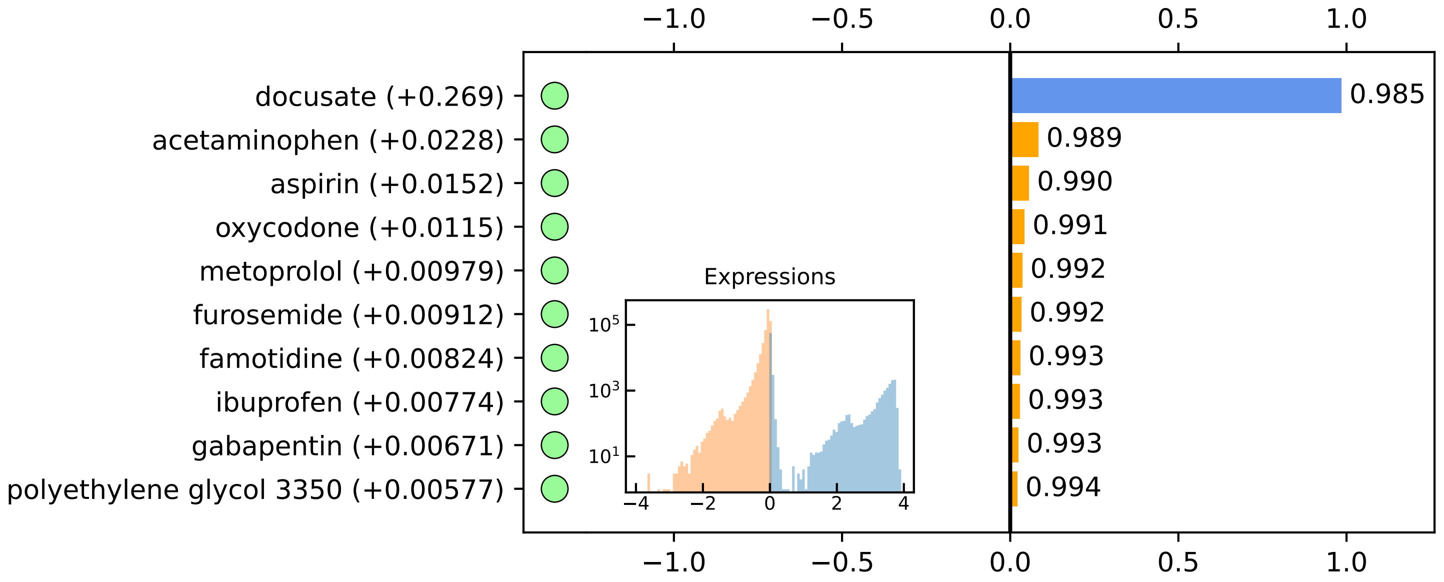

Signature [1119]; 7 To Reach 0.975, (10 Plotted)

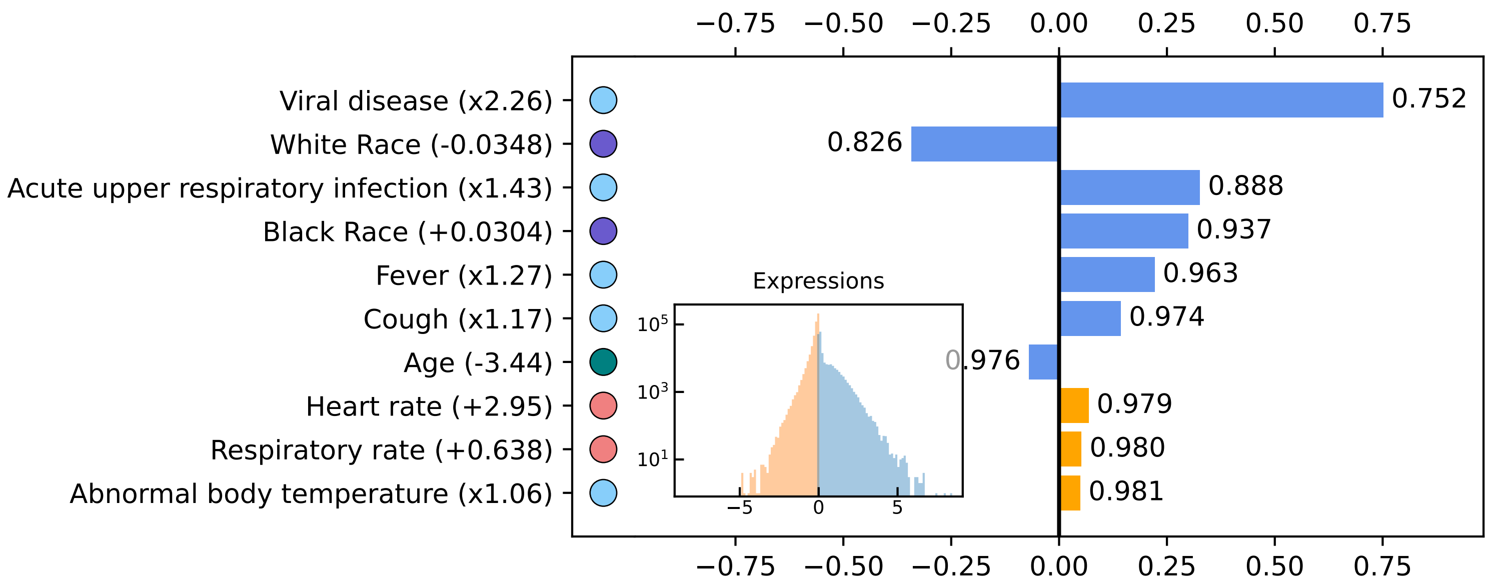

Signature [1120]; 122 To Reach 0.975, (100 Plotted)

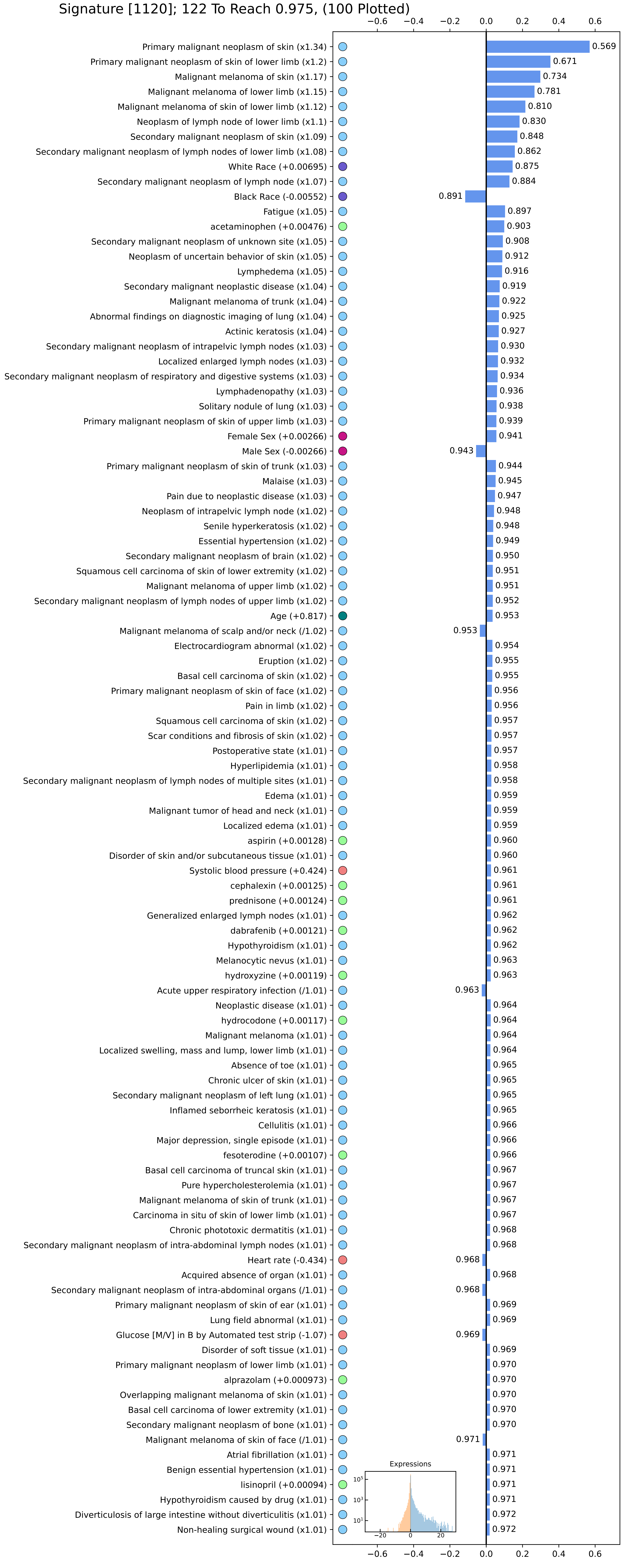

Signature [1121]; 1 To Reach 0.975, (10 Plotted)

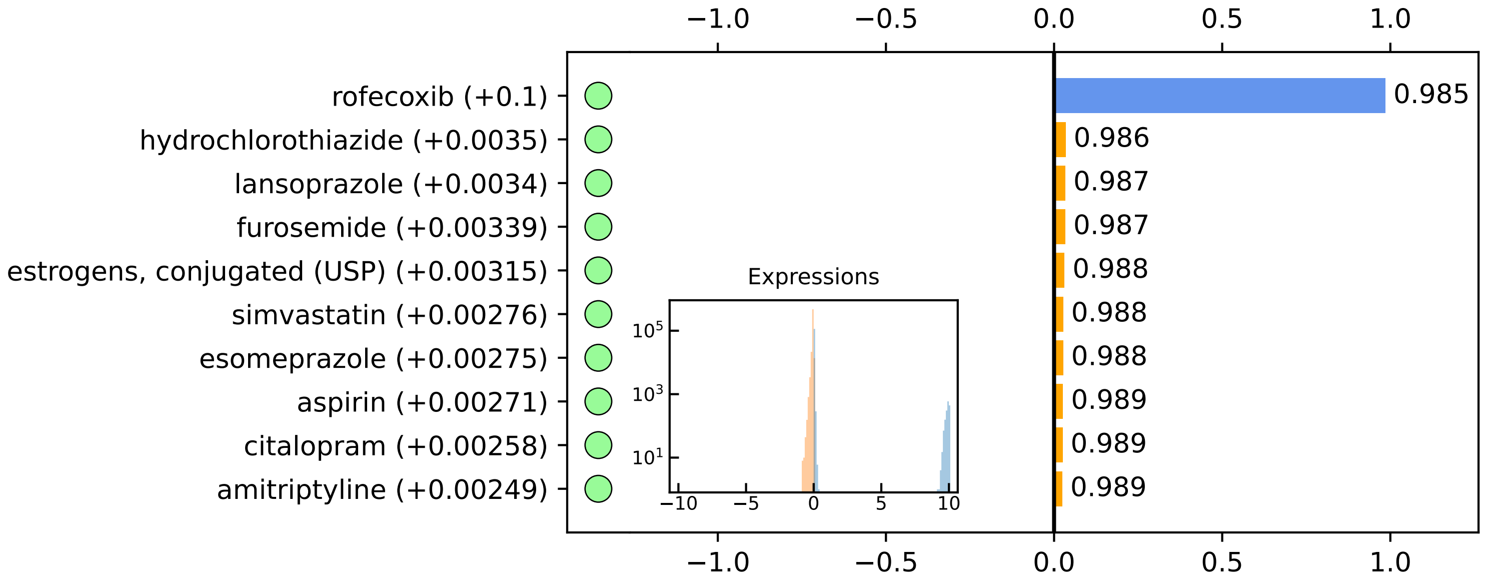

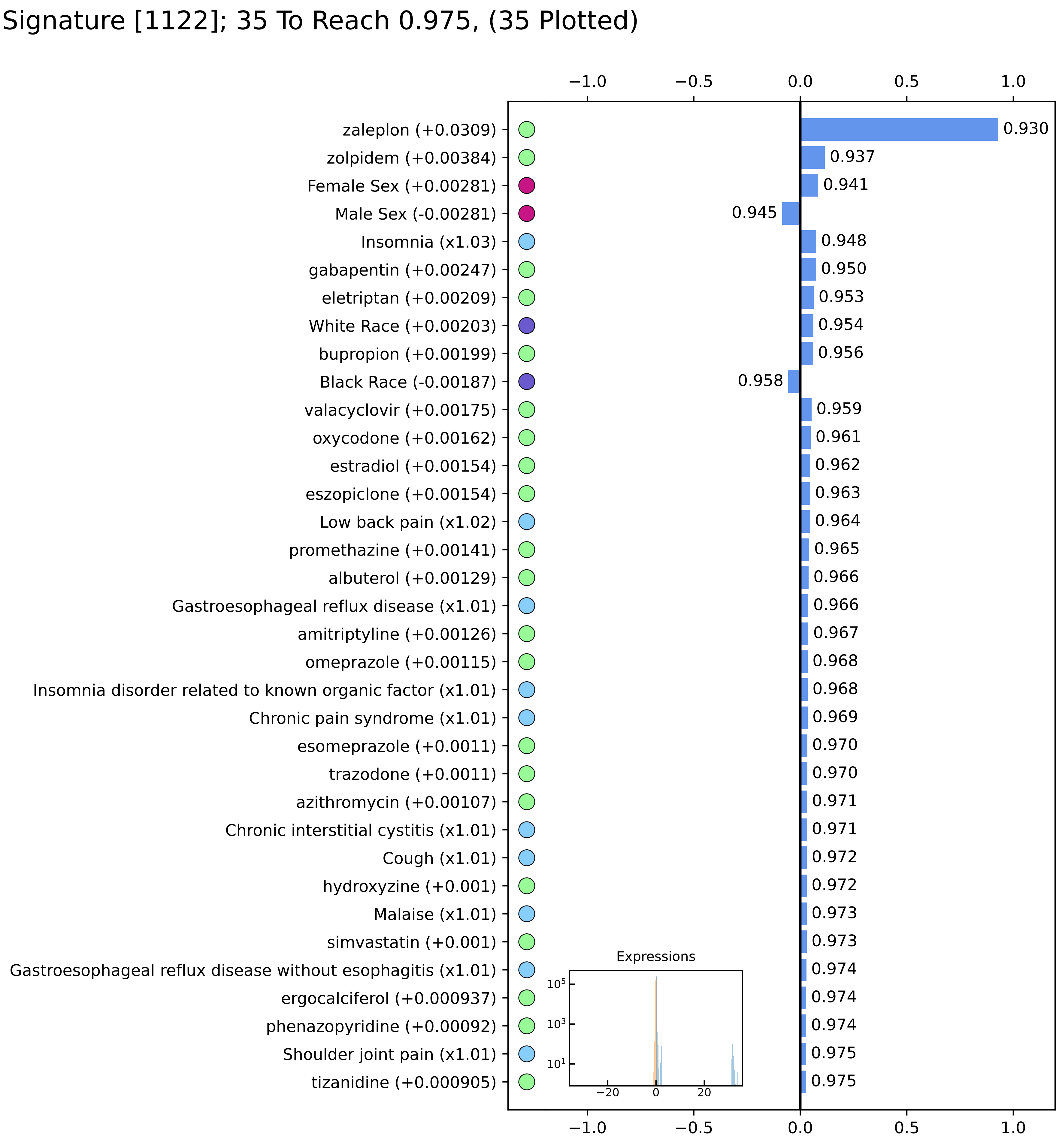

Signature [1122]; 35 To Reach 0.975, (35 Plotted)
−1.0
−0.5
0.0
0.5
1.0
zaleplon (+0.0309)
0.930
zolpidem (+0.00384)
0.937
Female Sex (+0.00281)
0.941
Male Sex (-0.00281)
0.945
Insomnia (x1.03)
0.948
gabapentin (+0.00247)
0.950
eletriptan (+0.00209)
0.953
White Race (+0.00203)
0.954
bupropion (+0.00199)
0.956
Black Race (-0.00187)
0.958
valacyclovir (+0.00175)
0.959
oxycodone (+0.00162)
0.961
estradiol (+0.00154)
0.962
eszopiclone (+0.00154)
0.963
Low back pain (x1.02)
0.964
promethazine (+0.00141)
0.965
albuterol (+0.00129)
0.966
Gastroesophageal reflux disease (x1.01)
0.966
amitriptyline (+0.00126)
0.967
omeprazole (+0.00115)
0.968
Insomnia disorder related to known organic factor (x1.01)
0.968
Chronic pain syndrome (x1.01)
0.969
esomeprazole (+0.0011)
0.970
trazodone (+0.0011)
0.970
azithromycin (+0.00107)
0.971
Chronic interstitial cystitis (x1.01)
0.971
Cough (x1.01)
0.972
hydroxyzine (+0.001)
0.972
Malaise (x1.01)
0.973
simvastatin (+0.001)
0.973
Gastroesophageal reflux disease without esophagitis (x1.01)
0.974
ergocalciferol (+0.000937)
0.974
phenazopyridine (+0.00092)
0.974
Shoulder joint pain (x1.01)
0.975
tizanidine (+0.000905)
0.975
Expressions
10^5
10^3
10^1
−20
0
20

Signature [1123]; 14 To Reach 0.975, (14 Plotted)

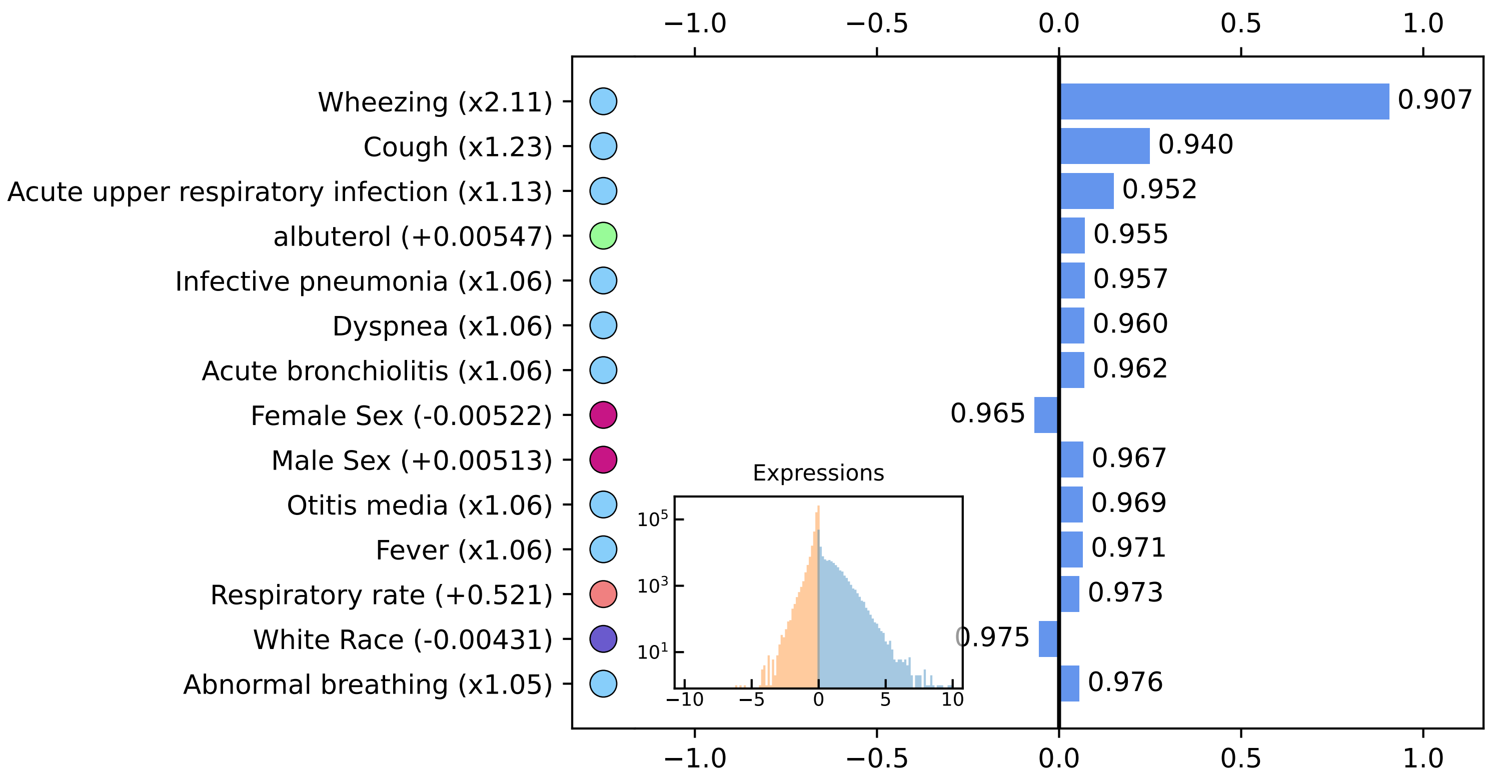

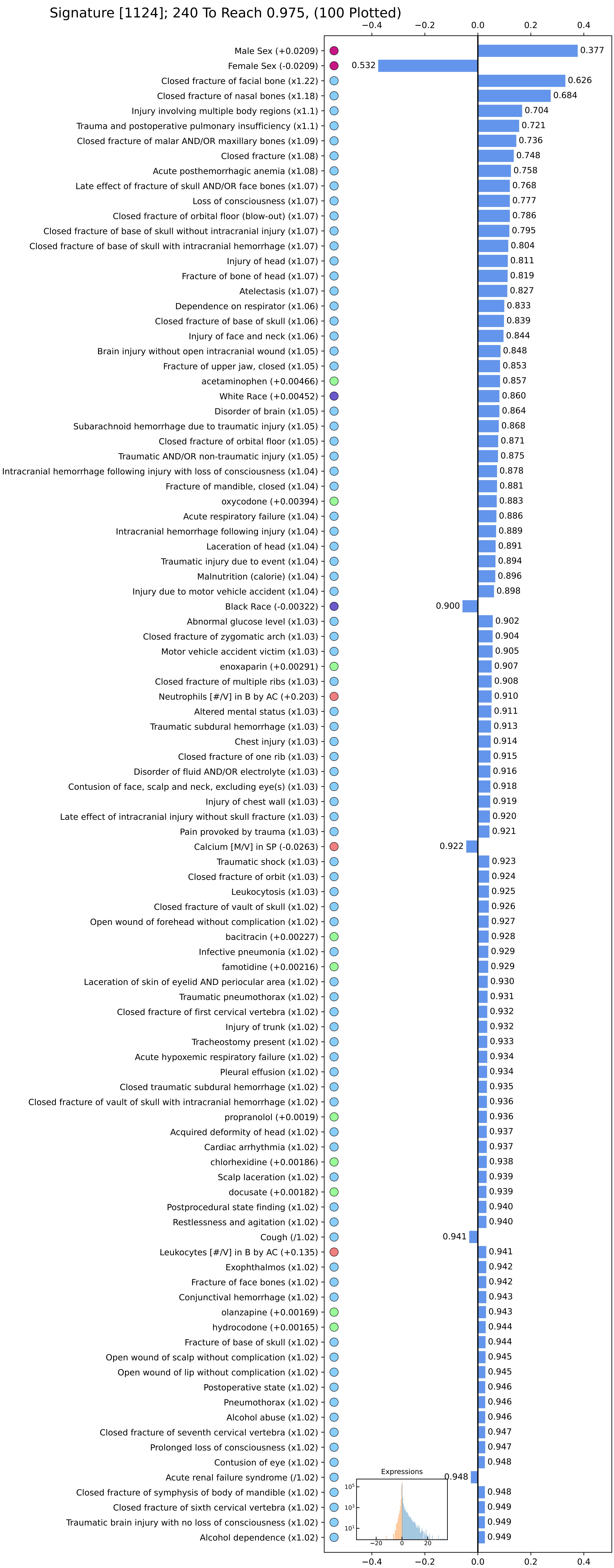

Signature [1124]; 240 To Reach 0.975, (100 Plotted)
−0.4
−0.2
0.0
0.2
0.4
Male Sex (+0.0209) 0.377
Female Sex (-0.0209) 0.532
Closed fracture of facial bone (x1.22) 0.626
Closed fracture of nasal bones (x1.18) 0.684
Injury involving multiple body regions (x1.1) 0.704
Trauma and postoperative pulmonary insufficiency (x1.1) 0.721
Closed fracture of malar AND/OR maxillary bones (x1.09) 0.736
Closed fracture (x1.08) 0.748
Acute posthemorrhagic anemia (x1.08) 0.758
Late effect of fracture of skull AND/OR face bones (x1.07) 0.768
Loss of consciousness (x1.07) 0.777
Closed fracture of orbital floor (blow-out) (x1.07) 0.786
Closed fracture of base of skull without intracranial injury (x1.07) 0.795
Closed fracture of base of skull with intracranial hemorrhage (x1.07) 0.804
Injury of head (x1.07) 0.811
Fracture of bone of head (x1.07) 0.819
Atelectasis (x1.07) 0.827
Dependence on respirator (x1.06) 0.833
Closed fracture of base of skull (x1.06) 0.839
Injury of face and neck (x1.06) 0.844
Brain injury without open intracranial wound (x1.05) 0.848
Fracture of upper jaw, closed (x1.05) 0.853
acetaminophen (+0.00466) 0.857
White Race (+0.00452) 0.860
Disorder of brain (x1.05) 0.864
Subarachnoid hemorrhage due to traumatic injury (x1.05) 0.868
Closed fracture of orbital floor (x1.05) 0.871
Traumatic AND/OR non-traumatic injury (x1.05) 0.875
Intracranial hemorrhage following injury with loss of consciousness (x1.04) 0.878
Fracture of mandible, closed (x1.04) 0.881
oxycodone (+0.00394) 0.883
Acute respiratory failure (x1.04) 0.886
Intracranial hemorrhage following injury (x1.04) 0.889
Laceration of head (x1.04) 0.891
Traumatic injury due to event (x1.04) 0.894
Malnutrition (calorie) (x1.04) 0.896
Injury due to motor vehicle accident (x1.04) 0.898
Black Race (-0.00322) 0.900
Abnormal glucose level (x1.03) 0.902
Closed fracture of zygomatic arch (x1.03) 0.904
Motor vehicle accident victim (x1.03) 0.905
enoxaparin (+0.00291) 0.907
Closed fracture of multiple ribs (x1.03) 0.908
Neutrophils [#/V] in B by AC (+0.203) 0.910
Altered mental status (x1.03) 0.911
Traumatic subdural hemorrhage (x1.03) 0.913
Chest injury (x1.03) 0.914
Closed fracture of one rib (x1.03) 0.915
Disorder of fluid AND/OR electrolyte (x1.03) 0.916
Contusion of face, scalp and neck, excluding eye(s) (x1.03) 0.918
Injury of chest wall (x1.03) 0.919
Late effect of intracranial injury without skull fracture (x1.03) 0.920
Pain provoked by trauma (x1.03) 0.921
Calcium [M/V] in SP (-0.0263) 0.922
Traumatic shock (x1.03) 0.923
Closed fracture of orbit (x1.03) 0.924
Leukocytosis (x1.03) 0.925
Closed fracture of vault of skull (x1.02) 0.926
Open wound of forehead without complication (x1.02) 0.927
bacitracin (+0.00227) 0.928
Infective pneumonia (x1.02) 0.929
famotidine (+0.00216) 0.929
Laceration of skin of eyelid AND periocular area (x1.02) 0.930
Traumatic pneumothorax (x1.02) 0.931
Closed fracture of first cervical vertebra (x1.02) 0.932
Injury of trunk (x1.02) 0.932
Tracheostomy present (x1.02) 0.933
Acute hypoxemic respiratory failure (x1.02) 0.934
Pleural effusion (x1.02) 0.934
Closed traumatic subdural hemorrhage (x1.02) 0.935
Closed fracture of vault of skull with intracranial hemorrhage (x1.02) 0.936
propranolol (+0.0019) 0.936
Acquired deformity of head (x1.02) 0.937
Cardiac arrhythmia (x1.02) 0.937
chlorhexidine (+0.00186) 0.938
Scalp laceration (x1.02) 0.939
docusate (+0.00182) 0.939
Postprocedural state finding (x1.02) 0.940
Restlessness and agitation (x1.02) 0.940
Cough (/1.02) 0.941
Leukocytes [#/V] in B by AC (+0.135) 0.941
Exophthalmos (x1.02) 0.942
Fracture of face bones (x1.02) 0.942
Conjunctival hemorrhage (x1.02) 0.943
olanzapine (+0.00169) 0.943
hydrocodone (+0.00165) 0.944
Fracture of base of skull (x1.02) 0.944
Open wound of scalp without complication (x1.02) 0.945
Open wound of lip without complication (x1.02) 0.945
Postoperative state (x1.02) 0.946
Pneumothorax (x1.02) 0.946
Alcohol abuse (x1.02) 0.946
Closed fracture of seventh cervical vertebra (x1.02) 0.947
Prolonged loss of consciousness (x1.02) 0.947
Contusion of eye (x1.02) 0.948
Acute renal failure syndrome (/1.02) 0.948
Closed fracture of symphysis of body of mandible (x1.02) 0.948
Closed fracture of sixth cervical vertebra (x1.02) 0.949
Traumatic brain injury with no loss of consciousness (x1.02) 0.949
Alcohol dependence (x1.02) 0.949
Expressions
10^5
10^3
10^1
−20
0
20

Signature [1125]; 477 To Reach 0.975, (100 Plotted)

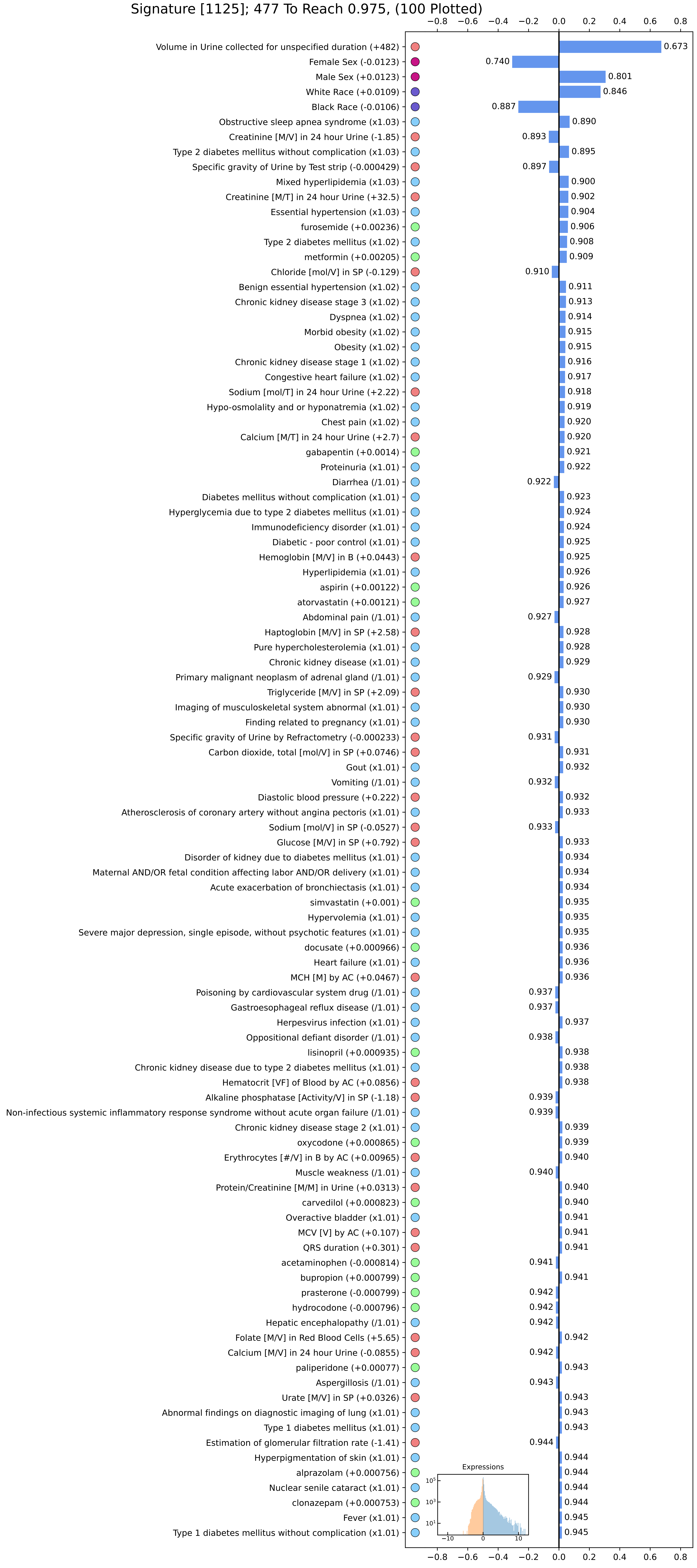

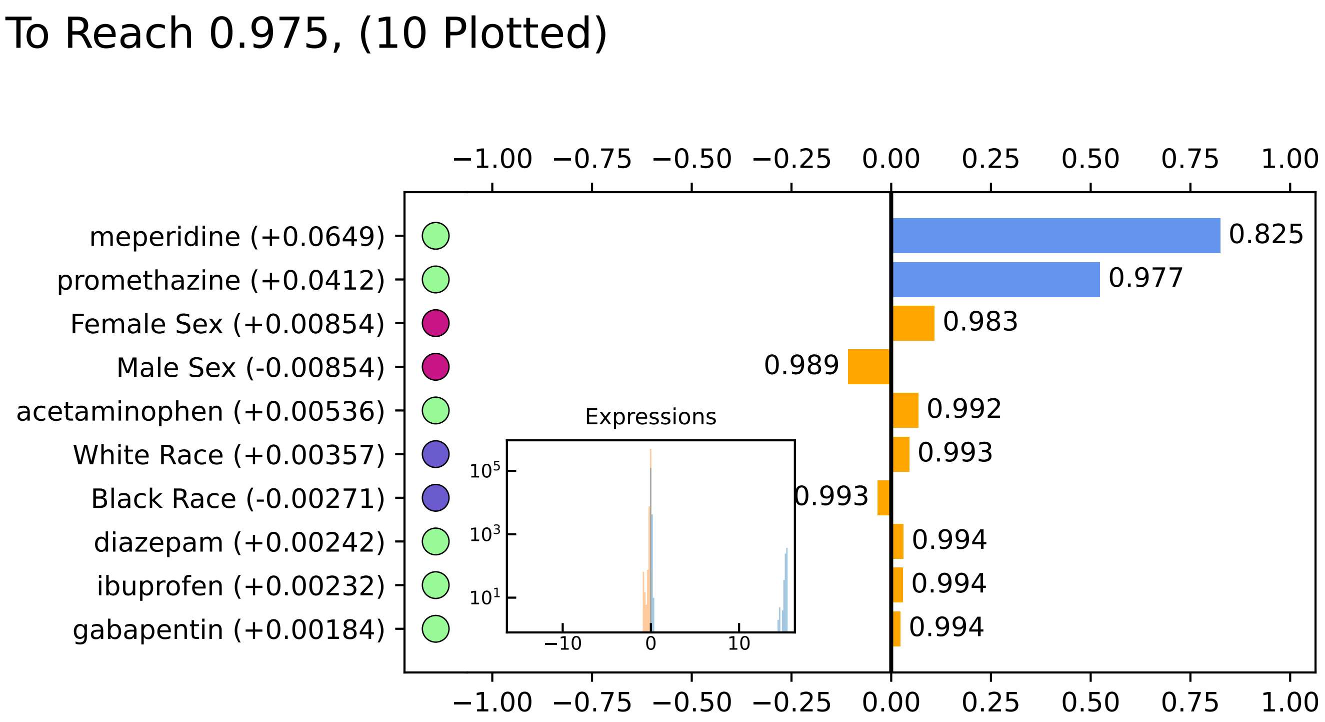
Signature [1126]; 2 To Reach 0.975, (10 Plotted)
meperidine (+0.0649)
promethazine (+0.0412)
Female Sex (+0.00854)
Male Sex (-0.00854)
acetaminophen (+0.00536)
White Race (+0.00357)
Black Race (-0.00271)
diazepam (+0.00242)
ibuprofen (+0.00232)
gabapentin (+0.00184)
0.825
0.977
0.983
0.989
0.992
0.993
0.993
0.994
0.994
0.994
Expressions
−1.00 −0.75 −0.50 −0.25 0.00 0.25 0.50 0.75 1.00

Signature [1127]; 9 To Reach 0.975, (10 Plotted)

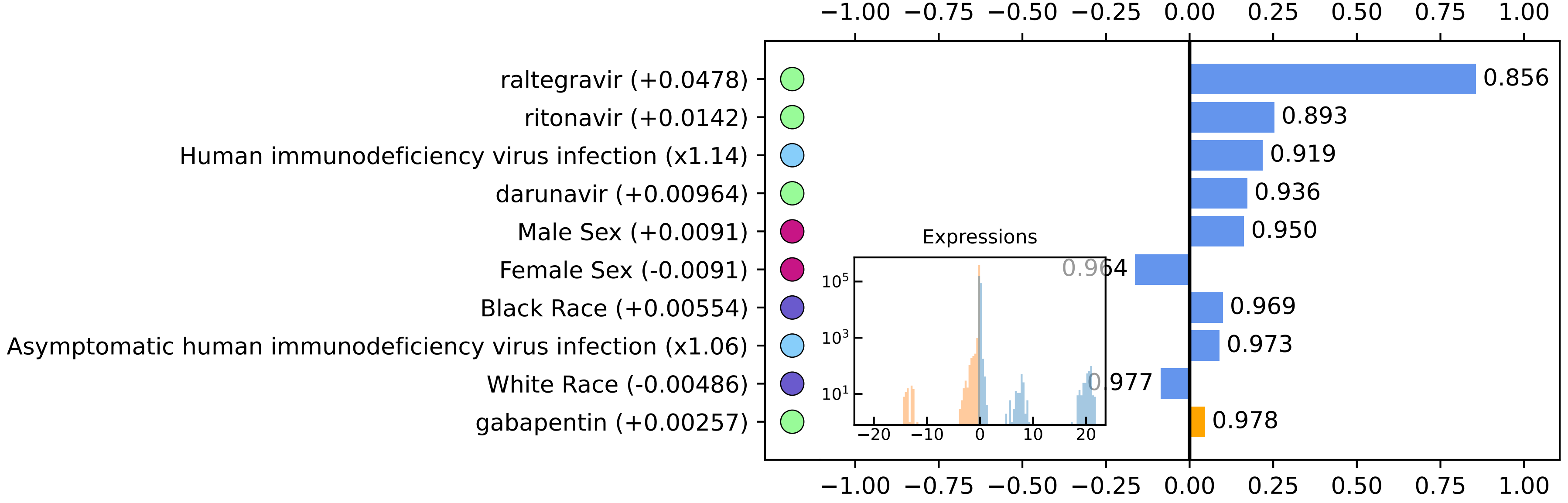

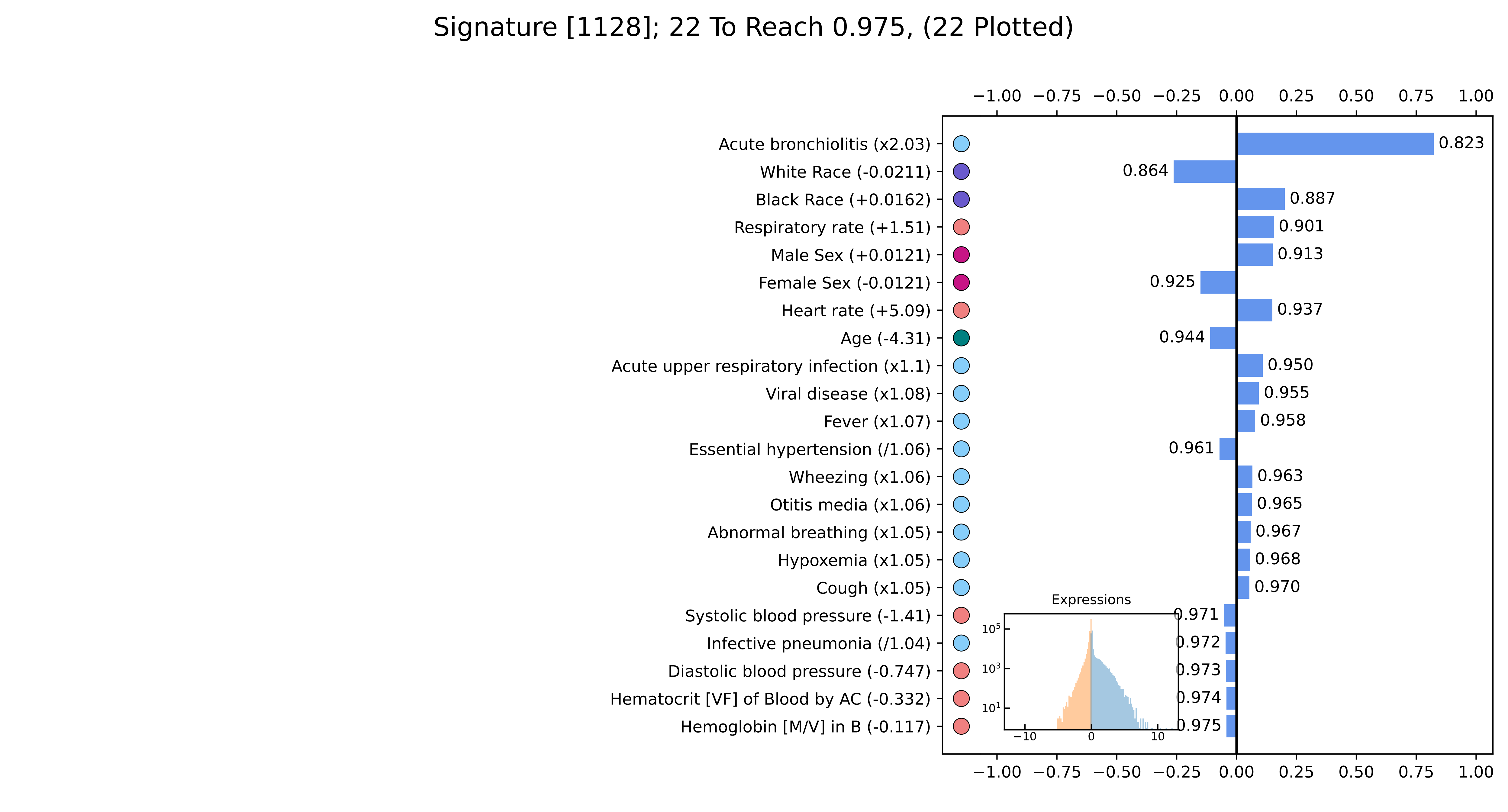
Signature [1128]; 22 To Reach 0.975, (22 Plotted)
Acute bronchiolitis (x2.03)
White Race (-0.0211)
Black Race (+0.0162)
Respiratory rate (+1.51)
Male Sex (+0.0121)
Female Sex (-0.0121)
Heart rate (+5.09)
Age (-4.31)
Acute upper respiratory infection (x1.1)
Viral disease (x1.08)
Fever (x1.07)
Essential hypertension (/1.06)
Wheezing (x1.06)
Otitis media (x1.06)
Abnormal breathing (x1.05)
Hypoxemia (x1.05)
Cough (x1.05)
Systolic blood pressure (-1.41)
Infective pneumonia (/1.04)
Diastolic blood pressure (-0.747)
Hematocrit [VF] of Blood by AC (-0.332)
Hemoglobin [M/V] in B (-0.117)
0.823
0.864
0.887
0.901
0.913
0.925
0.937
0.944
0.950
0.955
0.958
0.961
0.963
0.965
0.967
0.968
0.970
0.971
0.972
0.973
0.974
0.975
Expressions

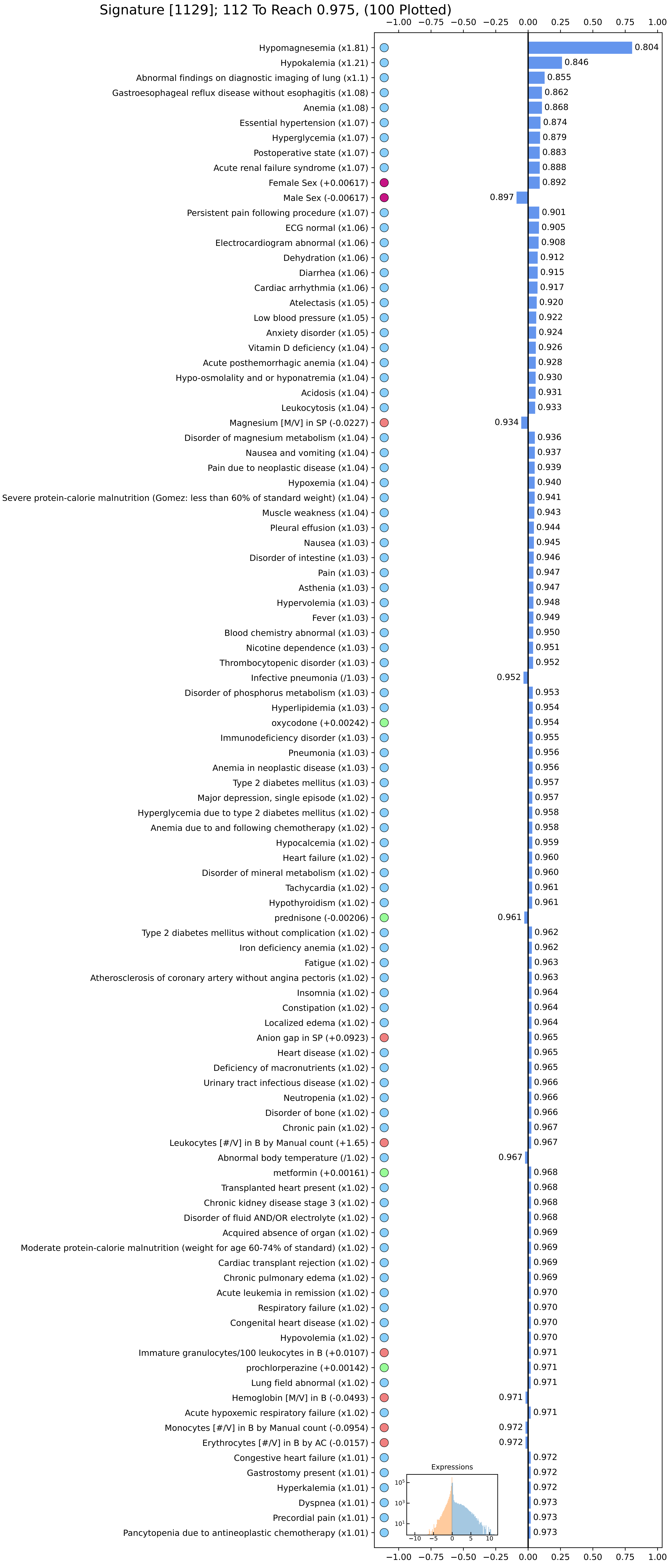

Signature [1129]; 112 To Reach 0.975, (100 Plotted)
Hypomagnesemia (x1.81) 0.804
Hypokalemia (x1.21) 0.846
Abnormal findings on diagnostic imaging of lung (x1.1) 0.855
Gastroesophageal reflux disease without esophagitis (x1.08) 0.862
Anemia (x1.08) 0.868
Essential hypertension (x1.07) 0.874
Hyperglycemia (x1.07) 0.879
Postoperative state (x1.07) 0.883
Acute renal failure syndrome (x1.07) 0.888
Female Sex (+0.00617) 0.892
Male Sex (-0.00617) 0.897
Persistent pain following procedure (x1.07) 0.901
ECG normal (x1.06) 0.905
Electrocardiogram abnormal (x1.06) 0.908
Dehydration (x1.06) 0.912
Diarrhea (x1.06) 0.915
Cardiac arrhythmia (x1.06) 0.917
Atelectasis (x1.05) 0.920
Low blood pressure (x1.05) 0.922
Anxiety disorder (x1.05) 0.924
Vitamin D deficiency (x1.04) 0.926
Acute posthemorrhagic anemia (x1.04) 0.928
Hypo-osmolality and or hyponatremia (x1.04) 0.930
Acidosis (x1.04) 0.931
Leukocytosis (x1.04) 0.933
Magnesium [M/V] in SP (-0.0227) 0.934
Disorder of magnesium metabolism (x1.04) 0.936
Nausea and vomiting (x1.04) 0.937
Pain due to neoplastic disease (x1.04) 0.939
Hypoxemia (x1.04) 0.940
Severe protein-calorie malnutrition (Gomez: less than 60% of standard weight) (x1.04) 0.941
Muscle weakness (x1.04) 0.943
Pleural effusion (x1.03) 0.944
Nausea (x1.03) 0.945
Disorder of intestine (x1.03) 0.946
Pain (x1.03) 0.947
Asthenia (x1.03) 0.947
Hypervolemia (x1.03) 0.948
Fever (x1.03) 0.949
Blood chemistry abnormal (x1.03) 0.950
Nicotine dependence (x1.03) 0.951
Thrombocytopenic disorder (x1.03) 0.952
Infective pneumonia (/1.03) 0.952
Disorder of phosphorus metabolism (x1.03) 0.953
Hyperlipidemia (x1.03) 0.954
oxycodone (+0.00242) 0.954
Immunodeficiency disorder (x1.03) 0.955
Pneumonia (x1.03) 0.956
Anemia in neoplastic disease (x1.03) 0.956
Type 2 diabetes mellitus (x1.03) 0.957
Major depression, single episode (x1.02) 0.957
Hyperglycemia due to type 2 diabetes mellitus (x1.02) 0.958
Anemia due to and following chemotherapy (x1.02) 0.958
Hypocalcemia (x1.02) 0.959
Heart failure (x1.02) 0.960
Disorder of mineral metabolism (x1.02) 0.960
Tachycardia (x1.02) 0.961
Hypothyroidism (x1.02) 0.961
prednisone (-0.00206) 0.961
Type 2 diabetes mellitus without complication (x1.02) 0.962
Iron deficiency anemia (x1.02) 0.962
Fatigue (x1.02) 0.963
Atherosclerosis of coronary artery without angina pectoris (x1.02) 0.963
Insomnia (x1.02) 0.964
Constipation (x1.02) 0.964
Localized edema (x1.02) 0.964
Anion gap in SP (+0.0923) 0.965
Heart disease (x1.02) 0.965
Deficiency of macronutrients (x1.02) 0.965
Urinary tract infectious disease (x1.02) 0.966
Neutropenia (x1.02) 0.966
Disorder of bone (x1.02) 0.966
Chronic pain (x1.02) 0.967
Leukocytes [#/V] in B by Manual count (+1.65) 0.967
Abnormal body temperature (/1.02) 0.967
metformin (+0.00161) 0.968
Transplanted heart present (x1.02) 0.968
Chronic kidney disease stage 3 (x1.02) 0.968
Disorder of fluid AND/OR electrolyte (x1.02) 0.968
Acquired absence of organ (x1.02) 0.969
Moderate protein-calorie malnutrition (weight for age 60-74% of standard) (x1.02) 0.969
Cardiac transplant rejection (x1.02) 0.969
Chronic pulmonary edema (x1.02) 0.969
Acute leukemia in remission (x1.02) 0.970
Respiratory failure (x1.02) 0.970
Congenital heart disease (x1.02) 0.970
Hypovolemia (x1.02) 0.970
Immature granulocytes/100 leukocytes in B (+0.0107) 0.971
prochlorperazine (+0.00142) 0.971
Lung field abnormal (x1.02) 0.971
Hemoglobin [M/V] in B (-0.0493) 0.971
Acute hypoxemic respiratory failure (x1.02) 0.971
Monocytes [#/V] in B by Manual count (-0.0954) 0.972
Erythrocytes [#/V] in B by AC (-0.0157) 0.972
Congestive heart failure (x1.01) 0.972
Gastrostomy present (x1.01) 0.972
Hyperkalemia (x1.01) 0.972
Dyspnea (x1.01) 0.973
Precordial pain (x1.01) 0.973
Pancytopenia due to antineoplastic chemotherapy (x1.01) 0.973
−1.00 −0.75 −0.50 −0.25 0.00 0.25 0.50 0.75 1.00
Expressions

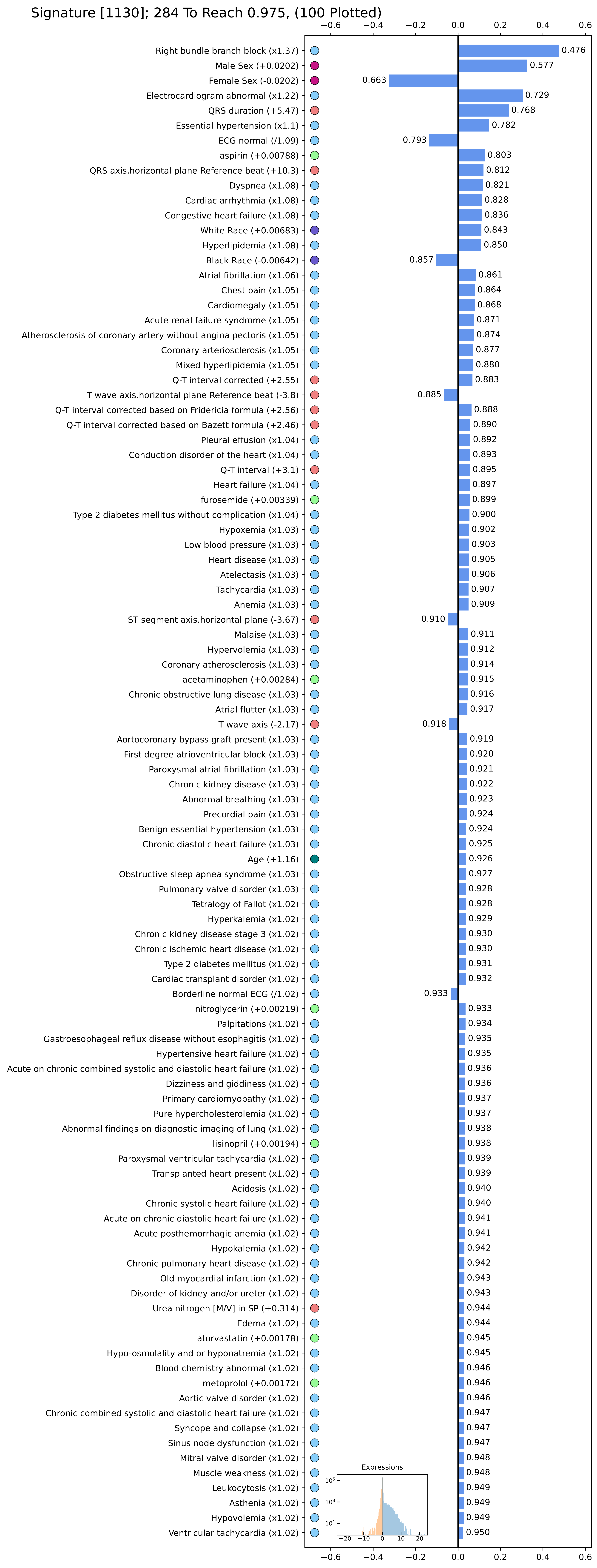
Signature [1130]; 284 To Reach 0.975, (100 Plotted)
−0.6
−0.4
−0.2
0.0
0.2
0.4
0.6
Right bundle branch block (x1.37) 0.476
Male Sex (+0.0202) 0.577
Female Sex (-0.0202) 0.663
Electrocardiogram abnormal (x1.22) 0.729
QRS duration (+5.47) 0.768
Essential hypertension (x1.1) 0.782
ECG normal (/1.09) 0.793
aspirin (+0.00788) 0.803
QRS axis.horizontal plane Reference beat (+10.3) 0.812
Dyspnea (x1.08) 0.821
Cardiac arrhythmia (x1.08) 0.828
Congestive heart failure (x1.08) 0.836
White Race (+0.00683) 0.843
Hyperlipidemia (x1.08) 0.850
Black Race (-0.00642) 0.857
Atrial fibrillation (x1.06) 0.861
Chest pain (x1.05) 0.864
Cardiomegaly (x1.05) 0.868
Acute renal failure syndrome (x1.05) 0.871
Atherosclerosis of coronary artery without angina pectoris (x1.05) 0.874
Coronary arteriosclerosis (x1.05) 0.877
Mixed hyperlipidemia (x1.05) 0.880
Q-T interval corrected (+2.55) 0.883
T wave axis.horizontal plane Reference beat (-3.8) 0.885
Q-T interval corrected based on Fridericia formula (+2.56) 0.888
Q-T interval corrected based on Bazett formula (+2.46) 0.890
Pleural effusion (x1.04) 0.892
Conduction disorder of the heart (x1.04) 0.893
Q-T interval (+3.1) 0.895
Heart failure (x1.04) 0.897
furosemide (+0.00339) 0.899
Type 2 diabetes mellitus without complication (x1.04) 0.900
Hypoxemia (x1.03) 0.902
Low blood pressure (x1.03) 0.903
Heart disease (x1.03) 0.905
Atelectasis (x1.03) 0.906
Tachycardia (x1.03) 0.907
Anemia (x1.03) 0.909
ST segment axis.horizontal plane (-3.67) 0.910
Malaise (x1.03) 0.911
Hypervolemia (x1.03) 0.912
Coronary atherosclerosis (x1.03) 0.914
acetaminophen (+0.00284) 0.915
Chronic obstructive lung disease (x1.03) 0.916
Atrial flutter (x1.03) 0.917
T wave axis (-2.17) 0.918
Aortocoronary bypass graft present (x1.03) 0.919
First degree atrioventricular block (x1.03) 0.920
Paroxysmal atrial fibrillation (x1.03) 0.921
Chronic kidney disease (x1.03) 0.922
Abnormal breathing (x1.03) 0.923
Precordial pain (x1.03) 0.924
Benign essential hypertension (x1.03) 0.924
Chronic diastolic heart failure (x1.03) 0.925
Age (+1.16) 0.926
Obstructive sleep apnea syndrome (x1.03) 0.927
Pulmonary valve disorder (x1.03) 0.928
Tetralogy of Fallot (x1.02) 0.928
Hyperkalemia (x1.02) 0.929
Chronic kidney disease stage 3 (x1.02) 0.930
Chronic ischemic heart disease (x1.02) 0.930
Type 2 diabetes mellitus (x1.02) 0.931
Cardiac transplant disorder (x1.02) 0.932
Borderline normal ECG (/1.02) 0.933
nitroglycerin (+0.00219) 0.933
Palpitations (x1.02) 0.934
Gastroesophageal reflux disease without esophagitis (x1.02) 0.935
Hypertensive heart failure (x1.02) 0.935
Acute on chronic combined systolic and diastolic heart failure (x1.02) 0.936
Dizziness and giddiness (x1.02) 0.936
Primary cardiomyopathy (x1.02) 0.937
Pure hypercholesterolemia (x1.02) 0.937
Abnormal findings on diagnostic imaging of lung (x1.02) 0.938
lisinopril (+0.00194) 0.938
Paroxysmal ventricular tachycardia (x1.02) 0.939
Transplanted heart present (x1.02) 0.939
Acidosis (x1.02) 0.940
Chronic systolic heart failure (x1.02) 0.940
Acute on chronic diastolic heart failure (x1.02) 0.941
Acute posthemorrhagic anemia (x1.02) 0.941
Hypokalemia (x1.02) 0.942
Chronic pulmonary heart disease (x1.02) 0.942
Old myocardial infarction (x1.02) 0.943
Disorder of kidney and/or ureter (x1.02) 0.943
Urea nitrogen [M/V] in SP (+0.314) 0.944
Edema (x1.02) 0.944
atorvastatin (+0.00178) 0.945
Hypo-osmolality and or hyponatremia (x1.02) 0.945
Blood chemistry abnormal (x1.02) 0.946
metoprolol (+0.00172) 0.946
Aortic valve disorder (x1.02) 0.946
Chronic combined systolic and diastolic heart failure (x1.02) 0.947
Syncope and collapse (x1.02) 0.947
Sinus node dysfunction (x1.02) 0.947
Mitral valve disorder (x1.02) 0.948
Muscle weakness (x1.02) 0.948
Leukocytosis (x1.02) 0.949
Asthenia (x1.02) 0.949
Hypovolemia (x1.02) 0.949
Ventricular tachycardia (x1.02) 0.950
Expressions
10^5
10^3
10^1
−20
−10
0
10
20

Signature [1131]; 2 To Reach 0.975, (10 Plotted)

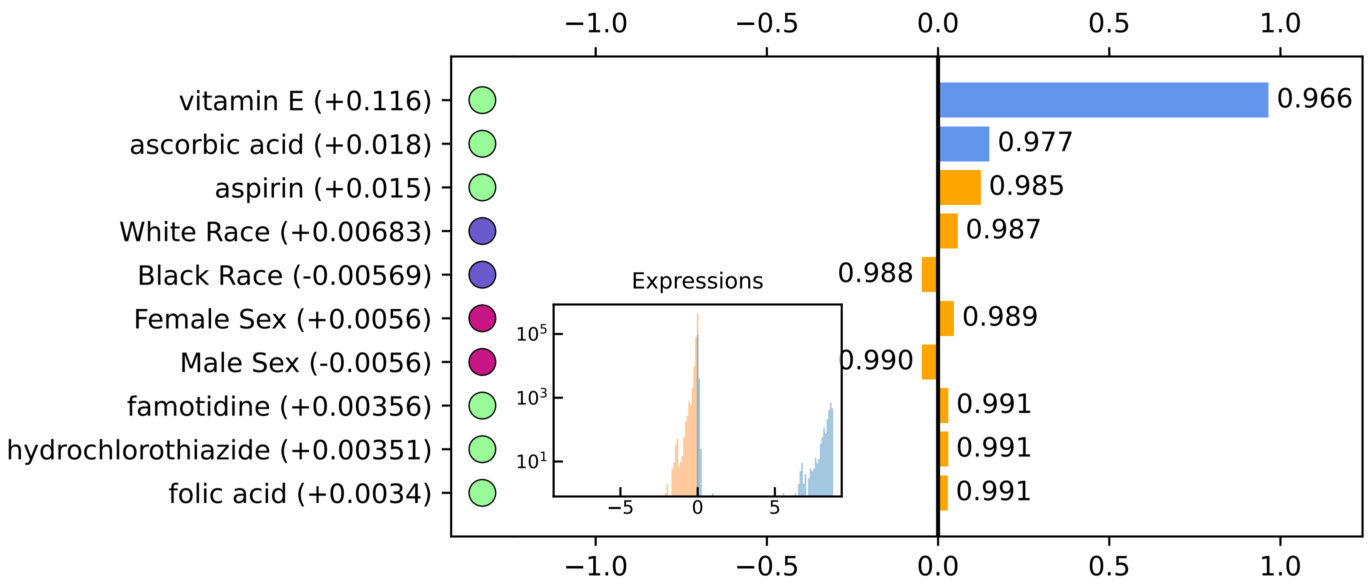

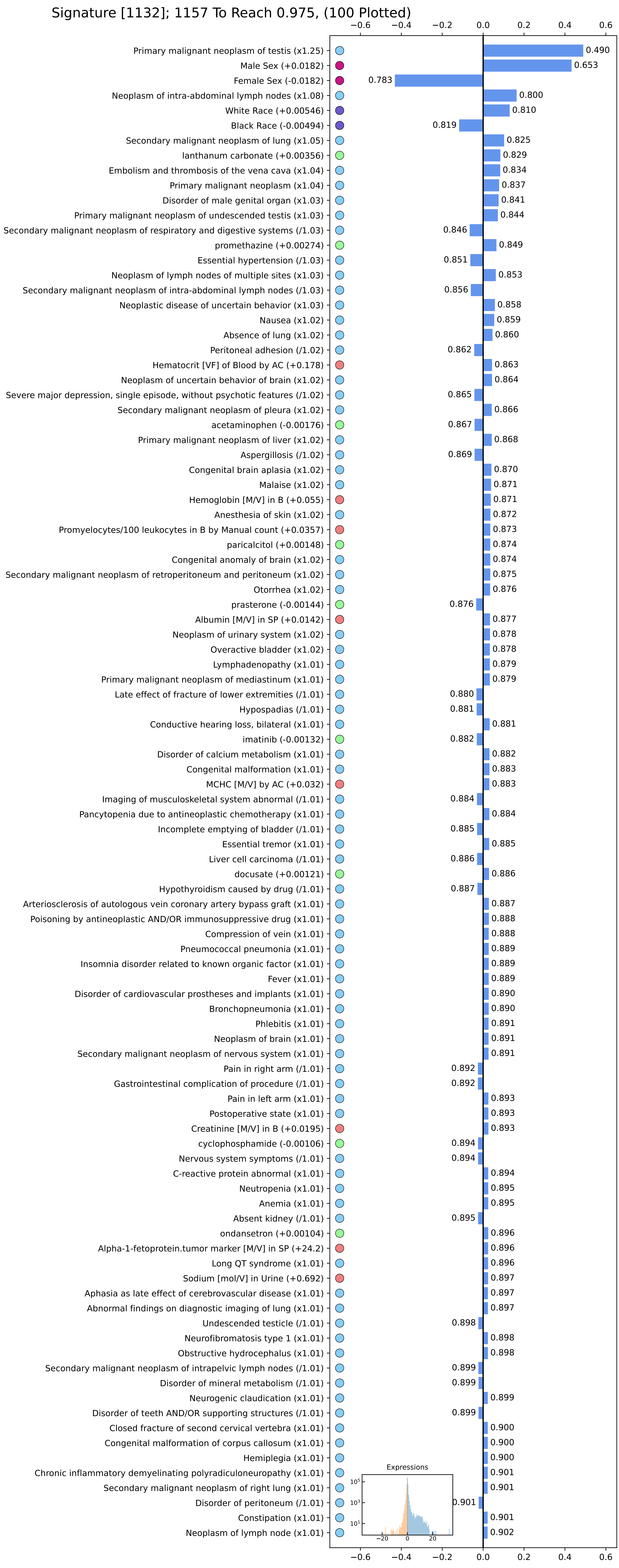

Signature [1132]; 1157 To Reach 0.975, (100 Plotted)
Primary malignant neoplasm of testis (x1.25)
Male Sex (+0.0182)
Female Sex (-0.0182)
Neoplasm of intra-abdominal lymph nodes (x1.08)
White Race (+0.00546)
Black Race (-0.00494)
Secondary malignant neoplasm of lung (x1.05)
lanthanum carbonate (+0.00356)
Embolism and thrombosis of the vena cava (x1.04)
Primary malignant neoplasm (x1.04)
Disorder of male genital organ (x1.03)
Primary malignant neoplasm of undescended testis (x1.03)
Secondary malignant neoplasm of respiratory and digestive systems (/1.03)
promethazine (+0.00274)
Essential hypertension (/1.03)
Neoplasm of lymph nodes of multiple sites (x1.03)
Secondary malignant neoplasm of intra-abdominal lymph nodes (/1.03)
Neoplastic disease of uncertain behavior (x1.03)
Nausea (x1.02)
Absence of lung (x1.02)
Peritoneal adhesion (/1.02)
Hematocrit [VF] of Blood by AC (+0.178)
Neoplasm of uncertain behavior of brain (x1.02)
Severe major depression, single episode, without psychotic features (/1.02)
Secondary malignant neoplasm of pleura (x1.02)
acetaminophen (-0.00176)
Primary malignant neoplasm of liver (x1.02)
Aspergillosis (/1.02)
Congenital brain aplasia (x1.02)
Malaise (x1.02)
Hemoglobin [M/V] in B (+0.055)
Anesthesia of skin (x1.02)
Promyelocytes/100 leukocytes in B by Manual count (+0.0357)
paricalcitol (+0.00148)
Congenital anomaly of brain (x1.02)
Secondary malignant neoplasm of retroperitoneum and peritoneum (x1.02)
Otorrhea (x1.02)
prasterone (-0.00144)
Albumin [M/V] in SP (+0.0142)
Neoplasm of urinary system (x1.02)
Overactive bladder (x1.02)
Lymphadenopathy (x1.01)
Primary malignant neoplasm of mediastinum (x1.01)
Late effect of fracture of lower extremities (/1.01)
Hypospadias (/1.01)
Conductive hearing loss, bilateral (x1.01)
imatinib (-0.00132)
Disorder of calcium metabolism (x1.01)
Congenital malformation (x1.01)
MCHC [M/V] by AC (+0.032)
Imaging of musculoskeletal system abnormal (/1.01)
Pancytopenia due to antineoplastic chemotherapy (x1.01)
Incomplete emptying of bladder (/1.01)
Essential tremor (x1.01)
Liver cell carcinoma (/1.01)
docusate (+0.00121)
Hypothyroidism caused by drug (/1.01)
Arteriosclerosis of autologous vein coronary artery bypass graft (x1.01)
Poisoning by antineoplastic AND/OR immunosuppressive drug (x1.01)
Compression of vein (x1.01)
Pneumococcal pneumonia (x1.01)
Insomnia disorder related to known organic factor (x1.01)
Fever (x1.01)
Disorder of cardiovascular prostheses and implants (x1.01)
Bronchopneumonia (x1.01)
Phlebitis (x1.01)
Neoplasm of brain (x1.01)
Secondary malignant neoplasm of nervous system (x1.01)
Pain in right arm (/1.01)
Gastrointestinal complication of procedure (/1.01)
Pain in left arm (x1.01)
Postoperative state (x1.01)
Creatinine [M/V] in B (+0.0195)
cyclophosphamide (-0.00106)
Nervous system symptoms (/1.01)
C-reactive protein abnormal (x1.01)
Neutropenia (x1.01)
Anemia (x1.01)
Absent kidney (/1.01)
ondansetron (+0.00104)
Alpha-1-fetoprotein.tumor marker [M/V] in SP (+24.2)
Long QT syndrome (x1.01)
Sodium [mol/V] in Urine (+0.692)
Aphasia as late effect of cerebrovascular disease (x1.01)
Abnormal findings on diagnostic imaging of lung (x1.01)
Undescended testicle (/1.01)
Neurofibromatosis type 1 (x1.01)
Obstructive hydrocephalus (x1.01)
Secondary malignant neoplasm of intrapelvic lymph nodes (/1.01)
Disorder of mineral metabolism (/1.01)
Neurogenic claudication (x1.01)
Disorder of teeth AND/OR supporting structures (/1.01)
Closed fracture of second cervical vertebra (x1.01)
Congenital malformation of corpus callosum (x1.01)
Hemiplegia (x1.01)
Chronic inflammatory demyelinating polyradiculoneuropathy (x1.01)
Secondary malignant neoplasm of right lung (x1.01)
Disorder of peritoneum (/1.01)
Constipation (x1.01)
Neoplasm of lymph node (x1.01)
0.490
0.653
0.783
0.800
0.810
0.819
0.825
0.829
0.834
0.837
0.841
0.844
0.846
0.849
0.851
0.853
0.856
0.858
0.859
0.860
0.862
0.863
0.864
0.865
0.866
0.867
0.868
0.869
0.870
0.871
0.871
0.872
0.873
0.874
0.874
0.875
0.876
0.876
0.877
0.878
0.878
0.879
0.879
0.880
0.881
0.881
0.882
0.882
0.883
0.883
0.884
0.884
0.885
0.885
0.886
0.886
0.887
0.887
0.888
0.888
0.889
0.889
0.889
0.890
0.890
0.891
0.891
0.891
0.892
0.892
0.893
0.893
0.893
0.894
0.894
0.894
0.895
0.895
0.895
0.896
0.896
0.896
0.897
0.897
0.897
0.898
0.898
0.898
0.899
0.899
0.899
0.899
0.900
0.900
0.900
0.901
0.901
0.901
0.901
0.902
−0.6 −0.4 −0.2 0.0 0.2 0.4 0.6
Expressions

Signature [1133]; 7 To Reach 0.975, (10 Plotted)

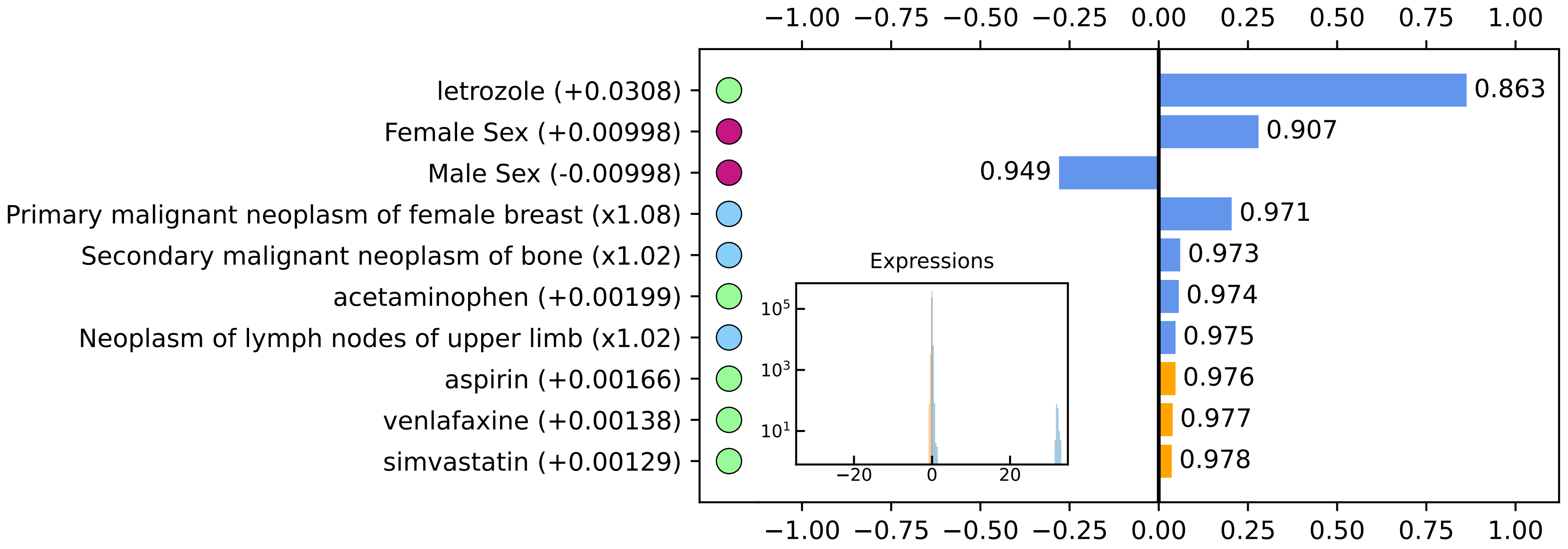

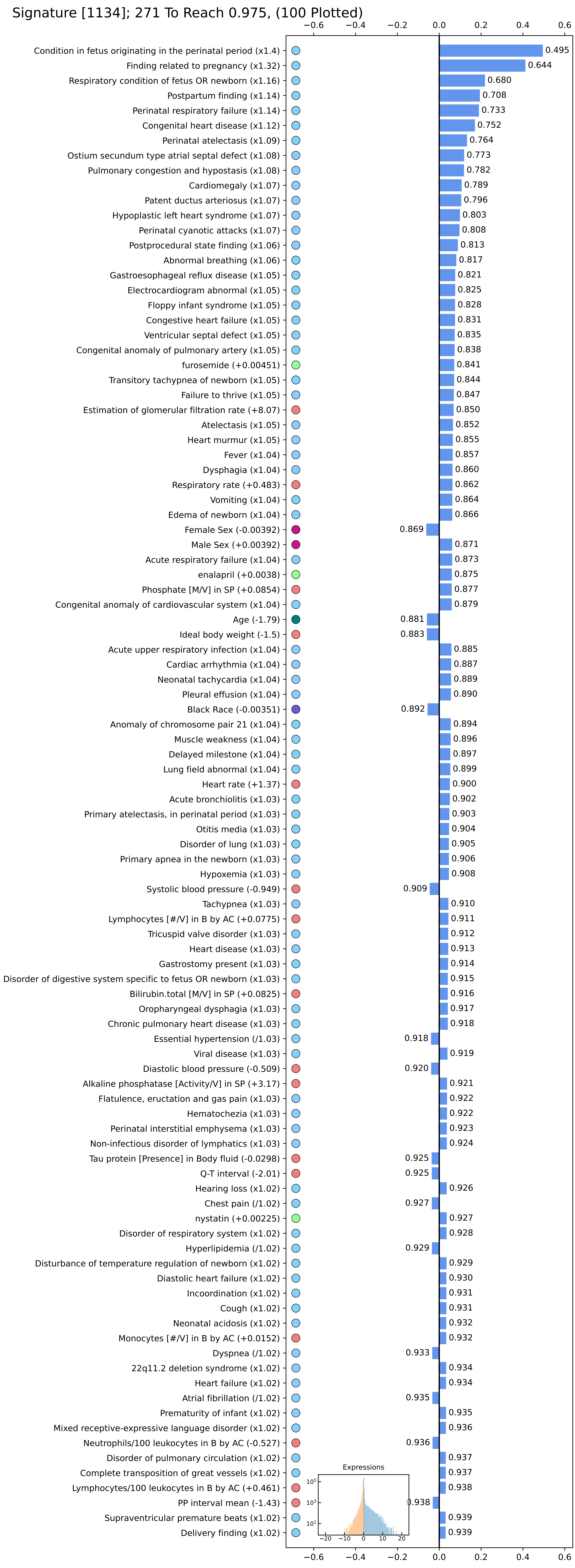

Signature [1134]; 271 To Reach 0.975, (100 Plotted)
Condition in fetus originating in the perinatal period (x1.4) 0.495
Finding related to pregnancy (x1.32) 0.644
Respiratory condition of fetus OR newborn (x1.16) 0.680
Postpartum finding (x1.14) 0.708
Perinatal respiratory failure (x1.14) 0.733
Congenital heart disease (x1.12) 0.752
Perinatal atelectasis (x1.09) 0.764
Ostium secundum type atrial septal defect (x1.08) 0.773
Pulmonary congestion and hypostasis (x1.08) 0.782
Cardiomegaly (x1.07) 0.789
Patent ductus arteriosus (x1.07) 0.796
Hypoplastic left heart syndrome (x1.07) 0.803
Perinatal cyanotic attacks (x1.07) 0.808
Postprocedural state finding (x1.06) 0.813
Abnormal breathing (x1.06) 0.817
Gastroesophageal reflux disease (x1.05) 0.821
Electrocardiogram abnormal (x1.05) 0.825
Floppy infant syndrome (x1.05) 0.828
Congestive heart failure (x1.05) 0.831
Ventricular septal defect (x1.05) 0.835
Congenital anomaly of pulmonary artery (x1.05) 0.838
furosemide (+0.00451) 0.841
Transitory tachypnea of newborn (x1.05) 0.844
Failure to thrive (x1.05) 0.847
Estimation of glomerular filtration rate (+8.07) 0.850
Atelectasis (x1.05) 0.852
Heart murmur (x1.05) 0.855
Fever (x1.04) 0.857
Dysphagia (x1.04) 0.860
Respiratory rate (+0.483) 0.862
Vomiting (x1.04) 0.864
Edema of newborn (x1.04) 0.866
Female Sex (-0.00392) 0.869
Male Sex (+0.00392) 0.871
Acute respiratory failure (x1.04) 0.873
enalapril (+0.0038) 0.875
Phosphate [M/V] in SP (+0.0854) 0.877
Congenital anomaly of cardiovascular system (x1.04) 0.879
Age (-1.79) 0.881
Ideal body weight (-1.5) 0.883
Acute upper respiratory infection (x1.04) 0.885
Cardiac arrhythmia (x1.04) 0.887
Neonatal tachycardia (x1.04) 0.889
Pleural effusion (x1.04) 0.890
Black Race (-0.00351) 0.892
Anomaly of chromosome pair 21 (x1.04) 0.894
Muscle weakness (x1.04) 0.896
Delayed milestone (x1.04) 0.897
Lung field abnormal (x1.04) 0.899
Heart rate (+1.37) 0.900
Acute bronchiolitis (x1.03) 0.902
Primary atelectasis, in perinatal period (x1.03) 0.903
Otitis media (x1.03) 0.904
Disorder of lung (x1.03) 0.905
Primary apnea in the newborn (x1.03) 0.906
Hypoxemia (x1.03) 0.908
Systolic blood pressure (-0.949) 0.909
Tachypnea (x1.03) 0.910
Lymphocytes [#/V] in B by AC (+0.0775) 0.911
Tricuspid valve disorder (x1.03) 0.912
Heart disease (x1.03) 0.913
Gastrostomy present (x1.03) 0.914
Disorder of digestive system specific to fetus OR newborn (x1.03) 0.915
Bilirubin.total [M/V] in SP (+0.0825) 0.916
Oropharyngeal dysphagia (x1.03) 0.917
Chronic pulmonary heart disease (x1.03) 0.918
Essential hypertension (/1.03) 0.918
Viral disease (x1.03) 0.919
Diastolic blood pressure (-0.509) 0.920
Alkaline phosphatase [Activity/V] in SP (+3.17) 0.921
Flatulence, eructation and gas pain (x1.03) 0.922
Hematochezia (x1.03) 0.922
Perinatal interstitial emphysema (x1.03) 0.923
Non-infectious disorder of lymphatics (x1.03) 0.924
Tau protein [Presence] in Body fluid (-0.0298) 0.925
Q-T interval (-2.01) 0.925
Hearing loss (x1.02) 0.926
Chest pain (/1.02) 0.927
nystatin (+0.00225) 0.927
Disorder of respiratory system (x1.02) 0.928
Hyperlipidemia (/1.02) 0.929
Disturbance of temperature regulation of newborn (x1.02) 0.929
Diastolic heart failure (x1.02) 0.930
Incoordination (x1.02) 0.931
Cough (x1.02) 0.931
Neonatal acidosis (x1.02) 0.932
Monocytes [#/V] in B by AC (+0.0152) 0.932
Dyspnea (/1.02) 0.933
22q11.2 deletion syndrome (x1.02) 0.934
Heart failure (x1.02) 0.934
Atrial fibrillation (/1.02) 0.935
Prematurity of infant (x1.02) 0.935
Mixed receptive-expressive language disorder (x1.02) 0.936
Neutrophils/100 leukocytes in B by AC (-0.527) 0.936
Disorder of pulmonary circulation (x1.02) 0.937
Complete transposition of great vessels (x1.02) 0.937
Lymphocytes/100 leukocytes in B by AC (+0.461) 0.938
PP interval mean (-1.43) 0.938
Supraventricular premature beats (x1.02) 0.939
Delivery finding (x1.02) 0.939
Expressions
−0.6 −0.4 −0.2 0.0 0.2 0.4 0.6

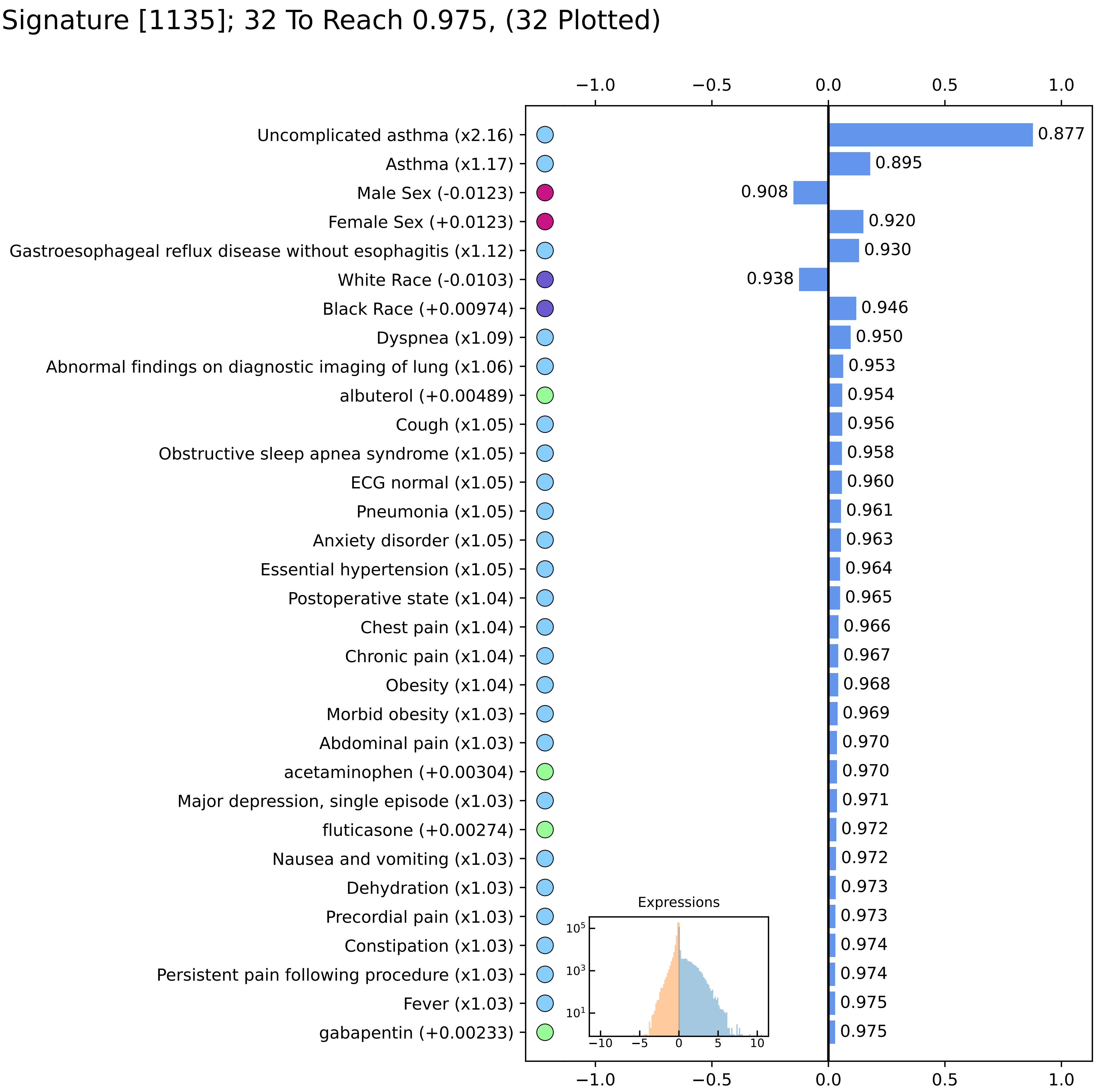
Signature [1135]; 32 To Reach 0.975, (32 Plotted)
Uncomplicated asthma (x2.16) 0.877
Asthma (x1.17) 0.895
Male Sex (-0.0123) 0.908
Female Sex (+0.0123) 0.920
Gastroesophageal reflux disease without esophagitis (x1.12) 0.930
White Race (-0.0103) 0.938
Black Race (+0.00974) 0.946
Dyspnea (x1.09) 0.950
Abnormal findings on diagnostic imaging of lung (x1.06) 0.953
albuterol (+0.00489) 0.954
Cough (x1.05) 0.956
Obstructive sleep apnea syndrome (x1.05) 0.958
ECG normal (x1.05) 0.960
Pneumonia (x1.05) 0.961
Anxiety disorder (x1.05) 0.963
Essential hypertension (x1.05) 0.964
Postoperative state (x1.04) 0.965
Chest pain (x1.04) 0.966
Chronic pain (x1.04) 0.967
Obesity (x1.04) 0.968
Morbid obesity (x1.03) 0.969
Abdominal pain (x1.03) 0.970
acetaminophen (+0.00304) 0.970
Major depression, single episode (x1.03) 0.971
fluticasone (+0.00274) 0.972
Nausea and vomiting (x1.03) 0.972
Dehydration (x1.03) 0.973
Precordial pain (x1.03) 0.973
Constipation (x1.03) 0.974
Persistent pain following procedure (x1.03) 0.974
Fever (x1.03) 0.975
gabapentin (+0.00233) 0.975
Expressions

Signature [1136]; 1 To Reach 0.975, (10 Plotted)

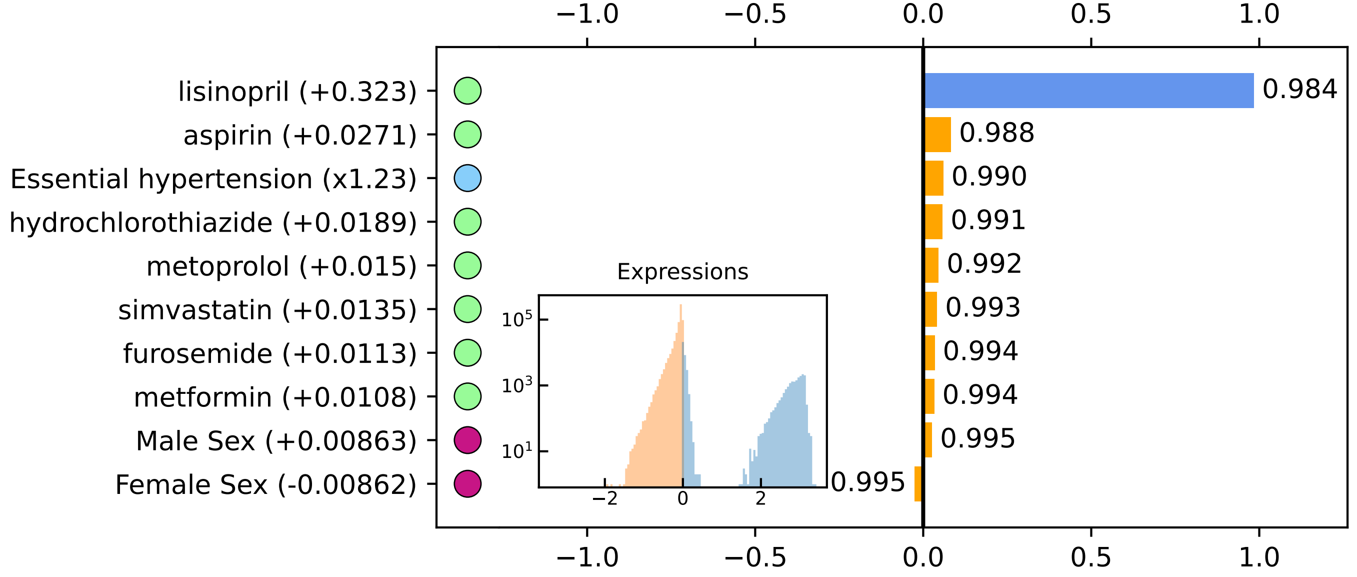

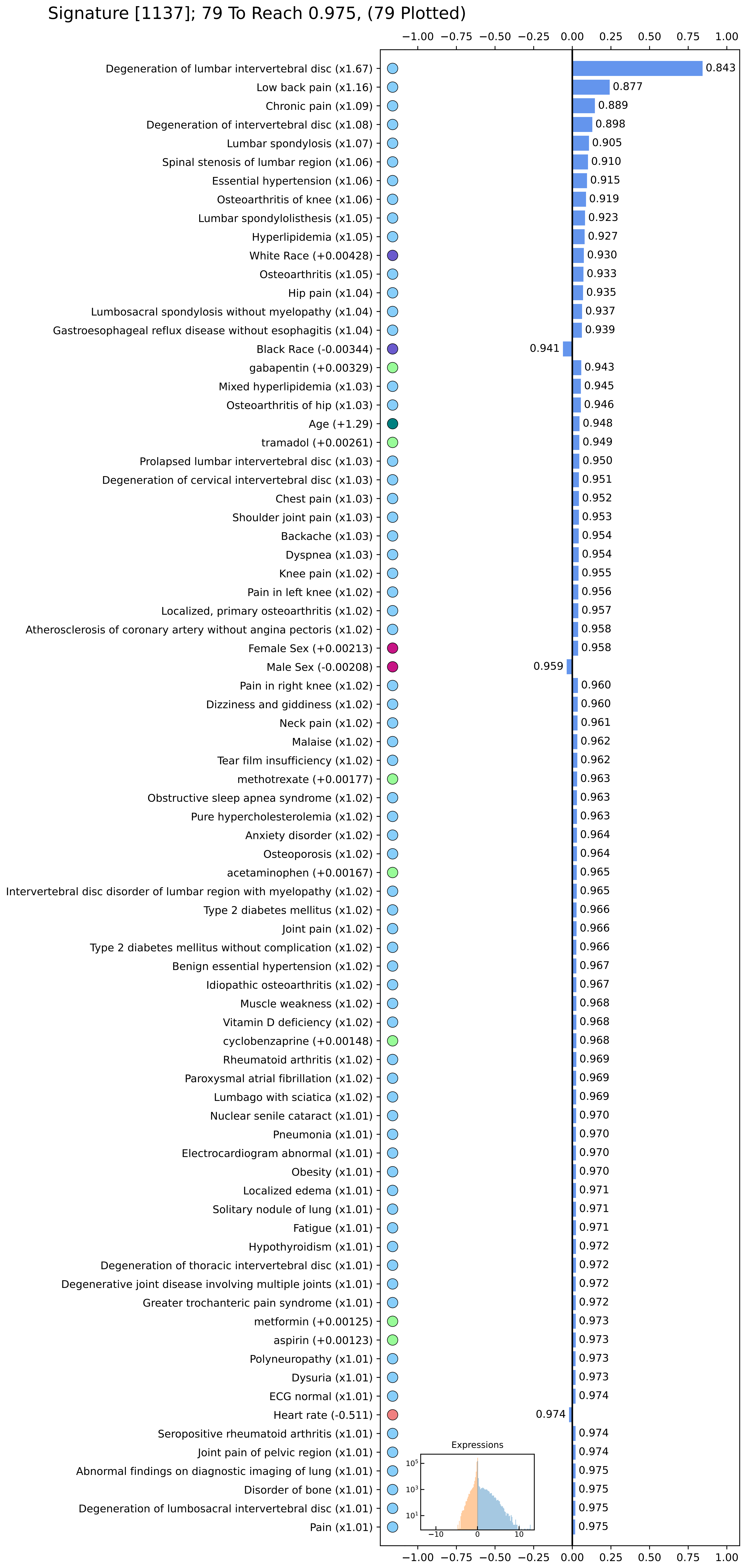
Signature [1137]; 79 To Reach 0.975, (79 Plotted)
Degeneration of lumbar intervertebral disc (x1.67) 0.843
Low back pain (x1.16) 0.877
Chronic pain (x1.09) 0.889
Degeneration of intervertebral disc (x1.08) 0.898
Lumbar spondylosis (x1.07) 0.905
Spinal stenosis of lumbar region (x1.06) 0.910
Essential hypertension (x1.06) 0.915
Osteoarthritis of knee (x1.06) 0.919
Lumbar spondylolisthesis (x1.05) 0.923
Hyperlipidemia (x1.05) 0.927
White Race (+0.00428) 0.930
Osteoarthritis (x1.05) 0.933
Hip pain (x1.04) 0.935
Lumbosacral spondylosis without myelopathy (x1.04) 0.937
Gastroesophageal reflux disease without esophagitis (x1.04) 0.939
Black Race (-0.00344) 0.941
gabapentin (+0.00329) 0.943
Mixed hyperlipidemia (x1.03) 0.945
Osteoarthritis of hip (x1.03) 0.946
Age (+1.29) 0.948
tramadol (+0.00261) 0.949
Prolapsed lumbar intervertebral disc (x1.03) 0.950
Degeneration of cervical intervertebral disc (x1.03) 0.951
Chest pain (x1.03) 0.952
Shoulder joint pain (x1.03) 0.953
Backache (x1.03) 0.954
Dyspnea (x1.03) 0.954
Knee pain (x1.02) 0.955
Pain in left knee (x1.02) 0.956
Localized, primary osteoarthritis (x1.02) 0.957
Atherosclerosis of coronary artery without angina pectoris (x1.02) 0.958
Female Sex (+0.00213) 0.958
Male Sex (-0.00208) 0.959
Pain in right knee (x1.02) 0.960
Dizziness and giddiness (x1.02) 0.960
Neck pain (x1.02) 0.961
Malaise (x1.02) 0.962
Tear film insufficiency (x1.02) 0.962
methotrexate (+0.00177) 0.963
Obstructive sleep apnea syndrome (x1.02) 0.963
Pure hypercholesterolemia (x1.02) 0.963
Anxiety disorder (x1.02) 0.964
Osteoporosis (x1.02) 0.964
acetaminophen (+0.00167) 0.965
Intervertebral disc disorder of lumbar region with myelopathy (x1.02) 0.965
Type 2 diabetes mellitus (x1.02) 0.966
Joint pain (x1.02) 0.966
Type 2 diabetes mellitus without complication (x1.02) 0.966
Benign essential hypertension (x1.02) 0.967
Idiopathic osteoarthritis (x1.02) 0.967
Muscle weakness (x1.02) 0.968
Vitamin D deficiency (x1.02) 0.968
cyclobenzaprine (+0.00148) 0.968
Rheumatoid arthritis (x1.02) 0.969
Paroxysmal atrial fibrillation (x1.02) 0.969
Lumbago with sciatica (x1.02) 0.969
Nuclear senile cataract (x1.01) 0.970
Pneumonia (x1.01) 0.970
Electrocardiogram abnormal (x1.01) 0.970
Obesity (x1.01) 0.970
Localized edema (x1.01) 0.971
Solitary nodule of lung (x1.01) 0.971
Fatigue (x1.01) 0.971
Hypothyroidism (x1.01) 0.972
Degeneration of thoracic intervertebral disc (x1.01) 0.972
Degenerative joint disease involving multiple joints (x1.01) 0.972
Greater trochanteric pain syndrome (x1.01) 0.972
metformin (+0.00125) 0.973
aspirin (+0.00123) 0.973
Polyneuropathy (x1.01) 0.973
Dysuria (x1.01) 0.973
ECG normal (x1.01) 0.974
Heart rate (-0.511) 0.974
Seropositive rheumatoid arthritis (x1.01) 0.974
Joint pain of pelvic region (x1.01) 0.974
Abnormal findings on diagnostic imaging of lung (x1.01) 0.975
Disorder of bone (x1.01) 0.975
Degeneration of lumbosacral intervertebral disc (x1.01) 0.975
Pain (x1.01) 0.975
−1.00 −0.75 −0.50 −0.25 0.00 0.25 0.50 0.75 1.00
Expressions

Signature [1138]; 1 To Reach 0.975, (10 Plotted)

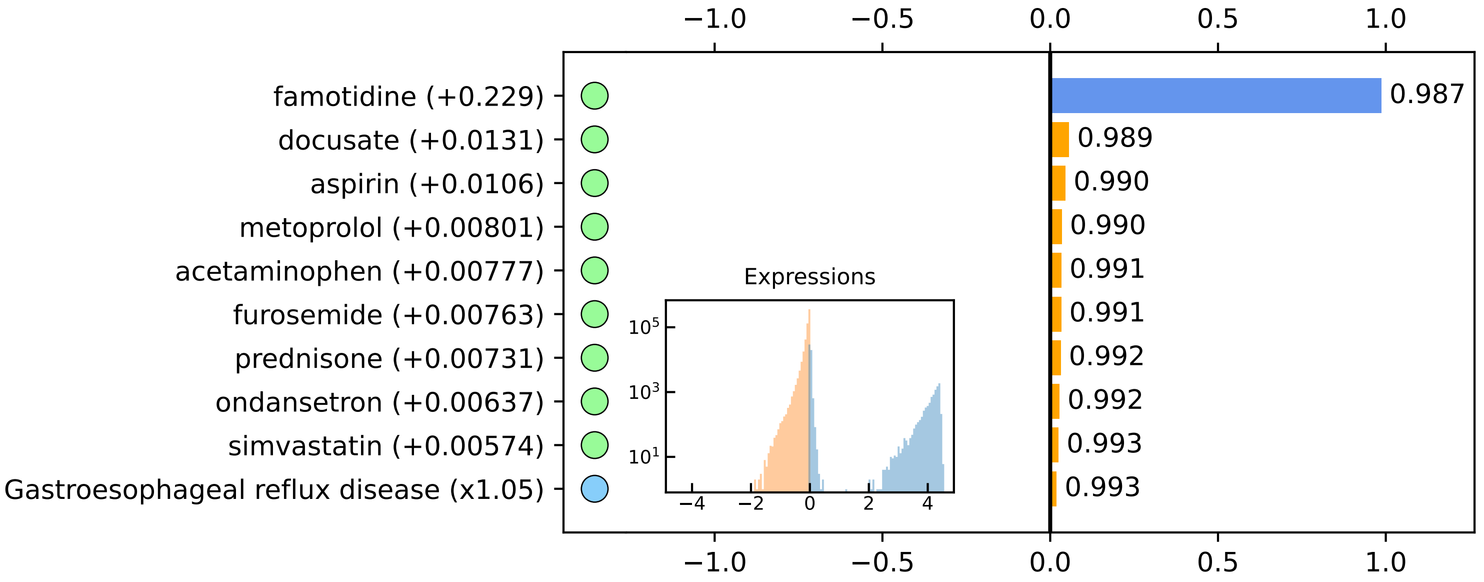

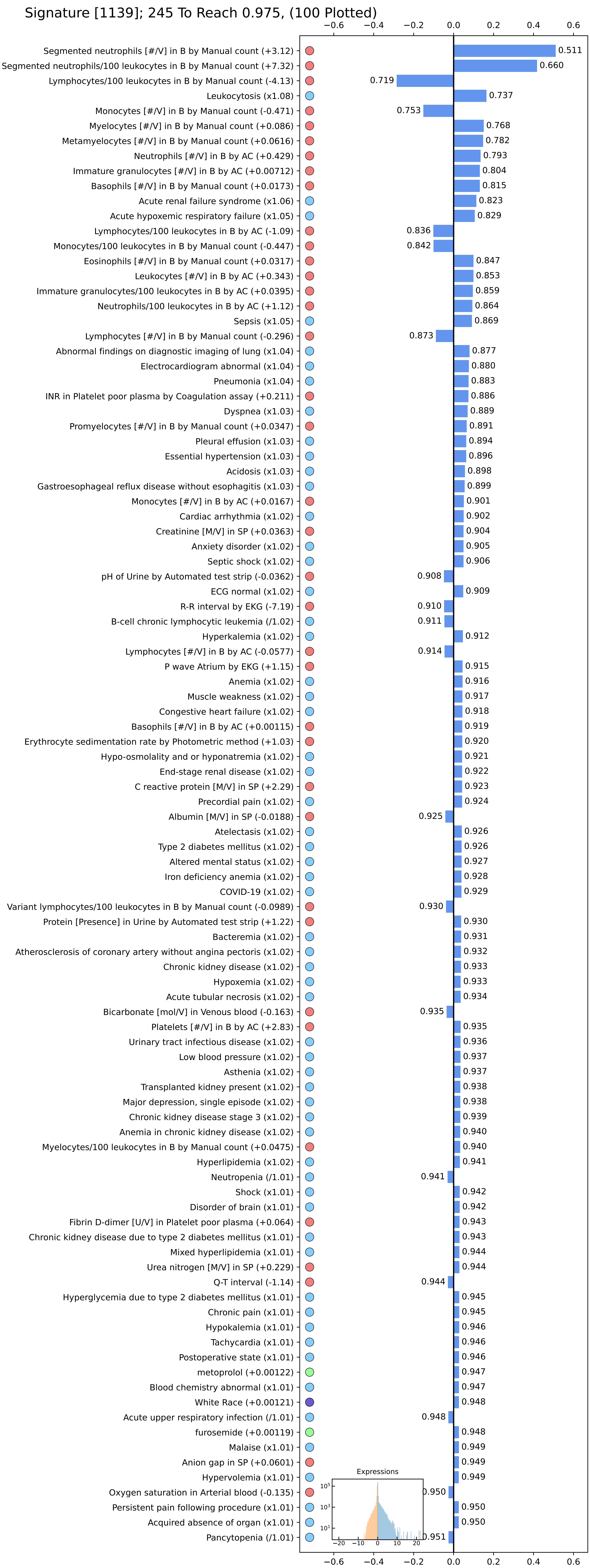

Signature [1139]; 245 To Reach 0.975, (100 Plotted)
Segmented neutrophils [#/V] in B by Manual count (+3.12) 0.511
Segmented neutrophils/100 leukocytes in B by Manual count (+7.32) 0.660
Lymphocytes/100 leukocytes in B by Manual count (-4.13) 0.719
Leukocytosis (x1.08) 0.737
Monocytes [#/V] in B by Manual count (-0.471) 0.753
Myelocytes [#/V] in B by Manual count (+0.086) 0.768
Metamyelocytes [#/V] in B by Manual count (+0.0616) 0.782
Neutrophils [#/V] in B by AC (+0.429) 0.793
Immature granulocytes [#/V] in B by AC (+0.00712) 0.804
Basophils [#/V] in B by Manual count (+0.0173) 0.815
Acute renal failure syndrome (x1.06) 0.823
Acute hypoxemic respiratory failure (x1.05) 0.829
Lymphocytes/100 leukocytes in B by AC (-1.09) 0.836
Monocytes/100 leukocytes in B by Manual count (-0.447) 0.842
Eosinophils [#/V] in B by Manual count (+0.0317) 0.847
Leukocytes [#/V] in B by AC (+0.343) 0.853
Immature granulocytes/100 leukocytes in B by AC (+0.0395) 0.859
Neutrophils/100 leukocytes in B by AC (+1.12) 0.864
Sepsis (x1.05) 0.869
Lymphocytes [#/V] in B by Manual count (-0.296) 0.873
Abnormal findings on diagnostic imaging of lung (x1.04) 0.877
Electrocardiogram abnormal (x1.04) 0.880
Pneumonia (x1.04) 0.883
INR in Platelet poor plasma by Coagulation assay (+0.211) 0.886
Dyspnea (x1.03) 0.889
Promyelocytes [#/V] in B by Manual count (+0.0347) 0.891
Pleural effusion (x1.03) 0.894
Essential hypertension (x1.03) 0.896
Acidosis (x1.03) 0.898
Gastroesophageal reflux disease without esophagitis (x1.03) 0.899
Monocytes [#/V] in B by AC (+0.0167) 0.901
Cardiac arrhythmia (x1.02) 0.902
Creatinine [M/V] in SP (+0.0363) 0.904
Anxiety disorder (x1.02) 0.905
Septic shock (x1.02) 0.906
pH of Urine by Automated test strip (-0.0362) 0.908
ECG normal (x1.02) 0.909
R-R interval by EKG (-7.19) 0.910
B-cell chronic lymphocytic leukemia (/1.02) 0.911
Hyperkalemia (x1.02) 0.912
Lymphocytes [#/V] in B by AC (-0.0577) 0.914
P wave Atrium by EKG (+1.15) 0.915
Anemia (x1.02) 0.916
Muscle weakness (x1.02) 0.917
Congestive heart failure (x1.02) 0.918
Basophils [#/V] in B by AC (+0.00115) 0.919
Erythrocyte sedimentation rate by Photometric method (+1.03) 0.920
Hypo-osmolality and or hyponatremia (x1.02) 0.921
End-stage renal disease (x1.02) 0.922
C reactive protein [M/V] in SP (+2.29) 0.923
Precordial pain (x1.02) 0.924
Albumin [M/V] in SP (-0.0188) 0.925
Atelectasis (x1.02) 0.926
Type 2 diabetes mellitus (x1.02) 0.926
Altered mental status (x1.02) 0.927
Iron deficiency anemia (x1.02) 0.928
COVID-19 (x1.02) 0.929
Variant lymphocytes/100 leukocytes in B by Manual count (-0.0989) 0.930
Protein [Presence] in Urine by Automated test strip (+1.22) 0.930
Bacteremia (x1.02) 0.931
Atherosclerosis of coronary artery without angina pectoris (x1.02) 0.932
Chronic kidney disease (x1.02) 0.933
Hypoxemia (x1.02) 0.933
Acute tubular necrosis (x1.02) 0.934
Bicarbonate [mol/V] in Venous blood (-0.163) 0.935
Platelets [#/V] in B by AC (+2.83) 0.935
Urinary tract infectious disease (x1.02) 0.936
Low blood pressure (x1.02) 0.937
Asthenia (x1.02) 0.937
Transplanted kidney present (x1.02) 0.938
Major depression, single episode (x1.02) 0.938
Chronic kidney disease stage 3 (x1.02) 0.939
Anemia in chronic kidney disease (x1.02) 0.940
Myelocytes/100 leukocytes in B by Manual count (+0.0475) 0.940
Hyperlipidemia (x1.02) 0.941
Neutropenia (/1.01) 0.941
Shock (x1.01) 0.942
Disorder of brain (x1.01) 0.942
Fibrin D-dimer [U/V] in Platelet poor plasma (+0.064) 0.943
Chronic kidney disease due to type 2 diabetes mellitus (x1.01) 0.943
Mixed hyperlipidemia (x1.01) 0.944
Urea nitrogen [M/V] in SP (+0.229) 0.944
Q-T interval (-1.14) 0.944
Hyperglycemia due to type 2 diabetes mellitus (x1.01) 0.945
Chronic pain (x1.01) 0.945
Hypokalemia (x1.01) 0.946
Tachycardia (x1.01) 0.946
Postoperative state (x1.01) 0.946
metoprolol (+0.00122) 0.947
Blood chemistry abnormal (x1.01) 0.947
White Race (+0.00121) 0.948
Acute upper respiratory infection (/1.01) 0.948
furosemide (+0.00119) 0.948
Malaise (x1.01) 0.949
Anion gap in SP (+0.0601) 0.949
Hypervolemia (x1.01) 0.949
Oxygen saturation in Arterial blood (-0.135) 0.950
Persistent pain following procedure (x1.01) 0.950
Acquired absence of organ (x1.01) 0.950
Pancytopenia (/1.01) 0.951
Expressions
−0.6 −0.4 −0.2 0.0 0.2 0.4 0.6

Signature [1140]; 1 To Reach 0.975, (10 Plotted)

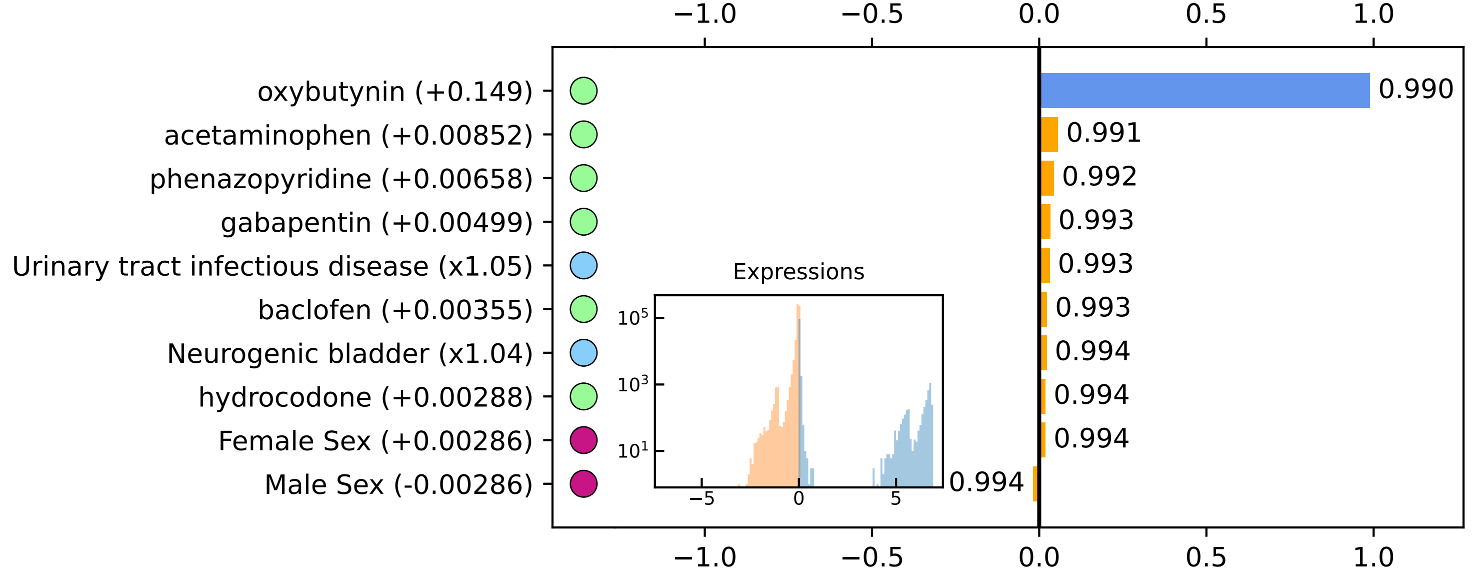

Signature [1141]; 1 To Reach 0.975, (10 Plotted)

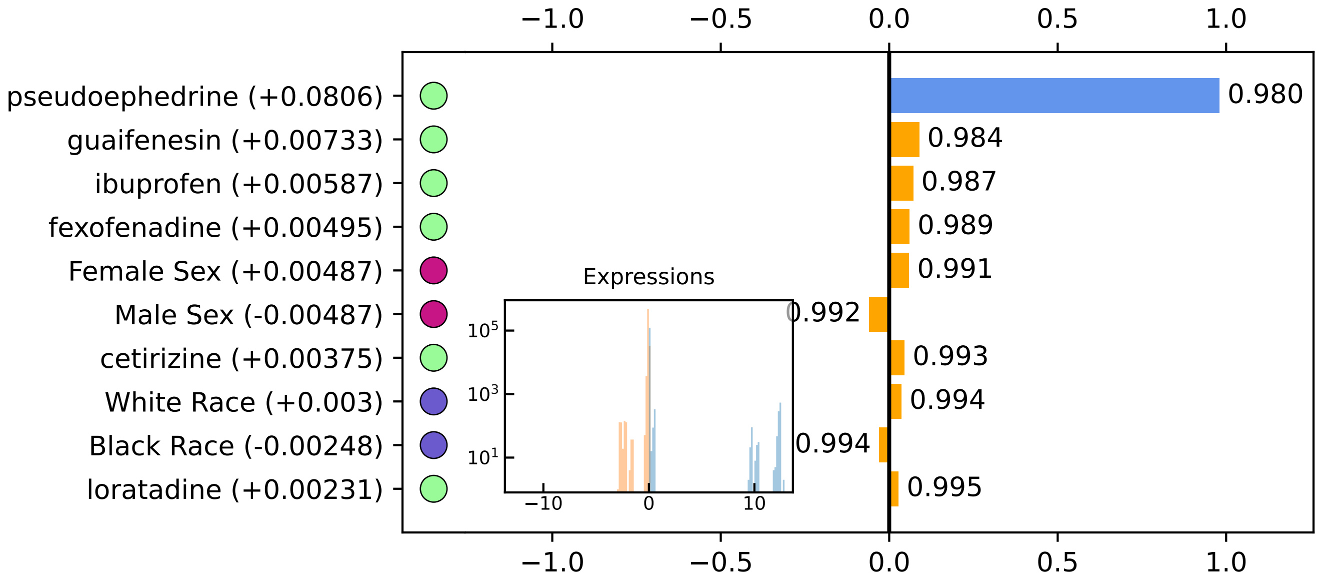

Signature [1142]; 3 To Reach 0.975, (10 Plotted)

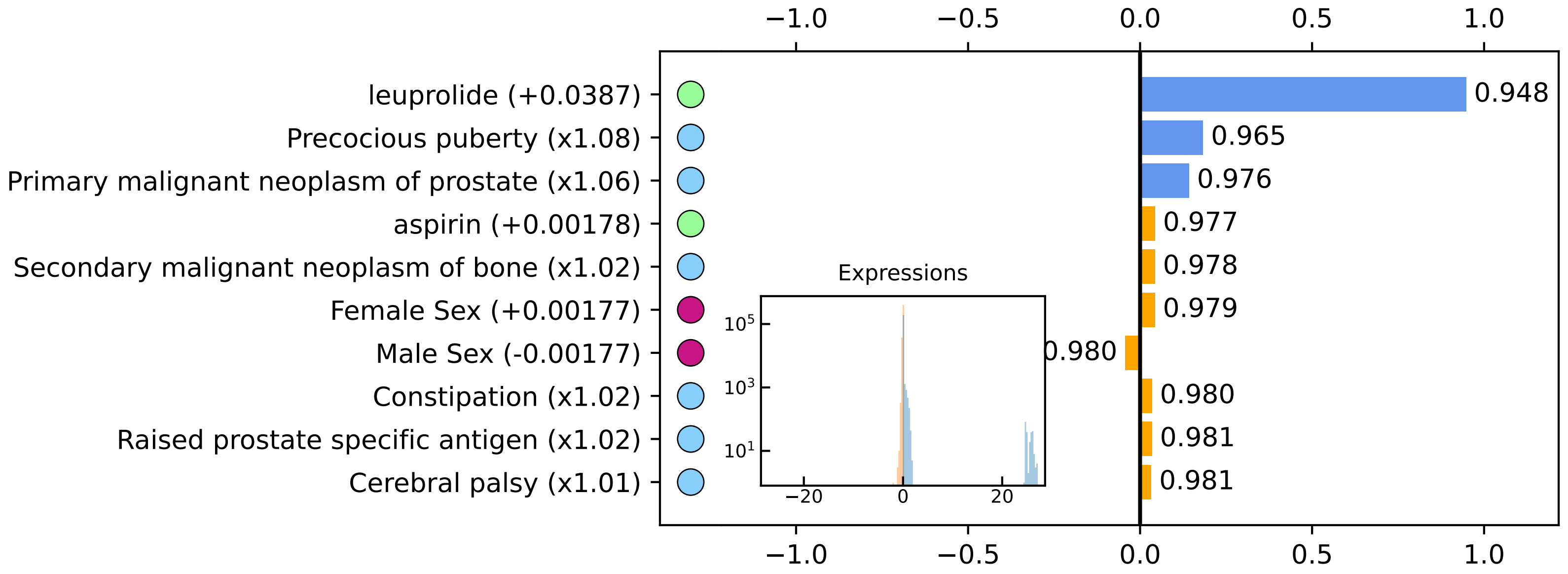

Signature [1143]; 1012 To Reach 0.975, (100 Plotted)

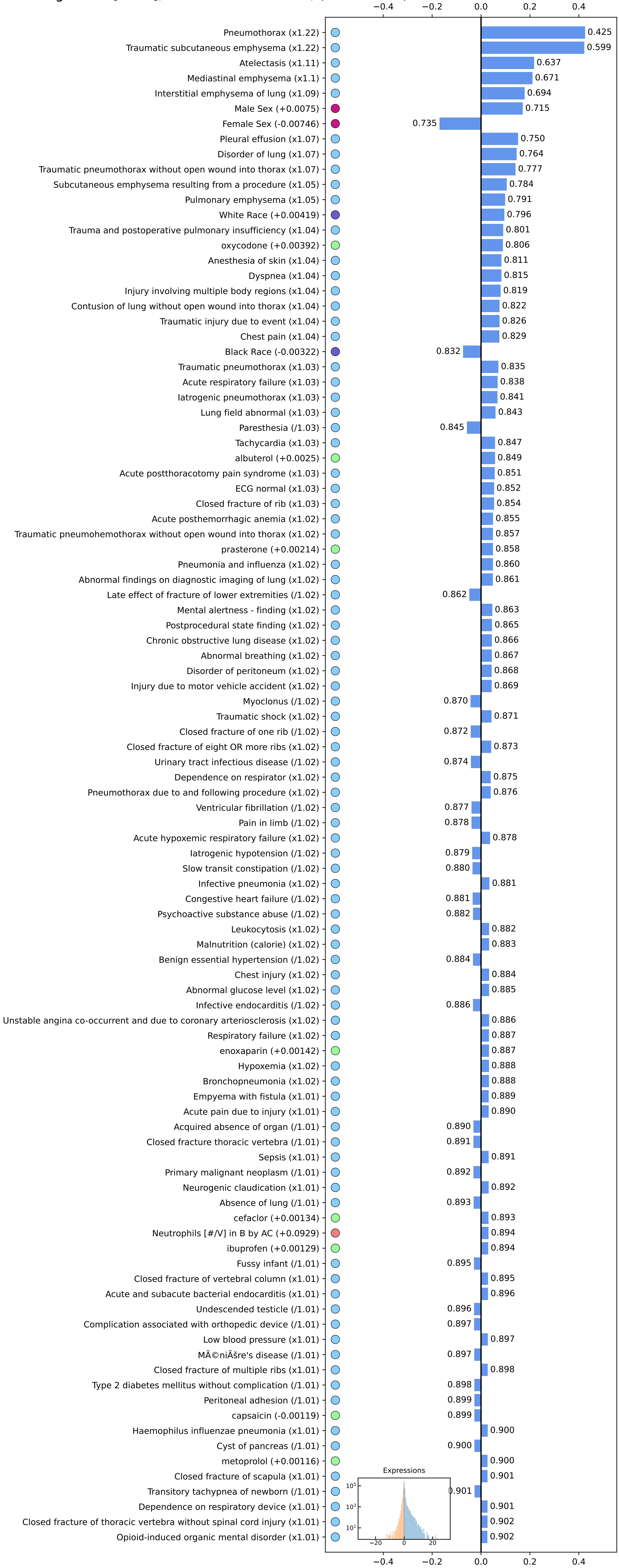

Signature [1144]; 2 To Reach 0.975, (10 Plotted)

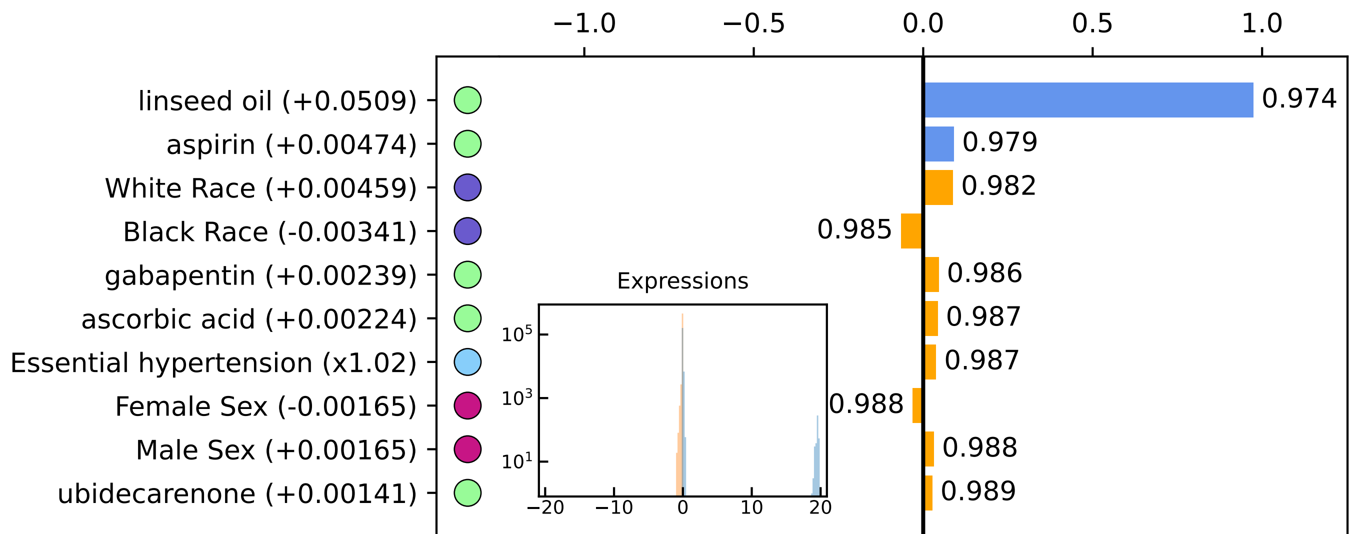

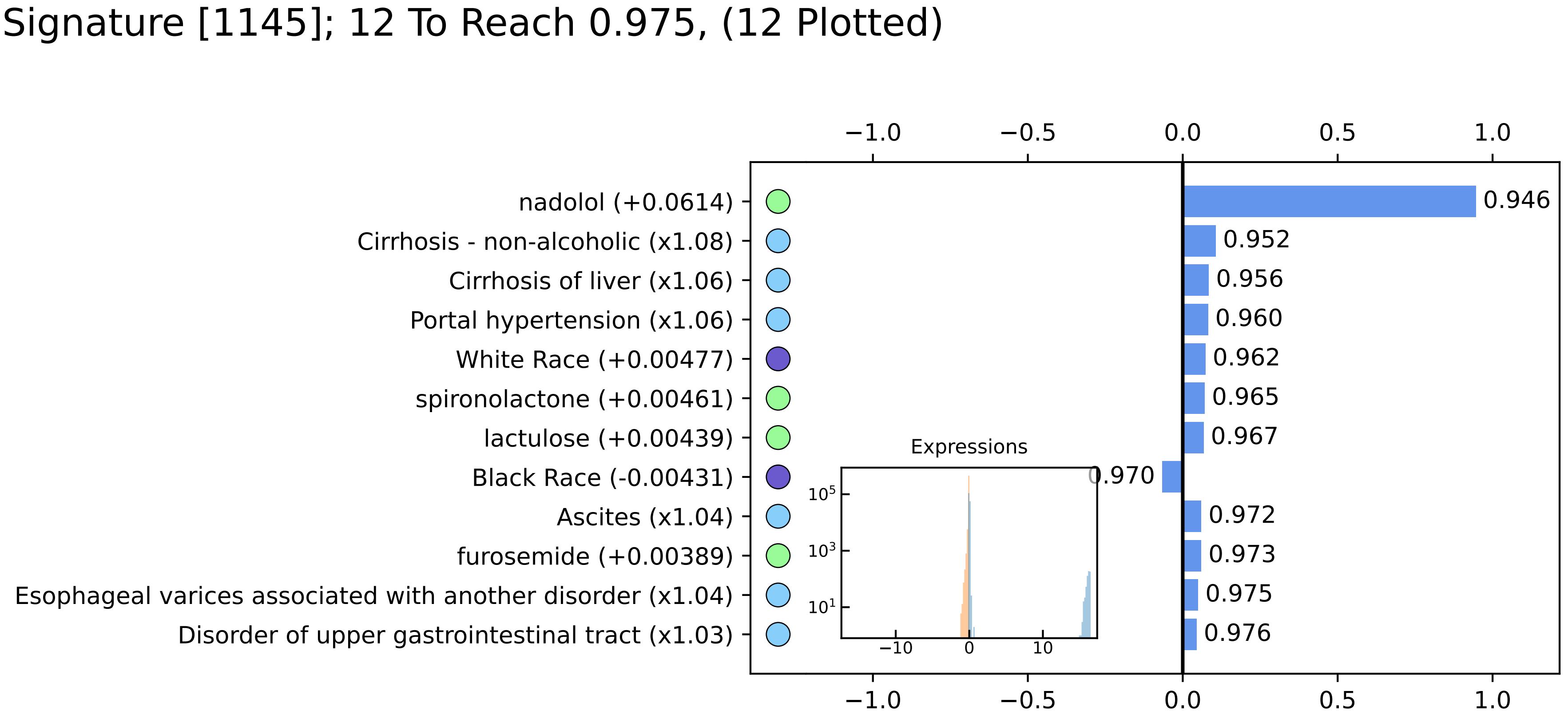

Signature [1145]; 12 To Reach 0.975, (12 Plotted)
nadolol (+0.0614)
Cirrhosis - non-alcoholic (x1.08)
Cirrhosis of liver (x1.06)
Portal hypertension (x1.06)
White Race (+0.00477)
spironolactone (+0.00461)
lactulose (+0.00439)
Black Race (-0.00431)
Ascites (x1.04)
furosemide (+0.00389)
Esophageal varices associated with another disorder (x1.04)
Disorder of upper gastrointestinal tract (x1.03)
0.946
0.952
0.956
0.960
0.962
0.965
0.967
0.970
0.972
0.973
0.975
0.976
−1.0
−0.5
0.0
0.5
1.0
Expressions
10^5
10^3
10^1
−10
0
10

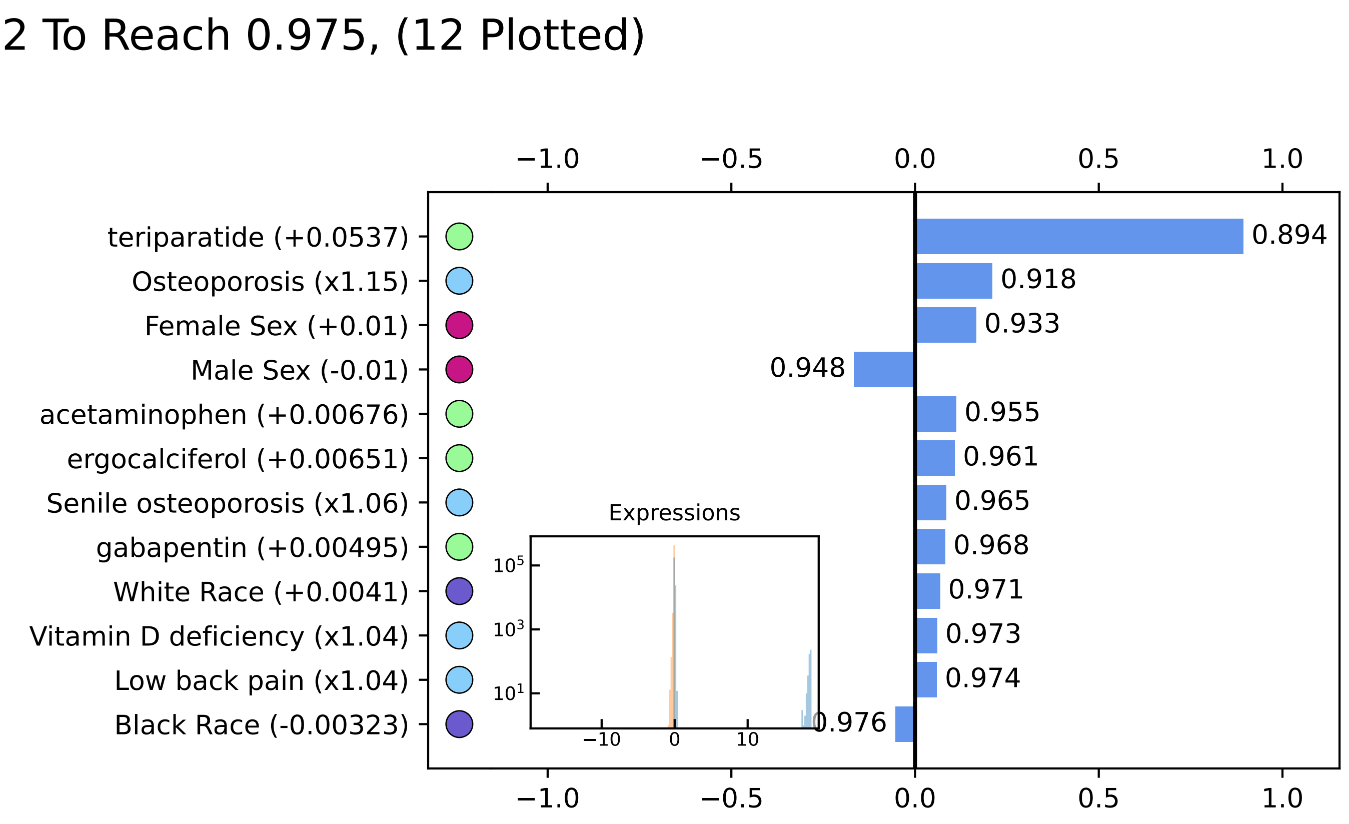

Signature [1146]; 12 To Reach 0.975, (12 Plotted)
teriparatide (+0.0537)
Osteoporosis (x1.15)
Female Sex (+0.01)
Male Sex (-0.01)
acetaminophen (+0.00676)
ergocalciferol (+0.00651)
Senile osteoporosis (x1.06)
gabapentin (+0.00495)
White Race (+0.0041)
Vitamin D deficiency (x1.04)
Low back pain (x1.04)
Black Race (-0.00323)
0.894
0.918
0.933
0.948
0.955
0.961
0.965
0.968
0.971
0.973
0.974
0.976
−1.0
−0.5
0.0
0.5
1.0
Expressions
10^5
10^3
10^1
−10
0
10

Signature [1147]; 86 To Reach 0.975, (86 Plotted)

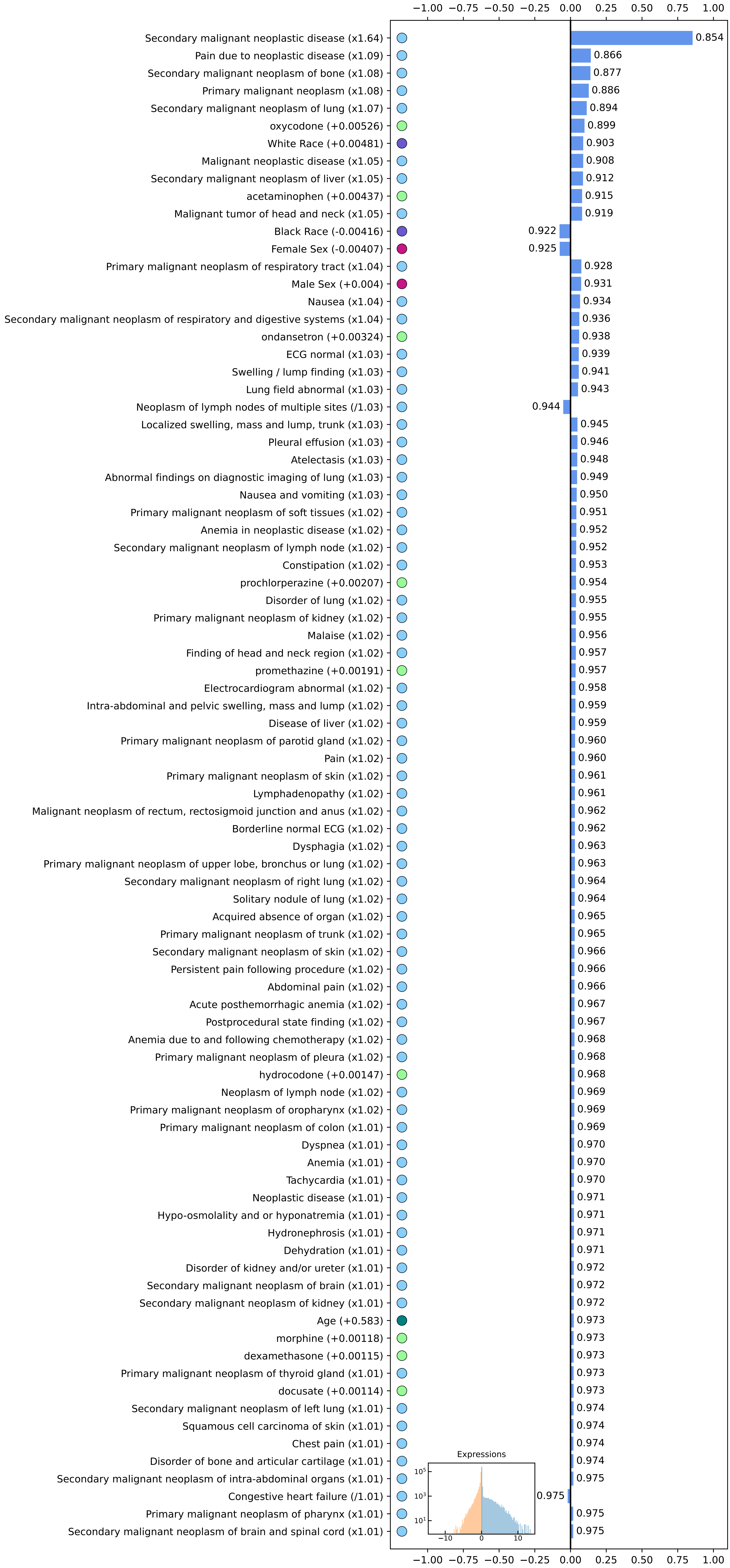

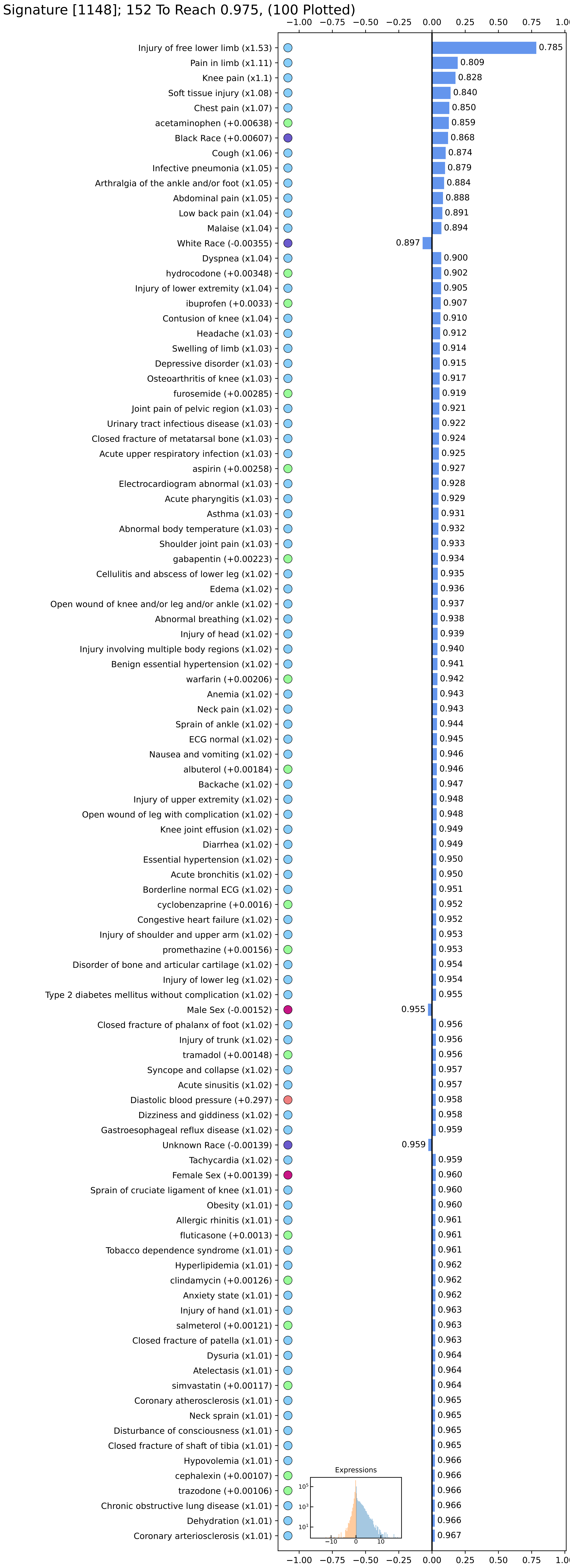
Signature [1148]; 152 To Reach 0.975, (100 Plotted)
−1.00 −0.75 −0.50 −0.25 0.00 0.25 0.50 0.75 1.00
Injury of free lower limb (x1.53) 0.785
Pain in limb (x1.11) 0.809
Knee pain (x1.1) 0.828
Soft tissue injury (x1.08) 0.840
Chest pain (x1.07) 0.850
acetaminophen (+0.00638) 0.859
Black Race (+0.00607) 0.868
Cough (x1.06) 0.874
Infective pneumonia (x1.05) 0.879
Arthralgia of the ankle and/or foot (x1.05) 0.884
Abdominal pain (x1.05) 0.888
Low back pain (x1.04) 0.891
Malaise (x1.04) 0.894
White Race (-0.00355) 0.897
Dyspnea (x1.04) 0.900
hydrocodone (+0.00348) 0.902
Injury of lower extremity (x1.04) 0.905
ibuprofen (+0.0033) 0.907
Contusion of knee (x1.04) 0.910
Headache (x1.03) 0.912
Swelling of limb (x1.03) 0.914
Depressive disorder (x1.03) 0.915
Osteoarthritis of knee (x1.03) 0.917
furosemide (+0.00285) 0.919
Joint pain of pelvic region (x1.03) 0.921
Urinary tract infectious disease (x1.03) 0.922
Closed fracture of metatarsal bone (x1.03) 0.924
Acute upper respiratory infection (x1.03) 0.925
aspirin (+0.00258) 0.927
Electrocardiogram abnormal (x1.03) 0.928
Acute pharyngitis (x1.03) 0.929
Asthma (x1.03) 0.931
Abnormal body temperature (x1.03) 0.932
Shoulder joint pain (x1.03) 0.933
gabapentin (+0.00223) 0.934
Cellulitis and abscess of lower leg (x1.02) 0.935
Edema (x1.02) 0.936
Open wound of knee and/or leg and/or ankle (x1.02) 0.937
Abnormal breathing (x1.02) 0.938
Injury of head (x1.02) 0.939
Injury involving multiple body regions (x1.02) 0.940
Benign essential hypertension (x1.02) 0.941
warfarin (+0.00206) 0.942
Anemia (x1.02) 0.943
Neck pain (x1.02) 0.943
Sprain of ankle (x1.02) 0.944
ECG normal (x1.02) 0.945
Nausea and vomiting (x1.02) 0.946
albuterol (+0.00184) 0.946
Backache (x1.02) 0.947
Injury of upper extremity (x1.02) 0.948
Open wound of leg with complication (x1.02) 0.948
Knee joint effusion (x1.02) 0.949
Diarrhea (x1.02) 0.949
Essential hypertension (x1.02) 0.950
Acute bronchitis (x1.02) 0.950
Borderline normal ECG (x1.02) 0.951
cyclobenzaprine (+0.0016) 0.952
Congestive heart failure (x1.02) 0.952
Injury of shoulder and upper arm (x1.02) 0.953
promethazine (+0.00156) 0.953
Disorder of bone and articular cartilage (x1.02) 0.954
Injury of lower leg (x1.02) 0.954
Type 2 diabetes mellitus without complication (x1.02) 0.955
Male Sex (-0.00152) 0.955
Closed fracture of phalanx of foot (x1.02) 0.956
Injury of trunk (x1.02) 0.956
tramadol (+0.00148) 0.956
Syncope and collapse (x1.02) 0.957
Acute sinusitis (x1.02) 0.957
Diastolic blood pressure (+0.297) 0.958
Dizziness and giddiness (x1.02) 0.958
Gastroesophageal reflux disease (x1.02) 0.959
Unknown Race (-0.00139) 0.959
Tachycardia (x1.02) 0.959
Female Sex (+0.00139) 0.960
Sprain of cruciate ligament of knee (x1.01) 0.960
Obesity (x1.01) 0.960
Allergic rhinitis (x1.01) 0.961
fluticasone (+0.0013) 0.961
Tobacco dependence syndrome (x1.01) 0.961
Hyperlipidemia (x1.01) 0.962
clindamycin (+0.00126) 0.962
Anxiety state (x1.01) 0.962
Injury of hand (x1.01) 0.963
salmeterol (+0.00121) 0.963
Closed fracture of patella (x1.01) 0.963
Dysuria (x1.01) 0.964
Atelectasis (x1.01) 0.964
simvastatin (+0.00117) 0.964
Coronary atherosclerosis (x1.01) 0.965
Neck sprain (x1.01) 0.965
Disturbance of consciousness (x1.01) 0.965
Closed fracture of shaft of tibia (x1.01) 0.965
Hypovolemia (x1.01) 0.966
cephalexin (+0.00107) 0.966
trazodone (+0.00106) 0.966
Chronic obstructive lung disease (x1.01) 0.966
Dehydration (x1.01) 0.966
Coronary arteriosclerosis (x1.01) 0.967
Expressions
10^5
10^3
10^1
−10
0
10
−1.00 −0.75 −0.50 −0.25 0.00 0.25 0.50 0.75 1.00

Signature [1149]; 1 To Reach 0.975, (10 Plotted)

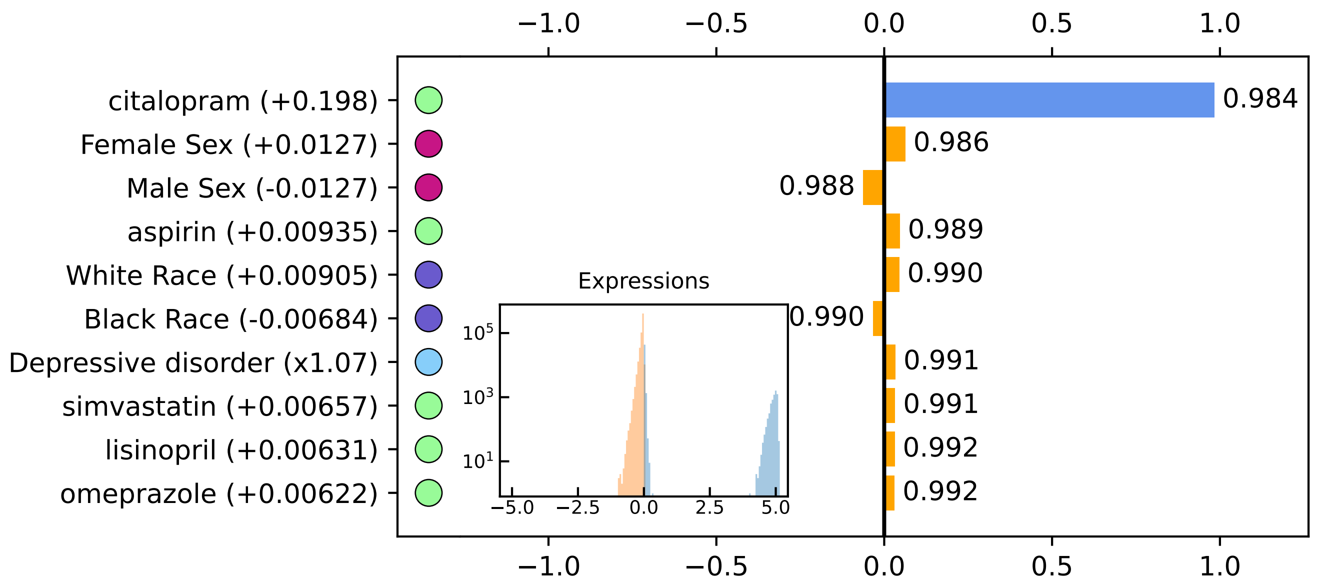

Signature [1150]; 8 To Reach 0.975, (10 Plotted)

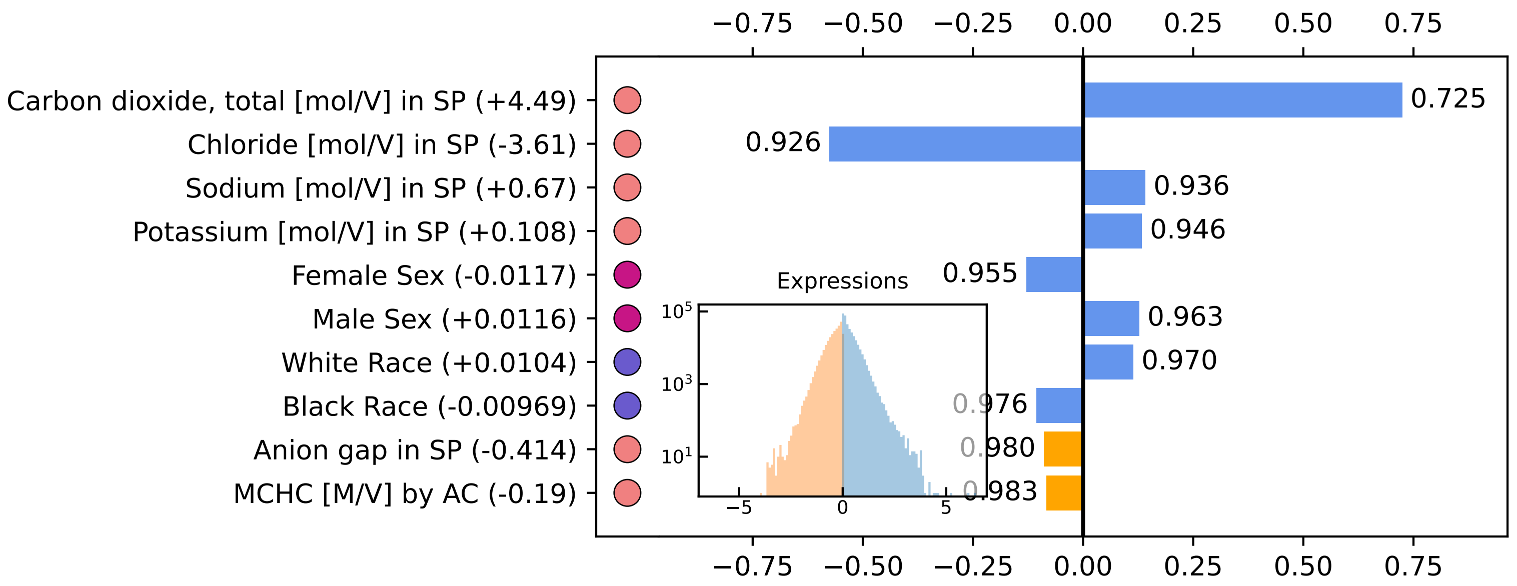

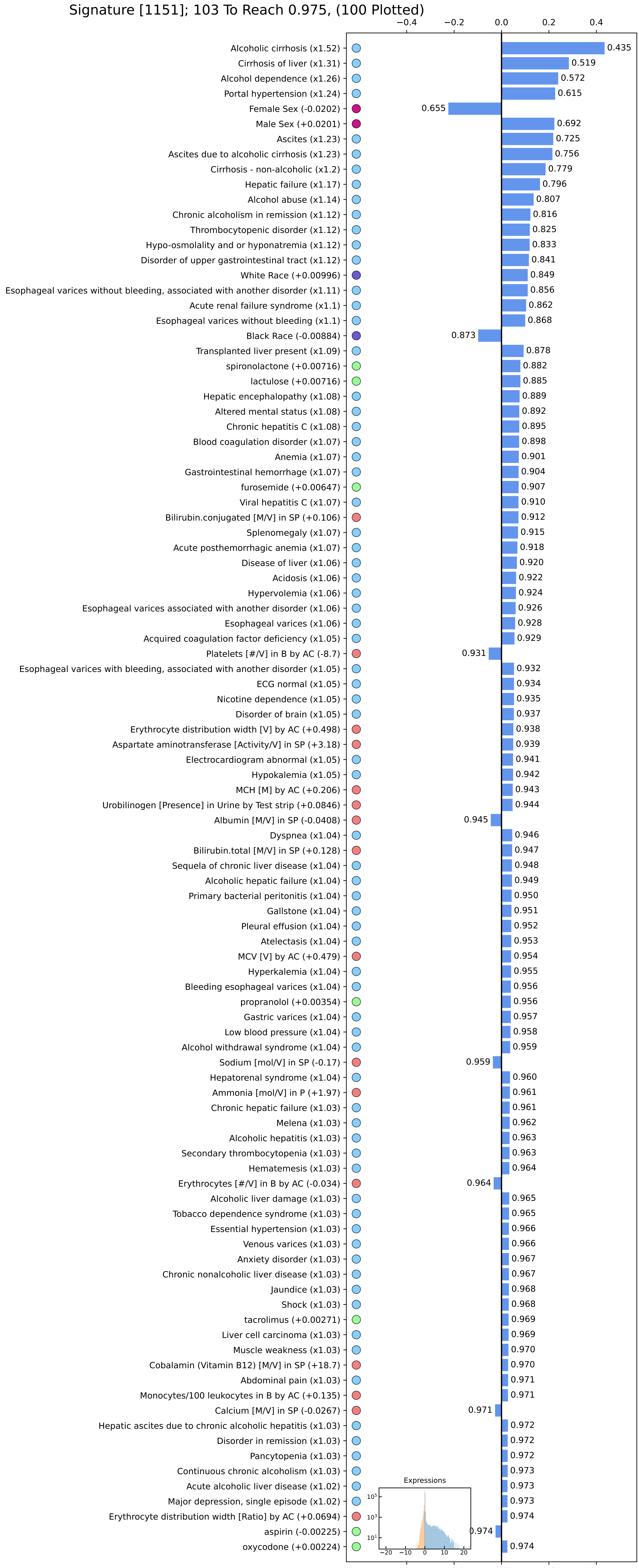

Signature [1151]; 103 To Reach 0.975, (100 Plotted)
Alcoholic cirrhosis (x1.52) 0.435
Cirrhosis of liver (x1.31) 0.519
Alcohol dependence (x1.26) 0.572
Portal hypertension (x1.24) 0.615
Female Sex (-0.0202) 0.655
Male Sex (+0.0201) 0.692
Ascites (x1.23) 0.725
Ascites due to alcoholic cirrhosis (x1.23) 0.756
Cirrhosis - non-alcoholic (x1.2) 0.779
Hepatic failure (x1.17) 0.796
Alcohol abuse (x1.14) 0.807
Chronic alcoholism in remission (x1.12) 0.816
Thrombocytopenic disorder (x1.12) 0.825
Hypo-osmolality and or hyponatremia (x1.12) 0.833
Disorder of upper gastrointestinal tract (x1.12) 0.841
White Race (+0.00996) 0.849
Esophageal varices without bleeding, associated with another disorder (x1.11) 0.856
Acute renal failure syndrome (x1.1) 0.862
Esophageal varices without bleeding (x1.1) 0.868
Black Race (-0.00884) 0.873
Transplanted liver present (x1.09) 0.878
spironolactone (+0.00716) 0.882
lactulose (+0.00716) 0.885
Hepatic encephalopathy (x1.08) 0.889
Altered mental status (x1.08) 0.892
Chronic hepatitis C (x1.08) 0.895
Blood coagulation disorder (x1.07) 0.898
Anemia (x1.07) 0.901
Gastrointestinal hemorrhage (x1.07) 0.904
furosemide (+0.00647) 0.907
Viral hepatitis C (x1.07) 0.910
Bilirubin.conjugated [M/V] in SP (+0.106) 0.912
Splenomegaly (x1.07) 0.915
Acute posthemorrhagic anemia (x1.07) 0.918
Disease of liver (x1.06) 0.920
Acidosis (x1.06) 0.922
Hypervolemia (x1.06) 0.924
Esophageal varices associated with another disorder (x1.06) 0.926
Esophageal varices (x1.06) 0.928
Acquired coagulation factor deficiency (x1.05) 0.929
Platelets [#/V] in B by AC (-8.7) 0.931
Esophageal varices with bleeding, associated with another disorder (x1.05) 0.932
ECG normal (x1.05) 0.934
Nicotine dependence (x1.05) 0.935
Disorder of brain (x1.05) 0.937
Erythrocyte distribution width [V] by AC (+0.498) 0.938
Aspartate aminotransferase [Activity/V] in SP (+3.18) 0.939
Electrocardiogram abnormal (x1.05) 0.941
Hypokalemia (x1.05) 0.942
MCH [M] by AC (+0.206) 0.943
Urobilinogen [Presence] in Urine by Test strip (+0.0846) 0.944
Albumin [M/V] in SP (-0.0408) 0.945
Dyspnea (x1.04) 0.946
Bilirubin.total [M/V] in SP (+0.128) 0.947
Sequela of chronic liver disease (x1.04) 0.948
Alcoholic hepatic failure (x1.04) 0.949
Primary bacterial peritonitis (x1.04) 0.950
Gallstone (x1.04) 0.951
Pleural effusion (x1.04) 0.952
Atelectasis (x1.04) 0.953
MCV [V] by AC (+0.479) 0.954
Hyperkalemia (x1.04) 0.955
Bleeding esophageal varices (x1.04) 0.956
propranolol (+0.00354) 0.956
Gastric varices (x1.04) 0.957
Low blood pressure (x1.04) 0.958
Alcohol withdrawal syndrome (x1.04) 0.959
Sodium [mol/V] in SP (-0.17) 0.959
Hepatorenal syndrome (x1.04) 0.960
Ammonia [mol/V] in P (+1.97) 0.961
Chronic hepatic failure (x1.03) 0.961
Melena (x1.03) 0.962
Alcoholic hepatitis (x1.03) 0.963
Secondary thrombocytopenia (x1.03) 0.963
Hematemesis (x1.03) 0.964
Erythrocytes [#/V] in B by AC (-0.034) 0.964
Alcoholic liver damage (x1.03) 0.965
Tobacco dependence syndrome (x1.03) 0.965
Essential hypertension (x1.03) 0.966
Venous varices (x1.03) 0.966
Anxiety disorder (x1.03) 0.967
Chronic nonalcoholic liver disease (x1.03) 0.967
Jaundice (x1.03) 0.968
Shock (x1.03) 0.968
tacrolimus (+0.00271) 0.969
Liver cell carcinoma (x1.03) 0.969
Muscle weakness (x1.03) 0.970
Cobalamin (Vitamin B12) [M/V] in SP (+18.7) 0.970
Abdominal pain (x1.03) 0.971
Monocytes/100 leukocytes in B by AC (+0.135) 0.971
Calcium [M/V] in SP (-0.0267) 0.971
Hepatic ascites due to chronic alcoholic hepatitis (x1.03) 0.972
Disorder in remission (x1.03) 0.972
Pancytopenia (x1.03) 0.972
Continuous chronic alcoholism (x1.03) 0.973
Acute alcoholic liver disease (x1.02) 0.973
Major depression, single episode (x1.02) 0.973
Erythrocyte distribution width [Ratio] by AC (+0.0694) 0.974
aspirin (-0.00225) 0.974
oxycodone (+0.00224) 0.974
Expressions

Signature [1152]; 1 To Reach 0.975, (10 Plotted)

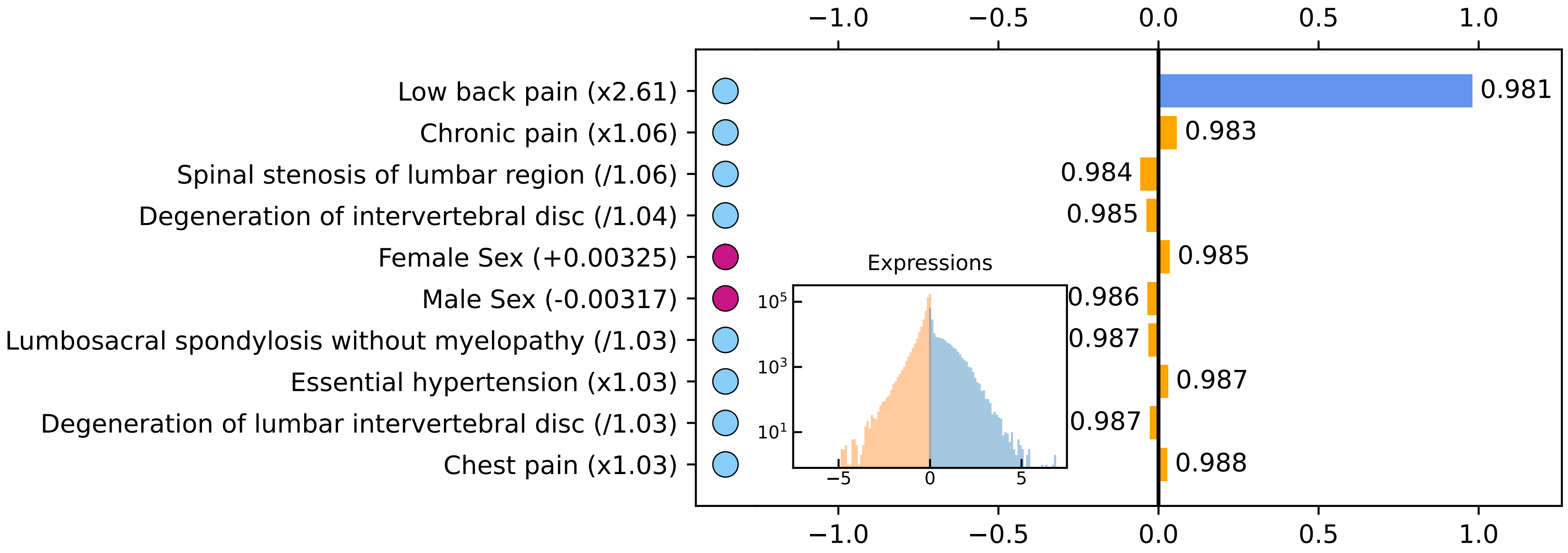

Signature [1153]; 29 To Reach 0.975, (29 Plotted)

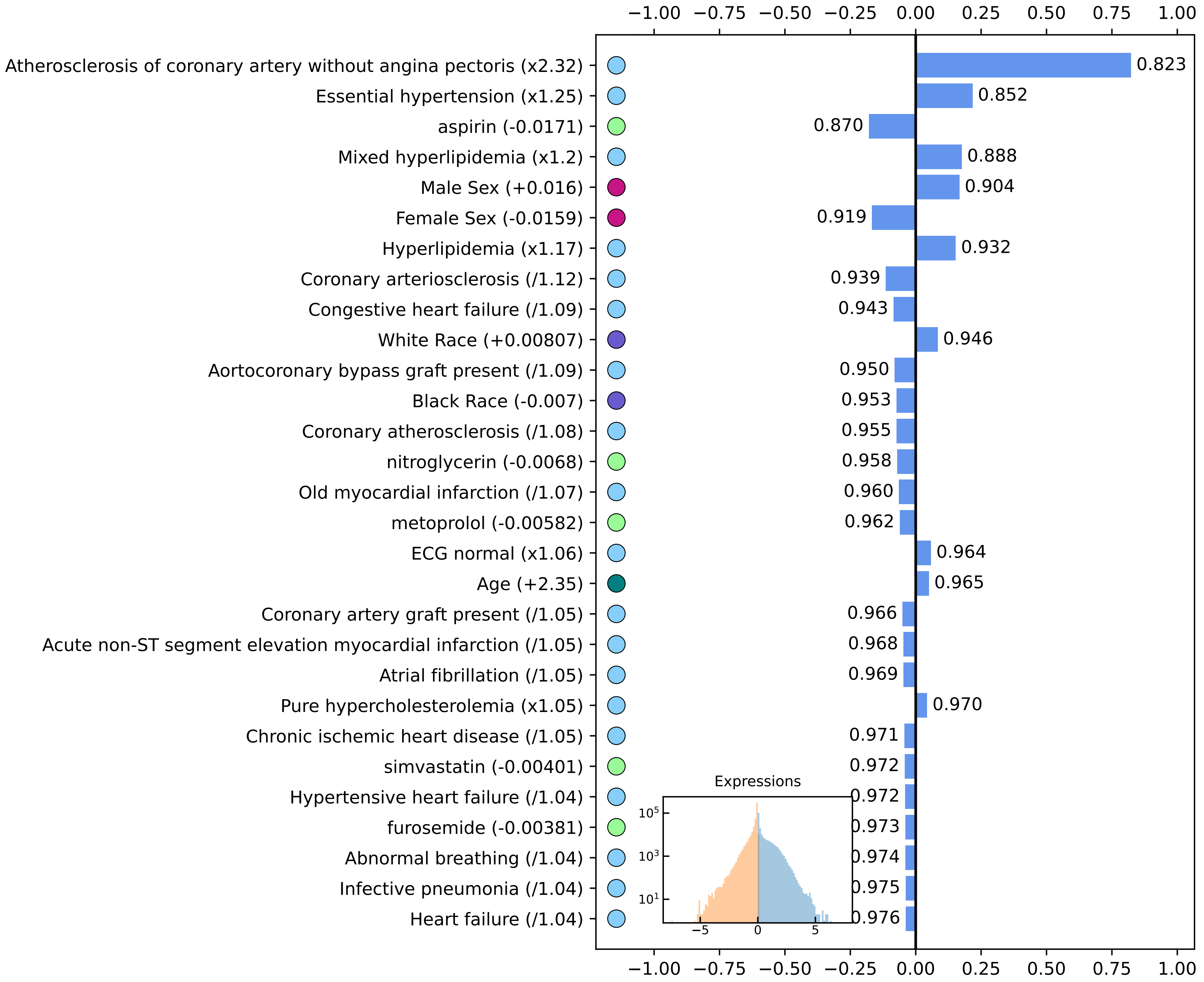

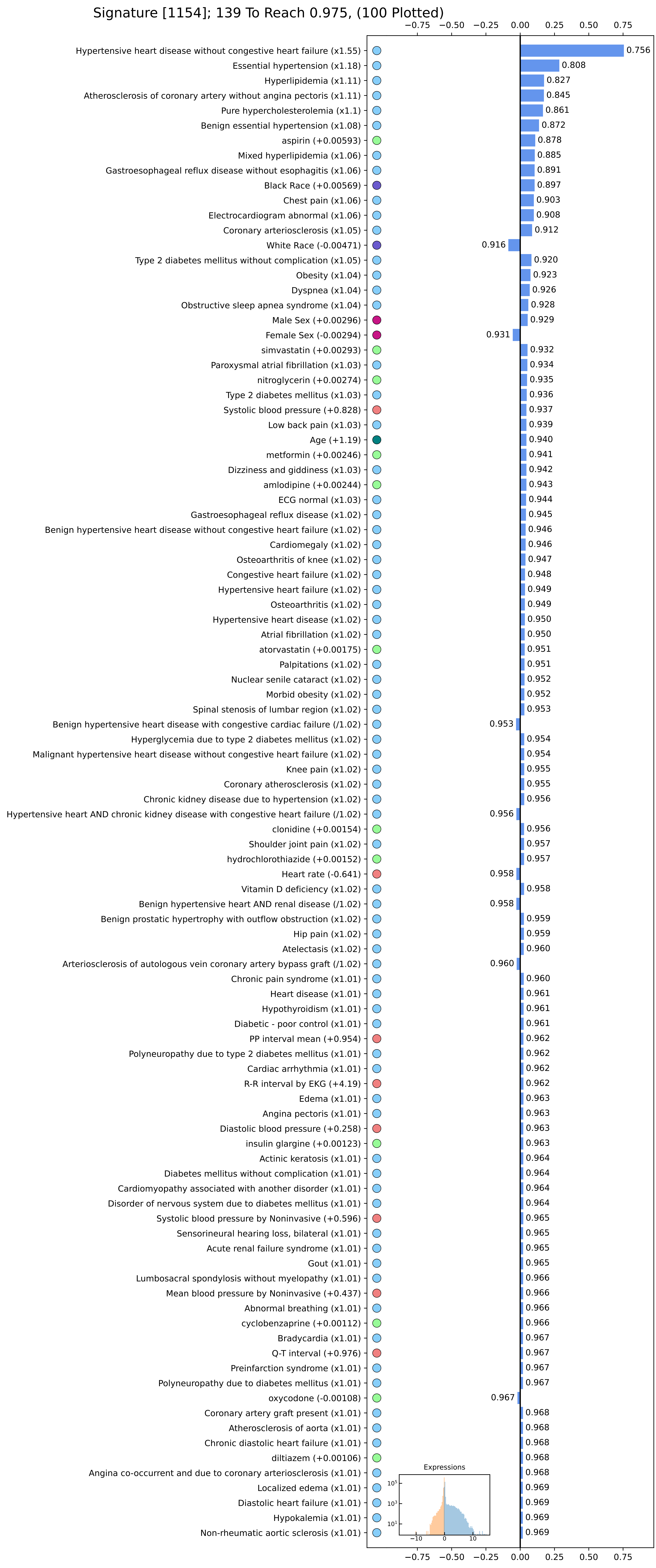

Signature [1154]; 139 To Reach 0.975, (100 Plotted)
Hypertensive heart disease without congestive heart failure (x1.55) 0.756
Essential hypertension (x1.18) 0.808
Hyperlipidemia (x1.11) 0.827
Atherosclerosis of coronary artery without angina pectoris (x1.11) 0.845
Pure hypercholesterolemia (x1.1) 0.861
Benign essential hypertension (x1.08) 0.872
aspirin (+0.00593) 0.878
Mixed hyperlipidemia (x1.06) 0.885
Gastroesophageal reflux disease without esophagitis (x1.06) 0.891
Black Race (+0.00569) 0.897
Chest pain (x1.06) 0.903
Electrocardiogram abnormal (x1.06) 0.908
Coronary arteriosclerosis (x1.05) 0.912
White Race (-0.00471) 0.916
Type 2 diabetes mellitus without complication (x1.05) 0.920
Obesity (x1.04) 0.923
Dyspnea (x1.04) 0.926
Obstructive sleep apnea syndrome (x1.04) 0.928
Male Sex (+0.00296) 0.929
Female Sex (-0.00294) 0.931
simvastatin (+0.00293) 0.932
Paroxysmal atrial fibrillation (x1.03) 0.934
nitroglycerin (+0.00274) 0.935
Type 2 diabetes mellitus (x1.03) 0.936
Systolic blood pressure (+0.828) 0.937
Low back pain (x1.03) 0.939
Age (+1.19) 0.940
metformin (+0.00246) 0.941
Dizziness and giddiness (x1.03) 0.942
amlodipine (+0.00244) 0.943
ECG normal (x1.03) 0.944
Gastroesophageal reflux disease (x1.02) 0.945
Benign hypertensive heart disease without congestive heart failure (x1.02) 0.946
Cardiomegaly (x1.02) 0.946
Osteoarthritis of knee (x1.02) 0.947
Congestive heart failure (x1.02) 0.948
Hypertensive heart failure (x1.02) 0.949
Osteoarthritis (x1.02) 0.949
Hypertensive heart disease (x1.02) 0.950
Atrial fibrillation (x1.02) 0.950
atorvastatin (+0.00175) 0.951
Palpitations (x1.02) 0.951
Nuclear senile cataract (x1.02) 0.952
Morbid obesity (x1.02) 0.952
Spinal stenosis of lumbar region (x1.02) 0.953
Benign hypertensive heart disease with congestive cardiac failure (/1.02) 0.953
Hyperglycemia due to type 2 diabetes mellitus (x1.02) 0.954
Malignant hypertensive heart disease without congestive heart failure (x1.02) 0.954
Knee pain (x1.02) 0.955
Coronary atherosclerosis (x1.02) 0.955
Chronic kidney disease due to hypertension (x1.02) 0.956
Hypertensive heart AND chronic kidney disease with congestive heart failure (/1.02) 0.956
clonidine (+0.00154) 0.956
Shoulder joint pain (x1.02) 0.957
hydrochlorothiazide (+0.00152) 0.957
Heart rate (-0.641) 0.958
Vitamin D deficiency (x1.02) 0.958
Benign hypertensive heart AND renal disease (/1.02) 0.958
Benign prostatic hypertrophy with outflow obstruction (x1.02) 0.959
Hip pain (x1.02) 0.959
Atelectasis (x1.02) 0.960
Arteriosclerosis of autologous vein coronary artery bypass graft (/1.02) 0.960
Chronic pain syndrome (x1.01) 0.960
Heart disease (x1.01) 0.961
Hypothyroidism (x1.01) 0.961
Diabetic - poor control (x1.01) 0.961
PP interval mean (+0.954) 0.962
Polyneuropathy due to type 2 diabetes mellitus (x1.01) 0.962
Cardiac arrhythmia (x1.01) 0.962
R-R interval by EKG (+4.19) 0.962
Edema (x1.01) 0.963
Angina pectoris (x1.01) 0.963
Diastolic blood pressure (+0.258) 0.963
insulin glargine (+0.00123) 0.963
Actinic keratosis (x1.01) 0.964
Diabetes mellitus without complication (x1.01) 0.964
Cardiomyopathy associated with another disorder (x1.01) 0.964
Disorder of nervous system due to diabetes mellitus (x1.01) 0.964
Systolic blood pressure by Noninvasive (+0.596) 0.965
Sensorineural hearing loss, bilateral (x1.01) 0.965
Acute renal failure syndrome (x1.01) 0.965
Gout (x1.01) 0.965
Lumbosacral spondylosis without myelopathy (x1.01) 0.966
Mean blood pressure by Noninvasive (+0.437) 0.966
Abnormal breathing (x1.01) 0.966
cyclobenzaprine (+0.00112) 0.966
Bradycardia (x1.01) 0.967
Q-T interval (+0.976) 0.967
Preinfarction syndrome (x1.01) 0.967
Polyneuropathy due to diabetes mellitus (x1.01) 0.967
oxycodone (-0.00108) 0.967
Coronary artery graft present (x1.01) 0.968
Atherosclerosis of aorta (x1.01) 0.968
Chronic diastolic heart failure (x1.01) 0.968
diltiazem (+0.00106) 0.968
Angina co-occurrent and due to coronary arteriosclerosis (x1.01) 0.968
Localized edema (x1.01) 0.969
Diastolic heart failure (x1.01) 0.969
Hypokalemia (x1.01) 0.969
Non-rheumatic aortic sclerosis (x1.01) 0.969
−0.75 −0.50 −0.25 0.00 0.25 0.50 0.75
Expressions

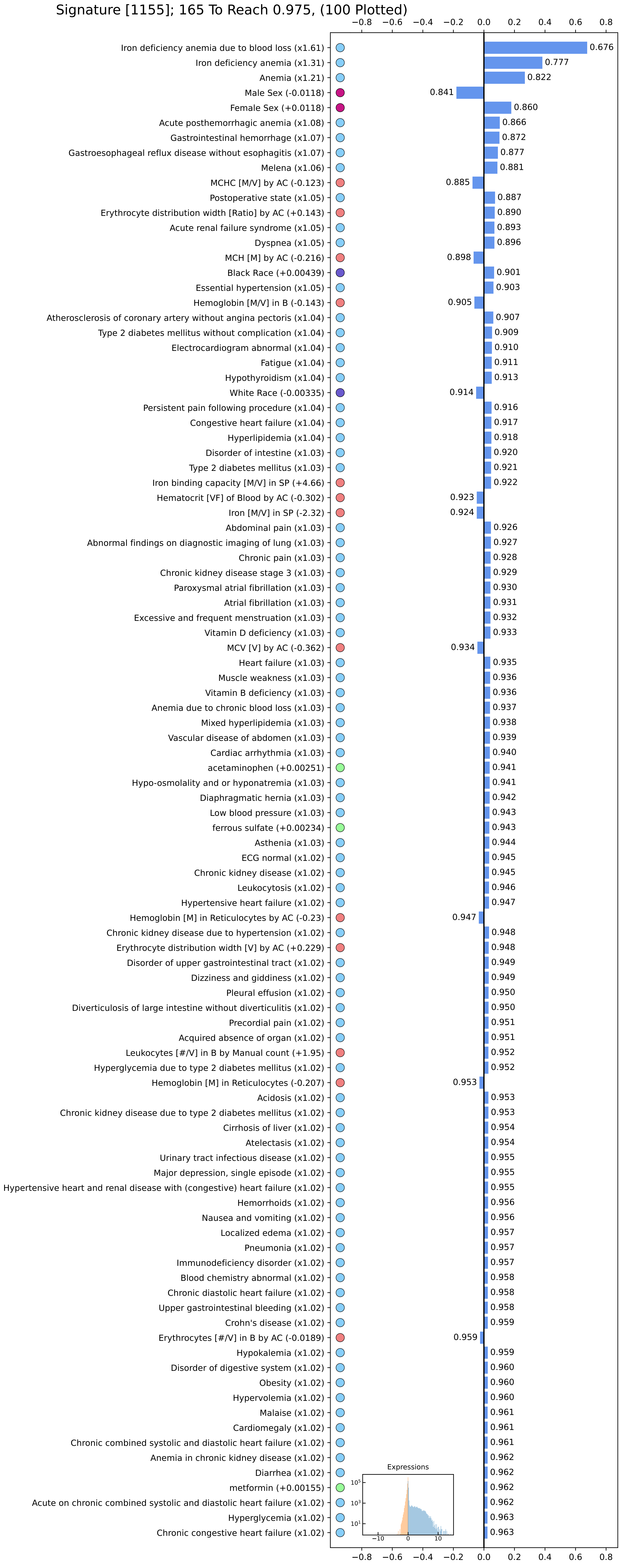
Signature [1155]; 165 To Reach 0.975, (100 Plotted)
Iron deficiency anemia due to blood loss (x1.61) 0.676
Iron deficiency anemia (x1.31) 0.777
Anemia (x1.21) 0.822
Male Sex (-0.0118) 0.841
Female Sex (+0.0118) 0.860
Acute posthemorrhagic anemia (x1.08) 0.866
Gastrointestinal hemorrhage (x1.07) 0.872
Gastroesophageal reflux disease without esophagitis (x1.07) 0.877
Melena (x1.06) 0.881
MCHC [M/V] by AC (-0.123) 0.885
Postoperative state (x1.05) 0.887
Erythrocyte distribution width [Ratio] by AC (+0.143) 0.890
Acute renal failure syndrome (x1.05) 0.893
Dyspnea (x1.05) 0.896
MCH [M] by AC (-0.216) 0.898
Black Race (+0.00439) 0.901
Essential hypertension (x1.05) 0.903
Hemoglobin [M/V] in B (-0.143) 0.905
Atherosclerosis of coronary artery without angina pectoris (x1.04) 0.907
Type 2 diabetes mellitus without complication (x1.04) 0.909
Electrocardiogram abnormal (x1.04) 0.910
Fatigue (x1.04) 0.911
Hypothyroidism (x1.04) 0.913
White Race (-0.00335) 0.914
Persistent pain following procedure (x1.04) 0.916
Congestive heart failure (x1.04) 0.917
Hyperlipidemia (x1.04) 0.918
Disorder of intestine (x1.03) 0.920
Type 2 diabetes mellitus (x1.03) 0.921
Iron binding capacity [M/V] in SP (+4.66) 0.922
Hematocrit [VF] of Blood by AC (-0.302) 0.923
Iron [M/V] in SP (-2.32) 0.924
Abdominal pain (x1.03) 0.926
Abnormal findings on diagnostic imaging of lung (x1.03) 0.927
Chronic pain (x1.03) 0.928
Chronic kidney disease stage 3 (x1.03) 0.929
Paroxysmal atrial fibrillation (x1.03) 0.930
Atrial fibrillation (x1.03) 0.931
Excessive and frequent menstruation (x1.03) 0.932
Vitamin D deficiency (x1.03) 0.933
MCV [V] by AC (-0.362) 0.934
Heart failure (x1.03) 0.935
Muscle weakness (x1.03) 0.936
Vitamin B deficiency (x1.03) 0.936
Anemia due to chronic blood loss (x1.03) 0.937
Mixed hyperlipidemia (x1.03) 0.938
Vascular disease of abdomen (x1.03) 0.939
Cardiac arrhythmia (x1.03) 0.940
acetaminophen (+0.00251) 0.941
Hypo-osmolality and or hyponatremia (x1.03) 0.941
Diaphragmatic hernia (x1.03) 0.942
Low blood pressure (x1.03) 0.943
ferrous sulfate (+0.00234) 0.943
Asthenia (x1.03) 0.944
ECG normal (x1.02) 0.945
Chronic kidney disease (x1.02) 0.945
Leukocytosis (x1.02) 0.946
Hypertensive heart failure (x1.02) 0.947
Hemoglobin [M] in Reticulocytes by AC (-0.23) 0.947
Chronic kidney disease due to hypertension (x1.02) 0.948
Erythrocyte distribution width [V] by AC (+0.229) 0.948
Disorder of upper gastrointestinal tract (x1.02) 0.949
Dizziness and giddiness (x1.02) 0.949
Pleural effusion (x1.02) 0.950
Diverticulosis of large intestine without diverticulitis (x1.02) 0.950
Precordial pain (x1.02) 0.951
Acquired absence of organ (x1.02) 0.951
Leukocytes [#/V] in B by Manual count (+1.95) 0.952
Hyperglycemia due to type 2 diabetes mellitus (x1.02) 0.952
Hemoglobin [M] in Reticulocytes (-0.207) 0.953
Acidosis (x1.02) 0.953
Chronic kidney disease due to type 2 diabetes mellitus (x1.02) 0.953
Cirrhosis of liver (x1.02) 0.954
Atelectasis (x1.02) 0.954
Urinary tract infectious disease (x1.02) 0.955
Major depression, single episode (x1.02) 0.955
Hypertensive heart and renal disease with (congestive) heart failure (x1.02) 0.955
Hemorrhoids (x1.02) 0.956
Nausea and vomiting (x1.02) 0.956
Localized edema (x1.02) 0.957
Pneumonia (x1.02) 0.957
Immunodeficiency disorder (x1.02) 0.957
Blood chemistry abnormal (x1.02) 0.958
Chronic diastolic heart failure (x1.02) 0.958
Upper gastrointestinal bleeding (x1.02) 0.958
Crohn's disease (x1.02) 0.959
Erythrocytes [#/V] in B by AC (-0.0189) 0.959
Hypokalemia (x1.02) 0.959
Disorder of digestive system (x1.02) 0.960
Obesity (x1.02) 0.960
Hypervolemia (x1.02) 0.960
Malaise (x1.02) 0.961
Cardiomegaly (x1.02) 0.961
Chronic combined systolic and diastolic heart failure (x1.02) 0.961
Anemia in chronic kidney disease (x1.02) 0.962
Diarrhea (x1.02) 0.962
metformin (+0.00155) 0.962
Acute on chronic combined systolic and diastolic heart failure (x1.02) 0.962
Hyperglycemia (x1.02) 0.963
Chronic congestive heart failure (x1.02) 0.963
−0.8 −0.6 −0.4 −0.2 0.0 0.2 0.4 0.6 0.8
Expressions
10^5
10^3
10^1
−10 0 10

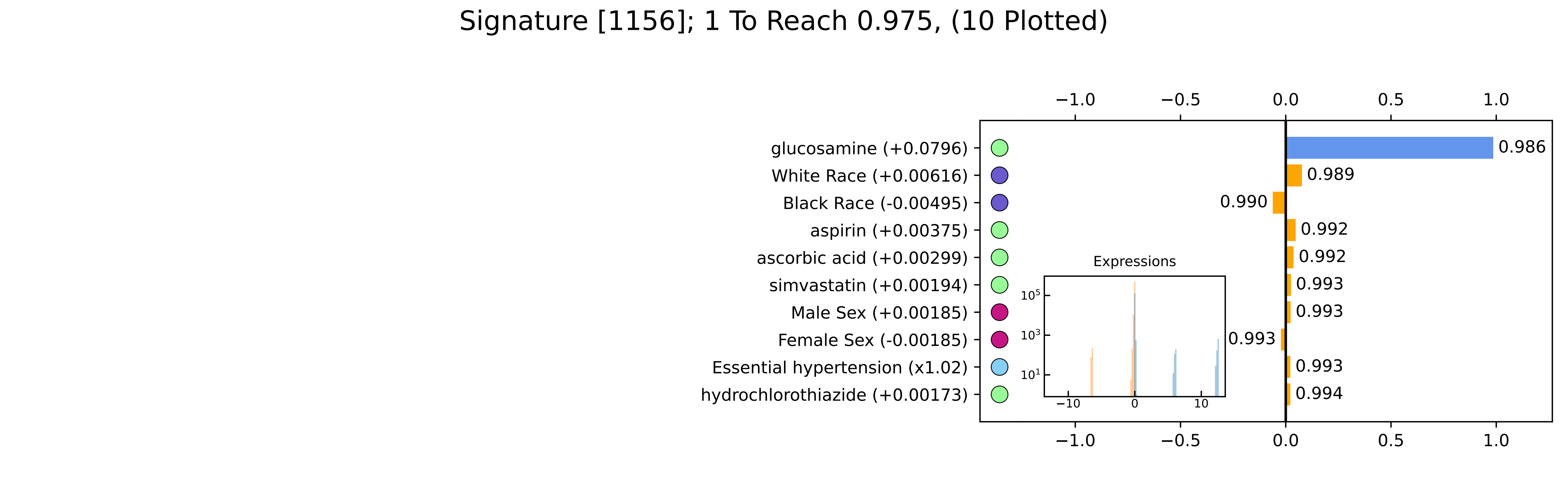

Signature [1156]; 1 To Reach 0.975, (10 Plotted)
glucosamine (+0.0796)
White Race (+0.00616)
Black Race (-0.00495)
aspirin (+0.00375)
ascorbic acid (+0.00299)
simvastatin (+0.00194)
Male Sex (+0.00185)
Female Sex (-0.00185)
Essential hypertension (x1.02)
hydrochlorothiazide (+0.00173)
0.986
0.989
0.990
0.992
0.992
0.993
0.993
0.993
0.993
0.994
−1.0
−0.5
0.0
0.5
1.0
Expressions
10^5
10^3
10^1
−10
0
10

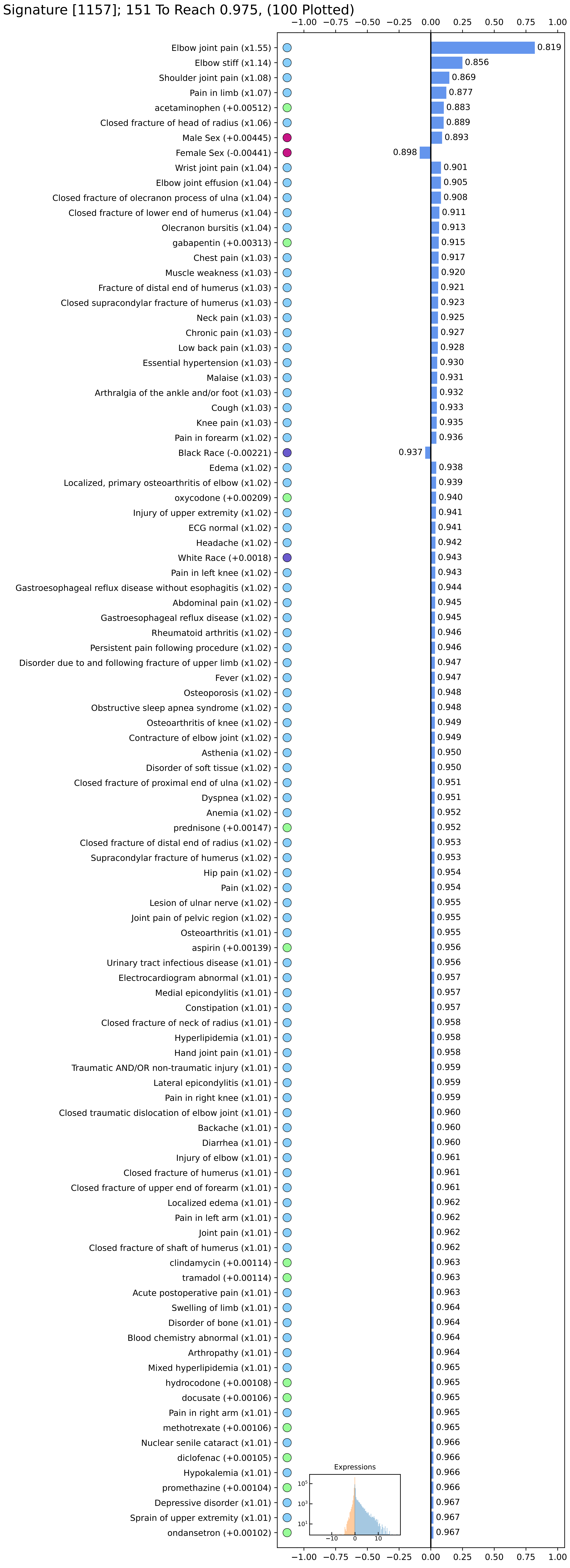

Signature [1157]; 151 To Reach 0.975, (100 Plotted)
Elbow joint pain (x1.55) 0.819
Elbow stiff (x1.14) 0.856
Shoulder joint pain (x1.08) 0.869
Pain in limb (x1.07) 0.877
acetaminophen (+0.00512) 0.883
Closed fracture of head of radius (x1.06) 0.889
Male Sex (+0.00445) 0.893
Female Sex (-0.00441) 0.898
Wrist joint pain (x1.04) 0.901
Elbow joint effusion (x1.04) 0.905
Closed fracture of olecranon process of ulna (x1.04) 0.908
Closed fracture of lower end of humerus (x1.04) 0.911
Olecranon bursitis (x1.04) 0.913
gabapentin (+0.00313) 0.915
Chest pain (x1.03) 0.917
Muscle weakness (x1.03) 0.920
Fracture of distal end of humerus (x1.03) 0.921
Closed supracondylar fracture of humerus (x1.03) 0.923
Neck pain (x1.03) 0.925
Chronic pain (x1.03) 0.927
Low back pain (x1.03) 0.928
Essential hypertension (x1.03) 0.930
Malaise (x1.03) 0.931
Arthralgia of the ankle and/or foot (x1.03) 0.932
Cough (x1.03) 0.933
Knee pain (x1.03) 0.935
Pain in forearm (x1.02) 0.936
Black Race (-0.00221) 0.937
Edema (x1.02) 0.938
Localized, primary osteoarthritis of elbow (x1.02) 0.939
oxycodone (+0.00209) 0.940
Injury of upper extremity (x1.02) 0.941
ECG normal (x1.02) 0.941
Headache (x1.02) 0.942
White Race (+0.0018) 0.943
Pain in left knee (x1.02) 0.943
Gastroesophageal reflux disease without esophagitis (x1.02) 0.944
Abdominal pain (x1.02) 0.945
Gastroesophageal reflux disease (x1.02) 0.945
Rheumatoid arthritis (x1.02) 0.946
Persistent pain following procedure (x1.02) 0.946
Disorder due to and following fracture of upper limb (x1.02) 0.947
Fever (x1.02) 0.947
Osteoporosis (x1.02) 0.948
Obstructive sleep apnea syndrome (x1.02) 0.948
Osteoarthritis of knee (x1.02) 0.949
Contracture of elbow joint (x1.02) 0.949
Asthenia (x1.02) 0.950
Disorder of soft tissue (x1.02) 0.950
Closed fracture of proximal end of ulna (x1.02) 0.951
Dyspnea (x1.02) 0.951
Anemia (x1.02) 0.952
prednisone (+0.00147) 0.952
Closed fracture of distal end of radius (x1.02) 0.953
Supracondylar fracture of humerus (x1.02) 0.953
Hip pain (x1.02) 0.954
Pain (x1.02) 0.954
Lesion of ulnar nerve (x1.02) 0.955
Joint pain of pelvic region (x1.02) 0.955
Osteoarthritis (x1.01) 0.955
aspirin (+0.00139) 0.956
Urinary tract infectious disease (x1.01) 0.956
Electrocardiogram abnormal (x1.01) 0.957
Medial epicondylitis (x1.01) 0.957
Constipation (x1.01) 0.957
Closed fracture of neck of radius (x1.01) 0.958
Hyperlipidemia (x1.01) 0.958
Hand joint pain (x1.01) 0.958
Traumatic AND/OR non-traumatic injury (x1.01) 0.959
Lateral epicondylitis (x1.01) 0.959
Pain in right knee (x1.01) 0.959
Closed traumatic dislocation of elbow joint (x1.01) 0.960
Backache (x1.01) 0.960
Diarrhea (x1.01) 0.960
Injury of elbow (x1.01) 0.961
Closed fracture of humerus (x1.01) 0.961
Closed fracture of upper end of forearm (x1.01) 0.961
Localized edema (x1.01) 0.962
Pain in left arm (x1.01) 0.962
Joint pain (x1.01) 0.962
Closed fracture of shaft of humerus (x1.01) 0.962
clindamycin (+0.00114) 0.963
tramadol (+0.00114) 0.963
Acute postoperative pain (x1.01) 0.963
Swelling of limb (x1.01) 0.964
Disorder of bone (x1.01) 0.964
Blood chemistry abnormal (x1.01) 0.964
Arthropathy (x1.01) 0.964
Mixed hyperlipidemia (x1.01) 0.965
hydrocodone (+0.00108) 0.965
docusate (+0.00106) 0.965
Pain in right arm (x1.01) 0.965
methotrexate (+0.00106) 0.965
Nuclear senile cataract (x1.01) 0.966
diclofenac (+0.00105) 0.966
Hypokalemia (x1.01) 0.966
promethazine (+0.00104) 0.966
Depressive disorder (x1.01) 0.967
Sprain of upper extremity (x1.01) 0.967
ondansetron (+0.00102) 0.967
Expressions
−1.00 −0.75 −0.50 −0.25 0.00 0.25 0.50 0.75 1.00

Signature [1158]; 1 To Reach 0.975, (10 Plotted)

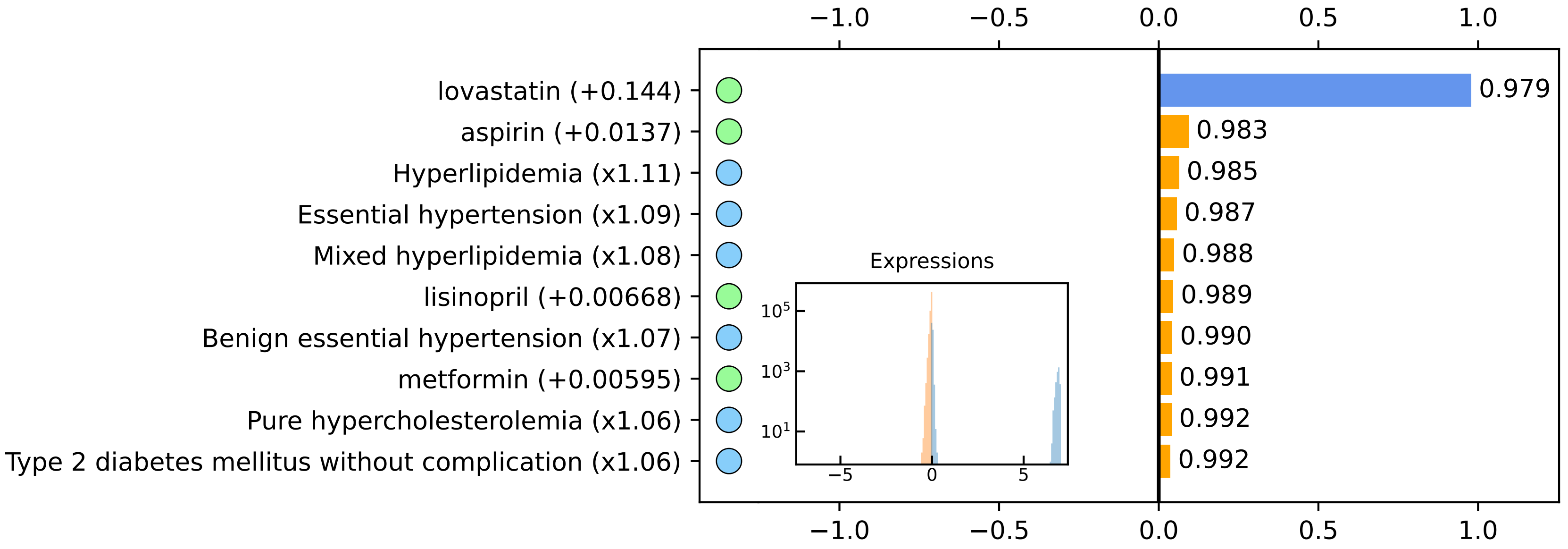

Signature [1159]; 18 To Reach 0.975, (18 Plotted)

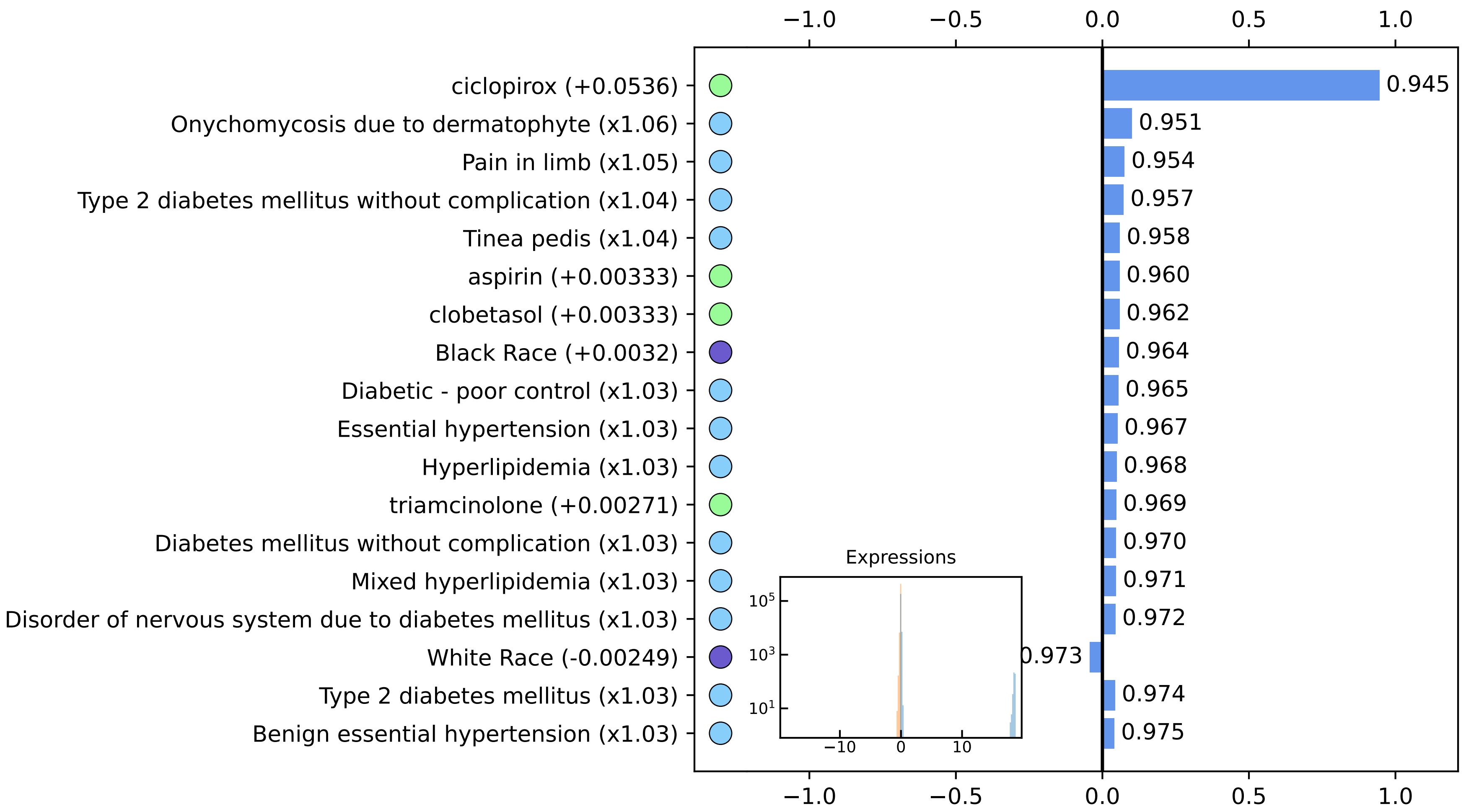

Signature [1160]; 407 To Reach 0.975, (100 Plotted)

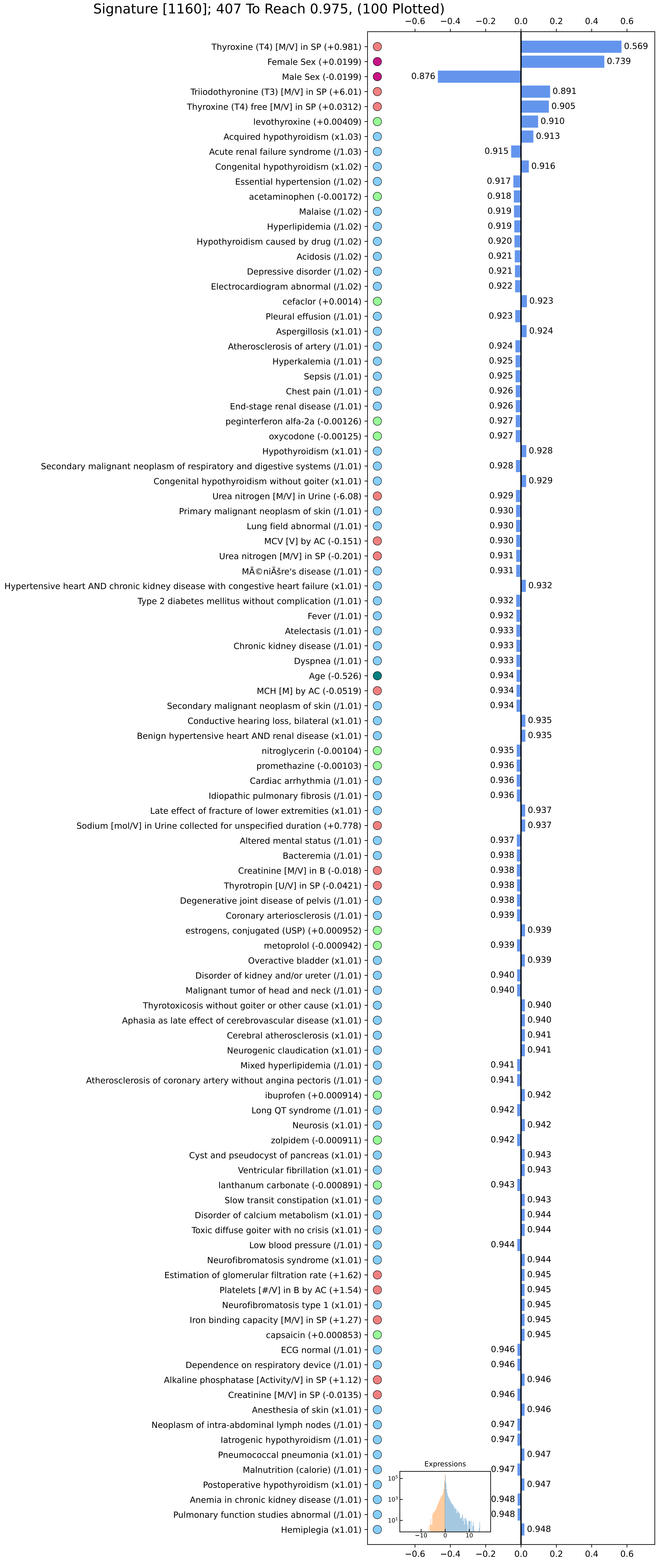

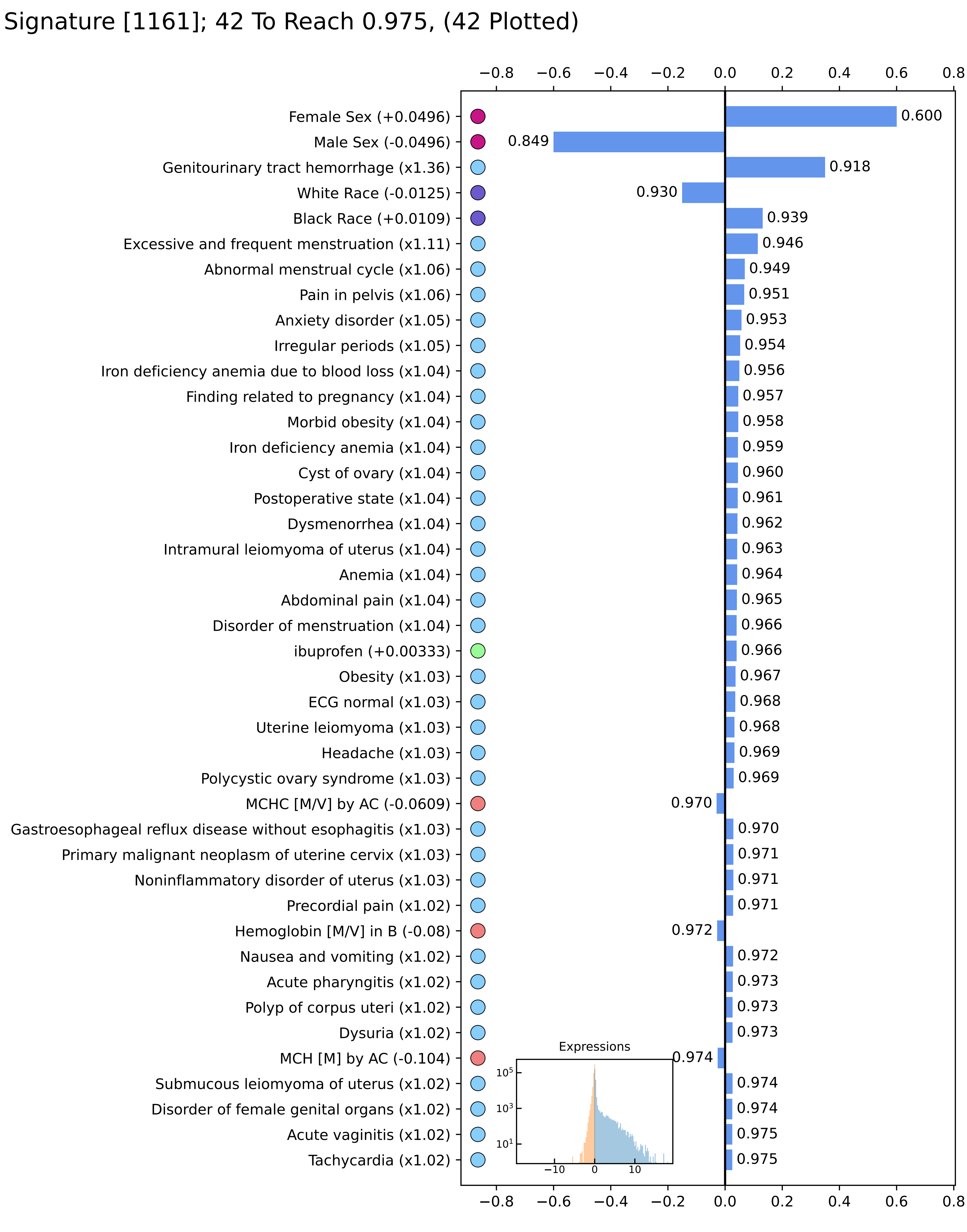

Signature [1161]; 42 To Reach 0.975, (42 Plotted)
Female Sex (+0.0496)
0.600
Male Sex (-0.0496)
0.849
Genitourinary tract hemorrhage (x1.36)
0.918
White Race (-0.0125)
0.930
Black Race (+0.0109)
0.939
Excessive and frequent menstruation (x1.11)
0.946
Abnormal menstrual cycle (x1.06)
0.949
Pain in pelvis (x1.06)
0.951
Anxiety disorder (x1.05)
0.953
Irregular periods (x1.05)
0.954
Iron deficiency anemia due to blood loss (x1.04)
0.956
Finding related to pregnancy (x1.04)
0.957
Morbid obesity (x1.04)
0.958
Iron deficiency anemia (x1.04)
0.959
Cyst of ovary (x1.04)
0.960
Postoperative state (x1.04)
0.961
Dysmenorrhea (x1.04)
0.962
Intramural leiomyoma of uterus (x1.04)
0.963
Anemia (x1.04)
0.964
Abdominal pain (x1.04)
0.965
Disorder of menstruation (x1.04)
0.966
ibuprofen (+0.00333)
0.966
Obesity (x1.03)
0.967
ECG normal (x1.03)
0.968
Uterine leiomyoma (x1.03)
0.968
Headache (x1.03)
0.969
Polycystic ovary syndrome (x1.03)
0.969
MCHC [M/V] by AC (-0.0609)
0.970
Gastroesophageal reflux disease without esophagitis (x1.03)
0.970
Primary malignant neoplasm of uterine cervix (x1.03)
0.971
Noninflammatory disorder of uterus (x1.03)
0.971
Precordial pain (x1.02)
0.971
Hemoglobin [M/V] in B (-0.08)
0.972
Nausea and vomiting (x1.02)
0.972
Acute pharyngitis (x1.02)
0.973
Polyp of corpus uteri (x1.02)
0.973
Dysuria (x1.02)
0.973
MCH [M] by AC (-0.104)
0.974
Submucous leiomyoma of uterus (x1.02)
0.974
Disorder of female genital organs (x1.02)
0.974
Acute vaginitis (x1.02)
0.975
Tachycardia (x1.02)
0.975
Expressions
−0.8 −0.6 −0.4 −0.2 0.0 0.2 0.4 0.6 0.8

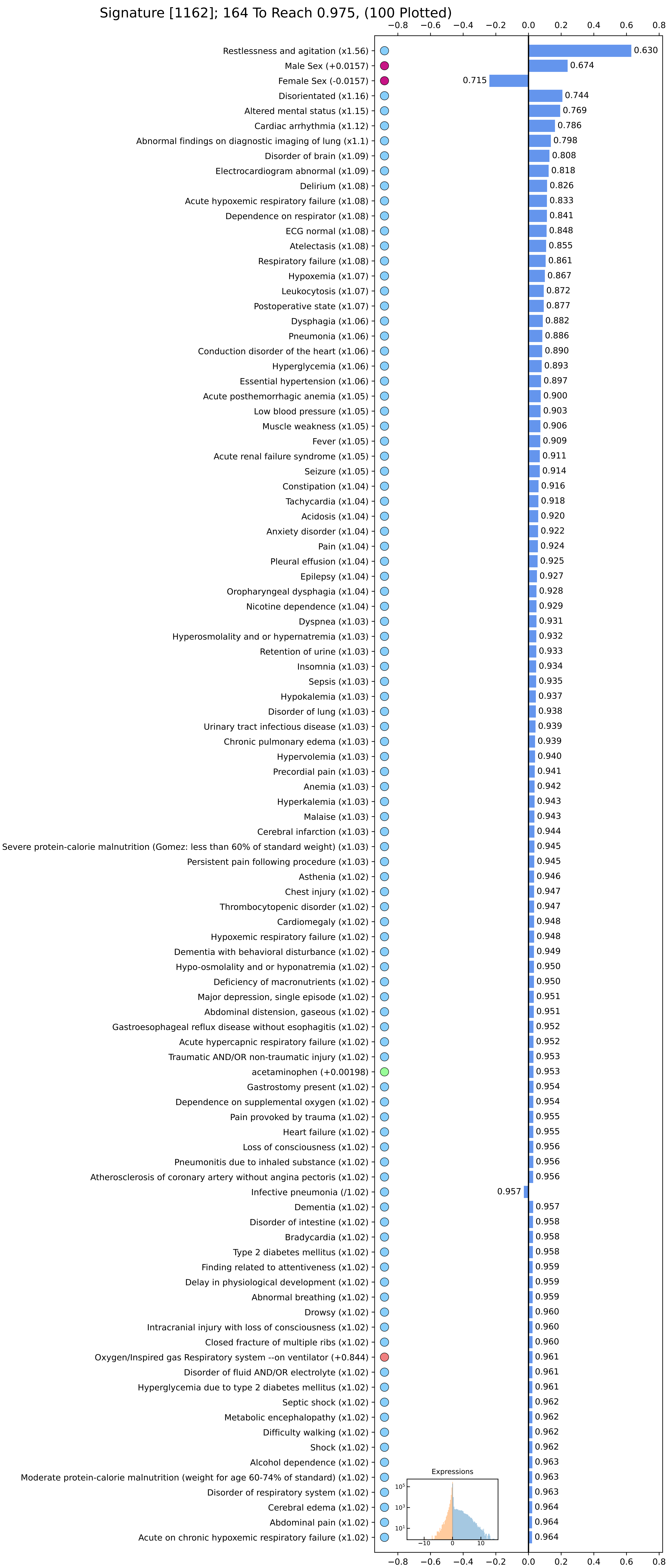
Signature [1162]; 164 To Reach 0.975, (100 Plotted)
Restlessness and agitation (x1.56) 0.630
Male Sex (+0.0157) 0.674
Female Sex (-0.0157) 0.715
Disorientated (x1.16) 0.744
Altered mental status (x1.15) 0.769
Cardiac arrhythmia (x1.12) 0.786
Abnormal findings on diagnostic imaging of lung (x1.1) 0.798
Disorder of brain (x1.09) 0.808
Electrocardiogram abnormal (x1.09) 0.818
Delirium (x1.08) 0.826
Acute hypoxemic respiratory failure (x1.08) 0.833
Dependence on respirator (x1.08) 0.841
ECG normal (x1.08) 0.848
Atelectasis (x1.08) 0.855
Respiratory failure (x1.08) 0.861
Hypoxemia (x1.07) 0.867
Leukocytosis (x1.07) 0.872
Postoperative state (x1.07) 0.877
Dysphagia (x1.06) 0.882
Pneumonia (x1.06) 0.886
Conduction disorder of the heart (x1.06) 0.890
Hyperglycemia (x1.06) 0.893
Essential hypertension (x1.06) 0.897
Acute posthemorrhagic anemia (x1.05) 0.900
Low blood pressure (x1.05) 0.903
Muscle weakness (x1.05) 0.906
Fever (x1.05) 0.909
Acute renal failure syndrome (x1.05) 0.911
Seizure (x1.05) 0.914
Constipation (x1.04) 0.916
Tachycardia (x1.04) 0.918
Acidosis (x1.04) 0.920
Anxiety disorder (x1.04) 0.922
Pain (x1.04) 0.924
Pleural effusion (x1.04) 0.925
Epilepsy (x1.04) 0.927
Oropharyngeal dysphagia (x1.04) 0.928
Nicotine dependence (x1.04) 0.929
Dyspnea (x1.03) 0.931
Hyperosmolality and or hypernatremia (x1.03) 0.932
Retention of urine (x1.03) 0.933
Insomnia (x1.03) 0.934
Sepsis (x1.03) 0.935
Hypokalemia (x1.03) 0.937
Disorder of lung (x1.03) 0.938
Urinary tract infectious disease (x1.03) 0.939
Chronic pulmonary edema (x1.03) 0.939
Hypervolemia (x1.03) 0.940
Precordial pain (x1.03) 0.941
Anemia (x1.03) 0.942
Hyperkalemia (x1.03) 0.943
Malaise (x1.03) 0.943
Cerebral infarction (x1.03) 0.944
Severe protein-calorie malnutrition (Gomez: less than 60% of standard weight) (x1.03) 0.945
Persistent pain following procedure (x1.03) 0.945
Asthenia (x1.02) 0.946
Chest injury (x1.02) 0.947
Thrombocytopenic disorder (x1.02) 0.947
Cardiomegaly (x1.02) 0.948
Hypoxemic respiratory failure (x1.02) 0.948
Dementia with behavioral disturbance (x1.02) 0.949
Hypo-osmolality and or hyponatremia (x1.02) 0.950
Deficiency of macronutrients (x1.02) 0.950
Major depression, single episode (x1.02) 0.951
Abdominal distension, gaseous (x1.02) 0.951
Gastroesophageal reflux disease without esophagitis (x1.02) 0.952
Acute hypercapnic respiratory failure (x1.02) 0.952
Traumatic AND/OR non-traumatic injury (x1.02) 0.953
acetaminophen (+0.00198) 0.953
Gastrostomy present (x1.02) 0.954
Dependence on supplemental oxygen (x1.02) 0.954
Pain provoked by trauma (x1.02) 0.955
Heart failure (x1.02) 0.955
Loss of consciousness (x1.02) 0.956
Pneumonitis due to inhaled substance (x1.02) 0.956
Atherosclerosis of coronary artery without angina pectoris (x1.02) 0.956
Infective pneumonia (/1.02) 0.957
Dementia (x1.02) 0.957
Disorder of intestine (x1.02) 0.958
Bradycardia (x1.02) 0.958
Type 2 diabetes mellitus (x1.02) 0.958
Finding related to attentiveness (x1.02) 0.959
Delay in physiological development (x1.02) 0.959
Abnormal breathing (x1.02) 0.959
Drowsy (x1.02) 0.960
Intracranial injury with loss of consciousness (x1.02) 0.960
Closed fracture of multiple ribs (x1.02) 0.960
Oxygen/Inspired gas Respiratory system --on ventilator (+0.844) 0.961
Disorder of fluid AND/OR electrolyte (x1.02) 0.961
Hyperglycemia due to type 2 diabetes mellitus (x1.02) 0.961
Septic shock (x1.02) 0.962
Metabolic encephalopathy (x1.02) 0.962
Difficulty walking (x1.02) 0.962
Shock (x1.02) 0.962
Alcohol dependence (x1.02) 0.963
Moderate protein-calorie malnutrition (weight for age 60-74% of standard) (x1.02) 0.963
Disorder of respiratory system (x1.02) 0.963
Cerebral edema (x1.02) 0.964
Abdominal pain (x1.02) 0.964
Acute on chronic hypoxemic respiratory failure (x1.02) 0.964
Expressions
−0.8 −0.6 −0.4 −0.2 0.0 0.2 0.4 0.6 0.8

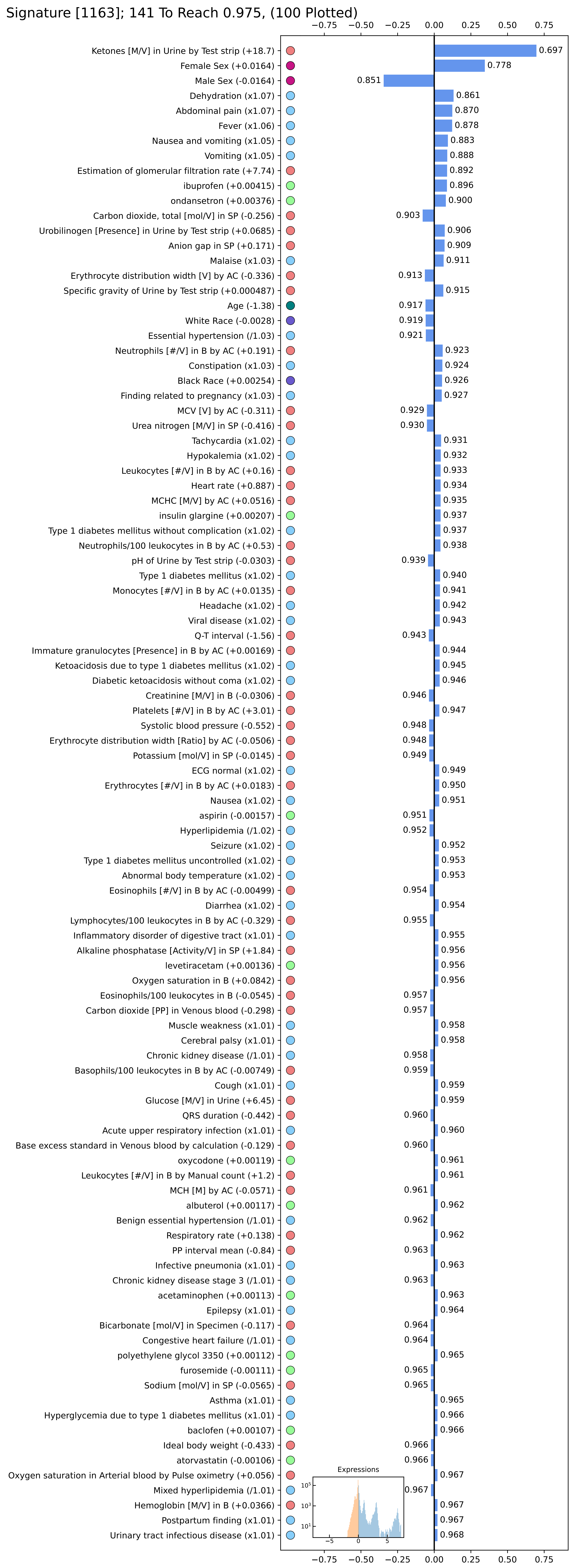

Signature [1163]; 141 To Reach 0.975, (100 Plotted)
−0.75 −0.50 −0.25 0.00 0.25 0.50 0.75
Ketones [M/V] in Urine by Test strip (+18.7) 0.697
Female Sex (+0.0164) 0.778
Male Sex (-0.0164) 0.851
Dehydration (x1.07) 0.861
Abdominal pain (x1.07) 0.870
Fever (x1.06) 0.878
Nausea and vomiting (x1.05) 0.883
Vomiting (x1.05) 0.888
Estimation of glomerular filtration rate (+7.74) 0.892
ibuprofen (+0.00415) 0.896
ondansetron (+0.00376) 0.900
Carbon dioxide, total [mol/V] in SP (-0.256) 0.903
Urobilinogen [Presence] in Urine by Test strip (+0.0685) 0.906
Anion gap in SP (+0.171) 0.909
Malaise (x1.03) 0.911
Erythrocyte distribution width [V] by AC (-0.336) 0.913
Specific gravity of Urine by Test strip (+0.000487) 0.915
Age (-1.38) 0.917
White Race (-0.0028) 0.919
Essential hypertension (/1.03) 0.921
Neutrophils [#/V] in B by AC (+0.191) 0.923
Constipation (x1.03) 0.924
Black Race (+0.00254) 0.926
Finding related to pregnancy (x1.03) 0.927
MCV [V] by AC (-0.311) 0.929
Urea nitrogen [M/V] in SP (-0.416) 0.930
Tachycardia (x1.02) 0.931
Hypokalemia (x1.02) 0.932
Leukocytes [#/V] in B by AC (+0.16) 0.933
Heart rate (+0.887) 0.934
MCHC [M/V] by AC (+0.0516) 0.935
insulin glargine (+0.00207) 0.937
Type 1 diabetes mellitus without complication (x1.02) 0.937
Neutrophils/100 leukocytes in B by AC (+0.53) 0.938
pH of Urine by Test strip (-0.0303) 0.939
Type 1 diabetes mellitus (x1.02) 0.940
Monocytes [#/V] in B by AC (+0.0135) 0.941
Headache (x1.02) 0.942
Viral disease (x1.02) 0.943
Q-T interval (-1.56) 0.943
Immature granulocytes [Presence] in B by AC (+0.00169) 0.944
Ketoacidosis due to type 1 diabetes mellitus (x1.02) 0.945
Diabetic ketoacidosis without coma (x1.02) 0.946
Creatinine [M/V] in B (-0.0306) 0.946
Platelets [#/V] in B by AC (+3.01) 0.947
Systolic blood pressure (-0.552) 0.948
Erythrocyte distribution width [Ratio] by AC (-0.0506) 0.948
Potassium [mol/V] in SP (-0.0145) 0.949
ECG normal (x1.02) 0.949
Erythrocytes [#/V] in B by AC (+0.0183) 0.950
Nausea (x1.02) 0.951
aspirin (-0.00157) 0.951
Hyperlipidemia (/1.02) 0.952
Seizure (x1.02) 0.952
Type 1 diabetes mellitus uncontrolled (x1.02) 0.953
Abnormal body temperature (x1.02) 0.953
Eosinophils [#/V] in B by AC (-0.00499) 0.954
Diarrhea (x1.02) 0.954
Lymphocytes/100 leukocytes in B by AC (-0.329) 0.955
Inflammatory disorder of digestive tract (x1.01) 0.955
Alkaline phosphatase [Activity/V] in SP (+1.84) 0.956
levetiracetam (+0.00136) 0.956
Oxygen saturation in B (+0.0842) 0.956
Eosinophils/100 leukocytes in B (-0.0545) 0.957
Carbon dioxide [PP] in Venous blood (-0.298) 0.957
Muscle weakness (x1.01) 0.958
Cerebral palsy (x1.01) 0.958
Chronic kidney disease (/1.01) 0.958
Basophils/100 leukocytes in B by AC (-0.00749) 0.959
Cough (x1.01) 0.959
Glucose [M/V] in Urine (+6.45) 0.959
QRS duration (-0.442) 0.960
Acute upper respiratory infection (x1.01) 0.960
Base excess standard in Venous blood by calculation (-0.129) 0.960
oxycodone (+0.00119) 0.961
Leukocytes [#/V] in B by Manual count (+1.2) 0.961
MCH [M] by AC (-0.0571) 0.961
albuterol (+0.00117) 0.962
Benign essential hypertension (/1.01) 0.962
Respiratory rate (+0.138) 0.962
PP interval mean (-0.84) 0.963
Infective pneumonia (x1.01) 0.963
Chronic kidney disease stage 3 (/1.01) 0.963
acetaminophen (+0.00113) 0.963
Epilepsy (x1.01) 0.964
Bicarbonate [mol/V] in Specimen (-0.117) 0.964
Congestive heart failure (/1.01) 0.964
polyethylene glycol 3350 (+0.00112) 0.965
furosemide (-0.00111) 0.965
Sodium [mol/V] in SP (-0.0565) 0.965
Asthma (x1.01) 0.965
Hyperglycemia due to type 1 diabetes mellitus (x1.01) 0.966
baclofen (+0.00107) 0.966
Ideal body weight (-0.433) 0.966
atorvastatin (-0.00106) 0.966
Oxygen saturation in Arterial blood by Pulse oximetry (+0.056) 0.967
Mixed hyperlipidemia (/1.01) 0.967
Hemoglobin [M/V] in B (+0.0366) 0.967
Postpartum finding (x1.01) 0.967
Urinary tract infectious disease (x1.01) 0.968
Expressions
10^5
10^3
10^1
−5 0 5
−0.75 −0.50 −0.25 0.00 0.25 0.50 0.75

Signature [1164]; 25 To Reach 0.975, (25 Plotted)

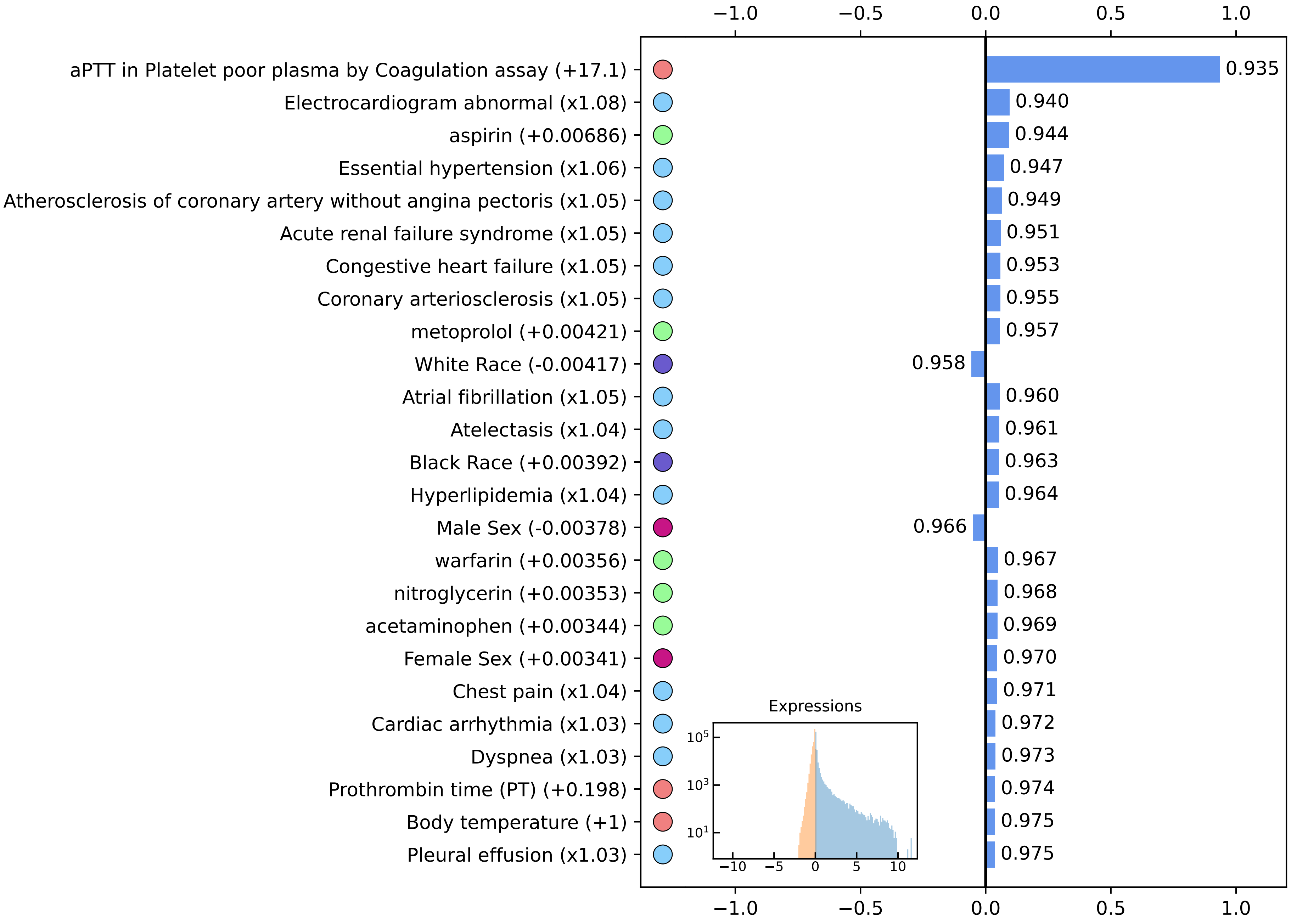

Signature [1165]; 171 To Reach 0.975, (100 Plotted)

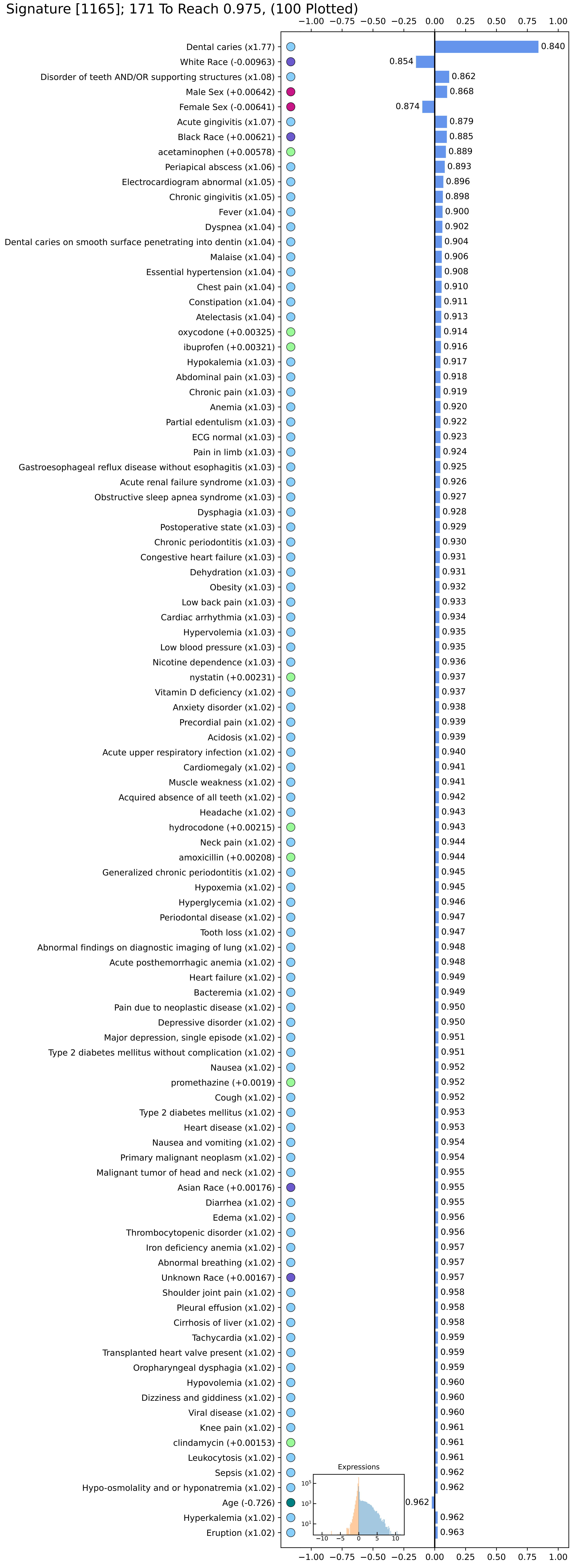

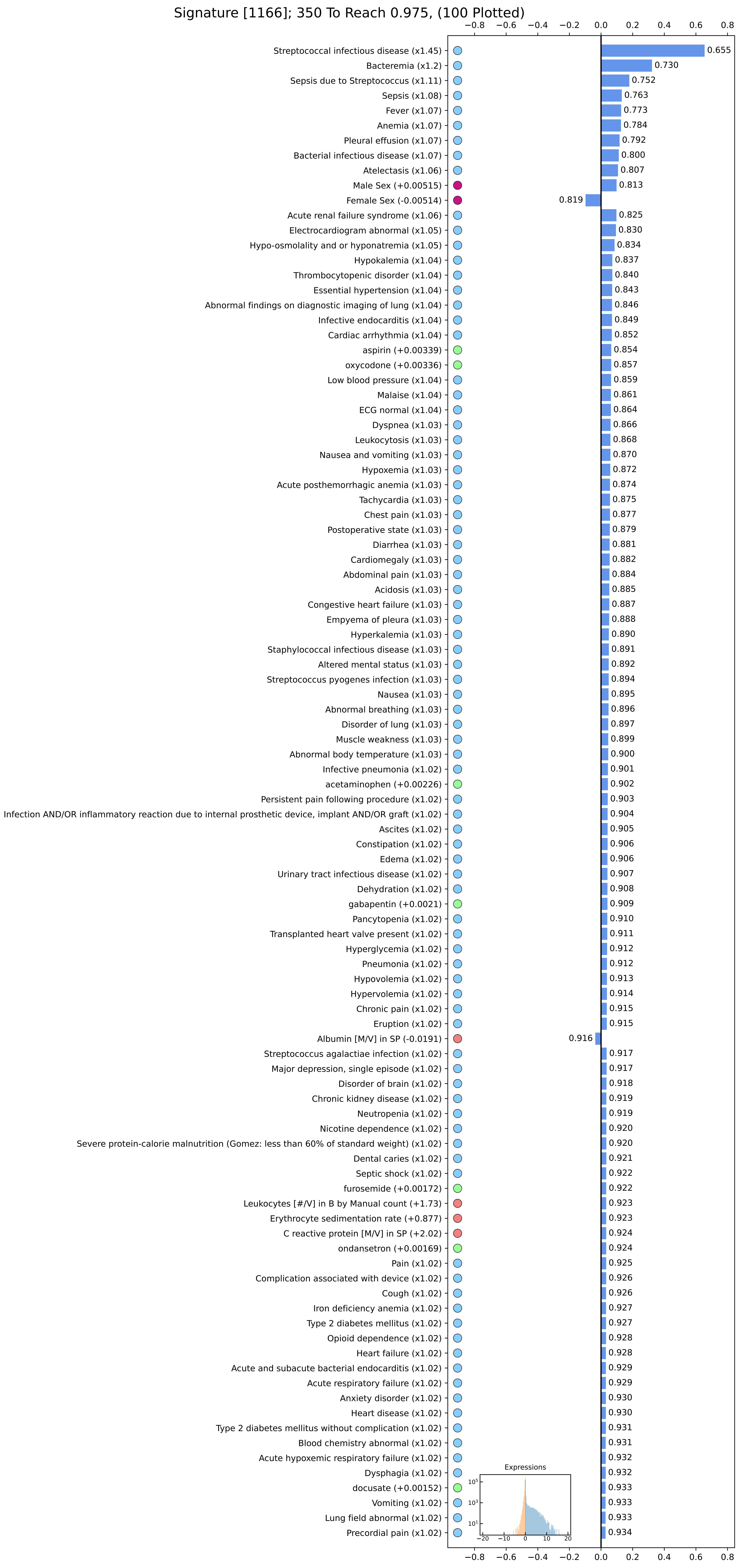
Signature [1166]; 350 To Reach 0.975, (100 Plotted)
−0.8 −0.6 −0.4 −0.2 0.0 0.2 0.4 0.6 0.8
Streptococcal infectious disease (x1.45) 0.655
Bacteremia (x1.2) 0.730
Sepsis due to Streptococcus (x1.11) 0.752
Sepsis (x1.08) 0.763
Fever (x1.07) 0.773
Anemia (x1.07) 0.784
Pleural effusion (x1.07) 0.792
Bacterial infectious disease (x1.07) 0.800
Atelectasis (x1.06) 0.807
Male Sex (+0.00515) 0.813
Female Sex (-0.00514) 0.819
Acute renal failure syndrome (x1.06) 0.825
Electrocardiogram abnormal (x1.05) 0.830
Hypo-osmolality and or hyponatremia (x1.05) 0.834
Hypokalemia (x1.04) 0.837
Thrombocytopenic disorder (x1.04) 0.840
Essential hypertension (x1.04) 0.843
Abnormal findings on diagnostic imaging of lung (x1.04) 0.846
Infective endocarditis (x1.04) 0.849
Cardiac arrhythmia (x1.04) 0.852
aspirin (+0.00339) 0.854
oxycodone (+0.00336) 0.857
Low blood pressure (x1.04) 0.859
Malaise (x1.04) 0.861
ECG normal (x1.04) 0.864
Dyspnea (x1.03) 0.866
Leukocytosis (x1.03) 0.868
Nausea and vomiting (x1.03) 0.870
Hypoxemia (x1.03) 0.872
Acute posthemorrhagic anemia (x1.03) 0.874
Tachycardia (x1.03) 0.875
Chest pain (x1.03) 0.877
Postoperative state (x1.03) 0.879
Diarrhea (x1.03) 0.881
Cardiomegaly (x1.03) 0.882
Abdominal pain (x1.03) 0.884
Acidosis (x1.03) 0.885
Congestive heart failure (x1.03) 0.887
Empyema of pleura (x1.03) 0.888
Hyperkalemia (x1.03) 0.890
Staphylococcal infectious disease (x1.03) 0.891
Altered mental status (x1.03) 0.892
Streptococcus pyogenes infection (x1.03) 0.894
Nausea (x1.03) 0.895
Abnormal breathing (x1.03) 0.896
Disorder of lung (x1.03) 0.897
Muscle weakness (x1.03) 0.899
Abnormal body temperature (x1.03) 0.900
Infective pneumonia (x1.02) 0.901
acetaminophen (+0.00226) 0.902
Persistent pain following procedure (x1.02) 0.903
Infection AND/OR inflammatory reaction due to internal prosthetic device, implant AND/OR graft (x1.02) 0.904
Ascites (x1.02) 0.905
Constipation (x1.02) 0.906
Edema (x1.02) 0.906
Urinary tract infectious disease (x1.02) 0.907
Dehydration (x1.02) 0.908
gabapentin (+0.0021) 0.909
Pancytopenia (x1.02) 0.910
Transplanted heart valve present (x1.02) 0.911
Hyperglycemia (x1.02) 0.912
Pneumonia (x1.02) 0.912
Hypovolemia (x1.02) 0.913
Hypervolemia (x1.02) 0.914
Chronic pain (x1.02) 0.915
Eruption (x1.02) 0.915
Albumin [M/V] in SP (-0.0191) 0.916
Streptococcus agalactiae infection (x1.02) 0.917
Major depression, single episode (x1.02) 0.917
Disorder of brain (x1.02) 0.918
Chronic kidney disease (x1.02) 0.919
Neutropenia (x1.02) 0.919
Nicotine dependence (x1.02) 0.920
Severe protein-calorie malnutrition (Gomez: less than 60% of standard weight) (x1.02) 0.920
Dental caries (x1.02) 0.921
Septic shock (x1.02) 0.922
furosemide (+0.00172) 0.922
Leukocytes [#/V] in B by Manual count (+1.73) 0.923
Erythrocyte sedimentation rate (+0.877) 0.923
C reactive protein [M/V] in SP (+2.02) 0.924
ondansetron (+0.00169) 0.924
Pain (x1.02) 0.925
Complication associated with device (x1.02) 0.926
Cough (x1.02) 0.926
Iron deficiency anemia (x1.02) 0.927
Type 2 diabetes mellitus (x1.02) 0.927
Opioid dependence (x1.02) 0.928
Heart failure (x1.02) 0.928
Acute and subacute bacterial endocarditis (x1.02) 0.929
Acute respiratory failure (x1.02) 0.929
Anxiety disorder (x1.02) 0.930
Heart disease (x1.02) 0.930
Type 2 diabetes mellitus without complication (x1.02) 0.931
Blood chemistry abnormal (x1.02) 0.931
Acute hypoxemic respiratory failure (x1.02) 0.932
Dysphagia (x1.02) 0.932
docusate (+0.00152) 0.933
Vomiting (x1.02) 0.933
Lung field abnormal (x1.02) 0.933
Precordial pain (x1.02) 0.934
Expressions
−20 −10 0 10 20
−0.8 −0.6 −0.4 −0.2 0.0 0.2 0.4 0.6 0.8

Signature [1167]; 1 To Reach 0.975, (10 Plotted)

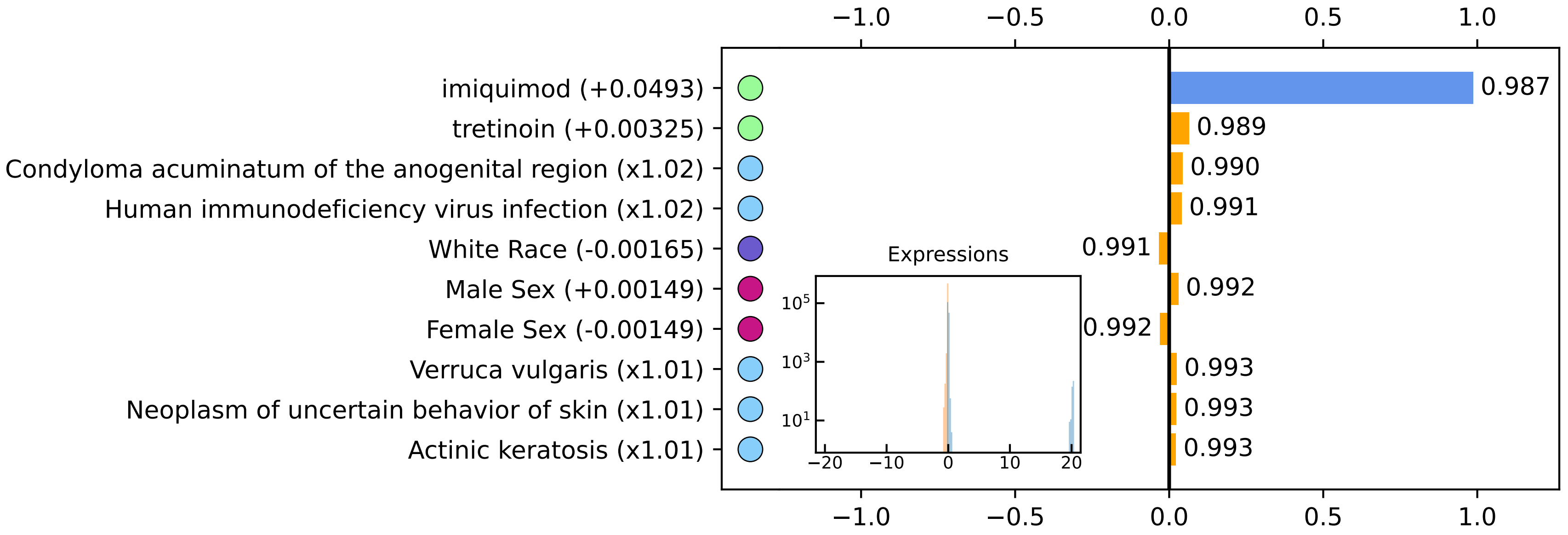

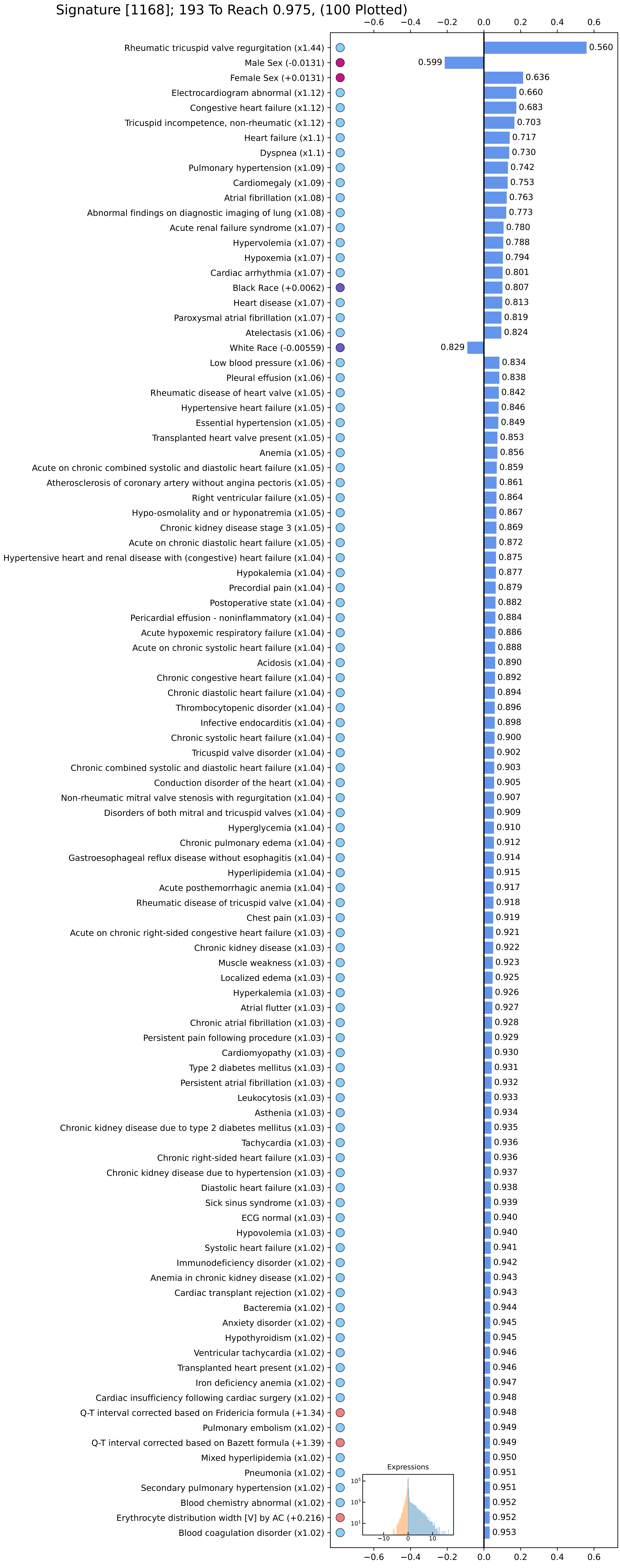
Signature [1168]; 193 To Reach 0.975, (100 Plotted)
Rheumatic tricuspid valve regurgitation (x1.44) 0.560
Male Sex (-0.0131) 0.599
Female Sex (+0.0131) 0.636
Electrocardiogram abnormal (x1.12) 0.660
Congestive heart failure (x1.12) 0.683
Tricuspid incompetence, non-rheumatic (x1.12) 0.703
Heart failure (x1.1) 0.717
Dyspnea (x1.1) 0.730
Pulmonary hypertension (x1.09) 0.742
Cardiomegaly (x1.09) 0.753
Atrial fibrillation (x1.08) 0.763
Abnormal findings on diagnostic imaging of lung (x1.08) 0.773
Acute renal failure syndrome (x1.07) 0.780
Hypervolemia (x1.07) 0.788
Hypoxemia (x1.07) 0.794
Cardiac arrhythmia (x1.07) 0.801
Black Race (+0.0062) 0.807
Heart disease (x1.07) 0.813
Paroxysmal atrial fibrillation (x1.07) 0.819
Atelectasis (x1.06) 0.824
White Race (-0.00559) 0.829
Low blood pressure (x1.06) 0.834
Pleural effusion (x1.06) 0.838
Rheumatic disease of heart valve (x1.05) 0.842
Hypertensive heart failure (x1.05) 0.846
Essential hypertension (x1.05) 0.849
Transplanted heart valve present (x1.05) 0.853
Anemia (x1.05) 0.856
Acute on chronic combined systolic and diastolic heart failure (x1.05) 0.859
Atherosclerosis of coronary artery without angina pectoris (x1.05) 0.861
Right ventricular failure (x1.05) 0.864
Hypo-osmolality and or hyponatremia (x1.05) 0.867
Chronic kidney disease stage 3 (x1.05) 0.869
Acute on chronic diastolic heart failure (x1.05) 0.872
Hypertensive heart and renal disease with (congestive) heart failure (x1.04) 0.875
Hypokalemia (x1.04) 0.877
Precordial pain (x1.04) 0.879
Postoperative state (x1.04) 0.882
Pericardial effusion - noninflammatory (x1.04) 0.884
Acute hypoxemic respiratory failure (x1.04) 0.886
Acute on chronic systolic heart failure (x1.04) 0.888
Acidosis (x1.04) 0.890
Chronic congestive heart failure (x1.04) 0.892
Chronic diastolic heart failure (x1.04) 0.894
Thrombocytopenic disorder (x1.04) 0.896
Infective endocarditis (x1.04) 0.898
Chronic systolic heart failure (x1.04) 0.900
Tricuspid valve disorder (x1.04) 0.902
Chronic combined systolic and diastolic heart failure (x1.04) 0.903
Conduction disorder of the heart (x1.04) 0.905
Non-rheumatic mitral valve stenosis with regurgitation (x1.04) 0.907
Disorders of both mitral and tricuspid valves (x1.04) 0.909
Hyperglycemia (x1.04) 0.910
Chronic pulmonary edema (x1.04) 0.912
Gastroesophageal reflux disease without esophagitis (x1.04) 0.914
Hyperlipidemia (x1.04) 0.915
Acute posthemorrhagic anemia (x1.04) 0.917
Rheumatic disease of tricuspid valve (x1.04) 0.918
Chest pain (x1.03) 0.919
Acute on chronic right-sided congestive heart failure (x1.03) 0.921
Chronic kidney disease (x1.03) 0.922
Muscle weakness (x1.03) 0.923
Localized edema (x1.03) 0.925
Hyperkalemia (x1.03) 0.926
Atrial flutter (x1.03) 0.927
Chronic atrial fibrillation (x1.03) 0.928
Persistent pain following procedure (x1.03) 0.929
Cardiomyopathy (x1.03) 0.930
Type 2 diabetes mellitus (x1.03) 0.931
Persistent atrial fibrillation (x1.03) 0.932
Leukocytosis (x1.03) 0.933
Asthenia (x1.03) 0.934
Chronic kidney disease due to type 2 diabetes mellitus (x1.03) 0.935
Tachycardia (x1.03) 0.936
Chronic right-sided heart failure (x1.03) 0.936
Chronic kidney disease due to hypertension (x1.03) 0.937
Diastolic heart failure (x1.03) 0.938
Sick sinus syndrome (x1.03) 0.939
ECG normal (x1.03) 0.940
Hypovolemia (x1.03) 0.940
Systolic heart failure (x1.02) 0.941
Immunodeficiency disorder (x1.02) 0.942
Anemia in chronic kidney disease (x1.02) 0.943
Cardiac transplant rejection (x1.02) 0.943
Bacteremia (x1.02) 0.944
Anxiety disorder (x1.02) 0.945
Hypothyroidism (x1.02) 0.945
Ventricular tachycardia (x1.02) 0.946
Transplanted heart present (x1.02) 0.946
Iron deficiency anemia (x1.02) 0.947
Cardiac insufficiency following cardiac surgery (x1.02) 0.948
Q-T interval corrected based on Fridericia formula (+1.34) 0.948
Pulmonary embolism (x1.02) 0.949
Q-T interval corrected based on Bazett formula (+1.39) 0.949
Mixed hyperlipidemia (x1.02) 0.950
Pneumonia (x1.02) 0.951
Secondary pulmonary hypertension (x1.02) 0.951
Blood chemistry abnormal (x1.02) 0.952
Erythrocyte distribution width [V] by AC (+0.216) 0.952
Blood coagulation disorder (x1.02) 0.953
−0.6 −0.4 −0.2 0.0 0.2 0.4 0.6
Expressions
10^5
10^3
10^1
−10 0 10

Signature [1169]; 245 To Reach 0.975, (100 Plotted)

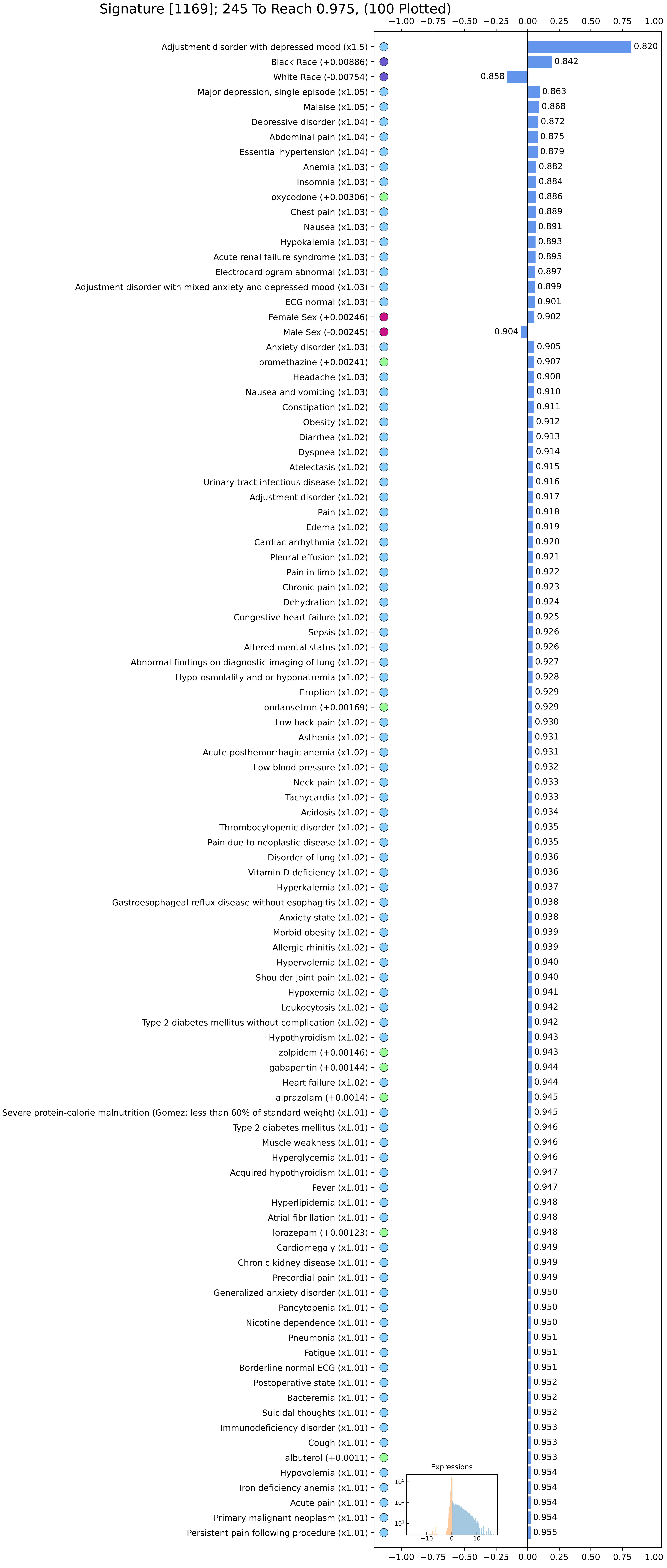

Signature [1170]; 1267 To Reach 0.975, (100 Plotted)

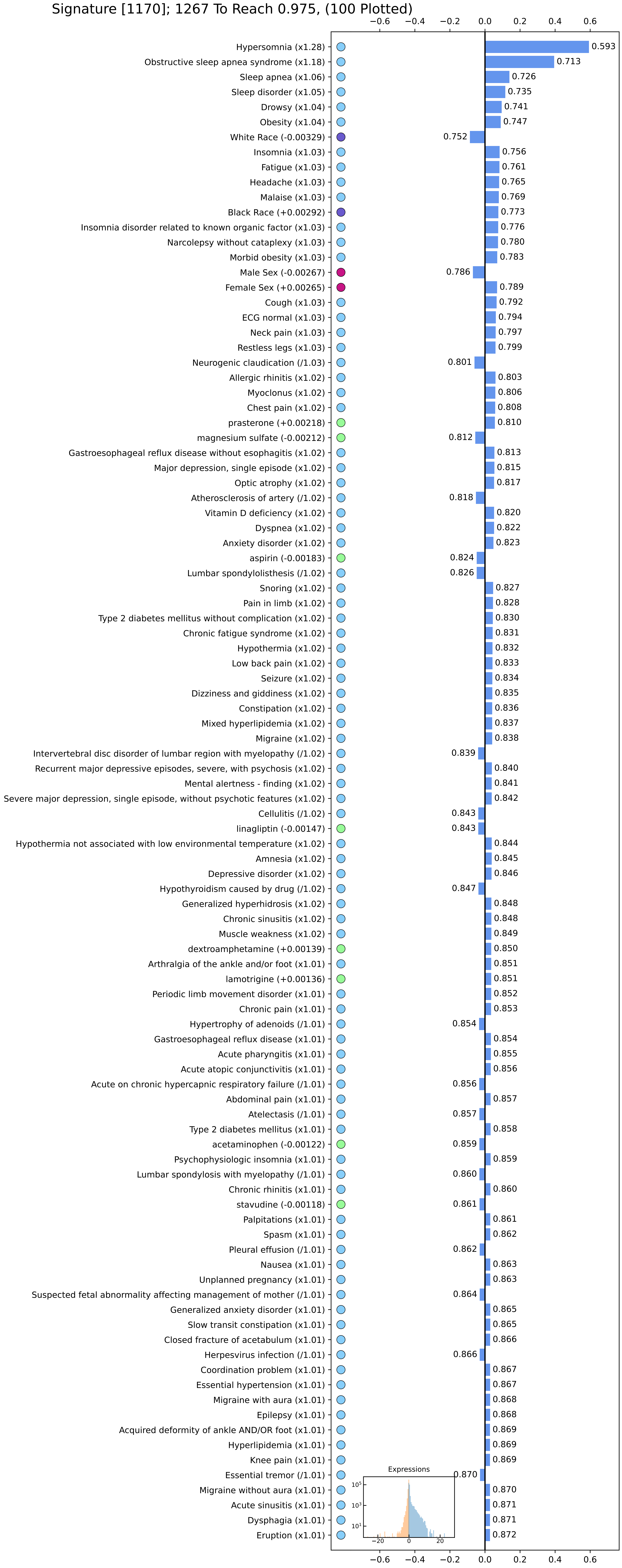

Signature [1171]; 230 To Reach 0.975, (100 Plotted)

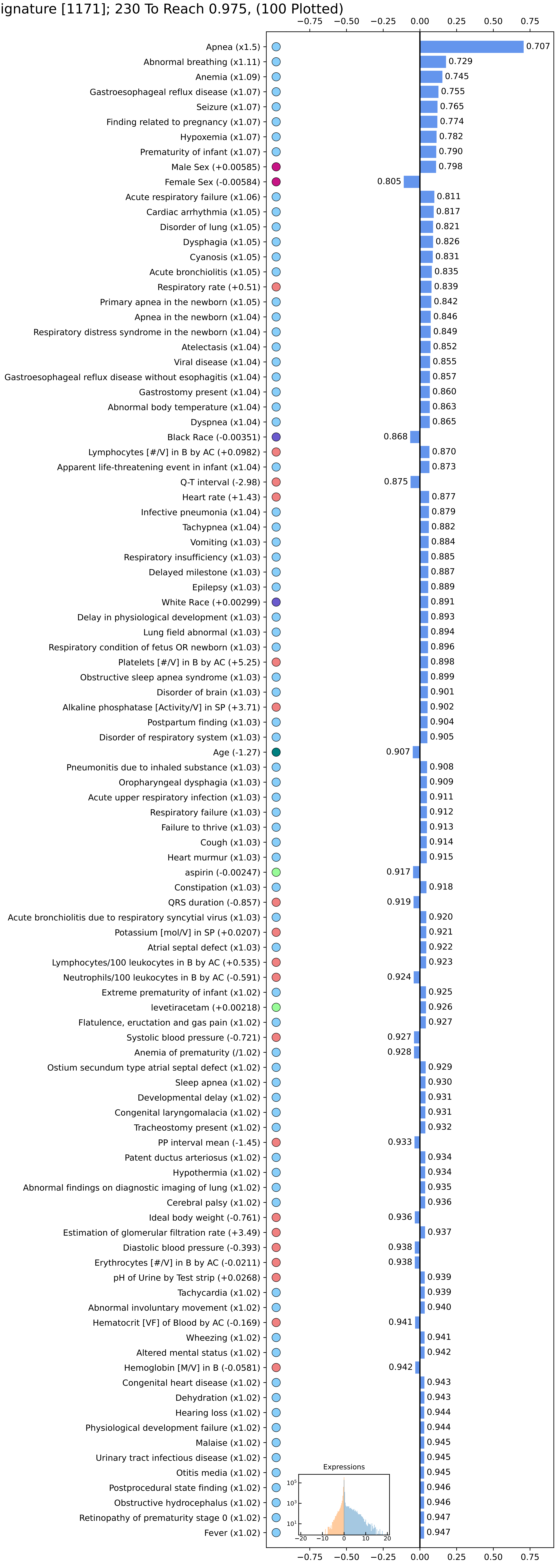

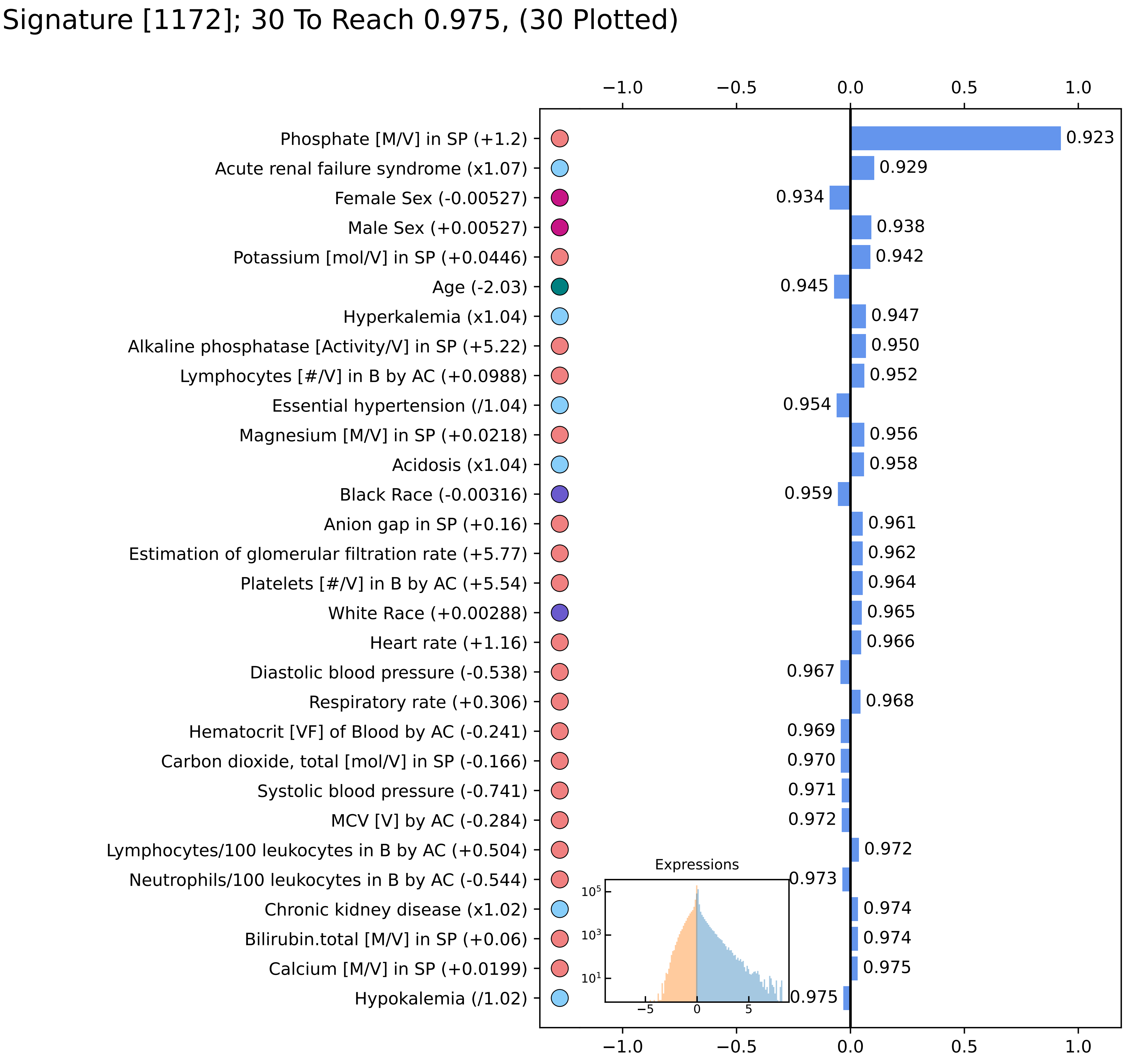
Signature [1172]; 30 To Reach 0.975, (30 Plotted)
Phosphate [M/V] in SP (+1.2)
Acute renal failure syndrome (x1.07)
Female Sex (-0.00527)
Male Sex (+0.00527)
Potassium [mol/V] in SP (+0.0446)
Age (-2.03)
Hyperkalemia (x1.04)
Alkaline phosphatase [Activity/V] in SP (+5.22)
Lymphocytes [#/V] in B by AC (+0.0988)
Essential hypertension (/1.04)
Magnesium [M/V] in SP (+0.0218)
Acidosis (x1.04)
Black Race (-0.00316)
Anion gap in SP (+0.16)
Estimation of glomerular filtration rate (+5.77)
Platelets [#/V] in B by AC (+5.54)
White Race (+0.00288)
Heart rate (+1.16)
Diastolic blood pressure (-0.538)
Respiratory rate (+0.306)
Hematocrit [VF] of Blood by AC (-0.241)
Carbon dioxide, total [mol/V] in SP (-0.166)
Systolic blood pressure (-0.741)
MCV [V] by AC (-0.284)
Lymphocytes/100 leukocytes in B by AC (+0.504)
Neutrophils/100 leukocytes in B by AC (-0.544)
Chronic kidney disease (x1.02)
Bilirubin.total [M/V] in SP (+0.06)
Calcium [M/V] in SP (+0.0199)
Hypokalemia (/1.02)
0.923
0.929
0.934
0.938
0.942
0.945
0.947
0.950
0.952
0.954
0.956
0.958
0.959
0.961
0.962
0.964
0.965
0.966
0.967
0.968
0.969
0.970
0.971
0.972
0.972
0.973
0.974
0.974
0.975
0.975
−1.0
−0.5
0.0
0.5
1.0
Expressions
10^5
10^3
10^1
−5
0
5

Signature [1173]; 157 To Reach 0.975, (100 Plotted)

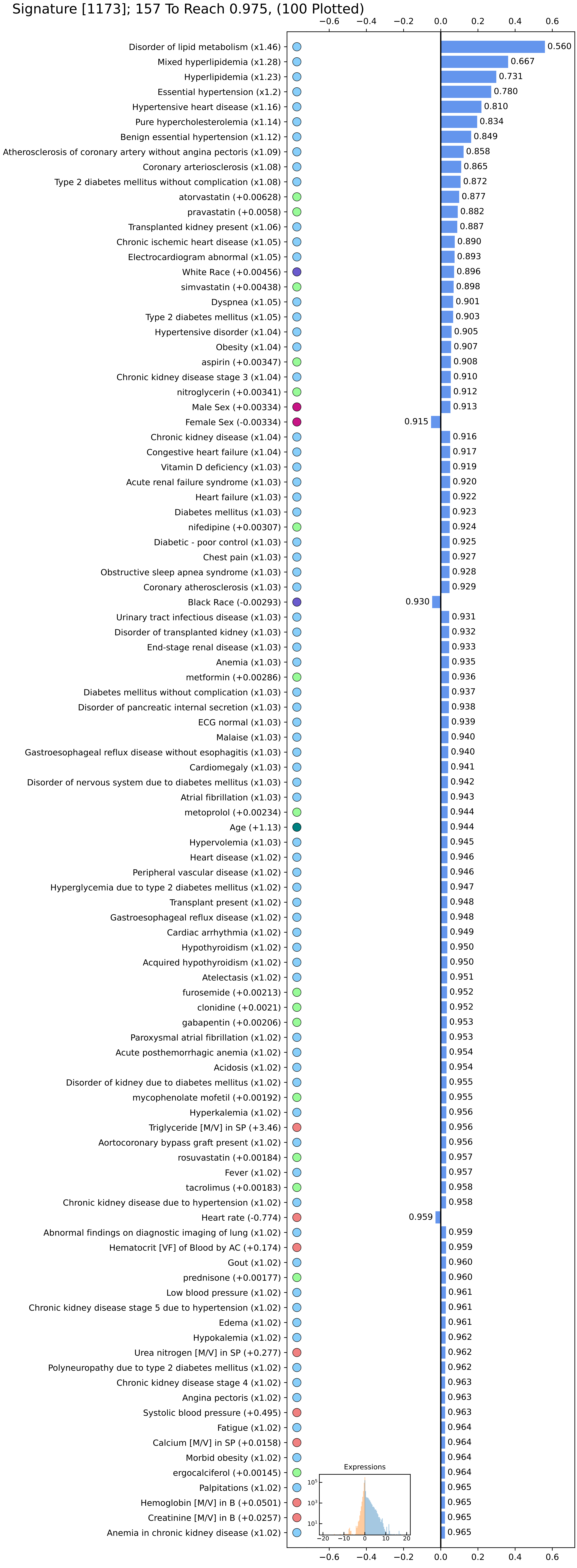

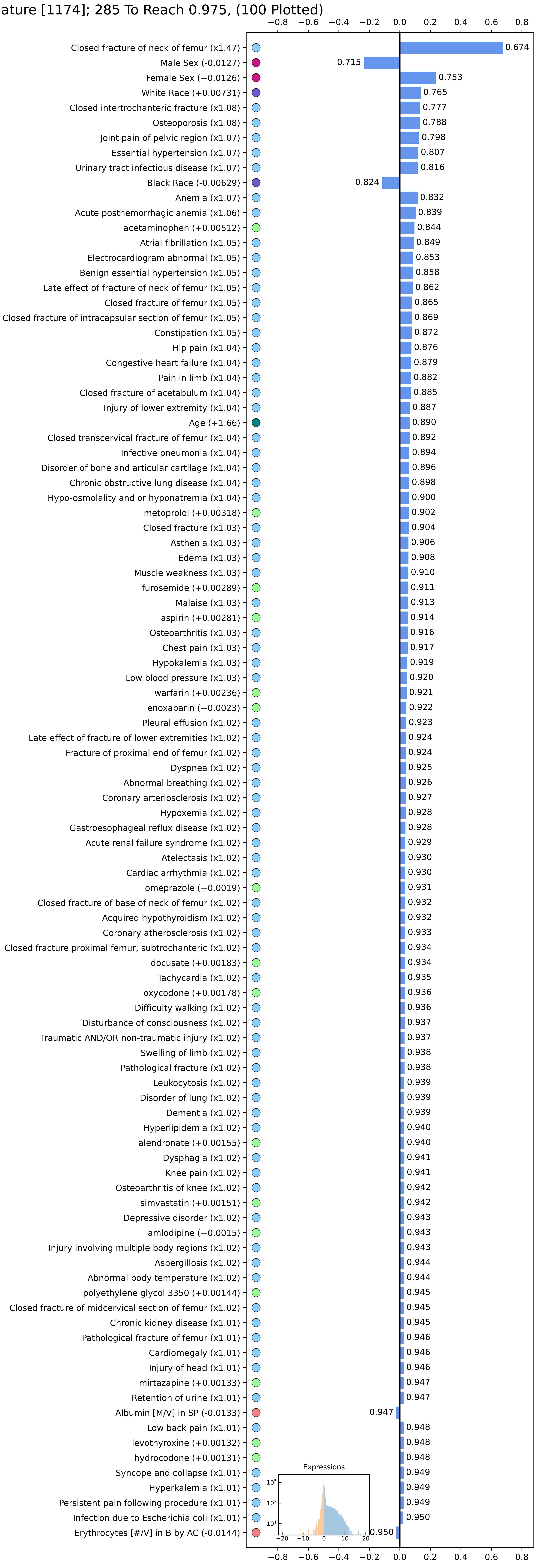

Signature [1174]; 285 To Reach 0.975, (100 Plotted)
Closed fracture of neck of femur (x1.47) 0.674
Male Sex (-0.0127) 0.715
Female Sex (+0.0126) 0.753
White Race (+0.00731) 0.765
Closed intertrochanteric fracture (x1.08) 0.777
Osteoporosis (x1.08) 0.788
Joint pain of pelvic region (x1.07) 0.798
Essential hypertension (x1.07) 0.807
Urinary tract infectious disease (x1.07) 0.816
Black Race (-0.00629) 0.824
Anemia (x1.07) 0.832
Acute posthemorrhagic anemia (x1.06) 0.839
acetaminophen (+0.00512) 0.844
Atrial fibrillation (x1.05) 0.849
Electrocardiogram abnormal (x1.05) 0.853
Benign essential hypertension (x1.05) 0.858
Late effect of fracture of neck of femur (x1.05) 0.862
Closed fracture of femur (x1.05) 0.865
Closed fracture of intracapsular section of femur (x1.05) 0.869
Constipation (x1.05) 0.872
Hip pain (x1.04) 0.876
Congestive heart failure (x1.04) 0.879
Pain in limb (x1.04) 0.882
Closed fracture of acetabulum (x1.04) 0.885
Injury of lower extremity (x1.04) 0.887
Age (+1.66) 0.890
Closed transcervical fracture of femur (x1.04) 0.892
Infective pneumonia (x1.04) 0.894
Disorder of bone and articular cartilage (x1.04) 0.896
Chronic obstructive lung disease (x1.04) 0.898
Hypo-osmolality and or hyponatremia (x1.04) 0.900
metoprolol (+0.00318) 0.902
Closed fracture (x1.03) 0.904
Asthenia (x1.03) 0.906
Edema (x1.03) 0.908
Muscle weakness (x1.03) 0.910
furosemide (+0.00289) 0.911
Malaise (x1.03) 0.913
aspirin (+0.00281) 0.914
Osteoarthritis (x1.03) 0.916
Chest pain (x1.03) 0.917
Hypokalemia (x1.03) 0.919
Low blood pressure (x1.03) 0.920
warfarin (+0.00236) 0.921
enoxaparin (+0.0023) 0.922
Pleural effusion (x1.02) 0.923
Late effect of fracture of lower extremities (x1.02) 0.924
Fracture of proximal end of femur (x1.02) 0.924
Dyspnea (x1.02) 0.925
Abnormal breathing (x1.02) 0.926
Coronary arteriosclerosis (x1.02) 0.927
Hypoxemia (x1.02) 0.928
Gastroesophageal reflux disease (x1.02) 0.928
Acute renal failure syndrome (x1.02) 0.929
Atelectasis (x1.02) 0.930
Cardiac arrhythmia (x1.02) 0.930
omeprazole (+0.0019) 0.931
Closed fracture of base of neck of femur (x1.02) 0.932
Acquired hypothyroidism (x1.02) 0.932
Coronary atherosclerosis (x1.02) 0.933
Closed fracture proximal femur, subtrochanteric (x1.02) 0.934
docusate (+0.00183) 0.934
Tachycardia (x1.02) 0.935
oxycodone (+0.00178) 0.936
Difficulty walking (x1.02) 0.936
Disturbance of consciousness (x1.02) 0.937
Traumatic AND/OR non-traumatic injury (x1.02) 0.937
Swelling of limb (x1.02) 0.938
Pathological fracture (x1.02) 0.938
Leukocytosis (x1.02) 0.939
Disorder of lung (x1.02) 0.939
Dementia (x1.02) 0.939
Hyperlipidemia (x1.02) 0.940
alendronate (+0.00155) 0.940
Dysphagia (x1.02) 0.941
Knee pain (x1.02) 0.941
Osteoarthritis of knee (x1.02) 0.942
simvastatin (+0.00151) 0.942
Depressive disorder (x1.02) 0.943
amlodipine (+0.0015) 0.943
Injury involving multiple body regions (x1.02) 0.943
Aspergillosis (x1.02) 0.944
Abnormal body temperature (x1.02) 0.944
polyethylene glycol 3350 (+0.00144) 0.945
Closed fracture of midcervical section of femur (x1.02) 0.945
Chronic kidney disease (x1.01) 0.945
Pathological fracture of femur (x1.01) 0.946
Cardiomegaly (x1.01) 0.946
Injury of head (x1.01) 0.946
mirtazapine (+0.00133) 0.947
Retention of urine (x1.01) 0.947
Albumin [M/V] in SP (-0.0133) 0.947
Low back pain (x1.01) 0.948
levothyroxine (+0.00132) 0.948
hydrocodone (+0.00131) 0.948
Syncope and collapse (x1.01) 0.949
Hyperkalemia (x1.01) 0.949
Persistent pain following procedure (x1.01) 0.949
Infection due to Escherichia coli (x1.01) 0.950
Erythrocytes [#/V] in B by AC (-0.0144) 0.950
−0.8 −0.6 −0.4 −0.2 0.0 0.2 0.4 0.6 0.8
Expressions
10^5
10^3
10^1
−20 −10 0 10 20

Signature [1175]; 3 To Reach 0.975, (10 Plotted)

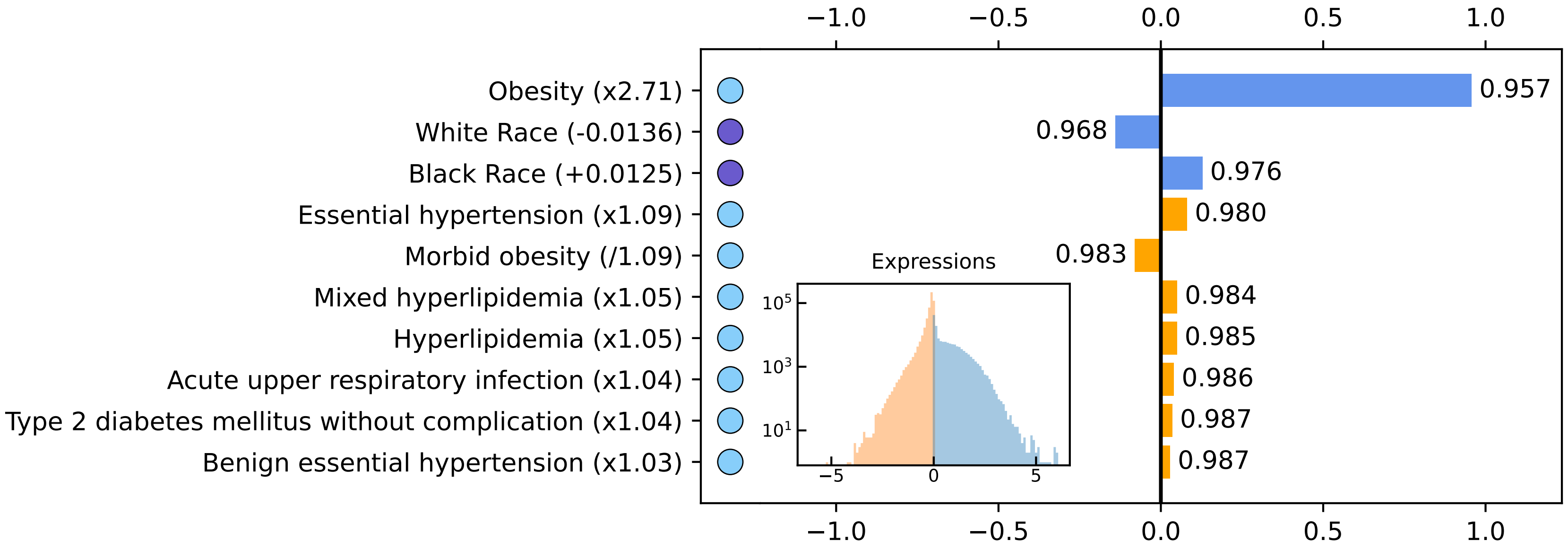

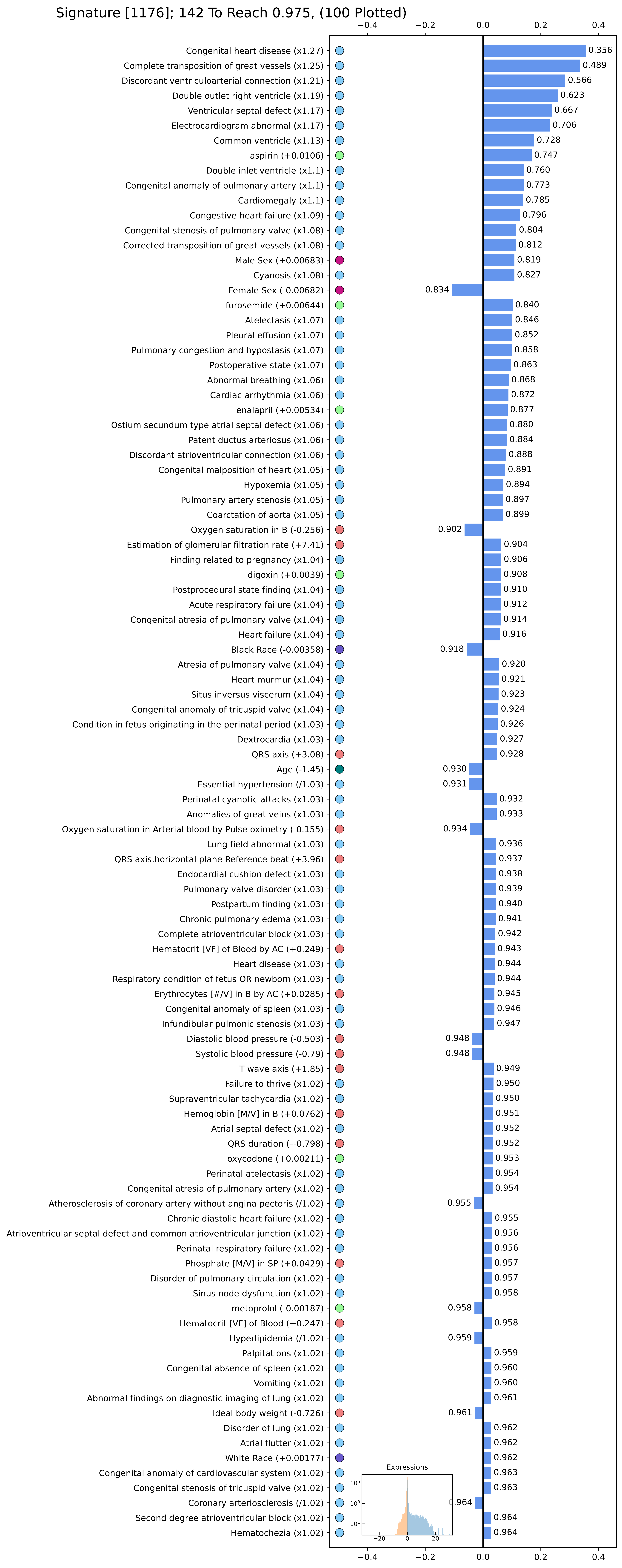
Signature [1176]; 142 To Reach 0.975, (100 Plotted)
Congenital heart disease (x1.27) 0.356
Complete transposition of great vessels (x1.25) 0.489
Discordant ventriculoarterial connection (x1.21) 0.566
Double outlet right ventricle (x1.19) 0.623
Ventricular septal defect (x1.17) 0.667
Electrocardiogram abnormal (x1.17) 0.706
Common ventricle (x1.13) 0.728
aspirin (+0.0106) 0.747
Double inlet ventricle (x1.1) 0.760
Congenital anomaly of pulmonary artery (x1.1) 0.773
Cardiomegaly (x1.1) 0.785
Congestive heart failure (x1.09) 0.796
Congenital stenosis of pulmonary valve (x1.08) 0.804
Corrected transposition of great vessels (x1.08) 0.812
Male Sex (+0.00683) 0.819
Cyanosis (x1.08) 0.827
Female Sex (-0.00682) 0.834
furosemide (+0.00644) 0.840
Atelectasis (x1.07) 0.846
Pleural effusion (x1.07) 0.852
Pulmonary congestion and hypostasis (x1.07) 0.858
Postoperative state (x1.07) 0.863
Abnormal breathing (x1.06) 0.868
Cardiac arrhythmia (x1.06) 0.872
enalapril (+0.00534) 0.877
Ostium secundum type atrial septal defect (x1.06) 0.880
Patent ductus arteriosus (x1.06) 0.884
Discordant atrioventricular connection (x1.06) 0.888
Congenital malposition of heart (x1.05) 0.891
Hypoxemia (x1.05) 0.894
Pulmonary artery stenosis (x1.05) 0.897
Coarctation of aorta (x1.05) 0.899
Oxygen saturation in B (-0.256) 0.902
Estimation of glomerular filtration rate (+7.41) 0.904
Finding related to pregnancy (x1.04) 0.906
digoxin (+0.0039) 0.908
Postprocedural state finding (x1.04) 0.910
Acute respiratory failure (x1.04) 0.912
Congenital atresia of pulmonary valve (x1.04) 0.914
Heart failure (x1.04) 0.916
Black Race (-0.00358) 0.918
Atresia of pulmonary valve (x1.04) 0.920
Heart murmur (x1.04) 0.921
Situs inversus viscerum (x1.04) 0.923
Congenital anomaly of tricuspid valve (x1.04) 0.924
Condition in fetus originating in the perinatal period (x1.03) 0.926
Dextrocardia (x1.03) 0.927
QRS axis (+3.08) 0.928
Age (-1.45) 0.930
Essential hypertension (/1.03) 0.931
Perinatal cyanotic attacks (x1.03) 0.932
Anomalies of great veins (x1.03) 0.933
Oxygen saturation in Arterial blood by Pulse oximetry (-0.155) 0.934
Lung field abnormal (x1.03) 0.936
QRS axis.horizontal plane Reference beat (+3.96) 0.937
Endocardial cushion defect (x1.03) 0.938
Pulmonary valve disorder (x1.03) 0.939
Postpartum finding (x1.03) 0.940
Chronic pulmonary edema (x1.03) 0.941
Complete atrioventricular block (x1.03) 0.942
Hematocrit [VF] of Blood by AC (+0.249) 0.943
Heart disease (x1.03) 0.944
Respiratory condition of fetus OR newborn (x1.03) 0.944
Erythrocytes [#/V] in B by AC (+0.0285) 0.945
Congenital anomaly of spleen (x1.03) 0.946
Infundibular pulmonic stenosis (x1.03) 0.947
Diastolic blood pressure (-0.503) 0.948
Systolic blood pressure (-0.79) 0.948
T wave axis (+1.85) 0.949
Failure to thrive (x1.02) 0.950
Supraventricular tachycardia (x1.02) 0.950
Hemoglobin [M/V] in B (+0.0762) 0.951
Atrial septal defect (x1.02) 0.952
QRS duration (+0.798) 0.952
oxycodone (+0.00211) 0.953
Perinatal atelectasis (x1.02) 0.954
Congenital atresia of pulmonary artery (x1.02) 0.954
Atherosclerosis of coronary artery without angina pectoris (/1.02) 0.955
Chronic diastolic heart failure (x1.02) 0.955
Atrioventricular septal defect and common atrioventricular junction (x1.02) 0.956
Perinatal respiratory failure (x1.02) 0.956
Phosphate [M/V] in SP (+0.0429) 0.957
Disorder of pulmonary circulation (x1.02) 0.957
Sinus node dysfunction (x1.02) 0.958
metoprolol (-0.00187) 0.958
Hematocrit [VF] of Blood (+0.247) 0.958
Hyperlipidemia (/1.02) 0.959
Palpitations (x1.02) 0.959
Congenital absence of spleen (x1.02) 0.960
Vomiting (x1.02) 0.960
Abnormal findings on diagnostic imaging of lung (x1.02) 0.961
Ideal body weight (-0.726) 0.961
Disorder of lung (x1.02) 0.962
Atrial flutter (x1.02) 0.962
White Race (+0.00177) 0.962
Congenital anomaly of cardiovascular system (x1.02) 0.963
Congenital stenosis of tricuspid valve (x1.02) 0.963
Coronary arteriosclerosis (/1.02) 0.964
Second degree atrioventricular block (x1.02) 0.964
Hematochezia (x1.02) 0.964
Expressions
−0.4 −0.2 0.0 0.2 0.4

Signature [1177]; 188 To Reach 0.975, (100 Plotted)

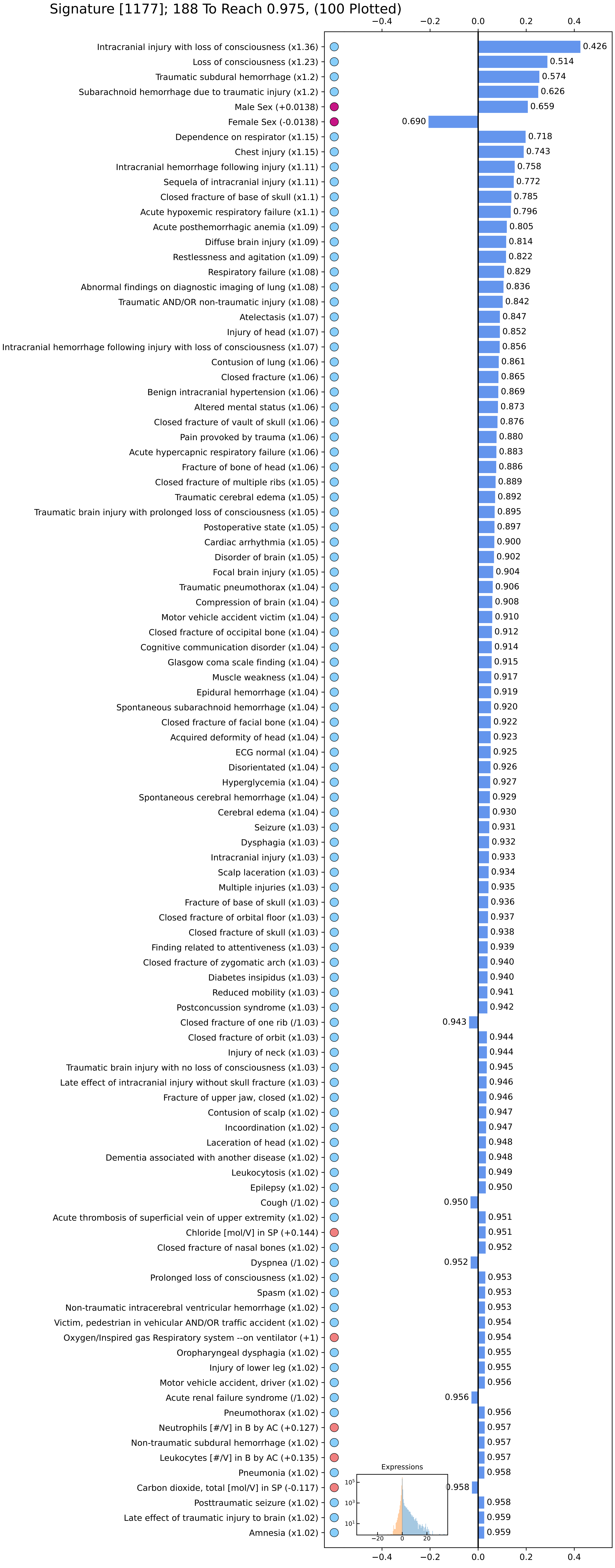

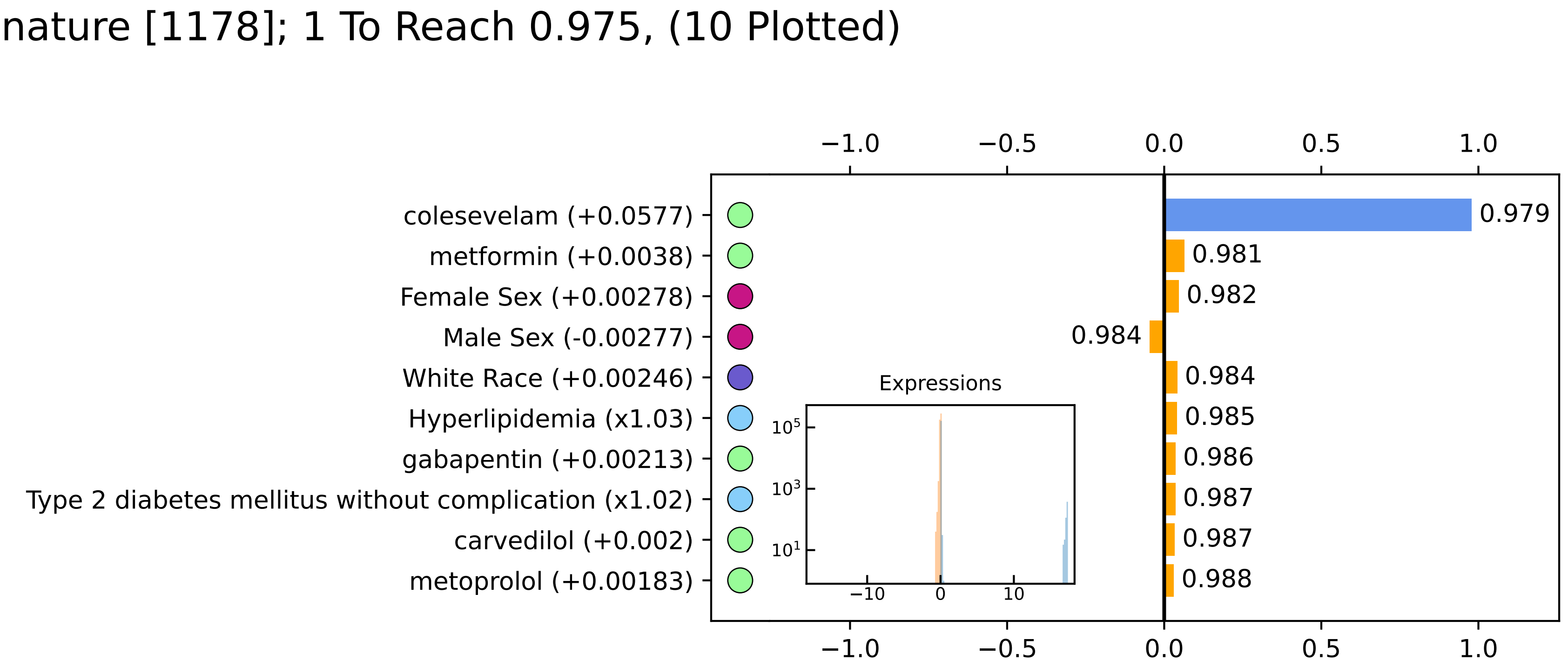
Signature [1178]; 1 To Reach 0.975, (10 Plotted)
colesevelam (+0.0577)
metformin (+0.0038)
Female Sex (+0.00278)
Male Sex (-0.00277)
White Race (+0.00246)
Hyperlipidemia (x1.03)
gabapentin (+0.00213)
Type 2 diabetes mellitus without complication (x1.02)
carvedilol (+0.002)
metoprolol (+0.00183)
0.979
0.981
0.982
0.984
0.984
0.985
0.986
0.987
0.987
0.988
Expressions
−1.0
−0.5
0.0
0.5
1.0

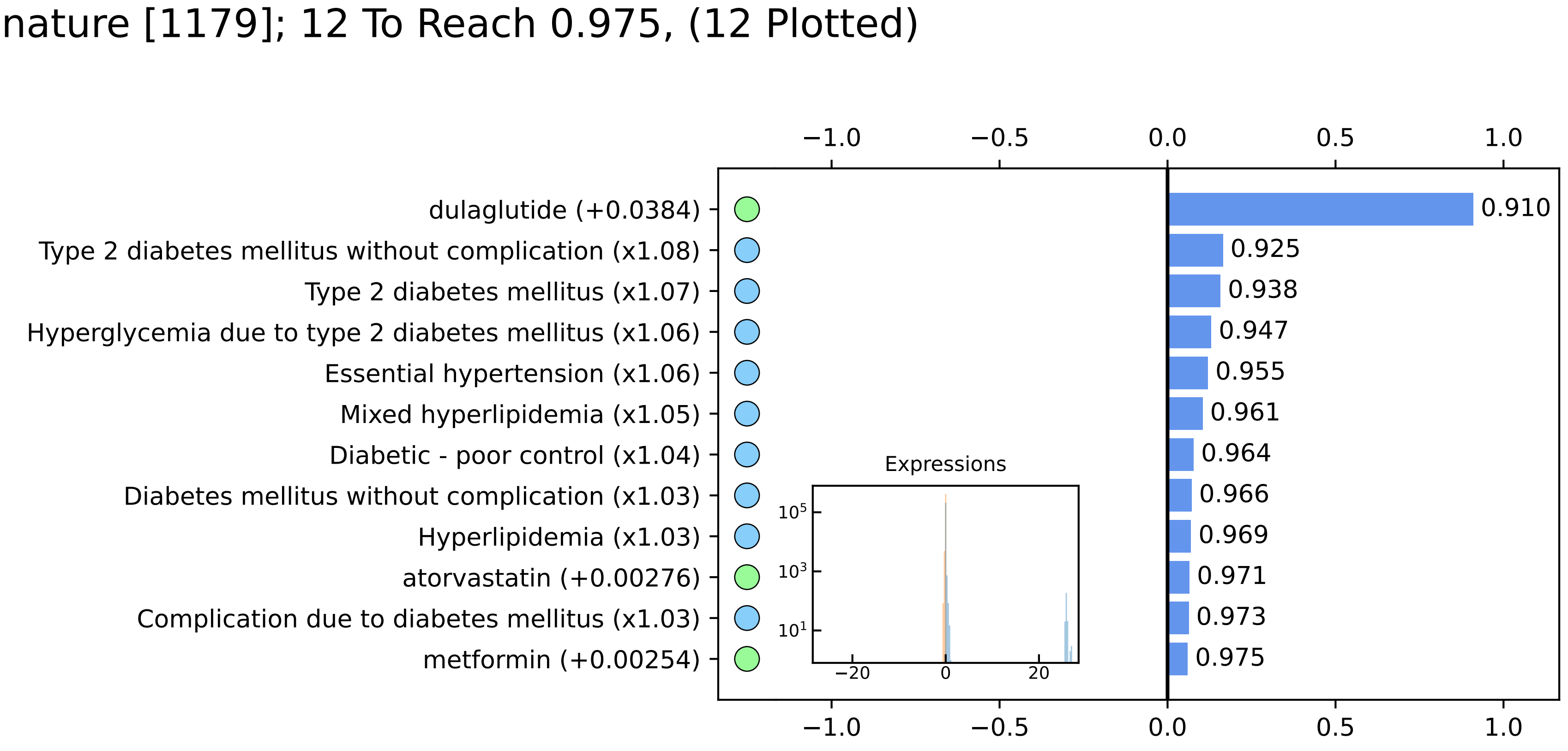
Signature [1179]; 12 To Reach 0.975, (12 Plotted)
dulaglutide (+0.0384)
Type 2 diabetes mellitus without complication (x1.08)
Type 2 diabetes mellitus (x1.07)
Hyperglycemia due to type 2 diabetes mellitus (x1.06)
Essential hypertension (x1.06)
Mixed hyperlipidemia (x1.05)
Diabetic - poor control (x1.04)
Diabetes mellitus without complication (x1.03)
Hyperlipidemia (x1.03)
atorvastatin (+0.00276)
Complication due to diabetes mellitus (x1.03)
metformin (+0.00254)
0.910
0.925
0.938
0.947
0.955
0.961
0.964
0.966
0.969
0.971
0.973
0.975
−1.0
−0.5
0.0
0.5
1.0
Expressions
10^5
10^3
10^1
−20
0
20

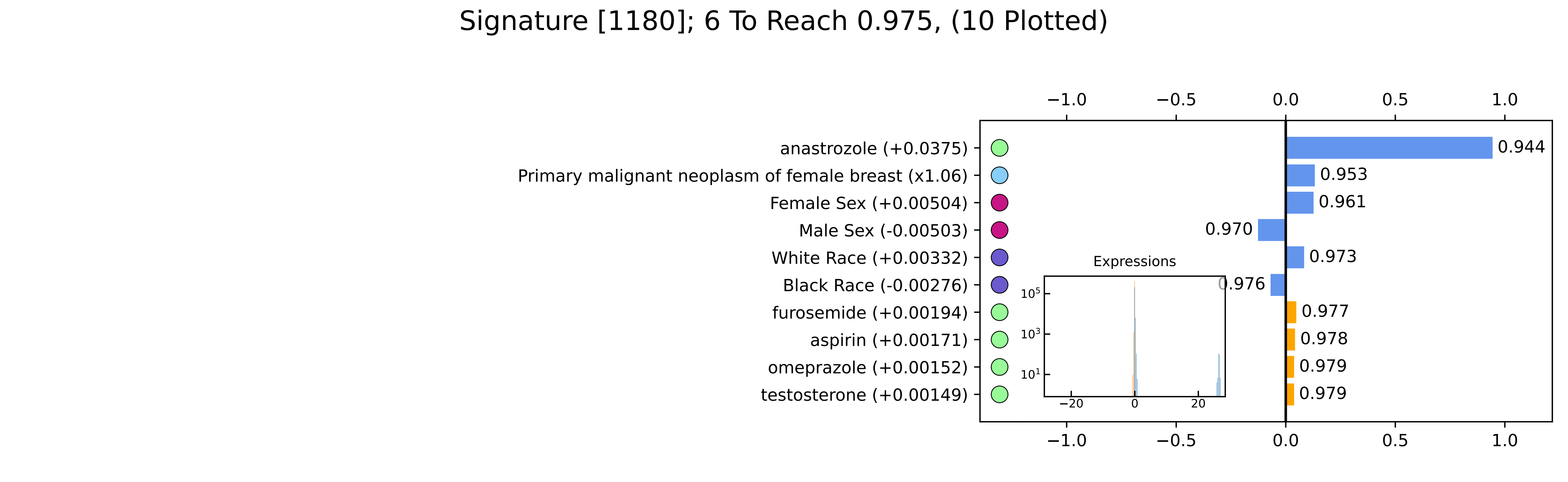

Signature [1180]; 6 To Reach 0.975, (10 Plotted)
anastrozole (+0.0375)
Primary malignant neoplasm of female breast (x1.06)
Female Sex (+0.00504)
Male Sex (-0.00503)
White Race (+0.00332)
Black Race (-0.00276)
furosemide (+0.00194)
aspirin (+0.00171)
omeprazole (+0.00152)
testosterone (+0.00149)
0.944
0.953
0.961
0.970
0.973
0.976
0.977
0.978
0.979
0.979
Expressions
−1.0
−0.5
0.0
0.5
1.0

Signature [1181]; 1 To Reach 0.975, (10 Plotted)

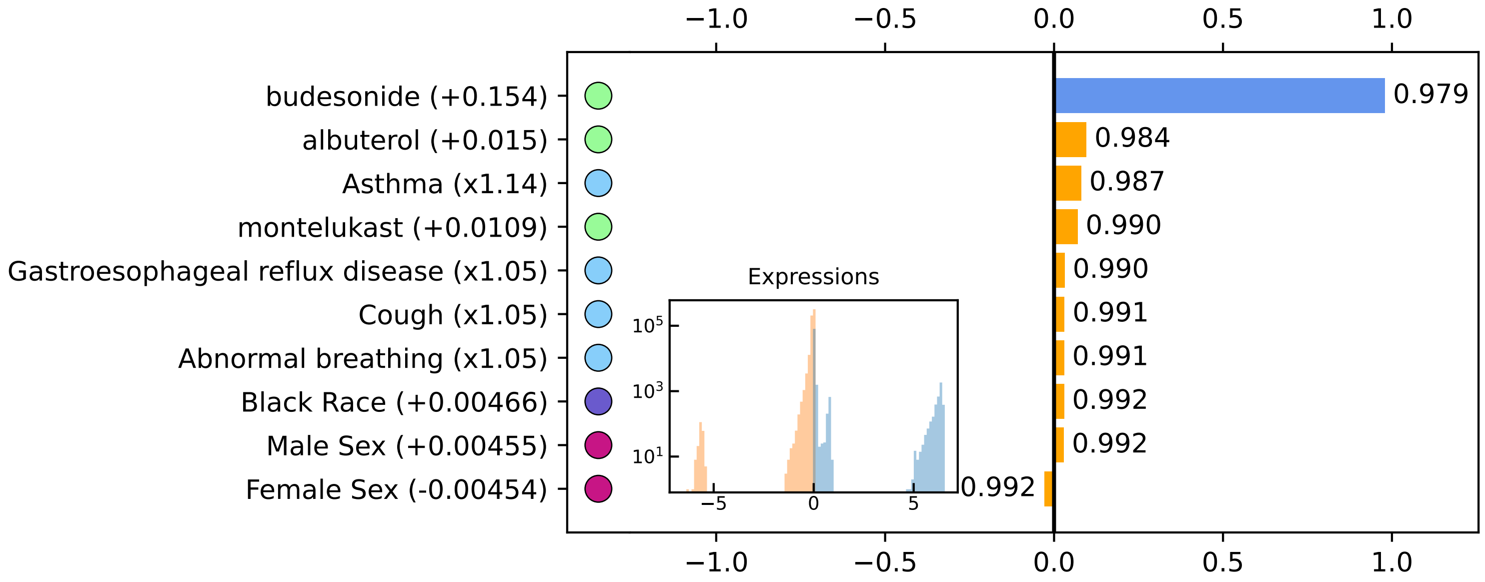

Signature [1182]; 4 To Reach 0.975, (10 Plotted)

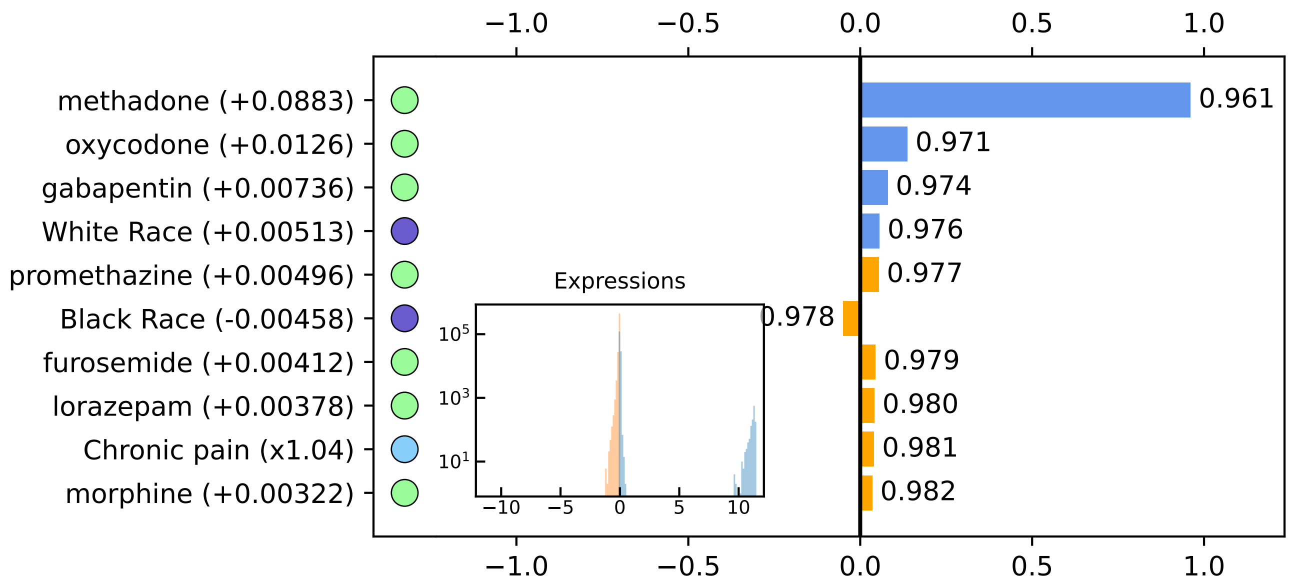

Signature [1183]; 2 To Reach 0.975, (10 Plotted)

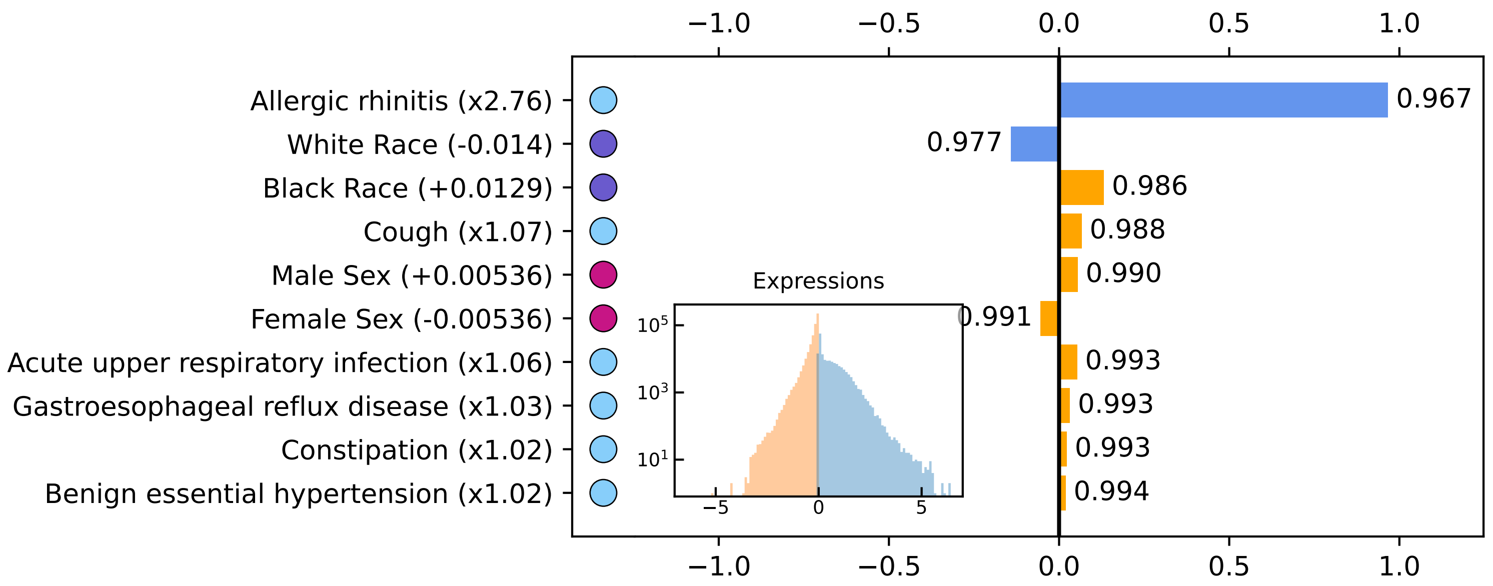

Signature [1184]; 70 To Reach 0.975, (70 Plotted)

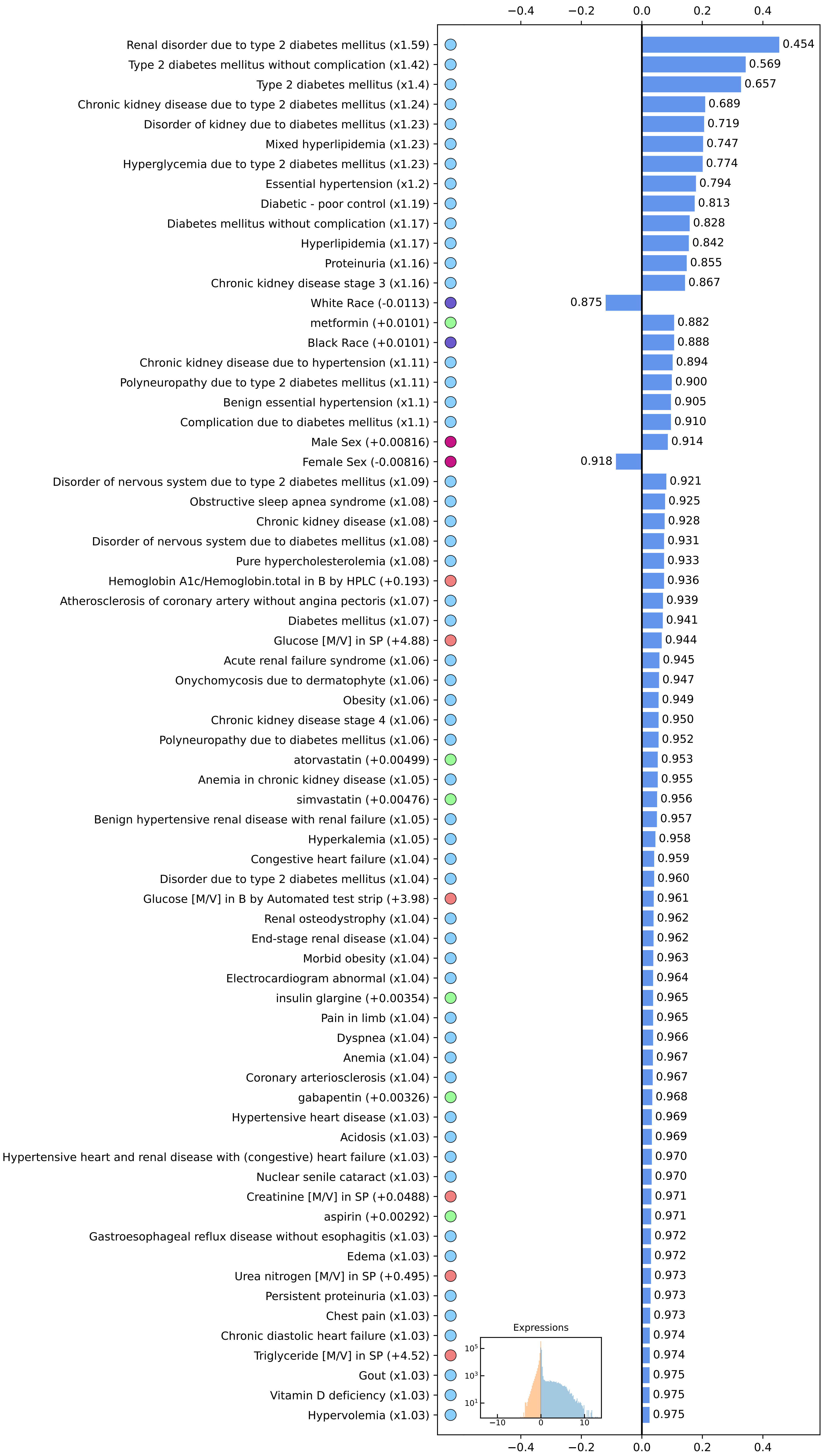

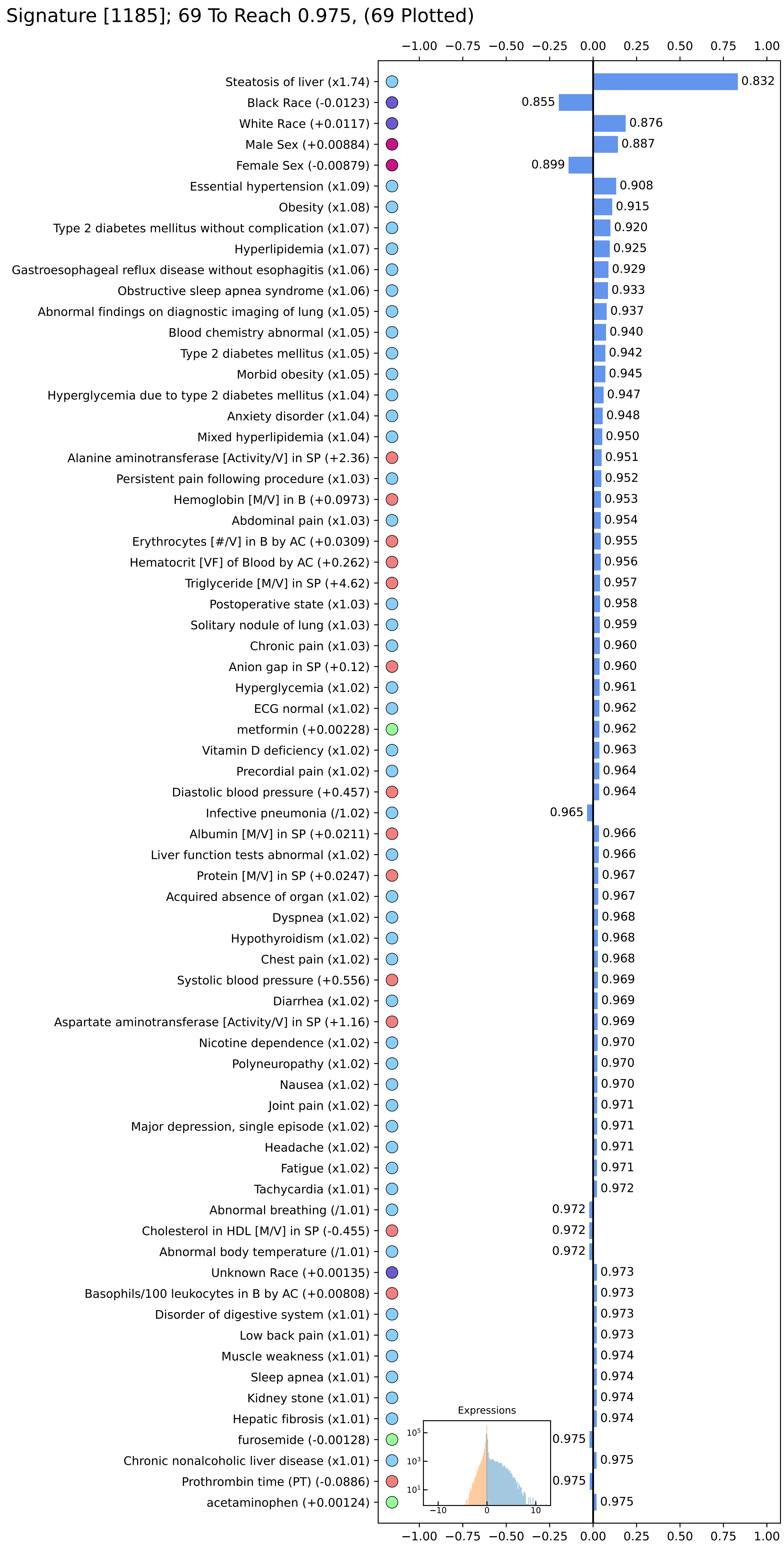

Signature [1185]; 69 To Reach 0.975, (69 Plotted)
−1.00 −0.75 −0.50 −0.25 0.00 0.25 0.50 0.75 1.00
Steatosis of liver (x1.74) 0.832
Black Race (-0.0123) 0.855
White Race (+0.0117) 0.876
Male Sex (+0.00884) 0.887
Female Sex (-0.00879) 0.899
Essential hypertension (x1.09) 0.908
Obesity (x1.08) 0.915
Type 2 diabetes mellitus without complication (x1.07) 0.920
Hyperlipidemia (x1.07) 0.925
Gastroesophageal reflux disease without esophagitis (x1.06) 0.929
Obstructive sleep apnea syndrome (x1.06) 0.933
Abnormal findings on diagnostic imaging of lung (x1.05) 0.937
Blood chemistry abnormal (x1.05) 0.940
Type 2 diabetes mellitus (x1.05) 0.942
Morbid obesity (x1.05) 0.945
Hyperglycemia due to type 2 diabetes mellitus (x1.04) 0.947
Anxiety disorder (x1.04) 0.948
Mixed hyperlipidemia (x1.04) 0.950
Alanine aminotransferase [Activity/V] in SP (+2.36) 0.951
Persistent pain following procedure (x1.03) 0.952
Hemoglobin [M/V] in B (+0.0973) 0.953
Abdominal pain (x1.03) 0.954
Erythrocytes [#/V] in B by AC (+0.0309) 0.955
Hematocrit [VF] of Blood by AC (+0.262) 0.956
Triglyceride [M/V] in SP (+4.62) 0.957
Postoperative state (x1.03) 0.958
Solitary nodule of lung (x1.03) 0.959
Chronic pain (x1.03) 0.960
Anion gap in SP (+0.12) 0.960
Hyperglycemia (x1.02) 0.961
ECG normal (x1.02) 0.962
metformin (+0.00228) 0.962
Vitamin D deficiency (x1.02) 0.963
Precordial pain (x1.02) 0.964
Diastolic blood pressure (+0.457) 0.964
Infective pneumonia (/1.02) 0.965
Albumin [M/V] in SP (+0.0211) 0.966
Liver function tests abnormal (x1.02) 0.966
Protein [M/V] in SP (+0.0247) 0.967
Acquired absence of organ (x1.02) 0.967
Dyspnea (x1.02) 0.968
Hypothyroidism (x1.02) 0.968
Chest pain (x1.02) 0.968
Systolic blood pressure (+0.556) 0.969
Diarrhea (x1.02) 0.969
Aspartate aminotransferase [Activity/V] in SP (+1.16) 0.969
Nicotine dependence (x1.02) 0.970
Polyneuropathy (x1.02) 0.970
Nausea (x1.02) 0.970
Joint pain (x1.02) 0.971
Major depression, single episode (x1.02) 0.971
Headache (x1.02) 0.971
Fatigue (x1.02) 0.971
Tachycardia (x1.01) 0.972
Abnormal breathing (/1.01) 0.972
Cholesterol in HDL [M/V] in SP (-0.455) 0.972
Abnormal body temperature (/1.01) 0.972
Unknown Race (+0.00135) 0.973
Basophils/100 leukocytes in B by AC (+0.00808) 0.973
Disorder of digestive system (x1.01) 0.973
Low back pain (x1.01) 0.973
Muscle weakness (x1.01) 0.974
Sleep apnea (x1.01) 0.974
Kidney stone (x1.01) 0.974
Hepatic fibrosis (x1.01) 0.974
furosemide (-0.00128) 0.975
Chronic nonalcoholic liver disease (x1.01) 0.975
Prothrombin time (PT) (-0.0886) 0.975
acetaminophen (+0.00124) 0.975
Expressions
10^5
10^3
10^1
−10 0 10

Signature [1186]; 186 To Reach 0.975, (100 Plotted)

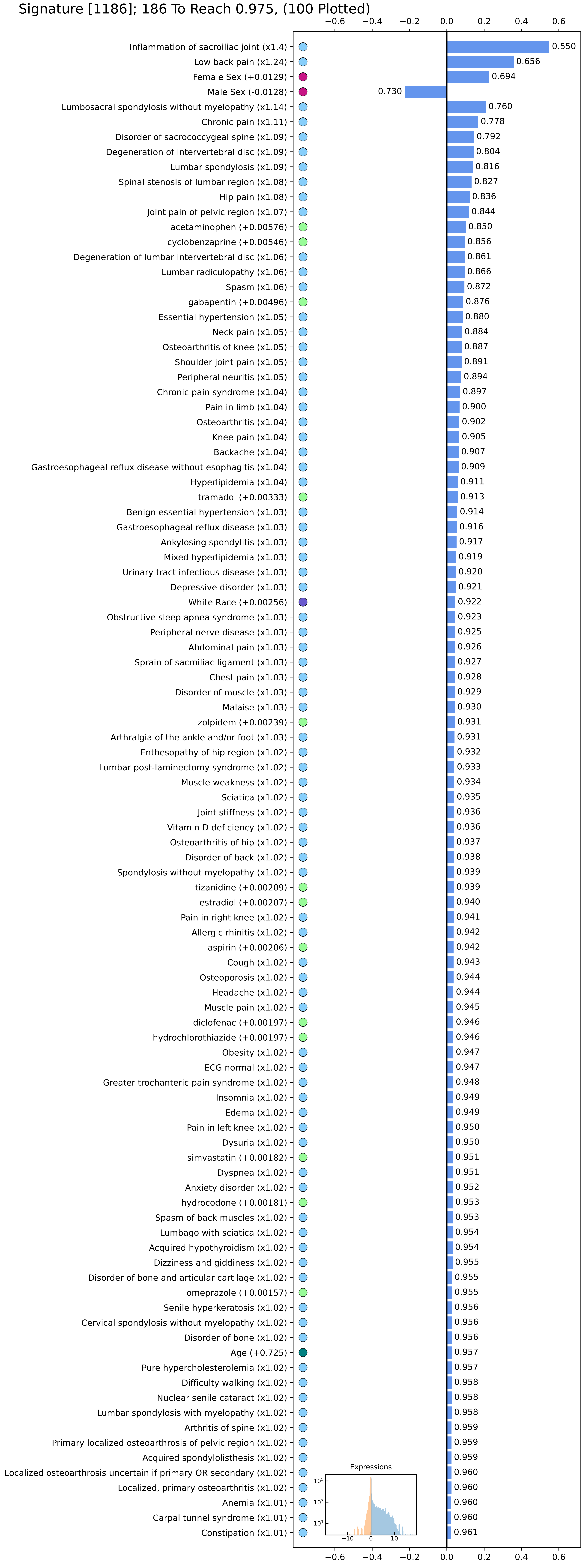

Signature [1187]; 149 To Reach 0.975, (100 Plotted)

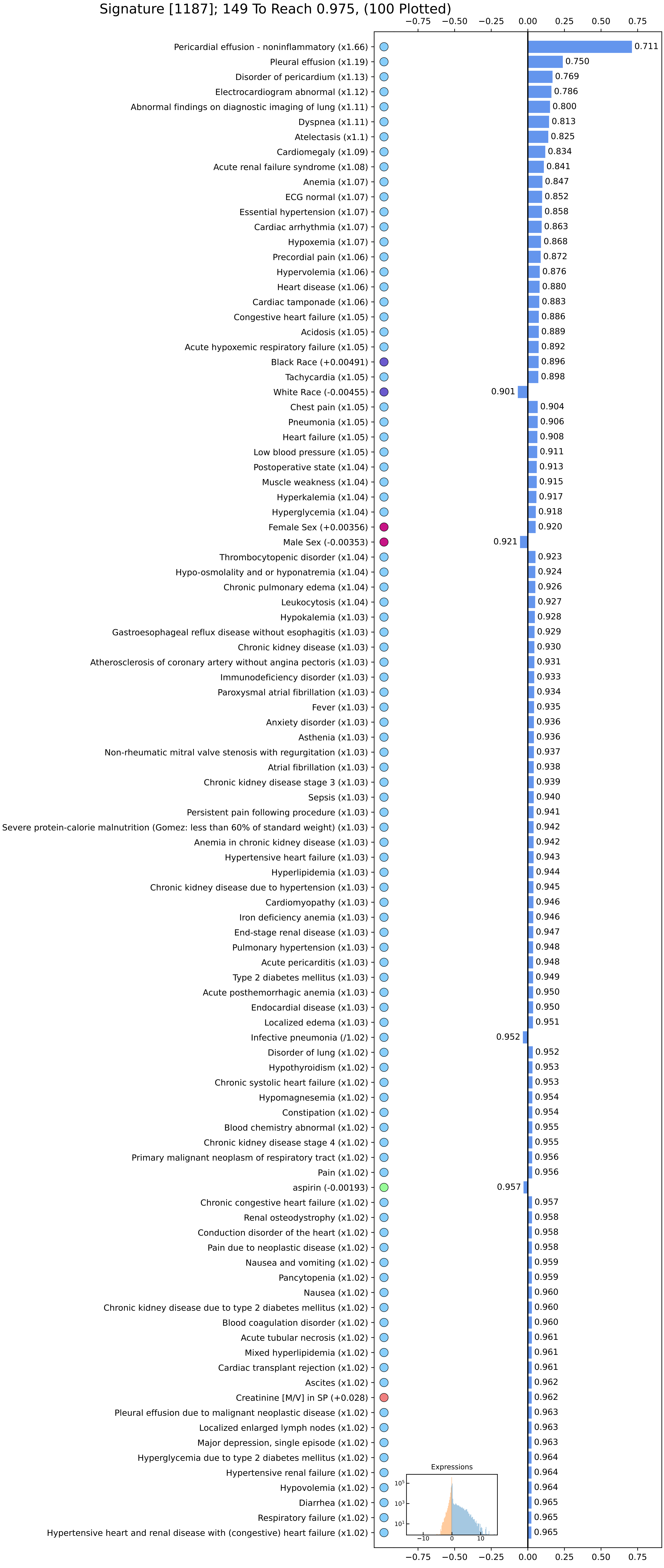

Signature [1188]; 3 To Reach 0.975, (10 Plotted)

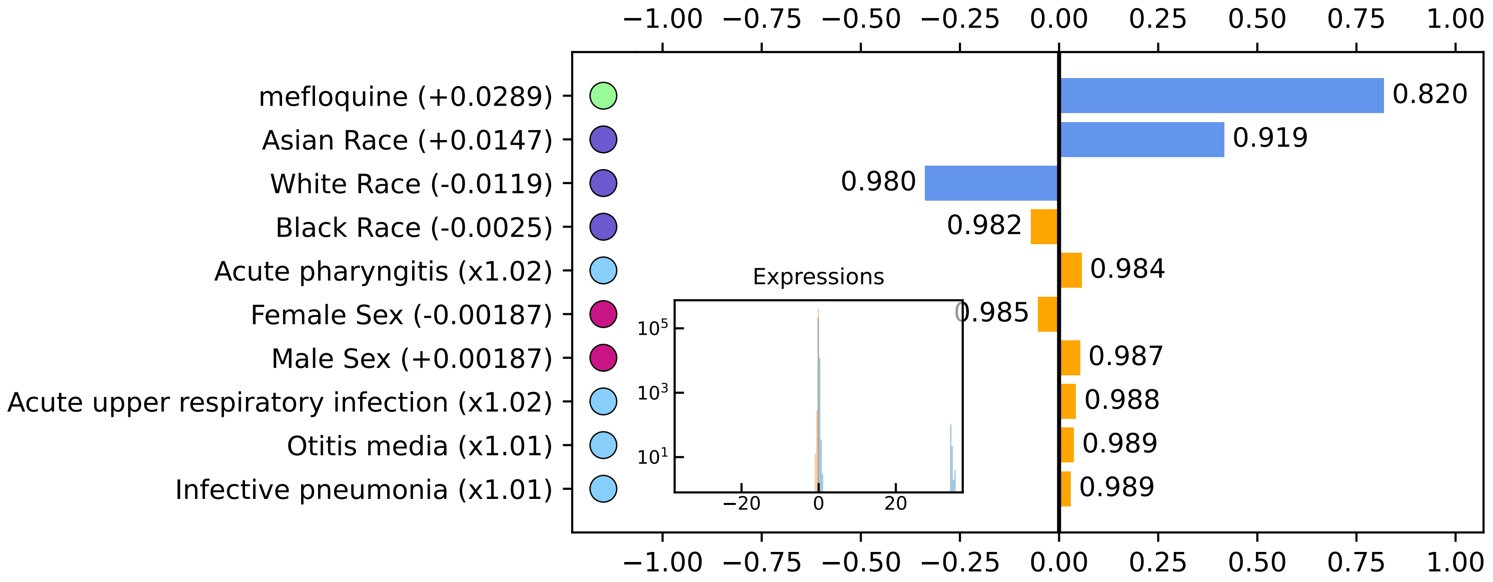

Signature [1189]; 2 To Reach 0.975, (10 Plotted)

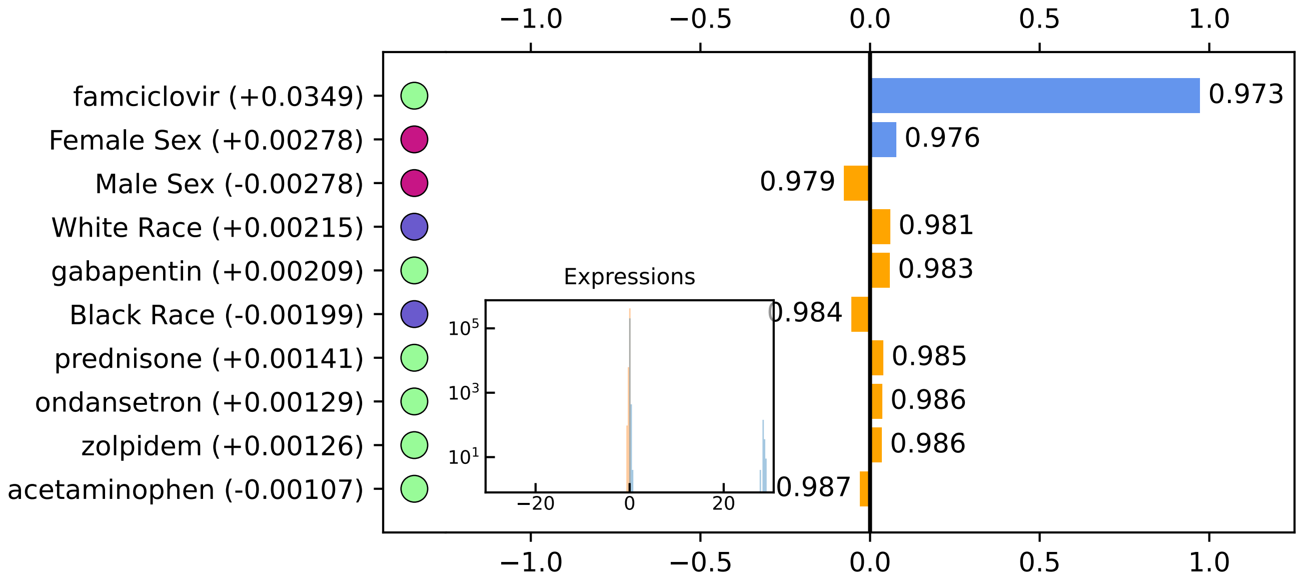

Signature [1190]; 74 To Reach 0.975, (74 Plotted)

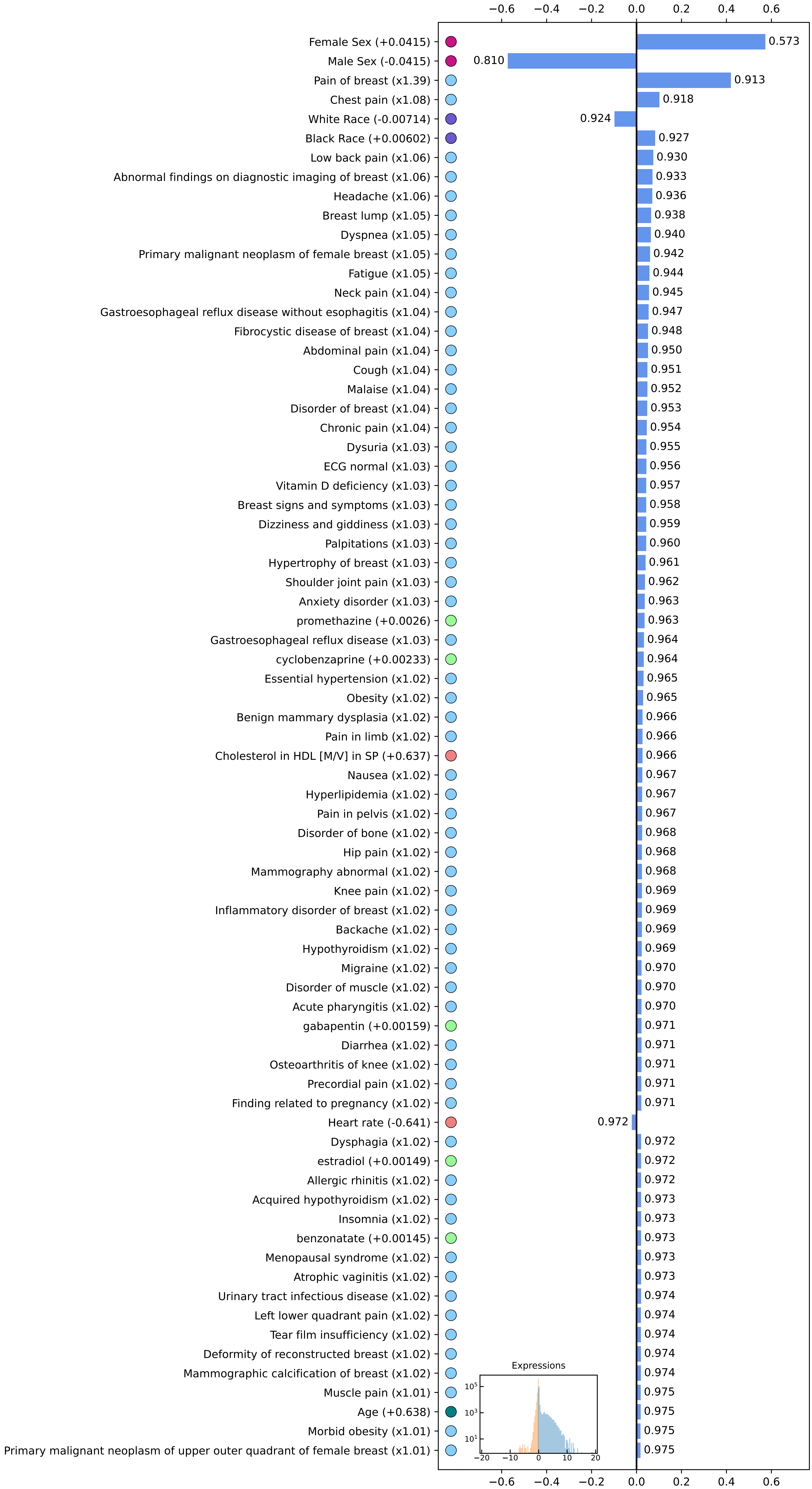

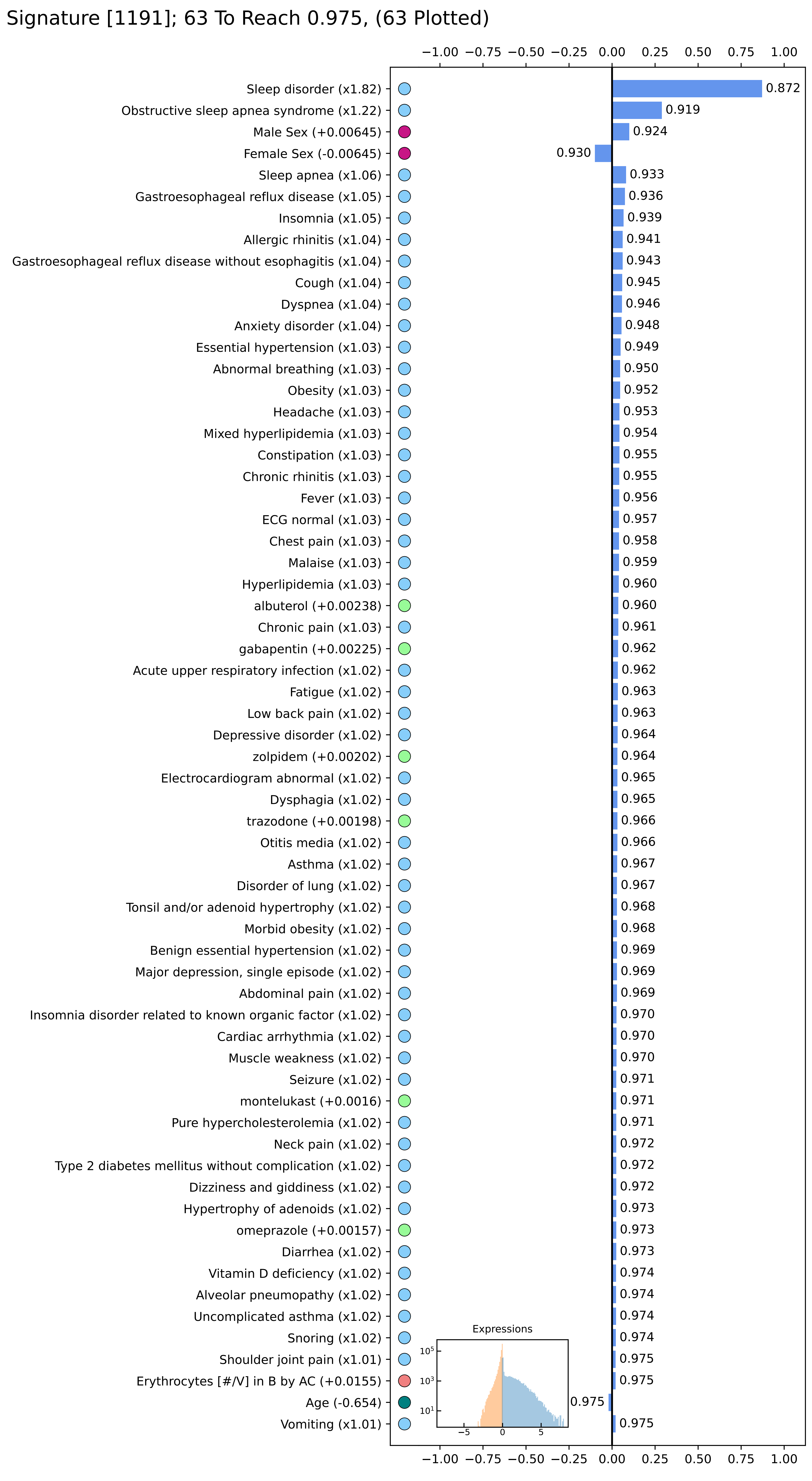

Signature [1191]; 63 To Reach 0.975, (63 Plotted)
Sleep disorder (x1.82) 0.872
Obstructive sleep apnea syndrome (x1.22) 0.919
Male Sex (+0.00645) 0.924
Female Sex (-0.00645) 0.930
Sleep apnea (x1.06) 0.933
Gastroesophageal reflux disease (x1.05) 0.936
Insomnia (x1.05) 0.939
Allergic rhinitis (x1.04) 0.941
Gastroesophageal reflux disease without esophagitis (x1.04) 0.943
Cough (x1.04) 0.945
Dyspnea (x1.04) 0.946
Anxiety disorder (x1.04) 0.948
Essential hypertension (x1.03) 0.949
Abnormal breathing (x1.03) 0.950
Obesity (x1.03) 0.952
Headache (x1.03) 0.953
Mixed hyperlipidemia (x1.03) 0.954
Constipation (x1.03) 0.955
Chronic rhinitis (x1.03) 0.955
Fever (x1.03) 0.956
ECG normal (x1.03) 0.957
Chest pain (x1.03) 0.958
Malaise (x1.03) 0.959
Hyperlipidemia (x1.03) 0.960
albuterol (+0.00238) 0.960
Chronic pain (x1.03) 0.961
gabapentin (+0.00225) 0.962
Acute upper respiratory infection (x1.02) 0.962
Fatigue (x1.02) 0.963
Low back pain (x1.02) 0.963
Depressive disorder (x1.02) 0.964
zolpidem (+0.00202) 0.964
Electrocardiogram abnormal (x1.02) 0.965
Dysphagia (x1.02) 0.965
trazodone (+0.00198) 0.966
Otitis media (x1.02) 0.966
Asthma (x1.02) 0.967
Disorder of lung (x1.02) 0.967
Tonsil and/or adenoid hypertrophy (x1.02) 0.968
Morbid obesity (x1.02) 0.968
Benign essential hypertension (x1.02) 0.969
Major depression, single episode (x1.02) 0.969
Abdominal pain (x1.02) 0.969
Insomnia disorder related to known organic factor (x1.02) 0.970
Cardiac arrhythmia (x1.02) 0.970
Muscle weakness (x1.02) 0.970
Seizure (x1.02) 0.971
montelukast (+0.0016) 0.971
Pure hypercholesterolemia (x1.02) 0.971
Neck pain (x1.02) 0.972
Type 2 diabetes mellitus without complication (x1.02) 0.972
Dizziness and giddiness (x1.02) 0.972
Hypertrophy of adenoids (x1.02) 0.973
omeprazole (+0.00157) 0.973
Diarrhea (x1.02) 0.973
Vitamin D deficiency (x1.02) 0.974
Alveolar pneumopathy (x1.02) 0.974
Uncomplicated asthma (x1.02) 0.974
Snoring (x1.02) 0.974
Shoulder joint pain (x1.01) 0.975
Erythrocytes [#/V] in B by AC (+0.0155) 0.975
Age (-0.654) 0.975
Vomiting (x1.01) 0.975
−1.00 −0.75 −0.50 −0.25 0.00 0.25 0.50 0.75 1.00
Expressions

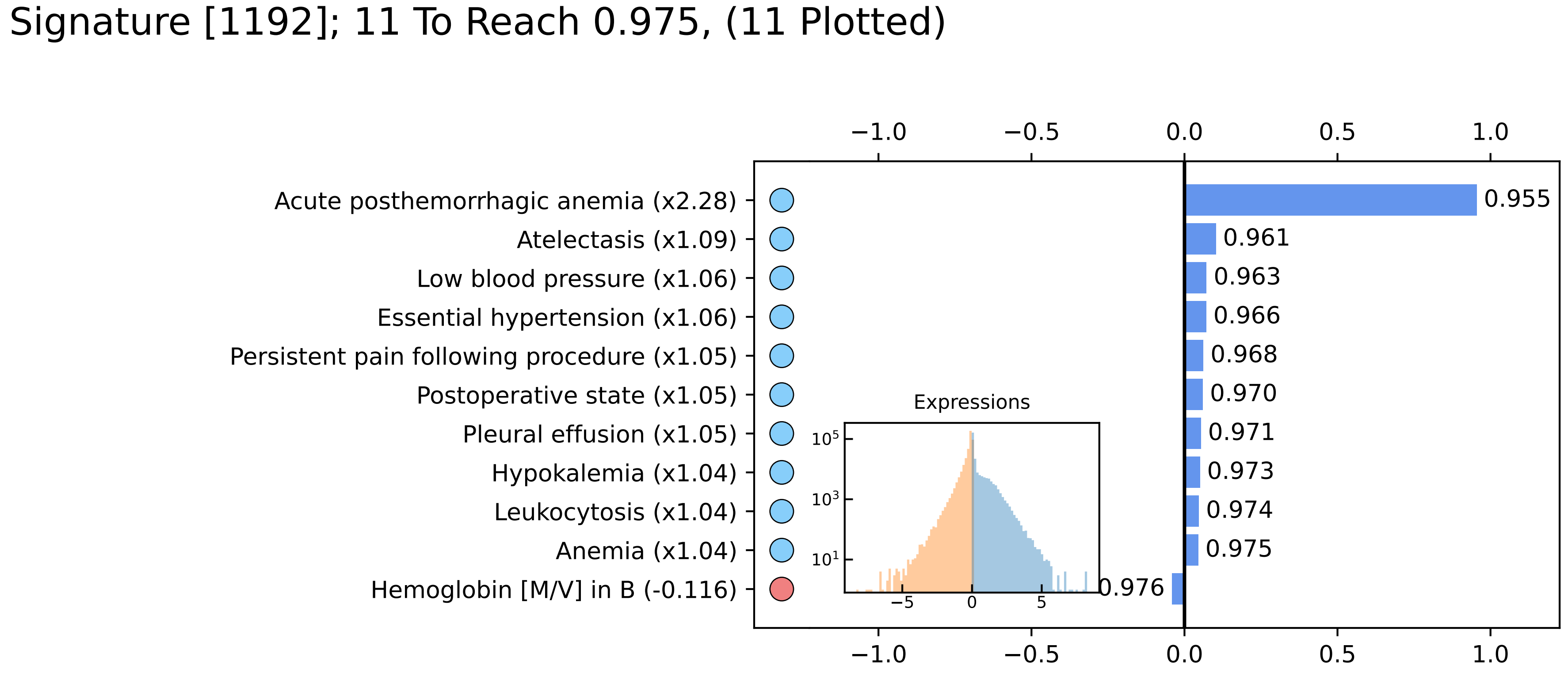
Signature [1192]; 11 To Reach 0.975, (11 Plotted)
Acute posthemorrhagic anemia (x2.28)
Atelectasis (x1.09)
Low blood pressure (x1.06)
Essential hypertension (x1.06)
Persistent pain following procedure (x1.05)
Postoperative state (x1.05)
Pleural effusion (x1.05)
Hypokalemia (x1.04)
Leukocytosis (x1.04)
Anemia (x1.04)
Hemoglobin [M/V] in B (-0.116)
0.955
0.961
0.963
0.966
0.968
0.970
0.971
0.973
0.974
0.975
0.976
Expressions
−1.0
−0.5
0.0
0.5
1.0

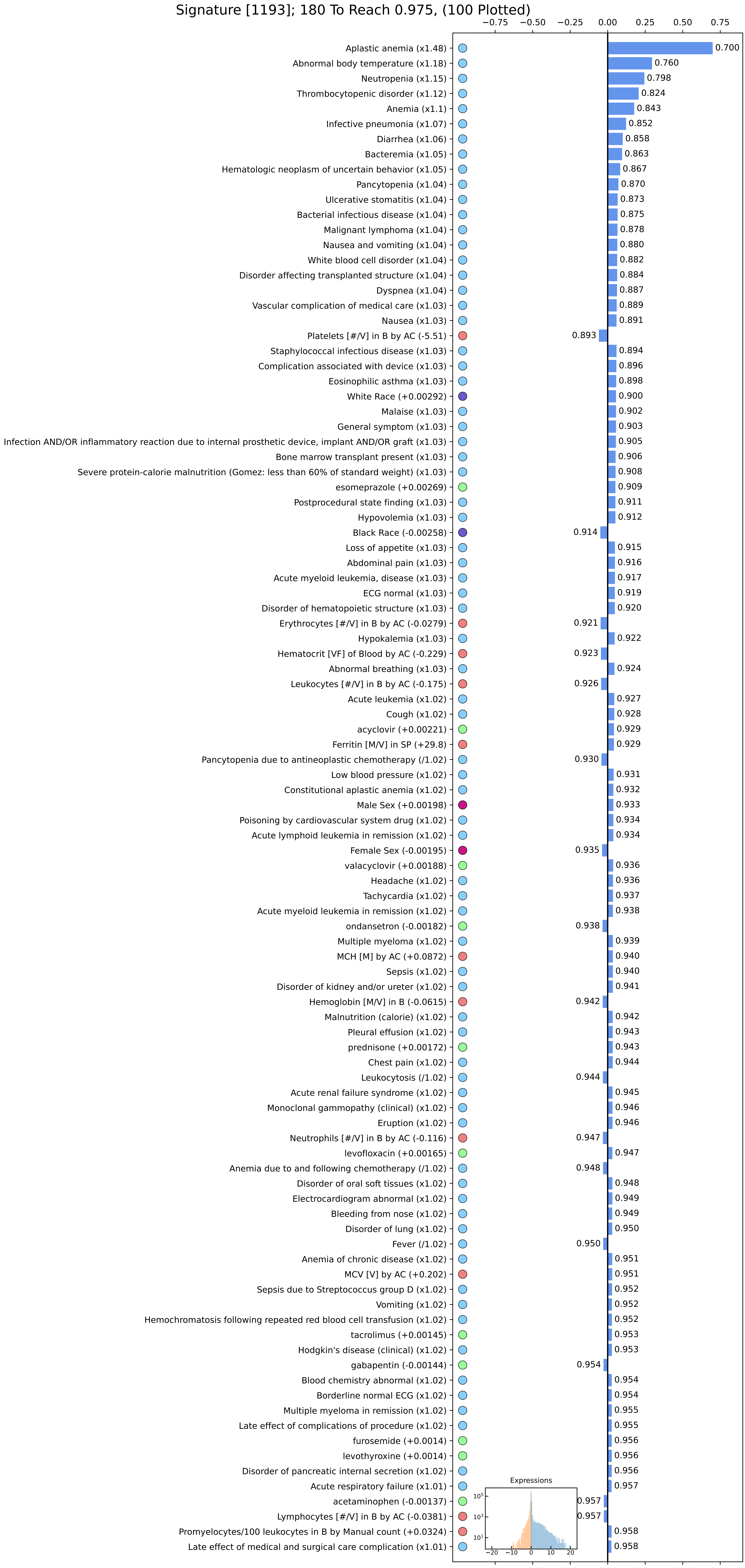

Signature [1193]; 180 To Reach 0.975, (100 Plotted)
Aplastic anemia (x1.48) 0.700
Abnormal body temperature (x1.18) 0.760
Neutropenia (x1.15) 0.798
Thrombocytopenic disorder (x1.12) 0.824
Anemia (x1.1) 0.843
Infective pneumonia (x1.07) 0.852
Diarrhea (x1.06) 0.858
Bacteremia (x1.05) 0.863
Hematologic neoplasm of uncertain behavior (x1.05) 0.867
Pancytopenia (x1.04) 0.870
Ulcerative stomatitis (x1.04) 0.873
Bacterial infectious disease (x1.04) 0.875
Malignant lymphoma (x1.04) 0.878
Nausea and vomiting (x1.04) 0.880
White blood cell disorder (x1.04) 0.882
Disorder affecting transplanted structure (x1.04) 0.884
Dyspnea (x1.04) 0.887
Vascular complication of medical care (x1.03) 0.889
Nausea (x1.03) 0.891
Platelets [#/V] in B by AC (-5.51) 0.893
Staphylococcal infectious disease (x1.03) 0.894
Complication associated with device (x1.03) 0.896
Eosinophilic asthma (x1.03) 0.898
White Race (+0.00292) 0.900
Malaise (x1.03) 0.902
General symptom (x1.03) 0.903
Infection AND/OR inflammatory reaction due to internal prosthetic device, implant AND/OR graft (x1.03) 0.905
Bone marrow transplant present (x1.03) 0.906
Severe protein-calorie malnutrition (Gomez: less than 60% of standard weight) (x1.03) 0.908
esomeprazole (+0.00269) 0.909
Postprocedural state finding (x1.03) 0.911
Hypovolemia (x1.03) 0.912
Black Race (-0.00258) 0.914
Loss of appetite (x1.03) 0.915
Abdominal pain (x1.03) 0.916
Acute myeloid leukemia, disease (x1.03) 0.917
ECG normal (x1.03) 0.919
Disorder of hematopoietic structure (x1.03) 0.920
Erythrocytes [#/V] in B by AC (-0.0279) 0.921
Hypokalemia (x1.03) 0.922
Hematocrit [VF] of Blood by AC (-0.229) 0.923
Abnormal breathing (x1.03) 0.924
Leukocytes [#/V] in B by AC (-0.175) 0.926
Acute leukemia (x1.02) 0.927
Cough (x1.02) 0.928
acyclovir (+0.00221) 0.929
Ferritin [M/V] in SP (+29.8) 0.929
Pancytopenia due to antineoplastic chemotherapy (/1.02) 0.930
Low blood pressure (x1.02) 0.931
Constitutional aplastic anemia (x1.02) 0.932
Male Sex (+0.00198) 0.933
Poisoning by cardiovascular system drug (x1.02) 0.934
Acute lymphoid leukemia in remission (x1.02) 0.934
Female Sex (-0.00195) 0.935
valacyclovir (+0.00188) 0.936
Headache (x1.02) 0.936
Tachycardia (x1.02) 0.937
Acute myeloid leukemia in remission (x1.02) 0.938
ondansetron (-0.00182) 0.938
Multiple myeloma (x1.02) 0.939
MCH [M] by AC (+0.0872) 0.940
Sepsis (x1.02) 0.940
Disorder of kidney and/or ureter (x1.02) 0.941
Hemoglobin [M/V] in B (-0.0615) 0.942
Malnutrition (calorie) (x1.02) 0.942
Pleural effusion (x1.02) 0.943
prednisone (+0.00172) 0.943
Chest pain (x1.02) 0.944
Leukocytosis (/1.02) 0.944
Acute renal failure syndrome (x1.02) 0.945
Monoclonal gammopathy (clinical) (x1.02) 0.946
Eruption (x1.02) 0.946
Neutrophils [#/V] in B by AC (-0.116) 0.947
levofloxacin (+0.00165) 0.947
Anemia due to and following chemotherapy (/1.02) 0.948
Disorder of oral soft tissues (x1.02) 0.948
Electrocardiogram abnormal (x1.02) 0.949
Bleeding from nose (x1.02) 0.949
Disorder of lung (x1.02) 0.950
Fever (/1.02) 0.950
Anemia of chronic disease (x1.02) 0.951
MCV [V] by AC (+0.202) 0.951
Sepsis due to Streptococcus group D (x1.02) 0.952
Vomiting (x1.02) 0.952
Hemochromatosis following repeated red blood cell transfusion (x1.02) 0.952
tacrolimus (+0.00145) 0.953
Hodgkin's disease (clinical) (x1.02) 0.953
gabapentin (-0.00144) 0.954
Blood chemistry abnormal (x1.02) 0.954
Borderline normal ECG (x1.02) 0.954
Multiple myeloma in remission (x1.02) 0.955
Late effect of complications of procedure (x1.02) 0.955
furosemide (+0.0014) 0.956
levothyroxine (+0.0014) 0.956
Disorder of pancreatic internal secretion (x1.02) 0.956
Acute respiratory failure (x1.01) 0.957
acetaminophen (-0.00137) 0.957
Lymphocytes [#/V] in B by AC (-0.0381) 0.957
Promyelocytes/100 leukocytes in B by Manual count (+0.0324) 0.958
Late effect of medical and surgical care complication (x1.01) 0.958
Expressions
−0.75 −0.50 −0.25 0.00 0.25 0.50 0.75

Signature [1194]; 3 To Reach 0.975, (10 Plotted)

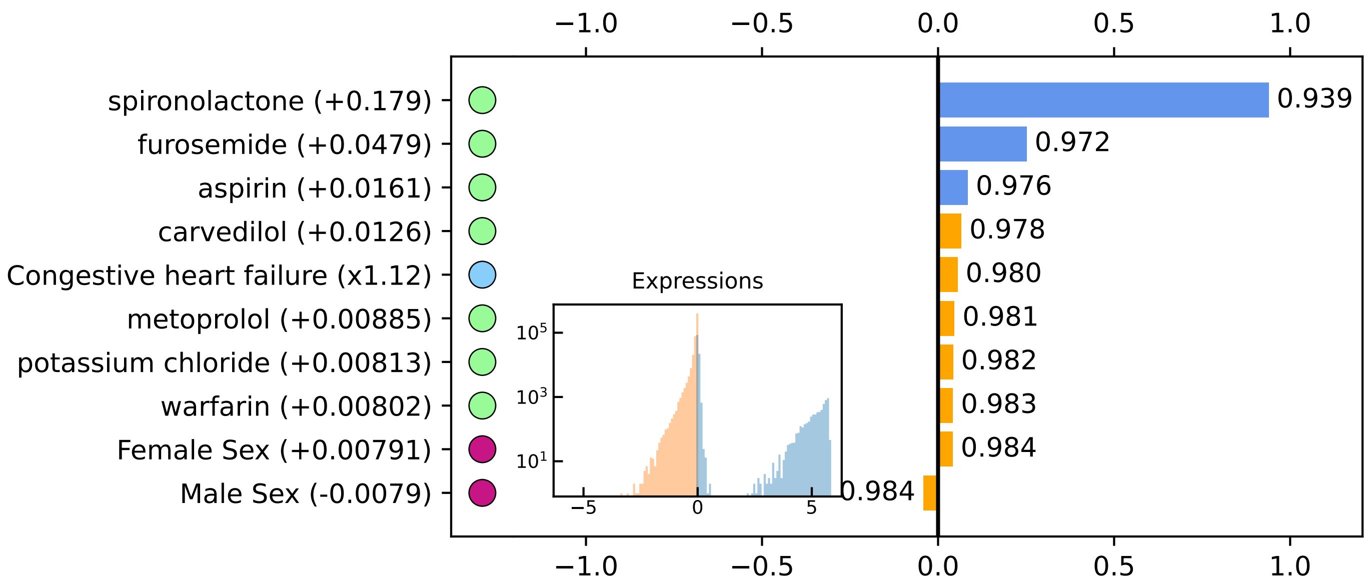

Signature [1195]; 188 To Reach 0.975, (100 Plotted)

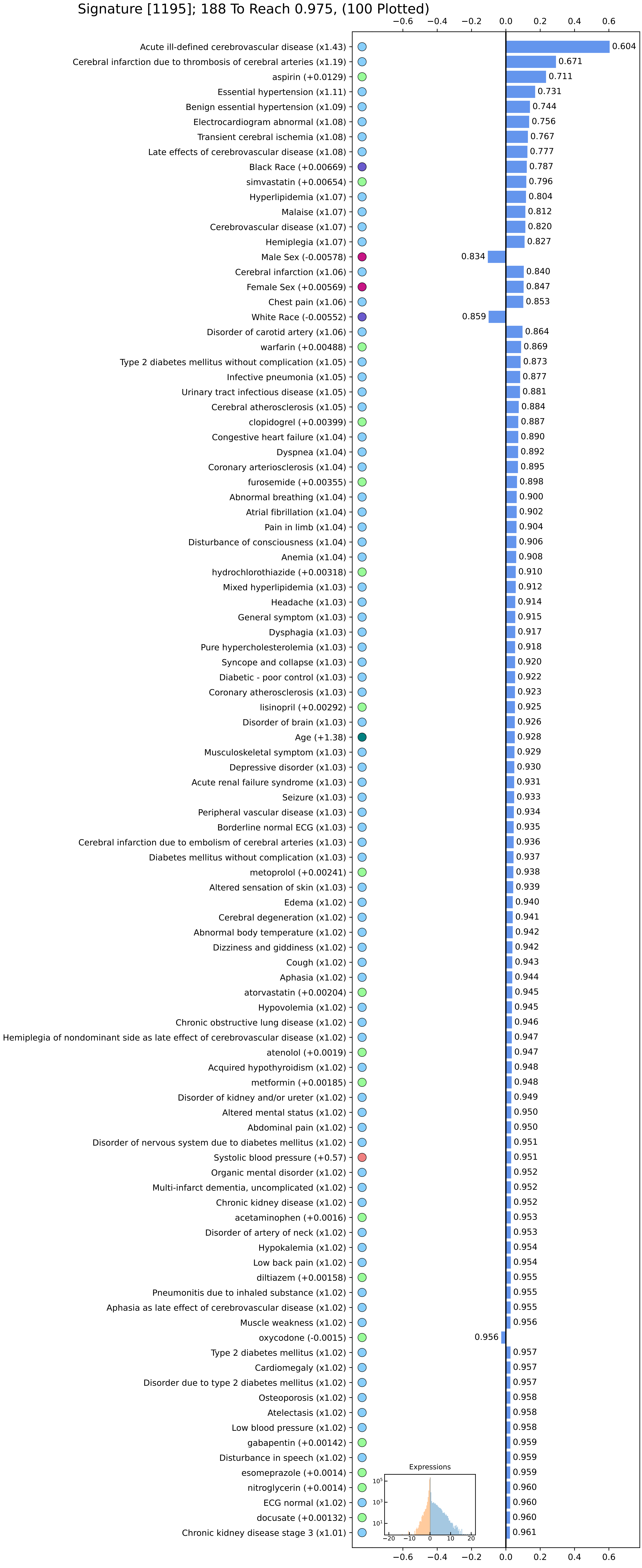

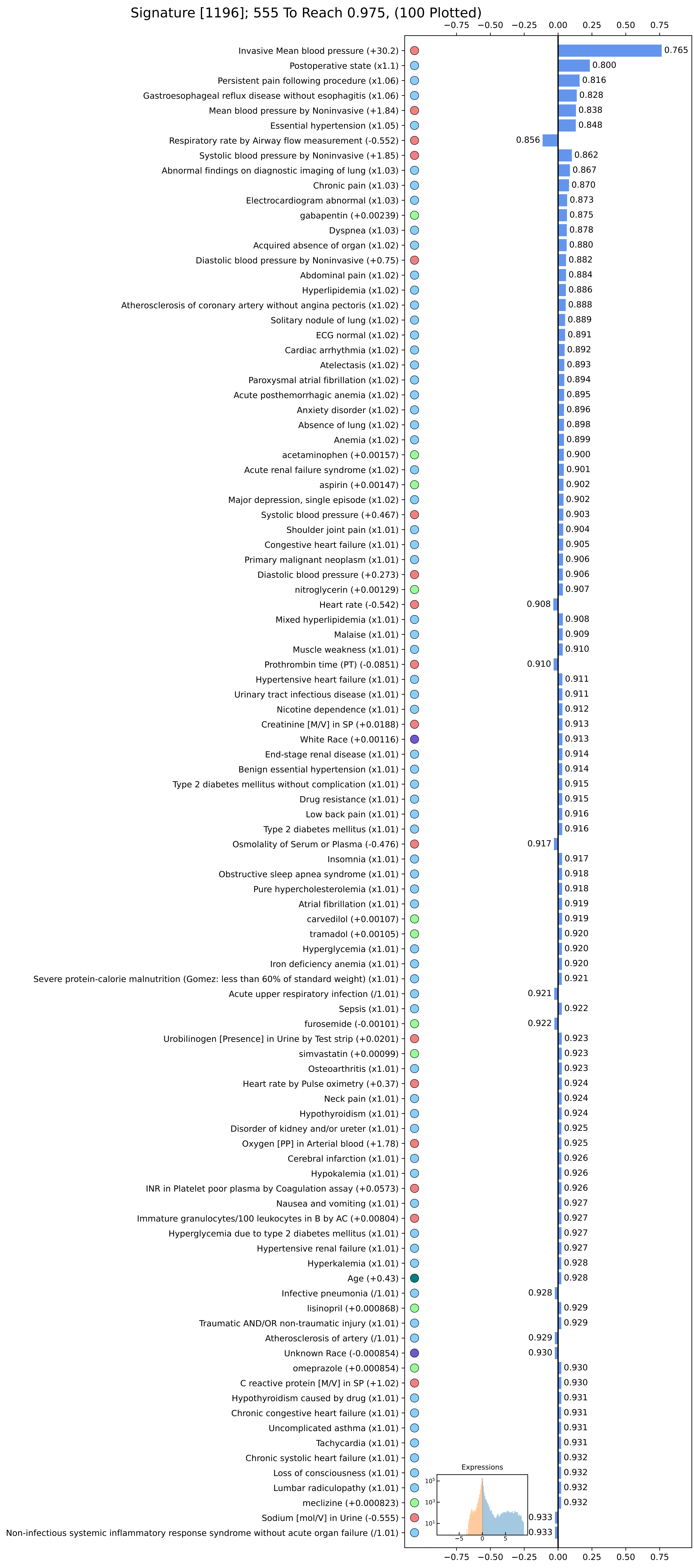
Signature [1196]; 555 To Reach 0.975, (100 Plotted)
−0.75 −0.50 −0.25 0.00 0.25 0.50 0.75
Invasive Mean blood pressure (+30.2)
0.765
Postoperative state (x1.1)
0.800
Persistent pain following procedure (x1.06)
0.816
Gastroesophageal reflux disease without esophagitis (x1.06)
0.828
Mean blood pressure by Noninvasive (+1.84)
0.838
Essential hypertension (x1.05)
0.848
Respiratory rate by Airway flow measurement (-0.552)
0.856
Systolic blood pressure by Noninvasive (+1.85)
0.862
Abnormal findings on diagnostic imaging of lung (x1.03)
0.867
Chronic pain (x1.03)
0.870
Electrocardiogram abnormal (x1.03)
0.873
gabapentin (+0.00239)
0.875
Dyspnea (x1.03)
0.878
Acquired absence of organ (x1.02)
0.880
Diastolic blood pressure by Noninvasive (+0.75)
0.882
Abdominal pain (x1.02)
0.884
Hyperlipidemia (x1.02)
0.886
Atherosclerosis of coronary artery without angina pectoris (x1.02)
0.888
Solitary nodule of lung (x1.02)
0.889
ECG normal (x1.02)
0.891
Cardiac arrhythmia (x1.02)
0.892
Atelectasis (x1.02)
0.893
Paroxysmal atrial fibrillation (x1.02)
0.894
Acute posthemorrhagic anemia (x1.02)
0.895
Anxiety disorder (x1.02)
0.896
Absence of lung (x1.02)
0.898
Anemia (x1.02)
0.899
acetaminophen (+0.00157)
0.900
Acute renal failure syndrome (x1.02)
0.901
aspirin (+0.00147)
0.902
Major depression, single episode (x1.02)
0.902
Systolic blood pressure (+0.467)
0.903
Shoulder joint pain (x1.01)
0.904
Congestive heart failure (x1.01)
0.905
Primary malignant neoplasm (x1.01)
0.906
Diastolic blood pressure (+0.273)
0.906
nitroglycerin (+0.00129)
0.907
Heart rate (-0.542)
0.908
Mixed hyperlipidemia (x1.01)
0.908
Malaise (x1.01)
0.909
Muscle weakness (x1.01)
0.910
Prothrombin time (PT) (-0.0851)
0.910
Hypertensive heart failure (x1.01)
0.911
Urinary tract infectious disease (x1.01)
0.911
Nicotine dependence (x1.01)
0.912
Creatinine [M/V] in SP (+0.0188)
0.913
White Race (+0.00116)
0.913
End-stage renal disease (x1.01)
0.914
Benign essential hypertension (x1.01)
0.914
Type 2 diabetes mellitus without complication (x1.01)
0.915
Drug resistance (x1.01)
0.915
Low back pain (x1.01)
0.916
Type 2 diabetes mellitus (x1.01)
0.916
Osmolality of Serum or Plasma (-0.476)
0.917
Insomnia (x1.01)
0.917
Obstructive sleep apnea syndrome (x1.01)
0.918
Pure hypercholesterolemia (x1.01)
0.918
Atrial fibrillation (x1.01)
0.919
carvedilol (+0.00107)
0.919
tramadol (+0.00105)
0.920
Hyperglycemia (x1.01)
0.920
Iron deficiency anemia (x1.01)
0.920
Severe protein-calorie malnutrition (Gomez: less than 60% of standard weight) (x1.01)
0.921
Acute upper respiratory infection (/1.01)
0.921
Sepsis (x1.01)
0.922
furosemide (-0.00101)
0.922
Urobilinogen [Presence] in Urine by Test strip (+0.0201)
0.923
simvastatin (+0.00099)
0.923
Osteoarthritis (x1.01)
0.923
Heart rate by Pulse oximetry (+0.37)
0.924
Neck pain (x1.01)
0.924
Hypothyroidism (x1.01)
0.924
Disorder of kidney and/or ureter (x1.01)
0.925
Oxygen [PP] in Arterial blood (+1.78)
0.925
Cerebral infarction (x1.01)
0.926
Hypokalemia (x1.01)
0.926
INR in Platelet poor plasma by Coagulation assay (+0.0573)
0.926
Nausea and vomiting (x1.01)
0.927
Immature granulocytes/100 leukocytes in B by AC (+0.00804)
0.927
Hyperglycemia due to type 2 diabetes mellitus (x1.01)
0.927
Hypertensive renal failure (x1.01)
0.927
Hyperkalemia (x1.01)
0.928
Age (+0.43)
0.928
Infective pneumonia (/1.01)
0.928
lisinopril (+0.000868)
0.929
Traumatic AND/OR non-traumatic injury (x1.01)
0.929
Atherosclerosis of artery (/1.01)
0.929
Unknown Race (-0.000854)
0.930
omeprazole (+0.000854)
0.930
C reactive protein [M/V] in SP (+1.02)
0.930
Hypothyroidism caused by drug (x1.01)
0.931
Chronic congestive heart failure (x1.01)
0.931
Uncomplicated asthma (x1.01)
0.931
Tachycardia (x1.01)
0.931
Chronic systolic heart failure (x1.01)
0.932
Loss of consciousness (x1.01)
0.932
Lumbar radiculopathy (x1.01)
0.932
meclizine (+0.000823)
0.932
Sodium [mol/V] in Urine (-0.555)
0.933
Non-infectious systemic inflammatory response syndrome without acute organ failure (/1.01)
0.933
Expressions
10^5
10^3
10^1
−5 0 5
−0.75 −0.50 −0.25 0.00 0.25 0.50 0.75

Signature [1197]; 51 To Reach 0.975, (51 Plotted)

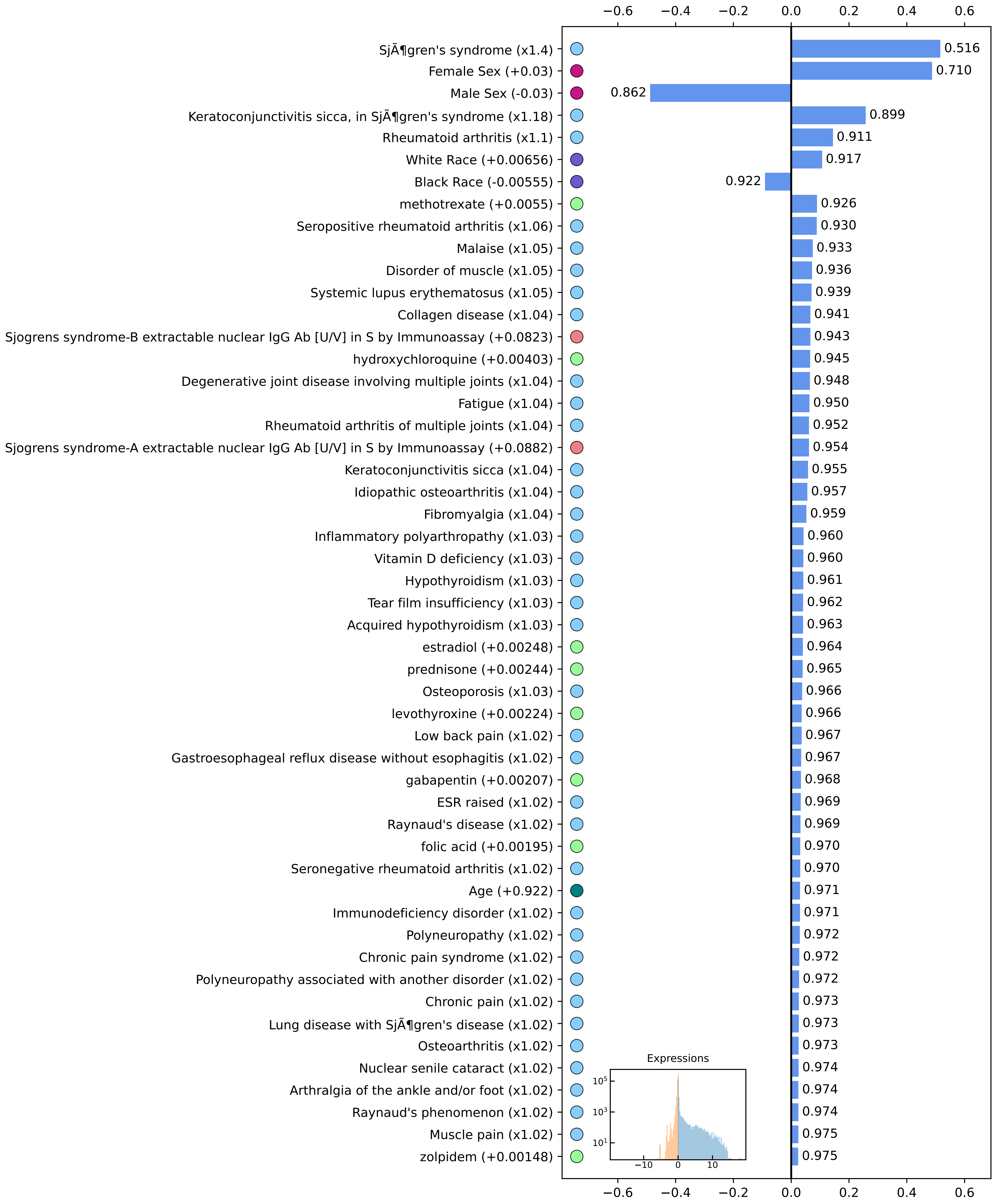

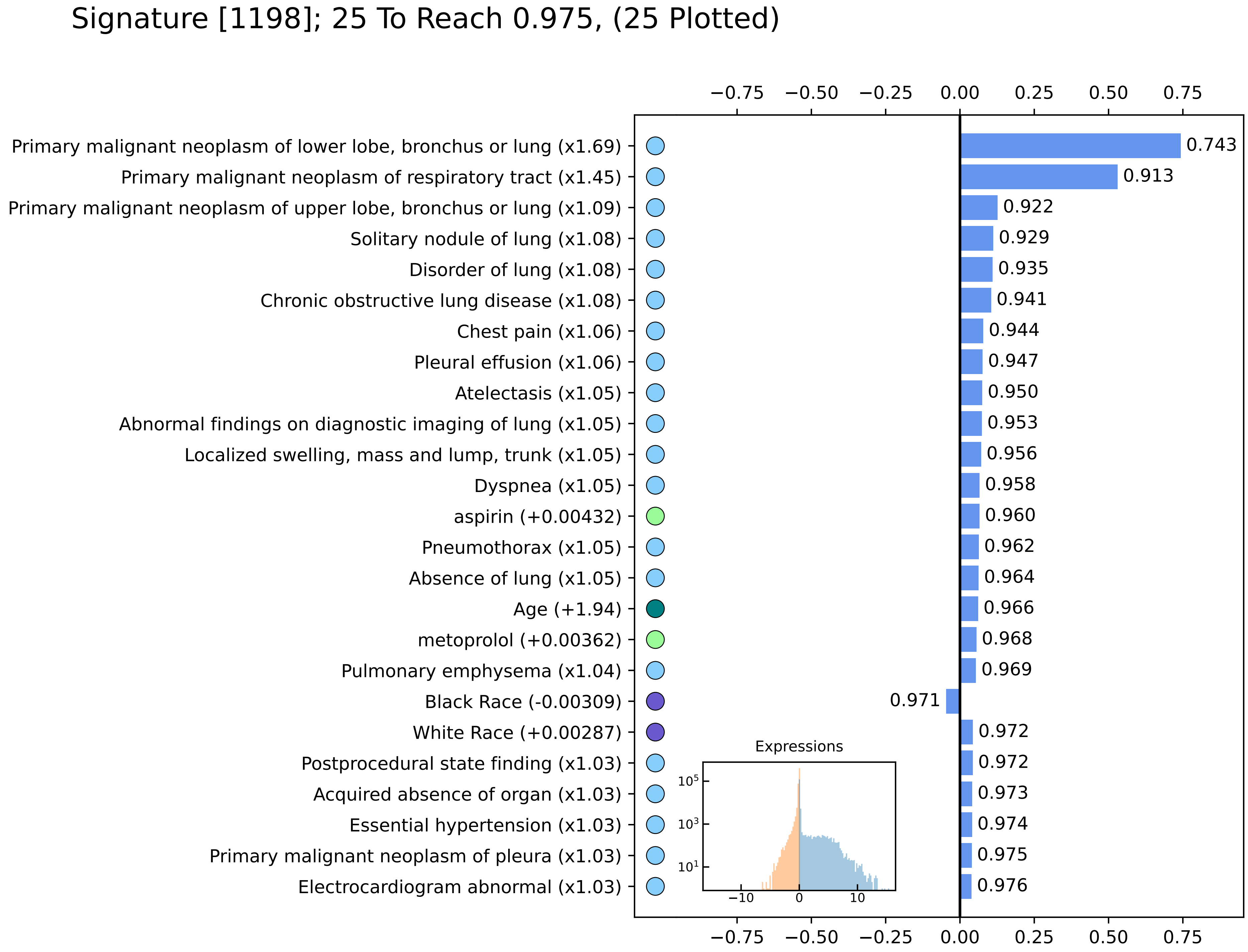
Signature [1198]; 25 To Reach 0.975, (25 Plotted)
Primary malignant neoplasm of lower lobe, bronchus or lung (x1.69)
0.743
Primary malignant neoplasm of respiratory tract (x1.45)
0.913
Primary malignant neoplasm of upper lobe, bronchus or lung (x1.09)
0.922
Solitary nodule of lung (x1.08)
0.929
Disorder of lung (x1.08)
0.935
Chronic obstructive lung disease (x1.08)
0.941
Chest pain (x1.06)
0.944
Pleural effusion (x1.06)
0.947
Atelectasis (x1.05)
0.950
Abnormal findings on diagnostic imaging of lung (x1.05)
0.953
Localized swelling, mass and lump, trunk (x1.05)
0.956
Dyspnea (x1.05)
0.958
aspirin (+0.00432)
0.960
Pneumothorax (x1.05)
0.962
Absence of lung (x1.05)
0.964
Age (+1.94)
0.966
metoprolol (+0.00362)
0.968
Pulmonary emphysema (x1.04)
0.969
Black Race (-0.00309)
0.971
White Race (+0.00287)
0.972
Postprocedural state finding (x1.03)
0.972
Acquired absence of organ (x1.03)
0.973
Essential hypertension (x1.03)
0.974
Primary malignant neoplasm of pleura (x1.03)
0.975
Electrocardiogram abnormal (x1.03)
0.976
−0.75
−0.50
−0.25
0.00
0.25
0.50
0.75
Expressions
10^5
10^3
10^1
−10
0
10

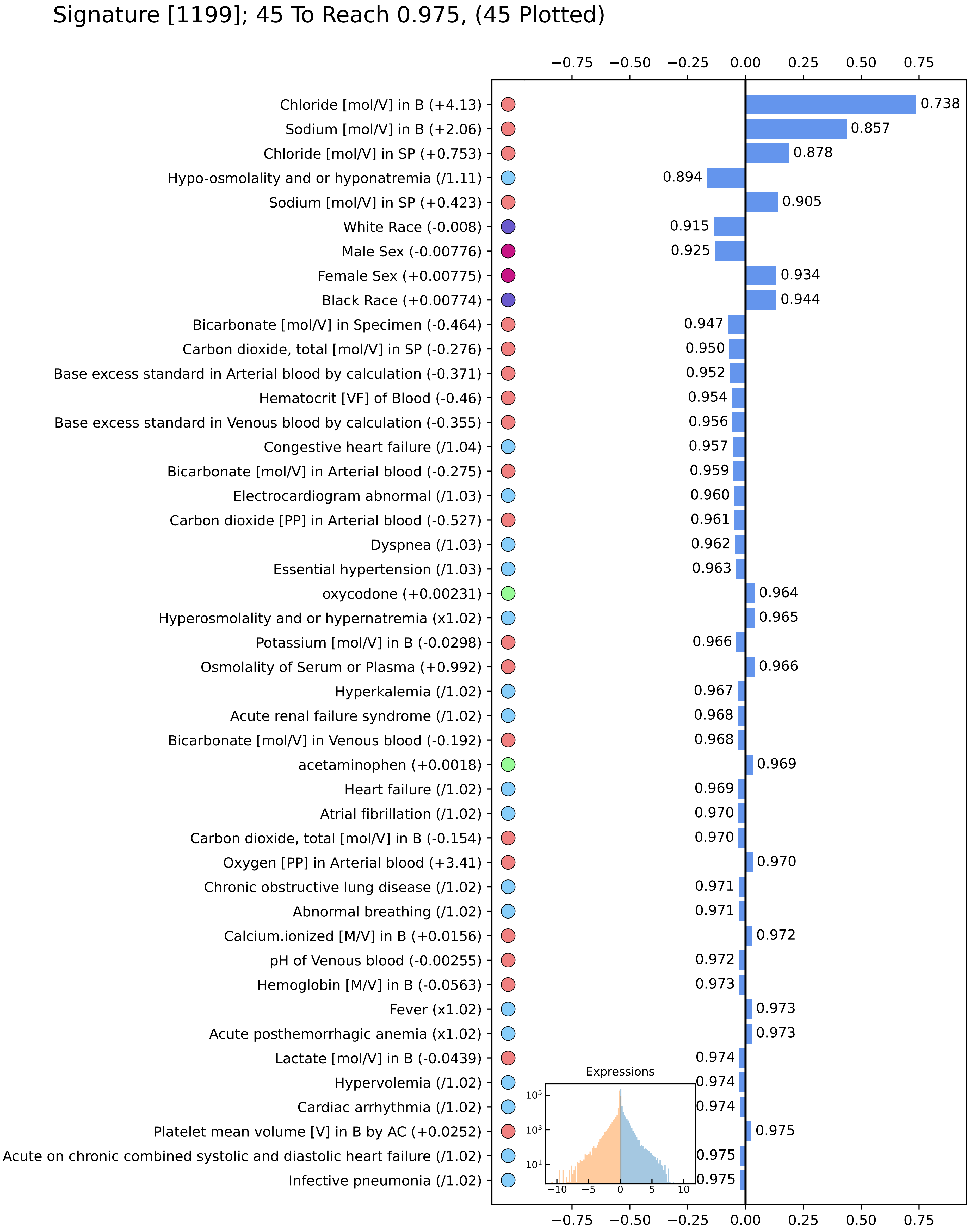
Signature [1199]; 45 To Reach 0.975, (45 Plotted)
Chloride [mol/V] in B (+4.13) 0.738
Sodium [mol/V] in B (+2.06) 0.857
Chloride [mol/V] in SP (+0.753) 0.878
Hypo-osmolality and or hyponatremia (/1.11) 0.894
Sodium [mol/V] in SP (+0.423) 0.905
White Race (-0.008) 0.915
Male Sex (-0.00776) 0.925
Female Sex (+0.00775) 0.934
Black Race (+0.00774) 0.944
Bicarbonate [mol/V] in Specimen (-0.464) 0.947
Carbon dioxide, total [mol/V] in SP (-0.276) 0.950
Base excess standard in Arterial blood by calculation (-0.371) 0.952
Hematocrit [VF] of Blood (-0.46) 0.954
Base excess standard in Venous blood by calculation (-0.355) 0.956
Congestive heart failure (/1.04) 0.957
Bicarbonate [mol/V] in Arterial blood (-0.275) 0.959
Electrocardiogram abnormal (/1.03) 0.960
Carbon dioxide [PP] in Arterial blood (-0.527) 0.961
Dyspnea (/1.03) 0.962
Essential hypertension (/1.03) 0.963
oxycodone (+0.00231) 0.964
Hyperosmolality and or hypernatremia (x1.02) 0.965
Potassium [mol/V] in B (-0.0298) 0.966
Osmolality of Serum or Plasma (+0.992) 0.966
Hyperkalemia (/1.02) 0.967
Acute renal failure syndrome (/1.02) 0.968
Bicarbonate [mol/V] in Venous blood (-0.192) 0.968
acetaminophen (+0.0018) 0.969
Heart failure (/1.02) 0.969
Atrial fibrillation (/1.02) 0.970
Carbon dioxide, total [mol/V] in B (-0.154) 0.970
Oxygen [PP] in Arterial blood (+3.41) 0.970
Chronic obstructive lung disease (/1.02) 0.971
Abnormal breathing (/1.02) 0.971
Calcium.ionized [M/V] in B (+0.0156) 0.972
pH of Venous blood (-0.00255) 0.972
Hemoglobin [M/V] in B (-0.0563) 0.973
Fever (x1.02) 0.973
Acute posthemorrhagic anemia (x1.02) 0.973
Lactate [mol/V] in B (-0.0439) 0.974
Hypervolemia (/1.02) 0.974
Cardiac arrhythmia (/1.02) 0.974
Platelet mean volume [V] in B by AC (+0.0252) 0.975
Acute on chronic combined systolic and diastolic heart failure (/1.02) 0.975
Infective pneumonia (/1.02) 0.975
−0.75 −0.50 −0.25 0.00 0.25 0.50 0.75
Expressions
10^5 10^3 10^1
−10 −5 0 5 10

Signature [1200]; 4 To Reach 0.975, (10 Plotted)

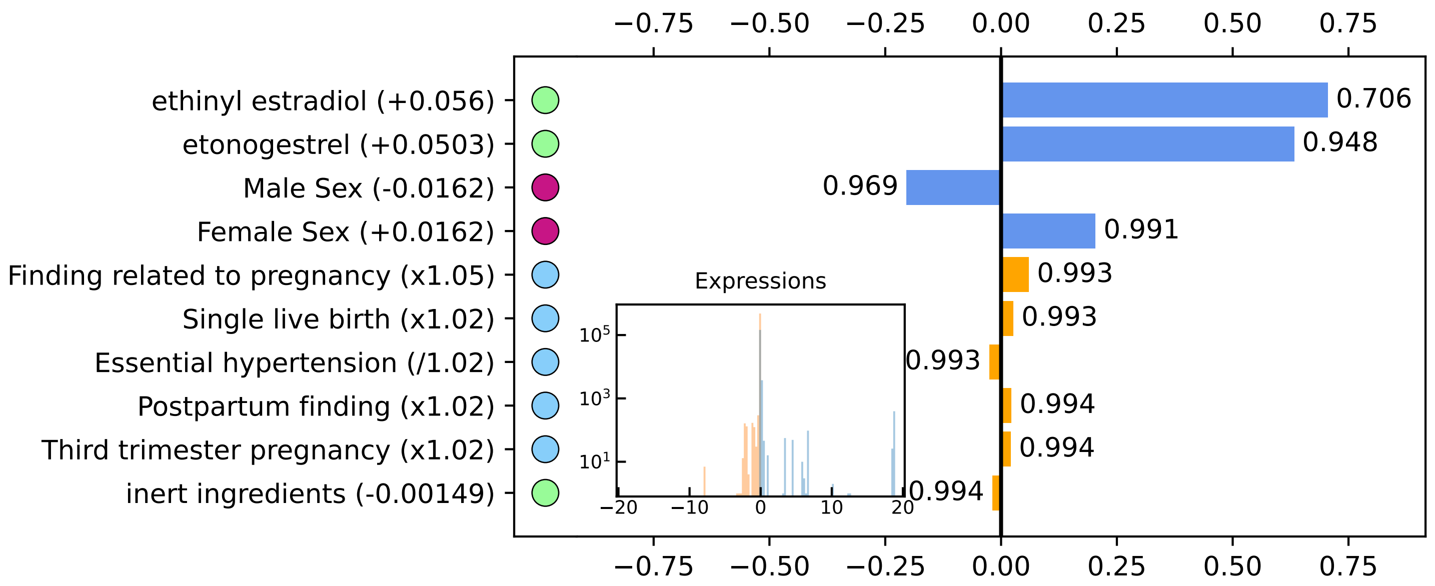

Signature [1201]; 1 To Reach 0.975, (10 Plotted)

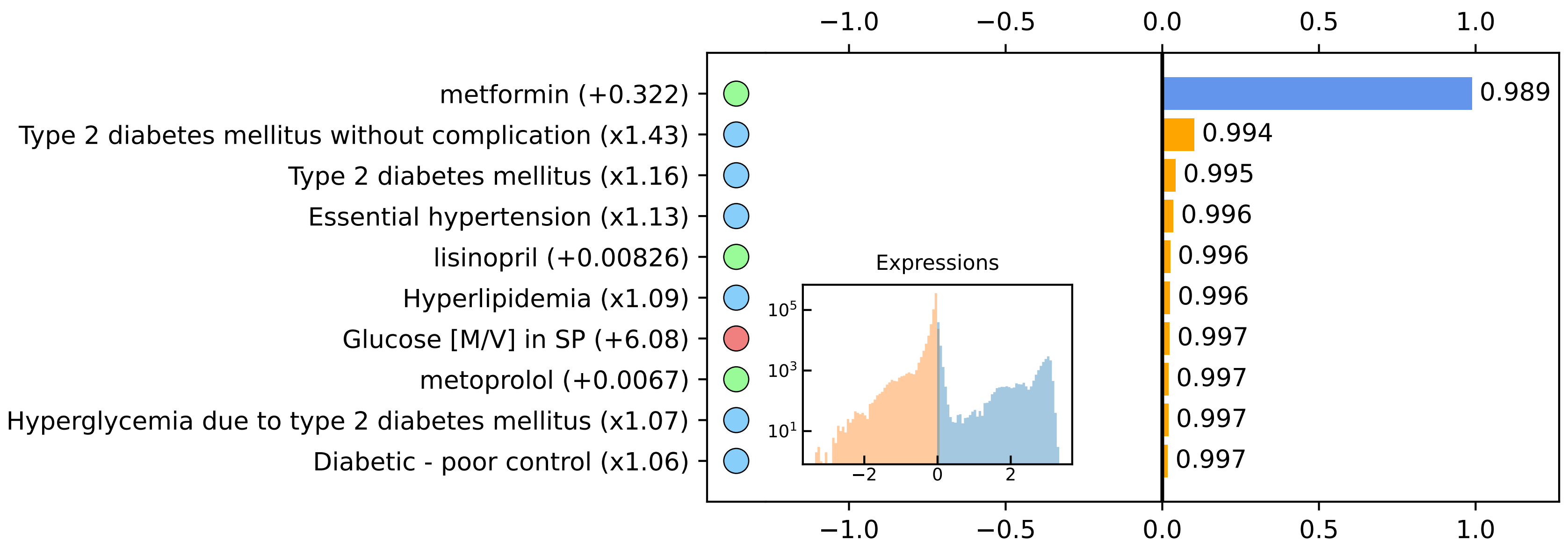

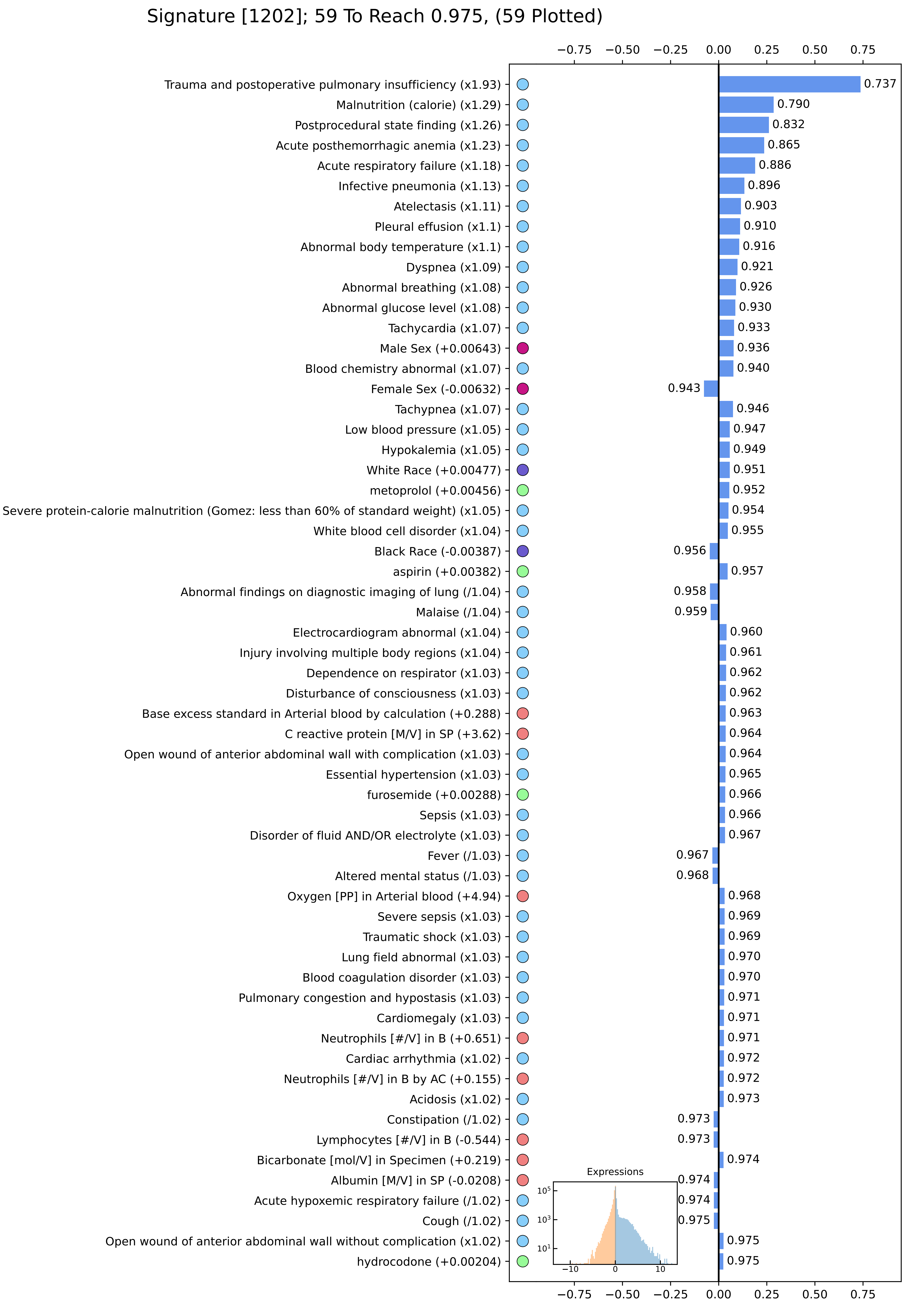

Signature [1202]; 59 To Reach 0.975, (59 Plotted)
Trauma and postoperative pulmonary insufficiency (x1.93) 0.737
Malnutrition (calorie) (x1.29) 0.790
Postprocedural state finding (x1.26) 0.832
Acute posthemorrhagic anemia (x1.23) 0.865
Acute respiratory failure (x1.18) 0.886
Infective pneumonia (x1.13) 0.896
Atelectasis (x1.11) 0.903
Pleural effusion (x1.1) 0.910
Abnormal body temperature (x1.1) 0.916
Dyspnea (x1.09) 0.921
Abnormal breathing (x1.08) 0.926
Abnormal glucose level (x1.08) 0.930
Tachycardia (x1.07) 0.933
Male Sex (+0.00643) 0.936
Blood chemistry abnormal (x1.07) 0.940
Female Sex (-0.00632) 0.943
Tachypnea (x1.07) 0.946
Low blood pressure (x1.05) 0.947
Hypokalemia (x1.05) 0.949
White Race (+0.00477) 0.951
metoprolol (+0.00456) 0.952
Severe protein-calorie malnutrition (Gomez: less than 60% of standard weight) (x1.05) 0.954
White blood cell disorder (x1.04) 0.955
Black Race (-0.00387) 0.956
aspirin (+0.00382) 0.957
Abnormal findings on diagnostic imaging of lung (/1.04) 0.958
Malaise (/1.04) 0.959
Electrocardiogram abnormal (x1.04) 0.960
Injury involving multiple body regions (x1.04) 0.961
Dependence on respirator (x1.03) 0.962
Disturbance of consciousness (x1.03) 0.962
Base excess standard in Arterial blood by calculation (+0.288) 0.963
C reactive protein [M/V] in SP (+3.62) 0.964
Open wound of anterior abdominal wall with complication (x1.03) 0.964
Essential hypertension (x1.03) 0.965
furosemide (+0.00288) 0.966
Sepsis (x1.03) 0.966
Disorder of fluid AND/OR electrolyte (x1.03) 0.967
Fever (/1.03) 0.967
Altered mental status (/1.03) 0.968
Oxygen [PP] in Arterial blood (+4.94) 0.968
Severe sepsis (x1.03) 0.969
Traumatic shock (x1.03) 0.969
Lung field abnormal (x1.03) 0.970
Blood coagulation disorder (x1.03) 0.970
Pulmonary congestion and hypostasis (x1.03) 0.971
Cardiomegaly (x1.03) 0.971
Neutrophils [#/V] in B (+0.651) 0.971
Cardiac arrhythmia (x1.02) 0.972
Neutrophils [#/V] in B by AC (+0.155) 0.972
Acidosis (x1.02) 0.973
Constipation (/1.02) 0.973
Lymphocytes [#/V] in B (-0.544) 0.973
Bicarbonate [mol/V] in Specimen (+0.219) 0.974
Albumin [M/V] in SP (-0.0208) 0.974
Acute hypoxemic respiratory failure (/1.02) 0.974
Cough (/1.02) 0.975
Open wound of anterior abdominal wall without complication (x1.02) 0.975
hydrocodone (+0.00204) 0.975
−0.75 −0.50 −0.25 0.00 0.25 0.50 0.75
Expressions

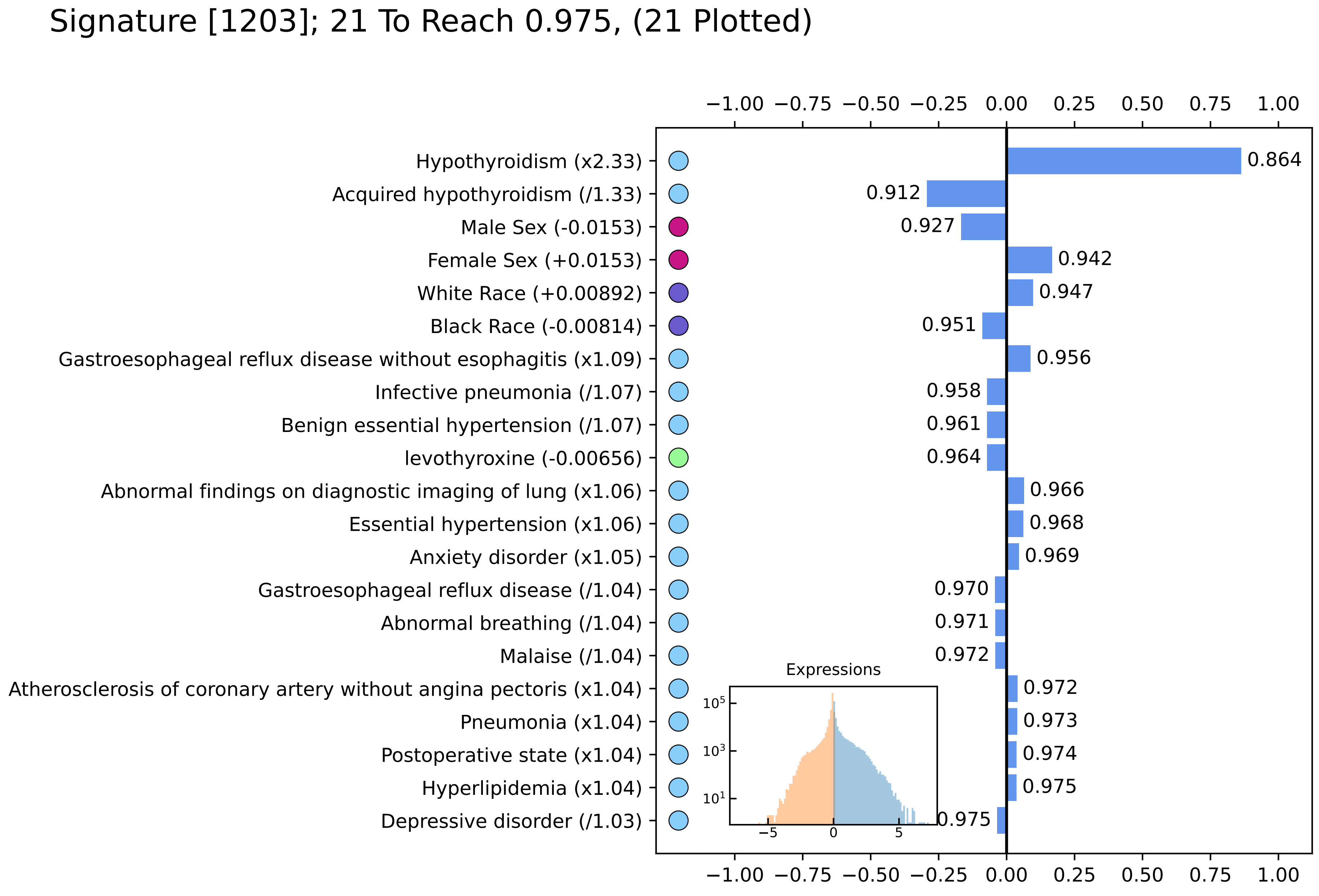

Signature [1203]; 21 To Reach 0.975, (21 Plotted)
Hypothyroidism (x2.33)
Acquired hypothyroidism (/1.33)
Male Sex (-0.0153)
Female Sex (+0.0153)
White Race (+0.00892)
Black Race (-0.00814)
Gastroesophageal reflux disease without esophagitis (x1.09)
Infective pneumonia (/1.07)
Benign essential hypertension (/1.07)
levothyroxine (-0.00656)
Abnormal findings on diagnostic imaging of lung (x1.06)
Essential hypertension (x1.06)
Anxiety disorder (x1.05)
Gastroesophageal reflux disease (/1.04)
Abnormal breathing (/1.04)
Malaise (/1.04)
Atherosclerosis of coronary artery without angina pectoris (x1.04)
Pneumonia (x1.04)
Postoperative state (x1.04)
Hyperlipidemia (x1.04)
Depressive disorder (/1.03)
0.864
0.912
0.927
0.942
0.947
0.951
0.956
0.958
0.961
0.964
0.966
0.968
0.969
0.970
0.971
0.972
0.972
0.973
0.974
0.975
0.975
−1.00 −0.75 −0.50 −0.25 0.00 0.25 0.50 0.75 1.00
Expressions
10^5
10^3
10^1
−5 0 5

Signature [1204]; 125 To Reach 0.975, (100 Plotted)

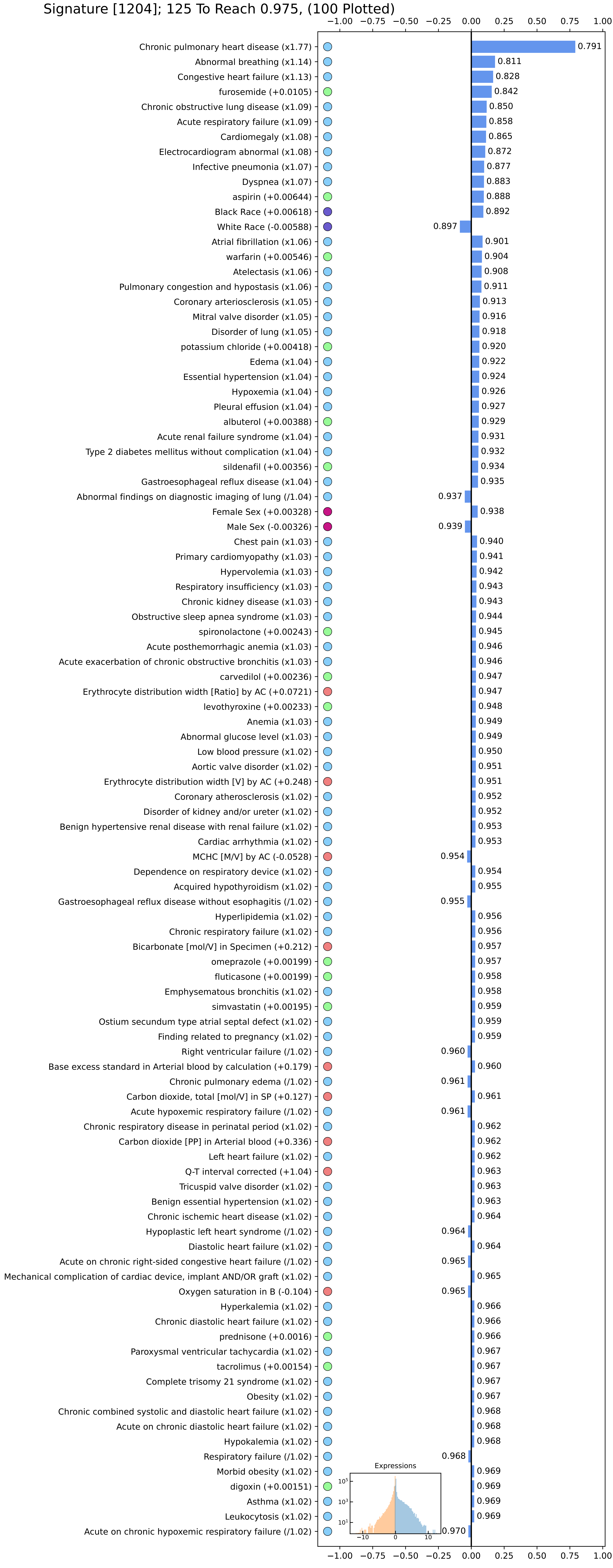

Signature [1205]; 141 To Reach 0.975, (100 Plotted)

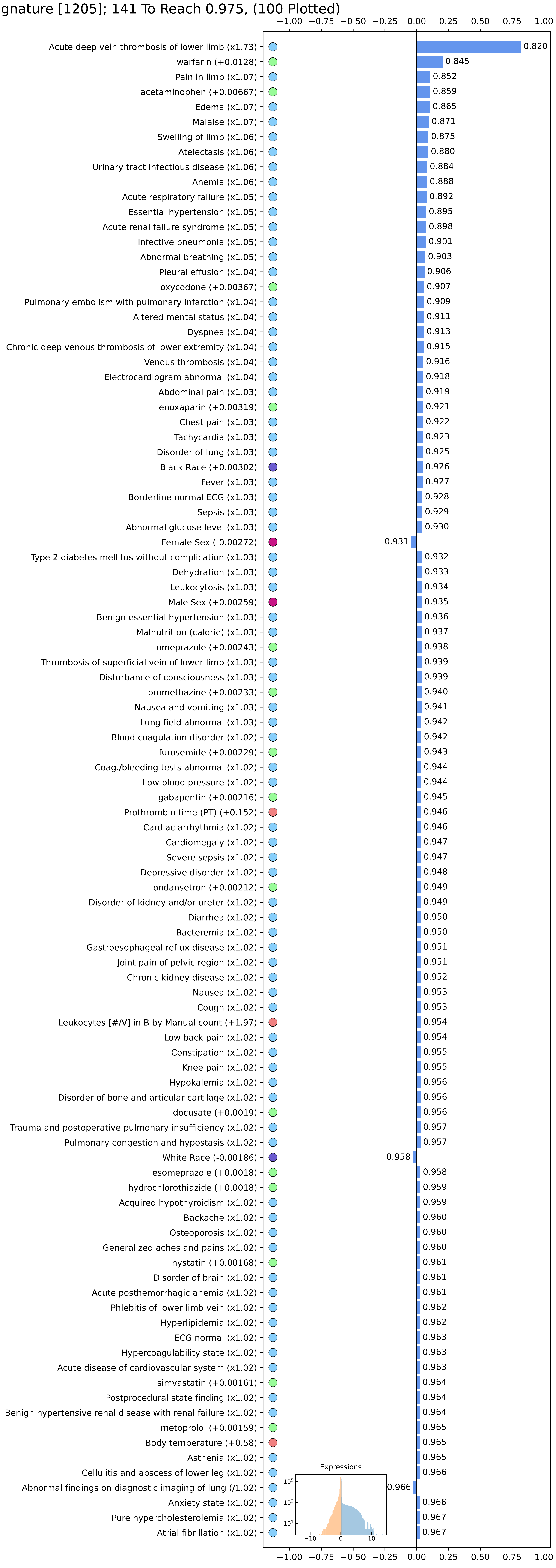

Signature [1206]; 3 To Reach 0.975, (10 Plotted)

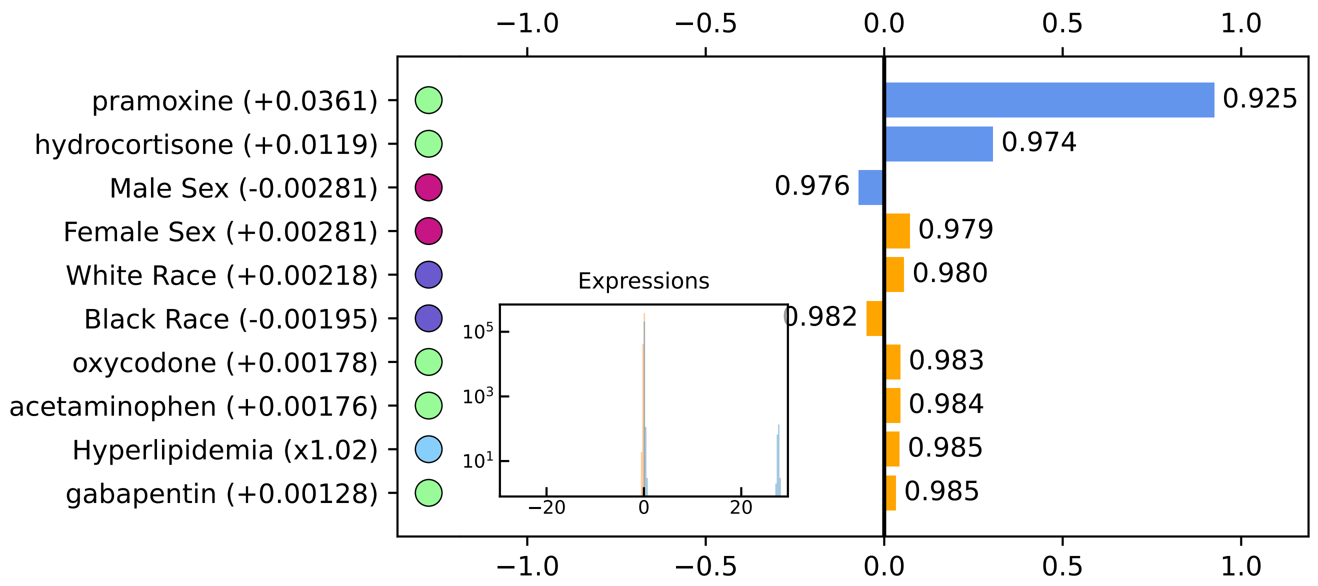

Signature [1207]; 16 To Reach 0.975, (16 Plotted)

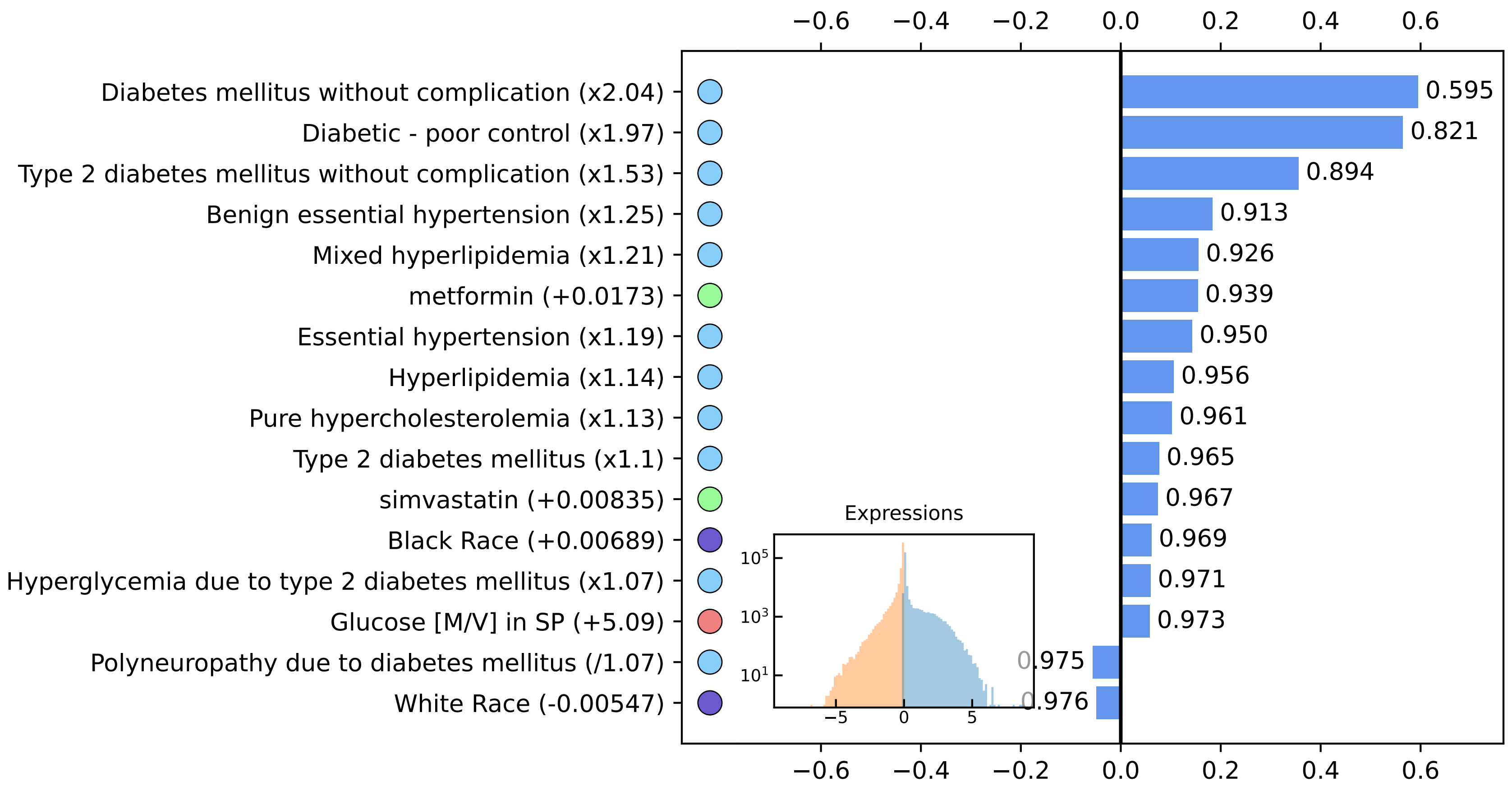

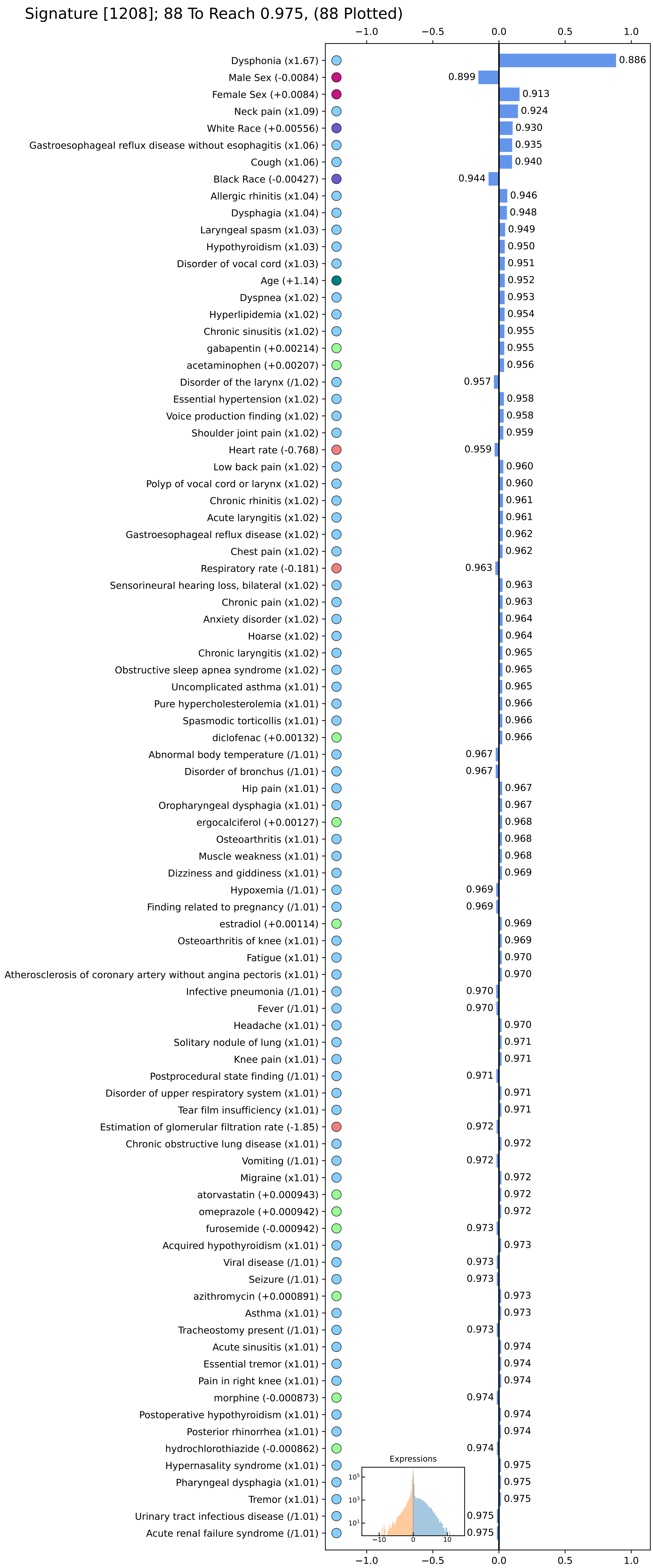

Signature [1208]; 88 To Reach 0.975, (88 Plotted)
−1.0
−0.5
0.0
0.5
1.0
Dysphonia (x1.67) 0.886
Male Sex (-0.0084) 0.899
Female Sex (+0.0084) 0.913
Neck pain (x1.09) 0.924
White Race (+0.00556) 0.930
Gastroesophageal reflux disease without esophagitis (x1.06) 0.935
Cough (x1.06) 0.940
Black Race (-0.00427) 0.944
Allergic rhinitis (x1.04) 0.946
Dysphagia (x1.04) 0.948
Laryngeal spasm (x1.03) 0.949
Hypothyroidism (x1.03) 0.950
Disorder of vocal cord (x1.03) 0.951
Age (+1.14) 0.952
Dyspnea (x1.02) 0.953
Hyperlipidemia (x1.02) 0.954
Chronic sinusitis (x1.02) 0.955
gabapentin (+0.00214) 0.955
acetaminophen (+0.00207) 0.956
Disorder of the larynx (/1.02) 0.957
Essential hypertension (x1.02) 0.958
Voice production finding (x1.02) 0.958
Shoulder joint pain (x1.02) 0.959
Heart rate (-0.768) 0.959
Low back pain (x1.02) 0.960
Polyp of vocal cord or larynx (x1.02) 0.960
Chronic rhinitis (x1.02) 0.961
Acute laryngitis (x1.02) 0.961
Gastroesophageal reflux disease (x1.02) 0.962
Chest pain (x1.02) 0.962
Respiratory rate (-0.181) 0.963
Sensorineural hearing loss, bilateral (x1.02) 0.963
Chronic pain (x1.02) 0.963
Anxiety disorder (x1.02) 0.964
Hoarse (x1.02) 0.964
Chronic laryngitis (x1.02) 0.965
Obstructive sleep apnea syndrome (x1.02) 0.965
Uncomplicated asthma (x1.01) 0.965
Pure hypercholesterolemia (x1.01) 0.966
Spasmodic torticollis (x1.01) 0.966
diclofenac (+0.00132) 0.966
Abnormal body temperature (/1.01) 0.967
Disorder of bronchus (/1.01) 0.967
Hip pain (x1.01) 0.967
Oropharyngeal dysphagia (x1.01) 0.967
ergocalciferol (+0.00127) 0.968
Osteoarthritis (x1.01) 0.968
Muscle weakness (x1.01) 0.968
Dizziness and giddiness (x1.01) 0.969
Hypoxemia (/1.01) 0.969
Finding related to pregnancy (/1.01) 0.969
estradiol (+0.00114) 0.969
Osteoarthritis of knee (x1.01) 0.969
Fatigue (x1.01) 0.970
Atherosclerosis of coronary artery without angina pectoris (x1.01) 0.970
Infective pneumonia (/1.01) 0.970
Fever (/1.01) 0.970
Headache (x1.01) 0.970
Solitary nodule of lung (x1.01) 0.971
Knee pain (x1.01) 0.971
Postprocedural state finding (/1.01) 0.971
Disorder of upper respiratory system (x1.01) 0.971
Tear film insufficiency (x1.01) 0.971
Estimation of glomerular filtration rate (-1.85) 0.972
Chronic obstructive lung disease (x1.01) 0.972
Vomiting (/1.01) 0.972
Migraine (x1.01) 0.972
atorvastatin (+0.000943) 0.972
omeprazole (+0.000942) 0.972
furosemide (-0.000942) 0.973
Acquired hypothyroidism (x1.01) 0.973
Viral disease (/1.01) 0.973
Seizure (/1.01) 0.973
azithromycin (+0.000891) 0.973
Asthma (x1.01) 0.973
Tracheostomy present (/1.01) 0.973
Acute sinusitis (x1.01) 0.974
Essential tremor (x1.01) 0.974
Pain in right knee (x1.01) 0.974
morphine (-0.000873) 0.974
Postoperative hypothyroidism (x1.01) 0.974
Posterior rhinorrhea (x1.01) 0.974
hydrochlorothiazide (-0.000862) 0.974
Hypernasality syndrome (x1.01) 0.975
Pharyngeal dysphagia (x1.01) 0.975
Tremor (x1.01) 0.975
Urinary tract infectious disease (/1.01) 0.975
Acute renal failure syndrome (/1.01) 0.975
Expressions

Signature [1209]; 6 To Reach 0.975, (10 Plotted)

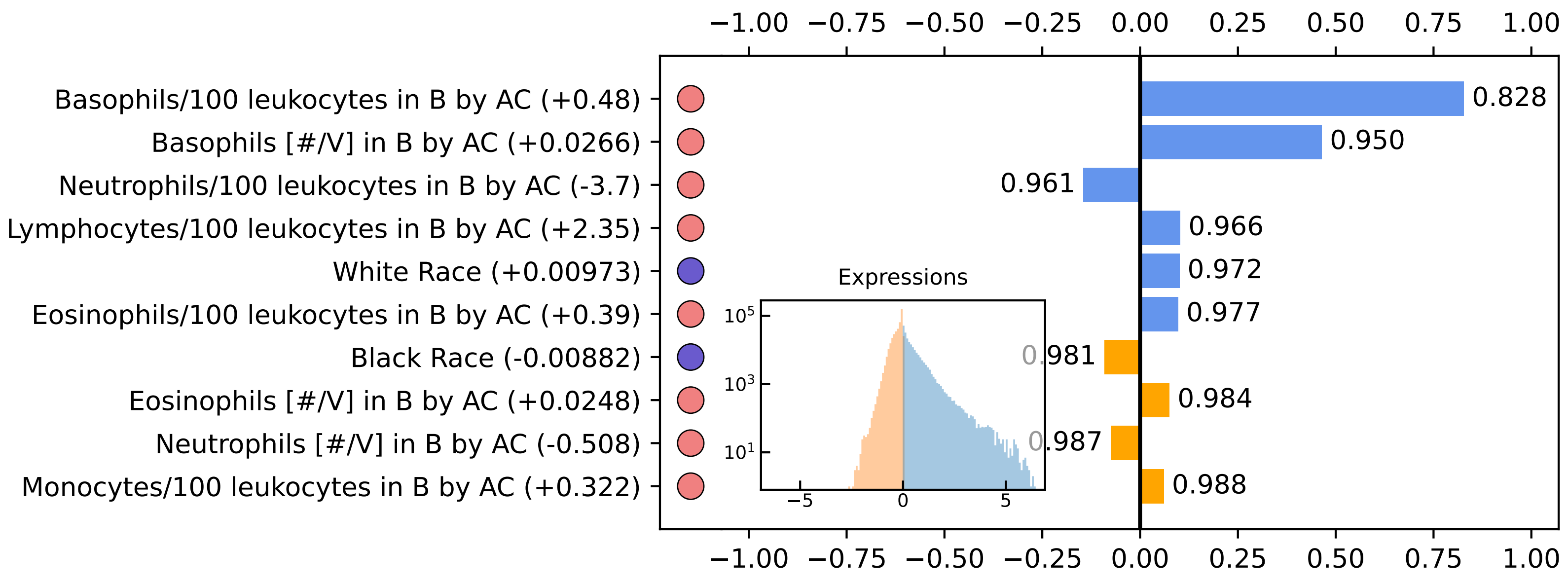

Signature [1210]; 197 To Reach 0.975, (100 Plotted)

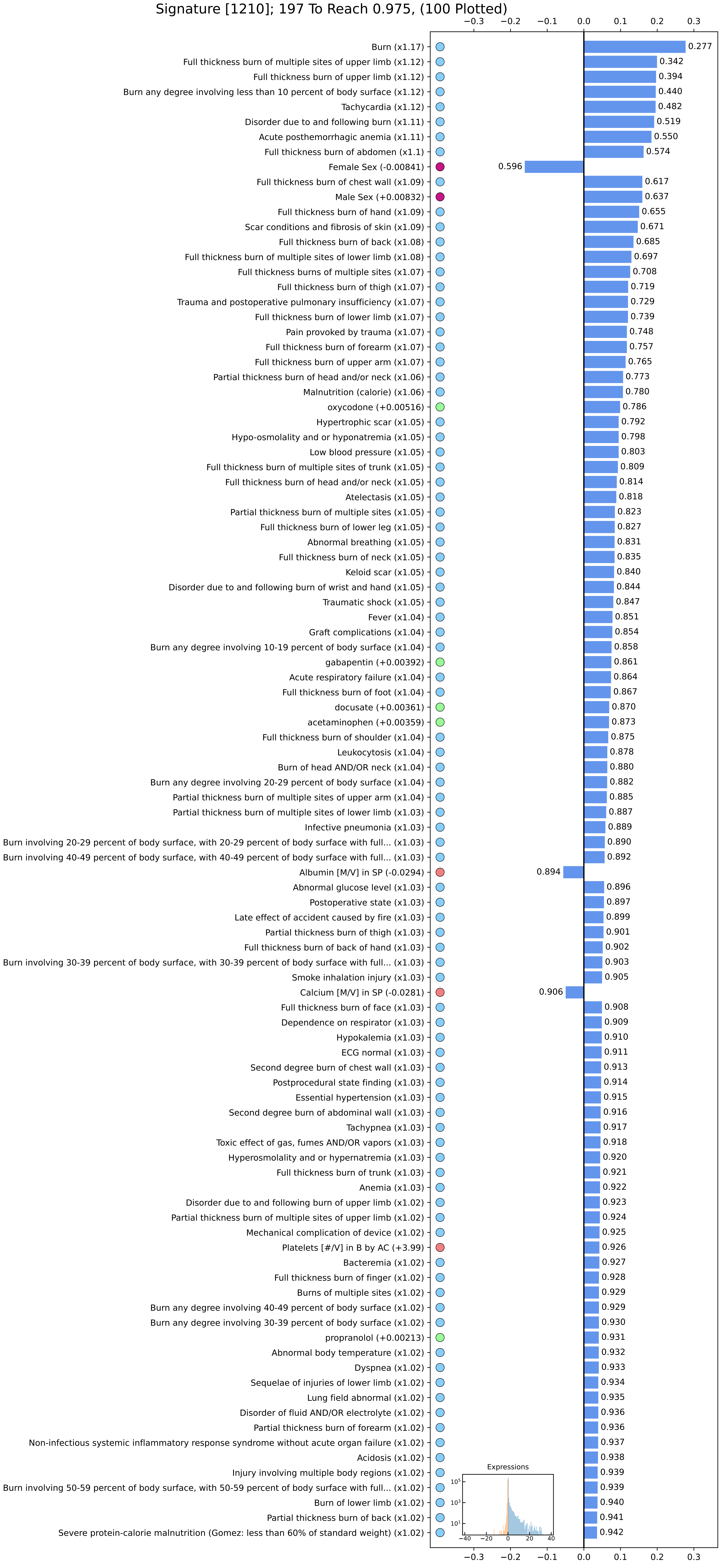

Signature [1211]; 1 To Reach 0.975, (10 Plotted)

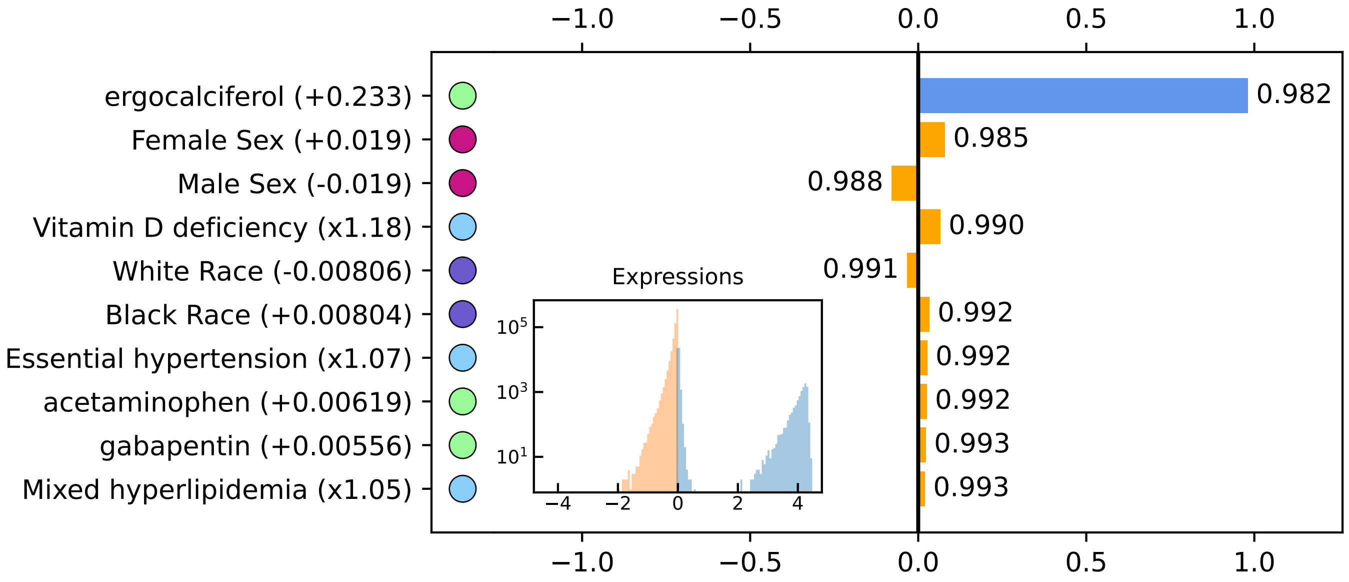

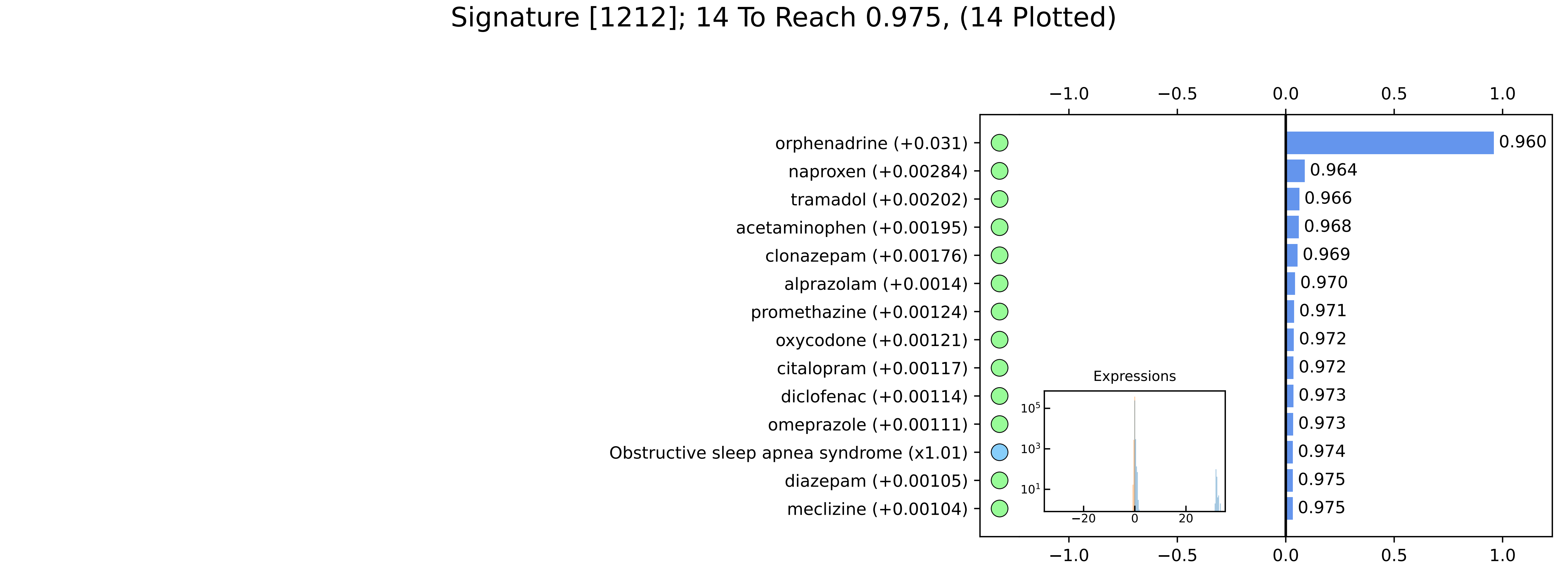

Signature [1212]; 14 To Reach 0.975, (14 Plotted)
orphenadrine (+0.031)
naproxen (+0.00284)
tramadol (+0.00202)
acetaminophen (+0.00195)
clonazepam (+0.00176)
alprazolam (+0.0014)
promethazine (+0.00124)
oxycodone (+0.00121)
citalopram (+0.00117)
diclofenac (+0.00114)
omeprazole (+0.00111)
Obstructive sleep apnea syndrome (x1.01)
diazepam (+0.00105)
meclizine (+0.00104)
0.960
0.964
0.966
0.968
0.969
0.970
0.971
0.972
0.972
0.973
0.973
0.974
0.975
0.975
−1.0
−0.5
0.0
0.5
1.0
Expressions

Signature [1213]; 1 To Reach 0.975, (10 Plotted)

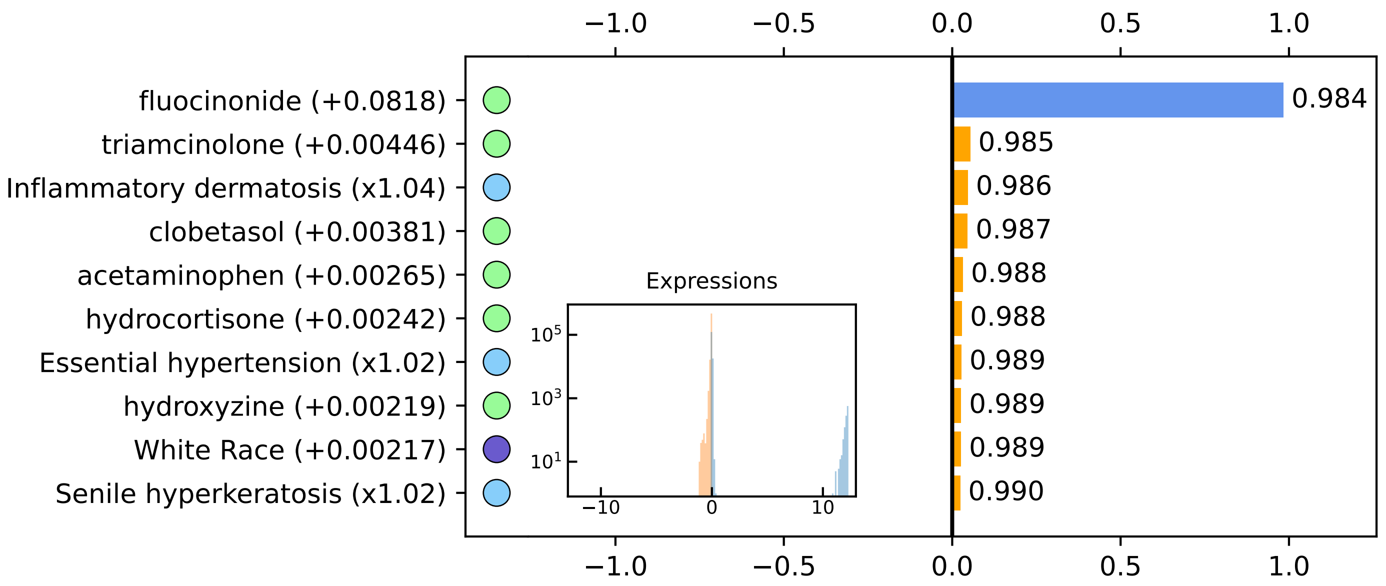

Signature [1214]; 103 To Reach 0.975, (100 Plotted)

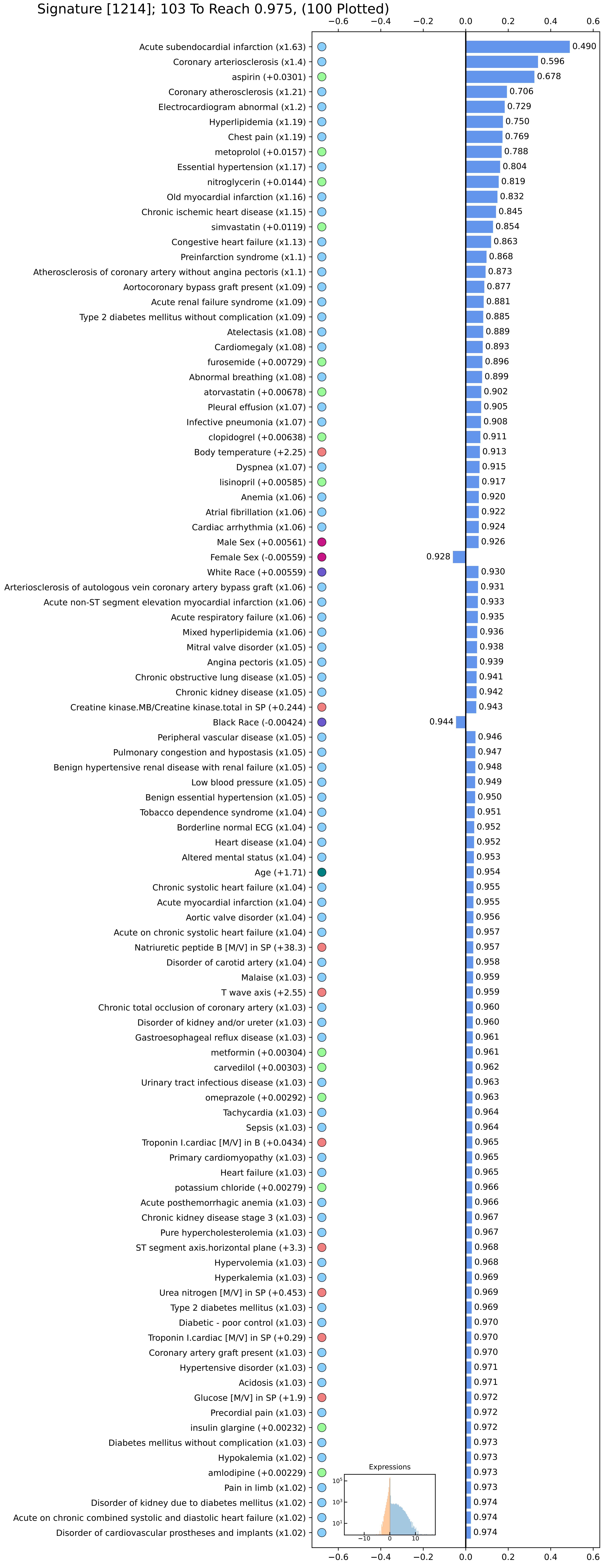

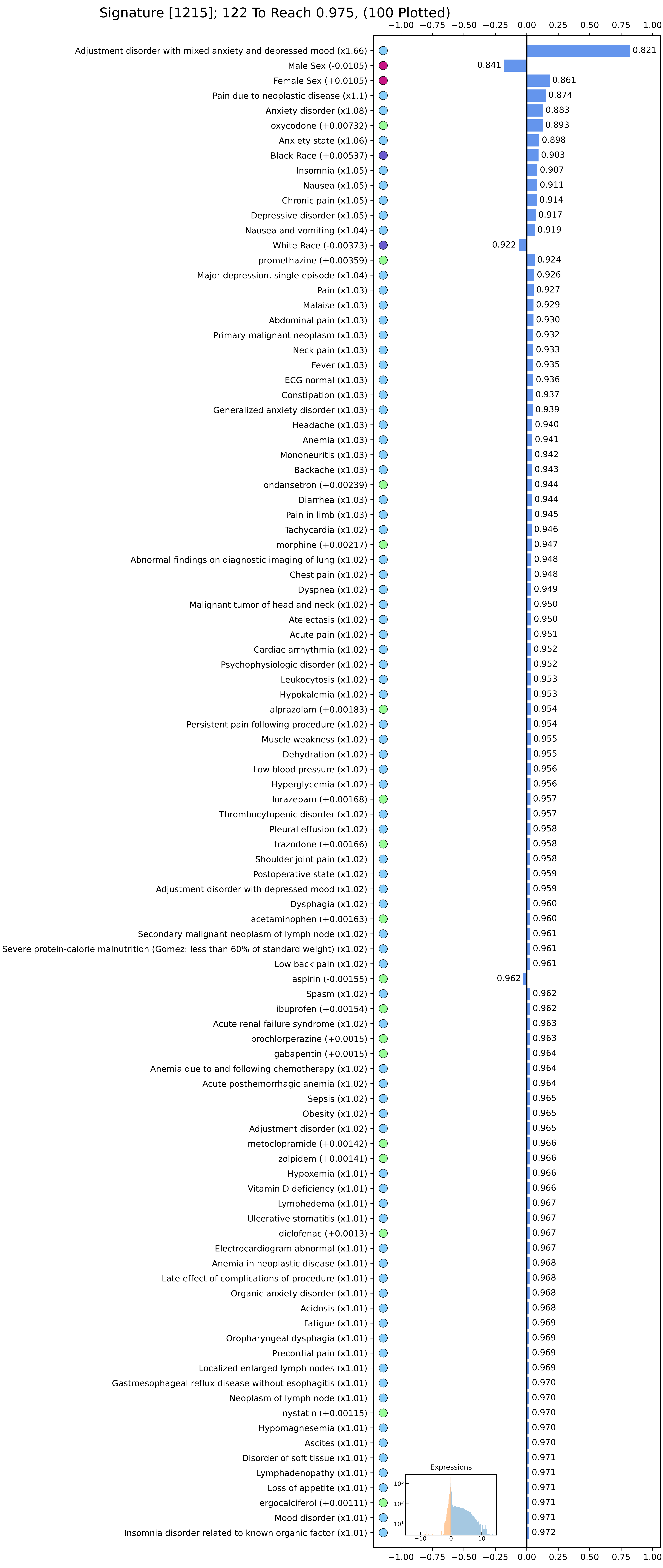
Signature [1215]; 122 To Reach 0.975, (100 Plotted)
−1.00 −0.75 −0.50 −0.25 0.00 0.25 0.50 0.75 1.00
Adjustment disorder with mixed anxiety and depressed mood (x1.66)
Male Sex (-0.0105)
Female Sex (+0.0105)
Pain due to neoplastic disease (x1.1)
Anxiety disorder (x1.08)
oxycodone (+0.00732)
Anxiety state (x1.06)
Black Race (+0.00537)
Insomnia (x1.05)
Nausea (x1.05)
Chronic pain (x1.05)
Depressive disorder (x1.05)
Nausea and vomiting (x1.04)
White Race (-0.00373)
promethazine (+0.00359)
Major depression, single episode (x1.04)
Pain (x1.03)
Malaise (x1.03)
Abdominal pain (x1.03)
Primary malignant neoplasm (x1.03)
Neck pain (x1.03)
Fever (x1.03)
ECG normal (x1.03)
Constipation (x1.03)
Generalized anxiety disorder (x1.03)
Headache (x1.03)
Anemia (x1.03)
Mononeuritis (x1.03)
Backache (x1.03)
ondansetron (+0.00239)
Diarrhea (x1.03)
Pain in limb (x1.03)
Tachycardia (x1.02)
morphine (+0.00217)
Abnormal findings on diagnostic imaging of lung (x1.02)
Chest pain (x1.02)
Dyspnea (x1.02)
Malignant tumor of head and neck (x1.02)
Atelectasis (x1.02)
Acute pain (x1.02)
Cardiac arrhythmia (x1.02)
Psychophysiologic disorder (x1.02)
Leukocytosis (x1.02)
Hypokalemia (x1.02)
alprazolam (+0.00183)
Persistent pain following procedure (x1.02)
Muscle weakness (x1.02)
Dehydration (x1.02)
Low blood pressure (x1.02)
Hyperglycemia (x1.02)
lorazepam (+0.00168)
Thrombocytopenic disorder (x1.02)
Pleural effusion (x1.02)
trazodone (+0.00166)
Shoulder joint pain (x1.02)
Postoperative state (x1.02)
Adjustment disorder with depressed mood (x1.02)
Dysphagia (x1.02)
acetaminophen (+0.00163)
Secondary malignant neoplasm of lymph node (x1.02)
Severe protein-calorie malnutrition (Gomez: less than 60% of standard weight) (x1.02)
Low back pain (x1.02)
aspirin (-0.00155)
Spasm (x1.02)
ibuprofen (+0.00154)
Acute renal failure syndrome (x1.02)
prochlorperazine (+0.0015)
gabapentin (+0.0015)
Anemia due to and following chemotherapy (x1.02)
Acute posthemorrhagic anemia (x1.02)
Sepsis (x1.02)
Obesity (x1.02)
Adjustment disorder (x1.02)
metoclopramide (+0.00142)
zolpidem (+0.00141)
Hypoxemia (x1.01)
Vitamin D deficiency (x1.01)
Lymphedema (x1.01)
Ulcerative stomatitis (x1.01)
diclofenac (+0.0013)
Electrocardiogram abnormal (x1.01)
Anemia in neoplastic disease (x1.01)
Late effect of complications of procedure (x1.01)
Organic anxiety disorder (x1.01)
Acidosis (x1.01)
Fatigue (x1.01)
Oropharyngeal dysphagia (x1.01)
Precordial pain (x1.01)
Localized enlarged lymph nodes (x1.01)
Gastroesophageal reflux disease without esophagitis (x1.01)
Neoplasm of lymph node (x1.01)
nystatin (+0.00115)
Hypomagnesemia (x1.01)
Ascites (x1.01)
Disorder of soft tissue (x1.01)
Lymphadenopathy (x1.01)
Loss of appetite (x1.01)
ergocalciferol (+0.00111)
Mood disorder (x1.01)
Insomnia disorder related to known organic factor (x1.01)
0.821
0.841
0.861
0.874
0.883
0.893
0.898
0.903
0.907
0.911
0.914
0.917
0.919
0.922
0.924
0.926
0.927
0.929
0.930
0.932
0.933
0.935
0.936
0.937
0.939
0.940
0.941
0.942
0.943
0.944
0.944
0.945
0.946
0.947
0.948
0.948
0.949
0.950
0.950
0.951
0.952
0.952
0.953
0.953
0.954
0.954
0.955
0.955
0.956
0.956
0.957
0.957
0.958
0.958
0.958
0.959
0.959
0.960
0.960
0.961
0.961
0.961
0.962
0.962
0.962
0.963
0.963
0.964
0.964
0.964
0.965
0.965
0.965
0.966
0.966
0.966
0.966
0.967
0.967
0.967
0.967
0.968
0.968
0.968
0.968
0.969
0.969
0.969
0.969
0.970
0.970
0.970
0.970
0.970
0.971
0.971
0.971
0.971
0.971
0.972
Expressions
−10
0
10
−1.00 −0.75 −0.50 −0.25 0.00 0.25 0.50 0.75 1.00

Signature [1216]; 327 To Reach 0.975, (100 Plotted)

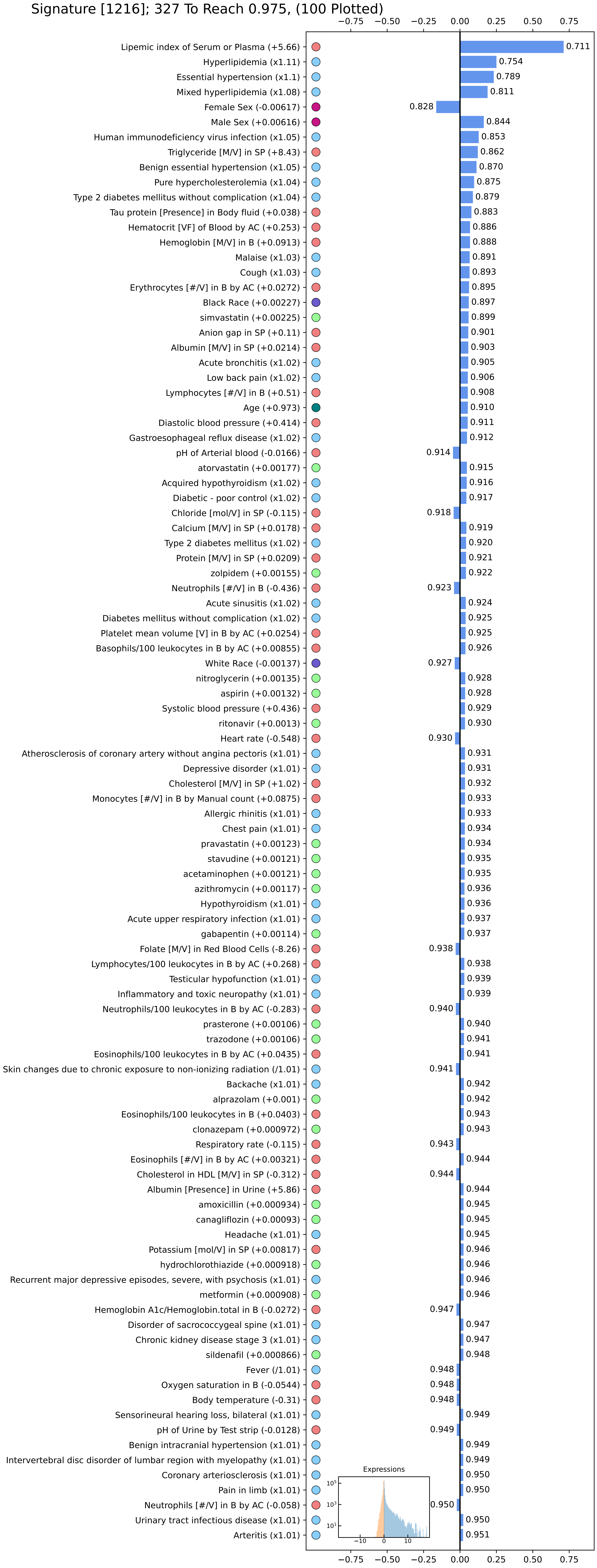

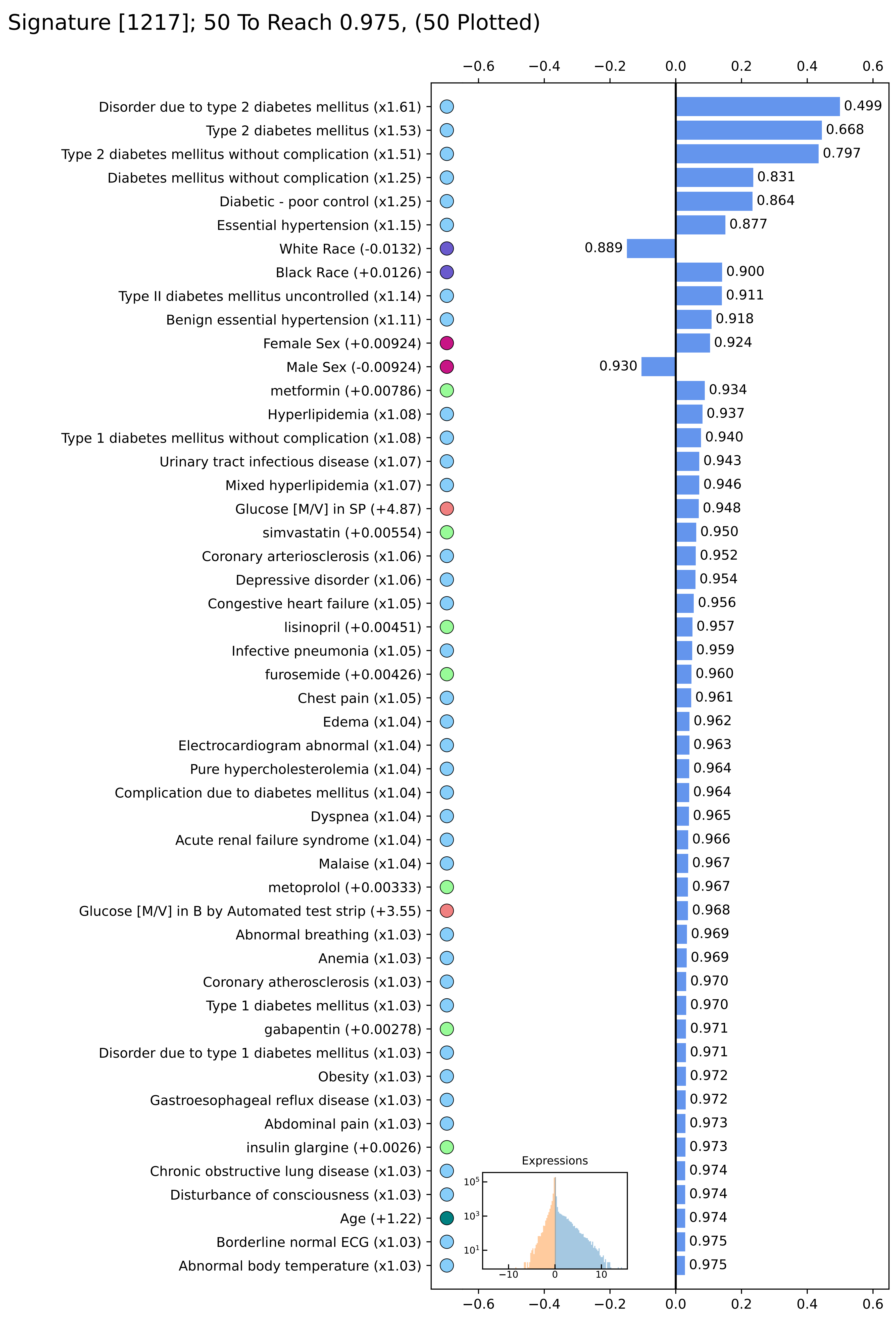
Signature [1217]; 50 To Reach 0.975, (50 Plotted)
Disorder due to type 2 diabetes mellitus (x1.61) 0.499
Type 2 diabetes mellitus (x1.53) 0.668
Type 2 diabetes mellitus without complication (x1.51) 0.797
Diabetes mellitus without complication (x1.25) 0.831
Diabetic - poor control (x1.25) 0.864
Essential hypertension (x1.15) 0.877
White Race (-0.0132) 0.889
Black Race (+0.0126) 0.900
Type II diabetes mellitus uncontrolled (x1.14) 0.911
Benign essential hypertension (x1.11) 0.918
Female Sex (+0.00924) 0.924
Male Sex (-0.00924) 0.930
metformin (+0.00786) 0.934
Hyperlipidemia (x1.08) 0.937
Type 1 diabetes mellitus without complication (x1.08) 0.940
Urinary tract infectious disease (x1.07) 0.943
Mixed hyperlipidemia (x1.07) 0.946
Glucose [M/V] in SP (+4.87) 0.948
simvastatin (+0.00554) 0.950
Coronary arteriosclerosis (x1.06) 0.952
Depressive disorder (x1.06) 0.954
Congestive heart failure (x1.05) 0.956
lisinopril (+0.00451) 0.957
Infective pneumonia (x1.05) 0.959
furosemide (+0.00426) 0.960
Chest pain (x1.05) 0.961
Edema (x1.04) 0.962
Electrocardiogram abnormal (x1.04) 0.963
Pure hypercholesterolemia (x1.04) 0.964
Complication due to diabetes mellitus (x1.04) 0.964
Dyspnea (x1.04) 0.965
Acute renal failure syndrome (x1.04) 0.966
Malaise (x1.04) 0.967
metoprolol (+0.00333) 0.967
Glucose [M/V] in B by Automated test strip (+3.55) 0.968
Abnormal breathing (x1.03) 0.969
Anemia (x1.03) 0.969
Coronary atherosclerosis (x1.03) 0.970
Type 1 diabetes mellitus (x1.03) 0.970
gabapentin (+0.00278) 0.971
Disorder due to type 1 diabetes mellitus (x1.03) 0.971
Obesity (x1.03) 0.972
Gastroesophageal reflux disease (x1.03) 0.972
Abdominal pain (x1.03) 0.973
insulin glargine (+0.0026) 0.973
Chronic obstructive lung disease (x1.03) 0.974
Disturbance of consciousness (x1.03) 0.974
Age (+1.22) 0.974
Borderline normal ECG (x1.03) 0.975
Abnormal body temperature (x1.03) 0.975
−0.6 −0.4 −0.2 0.0 0.2 0.4 0.6
Expressions
10^5
10^3
10^1
−10 0 10

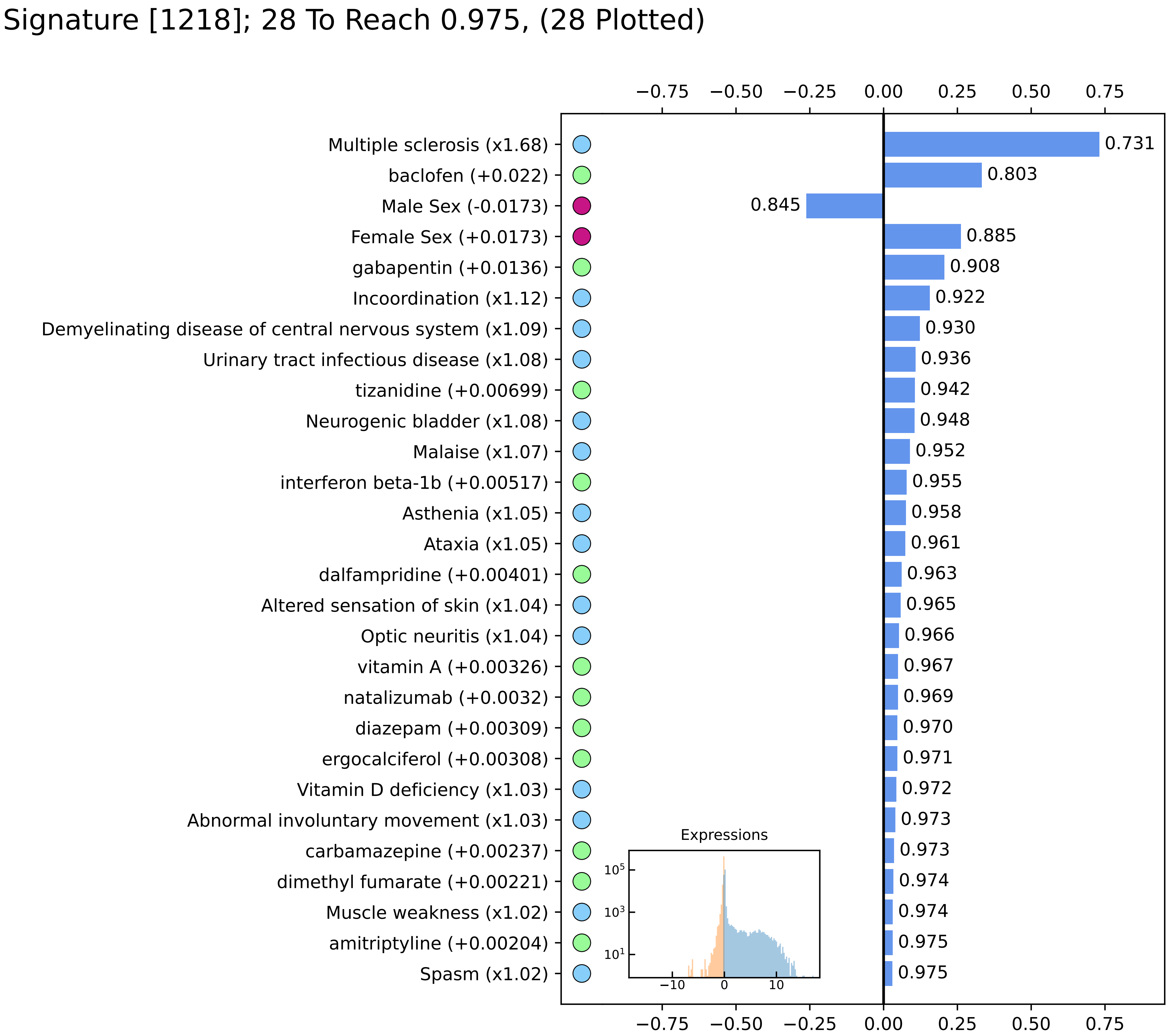
Signature [1218]; 28 To Reach 0.975, (28 Plotted)
Multiple sclerosis (x1.68) 0.731
baclofen (+0.022) 0.803
Male Sex (-0.0173) 0.845
Female Sex (+0.0173) 0.885
gabapentin (+0.0136) 0.908
Incoordination (x1.12) 0.922
Demyelinating disease of central nervous system (x1.09) 0.930
Urinary tract infectious disease (x1.08) 0.936
tizanidine (+0.00699) 0.942
Neurogenic bladder (x1.08) 0.948
Malaise (x1.07) 0.952
interferon beta-1b (+0.00517) 0.955
Asthenia (x1.05) 0.958
Ataxia (x1.05) 0.961
dalfampridine (+0.00401) 0.963
Altered sensation of skin (x1.04) 0.965
Optic neuritis (x1.04) 0.966
vitamin A (+0.00326) 0.967
natalizumab (+0.0032) 0.969
diazepam (+0.00309) 0.970
ergocalciferol (+0.00308) 0.971
Vitamin D deficiency (x1.03) 0.972
Abnormal involuntary movement (x1.03) 0.973
carbamazepine (+0.00237) 0.973
dimethyl fumarate (+0.00221) 0.974
Muscle weakness (x1.02) 0.974
amitriptyline (+0.00204) 0.975
Spasm (x1.02) 0.975
Expressions

Signature [1219]; 322 To Reach 0.975, (100 Plotted)

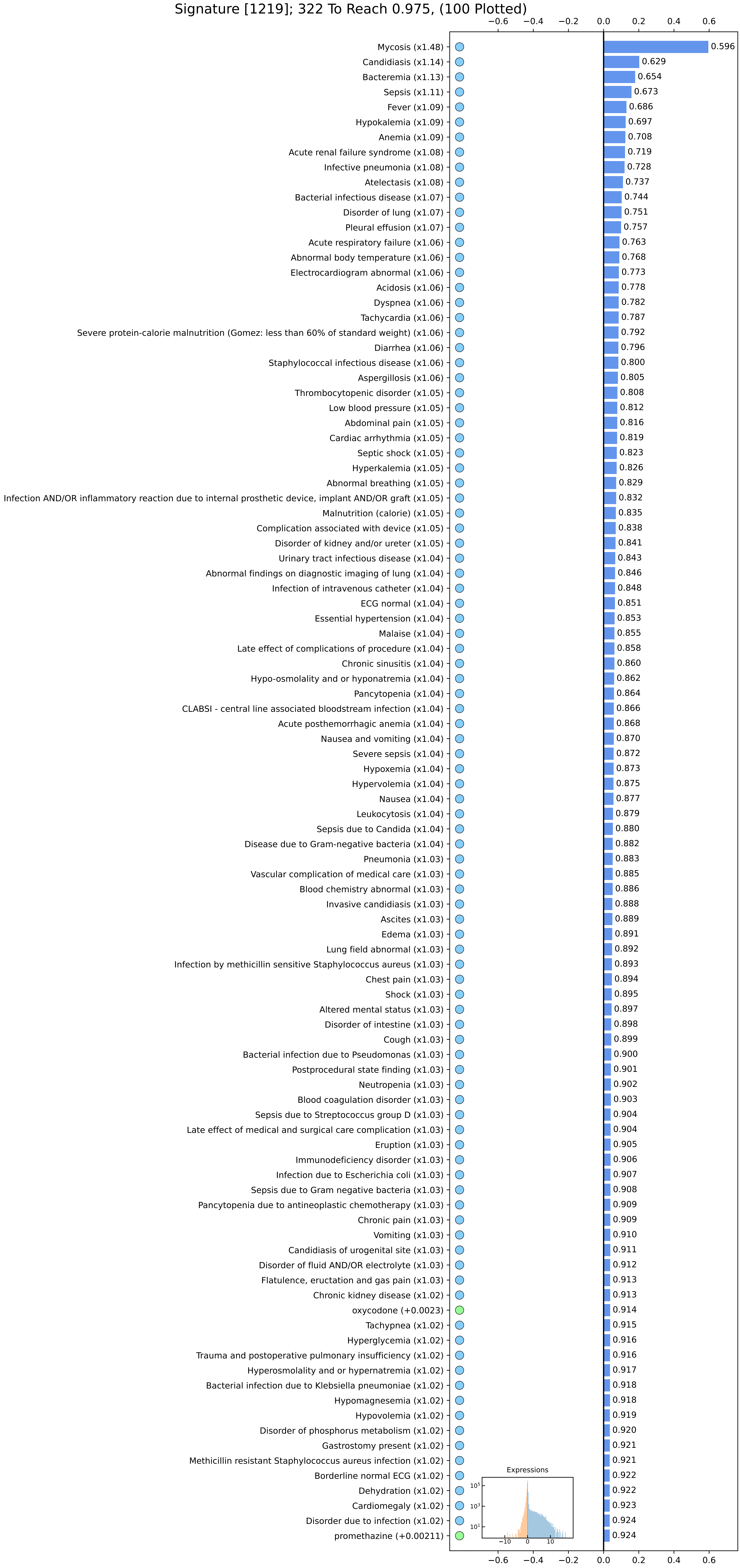

Signature [1220]; 1 To Reach 0.975, (10 Plotted)

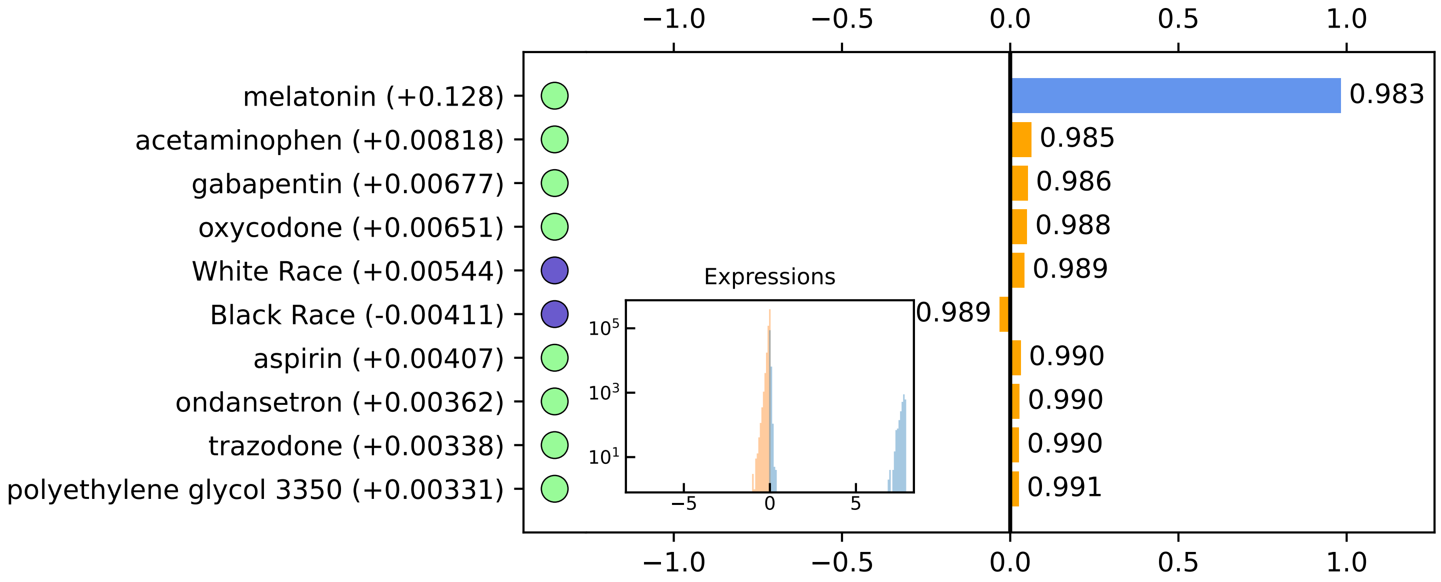

Signature [1221]; 1888 To Reach 0.975, (100 Plotted)

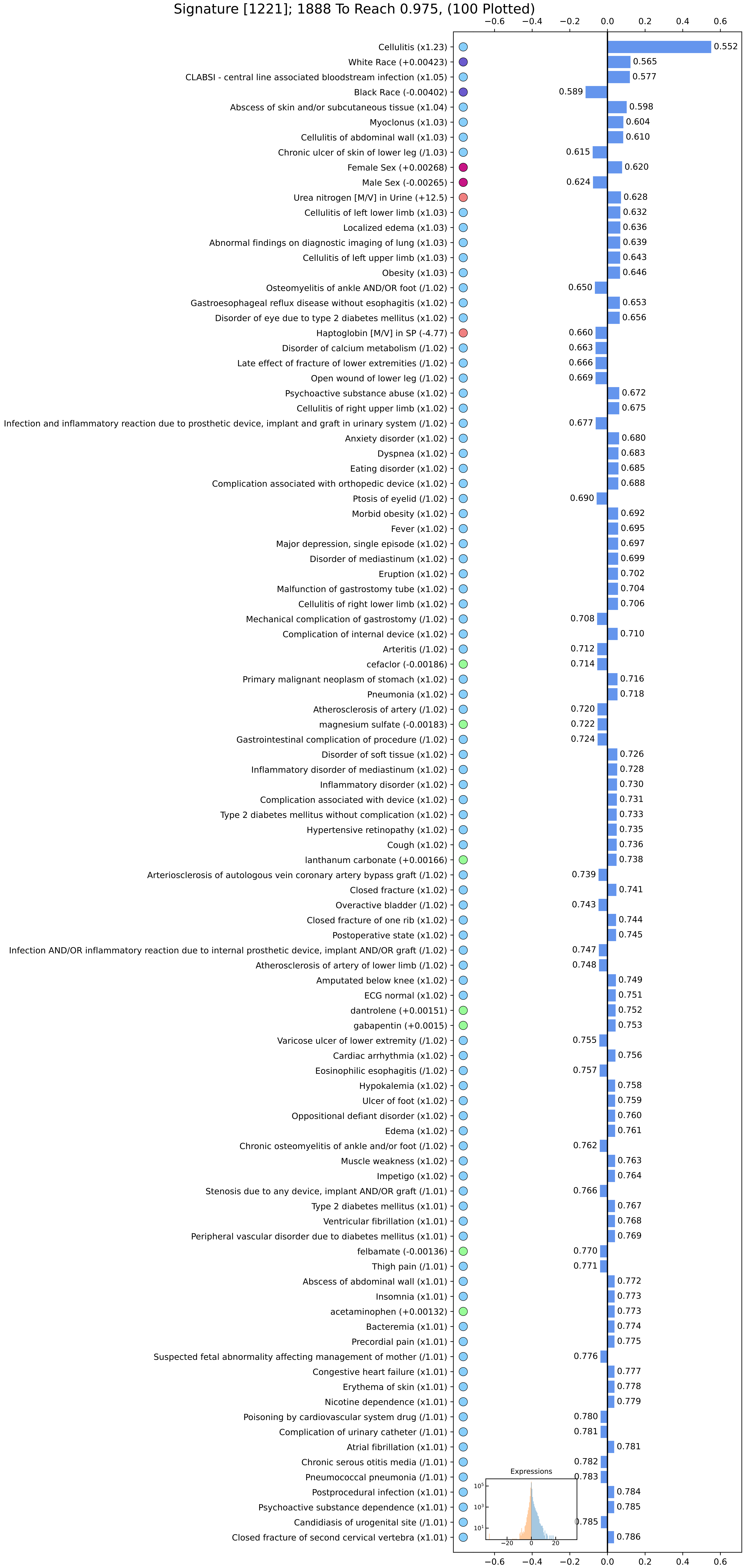

Signature [1222]; 167 To Reach 0.975, (100 Plotted)

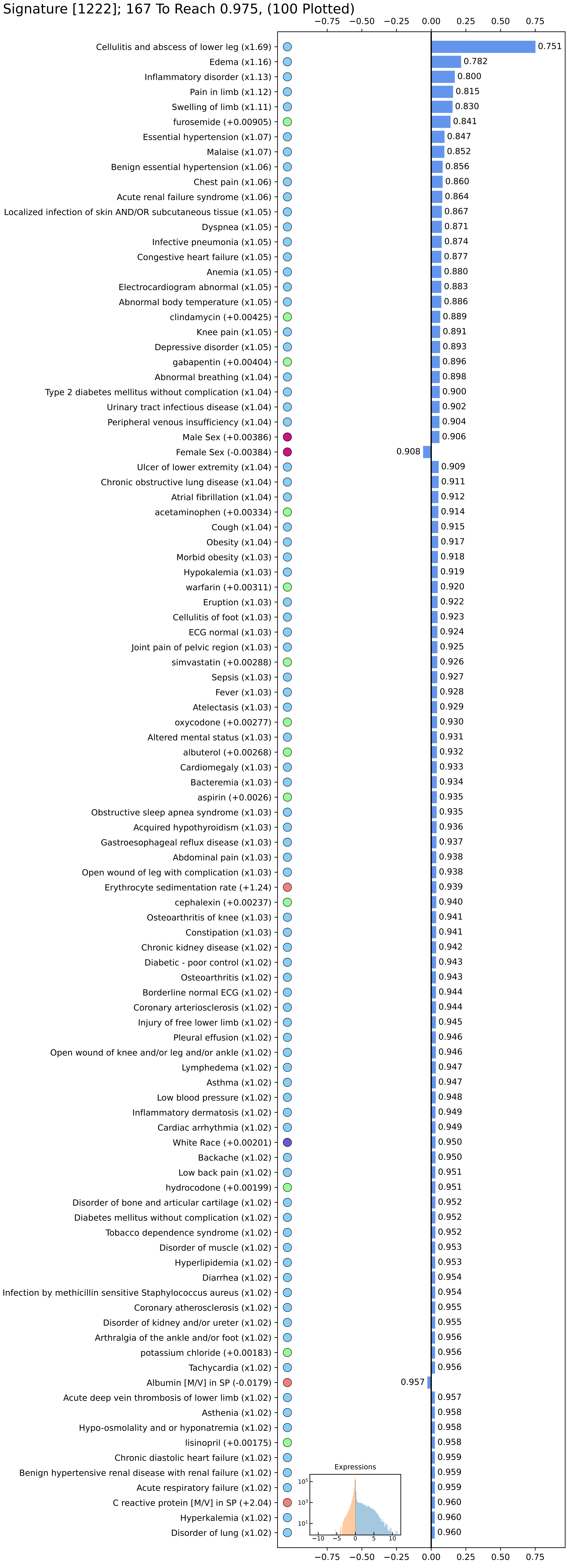

Signature [1223]; 20 To Reach 0.975, (20 Plotted)

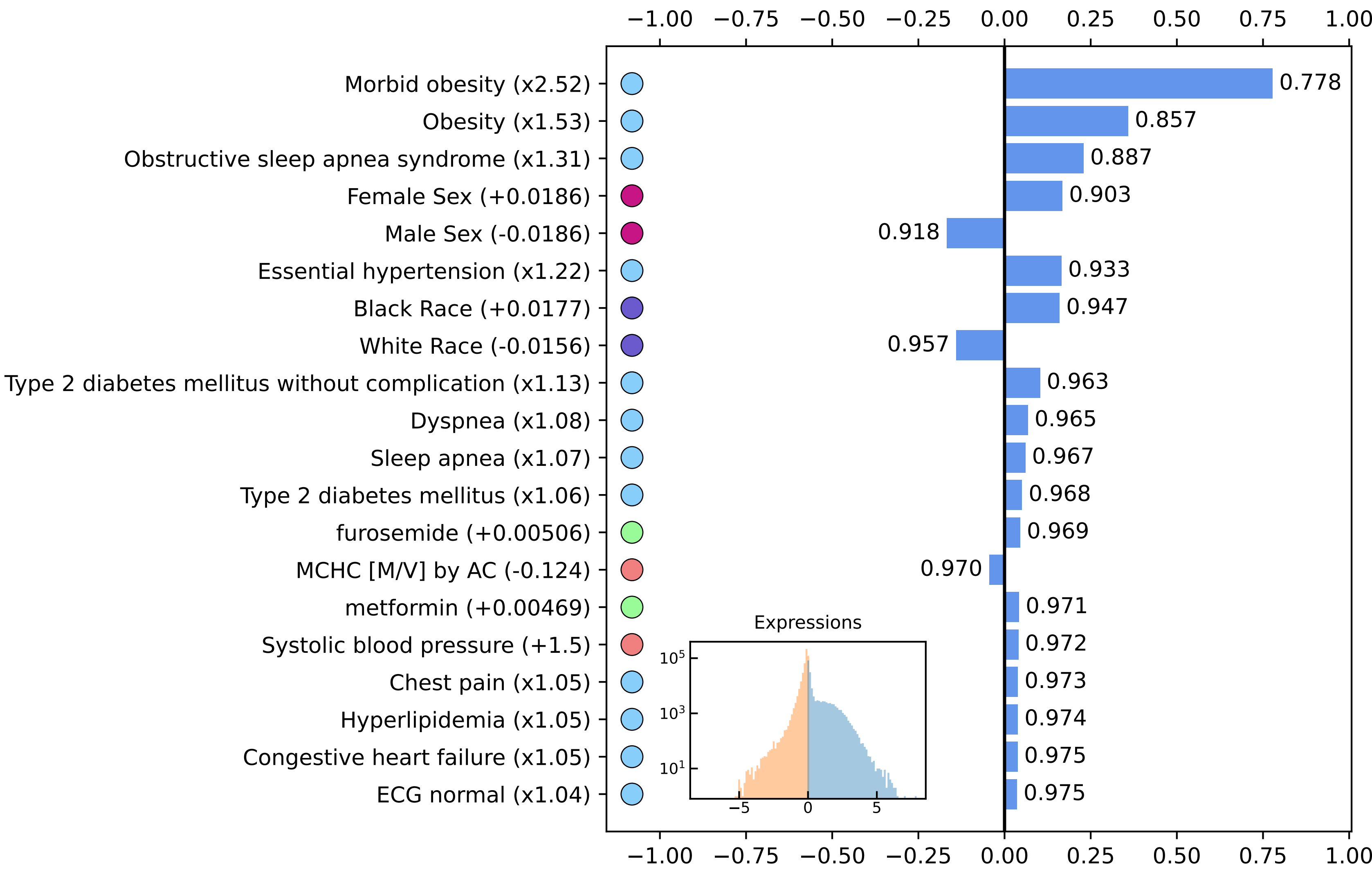

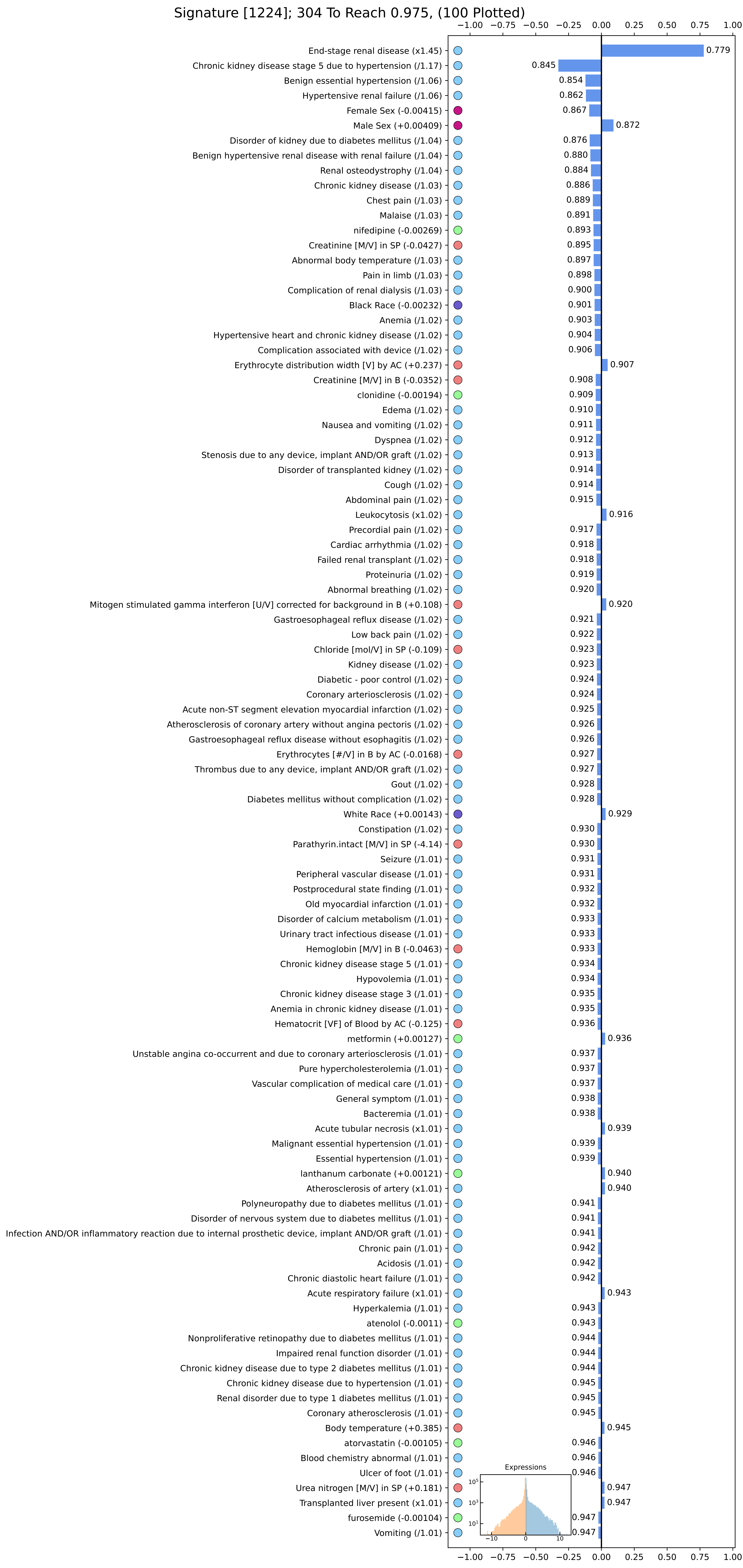

Signature [1224]; 304 To Reach 0.975, (100 Plotted)
−1.00 −0.75 −0.50 −0.25 0.00 0.25 0.50 0.75 1.00
End-stage renal disease (x1.45) 0.779
Chronic kidney disease stage 5 due to hypertension (/1.17) 0.845
Benign essential hypertension (/1.06) 0.854
Hypertensive renal failure (/1.06) 0.862
Female Sex (-0.00415) 0.867
Male Sex (+0.00409) 0.872
Disorder of kidney due to diabetes mellitus (/1.04) 0.876
Benign hypertensive renal disease with renal failure (/1.04) 0.880
Renal osteodystrophy (/1.04) 0.884
Chronic kidney disease (/1.03) 0.886
Chest pain (/1.03) 0.889
Malaise (/1.03) 0.891
nifedipine (-0.00269) 0.893
Creatinine [M/V] in SP (-0.0427) 0.895
Abnormal body temperature (/1.03) 0.897
Pain in limb (/1.03) 0.898
Complication of renal dialysis (/1.03) 0.900
Black Race (-0.00232) 0.901
Anemia (/1.02) 0.903
Hypertensive heart and chronic kidney disease (/1.02) 0.904
Complication associated with device (/1.02) 0.906
Erythrocyte distribution width [V] by AC (+0.237) 0.907
Creatinine [M/V] in B (-0.0352) 0.908
clonidine (-0.00194) 0.909
Edema (/1.02) 0.910
Nausea and vomiting (/1.02) 0.911
Dyspnea (/1.02) 0.912
Stenosis due to any device, implant AND/OR graft (/1.02) 0.913
Disorder of transplanted kidney (/1.02) 0.914
Cough (/1.02) 0.914
Abdominal pain (/1.02) 0.915
Leukocytosis (x1.02) 0.916
Precordial pain (/1.02) 0.917
Cardiac arrhythmia (/1.02) 0.918
Failed renal transplant (/1.02) 0.918
Proteinuria (/1.02) 0.919
Abnormal breathing (/1.02) 0.920
Mitogen stimulated gamma interferon [U/V] corrected for background in B (+0.108) 0.920
Gastroesophageal reflux disease (/1.02) 0.921
Low back pain (/1.02) 0.922
Chloride [mol/V] in SP (-0.109) 0.923
Kidney disease (/1.02) 0.923
Diabetic - poor control (/1.02) 0.924
Coronary arteriosclerosis (/1.02) 0.924
Acute non-ST segment elevation myocardial infarction (/1.02) 0.925
Atherosclerosis of coronary artery without angina pectoris (/1.02) 0.926
Gastroesophageal reflux disease without esophagitis (/1.02) 0.926
Erythrocytes [#/V] in B by AC (-0.0168) 0.927
Thrombus due to any device, implant AND/OR graft (/1.02) 0.927
Gout (/1.02) 0.928
Diabetes mellitus without complication (/1.02) 0.928
White Race (+0.00143) 0.929
Constipation (/1.02) 0.930
Parathyrin.intact [M/V] in SP (-4.14) 0.930
Seizure (/1.01) 0.931
Peripheral vascular disease (/1.01) 0.931
Postprocedural state finding (/1.01) 0.932
Old myocardial infarction (/1.01) 0.932
Disorder of calcium metabolism (/1.01) 0.933
Urinary tract infectious disease (/1.01) 0.933
Hemoglobin [M/V] in B (-0.0463) 0.933
Chronic kidney disease stage 5 (/1.01) 0.934
Hypovolemia (/1.01) 0.934
Chronic kidney disease stage 3 (/1.01) 0.935
Anemia in chronic kidney disease (/1.01) 0.935
Hematocrit [VF] of Blood by AC (-0.125) 0.936
metformin (+0.00127) 0.936
Unstable angina co-occurrent and due to coronary arteriosclerosis (/1.01) 0.937
Pure hypercholesterolemia (/1.01) 0.937
Vascular complication of medical care (/1.01) 0.937
General symptom (/1.01) 0.938
Bacteremia (/1.01) 0.938
Acute tubular necrosis (x1.01) 0.939
Malignant essential hypertension (/1.01) 0.939
Essential hypertension (/1.01) 0.939
lanthanum carbonate (+0.00121) 0.940
Atherosclerosis of artery (x1.01) 0.940
Polyneuropathy due to diabetes mellitus (/1.01) 0.941
Disorder of nervous system due to diabetes mellitus (/1.01) 0.941
Infection AND/OR inflammatory reaction due to internal prosthetic device, implant AND/OR graft (/1.01) 0.941
Chronic pain (/1.01) 0.942
Acidosis (/1.01) 0.942
Chronic diastolic heart failure (/1.01) 0.942
Acute respiratory failure (x1.01) 0.943
Hyperkalemia (/1.01) 0.943
atenolol (-0.0011) 0.943
Nonproliferative retinopathy due to diabetes mellitus (/1.01) 0.944
Impaired renal function disorder (/1.01) 0.944
Chronic kidney disease due to type 2 diabetes mellitus (/1.01) 0.944
Chronic kidney disease due to hypertension (/1.01) 0.945
Renal disorder due to type 1 diabetes mellitus (/1.01) 0.945
Coronary atherosclerosis (/1.01) 0.945
Body temperature (+0.385) 0.945
atorvastatin (-0.00105) 0.946
Blood chemistry abnormal (/1.01) 0.946
Ulcer of foot (/1.01) 0.946
Urea nitrogen [M/V] in SP (+0.181) 0.947
Transplanted liver present (x1.01) 0.947
furosemide (-0.00104) 0.947
Vomiting (/1.01) 0.947
Expressions
10^5
10^3
10^1
−10 0 10

Signature [1225]; 16 To Reach 0.975, (16 Plotted)

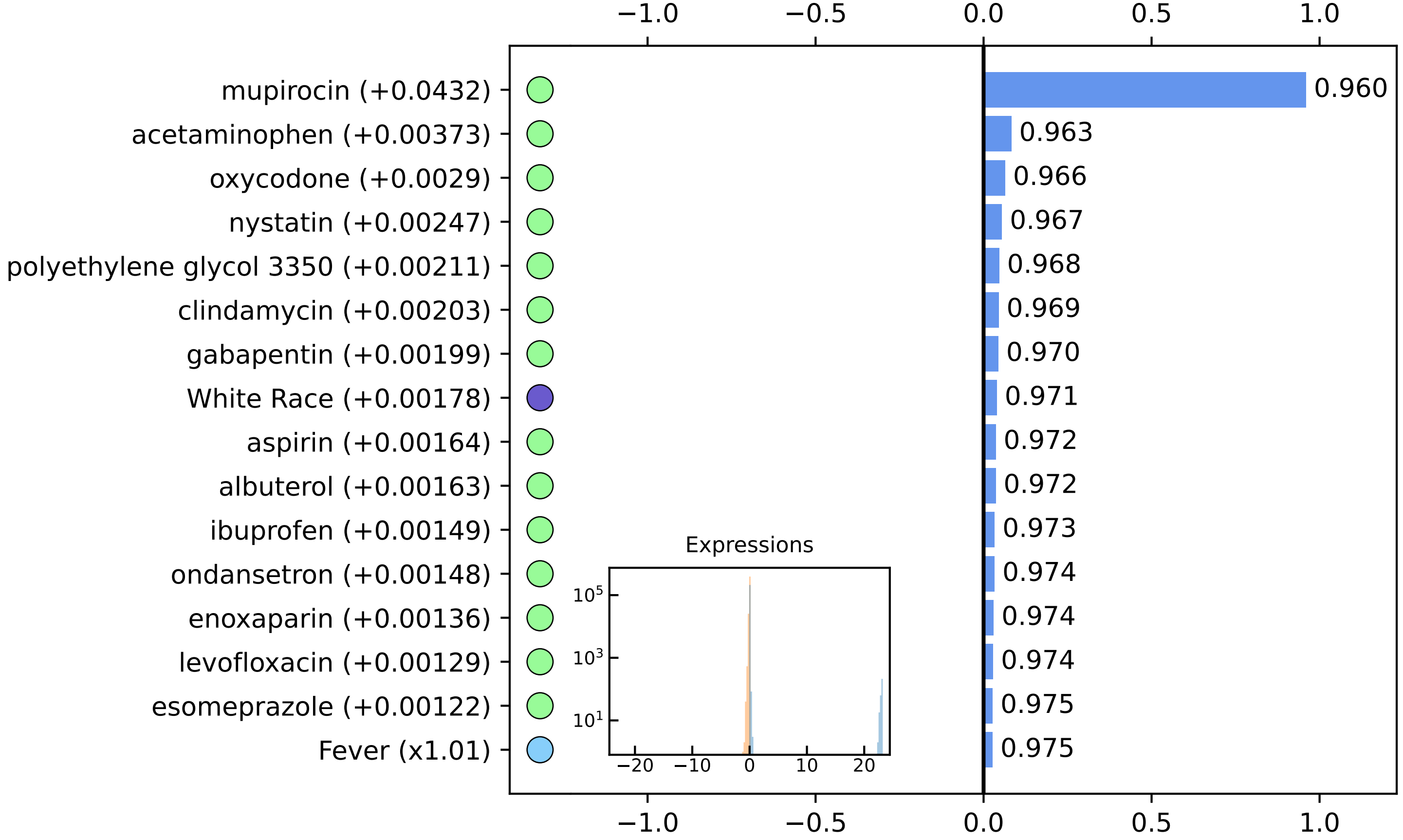

Signature [1226]; 1 To Reach 0.975, (10 Plotted)

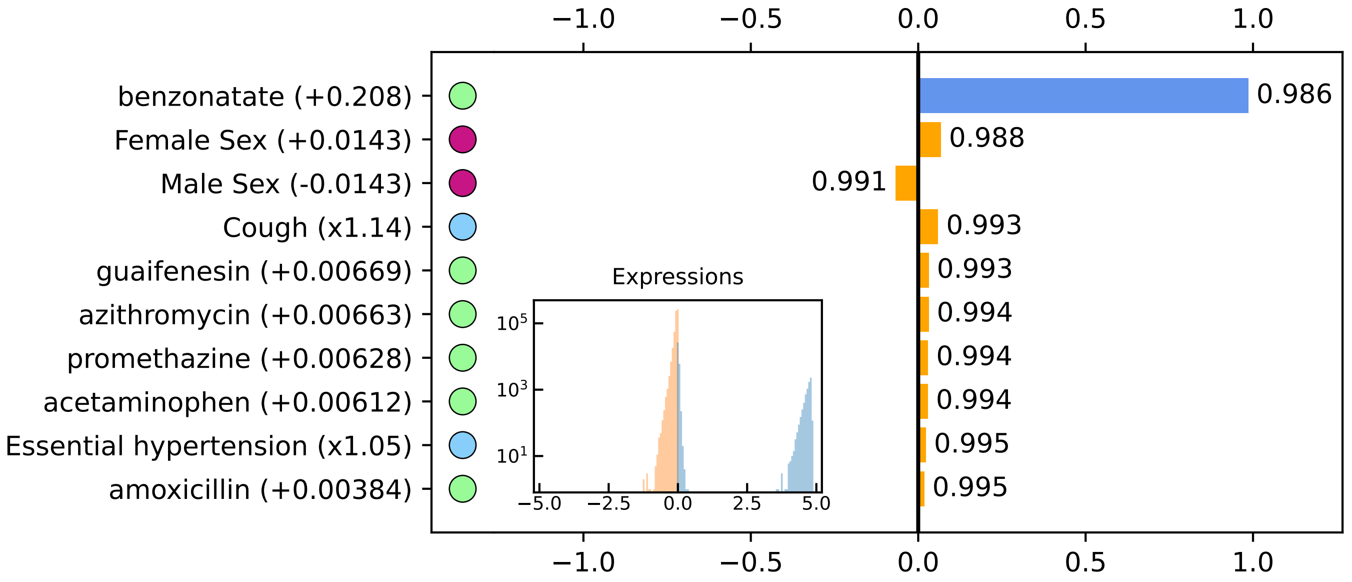

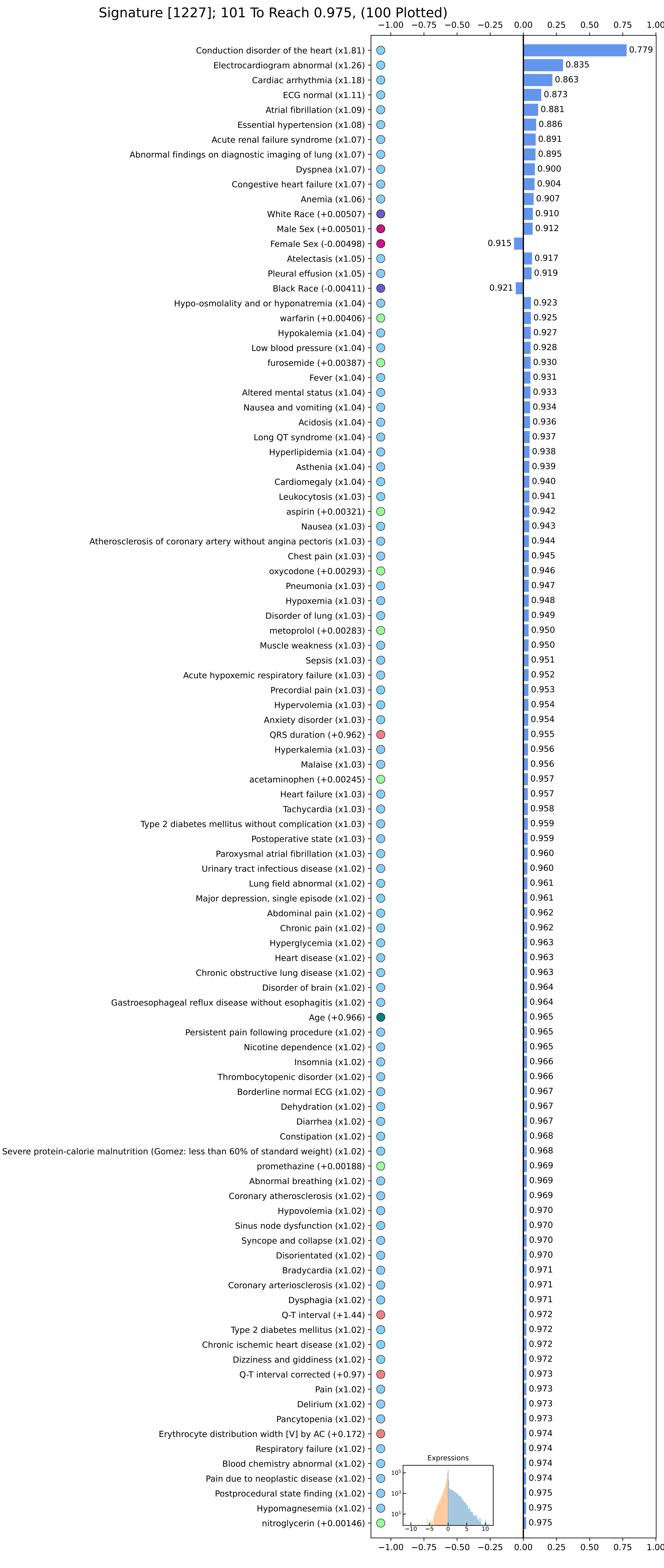

Signature [1227]; 101 To Reach 0.975, (100 Plotted)
−1.00 −0.75 −0.50 −0.25 0.00 0.25 0.50 0.75 1.00
Conduction disorder of the heart (x1.81)
0.779
Electrocardiogram abnormal (x1.26)
0.835
Cardiac arrhythmia (x1.18)
0.863
ECG normal (x1.11)
0.873
Atrial fibrillation (x1.09)
0.881
Essential hypertension (x1.08)
0.886
Acute renal failure syndrome (x1.07)
0.891
Abnormal findings on diagnostic imaging of lung (x1.07)
0.895
Dyspnea (x1.07)
0.900
Congestive heart failure (x1.07)
0.904
Anemia (x1.06)
0.907
White Race (+0.00507)
0.910
Male Sex (+0.00501)
0.912
Female Sex (-0.00498)
0.915
Atelectasis (x1.05)
0.917
Pleural effusion (x1.05)
0.919
Black Race (-0.00411)
0.921
Hypo-osmolality and or hyponatremia (x1.04)
0.923
warfarin (+0.00406)
0.925
Hypokalemia (x1.04)
0.927
Low blood pressure (x1.04)
0.928
furosemide (+0.00387)
0.930
Fever (x1.04)
0.931
Altered mental status (x1.04)
0.933
Nausea and vomiting (x1.04)
0.934
Acidosis (x1.04)
0.936
Long QT syndrome (x1.04)
0.937
Hyperlipidemia (x1.04)
0.938
Asthenia (x1.04)
0.939
Cardiomegaly (x1.04)
0.940
Leukocytosis (x1.03)
0.941
aspirin (+0.00321)
0.942
Nausea (x1.03)
0.943
Atherosclerosis of coronary artery without angina pectoris (x1.03)
0.944
Chest pain (x1.03)
0.945
oxycodone (+0.00293)
0.946
Pneumonia (x1.03)
0.947
Hypoxemia (x1.03)
0.948
Disorder of lung (x1.03)
0.949
metoprolol (+0.00283)
0.950
Muscle weakness (x1.03)
0.950
Sepsis (x1.03)
0.951
Acute hypoxemic respiratory failure (x1.03)
0.952
Precordial pain (x1.03)
0.953
Hypervolemia (x1.03)
0.954
Anxiety disorder (x1.03)
0.954
QRS duration (+0.962)
0.955
Hyperkalemia (x1.03)
0.956
Malaise (x1.03)
0.956
acetaminophen (+0.00245)
0.957
Heart failure (x1.03)
0.957
Tachycardia (x1.03)
0.958
Type 2 diabetes mellitus without complication (x1.03)
0.959
Postoperative state (x1.03)
0.959
Paroxysmal atrial fibrillation (x1.03)
0.960
Urinary tract infectious disease (x1.02)
0.960
Lung field abnormal (x1.02)
0.961
Major depression, single episode (x1.02)
0.961
Abdominal pain (x1.02)
0.962
Chronic pain (x1.02)
0.962
Hyperglycemia (x1.02)
0.963
Heart disease (x1.02)
0.963
Chronic obstructive lung disease (x1.02)
0.963
Disorder of brain (x1.02)
0.964
Gastroesophageal reflux disease without esophagitis (x1.02)
0.964
Age (+0.966)
0.965
Persistent pain following procedure (x1.02)
0.965
Nicotine dependence (x1.02)
0.965
Insomnia (x1.02)
0.966
Thrombocytopenic disorder (x1.02)
0.966
Borderline normal ECG (x1.02)
0.967
Dehydration (x1.02)
0.967
Diarrhea (x1.02)
0.967
Constipation (x1.02)
0.968
Severe protein-calorie malnutrition (Gomez: less than 60% of standard weight) (x1.02)
0.968
promethazine (+0.00188)
0.969
Abnormal breathing (x1.02)
0.969
Coronary atherosclerosis (x1.02)
0.969
Hypovolemia (x1.02)
0.970
Sinus node dysfunction (x1.02)
0.970
Syncope and collapse (x1.02)
0.970
Disorientated (x1.02)
0.970
Bradycardia (x1.02)
0.971
Coronary arteriosclerosis (x1.02)
0.971
Dysphagia (x1.02)
0.971
Q-T interval (+1.44)
0.972
Type 2 diabetes mellitus (x1.02)
0.972
Chronic ischemic heart disease (x1.02)
0.972
Dizziness and giddiness (x1.02)
0.972
Q-T interval corrected (+0.97)
0.973
Pain (x1.02)
0.973
Delirium (x1.02)
0.973
Pancytopenia (x1.02)
0.973
Erythrocyte distribution width [V] by AC (+0.172)
0.974
Respiratory failure (x1.02)
0.974
Blood chemistry abnormal (x1.02)
0.974
Pain due to neoplastic disease (x1.02)
0.974
Postprocedural state finding (x1.02)
0.975
Hypomagnesemia (x1.02)
0.975
nitroglycerin (+0.00146)
0.975
Expressions
10^5
10^3
10^1
−10 −5 0 5 10
−1.00 −0.75 −0.50 −0.25 0.00 0.25 0.50 0.75 1.00

Signature [1228]; 364 To Reach 0.975, (100 Plotted)

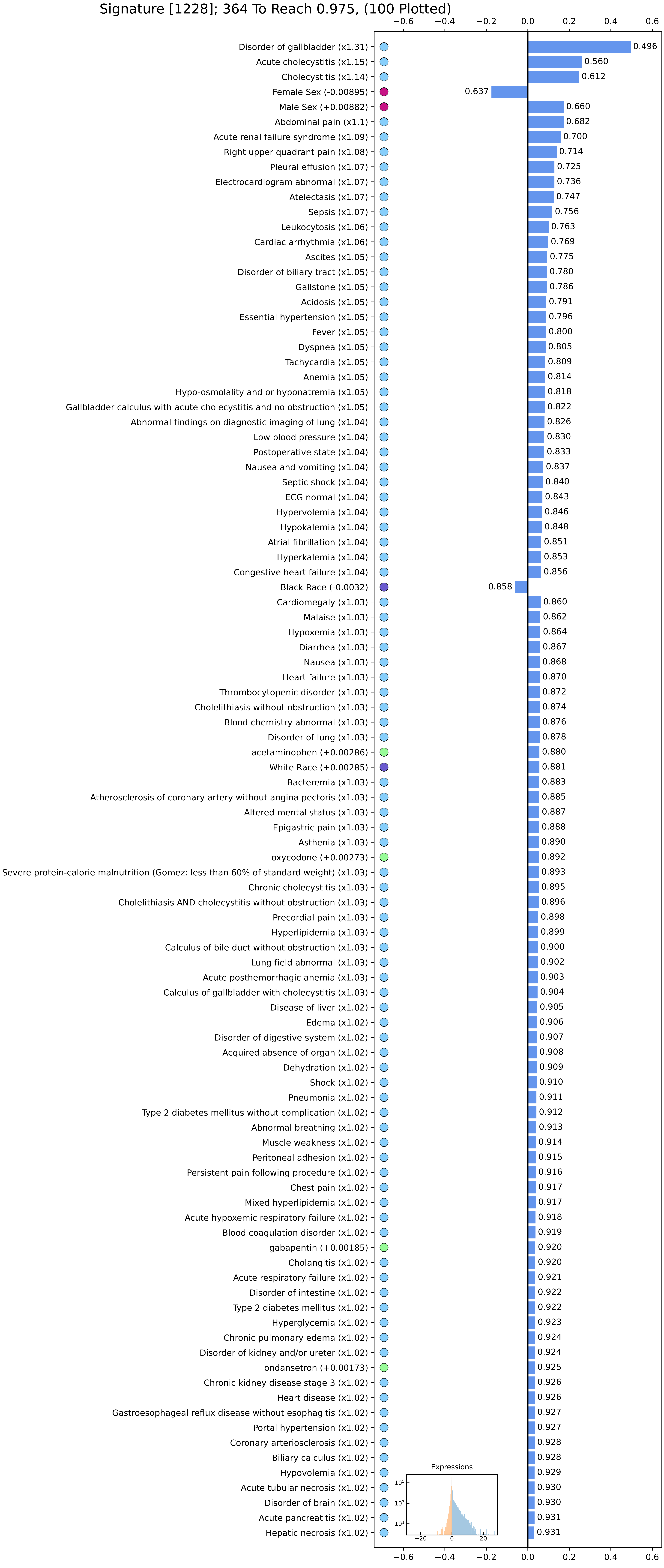

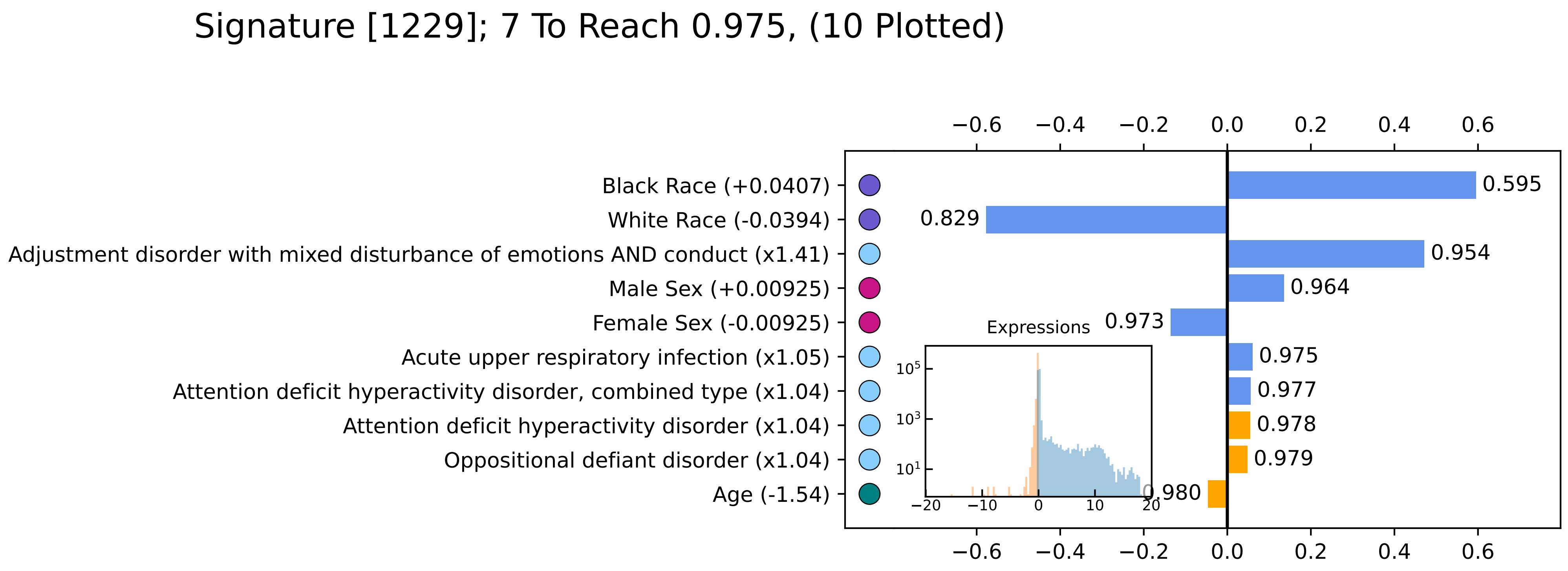
Signature [1229]; 7 To Reach 0.975, (10 Plotted)
Black Race (+0.0407)
White Race (-0.0394)
Adjustment disorder with mixed disturbance of emotions AND conduct (x1.41)
Male Sex (+0.00925)
Female Sex (-0.00925)
Acute upper respiratory infection (x1.05)
Attention deficit hyperactivity disorder, combined type (x1.04)
Attention deficit hyperactivity disorder (x1.04)
Oppositional defiant disorder (x1.04)
Age (-1.54)
0.595
0.829
0.954
0.964
0.973
0.975
0.977
0.978
0.979
0.980
Expressions
−0.6
−0.4
−0.2
0.0
0.2
0.4
0.6

Signature [1230]; 3 To Reach 0.975, (10 Plotted)

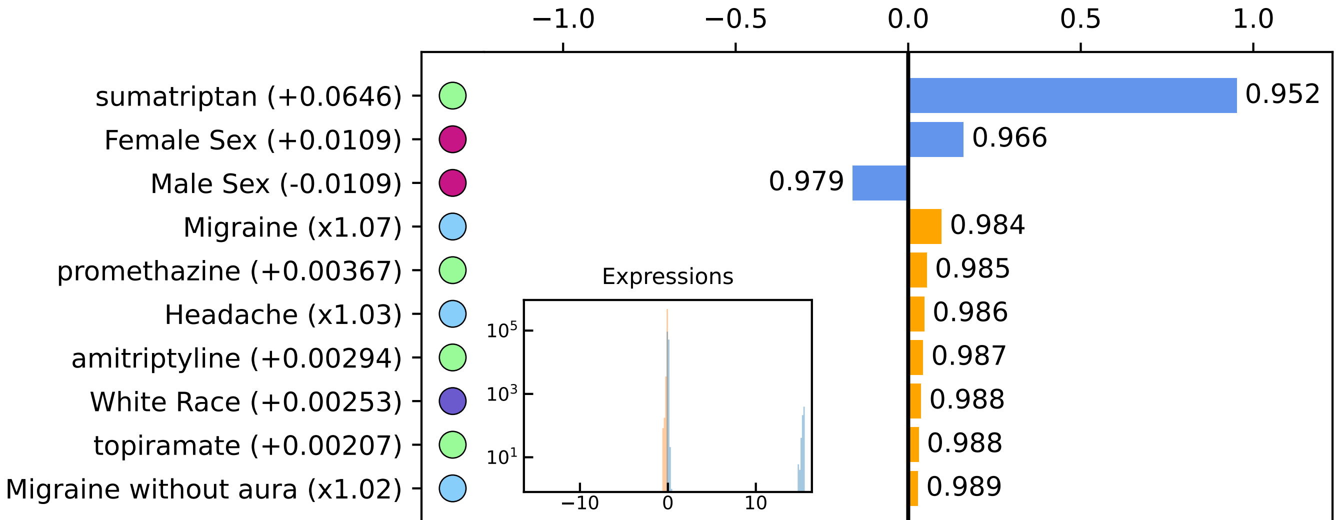

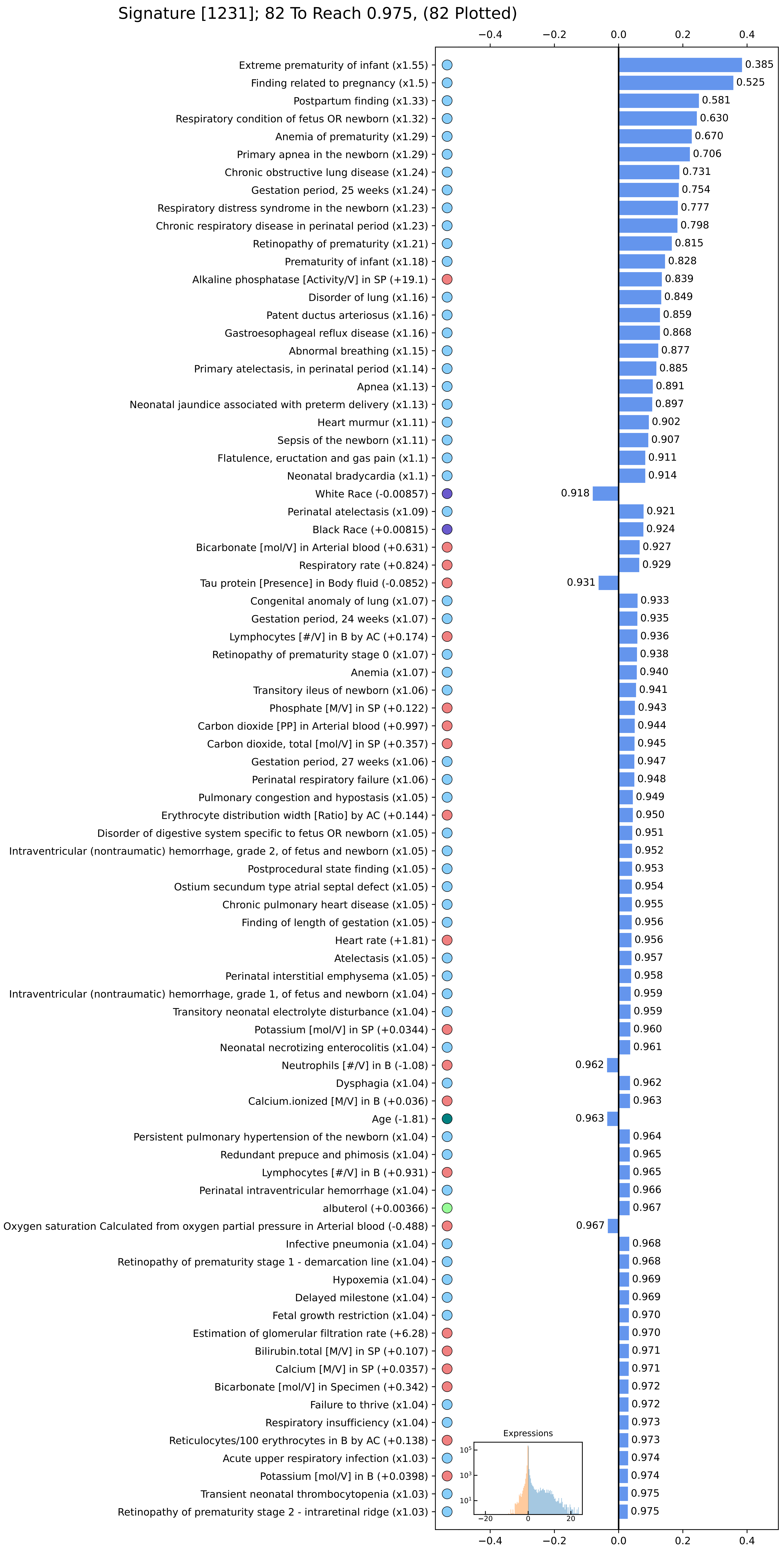
Signature [1231]; 82 To Reach 0.975, (82 Plotted)
−0.4
−0.2
0.0
0.2
0.4
Extreme prematurity of infant (x1.55)
0.385
Finding related to pregnancy (x1.5)
0.525
Postpartum finding (x1.33)
0.581
Respiratory condition of fetus OR newborn (x1.32)
0.630
Anemia of prematurity (x1.29)
0.670
Primary apnea in the newborn (x1.29)
0.706
Chronic obstructive lung disease (x1.24)
0.731
Gestation period, 25 weeks (x1.24)
0.754
Respiratory distress syndrome in the newborn (x1.23)
0.777
Chronic respiratory disease in perinatal period (x1.23)
0.798
Retinopathy of prematurity (x1.21)
0.815
Prematurity of infant (x1.18)
0.828
Alkaline phosphatase [Activity/V] in SP (+19.1)
0.839
Disorder of lung (x1.16)
0.849
Patent ductus arteriosus (x1.16)
0.859
Gastroesophageal reflux disease (x1.16)
0.868
Abnormal breathing (x1.15)
0.877
Primary atelectasis, in perinatal period (x1.14)
0.885
Apnea (x1.13)
0.891
Neonatal jaundice associated with preterm delivery (x1.13)
0.897
Heart murmur (x1.11)
0.902
Sepsis of the newborn (x1.11)
0.907
Flatulence, eructation and gas pain (x1.1)
0.911
Neonatal bradycardia (x1.1)
0.914
White Race (-0.00857)
0.918
Perinatal atelectasis (x1.09)
0.921
Black Race (+0.00815)
0.924
Bicarbonate [mol/V] in Arterial blood (+0.631)
0.927
Respiratory rate (+0.824)
0.929
Tau protein [Presence] in Body fluid (-0.0852)
0.931
Congenital anomaly of lung (x1.07)
0.933
Gestation period, 24 weeks (x1.07)
0.935
Lymphocytes [#/V] in B by AC (+0.174)
0.936
Retinopathy of prematurity stage 0 (x1.07)
0.938
Anemia (x1.07)
0.940
Transitory ileus of newborn (x1.06)
0.941
Phosphate [M/V] in SP (+0.122)
0.943
Carbon dioxide [PP] in Arterial blood (+0.997)
0.944
Carbon dioxide, total [mol/V] in SP (+0.357)
0.945
Gestation period, 27 weeks (x1.06)
0.947
Perinatal respiratory failure (x1.06)
0.948
Pulmonary congestion and hypostasis (x1.05)
0.949
Erythrocyte distribution width [Ratio] by AC (+0.144)
0.950
Disorder of digestive system specific to fetus OR newborn (x1.05)
0.951
Intraventricular (nontraumatic) hemorrhage, grade 2, of fetus and newborn (x1.05)
0.952
Postprocedural state finding (x1.05)
0.953
Ostium secundum type atrial septal defect (x1.05)
0.954
Chronic pulmonary heart disease (x1.05)
0.955
Finding of length of gestation (x1.05)
0.956
Heart rate (+1.81)
0.956
Atelectasis (x1.05)
0.957
Perinatal interstitial emphysema (x1.05)
0.958
Intraventricular (nontraumatic) hemorrhage, grade 1, of fetus and newborn (x1.04)
0.959
Transitory neonatal electrolyte disturbance (x1.04)
0.959
Potassium [mol/V] in SP (+0.0344)
0.960
Neonatal necrotizing enterocolitis (x1.04)
0.961
Neutrophils [#/V] in B (-1.08)
0.962
Dysphagia (x1.04)
0.962
Calcium.ionized [M/V] in B (+0.036)
0.963
Age (-1.81)
0.963
Persistent pulmonary hypertension of the newborn (x1.04)
0.964
Redundant prepuce and phimosis (x1.04)
0.965
Lymphocytes [#/V] in B (+0.931)
0.965
Perinatal intraventricular hemorrhage (x1.04)
0.966
albuterol (+0.00366)
0.967
Oxygen saturation Calculated from oxygen partial pressure in Arterial blood (-0.488)
0.967
Infective pneumonia (x1.04)
0.968
Retinopathy of prematurity stage 1 - demarcation line (x1.04)
0.968
Hypoxemia (x1.04)
0.969
Delayed milestone (x1.04)
0.969
Fetal growth restriction (x1.04)
0.970
Estimation of glomerular filtration rate (+6.28)
0.970
Bilirubin.total [M/V] in SP (+0.107)
0.971
Calcium [M/V] in SP (+0.0357)
0.971
Bicarbonate [mol/V] in Specimen (+0.342)
0.972
Failure to thrive (x1.04)
0.972
Respiratory insufficiency (x1.04)
0.973
Reticulocytes/100 erythrocytes in B by AC (+0.138)
0.973
Acute upper respiratory infection (x1.03)
0.974
Potassium [mol/V] in B (+0.0398)
0.974
Transient neonatal thrombocytopenia (x1.03)
0.975
Retinopathy of prematurity stage 2 - intraretinal ridge (x1.03)
0.975
Expressions
10^5
10^3
10^1
−20
0
20
−0.4
−0.2
0.0
0.2
0.4

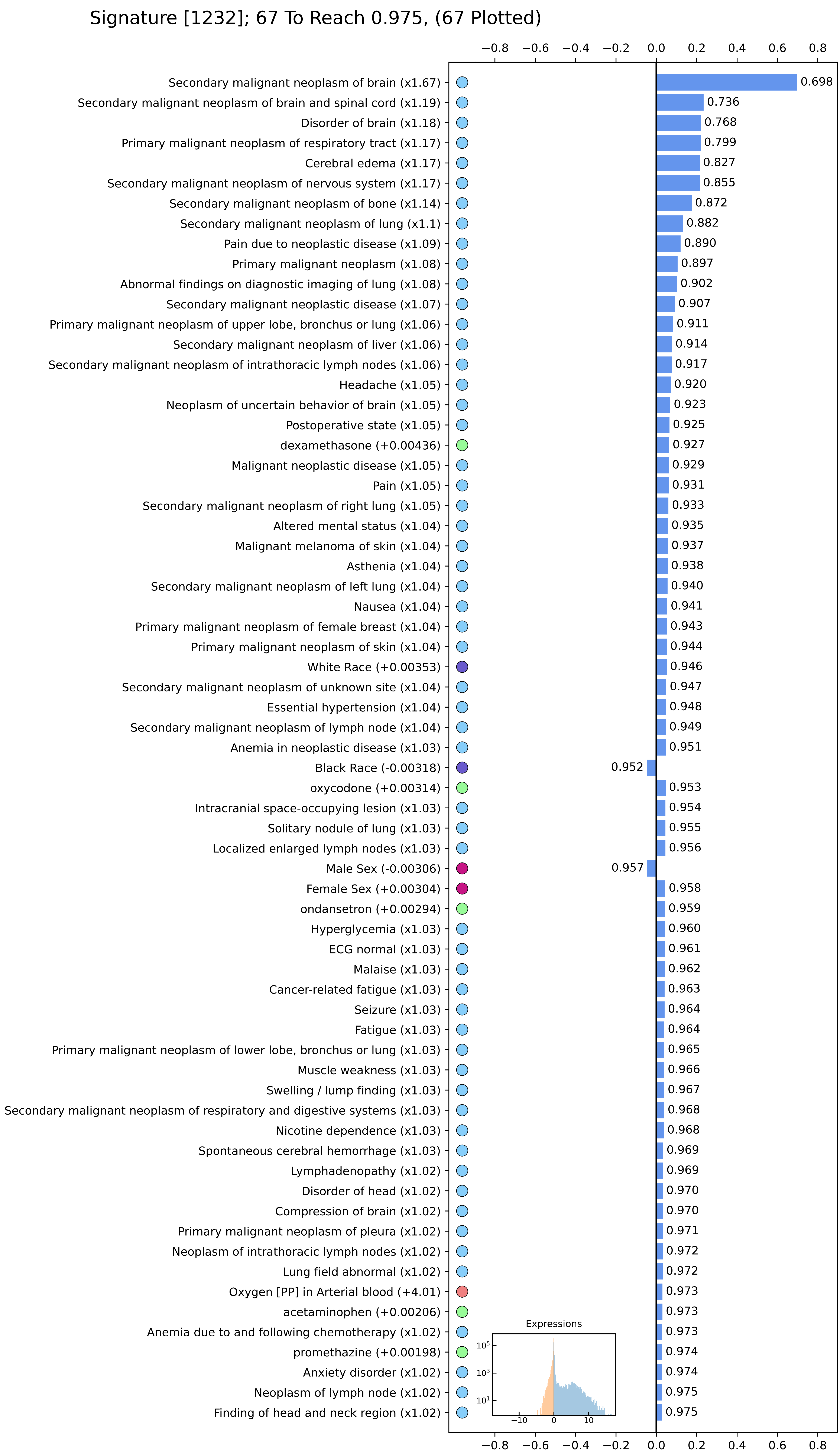

Signature [1232]; 67 To Reach 0.975, (67 Plotted)
Secondary malignant neoplasm of brain (x1.67) 0.698
Secondary malignant neoplasm of brain and spinal cord (x1.19) 0.736
Disorder of brain (x1.18) 0.768
Primary malignant neoplasm of respiratory tract (x1.17) 0.799
Cerebral edema (x1.17) 0.827
Secondary malignant neoplasm of nervous system (x1.17) 0.855
Secondary malignant neoplasm of bone (x1.14) 0.872
Secondary malignant neoplasm of lung (x1.1) 0.882
Pain due to neoplastic disease (x1.09) 0.890
Primary malignant neoplasm (x1.08) 0.897
Abnormal findings on diagnostic imaging of lung (x1.08) 0.902
Secondary malignant neoplastic disease (x1.07) 0.907
Primary malignant neoplasm of upper lobe, bronchus or lung (x1.06) 0.911
Secondary malignant neoplasm of liver (x1.06) 0.914
Secondary malignant neoplasm of intrathoracic lymph nodes (x1.06) 0.917
Headache (x1.05) 0.920
Neoplasm of uncertain behavior of brain (x1.05) 0.923
Postoperative state (x1.05) 0.925
dexamethasone (+0.00436) 0.927
Malignant neoplastic disease (x1.05) 0.929
Pain (x1.05) 0.931
Secondary malignant neoplasm of right lung (x1.05) 0.933
Altered mental status (x1.04) 0.935
Malignant melanoma of skin (x1.04) 0.937
Asthenia (x1.04) 0.938
Secondary malignant neoplasm of left lung (x1.04) 0.940
Nausea (x1.04) 0.941
Primary malignant neoplasm of female breast (x1.04) 0.943
Primary malignant neoplasm of skin (x1.04) 0.944
White Race (+0.00353) 0.946
Secondary malignant neoplasm of unknown site (x1.04) 0.947
Essential hypertension (x1.04) 0.948
Secondary malignant neoplasm of lymph node (x1.04) 0.949
Anemia in neoplastic disease (x1.03) 0.951
Black Race (-0.00318) 0.952
oxycodone (+0.00314) 0.953
Intracranial space-occupying lesion (x1.03) 0.954
Solitary nodule of lung (x1.03) 0.955
Localized enlarged lymph nodes (x1.03) 0.956
Male Sex (-0.00306) 0.957
Female Sex (+0.00304) 0.958
ondansetron (+0.00294) 0.959
Hyperglycemia (x1.03) 0.960
ECG normal (x1.03) 0.961
Malaise (x1.03) 0.962
Cancer-related fatigue (x1.03) 0.963
Seizure (x1.03) 0.964
Fatigue (x1.03) 0.964
Primary malignant neoplasm of lower lobe, bronchus or lung (x1.03) 0.965
Muscle weakness (x1.03) 0.966
Swelling / lump finding (x1.03) 0.967
Secondary malignant neoplasm of respiratory and digestive systems (x1.03) 0.968
Nicotine dependence (x1.03) 0.968
Spontaneous cerebral hemorrhage (x1.03) 0.969
Lymphadenopathy (x1.02) 0.969
Disorder of head (x1.02) 0.970
Compression of brain (x1.02) 0.970
Primary malignant neoplasm of pleura (x1.02) 0.971
Neoplasm of intrathoracic lymph nodes (x1.02) 0.972
Lung field abnormal (x1.02) 0.972
Oxygen [PP] in Arterial blood (+4.01) 0.973
acetaminophen (+0.00206) 0.973
Anemia due to and following chemotherapy (x1.02) 0.973
promethazine (+0.00198) 0.974
Anxiety disorder (x1.02) 0.974
Neoplasm of lymph node (x1.02) 0.975
Finding of head and neck region (x1.02) 0.975
−0.8 −0.6 −0.4 −0.2 0.0 0.2 0.4 0.6 0.8
Expressions
10^5
10^3
10^1
−10 0 10

Signature [1233]; 2074 To Reach 0.975, (100 Plotted)

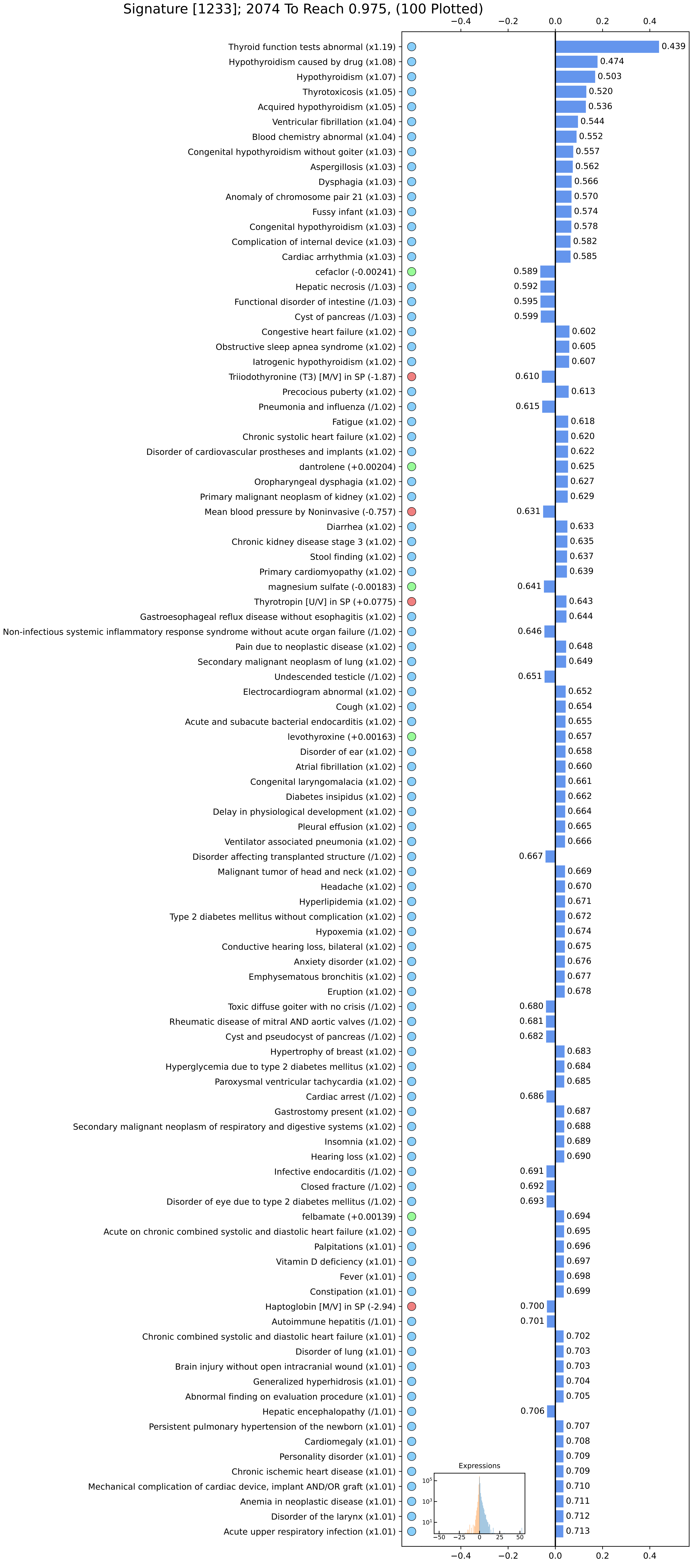

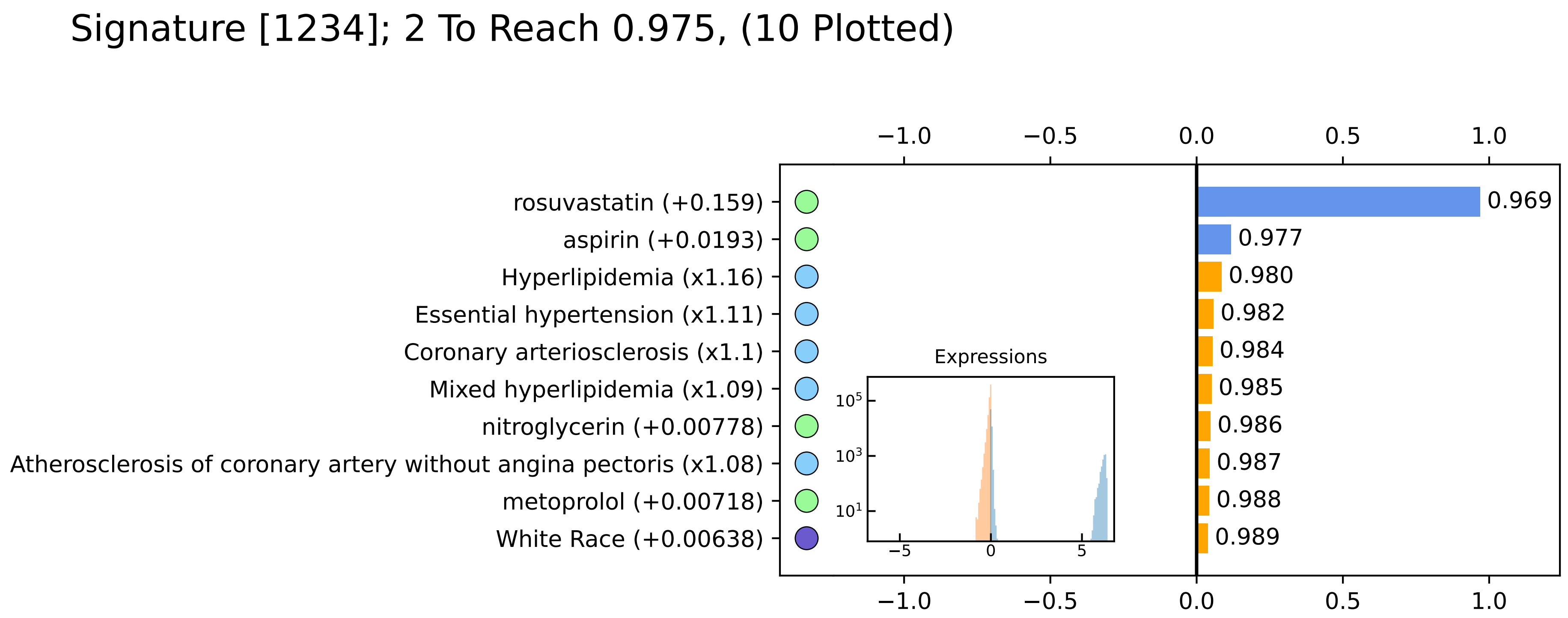
Signature [1234]; 2 To Reach 0.975, (10 Plotted)
rosuvastatin (+0.159)
aspirin (+0.0193)
Hyperlipidemia (x1.16)
Essential hypertension (x1.11)
Coronary arteriosclerosis (x1.1)
Mixed hyperlipidemia (x1.09)
nitroglycerin (+0.00778)
Atherosclerosis of coronary artery without angina pectoris (x1.08)
metoprolol (+0.00718)
White Race (+0.00638)
0.969
0.977
0.980
0.982
0.984
0.985
0.986
0.987
0.988
0.989
Expressions
−1.0
−0.5
0.0
0.5
1.0

Signature [1235]; 154 To Reach 0.975, (100 Plotted)

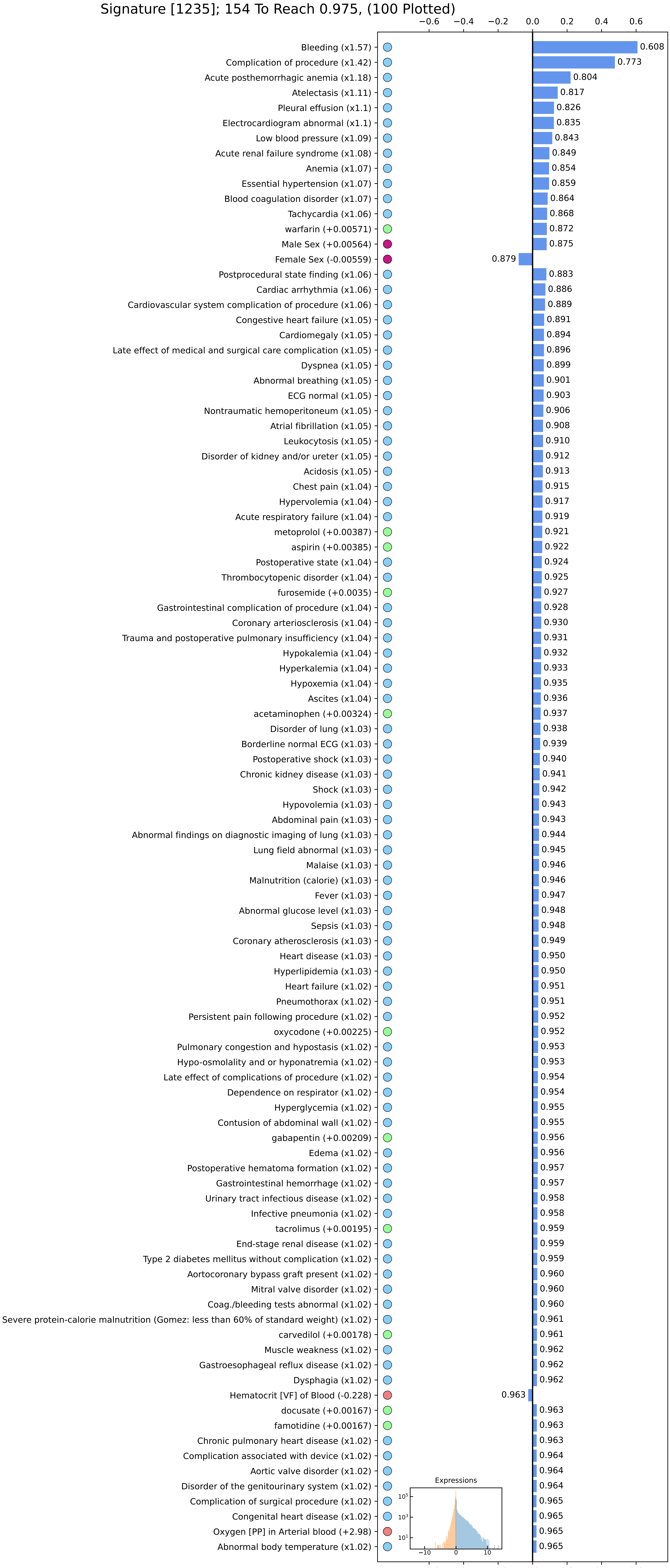

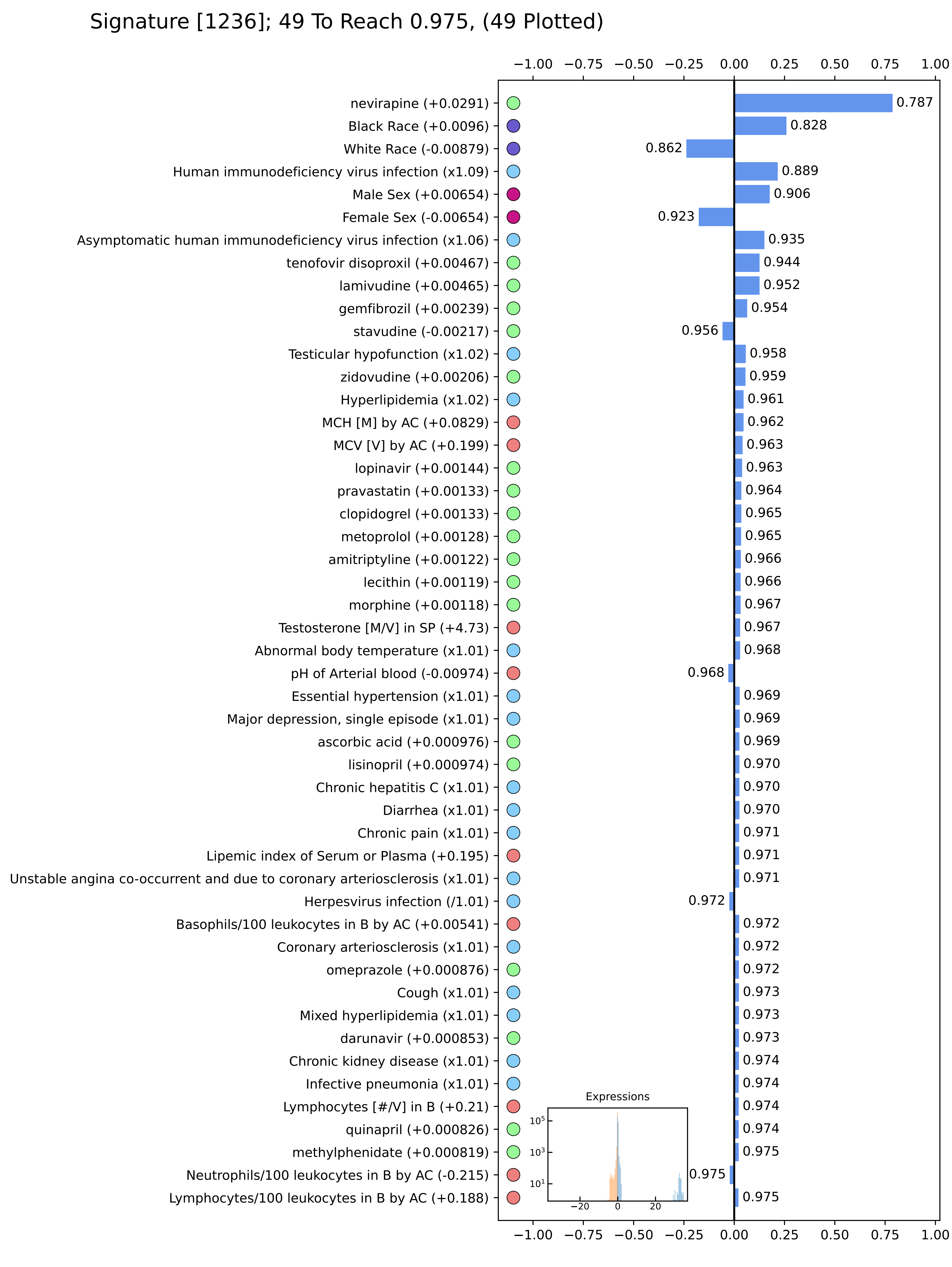

Signature [1236]; 49 To Reach 0.975, (49 Plotted)
nevirapine (+0.0291) 0.787
Black Race (+0.0096) 0.828
White Race (-0.00879) 0.862
Human immunodeficiency virus infection (x1.09) 0.889
Male Sex (+0.00654) 0.906
Female Sex (-0.00654) 0.923
Asymptomatic human immunodeficiency virus infection (x1.06) 0.935
tenofovir disoproxil (+0.00467) 0.944
lamivudine (+0.00465) 0.952
gemfibrozil (+0.00239) 0.954
stavudine (-0.00217) 0.956
Testicular hypofunction (x1.02) 0.958
zidovudine (+0.00206) 0.959
Hyperlipidemia (x1.02) 0.961
MCH [M] by AC (+0.0829) 0.962
MCV [V] by AC (+0.199) 0.963
lopinavir (+0.00144) 0.963
pravastatin (+0.00133) 0.964
clopidogrel (+0.00133) 0.965
metoprolol (+0.00128) 0.965
amitriptyline (+0.00122) 0.966
lecithin (+0.00119) 0.966
morphine (+0.00118) 0.967
Testosterone [M/V] in SP (+4.73) 0.967
Abnormal body temperature (x1.01) 0.968
pH of Arterial blood (-0.00974) 0.968
Essential hypertension (x1.01) 0.969
Major depression, single episode (x1.01) 0.969
ascorbic acid (+0.000976) 0.969
lisinopril (+0.000974) 0.970
Chronic hepatitis C (x1.01) 0.970
Diarrhea (x1.01) 0.970
Chronic pain (x1.01) 0.971
Lipemic index of Serum or Plasma (+0.195) 0.971
Unstable angina co-occurrent and due to coronary arteriosclerosis (x1.01) 0.971
Herpesvirus infection (/1.01) 0.972
Basophils/100 leukocytes in B by AC (+0.00541) 0.972
Coronary arteriosclerosis (x1.01) 0.972
omeprazole (+0.000876) 0.972
Cough (x1.01) 0.973
Mixed hyperlipidemia (x1.01) 0.973
darunavir (+0.000853) 0.973
Chronic kidney disease (x1.01) 0.974
Infective pneumonia (x1.01) 0.974
Lymphocytes [#/V] in B (+0.21) 0.974
quinapril (+0.000826) 0.974
methylphenidate (+0.000819) 0.975
Neutrophils/100 leukocytes in B by AC (-0.215) 0.975
Lymphocytes/100 leukocytes in B by AC (+0.188) 0.975
−1.00 −0.75 −0.50 −0.25 0.00 0.25 0.50 0.75 1.00
Expressions
10^5
10^3
10^1
−20 0 20

Signature [1237]; 84 To Reach 0.975, (84 Plotted)

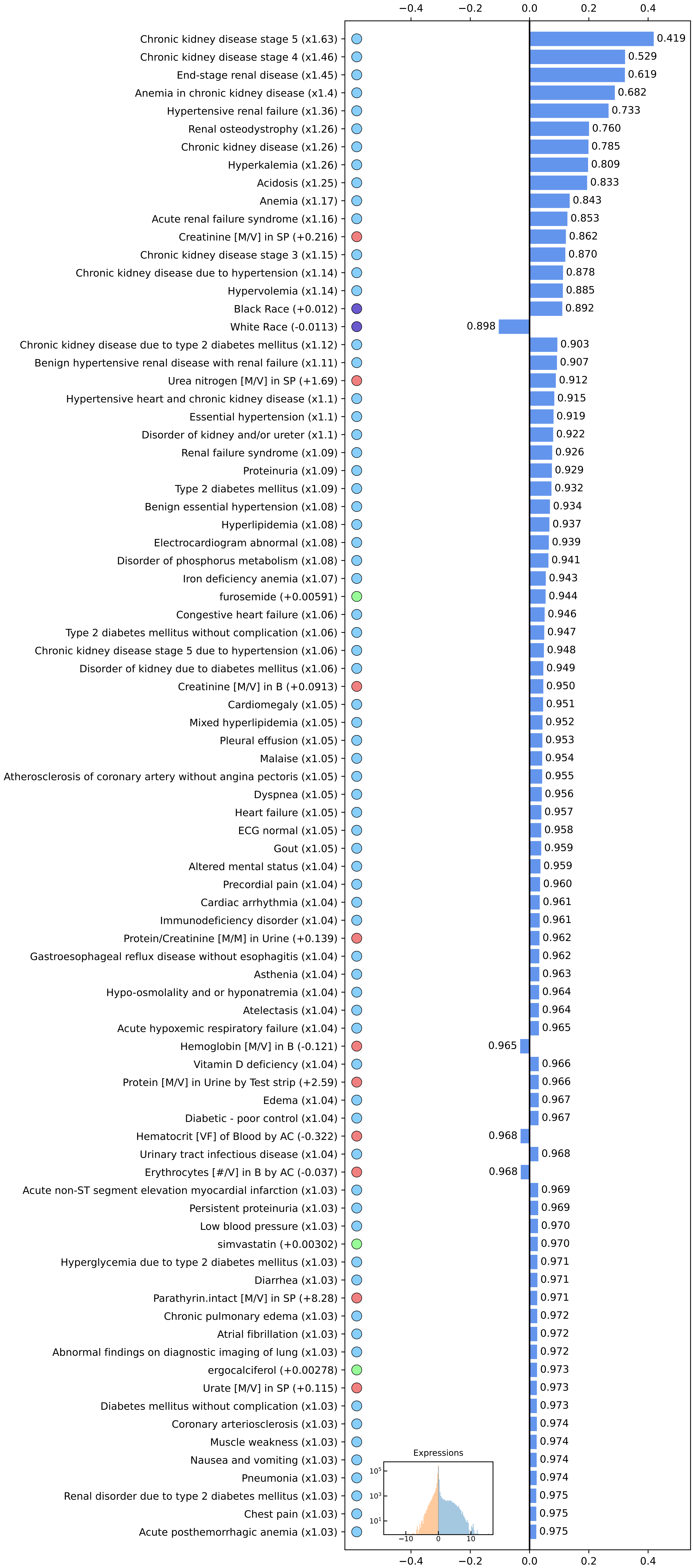

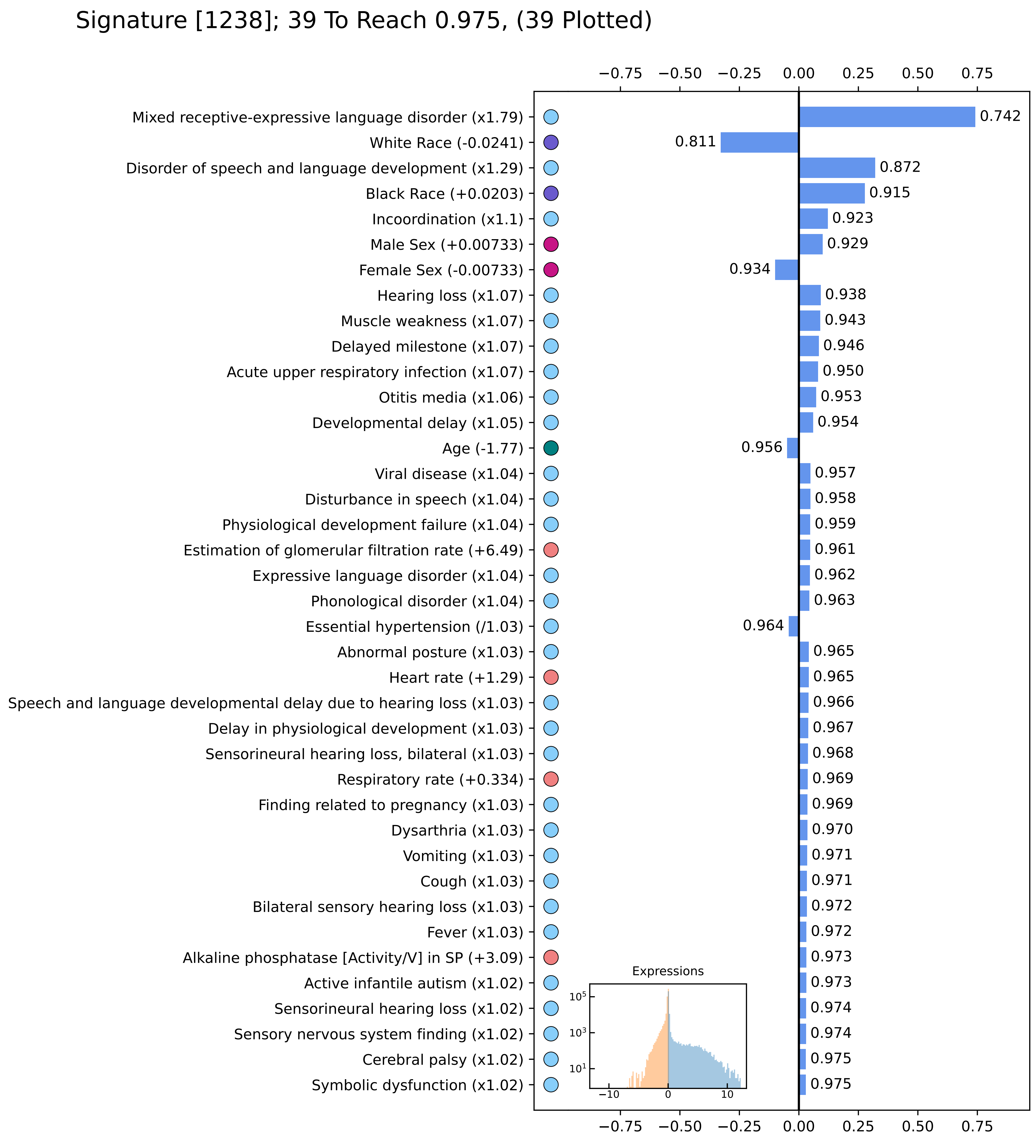
Signature [1238]; 39 To Reach 0.975, (39 Plotted)
Mixed receptive-expressive language disorder (x1.79) 0.742
White Race (-0.0241) 0.811
Disorder of speech and language development (x1.29) 0.872
Black Race (+0.0203) 0.915
Incoordination (x1.1) 0.923
Male Sex (+0.00733) 0.929
Female Sex (-0.00733) 0.934
Hearing loss (x1.07) 0.938
Muscle weakness (x1.07) 0.943
Delayed milestone (x1.07) 0.946
Acute upper respiratory infection (x1.07) 0.950
Otitis media (x1.06) 0.953
Developmental delay (x1.05) 0.954
Age (-1.77) 0.956
Viral disease (x1.04) 0.957
Disturbance in speech (x1.04) 0.958
Physiological development failure (x1.04) 0.959
Estimation of glomerular filtration rate (+6.49) 0.961
Expressive language disorder (x1.04) 0.962
Phonological disorder (x1.04) 0.963
Essential hypertension (/1.03) 0.964
Abnormal posture (x1.03) 0.965
Heart rate (+1.29) 0.965
Speech and language developmental delay due to hearing loss (x1.03) 0.966
Delay in physiological development (x1.03) 0.967
Sensorineural hearing loss, bilateral (x1.03) 0.968
Respiratory rate (+0.334) 0.969
Finding related to pregnancy (x1.03) 0.969
Dysarthria (x1.03) 0.970
Vomiting (x1.03) 0.971
Cough (x1.03) 0.971
Bilateral sensory hearing loss (x1.03) 0.972
Fever (x1.03) 0.972
Alkaline phosphatase [Activity/V] in SP (+3.09) 0.973
Active infantile autism (x1.02) 0.973
Sensorineural hearing loss (x1.02) 0.974
Sensory nervous system finding (x1.02) 0.974
Cerebral palsy (x1.02) 0.975
Symbolic dysfunction (x1.02) 0.975
−0.75 −0.50 −0.25 0.00 0.25 0.50 0.75
Expressions
10^5
10^3
10^1
−10 0 10

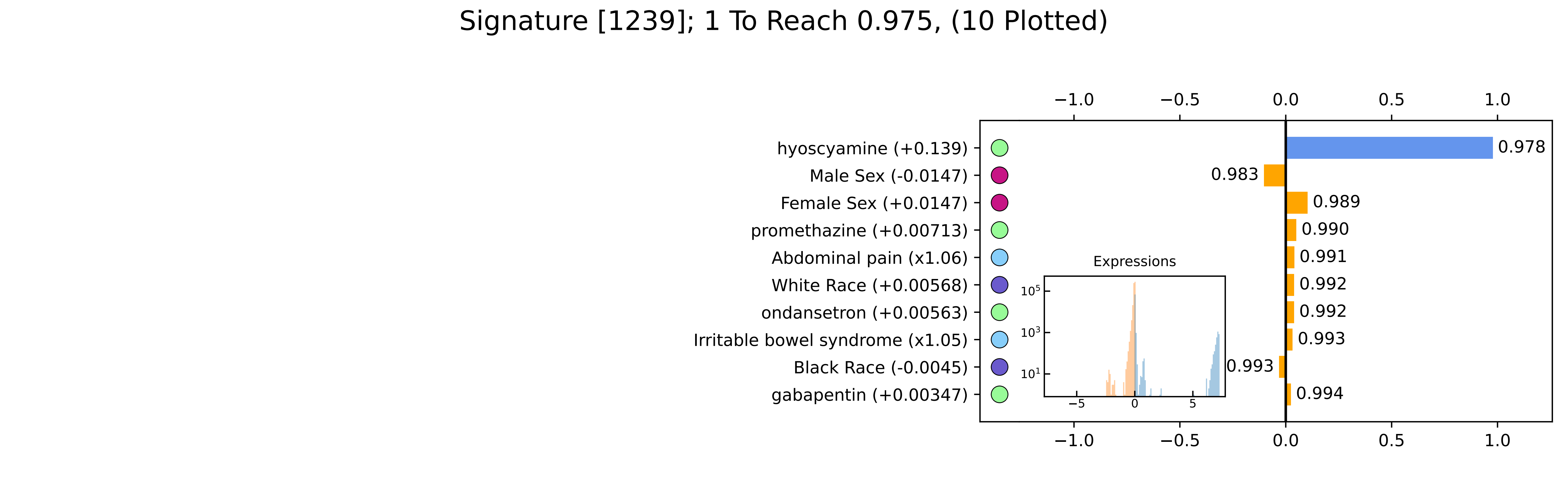
Signature [1239]; 1 To Reach 0.975, (10 Plotted)
hyoscyamine (+0.139)
Male Sex (-0.0147)
Female Sex (+0.0147)
promethazine (+0.00713)
Abdominal pain (x1.06)
White Race (+0.00568)
ondansetron (+0.00563)
Irritable bowel syndrome (x1.05)
Black Race (-0.0045)
gabapentin (+0.00347)
0.978
0.983
0.989
0.990
0.991
0.992
0.992
0.993
0.993
0.994
Expressions
−1.0
−0.5
0.0
0.5
1.0

Signature [1240]; 1 To Reach 0.975, (10 Plotted)

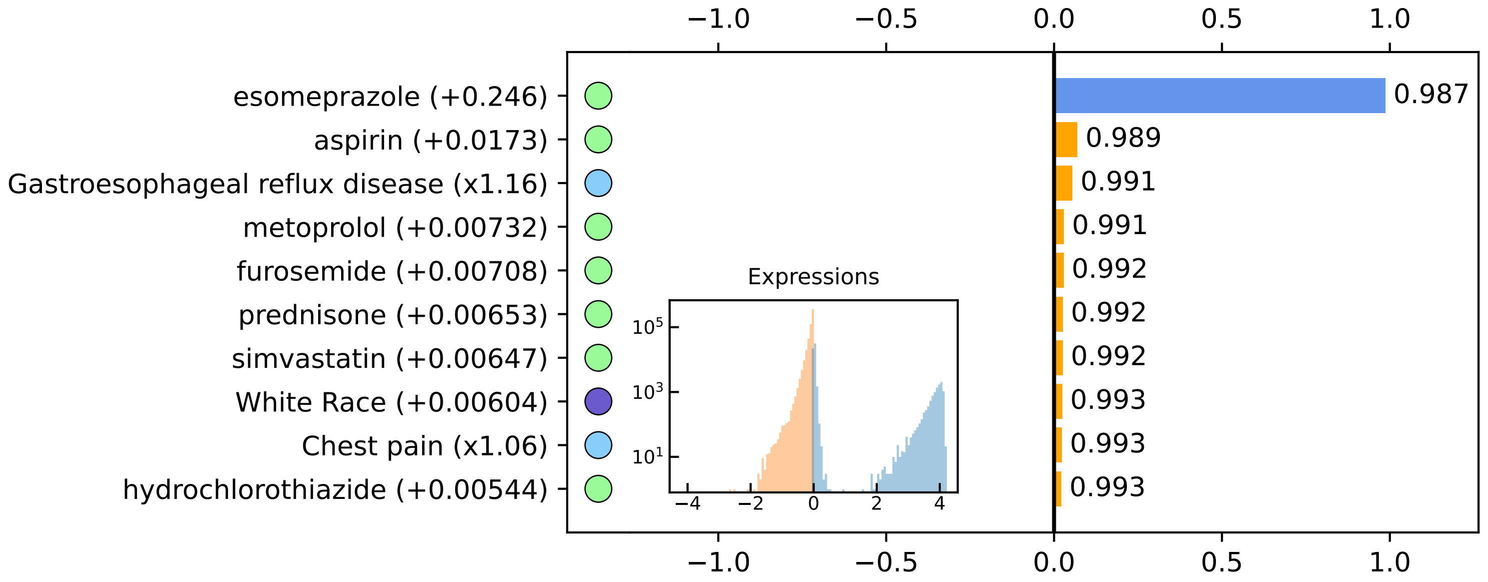

Signature [1241]; 59 To Reach 0.975, (59 Plotted)

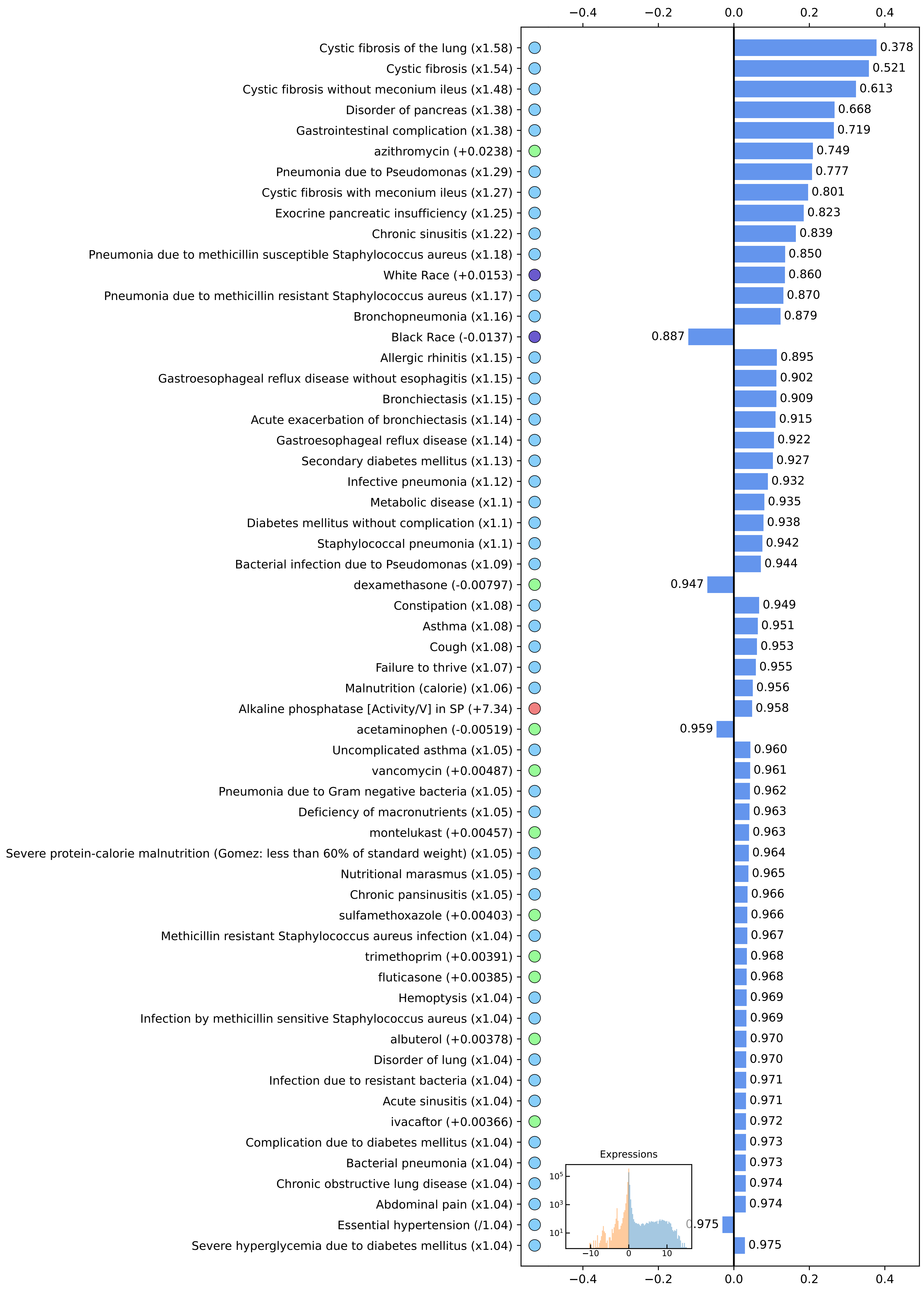

Signature [1242]; 53 To Reach 0.975, (53 Plotted)

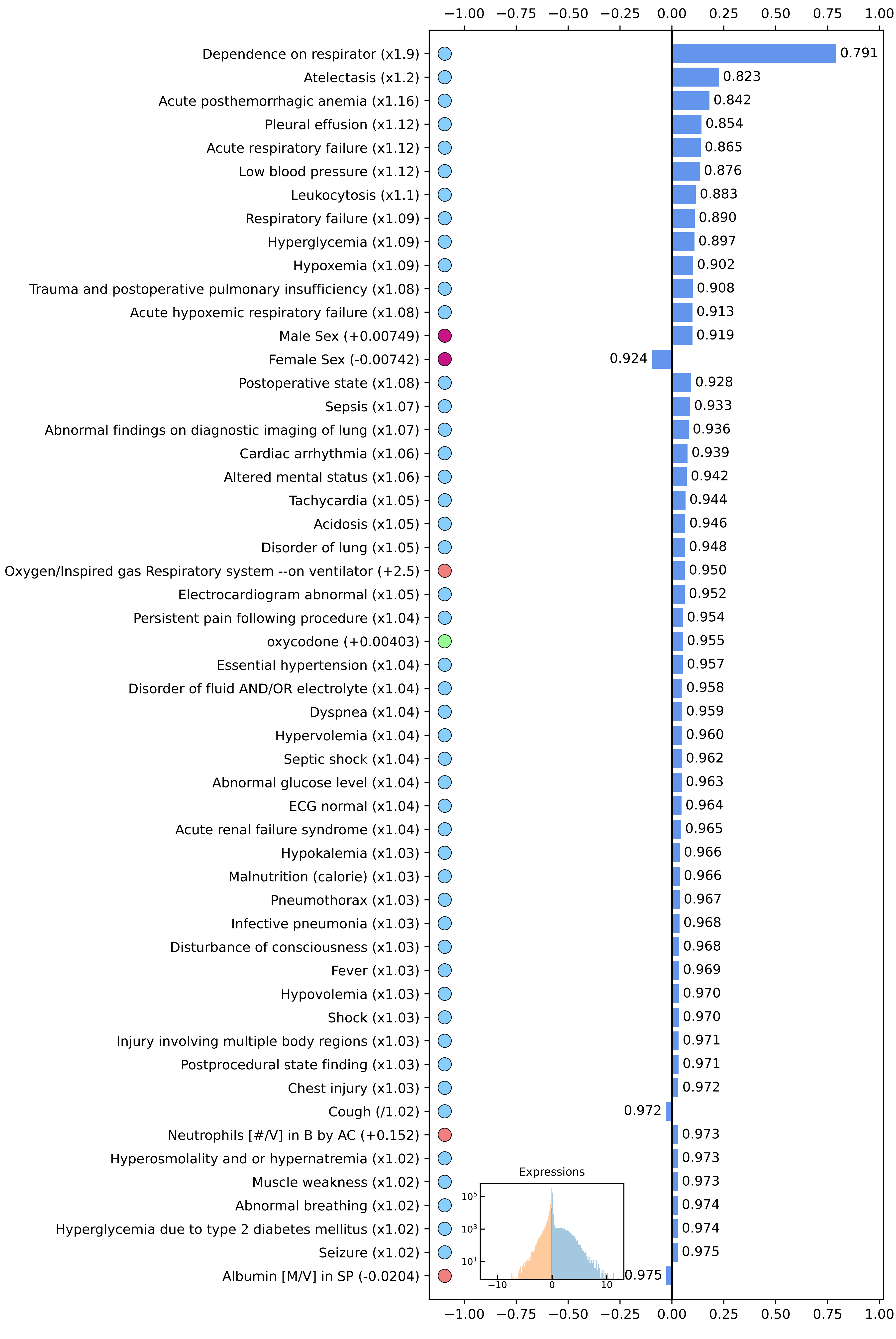

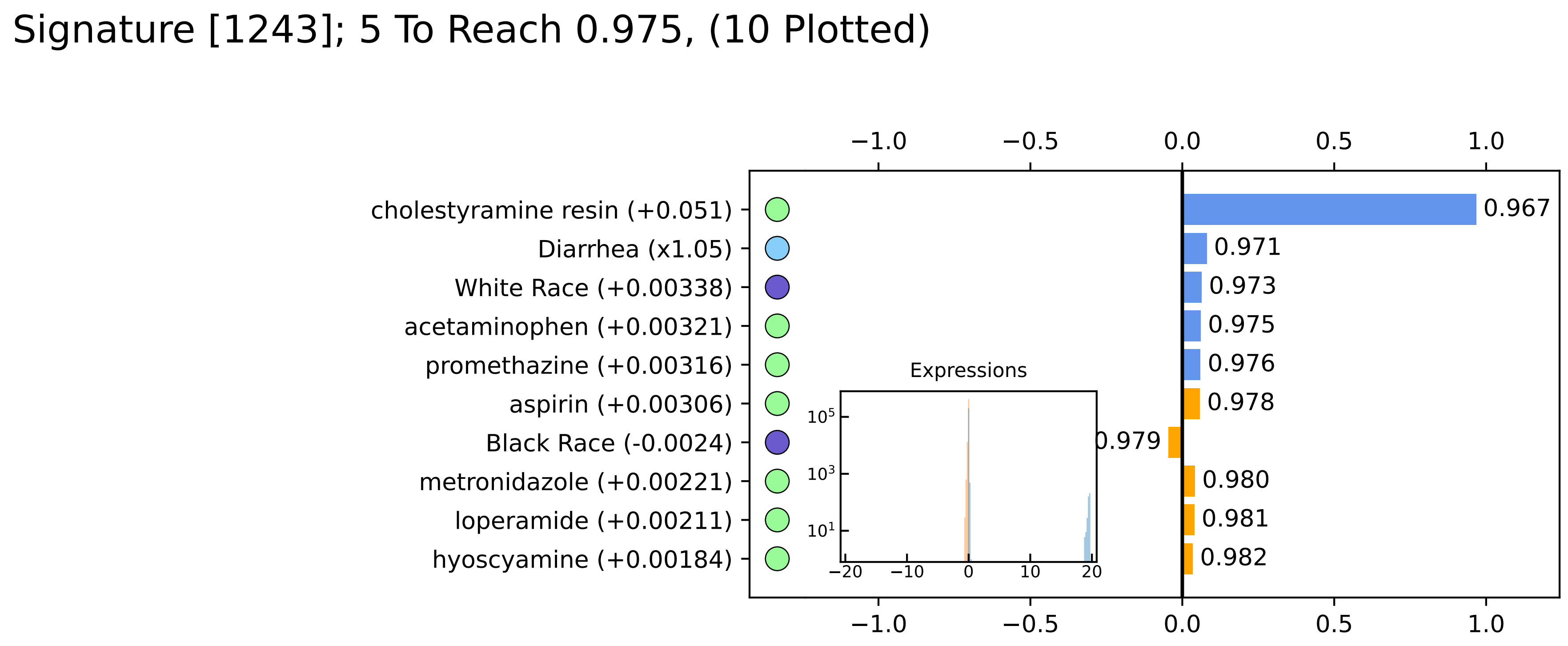

Signature [1243]; 5 To Reach 0.975, (10 Plotted)
cholestyramine resin (+0.051)
Diarrhea (x1.05)
White Race (+0.00338)
acetaminophen (+0.00321)
promethazine (+0.00316)
aspirin (+0.00306)
Black Race (-0.0024)
metronidazole (+0.00221)
loperamide (+0.00211)
hyoscyamine (+0.00184)
0.967
0.971
0.973
0.975
0.976
0.978
0.979
0.980
0.981
0.982
Expressions
−1.0
−0.5
0.0
0.5
1.0

Signature [1244]; 8 To Reach 0.975, (10 Plotted)

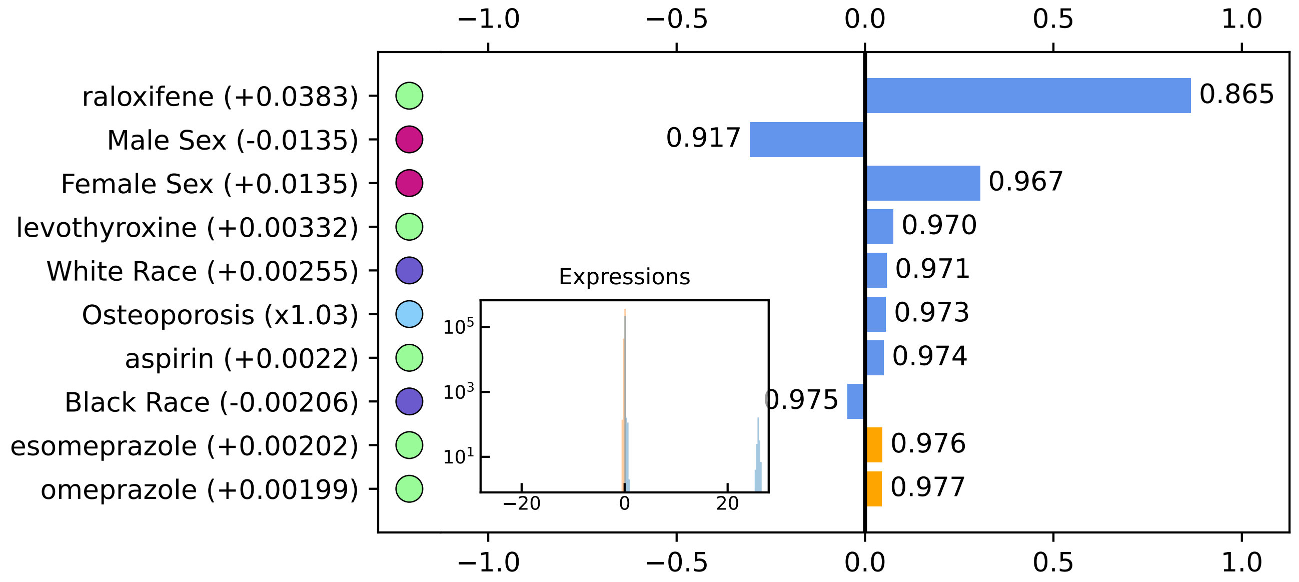

Signature [1245]; 175 To Reach 0.975, (100 Plotted)

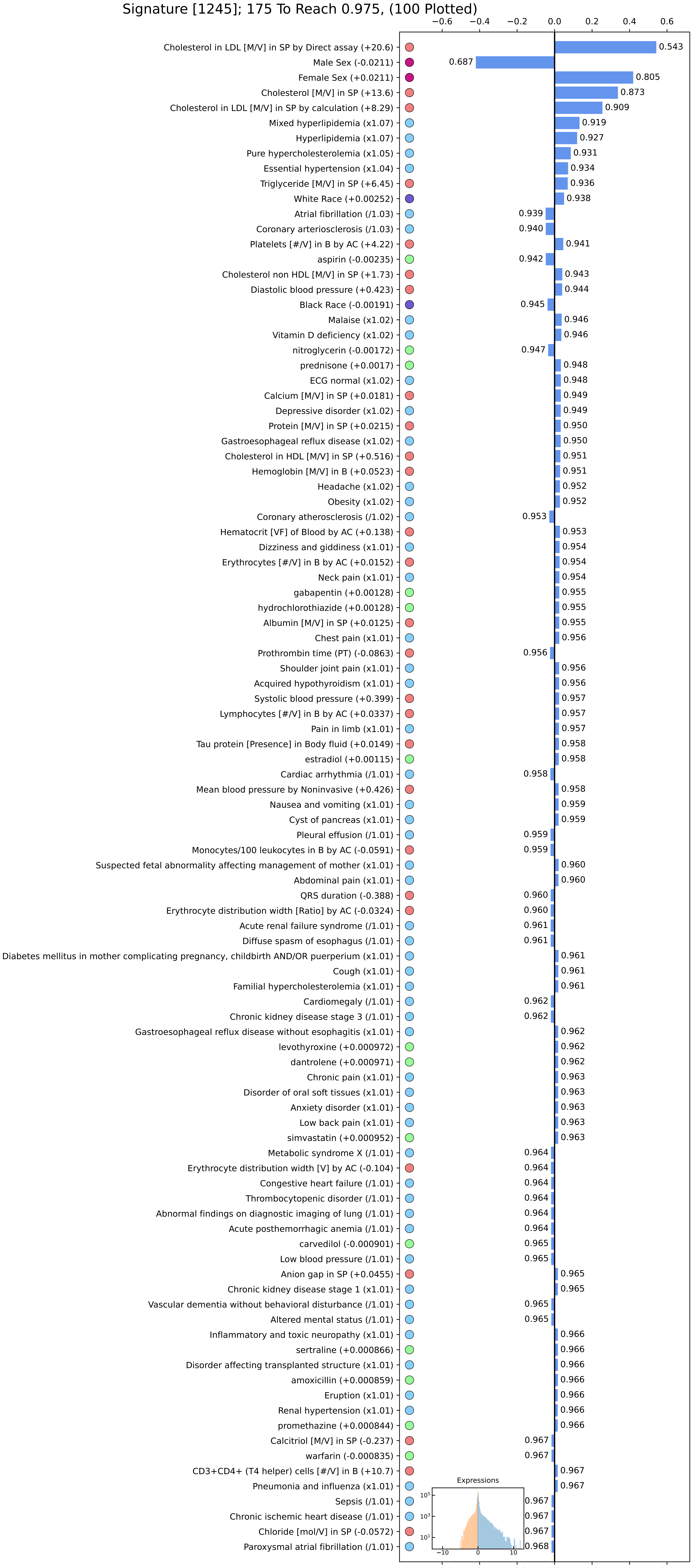

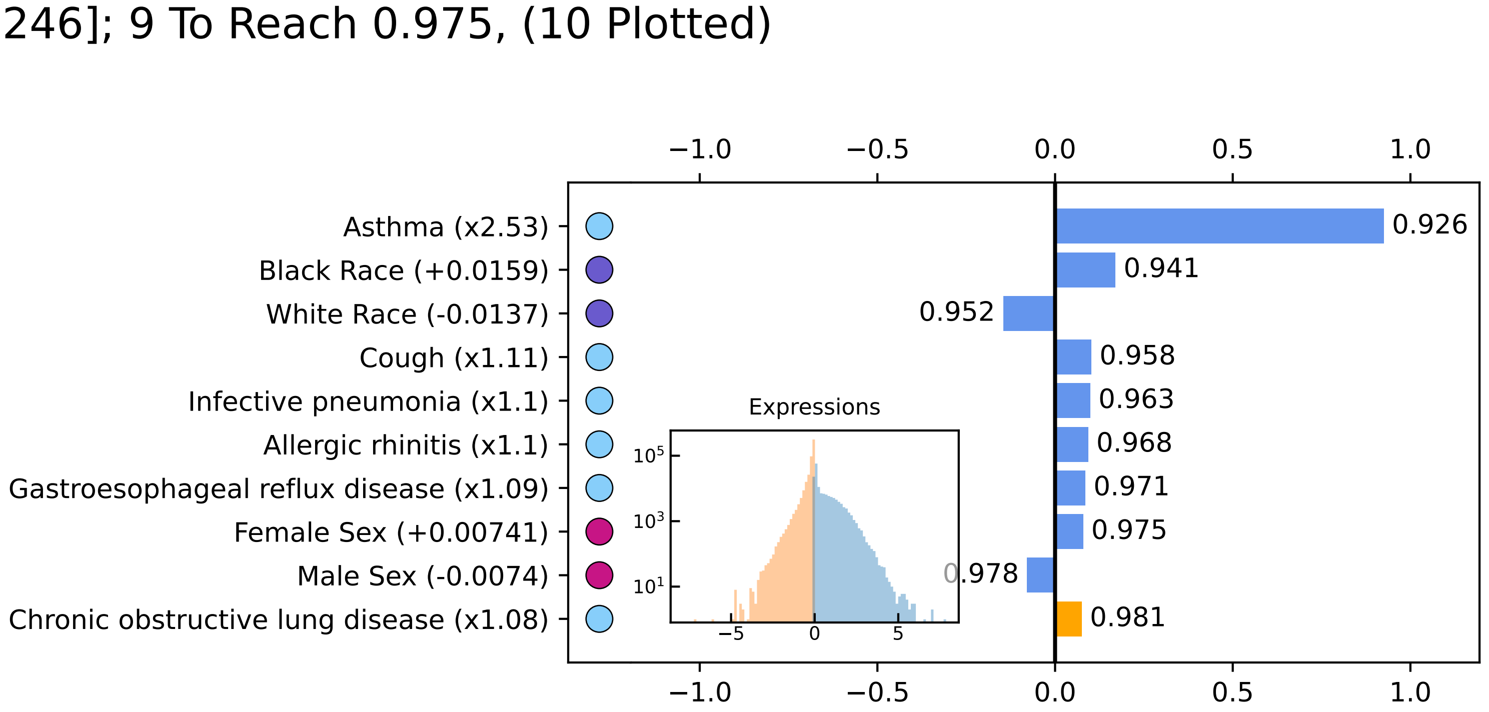

Signature [1246]; 9 To Reach 0.975, (10 Plotted)
−1.0
−0.5
0.0
0.5
1.0
Asthma (x2.53)
Black Race (+0.0159)
White Race (-0.0137)
Cough (x1.11)
Infective pneumonia (x1.1)
Allergic rhinitis (x1.1)
Gastroesophageal reflux disease (x1.09)
Female Sex (+0.00741)
Male Sex (-0.0074)
Chronic obstructive lung disease (x1.08)
0.926
0.941
0.952
0.958
0.963
0.968
0.971
0.975
0.978
0.981
Expressions
10^5
10^3
10^1
−5
0
5

Signature [1247]; 5 To Reach 0.975, (10 Plotted)

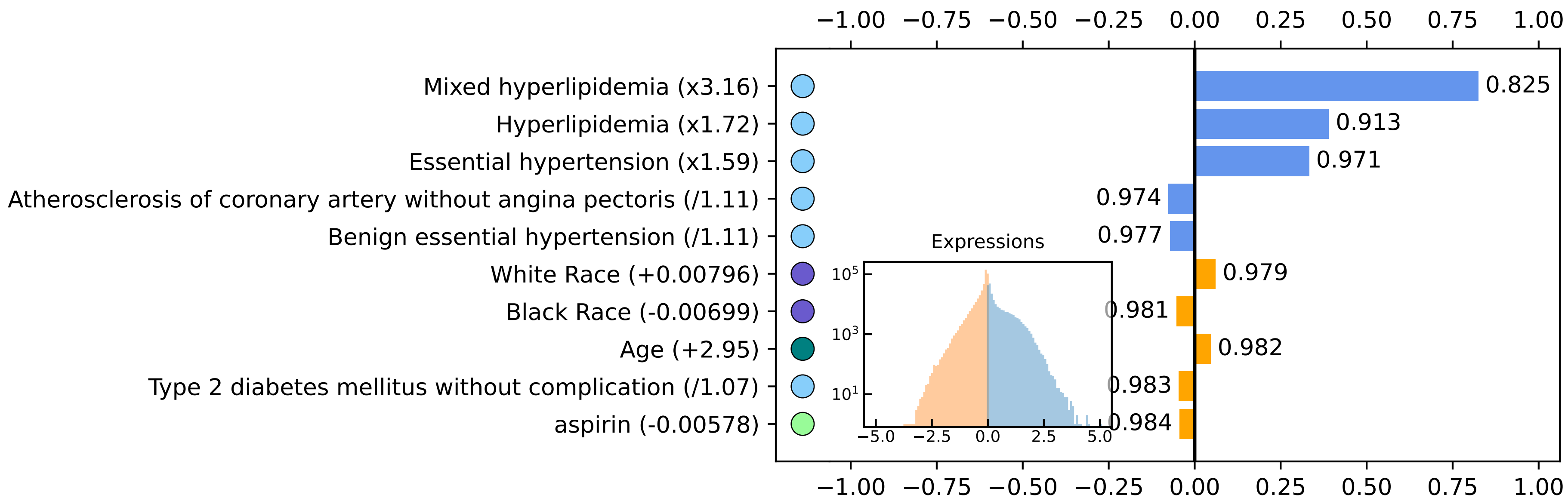

Signature [1248]; 257 To Reach 0.975, (100 Plotted)

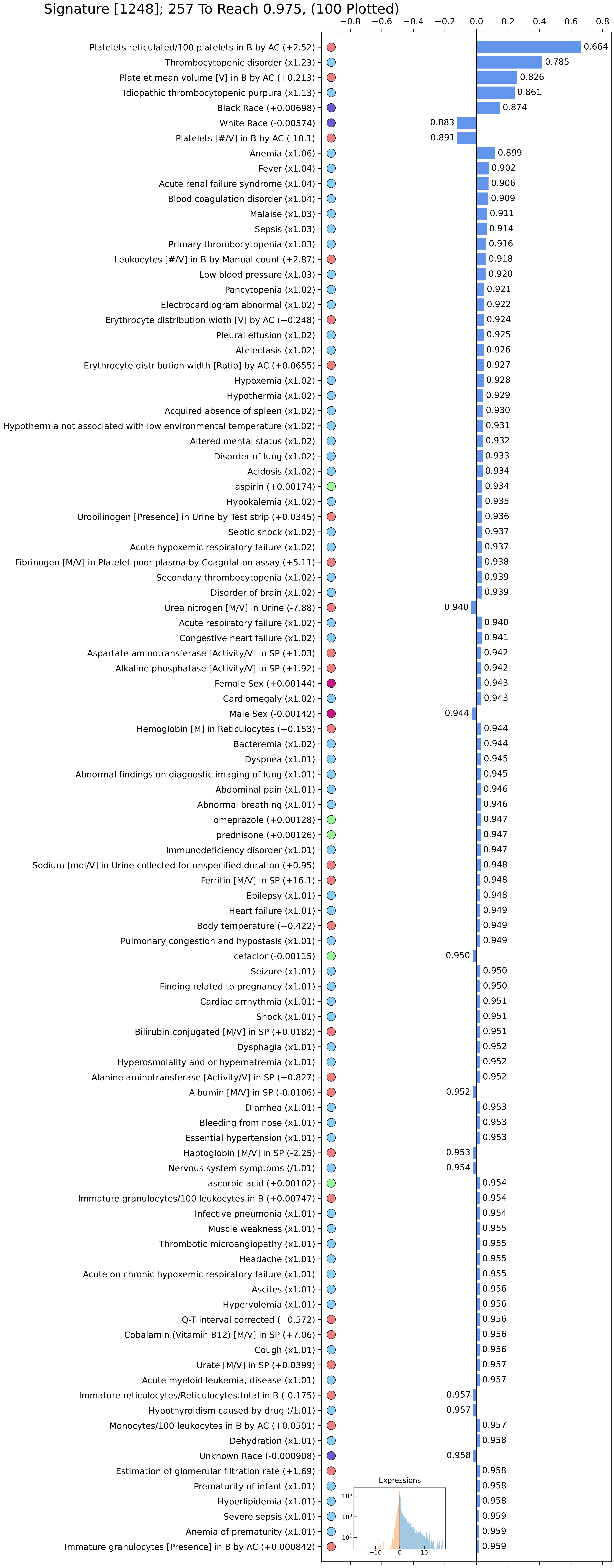

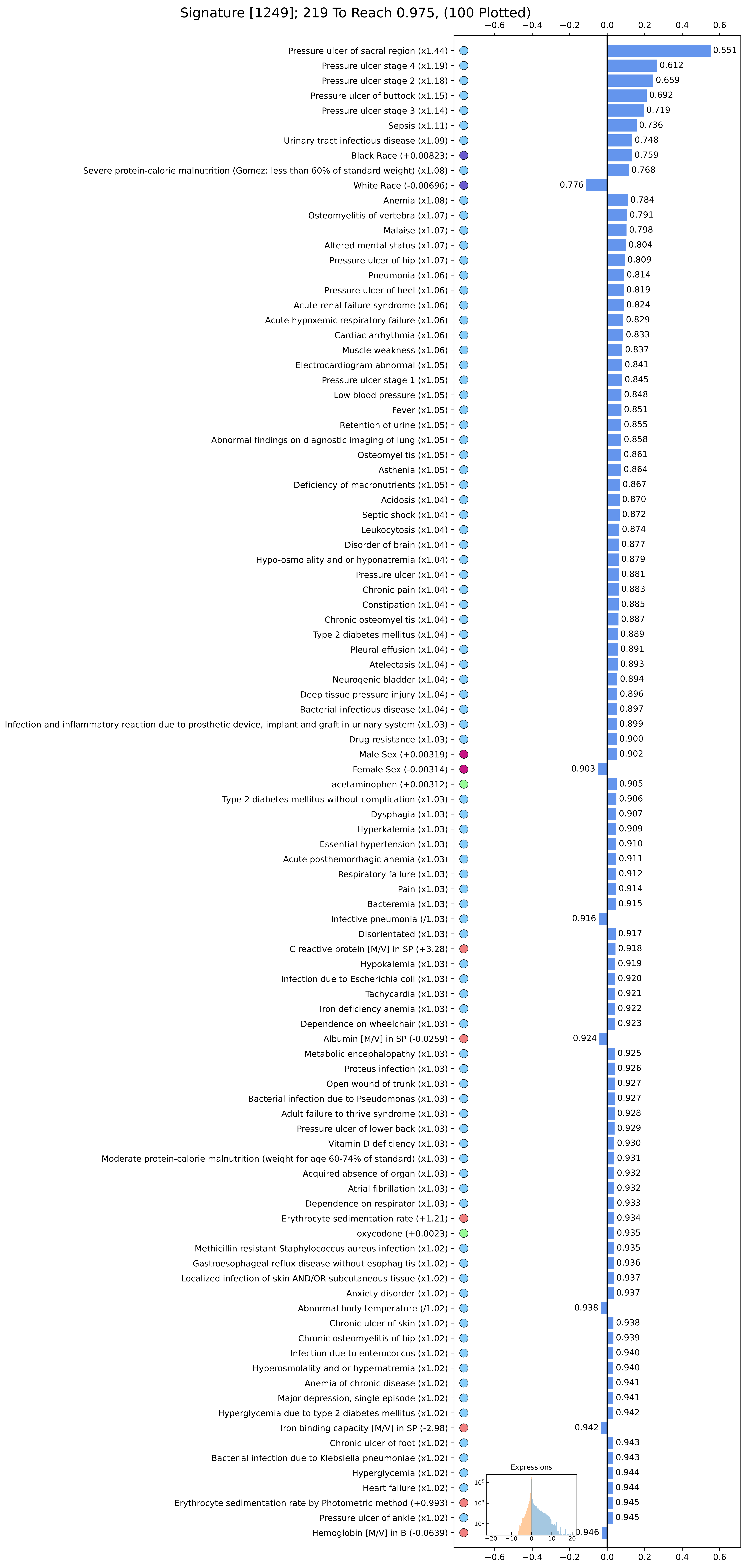
Signature [1249]; 219 To Reach 0.975, (100 Plotted)
Pressure ulcer of sacral region (x1.44) 0.551
Pressure ulcer stage 4 (x1.19) 0.612
Pressure ulcer stage 2 (x1.18) 0.659
Pressure ulcer of buttock (x1.15) 0.692
Pressure ulcer stage 3 (x1.14) 0.719
Sepsis (x1.11) 0.736
Urinary tract infectious disease (x1.09) 0.748
Black Race (+0.00823) 0.759
Severe protein-calorie malnutrition (Gomez: less than 60% of standard weight) (x1.08) 0.768
White Race (-0.00696) 0.776
Anemia (x1.08) 0.784
Osteomyelitis of vertebra (x1.07) 0.791
Malaise (x1.07) 0.798
Altered mental status (x1.07) 0.804
Pressure ulcer of hip (x1.07) 0.809
Pneumonia (x1.06) 0.814
Pressure ulcer of heel (x1.06) 0.819
Acute renal failure syndrome (x1.06) 0.824
Acute hypoxemic respiratory failure (x1.06) 0.829
Cardiac arrhythmia (x1.06) 0.833
Muscle weakness (x1.06) 0.837
Electrocardiogram abnormal (x1.05) 0.841
Pressure ulcer stage 1 (x1.05) 0.845
Low blood pressure (x1.05) 0.848
Fever (x1.05) 0.851
Retention of urine (x1.05) 0.855
Abnormal findings on diagnostic imaging of lung (x1.05) 0.858
Osteomyelitis (x1.05) 0.861
Asthenia (x1.05) 0.864
Deficiency of macronutrients (x1.05) 0.867
Acidosis (x1.04) 0.870
Septic shock (x1.04) 0.872
Leukocytosis (x1.04) 0.874
Disorder of brain (x1.04) 0.877
Hypo-osmolality and or hyponatremia (x1.04) 0.879
Pressure ulcer (x1.04) 0.881
Chronic pain (x1.04) 0.883
Constipation (x1.04) 0.885
Chronic osteomyelitis (x1.04) 0.887
Type 2 diabetes mellitus (x1.04) 0.889
Pleural effusion (x1.04) 0.891
Atelectasis (x1.04) 0.893
Neurogenic bladder (x1.04) 0.894
Deep tissue pressure injury (x1.04) 0.896
Bacterial infectious disease (x1.04) 0.897
Infection and inflammatory reaction due to prosthetic device, implant and graft in urinary system (x1.03) 0.899
Drug resistance (x1.03) 0.900
Male Sex (+0.00319) 0.902
Female Sex (-0.00314) 0.903
acetaminophen (+0.00312) 0.905
Type 2 diabetes mellitus without complication (x1.03) 0.906
Dysphagia (x1.03) 0.907
Hyperkalemia (x1.03) 0.909
Essential hypertension (x1.03) 0.910
Acute posthemorrhagic anemia (x1.03) 0.911
Respiratory failure (x1.03) 0.912
Pain (x1.03) 0.914
Bacteremia (x1.03) 0.915
Infective pneumonia (/1.03) 0.916
Disorientated (x1.03) 0.917
C reactive protein [M/V] in SP (+3.28) 0.918
Hypokalemia (x1.03) 0.919
Infection due to Escherichia coli (x1.03) 0.920
Tachycardia (x1.03) 0.921
Iron deficiency anemia (x1.03) 0.922
Dependence on wheelchair (x1.03) 0.923
Albumin [M/V] in SP (-0.0259) 0.924
Metabolic encephalopathy (x1.03) 0.925
Proteus infection (x1.03) 0.926
Open wound of trunk (x1.03) 0.927
Bacterial infection due to Pseudomonas (x1.03) 0.927
Adult failure to thrive syndrome (x1.03) 0.928
Pressure ulcer of lower back (x1.03) 0.929
Vitamin D deficiency (x1.03) 0.930
Moderate protein-calorie malnutrition (weight for age 60-74% of standard) (x1.03) 0.931
Acquired absence of organ (x1.03) 0.932
Atrial fibrillation (x1.03) 0.932
Dependence on respirator (x1.03) 0.933
Erythrocyte sedimentation rate (+1.21) 0.934
oxycodone (+0.0023) 0.935
Methicillin resistant Staphylococcus aureus infection (x1.02) 0.935
Gastroesophageal reflux disease without esophagitis (x1.02) 0.936
Localized infection of skin AND/OR subcutaneous tissue (x1.02) 0.937
Anxiety disorder (x1.02) 0.937
Abnormal body temperature (/1.02) 0.938
Chronic ulcer of skin (x1.02) 0.938
Chronic osteomyelitis of hip (x1.02) 0.939
Infection due to enterococcus (x1.02) 0.940
Hyperosmolality and or hypernatremia (x1.02) 0.940
Anemia of chronic disease (x1.02) 0.941
Major depression, single episode (x1.02) 0.941
Hyperglycemia due to type 2 diabetes mellitus (x1.02) 0.942
Iron binding capacity [M/V] in SP (-2.98) 0.942
Chronic ulcer of foot (x1.02) 0.943
Bacterial infection due to Klebsiella pneumoniae (x1.02) 0.943
Hyperglycemia (x1.02) 0.944
Heart failure (x1.02) 0.944
Erythrocyte sedimentation rate by Photometric method (+0.993) 0.945
Pressure ulcer of ankle (x1.02) 0.945
Hemoglobin [M/V] in B (-0.0639) 0.946
Expressions
−0.6 −0.4 −0.2 0.0 0.2 0.4 0.6

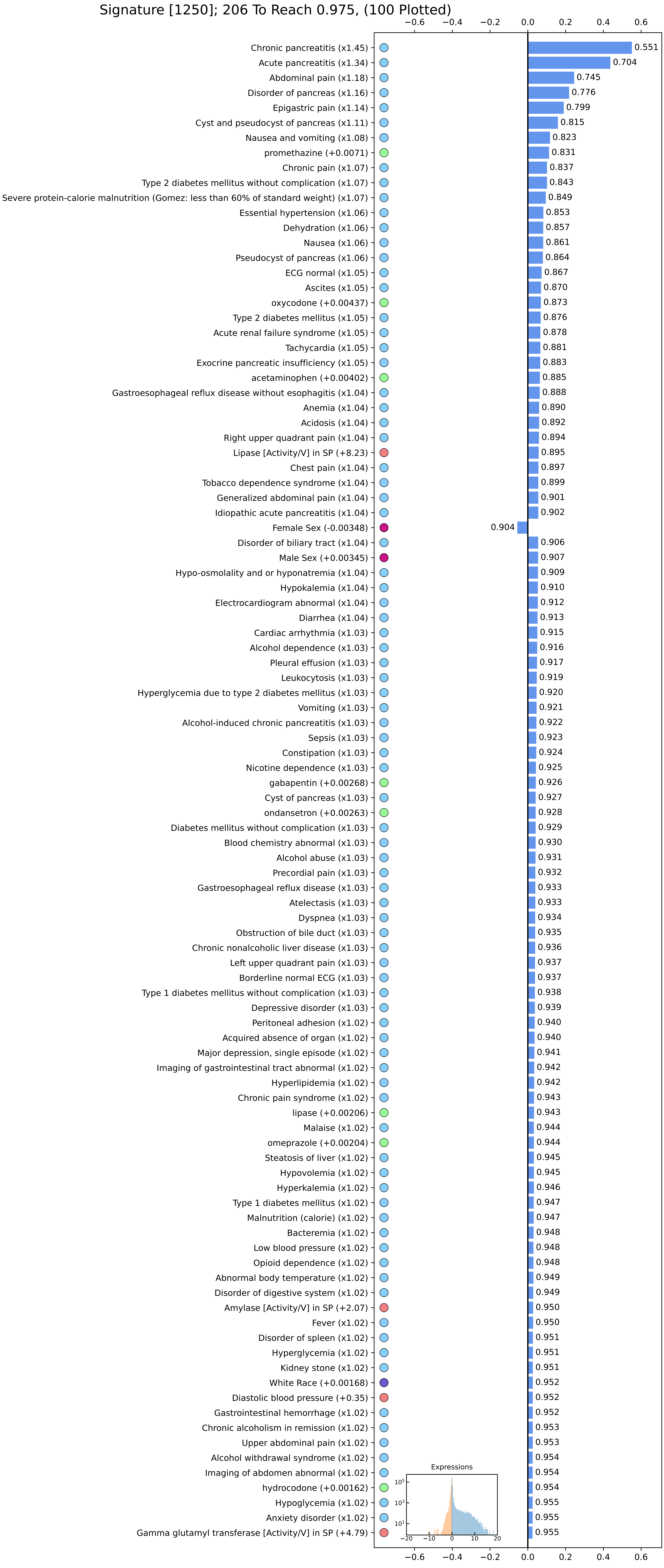
Signature [1250]; 206 To Reach 0.975, (100 Plotted)
−0.6 −0.4 −0.2 0.0 0.2 0.4 0.6
Chronic pancreatitis (x1.45) 0.551
Acute pancreatitis (x1.34) 0.704
Abdominal pain (x1.18) 0.745
Disorder of pancreas (x1.16) 0.776
Epigastric pain (x1.14) 0.799
Cyst and pseudocyst of pancreas (x1.11) 0.815
Nausea and vomiting (x1.08) 0.823
promethazine (+0.0071) 0.831
Chronic pain (x1.07) 0.837
Type 2 diabetes mellitus without complication (x1.07) 0.843
Severe protein-calorie malnutrition (Gomez: less than 60% of standard weight) (x1.07) 0.849
Essential hypertension (x1.06) 0.853
Dehydration (x1.06) 0.857
Nausea (x1.06) 0.861
Pseudocyst of pancreas (x1.06) 0.864
ECG normal (x1.05) 0.867
Ascites (x1.05) 0.870
oxycodone (+0.00437) 0.873
Type 2 diabetes mellitus (x1.05) 0.876
Acute renal failure syndrome (x1.05) 0.878
Tachycardia (x1.05) 0.881
Exocrine pancreatic insufficiency (x1.05) 0.883
acetaminophen (+0.00402) 0.885
Gastroesophageal reflux disease without esophagitis (x1.04) 0.888
Anemia (x1.04) 0.890
Acidosis (x1.04) 0.892
Right upper quadrant pain (x1.04) 0.894
Lipase [Activity/V] in SP (+8.23) 0.895
Chest pain (x1.04) 0.897
Tobacco dependence syndrome (x1.04) 0.899
Generalized abdominal pain (x1.04) 0.901
Idiopathic acute pancreatitis (x1.04) 0.902
Female Sex (-0.00348) 0.904
Disorder of biliary tract (x1.04) 0.906
Male Sex (+0.00345) 0.907
Hypo-osmolality and or hyponatremia (x1.04) 0.909
Hypokalemia (x1.04) 0.910
Electrocardiogram abnormal (x1.04) 0.912
Diarrhea (x1.04) 0.913
Cardiac arrhythmia (x1.03) 0.915
Alcohol dependence (x1.03) 0.916
Pleural effusion (x1.03) 0.917
Leukocytosis (x1.03) 0.919
Hyperglycemia due to type 2 diabetes mellitus (x1.03) 0.920
Vomiting (x1.03) 0.921
Alcohol-induced chronic pancreatitis (x1.03) 0.922
Sepsis (x1.03) 0.923
Constipation (x1.03) 0.924
Nicotine dependence (x1.03) 0.925
gabapentin (+0.00268) 0.926
Cyst of pancreas (x1.03) 0.927
ondansetron (+0.00263) 0.928
Diabetes mellitus without complication (x1.03) 0.929
Blood chemistry abnormal (x1.03) 0.930
Alcohol abuse (x1.03) 0.931
Precordial pain (x1.03) 0.932
Gastroesophageal reflux disease (x1.03) 0.933
Atelectasis (x1.03) 0.933
Dyspnea (x1.03) 0.934
Obstruction of bile duct (x1.03) 0.935
Chronic nonalcoholic liver disease (x1.03) 0.936
Left upper quadrant pain (x1.03) 0.937
Borderline normal ECG (x1.03) 0.937
Type 1 diabetes mellitus without complication (x1.03) 0.938
Depressive disorder (x1.03) 0.939
Peritoneal adhesion (x1.02) 0.940
Acquired absence of organ (x1.02) 0.940
Major depression, single episode (x1.02) 0.941
Imaging of gastrointestinal tract abnormal (x1.02) 0.942
Hyperlipidemia (x1.02) 0.942
Chronic pain syndrome (x1.02) 0.943
lipase (+0.00206) 0.943
Malaise (x1.02) 0.944
omeprazole (+0.00204) 0.944
Steatosis of liver (x1.02) 0.945
Hypovolemia (x1.02) 0.945
Hyperkalemia (x1.02) 0.946
Type 1 diabetes mellitus (x1.02) 0.947
Malnutrition (calorie) (x1.02) 0.947
Bacteremia (x1.02) 0.948
Low blood pressure (x1.02) 0.948
Opioid dependence (x1.02) 0.948
Abnormal body temperature (x1.02) 0.949
Disorder of digestive system (x1.02) 0.949
Amylase [Activity/V] in SP (+2.07) 0.950
Fever (x1.02) 0.950
Disorder of spleen (x1.02) 0.951
Hyperglycemia (x1.02) 0.951
Kidney stone (x1.02) 0.951
White Race (+0.00168) 0.952
Diastolic blood pressure (+0.35) 0.952
Gastrointestinal hemorrhage (x1.02) 0.952
Chronic alcoholism in remission (x1.02) 0.953
Upper abdominal pain (x1.02) 0.953
Alcohol withdrawal syndrome (x1.02) 0.954
Imaging of abdomen abnormal (x1.02) 0.954
hydrocodone (+0.00162) 0.954
Hypoglycemia (x1.02) 0.955
Anxiety disorder (x1.02) 0.955
Gamma glutamyl transferase [Activity/V] in SP (+4.79) 0.955
Expressions
10^5
10^3
10^1
−20 −10 0 10 20
−0.6 −0.4 −0.2 0.0 0.2 0.4 0.6

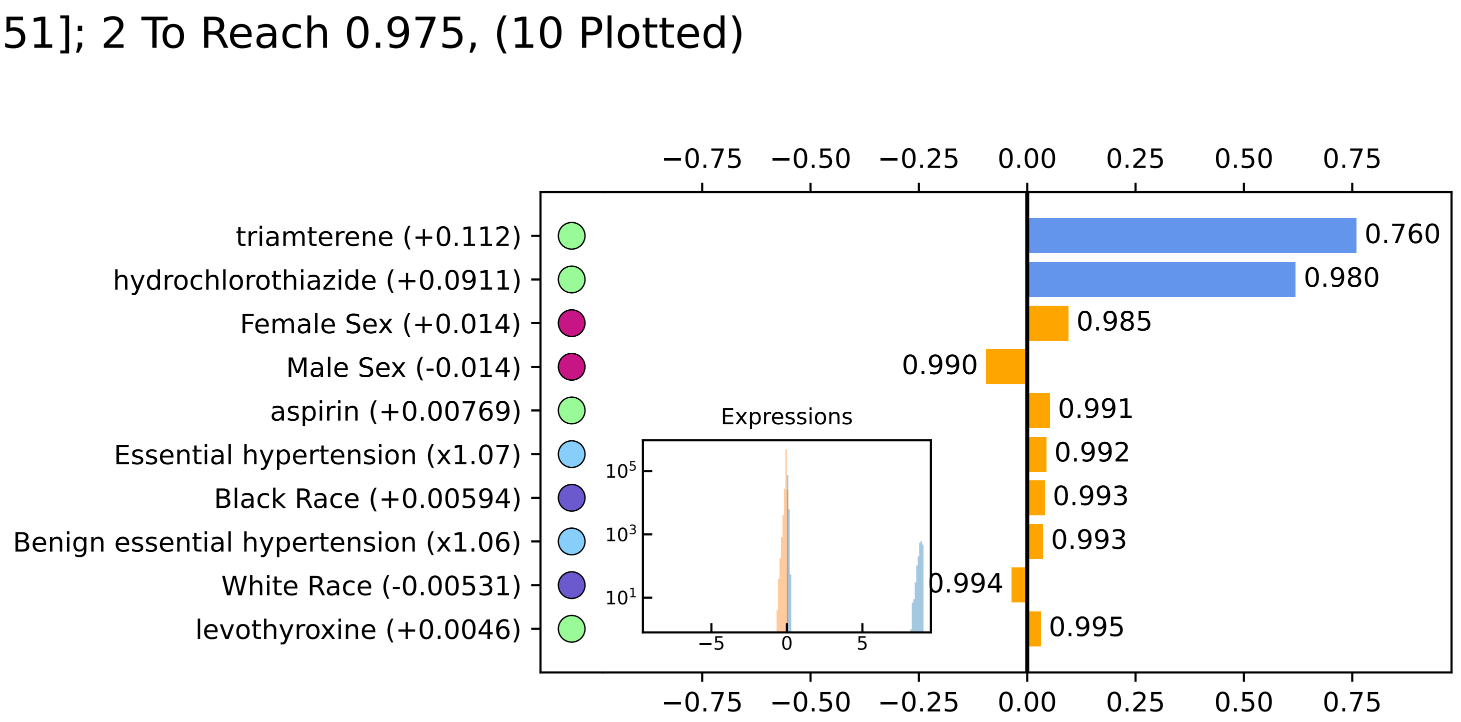
Signature [1251]; 2 To Reach 0.975, (10 Plotted)
triamterene (+0.112)
hydrochlorothiazide (+0.0911)
Female Sex (+0.014)
Male Sex (-0.014)
aspirin (+0.00769)
Essential hypertension (x1.07)
Black Race (+0.00594)
Benign essential hypertension (x1.06)
White Race (-0.00531)
levothyroxine (+0.0046)
0.760
0.980
0.985
0.990
0.991
0.992
0.993
0.993
0.994
0.995
−0.75 −0.50 −0.25 0.00 0.25 0.50 0.75
Expressions
−5 0 5

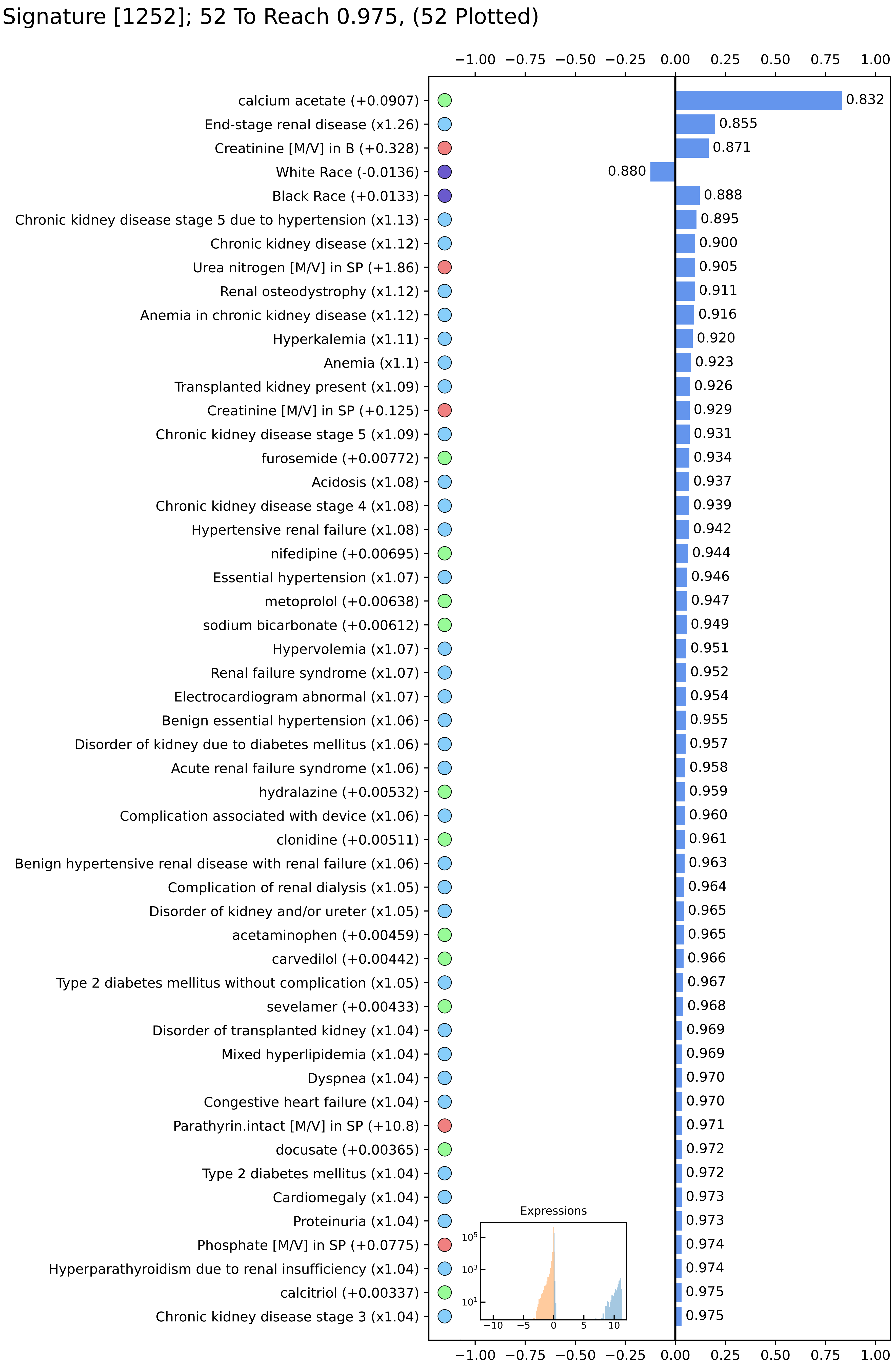

Signature [1252]; 52 To Reach 0.975, (52 Plotted)
calcium acetate (+0.0907)
0.832
End-stage renal disease (x1.26)
0.855
Creatinine [M/V] in B (+0.328)
0.871
White Race (-0.0136)
0.880
Black Race (+0.0133)
0.888
Chronic kidney disease stage 5 due to hypertension (x1.13)
0.895
Chronic kidney disease (x1.12)
0.900
Urea nitrogen [M/V] in SP (+1.86)
0.905
Renal osteodystrophy (x1.12)
0.911
Anemia in chronic kidney disease (x1.12)
0.916
Hyperkalemia (x1.11)
0.920
Anemia (x1.1)
0.923
Transplanted kidney present (x1.09)
0.926
Creatinine [M/V] in SP (+0.125)
0.929
Chronic kidney disease stage 5 (x1.09)
0.931
furosemide (+0.00772)
0.934
Acidosis (x1.08)
0.937
Chronic kidney disease stage 4 (x1.08)
0.939
Hypertensive renal failure (x1.08)
0.942
nifedipine (+0.00695)
0.944
Essential hypertension (x1.07)
0.946
metoprolol (+0.00638)
0.947
sodium bicarbonate (+0.00612)
0.949
Hypervolemia (x1.07)
0.951
Renal failure syndrome (x1.07)
0.952
Electrocardiogram abnormal (x1.07)
0.954
Benign essential hypertension (x1.06)
0.955
Disorder of kidney due to diabetes mellitus (x1.06)
0.957
Acute renal failure syndrome (x1.06)
0.958
hydralazine (+0.00532)
0.959
Complication associated with device (x1.06)
0.960
clonidine (+0.00511)
0.961
Benign hypertensive renal disease with renal failure (x1.06)
0.963
Complication of renal dialysis (x1.05)
0.964
Disorder of kidney and/or ureter (x1.05)
0.965
acetaminophen (+0.00459)
0.965
carvedilol (+0.00442)
0.966
Type 2 diabetes mellitus without complication (x1.05)
0.967
sevelamer (+0.00433)
0.968
Disorder of transplanted kidney (x1.04)
0.969
Mixed hyperlipidemia (x1.04)
0.969
Dyspnea (x1.04)
0.970
Congestive heart failure (x1.04)
0.970
Parathyrin.intact [M/V] in SP (+10.8)
0.971
docusate (+0.00365)
0.972
Type 2 diabetes mellitus (x1.04)
0.972
Cardiomegaly (x1.04)
0.973
Proteinuria (x1.04)
0.973
Phosphate [M/V] in SP (+0.0775)
0.974
Hyperparathyroidism due to renal insufficiency (x1.04)
0.974
calcitriol (+0.00337)
0.975
Chronic kidney disease stage 3 (x1.04)
0.975
Expressions
−1.00 −0.75 −0.50 −0.25 0.00 0.25 0.50 0.75 1.00

Signature [1253]; 126 To Reach 0.975, (100 Plotted)

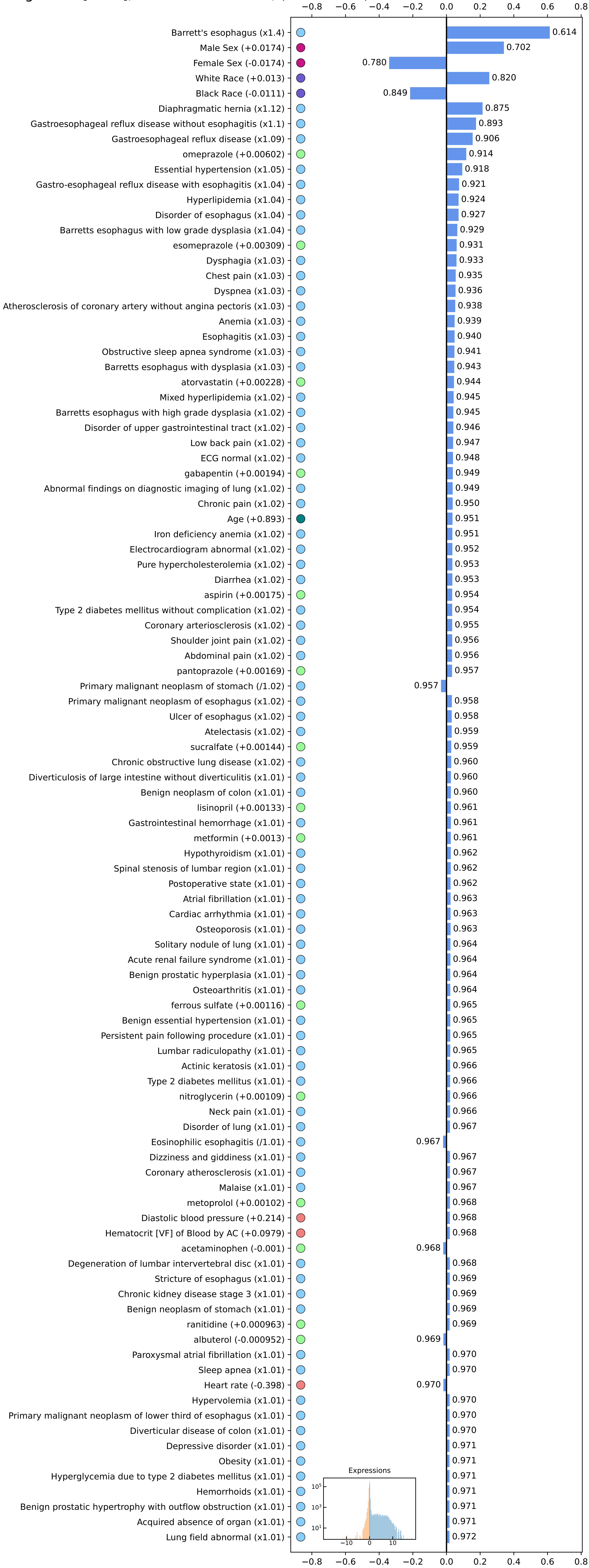

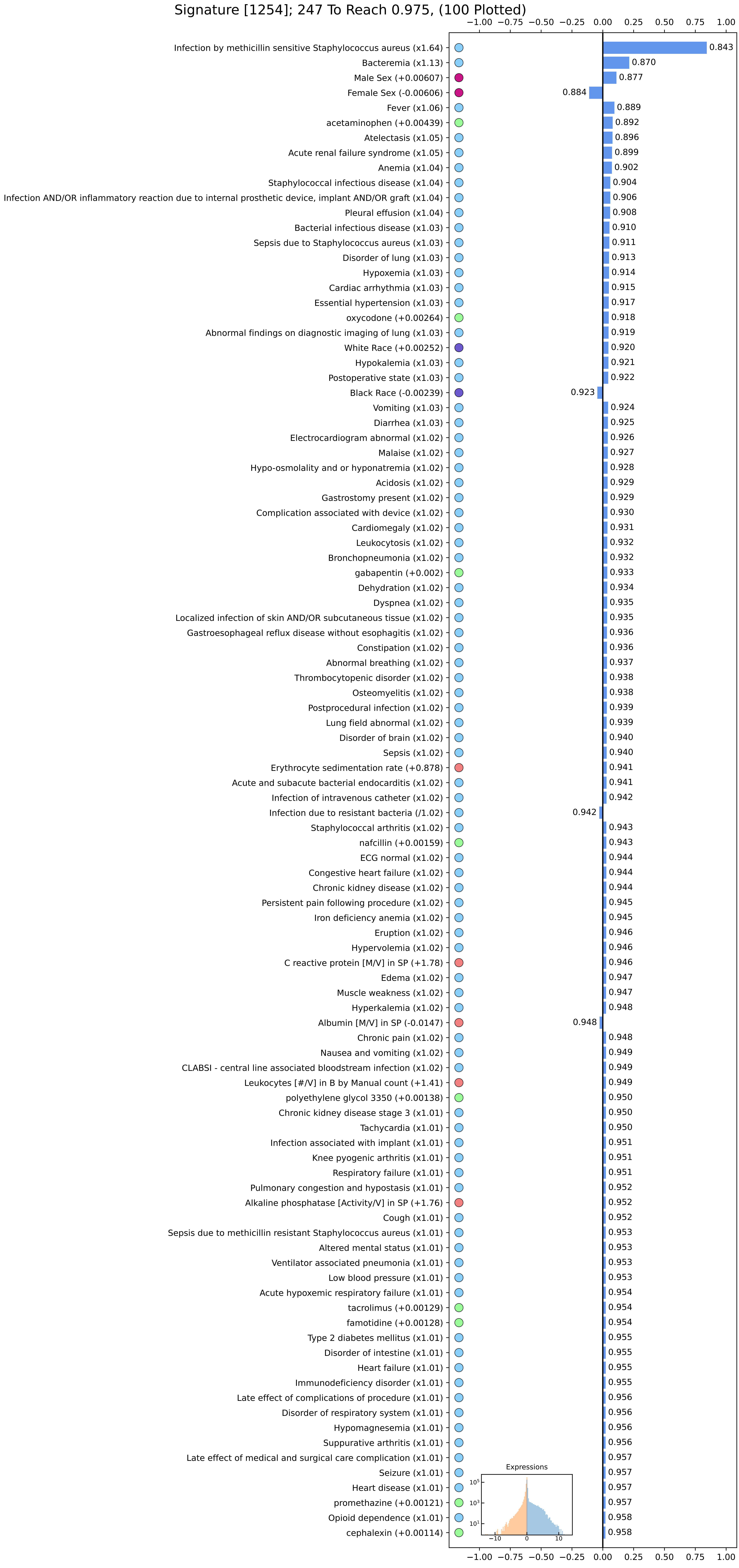

Signature [1254]; 247 To Reach 0.975, (100 Plotted)
Infection by methicillin sensitive Staphylococcus aureus (x1.64) 0.843
Bacteremia (x1.13) 0.870
Male Sex (+0.00607) 0.877
Female Sex (-0.00606) 0.884
Fever (x1.06) 0.889
acetaminophen (+0.00439) 0.892
Atelectasis (x1.05) 0.896
Acute renal failure syndrome (x1.05) 0.899
Anemia (x1.04) 0.902
Staphylococcal infectious disease (x1.04) 0.904
Infection AND/OR inflammatory reaction due to internal prosthetic device, implant AND/OR graft (x1.04) 0.906
Pleural effusion (x1.04) 0.908
Bacterial infectious disease (x1.03) 0.910
Sepsis due to Staphylococcus aureus (x1.03) 0.911
Disorder of lung (x1.03) 0.913
Hypoxemia (x1.03) 0.914
Cardiac arrhythmia (x1.03) 0.915
Essential hypertension (x1.03) 0.917
oxycodone (+0.00264) 0.918
Abnormal findings on diagnostic imaging of lung (x1.03) 0.919
White Race (+0.00252) 0.920
Hypokalemia (x1.03) 0.921
Postoperative state (x1.03) 0.922
Black Race (-0.00239) 0.923
Vomiting (x1.03) 0.924
Diarrhea (x1.03) 0.925
Electrocardiogram abnormal (x1.02) 0.926
Malaise (x1.02) 0.927
Hypo-osmolality and or hyponatremia (x1.02) 0.928
Acidosis (x1.02) 0.929
Gastrostomy present (x1.02) 0.929
Complication associated with device (x1.02) 0.930
Cardiomegaly (x1.02) 0.931
Leukocytosis (x1.02) 0.932
Bronchopneumonia (x1.02) 0.932
gabapentin (+0.002) 0.933
Dehydration (x1.02) 0.934
Dyspnea (x1.02) 0.935
Localized infection of skin AND/OR subcutaneous tissue (x1.02) 0.935
Gastroesophageal reflux disease without esophagitis (x1.02) 0.936
Constipation (x1.02) 0.936
Abnormal breathing (x1.02) 0.937
Thrombocytopenic disorder (x1.02) 0.938
Osteomyelitis (x1.02) 0.938
Postprocedural infection (x1.02) 0.939
Lung field abnormal (x1.02) 0.939
Disorder of brain (x1.02) 0.940
Sepsis (x1.02) 0.940
Erythrocyte sedimentation rate (+0.878) 0.941
Acute and subacute bacterial endocarditis (x1.02) 0.941
Infection of intravenous catheter (x1.02) 0.942
Infection due to resistant bacteria (/1.02) 0.942
Staphylococcal arthritis (x1.02) 0.943
nafcillin (+0.00159) 0.943
ECG normal (x1.02) 0.944
Congestive heart failure (x1.02) 0.944
Chronic kidney disease (x1.02) 0.944
Persistent pain following procedure (x1.02) 0.945
Iron deficiency anemia (x1.02) 0.945
Eruption (x1.02) 0.946
Hypervolemia (x1.02) 0.946
C reactive protein [M/V] in SP (+1.78) 0.946
Edema (x1.02) 0.947
Muscle weakness (x1.02) 0.947
Hyperkalemia (x1.02) 0.948
Albumin [M/V] in SP (-0.0147) 0.948
Chronic pain (x1.02) 0.948
Nausea and vomiting (x1.02) 0.949
CLABSI - central line associated bloodstream infection (x1.02) 0.949
Leukocytes [#/V] in B by Manual count (+1.41) 0.949
polyethylene glycol 3350 (+0.00138) 0.950
Chronic kidney disease stage 3 (x1.01) 0.950
Tachycardia (x1.01) 0.950
Infection associated with implant (x1.01) 0.951
Knee pyogenic arthritis (x1.01) 0.951
Respiratory failure (x1.01) 0.951
Pulmonary congestion and hypostasis (x1.01) 0.952
Alkaline phosphatase [Activity/V] in SP (+1.76) 0.952
Cough (x1.01) 0.952
Sepsis due to methicillin resistant Staphylococcus aureus (x1.01) 0.953
Altered mental status (x1.01) 0.953
Ventilator associated pneumonia (x1.01) 0.953
Low blood pressure (x1.01) 0.953
Acute hypoxemic respiratory failure (x1.01) 0.954
tacrolimus (+0.00129) 0.954
famotidine (+0.00128) 0.954
Type 2 diabetes mellitus (x1.01) 0.955
Disorder of intestine (x1.01) 0.955
Heart failure (x1.01) 0.955
Immunodeficiency disorder (x1.01) 0.955
Late effect of complications of procedure (x1.01) 0.956
Disorder of respiratory system (x1.01) 0.956
Hypomagnesemia (x1.01) 0.956
Suppurative arthritis (x1.01) 0.956
Late effect of medical and surgical care complication (x1.01) 0.957
Seizure (x1.01) 0.957
Heart disease (x1.01) 0.957
promethazine (+0.00121) 0.957
Opioid dependence (x1.01) 0.958
cephalexin (+0.00114) 0.958
−1.00 −0.75 −0.50 −0.25 0.00 0.25 0.50 0.75 1.00
Expressions

Signature [1255]; 5 To Reach 0.975, (10 Plotted)

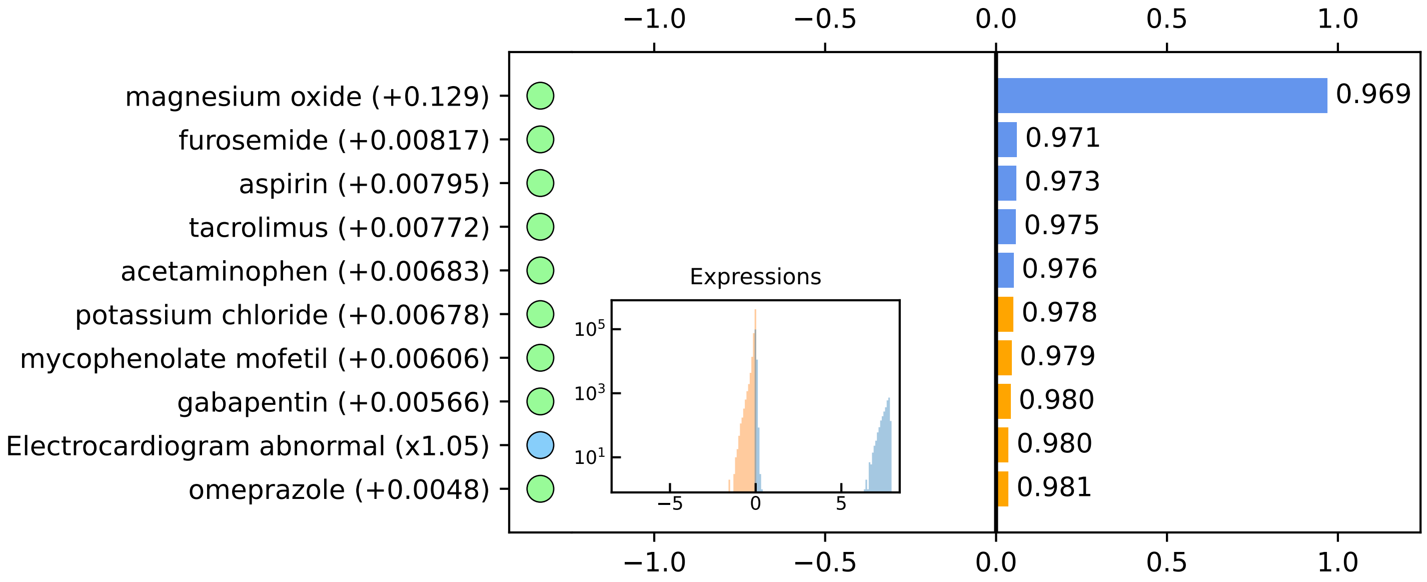

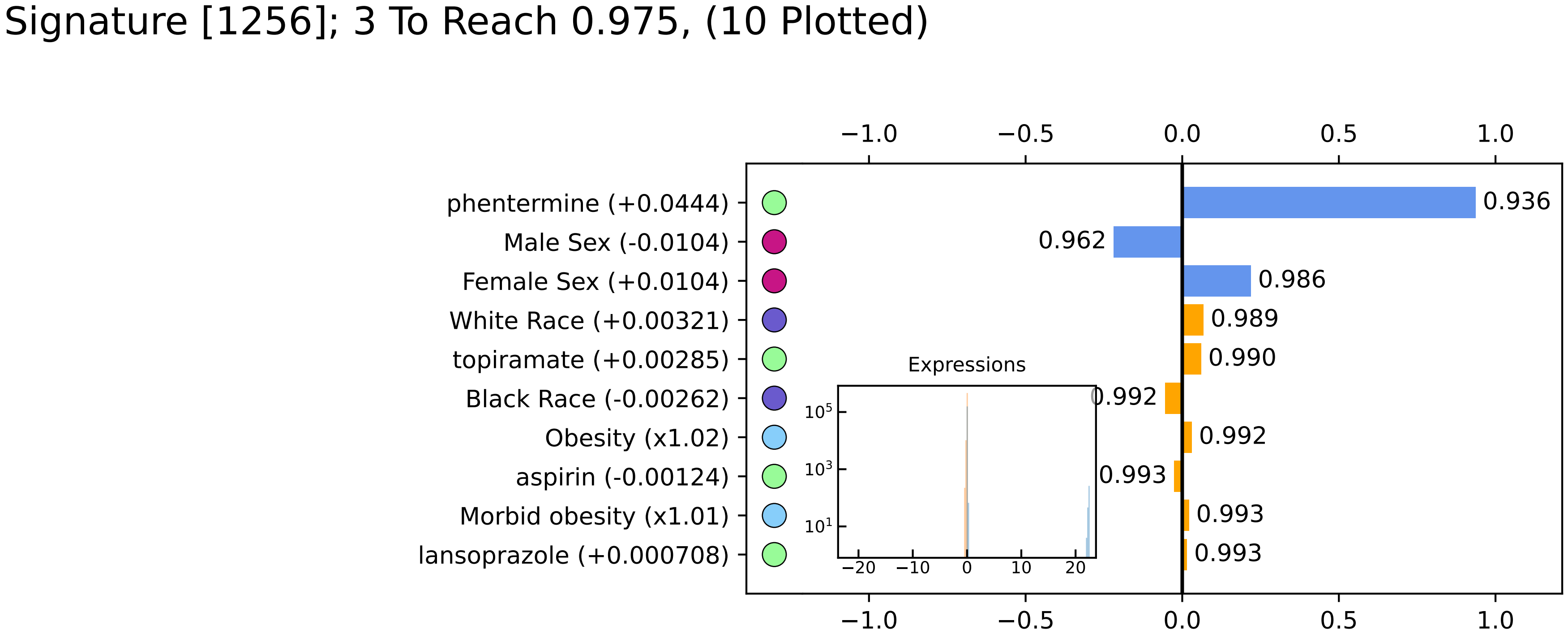
Signature [1256]; 3 To Reach 0.975, (10 Plotted)
phentermine (+0.0444)
Male Sex (-0.0104)
Female Sex (+0.0104)
White Race (+0.00321)
topiramate (+0.00285)
Black Race (-0.00262)
Obesity (x1.02)
aspirin (-0.00124)
Morbid obesity (x1.01)
lansoprazole (+0.000708)
0.936
0.962
0.986
0.989
0.990
0.992
0.992
0.993
0.993
0.993
Expressions
−1.0
−0.5
0.0
0.5
1.0

Signature [1257]; 100 To Reach 0.975, (100 Plotted)

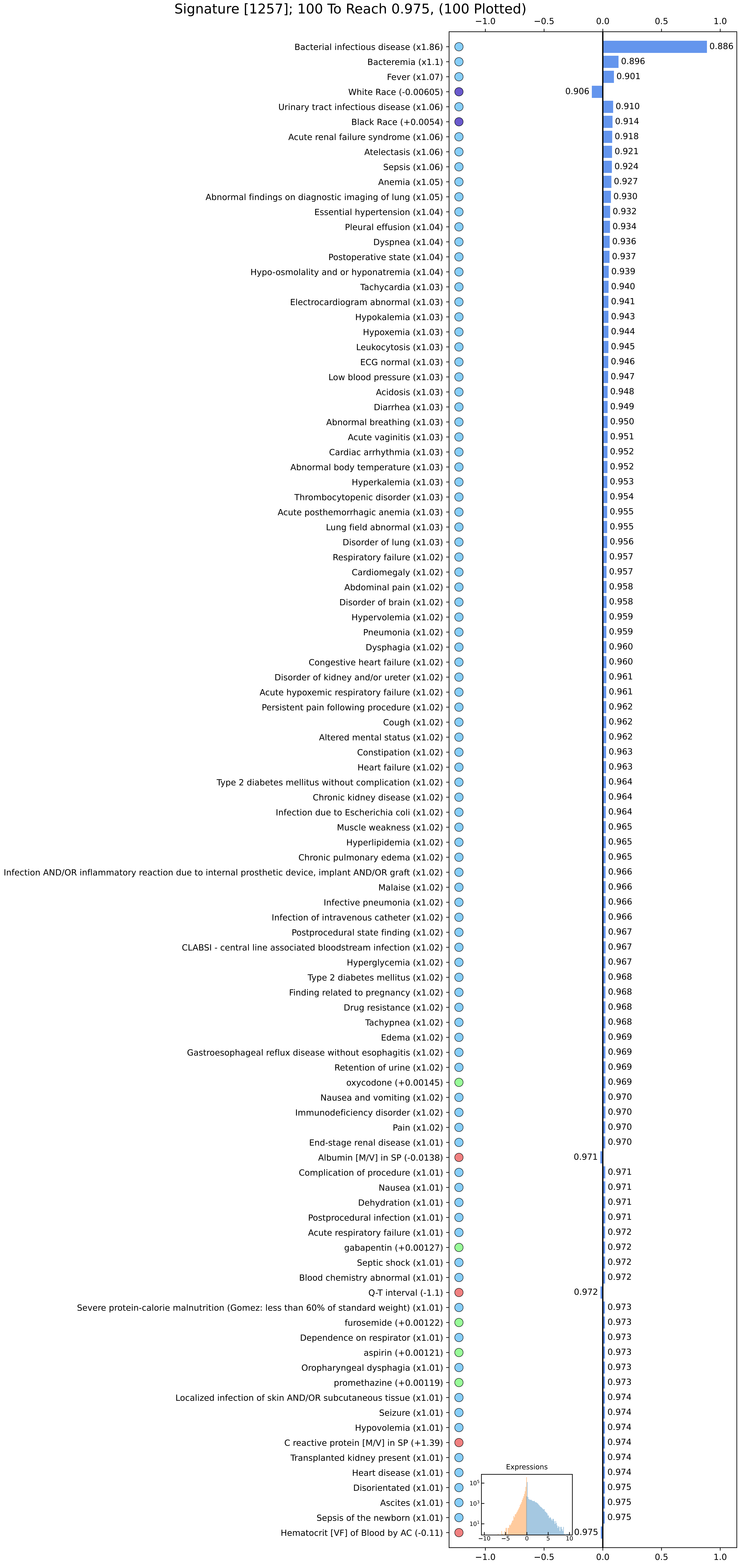

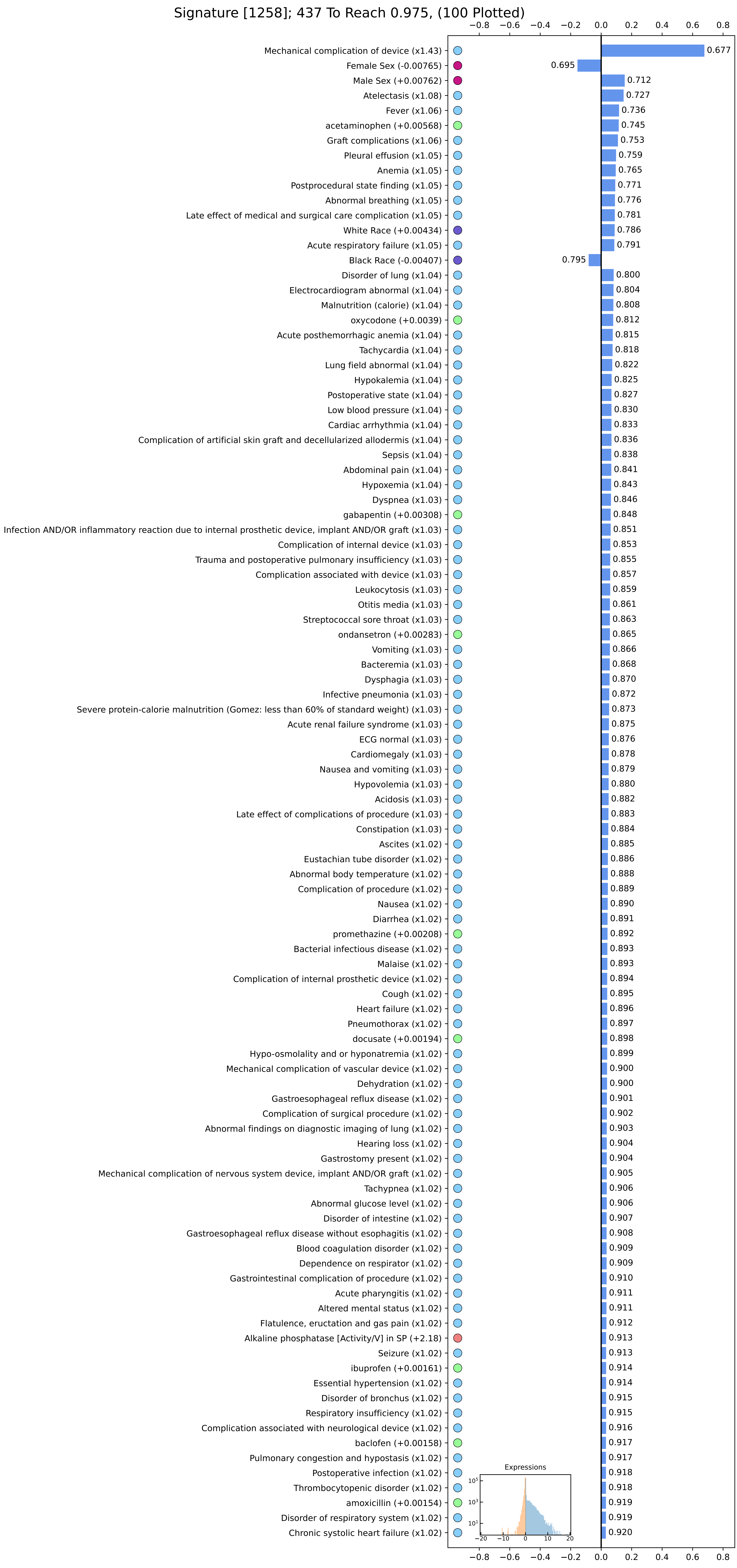

Signature [1258]; 437 To Reach 0.975, (100 Plotted)
Mechanical complication of device (x1.43) 0.677
Female Sex (-0.00765) 0.695
Male Sex (+0.00762) 0.712
Atelectasis (x1.08) 0.727
Fever (x1.06) 0.736
acetaminophen (+0.00568) 0.745
Graft complications (x1.06) 0.753
Pleural effusion (x1.05) 0.759
Anemia (x1.05) 0.765
Postprocedural state finding (x1.05) 0.771
Abnormal breathing (x1.05) 0.776
Late effect of medical and surgical care complication (x1.05) 0.781
White Race (+0.00434) 0.786
Acute respiratory failure (x1.05) 0.791
Black Race (-0.00407) 0.795
Disorder of lung (x1.04) 0.800
Electrocardiogram abnormal (x1.04) 0.804
Malnutrition (calorie) (x1.04) 0.808
oxycodone (+0.0039) 0.812
Acute posthemorrhagic anemia (x1.04) 0.815
Tachycardia (x1.04) 0.818
Lung field abnormal (x1.04) 0.822
Hypokalemia (x1.04) 0.825
Postoperative state (x1.04) 0.827
Low blood pressure (x1.04) 0.830
Cardiac arrhythmia (x1.04) 0.833
Complication of artificial skin graft and decellularized allodermis (x1.04) 0.836
Sepsis (x1.04) 0.838
Abdominal pain (x1.04) 0.841
Hypoxemia (x1.04) 0.843
Dyspnea (x1.03) 0.846
gabapentin (+0.00308) 0.848
Infection AND/OR inflammatory reaction due to internal prosthetic device, implant AND/OR graft (x1.03) 0.851
Complication of internal device (x1.03) 0.853
Trauma and postoperative pulmonary insufficiency (x1.03) 0.855
Complication associated with device (x1.03) 0.857
Leukocytosis (x1.03) 0.859
Otitis media (x1.03) 0.861
Streptococcal sore throat (x1.03) 0.863
ondansetron (+0.00283) 0.865
Vomiting (x1.03) 0.866
Bacteremia (x1.03) 0.868
Dysphagia (x1.03) 0.870
Infective pneumonia (x1.03) 0.872
Severe protein-calorie malnutrition (Gomez: less than 60% of standard weight) (x1.03) 0.873
Acute renal failure syndrome (x1.03) 0.875
ECG normal (x1.03) 0.876
Cardiomegaly (x1.03) 0.878
Nausea and vomiting (x1.03) 0.879
Hypovolemia (x1.03) 0.880
Acidosis (x1.03) 0.882
Late effect of complications of procedure (x1.03) 0.883
Constipation (x1.03) 0.884
Ascites (x1.02) 0.885
Eustachian tube disorder (x1.02) 0.886
Abnormal body temperature (x1.02) 0.888
Complication of procedure (x1.02) 0.889
Nausea (x1.02) 0.890
Diarrhea (x1.02) 0.891
promethazine (+0.00208) 0.892
Bacterial infectious disease (x1.02) 0.893
Malaise (x1.02) 0.893
Complication of internal prosthetic device (x1.02) 0.894
Cough (x1.02) 0.895
Heart failure (x1.02) 0.896
Pneumothorax (x1.02) 0.897
docusate (+0.00194) 0.898
Hypo-osmolality and or hyponatremia (x1.02) 0.899
Mechanical complication of vascular device (x1.02) 0.900
Dehydration (x1.02) 0.900
Gastroesophageal reflux disease (x1.02) 0.901
Complication of surgical procedure (x1.02) 0.902
Abnormal findings on diagnostic imaging of lung (x1.02) 0.903
Hearing loss (x1.02) 0.904
Gastrostomy present (x1.02) 0.904
Mechanical complication of nervous system device, implant AND/OR graft (x1.02) 0.905
Tachypnea (x1.02) 0.906
Abnormal glucose level (x1.02) 0.906
Disorder of intestine (x1.02) 0.907
Gastroesophageal reflux disease without esophagitis (x1.02) 0.908
Blood coagulation disorder (x1.02) 0.909
Dependence on respirator (x1.02) 0.909
Gastrointestinal complication of procedure (x1.02) 0.910
Acute pharyngitis (x1.02) 0.911
Altered mental status (x1.02) 0.911
Flatulence, eructation and gas pain (x1.02) 0.912
Alkaline phosphatase [Activity/V] in SP (+2.18) 0.913
Seizure (x1.02) 0.913
ibuprofen (+0.00161) 0.914
Essential hypertension (x1.02) 0.914
Disorder of bronchus (x1.02) 0.915
Respiratory insufficiency (x1.02) 0.915
Complication associated with neurological device (x1.02) 0.916
baclofen (+0.00158) 0.917
Pulmonary congestion and hypostasis (x1.02) 0.917
Postoperative infection (x1.02) 0.918
Thrombocytopenic disorder (x1.02) 0.918
amoxicillin (+0.00154) 0.919
Disorder of respiratory system (x1.02) 0.919
Chronic systolic heart failure (x1.02) 0.920
Expressions
−0.8 −0.6 −0.4 −0.2 0.0 0.2 0.4 0.6 0.8

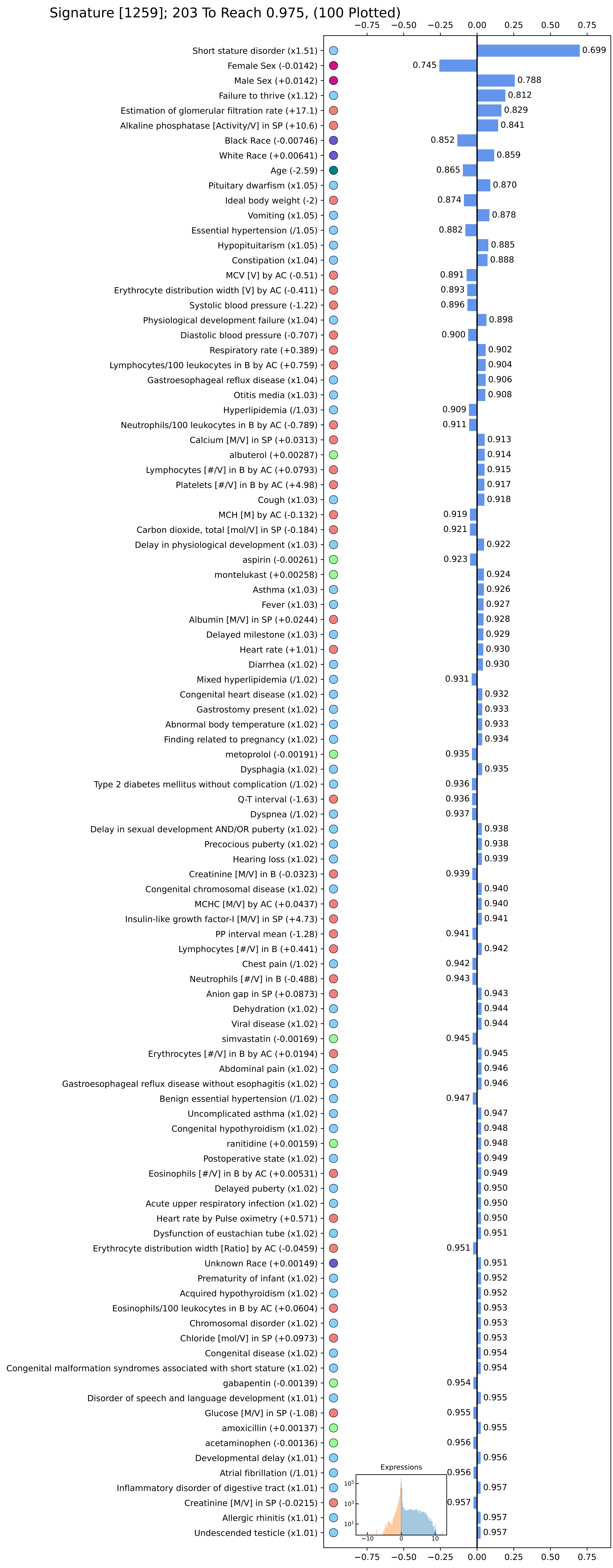

Signature [1259]; 203 To Reach 0.975, (100 Plotted)
Short stature disorder (x1.51) 0.699
Female Sex (-0.0142) 0.745
Male Sex (+0.0142) 0.788
Failure to thrive (x1.12) 0.812
Estimation of glomerular filtration rate (+17.1) 0.829
Alkaline phosphatase [Activity/V] in SP (+10.6) 0.841
Black Race (-0.00746) 0.852
White Race (+0.00641) 0.859
Age (-2.59) 0.865
Pituitary dwarfism (x1.05) 0.870
Ideal body weight (-2) 0.874
Vomiting (x1.05) 0.878
Essential hypertension (/1.05) 0.882
Hypopituitarism (x1.05) 0.885
Constipation (x1.04) 0.888
MCV [V] by AC (-0.51) 0.891
Erythrocyte distribution width [V] by AC (-0.411) 0.893
Systolic blood pressure (-1.22) 0.896
Physiological development failure (x1.04) 0.898
Diastolic blood pressure (-0.707) 0.900
Respiratory rate (+0.389) 0.902
Lymphocytes/100 leukocytes in B by AC (+0.759) 0.904
Gastroesophageal reflux disease (x1.04) 0.906
Otitis media (x1.03) 0.908
Hyperlipidemia (/1.03) 0.909
Neutrophils/100 leukocytes in B by AC (-0.789) 0.911
Calcium [M/V] in SP (+0.0313) 0.913
albuterol (+0.00287) 0.914
Lymphocytes [#/V] in B by AC (+0.0793) 0.915
Platelets [#/V] in B by AC (+4.98) 0.917
Cough (x1.03) 0.918
MCH [M] by AC (-0.132) 0.919
Carbon dioxide, total [mol/V] in SP (-0.184) 0.921
Delay in physiological development (x1.03) 0.922
aspirin (-0.00261) 0.923
montelukast (+0.00258) 0.924
Asthma (x1.03) 0.926
Fever (x1.03) 0.927
Albumin [M/V] in SP (+0.0244) 0.928
Delayed milestone (x1.03) 0.929
Heart rate (+1.01) 0.930
Diarrhea (x1.02) 0.930
Mixed hyperlipidemia (/1.02) 0.931
Congenital heart disease (x1.02) 0.932
Gastrostomy present (x1.02) 0.933
Abnormal body temperature (x1.02) 0.933
Finding related to pregnancy (x1.02) 0.934
metoprolol (-0.00191) 0.935
Dysphagia (x1.02) 0.935
Type 2 diabetes mellitus without complication (/1.02) 0.936
Q-T interval (-1.63) 0.936
Dyspnea (/1.02) 0.937
Delay in sexual development AND/OR puberty (x1.02) 0.938
Precocious puberty (x1.02) 0.938
Hearing loss (x1.02) 0.939
Creatinine [M/V] in B (-0.0323) 0.939
Congenital chromosomal disease (x1.02) 0.940
MCHC [M/V] by AC (+0.0437) 0.940
Insulin-like growth factor-I [M/V] in SP (+4.73) 0.941
PP interval mean (-1.28) 0.941
Lymphocytes [#/V] in B (+0.441) 0.942
Chest pain (/1.02) 0.942
Neutrophils [#/V] in B (-0.488) 0.943
Anion gap in SP (+0.0873) 0.943
Dehydration (x1.02) 0.944
Viral disease (x1.02) 0.944
simvastatin (-0.00169) 0.945
Erythrocytes [#/V] in B by AC (+0.0194) 0.945
Abdominal pain (x1.02) 0.946
Gastroesophageal reflux disease without esophagitis (x1.02) 0.946
Benign essential hypertension (/1.02) 0.947
Uncomplicated asthma (x1.02) 0.947
Congenital hypothyroidism (x1.02) 0.948
ranitidine (+0.00159) 0.948
Postoperative state (x1.02) 0.949
Eosinophils [#/V] in B by AC (+0.00531) 0.949
Delayed puberty (x1.02) 0.950
Acute upper respiratory infection (x1.02) 0.950
Heart rate by Pulse oximetry (+0.571) 0.950
Dysfunction of eustachian tube (x1.02) 0.951
Erythrocyte distribution width [Ratio] by AC (-0.0459) 0.951
Unknown Race (+0.00149) 0.951
Prematurity of infant (x1.02) 0.952
Acquired hypothyroidism (x1.02) 0.952
Eosinophils/100 leukocytes in B by AC (+0.0604) 0.953
Chromosomal disorder (x1.02) 0.953
Chloride [mol/V] in SP (+0.0973) 0.953
Congenital disease (x1.02) 0.954
Congenital malformation syndromes associated with short stature (x1.02) 0.954
gabapentin (-0.00139) 0.954
Disorder of speech and language development (x1.01) 0.955
Glucose [M/V] in SP (-1.08) 0.955
amoxicillin (+0.00137) 0.955
acetaminophen (-0.00136) 0.956
Developmental delay (x1.01) 0.956
Atrial fibrillation (/1.01) 0.956
Inflammatory disorder of digestive tract (x1.01) 0.957
Creatinine [M/V] in SP (-0.0215) 0.957
Allergic rhinitis (x1.01) 0.957
Undescended testicle (x1.01) 0.957
−0.75 −0.50 −0.25 0.00 0.25 0.50 0.75
Expressions

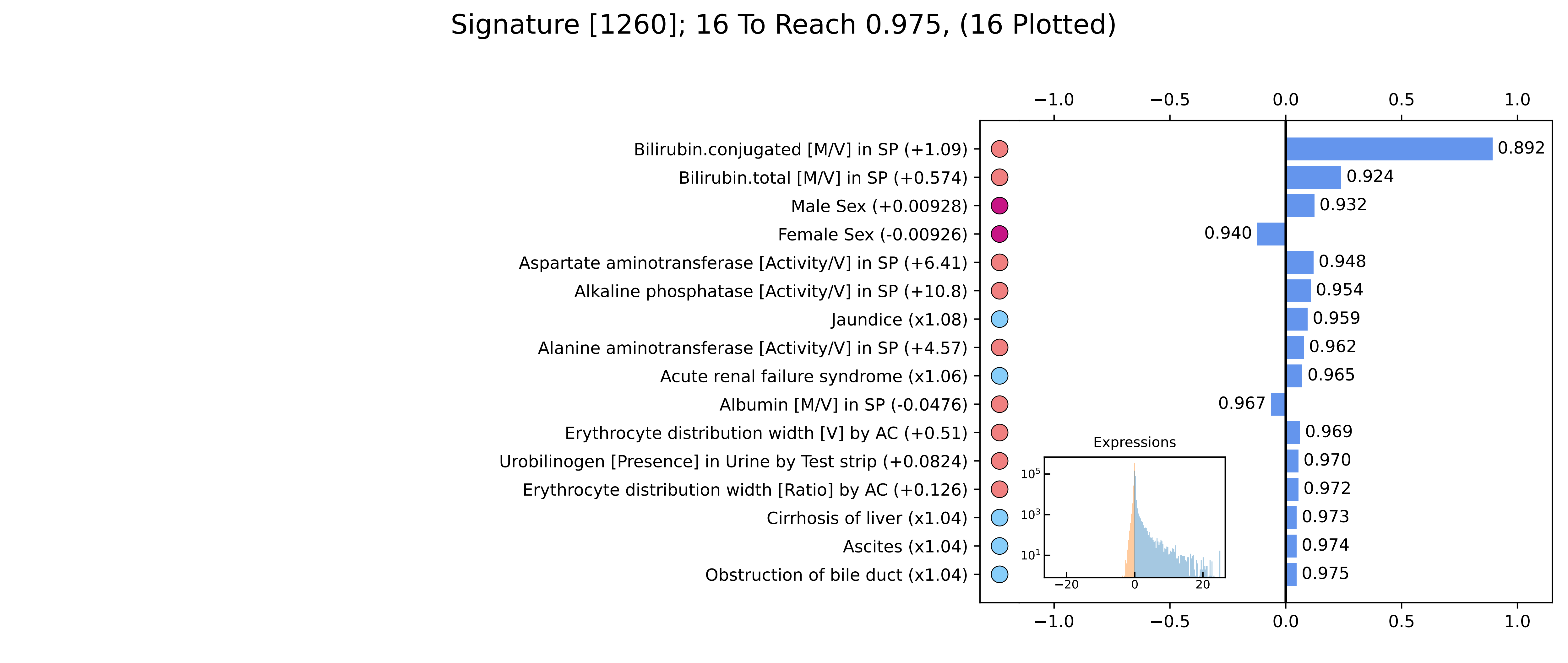
Signature [1260]; 16 To Reach 0.975, (16 Plotted)
Bilirubin.conjugated [M/V] in SP (+1.09)
0.892
Bilirubin.total [M/V] in SP (+0.574)
0.924
Male Sex (+0.00928)
0.932
Female Sex (-0.00926)
0.940
Aspartate aminotransferase [Activity/V] in SP (+6.41)
0.948
Alkaline phosphatase [Activity/V] in SP (+10.8)
0.954
Jaundice (x1.08)
0.959
Alanine aminotransferase [Activity/V] in SP (+4.57)
0.962
Acute renal failure syndrome (x1.06)
0.965
Albumin [M/V] in SP (-0.0476)
0.967
Erythrocyte distribution width [V] by AC (+0.51)
0.969
Urobilinogen [Presence] in Urine by Test strip (+0.0824)
0.970
Erythrocyte distribution width [Ratio] by AC (+0.126)
0.972
Cirrhosis of liver (x1.04)
0.973
Ascites (x1.04)
0.974
Obstruction of bile duct (x1.04)
0.975
Expressions
−1.0
−0.5
0.0
0.5
1.0

Signature [1261]; 202 To Reach 0.975, (100 Plotted)

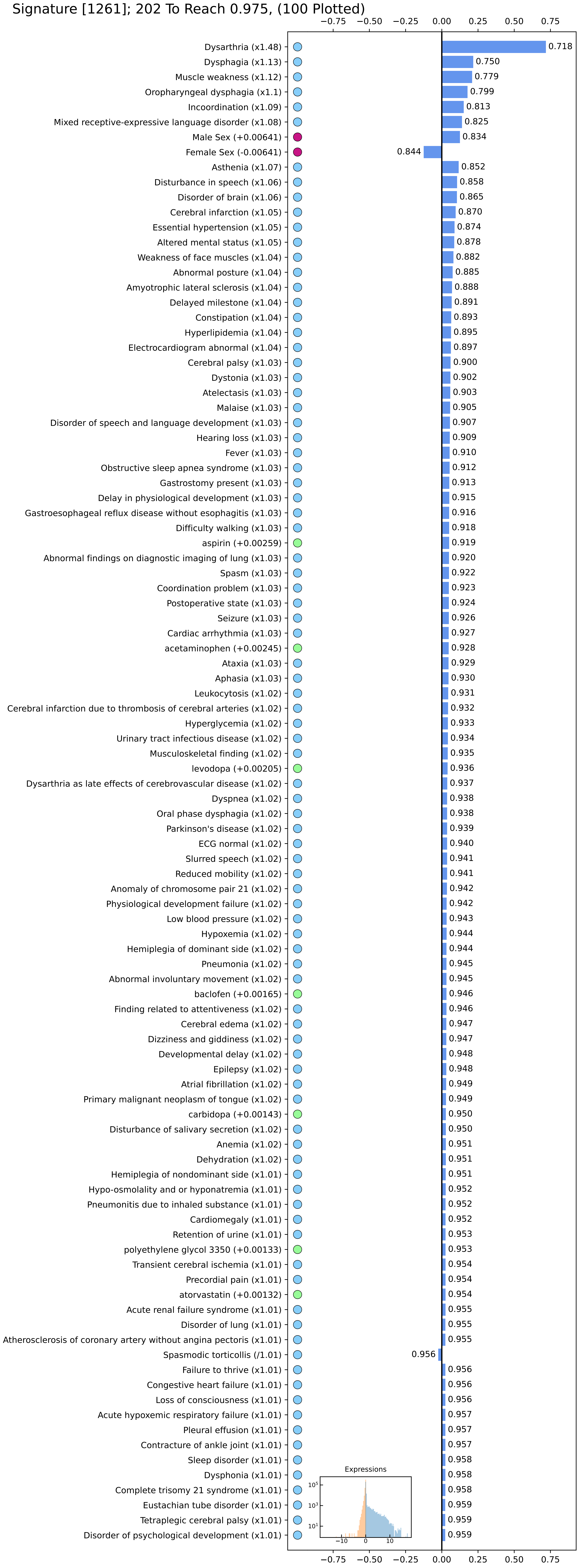

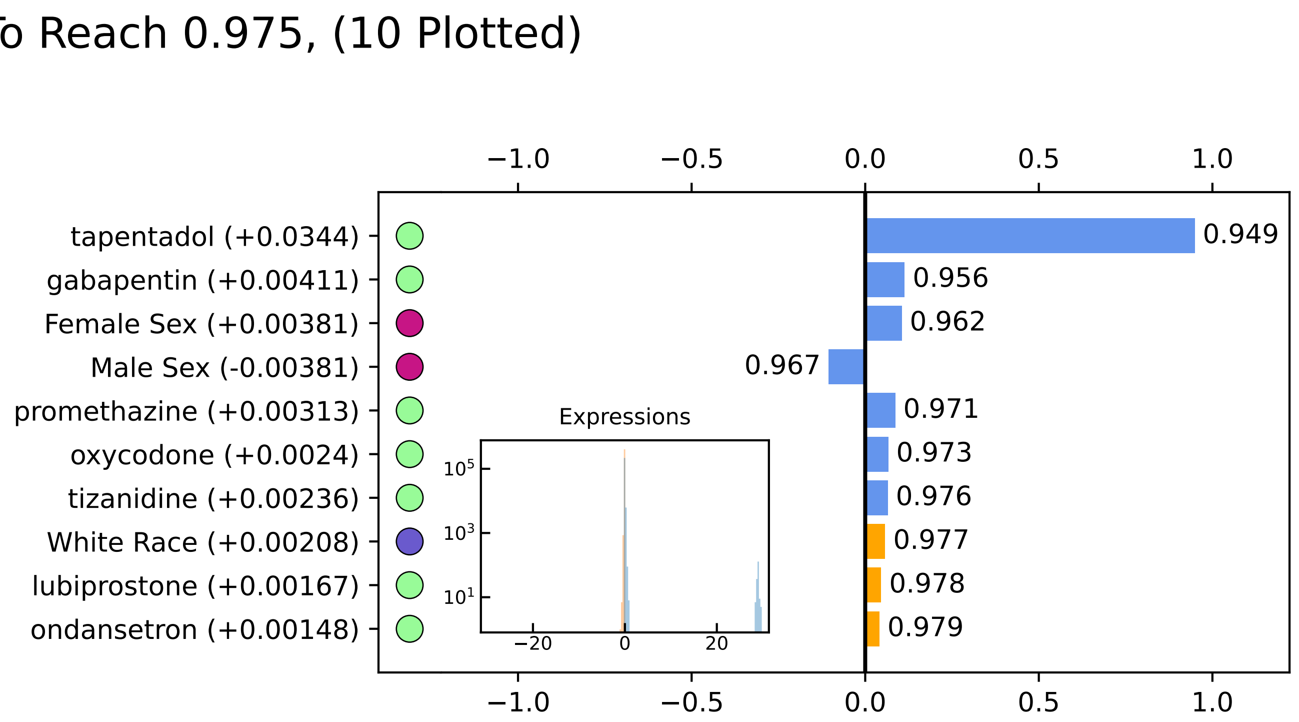
Signature [1262]; 7 To Reach 0.975, (10 Plotted)
tapentadol (+0.0344)
gabapentin (+0.00411)
Female Sex (+0.00381)
Male Sex (-0.00381)
promethazine (+0.00313)
oxycodone (+0.0024)
tizanidine (+0.00236)
White Race (+0.00208)
lubiprostone (+0.00167)
ondansetron (+0.00148)
0.949
0.956
0.962
0.967
0.971
0.973
0.976
0.977
0.978
0.979
−1.0
−0.5
0.0
0.5
1.0
Expressions
10^5
10^3
10^1
−20
0
20

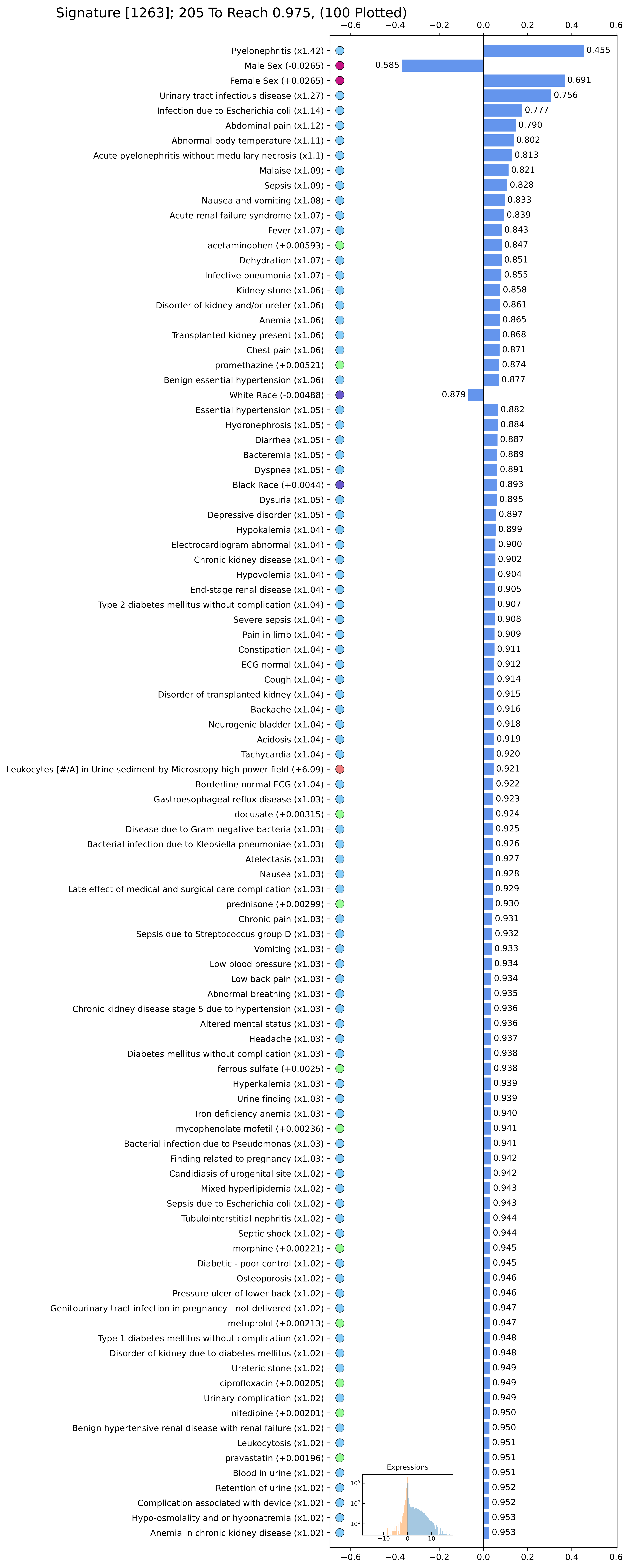

Signature [1263]; 205 To Reach 0.975, (100 Plotted)
Pyelonephritis (x1.42) 0.455
Male Sex (-0.0265) 0.585
Female Sex (+0.0265) 0.691
Urinary tract infectious disease (x1.27) 0.756
Infection due to Escherichia coli (x1.14) 0.777
Abdominal pain (x1.12) 0.790
Abnormal body temperature (x1.11) 0.802
Acute pyelonephritis without medullary necrosis (x1.1) 0.813
Malaise (x1.09) 0.821
Sepsis (x1.09) 0.828
Nausea and vomiting (x1.08) 0.833
Acute renal failure syndrome (x1.07) 0.839
Fever (x1.07) 0.843
acetaminophen (+0.00593) 0.847
Dehydration (x1.07) 0.851
Infective pneumonia (x1.07) 0.855
Kidney stone (x1.06) 0.858
Disorder of kidney and/or ureter (x1.06) 0.861
Anemia (x1.06) 0.865
Transplanted kidney present (x1.06) 0.868
Chest pain (x1.06) 0.871
promethazine (+0.00521) 0.874
Benign essential hypertension (x1.06) 0.877
White Race (-0.00488) 0.879
Essential hypertension (x1.05) 0.882
Hydronephrosis (x1.05) 0.884
Diarrhea (x1.05) 0.887
Bacteremia (x1.05) 0.889
Dyspnea (x1.05) 0.891
Black Race (+0.0044) 0.893
Dysuria (x1.05) 0.895
Depressive disorder (x1.05) 0.897
Hypokalemia (x1.04) 0.899
Electrocardiogram abnormal (x1.04) 0.900
Chronic kidney disease (x1.04) 0.902
Hypovolemia (x1.04) 0.904
End-stage renal disease (x1.04) 0.905
Type 2 diabetes mellitus without complication (x1.04) 0.907
Severe sepsis (x1.04) 0.908
Pain in limb (x1.04) 0.909
Constipation (x1.04) 0.911
ECG normal (x1.04) 0.912
Cough (x1.04) 0.914
Disorder of transplanted kidney (x1.04) 0.915
Backache (x1.04) 0.916
Neurogenic bladder (x1.04) 0.918
Acidosis (x1.04) 0.919
Tachycardia (x1.04) 0.920
Leukocytes [#/A] in Urine sediment by Microscopy high power field (+6.09) 0.921
Borderline normal ECG (x1.04) 0.922
Gastroesophageal reflux disease (x1.03) 0.923
docusate (+0.00315) 0.924
Disease due to Gram-negative bacteria (x1.03) 0.925
Bacterial infection due to Klebsiella pneumoniae (x1.03) 0.926
Atelectasis (x1.03) 0.927
Nausea (x1.03) 0.928
Late effect of medical and surgical care complication (x1.03) 0.929
prednisone (+0.00299) 0.930
Chronic pain (x1.03) 0.931
Sepsis due to Streptococcus group D (x1.03) 0.932
Vomiting (x1.03) 0.933
Low blood pressure (x1.03) 0.934
Low back pain (x1.03) 0.934
Abnormal breathing (x1.03) 0.935
Chronic kidney disease stage 5 due to hypertension (x1.03) 0.936
Altered mental status (x1.03) 0.936
Headache (x1.03) 0.937
Diabetes mellitus without complication (x1.03) 0.938
ferrous sulfate (+0.0025) 0.938
Hyperkalemia (x1.03) 0.939
Urine finding (x1.03) 0.939
Iron deficiency anemia (x1.03) 0.940
mycophenolate mofetil (+0.00236) 0.941
Bacterial infection due to Pseudomonas (x1.03) 0.941
Finding related to pregnancy (x1.03) 0.942
Candidiasis of urogenital site (x1.02) 0.942
Mixed hyperlipidemia (x1.02) 0.943
Sepsis due to Escherichia coli (x1.02) 0.943
Tubulointerstitial nephritis (x1.02) 0.944
Septic shock (x1.02) 0.944
morphine (+0.00221) 0.945
Diabetic - poor control (x1.02) 0.945
Osteoporosis (x1.02) 0.946
Pressure ulcer of lower back (x1.02) 0.946
Genitourinary tract infection in pregnancy - not delivered (x1.02) 0.947
metoprolol (+0.00213) 0.947
Type 1 diabetes mellitus without complication (x1.02) 0.948
Disorder of kidney due to diabetes mellitus (x1.02) 0.948
Ureteric stone (x1.02) 0.949
ciprofloxacin (+0.00205) 0.949
Urinary complication (x1.02) 0.949
nifedipine (+0.00201) 0.950
Benign hypertensive renal disease with renal failure (x1.02) 0.950
Leukocytosis (x1.02) 0.951
pravastatin (+0.00196) 0.951
Blood in urine (x1.02) 0.951
Retention of urine (x1.02) 0.952
Complication associated with device (x1.02) 0.952
Hypo-osmolality and or hyponatremia (x1.02) 0.953
Anemia in chronic kidney disease (x1.02) 0.953
−0.6 −0.4 −0.2 0.0 0.2 0.4 0.6
Expressions

Signature [1264]; 1 To Reach 0.975, (10 Plotted)

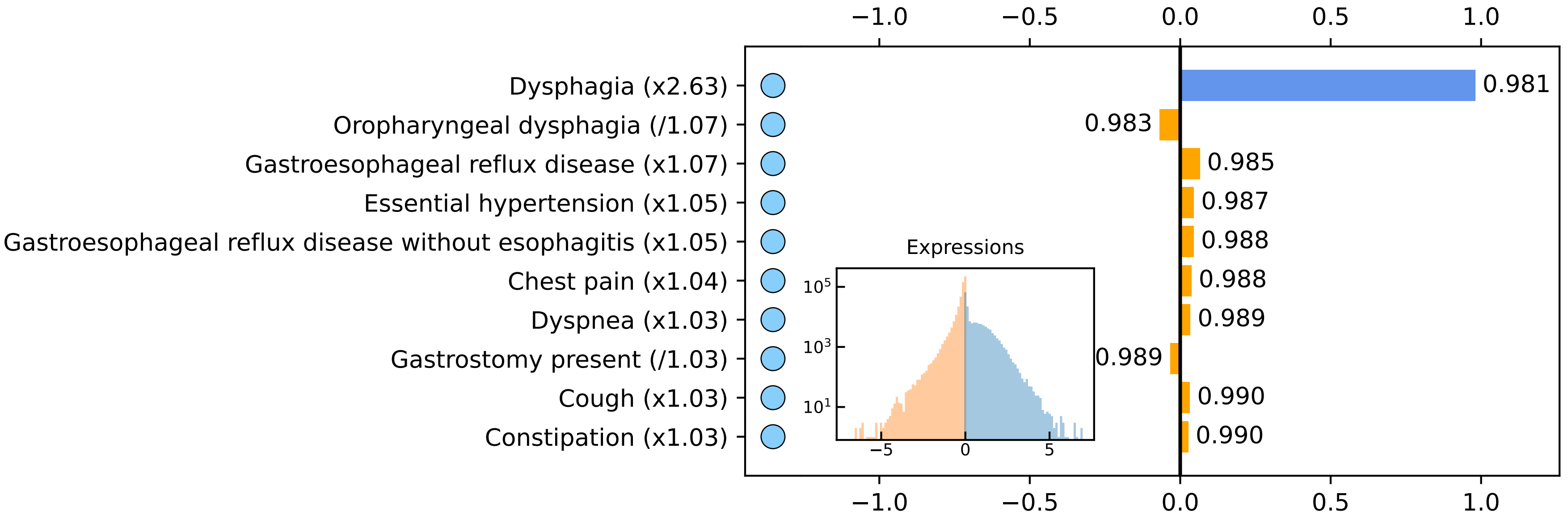

Signature [1265]; 1 To Reach 0.975, (10 Plotted)

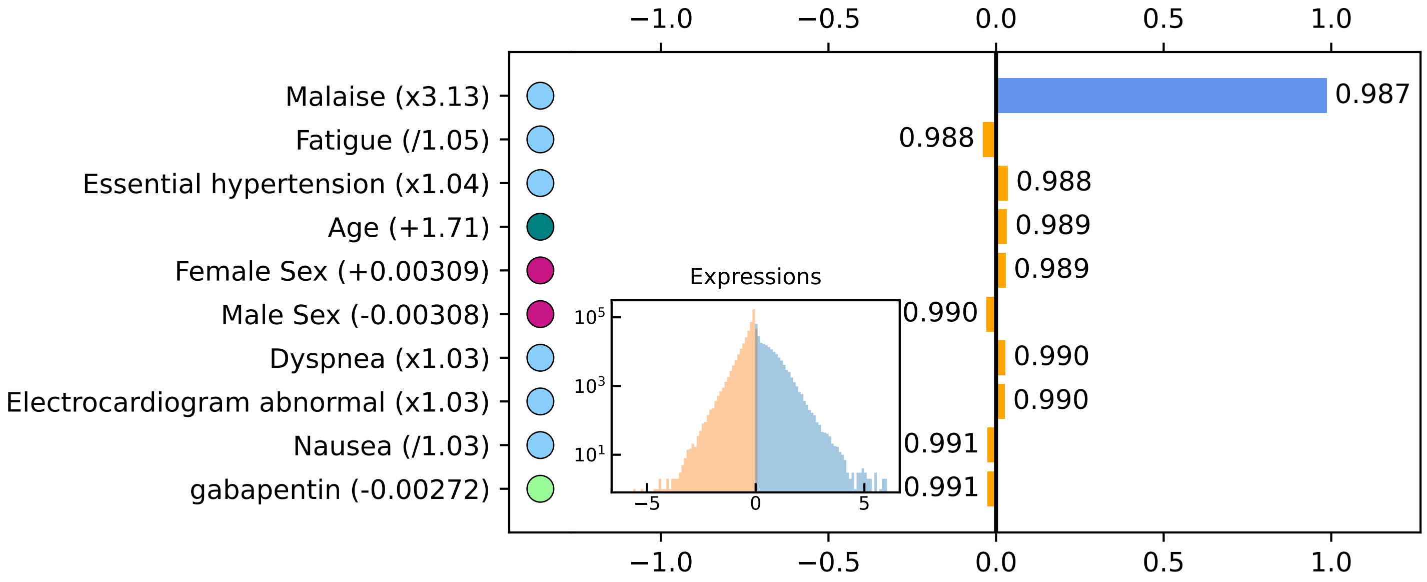

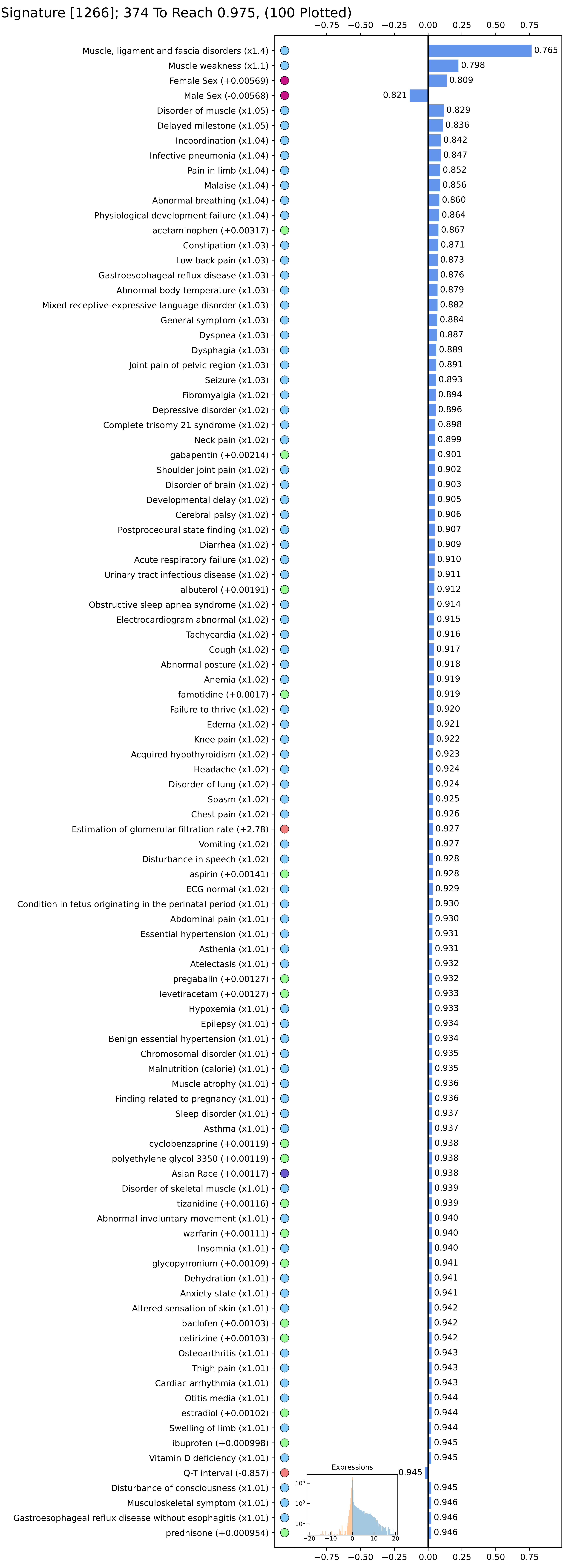
Signature [1266]; 374 To Reach 0.975, (100 Plotted)
Muscle, ligament and fascia disorders (x1.4) 0.765
Muscle weakness (x1.1) 0.798
Female Sex (+0.00569) 0.809
Male Sex (-0.00568) 0.821
Disorder of muscle (x1.05) 0.829
Delayed milestone (x1.05) 0.836
Incoordination (x1.04) 0.842
Infective pneumonia (x1.04) 0.847
Pain in limb (x1.04) 0.852
Malaise (x1.04) 0.856
Abnormal breathing (x1.04) 0.860
Physiological development failure (x1.04) 0.864
acetaminophen (+0.00317) 0.867
Constipation (x1.03) 0.871
Low back pain (x1.03) 0.873
Gastroesophageal reflux disease (x1.03) 0.876
Abnormal body temperature (x1.03) 0.879
Mixed receptive-expressive language disorder (x1.03) 0.882
General symptom (x1.03) 0.884
Dyspnea (x1.03) 0.887
Dysphagia (x1.03) 0.889
Joint pain of pelvic region (x1.03) 0.891
Seizure (x1.03) 0.893
Fibromyalgia (x1.02) 0.894
Depressive disorder (x1.02) 0.896
Complete trisomy 21 syndrome (x1.02) 0.898
Neck pain (x1.02) 0.899
gabapentin (+0.00214) 0.901
Shoulder joint pain (x1.02) 0.902
Disorder of brain (x1.02) 0.903
Developmental delay (x1.02) 0.905
Cerebral palsy (x1.02) 0.906
Postprocedural state finding (x1.02) 0.907
Diarrhea (x1.02) 0.909
Acute respiratory failure (x1.02) 0.910
Urinary tract infectious disease (x1.02) 0.911
albuterol (+0.00191) 0.912
Obstructive sleep apnea syndrome (x1.02) 0.914
Electrocardiogram abnormal (x1.02) 0.915
Tachycardia (x1.02) 0.916
Cough (x1.02) 0.917
Abnormal posture (x1.02) 0.918
Anemia (x1.02) 0.919
famotidine (+0.0017) 0.919
Failure to thrive (x1.02) 0.920
Edema (x1.02) 0.921
Knee pain (x1.02) 0.922
Acquired hypothyroidism (x1.02) 0.923
Headache (x1.02) 0.924
Disorder of lung (x1.02) 0.924
Spasm (x1.02) 0.925
Chest pain (x1.02) 0.926
Estimation of glomerular filtration rate (+2.78) 0.927
Vomiting (x1.02) 0.927
Disturbance in speech (x1.02) 0.928
aspirin (+0.00141) 0.928
ECG normal (x1.02) 0.929
Condition in fetus originating in the perinatal period (x1.01) 0.930
Abdominal pain (x1.01) 0.930
Essential hypertension (x1.01) 0.931
Asthenia (x1.01) 0.931
Atelectasis (x1.01) 0.932
pregabalin (+0.00127) 0.932
levetiracetam (+0.00127) 0.933
Hypoxemia (x1.01) 0.933
Epilepsy (x1.01) 0.934
Benign essential hypertension (x1.01) 0.934
Chromosomal disorder (x1.01) 0.935
Malnutrition (calorie) (x1.01) 0.935
Muscle atrophy (x1.01) 0.936
Finding related to pregnancy (x1.01) 0.936
Sleep disorder (x1.01) 0.937
Asthma (x1.01) 0.937
cyclobenzaprine (+0.00119) 0.938
polyethylene glycol 3350 (+0.00119) 0.938
Asian Race (+0.00117) 0.938
Disorder of skeletal muscle (x1.01) 0.939
tizanidine (+0.00116) 0.939
Abnormal involuntary movement (x1.01) 0.940
warfarin (+0.00111) 0.940
Insomnia (x1.01) 0.940
glycopyrronium (+0.00109) 0.941
Dehydration (x1.01) 0.941
Anxiety state (x1.01) 0.941
Altered sensation of skin (x1.01) 0.942
baclofen (+0.00103) 0.942
cetirizine (+0.00103) 0.942
Osteoarthritis (x1.01) 0.943
Thigh pain (x1.01) 0.943
Cardiac arrhythmia (x1.01) 0.943
Otitis media (x1.01) 0.944
estradiol (+0.00102) 0.944
Swelling of limb (x1.01) 0.944
ibuprofen (+0.000998) 0.945
Vitamin D deficiency (x1.01) 0.945
Q-T interval (-0.857) 0.945
Disturbance of consciousness (x1.01) 0.945
Musculoskeletal symptom (x1.01) 0.946
Gastroesophageal reflux disease without esophagitis (x1.01) 0.946
prednisone (+0.000954) 0.946
−0.75 −0.50 −0.25 0.00 0.25 0.50 0.75
Expressions
10^5 10^3 10^1
−20 −10 0 10 20

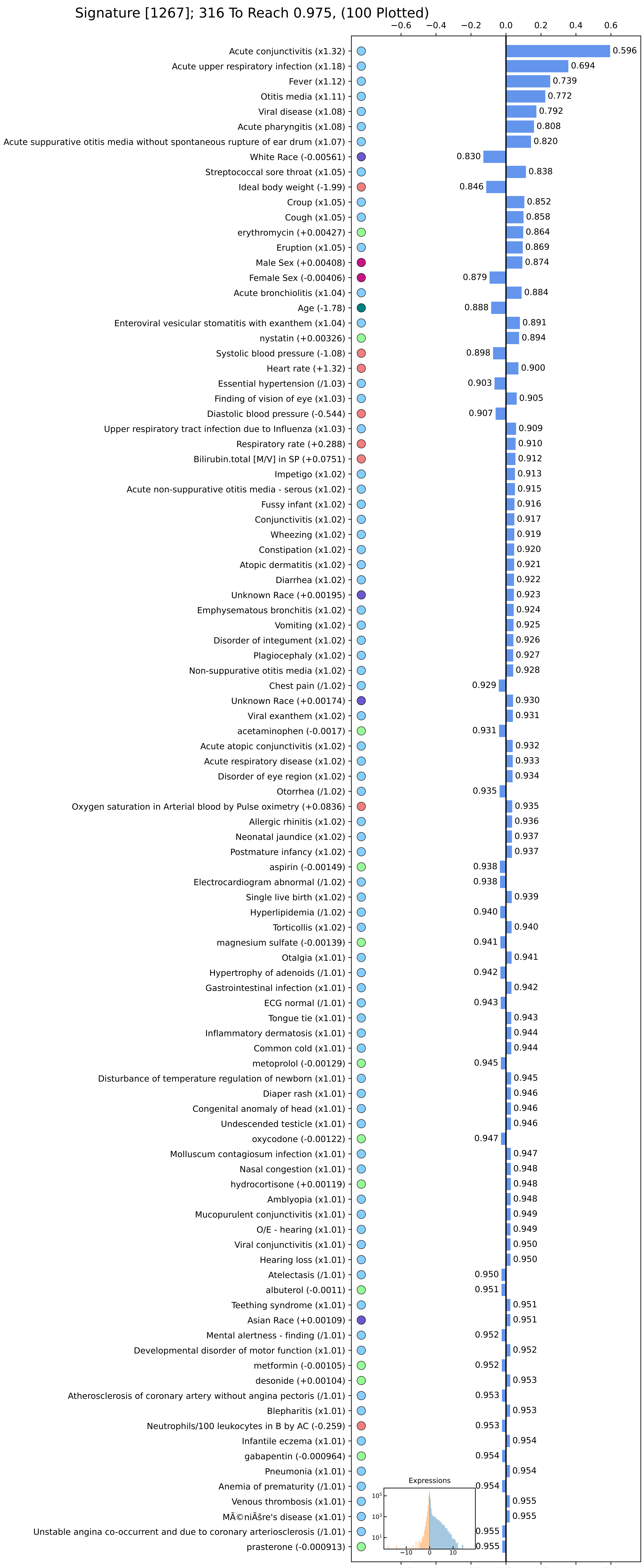

Signature [1267]; 316 To Reach 0.975, (100 Plotted)
Acute conjunctivitis (x1.32) 0.596
Acute upper respiratory infection (x1.18) 0.694
Fever (x1.12) 0.739
Otitis media (x1.11) 0.772
Viral disease (x1.08) 0.792
Acute pharyngitis (x1.08) 0.808
Acute suppurative otitis media without spontaneous rupture of ear drum (x1.07) 0.820
White Race (-0.00561) 0.830
Streptococcal sore throat (x1.05) 0.838
Ideal body weight (-1.99) 0.846
Croup (x1.05) 0.852
Cough (x1.05) 0.858
erythromycin (+0.00427) 0.864
Eruption (x1.05) 0.869
Male Sex (+0.00408) 0.874
Female Sex (-0.00406) 0.879
Acute bronchiolitis (x1.04) 0.884
Age (-1.78) 0.888
Enteroviral vesicular stomatitis with exanthem (x1.04) 0.891
nystatin (+0.00326) 0.894
Systolic blood pressure (-1.08) 0.898
Heart rate (+1.32) 0.900
Essential hypertension (/1.03) 0.903
Finding of vision of eye (x1.03) 0.905
Diastolic blood pressure (-0.544) 0.907
Upper respiratory tract infection due to Influenza (x1.03) 0.909
Respiratory rate (+0.288) 0.910
Bilirubin.total [M/V] in SP (+0.0751) 0.912
Impetigo (x1.02) 0.913
Acute non-suppurative otitis media - serous (x1.02) 0.915
Fussy infant (x1.02) 0.916
Conjunctivitis (x1.02) 0.917
Wheezing (x1.02) 0.919
Constipation (x1.02) 0.920
Atopic dermatitis (x1.02) 0.921
Diarrhea (x1.02) 0.922
Unknown Race (+0.00195) 0.923
Emphysematous bronchitis (x1.02) 0.924
Vomiting (x1.02) 0.925
Disorder of integument (x1.02) 0.926
Plagiocephaly (x1.02) 0.927
Non-suppurative otitis media (x1.02) 0.928
Chest pain (/1.02) 0.929
Unknown Race (+0.00174) 0.930
Viral exanthem (x1.02) 0.931
acetaminophen (-0.0017) 0.931
Acute atopic conjunctivitis (x1.02) 0.932
Acute respiratory disease (x1.02) 0.933
Disorder of eye region (x1.02) 0.934
Otorrhea (/1.02) 0.935
Oxygen saturation in Arterial blood by Pulse oximetry (+0.0836) 0.935
Allergic rhinitis (x1.02) 0.936
Neonatal jaundice (x1.02) 0.937
Postmature infancy (x1.02) 0.937
aspirin (-0.00149) 0.938
Electrocardiogram abnormal (/1.02) 0.938
Single live birth (x1.02) 0.939
Hyperlipidemia (/1.02) 0.940
Torticollis (x1.02) 0.940
magnesium sulfate (-0.00139) 0.941
Otalgia (x1.01) 0.941
Hypertrophy of adenoids (/1.01) 0.942
Gastrointestinal infection (x1.01) 0.942
ECG normal (/1.01) 0.943
Tongue tie (x1.01) 0.943
Inflammatory dermatosis (x1.01) 0.944
Common cold (x1.01) 0.944
metoprolol (-0.00129) 0.945
Disturbance of temperature regulation of newborn (x1.01) 0.945
Diaper rash (x1.01) 0.946
Congenital anomaly of head (x1.01) 0.946
Undescended testicle (x1.01) 0.946
oxycodone (-0.00122) 0.947
Molluscum contagiosum infection (x1.01) 0.947
Nasal congestion (x1.01) 0.948
hydrocortisone (+0.00119) 0.948
Amblyopia (x1.01) 0.948
Mucopurulent conjunctivitis (x1.01) 0.949
O/E - hearing (x1.01) 0.949
Viral conjunctivitis (x1.01) 0.950
Hearing loss (x1.01) 0.950
Atelectasis (/1.01) 0.950
albuterol (-0.0011) 0.951
Teething syndrome (x1.01) 0.951
Asian Race (+0.00109) 0.951
Mental alertness - finding (/1.01) 0.952
Developmental disorder of motor function (x1.01) 0.952
metformin (-0.00105) 0.952
desonide (+0.00104) 0.953
Atherosclerosis of coronary artery without angina pectoris (/1.01) 0.953
Blepharitis (x1.01) 0.953
Neutrophils/100 leukocytes in B by AC (-0.259) 0.953
Infantile eczema (x1.01) 0.954
gabapentin (-0.000964) 0.954
Pneumonia (x1.01) 0.954
Anemia of prematurity (/1.01) 0.954
Venous thrombosis (x1.01) 0.955
MÃ©niÃšre's disease (x1.01) 0.955
Unstable angina co-occurrent and due to coronary arteriosclerosis (/1.01) 0.955
prasterone (-0.000913) 0.955
−0.6 −0.4 −0.2 0.0 0.2 0.4 0.6
Expressions
10^5
10^3
10^1
−10 0 10

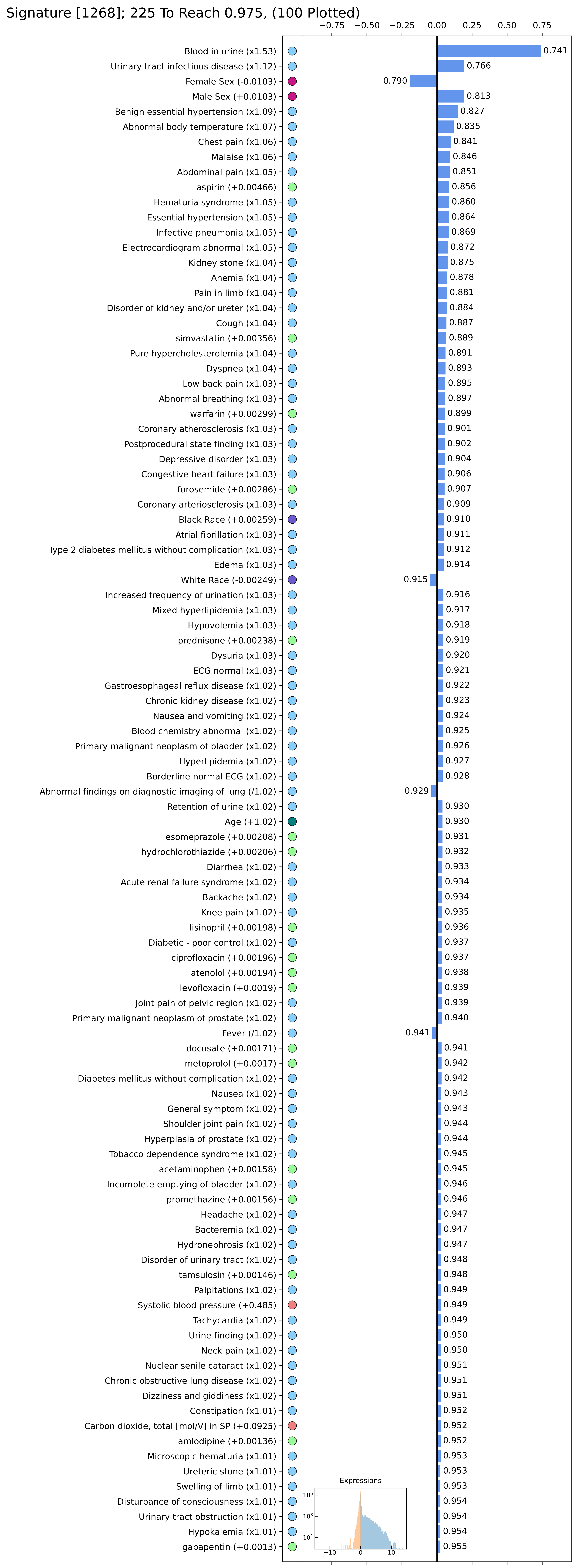

Signature [1268]; 225 To Reach 0.975, (100 Plotted)
Blood in urine (x1.53) 0.741
Urinary tract infectious disease (x1.12) 0.766
Female Sex (-0.0103) 0.790
Male Sex (+0.0103) 0.813
Benign essential hypertension (x1.09) 0.827
Abnormal body temperature (x1.07) 0.835
Chest pain (x1.06) 0.841
Malaise (x1.06) 0.846
Abdominal pain (x1.05) 0.851
aspirin (+0.00466) 0.856
Hematuria syndrome (x1.05) 0.860
Essential hypertension (x1.05) 0.864
Infective pneumonia (x1.05) 0.869
Electrocardiogram abnormal (x1.05) 0.872
Kidney stone (x1.04) 0.875
Anemia (x1.04) 0.878
Pain in limb (x1.04) 0.881
Disorder of kidney and/or ureter (x1.04) 0.884
Cough (x1.04) 0.887
simvastatin (+0.00356) 0.889
Pure hypercholesterolemia (x1.04) 0.891
Dyspnea (x1.04) 0.893
Low back pain (x1.03) 0.895
Abnormal breathing (x1.03) 0.897
warfarin (+0.00299) 0.899
Coronary atherosclerosis (x1.03) 0.901
Postprocedural state finding (x1.03) 0.902
Depressive disorder (x1.03) 0.904
Congestive heart failure (x1.03) 0.906
furosemide (+0.00286) 0.907
Coronary arteriosclerosis (x1.03) 0.909
Black Race (+0.00259) 0.910
Atrial fibrillation (x1.03) 0.911
Type 2 diabetes mellitus without complication (x1.03) 0.912
Edema (x1.03) 0.914
White Race (-0.00249) 0.915
Increased frequency of urination (x1.03) 0.916
Mixed hyperlipidemia (x1.03) 0.917
Hypovolemia (x1.03) 0.918
prednisone (+0.00238) 0.919
Dysuria (x1.03) 0.920
ECG normal (x1.03) 0.921
Gastroesophageal reflux disease (x1.02) 0.922
Chronic kidney disease (x1.02) 0.923
Nausea and vomiting (x1.02) 0.924
Blood chemistry abnormal (x1.02) 0.925
Primary malignant neoplasm of bladder (x1.02) 0.926
Hyperlipidemia (x1.02) 0.927
Borderline normal ECG (x1.02) 0.928
Abnormal findings on diagnostic imaging of lung (/1.02) 0.929
Retention of urine (x1.02) 0.930
Age (+1.02) 0.930
esomeprazole (+0.00208) 0.931
hydrochlorothiazide (+0.00206) 0.932
Diarrhea (x1.02) 0.933
Acute renal failure syndrome (x1.02) 0.934
Backache (x1.02) 0.934
Knee pain (x1.02) 0.935
lisinopril (+0.00198) 0.936
Diabetic - poor control (x1.02) 0.937
ciprofloxacin (+0.00196) 0.937
atenolol (+0.00194) 0.938
levofloxacin (+0.0019) 0.939
Joint pain of pelvic region (x1.02) 0.939
Primary malignant neoplasm of prostate (x1.02) 0.940
Fever (/1.02) 0.941
docusate (+0.00171) 0.941
metoprolol (+0.0017) 0.942
Diabetes mellitus without complication (x1.02) 0.942
Nausea (x1.02) 0.943
General symptom (x1.02) 0.943
Shoulder joint pain (x1.02) 0.944
Hyperplasia of prostate (x1.02) 0.944
Tobacco dependence syndrome (x1.02) 0.945
acetaminophen (+0.00158) 0.945
Incomplete emptying of bladder (x1.02) 0.946
promethazine (+0.00156) 0.946
Headache (x1.02) 0.947
Bacteremia (x1.02) 0.947
Hydronephrosis (x1.02) 0.947
Disorder of urinary tract (x1.02) 0.948
tamsulosin (+0.00146) 0.948
Palpitations (x1.02) 0.949
Systolic blood pressure (+0.485) 0.949
Tachycardia (x1.02) 0.949
Urine finding (x1.02) 0.950
Neck pain (x1.02) 0.950
Nuclear senile cataract (x1.02) 0.951
Chronic obstructive lung disease (x1.02) 0.951
Dizziness and giddiness (x1.02) 0.951
Constipation (x1.01) 0.952
Carbon dioxide, total [mol/V] in SP (+0.0925) 0.952
amlodipine (+0.00136) 0.952
Microscopic hematuria (x1.01) 0.953
Ureteric stone (x1.01) 0.953
Swelling of limb (x1.01) 0.953
Disturbance of consciousness (x1.01) 0.954
Urinary tract obstruction (x1.01) 0.954
Hypokalemia (x1.01) 0.954
gabapentin (+0.0013) 0.955
−0.75 −0.50 −0.25 0.00 0.25 0.50 0.75
Expressions

Signature [1269]; 87 To Reach 0.975, (87 Plotted)

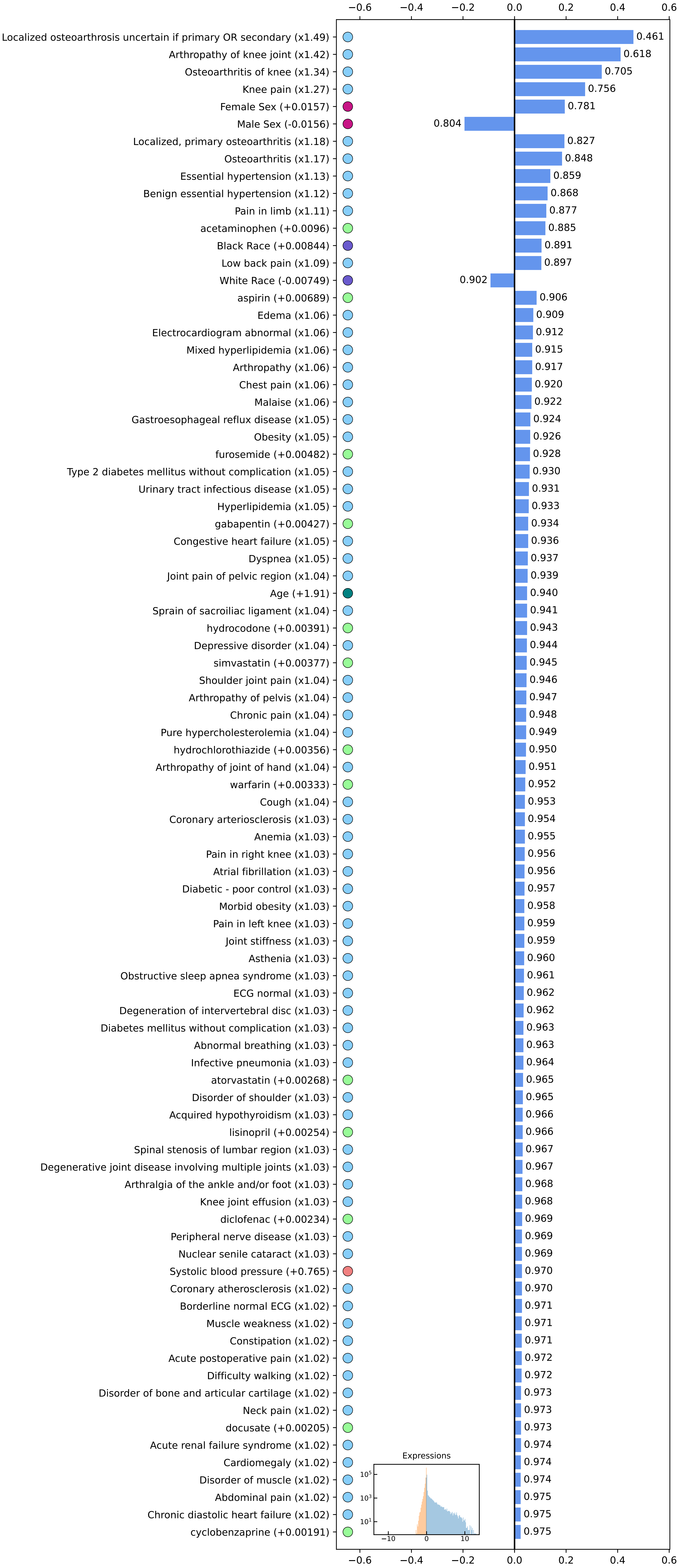

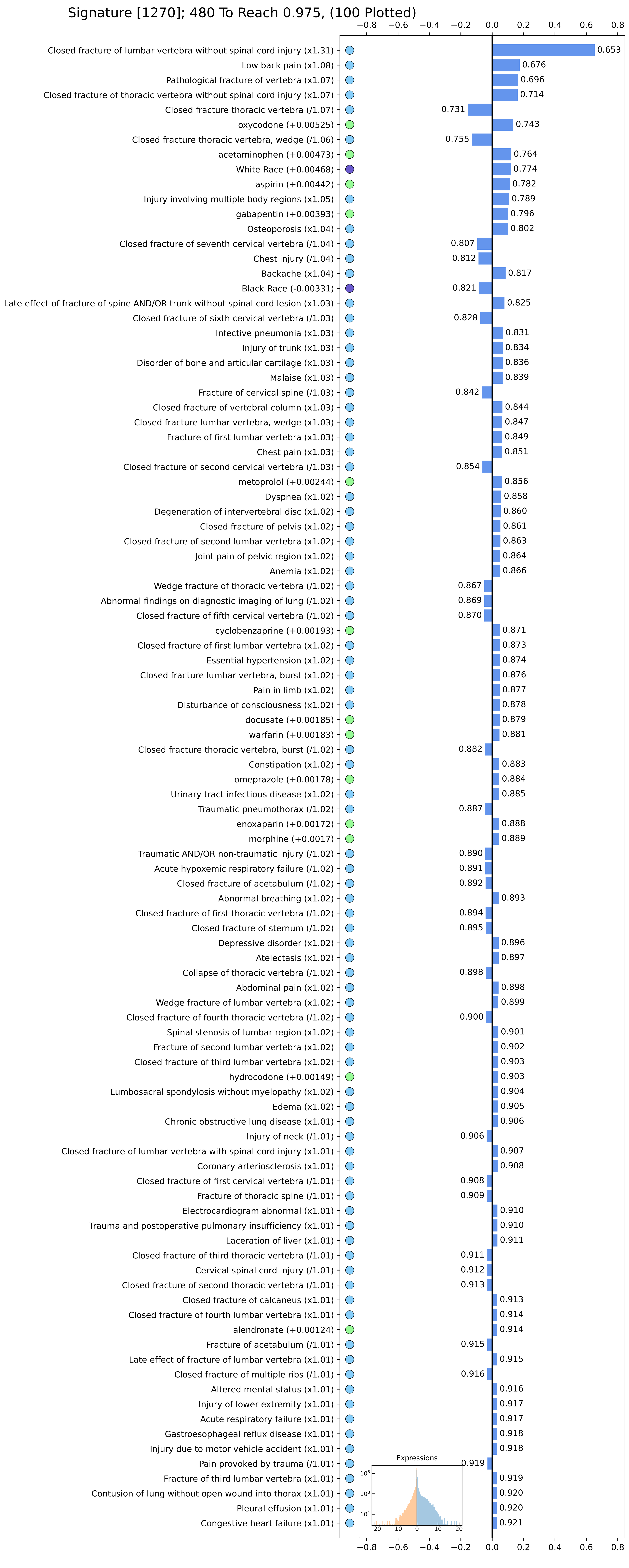
Signature [1270]; 480 To Reach 0.975, (100 Plotted)
−0.8 −0.6 −0.4 −0.2 0.0 0.2 0.4 0.6 0.8
Closed fracture of lumbar vertebra without spinal cord injury (x1.31) 0.653
Low back pain (x1.08) 0.676
Pathological fracture of vertebra (x1.07) 0.696
Closed fracture of thoracic vertebra without spinal cord injury (x1.07) 0.714
Closed fracture thoracic vertebra (/1.07) 0.731
oxycodone (+0.00525) 0.743
Closed fracture thoracic vertebra, wedge (/1.06) 0.755
acetaminophen (+0.00473) 0.764
White Race (+0.00468) 0.774
aspirin (+0.00442) 0.782
Injury involving multiple body regions (x1.05) 0.789
gabapentin (+0.00393) 0.796
Osteoporosis (x1.04) 0.802
Closed fracture of seventh cervical vertebra (/1.04) 0.807
Chest injury (/1.04) 0.812
Backache (x1.04) 0.817
Black Race (-0.00331) 0.821
Late effect of fracture of spine AND/OR trunk without spinal cord lesion (x1.03) 0.825
Closed fracture of sixth cervical vertebra (/1.03) 0.828
Infective pneumonia (x1.03) 0.831
Injury of trunk (x1.03) 0.834
Disorder of bone and articular cartilage (x1.03) 0.836
Malaise (x1.03) 0.839
Fracture of cervical spine (/1.03) 0.842
Closed fracture of vertebral column (x1.03) 0.844
Closed fracture lumbar vertebra, wedge (x1.03) 0.847
Fracture of first lumbar vertebra (x1.03) 0.849
Chest pain (x1.03) 0.851
Closed fracture of second cervical vertebra (/1.03) 0.854
metoprolol (+0.00244) 0.856
Dyspnea (x1.02) 0.858
Degeneration of intervertebral disc (x1.02) 0.860
Closed fracture of pelvis (x1.02) 0.861
Closed fracture of second lumbar vertebra (x1.02) 0.863
Joint pain of pelvic region (x1.02) 0.864
Anemia (x1.02) 0.866
Wedge fracture of thoracic vertebra (/1.02) 0.867
Abnormal findings on diagnostic imaging of lung (/1.02) 0.869
Closed fracture of fifth cervical vertebra (/1.02) 0.870
cyclobenzaprine (+0.00193) 0.871
Closed fracture of first lumbar vertebra (x1.02) 0.873
Essential hypertension (x1.02) 0.874
Closed fracture lumbar vertebra, burst (x1.02) 0.876
Pain in limb (x1.02) 0.877
Disturbance of consciousness (x1.02) 0.878
docusate (+0.00185) 0.879
warfarin (+0.00183) 0.881
Closed fracture thoracic vertebra, burst (/1.02) 0.882
Constipation (x1.02) 0.883
omeprazole (+0.00178) 0.884
Urinary tract infectious disease (x1.02) 0.885
Traumatic pneumothorax (/1.02) 0.887
enoxaparin (+0.00172) 0.888
morphine (+0.0017) 0.889
Traumatic AND/OR non-traumatic injury (/1.02) 0.890
Acute hypoxemic respiratory failure (/1.02) 0.891
Closed fracture of acetabulum (/1.02) 0.892
Abnormal breathing (x1.02) 0.893
Closed fracture of first thoracic vertebra (/1.02) 0.894
Closed fracture of sternum (/1.02) 0.895
Depressive disorder (x1.02) 0.896
Atelectasis (x1.02) 0.897
Collapse of thoracic vertebra (/1.02) 0.898
Abdominal pain (x1.02) 0.898
Wedge fracture of lumbar vertebra (x1.02) 0.899
Closed fracture of fourth thoracic vertebra (/1.02) 0.900
Spinal stenosis of lumbar region (x1.02) 0.901
Fracture of second lumbar vertebra (x1.02) 0.902
Closed fracture of third lumbar vertebra (x1.02) 0.903
hydrocodone (+0.00149) 0.903
Lumbosacral spondylosis without myelopathy (x1.02) 0.904
Edema (x1.02) 0.905
Chronic obstructive lung disease (x1.01) 0.906
Injury of neck (/1.01) 0.906
Closed fracture of lumbar vertebra with spinal cord injury (x1.01) 0.907
Coronary arteriosclerosis (x1.01) 0.908
Closed fracture of first cervical vertebra (/1.01) 0.908
Fracture of thoracic spine (/1.01) 0.909
Electrocardiogram abnormal (x1.01) 0.910
Trauma and postoperative pulmonary insufficiency (x1.01) 0.910
Laceration of liver (x1.01) 0.911
Closed fracture of third thoracic vertebra (/1.01) 0.911
Cervical spinal cord injury (/1.01) 0.912
Closed fracture of second thoracic vertebra (/1.01) 0.913
Closed fracture of calcaneus (x1.01) 0.913
Closed fracture of fourth lumbar vertebra (x1.01) 0.914
alendronate (+0.00124) 0.914
Fracture of acetabulum (/1.01) 0.915
Late effect of fracture of lumbar vertebra (x1.01) 0.915
Closed fracture of multiple ribs (/1.01) 0.916
Altered mental status (x1.01) 0.916
Injury of lower extremity (x1.01) 0.917
Acute respiratory failure (x1.01) 0.917
Gastroesophageal reflux disease (x1.01) 0.918
Injury due to motor vehicle accident (x1.01) 0.918
Pain provoked by trauma (/1.01) 0.919
Fracture of third lumbar vertebra (x1.01) 0.919
Contusion of lung without open wound into thorax (x1.01) 0.920
Pleural effusion (x1.01) 0.920
Congestive heart failure (x1.01) 0.921
Expressions
−20 −10 0 10 20
−0.8 −0.6 −0.4 −0.2 0.0 0.2 0.4 0.6 0.8

Signature [1271]; 83 To Reach 0.975, (83 Plotted)

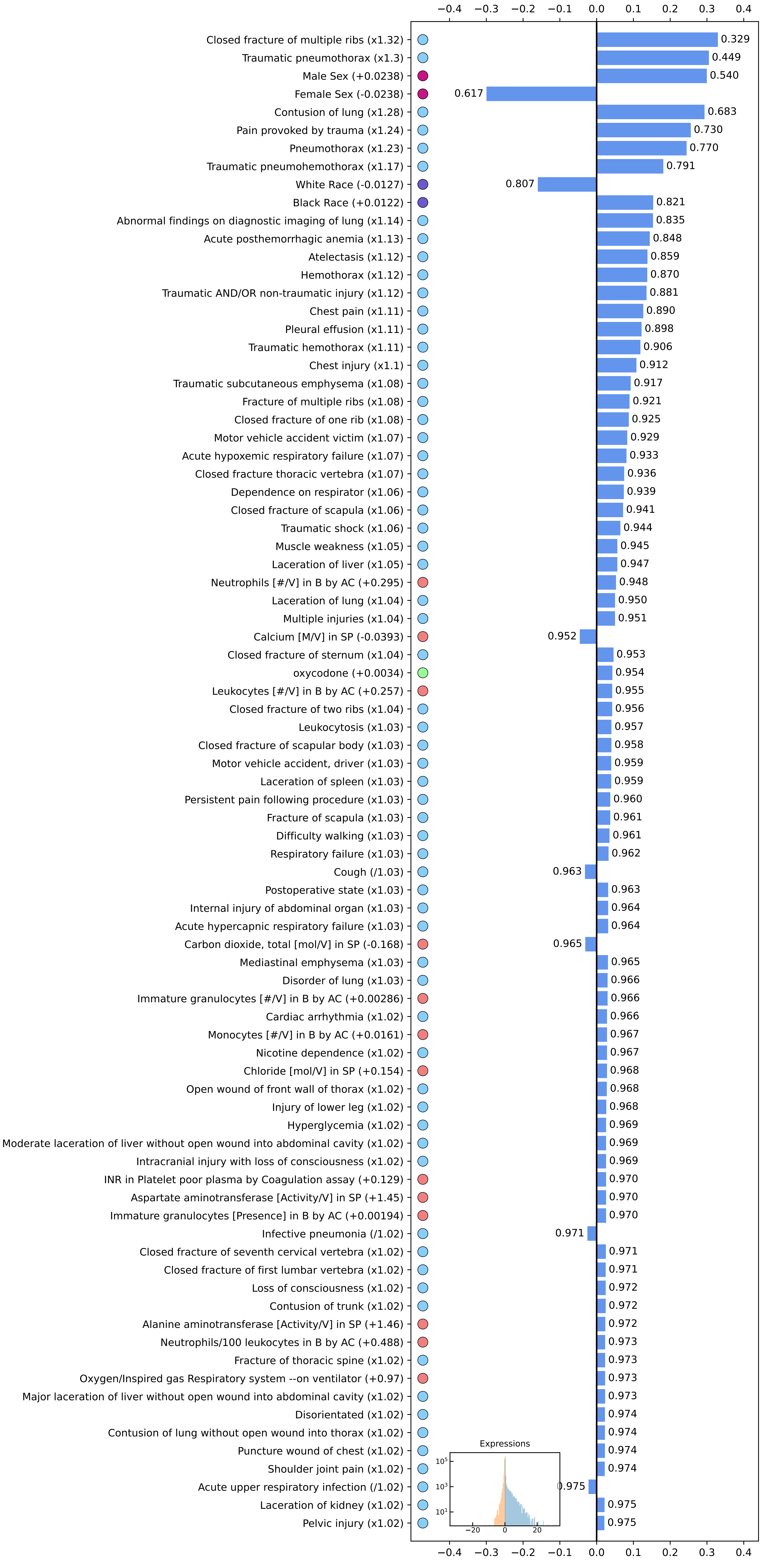

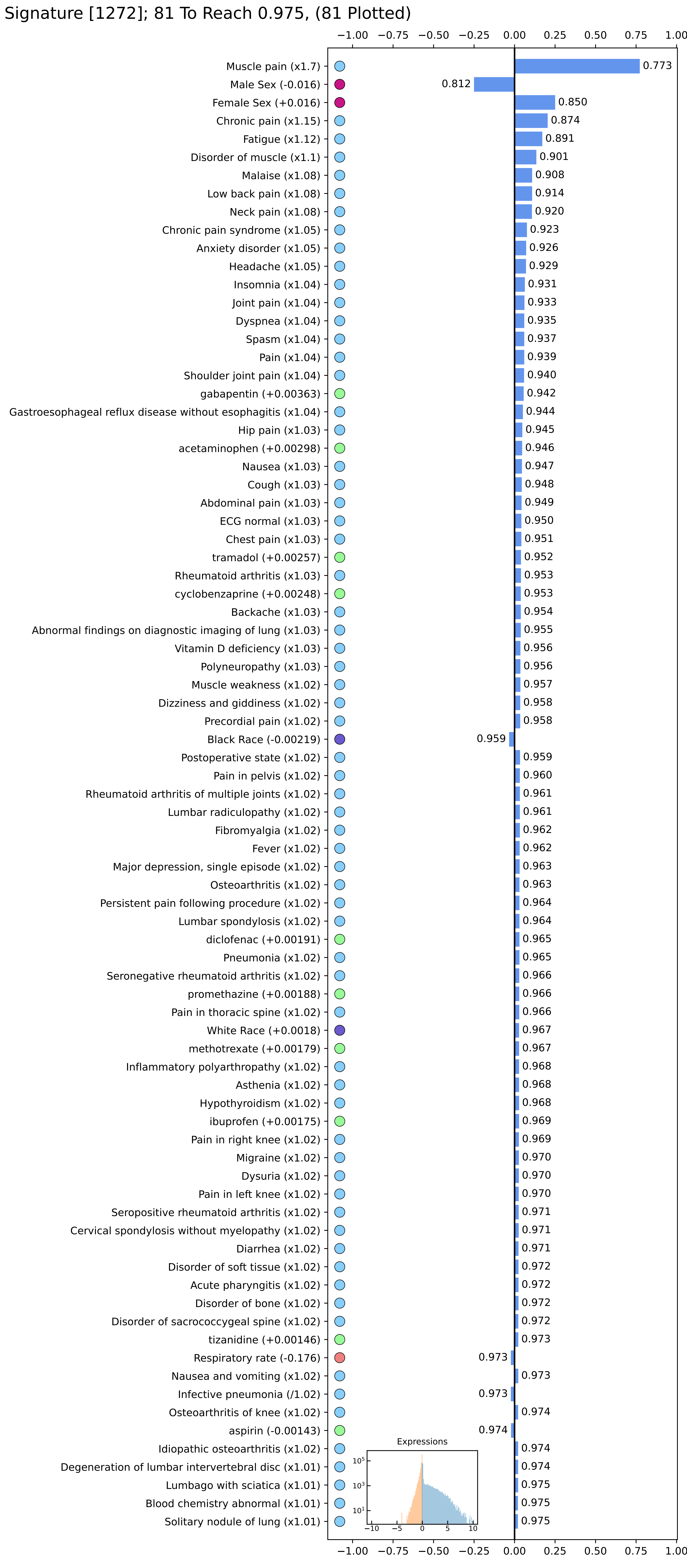

Signature [1272]; 81 To Reach 0.975, (81 Plotted)
Muscle pain (x1.7) 0.773
Male Sex (-0.016) 0.812
Female Sex (+0.016) 0.850
Chronic pain (x1.15) 0.874
Fatigue (x1.12) 0.891
Disorder of muscle (x1.1) 0.901
Malaise (x1.08) 0.908
Low back pain (x1.08) 0.914
Neck pain (x1.08) 0.920
Chronic pain syndrome (x1.05) 0.923
Anxiety disorder (x1.05) 0.926
Headache (x1.05) 0.929
Insomnia (x1.04) 0.931
Joint pain (x1.04) 0.933
Dyspnea (x1.04) 0.935
Spasm (x1.04) 0.937
Pain (x1.04) 0.939
Shoulder joint pain (x1.04) 0.940
gabapentin (+0.00363) 0.942
Gastroesophageal reflux disease without esophagitis (x1.04) 0.944
Hip pain (x1.03) 0.945
acetaminophen (+0.00298) 0.946
Nausea (x1.03) 0.947
Cough (x1.03) 0.948
Abdominal pain (x1.03) 0.949
ECG normal (x1.03) 0.950
Chest pain (x1.03) 0.951
tramadol (+0.00257) 0.952
Rheumatoid arthritis (x1.03) 0.953
cyclobenzaprine (+0.00248) 0.953
Backache (x1.03) 0.954
Abnormal findings on diagnostic imaging of lung (x1.03) 0.955
Vitamin D deficiency (x1.03) 0.956
Polyneuropathy (x1.03) 0.956
Muscle weakness (x1.02) 0.957
Dizziness and giddiness (x1.02) 0.958
Precordial pain (x1.02) 0.958
Black Race (-0.00219) 0.959
Postoperative state (x1.02) 0.959
Pain in pelvis (x1.02) 0.960
Rheumatoid arthritis of multiple joints (x1.02) 0.961
Lumbar radiculopathy (x1.02) 0.961
Fibromyalgia (x1.02) 0.962
Fever (x1.02) 0.962
Major depression, single episode (x1.02) 0.963
Osteoarthritis (x1.02) 0.963
Persistent pain following procedure (x1.02) 0.964
Lumbar spondylosis (x1.02) 0.964
diclofenac (+0.00191) 0.965
Pneumonia (x1.02) 0.965
Seronegative rheumatoid arthritis (x1.02) 0.966
promethazine (+0.00188) 0.966
Pain in thoracic spine (x1.02) 0.966
White Race (+0.0018) 0.967
methotrexate (+0.00179) 0.967
Inflammatory polyarthropathy (x1.02) 0.968
Asthenia (x1.02) 0.968
Hypothyroidism (x1.02) 0.968
ibuprofen (+0.00175) 0.969
Pain in right knee (x1.02) 0.969
Migraine (x1.02) 0.970
Dysuria (x1.02) 0.970
Pain in left knee (x1.02) 0.970
Seropositive rheumatoid arthritis (x1.02) 0.971
Cervical spondylosis without myelopathy (x1.02) 0.971
Diarrhea (x1.02) 0.971
Disorder of soft tissue (x1.02) 0.972
Acute pharyngitis (x1.02) 0.972
Disorder of bone (x1.02) 0.972
Disorder of sacrococcygeal spine (x1.02) 0.972
tizanidine (+0.00146) 0.973
Respiratory rate (-0.176) 0.973
Nausea and vomiting (x1.02) 0.973
Infective pneumonia (/1.02) 0.973
Osteoarthritis of knee (x1.02) 0.974
aspirin (-0.00143) 0.974
Idiopathic osteoarthritis (x1.02) 0.974
Degeneration of lumbar intervertebral disc (x1.01) 0.974
Lumbago with sciatica (x1.01) 0.975
Blood chemistry abnormal (x1.01) 0.975
Solitary nodule of lung (x1.01) 0.975
Expressions

Signature [1273]; 343 To Reach 0.975, (100 Plotted)

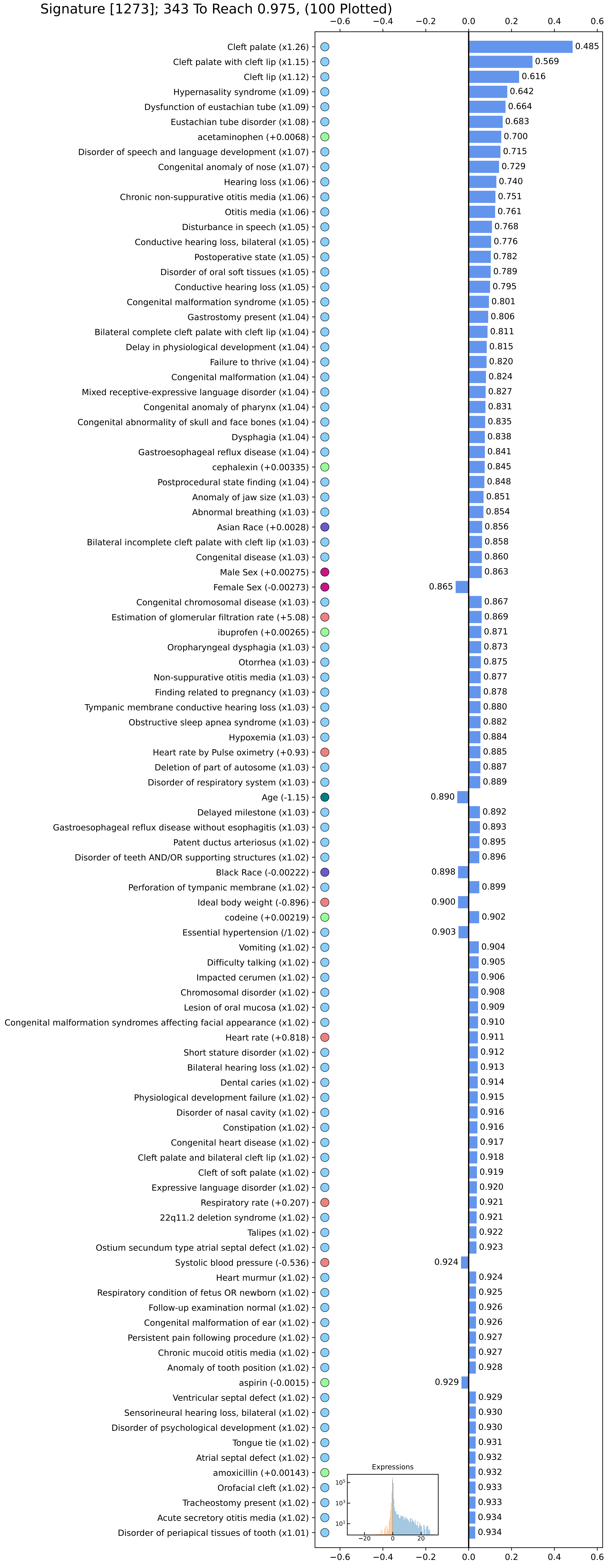

Signature [1274]; 28 To Reach 0.975, (28 Plotted)

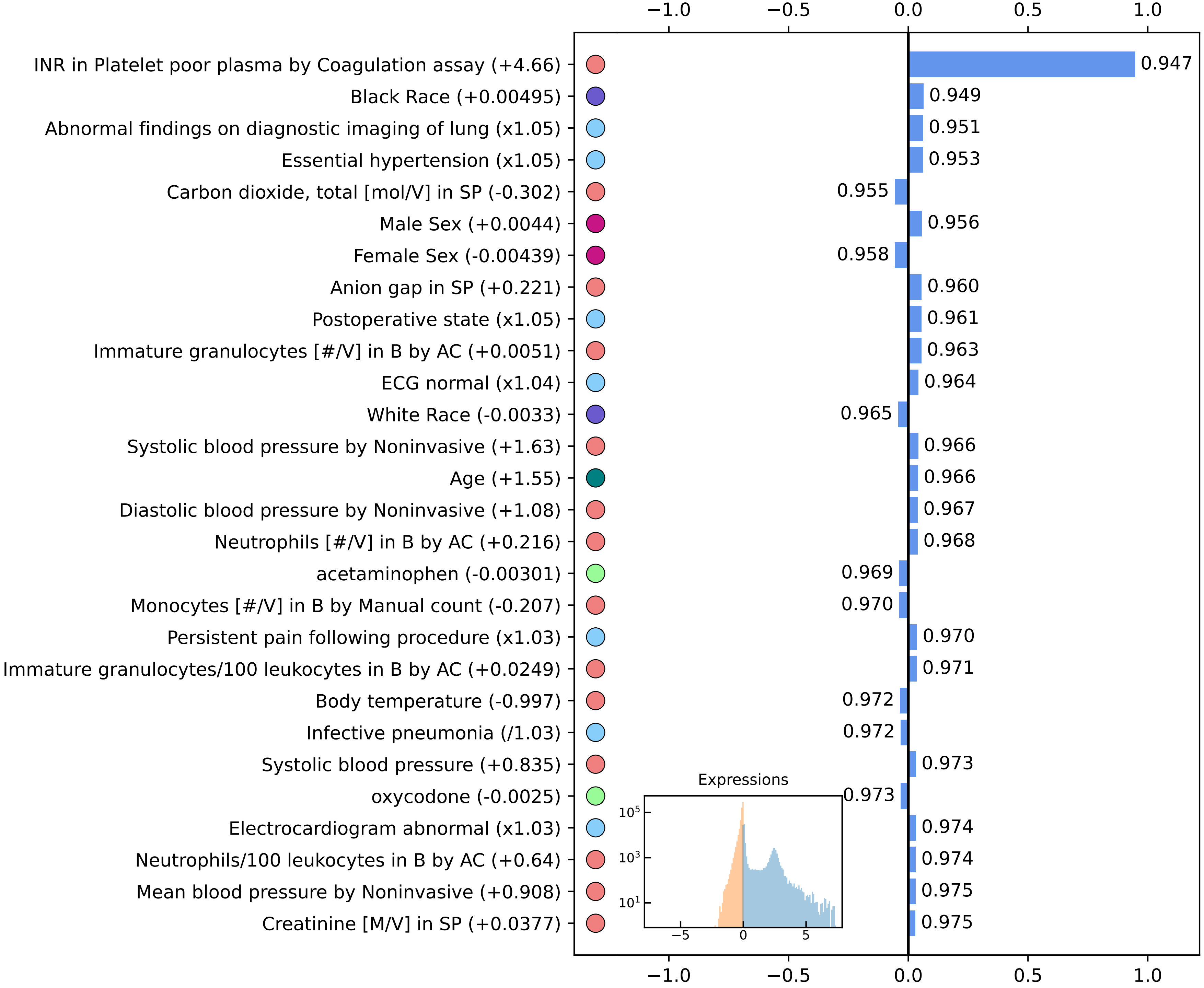

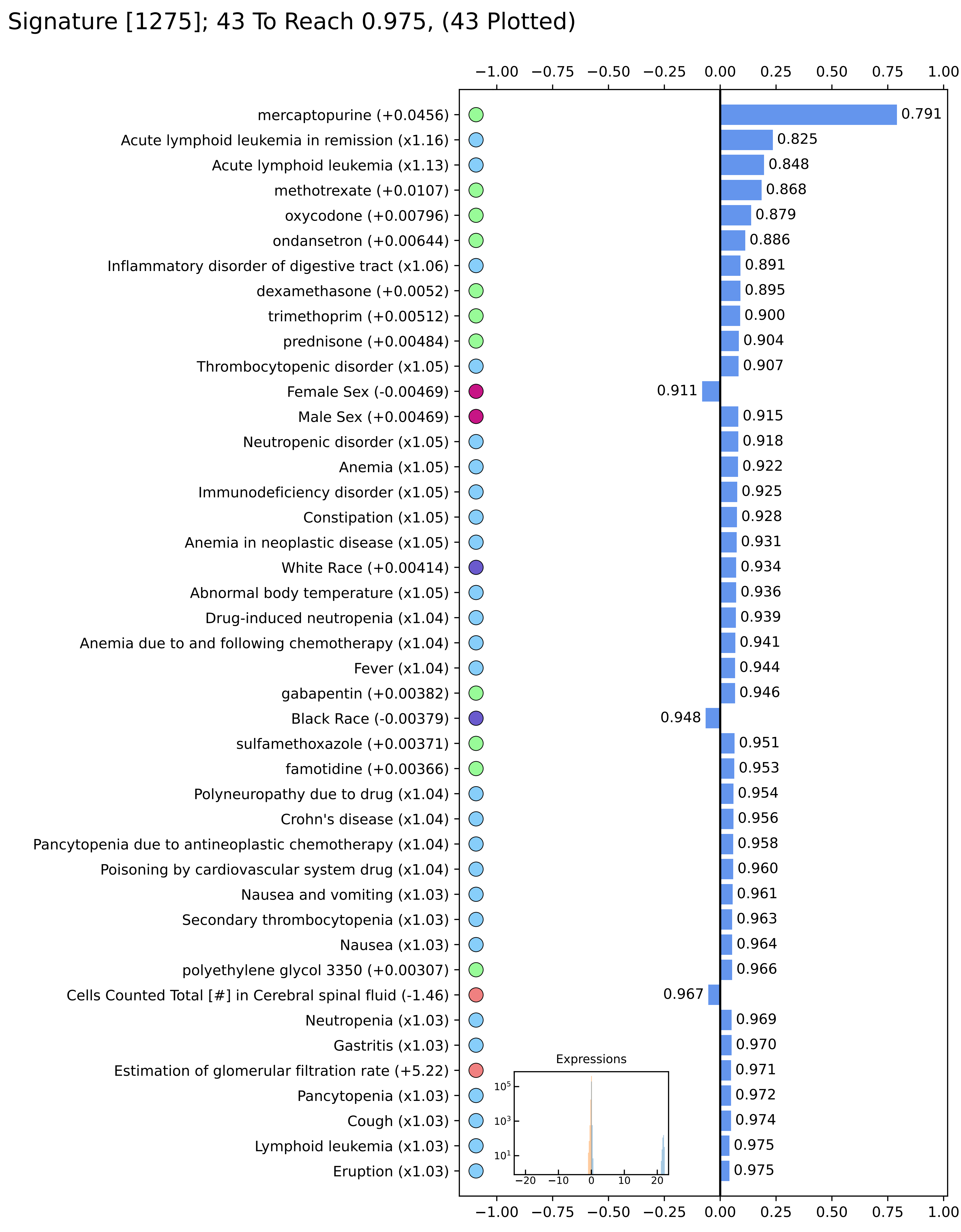

Signature [1275]; 43 To Reach 0.975, (43 Plotted)
mercaptopurine (+0.0456) 0.791
Acute lymphoid leukemia in remission (x1.16) 0.825
Acute lymphoid leukemia (x1.13) 0.848
methotrexate (+0.0107) 0.868
oxycodone (+0.00796) 0.879
ondansetron (+0.00644) 0.886
Inflammatory disorder of digestive tract (x1.06) 0.891
dexamethasone (+0.0052) 0.895
trimethoprim (+0.00512) 0.900
prednisone (+0.00484) 0.904
Thrombocytopenic disorder (x1.05) 0.907
Female Sex (-0.00469) 0.911
Male Sex (+0.00469) 0.915
Neutropenic disorder (x1.05) 0.918
Anemia (x1.05) 0.922
Immunodeficiency disorder (x1.05) 0.925
Constipation (x1.05) 0.928
Anemia in neoplastic disease (x1.05) 0.931
White Race (+0.00414) 0.934
Abnormal body temperature (x1.05) 0.936
Drug-induced neutropenia (x1.04) 0.939
Anemia due to and following chemotherapy (x1.04) 0.941
Fever (x1.04) 0.944
gabapentin (+0.00382) 0.946
Black Race (-0.00379) 0.948
sulfamethoxazole (+0.00371) 0.951
famotidine (+0.00366) 0.953
Polyneuropathy due to drug (x1.04) 0.954
Crohn's disease (x1.04) 0.956
Pancytopenia due to antineoplastic chemotherapy (x1.04) 0.958
Poisoning by cardiovascular system drug (x1.04) 0.960
Nausea and vomiting (x1.03) 0.961
Secondary thrombocytopenia (x1.03) 0.963
Nausea (x1.03) 0.964
polyethylene glycol 3350 (+0.00307) 0.966
Cells Counted Total [#] in Cerebral spinal fluid (-1.46) 0.967
Neutropenia (x1.03) 0.969
Gastritis (x1.03) 0.970
Estimation of glomerular filtration rate (+5.22) 0.971
Pancytopenia (x1.03) 0.972
Cough (x1.03) 0.974
Lymphoid leukemia (x1.03) 0.975
Eruption (x1.03) 0.975
−1.00 −0.75 −0.50 −0.25 0.00 0.25 0.50 0.75 1.00
Expressions
10^5 10^3 10^1
−20 −10 0 10 20

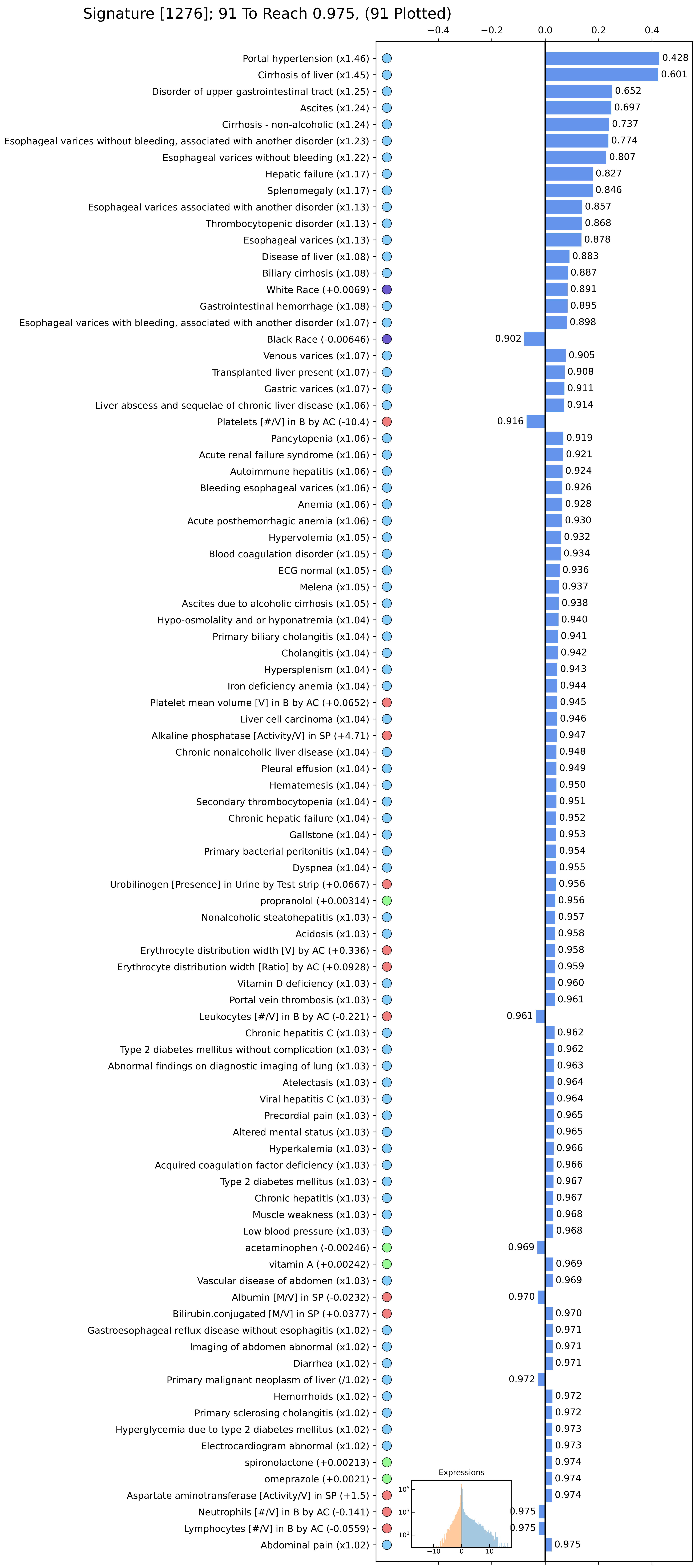
Signature [1276]; 91 To Reach 0.975, (91 Plotted)
Portal hypertension (x1.46) 0.428
Cirrhosis of liver (x1.45) 0.601
Disorder of upper gastrointestinal tract (x1.25) 0.652
Ascites (x1.24) 0.697
Cirrhosis - non-alcoholic (x1.24) 0.737
Esophageal varices without bleeding, associated with another disorder (x1.23) 0.774
Esophageal varices without bleeding (x1.22) 0.807
Hepatic failure (x1.17) 0.827
Splenomegaly (x1.17) 0.846
Esophageal varices associated with another disorder (x1.13) 0.857
Thrombocytopenic disorder (x1.13) 0.868
Esophageal varices (x1.13) 0.878
Disease of liver (x1.08) 0.883
Biliary cirrhosis (x1.08) 0.887
White Race (+0.0069) 0.891
Gastrointestinal hemorrhage (x1.08) 0.895
Esophageal varices with bleeding, associated with another disorder (x1.07) 0.898
Black Race (-0.00646) 0.902
Venous varices (x1.07) 0.905
Transplanted liver present (x1.07) 0.908
Gastric varices (x1.07) 0.911
Liver abscess and sequelae of chronic liver disease (x1.06) 0.914
Platelets [#/V] in B by AC (-10.4) 0.916
Pancytopenia (x1.06) 0.919
Acute renal failure syndrome (x1.06) 0.921
Autoimmune hepatitis (x1.06) 0.924
Bleeding esophageal varices (x1.06) 0.926
Anemia (x1.06) 0.928
Acute posthemorrhagic anemia (x1.06) 0.930
Hypervolemia (x1.05) 0.932
Blood coagulation disorder (x1.05) 0.934
ECG normal (x1.05) 0.936
Melena (x1.05) 0.937
Ascites due to alcoholic cirrhosis (x1.05) 0.938
Hypo-osmolality and or hyponatremia (x1.04) 0.940
Primary biliary cholangitis (x1.04) 0.941
Cholangitis (x1.04) 0.942
Hypersplenism (x1.04) 0.943
Iron deficiency anemia (x1.04) 0.944
Platelet mean volume [V] in B by AC (+0.0652) 0.945
Liver cell carcinoma (x1.04) 0.946
Alkaline phosphatase [Activity/V] in SP (+4.71) 0.947
Chronic nonalcoholic liver disease (x1.04) 0.948
Pleural effusion (x1.04) 0.949
Hematemesis (x1.04) 0.950
Secondary thrombocytopenia (x1.04) 0.951
Chronic hepatic failure (x1.04) 0.952
Gallstone (x1.04) 0.953
Primary bacterial peritonitis (x1.04) 0.954
Dyspnea (x1.04) 0.955
Urobilinogen [Presence] in Urine by Test strip (+0.0667) 0.956
propranolol (+0.00314) 0.956
Nonalcoholic steatohepatitis (x1.03) 0.957
Acidosis (x1.03) 0.958
Erythrocyte distribution width [V] by AC (+0.336) 0.958
Erythrocyte distribution width [Ratio] by AC (+0.0928) 0.959
Vitamin D deficiency (x1.03) 0.960
Portal vein thrombosis (x1.03) 0.961
Leukocytes [#/V] in B by AC (-0.221) 0.961
Chronic hepatitis C (x1.03) 0.962
Type 2 diabetes mellitus without complication (x1.03) 0.962
Abnormal findings on diagnostic imaging of lung (x1.03) 0.963
Atelectasis (x1.03) 0.964
Viral hepatitis C (x1.03) 0.964
Precordial pain (x1.03) 0.965
Altered mental status (x1.03) 0.965
Hyperkalemia (x1.03) 0.966
Acquired coagulation factor deficiency (x1.03) 0.966
Type 2 diabetes mellitus (x1.03) 0.967
Chronic hepatitis (x1.03) 0.967
Muscle weakness (x1.03) 0.968
Low blood pressure (x1.03) 0.968
acetaminophen (-0.00246) 0.969
vitamin A (+0.00242) 0.969
Vascular disease of abdomen (x1.03) 0.969
Albumin [M/V] in SP (-0.0232) 0.970
Bilirubin.conjugated [M/V] in SP (+0.0377) 0.970
Gastroesophageal reflux disease without esophagitis (x1.02) 0.971
Imaging of abdomen abnormal (x1.02) 0.971
Diarrhea (x1.02) 0.971
Primary malignant neoplasm of liver (/1.02) 0.972
Hemorrhoids (x1.02) 0.972
Primary sclerosing cholangitis (x1.02) 0.972
Hyperglycemia due to type 2 diabetes mellitus (x1.02) 0.973
Electrocardiogram abnormal (x1.02) 0.973
spironolactone (+0.00213) 0.974
omeprazole (+0.0021) 0.974
Aspartate aminotransferase [Activity/V] in SP (+1.5) 0.974
Neutrophils [#/V] in B by AC (-0.141) 0.975
Lymphocytes [#/V] in B by AC (-0.0559) 0.975
Abdominal pain (x1.02) 0.975
−0.4 −0.2 0.0 0.2 0.4
Expressions
−10 0 10

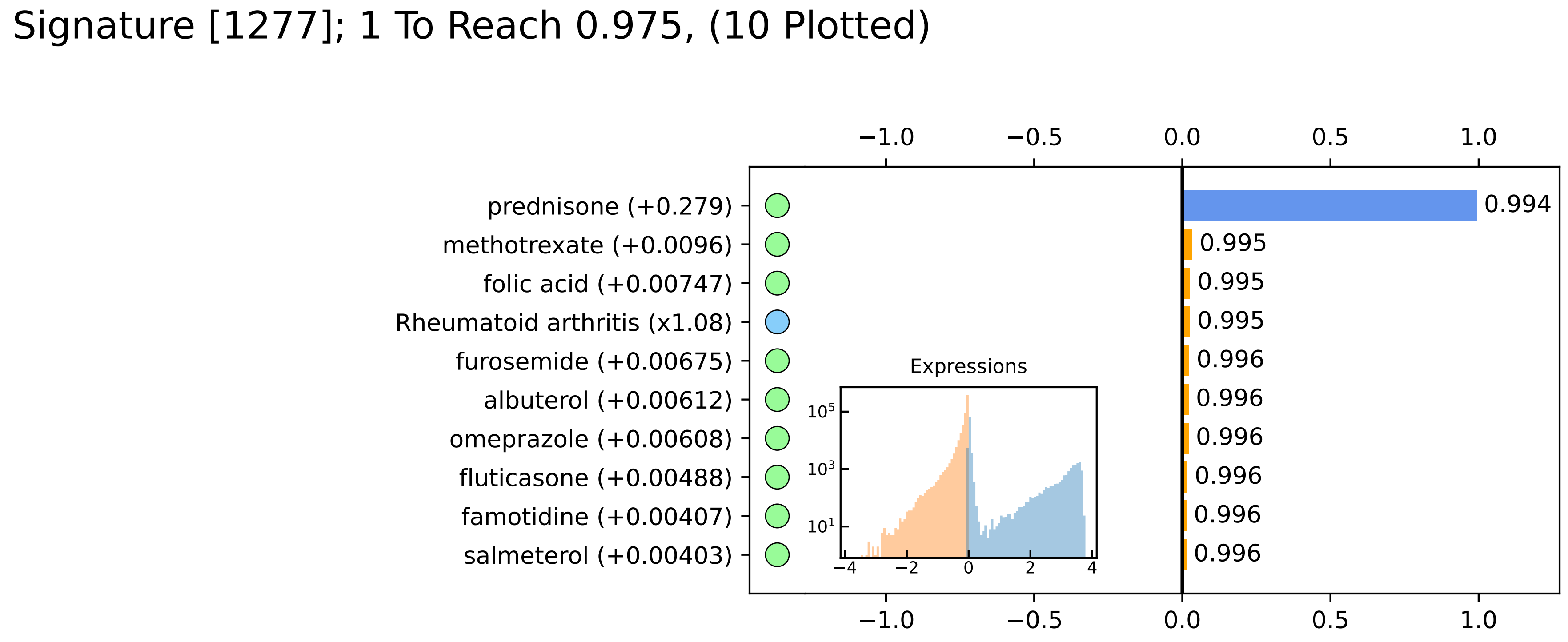

Signature [1277]; 1 To Reach 0.975, (10 Plotted)
prednisone (+0.279)
methotrexate (+0.0096)
folic acid (+0.00747)
Rheumatoid arthritis (x1.08)
furosemide (+0.00675)
albuterol (+0.00612)
omeprazole (+0.00608)
fluticasone (+0.00488)
famotidine (+0.00407)
salmeterol (+0.00403)
0.994
0.995
0.995
0.995
0.996
0.996
0.996
0.996
0.996
0.996
Expressions
−1.0
−0.5
0.0
0.5
1.0

Signature [1278]; 54 To Reach 0.975, (54 Plotted)

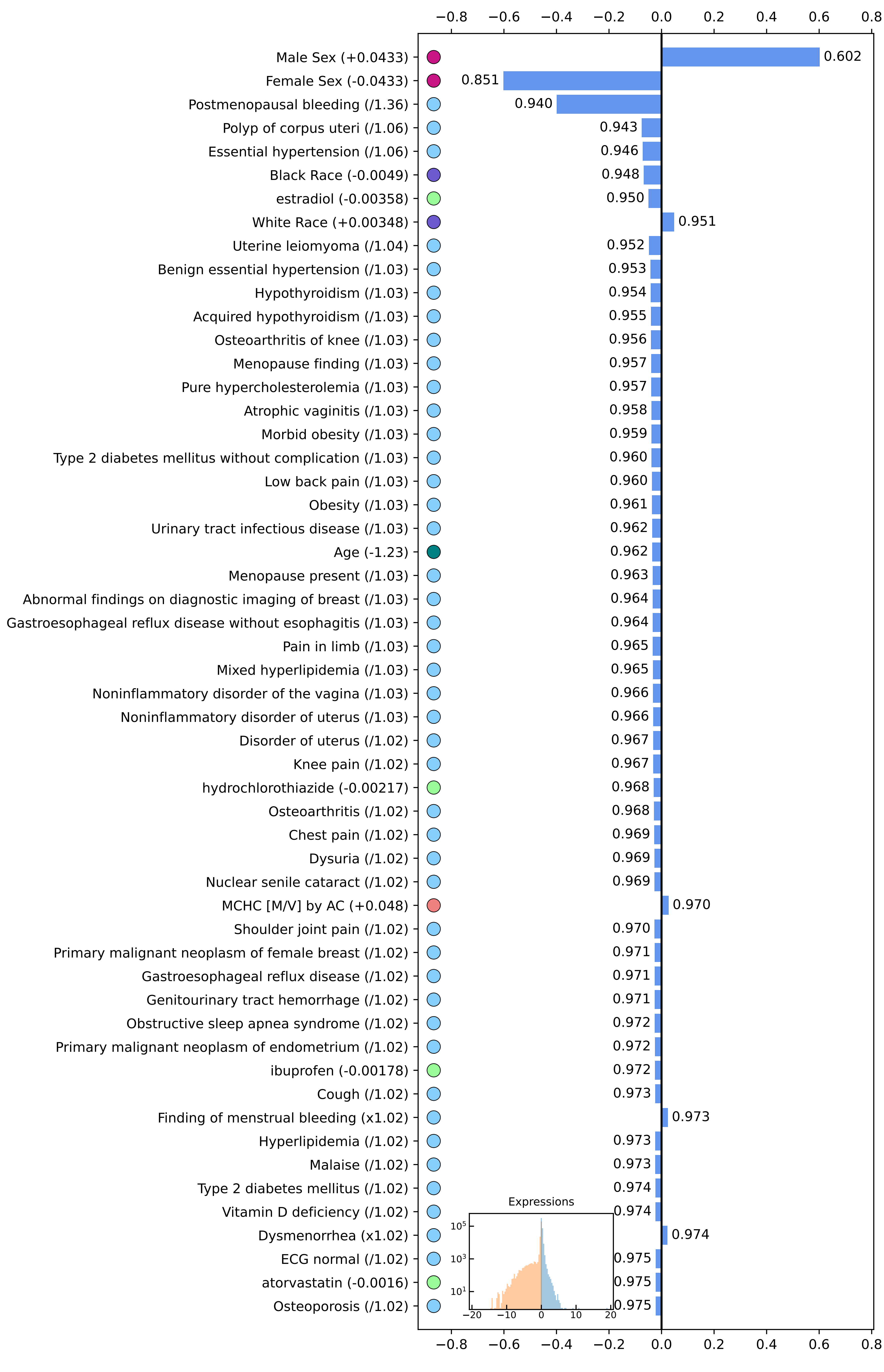

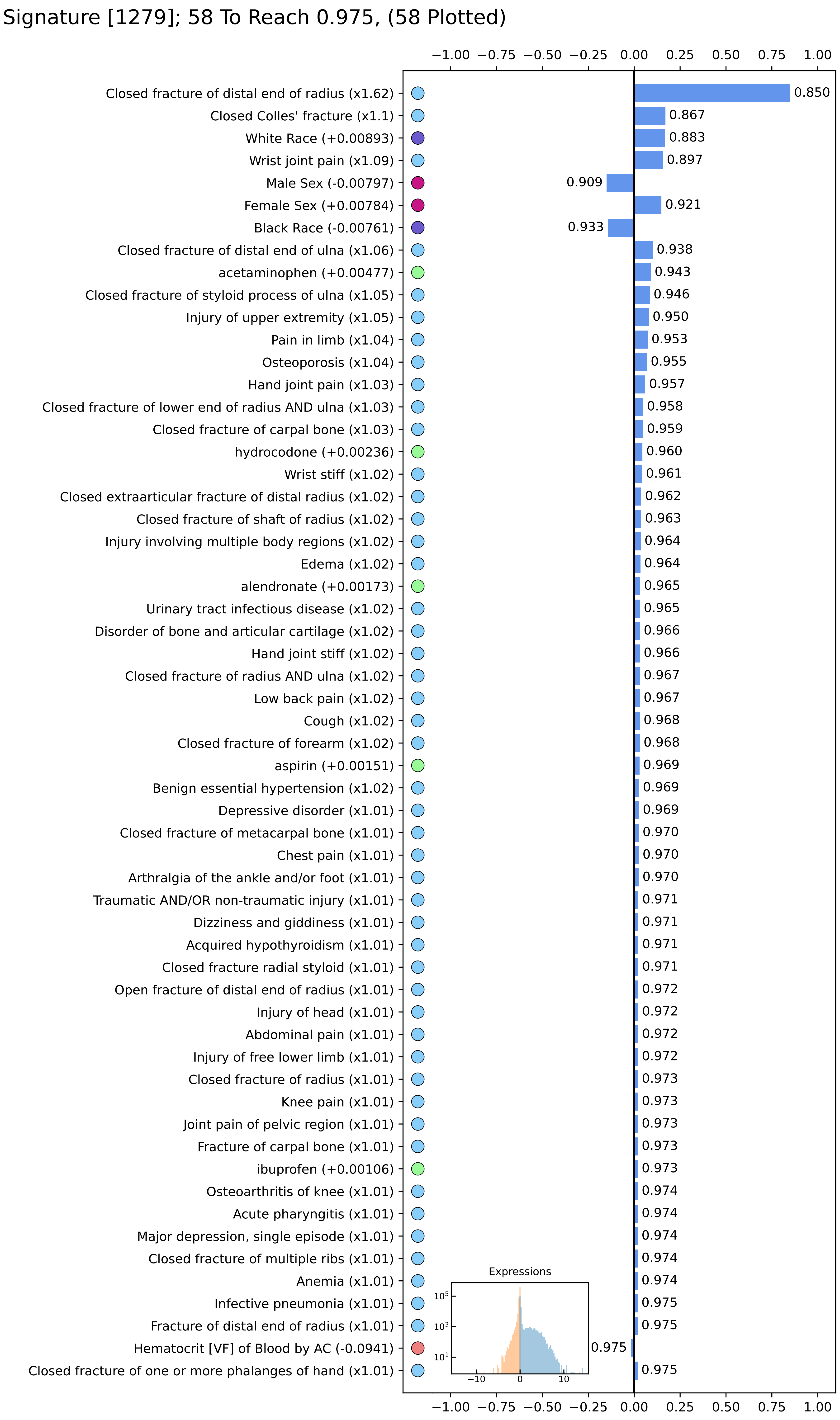

Signature [1279]; 58 To Reach 0.975, (58 Plotted)
Closed fracture of distal end of radius (x1.62) 0.850
Closed Colles' fracture (x1.1) 0.867
White Race (+0.00893) 0.883
Wrist joint pain (x1.09) 0.897
Male Sex (-0.00797) 0.909
Female Sex (+0.00784) 0.921
Black Race (-0.00761) 0.933
Closed fracture of distal end of ulna (x1.06) 0.938
acetaminophen (+0.00477) 0.943
Closed fracture of styloid process of ulna (x1.05) 0.946
Injury of upper extremity (x1.05) 0.950
Pain in limb (x1.04) 0.953
Osteoporosis (x1.04) 0.955
Hand joint pain (x1.03) 0.957
Closed fracture of lower end of radius AND ulna (x1.03) 0.958
Closed fracture of carpal bone (x1.03) 0.959
hydrocodone (+0.00236) 0.960
Wrist stiff (x1.02) 0.961
Closed extraarticular fracture of distal radius (x1.02) 0.962
Closed fracture of shaft of radius (x1.02) 0.963
Injury involving multiple body regions (x1.02) 0.964
Edema (x1.02) 0.964
alendronate (+0.00173) 0.965
Urinary tract infectious disease (x1.02) 0.965
Disorder of bone and articular cartilage (x1.02) 0.966
Hand joint stiff (x1.02) 0.966
Closed fracture of radius AND ulna (x1.02) 0.967
Low back pain (x1.02) 0.967
Cough (x1.02) 0.968
Closed fracture of forearm (x1.02) 0.968
aspirin (+0.00151) 0.969
Benign essential hypertension (x1.02) 0.969
Depressive disorder (x1.01) 0.969
Closed fracture of metacarpal bone (x1.01) 0.970
Chest pain (x1.01) 0.970
Arthralgia of the ankle and/or foot (x1.01) 0.970
Traumatic AND/OR non-traumatic injury (x1.01) 0.971
Dizziness and giddiness (x1.01) 0.971
Acquired hypothyroidism (x1.01) 0.971
Closed fracture radial styloid (x1.01) 0.971
Open fracture of distal end of radius (x1.01) 0.972
Injury of head (x1.01) 0.972
Abdominal pain (x1.01) 0.972
Injury of free lower limb (x1.01) 0.972
Closed fracture of radius (x1.01) 0.973
Knee pain (x1.01) 0.973
Joint pain of pelvic region (x1.01) 0.973
Fracture of carpal bone (x1.01) 0.973
ibuprofen (+0.00106) 0.973
Osteoarthritis of knee (x1.01) 0.974
Acute pharyngitis (x1.01) 0.974
Major depression, single episode (x1.01) 0.974
Closed fracture of multiple ribs (x1.01) 0.974
Anemia (x1.01) 0.974
Infective pneumonia (x1.01) 0.975
Fracture of distal end of radius (x1.01) 0.975
Hematocrit [VF] of Blood by AC (-0.0941) 0.975
Closed fracture of one or more phalanges of hand (x1.01) 0.975
−1.00 −0.75 −0.50 −0.25 0.00 0.25 0.50 0.75 1.00
Expressions
10^5
10^3
10^1
−10
0
10

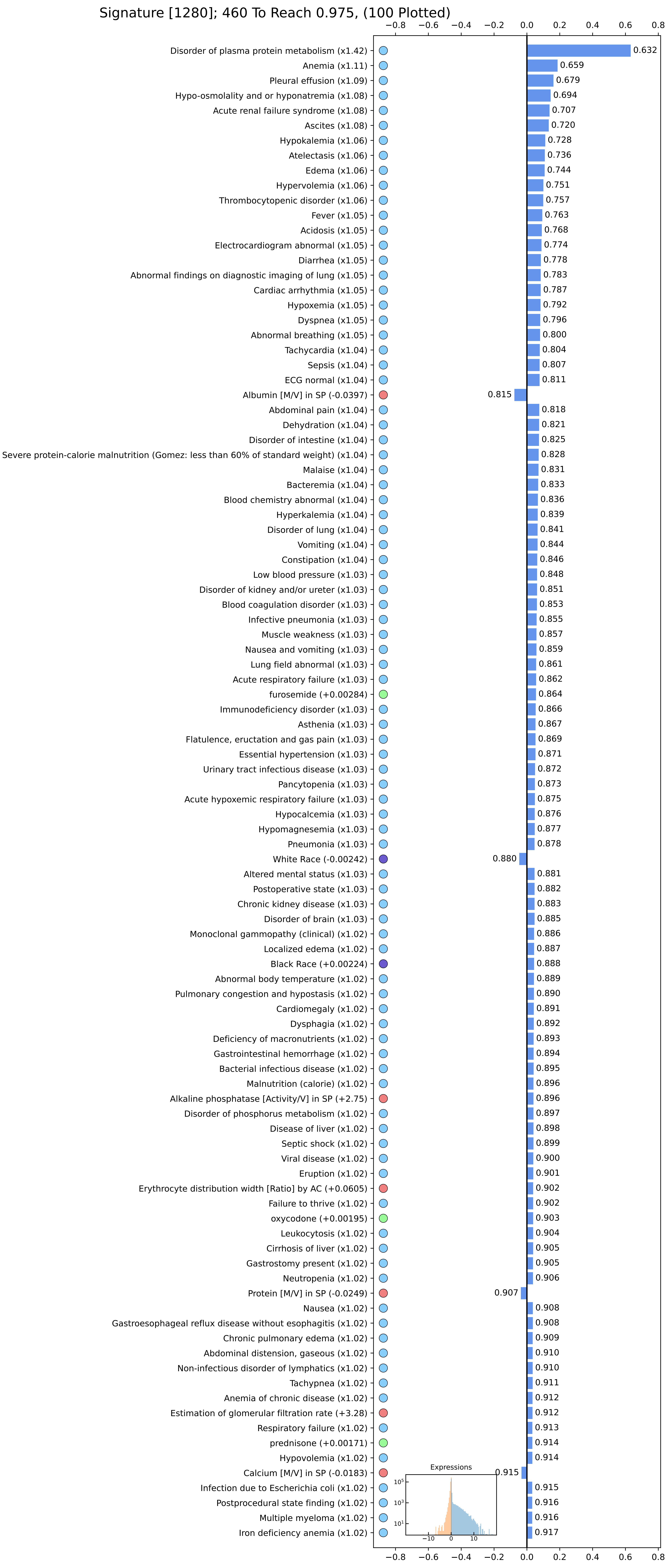
Signature [1280]; 460 To Reach 0.975, (100 Plotted)
Disorder of plasma protein metabolism (x1.42) 0.632
Anemia (x1.11) 0.659
Pleural effusion (x1.09) 0.679
Hypo-osmolality and or hyponatremia (x1.08) 0.694
Acute renal failure syndrome (x1.08) 0.707
Ascites (x1.08) 0.720
Hypokalemia (x1.06) 0.728
Atelectasis (x1.06) 0.736
Edema (x1.06) 0.744
Hypervolemia (x1.06) 0.751
Thrombocytopenic disorder (x1.06) 0.757
Fever (x1.05) 0.763
Acidosis (x1.05) 0.768
Electrocardiogram abnormal (x1.05) 0.774
Diarrhea (x1.05) 0.778
Abnormal findings on diagnostic imaging of lung (x1.05) 0.783
Cardiac arrhythmia (x1.05) 0.787
Hypoxemia (x1.05) 0.792
Dyspnea (x1.05) 0.796
Abnormal breathing (x1.05) 0.800
Tachycardia (x1.04) 0.804
Sepsis (x1.04) 0.807
ECG normal (x1.04) 0.811
Albumin [M/V] in SP (-0.0397) 0.815
Abdominal pain (x1.04) 0.818
Dehydration (x1.04) 0.821
Disorder of intestine (x1.04) 0.825
Severe protein-calorie malnutrition (Gomez: less than 60% of standard weight) (x1.04) 0.828
Malaise (x1.04) 0.831
Bacteremia (x1.04) 0.833
Blood chemistry abnormal (x1.04) 0.836
Hyperkalemia (x1.04) 0.839
Disorder of lung (x1.04) 0.841
Vomiting (x1.04) 0.844
Constipation (x1.04) 0.846
Low blood pressure (x1.03) 0.848
Disorder of kidney and/or ureter (x1.03) 0.851
Blood coagulation disorder (x1.03) 0.853
Infective pneumonia (x1.03) 0.855
Muscle weakness (x1.03) 0.857
Nausea and vomiting (x1.03) 0.859
Lung field abnormal (x1.03) 0.861
Acute respiratory failure (x1.03) 0.862
furosemide (+0.00284) 0.864
Immunodeficiency disorder (x1.03) 0.866
Asthenia (x1.03) 0.867
Flatulence, eructation and gas pain (x1.03) 0.869
Essential hypertension (x1.03) 0.871
Urinary tract infectious disease (x1.03) 0.872
Pancytopenia (x1.03) 0.873
Acute hypoxemic respiratory failure (x1.03) 0.875
Hypocalcemia (x1.03) 0.876
Hypomagnesemia (x1.03) 0.877
Pneumonia (x1.03) 0.878
White Race (-0.00242) 0.880
Altered mental status (x1.03) 0.881
Postoperative state (x1.03) 0.882
Chronic kidney disease (x1.03) 0.883
Disorder of brain (x1.03) 0.885
Monoclonal gammopathy (clinical) (x1.02) 0.886
Localized edema (x1.02) 0.887
Black Race (+0.00224) 0.888
Abnormal body temperature (x1.02) 0.889
Pulmonary congestion and hypostasis (x1.02) 0.890
Cardiomegaly (x1.02) 0.891
Dysphagia (x1.02) 0.892
Deficiency of macronutrients (x1.02) 0.893
Gastrointestinal hemorrhage (x1.02) 0.894
Bacterial infectious disease (x1.02) 0.895
Malnutrition (calorie) (x1.02) 0.896
Alkaline phosphatase [Activity/V] in SP (+2.75) 0.896
Disorder of phosphorus metabolism (x1.02) 0.897
Disease of liver (x1.02) 0.898
Septic shock (x1.02) 0.899
Viral disease (x1.02) 0.900
Eruption (x1.02) 0.901
Erythrocyte distribution width [Ratio] by AC (+0.0605) 0.902
Failure to thrive (x1.02) 0.902
oxycodone (+0.00195) 0.903
Leukocytosis (x1.02) 0.904
Cirrhosis of liver (x1.02) 0.905
Gastrostomy present (x1.02) 0.905
Neutropenia (x1.02) 0.906
Protein [M/V] in SP (-0.0249) 0.907
Nausea (x1.02) 0.908
Gastroesophageal reflux disease without esophagitis (x1.02) 0.908
Chronic pulmonary edema (x1.02) 0.909
Abdominal distension, gaseous (x1.02) 0.910
Non-infectious disorder of lymphatics (x1.02) 0.910
Tachypnea (x1.02) 0.911
Anemia of chronic disease (x1.02) 0.912
Estimation of glomerular filtration rate (+3.28) 0.912
Respiratory failure (x1.02) 0.913
prednisone (+0.00171) 0.914
Hypovolemia (x1.02) 0.914
Calcium [M/V] in SP (-0.0183) 0.915
Infection due to Escherichia coli (x1.02) 0.915
Postprocedural state finding (x1.02) 0.916
Multiple myeloma (x1.02) 0.916
Iron deficiency anemia (x1.02) 0.917
Expressions
−0.8 −0.6 −0.4 −0.2 0.0 0.2 0.4 0.6 0.8

Signature [1281]; 198 To Reach 0.975, (100 Plotted)

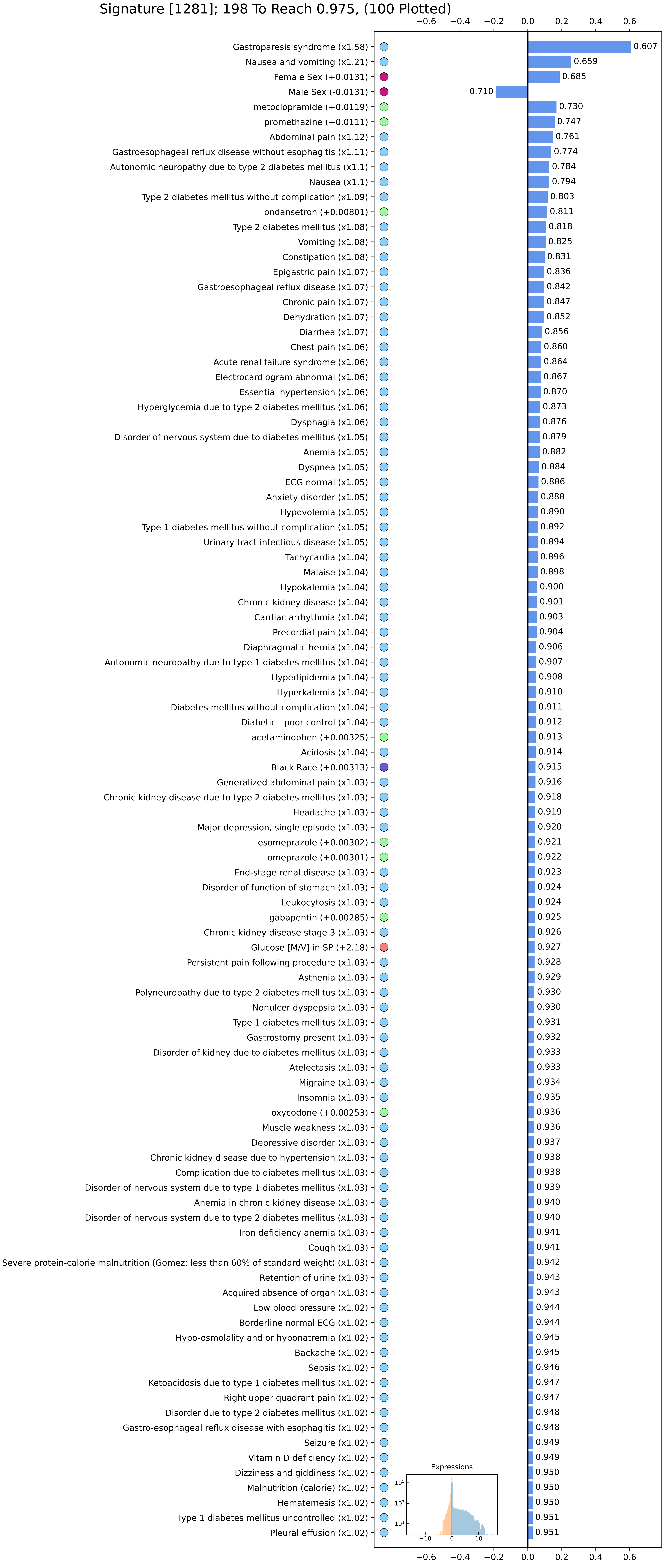

Signature [1282]; 8 To Reach 0.975, (10 Plotted)

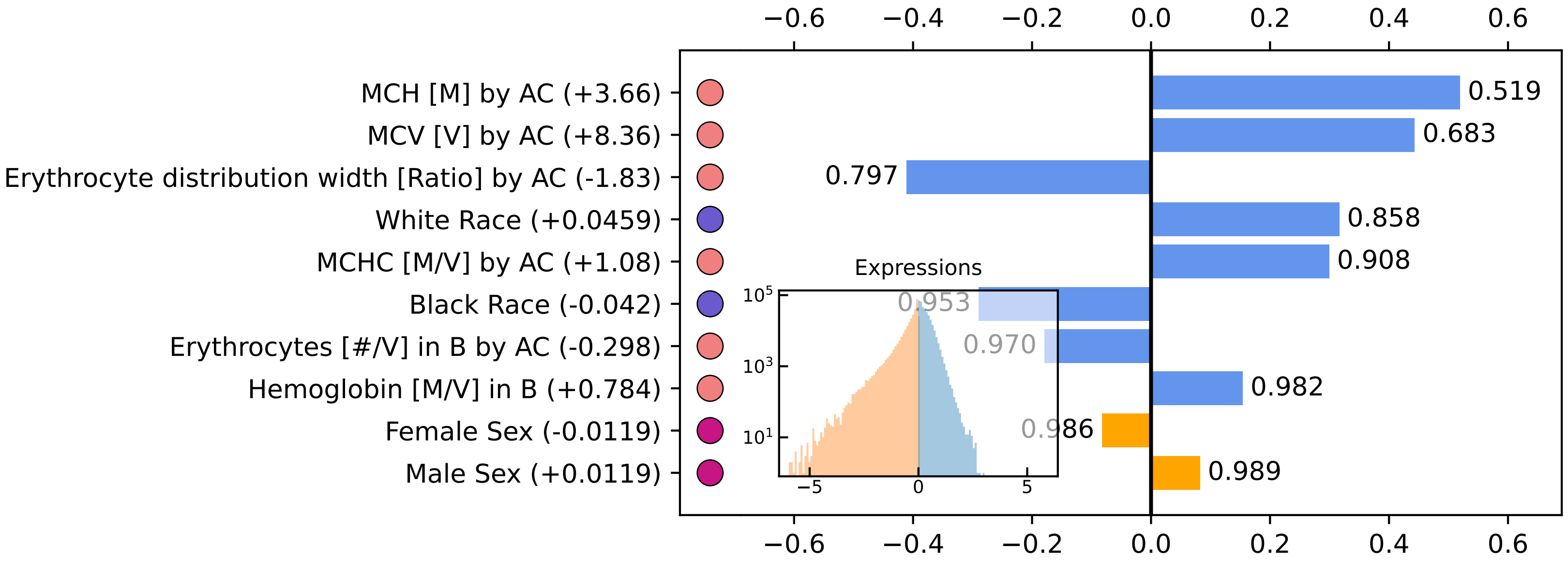

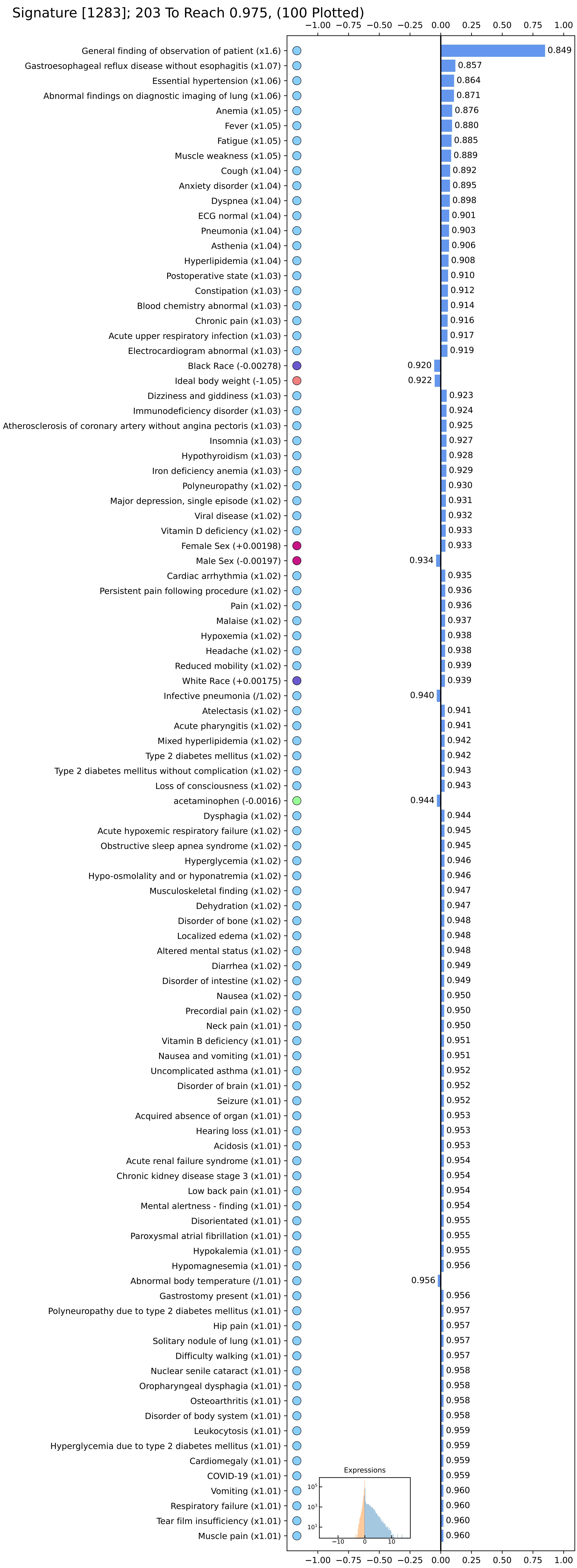

Signature [1283]; 203 To Reach 0.975, (100 Plotted)
General finding of observation of patient (x1.6) 0.849
Gastroesophageal reflux disease without esophagitis (x1.07) 0.857
Essential hypertension (x1.06) 0.864
Abnormal findings on diagnostic imaging of lung (x1.06) 0.871
Anemia (x1.05) 0.876
Fever (x1.05) 0.880
Fatigue (x1.05) 0.885
Muscle weakness (x1.05) 0.889
Cough (x1.04) 0.892
Anxiety disorder (x1.04) 0.895
Dyspnea (x1.04) 0.898
ECG normal (x1.04) 0.901
Pneumonia (x1.04) 0.903
Asthenia (x1.04) 0.906
Hyperlipidemia (x1.04) 0.908
Postoperative state (x1.03) 0.910
Constipation (x1.03) 0.912
Blood chemistry abnormal (x1.03) 0.914
Chronic pain (x1.03) 0.916
Acute upper respiratory infection (x1.03) 0.917
Electrocardiogram abnormal (x1.03) 0.919
Black Race (-0.00278) 0.920
Ideal body weight (-1.05) 0.922
Dizziness and giddiness (x1.03) 0.923
Immunodeficiency disorder (x1.03) 0.924
Atherosclerosis of coronary artery without angina pectoris (x1.03) 0.925
Insomnia (x1.03) 0.927
Hypothyroidism (x1.03) 0.928
Iron deficiency anemia (x1.03) 0.929
Polyneuropathy (x1.02) 0.930
Major depression, single episode (x1.02) 0.931
Viral disease (x1.02) 0.932
Vitamin D deficiency (x1.02) 0.933
Female Sex (+0.00198) 0.933
Male Sex (-0.00197) 0.934
Cardiac arrhythmia (x1.02) 0.935
Persistent pain following procedure (x1.02) 0.936
Pain (x1.02) 0.936
Malaise (x1.02) 0.937
Hypoxemia (x1.02) 0.938
Headache (x1.02) 0.938
Reduced mobility (x1.02) 0.939
White Race (+0.00175) 0.939
Infective pneumonia (/1.02) 0.940
Atelectasis (x1.02) 0.941
Acute pharyngitis (x1.02) 0.941
Mixed hyperlipidemia (x1.02) 0.942
Type 2 diabetes mellitus (x1.02) 0.942
Type 2 diabetes mellitus without complication (x1.02) 0.943
Loss of consciousness (x1.02) 0.943
acetaminophen (-0.0016) 0.944
Dysphagia (x1.02) 0.944
Acute hypoxemic respiratory failure (x1.02) 0.945
Obstructive sleep apnea syndrome (x1.02) 0.945
Hyperglycemia (x1.02) 0.946
Hypo-osmolality and or hyponatremia (x1.02) 0.946
Musculoskeletal finding (x1.02) 0.947
Dehydration (x1.02) 0.947
Disorder of bone (x1.02) 0.948
Localized edema (x1.02) 0.948
Altered mental status (x1.02) 0.948
Diarrhea (x1.02) 0.949
Disorder of intestine (x1.02) 0.949
Nausea (x1.02) 0.950
Precordial pain (x1.02) 0.950
Neck pain (x1.01) 0.950
Vitamin B deficiency (x1.01) 0.951
Nausea and vomiting (x1.01) 0.951
Uncomplicated asthma (x1.01) 0.952
Disorder of brain (x1.01) 0.952
Seizure (x1.01) 0.952
Acquired absence of organ (x1.01) 0.953
Hearing loss (x1.01) 0.953
Acidosis (x1.01) 0.953
Acute renal failure syndrome (x1.01) 0.954
Chronic kidney disease stage 3 (x1.01) 0.954
Low back pain (x1.01) 0.954
Mental alertness - finding (x1.01) 0.954
Disorientated (x1.01) 0.955
Paroxysmal atrial fibrillation (x1.01) 0.955
Hypokalemia (x1.01) 0.955
Hypomagnesemia (x1.01) 0.956
Abnormal body temperature (/1.01) 0.956
Gastrostomy present (x1.01) 0.956
Polyneuropathy due to type 2 diabetes mellitus (x1.01) 0.957
Hip pain (x1.01) 0.957
Solitary nodule of lung (x1.01) 0.957
Difficulty walking (x1.01) 0.957
Nuclear senile cataract (x1.01) 0.958
Oropharyngeal dysphagia (x1.01) 0.958
Osteoarthritis (x1.01) 0.958
Disorder of body system (x1.01) 0.958
Leukocytosis (x1.01) 0.959
Hyperglycemia due to type 2 diabetes mellitus (x1.01) 0.959
Cardiomegaly (x1.01) 0.959
COVID-19 (x1.01) 0.959
Vomiting (x1.01) 0.960
Respiratory failure (x1.01) 0.960
Tear film insufficiency (x1.01) 0.960
Muscle pain (x1.01) 0.960
Expressions
−1.00 −0.75 −0.50 −0.25 0.00 0.25 0.50 0.75 1.00

Signature [1284]; 16 To Reach 0.975, (16 Plotted)

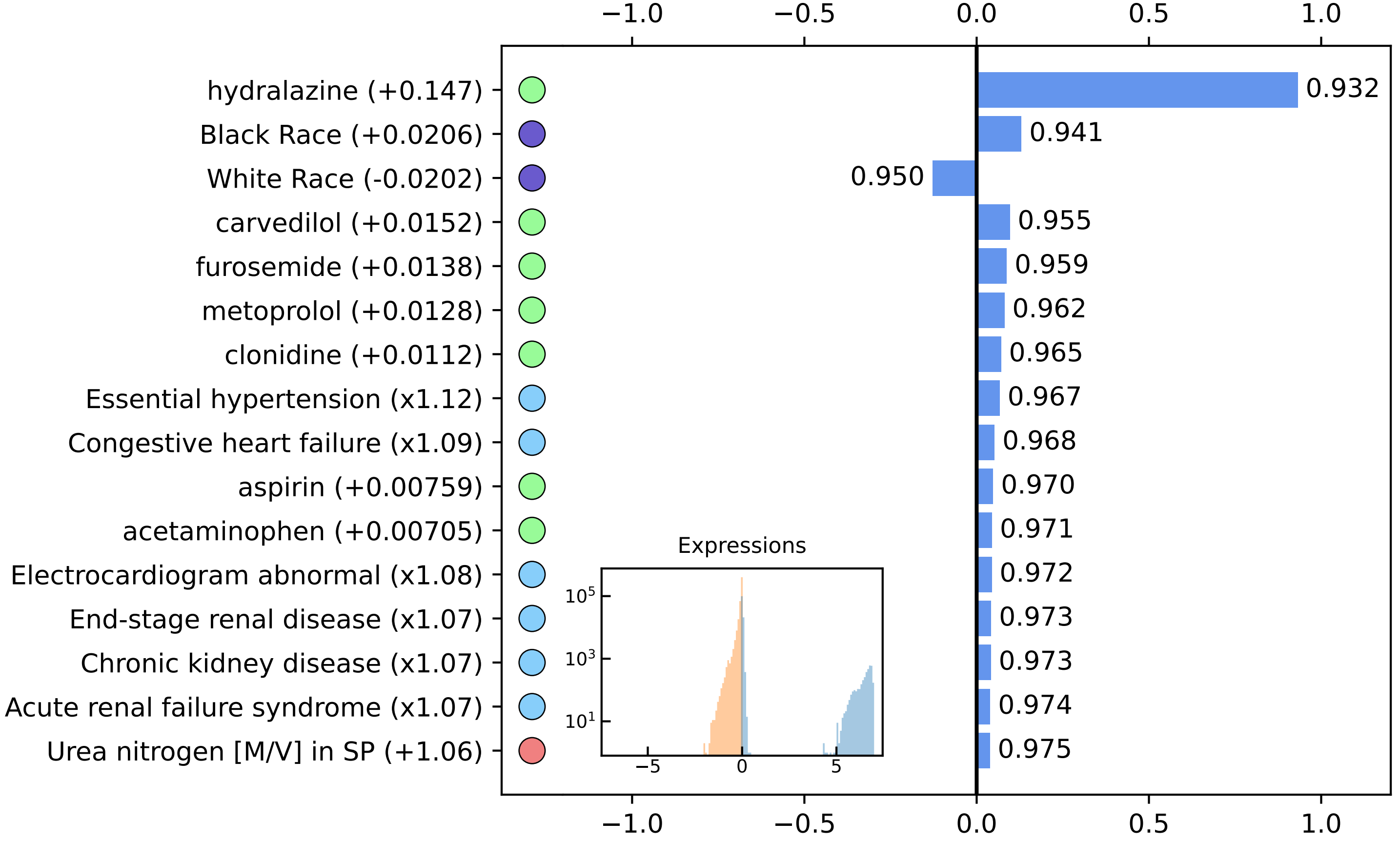

Signature [1285]; 71 To Reach 0.975, (71 Plotted)

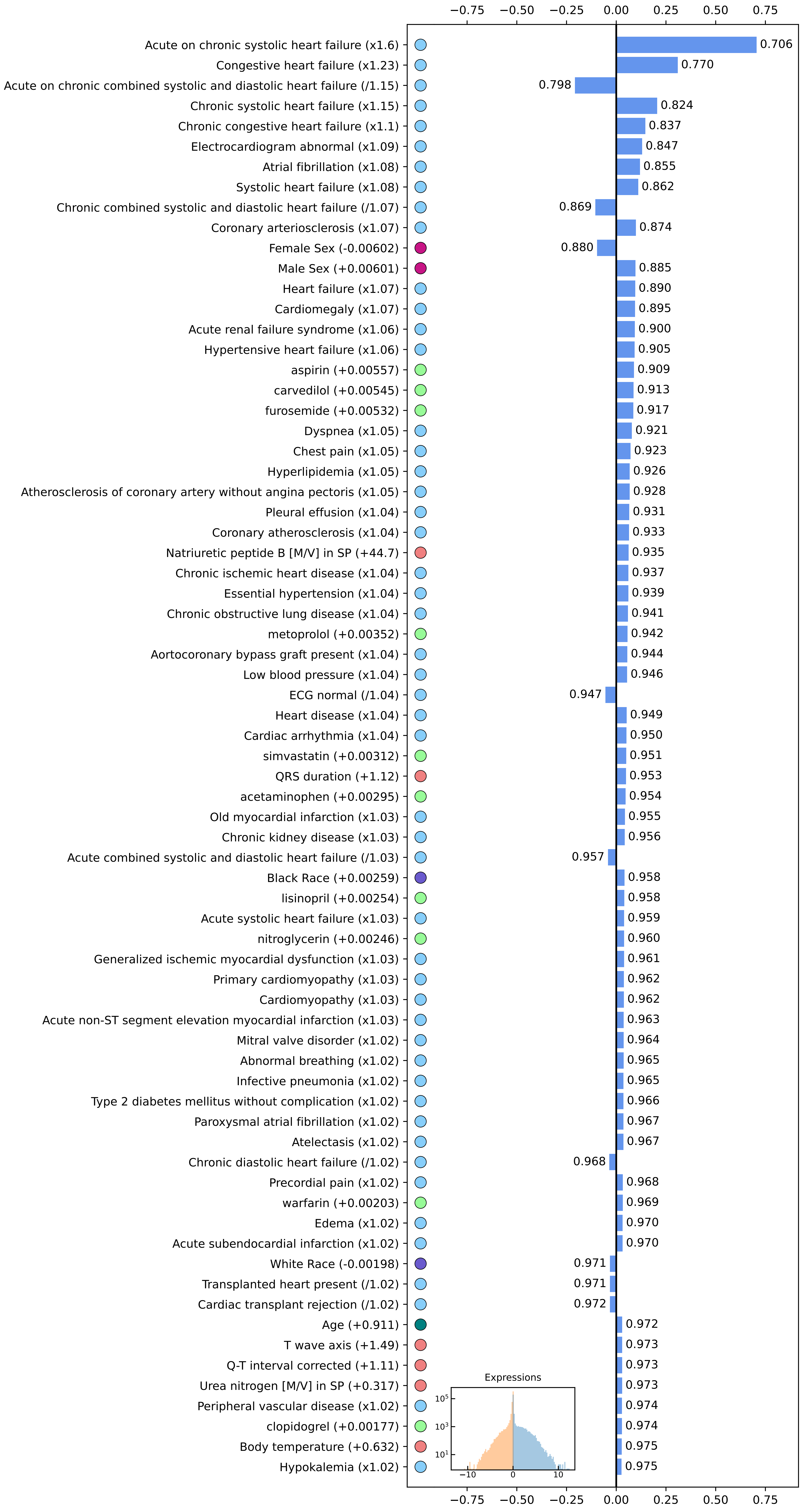

Signature [1286]; 186 To Reach 0.975, (100 Plotted)

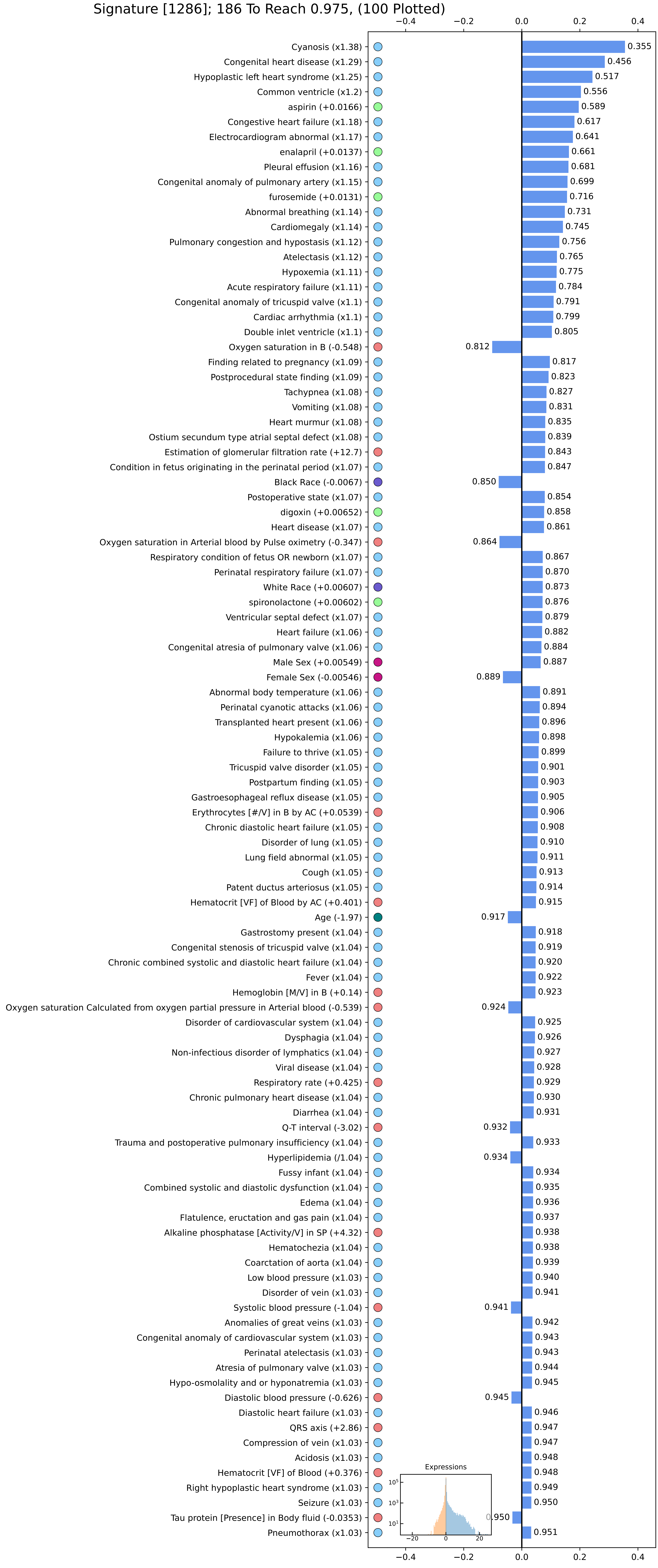

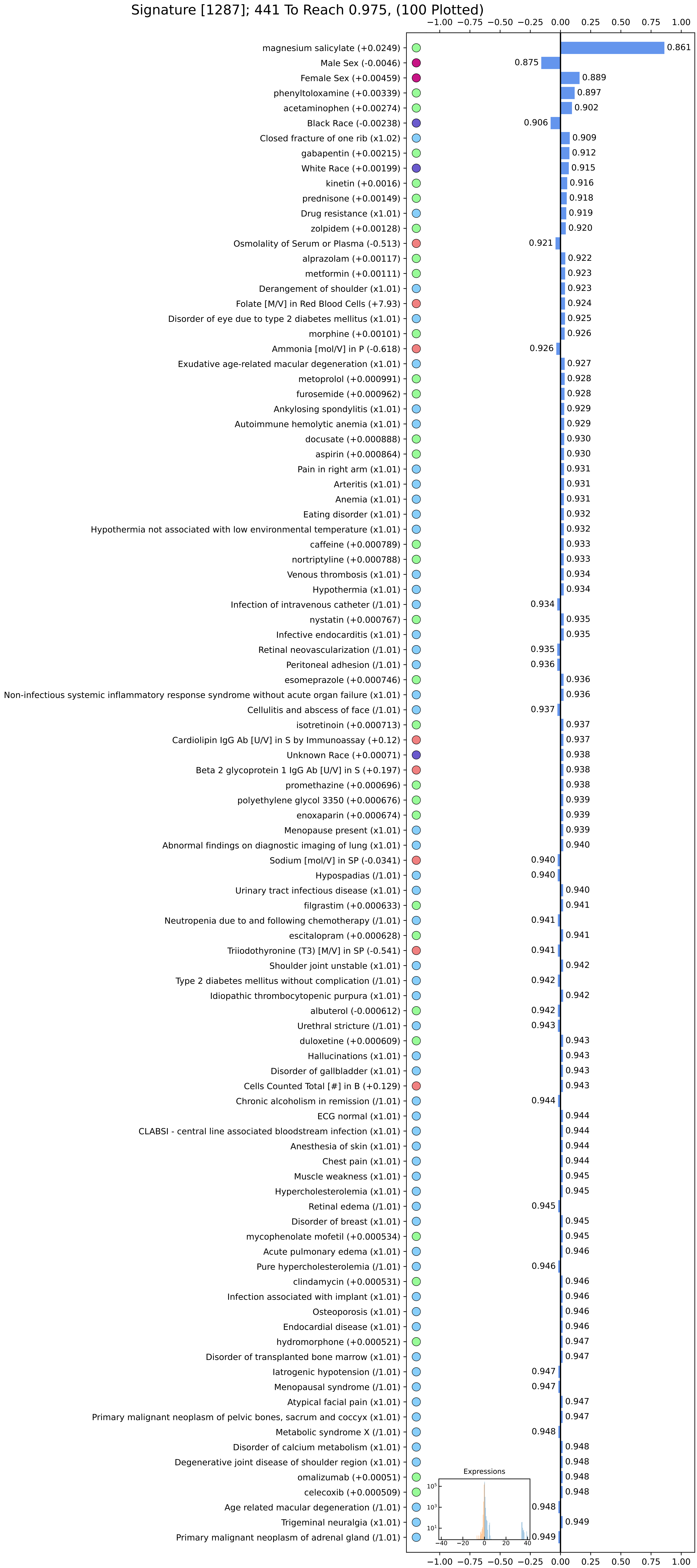
Signature [1287]; 441 To Reach 0.975, (100 Plotted)
magnesium salicylate (+0.0249) 0.861
Male Sex (-0.0046) 0.875
Female Sex (+0.00459) 0.889
phenyltoloxamine (+0.00339) 0.897
acetaminophen (+0.00274) 0.902
Black Race (-0.00238) 0.906
Closed fracture of one rib (x1.02) 0.909
gabapentin (+0.00215) 0.912
White Race (+0.00199) 0.915
kinetin (+0.0016) 0.916
prednisone (+0.00149) 0.918
Drug resistance (x1.01) 0.919
zolpidem (+0.00128) 0.920
Osmolality of Serum or Plasma (-0.513) 0.921
alprazolam (+0.00117) 0.922
metformin (+0.00111) 0.923
Derangement of shoulder (x1.01) 0.923
Folate [M/V] in Red Blood Cells (+7.93) 0.924
Disorder of eye due to type 2 diabetes mellitus (x1.01) 0.925
morphine (+0.00101) 0.926
Ammonia [mol/V] in P (-0.618) 0.926
Exudative age-related macular degeneration (x1.01) 0.927
metoprolol (+0.000991) 0.928
furosemide (+0.000962) 0.928
Ankylosing spondylitis (x1.01) 0.929
Autoimmune hemolytic anemia (x1.01) 0.929
docusate (+0.000888) 0.930
aspirin (+0.000864) 0.930
Pain in right arm (x1.01) 0.931
Arteritis (x1.01) 0.931
Anemia (x1.01) 0.931
Eating disorder (x1.01) 0.932
Hypothermia not associated with low environmental temperature (x1.01) 0.932
caffeine (+0.000789) 0.933
nortriptyline (+0.000788) 0.933
Venous thrombosis (x1.01) 0.934
Hypothermia (x1.01) 0.934
Infection of intravenous catheter (/1.01) 0.934
nystatin (+0.000767) 0.935
Infective endocarditis (x1.01) 0.935
Retinal neovascularization (/1.01) 0.935
Peritoneal adhesion (/1.01) 0.936
esomeprazole (+0.000746) 0.936
Non-infectious systemic inflammatory response syndrome without acute organ failure (x1.01) 0.936
Cellulitis and abscess of face (/1.01) 0.937
isotretinoin (+0.000713) 0.937
Cardiolipin IgG Ab [U/V] in S by Immunoassay (+0.12) 0.937
Unknown Race (+0.00071) 0.938
Beta 2 glycoprotein 1 IgG Ab [U/V] in S (+0.197) 0.938
promethazine (+0.000696) 0.938
polyethylene glycol 3350 (+0.000676) 0.939
enoxaparin (+0.000674) 0.939
Menopause present (x1.01) 0.939
Abnormal findings on diagnostic imaging of lung (x1.01) 0.940
Sodium [mol/V] in SP (-0.0341) 0.940
Hypospadias (/1.01) 0.940
Urinary tract infectious disease (x1.01) 0.940
filgrastim (+0.000633) 0.941
Neutropenia due to and following chemotherapy (/1.01) 0.941
escitalopram (+0.000628) 0.941
Triiodothyronine (T3) [M/V] in SP (-0.541) 0.941
Shoulder joint unstable (x1.01) 0.942
Type 2 diabetes mellitus without complication (/1.01) 0.942
Idiopathic thrombocytopenic purpura (x1.01) 0.942
albuterol (-0.000612) 0.942
Urethral stricture (/1.01) 0.943
duloxetine (+0.000609) 0.943
Hallucinations (x1.01) 0.943
Disorder of gallbladder (x1.01) 0.943
Cells Counted Total [#] in B (+0.129) 0.943
Chronic alcoholism in remission (/1.01) 0.944
ECG normal (x1.01) 0.944
CLABSI - central line associated bloodstream infection (x1.01) 0.944
Anesthesia of skin (x1.01) 0.944
Chest pain (x1.01) 0.944
Muscle weakness (x1.01) 0.945
Hypercholesterolemia (x1.01) 0.945
Retinal edema (/1.01) 0.945
Disorder of breast (x1.01) 0.945
mycophenolate mofetil (+0.000534) 0.945
Acute pulmonary edema (x1.01) 0.946
Pure hypercholesterolemia (/1.01) 0.946
clindamycin (+0.000531) 0.946
Infection associated with implant (x1.01) 0.946
Osteoporosis (x1.01) 0.946
Endocardial disease (x1.01) 0.946
hydromorphone (+0.000521) 0.947
Disorder of transplanted bone marrow (x1.01) 0.947
Iatrogenic hypotension (/1.01) 0.947
Menopausal syndrome (/1.01) 0.947
Atypical facial pain (x1.01) 0.947
Primary malignant neoplasm of pelvic bones, sacrum and coccyx (x1.01) 0.947
Metabolic syndrome X (/1.01) 0.948
Disorder of calcium metabolism (x1.01) 0.948
Degenerative joint disease of shoulder region (x1.01) 0.948
omalizumab (+0.00051) 0.948
celecoxib (+0.000509) 0.948
Age related macular degeneration (/1.01) 0.948
Trigeminal neuralgia (x1.01) 0.949
Primary malignant neoplasm of adrenal gland (/1.01) 0.949
−1.00 −0.75 −0.50 −0.25 0.00 0.25 0.50 0.75 1.00
Expressions
10^5
10^3
10^1
−40 −20 0 20 40

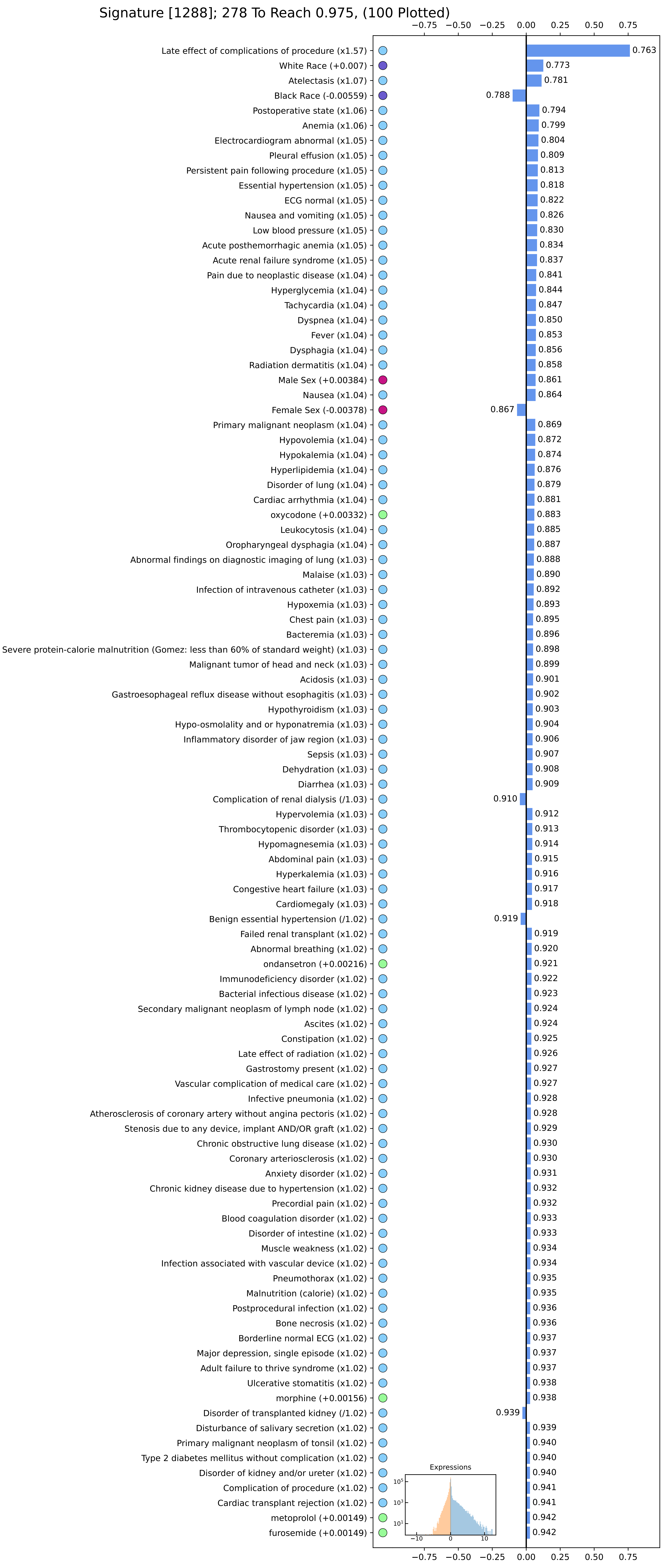

Signature [1288]; 278 To Reach 0.975, (100 Plotted)
−0.75 −0.50 −0.25 0.00 0.25 0.50 0.75
Late effect of complications of procedure (x1.57) 0.763
White Race (+0.007) 0.773
Atelectasis (x1.07) 0.781
Black Race (-0.00559) 0.788
Postoperative state (x1.06) 0.794
Anemia (x1.06) 0.799
Electrocardiogram abnormal (x1.05) 0.804
Pleural effusion (x1.05) 0.809
Persistent pain following procedure (x1.05) 0.813
Essential hypertension (x1.05) 0.818
ECG normal (x1.05) 0.822
Nausea and vomiting (x1.05) 0.826
Low blood pressure (x1.05) 0.830
Acute posthemorrhagic anemia (x1.05) 0.834
Acute renal failure syndrome (x1.05) 0.837
Pain due to neoplastic disease (x1.04) 0.841
Hyperglycemia (x1.04) 0.844
Tachycardia (x1.04) 0.847
Dyspnea (x1.04) 0.850
Fever (x1.04) 0.853
Dysphagia (x1.04) 0.856
Radiation dermatitis (x1.04) 0.858
Male Sex (+0.00384) 0.861
Nausea (x1.04) 0.864
Female Sex (-0.00378) 0.867
Primary malignant neoplasm (x1.04) 0.869
Hypovolemia (x1.04) 0.872
Hypokalemia (x1.04) 0.874
Hyperlipidemia (x1.04) 0.876
Disorder of lung (x1.04) 0.879
Cardiac arrhythmia (x1.04) 0.881
oxycodone (+0.00332) 0.883
Leukocytosis (x1.04) 0.885
Oropharyngeal dysphagia (x1.04) 0.887
Abnormal findings on diagnostic imaging of lung (x1.03) 0.888
Malaise (x1.03) 0.890
Infection of intravenous catheter (x1.03) 0.892
Hypoxemia (x1.03) 0.893
Chest pain (x1.03) 0.895
Bacteremia (x1.03) 0.896
Severe protein-calorie malnutrition (Gomez: less than 60% of standard weight) (x1.03) 0.898
Malignant tumor of head and neck (x1.03) 0.899
Acidosis (x1.03) 0.901
Gastroesophageal reflux disease without esophagitis (x1.03) 0.902
Hypothyroidism (x1.03) 0.903
Hypo-osmolality and or hyponatremia (x1.03) 0.904
Inflammatory disorder of jaw region (x1.03) 0.906
Sepsis (x1.03) 0.907
Dehydration (x1.03) 0.908
Diarrhea (x1.03) 0.909
Complication of renal dialysis (/1.03) 0.910
Hypervolemia (x1.03) 0.912
Thrombocytopenic disorder (x1.03) 0.913
Hypomagnesemia (x1.03) 0.914
Abdominal pain (x1.03) 0.915
Hyperkalemia (x1.03) 0.916
Congestive heart failure (x1.03) 0.917
Cardiomegaly (x1.03) 0.918
Benign essential hypertension (/1.02) 0.919
Failed renal transplant (x1.02) 0.919
Abnormal breathing (x1.02) 0.920
ondansetron (+0.00216) 0.921
Immunodeficiency disorder (x1.02) 0.922
Bacterial infectious disease (x1.02) 0.923
Secondary malignant neoplasm of lymph node (x1.02) 0.924
Ascites (x1.02) 0.924
Constipation (x1.02) 0.925
Late effect of radiation (x1.02) 0.926
Gastrostomy present (x1.02) 0.927
Vascular complication of medical care (x1.02) 0.927
Infective pneumonia (x1.02) 0.928
Atherosclerosis of coronary artery without angina pectoris (x1.02) 0.928
Stenosis due to any device, implant AND/OR graft (x1.02) 0.929
Chronic obstructive lung disease (x1.02) 0.930
Coronary arteriosclerosis (x1.02) 0.930
Anxiety disorder (x1.02) 0.931
Chronic kidney disease due to hypertension (x1.02) 0.932
Precordial pain (x1.02) 0.932
Blood coagulation disorder (x1.02) 0.933
Disorder of intestine (x1.02) 0.933
Muscle weakness (x1.02) 0.934
Infection associated with vascular device (x1.02) 0.934
Pneumothorax (x1.02) 0.935
Malnutrition (calorie) (x1.02) 0.935
Postprocedural infection (x1.02) 0.936
Bone necrosis (x1.02) 0.936
Borderline normal ECG (x1.02) 0.937
Major depression, single episode (x1.02) 0.937
Adult failure to thrive syndrome (x1.02) 0.937
Ulcerative stomatitis (x1.02) 0.938
morphine (+0.00156) 0.938
Disorder of transplanted kidney (/1.02) 0.939
Disturbance of salivary secretion (x1.02) 0.939
Primary malignant neoplasm of tonsil (x1.02) 0.940
Type 2 diabetes mellitus without complication (x1.02) 0.940
Disorder of kidney and/or ureter (x1.02) 0.940
Complication of procedure (x1.02) 0.941
Cardiac transplant rejection (x1.02) 0.941
metoprolol (+0.00149) 0.942
furosemide (+0.00149) 0.942
Expressions
−0.75 −0.50 −0.25 0.00 0.25 0.50 0.75

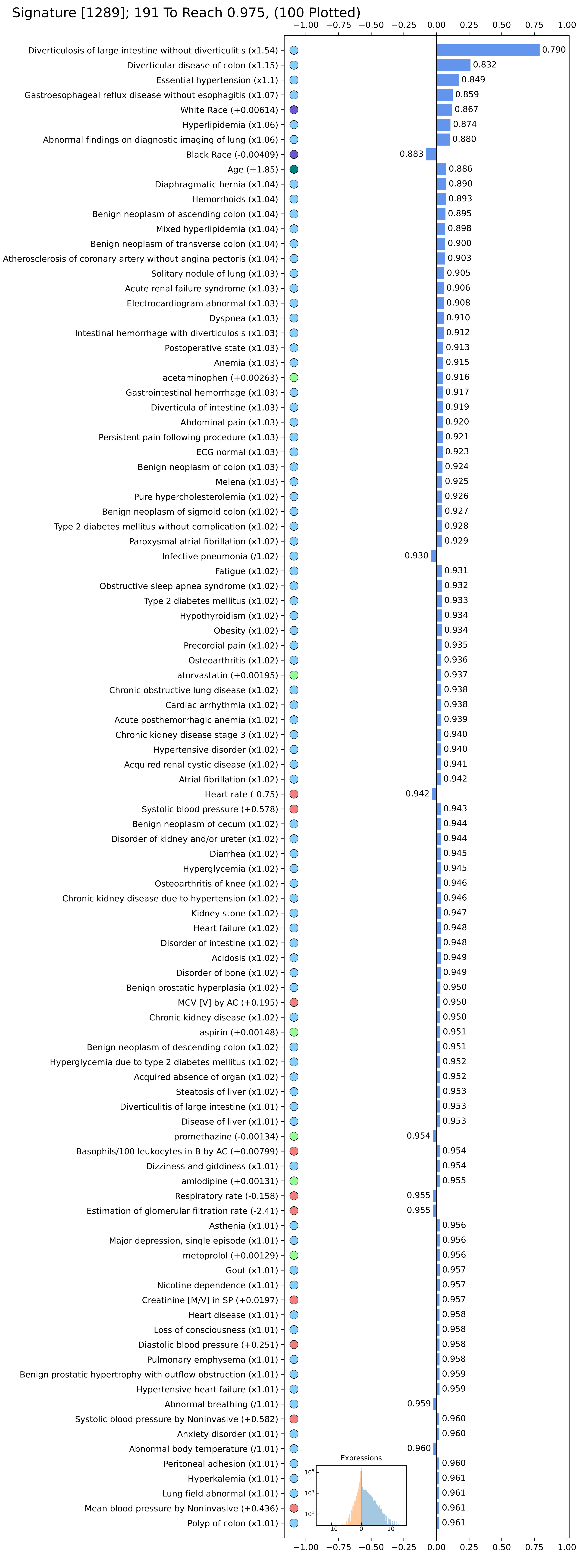

Signature [1289]; 191 To Reach 0.975, (100 Plotted)
−1.00 −0.75 −0.50 −0.25 0.00 0.25 0.50 0.75 1.00
Diverticulosis of large intestine without diverticulitis (x1.54) 0.790
Diverticular disease of colon (x1.15) 0.832
Essential hypertension (x1.1) 0.849
Gastroesophageal reflux disease without esophagitis (x1.07) 0.859
White Race (+0.00614) 0.867
Hyperlipidemia (x1.06) 0.874
Abnormal findings on diagnostic imaging of lung (x1.06) 0.880
Black Race (-0.00409) 0.883
Age (+1.85) 0.886
Diaphragmatic hernia (x1.04) 0.890
Hemorrhoids (x1.04) 0.893
Benign neoplasm of ascending colon (x1.04) 0.895
Mixed hyperlipidemia (x1.04) 0.898
Benign neoplasm of transverse colon (x1.04) 0.900
Atherosclerosis of coronary artery without angina pectoris (x1.04) 0.903
Solitary nodule of lung (x1.03) 0.905
Acute renal failure syndrome (x1.03) 0.906
Electrocardiogram abnormal (x1.03) 0.908
Dyspnea (x1.03) 0.910
Intestinal hemorrhage with diverticulosis (x1.03) 0.912
Postoperative state (x1.03) 0.913
Anemia (x1.03) 0.915
acetaminophen (+0.00263) 0.916
Gastrointestinal hemorrhage (x1.03) 0.917
Diverticula of intestine (x1.03) 0.919
Abdominal pain (x1.03) 0.920
Persistent pain following procedure (x1.03) 0.921
ECG normal (x1.03) 0.923
Benign neoplasm of colon (x1.03) 0.924
Melena (x1.03) 0.925
Pure hypercholesterolemia (x1.02) 0.926
Benign neoplasm of sigmoid colon (x1.02) 0.927
Type 2 diabetes mellitus without complication (x1.02) 0.928
Paroxysmal atrial fibrillation (x1.02) 0.929
Infective pneumonia (/1.02) 0.930
Fatigue (x1.02) 0.931
Obstructive sleep apnea syndrome (x1.02) 0.932
Type 2 diabetes mellitus (x1.02) 0.933
Hypothyroidism (x1.02) 0.934
Obesity (x1.02) 0.934
Precordial pain (x1.02) 0.935
Osteoarthritis (x1.02) 0.936
atorvastatin (+0.00195) 0.937
Chronic obstructive lung disease (x1.02) 0.938
Cardiac arrhythmia (x1.02) 0.938
Acute posthemorrhagic anemia (x1.02) 0.939
Chronic kidney disease stage 3 (x1.02) 0.940
Hypertensive disorder (x1.02) 0.940
Acquired renal cystic disease (x1.02) 0.941
Atrial fibrillation (x1.02) 0.942
Heart rate (-0.75) 0.942
Systolic blood pressure (+0.578) 0.943
Benign neoplasm of cecum (x1.02) 0.944
Disorder of kidney and/or ureter (x1.02) 0.944
Diarrhea (x1.02) 0.945
Hyperglycemia (x1.02) 0.945
Osteoarthritis of knee (x1.02) 0.946
Chronic kidney disease due to hypertension (x1.02) 0.946
Kidney stone (x1.02) 0.947
Heart failure (x1.02) 0.948
Disorder of intestine (x1.02) 0.948
Acidosis (x1.02) 0.949
Disorder of bone (x1.02) 0.949
Benign prostatic hyperplasia (x1.02) 0.950
MCV [V] by AC (+0.195) 0.950
Chronic kidney disease (x1.02) 0.950
aspirin (+0.00148) 0.951
Benign neoplasm of descending colon (x1.02) 0.951
Hyperglycemia due to type 2 diabetes mellitus (x1.02) 0.952
Acquired absence of organ (x1.02) 0.952
Steatosis of liver (x1.02) 0.953
Diverticulitis of large intestine (x1.01) 0.953
Disease of liver (x1.01) 0.953
promethazine (-0.00134) 0.954
Basophils/100 leukocytes in B by AC (+0.00799) 0.954
Dizziness and giddiness (x1.01) 0.954
amlodipine (+0.00131) 0.955
Respiratory rate (-0.158) 0.955
Estimation of glomerular filtration rate (-2.41) 0.955
Asthenia (x1.01) 0.956
Major depression, single episode (x1.01) 0.956
metoprolol (+0.00129) 0.956
Gout (x1.01) 0.957
Nicotine dependence (x1.01) 0.957
Creatinine [M/V] in SP (+0.0197) 0.957
Heart disease (x1.01) 0.958
Loss of consciousness (x1.01) 0.958
Diastolic blood pressure (+0.251) 0.958
Pulmonary emphysema (x1.01) 0.958
Benign prostatic hypertrophy with outflow obstruction (x1.01) 0.959
Hypertensive heart failure (x1.01) 0.959
Abnormal breathing (/1.01) 0.959
Systolic blood pressure by Noninvasive (+0.582) 0.960
Anxiety disorder (x1.01) 0.960
Abnormal body temperature (/1.01) 0.960
Peritoneal adhesion (x1.01) 0.960
Hyperkalemia (x1.01) 0.961
Lung field abnormal (x1.01) 0.961
Mean blood pressure by Noninvasive (+0.436) 0.961
Polyp of colon (x1.01) 0.961
Expressions
10^5
10^3
10^1
−10 0 10

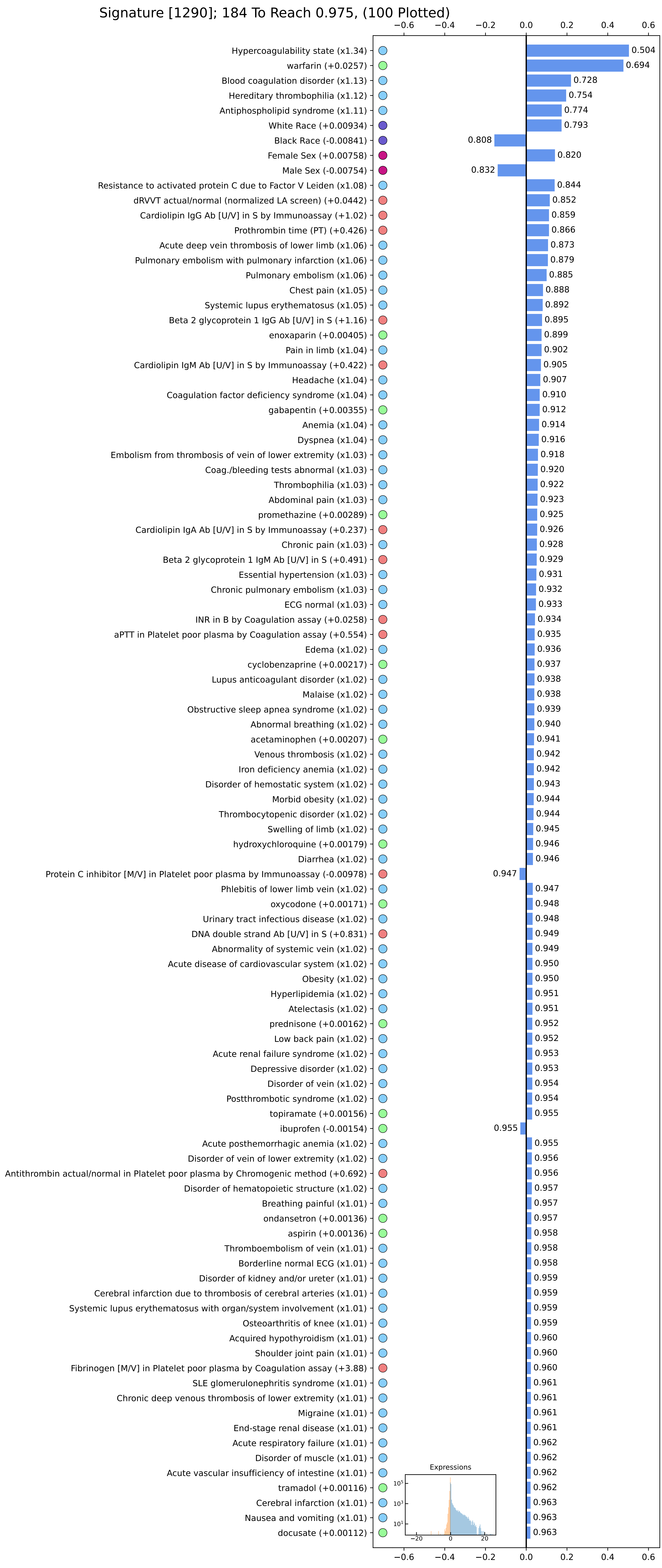

Signature [1290]; 184 To Reach 0.975, (100 Plotted)
Hypercoagulability state (x1.34) 0.504
warfarin (+0.0257) 0.694
Blood coagulation disorder (x1.13) 0.728
Hereditary thrombophilia (x1.12) 0.754
Antiphospholipid syndrome (x1.11) 0.774
White Race (+0.00934) 0.793
Black Race (-0.00841) 0.808
Female Sex (+0.00758) 0.820
Male Sex (-0.00754) 0.832
Resistance to activated protein C due to Factor V Leiden (x1.08) 0.844
dRVVT actual/normal (normalized LA screen) (+0.0442) 0.852
Cardiolipin IgG Ab [U/V] in S by Immunoassay (+1.02) 0.859
Prothrombin time (PT) (+0.426) 0.866
Acute deep vein thrombosis of lower limb (x1.06) 0.873
Pulmonary embolism with pulmonary infarction (x1.06) 0.879
Pulmonary embolism (x1.06) 0.885
Chest pain (x1.05) 0.888
Systemic lupus erythematosus (x1.05) 0.892
Beta 2 glycoprotein 1 IgG Ab [U/V] in S (+1.16) 0.895
enoxaparin (+0.00405) 0.899
Pain in limb (x1.04) 0.902
Cardiolipin IgM Ab [U/V] in S by Immunoassay (+0.422) 0.905
Headache (x1.04) 0.907
Coagulation factor deficiency syndrome (x1.04) 0.910
gabapentin (+0.00355) 0.912
Anemia (x1.04) 0.914
Dyspnea (x1.04) 0.916
Embolism from thrombosis of vein of lower extremity (x1.03) 0.918
Coag./bleeding tests abnormal (x1.03) 0.920
Thrombophilia (x1.03) 0.922
Abdominal pain (x1.03) 0.923
promethazine (+0.00289) 0.925
Cardiolipin IgA Ab [U/V] in S by Immunoassay (+0.237) 0.926
Chronic pain (x1.03) 0.928
Beta 2 glycoprotein 1 IgM Ab [U/V] in S (+0.491) 0.929
Essential hypertension (x1.03) 0.931
Chronic pulmonary embolism (x1.03) 0.932
ECG normal (x1.03) 0.933
INR in B by Coagulation assay (+0.0258) 0.934
aPTT in Platelet poor plasma by Coagulation assay (+0.554) 0.935
Edema (x1.02) 0.936
cyclobenzaprine (+0.00217) 0.937
Lupus anticoagulant disorder (x1.02) 0.938
Malaise (x1.02) 0.938
Obstructive sleep apnea syndrome (x1.02) 0.939
Abnormal breathing (x1.02) 0.940
acetaminophen (+0.00207) 0.941
Venous thrombosis (x1.02) 0.942
Iron deficiency anemia (x1.02) 0.942
Disorder of hemostatic system (x1.02) 0.943
Morbid obesity (x1.02) 0.944
Thrombocytopenic disorder (x1.02) 0.944
Swelling of limb (x1.02) 0.945
hydroxychloroquine (+0.00179) 0.946
Diarrhea (x1.02) 0.946
Protein C inhibitor [M/V] in Platelet poor plasma by Immunoassay (-0.00978) 0.947
Phlebitis of lower limb vein (x1.02) 0.947
oxycodone (+0.00171) 0.948
Urinary tract infectious disease (x1.02) 0.948
DNA double strand Ab [U/V] in S (+0.831) 0.949
Abnormality of systemic vein (x1.02) 0.949
Acute disease of cardiovascular system (x1.02) 0.950
Obesity (x1.02) 0.950
Hyperlipidemia (x1.02) 0.951
Atelectasis (x1.02) 0.951
prednisone (+0.00162) 0.952
Low back pain (x1.02) 0.952
Acute renal failure syndrome (x1.02) 0.953
Depressive disorder (x1.02) 0.953
Disorder of vein (x1.02) 0.954
Postthrombotic syndrome (x1.02) 0.954
topiramate (+0.00156) 0.955
ibuprofen (-0.00154) 0.955
Acute posthemorrhagic anemia (x1.02) 0.955
Disorder of vein of lower extremity (x1.02) 0.956
Antithrombin actual/normal in Platelet poor plasma by Chromogenic method (+0.692) 0.956
Disorder of hematopoietic structure (x1.02) 0.957
Breathing painful (x1.01) 0.957
ondansetron (+0.00136) 0.957
aspirin (+0.00136) 0.958
Thromboembolism of vein (x1.01) 0.958
Borderline normal ECG (x1.01) 0.958
Disorder of kidney and/or ureter (x1.01) 0.959
Cerebral infarction due to thrombosis of cerebral arteries (x1.01) 0.959
Systemic lupus erythematosus with organ/system involvement (x1.01) 0.959
Osteoarthritis of knee (x1.01) 0.959
Acquired hypothyroidism (x1.01) 0.960
Shoulder joint pain (x1.01) 0.960
Fibrinogen [M/V] in Platelet poor plasma by Coagulation assay (+3.88) 0.960
SLE glomerulonephritis syndrome (x1.01) 0.961
Chronic deep venous thrombosis of lower extremity (x1.01) 0.961
Migraine (x1.01) 0.961
End-stage renal disease (x1.01) 0.961
Acute respiratory failure (x1.01) 0.962
Disorder of muscle (x1.01) 0.962
Acute vascular insufficiency of intestine (x1.01) 0.962
tramadol (+0.00116) 0.962
Cerebral infarction (x1.01) 0.963
Nausea and vomiting (x1.01) 0.963
docusate (+0.00112) 0.963
−0.6 −0.4 −0.2 0.0 0.2 0.4 0.6
Expressions

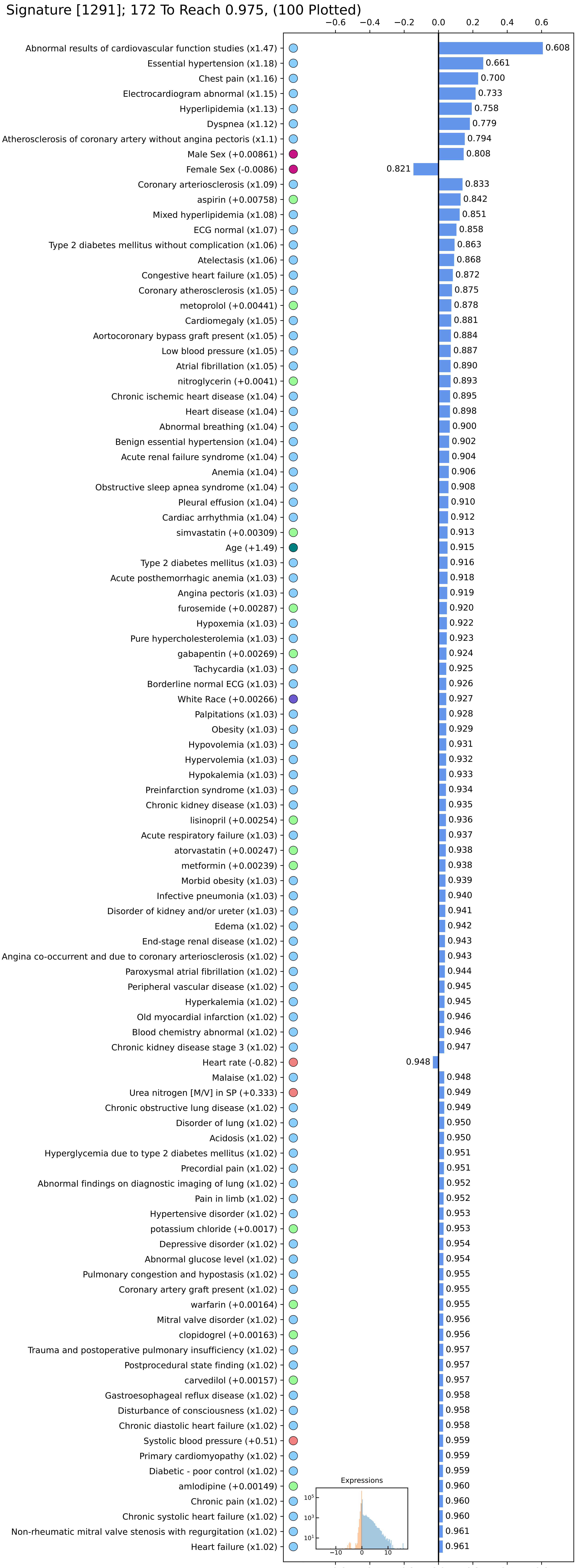

Signature [1291]; 172 To Reach 0.975, (100 Plotted)
Abnormal results of cardiovascular function studies (x1.47) 0.608
Essential hypertension (x1.18) 0.661
Chest pain (x1.16) 0.700
Electrocardiogram abnormal (x1.15) 0.733
Hyperlipidemia (x1.13) 0.758
Dyspnea (x1.12) 0.779
Atherosclerosis of coronary artery without angina pectoris (x1.1) 0.794
Male Sex (+0.00861) 0.808
Female Sex (-0.0086) 0.821
Coronary arteriosclerosis (x1.09) 0.833
aspirin (+0.00758) 0.842
Mixed hyperlipidemia (x1.08) 0.851
ECG normal (x1.07) 0.858
Type 2 diabetes mellitus without complication (x1.06) 0.863
Atelectasis (x1.06) 0.868
Congestive heart failure (x1.05) 0.872
Coronary atherosclerosis (x1.05) 0.875
metoprolol (+0.00441) 0.878
Cardiomegaly (x1.05) 0.881
Aortocoronary bypass graft present (x1.05) 0.884
Low blood pressure (x1.05) 0.887
Atrial fibrillation (x1.05) 0.890
nitroglycerin (+0.0041) 0.893
Chronic ischemic heart disease (x1.04) 0.895
Heart disease (x1.04) 0.898
Abnormal breathing (x1.04) 0.900
Benign essential hypertension (x1.04) 0.902
Acute renal failure syndrome (x1.04) 0.904
Anemia (x1.04) 0.906
Obstructive sleep apnea syndrome (x1.04) 0.908
Pleural effusion (x1.04) 0.910
Cardiac arrhythmia (x1.04) 0.912
simvastatin (+0.00309) 0.913
Age (+1.49) 0.915
Type 2 diabetes mellitus (x1.03) 0.916
Acute posthemorrhagic anemia (x1.03) 0.918
Angina pectoris (x1.03) 0.919
furosemide (+0.00287) 0.920
Hypoxemia (x1.03) 0.922
Pure hypercholesterolemia (x1.03) 0.923
gabapentin (+0.00269) 0.924
Tachycardia (x1.03) 0.925
Borderline normal ECG (x1.03) 0.926
White Race (+0.00266) 0.927
Palpitations (x1.03) 0.928
Obesity (x1.03) 0.929
Hypovolemia (x1.03) 0.931
Hypervolemia (x1.03) 0.932
Hypokalemia (x1.03) 0.933
Preinfarction syndrome (x1.03) 0.934
Chronic kidney disease (x1.03) 0.935
lisinopril (+0.00254) 0.936
Acute respiratory failure (x1.03) 0.937
atorvastatin (+0.00247) 0.938
metformin (+0.00239) 0.938
Morbid obesity (x1.03) 0.939
Infective pneumonia (x1.03) 0.940
Disorder of kidney and/or ureter (x1.03) 0.941
Edema (x1.02) 0.942
End-stage renal disease (x1.02) 0.943
Angina co-occurrent and due to coronary arteriosclerosis (x1.02) 0.943
Paroxysmal atrial fibrillation (x1.02) 0.944
Peripheral vascular disease (x1.02) 0.945
Hyperkalemia (x1.02) 0.945
Old myocardial infarction (x1.02) 0.946
Blood chemistry abnormal (x1.02) 0.946
Chronic kidney disease stage 3 (x1.02) 0.947
Heart rate (-0.82) 0.948
Malaise (x1.02) 0.948
Urea nitrogen [M/V] in SP (+0.333) 0.949
Chronic obstructive lung disease (x1.02) 0.949
Disorder of lung (x1.02) 0.950
Acidosis (x1.02) 0.950
Hyperglycemia due to type 2 diabetes mellitus (x1.02) 0.951
Precordial pain (x1.02) 0.951
Abnormal findings on diagnostic imaging of lung (x1.02) 0.952
Pain in limb (x1.02) 0.952
Hypertensive disorder (x1.02) 0.953
potassium chloride (+0.0017) 0.953
Depressive disorder (x1.02) 0.954
Abnormal glucose level (x1.02) 0.954
Pulmonary congestion and hypostasis (x1.02) 0.955
Coronary artery graft present (x1.02) 0.955
warfarin (+0.00164) 0.955
Mitral valve disorder (x1.02) 0.956
clopidogrel (+0.00163) 0.956
Trauma and postoperative pulmonary insufficiency (x1.02) 0.957
Postprocedural state finding (x1.02) 0.957
carvedilol (+0.00157) 0.957
Gastroesophageal reflux disease (x1.02) 0.958
Disturbance of consciousness (x1.02) 0.958
Chronic diastolic heart failure (x1.02) 0.958
Systolic blood pressure (+0.51) 0.959
Primary cardiomyopathy (x1.02) 0.959
Diabetic - poor control (x1.02) 0.959
amlodipine (+0.00149) 0.960
Chronic pain (x1.02) 0.960
Chronic systolic heart failure (x1.02) 0.960
Non-rheumatic mitral valve stenosis with regurgitation (x1.02) 0.961
Heart failure (x1.02) 0.961
−0.6 −0.4 −0.2 0.0 0.2 0.4 0.6
Expressions

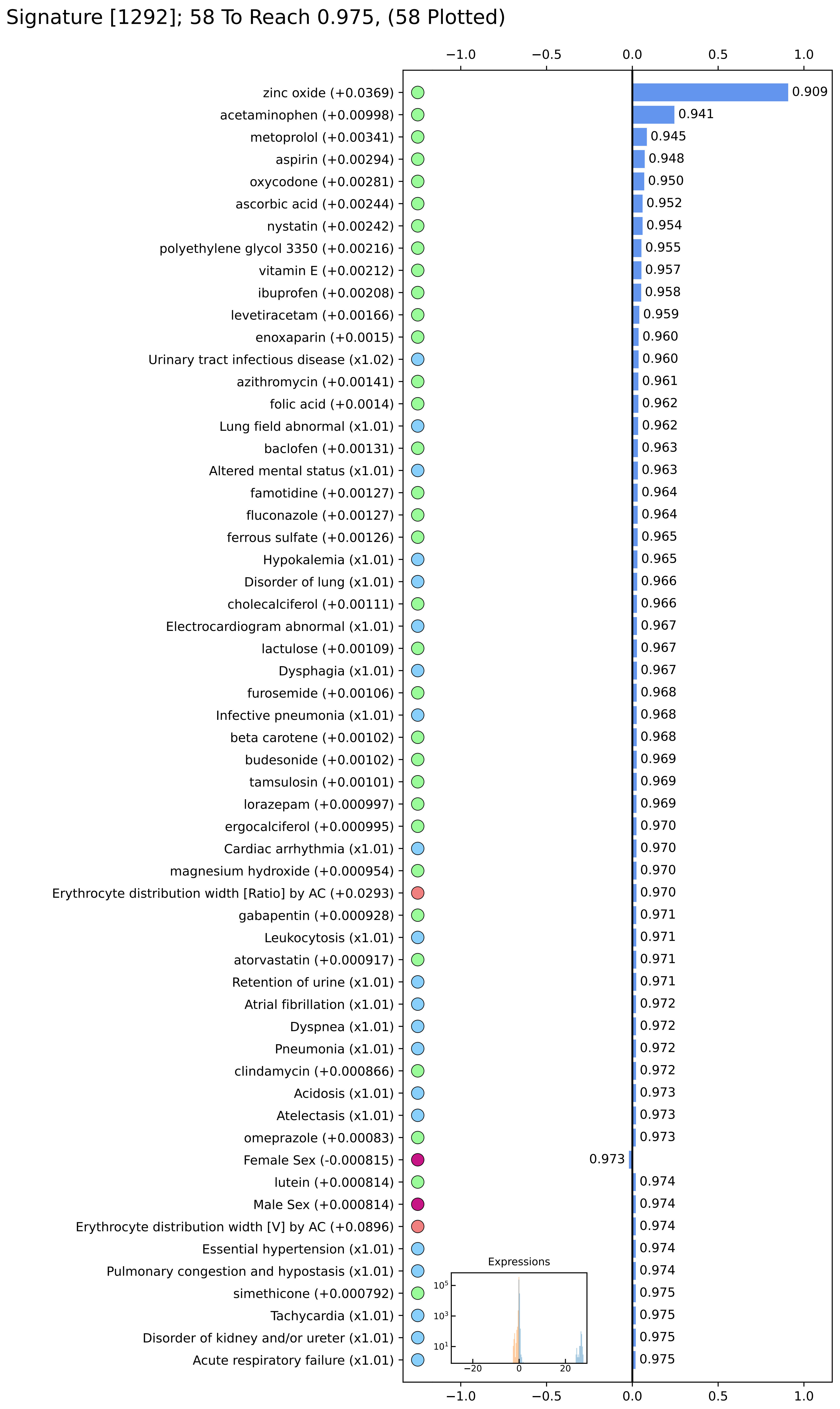

Signature [1292]; 58 To Reach 0.975, (58 Plotted)
zinc oxide (+0.0369) 0.909
acetaminophen (+0.00998) 0.941
metoprolol (+0.00341) 0.945
aspirin (+0.00294) 0.948
oxycodone (+0.00281) 0.950
ascorbic acid (+0.00244) 0.952
nystatin (+0.00242) 0.954
polyethylene glycol 3350 (+0.00216) 0.955
vitamin E (+0.00212) 0.957
ibuprofen (+0.00208) 0.958
levetiracetam (+0.00166) 0.959
enoxaparin (+0.0015) 0.960
Urinary tract infectious disease (x1.02) 0.960
azithromycin (+0.00141) 0.961
folic acid (+0.0014) 0.962
Lung field abnormal (x1.01) 0.962
baclofen (+0.00131) 0.963
Altered mental status (x1.01) 0.963
famotidine (+0.00127) 0.964
fluconazole (+0.00127) 0.964
ferrous sulfate (+0.00126) 0.965
Hypokalemia (x1.01) 0.965
Disorder of lung (x1.01) 0.966
cholecalciferol (+0.00111) 0.966
Electrocardiogram abnormal (x1.01) 0.967
lactulose (+0.00109) 0.967
Dysphagia (x1.01) 0.967
furosemide (+0.00106) 0.968
Infective pneumonia (x1.01) 0.968
beta carotene (+0.00102) 0.968
budesonide (+0.00102) 0.969
tamsulosin (+0.00101) 0.969
lorazepam (+0.000997) 0.969
ergocalciferol (+0.000995) 0.970
Cardiac arrhythmia (x1.01) 0.970
magnesium hydroxide (+0.000954) 0.970
Erythrocyte distribution width [Ratio] by AC (+0.0293) 0.970
gabapentin (+0.000928) 0.971
Leukocytosis (x1.01) 0.971
atorvastatin (+0.000917) 0.971
Retention of urine (x1.01) 0.971
Atrial fibrillation (x1.01) 0.972
Dyspnea (x1.01) 0.972
Pneumonia (x1.01) 0.972
clindamycin (+0.000866) 0.972
Acidosis (x1.01) 0.973
Atelectasis (x1.01) 0.973
omeprazole (+0.00083) 0.973
Female Sex (-0.000815) 0.973
lutein (+0.000814) 0.974
Male Sex (+0.000814) 0.974
Erythrocyte distribution width [V] by AC (+0.0896) 0.974
Essential hypertension (x1.01) 0.974
Pulmonary congestion and hypostasis (x1.01) 0.974
simethicone (+0.000792) 0.975
Tachycardia (x1.01) 0.975
Disorder of kidney and/or ureter (x1.01) 0.975
Acute respiratory failure (x1.01) 0.975
−1.0 −0.5 0.0 0.5 1.0
Expressions

Signature [1293]; 8 To Reach 0.975, (10 Plotted)

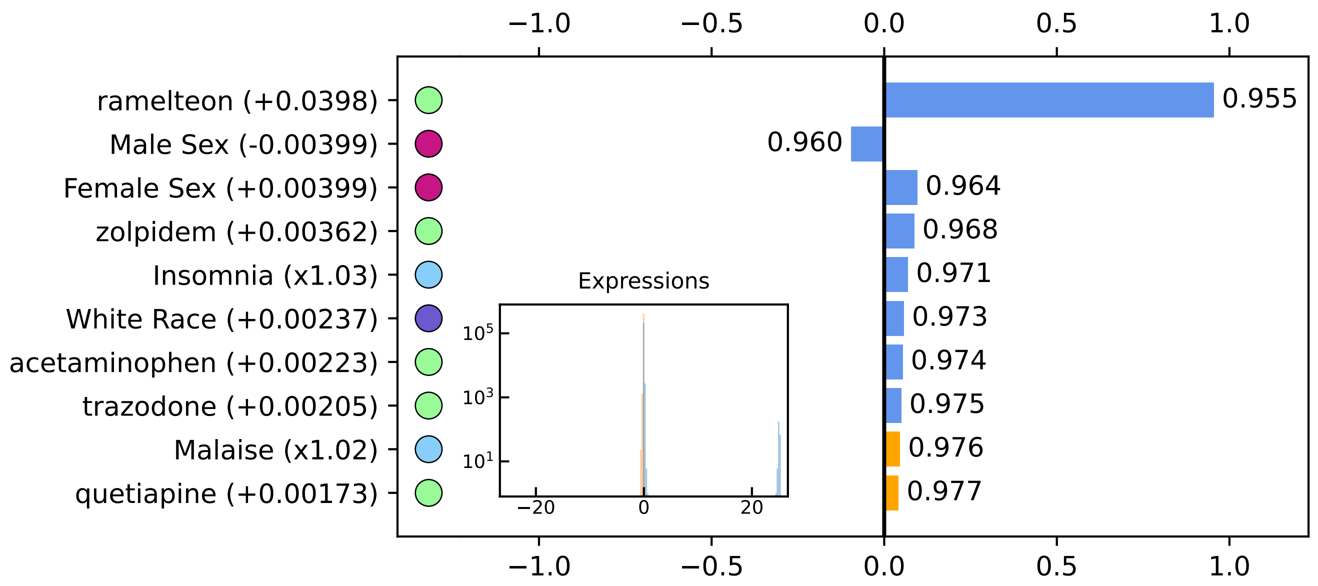

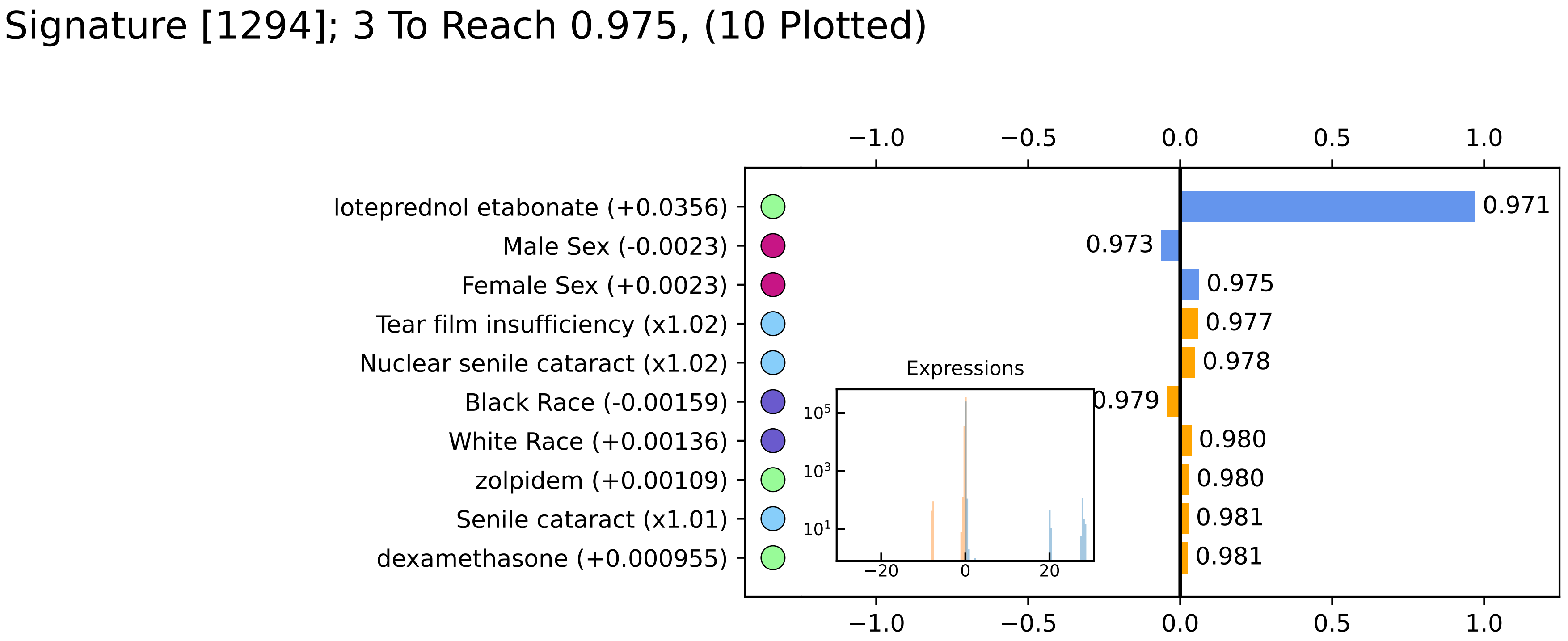

Signature [1294]; 3 To Reach 0.975, (10 Plotted)
loteprednol etabonate (+0.0356)
Male Sex (-0.0023)
Female Sex (+0.0023)
Tear film insufficiency (x1.02)
Nuclear senile cataract (x1.02)
Black Race (-0.00159)
White Race (+0.00136)
zolpidem (+0.00109)
Senile cataract (x1.01)
dexamethasone (+0.000955)
0.971
0.973
0.975
0.977
0.978
0.979
0.980
0.980
0.981
0.981
−1.0
−0.5
0.0
0.5
1.0
Expressions
10^5
10^3
10^1
−20
0
20

Signature [1295]; 5 To Reach 0.975, (10 Plotted)

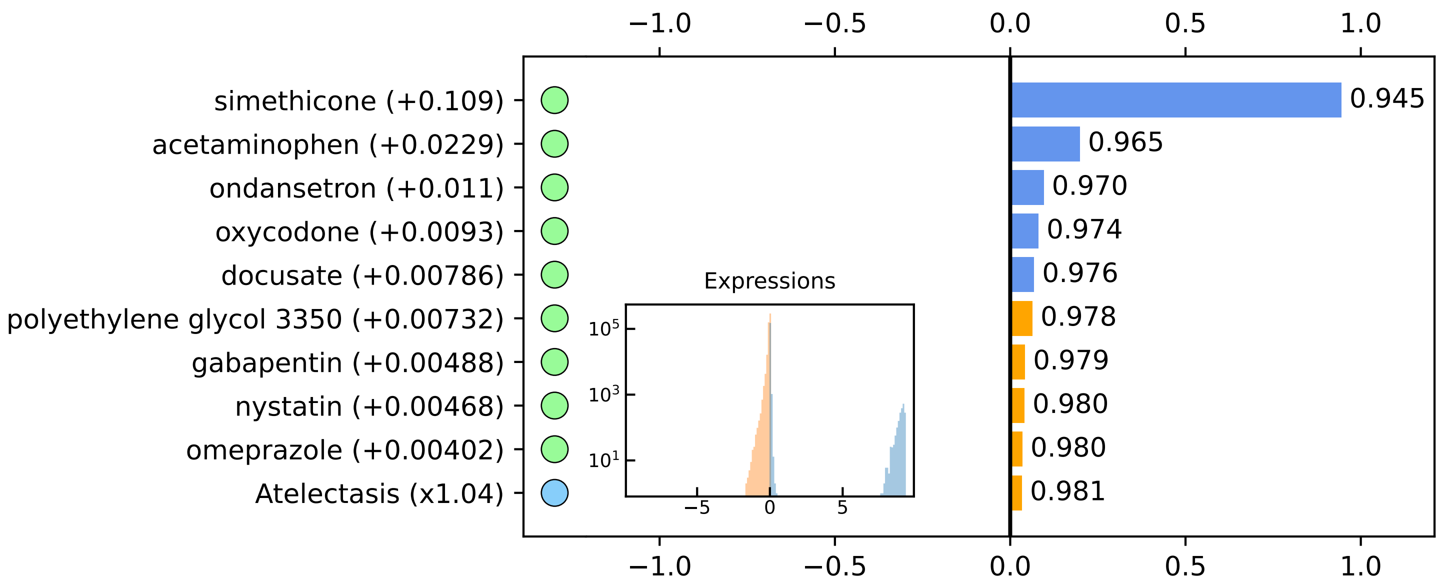

Signature [1296]; 14 To Reach 0.975, (14 Plotted)

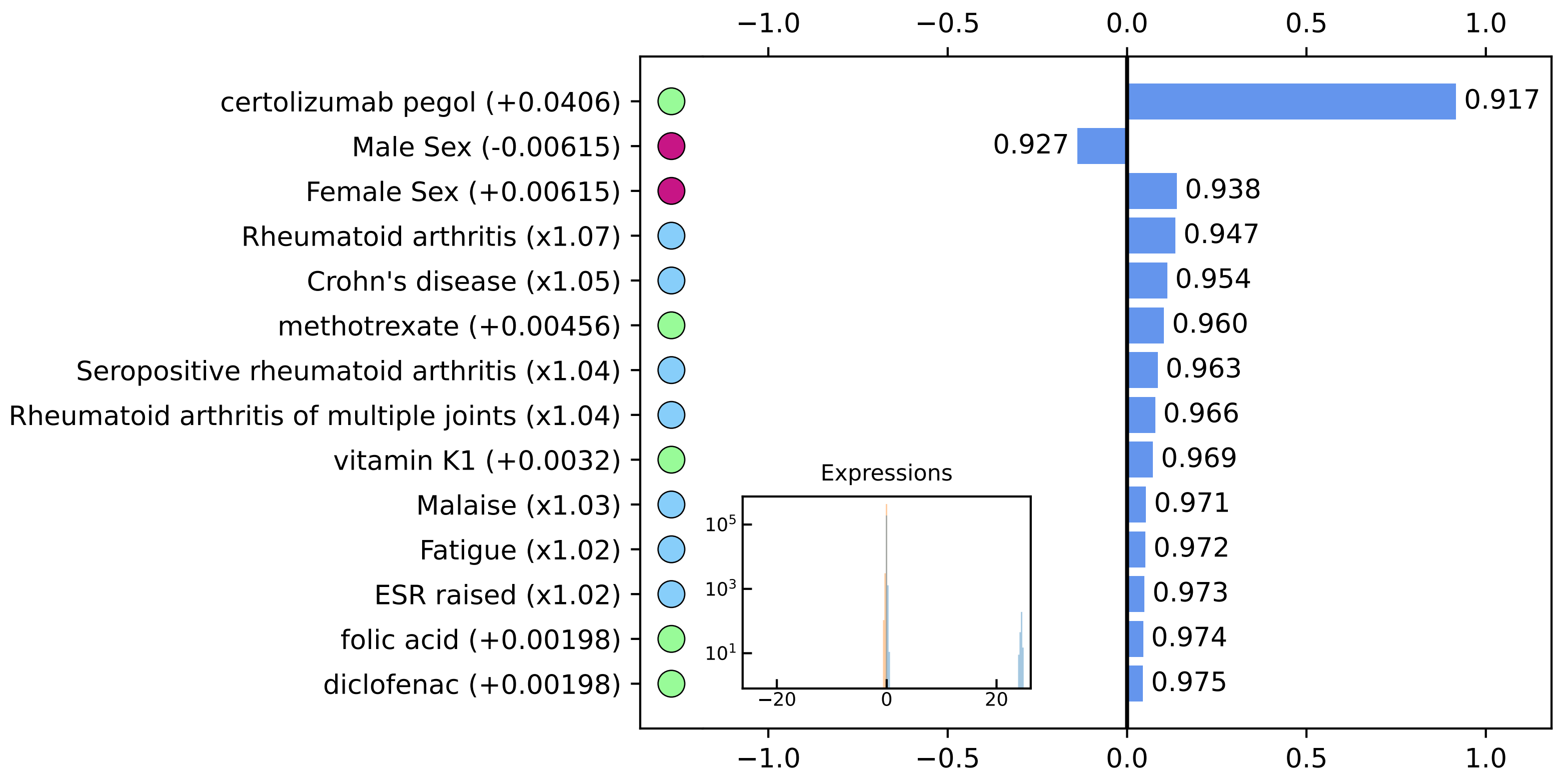

Signature [1297]; 178 To Reach 0.975, (100 Plotted)

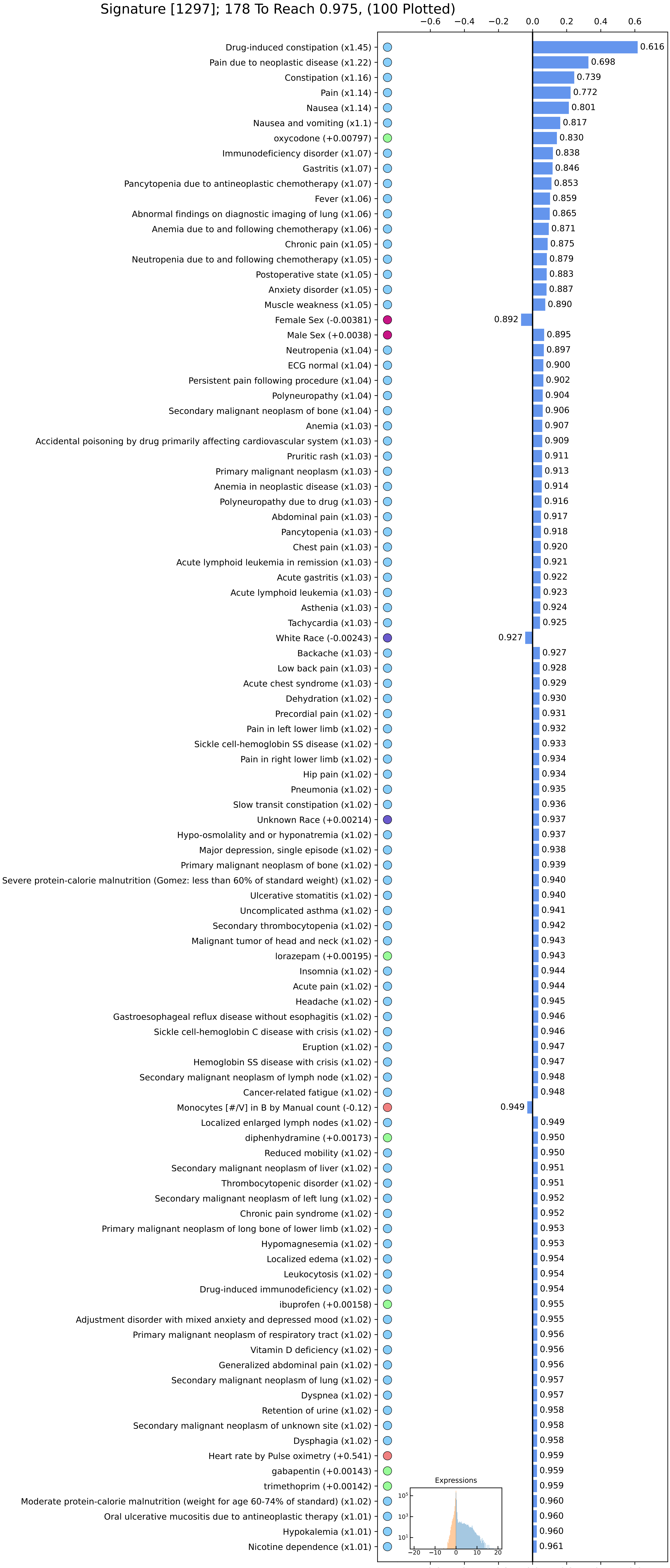

Signature [1298]; 266 To Reach 0.975, (100 Plotted)

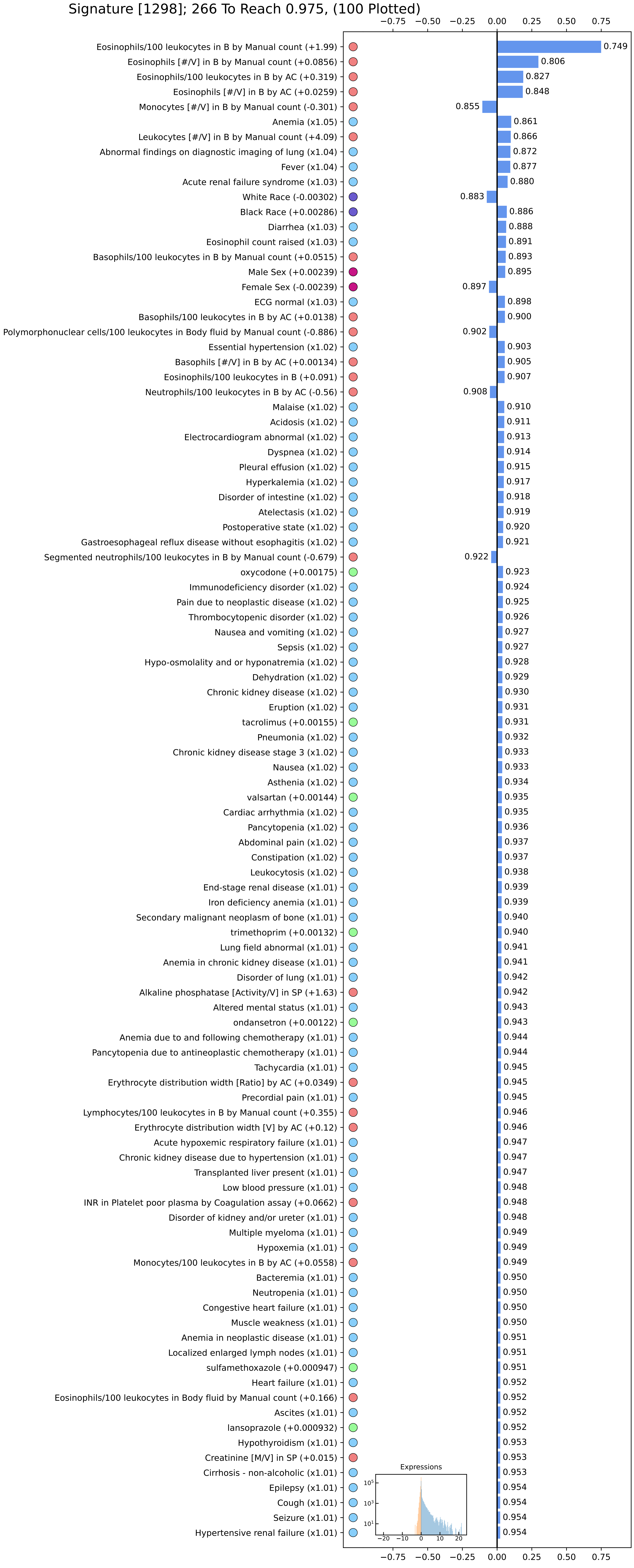

Signature [1299]; 9 To Reach 0.975, (10 Plotted)

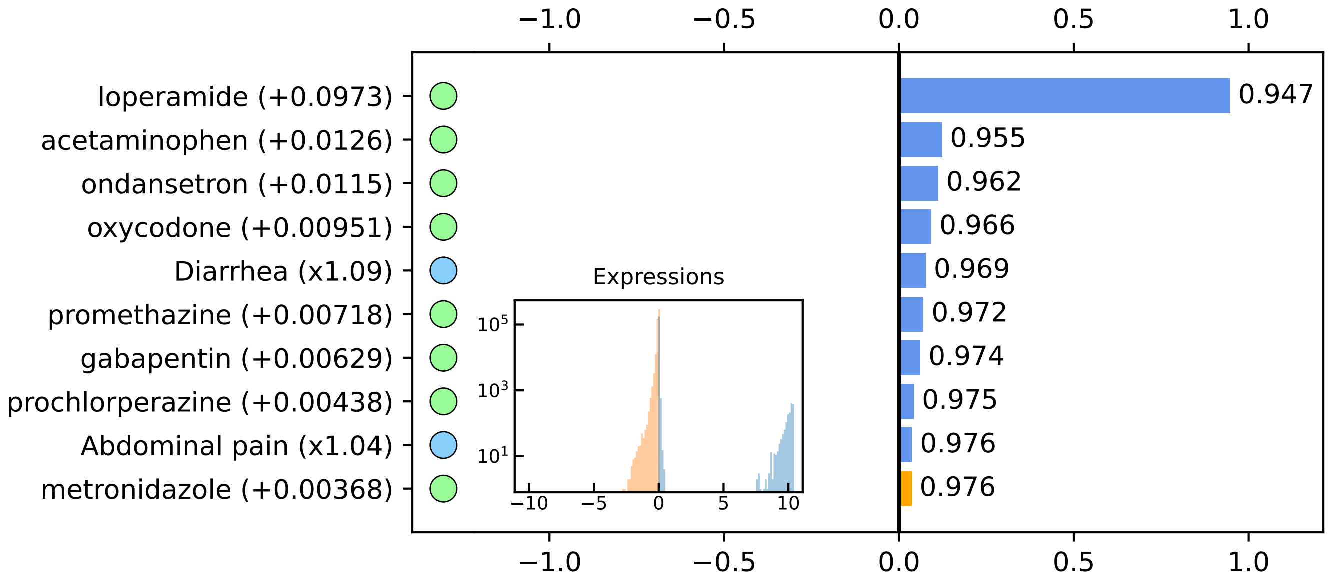

Signature [1300]; 21 To Reach 0.975, (21 Plotted)

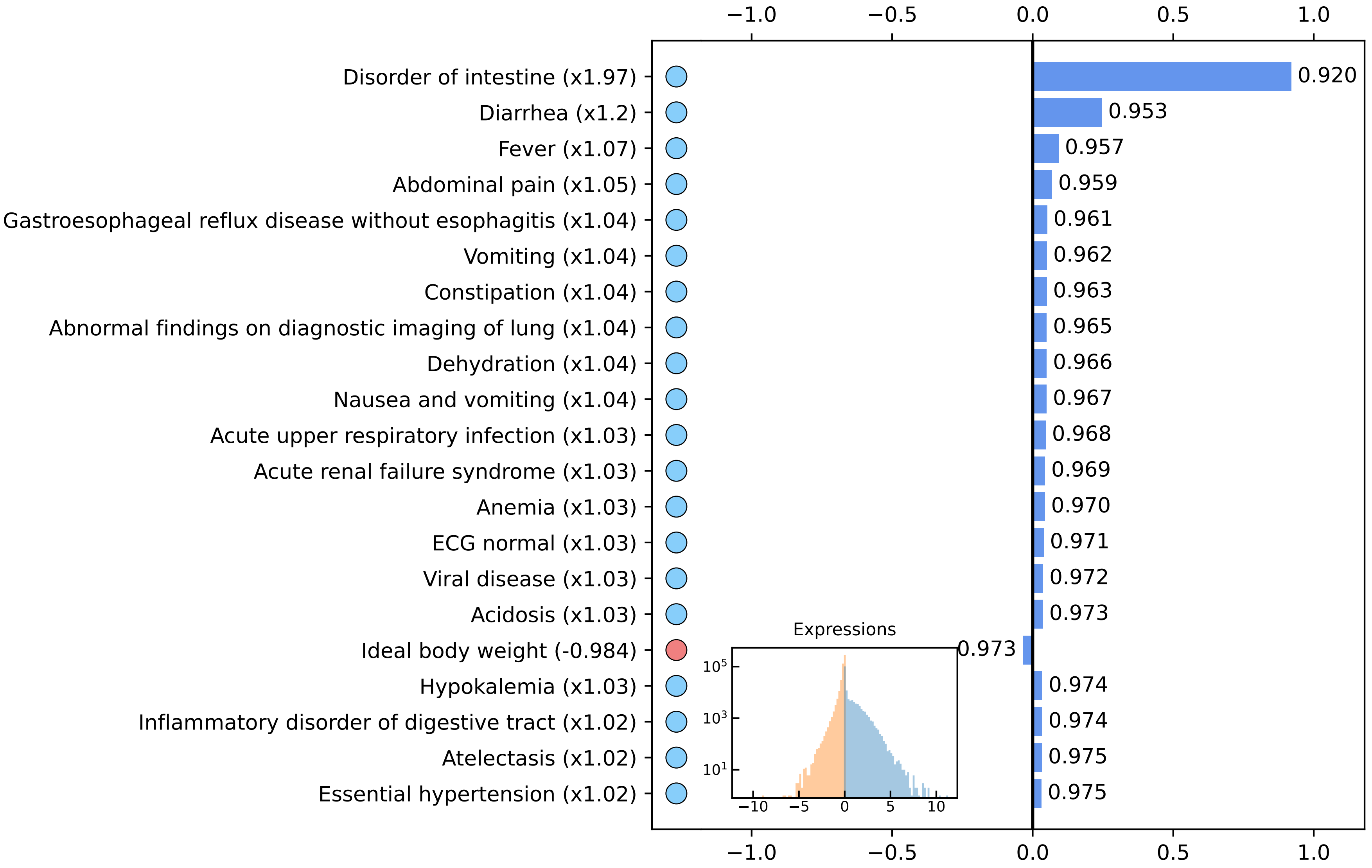

Signature [1301]; 102 To Reach 0.975, (100 Plotted)

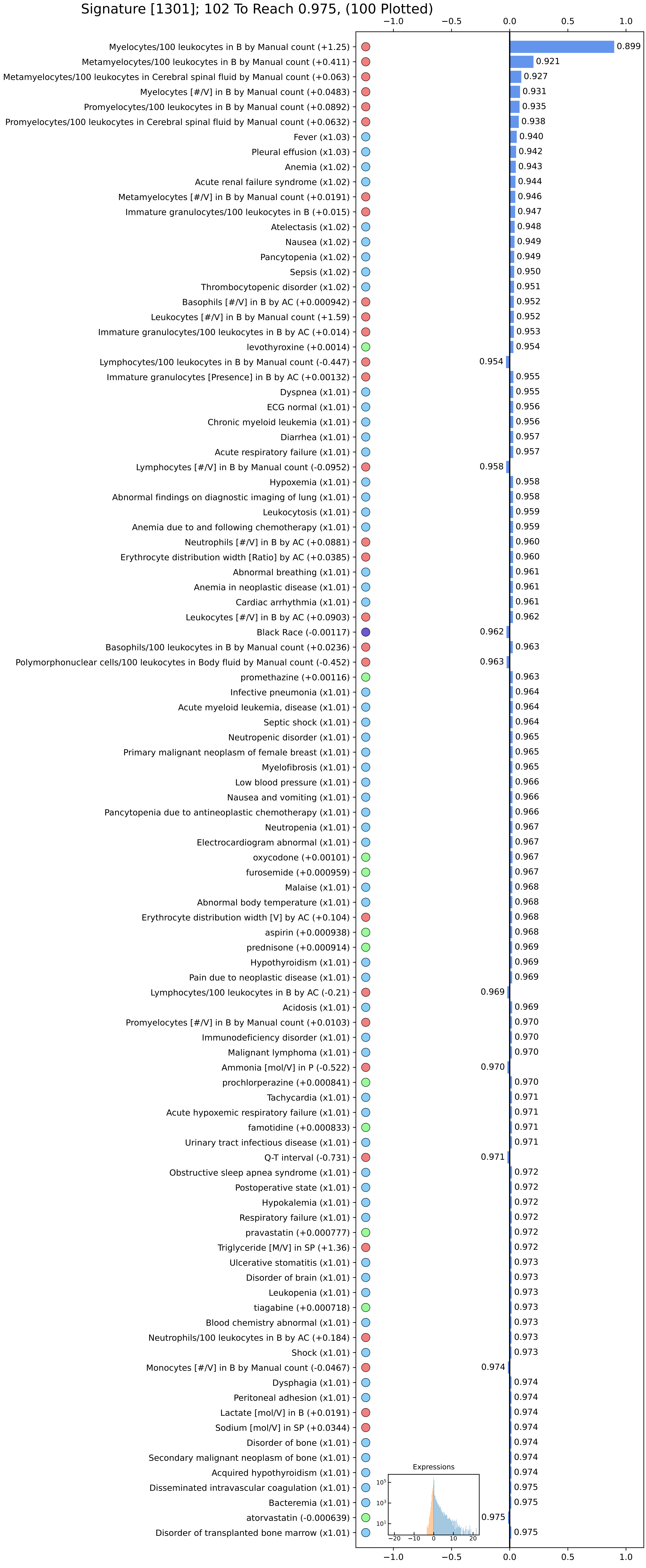

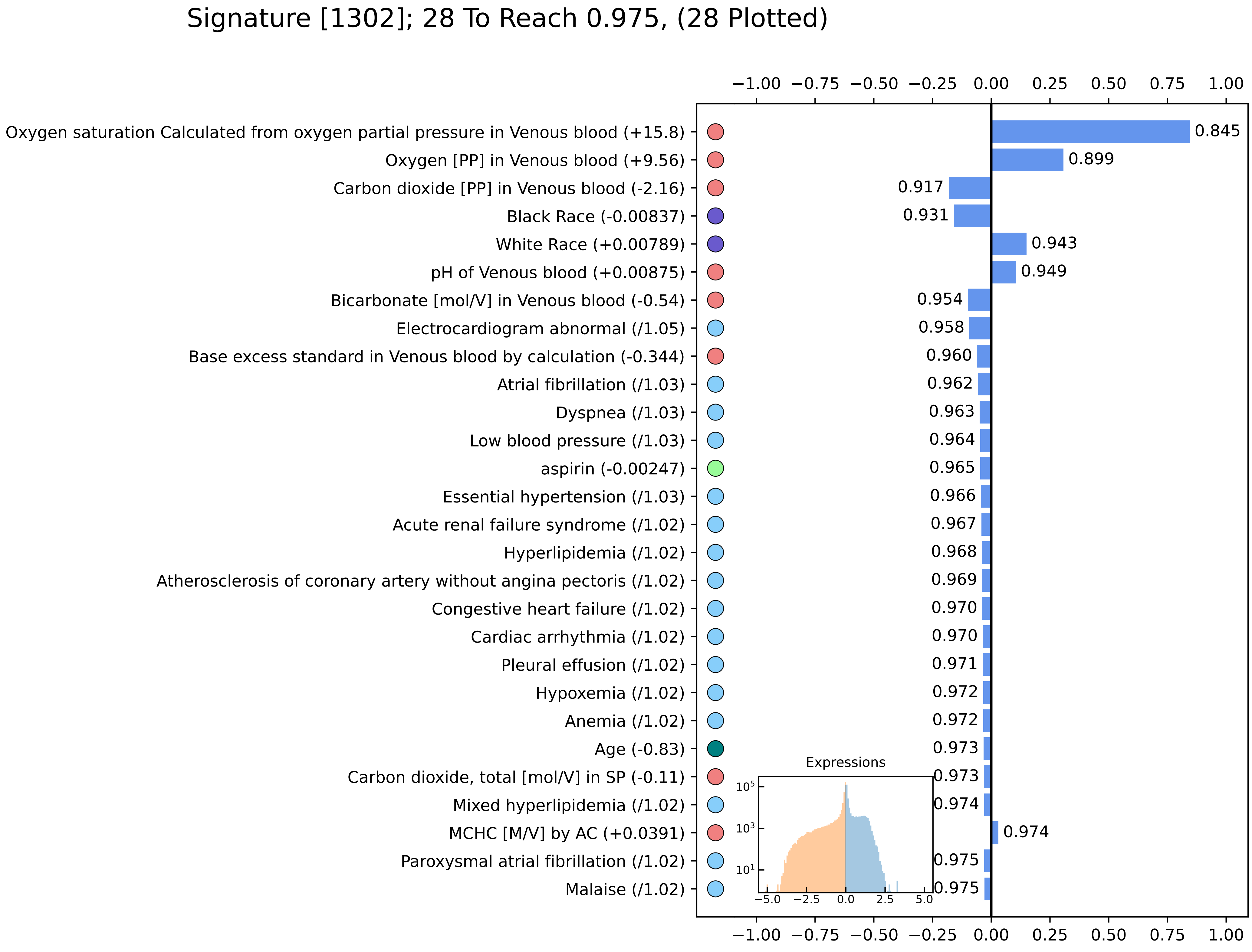

Signature [1302]; 28 To Reach 0.975, (28 Plotted)
Oxygen saturation Calculated from oxygen partial pressure in Venous blood (+15.8)
0.845
Oxygen [PP] in Venous blood (+9.56)
0.899
Carbon dioxide [PP] in Venous blood (-2.16)
0.917
Black Race (-0.00837)
0.931
White Race (+0.00789)
0.943
pH of Venous blood (+0.00875)
0.949
Bicarbonate [mol/V] in Venous blood (-0.54)
0.954
Electrocardiogram abnormal (/1.05)
0.958
Base excess standard in Venous blood by calculation (-0.344)
0.960
Atrial fibrillation (/1.03)
0.962
Dyspnea (/1.03)
0.963
Low blood pressure (/1.03)
0.964
aspirin (-0.00247)
0.965
Essential hypertension (/1.03)
0.966
Acute renal failure syndrome (/1.02)
0.967
Hyperlipidemia (/1.02)
0.968
Atherosclerosis of coronary artery without angina pectoris (/1.02)
0.969
Congestive heart failure (/1.02)
0.970
Cardiac arrhythmia (/1.02)
0.970
Pleural effusion (/1.02)
0.971
Hypoxemia (/1.02)
0.972
Anemia (/1.02)
0.972
Age (-0.83)
0.973
Carbon dioxide, total [mol/V] in SP (-0.11)
0.973
Mixed hyperlipidemia (/1.02)
0.974
MCHC [M/V] by AC (+0.0391)
0.974
Paroxysmal atrial fibrillation (/1.02)
0.975
Malaise (/1.02)
0.975
−1.00 −0.75 −0.50 −0.25 0.00 0.25 0.50 0.75 1.00
Expressions
10^5
10^3
10^1
−5.0 −2.5 0.0 2.5 5.0

Signature [1303]; 1 To Reach 0.975, (10 Plotted)

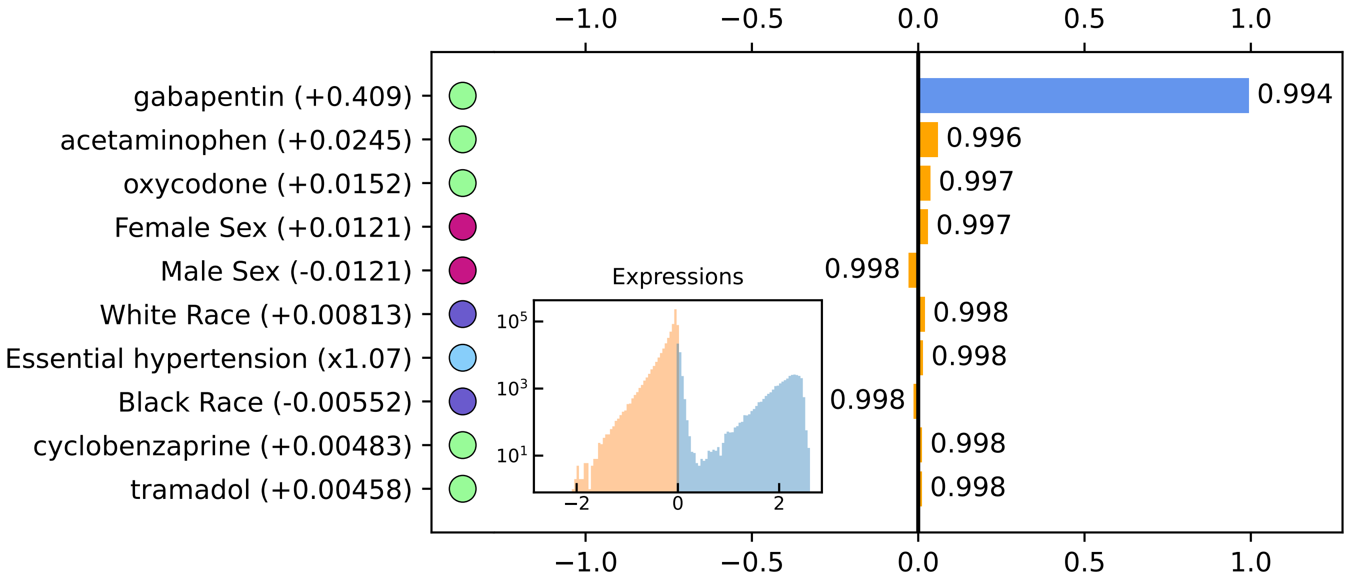

Signature [1304]; 338 To Reach 0.975, (100 Plotted)

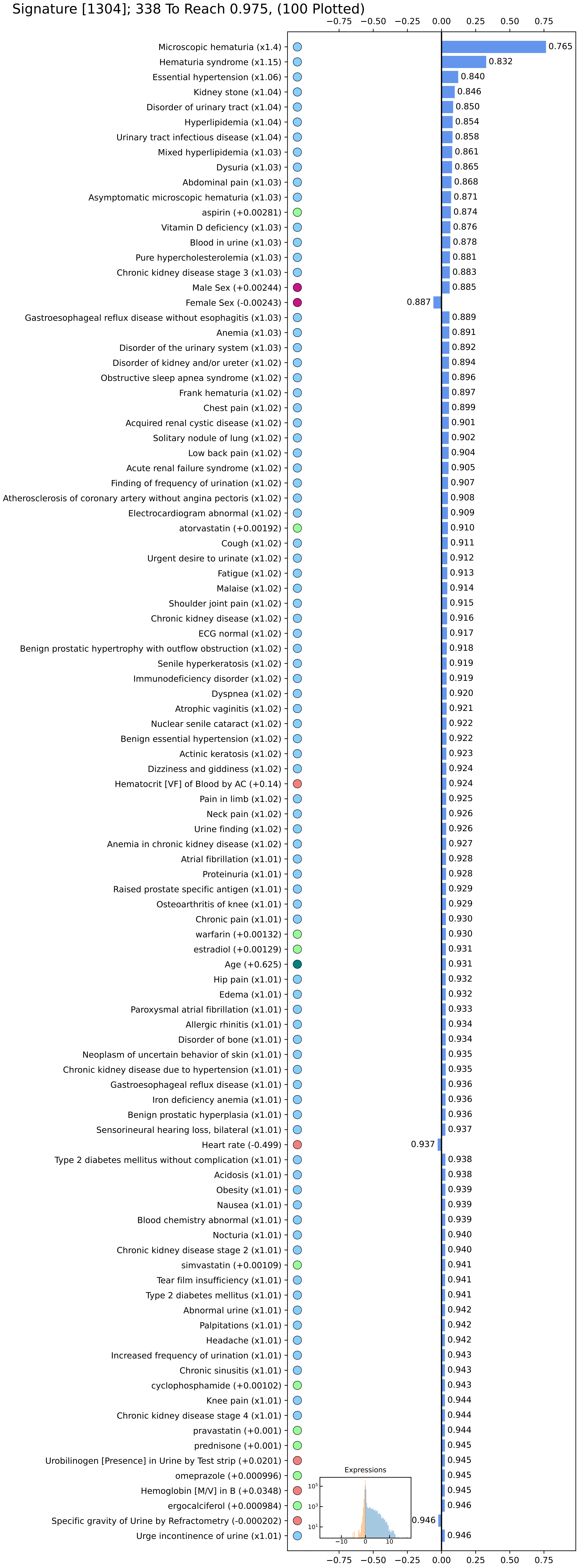

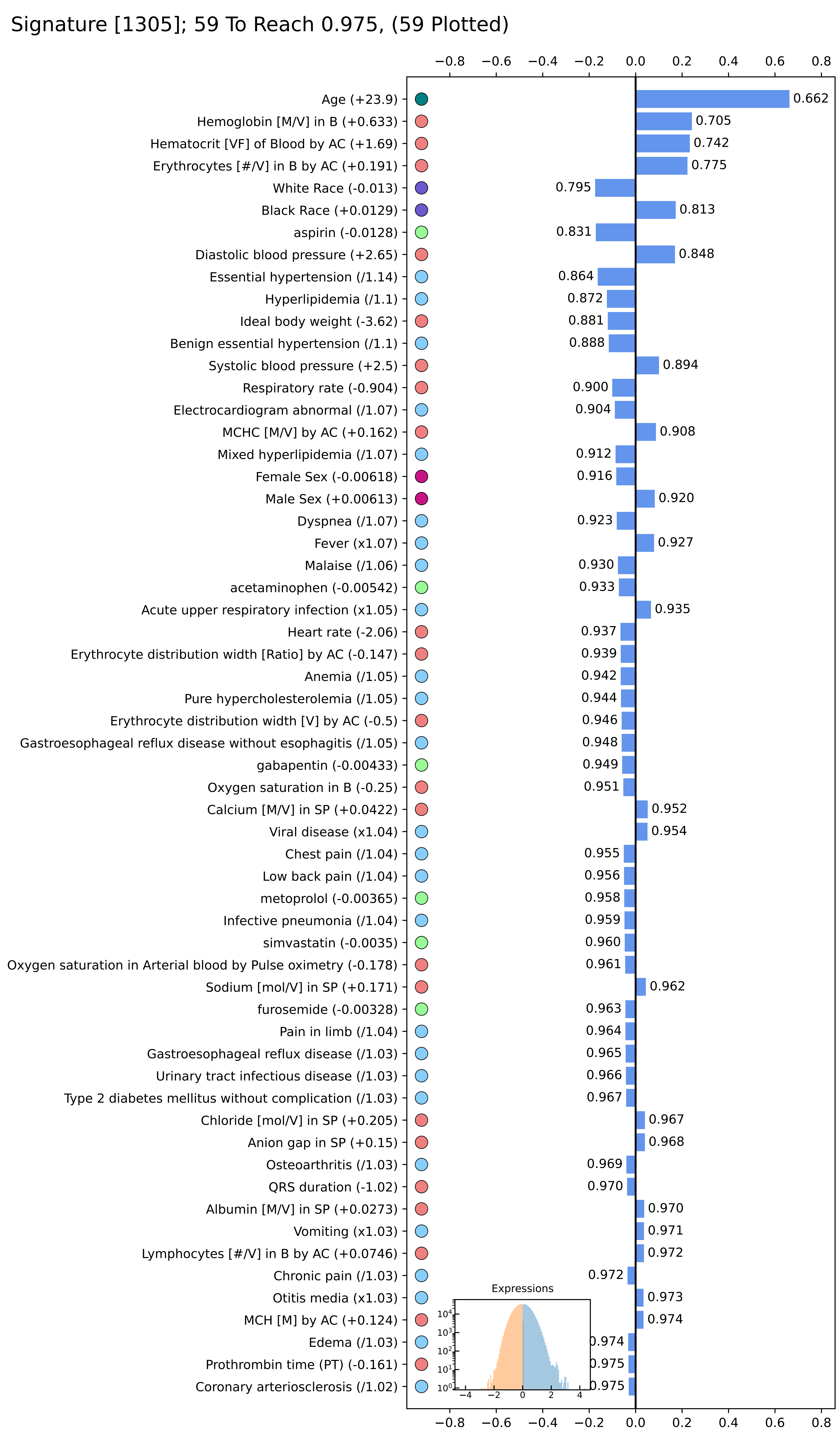
Signature [1305]; 59 To Reach 0.975, (59 Plotted)
Age (+23.9) 0.662
Hemoglobin [M/V] in B (+0.633) 0.705
Hematocrit [VF] of Blood by AC (+1.69) 0.742
Erythrocytes [#/V] in B by AC (+0.191) 0.775
White Race (-0.013) 0.795
Black Race (+0.0129) 0.813
aspirin (-0.0128) 0.831
Diastolic blood pressure (+2.65) 0.848
Essential hypertension (/1.14) 0.864
Hyperlipidemia (/1.1) 0.872
Ideal body weight (-3.62) 0.881
Benign essential hypertension (/1.1) 0.888
Systolic blood pressure (+2.5) 0.894
Respiratory rate (-0.904) 0.900
Electrocardiogram abnormal (/1.07) 0.904
MCHC [M/V] by AC (+0.162) 0.908
Mixed hyperlipidemia (/1.07) 0.912
Female Sex (-0.00618) 0.916
Male Sex (+0.00613) 0.920
Dyspnea (/1.07) 0.923
Fever (x1.07) 0.927
Malaise (/1.06) 0.930
acetaminophen (-0.00542) 0.933
Acute upper respiratory infection (x1.05) 0.935
Heart rate (-2.06) 0.937
Erythrocyte distribution width [Ratio] by AC (-0.147) 0.939
Anemia (/1.05) 0.942
Pure hypercholesterolemia (/1.05) 0.944
Erythrocyte distribution width [V] by AC (-0.5) 0.946
Gastroesophageal reflux disease without esophagitis (/1.05) 0.948
gabapentin (-0.00433) 0.949
Oxygen saturation in B (-0.25) 0.951
Calcium [M/V] in SP (+0.0422) 0.952
Viral disease (x1.04) 0.954
Chest pain (/1.04) 0.955
Low back pain (/1.04) 0.956
metoprolol (-0.00365) 0.958
Infective pneumonia (/1.04) 0.959
simvastatin (-0.0035) 0.960
Oxygen saturation in Arterial blood by Pulse oximetry (-0.178) 0.961
Sodium [mol/V] in SP (+0.171) 0.962
furosemide (-0.00328) 0.963
Pain in limb (/1.04) 0.964
Gastroesophageal reflux disease (/1.03) 0.965
Urinary tract infectious disease (/1.03) 0.966
Type 2 diabetes mellitus without complication (/1.03) 0.967
Chloride [mol/V] in SP (+0.205) 0.967
Anion gap in SP (+0.15) 0.968
Osteoarthritis (/1.03) 0.969
QRS duration (-1.02) 0.970
Albumin [M/V] in SP (+0.0273) 0.970
Vomiting (x1.03) 0.971
Lymphocytes [#/V] in B by AC (+0.0746) 0.972
Chronic pain (/1.03) 0.972
Otitis media (x1.03) 0.973
MCH [M] by AC (+0.124) 0.974
Edema (/1.03) 0.974
Prothrombin time (PT) (-0.161) 0.975
Coronary arteriosclerosis (/1.02) 0.975
Expressions
−0.8 −0.6 −0.4 −0.2 0.0 0.2 0.4 0.6 0.8

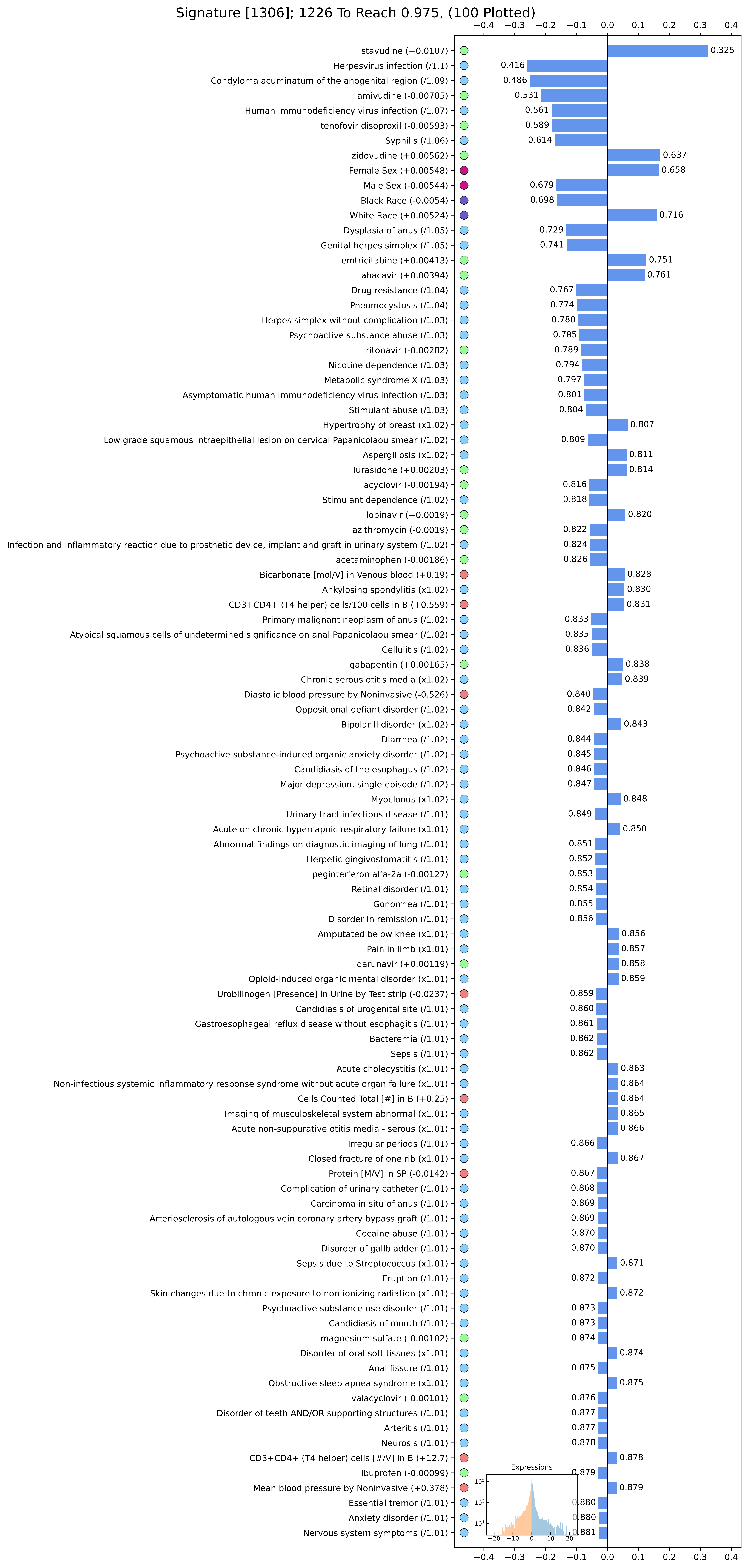

Signature [1306]; 1226 To Reach 0.975, (100 Plotted)
stavudine (+0.0107) 0.325
Herpesvirus infection (/1.1) 0.416
Condyloma acuminatum of the anogenital region (/1.09) 0.486
lamivudine (-0.00705) 0.531
Human immunodeficiency virus infection (/1.07) 0.561
tenofovir disoproxil (-0.00593) 0.589
Syphilis (/1.06) 0.614
zidovudine (+0.00562) 0.637
Female Sex (+0.00548) 0.658
Male Sex (-0.00544) 0.679
Black Race (-0.0054) 0.698
White Race (+0.00524) 0.716
Dysplasia of anus (/1.05) 0.729
Genital herpes simplex (/1.05) 0.741
emtricitabine (+0.00413) 0.751
abacavir (+0.00394) 0.761
Drug resistance (/1.04) 0.767
Pneumocystosis (/1.04) 0.774
Herpes simplex without complication (/1.03) 0.780
Psychoactive substance abuse (/1.03) 0.785
ritonavir (-0.00282) 0.789
Nicotine dependence (/1.03) 0.794
Metabolic syndrome X (/1.03) 0.797
Asymptomatic human immunodeficiency virus infection (/1.03) 0.801
Stimulant abuse (/1.03) 0.804
Hypertrophy of breast (x1.02) 0.807
Low grade squamous intraepithelial lesion on cervical Papanicolaou smear (/1.02) 0.809
Aspergillosis (x1.02) 0.811
lurasidone (+0.00203) 0.814
acyclovir (-0.00194) 0.816
Stimulant dependence (/1.02) 0.818
lopinavir (+0.0019) 0.820
azithromycin (-0.0019) 0.822
Infection and inflammatory reaction due to prosthetic device, implant and graft in urinary system (/1.02) 0.824
acetaminophen (-0.00186) 0.826
Bicarbonate [mol/V] in Venous blood (+0.19) 0.828
Ankylosing spondylitis (x1.02) 0.830
CD3+CD4+ (T4 helper) cells/100 cells in B (+0.559) 0.831
Primary malignant neoplasm of anus (/1.02) 0.833
Atypical squamous cells of undetermined significance on anal Papanicolaou smear (/1.02) 0.835
Cellulitis (/1.02) 0.836
gabapentin (+0.00165) 0.838
Chronic serous otitis media (x1.02) 0.839
Diastolic blood pressure by Noninvasive (-0.526) 0.840
Oppositional defiant disorder (/1.02) 0.842
Bipolar II disorder (x1.02) 0.843
Diarrhea (/1.02) 0.844
Psychoactive substance-induced organic anxiety disorder (/1.02) 0.845
Candidiasis of the esophagus (/1.02) 0.846
Major depression, single episode (/1.02) 0.847
Myoclonus (x1.02) 0.848
Urinary tract infectious disease (/1.01) 0.849
Acute on chronic hypercapnic respiratory failure (x1.01) 0.850
Abnormal findings on diagnostic imaging of lung (/1.01) 0.851
Herpetic gingivostomatitis (/1.01) 0.852
peginterferon alfa-2a (-0.00127) 0.853
Retinal disorder (/1.01) 0.854
Gonorrhea (/1.01) 0.855
Disorder in remission (/1.01) 0.856
Amputated below knee (x1.01) 0.856
Pain in limb (x1.01) 0.857
darunavir (+0.00119) 0.858
Opioid-induced organic mental disorder (x1.01) 0.859
Urobilinogen [Presence] in Urine by Test strip (-0.0237) 0.859
Candidiasis of urogenital site (/1.01) 0.860
Gastroesophageal reflux disease without esophagitis (/1.01) 0.861
Bacteremia (/1.01) 0.862
Sepsis (/1.01) 0.862
Acute cholecystitis (x1.01) 0.863
Non-infectious systemic inflammatory response syndrome without acute organ failure (x1.01) 0.864
Cells Counted Total [#] in B (+0.25) 0.864
Imaging of musculoskeletal system abnormal (x1.01) 0.865
Acute non-suppurative otitis media - serous (x1.01) 0.866
Irregular periods (/1.01) 0.866
Closed fracture of one rib (x1.01) 0.867
Protein [M/V] in SP (-0.0142) 0.867
Complication of urinary catheter (/1.01) 0.868
Carcinoma in situ of anus (/1.01) 0.869
Arteriosclerosis of autologous vein coronary artery bypass graft (/1.01) 0.869
Cocaine abuse (/1.01) 0.870
Disorder of gallbladder (/1.01) 0.870
Sepsis due to Streptococcus (x1.01) 0.871
Eruption (/1.01) 0.872
Skin changes due to chronic exposure to non-ionizing radiation (x1.01) 0.872
Psychoactive substance use disorder (/1.01) 0.873
Candidiasis of mouth (/1.01) 0.873
magnesium sulfate (-0.00102) 0.874
Disorder of oral soft tissues (x1.01) 0.874
Anal fissure (/1.01) 0.875
Obstructive sleep apnea syndrome (x1.01) 0.875
valacyclovir (-0.00101) 0.876
Disorder of teeth AND/OR supporting structures (/1.01) 0.877
Arteritis (/1.01) 0.877
Neurosis (/1.01) 0.878
CD3+CD4+ (T4 helper) cells [#/V] in B (+12.7) 0.878
ibuprofen (-0.00099) 0.879
Mean blood pressure by Noninvasive (+0.378) 0.879
Essential tremor (/1.01) 0.880
Anxiety disorder (/1.01) 0.880
Nervous system symptoms (/1.01) 0.881
−0.4 −0.3 −0.2 −0.1 0.0 0.1 0.2 0.3 0.4
Expressions
10^5 10^3 10^1
−20 −10 0 10 20

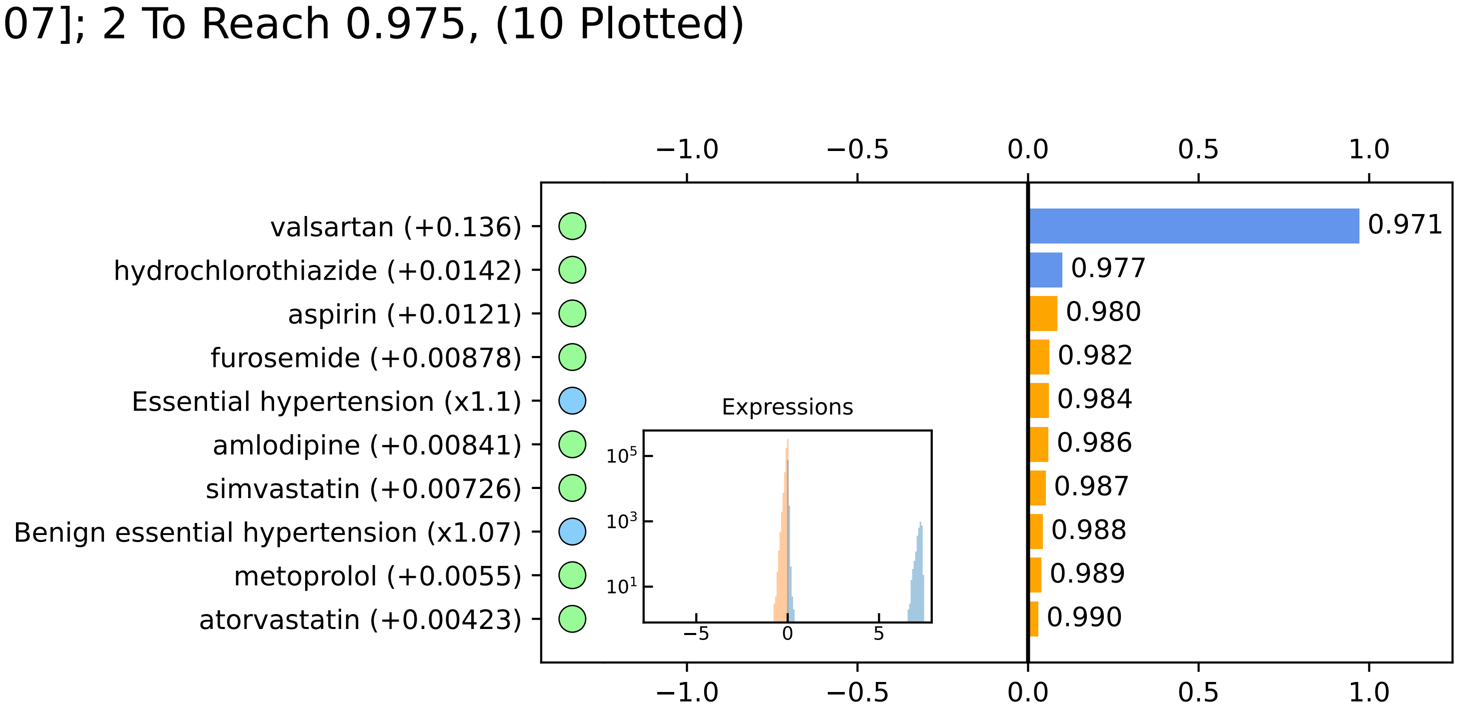

Signature [1307]; 2 To Reach 0.975, (10 Plotted)
valsartan (+0.136)
hydrochlorothiazide (+0.0142)
aspirin (+0.0121)
furosemide (+0.00878)
Essential hypertension (x1.1)
amlodipine (+0.00841)
simvastatin (+0.00726)
Benign essential hypertension (x1.07)
metoprolol (+0.0055)
atorvastatin (+0.00423)
0.971
0.977
0.980
0.982
0.984
0.986
0.987
0.988
0.989
0.990
Expressions
−1.0
−0.5
0.0
0.5
1.0

Signature [1308]; 6 To Reach 0.975, (10 Plotted)

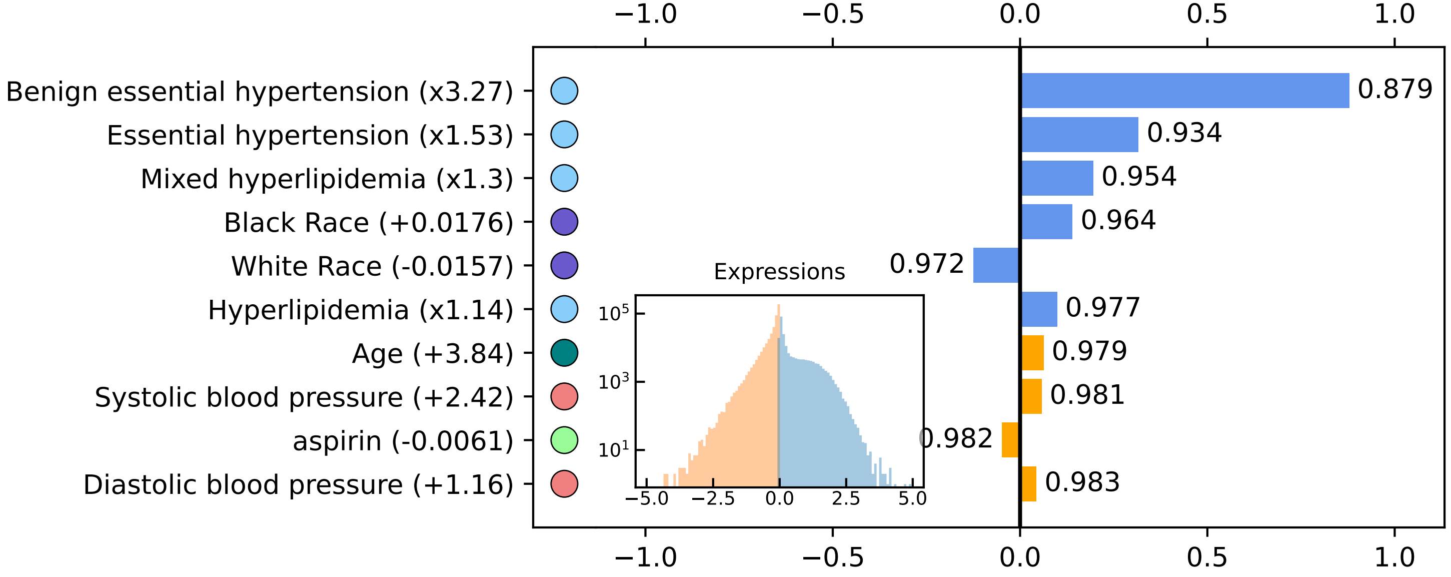

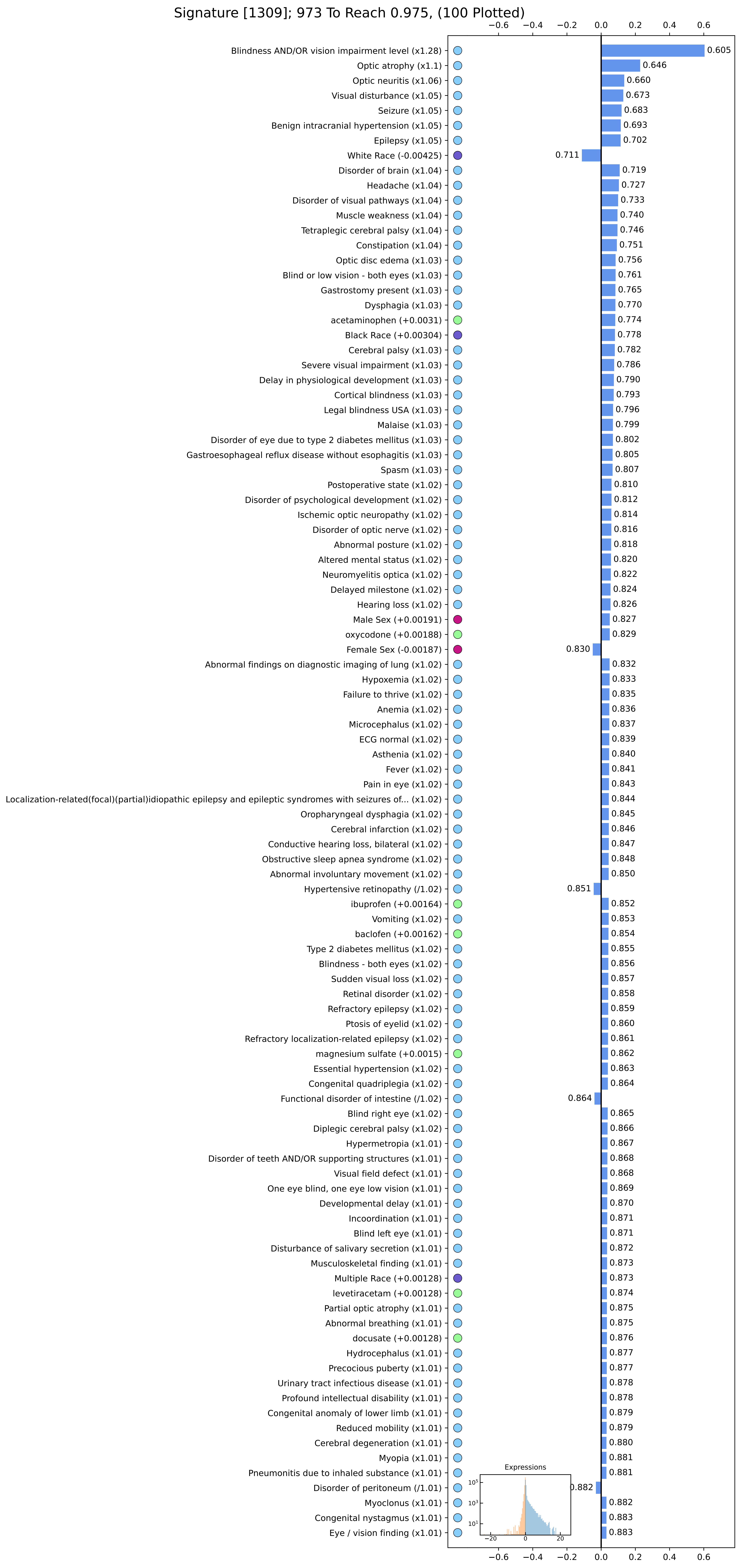

Signature [1309]; 973 To Reach 0.975, (100 Plotted)
Blindness AND/OR vision impairment level (x1.28) 0.605
Optic atrophy (x1.1) 0.646
Optic neuritis (x1.06) 0.660
Visual disturbance (x1.05) 0.673
Seizure (x1.05) 0.683
Benign intracranial hypertension (x1.05) 0.693
Epilepsy (x1.05) 0.702
White Race (-0.00425) 0.711
Disorder of brain (x1.04) 0.719
Headache (x1.04) 0.727
Disorder of visual pathways (x1.04) 0.733
Muscle weakness (x1.04) 0.740
Tetraplegic cerebral palsy (x1.04) 0.746
Constipation (x1.04) 0.751
Optic disc edema (x1.03) 0.756
Blind or low vision - both eyes (x1.03) 0.761
Gastrostomy present (x1.03) 0.765
Dysphagia (x1.03) 0.770
acetaminophen (+0.0031) 0.774
Black Race (+0.00304) 0.778
Cerebral palsy (x1.03) 0.782
Severe visual impairment (x1.03) 0.786
Delay in physiological development (x1.03) 0.790
Cortical blindness (x1.03) 0.793
Legal blindness USA (x1.03) 0.796
Malaise (x1.03) 0.799
Disorder of eye due to type 2 diabetes mellitus (x1.03) 0.802
Gastroesophageal reflux disease without esophagitis (x1.03) 0.805
Spasm (x1.03) 0.807
Postoperative state (x1.02) 0.810
Disorder of psychological development (x1.02) 0.812
Ischemic optic neuropathy (x1.02) 0.814
Disorder of optic nerve (x1.02) 0.816
Abnormal posture (x1.02) 0.818
Altered mental status (x1.02) 0.820
Neuromyelitis optica (x1.02) 0.822
Delayed milestone (x1.02) 0.824
Hearing loss (x1.02) 0.826
Male Sex (+0.00191) 0.827
oxycodone (+0.00188) 0.829
Female Sex (-0.00187) 0.830
Abnormal findings on diagnostic imaging of lung (x1.02) 0.832
Hypoxemia (x1.02) 0.833
Failure to thrive (x1.02) 0.835
Anemia (x1.02) 0.836
Microcephalus (x1.02) 0.837
ECG normal (x1.02) 0.839
Asthenia (x1.02) 0.840
Fever (x1.02) 0.841
Pain in eye (x1.02) 0.843
Localization-related(focal)(partial)idiopathic epilepsy and epileptic syndromes with seizures of... (x1.02) 0.844
Oropharyngeal dysphagia (x1.02) 0.845
Cerebral infarction (x1.02) 0.846
Conductive hearing loss, bilateral (x1.02) 0.847
Obstructive sleep apnea syndrome (x1.02) 0.848
Abnormal involuntary movement (x1.02) 0.850
Hypertensive retinopathy (/1.02) 0.851
ibuprofen (+0.00164) 0.852
Vomiting (x1.02) 0.853
baclofen (+0.00162) 0.854
Type 2 diabetes mellitus (x1.02) 0.855
Blindness - both eyes (x1.02) 0.856
Sudden visual loss (x1.02) 0.857
Retinal disorder (x1.02) 0.858
Refractory epilepsy (x1.02) 0.859
Ptosis of eyelid (x1.02) 0.860
Refractory localization-related epilepsy (x1.02) 0.861
magnesium sulfate (+0.0015) 0.862
Essential hypertension (x1.02) 0.863
Congenital quadriplegia (x1.02) 0.864
Functional disorder of intestine (/1.02) 0.864
Blind right eye (x1.02) 0.865
Diplegic cerebral palsy (x1.02) 0.866
Hypermetropia (x1.01) 0.867
Disorder of teeth AND/OR supporting structures (x1.01) 0.868
Visual field defect (x1.01) 0.868
One eye blind, one eye low vision (x1.01) 0.869
Developmental delay (x1.01) 0.870
Incoordination (x1.01) 0.871
Blind left eye (x1.01) 0.871
Disturbance of salivary secretion (x1.01) 0.872
Musculoskeletal finding (x1.01) 0.873
Multiple Race (+0.00128) 0.873
levetiracetam (+0.00128) 0.874
Partial optic atrophy (x1.01) 0.875
Abnormal breathing (x1.01) 0.875
docusate (+0.00128) 0.876
Hydrocephalus (x1.01) 0.877
Precocious puberty (x1.01) 0.877
Urinary tract infectious disease (x1.01) 0.878
Profound intellectual disability (x1.01) 0.878
Congenital anomaly of lower limb (x1.01) 0.879
Reduced mobility (x1.01) 0.879
Cerebral degeneration (x1.01) 0.880
Myopia (x1.01) 0.881
Pneumonitis due to inhaled substance (x1.01) 0.881
Disorder of peritoneum (/1.01) 0.882
Myoclonus (x1.01) 0.882
Congenital nystagmus (x1.01) 0.883
Eye / vision finding (x1.01) 0.883
Expressions
−0.6 −0.4 −0.2 0.0 0.2 0.4 0.6

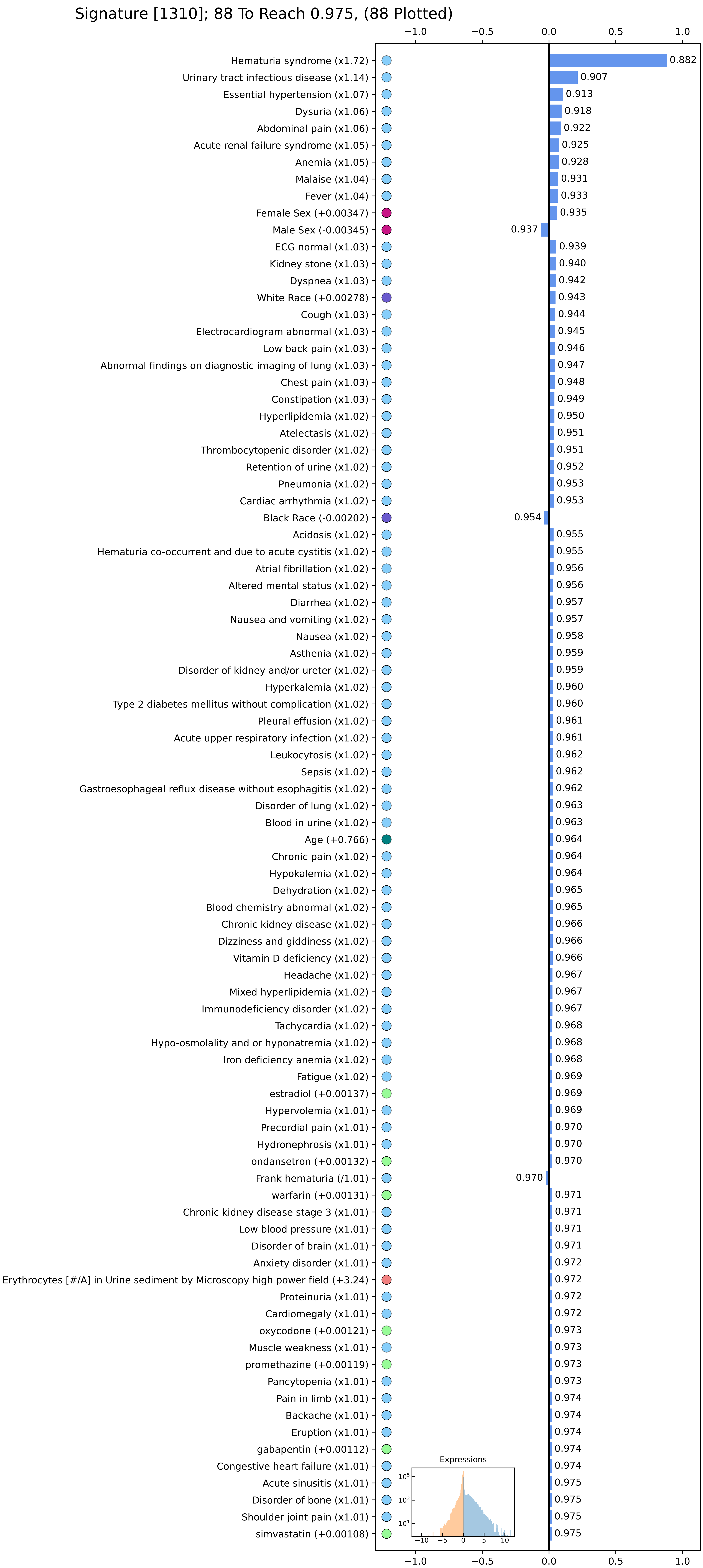

Signature [1310]; 88 To Reach 0.975, (88 Plotted)
Hematuria syndrome (x1.72) 0.882
Urinary tract infectious disease (x1.14) 0.907
Essential hypertension (x1.07) 0.913
Dysuria (x1.06) 0.918
Abdominal pain (x1.06) 0.922
Acute renal failure syndrome (x1.05) 0.925
Anemia (x1.05) 0.928
Malaise (x1.04) 0.931
Fever (x1.04) 0.933
Female Sex (+0.00347) 0.935
Male Sex (-0.00345) 0.937
ECG normal (x1.03) 0.939
Kidney stone (x1.03) 0.940
Dyspnea (x1.03) 0.942
White Race (+0.00278) 0.943
Cough (x1.03) 0.944
Electrocardiogram abnormal (x1.03) 0.945
Low back pain (x1.03) 0.946
Abnormal findings on diagnostic imaging of lung (x1.03) 0.947
Chest pain (x1.03) 0.948
Constipation (x1.03) 0.949
Hyperlipidemia (x1.02) 0.950
Atelectasis (x1.02) 0.951
Thrombocytopenic disorder (x1.02) 0.951
Retention of urine (x1.02) 0.952
Pneumonia (x1.02) 0.953
Cardiac arrhythmia (x1.02) 0.953
Black Race (-0.00202) 0.954
Acidosis (x1.02) 0.955
Hematuria co-occurrent and due to acute cystitis (x1.02) 0.955
Atrial fibrillation (x1.02) 0.956
Altered mental status (x1.02) 0.956
Diarrhea (x1.02) 0.957
Nausea and vomiting (x1.02) 0.957
Nausea (x1.02) 0.958
Asthenia (x1.02) 0.959
Disorder of kidney and/or ureter (x1.02) 0.959
Hyperkalemia (x1.02) 0.960
Type 2 diabetes mellitus without complication (x1.02) 0.960
Pleural effusion (x1.02) 0.961
Acute upper respiratory infection (x1.02) 0.961
Leukocytosis (x1.02) 0.962
Sepsis (x1.02) 0.962
Gastroesophageal reflux disease without esophagitis (x1.02) 0.962
Disorder of lung (x1.02) 0.963
Blood in urine (x1.02) 0.963
Age (+0.766) 0.964
Chronic pain (x1.02) 0.964
Hypokalemia (x1.02) 0.964
Dehydration (x1.02) 0.965
Blood chemistry abnormal (x1.02) 0.965
Chronic kidney disease (x1.02) 0.966
Dizziness and giddiness (x1.02) 0.966
Vitamin D deficiency (x1.02) 0.966
Headache (x1.02) 0.967
Mixed hyperlipidemia (x1.02) 0.967
Immunodeficiency disorder (x1.02) 0.967
Tachycardia (x1.02) 0.968
Hypo-osmolality and or hyponatremia (x1.02) 0.968
Iron deficiency anemia (x1.02) 0.968
Fatigue (x1.02) 0.969
estradiol (+0.00137) 0.969
Hypervolemia (x1.01) 0.969
Precordial pain (x1.01) 0.970
Hydronephrosis (x1.01) 0.970
ondansetron (+0.00132) 0.970
Frank hematuria (/1.01) 0.970
warfarin (+0.00131) 0.971
Chronic kidney disease stage 3 (x1.01) 0.971
Low blood pressure (x1.01) 0.971
Disorder of brain (x1.01) 0.971
Anxiety disorder (x1.01) 0.972
Erythrocytes [#/A] in Urine sediment by Microscopy high power field (+3.24) 0.972
Proteinuria (x1.01) 0.972
Cardiomegaly (x1.01) 0.972
oxycodone (+0.00121) 0.973
Muscle weakness (x1.01) 0.973
promethazine (+0.00119) 0.973
Pancytopenia (x1.01) 0.973
Pain in limb (x1.01) 0.974
Backache (x1.01) 0.974
Eruption (x1.01) 0.974
gabapentin (+0.00112) 0.974
Congestive heart failure (x1.01) 0.974
Acute sinusitis (x1.01) 0.975
Disorder of bone (x1.01) 0.975
Shoulder joint pain (x1.01) 0.975
simvastatin (+0.00108) 0.975
Expressions

Signature [1311]; 3 To Reach 0.975, (10 Plotted)

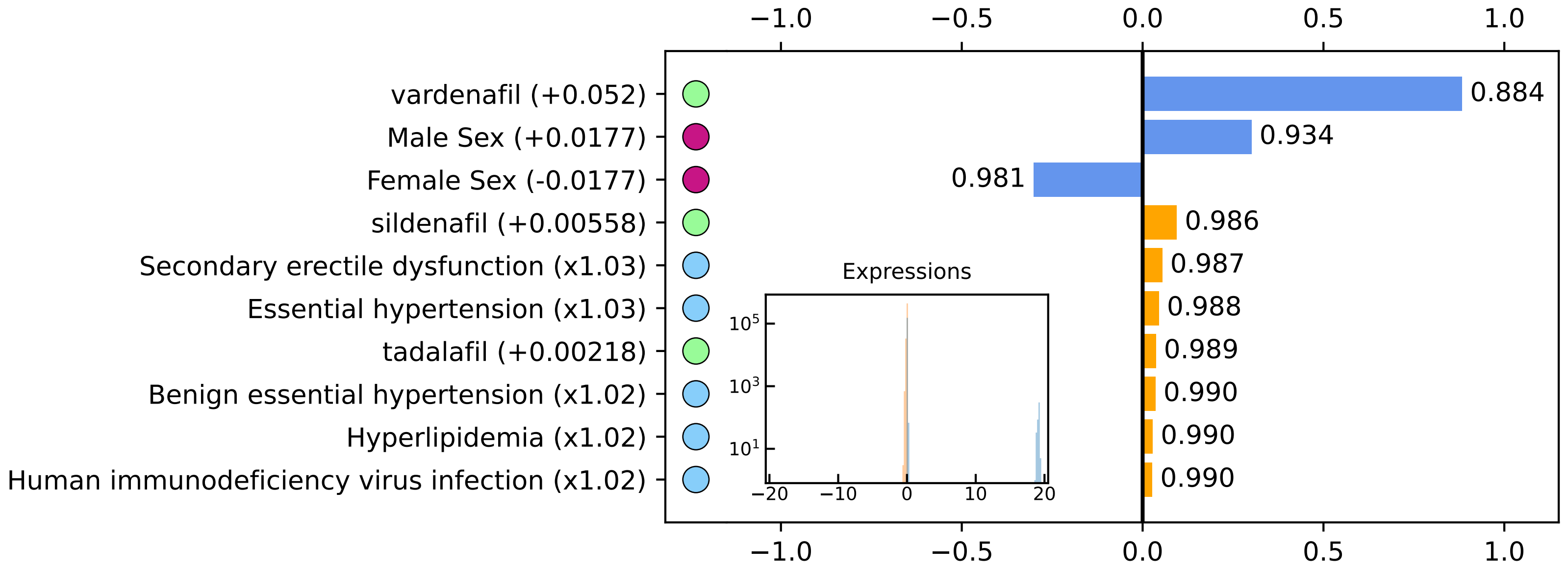

Signature [1312]; 4 To Reach 0.975, (10 Plotted)

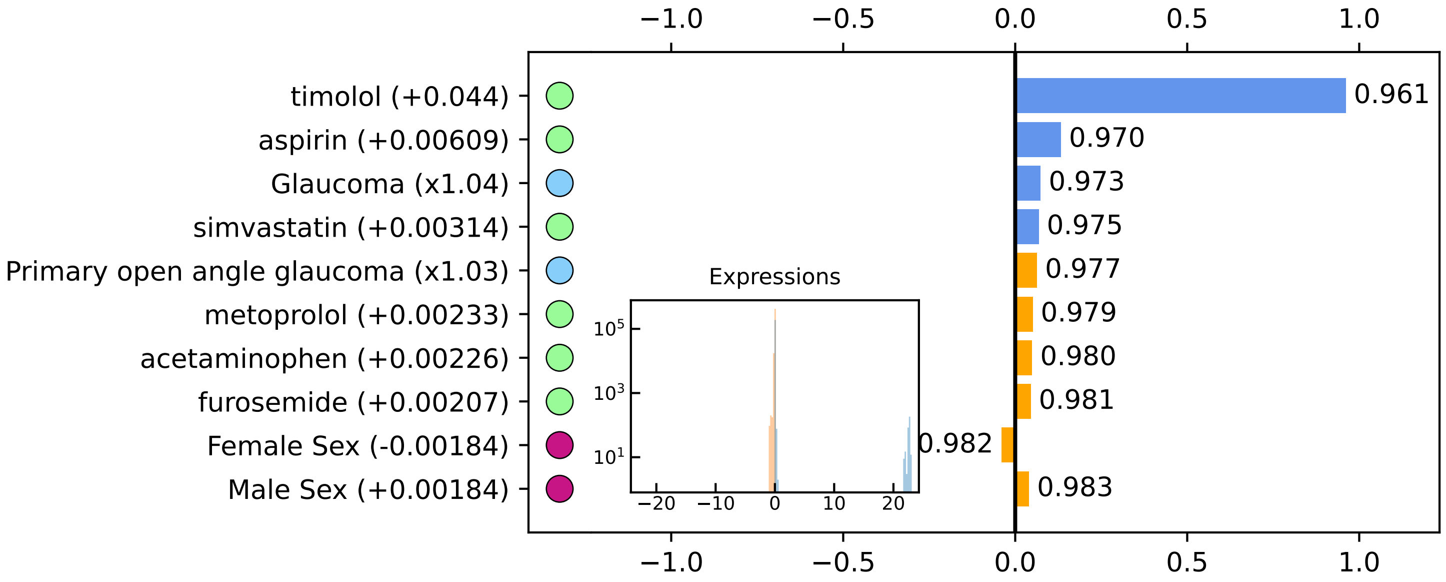

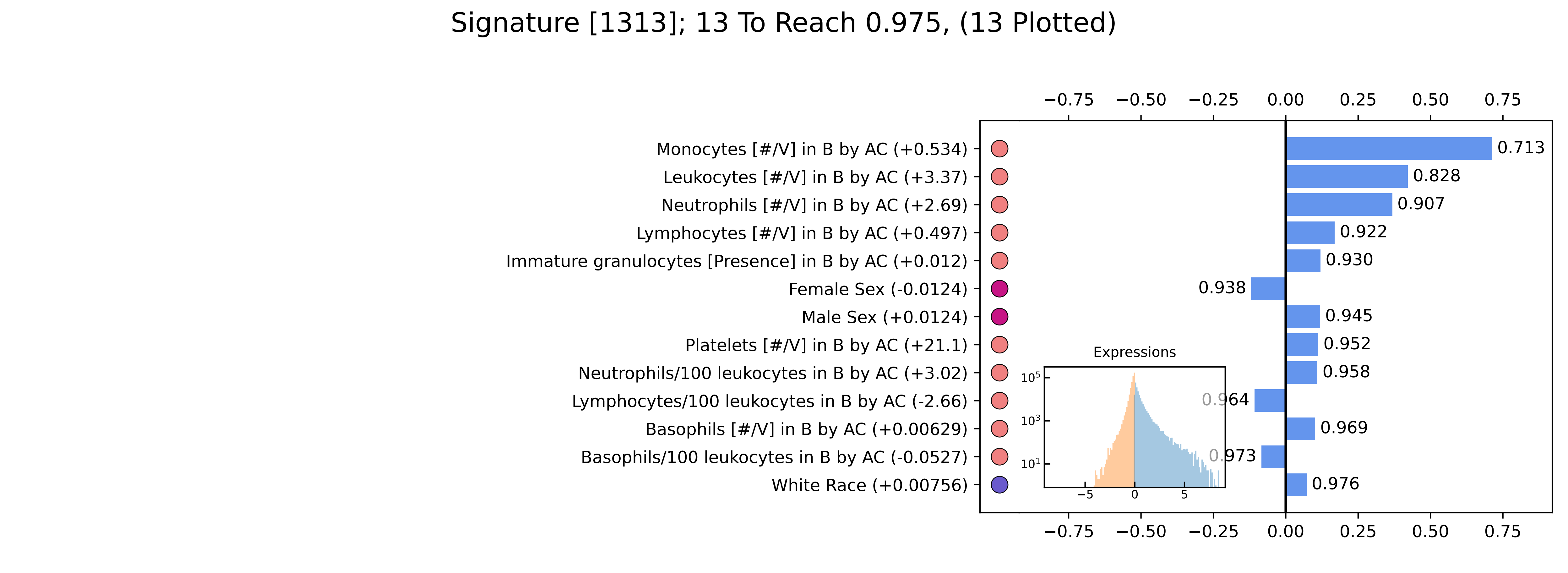

Signature [1313]; 13 To Reach 0.975, (13 Plotted)
Monocytes [#/V] in B by AC (+0.534)
Leukocytes [#/V] in B by AC (+3.37)
Neutrophils [#/V] in B by AC (+2.69)
Lymphocytes [#/V] in B by AC (+0.497)
Immature granulocytes [Presence] in B by AC (+0.012)
Female Sex (-0.0124)
Male Sex (+0.0124)
Platelets [#/V] in B by AC (+21.1)
Neutrophils/100 leukocytes in B by AC (+3.02)
Lymphocytes/100 leukocytes in B by AC (-2.66)
Basophils [#/V] in B by AC (+0.00629)
Basophils/100 leukocytes in B by AC (-0.0527)
White Race (+0.00756)
0.713
0.828
0.907
0.922
0.930
0.938
0.945
0.952
0.958
0.964
0.969
0.973
0.976
Expressions
−0.75
−0.50
−0.25
0.00
0.25
0.50
0.75

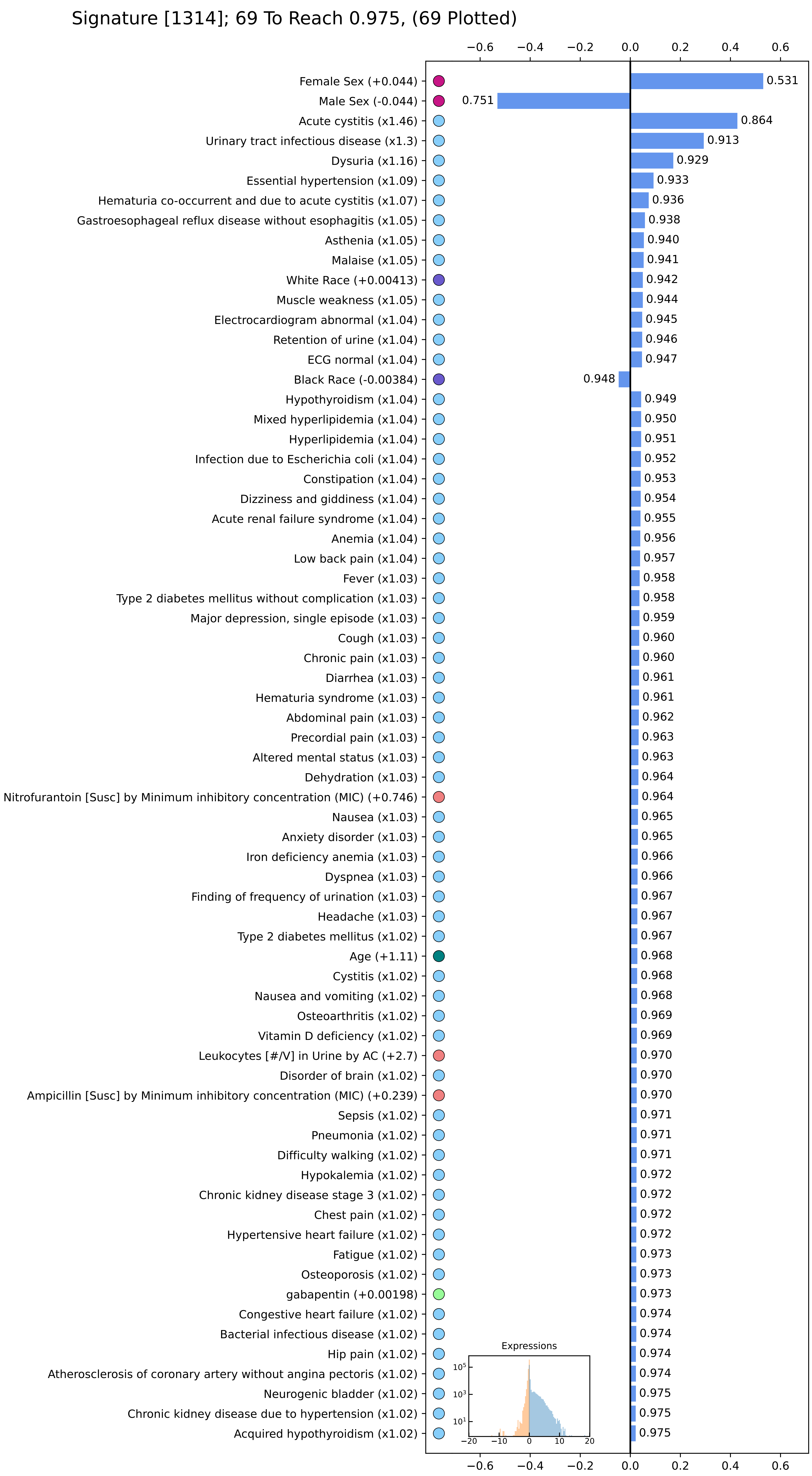

Signature [1314]; 69 To Reach 0.975, (69 Plotted)
Female Sex (+0.044) 0.531
Male Sex (-0.044) 0.751
Acute cystitis (x1.46) 0.864
Urinary tract infectious disease (x1.3) 0.913
Dysuria (x1.16) 0.929
Essential hypertension (x1.09) 0.933
Hematuria co-occurrent and due to acute cystitis (x1.07) 0.936
Gastroesophageal reflux disease without esophagitis (x1.05) 0.938
Asthenia (x1.05) 0.940
Malaise (x1.05) 0.941
White Race (+0.00413) 0.942
Muscle weakness (x1.05) 0.944
Electrocardiogram abnormal (x1.04) 0.945
Retention of urine (x1.04) 0.946
ECG normal (x1.04) 0.947
Black Race (-0.00384) 0.948
Hypothyroidism (x1.04) 0.949
Mixed hyperlipidemia (x1.04) 0.950
Hyperlipidemia (x1.04) 0.951
Infection due to Escherichia coli (x1.04) 0.952
Constipation (x1.04) 0.953
Dizziness and giddiness (x1.04) 0.954
Acute renal failure syndrome (x1.04) 0.955
Anemia (x1.04) 0.956
Low back pain (x1.04) 0.957
Fever (x1.03) 0.958
Type 2 diabetes mellitus without complication (x1.03) 0.958
Major depression, single episode (x1.03) 0.959
Cough (x1.03) 0.960
Chronic pain (x1.03) 0.960
Diarrhea (x1.03) 0.961
Hematuria syndrome (x1.03) 0.961
Abdominal pain (x1.03) 0.962
Precordial pain (x1.03) 0.963
Altered mental status (x1.03) 0.963
Dehydration (x1.03) 0.964
Nitrofurantoin [Susc] by Minimum inhibitory concentration (MIC) (+0.746) 0.964
Nausea (x1.03) 0.965
Anxiety disorder (x1.03) 0.965
Iron deficiency anemia (x1.03) 0.966
Dyspnea (x1.03) 0.966
Finding of frequency of urination (x1.03) 0.967
Headache (x1.03) 0.967
Type 2 diabetes mellitus (x1.02) 0.967
Age (+1.11) 0.968
Cystitis (x1.02) 0.968
Nausea and vomiting (x1.02) 0.968
Osteoarthritis (x1.02) 0.969
Vitamin D deficiency (x1.02) 0.969
Leukocytes [#/V] in Urine by AC (+2.7) 0.970
Disorder of brain (x1.02) 0.970
Ampicillin [Susc] by Minimum inhibitory concentration (MIC) (+0.239) 0.970
Sepsis (x1.02) 0.971
Pneumonia (x1.02) 0.971
Difficulty walking (x1.02) 0.971
Hypokalemia (x1.02) 0.972
Chronic kidney disease stage 3 (x1.02) 0.972
Chest pain (x1.02) 0.972
Hypertensive heart failure (x1.02) 0.972
Fatigue (x1.02) 0.973
Osteoporosis (x1.02) 0.973
gabapentin (+0.00198) 0.973
Congestive heart failure (x1.02) 0.974
Bacterial infectious disease (x1.02) 0.974
Hip pain (x1.02) 0.974
Atherosclerosis of coronary artery without angina pectoris (x1.02) 0.974
Neurogenic bladder (x1.02) 0.975
Chronic kidney disease due to hypertension (x1.02) 0.975
Acquired hypothyroidism (x1.02) 0.975
−0.6 −0.4 −0.2 0.0 0.2 0.4 0.6
Expressions
10^5 10^3 10^1
−20 −10 0 10 20

Signature [1315]; 14 To Reach 0.975, (14 Plotted)

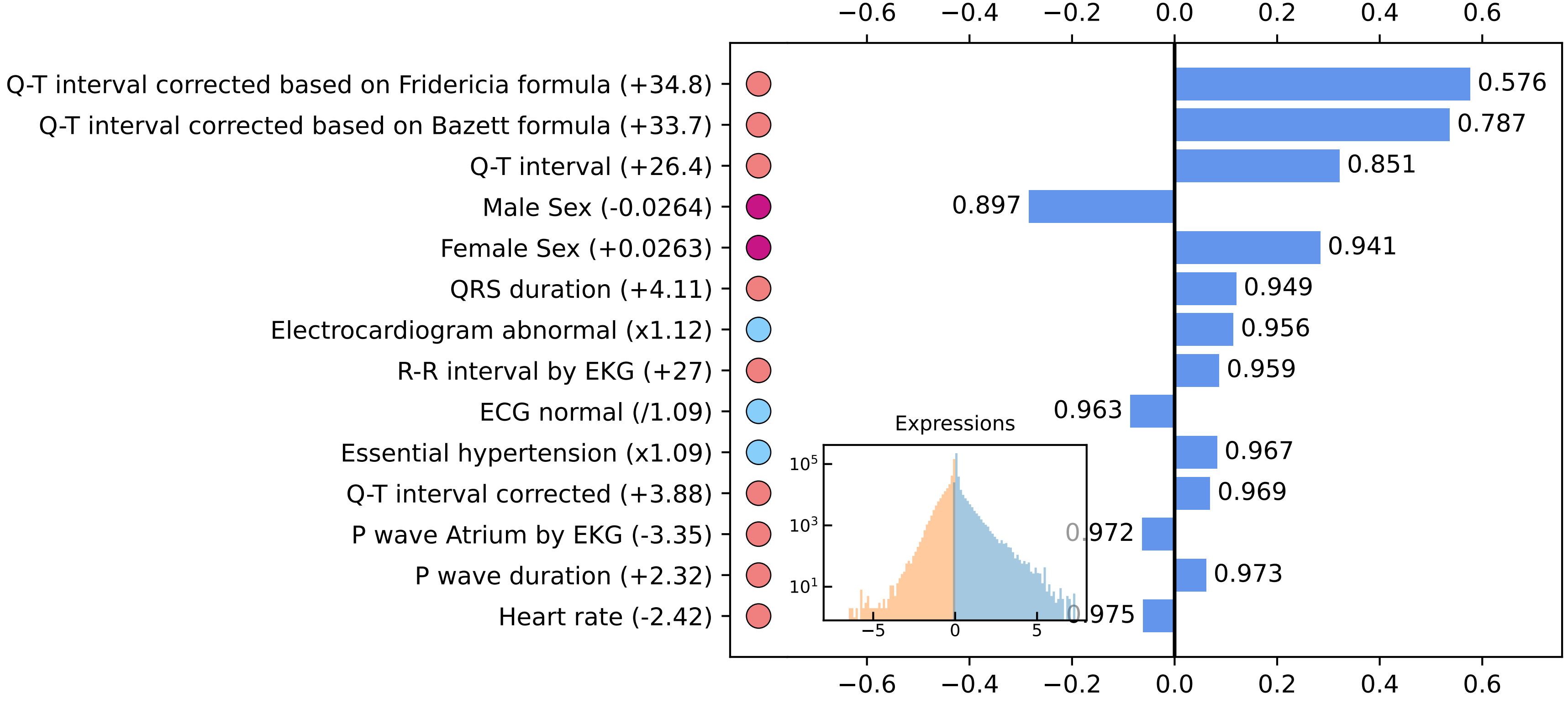

Signature [1316]; 20 To Reach 0.975, (20 Plotted)

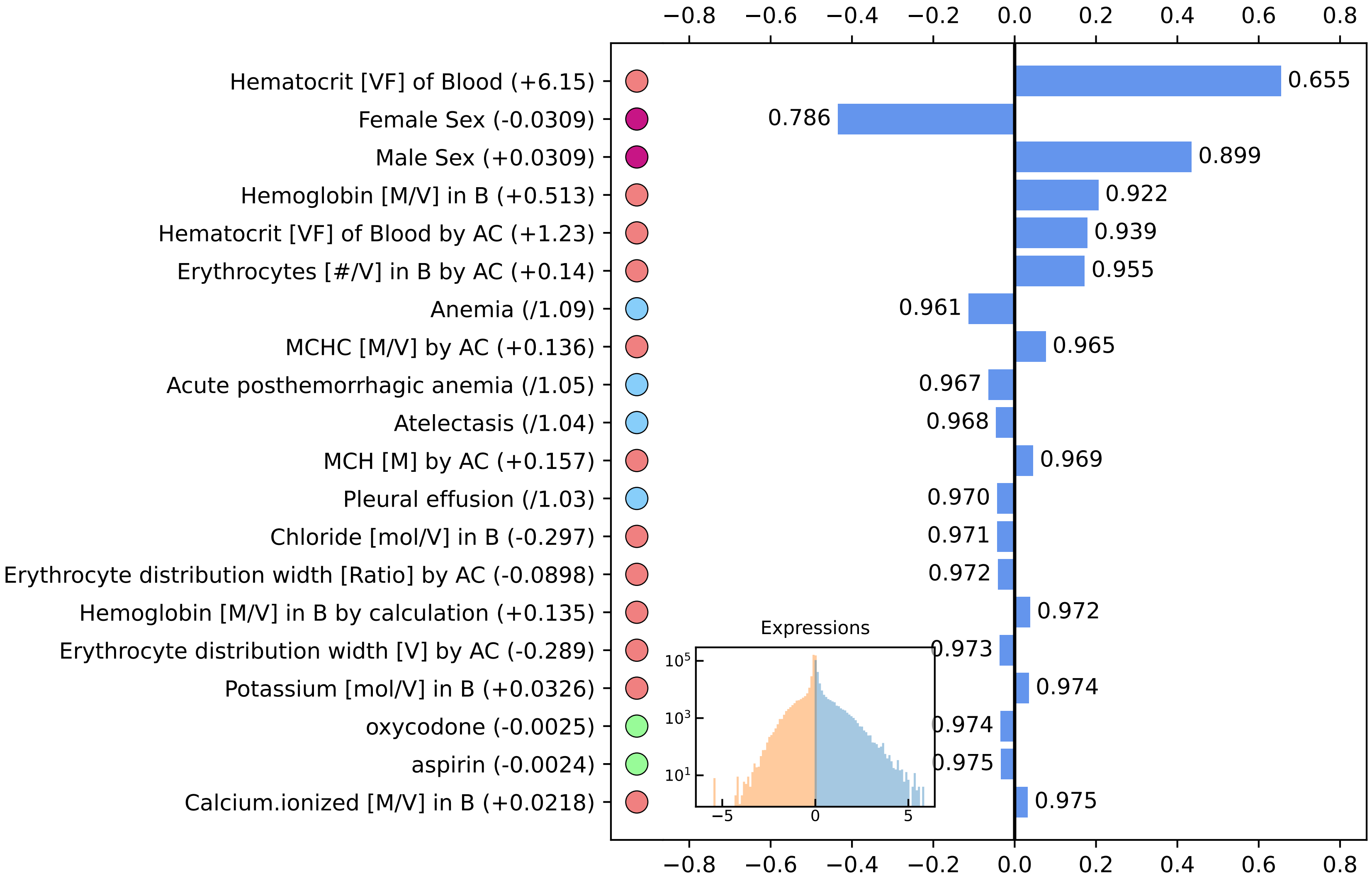

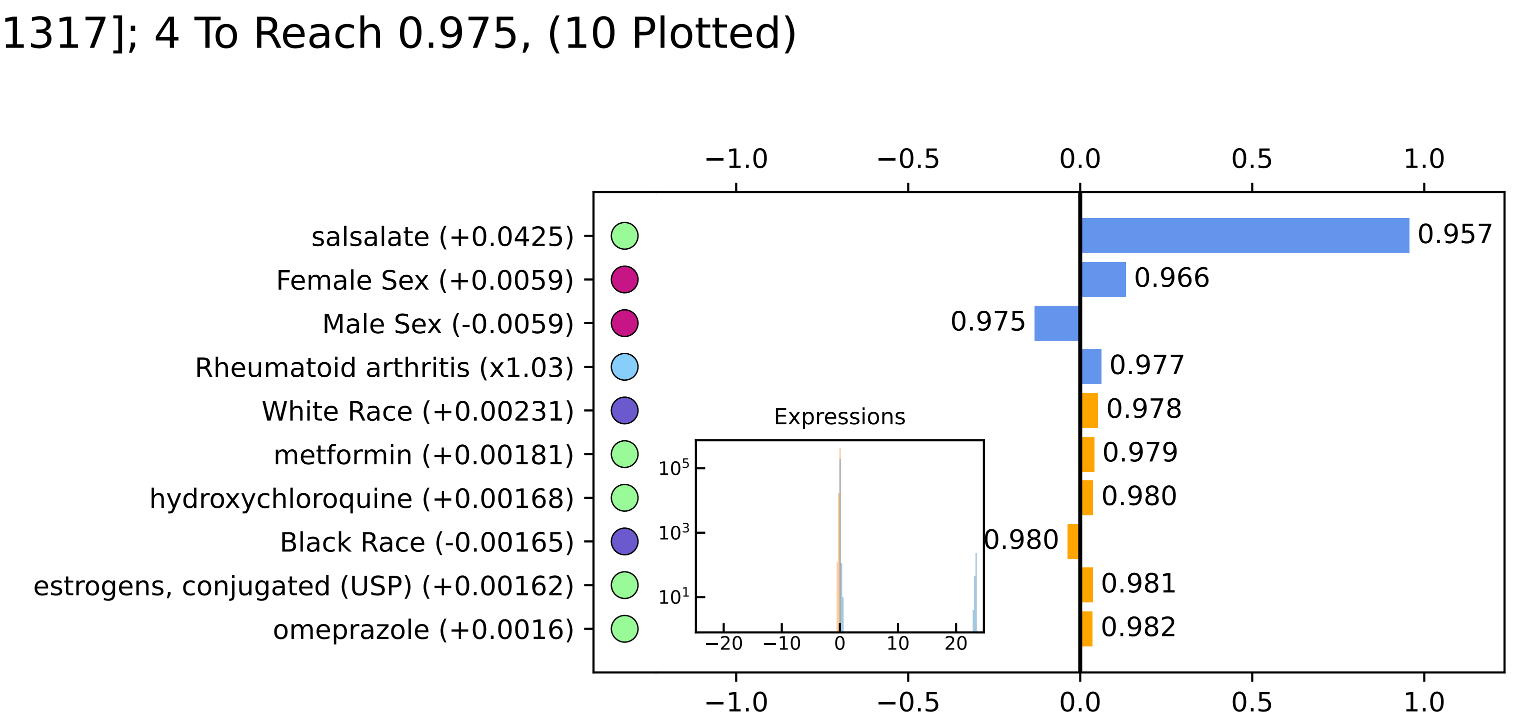

Signature [1317]; 4 To Reach 0.975, (10 Plotted)
salsalate (+0.0425)
Female Sex (+0.0059)
Male Sex (-0.0059)
Rheumatoid arthritis (x1.03)
White Race (+0.00231)
metformin (+0.00181)
hydroxychloroquine (+0.00168)
Black Race (-0.00165)
estrogens, conjugated (USP) (+0.00162)
omeprazole (+0.0016)
0.957
0.966
0.975
0.977
0.978
0.979
0.980
0.980
0.981
0.982
Expressions
−1.0
−0.5
0.0
0.5
1.0

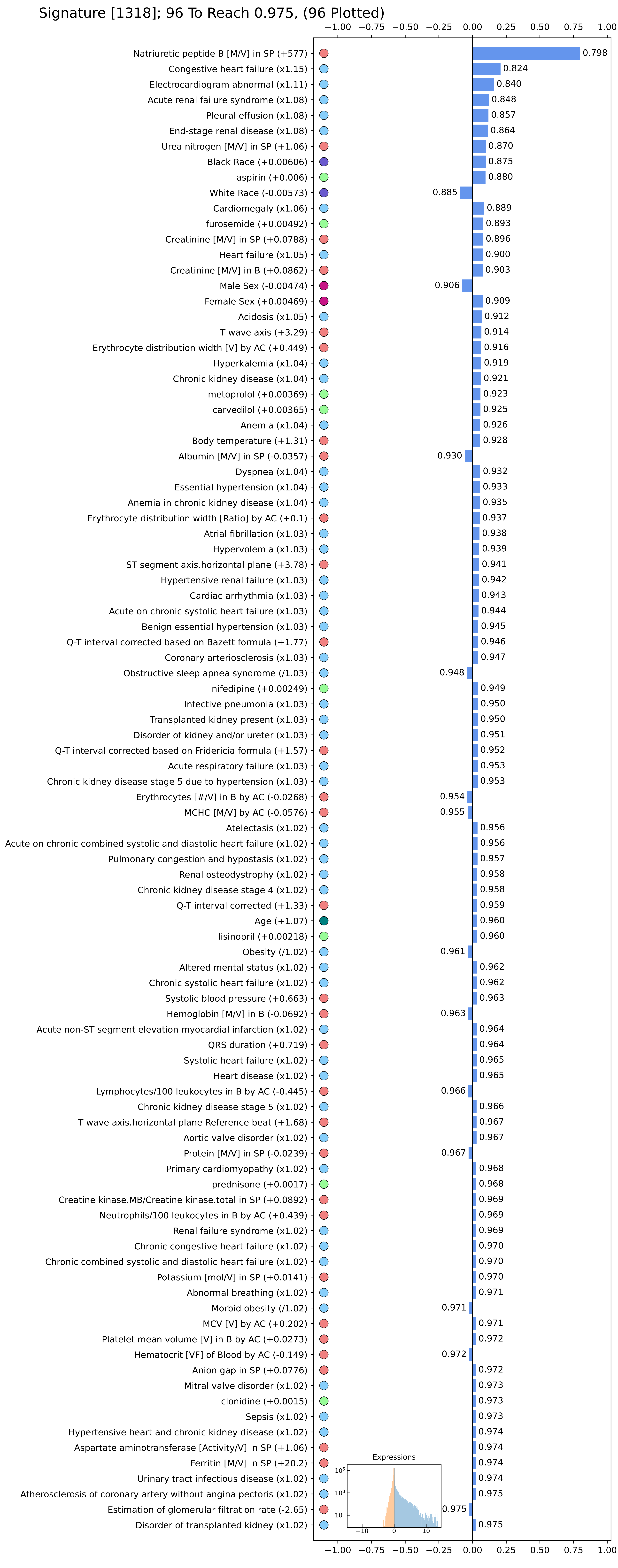
Signature [1318]; 96 To Reach 0.975, (96 Plotted)
−1.00 −0.75 −0.50 −0.25 0.00 0.25 0.50 0.75 1.00
Natriuretic peptide B [M/V] in SP (+577) 0.798
Congestive heart failure (x1.15) 0.824
Electrocardiogram abnormal (x1.11) 0.840
Acute renal failure syndrome (x1.08) 0.848
Pleural effusion (x1.08) 0.857
End-stage renal disease (x1.08) 0.864
Urea nitrogen [M/V] in SP (+1.06) 0.870
Black Race (+0.00606) 0.875
aspirin (+0.006) 0.880
White Race (-0.00573) 0.885
Cardiomegaly (x1.06) 0.889
furosemide (+0.00492) 0.893
Creatinine [M/V] in SP (+0.0788) 0.896
Heart failure (x1.05) 0.900
Creatinine [M/V] in B (+0.0862) 0.903
Male Sex (-0.00474) 0.906
Female Sex (+0.00469) 0.909
Acidosis (x1.05) 0.912
T wave axis (+3.29) 0.914
Erythrocyte distribution width [V] by AC (+0.449) 0.916
Hyperkalemia (x1.04) 0.919
Chronic kidney disease (x1.04) 0.921
metoprolol (+0.00369) 0.923
carvedilol (+0.00365) 0.925
Anemia (x1.04) 0.926
Body temperature (+1.31) 0.928
Albumin [M/V] in SP (-0.0357) 0.930
Dyspnea (x1.04) 0.932
Essential hypertension (x1.04) 0.933
Anemia in chronic kidney disease (x1.04) 0.935
Erythrocyte distribution width [Ratio] by AC (+0.1) 0.937
Atrial fibrillation (x1.03) 0.938
Hypervolemia (x1.03) 0.939
ST segment axis.horizontal plane (+3.78) 0.941
Hypertensive renal failure (x1.03) 0.942
Cardiac arrhythmia (x1.03) 0.943
Acute on chronic systolic heart failure (x1.03) 0.944
Benign essential hypertension (x1.03) 0.945
Q-T interval corrected based on Bazett formula (+1.77) 0.946
Coronary arteriosclerosis (x1.03) 0.947
Obstructive sleep apnea syndrome (/1.03) 0.948
nifedipine (+0.00249) 0.949
Infective pneumonia (x1.03) 0.950
Transplanted kidney present (x1.03) 0.950
Disorder of kidney and/or ureter (x1.03) 0.951
Q-T interval corrected based on Fridericia formula (+1.57) 0.952
Acute respiratory failure (x1.03) 0.953
Chronic kidney disease stage 5 due to hypertension (x1.03) 0.953
Erythrocytes [#/V] in B by AC (-0.0268) 0.954
MCHC [M/V] by AC (-0.0576) 0.955
Atelectasis (x1.02) 0.956
Acute on chronic combined systolic and diastolic heart failure (x1.02) 0.956
Pulmonary congestion and hypostasis (x1.02) 0.957
Renal osteodystrophy (x1.02) 0.958
Chronic kidney disease stage 4 (x1.02) 0.958
Q-T interval corrected (+1.33) 0.959
Age (+1.07) 0.960
lisinopril (+0.00218) 0.960
Obesity (/1.02) 0.961
Altered mental status (x1.02) 0.962
Chronic systolic heart failure (x1.02) 0.962
Systolic blood pressure (+0.663) 0.963
Hemoglobin [M/V] in B (-0.0692) 0.963
Acute non-ST segment elevation myocardial infarction (x1.02) 0.964
QRS duration (+0.719) 0.964
Systolic heart failure (x1.02) 0.965
Heart disease (x1.02) 0.965
Lymphocytes/100 leukocytes in B by AC (-0.445) 0.966
Chronic kidney disease stage 5 (x1.02) 0.966
T wave axis.horizontal plane Reference beat (+1.68) 0.967
Aortic valve disorder (x1.02) 0.967
Protein [M/V] in SP (-0.0239) 0.967
Primary cardiomyopathy (x1.02) 0.968
prednisone (+0.0017) 0.968
Creatine kinase.MB/Creatine kinase.total in SP (+0.0892) 0.969
Neutrophils/100 leukocytes in B by AC (+0.439) 0.969
Renal failure syndrome (x1.02) 0.969
Chronic congestive heart failure (x1.02) 0.970
Chronic combined systolic and diastolic heart failure (x1.02) 0.970
Potassium [mol/V] in SP (+0.0141) 0.970
Abnormal breathing (x1.02) 0.971
Morbid obesity (/1.02) 0.971
MCV [V] by AC (+0.202) 0.971
Platelet mean volume [V] in B by AC (+0.0273) 0.972
Hematocrit [VF] of Blood by AC (-0.149) 0.972
Anion gap in SP (+0.0776) 0.972
Mitral valve disorder (x1.02) 0.973
clonidine (+0.0015) 0.973
Sepsis (x1.02) 0.973
Hypertensive heart and chronic kidney disease (x1.02) 0.974
Aspartate aminotransferase [Activity/V] in SP (+1.06) 0.974
Ferritin [M/V] in SP (+20.2) 0.974
Urinary tract infectious disease (x1.02) 0.974
Atherosclerosis of coronary artery without angina pectoris (x1.02) 0.975
Estimation of glomerular filtration rate (-2.65) 0.975
Disorder of transplanted kidney (x1.02) 0.975
Expressions
10^5
10^3
10^1
−10 0 10
−1.00 −0.75 −0.50 −0.25 0.00 0.25 0.50 0.75 1.00

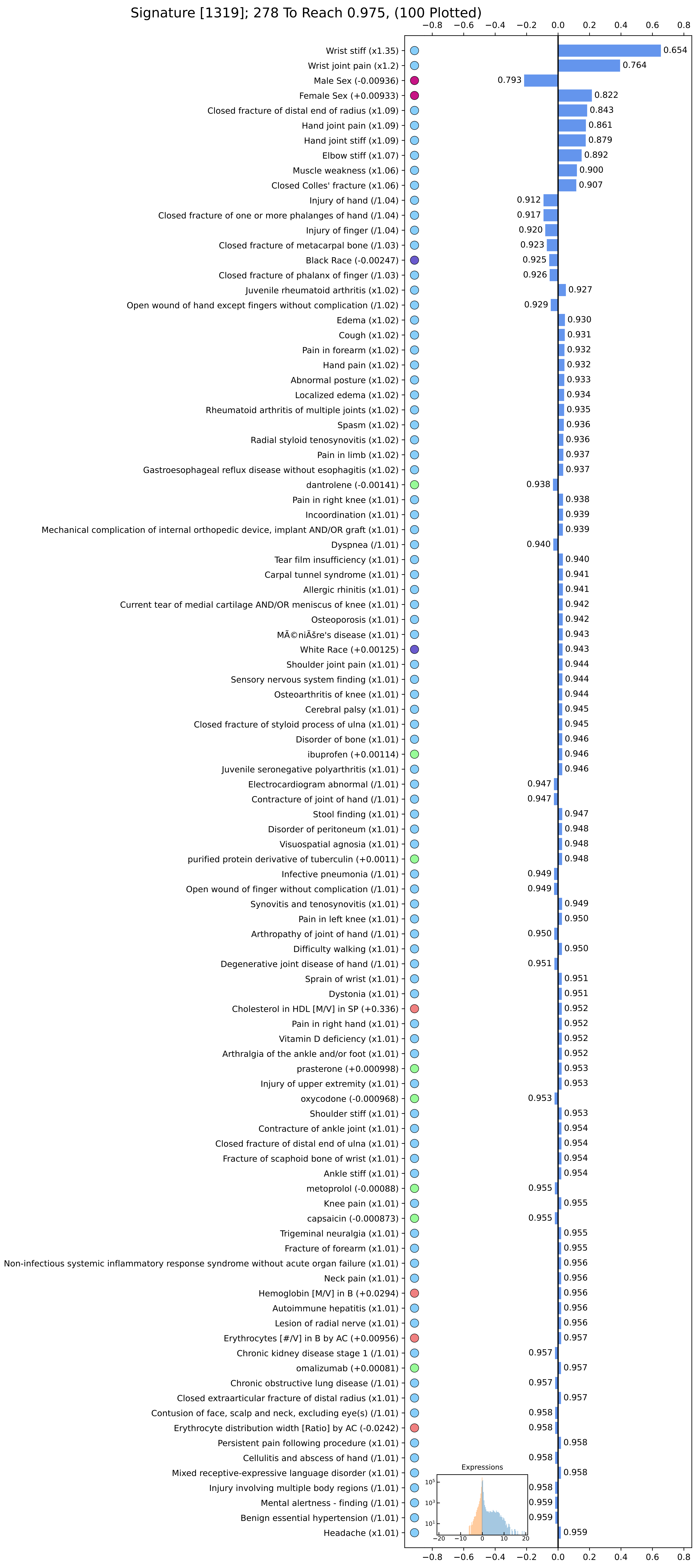

Signature [1319]; 278 To Reach 0.975, (100 Plotted)
−0.8 −0.6 −0.4 −0.2 0.0 0.2 0.4 0.6 0.8
Wrist stiff (x1.35) 0.654
Wrist joint pain (x1.2) 0.764
Male Sex (-0.00936) 0.793
Female Sex (+0.00933) 0.822
Closed fracture of distal end of radius (x1.09) 0.843
Hand joint pain (x1.09) 0.861
Hand joint stiff (x1.09) 0.879
Elbow stiff (x1.07) 0.892
Muscle weakness (x1.06) 0.900
Closed Colles' fracture (x1.06) 0.907
Injury of hand (/1.04) 0.912
Closed fracture of one or more phalanges of hand (/1.04) 0.917
Injury of finger (/1.04) 0.920
Closed fracture of metacarpal bone (/1.03) 0.923
Black Race (-0.00247) 0.925
Closed fracture of phalanx of finger (/1.03) 0.926
Juvenile rheumatoid arthritis (x1.02) 0.927
Open wound of hand except fingers without complication (/1.02) 0.929
Edema (x1.02) 0.930
Cough (x1.02) 0.931
Pain in forearm (x1.02) 0.932
Hand pain (x1.02) 0.932
Abnormal posture (x1.02) 0.933
Localized edema (x1.02) 0.934
Rheumatoid arthritis of multiple joints (x1.02) 0.935
Spasm (x1.02) 0.936
Radial styloid tenosynovitis (x1.02) 0.936
Pain in limb (x1.02) 0.937
Gastroesophageal reflux disease without esophagitis (x1.02) 0.937
dantrolene (-0.00141) 0.938
Pain in right knee (x1.01) 0.938
Incoordination (x1.01) 0.939
Mechanical complication of internal orthopedic device, implant AND/OR graft (x1.01) 0.939
Dyspnea (/1.01) 0.940
Tear film insufficiency (x1.01) 0.940
Carpal tunnel syndrome (x1.01) 0.941
Allergic rhinitis (x1.01) 0.941
Current tear of medial cartilage AND/OR meniscus of knee (x1.01) 0.942
Osteoporosis (x1.01) 0.942
MÃ©niÃšre's disease (x1.01) 0.943
White Race (+0.00125) 0.943
Shoulder joint pain (x1.01) 0.944
Sensory nervous system finding (x1.01) 0.944
Osteoarthritis of knee (x1.01) 0.944
Cerebral palsy (x1.01) 0.945
Closed fracture of styloid process of ulna (x1.01) 0.945
Disorder of bone (x1.01) 0.946
ibuprofen (+0.00114) 0.946
Juvenile seronegative polyarthritis (x1.01) 0.946
Electrocardiogram abnormal (/1.01) 0.947
Contracture of joint of hand (/1.01) 0.947
Stool finding (x1.01) 0.947
Disorder of peritoneum (x1.01) 0.948
Visuospatial agnosia (x1.01) 0.948
purified protein derivative of tuberculin (+0.0011) 0.948
Infective pneumonia (/1.01) 0.949
Open wound of finger without complication (/1.01) 0.949
Synovitis and tenosynovitis (x1.01) 0.949
Pain in left knee (x1.01) 0.950
Arthropathy of joint of hand (/1.01) 0.950
Difficulty walking (x1.01) 0.950
Degenerative joint disease of hand (/1.01) 0.951
Sprain of wrist (x1.01) 0.951
Dystonia (x1.01) 0.951
Cholesterol in HDL [M/V] in SP (+0.336) 0.952
Pain in right hand (x1.01) 0.952
Vitamin D deficiency (x1.01) 0.952
Arthralgia of the ankle and/or foot (x1.01) 0.952
prasterone (+0.000998) 0.953
Injury of upper extremity (x1.01) 0.953
oxycodone (-0.000968) 0.953
Shoulder stiff (x1.01) 0.953
Contracture of ankle joint (x1.01) 0.954
Closed fracture of distal end of ulna (x1.01) 0.954
Fracture of scaphoid bone of wrist (x1.01) 0.954
Ankle stiff (x1.01) 0.954
metoprolol (-0.00088) 0.955
Knee pain (x1.01) 0.955
capsaicin (-0.000873) 0.955
Trigeminal neuralgia (x1.01) 0.955
Fracture of forearm (x1.01) 0.955
Non-infectious systemic inflammatory response syndrome without acute organ failure (x1.01) 0.956
Neck pain (x1.01) 0.956
Hemoglobin [M/V] in B (+0.0294) 0.956
Autoimmune hepatitis (x1.01) 0.956
Lesion of radial nerve (x1.01) 0.956
Erythrocytes [#/V] in B by AC (+0.00956) 0.957
Chronic kidney disease stage 1 (/1.01) 0.957
omalizumab (+0.00081) 0.957
Chronic obstructive lung disease (/1.01) 0.957
Closed extraarticular fracture of distal radius (x1.01) 0.957
Contusion of face, scalp and neck, excluding eye(s) (/1.01) 0.958
Erythrocyte distribution width [Ratio] by AC (-0.0242) 0.958
Persistent pain following procedure (x1.01) 0.958
Cellulitis and abscess of hand (/1.01) 0.958
Mixed receptive-expressive language disorder (x1.01) 0.958
Injury involving multiple body regions (/1.01) 0.958
Mental alertness - finding (/1.01) 0.959
Benign essential hypertension (/1.01) 0.959
Headache (x1.01) 0.959
Expressions
10^5
10^3
10^1
−20 −10 0 10 20

Signature [1320]; 2 To Reach 0.975, (10 Plotted)

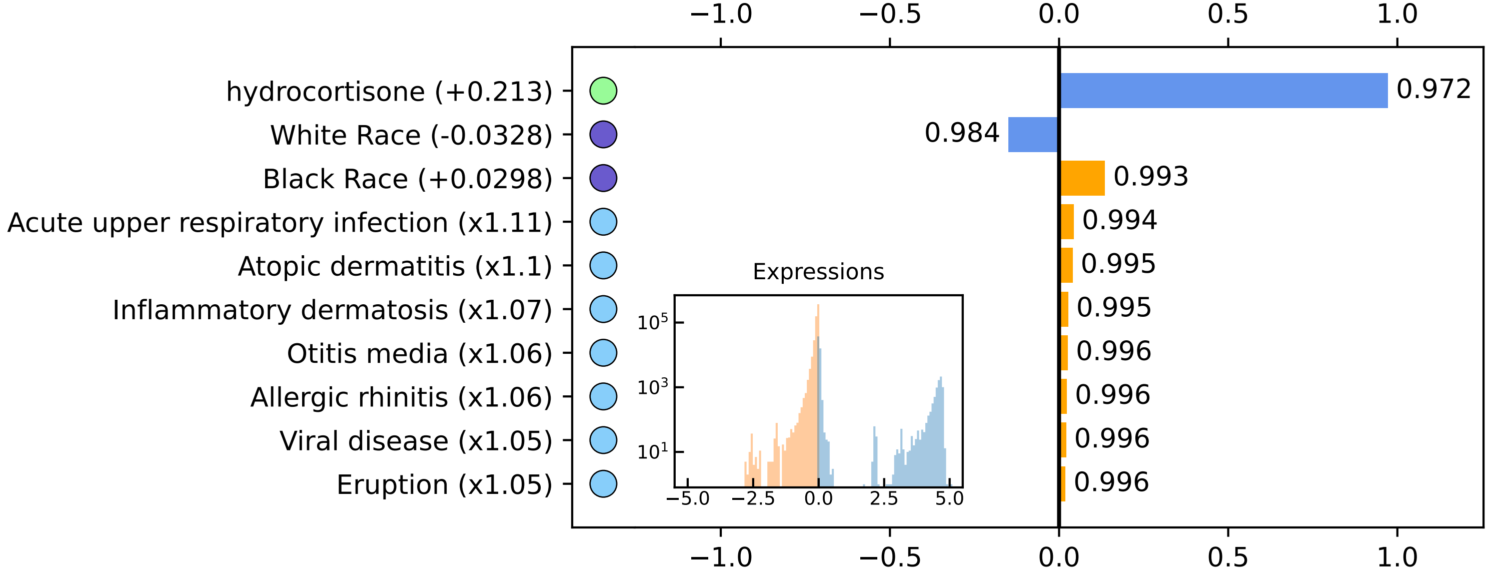

Signature [1321]; 7 To Reach 0.975, (10 Plotted)

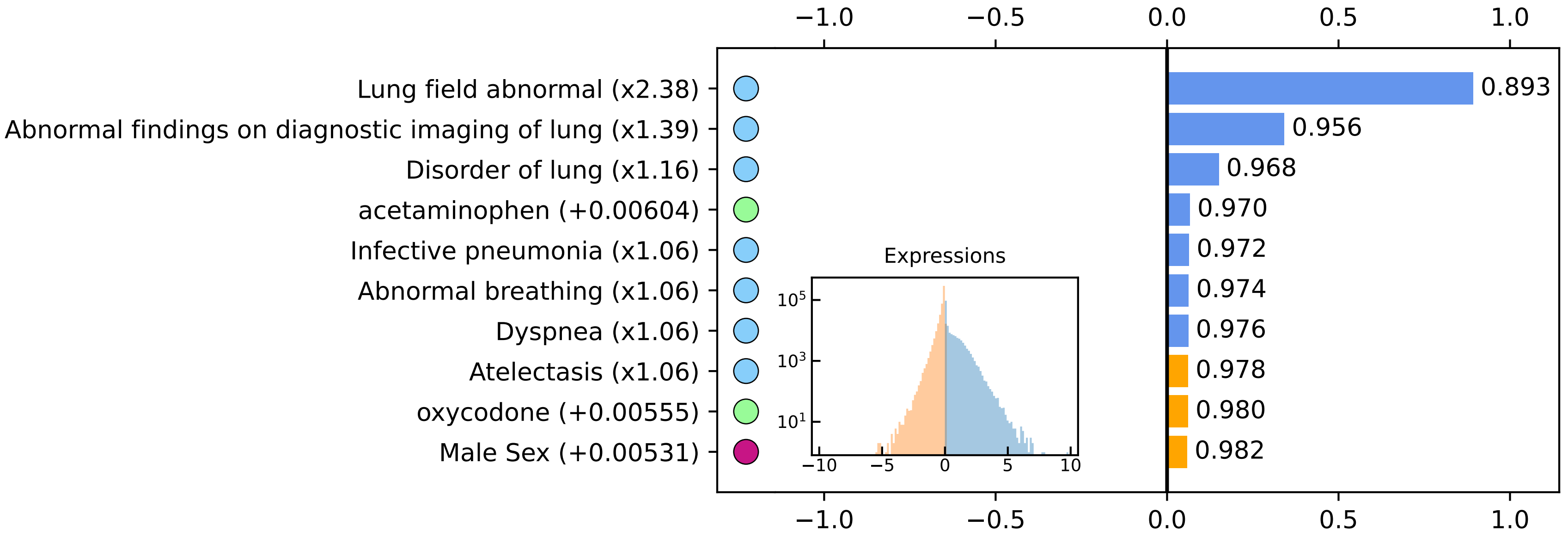

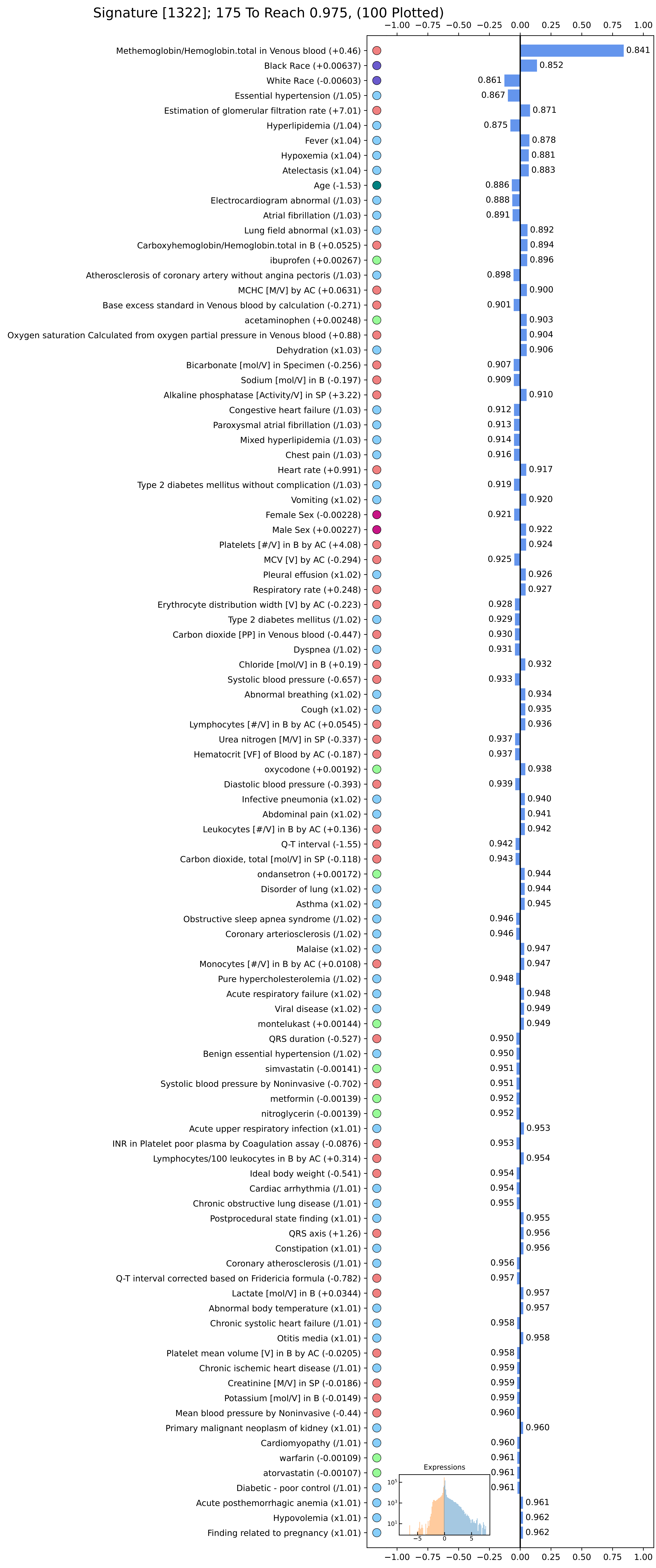

Signature [1322]; 175 To Reach 0.975, (100 Plotted)
Methemoglobin/Hemoglobin.total in Venous blood (+0.46)
Black Race (+0.00637)
White Race (-0.00603)
Essential hypertension (/1.05)
Estimation of glomerular filtration rate (+7.01)
Hyperlipidemia (/1.04)
Fever (x1.04)
Hypoxemia (x1.04)
Atelectasis (x1.04)
Age (-1.53)
Electrocardiogram abnormal (/1.03)
Atrial fibrillation (/1.03)
Lung field abnormal (x1.03)
Carboxyhemoglobin/Hemoglobin.total in B (+0.0525)
ibuprofen (+0.00267)
Atherosclerosis of coronary artery without angina pectoris (/1.03)
MCHC [M/V] by AC (+0.0631)
Base excess standard in Venous blood by calculation (-0.271)
acetaminophen (+0.00248)
Oxygen saturation Calculated from oxygen partial pressure in Venous blood (+0.88)
Dehydration (x1.03)
Bicarbonate [mol/V] in Specimen (-0.256)
Sodium [mol/V] in B (-0.197)
Alkaline phosphatase [Activity/V] in SP (+3.22)
Congestive heart failure (/1.03)
Paroxysmal atrial fibrillation (/1.03)
Mixed hyperlipidemia (/1.03)
Chest pain (/1.03)
Heart rate (+0.991)
Type 2 diabetes mellitus without complication (/1.03)
Vomiting (x1.02)
Female Sex (-0.00228)
Male Sex (+0.00227)
Platelets [#/V] in B by AC (+4.08)
MCV [V] by AC (-0.294)
Pleural effusion (x1.02)
Respiratory rate (+0.248)
Erythrocyte distribution width [V] by AC (-0.223)
Type 2 diabetes mellitus (/1.02)
Carbon dioxide [PP] in Venous blood (-0.447)
Dyspnea (/1.02)
Chloride [mol/V] in B (+0.19)
Systolic blood pressure (-0.657)
Abnormal breathing (x1.02)
Cough (x1.02)
Lymphocytes [#/V] in B by AC (+0.0545)
Urea nitrogen [M/V] in SP (-0.337)
Hematocrit [VF] of Blood by AC (-0.187)
oxycodone (+0.00192)
Diastolic blood pressure (-0.393)
Infective pneumonia (x1.02)
Abdominal pain (x1.02)
Leukocytes [#/V] in B by AC (+0.136)
Q-T interval (-1.55)
Carbon dioxide, total [mol/V] in SP (-0.118)
ondansetron (+0.00172)
Disorder of lung (x1.02)
Asthma (x1.02)
Obstructive sleep apnea syndrome (/1.02)
Coronary arteriosclerosis (/1.02)
Malaise (x1.02)
Monocytes [#/V] in B by AC (+0.0108)
Pure hypercholesterolemia (/1.02)
Acute respiratory failure (x1.02)
Viral disease (x1.02)
montelukast (+0.00144)
QRS duration (-0.527)
Benign essential hypertension (/1.02)
simvastatin (-0.00141)
Systolic blood pressure by Noninvasive (-0.702)
metformin (-0.00139)
nitroglycerin (-0.00139)
Acute upper respiratory infection (x1.01)
INR in Platelet poor plasma by Coagulation assay (-0.0876)
Lymphocytes/100 leukocytes in B by AC (+0.314)
Ideal body weight (-0.541)
Cardiac arrhythmia (/1.01)
Chronic obstructive lung disease (/1.01)
Postprocedural state finding (x1.01)
QRS axis (+1.26)
Constipation (x1.01)
Coronary atherosclerosis (/1.01)
Q-T interval corrected based on Fridericia formula (-0.782)
Lactate [mol/V] in B (+0.0344)
Abnormal body temperature (x1.01)
Chronic systolic heart failure (/1.01)
Otitis media (x1.01)
Platelet mean volume [V] in B by AC (-0.0205)
Chronic ischemic heart disease (/1.01)
Creatinine [M/V] in SP (-0.0186)
Potassium [mol/V] in B (-0.0149)
Mean blood pressure by Noninvasive (-0.44)
Primary malignant neoplasm of kidney (x1.01)
Cardiomyopathy (/1.01)
warfarin (-0.00109)
atorvastatin (-0.00107)
Diabetic - poor control (/1.01)
Acute posthemorrhagic anemia (x1.01)
Hypovolemia (x1.01)
Finding related to pregnancy (x1.01)
0.841
0.852
0.861
0.867
0.871
0.875
0.878
0.881
0.883
0.886
0.888
0.891
0.892
0.894
0.896
0.898
0.900
0.901
0.903
0.904
0.906
0.907
0.909
0.910
0.912
0.913
0.914
0.916
0.917
0.919
0.920
0.921
0.922
0.924
0.925
0.926
0.927
0.928
0.929
0.930
0.931
0.932
0.933
0.934
0.935
0.936
0.937
0.937
0.938
0.939
0.940
0.941
0.942
0.942
0.943
0.944
0.944
0.945
0.946
0.946
0.947
0.947
0.948
0.948
0.949
0.949
0.950
0.950
0.951
0.951
0.952
0.952
0.953
0.953
0.954
0.954
0.954
0.955
0.955
0.956
0.956
0.956
0.957
0.957
0.957
0.958
0.958
0.958
0.959
0.959
0.959
0.960
0.960
0.960
0.961
0.961
0.961
0.961
0.962
0.962
−1.00 −0.75 −0.50 −0.25 0.00 0.25 0.50 0.75 1.00
Expressions

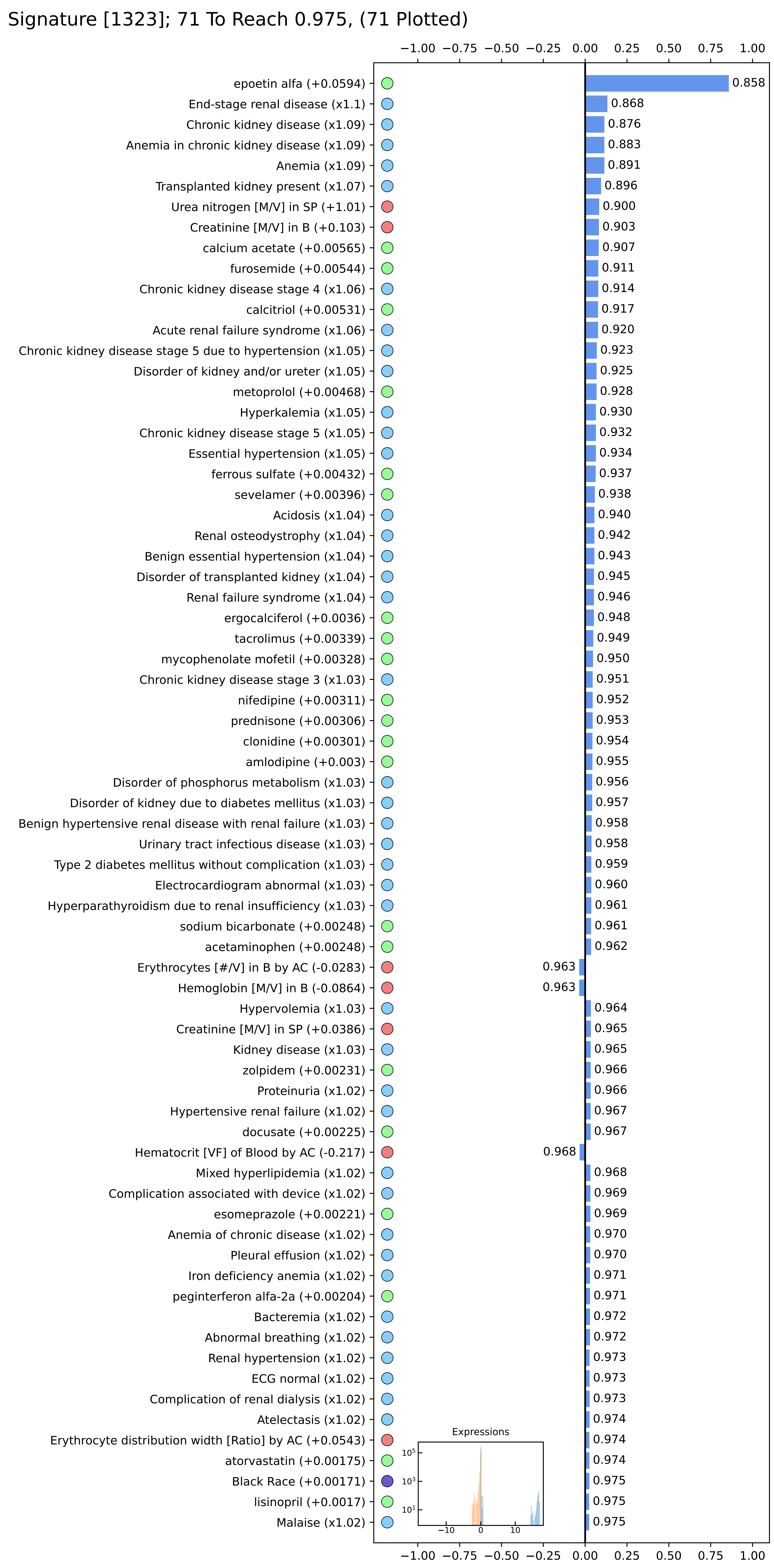

Signature [1323]; 71 To Reach 0.975, (71 Plotted)
epoetin alfa (+0.0594) 0.858
End-stage renal disease (x1.1) 0.868
Chronic kidney disease (x1.09) 0.876
Anemia in chronic kidney disease (x1.09) 0.883
Anemia (x1.09) 0.891
Transplanted kidney present (x1.07) 0.896
Urea nitrogen [M/V] in SP (+1.01) 0.900
Creatinine [M/V] in B (+0.103) 0.903
calcium acetate (+0.00565) 0.907
furosemide (+0.00544) 0.911
Chronic kidney disease stage 4 (x1.06) 0.914
calcitriol (+0.00531) 0.917
Acute renal failure syndrome (x1.06) 0.920
Chronic kidney disease stage 5 due to hypertension (x1.05) 0.923
Disorder of kidney and/or ureter (x1.05) 0.925
metoprolol (+0.00468) 0.928
Hyperkalemia (x1.05) 0.930
Chronic kidney disease stage 5 (x1.05) 0.932
Essential hypertension (x1.05) 0.934
ferrous sulfate (+0.00432) 0.937
sevelamer (+0.00396) 0.938
Acidosis (x1.04) 0.940
Renal osteodystrophy (x1.04) 0.942
Benign essential hypertension (x1.04) 0.943
Disorder of transplanted kidney (x1.04) 0.945
Renal failure syndrome (x1.04) 0.946
ergocalciferol (+0.0036) 0.948
tacrolimus (+0.00339) 0.949
mycophenolate mofetil (+0.00328) 0.950
Chronic kidney disease stage 3 (x1.03) 0.951
nifedipine (+0.00311) 0.952
prednisone (+0.00306) 0.953
clonidine (+0.00301) 0.954
amlodipine (+0.003) 0.955
Disorder of phosphorus metabolism (x1.03) 0.956
Disorder of kidney due to diabetes mellitus (x1.03) 0.957
Benign hypertensive renal disease with renal failure (x1.03) 0.958
Urinary tract infectious disease (x1.03) 0.958
Type 2 diabetes mellitus without complication (x1.03) 0.959
Electrocardiogram abnormal (x1.03) 0.960
Hyperparathyroidism due to renal insufficiency (x1.03) 0.961
sodium bicarbonate (+0.00248) 0.961
acetaminophen (+0.00248) 0.962
Erythrocytes [#/V] in B by AC (-0.0283) 0.963
Hemoglobin [M/V] in B (-0.0864) 0.963
Hypervolemia (x1.03) 0.964
Creatinine [M/V] in SP (+0.0386) 0.965
Kidney disease (x1.03) 0.965
zolpidem (+0.00231) 0.966
Proteinuria (x1.02) 0.966
Hypertensive renal failure (x1.02) 0.967
docusate (+0.00225) 0.967
Hematocrit [VF] of Blood by AC (-0.217) 0.968
Mixed hyperlipidemia (x1.02) 0.968
Complication associated with device (x1.02) 0.969
esomeprazole (+0.00221) 0.969
Anemia of chronic disease (x1.02) 0.970
Pleural effusion (x1.02) 0.970
Iron deficiency anemia (x1.02) 0.971
peginterferon alfa-2a (+0.00204) 0.971
Bacteremia (x1.02) 0.972
Abnormal breathing (x1.02) 0.972
Renal hypertension (x1.02) 0.973
ECG normal (x1.02) 0.973
Complication of renal dialysis (x1.02) 0.973
Atelectasis (x1.02) 0.974
Erythrocyte distribution width [Ratio] by AC (+0.0543) 0.974
atorvastatin (+0.00175) 0.974
Black Race (+0.00171) 0.975
lisinopril (+0.0017) 0.975
Malaise (x1.02) 0.975
Expressions
−1.00 −0.75 −0.50 −0.25 0.00 0.25 0.50 0.75 1.00

Signature [1324]; 179 To Reach 0.975, (100 Plotted)

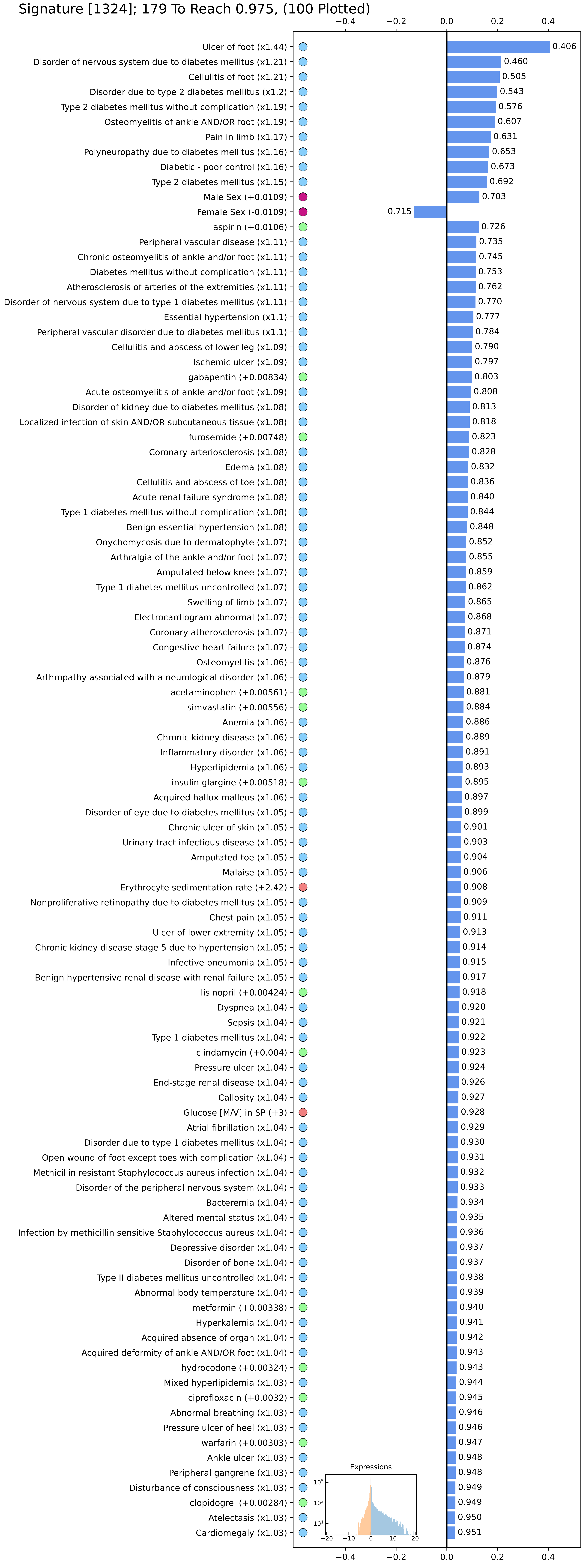

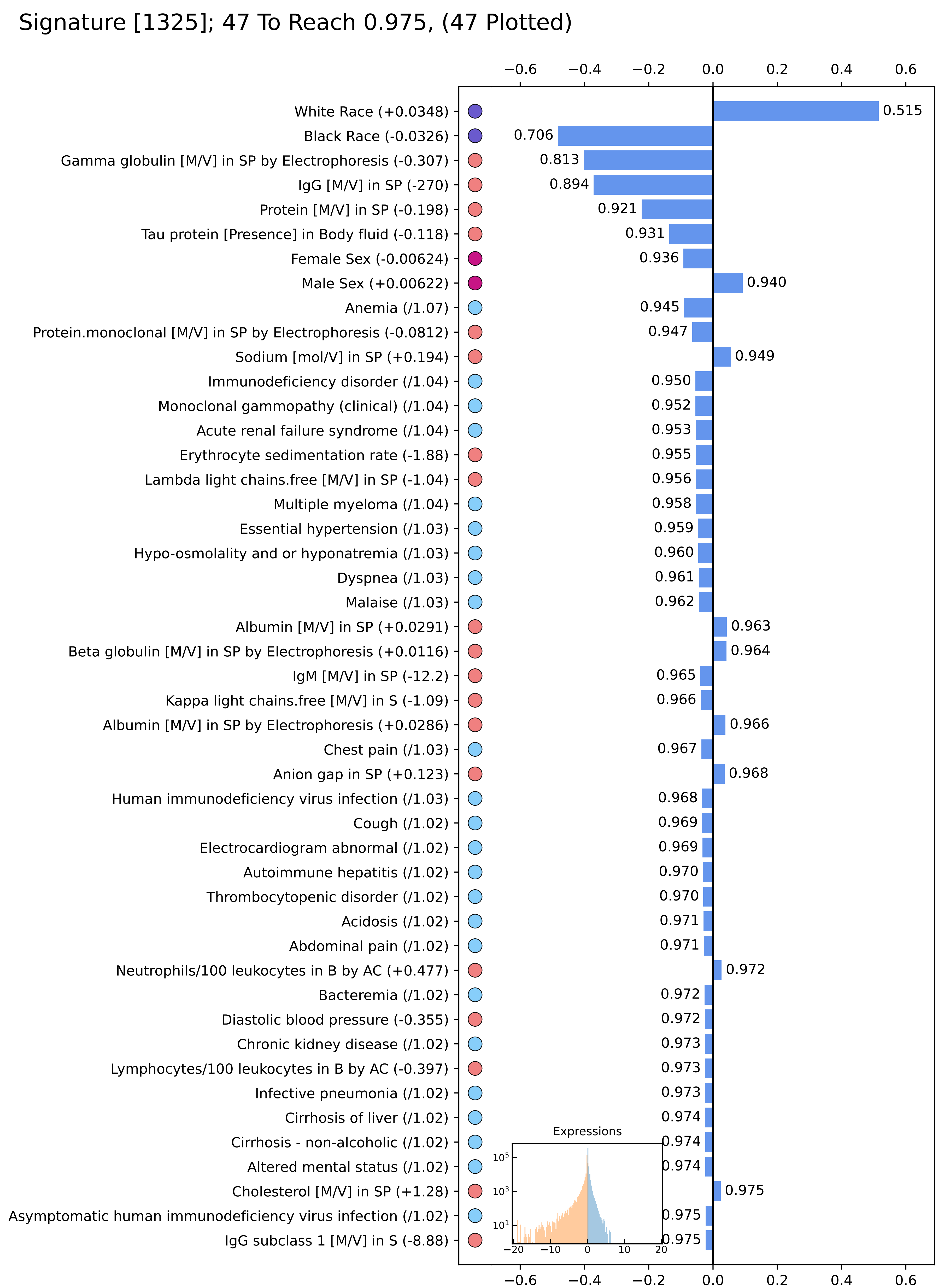

Signature [1325]; 47 To Reach 0.975, (47 Plotted)
White Race (+0.0348)
Black Race (-0.0326)
Gamma globulin [M/V] in SP by Electrophoresis (-0.307)
IgG [M/V] in SP (-270)
Protein [M/V] in SP (-0.198)
Tau protein [Presence] in Body fluid (-0.118)
Female Sex (-0.00624)
Male Sex (+0.00622)
Anemia (/1.07)
Protein.monoclonal [M/V] in SP by Electrophoresis (-0.0812)
Sodium [mol/V] in SP (+0.194)
Immunodeficiency disorder (/1.04)
Monoclonal gammopathy (clinical) (/1.04)
Acute renal failure syndrome (/1.04)
Erythrocyte sedimentation rate (-1.88)
Lambda light chains.free [M/V] in SP (-1.04)
Multiple myeloma (/1.04)
Essential hypertension (/1.03)
Hypo-osmolality and or hyponatremia (/1.03)
Dyspnea (/1.03)
Malaise (/1.03)
Albumin [M/V] in SP (+0.0291)
Beta globulin [M/V] in SP by Electrophoresis (+0.0116)
IgM [M/V] in SP (-12.2)
Kappa light chains.free [M/V] in S (-1.09)
Albumin [M/V] in SP by Electrophoresis (+0.0286)
Chest pain (/1.03)
Anion gap in SP (+0.123)
Human immunodeficiency virus infection (/1.03)
Cough (/1.02)
Electrocardiogram abnormal (/1.02)
Autoimmune hepatitis (/1.02)
Thrombocytopenic disorder (/1.02)
Acidosis (/1.02)
Abdominal pain (/1.02)
Neutrophils/100 leukocytes in B by AC (+0.477)
Bacteremia (/1.02)
Diastolic blood pressure (-0.355)
Chronic kidney disease (/1.02)
Lymphocytes/100 leukocytes in B by AC (-0.397)
Infective pneumonia (/1.02)
Cirrhosis of liver (/1.02)
Cirrhosis - non-alcoholic (/1.02)
Altered mental status (/1.02)
Cholesterol [M/V] in SP (+1.28)
Asymptomatic human immunodeficiency virus infection (/1.02)
IgG subclass 1 [M/V] in S (-8.88)
0.515
0.706
0.813
0.894
0.921
0.931
0.936
0.940
0.945
0.947
0.949
0.950
0.952
0.953
0.955
0.956
0.958
0.959
0.960
0.961
0.962
0.963
0.964
0.965
0.966
0.966
0.967
0.968
0.968
0.969
0.969
0.970
0.970
0.971
0.971
0.972
0.972
0.972
0.973
0.973
0.973
0.974
0.974
0.974
0.975
0.975
0.975
−0.6 −0.4 −0.2 0.0 0.2 0.4 0.6
Expressions

Signature [1326]; 1 To Reach 0.975, (10 Plotted)

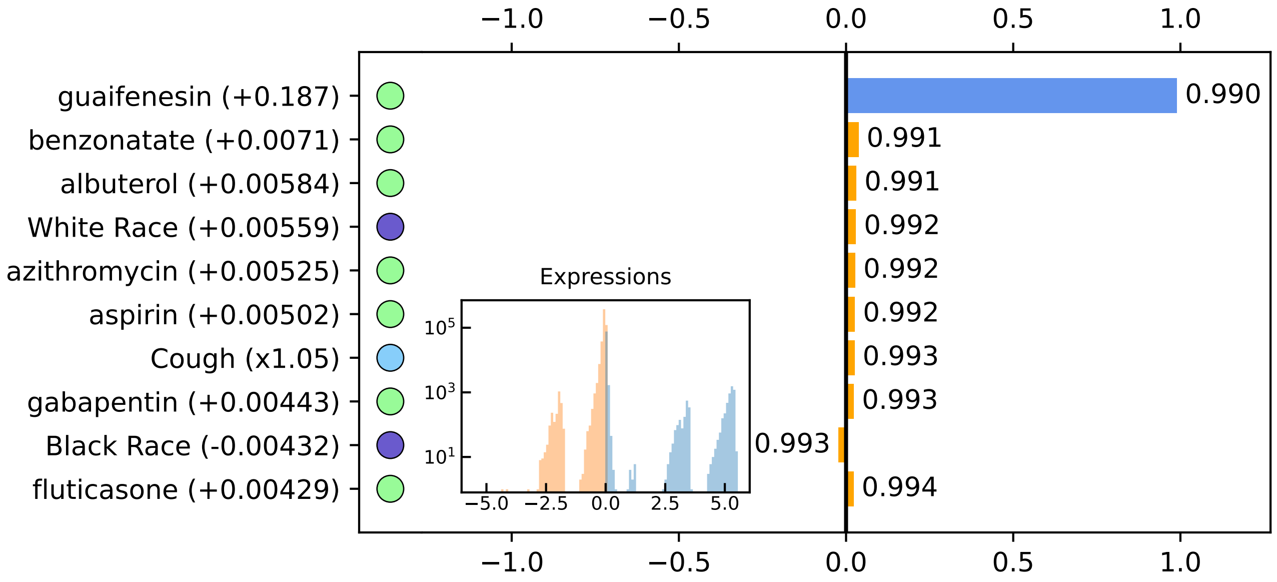

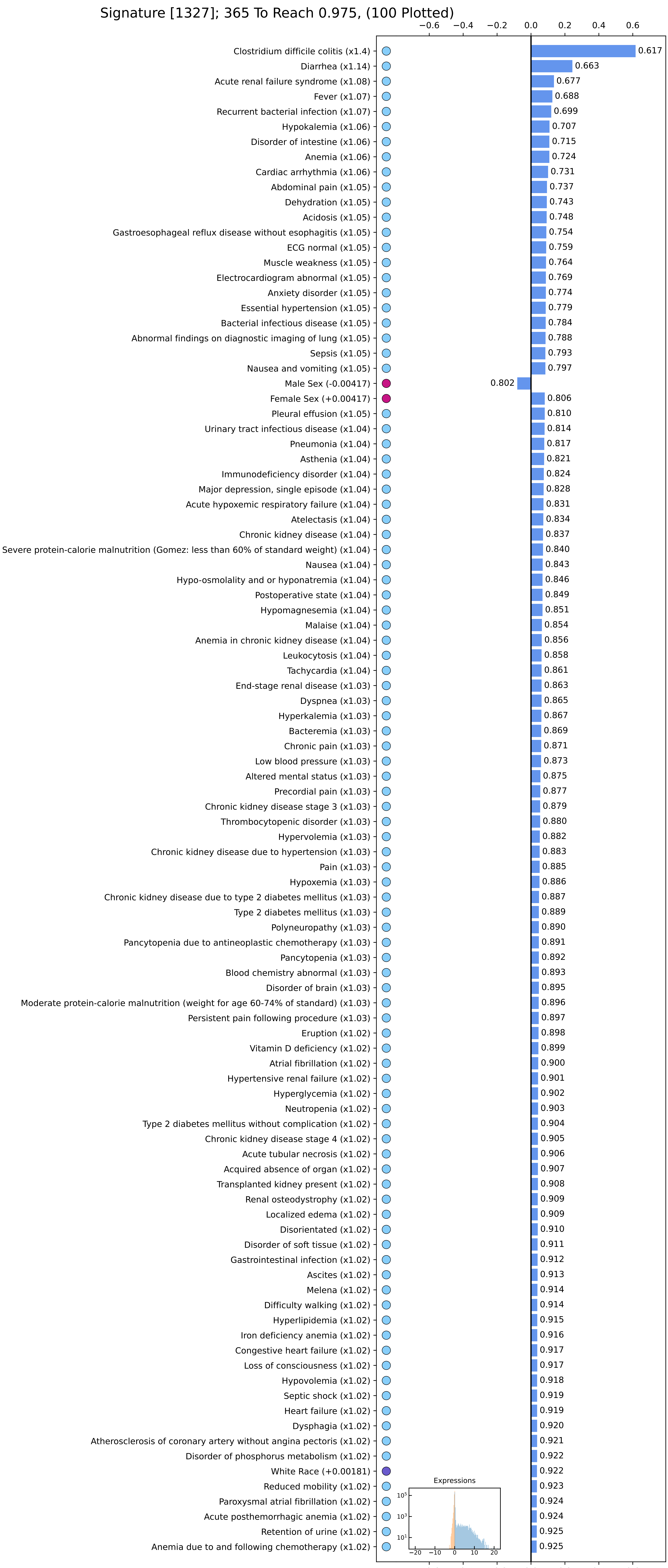
Signature [1327]; 365 To Reach 0.975, (100 Plotted)
Clostridium difficile colitis (x1.4) 0.617
Diarrhea (x1.14) 0.663
Acute renal failure syndrome (x1.08) 0.677
Fever (x1.07) 0.688
Recurrent bacterial infection (x1.07) 0.699
Hypokalemia (x1.06) 0.707
Disorder of intestine (x1.06) 0.715
Anemia (x1.06) 0.724
Cardiac arrhythmia (x1.06) 0.731
Abdominal pain (x1.05) 0.737
Dehydration (x1.05) 0.743
Acidosis (x1.05) 0.748
Gastroesophageal reflux disease without esophagitis (x1.05) 0.754
ECG normal (x1.05) 0.759
Muscle weakness (x1.05) 0.764
Electrocardiogram abnormal (x1.05) 0.769
Anxiety disorder (x1.05) 0.774
Essential hypertension (x1.05) 0.779
Bacterial infectious disease (x1.05) 0.784
Abnormal findings on diagnostic imaging of lung (x1.05) 0.788
Sepsis (x1.05) 0.793
Nausea and vomiting (x1.05) 0.797
Male Sex (-0.00417) 0.802
Female Sex (+0.00417) 0.806
Pleural effusion (x1.05) 0.810
Urinary tract infectious disease (x1.04) 0.814
Pneumonia (x1.04) 0.817
Asthenia (x1.04) 0.821
Immunodeficiency disorder (x1.04) 0.824
Major depression, single episode (x1.04) 0.828
Acute hypoxemic respiratory failure (x1.04) 0.831
Atelectasis (x1.04) 0.834
Chronic kidney disease (x1.04) 0.837
Severe protein-calorie malnutrition (Gomez: less than 60% of standard weight) (x1.04) 0.840
Nausea (x1.04) 0.843
Hypo-osmolality and or hyponatremia (x1.04) 0.846
Postoperative state (x1.04) 0.849
Hypomagnesemia (x1.04) 0.851
Malaise (x1.04) 0.854
Anemia in chronic kidney disease (x1.04) 0.856
Leukocytosis (x1.04) 0.858
Tachycardia (x1.04) 0.861
End-stage renal disease (x1.03) 0.863
Dyspnea (x1.03) 0.865
Hyperkalemia (x1.03) 0.867
Bacteremia (x1.03) 0.869
Chronic pain (x1.03) 0.871
Low blood pressure (x1.03) 0.873
Altered mental status (x1.03) 0.875
Precordial pain (x1.03) 0.877
Chronic kidney disease stage 3 (x1.03) 0.879
Thrombocytopenic disorder (x1.03) 0.880
Hypervolemia (x1.03) 0.882
Chronic kidney disease due to hypertension (x1.03) 0.883
Pain (x1.03) 0.885
Hypoxemia (x1.03) 0.886
Chronic kidney disease due to type 2 diabetes mellitus (x1.03) 0.887
Type 2 diabetes mellitus (x1.03) 0.889
Polyneuropathy (x1.03) 0.890
Pancytopenia due to antineoplastic chemotherapy (x1.03) 0.891
Pancytopenia (x1.03) 0.892
Blood chemistry abnormal (x1.03) 0.893
Disorder of brain (x1.03) 0.895
Moderate protein-calorie malnutrition (weight for age 60-74% of standard) (x1.03) 0.896
Persistent pain following procedure (x1.03) 0.897
Eruption (x1.02) 0.898
Vitamin D deficiency (x1.02) 0.899
Atrial fibrillation (x1.02) 0.900
Hypertensive renal failure (x1.02) 0.901
Hyperglycemia (x1.02) 0.902
Neutropenia (x1.02) 0.903
Type 2 diabetes mellitus without complication (x1.02) 0.904
Chronic kidney disease stage 4 (x1.02) 0.905
Acute tubular necrosis (x1.02) 0.906
Acquired absence of organ (x1.02) 0.907
Transplanted kidney present (x1.02) 0.908
Renal osteodystrophy (x1.02) 0.909
Localized edema (x1.02) 0.909
Disorientated (x1.02) 0.910
Disorder of soft tissue (x1.02) 0.911
Gastrointestinal infection (x1.02) 0.912
Ascites (x1.02) 0.913
Melena (x1.02) 0.914
Difficulty walking (x1.02) 0.914
Hyperlipidemia (x1.02) 0.915
Iron deficiency anemia (x1.02) 0.916
Congestive heart failure (x1.02) 0.917
Loss of consciousness (x1.02) 0.917
Hypovolemia (x1.02) 0.918
Septic shock (x1.02) 0.919
Heart failure (x1.02) 0.919
Dysphagia (x1.02) 0.920
Atherosclerosis of coronary artery without angina pectoris (x1.02) 0.921
Disorder of phosphorus metabolism (x1.02) 0.922
White Race (+0.00181) 0.922
Reduced mobility (x1.02) 0.923
Paroxysmal atrial fibrillation (x1.02) 0.924
Acute posthemorrhagic anemia (x1.02) 0.924
Retention of urine (x1.02) 0.925
Anemia due to and following chemotherapy (x1.02) 0.925
−0.6 −0.4 −0.2 0.0 0.2 0.4 0.6
Expressions

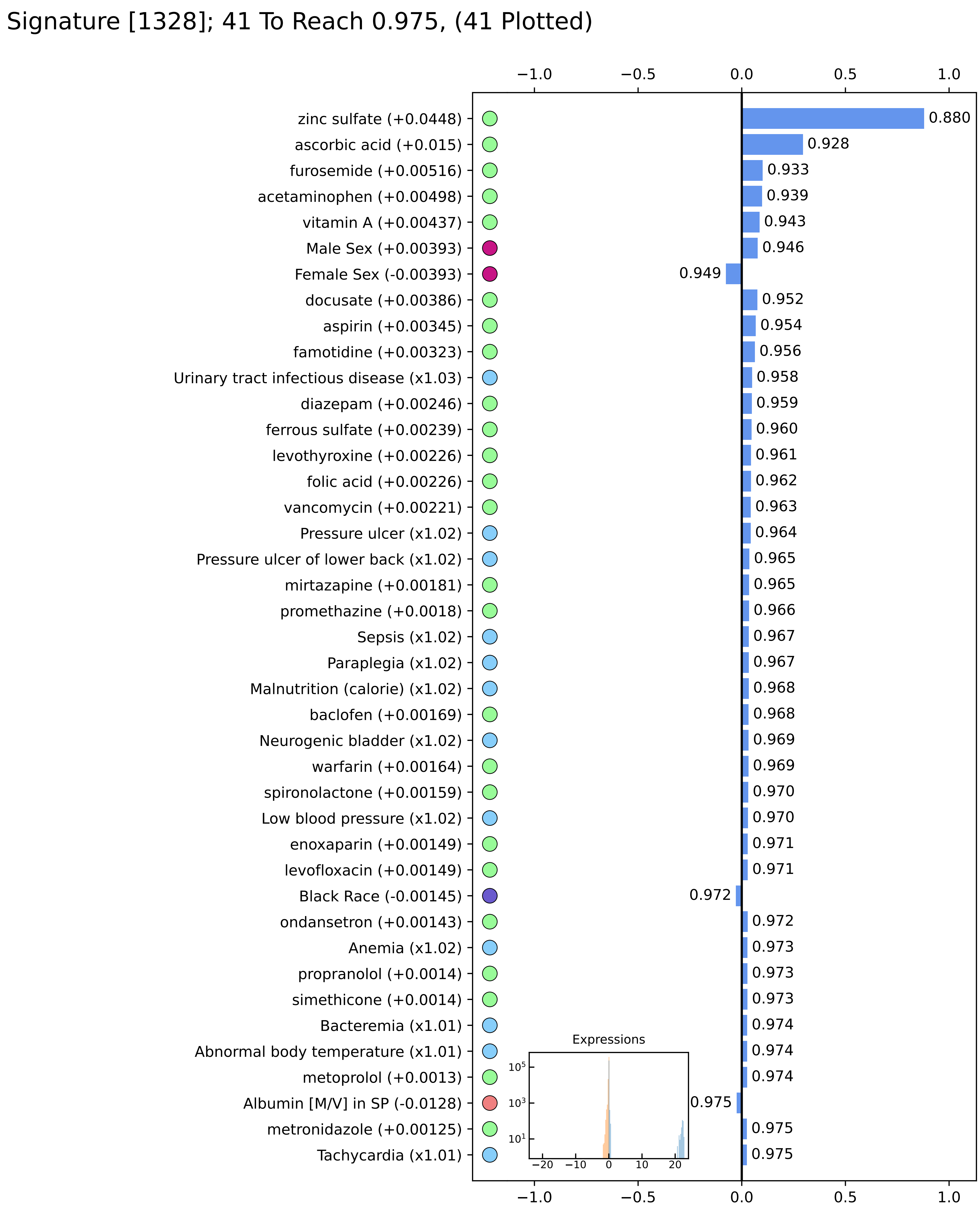

Signature [1328]; 41 To Reach 0.975, (41 Plotted)
−1.0
−0.5
0.0
0.5
1.0
zinc sulfate (+0.0448) 0.880
ascorbic acid (+0.015) 0.928
furosemide (+0.00516) 0.933
acetaminophen (+0.00498) 0.939
vitamin A (+0.00437) 0.943
Male Sex (+0.00393) 0.946
Female Sex (-0.00393) 0.949
docusate (+0.00386) 0.952
aspirin (+0.00345) 0.954
famotidine (+0.00323) 0.956
Urinary tract infectious disease (x1.03) 0.958
diazepam (+0.00246) 0.959
ferrous sulfate (+0.00239) 0.960
levothyroxine (+0.00226) 0.961
folic acid (+0.00226) 0.962
vancomycin (+0.00221) 0.963
Pressure ulcer (x1.02) 0.964
Pressure ulcer of lower back (x1.02) 0.965
mirtazapine (+0.00181) 0.965
promethazine (+0.0018) 0.966
Sepsis (x1.02) 0.967
Paraplegia (x1.02) 0.967
Malnutrition (calorie) (x1.02) 0.968
baclofen (+0.00169) 0.968
Neurogenic bladder (x1.02) 0.969
warfarin (+0.00164) 0.969
spironolactone (+0.00159) 0.970
Low blood pressure (x1.02) 0.970
enoxaparin (+0.00149) 0.971
levofloxacin (+0.00149) 0.971
Black Race (-0.00145) 0.972
ondansetron (+0.00143) 0.972
Anemia (x1.02) 0.973
propranolol (+0.0014) 0.973
simethicone (+0.0014) 0.973
Bacteremia (x1.01) 0.974
Abnormal body temperature (x1.01) 0.974
metoprolol (+0.0013) 0.974
Albumin [M/V] in SP (-0.0128) 0.975
metronidazole (+0.00125) 0.975
Tachycardia (x1.01) 0.975
Expressions
10^5
10^3
10^1
−20
−10
0
10
20

Signature [1329]; 536 To Reach 0.975, (100 Plotted)

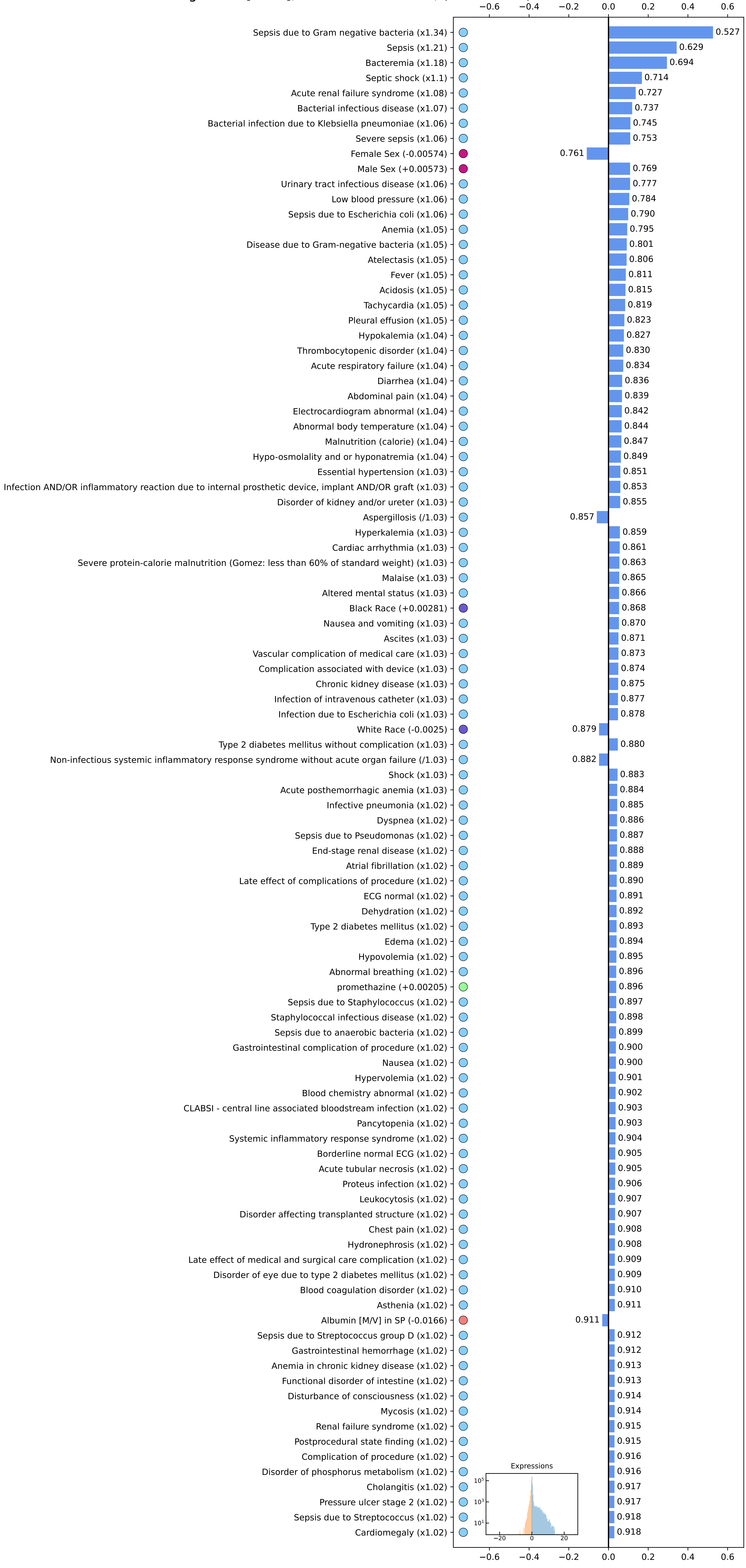

Signature [1330]; 2 To Reach 0.975, (10 Plotted)

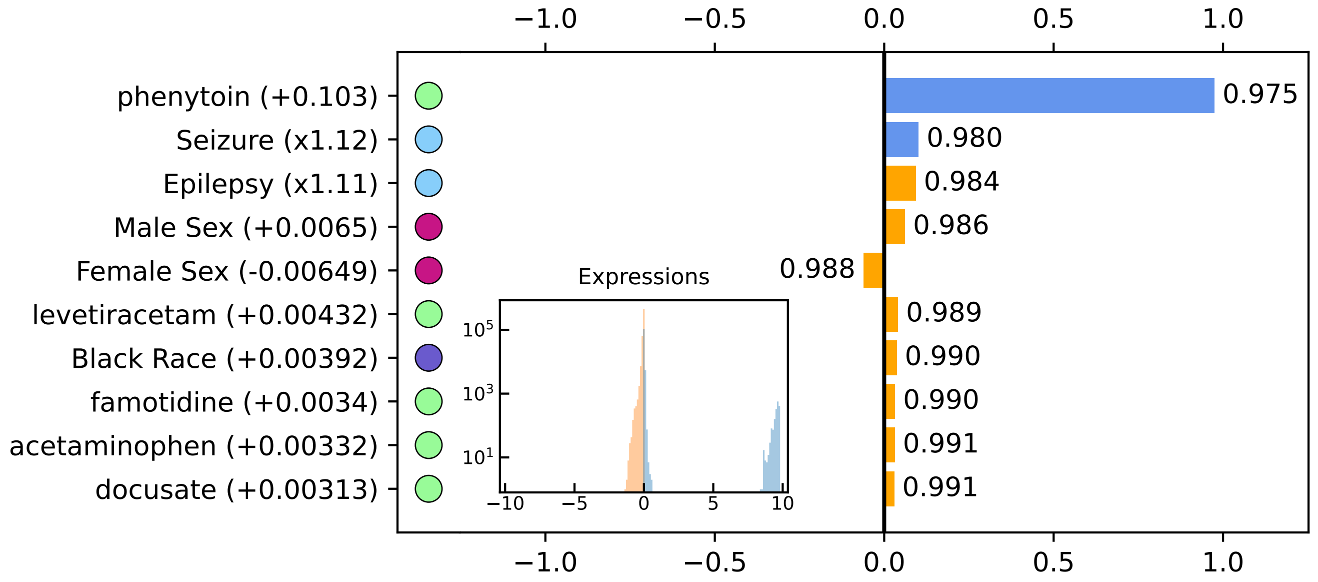

Signature [1331]; 239 To Reach 0.975, (100 Plotted)

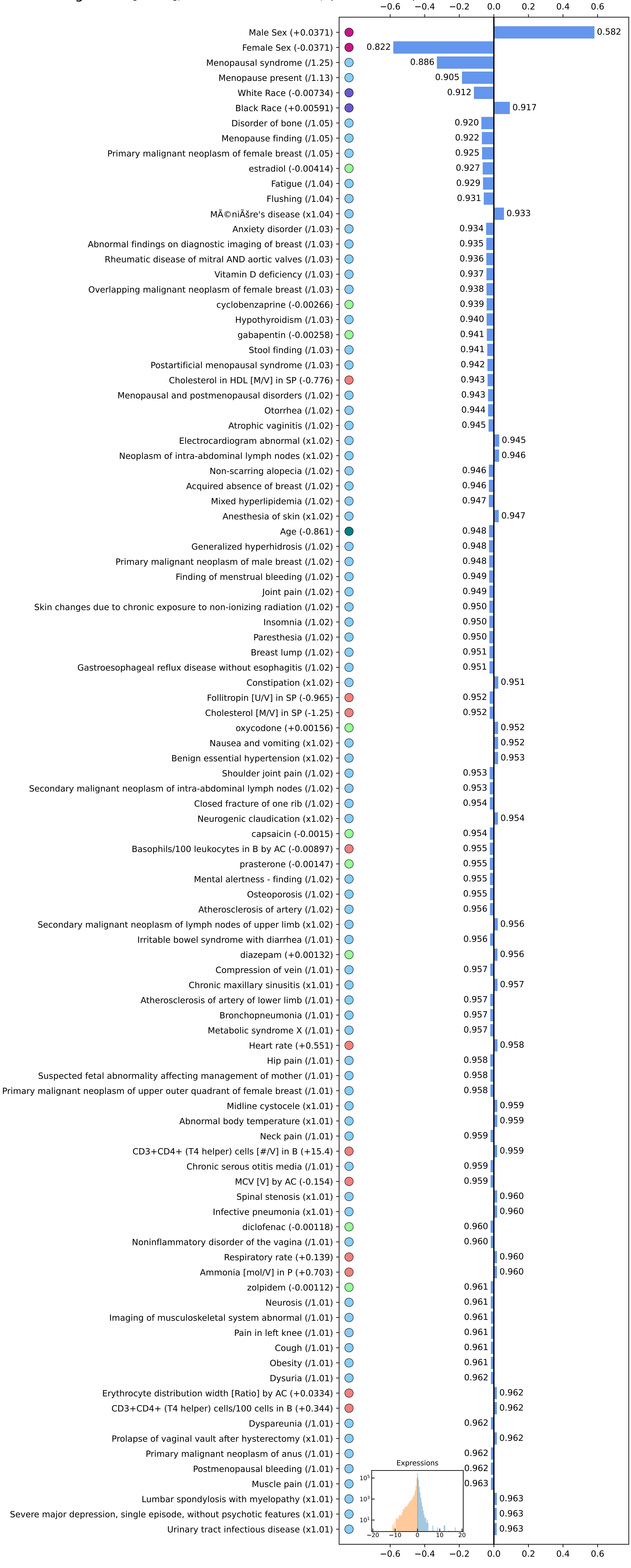

Signature [1332]; 507 To Reach 0.975, (100 Plotted)

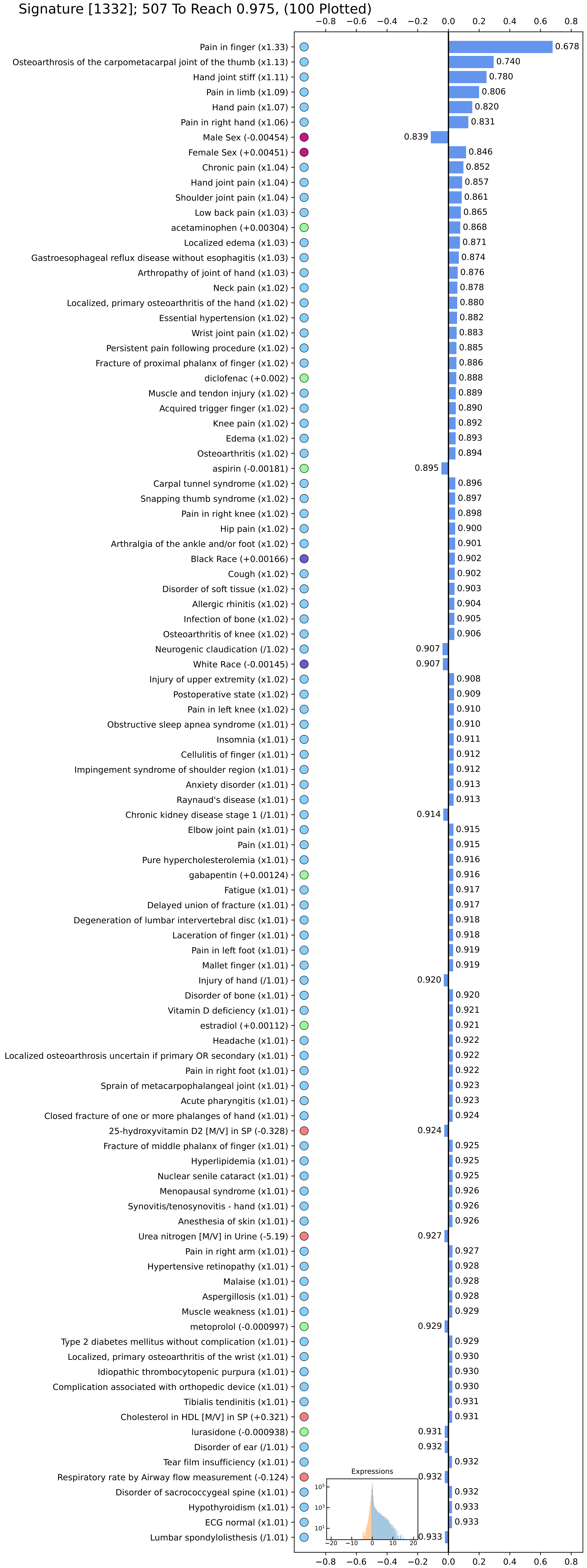

Signature [1333]; 2 To Reach 0.975, (10 Plotted)

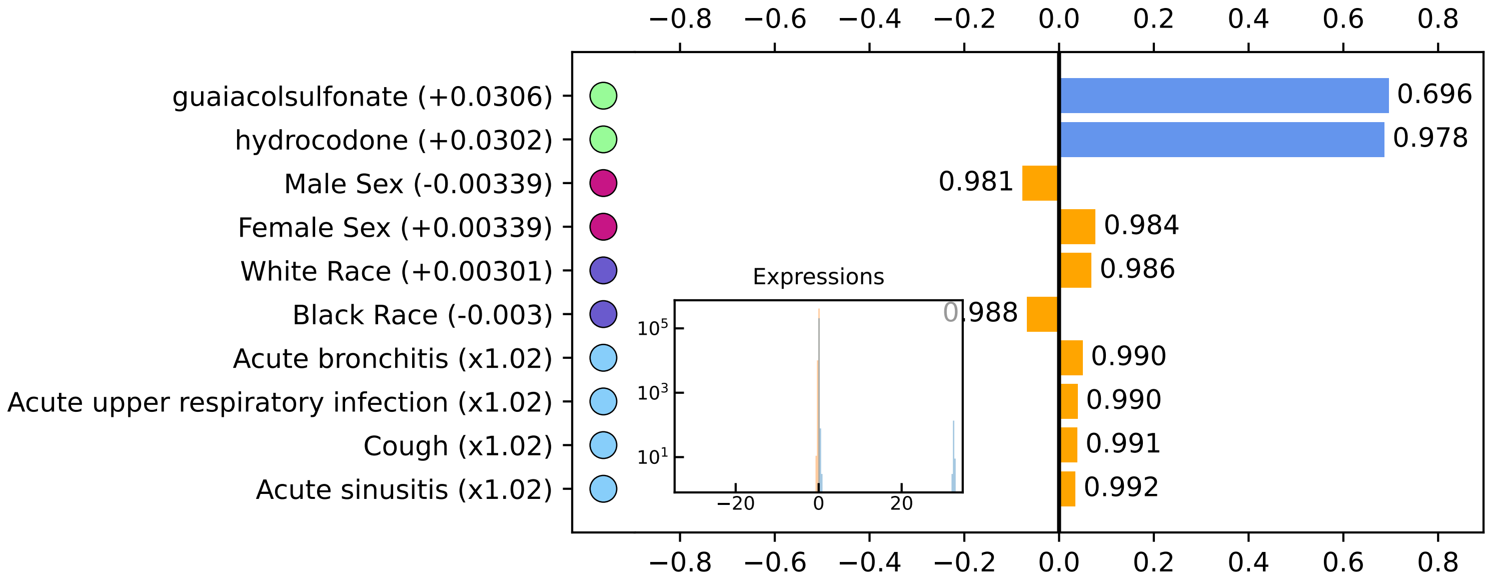

Signature [1334]; 183 To Reach 0.975, (100 Plotted)

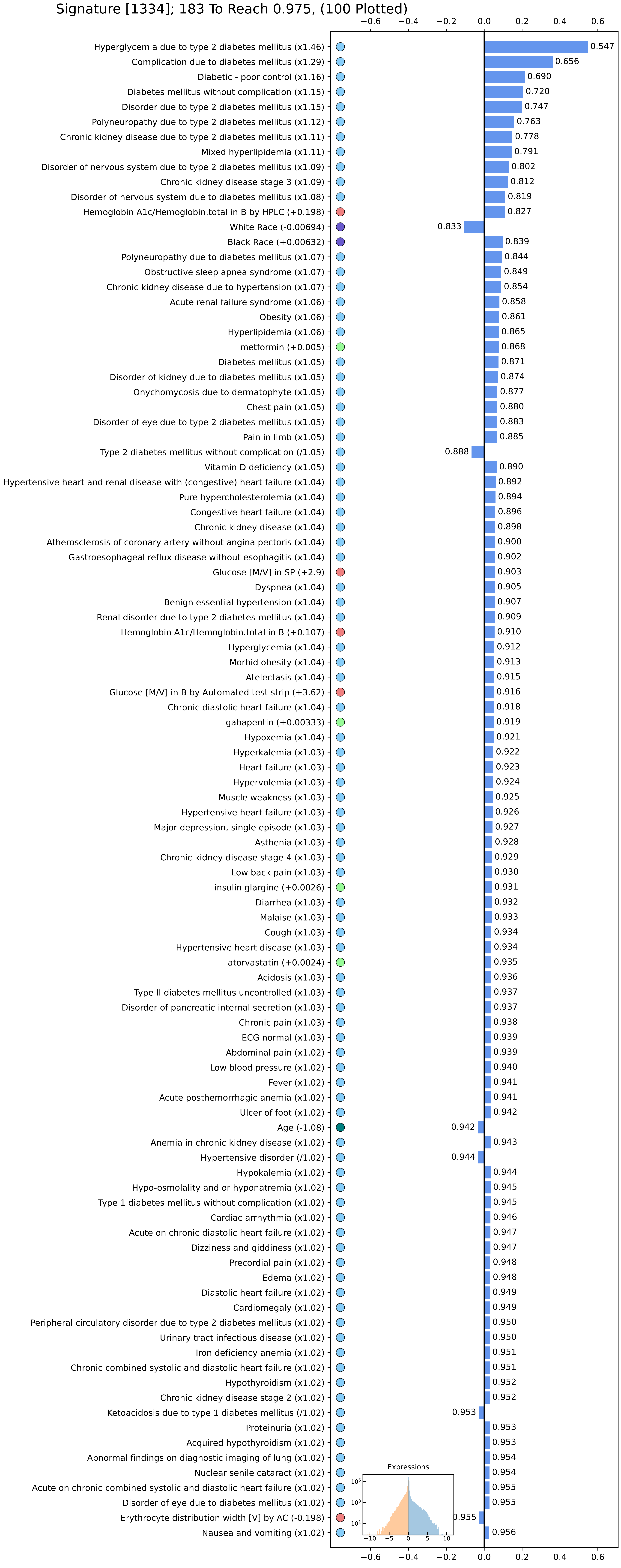

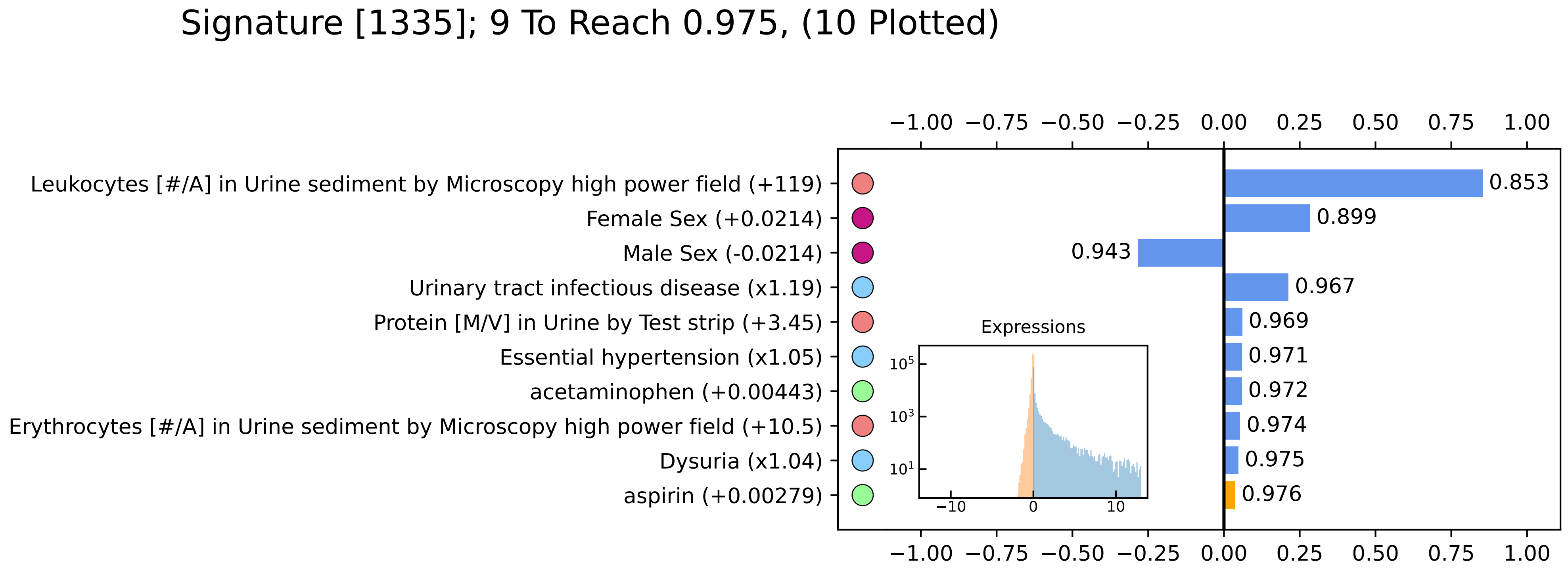

Signature [1335]; 9 To Reach 0.975, (10 Plotted)
Leukocytes [#/A] in Urine sediment by Microscopy high power field (+119)
Female Sex (+0.0214)
Male Sex (-0.0214)
Urinary tract infectious disease (x1.19)
Protein [M/V] in Urine by Test strip (+3.45)
Essential hypertension (x1.05)
acetaminophen (+0.00443)
Erythrocytes [#/A] in Urine sediment by Microscopy high power field (+10.5)
Dysuria (x1.04)
aspirin (+0.00279)
0.853
0.899
0.943
0.967
0.969
0.971
0.972
0.974
0.975
0.976
Expressions
−1.00 −0.75 −0.50 −0.25 0.00 0.25 0.50 0.75 1.00

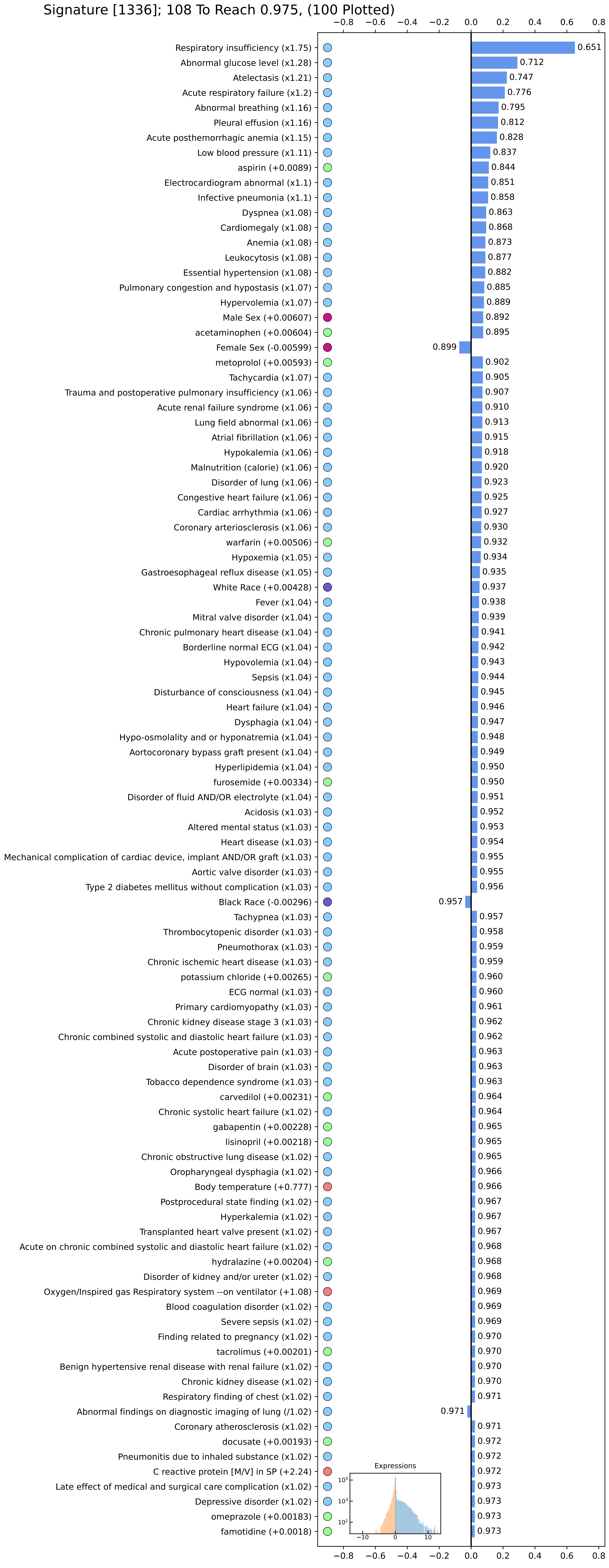

Signature [1336]; 108 To Reach 0.975, (100 Plotted)
−0.8 −0.6 −0.4 −0.2 0.0 0.2 0.4 0.6 0.8
Respiratory insufficiency (x1.75)
0.651
Abnormal glucose level (x1.28)
0.712
Atelectasis (x1.21)
0.747
Acute respiratory failure (x1.2)
0.776
Abnormal breathing (x1.16)
0.795
Pleural effusion (x1.16)
0.812
Acute posthemorrhagic anemia (x1.15)
0.828
Low blood pressure (x1.11)
0.837
aspirin (+0.0089)
0.844
Electrocardiogram abnormal (x1.1)
0.851
Infective pneumonia (x1.1)
0.858
Dyspnea (x1.08)
0.863
Cardiomegaly (x1.08)
0.868
Anemia (x1.08)
0.873
Leukocytosis (x1.08)
0.877
Essential hypertension (x1.08)
0.882
Pulmonary congestion and hypostasis (x1.07)
0.885
Hypervolemia (x1.07)
0.889
Male Sex (+0.00607)
0.892
acetaminophen (+0.00604)
0.895
Female Sex (-0.00599)
0.899
metoprolol (+0.00593)
0.902
Tachycardia (x1.07)
0.905
Trauma and postoperative pulmonary insufficiency (x1.06)
0.907
Acute renal failure syndrome (x1.06)
0.910
Lung field abnormal (x1.06)
0.913
Atrial fibrillation (x1.06)
0.915
Hypokalemia (x1.06)
0.918
Malnutrition (calorie) (x1.06)
0.920
Disorder of lung (x1.06)
0.923
Congestive heart failure (x1.06)
0.925
Cardiac arrhythmia (x1.06)
0.927
Coronary arteriosclerosis (x1.06)
0.930
warfarin (+0.00506)
0.932
Hypoxemia (x1.05)
0.934
Gastroesophageal reflux disease (x1.05)
0.935
White Race (+0.00428)
0.937
Fever (x1.04)
0.938
Mitral valve disorder (x1.04)
0.939
Chronic pulmonary heart disease (x1.04)
0.941
Borderline normal ECG (x1.04)
0.942
Hypovolemia (x1.04)
0.943
Sepsis (x1.04)
0.944
Disturbance of consciousness (x1.04)
0.945
Heart failure (x1.04)
0.946
Dysphagia (x1.04)
0.947
Hypo-osmolality and or hyponatremia (x1.04)
0.948
Aortocoronary bypass graft present (x1.04)
0.949
Hyperlipidemia (x1.04)
0.950
furosemide (+0.00334)
0.950
Disorder of fluid AND/OR electrolyte (x1.04)
0.951
Acidosis (x1.03)
0.952
Altered mental status (x1.03)
0.953
Heart disease (x1.03)
0.954
Mechanical complication of cardiac device, implant AND/OR graft (x1.03)
0.955
Aortic valve disorder (x1.03)
0.955
Type 2 diabetes mellitus without complication (x1.03)
0.956
Black Race (-0.00296)
0.957
Tachypnea (x1.03)
0.957
Thrombocytopenic disorder (x1.03)
0.958
Pneumothorax (x1.03)
0.959
Chronic ischemic heart disease (x1.03)
0.959
potassium chloride (+0.00265)
0.960
ECG normal (x1.03)
0.960
Primary cardiomyopathy (x1.03)
0.961
Chronic kidney disease stage 3 (x1.03)
0.962
Chronic combined systolic and diastolic heart failure (x1.03)
0.962
Acute postoperative pain (x1.03)
0.963
Disorder of brain (x1.03)
0.963
Tobacco dependence syndrome (x1.03)
0.963
carvedilol (+0.00231)
0.964
Chronic systolic heart failure (x1.02)
0.964
gabapentin (+0.00228)
0.965
lisinopril (+0.00218)
0.965
Chronic obstructive lung disease (x1.02)
0.965
Oropharyngeal dysphagia (x1.02)
0.966
Body temperature (+0.777)
0.966
Postprocedural state finding (x1.02)
0.967
Hyperkalemia (x1.02)
0.967
Transplanted heart valve present (x1.02)
0.967
Acute on chronic combined systolic and diastolic heart failure (x1.02)
0.968
hydralazine (+0.00204)
0.968
Disorder of kidney and/or ureter (x1.02)
0.968
Oxygen/Inspired gas Respiratory system --on ventilator (+1.08)
0.969
Blood coagulation disorder (x1.02)
0.969
Severe sepsis (x1.02)
0.969
Finding related to pregnancy (x1.02)
0.970
tacrolimus (+0.00201)
0.970
Benign hypertensive renal disease with renal failure (x1.02)
0.970
Chronic kidney disease (x1.02)
0.970
Respiratory finding of chest (x1.02)
0.971
Abnormal findings on diagnostic imaging of lung (/1.02)
0.971
Coronary atherosclerosis (x1.02)
0.971
docusate (+0.00193)
0.972
Pneumonitis due to inhaled substance (x1.02)
0.972
C reactive protein [M/V] in SP (+2.24)
0.972
Late effect of medical and surgical care complication (x1.02)
0.973
Depressive disorder (x1.02)
0.973
omeprazole (+0.00183)
0.973
famotidine (+0.0018)
0.973
Expressions
10^5
10^3
10^1
−10 0 10
−0.8 −0.6 −0.4 −0.2 0.0 0.2 0.4 0.6 0.8

Signature [1337]; 125 To Reach 0.975, (100 Plotted)

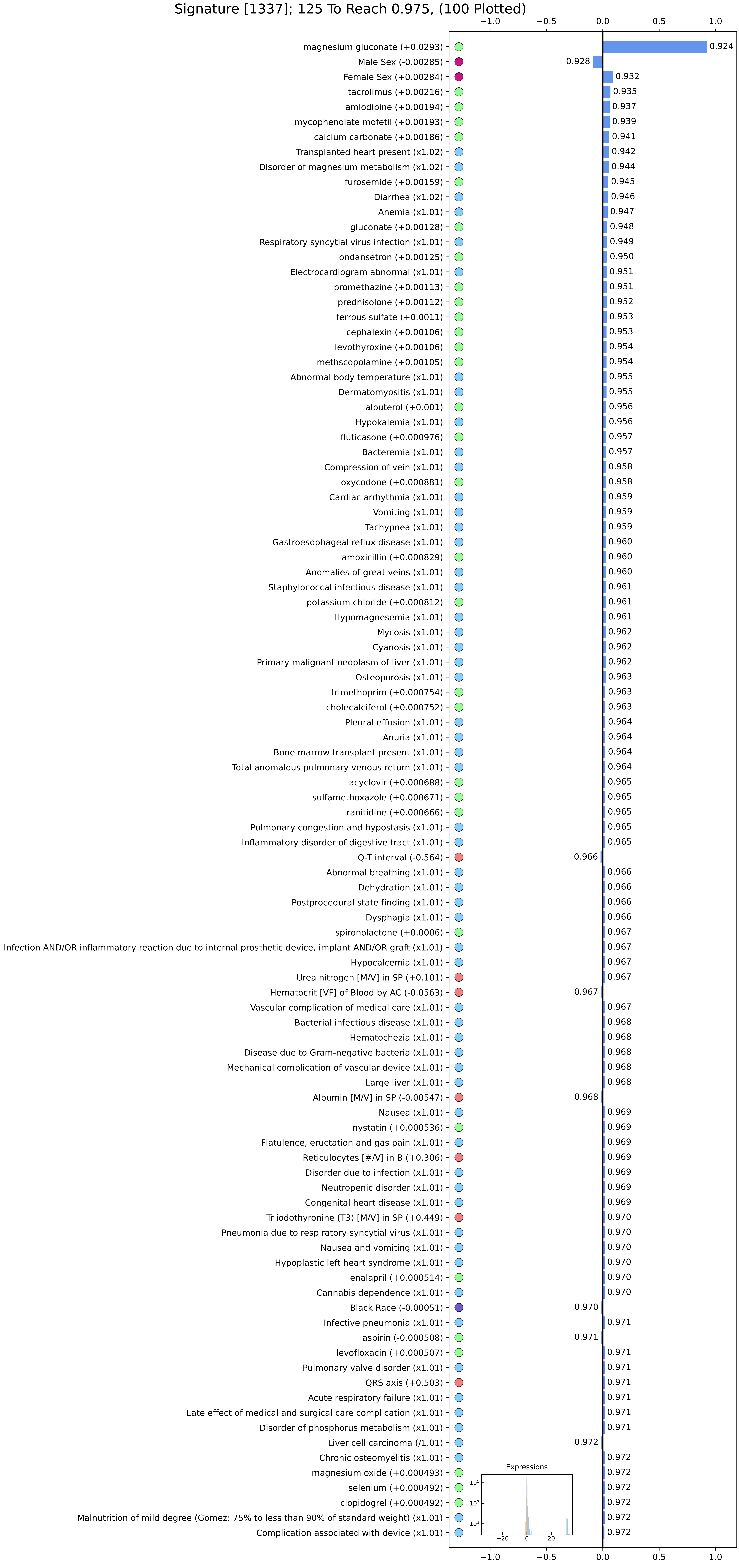

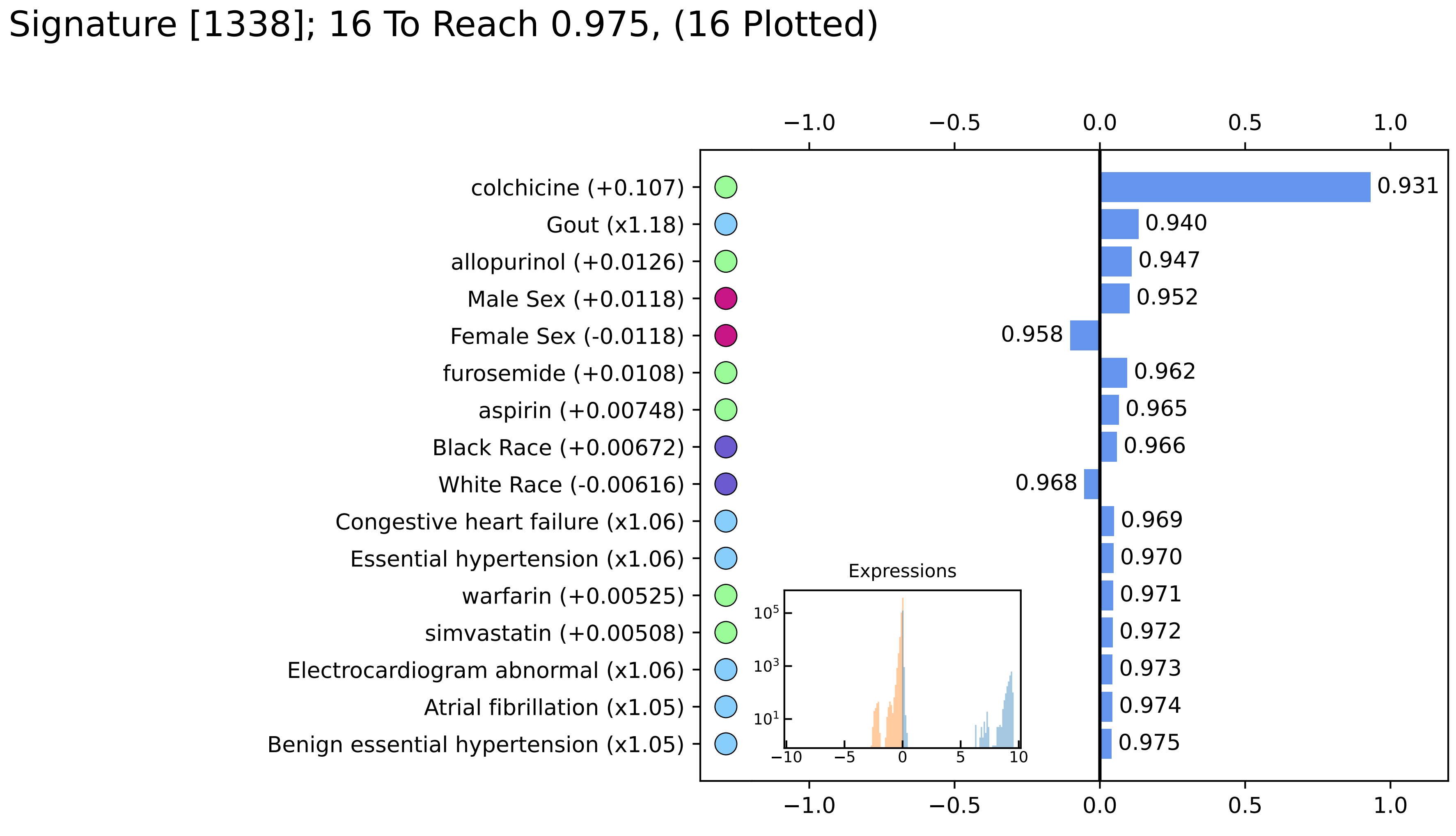

Signature [1338]; 16 To Reach 0.975, (16 Plotted)
colchicine (+0.107)
Gout (x1.18)
allopurinol (+0.0126)
Male Sex (+0.0118)
Female Sex (-0.0118)
furosemide (+0.0108)
aspirin (+0.00748)
Black Race (+0.00672)
White Race (-0.00616)
Congestive heart failure (x1.06)
Essential hypertension (x1.06)
warfarin (+0.00525)
simvastatin (+0.00508)
Electrocardiogram abnormal (x1.06)
Atrial fibrillation (x1.05)
Benign essential hypertension (x1.05)
0.931
0.940
0.947
0.952
0.958
0.962
0.965
0.966
0.968
0.969
0.970
0.971
0.972
0.973
0.974
0.975
−1.0
−0.5
0.0
0.5
1.0
Expressions

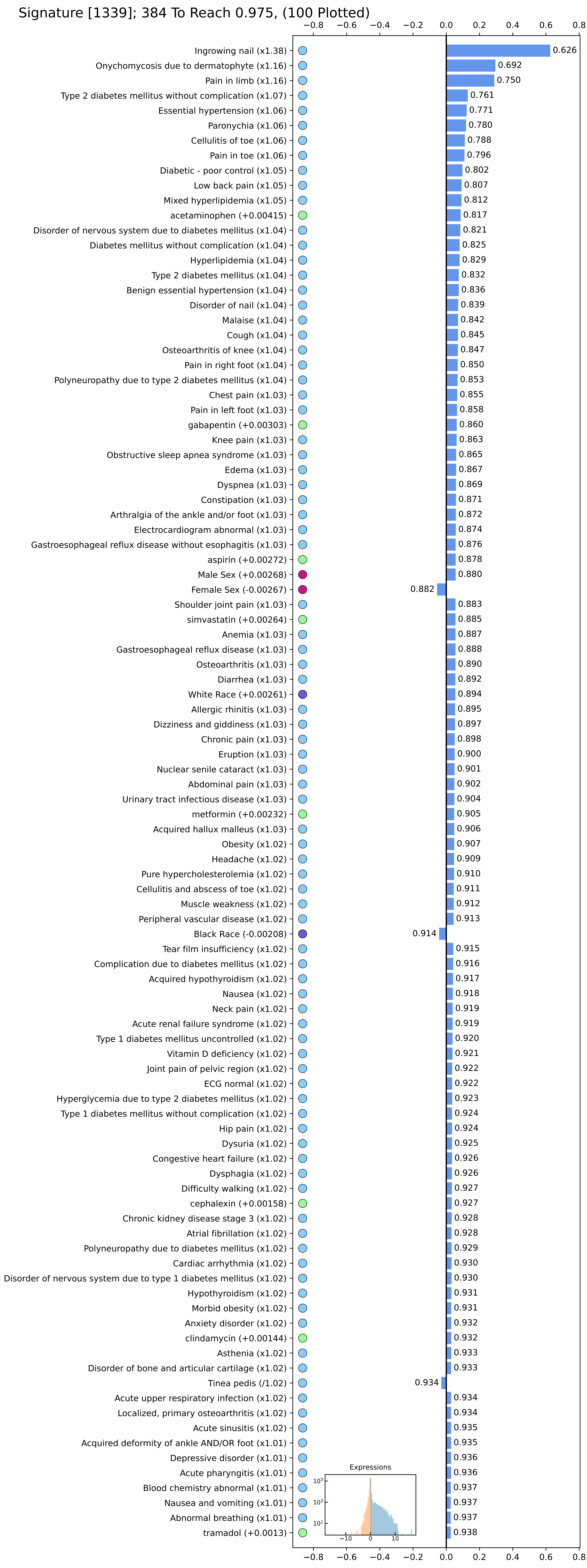

Signature [1339]; 384 To Reach 0.975, (100 Plotted)
Ingrowing nail (x1.38) 0.626
Onychomycosis due to dermatophyte (x1.16) 0.692
Pain in limb (x1.16) 0.750
Type 2 diabetes mellitus without complication (x1.07) 0.761
Essential hypertension (x1.06) 0.771
Paronychia (x1.06) 0.780
Cellulitis of toe (x1.06) 0.788
Pain in toe (x1.06) 0.796
Diabetic - poor control (x1.05) 0.802
Low back pain (x1.05) 0.807
Mixed hyperlipidemia (x1.05) 0.812
acetaminophen (+0.00415) 0.817
Disorder of nervous system due to diabetes mellitus (x1.04) 0.821
Diabetes mellitus without complication (x1.04) 0.825
Hyperlipidemia (x1.04) 0.829
Type 2 diabetes mellitus (x1.04) 0.832
Benign essential hypertension (x1.04) 0.836
Disorder of nail (x1.04) 0.839
Malaise (x1.04) 0.842
Cough (x1.04) 0.845
Osteoarthritis of knee (x1.04) 0.847
Pain in right foot (x1.04) 0.850
Polyneuropathy due to type 2 diabetes mellitus (x1.04) 0.853
Chest pain (x1.03) 0.855
Pain in left foot (x1.03) 0.858
gabapentin (+0.00303) 0.860
Knee pain (x1.03) 0.863
Obstructive sleep apnea syndrome (x1.03) 0.865
Edema (x1.03) 0.867
Dyspnea (x1.03) 0.869
Constipation (x1.03) 0.871
Arthralgia of the ankle and/or foot (x1.03) 0.872
Electrocardiogram abnormal (x1.03) 0.874
Gastroesophageal reflux disease without esophagitis (x1.03) 0.876
aspirin (+0.00272) 0.878
Male Sex (+0.00268) 0.880
Female Sex (-0.00267) 0.882
Shoulder joint pain (x1.03) 0.883
simvastatin (+0.00264) 0.885
Anemia (x1.03) 0.887
Gastroesophageal reflux disease (x1.03) 0.888
Osteoarthritis (x1.03) 0.890
Diarrhea (x1.03) 0.892
White Race (+0.00261) 0.894
Allergic rhinitis (x1.03) 0.895
Dizziness and giddiness (x1.03) 0.897
Chronic pain (x1.03) 0.898
Eruption (x1.03) 0.900
Nuclear senile cataract (x1.03) 0.901
Abdominal pain (x1.03) 0.902
Urinary tract infectious disease (x1.03) 0.904
metformin (+0.00232) 0.905
Acquired hallux malleus (x1.03) 0.906
Obesity (x1.02) 0.907
Headache (x1.02) 0.909
Pure hypercholesterolemia (x1.02) 0.910
Cellulitis and abscess of toe (x1.02) 0.911
Muscle weakness (x1.02) 0.912
Peripheral vascular disease (x1.02) 0.913
Black Race (-0.00208) 0.914
Tear film insufficiency (x1.02) 0.915
Complication due to diabetes mellitus (x1.02) 0.916
Acquired hypothyroidism (x1.02) 0.917
Nausea (x1.02) 0.918
Neck pain (x1.02) 0.919
Acute renal failure syndrome (x1.02) 0.919
Type 1 diabetes mellitus uncontrolled (x1.02) 0.920
Vitamin D deficiency (x1.02) 0.921
Joint pain of pelvic region (x1.02) 0.922
ECG normal (x1.02) 0.922
Hyperglycemia due to type 2 diabetes mellitus (x1.02) 0.923
Type 1 diabetes mellitus without complication (x1.02) 0.924
Hip pain (x1.02) 0.924
Dysuria (x1.02) 0.925
Congestive heart failure (x1.02) 0.926
Dysphagia (x1.02) 0.926
Difficulty walking (x1.02) 0.927
cephalexin (+0.00158) 0.927
Chronic kidney disease stage 3 (x1.02) 0.928
Atrial fibrillation (x1.02) 0.928
Polyneuropathy due to diabetes mellitus (x1.02) 0.929
Cardiac arrhythmia (x1.02) 0.930
Disorder of nervous system due to type 1 diabetes mellitus (x1.02) 0.930
Hypothyroidism (x1.02) 0.931
Morbid obesity (x1.02) 0.931
Anxiety disorder (x1.02) 0.932
clindamycin (+0.00144) 0.932
Asthenia (x1.02) 0.933
Disorder of bone and articular cartilage (x1.02) 0.933
Tinea pedis (/1.02) 0.934
Acute upper respiratory infection (x1.02) 0.934
Localized, primary osteoarthritis (x1.02) 0.934
Acute sinusitis (x1.02) 0.935
Acquired deformity of ankle AND/OR foot (x1.01) 0.935
Depressive disorder (x1.01) 0.936
Acute pharyngitis (x1.01) 0.936
Blood chemistry abnormal (x1.01) 0.937
Nausea and vomiting (x1.01) 0.937
Abnormal breathing (x1.01) 0.937
tramadol (+0.0013) 0.938
Expressions
−0.8 −0.6 −0.4 −0.2 0.0 0.2 0.4 0.6 0.8

Signature [1340]; 1 To Reach 0.975, (10 Plotted)

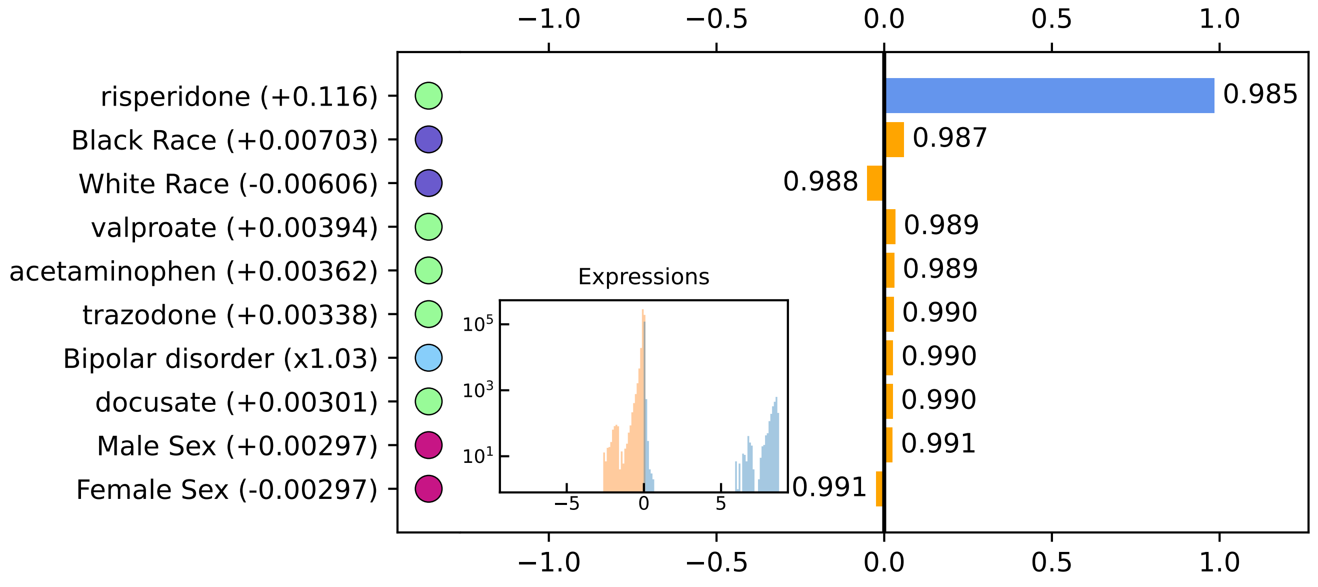

Signature [1341]; 68 To Reach 0.975, (68 Plotted)

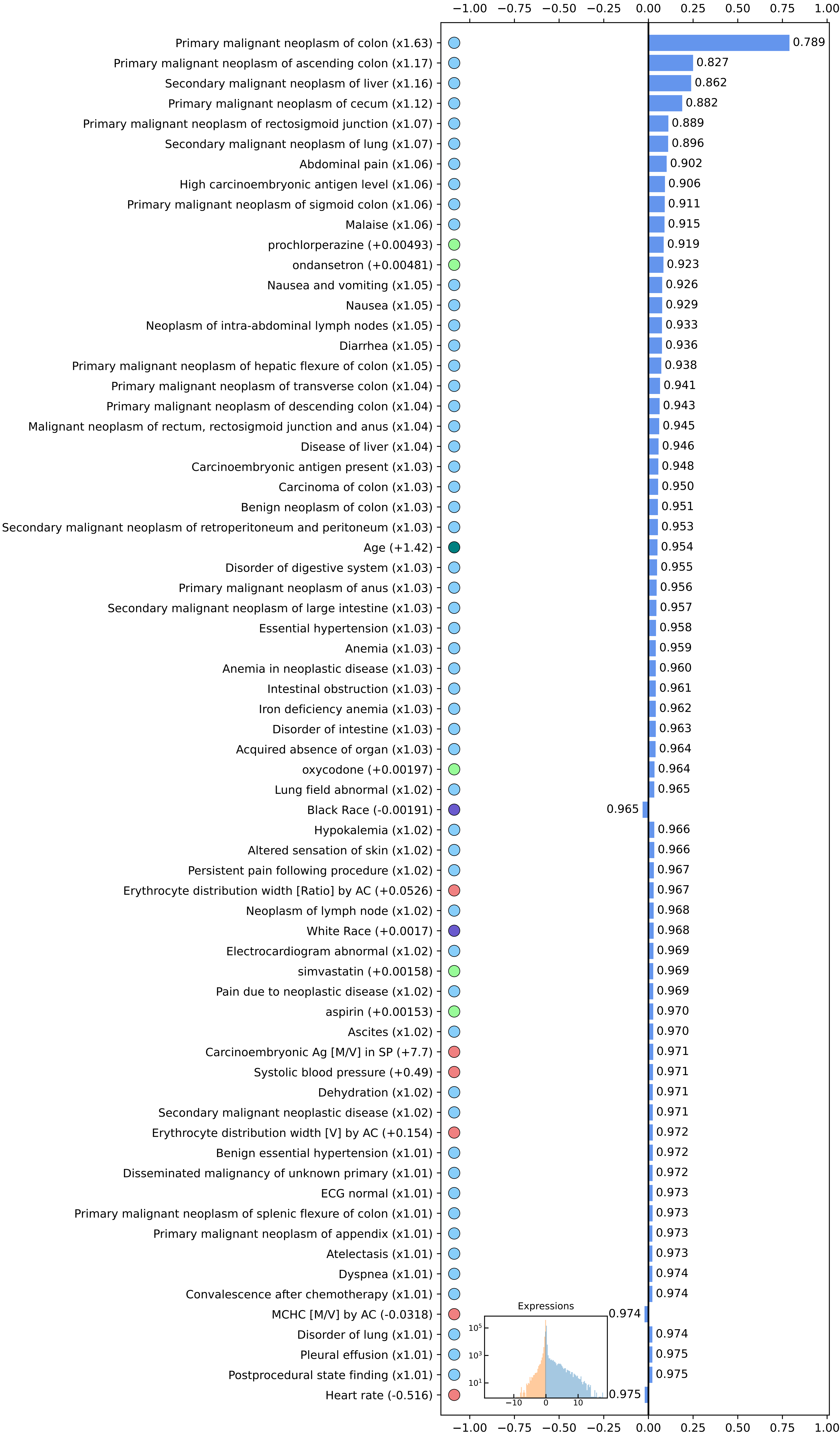

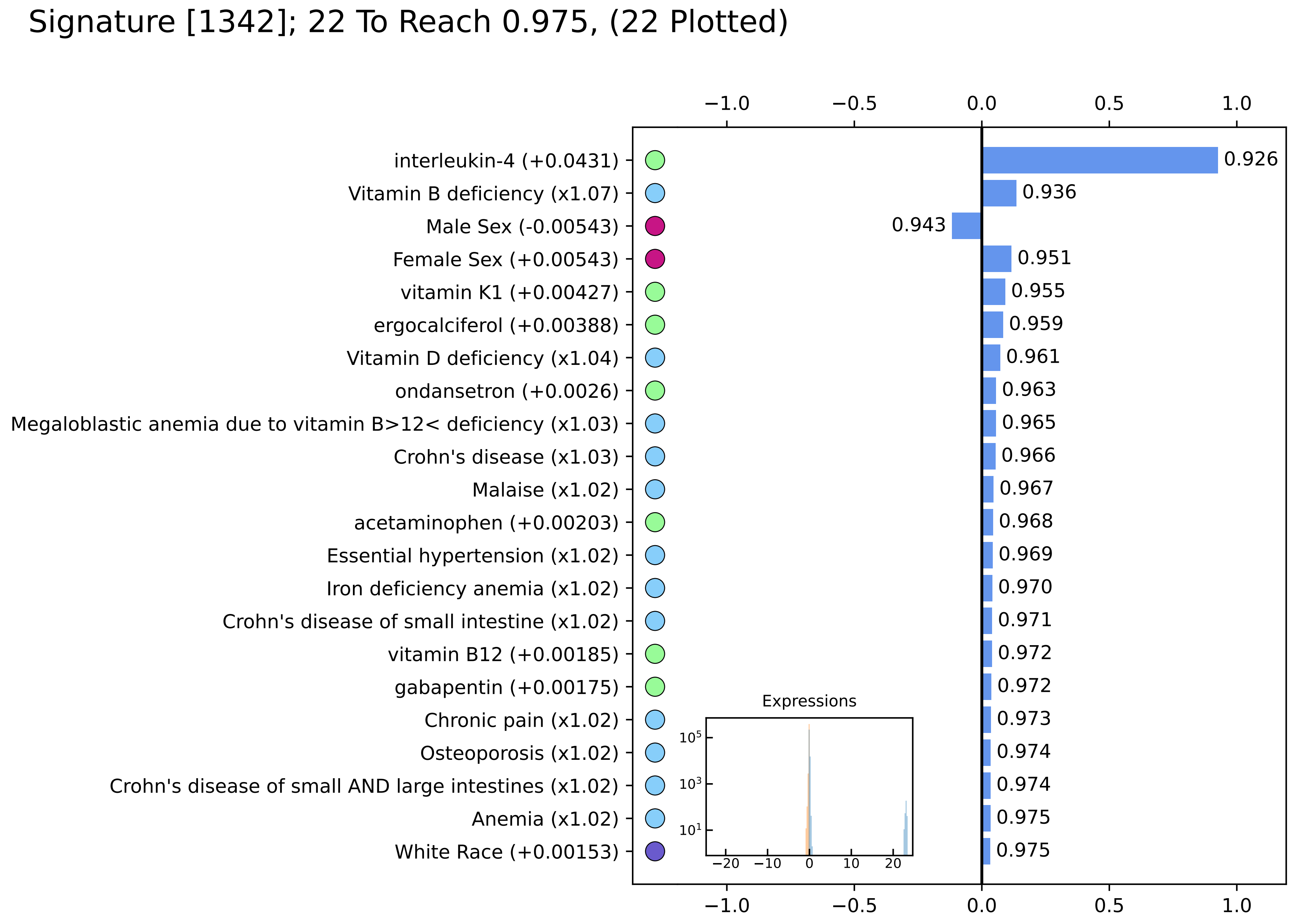

Signature [1342]; 22 To Reach 0.975, (22 Plotted)
interleukin-4 (+0.0431)
Vitamin B deficiency (x1.07)
Male Sex (-0.00543)
Female Sex (+0.00543)
vitamin K1 (+0.00427)
ergocalciferol (+0.00388)
Vitamin D deficiency (x1.04)
ondansetron (+0.0026)
Megaloblastic anemia due to vitamin B>12< deficiency (x1.03)
Crohn's disease (x1.03)
Malaise (x1.02)
acetaminophen (+0.00203)
Essential hypertension (x1.02)
Iron deficiency anemia (x1.02)
Crohn's disease of small intestine (x1.02)
vitamin B12 (+0.00185)
gabapentin (+0.00175)
Chronic pain (x1.02)
Osteoporosis (x1.02)
Crohn's disease of small AND large intestines (x1.02)
Anemia (x1.02)
White Race (+0.00153)
0.926
0.936
0.943
0.951
0.955
0.959
0.961
0.963
0.965
0.966
0.967
0.968
0.969
0.970
0.971
0.972
0.972
0.973
0.974
0.974
0.975
0.975
−1.0
−0.5
0.0
0.5
1.0
Expressions
10^5
10^3
10^1
−20
−10
0
10
20

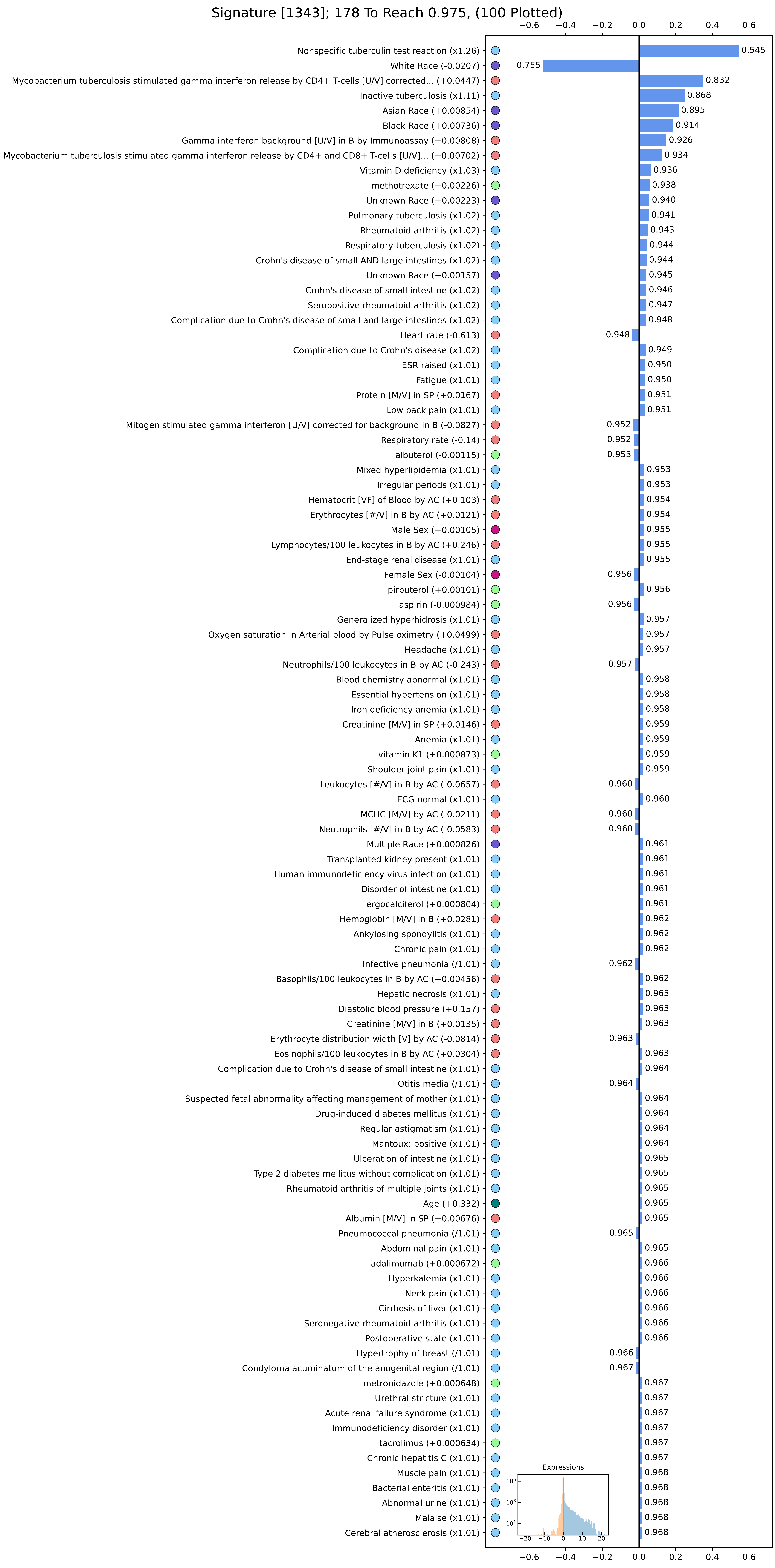

Signature [1343]; 178 To Reach 0.975, (100 Plotted)
Nonspecific tuberculin test reaction (x1.26) 0.545
White Race (-0.0207) 0.755
Mycobacterium tuberculosis stimulated gamma interferon release by CD4+ T-cells [U/V] corrected... (+0.0447) 0.832
Inactive tuberculosis (x1.11) 0.868
Asian Race (+0.00854) 0.895
Black Race (+0.00736) 0.914
Gamma interferon background [U/V] in B by Immunoassay (+0.00808) 0.926
Mycobacterium tuberculosis stimulated gamma interferon release by CD4+ and CD8+ T-cells [U/V]... (+0.00702) 0.934
Vitamin D deficiency (x1.03) 0.936
methotrexate (+0.00226) 0.938
Unknown Race (+0.00223) 0.940
Pulmonary tuberculosis (x1.02) 0.941
Rheumatoid arthritis (x1.02) 0.943
Respiratory tuberculosis (x1.02) 0.944
Crohn's disease of small AND large intestines (x1.02) 0.944
Unknown Race (+0.00157) 0.945
Crohn's disease of small intestine (x1.02) 0.946
Seropositive rheumatoid arthritis (x1.02) 0.947
Complication due to Crohn's disease of small and large intestines (x1.02) 0.948
Heart rate (-0.613) 0.948
Complication due to Crohn's disease (x1.02) 0.949
ESR raised (x1.01) 0.950
Fatigue (x1.01) 0.950
Protein [M/V] in SP (+0.0167) 0.951
Low back pain (x1.01) 0.951
Mitogen stimulated gamma interferon [U/V] corrected for background in B (-0.0827) 0.952
Respiratory rate (-0.14) 0.952
albuterol (-0.00115) 0.953
Mixed hyperlipidemia (x1.01) 0.953
Irregular periods (x1.01) 0.953
Hematocrit [VF] of Blood by AC (+0.103) 0.954
Erythrocytes [#/V] in B by AC (+0.0121) 0.954
Male Sex (+0.00105) 0.955
Lymphocytes/100 leukocytes in B by AC (+0.246) 0.955
End-stage renal disease (x1.01) 0.955
Female Sex (-0.00104) 0.956
pirbuterol (+0.00101) 0.956
aspirin (-0.000984) 0.956
Generalized hyperhidrosis (x1.01) 0.957
Oxygen saturation in Arterial blood by Pulse oximetry (+0.0499) 0.957
Headache (x1.01) 0.957
Neutrophils/100 leukocytes in B by AC (-0.243) 0.957
Blood chemistry abnormal (x1.01) 0.958
Essential hypertension (x1.01) 0.958
Iron deficiency anemia (x1.01) 0.958
Creatinine [M/V] in SP (+0.0146) 0.959
Anemia (x1.01) 0.959
vitamin K1 (+0.000873) 0.959
Shoulder joint pain (x1.01) 0.959
Leukocytes [#/V] in B by AC (-0.0657) 0.960
ECG normal (x1.01) 0.960
MCHC [M/V] by AC (-0.0211) 0.960
Neutrophils [#/V] in B by AC (-0.0583) 0.960
Multiple Race (+0.000826) 0.961
Transplanted kidney present (x1.01) 0.961
Human immunodeficiency virus infection (x1.01) 0.961
Disorder of intestine (x1.01) 0.961
ergocalciferol (+0.000804) 0.961
Hemoglobin [M/V] in B (+0.0281) 0.962
Ankylosing spondylitis (x1.01) 0.962
Chronic pain (x1.01) 0.962
Infective pneumonia (/1.01) 0.962
Basophils/100 leukocytes in B by AC (+0.00456) 0.962
Hepatic necrosis (x1.01) 0.963
Diastolic blood pressure (+0.157) 0.963
Creatinine [M/V] in B (+0.0135) 0.963
Erythrocyte distribution width [V] by AC (-0.0814) 0.963
Eosinophils/100 leukocytes in B by AC (+0.0304) 0.963
Complication due to Crohn's disease of small intestine (x1.01) 0.964
Otitis media (/1.01) 0.964
Suspected fetal abnormality affecting management of mother (x1.01) 0.964
Drug-induced diabetes mellitus (x1.01) 0.964
Regular astigmatism (x1.01) 0.964
Mantoux: positive (x1.01) 0.964
Ulceration of intestine (x1.01) 0.965
Type 2 diabetes mellitus without complication (x1.01) 0.965
Rheumatoid arthritis of multiple joints (x1.01) 0.965
Age (+0.332) 0.965
Albumin [M/V] in SP (+0.00676) 0.965
Pneumococcal pneumonia (/1.01) 0.965
Abdominal pain (x1.01) 0.965
adalimumab (+0.000672) 0.966
Hyperkalemia (x1.01) 0.966
Neck pain (x1.01) 0.966
Cirrhosis of liver (x1.01) 0.966
Seronegative rheumatoid arthritis (x1.01) 0.966
Postoperative state (x1.01) 0.966
Hypertrophy of breast (/1.01) 0.966
Condyloma acuminatum of the anogenital region (/1.01) 0.967
metronidazole (+0.000648) 0.967
Urethral stricture (x1.01) 0.967
Acute renal failure syndrome (x1.01) 0.967
Immunodeficiency disorder (x1.01) 0.967
tacrolimus (+0.000634) 0.967
Chronic hepatitis C (x1.01) 0.967
Muscle pain (x1.01) 0.968
Bacterial enteritis (x1.01) 0.968
Abnormal urine (x1.01) 0.968
Malaise (x1.01) 0.968
Cerebral atherosclerosis (x1.01) 0.968
−0.6 −0.4 −0.2 0.0 0.2 0.4 0.6
Expressions

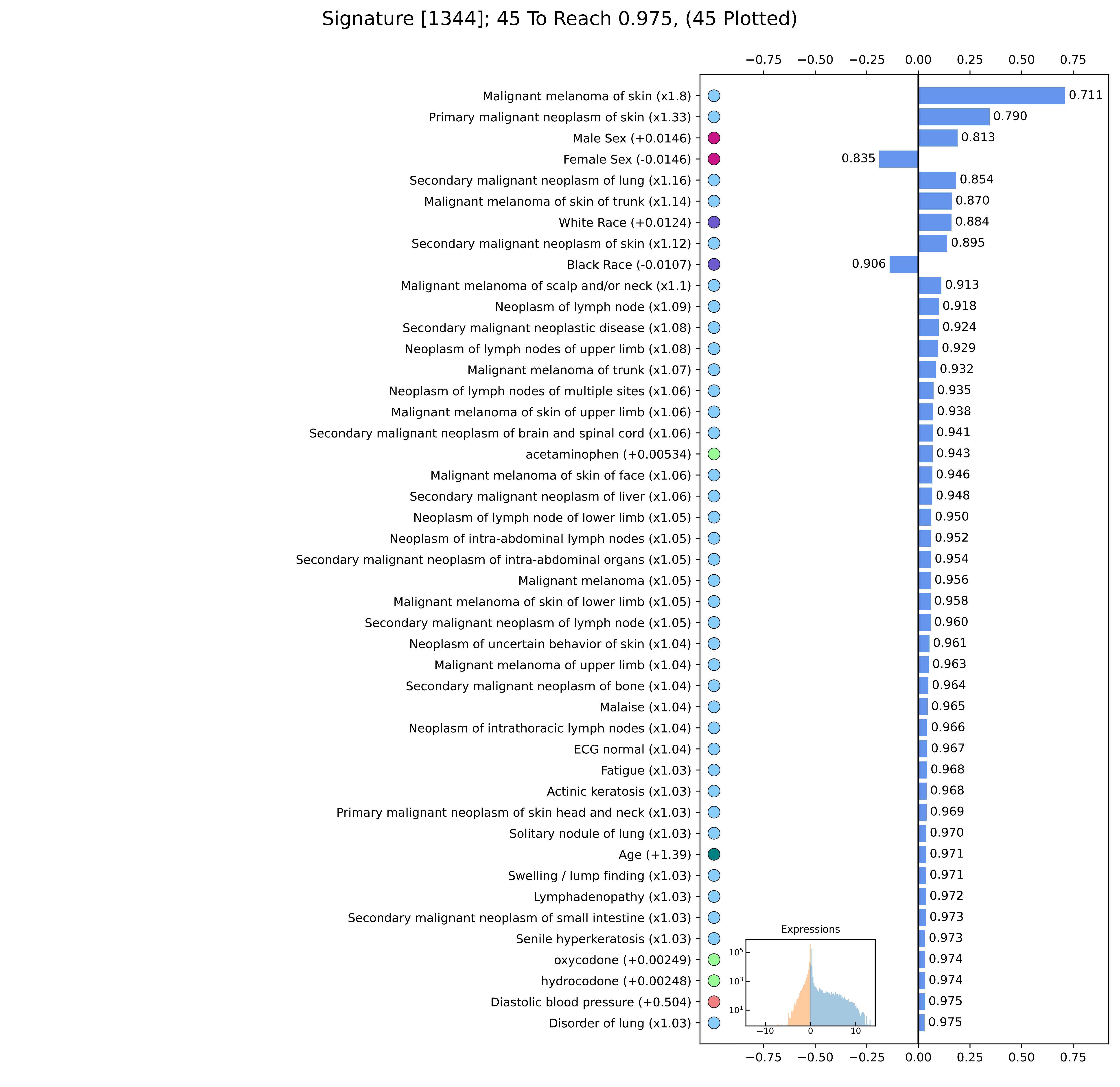
Signature [1344]; 45 To Reach 0.975, (45 Plotted)
Malignant melanoma of skin (x1.8) 0.711
Primary malignant neoplasm of skin (x1.33) 0.790
Male Sex (+0.0146) 0.813
Female Sex (-0.0146) 0.835
Secondary malignant neoplasm of lung (x1.16) 0.854
Malignant melanoma of skin of trunk (x1.14) 0.870
White Race (+0.0124) 0.884
Secondary malignant neoplasm of skin (x1.12) 0.895
Black Race (-0.0107) 0.906
Malignant melanoma of scalp and/or neck (x1.1) 0.913
Neoplasm of lymph node (x1.09) 0.918
Secondary malignant neoplastic disease (x1.08) 0.924
Neoplasm of lymph nodes of upper limb (x1.08) 0.929
Malignant melanoma of trunk (x1.07) 0.932
Neoplasm of lymph nodes of multiple sites (x1.06) 0.935
Malignant melanoma of skin of upper limb (x1.06) 0.938
Secondary malignant neoplasm of brain and spinal cord (x1.06) 0.941
acetaminophen (+0.00534) 0.943
Malignant melanoma of skin of face (x1.06) 0.946
Secondary malignant neoplasm of liver (x1.06) 0.948
Neoplasm of lymph node of lower limb (x1.05) 0.950
Neoplasm of intra-abdominal lymph nodes (x1.05) 0.952
Secondary malignant neoplasm of intra-abdominal organs (x1.05) 0.954
Malignant melanoma (x1.05) 0.956
Malignant melanoma of skin of lower limb (x1.05) 0.958
Secondary malignant neoplasm of lymph node (x1.05) 0.960
Neoplasm of uncertain behavior of skin (x1.04) 0.961
Malignant melanoma of upper limb (x1.04) 0.963
Secondary malignant neoplasm of bone (x1.04) 0.964
Malaise (x1.04) 0.965
Neoplasm of intrathoracic lymph nodes (x1.04) 0.966
ECG normal (x1.04) 0.967
Fatigue (x1.03) 0.968
Actinic keratosis (x1.03) 0.968
Primary malignant neoplasm of skin head and neck (x1.03) 0.969
Solitary nodule of lung (x1.03) 0.970
Age (+1.39) 0.971
Swelling / lump finding (x1.03) 0.971
Lymphadenopathy (x1.03) 0.972
Secondary malignant neoplasm of small intestine (x1.03) 0.973
Senile hyperkeratosis (x1.03) 0.973
oxycodone (+0.00249) 0.974
hydrocodone (+0.00248) 0.974
Diastolic blood pressure (+0.504) 0.975
Disorder of lung (x1.03) 0.975
−0.75 −0.50 −0.25 0.00 0.25 0.50 0.75
Expressions
10^5
10^3
10^1
−10 0 10

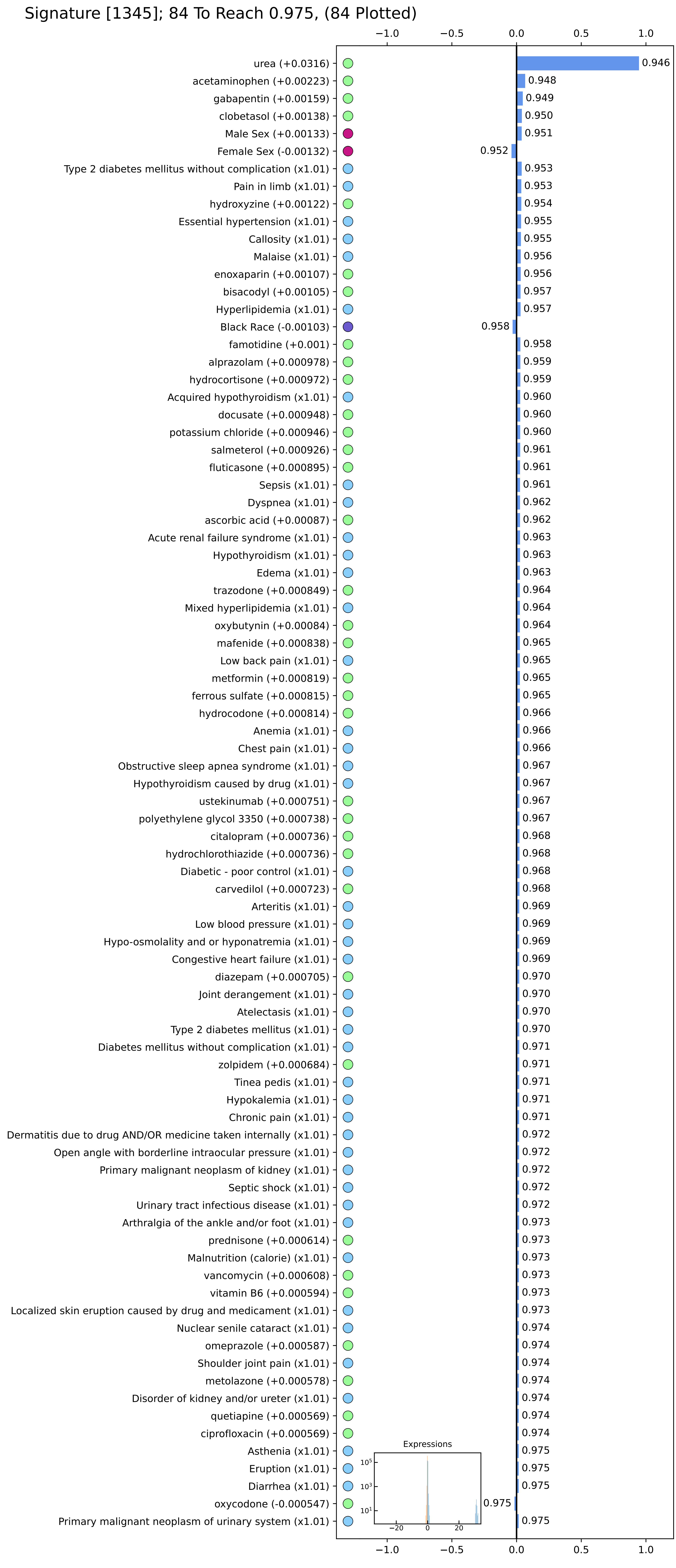

Signature [1345]; 84 To Reach 0.975, (84 Plotted)
−1.0
−0.5
0.0
0.5
1.0
urea (+0.0316) 0.946
acetaminophen (+0.00223) 0.948
gabapentin (+0.00159) 0.949
clobetasol (+0.00138) 0.950
Male Sex (+0.00133) 0.951
Female Sex (-0.00132) 0.952
Type 2 diabetes mellitus without complication (x1.01) 0.953
Pain in limb (x1.01) 0.953
hydroxyzine (+0.00122) 0.954
Essential hypertension (x1.01) 0.955
Callosity (x1.01) 0.955
Malaise (x1.01) 0.956
enoxaparin (+0.00107) 0.956
bisacodyl (+0.00105) 0.957
Hyperlipidemia (x1.01) 0.957
Black Race (-0.00103) 0.958
famotidine (+0.001) 0.958
alprazolam (+0.000978) 0.959
hydrocortisone (+0.000972) 0.959
Acquired hypothyroidism (x1.01) 0.960
docusate (+0.000948) 0.960
potassium chloride (+0.000946) 0.960
salmeterol (+0.000926) 0.961
fluticasone (+0.000895) 0.961
Sepsis (x1.01) 0.961
Dyspnea (x1.01) 0.962
ascorbic acid (+0.00087) 0.962
Acute renal failure syndrome (x1.01) 0.963
Hypothyroidism (x1.01) 0.963
Edema (x1.01) 0.963
trazodone (+0.000849) 0.964
Mixed hyperlipidemia (x1.01) 0.964
oxybutynin (+0.00084) 0.964
mafenide (+0.000838) 0.965
Low back pain (x1.01) 0.965
metformin (+0.000819) 0.965
ferrous sulfate (+0.000815) 0.965
hydrocodone (+0.000814) 0.966
Anemia (x1.01) 0.966
Chest pain (x1.01) 0.966
Obstructive sleep apnea syndrome (x1.01) 0.967
Hypothyroidism caused by drug (x1.01) 0.967
ustekinumab (+0.000751) 0.967
polyethylene glycol 3350 (+0.000738) 0.967
citalopram (+0.000736) 0.968
hydrochlorothiazide (+0.000736) 0.968
Diabetic - poor control (x1.01) 0.968
carvedilol (+0.000723) 0.968
Arteritis (x1.01) 0.969
Low blood pressure (x1.01) 0.969
Hypo-osmolality and or hyponatremia (x1.01) 0.969
Congestive heart failure (x1.01) 0.969
diazepam (+0.000705) 0.970
Joint derangement (x1.01) 0.970
Atelectasis (x1.01) 0.970
Type 2 diabetes mellitus (x1.01) 0.970
Diabetes mellitus without complication (x1.01) 0.971
zolpidem (+0.000684) 0.971
Tinea pedis (x1.01) 0.971
Hypokalemia (x1.01) 0.971
Chronic pain (x1.01) 0.971
Dermatitis due to drug AND/OR medicine taken internally (x1.01) 0.972
Open angle with borderline intraocular pressure (x1.01) 0.972
Primary malignant neoplasm of kidney (x1.01) 0.972
Septic shock (x1.01) 0.972
[truncated: 335,132 more chars]
